# Comprehensive analysis of chromothripsis in 2,658 human cancers using whole-genome sequencing

**DOI:** 10.1038/s41588-019-0576-7

**Published:** 2020-02-05

**Authors:** Isidro Cortés-Ciriano, Jake June-Koo Lee, Ruibin Xi, Dhawal Jain, Youngsook L. Jung, Lixing Yang, Dmitry Gordenin, Leszek J. Klimczak, Cheng-Zhong Zhang, David S. Pellman, PCAWG Structural Variation Working Group, Kadir C. Akdemir, Eva G. Alvarez, Adrian Baez-Ortega, Rameen Beroukhim, Paul C. Boutros, David D. L. Bowtell, Benedikt Brors, Kathleen H. Burns, Peter J. Campbell, Kin Chan, Ken Chen, Isidro Cortés-Ciriano, Ana Dueso-Barroso, Andrew J. Dunford, Paul A. Edwards, Xavier Estivill, Dariush Etemadmoghadam, Lars Feuerbach, J. Lynn Fink, Milana Frenkel-Morgenstern, Dale W. Garsed, Mark Gerstein, Dmitry A. Gordenin, David Haan, James E. Haber, Julian M. Hess, Barbara Hutter, Marcin Imielinski, David T. W. Jones, Young Seok Ju, Marat D. Kazanov, Leszek J. Klimczak, Youngil Koh, Jan O. Korbel, Kiran Kumar, Eunjung Alice Lee, Jake June-Koo Lee, Yilong Li, Andy G. Lynch, Geoff Macintyre, Florian Markowetz, Iñigo Martincorena, Alexander Martinez-Fundichely, Satoru Miyano, Hidewaki Nakagawa, Fabio C. P. Navarro, Stephan Ossowski, Peter J. Park, John V. Pearson, Montserrat Puiggròs, Karsten Rippe, Nicola D. Roberts, Steven A. Roberts, Bernardo Rodriguez-Martin, Steven E. Schumacher, Ralph Scully, Mark Shackleton, Nikos Sidiropoulos, Lina Sieverling, Chip Stewart, David Torrents, Jose M. C. Tubio, Izar Villasante, Nicola Waddell, Jeremiah A. Wala, Joachim Weischenfeldt, Lixing Yang, Xiaotong Yao, Sung-Soo Yoon, Jorge Zamora, Cheng-Zhong Zhang, Peter J. Park, PCAWG Consortium, Lauri A. Aaltonen, Federico Abascal, Adam Abeshouse, Hiroyuki Aburatani, David J. Adams, Nishant Agrawal, Keun Soo Ahn, Sung-Min Ahn, Hiroshi Aikata, Rehan Akbani, Kadir C. Akdemir, Hikmat Al-Ahmadie, Sultan T. Al-Sedairy, Fatima Al-Shahrour, Malik Alawi, Monique Albert, Kenneth Aldape, Ludmil B. Alexandrov, Adrian Ally, Kathryn Alsop, Eva G. Alvarez, Fernanda Amary, Samirkumar B. Amin, Brice Aminou, Ole Ammerpohl, Matthew J. Anderson, Yeng Ang, Davide Antonello, Pavana Anur, Samuel Aparicio, Elizabeth L. Appelbaum, Yasuhito Arai, Axel Aretz, Koji Arihiro, Shun-ichi Ariizumi, Joshua Armenia, Laurent Arnould, Sylvia Asa, Yassen Assenov, Gurnit Atwal, Sietse Aukema, J. Todd Auman, Miriam R. R. Aure, Philip Awadalla, Marta Aymerich, Gary D. Bader, Adrian Baez-Ortega, Matthew H. Bailey, Peter J. Bailey, Miruna Balasundaram, Saianand Balu, Pratiti Bandopadhayay, Rosamonde E. Banks, Stefano Barbi, Andrew P. Barbour, Jonathan Barenboim, Jill Barnholtz-Sloan, Hugh Barr, Elisabet Barrera, John Bartlett, Javier Bartolome, Claudio Bassi, Oliver F. Bathe, Daniel Baumhoer, Prashant Bavi, Stephen B. Baylin, Wojciech Bazant, Duncan Beardsmore, Timothy A. Beck, Sam Behjati, Andreas Behren, Beifang Niu, Cindy Bell, Sergi Beltran, Christopher Benz, Andrew Berchuck, Anke K. Bergmann, Erik N. Bergstrom, Benjamin P. Berman, Daniel M. Berney, Stephan H. Bernhart, Rameen Beroukhim, Mario Berrios, Samantha Bersani, Johanna Bertl, Miguel Betancourt, Vinayak Bhandari, Shriram G. Bhosle, Andrew V. Biankin, Matthias Bieg, Darell Bigner, Hans Binder, Ewan Birney, Michael Birrer, Nidhan K. Biswas, Bodil Bjerkehagen, Tom Bodenheimer, Lori Boice, Giada Bonizzato, Johann S. De Bono, Arnoud Boot, Moiz S. Bootwalla, Ake Borg, Arndt Borkhardt, Keith A. Boroevich, Ivan Borozan, Christoph Borst, Marcus Bosenberg, Mattia Bosio, Jacqueline Boultwood, Guillaume Bourque, Paul C. Boutros, G. Steven Bova, David T. Bowen, Reanne Bowlby, David D. L. Bowtell, Sandrine Boyault, Rich Boyce, Jeffrey Boyd, Alvis Brazma, Paul Brennan, Daniel S. Brewer, Arie B. Brinkman, Robert G. Bristow, Russell R. Broaddus, Jane E. Brock, Malcolm Brock, Annegien Broeks, Angela N. Brooks, Denise Brooks, Benedikt Brors, Søren Brunak, Timothy J. C. Bruxner, Alicia L. Bruzos, Alex Buchanan, Ivo Buchhalter, Christiane Buchholz, Susan Bullman, Hazel Burke, Birgit Burkhardt, Kathleen H. Burns, John Busanovich, Carlos D. Bustamante, Adam P. Butler, Atul J. Butte, Niall J. Byrne, Anne-Lise Børresen-Dale, Samantha J. Caesar-Johnson, Andy Cafferkey, Declan Cahill, Claudia Calabrese, Carlos Caldas, Fabien Calvo, Niedzica Camacho, Peter J. Campbell, Elias Campo, Cinzia Cantù, Shaolong Cao, Thomas E. Carey, Joana Carlevaro-Fita, Rebecca Carlsen, Ivana Cataldo, Mario Cazzola, Jonathan Cebon, Robert Cerfolio, Dianne E. Chadwick, Dimple Chakravarty, Don Chalmers, Calvin Wing Yiu Chan, Kin Chan, Michelle Chan-Seng-Yue, Vishal S. Chandan, David K. Chang, Stephen J. Chanock, Lorraine A. Chantrill, Aurélien Chateigner, Nilanjan Chatterjee, Kazuaki Chayama, Hsiao-Wei Chen, Jieming Chen, Ken Chen, Yiwen Chen, Zhaohong Chen, Andrew D. Cherniack, Jeremy Chien, Yoke-Eng Chiew, Suet-Feung Chin, Juok Cho, Sunghoon Cho, Jung Kyoon Choi, Wan Choi, Christine Chomienne, Zechen Chong, Su Pin Choo, Angela Chou, Angelika N. Christ, Elizabeth L. Christie, Eric Chuah, Carrie Cibulskis, Kristian Cibulskis, Sara Cingarlini, Peter Clapham, Alexander Claviez, Sean Cleary, Nicole Cloonan, Marek Cmero, Colin C. Collins, Ashton A. Connor, Susanna L. Cooke, Colin S. Cooper, Leslie Cope, Vincenzo Corbo, Matthew G. Cordes, Stephen M. Cordner, Isidro Cortés-Ciriano, Kyle Covington, Prue A. Cowin, Brian Craft, David Craft, Chad J. Creighton, Yupeng Cun, Erin Curley, Ioana Cutcutache, Karolina Czajka, Bogdan Czerniak, Rebecca A. Dagg, Ludmila Danilova, Maria Vittoria Davi, Natalie R. Davidson, Helen Davies, Ian J. Davis, Brandi N. Davis-Dusenbery, Kevin J. Dawson, Francisco M. De La Vega, Ricardo De Paoli-Iseppi, Timothy Defreitas, Angelo P. Dei Tos, Olivier Delaneau, John A. Demchok, Jonas Demeulemeester, German M. Demidov, Deniz Demircioğlu, Nening M. Dennis, Robert E. Denroche, Stefan C. Dentro, Nikita Desai, Vikram Deshpande, Amit G. Deshwar, Christine Desmedt, Jordi Deu-Pons, Noreen Dhalla, Neesha C. Dhani, Priyanka Dhingra, Rajiv Dhir, Anthony DiBiase, Klev Diamanti, Li Ding, Shuai Ding, Huy Q. Dinh, Luc Dirix, HarshaVardhan Doddapaneni, Nilgun Donmez, Michelle T. Dow, Ronny Drapkin, Oliver Drechsel, Ruben M. Drews, Serge Serge, Tim Dudderidge, Ana Dueso-Barroso, Andrew J. Dunford, Michael Dunn, Lewis Jonathan Dursi, Fraser R. Duthie, Ken Dutton-Regester, Jenna Eagles, Douglas F. Easton, Stuart Edmonds, Paul A. Edwards, Sandra E. Edwards, Rosalind A. Eeles, Anna Ehinger, Juergen Eils, Roland Eils, Adel El-Naggar, Matthew Eldridge, Kyle Ellrott, Serap Erkek, Georgia Escaramis, Shadrielle M. G. Espiritu, Xavier Estivill, Dariush Etemadmoghadam, Jorunn E. Eyfjord, Bishoy M. Faltas, Daiming Fan, Yu Fan, William C. Faquin, Claudiu Farcas, Matteo Fassan, Aquila Fatima, Francesco Favero, Nodirjon Fayzullaev, Ina Felau, Sian Fereday, Martin L. Ferguson, Vincent Ferretti, Lars Feuerbach, Matthew A. Field, J. Lynn Fink, Gaetano Finocchiaro, Cyril Fisher, Matthew W. Fittall, Anna Fitzgerald, Rebecca C. Fitzgerald, Adrienne M. Flanagan, Neil E. Fleshner, Paul Flicek, John A. Foekens, Kwun M. Fong, Nuno A. Fonseca, Christopher S. Foster, Natalie S. Fox, Michael Fraser, Scott Frazer, Milana Frenkel-Morgenstern, William Friedman, Joan Frigola, Catrina C. Fronick, Akihiro Fujimoto, Masashi Fujita, Masashi Fukayama, Lucinda A. Fulton, Robert S. Fulton, Mayuko Furuta, P. Andrew Futreal, Anja Füllgrabe, Stacey B. Gabriel, Steven Gallinger, Carlo Gambacorti-Passerini, Jianjiong Gao, Shengjie Gao, Levi Garraway, Øystein Garred, Erik Garrison, Dale W. Garsed, Nils Gehlenborg, Josep L. L. Gelpi, Joshy George, Daniela S. Gerhard, Clarissa Gerhauser, Jeffrey E. Gershenwald, Mark Gerstein, Moritz Gerstung, Gad Getz, Mohammed Ghori, Ronald Ghossein, Nasra H. Giama, Richard A. Gibbs, Bob Gibson, Anthony J. Gill, Pelvender Gill, Dilip D. Giri, Dominik Glodzik, Vincent J. Gnanapragasam, Maria Elisabeth Goebler, Mary J. Goldman, Carmen Gomez, Santiago Gonzalez, Abel Gonzalez-Perez, Dmitry A. Gordenin, James Gossage, Kunihito Gotoh, Ramaswamy Govindan, Dorthe Grabau, Janet S. Graham, Robert C. Grant, Anthony R. Green, Eric Green, Liliana Greger, Nicola Grehan, Sonia Grimaldi, Sean M. Grimmond, Robert L. Grossman, Adam Grundhoff, Gunes Gundem, Qianyun Guo, Manaswi Gupta, Shailja Gupta, Ivo G. Gut, Marta Gut, Jonathan Göke, Gavin Ha, Andrea Haake, David Haan, Siegfried Haas, Kerstin Haase, James E. Haber, Nina Habermann, Faraz Hach, Syed Haider, Natsuko Hama, Freddie C. Hamdy, Anne Hamilton, Mark P. Hamilton, Leng Han, George B. Hanna, Martin Hansmann, Nicholas J. Haradhvala, Olivier Harismendy, Ivon Harliwong, Arif O. Harmanci, Eoghan Harrington, Takanori Hasegawa, David Haussler, Steve Hawkins, Shinya Hayami, Shuto Hayashi, D. Neil Hayes, Stephen J. Hayes, Nicholas K. Hayward, Steven Hazell, Yao He, Allison P. Heath, Simon C. Heath, David Hedley, Apurva M. Hegde, David I. Heiman, Michael C. Heinold, Zachary Heins, Lawrence E. Heisler, Eva Hellstrom-Lindberg, Mohamed Helmy, Seong Gu Heo, Austin J. Hepperla, José María Heredia-Genestar, Carl Herrmann, Peter Hersey, Julian M. Hess, Holmfridur Hilmarsdottir, Jonathan Hinton, Satoshi Hirano, Nobuyoshi Hiraoka, Katherine A. Hoadley, Asger Hobolth, Ermin Hodzic, Jessica I. Hoell, Steve Hoffmann, Oliver Hofmann, Andrea Holbrook, Aliaksei Z. Holik, Michael A. Hollingsworth, Oliver Holmes, Robert A. Holt, Chen Hong, Eun Pyo Hong, Jongwhi H. Hong, Gerrit K. Hooijer, Henrik Hornshøj, Fumie Hosoda, Yong Hou, Volker Hovestadt, William Howat, Alan P. Hoyle, Ralph H. Hruban, Jianhong Hu, Taobo Hu, Xing Hua, Kuan-lin Huang, Mei Huang, Mi Ni Huang, Vincent Huang, Yi Huang, Wolfgang Huber, Thomas J. Hudson, Michael Hummel, Jillian A. Hung, David Huntsman, Ted R. Hupp, Jason Huse, Matthew R. Huska, Barbara Hutter, Carolyn M. Hutter, Daniel Hübschmann, Christine A. Iacobuzio-Donahue, Charles David Imbusch, Marcin Imielinski, Seiya Imoto, William B. Isaacs, Keren Isaev, Shumpei Ishikawa, Murat Iskar, S. M. Ashiqul Islam, Michael Ittmann, Sinisa Ivkovic, Jose M. G. Izarzugaza, Jocelyne Jacquemier, Valerie Jakrot, Nigel B. Jamieson, Gun Ho Jang, Se Jin Jang, Joy C. Jayaseelan, Reyka Jayasinghe, Stuart R. Jefferys, Karine Jegalian, Jennifer L. Jennings, Seung-Hyup Jeon, Lara Jerman, Yuan Ji, Wei Jiao, Peter A. Johansson, Amber L. Johns, Jeremy Johns, Rory Johnson, Todd A. Johnson, Clemency Jolly, Yann Joly, Jon G. Jonasson, Corbin D. Jones, David R. Jones, David T. W. Jones, Nic Jones, Steven J. M. Jones, Jos Jonkers, Young Seok Ju, Hartmut Juhl, Jongsun Jung, Malene Juul, Randi Istrup Juul, Sissel Juul, Natalie Jäger, Rolf Kabbe, Andre Kahles, Abdullah Kahraman, Vera B. Kaiser, Hojabr Kakavand, Sangeetha Kalimuthu, Christof von Kalle, Koo Jeong Kang, Katalin Karaszi, Beth Karlan, Rosa Karlić, Dennis Karsch, Katayoon Kasaian, Karin S. Kassahn, Hitoshi Katai, Mamoru Kato, Hiroto Katoh, Yoshiiku Kawakami, Jonathan D. Kay, Stephen H. Kazakoff, Marat D. Kazanov, Maria Keays, Electron Kebebew, Richard F. Kefford, Manolis Kellis, James G. Kench, Catherine J. Kennedy, Jules N. A. Kerssemakers, David Khoo, Vincent Khoo, Narong Khuntikeo, Ekta Khurana, Helena Kilpinen, Hark Kyun Kim, Hyung-Lae Kim, Hyung-Yong Kim, Hyunghwan Kim, Jaegil Kim, Jihoon Kim, Jong K. Kim, Youngwook Kim, Tari A. King, Wolfram Klapper, Kortine Kleinheinz, Leszek J. Klimczak, Stian Knappskog, Michael Kneba, Bartha M. Knoppers, Youngil Koh, Jan Komorowski, Daisuke Komura, Mitsuhiro Komura, Gu Kong, Marcel Kool, Jan O. Korbel, Viktoriya Korchina, Andrey Korshunov, Michael Koscher, Roelof Koster, Zsofia Kote-Jarai, Antonios Koures, Milena Kovacevic, Barbara Kremeyer, Helene Kretzmer, Markus Kreuz, Savitri Krishnamurthy, Dieter Kube, Kiran Kumar, Pardeep Kumar, Sushant Kumar, Yogesh Kumar, Ritika Kundra, Kirsten Kübler, Ralf Küppers, Jesper Lagergren, Phillip H. Lai, Peter W. Laird, Sunil R. Lakhani, Christopher M. Lalansingh, Emilie Lalonde, Fabien C. Lamaze, Adam Lambert, Eric Lander, Pablo Landgraf, Luca Landoni, Anita Langerød, Andrés Lanzós, Denis Larsimont, Erik Larsson, Mark Lathrop, Loretta M. S. Lau, Chris Lawerenz, Rita T. Lawlor, Michael S. Lawrence, Alexander J. Lazar, Ana Mijalkovic Lazic, Xuan Le, Darlene Lee, Donghoon Lee, Eunjung Alice Lee, Hee Jin Lee, Jake June-Koo Lee, Jeong-Yeon Lee, Juhee Lee, Ming Ta Michael Lee, Henry Lee-Six, Kjong-Van Lehmann, Hans Lehrach, Dido Lenze, Conrad R. Leonard, Daniel A. Leongamornlert, Ignaty Leshchiner, Louis Letourneau, Ivica Letunic, Douglas A. Levine, Lora Lewis, Tim Ley, Chang Li, Constance H. Li, Haiyan Irene Li, Jun Li, Lin Li, Shantao Li, Siliang Li, Xiaobo Li, Xiaotong Li, Xinyue Li, Yilong Li, Han Liang, Sheng-Ben Liang, Peter Lichter, Pei Lin, Ziao Lin, W. M. Linehan, Ole Christian Lingjærde, Dongbing Liu, Eric Minwei Liu, Fei-Fei Fei Liu, Fenglin Liu, Jia Liu, Xingmin Liu, Julie Livingstone, Dimitri Livitz, Naomi Livni, Lucas Lochovsky, Markus Loeffler, Georgina V. Long, Armando Lopez-Guillermo, Shaoke Lou, David N. Louis, Laurence B. Lovat, Yiling Lu, Yong-Jie Lu, Youyong Lu, Claudio Luchini, Ilinca Lungu, Xuemei Luo, Hayley J. Luxton, Andy G. Lynch, Lisa Lype, Cristina López, Carlos López-Otín, Eric Z. Ma, Yussanne Ma, Gaetan MacGrogan, Shona MacRae, Geoff Macintyre, Tobias Madsen, Kazuhiro Maejima, Andrea Mafficini, Dennis T. Maglinte, Arindam Maitra, Partha P. Majumder, Luca Malcovati, Salem Malikic, Giuseppe Malleo, Graham J. Mann, Luisa Mantovani-Löffler, Kathleen Marchal, Giovanni Marchegiani, Elaine R. Mardis, Adam A. Margolin, Maximillian G. Marin, Florian Markowetz, Julia Markowski, Jeffrey Marks, Tomas Marques-Bonet, Marco A. Marra, Luke Marsden, John W. M. Martens, Sancha Martin, Jose I. Martin-Subero, Iñigo Martincorena, Alexander Martinez-Fundichely, Yosef E. Maruvka, R. Jay Mashl, Charlie E. Massie, Thomas J. Matthew, Lucy Matthews, Erik Mayer, Simon Mayes, Michael Mayo, Faridah Mbabaali, Karen McCune, Ultan McDermott, Patrick D. McGillivray, Michael D. McLellan, John D. McPherson, John R. McPherson, Treasa A. McPherson, Samuel R. Meier, Alice Meng, Shaowu Meng, Andrew Menzies, Neil D. Merrett, Sue Merson, Matthew Meyerson, William Meyerson, Piotr A. Mieczkowski, George L. Mihaiescu, Sanja Mijalkovic, Tom Mikkelsen, Michele Milella, Linda Mileshkin, Christopher A. Miller, David K. Miller, Jessica K. Miller, Gordon B. Mills, Ana Milovanovic, Sarah Minner, Marco Miotto, Gisela Mir Arnau, Lisa Mirabello, Chris Mitchell, Thomas J. Mitchell, Satoru Miyano, Naoki Miyoshi, Shinichi Mizuno, Fruzsina Molnár-Gábor, Malcolm J. Moore, Richard A. Moore, Sandro Morganella, Quaid D. Morris, Carl Morrison, Lisle E. Mose, Catherine D. Moser, Ferran Muiños, Loris Mularoni, Andrew J. Mungall, Karen Mungall, Elizabeth A. Musgrove, Ville Mustonen, David Mutch, Francesc Muyas, Donna M. Muzny, Alfonso Muñoz, Jerome Myers, Ola Myklebost, Peter Möller, Genta Nagae, Adnan M. Nagrial, Hardeep K. Nahal-Bose, Hitoshi Nakagama, Hidewaki Nakagawa, Hiromi Nakamura, Toru Nakamura, Kaoru Nakano, Tannistha Nandi, Jyoti Nangalia, Mia Nastic, Arcadi Navarro, Fabio C. P. Navarro, David E. Neal, Gerd Nettekoven, Felicity Newell, Steven J. Newhouse, Yulia Newton, Alvin Wei Tian Ng, Anthony Ng, Jonathan Nicholson, David Nicol, Yongzhan Nie, G. Petur Nielsen, Morten Muhlig Nielsen, Serena Nik-Zainal, Michael S. Noble, Katia Nones, Paul A. Northcott, Faiyaz Notta, Brian D. O’Connor, Peter O’Donnell, Maria O’Donovan, Sarah O’Meara, Brian Patrick O’Neill, J. Robert O’Neill, David Ocana, Angelica Ochoa, Layla Oesper, Christopher Ogden, Hideki Ohdan, Kazuhiro Ohi, Lucila Ohno-Machado, Karin A. Oien, Akinyemi I. Ojesina, Hidenori Ojima, Takuji Okusaka, Larsson Omberg, Choon Kiat Ong, Stephan Ossowski, German Ott, B. F. Francis Ouellette, Christine P’ng, Marta Paczkowska, Salvatore Paiella, Chawalit Pairojkul, Marina Pajic, Qiang Pan-Hammarström, Elli Papaemmanuil, Irene Papatheodorou, Nagarajan Paramasivam, Ji Wan Park, Joong-Won Park, Keunchil Park, Kiejung Park, Peter J. Park, Joel S. Parker, Simon L. Parsons, Harvey Pass, Danielle Pasternack, Alessandro Pastore, Ann-Marie Patch, Iris Pauporté, Antonio Pea, John V. Pearson, Chandra Sekhar Pedamallu, Jakob Skou Pedersen, Paolo Pederzoli, Martin Peifer, Nathan A. Pennell, Charles M. Perou, Marc D. Perry, Gloria M. Petersen, Myron Peto, Nicholas Petrelli, Robert Petryszak, Stefan M. Pfister, Mark Phillips, Oriol Pich, Hilda A. Pickett, Todd D. Pihl, Nischalan Pillay, Sarah Pinder, Mark Pinese, Andreia V. Pinho, Esa Pitkänen, Xavier Pivot, Elena Piñeiro-Yáñez, Laura Planko, Christoph Plass, Paz Polak, Tirso Pons, Irinel Popescu, Olga Potapova, Aparna Prasad, Shaun R. Preston, Manuel Prinz, Antonia L. Pritchard, Stephenie D. Prokopec, Elena Provenzano, Xose S. Puente, Sonia Puig, Montserrat Puiggròs, Sergio Pulido-Tamayo, Gulietta M. Pupo, Colin A. Purdie, Michael C. Quinn, Raquel Rabionet, Janet S. Rader, Bernhard Radlwimmer, Petar Radovic, Benjamin Raeder, Keiran M. Raine, Manasa Ramakrishna, Kamna Ramakrishnan, Suresh Ramalingam, Benjamin J. Raphael, W. Kimryn Rathmell, Tobias Rausch, Guido Reifenberger, Jüri Reimand, Jorge Reis-Filho, Victor Reuter, Iker Reyes-Salazar, Matthew A. Reyna, Sheila M. Reynolds, Esther Rheinbay, Yasser Riazalhosseini, Andrea L. Richardson, Julia Richter, Matthew Ringel, Markus Ringnér, Yasushi Rino, Karsten Rippe, Jeffrey Roach, Lewis R. Roberts, Nicola D. Roberts, Steven A. Roberts, A. Gordon Robertson, Alan J. Robertson, Javier Bartolomé Rodriguez, Bernardo Rodriguez-Martin, F. Germán Rodríguez-González, Michael H. A. Roehrl, Marius Rohde, Hirofumi Rokutan, Gilles Romieu, Ilse Rooman, Tom Roques, Daniel Rosebrock, Mara Rosenberg, Philip C. Rosenstiel, Andreas Rosenwald, Edward W. Rowe, Romina Royo, Steven G. Rozen, Yulia Rubanova, Mark A. Rubin, Carlota Rubio-Perez, Vasilisa A. Rudneva, Borislav C. Rusev, Andrea Ruzzenente, Gunnar Rätsch, Radhakrishnan Sabarinathan, Veronica Y. Sabelnykova, Sara Sadeghi, S. Cenk Sahinalp, Natalie Saini, Mihoko Saito-Adachi, Gordon Saksena, Adriana Salcedo, Roberto Salgado, Leonidas Salichos, Richard Sallari, Charles Saller, Roberto Salvia, Michelle Sam, Jaswinder S. Samra, Francisco Sanchez-Vega, Chris Sander, Grant Sanders, Rajiv Sarin, Iman Sarrafi, Aya Sasaki-Oku, Torill Sauer, Guido Sauter, Robyn P. M. Saw, Maria Scardoni, Christopher J. Scarlett, Aldo Scarpa, Ghislaine Scelo, Dirk Schadendorf, Jacqueline E. Schein, Markus B. Schilhabel, Matthias Schlesner, Thorsten Schlomm, Heather K. Schmidt, Sarah-Jane Schramm, Stefan Schreiber, Nikolaus Schultz, Steven E. Schumacher, Roland F. Schwarz, Richard A. Scolyer, David Scott, Ralph Scully, Raja Seethala, Ayellet V. Segre, Iris Selander, Colin A. Semple, Yasin Senbabaoglu, Subhajit Sengupta, Elisabetta Sereni, Stefano Serra, Dennis C. Sgroi, Mark Shackleton, Nimish C. Shah, Sagedeh Shahabi, Catherine A. Shang, Ping Shang, Ofer Shapira, Troy Shelton, Ciyue Shen, Hui Shen, Rebecca Shepherd, Ruian Shi, Yan Shi, Yu-Jia Shiah, Tatsuhiro Shibata, Juliann Shih, Eigo Shimizu, Kiyo Shimizu, Seung Jun Shin, Yuichi Shiraishi, Tal Shmaya, Ilya Shmulevich, Solomon I. Shorser, Charles Short, Raunak Shrestha, Suyash S. Shringarpure, Craig Shriver, Shimin Shuai, Nikos Sidiropoulos, Reiner Siebert, Anieta M. Sieuwerts, Lina Sieverling, Sabina Signoretti, Katarzyna O. Sikora, Michele Simbolo, Ronald Simon, Janae V. Simons, Jared T. Simpson, Peter T. Simpson, Samuel Singer, Nasa Sinnott-Armstrong, Payal Sipahimalani, Tara J. Skelly, Marcel Smid, Jaclyn Smith, Karen Smith-McCune, Nicholas D. Socci, Heidi J. Sofia, Matthew G. Soloway, Lei Song, Anil K. Sood, Sharmila Sothi, Christos Sotiriou, Cameron M. Soulette, Paul N. Span, Paul T. Spellman, Nicola Sperandio, Andrew J. Spillane, Oliver Spiro, Jonathan Spring, Johan Staaf, Peter F. Stadler, Peter Staib, Stefan G. Stark, Lucy Stebbings, Ólafur Andri Stefánsson, Oliver Stegle, Lincoln D. Stein, Alasdair Stenhouse, Chip Stewart, Stephan Stilgenbauer, Miranda D. Stobbe, Michael R. Stratton, Jonathan R. Stretch, Adam J. Struck, Joshua M. Stuart, Henk G. Stunnenberg, Hong Su, Xiaoping Su, Ren X. Sun, Stephanie Sungalee, Hana Susak, Akihiro Suzuki, Fred Sweep, Monika Szczepanowski, Holger Sültmann, Takashi Yugawa, Angela Tam, David Tamborero, Benita Kiat Tee Tan, Donghui Tan, Patrick Tan, Hiroko Tanaka, Hirokazu Taniguchi, Tomas J. Tanskanen, Maxime Tarabichi, Roy Tarnuzzer, Patrick Tarpey, Morgan L. Taschuk, Kenji Tatsuno, Simon Tavaré, Darrin F. Taylor, Amaro Taylor-Weiner, Jon W. Teague, Bin Tean Teh, Varsha Tembe, Javier Temes, Kevin Thai, Sarah P. Thayer, Nina Thiessen, Gilles Thomas, Sarah Thomas, Alan Thompson, Alastair M. Thompson, John F. F. Thompson, R. Houston Thompson, Heather Thorne, Leigh B. Thorne, Adrian Thorogood, Grace Tiao, Nebojsa Tijanic, Lee E. Timms, Roberto Tirabosco, Marta Tojo, Stefania Tommasi, Christopher W. Toon, Umut H. Toprak, David Torrents, Giampaolo Tortora, Jörg Tost, Yasushi Totoki, David Townend, Nadia Traficante, Isabelle Treilleux, Jean-Rémi Trotta, Lorenz H. P. Trümper, Ming Tsao, Tatsuhiko Tsunoda, Jose M. C. Tubio, Olga Tucker, Richard Turkington, Daniel J. Turner, Andrew Tutt, Masaki Ueno, Naoto T. Ueno, Christopher Umbricht, Husen M. Umer, Timothy J. Underwood, Lara Urban, Tomoko Urushidate, Tetsuo Ushiku, Liis Uusküla-Reimand, Alfonso Valencia, David J. Van Den Berg, Steven Van Laere, Peter Van Loo, Erwin G. Van Meir, Gert G. Van den Eynden, Theodorus Van der Kwast, Naveen Vasudev, Miguel Vazquez, Ravikiran Vedururu, Umadevi Veluvolu, Shankar Vembu, Lieven P. C. Verbeke, Peter Vermeulen, Clare Verrill, Alain Viari, David Vicente, Caterina Vicentini, K. VijayRaghavan, Juris Viksna, Ricardo E. Vilain, Izar Villasante, Anne Vincent-Salomon, Tapio Visakorpi, Douglas Voet, Paresh Vyas, Ignacio Vázquez-García, Nick M. Waddell, Nicola Waddell, Claes Wadelius, Lina Wadi, Rabea Wagener, Jeremiah A. Wala, Jian Wang, Jiayin Wang, Linghua Wang, Qi Wang, Wenyi Wang, Yumeng Wang, Zhining Wang, Paul M. Waring, Hans-Jörg Warnatz, Jonathan Warrell, Anne Y. Warren, Sebastian M. Waszak, David C. Wedge, Dieter Weichenhan, Paul Weinberger, John N. Weinstein, Joachim Weischenfeldt, Daniel J. Weisenberger, Ian Welch, Michael C. Wendl, Johannes Werner, Justin P. Whalley, David A. Wheeler, Hayley C. Whitaker, Dennis Wigle, Matthew D. Wilkerson, Ashley Williams, James S. Wilmott, Gavin W. Wilson, Julie M. Wilson, Richard K. Wilson, Boris Winterhoff, Jeffrey A. Wintersinger, Maciej Wiznerowicz, Stephan Wolf, Bernice H. Wong, Tina Wong, Winghing Wong, Youngchoon Woo, Scott Wood, Bradly G. Wouters, Adam J. Wright, Derek W. Wright, Mark H. Wright, Chin-Lee Wu, Dai-Ying Wu, Guanming Wu, Jianmin Wu, Kui Wu, Yang Wu, Zhenggang Wu, Liu Xi, Tian Xia, Qian Xiang, Xiao Xiao, Rui Xing, Heng Xiong, Qinying Xu, Yanxun Xu, Hong Xue, Shinichi Yachida, Sergei Yakneen, Rui Yamaguchi, Takafumi N. Yamaguchi, Masakazu Yamamoto, Shogo Yamamoto, Hiroki Yamaue, Fan Yang, Huanming Yang, Jean Y. Yang, Liming Yang, Lixing Yang, Shanlin Yang, Tsun-Po Yang, Yang Yang, Xiaotong Yao, Marie-Laure Yaspo, Lucy Yates, Christina Yau, Chen Ye, Kai Ye, Venkata D. Yellapantula, Christopher J. Yoon, Sung-Soo Yoon, Fouad Yousif, Jun Yu, Kaixian Yu, Willie Yu, Yingyan Yu, Ke Yuan, Yuan Yuan, Denis Yuen, Christina K. Yung, Olga Zaikova, Jorge Zamora, Marc Zapatka, Jean C. Zenklusen, Thorsten Zenz, Nikolajs Zeps, Cheng-Zhong Zhang, Fan Zhang, Hailei Zhang, Hongwei Zhang, Hongxin Zhang, Jiashan Zhang, Jing Zhang, Junjun Zhang, Xiuqing Zhang, Xuanping Zhang, Yan Zhang, Zemin Zhang, Zhongming Zhao, Liangtao Zheng, Xiuqing Zheng, Wanding Zhou, Yong Zhou, Bin Zhu, Hongtu Zhu, Jingchun Zhu, Shida Zhu, Lihua Zou, Xueqing Zou, Anna deFazio, Nicholas van As, Carolien H. M. van Deurzen, Marc J. van de Vijver, L. van’t Veer, Christian von Mering

**Affiliations:** 1 Department of Biomedical Informatics, Harvard Medical School, Boston, MA USA; 2 Ludwig Center at Harvard, Boston, MA USA; 3 Centre for Molecular Science Informatics, Department of Chemistry, University of Cambridge, Cambridge, UK; 4 School of Mathematical Sciences and Center for Statistical Science, Peking University, Beijing, China; 5 Ben May Department for Cancer Research, University of Chicago, Chicago, IL USA; 6 Department of Human Genetics, The University of Chicago, Chicago, IL USA; 7 Genome Integrity and Structural Biology Laboratory, National Institute of Environmental Health Sciences, US National Institutes of Health, Durham, NC USA; 8 Integrative Bioinformatics Group, National Institute of Environmental Health Sciences, US National Institutes of Health, Durham, NC USA; 9 Broad Institute of MIT and Harvard, Cambridge, MA USA; 10 Department of Biostatistics and Computational Biology, Dana-Farber Cancer Institute and Harvard Medical School, Boston, MA USA; 11 Department of Pediatric Oncology, Dana-Farber Cancer Institute, Boston, MA USA; 12 Department of Cell Biology, Harvard Medical School, Blavatnik Institute, Boston, MA USA; 13 Howard Hughes Medical Institute, Boston, MA USA; 16 Present Address: European Molecular Biology Laboratory, European Bioinformatics Institute (EMBL-EBI), Cambridge, UK; 17 University of Texas MD Anderson Cancer Center, Houston, TX USA; 18 Department of Zoology, Genetics and Physical Anthropology, Universidade de Santiago de Compostela, Santiago de Compostela, Spain; 19 Centre for Research in Molecular Medicine and Chronic Diseases (CIMUS), Universidade de Santiago de Compostela, Santiago de Compostela, Spain; 20 The Biomedical Research Centre (CINBIO), Universidade de Vigo, Vigo, Spain; 21 Transmissible Cancer Group, Department of Veterinary Medicine, University of Cambridge, Cambridge, UK; 22 Department of Medical Oncology, Dana-Farber Cancer Institute, Boston, MA USA; 23 Harvard Medical School, Boston, MA USA; 24 Computational Biology Program, Ontario Institute for Cancer Research, Toronto, Ontario Canada; 25 Department of Medical Biophysics, University of Toronto, Toronto, Ontario Canada; 26 Department of Pharmacology, University of Toronto, Toronto, Ontario Canada; 27 University of California Los Angeles, Los Angeles, CA USA; 28 Peter MacCallum Cancer Centre, Melbourne, Victoria Australia; 29 Sir Peter MacCallum Department of Oncology, University of Melbourne, Melbourne, Victoria Australia; 30 Division of Applied Bioinformatics, German Cancer Research Center (DKFZ), Heidelberg, Germany; 31 German Cancer Genome Consortium (DKTK), German Cancer Research Center (DKFZ), Heidelberg, Germany; 32 National Center for Tumor Diseases (NCT) Heidelberg, Heidelberg, Germany; 33 Johns Hopkins School of Medicine, Baltimore, MD USA; 34 Department of Haematology, University of Cambridge, Cambridge, UK; 35 Wellcome Sanger Institute, Hinxton, UK; 36 Department of Biochemistry, Microbiology and Immunology, Faculty of Medicine, University of Ottawa, Ottawa, Ontario Canada; 37 Barcelona Supercomputing Center (BSC), Barcelona, Spain; 38 Cancer Research UK Cambridge Institute, University of Cambridge, Cambridge, UK; 39 University of Cambridge, Cambridge, UK; 40 Sidra Medicine, Doha, Qatar; 41 Queensland Centre for Medical Genomics, Institute for Molecular Bioscience, University of Queensland, St. Lucia, Brisbane, Queensland, Australia; 42 The Azrieli Faculty of Medicine, Bar-Ilan University, Safed, Israel; 43 Department of Computer Science, Princeton University, Princeton, NJ USA; 44 Department of Computer Science, Yale University, New Haven, CT USA; 45 Department of Molecular Biophysics and Biochemistry, Yale University, New Haven, CT USA; 46 Program in Computational Biology and Bioinformatics, Yale University, New Haven, CT USA; 47 Biomolecular Engineering Department, University of California, Santa Cruz, Santa Cruz, CA USA; 48 Brandeis University, Waltham, MA USA; 49 Massachusetts General Hospital Center for Cancer Research, Charlestown, MA USA; 50 Heidelberg Center for Personalized Oncology (DKFZ-HIPO), German Cancer Research Center (DKFZ), Heidelberg, Germany; 51 New York Genome Center, New York, NY USA; 52 Weill Cornell Medicine, New York, NY USA; 53 Hopp Children’s Cancer Center (KiTZ), Heidelberg, Germany; 54 Pediatric Glioma Research Group, German Cancer Research Center (DKFZ), Heidelberg, Germany; 55 Korea Advanced Institute of Science and Technology, Daejeon, South Korea; 56 Skolkovo Institute of Science and Technology, Moscow, Russia; 57 A. A. Kharkevich Institute of Information Transmission Problems, Moscow, Russia; 58 Dmitry Rogachev National Research Center of Pediatric Hematology, Oncology and Immunology, Moscow, Russia; 59 Center For Medical Innovation, Seoul National University Hospital, Seoul, South Korea; 60 Department of Internal Medicine, Seoul National University Hospital, Seoul, South Korea; 61 European Molecular Biology Laboratory, European Bioinformatics Institute (EMBL-EBI), Hinxton, UK; 62 Genome Biology Unit, European Molecular Biology Laboratory (EMBL), Heidelberg, Germany; 63 Division of Genetics and Genomics, Boston Children’s Hospital and Harvard Medical School, Boston, MA USA; 64 School of Medicine/School of Mathematics and Statistics, University of St. Andrews, St. Andrews, UK; 65 Department of Physiology and Biophysics, Weill Cornell Medicine, New York, NY USA; 66 Englander Institute for Precision Medicine, Weill Cornell Medicine, New York, NY USA; 67 Institute for Computational Biomedicine, Weill Cornell Medicine, New York, NY USA; 68 The Institute of Medical Science, The University of Tokyo, Tokyo, Japan; 69 RIKEN Center for Integrative Medical Sciences, Yokohama, Japan; 70 Centre for Genomic Regulation (CRG), The Barcelona Institute of Science and Technology, Barcelona, Spain; 71 Institute of Medical Genetics and Applied Genomics, University of Tübingen, Tübingen, Germany; 72 Universitat Pompeu Fabra (UPF), Barcelona, Spain; 73 Department of Genetics and Computational Biology, QIMR Berghofer Medical Research Institute, Brisbane, Queensland Australia; 74 Institute for Molecular Bioscience, University of Queensland, St. Lucia, Brisbane, Queensland Australia; 75 German Cancer Research Center (DKFZ), Heidelberg, Germany; 76 School of Molecular Biosciences and Center for Reproductive Biology, Washington State University, Pullman, WA USA; 77 Department of Cancer Biology, Dana-Farber Cancer Institute, Boston, MA USA; 78 Cancer Research Institute, Beth Israel Deaconess Medical Center, Boston, MA USA; 79 Biotech Research and Innovation Centre, Faculty of Health and Medical Sciences, University of Copenhagen, Copenhagen, Denmark; 80 Finsen Laboratory, Righospitalet, Copenhagen, Denmark; 81 Faculty of Biosciences, Heidelberg University, Heidelberg, Germany; 82 Institució Catalana de Recerca i Estudis Avançats (ICREA), Barcelona, Spain; 83 Institució Catalana de Recerca i Estudis Avançats (ICREA), Barcelona, Spain; 84 Department of Urology, Charité Universitätsmedizin Berlin, Berlin, Germany; 85 Tri-Institutional PhD Program of Computational Biology and Medicine, Weill Cornell Medicine, New York, NY USA; 200 Applied Tumor Genomics Research Program, Research Programs Unit, University of Helsinki, Helsinki, Finland; 201 Wellcome Sanger Institute, Wellcome Genome Campus, Hinxton, UK; 202 Memorial Sloan Kettering Cancer Center, New York, NY USA; 203 Genome Science Division, Research Center for Advanced Science and Technology, University of Tokyo, Tokyo, Japan; 204 Department of Surgery, University of Chicago, Chicago, IL USA; 205 Department of Surgery, Division of Hepatobiliary and Pancreatic Surgery, School of Medicine, Keimyung University Dongsan Medical Center, Daegu, South Korea; 206 Department of Oncology, Gil Medical Center, Gachon University, Incheon, South Korea; 207 Hiroshima University, Hiroshima, Japan; 208 Department of Bioinformatics and Computational Biology, The University of Texas MD Anderson Cancer Center, Houston, TX USA; 209 University of Texas MD Anderson Cancer Center, Houston, TX USA; 210 King Faisal Specialist Hospital and Research Centre, Al Maather, Riyadh, Saudi Arabia; 211 Bioinformatics Unit, Spanish National Cancer Research Centre (CNIO), Madrid, Spain; 212 Bioinformatics Core Facility, University Medical Center Hamburg, Hamburg, Germany; 213 Heinrich Pette Institute, Leibniz Institute for Experimental Virology, Hamburg, Germany; 214 Ontario Tumour Bank, Ontario Institute for Cancer Research, Toronto, ON Canada; 215 Department of Pathology, The University of Texas MD Anderson Cancer Center, Houston, TX USA; 216 Laboratory of Pathology, Center for Cancer Research, National Cancer Institute, Bethesda, MD USA; 217 Department of Cellular and Molecular Medicine and Department of Bioengineering, University of California San Diego, La Jolla, CA USA; 218 UC San Diego Moores Cancer Center, San Diego, CA USA; 219 Canada’s Michael Smith Genome Sciences Centre, BC Cancer, Vancouver, BC Canada; 220 Sir Peter MacCallum Department of Oncology, Peter MacCallum Cancer Centre, University of Melbourne, Melbourne, VIC Australia; 221 Centre for Research in Molecular Medicine and Chronic Diseases (CiMUS), Universidade de Santiago de Compostela, Santiago de Compostela, Spain; 222 Department of Zoology, Genetics and Physical Anthropology, (CiMUS), Universidade de Santiago de Compostela, Santiago de Compostela, Spain; 223 The Biomedical Research Centre (CINBIO), Universidade de Vigo, Vigo, Spain; 224 Royal National Orthopaedic Hospital - Bolsover, London, UK; 225 Department of Genomic Medicine, The University of Texas MD Anderson Cancer Center, Houston, TX USA; 226 Quantitative and Computational Biosciences Graduate Program, Baylor College of Medicine, Houston, TX USA; 227 The Jackson Laboratory for Genomic Medicine, Farmington, CT USA; 228 Genome Informatics Program, Ontario Institute for Cancer Research, Toronto, ON Canada; 229 Institute of Human Genetics, Christian-Albrechts-University, Kiel, Germany; 230 Institute of Human Genetics, Ulm University and Ulm University Medical Center, Ulm, Germany; 231 Queensland Centre for Medical Genomics, Institute for Molecular Bioscience, University of Queensland, St. Lucia, Brisbane, QLD Australia; 232 Salford Royal NHS Foundation Trust, Salford, UK; 233 Department of Surgery, Pancreas Institute, University and Hospital Trust of Verona, Verona, Italy; 234 Molecular and Medical Genetics, OHSU Knight Cancer Institute, Oregon Health and Science University, Portland, OR USA; 235 Department of Molecular Oncology, BC Cancer Research Centre, Vancouver, BC Canada; 236 The McDonnell Genome Institute at Washington University, St. Louis, MO USA; 237 University College London, London, UK; 238 Division of Cancer Genomics, National Cancer Center Research Institute, National Cancer Center, Tokyo, Japan; 239 DLR Project Management Agency, Bonn, Germany; 240 Tokyo Women’s Medical University, Tokyo, Japan; 241 Center for Molecular Oncology, Memorial Sloan Kettering Cancer Center, New York, NY USA; 242 Los Alamos National Laboratory, Los Alamos, NM USA; 243 Department of Pathology, University Health Network, Toronto General Hospital, Toronto, ON Canada; 244 Nottingham University Hospitals NHS Trust, Nottingham, UK; 245 Epigenomics and Cancer Risk Factors, German Cancer Research Center (DKFZ), Heidelberg, Germany; 246 Computational Biology Program, Ontario Institute for Cancer Research, Toronto, ON Canada; 247 Department of Molecular Genetics, University of Toronto, Toronto, ON Canada; 248 Vector Institute, Toronto, ON Canada; 249 Hematopathology Section, Institute of Pathology, Christian-Albrechts-University, Kiel, Germany; 250 Department of Pathology and Laboratory Medicine, School of Medicine, University of North Carolina at Chapel Hill, Chapel Hill, NC USA; 251 Department of Cancer Genetics, Institute for Cancer Research, Oslo University Hospital, The Norwegian Radium Hospital, Oslo, Norway; 252 Pathology, Hospital Clinic, Institut d’Investigacions Biomèdiques August Pi i Sunyer (IDIBAPS), University of Barcelona, Barcelona, Spain; 253 Department of Veterinary Medicine, Transmissible Cancer Group, University of Cambridge, Cambridge, UK; 254 Alvin J. Siteman Cancer Center, Washington University School of Medicine, St. Louis, MO USA; 255 Wolfson Wohl Cancer Research Centre, Institute of Cancer Sciences, University of Glasgow, Glasgow, UK; 256 Lineberger Comprehensive Cancer Center, University of North Carolina at Chapel Hill, Chapel Hill, NC USA; 257 Broad Institute of MIT and Harvard, Cambridge, MA USA; 258 Dana-Farber/Boston Children’s Cancer and Blood Disorders Center, Boston, MA USA; 259 Department of Pediatrics, Harvard Medical School, Boston, MA USA; 260 Leeds Institute of Medical Research @ St. James’s, University of Leeds, St. James’s University Hospital, Leeds, UK; 261 Department of Pathology and Diagnostics, University and Hospital Trust of Verona, Verona, Italy; 262 Department of Surgery, Princess Alexandra Hospital, Brisbane, QLD Australia; 263 Surgical Oncology Group, Diamantina Institute, University of Queensland, Brisbane, QLD Australia; 264 Department of Population and Quantitative Health Sciences, Case Western Reserve University School of Medicine, Cleveland, OH USA; 265 Research Health Analytics and Informatics, University Hospitals Cleveland Medical Center, Cleveland, OH USA; 266 Gloucester Royal Hospital, Gloucester, UK; 267 European Molecular Biology Laboratory, European Bioinformatics Institute (EMBL-EBI), Cambridge, UK; 268 Diagnostic Development, Ontario Institute for Cancer Research, Toronto, ON Canada; 269 Barcelona Supercomputing Center (BSC), Barcelona, Spain; 270 Arnie Charbonneau Cancer Institute, University of Calgary, Calgary, AB Canada; 271 Departments of Surgery and Oncology, University of Calgary, Calgary, AB Canada; 272 Department of Pathology, Oslo University Hospital, The Norwegian Radium Hospital, Oslo, Norway; 273 PanCuRx Translational Research Initiative, Ontario Institute for Cancer Research, Toronto, ON Canada; 274 Department of Oncology, Sidney Kimmel Comprehensive Cancer Center at Johns Hopkins University School of Medicine, Baltimore, MD USA; 275 University Hospital Southampton NHS Foundation Trust, Southampton, UK; 276 Royal Stoke University Hospital, Stoke-on-Trent, UK; 277 Genome Sequence Informatics, Ontario Institute for Cancer Research, Toronto, ON Canada; 278 Human Longevity Inc, San Diego, CA USA; 279 Olivia Newton-John Cancer Research Institute, La Trobe University, Heidelberg, VIC Australia; 280 Computer Network Information Center, Chinese Academy of Sciences, Beijing, China; 281 Genome Canada, Ottawa, ON Canada; 282 CNAG-CRG, Centre for Genomic Regulation (CRG), Barcelona Institute of Science and Technology (BIST), Barcelona, Spain; 283 Universitat Pompeu Fabra (UPF), Barcelona, Spain; 284 Buck Institute for Research on Aging, Novato, CA USA; 285 Duke University Medical Center, Durham, NC USA; 286 Department of Human Genetics, Hannover Medical School, Hannover, Germany; 287 Center for Bioinformatics and Functional Genomics, Cedars-Sinai Medical Center, Los Angeles, CA USA; 288 Department of Biomedical Sciences, Cedars-Sinai Medical Center, Los Angeles, CA USA; 289 The Hebrew University Faculty of Medicine, Jerusalem, Israel; 290 Barts Cancer Institute, Barts and the London School of Medicine and Dentistry, Queen Mary University of London, London, UK; 291 Department of Computer Science, Bioinformatics Group, University of Leipzig, Leipzig, Germany; 292 Interdisciplinary Center for Bioinformatics, University of Leipzig, Leipzig, Germany; 293 Transcriptome Bioinformatics, LIFE Research Center for Civilization Diseases, University of Leipzig, Leipzig, Germany; 294 Department of Medical Oncology, Dana-Farber Cancer Institute, Boston, MA USA; 295 Department of Cancer Biology, Dana-Farber Cancer Institute, Boston, MA USA; 296 Harvard Medical School, Boston, MA USA; 297 USC Norris Comprehensive Cancer Center, University of Southern California, Los Angeles, CA USA; 298 Department of Diagnostics and Public Health, University and Hospital Trust of Verona, Verona, Italy; 299 Department of Mathematics, Aarhus University, Aarhus, Denmark; 300 Department of Molecular Medicine (MOMA), Aarhus University Hospital, Aarhus N, Denmark; 301 Instituto Carlos Slim de la Salud, Mexico City, Mexico; 302 Department of Medical Biophysics, University of Toronto, Toronto, ON Canada; 303 Cancer Division, Garvan Institute of Medical Research, Kinghorn Cancer Centre, University of New South Wales (UNSW Sydney), Sydney, NSW Australia; 304 South Western Sydney Clinical School, Faculty of Medicine, University of New South Wales (UNSW Sydney), Liverpool, NSW Australia; 305 West of Scotland Pancreatic Unit, Glasgow Royal Infirmary, Glasgow, UK; 306 Center for Digital Health, Berlin Institute of Health and Charitè - Universitätsmedizin Berlin, Berlin, Germany; 307 Heidelberg Center for Personalized Oncology (DKFZ-HIPO), German Cancer Research Center (DKFZ), Heidelberg, Germany; 308 The Preston Robert Tisch Brain Tumor Center, Duke University Medical Center, Durham, NC USA; 309 Massachusetts General Hospital, Boston, MA USA; 310 National Institute of Biomedical Genomics, Kalyani, West Bengal India; 311 Institute of Clinical Medicine and Institute of Oral Biology, University of Oslo, Oslo, Norway; 312 University of North Carolina at Chapel Hill, Chapel Hill, NC USA; 313 ARC-Net Centre for Applied Research on Cancer, University and Hospital Trust of Verona, Verona, Italy; 314 The Institute of Cancer Research, London, UK; 315 Centre for Computational Biology, Duke-NUS Medical School, Singapore, Singapore; 316 Programme in Cancer and Stem Cell Biology, Duke-NUS Medical School, Singapore, Singapore; 317 Division of Oncology and Pathology, Department of Clinical Sciences Lund, Lund University, Lund, Sweden; 318 Department of Pediatric Oncology, Hematology and Clinical Immunology, Heinrich-Heine-University, Düsseldorf, Germany; 319 Laboratory for Medical Science Mathematics, RIKEN Center for Integrative Medical Sciences, Yokohama, Japan; 320 RIKEN Center for Integrative Medical Sciences, Yokohama, Japan; 321 Department of Internal Medicine/Hematology, Friedrich-Ebert-Hospital, Neumünster, Germany; 322 Departments of Dermatology and Pathology, Yale University, New Haven, CT USA; 323 Centre for Genomic Regulation (CRG), The Barcelona Institute of Science and Technology, Barcelona, Spain; 324 Radcliffe Department of Medicine, University of Oxford, Oxford, UK; 325 Canadian Center for Computational Genomics, McGill University, Montreal, QC Canada; 326 Department of Human Genetics, McGill University, Montreal, QC Canada; 327 Department of Human Genetics, University of California Los Angeles, Los Angeles, CA USA; 328 Department of Pharmacology, University of Toronto, Toronto, ON Canada; 329 Faculty of Medicine and Health Technology, Tampere University and Tays Cancer Center, Tampere University Hospital, Tampere, Finland; 330 Haematology, Leeds Teaching Hospitals NHS Trust, Leeds, UK; 331 Translational Research and Innovation, Centre Léon Bérard, Lyon, France; 332 Fox Chase Cancer Center, Philadelphia, PA USA; 333 International Agency for Research on Cancer, World Health Organization, Lyon, France; 334 Earlham Institute, Norwich, UK; 335 Norwich Medical School, University of East Anglia, Norwich, UK; 336 Department of Molecular Biology, Faculty of Science, Radboud Institute for Molecular Life Sciences, Radboud University, Nijmegen, HB The Netherlands; 337 CRUK Manchester Institute and Centre, Manchester, UK; 338 Department of Radiation Oncology, University of Toronto, Toronto, ON Canada; 339 Division of Cancer Sciences, Manchester Cancer Research Centre, University of Manchester, Manchester, UK; 340 Radiation Medicine Program, Princess Margaret Cancer Centre, Toronto, ON Canada; 341 Department of Pathology, Brigham and Women’s Hospital, Harvard Medical School, Boston, MA USA; 342 Department of Surgery, Division of Thoracic Surgery, The Johns Hopkins University School of Medicine, Baltimore, MD USA; 343 Division of Molecular Pathology, The Netherlands Cancer Institute, Oncode Institute, Amsterdam, CX The Netherlands; 344 Department of Biomolecular Engineering, University of California Santa Cruz, Santa Cruz, CA USA; 345 UC Santa Cruz Genomics Institute, University of California Santa Cruz, Santa Cruz, CA USA; 346 Division of Applied Bioinformatics, German Cancer Research Center (DKFZ), Heidelberg, Germany; 347 German Cancer Consortium (DKTK), German Cancer Research Center (DKFZ), Heidelberg, Germany; 348 National Center for Tumor Diseases (NCT) Heidelberg, Heidelberg, Germany; 349 Center for Biological Sequence Analysis, Department of Bio and Health Informatics, Technical University of Denmark, Lyngby, Denmark; 350 Novo Nordisk Foundation Center for Protein Research, University of Copenhagen, Copenhagen, Denmark; 351 Institute for Molecular Bioscience, University of Queensland, St. Lucia, Brisbane, QLD Australia; 352 Biomedical Engineering, Oregon Health and Science University, Portland, OR USA; 353 Division of Theoretical Bioinformatics, German Cancer Research Center (DKFZ), Heidelberg, Germany; 354 Institute of Pharmacy and Molecular Biotechnology and BioQuant, Heidelberg University, Heidelberg, Germany; 355 Federal Ministry of Education and Research, Berlin, Germany; 356 Melanoma Institute Australia, University of Sydney, Sydney, NSW Australia; 357 Pediatric Hematology and Oncology, University Hospital Muenster, Muenster, Germany; 358 Department of Pathology, Johns Hopkins University School of Medicine, Baltimore, MD USA; 359 McKusick-Nathans Institute of Genetic Medicine, Sidney Kimmel Comprehensive Cancer Center at Johns Hopkins University School of Medicine, Baltimore, MD USA; 360 Foundation Medicine, Inc, Cambridge, MA USA; 361 Department of Biomedical Data Science, Stanford University School of Medicine, Stanford, CA USA; 362 Department of Genetics, Stanford University School of Medicine, Stanford, CA USA; 363 Bakar Computational Health Sciences Institute and Department of Pediatrics, University of California, San Francisco, CA USA; 364 Institute of Clinical Medicine, Faculty of Medicine, University of Oslo, Oslo, Norway; 365 National Cancer Institute, National Institutes of Health, Bethesda, MD USA; 366 Royal Marsden NHS Foundation Trust, London and Sutton, UK; 367 Genome Biology Unit, European Molecular Biology Laboratory (EMBL), Heidelberg, Germany; 368 Department of Oncology, University of Cambridge, Cambridge, UK; 369 Li Ka Shing Centre, Cancer Research UK Cambridge Institute, University of Cambridge, Cambridge, UK; 370 Institut Gustave Roussy, Villejuif, France; 371 Cambridge University Hospitals NHS Foundation Trust, Cambridge, UK; 372 Department of Haematology, University of Cambridge, Cambridge, UK; 373 Anatomia Patológica, Hospital Clinic, Institut d’Investigacions Biomèdiques August Pi i Sunyer (IDIBAPS), University of Barcelona, Barcelona, Spain; 374 Spanish Ministry of Science and Innovation, Madrid, Spain; 375 University of Michigan Comprehensive Cancer Center, Ann Arbor, MI USA; 376 Department for BioMedical Research, University of Bern, Bern, Switzerland; 377 Department of Medical Oncology, Inselspital, University Hospital and University of Bern, Bern, Switzerland; 378 Graduate School for Cellular and Biomedical Sciences, University of Bern, Bern, Switzerland; 379 University of Pavia, Pavia, Italy; 380 University of Alabama at Birmingham, Birmingham, AL USA; 381 UHN Program in BioSpecimen Sciences, Toronto General Hospital, Toronto, ON Canada; 382 Department of Urology, Icahn School of Medicine at Mount Sinai, New York, NY USA; 383 Centre for Law and Genetics, University of Tasmania, Sandy Bay Campus, Hobart, TAS Australia; 384 Faculty of Biosciences, Heidelberg University, Heidelberg, Germany; 385 Department of Biochemistry, Microbiology and Immunology, Faculty of Medicine, University of Ottawa, Ottawa, ON Canada; 386 Division of Anatomic Pathology, Mayo Clinic, Rochester, MN USA; 387 Division of Cancer Epidemiology and Genetics, National Cancer Institute, National Institutes of Health, Bethesda, MD USA; 388 Illawarra Shoalhaven Local Health District L3 Illawarra Cancer Care Centre, Wollongong Hospital, Wollongong, NSW Australia; 389 BioForA, French National Institute for Agriculture, Food, and Environment (INRAE), ONF, Orléans, France; 390 Department of Biostatistics, Bloomberg School of Public Health, Johns Hopkins University, Baltimore, MD USA; 391 University of California San Diego, San Diego, CA USA; 392 Division of Experimental Pathology, Mayo Clinic, Rochester, MN USA; 393 Centre for Cancer Research, The Westmead Institute for Medical Research, University of Sydney, Sydney, NSW Australia; 394 Department of Gynaecological Oncology, Westmead Hospital, Sydney, NSW Australia; 395 PDXen Biosystems Inc, Seoul, South Korea; 396 Korea Advanced Institute of Science and Technology, Daejeon, South Korea; 397 Electronics and Telecommunications Research Institute, Daejeon, South Korea; 398 Institut National du Cancer (INCA), Boulogne-Billancourt, France; 399 Department of Genetics, Informatics Institute, University of Alabama at Birmingham, Birmingham, AL USA; 400 Division of Medical Oncology, National Cancer Centre, Singapore, Singapore; 401 Medical Oncology, University and Hospital Trust of Verona, Verona, Italy; 402 Department of Pediatrics, University Hospital Schleswig-Holstein, Kiel, Germany; 403 Hepatobiliary/Pancreatic Surgical Oncology Program, University Health Network, Toronto, ON Canada; 404 School of Biological Sciences, University of Auckland, Auckland, New Zealand; 405 Department of Surgery, University of Melbourne, Parkville, VIC Australia; 406 The Murdoch Children’s Research Institute, Royal Children’s Hospital, Parkville, VIC Australia; 407 Walter and Eliza Hall Institute, Parkville, VIC Australia; 408 Vancouver Prostate Centre, Vancouver, Canada; 409 Lunenfeld-Tanenbaum Research Institute, Mount Sinai Hospital, Toronto, ON Canada; 410 University of East Anglia, Norwich, UK; 411 Norfolk and Norwich University Hospital NHS Trust, Norwich, UK; 412 Victorian Institute of Forensic Medicine, Southbank, VIC Australia; 413 Department of Biomedical Informatics, Harvard Medical School, Boston, MA USA; 414 Department of Chemistry, Centre for Molecular Science Informatics, University of Cambridge, Cambridge, UK; 415 Ludwig Center at Harvard Medical School, Boston, MA USA; 416 Human Genome Sequencing Center, Baylor College of Medicine, Houston, TX USA; 417 Peter MacCallum Cancer Centre, University of Melbourne, Melbourne, VIC Australia; 418 Physics Division, Optimization and Systems Biology Lab, Massachusetts General Hospital, Boston, MA USA; 419 Department of Medicine, Baylor College of Medicine, Houston, TX USA; 420 University of Cologne, Cologne, Germany; 421 International Genomics Consortium, Phoenix, AZ USA; 422 Genomics Research Program, Ontario Institute for Cancer Research, Toronto, ON Canada; 423 Barking Havering and Redbridge University Hospitals NHS Trust, Romford, UK; 424 Children’s Hospital at Westmead, University of Sydney, Sydney, NSW Australia; 425 Department of Medicine, Section of Endocrinology, University and Hospital Trust of Verona, Verona, Italy; 426 Computational Biology Center, Memorial Sloan Kettering Cancer Center, New York, NY USA; 427 Department of Biology, ETH Zurich, Zürich, Switzerland; 428 Department of Computer Science, ETH Zurich, Zurich, Switzerland; 429 SIB Swiss Institute of Bioinformatics, Lausanne, Switzerland; 430 Weill Cornell Medical College, New York, NY USA; 431 Academic Department of Medical Genetics, University of Cambridge, Addenbrooke’s Hospital, Cambridge, UK; 432 MRC Cancer Unit, University of Cambridge, Cambridge, UK; 433 Departments of Pediatrics and Genetics, University of North Carolina at Chapel Hill, Chapel Hill, NC USA; 434 Seven Bridges Genomics, Charlestown, MA USA; 435 Annai Systems, Inc, Carlsbad, CA USA; 436 Department of Pathology, General Hospital of Treviso, Department of Medicine, University of Padua, Treviso, Italy; 437 Department of Computational Biology, University of Lausanne, Lausanne, Switzerland; 438 Department of Genetic Medicine and Development, University of Geneva Medical School, Geneva, CH Switzerland; 439 Swiss Institute of Bioinformatics, University of Geneva, Geneva, CH Switzerland; 440 The Francis Crick Institute, London, UK; 441 University of Leuven, Leuven, Belgium; 442 Institute of Medical Genetics and Applied Genomics, University of Tübingen, Tübingen, Germany; 443 Computational and Systems Biology, Genome Institute of Singapore, Singapore, Singapore; 444 School of Computing, National University of Singapore, Singapore, Singapore; 445 Big Data Institute, Li Ka Shing Centre, University of Oxford, Oxford, UK; 446 Biomedical Data Science Laboratory, Francis Crick Institute, London, UK; 447 Bioinformatics Group, Department of Computer Science, University College London, London, UK; 448 The Edward S. Rogers Sr. Department of Electrical and Computer Engineering, University of Toronto, Toronto, ON Canada; 449 Breast Cancer Translational Research Laboratory JC Heuson, Institut Jules Bordet, Brussels, Belgium; 450 Department of Oncology, Laboratory for Translational Breast Cancer Research, KU Leuven, Leuven, Belgium; 451 Institute for Research in Biomedicine (IRB Barcelona), The Barcelona Institute of Science and Technology, Barcelona, Spain; 452 Research Program on Biomedical Informatics, Universitat Pompeu Fabra, Barcelona, Spain; 453 Division of Medical Oncology, Princess Margaret Cancer Centre, Toronto, ON Canada; 454 Department of Physiology and Biophysics, Weill Cornell Medicine, New York, NY USA; 455 Institute for Computational Biomedicine, Weill Cornell Medicine, New York, NY USA; 456 Department of Pathology, UPMC Shadyside, Pittsburgh, PA USA; 457 Independent Consultant, Wellesley, USA; 458 Department of Cell and Molecular Biology, Science for Life Laboratory, Uppsala University, Uppsala, Sweden; 459 Department of Medicine and Department of Genetics, Washington University School of Medicine, St. Louis, St. Louis, MO USA; 460 Hefei University of Technology, Anhui, China; 461 Translational Cancer Research Unit, GZA Hospitals St.-Augustinus, Center for Oncological Research, Faculty of Medicine and Health Sciences, University of Antwerp, Antwerp, Belgium; 462 Simon Fraser University, Burnaby, BC Canada; 463 University of Pennsylvania, Philadelphia, PA USA; 464 Faculty of Science and Technology, University of Vic—Central University of Catalonia (UVic-UCC), Vic, Spain; 465 The Wellcome Trust, London, UK; 466 The Hospital for Sick Children, Toronto, ON Canada; 467 Department of Pathology, Queen Elizabeth University Hospital, Glasgow, UK; 468 Department of Genetics and Computational Biology, QIMR Berghofer Medical Research Institute, Brisbane, QLD Australia; 469 Department of Oncology, Centre for Cancer Genetic Epidemiology, University of Cambridge, Cambridge, UK; 470 Department of Public Health and Primary Care, Centre for Cancer Genetic Epidemiology, University of Cambridge, Cambridge, UK; 471 Prostate Cancer Canada, Toronto, ON Canada; 472 University of Cambridge, Cambridge, UK; 473 Department of Laboratory Medicine, Translational Cancer Research, Lund University Cancer Center at Medicon Village, Lund University, Lund, Sweden; 474 Heidelberg University, Heidelberg, Germany; 475 New BIH Digital Health Center, Berlin Institute of Health (BIH) and Charité - Universitätsmedizin Berlin, Berlin, Germany; 476 CIBER Epidemiología y Salud Pública (CIBERESP), Madrid, Spain; 477 Research Group on Statistics, Econometrics and Health (GRECS), UdG, Barcelona, Spain; 478 Quantitative Genomics Laboratories (qGenomics), Barcelona, Spain; 479 Icelandic Cancer Registry, Icelandic Cancer Society, Reykjavik, Iceland; 480 State Key Laboratory of Cancer Biology, and Xijing Hospital of Digestive Diseases, Fourth Military Medical University, Shaanxi, China; 481 Department of Medicine (DIMED), Surgical Pathology Unit, University of Padua, Padua, Italy; 482 Rigshospitalet, Copenhagen, Denmark; 483 Center for Cancer Genomics, National Cancer Institute, National Institutes of Health, Bethesda, MD USA; 484 Department of Biochemistry and Molecular Medicine, University of Montreal, Montreal, QC Canada; 485 Australian Institute of Tropical Health and Medicine, James Cook University, Douglas, QLD Australia; 486 Department of Neuro-Oncology, Istituto Neurologico Besta, Milano, Italy; 487 Bioplatforms Australia, North Ryde, NSW Australia; 488 Department of Pathology (Research), University College London Cancer Institute, London, UK; 489 Department of Surgical Oncology, Princess Margaret Cancer Centre, Toronto, ON Canada; 490 Department of Medical Oncology, Josephine Nefkens Institute and Cancer Genomics Centre, Erasmus Medical Center, Rotterdam, CN The Netherlands; 491 The University of Queensland Thoracic Research Centre, The Prince Charles Hospital, Brisbane, QLD Australia; 492 CIBIO/InBIO - Research Center in Biodiversity and Genetic Resources, Universidade do Porto, Vairão, Portugal; 493 HCA Laboratories, London, UK; 494 University of Liverpool, Liverpool, UK; 495 The Azrieli Faculty of Medicine, Bar-Ilan University, Safed, Israel; 496 Department of Neurosurgery, University of Florida, Gainesville, FL USA; 497 Department of Pathology, Graduate School of Medicine, University of Tokyo, Tokyo, Japan; 498 University of Milano Bicocca, Monza, Italy; 499 BGI-Shenzhen, Shenzhen, China; 500 Department of Pathology, Oslo University Hospital Ulleval, Oslo, Norway; 501 Center for Biomedical Informatics, Harvard Medical School, Boston, MA USA; 502 Department Biochemistry and Molecular Biomedicine, University of Barcelona, Barcelona, Spain; 503 Office of Cancer Genomics, National Cancer Institute, National Institutes of Health, Bethesda, MD USA; 504 Cancer Epigenomics, German Cancer Research Center (DKFZ), Heidelberg, Germany; 505 Department of Cancer Biology, The University of Texas MD Anderson Cancer Center, Houston, TX USA; 506 Department of Surgical Oncology, The University of Texas MD Anderson Cancer Center, Houston, TX USA; 507 Department of Computer Science, Yale University, New Haven, CT USA; 508 Department of Molecular Biophysics and Biochemistry, Yale University, New Haven, CT USA; 509 Program in Computational Biology and Bioinformatics, Yale University, New Haven, CT USA; 510 Center for Cancer Research, Massachusetts General Hospital, Boston, MA USA; 511 Department of Pathology, Massachusetts General Hospital, Boston, MA USA; 512 Department of Pathology, Memorial Sloan Kettering Cancer Center, New York, NY USA; 513 Division of Gastroenterology and Hepatology, Mayo Clinic, Rochester, MN USA; 514 University of Sydney, Sydney, NSW Australia; 515 University of Oxford, Oxford, UK; 516 Department of Surgery, Academic Urology Group, University of Cambridge, Cambridge, UK; 517 Department of Medicine II, University of Würzburg, Wuerzburg, Germany; 518 Sylvester Comprehensive Cancer Center, University of Miami, Miami, FL USA; 519 Institut Hospital del Mar d’Investigacions Mèdiques (IMIM), Barcelona, Spain; 520 Genome Integrity and Structural Biology Laboratory, National Institute of Environmental Health Sciences (NIEHS), Durham, NC USA; 521 St. Thomas’s Hospital, London, UK; 522 Osaka International Cancer Center, Osaka, Japan; 523 Department of Pathology, Skåne University Hospital, Lund University, Lund, Sweden; 524 Department of Medical Oncology, Beatson West of Scotland Cancer Centre, Glasgow, UK; 525 National Human Genome Research Institute, National Institutes of Health, Bethesda, MD USA; 526 Centre for Cancer Research, Victorian Comprehensive Cancer Centre, University of Melbourne, Melbourne, VIC Australia; 527 Department of Medicine, Section of Hematology/Oncology, University of Chicago, Chicago, IL USA; 528 German Center for Infection Research (DZIF), Partner Site Hamburg-Borstel-Lübeck-Riems, Hamburg, Germany; 529 Bioinformatics Research Centre (BiRC), Aarhus University, Aarhus, Denmark; 530 Department of Biotechnology, Ministry of Science and Technology, Government of India, New Delhi, Delhi India; 531 National Cancer Centre Singapore, Singapore, Singapore; 532 Brandeis University, Waltham, MA USA; 533 Department of Urologic Sciences, University of British Columbia, Vancouver, BC Canada; 534 Department of Internal Medicine, Stanford University, Stanford, CA USA; 535 The University of Texas Health Science Center at Houston, Houston, TX USA; 536 Imperial College NHS Trust, Imperial College, London, INY UK; 537 Senckenberg Institute of Pathology, University of Frankfurt Medical School, Frankfurt, Germany; 538 Department of Medicine, Division of Biomedical Informatics, UC San Diego School of Medicine, San Diego, CA USA; 539 Center for Precision Health, School of Biomedical Informatics, The University of Texas Health Science Center, Houston, TX USA; 540 Oxford Nanopore Technologies, New York, NY USA; 541 Institute of Medical Science, University of Tokyo, Tokyo, Japan; 542 Howard Hughes Medical Institute, University of California Santa Cruz, Santa Cruz, CA USA; 543 Wakayama Medical University, Wakayama, Japan; 544 Department of Internal Medicine, Division of Medical Oncology, Lineberger Comprehensive Cancer Center, University of North Carolina at Chapel Hill, Chapel Hill, NC USA; 545 University of Tennessee Health Science Center for Cancer Research, Memphis, TN USA; 546 Department of Histopathology, Salford Royal NHS Foundation Trust, Salford, UK; 547 Faculty of Biology, Medicine and Health, University of Manchester, Manchester, UK; 548 BIOPIC, ICG and College of Life Sciences, Peking University, Beijing, China; 549 Peking-Tsinghua Center for Life Sciences, Peking University, Beijing, China; 550 Children’s Hospital of Philadelphia, Philadelphia, PA USA; 551 Department of Bioinformatics and Computational Biology and Department of Systems Biology, The University of Texas MD Anderson Cancer Center, Houston, TX USA; 552 Karolinska Institute, Stockholm, Sweden; 553 The Donnelly Centre, University of Toronto, Toronto, ON Canada; 554 Department of Medical Genetics, College of Medicine, Hallym University, Chuncheon, South Korea; 555 Department of Experimental and Health Sciences, Institute of Evolutionary Biology (UPF-CSIC), Universitat Pompeu Fabra, Barcelona, Spain; 556 Health Data Science Unit, University Clinics, Heidelberg, Germany; 557 Massachusetts General Hospital Center for Cancer Research, Charlestown, MA USA; 558 Hokkaido University, Sapporo, Japan; 559 Department of Pathology and Clinical Laboratory, National Cancer Center Hospital, Tokyo, Japan; 560 Department of Genetics, University of North Carolina at Chapel Hill, Chapel Hill, NC USA; 561 Computational Biology, Leibniz Institute on Aging - Fritz Lipmann Institute (FLI), Jena, Germany; 562 University of Melbourne Centre for Cancer Research, Melbourne, VIC Australia; 563 University of Nebraska Medical Center, Omaha, NE USA; 564 Syntekabio Inc, Daejeon, South Korea; 565 Department of Pathology, Academic Medical Center, Amsterdam, AZ The Netherlands; 566 China National GeneBank-Shenzhen, Shenzhen, China; 567 Division of Molecular Genetics, German Cancer Research Center (DKFZ), Heidelberg, Germany; 568 Division of Life Science and Applied Genomics Center, Hong Kong University of Science and Technology, Clear Water Bay, Hong Kong, China; 569 Icahn School of Medicine at Mount Sinai, New York, NY USA; 570 Geneplus-Shenzhen, Shenzhen, China; 571 School of Computer Science and Technology, Xi’an Jiaotong University, Xi’an, China; 572 AbbVie, North Chicago, IL USA; 573 Institute of Pathology, Charité – University Medicine Berlin, Berlin, Germany; 574 Centre for Translational and Applied Genomics, British Columbia Cancer Agency, Vancouver, BC Canada; 575 Edinburgh Royal Infirmary, Edinburgh, UK; 576 Berlin Institute for Medical Systems Biology, Max Delbrück Center for Molecular Medicine, Berlin, Germany; 577 Department of Pediatric Immunology, Hematology and Oncology, University Hospital, Heidelberg, Germany; 578 German Cancer Research Center (DKFZ), Heidelberg, Germany; 579 Heidelberg Institute for Stem Cell Technology and Experimental Medicine (HI-STEM), Heidelberg, Germany; 580 Institute for Computational Biomedicine, Weill Cornell Medical College, New York, NY USA; 581 New York Genome Center, New York, NY USA; 582 Department of Urology, James Buchanan Brady Urological Institute, Johns Hopkins University School of Medicine, Baltimore, MD USA; 583 Department of Preventive Medicine, Graduate School of Medicine, The University of Tokyo, Tokyo, Japan; 584 Department of Molecular and Cellular Biology, Baylor College of Medicine, Houston, TX USA; 585 Department of Pathology and Immunology, Baylor College of Medicine, Houston, TX USA; 586 Michael E. DeBakey Veterans Affairs Medical Center, Houston, TX USA; 587 Technical University of Denmark, Lyngby, Denmark; 588 Department of Pathology, College of Medicine, Hanyang University, Seoul, South Korea; 589 Academic Unit of Surgery, School of Medicine, College of Medical, Veterinary and Life Sciences, University of Glasgow, Glasgow Royal Infirmary, Glasgow, UK; 590 Department of Pathology, Asan Medical Center, College of Medicine, Ulsan University, Songpa-gu, Seoul South Korea; 591 Science Writer, Garrett Park, MD USA; 592 International Cancer Genome Consortium (ICGC)/ICGC Accelerating Research in Genomic Oncology (ARGO) Secretariat, Ontario Institute for Cancer Research, Toronto, ON Canada; 593 University of Ljubljana, Ljubljana, Slovenia; 594 Department of Public Health Sciences, University of Chicago, Chicago, IL USA; 595 Research Institute, NorthShore University HealthSystem, Evanston, IL USA; 596 Department for Biomedical Research, University of Bern, Bern, Switzerland; 597 Centre of Genomics and Policy, McGill University and Génome Québec Innovation Centre, Montreal, QC Canada; 598 Carolina Center for Genome Sciences, University of North Carolina at Chapel Hill, Chapel Hill, NC USA; 599 Hopp Children’s Cancer Center (KiTZ), Heidelberg, Germany; 600 Pediatric Glioma Research Group, German Cancer Research Center (DKFZ), Heidelberg, Germany; 601 Cancer Research UK, London, UK; 602 Indivumed GmbH, Hamburg, Germany; 603 Genome Integration Data Center, Syntekabio, Inc, Daejeon, South Korea; 604 University Hospital Zurich, Zurich, Switzerland; 605 Clinical Bioinformatics, Swiss Institute of Bioinformatics, Geneva, Switzerland; 606 Institute for Pathology and Molecular Pathology, University Hospital Zurich, Zurich, Switzerland; 607 Institute of Molecular Life Sciences, University of Zurich, Zurich, Switzerland; 608 MRC Human Genetics Unit, MRC IGMM, University of Edinburgh, Edinburgh, UK; 609 Women’s Cancer Program at the Samuel Oschin Comprehensive Cancer Institute, Cedars-Sinai Medical Center, Los Angeles, CA USA; 610 Department of Biology, Bioinformatics Group, Division of Molecular Biology, Faculty of Science, University of Zagreb, Zagreb, Croatia; 611 Department for Internal Medicine II, University Hospital Schleswig-Holstein, Kiel, Germany; 612 Genetics and Molecular Pathology, SA Pathology, Adelaide, SA Australia; 613 Department of Gastric Surgery, National Cancer Center Hospital, Tokyo, Japan; 614 Department of Bioinformatics, Division of Cancer Genomics, National Cancer Center Research Institute, Tokyo, Japan; 615 A.A. Kharkevich Institute of Information Transmission Problems, Moscow, Russia; 616 Oncology and Immunology, Dmitry Rogachev National Research Center of Pediatric Hematology, Moscow, Russia; 617 Skolkovo Institute of Science and Technology, Moscow, Russia; 618 Department of Surgery, The George Washington University, School of Medicine and Health Science, Washington, DC USA; 619 Endocrine Oncology Branch, Center for Cancer Research, National Cancer Institute, National Institutes of Health, Bethesda, MD USA; 620 Melanoma Institute Australia, Macquarie University, Sydney, NSW Australia; 621 MIT Computer Science and Artificial Intelligence Laboratory, Massachusetts Institute of Technology, Cambridge, MA USA; 622 Tissue Pathology and Diagnostic Oncology, Royal Prince Alfred Hospital, Sydney, NSW Australia; 623 Cholangiocarcinoma Screening and Care Program and Liver Fluke and Cholangiocarcinoma Research Centre, Faculty of Medicine, Khon Kaen University, Khon Kaen, Thailand; 624 Controlled Department and Institution, New York, NY USA; 625 Englander Institute for Precision Medicine, Weill Cornell Medicine, New York, NY USA; 626 National Cancer Center, Gyeonggi, South Korea; 627 Department of Biochemistry, College of Medicine, Ewha Womans University, Seoul, South Korea; 628 Health Sciences Department of Biomedical Informatics, University of California San Diego, La Jolla, CA USA; 629 Research Core Center, National Cancer Centre Korea, Goyang-si, South Korea; 630 Department of Health Sciences and Technology, Sungkyunkwan University School of Medicine, Seoul, South Korea; 631 Samsung Genome Institute, Seoul, South Korea; 632 Breast Oncology Program, Dana-Farber/Brigham and Women’s Cancer Center, Boston, MA USA; 633 Department of Surgery, Memorial Sloan Kettering Cancer Center, New York, NY USA; 634 Division of Breast Surgery, Brigham and Women’s Hospital, Boston, MA USA; 635 Integrative Bioinformatics Support Group, National Institute of Environmental Health Sciences (NIEHS), Durham, NC USA; 636 Department of Clinical Science, University of Bergen, Bergen, Norway; 637 Center For Medical Innovation, Seoul National University Hospital, Seoul, South Korea; 638 Department of Internal Medicine, Seoul National University Hospital, Seoul, South Korea; 639 Institute of Computer Science, Polish Academy of Sciences, Warsawa, Poland; 640 Functional and Structural Genomics, German Cancer Research Center (DKFZ), Heidelberg, Germany; 641 Laboratory of Translational Genomics, Division of Cancer Epidemiology and Genetics, National Cancer Institute, , National Institutes of Health, Bethesda, MD USA; 642 Institute for Medical Informatics Statistics and Epidemiology, University of Leipzig, Leipzig, Germany; 643 Morgan Welch Inflammatory Breast Cancer Research Program and Clinic, The University of Texas MD Anderson Cancer Center, Houston, TX USA; 644 Department of Hematology and Oncology, Georg-Augusts-University of Göttingen, Göttingen, Germany; 645 Institute of Cell Biology (Cancer Research), University of Duisburg-Essen, Essen, Germany; 646 King’s College London and Guy’s and St. Thomas’ NHS Foundation Trust, London, UK; 647 Center for Epigenetics, Van Andel Research Institute, Grand Rapids, MI USA; 648 The University of Queensland Centre for Clinical Research, Royal Brisbane and Women’s Hospital, Herston, QLD Australia; 649 Department of Pediatric Oncology and Hematology, University of Cologne, Cologne, Germany; 650 University of Düsseldorf, Düsseldorf, Germany; 651 Department of Pathology, Institut Jules Bordet, Brussels, Belgium; 652 Institute of Biomedicine, Sahlgrenska Academy at University of Gothenburg, Gothenburg, Sweden; 653 Children’s Medical Research Institute, Sydney, NSW Australia; 654 ILSbio, LLC Biobank, Chestertown, MD USA; 655 Division of Genetics and Genomics, Boston Children’s Hospital, Harvard Medical School, Boston, MA USA; 656 Institute for Bioengineering and Biopharmaceutical Research (IBBR), Hanyang University, Seoul, South Korea; 657 Department of Statistics, University of California Santa Cruz, Santa Cruz, CA USA; 658 National Genotyping Center, Institute of Biomedical Sciences, Academia Sinica, Taipei, Taiwan; 659 Department of Vertebrate Genomics/Otto Warburg Laboratory Gene Regulation and Systems Biology of Cancer, Max Planck Institute for Molecular Genetics, Berlin, Germany; 660 McGill University and Genome Quebec Innovation Centre, Montreal, QC Canada; 661 biobyte solutions GmbH, Heidelberg, Germany; 662 Gynecologic Oncology, NYU Laura and Isaac Perlmutter Cancer Center, New York University, New York, NY USA; 663 Division of Oncology, Stem Cell Biology Section, Washington University School of Medicine, St. Louis, MO USA; 664 Department of Systems Biology, The University of Texas MD Anderson Cancer Center, Houston, TX USA; 665 Harvard University, Cambridge, MA USA; 666 Urologic Oncology Branch, Center for Cancer Research, National Cancer Institute, National Institutes of Health, Bethesda, MD USA; 667 University of Oslo, Oslo, Norway; 668 University of Toronto, Toronto, ON Canada; 669 Peking University, Beijing, China; 670 School of Life Sciences, Peking University, Beijing, China; 671 Leidos Biomedical Research, Inc, McLean, VA USA; 672 Hematology, Hospital Clinic, Institut d’Investigacions Biomèdiques August Pi i Sunyer (IDIBAPS), University of Barcelona, Barcelona, Spain; 673 Second Military Medical University, Shanghai, China; 674 Chinese Cancer Genome Consortium, Shenzhen, China; 675 Department of Medical Oncology, Beijing Hospital, Beijing, China; 676 Laboratory of Molecular Oncology, Key Laboratory of Carcinogenesis and Translational Research (Ministry of Education), Peking University Cancer Hospital and Institute, Beijing, China; 677 School of Medicine/School of Mathematics and Statistics, University of St. Andrews, St, Andrews, Fife UK; 678 Institute for Systems Biology, Seattle, WA USA; 679 Department of Biochemistry and Molecular Biology, Faculty of Medicine, University Institute of Oncology-IUOPA, Oviedo, Spain; 680 Institut Bergonié, Bordeaux, France; 681 Cancer Unit, MRC University of Cambridge, Cambridge, UK; 682 Department of Pathology and Laboratory Medicine, Center for Personalized Medicine, Children’s Hospital Los Angeles, Los Angeles, CA USA; 683 John Curtin School of Medical Research, Canberra, ACT Australia; 684 MVZ Department of Oncology, PraxisClinic am Johannisplatz, Leipzig, Germany; 685 Department of Information Technology, Ghent University, Ghent, Belgium; 686 Department of Plant Biotechnology and Bioinformatics, Ghent University, Ghent, Belgium; 687 Institute for Genomic Medicine, Nationwide Children’s Hospital, Columbus, OH USA; 688 Computational Biology Program, School of Medicine, Oregon Health and Science University, Portland, OR USA; 689 Department of Surgery, Duke University, Durham, NC USA; 690 Institució Catalana de Recerca i Estudis Avançats (ICREA), Barcelona, Spain; 691 Institut Català de Paleontologia Miquel Crusafont, Universitat Autònoma de Barcelona, Barcelona, Spain; 692 University of Glasgow, Glasgow, UK; 693 Institut d’Investigacions Biomèdiques August Pi i Sunyer (IDIBAPS), Barcelona, Spain; 694 Division of Oncology, Washington University School of Medicine, St. Louis, MO USA; 695 Department of Surgery and Cancer, Imperial College, London, INY UK; 696 Applications Department, Oxford Nanopore Technologies, Oxford, UK; 697 Department of Obstetrics, Gynecology and Reproductive Services, University of California San Francisco, San Francisco, CA USA; 698 Department of Biochemistry and Molecular Medicine, University California at Davis, Sacramento, CA USA; 699 STTARR Innovation Facility, Princess Margaret Cancer Centre, Toronto, ON Canada; 700 Discipline of Surgery, Western Sydney University, Penrith, NSW Australia; 701 Yale School of Medicine, Yale University, New Haven, CT USA; 702 Department of Genetics, Lineberger Comprehensive Cancer Center, University of North Carolina at Chapel Hill, Chapel Hill, NC USA; 703 Departments of Neurology and Neurosurgery, Henry Ford Hospital, Detroit, MI USA; 704 Precision Oncology, OHSU Knight Cancer Institute, Oregon Health and Science University, Portland, OR USA; 705 Institute of Pathology, University Medical Center Hamburg-Eppendorf, Hamburg, Germany; 706 Department of Health Sciences, Faculty of Medical Sciences, Kyushu University, Fukuoka, Japan; 707 Heidelberg Academy of Sciences and Humanities, Heidelberg, Germany; 708 Department of Clinical Pathology, University of Melbourne, Melbourne, VIC, Australia; 709 Department of Pathology, Roswell Park Cancer Institute, Buffalo, NY USA; 710 Department of Computer Science, University of Helsinki, Helsinki, Finland; 711 Institute of Biotechnology, University of Helsinki, Helsinki, Finland; 712 Organismal and Evolutionary Biology Research Programme, University of Helsinki, Helsinki, Finland; 713 Department of Obstetrics and Gynecology, Division of Gynecologic Oncology, Washington University School of Medicine, St. Louis, MO USA; 714 Penrose St. Francis Health Services, Colorado Springs, CO USA; 715 Institute of Pathology, Ulm University and University Hospital of Ulm, Ulm, Germany; 716 National Cancer Center, Tokyo, Japan; 717 Genome Institute of Singapore, Singapore, Singapore; 718 32Program in Computational Biology and Bioinformatics, Yale University, New Haven, CT USA; 719 German Cancer Aid, Bonn, Germany; 720 Programme in Cancer and Stem Cell Biology, Centre for Computational Biology, Duke-NUS Medical School, Singapore, Singapore; 721 The Chinese University of Hong Kong, Shatin, NT, Hong Kong China; 722 Fourth Military Medical University, Shaanxi, China; 723 The University of Cambridge School of Clinical Medicine, Cambridge, UK; 724 St. Jude Children’s Research Hospital, Memphis, TN USA; 725 University Health Network, Princess Margaret Cancer Centre, Toronto, ON Canada; 726 Center for Biomolecular Science and Engineering, University of California Santa Cruz, Santa Cruz, CA USA; 727 Department of Medicine, University of Chicago, Chicago, IL USA; 728 Department of Neurology, Mayo Clinic, Rochester, MN USA; 729 Cambridge Oesophagogastric Centre, Cambridge University Hospitals NHS Foundation Trust, Cambridge, UK; 730 Department of Computer Science, Carleton College, Northfield, MN USA; 731 Institute of Cancer Sciences, College of Medical Veterinary and Life Sciences, University of Glasgow, Glasgow, UK; 732 Department of Epidemiology, University of Alabama at Birmingham, Birmingham, AL USA; 733 HudsonAlpha Institute for Biotechnology, Huntsville, AL USA; 734 O’Neal Comprehensive Cancer Center, University of Alabama at Birmingham, Birmingham, AL USA; 735 Department of Pathology, Keio University School of Medicine, Tokyo, Japan; 736 Department of Hepatobiliary and Pancreatic Oncology, National Cancer Center Hospital, Tokyo, Japan; 737 Sage Bionetworks, Seattle, WA USA; 738 Lymphoma Genomic Translational Research Laboratory, National Cancer Centre, Singapore, Singapore; 739 Department of Clinical Pathology, Robert-Bosch-Hospital, Stuttgart, Germany; 740 Department of Cell and Systems Biology, University of Toronto, Toronto, ON Canada; 741 Department of Biosciences and Nutrition, Karolinska Institutet, Stockholm, Sweden; 742 Center for Liver Cancer, Research Institute and Hospital, National Cancer Center, Gyeonggi, South Korea; 743 Division of Hematology-Oncology, Samsung Medical Center, Sungkyunkwan University School of Medicine, Seoul, South Korea; 744 Samsung Advanced Institute for Health Sciences and Technology, Sungkyunkwan University School of Medicine, Seoul, South Korea; 745 Cheonan Industry-Academic Collaboration Foundation, Sangmyung University, Cheonan, South Korea; 746 NYU Langone Medical Center, New York, NY USA; 747 Department of Hematology and Medical Oncology, Cleveland Clinic, Cleveland, OH USA; 748 Department of Radiation Oncology, University of California San Francisco, San Francisco, CA USA; 749 Department of Health Sciences Research, Mayo Clinic, Rochester, MN USA; 750 Helen F. Graham Cancer Center at Christiana Care Health Systems, Newark, DE USA; 751 Heidelberg University Hospital, Heidelberg, Germany; 752 CSRA Incorporated, Fairfax, VA USA; 753 Research Department of Pathology, University College London Cancer Institute, London, UK; 754 Department of Research Oncology, Guy’s Hospital, King’s Health Partners AHSC, King’s College London School of Medicine, London, UK; 755 Faculty of Medicine and Health Sciences, Macquarie University, Sydney, NSW Australia; 756 University Hospital of Minjoz, INSERM UMR 1098, Besançon, France; 757 Spanish National Cancer Research Centre, Madrid, Spain; 758 Center of Digestive Diseases and Liver Transplantation, Fundeni Clinical Institute, Bucharest, Romania; 759 Cureline, Inc, South San Francisco, CA USA; 760 St. Luke’s Cancer Centre, Royal Surrey County Hospital NHS Foundation Trust, Guildford, UK; 761 Cambridge Breast Unit, Addenbrooke’s Hospital, Cambridge University Hospital NHS Foundation Trust and NIHR Cambridge Biomedical Research Centre, Cambridge, UK; 762 East of Scotland Breast Service, Ninewells Hospital, Aberdeen, UK; 763 Department of Genetics, Microbiology and Statistics, University of Barcelona, IRSJD, IBUB, Barcelona, Spain; 764 Department of Obstetrics and Gynecology, Medical College of Wisconsin, Milwaukee, WI USA; 765 Hematology and Medical Oncology, Winship Cancer Institute of Emory University, Atlanta, GA USA; 766 Department of Computer Science, Princeton University, Princeton, NJ USA; 767 Vanderbilt Ingram Cancer Center, Vanderbilt University, Nashville, TN USA; 768 Ohio State University College of Medicine and Arthur G. James Comprehensive Cancer Center, Columbus, OH USA; 769 Department of Surgery, Yokohama City University Graduate School of Medicine, Kanagawa, Japan; 770 Division of Chromatin Networks, German Cancer Research Center (DKFZ) and BioQuant, Heidelberg, Germany; 771 Research Computing Center, University of North Carolina at Chapel Hill, Chapel Hill, NC USA; 772 School of Molecular Biosciences and Center for Reproductive Biology, Washington State University, Pullman, WA USA; 773 Finsen Laboratory and Biotech Research and Innovation Centre (BRIC), University of Copenhagen, Copenhagen, Denmark; 774 Department of Laboratory Medicine and Pathobiology, University of Toronto, Toronto, ON Canada; 775 Department of Pathology, Human Oncology and Pathogenesis Program, Memorial Sloan Kettering Cancer Center, New York, NY USA; 776 University Hospital Giessen, Pediatric Hematology and Oncology, Giessen, Germany; 777 Oncologie Sénologie, ICM Institut Régional du Cancer, Montpellier, France; 778 Institute of Clinical Molecular Biology, Christian-Albrechts-University, Kiel, Germany; 779 Institute of Pathology, University of Wuerzburg, Wuerzburg, Germany; 780 Department of Urology, North Bristol NHS Trust, Bristol, UK; 781 SingHealth, Duke-NUS Institute of Precision Medicine, National Heart Centre Singapore, Singapore, Singapore; 782 Department of Computer Science, University of Toronto, Toronto, ON Canada; 783 Bern Center for Precision Medicine, University Hospital of Bern, University of Bern, Bern, Switzerland; 784 Englander Institute for Precision Medicine, Weill Cornell Medicine and New York Presbyterian Hospital, New York, NY USA; 785 Meyer Cancer Center, Weill Cornell Medicine, New York, NY USA; 786 Pathology and Laboratory, Weill Cornell Medical College, New York, NY USA; 787 Vall d’Hebron Institute of Oncology: VHIO, Barcelona, Spain; 788 General and Hepatobiliary-Biliary Surgery, Pancreas Institute, University and Hospital Trust of Verona, Verona, Italy; 789 National Centre for Biological Sciences, Tata Institute of Fundamental Research, Bangalore, India; 790 Indiana University, Bloomington, IN USA; 791 Department of Pathology, GZA-ZNA Hospitals, Antwerp, Belgium; 792 Analytical Biological Services, Inc, Wilmington, DE USA; 793 Sydney Medical School, University of Sydney, Sydney, NSW Australia; 794 cBio Center, Dana-Farber Cancer Institute, Harvard Medical School, Boston, MA USA; 795 Department of Cell Biology, Harvard Medical School, Boston, MA USA; 796 Advanced Centre for Treatment Research and Education in Cancer, Tata Memorial Centre, Navi Mumbai, Maharashtra India; 797 School of Environmental and Life Sciences, Faculty of Science, The University of Newcastle, Ourimbah, NSW Australia; 798 Department of Dermatology, University Hospital of Essen, Essen, Germany; 799 Bioinformatics and Omics Data Analytics, German Cancer Research Center (DKFZ), Heidelberg, Germany; 800 Department of Urology, Charité Universitätsmedizin Berlin, Berlin, Germany; 801 Martini-Clinic, Prostate Cancer Center, University Medical Center Hamburg-Eppendorf, Hamburg, Germany; 802 Department of General Internal Medicine, University of Kiel, Kiel, Germany; 803 German Cancer Consortium (DKTK), Partner site Berlin, Berlin, Germany; 804 Cancer Research Institute, Beth Israel Deaconess Medical Center, Boston, MA USA; 805 University of Pittsburgh, Pittsburgh, PA USA; 806 Department of Ophthalmology and Ocular Genomics Institute, Massachusetts Eye and Ear, Harvard Medical School, Boston, MA USA; 807 Center for Psychiatric Genetics, NorthShore University HealthSystem, Evanston, IL USA; 808 Van Andel Research Institute, Grand Rapids, MI USA; 809 Laboratory of Molecular Medicine, Human Genome Center, Institute of Medical Science, University of Tokyo, Tokyo, Japan; 810 Japan Agency for Medical Research and Development, Tokyo, Japan; 811 Korea University, Seoul, South Korea; 812 Murtha Cancer Center, Walter Reed National Military Medical Center, Bethesda, MD USA; 813 Human Genetics, University of Kiel, Kiel, Germany; 814 Department of Oncologic Pathology, Dana-Farber Cancer Institute, Harvard Medical School, Boston, MA USA; 815 Oregon Health and Science University, Portland, OR USA; 816 Center for RNA Interference and Noncoding RNA, The University of Texas MD Anderson Cancer Center, Houston, TX USA; 817 Department of Experimental Therapeutics, The University of Texas MD Anderson Cancer Center, Houston, TX USA; 818 Department of Gynecologic Oncology and Reproductive Medicine, The University of Texas MD Anderson Cancer Center, Houston, TX USA; 819 University Hospitals Coventry and Warwickshire NHS Trust, Coventry, UK; 820 Department of Radiation Oncology, Radboud University Nijmegen Medical Centre, Nijmegen, GA The Netherlands; 821 Institute for Genomics and Systems Biology, University of Chicago, Chicago, IL USA; 822 Clinic for Hematology and Oncology, St.-Antonius-Hospital, Eschweiler, Germany; 823 Computational and Systems Biology Program, Memorial Sloan Kettering Cancer Center, New York, NY USA; 824 University of Iceland, Reykjavik, Iceland; 825 Division of Computational Genomics and Systems Genetics, German Cancer Research Center (DKFZ), Heidelberg, Germany; 826 Dundee Cancer Centre, Ninewells Hospital, Dundee, UK; 827 Department for Internal Medicine III, University of Ulm and University Hospital of Ulm, Ulm, Germany; 828 Institut Curie, INSERM Unit 830, Paris, France; 829 Department of Gastroenterology and Hepatology, Yokohama City University Graduate School of Medicine, Kanagawa, Japan; 830 Department of Laboratory Medicine, Radboud University Nijmegen Medical Centre, Nijmegen, GA The Netherlands; 831 Division of Cancer Genome Research, German Cancer Research Center (DKFZ), Heidelberg, Germany; 832 Department of General Surgery, Singapore General Hospital, Singapore, Singapore; 833 Cancer Science Institute of Singapore, National University of Singapore, Singapore, Singapore; 834 Department of Medical and Clinical Genetics, Genome-Scale Biology Research Program, University of Helsinki, Helsinki, Finland; 835 East Anglian Medical Genetics Service, Cambridge University Hospitals NHS Foundation Trust, Cambridge, UK; 836 Irving Institute for Cancer Dynamics, Columbia University, New York, NY USA; 837 Institute of Molecular and Cell Biology, Singapore, Singapore; 838 Laboratory of Cancer Epigenome, Division of Medical Science, National Cancer Centre Singapore, Singapore, Singapore; 839 Universite Lyon, INCa-Synergie, Centre Léon Bérard, Lyon, France; 840 Department of Urology, Mayo Clinic, Rochester, MN USA; 841 Royal National Orthopaedic Hospital - Stanmore, Stanmore, Middlesex UK; 842 Department of Biochemistry, Genetics and Immunology, University of Vigo, Vigo, Spain; 843 Giovanni Paolo II / I.R.C.C.S. Cancer Institute, Bari, BA Italy; 844 Neuroblastoma Genomics, German Cancer Research Center (DKFZ), Heidelberg, Germany; 845 Fondazione Policlinico Universitario Gemelli IRCCS, Rome, Italy, Rome, Italy; 846 University of Verona, Verona, Italy; 847 Centre National de Génotypage, CEA - Institute de Génomique, Evry, France; 848 CAPHRI Research School, Maastricht University, Maastricht, ER The Netherlands; 849 Department of Biopathology, Centre Léon Bérard, Lyon, France; 850 Université Claude Bernard Lyon 1, Villeurbanne, France; 851 Core Research for Evolutional Science and Technology (CREST), JST, Tokyo, Japan; 852 Department of Biological Sciences, Laboratory for Medical Science Mathematics, Graduate School of Science, University of Tokyo, Yokohama, Japan; 853 Department of Medical Science Mathematics, Medical Research Institute, Tokyo Medical and Dental University (TMDU), Tokyo, Japan; 854 Cancer Ageing and Somatic Mutation Programme, Wellcome Sanger Institute, Hinxton, UK; 855 University Hospitals Birmingham NHS Foundation Trust, Birmingham, UK; 856 Centre for Cancer Research and Cell Biology, Queen’s University, Belfast, UK; 857 Breast Medical Oncology, The University of Texas MD Anderson Cancer Center, Houston, TX USA; 858 Department of Surgery, Johns Hopkins University School of Medicine, Baltimore, MD USA; 859 Department of Oncology-Pathology, Science for Life Laboratory, Karolinska Institute, Stockholm, Sweden; 860 School of Cancer Sciences, Faculty of Medicine, University of Southampton, Southampton, UK; 861 Department of Gene Technology, Tallinn University of Technology, Tallinn, Estonia; 862 Genetics and Genome Biology Program, SickKids Research Institute, The Hospital for Sick Children, Toronto, ON Canada; 863 Departments of Neurosurgery and Hematology and Medical Oncology, Winship Cancer Institute and School of Medicine, Emory University, Atlanta, GA USA; 864 Department of Clinical and Molecular Medicine, Faculty of Medicine and Health Sciences, Norwegian University of Science and Technology, Trondheim, Norway; 865 Argmix Consulting, North Vancouver, BC Canada; 866 Department of Information Technology, Ghent University, Interuniversitair Micro-Electronica Centrum (IMEC), Ghent, Belgium; 867 Nuffield Department of Surgical Sciences, John Radcliffe Hospital, University of Oxford, Oxford, UK; 868 Institute of Mathematics and Computer Science, University of Latvia, Riga, LV Latvia; 869 Discipline of Pathology, Sydney Medical School, University of Sydney, Sydney, NSW Australia; 870 Department of Applied Mathematics and Theoretical Physics, Centre for Mathematical Sciences, University of Cambridge, Cambridge, UK; 871 Department of Epidemiology and Biostatistics, Memorial Sloan Kettering Cancer Center, New York, NY USA; 872 Department of Statistics, Columbia University, New York, NY USA; 873 Department of Immunology, Genetics and Pathology, Science for Life Laboratory, Uppsala University, Uppsala, Sweden; 874 School of Electronic and Information Engineering, Xi’an Jiaotong University, Xi’an, China; 875 Department of Histopathology, Cambridge University Hospitals NHS Foundation Trust, Cambridge, UK; 876 Oxford NIHR Biomedical Research Centre, University of Oxford, Oxford, UK; 877 Georgia Regents University Cancer Center, Augusta, GA USA; 878 Wythenshawe Hospital, Manchester, UK; 879 Department of Genetics, Washington University School of Medicine, St.Louis, MO USA; 880 Department of Biological Oceanography, Leibniz Institute of Baltic Sea Research, Rostock, Germany; 881 Wellcome Centre for Human Genetics, University of Oxford, Oxford, UK; 882 Department of Molecular and Human Genetics, Baylor College of Medicine, Houston, TX USA; 883 Thoracic Oncology Laboratory, Mayo Clinic, Rochester, MN USA; 884 Institute for Genomic Medicine, Nationwide Children’s Hospital, Columbus, OH USA; 885 Department of Obstetrics and Gynecology, Division of Gynecologic Oncology, Mayo Clinic, Rochester, MN USA; 886 International Institute for Molecular Oncology, Poznań, Poland; 887 Poznan University of Medical Sciences, Poznań, Poland; 888 Genomics and Proteomics Core Facility High Throughput Sequencing Unit, German Cancer Research Center (DKFZ), Heidelberg, Germany; 889 NCCS-VARI Translational Research Laboratory, National Cancer Centre Singapore, Singapore, Singapore; 890 Edison Family Center for Genome Sciences and Systems Biology, Washington University, St. Louis, MO USA; 891 MRC-University of Glasgow Centre for Virus Research, Glasgow, UK; 892 Department of Medical Informatics and Clinical Epidemiology, Division of Bioinformatics and Computational Biology, OHSU Knight Cancer Institute, Oregon Health and Science University, Portland, OR USA; 893 School of Electronic Information and Communications, Huazhong University of Science and Technology, Wuhan, China; 894 Department of Applied Mathematics and Statistics, Johns Hopkins University, Baltimore, MD USA; 895 Department of Cancer Genome Informatics, Graduate School of Medicine, Osaka University, Osaka, Japan; 896 Institute of Computer Science, Heidelberg University, Heidelberg, Germany; 897 School of Mathematics and Statistics, University of Sydney, Sydney, NSW Australia; 898 Ben May Department for Cancer Research, University of Chicago, Chicago, IL USA; 899 Department of Human Genetics, University of Chicago, Chicago, IL USA; 900 Tri-Institutional PhD Program in Computational Biology and Medicine, Weill Cornell Medicine, New York, NY USA; 901 The First Affiliated Hospital, Xi’an Jiaotong University, Xi’an, China; 902 Department of Medicine and Therapeutics, The Chinese University of Hong Kong, Shatin, NT, Hong Kong China; 903 Department of Biostatistics, The University of Texas MD Anderson Cancer Center, Houston, TX USA; 904 Duke-NUS Medical School, Singapore, Singapore; 905 Department of Surgery, Ruijin Hospital, Shanghai Jiaotong University School of Medicine, Shanghai, China; 906 School of Computing Science, University of Glasgow, Glasgow, UK; 907 Division of Orthopaedic Surgery, Oslo University Hospital, Oslo, Norway; 908 Eastern Clinical School, Monash University, Melbourne, VIC Australia; 909 Epworth HealthCare, Richmond, VIC Australia; 910 Department of Biostatistics and Computational Biology, Dana-Farber Cancer Institute and Harvard Medical School, Boston, MA USA; 911 Department of Biomedical Informatics, College of Medicine, The Ohio State University, Columbus, OH USA; 912 The Ohio State University Comprehensive Cancer Center (OSUCCC – James), Columbus, OH USA; 913 The University of Texas School of Biomedical Informatics (SBMI) at Houston, Houston, TX USA; 914 Department of Biostatistics, University of North Carolina at Chapel Hill, Chapel Hill, NC USA; 915 Department of Biochemistry and Molecular Genetics, Feinberg School of Medicine, Northwestern University, Chicago, IL USA; 916 Faculty of Medicine and Health, University of Sydney, Sydney, NSW Australia; 917 Department of Pathology, Erasmus Medical Center Rotterdam, Rotterdam, GD The Netherlands; 918 Division of Molecular Carcinogenesis, The Netherlands Cancer Institute, Amsterdam, CX The Netherlands; 919 Institute of Molecular Life Sciences and Swiss Institute of Bioinformatics, University of Zurich, Zurich, Switzerland

**Keywords:** Cancer, Computational biology and bioinformatics, Genomics

## Abstract

Chromothripsis is a mutational phenomenon characterized by massive, clustered genomic rearrangements that occurs in cancer and other diseases. Recent studies in selected cancer types have suggested that chromothripsis may be more common than initially inferred from low-resolution copy-number data. Here, as part of the Pan-Cancer Analysis of Whole Genomes (PCAWG) Consortium of the International Cancer Genome Consortium (ICGC) and The Cancer Genome Atlas (TCGA), we analyze patterns of chromothripsis across 2,658 tumors from 38 cancer types using whole-genome sequencing data. We find that chromothripsis events are pervasive across cancers, with a frequency of more than 50% in several cancer types. Whereas canonical chromothripsis profiles display oscillations between two copy-number states, a considerable fraction of events involve multiple chromosomes and additional structural alterations. In addition to non-homologous end joining, we detect signatures of replication-associated processes and templated insertions. Chromothripsis contributes to oncogene amplification and to inactivation of genes such as mismatch-repair-related genes. These findings show that chromothripsis is a major process that drives genome evolution in human cancer.

## Abstract (web summary)

Analysis of whole-genome sequencing data across 2,658 tumors spanning 38 cancer types shows that chromothripsis is pervasive, with a frequency of more than 50% in several cancer types, contributing to oncogene amplification, gene inactivation and cancer genome evolution.

## Main

Chromothripsis is characterized by massive genomic rearrangements that are often generated in a single catastrophic event and localized to isolated chromosomal regions^1–4^. In contrast to the traditional view of tumorigenesis as the gradual process of the accumulation of mutations, chromothripsis provides a mechanism for the rapid accrual of hundreds of rearrangements in a few cell divisions. This phenomenon has been studied in primary tumors of diverse histological origins^5–10^, but similar random joining of chromosomal fragments has also been observed in the germline^11^. There has been considerable progress in elucidating the mechanisms by which chromothripsis may arise, including fragmentation and subsequent reassembly of a single chromatid in aberrant nuclear structures called micronuclei^2,12^ and the fragmentation of dicentric chromosomes during telomere crisis^13,14^. Chromothripsis is not specific to cancer as it can cause rare congenital human disease and can be transmitted through the germline^11,15^; it has also been described in plants, where it has been linked to micronucleation^16^. However, despite the recent rapid progress on elucidating the mechanisms of chromothripsis, much remains to be discovered regarding its cause, prevalence and consequences.

A hallmark of chromothripsis is multiple oscillations between two or three copy-number (CN) states^1,6^. Applying this criterion to CN profiles inferred from SNP arrays, chromothripsis was initially estimated to occur in at least 2–3% of human cancers^1^. Subsequent studies of large array-based datasets gave similar frequencies: 1.5% (124 out of 8,227 tumors across 30 cancer types)^17^ and 5% (918 out of 18,394 tumors)^18^, with the highest frequencies detected for soft-tissue tumors (54% for liposarcomas, 24% for fibrosarcomas and 23% for sarcomas)^18^. These estimates relied on the detection of CN oscillations that are more-densely clustered than expected by chance^8^.

Whole-genome sequencing (WGS) data provide a greatly enhanced view of structural variations (SVs) in the genome^19^, allowing us to generate a more nuanced set of criteria for chromothripsis and enhance detection specificity^3^. Our previous analysis of WGS data from cutaneous melanomas already found chromothripsis-like rearrangements in 38% of these tumors (45 out of 117)^10^; other studies using WGS data found 60–65% for pancreatic cancer^5^ and 32% for esophageal adenocarcinomas^7^. Whether these examples are outliers that reflect the unique biology of these tumors or whether they suggest a more general underestimation of the frequency of chromothripsis remained unclear.

Motivated by the importance of chromothripsis during tumor evolution and the need for more-comprehensive analyses, we determined the frequency and spectrum of chromothripsis events in the WGS data for 2,658 patients with cancer comprising 38 cancer types generated by the ICGC and TCGA projects, and aggregated by the PCAWG Consortium. These sequencing data were re-analyzed with standardized pipelines to align to the human genome (reference build hs37d5) and to identify germline variants and somatic mutations^20^. In addition to deriving more-accurate estimates of the per-tumor type prevalence of chromothripsis, we determined the size and genomic distribution of such events, examined their role in the amplification of oncogenes or loss of tumor-suppressor genes, described their relationship to genome ploidy and investigated whether their presence is correlated with patient survival. Our chromothripsis calls can be browsed at the accompanying website (http://compbio.med.harvard.edu/chromothripsis/).

## Results

### Prevalence of chromothripsis across cancer types

**Fig. 1:**
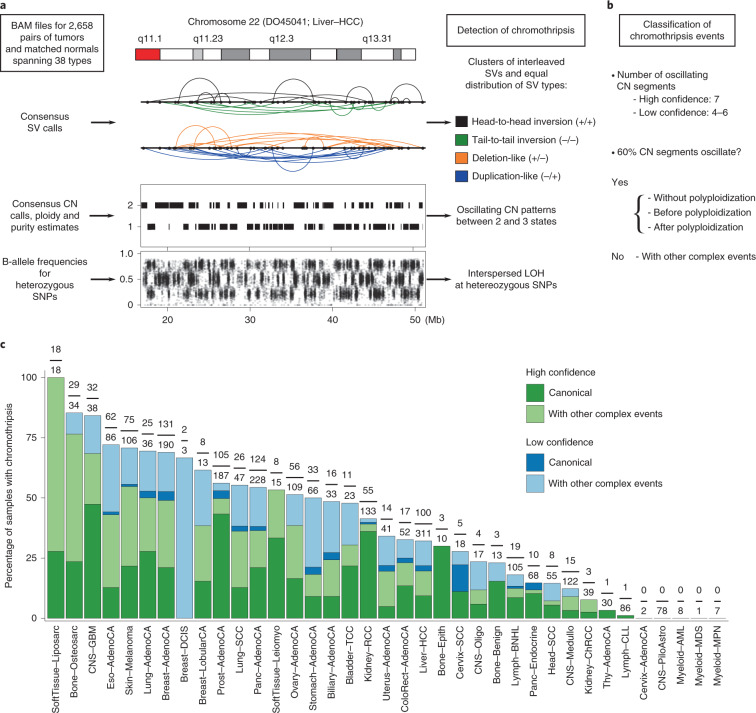
Overview of the chromothripsis-calling method and the frequency of events across 37 cancer types. **a**, Example of a region displaying the characteristic features of chromothripsis: cluster of interleaved SVs with equal proportions of SV types (that is, fragment joins), a CN profile that oscillates between two states and interspersed LOH. Details of the criteria are described in the Methods. Both the color scheme and the abbreviations shown in this figure are used throughout the manuscript. **b**, Classification of chromothripsis events. In a canonical event, more than 60% of the segments oscillate between two CN states; a tumor is classified as canonical if it showed at least one canonical chromothripsis event. **c**, Percentage of patients with chromothripsis events across the entire cohort. The fractions at the top of the bars are the number of tumors that showed high-confidence chromothripsis out of the total number of tumors of that type. The cancer type abbreviations used across the manuscript are as follows: Biliary-AdenoCA, biliary adenocarcinoma; Bladder-TCC, bladder transitional cell carcinoma; Bone-Benign, bone cartilaginous neoplasm, osteoblastoma and bone osteofibrous dysplasia; Bone-Epith, bone neoplasm, epithelioid; Bone-Osteosarc, sarcoma, bone; Breast-AdenoCA, breast adenocarcinoma; Breast-DCIS, breast ductal carcinoma in situ; Breast-LobularCA, breast lobular carcinoma; Cervix-AdenoCA, cervix adenocarcinoma; Cervix-SCC, cervix squamous cell carcinoma; CNS-GBM, central nervous system glioblastoma; CNS-Oligo, CNS oligodenroglioma; CNS-Medullo, CNS medulloblastoma; CNS-PiloAstro, CNS pilocytic astrocytoma; ColoRect-AdenoCA, colorectal adenocarcinoma; Eso-AdenoCA, esophagus adenocarcinoma; Head-SCC, head-and-neck squamous cell carcinoma; Kidney-ChRCC, kidney chromophobe renal cell carcinoma; Kidney-RCC, kidney renal cell carcinoma; Liver-HCC, liver hepatocellular carcinoma; Lung-AdenoCA, lung adenocarcinoma; Lung-SCC, lung squamous cell carcinoma; Lymph-CLL, lymphoid chronic lymphocytic leukemia; Lymph-BNHL, lymphoid mature B-cell lymphoma; Lymph-NOS, lymphoid not otherwise specified; Myeloid-AML, myeloid acute myeloid leukemia; Myeloid-MDS, myeloid myelodysplastic syndrome; Myeloid-MPN, myeloid myeloproliferative neoplasm; Ovary-AdenoCA, ovary adenocarcinoma; Panc-AdenoCA, pancreatic adenocarcinoma; Panc-Endocrine, pancreatic neuroendocrine tumor; Prost-AdenoCA, prostate adenocarcinoma; Skin-Melanoma, skin melanoma; SoftTissue-Leiomyo, leiomyosarcoma, soft tissue; SoftTissue-Liposarc, liposarcoma, soft tissue; Stomach-AdenoCA, stomach adenocarcinoma; Thy-AdenoCA, thyroid low-grade adenocarcinoma; and Uterus-AdenoCA, uterus adenocarcinoma.

We first sought to formulate a set of criteria for identifying chromothripsis events with varying complexities (Fig. 1a). The acknowledged model of chromothripsis posits that some of the DNA fragments generated by the shattering of the DNA are lost; thus, CN oscillations between two or three states^1,6^ are an obvious first criterion (Fig. 1a). Such deletions also lead to interspersed loss of heterozygosity (LOH) or altered haplotype ratios if there is only a single copy of the parental homolog of the fragmented chromatid. Although chromosome shattering and reassembly has been experimentally demonstrated to generate chromothripsis^2^, template-switching DNA-replication errors can generate a similar pattern^21^. Indeed, shattering and replication error models are not mutually exclusive and could co-occur^2^. Therefore, for the discussion below we will refer generally to ‘chromothripsis’ as encompassing both classes of models.

To detect chromothripsis in WGS data, we developed ShatterSeek (Methods and Supplementary Note). A key feature of our method is to identify clusters of breakpoints belonging to SVs that are interleaved—that is, the regions bridged by their breakpoints overlap instead of being nested (Fig. 1)—as is expected from random joining of genomic fragments. This encompasses the many cases that do not display simple oscillations (for example, partially oscillating CN profiles with interspersed amplifications) and oscillations that span multiple CN levels due to aneuploidy^5,22^. Rearrangements in chromothripsis should also follow a roughly even distribution for the different types of fragment joins (duplication-like, deletion-like, head-to-head and tail-to-tail inversions, which are shown in blue, orange, black and green, respectively, in Fig. 1a and throughout) and have breakpoints that are randomly distributed across the affected region^1–3^. Finally, we use interchromosomal SVs to identify chromothripsis events that involve multiple chromosomes. In the Supplementary Note, we have compiled the criteria that have been used in 27 major chromothripsis-related studies to date.

After removing low-quality samples using stringent quality control, we applied ShatterSeek to 2,543 tumor–normal pairs of 37 cancer types (Methods and Supplementary Table 1). Of those 2,543 pairs, 2,428 cases had SVs and were analyzed further. To tune the parameters in our method, we used statistical thresholds and visual inspection. For the minimum number of oscillating CN segments, we used two thresholds: high-confidence calls display oscillations between two states in at least seven adjacent segments, whereas low-confidence calls involve between four and six segments (Fig. 1b and Supplementary Note). The analyses described in the subsequent sections were performed using the high-confidence call set unless noted otherwise.

We first focused on the 1,427 nearly diploid genomes (ploidy ≤ 2.1; Supplementary Table 1), in which detection of chromothripsis is more straightforward. We defined as ‘canonical’ those events in which more than 60% of the CN segments in the affected region oscillated between two states (canonical events in polyploid tumors are described later). The frequency of canonical chromothripsis events is more than 40% for multiple cancer types, such as glioblastomas (50%) and lung adenocarcinomas (40%). These frequencies are much higher than previous estimates^17,18^.

When we extend our analysis to the entire cohort, we identify high-confidence events in 29% of the samples (734 out of 2,543), affecting 3.2% of all chromosomes (Fig. 1c and Supplementary Dataset 1). When low-confidence calls are included, the percentages increase to 40% and 5.3%, respectively (Supplementary Dataset 2).

The frequency varies markedly across cancer types. At the high end, we find that 100% of liposarcomas and 77% of osteosarcomas exhibit high-confidence chromothripsis (Fig. 1c and Supplementary Fig. 1). Although a higher susceptibility of these cancer types to chromothripsis has been described^1,22^, our estimated frequencies are substantially higher. Melanomas, glioblastomas and lung adenocarcinomas showed evidence of chromothripsis in more than 50% of cases (Fig. 1c). By contrast, the frequencies were lowest in thyroid adenocarcinomas (3.3%, *n* = 30), chronic lymphocytic leukemia (1.2%, *n* = 86) and pilocytic astrocytomas (0%, *n* = 78); in the other tumor types with low incidence, the sample sizes were too small to give meaningful estimates. Consistent with previous reports^23,24^, we find that chromothripsis is enriched in chromosomes 3 and 5 in kidney renal cell carcinomas and chromosome 12 in liposarcomas (Supplementary Fig. 1a). Overall, these results indicate a much greater prevalence of chromothripsis in a majority of human cancers than previously estimated^10,17,18^.

### Understanding the difference between our frequency estimates and previous ones

Our estimates are in accordance with recent analyses in specific tumor types^5,7^; however, they are considerably higher than those described in previous pan-cancer studies that used array-based platforms. With higher resolution from sequencing data, improved SV algorithms and refined criteria, we are able to provide more-accurate estimates.

To better understand the discrepancy between WGS-based studies, we carried out a detailed comparison using previously analyzed datasets. For 109 previously described prostate adenocarcinomas^25^, the authors used ShatterProof^26^ and found chromothripsis in 21% (23 out of 109). When we applied the same algorithm (with the same parameters) but using our CN and SV calls, the percentage more than doubled to 45% (49 out of 109). This indicates that the lower sensitivity of previous SV-detection methods is one of the main reasons for the discrepancy. Accurate SV detection remains challenging, especially for low-purity tumors. The SV calls that we used were generated by the PCAWG Structural Variation Working Group of the ICGC; each variant was required to be called by at least two of the four algorithms used in this analysis^27^.

Using ShatterSeek, we identified 11 additional cases for a total of 55% (60 out of 109). Of the 23 previously reported cases^25^, we missed four. The missed events are focal events comprising fewer than six SVs, which is the lowest number allowed in our criteria; the detected regions appear to be hypermutated regions characterized by tandem duplications or deletions. For the cases that we detect but that were missed previously^25^, visual inspection reveals that the differences are mostly due to the lower sensitivity of their SV calls (Supplementary Note). ShatterSeek has increased sensitivity by incorporating more complex patterns of oscillations and interchromosomal SVs while keeping the specificity high by imposing additional criteria on breakpoint homology to remove tandem duplications and those arising from breakage–fusion–bridge (BFB) cycles. Furthermore, we also compared our method against ChromAL^5^ for 76 pancreatic tumors. Both ChromAL and ShatterSeek detect chromothripsis in the same 41 tumors (54%).

Therefore, our estimates for the frequency of chromothripsis events are supported by the following: some tumor types such as thyroid adenocarcinoma, chronic lymphocytic leukemia and pilocytic astrocytomas have few or no events; diploid tumors, which have simpler configurations that are easier to reconstruct or verify visually, have high frequencies; the high-confidence cases were used for final estimates; more sensitive CN and SV calls result in higher frequencies for the same datasets; our estimates are in agreement with very recent analysis in specific tumor types; and our chromothripsis calls do not overlap with regions affected by chromoplexy (Supplementary Note).

### Frequent involvement of interchromosomal SVs

**Fig. 2:**
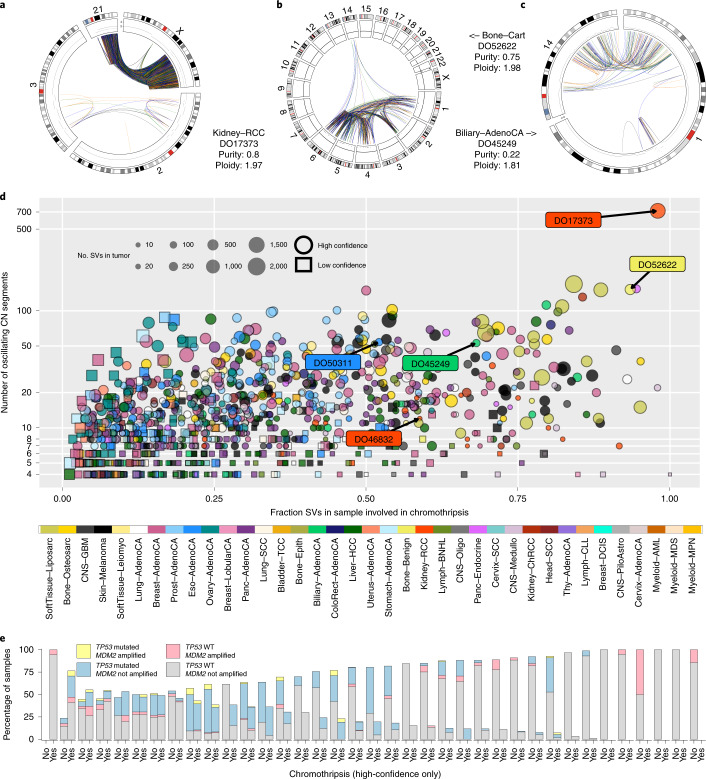
Heterogeneity of chromothripsis events. **a**–**c**, Examples of massive chromothripsis events on the background of quiescent genomes in samples from patients DO17373 (**a**), DO52622 (**b**) and DO45249 (**c**). **d**, The fraction of SVs involved in chromothripsis in each sample against the maximum number of contiguous oscillating CN segments for the high-confidence (circles) and low-confidence (squares) chromothripsis calls. **e**, Distribution of patients showing high-confidence chromothripsis, deleterious *TP53* mutations and *MDM2* amplification (CN ≥ 4). WT, wild-type allele.

An important feature of our approach is the incorporation of interchromosomal SVs to detect those events that involve multiple chromosomes. Chromothripsis affects only a single chromosome in 40% of the tumors with chromothripsis (Fig. 2a–c and Supplementary Figs. 1–3). A large number of chromosomes is frequently affected in some tumor types, for example, at least five chromosomes are affected in 61% osteosarcomas (Supplementary Figs. 1–4). In one extreme case, we found a single chromothripsis event that affected six chromosomes (Fig. 2b), with only seven of the 110 SVs on chromosome 5 being intrachromosomal. In another example (Supplementary Fig. 4d), an approximately 5-Mb region on chromosome 12 did not display CN oscillations, but it could be linked by interchromosomal SVs to another region that does show a clear chromothripsis pattern, suggesting that the amplification of *CCND2* on chromosome 12 may have originated from chromothripsis. Chromothripsis involving multiple chromosomes is likely to have arisen either from simultaneous fragmentation of multiple chromosomes (for example, in a micronucleus or in a chromosome bridge) or from fragmentation of a chromosome that had previously undergone a non-reciprocal translocation.

### Size and complexity of chromothripsis events are highly variable

Chromothripsis events span a wide range of genomic scale, with the number of breakpoints involved varying by two orders of magnitude within some tumor types (Supplementary Fig. 1c). We found that tumors had relatively focal chromothripsis events—usually a few megabases in size—that took place within an otherwise quiet genome (bottom-right quadrant in Fig. 2d). Although focal, these events can lead to the simultaneous amplification of multiple oncogenes located in different chromosomes (Supplementary Figs. 4c–e, 5a–c). Other focal events co-localize with other complex events in highly rearranged genomes (bottom-left quadrant in Fig. 2d). Overall, our analysis reveals that there is greater heterogeneity in chromothripsis patterns than previously appreciated, both in terms of the number of SVs and chromosomes involved.

### Relationship between chromothripsis and aneuploidy

Newly established polyploid cells have high rates of mitotic errors that generate lagging chromosomes^28,29^, which have been linked to chromothripsis in medulloblastomas and in vitro^2,12,14^. However, a causal relationship or even the frequency of association between polyploidy and chromothripsis has not been assessed in detail. To examine the sequence of events clearly, we focused on the canonical cases, for which we can infer whether chromothripsis occurred before or after polyploidization^30^. For example, if the CN oscillates between two and four copies in a tetraploid tumor, we infer that polyploidization occurred after chromothripsis; on the other hand, if the oscillation occurs between three and four copies, we infer that polyploidization occurred first^30^ (Supplementary Figs. 1, 2, 5d, 6 and Supplementary Note). Of the 194 cases in which we can distinguish the sequence of events, 74% show chromothripsis after polyploidization. This suggests that a large fraction of the canonical chromothripsis events in polyploid tumors are late events.

We observed canonical chromothripsis events in 26% of diploid-ranged tumors (431 out of 1,648) and in 40% of polyploid-ranged tumors (298 out of 748). After correcting for tumor type using the logistic regression, we estimate that, on average, the odds of chromothripsis occurring in a polyploid tumor (cases with ploidy ≥ 2.5) is 1.5 times larger than that in a diploid tumor (95% confidence interval, 1.20–1.85; *P* < 10^−3^). This increase may be due to the presence of more genomic material in polyploids, although polyploidy also reduces the sensitivity of CN and SV detection (due to a lower sequence coverage per copy) and makes it easier for the cell to lose the highly rearranged copy when intact copies are present^31^.

### Frequent co-localization of chromothripsis with other complex events

About half of the chromothripsis events co-localize with other genomic alterations (Fig. 1 and Supplementary Figs. 1, 2). There is evidence across multiple tumor types that chromothripsis might occur before or after additional layers of rearrangements^6–8,13,14,23^. For instance, BFB cycles have been mechanistically linked to chromothripsis and telomere attrition—which results in the formation of BFB cycles, has been identified as a predisposing factor for chromothripsis^6,13,32^.

Co-localization of APOBEC-mediated clustered hypermutation (kataegis) and rearrangements has been reported for multiple cancer types^33,34^, and has been linked to single-stranded DNA intermediates during break-induced replication^35^. To study the relationship between kataegis and chromothripsis, we examined the presence of clusters of APOBEC-induced mutations within the chromothripsis regions (Methods). Excluding melanoma samples (due to the overlap between the APOBEC and ultraviolet-light signatures^36^), we find that 28% of the 734 tumors with chromothripsis show at least five clustered APOBEC-induced mutations, and 9.3% display kataegis comprising more than 20 mutations. Previous analysis of liposarcomas has suggested that multiple BFB cycles on a derivative chromosome generated by chromothripsis underlie the formation of neochromosomes^23^. In agreement with this model, we observe variant allele fractions of 0.01–0.1 for APOBEC-induced mutations in chromothripsis regions that have high-level CN amplifications in soft-tissue liposarcomas, suggesting that they occurred at the late stages of tumor development, likely after chromothripsis (Supplementary Fig. 4e). Overall, although kataegis can co-occur with chromothripsis, this co-occurrence is not common. This is consistent with recent data that chromothriptic derivative chromosomes are mostly assembled by end-joining mechanisms that do not involve extensive DNA-end resection^37^.

### *TP53* mutation status and chromothripsis

Inactivating *TP53* mutations have been associated with chromothripsis in medulloblastomas^8^ and in pediatric cancers^38,39^, and *TP53*-deficient cells have been used as a model to generate chromothripsis in vitro^2,14^. Nevertheless, the relationship between deleterious *TP53* mutations and chromothripsis has not been examined comprehensively. In our data, 38% of the samples with inactivating *TP53* mutations show chromothripsis, whereas 24% of those with wild-type *TP53* have chromothripsis (Fig. 2e). After correcting for cancer type, this translates to an odds ratio of 1.54 (95% confidence interval, 1.21–1.95, *P* < 10^−3^) for chromothripsis in those with *TP53* mutations compared with *TP53* wild-type cancers. However, we note that 60% of the chromothripsis cases show neither *TP53* mutations nor *MDM2* amplifications (a regulator of TP53 by ubiquitination^40^), including those with massive cases of chromothripsis in diploid genomes (for example, DO25622 in Fig. 2b). This indicates that, although p53 malfunction and polyploidy are predisposing factors for chromothripsis, it still occurs frequently in diploid tumors with proficient p53.

### Signatures of repair mechanisms in chromothripsis regions

Although imprecise, it is possible to infer the predominant mechanisms responsible for the chromothripsis event based on the sequence homology at the breakpoints^41,42^. Previously, non-homologous end joining (NHEJ) has been implicated in the reassembly of DNA fragments generated by chromothripsis^2,37^, whereas alternative end joining (alt-EJ) has been proposed in constitutional chromothripsis and in glioblastomas^15,43^. In addition, short templated insertions suggestive of microhomology-mediated break-induced replication (MMBIR) or alt-EJ associated with polymerase theta have been detected in chromothripsis events that originated from DNA fragmentation in micronuclei^2,44–46^.

We analyzed the breakpoints involved in canonical chromothripsis events with interspersed LOH, as most SVs in such cases are related to chromothripsis (Fig. 1b). In 55% of these events, we only detected repair signatures that were concordant with NHEJ or alt-EJ (Supplementary Fig. 7). In 32%, we identified stretches of microhomology at two or more breakpoint junctions (mostly comprising 0–6 bp) and short insertions of 10–500 bp that map to distant locations within the affected region (Supplementary Fig. 7). For example, in the massive chromothripsis in Fig. 2a (1,394 SVs, hundreds of uninterrupted CN oscillations and interspersed LOH), we detect small nonrandom insertions of 10–379 bp at 60 breakpoints. Thus, NHEJ has a principal role in DNA repair, with partial contributions from MMBIR or alt-EJ.

**Fig. 3:**
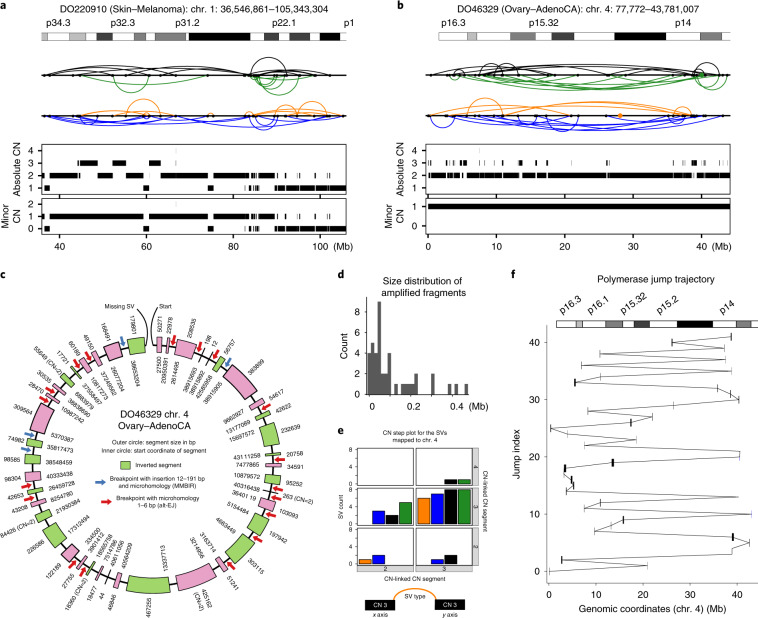
Example of canonical chromothripsis events displaying templated insertions and evidence of MMBIR. **a**, Evidence of chromothripsis in chromosome 1 in a skin-melanoma tumor with CN oscillations that span 3 CN levels and LOH. **b**, Example of a chromothripsis event in chromosome 4 involving low-level CN gains and absence of LOH in an ovarian adenocarcinoma. Segments at CN 3 correspond to templated insertions, as evidenced by their size, and breakpoint orientations at their edges. Breakpoints corresponding to interchromosomal SVs are depicted as colored dots in the SV profile, whereas intrachromosomal SVs are represented with black dots and colored arcs following the representation shown in Fig. 1. **c**, Reconstruction of the amplicon generated by the chromoanasynthesis event detected in chromosome 4 in tumor DO46329 (see **b**). Inverted segments are depicted in green. Red arrows highlight breakpoints with short microhomology tracts, whereas blue arrows indicate the presence of small insertions at the breakpoints. The CN for all segments is 3 unless otherwise indicated. **d**, Size distribution for the templated insertions forming the amplicon depicted in **c**. **e**, CN step plot for chromosome 4 indicating that most of the SVs mapped to chromosome 4 link genomic regions at CN 3. The *x* and *y* axes correspond to the CN level of the segments linked by a given SV. The color of the bars corresponds to the four types of SVs (that is, deletion-like, duplication-like, and head-to-head and tail-to-tail inversions) indicated in Fig. 1a and considered throughout the manuscript. **f**, Trajectory of the polymerase across chromosome 4 estimated from the template-switching events shown in **c**.

**Fig. 4:**
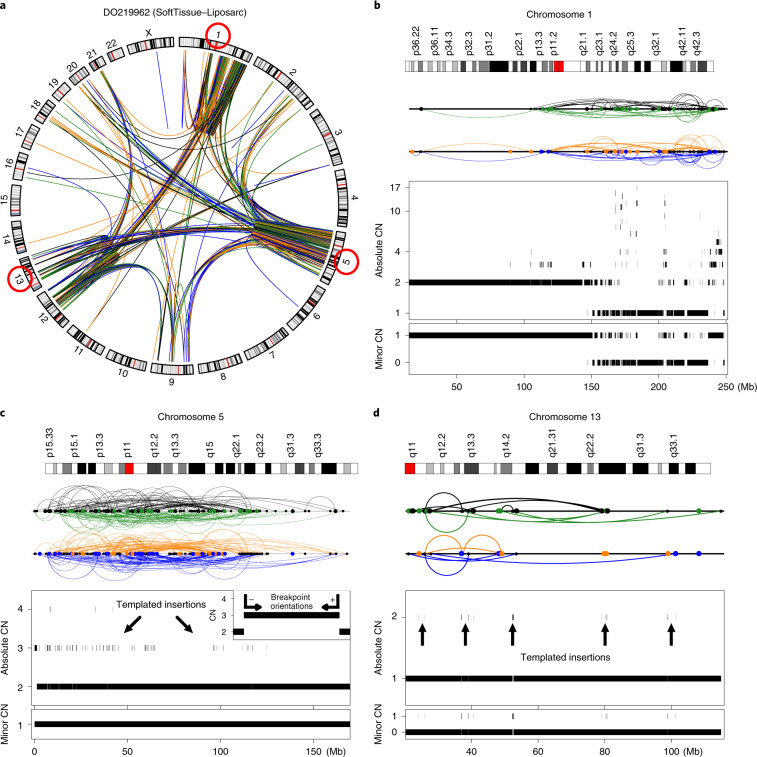
Example of a multichromosomal chromothripsis event in a soft-tissue liposarcoma co-localized with other complex events involving templated insertions. **a**, Scaled circos plot of the entire genome for this tumor except for chromosome Y. **b**–**d**, SV and CN profiles for chromosomes 1 (**b**), 5 (**c**) and 13 (**d**). Tens of CN oscillations and LOH in chromosome 1 are co-localized with additional rearrangements. The size, minor CN (from the allele with the lower number of copies) and orientation of the breakpoint junctions associated with the segments at CN 3 indicate that these are templated insertions. **c**, Inset: orientation of the breakpoint junctions at the edges of low-level CN gains originated from template switching (that is, − and + according to the annotation that we use in the manuscript).

By contrast, approximately 5% of the canonical events detected in diploid genomes show no evidence of LOH in part of the affected region or in the entire affected region, for example, oscillations between two and three CN, long stretches of microhomology and frequent evidence of template switching^27^ (Figs. 3, 4). For instance, in the case shown in Fig. 3b, both the size of the segments at CN 3 (mean of 45 kb) and the orientation of the breakpoints at their edges suggest that these are templated insertions^27^. In addition, multiple breakpoint junctions show features concordant with MMBIR. In this case, we could manually reconstruct part of the amplicon by following the polymerase trajectory across 42 template-switching events (Fig. 3c–f). This type of event might be more appropriately called chromoanasynthesis^21^, but systematically distinguishing chromoanasynthesis from chromothripsis is challenging due to their partially overlapping features (template switching events can generate LOH if the polymerase skips over segments of the template and LOH might not be present in chromothripsis events that occur in aneuploid genomes; Supplementary Note).

We also find features associated with replication-associated mechanisms in more-complex rearrangements involving multiple chromosomes. In an illustrative case (Fig. 4a), LOH is observed in some chromosomes (Fig. 4b) but absent in others, where the oscillations occur at higher CN states without LOH (Fig. 4c,d). There is evidence of templated insertions in chromosomes 5 and 13, which are linked to a chromothripsis event showing LOH in chromosome 1. Notably, the minor CN for the templated insertions in chromosome 13 is 1, whereas it is 0 for the rest of the chromosome. This suggests that one parental chromosome served as a template and was later lost.

Overall, these results indicate the involvement of template-switching events in the generation or repair of complex rearrangements, consistent with the observations of replication-associated processes in the formation of clustered rearrangements in congenital disorders and cancer^15,21,27,41,47^. Although further experimental evidence will be necessary, we suggest that the involvement of replication-associated mechanisms in the assembly of derivative chromosomes in chromothripsis might be substantial.

### Oncogene amplification and loss of tumor-suppressor genes in chromothripsis regions

Evidence of oncogene amplification in extrachromosomal circular DNA elements, termed double-minutes, generated as a consequence of chromothripsis has been reported for selected cancer types^1,2,8,43^. However, the extent to which chromothripsis contributes to double-minute formation has not been examined on a pan-cancer scale. Although reconstruction of a double-minute structure with discordant reads would present clear evidence for its extrachromosomal nature, this proves to be too difficult in general. Therefore, we rely on CN to make our inferences. We find that 15 patients (2% of tumors with chromothripsis) show CN oscillations between one low (CN ≤ 4) and one very high (CN ≥ 10) state, consistent with the presence of a double minute^8,43^. We detect known cancer drivers in these putative double minutes, including *MDM2* (four samples; Supplementary Figs. 4e, 5a and Supplementary Table 2) and *CDK4* (four samples). These amplifications lead to increased mRNA levels of, for example*, MDM2*, *NUP107* and *CDK4* in a glioblastoma sample (DO14049) compared to other glioblastoma tumors. In chromothripsis regions subject to additional rearrangements, it is difficult to discern, using bulk-sequencing data, whether highly amplified segments are part of double minutes or correspond to intrachromosomal amplification^48^. Furthermore, once a double minute has formed, the derivative chromosome showing chromothripsis may be lost if it has no other tumor-promoting mutations. Therefore, the contribution of chromothripsis to the formation of extrachromosomal DNA bodies is likely to be higher than estimated here.

Further analysis of focal amplifications, defined as regions with CN ≥ 4 and smaller than 6 Mb (ref. ^49^), in 1,268 tumors and 162 normal tissue samples with RNA-sequencing data reveals that 6,310 focal amplifications encompassing oncogenes (11.1%; or 20.5% when including low-confidence calls) localize to chromothripsis regions, often leading to increased expression (Supplementary Table 2). These include well-known cancer-associated genes, such as *CCND1* (25 tumors), *CDK4* (25 tumors), *MDM2* (23 tumors), *SETDB1* (23 tumors), *ERBB3* (11 tumors), *ERBB2* (11 tumors), *MYC* (10 tumors) and *MYCN* (five tumors). Therefore, chromothripsis—perhaps together with associated replication-based CN gains^22,50^—may make a substantial contribution to small-scale focal amplifications.

**Fig. 5:**
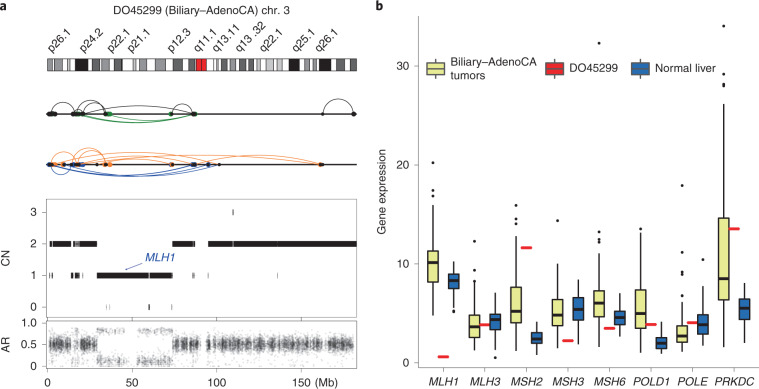
Chromothripsis-mediated depletion of *MLH1*. **a**, Chromothripsis event and expression levels of DNA MMR genes in the sample of patient DO45299 (biliary adenocarcinoma). **b**, Mean expression of DNA MMR genes in a panel of 16 biliary adenocarcinomas and 16 normal liver samples. Box plots in **b** show median, first and third quartiles (boxes), and the whiskers encompass observations within a distance of 1.5× the interquartile range from the first and third quartiles. AR, allelic ratio computed for heterozygous SNPs.

Expanding previous analyses^5,24^, we examined the extent to which chromothripsis contributes to the loss of tumor-suppressor genes across tumor types. We find that chromothripsis underlies 2.1% and 1.9% of the losses of tumor-suppressor and DNA-repair genes, respectively. These include *MLH1* (9 out of 301 tumors with *MLH1* deletions), *PTEN* (12 out of 358), *BRCA1* (8 out of 154), *BRCA2* (7 out of 270), *APC* (9 out of 201), *SMAD4* (10 out of 403) and *TP53* (8 out of 614) (Supplementary Fig. 8 and Supplementary Table 2). In 28 samples, both alleles were inactivated, one due to chromothripsis and the other due to a point mutation, including in *SMAD4*, *APC, TP53* and *CDKN2A*. In a biliary adenocarcinoma (Fig. 5), for instance, one *MLH1* allele was lost due to chromothripsis and the other allele was likely silenced due to promoter hypermethylation, as evidenced by low expression of *MLH1* and the microsatellite-instability phenotype in an otherwise mismatch repair (MMR)-proficient tumor^51^. Overall, these data illustrate the way in which chromothripsis can confer tumorigenic potential through the loss of key tumor-suppressor and DNA-repair genes. See Supplementary Note for additional analysis of the genes recurrently targeted by chromothripsis breakpoints, their role in the formation of gene fusions, enrichment of chromothripsis breakpoints in epigenomic marks and survival analyses.

## Discussion

Our analysis has revealed that chromothripsis plays a major part in shaping the architecture of cancer genomes across diverse cancers. We found that the prevalence and heterogeneity of chromothripsis was much higher than previously appreciated. Our approach enabled us to define more-nuanced criteria to detect chromothripsis events, including those that involve multiple chromosomes and those that were hard to detect previously due to the presence of other co-localized rearrangements.

We note that the estimated frequencies of chromothripsis depend on statistical thresholds. Although we chose conservative thresholds, we cannot exclude the possibility that some chromothripsis-like patterns might have arisen due to other sources of genomic instability. Conversely, it is also possible that we missed true chromothripsis events that have fewer than the required number of rearrangements; it is worth noting that such small-scale events are seen in experimentally generated chromothripsis^2^. Cases in which chromothripsis is followed by other complex rearrangements that mask the canonical CN pattern are especially difficult to detect, requiring additional criteria and in-depth manual inspection. Despite these limitations, we believe that our statistical approach is more sensitive than the reassembly-based approach in which one attempts to reconstruct the steps that led to the observed SV pattern. Most complex events are too complicated for reconstruction, especially when many breakpoints are undetected and some are incorrectly identified due to inherent limitations of short-read data, imperfect SV algorithms and insufficient sequencing coverage.

Given the pervasiveness of chromothripsis in human cancers and its association with poorer prognosis, another question that arises is whether chromothripsis itself constitutes an actionable molecular event that is amenable to therapy. This is of particular interest given the link between aneuploidy, depleted immune infiltration and reduced response to immunotherapy^52^. As more WGS data are linked to other data types including clinical information, it will become feasible to understand the influence of chromothripsis on tumorigenesis and its potential as a biomarker for diagnosis or treatment.

## Methods

### PCAWG whole-genome sequencing dataset

We integrated, using a common processing pipeline, whole-genome sequencing data from the TCGA and ICGC consortia for 2,658 tumor and matched normal pairs across 38 cancer types, of which 2,543 pairs from 37 cancer types that passed our quality-control criteria were selected for further analysis^53^. The list of samples is provided in Supplementary Table 1. Further information for all tumor samples and patients is provided in a separate study^20^. Sequencing reads were aligned using BWA-MEM v.0.7.8-r455, whereas BioBamBam v.0.0.138 was used to extract unpaired reads and mark duplicates^54,55^.

### Mutation calling

We used the consensus SNV and indel (insertions and deletions) call sets released by the PCAWG project (Supplementary Table 3). We used HaplotypeCaller v.3.4-46-gbc0262554 to call SNPs in both tumor and matched normal samples following the GATK best-practice guidelines. We retained only SNPs supported by at least ten reads. We processed a total of 210,021 nonsynonymous somatic mutations, of which 43,548 were predicted to be deleterious using the MetaLR score as implemented in Annovar^56^. To identify APOBEC mutagenesis, we followed a previously described procedure^36^. In brief, we considered as APOBEC-associated mutations those involving a change of (1) G within the sequence motif wGa to a C or A (where w is A or T) or (2) C in the sequence motif tCw to G or T (where w is A or T).

### Detection of SVs and CN alterations

The SVs were identified by the PCAWG Structural Variation Working Group, which applied four algorithms and selected those SVs found by at least two algorithms^20,27^. We used the consensus SV, CN, purity and ploidy call-sets generated by the PCAWG project (Supplementary Table 3). The calling pipelines are described in detail in associated papers^27,57^.

### RNA-seq data analysis

We processed RNA-seq data for a total of 162 normal and 1,268 and tumor samples. Sequencing reads were aligned using TopHat2 and STAR^58,59^. HTseq-count was subsequently used to calculate read counts for the genes encompassed in the PCAWG reference GTF set, namely Gencode v.19. Counts were normalized to UQ-FPKM (upper-quartile-normalized fragments per kb per million mapped reads) values using upper-quartile normalization. The expression values were averaged across the two alignments. The set of oncogenes was downloaded and curated from COSMIC (dominant genes) and IntOGen databases^60,61^, whereas the set of tumor suppressors was downloaded from TSGene v.2.0, COSMIC (recessive genes) and previous studies^62,63^. DNA-repair genes were extracted from a previous study^64^.

### Characterization of chromothripsis events using ShatterSeek

To identify and visualize chromothripsis-like patterns in the cancer genomes by using CN and SV data, we adapted the previously proposed set of statistical criteria^3^. The ShatterSeek code, the package documentation and a detailed tutorial are available at https://github.com/parklab/ShatterSeek. Interactive circos plots for all tumors in the PCAWG cohort analyzed in this study are provided at http://compbio.med.harvard.edu/chromothripsis/.

The values for the statistical criteria for all chromosomes across all samples are provided in Supplementary Table 1. Visual depictions of the high-confidence and low-confidence calls are provided in Supplementary Datasets 1 and 2. Visual depictions for the two sets of SV clusters not identified as chromothripsis by our method, namely (1) those involving clusters of duplications or deletions leading to CN oscillations, as well as oscillating CN profiles with few or no SVs mapped and (2) large clusters of interleaved SVs that did not display chromothripsis, are provided in Supplementary Datasets 3 and 4, respectively. In Supplementary Datasets 1–4 and in the main text (Figs. 1a, 3a,b, 4b–d and 5a), intrachromosomal SVs are depicted as arcs with the breakpoints represented by black points, whereas the breakpoints corresponding to interchromosomal SVs are depicted as colored points. Duplication-like SVs, deletion-like SVs, head-to-head and tail-to-tail inversions are depicted in blue, orange, black and green, respectively. The value for the statistical criteria described above for each event is provided underneath its representation.

### Reporting Summary

Further information on research design is available in the Nature Research Reporting Summary linked to this article.

## Online content

Any methods, additional references, Nature Research reporting summaries, source data, extended data, supplementary information, acknowledgements, peer review information; details of author contributions and competing interests; and statements of data and code availability are available at 10.1038/s41588-019-0576-7.

## Supplementary information

Supplementary Information Supplementary Figs. 1–8 and Supplementary Note

Reporting Summary

Supplementary Table 1 The results for the statistical criteria implemented to detect chromothripsis for 2,428 patients with SVs.

Supplementary Table 2 Genes detected in focally amplified and chromothripsis regions. **a**, Genes detected in focally amplified regions. **b**, Genes detected in chromothripsis regions with copy number of 1 or 0.

Supplementary Table 3 Summary of datasets used in this paper.

Supplementary Dataset 1 High-confidence chromothripsis calls.

Supplementary Dataset 2 Low-confidence chromothripsis calls.

Supplementary Dataset 3 Regions displaying CN oscillations not classified as chromothripsis. These include: (1) CN oscillating profiles characterized by clusters of tandem duplications or deletions, (2) candidate chromothripsis cases satisfying the statistical criteria but considered false positives by visual inspection, and (3) chromosomes displaying at least 7 CN oscillations with few or no SVs mapped.

Supplementary Dataset 4 Large clusters of interleaved SVs (>20) not identified as chromothripsis by our method.

## Data Availability

Descriptions and links to the datasets and variant calls used in the paper are listed in Supplementary Table 3. Information on accessing raw data can be found at https://docs.icgc.org/pcawg/data/; PCAWG analysis results are available at https://dcc.icgc.org/releases/PCAWG. Datasets marked ‘Controlled’ contain potentially identifiable information and require authorization from the ICGC and TCGA Data Access Committees. Further information regarding the availability of the data is provided in ref. ^20^. In accordance with the data access policies of the ICGC and TCGA projects, most data are in an open tier, which does not require access approval. To access potentially identifying information, researchers will need to apply to the TCGA Data Access Committee (DAC) via dbGaP (https://dbgap.ncbi.nlm.nih.gov/aa/wga.cgi?page=login) for access to the TCGA portion of the dataset, and to the ICGC Data Access Compliance Office (DACO; http://icgc.org/daco) for the ICGC portion.

## Code availability

The code for calling chromothripsis events is available at https://github.com/parklab/ShatterSeek. The core computational pipelines used by the PCAWG Consortium for alignment, quality control and variant calling are available to the public at https://dockstore.org/search?search=pcawg under a GNU General Public License v.3.0, which allows for reuse and distribution.
