# Supplementary material for: Comprehensive analysis of chromothripsis in 2,658 human cancers using whole-genome sequencing
Source: Nat Genet. 2020 Feb 5;52(3):331–41. doi: 10.1038/s41588-019-0576-7 (PMC7058534; doi:10.1038/s41588-019-0576-7)
Supplement: Supplementary file 1 — Supplementary Figs. 1–8 and Supplementary Note [file 41588_2019_576_MOESM1_ESM.pdf]

nature portfolio

https://doi.org/10.1038/s41588-019-0576-7

Supplementary information

Comprehensive analysis of chromothripsis in 2,658 human cancers using whole-genome sequencing

In the format provided by the authors and unedited

Supplementary Figures

Comprehensive analysis of chromothripsis in 2,658 human cancers using whole-genome sequencing

Isidro Cortés-Ciriano[1,2,3,†], Jake June-Koo Lee[1,2], Ruibin Xi[4], Dhawal Jain[1], Youngsook L. Jung[1], Lixing Yang[5], Dmitry Gordenin[6], Leszek J. Klimczak[7], Cheng-Zhong Zhang[1,8], David S. Pellman[9,10], PCAWG Structural Variation Working Group[11], Peter J. Park[1,2*] & PCAWG Consortium[12]

[1]Department of Biomedical Informatics, Harvard Medical School, Boston, MA, USA.

[2]Ludwig Center at Harvard, Boston, MA, USA.

[3]Centre for Molecular Science Informatics, Department of Chemistry, University of Cambridge, Cambridge, UK.

[4]School of Mathematical Sciences and Center for Statistical Science, Peking University, Beijing, China.

[5]The Ben May Department for Cancer Research and Department of Human Genetics, The University of Chicago, Chicago, IL, USA.

[6]Genome Integrity and Structural Biology Laboratory, National Institute of Environmental Health Sciences, US National Institutes of Health, Research Triangle Park, NC, USA.

[7]Integrative Bioinformatics Group, National Institute of Environmental Health Sciences, US National Institutes of Health, Research Triangle Park, NC, USA.

[8]Department of Data Sciences, Dana-Farber Cancer Institute, Boston, MA, USA.

[9]Department of Pediatric Oncology, Dana-Farber Cancer Institute, Boston, MA, USA.

[10]Howard Hughes Medical Institute and Department of Cell Biology, Harvard Medical School, Boston, MA, USA.

[11]The members of this working group are listed at the end of the paper.

[12]The members of this consortium are listed in the Supplementary Note.

†Present address: European Molecular Biology Laboratory, European Bioinformatics Institute, Wellcome Genome Campus, Hinxton, UK.

*e-mail: peter_park@hms.harvard.edu

Supplementary Figure 1

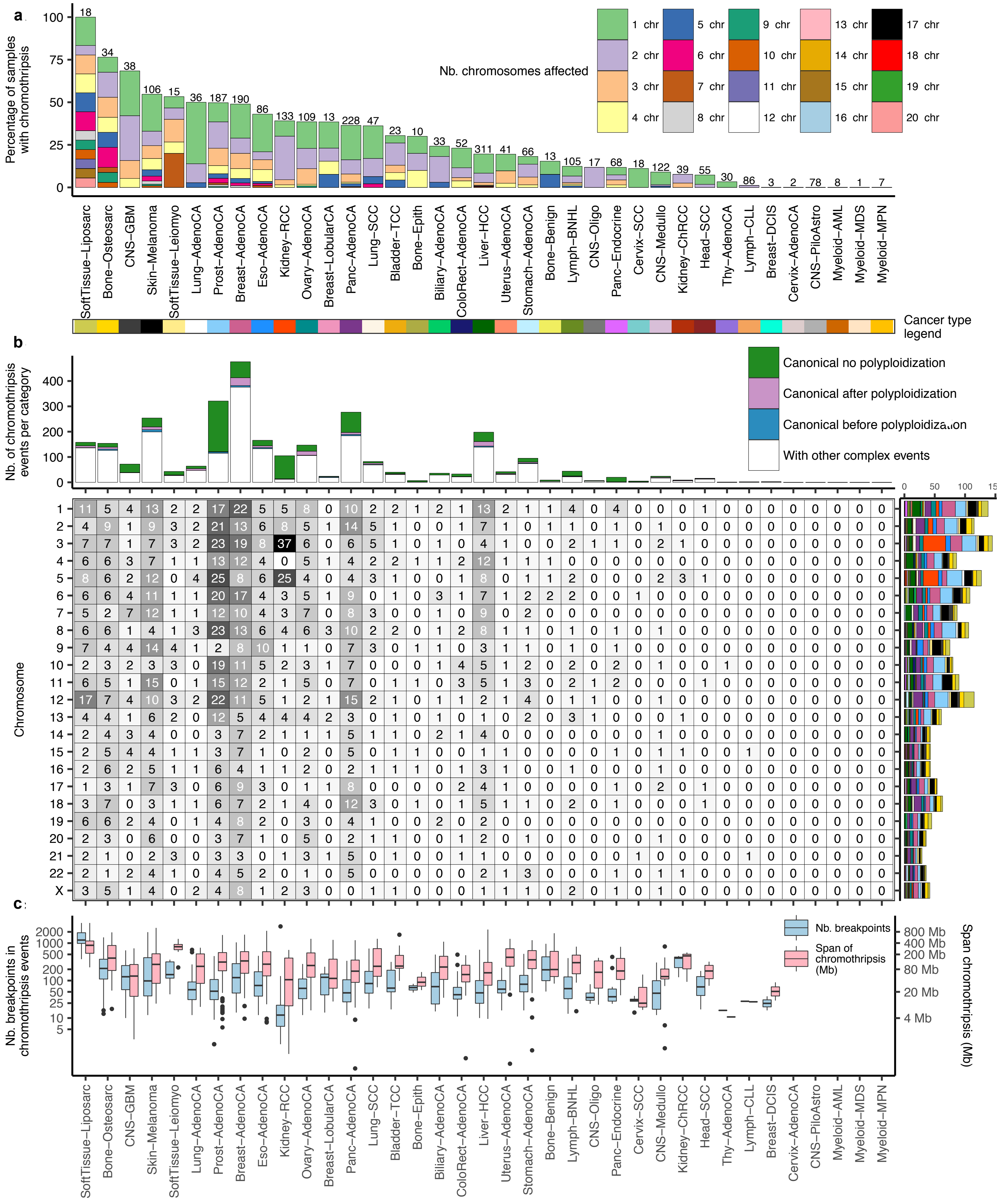

Supplementary Figure 1. Landscape of chromothripsis across 37 cancer types including high-confidence calls only. (**a**) Percentage of patients harboring chromothripsis events. The total number of tumors examined for each cancer type is indicated on top of the bars, whereas the colors of the bars indicate the number of chromosomes affected by chromothripsis. (**b**) Number of chromothripsis regions categorized on the basis of the oscillating copy number patterns and the temporal profile of the events with respect to polyploidization, and their distribution across the genome. (**c**) Distribution of the number of breakpoints (blue), and span of the chromothripsis events (pink). Box plots in (**c**) show median, first and third quartiles (boxes), and the whiskers encompass observations within a distance of 1.5 times the interquartile range from the first and third quartiles.

Supplementary Figure 2

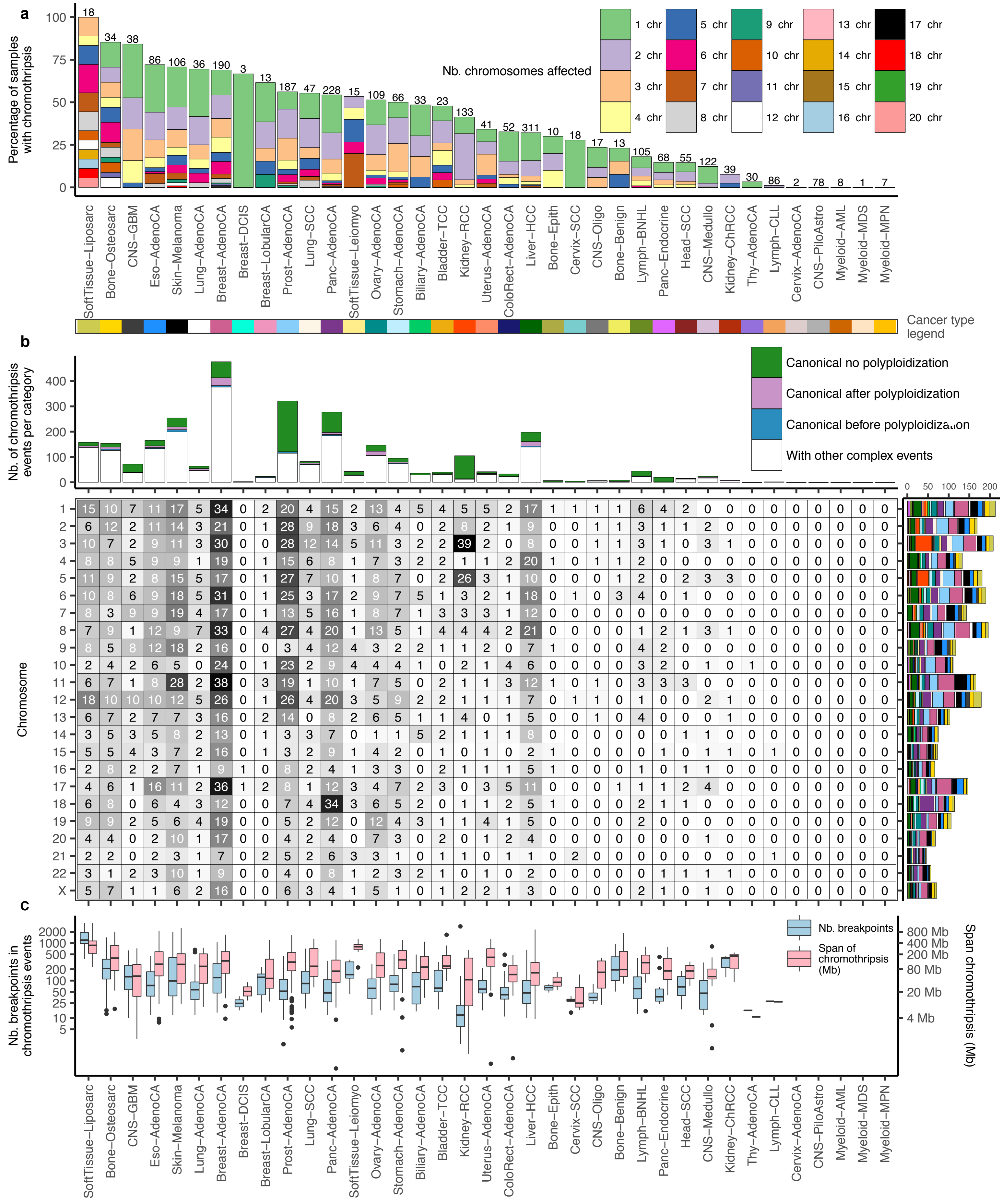

Supplementary Figure 2. Landscape of chromothripsis across 37 cancer types including the high- and low-confidence calls. (**a**) Percentage of patients harboring chromothripsis events. The total number of tumors examined for each cancer type is indicated on top of the bars, whereas the colors of the bars indicate the number of chromosomes affected by chromothripsis. (**b**) Number of chromothripsis regions categorized on the basis of the oscillating copy number patterns and the temporal profile of the events with respect to polyploidization, and their distribution across the genome. (**c**) Distribution of the number of breakpoints (blue), and span of the chromothripsis events (pink). Box plots in (**c**) show median, first and third quartiles (boxes), and the whiskers encompass observations within a distance of 1.5 times the interquartile range from the first and third quartiles.

Supplementary Figure 3

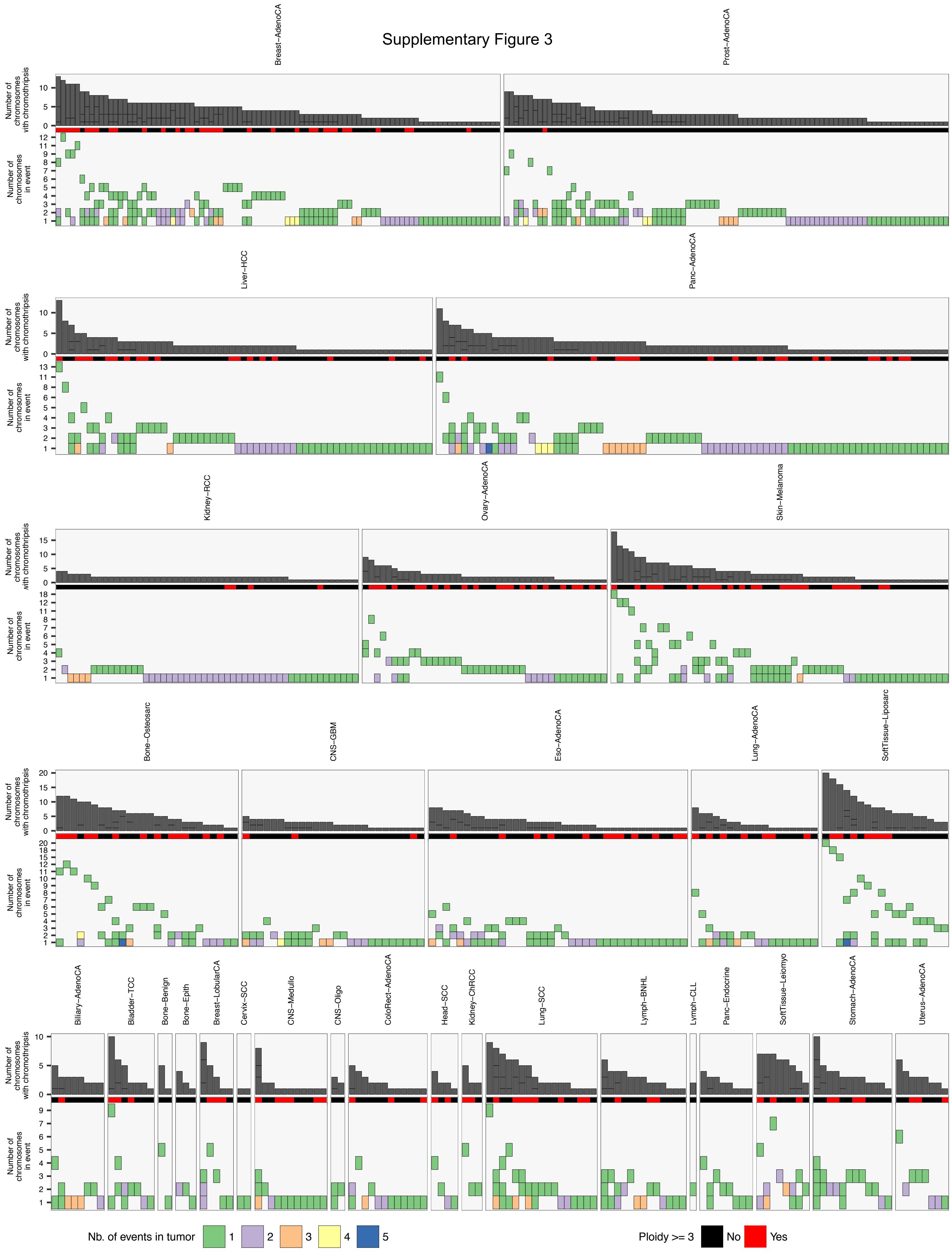

Supplementary Figure 3. Distribution of the number of chromosomes harboring high-confidence chromothripsis. The top barplot reports the total number of chromosomes with chromothripsis (including low- and high-confidence regions) per tumor (*x*-axis). The middle heatmap indicates whether the ploidy of the tumor was estimated to be >=3 (red) or not (black). The bottom heatmap reports the total number of independent chromothripsis events detected in a given tumor (*y*-axis). The colour of each cell represents the number of chromosomes involved in each independent event.

Supplementary Figure 4

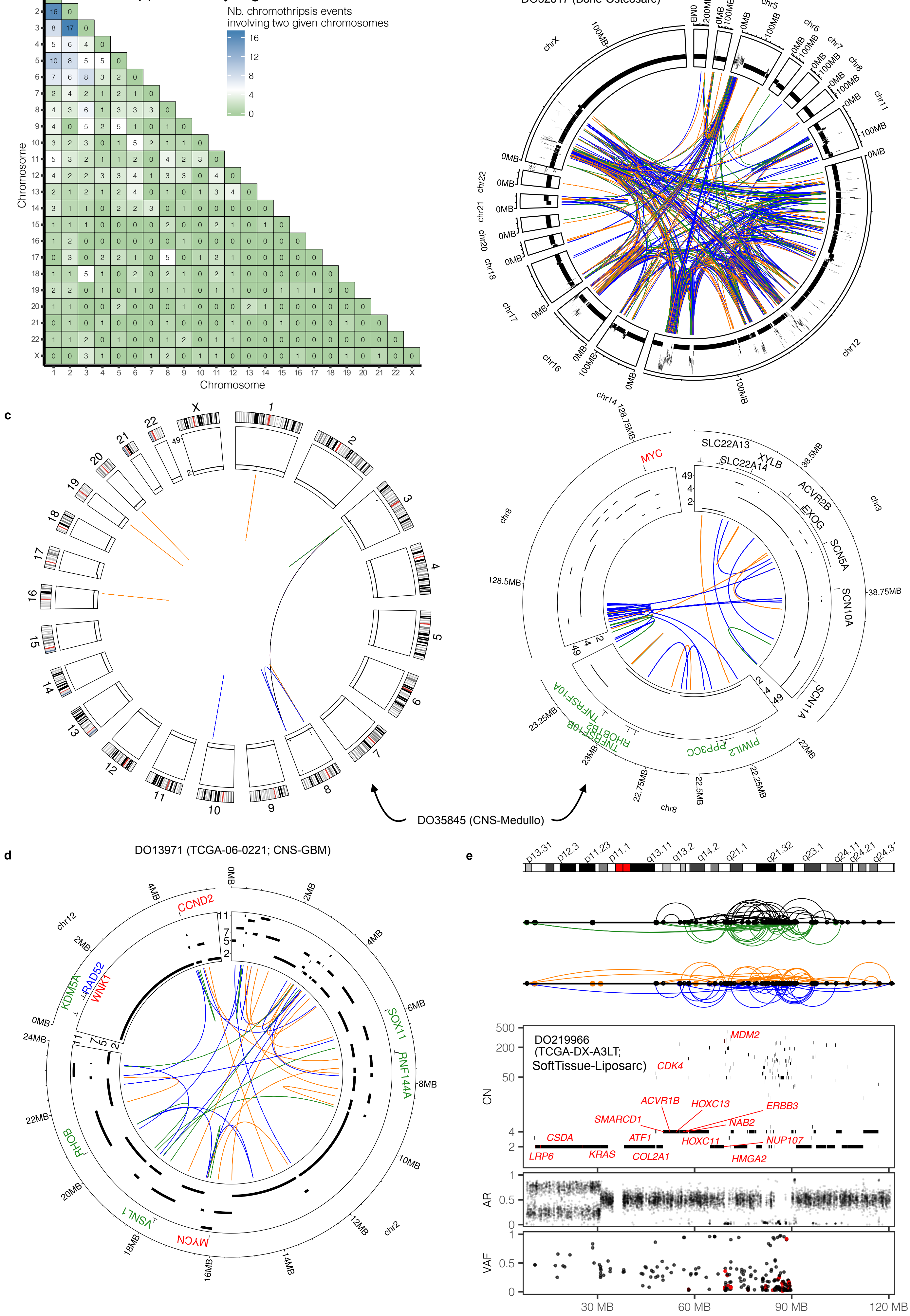

Supplementary Figure 4. (**a**) Number of chromothripsis events involving two given chromosomes across the entire set of high-confidence calls. (**b**) Unscaled Circos plot showing a massive chromothripsis event in patient DO52617 (Bone-Osteosarc) involving 16 chromosomes**.** (**c**) Example of a chromothripsis event in patient DO35845 (CNS-Medullo) affecting a few megabases in chromosomes 3 and 8, with an oscillating copy number pattern between states 4 and 49. This case also illustrates the amplification of *MYC*. Panel (**a**) shows the SVs detected across the entire genome, whereas panel (**b**) corresponds to the regions affected by chromothripsis (scaled Circos plot). (**d**) Scaled Circos plot representing the chromothripsis event in patient DO13971 (TCGA-06-0221; CNS-GBM) involving chromosomes 2 and 12, leading to the concomitant amplification of *MYCN* and *CCND2*. (**e**) Concomitant amplification of *CDK4* and *MDM2* in chromosome 12 in patient DO219966 (TCGA-DX-A3LT; SoftTissue-Liposarc). The bottom panel represents the variant allele fraction (VAF) for somatic SNVs. APOBEC-associated mutations are colored in red.

Supplementary Figure 5

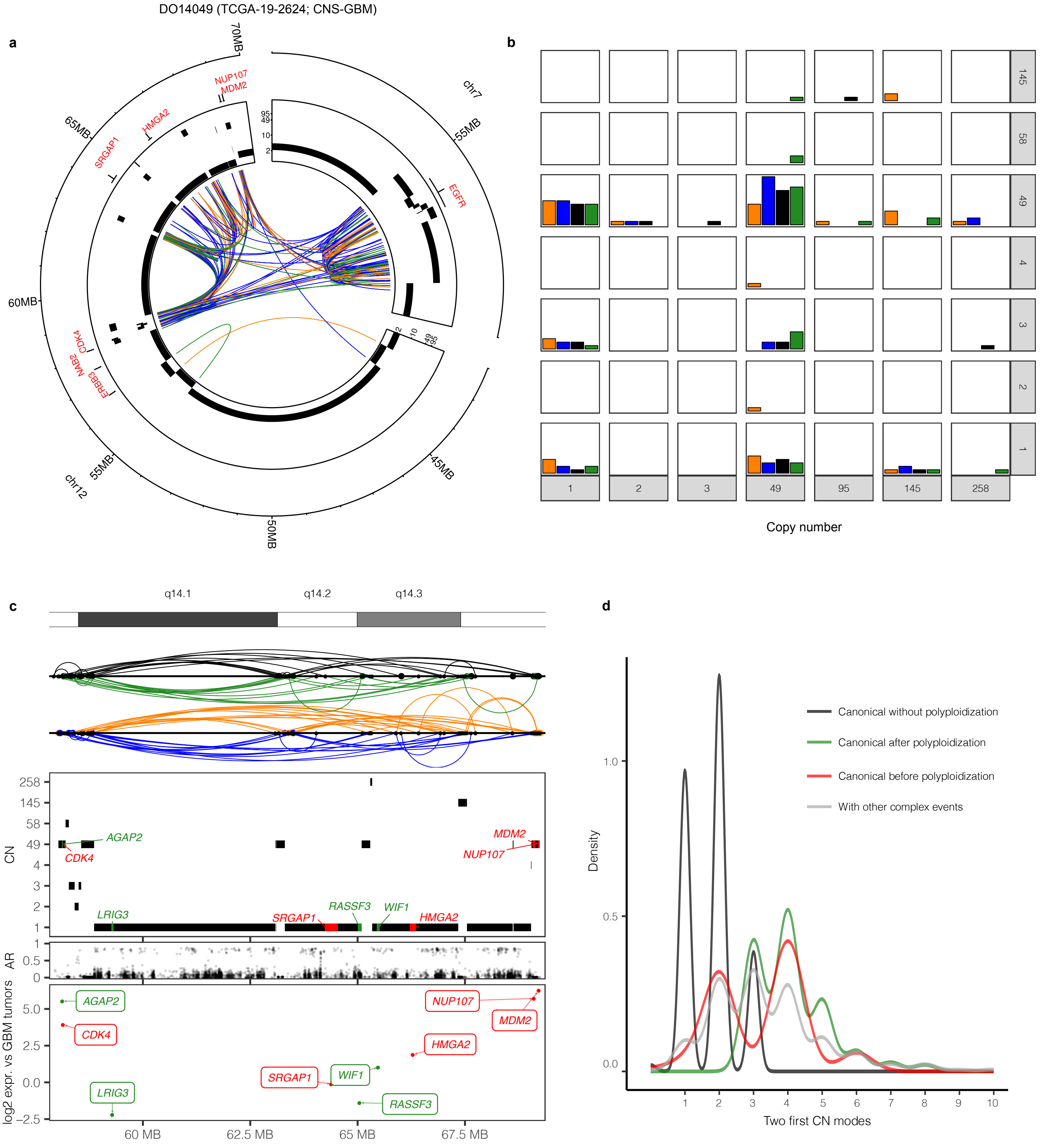

Supplementary Figure 5. (**a**) Example of a focal chromothripsis event in patient DO14049 (TCGA-19-2624; CNS-GBM) involving chromosomes 7 and 12 leading to the concomitant amplification of *CDK4*, *MDM2*, and *EGFR*. A previous study where this tumor was analysed using single-cell sequencing reported that genomic alterations in the *EGFR* locus defined different subclones[48]. These results are concordant with our calls in that the chromothripsis event affecting the *EGFR* locus presents CN oscillations across two states in the regions containing *MDM2* and *CDK4*, whereas the oscillating pattern is distorted at the *EGFR* locus, consistent with the presence of secondary subclonal rearrangements. The lack of a clear oscillation between two CN states would be expected from bulk sequencing data if secondary rearrangements had further altered the *EGFR* locus in different subclones. Although the canonical pattern of chromothripsis is not fully apparent at the *EGFR* locus when using bulk data, we could correctly detect chromothripsis at that locus by using interchromosomal SVs. (**b**) Copy-number step plot[6]. Each cell represents the distribution of types (see Fig. 1C) for those SVs connecting segments of the CN equal to the values indicated in the *x* and *y*-axis. (**c**)**.** Example of a chromothripsis event in chromosome 12 in patient DO14049 (TCGA-19-2624; CNS-GBM) leading to the concomitant amplification of *CDK4*, *MDM2*, and *NUP107* likely as a result of DM formation. (**d**) Distribution of the two first CN modes for the chromothripsis events categorized as canonical without polyploidization (black), canonical after polyploidization (green), canonical before polyploidization (red), and chromothripsis events in the context of other complex events (gray). Cases with CN modes higher than 10 were not depicted for clarity.

Supplementary Figure 6

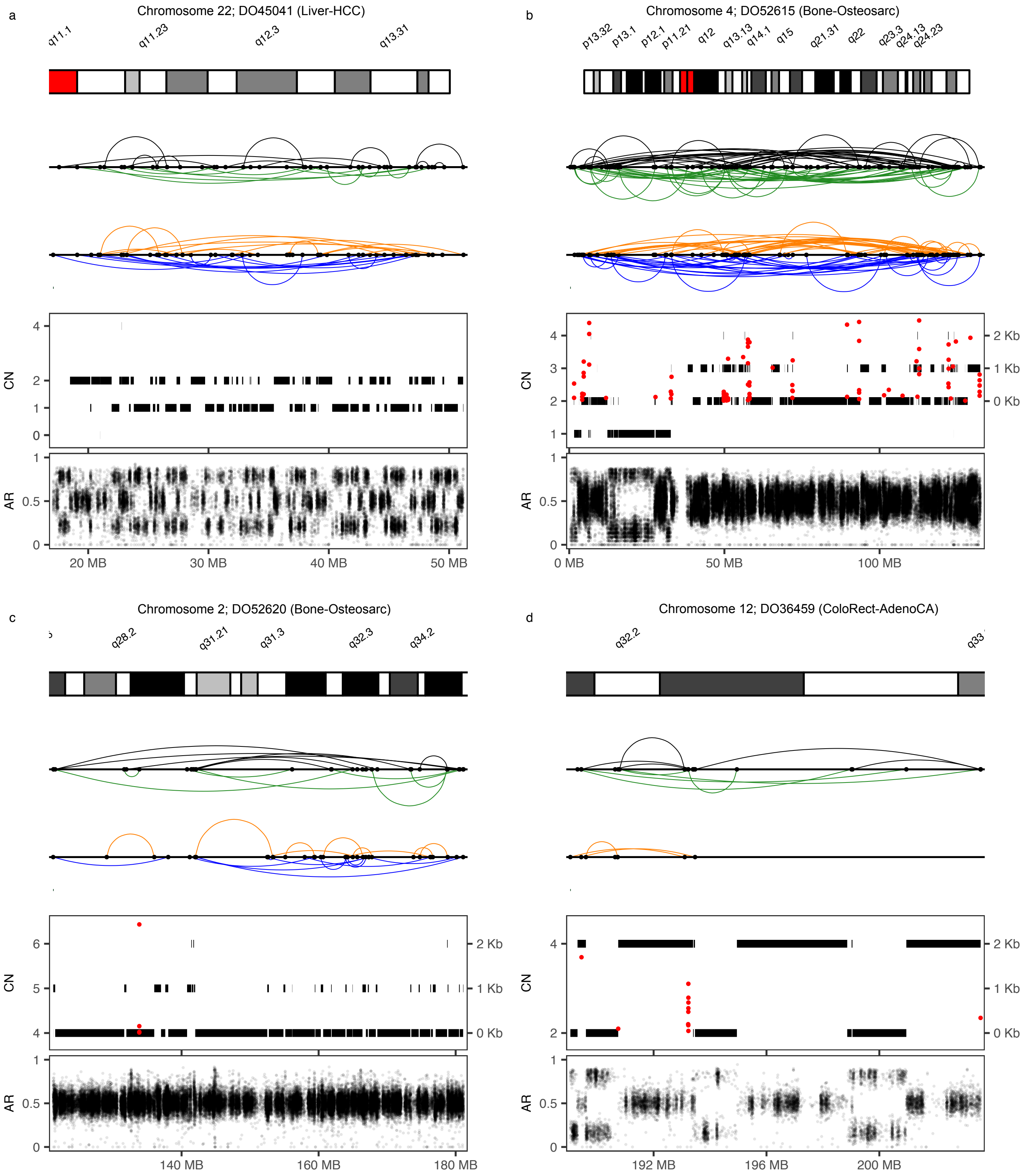

Supplementary Figure 6. Examples of chromothripsis regions. (**a**) canonical chromothripsis without polyploidization (chromosome 22; DO45041, Liver-HCC), (**b**) canonical without polyploidization displaying APOBEC-mediated kataegis (chromosome 4; DO52615, Bone-Osteosarc), (**c**) canonical chromothripsis after polyploidization (chromosome 2; DO52620, Bone-Osteosarc), and (**d**) canonical chromothripsis before polyploidization (chromosome 12; DO36459, ColoRect-AdenoCA). Clustered APOBEC-associated mutations are shown as red dots.

Supplementary Figure 7

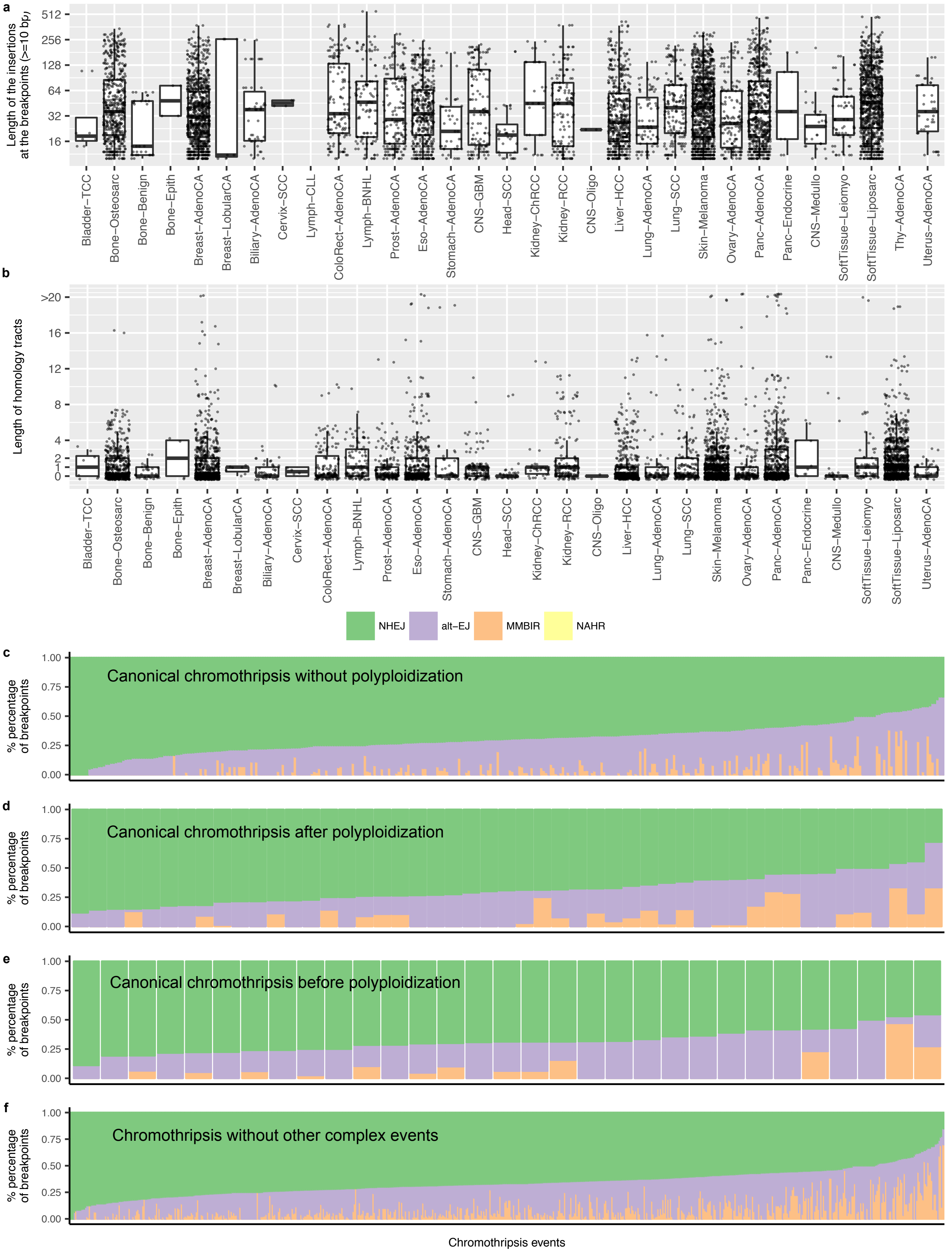

Supplementary Figure 7. Analysis of the DNA repair mechanisms for the high-confidence chromothripsis events**.** (**a,b**) Distribution of the length of the insertions and homology sequences, respectively, detected at the breakpoints for the high-confidence cases with at least 80% of the breakpoints detected pertaining to chromothripsis. Percentage of breakpoints for each DNA repair mechanism across all cases displaying canonical chromothripsis without polyploidization (**c**)**,** canonical chromothripsis after polyploidization (**d**)**,** canonical chromothripsis before polyploidization (**e**), and chromothripsis co-localized with other complex rearrangements (**f**). Box plots in (**a,b**) show median, first and third quartiles (boxes), and the whiskers encompass observations within a distance of 1.5 times the interquartile range from the first and third quartiles.

Supplementary Figure 8

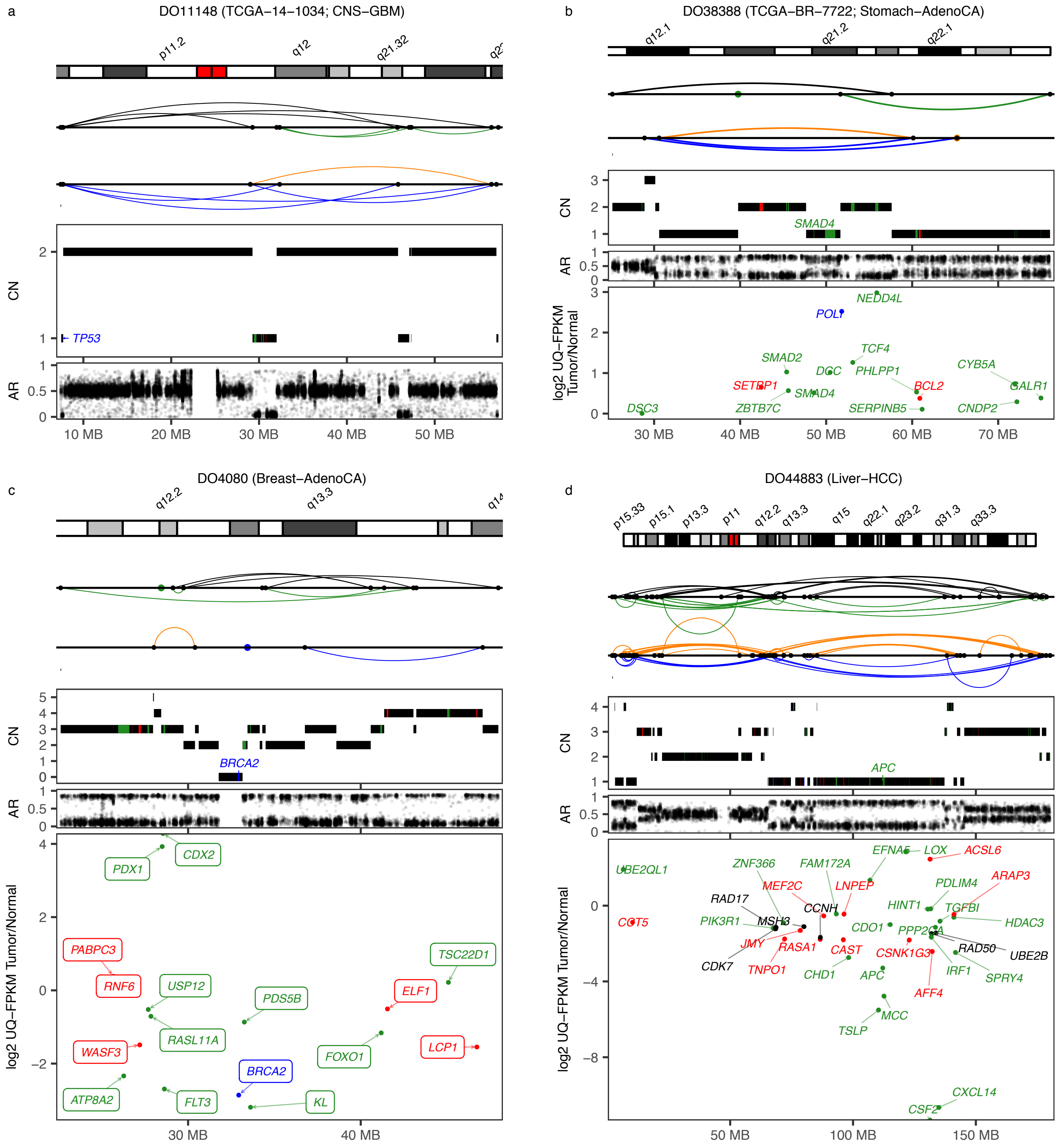

Supplementary Figure 8. Chromothripsis-mediated loss of tumor suppressors. (**a**) Evidence of chromothripsis-mediated loss of one *TP53* allele in patient DO11148 (TCGA-14-1034; CNS-GBM). The remaining allele harbors a deleterious mutation (pathogenic missense SNV, p.R337C; COSM11071). (**b**) Chromothripsis-mediated loss of one *SMAD4* allele in patient DO38388 (TCGA-BR-7722; Stomach-AdenoCA). The remaining allele harbors a deleterious mutation (pathogenic missense SNV, chr18: 48,591,925 G>A). (**c**) Biallelic *BRCA2* deletion in patient DO4080 (Breast-AdenoCA) confirmed by the low expression levels of *BRCA2*). This tumor also exhibited the mutational signature 3, which is known to be related to *BRCA1*/*BRCA2* deficiency. (**d**) Monoallelic loss of *APC* in patient DO44883 (Liver-HCC).

Supplementary Note

Comprehensive analysis of chromothripsis in 2,658 human cancers using whole-genome sequencing

Isidro Cortés-Ciriano[1,2,3,†], Jake June-Koo Lee[1,2], Ruibin Xi[4], Dhawal Jain[1], Youngsook L. Jung[1], Lixing Yang[5], Dmitry Gordenin[6], Leszek J. Klimczak[7], Cheng-Zhong Zhang[1,8], David S. Pellman[9,10], PCAWG Structural Variation Working Group[11], Peter J. Park[1,2*] & PCAWG Consortium[12]

[1]Department of Biomedical Informatics, Harvard Medical School, Boston, MA, USA.

[2]Ludwig Center at Harvard, Boston, MA, USA.

[3]Centre for Molecular Science Informatics, Department of Chemistry, University of Cambridge, Cambridge, UK.

[4]School of Mathematical Sciences and Center for Statistical Science, Peking University, Beijing, China.

[5]The Ben May Department for Cancer Research and Department of Human Genetics, The University of Chicago, Chicago, IL, USA.

[6]Genome Integrity and Structural Biology Laboratory, National Institute of Environmental Health Sciences, US National Institutes of Health, Research Triangle Park, NC, USA.

[7]Integrative Bioinformatics Group, National Institute of Environmental Health Sciences, US National Institutes of Health, Research Triangle Park, NC, USA.

[8]Department of Data Sciences, Dana-Farber Cancer Institute, Boston, MA, USA.

[9]Department of Pediatric Oncology, Dana-Farber Cancer Institute, Boston, MA, USA.

[10]Howard Hughes Medical Institute and Department of Cell Biology, Harvard Medical School, Boston, MA, USA.

[11]The members of this working group are listed at the end of the paper.

[12]The members of this consortium are listed in the Supplementary Note.

†Present address: European Molecular Biology Laboratory, European Bioinformatics Institute, Wellcome Genome Campus, Hinxton, UK.

*e-mail: peter_park@hms.harvard.edu

Table of contents

1. Chromothripsis detection using Shatterseek…3
   1.1 Algorithm description ... 3
   1.2 Confidence of chromothripsis calls ... 5
   1.3 False positive and false negative rate analysis ... 6
   1.4 Chromothripsis and chromoplexy ... 11
   1.5 Chromothripsis and chromoanasynthesis ... 13
   1.6 Defining the extent of chromothripsis events ... 13
   1.7 Classification of chromothripsis events ... 14
   1.8 Effect of tumor purity and heterogeneity ... 15
2. Comparison of ShatterSeek to other methods ... 15
   2.1 Comparison with previous calling criteria ... 15
   2.2 Validation in diploid tumors ... 19
   2.3 Comparison with ShatterProof ... 20
   2.4 Cases missed by ShatterSeek and called by ShatterProof ... 22
   2.5 Cases missed by ShatterProof but detected by ShatterSeek ... 23
   2.6 Comparing the chromothripsis rates for prostate tumors ... 24
   2.7 Chromothripsis rates as a function of the ShatterProof cut-off value ... 26
   2.8 Comparison against Chrom-AL ... 26
   2.9 Manual inspection of chromothripsis calls ... 26
3. Contribution of chromothripsis to gene fusion formation ... 27
4. Analysis of the association between chromothripsis-related breakpoints and epigenetic marks ... 28
5. Chromothripsis is prognostic of poor patient survival ... 30
6. References ... 33
7. Members of the PCAWG Consortium…35

1. Chromothripsis detection using ShatterSeek

1.1 Algorithm description

Here, we detail the chromothripsis detection pipeline implemented in ShatterSeek for identifying chromothripsis patterns from whole-genome sequencing data. The code can be found at https://github.com/parklab/ShatterSeek. Our chromothripsis calls for each PCAWG sample can be explored through interactive Circos plots at: http://compbio.med.harvard.edu/chromothripsis/.

A key feature of ShatterSeek is based on the fact that the regions affected by chromothripsis should be characterized by clusters of breakpoints belonging to SVs that are interleaved, *i.e.*, the regions bridged by their breakpoints overlap instead of being nested, as is expected from random joining of genomic fragments (Supplementary Note Fig. 1).

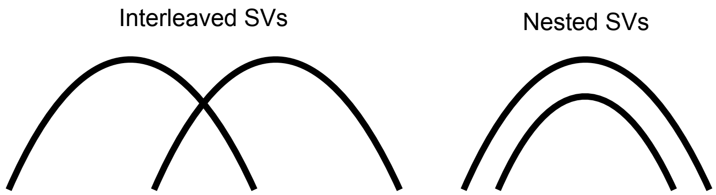

Supplementary Note Figure 1. Illustration of the pattern of interleaved SVs used in ShatterSeek to detect candidate chromothripsis regions displaying clusters of intrachromosomal SVs.

This approach allows us to encompass the many cases that do not display simple oscillations, *e.g.*, partially oscillating CN profiles with interspersed amplifications, and oscillations spanning multiple CN levels due to aneuploidy[1,2]. Therefore, we firstly scanned the cancer genomes for the presence of clusters of interwoven SVs. To find clusters within a given chromosome, ShatterSeek constructs an undirected graph whose nodes correspond to SVs and whose edges connect interleaved SVs. Thus, clusters of SVs were detected by finding the connected components in the graph. The connected component in each chromosome with the highest number of SVs was considered for further analysis. We confined our analyses to the entire mappable genome with the exception of chromosome Y. We use the term 'chromothripsis region' to refer to cases where chromothripsis affects only one chromosome, whereas we term 'chromothripsis event' the set of chromothripsis regions involved in a single catastrophic event. Thus, a chromothripsis event can affect a single chromosome, or encompass genomic regions from multiple chromosomes.

Rearrangements in chromothripsis should also follow a roughly even distribution for the different types of fragment joins (*i.e.*, duplication-like, deletion-like, head-to-head and tail-to-tail inversions, depicted in blue, orange, black, and green, respectively, in Fig. 1a, in the main text, throughout the entire article, and in the Supplementary Information) and have breakpoints randomly distributed across the affected region[3–5]. Hence, once the SV clusters were detected,

we tested whether the distribution of DNA fragment joins diverged from a multinomial distribution with equal probabilities for each category using the goodness-of-fit test for the multinomial distribution (chiq.test function from the R package *stats*), as described by Korbel and Campbell[4] (fragment joins test). In addition, we used the binomial test corrected for mappability to evaluate the enrichment of SVs in each chromosome (chromosomal enrichment test). We also evaluated whether the distribution of breakpoints differed from an exponential distribution as described by Korbel and Campbell[4] (exponential distribution of breakpoints test). The *P* values from these three tests were corrected using the FDR method. The level of significance was set to $q \leq 0.2$. Finally, the genomic regions delimited by the distal breakpoints composing the clusters of interleaved SVs were further examined for the presence of contiguous genomic segments oscillating between two CN states.

We decided to only consider the criteria of loss of heterozygosity proposed by Korbel and Campbell [4] when examining the set of chromothripsis calls detected in diploid genomes. We did not consider this criterion to call chromothripsis across the entire tumor cohort because we noticed that some *bona fide* chromothripsis cases identified in low-purity samples (*e.g.,* Fig. 2c in the main text) might not meet the cut-off value for statistical significance due to the infiltration of normal tissue. In such cases, the allelic ratios (*i.e.*, B allele frequencies) for heterozygous SNPs would not divert significantly from 0.5, and thus, would hamper the observation of alternating LOH patterns associated to copy losses[6]. This phenomenon is exacerbated when chromothripsis occurs in aneuploid tumors due to increased CN, and hence, chromothripsis events do not necessarily lead to LOH (see *e.g.,* Supplementary Fig. 6c).

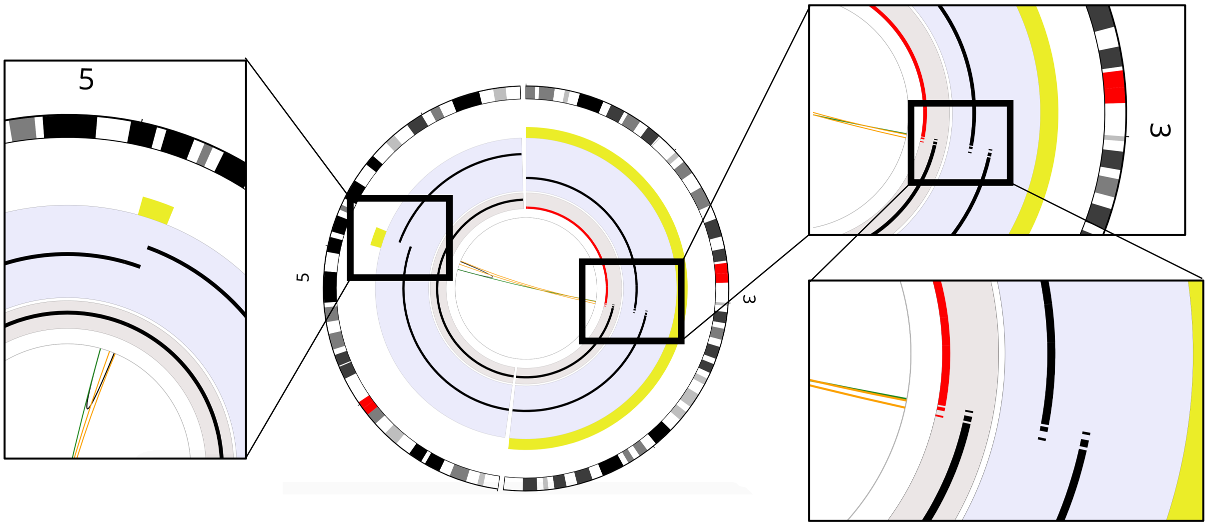

Supplementary Note Figure 2. Evidence of a chromothripsis event in tumor DO17178 (TCGA-AK-3454; Kidney-RCC) involving few interchromosomal SVs between chromosomes 3 and 5. Such type of chromothripsis events in Kidney-RCC tumors have been previously reported (Mitchell *et al.*, Cell, 2018). This case was missed by ShatterSeek because the SVs involved in the event do not generate a cluster of interleaved SVs. It was however identified through visual inspection.

Integration of SV and CN information was crucial for filtering candidate regions with clusters of breakpoints originating from tandem duplications or deletions rather than from chromothripsis (Supplementary Data Files 3-4). Whereas these cases often satisfied the CN-based criterion

used to call chromothripsis in some previous studies (*e.g.*, 10 segments oscillating between 2 CN states)[7–9], the predominance of one type of SV permitted us to dismiss chromothripsis as their generating mechanism. In addition, considering both SV and CN information resulted in higher sensitivity for detecting those chromothripsis events involving relatively few SVs or CN segments and/or are co-localized with other complex events (Supplementary Data Files 1-2).

1.2 Confidence of chromothripsis calls

After visual inspection of a number of chromothripsis calls, we considered as high-confidence chromothripsis those regions satisfying at least one of the following sets of cut-off values for the statistical criteria described above: (i) at least 6 interleaved intrachromosomal SVs, 7 adjacent segments oscillating between 2 CN states, the fragment joins test, and either the chromosomal enrichment or the exponential distribution of breakpoints test; and (ii) at least 3 interleaved intrachromosomal SVs and 4 or more interchromosomal SVs, 7 adjacent segments oscillating between 2 CN states and the fragment joins test. Given that the oscillating CN pattern is often only partly retained in chromothripsis events in Bone-Osteosarc, SoftTissue-Leiomyo, and SoftTissue-Liposarc tumors due to subsequent amplification events[1,10,11], we also considered as high-confidence chromothripsis regions those detected in these cancer types involving at least 40 intra- or interchromosomal SVs, and satisfying the fragments joins test (this change in criteria affects six samples, but, because many of them have multiple events, only one sample that previously was not identified as having chromothripsis was identified as having one event).

We classified as low-confidence those chromothripsis regions meeting the following cut-off values: at least 6 interleaved intrachromosomal SVs, 4, 5 or 6 adjacent segments oscillating between 2 CN states (following the criteria previously used[2]), the fragment joins test, and either the chromosomal enrichment or the exponential distribution of breakpoints test. Therefore, the main difference between the low- and high-confidence calls resides in the number of oscillating CN segments.

We chose 7 CN oscillations as the cut-off value to define high-confidence for the following reasons: 1) combined with other criteria, it gave estimates that are largely concordant with previous findings for diploid cases as well as rates given in two recent studies[2,12]; 2) it removed most of the tandem duplication (false positive) cases; 3) a minimum number of 4 CN oscillations (assuming that other criteria were satisfied) used previously[2] were too relaxed because many focal cases did not look sufficiently convincing when visualized; 4) we observed that longer oscillations are frequently interrupted by secondary CN alterations (as noted in multiple studies, *e.g.,* Behjati *et al.*[1]) and thus requiring too many consecutive oscillations removed cases that are convincingly chromothripsis except for the interruption.

One should note that a cut-off value of 7 might be too stringent, as some experimentally generated chromothripsis events would be missed[3]. However, our analysis showed that 7 represents a reasonable compromise between sensitivity and specificity. The high *vs* low confidence classification was performed to account for the possibility that we may have missed

many events; but the estimates discussed throughout the main text are for high-confidence calls unless otherwise stated.

The impact of the threshold for the required number of CN oscillations is shown in the heatmap below, where we display the percentage of tumors with a chromothripsis call as a function of the CN oscillation parameter. This sensitivity analysis reveals that the chromothripsis rates are much higher than previously anticipated even when requiring at least 10 uninterrupted CN oscillations (Supplementary Note Fig. 3). The rates of chromothripsis as a function of the number of breakpoints involved, number of chromosomes affected, and the minimum number of uninterrupted CN oscillations between 2 CN states can be explored interactively at http://compbio.med.harvard.edu/chromothripsis/.

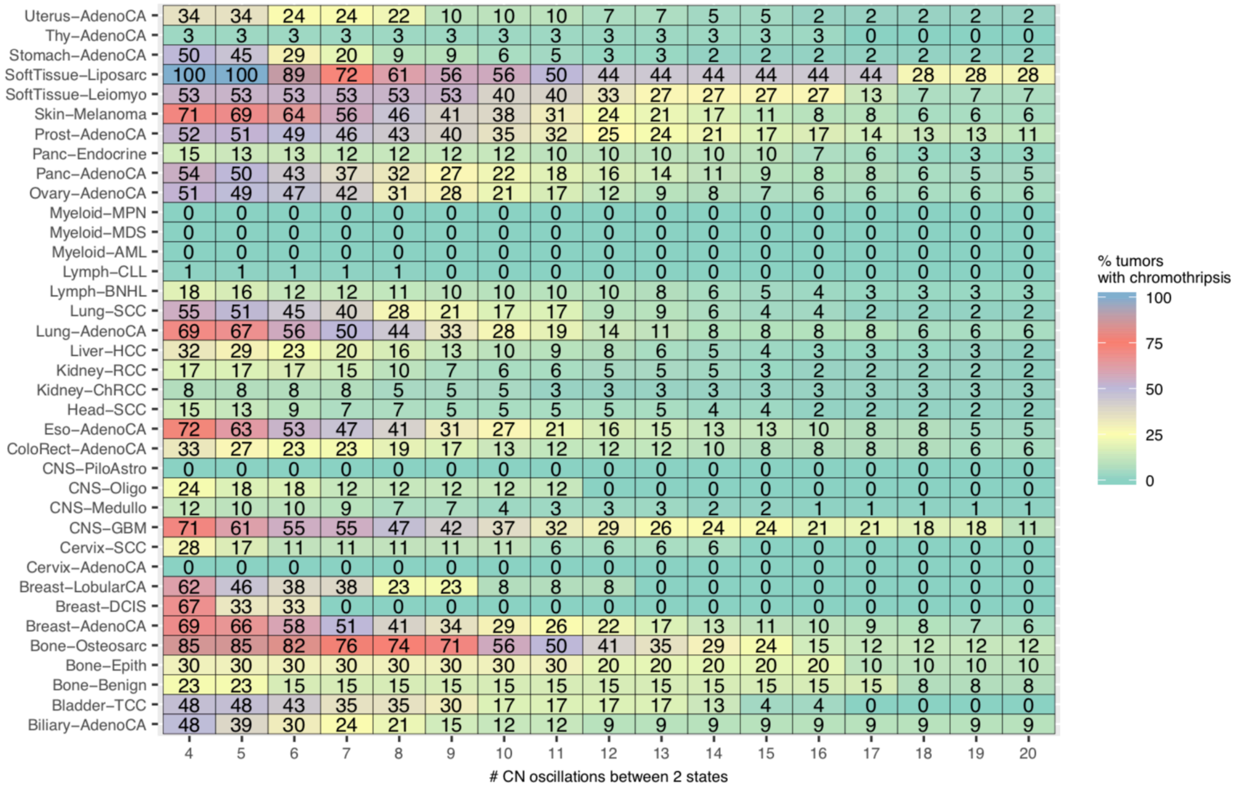

Supplementary Note Figure 3. Percentage of tumors with chromothripsis as a function of the minimum number of uninterrupted CN oscillations required to make a call.

1.3 False positive and false negative rate analysis

After visual inspection of all cases, we removed 165 high-confidence events (8.5% of high-confidence events; 106 tumors) and 136 low-confidence calls (10.4% of low-confidence events). We included 93 events across 45 tumors after visual inspection (*e.g.,* Supplementary Note Fig. 2). All these cases are labelled in the column “comment” in Supplementary Table 1 and in the accompanying website.

Supplementary Note Table 1. Confusion matrix for ShatterSeek.

		**Visual inspection**	
		High-confidence chromothripsis	No chromothripsis
**ShatterSeek**	High-confidence chromothripsis	689	106
	No chromothripsis	45	1703

We estimate a FP rate for our method when evaluated at the tumor level of 5.9% for high-confidence calls, which is comparable to that of previous studies (Notta *et al.*[2] reported a FP rate of 7% for Chrom-AL; Supplementary Note Table 2). The difference between the FP rate of Chrom-AL and our method is largely due to differences in SV rates across cancer types (Supplementary Note Figs. 4-5). We also note that we assembled a highly stringent chromothripsis call set to guarantee that we were not overcalling, and hence, it is possible that a considerable fraction of the events we removed might be true chromothripsis cases.

Our false negative rate was estimated to be 5.8%, which is also comparable to that reported by Notta *et al.*, *i.e.,* 8%. We detected 93 false negatives in 45 tumors (Supplementary Note Fig. 6). These cases were mainly events involving two or three chromosomes that generate few CN oscillations and often led to the deletion of one chromosome arm. Therefore, these cases were missed because they involved few interchromosomal SVs, and hence, ShatterSeek failed to detect a cluster of interleaved intrachromosomal SVs at the affected regions (see Supplementary Note Fig. 2 for an example).These events were common in Kidney-RCC, as other studies have reported recently[13].

We note that the false positive and false negative rates of Chrom-AL were determined by visual inspection[2], as we also do. Given that we detected chromothripsis in the same tumors as Notta *et al.*[2] (see section 2.8), we next examined the false positive and false negative rates on a per tumor type basis to better understand the differences in false positive rates.

We observe that for tumor types where other sources of hypermutation are frequent (*e.g.,* Homologous Recombination -HR- deficiency) our approach shows a higher false positive rate. This is expected because, although these hypermutated tumors show clusters of tandem deletions and duplications that are not interleaved, our algorithm sometimes fails to dismiss cases where other structural variations (generally inversions) occur on top of the tandem duplications or deletions. Identifying clusters of *interleaved SVs* is crucial to correctly dismiss false positive cases—clusters of breakpoints generated by other mechanisms but showing CN oscillations. Of the 2,591 chromosomes in the PCAWG cohort that show CN oscillations, we only called chromothripsis in 69% thereof (1787 events), *i.e.,* the interleaved SV criterion removes 31% of events. Visual inspection of these events revealed that the CN oscillations were consequence of tandem duplications and deletions. A visual representation for all these events is given in Supplementary Data File 3. Overall, these results indicate that the difference in the false positive rate of our algorithm and Chrom-AL[2] is due to tumor-type specific genomic instability patterns. Note that our false negative rate for Panc-AdenoCA, the tumor type studied by Notta *et al.*, is comparable, *i.e.,* 5.8%.

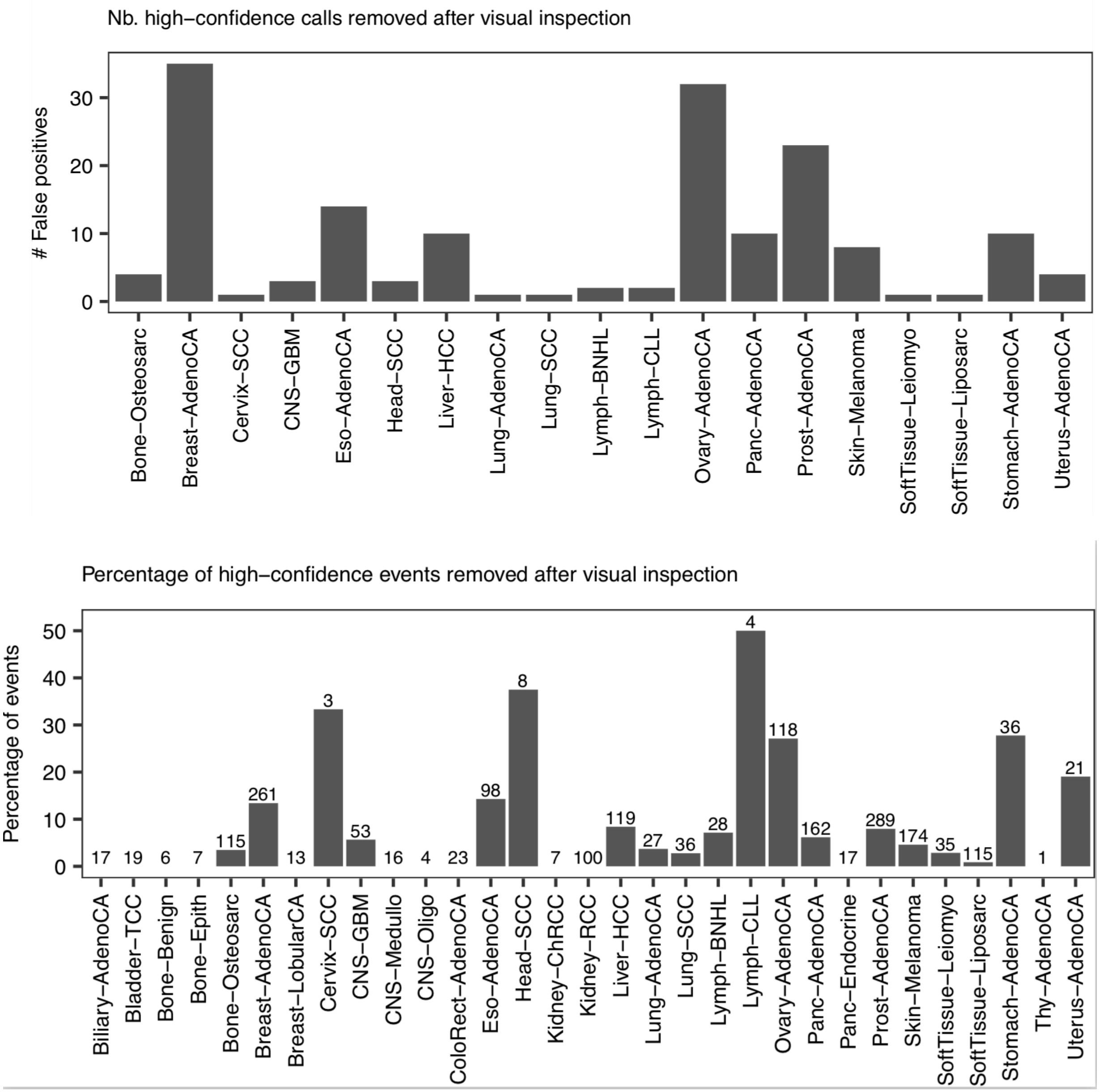

Supplementary Note Figure 4. Total number (top) and percentage (bottom) of high-confidence chromothripsis events (*e.g.,* chromosomes where chromothripsis was detected by ShatterSeek, not tumors) that were deemed false positives after visual inspection. Note that false positives are often detected in tumors that show true positive chromothripsis events in other chromosomes. In fact, of the 210 tumors where we detected false positives events, 103 thereof harbor true positive chromothripsis events in other genomic regions. The numbers on top of the bars on the bottom plot indicate the total number of high-confidence events detected in the tumors of the type indicated on the *x*-axis.

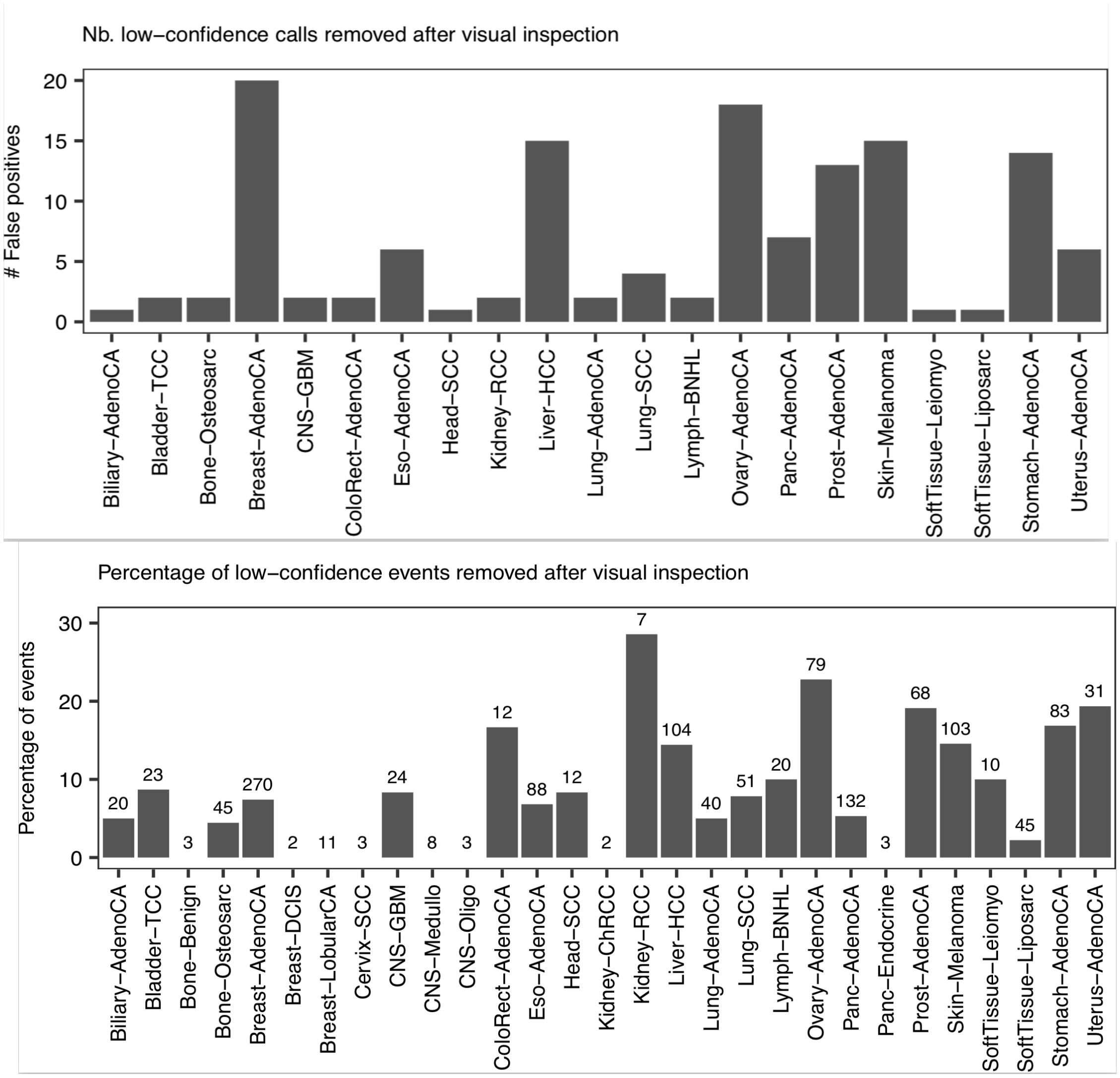

Supplementary Note Figure 5. Total number (top) and percentage (bottom) of low-confidence chromothripsis events (*e.g.,* chromosomes where chromothripsis was detected by ShatterSeek, not tumors) that were deemed false positives after visual inspection. The numbers on top of the bars on the bottom plot indicate the total number of low-confidence events detected in the tumors of the type indicated on the *x*-axis.

In total, 45 tumors were included (true negatives, left-hand side plot in Supplementary Note Fig. 6), and 108 were removed (false positives, right-hand plot in Supplementary Note Fig. 6) after visual inspection. For most cancer types, the percentage of false positive tumors is below 5%, and true negatives belonged to three tumor types only.

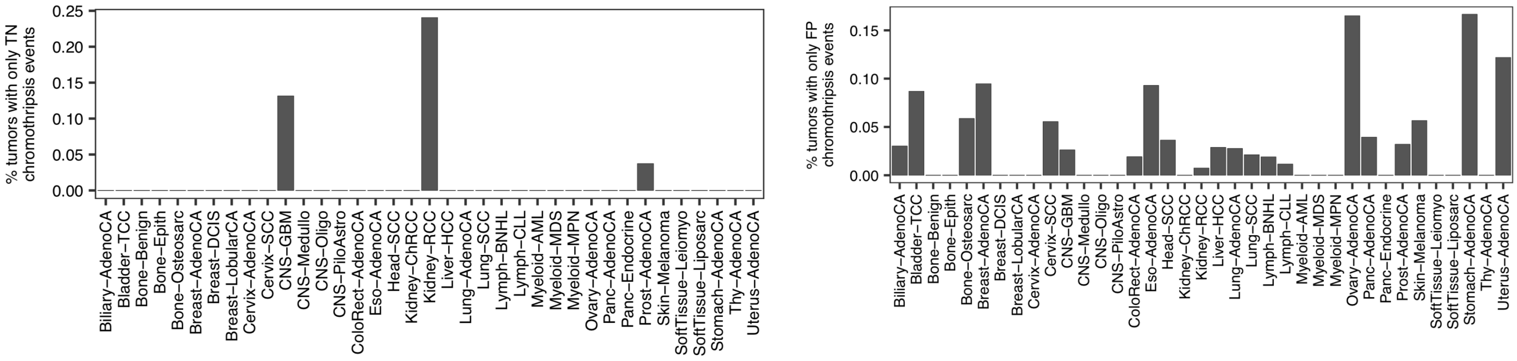

Supplementary Note Figure 6. Left. Percentage of tumors in the entire cohort with true negative events only. It can be seen that true negative events are enriched in Kidney-RCC, where chromothripsis events are recurrently formed by few interchromosomal SVs between chromosomes 3 and 5, thus leading to few CN oscillations (<10) and the loss of the *VHL* gene[13]. **Right.** Percentage of tumors in the PCAWG cohort with false positive calls only and that were not considered as harboring chromothripsis in this study. The chromothripsis rates are decreased by less than 5% in most tumor types after removing false positive cases by visual inspection. False positives are enriched in tumor types where hypermutation occurs frequently due to *e.g.,* HR deficiency, including Breast-AdenoCA and Ovary-AdenoCA.

We next examined whether the false positives we detect are characterized by a low number of CN oscillations, or by a low number of SVs (Supplementary Note Fig. 7). It can be seen that the false positives are evenly distributed across CN oscillations and number of SVs, indicating that false positives are not enriched for cases involving few SVs or displaying few oscillations.

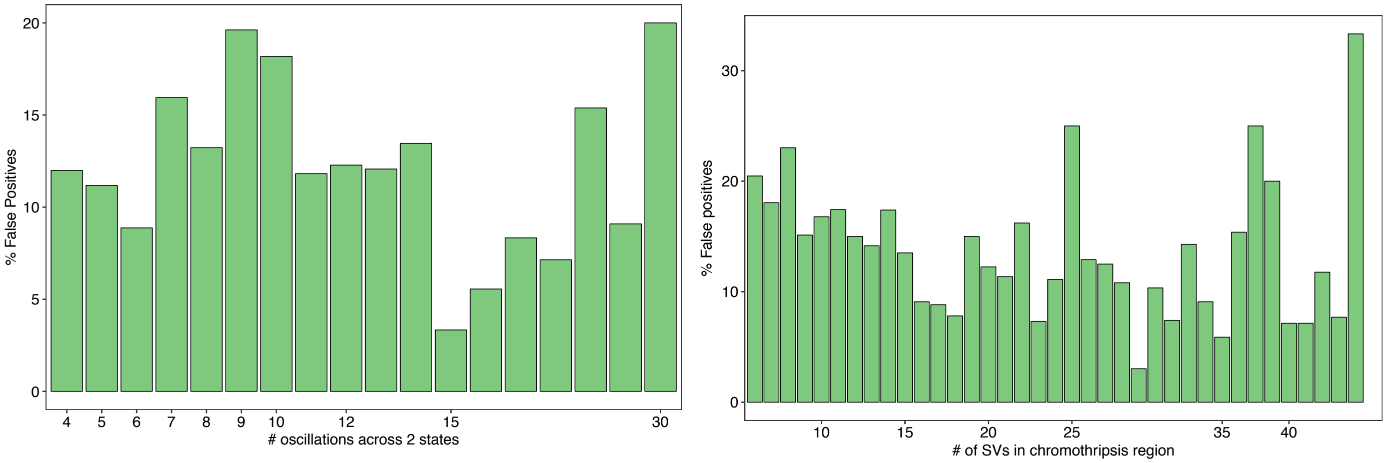

Supplementary Note Figure 7*.* Percentage of false positive events stratified on the basis of the number of CN oscillations in the event (left), and number of SVs involved in the event (right). The bars indicate the percentage of chromothripsis events called by ShatterSeek showing a given number of CN oscillations or SVs that were classified as false positives.

Finally, we assessed the fraction of regions showing clusters of SVs and a certain number of CN oscillations in the tumors examined where we make a chromothripsis call. We hypothesized that if our algorithm and visual inspection steps were not specific to chromothripsis, we would expect the algorithm to call a high rate of chromothripsis events for chromosomes displaying few CN oscillations. We found that the fraction of regions with 3-7 oscillations where we call chromothripsis is small, especially for those with 3-4 oscillations (Supplementary Note Fig. 8), indicating that our algorithm does not call chromothripsis arbitrarily in regions with few oscillations.

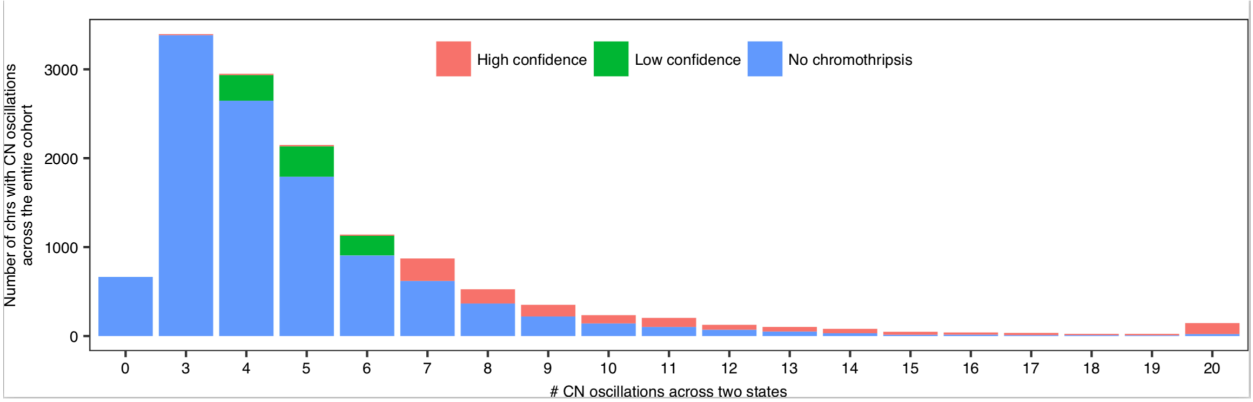

Supplementary Note Figure 8. Number of chromosomes harboring SV clusters across the entire cohort (13,120 regions across the 2,428 tumors with at least 1 SV that we examined; *y*-axis) showing the number of CN oscillations indicated in the *x*-axis. The x-axis indicates the number of CN oscillations in these regions. Regions with >20 oscillations were aggregated to the bar with the label "20".

1.4 Chromothripsis and chromoplexy

Chromoplexy is defined as a chain of balanced translocations, thus displaying balanced CN profiles rather than oscillating CN profiles, with the exception of those cases where DNA resection or segmental loss occurs[14]. Given that chromoplexy events where segmental losses occur might generate chromothripsis-like patterns, we next sought to analyse to what extent ShatterSeek misclassifies chromoplexy events as chromothripsis. To this, we detected chromoplexy events in all tumors considered using ChainFinder version 1.0.1.[14] We required at least three chromosomes involved in a chain to make a chromoplexy call. We detected 306 chromoplexy chains in 253 tumors (10% of tumors; Supplementary Note Fig. 9). We found that chromothripsis and chromoplexy occurred frequently in different genomic regions of the same tumor; only 12 tumors had regions where both ShatterSeek and ChainFinder made a call. This represents 3.2% (23/734) of the tumors with high-confidence chromothripsis events. We detected both chromoplexy and chromothripsis in the same region in 1 Biliary-AdenoCA, 1 Bone-Epith, 1 Breast-AdenoCA, 1 Breast-LobularCA, 1 ColoRect-AdenoCA, 1 Eso-AdenoCA, 2 Liver-HCC, 2 Panc-AdenoCA, and 2 Prost-AdenoCA tumors. Visual inspection of these cases revealed that both chromoplexy and chromothripsis events occurred in these tumors (see

Supplementary Note Fig. 10 for an example), and that both algorithms made correct calls even in cases where both mechanisms overlapped. By analizing the SVs in chromoplexy chains, we found that only 1% (when considering high-confidence chromothripsis regions only) of the SVs involved in chromoplexy mapped to regions affected by chromothripsis; 4.5% when also including low-confidence calls.

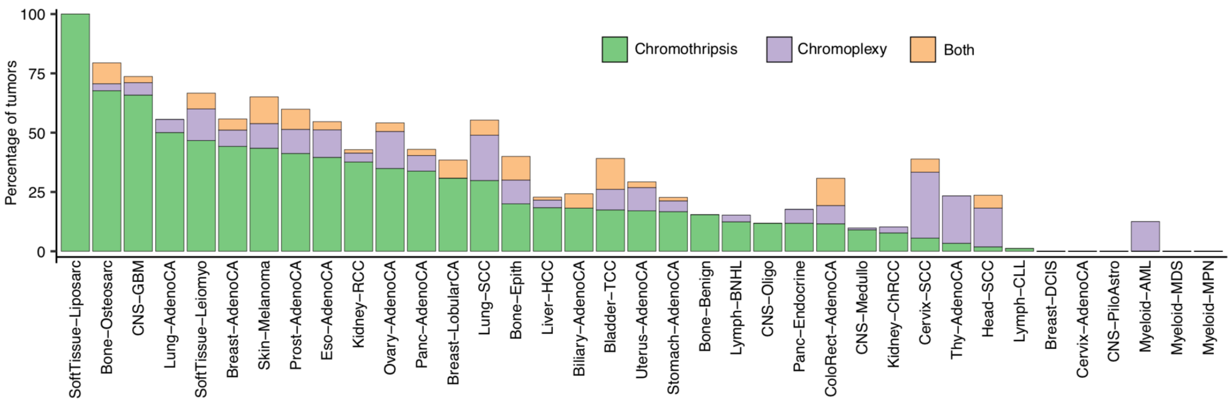

Supplementary Note Figure 9. Percentage of tumors harboring chromothripsis events only (green), both chromoplexy and chromothripsis in different regions of their genomes (orange), and chromoplexy events only (purple).

Given these results, coupled to the fact that we used high thresholds for the minimum number of chromosomal segments exhibiting CN oscillations, we are confident that there is not a substantial contamination of chromoplexy events in our chromothripsis call set. However, we cannot completely exclude the possibility that chromothripsis might have occurred in some

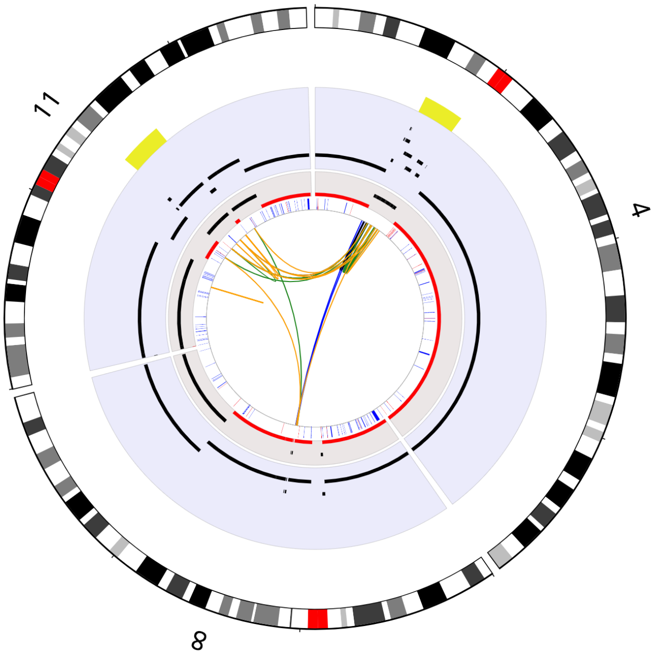

Supplementary Note Figure 10. Example of a tumor (Bone-Epith, CGP_donor_1602529) where both chromothripsis and chromoplexy affected the same region. An interactive version of this plot can be found at: http://compbio.med.harvard.edu/chromothripsis/

cases on already-rearranged chromosomes by chromoplexy and *vice versa*. These cases would belong to the “with other complex events” category in our classification of chromothripsis events.

1.5 Chromothripsis and chromoanasynthesis

Another mechanism that often generates chromothripsis-like patterns is chromoanasynthesis[15]. We note that the distinction between chromothripsis and chromoanasynthesis is often simple for diploid genomes in the absence of secondary rearrangements. When there is aneuploidy, the distinction is less clear because the CN and LOH patterns for a chromothripsis event in an aneuploid genome are comparable to that of a chromoanasynthesis even in a diploid tumor. Hence, it is not always possible to assess systematically the difference between these two scenarios (with the genome-wide ploidy and microhomology at breakpoints and SV connections, it may be possible to make educated guesses in some situations, but not always very reliably). Another complication is that some genomes appear to contain both chromothripsis and chromoanasynthesis events, often connected to each other (see Fig. 4 in the main text). Therefore, we used the canonical cases to estimate the fraction of chromoanasynthesis cases in our call set, which was found to be in the order of ~5% (see the main text for further details).

To identify templated insertions, we followed the set of criteria described by Li *et al.*[16], namely: orientation and microhomology at the breakpoints, connections between amplified segments, and the size of the amplified segments.

1.6 Defining the extent of chromothripsis events

We considered that two or more intrachromosomal chromothripsis regions belong to the same catastrophic event if these regions +/- 10Kb are linked by at least two interchromosomal SVs. Given that we did not always detect rearrangements between all regions belonging to the same chromothripsis event in cases where at least three chromosomes were involved, we applied transitive reasoning to identify the full extent thereof. For instance, if the chromothripsis regions in chromosome 1 and 2 are linked, and those detected in chromosome 2 are also linked to a chromothripsis region in chromosome 3, we consider that the chromothripsis patterns detected in these three chromosomes belong to the same event.

In each chromosome, the genomic region encompassed between the breakpoints at both ends of the complex rearrangement was considered as the region affected by chromothripsis. However, we note that manual review is required in cases where a chromothripsis event leads to the loss of the entire genomic region upstream or downstream the SV cluster till the chromosome ends, as in these cases those regions are not comprised within the SV cluster (see two examples in Supplementary Note Figs. 2 and 11)[13].

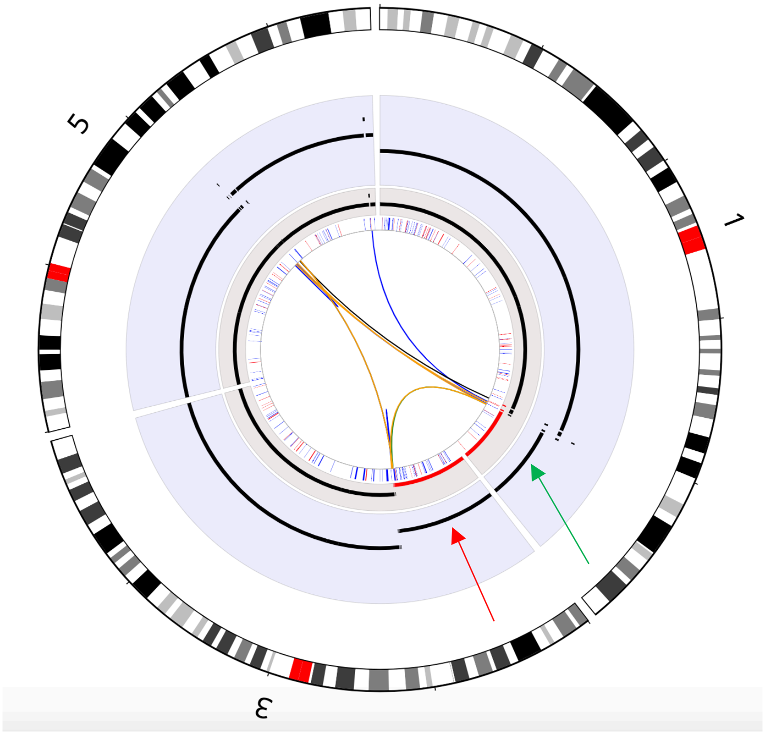

Supplementary Note Figure 11. Example of an event that required manual inspection to determine that the regions downstream (chromosome 1; green arrow) and upstream (chromosome 3; red arrow) of the cluster of SVs generated by a multichromosomal event were lost due to chromothripsis. Tumor DO19733 (Kidney-RCC; TCGA-CW-5585). An interactive version of this plot can be found at: http://compbio.med.harvard.edu/chromothripsis/

1.7 Classification of chromothripsis events

Chromothripsis events were classified as canonical if at least 60% of contiguous CN segments alternate between two copy number states. We inferred the mutational timing using the pattern of the copy number variation and the loss of heterozygosity as previously proposed[17], and used by others[2]. The basic idea is that if chromothripsis occurs before genome doubling, the copy number oscillations, for instance, would go from 1-2-1-2-1-2 to 2-4-2-4-2-4; if after genome doubling, it would be 3-4-3-4-3-4-3 because it would affect only one chromosome[17]. Canonical chromothripsis events with alternating CN profiles between CN states ≥ 3 were considered to have occurred after polyploidization. Canonical events with CN profiles alternating between two CN states encompassed in the set {0,1,2,3} were labeled as canonical without polyploidization. For the canonical chromothripsis events occurring in polyploid tumors, we can determine whether chromothripsis occurred before or after polyploidization. Chromothripsis events that preceded polyploidization display a CN pattern that oscillates between CN states that are multiple of 2 (*e.g.*, 2 and 4 in the case of whole genome duplication; Supplementary Fig. 6d). For example, if the CN oscillation occurs between 2 and 4 copies in a tetraploid tumor, we infer that polyploidization occurred after chromothripsis. Chromothripsis patterns detected in complex

rearrangements where less than 60% of adjacent copy number segments alternate between two copy number states were considered to colocalize with other complex events likely generated by mechanisms other than chromothripsis (see Fig. 1 in main text).

1.8 Effect of tumor purity and heterogeneity

Tumor purity and intra-tumor heterogeneity are potential confounding factors in the estimation of chromothripsis rates as they reduce the sensitivity of SV and CN detection. Tumor purity is highly variable across cancer types (Supplementary Table 1). However, we found that the chromothripsis rates we estimate are not correlated with average tumor purities across cancer types (Supplementary Figs. 1-2, and Supplementary Table 1). This is probably because most chromothripsis events generate many CN alterations and SVs, and the probability of missing the majority of these events is low even with relatively low purity. In many cases, we were able to detect chromothripsis events in low-purity samples (see Fig. 2c in the main text, where the purity was 0.22) or in tumors with multiple subclonal populations (Supplementary Fig. 5a). However, it is possible that chromothripsis events present in a reduced fraction of cancer cells are missed by our pipeline, resulting in under-estimation of the chromothripsis frequency.

2. Comparison of ShatterSeek to other methods

2.1 Comparison with previous calling criteria

Supplementary Note Table 2. The table below lists the major papers that involved chromothripsis calls, along with the algorithms and criteria used for its detection.

	Title	Year	Journal	Authors (first, senior)	Algorithm
Original chromothripsis paper	Massive genomic rearrangement acquired in a single catastrophic event during cancer development	2011	Cell	Stephens, Campbell	Custom (simulations)
Multiple myeloma	Chromothripsis identifies a rare and aggressive entity among newly diagnosed multiple myeloma patients	2011	Blood	Magrangeas, Minville	Custom software. Criteria based on CN oscillations
Colorectal cancer	Chromothripsis is a common mechanism driving genomic rearrangements in primary and metastatic colorectal cancer	2011	Genome Biology	Kloosterman, Cuppen	Custom software. CN oscillations; comparison w.r.t. Stephens *et al*. cases (Cell, 2011)

	Title	Year	Journal	Authors (first, senior)	Algorithm
Germline chromothripsis	Chromothripsis as a mechanism driving complex de novo structural rearrangements in the germline	2011	Human Molecular Genetics	Kloosterman, Cuppen	Custom software. CN oscillations; comparison w.r.t. Stephens *et al*. cases (Cell, 2011)
Medulloblastoma	Genome sequencing of pediatric medulloblastoma links catastrophic DNA rearrangements with *TP53* mutations	2012	Cell	Rausch, Korbel	10 CN oscillations. According to Korbel (personal communication), this approach works well for medulloblastomas, but it might not work for other cancer types.
Medulloblastoma	Sequencing of neuroblastoma identifies chromothripsis and defects in neuritogenesis genes	2012	Nature	Molenaar, Versteeg	"Genomes were annotated as having chromothripsis-like characteristics when the sum of intra-chromosomal somatic junctions (as reported by JunctionDiff and filtered as above) within a single chromosome was larger or equal to 20. Focused amplified regions (cgCGH scores ≥3) within a chromosome were excluded from this sum. Using these characteristics, we annotated 10 out of the 87 patients as chromothripsis-like"
Prostate cancer	Poly-gene fusion transcripts and chromothripsis in prostate cancer	2012	Genes Chromosomes Cancer	Wu, Collins	Custom code based on Stephens *et al*. (Cell, 2011)
Criteria inference chromothripsis	Criteria for inference of chromothripsis in cancer genomes	2013	Cell	Korbel & Campbell	Criteria for inference of chromothripsis are proposed
Pan-Cancer analysis array data	Functional genomic analysis of chromosomal aberrations in a	2013	Genome Research	Kim, Park	Custom code partly based on Korbel and Campbell (Cell, 2013)

	Title	Year	Journal	Authors (first, senior)	Algorithm
	compendium of 8000 cancer genomes				
Esophageal adenocarcinomas	Genomic catastrophes frequently arise in esophageal adenocarcinoma and drive tumorigenesis	2014	Nature Communications	Nones, Barbour	Custom code based on Korbel and Campbell (Cell, 2013)
Leukemia	Constitutional and somatic rearrangement of chromosome 21 in acute lymphoblastic leukaemia	2014	Nature	Li, Harrison	Custom code based on Korbel and Campbell (Cell, 2013)
GBM	Whole genome sequence analysis links chromothripsis to *EGFR, MDM2, MDM4*, and *CDK4* amplification in glioblastoma	2015	Oncoscience	Furgason, Bahassi	ShatterProof (score cut-off value 0.37)
Pancreatic cancer	Whole genomes redefine the mutational landscape of pancreatic cancer	2015	Nature	Waddell, Grimmond	Custom (not specified); only reference to Campbell and Korbel (Cell, 2013)
CAST *in vitro*	A cell-based model system links chromothripsis with hyperploidy	2015	Molecular Systems Biology	Marvin, Korbel	Custom code based on Korbel and Campbell (Cell, 2013)
Chromothripsis and kataegis in vitro	Chromothripsis and kataegis induced by telomere crisis	2015	Cell	Maciejowski, de Lange	Custom based on Korbel and Campbell (Cell, 2013)
Look-Seq: Chromothripsis and micronuclei	Chromothripsis from DNA damage in micronuclei	2015	Nature	Zhang, Pellman	Look-seq: custom analysis of single-cell sequencing data
Renal cell carcinoma	Multilevel genomics-based taxonomy of renal cell carcinoma	2015	Cancer Cell	TCGA	Not specified
Melanoma	Genomic classification of cutaneous melanoma	2016	Cell	TCGA	ShatterSeek (score cut-off value 0.517)
Acute Myeloid Leukemia	Prognosis and *TP53* alterations	2016	Blood	Fontana, Martinelli	CTLP scanner

	Title	Year	Journal	Authors (first, senior)	Algorithm
Prostate cancer	Genomic hallmarks of localized, non-indolent prostate cancer	2017	Nature	Fraser, Boutros	ShatterProof (score cut-off value 0.517)
Pancreatic cancer	A renewed model of pancreatic cancer evolution based on genomic rearrangement patterns	2017	Nature	Notta, Gallinger	Custom (Chrom-AL) based on Korbel and Campbell (Cell, 2013)
Liposarcomas	Recurrent mutation of IGF signalling genes and distinct patterns of genomic rearrangement in osteosarcoma	2017	Nature Communications	Behjati, Campbell	Custom based on Korbel and Campbell (Cell, 2013)
Prostate cancer	Germline *BRCA2* mutations drive prostate cancers with distinct evolutionary trajectories	2017	Nature Communications	Renea, Bristow (including Boutros)	ShatterProof (score cut-off value 0.517)
Leiomyosarcoma	Integrative genomic and transcriptomic analysis of leiomyosarcoma	2018	Nature Communications	Chudasama, Frohling	Custom based on Korbel and Campbell (Cell, 2013)
Pediatric cancers	Pan-cancer genome and transcriptome analyses of 1,699 pediatric leukaemias and solid tumours	2018	Nature	Ma, Zhang	Custom based on Korbel and Campbell (Cell, 2013)
Pediatric cancers	The landscape of genomic alterations across childhood cancers	2018	Nature	Grobner, Pfister	Custom based on Korbel and Campbell (Cell, 2013) and visual inspection
Clear cell renal cell cancer	Timing the landmark events in the evolution of clear cell renal cell cancer: TRACERx Renal	2018	Cell	Mitchell, Campbell	Custom based on Korbel and Campbell (Cell, 2013) and visual inspection

Overall, most of the studies where array or sequencing data was used to detect chromothripsis followed the statistical criteria described by Korbel and Campbell[4]. In addition to these, other metrics to detect simultaneous rearrangements in cancer genomes have been proposed[18–20]. However, these methods are very sensitive to missing SVs, as acknowledged, *e.g.,* in Oesper *et al.* [18], and based on our experience of trying to reconstruct complex regions[21]. In practice, a substantial fraction of the SVs present in a tumor sample are missed in WGS analysis due to intra-tumor heterogeneity, breakpoints in repetitive sequences, low tumor purity, alignment artifacts, etc. Moreover, the PCAWG SV calling pipeline was designed to maximize specificity over sensitivity (by requiring overlap in two or more algorithms). Hence, we believe that our

statistical approach, followed by visual inspection of all calls, represents a more suitable approach for the detection of chromothripsis from our current data. Note that one of the six criteria in the review by Korbel and Campbell includes the “ability to walk the derivative chromosome”, but this criterion is not used in practice (see *e.g.,* Li *et al.*[22]).

CTLPScanner[8,23] is designed to use CN profiles derived from SNP array data as input, and thus lacks sensitivity that comes from WGS data; this makes a comparison between CTLPScanner and our method difficult to interpret. Therefore, we have focused on the comparison of our method with ShatterProof[24], which is the only available package that takes both CN and SV information as input, and with the results provided for Chrom-AL[2].

2.2 Validation in diploid tumors

We first carried out an analysis for the ‘canonical’ chromothripsis events in 1,427 diploid tumors, which are much easier to handle, do not generally show secondary events (*e.g.,* high-level amplifications following chromothripsis), and where we could examine all the criteria used for chromothripsis detection (analysis of loss of heterozygosity is not always possible in aneuploid tumors). As can be seen in Supplementary Note Fig. 12, the rates of chromothripsis based only on canonical events are still much higher than previously reported. Three cancer types exhibit chromothripsis rates over 40% (CNS−GBM, Ovary-AdenoCA, and Prost-AdenoCA), and over half of the cancer types examined display rates over 20%.

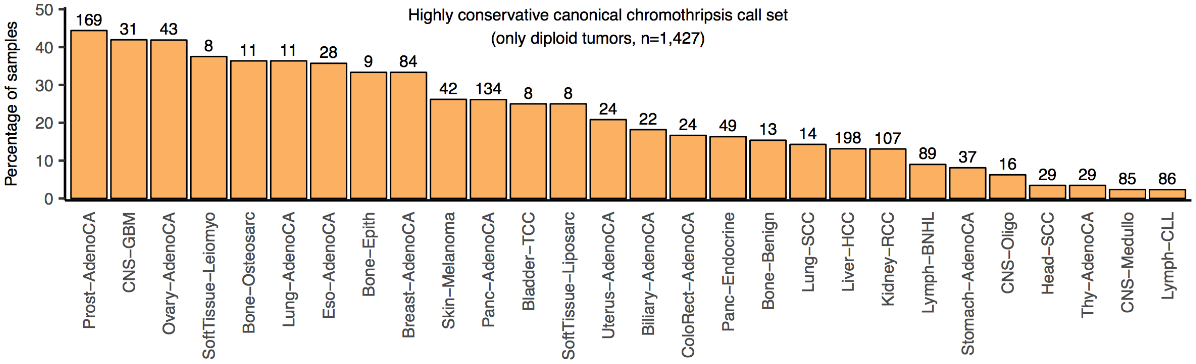

Supplementary Note Figure 12. Rates of chromothripsis in diploid tumors (ploidy < 2.1). the number on top of the bars indicates the number of tumors analysed.

Our calls in these cases are stringent: as a reminder, we require interleaved SVs, >6 SV breakpoints, 7 contiguous segments oscillating between two states (4 for low-confidence), and equal distribution of SVs types (to remove tandem duplications/deletions, Breakage-Fusion-Bridge (BFB)-only events, etc..). Two examples that appear to be chromothripsis by visual inspection but are not called as canonical chromothripsis cases are shown in Supplementary Note Fig. 13. Therefore, the rates estimated using only diploid cases are likely to be an underestimation of the true chromothripsis rates.

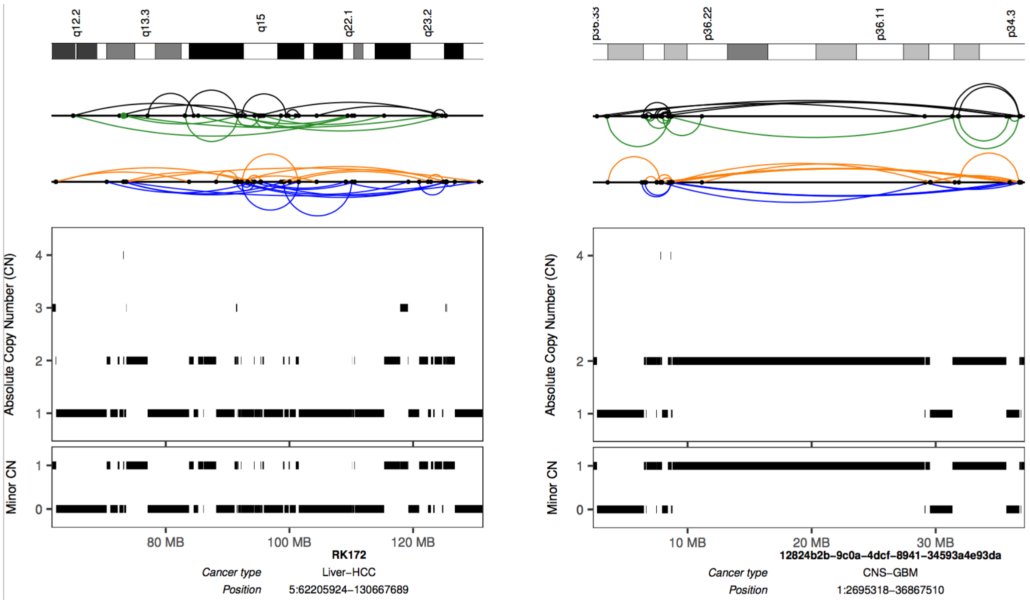

Supplementary Note Figure 13. Example of two events that are not detected as canonical chromothripsis in the diploid analysis but appear to be chromothripsis by visual inspection.

2.3 Comparison with ShatterProof

To benchmark our tool against the state of the art, we used ShatterProof to detect chromothripsis across the entire PCAWG cohort.

We used ShatterProof to detect chromothripsis across the 2,428 tumors that have at least one rearrangement (Supplementary Note Fig. 14). In the ShatterProof publication[24], the authors suggest a cut-off value for the ShatterProof score of 0.37 based on their analysis of 20 whole genomes. In a more recent study[12], the authors used a cut-off value of 0.517. We did not find any justification in that study as to why 0.517 should be used instead of 0.37. Thus, we have used both thresholds for our comparisons. The rates estimated using a cut-off value of 0.37 and 0.517 are shown in Supplementary Note Fig. 14. Overall, the rates of chromothripsis estimated using ShatterProof are substantially higher than ours (Supplementary Note Fig. 15) when using a cut-off value of 0.37 (over 95% of tumors for 12 cancer types). The rates are a bit lower but correlated with ours when using the more stringent cut-off value of 0.517.

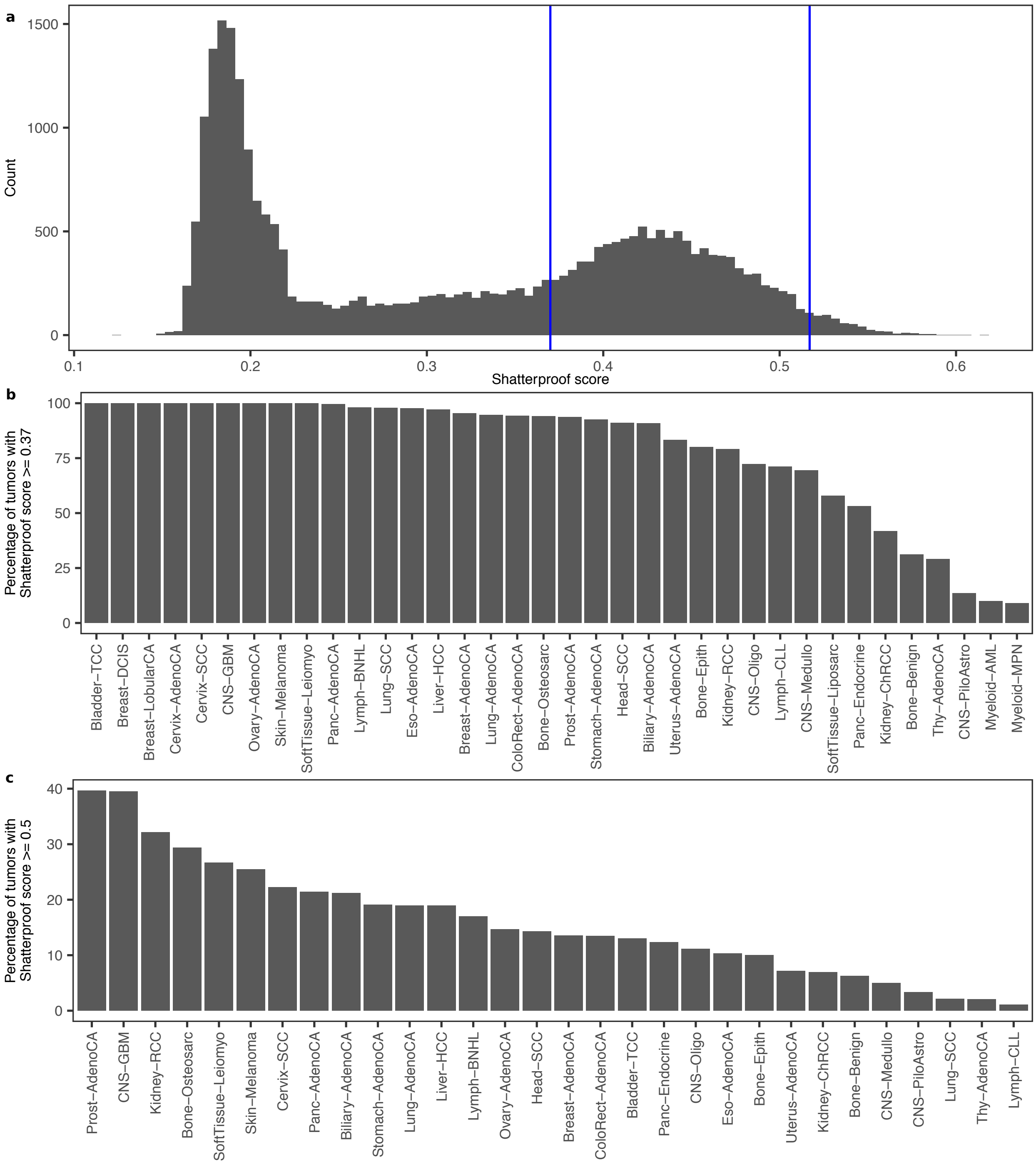

Supplementary Note Figure 14. Chromothripsis rates estimated with ShatterProof. (**a**) Distribution of non-zero ShatterProof scores calculated for the PCAWG cohort. The two blue lines indicate the cut-off values used by Govind *et al.* (*i.e.*, 0.37), and by Fraser *et al.* (*i.e.*, 0.517). (**b-c**) Rates of chromothripsis estimated using ShatterProof and a cut-off value of 0.37 (**b**), and 0.517 (**c**), respectively, for the 2,428 tumors we analysed.

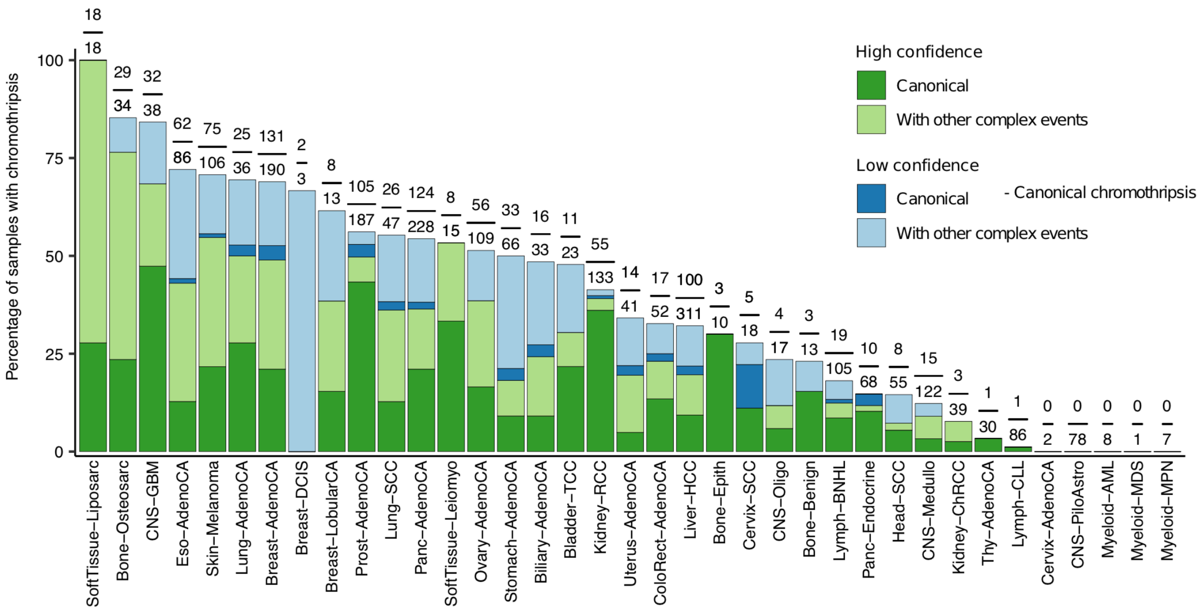

Supplementary Note Figure 15. Rates of chromothripsis estimated using ShatterSeek (Fig. 1c in the main text).

To better understand the differences between ShatterProof and ShatterSeek, we have visually inspected all the chromothripsis regions that were detected by only one approach but not the other.

2.4 Cases missed by ShatterSeek and called by ShatterProof

The regions detected by ShatterProof but not by our method are mostly cases characterized by CN oscillations corresponding to tandem deletions or duplications (two examples are shown in Supplementary Note Fig. 16). For instance, BRCA-deficient breast tumors display many tandem duplications; these should not be called as chromothripsis events, but they are often mistakenly called by ShatterProof. Our method has filtering criteria to avoid calling such events (*i.e.,* the requirement for equal distribution of SV types and the presence of interleaved SVs). In addition, we also removed those cases by visual inspection when necessary.

We note that we do miss to call a small subset of the regions with high ShatterProof scores and are likely to have originated by chromothripsis. These are mostly focal events with less than 6 SVs mapped (the minimum number we require to make a call). We decided to use 6 SVs as a

threshold as this value was used in previous work[2], and because we found through visual inspection of hundreds of cases that requiring less than 6 SVs increased the false positive rate, as clusters of inversions or tandem duplications with overlapping inversions might be included. Another reason to consider at least 6 SVs was to have enough statistical power to assess the even distribution of SV types in order not to call as chromothripsis other mutation types characterized by one SV type (*e.g.,* BFB cycles).

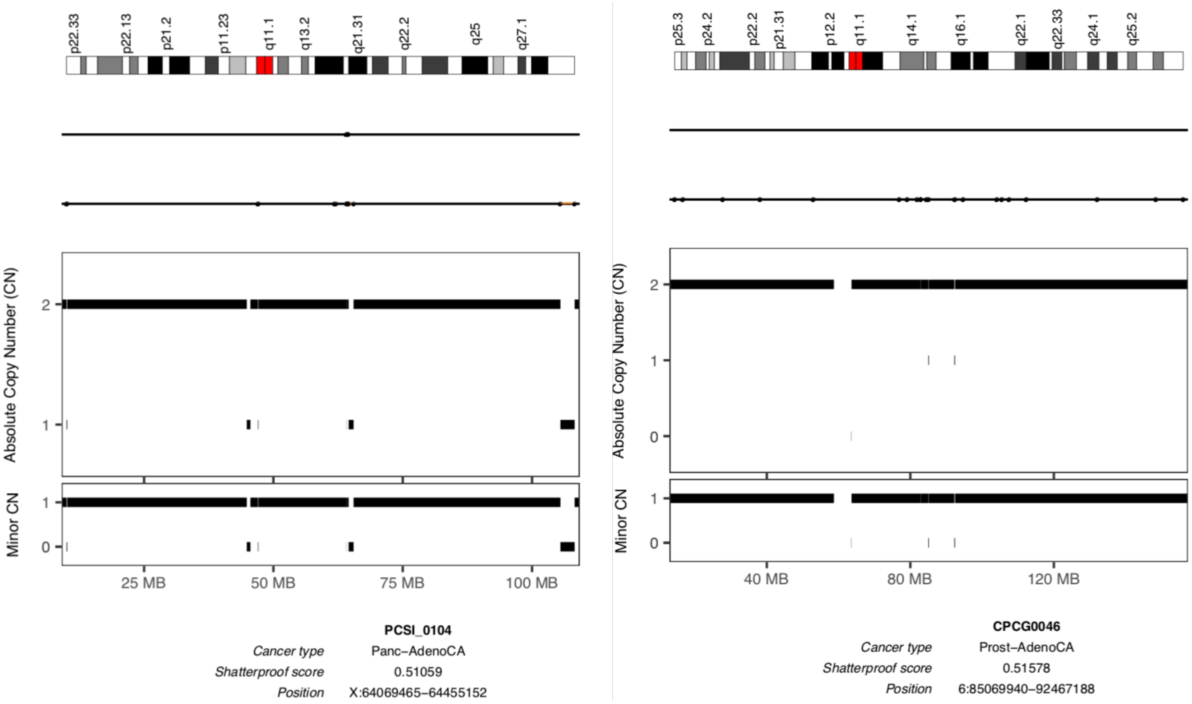

Supplementary Note Figure 16*.* Examples of regions with a high ShatterProof score and not detected by our method. These two examples show two regions with clusters of tandem deletions (each black dot on the second horizontal line below the chromosome ideogram represents a deletion) that generate CN oscillations. Our method correctly detected that these oscillations are not due to chromothripsis events. However, ShatterProof assigns a score over 0.5 for both, and hence, erroneously calls these two as chromothripsis.

2.5 Cases missed by ShatterProof but detected by ShatterSeek

In numerous cases, ShatterProof misses regions clearly showing the hallmarks of chromothripsis. We suspect that this might be due to the fact that ShatterProof only calculates scores for highly mutated regions (from Govind *et al.*[24]: "*The metrics produced from the sliding window analysis are used to identify highly mutated regions by comparing region-specific SV density to genome-wide density using a z-scale approach*"). In addition, some of the components of the ShatterProof score also depend on the SV density in other chromosomes: for instance, the 'genome localization' score in ShatterProof is only calculated for chromosomes with more SVs than the average number of SVs across all chromosomes. Therefore, ShatterProof will not consider for further analysis regions showing an SV rate lower than the genome-wide SV rate.

An illustrative example of this is shown in Supplementary Note Fig. 17. The two chromothripsis events in chromosomes 21 and X lead to high estimates of genome-wide SV rates, thus not permitting ShatterProof to detect the two chromothripsis regions in chromosomes 2 and 3 involving fewer SVs; the ShatterProof scores for these two chromosomes are 0. This illustrates that methods that rely on increased SV density with respect to the genome-wide rate to detect chromothripsis are likely to miss events in hypermutated tumors (*e.g., BRCA* deficient tumors), or focal events comprising less SVs than the average SV rate in other chromosomes. Based on these observations, we tuned our calling criteria to account for this issue.

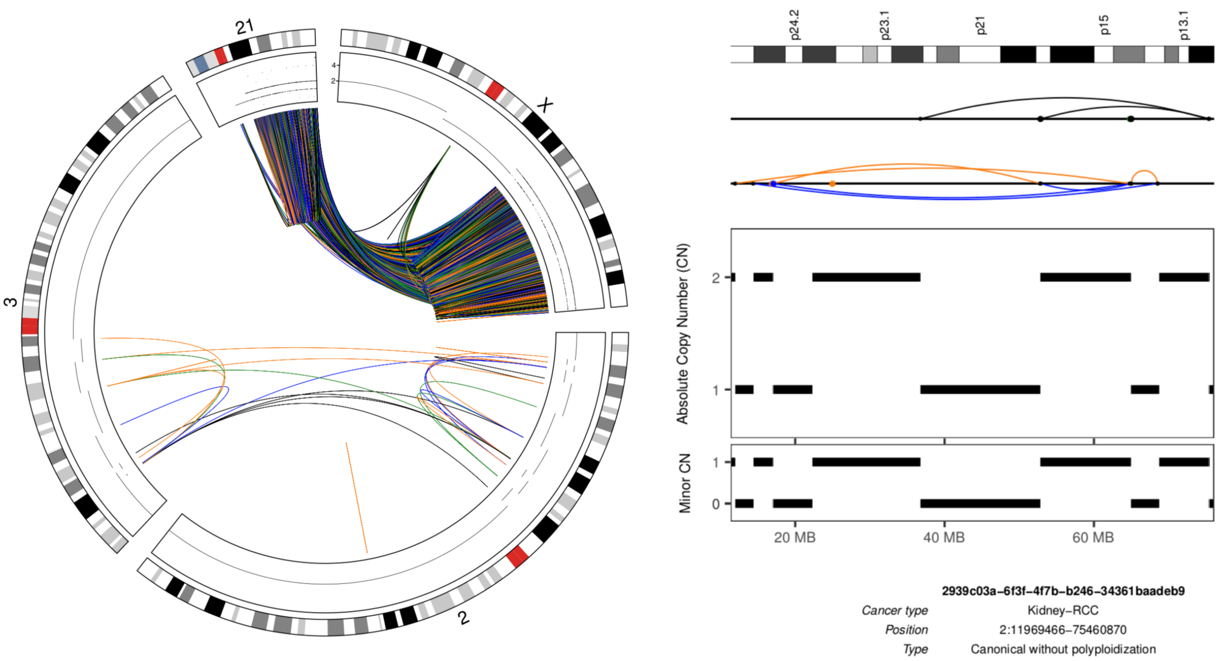

Supplementary Note Figure 17. Circos plot including chromosomes 2, 3, 21 and X for the Kidney-RCC tumor DO17373. The plot on the right-hand side corresponds to the chromothripsis event we detect in chromosome 2.

2.6 Comparing the chromothripsis rates for prostate tumors

Next, we compared our calls against those previously reported for 109 prostate adenocarcinomas also present in our cohort[12]. Using ShatterProof, the authors detected chromothripsis in 23 prostate tumors (21%). Using our CN and SV calls and ShatterProof version 0.14[24] with default settings we detect chromothripsis in 49 (45%), and in 60 (55%) with our tool, *i.e.,* ShatterSeek. Of the 23 cases reported by Fraser *et al.*[12], we only miss 4, which are focal events involving less than 6 SVs. It is paramount to note that when ShatterProof is run with the default parameter of 0.37 suggested in the original publication, 97% (106/109) of the tumors are annotated as harboring chromothripsis events.

Visual inspection of the cases we detect but are missed by Fraser *et al.*[12] reveals that the lower sensitivity of the SV and CN calls used in that study underlies these differences, as they do not

detect any SVs in some chromosomes where we find clear evidence of chromothripsis. Two examples are shown below in Supplementary Note Figs. 18-19.

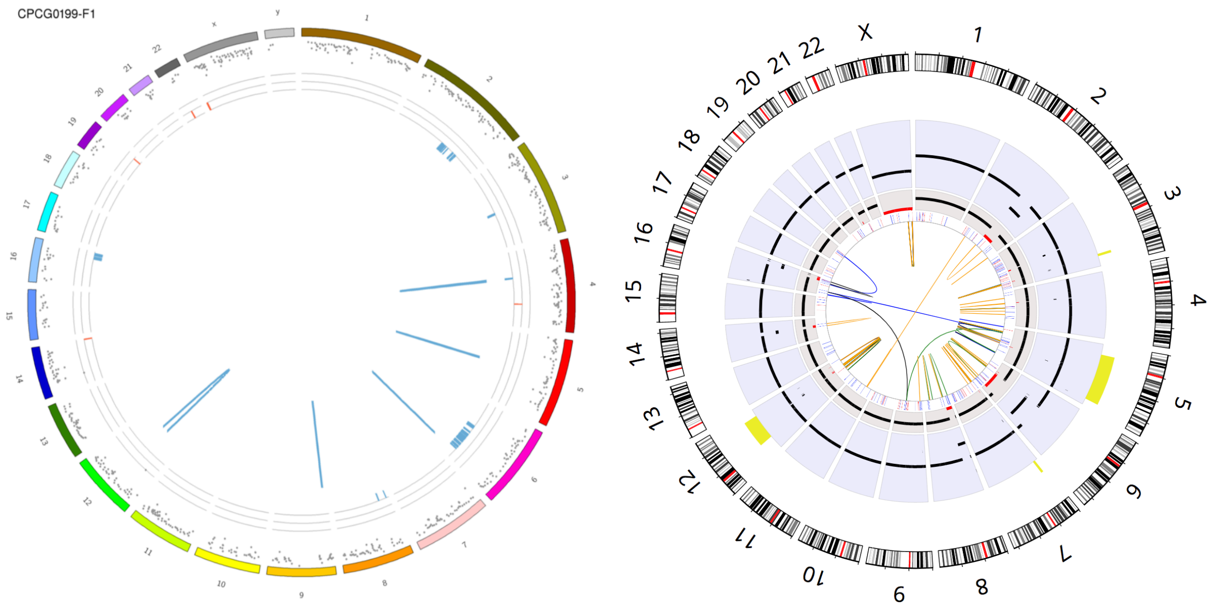

Supplementary Note Figure 18. Example of two focal chromothripsis events missed by ShatterProof in Fraser *et al.* (Circos plot on the left) that we manage to detect (Circos plot on the right) in tumor CPCG0199.

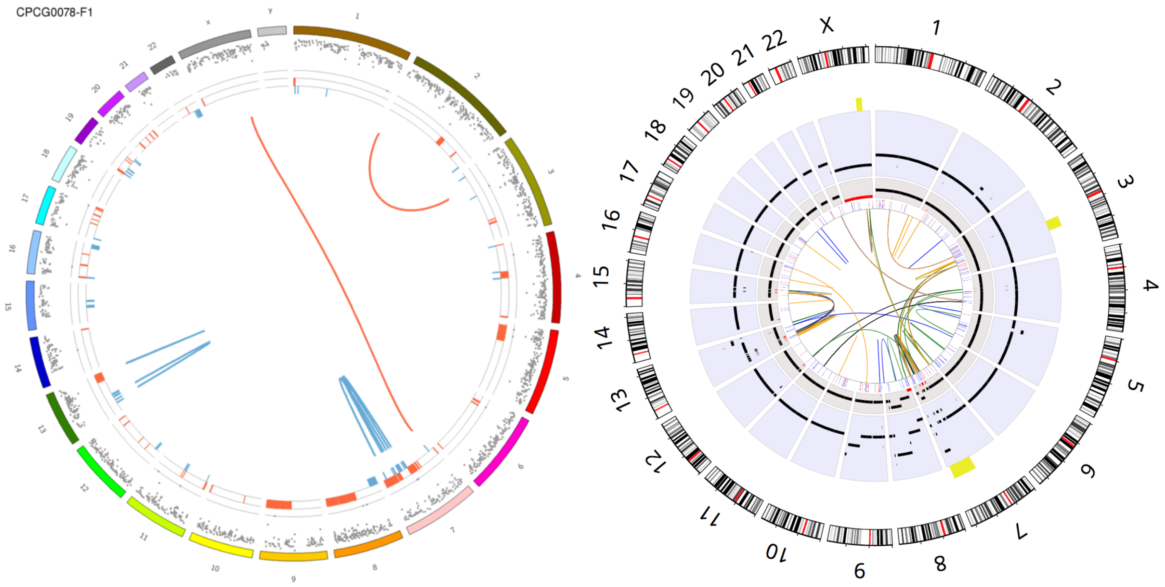

Supplementary Note Figure 19. Example of a chromothripsis case in chromosome 7 in tumor CPCG0078 not reported by Fraser *et al.* (Circos plot on the left).

2.7 Chromothripsis rates as a function of the ShatterProof cut-off value

Finally, we calculated the pan-cancer rate of chromothripsis as a function of the ShatterProof score. As can be seen below (Supplementary Note Fig. 20), the estimated rates vary strongly depending on the cut-off value used and do not converge to a stable value. Hence, we could not determine a reliable cut-off for ShatterProof in our analysis.

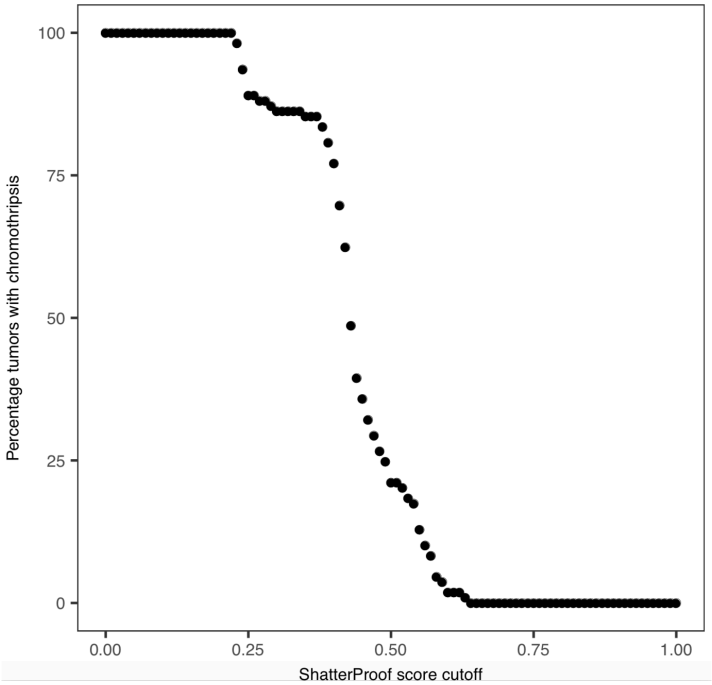

Supplementary Note Figure 20. Pan-cancer chromothripsis rates estimated using increasingly higher values for the ShatterProof cut-off score.

2.8 Comparison against Chrom-AL

We also compared our method against Chrom-AL[2] for 76 pancreatic tumors also present in our cohort. Both Chrom-AL and our method detect chromothripsis in the same 41 tumors.

2.9 Manual inspection of chromothripsis calls

Finally, we note that none of the statistical criteria proposed and used in the literature can give a definitive result about whether a region is subject to chromothripsis or not. Hence, visual inspection of all cases remains essential (as was done in, *e.g.,* Notta *et al.*[2]). We have visually inspected all our chromothripsis calls, which amounts to analyzing over 55,000 pictures (one picture per chromosome and tumor) of CN and SV profiles.

3. Contribution of chromothripsis to gene fusion formation

We also examined those genes targeted by chromothripsis-related SVs. We find that 61% of the breakpoints in high confidence chromothripsis regions map to exonic or intronic regions. This frequency was comparable for canonical events and those co-localized with other complex events. Analysis of these genes reveals that most of them are not recurrently targeted by chromothripsis (*e.g.,* they are targeted in few tumors; Supplementary Note Fig. 21a), in contrast to chromoplexy, as some genes, *e.g., TMPRSS2*, are affected in multiple tumors (Supplementary Note Fig. 21b)[5,14].

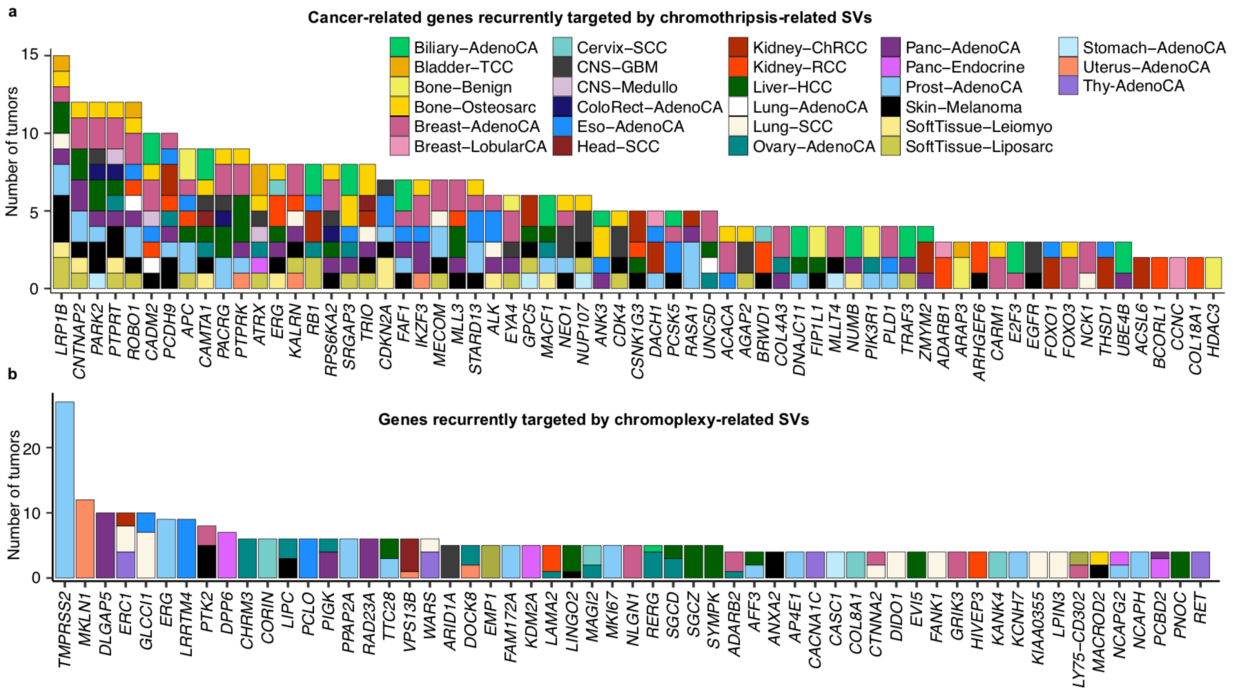

Supplementary Note Figure 21. (**a**) Cancer-related genes recurrently targeted by SVs involved in chromothripsis. (**b**) Genes recurrently involved in chromoplexy chains.

The rejoining of DNA fragments in chromothripsis is a potential source of gene fusions[5]. To examine the extent to which chromothripsis contributes to the generation of gene fusions across diverse cancer types, we considered 2,493 gene fusions previously detected using RNA-seq data for the 455 TCGA tumors also present in our cohort that had at least 1 fusion (median=3)[25]. We find that 1,116 fusions (44.8%; Supplementary Figure 22) have at least one gene partner in a chromothripsis region, with the highest frequencies observed for complex events in SoftTissue−Liposarc, Breast-AdenoCA, Skin-Melanoma, and CNS-GBM. Chromoplexy generates gene fusions in CNS-GBM (1 fusion), Kidney-RCC (1), Thy-AdenoCA (4), and, as previously reported[14], in Prost-AdenoCA (2).

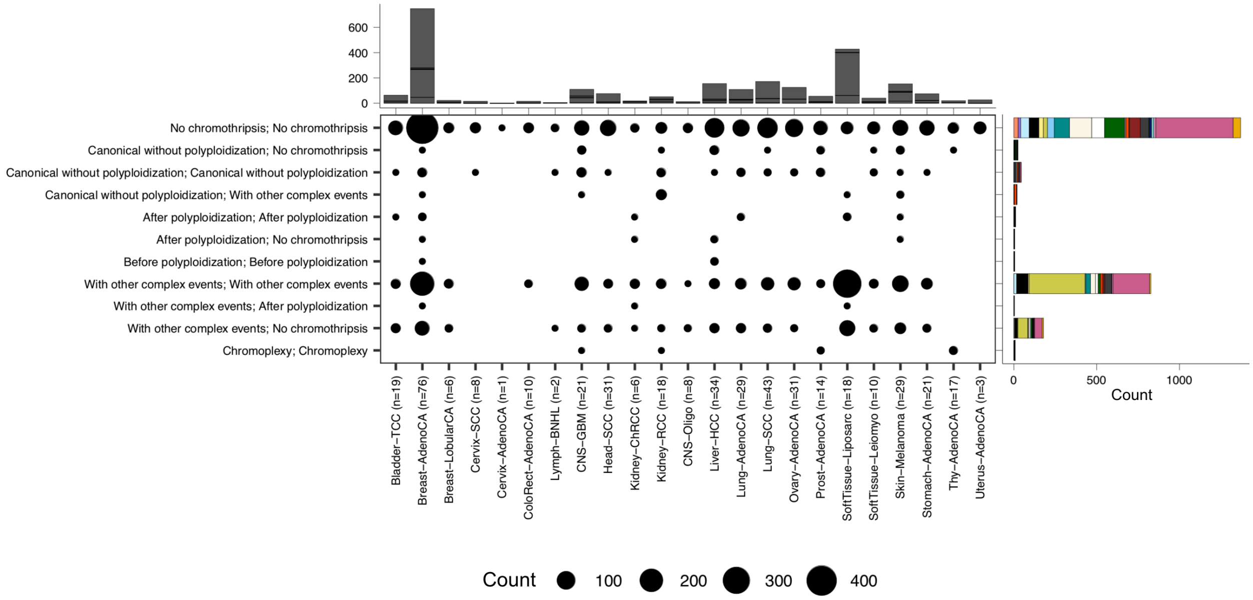

Supplementary Note Figure 22. Gene fusion landscape across 455 tumors. The top barplot shows the number of fusions detected in the tumors corresponding to the cancer type indicated in the *x*-axis. The dot plot represents the number of fusions with breakpoints mapped to regions of the categories indicated on the *y*-axis. The barplot on the right-hand side represents the total count of fusions for each type of event.

4. Analysis of the association between chromothripsis-related breakpoints and epigenetic marks

To examine the association between chromothripsis-related SVs and epigenomic features, we compared the SV density with the 25-state chromatin state maps developed by the Roadmap Epigenomics project[26]. For best estimates, the chromatin state map from the most 'similar' tissue type was used for each tumor type. Specifically, we downloaded the coordinates of the 25-state chromatin state map defined using 12 marks (H3K4me1, H3K4me2, H3K4me3, H3K9ac, H3K27ac, H4K20me1, H3K79me2, H3K36me3, H3K9me3, H3K27me3, H2A.Z and DNase) across 127 reference epigenomes from the Epigenome Roadmap project. The list of chromothripsis-related breakpoints for each cancer type was intersected with the chromatin state maps defined using cell lines from the same anatomical location as the tumor types. We used the chromatin state maps defined using the epigenomes E092, E094, E0110 and E0111 for Stomach-AdenoCA (ICGC projects STAD-US and GACA-CN), E117 and E076 for Uterus-AdenoCA (UCEC-US), E106 and E075 for ColoRect-AdenoCA (COAD-US and READ-US), E086 for Kidney-ChRCC (KICH-US and KIRP-US), E027, E028 and E119 for Breast-AdenoCA, Breast-LobularCA and Breast-DCIS (BRCA-US), E053, E054, E067, E068, E069, E070, E071, E072, E073, E074, E081, E082 and E0125 for CNS-GBM (GBM-US), E097 for Ovary-AdenoCA (OV-US), E088, E096, E114 and E128 for Lung-SCC (LUSC-US), E055, E056, E057, E059, E061, E126, E127 and E058 for Head-SCC (HNSC-US), E086 for Kidney-ChRCC (KICH-US), E115 for CNS-Medullo and CNS-PiloAstro (PBCA-DE), E115 for CNS-Oligo (LGG-US), E066 and E118 for Liver-HCC (LICA-FR, LIHC-US, LINC-JP and LIRI-JP), and E061 for Skin-

Melanoma (SKCM-US). Subsequently, the percentage of chromothripsis SVs detected in all tumors of a given cancer type overlapping each chromatin state was calculated for each matched epigenome, and compared to the fraction of the mappable genome annotated with each chromatin state. The two-tailed Fisher's exact test was used to assess the significance of the enrichment for chromothripsis-related SVs of each chromatin state in each of the cancer genomes. The significance level was set to 0.01 after Bonferroni correction of raw *P* values.

Overall, our analysis indicates that chromothripsis SVs are enriched in actively-transcribed regions, promoters and enhancers in CNS-GBM, ColoRect-AdenoCA, Skin-Melanoma, Uterus-AdenoCA and Stomach-AdenoCA (Supplementary Note Fig. 23), and quiescent chromatin is depleted for chromothripsis SVs in CNS-GBM and Breast-AdenoCA. Together, these results show the over-representation of chromothripsis SVs in functionally important, typically open-chromatin regions, consistent with previous work[27]. Larger patient cohorts will be needed to increase our power to assess whether the lack of enrichment in certain regions is significant.

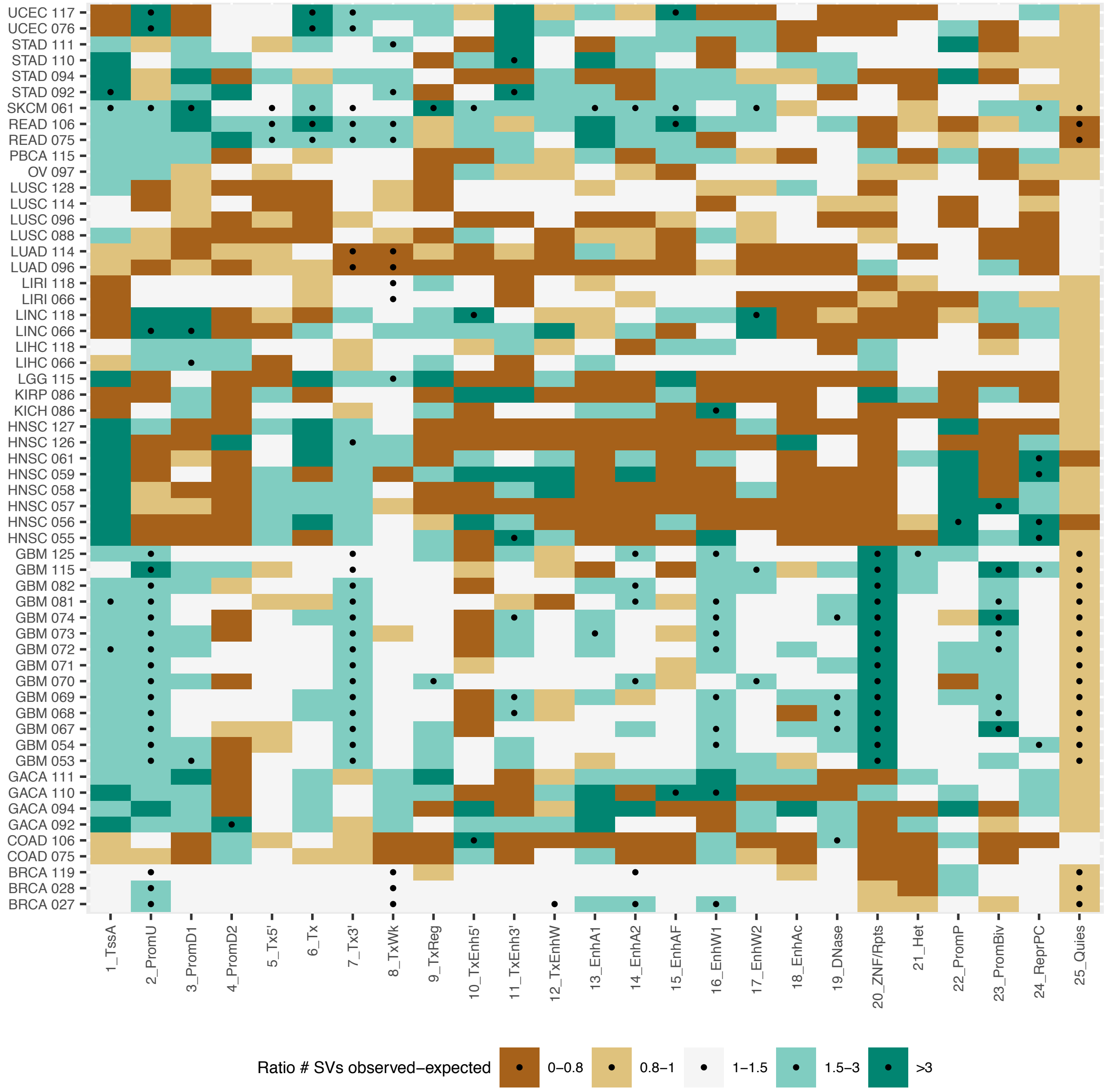

Supplementary Note Figure 23. Enrichment of chromothripsis breakpoints in chromatin states. The colors indicate the ratio between the observed percentage of SVs in each mark and the expected number. Black dots denote statistical significance.

5. Chromothripsis is prognostic of poor patient survival

Chromothripsis has been associated with poor prognosis for several cancer types[2,7,28,29]. Here, the increased sensitivity of our approach and the larger cohort permits us to evaluate the impact of chromothripsis on patient survival in greater detail, and to determine the biological contexts in which chromothripsis leads to more aggressive tumors.

We performed multivariate analysis using the Cox proportional hazards model corrected for

confounding factors known to influence survival rates, namely: age at the time of diagnosis, sex, tumor stage, radiation therapy, the presence of metastasis, and cancer type. Survival analysis was conducted using the *coxph* function from the R package survival version 2.30. Significance was assessed by the likelihood ratio and Wald tests. Tumor stages for all cancer types were manually curated and grouped into four categories. In the pan-cancer analysis, we considered only cancer types with clinical data available for at least 20 patients. The clinical data are listed in Supplementary Table 1. We did not find a significant association between chromothripsis and survival in a multivariate pan-cancer analysis when stratifying the patients into two categories with respect to the presence or absence of chromothripsis (Wald and likelihood ratio tests (LRT)<10^{-15}; hazard ratio (HR): 1.07, *P*=0.24). However, we find a significant association in a stratified analysis when the samples are ordered by the percentage of SVs involved in

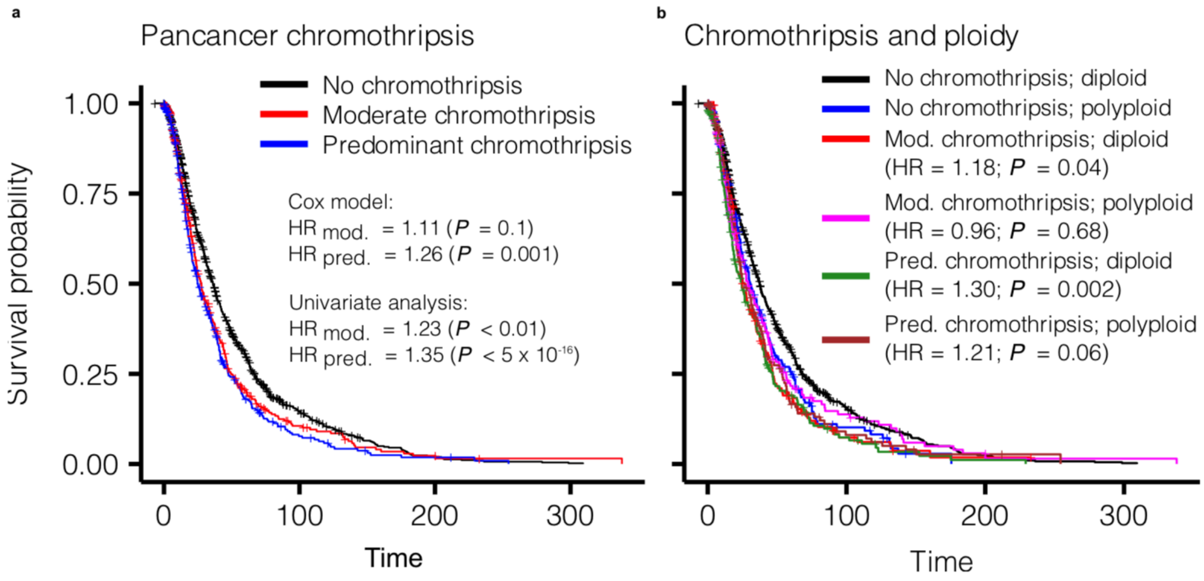

Supplementary Note Figure 24*.* Association between chromothripsis and patient survival. Samples were stratified into three categories based on the fraction of SVs that map to chromothripsis regions: absent (black; no chromothripsis), moderate ("mod."; red; the fraction of chromothripsis-related SVs is smaller than the median within the same cancer type), and predominant ("pred."; blue; the fraction of chromothripsis-related SVs is higher than the median within the same cancer type). Kaplan-Meier plots and estimated Hazard Ratios (HR; 95% confidence intervals) for (**a**) all samples stratified on the basis of chromothripsis. Even after accounting for other variables (Cox model), the predominantly chromothripsis samples have significantly worse survival than those without chromothripsis (HR=1.26, *P*=0.001). (**b**) Chromothripsis and ploidy. For the diploid samples, the negative impact of chromothripsis is stronger, with both predominant and moderate groups reaching statistical significance (*cf*. (**a**))**.**

chromothripsis in each tumor type and classified into three equal-sized groups 'absent', 'moderate', and 'predominant' (Wald and LTR<10^{-15}; Supplementary Note Fig. 24a). The HR for the samples in the predominant and moderate chromothripsis categories are 1.26 (*P*=0.001) and 1.11 (*P*=0.12), respectively. This trend is also observed when also including the low-confidence calls in the analysis, namely 1.26 (*P*<0.001) and 1.12 (*P*=0.10), respectively.

Several factors could influence whether chromothripsis is more prevalent in diploid or polyploid

tumors. In a cancer primarily driven by tumor suppressor loss, the growth-promoting effects should be more penetrant in a diploid tumor relative to a polyploid tumor if chromothripsis happened after the genome doubling. To address the relationship between chromothripsis and polyploidy, we divided the patients into 6 categories depending on the penetrance of chromothripsis (*i.e.,* absent, moderate and predominant) and ploidy, *i.e.,* diploids *vs* polyploids (ploidy ≥ 2.5). We find that the association between chromothripsis and poor prognosis is stronger when it occurs in diploid tumors harboring either moderate ($HR_{diploids}$: 1.18, *P*=0.04; $HR_{polyploids}$: 0.96, *P*=0.68) or predominant chromothripsis ($HR_{diploids}$: 1.30, *P*=0.002; $HR_{polyploids}$: 1.21, *P*=0.06; Supplementary Note Fig. 24b).

We finally evaluated the association between chromothripsis and the infiltration of immune cell types by regressing the average gene expression of immune gene sets, as well as that of individual genes, on the chromothripsis category and other covariates known to influence the degree of tumor immune cell infiltration [30]. We find that tumors displaying predominant chromothripsis show a significant depletion of cytolytic markers (*PRF1*; $P < 0.05$), natural killer cells, cytolytic T cells (*CD8A*; $P < 0.05$), and tumor antigen presentation (MHC Class I: *B2M* and *TAP1*; $P < 0.05$). This may suggest a non-immunogenic niche for the tumors showing chromothripsis, which warrants clinical considerations and further investigation.

6. References

1. Behjati, S. *et al.* Recurrent mutation of IGF signalling genes and distinct patterns of genomic rearrangement in osteosarcoma. *Nat. Commun.* **8**, 15936 (2017).

2. Notta, F. *et al.* A renewed model of pancreatic cancer evolution based on genomic rearrangement patterns. *Nature* **538**, 378–382 (2016).

3. Zhang, C.-Z. *et al.* Chromothripsis from DNA damage in micronuclei. *Nature* **522**, 179–184 (2015).

4. Korbel, J. O. & Campbell, P. J. Criteria for inference of chromothripsis in cancer genomes. *Cell* **152**, 1226–36 (2013).

5. Stephens, P. J. *et al.* Massive genomic rearrangement acquired in a single catastrophic event during cancer development. *Cell* **144**, 27–40 (2011).

6. Song, S. *et al.* qpure: A tool to estimate tumor cellularity from genome-wide single-nucleotide polymorphism profiles. *PLoS One* **7**, e45835 (2012).

7. Rausch, T. *et al.* Genome sequencing of pediatric medulloblastoma links catastrophic DNA rearrangements with TP53 mutations. *Cell* **148**, 59–71 (2012).

8. Cai, H. *et al.* Chromothripsis-like patterns are recurring but heterogeneously distributed features in a survey of 22,347 cancer genome screens. *BMC Genomics* **15**, 82 (2014).

9. Kim, T.-M. *et al.* Functional genomic analysis of chromosomal aberrations in a compendium of 8000 cancer genomes. *Genome Res.* **23**, 217–27 (2013).

10. Garsed, D. W. *et al.* The architecture and evolution of cancer neochromosomes. *Cancer Cell* **26**, 653–667 (2014).

11. Chudasama, P. *et al.* Integrative genomic and transcriptomic analysis of leiomyosarcoma. *Nat. Commun.* **9**, 144 (2018).

12. Fraser, M. *et al.* Genomic hallmarks of localized, non-indolent prostate cancer. *Nature* **541**, 359–364 (2017).

13. Mitchell, T. J. *et al.* Timing the landmark events in the evolution of clear cell renal cell cancer: TRACERx Renal. *Cell* **173**, 611-623.e17 (2018).

14. Baca, S. C. *et al.* Punctuated evolution of prostate cancer genomes. *Cell* **153**, 666–677 (2013).

15. Liu, P. *et al.* Chromosome catastrophes involve replication mechanisms generating complex genomic rearrangements. *Cell* **146**, 889–903 (2011).

16. Li, Y. *et al.* Patterns of somatic structural variation in human cancer. *Nature* (2019).

17. Zack, T. I. *et al.* Pan-cancer patterns of somatic copy number alteration. *Nat. Genet.* **45**, 1134–1140 (2013).

18. Oesper, L., Dantas, S. & Raphael, B. J. Identifying simultaneous rearrangements in cancer genomes. *Bioinformatics* **34**, 346–352 (2018).

19. Weinreb, C., Oesper, L. & Raphael, B. J. Open adjacencies and k-breaks: detecting simultaneous rearrangements in cancer genomes. *BMC Genomics* **15 Suppl 6**, S4 (2014).

20. Alexeev, N., Pologova, A. & Alekseyev, M. A. Generalized hultman numbers and cycle structures of breakpoint graphs. *J. Comput. Biol.* **24**, 93–105 (2017).

21. Yang, L. *et al.* Diverse mechanisms of somatic structural variations in human cancer genomes. *Cell* **153**, 919–29 (2013).

22. Li, Y. *et al.* Constitutional and somatic rearrangement of chromosome 21 in acute lymphoblastic leukaemia. *Nature* **508**, 98–102 (2014).

23. Yang, J. *et al.* CTLPScanner: a web server for chromothripsis-like pattern detection. *Nucleic Acids Res.* **44**, W252–W258 (2016).

24. Govind, S. K. *et al.* ShatterProof: operational detection and quantification of chromothripsis. *BMC Bioinformatics* **15**, 78 (2014).

25. Gao, Q. *et al.* Driver fusions and their implications in the development and treatment of human cancers. *Cell Rep.* **23**, 227-238.e3 (2018).

26. Roadmap Epigenomics Consortium *et al.* Integrative analysis of 111 reference human epigenomes. *Nature* **518**, 317–330 (2015).

27. Rheinbay, E. *et al.* Analyses of non-coding somatic drivers in 2,693 cancer whole genomes. *Nature* (2019).

28. Molenaar, J. J. *et al.* Sequencing of neuroblastoma identifies chromothripsis and defects in neuritogenesis genes. *Nature* **483**, 589–593 (2012).

29. Fontana, M. C. *et al.* Chromothripsis in acute myeloid leukemia: Biological features and impact on survival. *Leukemia* **32**, 1609–1620 (2018).

30. Rooney, M. S., Shukla, S. A., Wu, C. J., Getz, G. & Hacohen, N. Molecular and genetic properties of tumors associated with local immune cytolytic activity. *Cell* **160**, 48–61 (2015).

The ICGC/TCGA Pan-Cancer Analysis of Whole Genomes (PCAWG) Consortium Working Groups

PCAWG Steering committee

Peter J Campbell#[1,2], **Gad Getz#**[3,4,5,6], **Jan O Korbel#**[7,8], **Lincoln D Stein#**[9,10] and **Joshua M Stuart#**[11,12]

PCAWG Head of project management

Jennifer L Jennings[13]

PCAWG Executive committee

Sultan T Al-Sedairy[14], Axel Aretz[15], Cindy Bell[16], Miguel Betancourt[17], Christiane Buchholz[18], Fabien Calvo[19], Christine Chomienne[20], Michael Dunn[21], Stuart Edmonds[22], Eric Green[23], Shailja Gupta[24], Carolyn M Hutter[23], Karine Jegalian[25], Jennifer L Jennings[13], Nic Jones[26], Hyung-Lae Kim[27], Youyong Lu[28,29,30], Hitoshi Nakagama[31], Gerd Nettekoven[32], Laura Planko[32], David Scott[26], Tatsuhiro Shibata[33,34], Kiyo Shimizu[35], **Lincoln D Stein#**[9,10], Michael R Stratton[1], Takashi Yugawa[35], Giampaolo Tortora[36,37], K VijayRaghavan[24], Huanming Yang[38] and Jean C Zenklusen[39]

PCAWG Ethics and Legal Working Group

Don Chalmers#[40], Yann Joly[41], **Bartha M Knoppers#**[41], Fruzsina Molnár-Gábor[42], Mark Phillips[41], Adrian Thorogood[41] and David Townend[43]

PCAWG Technical Working Group

Brice Aminou[44], Javier Bartolome[45], Keith A Boroevich[46,47], Rich Boyce[7], Alvis Brazma[7], Angela N Brooks[3,11,12,48], Alex Buchanan[49], Ivo Buchhalter[50,51,52], Adam P Butler[1], Niall J Byrne[44], Andy Cafferkey[7], Peter J Campbell[1,2], Zhaohong Chen[53], Sunghoon Cho[54], Wan Choi[55], Peter Clapham[1], Brandi N Davis-Dusenbery[56], Francisco M De La Vega[57,58,59], Jonas Demeulemeester[60,61], Michelle T Dow[53], Lewis Jonathan Dursi[9,62], Juergen Eils[63,64], Roland Eils[50,52,63,64], Kyle Ellrott[49], Claudiu Farcas[53], Nodirjon Fayzullaev[44], Vincent Ferretti[44,65], Paul Flicek[7], Nuno A Fonseca[7,66], Josep L L Gelpi[45,67], Gad Getz[3,4,5,6], Robert L Grossman[68], Olivier Harismendy[69,70], Allison P Heath[71], Michael C Heinold[50,52], Julian M Hess[3,72], Oliver Hofmann[73], Jongwhi H Hong[74], Thomas J Hudson[75,76], Barbara Hutter[77,78,79], Carolyn M Hutter[23], Daniel Hübschmann[52,63,80,81,82], Seiya Imoto[83], Sinisa Ivkovic[56], Seung-Hyup Jeon[55], Wei Jiao[9], Jongsun Jung[84], Rolf Kabbe[50], Andre Kahles[85,86,87,88,89], Jules NA Kerssemakers[50], Hyung-Lae Kim[27], Hyunghwan Kim[55], Jihoon Kim[90], Youngwook Kim[91,92],

Kortine Kleinheinz[50,52], Jan O Korbel[7,8], Michael Koscher[93], Antonios Koures[53], Milena Kovacevic[56], Chris Lawerenz[64], Ignaty Leshchiner[3], Jia Liu[94], Dimitri Livitz[3], George L Mihaiescu[44], Sanja Mijalkovic[56], Ana Mijalkovic Mijalkovic-Lazic[56], Satoru Miyano[83], Naoki Miyoshi[83], Hardeep K Nahal-Bose[44], Hidewaki Nakagawa[47], Mia Nastic[56], Steven J Newhouse[7], Jonathan Nicholson[1], **Brian D O'Connor#**[44,95], David Ocana[7], Kazuhiro Ohi[83], Lucila Ohno-Machado[53], Larsson Omberg[96], BF Francis Ouellette[44,97], Nagarajan Paramasivam[50,78], Marc D Perry[44,98], Todd D Pihl[99], Manuel Prinz[50], Montserrat Puiggròs[45], Petar Radovic[56], Keiran M Raine[1], Esther Rheinbay[3,6,100], Mara Rosenberg[3,100], Romina Royo[45], Gunnar Rätsch[85,86,87,88,89,101], Gordon Saksena[3], Matthias Schlesner[50,102], Solomon I Shorser[9], Charles Short[7], Heidi J Sofia[23], Jonathan Spring[103], **Lincoln D Stein#**[9,10], Adam J Struck[104], Grace Tiao[3], Nebojsa Tijanic[56], David Torrents[45,105], Peter Van Loo[60,61], Miguel Vazquez[45,106], David Vicente[45], Jeremiah A Wala[3,6,48], Zhining Wang[39], Sebastian M Waszak[8], Joachim Weischenfeldt[8,107,108], Johannes Werner[50,109], Ashley Williams[53], Youngchoon Woo[55], Adam J Wright[9], Qian Xiang[44], **Sergei Yakneen#**[8], Liming Yang[39], Denis Yuen[9], **Christina K Yung#**[44] and **Junjun Zhang#**[44]

PCAWG Reference Annotations Working Group

Angela N Brooks[3,11,12,48], Ivo Buchhalter[50,51,52], Peter J Campbell[1,2], Priyanka Dhingra[110,111], Lars Feuerbach[112], Mark Gerstein[113,114,115], Gad Getz[3,4,5,6], Mark P Hamilton[116], Henrik Hornshøj[117], Todd A Johnson[46], Andre Kahles[85,86,87,88,89], Abdullah Kahraman[118,119,120], Manolis Kellis[3,121], **Ekta Khurana#**[110,111,122,123], Jan O Korbel[7,8], Morten Muhlig Nielsen[117], Jakob Skou Pedersen[117,124], Paz Polak[3,4,6], Jüri Reimand[9,125], Esther Rheinbay[3,6,100], Nicola D Roberts[1], Gunnar Rätsch[85,86,87,88,89,101], Richard Sallari[3], Nasa Sinnott-Armstrong[3,59], Alfonso Valencia[45,105], Miguel Vazquez[45,106], Sebastian M Waszak[8], Joachim Weischenfeldt[8,107,108] and Christian von Mering[120,126]

PCAWG Quality-Control Working Group

Sergi Beltran[127,128], Ivo Buchhalter[50,51,52], Peter J Campbell[1,2], Roland Eils[50,52,63,64], Daniela S Gerhard[129], Gad Getz[3,4,5,6], **Ivo G Gut#**[127,128], Marta Gut[127,128], Barbara Hutter[77,78,79], Daniel Hübschmann[52,63,80,81,82], Kortine Kleinheinz[50,52], Jan O Korbel[7,8], Dimitri Livitz[3], Marc D Perry[44,98], Keiran M Raine[1], Esther Rheinbay[3,6,100], Mara Rosenberg[3,100], Gordon Saksena[3], Matthias Schlesner[50,102], Miranda D Stobbe[127,128], Jean-Rémi Trotta[127], Johannes Werner[50,109] and Justin P Whalley[127]

PCAWG SNV Calling Working Group

Matthew H Bailey[130,131], Beifang Niu[132], Matthias Bieg[78,133], Paul C Boutros[9,125,134,135], Ivo Buchhalter[50,51,52], Adam P Butler[1], Ken Chen[136], Zechen Chong[137], **Li Ding#**[130,131,138], Oliver Drechsel[128,139], Lewis Jonathan Dursi[9,62], Roland Eils[50,52,63,64], Kyle Ellrott[49], Shadrielle MG Espiritu[9], Yu Fan[140], Robert S Fulton[130,131,138], Shengjie Gao[38], Josep L L Gelpi[45,67], Mark Gerstein[113,114,115], Gad Getz[3,4,5,6], Santiago Gonzalez[7,8], Ivo G Gut[127,128], Faraz Hach[141,142], Michael C Heinold[50,52], Julian M Hess[3,72], Jonathan Hinton[1], Taobo Hu[143], Vincent Huang[9], Yi Huang[144,145], Barbara Hutter[77,78,79],

David R Jones[1], Jongsun Jung[84], Natalie Jäger[50], Hyung-Lae Kim[27], Kortine Kleinheinz[50,52], Sushant Kumar[114,115], Yogesh Kumar[143], Christopher M Lalansingh[9], Ignaty Leshchiner[3], Ivica Letunic[146], Dimitri Livitz[3], Eric Z Ma[143], Yosef E Maruvka[3,72,100], R Jay Mashl[131,147], Michael D McLellan[130,131,138], Andrew Menzies[1], Ana Milovanovic[45], Morten Muhlig Nielsen[117], Stephan Ossowski[128,139,148], Nagarajan Paramasivam[50,78], Jakob Skou Pedersen[117,124], Marc D Perry[44,98], Montserrat Puiggròs[45], Keiran M Raine[1], Esther Rheinbay[3,6,100], Romina Royo[45], S Cenk Sahinalp[142,149,150], Gordon Saksena[3], Iman Sarrafi[142,150], Matthias Schlesner[50,102], **Jared T Simpson#**[9,151], Lucy Stebbings[1], Chip Stewart[3], Miranda D Stobbe[127,128], Jon W Teague[1], Grace Tiao[3], David Torrents[45,105], Jeremiah A Wala[3,6,48], Jiayin Wang[131,145,152], Wenyi Wang[140], Sebastian M Waszak[8], Joachim Weischenfeldt[8,107,108], Michael C Wendl[131,138,153], Johannes Werner[50,109], David A Wheeler[154,155], Zhenggang Wu[143], Hong Xue[143], Sergei Yakneen[8], Takafumi N Yamaguchi[9], Kai Ye[152,156], Venkata D Yellapantula[138,157], Christina K Yung[44] and Junjun Zhang[44]

PCAWG Drivers and Functional Interpretation Working Group

Federico Abascal[1], Samirkumar B Amin[158,159,160], Gary D Bader[10], Pratiti Bandopadhayay[3,161,162], Jonathan Barenboim[9], Rameen Beroukhim[3,6,163], Johanna Bertl[117,164], Keith A Boroevich[46,47], Søren Brunak[165,166], Peter J Campbell[1,2], Joana Carlevaro-Fita[167,168,169], Dimple Chakravarty[170], Calvin Wing Yiu Chan[50,171], Ken Chen[136], Jung Kyoon Choi[172], Jordi Deu-Pons[173,174], Priyanka Dhingra[110,111], Klev Diamanti[175], Lars Feuerbach[112], J Lynn Fink[45,176], Nuno A Fonseca[7,66], Joan Frigola[173], Carlo Gambacorti-Passerini[177], Dale W Garsed[178], **Mark Gerstein#**[113,114,115], **Gad Getz#**[3,4,5,6], Qianyun Guo[124], Ivo G Gut[127,128], David Haan[11], Mark P Hamilton[116], Nicholas J Haradhvala[3,100], Arif O Harmanci[115,179], Mohamed Helmy[180], Carl Herrmann[50,52,181], Julian M Hess[3,72], Asger Hobolth[124,164], Ermin Hodzic[150], Chen Hong[112,171], Henrik Hornshøj[117], Keren Isaev[9,125], Jose MG Izarzugaza[182], Rory Johnson[168,183], Todd A Johnson[46], Malene Juul[117], Randi Istrup Juul[117], Andre Kahles[85,86,87,88,89], Abdullah Kahraman[118,119,120], Manolis Kellis[3,121], Ekta Khurana[110,111,122,123], Jaegil Kim[3], Jong K Kim[184], Youngwook Kim[91,92], Jan Komorowski[175,185], Jan O Korbel[7,8], Sushant Kumar[114,115], Andrés Lanzós[168,169,183], Erik Larsson[85], **Michael S Lawrence#**[3,46,100], Donghoon Lee[115], Kjong-Van Lehmann[85,86,87,88,89], Shantao Li[115], Xiaotong Li[115], Ziao Lin[3,186], Eric Minwei Liu[110,111,187], Lucas Lochovsky[114,115,160], Shaoke Lou[114,115], Tobias Madsen[117], Kathleen Marchal[188,189], Iñigo Martincorena[1], Alexander Martinez-Fundichely[110,111,123], Yosef E Maruvka[3,72,100], Patrick D McGillivray[114], William Meyerson[115,190], Ferran Muiños[174,191], Loris Mularoni[174,191], Hidewaki Nakagawa[47], Morten Muhlig Nielsen[117], Marta Paczkowska[9], Keunchil Park[192,193], Kiejung Park[194], **Jakob Skou Pedersen#**[117,124], Tirso Pons[195], Sergio Pulido-Tamayo[188,189], **Benjamin J Raphael#**[196], Jüri Reimand[9,125], Iker Reyes-Salazar[191], Matthew A Reyna[196], Esther Rheinbay[3,6,100], Mark A Rubin[183,197,198,199,200], Carlota Rubio-Perez[174,191,201], S Cenk Sahinalp[142,149,150], Gordon Saksena[3], Leonidas Salichos[114,115], Chris Sander[85,202,203], Steven E Schumacher[3,204], Mark Shackleton[178], Ofer Shapira[3,205], Ciyue Shen[203,206], Raunak Shrestha[142], Shimin Shuai[9,10], Nikos Sidiropoulos[108], Lina Sieverling[112,171], Nasa Sinnott-Armstrong[3,59], Lincoln D Stein[9,10], **Joshua M Stuart#**[11,12], David Tamborero[174,191], Grace Tiao[3], Tatsuhiko Tsunoda[46,207,208,209], Husen M Umer[175,210], Liis Uusküla-Reimand[211,212], Alfonso Valencia[45,105], Miguel Vazquez[45,106], Lieven PC Verbeke[189,213], Claes Wadelius[214], Lina Wadi[9], Jiayin Wang[131,145,152], Jonathan Warrell[114,115], Sebastian M Waszak[8], Joachim Weischenfeldt[8,107,108], **David A Wheeler#**[154,155], Guanming Wu[215], Jun Yu[216], Jing Zhang[115], Xuanping Zhang[145,217], Yan Zhang[115,218,219], Zhongming Zhao[220], Lihua Zou[221] and Christian von

Mering[120,126]

PCAWG Transcriptome Working Group

Samirkumar B Amin[158,159,160], Philip Awadalla[9,10], Peter J Bailey[222], **Alvis Brazma#**[7], **Angela N Brooks#**[3,11,12,48], Claudia Calabrese[7,8], Aurélien Chateigner[44], Isidro Cortés-Ciriano[223,224,225], Brian Craft[12], David Craft[3,226], Chad J Creighton[227], Natalie R Davidson[85,86,87,88,101], Deniz Demircioğlu[228,229], Serap Erkek[8], Nuno A Fonseca[7,66], Milana Frenkel-Morgenstern[230], Mary J Goldman[12], Liliana Greger[7], Jonathan Göke[228,231], Yao He[232], Katherine A Hoadley[233,234], Yong Hou[38,235], Matthew R Huska[236], Andre Kahles[85,86,87,88,89], Ekta Khurana[110,111,122,123], Helena Kilpinen[237], Jan O Korbel[7,8], Fabien C Lamaze[9], Kjong-Van Lehmann[85,86,87,88,89], Chang Li[38,235], Siliang Li[38,235], Xiaobo Li[38,235], Xinyue Li[38], Dongbing Liu[38,235], Fenglin Liu[232,238], Xingmin Liu[38,235], Maximillian G Marin[11], Julia Markowski[236], Matthew Meyerson[3,6,48], Tannistha Nandi[239], Morten Muhlig Nielsen[117], Akinyemi I Ojesina[240,241,242], BF Francis Ouellette[44,97], Qiang Pan-Hammarström[38,243], Peter J Park[223,225], Chandra Sekhar Pedamallu[3,6,163], Jakob Skou Pedersen[117,124], Marc D Perry[44,98], **Gunnar Rätsch#**[85,86,87,88,89,101], Roland F Schwarz[7,81,236,244], Yuichi Shiraishi[83], Reiner Siebert[245,246], Cameron M Soulette[11], Stefan G Stark[86,88,247,248], Oliver Stegle[7,8,249], Hong Su[38,235], Patrick Tan[239,250,251,252], Bin Tean Teh[250,251,252,253,254], Lara Urban[7,8], Jian Wang[38], Sebastian M Waszak[8], Kui Wu[38,235], Qian Xiang[44], Heng Xiong[38,235], Sergei Yakneen[8], Huanming Yang[38], Chen Ye[38,235], Christina K Yung[44], Fan Zhang[232], Junjun Zhang[44], Xiuqing Zhang[38], Zemin Zhang[232,255], Liangtao Zheng[232], Jingchun Zhu[12] and Shida Zhu[38,235]

PCAWG Epigenome Working Group

Hiroyuki Aburatani[256], **Benjamin P Berman#**[257,258,259], Hans Binder[260,261], **Benedikt Brors#**[79,112,262], Huy Q Dinh[257], Lars Feuerbach[112], Shengjie Gao[38], Ivo G Gut[127,128], Simon C Heath[127,128], Steve Hoffmann[260,261,263,264], Charles David Imbusch[112], Ekta Khurana[110,111,122,123], Helene Kretzmer[261,264], Peter W Laird[265], Jose I Martin-Subero[105,266], Genta Nagae[256,267], **Christoph Plass#**[268], Paz Polak[3,4,6], Hui Shen[269], Reiner Siebert[245,246], Nasa Sinnott-Armstrong[3,59], Miranda D Stobbe[127,128], Qi Wang[93], Dieter Weichenhan[268], Sergei Yakneen[8] and Wanding Zhou[269]

PCAWG Structural Variation Working Group

Kadir C Akdemir[136], Eva G Alvarez[270,271,272], Adrian Baez-Ortega[273], **Rameen Beroukhim#**[3,6,163], Paul C Boutros[9,125,134,135], David D L Bowtell[178], Benedikt Brors[79,112,262], Kathleen H Burns[274,275], John Busanovich[3,276], **Peter J Campbell#**[1,2], Kin Chan[277], Ken Chen[136], Isidro Cortés-Ciriano[223,224,225], Ana Dueso-Barroso[45], Andrew J Dunford[3], Paul A Edwards[278,279], Xavier Estivill[139,280], Dariush Etemadmoghadam[178], Lars Feuerbach[112], J Lynn Fink[45,176], Milana Frenkel-Morgenstern[230], Dale W Garsed[178], Mark Gerstein[113,114,115], Dmitry A Gordenin[281], David Haan[11], James E Haber[282], Julian M Hess[3,72], Barbara Hutter[77,78,79], Marcin Imielinski[283,284], David TW Jones[285,286], Young Seok Ju[1,172], Marat D Kazanov[287,288,289], Leszek J Klimczak[290], Youngil Koh[291,292], Jan O Korbel[7,8], Kiran Kumar[3], Eunjung Alice Lee[293], Jake June-Koo Lee[223,225], Yilong Li[1], Andy G Lynch[278,279,294], Geoff

Macintyre[278], Florian Markowetz[278,279], Iñigo Martincorena[1], Alexander Martinez-Fundichely[110,111,123], Satoru Miyano[83], Hidewaki Nakagawa[47], Fabio CP Navarro[114], Stephan Ossowski[128,139,148], Peter J Park[223,225], John V Pearson[295,296], Montserrat Puiggròs[45], Karsten Rippe[81], Nicola D Roberts[1], Steven A Roberts[297], Bernardo Rodriguez-Martin[270,271,272], Steven E Schumacher[3,204], Ralph Scully[298], Mark Shackleton[178], Nikos Sidiropoulos[108], Lina Sieverling[112,171], Chip Stewart[3], David Torrents[45,105], Jose MC Tubio[270,271,272], Izar Villasante[45], Nicola Waddell[295,296], Jeremiah A Wala[3,6,48], Joachim Weischenfeldt[8,107,108], Lixing Yang[299], Xiaotong Yao[284,300], Sung-Soo Yoon[292], Jorge Zamora[1,270,271,272] and Cheng-Zhong Zhang[3,301]

PCAWG Mutational Signatures Working Group

Ludmil B Alexandrov[1,70,302], Erik N Bergstrom[70,303], Arnoud Boot[251,304], Paul C Boutros[9,125,134,135], Kin Chan[277], Kyle Covington[155], Akihiro Fujimoto[47], Gad Getz[3,4,5,6], Dmitry A Gordenin[281], Nicholas J Haradhvala[3,100], Mi Ni Huang[251,304], S. M. Ashiqul Islam[53], Marat D Kazanov[287,288,289], Jaegil Kim[3], Leszek J Klimczak[290], Michael S Lawrence[3,46,100], Iñigo Martincorena[1], John R McPherson[251,304], Sandro Morganella[1], Ville Mustonen[305,306,307], Hidewaki Nakagawa[47], Alvin Wei Tian Ng[308], Serena Nik-Zainal[1,309,310,311], Paz Polak[3,4,6], Stephenie D Prokopec[9], Steven A Roberts[297], **Steven G Rozen#**[251,252,304], Radhakrishnan Sabarinathan[174,191,312], Natalie Saini[281], Tatsuhiro Shibata[33,34], Yuichi Shiraishi[83], **Michael R Stratton#**[1], **Bin Tean Teh#**[250,251,252,253,254], Ignacio Vázquez-García[1,157,313,314], Yang Wu[251,304], Fouad Yousif[9] and Willie Yu[315]

PCAWG Germline Cancer Genome Working Group

Ludmil B Alexandrov[1,70,302], Eva G Alvarez[270,271,272], Adrian Baez-Ortega[273], Matthew H Bailey[130,131], Mattia Bosio[45,128,139], G Steven Bova[316], Alvis Brazma[7], Alicia L Bruzos[270,271,272], Ivo Buchhalter[50,51,52], Carlos D Bustamante[58,59], Atul J Butte[317], Andy Cafferkey[7], Claudia Calabrese[7,8], Peter J Campbell[1,2], Stephen J Chanock[318], Nilanjan Chatterjee[319,320], Jieming Chen[115,321], Francisco M De La Vega[57,58,59], Olivier Delaneau[322,323,324], German M Demidov[128,139,148], Anthony DiBiase[325], Li Ding[130,131,138], Oliver Drechsel[128,139], Lewis Jonathan Dursi[9,62], Douglas F Easton[326,327], Serap Erkek[8], Georgia Escaramis[139,328,329], **Xavier Estivill#**[139,280], Erik Garrison[1], Mark Gerstein[113,114,115], Gad Getz[3,4,5,6], Dmitry A Gordenin[281], Nina Habermann[8], Olivier Harismendy[69,70], Eoghan Harrington[330], Shuto Hayashi[83], Seong Gu Heo[331], José María Heredia-Genestar[332], Aliaksei Z Holik[139], Eun Pyo Hong[331], Xing Hua[318], Kuan-lin Huang[131,333], Seiya Imoto[83], Sissel Juul[330], Ekta Khurana[110,111,122,123], Hyung-Lae Kim[27], Youngwook Kim[91,92], Leszek J Klimczak[290], **Jan O Korbel#**[7,8], Roelof Koster[334], Sushant Kumar[114,115], Ivica Letunic[146], Yilong Li[1], Tomas Marques-Bonet[105,127,332,335], R Jay Mashl[131,147], Simon Mayes[336], Michael D McLellan[130,131,138], Lisa Mirabello[318], Francesc Muyas[128,139,148], Hidewaki Nakagawa[47], Arcadi Navarro[105,127,332], Steven J Newhouse[7], Stephan Ossowski[128,139,148], Ji Wan Park[331], Esa Pitkänen[8], Aparna Prasad[128], Raquel Rabionet[128,139,337], Benjamin Raeder[8], Tobias Rausch[8], Steven A Roberts[297], Bernardo Rodriguez-Martin[270,271,272], Vasilisa A Rudneva[8], Gunnar Rätsch[85,86,87,88,89,101], Natalie Saini[281], Matthias Schlesner[50,102], Roland F Schwarz[7,81,236,244], Ayellet V Segre[3,338], Tal Shmaya[57], Suyash S Shringarpure[59], Nikos Sidiropoulos[108], Reiner Siebert[245,246], Jared T Simpson[9,151], Lei Song[318], Oliver Stegle[7,8,249], Hana Susak[128,139], Tomas J Tanskanen[339], Grace Tiao[3], Marta Tojo[272], Jose MC

Tubio[270,271,272], Daniel J Turner[336], Lara Urban[7,8], Sebastian M Waszak[8], David C Wedge[1,340,341], Joachim Weischenfeldt[8,107,108], David A Wheeler[154,155], Mark H Wright[59], Dai-Ying Wu[57], Tian Xia[342], Sergei Yakneen[8], Kai Ye[152,156], Venkata D Yellapantula[138,157], Jorge Zamora[1,270,271,272] and Bin Zhu[318]

PCAWG Pathology and Clinical Correlates Working Group

Fatima Al-Shahrour[343], Gurnit Atwal[9,10,344], Peter J Bailey[222], **Andrew V Biankin#**[222,345,346,347], Paul C Boutros[9,125,134,135], Peter J Campbell[1,2], David K Chang[222,345], Susanna L Cooke[222], Vikram Deshpande[100], Bishoy M Faltas[101], William C Faquin[100], **Levi Garraway#**[48], Gad Getz[3,4,5,6], **Sean M Grimmond#**[348], Syed Haider[9], **Katherine A Hoadley#**[233,234], Wei Jiao[9], Vera B Kaiser[349], Rosa Karlić[350], Mamoru Kato[351], Kirsten Kübler[3,6,100], Alexander J Lazar[158,352], Constance H Li[9,125], David N Louis[100], Adam A Margolin[104], Sancha Martin[1,353], Hardeep K Nahal-Bose[44], G Petur Nielsen[100], Serena Nik-Zainal[1,309,310,311], Larsson Omberg[96], Christine P'ng[9], Marc D Perry[44,98], Paz Polak[3,4,6], Esther Rheinbay[3,6,100], Mark A Rubin[183,197,198,199,200], Colin A Semple[349], Dennis C Sgroi[100], Tatsuhiro Shibata[33,34], Reiner Siebert[245,246], Jaclyn Smith[354], **Lincoln D Stein#**[9,10], Miranda D Stobbe[127,128], Ren X Sun[9], Kevin Thai[44], Derek W Wright[222,355], Chin-Lee Wu[100], Ke Yuan[278,353,356] and Junjun Zhang[44]

PCAWG Evolution & Heterogeneity Working Group

David J Adams[1], Pavana Anur[357], Rameen Beroukhim[3,6,163], Paul C Boutros[9,125,134,135], David D L Bowtell[178], Peter J Campbell[1,2], Shaolong Cao[140], Elizabeth L Christie[178], Marek Cmero[358,359,360], Yupeng Cun[361], Kevin J Dawson[1], Jonas Demeulemeester[60,61], Stefan C Dentro[1,60,340], Amit G Deshwar[362], Nilgun Donmez[142,150], Ruben M Drews[278], Roland Eils[50,52,63,64], Yu Fan[140], Matthew W Fittall[60], Dale W Garsed[178], Moritz Gerstung[7,8], Gad Getz[3,4,5,6], Santiago Gonzalez[7,8], Gavin Ha[3], Kerstin Haase[60], Marcin Imielinski[283,284], Lara Jerman[8,363], Yuan Ji[364,365], Clemency Jolly[60], Kortine Kleinheinz[50,52], Juhee Lee[366], Henry Lee-Six[1], Ignaty Leshchiner[3], Dimitri Livitz[3], Geoff Macintyre[278], Salem Malikic[142,150], Florian Markowetz[278,279], Iñigo Martincorena[1], Thomas J Mitchell[1,279,367], Quaid D Morris[344,368], Ville Mustonen[305,306,307], Layla Oesper[369], Martin Peifer[361], Myron Peto[357], Benjamin J Raphael[196], Daniel Rosebrock[3], Yulia Rubanova[151,344], S Cenk Sahinalp[142,149,150], Adriana Salcedo[9], Matthias Schlesner[50,102], Steven E Schumacher[3,204], Subhajit Sengupta[370], Ruian Shi[368], Seung Jun Shin[248], **Paul T Spellman#**[357], Oliver Spiro[3], Lincoln D Stein[9,10], Maxime Tarabichi[1,60], **Peter Van Loo#**[60,61], Shankar Vembu[368,371], Ignacio Vázquez-García[1,157,313,314], Wenyi Wang[140], **David C Wedge#**[1,340,341], David A Wheeler[154,155], Jeffrey A Wintersinger[151,180,344], Tsun-Po Yang[361], Xiaotong Yao[284,300], Kaixian Yu[372], Ke Yuan[278,353,356] and Hongtu Zhu[372,373]

PCAWG Portals and Visualization Working Group

Fatima Al-Shahrour[343], Elisabet Barrera[7], Wojciech Bazant[7], Alvis Brazma[7], Isidro Cortés-Ciriano[223,224,225], Brian Craft[12], David Craft[3,226], Vincent Ferretti[44,65], Nuno A Fonseca[7,66],

Anja Füllgrabe[7], Mary J Goldman[12], **David Haussler#**[12,374], Wolfgang Huber[8], Maria Keays[7], Alfonso Muñoz[7], Brian D O'Connor[44,95], Irene Papatheodorou[7], Robert Petryszak[7], Elena Piñeiro-Yáñez[343], Alfonso Valencia[45,105], **Miguel Vazquez#**[45,106], John N Weinstein[375,376], Qian Xiang[44], Junjun Zhang[44] and **Jingchun Zhu#**[12]

PCAWG Mitochondrial Genome and Immunogenomics Working Group

Peter J Campbell[1,2], Yiwen Chen[140], Chad J Creighton[227], Li Ding[130,131,138], Akihiro Fujimoto[47], Masashi Fujita[47], Gad Getz[3,4,5,6], Leng Han[217], Takanori Hasegawa[83], Shuto Hayashi[83], Seiya Imoto[83], Young Seok Ju[1,172], Hyung-Lae Kim[27], Youngwook Kim[91,92], Youngil Koh[291,292], Mitsuhiro Komura[83], Jun Li[140], **Han Liang#**[140], Iñigo Martincorena[1], Satoru Miyano[83], Shinichi Mizuno[377], **Hidewaki Nakagawa#**[47], Keunchil Park[192,193], Eigo Shimizu[83], Yumeng Wang[140], John N Weinstein[375,376], Yanxun Xu[378], Rui Yamaguchi[83], Fan Yang[368], Yang Yang[217], Christopher J Yoon[172], Sung-Soo Yoon[292], Yuan Yuan[140], Fan Zhang[232] and Zemin Zhang[232,255]

PCAWG Pathogens Working Group

Malik Alawi[379,380], Ivan Borozan[9], Daniel S Brewer[381,382], Colin S Cooper[382,383,384], Nikita Desai[44], Roland Eils[50,52,63,64], Vincent Ferretti[44,65], Adam Grundhoff[380,385], Murat Iskar[386], Kortine Kleinheinz[50,52], **Peter Lichter#**[77,386], Hidewaki Nakagawa[47], Akinyemi I Ojesina[240,241,242], Chandra Sekhar Pedamallu[3,6,163], Matthias Schlesner[50,102], Xiaoping Su[352] and **Marc Zapatka#**[386]

Providers of tumour-sequencing data

PCAWG Tumour-specific providers (ovarian cancer) in Australia

Kathryn Alsop[178], Australian Ovarian Cancer Study Group[295,387,388], **David D L Bowtell#**[178], Timothy JC Bruxner[176], Angelika N Christ[176], Elizabeth L Christie[178], Stephen M Cordner[389], Prue A Cowin[387], Ronny Drapkin[390], Dariush Etemadmoghadam[178], Sian Fereday[178], Dale W Garsed[178], Joshy George[160], Sean M Grimmond[348], Anne Hamilton[387], Oliver Holmes[295,296], Jillian A Hung[391], Karin S Kassahn[176,392], Stephen H Kazakoff[295,296], Catherine J Kennedy[391,393], Conrad R Leonard[295,296], Linda Mileshkin[178], David K Miller[176,345], Gisela Mir Arnau[387], Chris Mitchell[178], Felicity Newell[295,296], Katia Nones[295,296], Ann-Marie Patch[295,296], John V Pearson[295,296], Michael C Quinn[295,296], Mark Shackleton[178], Darrin F Taylor[176], Heather Thorne[178], Nadia Traficante[178], Ravikiran Vedururu[387], Nick M Waddell[296], Nicola Waddell[295,296], Paul M Waring[394], Scott Wood[295,296], Qinying Xu[295,296] and Anna deFazio[391,393,395]

PCAWG Tumour-specific providers (pancreatic cancer) in Australia

Matthew J Anderson[176], Davide Antonello[396], Andrew P Barbour[397,398], Claudio Bassi[396], Samantha Bersani[399], **Andrew V Biankin#**[222,345,346,347], Timothy JC Bruxner[176], Ivana Cataldo[399,400], David K Chang[222,345], Lorraine A Chantrill[345,401], Yoke-Eng Chiew[391,393], Angela Chou[345,393], Angelika N Christ[176], Sara Cingarlini[36], Nicole Cloonan[402], Vincenzo Corbo[400,403], Maria Vittoria Davi[404], Fraser R Duthie[222,405], J Lynn Fink[45,176], Anthony J Gill[345,406], Janet S Graham[222,407], **Sean M Grimmond#**[348], Ivon Harliwong[176], Oliver Holmes[295,296], Nigel B Jamieson[222,347,408], Amber L Johns[345], Karin S Kassahn[176,392], Stephen H Kazakoff[295,296], James G Kench[345,406,409], Luca Landoni[396], Rita T Lawlor[400], Conrad R Leonard[295,296], Andrea Mafficini[400], Neil D Merrett[396,410], David K Miller[176,345], Marco Miotto[396], Elizabeth A Musgrove[222], Adnan M Nagrial[345], Felicity Newell[295,296], Katia Nones[295,296], Karin A Oien[394,411], Marina Pajic[345], Ann-Marie Patch[295,296], John V Pearson[295,296], Mark Pinese[345], Andreia V Pinho[412], Michael C Quinn[295,296], Alan J Robertson[176], Ilse Rooman[345], Borislav C Rusev[400], Jaswinder S Samra[396,413], Maria Scardoni[399], Christopher J Scarlett[345,414], Aldo Scarpa[400], Elisabetta Sereni[396], Katarzyna O Sikora[400], Michele Simbolo[403], Morgan L Taschuk[44], Christopher W Toon[345], Giampaolo Tortora[36,37], Caterina Vicentini[400], Nick M Waddell[296], Nicola Waddell[295,296], Scott Wood[295,296], Jianmin Wu[345], Qinying Xu[295,296] and Nikolajs Zeps[415,416]

PCAWG Tumour-specific providers (skin cancer) in Australia

Lauri A Aaltonen[417], Andreas Behren[418], Hazel Burke[419], Jonathan Cebon[418], Rebecca A Dagg[420], Ricardo De Paoli-Iseppi[419], Ken Dutton-Regester[295], Matthew A Field[421], Anna Fitzgerald[422], Sean M Grimmond[348], **Nicholas K Hayward#**[295,419], Peter Hersey[419], Oliver Holmes[295,296], Valerie Jakrot[419], Peter A Johansson[295], Hojabr Kakavand[419], Stephen H Kazakoff[295,296], Richard F Kefford[423], Loretta MS Lau[424], Conrad R Leonard[295,296], Georgina V Long[419], **Graham J Mann#**[393,419,419], Felicity Newell[295,296], Katia Nones[295,296], Ann-Marie Patch[295,296], John V Pearson[295,296], Hilda A Pickett[424], Antonia L Pritchard[295], Gulietta M Pupo[393], Robyn PM Saw[419], Sarah-Jane Schramm[393], **Richard A Scolyer#**[409,413,419], Mark Shackleton[178], Catherine A Shang[422], Ping Shang[419], Andrew J Spillane[419], Jonathan R Stretch[419], Varsha Tembe[393], John F Thompson[419], Ricardo E Vilain[425], Nick M Waddell[296], Nicola Waddell[295,296], James S Wilmott[419], Scott Wood[295,296], Qinying Xu[295,296] and Jean Y Yang[426]

PCAWG Tumour-specific providers (pancreatic cancer) in Canada

John Bartlett[427,428], Prashant Bavi[429], Ivan Borozan[9], Dianne E Chadwick[430], Michelle Chan-Seng-Yue[429], Sean Cleary[429,431], Ashton A Connor[431,432], Karolina Czajka[76], Robert E Denroche[429], Neesha C Dhani[433], Jenna Eagles[76], Vincent Ferretti[44,65], Steven Gallinger[429,431,432], Robert C Grant[429,432], David Hedley[433], Michael A Hollingsworth[434], **Thomas J Hudson#**[75,76], Gun Ho Jang[429], Jeremy Johns[76], Sangeetha Kalimuthu[429], Sheng-Ben Liang[430], Ilinca Lungu[429,435], Xuemei Luo[9], Faridah Mbabaali[76], **John D McPherson#**[76,429,436], Treasa A McPherson[432], Jessica K Miller[76], Malcolm J Moore[433], Faiyaz Notta[429,437], Danielle Pasternack[76], Gloria M Petersen[438], Michael H A Roehrl[125,429,430,439,440,441], Michelle Sam[76], Iris Selander[432], Stefano Serra[394], Sagedeh Shahabi[430], **Lincoln D Stein#**[9,10], Morgan L Taschuk[44], Sarah P Thayer[434], Lee E Timms[76], Gavin W Wilson[9,429], Julie M Wilson[429] and Bradly G Wouters[125]

PCAWG Tumour-specific providers (prostate cancer) in Canada

Timothy A Beck[44,442], Vinayak Bhandari[9], **Paul C Boutros#**[9,125,134,135], **Robert G Bristow#**[125,443,444,445,446], Colin C Collins[142], Shadrielle MG Espiritu[9], Neil E Fleshner[447], Natalie S Fox[9], Michael Fraser[9], Syed Haider[9], Lawrence E Heisler[44], Vincent Huang[9], Emilie Lalonde[9], Julie Livingstone[9], John D McPherson[76,429,436], Alice Meng[448], Veronica Y Sabelnykova[9], Adriana Salcedo[9], Yu-Jia Shiah[9], Theodorus Van der Kwast[441] and Takafumi N Yamaguchi[9]

PCAWG Tumour-specific providers (gastric cancer) in China

Shuai Ding[449], Daiming Fan[450], Yong Hou[38,235], Yi Huang[144,145], Lin Li[38], Siliang Li[38,235], Dongbing Liu[38,235], Xingmin Liu[38,235], **Youyong Lu#**[28,29,30], Yongzhan Nie[450,451], Hong Su[38,235], Jian Wang[38], Kui Wu[38,235], Xiao Xiao[145], Rui Xing[30], **Huanming Yang#**[38], Shanlin Yang[449], Yingyan Yu[452], Xiuqing Zhang[38], Yong Zhou[38] and Shida Zhu[38,235]

PCAWG Tumour-specific providers (renal cancer) in the EU & France

Rosamonde E Banks[453], Guillaume Bourque[454,455], Alvis Brazma[7], Paul Brennan[456], **Mark Lathrop#**[455], Louis Letourneau[457], Yasser Riazalhosseini[455], Ghislaine Scelo[456], **Jörg Tost#**[458], Naveen Vasudev[459] and Juris Viksna[460]

PCAWG Tumour-specific providers (breast cancer) in the EU & United Kingdom

Sung-Min Ahn[461], Ludmil B Alexandrov[1,70,302], Samuel Aparicio[462], Laurent Arnould[463], MR Aure[464], Shriram G Bhosle[1], Ewan Birney[7], Ake Borg[465], Sandrine Boyault[466], Arie B Brinkman[467], Jane E Brock[468], Annegien Broeks[469], Adam P Butler[1], Anne-Lise Børresen-Dale[464,470], Carlos Caldas[278,471], Peter J Campbell[1,2], Suet-Feung Chin[278,471], Helen Davies[1,309,310], Christine Desmedt[472,473], Luc Dirix[474], Serge Serge[1], Anna Ehinger[475], Jorunn E Eyfjord[476], Aquila Fatima[204], John A Foekens[477], P Andrew Futreal[478], Øystein Garred[479,480], Moritz Gerstung[7,8], Dilip D Giri[481], Dominik Glodzik[1], Dorthe Grabau[482], Holmfridur Hilmarsdottir[476], Gerrit K Hooijer[483], Jocelyne Jacquemier[484], Se Jin Jang[485], Jon G Jonasson[476], Jos Jonkers[486], Hyung-Yong Kim[484], Tari A King[487,488,489], Stian Knappskog[1,490], Gu Kong[484], Savitri Krishnamurthy[352,491], Sunil R Lakhani[492], Anita Langerød[464], Denis Larsimont[493], Hee Jin Lee[485], Jeong-Yeon Lee[494], Ming Ta Michael Lee[478], Yilong Li[1], Ole Christian Lingjærde[495], Gaetan MacGrogan[496], John WM Martens[477], Sancha Martin[1,353], Iñigo Martincorena[1], Andrew Menzies[1], Sandro Morganella[1], Ville Mustonen[305,306,307], Serena Nik-Zainal[1,309,310,311], Sarah O'Meara[1], Iris Pauporté[20], Sarah Pinder[497], Xavier Pivot[498], Elena Provenzano[499], Colin A Purdie[500], Keiran M Raine[1], Manasa Ramakrishna[1], Kamna Ramakrishnan[1], Jorge Reis-Filho[481], Andrea L Richardson[204], Markus Ringnér[501], Javier Bartolomé

Rodriguez[45], F Germán Rodríguez-González[502], Gilles Romieu[503], Roberto Salgado[394], Torill Sauer[495], Rebecca Shepherd[1], Anieta M Sieuwerts[477], Peter T Simpson[492], Marcel Smid[477], Christos Sotiriou[53], Paul N Span[504], Lucy Stebbings[1], Ólafur Andri Stefánsson[505], Alasdair Stenhouse[506], **Michael R Stratton#**[1], Henk G Stunnenberg[235,507], Fred Sweep[508], Benita Kiat Tee Tan[509], Jon W Teague[1], Gilles Thomas[510], Alastair M Thompson[506], Stefania Tommasi[511], Isabelle Treilleux[512,513], Andrew Tutt[204], Naoto T Ueno[514], Steven Van Laere[474], Peter Van Loo[60,61], Gert G Van den Eynden[474], Peter Vermeulen[474], Alain Viari[400], Anne Vincent-Salomon[507], David C Wedge[1,340,341], Bernice H Wong[515], Lucy Yates[1], Xueqing Zou[1], Carolien HM van Deurzen[516], Marc J van de Vijver[394] and L van't Veer[517]

PCAWG Tumour-specific providers (malignant lymphoma) in Germany

Ole Ammerpohl[518,519], Sietse Aukema[520,521], Anke K Bergmann[522], Stephan H Bernhart[260,261,264], Hans Binder[260,261], Arndt Borkhardt[523], Christoph Borst[524], Benedikt Brors[79,112,262], Birgit Burkhardt[525], Alexander Claviez[526], Roland Eils[50,52,63,64], Maria Elisabeth Goebler[527], Andrea Haake[518], Siegfried Haas[524], Martin Hansmann[528], Jessica I Hoell[523], Steve Hoffmann[260,261,263,264], Michael Hummel[529], Daniel Hübschmann[52,63,80,81,82], Dennis Karsch[530], Wolfram Klapper[520], Kortine Kleinheinz[50,52], Michael Kneba[530], Jan O Korbel[7,8], Helene Kretzmer[261,264], Markus Kreuz[531], Dieter Kube[532], Ralf Küppers[533], Chris Lawerenz[64], Dido Lenze[529], Peter Lichter[77,386], Markus Loeffler[531], Cristina López[246,518], Luisa Mantovani-Löffler[534], Peter Möller[535], German Ott[536], Bernhard Radlwimmer[386], Julia Richter[518,520], Marius Rohde[537], Philip C Rosenstiel[538], Andreas Rosenwald[539], Markus B Schilhabel[538], Matthias Schlesner[50,102], Stefan Schreiber[540], **Reiner Siebert#**[245,246], Peter F Stadler[260,261,264], Peter Staib[541], Stephan Stilgenbauer[542], Stephanie Sungalee[8], Monika Szczepanowski[520], Umut H Toprak[52,543], Lorenz HP Trümper[532], Rabea Wagener[246,518] and Thorsten Zenz[79]

PCAWG Tumour-specific providers (paediatric brain cancer) in Germany

Ivo Buchhalter[50,51,52], Juergen Eils[63,64], Roland Eils[50,52,63,64], Volker Hovestadt[386], Barbara Hutter[77,78,79], David TW Jones[285,286], Natalie Jäger[50], Christof von Kalle[81], Marcel Kool[93,285], Jan O Korbel[7,8], Andrey Korshunov[93], Pablo Landgraf[544,545], Chris Lawerenz[64], Hans Lehrach[546], **Peter Lichter#**[77,386], Paul A Northcott[547], Stefan M Pfister[93,285,548], Bernhard Radlwimmer[386], Guido Reifenberger[545], Matthias Schlesner[50,102], Hans-Jörg Warnatz[546], Joachim Weischenfeldt[8,107,108], Stephan Wolf[549], Marie-Laure Yaspo[546] and Marc Zapatka[386]

PCAWG Tumour-specific providers (prostate cancer) in Germany

Yassen Assenov[550], Benedikt Brors[79,112,262], Juergen Eils[63,64], Roland Eils[50,52,63,64], Lars Feuerbach[112], Clarissa Gerhauser[268], Jan O Korbel[7,8], Chris Lawerenz[64], Hans Lehrach[546], Sarah Minner[551],

Christoph Plass[268], **Guido Sauter#**[552], Thorsten Schlomm[107,553], Nikos Sidiropoulos[108], Ronald Simon[552], **Holger Sültmann#**[79,554], Hans-Jörg Warnatz[546], Dieter Weichenhan[268], Joachim Weischenfeldt[8,107,108] and Marie-Laure Yaspo[546]

PCAWG Tumour-specific providers (oral cancer) in India

Nidhan K Biswas[555], Luca Landoni[396], Arindam Maitra[555], **Partha P Majumder#**[555] and **Rajiv Sarin#**[556]

PCAWG Tumour-specific providers (pancreatic cancer) in Italy

Davide Antonello[396], Stefano Barbi[403], Claudio Bassi[396], Samantha Bersani[399], Giada Bonizzato[400], Cinzia Cantù[400], Ivana Cataldo[399,400], Sara Cingarlini[36], Vincenzo Corbo[400,403], Maria Vittoria Davi[404], Angelo P Dei Tos[557], Matteo Fassan[558], Sonia Grimaldi[400], Luca Landoni[396], Rita T Lawlor[400], Claudio Luchini[399], Andrea Mafficini[400], Giuseppe Malleo[396], Giovanni Marchegiani[396], Michele Milella[36], Marco Miotto[396], Salvatore Paiella[396], Antonio Pea[396], Paolo Pederzoli[396], Borislav C Rusev[400], Andrea Ruzzenente[396], Roberto Salvia[396], Maria Scardoni[399], **Aldo Scarpa#**[400], Elisabetta Sereni[396], Michele Simbolo[403], Nicola Sperandio[400], Giampaolo Tortora[36,37] and Caterina Vicentini[400]

PCAWG Tumour-specific providers (biliary tract cancer) in Japan

Yasuhito Arai[33], Natsuko Hama[33], Nobuyoshi Hiraoka[559], Fumie Hosoda[33], Mamoru Kato[351], Hiromi Nakamura[33], Hidenori Ojima[560], Takuji Okusaka[561], **Tatsuhiro Shibata#**[33,34], Yasushi Totoki[33] and Tomoko Urushidate[34]

PCAWG Tumour-specific providers (gastric cancer) in Japan

Hiroyuki Aburatani#[256], Yasuhito Arai[33], Masashi Fukayama[562], Natsuko Hama[33], Fumie Hosoda[33], Shumpei Ishikawa[563], Hitoshi Katai[564], Mamoru Kato[351], Hiroto Katoh[563], Daisuke Komura[563], Genta Nagae[256,267], Hiromi Nakamura[33], Hirofumi Rokutan[351], Mihoko Saito-Adachi[351], **Tatsuhiro Shibata#**[33,34], Akihiro Suzuki[256,565], Hirokazu Taniguchi[34], Kenji Tatsuno[256], Yasushi Totoki[33], Tetsuo Ushiku[562], Shinichi Yachida[33,566] and Shogo Yamamoto[256]

PCAWG Tumour-specific providers (liver cancer) in Japan

Hiroyuki Aburatani[256], Hiroshi Aikata[567], Koji Arihiro[567], Shun-ichi Ariizumi[568], Keith A Boroevich[46,47], Kazuaki Chayama[567], Akihiro Fujimoto[47], Masashi Fujita[47], Mayuko Furuta[47], Kunihito Gotoh[569], Natsuko Hama[33], Takanori Hasegawa[83], Shinya Hayami[570], Shuto Hayashi[83], Satoshi Hirano[571], Seiya Imoto[83], Mamoru Kato[351], Yoshiiku Kawakami[567], Kazuhiro Maejima[47],

Satoru Miyano[83], Genta Nagae[256,267], **Hidewaki Nakagawa#**[47], Hiromi Nakamura[33], Toru Nakamura[571], Kaoru Nakano[47], Hideki Ohdan[567], Yasushi Rino[572], Aya Sasaki-Oku[47], **Tatsuhiro Shibata#**[33,34], Yuichi Shiraishi[83], Hiroko Tanaka[83], Yasushi Totoki[33], Tatsuhiko Tsunoda[46,207,208,209], Masaki Ueno[570], Rui Yamaguchi[83], Masakazu Yamamoto[568] and Hiroki Yamaue[570]

PCAWG Tumour-specific providers (biliary tract cancer) in Singapore

Su Pin Choo[573], Ioana Cutcutache[251,304], Narong Khuntikeo[396,574], John R McPherson[251,304], Choon Kiat Ong[575], Chawalit Pairojkul[394], Irinel Popescu[576], **Steven G Rozen#**[251,252,304], **Patrick Tan#**[239,250,251,252] and **Bin Tean Teh#**[250,251,252,253,254]

PCAWG Tumour-specific providers (blood cancer) in South Korea

Keun Soo Ahn[577], Hyung-Lae Kim[27], Youngil Koh[291,292] and **Sung-Soo Yoon#**[292]

PCAWG Tumour-specific providers (chronic lymphocytic leukaemia) in Spain

Marta Aymerich[578], **Elias Campo#**[579,580], Josep L L Gelpi[45,67], Ivo G Gut[127,128], Marta Gut[127,128], Armando Lopez-Guillermo[581], Carlos López-Otín[582], Xose S Puente[582], Romina Royo[45] and David Torrents[45,105]

PCAWG Tumour-specific providers (bone cancer) in the United Kingdom

Fernanda Amary[583], Daniel Baumhoer[584], Sam Behjati[1], Bodil Bjerkehagen[584,585], **Peter J Campbell#**[1,2], **Adrienne M Flanagan#**[586], P Andrew Futreal[478], Ola Myklebost[490], Nischalan Pillay[587], Patrick Tarpey[588], Roberto Tirabosco[589] and Olga Zaikova[590]

PCAWG Tumour-specific providers (chronic myeloid disorders) in the United Kingdom

Jacqueline Boultwood[591], David T Bowen[1], Adam P Butler[1], **Peter J Campbell#**[1,2], Mario Cazzola[592], Carlo Gambacorti-Passerini[177], Anthony R Green[279], Eva Hellstrom-Lindberg[593], Luca Malcovati[592], Sancha Martin[1,353], Jyoti Nangalia[1], Elli Papaemmanuil[2] and Paresh Vyas[295,594]

PCAWG Tumour-specific providers (oesophageal cancer) in the

United Kingdom

Yeng Ang[595], Hugh Barr[596], Duncan Beardsmore[597], Matthew Eldridge[278], **Rebecca C Fitzgerald#**[310], James Gossage[598], Nicola Grehan[310], George B Hanna[599], Stephen J Hayes[600,601], Ted R Hupp[602], David Khoo[603], Jesper Lagergren[593,604], Laurence B Lovat[237], Shona MacRae[375], Maria O'Donovan[310], J Robert O'Neill[605], Simon L Parsons[606], Shaun R Preston[607], Sonia Puig[608], Tom Roques[609], Grant Sanders[234], Sharmila Sothi[610], Simon Tavaré[278], Olga Tucker[611], Richard Turkington[612], Timothy J Underwood[613] and Ian Welch[614]

PCAWG Tumour-specific providers (prostate cancer) in the United Kingdom

Daniel M Berney[615], Johann S De Bono[383], G Steven Bova[316], Daniel S Brewer[381,382], Adam P Butler[1], Declan Cahill[616], Niedzica Camacho[383], **Colin S Cooper#**[382,383,384], Nening M Dennis[616], Tim Dudderidge[616,617], Sandra E Edwards[383], **Rosalind A Eeles#**[383,616], Cyril Fisher[616], Christopher S Foster[618,619], Mohammed Ghori[1], Pelvender Gill[594], Vincent J Gnanapragasam[367,620], Gunes Gundem[187], Freddie C Hamdy[594], Steve Hawkins[278], Steven Hazell[616], William Howat[367], William B Isaacs[621], Katalin Karaszi[594], Jonathan D Kay[237], Vincent Khoo[616], Zsofia Kote-Jarai[383], Barbara Kremeyer[1], Pardeep Kumar[616], Adam Lambert[594], Daniel A Leongamornlert[1,383], Naomi Livni[616], Yong-Jie Lu[615,622], Hayley J Luxton[237], Andy G Lynch[278,279,294], Luke Marsden[594], Charlie E Massie[278], Lucy Matthews[383], Erik Mayer[616,623], Ultan McDermott[1], Sue Merson[383], Thomas J Mitchell[1,279,367], David E Neal[278,367], Anthony Ng[624], David Nicol[616], Christopher Ogden[616], Edward W Rowe[616], Nimish C Shah[367], Jon W Teague[1], Sarah Thomas[616], Alan Thompson[616], Peter Van Loo[60,61], Clare Verrill[594,625], Tapio Visakorpi[316], Anne Y Warren[367,626], David C Wedge[1,340,341], Hayley C Whitaker[237], Jorge Zamora[1,270,271,272], Hongwei Zhang[622] and Nicholas van As[616]

PCAWG Tumour-specific providers (TCGA) in the United States

Adam Abeshouse[187], Nishant Agrawal[627], Rehan Akbani[310,376], Hikmat Al-Ahmadie[187], Monique Albert[428], Kenneth Aldape[352,628], Adrian Ally[629], Yeng Ang[595], Elizabeth L Appelbaum[131,237], Joshua Armenia[630], Sylvia Asa[441,606], J Todd Auman[631], Matthew H Bailey[130,131], Miruna Balasundaram[629], Saianand Balu[234], Jill Barnholtz-Sloan[632,633], Hugh Barr[596], John Bartlett[427,428], Oliver F Bathe[634,635], Stephen B Baylin[320,617], Duncan Beardsmore[597], Christopher Benz[636], Andrew Berchuck[637], Benjamin P Berman[257,258,259], Rameen Beroukhim[3,6,163], Mario Berrios[638], Darell Bigner[639], Michael Birrer[100], Tom Bodenheimer[234], Lori Boice[608], Moiz S Bootwalla[638], Marcus Bosenberg[640], Reanne Bowlby[629], Jeffrey Boyd[641], Russell R Broaddus[352], Malcolm Brock[642], Denise Brooks[629], Susan Bullman[3,163], Samantha J Caesar-Johnson[39], Thomas E Carey[643], Rebecca Carlsen[629], Robert Cerfolio[644], Vishal S Chandan[645], Hsiao-Wei Chen[595,630], Andrew D Cherniack[3,48,163], Jeremy Chien[646], Juok Cho[3], Eric Chuah[629], Carrie Cibulskis[3], Kristian Cibulskis[3], Leslie Cope[320], Matthew G Cordes[131,609], Kyle Covington[155], Erin Curley[647], Bogdan Czerniak[352,603], Ludmila Danilova[320], Ian J Davis[648], Timothy Defreitas[3], John A Demchok[39], Noreen Dhalla[629], Rajiv Dhir[649], Li Ding[130,131,138], HarshaVardhan Doddapaneni[155], Adel El-Naggar[352,603], Ina Felau[39], Martin L Ferguson[650], Gaetano

Finocchiaro[651], Kwun M Fong[652], Scott Frazer[3], William Friedman[653], Catrina C Fronick[131,609], Lucinda A Fulton[131], Robert S Fulton[130,131,138], Stacey B Gabriel[3], Jianjiong Gao[630], Nils Gehlenborg[3,654], Jeffrey E Gershenwald[655,656], Gad Getz[3,4,5,6], Ronald Ghossein[481], Nasra H Giama[657], Richard A Gibbs[155], Carmen Gomez[658], James Gossage[598], Ramaswamy Govindan[130], Nicola Grehan[310], George B Hanna[599], D Neil Hayes[234,659,660], Stephen J Hayes[600,601], Apurva M Hegde[375,376], David I Heiman[3], Zachary Heins[187], Austin J Hepperla[234], Katherine A Hoadley[233,234], Andrea Holbrook[638], Robert A Holt[629], Alan P Hoyle[234], Ralph H Hruban[320], Jianhong Hu[155], Mei Huang[608], David Huntsman[661], Ted R Hupp[602], Jason Huse[187], **Carolyn M Hutter#**[23], Christine A Iacobuzio-Donahue[481], Michael Ittmann[662,663,664], Joy C Jayaseelan[155], Stuart R Jefferys[234], Corbin D Jones[665], Steven JM Jones[629], Hartmut Juhl[666], Koo Jeong Kang[667], Beth Karlan[668], Katayoon Kasaian[629], Electron Kebebew[669,670], David Khoo[603], Hark Kyun Kim[671], Jaegil Kim[3], Tari A King[487,488,489], Viktoriya Korchina[155], Ritika Kundra[595,630], Jesper Lagergren[593,604], Phillip H Lai[638], Peter W Laird[265], Eric Lander[3], Michael S Lawrence[3,46,100], Alexander J Lazar[158,352], Xuan Le[672], Darlene Lee[673], Douglas A Levine[187,674], Lora Lewis[155], Tim Ley[675], Haiyan Irene Li[673], Pei Lin[3], W M Linehan[676], Eric Minwei Liu[110,111,187], Fei Fei Liu[368], Laurence B Lovat[237], Yiling Lu[376], Lisa Lype[677], Yussanne Ma[673], Shona MacRae[375], Dennis T Maglinte[638,678], Elaine R Mardis[131,641,679], Jeffrey Marks[396,680], Marco A Marra[673], Thomas J Matthew[11], Michael Mayo[673], Karen McCune[681], Michael D McLellan[130,131,138], Samuel R Meier[3], Shaowu Meng[234], Matthew Meyerson[3,6,48], Piotr A Mieczkowski[233], Tom Mikkelsen[682], Christopher A Miller[131], Gordon B Mills[683], Richard A Moore[673], Carl Morrison[394,684], Lisle E Mose[234], Catherine D Moser[657], Andrew J Mungall[673], Karen Mungall[673], David Mutch[685], Donna M Muzny[155], Jerome Myers[686], Yulia Newton[11], Michael S Noble[3], Peter O'Donnell[687], Brian Patrick O'Neill[688], Angelica Ochoa[187], Akinyemi I Ojesina[240,241,242], Joong-Won Park[689], Joel S Parker[690], Simon L Parsons[606], Harvey Pass[691], Alessandro Pastore[85], Chandra Sekhar Pedamallu[3,6,163], Nathan A Pennell[692], Charles M Perou[234,690,693], Gloria M Petersen[438], Nicholas Petrelli[694], Olga Potapova[695], Shaun R Preston[607], Sonia Puig[608], Janet S Rader[696], Suresh Ramalingam[697], W Kimryn Rathmell[698], Victor Reuter[481], Sheila M Reynolds[677], Matthew Ringel[699], Jeffrey Roach[700], Lewis R Roberts[657], A Gordon Robertson[673], Tom Roques[609], Mark A Rubin[183,197,198,199,200], Sara Sadeghi[673], Gordon Saksena[3], Charles Saller[701], Francisco Sanchez-Vega[595,630], Chris Sander[85,202,203], Grant Sanders[234], Dirk Schadendorf[77,702], Jacqueline E Schein[673], Heather K Schmidt[131], Nikolaus Schultz[630], Steven E Schumacher[3,204], Richard A Scolyer[409,413,419], Raja Seethala[703], Yasin Senbabaoglu[85], Troy Shelton[647], Yan Shi[234], Juliann Shih[3,163], Ilya Shmulevich[677], Craig Shriver[704], Sabina Signoretti[163,168,705], Janae V Simons[234], Samuel Singer[396,706], Payal Sipahimalani[673], Tara J Skelly[233], Karen Smith-McCune[681], Nicholas D Socci[85], Heidi J Sofia[23], Matthew G Soloway[690], Anil K Sood[707,708,709], Sharmila Sothi[610], Angela Tam[673], Donghui Tan[233], Roy Tarnuzzer[39], Nina Thiessen[673], R Houston Thompson[710], Leigh B Thorne[608], Ming Tsao[437,606], Olga Tucker[611], Richard Turkington[612], Christopher Umbricht[274,597,711], Timothy J Underwood[613], David J Van Den Berg[638], Erwin G Van Meir[712], Umadevi Veluvolu[233], Douglas Voet[3], Jiayin Wang[131,145,152], Linghua Wang[155], Zhining Wang[39], Paul Weinberger[713], John N Weinstein[375,376], Daniel J Weisenberger[638], Ian Welch[614], David A Wheeler[154,155], Dennis Wigle[714], Matthew D Wilkerson[233], Richard K Wilson[131,715], Boris Winterhoff[716], Maciej Wiznerowicz[717,718], Tina Wong[131,673], Winghing Wong[719], Liu Xi[155], Liming Yang[39], Christina Yau[636], Venkata D Yellapantula[138,157], **Jean C Zenklusen#**[39], Hailei Zhang[3], Hongxin Zhang[630] and Jiashan Zhang[39]

# Denotes **working group or project co-leader**

Author Affiliations

1. Wellcome Sanger Institute, Wellcome Genome Campus, Hinxton CB10 1SA, UK.
2. Department of Haematology, University of Cambridge, Cambridge CB2 2XY, UK.
3. Broad Institute of MIT and Harvard, Cambridge, MA 02142, USA.
4. Center for Cancer Research, Massachusetts General Hospital, Boston, MA 02129, USA.
5. Department of Pathology, Massachusetts General Hospital, Boston, MA 02115, USA.
6. Harvard Medical School, Boston, MA 02115, USA.
7. European Molecular Biology Laboratory, European Bioinformatics Institute (EMBL-EBI), Cambridge CB10 1SD, UK.
8. Genome Biology Unit, European Molecular Biology Laboratory (EMBL), Heidelberg 69117, Germany.
9. Computational Biology Program, Ontario Institute for Cancer Research, Toronto, ON M5G 0A3, Canada.
10. Department of Molecular Genetics, University of Toronto, Toronto, ON M5S 1A8, Canada.
11. Department of Biomolecular Engineering, University of California Santa Cruz, Santa Cruz, CA 95064, USA.
12. UC Santa Cruz Genomics Institute, University of California Santa Cruz, Santa Cruz, CA 95064, USA.
13. International Cancer Genome Consortium (ICGC)/ICGC Accelerating Research in Genomic Oncology (ARGO) Secretariat, Ontario Institute for Cancer Research, Toronto, ON M5G 0A3, Canada.
14. King Faisal Specialist Hospital and Research Centre, Al Maather, Riyadh 12713, Saudi Arabia.
15. DLR Project Management Agency, Bonn 53227, Germany.
16. Genome Canada, Ottawa, ON K2P 1P1, Canada.
17. Instituto Carlos Slim de la Salud, Mexico City, Mexico.
18. Federal Ministry of Education and Research, Berlin 10117, Germany.
19. Institut Gustave Roussy, Villejuif 94805, France.
20. Institut National du Cancer (INCA), Boulogne-Billancourt 92100, France.
21. The Wellcome Trust, London NW1 2BE, UK.
22. Prostate Cancer Canada, Toronto, ON M5C 1M1, Canada.
23. National Human Genome Research Institute, National Institutes of Health, Bethesda, MD 20892, USA.
24. Department of Biotechnology, Ministry of Science and Technology, Government of India, New Delhi, Delhi 110003, India.
25. Science Writer, Garrett Park, MD 20896, USA.
26. Cancer Research UK, London EC1V 4AD, UK.
27. Department of Biochemistry, College of Medicine, Ewha Womans University, Seoul 07895, South Korea.
28. Chinese Cancer Genome Consortium, Shenzhen 518083, China.
29. Department of Medical Oncology, Beijing Hospital, Beijing 100730, China.
30. Laboratory of Molecular Oncology, Key Laboratory of Carcinogenesis and Translational Research (Ministry of Education), Peking University Cancer Hospital and Institute, Beijing 100142, China.
31. National Cancer Center, Tokyo 104-0045, Japan.

32. German Cancer Aid, Bonn 53113, Germany.
33. Division of Cancer Genomics, National Cancer Center Research Institute, National Cancer Center, Tokyo 104-0045, Japan.
34. Laboratory of Molecular Medicine, Human Genome Center, Institute of Medical Science, University of Tokyo, Tokyo 108-8639, Japan.
35. Japan Agency for Medical Research and Development, Tokyo 100-0004, Japan.
36. Medical Oncology, University and Hospital Trust of Verona, Verona 37134, Italy.
37. University of Verona, Verona 37129, Italy.
38. BGI-Shenzhen, Shenzhen 518083, China.
39. National Cancer Institute, National Institutes of Health, Bethesda, MD 20892, USA.
40. Centre for Law and Genetics, University of Tasmania, Sandy Bay Campus, Hobart, TAS 7001, Australia.
41. Centre of Genomics and Policy, McGill University and Génome Québec Innovation Centre, Montreal, QC H3A 1A4, Canada.
42. Heidelberg Academy of Sciences and Humanities, Heidelberg 69120, Germany.
43. CAPHRI Research School, Maastricht University, Maastricht, ER 6200MD, The Netherlands.
44. Genome Informatics Program, Ontario Institute for Cancer Research, Toronto, ON M5G 0A3, Canada.
45. Barcelona Supercomputing Center (BSC), Barcelona 08034, Spain.
46. Laboratory for Medical Science Mathematics, RIKEN Center for Integrative Medical Sciences, Yokohama 230-0045, Japan.
47. RIKEN Center for Integrative Medical Sciences, Yokohama 230-0045, Japan.
48. Department of Medical Oncology, Dana-Farber Cancer Institute, Boston, MA 02215, USA.
49. Biomedical Engineering, Oregon Health and Science University, Portland, OR 97239, USA.
50. Division of Theoretical Bioinformatics, German Cancer Research Center (DKFZ), Heidelberg 69120, Germany.
51. Heidelberg Center for Personalized Oncology (DKFZ-HIPO), German Cancer Research Center, Heidelberg 69120, Germany.
52. Institute of Pharmacy and Molecular Biotechnology and BioQuant, Heidelberg University, Heidelberg 69120, Germany.
53. University of California San Diego, San Diego, CA 92093, USA.
54. PDXen Biosystems Inc, Seoul 4900, South Korea.
55. Electronics and Telecommunications Research Institute, Daejeon 34129, South Korea.
56. Seven Bridges Genomics, Charlestown, MA 02129, USA.
57. Annai Systems, Inc, Carlsbad, CA 92013, USA.
58. Department of Biomedical Data Science, Stanford University School of Medicine, Stanford, CA 94305, USA.
59. Department of Genetics, Stanford University School of Medicine, Stanford, CA 94305, USA.
60. The Francis Crick Institute, London NW1 1AT, UK.
61. University of Leuven, Leuven B-3000, Belgium.
62. The Hospital for Sick Children, Toronto, ON M5G 0A4, Canada.
63. Heidelberg University, Heidelberg 69120, Germany.
64. New BIH Digital Health Center, Berlin Institute of Health (BIH) and Charité - Universitätsmedizin Berlin, Berlin 10117, Germany.
65. Department of Biochemistry and Molecular Medicine, University of Montreal, Montreal, QC

H3C 3J7, Canada.
66. CIBIO/InBIO - Research Center in Biodiversity and Genetic Resources, Universidade do Porto, Vairão 4485-601, Portugal.
67. Department Biochemistry and Molecular Biomedicine, University of Barcelona, Barcelona 08028, Spain.
68. Department of Medicine, Section of Hematology/Oncology, University of Chicago, Chicago, IL 60637, USA.
69. Department of Medicine, Division of Biomedical Informatics, UC San Diego School of Medicine, San Diego, CA 92093, USA.
70. UC San Diego Moores Cancer Center, San Diego, CA 92093, USA.
71. Children's Hospital of Philadelphia, Philadelphia, PA 19146, USA.
72. Massachusetts General Hospital Center for Cancer Research, Charlestown, MA 02129, USA.
73. University of Melbourne Centre for Cancer Research, Melbourne, VIC 3010, Australia.
74. Syntekabio Inc, Daejon 34025, South Korea.
75. AbbVie, North Chicago, IL 60064, USA.
76. Genomics Research Program, Ontario Institute for Cancer Research, Toronto, ON M5G 0A3, Canada.
77. German Cancer Consortium (DKTK), Heidelberg 69120, Germany.
78. Heidelberg Center for Personalized Oncology (DKFZ-HIPO), German Cancer Research Center (DKFZ), Heidelberg 69120, Germany.
79. National Center for Tumor Diseases (NCT) Heidelberg, Heidelberg 69120, Germany.
80. Department of Pediatric Immunology, Hematology and Oncology, University Hospital, Heidelberg 69120, Germany.
81. German Cancer Research Center (DKFZ), Heidelberg 69120, Germany.
82. Heidelberg Institute for Stem Cell Technology and Experimental Medicine (HI-STEM), Heidelberg 69120, Germany.
83. Institute of Medical Science, University of Tokyo, Tokyo 108-8639, Japan.
84. Genome Integration Data Center, Syntekabio, Inc, Daejon, 34025, South Korea.
85. Computational Biology Center, Memorial Sloan Kettering Cancer Center, New York, NY 10065, USA.
86. Department of Biology, ETH Zurich, Zürich 8093, Switzerland.
87. Department of Computer Science, ETH Zurich, Zurich 8092, Switzerland.
88. SIB Swiss Institute of Bioinformatics, Lausanne 1015, Switzerland.
89. University Hospital Zurich, Zurich 8091, Switzerland.
90. Health Sciences Department of Biomedical Informatics, University of California San Diego, La Jolla, CA 92093, USA.
91. Department of Health Sciences and Technology, Sungkyunkwan University School of Medicine, Seoul 06351, South Korea.
92. Samsung Genome Institute, Seoul 06351, South Korea.
93. Functional and Structural Genomics, German Cancer Research Center (DKFZ), Heidelberg 69120, Germany.
94. Leidos Biomedical Research, Inc, McLean, VA 22102, USA.
95. Center for Biomolecular Science and Engineering, University of California Santa Cruz, Santa Cruz, CA 95064, USA.
96. Sage Bionetworks, Seattle, WA 98109, USA.

97. Department of Cell and Systems Biology, University of Toronto, Toronto, ON M5S 3G5, Canada.
98. Department of Radiation Oncology, University of California San Francisco, San Francisco, CA 94518, USA.
99. CSRA Incorporated, Fairfax, VA 22042, USA.
100. Massachusetts General Hospital, Boston, MA 02114, USA.
101. Weill Cornell Medical College, New York, NY 10065, USA.
102. Bioinformatics and Omics Data Analytics, German Cancer Research Center (DKFZ), Heidelberg 69120, Germany.
103. Institute for Genomics and Systems Biology, University of Chicago, Chicago, IL 60637, USA.
104. Computational Biology Program, School of Medicine, Oregon Health and Science University, Portland, OR 97239, USA.
105. Institució Catalana de Recerca i Estudis Avançats (ICREA), Barcelona 08010, Spain.
106. Department of Clinical and Molecular Medicine, Faculty of Medicine and Health Sciences, Norwegian University of Science and Technology, Trondheim 7030, Norway.
107. Department of Urology, Charité Universitätsmedizin Berlin, Berlin 10117, Germany.
108. Finsen Laboratory and Biotech Research and Innovation Centre (BRIC), University of Copenhagen, Copenhagen 2200, Denmark.
109. Department of Biological Oceanography, Leibniz Institute of Baltic Sea Research, Rostock 18119, Germany.
110. Department of Physiology and Biophysics, Weill Cornell Medicine, New York, NY 10065, USA.
111. Institute for Computational Biomedicine, Weill Cornell Medicine, New York, NY 10021, USA.
112. Division of Applied Bioinformatics, German Cancer Research Center (DKFZ), Heidelberg 69120, Germany.
113. Department of Computer Science, Yale University, New Haven, CT 06520, USA.
114. Department of Molecular Biophysics and Biochemistry, Yale University, New Haven, CT 06520, USA.
115. Program in Computational Biology and Bioinformatics, Yale University, New Haven, CT 06520, USA.
116. Department of Internal Medicine, Stanford University, Stanford, CA 94305, USA.
117. Department of Molecular Medicine (MOMA), Aarhus University Hospital, Aarhus N 8200, Denmark.
118. Clinical Bioinformatics, Swiss Institute of Bioinformatics, Geneva 1202, Switzerland.
119. Institute for Pathology and Molecular Pathology, University Hospital Zurich, Zurich 8091, Switzerland.
120. Institute of Molecular Life Sciences, University of Zurich, Zurich 8057, Switzerland.
121. MIT Computer Science and Artificial Intelligence Laboratory, Massachusetts Institute of Technology, Cambridge, MA 02139, USA.
122. Controlled Department and Institution, New York, NY 10065, USA.
123. Englander Institute for Precision Medicine, Weill Cornell Medicine, New York, NY 10065, USA.
124. Bioinformatics Research Centre (BiRC), Aarhus University, Aarhus 8000, Denmark.
125. Department of Medical Biophysics, University of Toronto, Toronto, ON M5S 1A8, Canada.

126. Institute of Molecular Life Sciences and Swiss Institute of Bioinformatics, University of Zurich, Zurich 8057, Switzerland.
127. CNAG-CRG, Centre for Genomic Regulation (CRG), Barcelona Institute of Science and Technology (BIST), Barcelona 08028, Spain.
128. Universitat Pompeu Fabra (UPF), Barcelona 08003, Spain.
129. Office of Cancer Genomics, National Cancer Institute, National Institutes of Health, Bethesda, MD 20892, USA.
130. Alvin J. Siteman Cancer Center, Washington University School of Medicine, St Louis, MO 63110, USA.
131. The McDonnell Genome Institute at Washington University, St Louis, MO 63108, USA.
132. Computer Network Information Center, Chinese Academy of Sciences, Beijing 100190, China.
133. Center for Digital Health, Berlin Institute of Health and Charitè - Universitätsmedizin Berlin, Berlin 10117, Germany.
134. Department of Human Genetics, University of California Los Angeles, Los Angeles, CA 90095, USA.
135. Department of Pharmacology, University of Toronto, Toronto, ON M5S 1A8, Canada.
136. University of Texas MD Anderson Cancer Center, Houston, TX 77030, USA.
137. Department of Genetics, Informatics Institute, University of Alabama at Birmingham, Birmingham, AL 35294, USA.
138. Department of Medicine and Department of Genetics, Washington University School of Medicine, St. Louis, St Louis, MO 63110, USA.
139. Centre for Genomic Regulation (CRG), The Barcelona Institute of Science and Technology, Barcelona 08003, Spain.
140. Department of Bioinformatics and Computational Biology, The University of Texas MD Anderson Cancer Center, Houston, TX 77030, USA.
141. Department of Urologic Sciences, University of British Columbia, Vancouver, BC V5Z 1M9, Canada.
142. Vancouver Prostate Centre, Vancouver, BC V6H 3Z6, Canada.
143. Division of Life Science and Applied Genomics Center, Hong Kong University of Science and Technology, Clear Water Bay, Hong Kong, China.
144. Geneplus-Shenzhen, Shenzhen 518122, China.
145. School of Computer Science and Technology, Xi'an Jiaotong University, Xi'an 710048, China.
146. Biobyte solutions GmbH, Heidelberg 69126, Germany.
147. Division of Oncology, Washington University School of Medicine, St Louis, MO 63110, USA.
148. Institute of Medical Genetics and Applied Genomics, University of Tübingen, Tübingen 72076, Germany.
149. Indiana University, Bloomington, IN 47405, USA.
150. Simon Fraser University, Burnaby, BC V5A 1S6, Canada.
151. Department of Computer Science, University of Toronto, Toronto, ON M5S 1A8, Canada.
152. School of Electronic and Information Engineering, Xi'an Jiaotong University, Xi'an 710048, China.
153. Department of Genetics, Washington University School of Medicine, St Louis, MO 63110, USA.

154. Department of Molecular and Human Genetics, Baylor College of Medicine, Houston, TX 77030, USA.
155. Human Genome Sequencing Center, Baylor College of Medicine, Houston, TX 77030, USA.
156. The First Affiliated Hospital, Xi'an Jiaotong University, Xi'an 710048, China.
157. Department of Epidemiology and Biostatistics, Memorial Sloan Kettering Cancer Center, New York, NY 10065, USA.
158. Department of Genomic Medicine, The University of Texas MD Anderson Cancer Center, Houston, TX 77030, USA.
159. Quantitative and Computational Biosciences Graduate Program, Baylor College of Medicine, Houston, TX 77030, USA.
160. The Jackson Laboratory for Genomic Medicine, Farmington, CT 06032, USA.
161. Dana-Farber/Boston Children's Cancer and Blood Disorders Center, Boston, MA 02215, USA.
162. Department of Pediatrics, Harvard Medical School, Boston, MA 02115, USA.
163. Department of Medical Oncology, Dana-Farber Cancer Institute, Boston, MA 02115, USA.
164. Department of Mathematics, Aarhus University, Aarhus 8000, Denmark.
165. Center for Biological Sequence Analysis, Department of Bio and Health Informatics, Technical University of Denmark, Lyngby 2800, Denmark.
166. Novo Nordisk Foundation Center for Protein Research, University of Copenhagen, Copenhagen 2200, Denmark.
167. Department for BioMedical Research, University of Bern, Bern 3008, Switzerland.
168. Department of Medical Oncology, Inselspital, University Hospital and University of Bern, Bern 3010, Switzerland.
169. Graduate School for Cellular and Biomedical Sciences, University of Bern, Bern 3012, Switzerland.
170. Department of Urology, Icahn School of Medicine at Mount Sinai, New York, NY 10029, USA.
171. Faculty of Biosciences, Heidelberg University, Heidelberg 69120, Germany.
172. Korea Advanced Institute of Science and Technology, Daejeon 34141, South Korea.
173. Institute for Research in Biomedicine (IRB Barcelona), The Barcelona Institute of Science and Technology, Barcelona 8003, Spain.
174. Research Program on Biomedical Informatics, Universitat Pompeu Fabra, Barcelona 08002, Spain.
175. Department of Cell and Molecular Biology, Science for Life Laboratory, Uppsala University, Uppsala SE-75124, Sweden.
176. Queensland Centre for Medical Genomics, Institute for Molecular Bioscience, University of Queensland, St Lucia, Brisbane, QLD 4072, Australia.
177. University of Milano Bicocca, Monza 20052, Italy.
178. Sir Peter MacCallum Department of Oncology, Peter MacCallum Cancer Centre, University of Melbourne, Melbourne, VIC 3000, Australia.
179. Center for Precision Health, School of Biomedical Informatics, The University of Texas Health Science Center, Houston, TX 77030, USA.
180. The Donnelly Centre, University of Toronto, Toronto, ON M5S 3E1, Canada.
181. Health Data Science Unit, University Clinics, Heidelberg 69120, Germany.
182. Technical University of Denmark, Lyngby 2800, Denmark.

183. Department for Biomedical Research, University of Bern, Bern 3008, Switzerland.
184. Research Core Center, National Cancer Centre Korea, Goyang-si 410-769, South Korea.
185. Institute of Computer Science, Polish Academy of Sciences, Warsawa 01-248, Poland.
186. Harvard University, Cambridge, MA 02138, USA.
187. Memorial Sloan Kettering Cancer Center, New York, NY 10065, USA.
188. Department of Information Technology, Ghent University, Ghent B-9000, Belgium.
189. Department of Plant Biotechnology and Bioinformatics, Ghent University, Ghent B-9000, Belgium.
190. Yale School of Medicine, Yale University, New Haven, CT 06520, USA.
191. Institute for Research in Biomedicine (IRB Barcelona), Barcelona 08028, Spain.
192. Division of Hematology-Oncology, Samsung Medical Center, Sungkyunkwan University School of Medicine, Seoul 06351, South Korea.
193. Samsung Advanced Institute for Health Sciences and Technology, Sungkyunkwan University School of Medicine, Seoul 06351, South Korea.
194. Cheonan Industry-Academic Collaboration Foundation, Sangmyung University, Cheonan 31066, South Korea.
195. Spanish National Cancer Research Centre, Madrid 28029, Spain.
196. Department of Computer Science, Princeton University, Princeton, NJ 08540, USA.
197. Bern Center for Precision Medicine, University Hospital of Bern, University of Bern, Bern 3008, Switzerland.
198. Englander Institute for Precision Medicine, Weill Cornell Medicine and New York Presbyterian Hospital, New York, NY 10021, USA.
199. Meyer Cancer Center, Weill Cornell Medicine, New York, NY 10065, USA.
200. Pathology and Laboratory, Weill Cornell Medical College, New York, NY 10021, USA.
201. Vall d'Hebron Institute of Oncology: VHIO, Barcelona 08035, Spain.
202. cBio Center, Dana-Farber Cancer Institute, Harvard Medical School, Boston, MA 02115, USA.
203. Department of Cell Biology, Harvard Medical School, Boston, MA 02115, USA.
204. Department of Cancer Biology, Dana-Farber Cancer Institute, Boston, MA 02215, USA.
205. Dana-Farber Cancer Institute, Boston, MA 02215, USA.
206. cBio Center, Dana-Farber Cancer Institute, Harvard Medical School, Boston, MA 02215, USA.
207. Core Research for Evolutional Science and Technology (CREST), JST, Tokyo 102-8666, Japan.
208. Department of Biological Sciences, Laboratory for Medical Science Mathematics, Graduate School of Science, University of Tokyo, Yokohama 230-0045, Japan.
209. Department of Medical Science Mathematics, Medical Research Institute, Tokyo Medical and Dental University (TMDU), Tokyo 113-8510, Japan.
210. Department of Oncology-Pathology, Science for Life Laboratory, Karolinska Institutet, Stockholm 17121, Sweden.
211. Department of Gene Technology, Tallinn University of Technology, Tallinn 12616, Estonia.
212. Genetics and Genome Biology Program, SickKids Research Institute, The Hospital for Sick Children, Toronto, ON M5G 1X8, Canada.
213. Department of Information Technology, Ghent University, Interuniversitair Micro-Electronica Centrum (IMEC), Ghent B-9000, Belgium.

214. Department of Immunology, Genetics and Pathology, Science for Life Laboratory, Uppsala University, Uppsala SE-75108, Sweden.
215. Department of Medical Informatics and Clinical Epidemiology, Division of Bioinformatics and Computational Biology, OHSU Knight Cancer Institute, Oregon Health and Science University, Portland, OR 97239, USA.
216. Department of Medicine and Therapeutics, The Chinese University of Hong Kong, Shatin, NT, Hong Kong, China.
217. The University of Texas Health Science Center at Houston, Houston, TX 77030, USA.
218. Department of Biomedical Informatics, College of Medicine, The Ohio State University, Columbus, OH 43210, USA.
219. The Ohio State University Comprehensive Cancer Center (OSUCCC – James), Columbus, OH 43210, USA.
220. The University of Texas School of Biomedical Informatics (SBMI) at Houston, Houston, TX 77030, USA.
221. Department of Biochemistry and Molecular Genetics, Feinberg School of Medicine, Northwestern University, Chicago, IL 60637, USA.
222. Wolfson Wohl Cancer Research Centre, Institute of Cancer Sciences, University of Glasgow, Glasgow G61 1BD, UK.
223. Department of Biomedical Informatics, Harvard Medical School, Boston, MA 02115, USA.
224. Department of Chemistry, Centre for Molecular Science Informatics, University of Cambridge, Cambridge CB2 1EW, UK.
225. Ludwig Center at Harvard Medical School, Boston, MA 02115, USA.
226. Physics Division, Optimization and Systems Biology Lab, Massachusetts General Hospital, Boston, MA 02114, USA.
227. Department of Medicine, Baylor College of Medicine, Houston, TX 77030, USA.
228. Computational and Systems Biology, Genome Institute of Singapore, Singapore 138672, Singapore.
229. School of Computing, National University of Singapore, Singapore 117417, Singapore.
230. The Azrieli Faculty of Medicine, Bar-Ilan University, Safed 13195, Israel.
231. National Cancer Centre Singapore, Singapore 169610, Singapore.
232. Peking University, Beijing 100871, China.
233. Department of Genetics, University of North Carolina at Chapel Hill, Chapel Hill, NC 27599, USA.
234. Lineberger Comprehensive Cancer Center, University of North Carolina at Chapel Hill, Chapel Hill, NC 27599, USA.
235. China National GeneBank-Shenzhen, Shenzhen 518083, China.
236. Berlin Institute for Medical Systems Biology, Max Delbrück Center for Molecular Medicine, Berlin 13125, Germany.
237. University College London, London WC1E 6BT, UK.
238. School of Life Sciences, Peking University, Beijing 100180, China.
239. Genome Institute of Singapore, Singapore 138672, Singapore.
240. Department of Epidemiology, University of Alabama at Birmingham, Birmingham, AL 35294, USA.
241. HudsonAlpha Institute for Biotechnology, Huntsville, AL 35806, USA.
242. O'Neal Comprehensive Cancer Center, University of Alabama at Birmingham, Birmingham,

AL 35294, USA.
243. Department of Biosciences and Nutrition, Karolinska Institutet, Stockholm 14183, Sweden.
244. German Cancer Consortium (DKTK), Partner site Berlin.
245. Human Genetics, University of Kiel, Kiel 24118, Germany.
246. Institute of Human Genetics, Ulm University and Ulm University Medical Center, Ulm 89081, Germany.
247. Computational and Systems Biology Program, Memorial Sloan Kettering Cancer Center, New York, NY 10065, USA.
248. Korea University, Seoul 02481, South Korea.
249. Division of Computational Genomics and Systems Genetics, German Cancer Research Center (DKFZ), Heidelberg 69120, Germany.
250. Cancer Science Institute of Singapore, National University of Singapore, Singapore 169609, Singapore.
251. Programme in Cancer and Stem Cell Biology, Duke-NUS Medical School, Singapore 169857, Singapore.
252. SingHealth, Duke-NUS Institute of Precision Medicine, National Heart Centre Singapore, Singapore 169609, Singapore.
253. Institute of Molecular and Cell Biology, Singapore 169609, Singapore.
254. Laboratory of Cancer Epigenome, Division of Medical Science, National Cancer Centre Singapore, Singapore 169610, Singapore.
255. BIOPIC, ICG and College of Life Sciences, Peking University, Beijing 100871, China.
256. Genome Science Division, Research Center for Advanced Science and Technology, University of Tokyo, Tokyo 153-8904, Japan.
257. Center for Bioinformatics and Functional Genomics, Cedars-Sinai Medical Center, Los Angeles, CA 90048, USA.
258. Department of Biomedical Sciences, Cedars-Sinai Medical Center, Los Angeles, CA 90048, USA.
259. The Hebrew University Faculty of Medicine, Jerusalem 91120, Israel.
260. Department of Computer Science, Bioinformatics Group, University of Leipzig, Leipzig 04109, Germany.
261. Interdisciplinary Center for Bioinformatics, University of Leipzig, Leipzig 04109, Germany.
262. German Cancer Consortium (DKTK), German Cancer Research Center (DKFZ), Heidelberg 69120, Germany.
263. Computational Biology, Leibniz Institute on Aging - Fritz Lipmann Institute (FLI), Jena 07745, Germany.
264. Transcriptome Bioinformatics, LIFE Research Center for Civilization Diseases, University of Leipzig, Leipzig 04109, Germany.
265. Center for Epigenetics, Van Andel Research Institute, Grand Rapids, MI 49503, USA.
266. Institut d'Investigacions Biomèdiques August Pi i Sunyer (IDIBAPS), Barcelona 08036, Spain.
267. Research Center for Advanced Science and Technology, University of Tokyo, Tokyo 108-8639, Japan.
268. Cancer Epigenomics, German Cancer Research Center (DKFZ), Heidelberg 69120, Germany.
269. Van Andel Research Institute, Grand Rapids, MI 49503, USA.
270. Centre for Research in Molecular Medicine and Chronic Diseases (CiMUS), Universidade de Santiago de Compostela, Santiago de Compostela 15706, Spain.

271. Department of Zoology, Genetics and Physical Anthropology, (CiMUS), Universidade de Santiago de Compostela, Santiago de Compostela 15706, Spain.
272. The Biomedical Research Centre (CINBIO), Universidade de Vigo, Vigo 36310, Spain.
273. Department of Veterinary Medicine, Transmissible Cancer Group, University of Cambridge, Cambridge CB3 0ES, UK.
274. Department of Pathology, Johns Hopkins University School of Medicine, Baltimore, MD 21205, USA.
275. McKusick-Nathans Institute of Genetic Medicine, Sidney Kimmel Comprehensive Cancer Center at Johns Hopkins University School of Medicine, Baltimore, MD 21287, USA.
276. Foundation Medicine, Inc, Cambridge, MA 02141, USA.
277. Department of Biochemistry, Microbiology and Immunology, Faculty of Medicine, University of Ottawa, Ottawa, ON K1H 8M5, Canada.
278. Li Ka Shing Centre, Cancer Research UK Cambridge Institute, University of Cambridge, Cambridge CB2 0RE, UK.
279. University of Cambridge, Cambridge CB2 1TN, UK.
280. Quantitative Genomics Laboratories (qGenomics), Barcelona 08950, Spain.
281. Genome Integrity and Structural Biology Laboratory, National Institute of Environmental Health Sciences (NIEHS), Durham, NC 27709, USA.
282. Brandeis University, Waltham, MA 02254, USA.
283. Institute for Computational Biomedicine, Weill Cornell Medical College, New York, NY 10065, USA.
284. New York Genome Center, New York, NY 10013, USA.
285. Hopp Children's Cancer Center (KiTZ), Heidelberg 69120, Germany.
286. Pediatric Glioma Research Group, German Cancer Research Center (DKFZ), Heidelberg 69120, Germany.
287. A.A. Kharkevich Institute of Information Transmission Problems, Moscow 127051, Russia.
288. Oncology and Immunology, Dmitry Rogachev National Research Center of Pediatric Hematology, Moscow 117997, Russia.
289. Skolkovo Institute of Science and Technology, Moscow 121205, Russia.
290. Integrative Bioinformatics Support Group, National Institute of Environmental Health Sciences (NIEHS), Durham, NC 27709, USA.
291. Center For Medical Innovation, Seoul National University Hospital, Seoul 03080, South Korea.
292. Department of Internal Medicine, Seoul National University Hospital, Seoul 03080, South Korea.
293. Division of Genetics and Genomics, Boston Children's Hospital, Harvard Medical School, Boston, MA 02115, USA.
294. School of Medicine/School of Mathematics and Statistics, University of St Andrews, St Andrews, Fife KY16 9SS, UK.
295. Department of Genetics and Computational Biology, QIMR Berghofer Medical Research Institute, Brisbane, QLD 4006, Australia.
296. Institute for Molecular Bioscience, University of Queensland, St Lucia, Brisbane, QLD 4072, Australia.
297. School of Molecular Biosciences and Center for Reproductive Biology, Washington State University, Pullman, WA 99164, USA.

298. Cancer Research Institute, Beth Israel Deaconess Medical Center, Boston, MA 02215, USA.
299. Ben May Department for Cancer Research and Department of Human Genetics, University of Chicago, Chicago, IL 60637, USA.
300. Tri-Institutional PhD Program in Computational Biology and Medicine, Weill Cornell Medicine, New York, NY 10065, USA.
301. Department of Biostatistics and Computational Biology, Dana-Farber Cancer Institute and Harvard Medical School, Boston, MA 02215, USA.
302. Department of Cellular and Molecular Medicine and Department of Bioengineering, University of California San Diego, La Jolla, CA 92093, USA.
303. Department of Cellular and Molecular Medicine and Department of Bioengineering, University of California, San Diego, La Jolla, CA 92093, USA.
304. Centre for Computational Biology, Duke-NUS Medical School, Singapore 169857, Singapore.
305. Department of Computer Science, University of Helsinki, Helsinki 00014, Finland.
306. Institute of Biotechnology, University of Helsinki, Helsinki 00014, Finland.
307. Organismal and Evolutionary Biology Research Programme, University of Helsinki, Helsinki 00014, Finland.
308. Programme in Cancer and Stem Cell Biology, Centre for Computational Biology, Duke-NUS Medical School, Singapore 169857, Singapore.
309. Academic Department of Medical Genetics, University of Cambridge, Addenbrooke's Hospital, Cambridge CB2 0QQ, UK.
310. MRC Cancer Unit, University of Cambridge, Cambridge CB2 0XZ, UK.
311. The University of Cambridge School of Clinical Medicine, Cambridge CB2 0SP, UK.
312. National Centre for Biological Sciences, Tata Institute of Fundamental Research, Bangalore 560065, India.
313. Department of Applied Mathematics and Theoretical Physics, Centre for Mathematical Sciences, University of Cambridge, Cambridge CB3 0WA, UK.
314. Department of Statistics, Columbia University, New York, NY 10027, USA.
315. Duke-NUS Medical School, Singapore 169857, Singapore.
316. Faculty of Medicine and Health Technology, Tampere University and Tays Cancer Center, Tampere University Hospital, Tampere Fl-33014, Finland.
317. Bakar Computational Health Sciences Institute and Department of Pediatrics, University of California, San Francisco, CA 94158-2549, USA.
318. Division of Cancer Epidemiology and Genetics, National Cancer Institute, National Institutes of Health, Bethesda, MD 20892, USA.
319. Department of Biostatistics, Bloomberg School of Public Health, Johns Hopkins University, Baltimore, MD 21205, USA.
320. Department of Oncology, Sidney Kimmel Comprehensive Cancer Center at Johns Hopkins University School of Medicine, Baltimore, MD 21287, USA.
321. Integrated Graduate Program in Physical and Engineering Biology, Yale University, New Haven, CT 06520, USA.
322. Department of Computational Biology, University of Lausanne, Lausanne 1015, Switzerland.
323. Department of Genetic Medicine and Development, University of Geneva Medical School, Geneva CH 1211, Switzerland.

324. Swiss Institute of Bioinformatics, University of Geneva, Geneva CH 1211, Switzerland.
325. Independent Consultant, Wellesley 02481, USA.
326. Department of Oncology, Centre for Cancer Genetic Epidemiology, University of Cambridge, Cambridge CB1 8RN, UK.
327. Department of Public Health and Primary Care, Centre for Cancer Genetic Epidemiology, University of Cambridge, Cambridge CB1 8RN, UK.
328. CIBER Epidemiología y Salud Pública (CIBERESP), Madrid 28029, Spain.
329. Research Group on Statistics, Econometrics and Health (GRECS), UdG, Barcelona 8041, Spain.
330. Oxford Nanopore Technologies, New York, NY 10013, USA.
331. Department of Medical Genetics, College of Medicine, Hallym University, Chuncheon 24252, South Korea.
332. Department of Experimental and Health Sciences, Institute of Evolutionary Biology (UPF-CSIC), Universitat Pompeu Fabra, Barcelona 08003, Spain.
333. Icahn School of Medicine at Mount Sinai, New York, NY 10029, USA.
334. Laboratory of Translational Genomics, Division of Cancer Epidemiology and Genetics, National Cancer Institute, National Institutes of Health, Bethesda, MD 20892, USA.
335. Institut Català de Paleontologia Miquel Crusafont, Universitat Autònoma de Barcelona, Barcelona 08193, Spain.
336. Applications Department, Oxford Nanopore Technologies, Oxford OX4 4DQ, UK.
337. Department of Genetics, Microbiology and Statistics, University of Barcelona, IRSJD, IBUB, Barcelona 08028, Spain.
338. Department of Ophthalmology and Ocular Genomics Institute, Massachusetts Eye and Ear, Harvard Medical School, Boston, MA 02114, USA.
339. Department of Medical and Clinical Genetics, Genome-Scale Biology Research Program, University of Helsinki, Helsinki 00100, Finland.
340. Big Data Institute, Li Ka Shing Centre, University of Oxford, Oxford OX3 7LF, UK.
341. Oxford NIHR Biomedical Research Centre, University of Oxford, Oxford OX4 2PG, UK.
342. School of Electronic Information and Communications, Huazhong University of Science and Technology, Wuhan 430074, China.
343. Bioinformatics Unit, Spanish National Cancer Research Centre (CNIO), Madrid 28029, Spain.
344. Vector Institute, Toronto, ON M5G 0A3, Canada.
345. Cancer Division, Garvan Institute of Medical Research, Kinghorn Cancer Centre, University of New South Wales (UNSW Sydney), Sydney, NSW 2010, Australia.
346. South Western Sydney Clinical School, Faculty of Medicine, University of New South Wales (UNSW Sydney), Liverpool, NSW 2170, Australia.
347. West of Scotland Pancreatic Unit, Glasgow Royal Infirmary, Glasgow G31 2ER, UK.
348. Centre for Cancer Research, Victorian Comprehensive Cancer Centre, University of Melbourne, Melbourne, VIC 3010, Australia.
349. MRC Human Genetics Unit, MRC IGMM, University of Edinburgh, Edinburgh EH4 2XU, UK.
350. Department of Biology, Bioinformatics Group, Division of Molecular Biology, Faculty of Science, University of Zagreb, Zagreb 10000, Croatia.
351. Department of Bioinformatics, Division of Cancer Genomics, National Cancer Center Research Institute, Tokyo 104-0045, Japan.

352. Department of Pathology, The University of Texas MD Anderson Cancer Center, Houston, TX 77030, USA.
353. University of Glasgow, Glasgow G61 1BD, UK.
354. Oregon Health and Science University, Portland, OR 97239, USA.
355. MRC-University of Glasgow Centre for Virus Research, Glasgow G61 1QH, UK.
356. School of Computing Science, University of Glasgow, Glasgow G12 8RZ, UK.
357. Molecular and Medical Genetics, OHSU Knight Cancer Institute, Oregon Health and Science University, Portland, OR 97239, USA.
358. Department of Surgery, University of Melbourne, Parkville, VIC 3010, Australia.
359. The Murdoch Children's Research Institute, Royal Children's Hospital, Parkville, VIC 3052, Australia.
360. Walter and Eliza Hall Institute, Parkville, VIC 3052, Australia.
361. University of Cologne, Cologne 50931, Germany.
362. The Edward S. Rogers Sr. Department of Electrical and Computer Engineering, University of Toronto, Toronto, ON M5S 3G4, Canada.
363. University of Ljubljana, Ljubljana 1000, Slovenia.
364. Department of Public Health Sciences, University of Chicago, Chicago, IL 60637, USA.
365. Research Institute, NorthShore University HealthSystem, Evanston, IL 60201, USA.
366. Department of Statistics, University of California Santa Cruz, Santa Cruz, CA 95064, USA.
367. Cambridge University Hospitals NHS Foundation Trust, Cambridge CB2 0QQ, UK.
368. University of Toronto, Toronto, ON M5G 2M9, Canada.
369. Department of Computer Science, Carleton College, Northfield, MN 55057, USA.
370. Center for Psychiatric Genetics, NorthShore University HealthSystem, Evanston, IL 60201, USA.
371. Argmix Consulting, North Vancouver, BC V7M 2J5, Canada.
372. Department of Biostatistics, The University of Texas MD Anderson Cancer Center, Houston, TX 77030, USA.
373. Department of Biostatistics, University of North Carolina at Chapel Hill, Chapel Hill, NC 27599, USA.
374. Howard Hughes Medical Institute, University of California Santa Cruz, Santa Cruz, CA 95064, USA.
375. Cancer Unit, MRC University of Cambridge, Cambridge CB2 0XZ, UK.
376. Department of Bioinformatics and Computational Biology and Department of Systems Biology, The University of Texas MD Anderson Cancer Center, Houston, TX 77030, USA.
377. Department of Health Sciences, Faculty of Medical Sciences, Kyushu University, Fukuoka 812-8582, Japan.
378. Department of Applied Mathematics and Statistics, Johns Hopkins University, Baltimore, MD 21218, USA.
379. Bioinformatics Core Facility, University Medical Center Hamburg, Hamburg 20246, Germany.
380. Heinrich Pette Institute, Leibniz Institute for Experimental Virology, Hamburg 20251, Germany.
381. Earlham Institute, Norwich NR4 7UZ, UK.
382. Norwich Medical School, University of East Anglia, Norwich NR4 7TJ, UK.
383. The Institute of Cancer Research, London SW7 3RP, UK.

384. University of East Anglia, Norwich NR4 7TJ, UK.
385. German Center for Infection Research (DZIF), Partner Site Hamburg-Borstel-Lübeck-Riems, Hamburg, Germany.
386. Division of Molecular Genetics, German Cancer Research Center (DKFZ), Heidelberg 69120, Germany.
387. Peter MacCallum Cancer Centre, University of Melbourne, Melbourne, VIC 3000, Australia.
388. QIMR Berghofer Medical Research Institute, Brisbane, QLD 4006, Australia.
389. Victorian Institute of Forensic Medicine, Southbank, VIC 3006, Australia.
390. University of Pennsylvania, Philadelphia, PA 19104, USA.
391. Department of Gynaecological Oncology, Westmead Hospital, Sydney, NSW 2145, Australia.
392. Genetics and Molecular Pathology, SA Pathology, Adelaide, SA 5000, Australia.
393. Centre for Cancer Research, Westmead Institute for Medical Research, University of Sydney, Sydney, NSW 2145, Australia.
394. Department of Clinical Pathology, University of Melbourne, Melbourne, VIC 3052, Australia.
395. Faculty of Medicine and Health, University of Sydney, Sydney, NSW 2145, Australia.
396. Department of Surgery, Pancreas Institute, University and Hospital Trust of Verona, Verona 37134, Italy.
397. Department of Surgery, Princess Alexandra Hospital, Brisbane, QLD 4102, Australia.
398. Surgical Oncology Group, Diamantina Institute, University of Queensland, Brisbane, QLD 4102, Australia.
399. Department of Diagnostics and Public Health, University and Hospital Trust of Verona, Verona 37134, Italy.
400. ARC-Net Centre for Applied Research on Cancer, University and Hospital Trust of Verona, Verona 37134, Italy.
401. Illawarra Shoalhaven Local Health District L3 Illawarra Cancer Care Centre, Wollongong Hospital, Wollongong, NSW 2500, Australia.
402. School of Biological Sciences, University of Auckland, Auckland 1010, New Zealand.
403. Department of Pathology and Diagnostics, University and Hospital Trust of Verona, Verona 37134, Italy.
404. Department of Medicine, Section of Endocrinology, University and Hospital Trust of Verona, Verona 37134, Italy.
405. Department of Pathology, Queen Elizabeth University Hospital, Glasgow G51 4TF, UK.
406. University of Sydney, Sydney, NSW 2006, Australia.
407. Department of Medical Oncology, Beatson West of Scotland Cancer Centre, Glasgow G12 0YN, UK.
408. Academic Unit of Surgery, School of Medicine, College of Medical, Veterinary and Life Sciences, University of Glasgow, Glasgow Royal Infirmary, Glasgow G4 OSF, UK.
409. Tissue Pathology and Diagnostic Oncology, Royal Prince Alfred Hospital, Sydney, NSW 2050, Australia.
410. Discipline of Surgery, Western Sydney University, Penrith, NSW 2751, Australia.
411. Institute of Cancer Sciences, College of Medical Veterinary and Life Sciences, University of Glasgow, Glasgow G12 8QQ, UK.
412. Faculty of Medicine and Health Sciences, Macquarie University, Sydney, NSW 2109,

Australia.
413. Sydney Medical School, University of Sydney, Sydney, NSW 2050, Australia.
414. School of Environmental and Life Sciences, Faculty of Science, The University of Newcastle, Ourimbah, NSW 2258, Australia.
415. Eastern Clinical School, Monash University, Melbourne, VIC 3128, Australia.
416. Epworth HealthCare, Richmond, VIC 3121, Australia.
417. Applied Tumor Genomics Research Program, Research Programs Unit, University of Helsinki, Helsinki 00290, Finland.
418. Olivia Newton-John Cancer Research Institute, La Trobe University, Heidelberg, VIC 3084, Australia.
419. Melanoma Institute Australia, University of Sydney, Sydney, NSW 2065, Australia.
420. Children's Hospital at Westmead, University of Sydney, Sydney, NSW 2145, Australia.
421. Australian Institute of Tropical Health and Medicine, James Cook University, Douglas, QLD 4814, Australia.
422. Bioplatforms Australia, North Ryde, NSW 2109, Australia.
423. Melanoma Institute Australia, Macquarie University, Sydney, NSW 2109, Australia.
424. Children's Medical Research Institute, Sydney, NSW 2145, Australia.
425. Discipline of Pathology, Sydney Medical School, University of Sydney, Sydney, NSW 2065, Australia.
426. School of Mathematics and Statistics, University of Sydney, Sydney, NSW 2006, Australia.
427. Diagnostic Development, Ontario Institute for Cancer Research, Toronto, ON M5G 0A3, Canada.
428. Ontario Tumour Bank, Ontario Institute for Cancer Research, Toronto, ON M5G 0A3, Canada.
429. PanCuRx Translational Research Initiative, Ontario Institute for Cancer Research, Toronto, ON M5G 0A3, Canada.
430. UHN Program in BioSpecimen Sciences, Toronto General Hospital, Toronto, ON M5G 2C4, Canada.
431. Hepatobiliary/Pancreatic Surgical Oncology Program, University Health Network, Toronto, ON M5G 2C4, Canada.
432. Lunenfeld-Tanenbaum Research Institute, Mount Sinai Hospital, Toronto, ON M5G 1X5, Canada.
433. Division of Medical Oncology, Princess Margaret Cancer Centre, Toronto, ON M5G 2M9, Canada.
434. University of Nebraska Medical Center, Omaha, NE 68198-6880, USA.
435. Transformative Pathology, Ontario Institute for Cancer Research, Toronto, ON M5G 0A3, Canada.
436. Department of Biochemistry and Molecular Medicine, University California at Davis, Sacramento, CA 95817, USA.
437. University Health Network, Princess Margaret Cancer Centre, Toronto, ON M5G 1L7, Canada.
438. Department of Health Sciences Research, Mayo Clinic, Rochester, MN 55905, USA.
439. Department of Laboratory Medicine and Pathobiology, University of Toronto, Toronto, ON M5S 1A8, Canada.
440. Department of Pathology, Human Oncology and Pathogenesis Program, Memorial Sloan

Kettering Cancer Center, New York, NY 10053, USA.
441. Department of Pathology, University Health Network, Toronto General Hospital, Toronto, ON M5G 2C4, Canada.
442. Human Longevity Inc, San Diego, CA 92121, USA.
443. CRUKManchester Institute and Centre, Manchester M20 4GJ, UK.
444. Department of Radiation Oncology, University of Toronto, Toronto, ON M5S 1A8, Canada.
445. Division of Cancer Sciences, Manchester Cancer Research Centre, University of Manchester, Manchester M20 4GJ, UK.
446. Radiation Medicine Program, Princess Margaret Cancer Centre, Toronto, ON M5G 2M9, Canada.
447. Department of Surgical Oncology, Princess Margaret Cancer Centre, Toronto, ON M5G 2M9, Canada.
448. STTARR Innovation Facility, Princess Margaret Cancer Centre, Toronto, ON M5G 1L7, Canada.
449. Hefei University of Technology, Anhui 230009, China.
450. State Key Laboratory of Cancer Biology, and Xijing Hospital of Digestive Diseases, Fourth Military Medical University, Shaanxi 710032, China.
451. Fourth Military Medical University, Shaanxi 710032, China.
452. Department of Surgery, Ruijin Hospital, Shanghai Jiaotong University School of Medicine, Shanghai 200025, China.
453. Leeds Institute of Medical Research, University of Leeds, St James's University Hospital, Leeds LS9 7TF, UK.
454. Canadian Center for Computational Genomics, McGill University, Montreal, QC H3A 0G1, Canada.
455. Department of Human Genetics, McGill University, Montreal, QC H3A 1B1, Canada.
456. International Agency for Research on Cancer, Lyon 69008, France.
457. McGill University and Genome Quebec Innovation Centre, Montreal, QC H3A 0G1, Canada.
458. Centre National de Génotypage, CEA - Institute de Génomique, Evry 91000, France.
459. Leeds Institute of Medical Research @ St James's, University of Leeds, St James's University Hospital, Leeds LS9 7TF, UK.
460. Institute of Mathematics and Computer Science, University of Latvia, Riga LV 1459, Latvia.
461. Department of Oncology, Gil Medical Center, Gachon University, Incheon 405-760, South Korea.
462. Department of Molecular Oncology, BC Cancer Research Centre, Vancouver, BC V5Z 1L3, Canada.
463. Los Alamos National Laboratory, Los Alamos, NM 87545, USA.
464. Department of Cancer Genetics, Institute for Cancer Research, Oslo University Hospital-Radiumhospitalet, Oslo O310, Norway.
465. Department of Clinical Sciences, Lund, Division of Oncology and Pathology, Skåne University Hospital, Lund University, Lund 223 62, Sweden.
466. Translational Research Lab, Centre Léon Bérard, Lyon 69373, France.
467. Department of Molecular Biology, Faculty of Science, Radboud Institute for Molecular Life Sciences, Radboud University, Nijmegen 6500 HB, The Netherlands.
468. Department of Pathology, Brigham and Women's Hospital, Harvard Medical School, Boston, MA 02115, USA.

469. Department of Molecular Pathology, The Netherlands Cancer Institute, Amsterdam 1066 CX, The Netherlands.
470. Institute of Clinical Medicine, Faculty of Medicine, University of Oslo, Oslo O310, Norway.
471. Department of Oncology, University of Cambridge, Cambridge CB2 1TN, UK.
472. Breast Cancer Translational Research Laboratory JC Heuson, Institut Jules Bordet, Brussels 1000, Belgium.
473. Department of Oncology, Laboratory for Translational Breast Cancer Research, KU Leuven, Leuven 3000, Belgium.
474. Translational Cancer Research Unit, GZA Hospitals St.-Augustinus, Center for Oncological Research, Faculty of Medicine and Health Sciences, University of Antwerp, Antwerp 2000, Belgium.
475. Department of Laboratory Medicine, Translational Cancer Research, Lund University Cancer Center at Medicon Village, Lund University, Lund SE-221 85, Sweden.
476. Icelandic Cancer Registry, Icelandic Cancer Society, Reykjavik 125, Iceland.
477. Department of Medical Oncology, Josephine Nefkens Institute and Cancer Genomics Centre, Erasmus Medical Center, Rotterdam 3015 CN, The Netherlands.
478. National Genotyping Center, Institute of Biomedical Sciences, Academia Sinica, Taipei 115, Taiwan.
479. Department of Pathology, Oslo University Hospital Ulleval, Oslo 0450, Norway.
480. Faculty of Medicine and Institute of Clinical Medicine, University of Oslo, Oslo NO-0316, Norway.
481. Department of Pathology, Memorial Sloan Kettering Cancer Center, New York, NY 10065, USA.
482. Department of Pathology, Skåne University Hospital, Lund University, Lund SE-221 85, Sweden.
483. Department of Pathology, Academic Medical Center, Amsterdam 1105 AZ, The Netherlands.
484. Department of Pathology, College of Medicine, Hanyang University, Seoul 133-791, South Korea.
485. Department of Pathology, Asan Medical Center, College of Medicine, Ulsan University, Songpa-gu, Seoul 05505, South Korea.
486. The Netherlands Cancer Institute, Amsterdam 1066 CX, The Netherlands.
487. Breast Oncology Program, Dana-Farber/Brigham and Women's Cancer Center, Boston, MA 02115, USA.
488. Department of Surgery, Memorial Sloan-Kettering Cancer Center, New York, NY 10065, USA.
489. Division of Breast Surgery, Brigham and Women's Hospital, Boston, MA 02115, USA.
490. Department of Clinical Science, University of Bergen, Bergen 5020, Norway.
491. Morgan Welch Inflammatory Breast Cancer Research Program and Clinic, The University of Texas MD Anderson Cancer Center, Houston, TX 77030, USA.
492. The University of Queensland Centre for Clinical Research, Royal Brisbane and Women's Hospital, Herston, QLD 4029, Australia.
493. Department of Pathology, Institut Jules Bordet, Brussels 1000, Belgium.
494. Institute for Bioengineering and Biopharmaceutical Research (IBBR), Hanyang University, Seoul 133-791, South Korea.

495. University of Oslo, Oslo 0316, Norway.
496. Institut Bergonié, Bordeaux 33076, France.
497. Department of Research Oncology, Guy's Hospital, King's Health Partners AHSC, King's College London School of Medicine, London SE1 9RT, UK.
498. University Hospital of Minjoz, INSERM UMR 1098, Besançon 25000, France.
499. Cambridge Breast Unit, Addenbrooke's Hospital, Cambridge University Hospital NHS Foundation Trust and NIHR Cambridge Biomedical Research Centre, Cambridge CB2 2QQ, UK.
500. East of Scotland Breast Service, Ninewells Hospital, Aberdeen AB25 2XF, UK.
501. Department of Clinical Sciences, Lund, Division of Oncology and Pathology, Lund University, Lund 223 62, Sweden.
502. University of Copenhagen, Copenhagen 2200, Denmark.
503. Oncologie Sénologie, ICM Institut Régional du Cancer, Montpellier 34298, France.
504. Department of Radiation Oncology, Radboud University Nijmegen Medical Centre, Nijmegen 6525 GA, The Netherlands.
505. University of Iceland, Reykjavik 101, Iceland.
506. Dundee Cancer Centre, Ninewells Hospital, Dundee DD2 1SY, UK.
507. Institut Curie, INSERM Unit 830, Paris 75248, France.
508. Department of Laboratory Medicine, Radboud University Nijmegen Medical Centre, Nijmegen 6525 GA, The Netherlands.
509. Department of General Surgery, Singapore General Hospital, Singapore 169608, Singapore.
510. Universite Lyon, INCa-Synergie, Centre Léon Bérard, Lyon 69008, France.
511. Giovanni Paolo II / I.R.C.C.S. Cancer Institute, Bari BA 70124, Italy.
512. Department of Biopathology, Centre Léon Bérard, Lyon 69008, France.
513. Université Claude Bernard Lyon 1, Villeurbanne 69100, France.
514. Breast Medical Oncology, The University of Texas MD Anderson Cancer Center, Houston, TX 77030, USA.
515. NCCS-VARI Translational Research Laboratory, National Cancer Centre Singapore, Singapore 169610, Singapore.
516. Department of Pathology, Erasmus Medical Center Rotterdam, Rotterdam 3015 GD, The Netherlands.
517. Division of Molecular Carcinogenesis, The Netherlands Cancer Institute, Amsterdam 1066 CX, The Netherlands.
518. Institute of Human Genetics, Christian-Albrechts-University, Kiel 24118, Germany.
519. Institute of Human Genetics, Ulm University and Ulm University Medical Center of Ulm, Ulm 89081, Germany.
520. Hematopathology Section, Institute of Pathology, Christian-Albrechts-University, Kiel 24118, Germany.
521. Institute of Human Genetics, University of Ulm and University Hospital of Ulm, Ulm 89081, Germany.
522. Department of Human Genetics, Hannover Medical School, Hannover 30625, Germany.
523. Department of Pediatric Oncology, Hematology and Clinical Immunology, Heinrich-Heine-University, Düsseldorf 40225, Germany.
524. Department of Internal Medicine/Hematology, Friedrich-Ebert-Hospital, Neumünster 24534, Germany.
525. Pediatric Hematology and Oncology, University Hospital Muenster, Muenster 24534,

Germany.
526. Department of Pediatrics, University Hospital Schleswig-Holstein, Kiel 24105, Germany.
527. Department of Medicine II, University of Würzburg, Wuerzburg 97070, Germany.
528. Senckenberg Institute of Pathology, University of Frankfurt Medical School, Frankfurt 60596, Germany.
529. Institute of Pathology, Charité – University Medicine Berlin, Berlin 10117, Germany.
530. Department for Internal Medicine II, University Hospital Schleswig-Holstein, Kiel 24105, Germany.
531. Institute for Medical Informatics Statistics and Epidemiology, University of Leipzig, Leipzig 04109, Germany.
532. Department of Hematology and Oncology, Georg-Augusts-University of Göttingen, Göttingen 37073, Germany.
533. Institute of Cell Biology (Cancer Research), University of Duisburg-Essen, Essen D-45147, Germany.
534. MVZ Department of Oncology, PraxisClinic am Johannisplatz, Leipzig 04109, Germany.
535. Institute of Pathology, Ulm University and University Hospital of Ulm, Ulm 89081, Germany.
536. Department of Pathology, Robert-Bosch-Hospital, Stuttgart, Germany, Stuttgart 70376, Germany.
537. University Hospital Giessen, Pediatric Hematology and Oncology, Giessen 35392, Germany.
538. Institute of Clinical Molecular Biology, Christian-Albrechts-University, Kiel 24118, Germany.
539. Institute of Pathology, University of Wuerzburg, Wuerzburg 97070, Germany.
540. Department of General Internal Medicine, University Kiel, Kiel 24118, Germany.
541. Clinic for Hematology and Oncology, St.-Antonius-Hospital, Eschweiler D-52249, Germany.
542. Department for Internal Medicine III, University of Ulm and University Hospital of Ulm, Ulm 89081, Germany.
543. Neuroblastoma Genomics, German Cancer Research Center (DKFZ), Heidelberg 69120, Germany.
544. Department of Pediatric Oncology and Hematology, University of Cologne, Cologne 50937, Germany.
545. University of Düsseldorf, Düsseldorf 40225, Germany.
546. Department of Vertebrate Genomics/Otto Warburg Laboratory Gene Regulation and Systems Biology of Cancer, Max Planck Institute for Molecular Genetics, Berlin 14195, Germany.
547. St. Jude Children's Research Hospital, Memphis, TN 38105-3678, USA.
548. Heidelberg University Hospital, Heidelberg 69120, Germany.
549. Genomics and Proteomics Core Facility High Throughput Sequencing Unit, German Cancer Research Center (DKFZ), Heidelberg 69120, Germany.
550. Epigenomics and Cancer Risk Factors, German Cancer Research Center (DKFZ), Heidelberg 69120, Germany.
551. Institute of Pathology, University Medical Center Hamburg-Eppendorf, Hamburg 20246, Germany.
552. Institute of Pathology, University Medical Center Hamburg-Eppendorf, Hamburg 20251, Germany.
553. Martini-Clinic, Prostate Cancer Center, University Medical Center Hamburg-Eppendorf, Hamburg 20095, Germany.

554. Division of Cancer Genome Research, German Cancer Research Center (DKFZ), Heidelberg 69120, Germany.
555. National Institute of Biomedical Genomics, Kalyani 741235, West Bengal, India.
556. Advanced Centre for Treatment Research and Education in Cancer, Tata Memorial Centre, Navi Mumbai, Maharashtra 410210, India.
557. Department of Pathology, General Hospital of Treviso, Department of Medicine, University of Padua, Treviso 31100, Italy.
558. Department of Medicine (DIMED), Surgical Pathology Unit, University of Padua, Padua 35121, Italy.
559. Department of Pathology and Clinical Laboratory, National Cancer Center Hospital, Tokyo 104-0045, Japan.
560. Department of Pathology, Keio University School of Medicine, Tokyo 160-8582, Japan.
561. Department of Hepatobiliary and Pancreatic Oncology, National Cancer Center Hospital, Tokyo 104-0045, Japan.
562. Department of Pathology, Graduate School of Medicine, University of Tokyo, Tokyo 113-0033, Japan.
563. Preventive Medicine, Graduate School of Medicine, University of Tokyo, Tokyo 113-0033, Japan.
564. Department of Gastric Surgery, National Cancer Center Hospital, Tokyo 104-0045, Japan.
565. Department of Gastroenterology and Hepatology, Yokohama City University Graduate School of Medicine, Kanagawa 236-0004, Japan.
566. Department of Cancer Genome Informatics, Graduate School of Medicine, Osaka University, Osaka 565-0871, Japan.
567. Hiroshima University, Hiroshima 734-8553, Japan.
568. Tokyo Women’s Medical University, Tokyo 162-8666, Japan.
569. Osaka International Cancer Center, Osaka 541-8567, Japan.
570. Wakayama Medical University, Wakayama 641-8509, Japan.
571. Hokkaido University, Sapporo 060-8648, Japan.
572. Department of Surgery, Yokohama City University Graduate School of Medicine, Kanagawa 236-0004, Japan.
573. Division of Medical Oncology, National Cancer Centre, Singapore 169610, Singapore.
574. Cholangiocarcinoma Screening and Care Program and Liver Fluke and Cholangiocarcinoma Research Centre, Faculty of Medicine, Khon Kaen University, Khon Kaen 40002, Thailand.
575. Lymphoma Genomic Translational Research Laboratory, National Cancer Centre, Singapore 169610, Singapore.
576. Center of Digestive Diseases and Liver Transplantation, Fundeni Clinical Institute, Bucharest 022328, Romania.
577. Department of Surgery, Division of Hepatobiliary and Pancreatic Surgery, School of Medicine, Keimyung University Dongsan Medical Center, Daegu 41931, South Korea.
578. Pathology, Hospital Clinic, Institut d'Investigacions Biomèdiques August Pi i Sunyer (IDIBAPS), University of Barcelona, Barcelona 8034, Spain.
579. Anatomia Patológica, Hospital Clinic, Institut d'Investigacions Biomèdiques August Pi i Sunyer (IDIBAPS), University of Barcelona, Barcelona 8036, Spain.
580. Spanish Ministry of Science and Innovation, Madrid 28046, Spain.
581. Hematology, Hospital Clinic, Institut d'Investigacions Biomèdiques August Pi i Sunyer

(IDIBAPS), University of Barcelona, Barcelona 8034, Spain.
582. Department of Biochemistry and Molecular Biology, Faculty of Medicine, University Institute of Oncology-IUOPA, Oviedo 33006, Spain.
583. Royal National Orthopaedic Hospital - Bolsover, London W1W 5AQ, UK.
584. Department of Pathology, Oslo University Hospital, The Norwegian Radium Hospital, Oslo O310, Norway.
585. Institute of Clinical Medicine and Institute of Oral Biology, University of Oslo, Oslo O310, Norway.
586. Department of Pathology (Research), University College London Cancer Institute, London WC1E 6BT, UK.
587. Research Department of Pathology, University College London Cancer Institute, London WC1E 6BT, UK.
588. East Anglian Medical Genetics Service, Cambridge University Hospitals NHS Foundation Trust, Cambridge CB2 0QQ, UK.
589. Royal National Orthopaedic Hospital - Stanmore, Stanmore, Middlesex HA7 4LP, UK.
590. Division of Orthopaedic Surgery, Oslo University Hospital, Oslo 0379, Norway.
591. Radcliffe Department of Medicine, University of Oxford, Oxford OX3 9DU, UK.
592. University of Pavia, Pavia 27100, Italy.
593. Karolinska Institute, Stockholm SE-171 76, Sweden.
594. University of Oxford, Oxford OX3 9DU, UK.
595. Salford Royal NHS Foundation Trust, Salford M6 8HD, UK.
596. Gloucester Royal Hospital, Gloucester GL1 3NL, UK.
597. Royal Stoke University Hospital, Stoke-on-Trent ST4 6QG, UK.
598. St Thomas's Hospital, London SE1 7EH, UK.
599. Imperial College NHS Trust, Imperial College, London W2 INY, UK.
600. Department of Histopathology, Salford Royal NHS Foundation Trust, Salford M6 8HD, UK.
601. Faculty of Biology, Medicine and Health, University of Manchester, Manchester M13 9PL, UK.
602. Edinburgh Royal Infirmary, Edinburgh EH16 4SA, UK.
603. Barking Havering and Redbridge University Hospitals NHS Trust, Romford RM7 0AG, UK.
604. King's College London and Guy's and St Thomas' NHS Foundation Trust, London SE1 7EH, UK.
605. Cambridge Oesophagogastric Centre, Cambridge University Hospitals NHS Foundation Trust, Cambridge CB2 0QQ, UK.
606. Nottingham University Hospitals NHS Trust, Nottingham NG7 2UH, UK.
607. St Luke's Cancer Centre, Royal Surrey County Hospital NHS Foundation Trust, Guildford GU2 7XX, UK.
608. University of North Carolina at Chapel Hill, Chapel Hill, NC 27599, USA.
609. Norfolk and Norwich University Hospital NHS Trust, Norwich NR4 7UY, UK.
610. University Hospitals Coventry and Warwickshire NHS Trust, Coventry CV2 2DX, UK.
611. University Hospitals Birmingham NHS Foundation Trust, Birmingham B15 2GW, UK.
612. Centre for Cancer Research and Cell Biology, Queen's University, Belfast BT9 7AB, UK.
613. School of Cancer Sciences, Faculty of Medicine, University of Southampton, Southampton SO17 1BJ, UK.
614. Wythenshawe Hospital, Manchester M23 9LT, UK.

615. Barts Cancer Institute, Barts and the London School of Medicine and Dentistry, Queen Mary University of London, London EC1M 6BQ, UK.
616. Royal Marsden NHS Foundation Trust, London and Sutton SW3 6JJ, UK.
617. University Hospital Southampton NHS Foundation Trust, Southampton SO16 6YD, UK.
618. HCA Laboratories, London W1G 8AQ, UK.
619. University of Liverpool, Liverpool L69 3BX, UK.
620. Department of Surgery, Academic Urology Group, University of Cambridge, Cambridge CB2 0QQ, UK.
621. Department of Urology, James Buchanan Brady Urological Institute, Johns Hopkins University School of Medicine, Baltimore, MD 21287, USA.
622. Second Military Medical University, Shanghai 200433, China.
623. Department of Surgery and Cancer, Imperial College, London W2 INY, UK.
624. The Chinese University of Hong Kong, Shatin, NT, Hong Kong, China.
625. Nuffield Department of Surgical Sciences, John Radcliffe Hospital, University of Oxford, Oxford OX3 9DU, UK.
626. Department of Histopathology, Cambridge University Hospitals NHS Foundation Trust, Cambridge CB2 0QQ, UK.
627. Department of Surgery, University of Chicago, Chicago, IL 60637, USA.
628. Laboratory of Pathology, Center for Cancer Research, National Cancer Institute, Bethesda, MD 20892, USA.
629. Canada's Michael Smith Genome Sciences Centre, BC Cancer Agency, Vancouver, BC V5Z 4S6, Canada.
630. Center for Molecular Oncology, Memorial Sloan Kettering Cancer Center, New York, NY 10065, USA.
631. Department of Pathology and Laboratory Medicine, School of Medicine, University of North Carolina at Chapel Hill, Chapel Hill, NC 27599, USA.
632. Department of Population and Quantitative Health Sciences, Case Western Reserve University School of Medicine, Cleveland, OH 44016, USA.
633. Research Health Analytics and Informatics, University Hospitals Cleveland Medical Center, Cleveland, OH 44106, USA.
634. Arnie Charbonneau Cancer Institute, University of Calgary, Calgary, AB T2N 4N2, Canada.
635. Departments of Surgery and Oncology, University of Calgary, Calgary, AB T2N 4N2, Canada.
636. Buck Institute for Research on Aging, Novato, CA 94945, USA.
637. Duke University Medical Center, Durham, NC 27710, USA.
638. USC Norris Comprehensive Cancer Center, University of Southern California, Los Angeles, CA 90033, USA.
639. The Preston Robert Tisch Brain Tumor Center, Duke University Medical Center, Durham, NC 27710, USA.
640. Departments of Dermatology and Pathology, Yale University, New Haven, CT 06510, USA.
641. Fox Chase Cancer Center, Philadelphia, PA 19111, USA.
642. Department of Surgery, Division of Thoracic Surgery, The Johns Hopkins University School of Medicine, Baltimore, MD 21287, USA.
643. University of Michigan Comprehensive Cancer Center, Ann Arbor, MI 48109, USA.
644. University of Alabama at Birmingham, Birmingham, AL 35294, USA.
645. Division of Anatomic Pathology, Mayo Clinic, Rochester, MN 55905, USA.

646. Division of Experimental Pathology, Mayo Clinic, Rochester, MN 55905, USA.
647. International Genomics Consortium, Phoenix, AZ 85004, USA.
648. Departments of Pediatrics and Genetics, University of North Carolina at Chapel Hill, Chapel Hill, NC 27599, USA.
649. Department of Pathology, UPMC Shadyside, Pittsburgh, PA 15232, USA.
650. Center for Cancer Genomics, National Cancer Institute, National Institutes of Health, Bethesda, MD 20892, USA.
651. Department of Neuro-Oncology, Istituto Neurologico Besta, Milano 20133, Italy.
652. The University of Queensland Thoracic Research Centre, The Prince Charles Hospital, Brisbane, QLD 4032, Australia.
653. Department of Neurosurgery, University of Florida, Gainesville, FL 32610, USA.
654. Center for Biomedical Informatics, Harvard Medical School, Boston, MA 02115, USA.
655. Department of Cancer Biology, The University of Texas MD Anderson Cancer Center, Houston, TX 77030, USA.
656. Department of Surgical Oncology, The University of Texas MD Anderson Cancer Center, Houston, TX 77030, USA.
657. Division of Gastroenterology and Hepatology, Mayo Clinic, Rochester, MN 55905, USA.
658. Sylvester Comprehensive Cancer Center, University of Miami, Miami, FL 33136, USA.
659. Department of Internal Medicine, Division of Medical Oncology, Lineberger Comprehensive Cancer Center, University of North Carolina at Chapel Hill, Chapel Hill, NC 27599, USA.
660. University of Tennessee Health Science Center for Cancer Research, Memphis, TN 38163, USA.
661. Centre for Translational and Applied Genomics, British Columbia Cancer Agency, Vancouver, BC V5Z 1L3, Canada.
662. Department of Molecular and Cellular Biology, Baylor College of Medicine, Houston, TX 77030, USA.
663. Department of Pathology and Immunology, Baylor College of Medicine, Houston, TX 77030, USA.
664. Michael E. DeBakey Veterans Affairs Medical Center, Houston, TX 77030, USA.
665. Carolina Center for Genome Sciences, University of North Carolina at Chapel Hill, Chapel Hill, NC 27599, USA.
666. Indivumed GmbH, Hamburg 20251, Germany.
667. Department of Surgery, Division of Hepatobiliary and Pancreatic Surgery, School of Medicine, Keimyung University Dong-san Medical Center, Daegu 41931, South Korea.
668. Women's Cancer Program at the Samuel Oschin Comprehensive Cancer Institute, Cedars-Sinai Medical Center, Los Angeles, CA 90048, USA.
669. Department of Surgery, The George Washington University, School of Medicine and Health Science, Washington, DC 20052, USA.
670. Endocrine Oncology Branch, Center for Cancer Research, National Cancer Institute, National Institutes of Health, Bethesda, MD 20892, USA.
671. National Cancer Center, Gyeonggi 10408, South Korea.
672. ILSbio, LLC Biobank, Chestertown, MD 21620, USA.
673. Canada's Michael Smith Genome Sciences Centre, BC Cancer Agency, Vancouver, BC V5Z 4S6, Canada.

674. Gynecologic Oncology, NYU Laura and Isaac Perlmutter Cancer Center, New York University, New York, NY 10016, USA.
675. Division of Oncology, Stem Cell Biology Section, Washington University School of Medicine, St. Louis, MO 63110, USA.
676. Urologic Oncology Branch, Center for Cancer Research, National Cancer Institute, National Institutes of Health, Bethesda, MD 20892, USA.
677. Institute for Systems Biology, Seattle, WA 98109, USA.
678. Department of Pathology and Laboratory Medicine, Center for Personalized Medicine, Children's Hospital Los Angeles, Los Angeles, CA 90027, USA.
679. Institute for Genomic Medicine, Nationwide Children's Hospital, Columbus, OH 43215, USA.
680. Department of Surgery, Duke University, Durham, NC 27710, USA.
681. Department of Obstetrics, Gynecology and Reproductive Services, University of California San Francisco, San Francisco, CA 94143, USA.
682. Departments of Neurology and Neurosurgery, Henry Ford Hospital, Detroit, MI 48202, USA.
683. Precision Oncology, OHSU Knight Cancer Institute, Oregon Health and Science University, Portland, OR 97239, USA.
684. Department of Pathology, Roswell Park Cancer Institute, Buffalo, NY 14263, USA.
685. Department of Obstetrics and Gynecology, Division of Gynecologic Oncology, Washington University School of Medicine, St. Louis, MO 63110, USA.
686. Penrose St. Francis Health Services, Colorado Springs, CO 80907, USA.
687. Department of Medicine, University of Chicago, Chicago, IL 60637, USA.
688. Department of Neurology, Mayo Clinic, Rochester, MN 55905, USA.
689. Center for Liver Cancer, Research Institute and Hospital, National Cancer Center, Gyeonggi 410-769, South Korea.
690. Department of Genetics, Lineberger Comprehensive Cancer Center, University of North Carolina at Chapel Hill, Chapel Hill, NC 27599, USA.
691. NYU Langone Medical Center, New York, NY 10016, USA.
692. Department of Hematology and Medical Oncology, Cleveland Clinic, Cleveland, OH 44195, USA.
693. Department of Pathology and Laboratory Medicine, University of North Carolina, Chapel Hill, NC 27599, USA.
694. Helen F. Graham Cancer Center at Christiana Care Health Systems, Newark, DE 19713, USA.
695. Cureline, Inc, South San Francisco, CA 94080, USA.
696. Department of Obstetrics and Gynecology, Medical College of Wisconsin, Milwaukee, WI 53226, USA.
697. Hematology and Medical Oncology, Winship Cancer Institute of Emory University, Atlanta, GA 30322, USA.
698. Vanderbilt Ingram Cancer Center, Vanderbilt University, Nashville, TN 37232, USA.
699. Ohio State University College of Medicine and Arthur G. James Comprehensive Cancer Center, Columbus, OH 43210, USA.
700. Research Computing Center, University of North Carolina at Chapel Hill, Chapel Hill, NC 27599, USA.

701. Analytical Biological Services, Inc, Wilmington, DE 19801, USA.
702. Department of Dermatology, University Hospital of Essen, Essen 45122, Germany.
703. University of Pittsburgh, Pittsburgh, PA 15213, USA.
704. Murtha Cancer Center, Walter Reed National Military Medical Center, Bethesda, MD 20889, USA.
705. Brigham and Women's Hospital, Harvard Medical School, Boston, MA 02115, USA.
706. Department of Surgery, Memorial Sloan Kettering Cancer Center, New York, NY 10065, USA.
707. Center for RNA Interference and Noncoding RNA, The University of Texas MD Anderson Cancer Center, Houston, TX 77030, USA.
708. Department of Experimental Therapeutics, The University of Texas MD Anderson Cancer Center, Houston, TX 77030, USA.
709. Department of Gynecologic Oncology and Reproductive Medicine, The University of Texas MD Anderson Cancer Center, Houston, TX 77030, USA.
710. Department of Urology, Mayo Clinic, Rochester, MN 55905, USA.
711. Department of Surgery, Johns Hopkins University School of Medicine, Baltimore, MD 21205, USA.
712. Departments of Neurosurgery and Hematology and Medical Oncology, Winship Cancer Institute and School of Medicine, Emory University, Atlanta, GA 30322, USA.
713. Georgia Regents University Cancer Center, Augusta, GA 30912, USA.
714. Thoracic Oncology Laboratory, Mayo Clinic, Rochester, MN 55905, USA.
715. Institute for Genomic Medicine, Nationwide Children's Hospital, Columbus, OH 43205, USA.
716. Department of Obstetrics and Gynecology, Division of Gynecologic Oncology, Mayo Clinic, Rochester, MN 55905, USA.
717. International Institute for Molecular Oncology, Poznań 60-203, Poland.
718. Poznan University of Medical Sciences, Poznań 61-701, Poland.
719. Edison Family Center for Genome Sciences and Systems Biology, Washington University, St. Louis, MO 63110, USA.
