# Supplementary material for: Comprehensive analysis of chromothripsis in 2,658 human cancers using whole-genome sequencing
Source: Nat Genet. 2020 Feb 5;52(3):331–41. doi: 10.1038/s41588-019-0576-7 (PMC7058534; doi:10.1038/s41588-019-0576-7)
Supplement: Supplementary file 6 — High-confidence chromothripsis calls. [file 41588_2019_576_MOESM6_ESM.pdf]

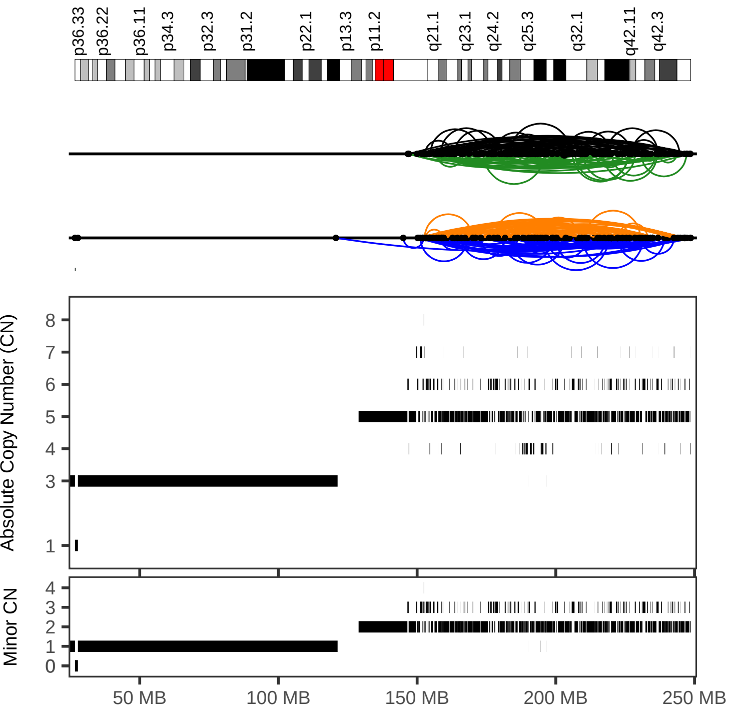

	BTCA_donor_A096
Cancer type	Biliary−AdenoCA
Position	1:120690998−248630158
Type	With other complex events
Interleaved intrachr. SVs	214
Total SVs (intrachr. + transl.)	215
SV types	DEL: 47; DUP: 56; h2hINV: 62; t2tINV: 49; TRA: 1
SVs in sample	292
Oscillating CN (2 and 3 states)	21, 25
CN segments	291
FDR fragment joints	0.64
FDR chr. breakp. enrich.	0
Linked to chrs	
Purity, ploidy	0.63, 2.77

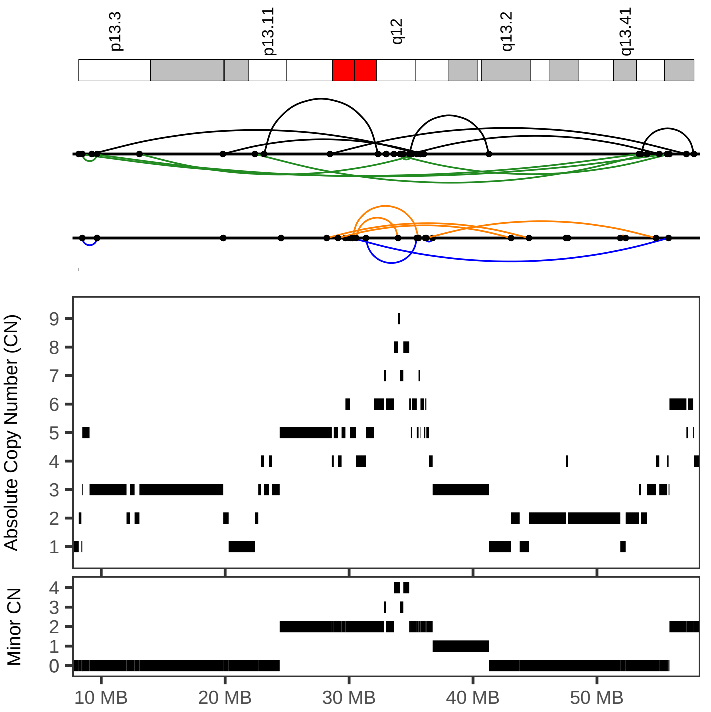

	BTCA_donor_B070
Cancer type	Biliary−AdenoCA
Position	19:8183835−57827516
Type	With other complex events
Interleaved intrachr. SVs	25
Total SVs (intrachr. + transl.)	25
SV types	DEL: 5; DUP: 4; h2hINV: 9; t2tINV: 7; TRA: 0
SVs in sample	166
Oscillating CN (2 and 3 states)	8, 15
CN segments	67
FDR fragment joints	0.64
FDR chr. breakp. enrich.	0
Linked to chrs	
Purity, ploidy	0.56, 2.93

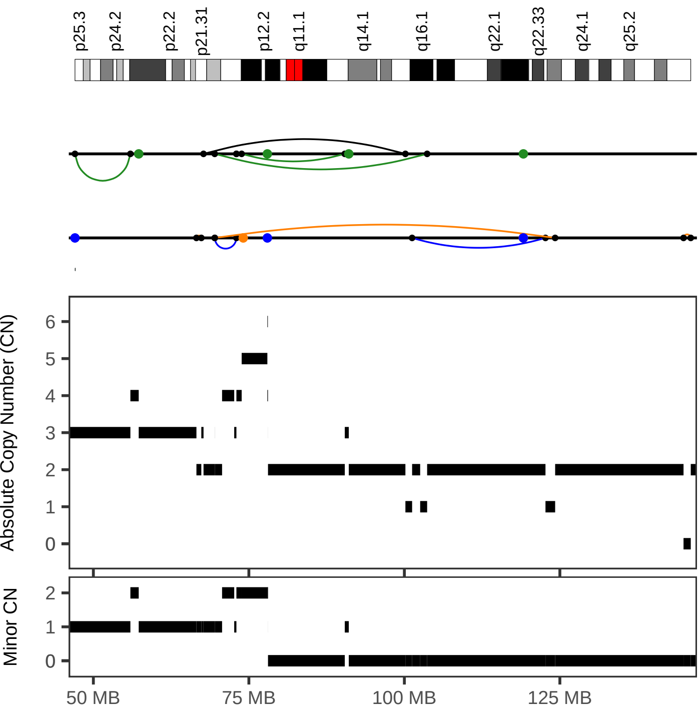

	BTCA_donor_C080
Cancer type	Biliary−AdenoCA
Position	6:67710168−124238025
Type	With other complex events
Interleaved intrachr. SVs	4
Total SVs (intrachr. + transl.)	11
SV types	DEL: 1; DUP: 1; h2hINV: 1; t2tINV: 1; TRA: 7
SVs in sample	142
Oscillating CN (2 and 3 states)	7, 11
CN segments	19
FDR fragment joints	1
FDR chr. breakp. enrich.	0
Linked to chrs	
Purity, ploidy	0.6, 2.79

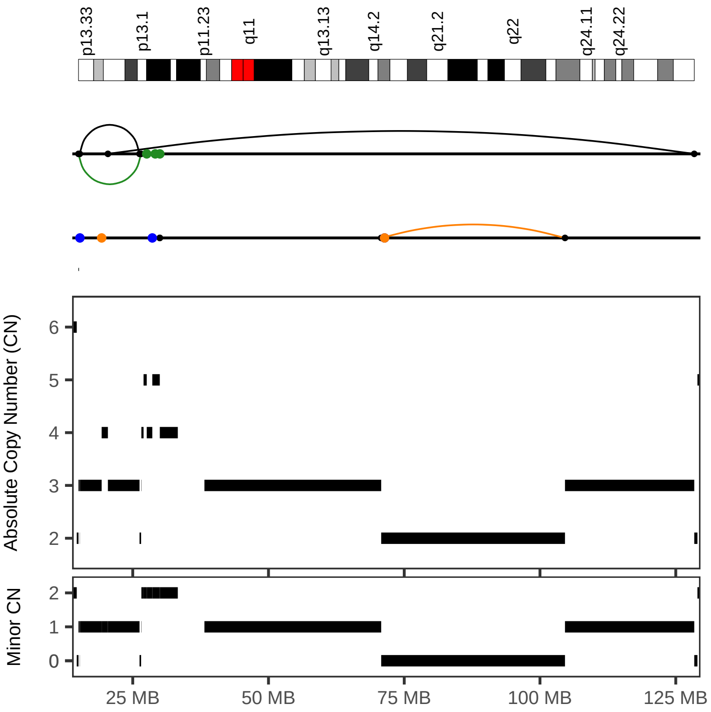

	BTCA_donor_C080
Cancer type	Biliary−AdenoCA
Position	12:15005593−128406118
Type	With other complex events
Interleaved intrachr. SVs	3
Total SVs (intrachr. + transl.)	11
SV types	DEL: 0; DUP: 0; h2hINV: 2; t2tINV: 1; TRA: 8
SVs in sample	142
Oscillating CN (2 and 3 states)	7, 18
CN segments	18
FDR fragment joints	0.59
FDR chr. breakp. enrich.	0.02
Linked to chrs	
Purity, ploidy	0.6, 2.79

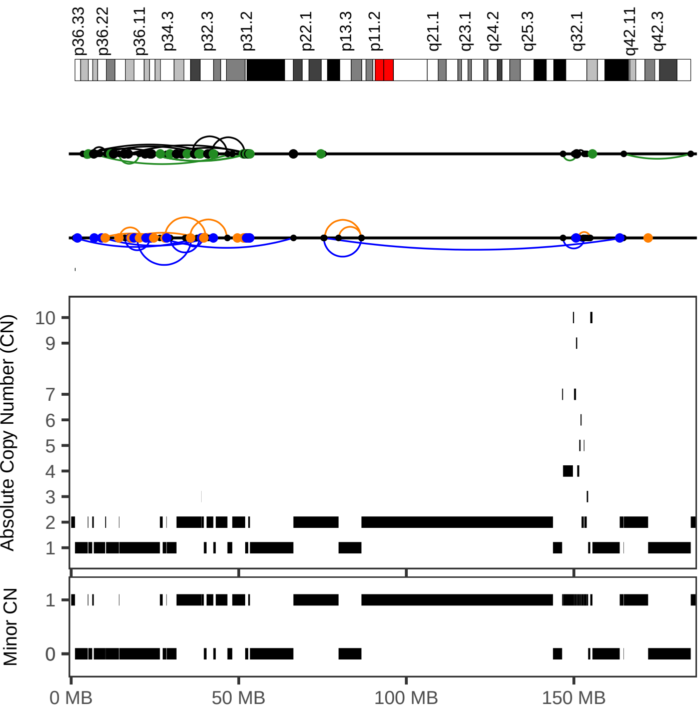

	**RK142**
Cancer type	Biliary−AdenoCA
Position	1:1138581−66347938
Type	With other complex events
Interleaved intrachr. SVs	34
Total SVs (intrachr. + transl.)	86
SV types	DEL: 7; DUP: 10; h2hINV: 11; t2tINV: 6; TRA: 52
SVs in sample	168
Oscillating CN (2 and 3 states)	14, 26
CN segments	26
FDR fragment joints	0.64
FDR chr. breakp. enrich.	0
Linked to chrs	
Purity, ploidy	0.22, 1.81

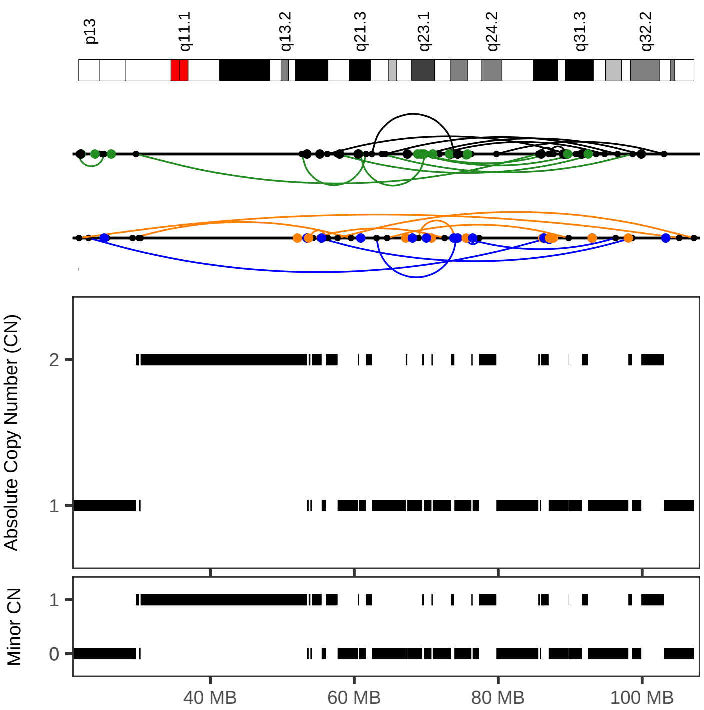

	**RK142**
Cancer type	Biliary−AdenoCA
Position	14:21707781−107204434
Type	Canonical without polyploidization
Interleaved intrachr. SVs	30
Total SVs (intrachr. + transl.)	79
SV types	DEL: 7; DUP: 6; h2hINV: 8; t2tINV: 9; TRA: 49
SVs in sample	168
Oscillating CN (2 and 3 states)	41, 41
CN segments	41
FDR fragment joints	0.91
FDR chr. breakp. enrich.	0
Linked to chrs	1:1138581−66347937;
Purity, ploidy	0.22, 1.81

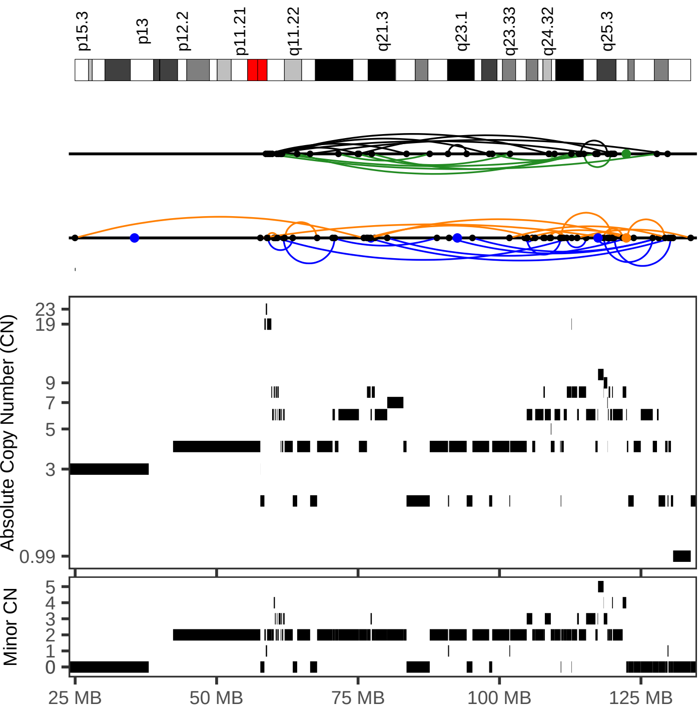

	**RK208**
Cancer type	Biliary−AdenoCA
Position	10:24963425−133800853
Type	With other complex events
Interleaved intrachr. SVs	50
Total SVs (intrachr. + transl.)	55
SV types	DEL: 15; DUP: 15; h2hINV: 11; t2tINV: 9; TRA: 5
SVs in sample	294
Oscillating CN (2 and 3 states)	11, 21
CN segments	89
FDR fragment joints	0.64
FDR chr. breakp. enrich.	0
Linked to chrs	
Purity, ploidy	0.2, 3.83

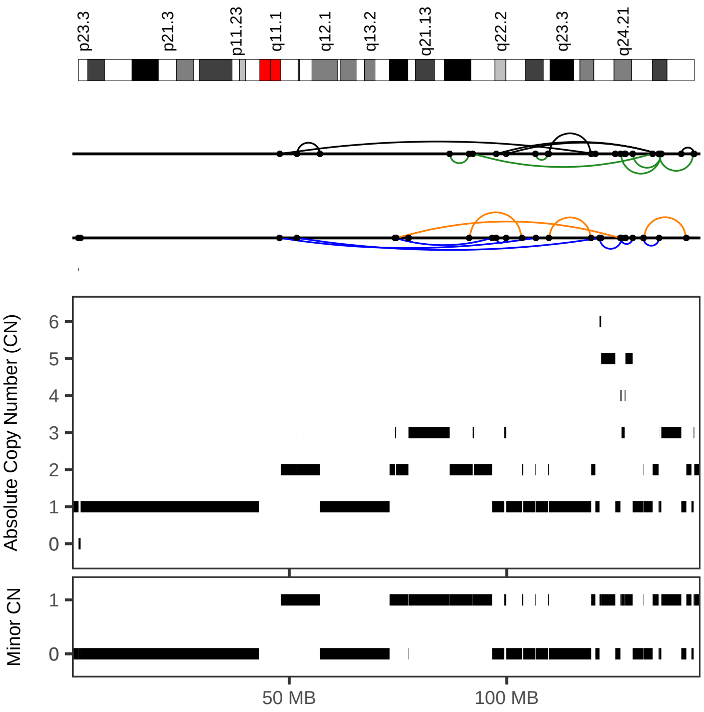

	**RK307**
Cancer type	Biliary−AdenoCA
Position	8:47769789−143083576
Type	With other complex events
Interleaved intrachr. SVs	19
Total SVs (intrachr. + transl.)	19
SV types	DEL: 3; DUP: 7; h2hINV: 4; t2tINV: 5; TRA: 0
SVs in sample	89
Oscillating CN (2 and 3 states)	9, 13
CN segments	45
FDR fragment joints	0.68
FDR chr. breakp. enrich.	0
Linked to chrs	
Purity, ploidy	0.37, 1.79

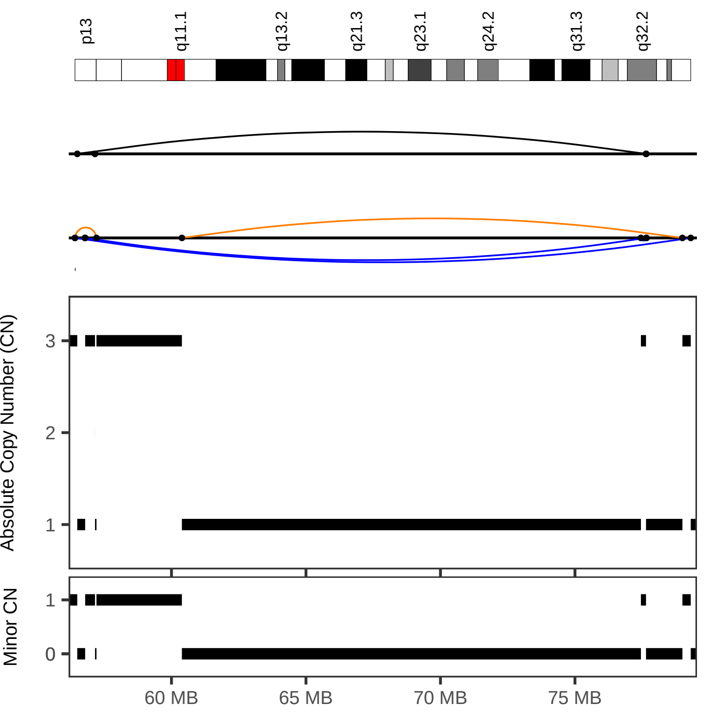

RK307	
Cancer type	Biliary−AdenoCA
Position	14:56412147−79304555
Type	Canonical without polyploidization
Interleaved intrachr. SVs	6
Total SVs (intrachr. + transl.)	6
SV types	DEL: 2; DUP: 3; h2hINV: 1; t2tINV: 0; TRA: 0
SVs in sample	89
Oscillating CN (2 and 3 states)	7, 8
CN segments	11
FDR fragment joints	0.59
FDR chr. breakp. enrich.	0.01
Linked to chrs	
Purity, ploidy	0.37, 1.79

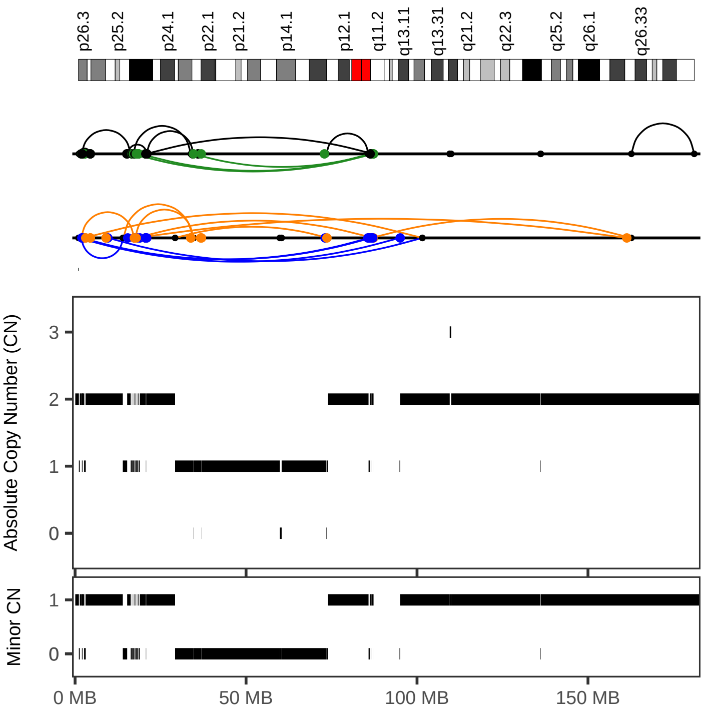

RK308	
Cancer type	Biliary−AdenoCA
Position	3:999520−181068319
Type	Canonical without polyploidization
Interleaved intrachr. SVs	35
Total SVs (intrachr. + transl.)	127
SV types	DEL: 12; DUP: 6; h2hINV: 12; t2tINV: 5; TRA: 92
SVs in sample	290
Oscillating CN (2 and 3 states)	42, 65
CN segments	65
FDR fragment joints	0.59
FDR chr. breakp. enrich.	0
Linked to chrs	13:20391473−54701210;6:4291463−102377482
Purity, ploidy	0.86, 1.98

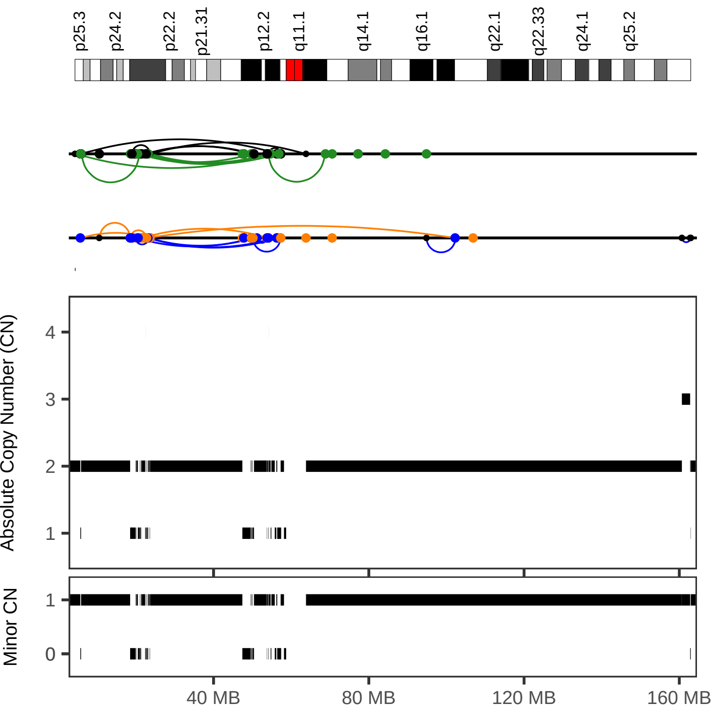

RK308	
Cancer type	Biliary−AdenoCA
Position	6:4291463−102377483
Type	With other complex events
Interleaved intrachr. SVs	27
Total SVs (intrachr. + transl.)	131
SV types	DEL: 5; DUP: 8; h2hINV: 6; t2tINV: 8; TRA: 104
SVs in sample	290
Oscillating CN (2 and 3 states)	21, 35
CN segments	54
FDR fragment joints	0.84
FDR chr. breakp. enrich.	0
Linked to chrs	13:20391473−54701210;3:999520−181068318
Purity, ploidy	0.86, 1.98

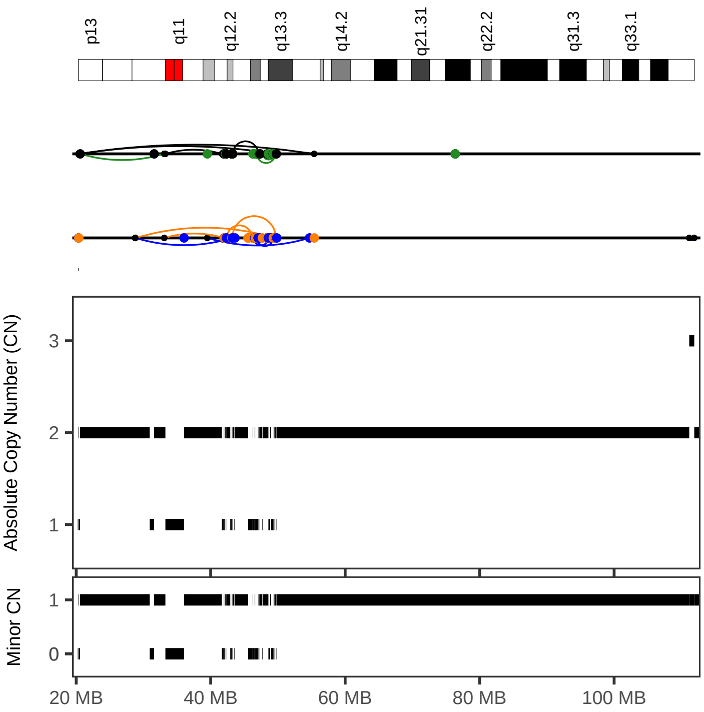

RK308	
Cancer type	Biliary−AdenoCA
Position	13:20391473−54701211
Type	Canonical without polyploidization
Interleaved intrachr. SVs	17
Total SVs (intrachr. + transl.)	89
SV types	DEL: 5; DUP: 5; h2hINV: 3; t2tINV: 4; TRA: 72
SVs in sample	290
Oscillating CN (2 and 3 states)	38, 38
CN segments	38
FDR fragment joints	0.92
FDR chr. breakp. enrich.	0
Linked to chrs	3:999520−181068318;6:4291463−102377482
Purity, ploidy	0.86, 1.98

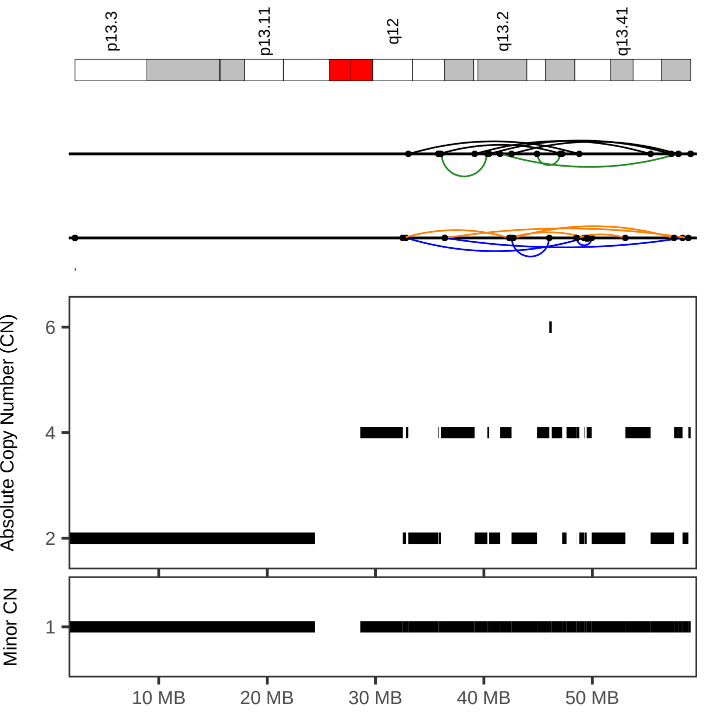

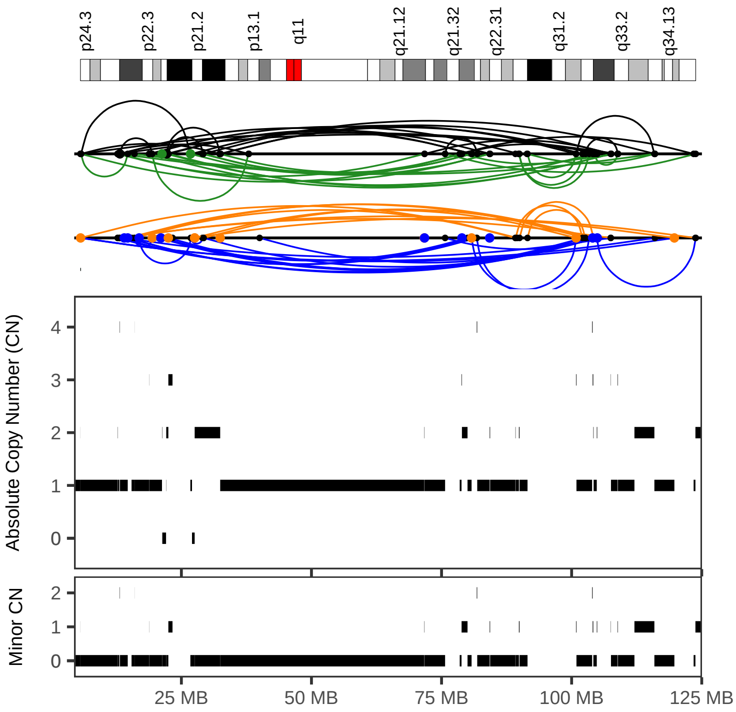

	RK308
Cancer type	Biliary−AdenoCA
Position	19:32501825−58859068
Type	With other complex events
Interleaved intrachr. SVs	18
Total SVs (intrachr. + transl.)	18
SV types	DEL: 5; DUP: 5; h2hINV: 5; t2tINV: 3; TRA: 0
SVs in sample	290
Oscillating CN (2 and 3 states)	17, 30
CN segments	30
FDR fragment joints	0.91
FDR chr. breakp. enrich.	0
Linked to chrs	
Purity, ploidy	0.86, 1.98

	RK316
Cancer type	Biliary−AdenoCA
Position	9:5597836−123851444
Type	With other complex events
Interleaved intrachr. SVs	79
Total SVs (intrachr. + transl.)	102
SV types	DEL: 13; DUP: 20; h2hINV: 23; t2tINV: 23; TRA: 23
SVs in sample	128
Oscillating CN (2 and 3 states)	7, 10
CN segments	50
FDR fragment joints	0.59
FDR chr. breakp. enrich.	0
Linked to chrs	6:108425970−158281848;
Purity, ploidy	0.33, 2.11

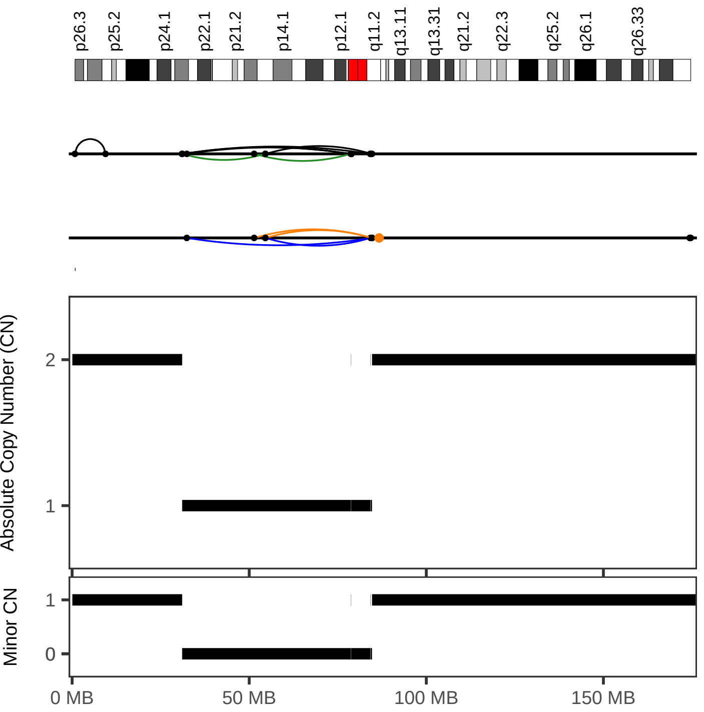

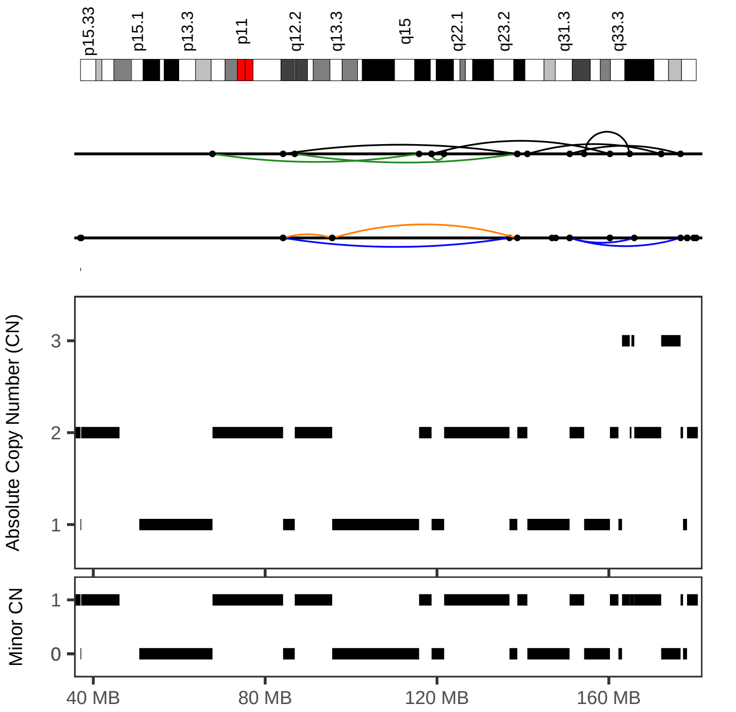

	096b4f32−10c1−4737−a0dd−cae04c54ee33
Cancer type	Bladder−TCC
Position	3:31044413−84685234
Type	Canonical without polyploidization
Interleaved intrachr. SVs	12
Total SVs (intrachr. + transl.)	12
SV types	DEL: 2; DUP: 2; h2hINV: 4; t2tINV: 4; TRA: 0
SVs in sample	136
Oscillating CN (2 and 3 states)	12, 12
CN segments	12
FDR fragment joints	0.8
FDR chr. breakp. enrich.	0.14
Linked to chrs	
Purity, ploidy	0.74, 1.77

	096b4f32−10c1−4737−a0dd−cae04c54ee33
Cancer type	Bladder−TCC
Position	5:67743249−176695147
Type	Canonical without polyploidization
Interleaved intrachr. SVs	14
Total SVs (intrachr. + transl.)	14
SV types	DEL: 2; DUP: 4; h2hINV: 5; t2tINV: 3; TRA: 0
SVs in sample	136
Oscillating CN (2 and 3 states)	14, 20
CN segments	20
FDR fragment joints	0.78
FDR chr. breakp. enrich.	0
Linked to chrs	
Purity, ploidy	0.74, 1.77

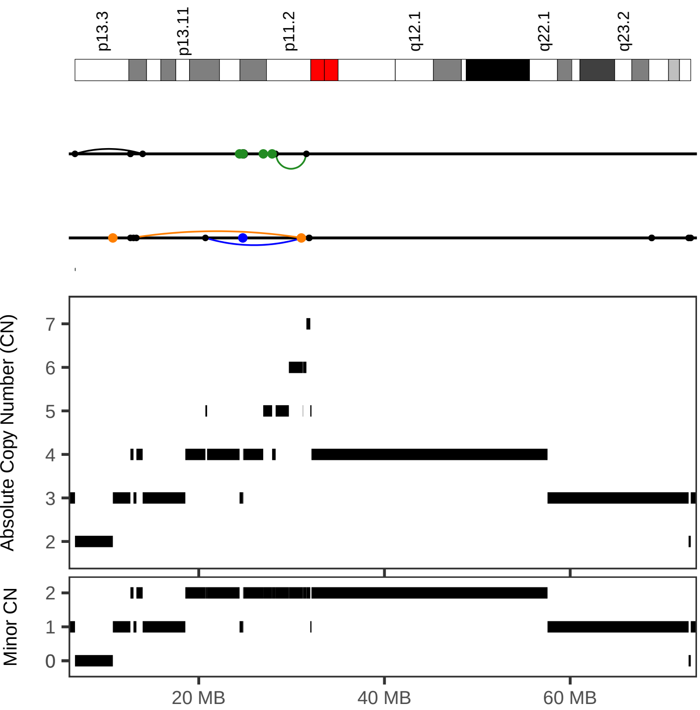

	3ed614e7−f356−4d87−985b−d3bbbae3bb40
Cancer type	Bladder−TCC
Position	16:6669849−31591597
Type	With other complex events
Interleaved intrachr. SVs	5
Total SVs (intrachr. + transl.)	13
SV types	DEL: 1; DUP: 1; h2hINV: 1;<br>t2tINV: 2; TRA: 8
SVs in sample	449
Oscillating CN (2 and 3 states)	8, 22
CN segments	22
FDR fragment joints	0.92
FDR chr. breakp. enrich.	0.27
Linked to chrs	11:8965507−99216922;X:3858348−76848684
Purity, ploidy	0.74, 3.29

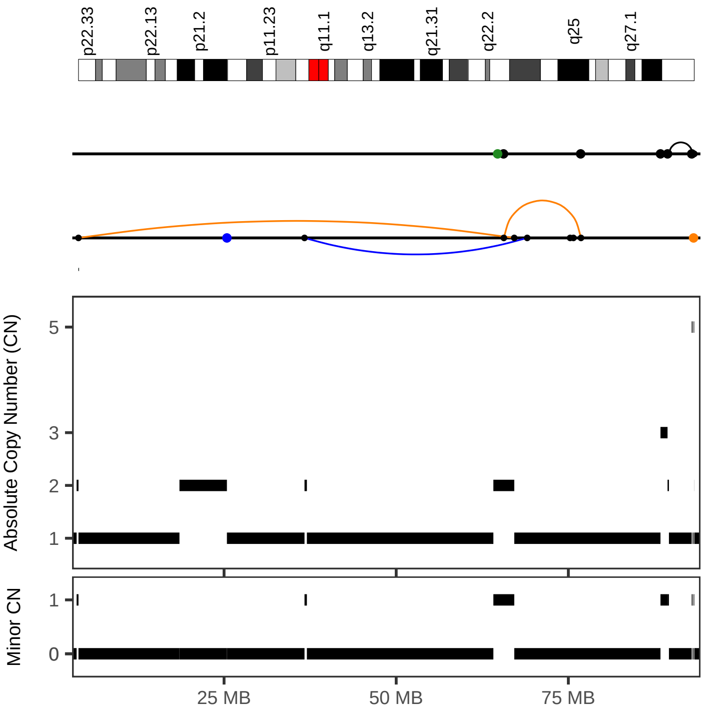

	3ed614e7−f356−4d87−985b−d3bbbae3bb40
Cancer type	Bladder−TCC
Position	X:3858348−76848685
Type	Canonical without polyploidization
Interleaved intrachr. SVs	4
Total SVs (intrachr. + transl.)	8
SV types	DEL: 2; DUP: 1; h2hINV: 1;<br>t2tINV: 0; TRA: 4
SVs in sample	449
Oscillating CN (2 and 3 states)	7, 7
CN segments	7
FDR fragment joints	0.64
FDR chr. breakp. enrich.	0.28
Linked to chrs	16:6669849−31591596;
Purity, ploidy	0.74, 3.29

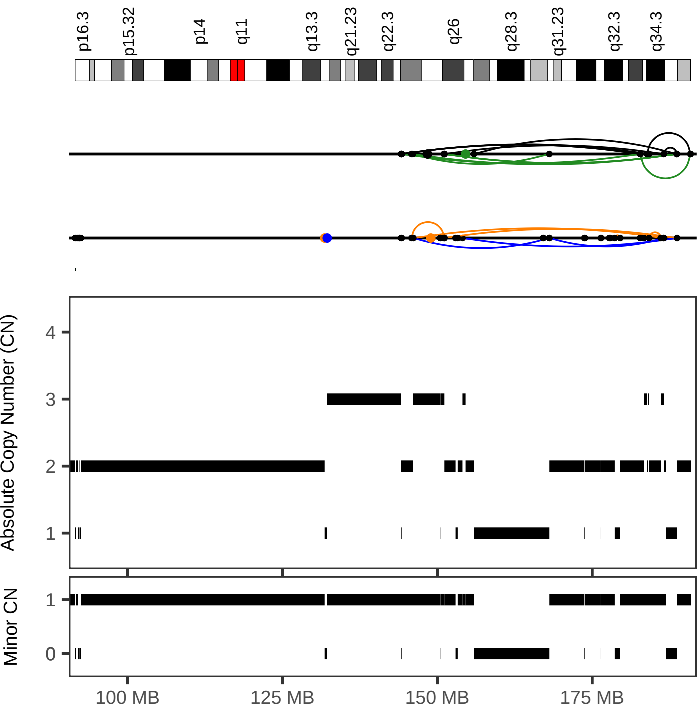

	493a4ff2−37a5−4b79−928d−83dbfe534556
Cancer type	Bladder−TCC
Position	4:144150885−190874076
Type	With other complex events
Interleaved intrachr. SVs	24
Total SVs (intrachr. + transl.)	27
SV types	DEL: 6; DUP: 3; h2hINV: 7;<br>t2tINV: 8; TRA: 3
SVs in sample	117
Oscillating CN (2 and 3 states)	9, 16
CN segments	29
FDR fragment joints	0.64
FDR chr. breakp. enrich.	0
Linked to chrs	
Purity, ploidy	0.85, 2

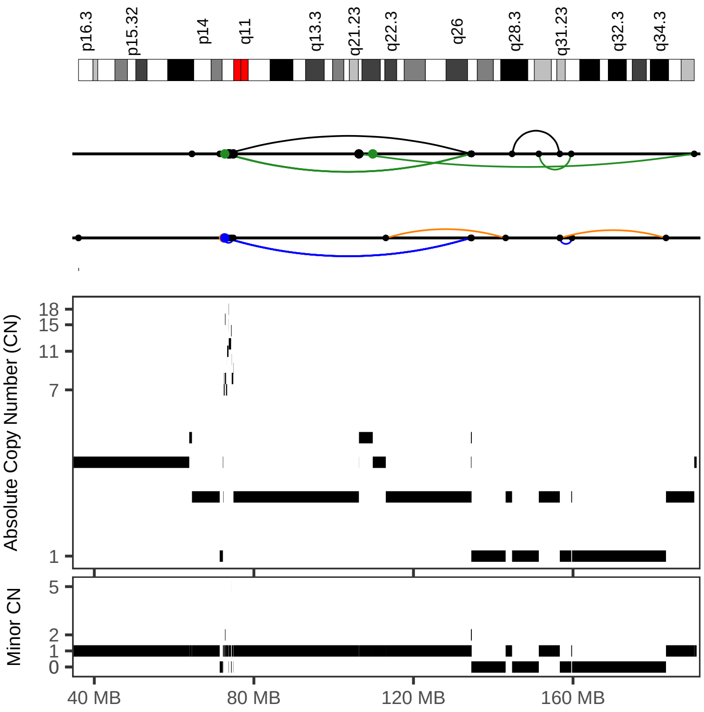

	5d54c742−5a8e−4c40−8d62−95e75e210ab8
Cancer type	Bladder−TCC
Position	4:72403638−190385402
Type	With other complex events
Interleaved intrachr. SVs	20
Total SVs (intrachr. + transl.)	29
SV types	DEL: 2; DUP: 6; h2hINV: 6;<br>t2tINV: 6; TRA: 9
SVs in sample	175
Oscillating CN (2 and 3 states)	9, 10
CN segments	37
FDR fragment joints	0.64
FDR chr. breakp. enrich.	0
Linked to chrs	14:53927912−83098831;
Purity, ploidy	0.6, 3.23

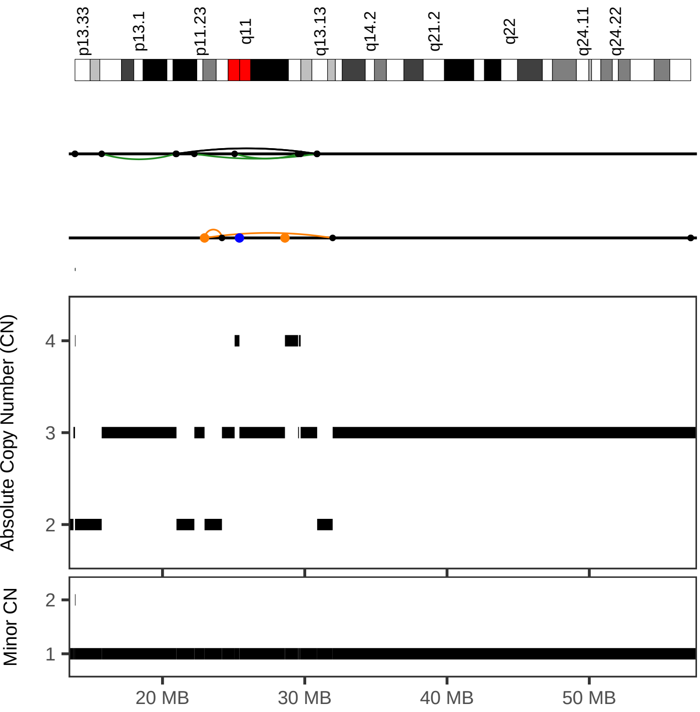

	5d54c742−5a8e−4c40−8d62−95e75e210ab8
Cancer type	Bladder−TCC
Position	12:15725603−31961309
Type	With other complex events
Interleaved intrachr. SVs	5
Total SVs (intrachr. + transl.)	8
SV types	DEL: 1; DUP: 0; h2hINV: 2; t2tINV: 2; TRA: 3
SVs in sample	175
Oscillating CN (2 and 3 states)	7, 13
CN segments	13
FDR fragment joints	0.64
FDR chr. breakp. enrich.	0.05
Linked to chrs	
Purity, ploidy	0.6, 3.23

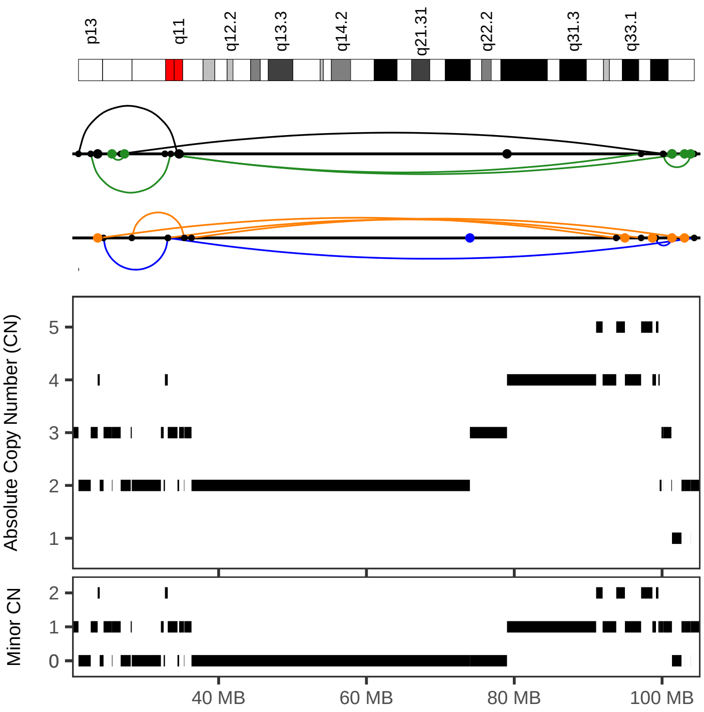

	797a71ee−372d−41e5−aeee−5ab3c4661110
Cancer type	Bladder−TCC
Position	13:21033268−104354353
Type	With other complex events
Interleaved intrachr. SVs	14
Total SVs (intrachr. + transl.)	29
SV types	DEL: 4; DUP: 3; h2hINV: 2; t2tINV: 5; TRA: 15
SVs in sample	145
Oscillating CN (2 and 3 states)	10, 27
CN segments	41
FDR fragment joints	0.78
FDR chr. breakp. enrich.	0
Linked to chrs	20:24187366−56783858;
Purity, ploidy	0.93, 3.26

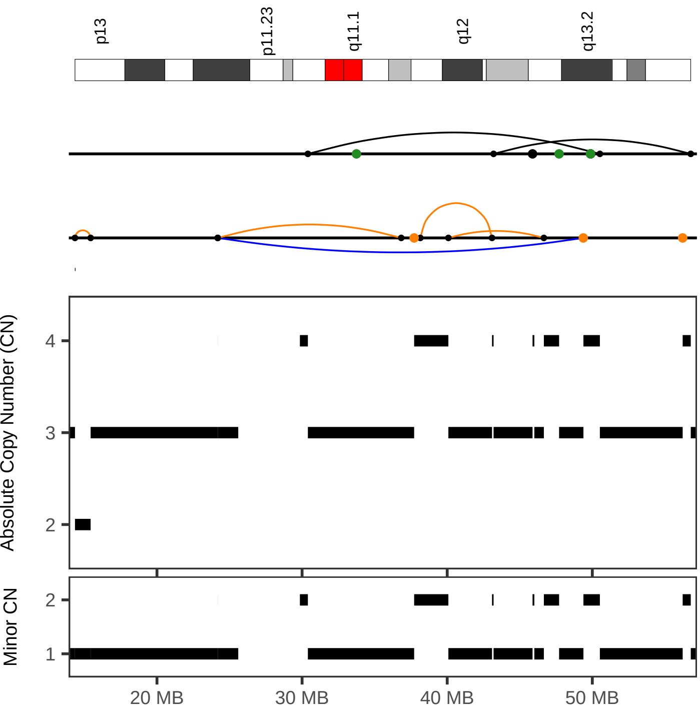

	797a71ee−372d−41e5−aeee−5ab3c4661110
Cancer type	Bladder−TCC
Position	20:24187366−56783859
Type	After polyploidization
Interleaved intrachr. SVs	6
Total SVs (intrachr. + transl.)	14
SV types	DEL: 3; DUP: 1; h2hINV: 2; t2tINV: 0; TRA: 8
SVs in sample	145
Oscillating CN (2 and 3 states)	16, 16
CN segments	16
FDR fragment joints	0.59
FDR chr. breakp. enrich.	0
Linked to chrs	13:21033268−104354352;
Purity, ploidy	0.93, 3.26

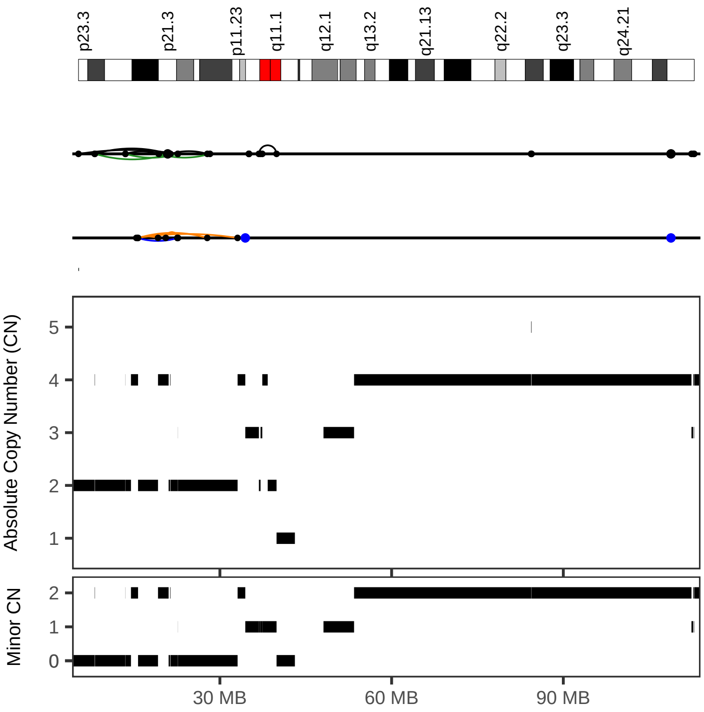

	904a8757−e0c5−41ef−b583−c8f170caaac1
Cancer type	Bladder−TCC
Position	8:5291228−33089595
Type	Before polyploidization
Interleaved intrachr. SVs	12
Total SVs (intrachr. + transl.)	13
SV types	DEL: 4; DUP: 1; h2hINV: 4; t2tINV: 3; TRA: 1
SVs in sample	274
Oscillating CN (2 and 3 states)	11, 14
CN segments	14
FDR fragment joints	0.64
FDR chr. breakp. enrich.	0.03
Linked to chrs	
Purity, ploidy	0.6, 2.92

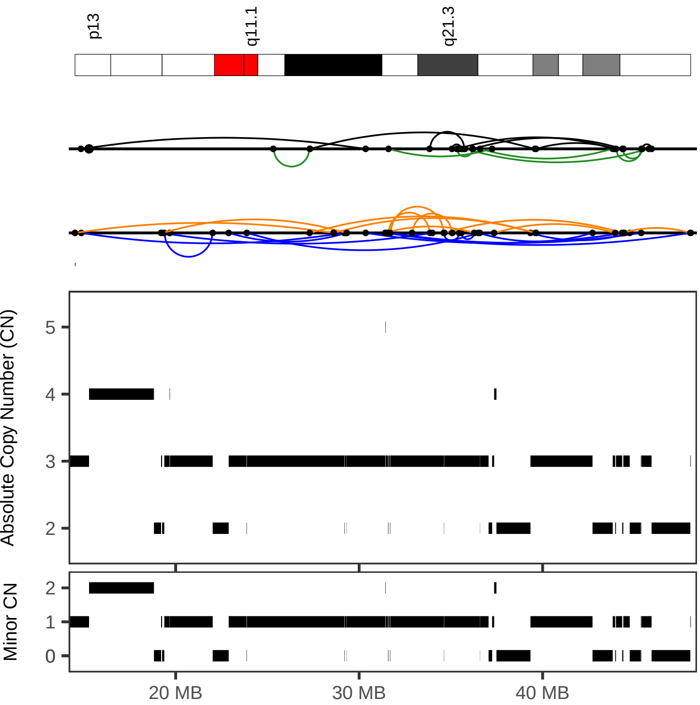

	904a8757−e0c5−41ef−b583−c8f170caaac1
Cancer type	Bladder−TCC
Position	21:14511470−48074038
Type	With other complex events
Interleaved intrachr. SVs	39
Total SVs (intrachr. + transl.)	40
SV types	DEL: 12; DUP: 11; h2hINV: 8; t2tINV: 8; TRA: 1
SVs in sample	274
Oscillating CN (2 and 3 states)	14, 16
CN segments	43
FDR fragment joints	0.81
FDR chr. breakp. enrich.	0
Linked to chrs	
Purity, ploidy	0.6, 2.92

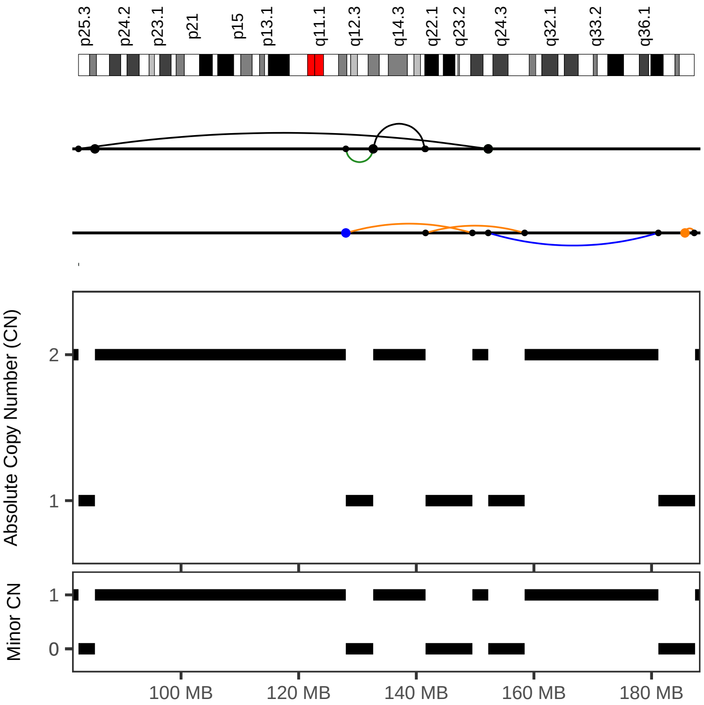

	f48937ed−e294−41f0−8872−7010391167a9
Cancer type	Bladder−TCC
Position	2:82560680−181154512
Type	Canonical without polyploidization
Interleaved intrachr. SVs	4
Total SVs (intrachr. + transl.)	9
SV types	DEL: 2; DUP: 1; h2hINV: 1; t2tINV: 0; TRA: 5
SVs in sample	78
Oscillating CN (2 and 3 states)	9, 9
CN segments	9
FDR fragment joints	0.64
FDR chr. breakp. enrich.	0
Linked to chrs	
Purity, ploidy	0.92, 2.07

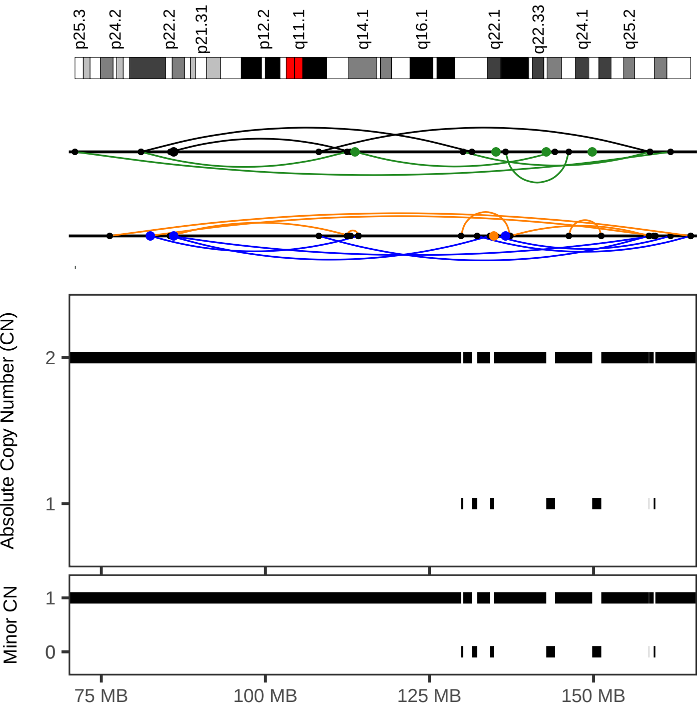

	CGP_donor_1437410
Cancer type	Bone−Benign
Position	6:70986165−164827209
Type	Canonical without polyploidization
Interleaved intrachr. SVs	21
Total SVs (intrachr. + transl.)	30
SV types	DEL: 7; DUP: 6; h2hINV: 3; t2tINV: 5; TRA: 9
SVs in sample	33
Oscillating CN (2 and 3 states)	17, 17
CN segments	17
FDR fragment joints	0.72
FDR chr. breakp. enrich.	0
Linked to chrs	
Purity, ploidy	0.72, 2

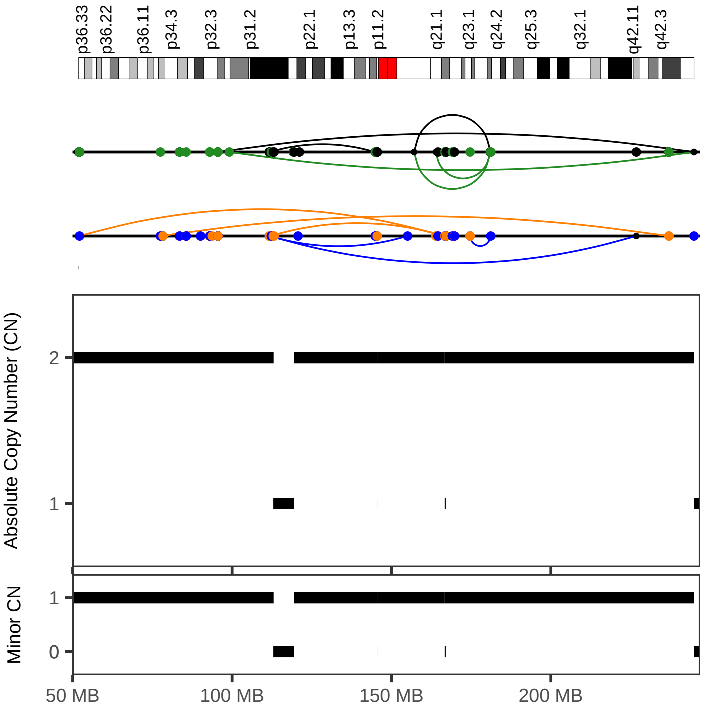

	CGP_donor_1437411
Cancer type	Bone−Benign
Position	1:51871319−244912139
Type	Canonical without polyploidization
Interleaved intrachr. SVs	12
Total SVs (intrachr. + transl.)	130
SV types	DEL: 3; DUP: 3; h2hINV: 3; t2tINV: 3; TRA: 118
SVs in sample	513
Oscillating CN (2 and 3 states)	18, 18
CN segments	18
FDR fragment joints	1
FDR chr. breakp. enrich.	0
Linked to chrs	2:8888556−242818177;4:3484005−186431923
Purity, ploidy	0.75, 1.98

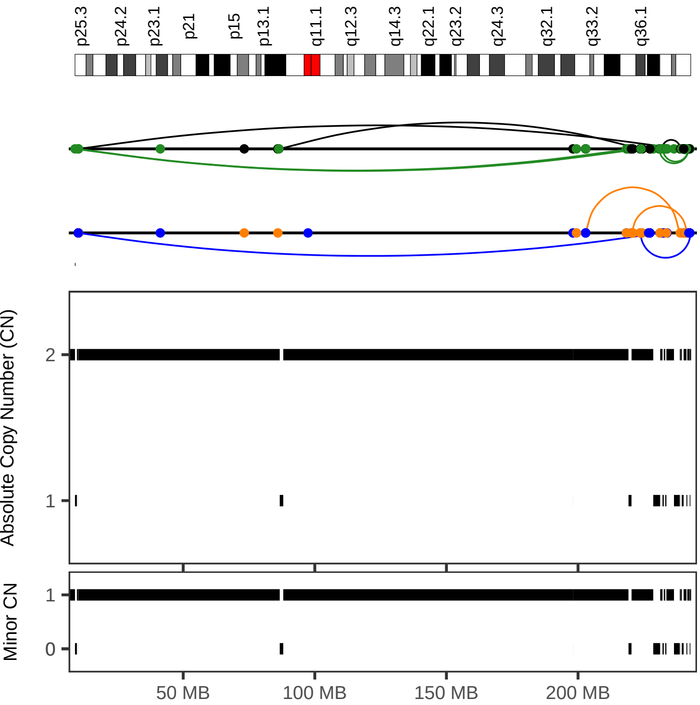

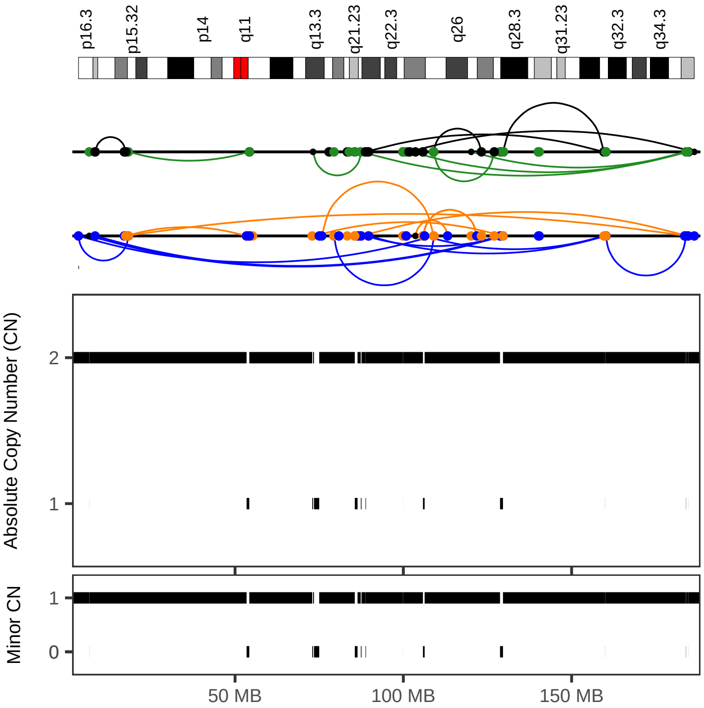

	CGP_donor_1437411
Cancer type	Bone−Benign
Position	2:8888556−242818178
Type	Canonical without polyploidization
Interleaved intrachr. SVs	13
Total SVs (intrachr. + transl.)	134
SV types	DEL: 2; DUP: 2; h2hINV: 3; t2tINV: 6; TRA: 121
SVs in sample	513
Oscillating CN (2 and 3 states)	37, 37
CN segments	37
FDR fragment joints	0.59
FDR chr. breakp. enrich.	0
Linked to chrs	1:51871319−244912138;4:3484005−186431923 5:67575508−163331316;
Purity, ploidy	0.75, 1.98

	CGP_donor_1437411
Cancer type	Bone−Benign
Position	4:3484005−186431924
Type	Canonical without polyploidization
Interleaved intrachr. SVs	30
Total SVs (intrachr. + transl.)	195
SV types	DEL: 8; DUP: 9; h2hINV: 6; t2tINV: 7; TRA: 165
SVs in sample	513
Oscillating CN (2 and 3 states)	37, 37
CN segments	37
FDR fragment joints	0.91
FDR chr. breakp. enrich.	0
Linked to chrs	1:51871319−244912138;2:8888556−242818177
Purity, ploidy	0.75, 1.98

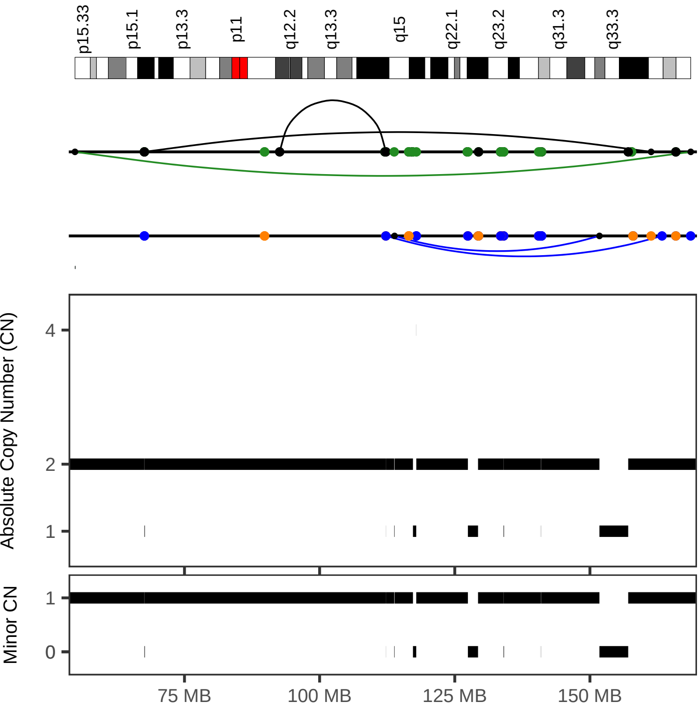

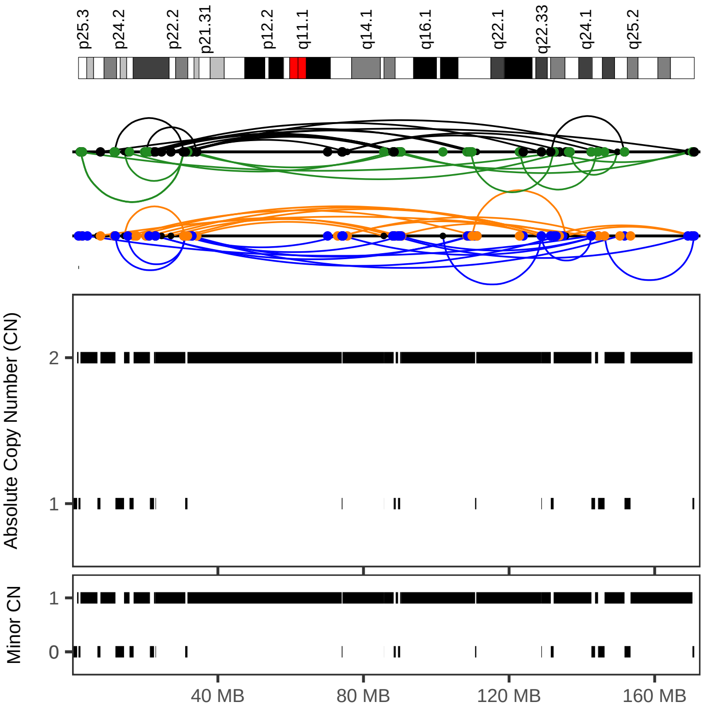

	CGP_donor_1437411
Cancer type	Bone−Benign
Position	5:67575508−163331317
Type	With other complex events
Interleaved intrachr. SVs	3
Total SVs (intrachr. + transl.)	93
SV types	DEL: 0; DUP: 1; h2hINV: 2; t2tINV: 0; TRA: 90
SVs in sample	513
Oscillating CN (2 and 3 states)	11, 11
CN segments	20
FDR fragment joints	0.59
FDR chr. breakp. enrich.	0
Linked to chrs	1:51871319−244912138;2:8888556−242818177 4:3484005−186431923;
Purity, ploidy	0.75, 1.98

	CGP_donor_1437411
Cancer type	Bone−Benign
Position	6:1694508−170873562
Type	Canonical without polyploidization
Interleaved intrachr. SVs	65
Total SVs (intrachr. + transl.)	284
SV types	DEL: 16; DUP: 15; h2hINV: 16; t2tINV: 18; TRA: 219
SVs in sample	513
Oscillating CN (2 and 3 states)	57, 57
CN segments	57
FDR fragment joints	0.98
FDR chr. breakp. enrich.	0
Linked to chrs	1:51871319−244912138;2:8888556−242818177 4:3484005−186431923;
Purity, ploidy	0.75, 1.98

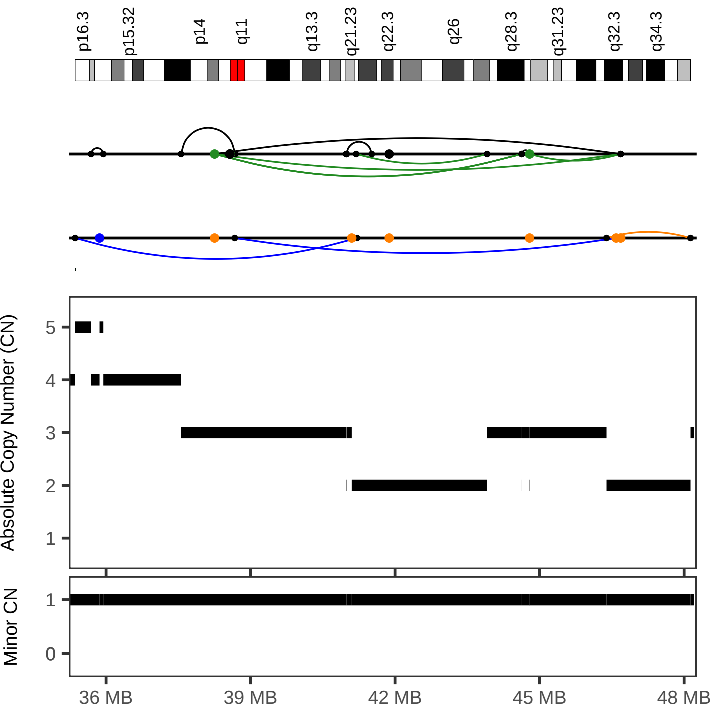

	**CGP_donor_1602529**
Cancer type	Bone−Epith
Position	4:35358971−48132846
Type	Canonical without polyploidization
Interleaved intrachr. SVs	11
Total SVs (intrachr. + transl.)	24
SV types	DEL: 1; DUP: 2; h2hINV: 3; t2tINV: 5; TRA: 13
SVs in sample	71
Oscillating CN (2 and 3 states)	11, 15
CN segments	15
FDR fragment joints	0.59
FDR chr. breakp. enrich.	0
Linked to chrs	
Purity, ploidy	0.77, 1.64

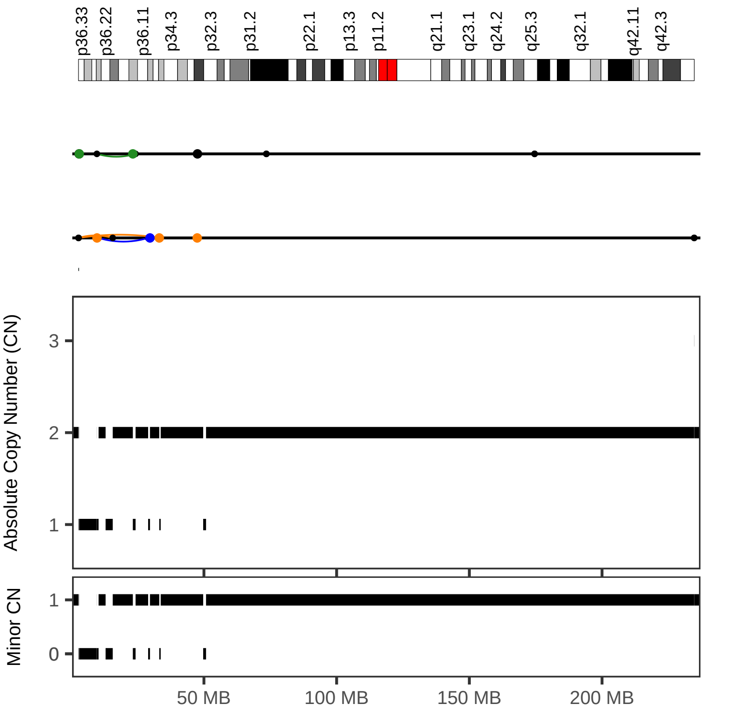

	**CGP_donor_1691150**
Cancer type	Bone−Epith
Position	1:2737096−33713570
Type	Canonical without polyploidization
Interleaved intrachr. SVs	4
Total SVs (intrachr. + transl.)	10
SV types	DEL: 2; DUP: 1; h2hINV: 0; t2tINV: 1; TRA: 6
SVs in sample	103
Oscillating CN (2 and 3 states)	15, 15
CN segments	15
FDR fragment joints	0.64
FDR chr. breakp. enrich.	0.07
Linked to chrs	15:33454042−35471243;
Purity, ploidy	0.76, 1.72

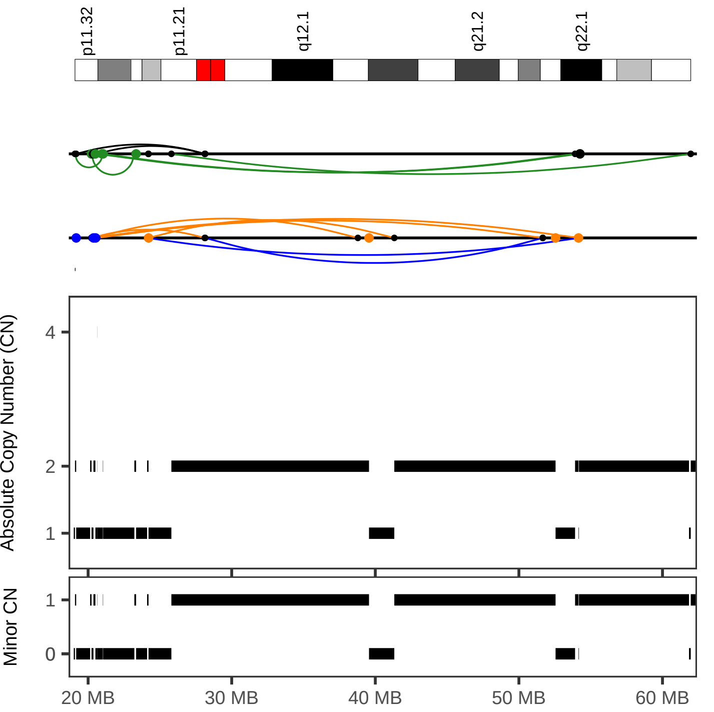

	**CGP_donor_1691150**
Cancer type	Bone−Epith
Position	18:19084928−61967083
Type	With other complex events
Interleaved intrachr. SVs	16
Total SVs (intrachr. + transl.)	32
SV types	DEL: 5; DUP: 2; h2hINV: 2; t2tINV: 7; TRA: 16
SVs in sample	103
Oscillating CN (2 and 3 states)	16, 27
CN segments	27
FDR fragment joints	0.59
FDR chr. breakp. enrich.	0
Linked to chrs	
Purity, ploidy	0.76, 1.72

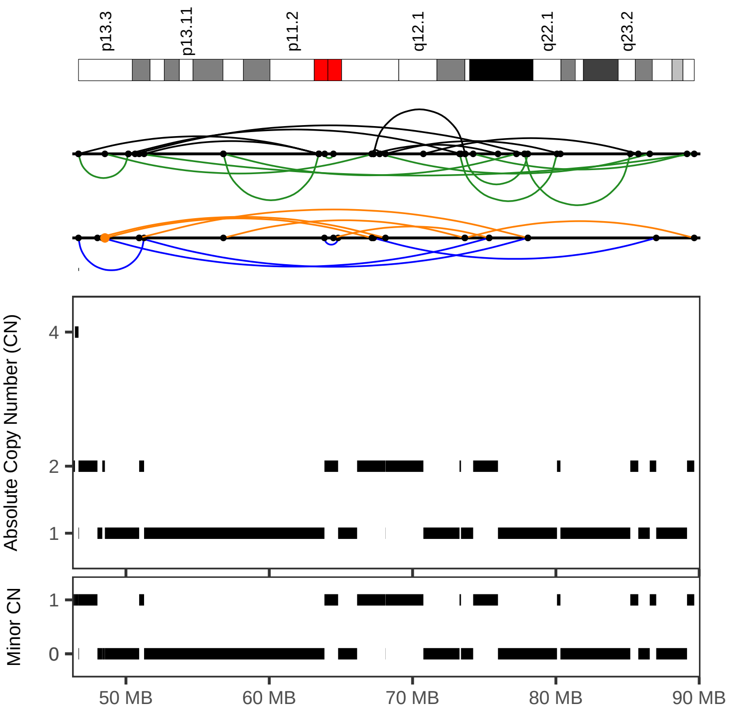

	**CGP_donor_1841267**
Cancer type	Bone−Epith
Position	16:46689644−89652038
Type	Canonical without polyploidization
Interleaved intrachr. SVs	32
Total SVs (intrachr. + transl.)	33
SV types	DEL: 6; DUP: 5; h2hINV: 9; t2tINV: 12; TRA: 1
SVs in sample	59
Oscillating CN (2 and 3 states)	24, 24
CN segments	24
FDR fragment joints	0.59
FDR chr. breakp. enrich.	0
Linked to chrs	
Purity, ploidy	0.32, 1.8

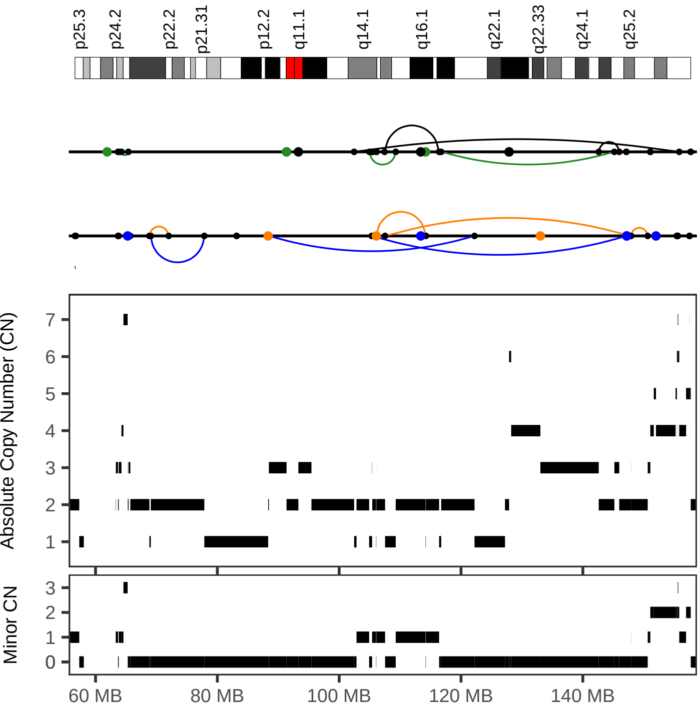

**CGP_donor_1397077**	
Cancer type	Bone−Osteosarc
Position	6:88458092−155828131
Type	With other complex events
Interleaved intrachr. SVs	11
Total SVs (intrachr. + transl.)	24
SV types	DEL: 2; DUP: 2; h2hINV: 4; t2tINV: 3; TRA: 13
SVs in sample	379
Oscillating CN (2 and 3 states)	9, 13
CN segments	40
FDR fragment joints	0.84
FDR chr. breakp. enrich.	0
Linked to chrs	20:14754035−59064330;5:759551−44322946
Purity, ploidy	0.47, 2.53

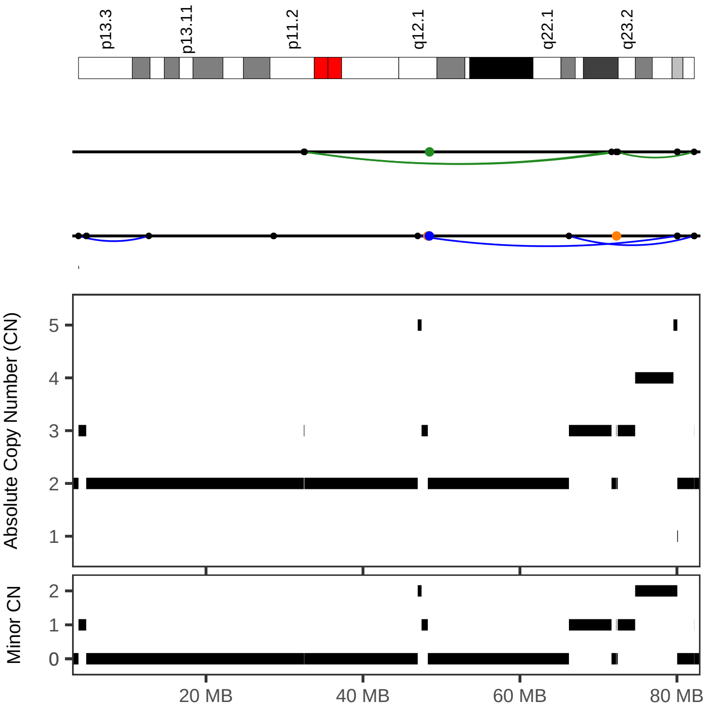

**CGP_donor_1397083**	
Cancer type	Bone−Osteosarc
Position	16:32468669−82206220
Type	With other complex events
Interleaved intrachr. SVs	5
Total SVs (intrachr. + transl.)	9
SV types	DEL: 0; DUP: 2; h2hINV: 0; t2tINV: 3; TRA: 4
SVs in sample	142
Oscillating CN (2 and 3 states)	7, 10
CN segments	19
FDR fragment joints	0.59
FDR chr. breakp. enrich.	0
Linked to chrs	
Purity, ploidy	0.57, 3.02

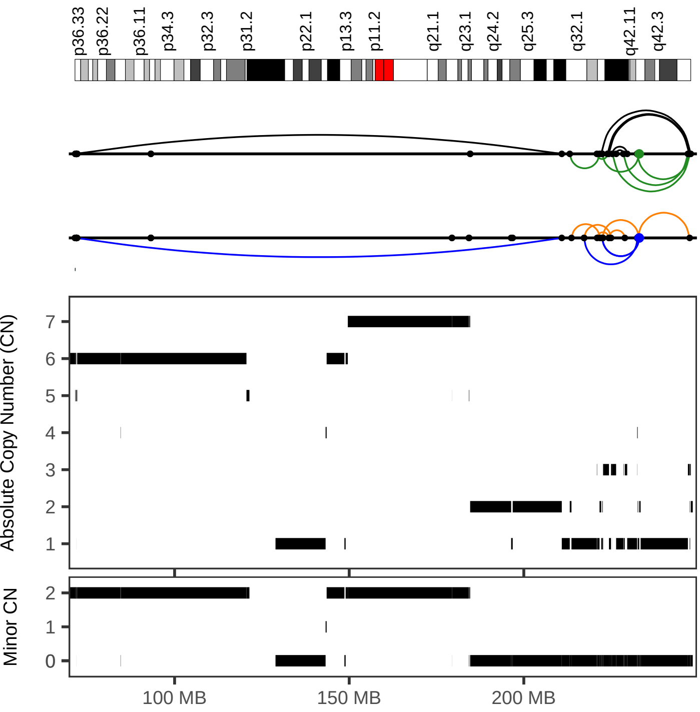

**CGP_donor_1397084**	
Cancer type	Bone−Osteosarc
Position	1:213162214−247806789
Type	With other complex events
Interleaved intrachr. SVs	21
Total SVs (intrachr. + transl.)	24
SV types	DEL: 6; DUP: 4; h2hINV: 5; t2tINV: 6; TRA: 3
SVs in sample	195
Oscillating CN (2 and 3 states)	13, 14
CN segments	30
FDR fragment joints	0.94
FDR chr. breakp. enrich.	0
Linked to chrs	
Purity, ploidy	0.7, 3.46

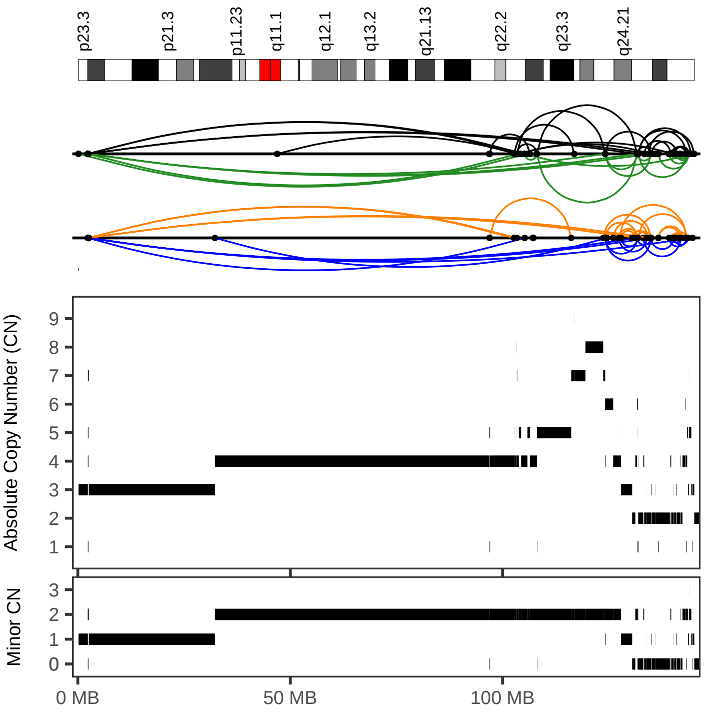

**CGP_donor_1397084**	
Cancer type	Bone−Osteosarc
Position	8:155450−145113709
Type	With other complex events
Interleaved intrachr. SVs	83
Total SVs (intrachr. + transl.)	83
SV types	DEL: 21; DUP: 16; h2hINV: 24; t2tINV: 22; TRA: 0
SVs in sample	195
Oscillating CN (2 and 3 states)	8, 20
CN segments	90
FDR fragment joints	0.72
FDR chr. breakp. enrich.	0
Linked to chrs	
Purity, ploidy	0.7, 3.46

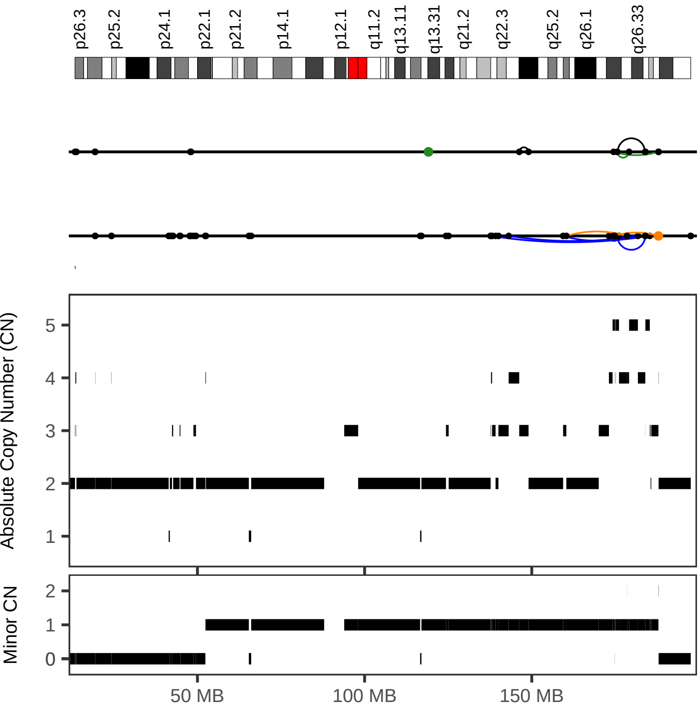

	**CGP_donor_1437403**
Cancer type	Bone−Osteosarc
Position	3:137693454−187956215
Type	With other complex events
Interleaved intrachr. SVs	11
Total SVs (intrachr. + transl.)	12
SV types	DEL: 2; DUP: 6; h2hINV: 1; t2tINV: 2; TRA: 1
SVs in sample	445
Oscillating CN (2 and 3 states)	11, 22
CN segments	30
FDR fragment joints	0.59
FDR chr. breakp. enrich.	0.16
Linked to chrs	
Purity, ploidy	0.8, 2.57

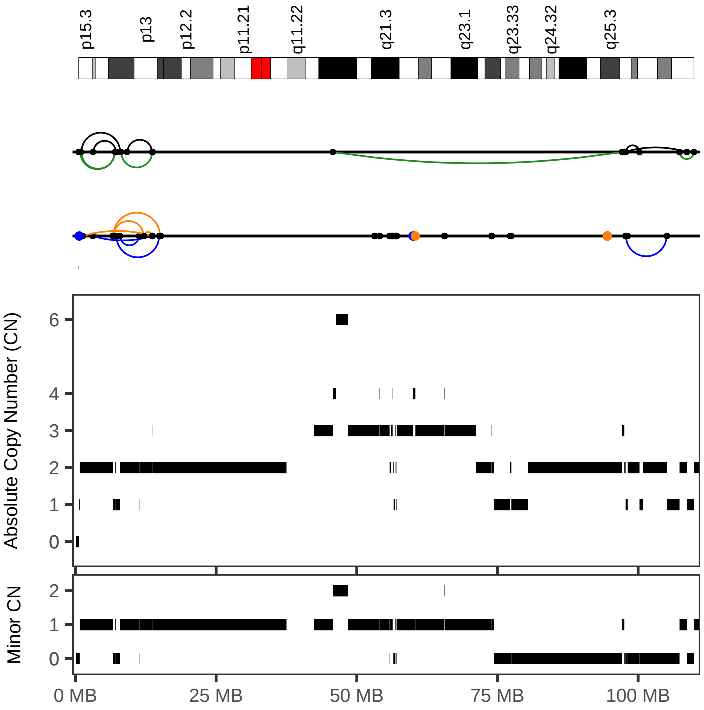

	**CGP_donor_1437403**
Cancer type	Bone−Osteosarc
Position	10:757620−15159495
Type	Canonical without polyploidization
Interleaved intrachr. SVs	14
Total SVs (intrachr. + transl.)	14
SV types	DEL: 4; DUP: 3; h2hINV: 4; t2tINV: 3; TRA: 0
SVs in sample	445
Oscillating CN (2 and 3 states)	9, 11
CN segments	11
FDR fragment joints	0.98
FDR chr. breakp. enrich.	0
Linked to chrs	
Purity, ploidy	0.8, 2.57

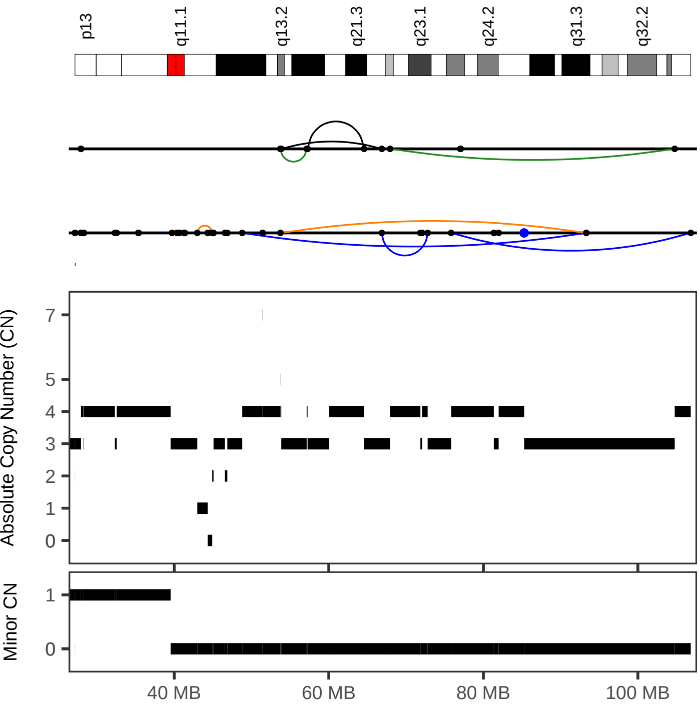

	**CGP_donor_1437403**
Cancer type	Bone−Osteosarc
Position	14:48802183−106839331
Type	After polyploidization
Interleaved intrachr. SVs	7
Total SVs (intrachr. + transl.)	8
SV types	DEL: 1; DUP: 3; h2hINV: 1; t2tINV: 2; TRA: 1
SVs in sample	445
Oscillating CN (2 and 3 states)	15, 19
CN segments	19
FDR fragment joints	0.74
FDR chr. breakp. enrich.	0.03
Linked to chrs	
Purity, ploidy	0.8, 2.57

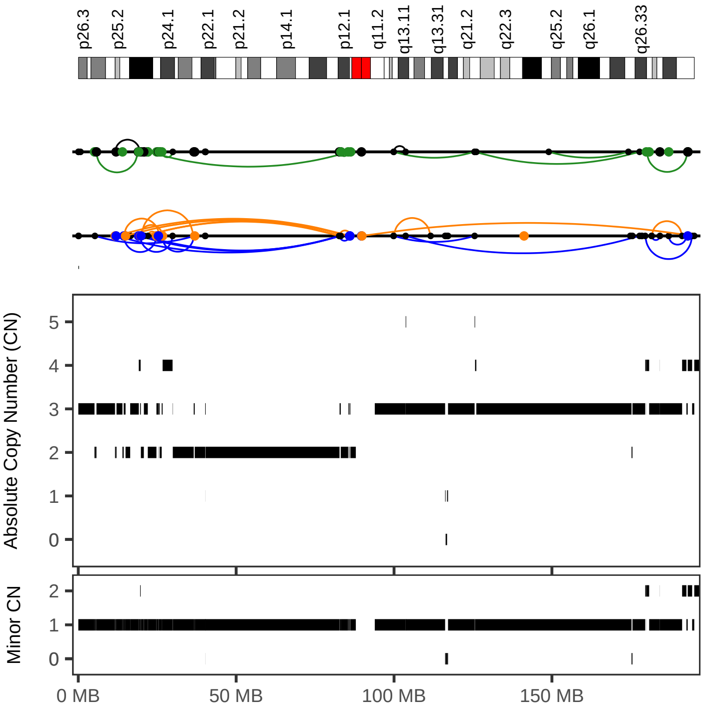

	**CGP_donor_1437404**
Cancer type	Bone−Osteosarc
Position	3:5273444−86230794
Type	With other complex events
Interleaved intrachr. SVs	25
Total SVs (intrachr. + transl.)	68
SV types	DEL: 9; DUP: 9; h2hINV: 3; t2tINV: 4; TRA: 43
SVs in sample	597
Oscillating CN (2 and 3 states)	19, 25
CN segments	55
FDR fragment joints	0.59
FDR chr. breakp. enrich.	0
Linked to chrs	6:40585455−137670951;
Purity, ploidy	0.78, 3.61

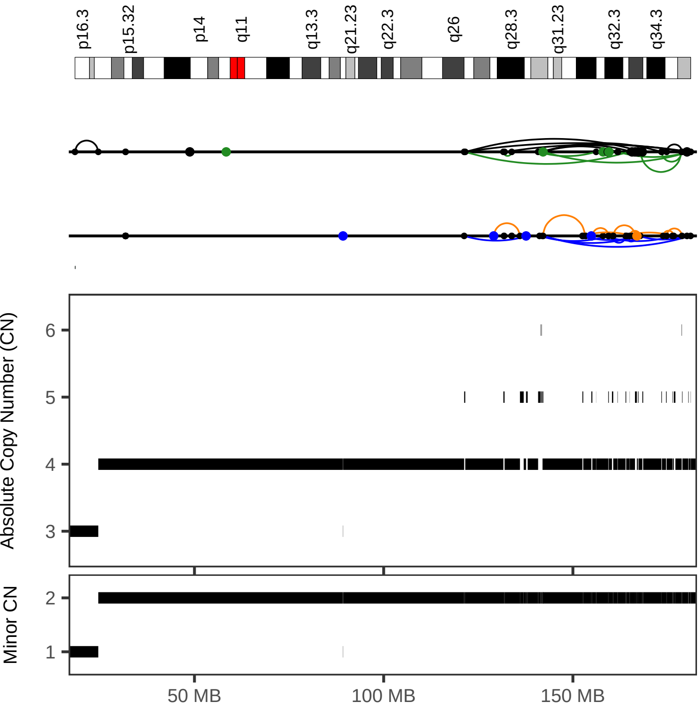

CGP_donor_1437404	
Cancer type	Bone−Osteosarc
Position	4:121261720−181140074
Type	After polyploidization
Interleaved intrachr. SVs	36
Total SVs (intrachr. + transl.)	49
SV types	DEL: 9; DUP: 11; h2hINV: 7; t2tINV: 9; TRA: 13
SVs in sample	597
Oscillating CN (2 and 3 states)	36, 50
CN segments	57
FDR fragment joints	0.87
FDR chr. breakp. enrich.	0
Linked to chrs	6:40585455−137670951;
Purity, ploidy	0.78, 3.61

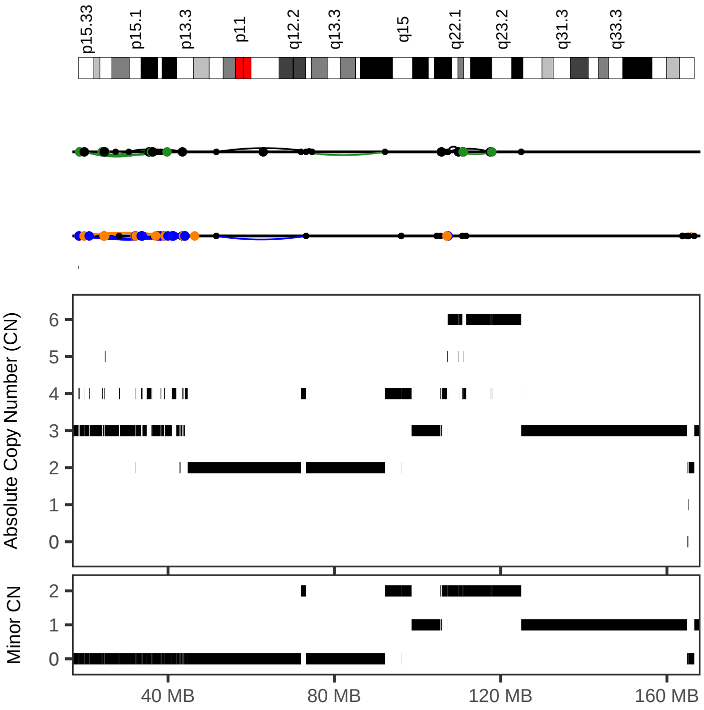

CGP_donor_1437404	
Cancer type	Bone−Osteosarc
Position	5:18472606−43606863
Type	With other complex events
Interleaved intrachr. SVs	19
Total SVs (intrachr. + transl.)	59
SV types	DEL: 3; DUP: 9; h2hINV: 3; t2tINV: 4; TRA: 40
SVs in sample	597
Oscillating CN (2 and 3 states)	14, 16
CN segments	33
FDR fragment joints	0.59
FDR chr. breakp. enrich.	0
Linked to chrs	16:956773−33610119;6:40585455−137670951
Purity, ploidy	0.78, 3.61

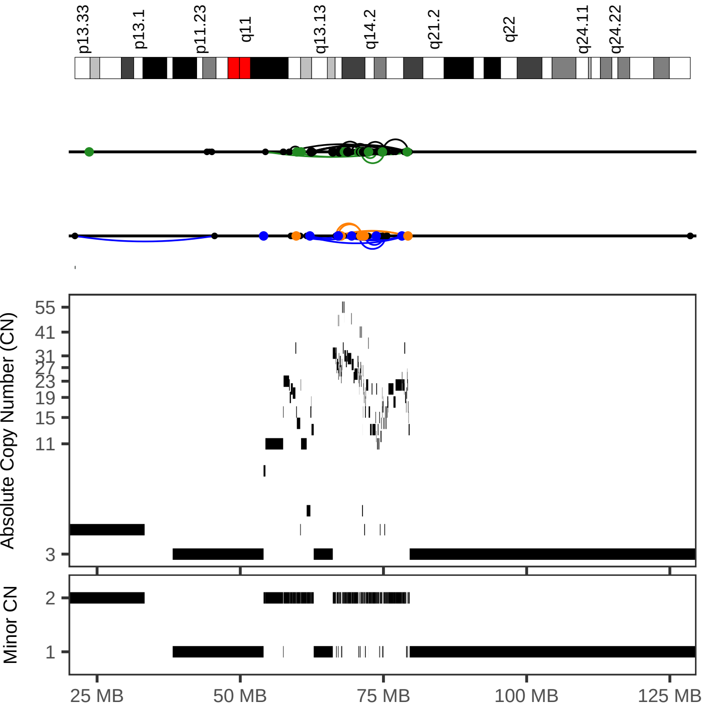

CGP_donor_1437404	
Cancer type	Bone−Osteosarc
Position	12:54388545−79574150
Type	With other complex events
Interleaved intrachr. SVs	58
Total SVs (intrachr. + transl.)	85
SV types	DEL: 16; DUP: 12; h2hINV: 16; t2tINV: 14; TRA: 27
SVs in sample	597
Oscillating CN (2 and 3 states)	3, 4
CN segments	127
FDR fragment joints	0.9
FDR chr. breakp. enrich.	0
Linked to chrs	2:41203222−217343687;20:35368877−35493653 6:40585455−137670951;
Purity, ploidy	0.78, 3.61

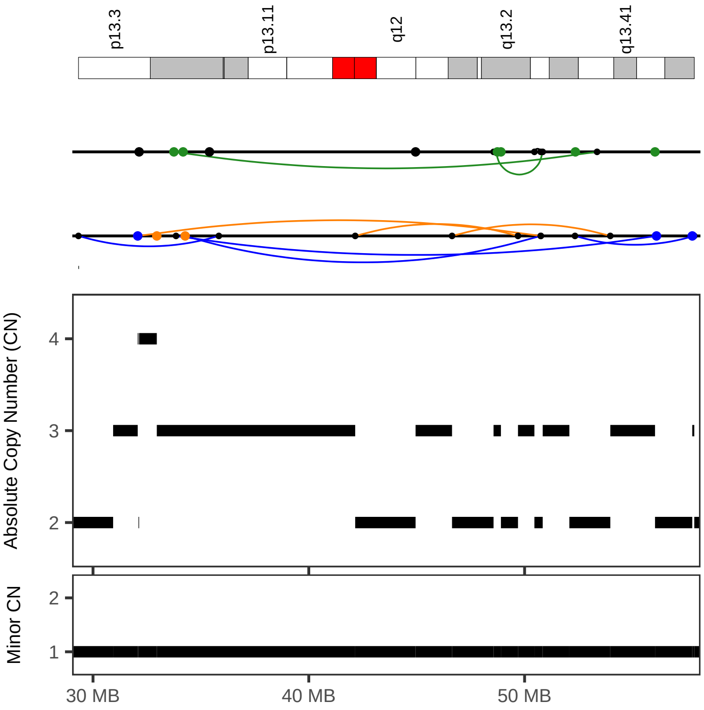

CGP_donor_1437404	
Cancer type	Bone−Osteosarc
Position	19:29326898−57862516
Type	Canonical without polyploidization
Interleaved intrachr. SVs	10
Total SVs (intrachr. + transl.)	24
SV types	DEL: 3; DUP: 4; h2hINV: 0; t2tINV: 3; TRA: 14
SVs in sample	597
Oscillating CN (2 and 3 states)	14, 14
CN segments	19
FDR fragment joints	0.59
FDR chr. breakp. enrich.	0
Linked to chrs	6:40585455−137670951;
Purity, ploidy	0.78, 3.61

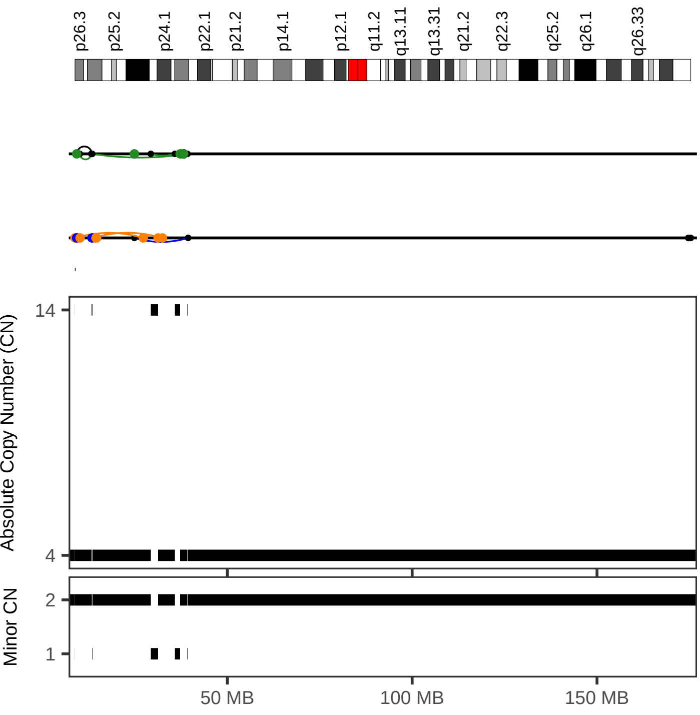

CGP_donor_1437406	
Cancer type	Bone−Osteosarc
Position	3:8793061−39393867
Type	After polyploidization
Interleaved intrachr. SVs	7
Total SVs (intrachr. + transl.)	23
SV types	DEL: 3; DUP: 1; h2hINV: 1; t2tINV: 2; TRA: 16
SVs in sample	871
Oscillating CN (2 and 3 states)	11, 11
CN segments	11
FDR fragment joints	0.74
FDR chr. breakp. enrich.	0
Linked to chrs	5:55072−32110729;
Purity, ploidy	0.52, 4.48

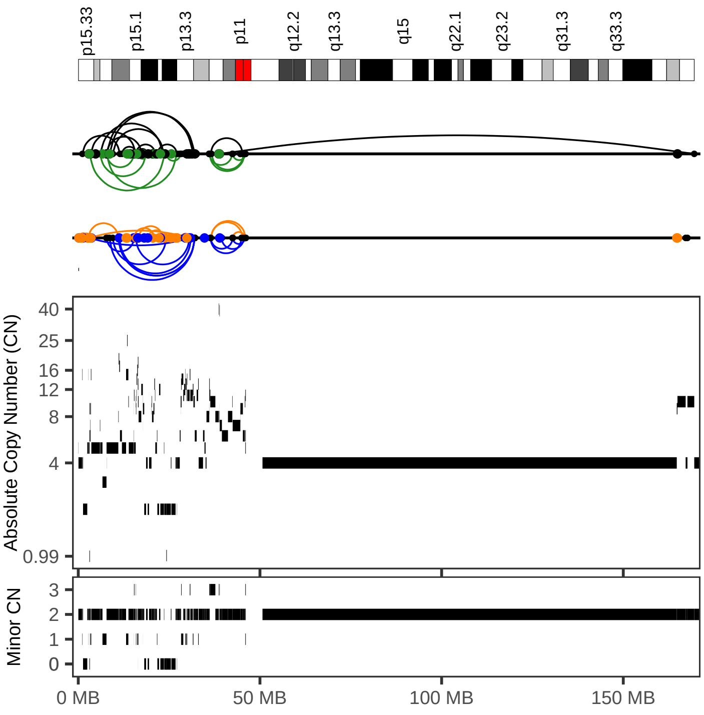

CGP_donor_1437406	
Cancer type	Bone−Osteosarc
Position	5:55072−32110730
Type	With other complex events
Interleaved intrachr. SVs	55
Total SVs (intrachr. + transl.)	130
SV types	DEL: 12; DUP: 17; h2hINV: 16; t2tINV: 10; TRA: 75
SVs in sample	871
Oscillating CN (2 and 3 states)	6, 6
CN segments	99
FDR fragment joints	0.64
FDR chr. breakp. enrich.	0
Linked to chrs	3:8793061−39393866;
Purity, ploidy	0.52, 4.48

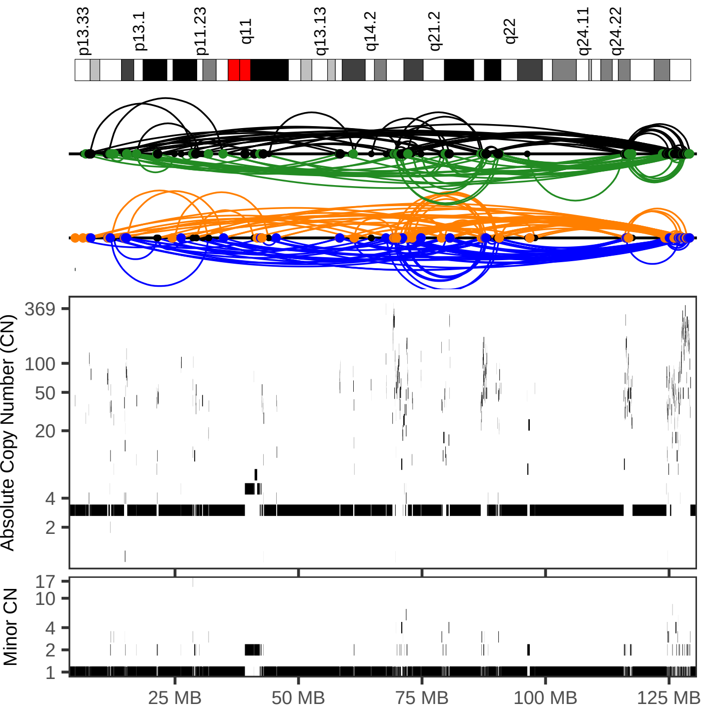

CGP_donor_1437406	
Cancer type	Bone−Osteosarc
Position	12:4757456−129380238
Type	With other complex events
Interleaved intrachr. SVs	340
Total SVs (intrachr. + transl.)	560
SV types	DEL: 90; DUP: 99; h2hINV: 72; t2tINV: 79; TRA: 220
SVs in sample	871
Oscillating CN (2 and 3 states)	4, 5
CN segments	551
FDR fragment joints	0.59
FDR chr. breakp. enrich.	0
Linked to chrs	5:55072−32110729;
Purity, ploidy	0.52, 4.48

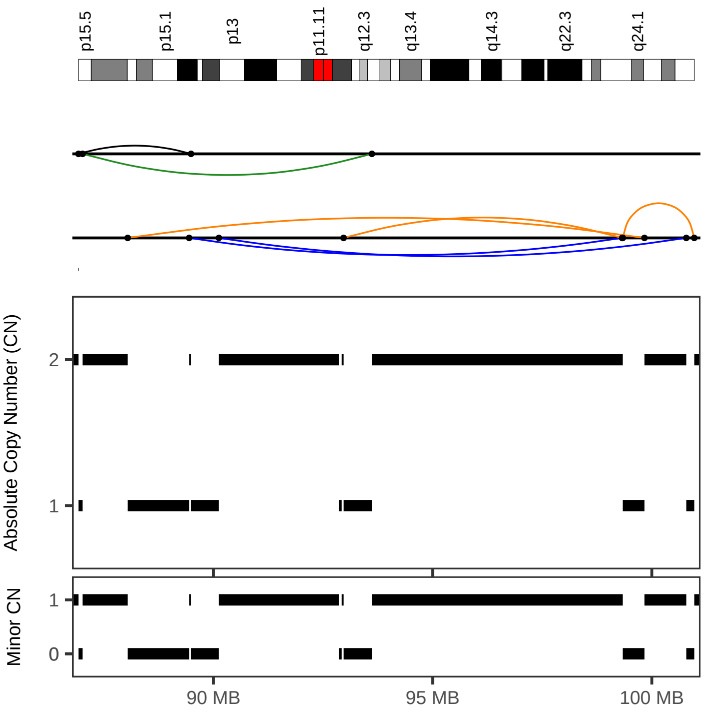

CGP_donor_1437407	
Cancer type	Bone−Osteosarc
Position	11:86917622−100968022
Type	Canonical without polyploidization
Interleaved intrachr. SVs	7
Total SVs (intrachr. + transl.)	7
SV types	DEL: 3; DUP: 2; h2hINV: 1; t2tINV: 1; TRA: 0
SVs in sample	92
Oscillating CN (2 and 3 states)	14, 14
CN segments	14
FDR fragment joints	0.74
FDR chr. breakp. enrich.	0.43
Linked to chrs	
Purity, ploidy	0.83, 1.77

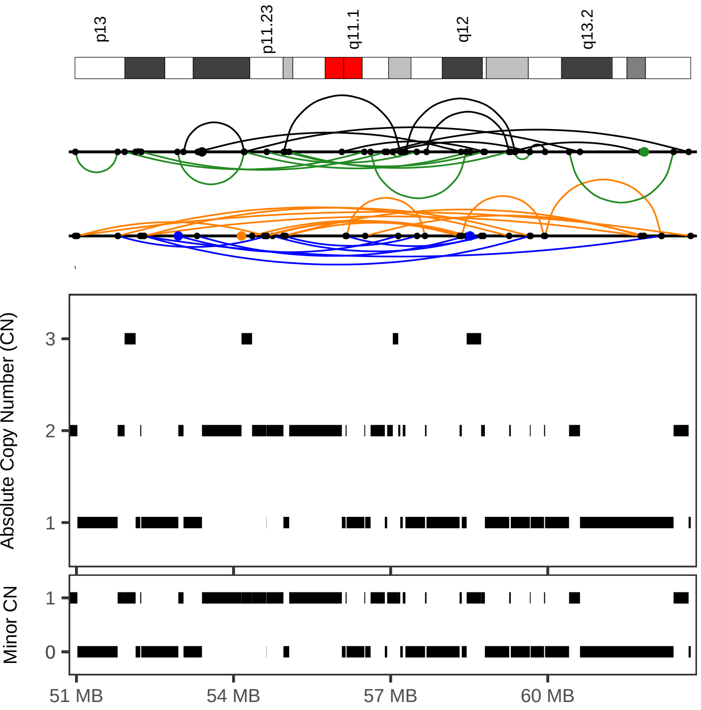

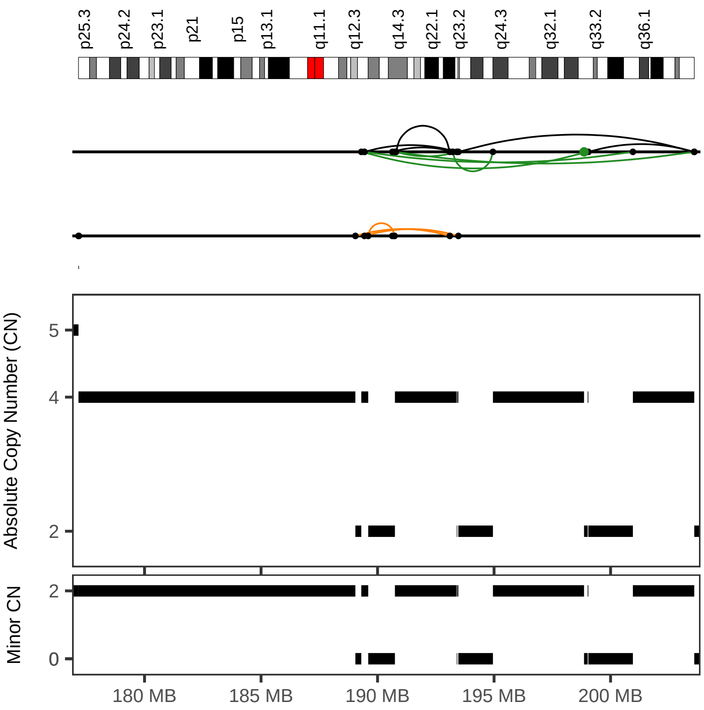

CGP_donor_1437407	
Cancer type	Bone−Osteosarc
Position	20:50980028−62689311
Type	With other complex events
Interleaved intrachr. SVs	45
Total SVs (intrachr. + transl.)	51
SV types	DEL: 13; DUP: 8; h2hINV: 12; t2tINV: 12; TRA: 6
SVs in sample	92
Oscillating CN (2 and 3 states)	13, 29
CN segments	46
FDR fragment joints	0.8
FDR chr. breakp. enrich.	0
Linked to chrs	
Purity, ploidy	0.83, 1.77

CGP_donor_1437409	
Cancer type	Bone−Osteosarc
Position	2:189048530−203590444
Type	Before polyploidization
Interleaved intrachr. SVs	15
Total SVs (intrachr. + transl.)	16
SV types	DEL: 3; DUP: 1; h2hINV: 6; t2tINV: 5; TRA: 1
SVs in sample	118
Oscillating CN (2 and 3 states)	13, 13
CN segments	13
FDR fragment joints	0.59
FDR chr. breakp. enrich.	0.05
Linked to chrs	
Purity, ploidy	0.67, 3.41

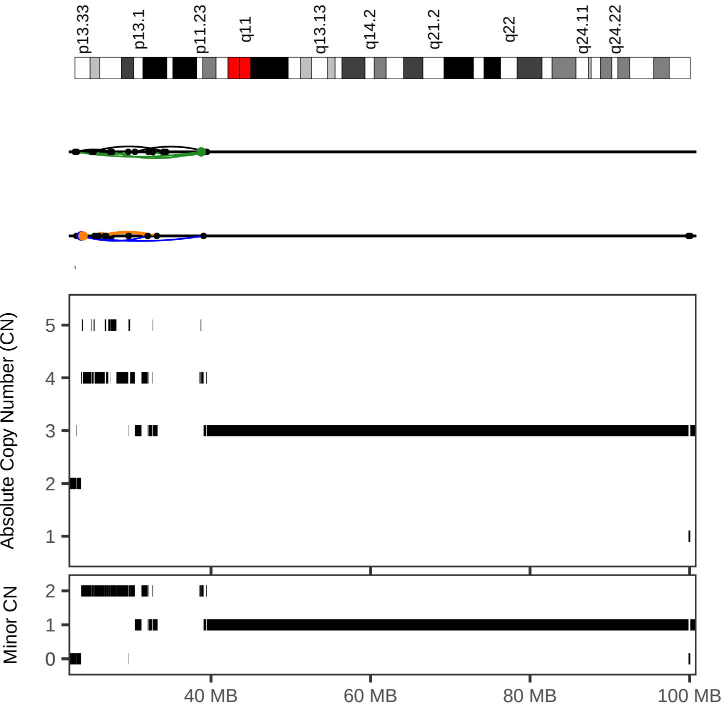

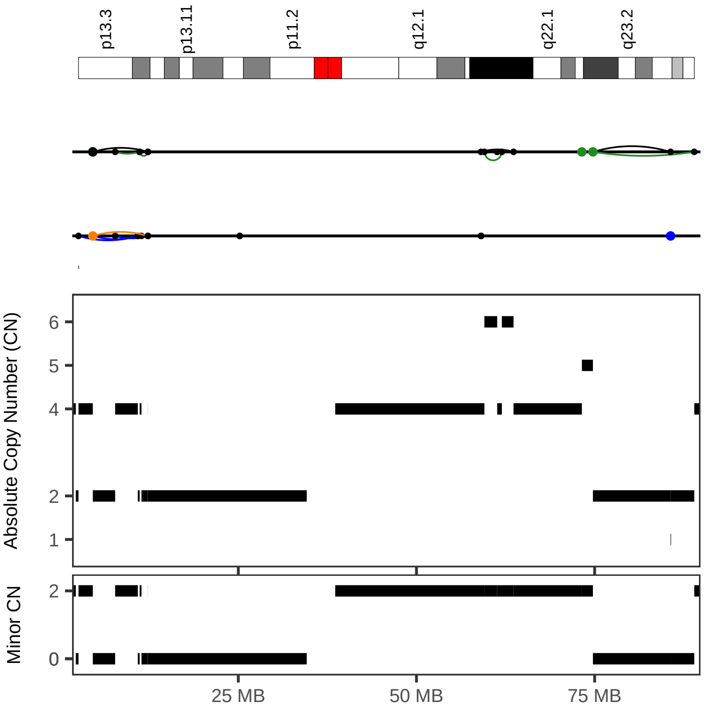

CGP_donor_1437409	
Cancer type	Bone−Osteosarc
Position	12:22931670−39481564
Type	With other complex events
Interleaved intrachr. SVs	14
Total SVs (intrachr. + transl.)	17
SV types	DEL: 3; DUP: 2; h2hINV: 4; t2tINV: 5; TRA: 3
SVs in sample	118
Oscillating CN (2 and 3 states)	14, 17
CN segments	34
FDR fragment joints	0.78
FDR chr. breakp. enrich.	0
Linked to chrs	
Purity, ploidy	0.67, 3.41

CGP_donor_1437409	
Cancer type	Bone−Osteosarc
Position	16:2570177−12331926
Type	Before polyploidization
Interleaved intrachr. SVs	8
Total SVs (intrachr. + transl.)	11
SV types	DEL: 1; DUP: 4; h2hINV: 1; t2tINV: 2; TRA: 3
SVs in sample	118
Oscillating CN (2 and 3 states)	8, 8
CN segments	8
FDR fragment joints	0.59
FDR chr. breakp. enrich.	0
Linked to chrs	11:79173759−84202447;
Purity, ploidy	0.67, 3.41

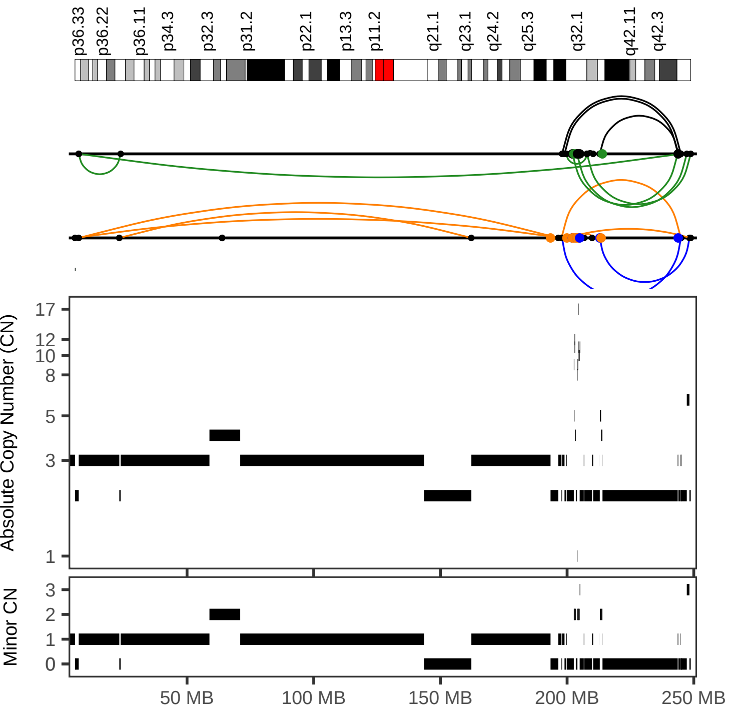

**CGP_donor_1490908**	
Cancer type	Bone−Osteosarc
Position	1:5777564−248793247
Type	With other complex events
Interleaved intrachr. SVs	23
Total SVs (intrachr. + transl.)	46
SV types	DEL: 6; DUP: 5; h2hINV: 6; t2tINV: 6; TRA: 23
SVs in sample	216
Oscillating CN (2 and 3 states)	12, 21
CN segments	52
FDR fragment joints	1
FDR chr. breakp. enrich.	0
Linked to chrs	14:60585562−102848228;
Purity, ploidy	0.67, 3.06

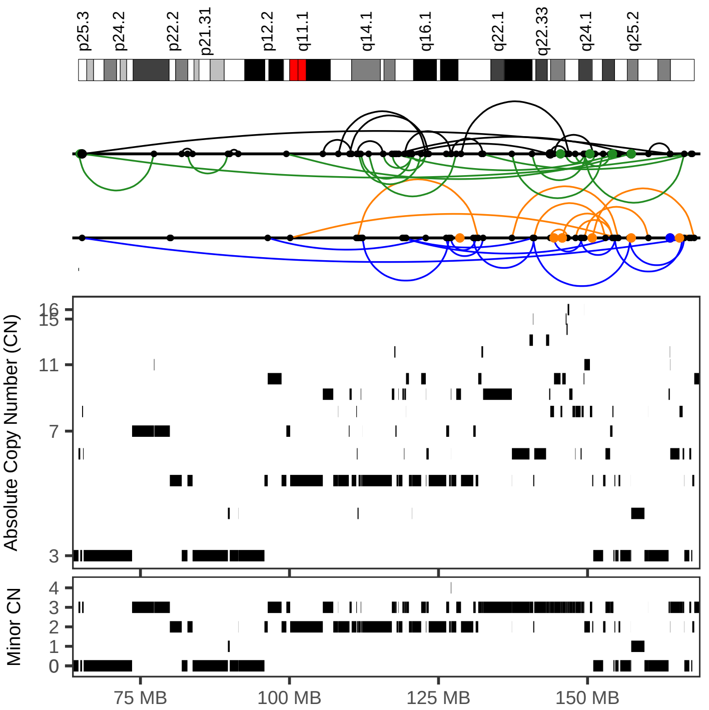

**CGP_donor_1490908**	
Cancer type	Bone−Osteosarc
Position	6:64603046−167895820
Type	With other complex events
Interleaved intrachr. SVs	58
Total SVs (intrachr. + transl.)	75
SV types	DEL: 10; DUP: 15; h2hINV: 15; t2tINV: 18; TRA: 17
SVs in sample	216
Oscillating CN (2 and 3 states)	11, 13
CN segments	130
FDR fragment joints	0.64
FDR chr. breakp. enrich.	0
Linked to chrs	
Purity, ploidy	0.67, 3.06

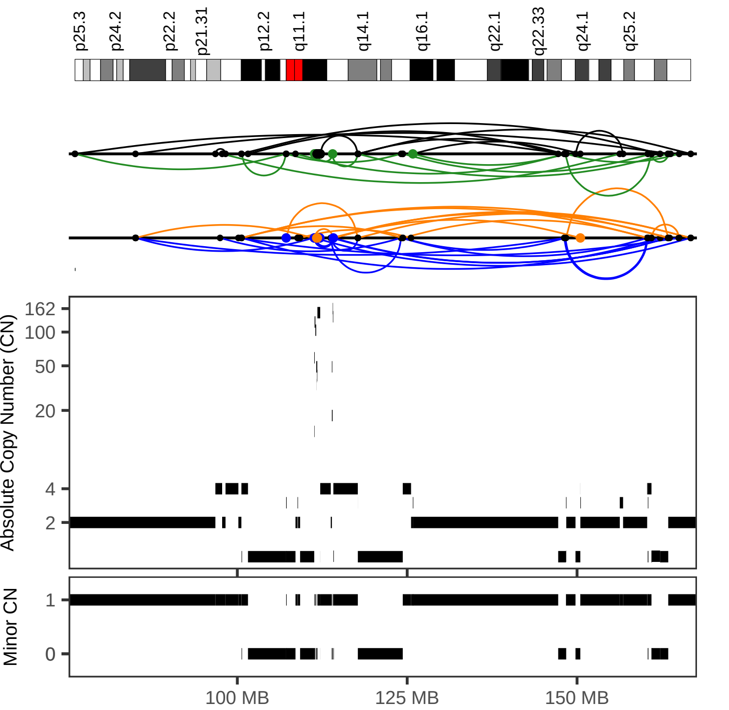

**CGP_donor_1490914**	
Cancer type	Bone−Osteosarc
Position	6:76106264−166727720
Type	With other complex events
Interleaved intrachr. SVs	61
Total SVs (intrachr. + transl.)	91
SV types	DEL: 20; DUP: 16; h2hINV: 12; t2tINV: 13; TRA: 30
SVs in sample	462
Oscillating CN (2 and 3 states)	6, 9
CN segments	65
FDR fragment joints	0.64
FDR chr. breakp. enrich.	0
Linked to chrs	11:101720341−105872168;
Purity, ploidy	0.64, 2.03

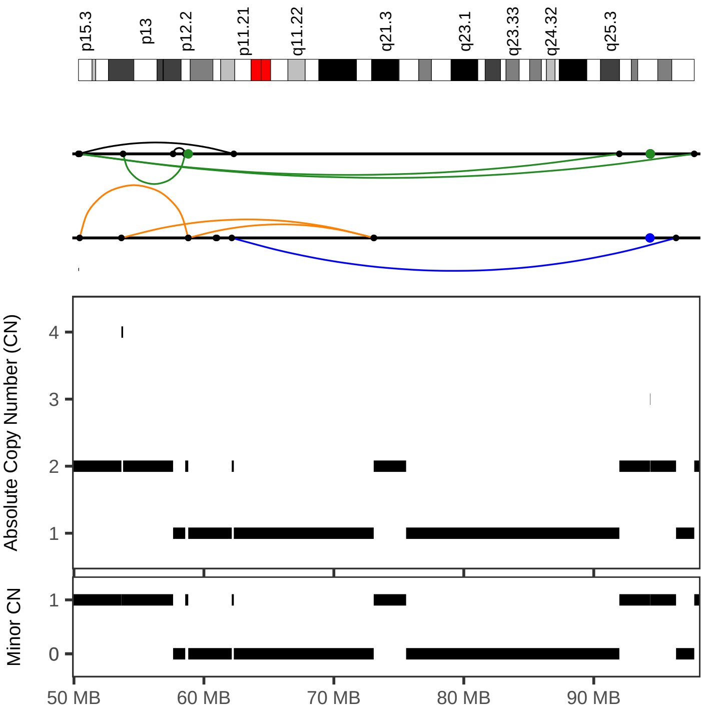

**CGP_donor_1490914**	
Cancer type	Bone−Osteosarc
Position	10:50348934−97731679
Type	Canonical without polyploidization
Interleaved intrachr. SVs	7
Total SVs (intrachr. + transl.)	12
SV types	DEL: 3; DUP: 1; h2hINV: 1; t2tINV: 2; TRA: 5
SVs in sample	462
Oscillating CN (2 and 3 states)	9, 13
CN segments	15
FDR fragment joints	0.74
FDR chr. breakp. enrich.	0.68
Linked to chrs	5:343444−40967399;
Purity, ploidy	0.64, 2.03

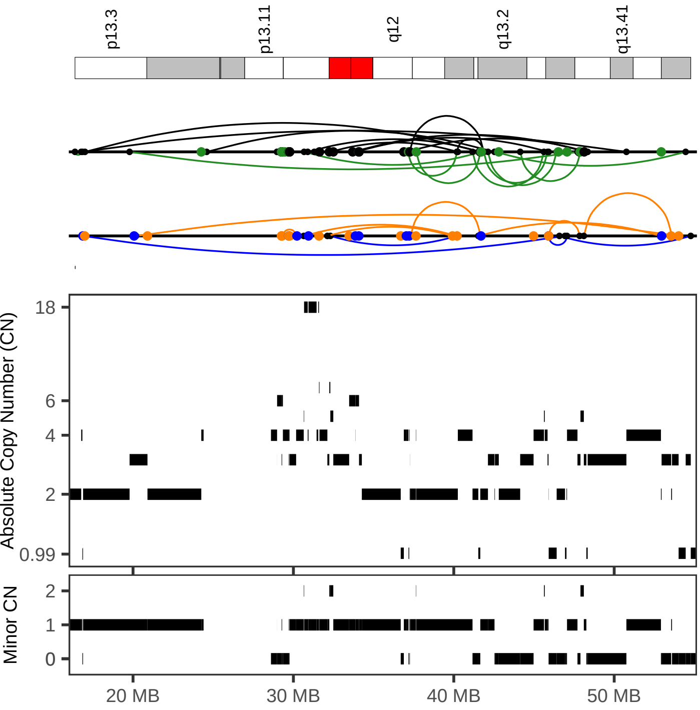

CGP_donor_1490914

Cancer type	Bone−Osteosarc
Position	19:16843361−54772913
Type	With other complex events
Interleaved intrachr. SVs	31
Total SVs (intrachr. + transl.)	100
SV types	DEL: 8; DUP: 4; h2hINV: 10; t2tINV: 9; TRA: 69
SVs in sample	462
Oscillating CN (2 and 3 states)	7, 13
CN segments	78
FDR fragment joints	0.64
FDR chr. breakp. enrich.	0
Linked to chrs	X:97627888−151461839;
Purity, ploidy	0.64, 2.03

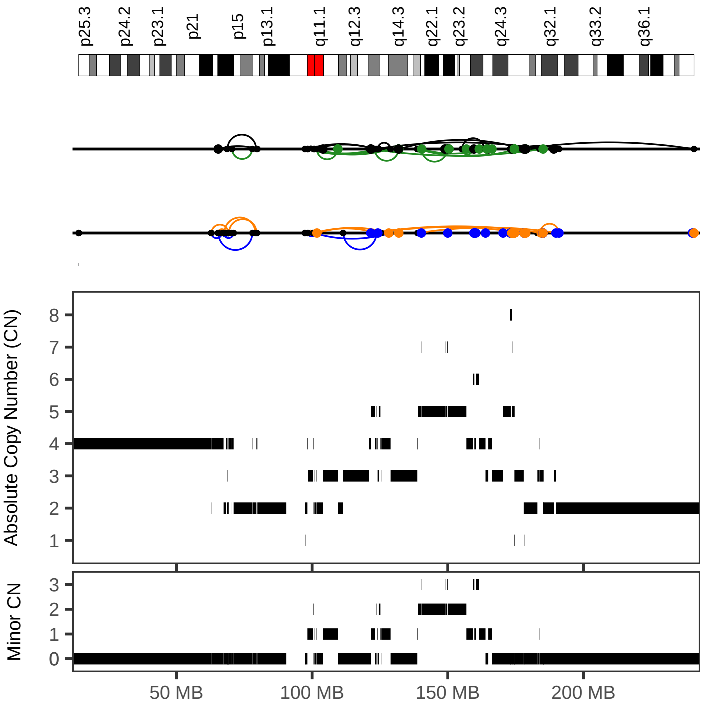

CGP_donor_1528364

Cancer type	Bone−Osteosarc
Position	2:98265301−240710956
Type	With other complex events
Interleaved intrachr. SVs	30
Total SVs (intrachr. + transl.)	95
SV types	DEL: 8; DUP: 5; h2hINV: 8; t2tINV: 9; TRA: 65
SVs in sample	927
Oscillating CN (2 and 3 states)	10, 16
CN segments	81
FDR fragment joints	0.83
FDR chr. breakp. enrich.	0
Linked to chrs	1:2249362−182869103;10:44478896−86379720 18:63082505−77710469;3:39705096−116872806
Purity, ploidy	0.79, 3.29

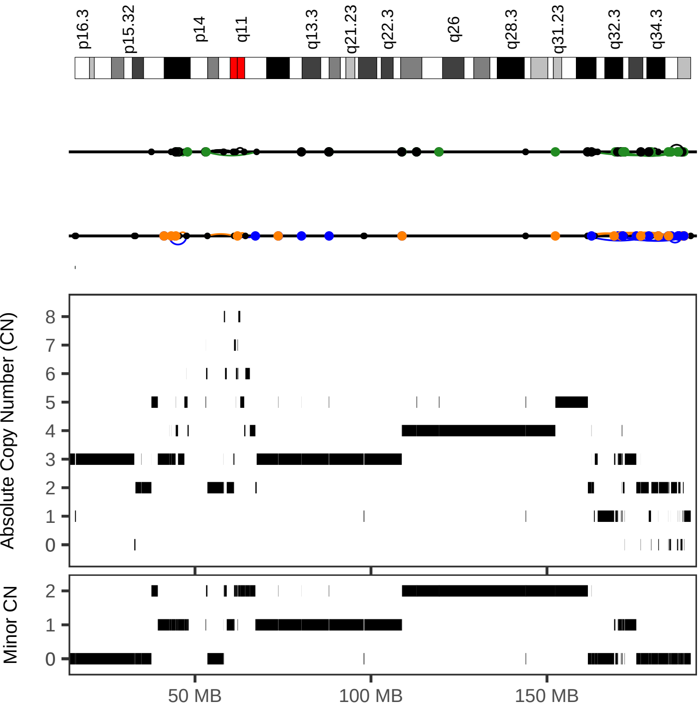

CGP_donor_1528364

Cancer type	Bone−Osteosarc
Position	4:161515724−190840263
Type	With other complex events
Interleaved intrachr. SVs	17
Total SVs (intrachr. + transl.)	84
SV types	DEL: 4; DUP: 7; h2hINV: 2; t2tINV: 4; TRA: 67
SVs in sample	927
Oscillating CN (2 and 3 states)	11, 12
CN segments	65
FDR fragment joints	0.59
FDR chr. breakp. enrich.	0
Linked to chrs	10:44478896−86379720;18:63082505−77710469 5:878141−41078796;9:1299761−9380244
Purity, ploidy	0.79, 3.29

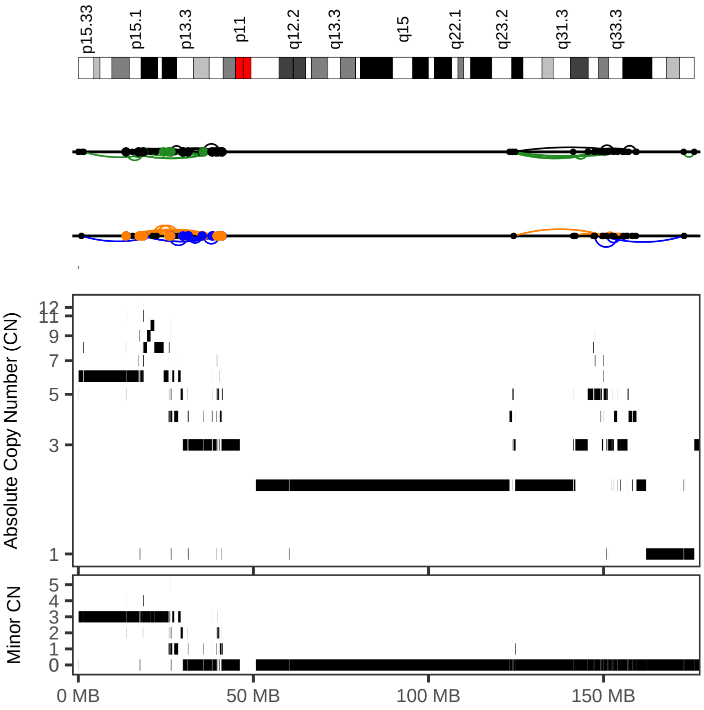

CGP_donor_1528364

Cancer type	Bone−Osteosarc
Position	5:878141−41078797
Type	With other complex events
Interleaved intrachr. SVs	57
Total SVs (intrachr. + transl.)	143
SV types	DEL: 16; DUP: 14; h2hINV: 16; t2tINV: 11; TRA: 86
SVs in sample	927
Oscillating CN (2 and 3 states)	12, 12
CN segments	126
FDR fragment joints	0.84
FDR chr. breakp. enrich.	0
Linked to chrs	10:44478896−86379720;18:63082505−77710469 9:1299761−9380244;
Purity, ploidy	0.79, 3.29

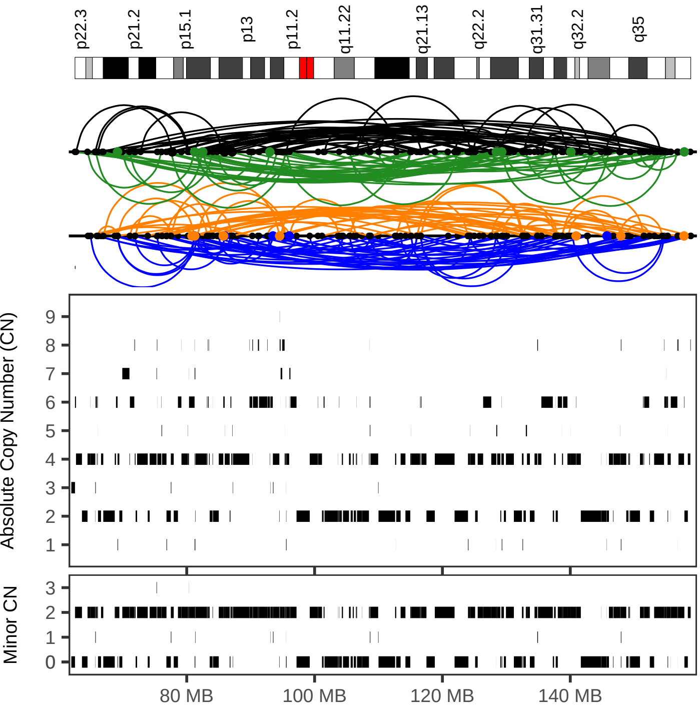

	CGP_donor_1528364
Cancer type	Bone−Osteosarc
Position	7:62514967−158846143
Type	With other complex events
Interleaved intrachr. SVs	233
Total SVs (intrachr. + transl.)	257
SV types	DEL: 56; DUP: 62; h2hINV: 56; t2tINV: 59; TRA: 24
SVs in sample	927
Oscillating CN (2 and 3 states)	8, 15
CN segments	274
FDR fragment joints	0.95
FDR chr. breakp. enrich.	0
Linked to chrs	2:98265301−240710955;
Purity, ploidy	0.79, 3.29

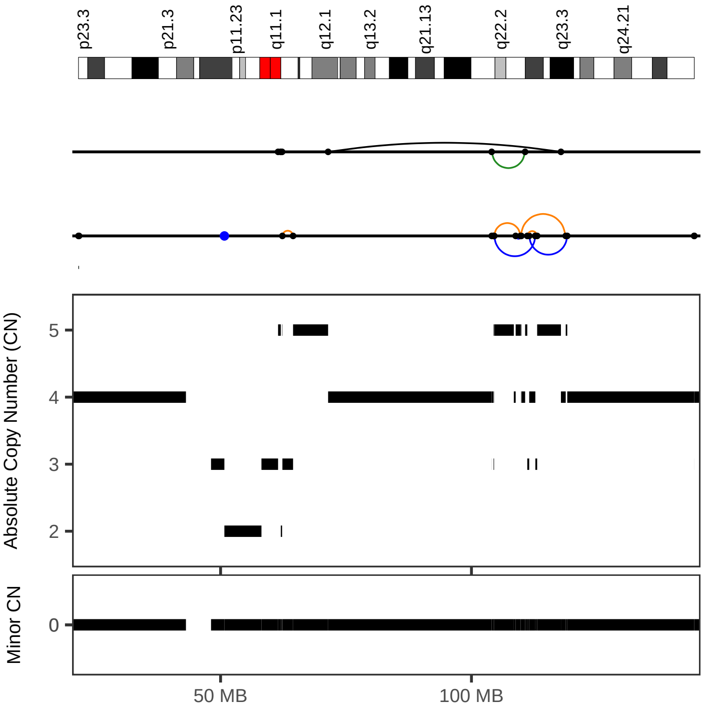

	CGP_donor_1528364
Cancer type	Bone−Osteosarc
Position	8:71431403−119090655
Type	With other complex events
Interleaved intrachr. SVs	8
Total SVs (intrachr. + transl.)	8
SV types	DEL: 3; DUP: 3; h2hINV: 1; t2tINV: 1; TRA: 0
SVs in sample	927
Oscillating CN (2 and 3 states)	9, 22
CN segments	22
FDR fragment joints	0.64
FDR chr. breakp. enrich.	0
Linked to chrs	
Purity, ploidy	0.79, 3.29

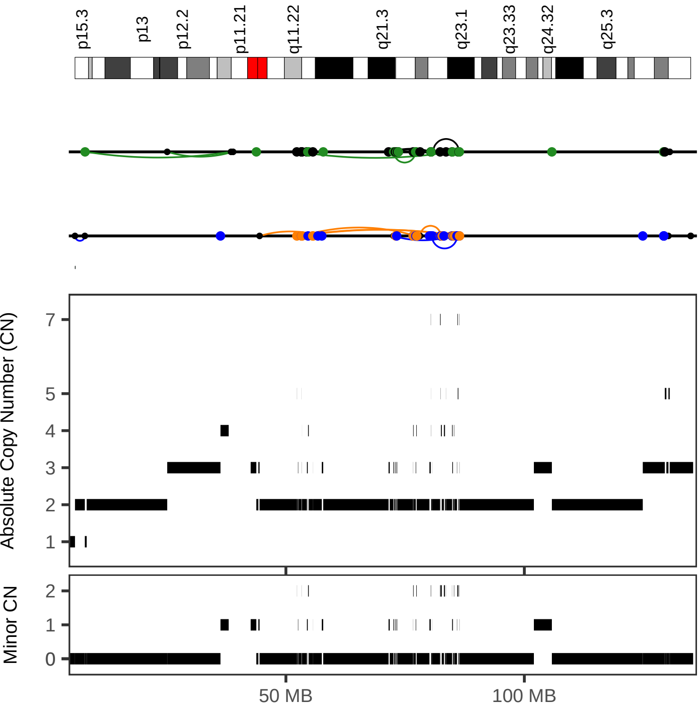

	CGP_donor_1528364
Cancer type	Bone−Osteosarc
Position	10:44478896−86379721
Type	With other complex events
Interleaved intrachr. SVs	13
Total SVs (intrachr. + transl.)	85
SV types	DEL: 6; DUP: 2; h2hINV: 3; t2tINV: 2; TRA: 72
SVs in sample	927
Oscillating CN (2 and 3 states)	15, 30
CN segments	70
FDR fragment joints	0.59
FDR chr. breakp. enrich.	0
Linked to chrs	18:63082505−77710469;5:878141−41078796
Purity, ploidy	0.79, 3.29

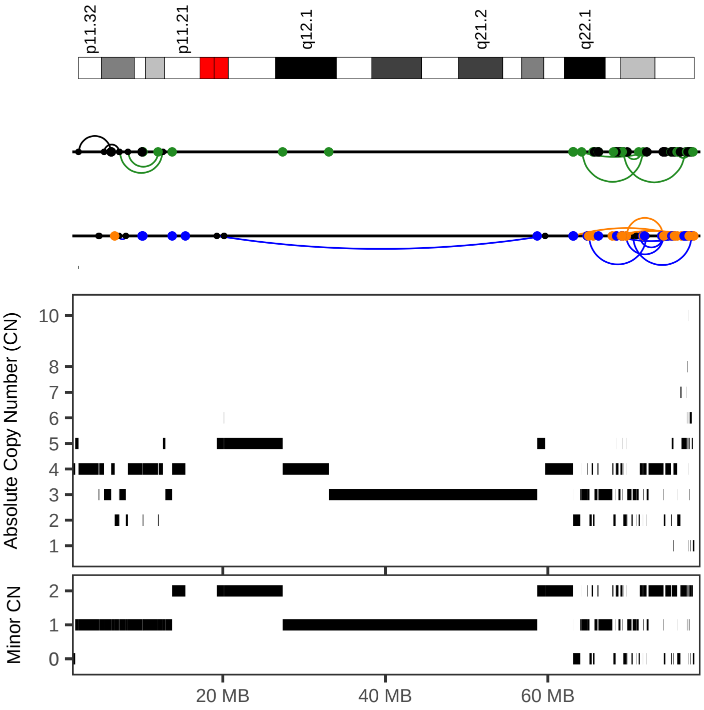

	CGP_donor_1528364
Cancer type	Bone−Osteosarc
Position	18:63082505−77710470
Type	With other complex events
Interleaved intrachr. SVs	16
Total SVs (intrachr. + transl.)	120
SV types	DEL: 4; DUP: 6; h2hINV: 1; t2tINV: 5; TRA: 104
SVs in sample	927
Oscillating CN (2 and 3 states)	7, 17
CN segments	83
FDR fragment joints	0.59
FDR chr. breakp. enrich.	0
Linked to chrs	10:44478896−86379720;5:878141−41078796
Purity, ploidy	0.79, 3.29

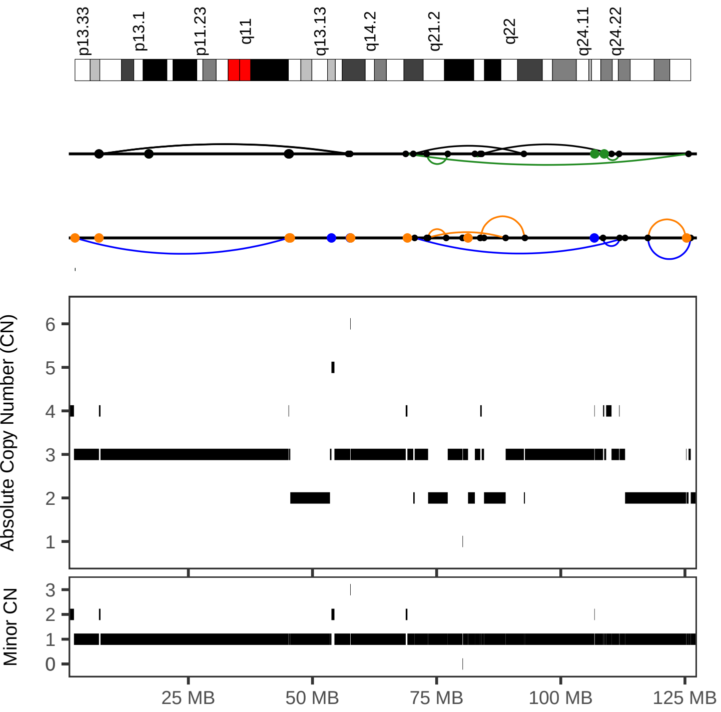

CGP_donor_1528371	
Cancer type	Bone−Osteosarc
Position	12:70291799−112943997
Type	With other complex events
Interleaved intrachr. SVs	9
Total SVs (intrachr. + transl.)	13
SV types	DEL: 2; DUP: 3; h2hINV: 2; t2tINV: 2; TRA: 4
SVs in sample	349
Oscillating CN (2 and 3 states)	9, 18
CN segments	23
FDR fragment joints	0.97
FDR chr. breakp. enrich.	0
Linked to chrs	
Purity, ploidy	0.33, 3.49

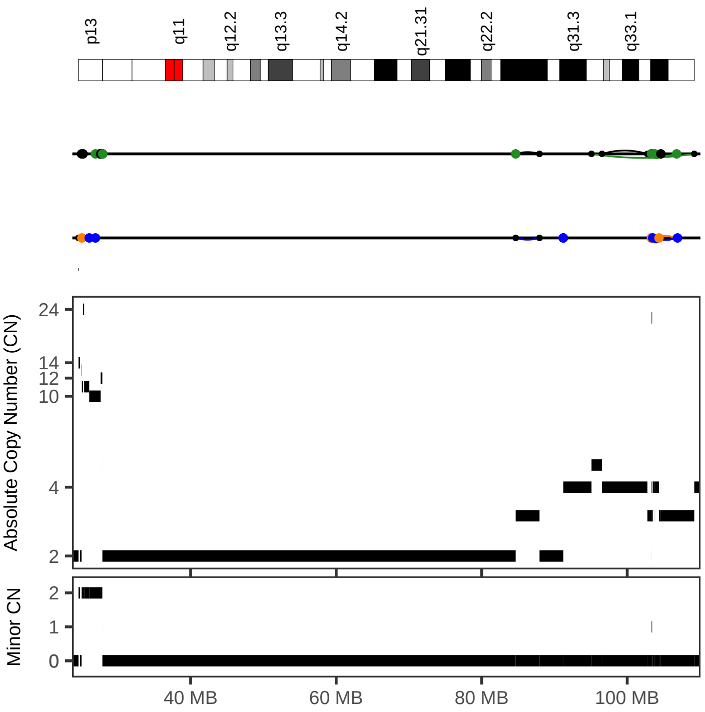

CGP_donor_1528371	
Cancer type	Bone−Osteosarc
Position	13:103350092−106906740
Type	With other complex events
Interleaved intrachr. SVs	11
Total SVs (intrachr. + transl.)	39
SV types	DEL: 4; DUP: 4; h2hINV: 0; t2tINV: 3; TRA: 28
SVs in sample	349
Oscillating CN (2 and 3 states)	7, 7
CN segments	12
FDR fragment joints	0.59
FDR chr. breakp. enrich.	0
Linked to chrs	
Purity, ploidy	0.33, 3.49

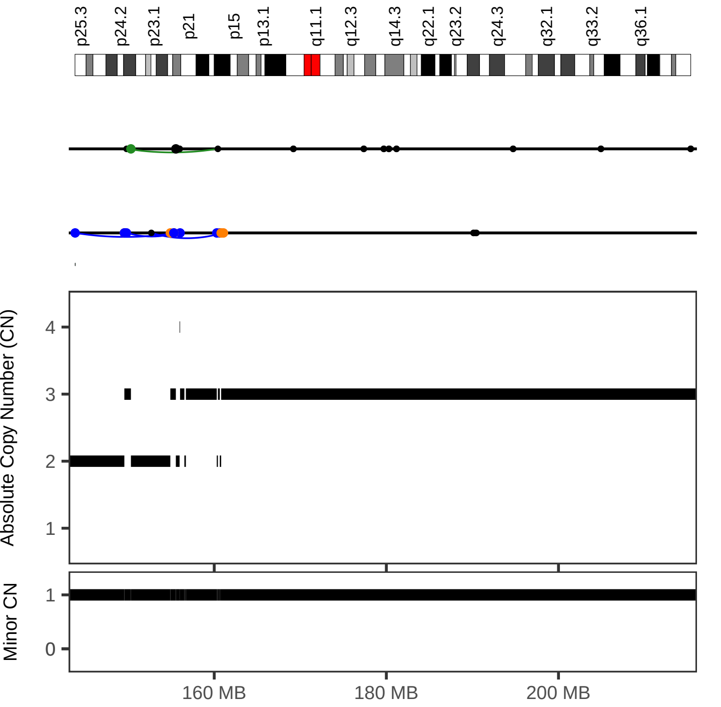

CGP_donor_1528381	
Cancer type	Bone−Osteosarc
Position	2:143820724−160979294
Type	With other complex events
Interleaved intrachr. SVs	5
Total SVs (intrachr. + transl.)	18
SV types	DEL: 0; DUP: 4; h2hINV: 0; t2tINV: 1; TRA: 13
SVs in sample	247
Oscillating CN (2 and 3 states)	7, 7
CN segments	13
FDR fragment joints	0.36
FDR chr. breakp. enrich.	0
Linked to chrs	17:7586844−41815158;19:5000187−56724968
Purity, ploidy	0.76, 1.75

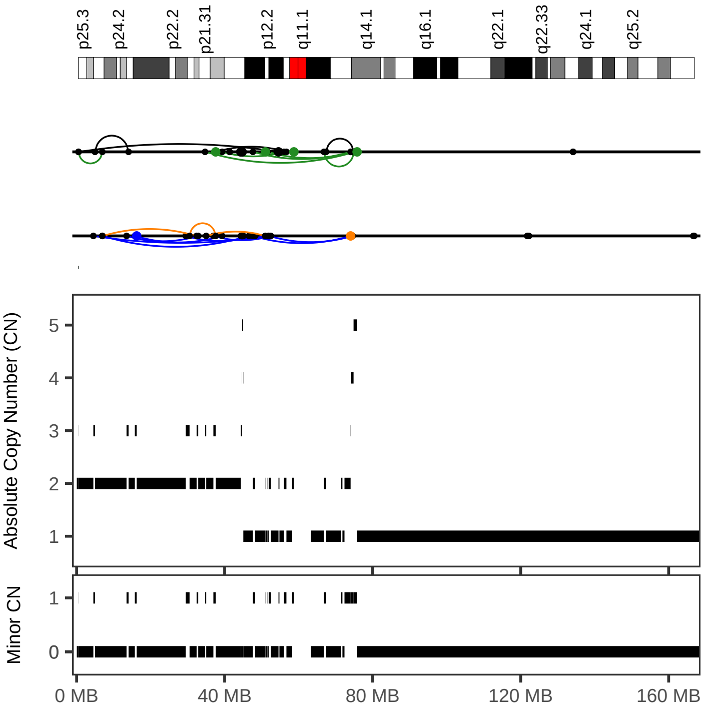

CGP_donor_1528381	
Cancer type	Bone−Osteosarc
Position	6:494867−75444947
Type	With other complex events
Interleaved intrachr. SVs	21
Total SVs (intrachr. + transl.)	29
SV types	DEL: 3; DUP: 8; h2hINV: 4; t2tINV: 6; TRA: 8
SVs in sample	247
Oscillating CN (2 and 3 states)	20, 24
CN segments	43
FDR fragment joints	0.63
FDR chr. breakp. enrich.	0
Linked to chrs	17:7586844−41815158;
Purity, ploidy	0.76, 1.75

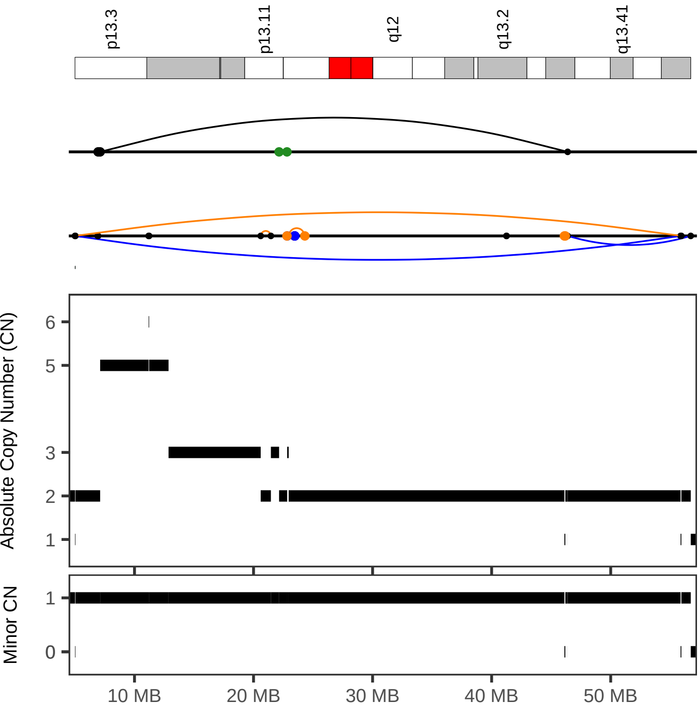

	**CGP_donor_1528381**
Cancer type	Bone−Osteosarc
Position	19:5000187−56724969
Type	With other complex events
Interleaved intrachr. SVs	3
Total SVs (intrachr. + transl.)	18
SV types	DEL: 1; DUP: 2; h2hINV: 0; t2tINV: 0; TRA: 15
SVs in sample	247
Oscillating CN (2 and 3 states)	8, 9
CN segments	18
FDR fragment joints	0.59
FDR chr. breakp. enrich.	0
Linked to chrs	17:7586844−41815158;2:143820724−160979293
Purity, ploidy	0.76, 1.75

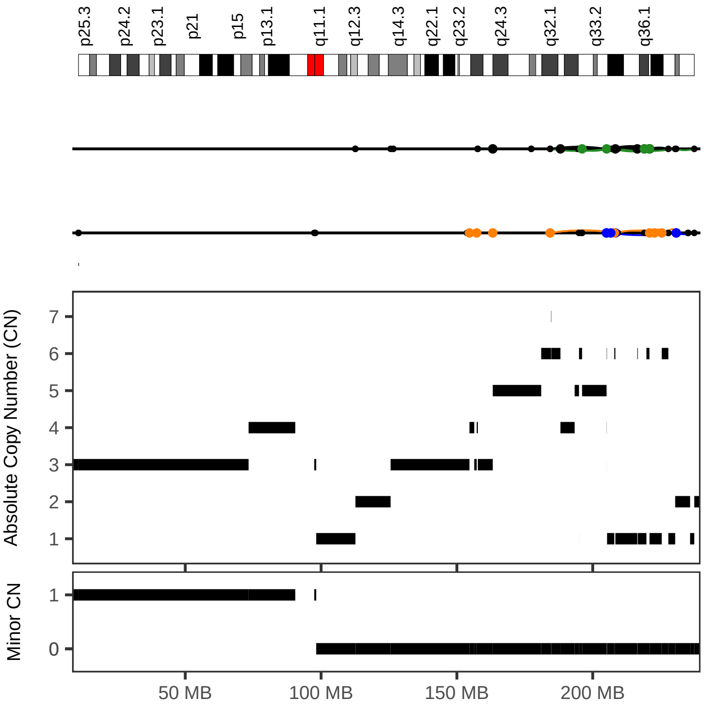

	**CGP_donor_1691133**
Cancer type	Bone−Osteosarc
Position	2:184287853−237379430
Type	With other complex events
Interleaved intrachr. SVs	22
Total SVs (intrachr. + transl.)	44
SV types	DEL: 8; DUP: 5; h2hINV: 4; t2tINV: 5; TRA: 22
SVs in sample	325
Oscillating CN (2 and 3 states)	11, 12
CN segments	27
FDR fragment joints	0.73
FDR chr. breakp. enrich.	0
Linked to chrs	18:20691527−64759311;
Purity, ploidy	0.68, 2.87

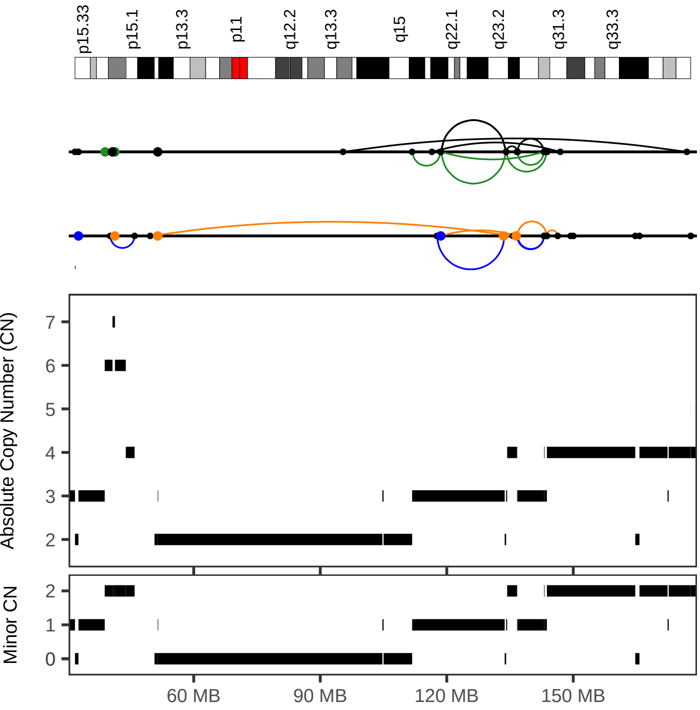

	**CGP_donor_1691133**
Cancer type	Bone−Osteosarc
Position	5:49680325−176939873
Type	With other complex events
Interleaved intrachr. SVs	24
Total SVs (intrachr. + transl.)	29
SV types	DEL: 5; DUP: 4; h2hINV: 9; t2tINV: 6; TRA: 5
SVs in sample	325
Oscillating CN (2 and 3 states)	8, 12
CN segments	20
FDR fragment joints	0.64
FDR chr. breakp. enrich.	0
Linked to chrs	11:9575691−134135388;
Purity, ploidy	0.68, 2.87

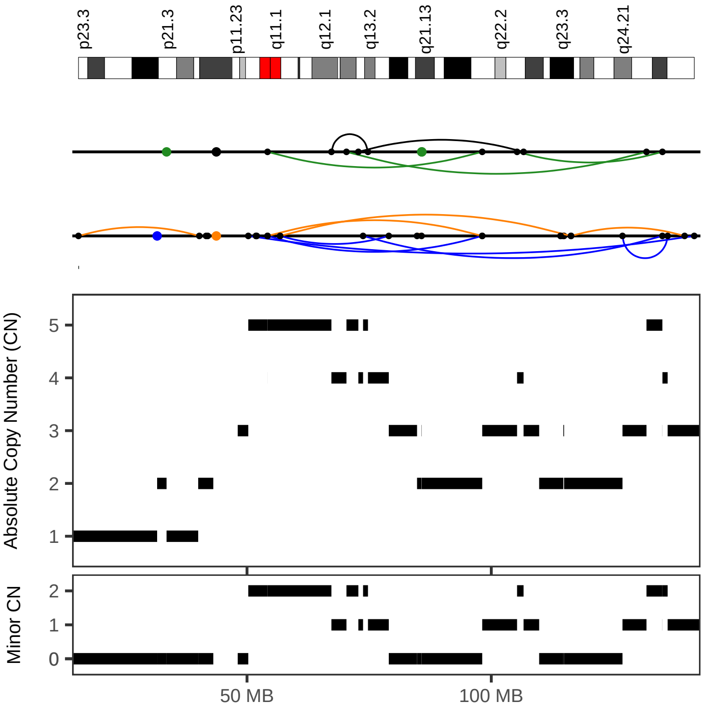

	**CGP_donor_1691133**
Cancer type	Bone−Osteosarc
Position	8:54201065−139568174
Type	With other complex events
Interleaved intrachr. SVs	12
Total SVs (intrachr. + transl.)	14
SV types	DEL: 3; DUP: 4; h2hINV: 2; t2tINV: 3; TRA: 2
SVs in sample	325
Oscillating CN (2 and 3 states)	8, 19
CN segments	23
FDR fragment joints	0.91
FDR chr. breakp. enrich.	0.09
Linked to chrs	11:9575691−134135388;
Purity, ploidy	0.68, 2.87

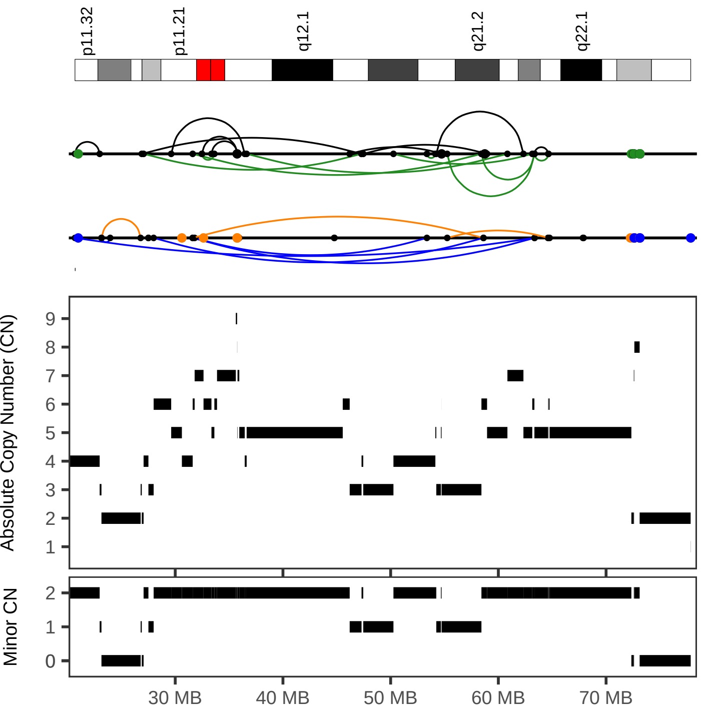

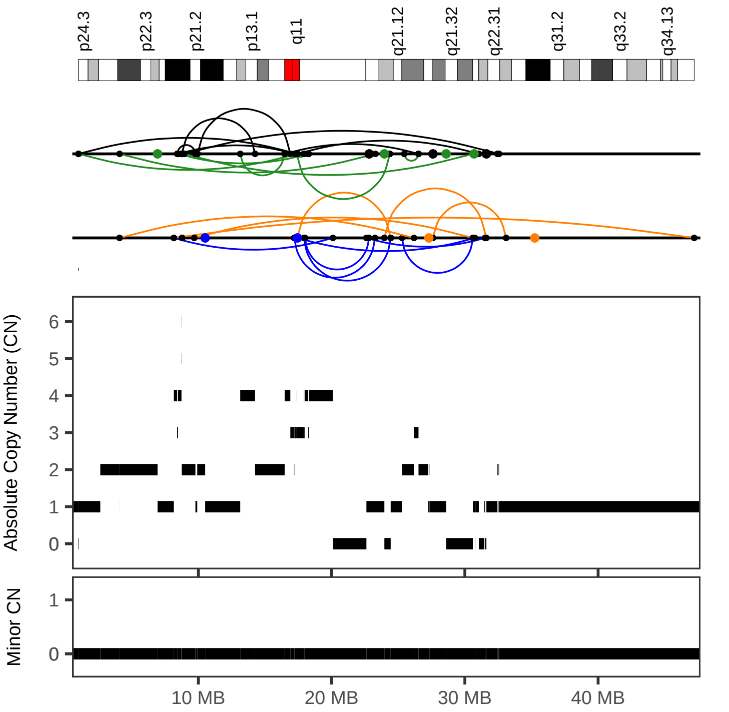

**CGP_donor_1691133**	
Cancer type	Bone−Osteosarc
Position	18:20691527−64759312
Type	With other complex events
Interleaved intrachr. SVs	22
Total SVs (intrachr. + transl.)	31
SV types	DEL: 2; DUP: 5; h2hINV: 7; t2tINV: 8; TRA: 9
SVs in sample	325
Oscillating CN (2 and 3 states)	7, 9
CN segments	43
FDR fragment joints	0.59
FDR chr. breakp. enrich.	0
Linked to chrs	2:184287853−237379429;
Purity, ploidy	0.68, 2.87

**CGP_donor_1691135**	
Cancer type	Bone−Osteosarc
Position	9:1002337−47212849
Type	With other complex events
Interleaved intrachr. SVs	32
Total SVs (intrachr. + transl.)	43
SV types	DEL: 7; DUP: 7; h2hINV: 9; t2tINV: 9; TRA: 11
SVs in sample	136
Oscillating CN (2 and 3 states)	9, 24
CN segments	54
FDR fragment joints	0.94
FDR chr. breakp. enrich.	0
Linked to chrs	
Purity, ploidy	0.73, 1.66

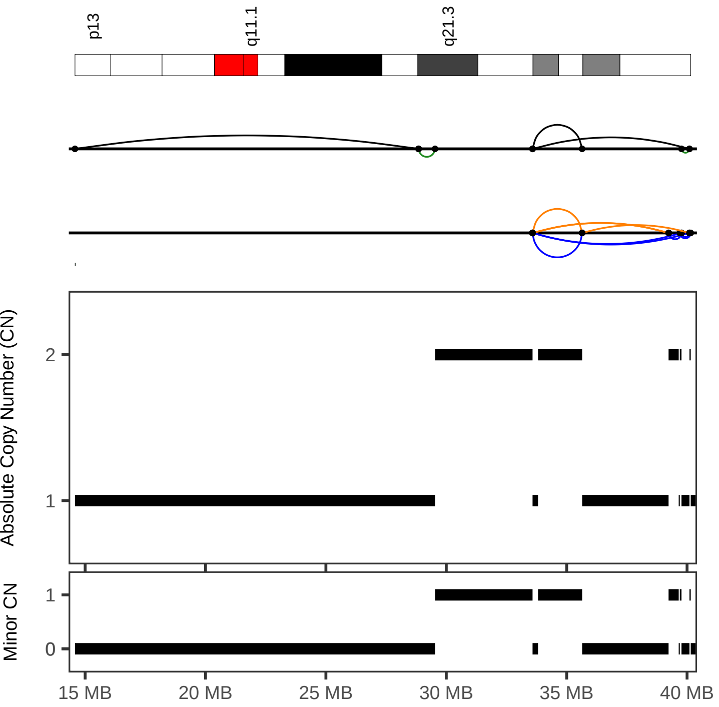

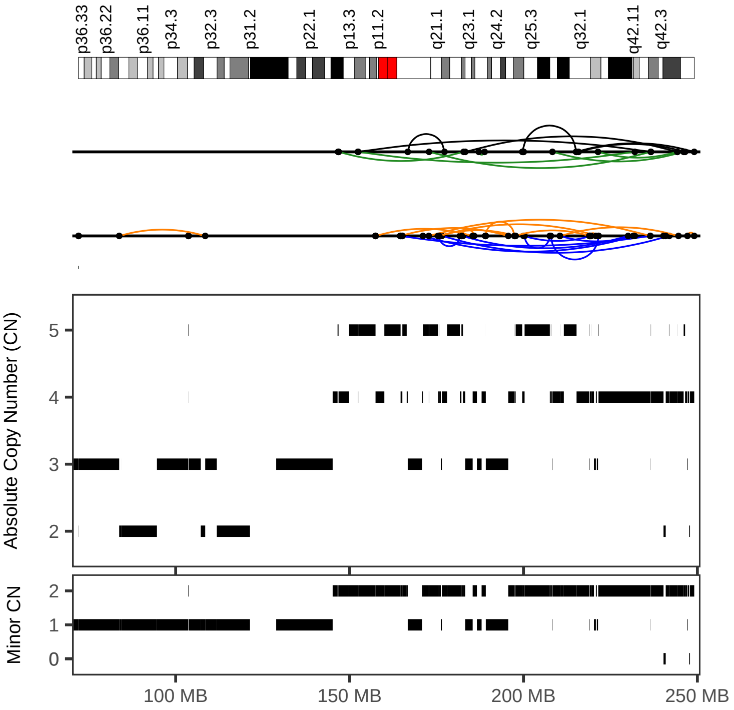

**CGP_donor_1691135**	
Cancer type	Bone−Osteosarc
Position	21:33576946−40152008
Type	Canonical without polyploidization
Interleaved intrachr. SVs	11
Total SVs (intrachr. + transl.)	11
SV types	DEL: 4; DUP: 5; h2hINV: 2; t2tINV: 0; TRA: 0
SVs in sample	136
Oscillating CN (2 and 3 states)	10, 10
CN segments	10
FDR fragment joints	0.59
FDR chr. breakp. enrich.	0
Linked to chrs	
Purity, ploidy	0.73, 1.66

**CGP_donor_1691147**	
Cancer type	Bone−Osteosarc
Position	1:146691688−249090423
Type	With other complex events
Interleaved intrachr. SVs	35
Total SVs (intrachr. + transl.)	35
SV types	DEL: 12; DUP: 10; h2hINV: 8; t2tINV: 5; TRA: 0
SVs in sample	279
Oscillating CN (2 and 3 states)	13, 67
CN segments	79
FDR fragment joints	0.59
FDR chr. breakp. enrich.	0
Linked to chrs	
Purity, ploidy	0.72, 2.91

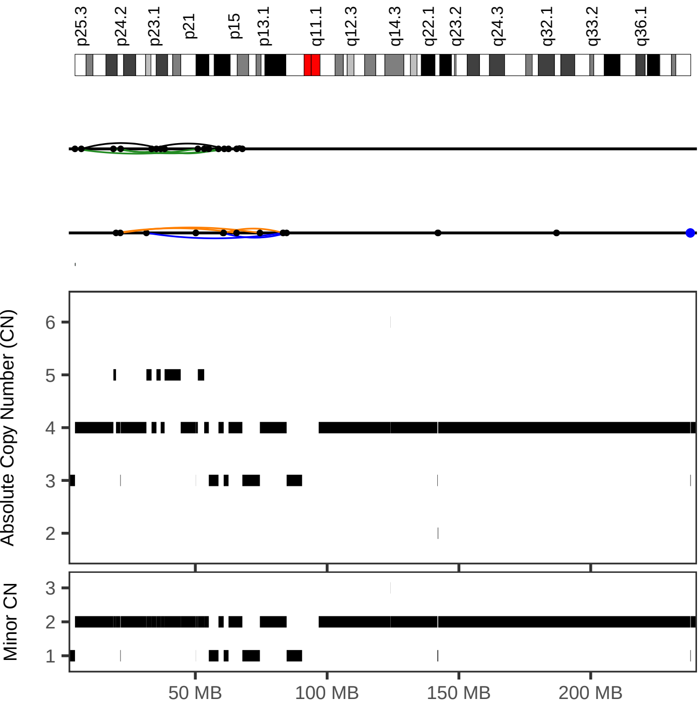

	CGP_donor_1691147
Cancer type	Bone−Osteosarc
Position	2:4339643−84633644
Type	With other complex events
Interleaved intrachr. SVs	11
Total SVs (intrachr. + transl.)	11
SV types	DEL: 3; DUP: 2; h2hINV: 2; t2tINV: 4; TRA: 0
SVs in sample	279
Oscillating CN (2 and 3 states)	8, 22
CN segments	22
FDR fragment joints	0.84
FDR chr. breakp. enrich.	0.76
Linked to chrs	
Purity, ploidy	0.72, 2.91

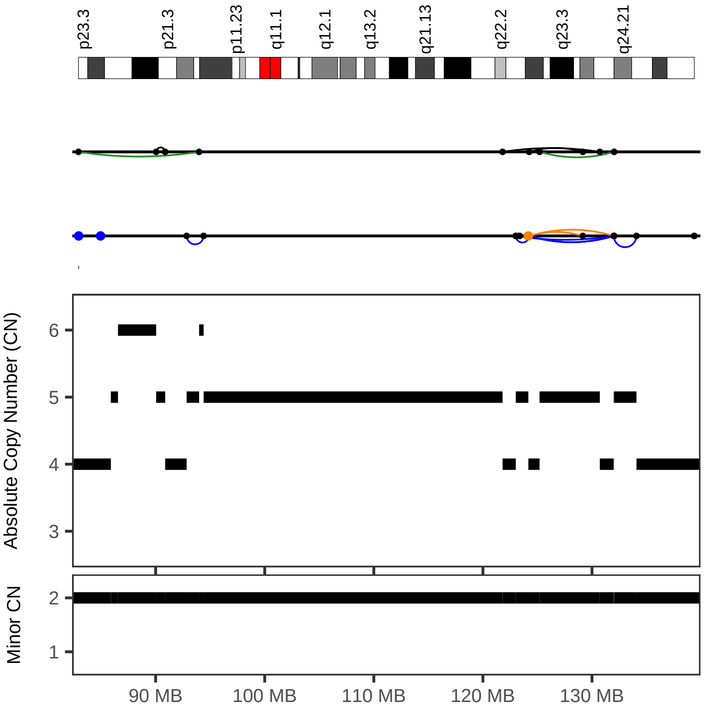

	CGP_donor_1691147
Cancer type	Bone−Osteosarc
Position	8:121801782−134076197
Type	After polyploidization
Interleaved intrachr. SVs	10
Total SVs (intrachr. + transl.)	11
SV types	DEL: 2; DUP: 5; h2hINV: 2; t2tINV: 1; TRA: 1
SVs in sample	279
Oscillating CN (2 and 3 states)	9, 9
CN segments	9
FDR fragment joints	0.59
FDR chr. breakp. enrich.	0.32
Linked to chrs	
Purity, ploidy	0.72, 2.91

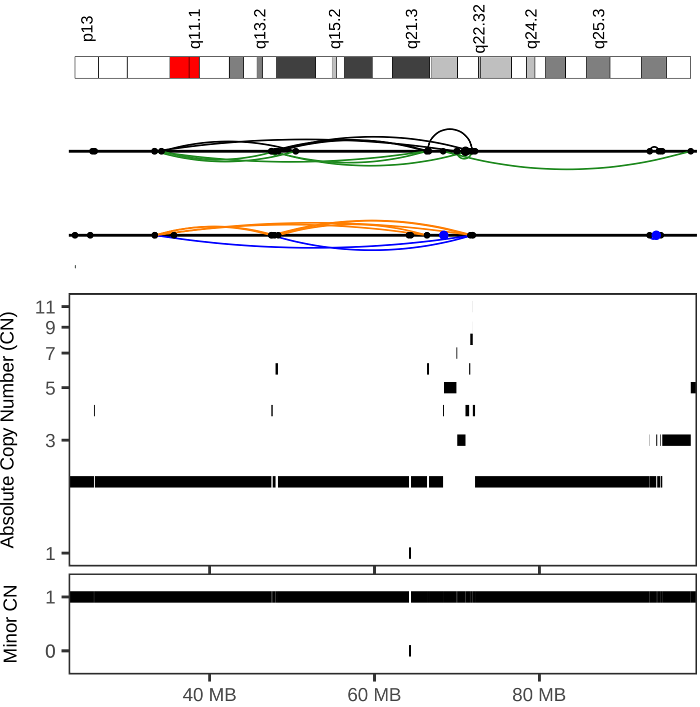

	CGP_donor_1691147
Cancer type	Bone−Osteosarc
Position	15:33299268−98372536
Type	With other complex events
Interleaved intrachr. SVs	23
Total SVs (intrachr. + transl.)	26
SV types	DEL: 7; DUP: 2; h2hINV: 6; t2tINV: 8; TRA: 3
SVs in sample	279
Oscillating CN (2 and 3 states)	8, 9
CN segments	29
FDR fragment joints	0.59
FDR chr. breakp. enrich.	0
Linked to chrs	
Purity, ploidy	0.72, 2.91

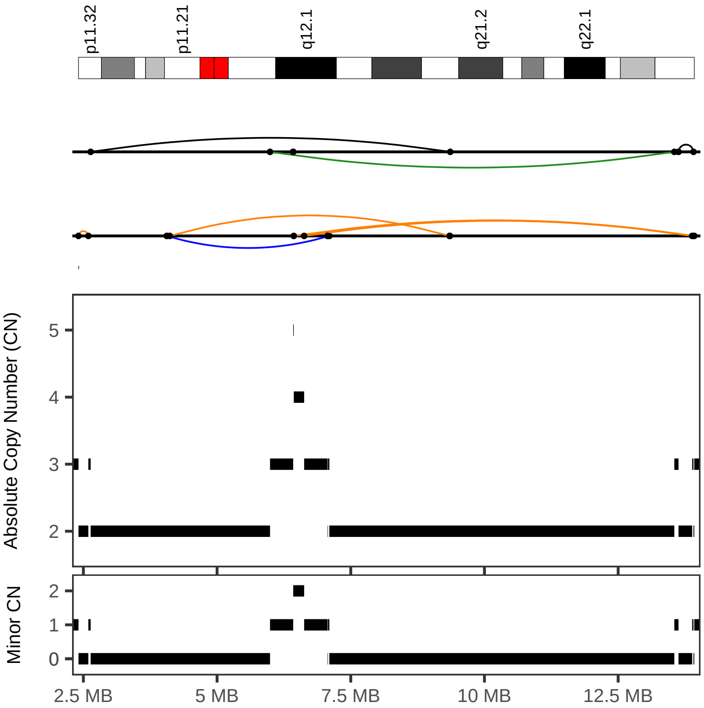

	CGP_donor_1691147
Cancer type	Bone−Osteosarc
Position	18:2636202−13923208
Type	Canonical without polyploidization
Interleaved intrachr. SVs	7
Total SVs (intrachr. + transl.)	7
SV types	DEL: 3; DUP: 1; h2hINV: 2; t2tINV: 1; TRA: 0
SVs in sample	279
Oscillating CN (2 and 3 states)	9, 11
CN segments	13
FDR fragment joints	0.74
FDR chr. breakp. enrich.	0.06
Linked to chrs	
Purity, ploidy	0.72, 2.91

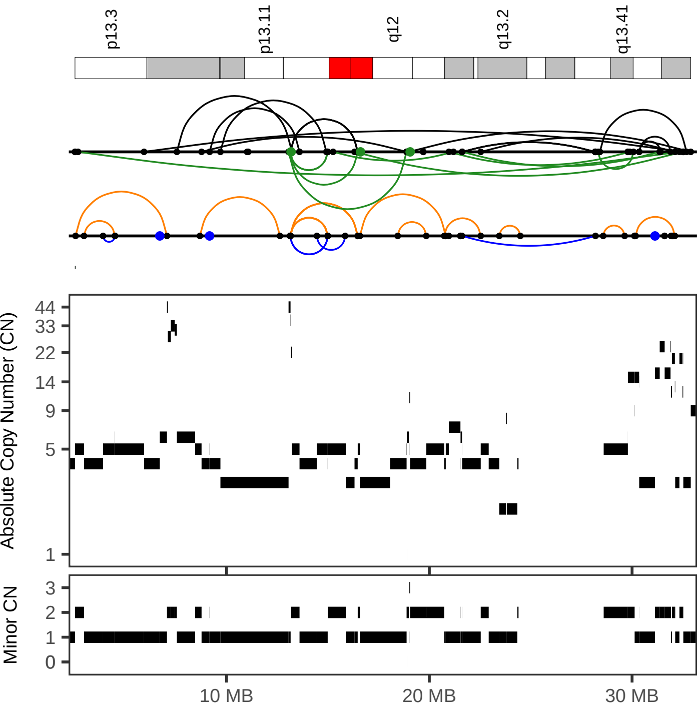

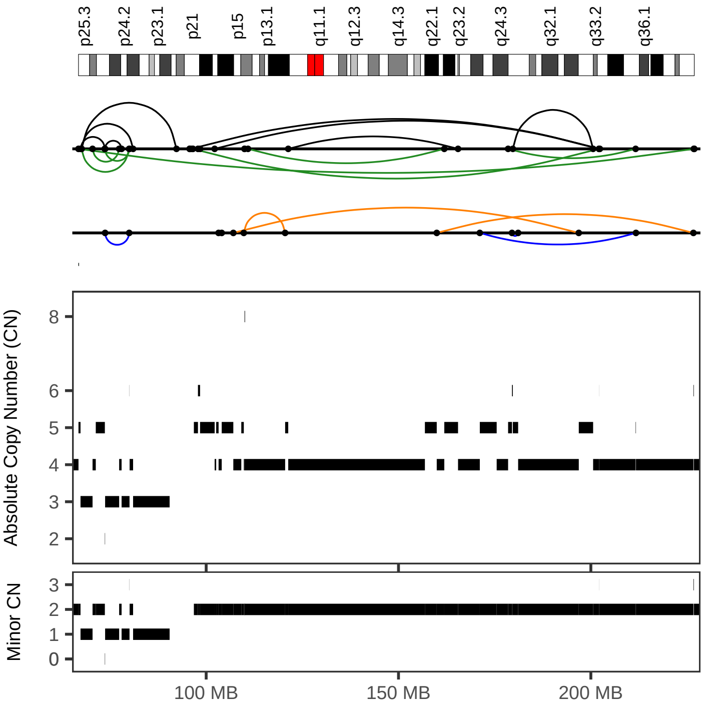

CGP_donor_1691147

Cancer type	Bone−Osteosarc
Position	19:2553855−32893078
Type	With other complex events
Interleaved intrachr. SVs	41
Total SVs (intrachr. + transl.)	47
SV types	DEL: 12; DUP: 4; h2hINV: 15; t2tINV: 10; TRA: 6
SVs in sample	279
Oscillating CN (2 and 3 states)	5, 7
CN segments	72
FDR fragment joints	0.48
FDR chr. breakp. enrich.	0
Linked to chrs	
Purity, ploidy	0.72, 2.91

CGP_donor_1691149

Cancer type	Bone−Osteosarc
Position	2:95756719−226666706
Type	With other complex events
Interleaved intrachr. SVs	13
Total SVs (intrachr. + transl.)	13
SV types	DEL: 3; DUP: 2; h2hINV: 4; t2tINV: 4; TRA: 0
SVs in sample	93
Oscillating CN (2 and 3 states)	10, 10
CN segments	32
FDR fragment joints	0.88
FDR chr. breakp. enrich.	0
Linked to chrs	
Purity, ploidy	0.84, 4.27

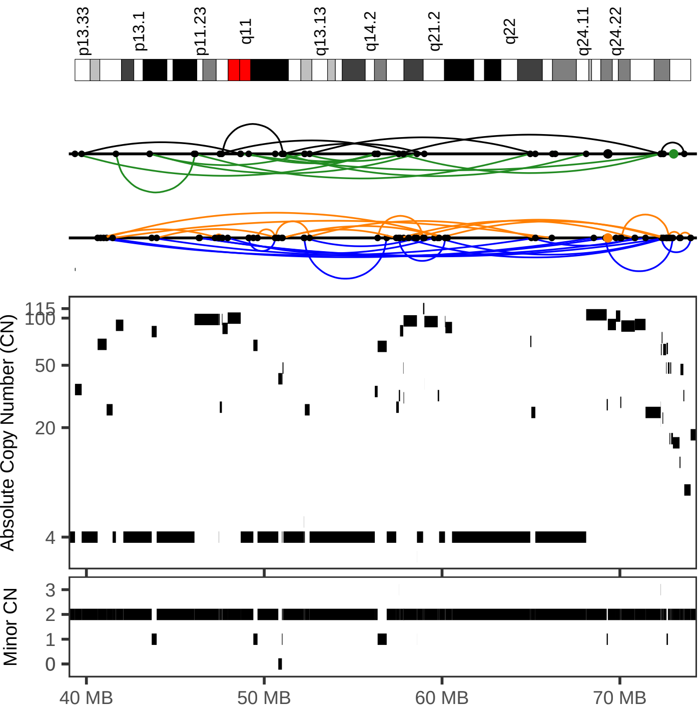

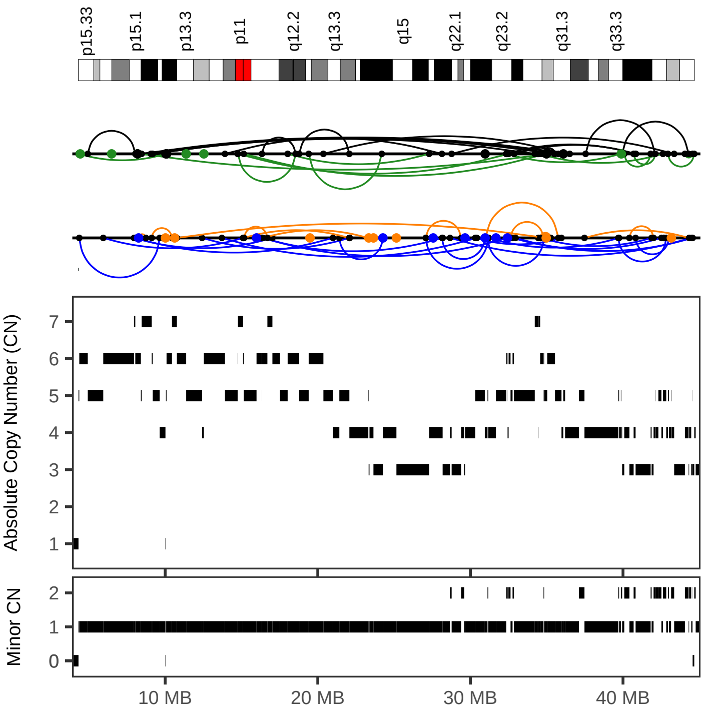

CGP_donor_1691149

Cancer type	Bone−Osteosarc
Position	12:39356696−73985137
Type	With other complex events
Interleaved intrachr. SVs	53
Total SVs (intrachr. + transl.)	56
SV types	DEL: 17; DUP: 16; h2hINV: 8; t2tINV: 12; TRA: 3
SVs in sample	93
Oscillating CN (2 and 3 states)	4, 5
CN segments	77
FDR fragment joints	0.59
FDR chr. breakp. enrich.	0
Linked to chrs	
Purity, ploidy	0.84, 4.27

CGP_donor_1691207

Cancer type	Bone−Osteosarc
Position	5:4320966−44667330
Type	With other complex events
Interleaved intrachr. SVs	63
Total SVs (intrachr. + transl.)	92
SV types	DEL: 12; DUP: 21; h2hINV: 16; t2tINV: 14; TRA: 29
SVs in sample	415
Oscillating CN (2 and 3 states)	14, 43
CN segments	112
FDR fragment joints	0.62
FDR chr. breakp. enrich.	0
Linked to chrs	
Purity, ploidy	0.71, 2.58

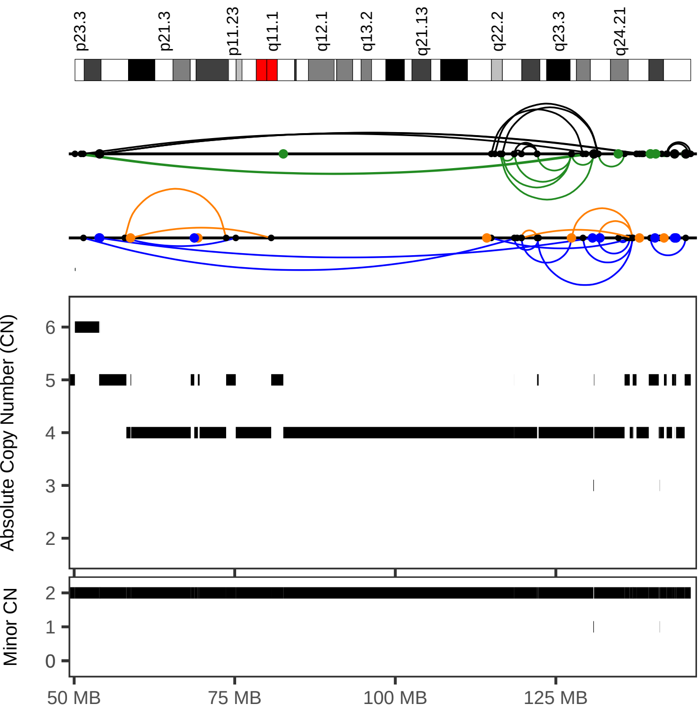

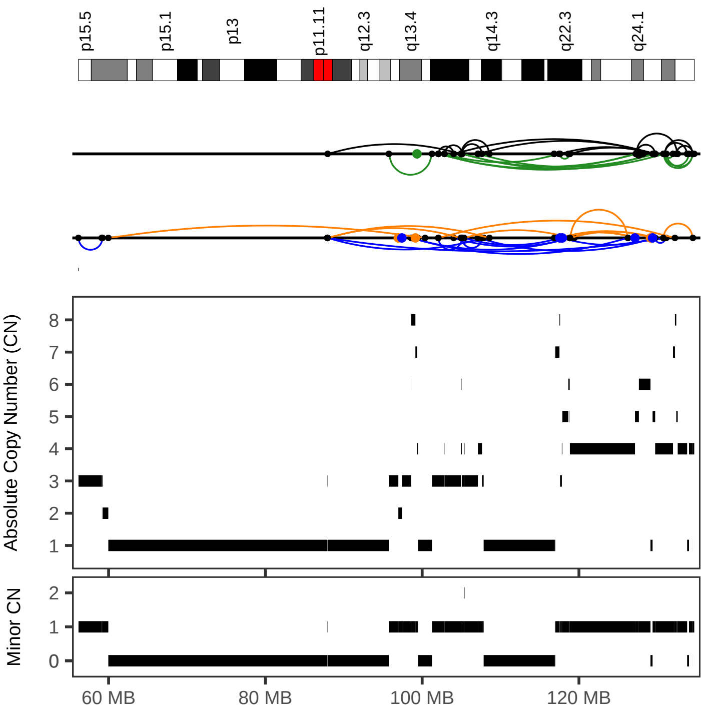

	CGP_donor_1691207
Cancer type	Bone−Osteosarc
Position	8:50128153−138434525
Type	With other complex events
Interleaved intrachr. SVs	31
Total SVs (intrachr. + transl.)	45
SV types	DEL: 4; DUP: 9; h2hINV: 9; t2tINV: 9; TRA: 14
SVs in sample	415
Oscillating CN (2 and 3 states)	16, 16
CN segments	30
FDR fragment joints	0.64
FDR chr. breakp. enrich.	0
Linked to chrs	5:4320966−44667329;7:3613250−157967148
Purity, ploidy	0.71, 2.58

	CGP_donor_1691208
Cancer type	Bone−Osteosarc
Position	11:59962284−134707313
Type	With other complex events
Interleaved intrachr. SVs	51
Total SVs (intrachr. + transl.)	61
SV types	DEL: 11; DUP: 12; h2hINV: 15; t2tINV: 13; TRA: 10
SVs in sample	124
Oscillating CN (2 and 3 states)	6, 7
CN segments	47
FDR fragment joints	0.91
FDR chr. breakp. enrich.	0
Linked to chrs	18:329522−9437571;4:27990522−39404507
Purity, ploidy	0.94, 1.69

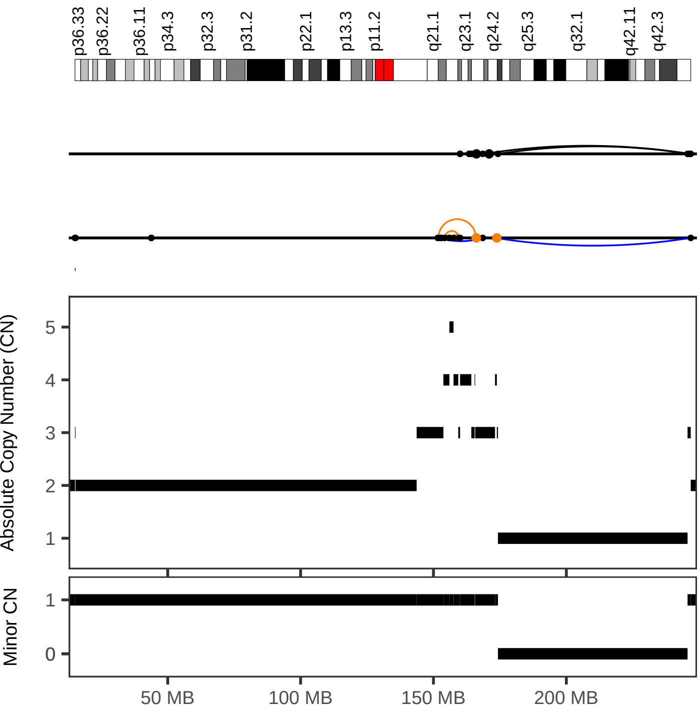

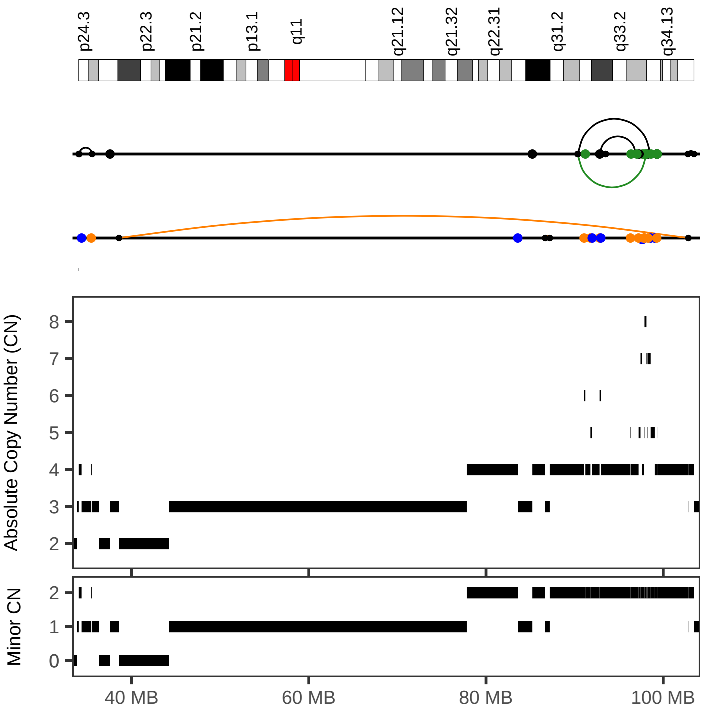

	CGP_donor_1691209
Cancer type	Bone−Osteosarc
Position	1:151613490−246812975
Type	With other complex events
Interleaved intrachr. SVs	3
Total SVs (intrachr. + transl.)	10
SV types	DEL: 3; DUP: 1; h2hINV: 1; t2tINV: 1; TRA: 4
SVs in sample	66
Oscillating CN (2 and 3 states)	8, 11
CN segments	14
FDR fragment joints	0.64
FDR chr. breakp. enrich.	0
Linked to chrs	
Purity, ploidy	0.7, 1.54

	CGP_donor_1691210
Cancer type	Bone−Osteosarc
Position	9:90338614−98608467
Type	With other complex events
Interleaved intrachr. SVs	6
Total SVs (intrachr. + transl.)	26
SV types	DEL: 1; DUP: 1; h2hINV: 2; t2tINV: 2; TRA: 20
SVs in sample	183
Oscillating CN (2 and 3 states)	10, 18
CN segments	28
FDR fragment joints	0.91
FDR chr. breakp. enrich.	0
Linked to chrs	
Purity, ploidy	0.85, 3.59

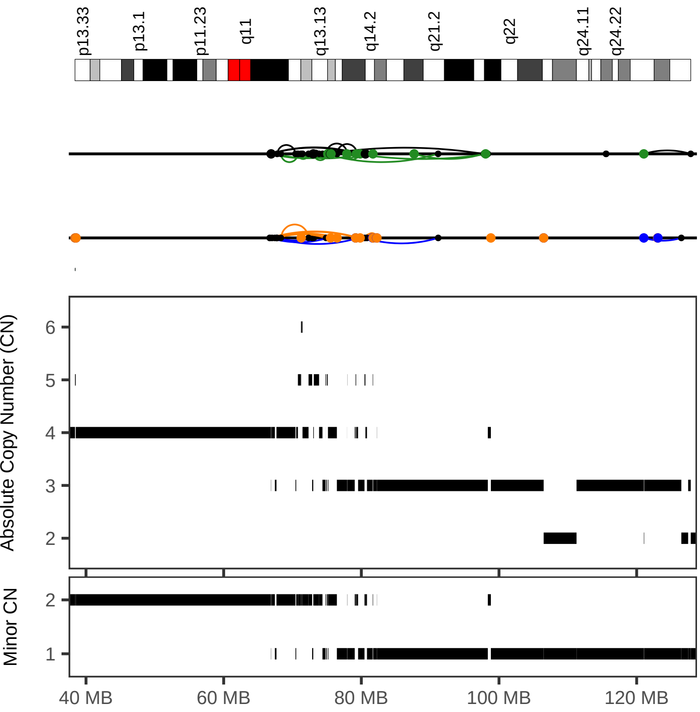

	CGP_donor_1691210
Cancer type	Bone−Osteosarc
Position	12:66698784−98376745
Type	With other complex events
Interleaved intrachr. SVs	32
Total SVs (intrachr. + transl.)	56
SV types	DEL: 6; DUP: 5; h2hINV: 9; t2tINV: 12; TRA: 24
SVs in sample	183
Oscillating CN (2 and 3 states)	11, 21
CN segments	55
FDR fragment joints	0.59
FDR chr. breakp. enrich.	0
Linked to chrs	9:90338614−98608466;
Purity, ploidy	0.85, 3.59

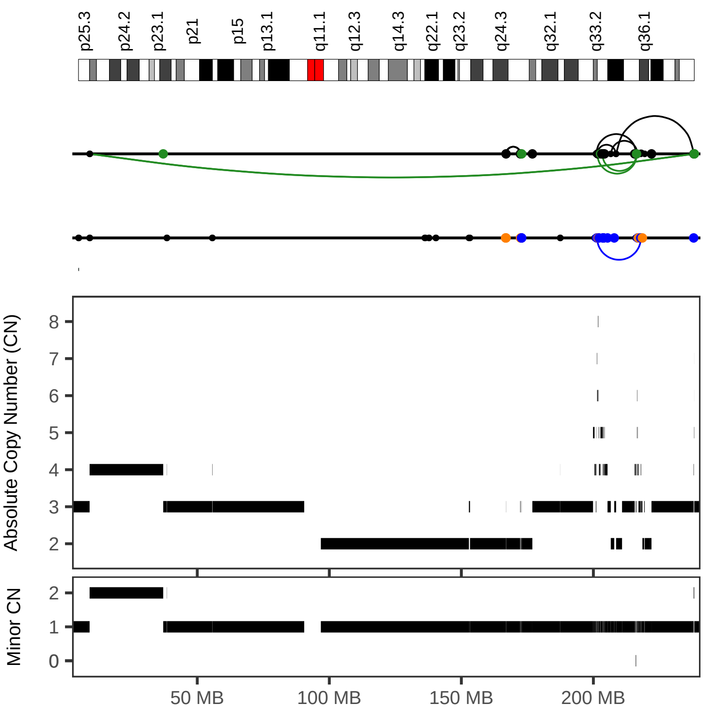

	CGP_donor_1691211
Cancer type	Bone−Osteosarc
Position	2:9233757−238199400
Type	With other complex events
Interleaved intrachr. SVs	15
Total SVs (intrachr. + transl.)	52
SV types	DEL: 1; DUP: 4; h2hINV: 5; t2tINV: 5; TRA: 37
SVs in sample	606
Oscillating CN (2 and 3 states)	15, 42
CN segments	76
FDR fragment joints	0.62
FDR chr. breakp. enrich.	0.04
Linked to chrs	17:996864−13569640;9:72321762−137935608
Purity, ploidy	0.62, 3.16

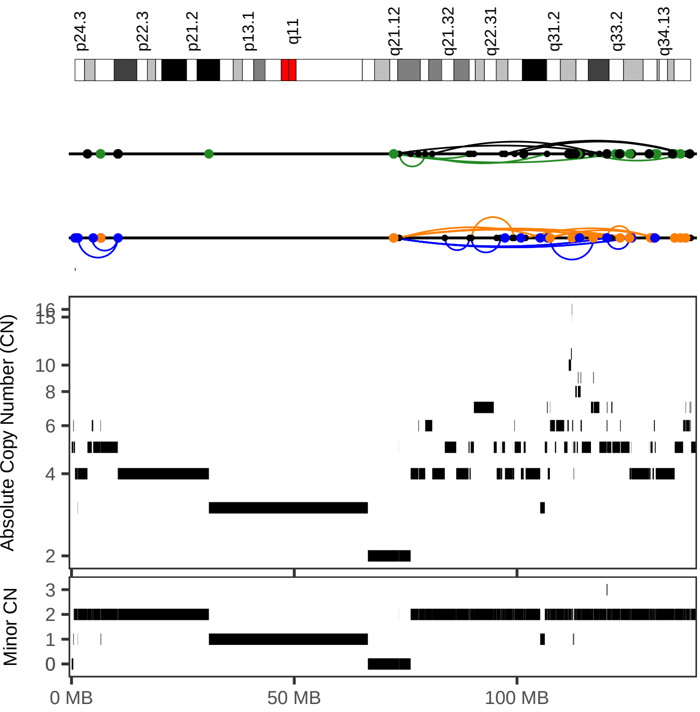

	CGP_donor_1691211
Cancer type	Bone−Osteosarc
Position	9:72321762−137935609
Type	With other complex events
Interleaved intrachr. SVs	28
Total SVs (intrachr. + transl.)	91
SV types	DEL: 11; DUP: 8; h2hINV: 4; t2tINV: 5; TRA: 63
SVs in sample	606
Oscillating CN (2 and 3 states)	9, 10
CN segments	86
FDR fragment joints	0.59
FDR chr. breakp. enrich.	0
Linked to chrs	1:20463312−245809115;2:9233757−238199399
Purity, ploidy	0.62, 3.16

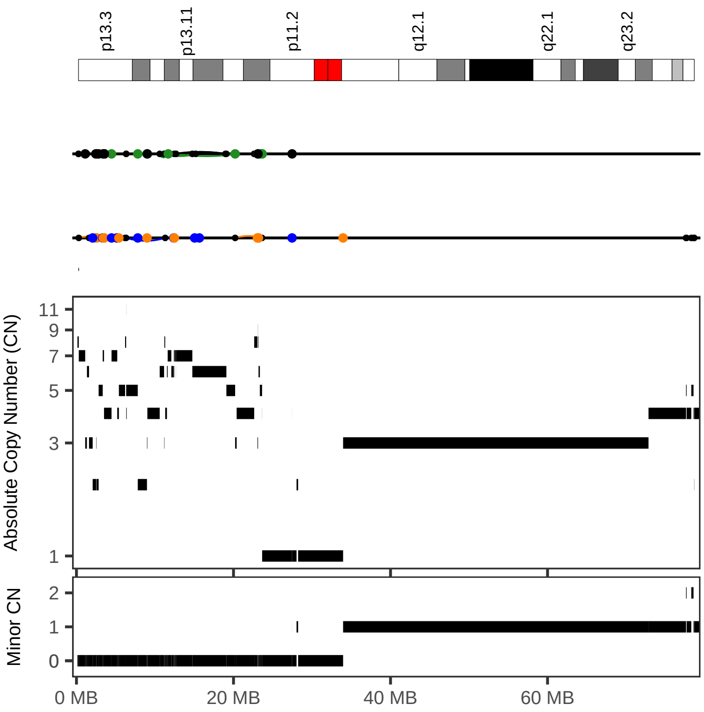

	CGP_donor_1691211
Cancer type	Bone−Osteosarc
Position	16:6326850−23636186
Type	With other complex events
Interleaved intrachr. SVs	9
Total SVs (intrachr. + transl.)	26
SV types	DEL: 1; DUP: 1; h2hINV: 3; t2tINV: 4; TRA: 17
SVs in sample	606
Oscillating CN (2 and 3 states)	9, 10
CN segments	28
FDR fragment joints	0.59
FDR chr. breakp. enrich.	0
Linked to chrs	
Purity, ploidy	0.62, 3.16

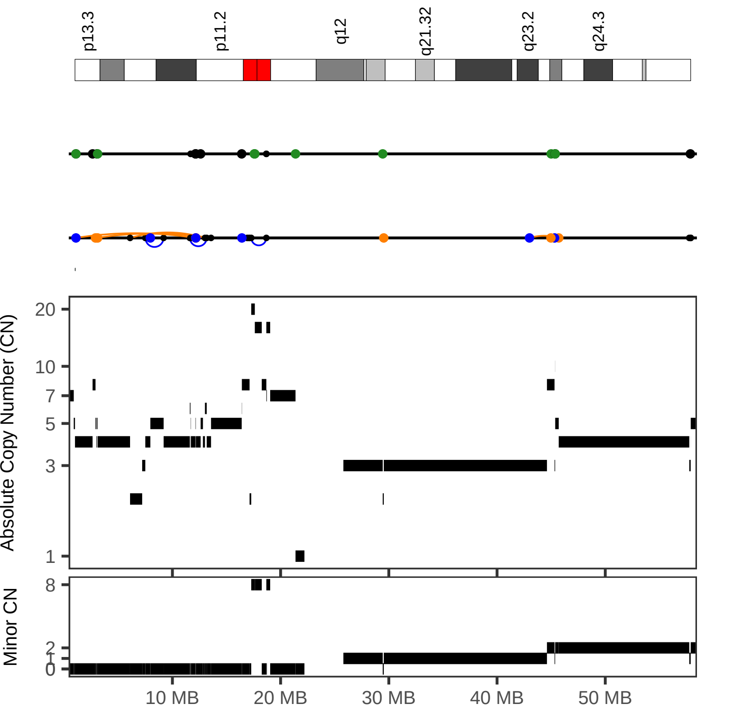

CGP_donor_1691211	
Cancer type	Bone−Osteosarc
Position	17:996864−13569641
Type	With other complex events
Interleaved intrachr. SVs	4
Total SVs (intrachr. + transl.)	19
SV types	DEL: 3; DUP: 1; h2hINV: 0; t2tINV: 0; TRA: 15
SVs in sample	606
Oscillating CN (2 and 3 states)	8, 11
CN segments	23
FDR fragment joints	0.48
FDR chr. breakp. enrich.	0
Linked to chrs	2:9233757−238199399;
Purity, ploidy	0.62, 3.16

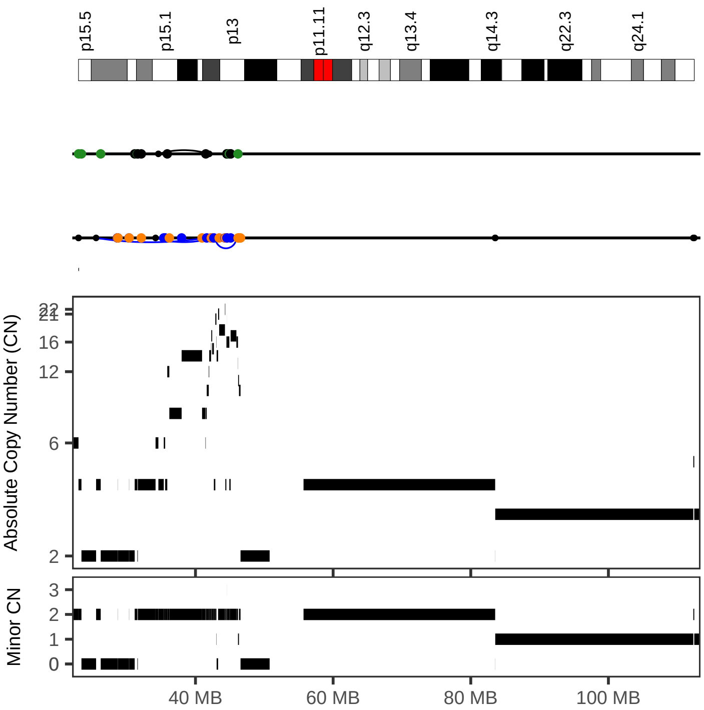

CGP_donor_1691213	
Cancer type	Bone−Osteosarc
Position	11:25558833−42430376
Type	With other complex events
Interleaved intrachr. SVs	4
Total SVs (intrachr. + transl.)	26
SV types	DEL: 1; DUP: 2; h2hINV: 1; t2tINV: 0; TRA: 22
SVs in sample	596
Oscillating CN (2 and 3 states)	9, 13
CN segments	25
FDR fragment joints	0.64
FDR chr. breakp. enrich.	0
Linked to chrs	12:38831735−119704558;19:7659219−23705866 8:98131760−131862130;
Purity, ploidy	0.87, 3.53

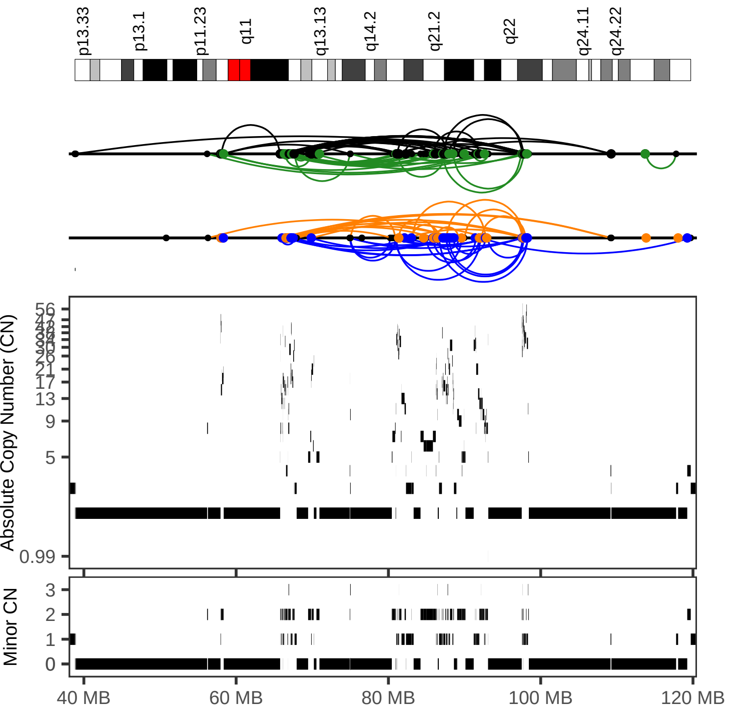

CGP_donor_1691213	
Cancer type	Bone−Osteosarc
Position	12:38831735−119704559
Type	With other complex events
Interleaved intrachr. SVs	111
Total SVs (intrachr. + transl.)	234
SV types	DEL: 22; DUP: 26; h2hINV: 34; t2tINV: 29; TRA: 123
SVs in sample	596
Oscillating CN (2 and 3 states)	5, 8
CN segments	232
FDR fragment joints	0.64
FDR chr. breakp. enrich.	0
Linked to chrs	11:25558833−42430375;13:24259765−79982209 4:57215180−77754048;8:98131760−131862130
Purity, ploidy	0.87, 3.53

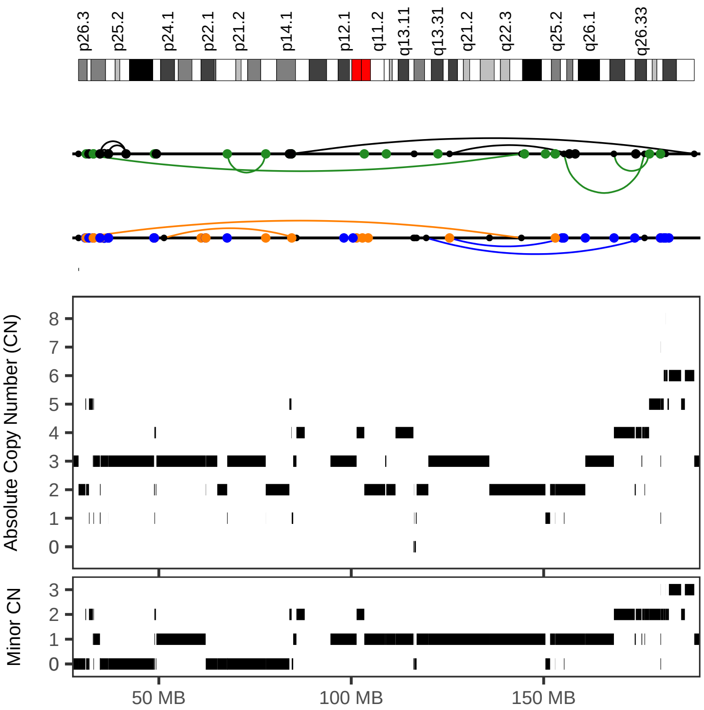

CGP_donor_1691214	
Cancer type	Bone−Osteosarc
Position	3:29117586−189128945
Type	With other complex events
Interleaved intrachr. SVs	10
Total SVs (intrachr. + transl.)	84
SV types	DEL: 2; DUP: 2; h2hINV: 3; t2tINV: 3; TRA: 74
SVs in sample	652
Oscillating CN (2 and 3 states)	7, 17
CN segments	82
FDR fragment joints	0.96
FDR chr. breakp. enrich.	0
Linked to chrs	1:155935299−166050472;13:33323085−107534871 18:5480934−11055616;X:48348455−150018346
Purity, ploidy	0.49, 3.33

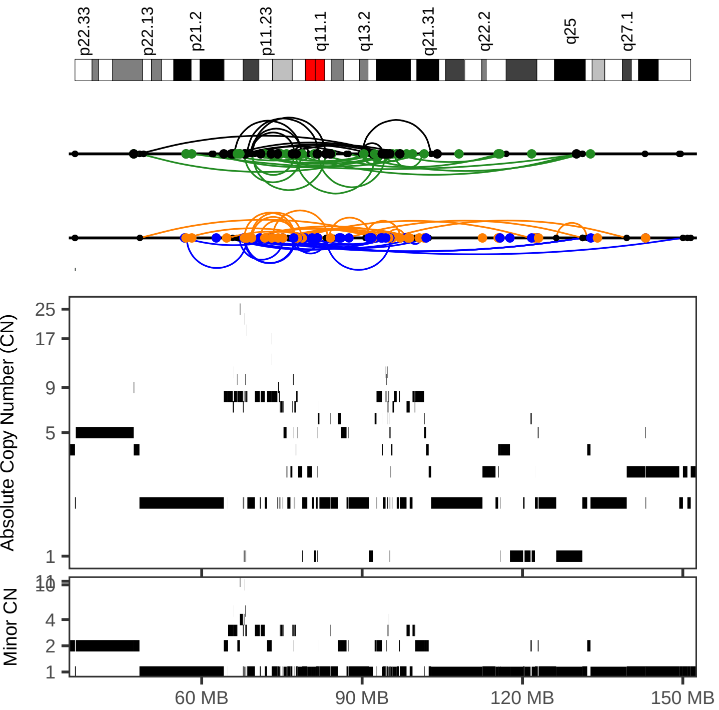

	**CGP_donor_1691214**
Cancer type	Bone−Osteosarc
Position	X:48348455−150018347
Type	With other complex events
Interleaved intrachr. SVs	98
Total SVs (intrachr. + transl.)	229
SV types	DEL: 24; DUP: 27; h2hINV: 19; t2tINV: 28; TRA: 131
SVs in sample	652
Oscillating CN (2 and 3 states)	7, 8
CN segments	166
FDR fragment joints	0.64
FDR chr. breakp. enrich.	0
Linked to chrs	14:41964680−78837997;18:5480934−11055616 2:11400−232123608;3:29117586−189128944
Purity, ploidy	0.49, 3.33

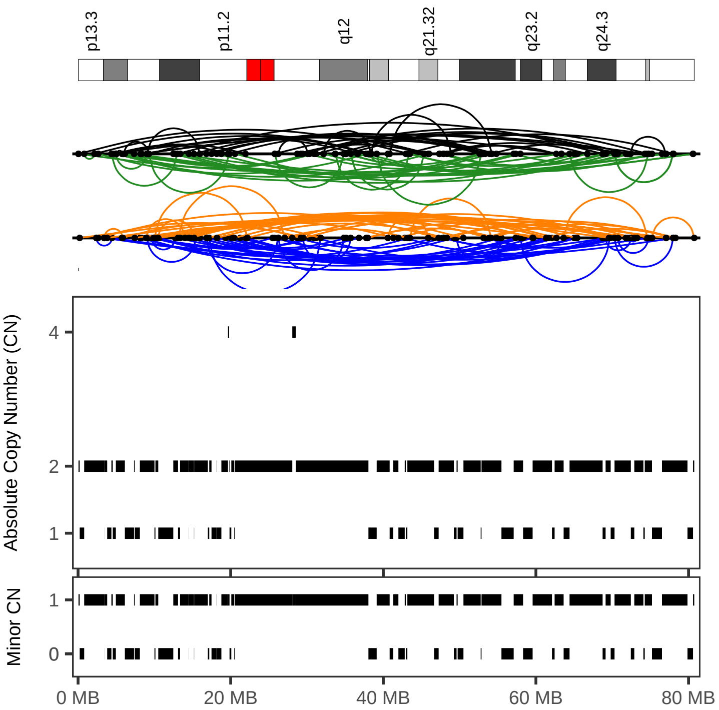

	**CGP_donor_1691215**
Cancer type	Bone−Osteosarc
Position	17:54416−80744029
Type	With other complex events
Interleaved intrachr. SVs	158
Total SVs (intrachr. + transl.)	158
SV types	DEL: 40; DUP: 44; h2hINV: 34; t2tINV: 40; TRA: 0
SVs in sample	166
Oscillating CN (2 and 3 states)	37, 73
CN segments	73
FDR fragment joints	0.81
FDR chr. breakp. enrich.	0
Linked to chrs	
Purity, ploidy	0.4, 2.01

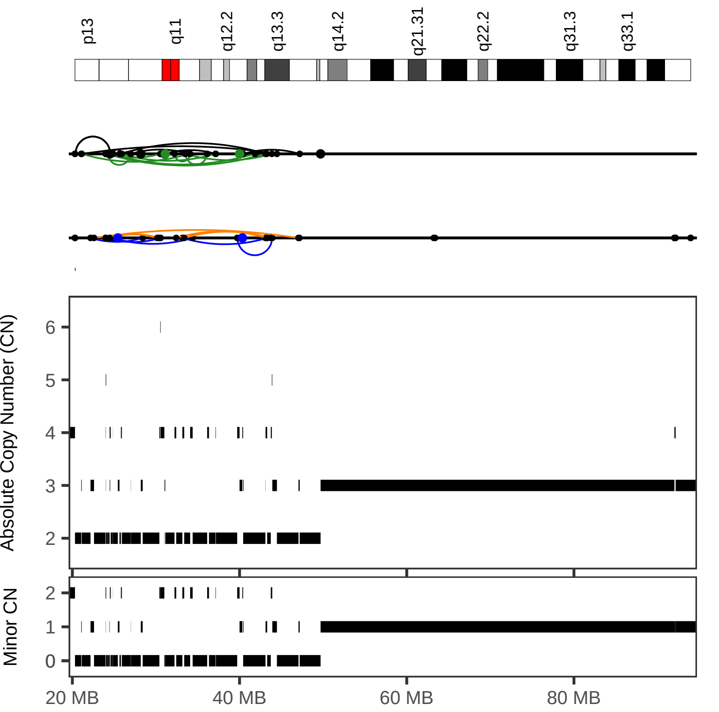

	**CGP_donor_1691217**
Cancer type	Bone−Osteosarc
Position	13:20312035−47194343
Type	With other complex events
Interleaved intrachr. SVs	32
Total SVs (intrachr. + transl.)	39
SV types	DEL: 6; DUP: 7; h2hINV: 10; t2tINV: 9; TRA: 7
SVs in sample	380
Oscillating CN (2 and 3 states)	12, 18
CN segments	56
FDR fragment joints	0.82
FDR chr. breakp. enrich.	0
Linked to chrs	4:90398285−117066510;6:109694663−128848926
Purity, ploidy	0.53, 2.89

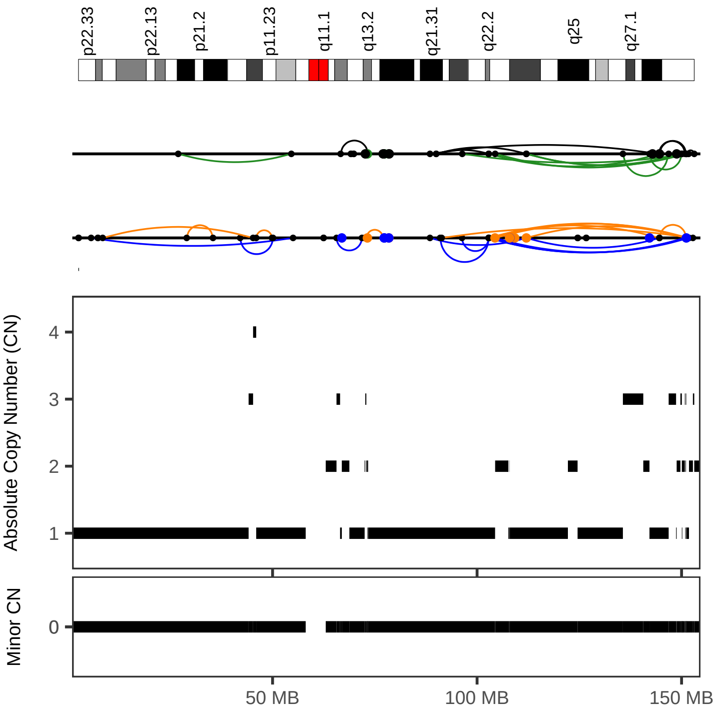

	**CGP_donor_1691217**
Cancer type	Bone−Osteosarc
Position	X:88486379−153095441
Type	With other complex events
Interleaved intrachr. SVs	28
Total SVs (intrachr. + transl.)	38
SV types	DEL: 7; DUP: 7; h2hINV: 6; t2tINV: 8; TRA: 10
SVs in sample	380
Oscillating CN (2 and 3 states)	9, 11
CN segments	29
FDR fragment joints	0.98
FDR chr. breakp. enrich.	0
Linked to chrs	19:43092652−56632953;6:109694663−128848926
Purity, ploidy	0.53, 2.89

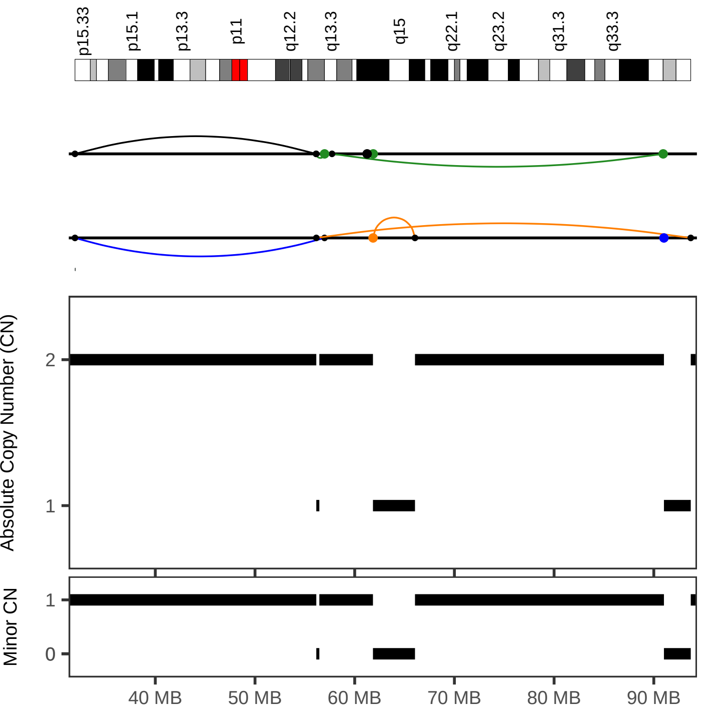

	**CGP_donor_1230755**
Cancer type	Breast−AdenoCA
Position	5:31942233−93707021
Type	Canonical without polyploidization
Interleaved intrachr. SVs	3
Total SVs (intrachr. + transl.)	9
SV types	DEL: 1; DUP: 1; h2hINV: 0; t2tINV: 1; TRA: 6
SVs in sample	54
Oscillating CN (2 and 3 states)	7, 7
CN segments	7
FDR fragment joints	0.84
FDR chr. breakp. enrich.	0
Linked to chrs	1:16076342−181848575;
Purity, ploidy	0.48, 2.12

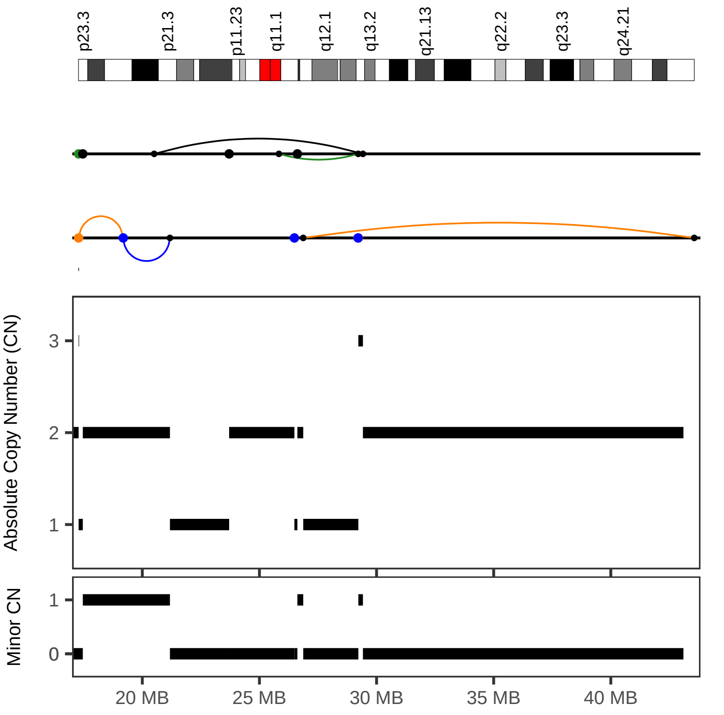

	**CGP_donor_1230755**
Cancer type	Breast−AdenoCA
Position	8:17274088−43561752
Type	With other complex events
Interleaved intrachr. SVs	5
Total SVs (intrachr. + transl.)	15
SV types	DEL: 2; DUP: 1; h2hINV: 1; t2tINV: 1; TRA: 10
SVs in sample	54
Oscillating CN (2 and 3 states)	7, 12
CN segments	12
FDR fragment joints	0.92
FDR chr. breakp. enrich.	0
Linked to chrs	1:16076342−181848575;6:21024781−109641657
Purity, ploidy	0.48, 2.12

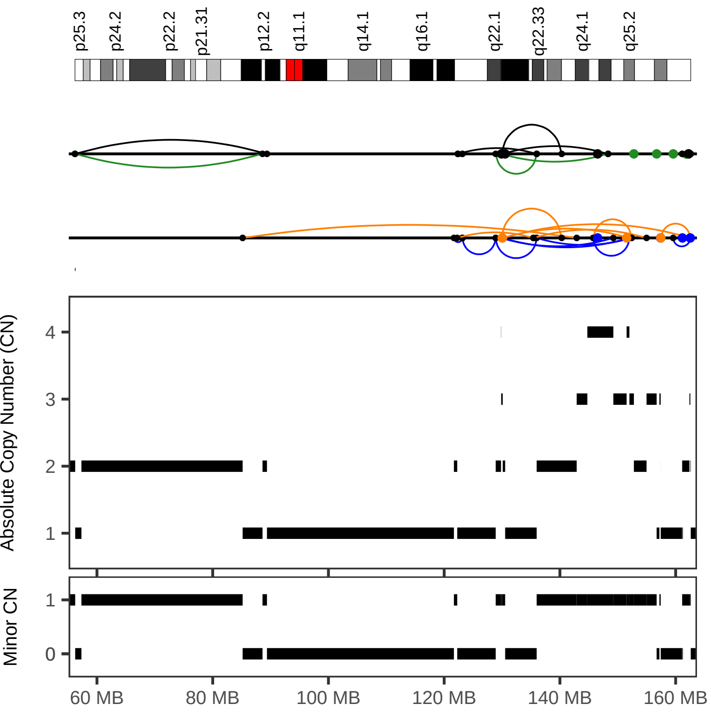

	**CGP_donor_1232859**
Cancer type	Breast−AdenoCA
Position	6:56201574−162603780
Type	With other complex events
Interleaved intrachr. SVs	22
Total SVs (intrachr. + transl.)	38
SV types	DEL: 7; DUP: 7; h2hINV: 4; t2tINV: 4; TRA: 16
SVs in sample	316
Oscillating CN (2 and 3 states)	9, 12
CN segments	31
FDR fragment joints	0.73
FDR chr. breakp. enrich.	0
Linked to chrs	20:33258582−62543744;
Purity, ploidy	0.54, 2.17

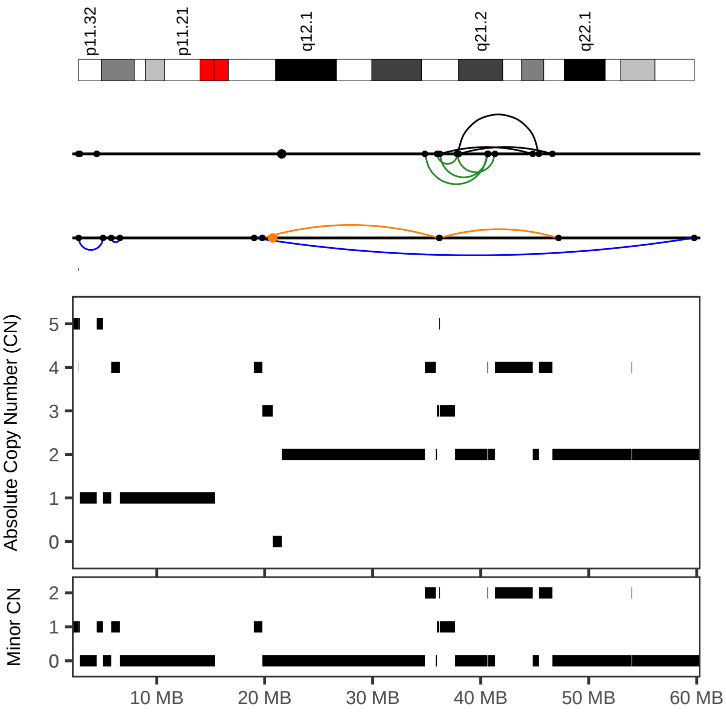

	**CGP_donor_1234121**
Cancer type	Breast−AdenoCA
Position	18:19776092−47210113
Type	With other complex events
Interleaved intrachr. SVs	9
Total SVs (intrachr. + transl.)	11
SV types	DEL: 2; DUP: 0; h2hINV: 3; t2tINV: 4; TRA: 2
SVs in sample	149
Oscillating CN (2 and 3 states)	7, 8
CN segments	15
FDR fragment joints	0.59
FDR chr. breakp. enrich.	0
Linked to chrs	
Purity, ploidy	0.4, 3.56

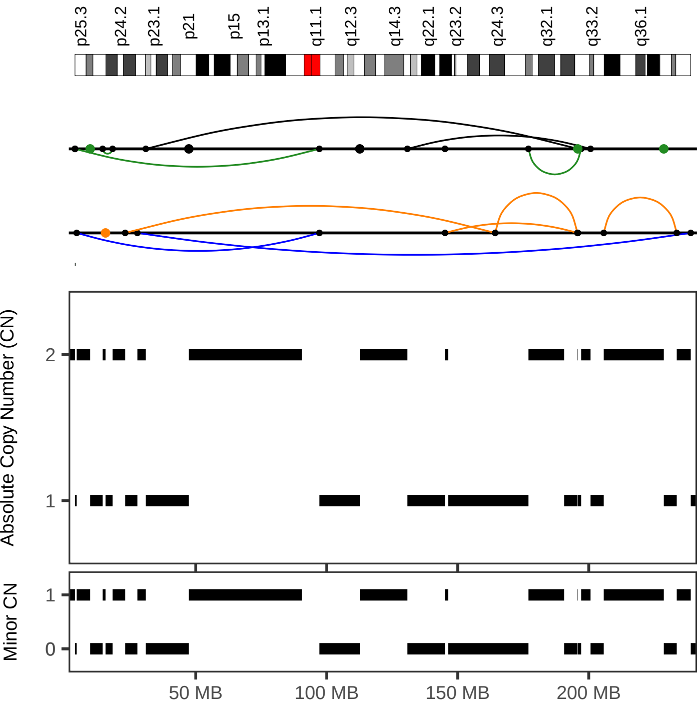

	CGP_donor_1234122
Cancer type	Breast−AdenoCA
Position	2:3892443−238924020
Type	Canonical without polyploidization
Interleaved intrachr. SVs	10
Total SVs (intrachr. + transl.)	16
SV types	DEL: 3; DUP: 2; h2hINV: 2; t2tINV: 3; TRA: 6
SVs in sample	83
Oscillating CN (2 and 3 states)	26, 26
CN segments	26
FDR fragment joints	0.96
FDR chr. breakp. enrich.	0
Linked to chrs	
Purity, ploidy	0.7, 1.77

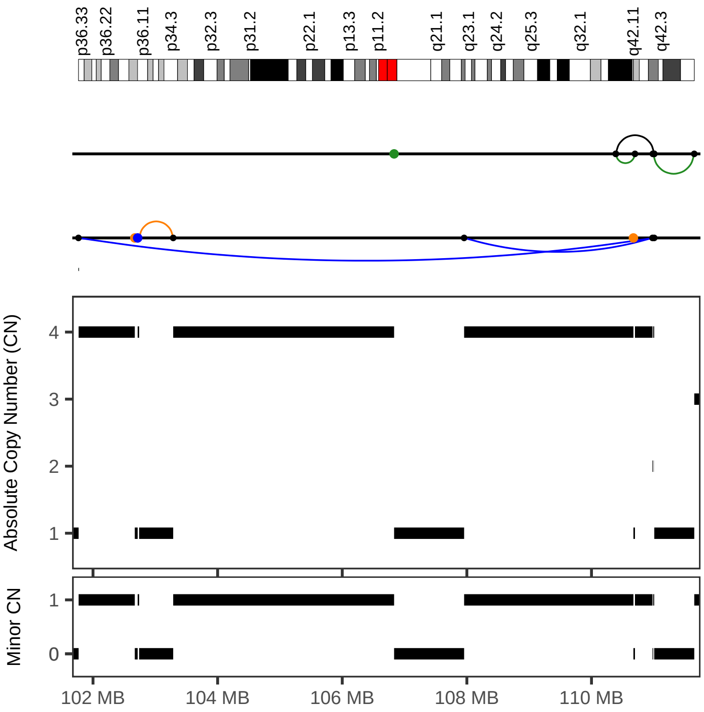

	CGP_donor_1234123
Cancer type	Breast−AdenoCA
Position	1:101766772−111649270
Type	Before polyploidization
Interleaved intrachr. SVs	5
Total SVs (intrachr. + transl.)	9
SV types	DEL: 0; DUP: 2; h2hINV: 1; t2tINV: 2; TRA: 4
SVs in sample	29
Oscillating CN (2 and 3 states)	10, 13
CN segments	15
FDR fragment joints	0.64
FDR chr. breakp. enrich.	0
Linked to chrs	
Purity, ploidy	0.35, 2.06

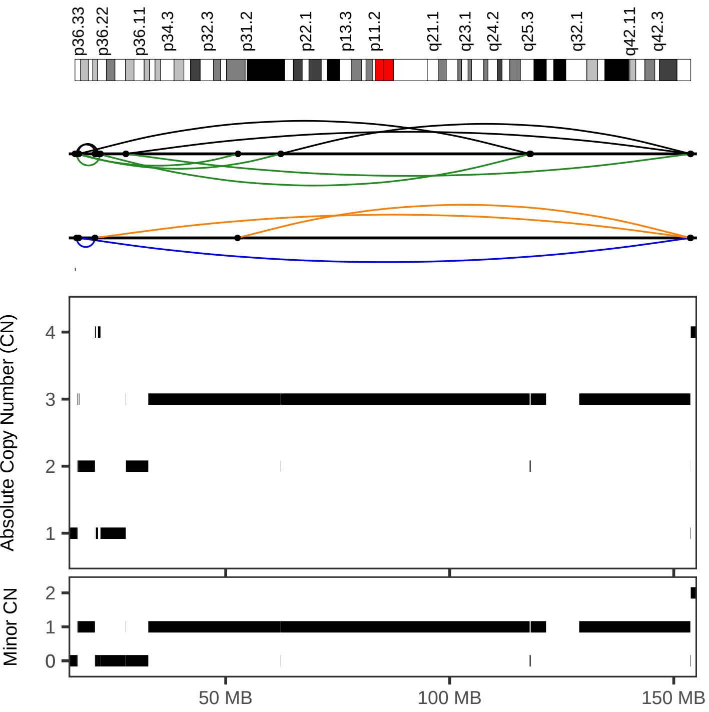

	CGP_donor_1234124
Cancer type	Breast−AdenoCA
Position	1:16353677−153794553
Type	With other complex events
Interleaved intrachr. SVs	17
Total SVs (intrachr. + transl.)	17
SV types	DEL: 2; DUP: 2; h2hINV: 7; t2tINV: 6; TRA: 0
SVs in sample	95
Oscillating CN (2 and 3 states)	7, 13
CN segments	24
FDR fragment joints	0.59
FDR chr. breakp. enrich.	0.01
Linked to chrs	
Purity, ploidy	0.59, 3

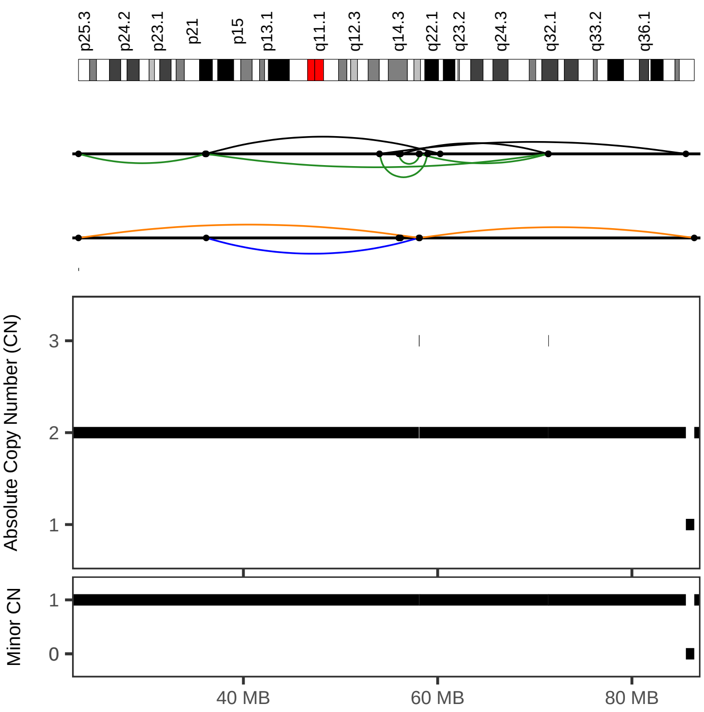

	CGP_donor_1234124
Cancer type	Breast−AdenoCA
Position	2:23018733−86416994
Type	Canonical without polyploidization
Interleaved intrachr. SVs	12
Total SVs (intrachr. + transl.)	12
SV types	DEL: 3; DUP: 1; h2hINV: 3; t2tINV: 5; TRA: 0
SVs in sample	95
Oscillating CN (2 and 3 states)	7, 9
CN segments	9
FDR fragment joints	0.64
FDR chr. breakp. enrich.	0.25
Linked to chrs	
Purity, ploidy	0.59, 3

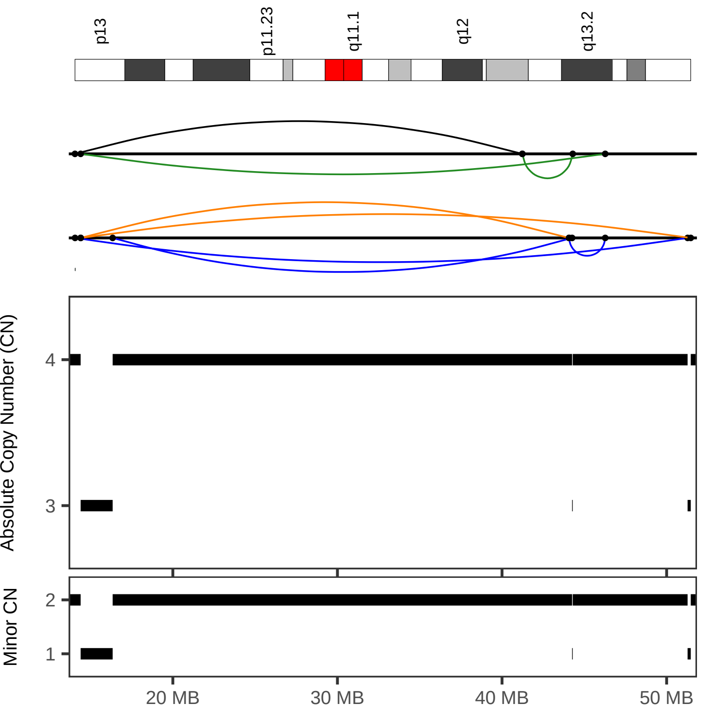

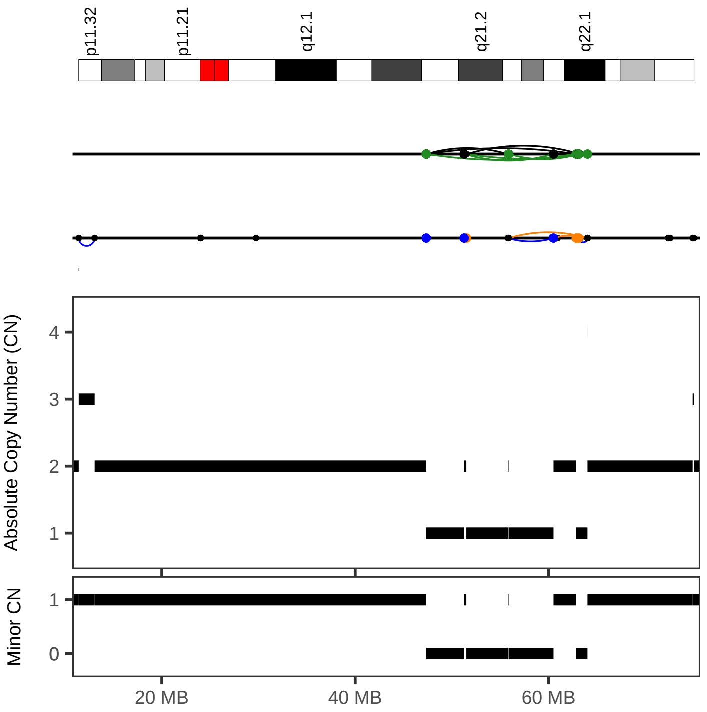

**CGP_donor_1234124**	
Cancer type	Breast−AdenoCA
Position	20:14054448−51465273
Type	After polyploidization
Interleaved intrachr. SVs	8
Total SVs (intrachr. + transl.)	8
SV types	DEL: 2; DUP: 3; h2hINV: 1; t2tINV: 2; TRA: 0
SVs in sample	95
Oscillating CN (2 and 3 states)	7, 7
CN segments	7
FDR fragment joints	0.84
FDR chr. breakp. enrich.	0.01
Linked to chrs	
Purity, ploidy	0.59, 3

**CGP_donor_1333047**	
Cancer type	Breast−AdenoCA
Position	18:47340927−64019671
Type	Canonical without polyploidization
Interleaved intrachr. SVs	12
Total SVs (intrachr. + transl.)	29
SV types	DEL: 2; DUP: 3; h2hINV: 3; t2tINV: 4; TRA: 17
SVs in sample	249
Oscillating CN (2 and 3 states)	7, 7
CN segments	9
FDR fragment joints	0.91
FDR chr. breakp. enrich.	0
Linked to chrs	4:57932328−158974416;
Purity, ploidy	0.39, 1.98

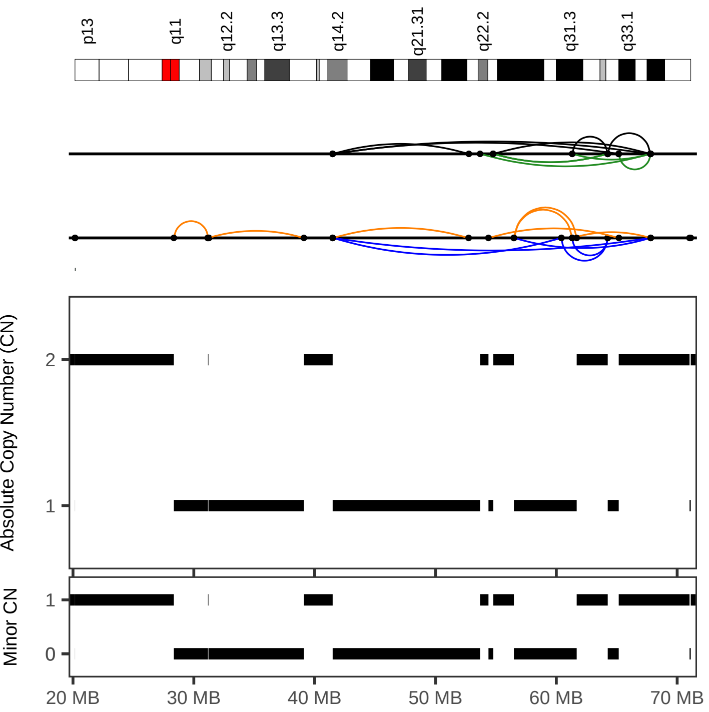

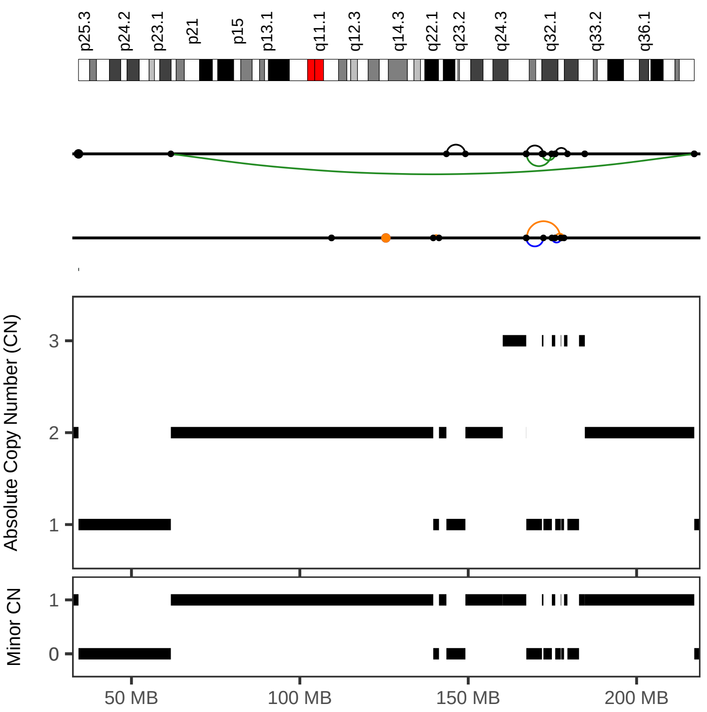

**CGP_donor_1333048**	
Cancer type	Breast−AdenoCA
Position	13:41489453−67817353
Type	Canonical without polyploidization
Interleaved intrachr. SVs	20
Total SVs (intrachr. + transl.)	20
SV types	DEL: 4; DUP: 5; h2hINV: 6; t2tINV: 5; TRA: 0
SVs in sample	178
Oscillating CN (2 and 3 states)	9, 9
CN segments	9
FDR fragment joints	0.96
FDR chr. breakp. enrich.	0
Linked to chrs	
Purity, ploidy	0.54, 2.02

**CGP_donor_1337214**	
Cancer type	Breast−AdenoCA
Position	2:167215273−179453980
Type	Canonical without polyploidization
Interleaved intrachr. SVs	8
Total SVs (intrachr. + transl.)	8
SV types	DEL: 2; DUP: 2; h2hINV: 2; t2tINV: 2; TRA: 0
SVs in sample	161
Oscillating CN (2 and 3 states)	9, 10
CN segments	11
FDR fragment joints	1
FDR chr. breakp. enrich.	0.3
Linked to chrs	
Purity, ploidy	0.63, 1.91

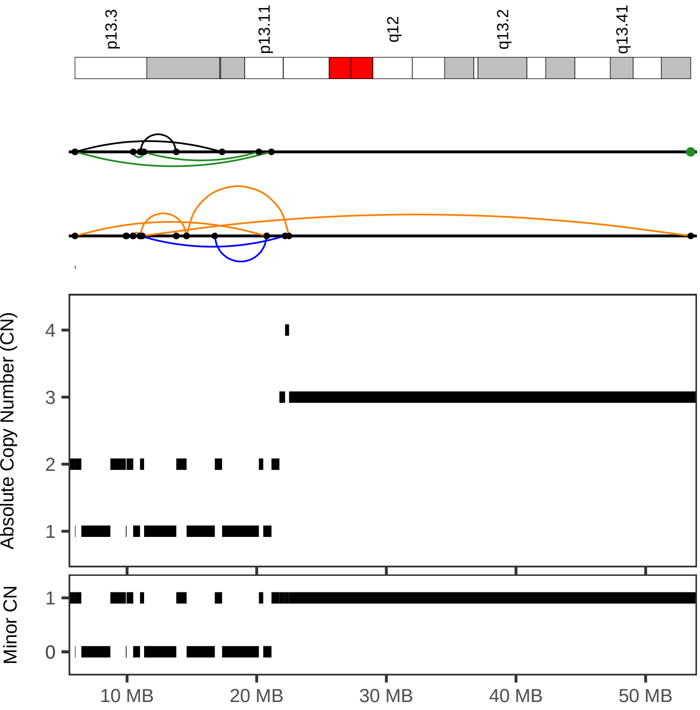

	CGP_donor_1337222
Cancer type	Breast−AdenoCA
Position	19:5982144−53477754
Type	Canonical without polyploidization
Interleaved intrachr. SVs	13
Total SVs (intrachr. + transl.)	14
SV types	DEL: 5; DUP: 3; h2hINV: 2; t2tINV: 3; TRA: 1
SVs in sample	23
Oscillating CN (2 and 3 states)	19, 22
CN segments	22
FDR fragment joints	0.77
FDR chr. breakp. enrich.	0
Linked to chrs	
Purity, ploidy	0.31, 1.83

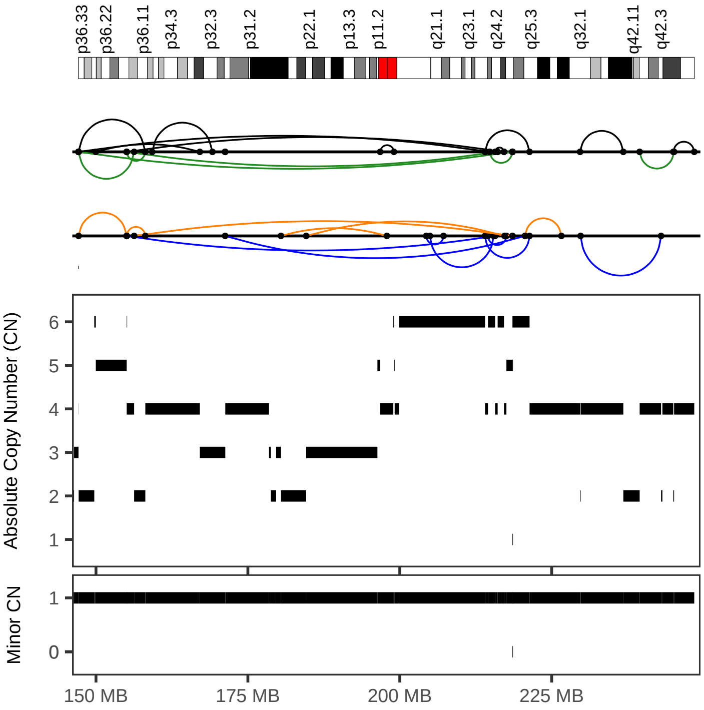

	CGP_donor_1337225
Cancer type	Breast−AdenoCA
Position	1:147121037−226598450
Type	With other complex events
Interleaved intrachr. SVs	26
Total SVs (intrachr. + transl.)	26
SV types	DEL: 6; DUP: 6; h2hINV: 8; t2tINV: 6; TRA: 0
SVs in sample	53
Oscillating CN (2 and 3 states)	7, 9
CN segments	31
FDR fragment joints	0.95
FDR chr. breakp. enrich.	0
Linked to chrs	
Purity, ploidy	0.47, 2.32

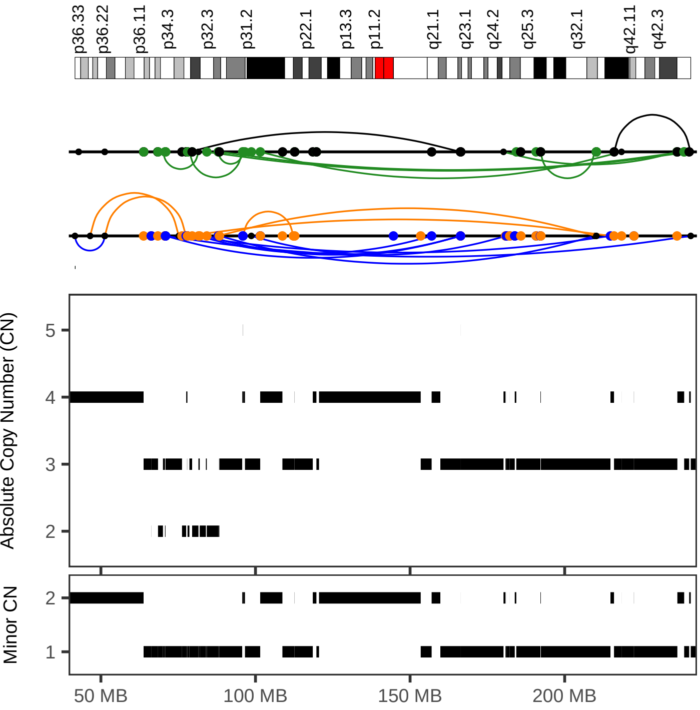

	CGP_donor_1337226
Cancer type	Breast−AdenoCA
Position	1:41613153−240778773
Type	With other complex events
Interleaved intrachr. SVs	23
Total SVs (intrachr. + transl.)	128
SV types	DEL: 5; DUP: 8; h2hINV: 2; t2tINV: 8; TRA: 105
SVs in sample	473
Oscillating CN (2 and 3 states)	21, 24
CN segments	59
FDR fragment joints	0.59
FDR chr. breakp. enrich.	0
Linked to chrs	2:26938396−230264705;3:88027440−192058994 6:35204768−167887450;
Purity, ploidy	0.7, 3.48

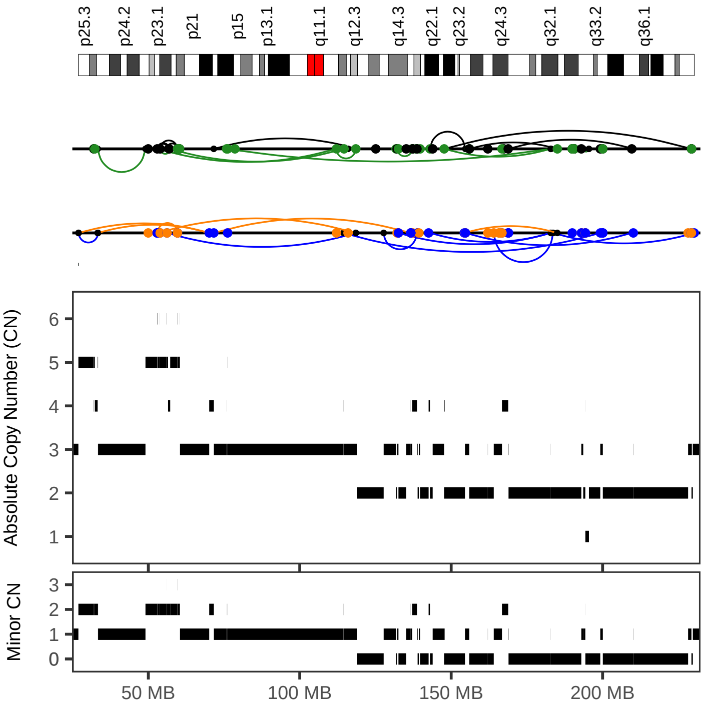

	CGP_donor_1337226
Cancer type	Breast−AdenoCA
Position	2:26938396−230264706
Type	With other complex events
Interleaved intrachr. SVs	40
Total SVs (intrachr. + transl.)	150
SV types	DEL: 6; DUP: 13; h2hINV: 12; t2tINV: 9; TRA: 110
SVs in sample	473
Oscillating CN (2 and 3 states)	12, 20
CN segments	86
FDR fragment joints	0.59
FDR chr. breakp. enrich.	0
Linked to chrs	1:41613153−240778772;3:88027440−192058994 6:35204768−167887450;
Purity, ploidy	0.7, 3.48

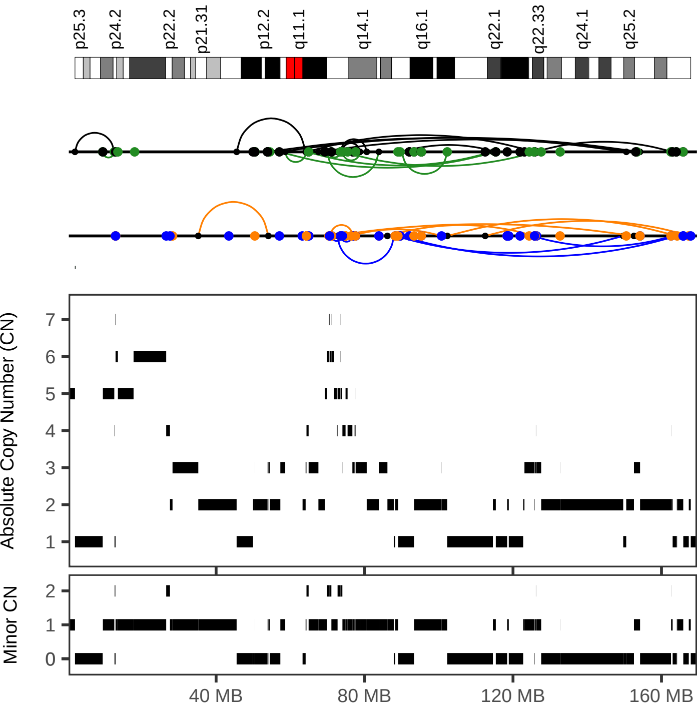

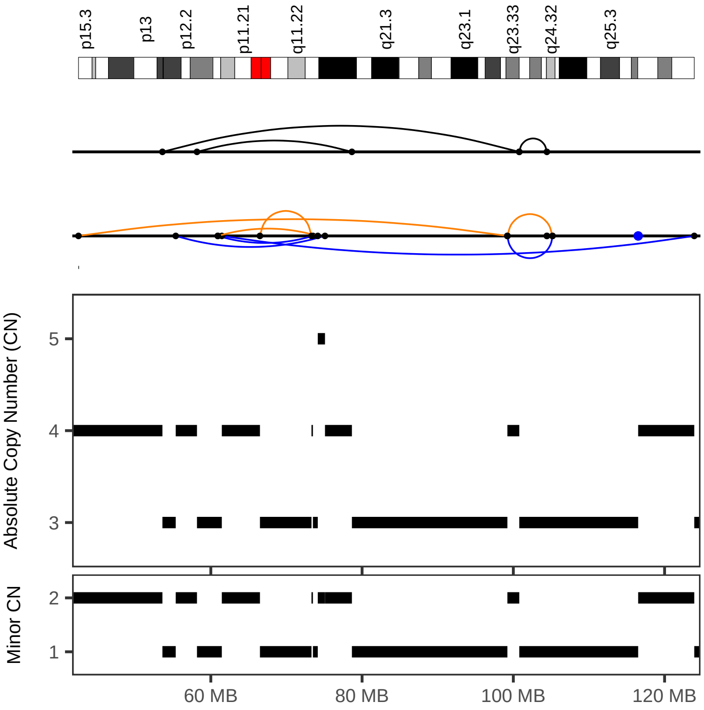

	CGP_donor_1337226
Cancer type	Breast−AdenoCA
Position	6:35204768−167887451
Type	With other complex events
Interleaved intrachr. SVs	31
Total SVs (intrachr. + transl.)	157
SV types	DEL: 7; DUP: 7; h2hINV: 9; t2tINV: 8; TRA: 126
SVs in sample	473
Oscillating CN (2 and 3 states)	12, 46
CN segments	89
FDR fragment joints	0.96
FDR chr. breakp. enrich.	0
Linked to chrs	1:41613153−240778772;2:26938396−230264705 3:88027440−192058994;
Purity, ploidy	0.7, 3.48

	CGP_donor_1337226
Cancer type	Breast−AdenoCA
Position	10:42502102−123920853
Type	With other complex events
Interleaved intrachr. SVs	10
Total SVs (intrachr. + transl.)	11
SV types	DEL: 3; DUP: 4; h2hINV: 3; t2tINV: 0; TRA: 1
SVs in sample	473
Oscillating CN (2 and 3 states)	8, 10
CN segments	15
FDR fragment joints	0.59
FDR chr. breakp. enrich.	0.19
Linked to chrs	
Purity, ploidy	0.7, 3.48

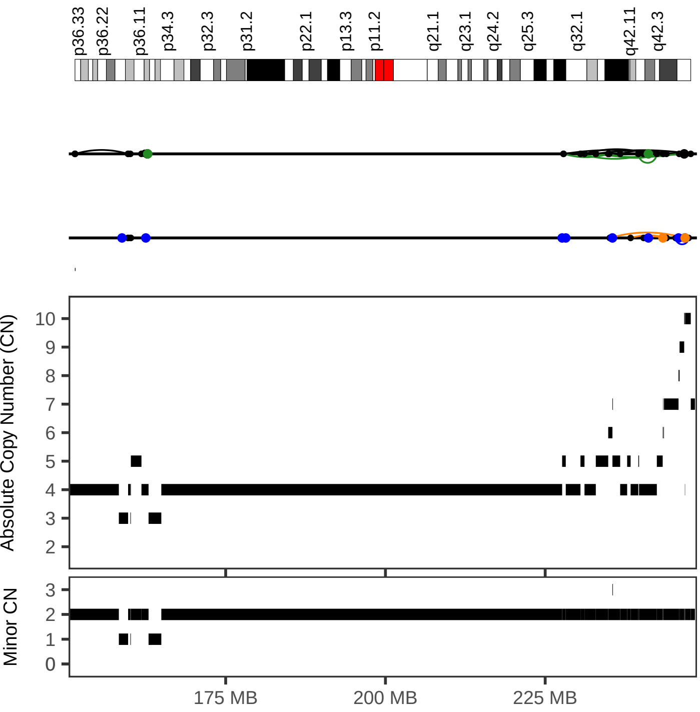

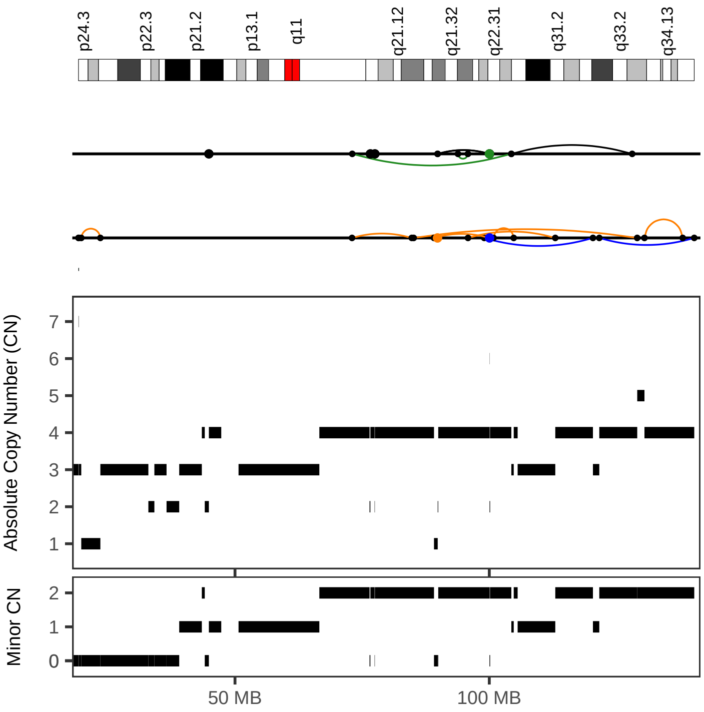

	CGP_donor_1337231
Cancer type	Breast−AdenoCA
Position	1:227864992−247779692
Type	With other complex events
Interleaved intrachr. SVs	16
Total SVs (intrachr. + transl.)	26
SV types	DEL: 4; DUP: 3; h2hINV: 4; t2tINV: 5; TRA: 10
SVs in sample	213
Oscillating CN (2 and 3 states)	7, 14
CN segments	24
FDR fragment joints	0.94
FDR chr. breakp. enrich.	0
Linked to chrs	19:44287113−54175477;
Purity, ploidy	0.74, 3

	CGP_donor_1337231
Cancer type	Breast−AdenoCA
Position	9:73011782−140301664
Type	With other complex events
Interleaved intrachr. SVs	11
Total SVs (intrachr. + transl.)	17
SV types	DEL: 5; DUP: 2; h2hINV: 3; t2tINV: 1; TRA: 6
SVs in sample	213
Oscillating CN (2 and 3 states)	7, 9
CN segments	20
FDR fragment joints	0.59
FDR chr. breakp. enrich.	0
Linked to chrs	
Purity, ploidy	0.74, 3

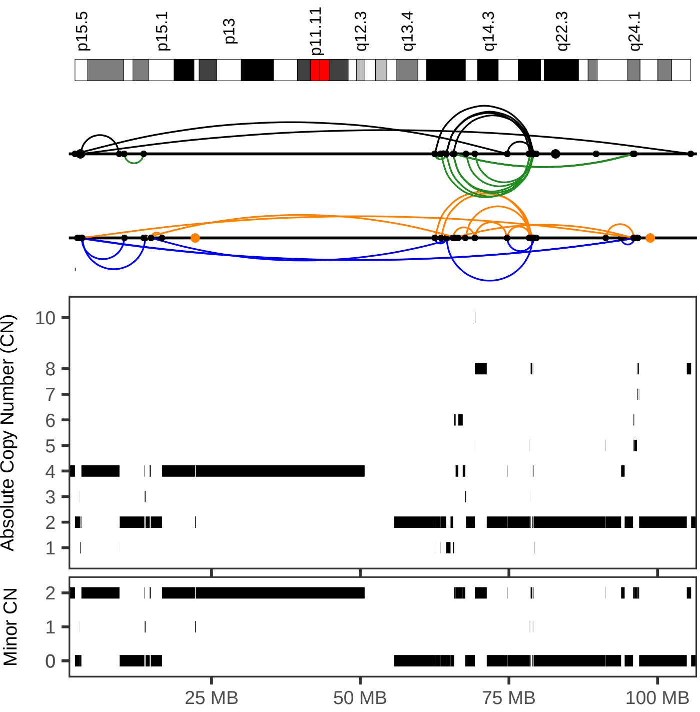

**CGP_donor_1337231**	
Cancer type	Breast−AdenoCA
Position	11:2020871−105572582
Type	With other complex events
Interleaved intrachr. SVs	48
Total SVs (intrachr. + transl.)	52
SV types	DEL: 14; DUP: 10; h2hINV: 10; t2tINV: 14; TRA: 4
SVs in sample	213
Oscillating CN (2 and 3 states)	8, 8
CN segments	67
FDR fragment joints	0.8
FDR chr. breakp. enrich.	0
Linked to chrs	
Purity, ploidy	0.74, 3

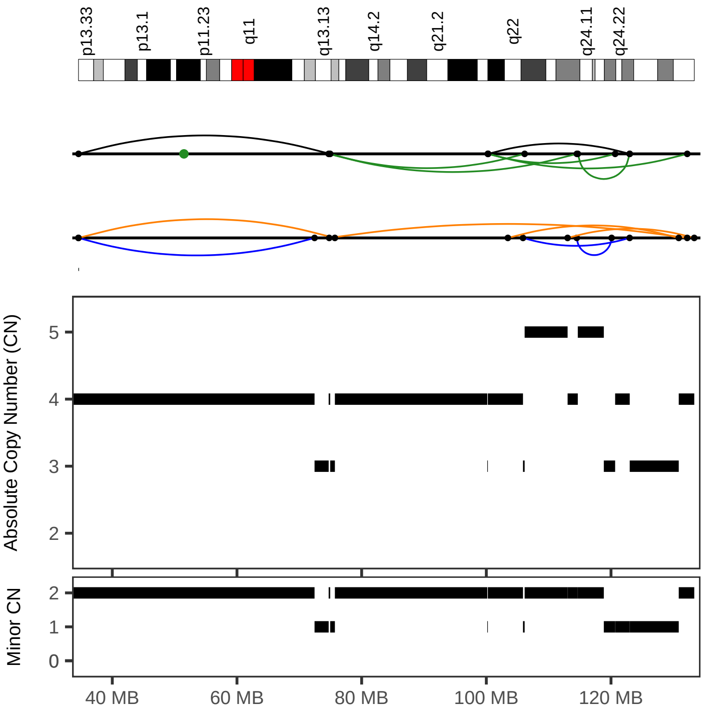

**CGP_donor_1337231**	
Cancer type	Breast−AdenoCA
Position	12:34572727−133356868
Type	With other complex events
Interleaved intrachr. SVs	14
Total SVs (intrachr. + transl.)	15
SV types	DEL: 4; DUP: 3; h2hINV: 2; t2tINV: 5; TRA: 1
SVs in sample	213
Oscillating CN (2 and 3 states)	10, 17
CN segments	17
FDR fragment joints	0.78
FDR chr. breakp. enrich.	0.21
Linked to chrs	
Purity, ploidy	0.74, 3

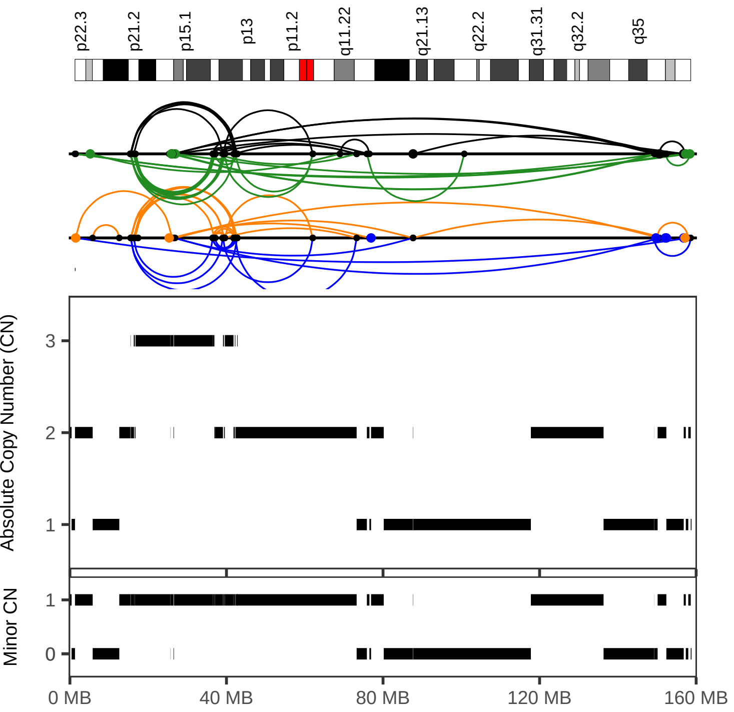

**CGP_donor_1347731**	
Cancer type	Breast−AdenoCA
Position	7:1295015−158613922
Type	Canonical without polyploidization
Interleaved intrachr. SVs	112
Total SVs (intrachr. + transl.)	130
SV types	DEL: 28; DUP: 25; h2hINV: 29; t2tINV: 30; TRA: 18
SVs in sample	155
Oscillating CN (2 and 3 states)	43, 62
CN segments	62
FDR fragment joints	0.94
FDR chr. breakp. enrich.	0
Linked to chrs	
Purity, ploidy	0.84, 1.96

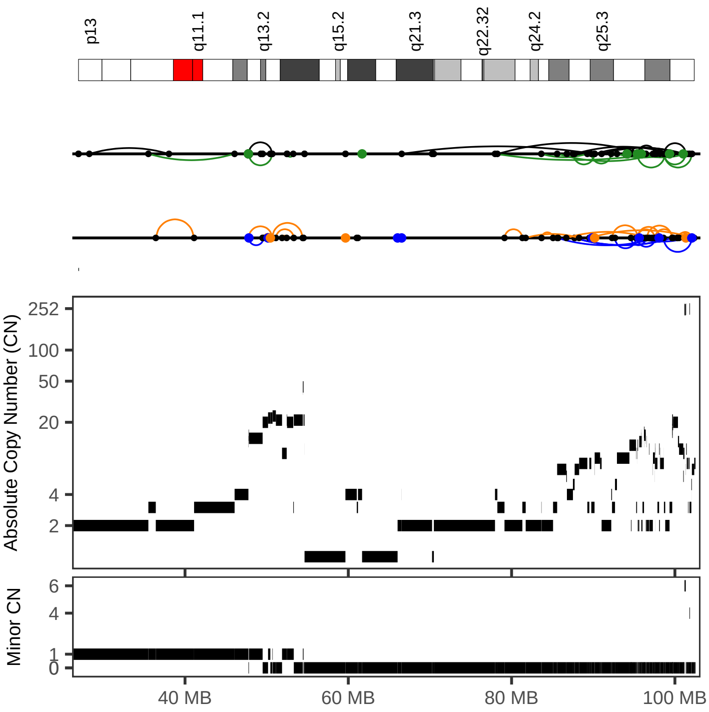

**CGP_donor_1347742**	
Cancer type	Breast−AdenoCA
Position	15:66537420−102369380
Type	With other complex events
Interleaved intrachr. SVs	54
Total SVs (intrachr. + transl.)	66
SV types	DEL: 14; DUP: 12; h2hINV: 13; t2tINV: 15; TRA: 12
SVs in sample	230
Oscillating CN (2 and 3 states)	7, 7
CN segments	110
FDR fragment joints	0.96
FDR chr. breakp. enrich.	0
Linked to chrs	1:221700044−232053720;
Purity, ploidy	0.52, 2.12

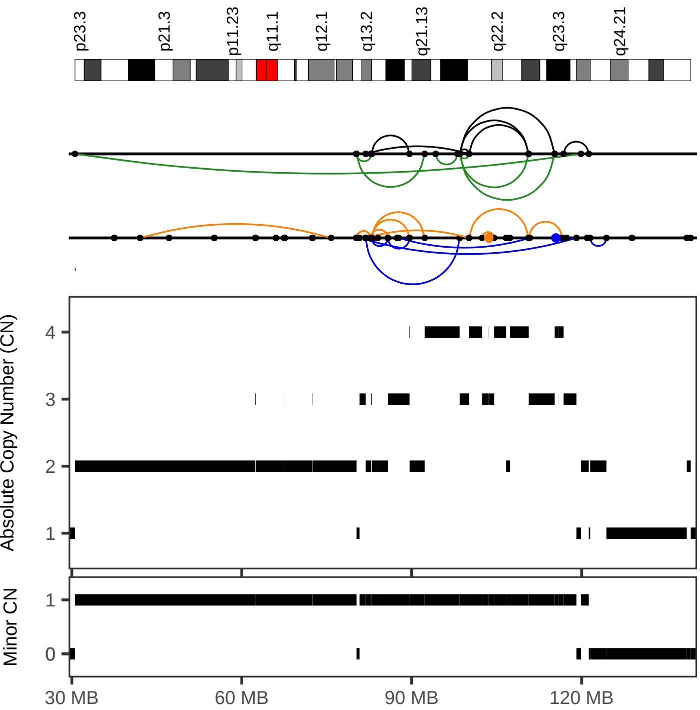

	**CGP_donor_1347751**
Cancer type	Breast−AdenoCA
Position	8:30557729−121251839
Type	With other complex events
Interleaved intrachr. SVs	28
Total SVs (intrachr. + transl.)	31
SV types	DEL: 8; DUP: 6; h2hINV: 8; t2tINV: 6; TRA: 3
SVs in sample	828
Oscillating CN (2 and 3 states)	11, 20
CN segments	41
FDR fragment joints	0.93
FDR chr. breakp. enrich.	0.12
Linked to chrs	
Purity, ploidy	0.9, 1.96

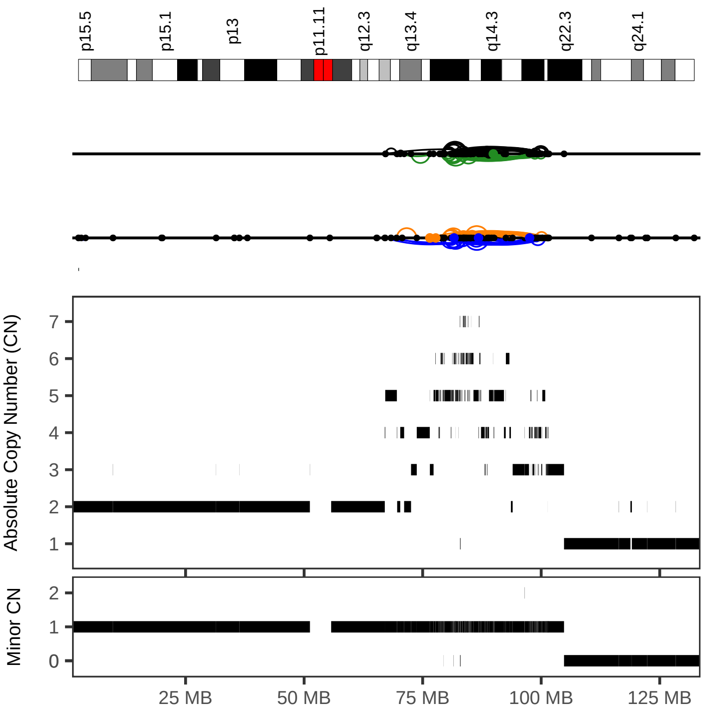

	**CGP_donor_1347751**
Cancer type	Breast−AdenoCA
Position	11:67012799−101615430
Type	With other complex events
Interleaved intrachr. SVs	204
Total SVs (intrachr. + transl.)	212
SV types	DEL: 45; DUP: 64; h2hINV: 47; t2tINV: 48; TRA: 8
SVs in sample	828
Oscillating CN (2 and 3 states)	18, 38
CN segments	175
FDR fragment joints	0.59
FDR chr. breakp. enrich.	0
Linked to chrs	18:20621968−77903677;9:1126285−125671727
Purity, ploidy	0.9, 1.96

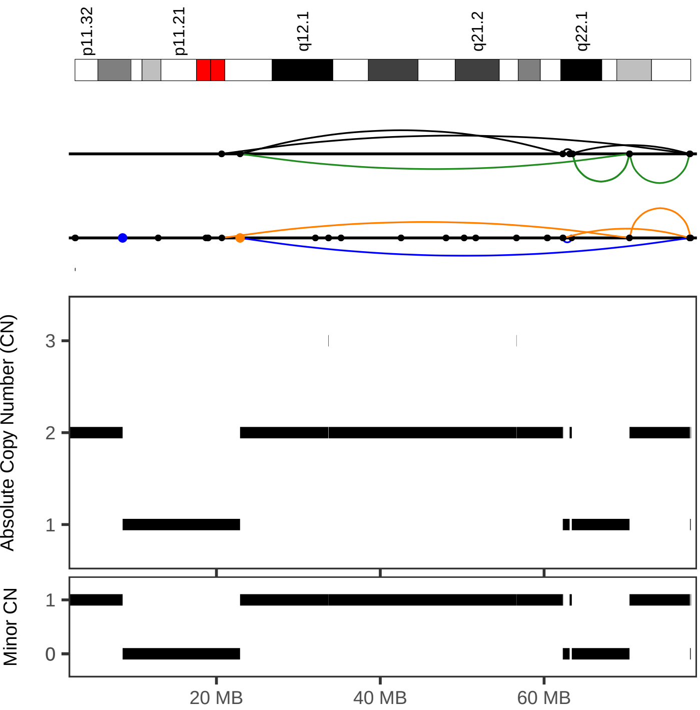

	**CGP_donor_1347751**
Cancer type	Breast−AdenoCA
Position	18:20621968−77903678
Type	Canonical without polyploidization
Interleaved intrachr. SVs	15
Total SVs (intrachr. + transl.)	16
SV types	DEL: 3; DUP: 2; h2hINV: 4; t2tINV: 6; TRA: 1
SVs in sample	828
Oscillating CN (2 and 3 states)	11, 16
CN segments	16
FDR fragment joints	0.64
FDR chr. breakp. enrich.	0.27
Linked to chrs	
Purity, ploidy	0.9, 1.96

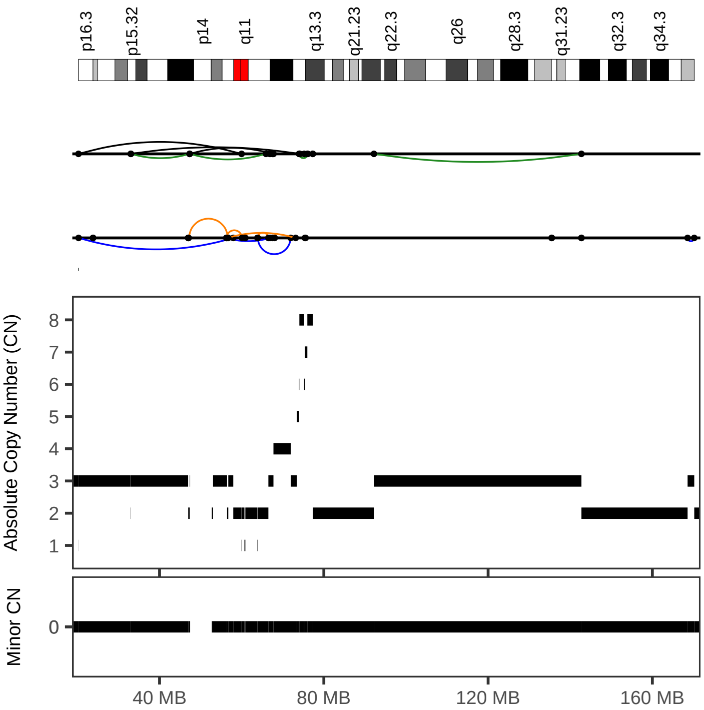

	**CGP_donor_1353427**
Cancer type	Breast−AdenoCA
Position	4:20249209−76099019
Type	With other complex events
Interleaved intrachr. SVs	16
Total SVs (intrachr. + transl.)	16
SV types	DEL: 4; DUP: 4; h2hINV: 4; t2tINV: 4; TRA: 0
SVs in sample	267
Oscillating CN (2 and 3 states)	12, 21
CN segments	28
FDR fragment joints	1
FDR chr. breakp. enrich.	0.13
Linked to chrs	
Purity, ploidy	0.29, 2.76

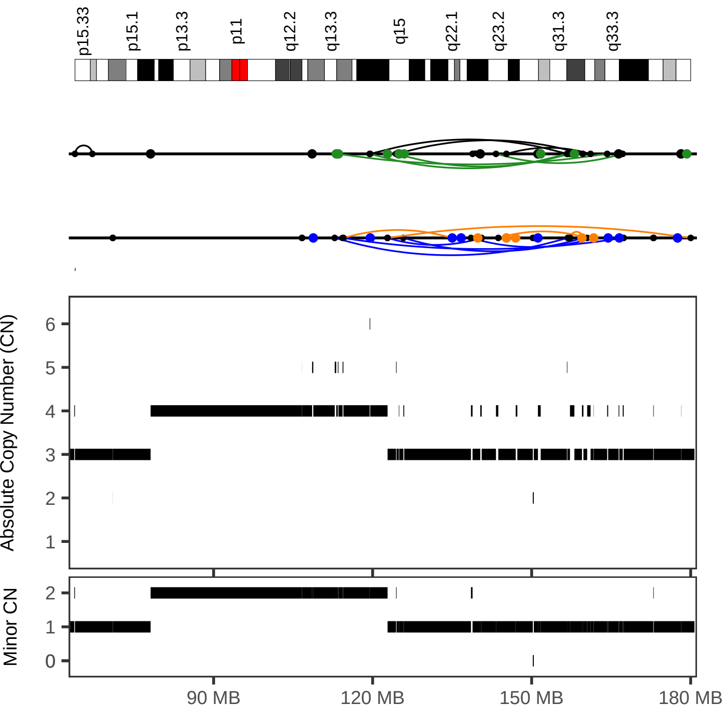

CGP_donor_1353427

Cancer type	Breast−AdenoCA
Position	5:112844490−180014178
Type	With other complex events
Interleaved intrachr. SVs	16
Total SVs (intrachr. + transl.)	43
SV types	DEL: 4; DUP: 5; h2hINV: 3; t2tINV: 4; TRA: 27
SVs in sample	267
Oscillating CN (2 and 3 states)	19, 23
CN segments	47
FDR fragment joints	0.94
FDR chr. breakp. enrich.	0
Linked to chrs	14:41756381−106200524;
Purity, ploidy	0.29, 2.76

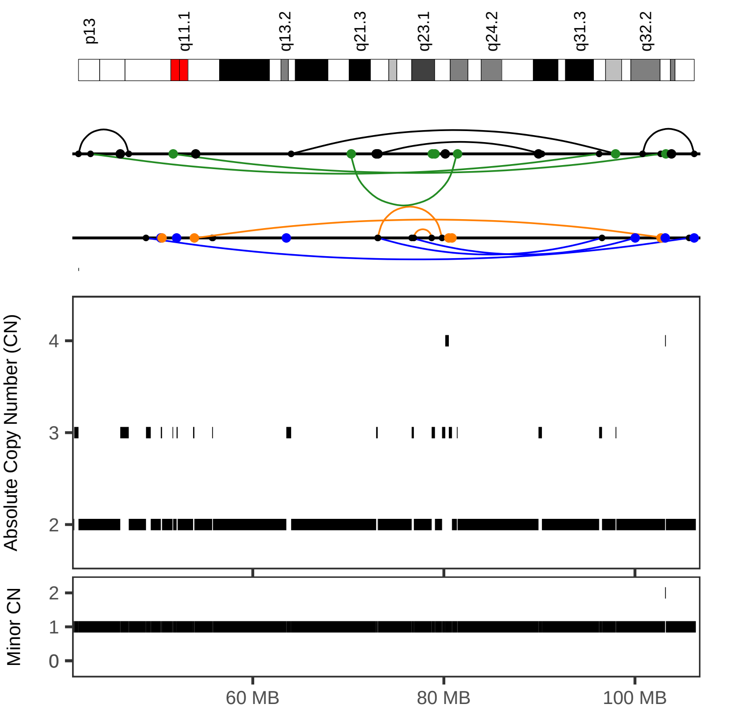

CGP_donor_1353427

Cancer type	Breast−AdenoCA
Position	14:41756381−106200525
Type	Canonical without polyploidization
Interleaved intrachr. SVs	12
Total SVs (intrachr. + transl.)	37
SV types	DEL: 2; DUP: 3; h2hINV: 4; t2tINV: 3; TRA: 25
SVs in sample	267
Oscillating CN (2 and 3 states)	26, 37
CN segments	39
FDR fragment joints	0.91
FDR chr. breakp. enrich.	0
Linked to chrs	5:112844490−180014177;
Purity, ploidy	0.29, 2.76

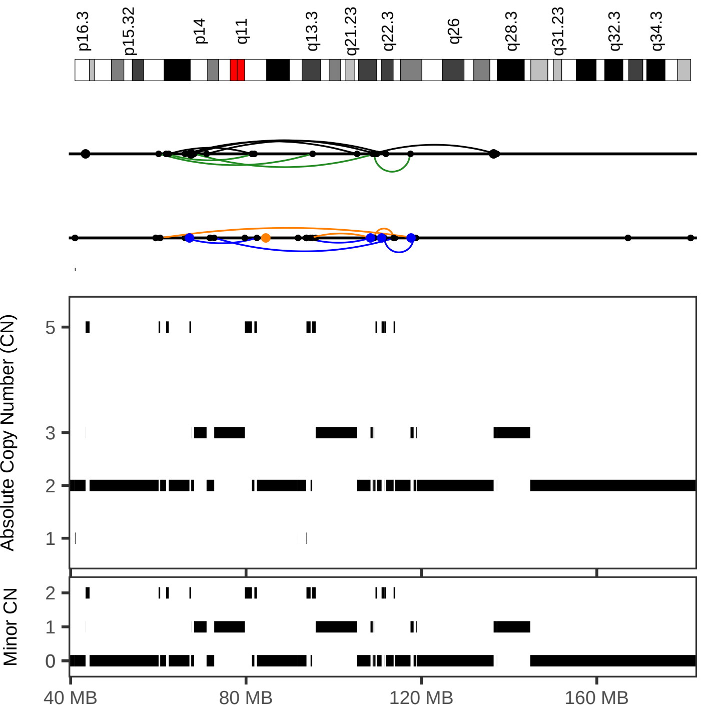

CGP_donor_1353434

Cancer type	Breast−AdenoCA
Position	4:60055635−137193613
Type	With other complex events
Interleaved intrachr. SVs	18
Total SVs (intrachr. + transl.)	25
SV types	DEL: 4; DUP: 5; h2hINV: 5; t2tINV: 4; TRA: 7
SVs in sample	291
Oscillating CN (2 and 3 states)	9, 9
CN segments	42
FDR fragment joints	0.98
FDR chr. breakp. enrich.	0.02
Linked to chrs	
Purity, ploidy	0.5, 2.94

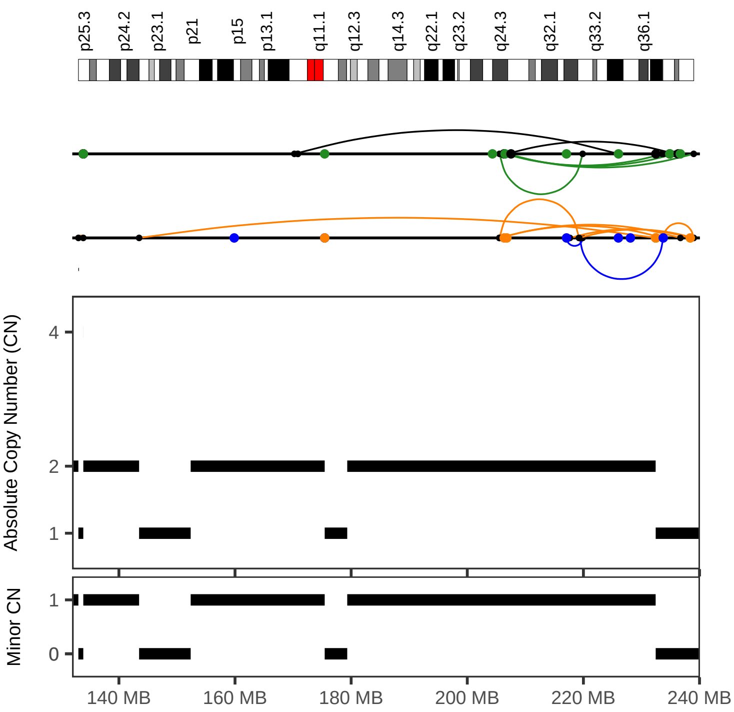

CGP_donor_1364028

Cancer type	Breast−AdenoCA
Position	2:143479926−239003903
Type	Canonical without polyploidization
Interleaved intrachr. SVs	17
Total SVs (intrachr. + transl.)	43
SV types	DEL: 9; DUP: 2; h2hINV: 2; t2tINV: 4; TRA: 26
SVs in sample	203
Oscillating CN (2 and 3 states)	7, 7
CN segments	7
FDR fragment joints	0.48
FDR chr. breakp. enrich.	0
Linked to chrs	3:11137338−196396671;
Purity, ploidy	0.68, 2.2

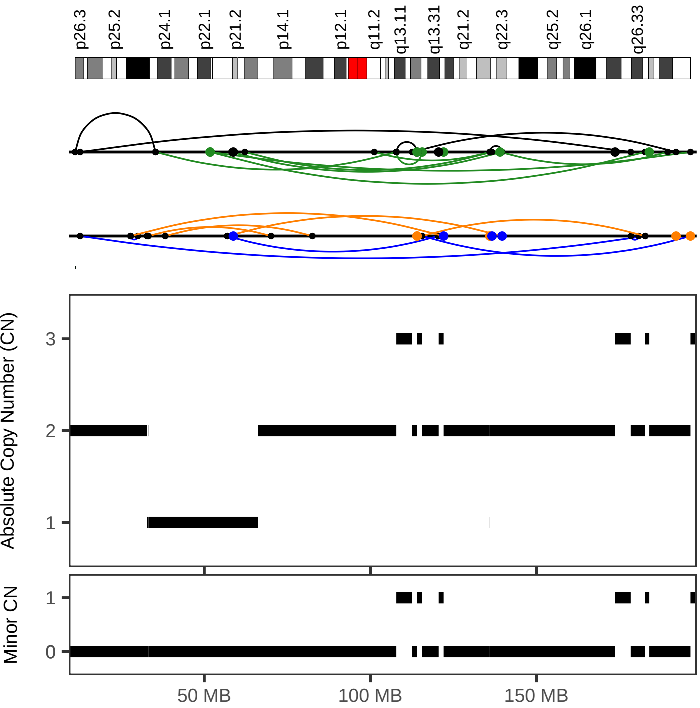

CGP_donor_1364028	
Cancer type	Breast−AdenoCA
Position	3:11137338−196396672
Type	With other complex events
Interleaved intrachr. SVs	22
Total SVs (intrachr. + transl.)	42
SV types	DEL: 5; DUP: 4; h2hINV: 5; t2tINV: 8; TRA: 20
SVs in sample	203
Oscillating CN (2 and 3 states)	9, 22
CN segments	22
FDR fragment joints	0.73
FDR chr. breakp. enrich.	0
Linked to chrs	2:143479926−239003902;
Purity, ploidy	0.68, 2.2

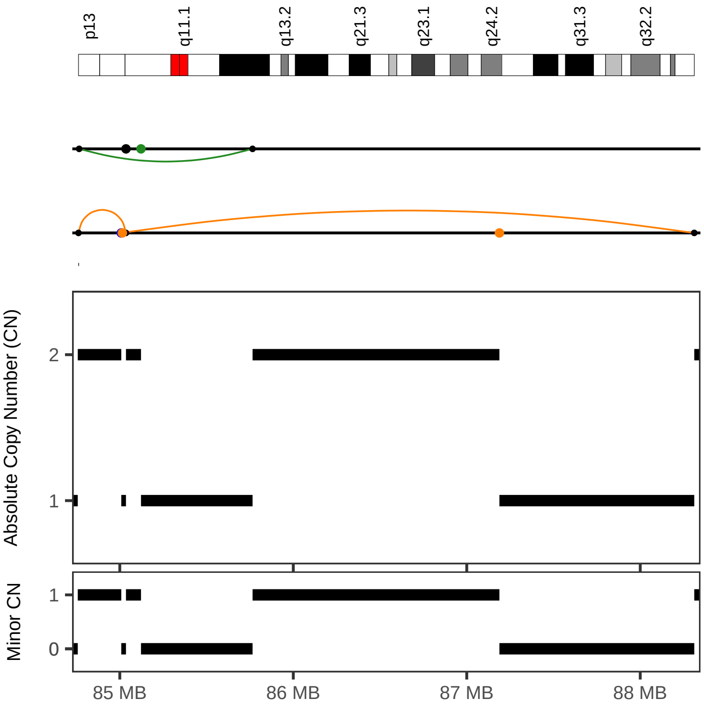

CGP_donor_1364028	
Cancer type	Breast−AdenoCA
Position	14:84761805−88311296
Type	Canonical without polyploidization
Interleaved intrachr. SVs	3
Total SVs (intrachr. + transl.)	8
SV types	DEL: 2; DUP: 0; h2hINV: 0; t2tINV: 1; TRA: 5
SVs in sample	203
Oscillating CN (2 and 3 states)	7, 7
CN segments	7
FDR fragment joints	0.59
FDR chr. breakp. enrich.	0.01
Linked to chrs	20:13487327−62399710;6:2192497−33282946
Purity, ploidy	0.68, 2.2

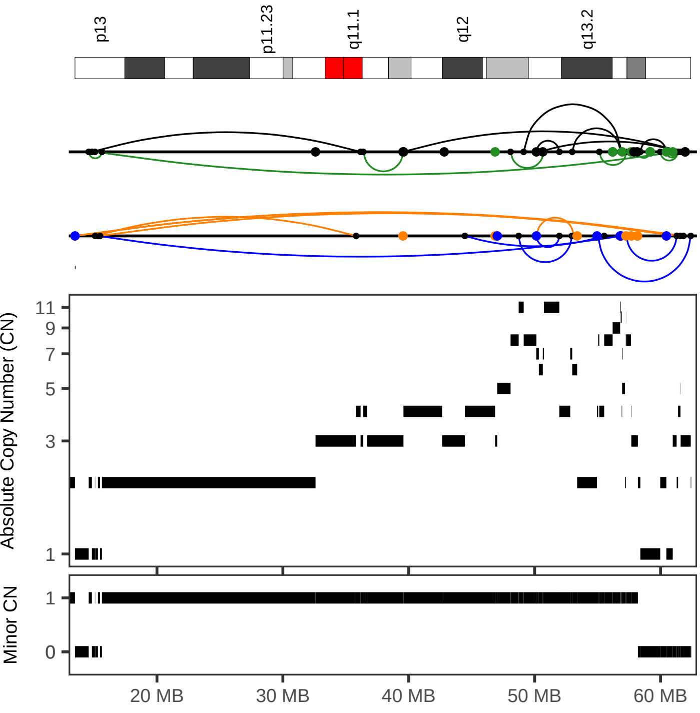

CGP_donor_1364028	
Cancer type	Breast−AdenoCA
Position	20:13487327−62399711
Type	With other complex events
Interleaved intrachr. SVs	27
Total SVs (intrachr. + transl.)	63
SV types	DEL: 5; DUP: 6; h2hINV: 8; t2tINV: 8; TRA: 36
SVs in sample	203
Oscillating CN (2 and 3 states)	9, 10
CN segments	59
FDR fragment joints	0.84
FDR chr. breakp. enrich.	0
Linked to chrs	14:84761805−88311295;6:2192497−33282946
Purity, ploidy	0.68, 2.2

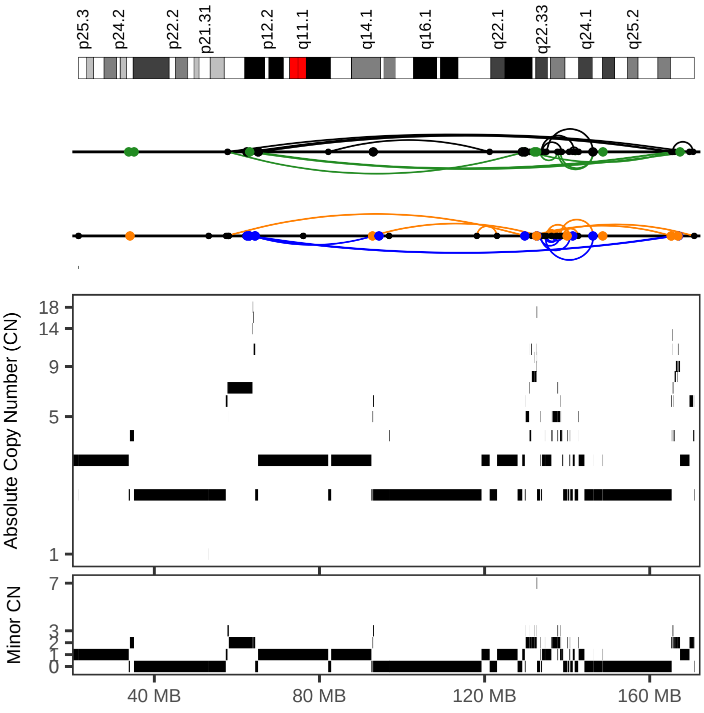

CGP_donor_1364033	
Cancer type	Breast−AdenoCA
Position	6:57399664−170784376
Type	With other complex events
Interleaved intrachr. SVs	47
Total SVs (intrachr. + transl.)	80
SV types	DEL: 13; DUP: 13; h2hINV: 10; t2tINV: 11; TRA: 33
SVs in sample	529
Oscillating CN (2 and 3 states)	7, 14
CN segments	94
FDR fragment joints	0.92
FDR chr. breakp. enrich.	0
Linked to chrs	17:15573740−80067669;20:23008810−61019045
Purity, ploidy	0.32, 3.04

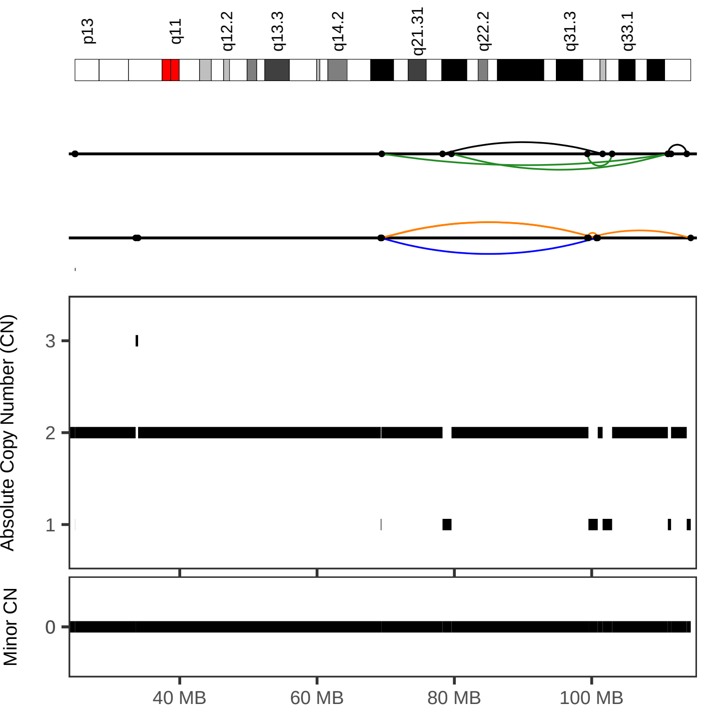

	**CGP_donor_1364033**
Cancer type	Breast−AdenoCA
Position	13:69277093−114427555
Type	Canonical without polyploidization
Interleaved intrachr. SVs	10
Total SVs (intrachr. + transl.)	10
SV types	DEL: 4; DUP: 1; h2hINV: 2; t2tINV: 3; TRA: 0
SVs in sample	529
Oscillating CN (2 and 3 states)	12, 12
CN segments	12
FDR fragment joints	0.64
FDR chr. breakp. enrich.	0.65
Linked to chrs	
Purity, ploidy	0.32, 3.04

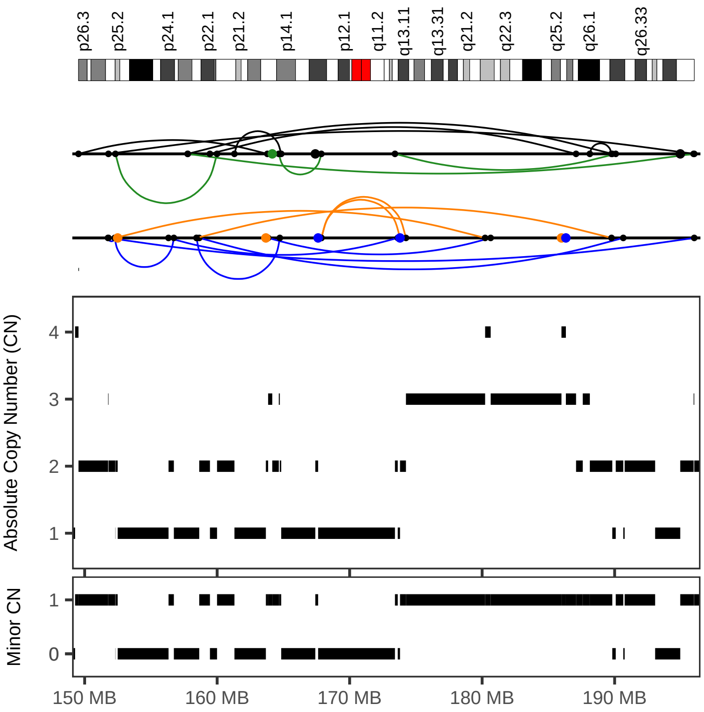

	**CGP_donor_1374616**
Cancer type	Breast−AdenoCA
Position	3:149533512−196009872
Type	With other complex events
Interleaved intrachr. SVs	20
Total SVs (intrachr. + transl.)	29
SV types	DEL: 4; DUP: 7; h2hINV: 5; t2tINV: 4; TRA: 9
SVs in sample	144
Oscillating CN (2 and 3 states)	11, 39
CN segments	39
FDR fragment joints	0.83
FDR chr. breakp. enrich.	0
Linked to chrs	X:3867520−67168966;
Purity, ploidy	0.5, 1.95

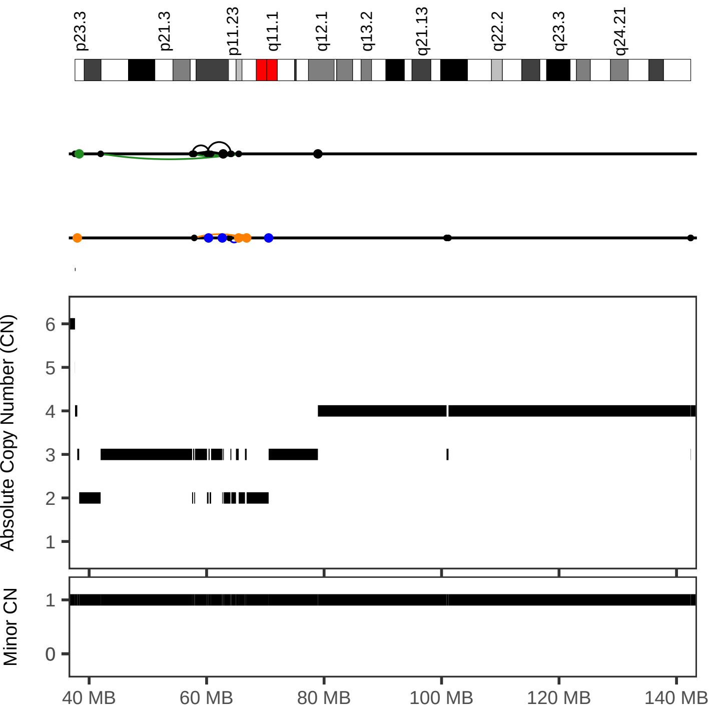

	**CGP_donor_1374616**
Cancer type	Breast−AdenoCA
Position	8:41958285−66532612
Type	Canonical without polyploidization
Interleaved intrachr. SVs	9
Total SVs (intrachr. + transl.)	13
SV types	DEL: 2; DUP: 1; h2hINV: 3; t2tINV: 3; TRA: 4
SVs in sample	144
Oscillating CN (2 and 3 states)	19, 19
CN segments	19
FDR fragment joints	0.83
FDR chr. breakp. enrich.	0
Linked to chrs	X:3867520−67168966;
Purity, ploidy	0.5, 1.95

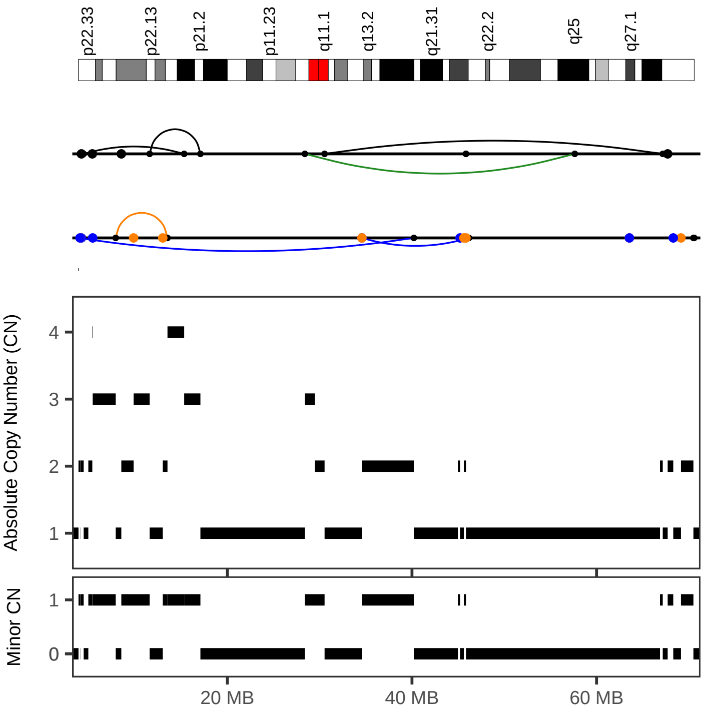

	**CGP_donor_1374616**
Cancer type	Breast−AdenoCA
Position	X:3867520−67168967
Type	With other complex events
Interleaved intrachr. SVs	4
Total SVs (intrachr. + transl.)	20
SV types	DEL: 0; DUP: 2; h2hINV: 1; t2tINV: 1; TRA: 16
SVs in sample	144
Oscillating CN (2 and 3 states)	10, 10
CN segments	26
FDR fragment joints	0.64
FDR chr. breakp. enrich.	0
Linked to chrs	
Purity, ploidy	0.5, 1.95

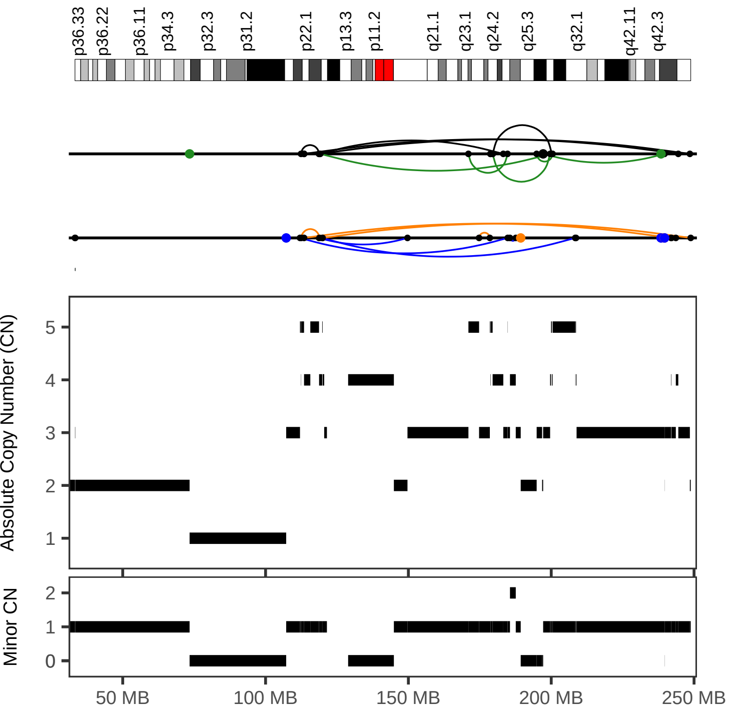

CGP_donor_1374617	
Cancer type	Breast−AdenoCA
Position	1:112053543−248836624
Type	With other complex events
Interleaved intrachr. SVs	18
Total SVs (intrachr. + transl.)	24
SV types	DEL: 3; DUP: 3; h2hINV: 6; t2tINV: 6; TRA: 6
SVs in sample	85
Oscillating CN (2 and 3 states)	10, 22
CN segments	44
FDR fragment joints	0.64
FDR chr. breakp. enrich.	0
Linked to chrs	3:160434199−161100668;8:809654−92053761
Purity, ploidy	0.5, 2.01

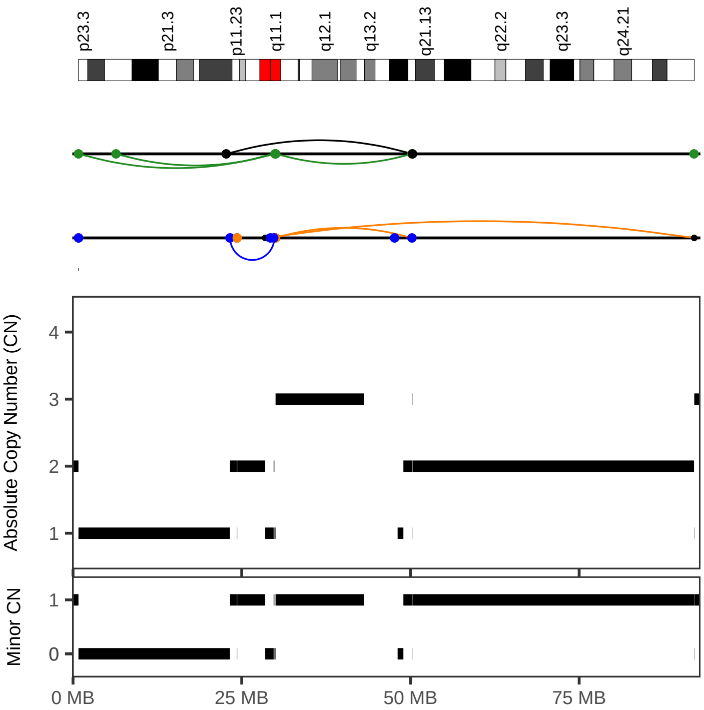

CGP_donor_1374617	
Cancer type	Breast−AdenoCA
Position	8:809654−92053762
Type	With other complex events
Interleaved intrachr. SVs	8
Total SVs (intrachr. + transl.)	30
SV types	DEL: 3; DUP: 1; h2hINV: 1; t2tINV: 3; TRA: 22
SVs in sample	85
Oscillating CN (2 and 3 states)	11, 23
CN segments	23
FDR fragment joints	0.64
FDR chr. breakp. enrich.	0
Linked to chrs	3:160434199−161100668;
Purity, ploidy	0.5, 2.01

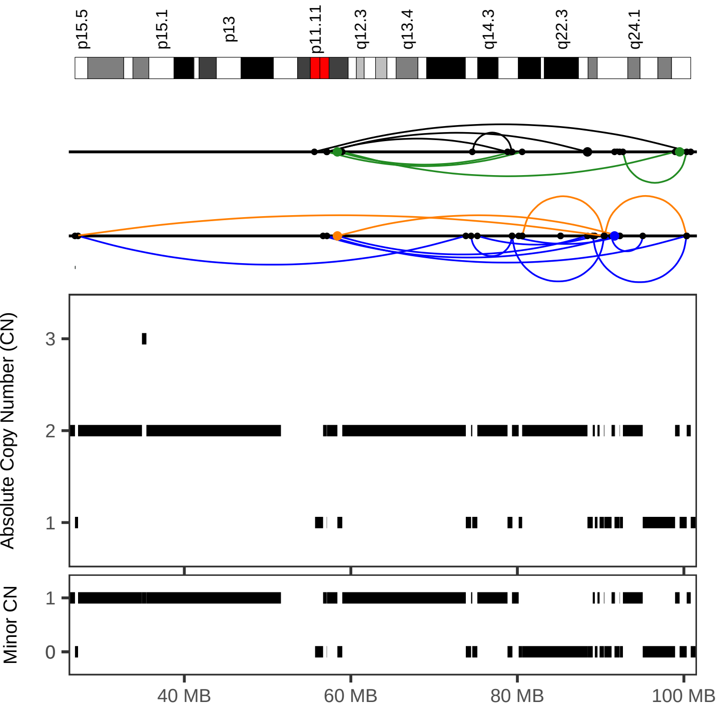

CGP_donor_1374618	
Cancer type	Breast−AdenoCA
Position	11:26862299−100822979
Type	Canonical without polyploidization
Interleaved intrachr. SVs	23
Total SVs (intrachr. + transl.)	28
SV types	DEL: 5; DUP: 10; h2hINV: 4; t2tINV: 4; TRA: 5
SVs in sample	51
Oscillating CN (2 and 3 states)	36, 39
CN segments	39
FDR fragment joints	0.59
FDR chr. breakp. enrich.	0
Linked to chrs	19:293047−16895003;
Purity, ploidy	0.62, 2.1

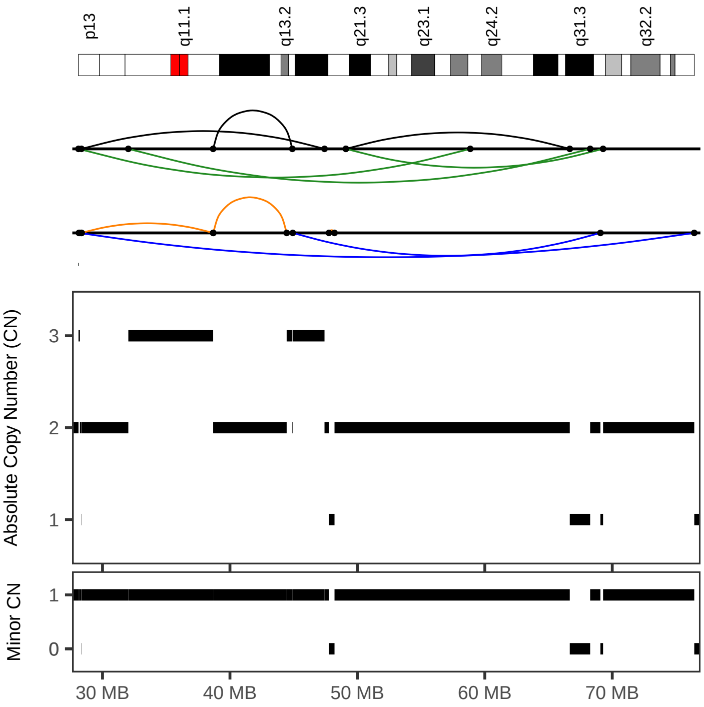

CGP_donor_1374618	
Cancer type	Breast−AdenoCA
Position	14:28112043−76435431
Type	With other complex events
Interleaved intrachr. SVs	10
Total SVs (intrachr. + transl.)	10
SV types	DEL: 2; DUP: 2; h2hINV: 3; t2tINV: 3; TRA: 0
SVs in sample	51
Oscillating CN (2 and 3 states)	8, 17
CN segments	17
FDR fragment joints	0.96
FDR chr. breakp. enrich.	0
Linked to chrs	
Purity, ploidy	0.62, 2.1

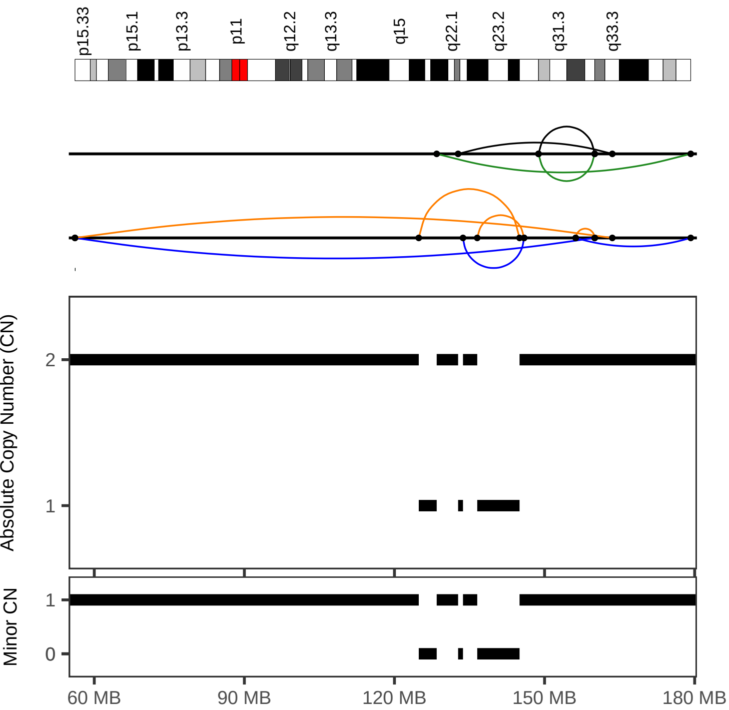

	**CGP_donor_1397088**
Cancer type	Breast−AdenoCA
Position	5:56141249−179216352
Type	Canonical without polyploidization
Interleaved intrachr. SVs	11
Total SVs (intrachr. + transl.)	11
SV types	DEL: 4; DUP: 3; h2hINV: 2; t2tINV: 2; TRA: 0
SVs in sample	16
Oscillating CN (2 and 3 states)	7, 7
CN segments	7
FDR fragment joints	0.84
FDR chr. breakp. enrich.	0
Linked to chrs	
Purity, ploidy	0.65, 1.97

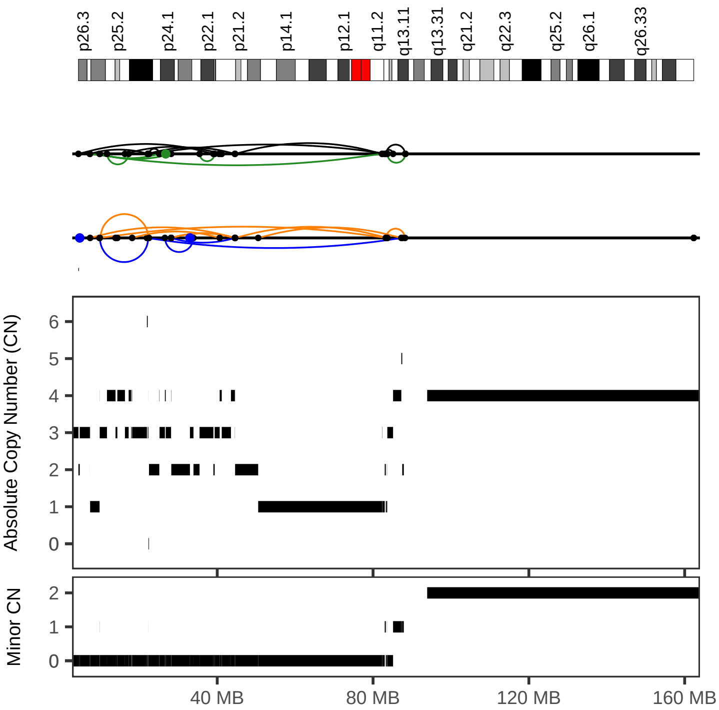

	**CGP_donor_1397260**
Cancer type	Breast−AdenoCA
Position	3:4365807−88334064
Type	With other complex events
Interleaved intrachr. SVs	31
Total SVs (intrachr. + transl.)	34
SV types	DEL: 10; DUP: 4; h2hINV: 10; t2tINV: 7; TRA: 3
SVs in sample	160
Oscillating CN (2 and 3 states)	11, 14
CN segments	56
FDR fragment joints	0.59
FDR chr. breakp. enrich.	0
Linked to chrs	6:38510448−42541218;
Purity, ploidy	0.41, 3.03

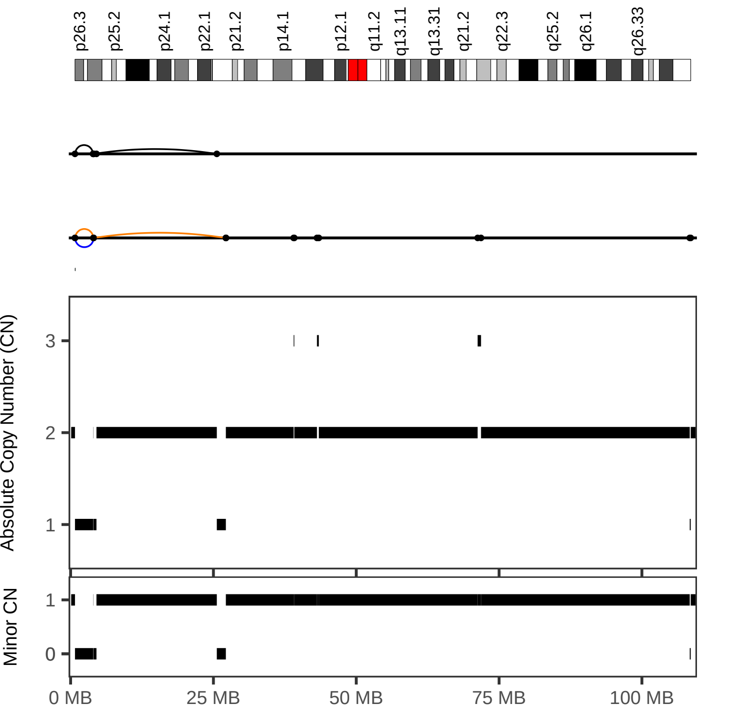

	**CGP_donor_1397282**
Cancer type	Breast−AdenoCA
Position	3:763757−27175998
Type	Canonical without polyploidization
Interleaved intrachr. SVs	7
Total SVs (intrachr. + transl.)	7
SV types	DEL: 2; DUP: 1; h2hINV: 2; t2tINV: 2; TRA: 0
SVs in sample	91
Oscillating CN (2 and 3 states)	8, 8
CN segments	8
FDR fragment joints	0.95
FDR chr. breakp. enrich.	0.13
Linked to chrs	
Purity, ploidy	0.35, 1.93

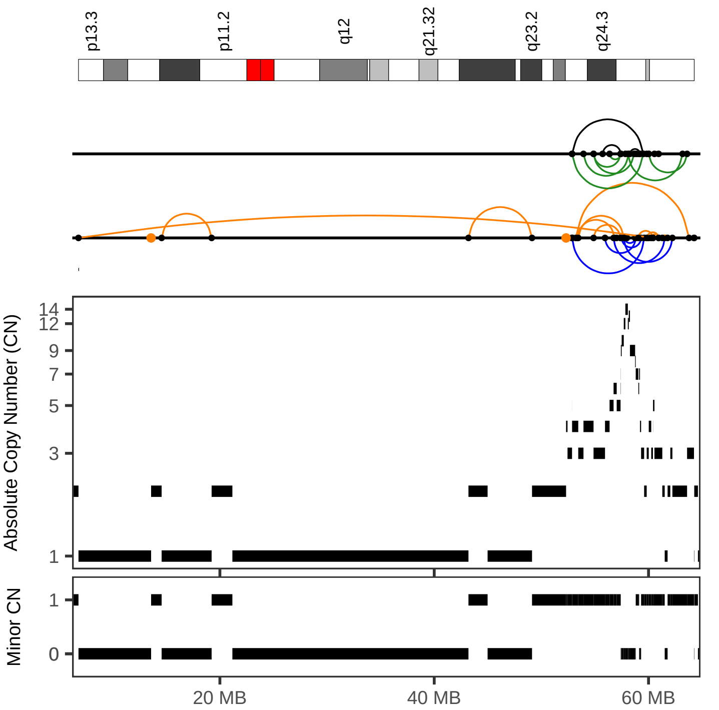

	**CGP_donor_1397282**
Cancer type	Breast−AdenoCA
Position	17:6803218−63783380
Type	With other complex events
Interleaved intrachr. SVs	31
Total SVs (intrachr. + transl.)	33
SV types	DEL: 10; DUP: 7; h2hINV: 4; t2tINV: 10; TRA: 2
SVs in sample	91
Oscillating CN (2 and 3 states)	8, 14
CN segments	48
FDR fragment joints	0.59
FDR chr. breakp. enrich.	0
Linked to chrs	
Purity, ploidy	0.35, 1.93

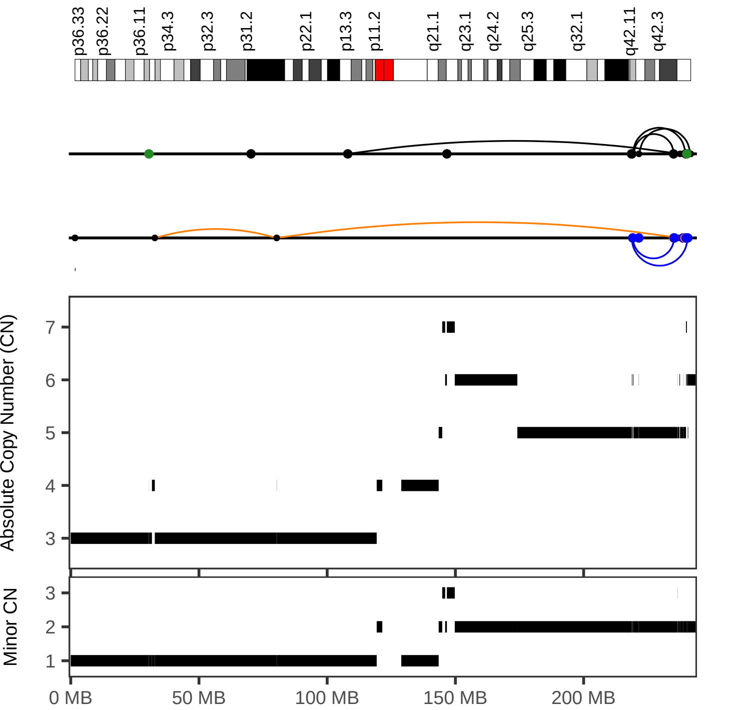

	**CGP_donor_1475202**
Cancer type	Breast−AdenoCA
Position	1:80341830−241765599
Type	With other complex events
Interleaved intrachr. SVs	10
Total SVs (intrachr. + transl.)	31
SV types	DEL: 1; DUP: 2; h2hINV: 6; t2tINV: 1; TRA: 21
SVs in sample	217
Oscillating CN (2 and 3 states)	14, 18
CN segments	27
FDR fragment joints	0.48
FDR chr. breakp. enrich.	0
Linked to chrs	
Purity, ploidy	0.87, 3.64

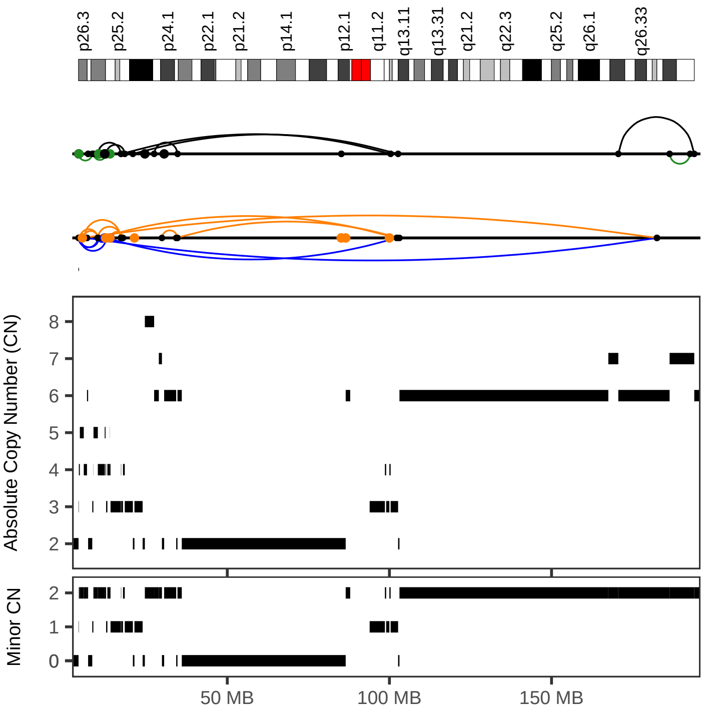

	**CGP_donor_1475202**
Cancer type	Breast−AdenoCA
Position	3:4105086−194006757
Type	With other complex events
Interleaved intrachr. SVs	31
Total SVs (intrachr. + transl.)	58
SV types	DEL: 15; DUP: 7; h2hINV: 6; t2tINV: 3; TRA: 27
SVs in sample	217
Oscillating CN (2 and 3 states)	7, 21
CN segments	48
FDR fragment joints	0.31
FDR chr. breakp. enrich.	0
Linked to chrs	19:4796337−21735945;
Purity, ploidy	0.87, 3.64

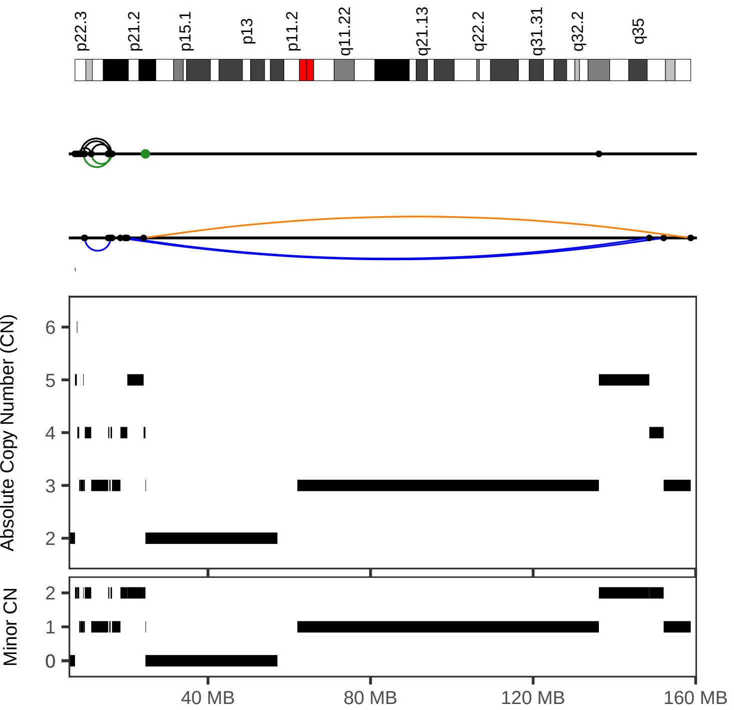

	**CGP_donor_1475202**
Cancer type	Breast−AdenoCA
Position	7:8316244−16409139
Type	After polyploidization
Interleaved intrachr. SVs	12
Total SVs (intrachr. + transl.)	12
SV types	DEL: 0; DUP: 3; h2hINV: 5; t2tINV: 4; TRA: 0
SVs in sample	217
Oscillating CN (2 and 3 states)	9, 13
CN segments	13
FDR fragment joints	0.59
FDR chr. breakp. enrich.	0.01
Linked to chrs	
Purity, ploidy	0.87, 3.64

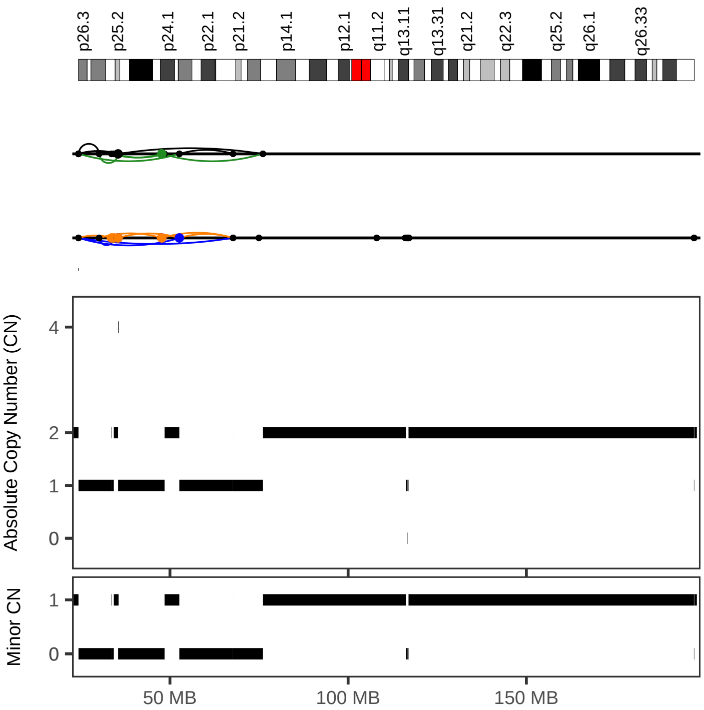

	**CGP_donor_1503014**
Cancer type	Breast−AdenoCA
Position	3:24341426−76090407
Type	With other complex events
Interleaved intrachr. SVs	19
Total SVs (intrachr. + transl.)	29
SV types	DEL: 6; DUP: 3; h2hINV: 5; t2tINV: 5; TRA: 10
SVs in sample	161
Oscillating CN (2 and 3 states)	7, 7
CN segments	13
FDR fragment joints	0.84
FDR chr. breakp. enrich.	0
Linked to chrs	7:88812878−106064287;
Purity, ploidy	0.48, 2.97

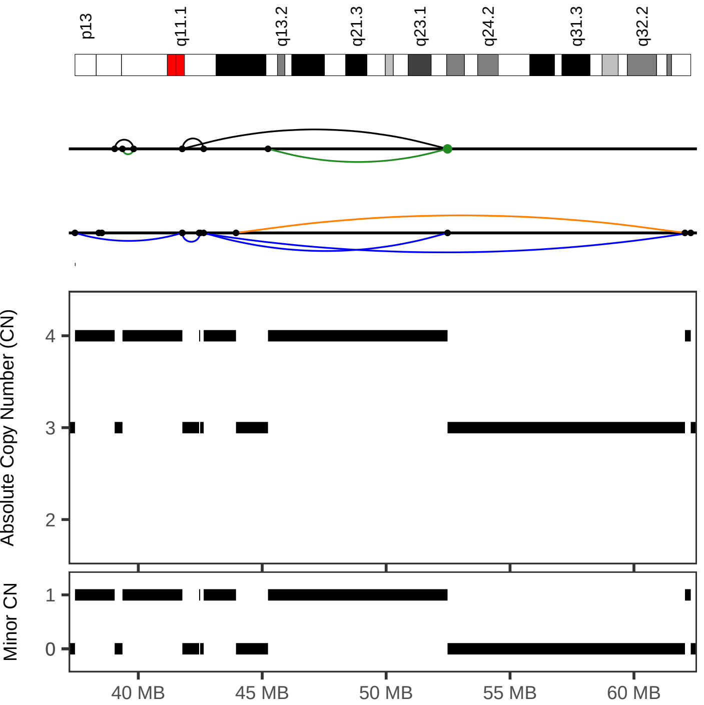

CGP_donor_1503014

Cancer type	Breast−AdenoCA
Position	14:37444009−62292108
Type	After polyploidization
Interleaved intrachr. SVs	7
Total SVs (intrachr. + transl.)	8
SV types	DEL: 1; DUP: 4; h2hINV: 2; t2tINV: 0; TRA: 1
SVs in sample	161
Oscillating CN (2 and 3 states)	12, 12
CN segments	12
FDR fragment joints	0.59
FDR chr. breakp. enrich.	0.03
Linked to chrs	
Purity, ploidy	0.48, 2.97

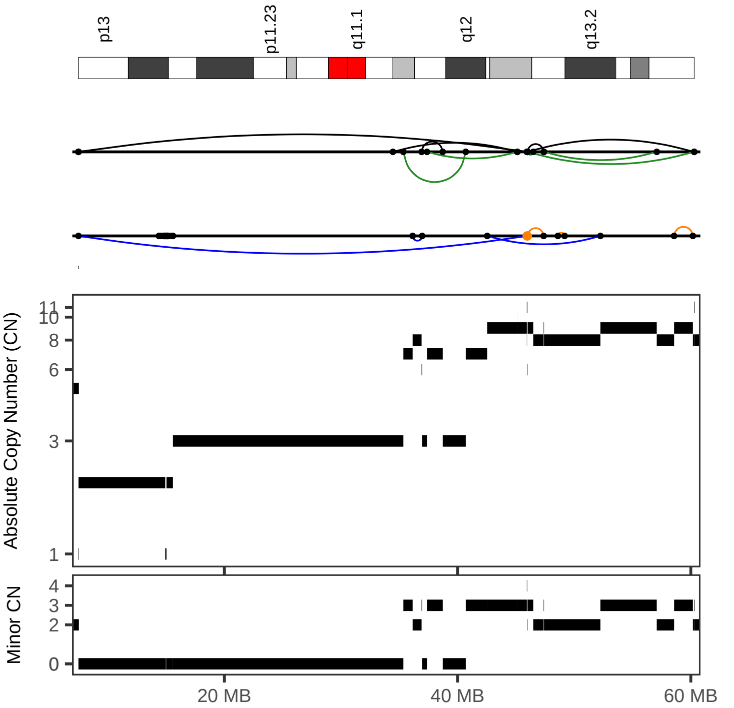

CGP_donor_1503014

Cancer type	Breast−AdenoCA
Position	20:7490391−60291793
Type	With other complex events
Interleaved intrachr. SVs	12
Total SVs (intrachr. + transl.)	13
SV types	DEL: 0; DUP: 4; h2hINV: 4; t2tINV: 4; TRA: 1
SVs in sample	161
Oscillating CN (2 and 3 states)	8, 8
CN segments	31
FDR fragment joints	0.59
FDR chr. breakp. enrich.	0
Linked to chrs	
Purity, ploidy	0.48, 2.97

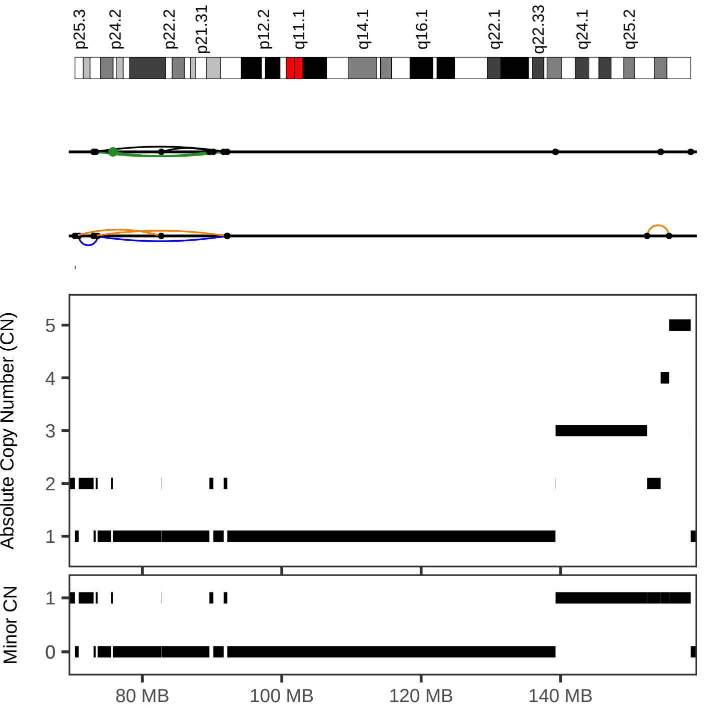

CGP_donor_1503016

Cancer type	Breast−AdenoCA
Position	6:70321603−92182155
Type	Canonical without polyploidization
Interleaved intrachr. SVs	8
Total SVs (intrachr. + transl.)	9
SV types	DEL: 2; DUP: 2; h2hINV: 2; t2tINV: 2; TRA: 1
SVs in sample	72
Oscillating CN (2 and 3 states)	13, 13
CN segments	13
FDR fragment joints	1
FDR chr. breakp. enrich.	0
Linked to chrs	
Purity, ploidy	0.54, 2.1

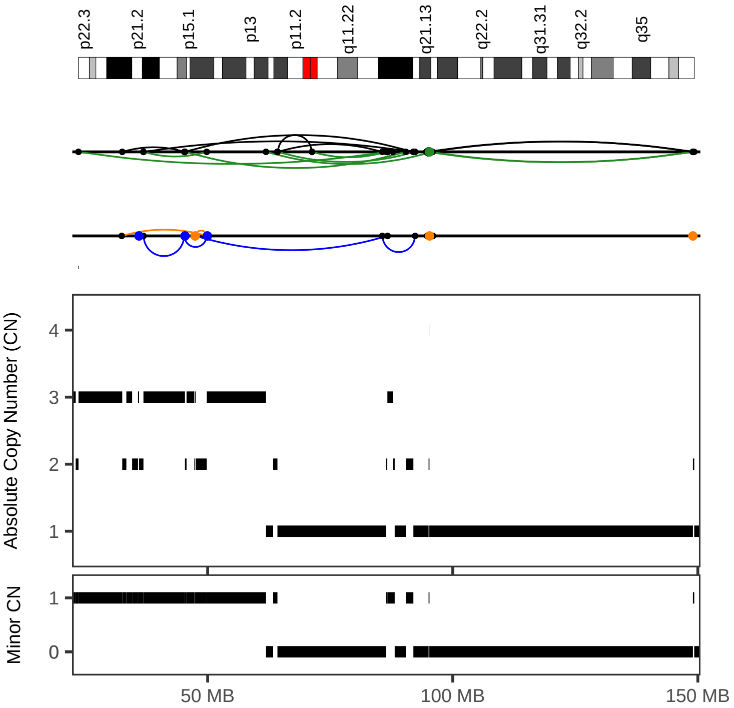

CGP_donor_1503020

Cancer type	Breast−AdenoCA
Position	7:23638008−149260298
Type	With other complex events
Interleaved intrachr. SVs	25
Total SVs (intrachr. + transl.)	33
SV types	DEL: 3; DUP: 5; h2hINV: 8; t2tINV: 9; TRA: 8
SVs in sample	45
Oscillating CN (2 and 3 states)	13, 17
CN segments	34
FDR fragment joints	0.59
FDR chr. breakp. enrich.	0
Linked to chrs	
Purity, ploidy	0.74, 1.91

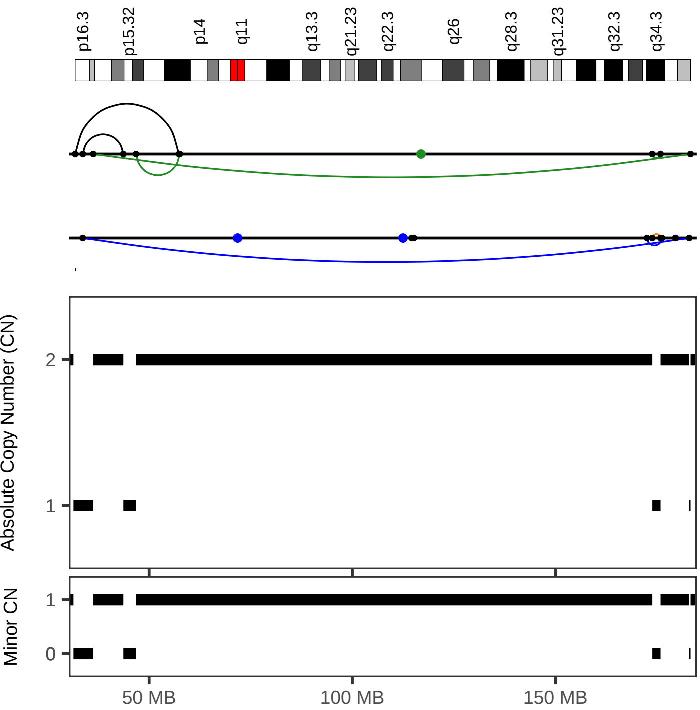

CGP_donor_1503150

Cancer type	Breast−AdenoCA
Position	4:31802737−183234503
Type	Canonical without polyploidization
Interleaved intrachr. SVs	5
Total SVs (intrachr. + transl.)	8
SV types	DEL: 0; DUP: 1; h2hINV: 2; t2tINV: 2; TRA: 3
SVs in sample	180
Oscillating CN (2 and 3 states)	8, 8
CN segments	8
FDR fragment joints	0.64
FDR chr. breakp. enrich.	0.93
Linked to chrs	
Purity, ploidy	0.82, 1.98

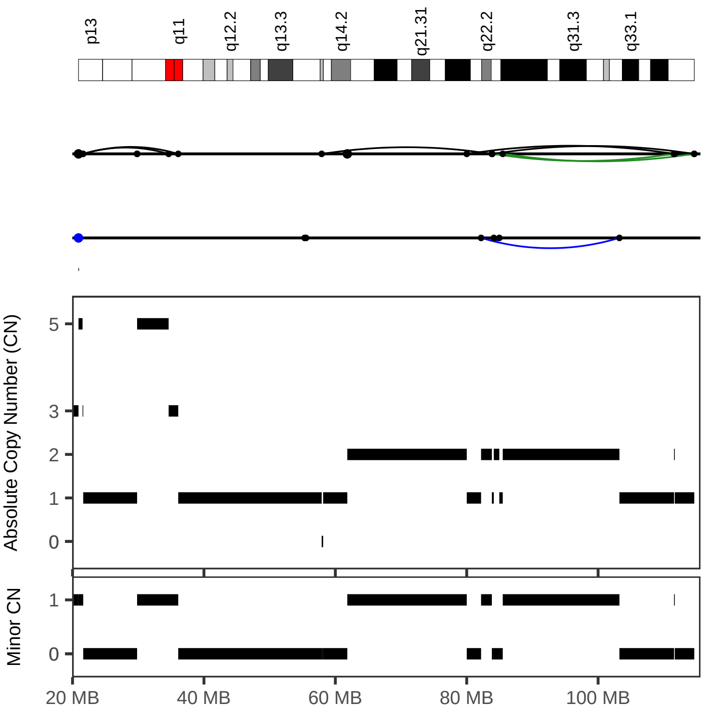

CGP_donor_1503150

Cancer type	Breast−AdenoCA
Position	13:57920742−114632856
Type	Canonical without polyploidization
Interleaved intrachr. SVs	6
Total SVs (intrachr. + transl.)	7
SV types	DEL: 0; DUP: 1; h2hINV: 3; t2tINV: 2; TRA: 1
SVs in sample	180
Oscillating CN (2 and 3 states)	11, 12
CN segments	12
FDR fragment joints	0.59
FDR chr. breakp. enrich.	0
Linked to chrs	
Purity, ploidy	0.82, 1.98

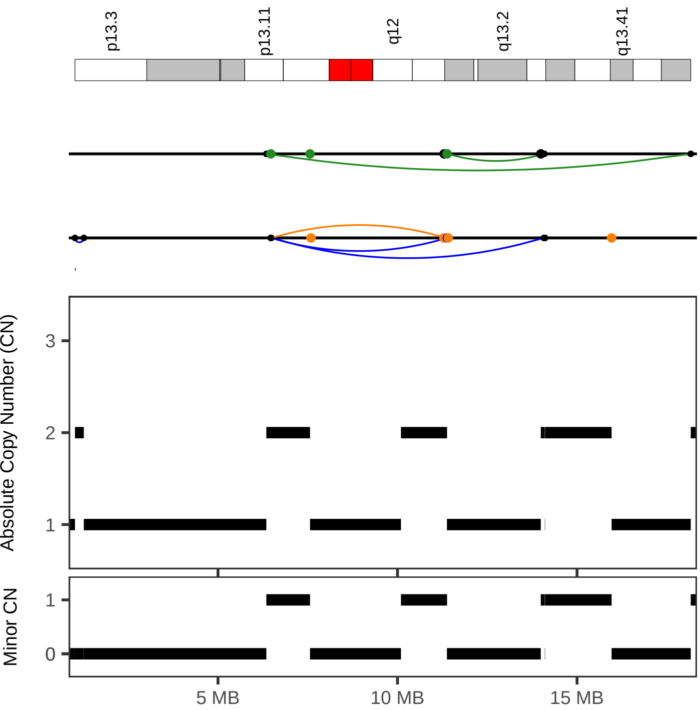

CGP_donor_1503150

Cancer type	Breast−AdenoCA
Position	19:6471749−14092495
Type	Canonical without polyploidization
Interleaved intrachr. SVs	5
Total SVs (intrachr. + transl.)	15
SV types	DEL: 1; DUP: 2; h2hINV: 1; t2tINV: 1; TRA: 10
SVs in sample	180
Oscillating CN (2 and 3 states)	7, 7
CN segments	7
FDR fragment joints	0.92
FDR chr. breakp. enrich.	0
Linked to chrs	
Purity, ploidy	0.82, 1.98

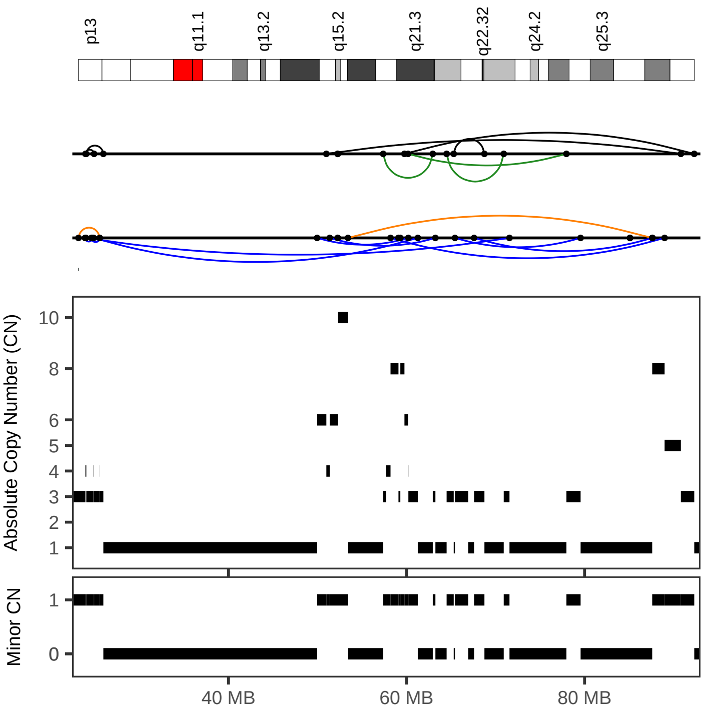

CGP_donor_1503156

Cancer type	Breast−AdenoCA
Position	15:23149223−92319473
Type	With other complex events
Interleaved intrachr. SVs	19
Total SVs (intrachr. + transl.)	19
SV types	DEL: 2; DUP: 9; h2hINV: 5; t2tINV: 3; TRA: 0
SVs in sample	66
Oscillating CN (2 and 3 states)	14, 16
CN segments	40
FDR fragment joints	0.48
FDR chr. breakp. enrich.	0
Linked to chrs	
Purity, ploidy	0.54, 1.82

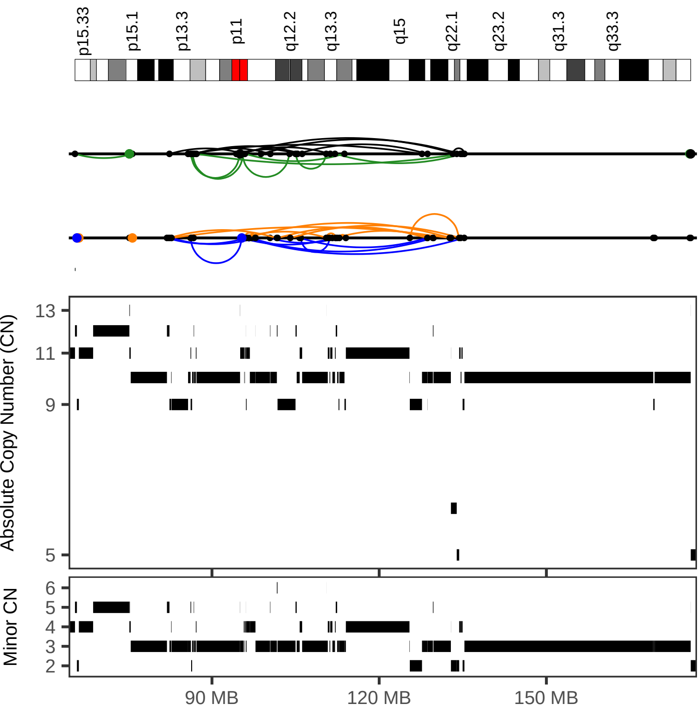

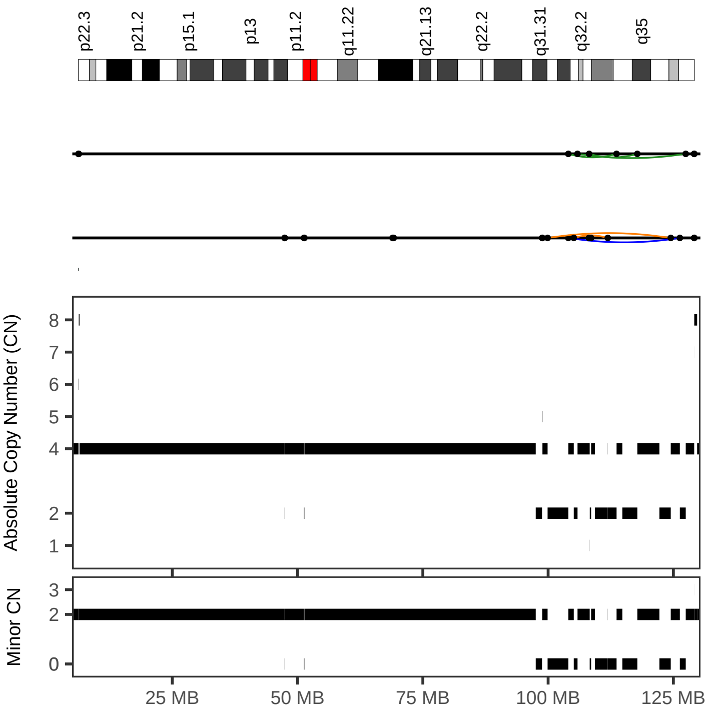

**CGP_donor_1187030**	
Cancer type	Breast−AdenoCA
Position	5:81925457−135300603
Type	With other complex events
Interleaved intrachr. SVs	40
Total SVs (intrachr. + transl.)	42
SV types	DEL: 13; DUP: 10; h2hINV: 10; t2tINV: 7; TRA: 2
SVs in sample	98
Oscillating CN (2 and 3 states)	7, 20
CN segments	63
FDR fragment joints	0.69
FDR chr. breakp. enrich.	0
Linked to chrs	1:63616590−107970984;
Purity, ploidy	0.54, 6.22

**CGP_donor_1187031**	
Cancer type	Breast−AdenoCA
Position	7:99895066−127463712
Type	Before polyploidization
Interleaved intrachr. SVs	7
Total SVs (intrachr. + transl.)	7
SV types	DEL: 3; DUP: 1; h2hINV: 0; t2tINV: 3; TRA: 0
SVs in sample	89
Oscillating CN (2 and 3 states)	13, 18
CN segments	18
FDR fragment joints	0.59
FDR chr. breakp. enrich.	0
Linked to chrs	
Purity, ploidy	0.36, 2.92

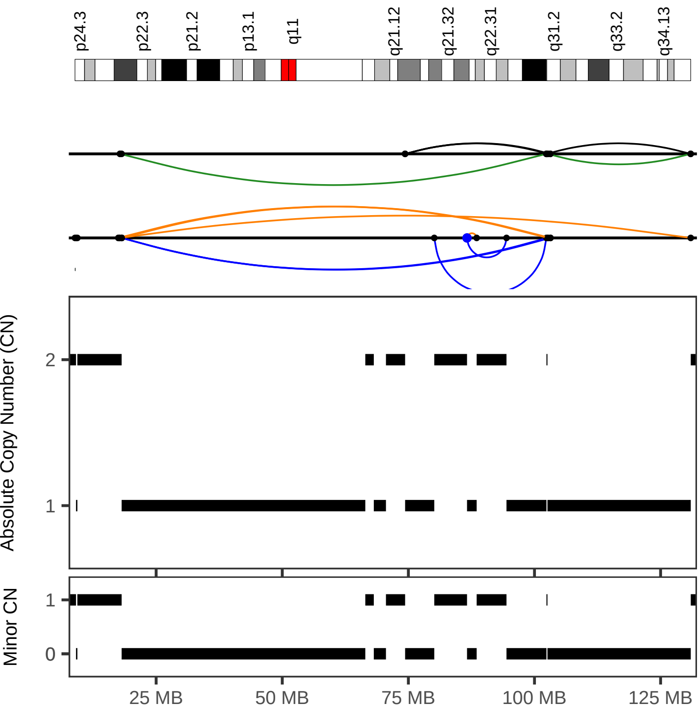

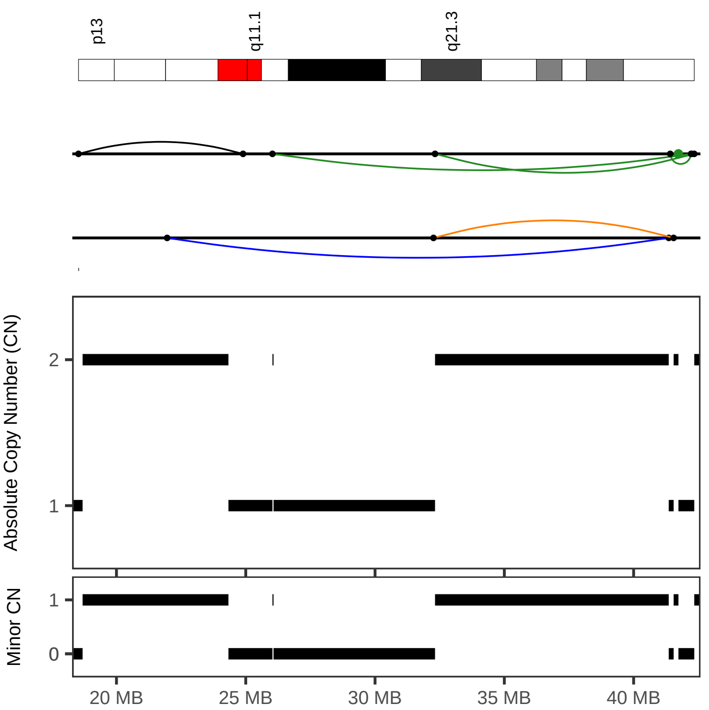

**CGP_donor_1187033**	
Cancer type	Breast−AdenoCA
Position	9:17517806−130978055
Type	Canonical without polyploidization
Interleaved intrachr. SVs	15
Total SVs (intrachr. + transl.)	16
SV types	DEL: 4; DUP: 4; h2hINV: 4; t2tINV: 3; TRA: 1
SVs in sample	228
Oscillating CN (2 and 3 states)	13, 13
CN segments	13
FDR fragment joints	0.99
FDR chr. breakp. enrich.	0
Linked to chrs	
Purity, ploidy	0.59, 1.81

**CGP_donor_1187033**	
Cancer type	Breast−AdenoCA
Position	21:18531210−42344561
Type	Canonical without polyploidization
Interleaved intrachr. SVs	6
Total SVs (intrachr. + transl.)	7
SV types	DEL: 1; DUP: 1; h2hINV: 1; t2tINV: 3; TRA: 1
SVs in sample	228
Oscillating CN (2 and 3 states)	10, 10
CN segments	10
FDR fragment joints	0.64
FDR chr. breakp. enrich.	0.04
Linked to chrs	
Purity, ploidy	0.59, 1.81

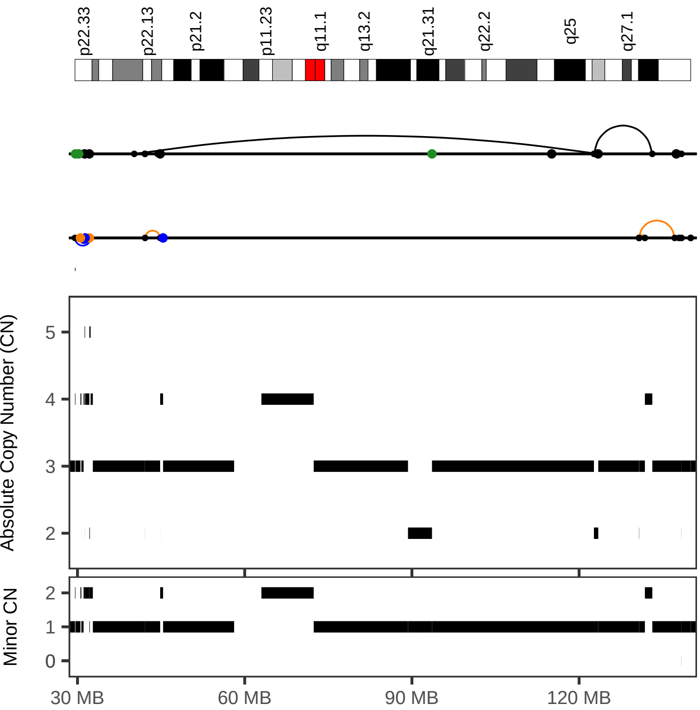

CGP_donor_1199104	
Cancer type	Breast−AdenoCA
Position	X:30692107−137148702
Type	With other complex events
Interleaved intrachr. SVs	3
Total SVs (intrachr. + transl.)	15
SV types	DEL: 2; DUP: 1; h2hINV: 2; t2tINV: 1; TRA: 9
SVs in sample	803
Oscillating CN (2 and 3 states)	7, 12
CN segments	25
FDR fragment joints	0.91
FDR chr. breakp. enrich.	0.28
Linked to chrs	4:53118926−163564695;
Purity, ploidy	0.53, 3.83

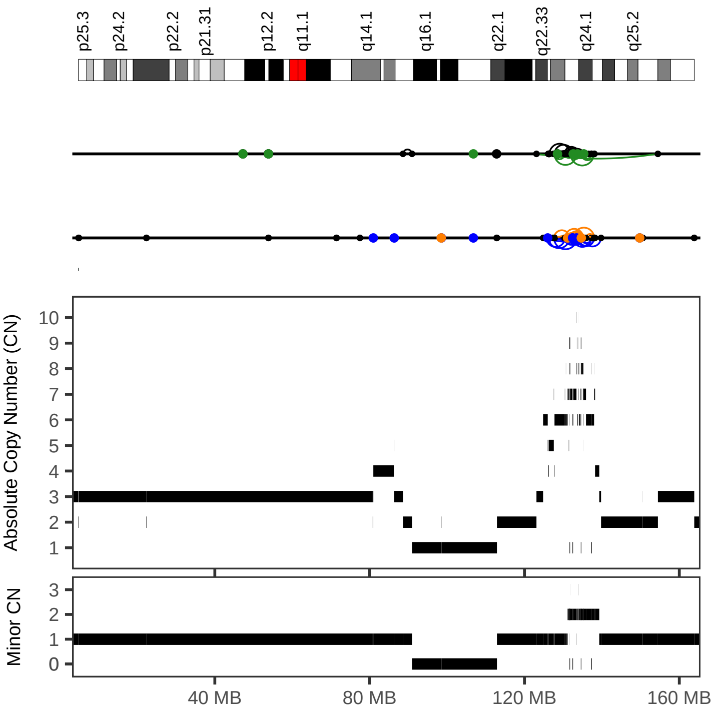

CGP_donor_1199129	
Cancer type	Breast−AdenoCA
Position	6:124809960−139777060
Type	With other complex events
Interleaved intrachr. SVs	80
Total SVs (intrachr. + transl.)	91
SV types	DEL: 14; DUP: 25; h2hINV: 21; t2tINV: 20; TRA: 11
SVs in sample	416
Oscillating CN (2 and 3 states)	9, 10
CN segments	80
FDR fragment joints	0.59
FDR chr. breakp. enrich.	0
Linked to chrs	1:7883677−218951308;
Purity, ploidy	0.56, 2.88

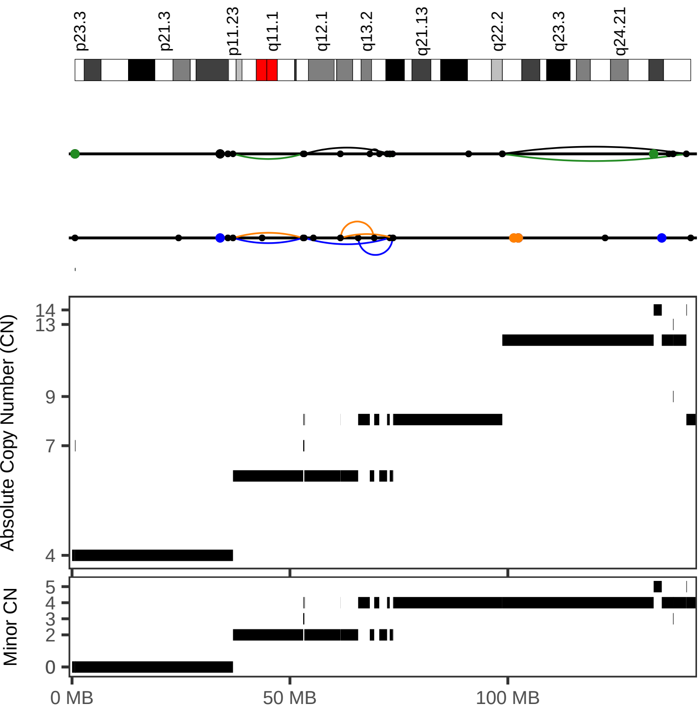

CGP_donor_1199137	
Cancer type	Breast−AdenoCA
Position	8:53301916−73691310
Type	After polyploidization
Interleaved intrachr. SVs	8
Total SVs (intrachr. + transl.)	8
SV types	DEL: 2; DUP: 2; h2hINV: 2; t2tINV: 2; TRA: 0
SVs in sample	249
Oscillating CN (2 and 3 states)	15, 15
CN segments	15
FDR fragment joints	1
FDR chr. breakp. enrich.	0
Linked to chrs	
Purity, ploidy	0.66, 3.91

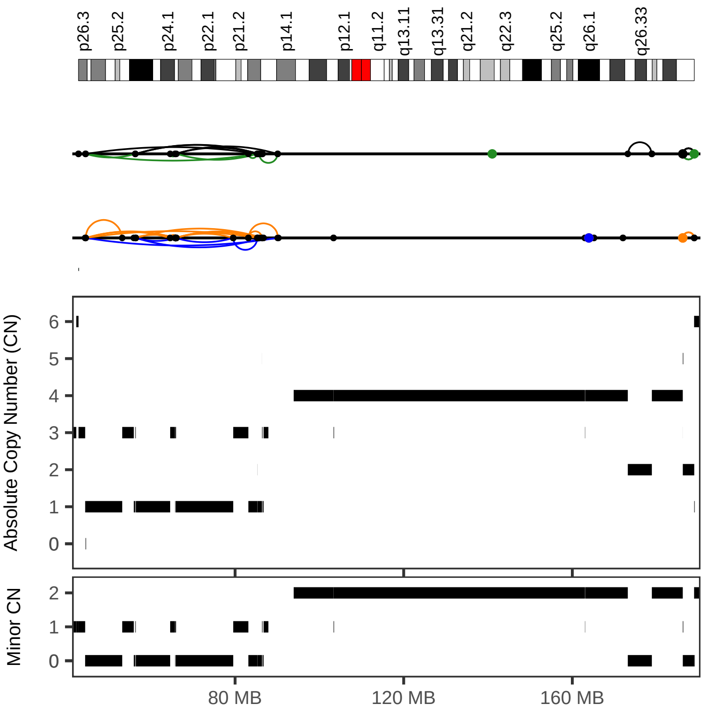

CGP_donor_1199138	
Cancer type	Breast−AdenoCA
Position	3:44425923−90280177
Type	Canonical without polyploidization
Interleaved intrachr. SVs	36
Total SVs (intrachr. + transl.)	36
SV types	DEL: 14; DUP: 8; h2hINV: 8; t2tINV: 6; TRA: 0
SVs in sample	426
Oscillating CN (2 and 3 states)	14, 17
CN segments	23
FDR fragment joints	0.59
FDR chr. breakp. enrich.	0
Linked to chrs	
Purity, ploidy	0.72, 3.18

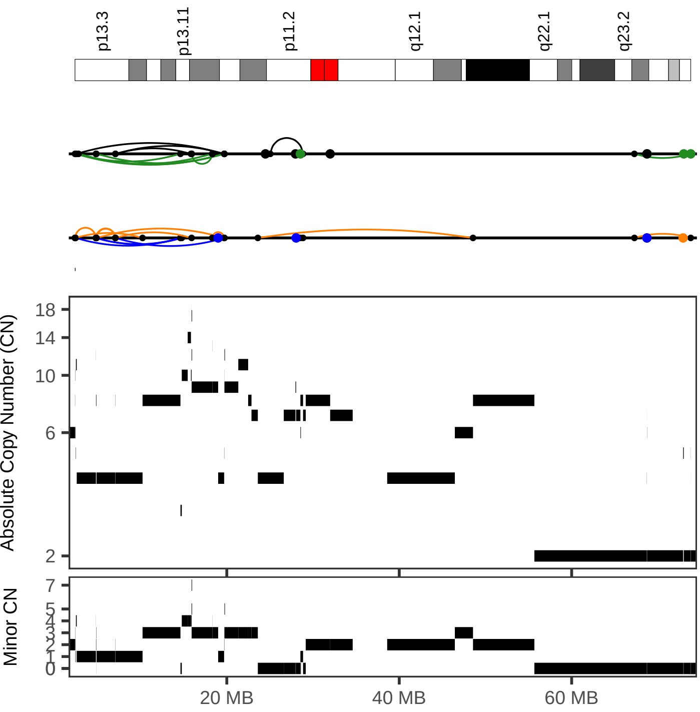

	**CGP_donor_1230728**
Cancer type	Breast−AdenoCA
Position	16:2388599−19726671
Type	With other complex events
Interleaved intrachr. SVs	21
Total SVs (intrachr. + transl.)	22
SV types	DEL: 7; DUP: 5; h2hINV: 3; t2tINV: 6; TRA: 1
SVs in sample	329
Oscillating CN (2 and 3 states)	8, 11
CN segments	37
FDR fragment joints	0.72
FDR chr. breakp. enrich.	0
Linked to chrs	
Purity, ploidy	0.27, 3.5

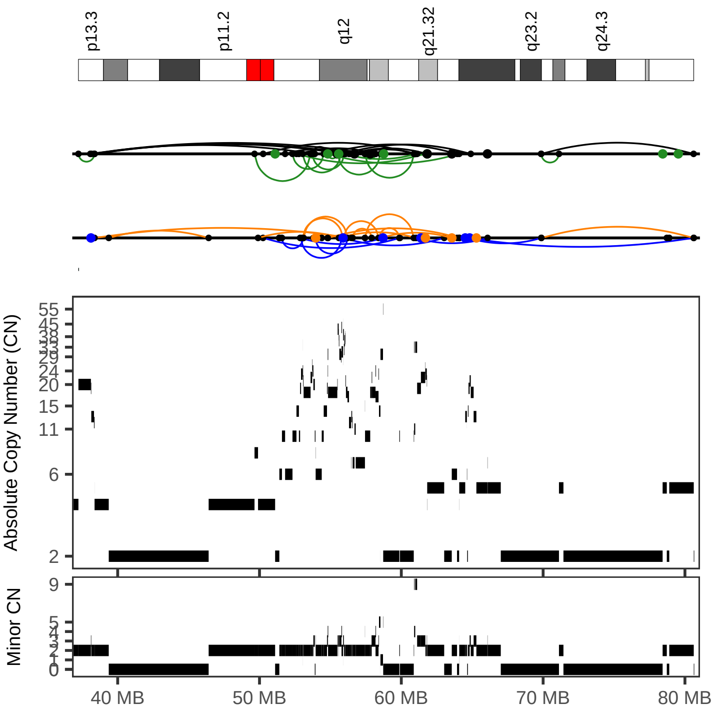

	**CGP_donor_1230728**
Cancer type	Breast−AdenoCA
Position	17:37232274−80621815
Type	With other complex events
Interleaved intrachr. SVs	55
Total SVs (intrachr. + transl.)	78
SV types	DEL: 15; DUP: 13; h2hINV: 14; t2tINV: 13; TRA: 23
SVs in sample	329
Oscillating CN (2 and 3 states)	8, 8
CN segments	116
FDR fragment joints	0.99
FDR chr. breakp. enrich.	0
Linked to chrs	18:2681027−74577800;
Purity, ploidy	0.27, 3.5

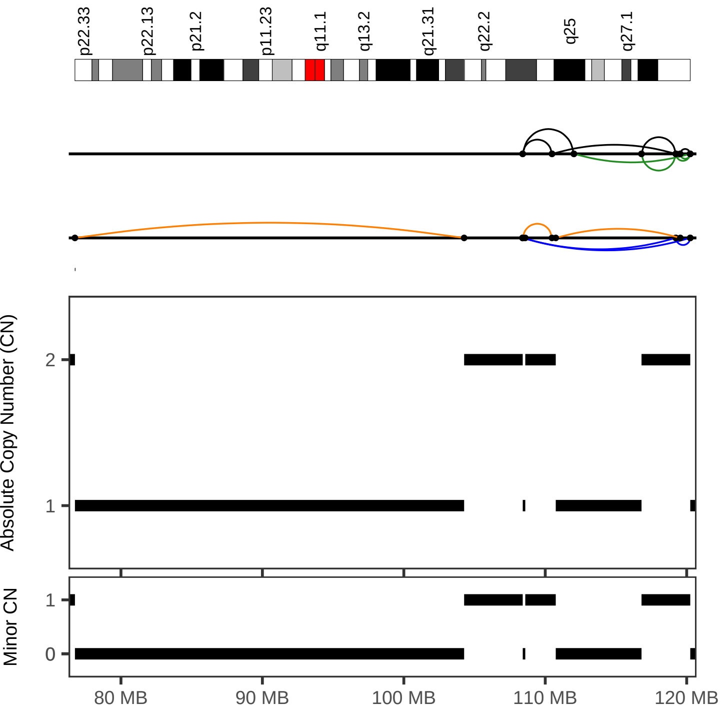

	**CGP_donor_1230729**
Cancer type	Breast−AdenoCA
Position	X:108395802−120251740
Type	Canonical without polyploidization
Interleaved intrachr. SVs	15
Total SVs (intrachr. + transl.)	15
SV types	DEL: 3; DUP: 3; h2hINV: 5; t2tINV: 4; TRA: 0
SVs in sample	107
Oscillating CN (2 and 3 states)	7, 7
CN segments	7
FDR fragment joints	0.9
FDR chr. breakp. enrich.	0
Linked to chrs	
Purity, ploidy	0.52, 1.7

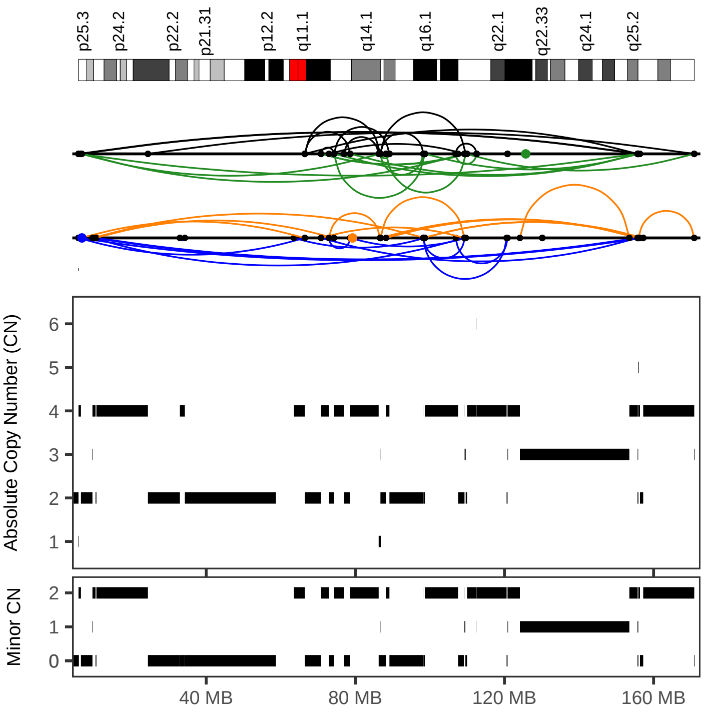

	**CGP_donor_1230778**
Cancer type	Breast−AdenoCA
Position	6:5750165−170904138
Type	With other complex events
Interleaved intrachr. SVs	54
Total SVs (intrachr. + transl.)	57
SV types	DEL: 11; DUP: 13; h2hINV: 15; t2tINV: 15; TRA: 3
SVs in sample	182
Oscillating CN (2 and 3 states)	12, 13
CN segments	54
FDR fragment joints	0.89
FDR chr. breakp. enrich.	0
Linked to chrs	
Purity, ploidy	0.24, 3.39

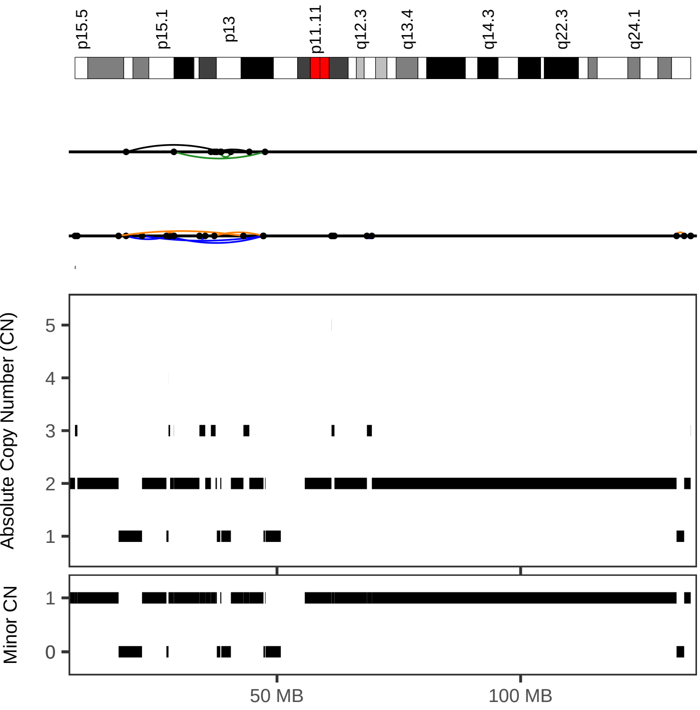

	CGP_donor_1337235
Cancer type	Breast−AdenoCA
Position	11:17489408−47547069
Type	With other complex events
Interleaved intrachr. SVs	13
Total SVs (intrachr. + transl.)	13
SV types	DEL: 3; DUP: 5; h2hINV: 2; t2tINV: 3; TRA: 0
SVs in sample	116
Oscillating CN (2 and 3 states)	8, 20
CN segments	20
FDR fragment joints	0.77
FDR chr. breakp. enrich.	0
Linked to chrs	
Purity, ploidy	0.64, 1.6

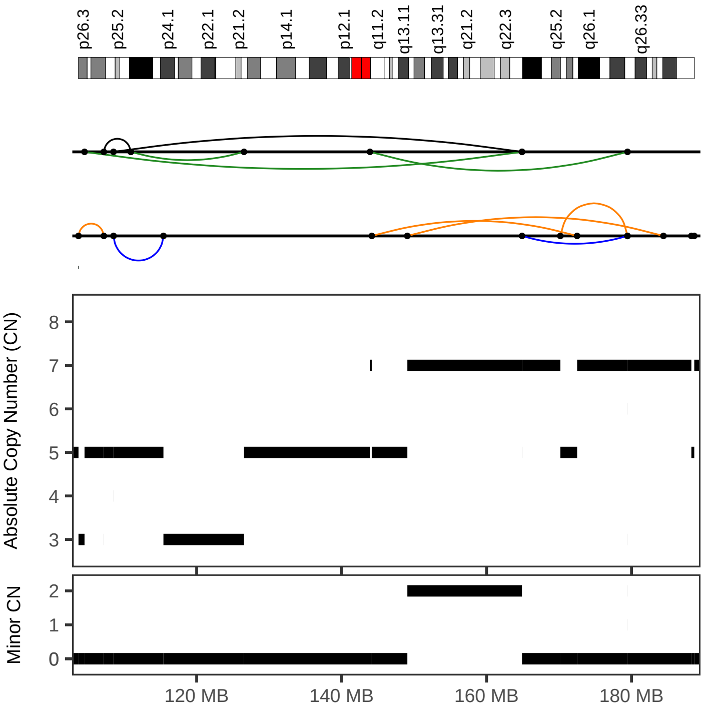

	CGP_donor_1347739
Cancer type	Breast−AdenoCA
Position	3:103694800−184410665
Type	With other complex events
Interleaved intrachr. SVs	11
Total SVs (intrachr. + transl.)	11
SV types	DEL: 4; DUP: 2; h2hINV: 2; t2tINV: 3; TRA: 0
SVs in sample	36
Oscillating CN (2 and 3 states)	8, 8
CN segments	18
FDR fragment joints	0.84
FDR chr. breakp. enrich.	0
Linked to chrs	
Purity, ploidy	0.32, 3.81

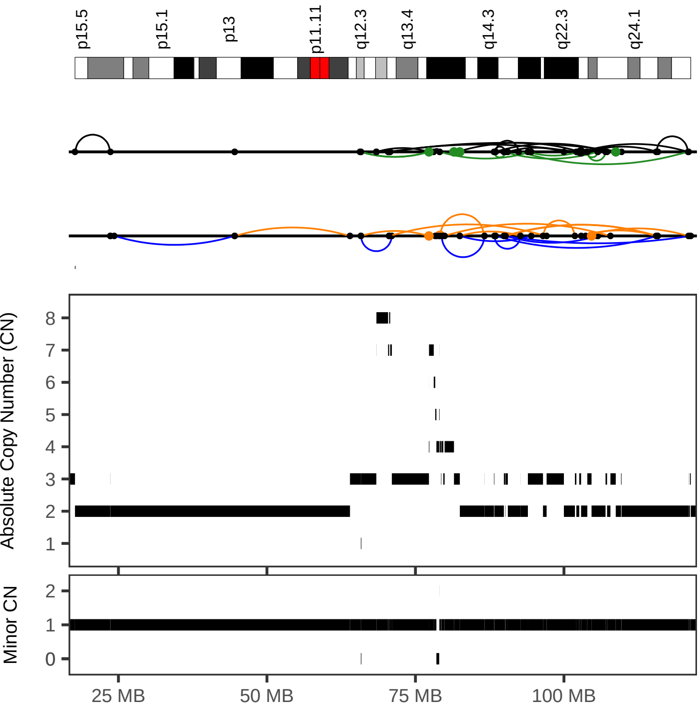

	CGP_donor_1353431
Cancer type	Breast−AdenoCA
Position	11:65669076−121347315
Type	With other complex events
Interleaved intrachr. SVs	42
Total SVs (intrachr. + transl.)	49
SV types	DEL: 11; DUP: 8; h2hINV: 13; t2tINV: 10; TRA: 7
SVs in sample	276
Oscillating CN (2 and 3 states)	34, 40
CN segments	59
FDR fragment joints	0.82
FDR chr. breakp. enrich.	0
Linked to chrs	5:164714637−176162290;9:77800927−139754034
Purity, ploidy	0.51, 3.26

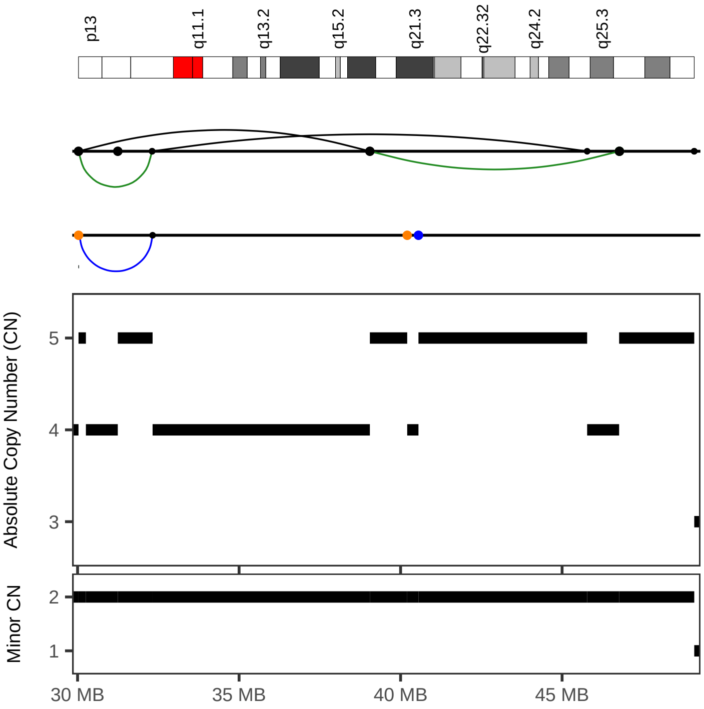

	CGP_donor_1353431
Cancer type	Breast−AdenoCA
Position	15:30027396−46766066
Type	After polyploidization
Interleaved intrachr. SVs	5
Total SVs (intrachr. + transl.)	11
SV types	DEL: 0; DUP: 1; h2hINV: 2; t2tINV: 2; TRA: 6
SVs in sample	276
Oscillating CN (2 and 3 states)	10, 10
CN segments	10
FDR fragment joints	0.64
FDR chr. breakp. enrich.	0.1
Linked to chrs	3:134692089−136270338;
Purity, ploidy	0.51, 3.26

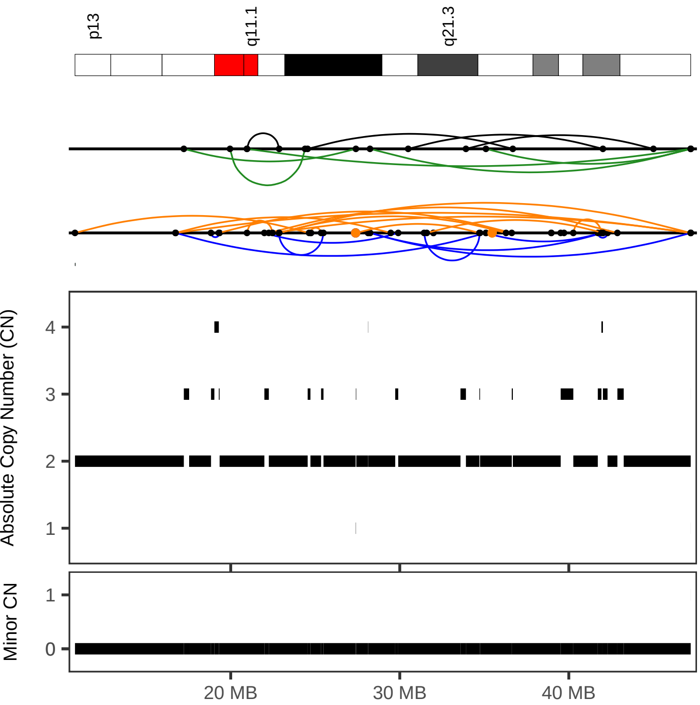

	**CGP_donor_1363969**
Cancer type	Breast−AdenoCA
Position	21:10785441−47214482
Type	With other complex events
Interleaved intrachr. SVs	30
Total SVs (intrachr. + transl.)	33
SV types	DEL: 12; DUP: 9; h2hINV: 4; t2tINV: 5; TRA: 3
SVs in sample	680
Oscillating CN (2 and 3 states)	12, 18
CN segments	36
FDR fragment joints	0.59
FDR chr. breakp. enrich.	0
Linked to chrs	
Purity, ploidy	0.45, 2.61

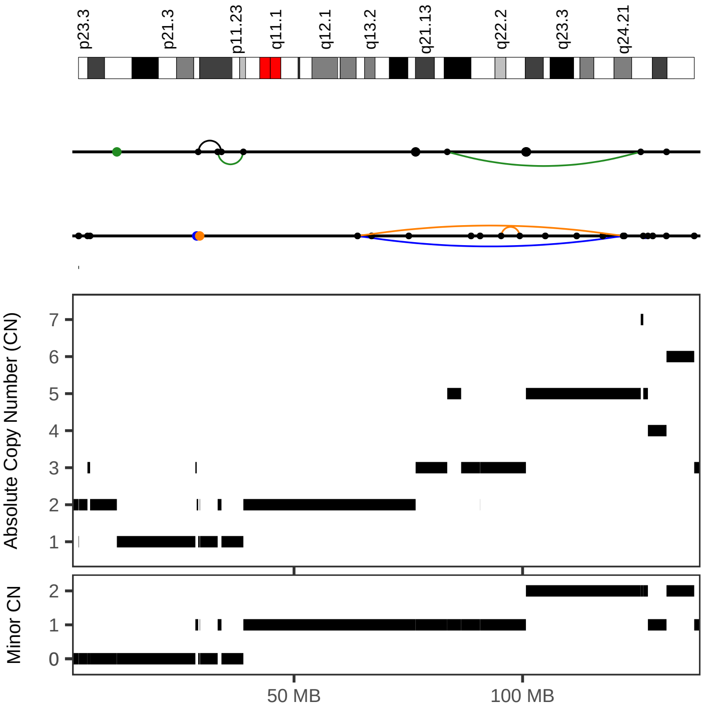

	**CGP_donor_1410205**
Cancer type	Breast−AdenoCA
Position	8:28416833−125849825
Type	With other complex events
Interleaved intrachr. SVs	3
Total SVs (intrachr. + transl.)	11
SV types	DEL: 1; DUP: 2; h2hINV: 1; t2tINV: 2; TRA: 5
SVs in sample	341
Oscillating CN (2 and 3 states)	7, 7
CN segments	15
FDR fragment joints	0.91
FDR chr. breakp. enrich.	0.06
Linked to chrs	
Purity, ploidy	0.35, 1.83

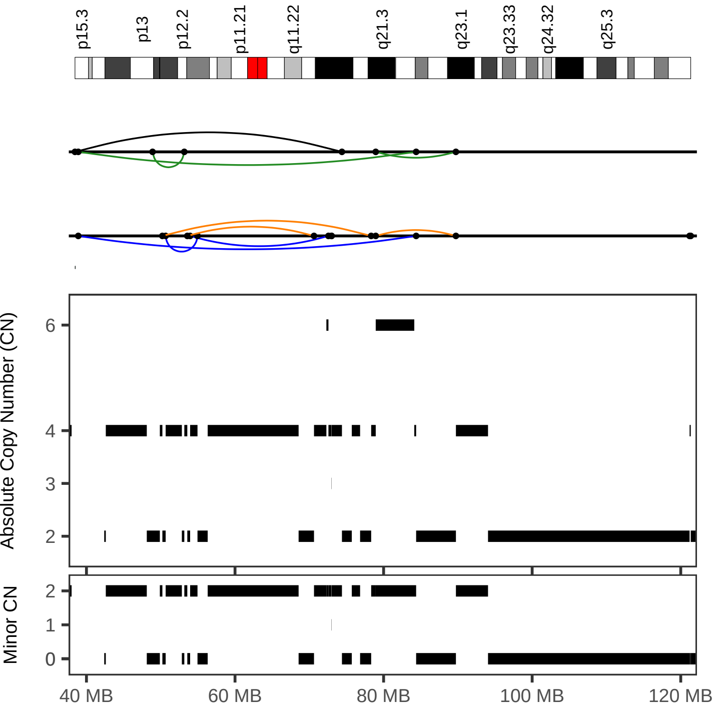

	**CGP_donor_1472395**
Cancer type	Breast−AdenoCA
Position	10:38453757−89733903
Type	With other complex events
Interleaved intrachr. SVs	10
Total SVs (intrachr. + transl.)	10
SV types	DEL: 3; DUP: 3; h2hINV: 1; t2tINV: 3; TRA: 0
SVs in sample	132
Oscillating CN (2 and 3 states)	15, 17
CN segments	27
FDR fragment joints	0.83
FDR chr. breakp. enrich.	0.08
Linked to chrs	
Purity, ploidy	0.5, 3.23

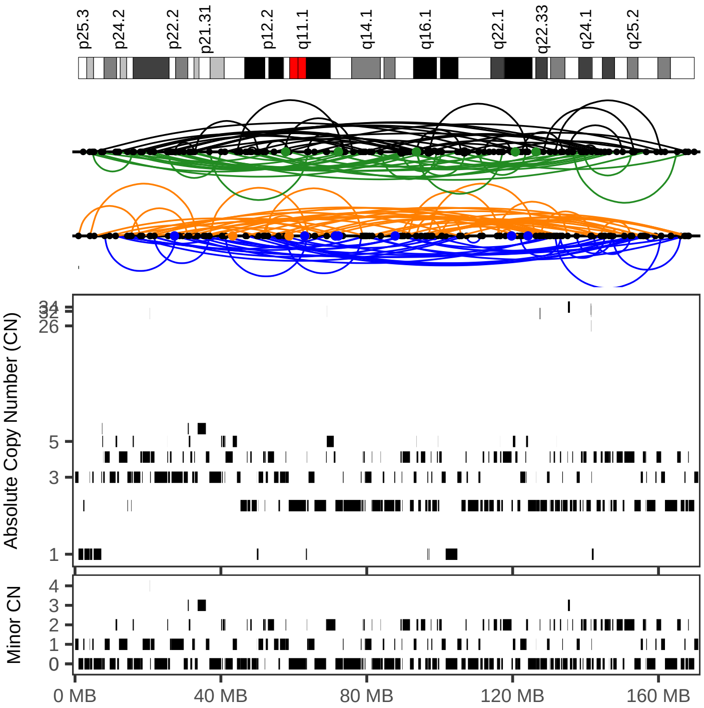

	**CGP_donor_1606179**
Cancer type	Breast−AdenoCA
Position	6:947294−169801583
Type	With other complex events
Interleaved intrachr. SVs	171
Total SVs (intrachr. + transl.)	190
SV types	DEL: 49; DUP: 42; h2hINV: 40; t2tINV: 40; TRA: 19
SVs in sample	372
Oscillating CN (2 and 3 states)	14, 19
CN segments	224
FDR fragment joints	0.81
FDR chr. breakp. enrich.	0
Linked to chrs	
Purity, ploidy	0.52, 2.83

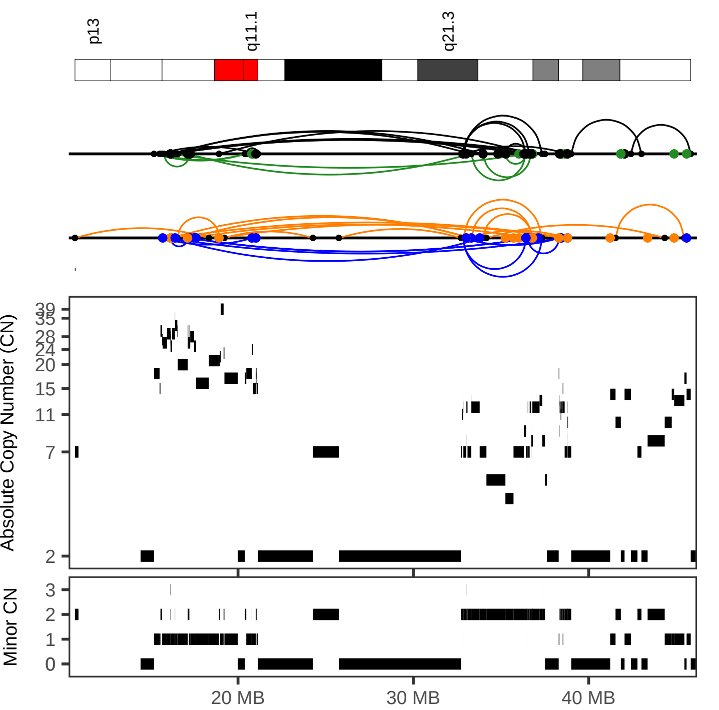

	**CGP_donor_1654385**
Cancer type	Breast−AdenoCA
Position	21:10701912−45821258
Type	With other complex events
Interleaved intrachr. SVs	55
Total SVs (intrachr. + transl.)	147
SV types	DEL: 15; DUP: 13; h2hINV: 18; t2tINV: 9; TRA: 92
SVs in sample	372
Oscillating CN (2 and 3 states)	7, 7
CN segments	112
FDR fragment joints	0.59
FDR chr. breakp. enrich.	0
Linked to chrs	
Purity, ploidy	0.72, 4.42

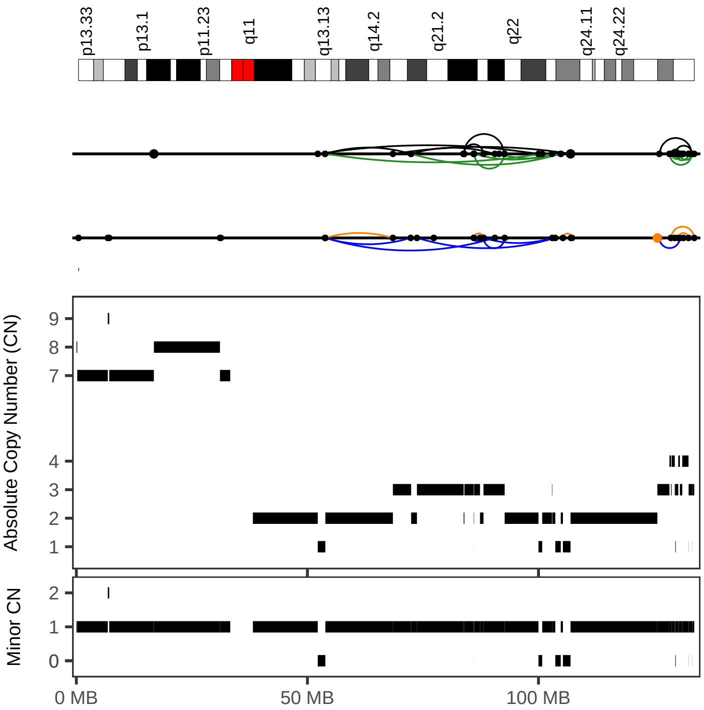

	**CGP_donor_1701345**
Cancer type	Breast−AdenoCA
Position	12:52240971−107248205
Type	With other complex events
Interleaved intrachr. SVs	20
Total SVs (intrachr. + transl.)	21
SV types	DEL: 2; DUP: 6; h2hINV: 6; t2tINV: 6; TRA: 1
SVs in sample	368
Oscillating CN (2 and 3 states)	8, 12
CN segments	22
FDR fragment joints	0.64
FDR chr. breakp. enrich.	0
Linked to chrs	
Purity, ploidy	0.43, 2.84

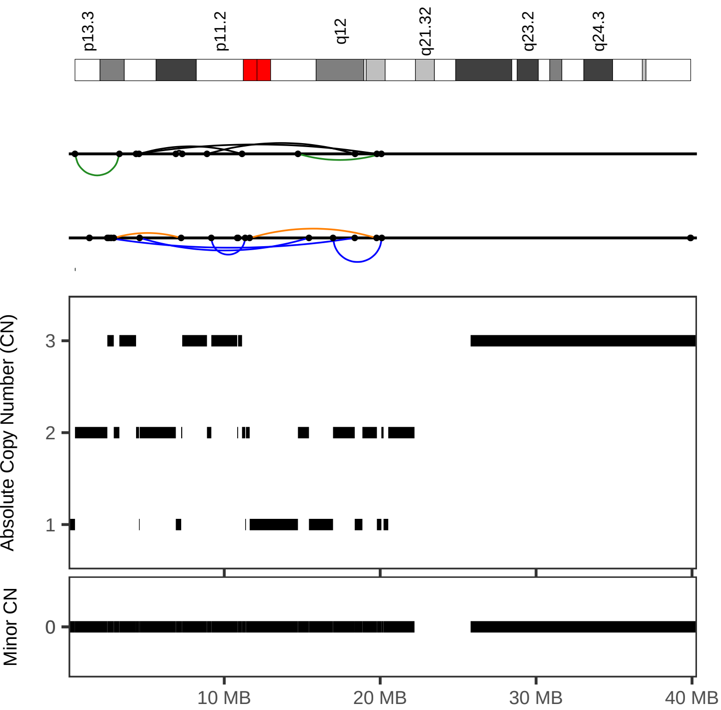

	**CGP_donor_1701345**
Cancer type	Breast−AdenoCA
Position	17:424593−20104085
Type	With other complex events
Interleaved intrachr. SVs	12
Total SVs (intrachr. + transl.)	12
SV types	DEL: 2; DUP: 4; h2hINV: 4; t2tINV: 2; TRA: 0
SVs in sample	368
Oscillating CN (2 and 3 states)	11, 25
CN segments	25
FDR fragment joints	0.8
FDR chr. breakp. enrich.	0.21
Linked to chrs	
Purity, ploidy	0.43, 2.84

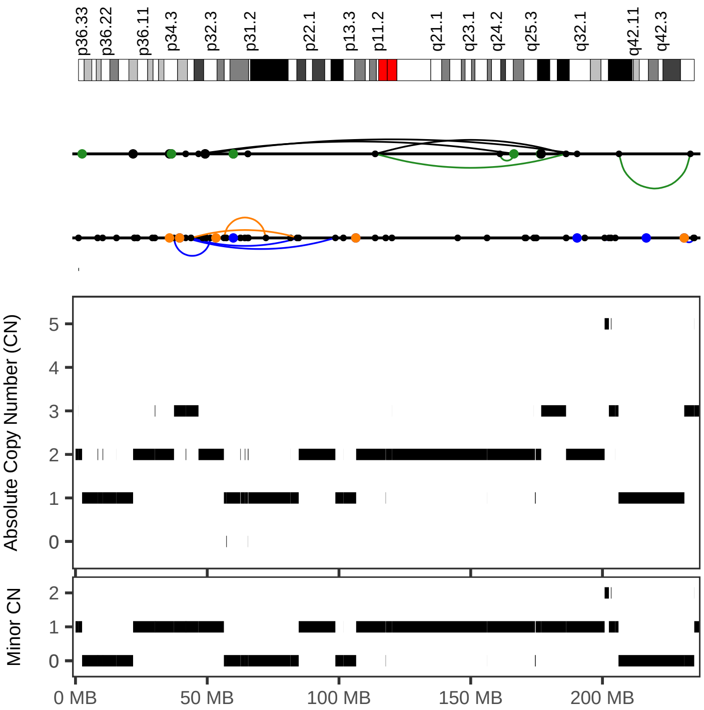

	**05506f4c−e701−4a9d−ae06−97f066aade43**
Cancer type	Breast−AdenoCA
Position	1:37390229−190370478
Type	With other complex events
Interleaved intrachr. SVs	8
Total SVs (intrachr. + transl.)	23
SV types	DEL: 1; DUP: 3; h2hINV: 3; t2tINV: 1; TRA: 15
SVs in sample	742
Oscillating CN (2 and 3 states)	12, 23
CN segments	35
FDR fragment joints	0.64
FDR chr. breakp. enrich.	0.03
Linked to chrs	18:3550118−39564907;3:112462046−195019170
Purity, ploidy	0.66, 1.78

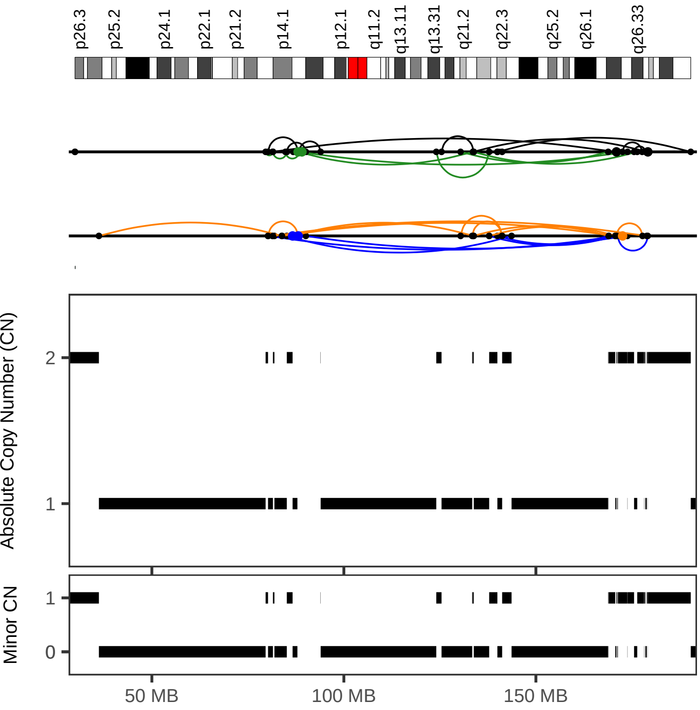

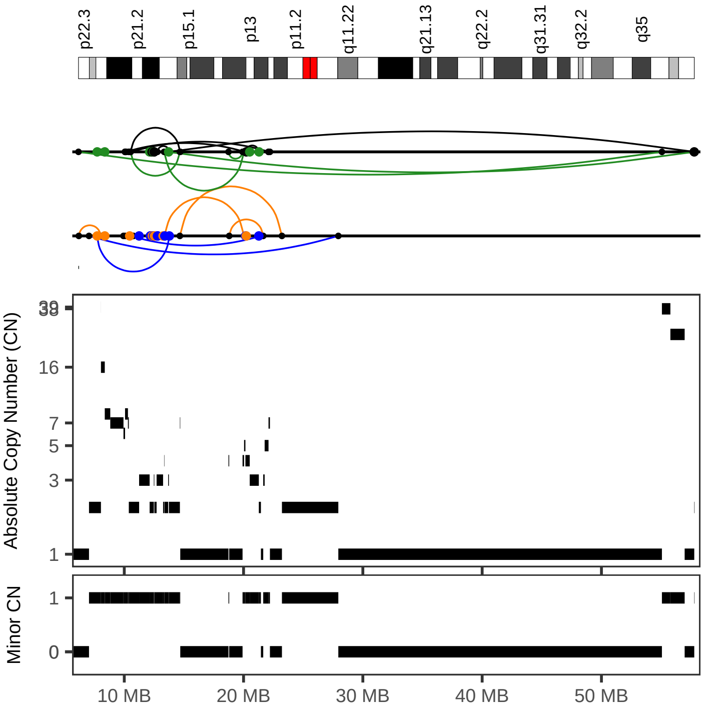

	084c6aec−94f7−4090−8f3f−59fa9e89721a
Cancer type	Breast−AdenoCA
Position	3:36209435−190323857
Type	Canonical without polyploidization
Interleaved intrachr. SVs	36
Total SVs (intrachr. + transl.)	43
SV types	DEL: 10; DUP: 7; h2hINV: 10; t2tINV: 9; TRA: 7
SVs in sample	257
Oscillating CN (2 and 3 states)	37, 37
CN segments	37
FDR fragment joints	0.91
FDR chr. breakp. enrich.	0
Linked to chrs	18:11570704−36396525;
Purity, ploidy	0.88, 1.98

	084c6aec−94f7−4090−8f3f−59fa9e89721a
Cancer type	Breast−AdenoCA
Position	7:6161338−57778540
Type	With other complex events
Interleaved intrachr. SVs	19
Total SVs (intrachr. + transl.)	48
SV types	DEL: 6; DUP: 3; h2hINV: 5; t2tINV: 5; TRA: 29
SVs in sample	257
Oscillating CN (2 and 3 states)	9, 13
CN segments	42
FDR fragment joints	0.84
FDR chr. breakp. enrich.	0
Linked to chrs	8:33053387−75087726;
Purity, ploidy	0.88, 1.98

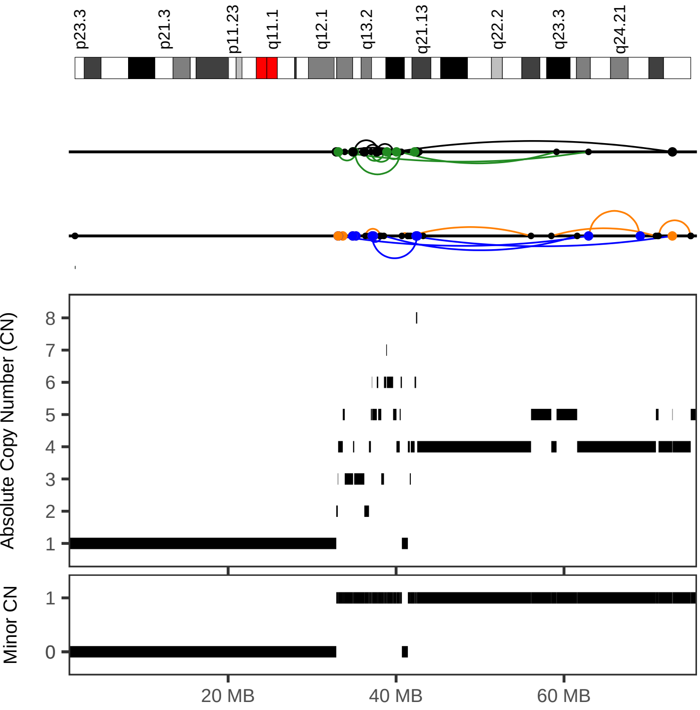

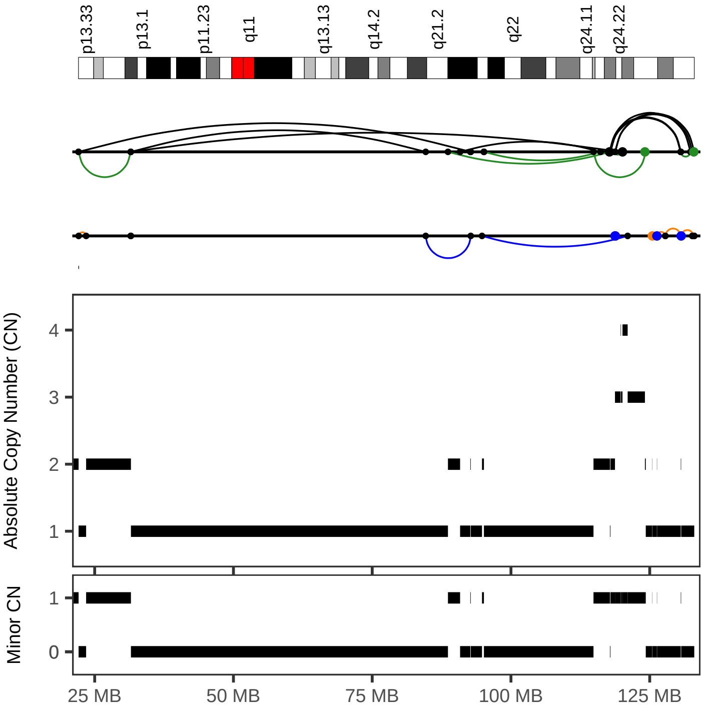

	084c6aec−94f7−4090−8f3f−59fa9e89721a
Cancer type	Breast−AdenoCA
Position	8:33053387−75087727
Type	With other complex events
Interleaved intrachr. SVs	25
Total SVs (intrachr. + transl.)	53
SV types	DEL: 6; DUP: 6; h2hINV: 5; t2tINV: 8; TRA: 28
SVs in sample	257
Oscillating CN (2 and 3 states)	9, 9
CN segments	36
FDR fragment joints	0.9
FDR chr. breakp. enrich.	0
Linked to chrs	7:6161338−57778539;
Purity, ploidy	0.88, 1.98

	084c6aec−94f7−4090−8f3f−59fa9e89721a
Cancer type	Breast−AdenoCA
Position	12:22079238−133021689
Type	With other complex events
Interleaved intrachr. SVs	23
Total SVs (intrachr. + transl.)	32
SV types	DEL: 2; DUP: 4; h2hINV: 9; t2tINV: 8; TRA: 9
SVs in sample	257
Oscillating CN (2 and 3 states)	13, 28
CN segments	28
FDR fragment joints	0.54
FDR chr. breakp. enrich.	0
Linked to chrs	
Purity, ploidy	0.88, 1.98

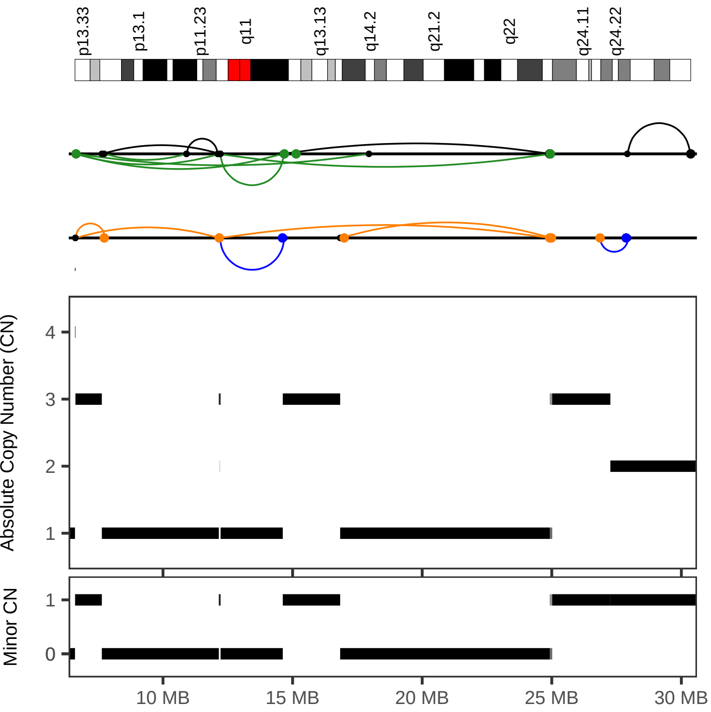

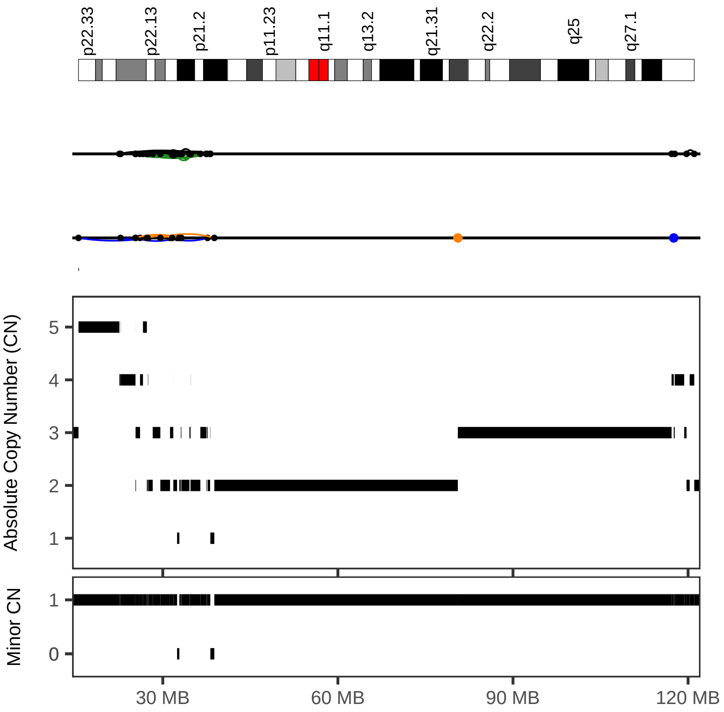

	0bc5744c−5fa3−45bb−87d0−70a02068b392
Cancer type	Breast−AdenoCA
Position	12:6603538−25009802
Type	Canonical without polyploidization
Interleaved intrachr. SVs	15
Total SVs (intrachr. + transl.)	26
SV types	DEL: 4; DUP: 2; h2hINV: 3; t2tINV: 6; TRA: 11
SVs in sample	487
Oscillating CN (2 and 3 states)	9, 13
CN segments	14
FDR fragment joints	0.64
FDR chr. breakp. enrich.	0.14
Linked to chrs	
Purity, ploidy	0.43, 2.16

	0bc5744c−5fa3−45bb−87d0−70a02068b392
Cancer type	Breast−AdenoCA
Position	X:15557457−38830595
Type	With other complex events
Interleaved intrachr. SVs	31
Total SVs (intrachr. + transl.)	32
SV types	DEL: 4; DUP: 5; h2hINV: 10; t2tINV: 12; TRA: 1
SVs in sample	487
Oscillating CN (2 and 3 states)	8, 18
CN segments	40
FDR fragment joints	0.53
FDR chr. breakp. enrich.	0.01
Linked to chrs	
Purity, ploidy	0.43, 2.16

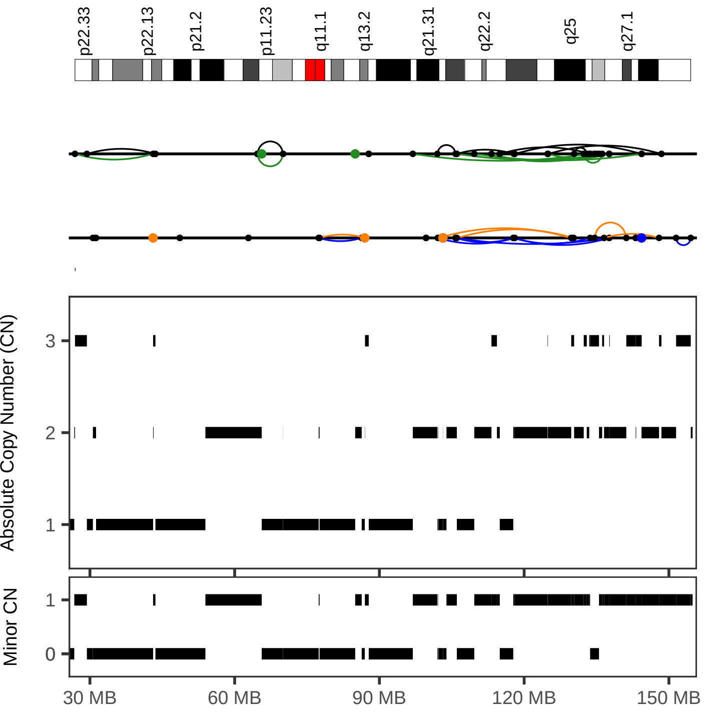

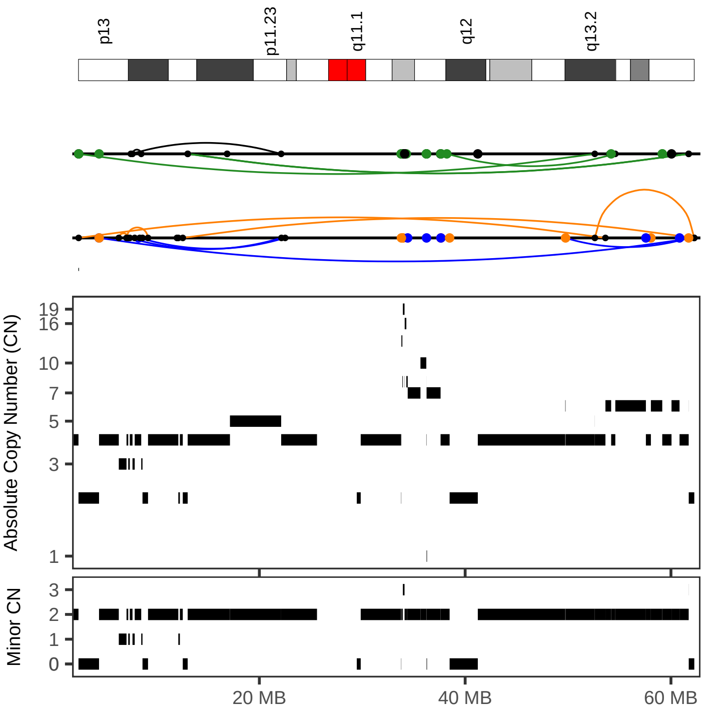

	0dca98b0−f43e−45b6−9a02−00092c78678c
Cancer type	Breast−AdenoCA
Position	X:96927939−148453406
Type	Canonical without polyploidization
Interleaved intrachr. SVs	22
Total SVs (intrachr. + transl.)	25
SV types	DEL: 4; DUP: 5; h2hINV: 7; t2tINV: 6; TRA: 3
SVs in sample	552
Oscillating CN (2 and 3 states)	23, 37
CN segments	37
FDR fragment joints	0.86
FDR chr. breakp. enrich.	0.01
Linked to chrs	16:695403−88774414;
Purity, ploidy	0.76, 2.13

	1174f6e4−ffbe−4e59−a000−8d861c968369
Cancer type	Breast−AdenoCA
Position	20:2418957−62264410
Type	With other complex events
Interleaved intrachr. SVs	16
Total SVs (intrachr. + transl.)	54
SV types	DEL: 5; DUP: 5; h2hINV: 2; t2tINV: 4; TRA: 38
SVs in sample	357
Oscillating CN (2 and 3 states)	10, 12
CN segments	51
FDR fragment joints	0.76
FDR chr. breakp. enrich.	0
Linked to chrs	3:95813661−124485879;
Purity, ploidy	0.49, 3.26

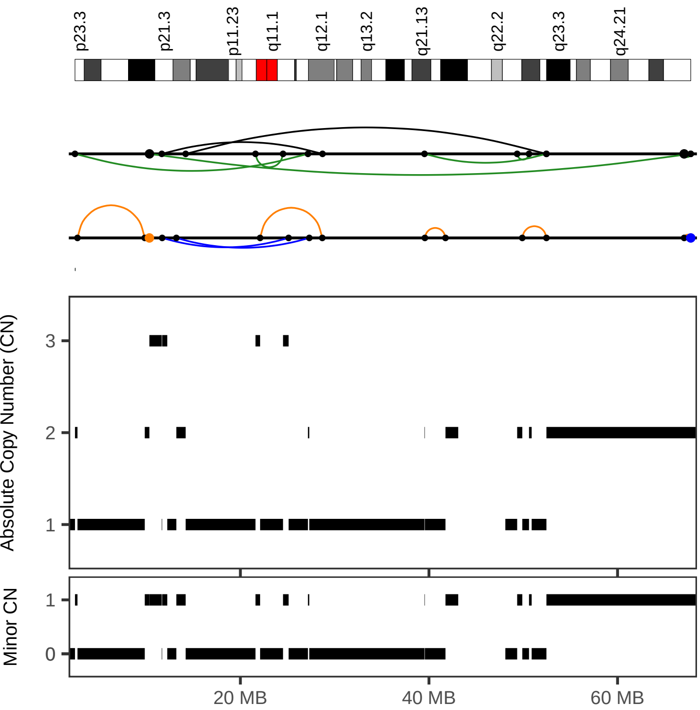

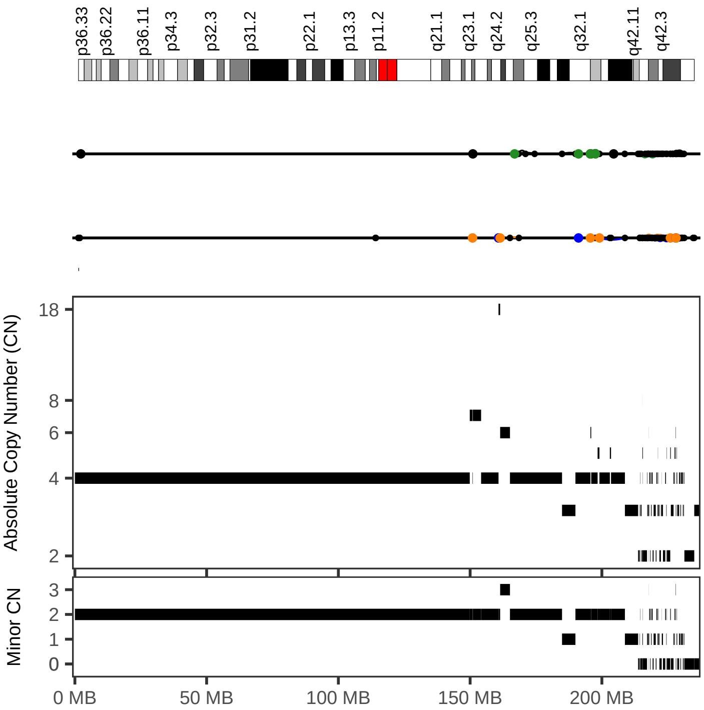

	17c1d42c−cb84−4655−a4cd−b54bae17ecaf
Cancer type	Breast−AdenoCA
Position	8:2456699−67762073
Type	With other complex events
Interleaved intrachr. SVs	11
Total SVs (intrachr. + transl.)	15
SV types	DEL: 2; DUP: 2; h2hINV: 2; t2tINV: 5; TRA: 4
SVs in sample	44
Oscillating CN (2 and 3 states)	12, 22
CN segments	24
FDR fragment joints	0.64
FDR chr. breakp. enrich.	0
Linked to chrs	
Purity, ploidy	0.93, 2

	1d27253f−b036−44e7−a04d−8da5bbf57419
Cancer type	Breast−AdenoCA
Position	1:214161940−231274267
Type	With other complex events
Interleaved intrachr. SVs	71
Total SVs (intrachr. + transl.)	74
SV types	DEL: 18; DUP: 20; h2hINV: 16; t2tINV: 17; TRA: 3
SVs in sample	256
Oscillating CN (2 and 3 states)	11, 13
CN segments	62
FDR fragment joints	0.94
FDR chr. breakp. enrich.	0
Linked to chrs	
Purity, ploidy	0.72, 4.37

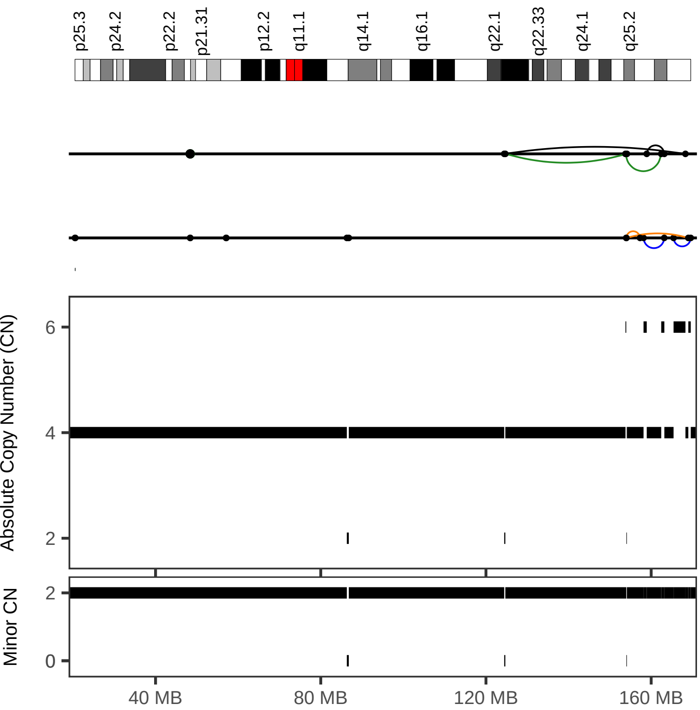

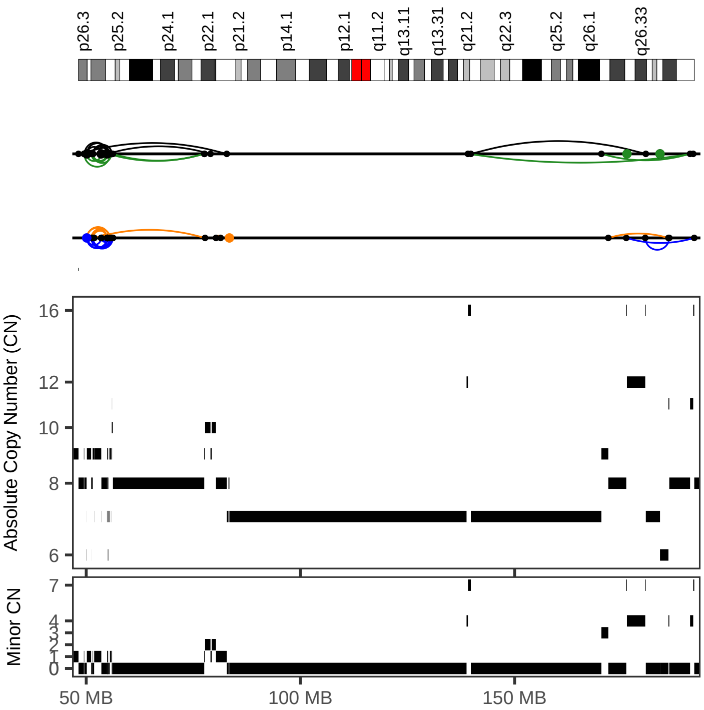

	1d27253f−b036−44e7−a04d−8da5bbf57419
Cancer type	Breast−AdenoCA
Position	6:124419945−169580563
Type	Before polyploidization
Interleaved intrachr. SVs	8
Total SVs (intrachr. + transl.)	8
SV types	DEL: 2; DUP: 2; h2hINV: 2; t2tINV: 2; TRA: 0
SVs in sample	256
Oscillating CN (2 and 3 states)	9, 14
CN segments	14
FDR fragment joints	1
FDR chr. breakp. enrich.	1
Linked to chrs	
Purity, ploidy	0.72, 4.37

	233b02f3−c4f0−4a67−9db5−e68d5cdaccb6
Cancer type	Breast−AdenoCA
Position	3:49458810−79003885
Type	With other complex events
Interleaved intrachr. SVs	51
Total SVs (intrachr. + transl.)	55
SV types	DEL: 13; DUP: 10; h2hINV: 17; t2tINV: 11; TRA: 4
SVs in sample	618
Oscillating CN (2 and 3 states)	7, 13
CN segments	48
FDR fragment joints	0.64
FDR chr. breakp. enrich.	0
Linked to chrs	
Purity, ploidy	0.7, 5.48

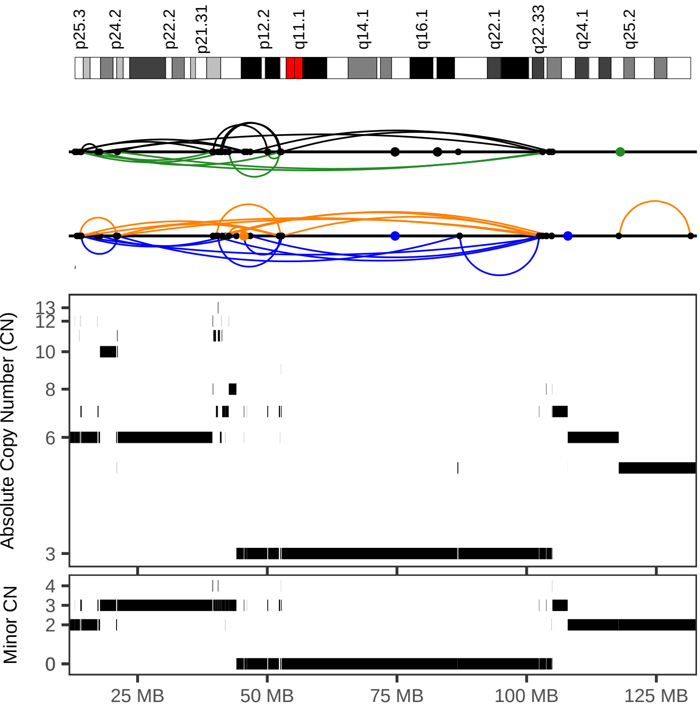

	**233b02f3−c4f0−4a67−9db5−e68d5cdaccb6**
Cancer type	Breast−AdenoCA
Position	6:12923765−104967173
Type	With other complex events
Interleaved intrachr. SVs	40
Total SVs (intrachr. + transl.)	44
SV types	DEL: 10; DUP: 11; h2hINV: 10; t2tINV: 9; TRA: 4
SVs in sample	618
Oscillating CN (2 and 3 states)	7, 12
CN segments	62
FDR fragment joints	0.99
FDR chr. breakp. enrich.	0.09
Linked to chrs	4:103236038−155236187;
Purity, ploidy	0.7, 5.48

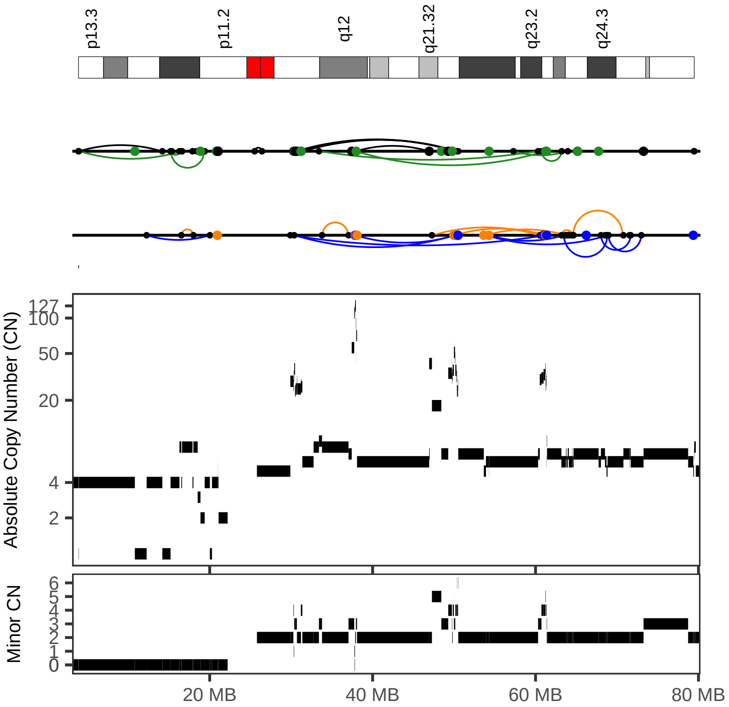

	**233b02f3−c4f0−4a67−9db5−e68d5cdaccb6**
Cancer type	Breast−AdenoCA
Position	17:29900854−72983271
Type	With other complex events
Interleaved intrachr. SVs	24
Total SVs (intrachr. + transl.)	59
SV types	DEL: 7; DUP: 9; h2hINV: 3; t2tINV: 5; TRA: 35
SVs in sample	618
Oscillating CN (2 and 3 states)	12, 18
CN segments	77
FDR fragment joints	0.59
FDR chr. breakp. enrich.	0
Linked to chrs	2:181721293−233322093;4:103236038−155236187 8:63956033−143798716;X:3683303−142419843
Purity, ploidy	0.7, 5.48

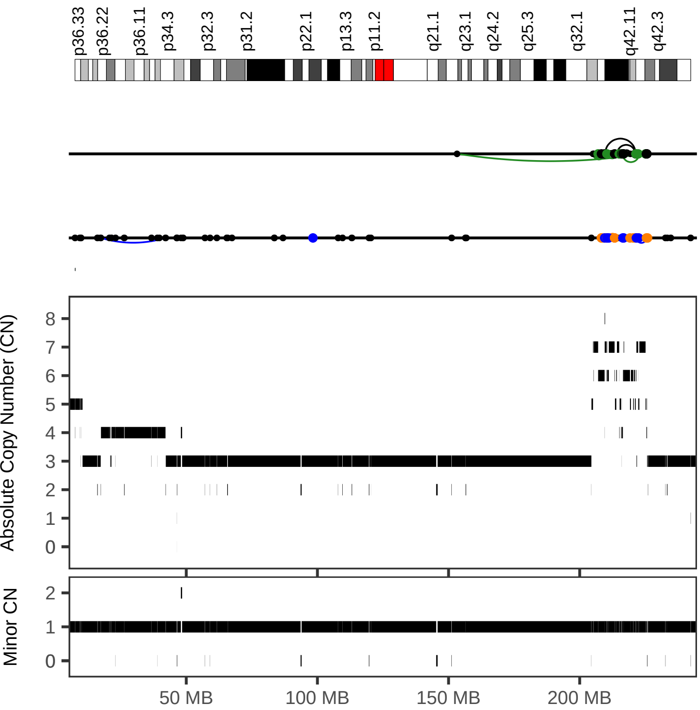

	**2a84997d−ccee−4f46−bea2−752534f26416**
Cancer type	Breast−AdenoCA
Position	1:153229764−225437151
Type	With other complex events
Interleaved intrachr. SVs	16
Total SVs (intrachr. + transl.)	49
SV types	DEL: 4; DUP: 1; h2hINV: 6; t2tINV: 5; TRA: 33
SVs in sample	596
Oscillating CN (2 and 3 states)	9, 12
CN segments	49
FDR fragment joints	0.59
FDR chr. breakp. enrich.	0
Linked to chrs	14:56204105−67427195;22:17885241−46307015
Purity, ploidy	0.46, 2.83

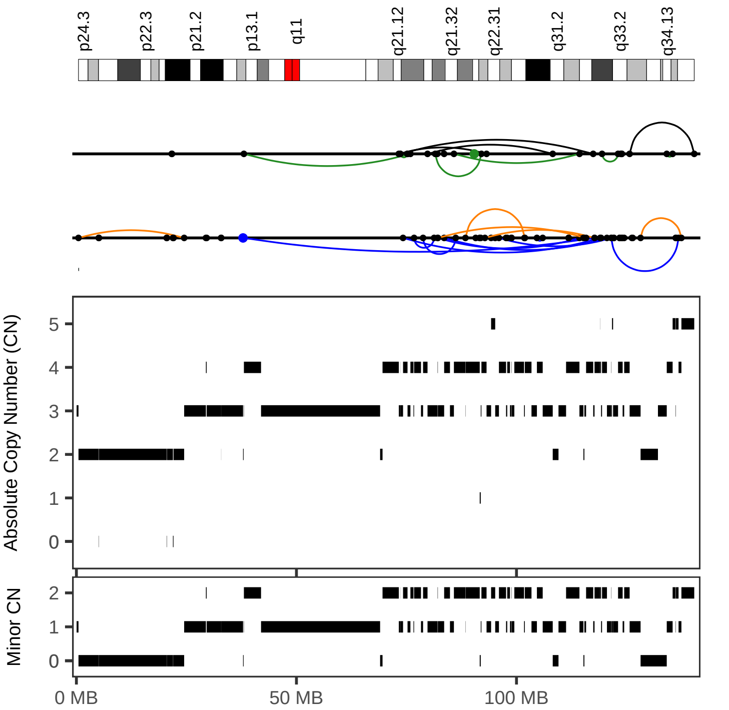

	**2a84997d−ccee−4f46−bea2−752534f26416**
Cancer type	Breast−AdenoCA
Position	9:38002140−140385138
Type	With other complex events
Interleaved intrachr. SVs	24
Total SVs (intrachr. + transl.)	25
SV types	DEL: 4; DUP: 9; h2hINV: 6; t2tINV: 5; TRA: 1
SVs in sample	596
Oscillating CN (2 and 3 states)	21, 29
CN segments	73
FDR fragment joints	0.64
FDR chr. breakp. enrich.	0
Linked to chrs	
Purity, ploidy	0.46, 2.83

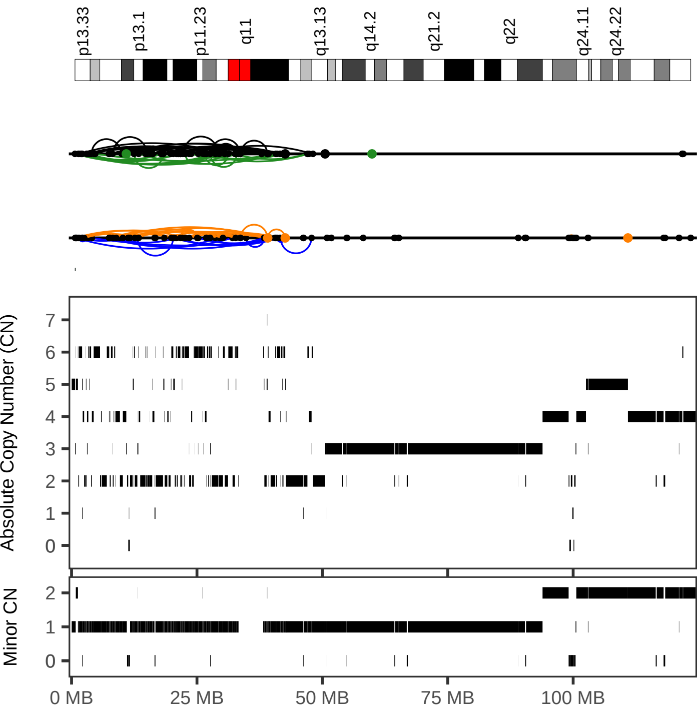

2a84997d−ccee−4f46−bea2−752534f26416

Cancer type	Breast−AdenoCA
Position	12:677748−48135242
Type	With other complex events
Interleaved intrachr. SVs	80
Total SVs (intrachr. + transl.)	84
SV types	DEL: 17; DUP: 16; h2hINV: 29; t2tINV: 18; TRA: 4
SVs in sample	596
Oscillating CN (2 and 3 states)	7, 11
CN segments	161
FDR fragment joints	0.59
FDR chr. breakp. enrich.	0
Linked to chrs	10:23170902−117935906;
Purity, ploidy	0.46, 2.83

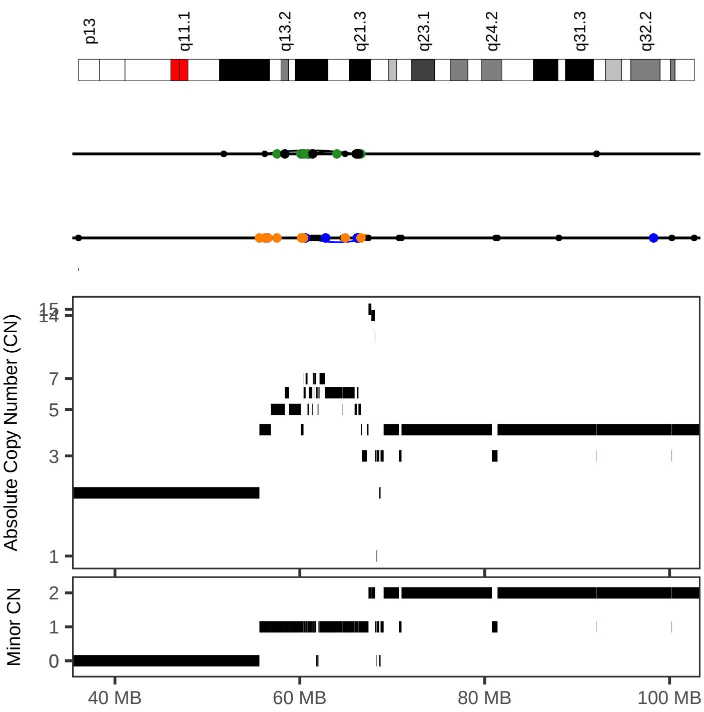

2a84997d−ccee−4f46−bea2−752534f26416

Cancer type	Breast−AdenoCA
Position	14:56204105−67427196
Type	With other complex events
Interleaved intrachr. SVs	5
Total SVs (intrachr. + transl.)	32
SV types	DEL: 1; DUP: 1; h2hINV: 2; t2tINV: 1; TRA: 27
SVs in sample	596
Oscillating CN (2 and 3 states)	8, 19
CN segments	32
FDR fragment joints	0.92
FDR chr. breakp. enrich.	0
Linked to chrs	1:153229764−225437150;22:17885241−46307015
Purity, ploidy	0.46, 2.83

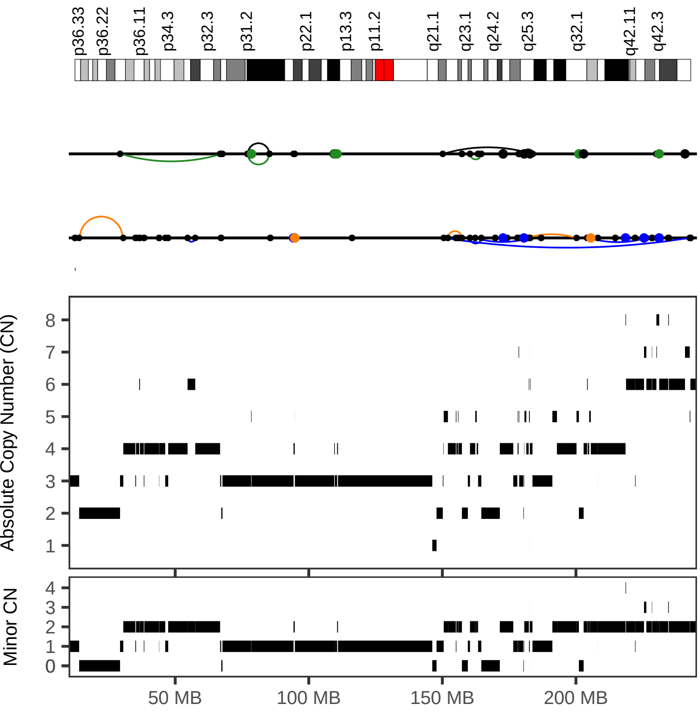

324bcba2−f6a4−45a6−807c−215bdffcca21

Cancer type	Breast−AdenoCA
Position	1:150164066−242610593
Type	With other complex events
Interleaved intrachr. SVs	11
Total SVs (intrachr. + transl.)	26
SV types	DEL: 1; DUP: 6; h2hINV: 2; t2tINV: 2; TRA: 15
SVs in sample	429
Oscillating CN (2 and 3 states)	7, 7
CN segments	61
FDR fragment joints	0.59
FDR chr. breakp. enrich.	0
Linked to chrs	2:42932222−160908995;
Purity, ploidy	0.67, 2.85

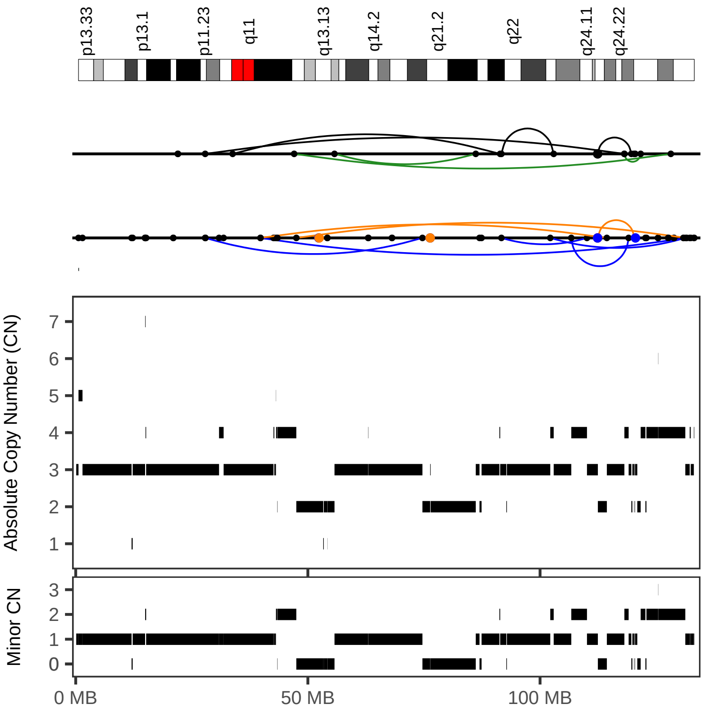

324bcba2−f6a4−45a6−807c−215bdffcca21

Cancer type	Breast−AdenoCA
Position	12:27880056−131551013
Type	With other complex events
Interleaved intrachr. SVs	16
Total SVs (intrachr. + transl.)	24
SV types	DEL: 3; DUP: 5; h2hINV: 5; t2tINV: 3; TRA: 8
SVs in sample	429
Oscillating CN (2 and 3 states)	8, 33
CN segments	48
FDR fragment joints	0.84
FDR chr. breakp. enrich.	0
Linked to chrs	10:8421238−126684699;
Purity, ploidy	0.67, 2.85

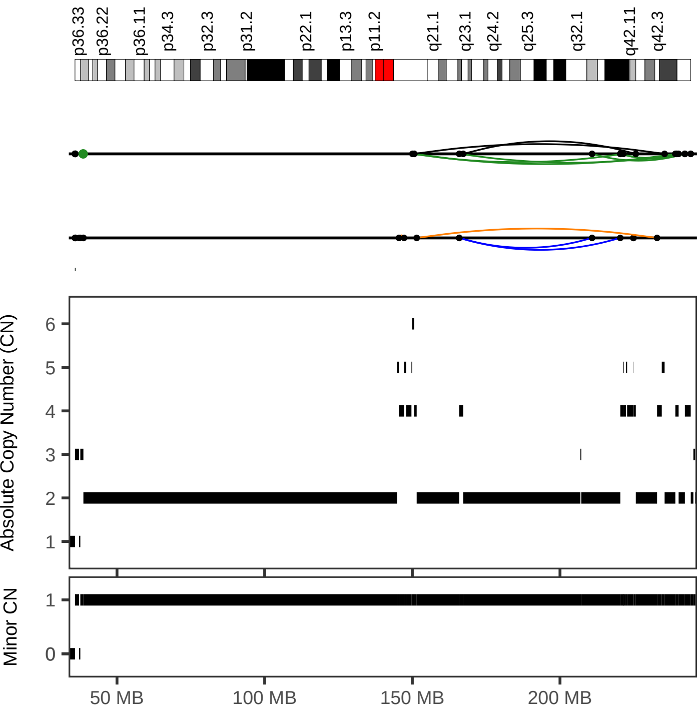

359f12f9−5c41−48a4−85bc−fd7e307bf7d8	
Cancer type	Breast−AdenoCA
Position	1:150066880−242290225
Type	With other complex events
Interleaved intrachr. SVs	12
Total SVs (intrachr. + transl.)	12
SV types	DEL: 1; DUP: 2; h2hINV: 3; t2tINV: 6; TRA: 0
SVs in sample	83
Oscillating CN (2 and 3 states)	7, 7
CN segments	21
FDR fragment joints	0.59
FDR chr. breakp. enrich.	0
Linked to chrs	
Purity, ploidy	0.68, 1.53

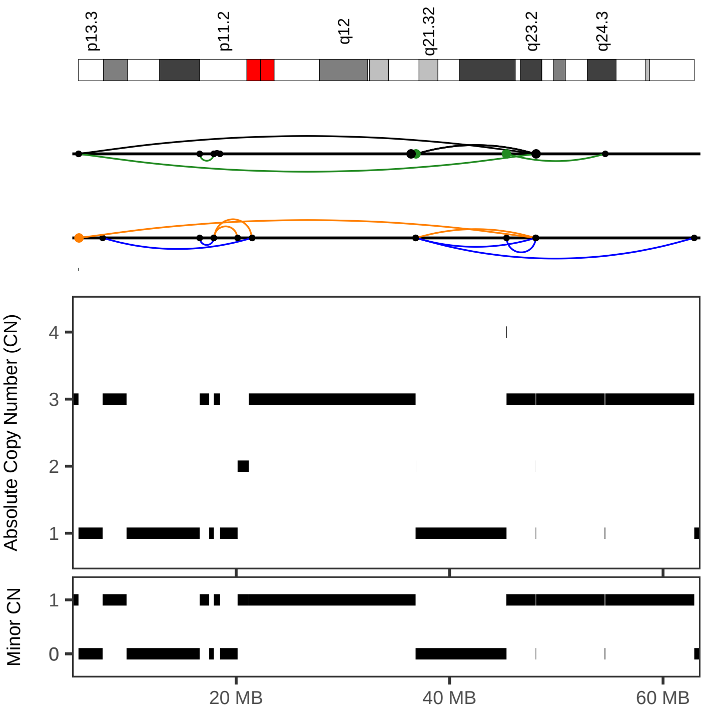

3fc3755d−a3f8−4e2c−813f−ff124f2a75c1	
Cancer type	Breast−AdenoCA
Position	17:5222718−62920805
Type	With other complex events
Interleaved intrachr. SVs	10
Total SVs (intrachr. + transl.)	15
SV types	DEL: 2; DUP: 3; h2hINV: 3; t2tINV: 2; TRA: 5
SVs in sample	35
Oscillating CN (2 and 3 states)	7, 13
CN segments	25
FDR fragment joints	0.96
FDR chr. breakp. enrich.	0
Linked to chrs	
Purity, ploidy	0.43, 2.15

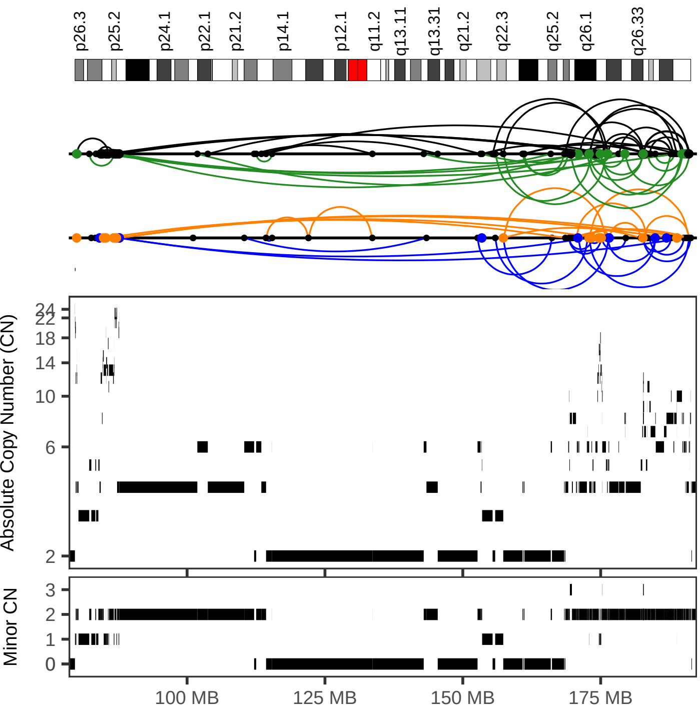

44bec761−b603−49c0−8634−f6bfe0319bb1	
Cancer type	Breast−AdenoCA
Position	3:79667212−191335908
Type	With other complex events
Interleaved intrachr. SVs	103
Total SVs (intrachr. + transl.)	158
SV types	DEL: 18; DUP: 22; h2hINV: 35; t2tINV: 28; TRA: 55
SVs in sample	320
Oscillating CN (2 and 3 states)	8, 8
CN segments	189
FDR fragment joints	0.48
FDR chr. breakp. enrich.	0
Linked to chrs	17:47241742−74862784;
Purity, ploidy	0.59, 3.37

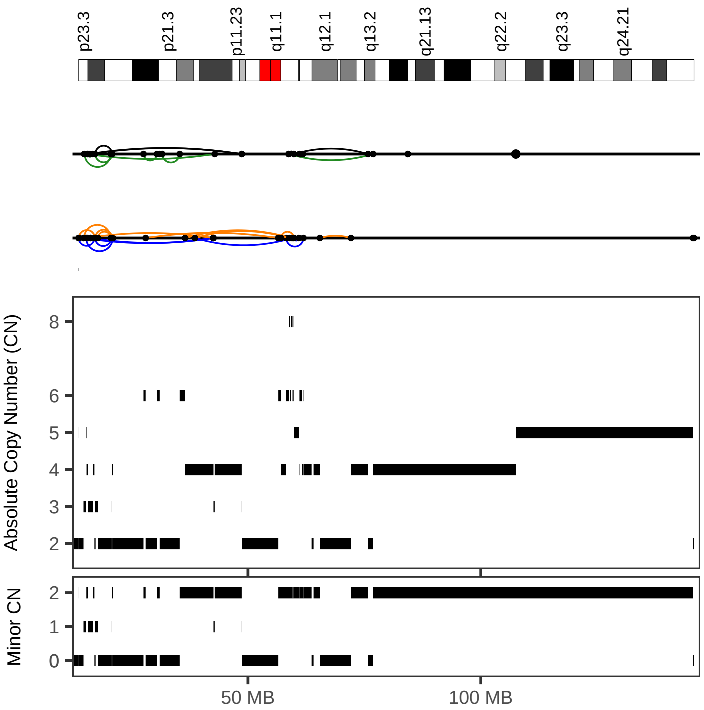

44bec761−b603−49c0−8634−f6bfe0319bb1	
Cancer type	Breast−AdenoCA
Position	8:13658370−76896511
Type	With other complex events
Interleaved intrachr. SVs	29
Total SVs (intrachr. + transl.)	29
SV types	DEL: 10; DUP: 8; h2hINV: 5; t2tINV: 6; TRA: 0
SVs in sample	320
Oscillating CN (2 and 3 states)	7, 13
CN segments	54
FDR fragment joints	0.64
FDR chr. breakp. enrich.	0
Linked to chrs	
Purity, ploidy	0.59, 3.37

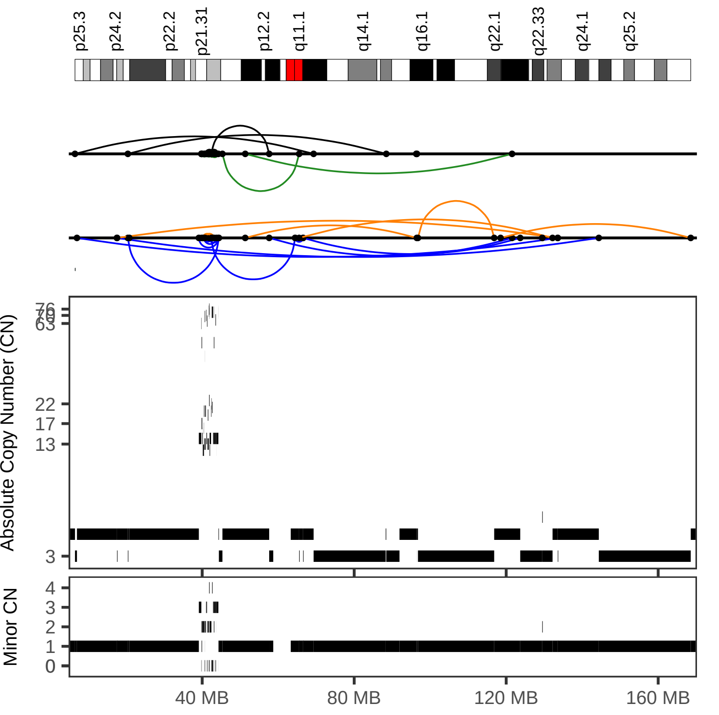

	53886143−c1c6−40e9−88e6−e4e5e0271fc8
Cancer type	Breast−AdenoCA
Position	6:6520338−168564355
Type	With other complex events
Interleaved intrachr. SVs	40
Total SVs (intrachr. + transl.)	40
SV types	DEL: 8; DUP: 12; h2hINV: 11; t2tINV: 9; TRA: 0
SVs in sample	113
Oscillating CN (2 and 3 states)	20, 27
CN segments	78
FDR fragment joints	0.84
FDR chr. breakp. enrich.	0
Linked to chrs	
Purity, ploidy	0.54, 2.98

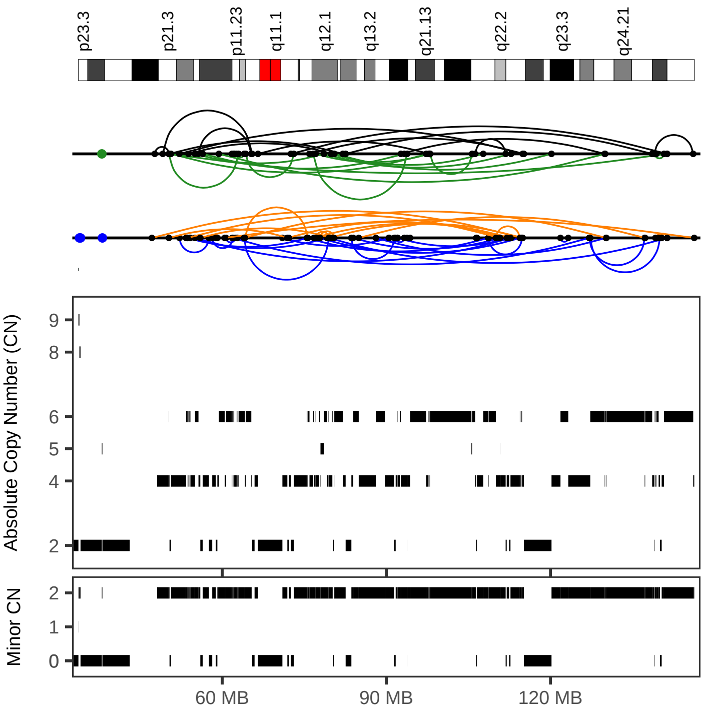

	5580b21a−2cdb−4777−ad79−6e06654144f5
Cancer type	Breast−AdenoCA
Position	8:47141968−146282239
Type	With other complex events
Interleaved intrachr. SVs	72
Total SVs (intrachr. + transl.)	72
SV types	DEL: 17; DUP: 23; h2hINV: 16; t2tINV: 16; TRA: 0
SVs in sample	252
Oscillating CN (2 and 3 states)	17, 54
CN segments	129
FDR fragment joints	0.67
FDR chr. breakp. enrich.	0
Linked to chrs	
Purity, ploidy	0.55, 3.56

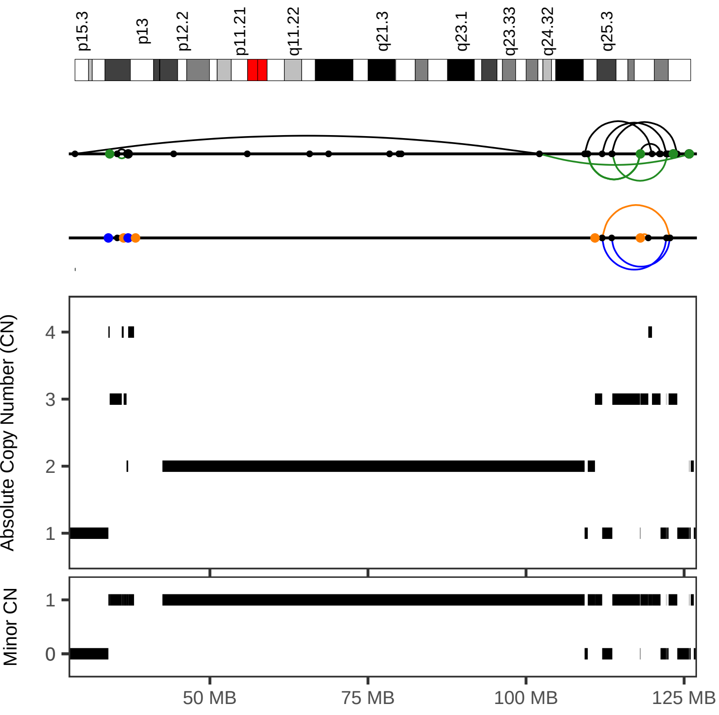

	566792ae−f853−4a47−856d−f02cdcfcb18a
Cancer type	Breast−AdenoCA
Position	10:109264246−123915303
Type	With other complex events
Interleaved intrachr. SVs	17
Total SVs (intrachr. + transl.)	22
SV types	DEL: 2; DUP: 3; h2hINV: 5; t2tINV: 7; TRA: 5
SVs in sample	579
Oscillating CN (2 and 3 states)	8, 14
CN segments	16
FDR fragment joints	0.59
FDR chr. breakp. enrich.	0
Linked to chrs	1:35669387−215888509;
Purity, ploidy	0.72, 2.01

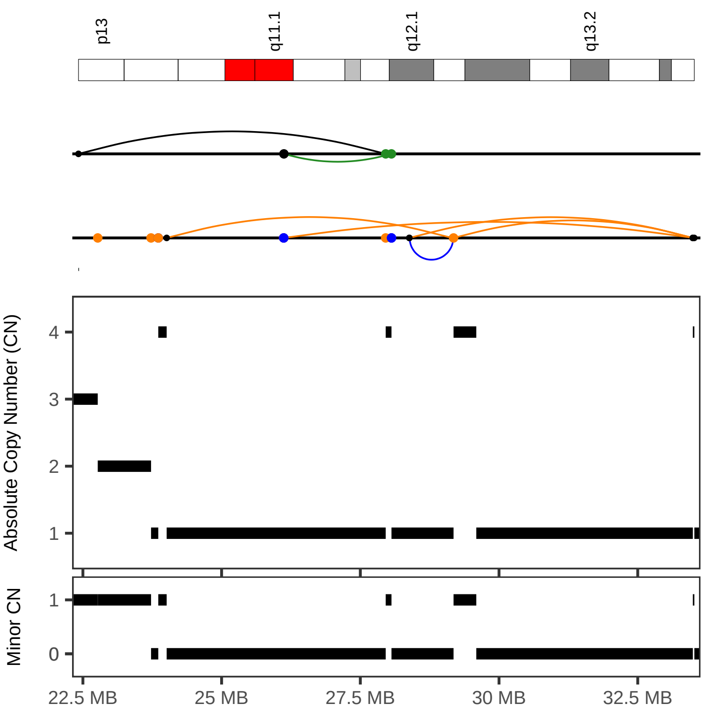

	566792ae−f853−4a47−856d−f02cdcfcb18a
Cancer type	Breast−AdenoCA
Position	22:22420637−33518317
Type	Before polyploidization
Interleaved intrachr. SVs	6
Total SVs (intrachr. + transl.)	20
SV types	DEL: 3; DUP: 1; h2hINV: 1; t2tINV: 1; TRA: 14
SVs in sample	579
Oscillating CN (2 and 3 states)	8, 10
CN segments	10
FDR fragment joints	0.64
FDR chr. breakp. enrich.	0
Linked to chrs	11:76728327−84744116;
Purity, ploidy	0.72, 2.01

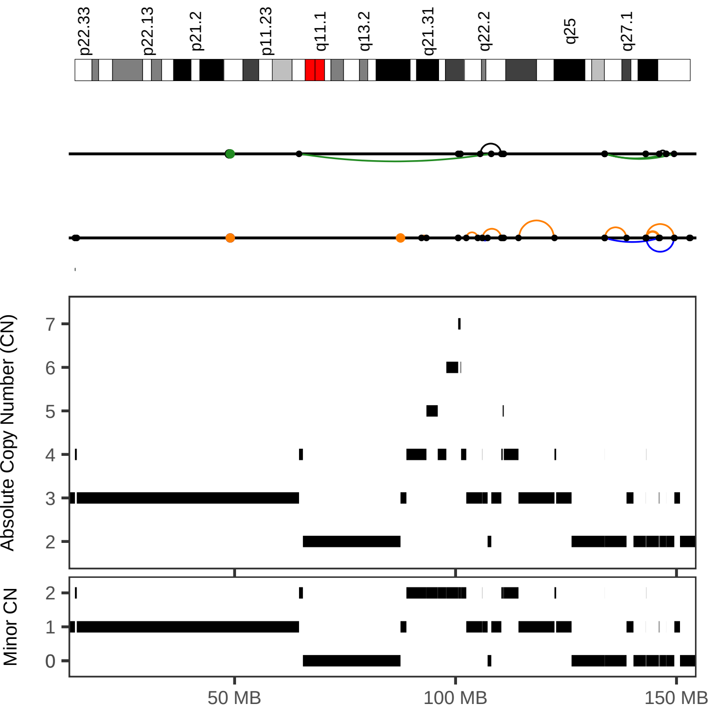

5dd423e8−feaa−4568−a750−500948c41d6c	
Cancer type	Breast−AdenoCA
Position	X:133728817−149525449
Type	With other complex events
Interleaved intrachr. SVs	10
Total SVs (intrachr. + transl.)	10
SV types	DEL: 3; DUP: 3; h2hINV: 2; t2tINV: 2; TRA: 0
SVs in sample	255
Oscillating CN (2 and 3 states)	10, 17
CN segments	17
FDR fragment joints	0.96
FDR chr. breakp. enrich.	0
Linked to chrs	
Purity, ploidy	0.8, 2.15

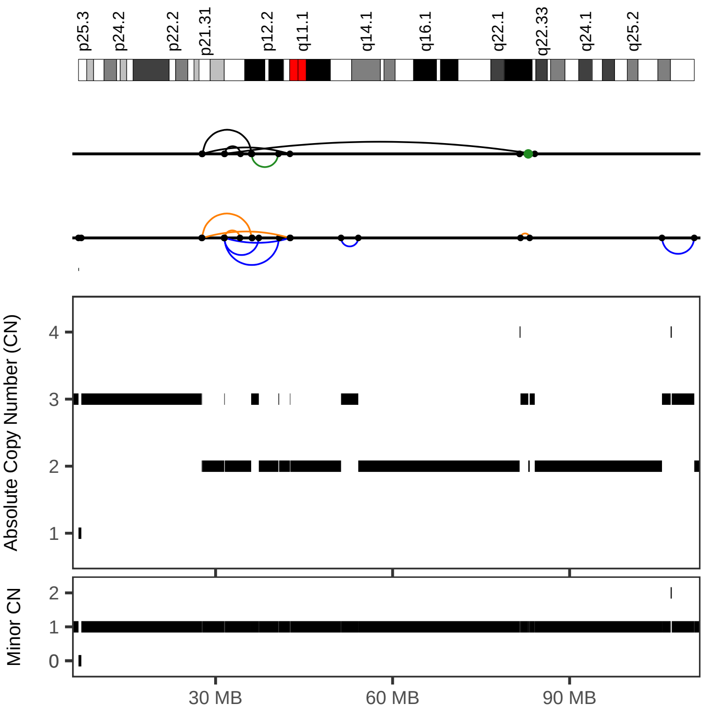

5fd9552a−c742−4388−940d−295d1107ae00	
Cancer type	Breast−AdenoCA
Position	6:27642577−84092516
Type	Canonical without polyploidization
Interleaved intrachr. SVs	10
Total SVs (intrachr. + transl.)	11
SV types	DEL: 3; DUP: 3; h2hINV: 3; t2tINV: 1; TRA: 1
SVs in sample	97
Oscillating CN (2 and 3 states)	17, 19
CN segments	22
FDR fragment joints	0.83
FDR chr. breakp. enrich.	0
Linked to chrs	
Purity, ploidy	0.78, 1.75

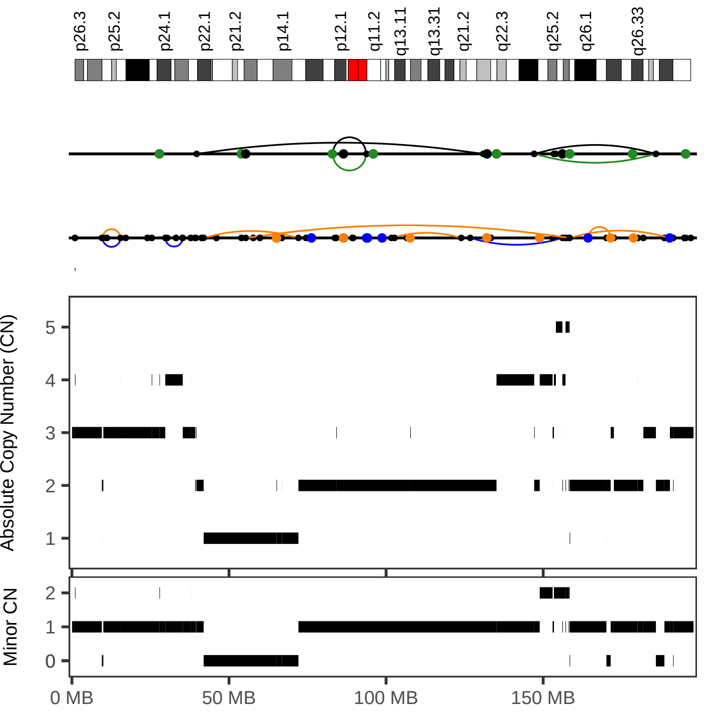

65cac997−4d39−4501−85ec−4fcb328a8eb5	
Cancer type	Breast−AdenoCA
Position	3:39702805−190330615
Type	With other complex events
Interleaved intrachr. SVs	7
Total SVs (intrachr. + transl.)	42
SV types	DEL: 3; DUP: 1; h2hINV: 2; t2tINV: 1; TRA: 35
SVs in sample	909
Oscillating CN (2 and 3 states)	7, 10
CN segments	40
FDR fragment joints	0.74
FDR chr. breakp. enrich.	0
Linked to chrs	15:38084470−80918007;4:36731786−170830419 8:22900664−128813813;9:31378526−139868175 X:9604512−99744773;
Purity, ploidy	0.66, 1.85

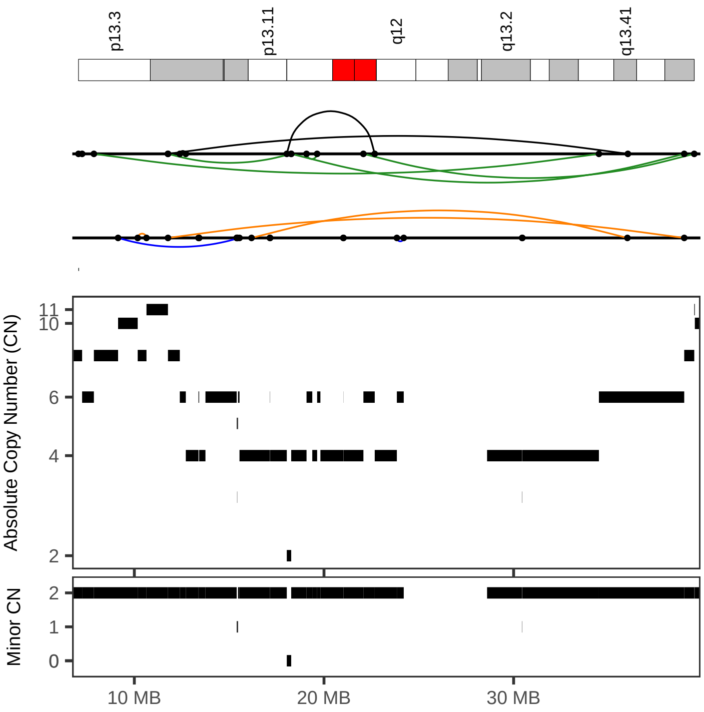

6fa2a667−9c36−4526−8a58−1975e863a806	
Cancer type	Breast−AdenoCA
Position	19:7866667−39524085
Type	With other complex events
Interleaved intrachr. SVs	9
Total SVs (intrachr. + transl.)	9
SV types	DEL: 2; DUP: 1; h2hINV: 2; t2tINV: 4; TRA: 0
SVs in sample	259
Oscillating CN (2 and 3 states)	11, 14
CN segments	34
FDR fragment joints	0.64
FDR chr. breakp. enrich.	0
Linked to chrs	
Purity, ploidy	0.88, 3.98

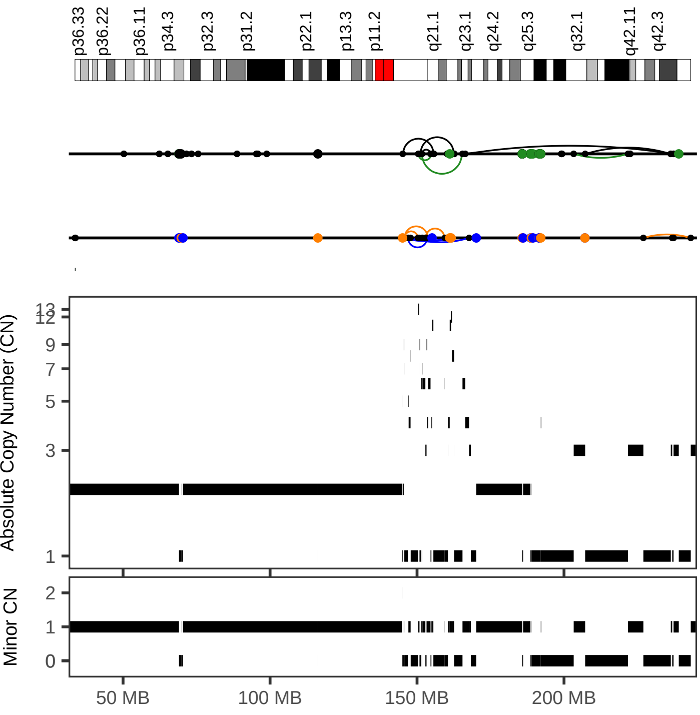

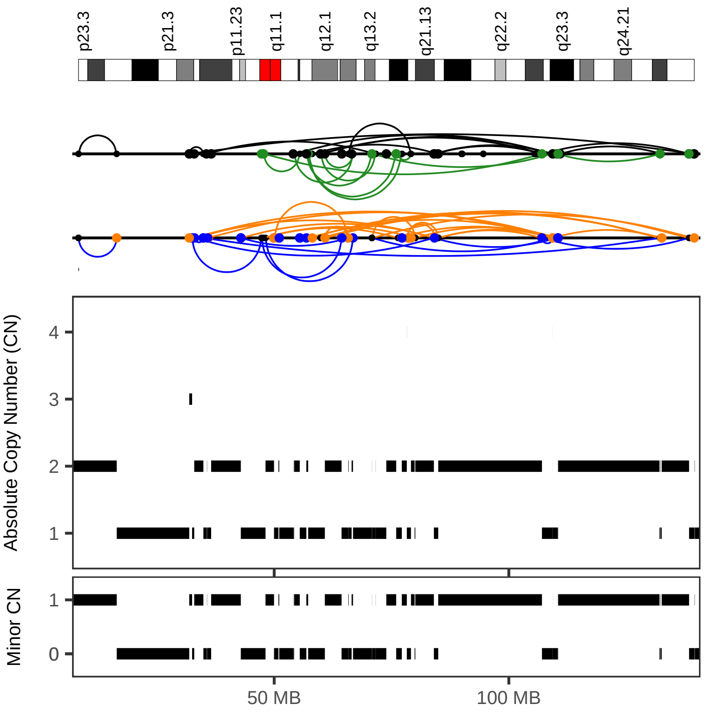

	733a2a0f−b37a−4b81−b49e−3c0f30d1eb37
Cancer type	Breast−AdenoCA
Position	1:145117775−243098983
Type	With other complex events
Interleaved intrachr. SVs	22
Total SVs (intrachr. + transl.)	54
SV types	DEL: 7; DUP: 4; h2hINV: 6; t2tINV: 5; TRA: 32
SVs in sample	403
Oscillating CN (2 and 3 states)	10, 10
CN segments	57
FDR fragment joints	0.86
FDR chr. breakp. enrich.	0
Linked to chrs	17:34384789−59566710;8:31896789−139505358
Purity, ploidy	0.6, 1.93

	733a2a0f−b37a−4b81−b49e−3c0f30d1eb37
Cancer type	Breast−AdenoCA
Position	8:31896789−139505359
Type	Canonical without polyploidization
Interleaved intrachr. SVs	58
Total SVs (intrachr. + transl.)	139
SV types	DEL: 23; DUP: 11; h2hINV: 13; t2tINV: 11; TRA: 81
SVs in sample	403
Oscillating CN (2 and 3 states)	36, 36
CN segments	58
FDR fragment joints	0.48
FDR chr. breakp. enrich.	0
Linked to chrs	1:145117775−243098982;17:34384789−59566710
Purity, ploidy	0.6, 1.93

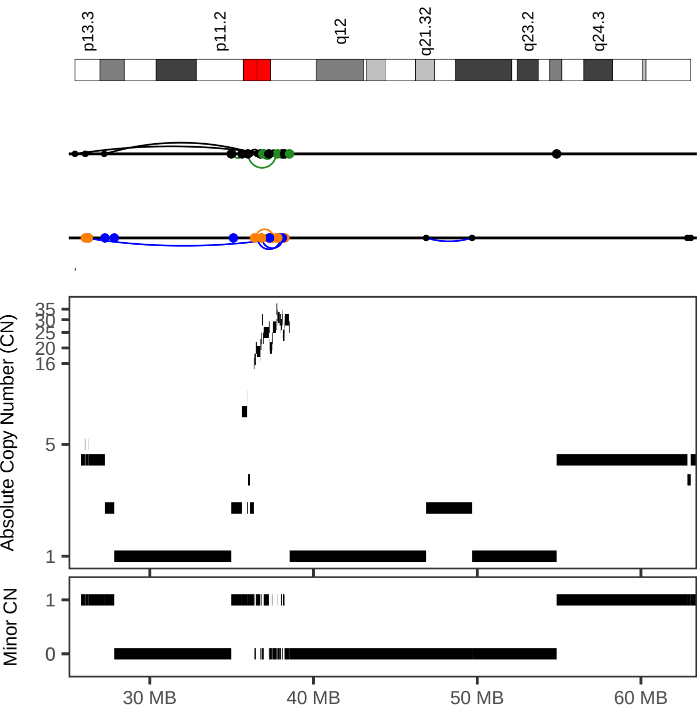

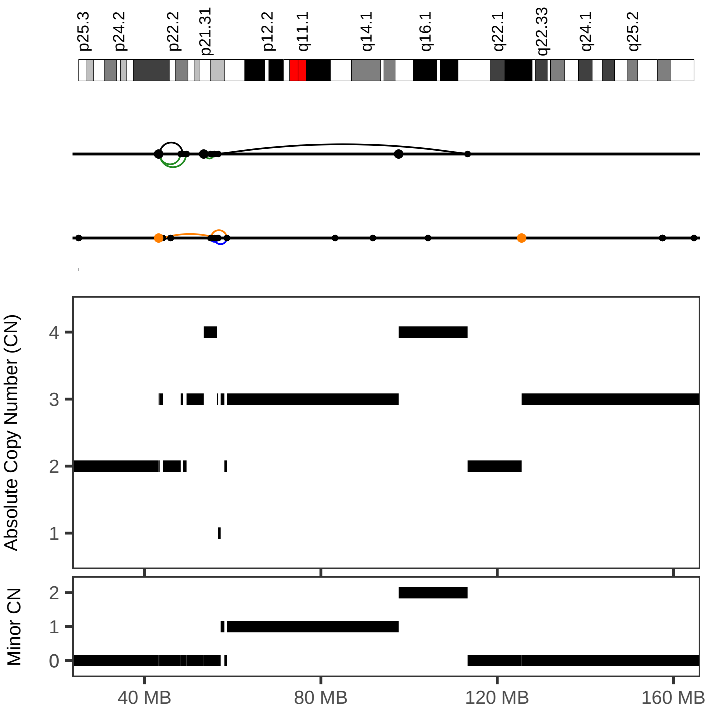

	784de7ac−8424−42eb−83d4−a1bebaa42b97
Cancer type	Breast−AdenoCA
Position	17:25428583−38145015
Type	With other complex events
Interleaved intrachr. SVs	12
Total SVs (intrachr. + transl.)	42
SV types	DEL: 2; DUP: 3; h2hINV: 5; t2tINV: 2; TRA: 30
SVs in sample	106
Oscillating CN (2 and 3 states)	7, 7
CN segments	48
FDR fragment joints	0.64
FDR chr. breakp. enrich.	0
Linked to chrs	11:48231454−78887151;
Purity, ploidy	0.6, 2.04

	8bd68696−5eeb−46d7−9dc6−c39c849029a4
Cancer type	Breast−AdenoCA
Position	6:43335892−113274184
Type	With other complex events
Interleaved intrachr. SVs	10
Total SVs (intrachr. + transl.)	12
SV types	DEL: 2; DUP: 2; h2hINV: 3; t2tINV: 3; TRA: 2
SVs in sample	343
Oscillating CN (2 and 3 states)	11, 11
CN segments	21
FDR fragment joints	0.96
FDR chr. breakp. enrich.	0.55
Linked to chrs	
Purity, ploidy	0.75, 1.87

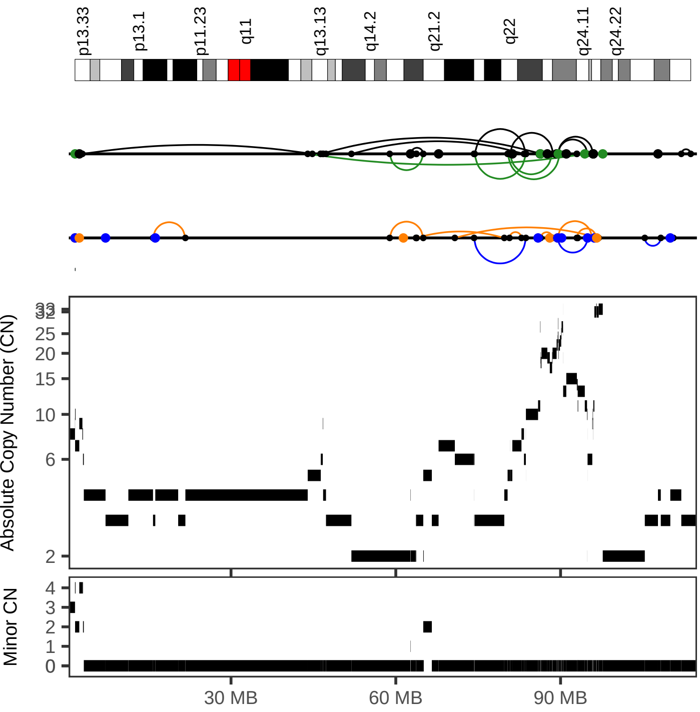

	8cf54607−01ce−42b0−9bd9−8627edd9f3b7
Cancer type	Breast−AdenoCA
Position	12:2943203−96943878
Type	With other complex events
Interleaved intrachr. SVs	29
Total SVs (intrachr. + transl.)	54
SV types	DEL: 7; DUP: 3; h2hINV: 10; t2tINV: 9; TRA: 25
SVs in sample	500
Oscillating CN (2 and 3 states)	9, 10
CN segments	88
FDR fragment joints	0.59
FDR chr. breakp. enrich.	0
Linked to chrs	6:66019356−135278032;
Purity, ploidy	0.55, 3.76

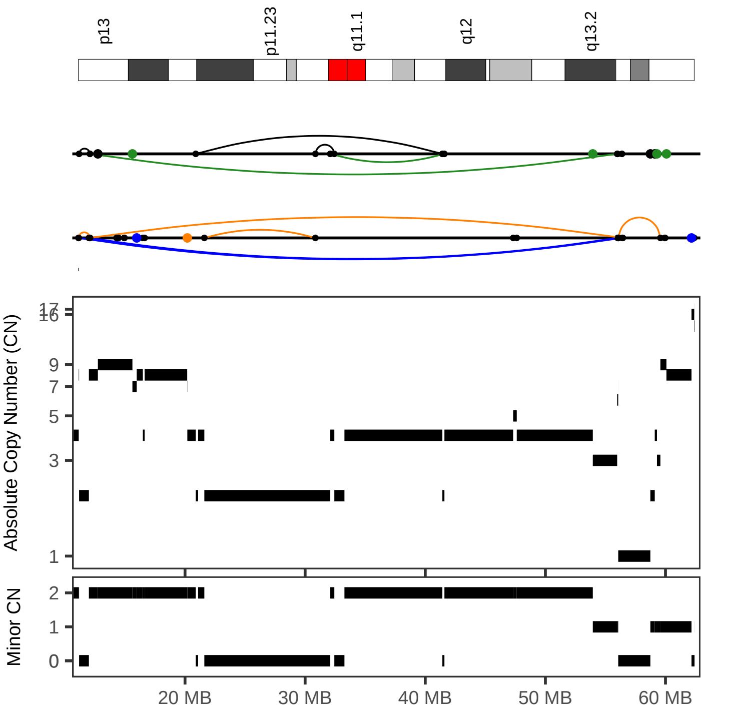

	8cf54607−01ce−42b0−9bd9−8627edd9f3b7
Cancer type	Breast−AdenoCA
Position	20:11131846−59577547
Type	With other complex events
Interleaved intrachr. SVs	7
Total SVs (intrachr. + transl.)	15
SV types	DEL: 3; DUP: 2; h2hINV: 1; t2tINV: 1; TRA: 8
SVs in sample	500
Oscillating CN (2 and 3 states)	9, 11
CN segments	29
FDR fragment joints	0.74
FDR chr. breakp. enrich.	0
Linked to chrs	21:35999819−47786447;
Purity, ploidy	0.55, 3.76

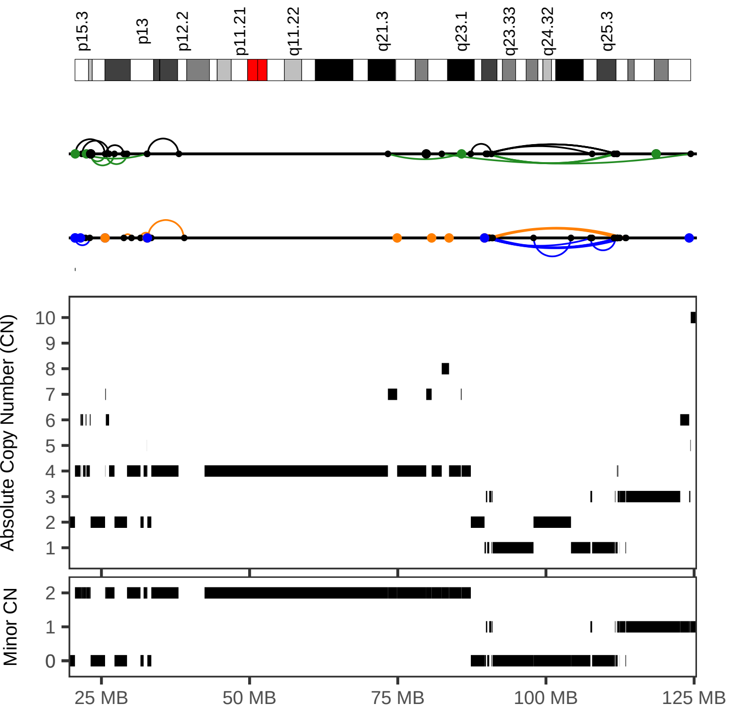

	9435447e−d65f−408b−863b−6576b1d652dd
Cancer type	Breast−AdenoCA
Position	10:87322920−113509134
Type	With other complex events
Interleaved intrachr. SVs	28
Total SVs (intrachr. + transl.)	30
SV types	DEL: 8; DUP: 10; h2hINV: 5; t2tINV: 5; TRA: 2
SVs in sample	137
Oscillating CN (2 and 3 states)	7, 21
CN segments	26
FDR fragment joints	0.64
FDR chr. breakp. enrich.	0
Linked to chrs	
Purity, ploidy	0.63, 3.27

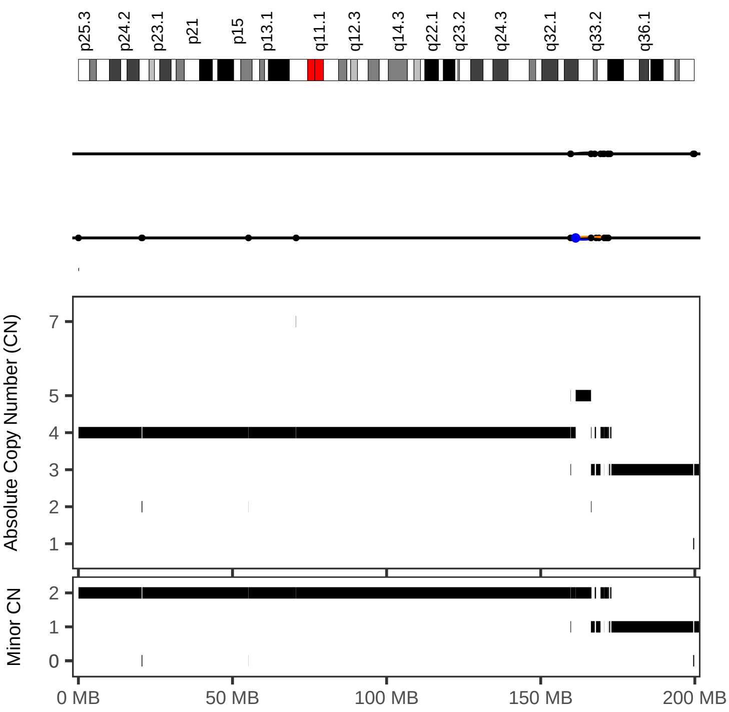

	993103b1−e5a1−4c33−8629−be53ebc41d64
Cancer type	Breast−AdenoCA
Position	2:159664646−172537729
Type	After polyploidization
Interleaved intrachr. SVs	11
Total SVs (intrachr. + transl.)	12
SV types	DEL: 2; DUP: 3; h2hINV: 1; t2tINV: 5; TRA: 1
SVs in sample	202
Oscillating CN (2 and 3 states)	11, 13
CN segments	17
FDR fragment joints	0.59
FDR chr. breakp. enrich.	0.69
Linked to chrs	
Purity, ploidy	0.41, 3.34

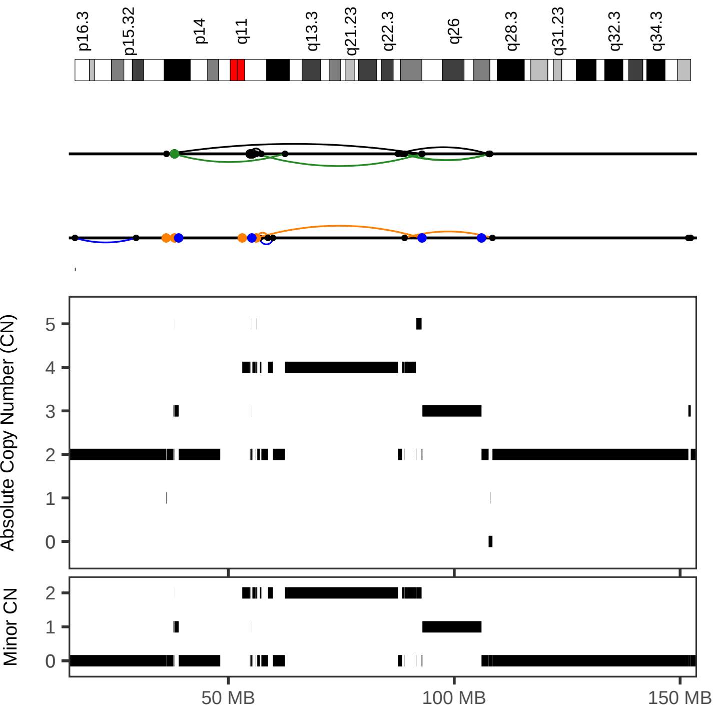

	993103b1−e5a1−4c33−8629−be53ebc41d64
Cancer type	Breast−AdenoCA
Position	4:36303099−108458117
Type	With other complex events
Interleaved intrachr. SVs	16
Total SVs (intrachr. + transl.)	27
SV types	DEL: 3; DUP: 1; h2hINV: 6; t2tINV: 6; TRA: 11
SVs in sample	202
Oscillating CN (2 and 3 states)	12, 14
CN segments	41
FDR fragment joints	0.59
FDR chr. breakp. enrich.	0
Linked to chrs	11:16582735−78899878;
Purity, ploidy	0.41, 3.34

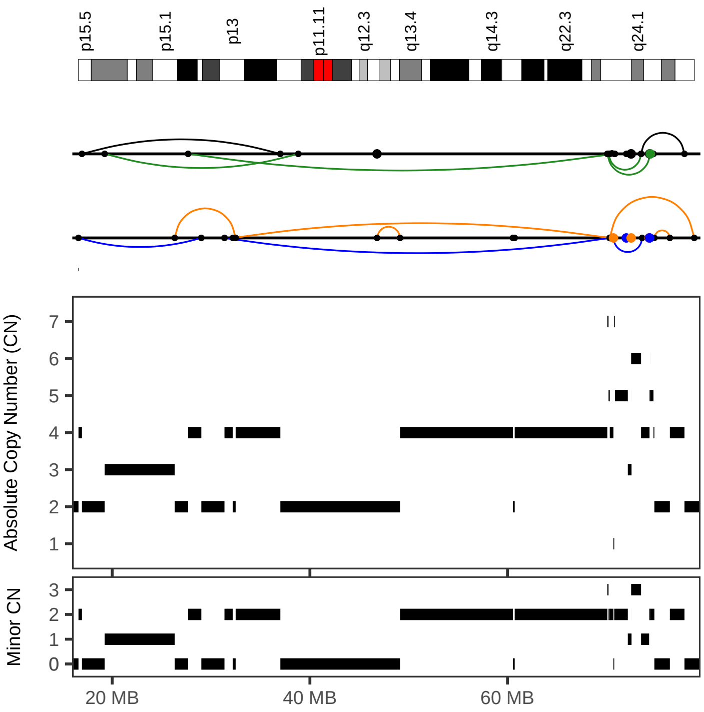

	993103b1−e5a1−4c33−8629−be53ebc41d64
Cancer type	Breast−AdenoCA
Position	11:16582735−78899879
Type	With other complex events
Interleaved intrachr. SVs	15
Total SVs (intrachr. + transl.)	26
SV types	DEL: 4; DUP: 3; h2hINV: 4; t2tINV: 4; TRA: 11
SVs in sample	202
Oscillating CN (2 and 3 states)	10, 13
CN segments	33
FDR fragment joints	0.99
FDR chr. breakp. enrich.	0
Linked to chrs	4:36303099−108458116;
Purity, ploidy	0.41, 3.34

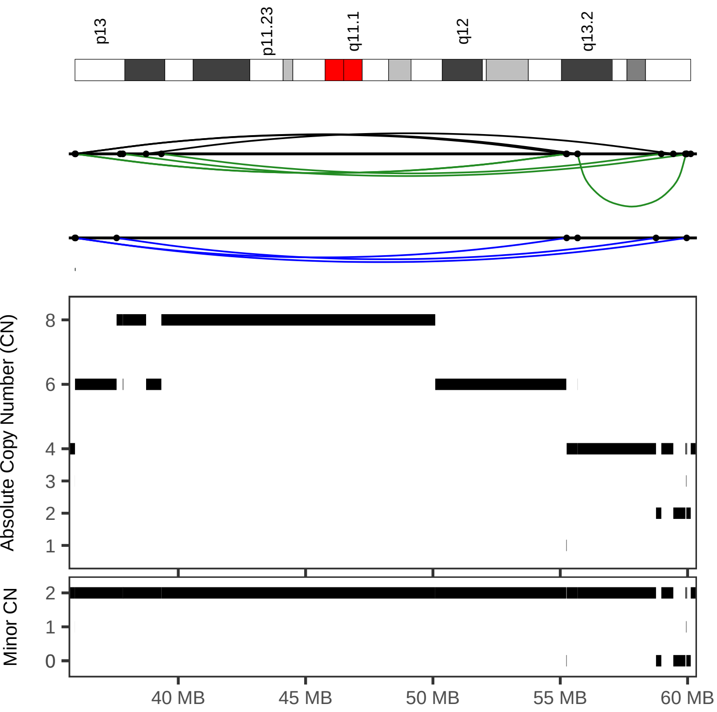

	993103b1−e5a1−4c33−8629−be53ebc41d64
Cancer type	Breast−AdenoCA
Position	20:35943756−60123567
Type	With other complex events
Interleaved intrachr. SVs	14
Total SVs (intrachr. + transl.)	14
SV types	DEL: 0; DUP: 4; h2hINV: 4; t2tINV: 6; TRA: 0
SVs in sample	202
Oscillating CN (2 and 3 states)	7, 9
CN segments	24
FDR fragment joints	0.59
FDR chr. breakp. enrich.	0
Linked to chrs	
Purity, ploidy	0.41, 3.34

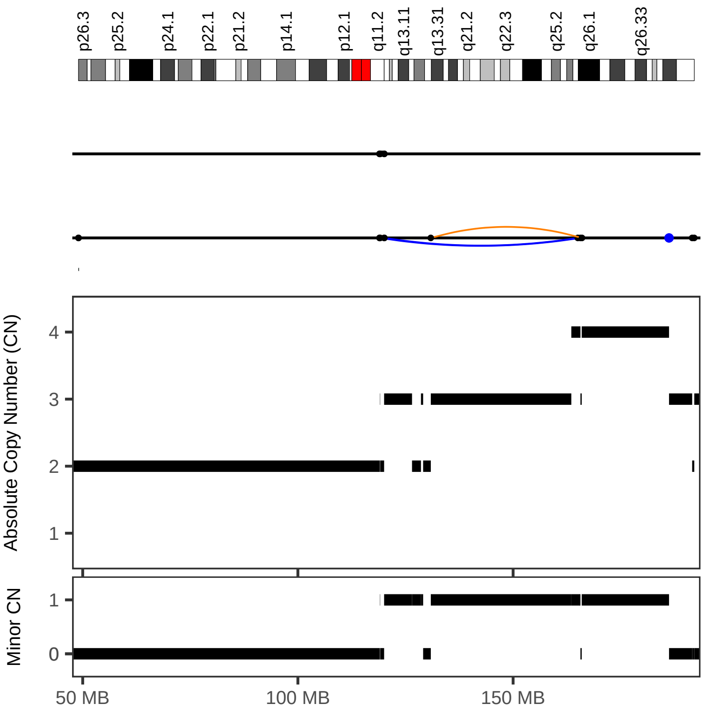

	9d95a65b−e41d−4f93−92d4−99dce29ff40d
Cancer type	Breast−AdenoCA
Position	3:118944666−165998674
Type	Canonical without polyploidization
Interleaved intrachr. SVs	8
Total SVs (intrachr. + transl.)	8
SV types	DEL: 3; DUP: 2; h2hINV: 2; t2tINV: 1; TRA: 0
SVs in sample	143
Oscillating CN (2 and 3 states)	8, 11
CN segments	11
FDR fragment joints	0.84
FDR chr. breakp. enrich.	0.75
Linked to chrs	
Purity, ploidy	0.72, 2.81

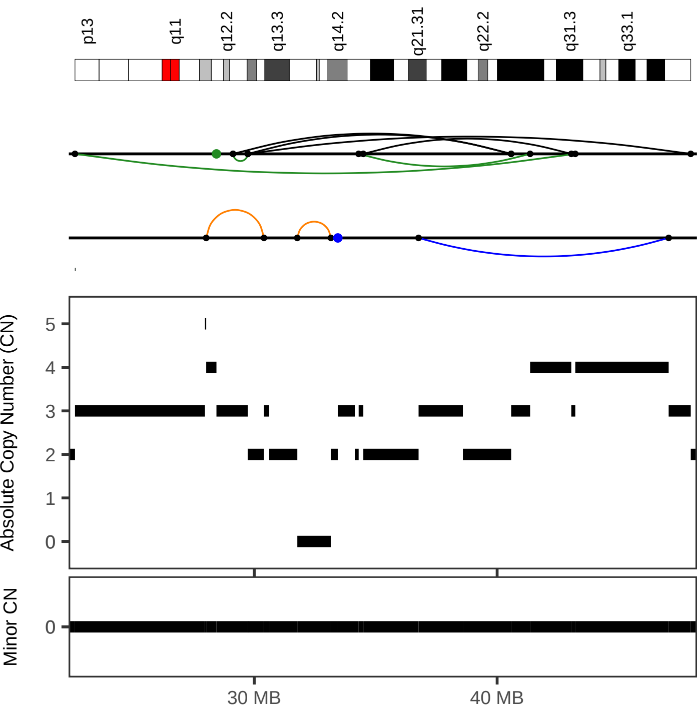

	9d95a65b−e41d−4f93−92d4−99dce29ff40d
Cancer type	Breast−AdenoCA
Position	13:22617012−47972350
Type	With other complex events
Interleaved intrachr. SVs	9
Total SVs (intrachr. + transl.)	11
SV types	DEL: 1; DUP: 1; h2hINV: 4; t2tINV: 3; TRA: 2
SVs in sample	143
Oscillating CN (2 and 3 states)	8, 13
CN segments	23
FDR fragment joints	0.59
FDR chr. breakp. enrich.	0
Linked to chrs	
Purity, ploidy	0.72, 2.81

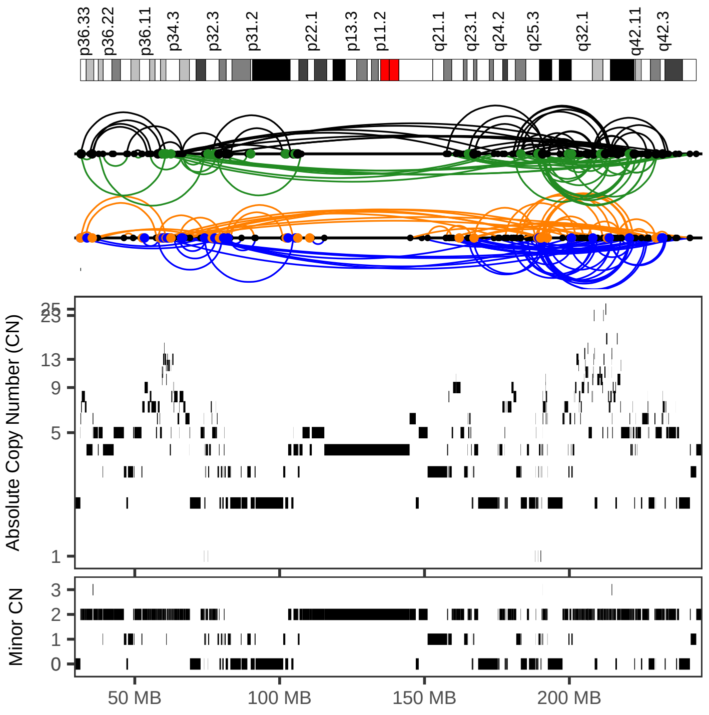

	9fefbe7c−f66a−4940−843e−285cb7b392c1
Cancer type	Breast−AdenoCA
Position	1:31254630−243789937
Type	With other complex events
Interleaved intrachr. SVs	254
Total SVs (intrachr. + transl.)	351
SV types	DEL: 64; DUP: 65; h2hINV: 60; t2tINV: 65; TRA: 97
SVs in sample	740
Oscillating CN (2 and 3 states)	16, 19
CN segments	345
FDR fragment joints	0.98
FDR chr. breakp. enrich.	0
Linked to chrs	6:5413201−164420805;
Purity, ploidy	0.83, 3.26

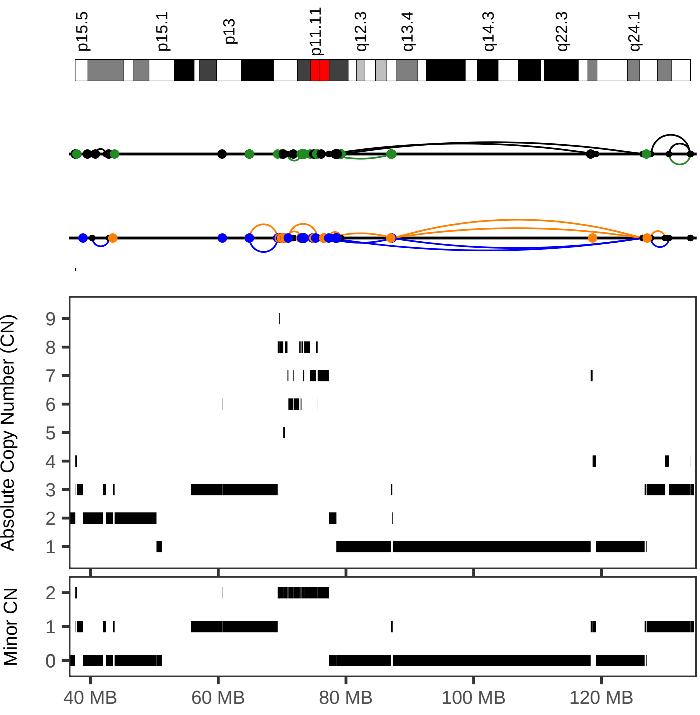

	9fefbe7c−f66a−4940−843e−285cb7b392c1
Cancer type	Breast−AdenoCA
Position	11:77294848−126487192
Type	With other complex events
Interleaved intrachr. SVs	12
Total SVs (intrachr. + transl.)	41
SV types	DEL: 4; DUP: 4; h2hINV: 2; t2tINV: 2; TRA: 29
SVs in sample	740
Oscillating CN (2 and 3 states)	10, 14
CN segments	20
FDR fragment joints	0.8
FDR chr. breakp. enrich.	0
Linked to chrs	1:31254630−243789936;6:5413201−164420805
Purity, ploidy	0.83, 3.26

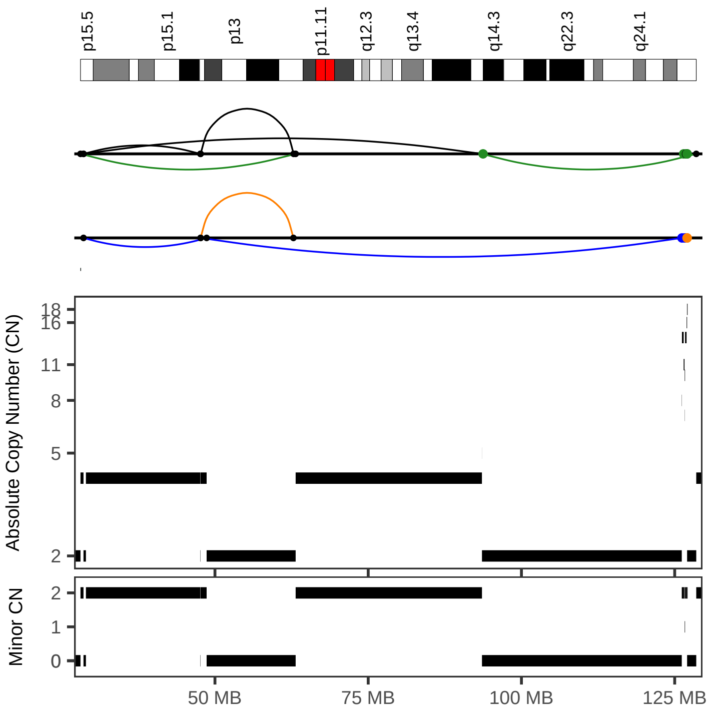

	a8015490−9740−45c9−8bd2−eb6d1beefc2e
Cancer type	Breast−AdenoCA
Position	11:28062319−128514909
Type	With other complex events
Interleaved intrachr. SVs	8
Total SVs (intrachr. + transl.)	18
SV types	DEL: 1; DUP: 2; h2hINV: 3; t2tINV: 2; TRA: 10
SVs in sample	76
Oscillating CN (2 and 3 states)	7, 8
CN segments	20
FDR fragment joints	0.84
FDR chr. breakp. enrich.	0
Linked to chrs	
Purity, ploidy	0.55, 3.31

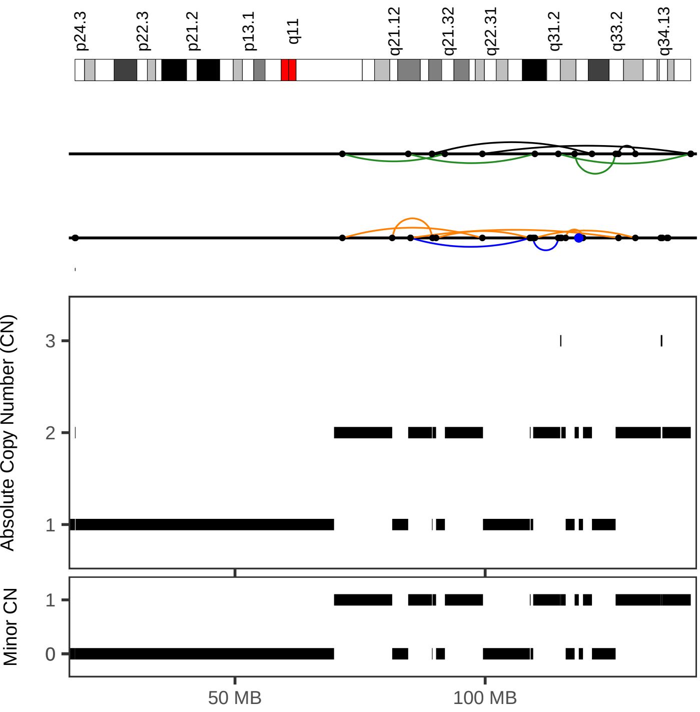

	a824b3bd−34d5−4cc1−a92f−f9d6ac0f1814
Cancer type	Breast−AdenoCA
Position	9:71455580−141089210
Type	With other complex events
Interleaved intrachr. SVs	15
Total SVs (intrachr. + transl.)	16
SV types	DEL: 6; DUP: 2; h2hINV: 3;<br>t2tINV: 4; TRA: 1
SVs in sample	463
Oscillating CN (2 and 3 states)	11, 21
CN segments	21
FDR fragment joints	0.64
FDR chr. breakp. enrich.	0.72
Linked to chrs	
Purity, ploidy	0.51, 2.1

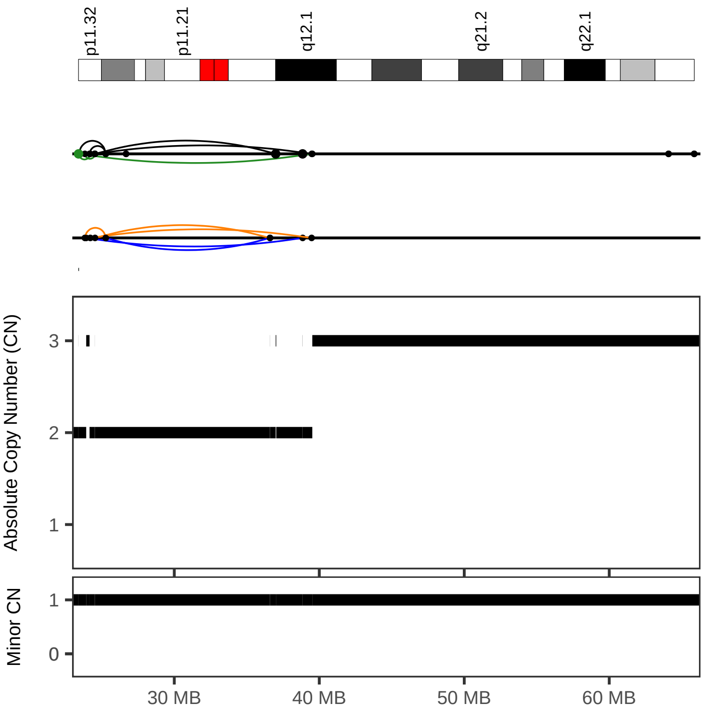

	aeb6a3a6−b57a−4c53−90e6−e833777dd2a1
Cancer type	Breast−AdenoCA
Position	18:23409571−39483311
Type	Canonical without polyploidization
Interleaved intrachr. SVs	11
Total SVs (intrachr. + transl.)	13
SV types	DEL: 3; DUP: 2; h2hINV: 4;<br>t2tINV: 2; TRA: 2
SVs in sample	117
Oscillating CN (2 and 3 states)	13, 13
CN segments	13
FDR fragment joints	0.84
FDR chr. breakp. enrich.	0
Linked to chrs	
Purity, ploidy	0.77, 2.2

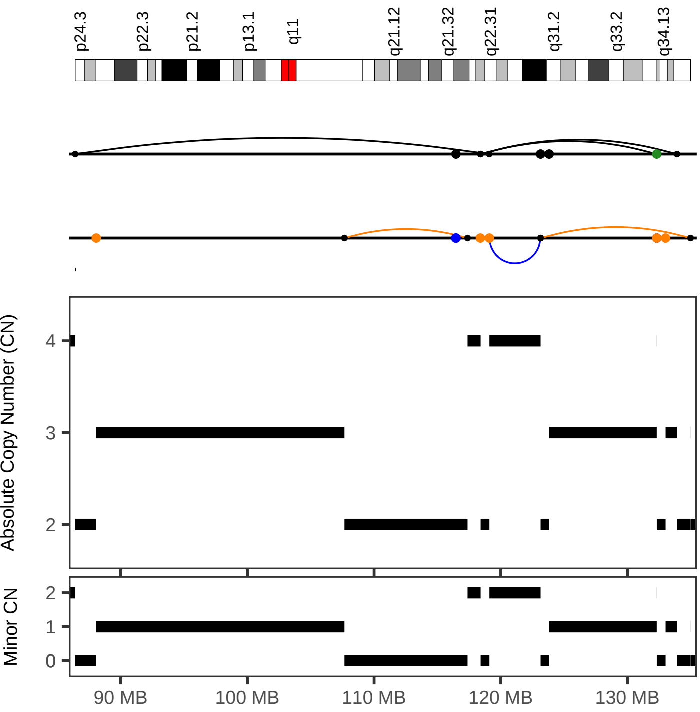

	b43e41af−1d82−4b5f−b8f1−add0510e6b86
Cancer type	Breast−AdenoCA
Position	9:86410803−134980826
Type	With other complex events
Interleaved intrachr. SVs	5
Total SVs (intrachr. + transl.)	17
SV types	DEL: 1; DUP: 1; h2hINV: 3;<br>t2tINV: 0; TRA: 12
SVs in sample	296
Oscillating CN (2 and 3 states)	7, 10
CN segments	15
FDR fragment joints	0.59
FDR chr. breakp. enrich.	0.05
Linked to chrs	2:9513684−64192181;
Purity, ploidy	0.72, 4.04

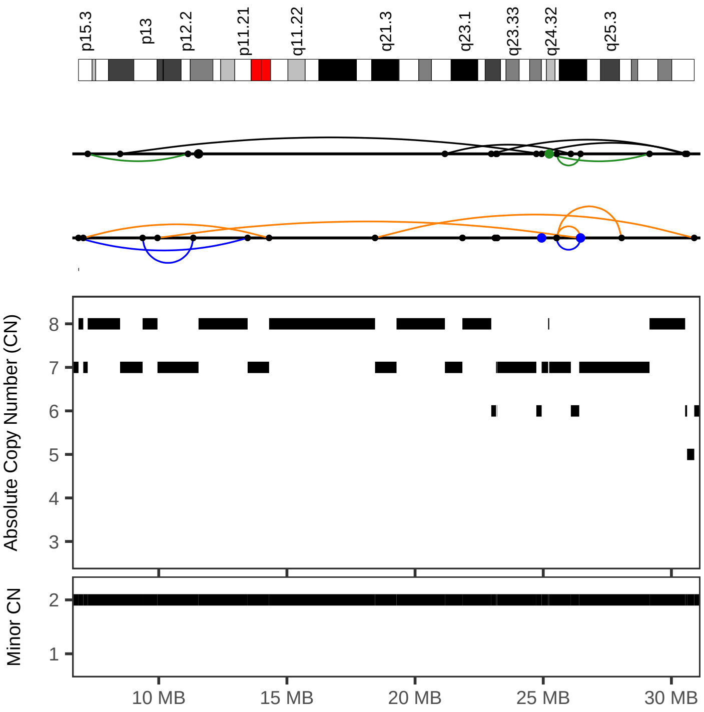

	b43e41af−1d82−4b5f−b8f1−add0510e6b86
Cancer type	Breast−AdenoCA
Position	10:6867995−30890977
Type	With other complex events
Interleaved intrachr. SVs	15
Total SVs (intrachr. + transl.)	19
SV types	DEL: 5; DUP: 3; h2hINV: 4;<br>t2tINV: 3; TRA: 4
SVs in sample	296
Oscillating CN (2 and 3 states)	13, 19
CN segments	27
FDR fragment joints	0.9
FDR chr. breakp. enrich.	0.01
Linked to chrs	
Purity, ploidy	0.72, 4.04

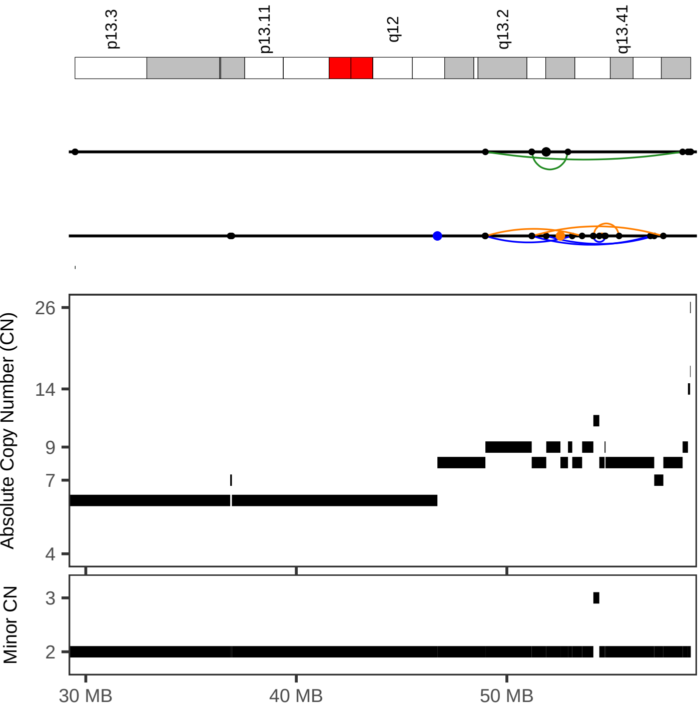

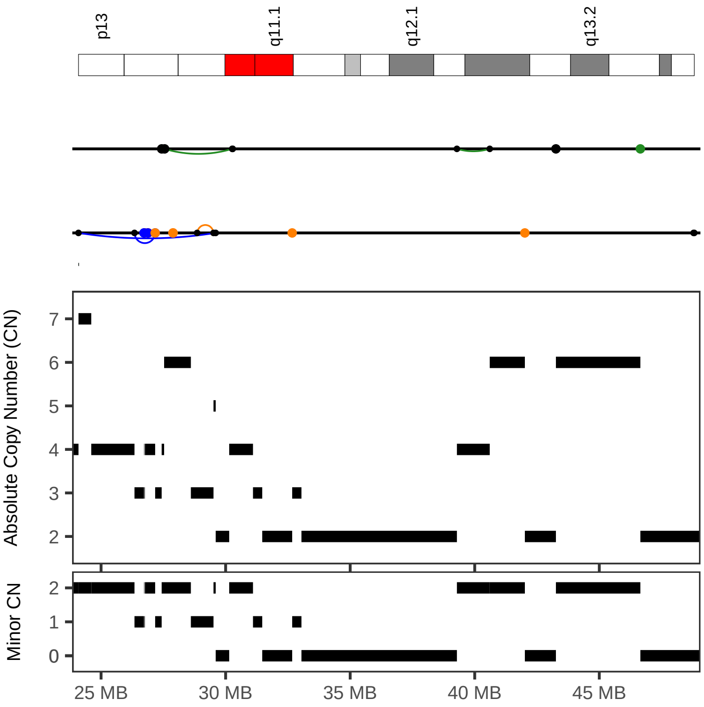

	**b43e41af−1d82−4b5f−b8f1−add0510e6b86**
Cancer type	Breast−AdenoCA
Position	19:48961354−58347531
Type	With other complex events
Interleaved intrachr. SVs	7
Total SVs (intrachr. + transl.)	9
SV types	DEL: 2; DUP: 3; h2hINV: 0; t2tINV: 2; TRA: 2
SVs in sample	296
Oscillating CN (2 and 3 states)	8, 8
CN segments	15
FDR fragment joints	0.64
FDR chr. breakp. enrich.	0
Linked to chrs	
Purity, ploidy	0.72, 4.04

	**b43e41af−1d82−4b5f−b8f1−add0510e6b86**
Cancer type	Breast−AdenoCA
Position	22:24093006−30283046
Type	With other complex events
Interleaved intrachr. SVs	3
Total SVs (intrachr. + transl.)	15
SV types	DEL: 0; DUP: 1; h2hINV: 1; t2tINV: 1; TRA: 12
SVs in sample	296
Oscillating CN (2 and 3 states)	7, 7
CN segments	13
FDR fragment joints	0.84
FDR chr. breakp. enrich.	0
Linked to chrs	20:4186490−34159089;
Purity, ploidy	0.72, 4.04

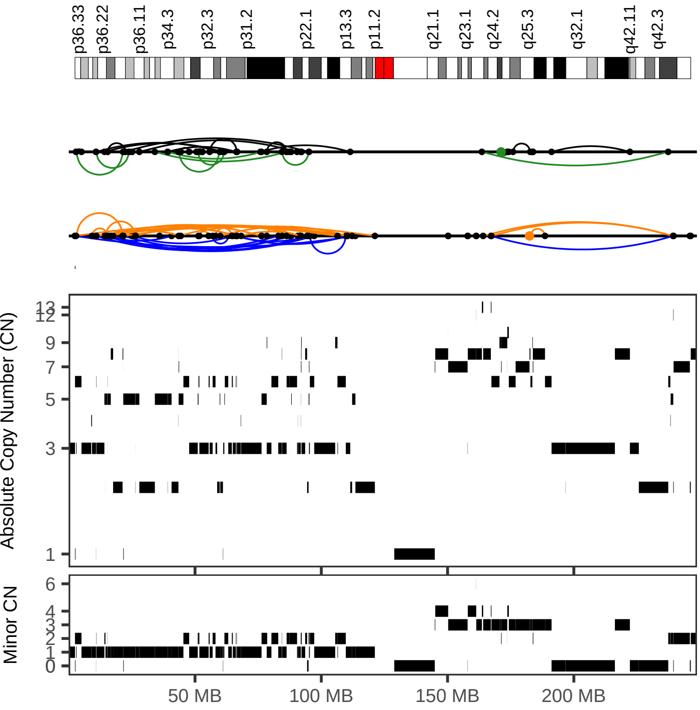

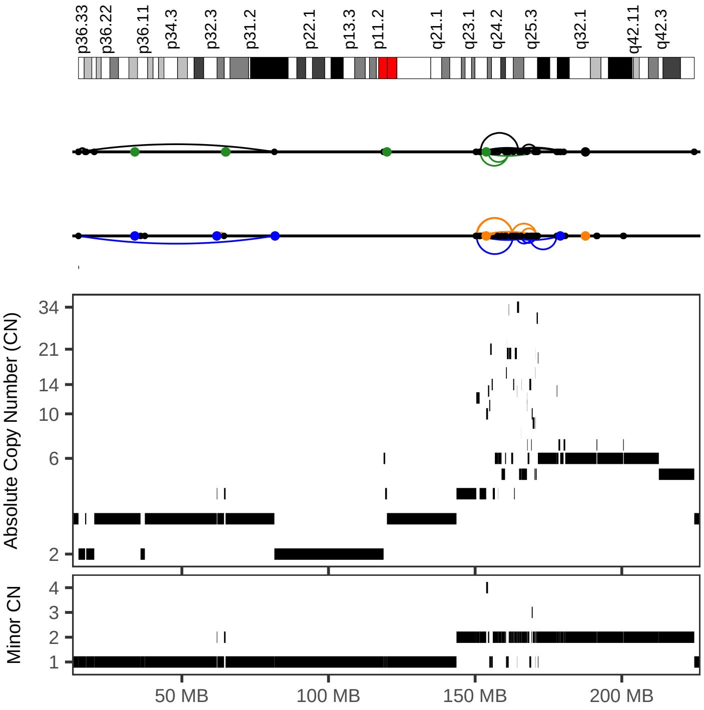

	**b97bf89a−7a85−4eef−ae7e−f787aead1f0a**
Cancer type	Breast−AdenoCA
Position	1:2468572−121208979
Type	With other complex events
Interleaved intrachr. SVs	54
Total SVs (intrachr. + transl.)	54
SV types	DEL: 19; DUP: 12; h2hINV: 14; t2tINV: 9; TRA: 0
SVs in sample	194
Oscillating CN (2 and 3 states)	8, 13
CN segments	99
FDR fragment joints	0.59
FDR chr. breakp. enrich.	0
Linked to chrs	
Purity, ploidy	0.47, 3.8

	**c0892598−1f7b−4f23−9cd8−731f797753d5**
Cancer type	Breast−AdenoCA
Position	1:150239597−180719223
Type	With other complex events
Interleaved intrachr. SVs	38
Total SVs (intrachr. + transl.)	41
SV types	DEL: 11; DUP: 12; h2hINV: 8; t2tINV: 7; TRA: 3
SVs in sample	358
Oscillating CN (2 and 3 states)	9, 9
CN segments	59
FDR fragment joints	0.69
FDR chr. breakp. enrich.	0
Linked to chrs	
Purity, ploidy	0.61, 3.55

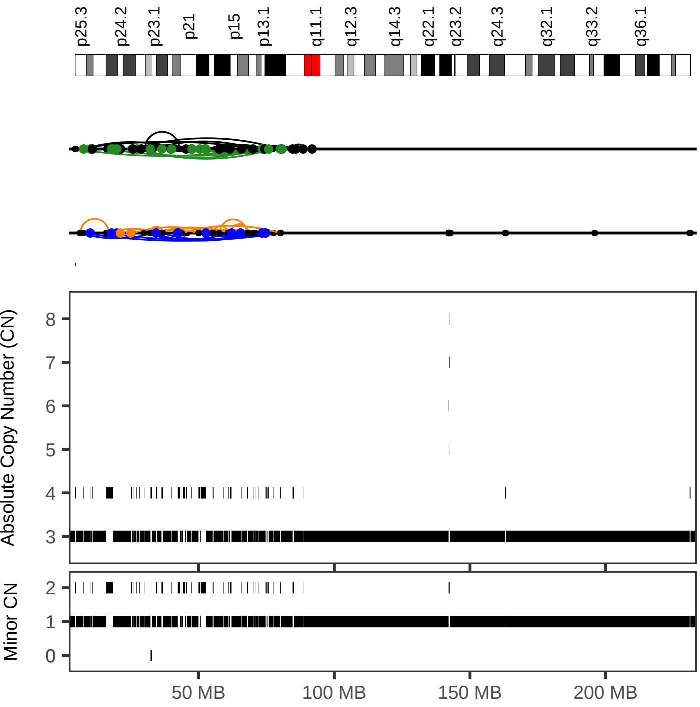

	**c0892598−1f7b−4f23−9cd8−731f797753d5**
Cancer type	Breast−AdenoCA
Position	2:4509988−88587826
Type	After polyploidization
Interleaved intrachr. SVs	39
Total SVs (intrachr. + transl.)	89
SV types	DEL: 12; DUP: 8; h2hINV: 12; t2tINV: 7; TRA: 50
SVs in sample	358
Oscillating CN (2 and 3 states)	90, 90
CN segments	90
FDR fragment joints	0.64
FDR chr. breakp. enrich.	0
Linked to chrs	19:12064068−51987941;6:107014877−166856041
Purity, ploidy	0.61, 3.55

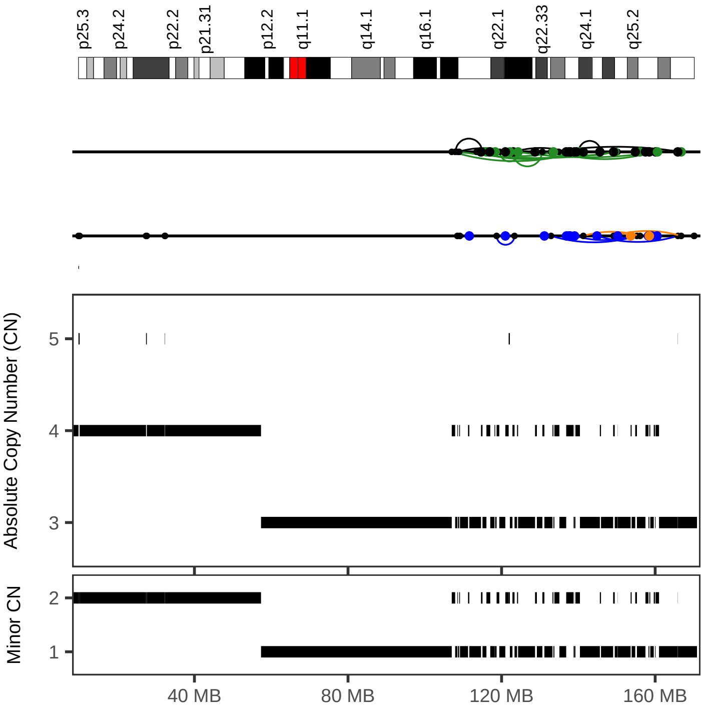

	**c0892598−1f7b−4f23−9cd8−731f797753d5**
Cancer type	Breast−AdenoCA
Position	6:107014877−166856042
Type	After polyploidization
Interleaved intrachr. SVs	23
Total SVs (intrachr. + transl.)	72
SV types	DEL: 2; DUP: 5; h2hINV: 6; t2tINV: 10; TRA: 49
SVs in sample	358
Oscillating CN (2 and 3 states)	37, 41
CN segments	57
FDR fragment joints	0.54
FDR chr. breakp. enrich.	0
Linked to chrs	2:4509988−88587825;
Purity, ploidy	0.61, 3.55

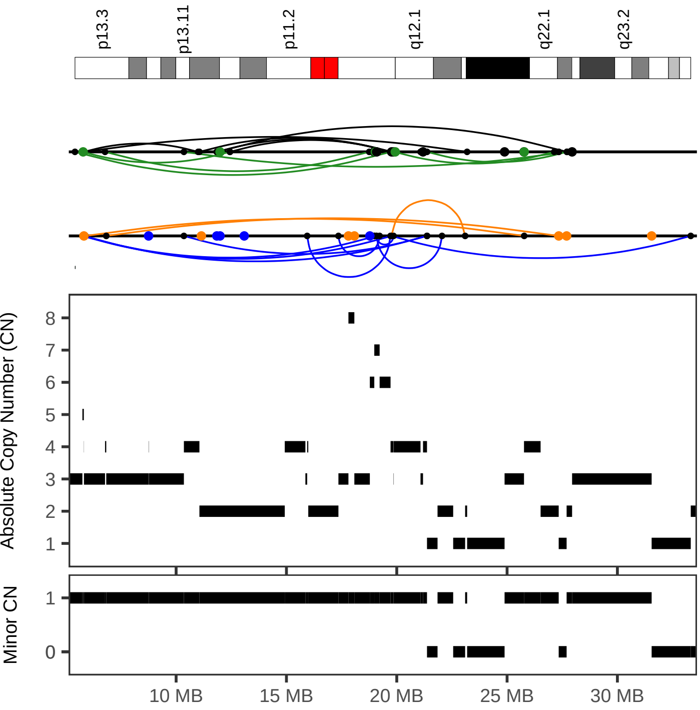

	**c364e81c−eb1e−4870−ab37−9c661f5f2e3d**
Cancer type	Breast−AdenoCA
Position	16:5415371−33325043
Type	With other complex events
Interleaved intrachr. SVs	25
Total SVs (intrachr. + transl.)	50
SV types	DEL: 3; DUP: 9; h2hINV: 6; t2tINV: 7; TRA: 25
SVs in sample	397
Oscillating CN (2 and 3 states)	7, 13
CN segments	38
FDR fragment joints	0.59
FDR chr. breakp. enrich.	0
Linked to chrs	12:56913152−101125707;20:24138933−61546428
Purity, ploidy	0.41, 2.02

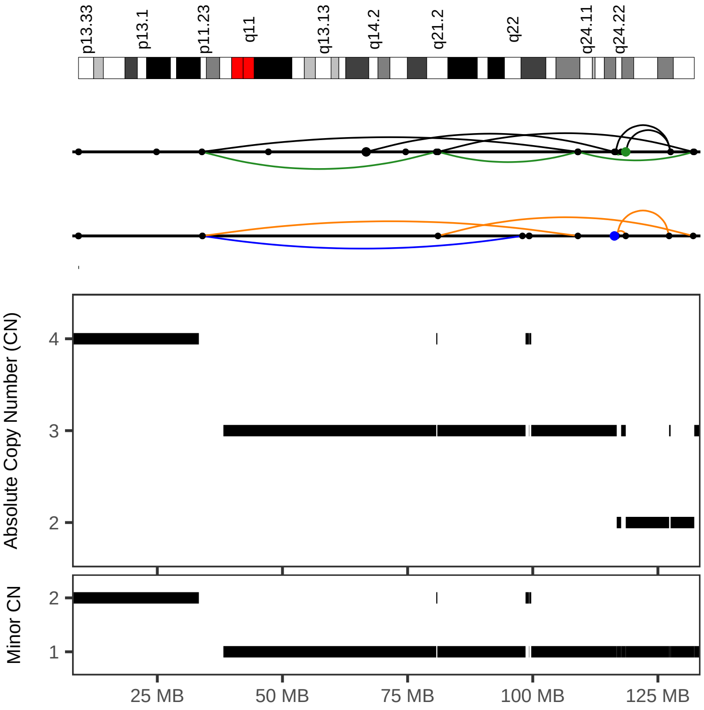

	**d67cd793−2931−429a−9084−2f3c4c8be7ad**
Cancer type	Breast−AdenoCA
Position	12:33908610−132258021
Type	With other complex events
Interleaved intrachr. SVs	10
Total SVs (intrachr. + transl.)	13
SV types	DEL: 2; DUP: 1; h2hINV: 4; t2tINV: 3; TRA: 3
SVs in sample	427
Oscillating CN (2 and 3 states)	8, 14
CN segments	14
FDR fragment joints	0.64
FDR chr. breakp. enrich.	0.28
Linked to chrs	
Purity, ploidy	0.54, 3.31

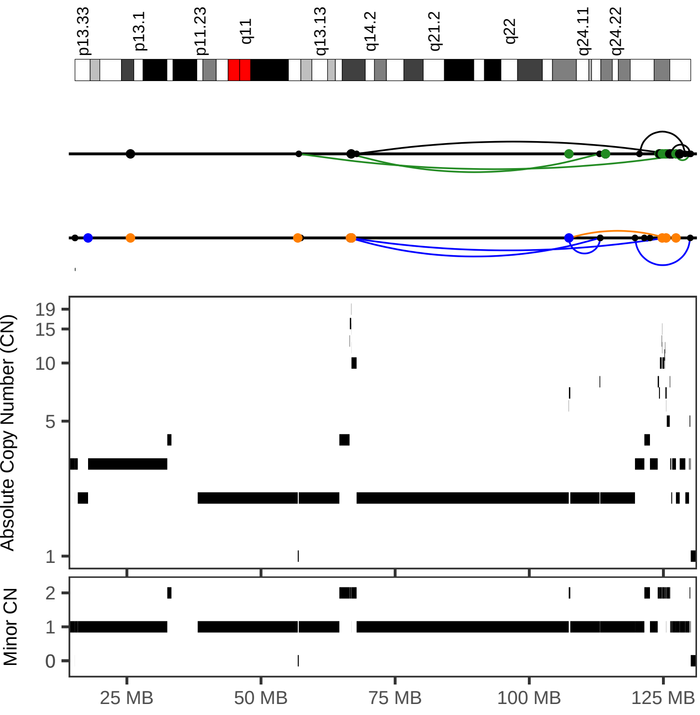

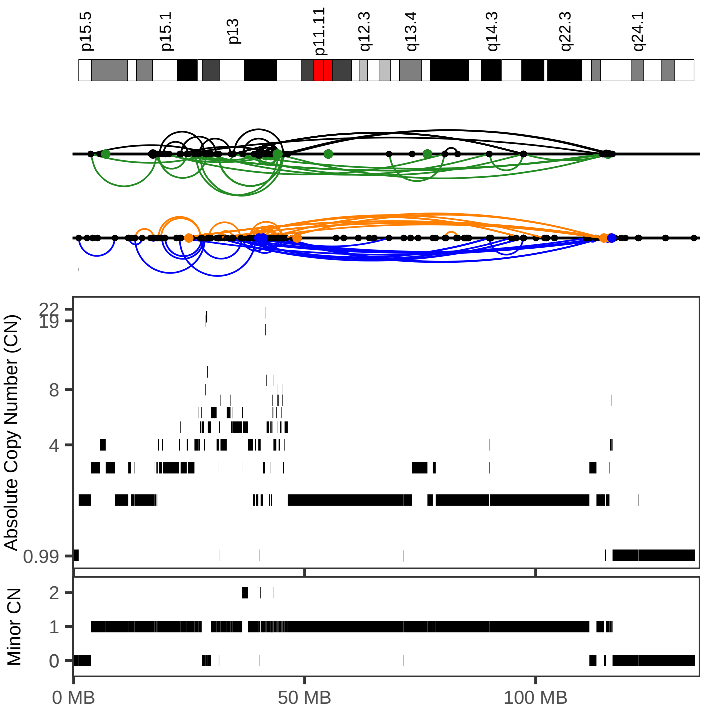

	d6f7afc0−1558−43ad−acb1−2b5311ed2264
Cancer type	Breast−AdenoCA
Position	12:66502753−130003739
Type	With other complex events
Interleaved intrachr. SVs	11
Total SVs (intrachr. + transl.)	32
SV types	DEL: 1; DUP: 5; h2hINV: 3; t2tINV: 2; TRA: 21
SVs in sample	304
Oscillating CN (2 and 3 states)	7, 7
CN segments	39
FDR fragment joints	0.59
FDR chr. breakp. enrich.	0
Linked to chrs	6:105302877−111874189;
Purity, ploidy	0.27, 2.34

	d9dc3b59−613d−469e−8b4f−6c5a557eb26a
Cancer type	Breast−AdenoCA
Position	11:1073009−116625595
Type	With other complex events
Interleaved intrachr. SVs	159
Total SVs (intrachr. + transl.)	176
SV types	DEL: 38; DUP: 51; h2hINV: 33; t2tINV: 37; TRA: 17
SVs in sample	1222
Oscillating CN (2 and 3 states)	10, 15
CN segments	139
FDR fragment joints	0.59
FDR chr. breakp. enrich.	0
Linked to chrs	3:83571470−194744513;9:100142774−123611054
Purity, ploidy	0.41, 2.07

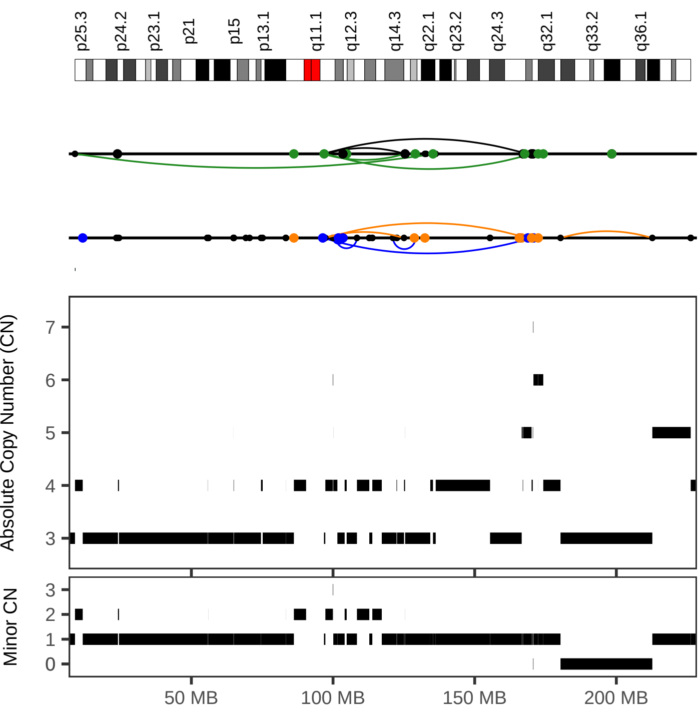

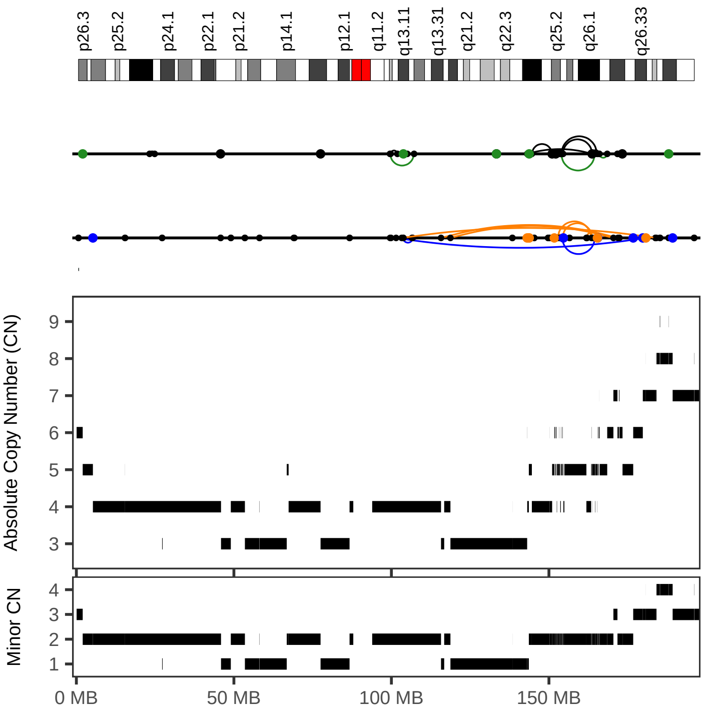

	eadf8482−e60e−4307−adb7−d5c3b9fa6cae
Cancer type	Breast−AdenoCA
Position	2:8912100−170495188
Type	With other complex events
Interleaved intrachr. SVs	9
Total SVs (intrachr. + transl.)	33
SV types	DEL: 2; DUP: 2; h2hINV: 2; t2tINV: 3; TRA: 24
SVs in sample	662
Oscillating CN (2 and 3 states)	11, 21
CN segments	46
FDR fragment joints	0.97
FDR chr. breakp. enrich.	0.76
Linked to chrs	
Purity, ploidy	0.65, 4.04

	eadf8482−e60e−4307−adb7−d5c3b9fa6cae
Cancer type	Breast−AdenoCA
Position	3:143115106−168498933
Type	With other complex events
Interleaved intrachr. SVs	17
Total SVs (intrachr. + transl.)	27
SV types	DEL: 5; DUP: 3; h2hINV: 6; t2tINV: 3; TRA: 10
SVs in sample	662
Oscillating CN (2 and 3 states)	8, 19
CN segments	43
FDR fragment joints	0.74
FDR chr. breakp. enrich.	0
Linked to chrs	11:55709941−81062380;
Purity, ploidy	0.65, 4.04

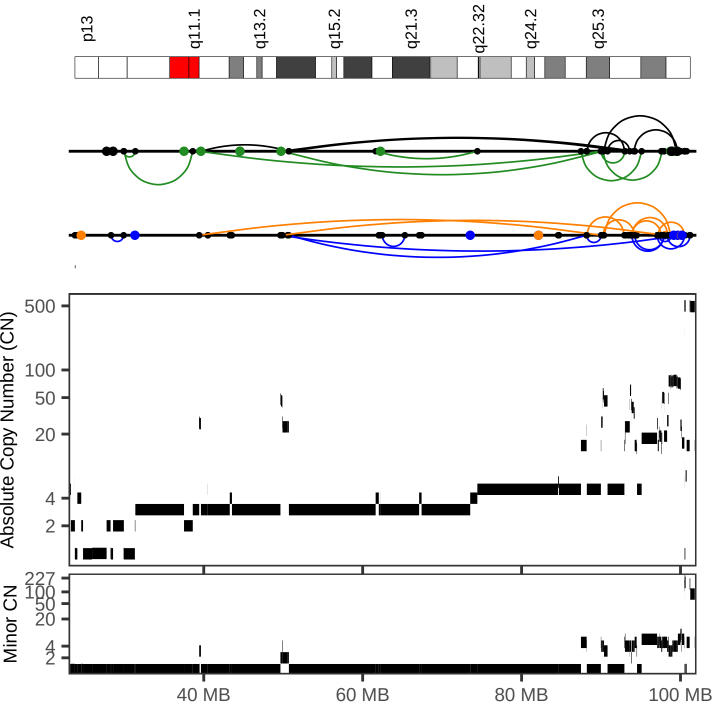

eadf8482−e60e−4307−adb7−d5c3b9fa6cae	
Cancer type	Breast−AdenoCA
Position	15:39425396−101227081
Type	With other complex events
Interleaved intrachr. SVs	37
Total SVs (intrachr. + transl.)	55
SV types	DEL: 11; DUP: 9; h2hINV: 10; t2tINV: 7; TRA: 18
SVs in sample	662
Oscillating CN (2 and 3 states)	8, 11
CN segments	107
FDR fragment joints	0.86
FDR chr. breakp. enrich.	0
Linked to chrs	13:37775397−48509222;14:20532445−36857209 17:46099635−77801969;20:2682796−61152781 7:49989231−144882102;
Purity, ploidy	0.65, 4.04

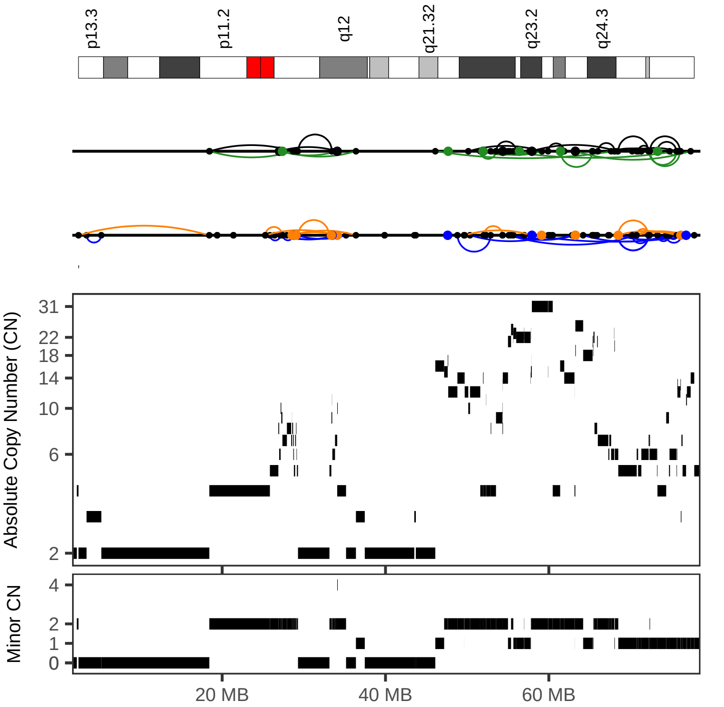

eadf8482−e60e−4307−adb7−d5c3b9fa6cae	
Cancer type	Breast−AdenoCA
Position	17:46099635−77801970
Type	With other complex events
Interleaved intrachr. SVs	47
Total SVs (intrachr. + transl.)	65
SV types	DEL: 7; DUP: 15; h2hINV: 14; t2tINV: 11; TRA: 18
SVs in sample	662
Oscillating CN (2 and 3 states)	8, 8
CN segments	91
FDR fragment joints	0.59
FDR chr. breakp. enrich.	0
Linked to chrs	13:37775397−48509222;15:39425396−101227080 2:8912100−170495187;3:143115106−168498932 6:6754753−166144214;
Purity, ploidy	0.65, 4.04

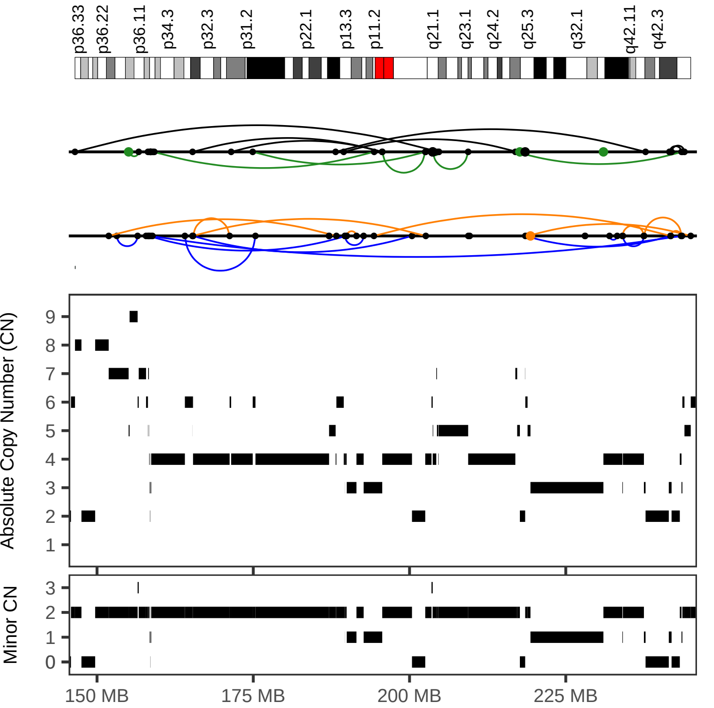

f0787165−6f58−4d67−b510−928eea2c4882	
Cancer type	Breast−AdenoCA
Position	1:146473725−244991894
Type	With other complex events
Interleaved intrachr. SVs	38
Total SVs (intrachr. + transl.)	45
SV types	DEL: 10; DUP: 9; h2hINV: 10; t2tINV: 9; TRA: 7
SVs in sample	421
Oscillating CN (2 and 3 states)	9, 10
CN segments	70
FDR fragment joints	1
FDR chr. breakp. enrich.	0
Linked to chrs	
Purity, ploidy	0.69, 3.99

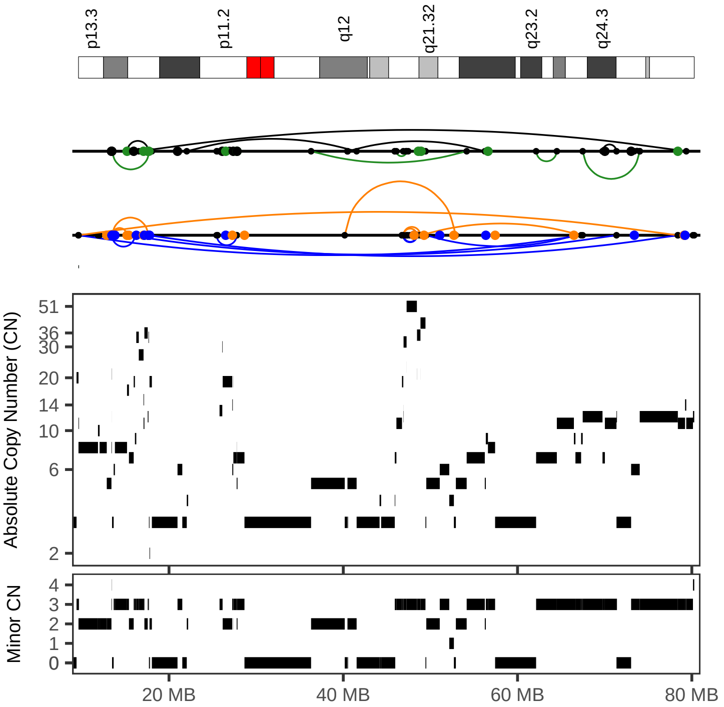

f0787165−6f58−4d67−b510−928eea2c4882	
Cancer type	Breast−AdenoCA
Position	17:22038970−67294772
Type	With other complex events
Interleaved intrachr. SVs	20
Total SVs (intrachr. + transl.)	39
SV types	DEL: 6; DUP: 5; h2hINV: 2; t2tINV: 7; TRA: 19
SVs in sample	421
Oscillating CN (2 and 3 states)	7, 10
CN segments	52
FDR fragment joints	0.63
FDR chr. breakp. enrich.	0
Linked to chrs	
Purity, ploidy	0.69, 3.99

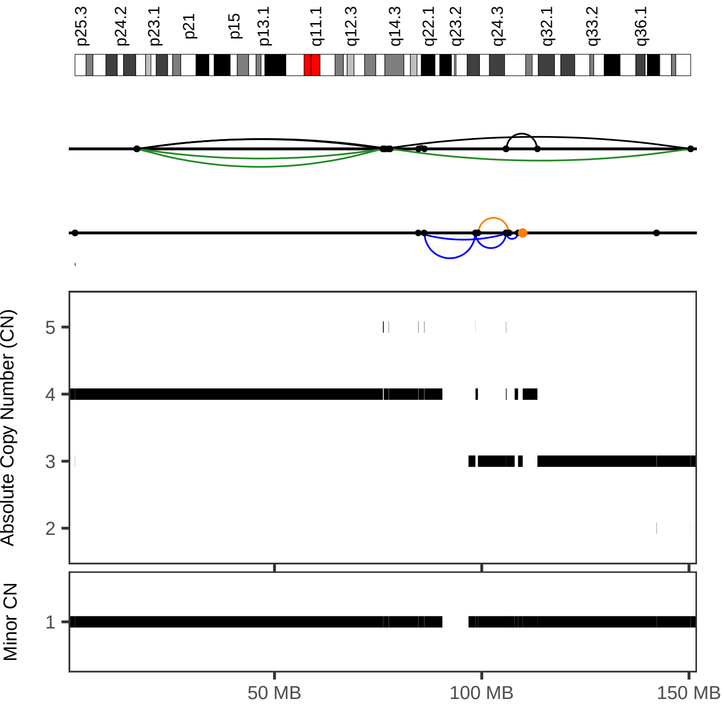

	f45210d3−9e66−4f5e−bef1−5ee5547cc893
Cancer type	Breast−AdenoCA
Position	2:16757311−150416613
Type	With other complex events
Interleaved intrachr. SVs	8
Total SVs (intrachr. + transl.)	9
SV types	DEL: 0; DUP: 0; h2hINV: 4; t2tINV: 4; TRA: 1
SVs in sample	179
Oscillating CN (2 and 3 states)	13, 13
CN segments	31
FDR fragment joints	0.46
FDR chr. breakp. enrich.	0.39
Linked to chrs	
Purity, ploidy	0.84, 3.82

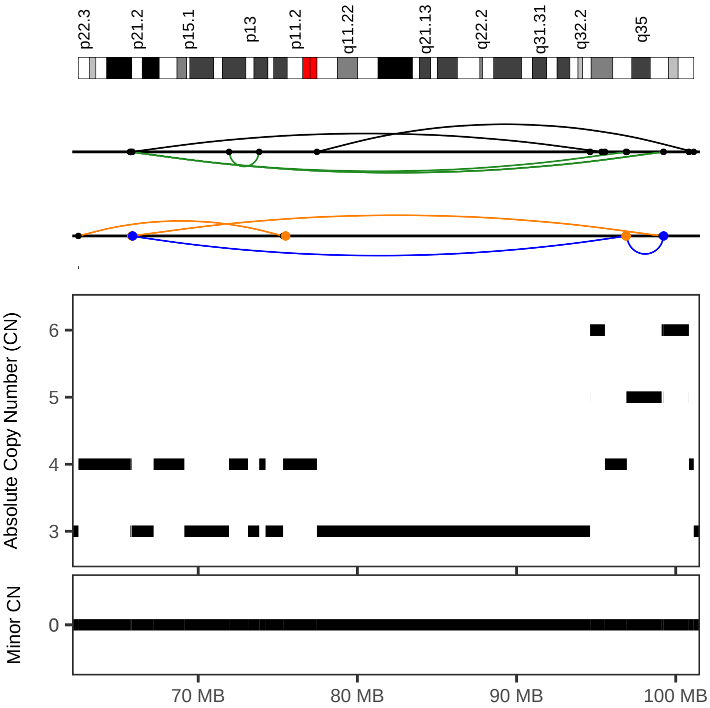

	f45210d3−9e66−4f5e−bef1−5ee5547cc893
Cancer type	Breast−AdenoCA
Position	7:62474285−101131925
Type	After polyploidization
Interleaved intrachr. SVs	11
Total SVs (intrachr. + transl.)	17
SV types	DEL: 2; DUP: 2; h2hINV: 3; t2tINV: 4; TRA: 6
SVs in sample	179
Oscillating CN (2 and 3 states)	18, 19
CN segments	30
FDR fragment joints	0.84
FDR chr. breakp. enrich.	0
Linked to chrs	
Purity, ploidy	0.84, 3.82

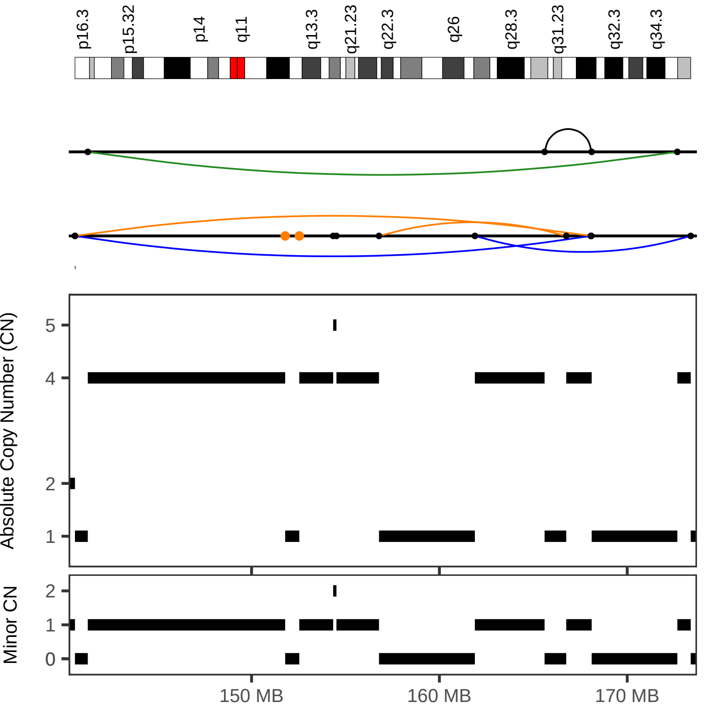

	f6e1ec78−5ad9−4879−9b7e−262d17b166ad
Cancer type	Breast−AdenoCA
Position	4:140599465−173380457
Type	Before polyploidization
Interleaved intrachr. SVs	6
Total SVs (intrachr. + transl.)	8
SV types	DEL: 2; DUP: 2; h2hINV: 1; t2tINV: 1; TRA: 2
SVs in sample	320
Oscillating CN (2 and 3 states)	8, 13
CN segments	13
FDR fragment joints	0.91
FDR chr. breakp. enrich.	0.07
Linked to chrs	
Purity, ploidy	0.53, 2.23

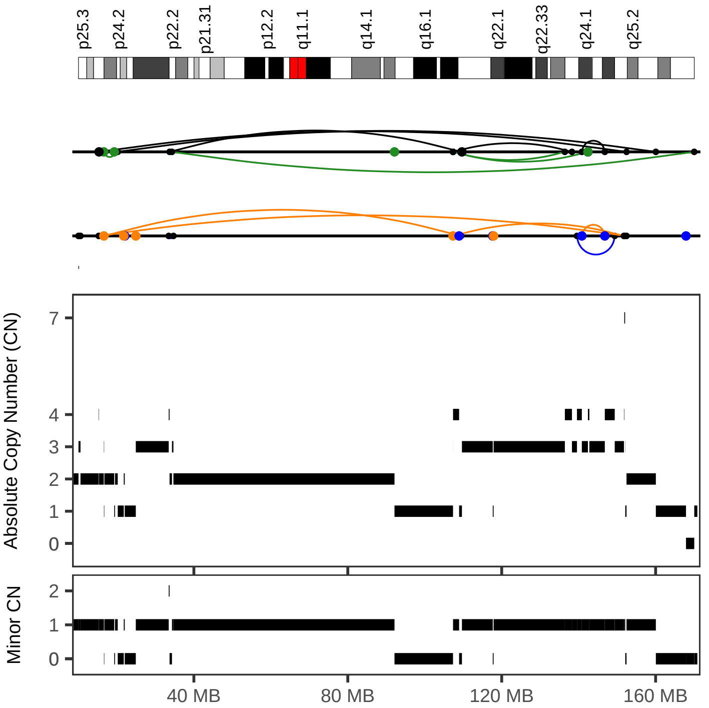

	f6e1ec78−5ad9−4879−9b7e−262d17b166ad
Cancer type	Breast−AdenoCA
Position	6:15272594−170042856
Type	With other complex events
Interleaved intrachr. SVs	14
Total SVs (intrachr. + transl.)	32
SV types	DEL: 4; DUP: 2; h2hINV: 5; t2tINV: 3; TRA: 18
SVs in sample	320
Oscillating CN (2 and 3 states)	10, 11
CN segments	41
FDR fragment joints	0.78
FDR chr. breakp. enrich.	0.01
Linked to chrs	
Purity, ploidy	0.53, 2.23

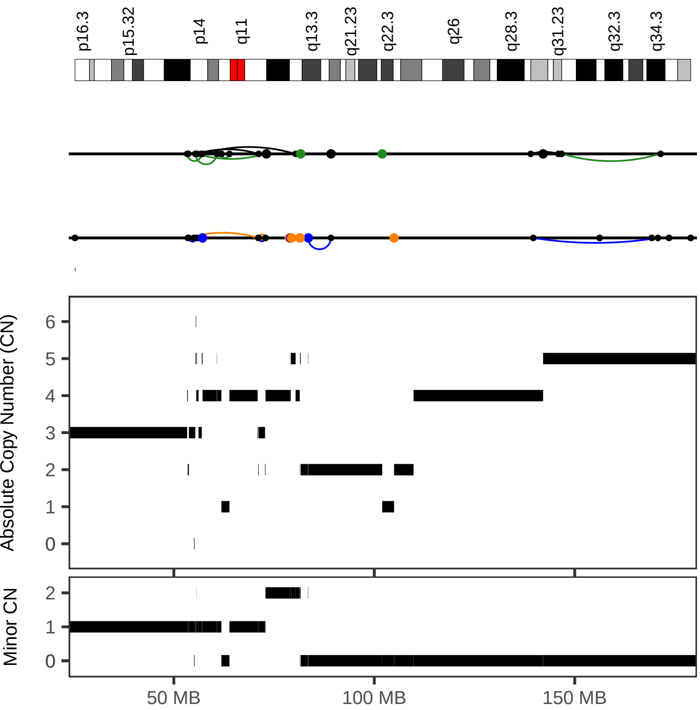

CGP_donor_1234120	
Cancer type	Breast−LobularCA
Position	4:53312523−80369667
Type	With other complex events
Interleaved intrachr. SVs	13
Total SVs (intrachr. + transl.)	18
SV types	DEL: 2; DUP: 3; h2hINV: 4; t2tINV: 4; TRA: 5
SVs in sample	344
Oscillating CN (2 and 3 states)	7, 7
CN segments	29
FDR fragment joints	0.88
FDR chr. breakp. enrich.	0.01
Linked to chrs	
Purity, ploidy	0.39, 2.94

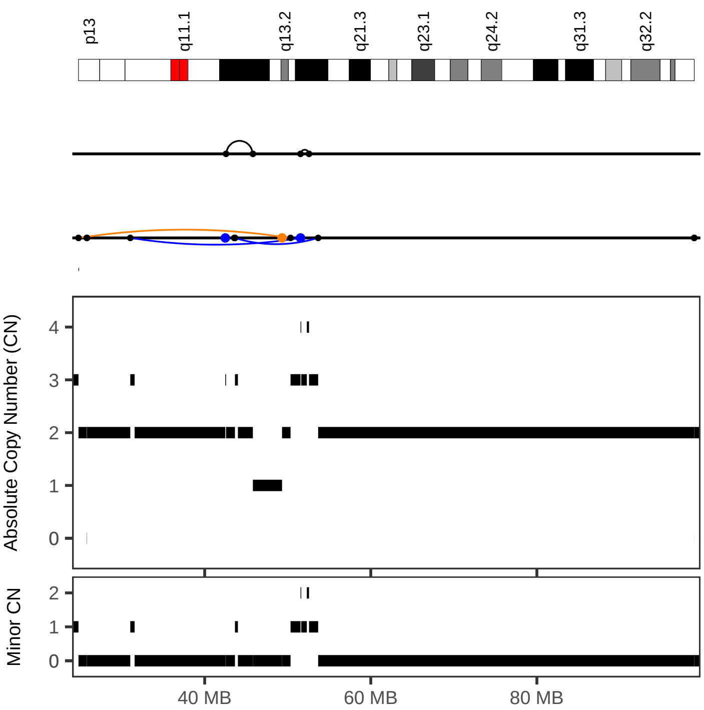

CGP_donor_1234120	
Cancer type	Breast−LobularCA
Position	14:24801053−53663407
Type	With other complex events
Interleaved intrachr. SVs	5
Total SVs (intrachr. + transl.)	8
SV types	DEL: 1; DUP: 2; h2hINV: 2; t2tINV: 0; TRA: 3
SVs in sample	344
Oscillating CN (2 and 3 states)	7, 15
CN segments	17
FDR fragment joints	0.64
FDR chr. breakp. enrich.	1
Linked to chrs	
Purity, ploidy	0.39, 2.94

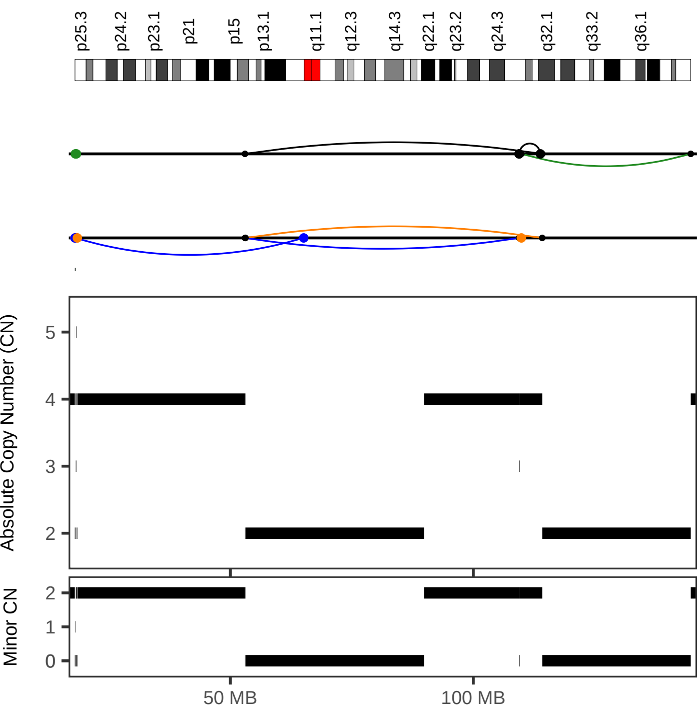

CGP_donor_1347756	
Cancer type	Breast−LobularCA
Position	2:18030650−144747582
Type	With other complex events
Interleaved intrachr. SVs	6
Total SVs (intrachr. + transl.)	19
SV types	DEL: 1; DUP: 2; h2hINV: 2; t2tINV: 1; TRA: 13
SVs in sample	114
Oscillating CN (2 and 3 states)	7, 11
CN segments	17
FDR fragment joints	0.91
FDR chr. breakp. enrich.	0
Linked to chrs	6:99814201−149683676;8:11620647−85317946 13:72316685−73246808;
Purity, ploidy	0.37, 3.69

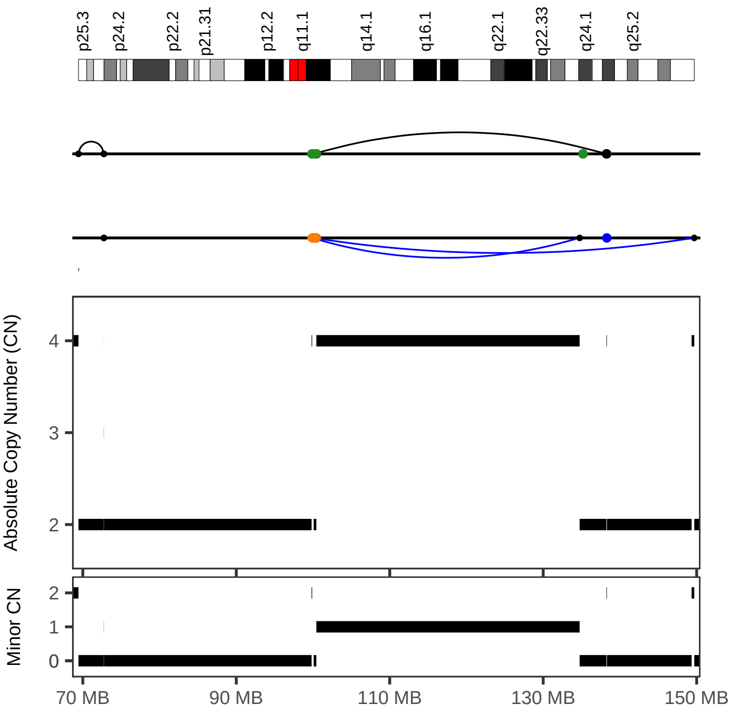

CGP_donor_1347756	
Cancer type	Breast−LobularCA
Position	6:99814201−149683677
Type	Before polyploidization
Interleaved intrachr. SVs	3
Total SVs (intrachr. + transl.)	18
SV types	DEL: 0; DUP: 2; h2hINV: 1; t2tINV: 0; TRA: 15
SVs in sample	114
Oscillating CN (2 and 3 states)	10, 10
CN segments	10
FDR fragment joints	0.59
FDR chr. breakp. enrich.	0
Linked to chrs	2:18030650−144747581;8:11620647−85317946
Purity, ploidy	0.37, 3.69

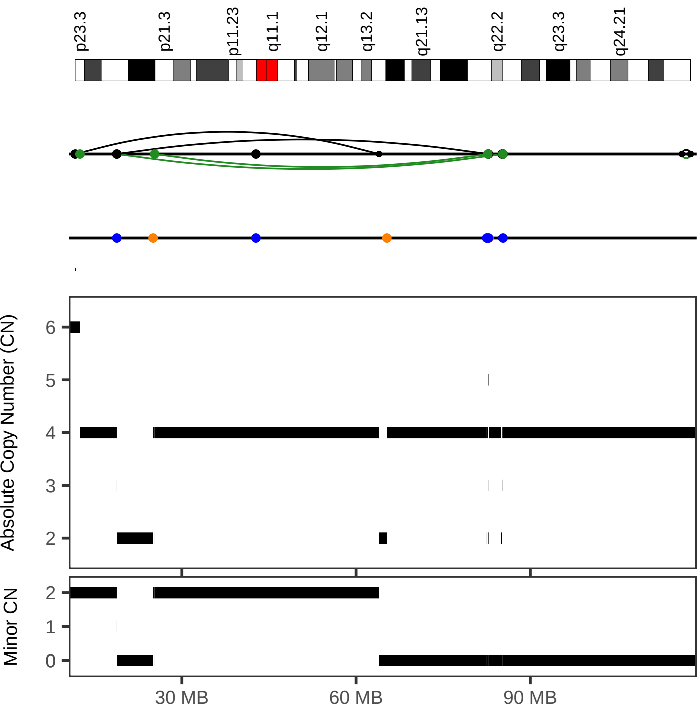

	**CGP_donor_1347756**
Cancer type	Breast−LobularCA
Position	8:11620647−85317947
Type	With other complex events
Interleaved intrachr. SVs	5
Total SVs (intrachr. + transl.)	27
SV types	DEL: 1; DUP: 0; h2hINV: 2; t2tINV: 2; TRA: 22
SVs in sample	114
Oscillating CN (2 and 3 states)	9, 11
CN segments	25
FDR fragment joints	0.64
FDR chr. breakp. enrich.	0
Linked to chrs	2:18030650−144747581;6:99814201−149683676 10:47144724−120650613;13:72316685−73246808
Purity, ploidy	0.37, 3.69

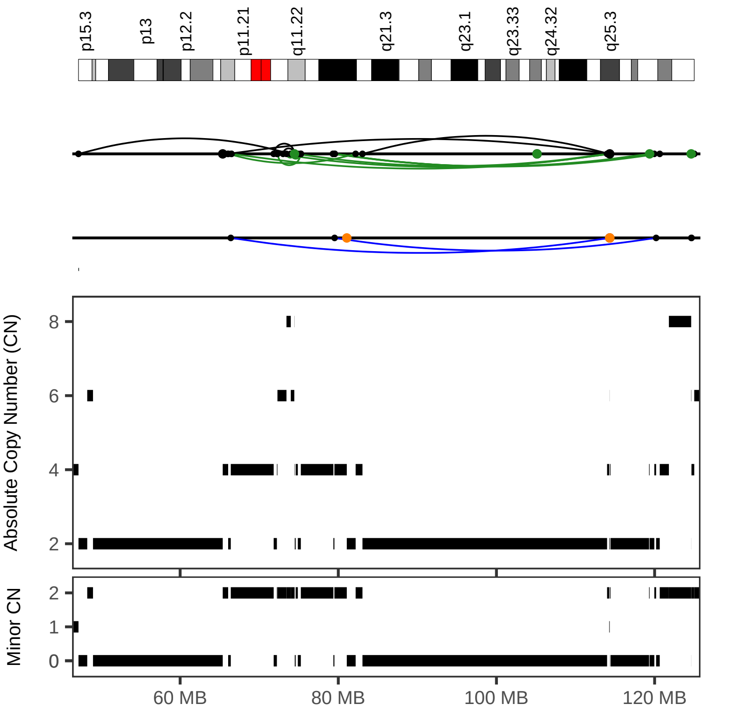

	**CGP_donor_1347756**
Cancer type	Breast−LobularCA
Position	10:47144724−120650614
Type	With other complex events
Interleaved intrachr. SVs	22
Total SVs (intrachr. + transl.)	32
SV types	DEL: 0; DUP: 4; h2hINV: 8; t2tINV: 10; TRA: 10
SVs in sample	114
Oscillating CN (2 and 3 states)	12, 21
CN segments	33
FDR fragment joints	0.24
FDR chr. breakp. enrich.	0
Linked to chrs	
Purity, ploidy	0.37, 3.69

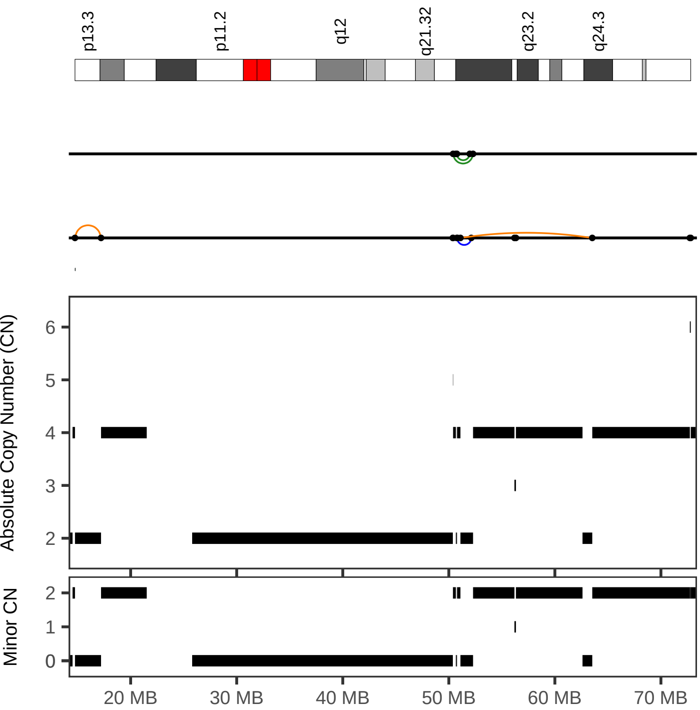

	**27b05b15−a44b−45ed−a6e3−e7d1ca488ea9**
Cancer type	Breast−LobularCA
Position	17:50377400−63525147
Type	Before polyploidization
Interleaved intrachr. SVs	7
Total SVs (intrachr. + transl.)	7
SV types	DEL: 2; DUP: 2; h2hINV: 1; t2tINV: 2; TRA: 0
SVs in sample	116
Oscillating CN (2 and 3 states)	9, 13
CN segments	14
FDR fragment joints	0.95
FDR chr. breakp. enrich.	0.01
Linked to chrs	
Purity, ploidy	0.31, 3.39

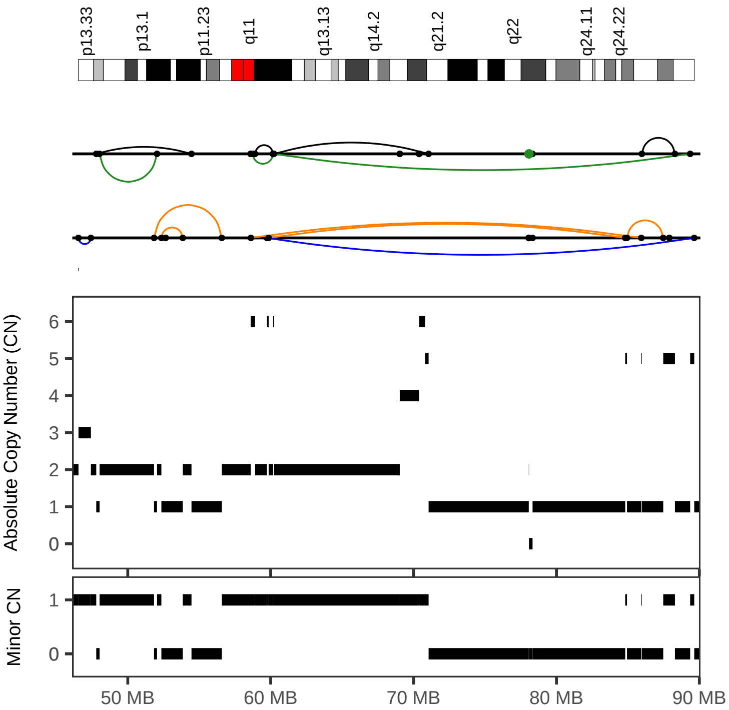

	**a482fc4c−ff1d−46a0−a968−7df06422cc4b**
Cancer type	Breast−LobularCA
Position	12:58617819−89640811
Type	With other complex events
Interleaved intrachr. SVs	8
Total SVs (intrachr. + transl.)	9
SV types	DEL: 3; DUP: 1; h2hINV: 2; t2tINV: 2; TRA: 1
SVs in sample	86
Oscillating CN (2 and 3 states)	9, 9
CN segments	21
FDR fragment joints	0.84
FDR chr. breakp. enrich.	0
Linked to chrs	
Purity, ploidy	0.51, 1.84

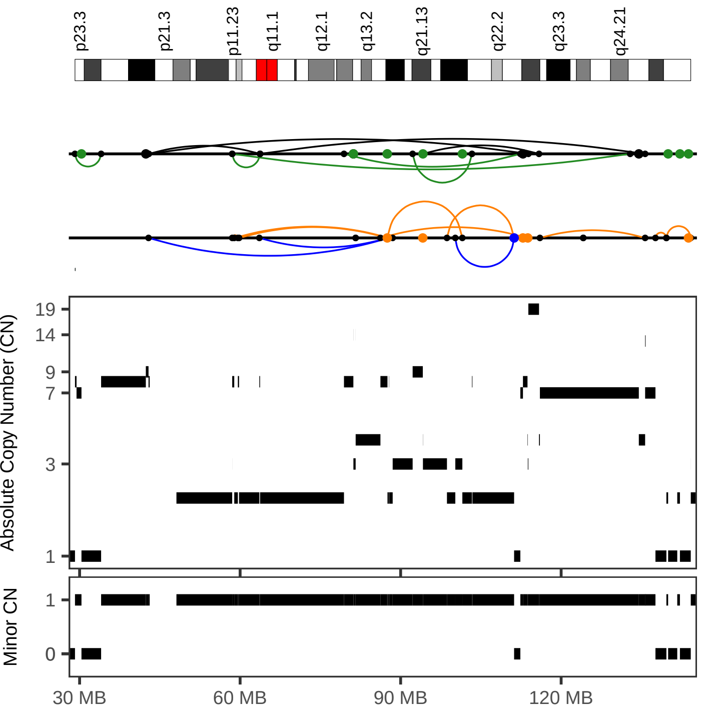

	**ef4cbd38−bc79−4d60−a715−647edd2ebe9e**
Cancer type	Breast−LobularCA
Position	8:42881572−135695930
Type	With other complex events
Interleaved intrachr. SVs	19
Total SVs (intrachr. + transl.)	32
SV types	DEL: 8; DUP: 3; h2hINV: 4;<br>t2tINV: 4; TRA: 13
SVs in sample	134
Oscillating CN (2 and 3 states)	7, 7
CN segments	41
FDR fragment joints	0.59
FDR chr. breakp. enrich.	0
Linked to chrs	13:19943737−57122358;
Purity, ploidy	0.77, 3.15

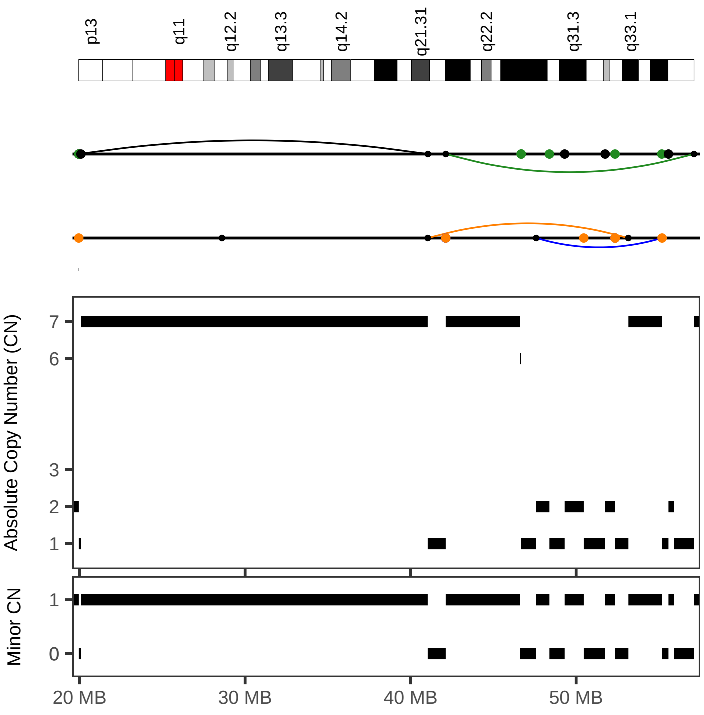

	**ef4cbd38−bc79−4d60−a715−647edd2ebe9e**
Cancer type	Breast−LobularCA
Position	13:19943737−57122359
Type	With other complex events
Interleaved intrachr. SVs	4
Total SVs (intrachr. + transl.)	23
SV types	DEL: 1; DUP: 1; h2hINV: 1;<br>t2tINV: 1; TRA: 19
SVs in sample	134
Oscillating CN (2 and 3 states)	7, 8
CN segments	21
FDR fragment joints	1
FDR chr. breakp. enrich.	0
Linked to chrs	8:42881572−135695929;
Purity, ploidy	0.77, 3.15

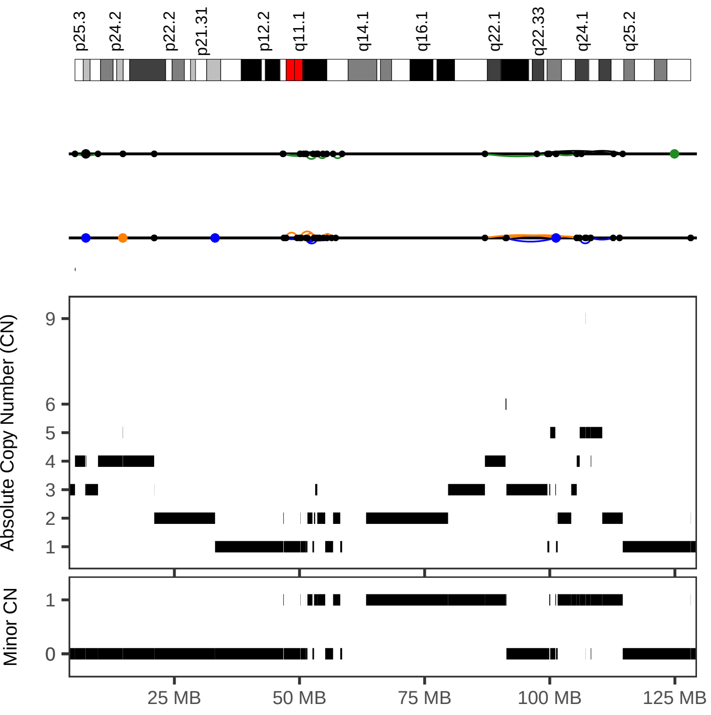

	**7c93642d−7e05−40f8−b1ef−a014edcfba42**
Cancer type	Cervix−SCC
Position	6:46659231−58507397
Type	Canonical without polyploidization
Interleaved intrachr. SVs	16
Total SVs (intrachr. + transl.)	16
SV types	DEL: 3; DUP: 6; h2hINV: 1;<br>t2tINV: 6; TRA: 0
SVs in sample	138
Oscillating CN (2 and 3 states)	10, 16
CN segments	16
FDR fragment joints	0.59
FDR chr. breakp. enrich.	0
Linked to chrs	
Purity, ploidy	0.62, 1.67

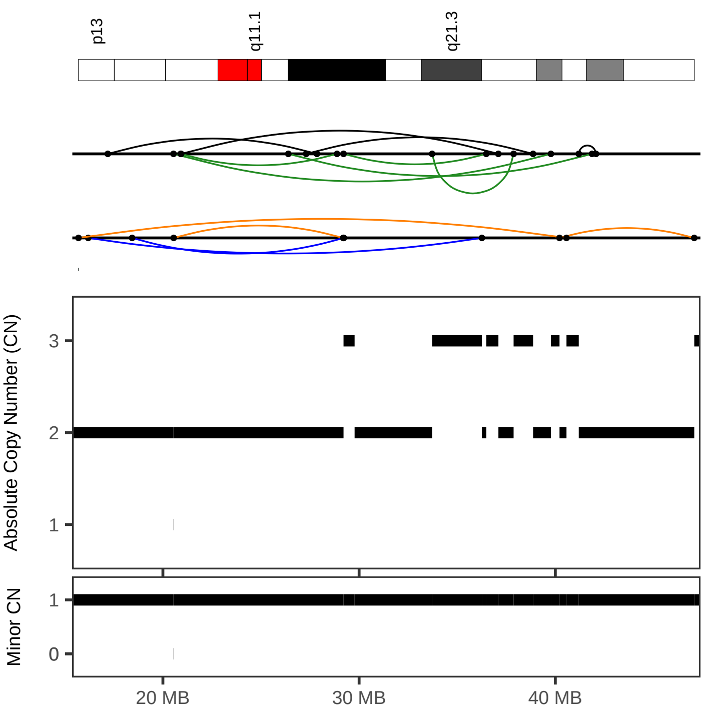

	**dfd30f94−19e3−4838−95df−14a3763419af**
Cancer type	Cervix−SCC
Position	21:15698147−47079851
Type	Canonical without polyploidization
Interleaved intrachr. SVs	14
Total SVs (intrachr. + transl.)	14
SV types	DEL: 3; DUP: 2; h2hINV: 4;<br>t2tINV: 5; TRA: 0
SVs in sample	48
Oscillating CN (2 and 3 states)	14, 16
CN segments	16
FDR fragment joints	0.78
FDR chr. breakp. enrich.	0
Linked to chrs	
Purity, ploidy	0.66, 2.12

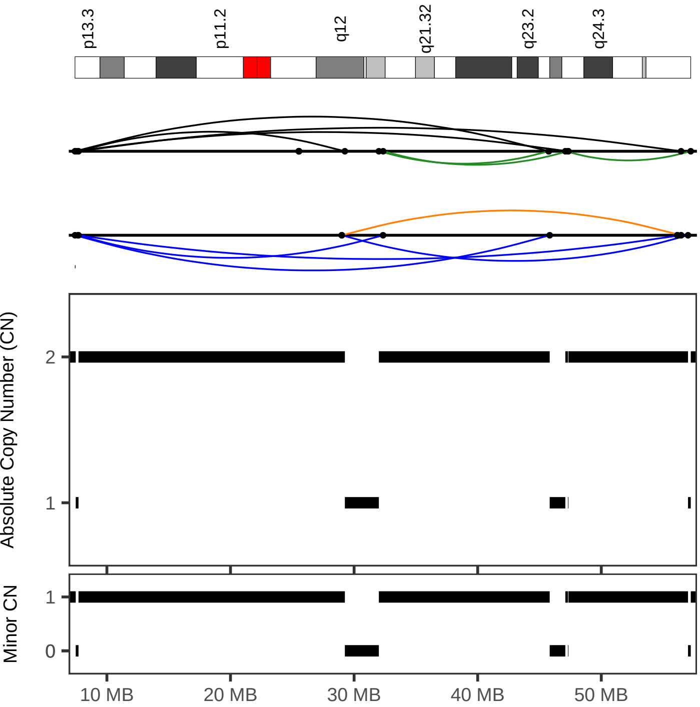

	0078b0c4−68a9−483b−9aab−61156d263213
Cancer type	CNS−GBM
Position	17:7417765−57239409
Type	Canonical without polyploidization
Interleaved intrachr. SVs	12
Total SVs (intrachr. + transl.)	12
SV types	DEL: 1; DUP: 4; h2hINV: 4; t2tINV: 3; TRA: 0
SVs in sample	78
Oscillating CN (2 and 3 states)	11, 11
CN segments	11
FDR fragment joints	0.64
FDR chr. breakp. enrich.	0
Linked to chrs	
Purity, ploidy	0.95, 1.78

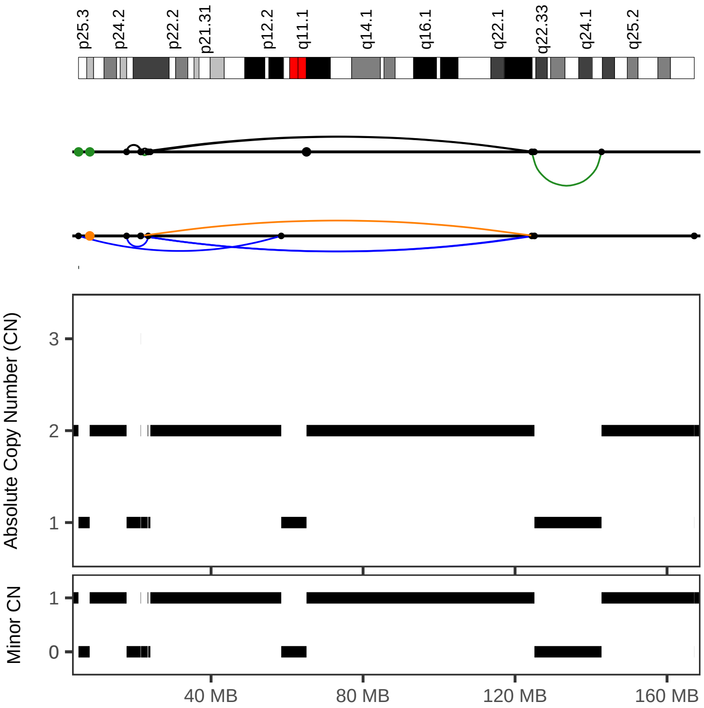

	01a92062−967a−4900−8dc7−a5ecd3b3f8e2
Cancer type	CNS−GBM
Position	6:5106696−142756791
Type	With other complex events
Interleaved intrachr. SVs	15
Total SVs (intrachr. + transl.)	20
SV types	DEL: 1; DUP: 4; h2hINV: 5; t2tINV: 5; TRA: 5
SVs in sample	98
Oscillating CN (2 and 3 states)	10, 17
CN segments	17
FDR fragment joints	0.62
FDR chr. breakp. enrich.	0
Linked to chrs	5:30169917−33114490;
Purity, ploidy	0.2, 2.05

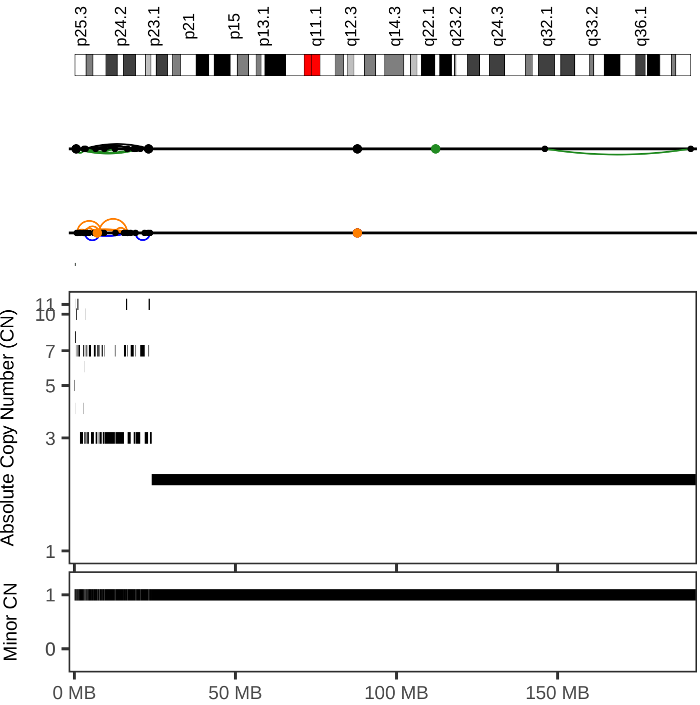

	054f472f−98cb−4559−b2e2−b5f800fc8eef
Cancer type	CNS−GBM
Position	2:176323−23468097
Type	With other complex events
Interleaved intrachr. SVs	22
Total SVs (intrachr. + transl.)	25
SV types	DEL: 10; DUP: 4; h2hINV: 4; t2tINV: 4; TRA: 3
SVs in sample	85
Oscillating CN (2 and 3 states)	19, 32
CN segments	49
FDR fragment joints	0.59
FDR chr. breakp. enrich.	0
Linked to chrs	
Purity, ploidy	0.82, 1.89

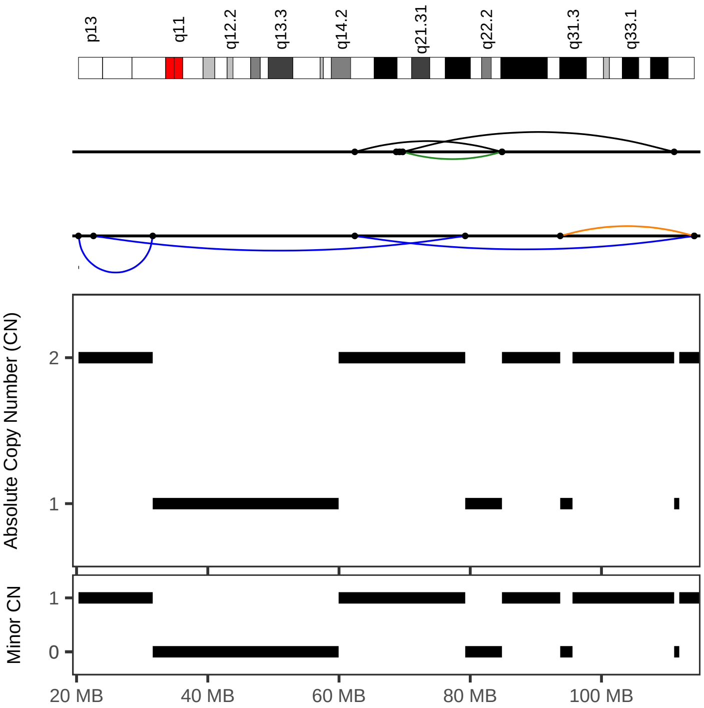

	054f472f−98cb−4559−b2e2−b5f800fc8eef
Cancer type	CNS−GBM
Position	13:20289812−114141768
Type	Canonical without polyploidization
Interleaved intrachr. SVs	7
Total SVs (intrachr. + transl.)	7
SV types	DEL: 1; DUP: 3; h2hINV: 2; t2tINV: 1; TRA: 0
SVs in sample	85
Oscillating CN (2 and 3 states)	9, 9
CN segments	9
FDR fragment joints	0.74
FDR chr. breakp. enrich.	0.02
Linked to chrs	
Purity, ploidy	0.82, 1.89

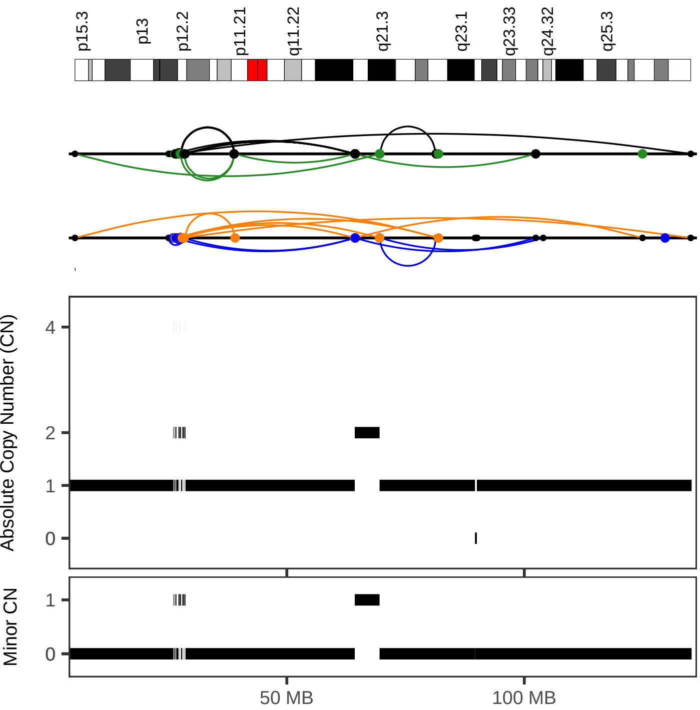

	**0798dbe2−1914−427c−a2fe−2a865d0d6eda**
Cancer type	CNS−GBM
Position	10:5388962−135052041
Type	With other complex events
Interleaved intrachr. SVs	68
Total SVs (intrachr. + transl.)	116
SV types	DEL: 20; DUP: 17; h2hINV: 16; t2tINV: 15; TRA: 48
SVs in sample	209
Oscillating CN (2 and 3 states)	19, 29
CN segments	59
FDR fragment joints	0.88
FDR chr. breakp. enrich.	0
Linked to chrs	9:17843117−36219342;
Purity, ploidy	0.8, 2.05

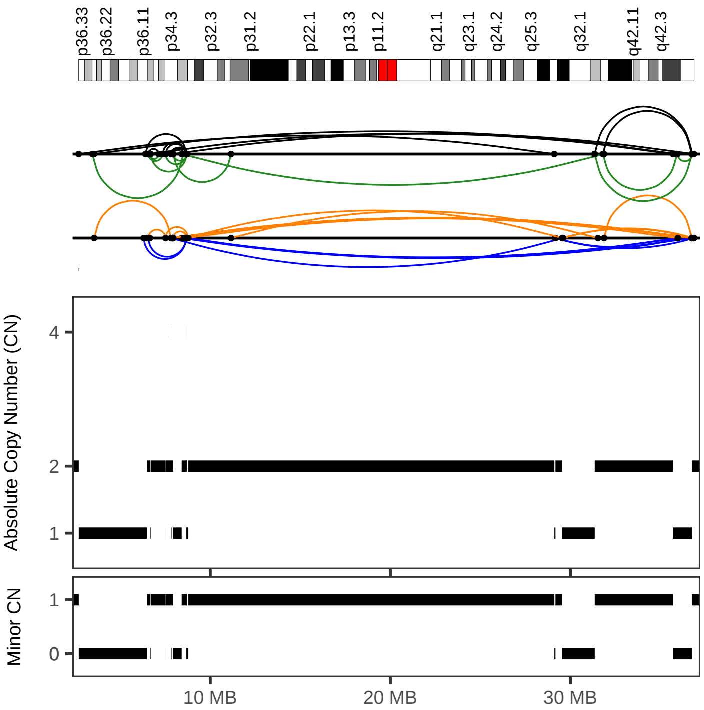

	**12824b2b−9c0a−4dcf−8941−34593a4e93da**
Cancer type	CNS−GBM
Position	1:2695318−36867510
Type	With other complex events
Interleaved intrachr. SVs	54
Total SVs (intrachr. + transl.)	54
SV types	DEL: 16; DUP: 11; h2hINV: 14; t2tINV: 13; TRA: 0
SVs in sample	92
Oscillating CN (2 and 3 states)	13, 20
CN segments	27
FDR fragment joints	0.85
FDR chr. breakp. enrich.	0
Linked to chrs	
Purity, ploidy	0.88, 2

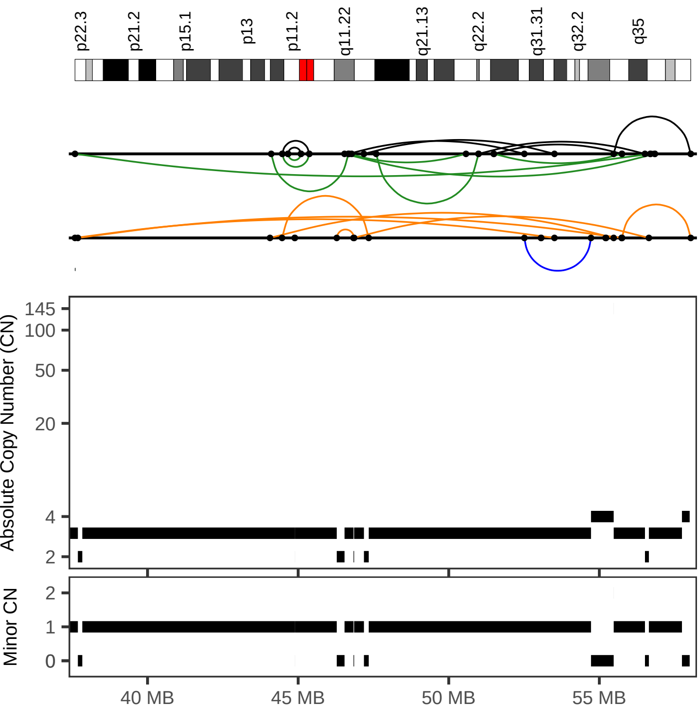

	**12824b2b−9c0a−4dcf−8941−34593a4e93da**
Cancer type	CNS−GBM
Position	7:37590136−58035420
Type	Canonical without polyploidization
Interleaved intrachr. SVs	21
Total SVs (intrachr. + transl.)	21
SV types	DEL: 7; DUP: 1; h2hINV: 7; t2tINV: 6; TRA: 0
SVs in sample	92
Oscillating CN (2 and 3 states)	11, 12
CN segments	18
FDR fragment joints	0.59
FDR chr. breakp. enrich.	0
Linked to chrs	
Purity, ploidy	0.88, 2

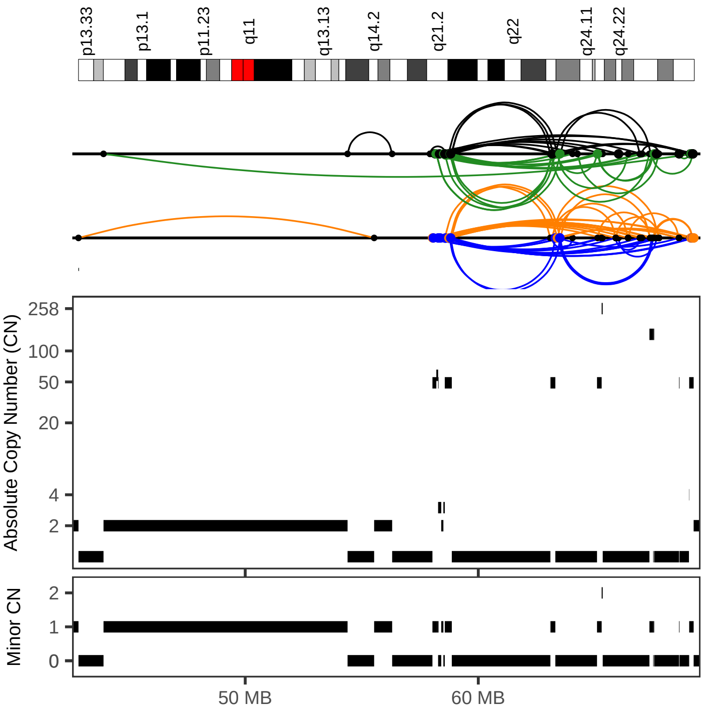

	**2f4a127f−101a−4192−b3e8−f9be2c8648bc**
Cancer type	CNS−GBM
Position	12:57931744−69260398
Type	With other complex events
Interleaved intrachr. SVs	100
Total SVs (intrachr. + transl.)	138
SV types	DEL: 29; DUP: 25; h2hINV: 22; t2tINV: 24; TRA: 38
SVs in sample	212
Oscillating CN (2 and 3 states)	7, 8
CN segments	25
FDR fragment joints	0.84
FDR chr. breakp. enrich.	0
Linked to chrs	
Purity, ploidy	0.94, 1.98

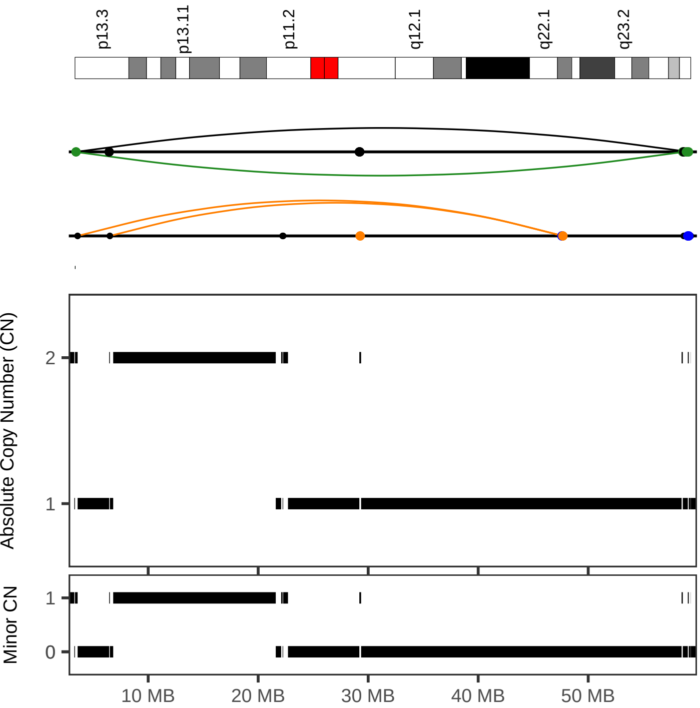

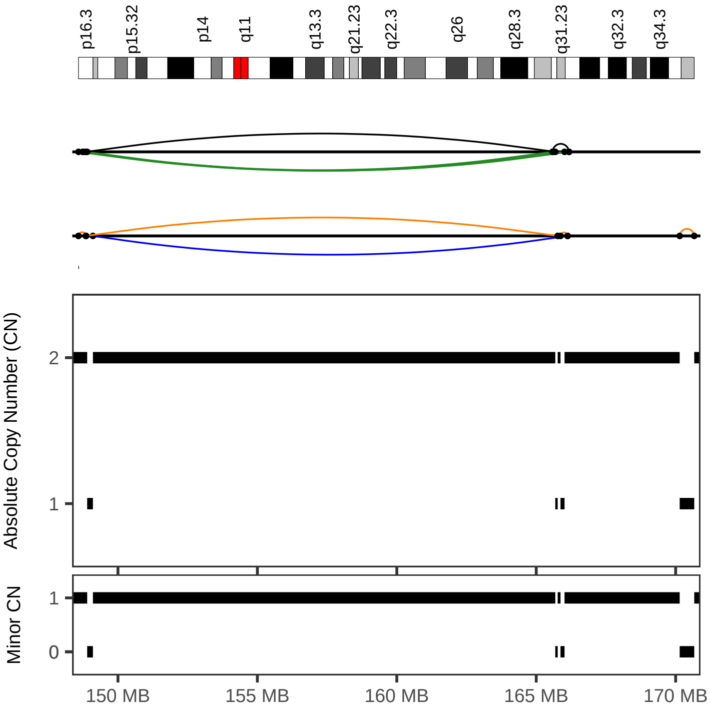

	309005a2−93a8−4566−b8d3−6b9310144266
Cancer type	CNS−GBM
Position	16:3345948−59322371
Type	Canonical without polyploidization
Interleaved intrachr. SVs	4
Total SVs (intrachr. + transl.)	17
SV types	DEL: 2; DUP: 0; h2hINV: 1; t2tINV: 1; TRA: 13
SVs in sample	46
Oscillating CN (2 and 3 states)	17, 17
CN segments	17
FDR fragment joints	0.64
FDR chr. breakp. enrich.	0
Linked to chrs	9:17888570−29115473;
Purity, ploidy	0.59, 1.84

	352768f9−3ce1−419c−beef−6515c78f5d7a
Cancer type	CNS−GBM
Position	4:148584038−166176935
Type	Canonical without polyploidization
Interleaved intrachr. SVs	10
Total SVs (intrachr. + transl.)	10
SV types	DEL: 3; DUP: 1; h2hINV: 3; t2tINV: 3; TRA: 0
SVs in sample	65
Oscillating CN (2 and 3 states)	7, 7
CN segments	7
FDR fragment joints	0.83
FDR chr. breakp. enrich.	0.02
Linked to chrs	
Purity, ploidy	0.67, 1.91

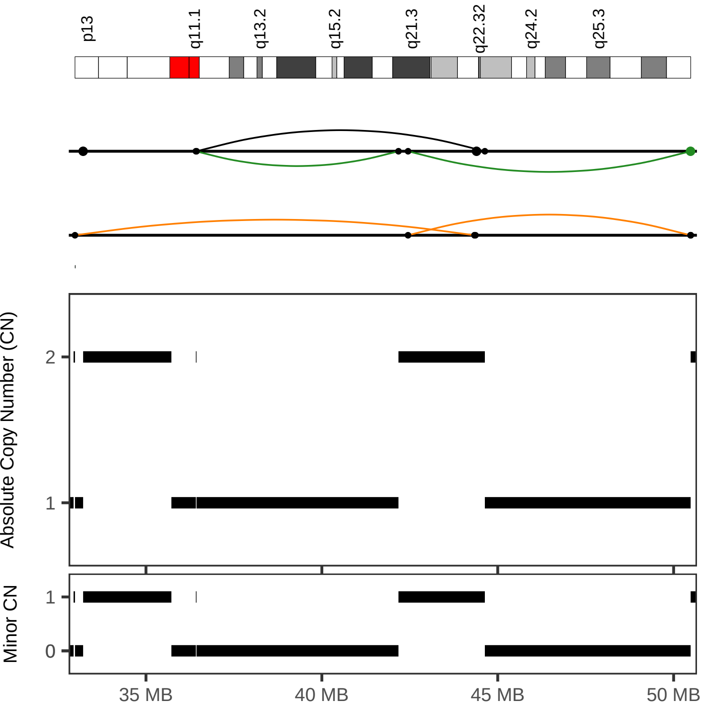

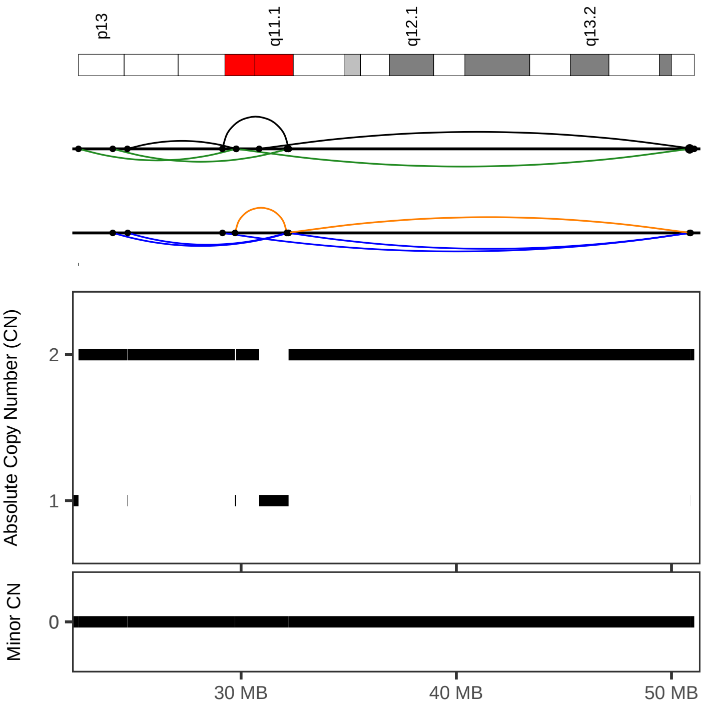

	386b629e−fab1−4033−b088−45d6eeb4a13e
Cancer type	CNS−GBM
Position	15:32981635−50483790
Type	Canonical without polyploidization
Interleaved intrachr. SVs	7
Total SVs (intrachr. + transl.)	10
SV types	DEL: 3; DUP: 0; h2hINV: 2; t2tINV: 2; TRA: 3
SVs in sample	114
Oscillating CN (2 and 3 states)	8, 8
CN segments	8
FDR fragment joints	0.64
FDR chr. breakp. enrich.	0
Linked to chrs	
Purity, ploidy	0.82, 1.88

	386b629e−fab1−4033−b088−45d6eeb4a13e
Cancer type	CNS−GBM
Position	22:22445020−51056576
Type	Canonical without polyploidization
Interleaved intrachr. SVs	13
Total SVs (intrachr. + transl.)	14
SV types	DEL: 2; DUP: 4; h2hINV: 3; t2tINV: 4; TRA: 1
SVs in sample	114
Oscillating CN (2 and 3 states)	9, 9
CN segments	9
FDR fragment joints	0.88
FDR chr. breakp. enrich.	0
Linked to chrs	
Purity, ploidy	0.82, 1.88

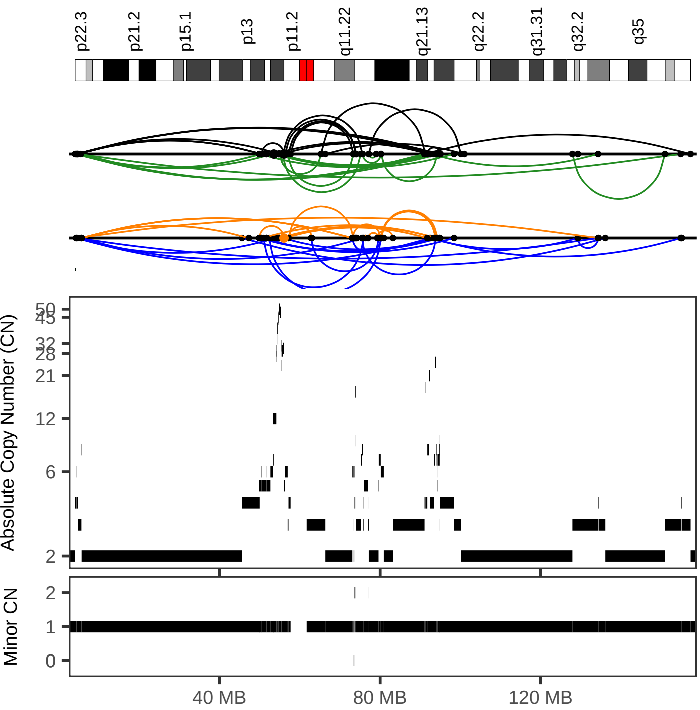

6c28f086−6a25−40b6−93eb−bba0014acda6	
Cancer type	CNS−GBM
Position	7:4042990−157318473
Type	With other complex events
Interleaved intrachr. SVs	103
Total SVs (intrachr. + transl.)	106
SV types	DEL: 23; DUP: 26; h2hINV: 27; t2tINV: 27; TRA: 3
SVs in sample	143
Oscillating CN (2 and 3 states)	8, 13
CN segments	109
FDR fragment joints	0.95
FDR chr. breakp. enrich.	0
Linked to chrs	
Purity, ploidy	0.71, 1.97

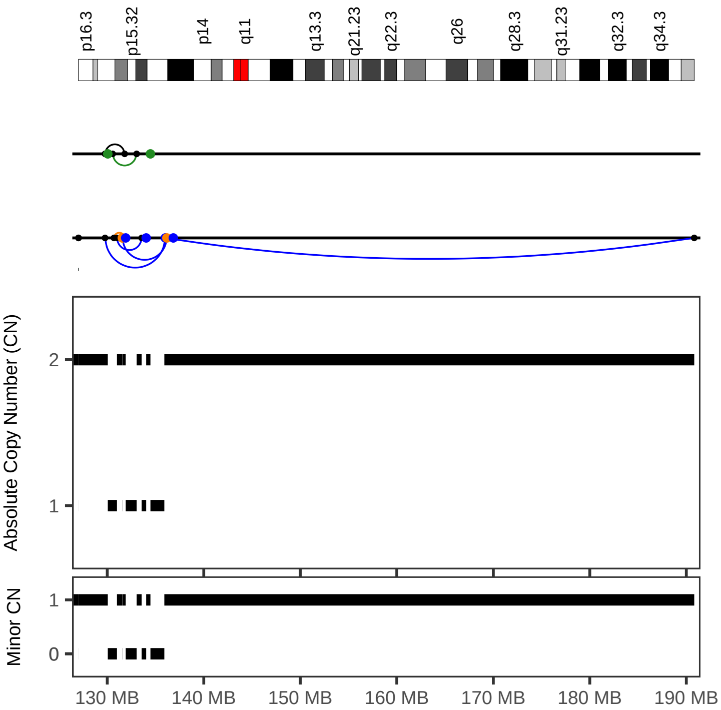

6c5154d2−af36−492f−b520−d925528824e4	
Cancer type	CNS−GBM
Position	4:129768712−190820043
Type	Canonical without polyploidization
Interleaved intrachr. SVs	7
Total SVs (intrachr. + transl.)	15
SV types	DEL: 1; DUP: 4; h2hINV: 1; t2tINV: 1; TRA: 8
SVs in sample	351
Oscillating CN (2 and 3 states)	11, 11
CN segments	11
FDR fragment joints	0.59
FDR chr. breakp. enrich.	0.42
Linked to chrs	3:172994395−174625575;7:25188974−57134154 19:32306219−58943997;
Purity, ploidy	0.86, 2

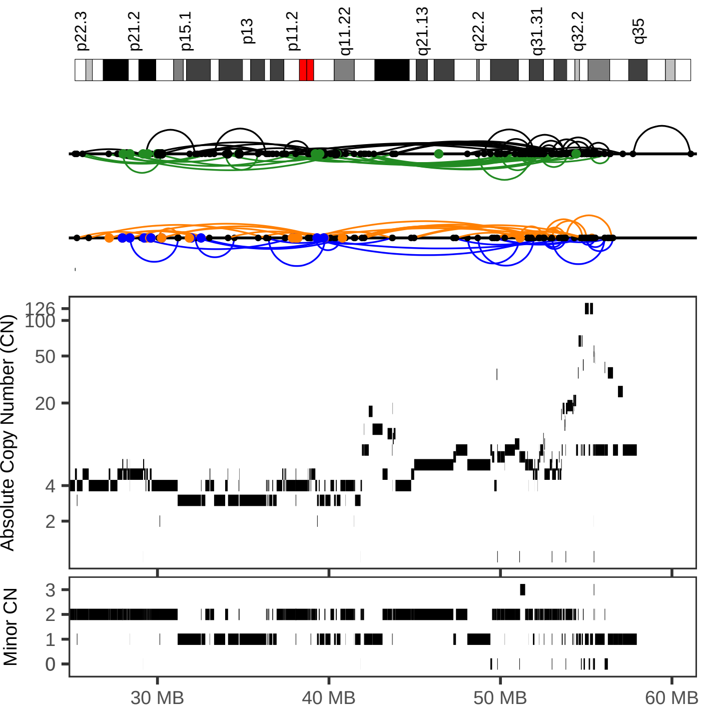

6c5154d2−af36−492f−b520−d925528824e4	
Cancer type	CNS−GBM
Position	7:25188974−57134155
Type	With other complex events
Interleaved intrachr. SVs	152
Total SVs (intrachr. + transl.)	204
SV types	DEL: 38; DUP: 32; h2hINV: 44; t2tINV: 38; TRA: 52
SVs in sample	351
Oscillating CN (2 and 3 states)	12, 15
CN segments	207
FDR fragment joints	0.67
FDR chr. breakp. enrich.	0
Linked to chrs	19:32306219−58943997;
Purity, ploidy	0.86, 2

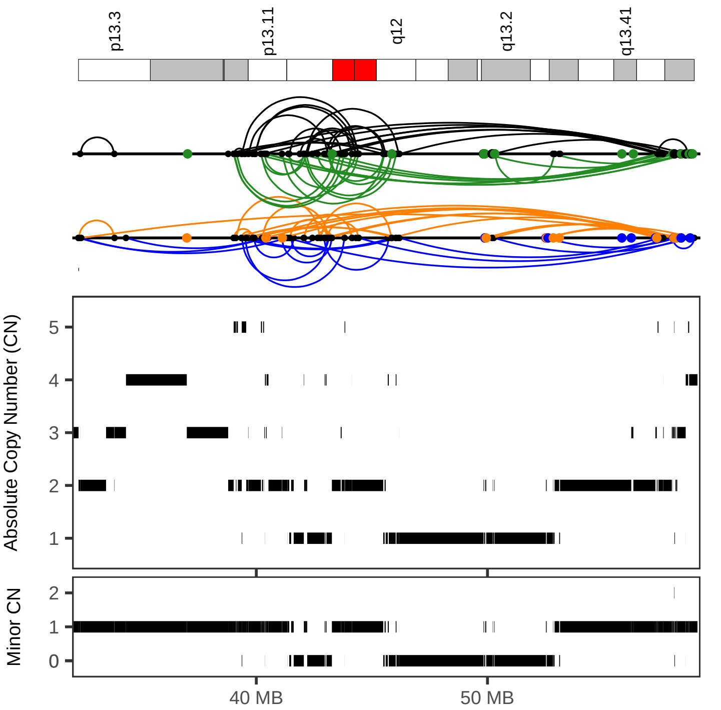

6c5154d2−af36−492f−b520−d925528824e4	
Cancer type	CNS−GBM
Position	19:32306219−58943998
Type	With other complex events
Interleaved intrachr. SVs	100
Total SVs (intrachr. + transl.)	150
SV types	DEL: 27; DUP: 21; h2hINV: 26; t2tINV: 26; TRA: 50
SVs in sample	351
Oscillating CN (2 and 3 states)	24, 30
CN segments	114
FDR fragment joints	0.87
FDR chr. breakp. enrich.	0
Linked to chrs	3:172994395−174625575;7:25188974−57134154
Purity, ploidy	0.86, 2

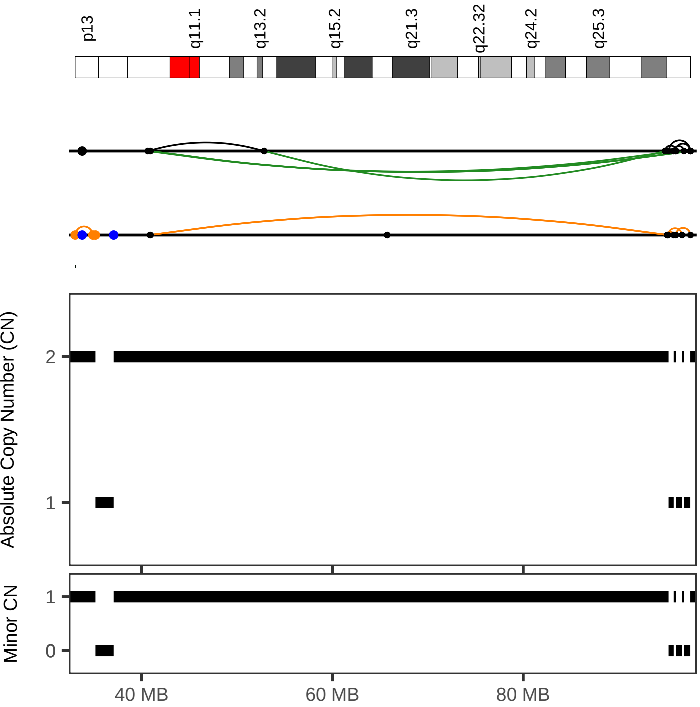

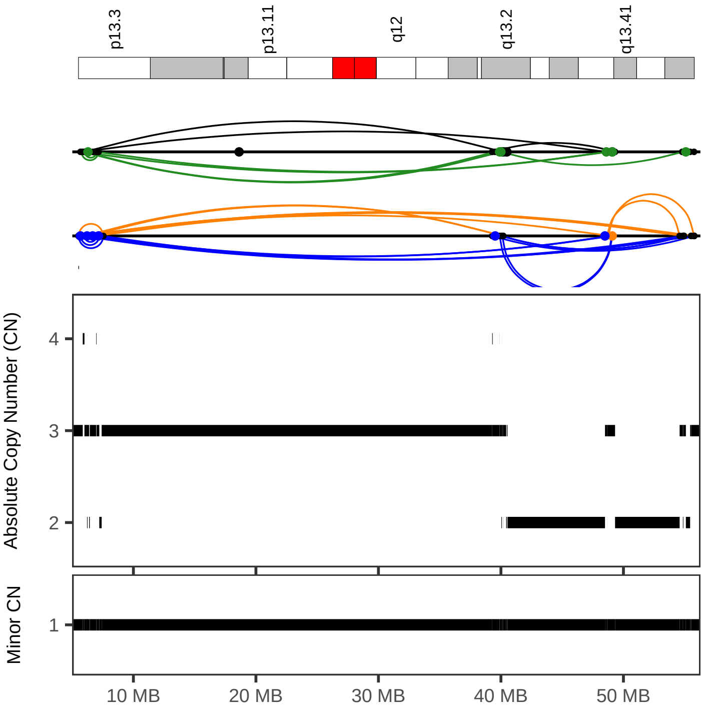

	713190ed−c6c1−4695−814b−85ca9b95e6a0	713190ed−c6c1−4695−814b−85ca9b95e6a0
Cancer type	CNS−GBM	CNS−GBM
Position	15:40649203−97539849	19:5516134−55780057
Type	Canonical without polyploidization	With other complex events
Interleaved intrachr. SVs	15	39
Total SVs (intrachr. + transl.)	15	60
SV types	DEL: 5; DUP: 0; h2hINV: 5; t2tINV: 5; TRA: 0	DEL: 9; DUP: 15; h2hINV: 4; t2tINV: 11; TRA: 21
SVs in sample	147	147
Oscillating CN (2 and 3 states)	7, 7	19, 37
CN segments	7	37
FDR fragment joints	0.59	0.48
FDR chr. breakp. enrich.	0	0
Linked to chrs		
Purity, ploidy	0.82, 1.98	0.82, 1.98

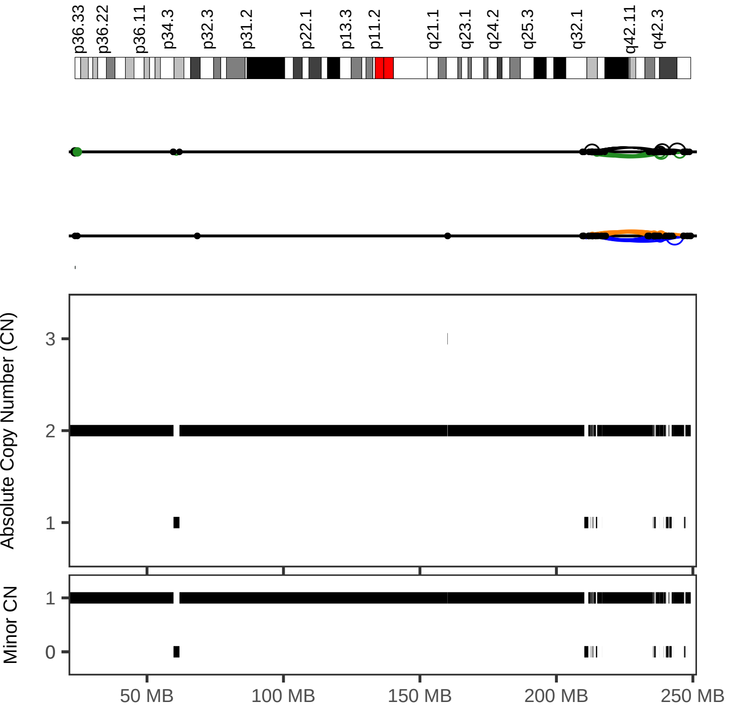

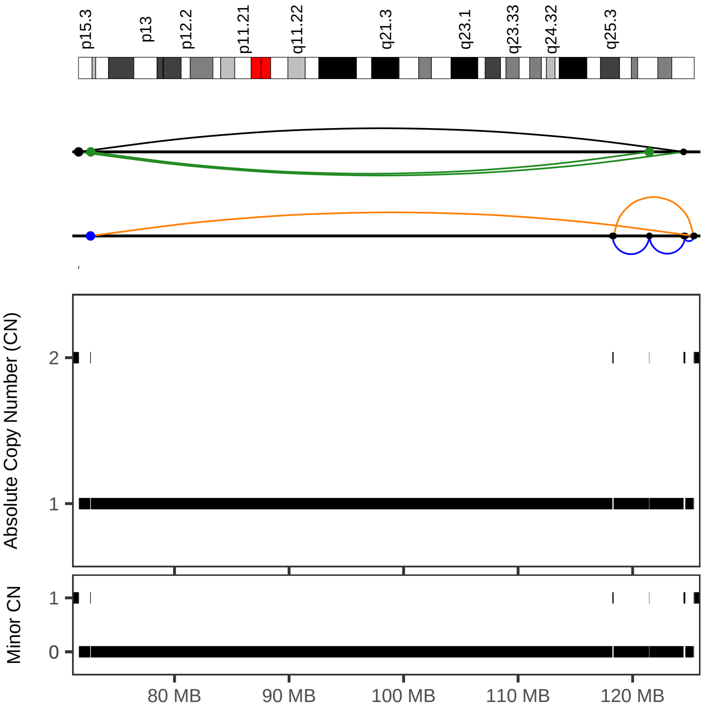

	737b35e1−d668−4fce−9b6e−76946c7952b6	737b35e1−d668−4fce−9b6e−76946c7952b6
Cancer type	CNS−GBM	CNS−GBM
Position	1:209530581−249214157	10:71615252−125378628
Type	Canonical without polyploidization	Canonical without polyploidization
Interleaved intrachr. SVs	103	8
Total SVs (intrachr. + transl.)	103	12
SV types	DEL: 26; DUP: 30; h2hINV: 24; t2tINV: 23; TRA: 0	DEL: 2; DUP: 3; h2hINV: 1; t2tINV: 2; TRA: 4
SVs in sample	173	173
Oscillating CN (2 and 3 states)	51, 51	14, 14
CN segments	51	14
FDR fragment joints	0.84	0.84
FDR chr. breakp. enrich.	0	0.07
Linked to chrs		
Purity, ploidy	0.93, 1.97	0.93, 1.97

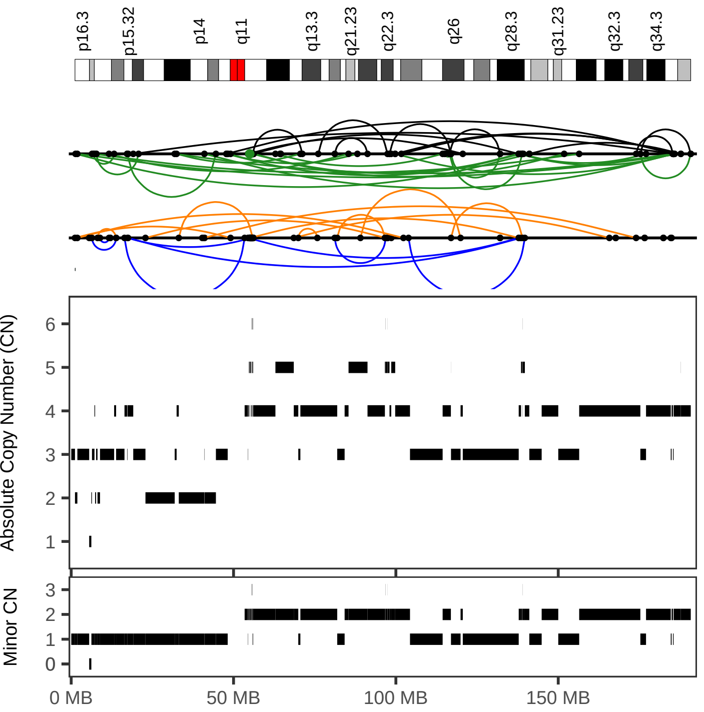

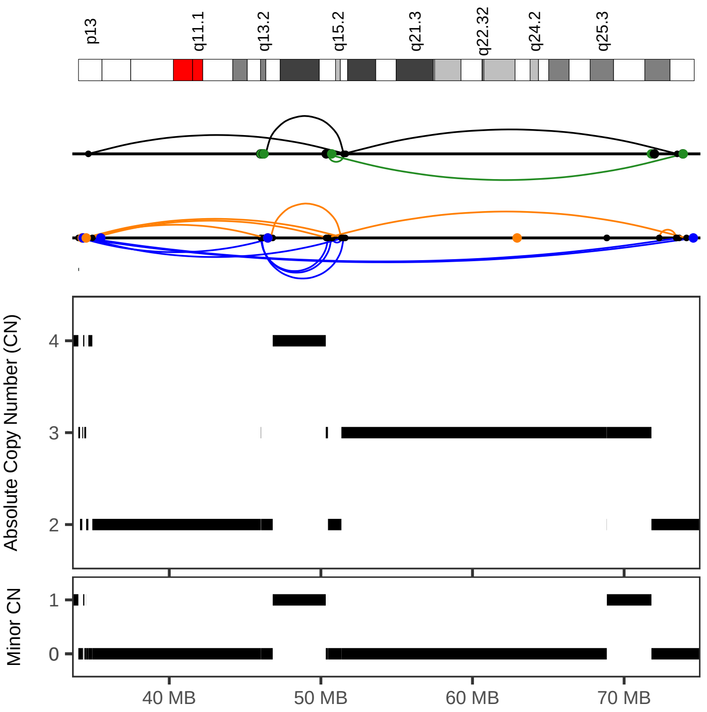

74139255−a635−4c87−814d−3dd04ed630a8	
Cancer type	CNS−GBM
Position	4:1163865−190815012
Type	With other complex events
Interleaved intrachr. SVs	94
Total SVs (intrachr. + transl.)	95
SV types	DEL: 21; DUP: 17; h2hINV: 27; t2tINV: 29; TRA: 1
SVs in sample	291
Oscillating CN (2 and 3 states)	12, 20
CN segments	90
FDR fragment joints	0.59
FDR chr. breakp. enrich.	0
Linked to chrs	
Purity, ploidy	0.38, 3.59

74139255−a635−4c87−814d−3dd04ed630a8	
Cancer type	CNS−GBM
Position	15:34114739−74116044
Type	With other complex events
Interleaved intrachr. SVs	17
Total SVs (intrachr. + transl.)	35
SV types	DEL: 5; DUP: 7; h2hINV: 3; t2tINV: 2; TRA: 18
SVs in sample	291
Oscillating CN (2 and 3 states)	8, 11
CN segments	19
FDR fragment joints	0.59
FDR chr. breakp. enrich.	0
Linked to chrs	9:21130542−26979039;
Purity, ploidy	0.38, 3.59

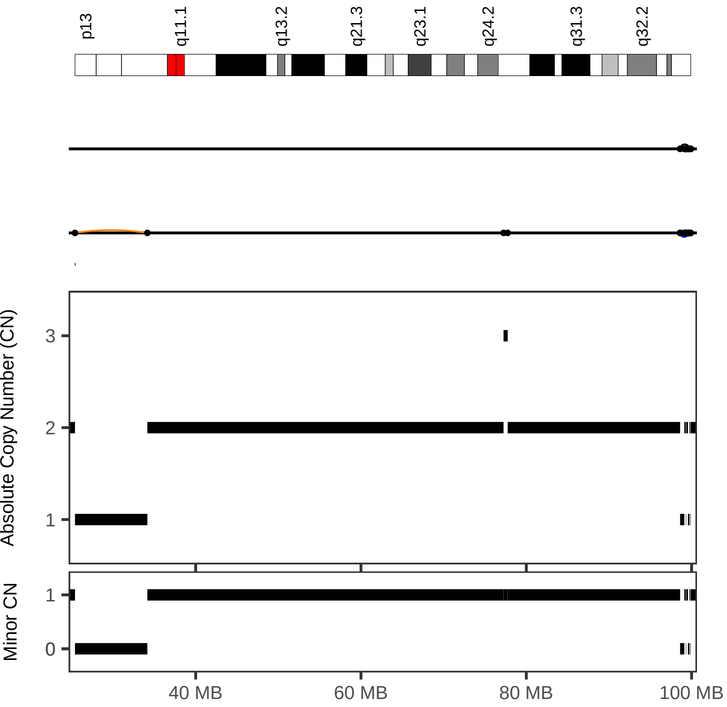

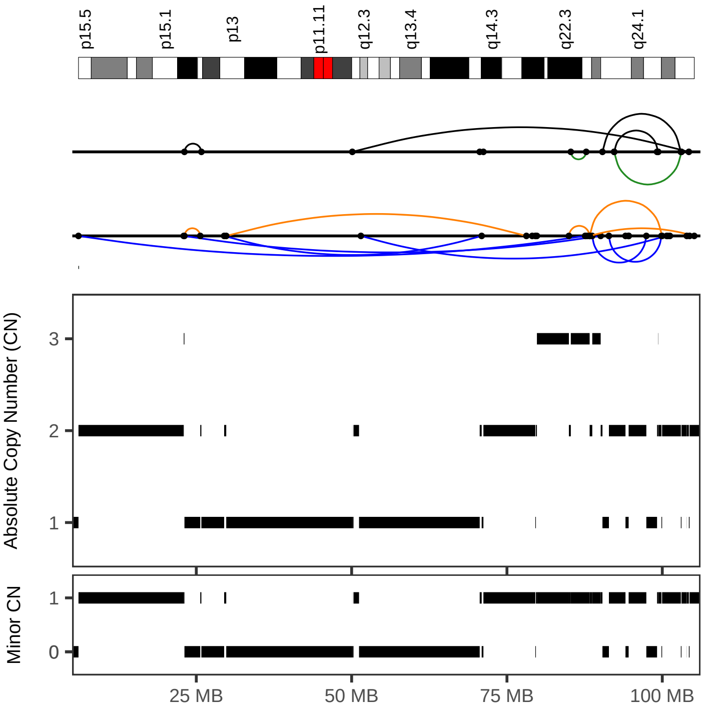

8da3103e−3e6c−4176−a583−d5fe5e60601e	
Cancer type	CNS−GBM
Position	14:98609565−99894680
Type	Canonical without polyploidization
Interleaved intrachr. SVs	36
Total SVs (intrachr. + transl.)	36
SV types	DEL: 10; DUP: 9; h2hINV: 10; t2tINV: 7; TRA: 0
SVs in sample	131
Oscillating CN (2 and 3 states)	10, 10
CN segments	10
FDR fragment joints	0.91
FDR chr. breakp. enrich.	0
Linked to chrs	
Purity, ploidy	0.59, 2.03

93ed7a2b−b0cb−4a84−871f−5c34a0b6a640	
Cancer type	CNS−GBM
Position	11:6033888−105143674
Type	With other complex events
Interleaved intrachr. SVs	18
Total SVs (intrachr. + transl.)	18
SV types	DEL: 5; DUP: 7; h2hINV: 3; t2tINV: 3; TRA: 0
SVs in sample	175
Oscillating CN (2 and 3 states)	15, 39
CN segments	39
FDR fragment joints	0.64
FDR chr. breakp. enrich.	0
Linked to chrs	
Purity, ploidy	0.92, 1.83

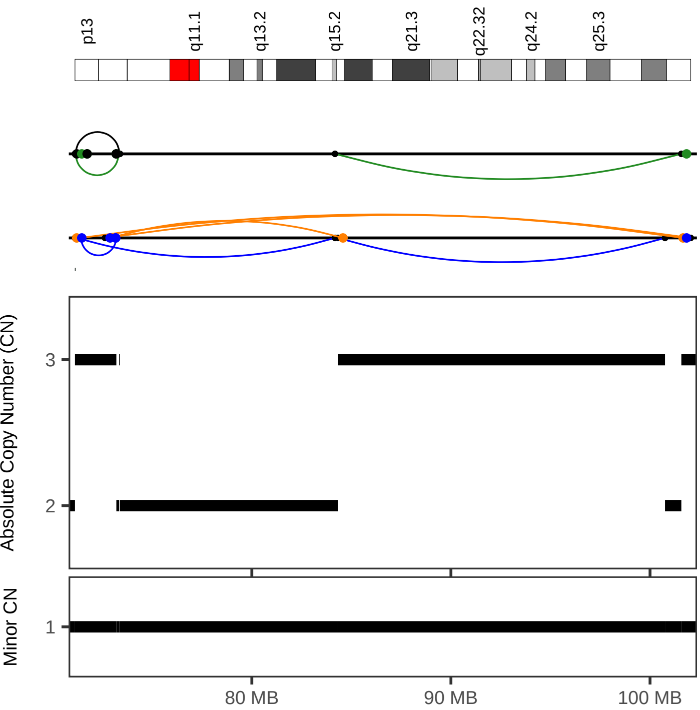

	**b37d944c−c65c−46d6−bb8b−3eb37cb85b68**
Cancer type	CNS−GBM
Position	15:71116986−102044792
Type	Canonical without polyploidization
Interleaved intrachr. SVs	10
Total SVs (intrachr. + transl.)	25
SV types	DEL: 3; DUP: 4; h2hINV: 1; t2tINV: 2; TRA: 15
SVs in sample	130
Oscillating CN (2 and 3 states)	7, 7
CN segments	7
FDR fragment joints	0.64
FDR chr. breakp. enrich.	0
Linked to chrs	
Purity, ploidy	0.89, 1.95

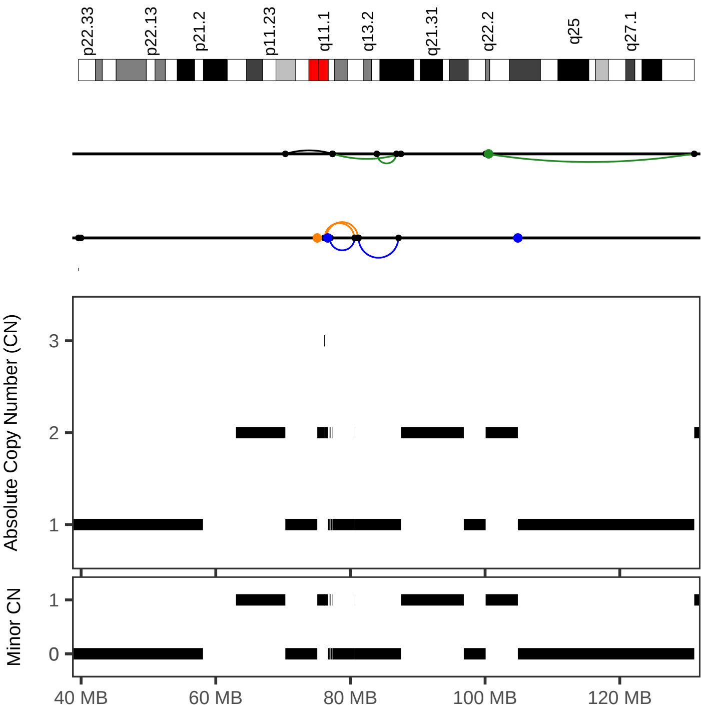

	**b37d944c−c65c−46d6−bb8b−3eb37cb85b68**
Cancer type	CNS−GBM
Position	X:70334273−87509185
Type	Canonical without polyploidization
Interleaved intrachr. SVs	8
Total SVs (intrachr. + transl.)	10
SV types	DEL: 2; DUP: 4; h2hINV: 1; t2tINV: 1; TRA: 2
SVs in sample	130
Oscillating CN (2 and 3 states)	9, 12
CN segments	12
FDR fragment joints	0.59
FDR chr. breakp. enrich.	0.02
Linked to chrs	
Purity, ploidy	0.89, 1.95

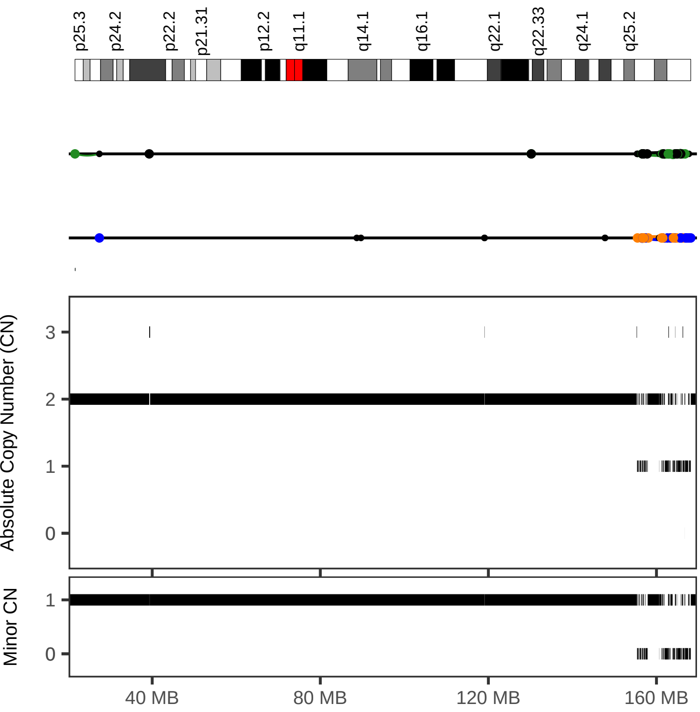

	**bf339349−062f−4ea9−a0b2−d87d3a21099e**
Cancer type	CNS−GBM
Position	6:155280936−168158297
Type	With other complex events
Interleaved intrachr. SVs	136
Total SVs (intrachr. + transl.)	186
SV types	DEL: 32; DUP: 34; h2hINV: 35; t2tINV: 35; TRA: 50
SVs in sample	338
Oscillating CN (2 and 3 states)	42, 43
CN segments	89
FDR fragment joints	0.99
FDR chr. breakp. enrich.	0
Linked to chrs	
Purity, ploidy	0.83, 1.97

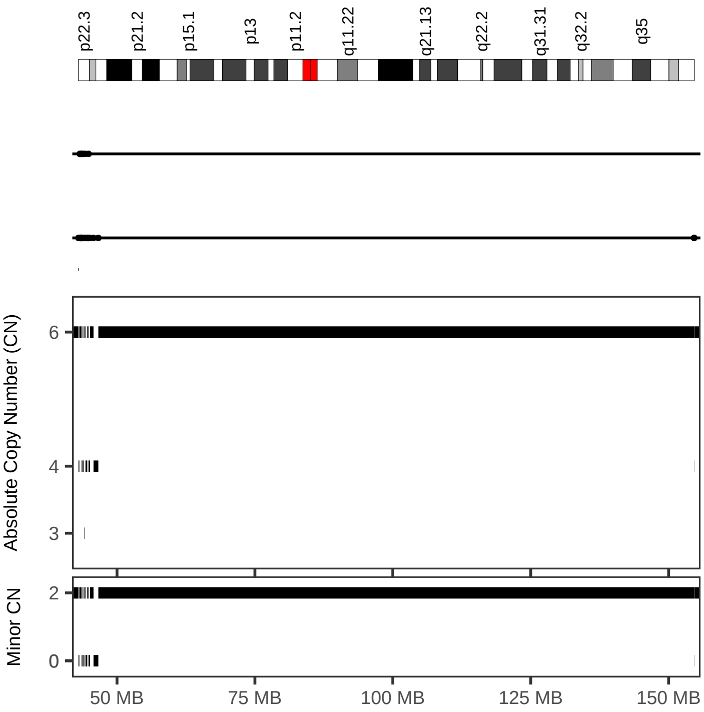

	**de7b7cac−f094−4d59−8651−e991e34ea093**
Cancer type	CNS−GBM
Position	7:43019383−45110155
Type	With other complex events
Interleaved intrachr. SVs	10
Total SVs (intrachr. + transl.)	10
SV types	DEL: 4; DUP: 3; h2hINV: 1; t2tINV: 2; TRA: 0
SVs in sample	105
Oscillating CN (2 and 3 states)	7, 7
CN segments	13
FDR fragment joints	0.64
FDR chr. breakp. enrich.	0
Linked to chrs	
Purity, ploidy	0.56, 3.59

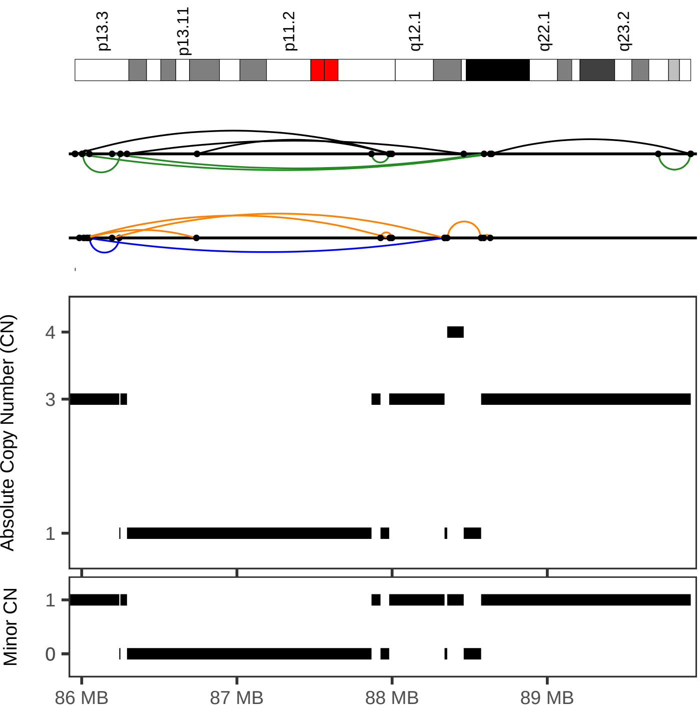

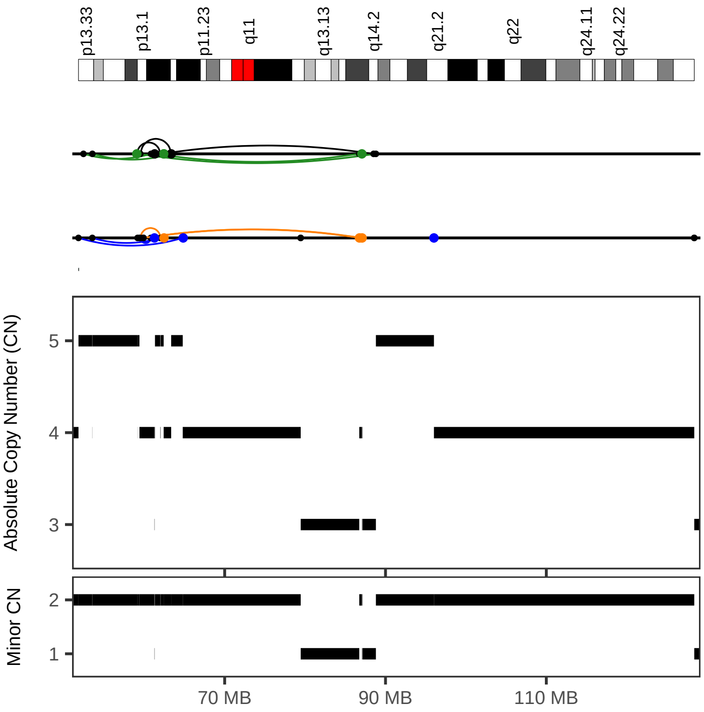

	**de7b7cac−f094−4d59−8651−e991e34ea093**
Cancer type	CNS−GBM
Position	16:85956549−89924266
Type	Canonical without polyploidization
Interleaved intrachr. SVs	19
Total SVs (intrachr. + transl.)	19
SV types	DEL: 7; DUP: 2; h2hINV: 5; t2tINV: 5; TRA: 0
SVs in sample	105
Oscillating CN (2 and 3 states)	8, 11
CN segments	11
FDR fragment joints	0.64
FDR chr. breakp. enrich.	0
Linked to chrs	
Purity, ploidy	0.56, 3.59

	**e17a6048−7a72−42c0−ad3f−97cbff02bc9f**
Cancer type	CNS−GBM
Position	12:51821337−88808438
Type	With other complex events
Interleaved intrachr. SVs	16
Total SVs (intrachr. + transl.)	27
SV types	DEL: 4; DUP: 4; h2hINV: 4; t2tINV: 4; TRA: 11
SVs in sample	65
Oscillating CN (2 and 3 states)	7, 19
CN segments	20
FDR fragment joints	1
FDR chr. breakp. enrich.	0
Linked to chrs	
Purity, ploidy	0.61, 3.36

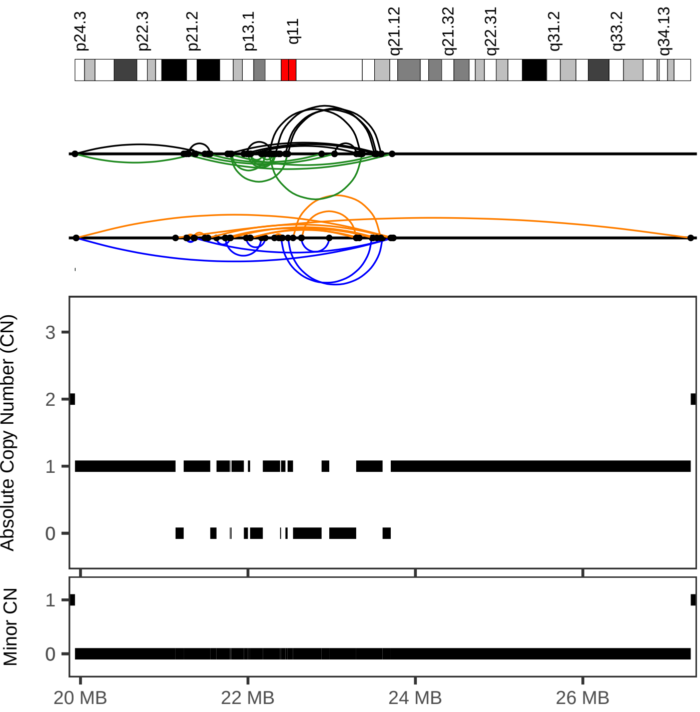

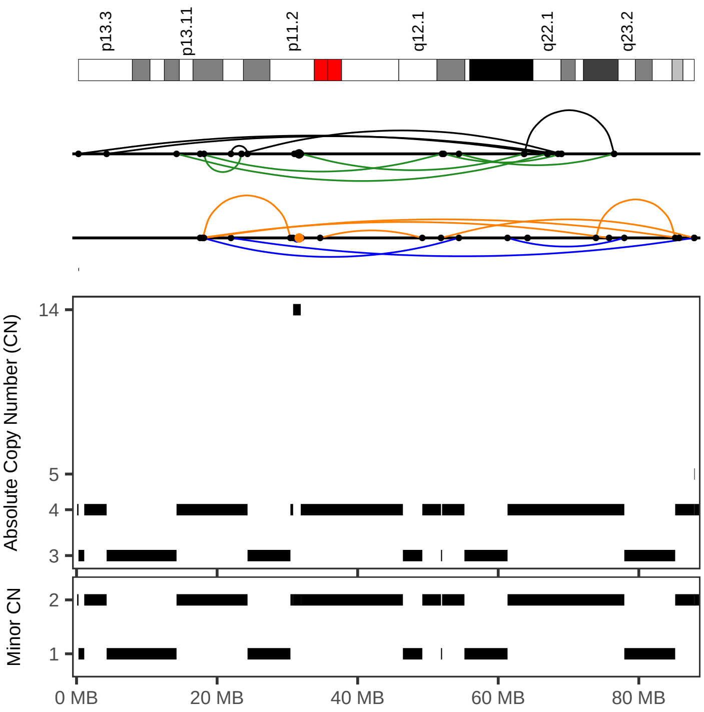

	**f45f4a30−6b4c−4f15−9140−959d6a25a45f**
Cancer type	CNS−GBM
Position	9:19934041−27288375
Type	Canonical without polyploidization
Interleaved intrachr. SVs	48
Total SVs (intrachr. + transl.)	48
SV types	DEL: 12; DUP: 11; h2hINV: 13; t2tINV: 12; TRA: 0
SVs in sample	119
Oscillating CN (2 and 3 states)	23, 25
CN segments	25
FDR fragment joints	0.99
FDR chr. breakp. enrich.	0
Linked to chrs	
Purity, ploidy	0.7, 2

	**ICGC_MB110**
Cancer type	CNS−Medullo
Position	16:275259−87888760
Type	With other complex events
Interleaved intrachr. SVs	22
Total SVs (intrachr. + transl.)	24
SV types	DEL: 5; DUP: 5; h2hINV: 6; t2tINV: 6; TRA: 2
SVs in sample	77
Oscillating CN (2 and 3 states)	9, 16
CN segments	18
FDR fragment joints	0.99
FDR chr. breakp. enrich.	0
Linked to chrs	
Purity, ploidy	0.92, 4.18

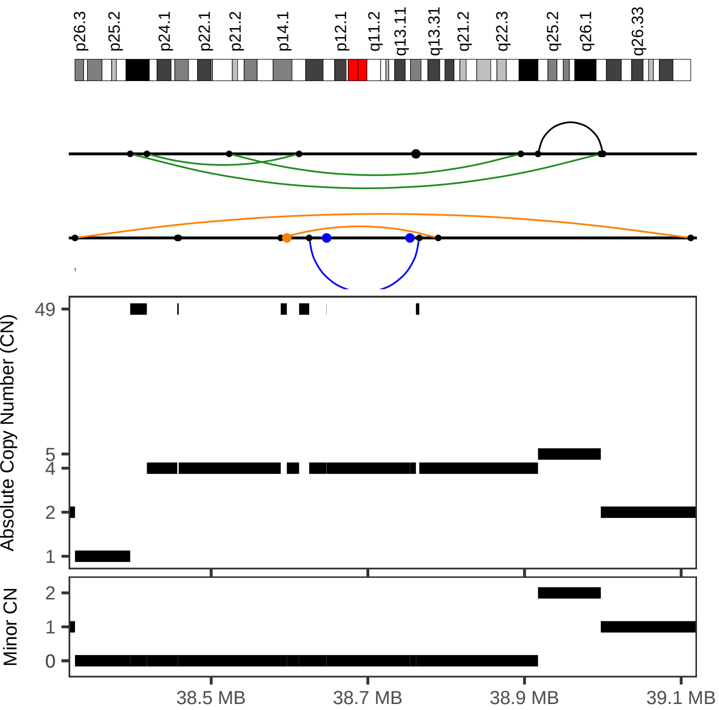

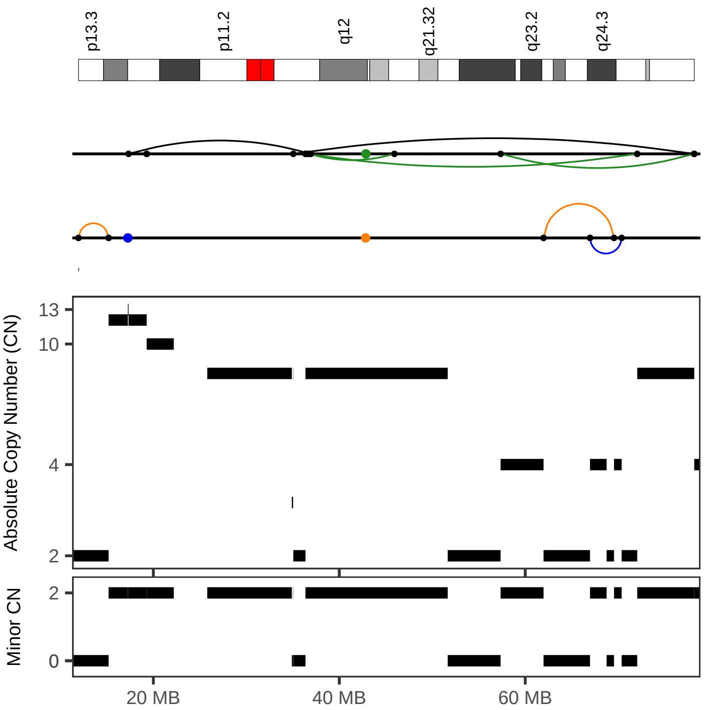

**ICGC_MB131**	
Cancer type	CNS−Medullo
Position	3:38417904−38895251
Type	After polyploidization
Interleaved intrachr. SVs	3
Total SVs (intrachr. + transl.)	9
SV types	DEL: 1; DUP: 0; h2hINV: 0; t2tINV: 2; TRA: 6
SVs in sample	39
Oscillating CN (2 and 3 states)	13, 13
CN segments	13
FDR fragment joints	0.59
FDR chr. breakp. enrich.	0
Linked to chrs	8:22113923−128760776;
Purity, ploidy	0.96, 2.05

**ICGC_MB145**	
Cancer type	CNS−Medullo
Position	17:17334828−78175336
Type	With other complex events
Interleaved intrachr. SVs	6
Total SVs (intrachr. + transl.)	8
SV types	DEL: 0; DUP: 0; h2hINV: 2; t2tINV: 4; TRA: 2
SVs in sample	61
Oscillating CN (2 and 3 states)	7, 11
CN segments	16
FDR fragment joints	0.48
FDR chr. breakp. enrich.	0
Linked to chrs	
Purity, ploidy	0.94, 4.27

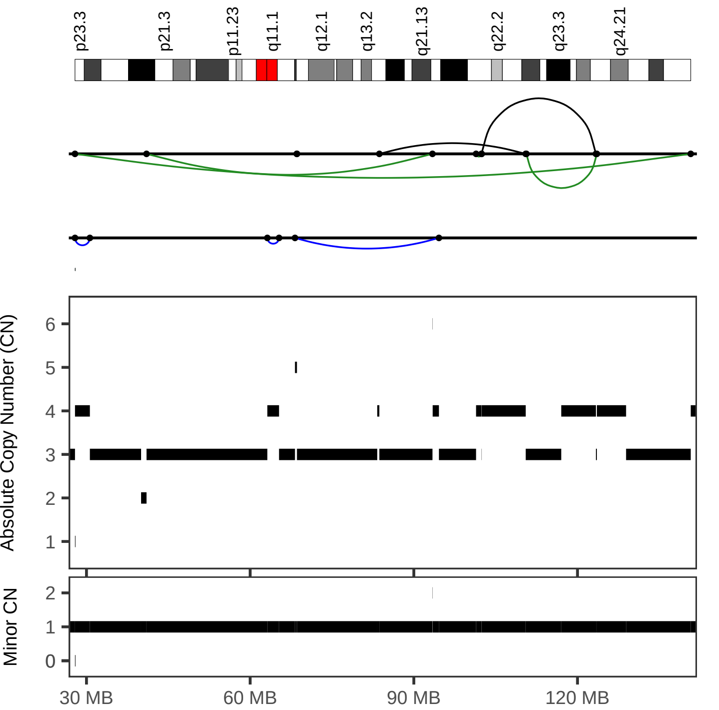

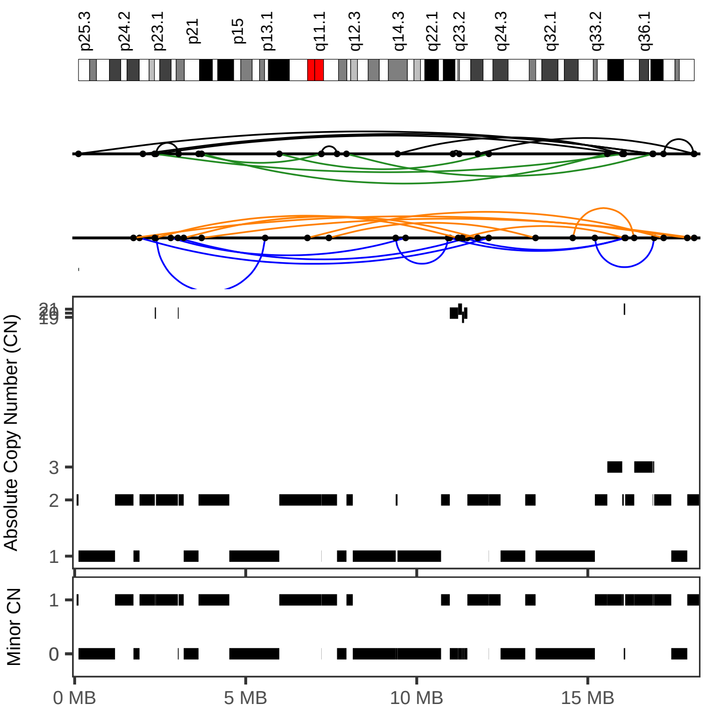

**ICGC_MB160**	
Cancer type	CNS−Medullo
Position	8:41002272−123551081
Type	With other complex events
Interleaved intrachr. SVs	6
Total SVs (intrachr. + transl.)	6
SV types	DEL: 0; DUP: 1; h2hINV: 2; t2tINV: 3; TRA: 0
SVs in sample	16
Oscillating CN (2 and 3 states)	9, 9
CN segments	17
FDR fragment joints	0.59
FDR chr. breakp. enrich.	0
Linked to chrs	
Purity, ploidy	0.67, 3.9

**ICGC_MB165**	
Cancer type	CNS−Medullo
Position	2:111074−18111365
Type	With other complex events
Interleaved intrachr. SVs	30
Total SVs (intrachr. + transl.)	30
SV types	DEL: 8; DUP: 8; h2hINV: 9; t2tINV: 5; TRA: 0
SVs in sample	98
Oscillating CN (2 and 3 states)	13, 22
CN segments	41
FDR fragment joints	0.83
FDR chr. breakp. enrich.	0
Linked to chrs	
Purity, ploidy	0.39, 1.87

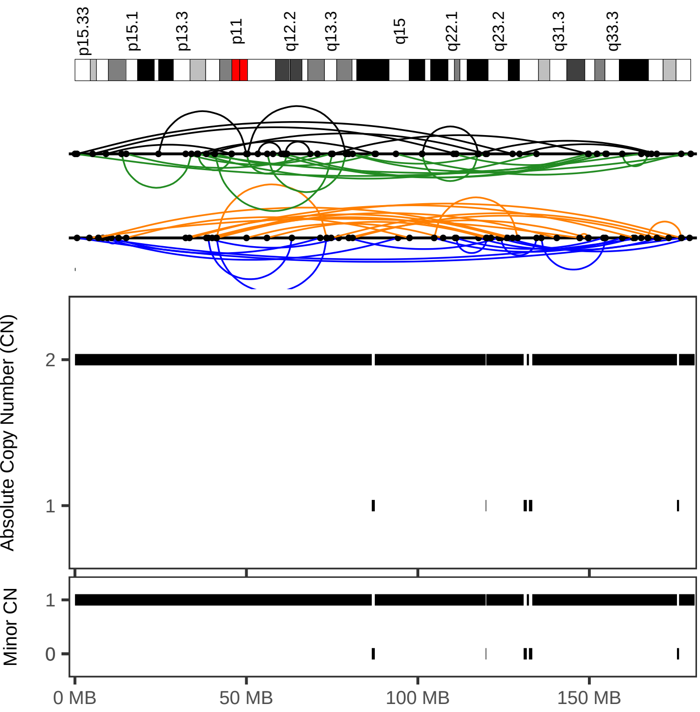

	ICGC_MB165
Cancer type	CNS−Medullo
Position	5:14327−179565981
Type	Canonical without polyploidization
Interleaved intrachr. SVs	66
Total SVs (intrachr. + transl.)	66
SV types	DEL: 17; DUP: 17; h2hINV: 13; t2tINV: 19; TRA: 0
SVs in sample	98
Oscillating CN (2 and 3 states)	11, 11
CN segments	11
FDR fragment joints	0.84
FDR chr. breakp. enrich.	0
Linked to chrs	
Purity, ploidy	0.39, 1.87

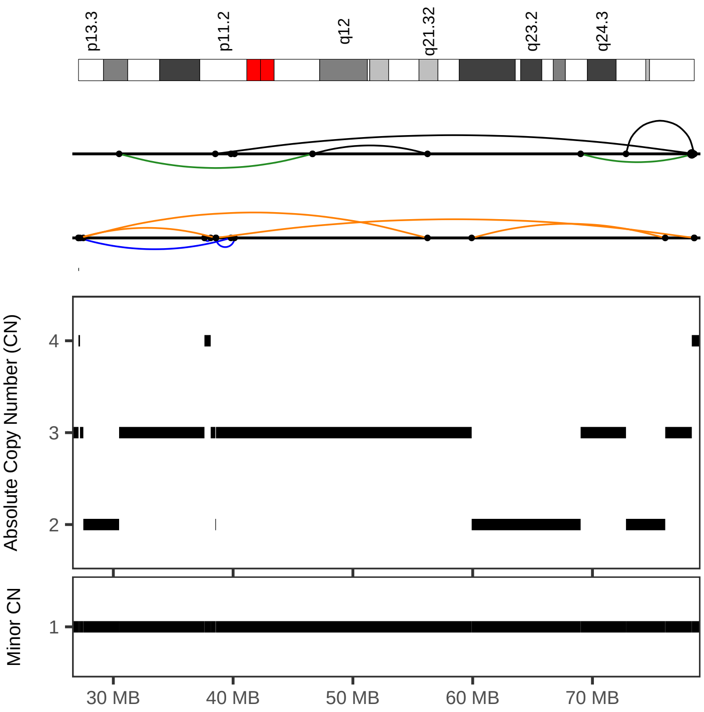

	ICGC_MB224
Cancer type	CNS−Medullo
Position	17:27090584−78497663
Type	With other complex events
Interleaved intrachr. SVs	10
Total SVs (intrachr. + transl.)	11
SV types	DEL: 3; DUP: 3; h2hINV: 2; t2tINV: 2; TRA: 1
SVs in sample	48
Oscillating CN (2 and 3 states)	7, 14
CN segments	14
FDR fragment joints	0.96
FDR chr. breakp. enrich.	0
Linked to chrs	
Purity, ploidy	0.99, 2.04

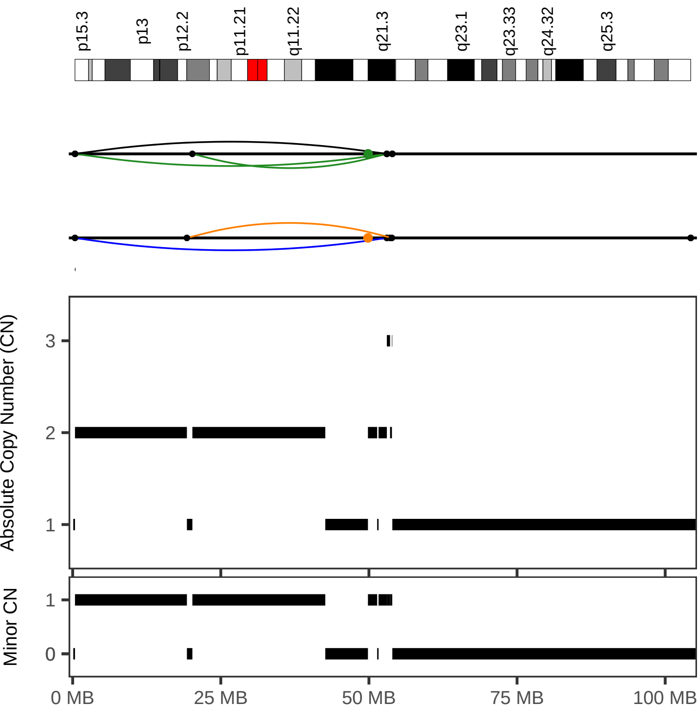

	ICGC_MB23
Cancer type	CNS−Medullo
Position	10:388457−53879148
Type	Canonical without polyploidization
Interleaved intrachr. SVs	5
Total SVs (intrachr. + transl.)	9
SV types	DEL: 1; DUP: 2; h2hINV: 1; t2tINV: 1; TRA: 4
SVs in sample	36
Oscillating CN (2 and 3 states)	8, 11
CN segments	11
FDR fragment joints	0.92
FDR chr. breakp. enrich.	0
Linked to chrs	
Purity, ploidy	0.98, 1.88

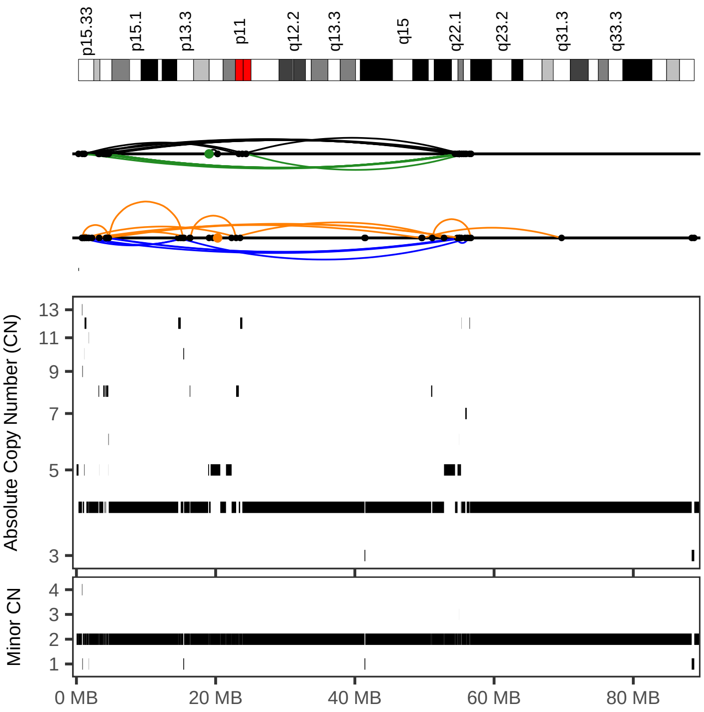

	ICGC_MB34
Cancer type	CNS−Medullo
Position	5:292496−69696821
Type	With other complex events
Interleaved intrachr. SVs	46
Total SVs (intrachr. + transl.)	48
SV types	DEL: 14; DUP: 9; h2hINV: 12; t2tINV: 11; TRA: 2
SVs in sample	242
Oscillating CN (2 and 3 states)	7, 9
CN segments	55
FDR fragment joints	0.84
FDR chr. breakp. enrich.	0
Linked to chrs	
Purity, ploidy	0.97, 3.62

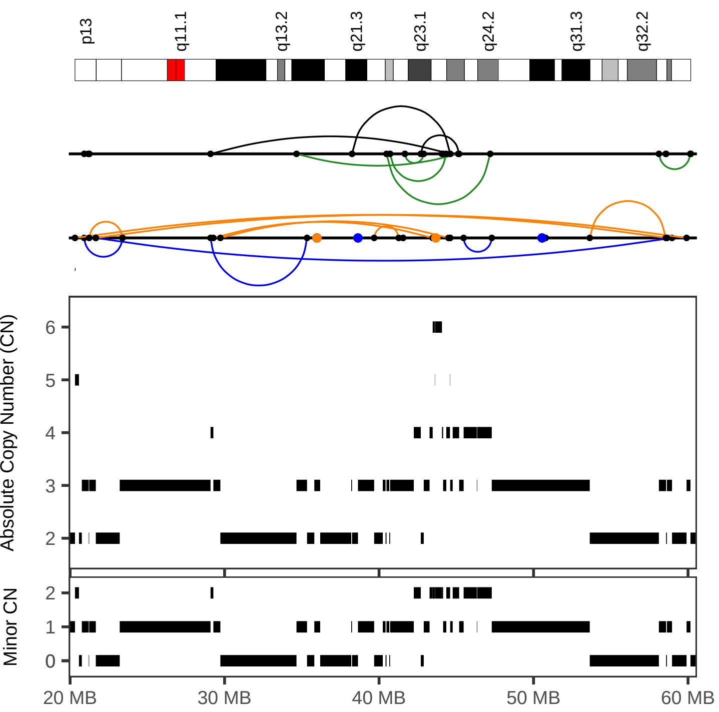

	ICGC_MB34
Cancer type	CNS−Medullo
Position	14:29090292−47295794
Type	With other complex events
Interleaved intrachr. SVs	13
Total SVs (intrachr. + transl.)	17
SV types	DEL: 4; DUP: 2; h2hINV: 3;<br>t2tINV: 4; TRA: 4
SVs in sample	242
Oscillating CN (2 and 3 states)	15, 15
CN segments	35
FDR fragment joints	0.88
FDR chr. breakp. enrich.	0
Linked to chrs	22:18004707−43092533;
Purity, ploidy	0.97, 3.62

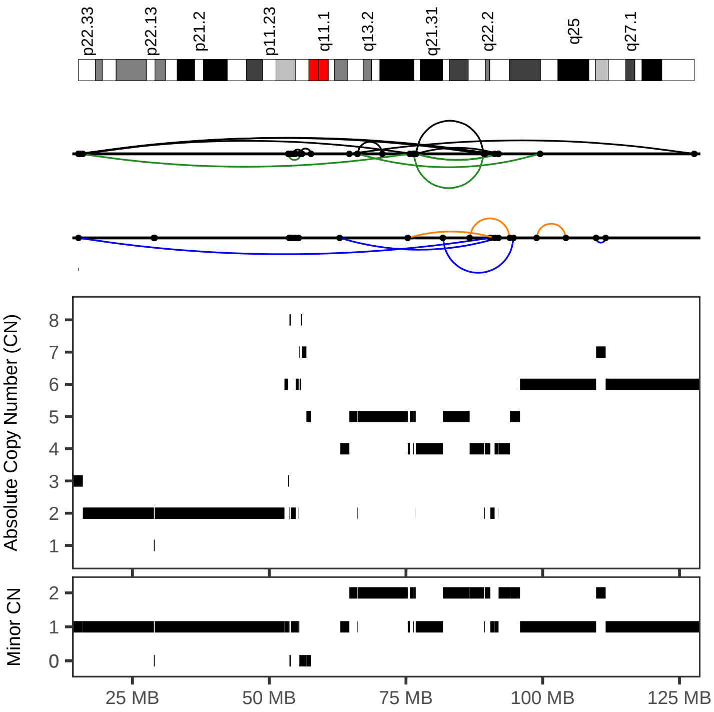

	ICGC_MB34
Cancer type	CNS−Medullo
Position	X:15128807−127701065
Type	With other complex events
Interleaved intrachr. SVs	17
Total SVs (intrachr. + transl.)	17
SV types	DEL: 3; DUP: 3; h2hINV: 6;<br>t2tINV: 5; TRA: 0
SVs in sample	242
Oscillating CN (2 and 3 states)	7, 10
CN segments	41
FDR fragment joints	0.74
FDR chr. breakp. enrich.	0
Linked to chrs	
Purity, ploidy	0.97, 3.62

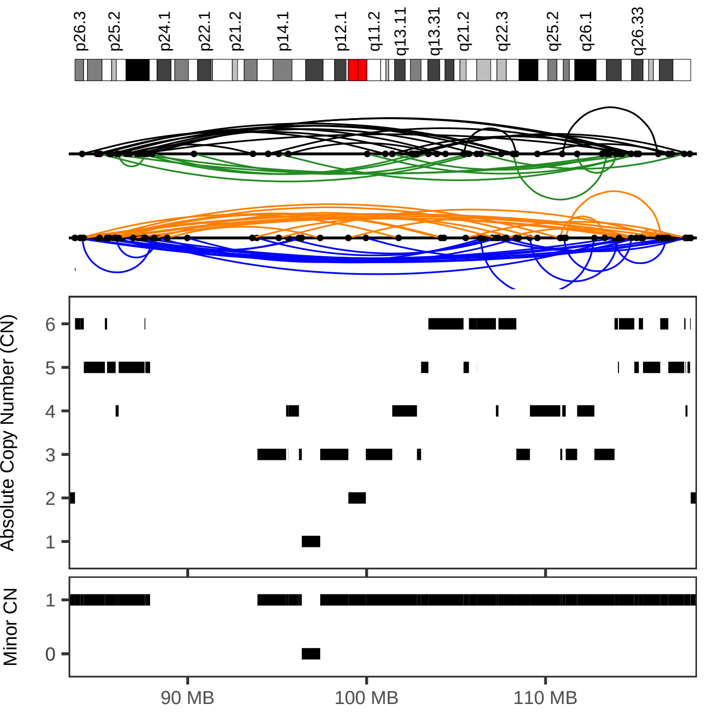

	ICGC_MB81
Cancer type	CNS−Medullo
Position	3:83993082−118079412
Type	With other complex events
Interleaved intrachr. SVs	74
Total SVs (intrachr. + transl.)	74
SV types	DEL: 20; DUP: 21; h2hINV: 20;<br>t2tINV: 13; TRA: 0
SVs in sample	91
Oscillating CN (2 and 3 states)	10, 21
CN segments	48
FDR fragment joints	0.64
FDR chr. breakp. enrich.	0
Linked to chrs	
Purity, ploidy	0.97, 2.02

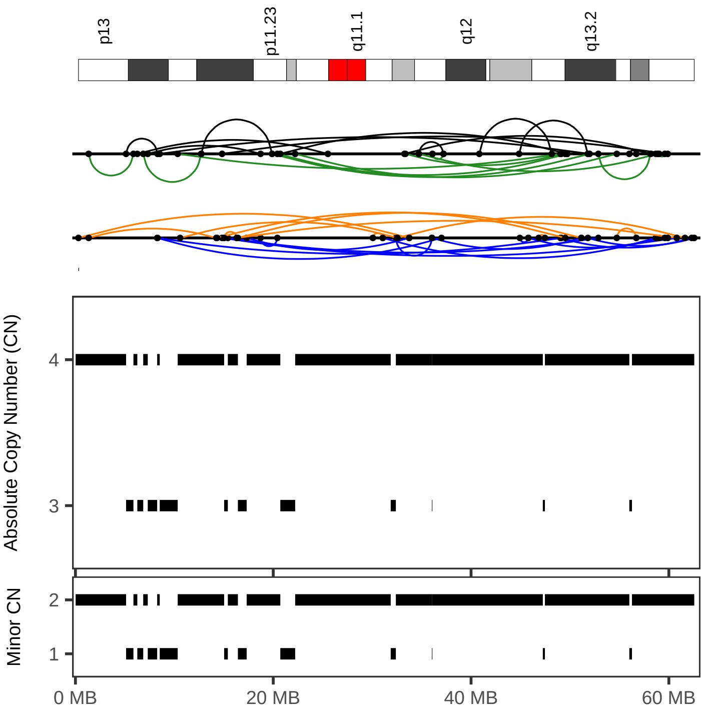

	ICGC_MB83
Cancer type	CNS−Medullo
Position	20:305936−62568476
Type	After polyploidization
Interleaved intrachr. SVs	47
Total SVs (intrachr. + transl.)	47
SV types	DEL: 11; DUP: 12; h2hINV: 13;<br>t2tINV: 11; TRA: 0
SVs in sample	58
Oscillating CN (2 and 3 states)	23, 23
CN segments	23
FDR fragment joints	0.98
FDR chr. breakp. enrich.	0
Linked to chrs	
Purity, ploidy	0.8, 4.45

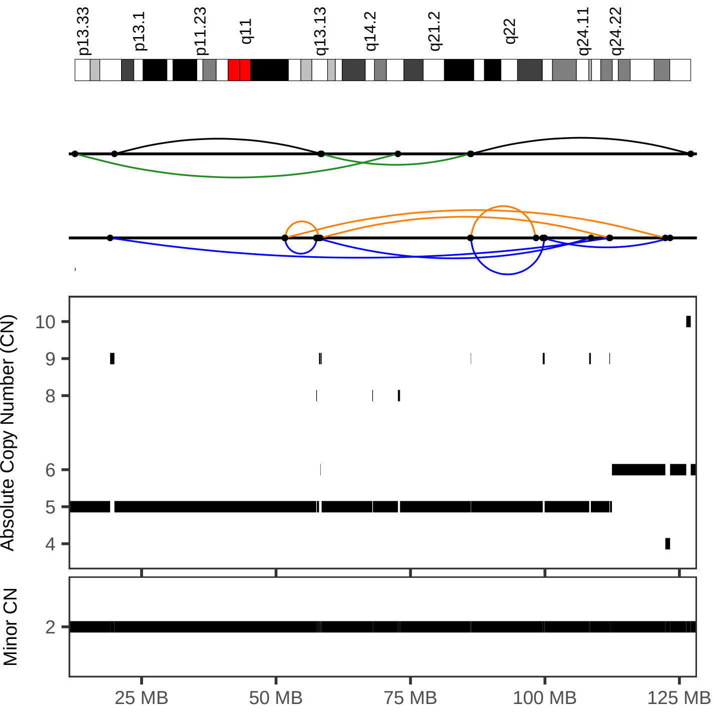

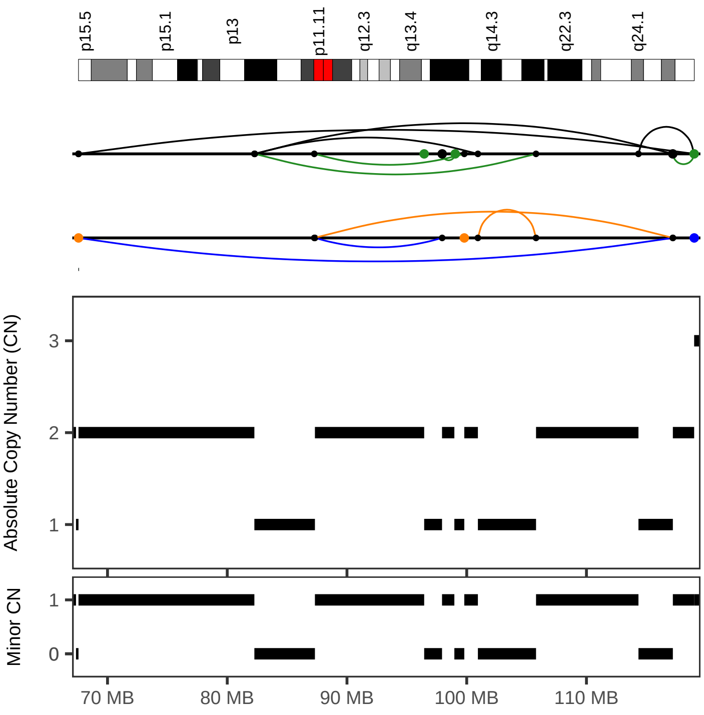

	ICGC_MB94
Cancer type	CNS−Medullo
Position	12:12616089−127105460
Type	With other complex events
Interleaved intrachr. SVs	13
Total SVs (intrachr. + transl.)	13
SV types	DEL: 4; DUP: 5; h2hINV: 2; t2tINV: 2; TRA: 0
SVs in sample	30
Oscillating CN (2 and 3 states)	9, 21
CN segments	26
FDR fragment joints	0.64
FDR chr. breakp. enrich.	0
Linked to chrs	
Purity, ploidy	0.95, 3.96

	70939245−41e0−4845−a473−5fef719b9828
Cancer type	CNS−Oligo
Position	11:67579787−119004447
Type	Canonical without polyploidization
Interleaved intrachr. SVs	10
Total SVs (intrachr. + transl.)	18
SV types	DEL: 2; DUP: 1; h2hINV: 3; t2tINV: 4; TRA: 8
SVs in sample	62
Oscillating CN (2 and 3 states)	11, 12
CN segments	12
FDR fragment joints	0.64
FDR chr. breakp. enrich.	0
Linked to chrs	
Purity, ploidy	0.8, 2.06

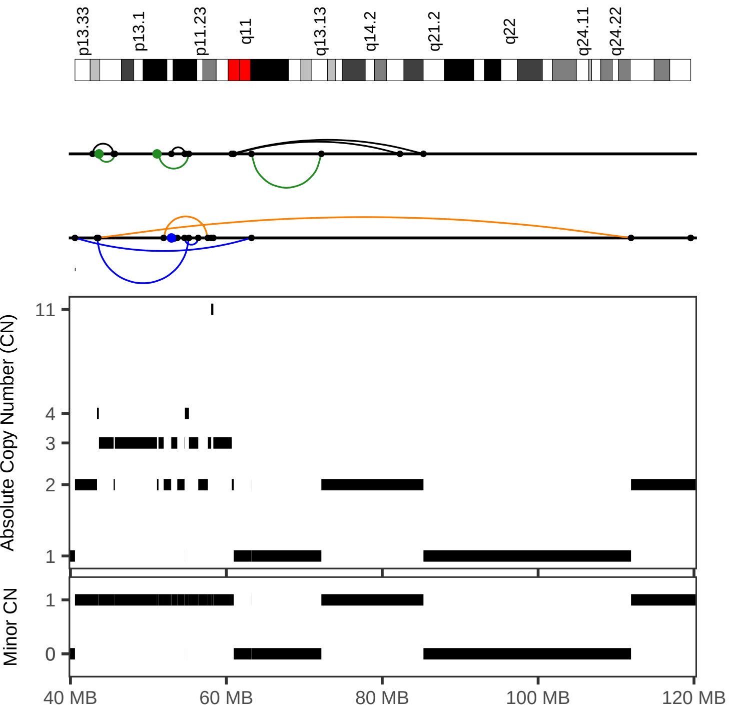

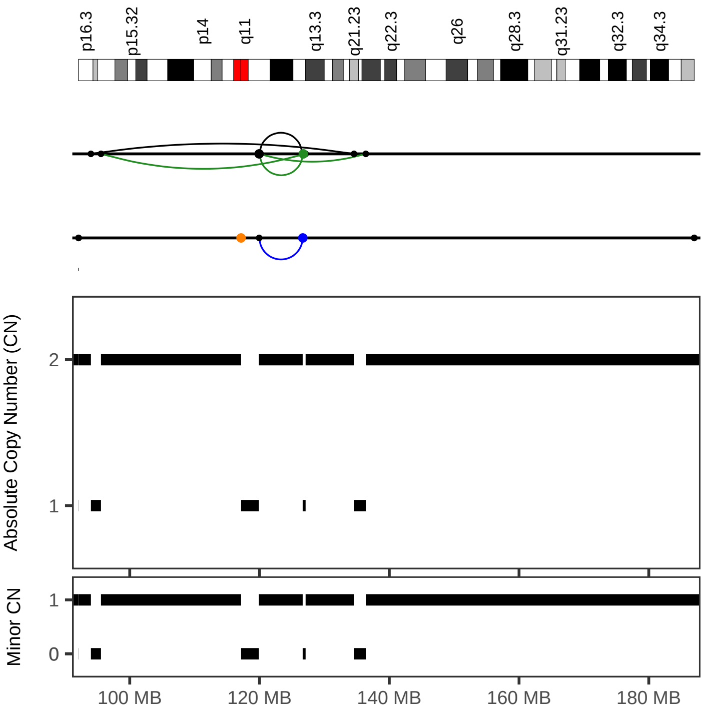

	a5f851c5−6130−4ea1−9f86−be59331a92f7
Cancer type	CNS−Oligo
Position	12:40577459−111918430
Type	With other complex events
Interleaved intrachr. SVs	13
Total SVs (intrachr. + transl.)	16
SV types	DEL: 2; DUP: 4; h2hINV: 4; t2tINV: 3; TRA: 3
SVs in sample	48
Oscillating CN (2 and 3 states)	11, 14
CN segments	29
FDR fragment joints	0.88
FDR chr. breakp. enrich.	0
Linked to chrs	
Purity, ploidy	0.93, 1.83

	300fff5a−0f61−402d−b845−3938ba7b1294
Cancer type	ColoRect−AdenoCA
Position	4:94012474−136381345
Type	Canonical without polyploidization
Interleaved intrachr. SVs	3
Total SVs (intrachr. + transl.)	10
SV types	DEL: 0; DUP: 1; h2hINV: 2; t2tINV: 3; TRA: 4
SVs in sample	51
Oscillating CN (2 and 3 states)	8, 8
CN segments	8
FDR fragment joints	0.59
FDR chr. breakp. enrich.	0
Linked to chrs	
Purity, ploidy	0.85, 2.14

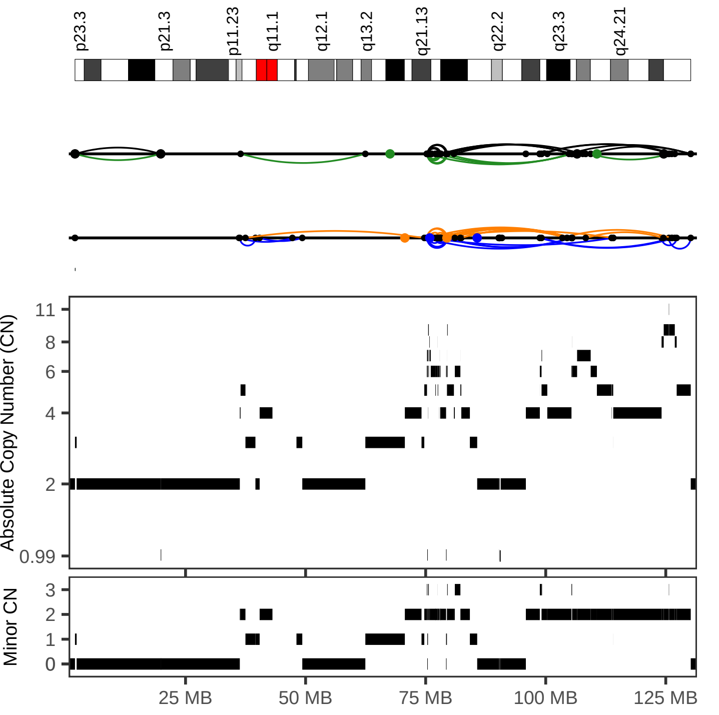

	**5116e3b4−2bac−40f5−8046−b9c1783faaa5**
Cancer type	ColoRect−AdenoCA
Position	8:75252026−130197825
Type	With other complex events
Interleaved intrachr. SVs	77
Total SVs (intrachr. + transl.)	83
SV types	DEL: 25; DUP: 17; h2hINV: 19; t2tINV: 16; TRA: 6
SVs in sample	322
Oscillating CN (2 and 3 states)	7, 8
CN segments	78
FDR fragment joints	0.64
FDR chr. breakp. enrich.	0
Linked to chrs	
Purity, ploidy	0.5, 3.09

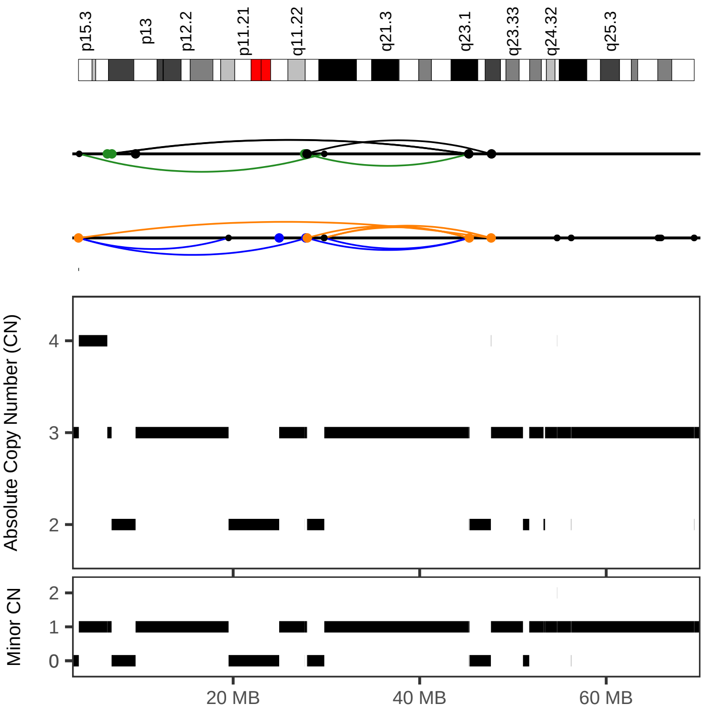

	**5116e3b4−2bac−40f5−8046−b9c1783faaa5**
Cancer type	ColoRect−AdenoCA
Position	10:3416561−47660152
Type	Canonical without polyploidization
Interleaved intrachr. SVs	15
Total SVs (intrachr. + transl.)	27
SV types	DEL: 5; DUP: 5; h2hINV: 3; t2tINV: 2; TRA: 12
SVs in sample	322
Oscillating CN (2 and 3 states)	18, 20
CN segments	22
FDR fragment joints	0.69
FDR chr. breakp. enrich.	0
Linked to chrs	11:4624293−78623113;
Purity, ploidy	0.5, 3.09

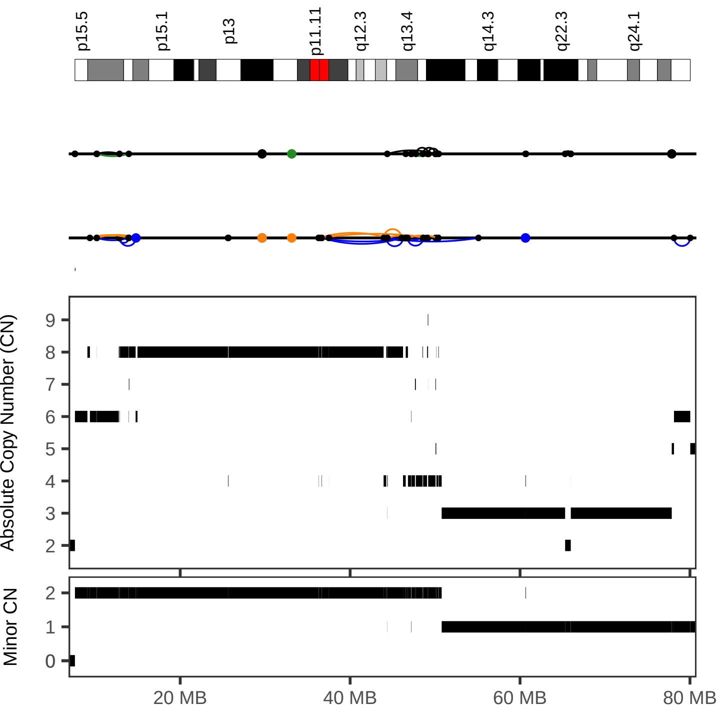

	**74a0264d−1d31−430d−9a88−e7334c8aa96c**
Cancer type	ColoRect−AdenoCA
Position	11:36286027−55106614
Type	With other complex events
Interleaved intrachr. SVs	22
Total SVs (intrachr. + transl.)	22
SV types	DEL: 6; DUP: 9; h2hINV: 4; t2tINV: 3; TRA: 0
SVs in sample	184
Oscillating CN (2 and 3 states)	9, 10
CN segments	38
FDR fragment joints	0.59
FDR chr. breakp. enrich.	0
Linked to chrs	
Purity, ploidy	0.71, 3.06

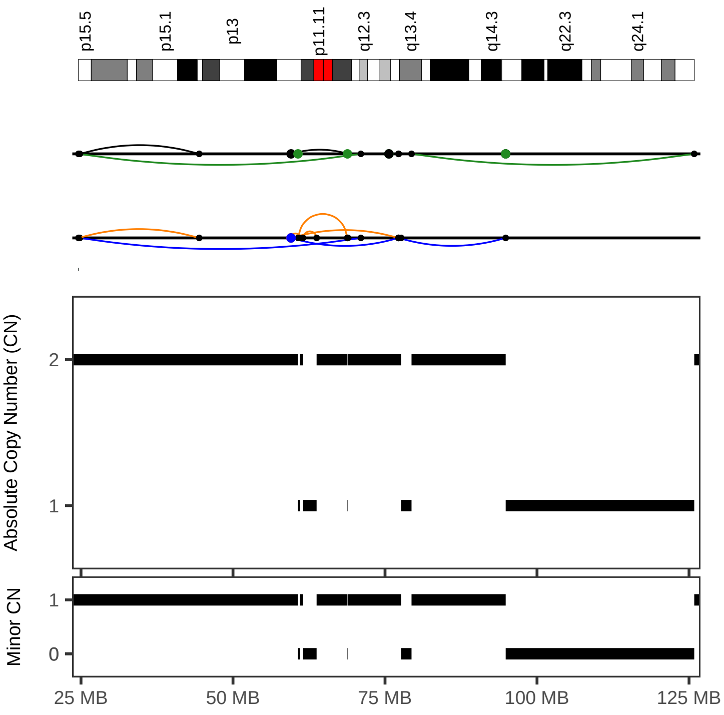

	**7d8eab0a−e6c8−4449−9ebf−50c41db94a06**
Cancer type	ColoRect−AdenoCA
Position	11:24587264−77217999
Type	Canonical without polyploidization
Interleaved intrachr. SVs	10
Total SVs (intrachr. + transl.)	18
SV types	DEL: 5; DUP: 2; h2hINV: 2; t2tINV: 1; TRA: 8
SVs in sample	170
Oscillating CN (2 and 3 states)	7, 7
CN segments	7
FDR fragment joints	0.59
FDR chr. breakp. enrich.	0
Linked to chrs	16:6213112−6590911;17:18897943−41926222
Purity, ploidy	0.7, 2.02

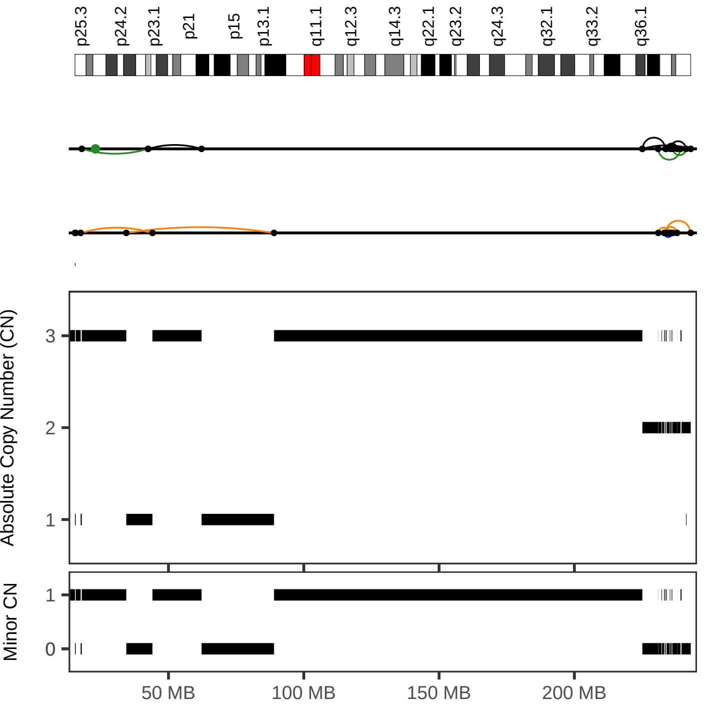

	84e34cf2−b3bb−4a1a−8fb7−6c2c604c4008
Cancer type	ColoRect−AdenoCA
Position	2:225114384−242999441
Type	Canonical without polyploidization
Interleaved intrachr. SVs	12
Total SVs (intrachr. + transl.)	12
SV types	DEL: 3; DUP: 3; h2hINV: 3; t2tINV: 3; TRA: 0
SVs in sample	96
Oscillating CN (2 and 3 states)	23, 25
CN segments	25
FDR fragment joints	1
FDR chr. breakp. enrich.	0
Linked to chrs	
Purity, ploidy	0.88, 2.09

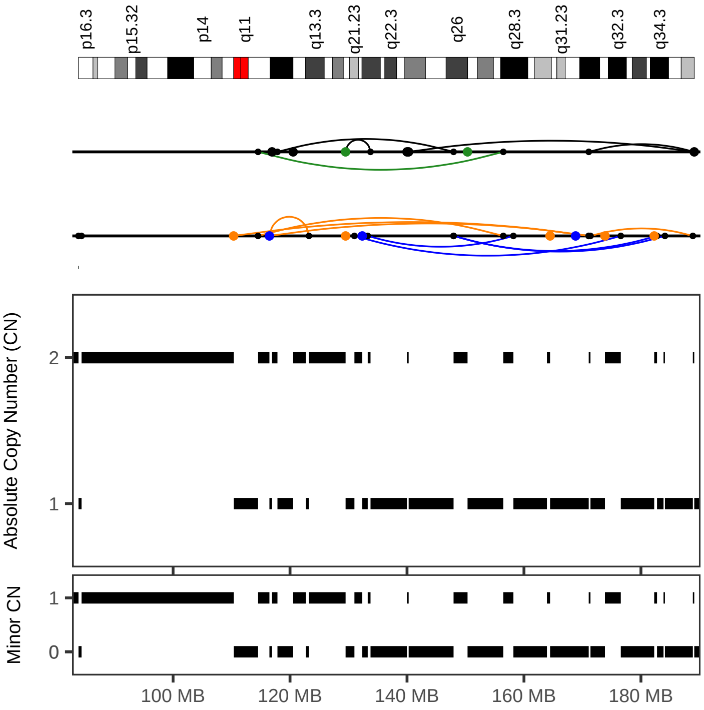

	84e34cf2−b3bb−4a1a−8fb7−6c2c604c4008
Cancer type	ColoRect−AdenoCA
Position	4:110377459−189112406
Type	Canonical without polyploidization
Interleaved intrachr. SVs	14
Total SVs (intrachr. + transl.)	28
SV types	DEL: 5; DUP: 4; h2hINV: 4; t2tINV: 1; TRA: 14
SVs in sample	96
Oscillating CN (2 and 3 states)	31, 31
CN segments	31
FDR fragment joints	0.64
FDR chr. breakp. enrich.	0
Linked to chrs	
Purity, ploidy	0.88, 2.09

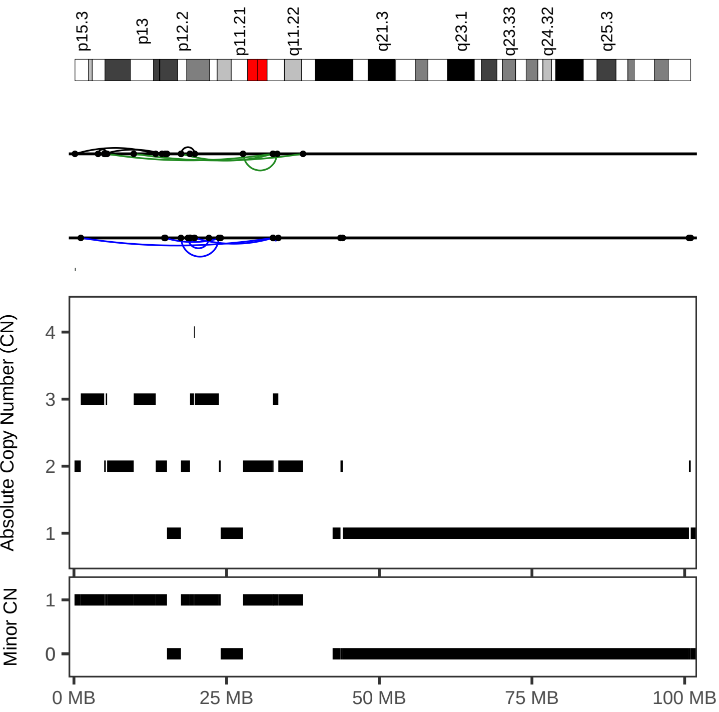

	857a5889−19e7−4208−8362−f1377c408037
Cancer type	ColoRect−AdenoCA
Position	10:170347−37518119
Type	With other complex events
Interleaved intrachr. SVs	16
Total SVs (intrachr. + transl.)	16
SV types	DEL: 0; DUP: 7; h2hINV: 5; t2tINV: 4; TRA: 0
SVs in sample	47
Oscillating CN (2 and 3 states)	7, 20
CN segments	20
FDR fragment joints	0.48
FDR chr. breakp. enrich.	0
Linked to chrs	
Purity, ploidy	0.73, 1.75

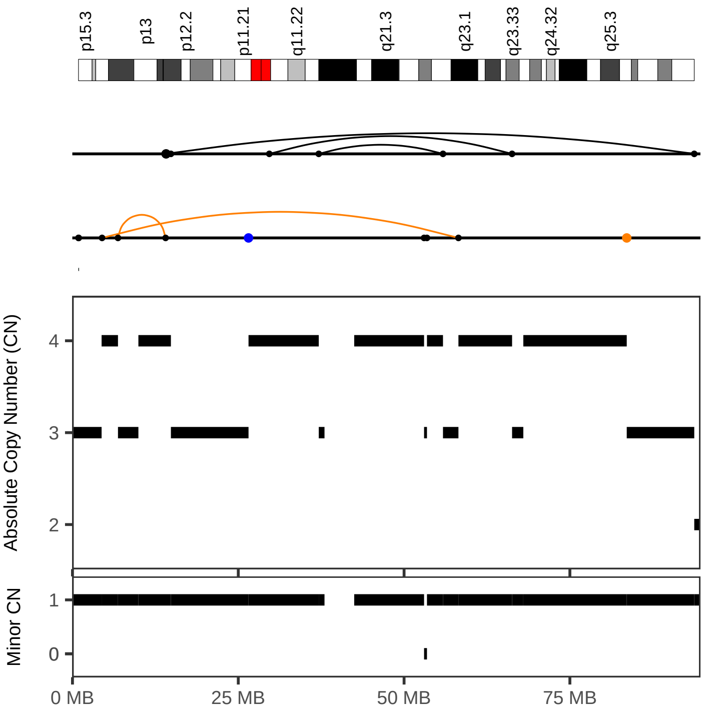

	a512701f−88bb−4b05−8c94−fd285b9dd13e
Cancer type	ColoRect−AdenoCA
Position	10:4459777−93753340
Type	After polyploidization
Interleaved intrachr. SVs	4
Total SVs (intrachr. + transl.)	7
SV types	DEL: 2; DUP: 0; h2hINV: 2; t2tINV: 0; TRA: 3
SVs in sample	134
Oscillating CN (2 and 3 states)	14, 15
CN segments	15
FDR fragment joints	0.59
FDR chr. breakp. enrich.	0.18
Linked to chrs	
Purity, ploidy	0.8, 2.77

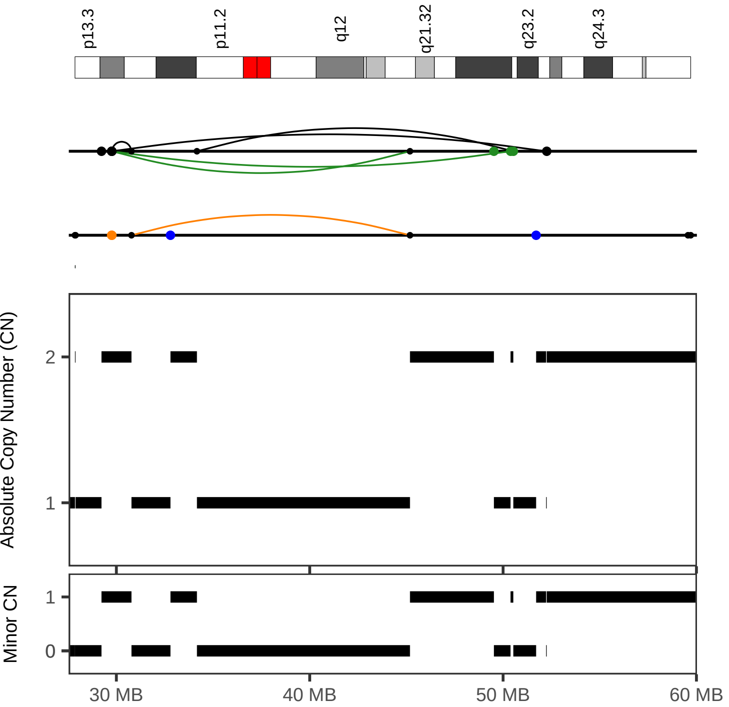

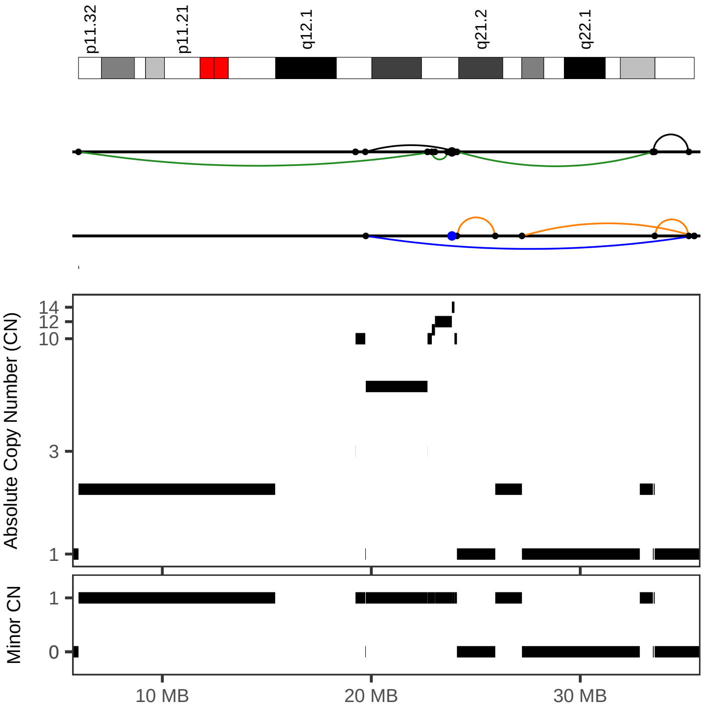

	d3b7ada6−5395−4212−a921−8e97c92b605d
Cancer type	ColoRect−AdenoCA
Position	17:29755964−52225944
Type	Canonical without polyploidization
Interleaved intrachr. SVs	6
Total SVs (intrachr. + transl.)	14
SV types	DEL: 1; DUP: 0; h2hINV: 2; t2tINV: 3; TRA: 8
SVs in sample	73
Oscillating CN (2 and 3 states)	10, 10
CN segments	10
FDR fragment joints	0.59
FDR chr. breakp. enrich.	0
Linked to chrs	
Purity, ploidy	0.61, 1.85

	d3b7ada6−5395−4212−a921−8e97c92b605d
Cancer type	ColoRect−AdenoCA
Position	18:5986794−35455161
Type	With other complex events
Interleaved intrachr. SVs	9
Total SVs (intrachr. + transl.)	11
SV types	DEL: 2; DUP: 1; h2hINV: 3; t2tINV: 3; TRA: 2
SVs in sample	73
Oscillating CN (2 and 3 states)	7, 7
CN segments	18
FDR fragment joints	0.83
FDR chr. breakp. enrich.	0
Linked to chrs	
Purity, ploidy	0.61, 1.85

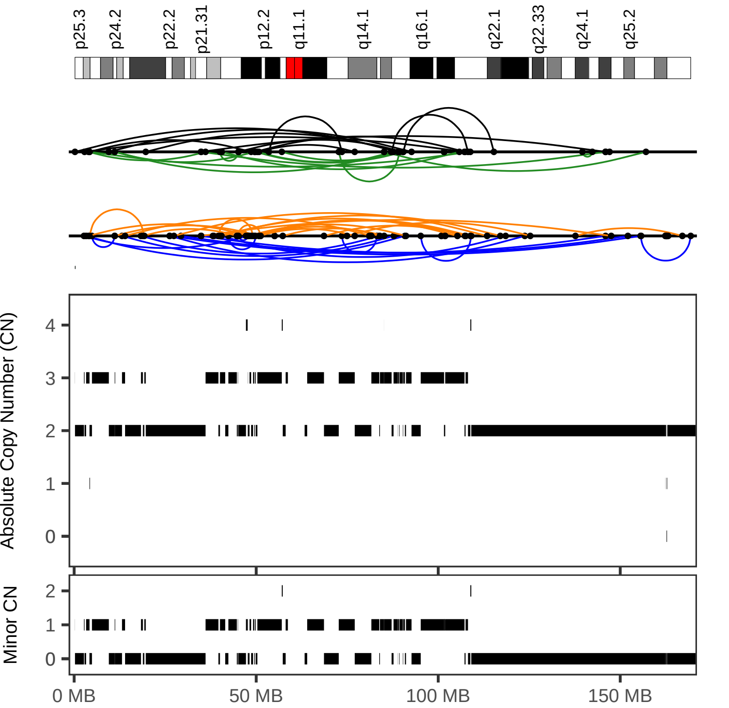

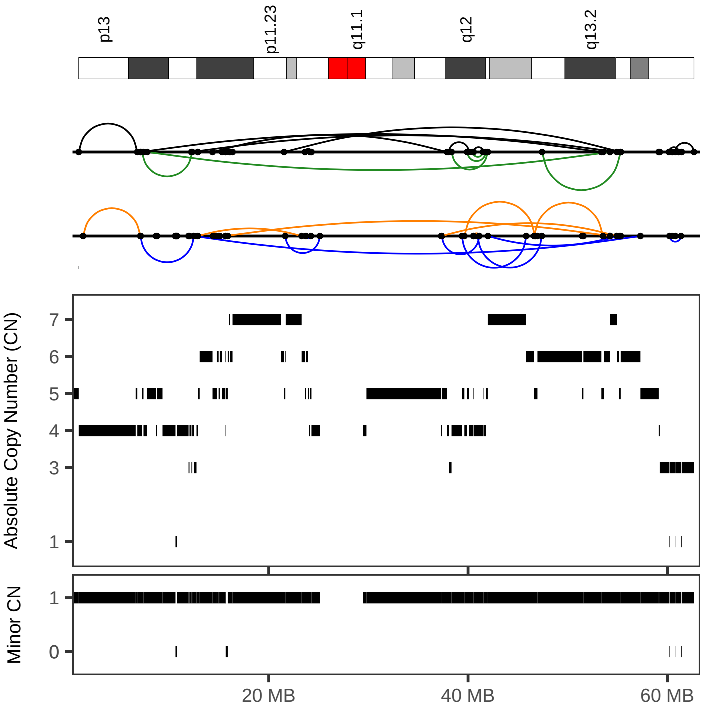

	dca004bf−9c14−45a1−b186−ab3759f6c7fe
Cancer type	ColoRect−AdenoCA
Position	6:221993−169360831
Type	With other complex events
Interleaved intrachr. SVs	63
Total SVs (intrachr. + transl.)	63
SV types	DEL: 22; DUP: 15; h2hINV: 14; t2tINV: 12; TRA: 0
SVs in sample	153
Oscillating CN (2 and 3 states)	23, 36
CN segments	87
FDR fragment joints	0.59
FDR chr. breakp. enrich.	0
Linked to chrs	
Purity, ploidy	0.62, 2.64

	e8ec26bd−c88d−42b9−ac8a−ab121b8140f8
Cancer type	ColoRect−AdenoCA
Position	20:7138898−57297995
Type	With other complex events
Interleaved intrachr. SVs	32
Total SVs (intrachr. + transl.)	32
SV types	DEL: 7; DUP: 9; h2hINV: 9; t2tINV: 7; TRA: 0
SVs in sample	94
Oscillating CN (2 and 3 states)	13, 19
CN segments	80
FDR fragment joints	0.94
FDR chr. breakp. enrich.	0
Linked to chrs	
Purity, ploidy	0.82, 2.5

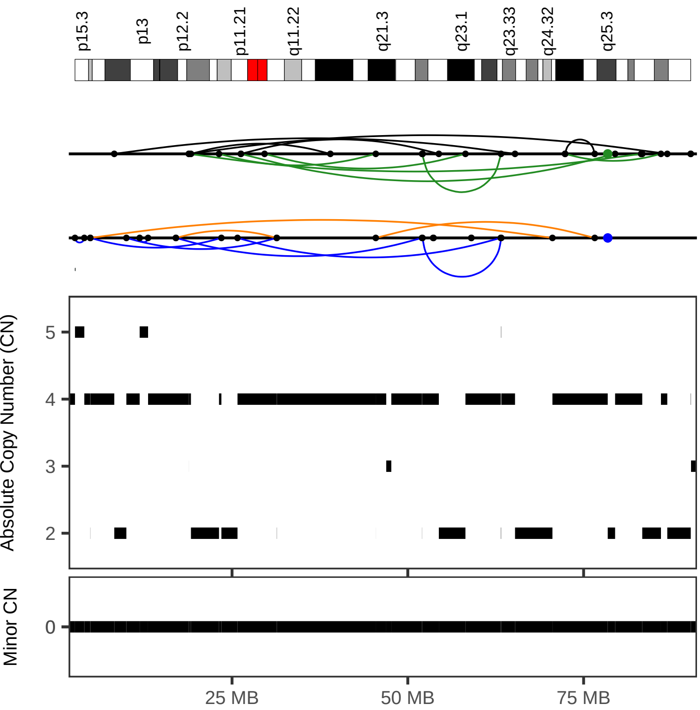

f87c6dcc−1a9e−4df5−962c−1f946e2611f8	
Cancer type	ColoRect−AdenoCA
Position	10:4835843−86898602
Type	With other complex events
Interleaved intrachr. SVs	20
Total SVs (intrachr. + transl.)	22
SV types	DEL: 3; DUP: 5; h2hINV: 6; t2tINV: 6; TRA: 2
SVs in sample	116
Oscillating CN (2 and 3 states)	9, 19
CN segments	32
FDR fragment joints	0.83
FDR chr. breakp. enrich.	0
Linked to chrs	
Purity, ploidy	0.71, 3.69

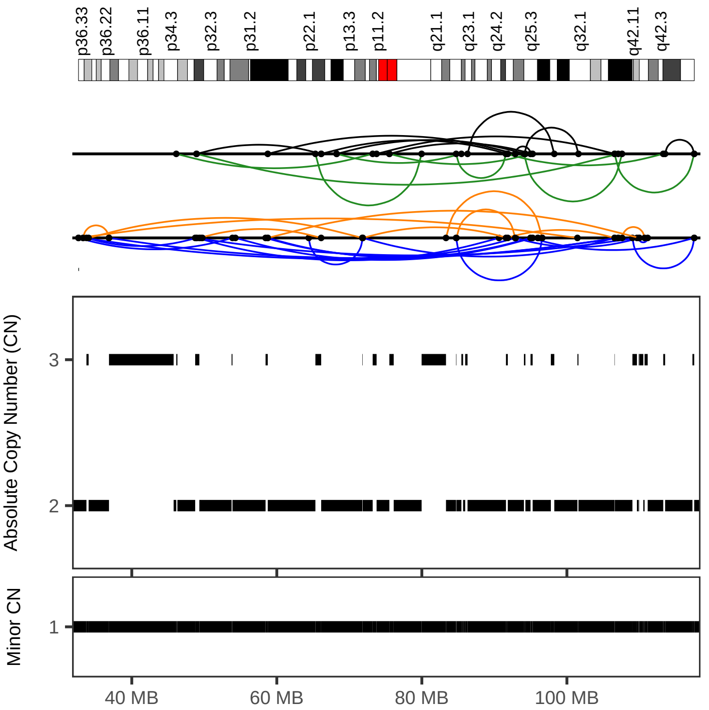

1388bba1−02ba−40df−93e5−c3d58b7bbae0	
Cancer type	ColoRect−AdenoCA
Position	1:32651575−117559110
Type	Canonical without polyploidization
Interleaved intrachr. SVs	44
Total SVs (intrachr. + transl.)	44
SV types	DEL: 10; DUP: 16; h2hINV: 9; t2tINV: 9; TRA: 0
SVs in sample	306
Oscillating CN (2 and 3 states)	53, 53
CN segments	53
FDR fragment joints	0.59
FDR chr. breakp. enrich.	0
Linked to chrs	
Purity, ploidy	0.82, 2.94

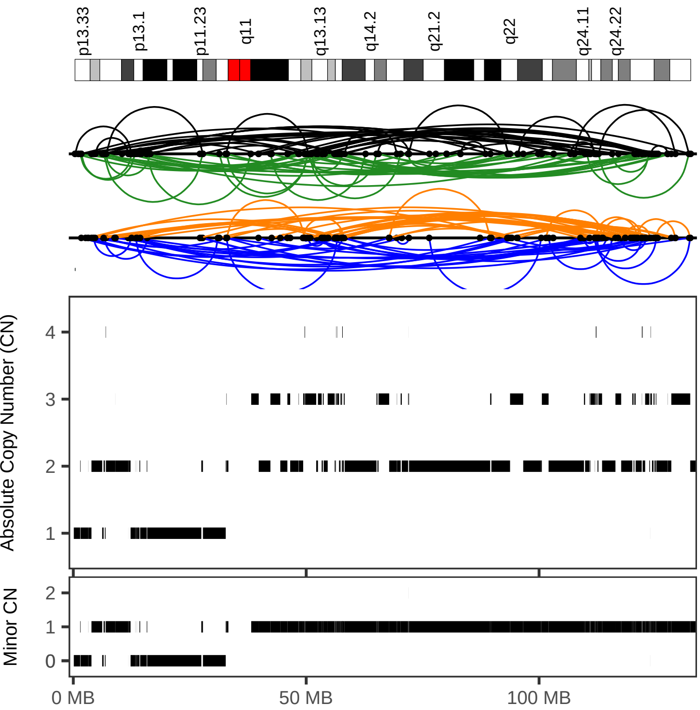

1388bba1−02ba−40df−93e5−c3d58b7bbae0	
Cancer type	ColoRect−AdenoCA
Position	12:361015−132561817
Type	With other complex events
Interleaved intrachr. SVs	179
Total SVs (intrachr. + transl.)	179
SV types	DEL: 42; DUP: 44; h2hINV: 46; t2tINV: 47; TRA: 0
SVs in sample	306
Oscillating CN (2 and 3 states)	17, 49
CN segments	129
FDR fragment joints	0.97
FDR chr. breakp. enrich.	0
Linked to chrs	
Purity, ploidy	0.82, 2.94

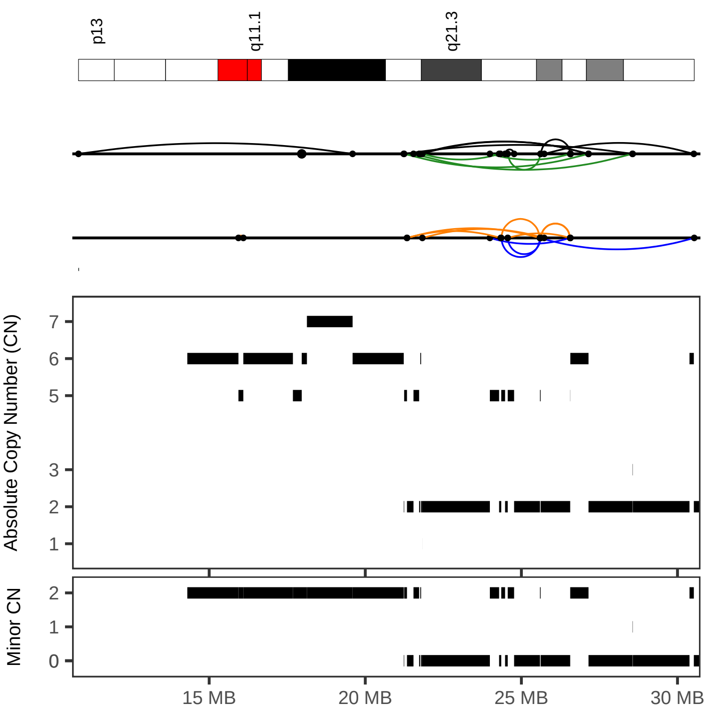

1388bba1−02ba−40df−93e5−c3d58b7bbae0	
Cancer type	ColoRect−AdenoCA
Position	21:21231971−30536463
Type	With other complex events
Interleaved intrachr. SVs	23
Total SVs (intrachr. + transl.)	23
SV types	DEL: 7; DUP: 4; h2hINV: 7; t2tINV: 5; TRA: 0
SVs in sample	306
Oscillating CN (2 and 3 states)	10, 10
CN segments	24
FDR fragment joints	0.84
FDR chr. breakp. enrich.	0
Linked to chrs	
Purity, ploidy	0.82, 2.94

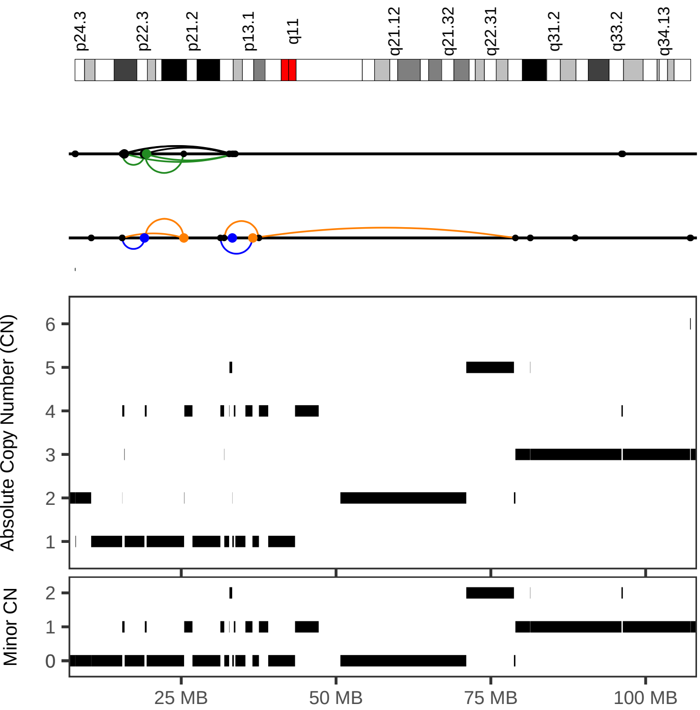

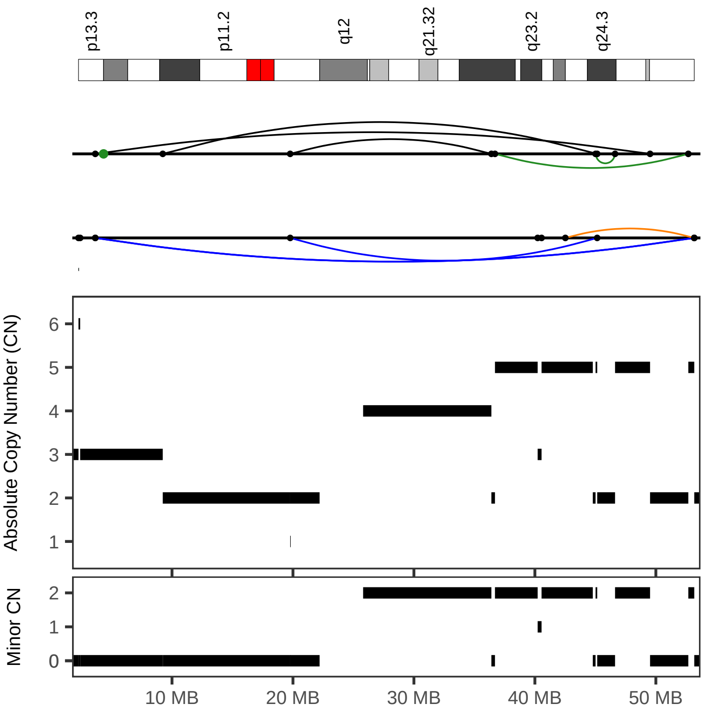

	OCCAMS−AH−014
Cancer type	Eso−AdenoCA
Position	9:15450269−78965593
Type	With other complex events
Interleaved intrachr. SVs	14
Total SVs (intrachr. + transl.)	21
SV types	DEL: 5; DUP: 2; h2hINV: 3; t2tINV: 4; TRA: 7
SVs in sample	299
Oscillating CN (2 and 3 states)	8, 9
CN segments	29
FDR fragment joints	0.78
FDR chr. breakp. enrich.	0
Linked to chrs	
Purity, ploidy	0.49, 2.86

	OCCAMS−AH−046
Cancer type	Eso−AdenoCA
Position	17:3658212−53172475
Type	With other complex events
Interleaved intrachr. SVs	9
Total SVs (intrachr. + transl.)	10
SV types	DEL: 1; DUP: 3; h2hINV: 3; t2tINV: 2; TRA: 1
SVs in sample	145
Oscillating CN (2 and 3 states)	8, 13
CN segments	16
FDR fragment joints	0.83
FDR chr. breakp. enrich.	0
Linked to chrs	
Purity, ploidy	0.37, 3.02

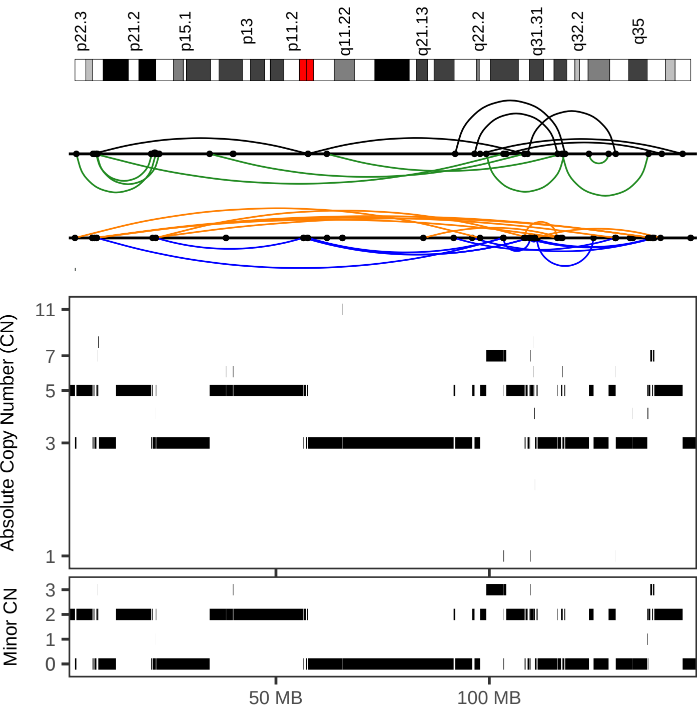

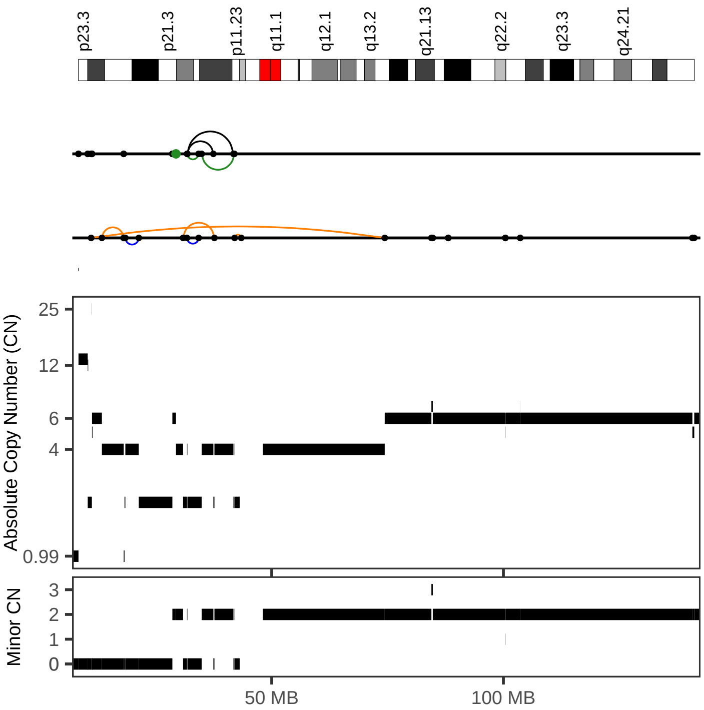

	OCCAMS−AH−048
Cancer type	Eso−AdenoCA
Position	7:2901227−145317445
Type	With other complex events
Interleaved intrachr. SVs	41
Total SVs (intrachr. + transl.)	41
SV types	DEL: 10; DUP: 12; h2hINV: 10; t2tINV: 9; TRA: 0
SVs in sample	142
Oscillating CN (2 and 3 states)	12, 12
CN segments	89
FDR fragment joints	0.95
FDR chr. breakp. enrich.	0
Linked to chrs	
Purity, ploidy	0.38, 3.19

	OCCAMS−AH−063
Cancer type	Eso−AdenoCA
Position	8:30876019−41958223
Type	Before polyploidization
Interleaved intrachr. SVs	6
Total SVs (intrachr. + transl.)	6
SV types	DEL: 1; DUP: 1; h2hINV: 2; t2tINV: 2; TRA: 0
SVs in sample	101
Oscillating CN (2 and 3 states)	8, 8
CN segments	8
FDR fragment joints	0.91
FDR chr. breakp. enrich.	0
Linked to chrs	
Purity, ploidy	0.55, 3.2

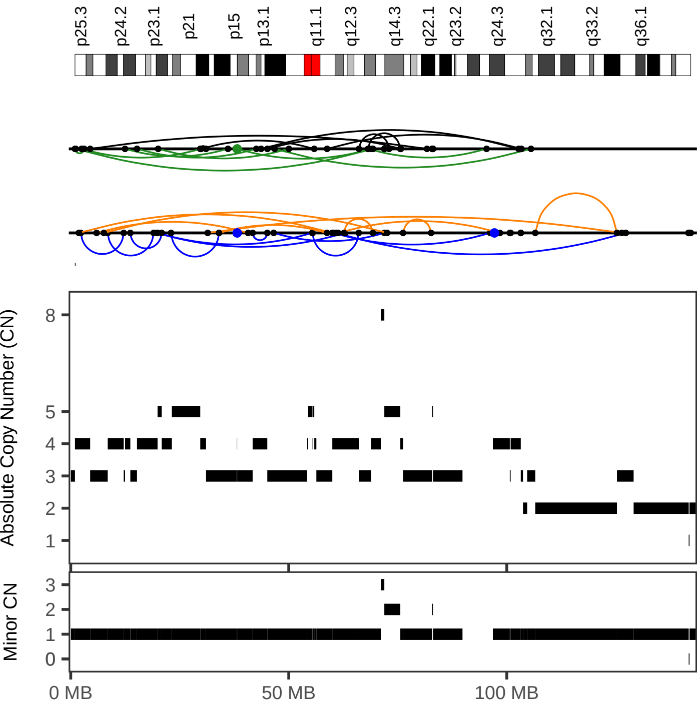

	**OCCAMS−AH−064**
Cancer type	Eso−AdenoCA
Position	2:968502−127296039
Type	With other complex events
Interleaved intrachr. SVs	36
Total SVs (intrachr. + transl.)	39
SV types	DEL: 8; DUP: 11; h2hINV: 8; t2tINV: 9; TRA: 3
SVs in sample	448
Oscillating CN (2 and 3 states)	7, 25
CN segments	39
FDR fragment joints	0.91
FDR chr. breakp. enrich.	0.25
Linked to chrs	
Purity, ploidy	0.65, 2.65

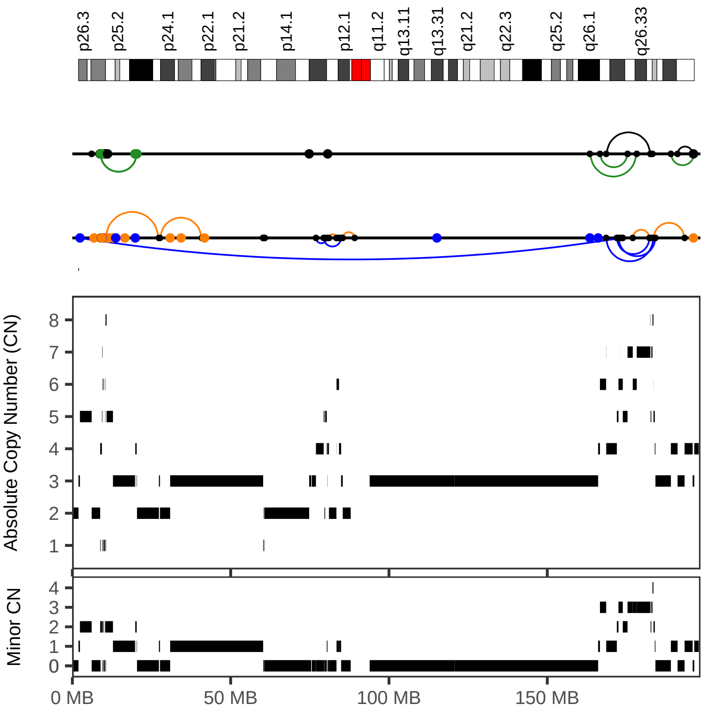

	**OCCAMS−AH−064**
Cancer type	Eso−AdenoCA
Position	3:1926043−196423191
Type	With other complex events
Interleaved intrachr. SVs	13
Total SVs (intrachr. + transl.)	53
SV types	DEL: 2; DUP: 5; h2hINV: 2; t2tINV: 4; TRA: 40
SVs in sample	448
Oscillating CN (2 and 3 states)	7, 9
CN segments	75
FDR fragment joints	0.64
FDR chr. breakp. enrich.	0
Linked to chrs	
Purity, ploidy	0.65, 2.65

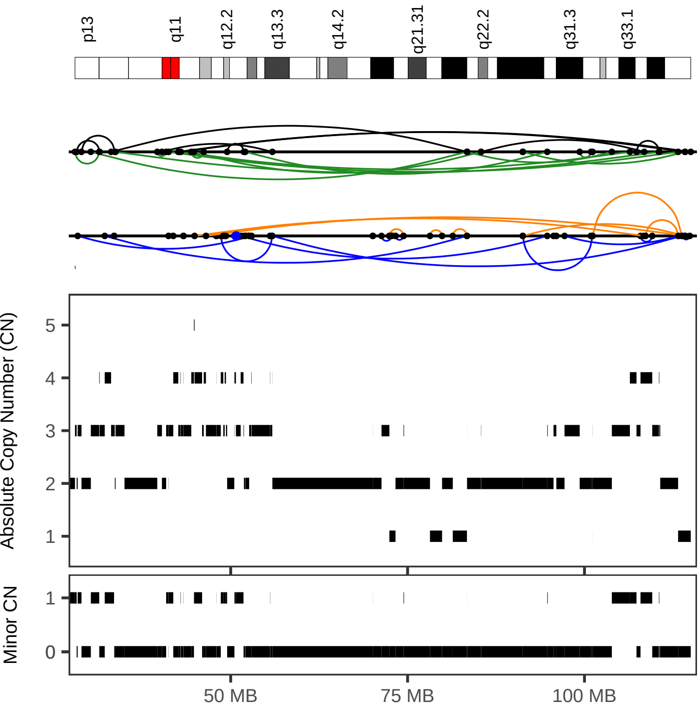

	**OCCAMS−AH−064**
Cancer type	Eso−AdenoCA
Position	13:27991549−115019710
Type	With other complex events
Interleaved intrachr. SVs	39
Total SVs (intrachr. + transl.)	40
SV types	DEL: 10; DUP: 9; h2hINV: 9; t2tINV: 11; TRA: 1
SVs in sample	448
Oscillating CN (2 and 3 states)	10, 34
CN segments	88
FDR fragment joints	0.98
FDR chr. breakp. enrich.	0
Linked to chrs	
Purity, ploidy	0.65, 2.65

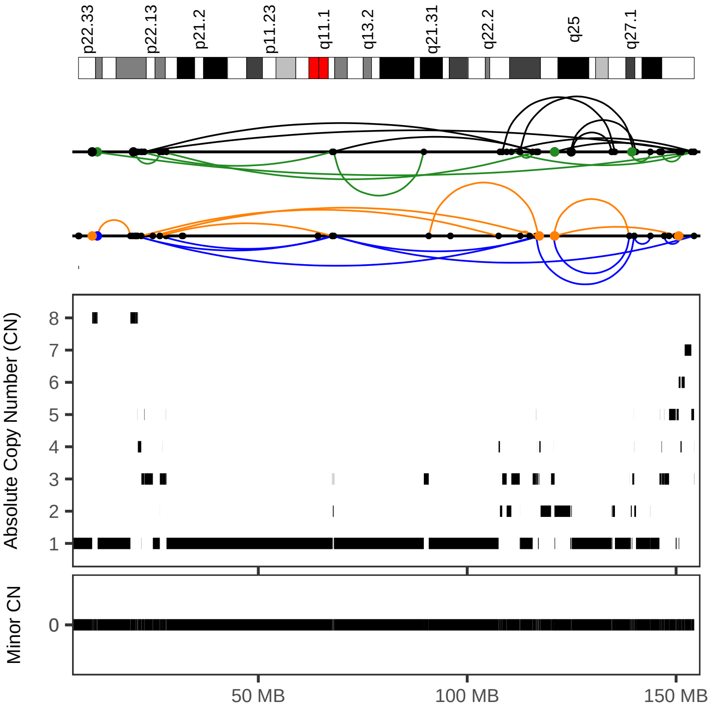

	**OCCAMS−AH−064**
Cancer type	Eso−AdenoCA
Position	X:10521537−154343000
Type	With other complex events
Interleaved intrachr. SVs	36
Total SVs (intrachr. + transl.)	47
SV types	DEL: 7; DUP: 9; h2hINV: 10; t2tINV: 10; TRA: 11
SVs in sample	448
Oscillating CN (2 and 3 states)	10, 15
CN segments	91
FDR fragment joints	0.91
FDR chr. breakp. enrich.	0
Linked to chrs	
Purity, ploidy	0.65, 2.65

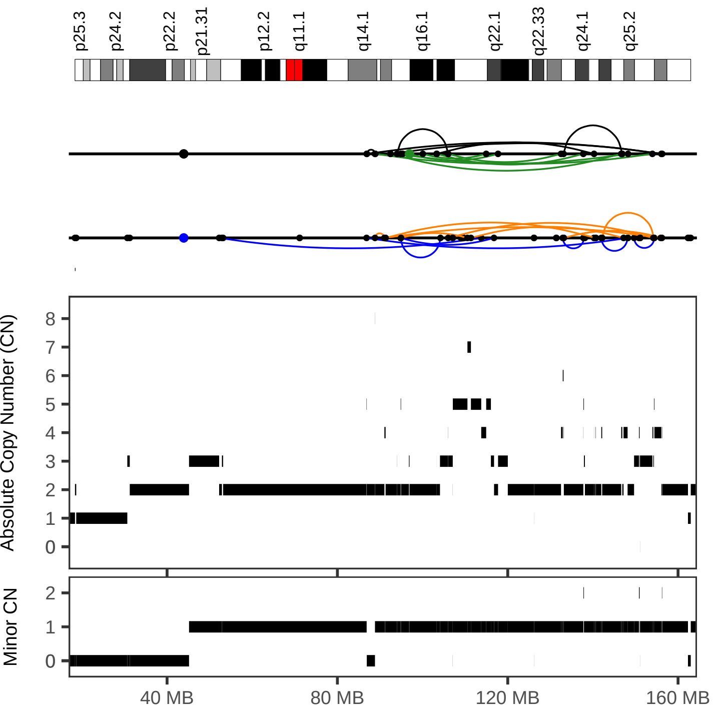

	OCCAMS−AH−082
Cancer type	Eso−AdenoCA
Position	6:52224981−156272848
Type	With other complex events
Interleaved intrachr. SVs	31
Total SVs (intrachr. + transl.)	32
SV types	DEL: 8; DUP: 9; h2hINV: 6; t2tINV: 8; TRA: 1
SVs in sample	433
Oscillating CN (2 and 3 states)	11, 14
CN segments	62
FDR fragment joints	0.92
FDR chr. breakp. enrich.	0
Linked to chrs	
Purity, ploidy	0.18, 2.48

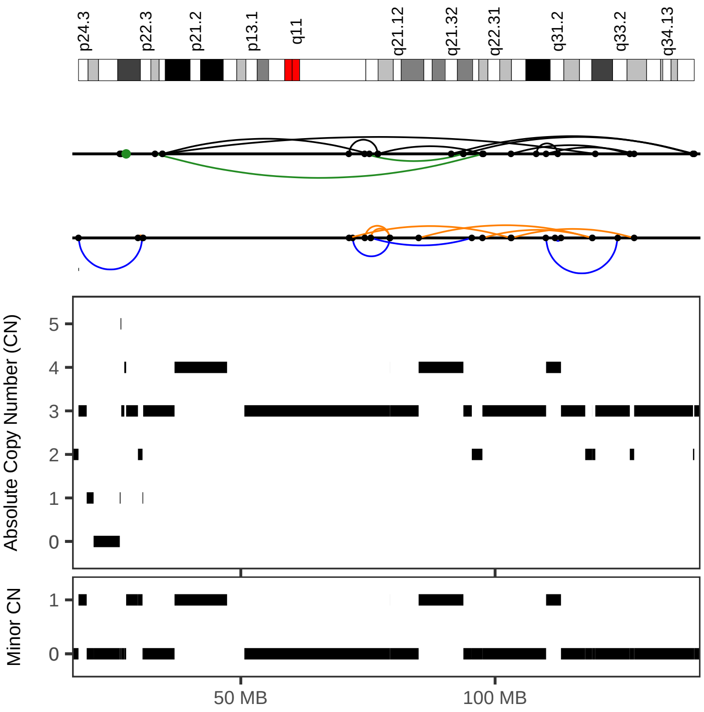

	OCCAMS−AH−085
Cancer type	Eso−AdenoCA
Position	9:33132354−139158900
Type	With other complex events
Interleaved intrachr. SVs	21
Total SVs (intrachr. + transl.)	21
SV types	DEL: 6; DUP: 4; h2hINV: 9; t2tINV: 2; TRA: 0
SVs in sample	170
Oscillating CN (2 and 3 states)	9, 19
CN segments	19
FDR fragment joints	0.59
FDR chr. breakp. enrich.	0
Linked to chrs	
Purity, ploidy	0.6, 4.06

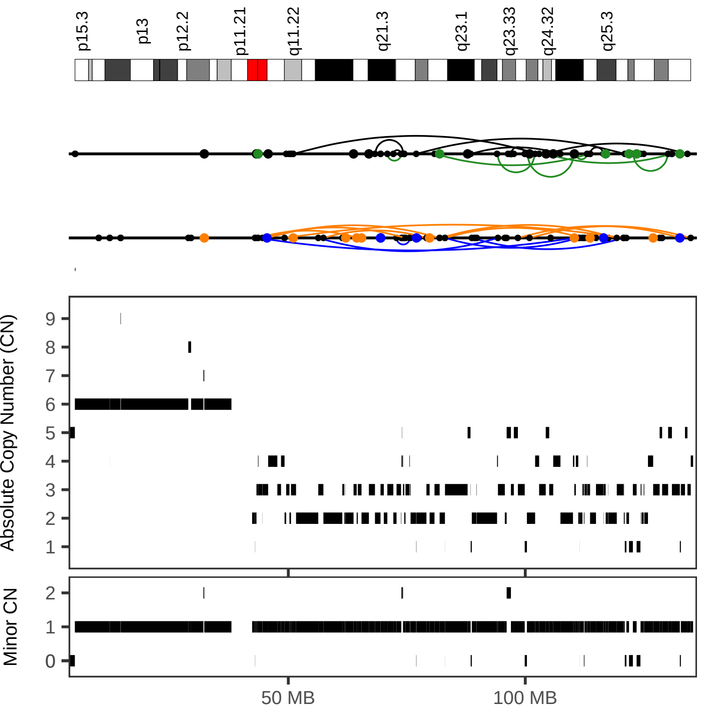

	OCCAMS−AH−086
Cancer type	Eso−AdenoCA
Position	10:43601978−134914515
Type	With other complex events
Interleaved intrachr. SVs	35
Total SVs (intrachr. + transl.)	65
SV types	DEL: 11; DUP: 7; h2hINV: 9; t2tINV: 8; TRA: 30
SVs in sample	619
Oscillating CN (2 and 3 states)	25, 30
CN segments	113
FDR fragment joints	0.84
FDR chr. breakp. enrich.	0
Linked to chrs	
Purity, ploidy	0.9, 2.18

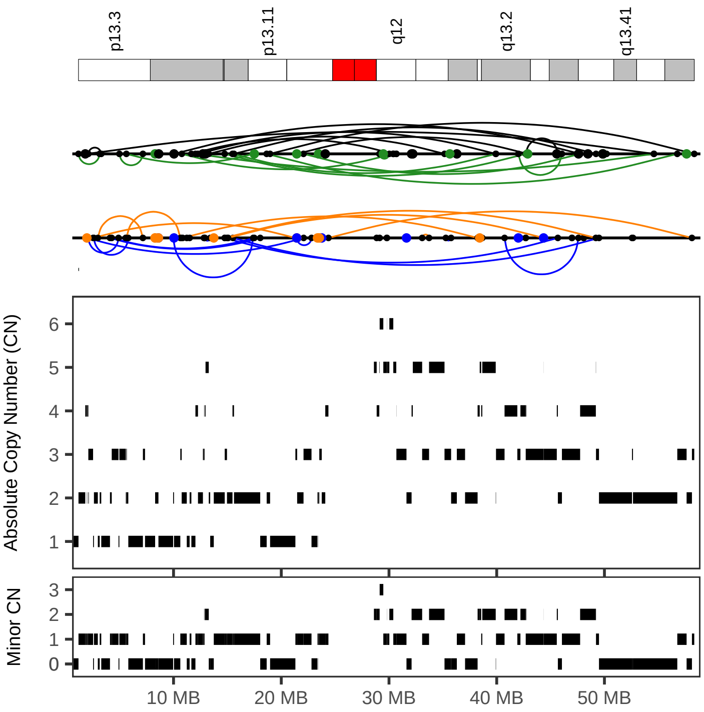

	OCCAMS−AH−086
Cancer type	Eso−AdenoCA
Position	19:1174502−58337393
Type	With other complex events
Interleaved intrachr. SVs	40
Total SVs (intrachr. + transl.)	80
SV types	DEL: 9; DUP: 11; h2hINV: 10; t2tINV: 10; TRA: 40
SVs in sample	619
Oscillating CN (2 and 3 states)	7, 12
CN segments	103
FDR fragment joints	0.99
FDR chr. breakp. enrich.	0
Linked to chrs	10:43601978−134914514;12:1246641−123710274
Purity, ploidy	0.9, 2.18

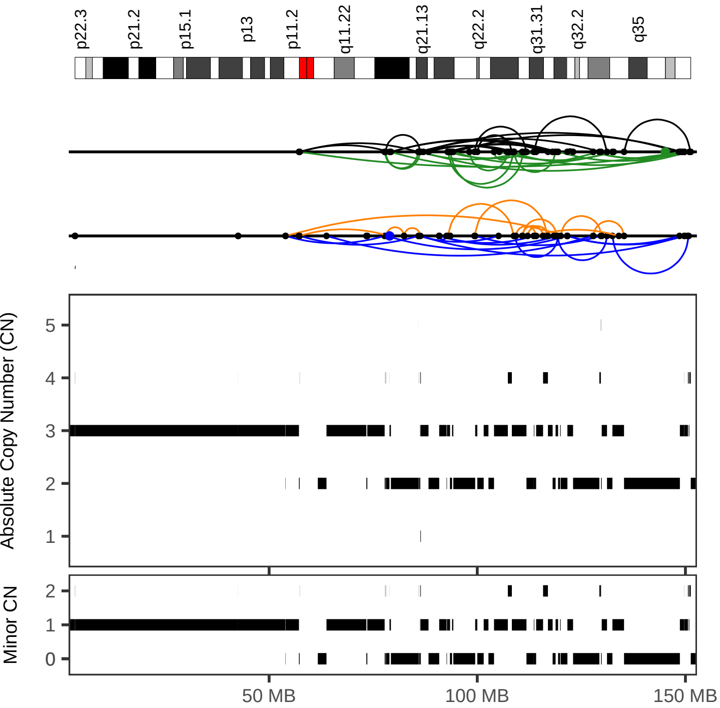

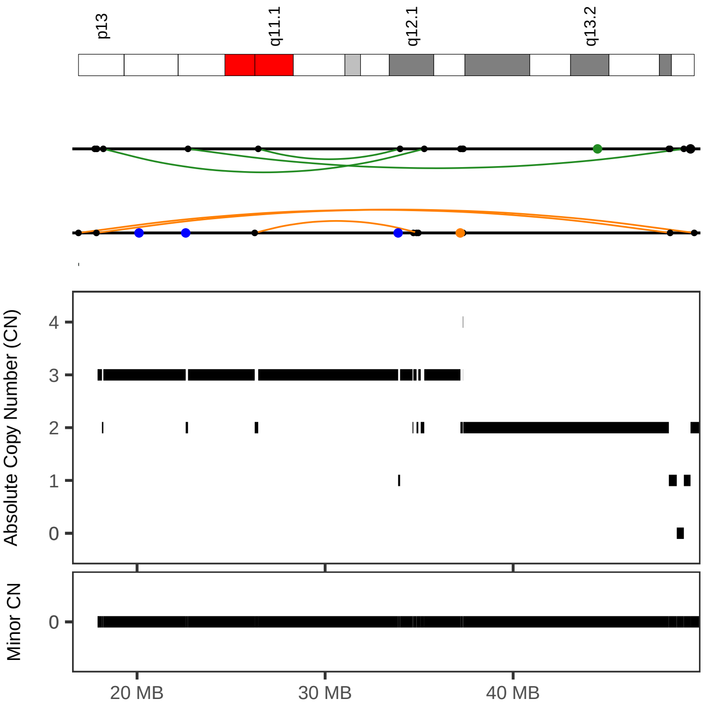

	OCCAMS−AH−088
Cancer type	Eso−AdenoCA
Position	7:53921918−151275079
Type	With other complex events
Interleaved intrachr. SVs	68
Total SVs (intrachr. + transl.)	70
SV types	DEL: 14; DUP: 15; h2hINV: 19; t2tINV: 20; TRA: 2
SVs in sample	167
Oscillating CN (2 and 3 states)	15, 33
CN segments	69
FDR fragment joints	0.75
FDR chr. breakp. enrich.	0
Linked to chrs	
Purity, ploidy	0.46, 2.71

	OCCAMS−AH−096
Cancer type	Eso−AdenoCA
Position	22:16884779−49630408
Type	With other complex events
Interleaved intrachr. SVs	6
Total SVs (intrachr. + transl.)	12
SV types	DEL: 2; DUP: 0; h2hINV: 1; t2tINV: 3; TRA: 6
SVs in sample	326
Oscillating CN (2 and 3 states)	8, 21
CN segments	25
FDR fragment joints	0.59
FDR chr. breakp. enrich.	0
Linked to chrs	
Purity, ploidy	0.26, 2.74

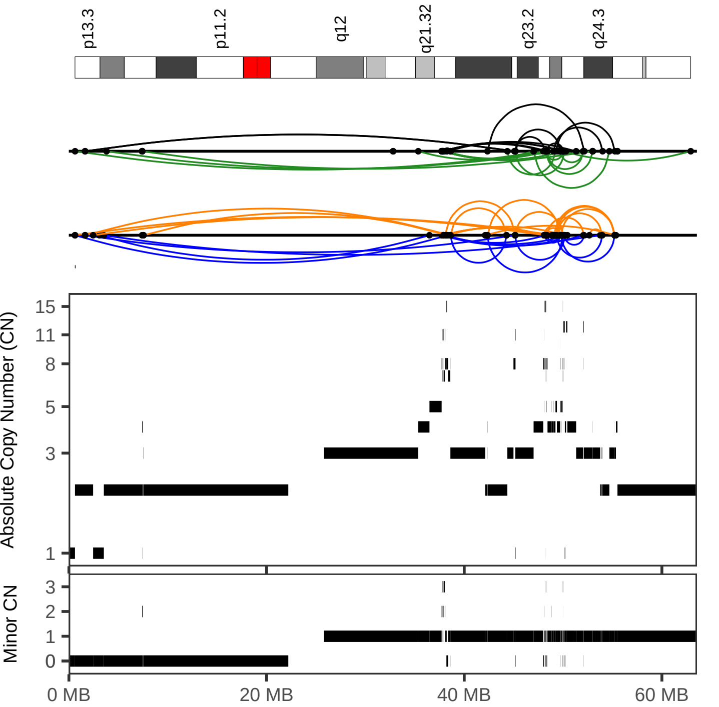

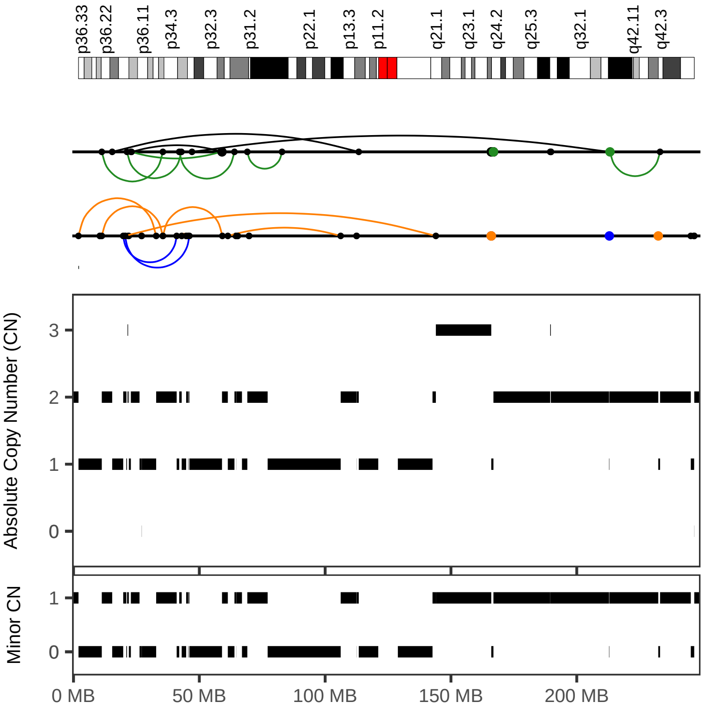

	OCCAMS−AH−108
Cancer type	Eso−AdenoCA
Position	17:614159−62917745
Type	With other complex events
Interleaved intrachr. SVs	80
Total SVs (intrachr. + transl.)	80
SV types	DEL: 22; DUP: 19; h2hINV: 19; t2tINV: 20; TRA: 0
SVs in sample	162
Oscillating CN (2 and 3 states)	7, 7
CN segments	102
FDR fragment joints	0.97
FDR chr. breakp. enrich.	0
Linked to chrs	
Purity, ploidy	0.47, 2.83

	OCCAMS−AH−112
Cancer type	Eso−AdenoCA
Position	1:1979872−212827045
Type	With other complex events
Interleaved intrachr. SVs	14
Total SVs (intrachr. + transl.)	24
SV types	DEL: 5; DUP: 2; h2hINV: 3; t2tINV: 4; TRA: 10
SVs in sample	221
Oscillating CN (2 and 3 states)	21, 33
CN segments	41
FDR fragment joints	0.78
FDR chr. breakp. enrich.	0
Linked to chrs	12:8323902−14885237;4:5188482−185655428
Purity, ploidy	0.71, 1.82

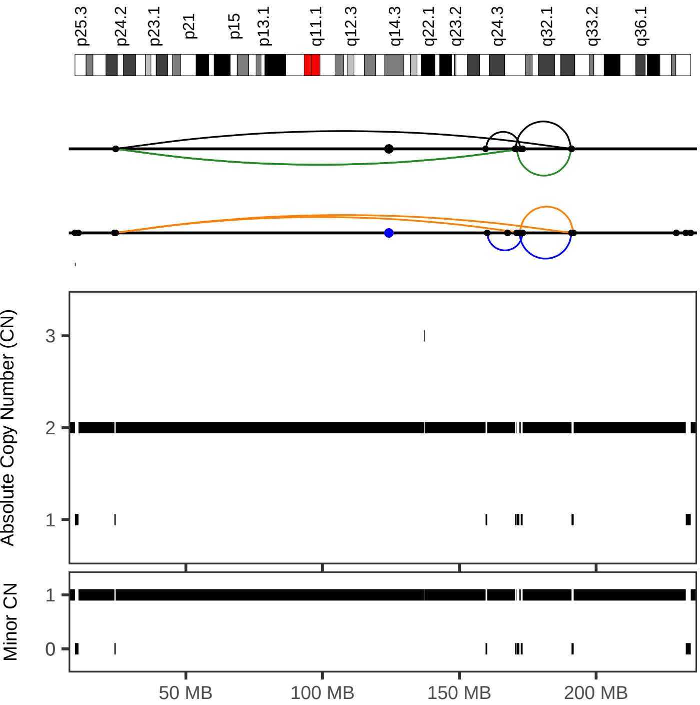

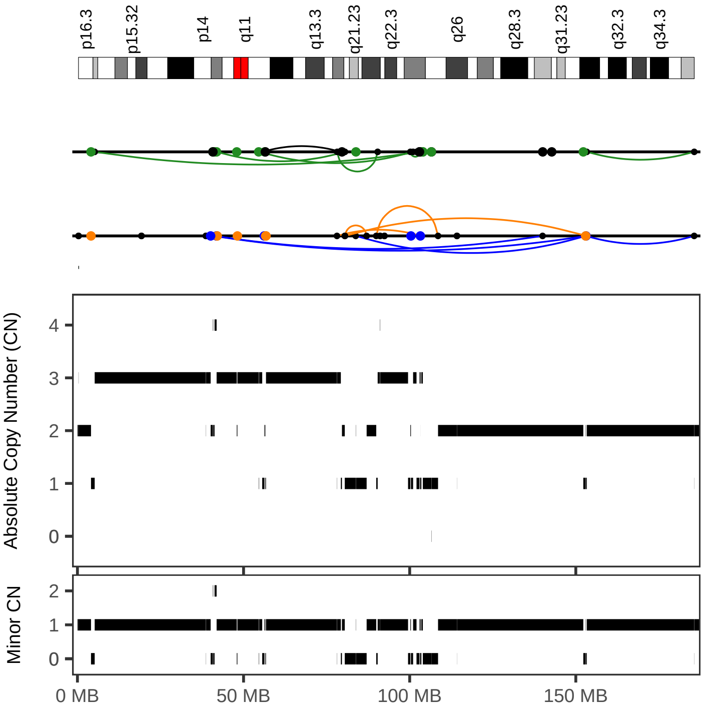

	**OCCAMS−AH−112**
Cancer type	Eso−AdenoCA
Position	2:23859234−191789699
Type	Canonical without polyploidization
Interleaved intrachr. SVs	11
Total SVs (intrachr. + transl.)	14
SV types	DEL: 3; DUP: 2; h2hINV: 3; t2tINV: 3; TRA: 3
SVs in sample	221
Oscillating CN (2 and 3 states)	11, 14
CN segments	14
FDR fragment joints	0.98
FDR chr. breakp. enrich.	1
Linked to chrs	
Purity, ploidy	0.71, 1.82

	**OCCAMS−AH−112**
Cancer type	Eso−AdenoCA
Position	4:5188482−185655429
Type	With other complex events
Interleaved intrachr. SVs	16
Total SVs (intrachr. + transl.)	48
SV types	DEL: 5; DUP: 4; h2hINV: 1; t2tINV: 6; TRA: 32
SVs in sample	221
Oscillating CN (2 and 3 states)	10, 21
CN segments	54
FDR fragment joints	0.59
FDR chr. breakp. enrich.	0
Linked to chrs	1:1979872−212827044;10:23310943−61673311 12:8323902−14885237;5:110689915−118671115 9:2688258−130163537;
Purity, ploidy	0.71, 1.82

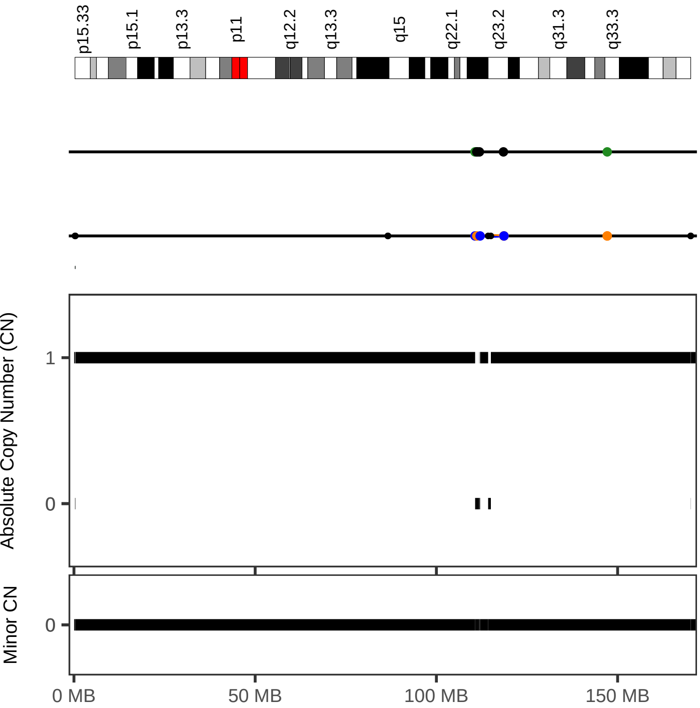

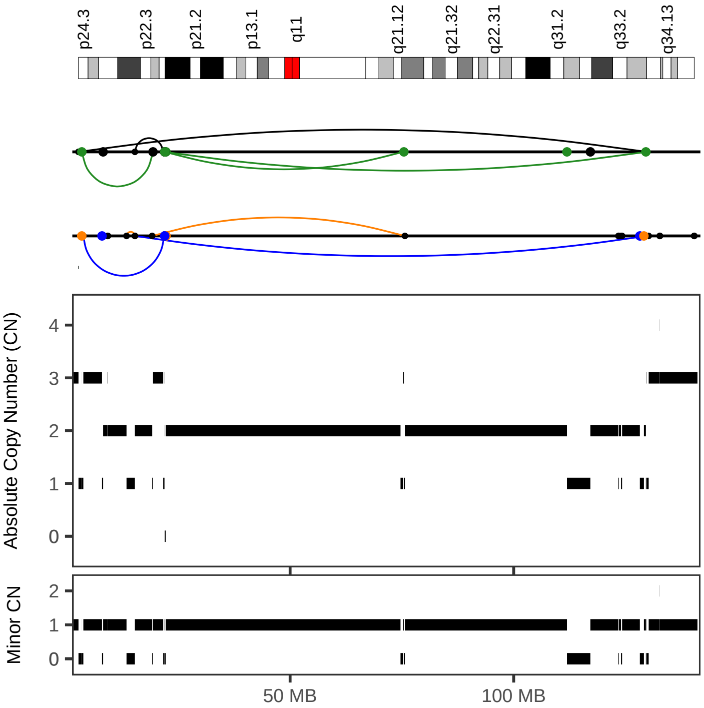

	**OCCAMS−AH−112**
Cancer type	Eso−AdenoCA
Position	5:110689915−118671116
Type	Canonical without polyploidization
Interleaved intrachr. SVs	6
Total SVs (intrachr. + transl.)	16
SV types	DEL: 1; DUP: 1; h2hINV: 3; t2tINV: 1; TRA: 10
SVs in sample	221
Oscillating CN (2 and 3 states)	8, 8
CN segments	8
FDR fragment joints	0.64
FDR chr. breakp. enrich.	0.01
Linked to chrs	9:2688258−130163537;
Purity, ploidy	0.71, 1.82

	**OCCAMS−AH−112**
Cancer type	Eso−AdenoCA
Position	9:2688258−130163538
Type	With other complex events
Interleaved intrachr. SVs	10
Total SVs (intrachr. + transl.)	34
SV types	DEL: 3; DUP: 2; h2hINV: 2; t2tINV: 3; TRA: 24
SVs in sample	221
Oscillating CN (2 and 3 states)	11, 19
CN segments	35
FDR fragment joints	0.96
FDR chr. breakp. enrich.	0
Linked to chrs	4:5188482−185655428;5:110689915−118671115
Purity, ploidy	0.71, 1.82

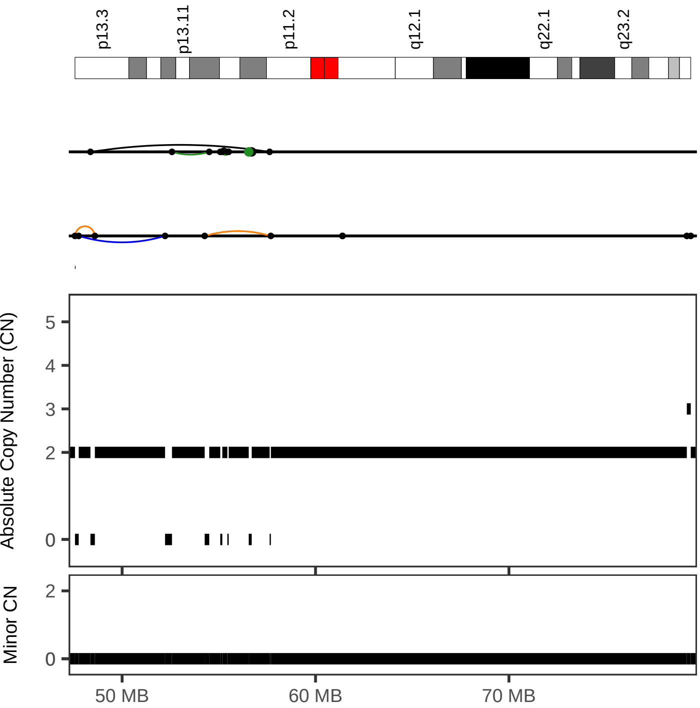

	**OCCAMS−AH−136**
Cancer type	Eso−AdenoCA
Position	16:47562340−57689371
Type	Canonical without polyploidization
Interleaved intrachr. SVs	5
Total SVs (intrachr. + transl.)	7
SV types	DEL: 2; DUP: 1; h2hINV: 1; t2tINV: 1; TRA: 2
SVs in sample	67
Oscillating CN (2 and 3 states)	16, 16
CN segments	16
FDR fragment joints	0.92
FDR chr. breakp. enrich.	0
Linked to chrs	
Purity, ploidy	0.25, 3.12

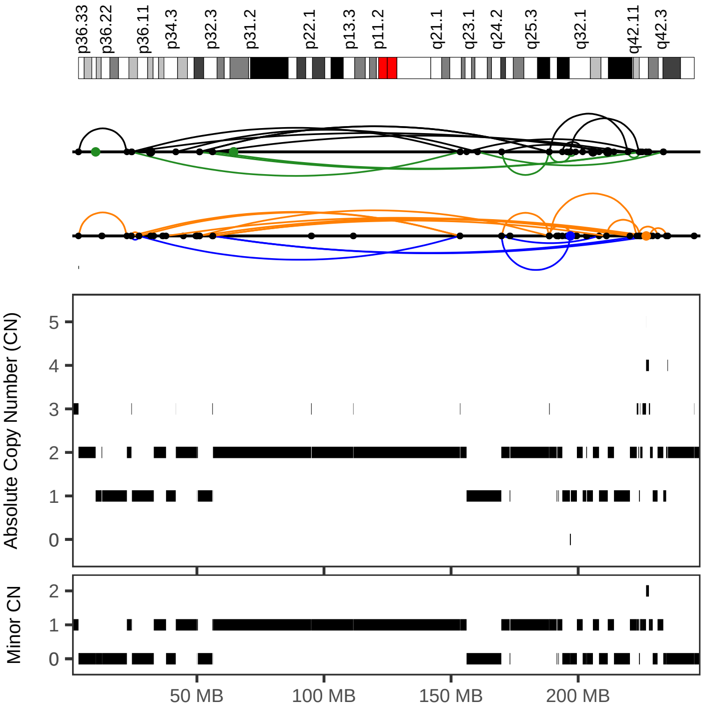

	**OCCAMS−AH−173**
Cancer type	Eso−AdenoCA
Position	1:24204431−234619940
Type	With other complex events
Interleaved intrachr. SVs	44
Total SVs (intrachr. + transl.)	51
SV types	DEL: 16; DUP: 8; h2hINV: 11; t2tINV: 9; TRA: 7
SVs in sample	410
Oscillating CN (2 and 3 states)	10, 37
CN segments	66
FDR fragment joints	0.59
FDR chr. breakp. enrich.	0
Linked to chrs	11:17492995−133541100;14:64173909−64406103
Purity, ploidy	0.6, 1.64

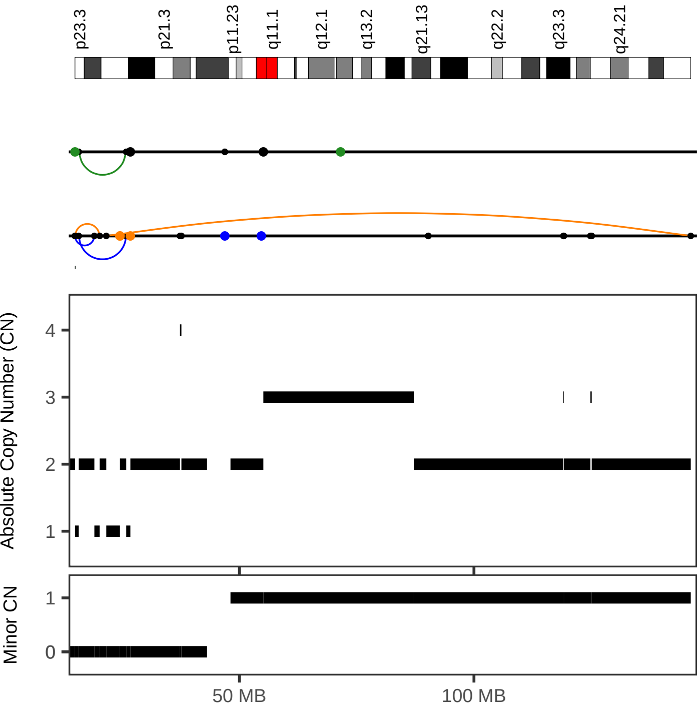

	**OCCAMS−AH−173**
Cancer type	Eso−AdenoCA
Position	8:14950710−146210675
Type	With other complex events
Interleaved intrachr. SVs	5
Total SVs (intrachr. + transl.)	14
SV types	DEL: 2; DUP: 2; h2hINV: 0; t2tINV: 1; TRA: 9
SVs in sample	410
Oscillating CN (2 and 3 states)	9, 9
CN segments	18
FDR fragment joints	0.64
FDR chr. breakp. enrich.	1
Linked to chrs	
Purity, ploidy	0.6, 1.64

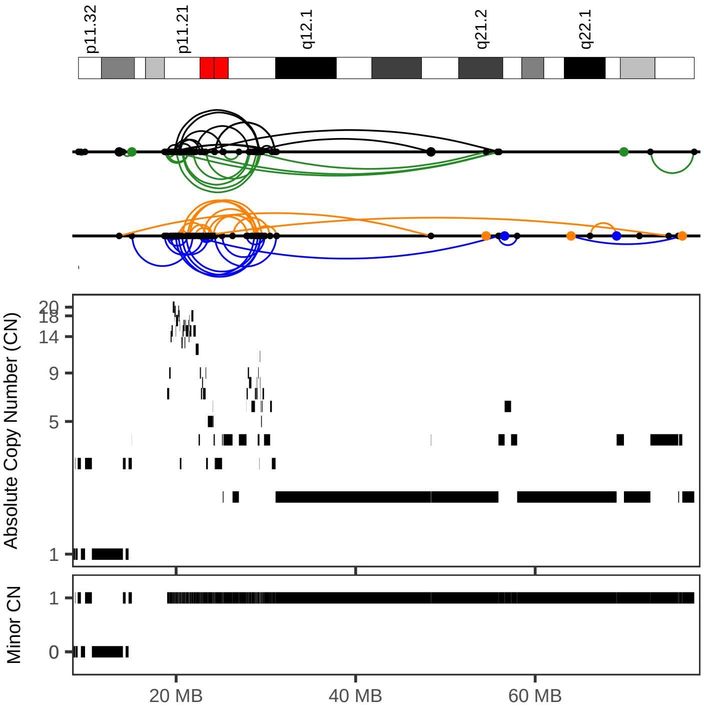

	**OCCAMS−AH−196**
Cancer type	Eso−AdenoCA
Position	18:13655503−77718608
Type	With other complex events
Interleaved intrachr. SVs	82
Total SVs (intrachr. + transl.)	90
SV types	DEL: 18; DUP: 25; h2hINV: 19; t2tINV: 20; TRA: 8
SVs in sample	216
Oscillating CN (2 and 3 states)	8, 9
CN segments	92
FDR fragment joints	0.78
FDR chr. breakp. enrich.	0
Linked to chrs	
Purity, ploidy	0.68, 2.13

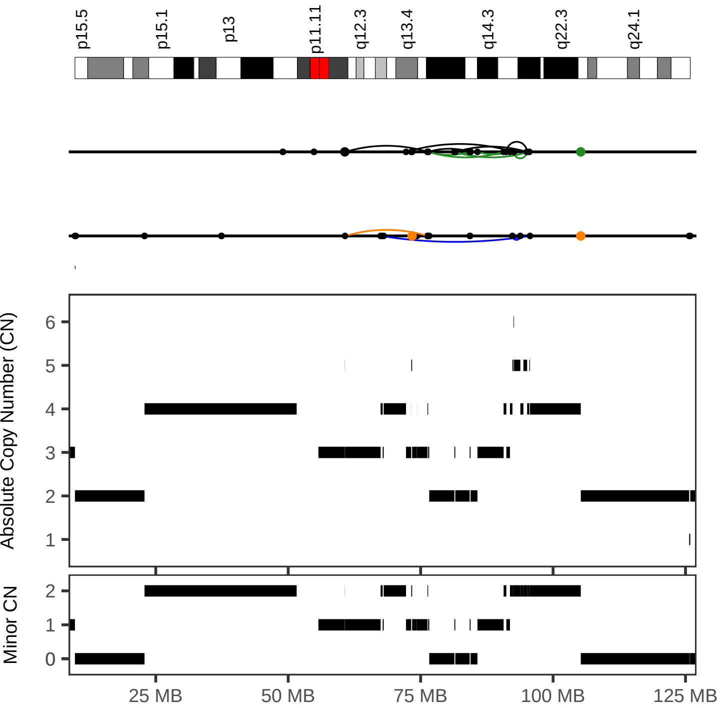

**OCCAMS−AH−197**	
Cancer type	Eso−AdenoCA
Position	11:60717275−95643409
Type	With other complex events
Interleaved intrachr. SVs	16
Total SVs (intrachr. + transl.)	17
SV types	DEL: 1; DUP: 4; h2hINV: 6; t2tINV: 5; TRA: 1
SVs in sample	269
Oscillating CN (2 and 3 states)	9, 24
CN segments	31
FDR fragment joints	0.59
FDR chr. breakp. enrich.	0
Linked to chrs	
Purity, ploidy	0.3, 3.09

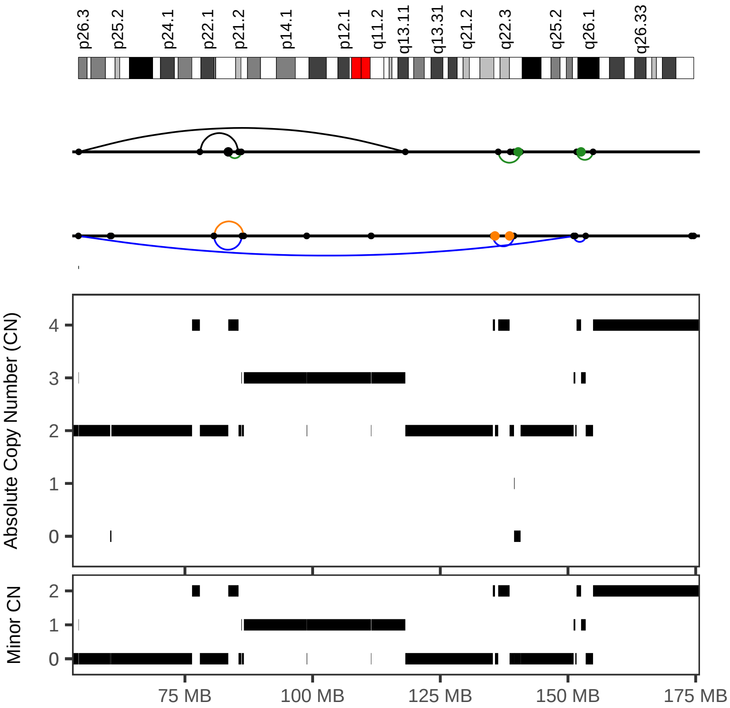

**OCCAMS−ED−003**	
Cancer type	Eso−AdenoCA
Position	3:77926901−140720659
Type	With other complex events
Interleaved intrachr. SVs	4
Total SVs (intrachr. + transl.)	12
SV types	DEL: 1; DUP: 2; h2hINV: 2; t2tINV: 3; TRA: 4
SVs in sample	117
Oscillating CN (2 and 3 states)	9, 15
CN segments	19
FDR fragment joints	0.84
FDR chr. breakp. enrich.	0
Linked to chrs	
Purity, ploidy	0.44, 2.99

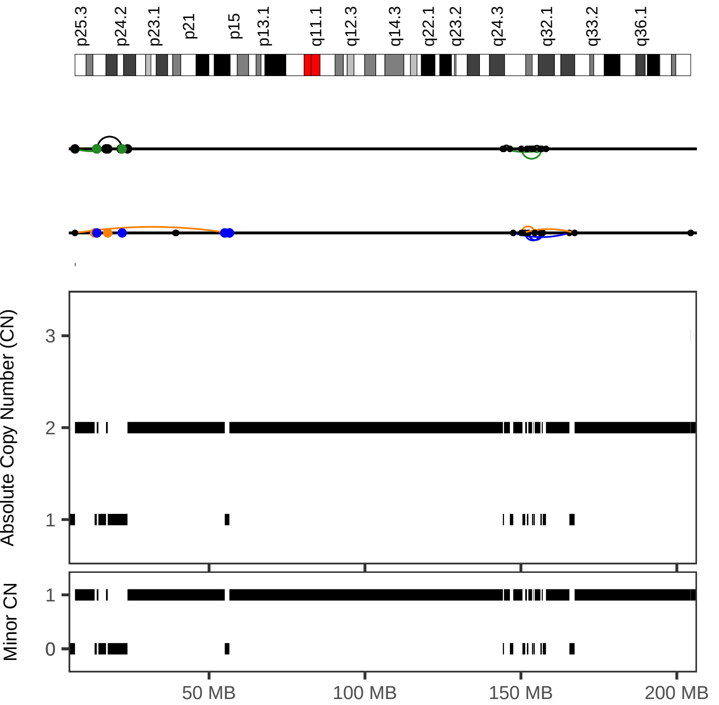

**OCCAMS−ED−041**	
Cancer type	Eso−AdenoCA
Position	2:144209346−167223868
Type	Canonical without polyploidization
Interleaved intrachr. SVs	11
Total SVs (intrachr. + transl.)	11
SV types	DEL: 2; DUP: 3; h2hINV: 3; t2tINV: 3; TRA: 0
SVs in sample	125
Oscillating CN (2 and 3 states)	18, 18
CN segments	18
FDR fragment joints	0.98
FDR chr. breakp. enrich.	0
Linked to chrs	
Purity, ploidy	0.72, 1.9

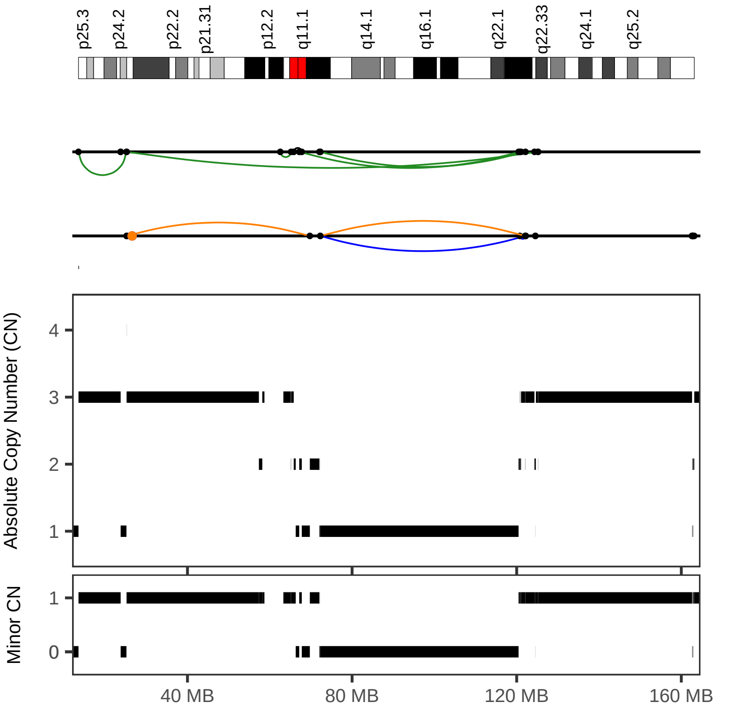

**OCCAMS−GS−002**	
Cancer type	Eso−AdenoCA
Position	6:25217251−125203324
Type	With other complex events
Interleaved intrachr. SVs	13
Total SVs (intrachr. + transl.)	15
SV types	DEL: 3; DUP: 3; h2hINV: 3; t2tINV: 4; TRA: 2
SVs in sample	134
Oscillating CN (2 and 3 states)	9, 23
CN segments	28
FDR fragment joints	0.98
FDR chr. breakp. enrich.	0
Linked to chrs	3:60205458−106286281;
Purity, ploidy	0.69, 1.97

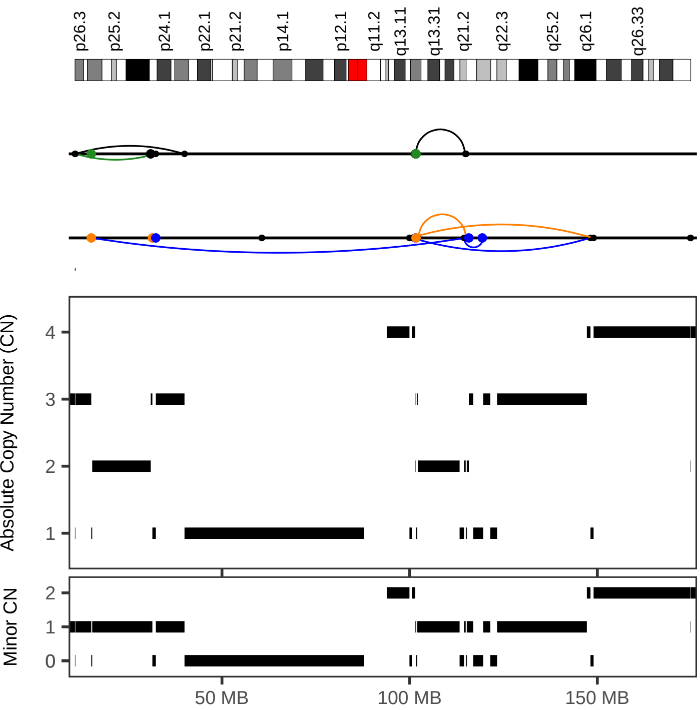

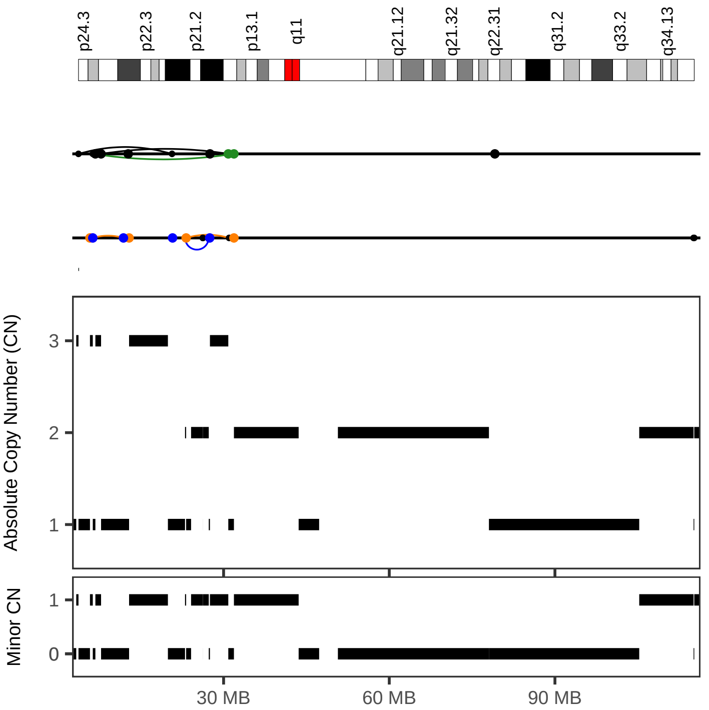

**OCCAMS−PS−014**	
Cancer type	Eso−AdenoCA
Position	3:10840533−149005314
Type	With other complex events
Interleaved intrachr. SVs	9
Total SVs (intrachr. + transl.)	21
SV types	DEL: 2; DUP: 3; h2hINV: 2; t2tINV: 2; TRA: 12
SVs in sample	53
Oscillating CN (2 and 3 states)	7, 13
CN segments	30
FDR fragment joints	0.97
FDR chr. breakp. enrich.	0
Linked to chrs	9:3720535−31869664;
Purity, ploidy	0.22, 1.96

**OCCAMS−PS−014**	
Cancer type	Eso−AdenoCA
Position	9:3720535−31869665
Type	With other complex events
Interleaved intrachr. SVs	4
Total SVs (intrachr. + transl.)	16
SV types	DEL: 1; DUP: 0; h2hINV: 2; t2tINV: 1; TRA: 12
SVs in sample	53
Oscillating CN (2 and 3 states)	7, 15
CN segments	15
FDR fragment joints	0.64
FDR chr. breakp. enrich.	0
Linked to chrs	3:10840533−149005313;
Purity, ploidy	0.22, 1.96

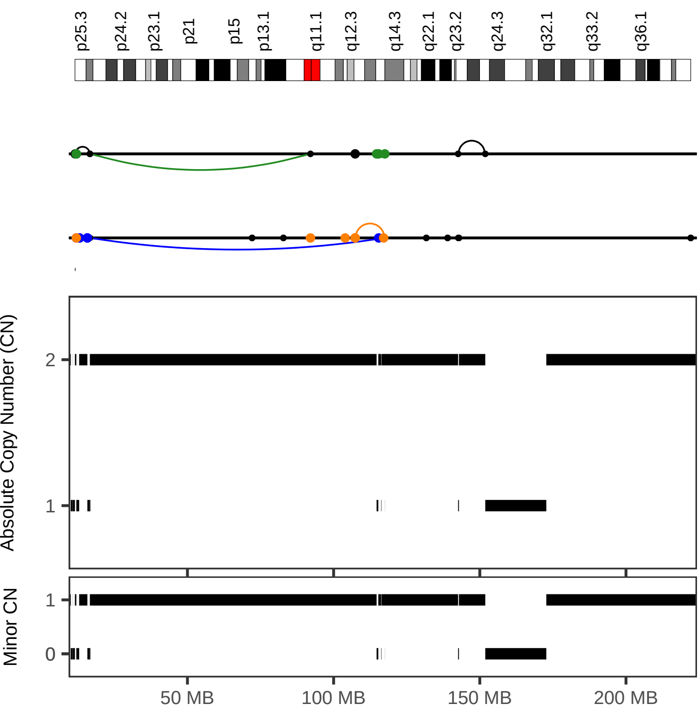

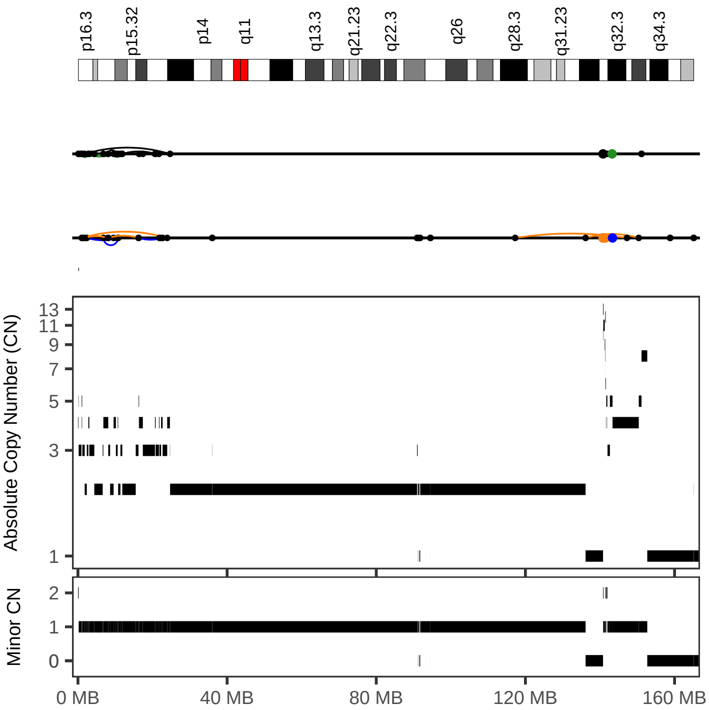

**OCCAMS−RS−006**	
Cancer type	Eso−AdenoCA
Position	2:11537277−117535028
Type	Canonical without polyploidization
Interleaved intrachr. SVs	6
Total SVs (intrachr. + transl.)	25
SV types	DEL: 2; DUP: 1; h2hINV: 1; t2tINV: 2; TRA: 19
SVs in sample	198
Oscillating CN (2 and 3 states)	15, 15
CN segments	15
FDR fragment joints	0.91
FDR chr. breakp. enrich.	0
Linked to chrs	13:31550607−112847785;7:39659225−139835184
Purity, ploidy	0.49, 1.67

**OCCAMS−RS−007**	
Cancer type	Eso−AdenoCA
Position	4:124056−24705189
Type	With other complex events
Interleaved intrachr. SVs	17
Total SVs (intrachr. + transl.)	17
SV types	DEL: 3; DUP: 5; h2hINV: 4; t2tINV: 5; TRA: 0
SVs in sample	242
Oscillating CN (2 and 3 states)	10, 17
CN segments	37
FDR fragment joints	0.92
FDR chr. breakp. enrich.	0
Linked to chrs	
Purity, ploidy	0.48, 2.58

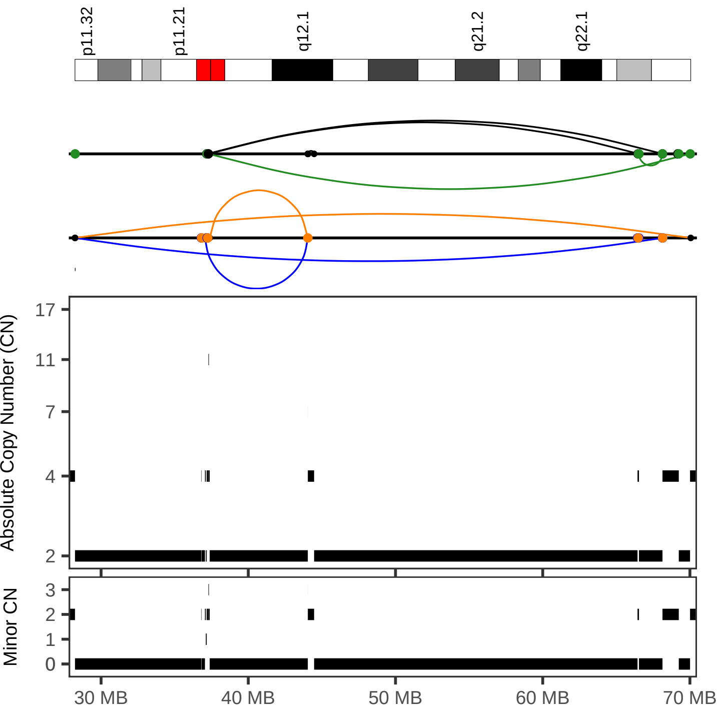

OCCAMS−RS−008

Cancer type	Eso−AdenoCA
Position	18:28233723−70007646
Type	With other complex events
Interleaved intrachr. SVs	7
Total SVs (intrachr. + transl.)	34
SV types	DEL: 1; DUP: 2; h2hINV: 2; t2tINV: 2; TRA: 27
SVs in sample	303
Oscillating CN (2 and 3 states)	8, 11
CN segments	19
FDR fragment joints	0.95
FDR chr. breakp. enrich.	0
Linked to chrs	6:55525409−88702536;
Purity, ploidy	0.19, 2.9

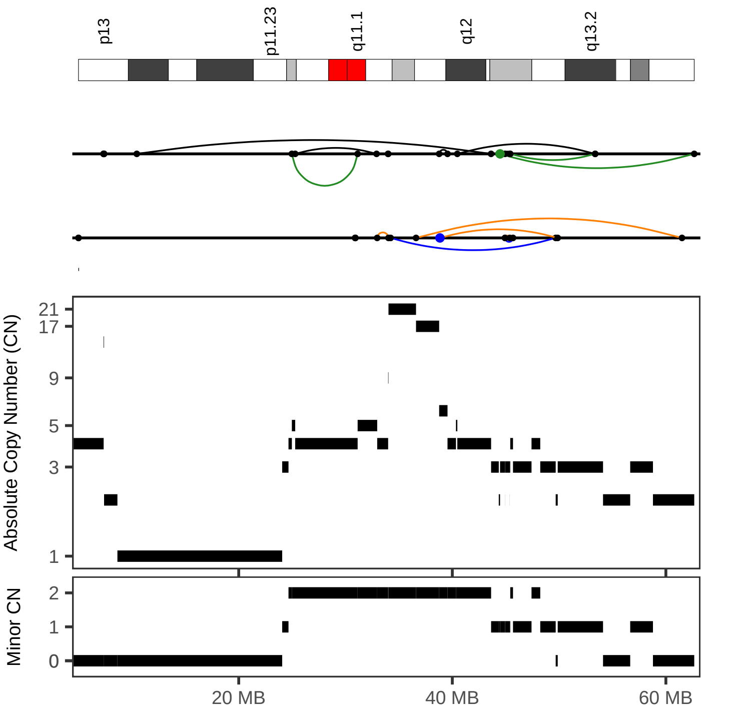

OCCAMS−RS−008

Cancer type	Eso−AdenoCA
Position	20:10454766−62655650
Type	With other complex events
Interleaved intrachr. SVs	11
Total SVs (intrachr. + transl.)	13
SV types	DEL: 3; DUP: 2; h2hINV: 4; t2tINV: 2; TRA: 2
SVs in sample	303
Oscillating CN (2 and 3 states)	7, 19
CN segments	30
FDR fragment joints	0.84
FDR chr. breakp. enrich.	0
Linked to chrs	1:200693009−220848035;
Purity, ploidy	0.19, 2.9

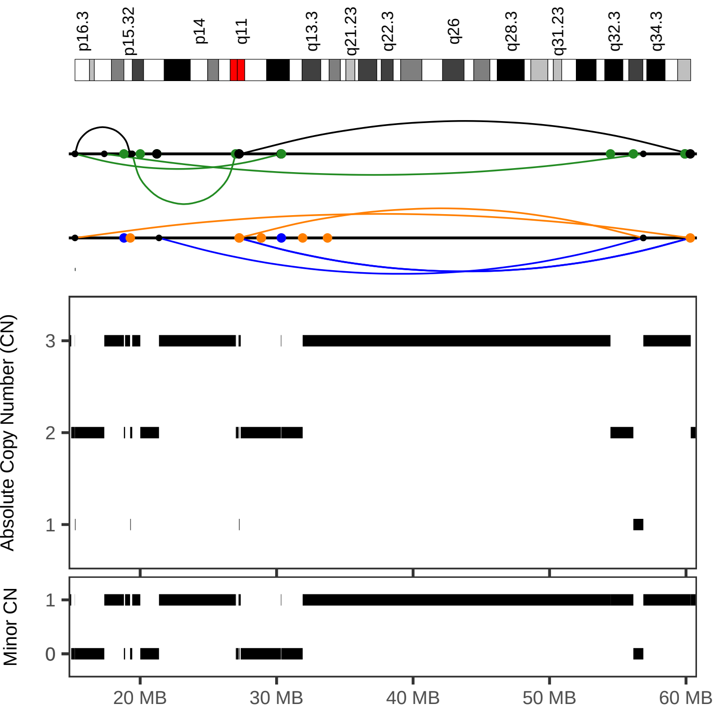

OCCAMS−RS−024

Cancer type	Eso−AdenoCA
Position	4:15222955−60346330
Type	With other complex events
Interleaved intrachr. SVs	12
Total SVs (intrachr. + transl.)	36
SV types	DEL: 2; DUP: 3; h2hINV: 3; t2tINV: 4; TRA: 24
SVs in sample	218
Oscillating CN (2 and 3 states)	8, 15
CN segments	28
FDR fragment joints	0.91
FDR chr. breakp. enrich.	0
Linked to chrs	2:105994075−107895375;5:61100033−179629172
Purity, ploidy	0.71, 2.93

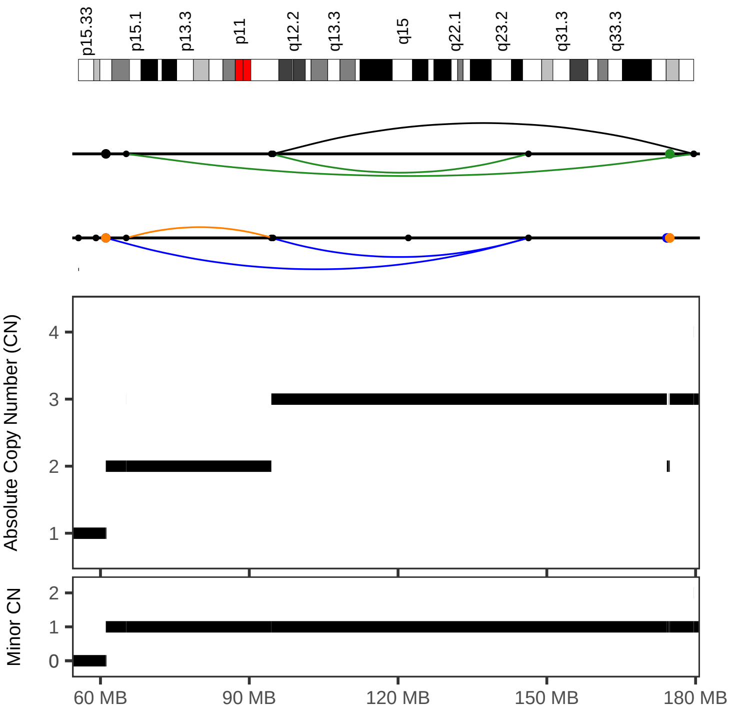

OCCAMS−RS−024

Cancer type	Eso−AdenoCA
Position	5:61100033−179629173
Type	Canonical without polyploidization
Interleaved intrachr. SVs	6
Total SVs (intrachr. + transl.)	16
SV types	DEL: 1; DUP: 2; h2hINV: 1; t2tINV: 2; TRA: 10
SVs in sample	218
Oscillating CN (2 and 3 states)	8, 11
CN segments	11
FDR fragment joints	0.91
FDR chr. breakp. enrich.	0.07
Linked to chrs	2:105994075−107895375;4:15222955−60346329
Purity, ploidy	0.71, 2.93

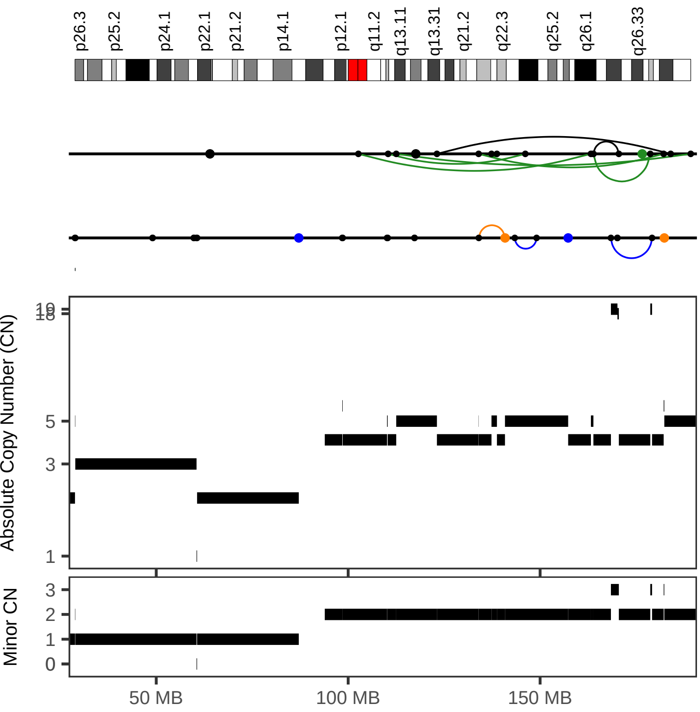

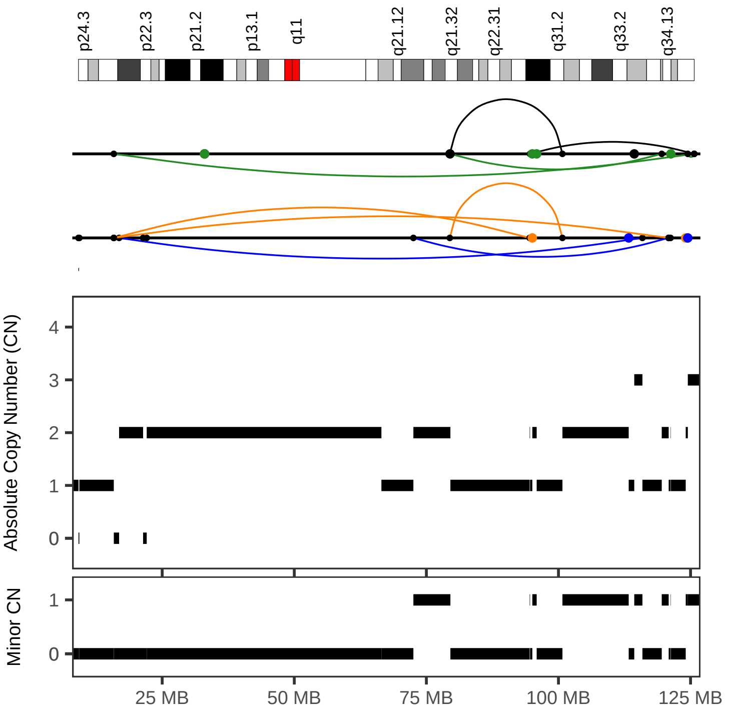

**OCCAMS−RS−031**	
Cancer type	Eso−AdenoCA
Position	3:102654747−189241556
Type	After polyploidization
Interleaved intrachr. SVs	6
Total SVs (intrachr. + transl.)	11
SV types	DEL: 0; DUP: 1; h2hINV: 1; t2tINV: 4; TRA: 5
SVs in sample	156
Oscillating CN (2 and 3 states)	13, 13
CN segments	20
FDR fragment joints	0.48
FDR chr. breakp. enrich.	0
Linked to chrs	4:8256829−162969785;
Purity, ploidy	0.4, 3.7

**OCCAMS−RS−032**	
Cancer type	Eso−AdenoCA
Position	9:15830088−125695294
Type	With other complex events
Interleaved intrachr. SVs	10
Total SVs (intrachr. + transl.)	22
SV types	DEL: 3; DUP: 2; h2hINV: 2; t2tINV: 3; TRA: 12
SVs in sample	159
Oscillating CN (2 and 3 states)	10, 24
CN segments	25
FDR fragment joints	0.96
FDR chr. breakp. enrich.	0
Linked to chrs	3:6793668−181259418;8:32815344−128323683
Purity, ploidy	0.61, 1.96

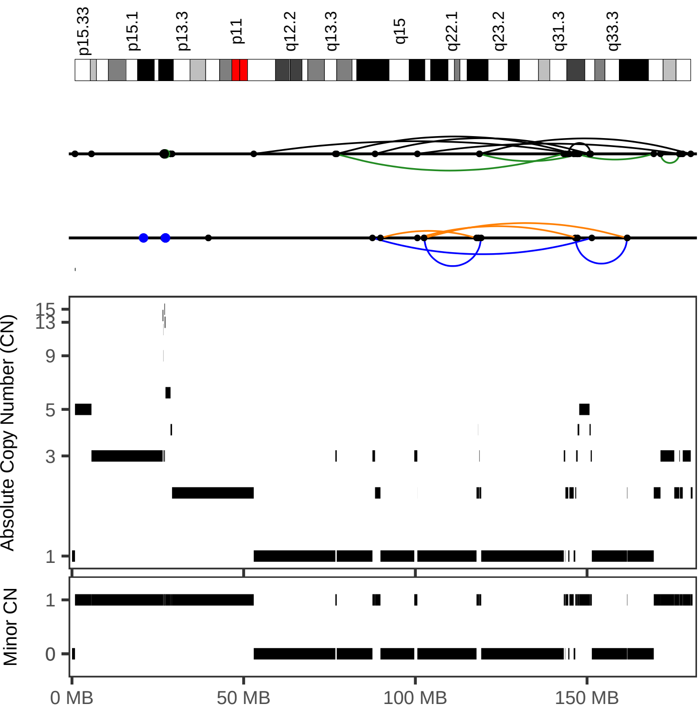

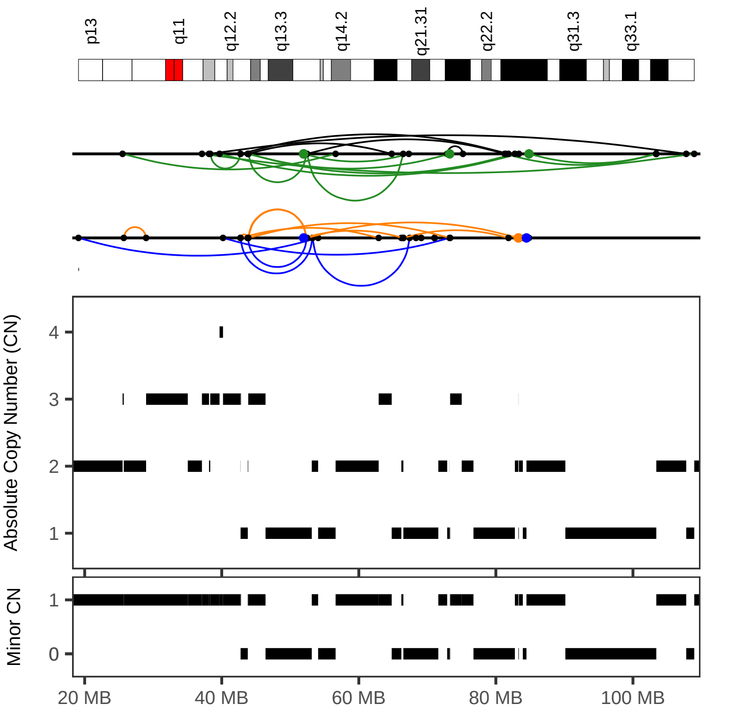

**OCCAMS−RS−036**	
Cancer type	Eso−AdenoCA
Position	5:52934089−180192620
Type	With other complex events
Interleaved intrachr. SVs	17
Total SVs (intrachr. + transl.)	17
SV types	DEL: 3; DUP: 3; h2hINV: 6; t2tINV: 5; TRA: 0
SVs in sample	332
Oscillating CN (2 and 3 states)	7, 11
CN segments	37
FDR fragment joints	0.74
FDR chr. breakp. enrich.	0.01
Linked to chrs	
Purity, ploidy	0.73, 2.22

**OCCAMS−RS−036**	
Cancer type	Eso−AdenoCA
Position	13:19096905−108942210
Type	With other complex events
Interleaved intrachr. SVs	28
Total SVs (intrachr. + transl.)	36
SV types	DEL: 7; DUP: 6; h2hINV: 5; t2tINV: 10; TRA: 8
SVs in sample	332
Oscillating CN (2 and 3 states)	8, 11
CN segments	41
FDR fragment joints	0.64
FDR chr. breakp. enrich.	0
Linked to chrs	9:82647126−121561548;
Purity, ploidy	0.73, 2.22

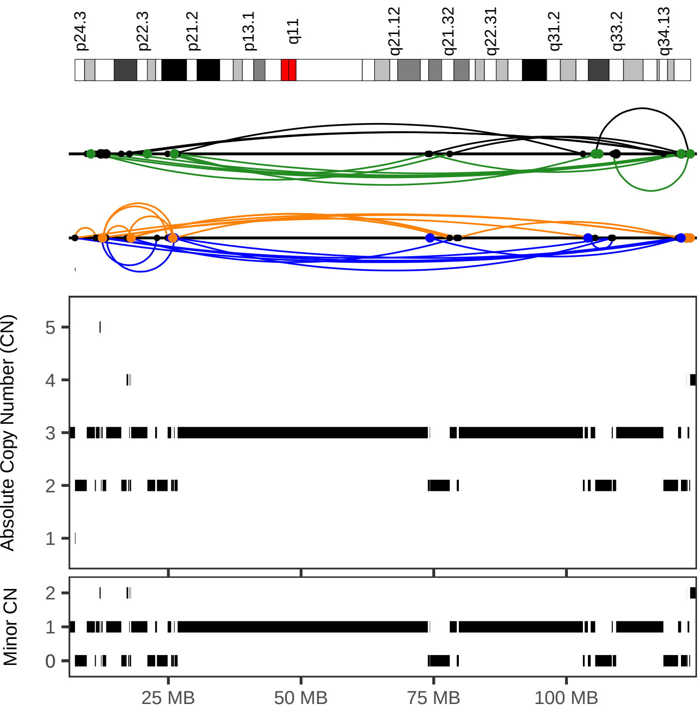

OCCAMS−SH−003	
Cancer type	Eso−AdenoCA
Position	9:7402627−123306880
Type	With other complex events
Interleaved intrachr. SVs	43
Total SVs (intrachr. + transl.)	67
SV types	DEL: 12; DUP: 14; h2hINV: 8; t2tINV: 9; TRA: 24
SVs in sample	250
Oscillating CN (2 and 3 states)	31, 31
CN segments	56
FDR fragment joints	0.64
FDR chr. breakp. enrich.	0
Linked to chrs	
Purity, ploidy	0.53, 3.64

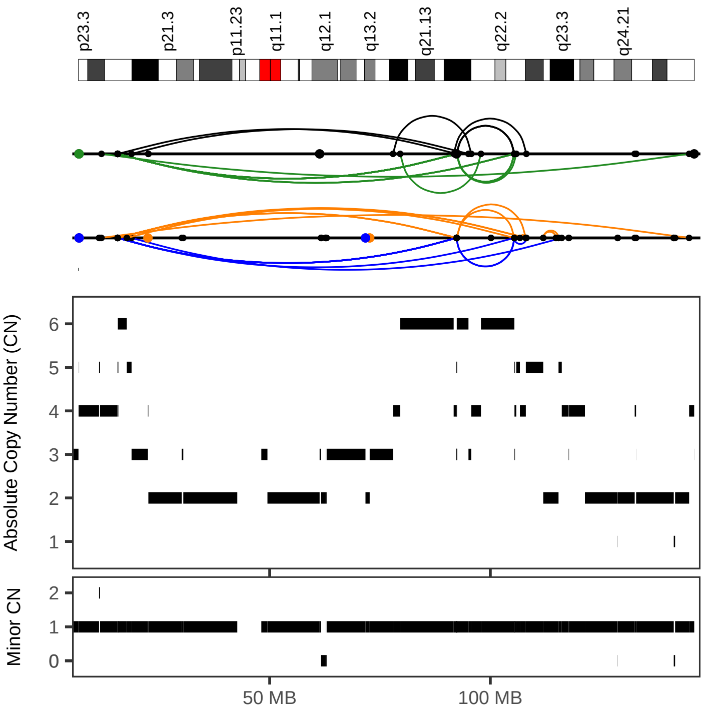

OCCAMS−SH−051	
Cancer type	Eso−AdenoCA
Position	8:15493178−116198864
Type	With other complex events
Interleaved intrachr. SVs	48
Total SVs (intrachr. + transl.)	53
SV types	DEL: 8; DUP: 13; h2hINV: 8; t2tINV: 19; TRA: 5
SVs in sample	450
Oscillating CN (2 and 3 states)	12, 13
CN segments	50
FDR fragment joints	0.48
FDR chr. breakp. enrich.	0
Linked to chrs	14:44423329−81442644;
Purity, ploidy	0.69, 2.44

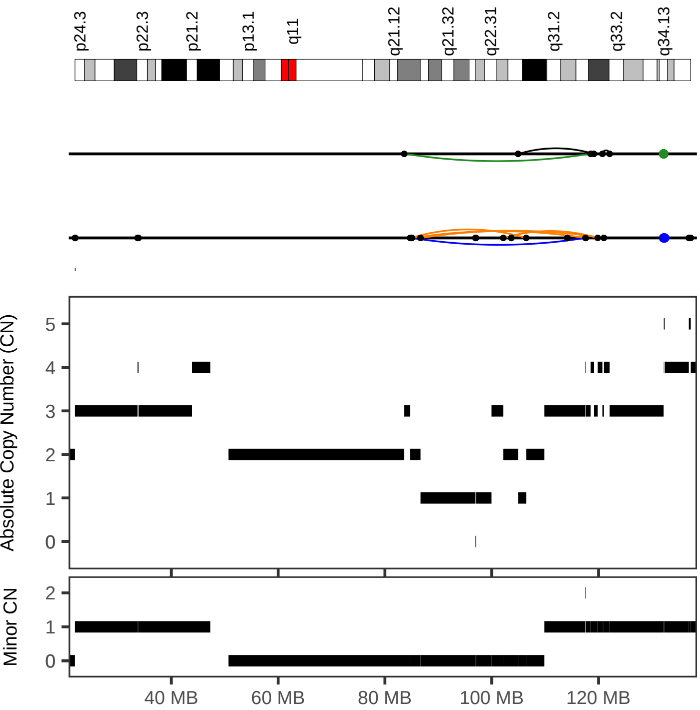

OCCAMS−SH−051	
Cancer type	Eso−AdenoCA
Position	9:83628603−122095709
Type	With other complex events
Interleaved intrachr. SVs	8
Total SVs (intrachr. + transl.)	8
SV types	DEL: 4; DUP: 1; h2hINV: 2; t2tINV: 1; TRA: 0
SVs in sample	450
Oscillating CN (2 and 3 states)	9, 13
CN segments	18
FDR fragment joints	0.59
FDR chr. breakp. enrich.	0.24
Linked to chrs	
Purity, ploidy	0.69, 2.44

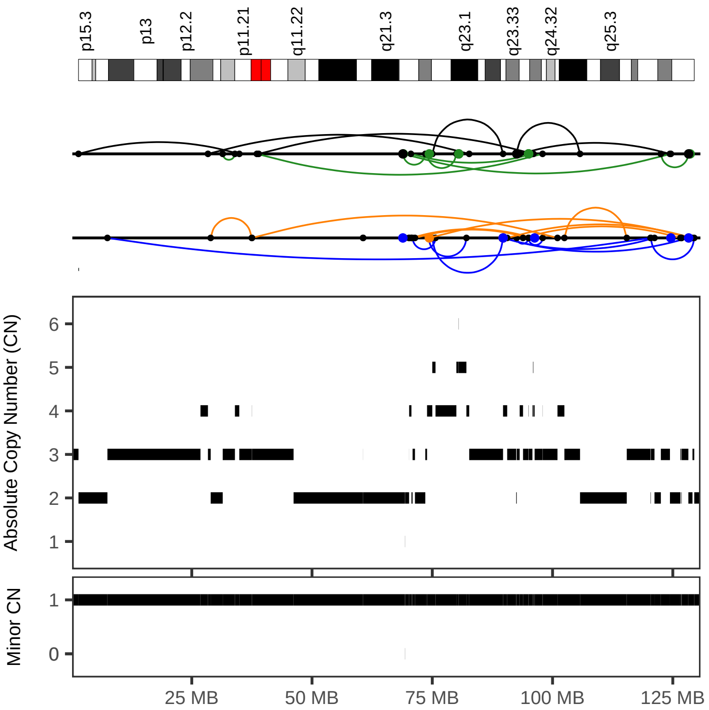

OCCAMS−SH−051	
Cancer type	Eso−AdenoCA
Position	10:1449967−129457144
Type	With other complex events
Interleaved intrachr. SVs	30
Total SVs (intrachr. + transl.)	48
SV types	DEL: 8; DUP: 10; h2hINV: 6; t2tINV: 6; TRA: 18
SVs in sample	450
Oscillating CN (2 and 3 states)	16, 63
CN segments	63
FDR fragment joints	0.77
FDR chr. breakp. enrich.	0
Linked to chrs	
Purity, ploidy	0.69, 2.44

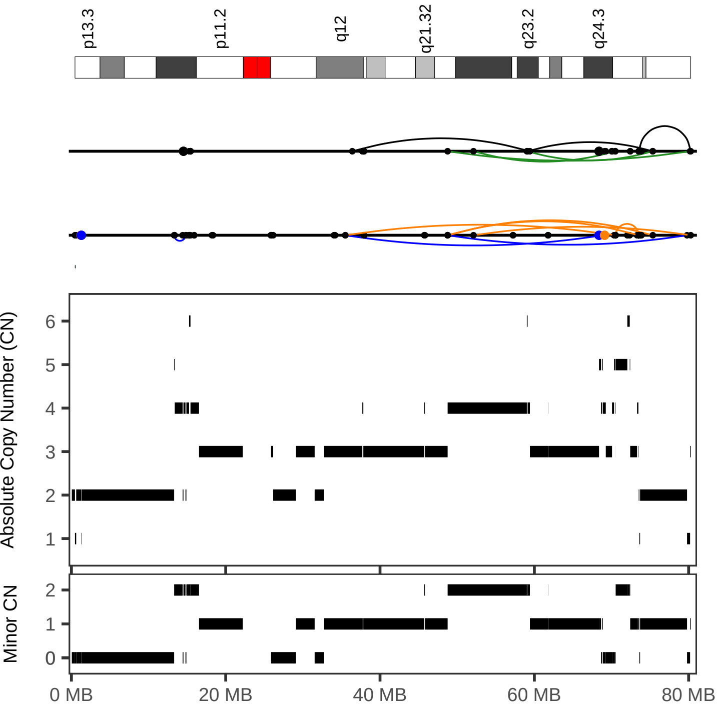

	OCCAMS−SH−051
Cancer type	Eso−AdenoCA
Position	17:35498058−80272573
Type	With other complex events
Interleaved intrachr. SVs	17
Total SVs (intrachr. + transl.)	20
SV types	DEL: 7; DUP: 3; h2hINV: 4; t2tINV: 3; TRA: 3
SVs in sample	450
Oscillating CN (2 and 3 states)	8, 13
CN segments	34
FDR fragment joints	0.64
FDR chr. breakp. enrich.	0
Linked to chrs	
Purity, ploidy	0.69, 2.44

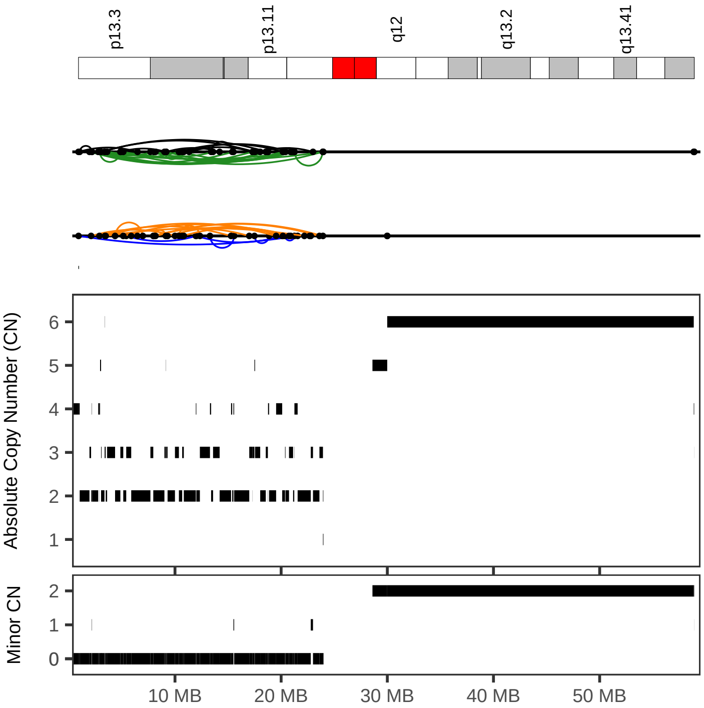

	OCCAMS−SH−071
Cancer type	Eso−AdenoCA
Position	19:910192−23977386
Type	With other complex events
Interleaved intrachr. SVs	49
Total SVs (intrachr. + transl.)	49
SV types	DEL: 14; DUP: 6; h2hINV: 12; t2tINV: 17; TRA: 0
SVs in sample	284
Oscillating CN (2 and 3 states)	11, 13
CN segments	71
FDR fragment joints	0.59
FDR chr. breakp. enrich.	0
Linked to chrs	
Purity, ploidy	0.42, 3.56

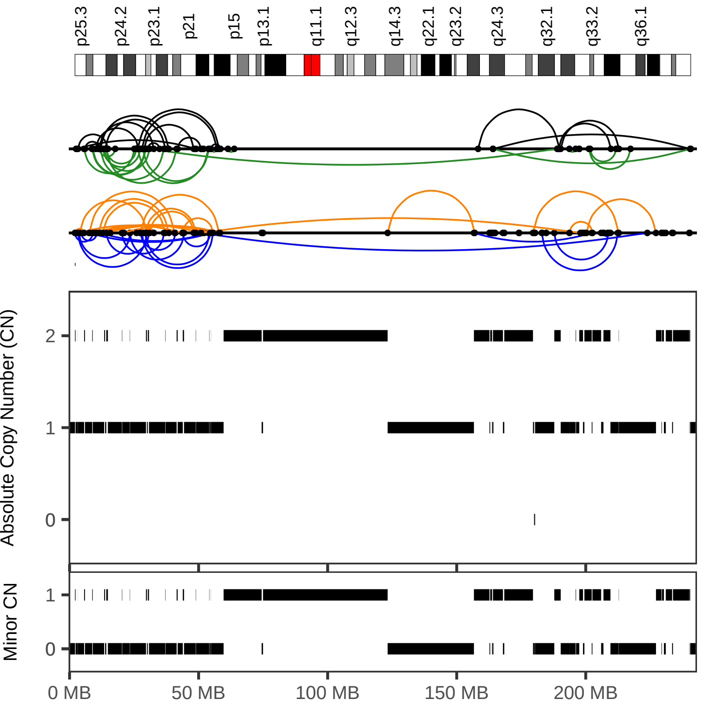

	OCCAMS−ST−020
Cancer type	Eso−AdenoCA
Position	2:2097136−240675922
Type	Canonical without polyploidization
Interleaved intrachr. SVs	80
Total SVs (intrachr. + transl.)	80
SV types	DEL: 25; DUP: 19; h2hINV: 18; t2tINV: 18; TRA: 0
SVs in sample	165
Oscillating CN (2 and 3 states)	50, 78
CN segments	78
FDR fragment joints	0.71
FDR chr. breakp. enrich.	0
Linked to chrs	
Purity, ploidy	0.43, 1.76

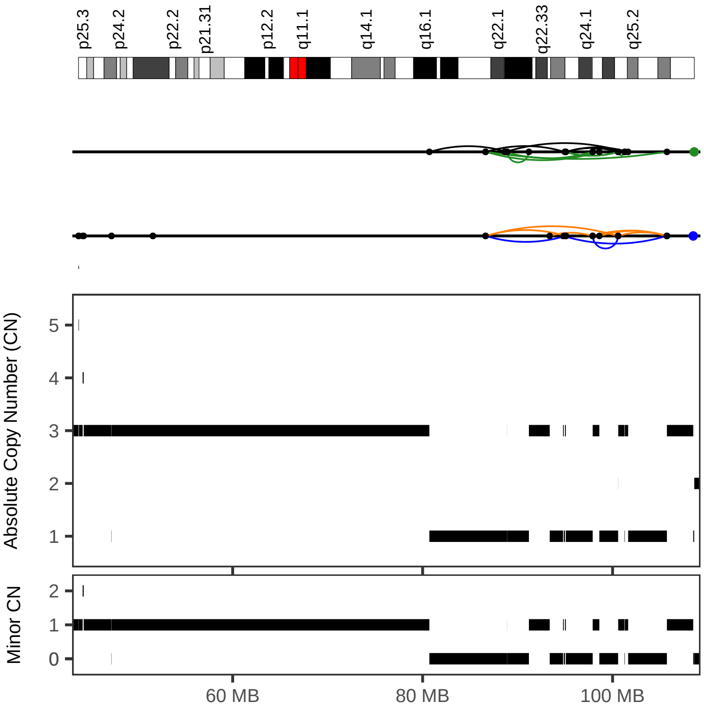

	OCCAMS−ST−029
Cancer type	Eso−AdenoCA
Position	6:80712446−105721586
Type	Canonical without polyploidization
Interleaved intrachr. SVs	23
Total SVs (intrachr. + transl.)	23
SV types	DEL: 7; DUP: 3; h2hINV: 6; t2tINV: 7; TRA: 0
SVs in sample	286
Oscillating CN (2 and 3 states)	13, 20
CN segments	20
FDR fragment joints	0.67
FDR chr. breakp. enrich.	0.02
Linked to chrs	
Purity, ploidy	0.35, 3.04

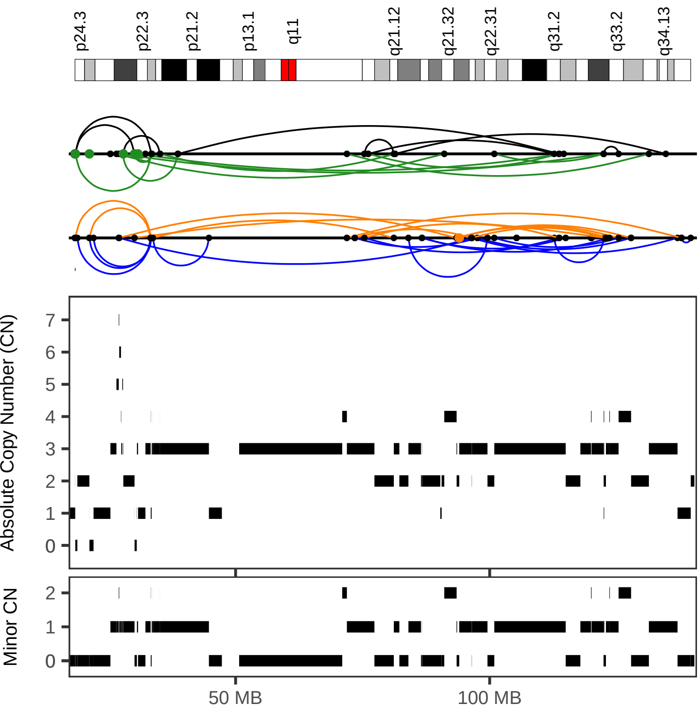

OCCAMS−ST−029

Cancer type	Eso−AdenoCA
Position	9:18394399−139562954
Type	With other complex events
Interleaved intrachr. SVs	49
Total SVs (intrachr. + transl.)	57
SV types	DEL: 13; DUP: 17; h2hINV: 8; t2tINV: 11; TRA: 8
SVs in sample	286
Oscillating CN (2 and 3 states)	11, 11
CN segments	73
FDR fragment joints	0.59
FDR chr. breakp. enrich.	0
Linked to chrs	12:13909019−15158939;
Purity, ploidy	0.35, 3.04

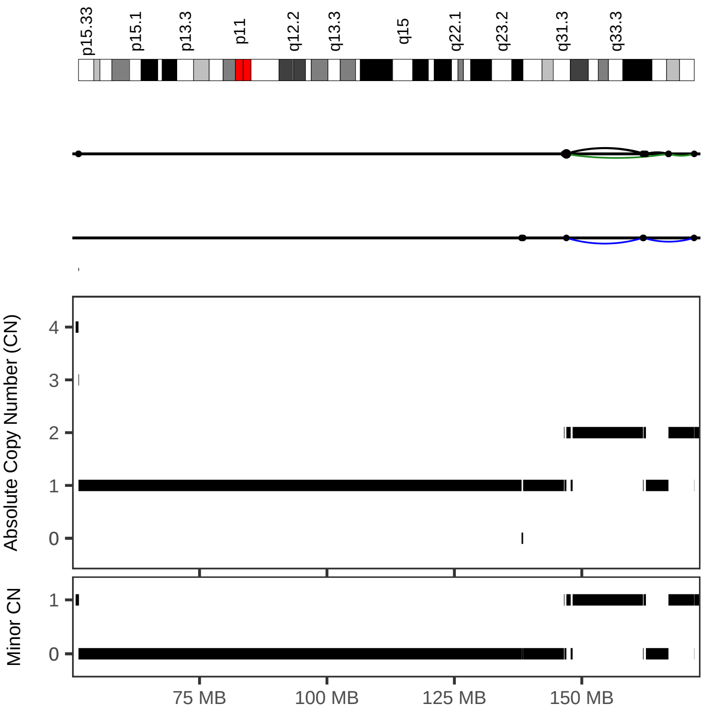

OCCAMS−WG−002

Cancer type	Eso−AdenoCA
Position	5:146494000−172062115
Type	Canonical without polyploidization
Interleaved intrachr. SVs	5
Total SVs (intrachr. + transl.)	7
SV types	DEL: 0; DUP: 1; h2hINV: 2; t2tINV: 2; TRA: 2
SVs in sample	138
Oscillating CN (2 and 3 states)	11, 11
CN segments	11
FDR fragment joints	0.64
FDR chr. breakp. enrich.	0.36
Linked to chrs	
Purity, ploidy	0.5, 1.84

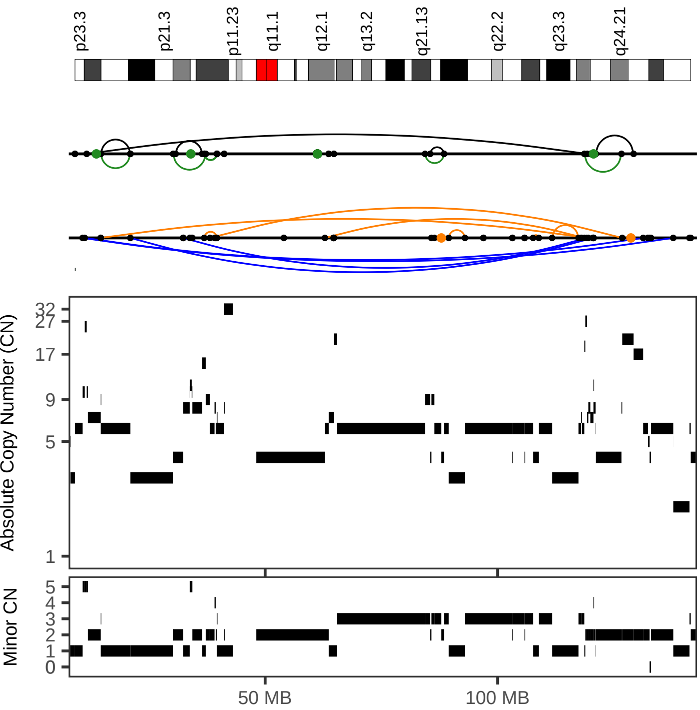

OCCAMS−WG−019

Cancer type	Eso−AdenoCA
Position	8:11621919−129269031
Type	With other complex events
Interleaved intrachr. SVs	20
Total SVs (intrachr. + transl.)	27
SV types	DEL: 5; DUP: 3; h2hINV: 5; t2tINV: 7; TRA: 7
SVs in sample	263
Oscillating CN (2 and 3 states)	7, 13
CN segments	56
FDR fragment joints	0.74
FDR chr. breakp. enrich.	0
Linked to chrs	
Purity, ploidy	0.25, 3.97

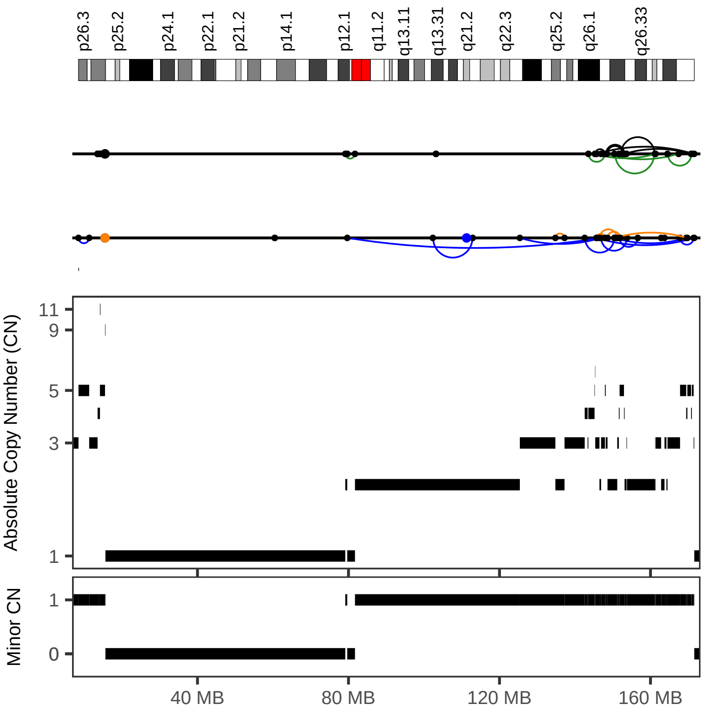

OCCAMS−ZZ−004

Cancer type	Eso−AdenoCA
Position	3:79151724−171586622
Type	With other complex events
Interleaved intrachr. SVs	25
Total SVs (intrachr. + transl.)	26
SV types	DEL: 4; DUP: 8; h2hINV: 6; t2tINV: 7; TRA: 1
SVs in sample	323
Oscillating CN (2 and 3 states)	10, 12
CN segments	38
FDR fragment joints	0.78
FDR chr. breakp. enrich.	0
Linked to chrs	
Purity, ploidy	0.56, 2.11

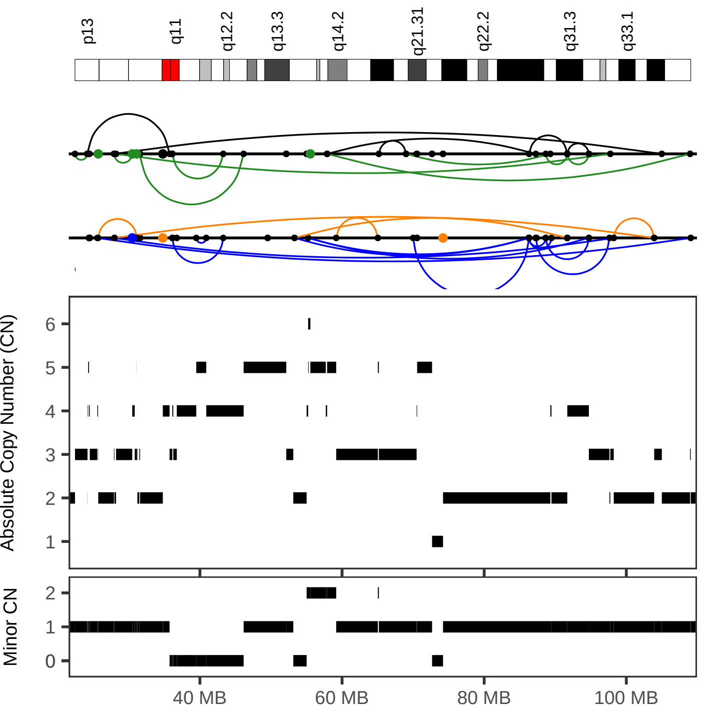

OCCAMS−ZZ−004	
Cancer type	Eso−AdenoCA
Position	13:22419265−109068331
Type	With other complex events
Interleaved intrachr. SVs	32
Total SVs (intrachr. + transl.)	41
SV types	DEL: 4; DUP: 12; h2hINV: 8; t2tINV: 8; TRA: 9
SVs in sample	323
Oscillating CN (2 and 3 states)	8, 15
CN segments	56
FDR fragment joints	0.59
FDR chr. breakp. enrich.	0
Linked to chrs	10:364377−35292357;
Purity, ploidy	0.56, 2.11

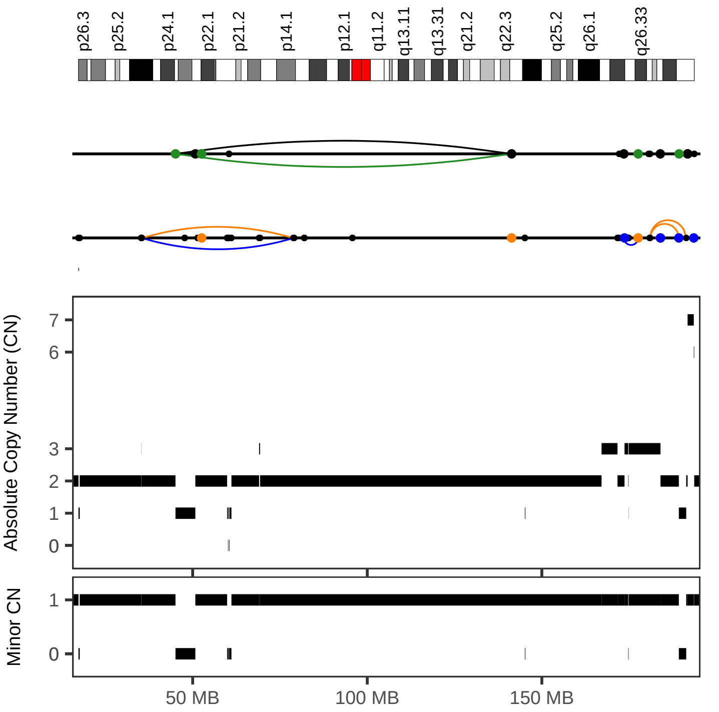

OCCAMS−ZZ−016	
Cancer type	Eso−AdenoCA
Position	3:35278145−141335185
Type	With other complex events
Interleaved intrachr. SVs	4
Total SVs (intrachr. + transl.)	11
SV types	DEL: 1; DUP: 1; h2hINV: 1; t2tINV: 1; TRA: 7
SVs in sample	286
Oscillating CN (2 and 3 states)	7, 14
CN segments	14
FDR fragment joints	1
FDR chr. breakp. enrich.	0
Linked to chrs	
Purity, ploidy	0.5, 2.45

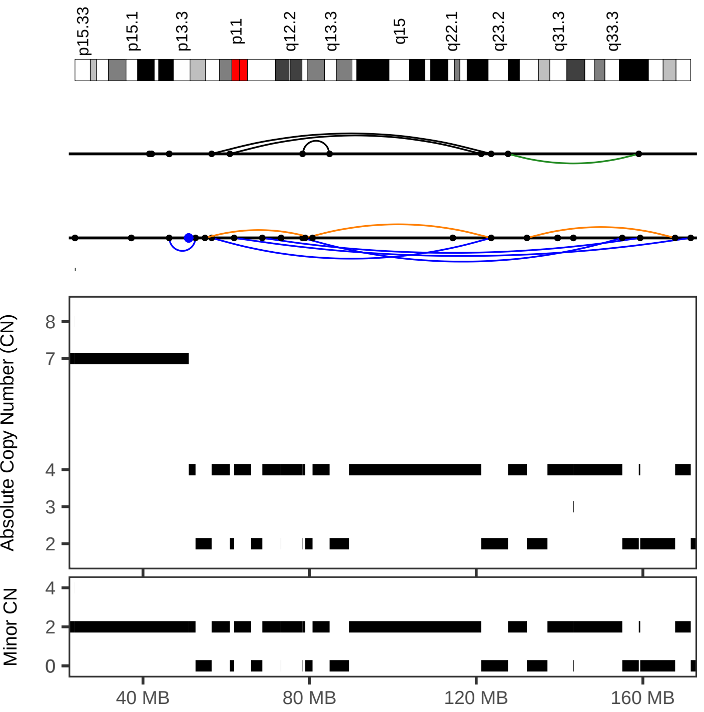

OCCAMS−ZZ−019	
Cancer type	Eso−AdenoCA
Position	5:54890695−171491811
Type	Before polyploidization
Interleaved intrachr. SVs	11
Total SVs (intrachr. + transl.)	11
SV types	DEL: 3; DUP: 4; h2hINV: 3; t2tINV: 1; TRA: 0
SVs in sample	190
Oscillating CN (2 and 3 states)	18, 25
CN segments	25
FDR fragment joints	0.71
FDR chr. breakp. enrich.	0.03
Linked to chrs	
Purity, ploidy	0.29, 3.21

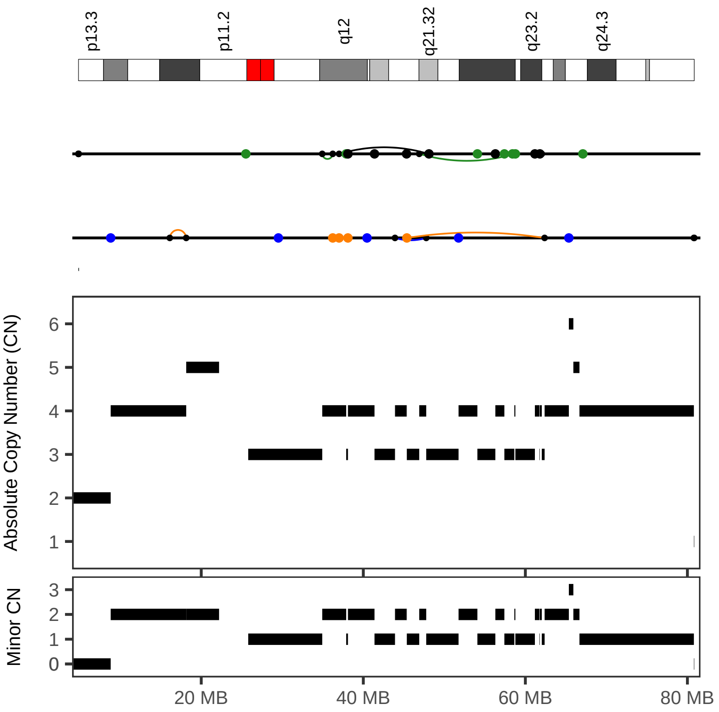

3e5f451a−5882−4914−ae1a−95c898c2bcca	
Cancer type	Head−SCC
Position	17:37011270−62375856
Type	After polyploidization
Interleaved intrachr. SVs	4
Total SVs (intrachr. + transl.)	21
SV types	DEL: 1; DUP: 1; h2hINV: 1; t2tINV: 1; TRA: 17
SVs in sample	362
Oscillating CN (2 and 3 states)	21, 21
CN segments	21
FDR fragment joints	1
FDR chr. breakp. enrich.	0
Linked to chrs	2:164623105−179954500;
Purity, ploidy	0.58, 3.09

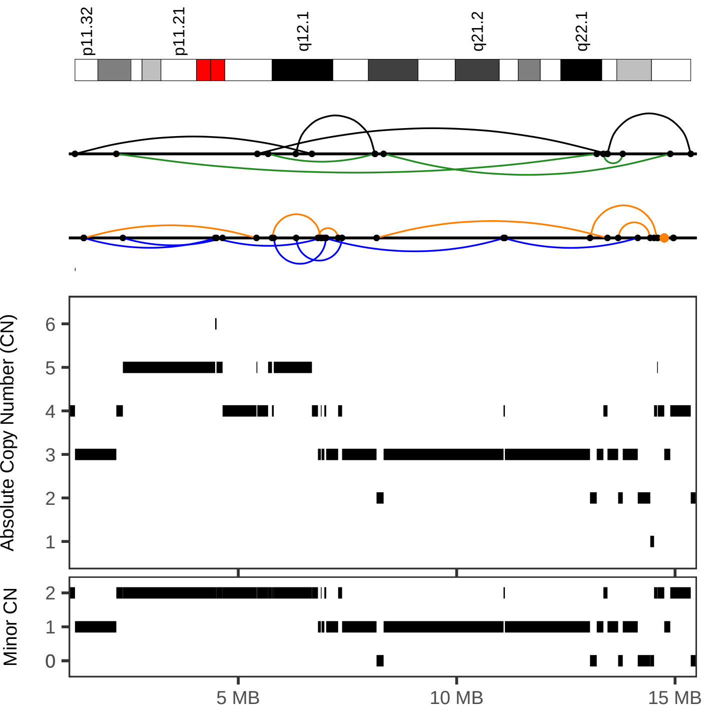

	ad6c9d09−2c03−4786−a72d−dd2aa5f603d4
Cancer type	Head−SCC
Position	18:1261751−15359143
Type	With other complex events
Interleaved intrachr. SVs	22
Total SVs (intrachr. + transl.)	23
SV types	DEL: 6; DUP: 8; h2hINV: 4; t2tINV: 4; TRA: 1
SVs in sample	360
Oscillating CN (2 and 3 states)	8, 32
CN segments	37
FDR fragment joints	0.64
FDR chr. breakp. enrich.	0
Linked to chrs	
Purity, ploidy	0.65, 3.12

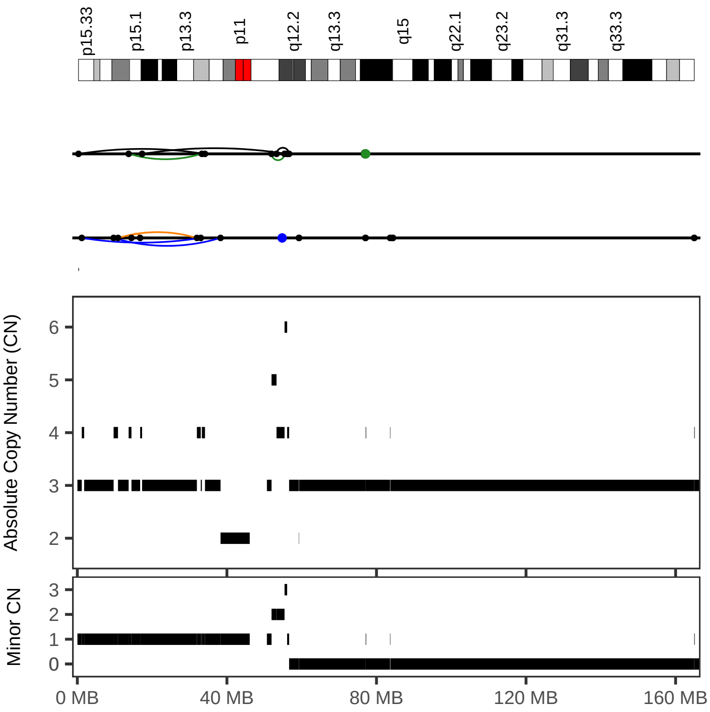

	cb99fb14−641c−4fb3−bf1d−17efb8cd982a
Cancer type	Head−SCC
Position	5:301373−56638638
Type	After polyploidization
Interleaved intrachr. SVs	8
Total SVs (intrachr. + transl.)	9
SV types	DEL: 1; DUP: 2; h2hINV: 3; t2tINV: 2; TRA: 1
SVs in sample	96
Oscillating CN (2 and 3 states)	13, 13
CN segments	20
FDR fragment joints	0.84
FDR chr. breakp. enrich.	0
Linked to chrs	
Purity, ploidy	0.52, 2.94

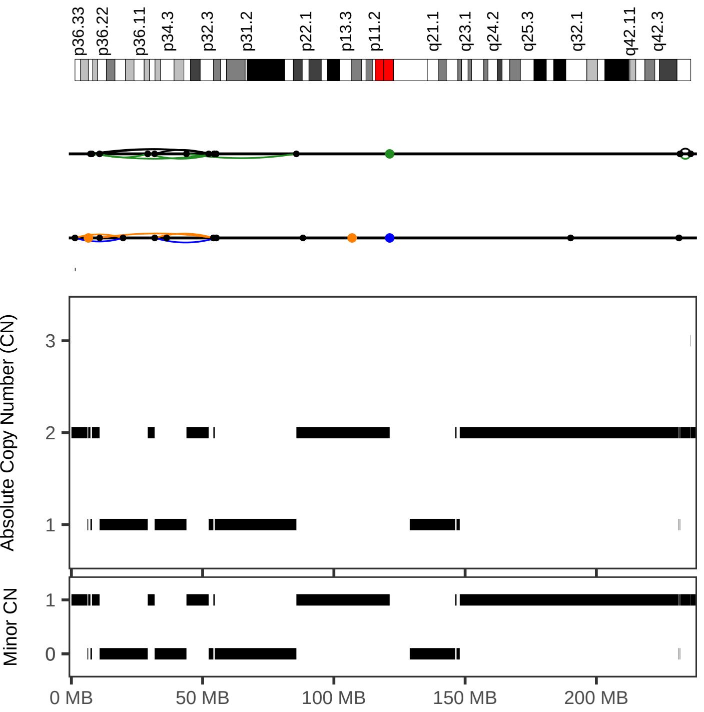

	e303b8c4−6428−4134−bb9d−a95dc1d8e35e
Cancer type	Head−SCC
Position	1:1362882−85717387
Type	Canonical without polyploidization
Interleaved intrachr. SVs	13
Total SVs (intrachr. + transl.)	14
SV types	DEL: 3; DUP: 3; h2hINV: 3; t2tINV: 4; TRA: 1
SVs in sample	224
Oscillating CN (2 and 3 states)	15, 15
CN segments	15
FDR fragment joints	0.98
FDR chr. breakp. enrich.	0.33
Linked to chrs	
Purity, ploidy	0.85, 2

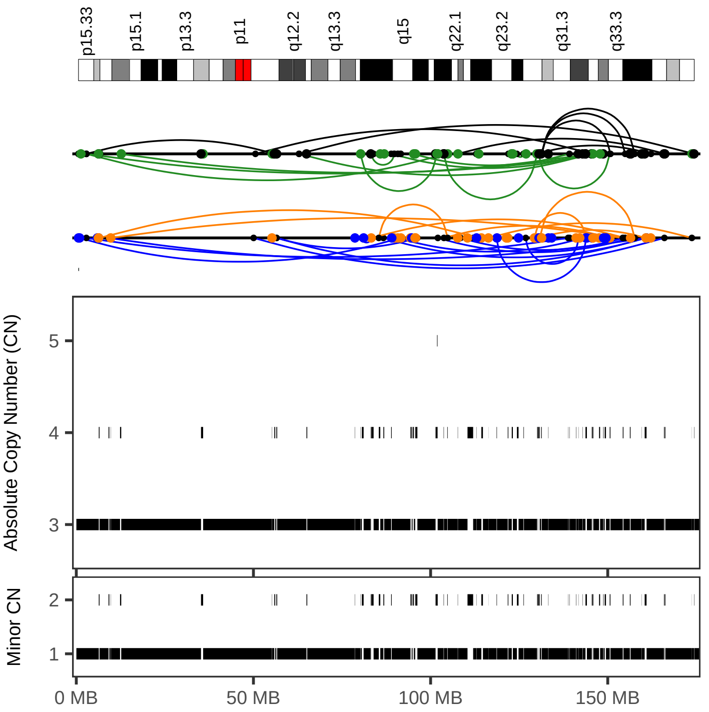

	2f1c4db6−88a7−4a5d−a5fe−94d85f569440
Cancer type	Kidney−ChRCC
Position	5:928958−174411985
Type	After polyploidization
Interleaved intrachr. SVs	40
Total SVs (intrachr. + transl.)	141
SV types	DEL: 9; DUP: 13; h2hINV: 8; t2tINV: 10; TRA: 101
SVs in sample	233
Oscillating CN (2 and 3 states)	82, 129
CN segments	129
FDR fragment joints	0.78
FDR chr. breakp. enrich.	0
Linked to chrs	13:20416020−113987909;
Purity, ploidy	0.9, 3.05

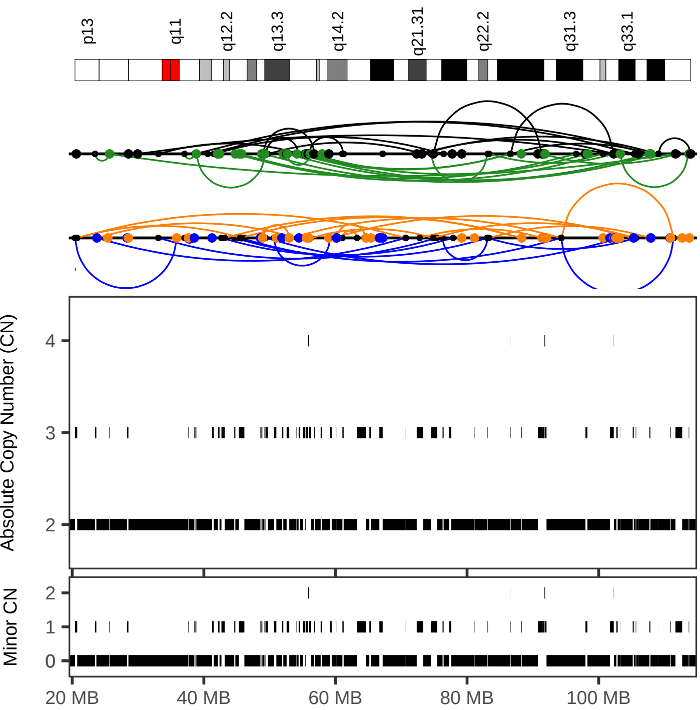

	2f1c4db6−88a7−4a5d−a5fe−94d85f569440
Cancer type	Kidney−ChRCC
Position	13:20416020−113987910
Type	With other complex events
Interleaved intrachr. SVs	58
Total SVs (intrachr. + transl.)	160
SV types	DEL: 15; DUP: 12; h2hINV: 15;<br>t2tINV: 16; TRA: 102
SVs in sample	233
Oscillating CN (2 and 3 states)	51, 138
CN segments	138
FDR fragment joints	0.92
FDR chr. breakp. enrich.	0
Linked to chrs	
Purity, ploidy	0.9, 3.05

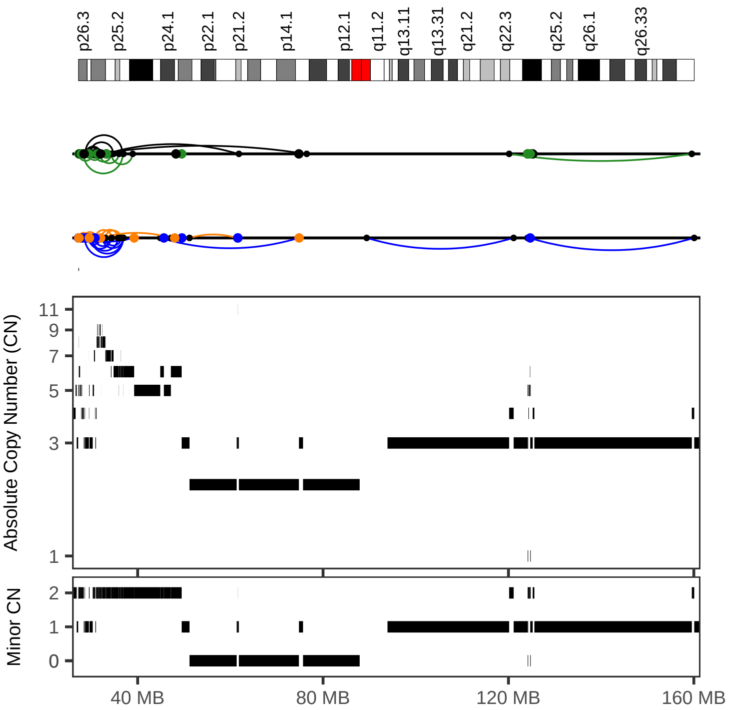

	70e306ee−9584−49d6−81a1−a49d837ab6aa
Cancer type	Kidney−ChRCC
Position	3:27225774−76470709
Type	With other complex events
Interleaved intrachr. SVs	44
Total SVs (intrachr. + transl.)	89
SV types	DEL: 10; DUP: 13; h2hINV: 12;<br>t2tINV: 9; TRA: 45
SVs in sample	305
Oscillating CN (2 and 3 states)	9, 22
CN segments	64
FDR fragment joints	0.86
FDR chr. breakp. enrich.	0
Linked to chrs	15:40179423−68153781;8:48323075−117143869
Purity, ploidy	0.89, 3.06

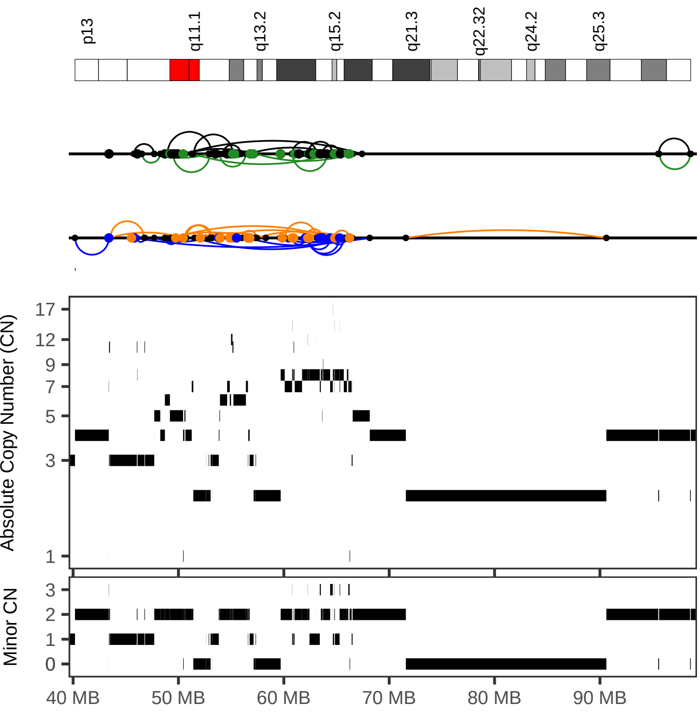

	70e306ee−9584−49d6−81a1−a49d837ab6aa
Cancer type	Kidney−ChRCC
Position	15:40179423−68153782
Type	With other complex events
Interleaved intrachr. SVs	54
Total SVs (intrachr. + transl.)	119
SV types	DEL: 13; DUP: 18; h2hINV: 12;<br>t2tINV: 11; TRA: 65
SVs in sample	305
Oscillating CN (2 and 3 states)	10, 10
CN segments	94
FDR fragment joints	0.64
FDR chr. breakp. enrich.	0
Linked to chrs	8:48323075−117143869;
Purity, ploidy	0.89, 3.06

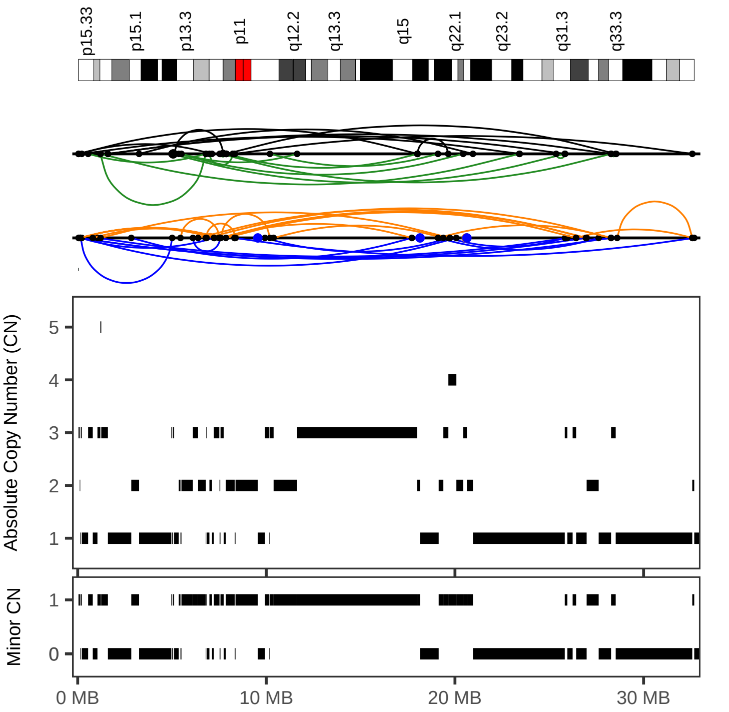

	f2b58595−dec0−4194−ab33−7c7dc6c058a6
Cancer type	Kidney−ChRCC
Position	5:40655−32685928
Type	With other complex events
Interleaved intrachr. SVs	56
Total SVs (intrachr. + transl.)	61
SV types	DEL: 18; DUP: 12; h2hINV: 12;<br>t2tINV: 14; TRA: 5
SVs in sample	81
Oscillating CN (2 and 3 states)	7, 15
CN segments	65
FDR fragment joints	0.71
FDR chr. breakp. enrich.	0
Linked to chrs	
Purity, ploidy	0.94, 1.59

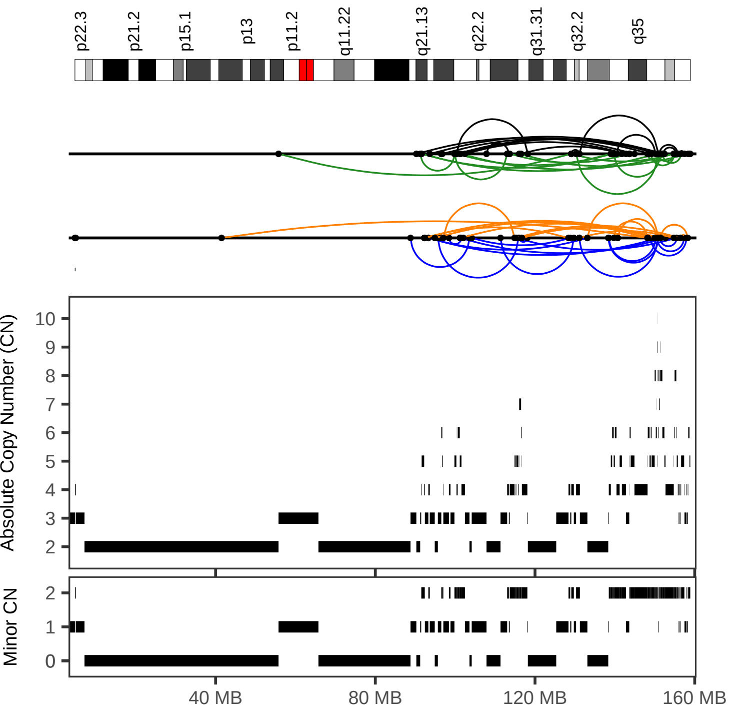

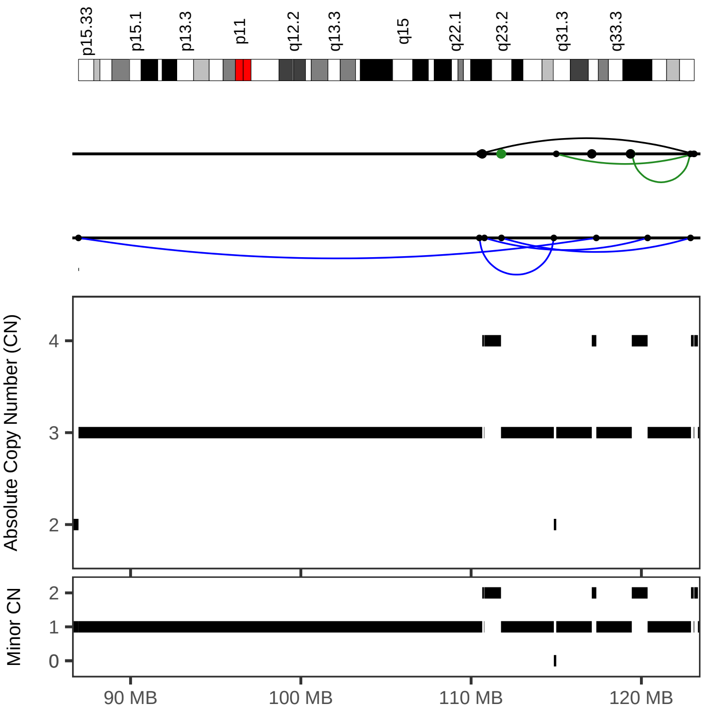

	659294b9−ded9−498a−bc00−c1d4d456aa4a
Cancer type	Kidney−RCC
Position	7:41484984−158377609
Type	With other complex events
Interleaved intrachr. SVs	66
Total SVs (intrachr. + transl.)	66
SV types	DEL: 15; DUP: 18; h2hINV: 16; t2tINV: 17; TRA: 0
SVs in sample	72
Oscillating CN (2 and 3 states)	7, 21
CN segments	110
FDR fragment joints	0.97
FDR chr. breakp. enrich.	0
Linked to chrs	
Purity, ploidy	0.82, 1.98

	bbbce1ba−c739−43ba−b9cf−a4f746491ae3
Cancer type	Kidney−RCC
Position	5:86939720−123103947
Type	With other complex events
Interleaved intrachr. SVs	7
Total SVs (intrachr. + transl.)	13
SV types	DEL: 0; DUP: 4; h2hINV: 1; t2tINV: 2; TRA: 6
SVs in sample	15
Oscillating CN (2 and 3 states)	8, 14
CN segments	14
FDR fragment joints	0.59
FDR chr. breakp. enrich.	0
Linked to chrs	
Purity, ploidy	0.48, 1.91

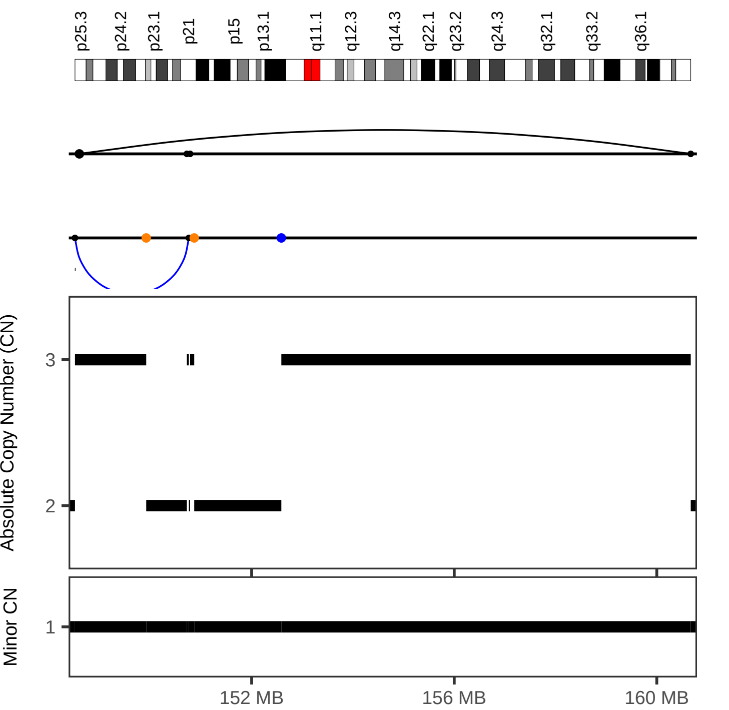

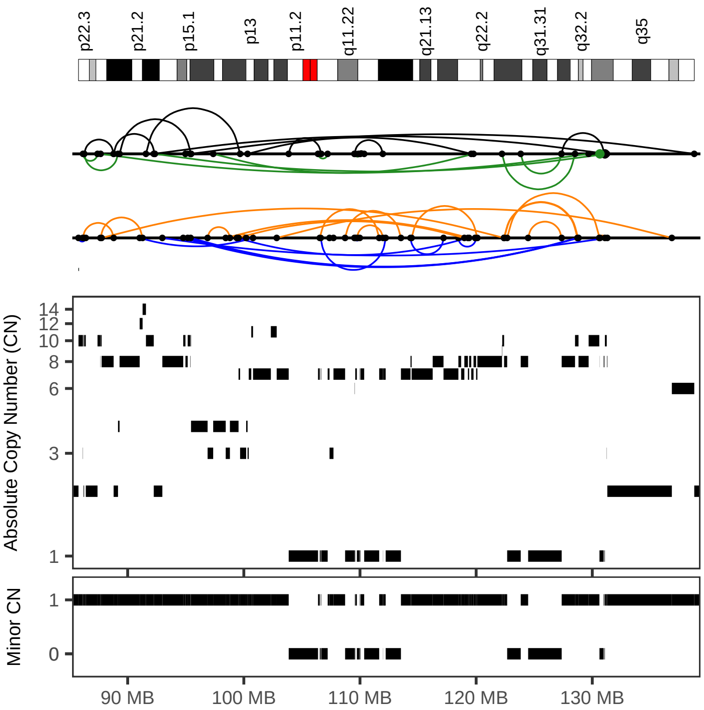

	db058eb4−9c5e−4226−92d1−de24aea83630
Cancer type	Kidney−RCC
Position	2:148510874−160670122
Type	Canonical without polyploidization
Interleaved intrachr. SVs	3
Total SVs (intrachr. + transl.)	7
SV types	DEL: 0; DUP: 1; h2hINV: 1; t2tINV: 1; TRA: 4
SVs in sample	9
Oscillating CN (2 and 3 states)	8, 8
CN segments	8
FDR fragment joints	0.84
FDR chr. breakp. enrich.	0
Linked to chrs	
Purity, ploidy	0.64, 2.03

	4edd92e1−5da5−49a3−80e4−fa456aa425b8
Cancer type	Kidney−RCC
Position	7:85764754−138776191
Type	With other complex events
Interleaved intrachr. SVs	43
Total SVs (intrachr. + transl.)	45
SV types	DEL: 16; DUP: 9; h2hINV: 10; t2tINV: 8; TRA: 2
SVs in sample	78
Oscillating CN (2 and 3 states)	14, 16
CN segments	93
FDR fragment joints	0.59
FDR chr. breakp. enrich.	0
Linked to chrs	
Purity, ploidy	0.76, 1.99

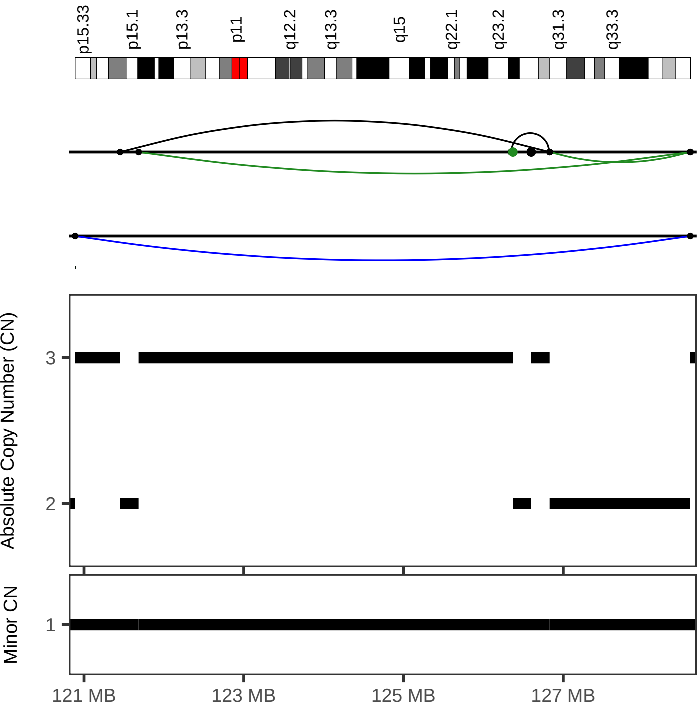

	C0005
Cancer type	Kidney−RCC
Position	5:120889469−128593853
Type	Canonical without polyploidization
Interleaved intrachr. SVs	5
Total SVs (intrachr. + transl.)	7
SV types	DEL: 0; DUP: 1; h2hINV: 2; t2tINV: 2; TRA: 2
SVs in sample	17
Oscillating CN (2 and 3 states)	7, 7
CN segments	7
FDR fragment joints	0.64
FDR chr. breakp. enrich.	0
Linked to chrs	3:62575892−70085334;
Purity, ploidy	0.33, 1.86

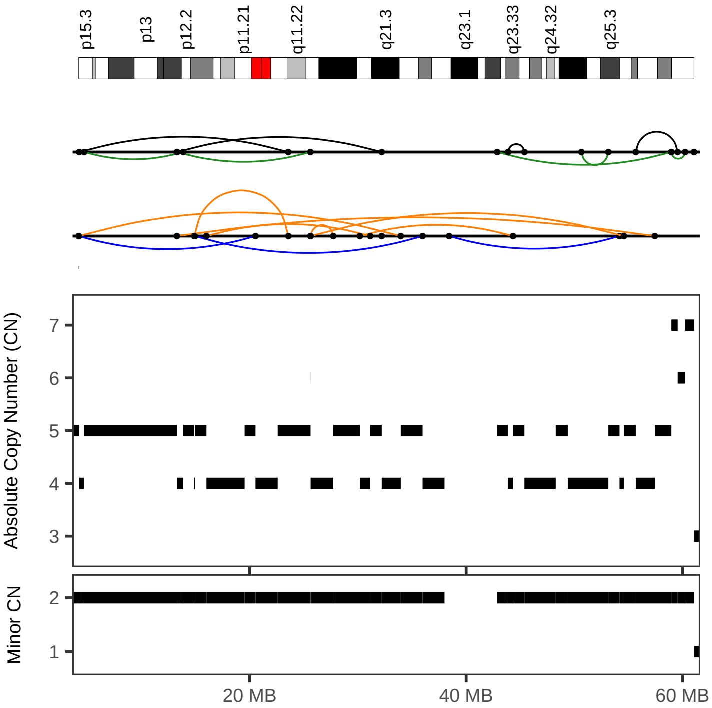

	C0012
Cancer type	Kidney−RCC
Position	10:4199382−60236974
Type	With other complex events
Interleaved intrachr. SVs	17
Total SVs (intrachr. + transl.)	17
SV types	DEL: 6; DUP: 3; h2hINV: 4; t2tINV: 4; TRA: 0
SVs in sample	49
Oscillating CN (2 and 3 states)	19, 31
CN segments	34
FDR fragment joints	0.84
FDR chr. breakp. enrich.	0
Linked to chrs	
Purity, ploidy	0.4, 4.08

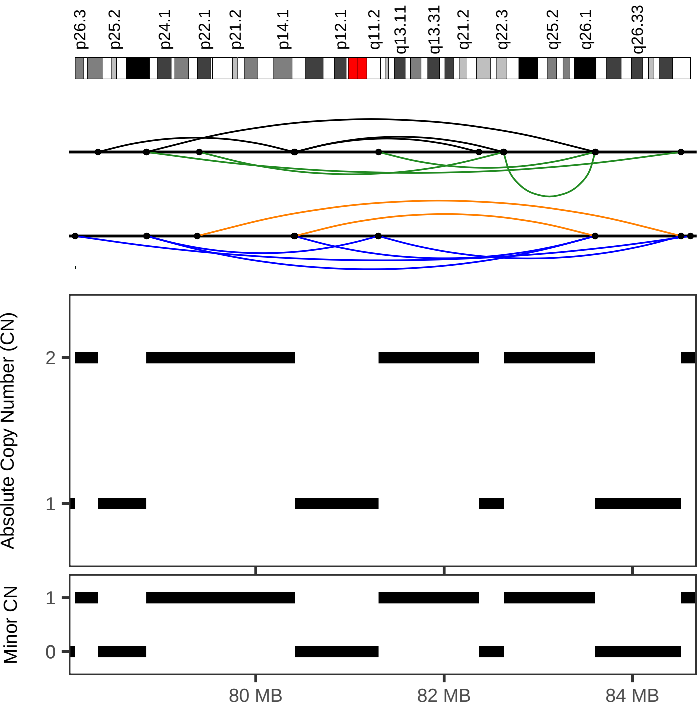

	C0014
Cancer type	Kidney−RCC
Position	3:78323577−84515937
Type	Canonical without polyploidization
Interleaved intrachr. SVs	14
Total SVs (intrachr. + transl.)	14
SV types	DEL: 2; DUP: 4; h2hINV: 4; t2tINV: 4; TRA: 0
SVs in sample	17
Oscillating CN (2 and 3 states)	8, 8
CN segments	8
FDR fragment joints	0.88
FDR chr. breakp. enrich.	0
Linked to chrs	
Purity, ploidy	0.61, 1.97

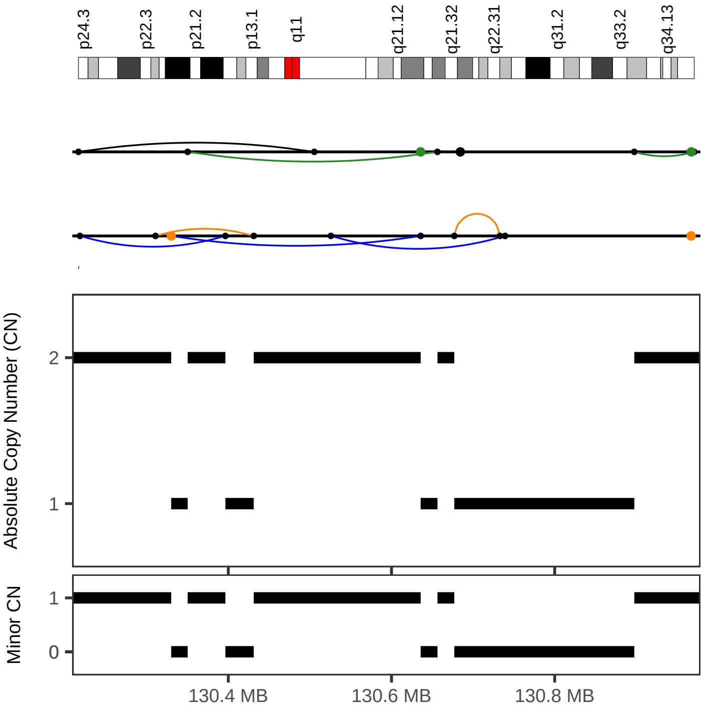

	C0020
Cancer type	Kidney−RCC
Position	9:130216384−130739182
Type	Canonical without polyploidization
Interleaved intrachr. SVs	6
Total SVs (intrachr. + transl.)	9
SV types	DEL: 1; DUP: 3; h2hINV: 1; t2tINV: 1; TRA: 3
SVs in sample	32
Oscillating CN (2 and 3 states)	8, 8
CN segments	8
FDR fragment joints	0.64
FDR chr. breakp. enrich.	0
Linked to chrs	
Purity, ploidy	0.65, 1.9

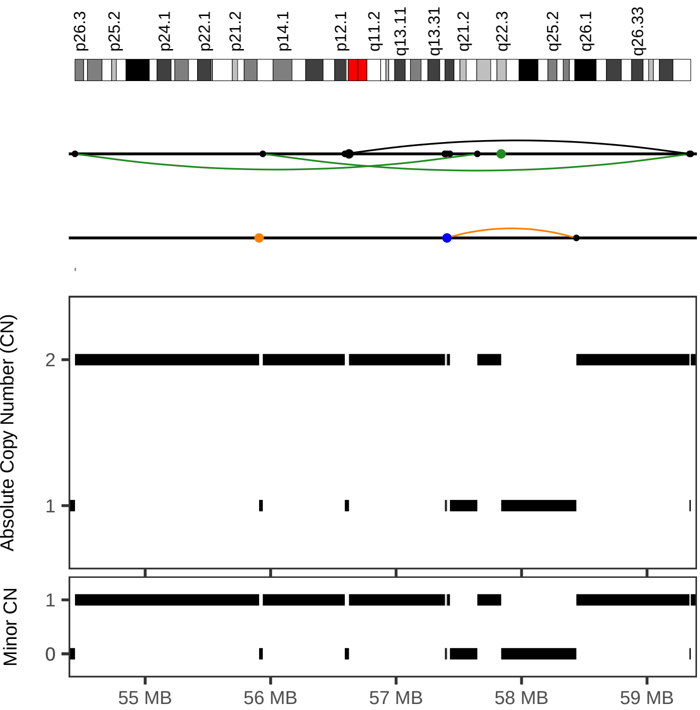

	C0021
Cancer type	Kidney−RCC
Position	3:54440509−59348361
Type	Canonical without polyploidization
Interleaved intrachr. SVs	5
Total SVs (intrachr. + transl.)	9
SV types	DEL: 1; DUP: 0; h2hINV: 2; t2tINV: 2; TRA: 4
SVs in sample	16
Oscillating CN (2 and 3 states)	15, 15
CN segments	15
FDR fragment joints	0.64
FDR chr. breakp. enrich.	0
Linked to chrs	5:75100143−77822720;
Purity, ploidy	0.61, 1.92

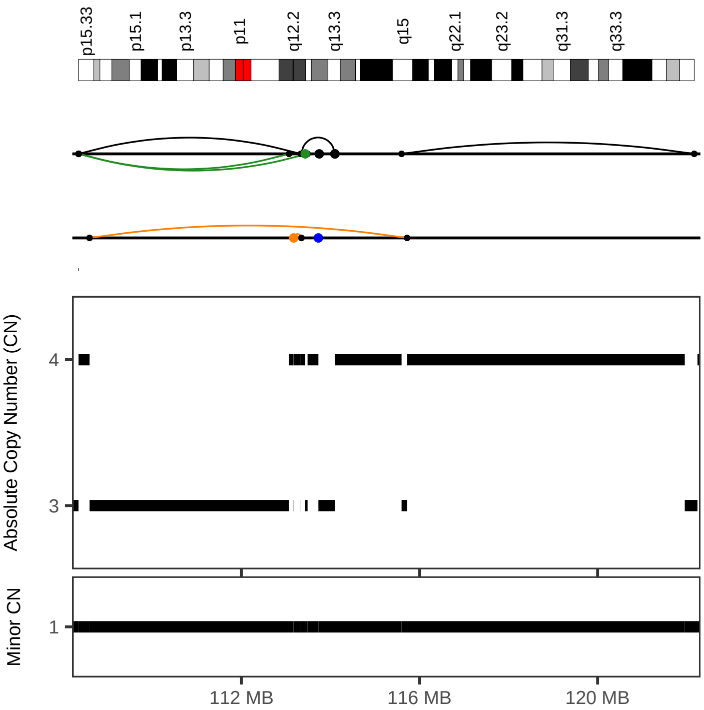

	C0028
Cancer type	Kidney−RCC
Position	5:108336748−122172505
Type	After polyploidization
Interleaved intrachr. SVs	7
Total SVs (intrachr. + transl.)	13
SV types	DEL: 2; DUP: 0; h2hINV: 3; t2tINV: 2; TRA: 6
SVs in sample	22
Oscillating CN (2 and 3 states)	14, 14
CN segments	14
FDR fragment joints	0.64
FDR chr. breakp. enrich.	0
Linked to chrs	3:53741890−60601042;
Purity, ploidy	0.65, 2.21

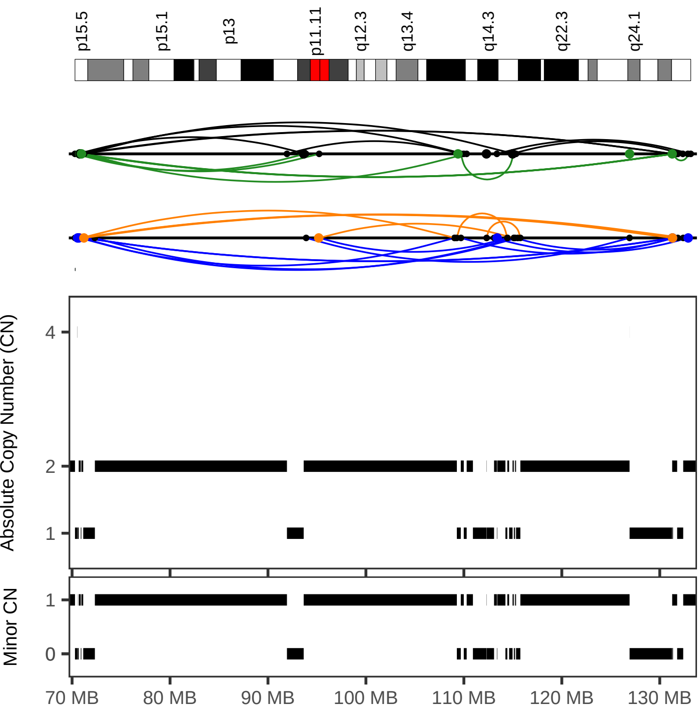

	C0038
Cancer type	Kidney−RCC
Position	11:70294810−133163692
Type	Canonical without polyploidization
Interleaved intrachr. SVs	39
Total SVs (intrachr. + transl.)	57
SV types	DEL: 9; DUP: 11; h2hINV: 10; t2tINV: 9; TRA: 18
SVs in sample	77
Oscillating CN (2 and 3 states)	30, 32
CN segments	41
FDR fragment joints	0.98
FDR chr. breakp. enrich.	0
Linked to chrs	5:1324772−162595017;
Purity, ploidy	0.52, 2.2

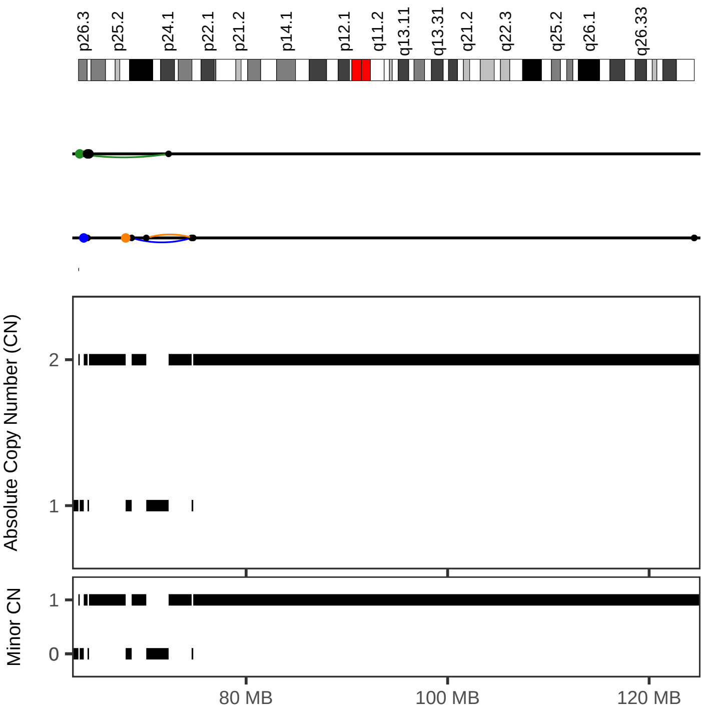

	C0042
Cancer type	Kidney−RCC
Position	3:63359585−74750084
Type	Canonical without polyploidization
Interleaved intrachr. SVs	4
Total SVs (intrachr. + transl.)	9
SV types	DEL: 1; DUP: 1; h2hINV: 1; t2tINV: 1; TRA: 5
SVs in sample	15
Oscillating CN (2 and 3 states)	11, 11
CN segments	11
FDR fragment joints	1
FDR chr. breakp. enrich.	0
Linked to chrs	
Purity, ploidy	0.44, 2.03

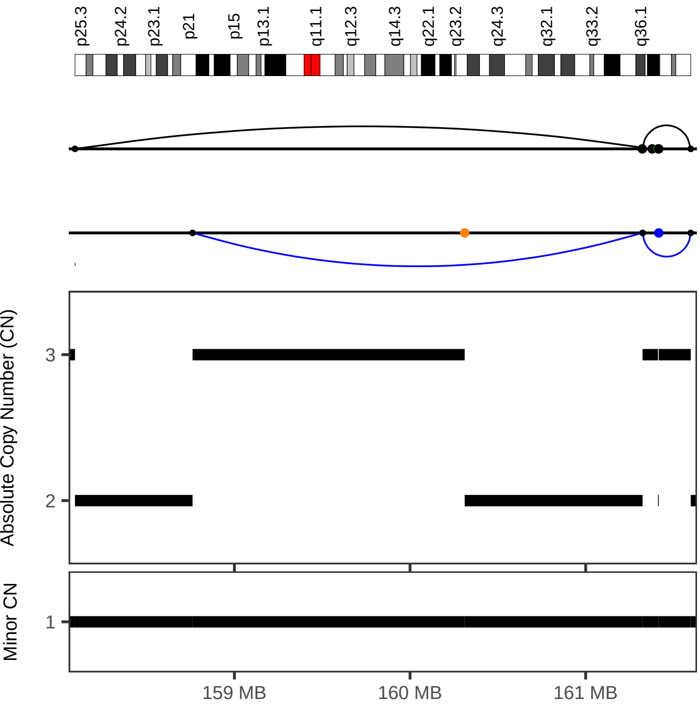

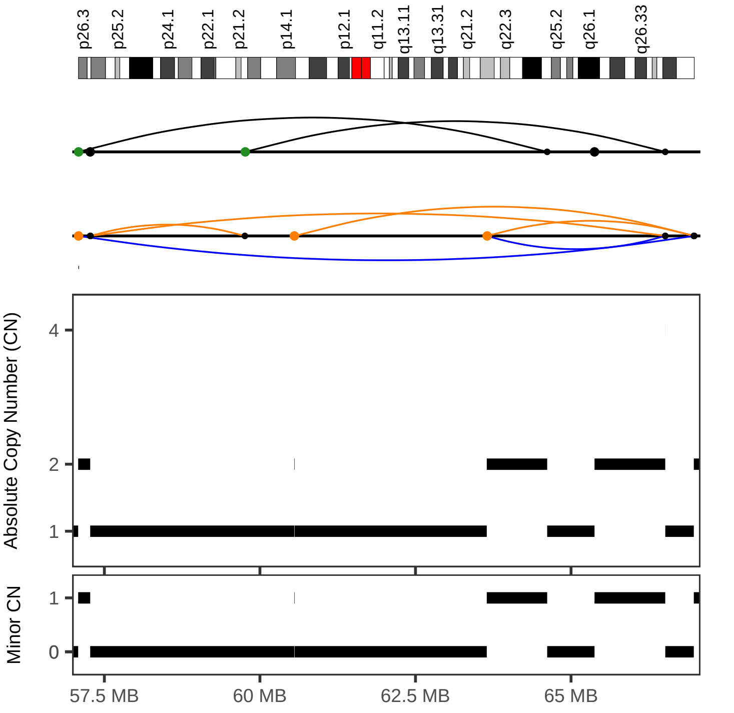

	**C0048**
Cancer type	Kidney−RCC
Position	2:158092242−161598269
Type	Canonical without polyploidization
Interleaved intrachr. SVs	4
Total SVs (intrachr. + transl.)	10
SV types	DEL: 0; DUP: 2; h2hINV: 2; t2tINV: 0; TRA: 6
SVs in sample	30
Oscillating CN (2 and 3 states)	7, 7
CN segments	7
FDR fragment joints	0.59
FDR chr. breakp. enrich.	0
Linked to chrs	
Purity, ploidy	0.57, 1.91

	**C0050**
Cancer type	Kidney−RCC
Position	3:57083918−66979704
Type	Canonical without polyploidization
Interleaved intrachr. SVs	8
Total SVs (intrachr. + transl.)	15
SV types	DEL: 4; DUP: 2; h2hINV: 2; t2tINV: 0; TRA: 7
SVs in sample	20
Oscillating CN (2 and 3 states)	7, 11
CN segments	11
FDR fragment joints	0.59
FDR chr. breakp. enrich.	0
Linked to chrs	13:87973980−95150380;
Purity, ploidy	0.47, 1.97

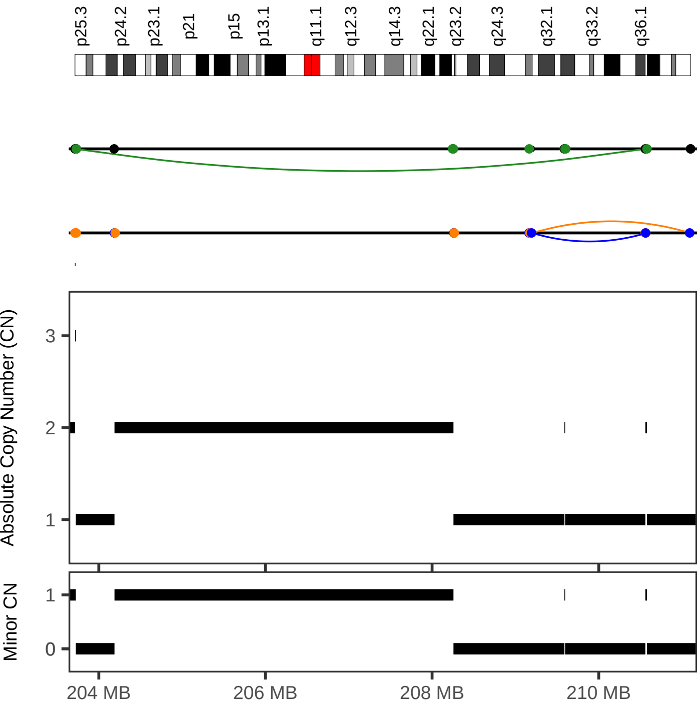

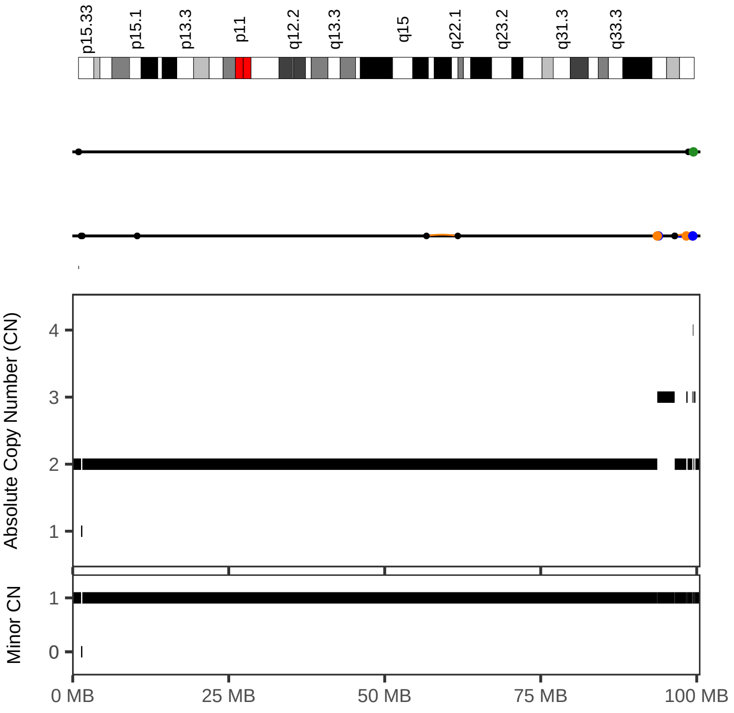

	**C0073**
Cancer type	Kidney−RCC
Position	2:203714228−211103949
Type	Canonical without polyploidization
Interleaved intrachr. SVs	3
Total SVs (intrachr. + transl.)	37
SV types	DEL: 1; DUP: 1; h2hINV: 0; t2tINV: 1; TRA: 34
SVs in sample	90
Oscillating CN (2 and 3 states)	7, 9
CN segments	9
FDR fragment joints	0.84
FDR chr. breakp. enrich.	0
Linked to chrs	5:89519689−113305324;
Purity, ploidy	0.47, 1.89

	**C0077**
Cancer type	Kidney−RCC
Position	5:96447966−99601957
Type	Canonical without polyploidization
Interleaved intrachr. SVs	5
Total SVs (intrachr. + transl.)	9
SV types	DEL: 3; DUP: 1; h2hINV: 0; t2tINV: 1; TRA: 4
SVs in sample	19
Oscillating CN (2 and 3 states)	7, 9
CN segments	11
FDR fragment joints	0.59
FDR chr. breakp. enrich.	0
Linked to chrs	
Purity, ploidy	0.54, 1.91

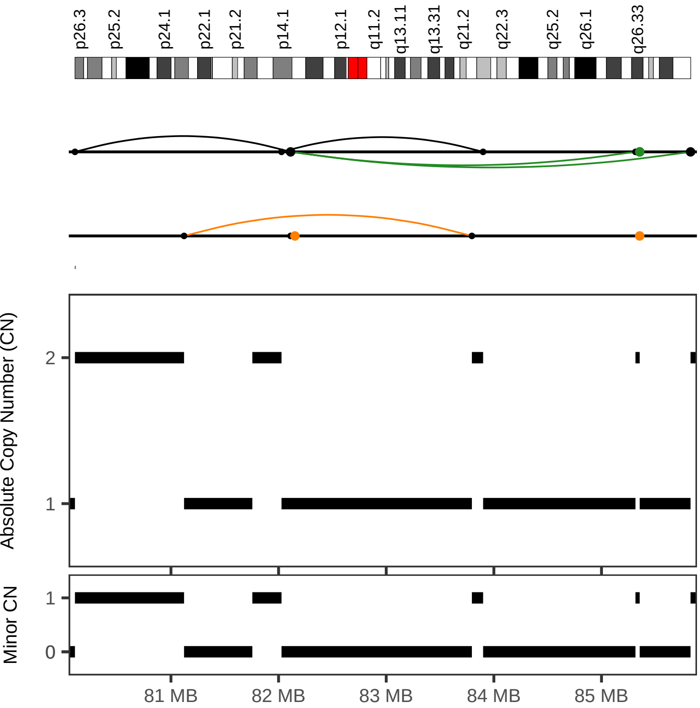

	**C0085**
Cancer type	Kidney−RCC
Position	3:80108501−85829127
Type	Canonical without polyploidization
Interleaved intrachr. SVs	6
Total SVs (intrachr. + transl.)	12
SV types	DEL: 2; DUP: 0; h2hINV: 2; t2tINV: 2; TRA: 6
SVs in sample	17
Oscillating CN (2 and 3 states)	9, 9
CN segments	9
FDR fragment joints	0.64
FDR chr. breakp. enrich.	0
Linked to chrs	
Purity, ploidy	0.37, 2.05

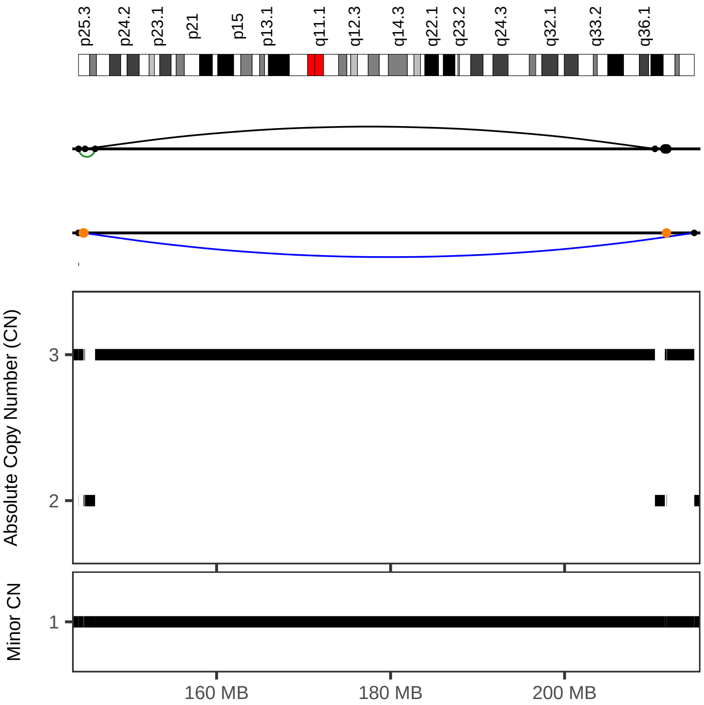

	**C0099**
Cancer type	Kidney−RCC
Position	2:144120765−214902326
Type	Canonical without polyploidization
Interleaved intrachr. SVs	4
Total SVs (intrachr. + transl.)	9
SV types	DEL: 1; DUP: 1; h2hINV: 1; t2tINV: 1; TRA: 5
SVs in sample	18
Oscillating CN (2 and 3 states)	13, 13
CN segments	13
FDR fragment joints	1
FDR chr. breakp. enrich.	0
Linked to chrs	3:95657165−111808748;
Purity, ploidy	0.37, 1.9

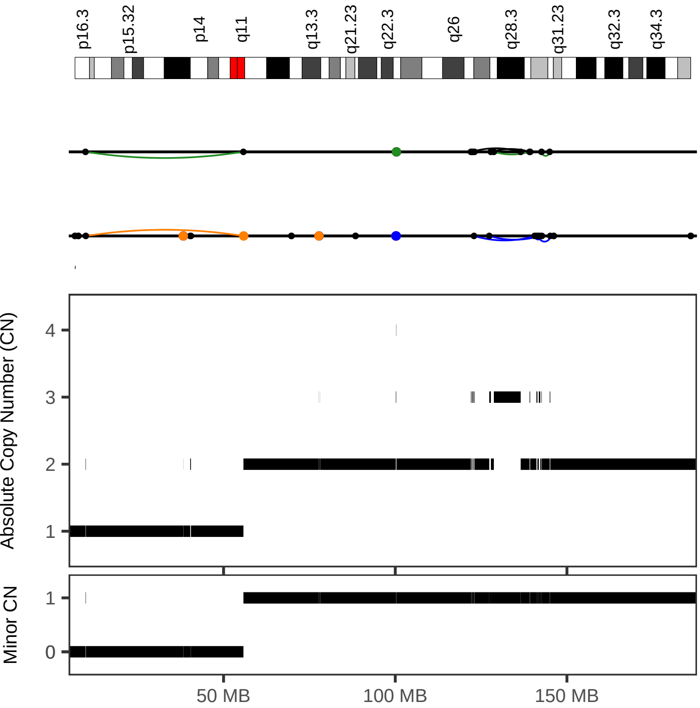

	**1987b453−f97d−45c7−9c89−b9a33313d645**
Cancer type	Liver−HCC
Position	4:122039841−145149274
Type	Canonical without polyploidization
Interleaved intrachr. SVs	10
Total SVs (intrachr. + transl.)	10
SV types	DEL: 0; DUP: 4; h2hINV: 3; t2tINV: 3; TRA: 0
SVs in sample	524
Oscillating CN (2 and 3 states)	20, 20
CN segments	20
FDR fragment joints	0.59
FDR chr. breakp. enrich.	1
Linked to chrs	
Purity, ploidy	0.74, 1.99

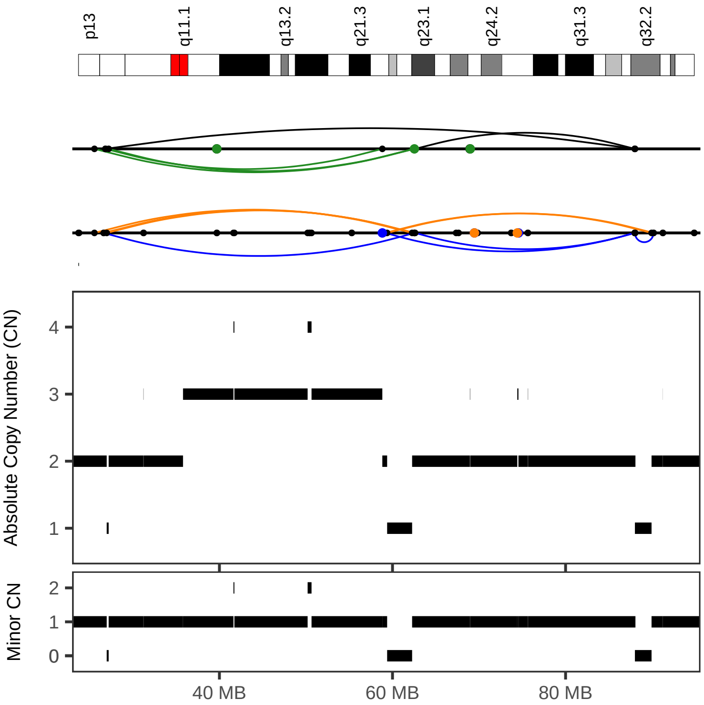

	**1987b453−f97d−45c7−9c89−b9a33313d645**
Cancer type	Liver−HCC
Position	14:25556654−90170623
Type	With other complex events
Interleaved intrachr. SVs	19
Total SVs (intrachr. + transl.)	29
SV types	DEL: 7; DUP: 5; h2hINV: 3; t2tINV: 4; TRA: 10
SVs in sample	524
Oscillating CN (2 and 3 states)	7, 23
CN segments	23
FDR fragment joints	0.68
FDR chr. breakp. enrich.	0
Linked to chrs	1:9273332−226103049;7:28246412−155112625
Purity, ploidy	0.74, 1.99

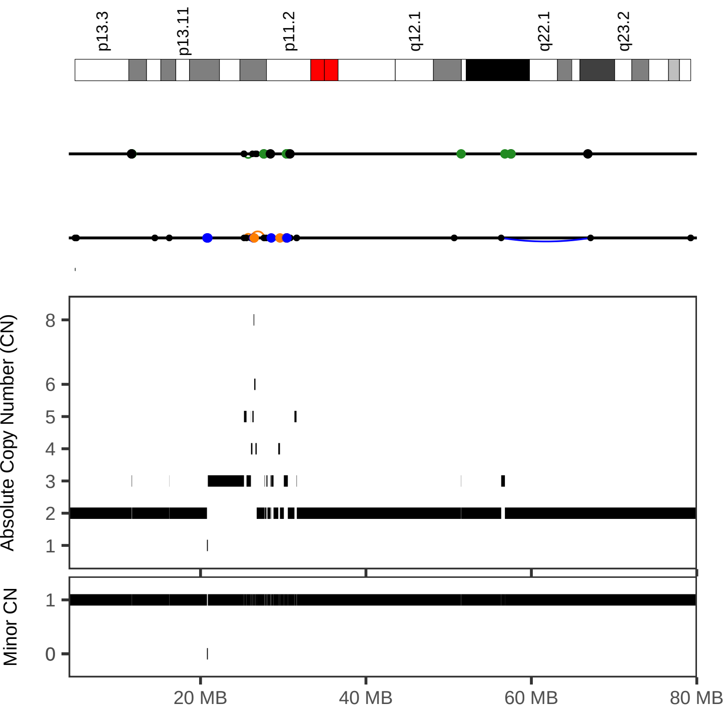

1987b453−f97d−45c7−9c89−b9a33313d645	
Cancer type	Liver−HCC
Position	16:25250822−30860384
Type	With other complex events
Interleaved intrachr. SVs	6
Total SVs (intrachr. + transl.)	21
SV types	DEL: 3; DUP: 1; h2hINV: 1; t2tINV: 1; TRA: 15
SVs in sample	524
Oscillating CN (2 and 3 states)	9, 14
CN segments	22
FDR fragment joints	0.64
FDR chr. breakp. enrich.	0
Linked to chrs	20:20959449−62129527;
Purity, ploidy	0.74, 1.99

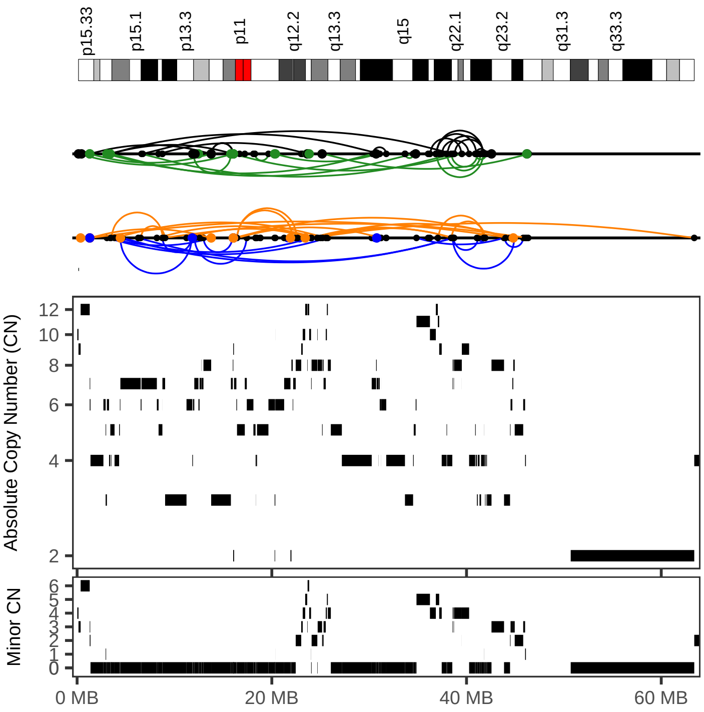

3acf7438−fd65−442c−8a30−68b6714537f3	
Cancer type	Liver−HCC
Position	5:141997−63388893
Type	With other complex events
Interleaved intrachr. SVs	71
Total SVs (intrachr. + transl.)	114
SV types	DEL: 23; DUP: 17; h2hINV: 17; t2tINV: 14; TRA: 43
SVs in sample	222
Oscillating CN (2 and 3 states)	7, 14
CN segments	138
FDR fragment joints	0.64
FDR chr. breakp. enrich.	0
Linked to chrs	
Purity, ploidy	0.36, 3.55

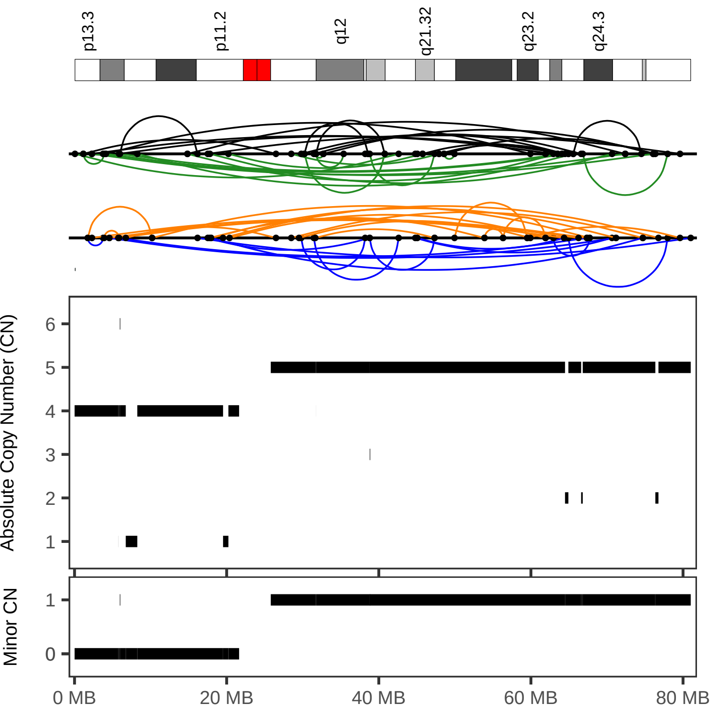

457cca7c−a8b4−414e−a3c0−7fccf0ee2034	
Cancer type	Liver−HCC
Position	17:57017−81011331
Type	With other complex events
Interleaved intrachr. SVs	66
Total SVs (intrachr. + transl.)	66
SV types	DEL: 18; DUP: 13; h2hINV: 17; t2tINV: 18; TRA: 0
SVs in sample	137
Oscillating CN (2 and 3 states)	7, 12
CN segments	20
FDR fragment joints	0.84
FDR chr. breakp. enrich.	0
Linked to chrs	
Purity, ploidy	0.48, 3.87

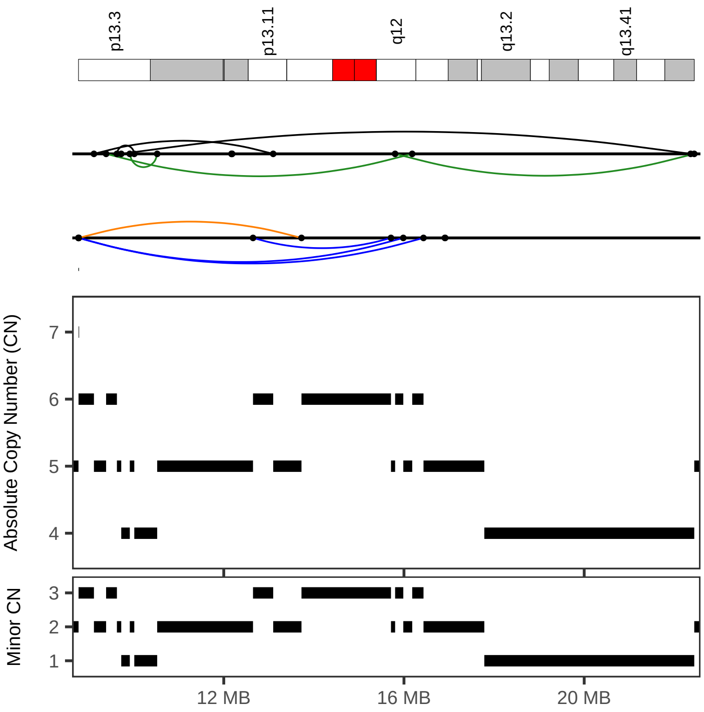

457cca7c−a8b4−414e−a3c0−7fccf0ee2034	
Cancer type	Liver−HCC
Position	19:8778812−22442312
Type	With other complex events
Interleaved intrachr. SVs	10
Total SVs (intrachr. + transl.)	10
SV types	DEL: 1; DUP: 3; h2hINV: 3; t2tINV: 3; TRA: 0
SVs in sample	137
Oscillating CN (2 and 3 states)	9, 19
CN segments	19
FDR fragment joints	0.83
FDR chr. breakp. enrich.	0
Linked to chrs	
Purity, ploidy	0.48, 3.87

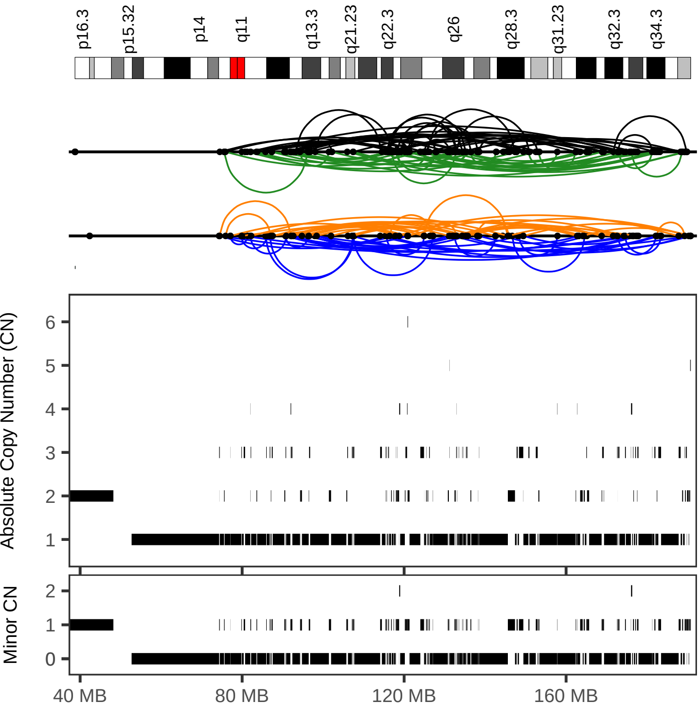

56403463−1727−4c23−a709−de5f168c4073	
Cancer type	Liver−HCC
Position	4:74309555−190780625
Type	With other complex events
Interleaved intrachr. SVs	157
Total SVs (intrachr. + transl.)	157
SV types	DEL: 31; DUP: 36; h2hINV: 45; t2tINV: 45; TRA: 0
SVs in sample	309
Oscillating CN (2 and 3 states)	13, 26
CN segments	204
FDR fragment joints	0.59
FDR chr. breakp. enrich.	0
Linked to chrs	
Purity, ploidy	0.69, 1.88

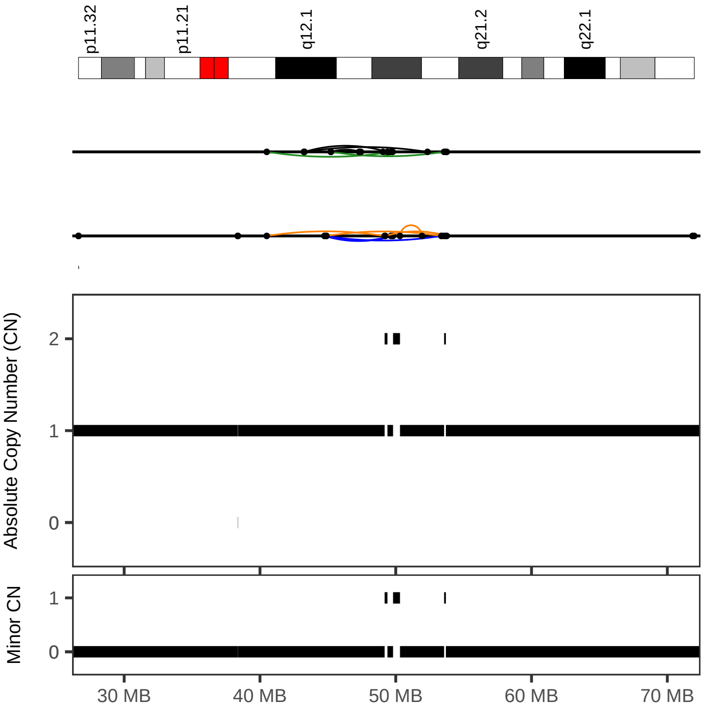

56403463−1727−4c23−a709−de5f168c4073	
Cancer type	Liver−HCC
Position	18:40499139−53757981
Type	Canonical without polyploidization
Interleaved intrachr. SVs	14
Total SVs (intrachr. + transl.)	14
SV types	DEL: 4; DUP: 3; h2hINV: 4; t2tINV: 3; TRA: 0
SVs in sample	309
Oscillating CN (2 and 3 states)	7, 7
CN segments	7
FDR fragment joints	0.98
FDR chr. breakp. enrich.	0
Linked to chrs	
Purity, ploidy	0.69, 1.88

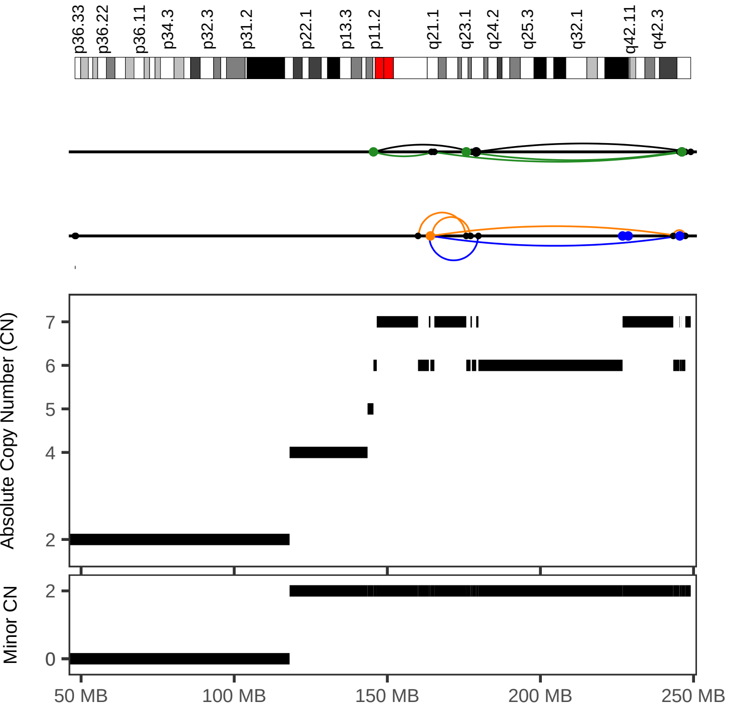

56a82a56−0241−4d3d−9de2−696b0c36df91	
Cancer type	Liver−HCC
Position	1:145474628−249083131
Type	After polyploidization
Interleaved intrachr. SVs	13
Total SVs (intrachr. + transl.)	24
SV types	DEL: 4; DUP: 2; h2hINV: 4; t2tINV: 3; TRA: 11
SVs in sample	109
Oscillating CN (2 and 3 states)	18, 19
CN segments	19
FDR fragment joints	0.88
FDR chr. breakp. enrich.	0
Linked to chrs	14:29177804−36937609;3:23541316−30879079
Purity, ploidy	0.88, 3.52

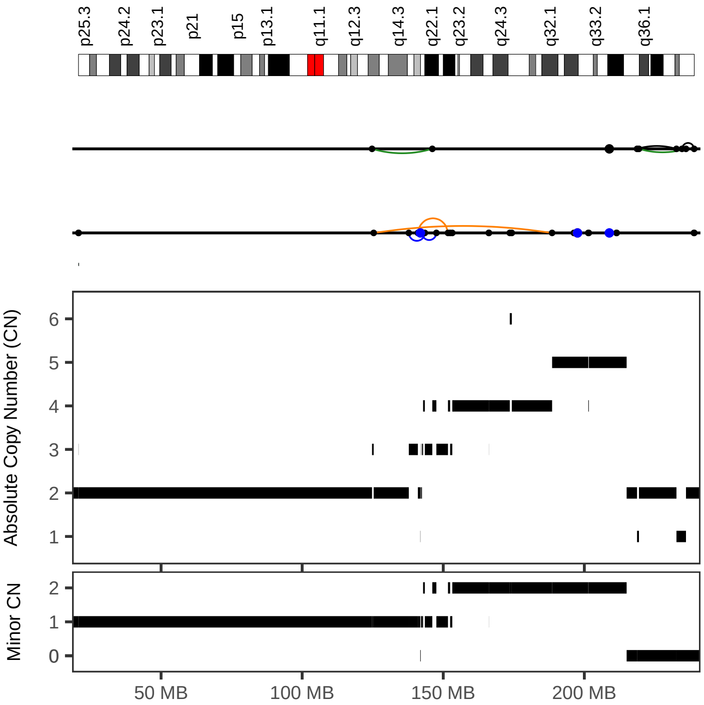

614c0ecf−6a84−4c86−8706−79be5bb00991	
Cancer type	Liver−HCC
Position	2:124760110−188573775
Type	With other complex events
Interleaved intrachr. SVs	6
Total SVs (intrachr. + transl.)	7
SV types	DEL: 3; DUP: 2; h2hINV: 0; t2tINV: 1; TRA: 1
SVs in sample	171
Oscillating CN (2 and 3 states)	10, 18
CN segments	21
FDR fragment joints	0.59
FDR chr. breakp. enrich.	0.02
Linked to chrs	
Purity, ploidy	0.82, 2.44

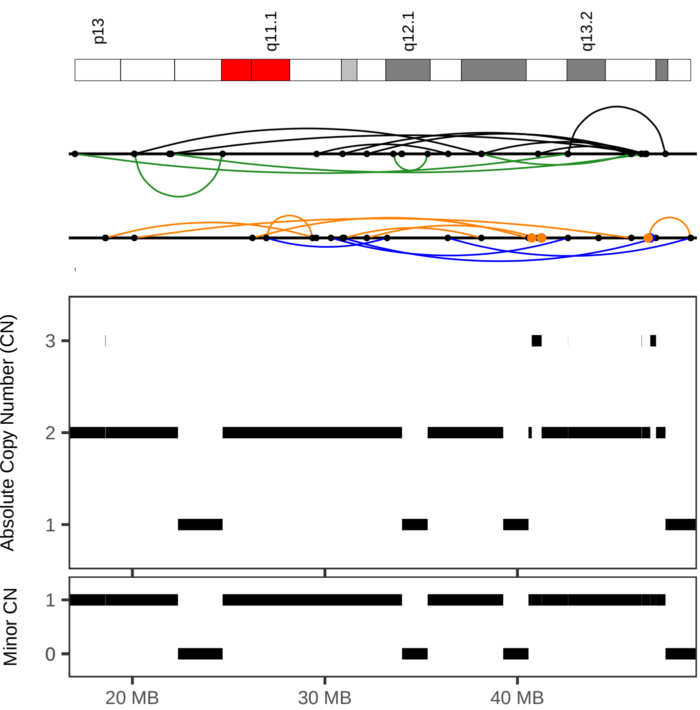

	614c0ecf−6a84−4c86−8706−79be5bb00991
Cancer type	Liver−HCC
Position	22:17018899−48999964
Type	With other complex events
Interleaved intrachr. SVs	25
Total SVs (intrachr. + transl.)	31
SV types	DEL: 7; DUP: 5; h2hINV: 8; t2tINV: 5; TRA: 6
SVs in sample	171
Oscillating CN (2 and 3 states)	9, 18
CN segments	18
FDR fragment joints	0.84
FDR chr. breakp. enrich.	0
Linked to chrs	
Purity, ploidy	0.82, 2.44

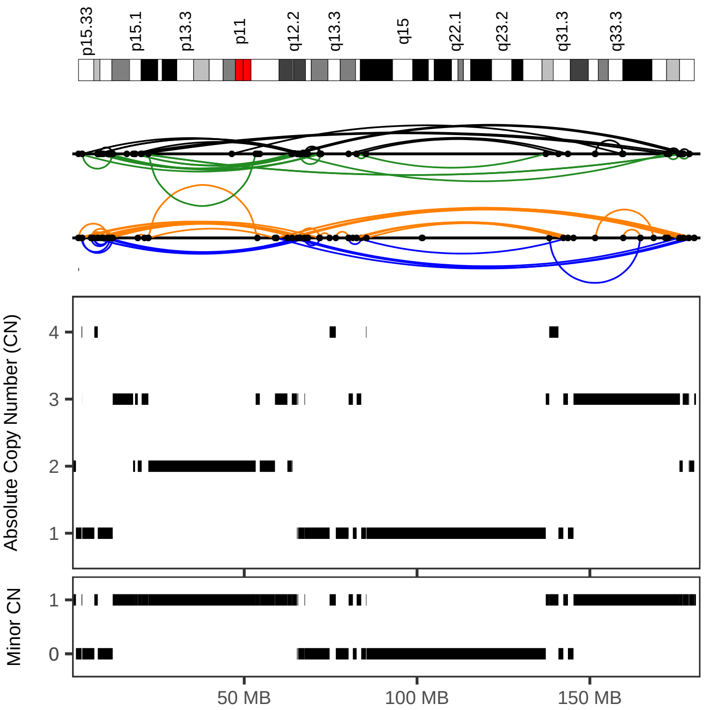

	69d6881b−9e9a−407b−8b02−bad5fc2455bf
Cancer type	Liver−HCC
Position	5:2046664−180200470
Type	With other complex events
Interleaved intrachr. SVs	88
Total SVs (intrachr. + transl.)	88
SV types	DEL: 28; DUP: 17; h2hINV: 22; t2tINV: 21; TRA: 0
SVs in sample	102
Oscillating CN (2 and 3 states)	13, 35
CN segments	50
FDR fragment joints	0.63
FDR chr. breakp. enrich.	0
Linked to chrs	
Purity, ploidy	0.78, 2.04

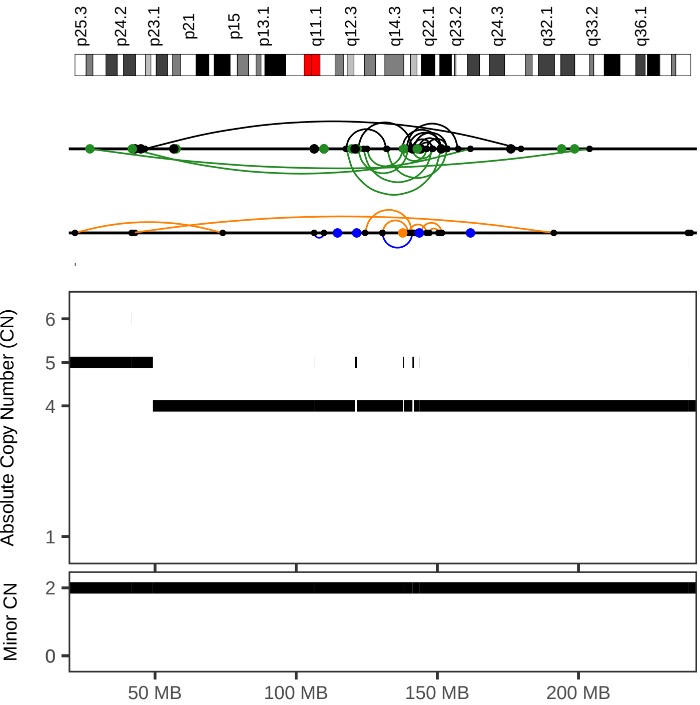

	6c79f64b−63fe−46f5−bf7b−77208231b0f2
Cancer type	Liver−HCC
Position	2:117574279−157367750
Type	After polyploidization
Interleaved intrachr. SVs	27
Total SVs (intrachr. + transl.)	39
SV types	DEL: 7; DUP: 2; h2hINV: 10; t2tINV: 8; TRA: 12
SVs in sample	125
Oscillating CN (2 and 3 states)	7, 11
CN segments	11
FDR fragment joints	0.59
FDR chr. breakp. enrich.	0
Linked to chrs	1:16944681−155955763;11:41208203−71433169
Purity, ploidy	0.48, 3.89

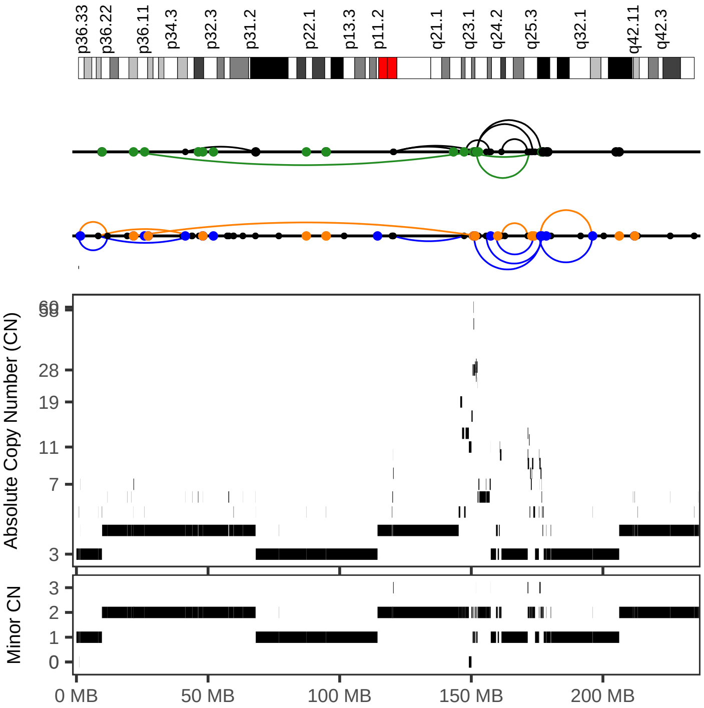

	6dcfc418−ab28−4365−95ea−c1ae254f2341
Cancer type	Liver−HCC
Position	1:774798−196124730
Type	With other complex events
Interleaved intrachr. SVs	35
Total SVs (intrachr. + transl.)	93
SV types	DEL: 6; DUP: 11; h2hINV: 10; t2tINV: 8; TRA: 58
SVs in sample	572
Oscillating CN (2 and 3 states)	11, 30
CN segments	128
FDR fragment joints	0.72
FDR chr. breakp. enrich.	0
Linked to chrs	10:35522520−95427338;14:70988852−75703456 19:1843528−50016660;2:64246510−172438745 3:170057914−194756878;6:1255518−166814605 7:8218986−100523823;8:8789701−145595243
Purity, ploidy	0.85, 3.57

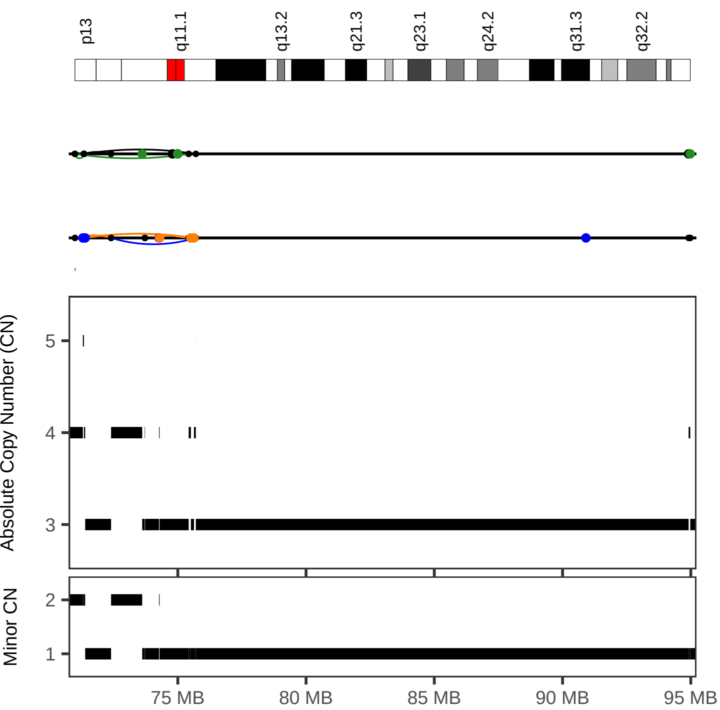

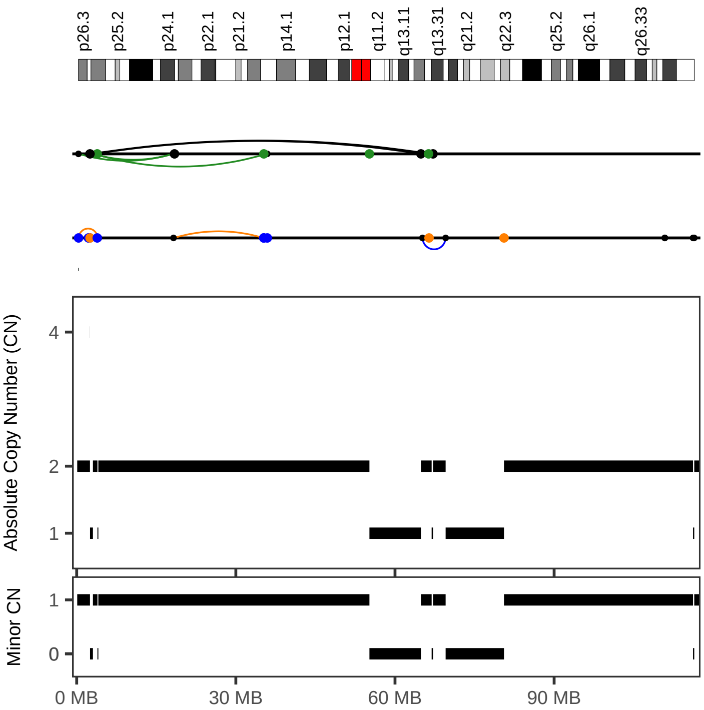

6dcfc418−ab28−4365−95ea−c1ae254f2341

Cancer type	Liver−HCC
Position	14:70988852−75703457
Type	After polyploidization
Interleaved intrachr. SVs	8
Total SVs (intrachr. + transl.)	19
SV types	DEL: 3; DUP: 1; h2hINV: 2; t2tINV: 2; TRA: 11
SVs in sample	572
Oscillating CN (2 and 3 states)	11, 14
CN segments	15
FDR fragment joints	0.84
FDR chr. breakp. enrich.	0.01
Linked to chrs	18:9039200−46434123;9:71361828−139860148
Purity, ploidy	0.85, 3.57

89791f8f−dc06−4a04−b166−afbfd6cf53de

Cancer type	Liver−HCC
Position	3:333940−69546080
Type	Canonical without polyploidization
Interleaved intrachr. SVs	9
Total SVs (intrachr. + transl.)	26
SV types	DEL: 2; DUP: 1; h2hINV: 3; t2tINV: 3; TRA: 17
SVs in sample	65
Oscillating CN (2 and 3 states)	11, 11
CN segments	13
FDR fragment joints	0.83
FDR chr. breakp. enrich.	0
Linked to chrs	
Purity, ploidy	0.7, 2.01

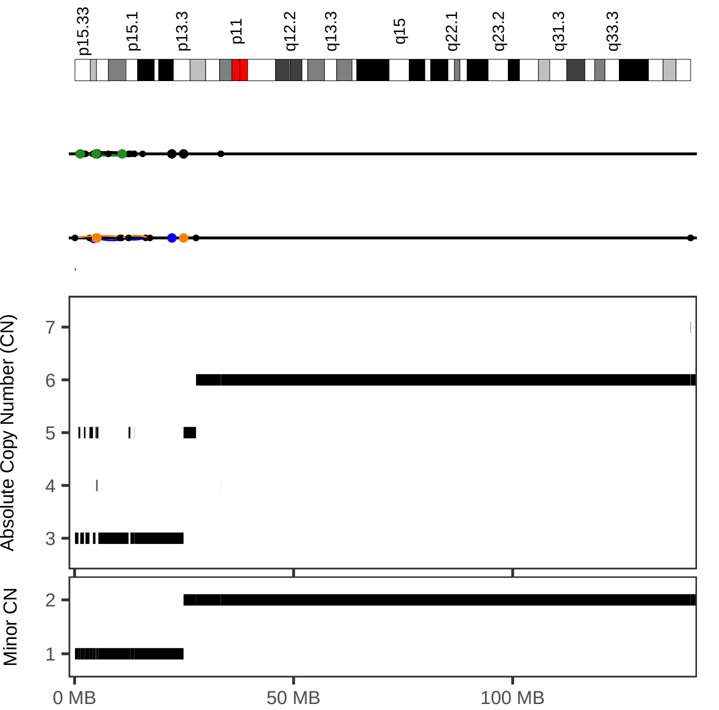

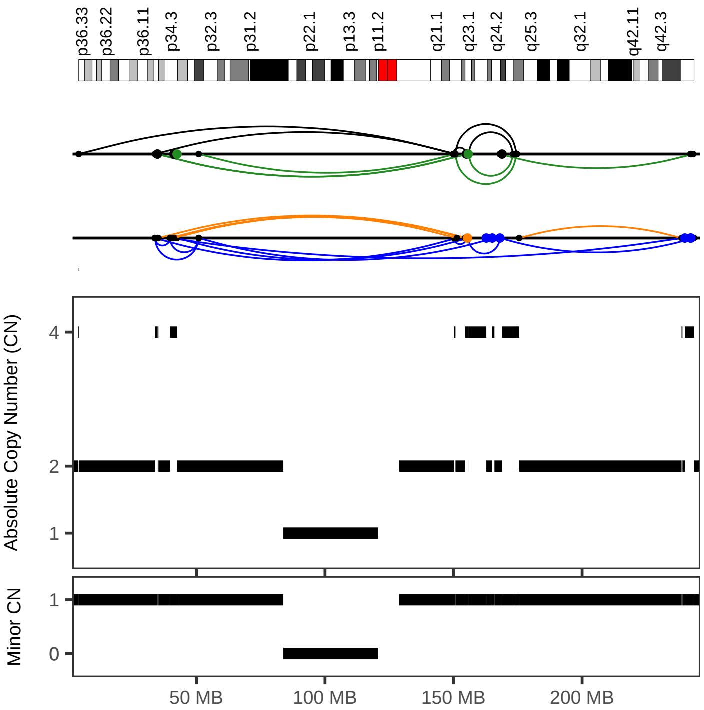

b865dec4−f051−4fbe−9405−f832ff2010d7

Cancer type	Liver−HCC
Position	5:90736−17223234
Type	With other complex events
Interleaved intrachr. SVs	12
Total SVs (intrachr. + transl.)	22
SV types	DEL: 2; DUP: 3; h2hINV: 3; t2tINV: 4; TRA: 10
SVs in sample	245
Oscillating CN (2 and 3 states)	9, 21
CN segments	21
FDR fragment joints	0.91
FDR chr. breakp. enrich.	0
Linked to chrs	7:29593133−29728788;
Purity, ploidy	0.78, 3.47

bce25281−502e−4599−9679−32dc8462ffb1

Cancer type	Liver−HCC
Position	1:4234113−243543953
Type	Before polyploidization
Interleaved intrachr. SVs	28
Total SVs (intrachr. + transl.)	42
SV types	DEL: 5; DUP: 10; h2hINV: 7; t2tINV: 6; TRA: 14
SVs in sample	154
Oscillating CN (2 and 3 states)	19, 31
CN segments	31
FDR fragment joints	0.64
FDR chr. breakp. enrich.	0
Linked to chrs	
Purity, ploidy	0.89, 1.98

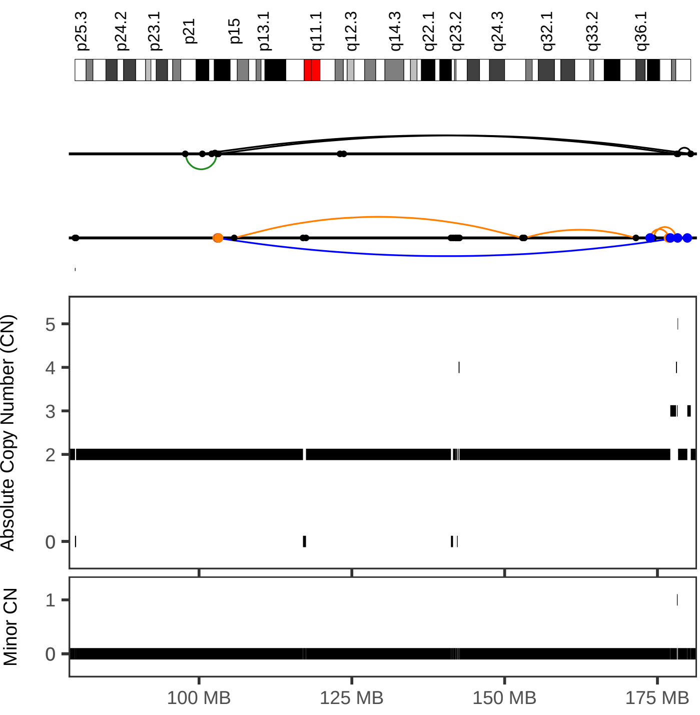

	bfd20d71−18a5−4f01−8d56−84378ad92359
Cancer type	Liver−HCC
Position	2:97744377−180446202
Type	With other complex events
Interleaved intrachr. SVs	6
Total SVs (intrachr. + transl.)	14
SV types	DEL: 0; DUP: 1; h2hINV: 4;<br>t2tINV: 1; TRA: 8
SVs in sample	96
Oscillating CN (2 and 3 states)	9, 9
CN segments	18
FDR fragment joints	0.48
FDR chr. breakp. enrich.	0
Linked to chrs	7:57254259−125189467;
Purity, ploidy	0.68, 1.98

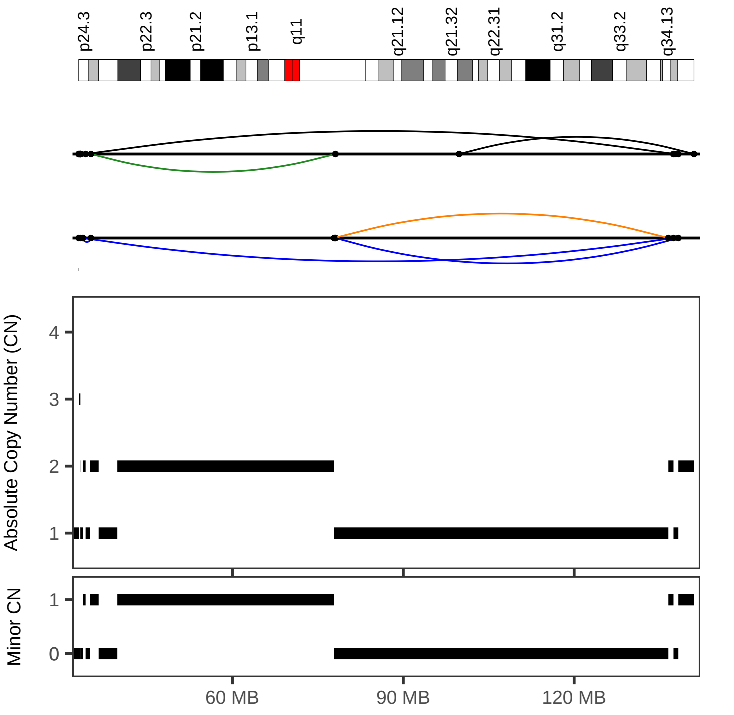

	d680df09−368e−42b5−b540−45c41ed31042
Cancer type	Liver−HCC
Position	9:33778034−141035750
Type	Canonical without polyploidization
Interleaved intrachr. SVs	9
Total SVs (intrachr. + transl.)	9
SV types	DEL: 1; DUP: 4; h2hINV: 2;<br>t2tINV: 2; TRA: 0
SVs in sample	82
Oscillating CN (2 and 3 states)	9, 10
CN segments	10
FDR fragment joints	0.64
FDR chr. breakp. enrich.	0
Linked to chrs	
Purity, ploidy	0.64, 1.94

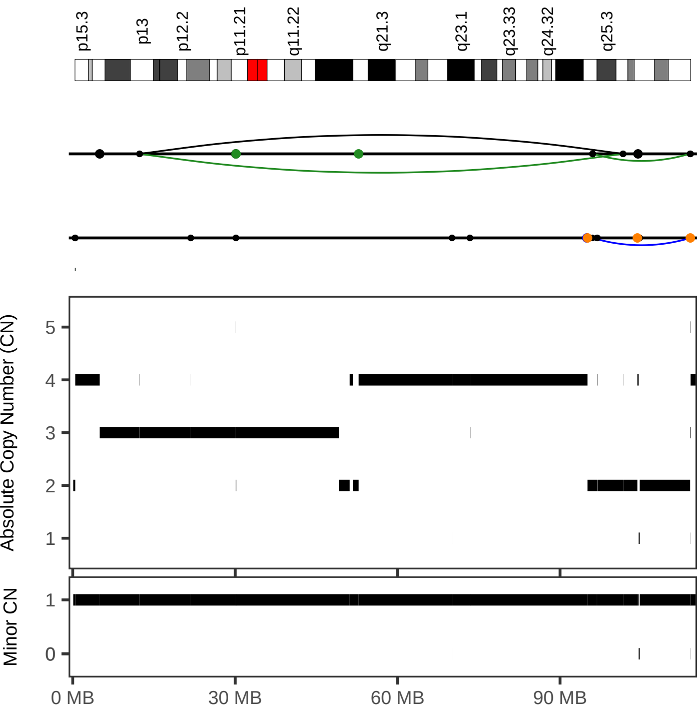

	ee63c44b−91f4−4004−8dea−192eab527036
Cancer type	Liver−HCC
Position	10:12324933−114133790
Type	With other complex events
Interleaved intrachr. SVs	7
Total SVs (intrachr. + transl.)	15
SV types	DEL: 0; DUP: 2; h2hINV: 3;<br>t2tINV: 2; TRA: 8
SVs in sample	405
Oscillating CN (2 and 3 states)	7, 10
CN segments	28
FDR fragment joints	0.64
FDR chr. breakp. enrich.	0.41
Linked to chrs	17:19411292−80161264;
Purity, ploidy	0.67, 2.88

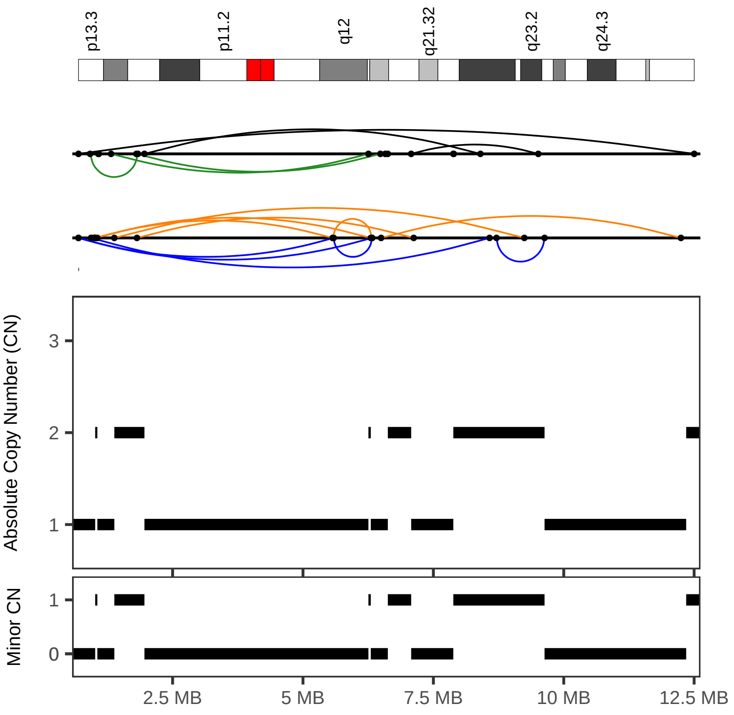

	HX10
Cancer type	Liver−HCC
Position	17:694982−12501666
Type	Canonical without polyploidization
Interleaved intrachr. SVs	18
Total SVs (intrachr. + transl.)	18
SV types	DEL: 7; DUP: 5; h2hINV: 3;<br>t2tINV: 3; TRA: 0
SVs in sample	51
Oscillating CN (2 and 3 states)	12, 12
CN segments	12
FDR fragment joints	0.64
FDR chr. breakp. enrich.	0
Linked to chrs	
Purity, ploidy	0.51, 2.02

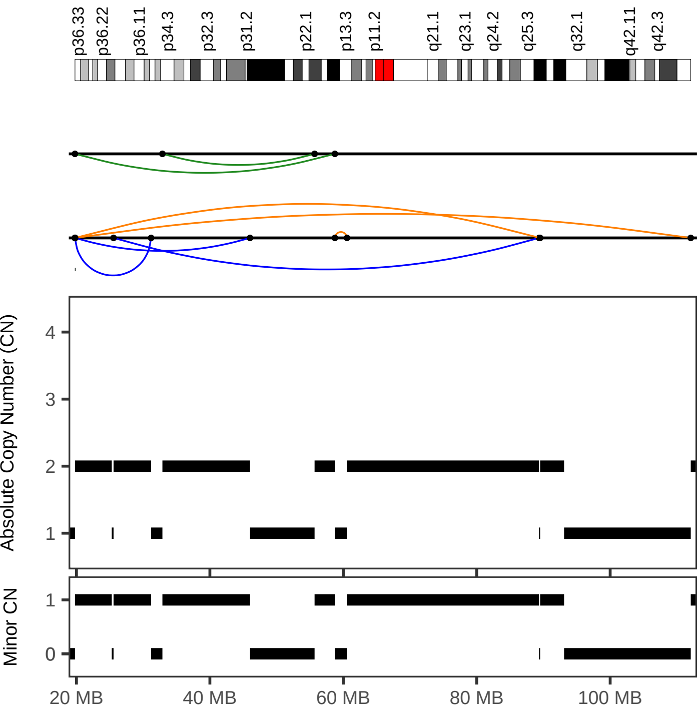

	**HX11**
Cancer type	Liver−HCC
Position	1:19776949−112077449
Type	Canonical without polyploidization
Interleaved intrachr. SVs	8
Total SVs (intrachr. + transl.)	8
SV types	DEL: 3; DUP: 3; h2hINV: 0; t2tINV: 2; TRA: 0
SVs in sample	42
Oscillating CN (2 and 3 states)	14, 14
CN segments	14
FDR fragment joints	0.59
FDR chr. breakp. enrich.	0.07
Linked to chrs	
Purity, ploidy	0.36, 1.9

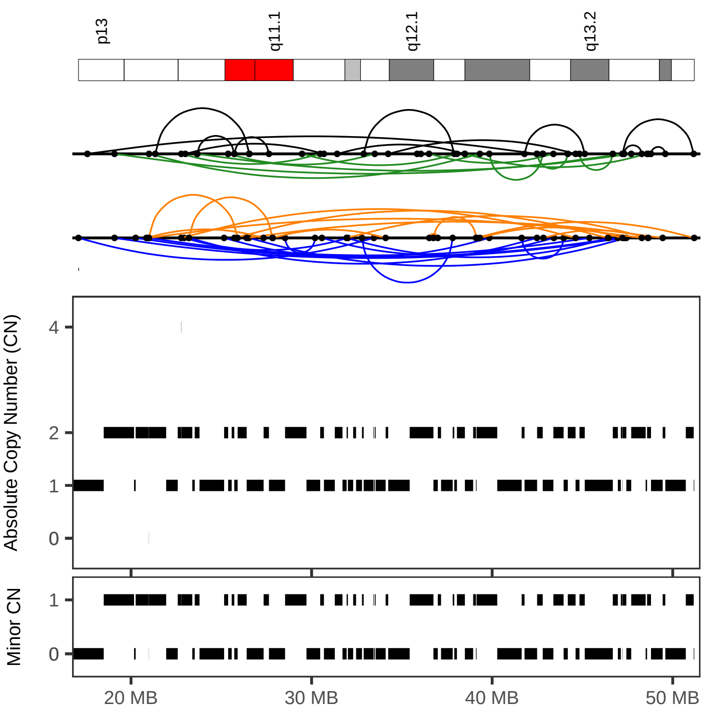

	**HX13**
Cancer type	Liver−HCC
Position	22:17088659−51192718
Type	Canonical without polyploidization
Interleaved intrachr. SVs	50
Total SVs (intrachr. + transl.)	50
SV types	DEL: 14; DUP: 13; h2hINV: 12; t2tINV: 11; TRA: 0
SVs in sample	70
Oscillating CN (2 and 3 states)	66, 66
CN segments	75
FDR fragment joints	0.96
FDR chr. breakp. enrich.	0
Linked to chrs	
Purity, ploidy	0.74, 1.97

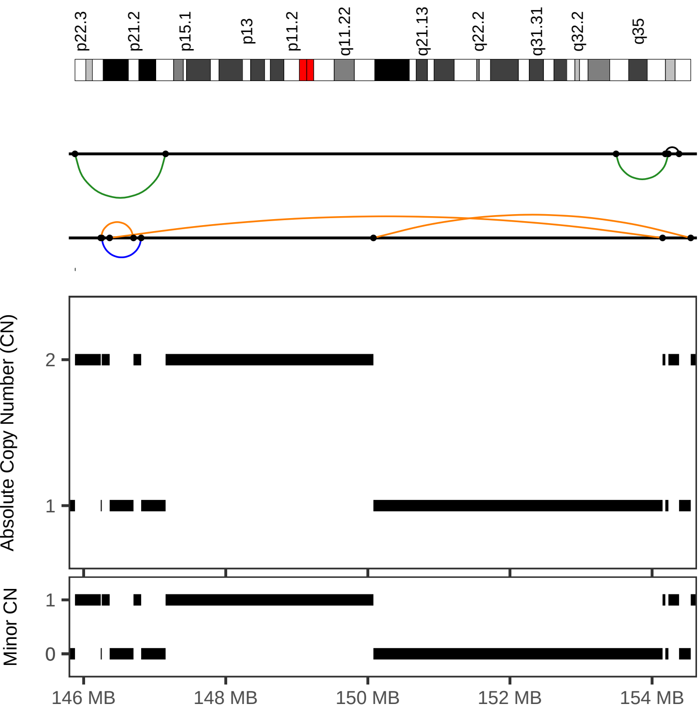

	**HX23**
Cancer type	Liver−HCC
Position	7:145877960−154544153
Type	Canonical without polyploidization
Interleaved intrachr. SVs	7
Total SVs (intrachr. + transl.)	7
SV types	DEL: 3; DUP: 1; h2hINV: 1; t2tINV: 2; TRA: 0
SVs in sample	48
Oscillating CN (2 and 3 states)	13, 13
CN segments	13
FDR fragment joints	0.74
FDR chr. breakp. enrich.	0.01
Linked to chrs	
Purity, ploidy	0.67, 1.82

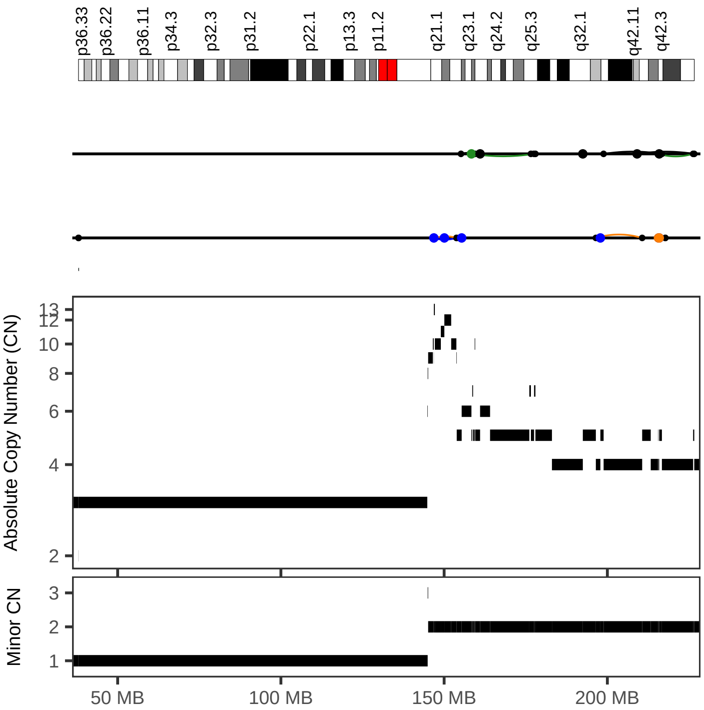

	**HX28**
Cancer type	Liver−HCC
Position	1:196470863−226612961
Type	After polyploidization
Interleaved intrachr. SVs	4
Total SVs (intrachr. + transl.)	10
SV types	DEL: 1; DUP: 0; h2hINV: 2; t2tINV: 1; TRA: 6
SVs in sample	160
Oscillating CN (2 and 3 states)	11, 11
CN segments	11
FDR fragment joints	0.64
FDR chr. breakp. enrich.	0.02
Linked to chrs	2:3659186−184750658;
Purity, ploidy	0.65, 3.46

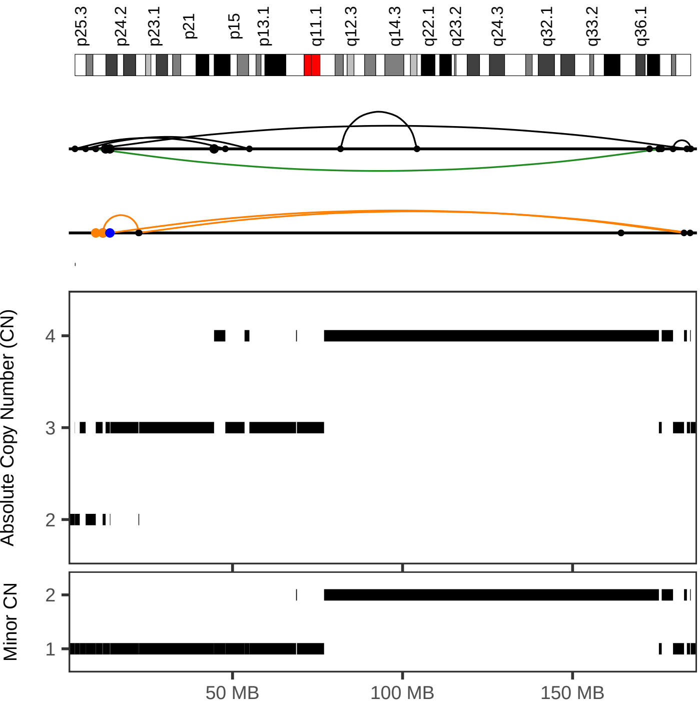

	**HX28**
Cancer type	Liver−HCC
Position	2:3659186−184750659
Type	With other complex events
Interleaved intrachr. SVs	8
Total SVs (intrachr. + transl.)	15
SV types	DEL: 3; DUP: 0; h2hINV: 4; t2tINV: 1; TRA: 7
SVs in sample	160
Oscillating CN (2 and 3 states)	15, 26
CN segments	26
FDR fragment joints	0.59
FDR chr. breakp. enrich.	0.15
Linked to chrs	1:196470863−226612960;
Purity, ploidy	0.65, 3.46

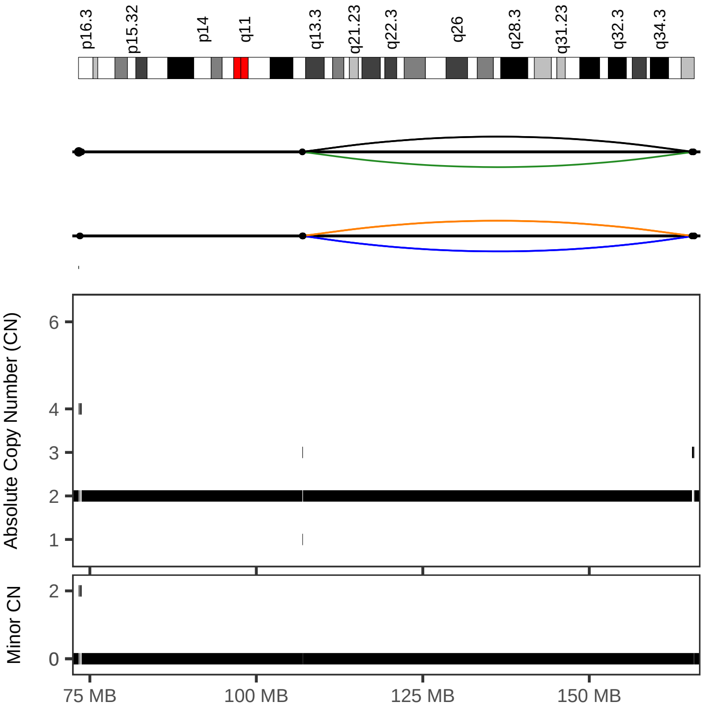

	**HX28**
Cancer type	Liver−HCC
Position	4:106905232−165763983
Type	With other complex events
Interleaved intrachr. SVs	11
Total SVs (intrachr. + transl.)	11
SV types	DEL: 4; DUP: 3; h2hINV: 2; t2tINV: 2; TRA: 0
SVs in sample	160
Oscillating CN (2 and 3 states)	7, 7
CN segments	12
FDR fragment joints	0.84
FDR chr. breakp. enrich.	0.13
Linked to chrs	
Purity, ploidy	0.65, 3.46

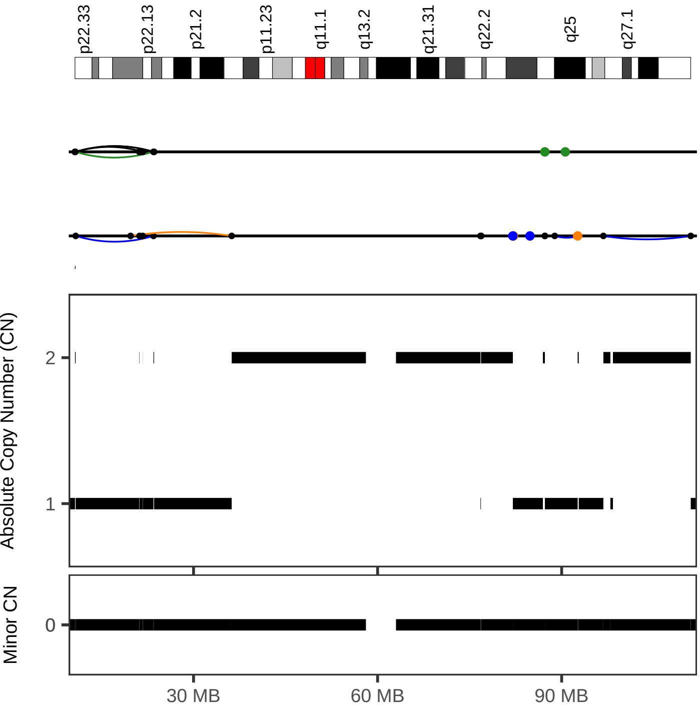

	**HX28**
Cancer type	Liver−HCC
Position	X:10674156−36219064
Type	Canonical without polyploidization
Interleaved intrachr. SVs	7
Total SVs (intrachr. + transl.)	7
SV types	DEL: 1; DUP: 1; h2hINV: 3; t2tINV: 2; TRA: 0
SVs in sample	160
Oscillating CN (2 and 3 states)	9, 9
CN segments	9
FDR fragment joints	0.74
FDR chr. breakp. enrich.	0
Linked to chrs	
Purity, ploidy	0.65, 3.46

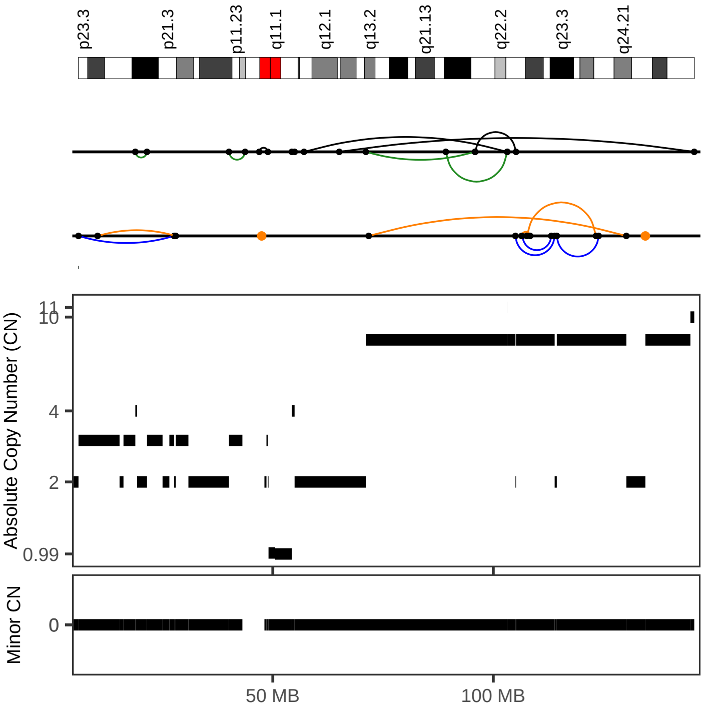

	**RK003**
Cancer type	Liver−HCC
Position	8:57075497−145572220
Type	With other complex events
Interleaved intrachr. SVs	11
Total SVs (intrachr. + transl.)	12
SV types	DEL: 3; DUP: 3; h2hINV: 3; t2tINV: 2; TRA: 1
SVs in sample	51
Oscillating CN (2 and 3 states)	7, 10
CN segments	11
FDR fragment joints	0.98
FDR chr. breakp. enrich.	0
Linked to chrs	
Purity, ploidy	0.54, 3.3

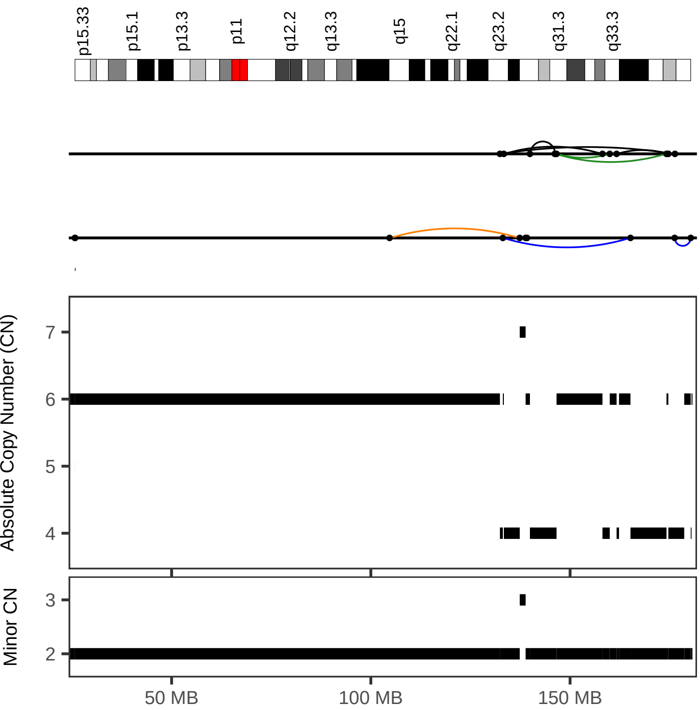

RK014	
Cancer type	Liver−HCC
Position	5:104702072−180283231
Type	Before polyploidization
Interleaved intrachr. SVs	9
Total SVs (intrachr. + transl.)	9
SV types	DEL: 1; DUP: 2; h2hINV: 4; t2tINV: 2; TRA: 0
SVs in sample	56
Oscillating CN (2 and 3 states)	12, 12
CN segments	17
FDR fragment joints	0.64
FDR chr. breakp. enrich.	0
Linked to chrs	
Purity, ploidy	0.38, 3.38

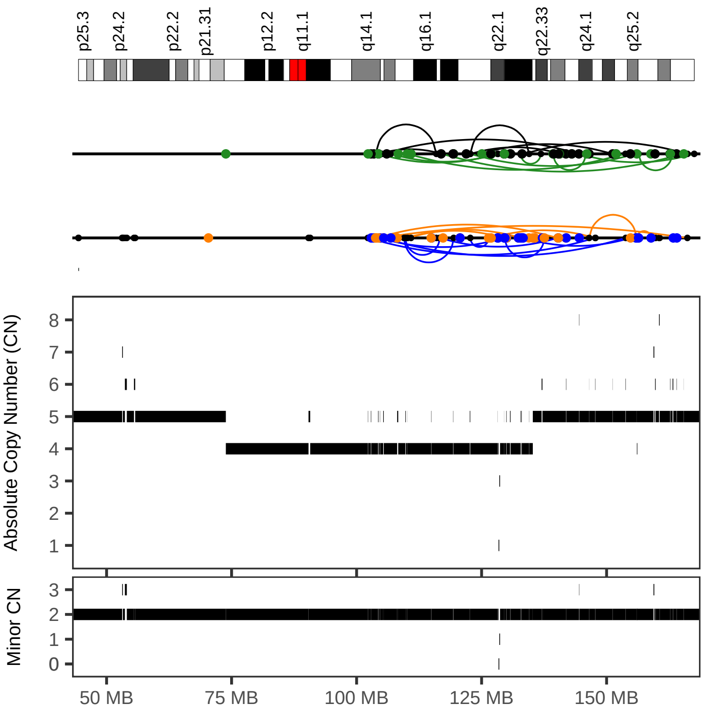

RK019	
Cancer type	Liver−HCC
Position	6:102249565−167532689
Type	With other complex events
Interleaved intrachr. SVs	32
Total SVs (intrachr. + transl.)	126
SV types	DEL: 7; DUP: 10; h2hINV: 7; t2tINV: 8; TRA: 94
SVs in sample	323
Oscillating CN (2 and 3 states)	28, 31
CN segments	74
FDR fragment joints	0.9
FDR chr. breakp. enrich.	0
Linked to chrs	10:4609618−130110174;
Purity, ploidy	0.63, 3.51

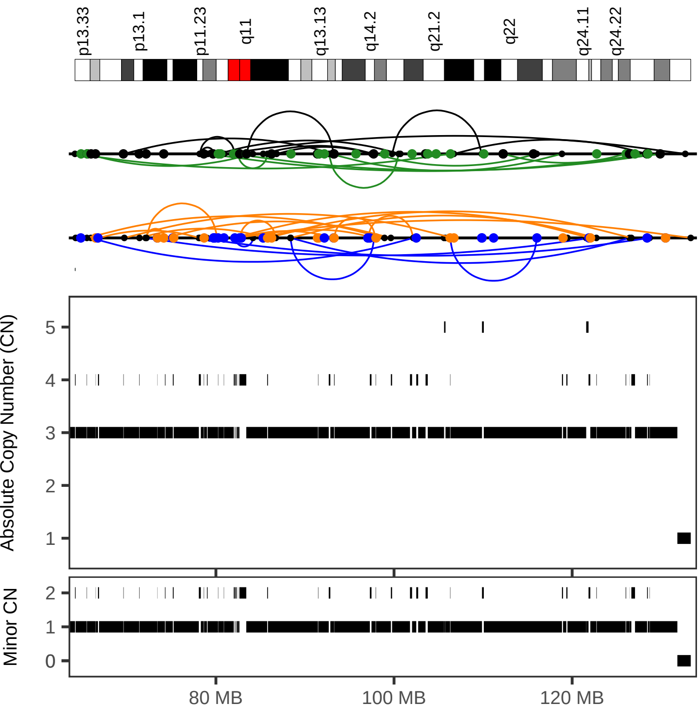

RK019	
Cancer type	Liver−HCC
Position	12:64174368−133313418
Type	After polyploidization
Interleaved intrachr. SVs	44
Total SVs (intrachr. + transl.)	133
SV types	DEL: 16; DUP: 8; h2hINV: 11; t2tINV: 9; TRA: 89
SVs in sample	323
Oscillating CN (2 and 3 states)	60, 74
CN segments	88
FDR fragment joints	0.59
FDR chr. breakp. enrich.	0
Linked to chrs	6:102249565−167532688;
Purity, ploidy	0.63, 3.51

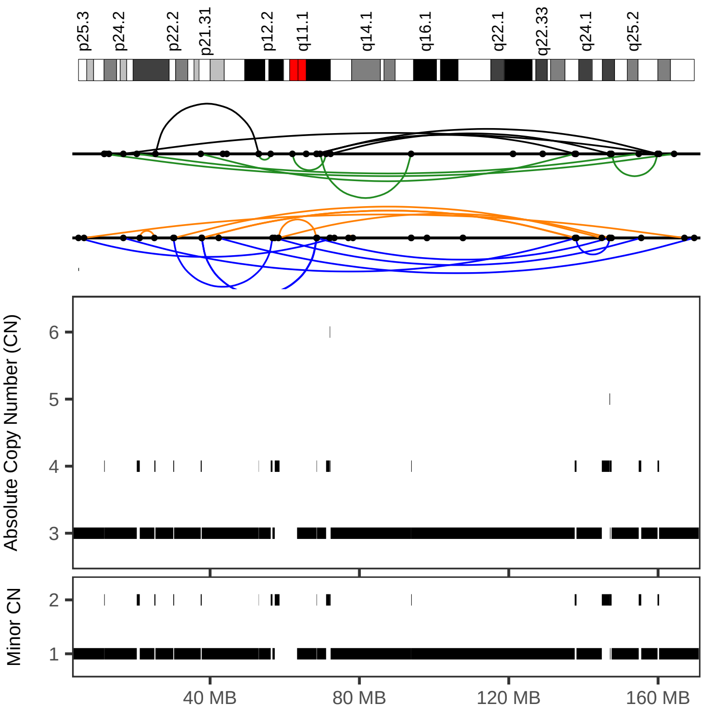

RK030	
Cancer type	Liver−HCC
Position	6:4750739−169684690
Type	With other complex events
Interleaved intrachr. SVs	30
Total SVs (intrachr. + transl.)	30
SV types	DEL: 6; DUP: 10; h2hINV: 7; t2tINV: 7; TRA: 0
SVs in sample	134
Oscillating CN (2 and 3 states)	20, 20
CN segments	38
FDR fragment joints	0.83
FDR chr. breakp. enrich.	0
Linked to chrs	
Purity, ploidy	0.88, 2.82

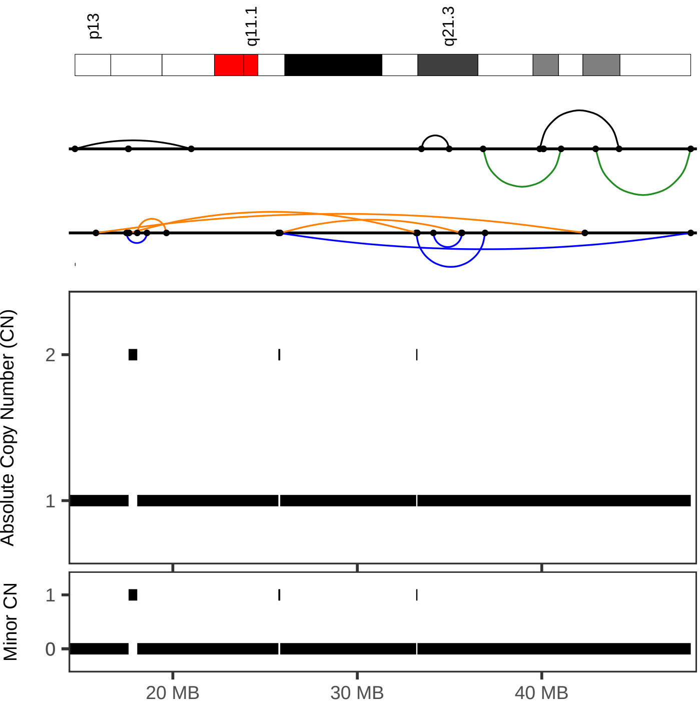

	RK038
Cancer type	Liver−HCC
Position	21:14694052−48076206
Type	Canonical without polyploidization
Interleaved intrachr. SVs	13
Total SVs (intrachr. + transl.)	13
SV types	DEL: 4; DUP: 4; h2hINV: 3; t2tINV: 2; TRA: 0
SVs in sample	99
Oscillating CN (2 and 3 states)	7, 7
CN segments	7
FDR fragment joints	0.88
FDR chr. breakp. enrich.	0
Linked to chrs	
Purity, ploidy	0.69, 1.92

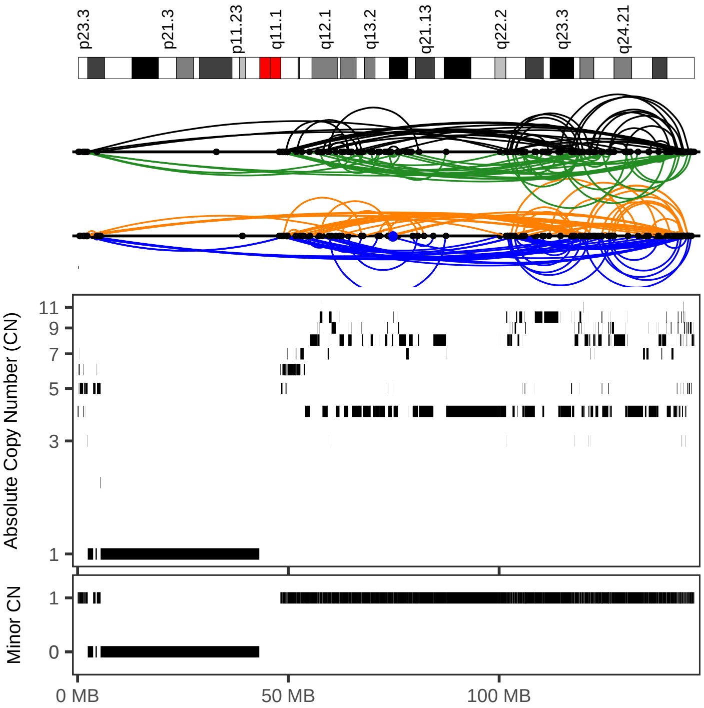

	RK056
Cancer type	Liver−HCC
Position	8:197777−146291756
Type	With other complex events
Interleaved intrachr. SVs	213
Total SVs (intrachr. + transl.)	214
SV types	DEL: 53; DUP: 57; h2hINV: 48; t2tINV: 55; TRA: 1
SVs in sample	351
Oscillating CN (2 and 3 states)	7, 17
CN segments	265
FDR fragment joints	0.88
FDR chr. breakp. enrich.	0
Linked to chrs	
Purity, ploidy	0.79, 2.25

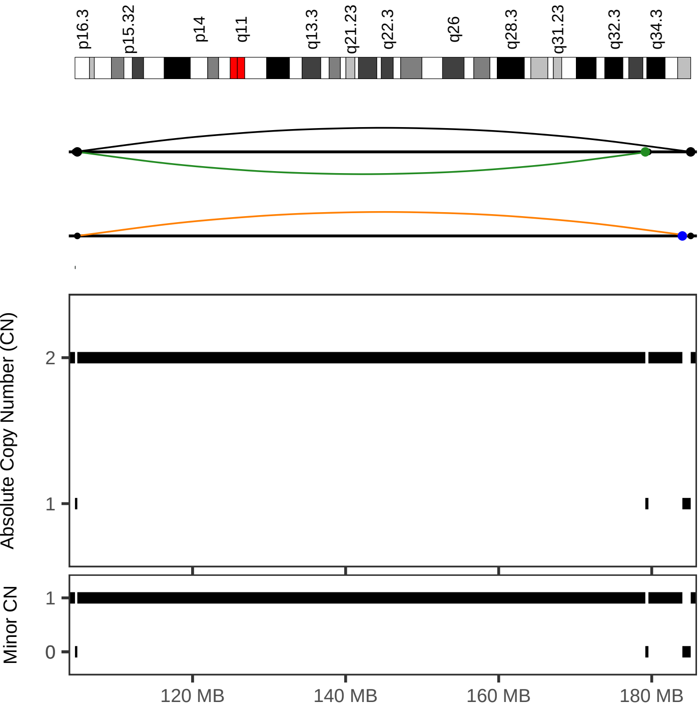

	RK062
Cancer type	Liver−HCC
Position	4:104630720−185100401
Type	Canonical without polyploidization
Interleaved intrachr. SVs	3
Total SVs (intrachr. + transl.)	7
SV types	DEL: 1; DUP: 0; h2hINV: 1; t2tINV: 1; TRA: 4
SVs in sample	31
Oscillating CN (2 and 3 states)	8, 8
CN segments	8
FDR fragment joints	0.84
FDR chr. breakp. enrich.	0.02
Linked to chrs	
Purity, ploidy	0.88, 1.94

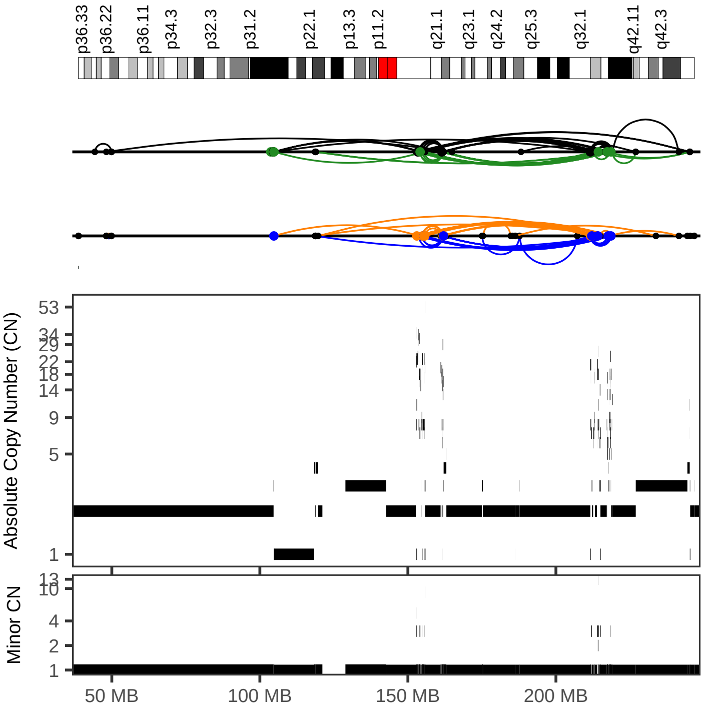

	RK067
Cancer type	Liver−HCC
Position	1:44269676−245339193
Type	With other complex events
Interleaved intrachr. SVs	141
Total SVs (intrachr. + transl.)	169
SV types	DEL: 30; DUP: 32; h2hINV: 45; t2tINV: 34; TRA: 28
SVs in sample	260
Oscillating CN (2 and 3 states)	10, 10
CN segments	224
FDR fragment joints	0.59
FDR chr. breakp. enrich.	0
Linked to chrs	
Purity, ploidy	0.38, 1.86

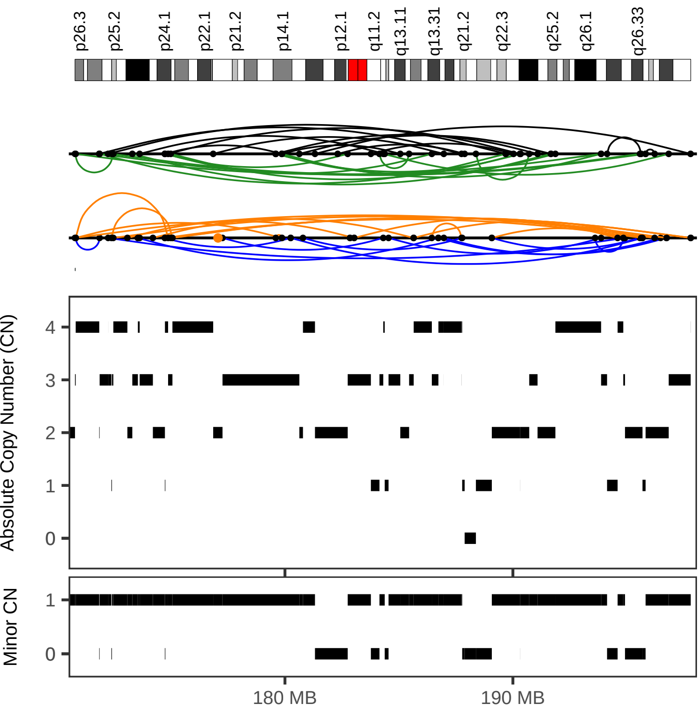

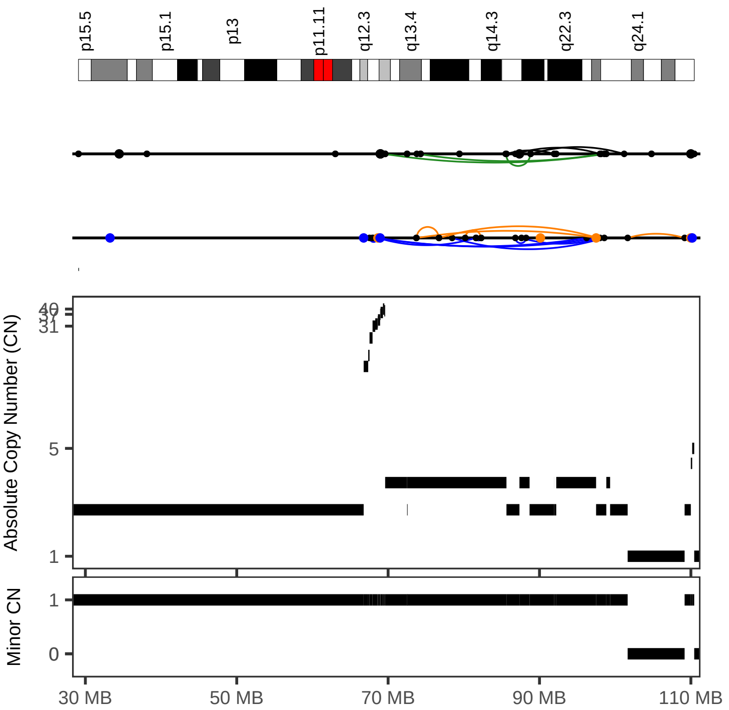

RK069	
Cancer type	Liver−HCC
Position	3:170788309−197801947
Type	With other complex events
Interleaved intrachr. SVs	57
Total SVs (intrachr. + transl.)	58
SV types	DEL: 16; DUP: 12; h2hINV: 14; t2tINV: 15; TRA: 1
SVs in sample	81
Oscillating CN (2 and 3 states)	8, 12
CN segments	57
FDR fragment joints	0.92
FDR chr. breakp. enrich.	0
Linked to chrs	
Purity, ploidy	0.84, 1.9

RK071	
Cancer type	Liver−HCC
Position	11:67544036−101188725
Type	With other complex events
Interleaved intrachr. SVs	19
Total SVs (intrachr. + transl.)	26
SV types	DEL: 4; DUP: 8; h2hINV: 3; t2tINV: 4; TRA: 7
SVs in sample	288
Oscillating CN (2 and 3 states)	12, 12
CN segments	21
FDR fragment joints	0.59
FDR chr. breakp. enrich.	0
Linked to chrs	
Purity, ploidy	0.79, 1.96

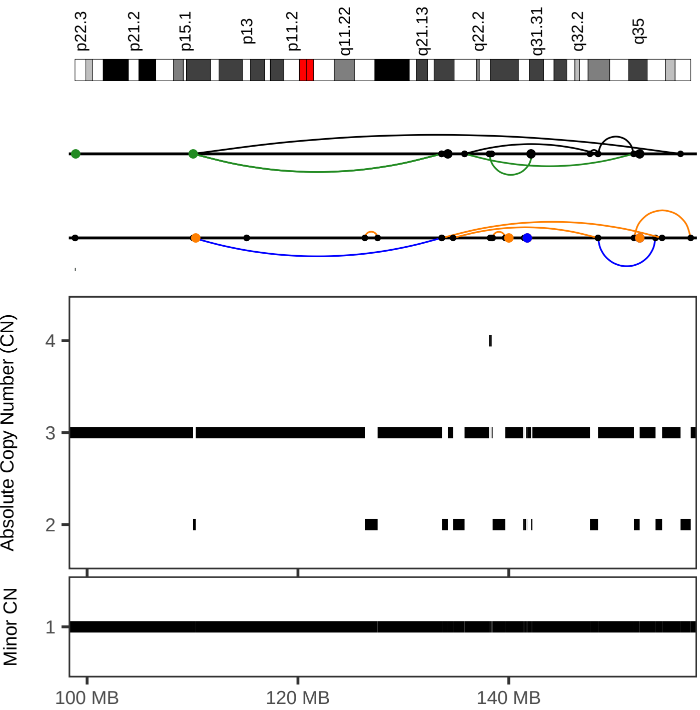

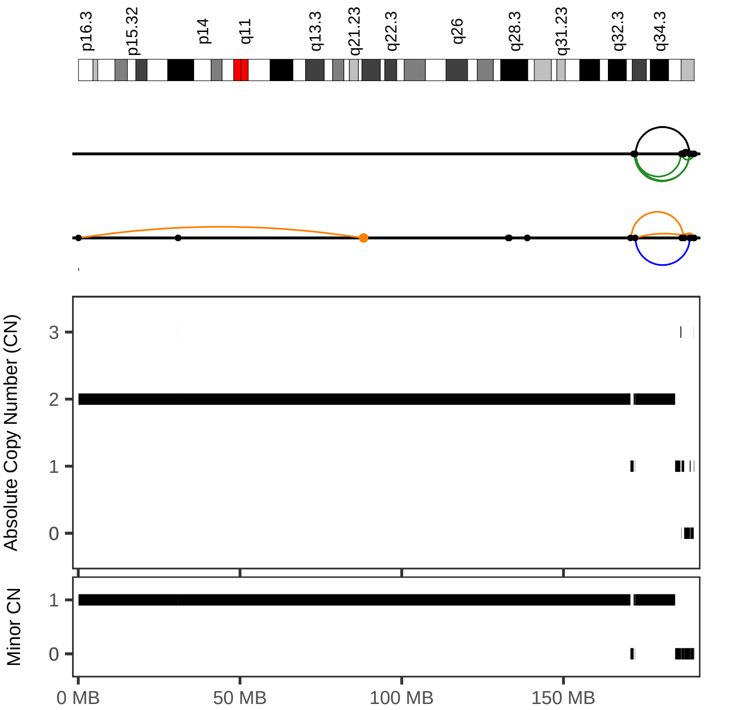

RK075	
Cancer type	Liver−HCC
Position	7:110056465−157254694
Type	With other complex events
Interleaved intrachr. SVs	8
Total SVs (intrachr. + transl.)	18
SV types	DEL: 3; DUP: 1; h2hINV: 3; t2tINV: 1; TRA: 10
SVs in sample	122
Oscillating CN (2 and 3 states)	19, 32
CN segments	32
FDR fragment joints	0.64
FDR chr. breakp. enrich.	0
Linked to chrs	2:70315462−240893779;
Purity, ploidy	0.39, 2.02

RK079	
Cancer type	Liver−HCC
Position	4:170703373−190415562
Type	With other complex events
Interleaved intrachr. SVs	16
Total SVs (intrachr. + transl.)	16
SV types	DEL: 4; DUP: 2; h2hINV: 5; t2tINV: 5; TRA: 0
SVs in sample	80
Oscillating CN (2 and 3 states)	9, 9
CN segments	17
FDR fragment joints	0.76
FDR chr. breakp. enrich.	0
Linked to chrs	
Purity, ploidy	0.76, 1.92

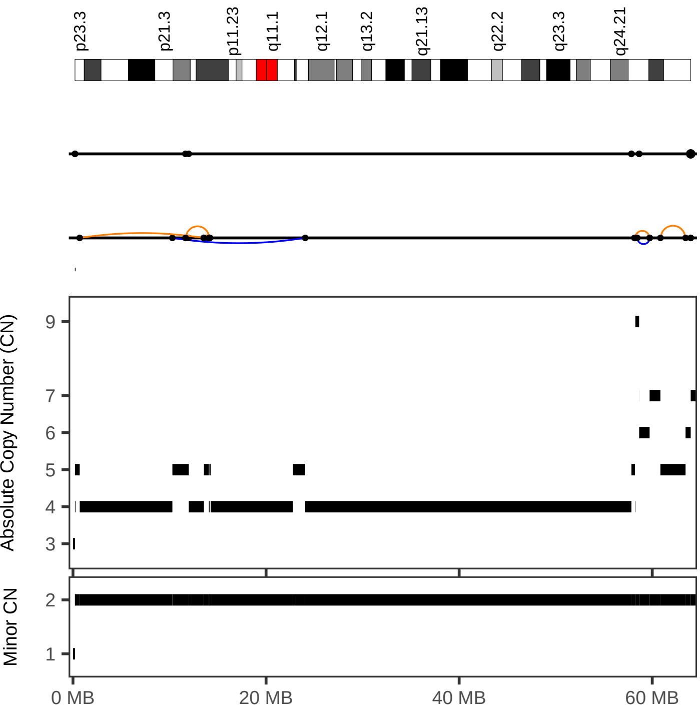

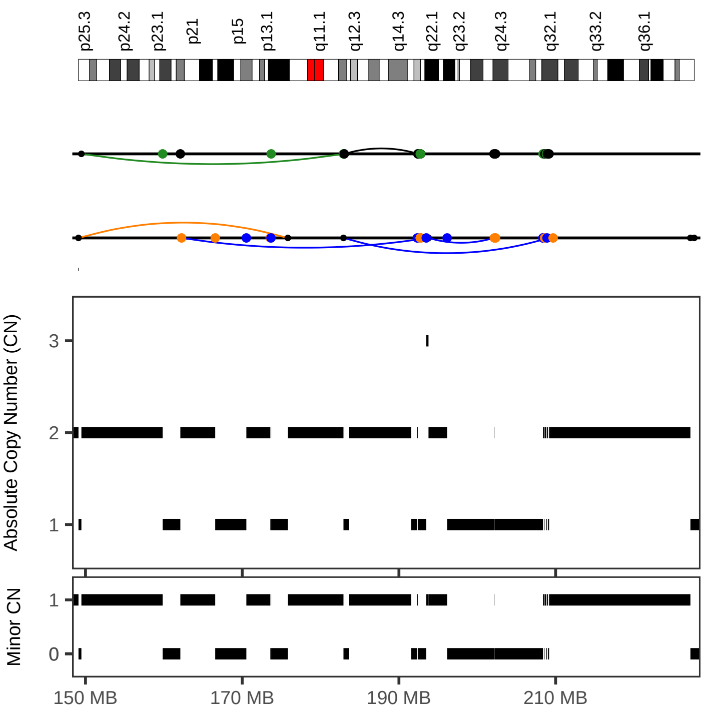

	RK088
Cancer type	Liver−HCC
Position	8:696677−24055234
Type	After polyploidization
Interleaved intrachr. SVs	7
Total SVs (intrachr. + transl.)	7
SV types	DEL: 4; DUP: 2; h2hINV: 0; t2tINV: 1; TRA: 0
SVs in sample	75
Oscillating CN (2 and 3 states)	13, 13
CN segments	13
FDR fragment joints	0.59
FDR chr. breakp. enrich.	0
Linked to chrs	
Purity, ploidy	0.78, 3.47

	RK091
Cancer type	Liver−HCC
Position	2:149101886−209165492
Type	With other complex events
Interleaved intrachr. SVs	5
Total SVs (intrachr. + transl.)	55
SV types	DEL: 1; DUP: 2; h2hINV: 1; t2tINV: 1; TRA: 50
SVs in sample	427
Oscillating CN (2 and 3 states)	17, 19
CN segments	29
FDR fragment joints	0.92
FDR chr. breakp. enrich.	0
Linked to chrs	4:13542462−186127249;
Purity, ploidy	0.74, 1.97

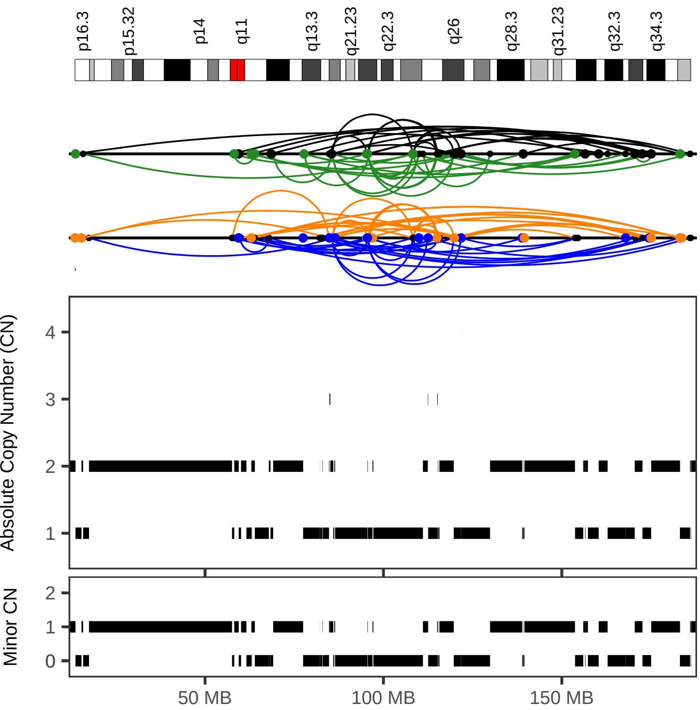

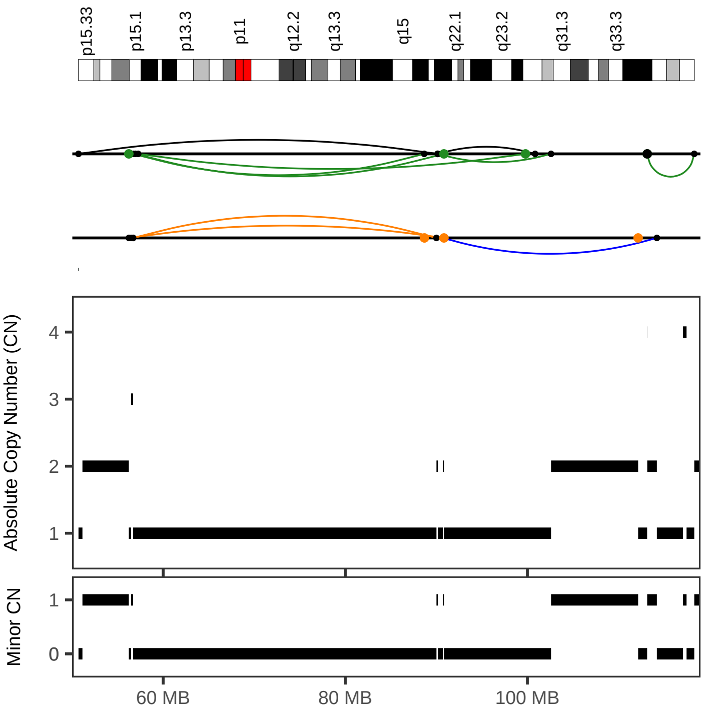

	RK091
Cancer type	Liver−HCC
Position	4:13542462−186127250
Type	With other complex events
Interleaved intrachr. SVs	108
Total SVs (intrachr. + transl.)	161
SV types	DEL: 29; DUP: 26; h2hINV: 23; t2tINV: 30; TRA: 53
SVs in sample	427
Oscillating CN (2 and 3 states)	31, 37
CN segments	75
FDR fragment joints	0.84
FDR chr. breakp. enrich.	0
Linked to chrs	
Purity, ploidy	0.74, 1.97

	RK091
Cancer type	Liver−HCC
Position	5:50697540−118326639
Type	With other complex events
Interleaved intrachr. SVs	10
Total SVs (intrachr. + transl.)	18
SV types	DEL: 2; DUP: 1; h2hINV: 2; t2tINV: 5; TRA: 8
SVs in sample	427
Oscillating CN (2 and 3 states)	7, 12
CN segments	18
FDR fragment joints	0.59
FDR chr. breakp. enrich.	0.52
Linked to chrs	6:41192505−161744823;
Purity, ploidy	0.74, 1.97

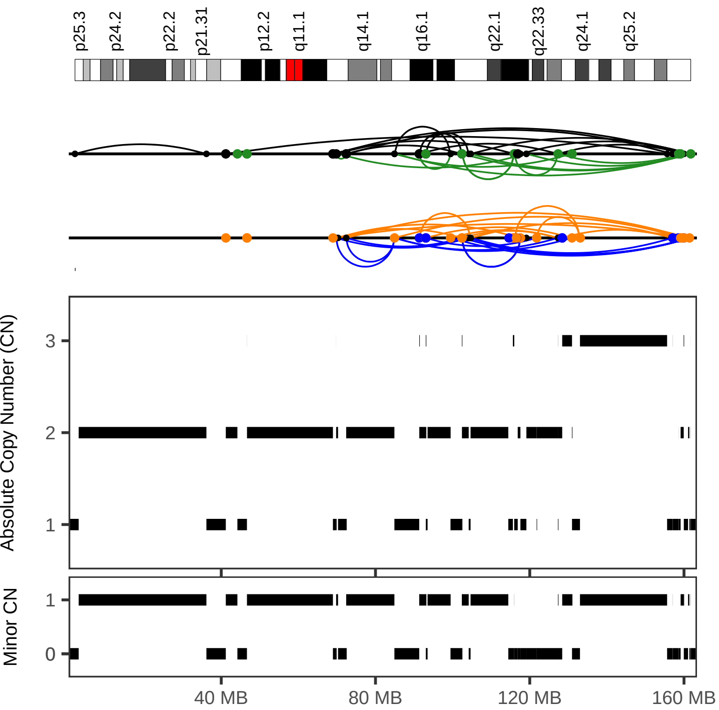

RK091	
Cancer type	Liver−HCC
Position	6:41192505−161744824
Type	With other complex events
Interleaved intrachr. SVs	54
Total SVs (intrachr. + transl.)	108
SV types	DEL: 13; DUP: 16; h2hINV: 13; t2tINV: 12; TRA: 54
SVs in sample	427
Oscillating CN (2 and 3 states)	8, 31
CN segments	71
FDR fragment joints	0.91
FDR chr. breakp. enrich.	0
Linked to chrs	10:59340268−108356079;5:50697540−118326638 7:70340834−157389137;
Purity, ploidy	0.74, 1.97

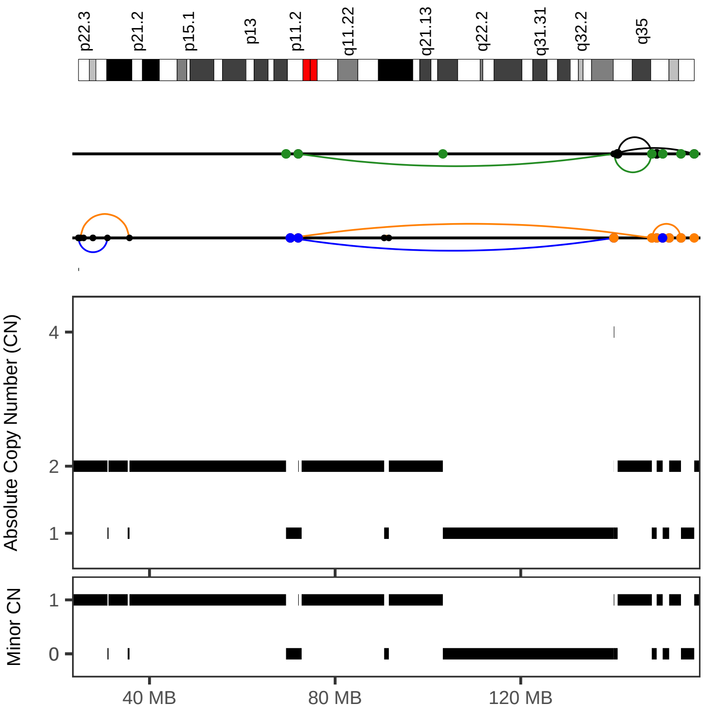

RK091	
Cancer type	Liver−HCC
Position	7:70340834−157389138
Type	Canonical without polyploidization
Interleaved intrachr. SVs	6
Total SVs (intrachr. + transl.)	42
SV types	DEL: 2; DUP: 1; h2hINV: 2; t2tINV: 1; TRA: 36
SVs in sample	427
Oscillating CN (2 and 3 states)	12, 20
CN segments	20
FDR fragment joints	0.91
FDR chr. breakp. enrich.	0
Linked to chrs	6:41192505−161744823;
Purity, ploidy	0.74, 1.97

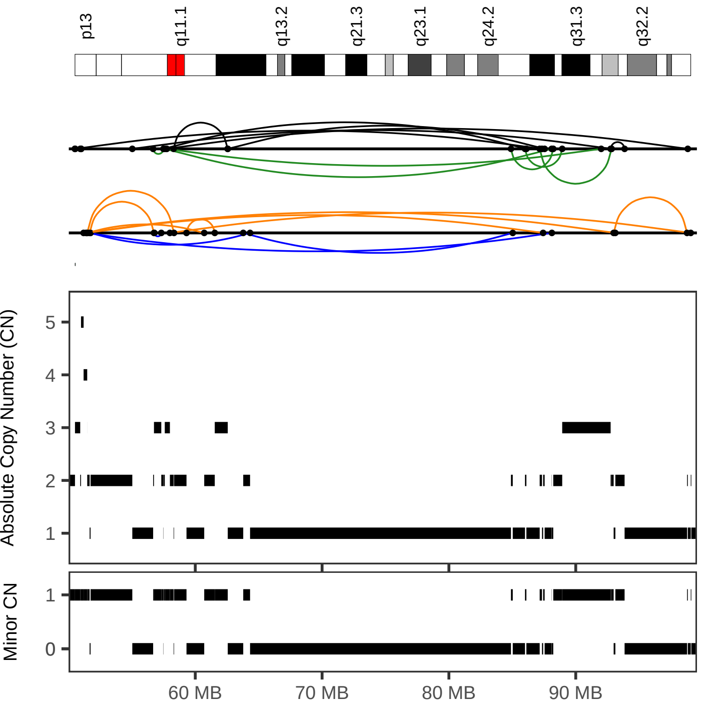

RK095	
Cancer type	Liver−HCC
Position	14:50525724−99068328
Type	With other complex events
Interleaved intrachr. SVs	24
Total SVs (intrachr. + transl.)	24
SV types	DEL: 8; DUP: 3; h2hINV: 7; t2tINV: 6; TRA: 0
SVs in sample	46
Oscillating CN (2 and 3 states)	14, 24
CN segments	45
FDR fragment joints	0.64
FDR chr. breakp. enrich.	0
Linked to chrs	
Purity, ploidy	0.61, 1.95

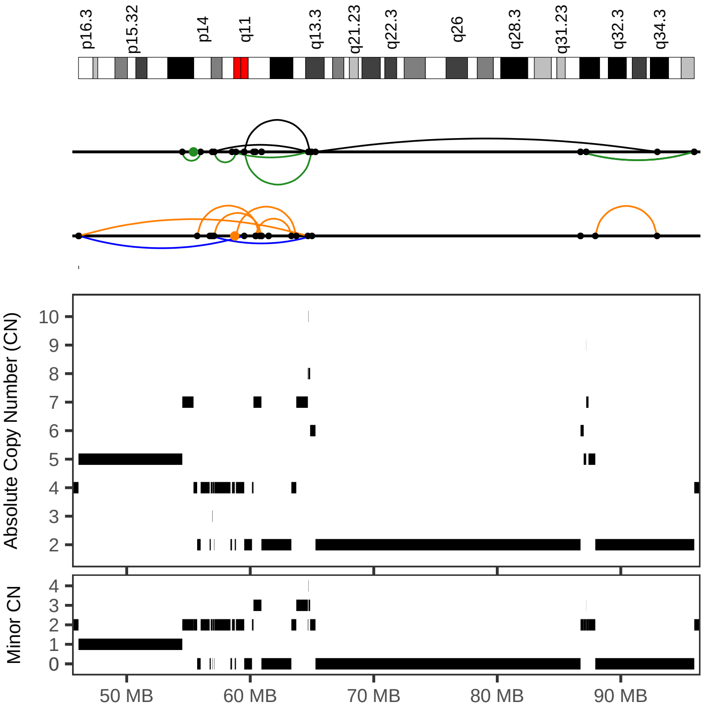

RK098	
Cancer type	Liver−HCC
Position	4:46094650−65000200
Type	With other complex events
Interleaved intrachr. SVs	16
Total SVs (intrachr. + transl.)	18
SV types	DEL: 7; DUP: 2; h2hINV: 2; t2tINV: 5; TRA: 2
SVs in sample	145
Oscillating CN (2 and 3 states)	9, 15
CN segments	25
FDR fragment joints	0.59
FDR chr. breakp. enrich.	0
Linked to chrs	X:63781853−76077024;
Purity, ploidy	0.38, 3.78

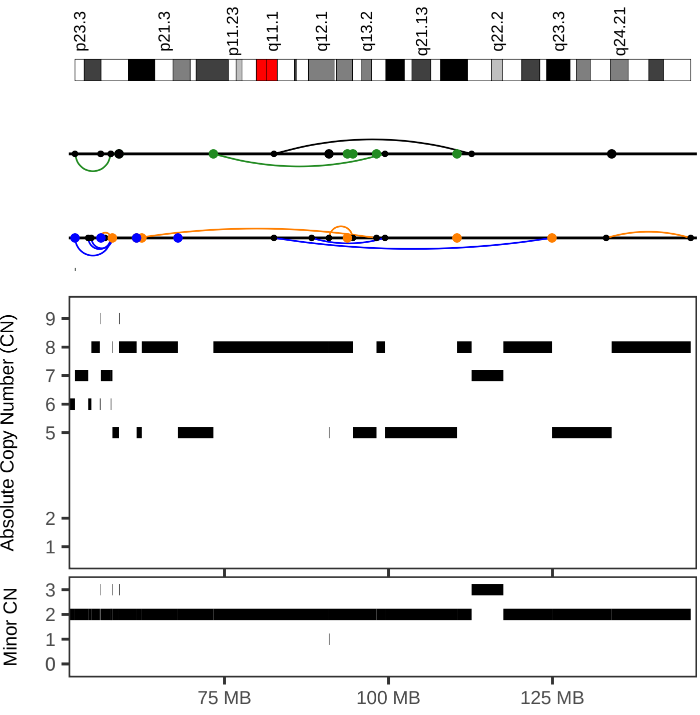

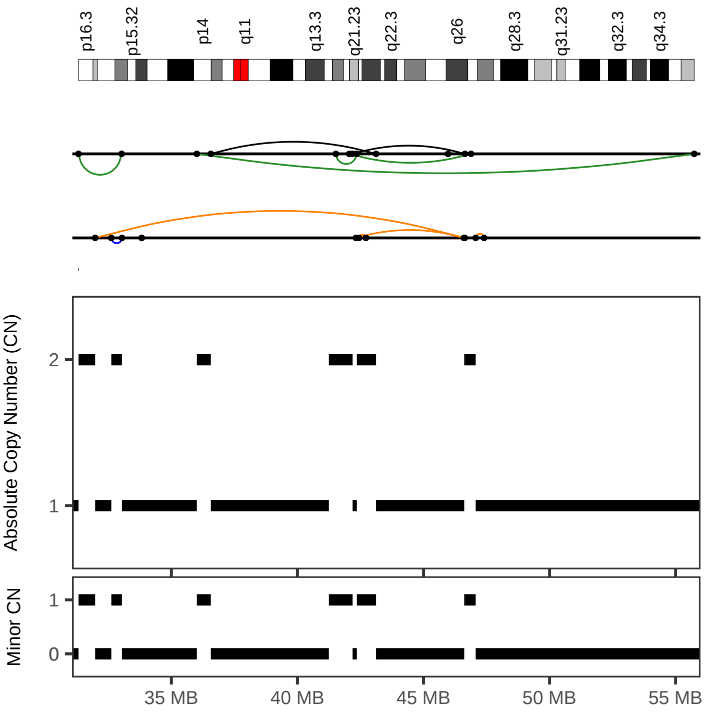

	**RK098**
Cancer type	Liver−HCC
Position	8:61570001−124934583
Type	After polyploidization
Interleaved intrachr. SVs	5
Total SVs (intrachr. + transl.)	17
SV types	DEL: 1; DUP: 2; h2hINV: 1; t2tINV: 1; TRA: 12
SVs in sample	145
Oscillating CN (2 and 3 states)	11, 13
CN segments	13
FDR fragment joints	0.92
FDR chr. breakp. enrich.	0
Linked to chrs	11:2705633−93356458;
Purity, ploidy	0.38, 3.78

	**RK099**
Cancer type	Liver−HCC
Position	4:31313801−55741627
Type	Canonical without polyploidization
Interleaved intrachr. SVs	9
Total SVs (intrachr. + transl.)	9
SV types	DEL: 2; DUP: 1; h2hINV: 2; t2tINV: 4; TRA: 0
SVs in sample	56
Oscillating CN (2 and 3 states)	14, 14
CN segments	14
FDR fragment joints	0.64
FDR chr. breakp. enrich.	0
Linked to chrs	
Purity, ploidy	0.56, 1.7

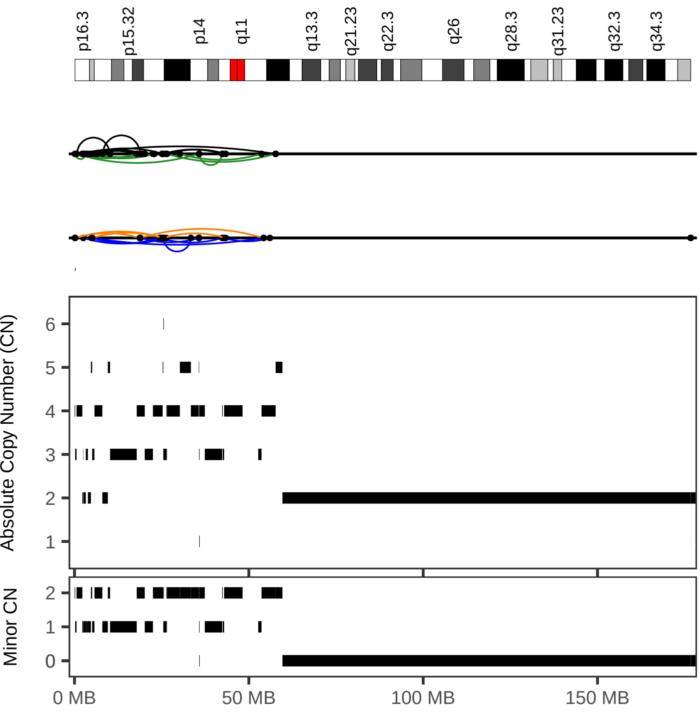

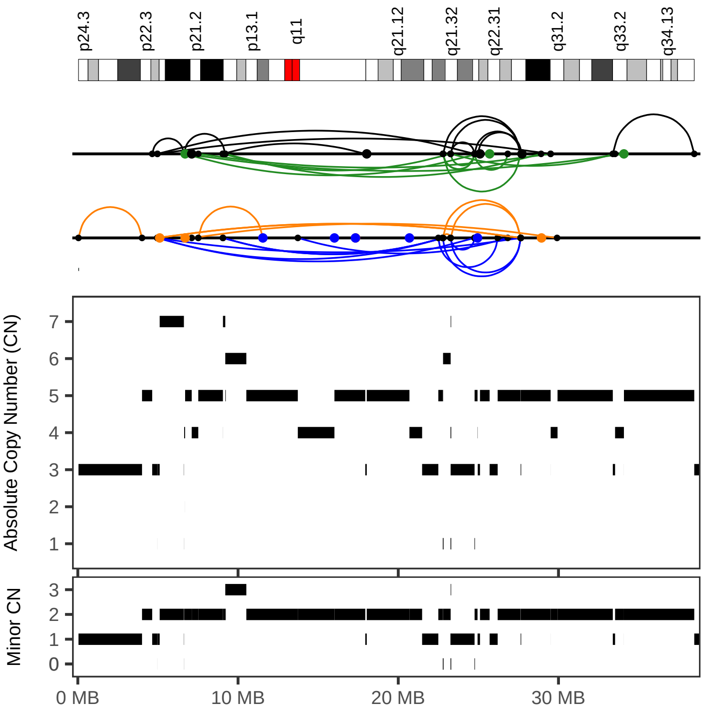

	**RK100**
Cancer type	Liver−HCC
Position	4:138117−57689386
Type	With other complex events
Interleaved intrachr. SVs	31
Total SVs (intrachr. + transl.)	31
SV types	DEL: 5; DUP: 8; h2hINV: 9; t2tINV: 9; TRA: 0
SVs in sample	104
Oscillating CN (2 and 3 states)	7, 9
CN segments	39
FDR fragment joints	0.79
FDR chr. breakp. enrich.	0
Linked to chrs	
Purity, ploidy	0.56, 3.33

	**RK101**
Cancer type	Liver−HCC
Position	9:4642656−38474066
Type	With other complex events
Interleaved intrachr. SVs	40
Total SVs (intrachr. + transl.)	56
SV types	DEL: 7; DUP: 10; h2hINV: 12; t2tINV: 11; TRA: 16
SVs in sample	218
Oscillating CN (2 and 3 states)	9, 11
CN segments	53
FDR fragment joints	0.78
FDR chr. breakp. enrich.	0
Linked to chrs	11:69040190−120528007;
Purity, ploidy	0.81, 3.69

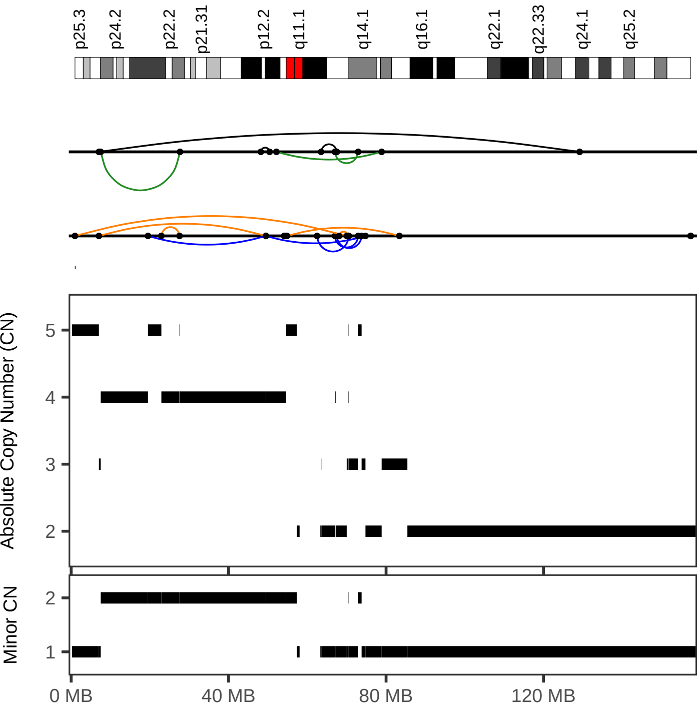

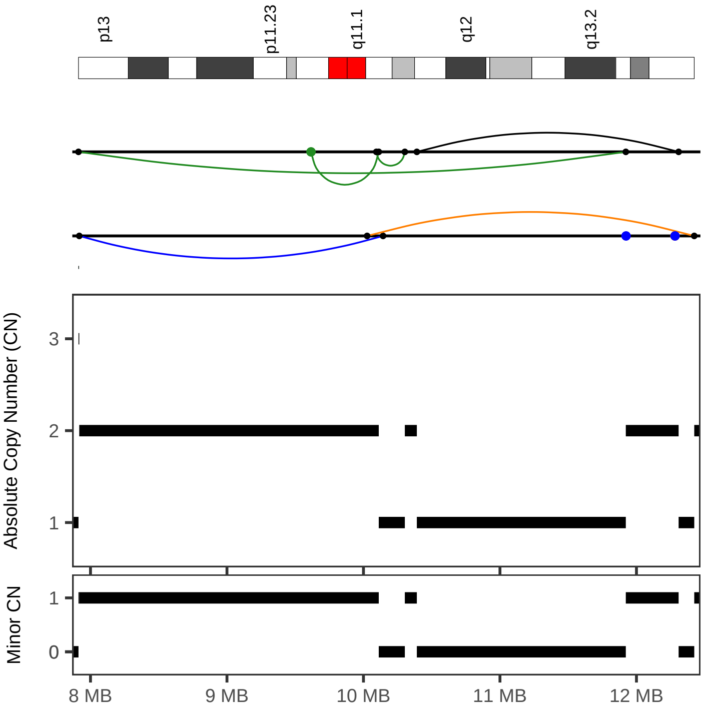

RK106

Cancer type	Liver−HCC
Position	6:966582−129186137
Type	With other complex events
Interleaved intrachr. SVs	15
Total SVs (intrachr. + transl.)	15
SV types	DEL: 4; DUP: 5; h2hINV: 3; t2tINV: 3; TRA: 0
SVs in sample	59
Oscillating CN (2 and 3 states)	8, 8
CN segments	26
FDR fragment joints	0.9
FDR chr. breakp. enrich.	0
Linked to chrs	
Purity, ploidy	0.3, 2.42

RK112

Cancer type	Liver−HCC
Position	20:7912024−12423476
Type	Canonical without polyploidization
Interleaved intrachr. SVs	6
Total SVs (intrachr. + transl.)	10
SV types	DEL: 1; DUP: 1; h2hINV: 1; t2tINV: 3; TRA: 4
SVs in sample	43
Oscillating CN (2 and 3 states)	7, 8
CN segments	8
FDR fragment joints	0.64
FDR chr. breakp. enrich.	0
Linked to chrs	
Purity, ploidy	0.3, 1.93

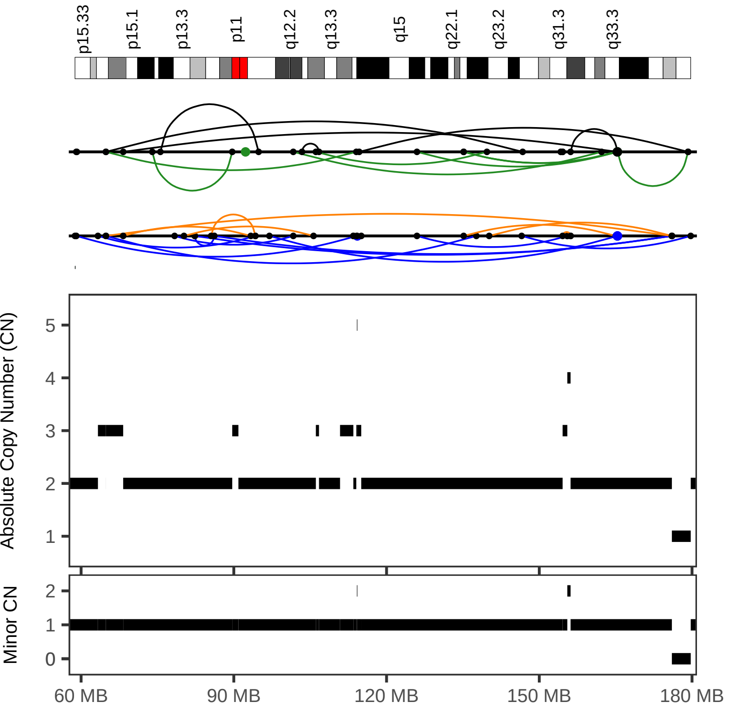

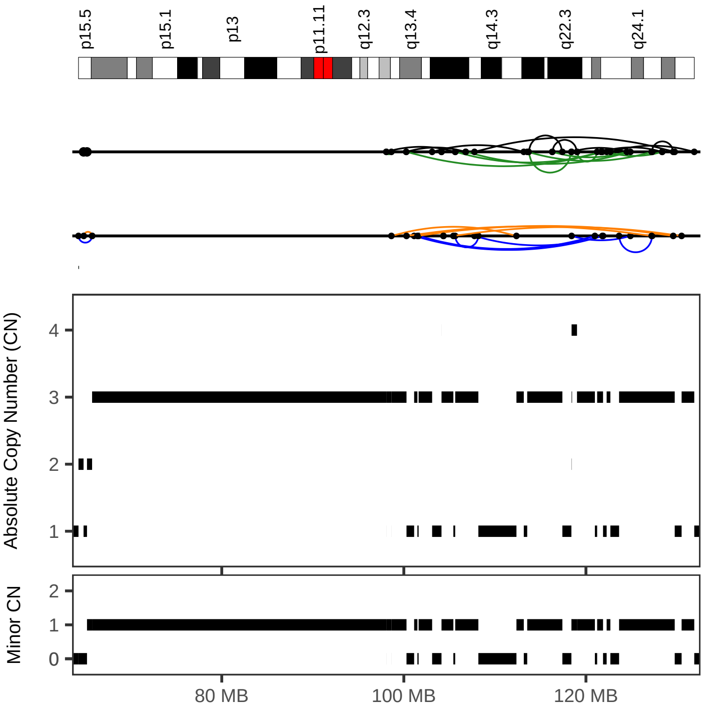

RK119

Cancer type	Liver−HCC
Position	5:59053606−179748409
Type	Canonical without polyploidization
Interleaved intrachr. SVs	36
Total SVs (intrachr. + transl.)	42
SV types	DEL: 8; DUP: 12; h2hINV: 8; t2tINV: 8; TRA: 6
SVs in sample	110
Oscillating CN (2 and 3 states)	12, 16
CN segments	20
FDR fragment joints	0.8
FDR chr. breakp. enrich.	0
Linked to chrs	2:152075233−182020725;
Purity, ploidy	0.7, 2.09

RK130

Cancer type	Liver−HCC
Position	11:98073900−131896731
Type	With other complex events
Interleaved intrachr. SVs	29
Total SVs (intrachr. + transl.)	29
SV types	DEL: 4; DUP: 6; h2hINV: 10; t2tINV: 9; TRA: 0
SVs in sample	68
Oscillating CN (2 and 3 states)	10, 10
CN segments	33
FDR fragment joints	0.59
FDR chr. breakp. enrich.	0
Linked to chrs	
Purity, ploidy	0.79, 1.93

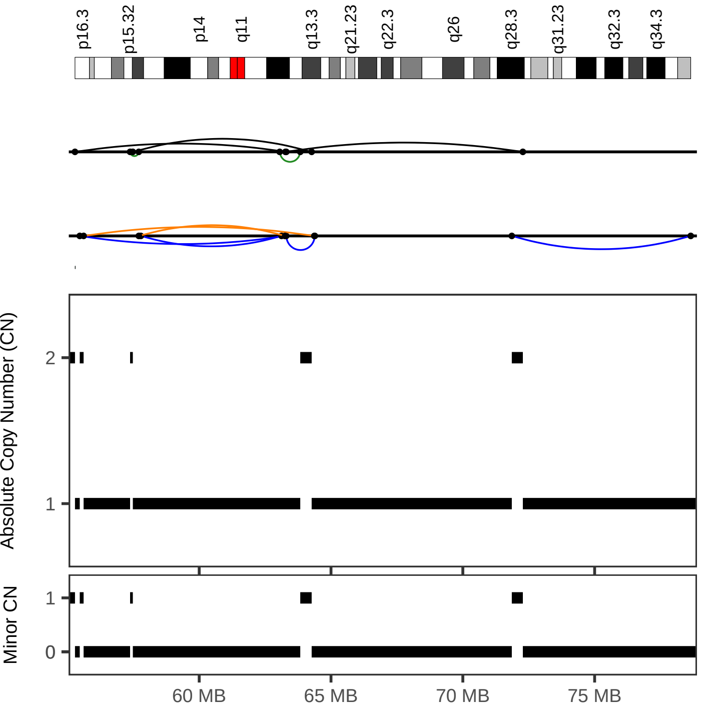

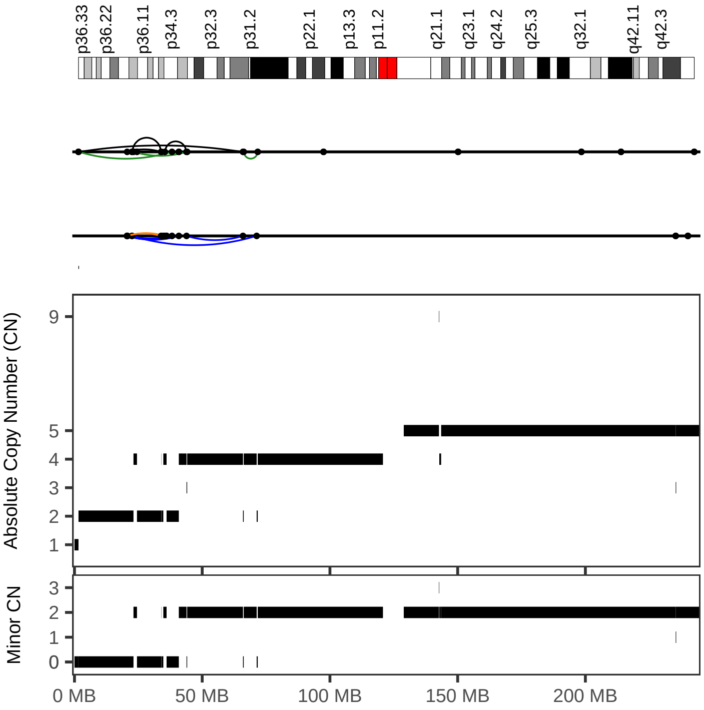

	RK163
Cancer type	Liver−HCC
Position	4:55287724−78645421
Type	Canonical without polyploidization
Interleaved intrachr. SVs	11
Total SVs (intrachr. + transl.)	11
SV types	DEL: 2; DUP: 4; h2hINV: 3; t2tINV: 2; TRA: 0
SVs in sample	38
Oscillating CN (2 and 3 states)	9, 9
CN segments	9
FDR fragment joints	0.84
FDR chr. breakp. enrich.	0
Linked to chrs	
Purity, ploidy	0.48, 2

	RK167
Cancer type	Liver−HCC
Position	1:1562933−71739261
Type	With other complex events
Interleaved intrachr. SVs	16
Total SVs (intrachr. + transl.)	16
SV types	DEL: 2; DUP: 6; h2hINV: 5; t2tINV: 3; TRA: 0
SVs in sample	81
Oscillating CN (2 and 3 states)	8, 14
CN segments	14
FDR fragment joints	0.64
FDR chr. breakp. enrich.	0
Linked to chrs	
Purity, ploidy	0.54, 3.68

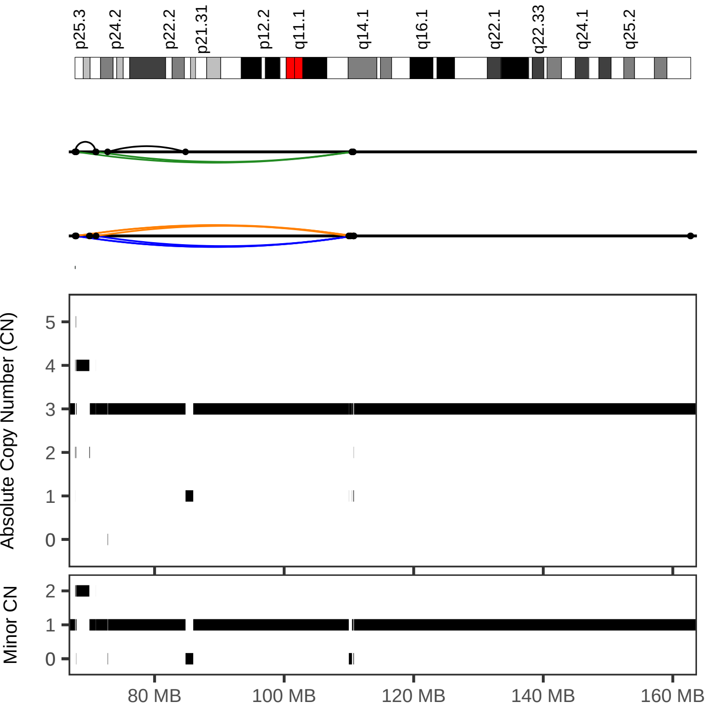

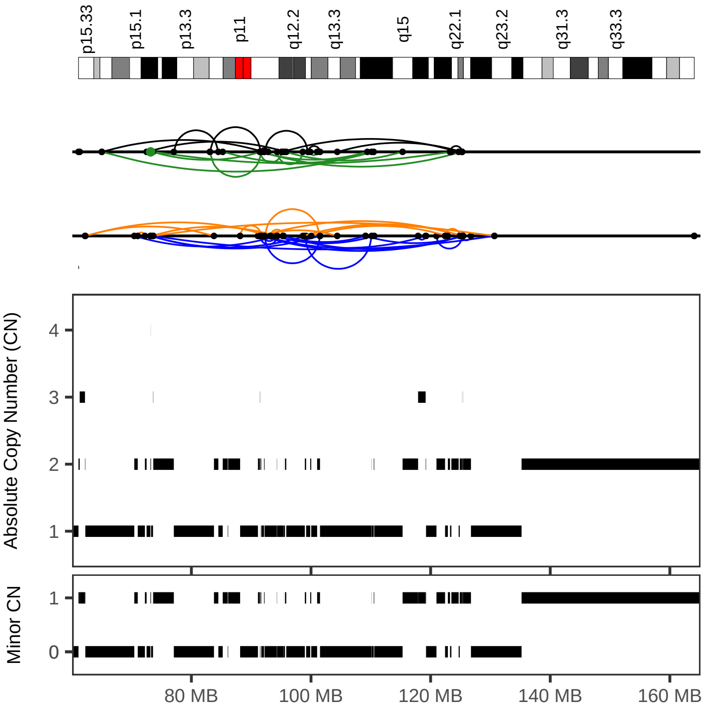

	RK169
Cancer type	Liver−HCC
Position	6:67707659−110782332
Type	With other complex events
Interleaved intrachr. SVs	12
Total SVs (intrachr. + transl.)	12
SV types	DEL: 4; DUP: 4; h2hINV: 2; t2tINV: 2; TRA: 0
SVs in sample	75
Oscillating CN (2 and 3 states)	8, 13
CN segments	24
FDR fragment joints	0.8
FDR chr. breakp. enrich.	0
Linked to chrs	
Purity, ploidy	0.24, 3.37

	RK172
Cancer type	Liver−HCC
Position	5:62205924−130667689
Type	With other complex events
Interleaved intrachr. SVs	54
Total SVs (intrachr. + transl.)	55
SV types	DEL: 13; DUP: 18; h2hINV: 12; t2tINV: 11; TRA: 1
SVs in sample	168
Oscillating CN (2 and 3 states)	25, 43
CN segments	64
FDR fragment joints	0.64
FDR chr. breakp. enrich.	0
Linked to chrs	
Purity, ploidy	0.86, 1.72

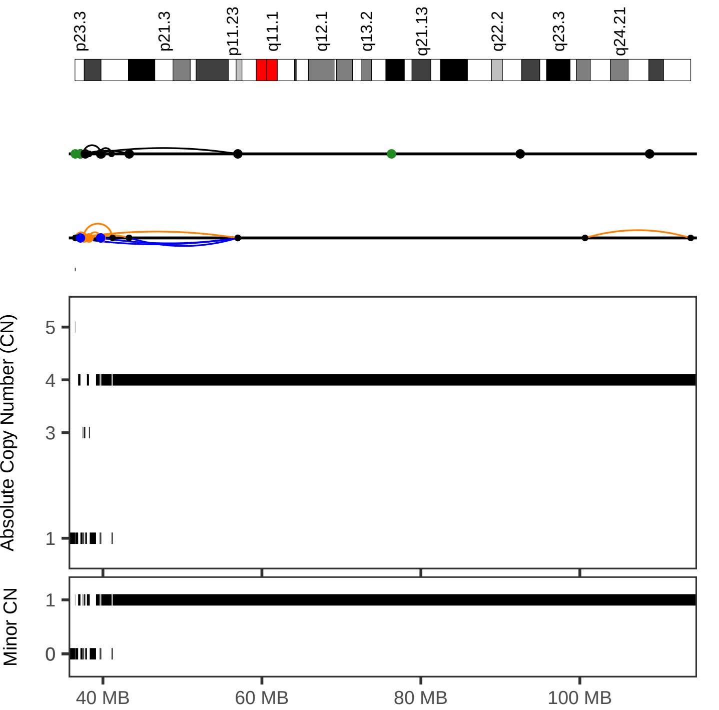

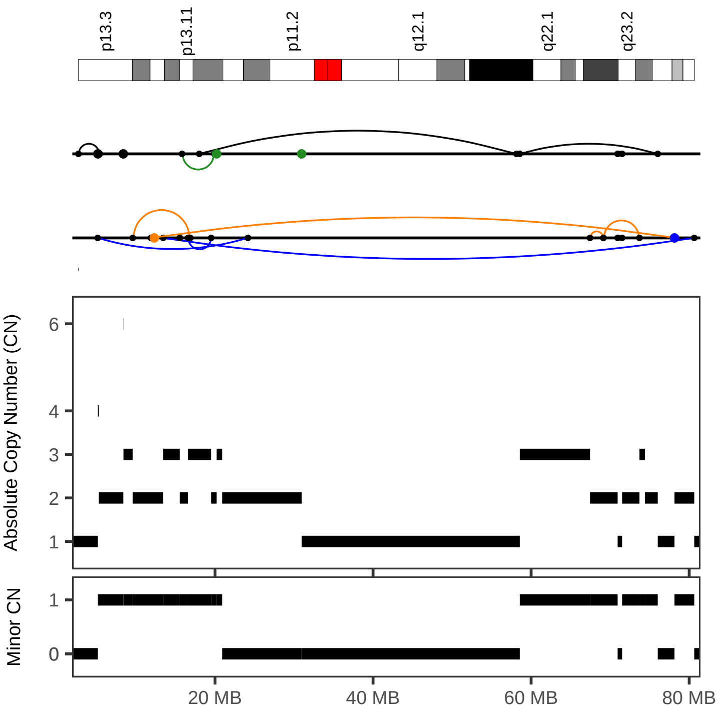

	RK176
Cancer type	Liver−HCC
Position	8:36542144−56972621
Type	With other complex events
Interleaved intrachr. SVs	19
Total SVs (intrachr. + transl.)	31
SV types	DEL: 7; DUP: 5; h2hINV: 5; t2tINV: 2; TRA: 12
SVs in sample	94
Oscillating CN (2 and 3 states)	8, 9
CN segments	17
FDR fragment joints	0.64
FDR chr. breakp. enrich.	0
Linked to chrs	
Purity, ploidy	0.62, 2.03

	RK177
Cancer type	Liver−HCC
Position	16:2729647−80636590
Type	With other complex events
Interleaved intrachr. SVs	9
Total SVs (intrachr. + transl.)	16
SV types	DEL: 2; DUP: 3; h2hINV: 2; t2tINV: 2; TRA: 7
SVs in sample	139
Oscillating CN (2 and 3 states)	8, 9
CN segments	22
FDR fragment joints	0.97
FDR chr. breakp. enrich.	0
Linked to chrs	8:50712276−144686188;
Purity, ploidy	0.72, 2

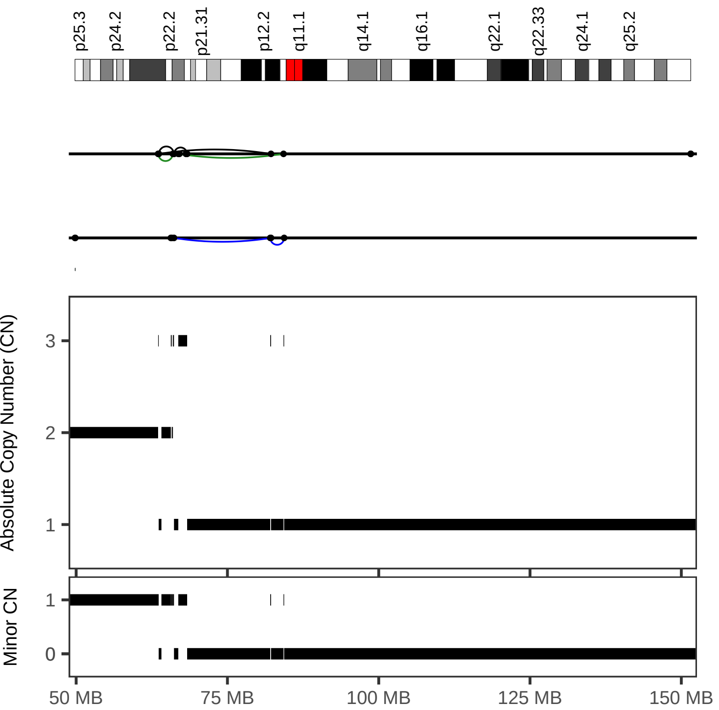

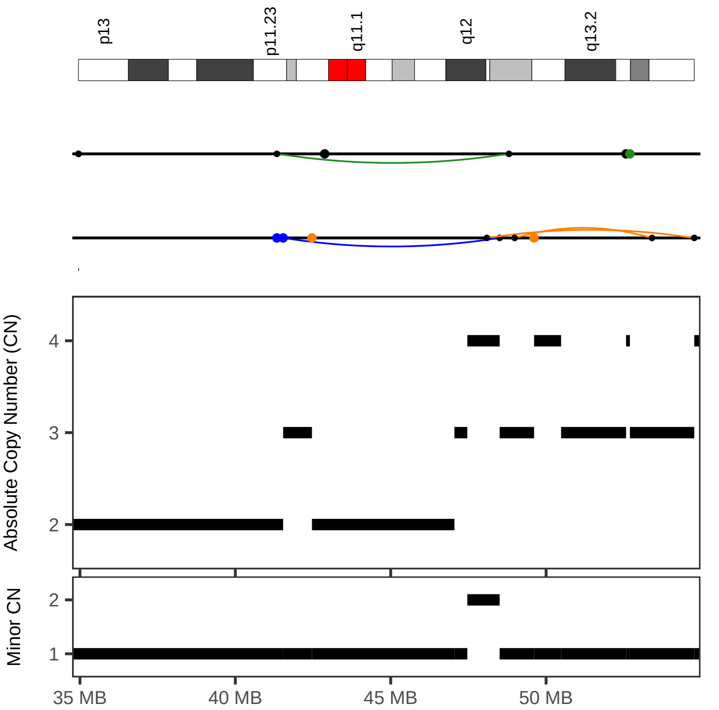

	RK178
Cancer type	Liver−HCC
Position	6:63544988−84372030
Type	Canonical without polyploidization
Interleaved intrachr. SVs	7
Total SVs (intrachr. + transl.)	7
SV types	DEL: 0; DUP: 2; h2hINV: 3; t2tINV: 2; TRA: 0
SVs in sample	63
Oscillating CN (2 and 3 states)	8, 11
CN segments	13
FDR fragment joints	0.64
FDR chr. breakp. enrich.	0.01
Linked to chrs	
Purity, ploidy	0.47, 2.18

	RK198
Cancer type	Liver−HCC
Position	20:41335728−54766828
Type	After polyploidization
Interleaved intrachr. SVs	3
Total SVs (intrachr. + transl.)	10
SV types	DEL: 1; DUP: 1; h2hINV: 0; t2tINV: 1; TRA: 7
SVs in sample	51
Oscillating CN (2 and 3 states)	8, 11
CN segments	11
FDR fragment joints	0.84
FDR chr. breakp. enrich.	0
Linked to chrs	
Purity, ploidy	0.3, 2

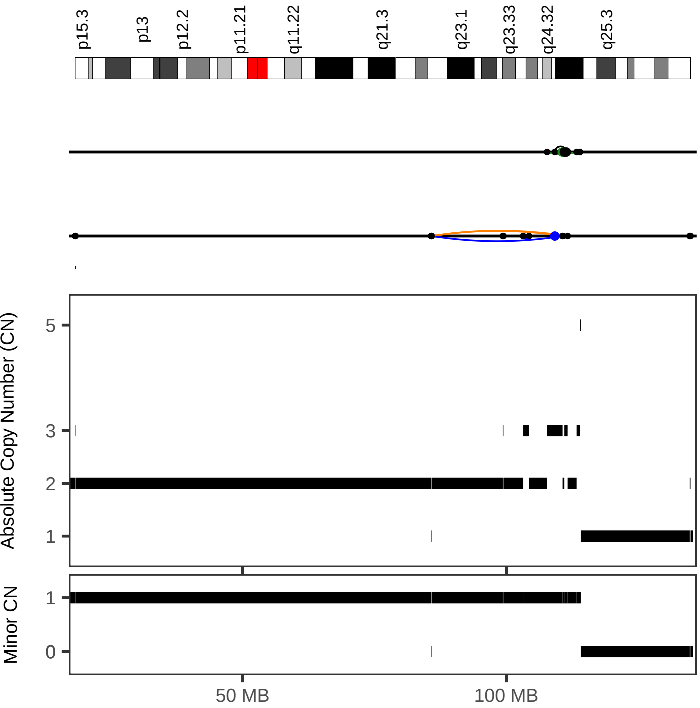

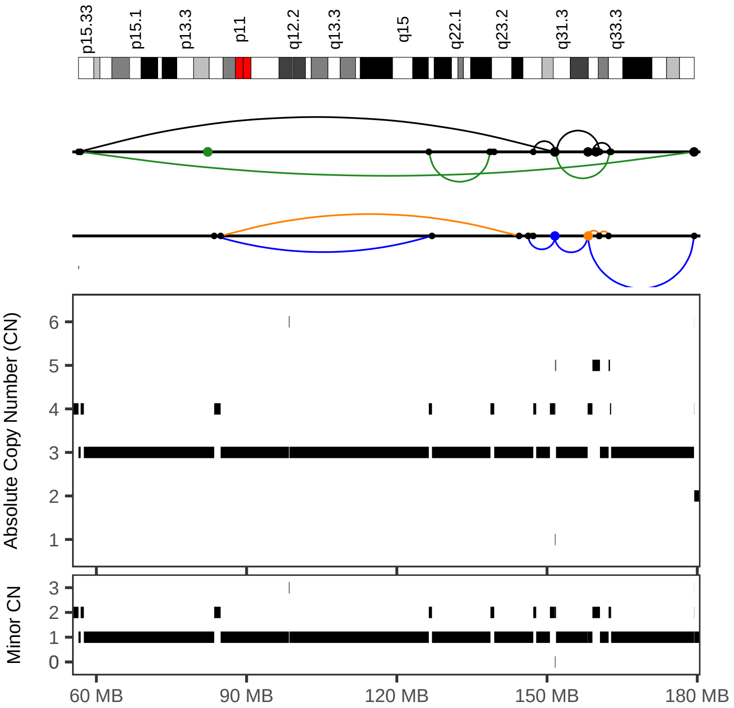

	RK211
Cancer type	Liver−HCC
Position	10:85728073−113319897
Type	Canonical without polyploidization
Interleaved intrachr. SVs	5
Total SVs (intrachr. + transl.)	9
SV types	DEL: 2; DUP: 1; h2hINV: 1; t2tINV: 1; TRA: 4
SVs in sample	227
Oscillating CN (2 and 3 states)	12, 13
CN segments	13
FDR fragment joints	0.92
FDR chr. breakp. enrich.	0.35
Linked to chrs	4:20275834−163873073;
Purity, ploidy	0.84, 1.82

	RK224
Cancer type	Liver−HCC
Position	5:56427433−179390593
Type	With other complex events
Interleaved intrachr. SVs	13
Total SVs (intrachr. + transl.)	22
SV types	DEL: 3; DUP: 3; h2hINV: 5; t2tINV: 2; TRA: 9
SVs in sample	138
Oscillating CN (2 and 3 states)	8, 8
CN segments	30
FDR fragment joints	0.77
FDR chr. breakp. enrich.	0
Linked to chrs	
Purity, ploidy	0.42, 3.24

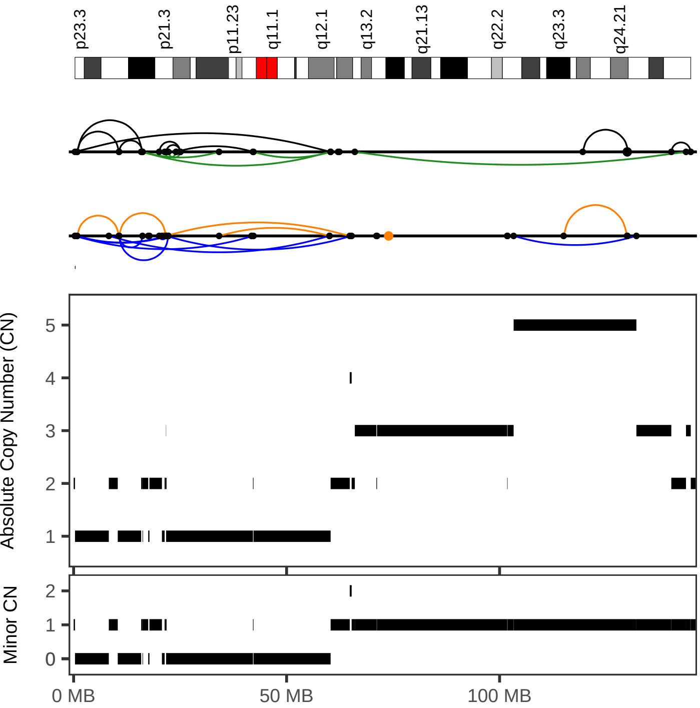

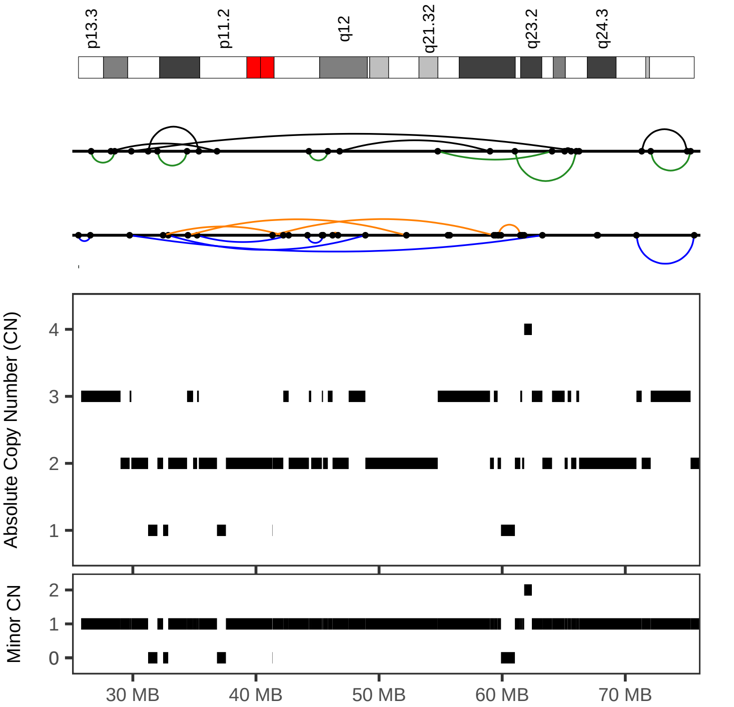

	RK236
Cancer type	Liver−HCC
Position	8:317288−65269814
Type	With other complex events
Interleaved intrachr. SVs	25
Total SVs (intrachr. + transl.)	25
SV types	DEL: 5; DUP: 8; h2hINV: 7; t2tINV: 5; TRA: 0
SVs in sample	148
Oscillating CN (2 and 3 states)	11, 11
CN segments	20
FDR fragment joints	0.84
FDR chr. breakp. enrich.	0
Linked to chrs	
Purity, ploidy	0.76, 2.01

	RK236
Cancer type	Liver−HCC
Position	17:26612392−66246801
Type	With other complex events
Interleaved intrachr. SVs	16
Total SVs (intrachr. + transl.)	16
SV types	DEL: 4; DUP: 4; h2hINV: 4; t2tINV: 4; TRA: 0
SVs in sample	148
Oscillating CN (2 and 3 states)	15, 34
CN segments	43
FDR fragment joints	1
FDR chr. breakp. enrich.	0
Linked to chrs	
Purity, ploidy	0.76, 2.01

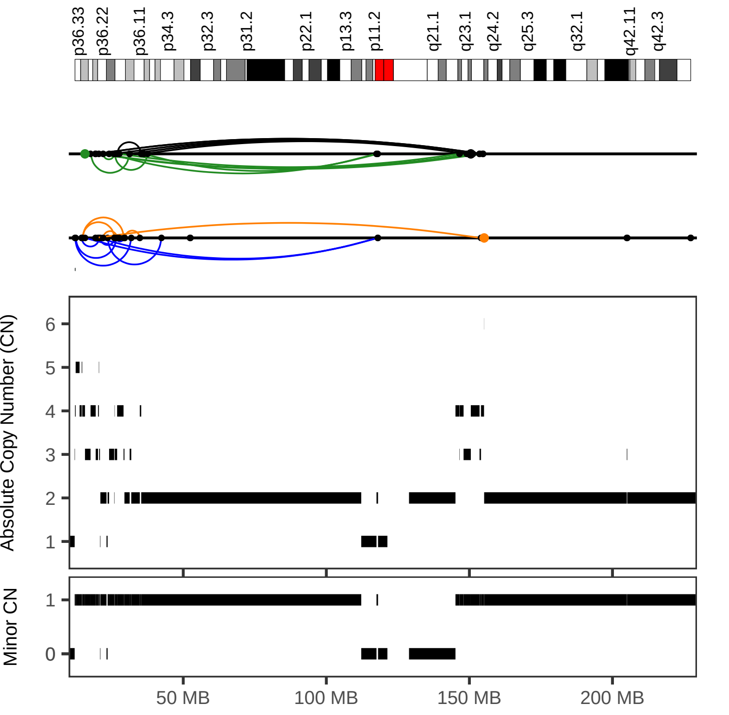

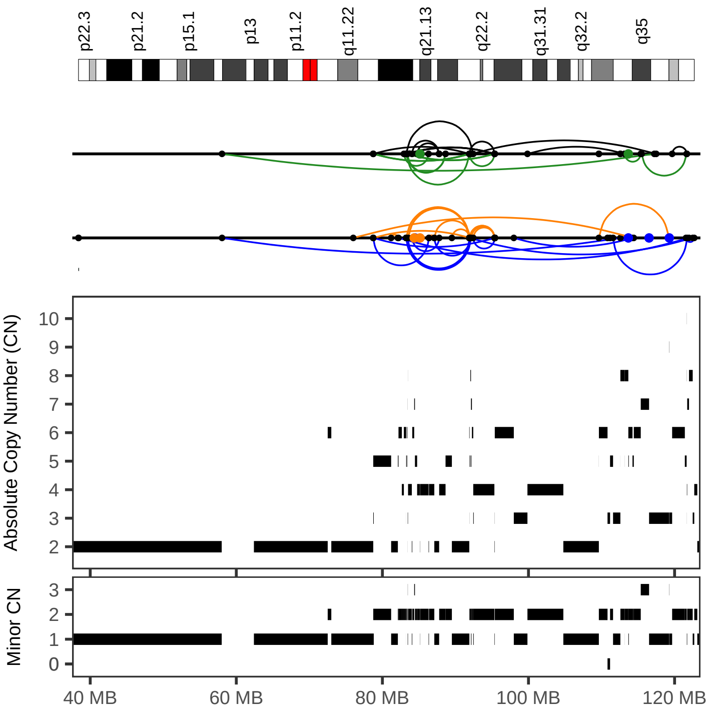

RK237	
Cancer type	Liver−HCC
Position	1:12167222−154761279
Type	With other complex events
Interleaved intrachr. SVs	29
Total SVs (intrachr. + transl.)	31
SV types	DEL: 7; DUP: 8; h2hINV: 6; t2tINV: 8; TRA: 2
SVs in sample	68
Oscillating CN (2 and 3 states)	7, 14
CN segments	38
FDR fragment joints	0.96
FDR chr. breakp. enrich.	0
Linked to chrs	
Purity, ploidy	0.49, 1.96

RK254	
Cancer type	Liver−HCC
Position	7:58042074−122691771
Type	With other complex events
Interleaved intrachr. SVs	57
Total SVs (intrachr. + transl.)	66
SV types	DEL: 15; DUP: 17; h2hINV: 12; t2tINV: 13; TRA: 9
SVs in sample	168
Oscillating CN (2 and 3 states)	9, 9
CN segments	79
FDR fragment joints	0.84
FDR chr. breakp. enrich.	0
Linked to chrs	
Purity, ploidy	0.45, 2.71

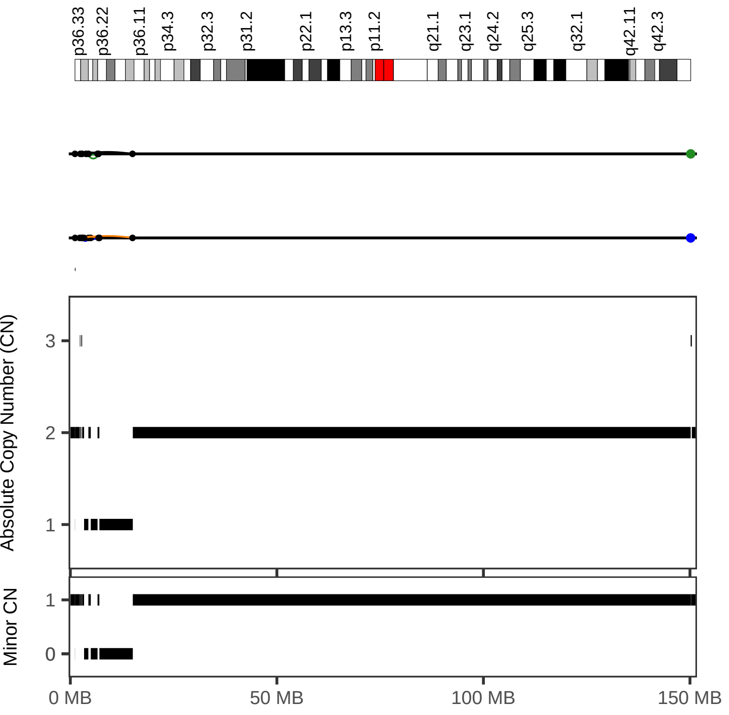

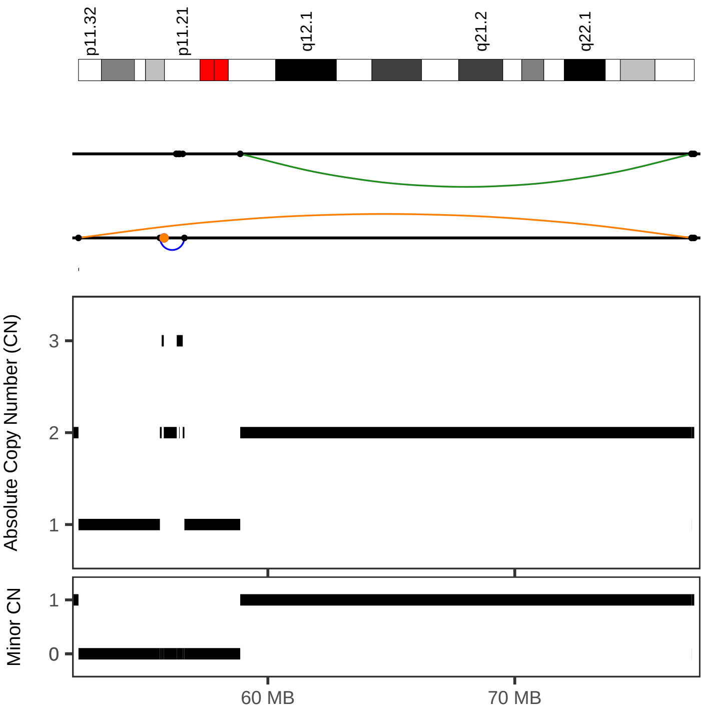

RK256	
Cancer type	Liver−HCC
Position	1:1119435−15049596
Type	Canonical without polyploidization
Interleaved intrachr. SVs	12
Total SVs (intrachr. + transl.)	12
SV types	DEL: 3; DUP: 4; h2hINV: 3; t2tINV: 2; TRA: 0
SVs in sample	91
Oscillating CN (2 and 3 states)	12, 15
CN segments	20
FDR fragment joints	0.91
FDR chr. breakp. enrich.	0
Linked to chrs	
Purity, ploidy	0.89, 1.86

RK259	
Cancer type	Liver−HCC
Position	18:52328461−77268592
Type	With other complex events
Interleaved intrachr. SVs	4
Total SVs (intrachr. + transl.)	10
SV types	DEL: 2; DUP: 0; h2hINV: 3; t2tINV: 3; TRA: 2
SVs in sample	45
Oscillating CN (2 and 3 states)	7, 12
CN segments	12
FDR fragment joints	0.59
FDR chr. breakp. enrich.	0
Linked to chrs	
Purity, ploidy	0.95, 2.26

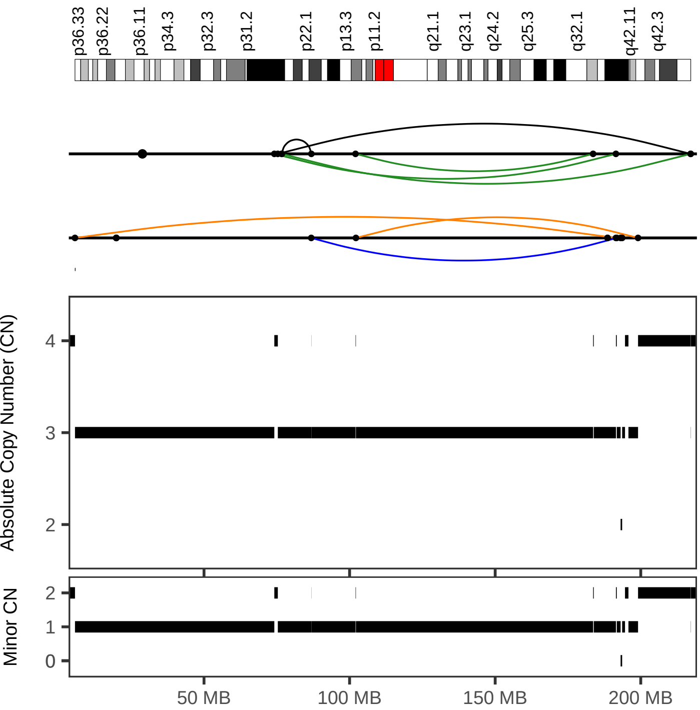

RK262	
Cancer type	Liver−HCC
Position	1:5685916−217171836
Type	After polyploidization
Interleaved intrachr. SVs	8
Total SVs (intrachr. + transl.)	9
SV types	DEL: 2; DUP: 1; h2hINV: 2; t2tINV: 3; TRA: 1
SVs in sample	76
Oscillating CN (2 and 3 states)	11, 18
CN segments	18
FDR fragment joints	0.84
FDR chr. breakp. enrich.	0.04
Linked to chrs	
Purity, ploidy	0.66, 3.46

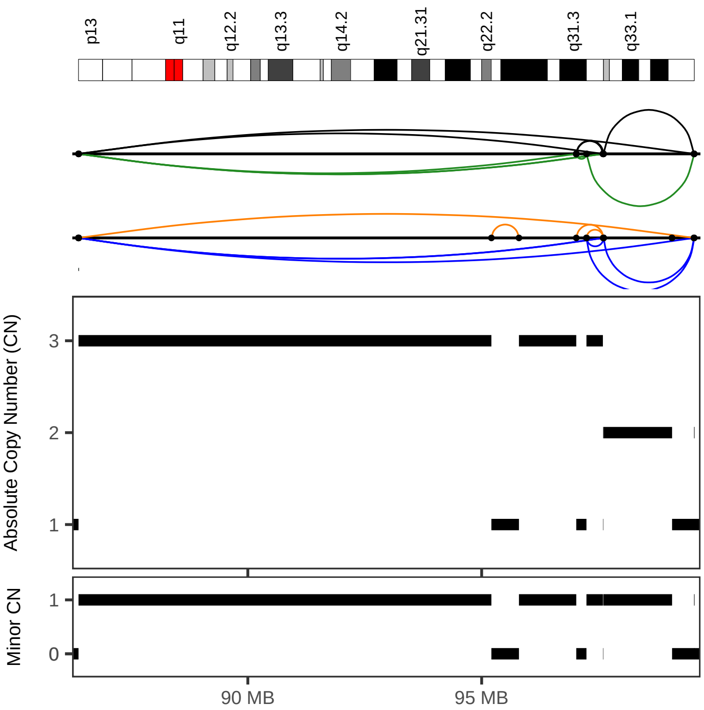

RK275	
Cancer type	Liver−HCC
Position	13:86378471−99546315
Type	With other complex events
Interleaved intrachr. SVs	20
Total SVs (intrachr. + transl.)	20
SV types	DEL: 3; DUP: 6; h2hINV: 6; t2tINV: 5; TRA: 0
SVs in sample	69
Oscillating CN (2 and 3 states)	8, 8
CN segments	14
FDR fragment joints	0.83
FDR chr. breakp. enrich.	0
Linked to chrs	
Purity, ploidy	0.64, 1.96

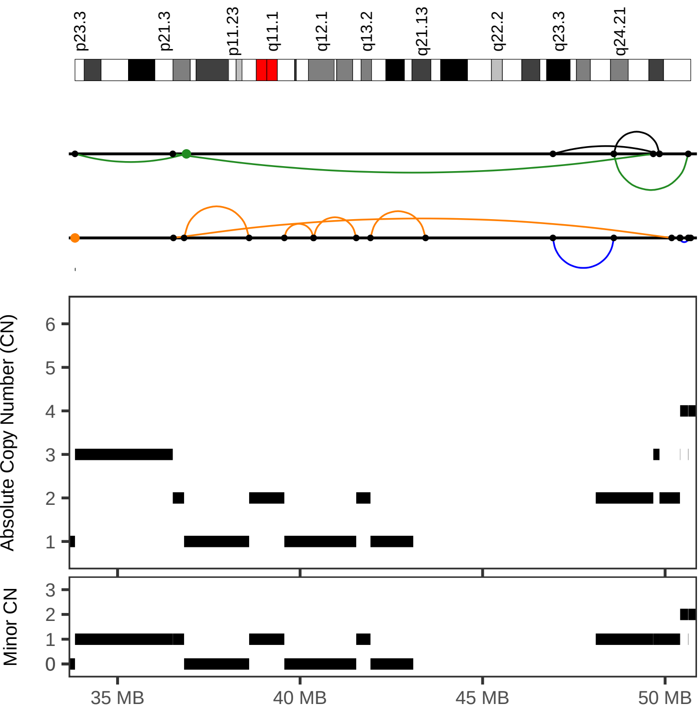

RK306	
Cancer type	Liver−HCC
Position	8:33832982−50702008
Type	With other complex events
Interleaved intrachr. SVs	10
Total SVs (intrachr. + transl.)	12
SV types	DEL: 3; DUP: 2; h2hINV: 2; t2tINV: 3; TRA: 2
SVs in sample	42
Oscillating CN (2 and 3 states)	7, 14
CN segments	14
FDR fragment joints	0.96
FDR chr. breakp. enrich.	0
Linked to chrs	
Purity, ploidy	0.86, 2.02

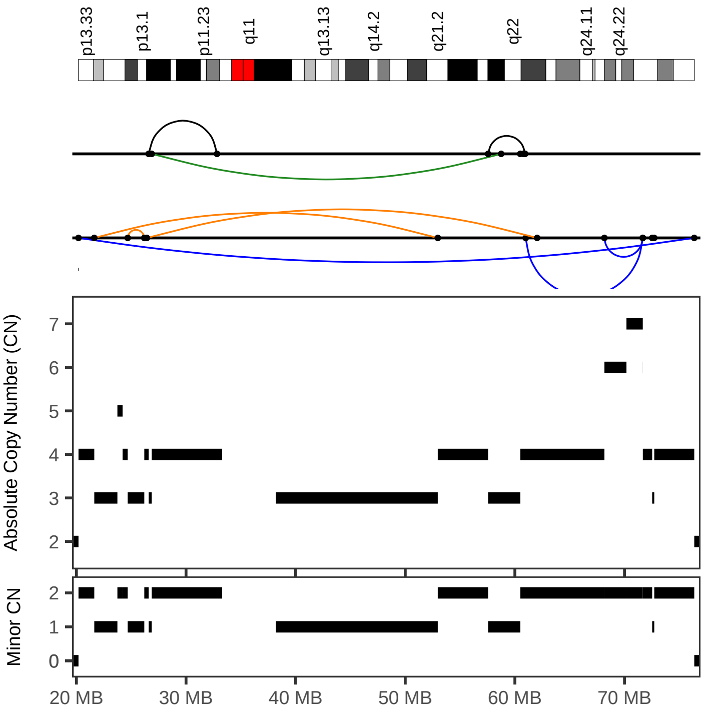

028e99e9−5b9a−4954−bb6e−6d4709a3cea8	
Cancer type	Lung−AdenoCA
Position	12:21630873−71673041
Type	After polyploidization
Interleaved intrachr. SVs	6
Total SVs (intrachr. + transl.)	6
SV types	DEL: 2; DUP: 1; h2hINV: 2; t2tINV: 1; TRA: 0
SVs in sample	20
Oscillating CN (2 and 3 states)	9, 13
CN segments	15
FDR fragment joints	0.91
FDR chr. breakp. enrich.	0
Linked to chrs	
Purity, ploidy	0.24, 3.52

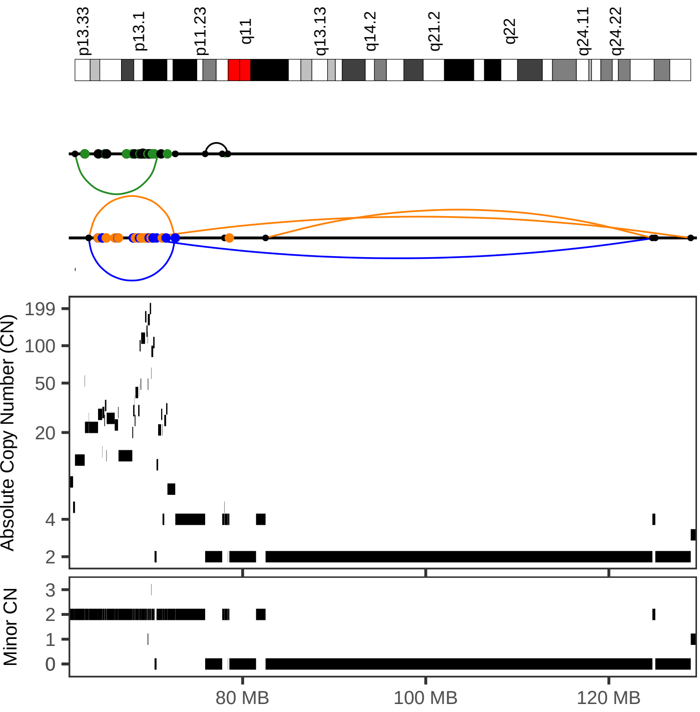

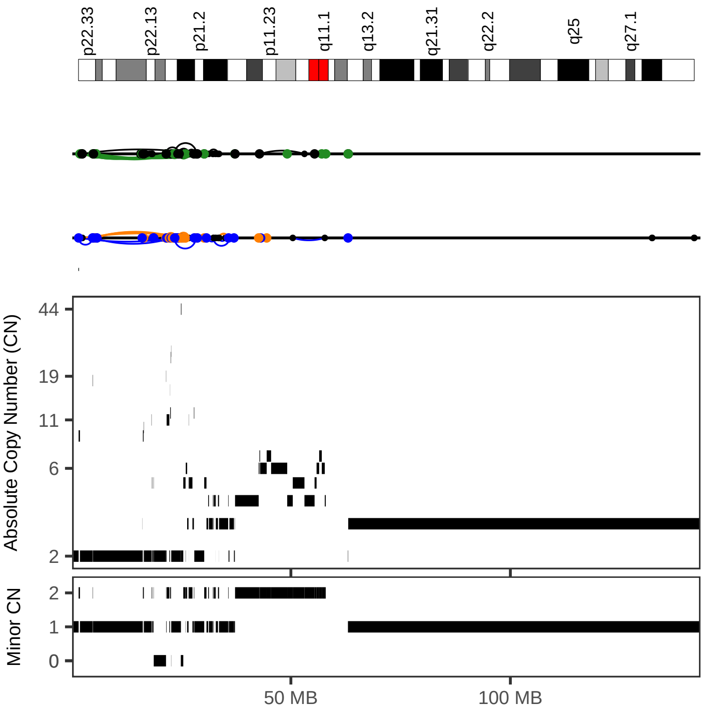

	0d66bf6c−eed0−4726−bd5b−3bf6d610b4e0
Cancer type	Lung−AdenoCA
Position	12:61686111−128942893
Type	With other complex events
Interleaved intrachr. SVs	10
Total SVs (intrachr. + transl.)	81
SV types	DEL: 3; DUP: 3; h2hINV: 2; t2tINV: 2; TRA: 71
SVs in sample	414
Oscillating CN (2 and 3 states)	8, 8
CN segments	59
FDR fragment joints	0.96
FDR chr. breakp. enrich.	0
Linked to chrs	8:89155199−132576363;9:524316−133004750 X:1621688−36004056;
Purity, ploidy	0.48, 3.84

	0d66bf6c−eed0−4726−bd5b−3bf6d610b4e0
Cancer type	Lung−AdenoCA
Position	X:1621688−36004057
Type	With other complex events
Interleaved intrachr. SVs	42
Total SVs (intrachr. + transl.)	122
SV types	DEL: 11; DUP: 10; h2hINV: 10; t2tINV: 11; TRA: 80
SVs in sample	414
Oscillating CN (2 and 3 states)	8, 16
CN segments	56
FDR fragment joints	1
FDR chr. breakp. enrich.	0
Linked to chrs	12:61686111−128942892;8:89155199−132576363 9:524316−133004750;
Purity, ploidy	0.48, 3.84

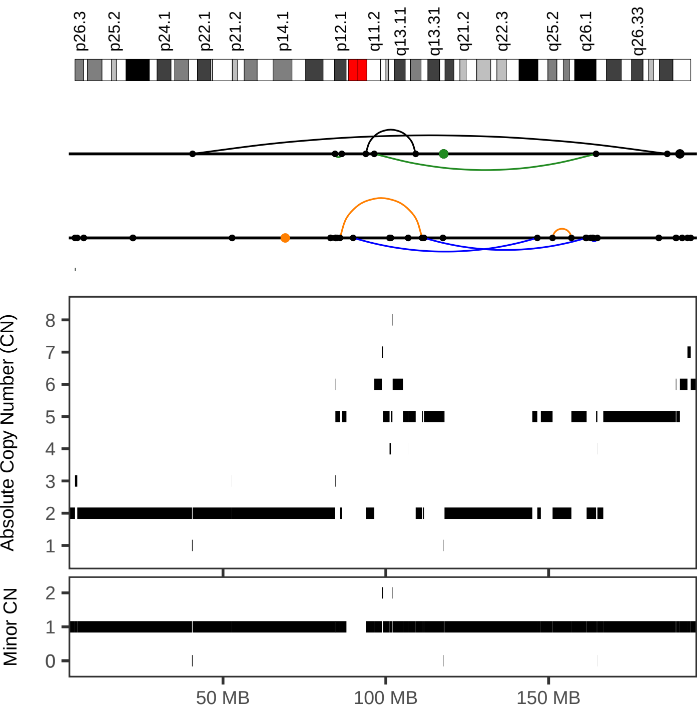

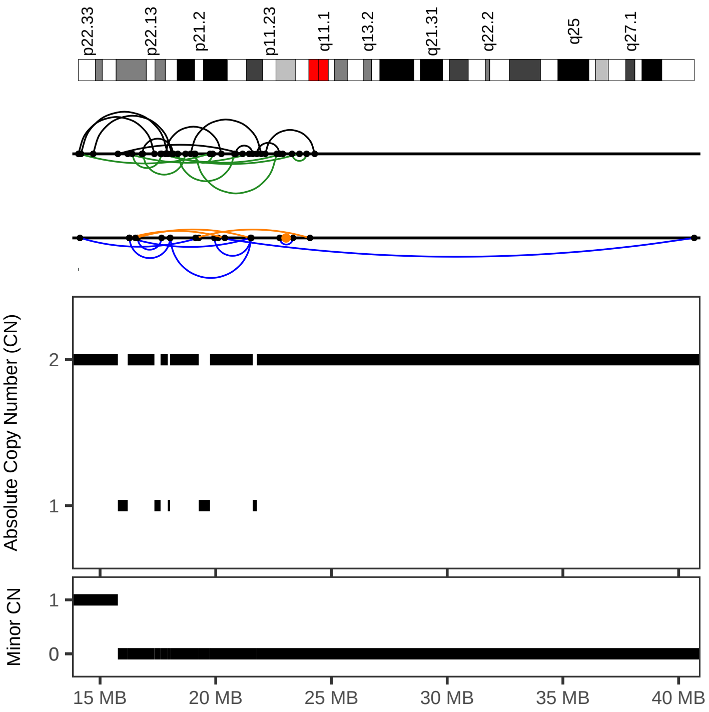

	0df573ee−28f0−4244−b434−09e6ca59fbf0
Cancer type	Lung−AdenoCA
Position	3:84403892−164988872
Type	With other complex events
Interleaved intrachr. SVs	7
Total SVs (intrachr. + transl.)	9
SV types	DEL: 1; DUP: 3; h2hINV: 1; t2tINV: 2; TRA: 2
SVs in sample	350
Oscillating CN (2 and 3 states)	9, 15
CN segments	33
FDR fragment joints	0.74
FDR chr. breakp. enrich.	0.49
Linked to chrs	
Purity, ploidy	0.42, 2.67

	2cd8ea73−cb3f−4b17−9b97−fa12eb03b85b
Cancer type	Lung−AdenoCA
Position	X:14068417−40674197
Type	Canonical without polyploidization
Interleaved intrachr. SVs	31
Total SVs (intrachr. + transl.)	32
SV types	DEL: 3; DUP: 8; h2hINV: 10; t2tINV: 10; TRA: 1
SVs in sample	59
Oscillating CN (2 and 3 states)	11, 11
CN segments	11
FDR fragment joints	0.59
FDR chr. breakp. enrich.	0
Linked to chrs	
Purity, ploidy	0.17, 2

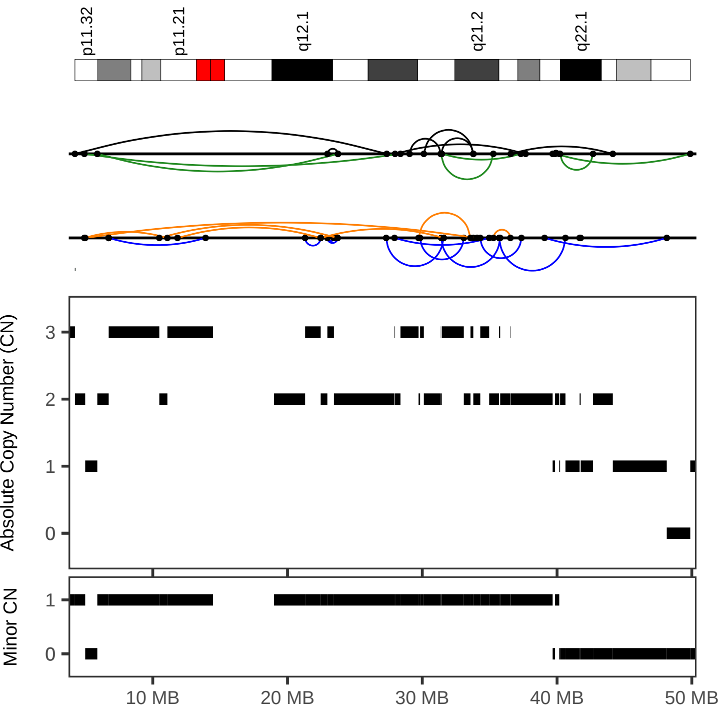

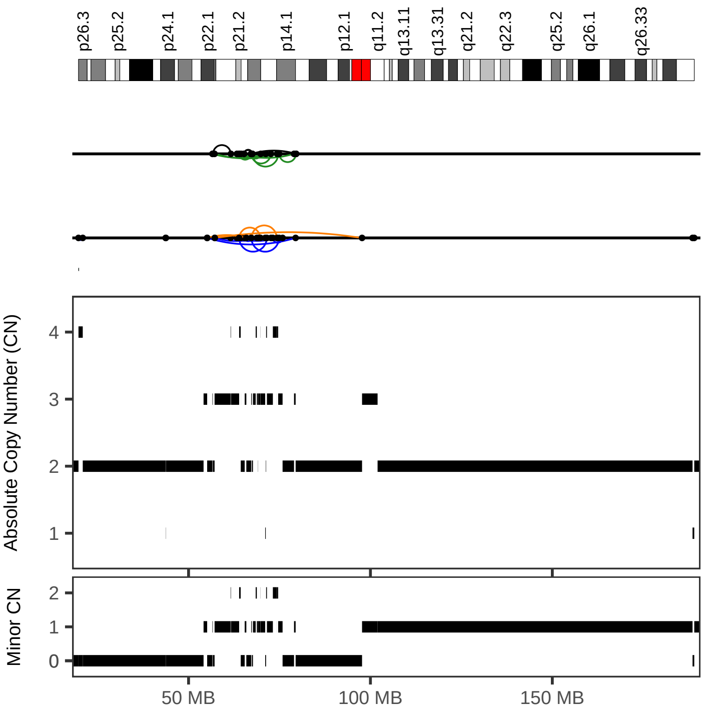

	35cb7841−9b09−465a−90c5−e3b8a9faad49
Cancer type	Lung−AdenoCA
Position	18:4223387−49884307
Type	Canonical without polyploidization
Interleaved intrachr. SVs	32
Total SVs (intrachr. + transl.)	32
SV types	DEL: 7; DUP: 11; h2hINV: 8; t2tINV: 6; TRA: 0
SVs in sample	208
Oscillating CN (2 and 3 states)	27, 40
CN segments	40
FDR fragment joints	0.7
FDR chr. breakp. enrich.	0
Linked to chrs	
Purity, ploidy	0.26, 2.72

	368e23f0−e573−4547−bf5a−14080baf737b
Cancer type	Lung−AdenoCA
Position	3:55114482−97697310
Type	With other complex events
Interleaved intrachr. SVs	27
Total SVs (intrachr. + transl.)	27
SV types	DEL: 5; DUP: 9; h2hINV: 5; t2tINV: 8; TRA: 0
SVs in sample	170
Oscillating CN (2 and 3 states)	10, 16
CN segments	35
FDR fragment joints	0.67
FDR chr. breakp. enrich.	0
Linked to chrs	
Purity, ploidy	0.36, 2.06

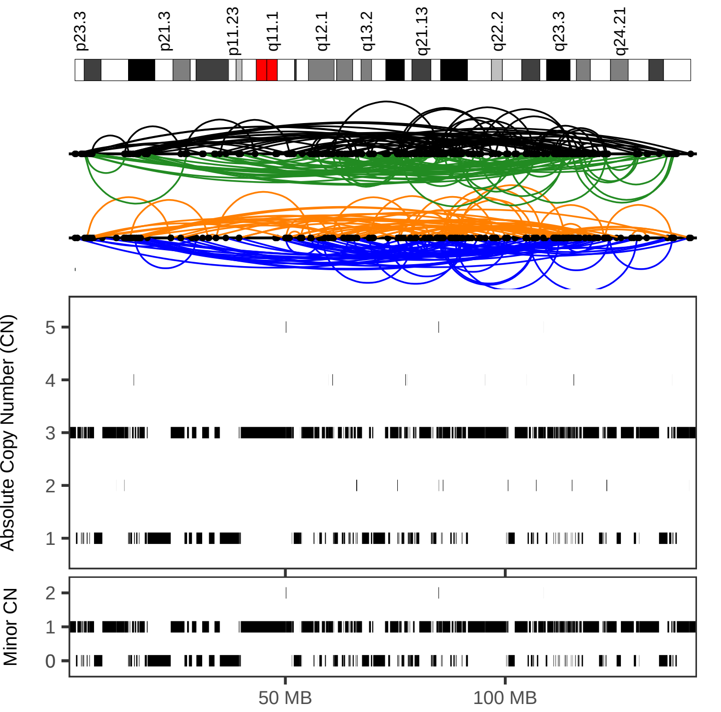

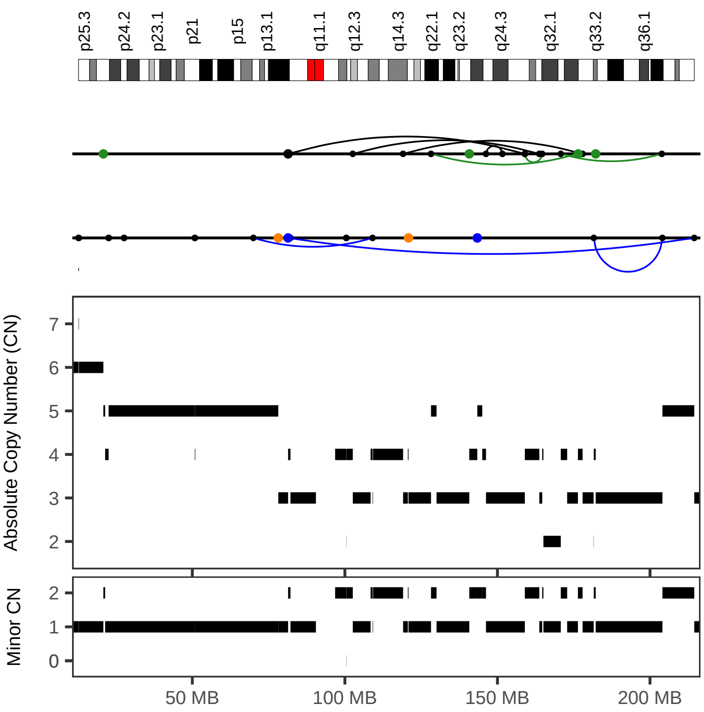

	397d3f69−1453−4057−b177−8723eec923d1
Cancer type	Lung−AdenoCA
Position	8:2155797−142175861
Type	With other complex events
Interleaved intrachr. SVs	331
Total SVs (intrachr. + transl.)	331
SV types	DEL: 81; DUP: 83; h2hINV: 83; t2tINV: 84; TRA: 0
SVs in sample	356
Oscillating CN (2 and 3 states)	26, 29
CN segments	256
FDR fragment joints	1
FDR chr. breakp. enrich.	0
Linked to chrs	
Purity, ploidy	0.91, 2.16

	3d2aa654−1b5f−4eb4−a1c2−af31f5760069
Cancer type	Lung−AdenoCA
Position	2:70005417−214498426
Type	With other complex events
Interleaved intrachr. SVs	10
Total SVs (intrachr. + transl.)	19
SV types	DEL: 0; DUP: 4; h2hINV: 3; t2tINV: 3; TRA: 9
SVs in sample	166
Oscillating CN (2 and 3 states)	8, 17
CN segments	33
FDR fragment joints	0.59
FDR chr. breakp. enrich.	0.01
Linked to chrs	
Purity, ploidy	0.62, 3.34

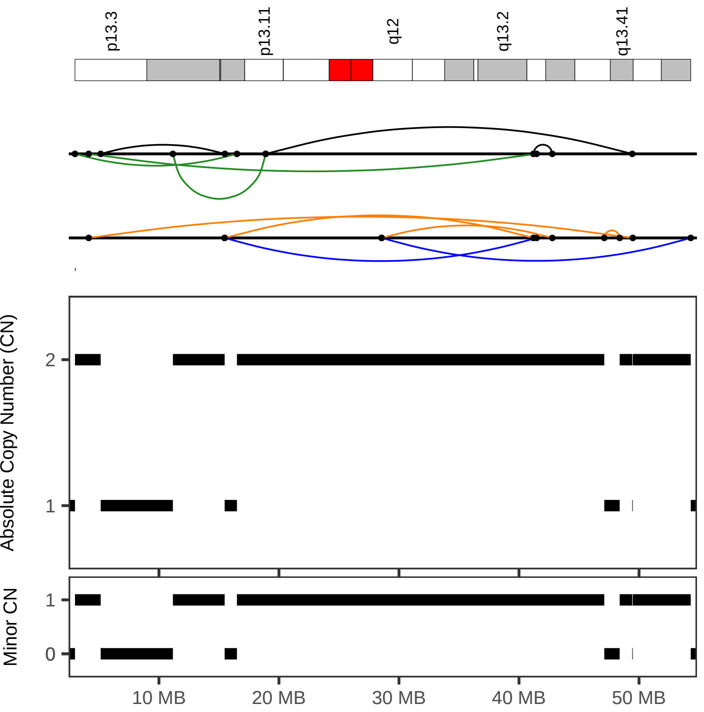

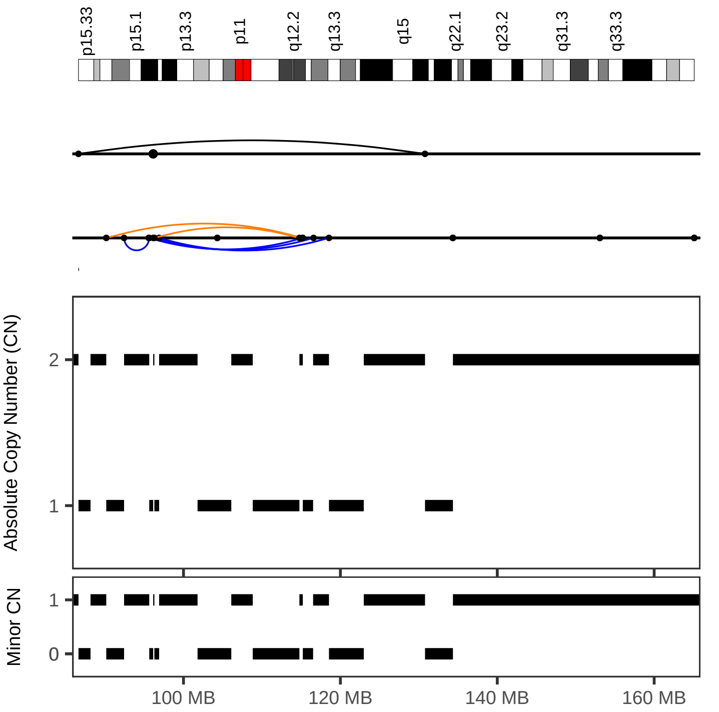

	61c655ec−52b5−453f−a6cc−b2aba445b027
Cancer type	Lung−AdenoCA
Position	19:3005321−54308857
Type	Canonical without polyploidization
Interleaved intrachr. SVs	11
Total SVs (intrachr. + transl.)	11
SV types	DEL: 3; DUP: 2; h2hINV: 3; t2tINV: 3; TRA: 0
SVs in sample	44
Oscillating CN (2 and 3 states)	10, 10
CN segments	10
FDR fragment joints	0.98
FDR chr. breakp. enrich.	0
Linked to chrs	
Purity, ploidy	0.72, 2.02

	6dfd47d2−831a−4386−9051−f78199a16bb5
Cancer type	Lung−AdenoCA
Position	5:90154962−118543958
Type	Canonical without polyploidization
Interleaved intrachr. SVs	6
Total SVs (intrachr. + transl.)	7
SV types	DEL: 2; DUP: 4; h2hINV: 0; t2tINV: 0; TRA: 1
SVs in sample	113
Oscillating CN (2 and 3 states)	13, 13
CN segments	13
FDR fragment joints	0.48
FDR chr. breakp. enrich.	0.22
Linked to chrs	
Purity, ploidy	0.86, 1.97

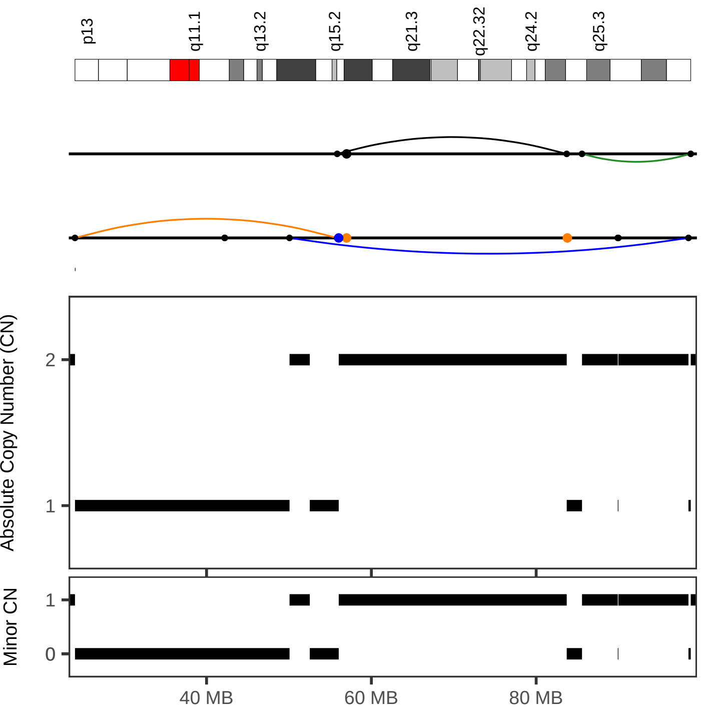

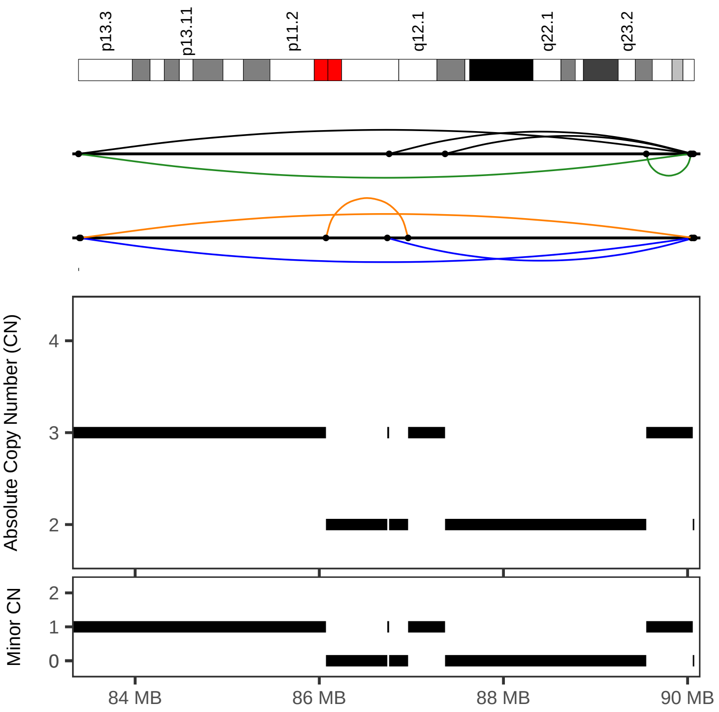

	6dfd47d2−831a−4386−9051−f78199a16bb5
Cancer type	Lung−AdenoCA
Position	15:24028054−98760287
Type	Canonical without polyploidization
Interleaved intrachr. SVs	3
Total SVs (intrachr. + transl.)	7
SV types	DEL: 1; DUP: 1; h2hINV: 0; t2tINV: 1; TRA: 4
SVs in sample	113
Oscillating CN (2 and 3 states)	10, 10
CN segments	10
FDR fragment joints	0.84
FDR chr. breakp. enrich.	0
Linked to chrs	
Purity, ploidy	0.86, 1.97

	9b132e4f−7e35−4cc5−8711−43ad62b906d0
Cancer type	Lung−AdenoCA
Position	16:83384632−90071935
Type	Canonical without polyploidization
Interleaved intrachr. SVs	9
Total SVs (intrachr. + transl.)	9
SV types	DEL: 2; DUP: 2; h2hINV: 3; t2tINV: 2; TRA: 0
SVs in sample	209
Oscillating CN (2 and 3 states)	8, 8
CN segments	8
FDR fragment joints	0.97
FDR chr. breakp. enrich.	0.02
Linked to chrs	
Purity, ploidy	0.9, 4.33

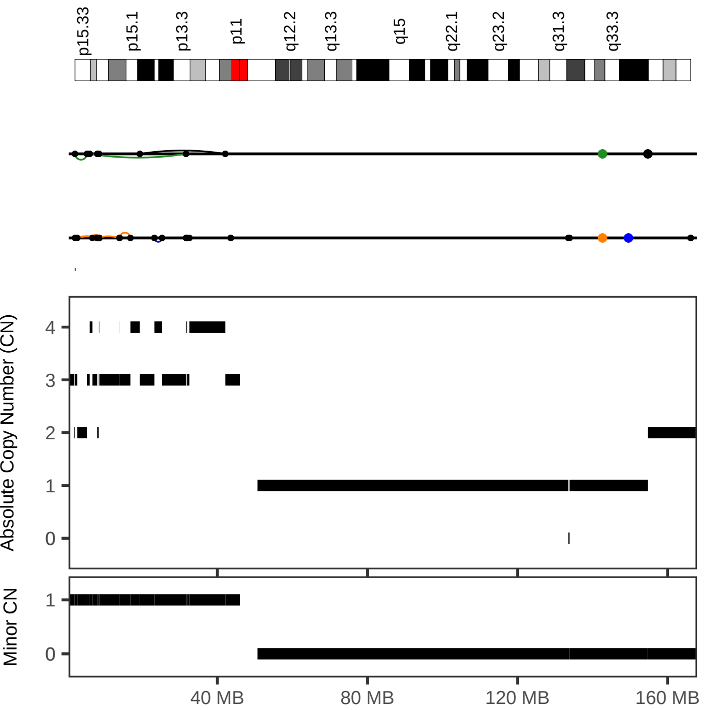

	9f81c602−8afa−4588−b0b6−6e5a1a128d5a
Cancer type	Lung−AdenoCA
Position	5:2066631−42135112
Type	After polyploidization
Interleaved intrachr. SVs	8
Total SVs (intrachr. + transl.)	8
SV types	DEL: 4; DUP: 0; h2hINV: 2; t2tINV: 2; TRA: 0
SVs in sample	84
Oscillating CN (2 and 3 states)	17, 23
CN segments	23
FDR fragment joints	0.59
FDR chr. breakp. enrich.	0
Linked to chrs	
Purity, ploidy	0.77, 1.79

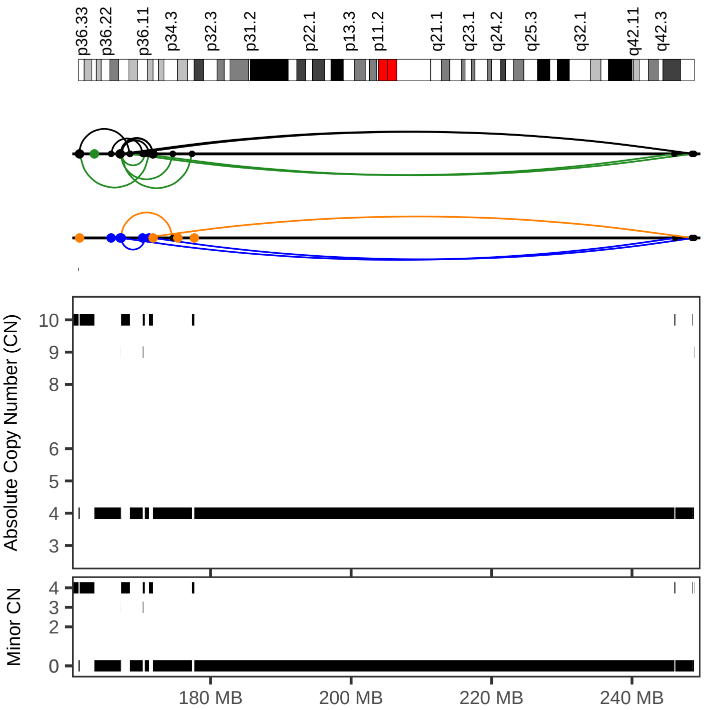

	a1e65587−24c1−4b41−92a7−4e1f15fffd78
Cancer type	Lung−AdenoCA
Position	1:161178683−248860526
Type	With other complex events
Interleaved intrachr. SVs	18
Total SVs (intrachr. + transl.)	31
SV types	DEL: 2; DUP: 3; h2hINV: 7; t2tINV: 6; TRA: 13
SVs in sample	107
Oscillating CN (2 and 3 states)	10, 11
CN segments	21
FDR fragment joints	0.59
FDR chr. breakp. enrich.	0
Linked to chrs	
Purity, ploidy	0.32, 4.75

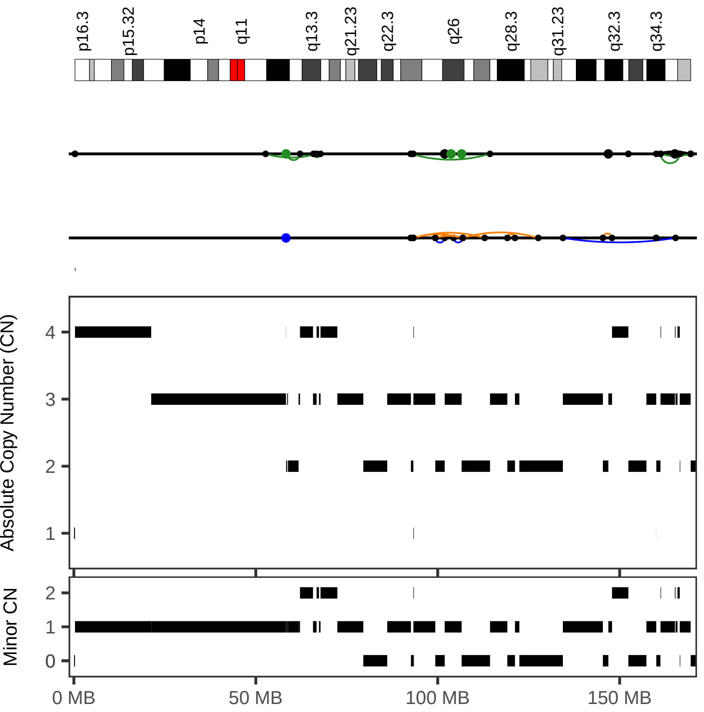

	a3de401d−91fe−49a2−bb07−81c1a06506e6
Cancer type	Lung−AdenoCA
Position	4:92565955−127635647
Type	Canonical without polyploidization
Interleaved intrachr. SVs	7
Total SVs (intrachr. + transl.)	10
SV types	DEL: 5; DUP: 1; h2hINV: 0; t2tINV: 1; TRA: 3
SVs in sample	94
Oscillating CN (2 and 3 states)	8, 8
CN segments	13
FDR fragment joints	0.38
FDR chr. breakp. enrich.	0
Linked to chrs	
Purity, ploidy	0.44, 3.2

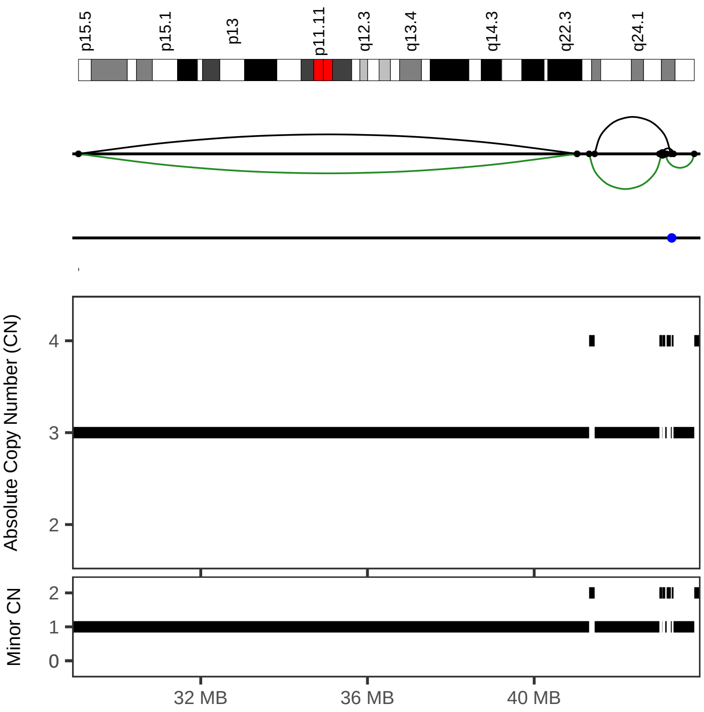

	a3de401d−91fe−49a2−bb07−81c1a06506e6
Cancer type	Lung−AdenoCA
Position	11:41318012−43840319
Type	After polyploidization
Interleaved intrachr. SVs	6
Total SVs (intrachr. + transl.)	8
SV types	DEL: 0; DUP: 0; h2hINV: 3; t2tINV: 3; TRA: 2
SVs in sample	94
Oscillating CN (2 and 3 states)	11, 11
CN segments	11
FDR fragment joints	0.48
FDR chr. breakp. enrich.	0.01
Linked to chrs	
Purity, ploidy	0.44, 3.2

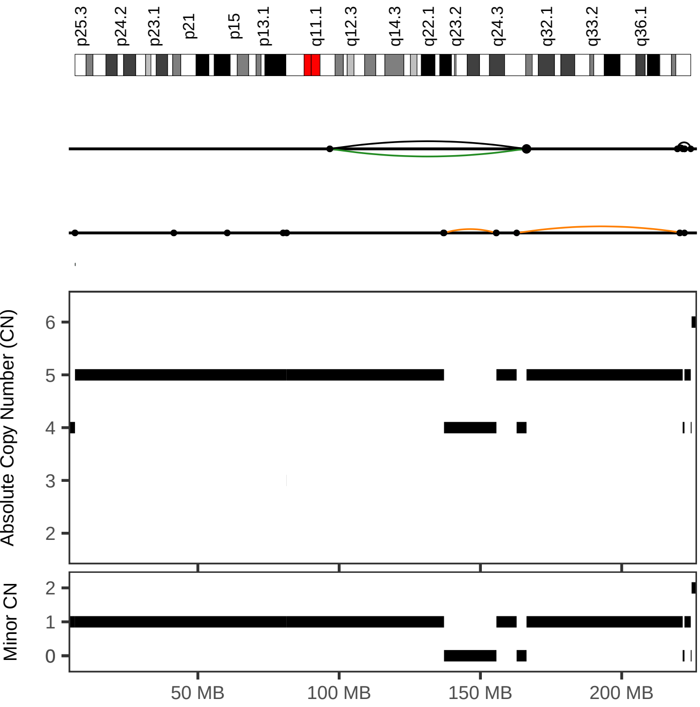

	cc4bd56a−25c5−4c48−b583−ac3aeb778ca6
Cancer type	Lung−AdenoCA
Position	2:96640631−224440317
Type	After polyploidization
Interleaved intrachr. SVs	6
Total SVs (intrachr. + transl.)	7
SV types	DEL: 1; DUP: 0; h2hINV: 4; t2tINV: 1; TRA: 1
SVs in sample	237
Oscillating CN (2 and 3 states)	8, 8
CN segments	8
FDR fragment joints	0.48
FDR chr. breakp. enrich.	1
Linked to chrs	
Purity, ploidy	0.5, 5.16

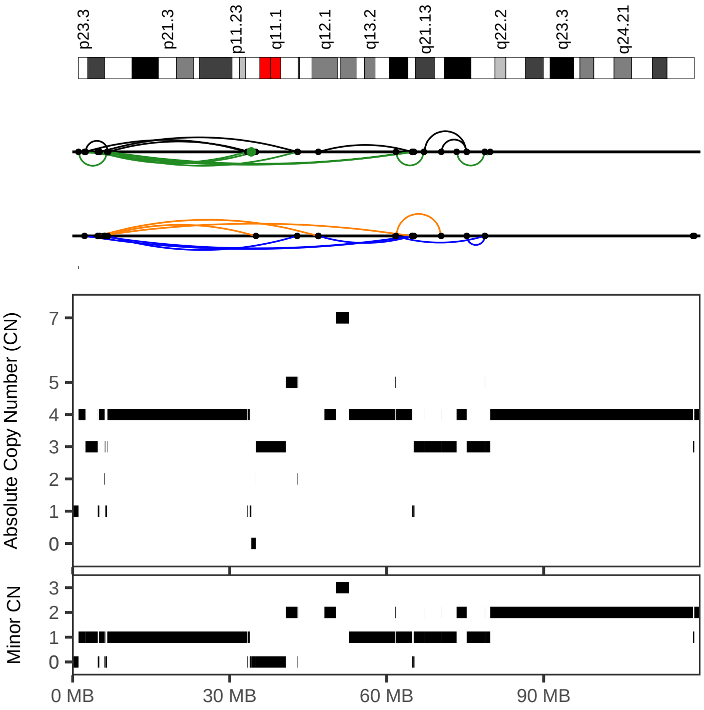

	d5326429−9805−47f9−97b0−fbda658e3f01
Cancer type	Lung−AdenoCA
Position	8:1104196−79784584
Type	With other complex events
Interleaved intrachr. SVs	29
Total SVs (intrachr. + transl.)	30
SV types	DEL: 5; DUP: 7; h2hINV: 7; t2tINV: 10; TRA: 1
SVs in sample	209
Oscillating CN (2 and 3 states)	7, 10
CN segments	45
FDR fragment joints	0.7
FDR chr. breakp. enrich.	0
Linked to chrs	
Purity, ploidy	0.63, 2.78

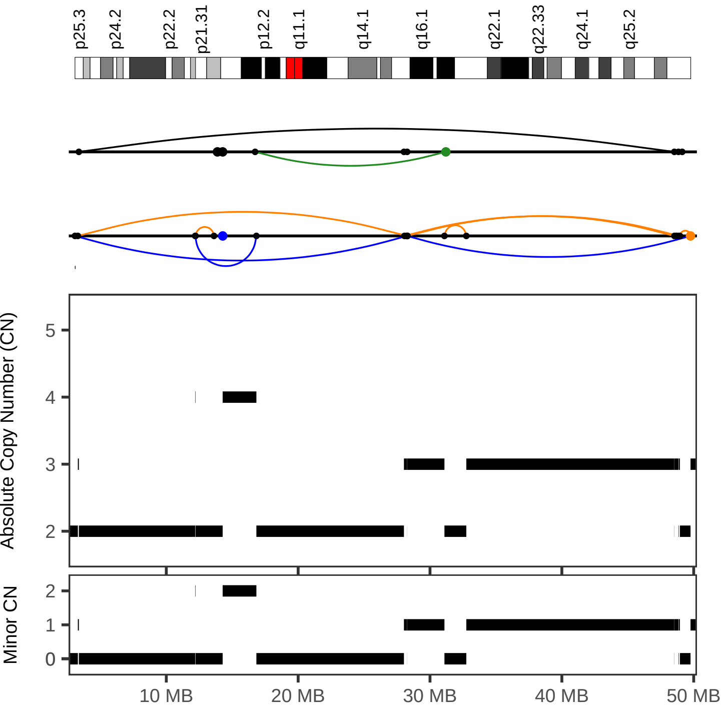

	dbd5b0de−94c9−45dd−afb3−6820a7ecaca2
Cancer type	Lung−AdenoCA
Position	6:3073059−49773930
Type	Canonical without polyploidization
Interleaved intrachr. SVs	13
Total SVs (intrachr. + transl.)	18
SV types	DEL: 6; DUP: 3; h2hINV: 2; t2tINV: 2; TRA: 5
SVs in sample	40
Oscillating CN (2 and 3 states)	12, 18
CN segments	18
FDR fragment joints	0.59
FDR chr. breakp. enrich.	0
Linked to chrs	17:9065515−76403932;
Purity, ploidy	0.72, 2.22

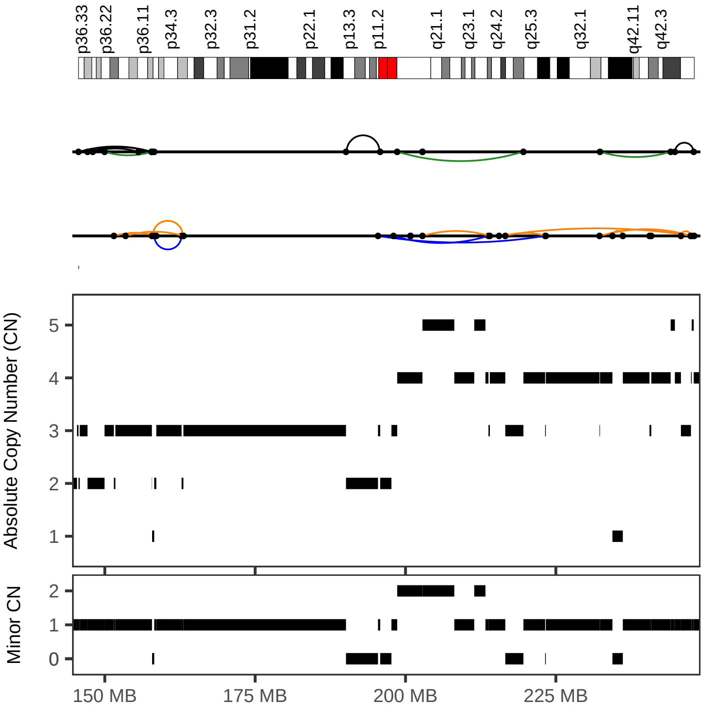

	ebcba7f2−ce13−4bae−97cd−91a6b1dcd465
Cancer type	Lung−AdenoCA
Position	1:145625856−163083972
Type	With other complex events
Interleaved intrachr. SVs	10
Total SVs (intrachr. + transl.)	10
SV types	DEL: 3; DUP: 2; h2hINV: 4; t2tINV: 1; TRA: 0
SVs in sample	52
Oscillating CN (2 and 3 states)	7, 12
CN segments	12
FDR fragment joints	0.64
FDR chr. breakp. enrich.	0
Linked to chrs	
Purity, ploidy	0.85, 1.76

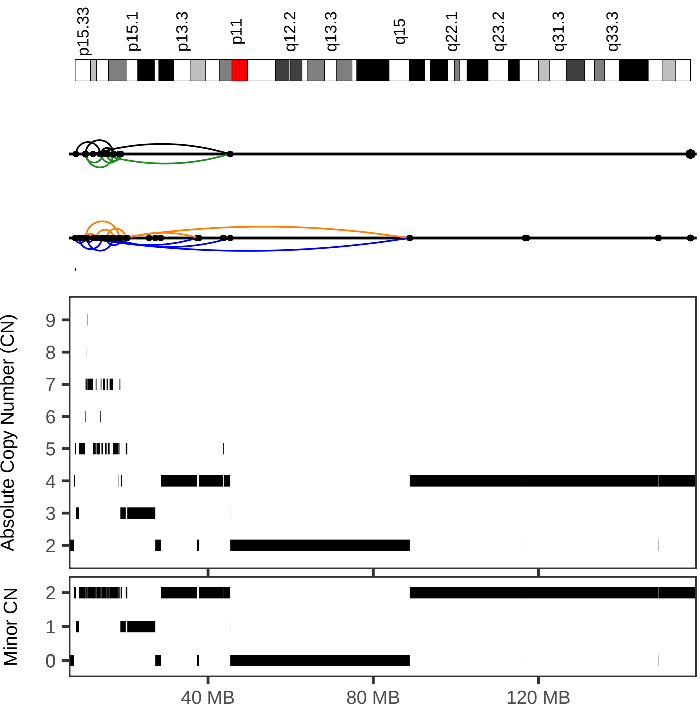

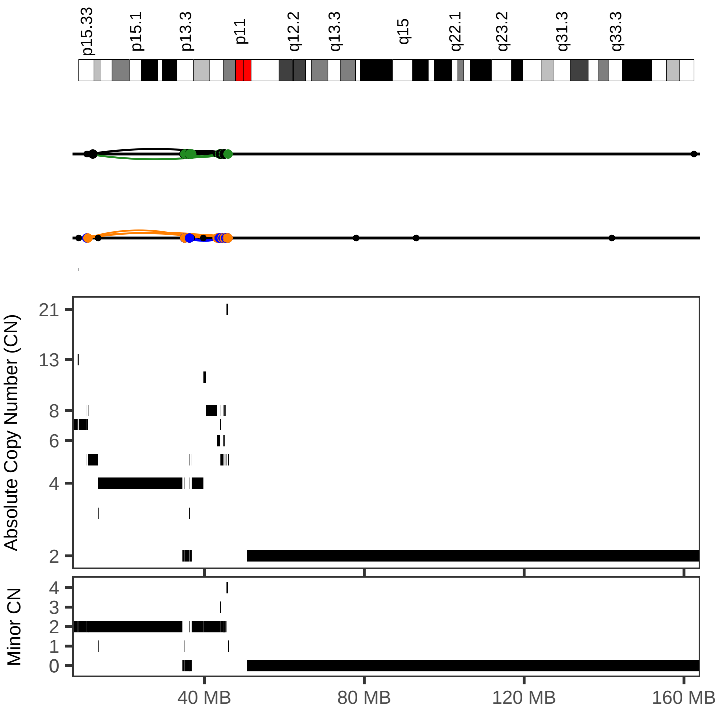

	0398eae1−7216−4595−80a5−6b117d96e070
Cancer type	Lung−SCC
Position	5:7830470−88829799
Type	With other complex events
Interleaved intrachr. SVs	28
Total SVs (intrachr. + transl.)	28
SV types	DEL: 7; DUP: 10; h2hINV: 5; t2tINV: 6; TRA: 0
SVs in sample	209
Oscillating CN (2 and 3 states)	9, 10
CN segments	45
FDR fragment joints	0.64
FDR chr. breakp. enrich.	0
Linked to chrs	
Purity, ploidy	0.57, 3.08

	1ee543d5−b8c0−4f79−8373−6bb6319f2ee2
Cancer type	Lung−SCC
Position	5:8531769−45801499
Type	With other complex events
Interleaved intrachr. SVs	36
Total SVs (intrachr. + transl.)	79
SV types	DEL: 8; DUP: 9; h2hINV: 11; t2tINV: 8; TRA: 43
SVs in sample	287
Oscillating CN (2 and 3 states)	8, 13
CN segments	41
FDR fragment joints	0.91
FDR chr. breakp. enrich.	0
Linked to chrs	
Purity, ploidy	0.4, 2.43

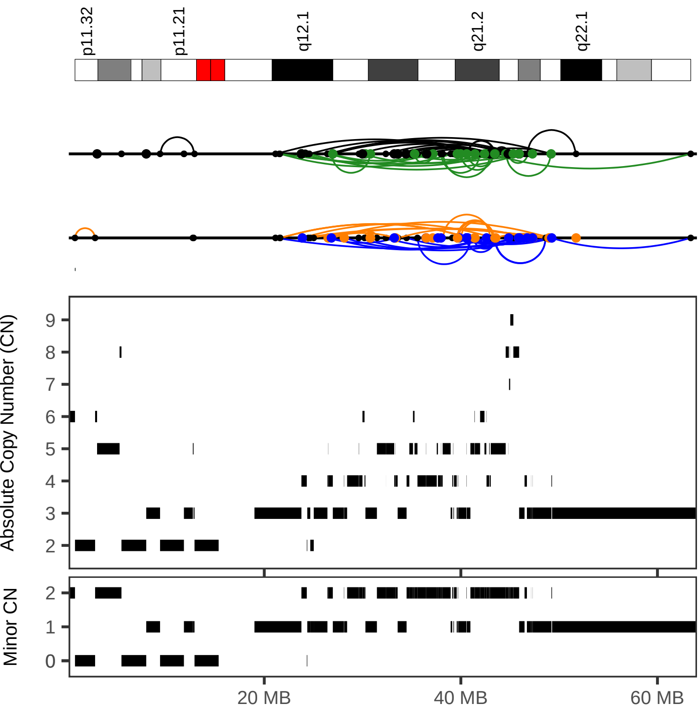

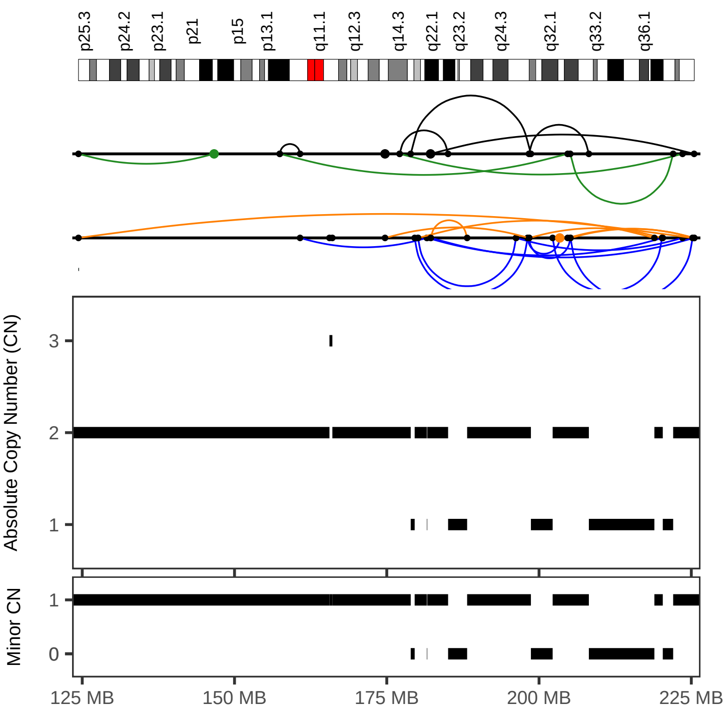

	1f6b2aca−7357−40d1−ba7a−99227d9900a2
Cancer type	Lung−SCC
Position	18:21140095−63388413
Type	With other complex events
Interleaved intrachr. SVs	74
Total SVs (intrachr. + transl.)	159
SV types	DEL: 15; DUP: 19; h2hINV: 20; t2tINV: 20; TRA: 85
SVs in sample	602
Oscillating CN (2 and 3 states)	11, 19
CN segments	81
FDR fragment joints	0.86
FDR chr. breakp. enrich.	0
Linked to chrs	11:46508302−111537991;12:4696888−43872964
Purity, ploidy	0.86, 2.97

	305f72a8−069b−410b−bbe4−4ecb761c748d
Cancer type	Lung−SCC
Position	2:124378520−225481223
Type	Canonical without polyploidization
Interleaved intrachr. SVs	26
Total SVs (intrachr. + transl.)	30
SV types	DEL: 7; DUP: 10; h2hINV: 5; t2tINV: 4; TRA: 4
SVs in sample	92
Oscillating CN (2 and 3 states)	13, 15
CN segments	15
FDR fragment joints	0.59
FDR chr. breakp. enrich.	0
Linked to chrs	
Purity, ploidy	0.72, 1.92

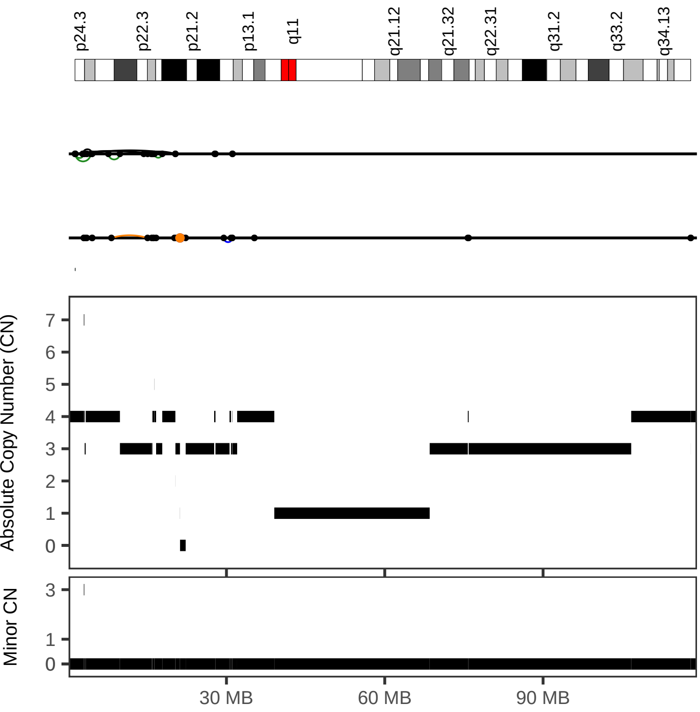

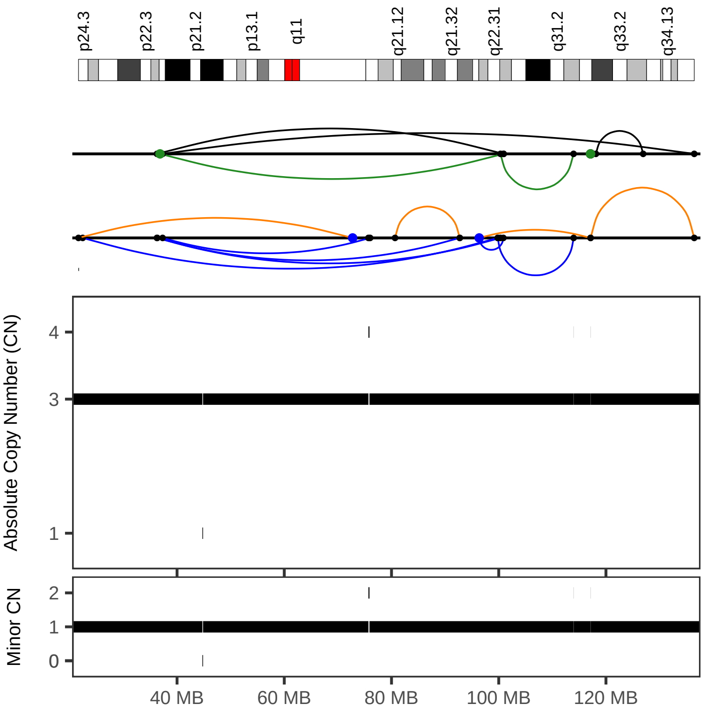

	376dfd27−68e8−4a1a−9c4f−5064279b2a9e
Cancer type	Lung−SCC
Position	9:1296608−20464401
Type	With other complex events
Interleaved intrachr. SVs	13
Total SVs (intrachr. + transl.)	13
SV types	DEL: 3; DUP: 1; h2hINV: 6; t2tINV: 3; TRA: 0
SVs in sample	234
Oscillating CN (2 and 3 states)	7, 7
CN segments	15
FDR fragment joints	0.59
FDR chr. breakp. enrich.	0
Linked to chrs	
Purity, ploidy	0.44, 3.03

	395babb3−3f5d−4e71−a675−af4443f23028
Cancer type	Lung−SCC
Position	9:21616519−136467696
Type	After polyploidization
Interleaved intrachr. SVs	12
Total SVs (intrachr. + transl.)	17
SV types	DEL: 2; DUP: 6; h2hINV: 2; t2tINV: 2; TRA: 5
SVs in sample	113
Oscillating CN (2 and 3 states)	7, 9
CN segments	9
FDR fragment joints	0.59
FDR chr. breakp. enrich.	0
Linked to chrs	
Purity, ploidy	0.38, 3.13

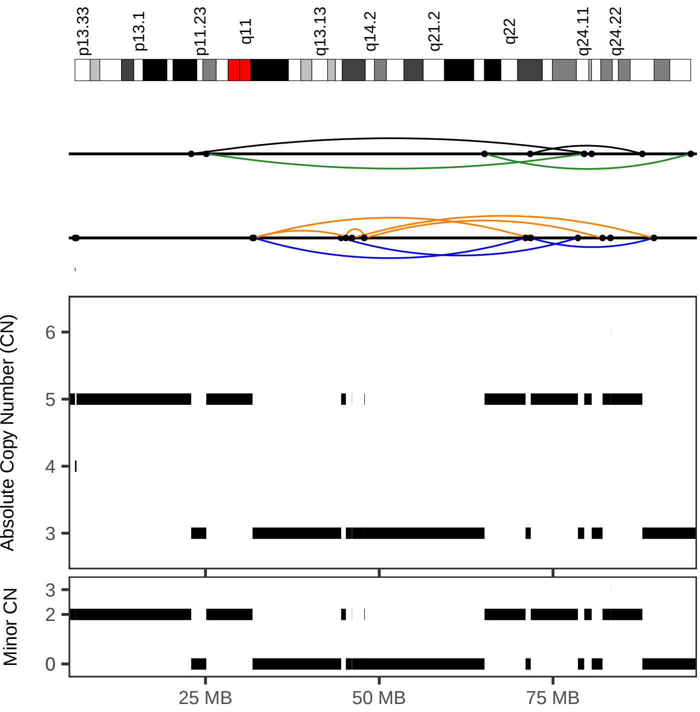

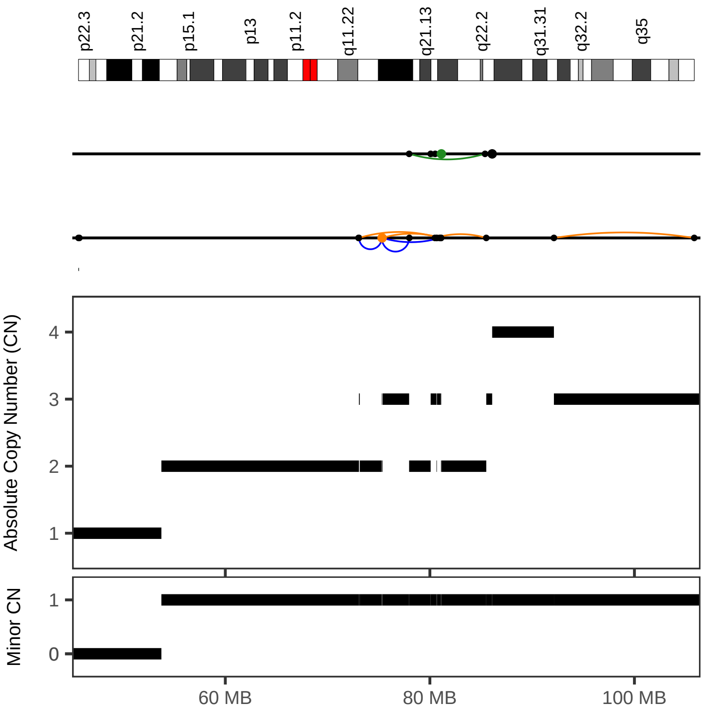

	3edf933a−be1a−46e8−bfb0−acdff30e64a0
Cancer type	Lung−SCC
Position	12:22945549−94815954
Type	After polyploidization
Interleaved intrachr. SVs	13
Total SVs (intrachr. + transl.)	13
SV types	DEL: 6; DUP: 3; h2hINV: 2; t2tINV: 2; TRA: 0
SVs in sample	149
Oscillating CN (2 and 3 states)	16, 19
CN segments	19
FDR fragment joints	0.59
FDR chr. breakp. enrich.	0.03
Linked to chrs	
Purity, ploidy	0.83, 3.06

	422a46b2−a67c−4a7e−923f−9b651ced96f8
Cancer type	Lung−SCC
Position	7:73026297−85512823
Type	Canonical without polyploidization
Interleaved intrachr. SVs	10
Total SVs (intrachr. + transl.)	12
SV types	DEL: 4; DUP: 4; h2hINV: 0; t2tINV: 2; TRA: 2
SVs in sample	76
Oscillating CN (2 and 3 states)	14, 14
CN segments	14
FDR fragment joints	0.59
FDR chr. breakp. enrich.	0
Linked to chrs	2:65460344−182013218;
Purity, ploidy	0.55, 1.97

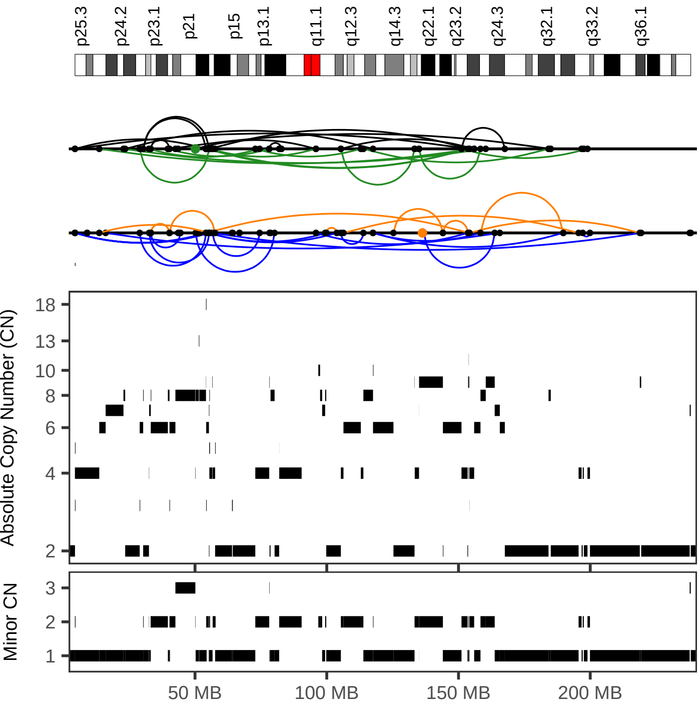

	43abe847−4ba7−466e−8283−5d7b80b999a7
Cancer type	Lung−SCC
Position	2:4435309−219331448
Type	With other complex events
Interleaved intrachr. SVs	63
Total SVs (intrachr. + transl.)	65
SV types	DEL: 13; DUP: 18; h2hINV: 15; t2tINV: 17; TRA: 2
SVs in sample	500
Oscillating CN (2 and 3 states)	7, 9
CN segments	92
FDR fragment joints	0.86
FDR chr. breakp. enrich.	0
Linked to chrs	
Purity, ploidy	0.69, 3.74

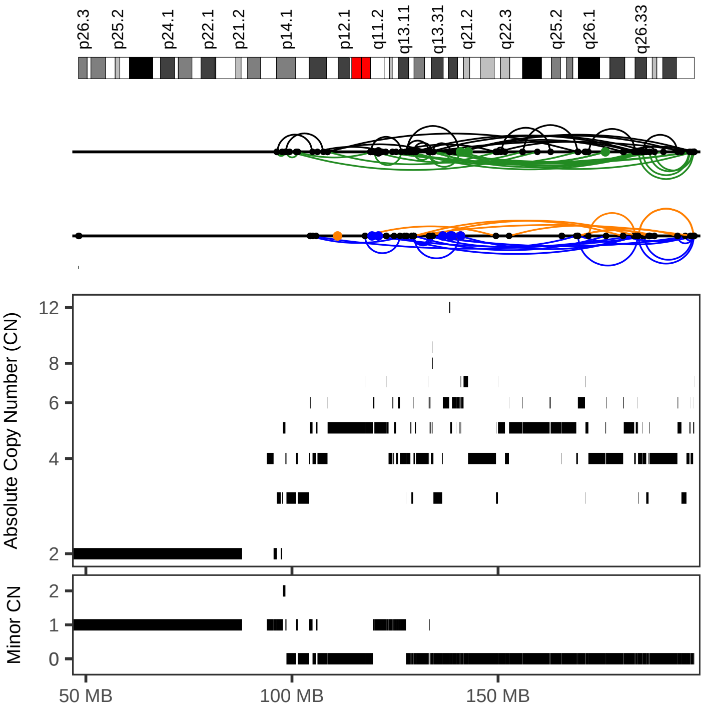

	43abe847−4ba7−466e−8283−5d7b80b999a7
Cancer type	Lung−SCC
Position	3:96334427−197597663
Type	With other complex events
Interleaved intrachr. SVs	95
Total SVs (intrachr. + transl.)	109
SV types	DEL: 13; DUP: 25; h2hINV: 30; t2tINV: 27; TRA: 14
SVs in sample	500
Oscillating CN (2 and 3 states)	7, 12
CN segments	122
FDR fragment joints	0.48
FDR chr. breakp. enrich.	0
Linked to chrs	5:932078−55824518;
Purity, ploidy	0.69, 3.74

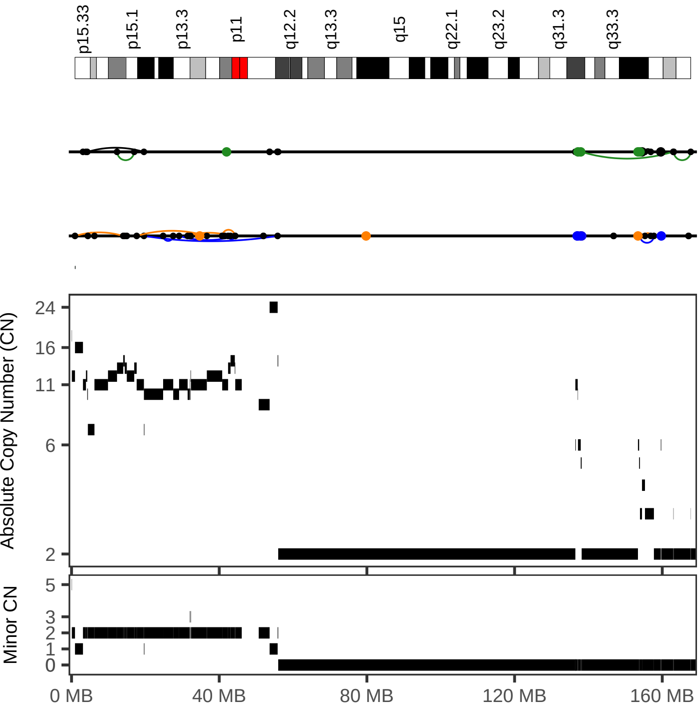

	43abe847−4ba7−466e−8283−5d7b80b999a7
Cancer type	Lung−SCC
Position	5:932078−55824519
Type	With other complex events
Interleaved intrachr. SVs	9
Total SVs (intrachr. + transl.)	11
SV types	DEL: 4; DUP: 3; h2hINV: 1; t2tINV: 1; TRA: 2
SVs in sample	500
Oscillating CN (2 and 3 states)	7, 12
CN segments	32
FDR fragment joints	0.59
FDR chr. breakp. enrich.	0.01
Linked to chrs	
Purity, ploidy	0.69, 3.74

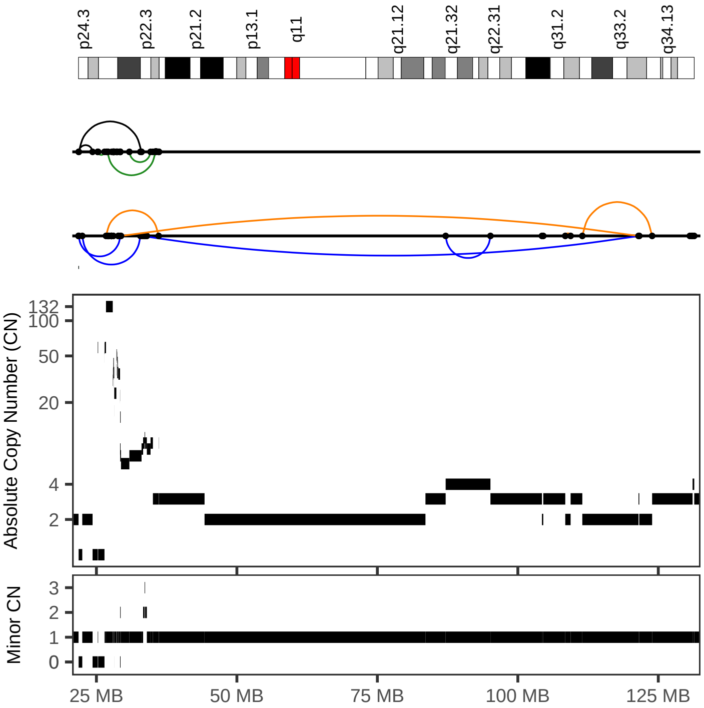

	43abe847−4ba7−466e−8283−5d7b80b999a7
Cancer type	Lung−SCC
Position	9:21846780−123901906
Type	With other complex events
Interleaved intrachr. SVs	14
Total SVs (intrachr. + transl.)	14
SV types	DEL: 3; DUP: 3; h2hINV: 4; t2tINV: 4; TRA: 0
SVs in sample	500
Oscillating CN (2 and 3 states)	9, 13
CN segments	59
FDR fragment joints	0.98
FDR chr. breakp. enrich.	0
Linked to chrs	
Purity, ploidy	0.69, 3.74

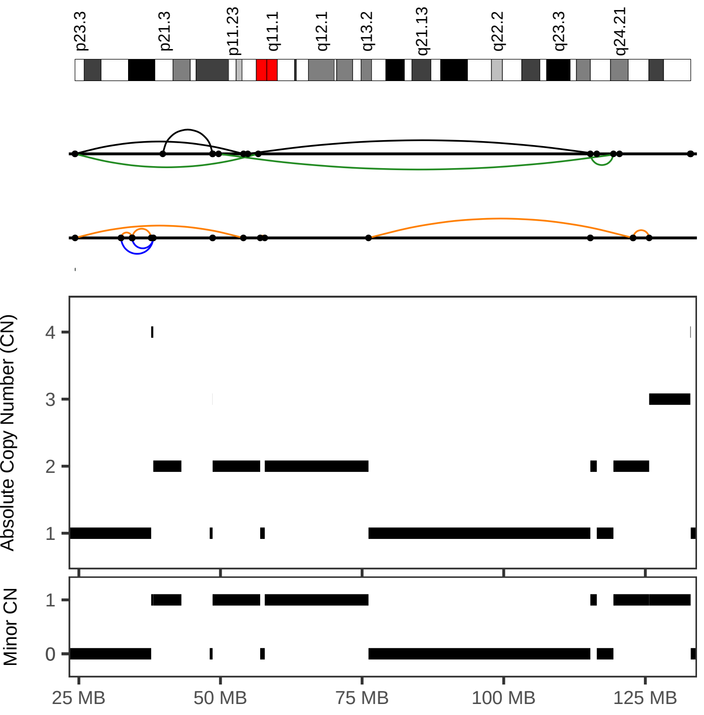

	4ca13c92−84b4−4edf−842a−b20b7e713415
Cancer type	Lung−SCC
Position	8:24315943−122822158
Type	With other complex events
Interleaved intrachr. SVs	7
Total SVs (intrachr. + transl.)	7
SV types	DEL: 2; DUP: 0; h2hINV: 2; t2tINV: 3; TRA: 0
SVs in sample	103
Oscillating CN (2 and 3 states)	7, 11
CN segments	13
FDR fragment joints	0.64
FDR chr. breakp. enrich.	0
Linked to chrs	
Purity, ploidy	0.4, 1.72

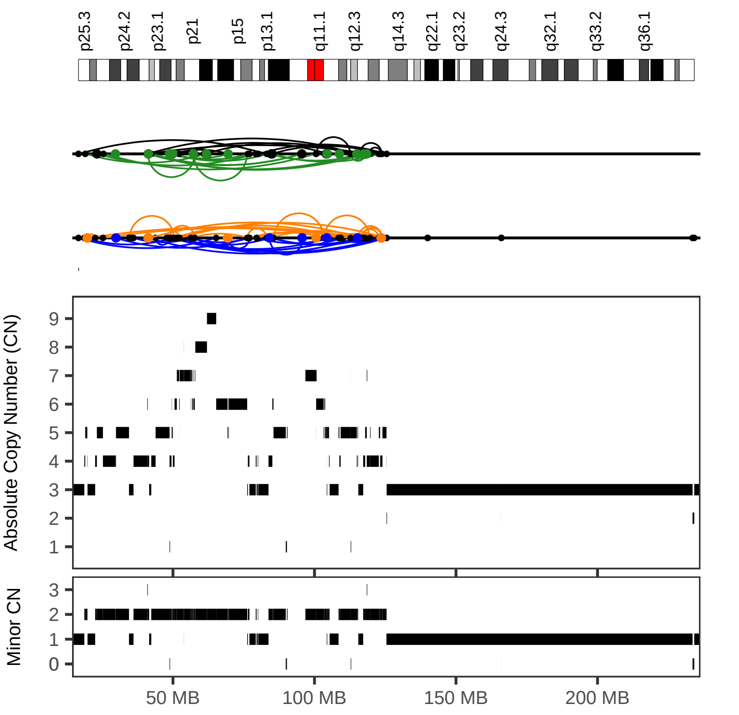

	7b982d5e−3a7d−40ac−bd25−6044c62879b6
Cancer type	Lung−SCC
Position	2:16626479−125491838
Type	With other complex events
Interleaved intrachr. SVs	70
Total SVs (intrachr. + transl.)	110
SV types	DEL: 21; DUP: 20; h2hINV: 11; t2tINV: 18; TRA: 40
SVs in sample	349
Oscillating CN (2 and 3 states)	8, 14
CN segments	97
FDR fragment joints	0.59
FDR chr. breakp. enrich.	0
Linked to chrs	18:5099437−27048292;3:30056583−192447181
Purity, ploidy	0.45, 2.92

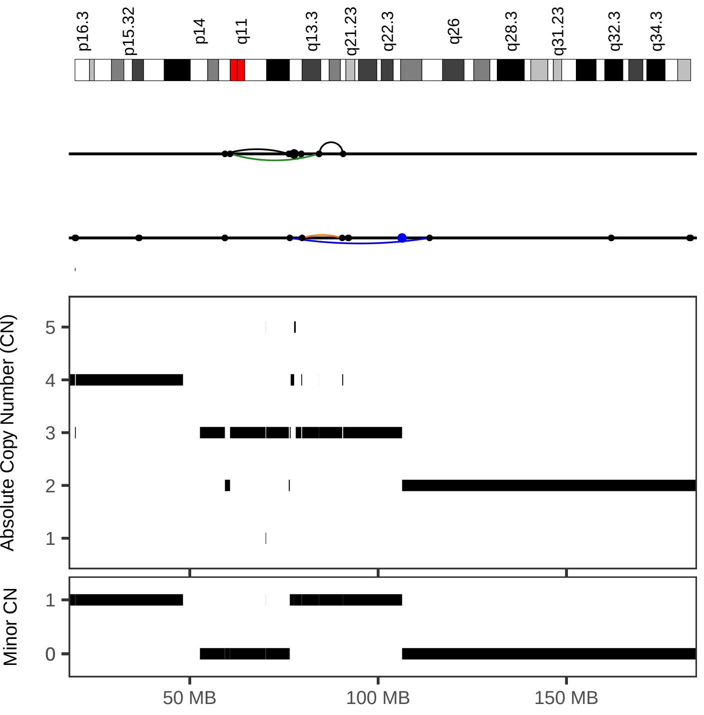

	7b982d5e−3a7d−40ac−bd25−6044c62879b6
Cancer type	Lung−SCC
Position	4:59343654−113670691
Type	With other complex events
Interleaved intrachr. SVs	5
Total SVs (intrachr. + transl.)	7
SV types	DEL: 1; DUP: 1; h2hINV: 2; t2tINV: 1; TRA: 2
SVs in sample	349
Oscillating CN (2 and 3 states)	7, 8
CN segments	17
FDR fragment joints	0.92
FDR chr. breakp. enrich.	0.24
Linked to chrs	
Purity, ploidy	0.45, 2.92

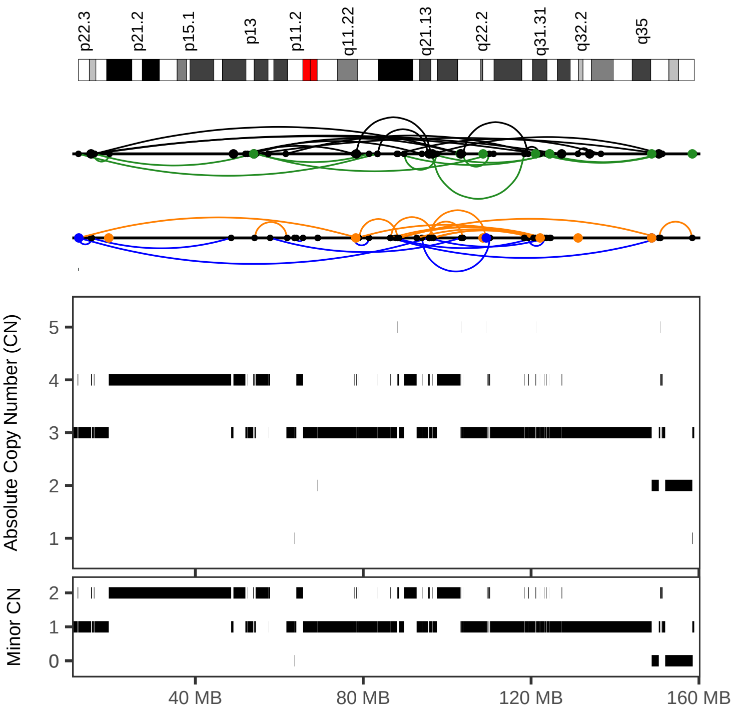

	7b982d5e−3a7d−40ac−bd25−6044c62879b6
Cancer type	Lung−SCC
Position	7:12149298−158873626
Type	With other complex events
Interleaved intrachr. SVs	53
Total SVs (intrachr. + transl.)	80
SV types	DEL: 14; DUP: 12; h2hINV: 14; t2tINV: 13; TRA: 27
SVs in sample	349
Oscillating CN (2 and 3 states)	26, 26
CN segments	100
FDR fragment joints	0.99
FDR chr. breakp. enrich.	0
Linked to chrs	18:5099437−27048292;
Purity, ploidy	0.45, 2.92

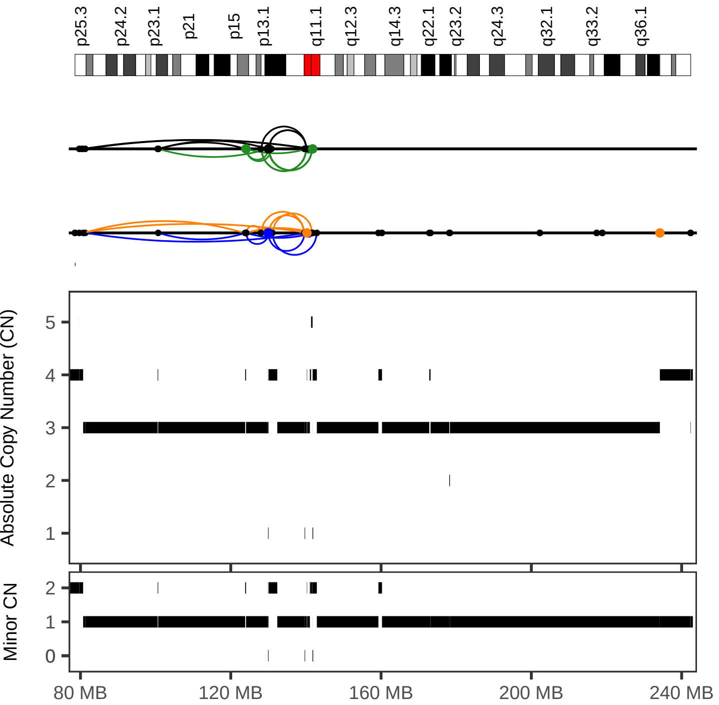

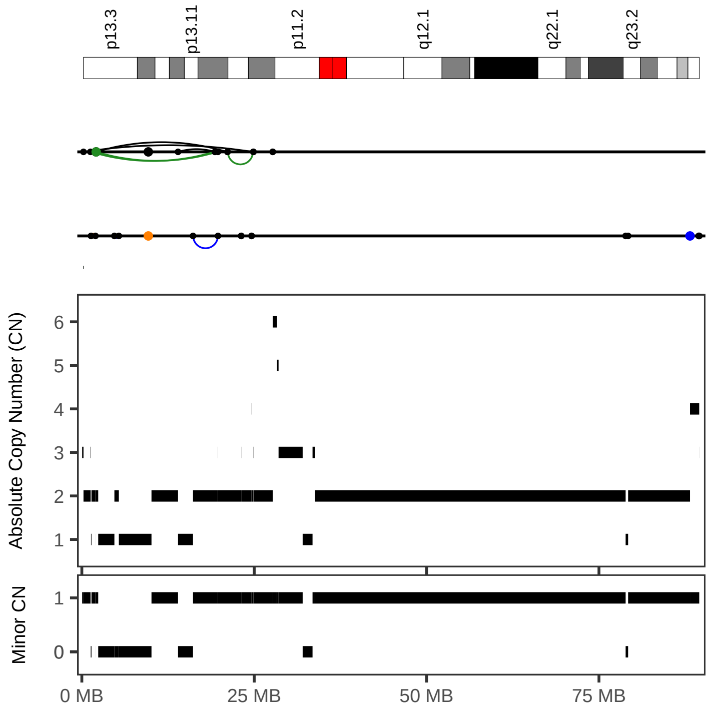

7d905f8f−3967−4ea8−96c8−17b1b03fbec3

Cancer type	Lung−SCC
Position	2:79670598−142912803
Type	With other complex events
Interleaved intrachr. SVs	39
Total SVs (intrachr. + transl.)	47
SV types	DEL: 12; DUP: 8; h2hINV: 8; t2tINV: 11; TRA: 8
SVs in sample	385
Oscillating CN (2 and 3 states)	8, 9
CN segments	26
FDR fragment joints	0.81
FDR chr. breakp. enrich.	0
Linked to chrs	20:49249923−49538223;
Purity, ploidy	0.57, 2.6

7d905f8f−3967−4ea8−96c8−17b1b03fbec3

Cancer type	Lung−SCC
Position	16:1242659−24867894
Type	With other complex events
Interleaved intrachr. SVs	7
Total SVs (intrachr. + transl.)	11
SV types	DEL: 1; DUP: 1; h2hINV: 2; t2tINV: 3; TRA: 4
SVs in sample	385
Oscillating CN (2 and 3 states)	11, 11
CN segments	19
FDR fragment joints	0.74
FDR chr. breakp. enrich.	0.01
Linked to chrs	
Purity, ploidy	0.57, 2.6

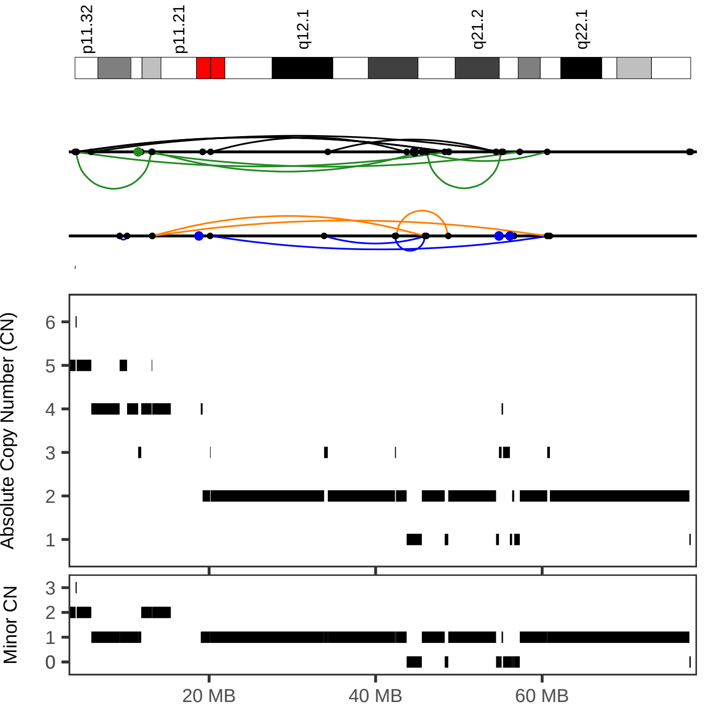

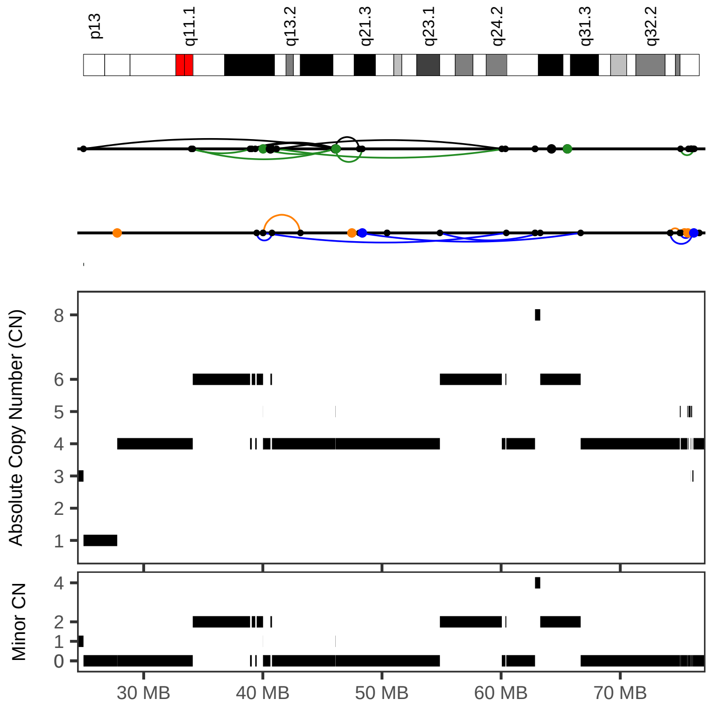

7d905f8f−3967−4ea8−96c8−17b1b03fbec3

Cancer type	Lung−SCC
Position	18:3889958−60926976
Type	With other complex events
Interleaved intrachr. SVs	18
Total SVs (intrachr. + transl.)	24
SV types	DEL: 3; DUP: 3; h2hINV: 6; t2tINV: 6; TRA: 6
SVs in sample	385
Oscillating CN (2 and 3 states)	7, 12
CN segments	32
FDR fragment joints	0.64
FDR chr. breakp. enrich.	0
Linked to chrs	
Purity, ploidy	0.57, 2.6

9af6ed4e−8cdc−4f49−84e9−ba1053b5b3ca

Cancer type	Lung−SCC
Position	14:24946194−66674963
Type	With other complex events
Interleaved intrachr. SVs	15
Total SVs (intrachr. + transl.)	25
SV types	DEL: 1; DUP: 4; h2hINV: 5; t2tINV: 5; TRA: 10
SVs in sample	428
Oscillating CN (2 and 3 states)	7, 17
CN segments	21
FDR fragment joints	0.62
FDR chr. breakp. enrich.	0
Linked to chrs	3:48477064−188733552;
Purity, ploidy	0.82, 3.26

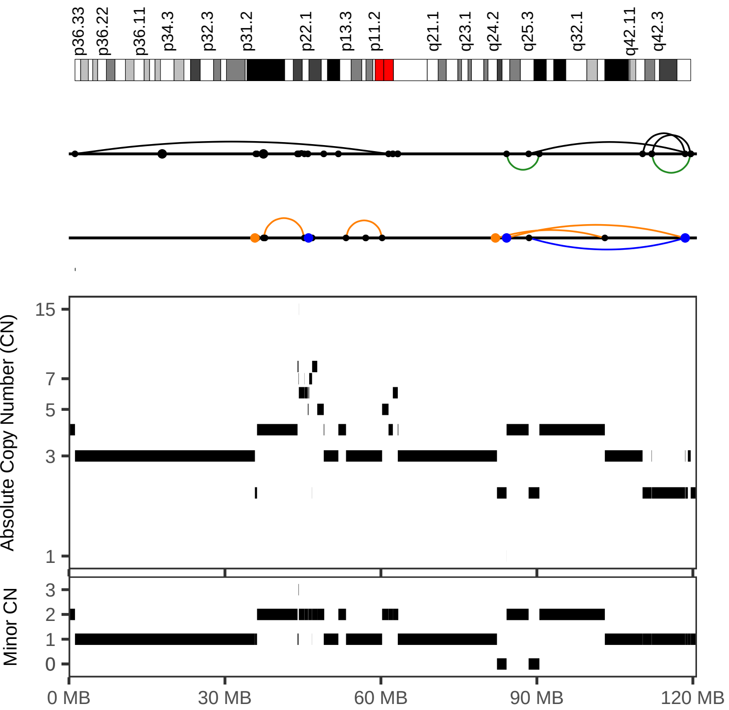

	adc853b1−b8bf−488c−a1e2−e95603459b55
Cancer type	Lung−SCC
Position	1:82323412−119584894
Type	Canonical without polyploidization
Interleaved intrachr. SVs	8
Total SVs (intrachr. + transl.)	10
SV types	DEL: 2; DUP: 1; h2hINV: 3; t2tINV: 2; TRA: 2
SVs in sample	176
Oscillating CN (2 and 3 states)	8, 8
CN segments	13
FDR fragment joints	0.84
FDR chr. breakp. enrich.	0
Linked to chrs	
Purity, ploidy	0.41, 3.04

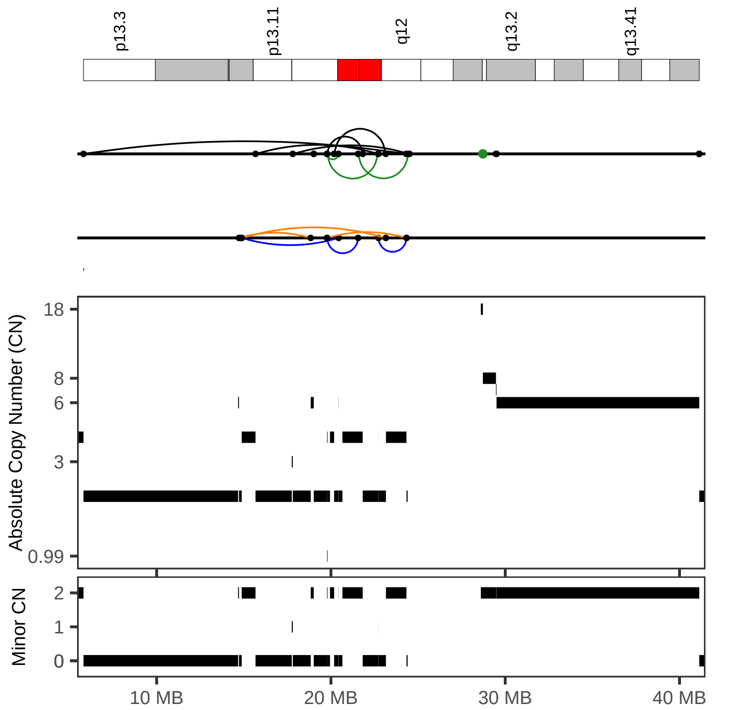

	ce8612ab−3149−4a6a−b424−29c0c21c9b8b
Cancer type	Lung−SCC
Position	19:5801908−24509732
Type	With other complex events
Interleaved intrachr. SVs	14
Total SVs (intrachr. + transl.)	14
SV types	DEL: 3; DUP: 3; h2hINV: 5; t2tINV: 3; TRA: 0
SVs in sample	213
Oscillating CN (2 and 3 states)	11, 18
CN segments	27
FDR fragment joints	0.88
FDR chr. breakp. enrich.	0
Linked to chrs	
Purity, ploidy	0.59, 3.12

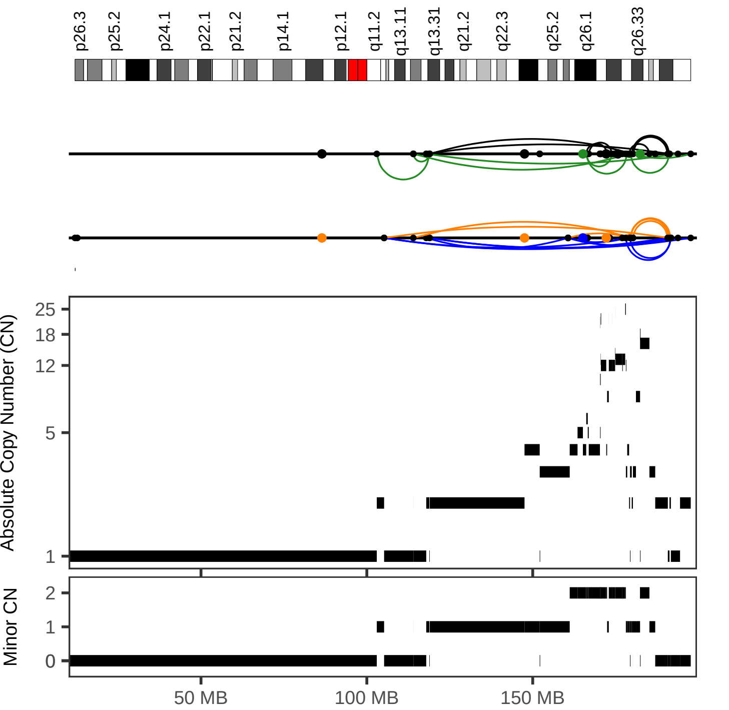

	d4bc755a−2585−4529−ae36−7e1d88bdecfe
Cancer type	Lung−SCC
Position	3:102997353−197639661
Type	With other complex events
Interleaved intrachr. SVs	39
Total SVs (intrachr. + transl.)	47
SV types	DEL: 8; DUP: 11; h2hINV: 9; t2tINV: 11; TRA: 8
SVs in sample	96
Oscillating CN (2 and 3 states)	7, 7
CN segments	56
FDR fragment joints	0.91
FDR chr. breakp. enrich.	0
Linked to chrs	
Purity, ploidy	0.74, 1.93

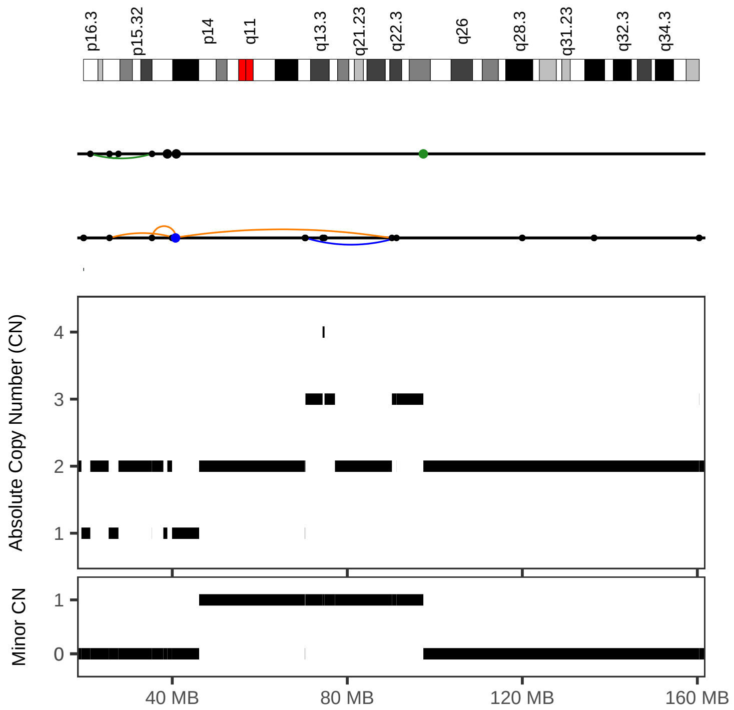

	fd9ee494−65fe−4de4−adff−7952a059b17f
Cancer type	Lung−SCC
Position	4:21261215−91243140
Type	Canonical without polyploidization
Interleaved intrachr. SVs	6
Total SVs (intrachr. + transl.)	9
SV types	DEL: 3; DUP: 1; h2hINV: 0; t2tINV: 2; TRA: 3
SVs in sample	273
Oscillating CN (2 and 3 states)	11, 17
CN segments	17
FDR fragment joints	0.59
FDR chr. breakp. enrich.	0.9
Linked to chrs	12:6510050−132119133;
Purity, ploidy	0.42, 2.74

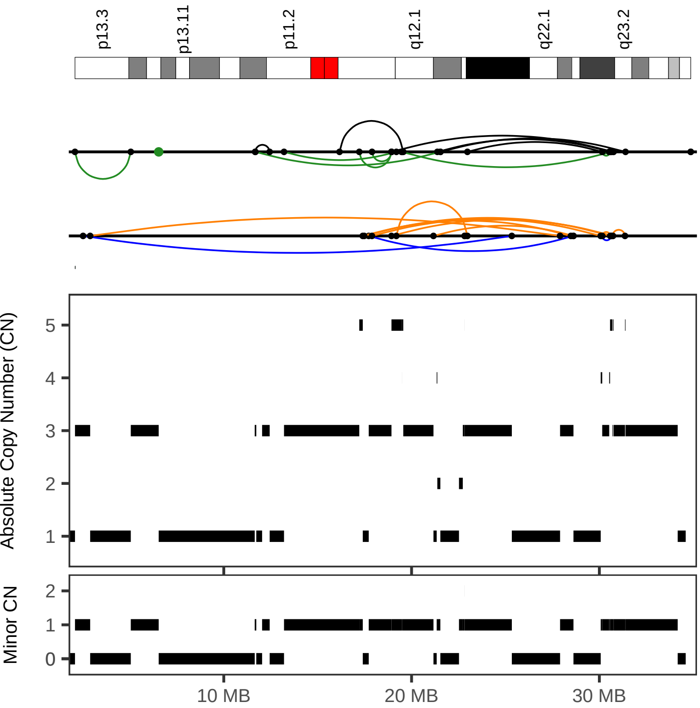

	**a8e2df1e−4042−42af−9231−3a00e83489f0**
Cancer type	Lymph−BNHL
Position	16:2075019−31393909
Type	With other complex events
Interleaved intrachr. SVs	28
Total SVs (intrachr. + transl.)	29
SV types	DEL: 9; DUP: 4; h2hINV: 7; t2tINV: 8; TRA: 1
SVs in sample	112
Oscillating CN (2 and 3 states)	9, 9
CN segments	36
FDR fragment joints	0.64
FDR chr. breakp. enrich.	0
Linked to chrs	
Purity, ploidy	0.88, 1.98

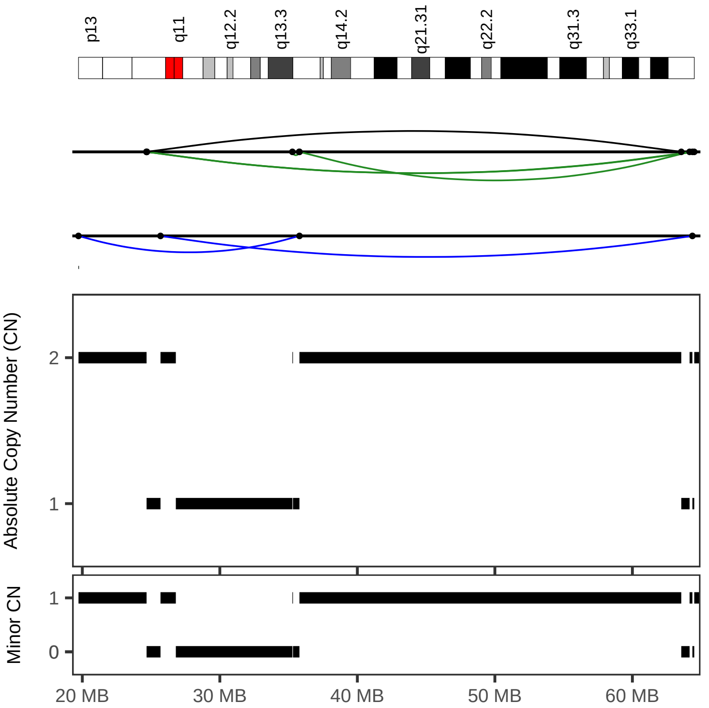

	**f0a326d2−1f3e−4a5d−bca8−32aaccc52338**
Cancer type	Lymph−BNHL
Position	13:19719472−64497953
Type	Canonical without polyploidization
Interleaved intrachr. SVs	7
Total SVs (intrachr. + transl.)	7
SV types	DEL: 0; DUP: 2; h2hINV: 2; t2tINV: 3; TRA: 0
SVs in sample	194
Oscillating CN (2 and 3 states)	11, 11
CN segments	11
FDR fragment joints	0.64
FDR chr. breakp. enrich.	0.78
Linked to chrs	
Purity, ploidy	0.8, 2.27

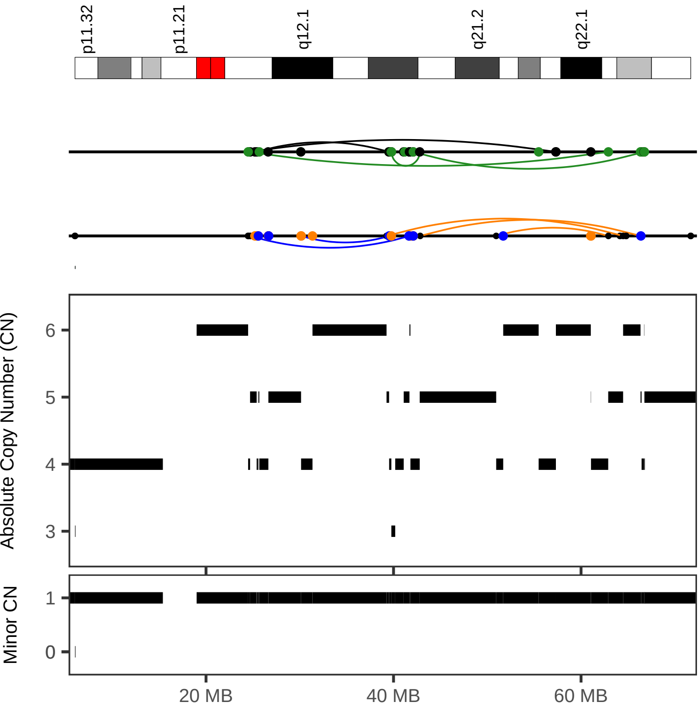

	**f0a326d2−1f3e−4a5d−bca8−32aaccc52338**
Cancer type	Lymph−BNHL
Position	18:24492930−66759172
Type	With other complex events
Interleaved intrachr. SVs	11
Total SVs (intrachr. + transl.)	68
SV types	DEL: 3; DUP: 3; h2hINV: 2; t2tINV: 3; TRA: 57
SVs in sample	194
Oscillating CN (2 and 3 states)	13, 15
CN segments	37
FDR fragment joints	0.98
FDR chr. breakp. enrich.	0
Linked to chrs	9:8189397−96357053;
Purity, ploidy	0.8, 2.27

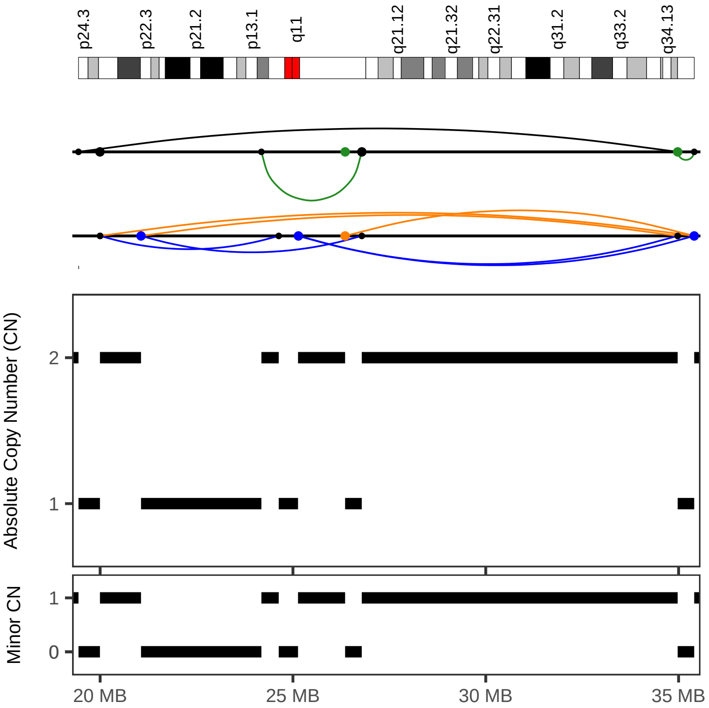

	**4108992**
Cancer type	Lymph−BNHL
Position	9:19438790−35406023
Type	Canonical without polyploidization
Interleaved intrachr. SVs	10
Total SVs (intrachr. + transl.)	18
SV types	DEL: 3; DUP: 4; h2hINV: 1; t2tINV: 2; TRA: 8
SVs in sample	244
Oscillating CN (2 and 3 states)	10, 10
CN segments	10
FDR fragment joints	0.64
FDR chr. breakp. enrich.	0.05
Linked to chrs	3:10293357−118705636;
Purity, ploidy	0.77, 1.88

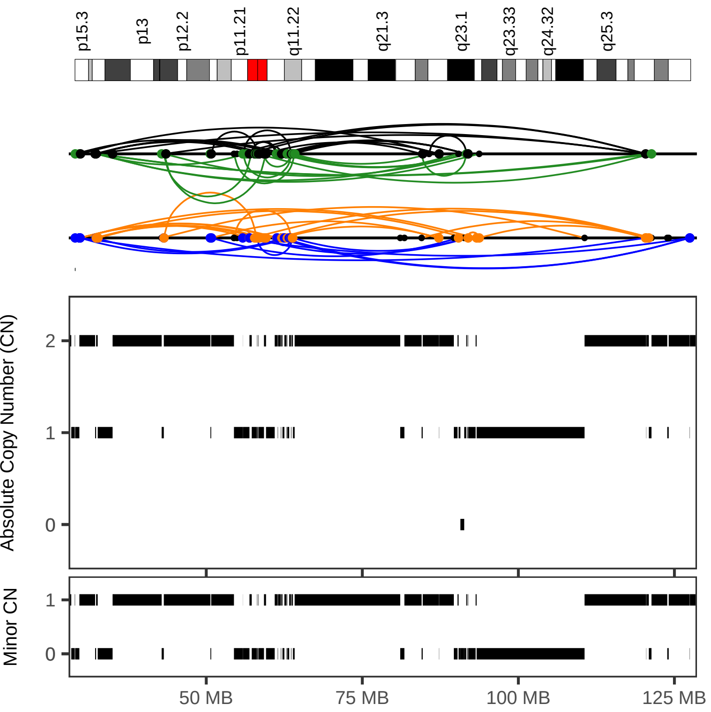

	**4108992**
Cancer type	Lymph−BNHL
Position	10:28967917−127614040
Type	Canonical without polyploidization
Interleaved intrachr. SVs	76
Total SVs (intrachr. + transl.)	171
SV types	DEL: 20; DUP: 13; h2hINV: 22; t2tINV: 21; TRA: 95
SVs in sample	244
Oscillating CN (2 and 3 states)	57, 76
CN segments	76
FDR fragment joints	0.64
FDR chr. breakp. enrich.	0
Linked to chrs	
Purity, ploidy	0.77, 1.88

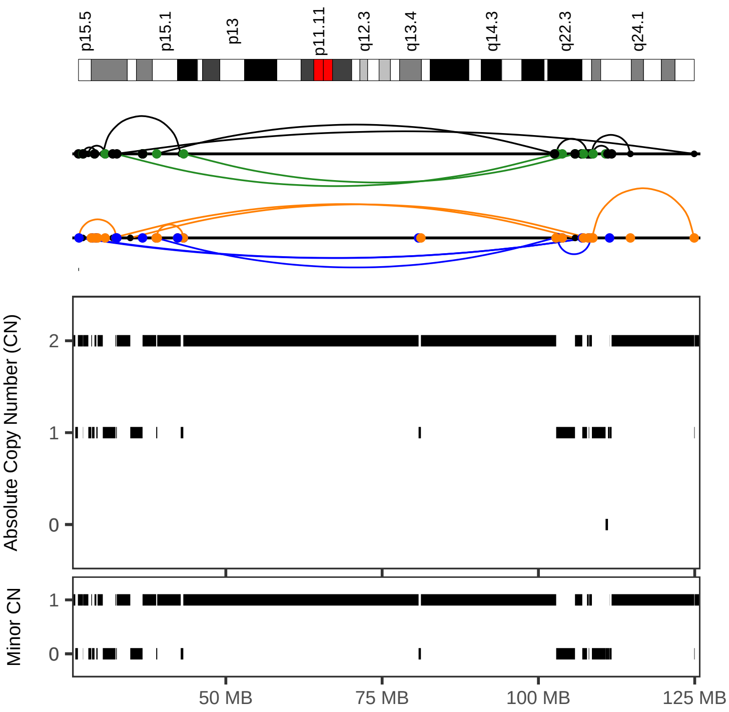

	**4108992**
Cancer type	Lymph−BNHL
Position	11:26426720−124921346
Type	Canonical without polyploidization
Interleaved intrachr. SVs	20
Total SVs (intrachr. + transl.)	91
SV types	DEL: 6; DUP: 5; h2hINV: 7; t2tINV: 2; TRA: 71
SVs in sample	244
Oscillating CN (2 and 3 states)	30, 36
CN segments	36
FDR fragment joints	0.63
FDR chr. breakp. enrich.	0
Linked to chrs	10:28967917−127614039;
Purity, ploidy	0.77, 1.88

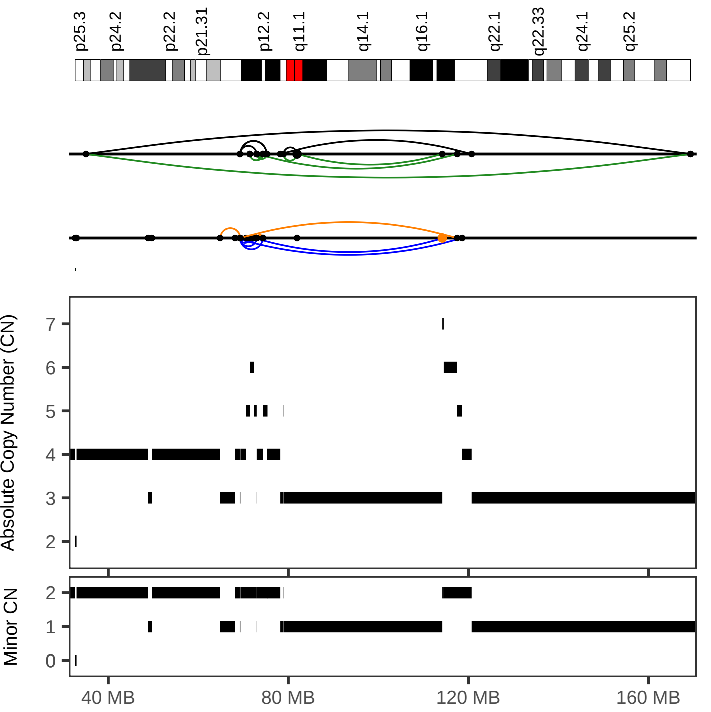

	**4111337**
Cancer type	Lymph−BNHL
Position	6:64848966−120732911
Type	With other complex events
Interleaved intrachr. SVs	21
Total SVs (intrachr. + transl.)	23
SV types	DEL: 4; DUP: 7; h2hINV: 5; t2tINV: 5; TRA: 2
SVs in sample	226
Oscillating CN (2 and 3 states)	7, 9
CN segments	27
FDR fragment joints	0.87
FDR chr. breakp. enrich.	0
Linked to chrs	1:152168528−204980294;
Purity, ploidy	0.62, 4.03

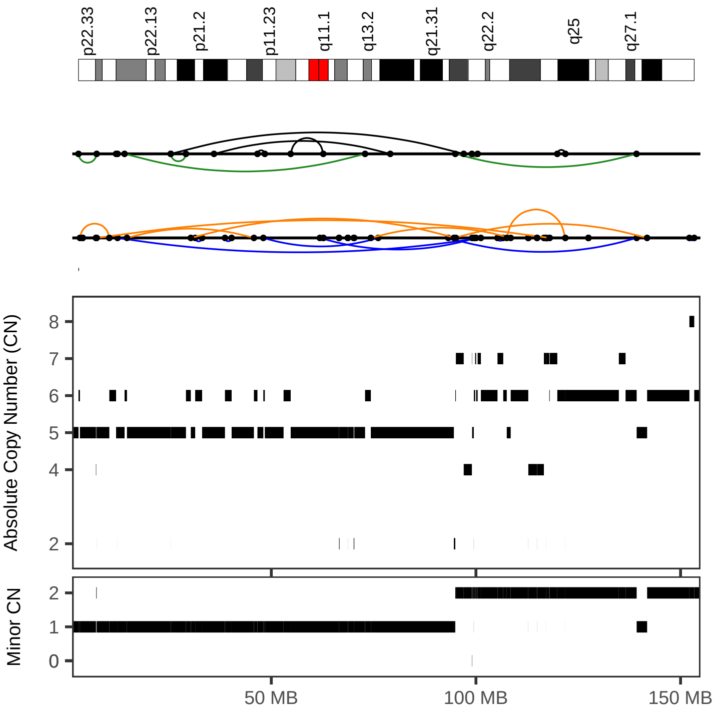

	**4111337**
Cancer type	Lymph−BNHL
Position	X:2879067−141829230
Type	With other complex events
Interleaved intrachr. SVs	23
Total SVs (intrachr. + transl.)	23
SV types	DEL: 7; DUP: 5; h2hINV: 6; t2tINV: 5; TRA: 0
SVs in sample	226
Oscillating CN (2 and 3 states)	13, 32
CN segments	66
FDR fragment joints	0.94
FDR chr. breakp. enrich.	0
Linked to chrs	
Purity, ploidy	0.62, 4.03

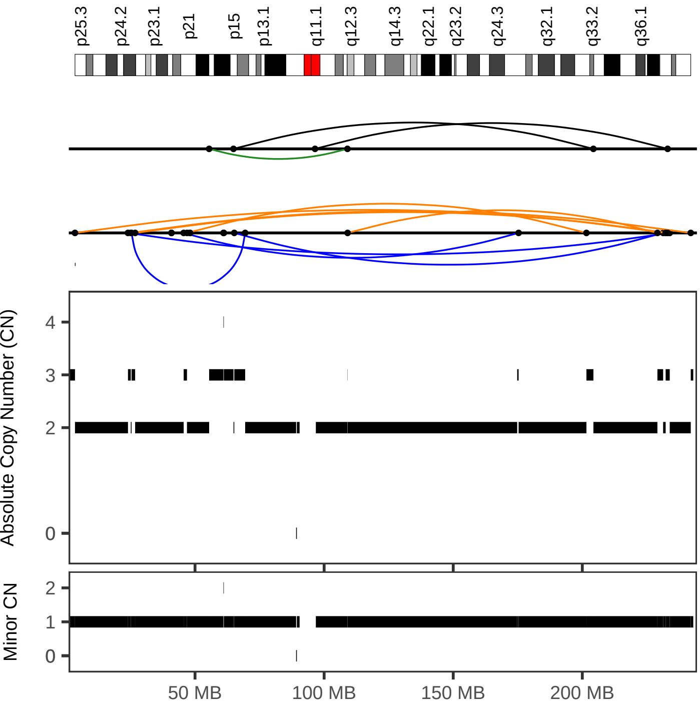

	**4120193**
Cancer type	Lymph−BNHL
Position	2:3501118−241999895
Type	With other complex events
Interleaved intrachr. SVs	12
Total SVs (intrachr. + transl.)	12
SV types	DEL: 5; DUP: 4; h2hINV: 2; t2tINV: 1; TRA: 0
SVs in sample	101
Oscillating CN (2 and 3 states)	12, 13
CN segments	27
FDR fragment joints	0.59
FDR chr. breakp. enrich.	0.11
Linked to chrs	
Purity, ploidy	0.47, 2.12

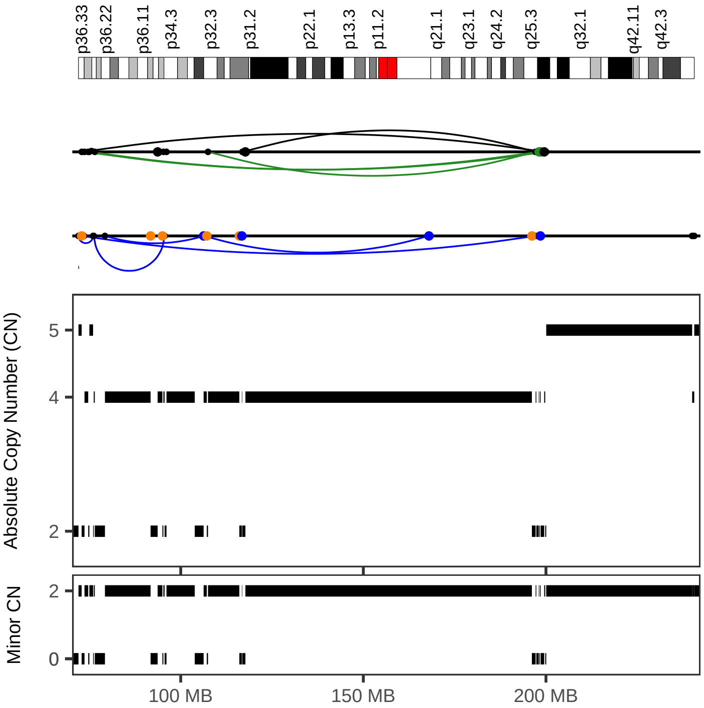

	**4134434**
Cancer type	Lymph−BNHL
Position	1:72008571−200073394
Type	Before polyploidization
Interleaved intrachr. SVs	13
Total SVs (intrachr. + transl.)	29
SV types	DEL: 0; DUP: 5; h2hINV: 3; t2tINV: 5; TRA: 16
SVs in sample	88
Oscillating CN (2 and 3 states)	27, 33
CN segments	33
FDR fragment joints	0.59
FDR chr. breakp. enrich.	0
Linked to chrs	5:99083900−158291676;
Purity, ploidy	0.82, 3.31

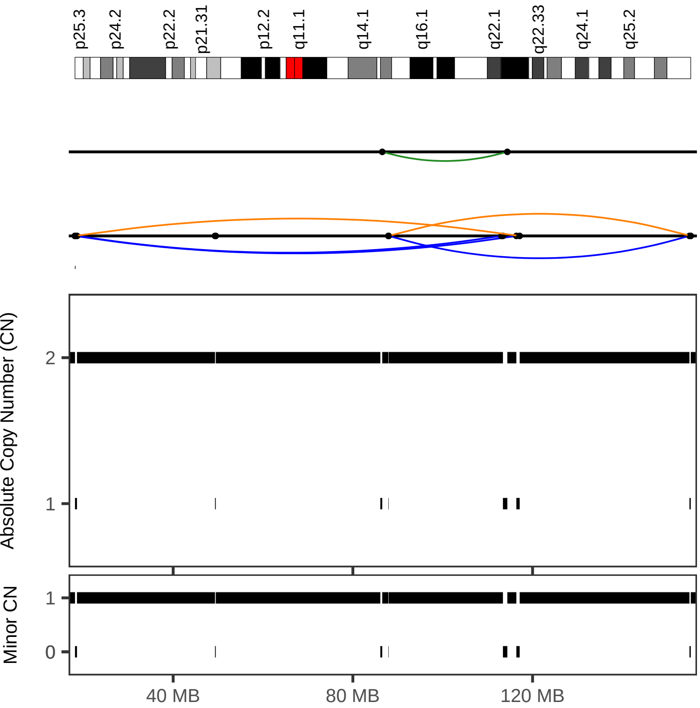

	**4139696**
Cancer type	Lymph−BNHL
Position	6:18142908−155212520
Type	Canonical without polyploidization
Interleaved intrachr. SVs	6
Total SVs (intrachr. + transl.)	6
SV types	DEL: 2; DUP: 3; h2hINV: 0; t2tINV: 1; TRA: 0
SVs in sample	33
Oscillating CN (2 and 3 states)	15, 15
CN segments	15
FDR fragment joints	0.59
FDR chr. breakp. enrich.	0
Linked to chrs	
Purity, ploidy	0.44, 2.02

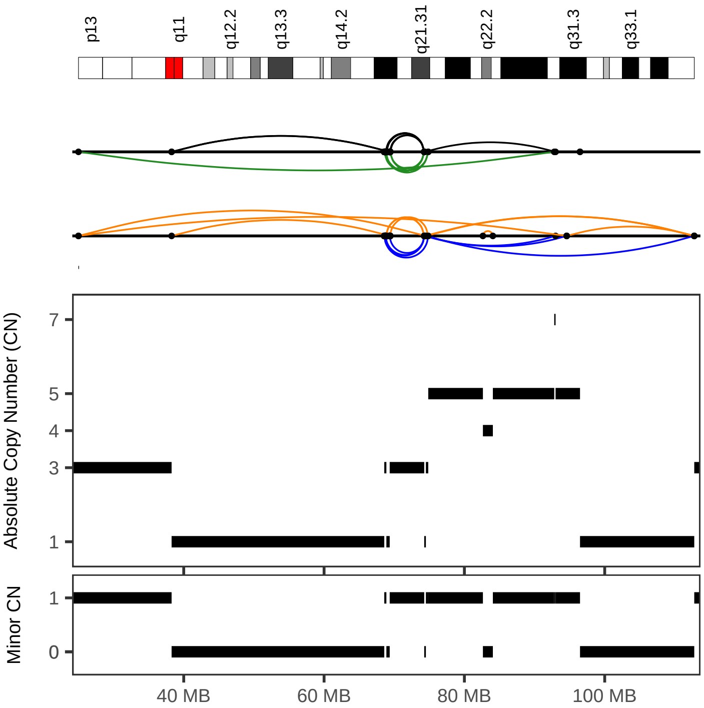

	**4144951**
Cancer type	Lymph−BNHL
Position	13:24997688−112761208
Type	With other complex events
Interleaved intrachr. SVs	77
Total SVs (intrachr. + transl.)	77
SV types	DEL: 18; DUP: 22; h2hINV: 18; t2tINV: 19; TRA: 0
SVs in sample	103
Oscillating CN (2 and 3 states)	7, 7
CN segments	14
FDR fragment joints	0.93
FDR chr. breakp. enrich.	0
Linked to chrs	
Purity, ploidy	0.72, 2.09

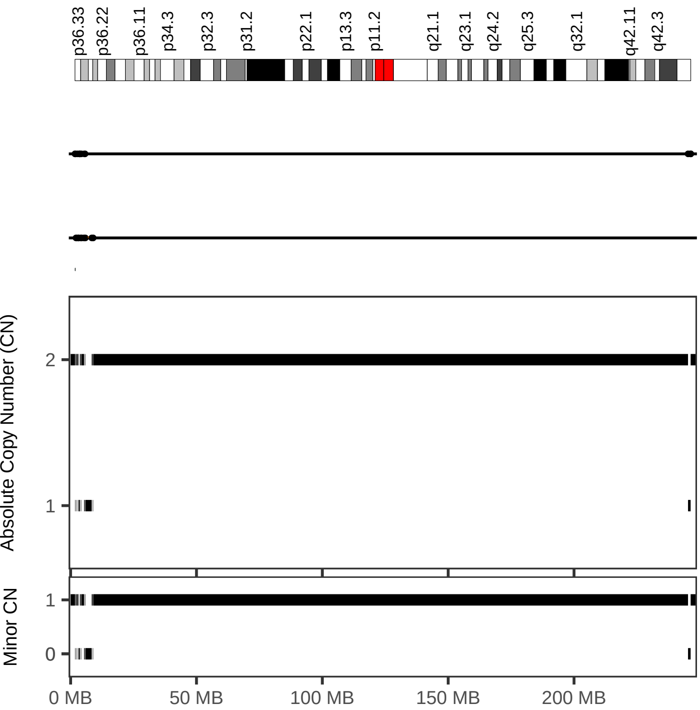

	4145177
Cancer type	Lymph−BNHL
Position	1:1727046−8960415
Type	Canonical without polyploidization
Interleaved intrachr. SVs	13
Total SVs (intrachr. + transl.)	13
SV types	DEL: 4; DUP: 3; h2hINV: 3; t2tINV: 3; TRA: 0
SVs in sample	55
Oscillating CN (2 and 3 states)	26, 26
CN segments	26
FDR fragment joints	0.98
FDR chr. breakp. enrich.	0
Linked to chrs	
Purity, ploidy	0.8, 2

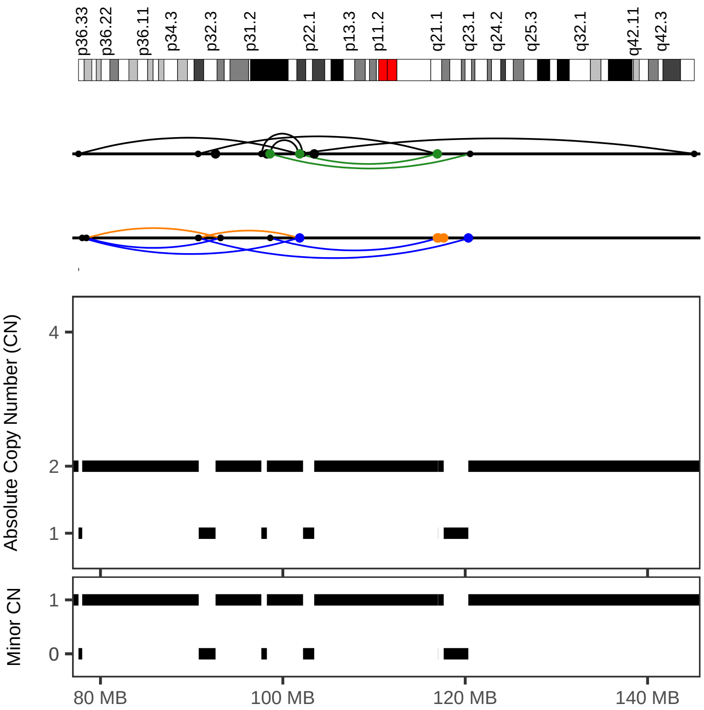

	4147081
Cancer type	Lymph−BNHL
Position	1:77588781−145118488
Type	Canonical without polyploidization
Interleaved intrachr. SVs	13
Total SVs (intrachr. + transl.)	25
SV types	DEL: 2; DUP: 4; h2hINV: 5; t2tINV: 2; TRA: 12
SVs in sample	107
Oscillating CN (2 and 3 states)	12, 12
CN segments	12
FDR fragment joints	0.64
FDR chr. breakp. enrich.	0
Linked to chrs	5:149829718−164575330;
Purity, ploidy	0.78, 1.94

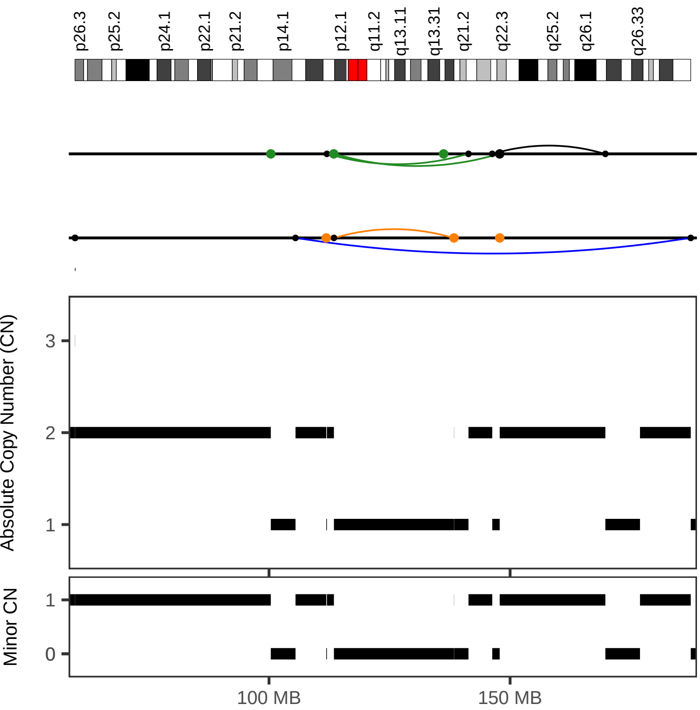

	4147081
Cancer type	Lymph−BNHL
Position	3:112007049−169759318
Type	Canonical without polyploidization
Interleaved intrachr. SVs	3
Total SVs (intrachr. + transl.)	8
SV types	DEL: 0; DUP: 0; h2hINV: 1; t2tINV: 2; TRA: 5
SVs in sample	107
Oscillating CN (2 and 3 states)	8, 8
CN segments	8
FDR fragment joints	0.59
FDR chr. breakp. enrich.	0.08
Linked to chrs	
Purity, ploidy	0.78, 1.94

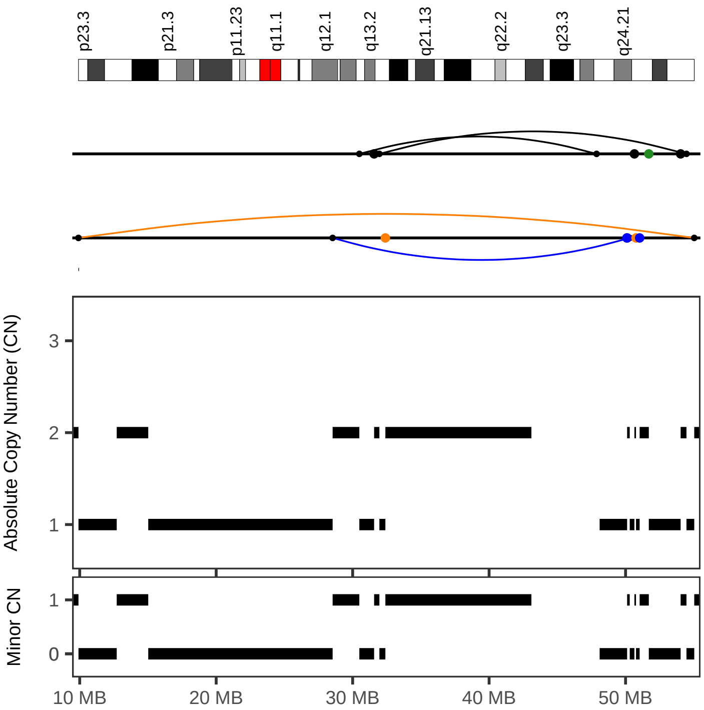

	4147081
Cancer type	Lymph−BNHL
Position	8:28532675−54463876
Type	Canonical without polyploidization
Interleaved intrachr. SVs	3
Total SVs (intrachr. + transl.)	12
SV types	DEL: 0; DUP: 1; h2hINV: 2; t2tINV: 0; TRA: 9
SVs in sample	107
Oscillating CN (2 and 3 states)	14, 14
CN segments	14
FDR fragment joints	0.59
FDR chr. breakp. enrich.	0
Linked to chrs	X:10911003−81430404;
Purity, ploidy	0.78, 1.94

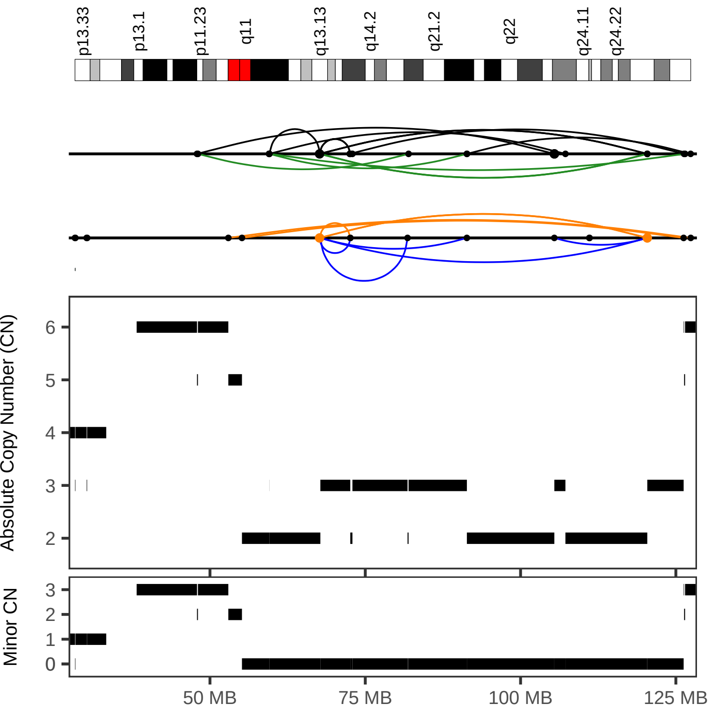

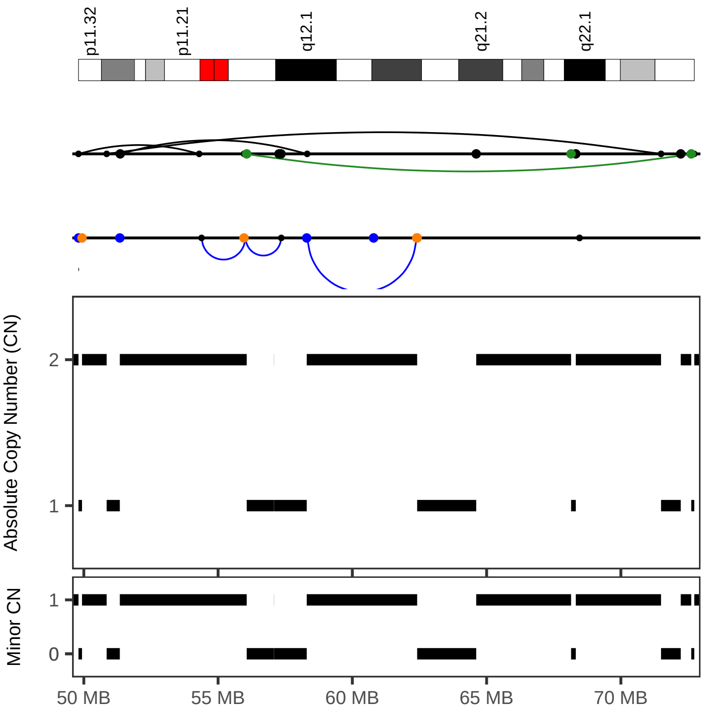

	**4147968**
Cancer type	Lymph−BNHL
Position	12:47913057−127394426
Type	Canonical without polyploidization
Interleaved intrachr. SVs	25
Total SVs (intrachr. + transl.)	30
SV types	DEL: 5; DUP: 5; h2hINV: 9; t2tINV: 6; TRA: 5
SVs in sample	140
Oscillating CN (2 and 3 states)	12, 13
CN segments	18
FDR fragment joints	0.71
FDR chr. breakp. enrich.	0
Linked to chrs	
Purity, ploidy	0.67, 3.49

	**4158726**
Cancer type	Lymph−BNHL
Position	18:49801362−72732256
Type	Canonical without polyploidization
Interleaved intrachr. SVs	5
Total SVs (intrachr. + transl.)	26
SV types	DEL: 0; DUP: 1; h2hINV: 3; t2tINV: 1; TRA: 21
SVs in sample	47
Oscillating CN (2 and 3 states)	16, 16
CN segments	16
FDR fragment joints	0.59
FDR chr. breakp. enrich.	0
Linked to chrs	9:20938863−81861601;13:29727063−43934816
Purity, ploidy	0.45, 1.93

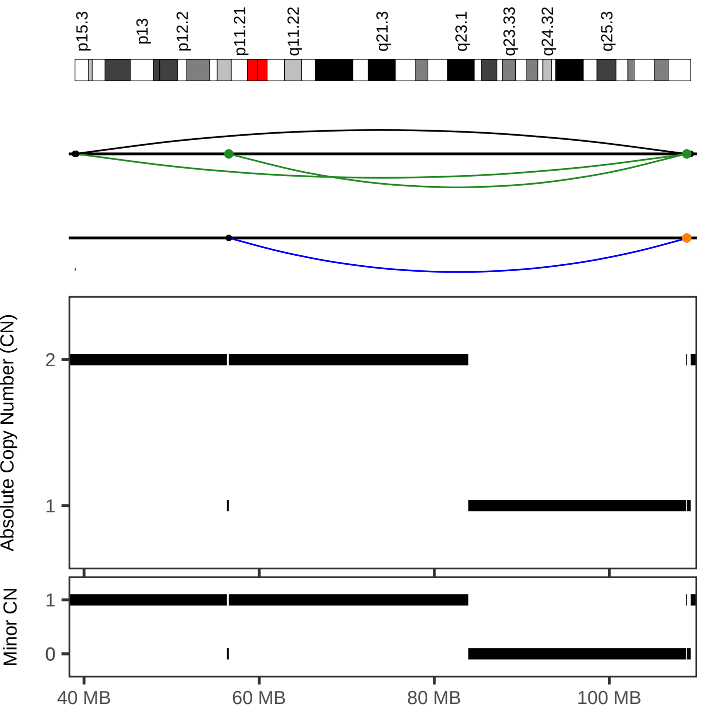

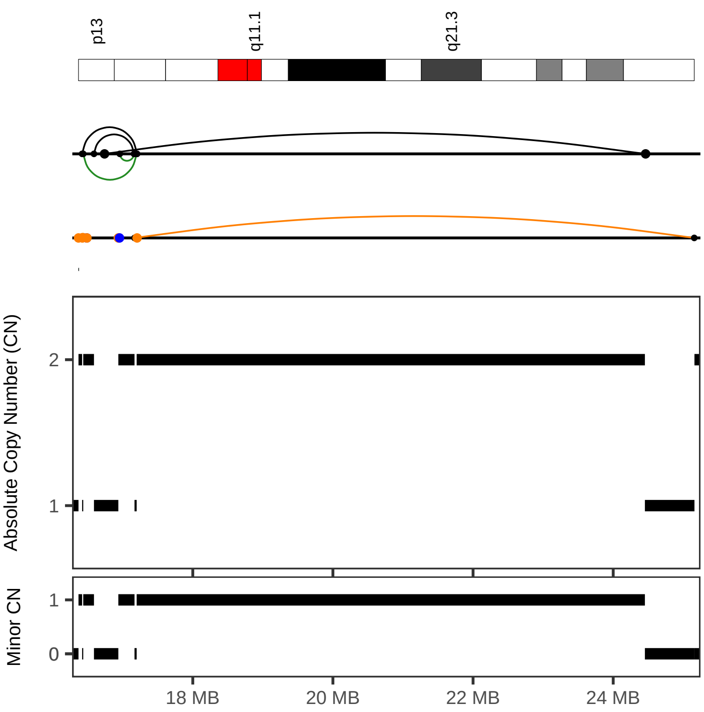

	**4177987**
Cancer type	Lymph−BNHL
Position	10:38964812−109297003
Type	Canonical without polyploidization
Interleaved intrachr. SVs	5
Total SVs (intrachr. + transl.)	8
SV types	DEL: 0; DUP: 1; h2hINV: 2; t2tINV: 2; TRA: 3
SVs in sample	15
Oscillating CN (2 and 3 states)	8, 8
CN segments	8
FDR fragment joints	0.64
FDR chr. breakp. enrich.	0
Linked to chrs	
Purity, ploidy	0.38, 1.92

	**141**
Cancer type	Lymph−CLL
Position	21:16368954−25156515
Type	Canonical without polyploidization
Interleaved intrachr. SVs	7
Total SVs (intrachr. + transl.)	16
SV types	DEL: 2; DUP: 0; h2hINV: 3; t2tINV: 2; TRA: 9
SVs in sample	31
Oscillating CN (2 and 3 states)	8, 8
CN segments	8
FDR fragment joints	0.64
FDR chr. breakp. enrich.	0
Linked to chrs	
Purity, ploidy	0.99, 1.92

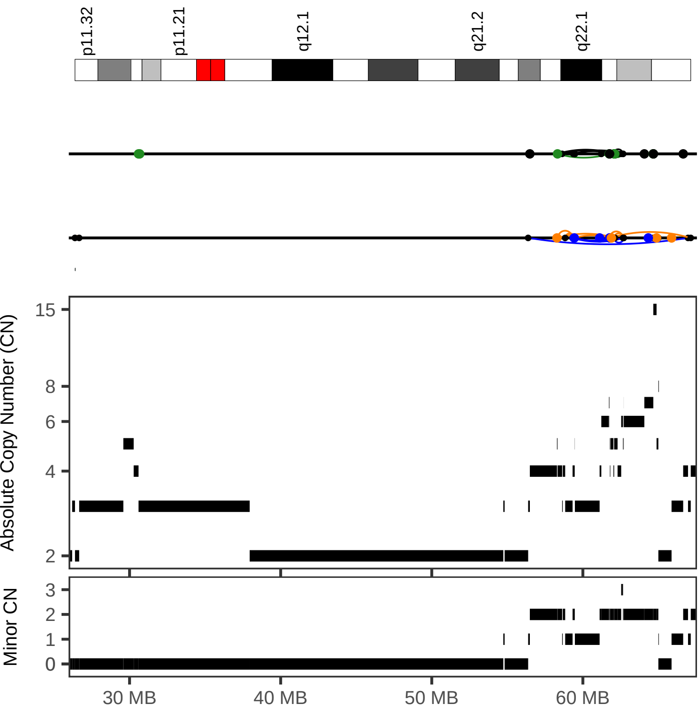

	AOCS−005
Cancer type	Ovary−AdenoCA
Position	18:56378430−67139124
Type	With other complex events
Interleaved intrachr. SVs	27
Total SVs (intrachr. + transl.)	49
SV types	DEL: 8; DUP: 7; h2hINV: 8; t2tINV: 4; TRA: 22
SVs in sample	399
Oscillating CN (2 and 3 states)	10, 17
CN segments	48
FDR fragment joints	0.74
FDR chr. breakp. enrich.	0
Linked to chrs	
Purity, ploidy	0.71, 3.06

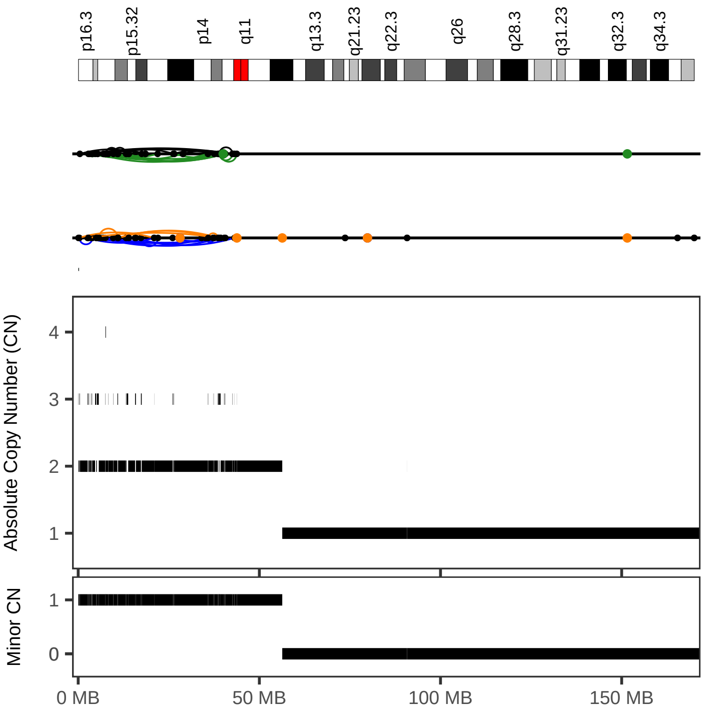

	AOCS−034
Cancer type	Ovary−AdenoCA
Position	4:77772−43781007
Type	Canonical without polyploidization
Interleaved intrachr. SVs	43
Total SVs (intrachr. + transl.)	47
SV types	DEL: 6; DUP: 12; h2hINV: 12; t2tINV: 13; TRA: 4
SVs in sample	308
Oscillating CN (2 and 3 states)	49, 66
CN segments	66
FDR fragment joints	0.62
FDR chr. breakp. enrich.	0
Linked to chrs	2:43382931−228657975;7:25135946−121567350
Purity, ploidy	0.79, 1.71

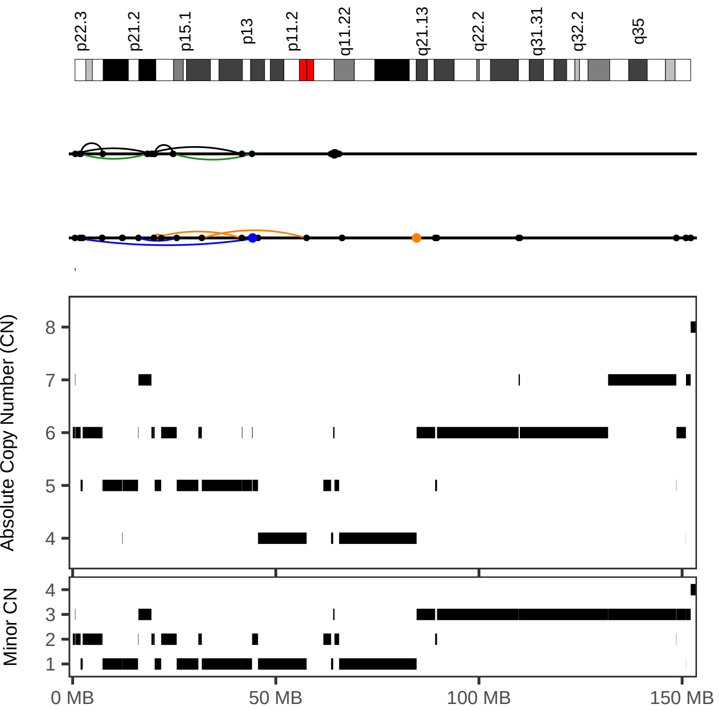

	AOCS−055
Cancer type	Ovary−AdenoCA
Position	7:567398−57567811
Type	With other complex events
Interleaved intrachr. SVs	13
Total SVs (intrachr. + transl.)	14
SV types	DEL: 4; DUP: 2; h2hINV: 4; t2tINV: 3; TRA: 1
SVs in sample	362
Oscillating CN (2 and 3 states)	12, 25
CN segments	25
FDR fragment joints	0.88
FDR chr. breakp. enrich.	0.32
Linked to chrs	
Purity, ploidy	0.5, 4.18

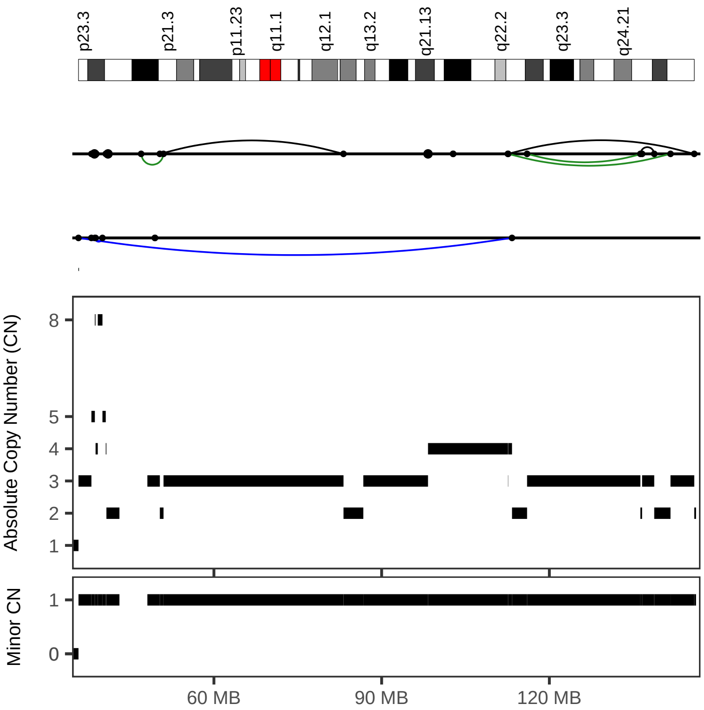

	AOCS−056
Cancer type	Ovary−AdenoCA
Position	8:35777050−145916683
Type	With other complex events
Interleaved intrachr. SVs	3
Total SVs (intrachr. + transl.)	9
SV types	DEL: 0; DUP: 3; h2hINV: 1; t2tINV: 2; TRA: 3
SVs in sample	110
Oscillating CN (2 and 3 states)	7, 11
CN segments	23
FDR fragment joints	0.59
FDR chr. breakp. enrich.	0
Linked to chrs	
Purity, ploidy	0.86, 1.96

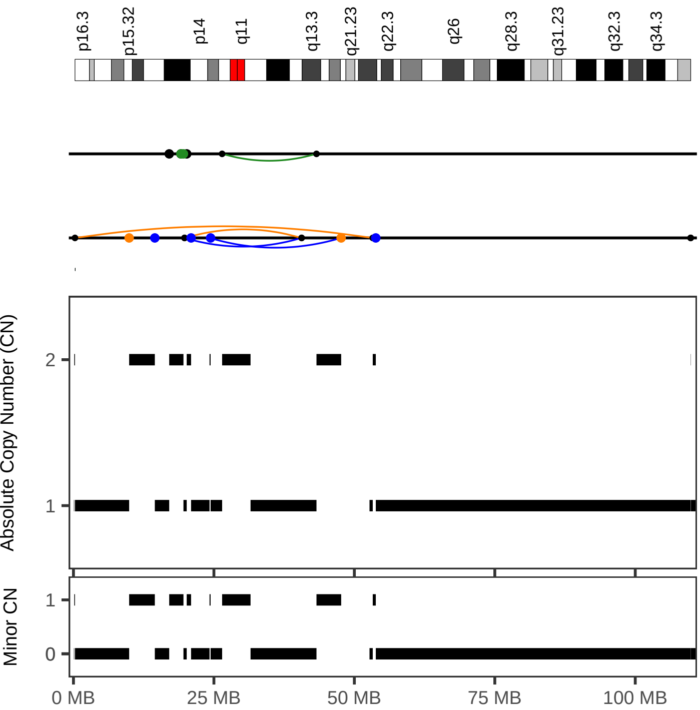

AOCS−058	
Cancer type	Ovary−AdenoCA
Position	4:19768980−47652511
Type	Canonical without polyploidization
Interleaved intrachr. SVs	4
Total SVs (intrachr. + transl.)	8
SV types	DEL: 1; DUP: 2; h2hINV: 0; t2tINV: 1; TRA: 4
SVs in sample	243
Oscillating CN (2 and 3 states)	9, 9
CN segments	9
FDR fragment joints	0.64
FDR chr. breakp. enrich.	0.55
Linked to chrs	21:31989442−43599046;
Purity, ploidy	0.83, 1.8

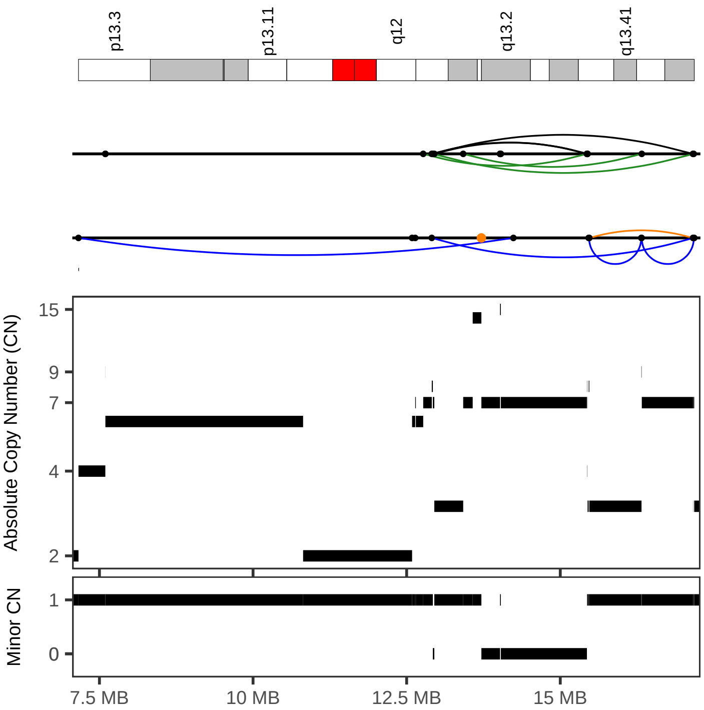

AOCS−059	
Cancer type	Ovary−AdenoCA
Position	19:7159079−17180659
Type	With other complex events
Interleaved intrachr. SVs	11
Total SVs (intrachr. + transl.)	12
SV types	DEL: 1; DUP: 4; h2hINV: 3; t2tINV: 3; TRA: 1
SVs in sample	43
Oscillating CN (2 and 3 states)	7, 7
CN segments	31
FDR fragment joints	0.71
FDR chr. breakp. enrich.	0
Linked to chrs	
Purity, ploidy	0.47, 2.94

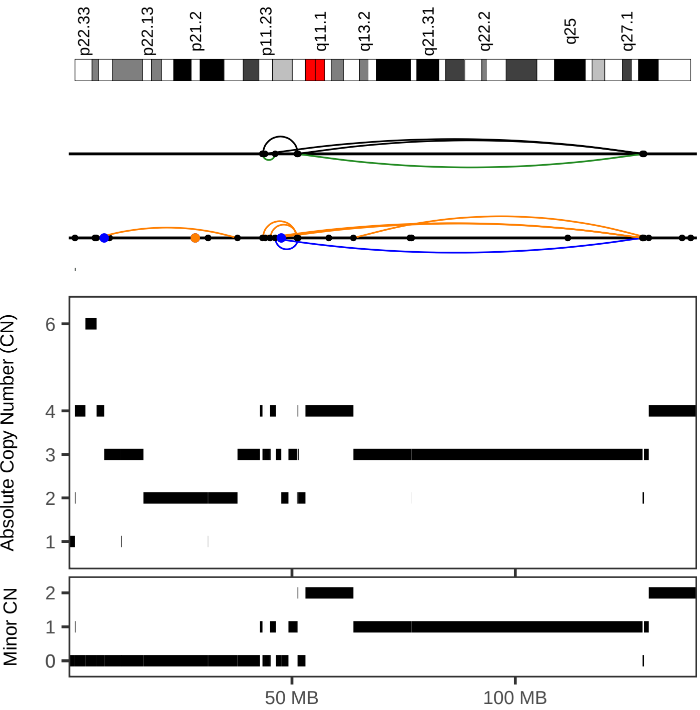

AOCS−064	
Cancer type	Ovary−AdenoCA
Position	X:43392645−129894760
Type	With other complex events
Interleaved intrachr. SVs	14
Total SVs (intrachr. + transl.)	15
SV types	DEL: 5; DUP: 2; h2hINV: 5; t2tINV: 2; TRA: 1
SVs in sample	402
Oscillating CN (2 and 3 states)	8, 8
CN segments	25
FDR fragment joints	0.64
FDR chr. breakp. enrich.	0.05
Linked to chrs	
Purity, ploidy	0.67, 3.2

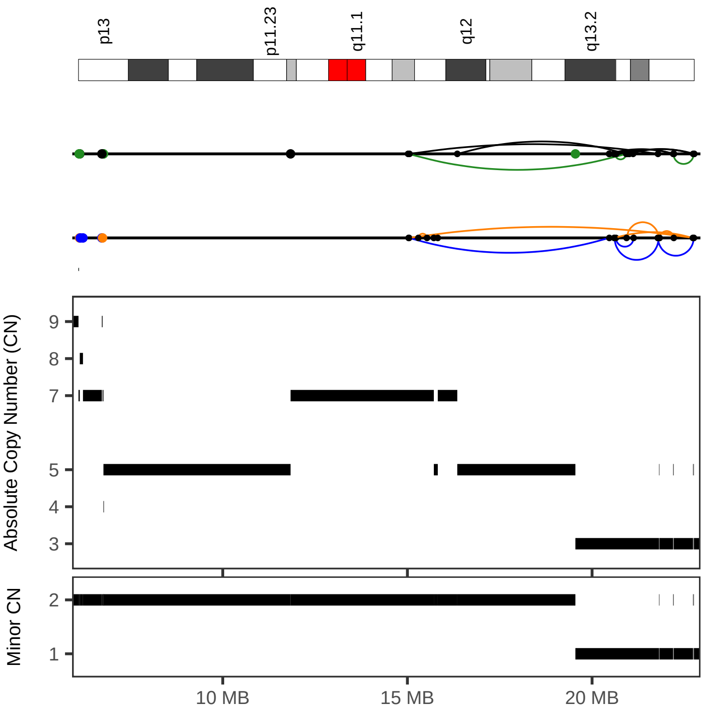

AOCS−080	
Cancer type	Ovary−AdenoCA
Position	20:15018409−22766307
Type	After polyploidization
Interleaved intrachr. SVs	21
Total SVs (intrachr. + transl.)	22
SV types	DEL: 4; DUP: 6; h2hINV: 6; t2tINV: 5; TRA: 1
SVs in sample	207
Oscillating CN (2 and 3 states)	10, 13
CN segments	13
FDR fragment joints	0.94
FDR chr. breakp. enrich.	0
Linked to chrs	
Purity, ploidy	0.92, 3.05

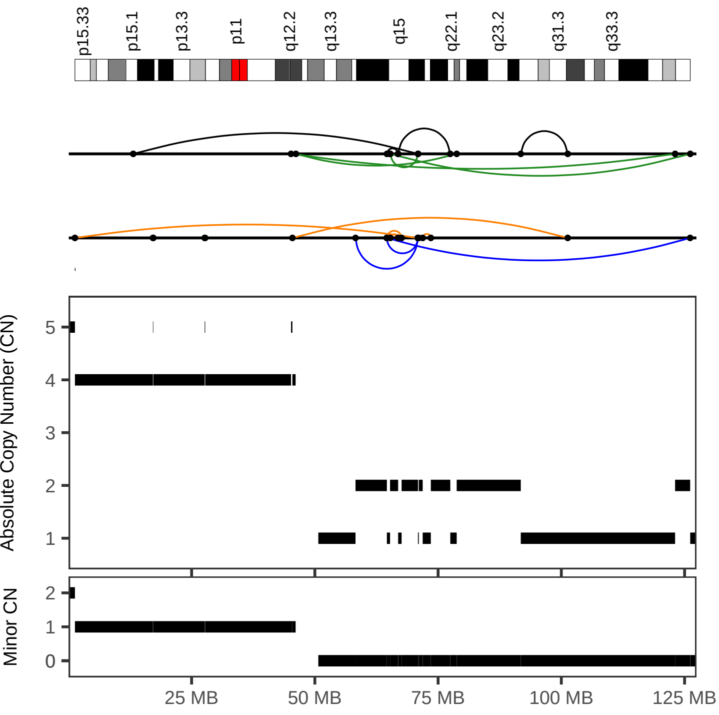

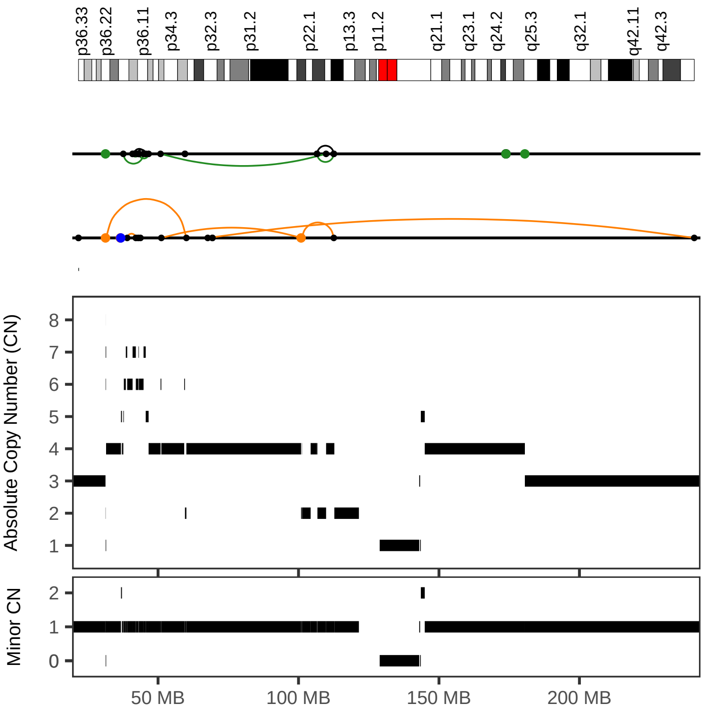

**AOCS−081**	
Cancer type	Ovary−AdenoCA
Position	5:1326917−126150912
Type	Canonical without polyploidization
Interleaved intrachr. SVs	16
Total SVs (intrachr. + transl.)	16
SV types	DEL: 5; DUP: 3; h2hINV: 4; t2tINV: 4; TRA: 0
SVs in sample	150
Oscillating CN (2 and 3 states)	20, 21
CN segments	27
FDR fragment joints	0.94
FDR chr. breakp. enrich.	0.02
Linked to chrs	
Purity, ploidy	0.68, 2.59

**AOCS−084**	
Cancer type	Ovary−AdenoCA
Position	1:37638433−46640509
Type	After polyploidization
Interleaved intrachr. SVs	8
Total SVs (intrachr. + transl.)	8
SV types	DEL: 1; DUP: 1; h2hINV: 3; t2tINV: 3; TRA: 0
SVs in sample	146
Oscillating CN (2 and 3 states)	8, 9
CN segments	11
FDR fragment joints	0.64
FDR chr. breakp. enrich.	0
Linked to chrs	
Purity, ploidy	0.64, 3.09

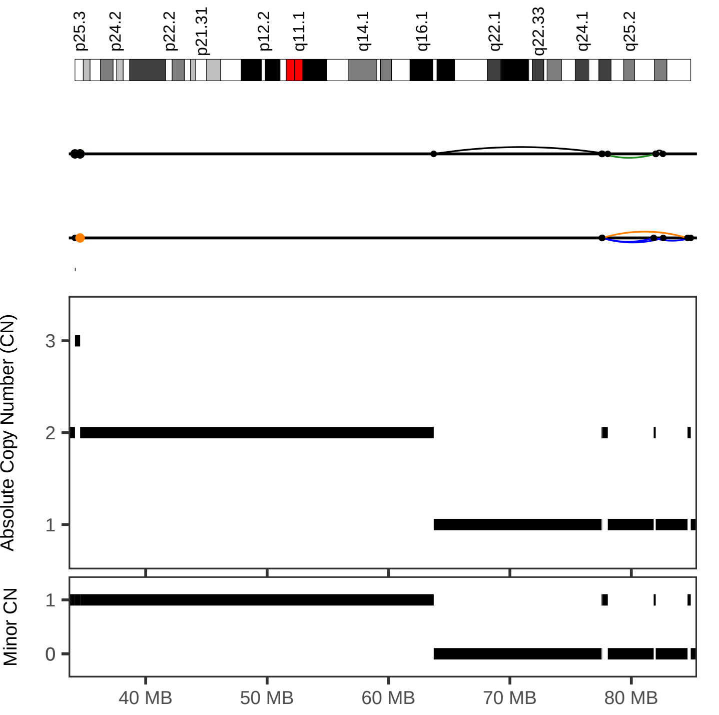

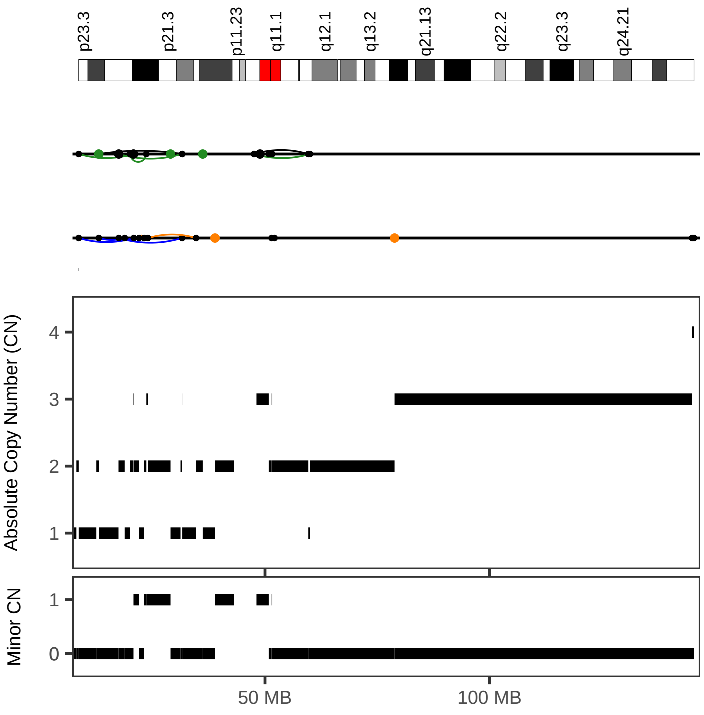

**AOCS−085**	
Cancer type	Ovary−AdenoCA
Position	6:63724002−84893098
Type	Canonical without polyploidization
Interleaved intrachr. SVs	10
Total SVs (intrachr. + transl.)	10
SV types	DEL: 1; DUP: 3; h2hINV: 4; t2tINV: 2; TRA: 0
SVs in sample	95
Oscillating CN (2 and 3 states)	9, 9
CN segments	9
FDR fragment joints	0.64
FDR chr. breakp. enrich.	0
Linked to chrs	
Purity, ploidy	0.62, 1.89

**AOCS−090**	
Cancer type	Ovary−AdenoCA
Position	8:8519924−34671479
Type	With other complex events
Interleaved intrachr. SVs	8
Total SVs (intrachr. + transl.)	12
SV types	DEL: 1; DUP: 3; h2hINV: 1; t2tINV: 3; TRA: 4
SVs in sample	334
Oscillating CN (2 and 3 states)	7, 16
CN segments	18
FDR fragment joints	0.64
FDR chr. breakp. enrich.	0.44
Linked to chrs	13:20915954−32359354;5:6366275−168079249
Purity, ploidy	0.78, 2.65

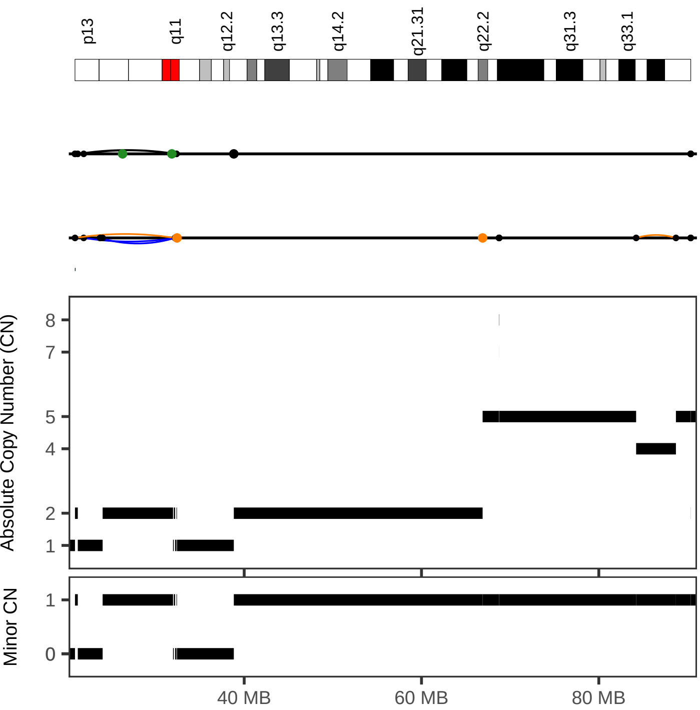

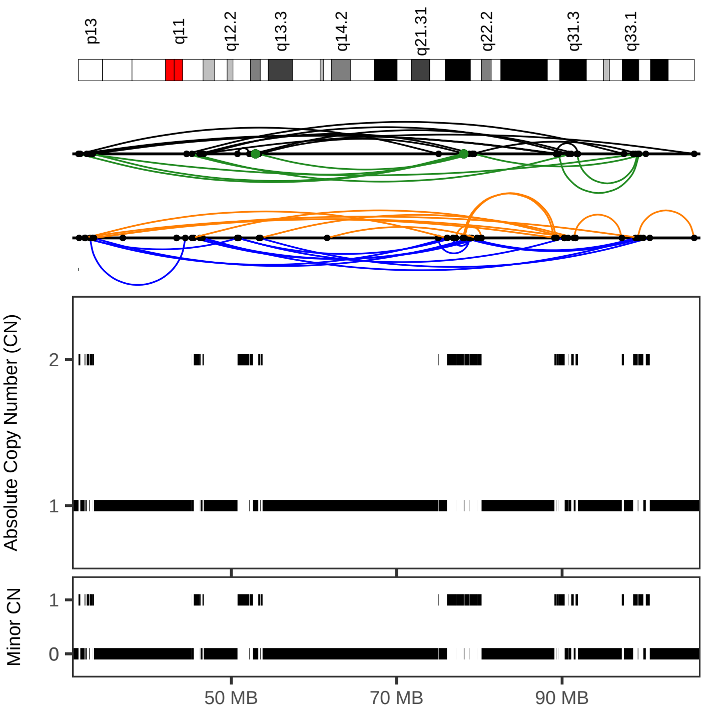

	**AOCS−090**
Cancer type	Ovary−AdenoCA
Position	13:20915954−32359355
Type	Canonical without polyploidization
Interleaved intrachr. SVs	7
Total SVs (intrachr. + transl.)	9
SV types	DEL: 1; DUP: 2; h2hINV: 2; t2tINV: 2; TRA: 2
SVs in sample	334
Oscillating CN (2 and 3 states)	9, 9
CN segments	9
FDR fragment joints	0.95
FDR chr. breakp. enrich.	0.07
Linked to chrs	
Purity, ploidy	0.78, 2.65

	**AOCS−093**
Cancer type	Ovary−AdenoCA
Position	13:31521128−105978091
Type	Canonical without polyploidization
Interleaved intrachr. SVs	49
Total SVs (intrachr. + transl.)	53
SV types	DEL: 12; DUP: 14; h2hINV: 13; t2tINV: 10; TRA: 4
SVs in sample	297
Oscillating CN (2 and 3 states)	70, 70
CN segments	70
FDR fragment joints	0.91
FDR chr. breakp. enrich.	0
Linked to chrs	2:125326527−209102159;
Purity, ploidy	0.47, 1.78

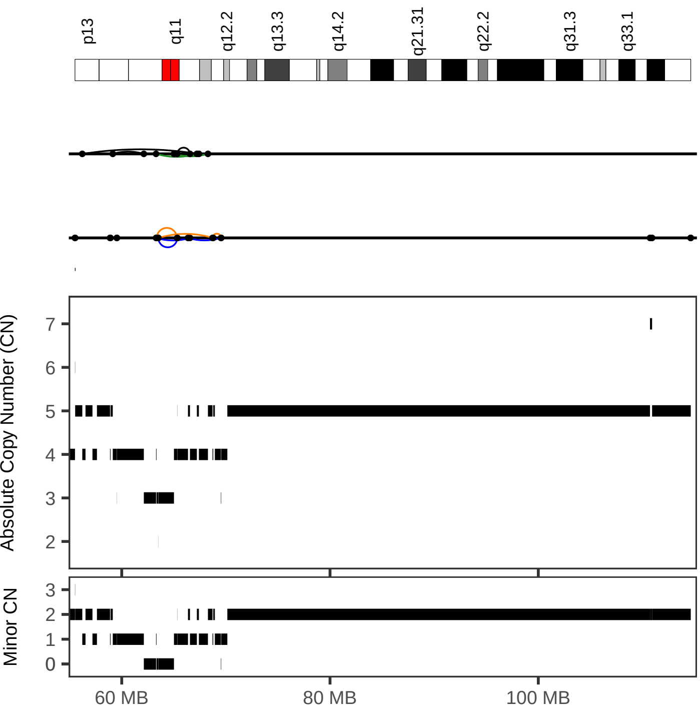

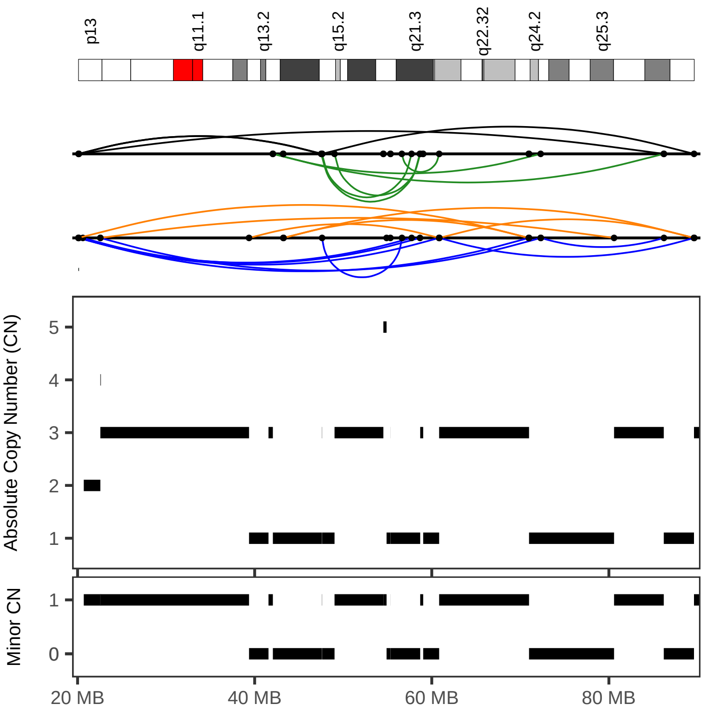

	**AOCS−096**
Cancer type	Ovary−AdenoCA
Position	13:56212396−69555802
Type	With other complex events
Interleaved intrachr. SVs	10
Total SVs (intrachr. + transl.)	10
SV types	DEL: 3; DUP: 3; h2hINV: 2; t2tINV: 2; TRA: 0
SVs in sample	197
Oscillating CN (2 and 3 states)	11, 27
CN segments	27
FDR fragment joints	0.96
FDR chr. breakp. enrich.	0.01
Linked to chrs	
Purity, ploidy	0.57, 3.38

	**AOCS−097**
Cancer type	Ovary−AdenoCA
Position	15:20101979−89639810
Type	With other complex events
Interleaved intrachr. SVs	25
Total SVs (intrachr. + transl.)	25
SV types	DEL: 6; DUP: 8; h2hINV: 5; t2tINV: 6; TRA: 0
SVs in sample	51
Oscillating CN (2 and 3 states)	10, 12
CN segments	21
FDR fragment joints	0.9
FDR chr. breakp. enrich.	0
Linked to chrs	
Purity, ploidy	0.94, 1.81

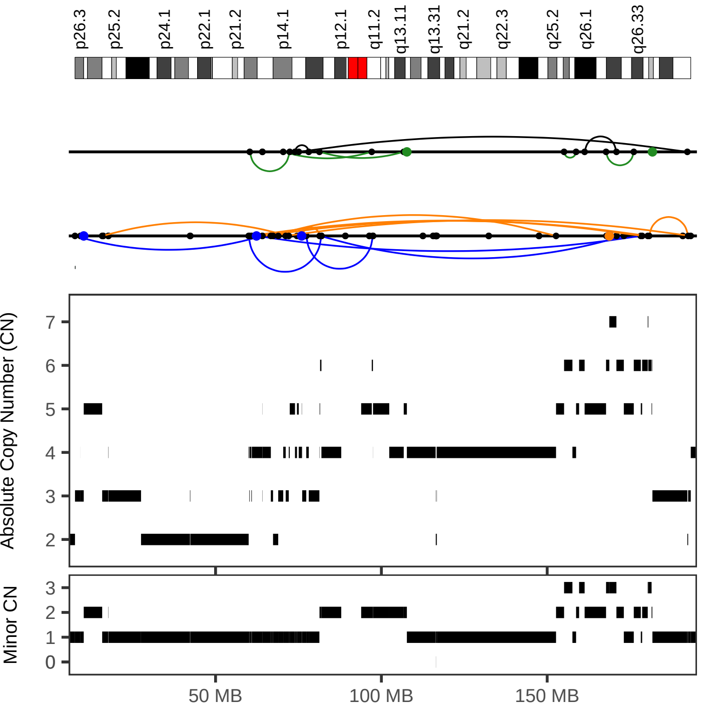

	AOCS−108
Cancer type	Ovary−AdenoCA
Position	3:7603269−193289152
Type	With other complex events
Interleaved intrachr. SVs	20
Total SVs (intrachr. + transl.)	27
SV types	DEL: 7; DUP: 6; h2hINV: 3; t2tINV: 4; TRA: 7
SVs in sample	415
Oscillating CN (2 and 3 states)	7, 27
CN segments	77
FDR fragment joints	0.64
FDR chr. breakp. enrich.	0
Linked to chrs	
Purity, ploidy	0.7, 3.64

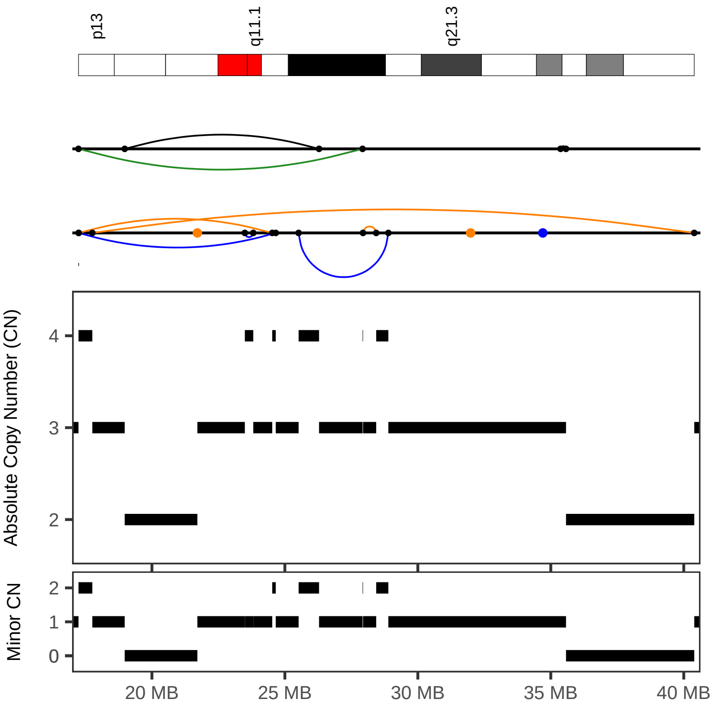

	AOCS−111
Cancer type	Ovary−AdenoCA
Position	21:17237944−40394460
Type	After polyploidization
Interleaved intrachr. SVs	6
Total SVs (intrachr. + transl.)	9
SV types	DEL: 2; DUP: 2; h2hINV: 1; t2tINV: 1; TRA: 3
SVs in sample	106
Oscillating CN (2 and 3 states)	11, 16
CN segments	16
FDR fragment joints	0.91
FDR chr. breakp. enrich.	0
Linked to chrs	19:28894477−56155201;
Purity, ploidy	0.62, 3.09

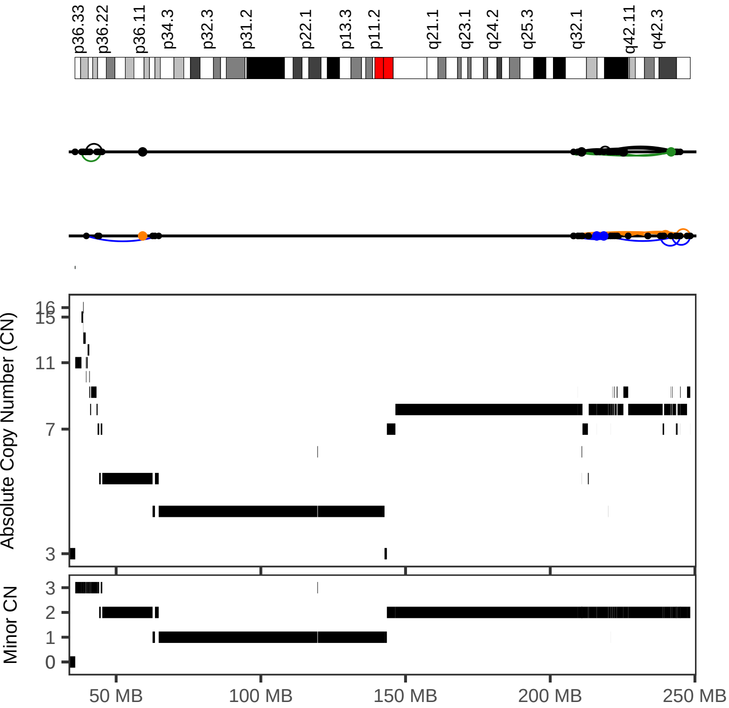

	AOCS−113
Cancer type	Ovary−AdenoCA
Position	1:208032015−248380796
Type	With other complex events
Interleaved intrachr. SVs	36
Total SVs (intrachr. + transl.)	41
SV types	DEL: 14; DUP: 6; h2hINV: 9; t2tINV: 7; TRA: 5
SVs in sample	163
Oscillating CN (2 and 3 states)	9, 24
CN segments	41
FDR fragment joints	0.59
FDR chr. breakp. enrich.	0
Linked to chrs	
Purity, ploidy	0.72, 4.33

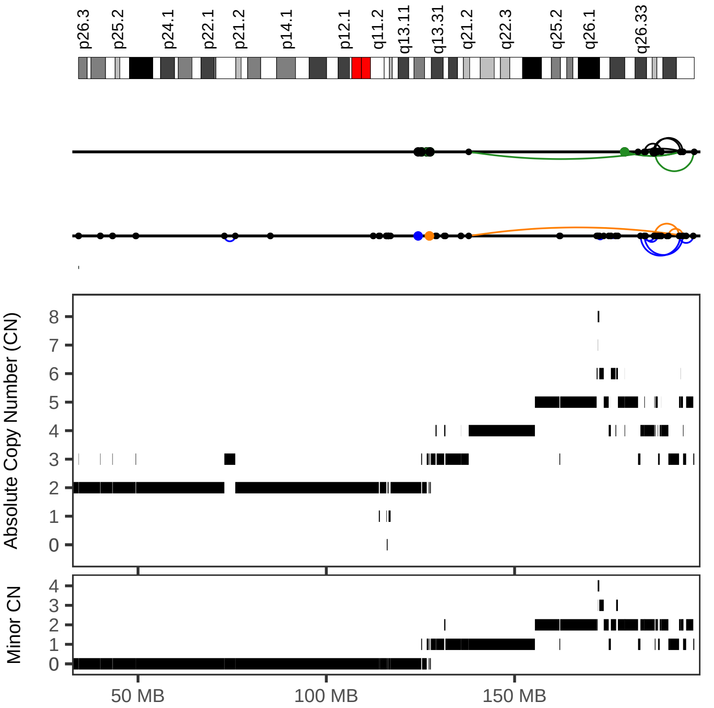

	AOCS−116
Cancer type	Ovary−AdenoCA
Position	3:137813498−197700924
Type	With other complex events
Interleaved intrachr. SVs	21
Total SVs (intrachr. + transl.)	24
SV types	DEL: 5; DUP: 6; h2hINV: 5; t2tINV: 5; TRA: 3
SVs in sample	481
Oscillating CN (2 and 3 states)	7, 10
CN segments	42
FDR fragment joints	1
FDR chr. breakp. enrich.	0
Linked to chrs	
Purity, ploidy	0.67, 2.63

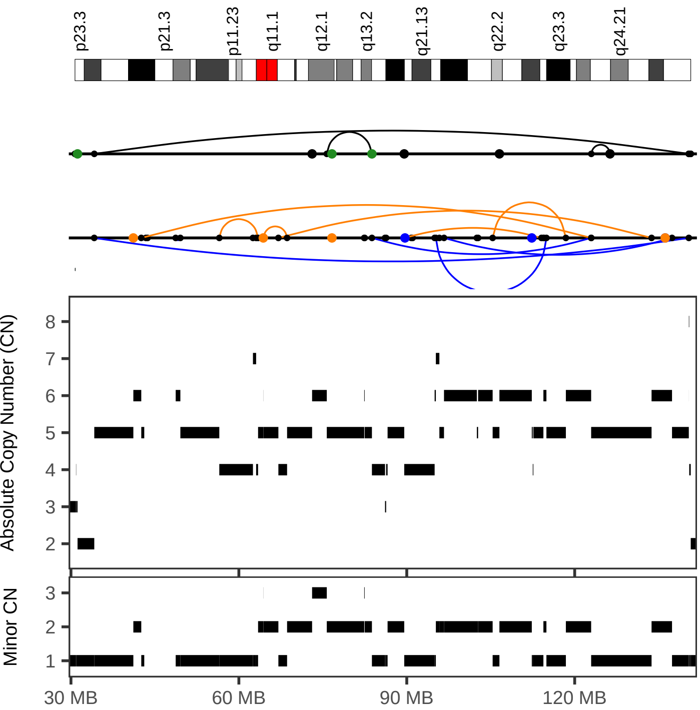

	AOCS−117
Cancer type	Ovary−AdenoCA
Position	8:42562155−137393245
Type	With other complex events
Interleaved intrachr. SVs	10
Total SVs (intrachr. + transl.)	22
SV types	DEL: 5; DUP: 4; h2hINV: 1; t2tINV: 0; TRA: 12
SVs in sample	333
Oscillating CN (2 and 3 states)	7, 20
CN segments	37
FDR fragment joints	0.48
FDR chr. breakp. enrich.	0
Linked to chrs	2:22291212−238932055;20:34757165−60260320 3:202871−63948255;
Purity, ploidy	0.88, 3.36

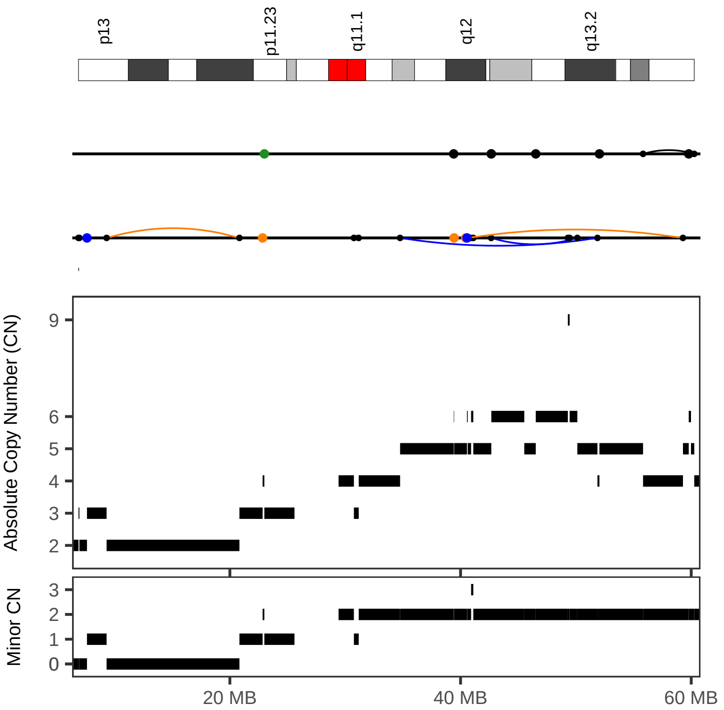

	AOCS−117
Cancer type	Ovary−AdenoCA
Position	20:34757165−60260321
Type	With other complex events
Interleaved intrachr. SVs	3
Total SVs (intrachr. + transl.)	12
SV types	DEL: 1; DUP: 1; h2hINV: 1; t2tINV: 0; TRA: 9
SVs in sample	333
Oscillating CN (2 and 3 states)	10, 10
CN segments	20
FDR fragment joints	0.84
FDR chr. breakp. enrich.	0
Linked to chrs	
Purity, ploidy	0.88, 3.36

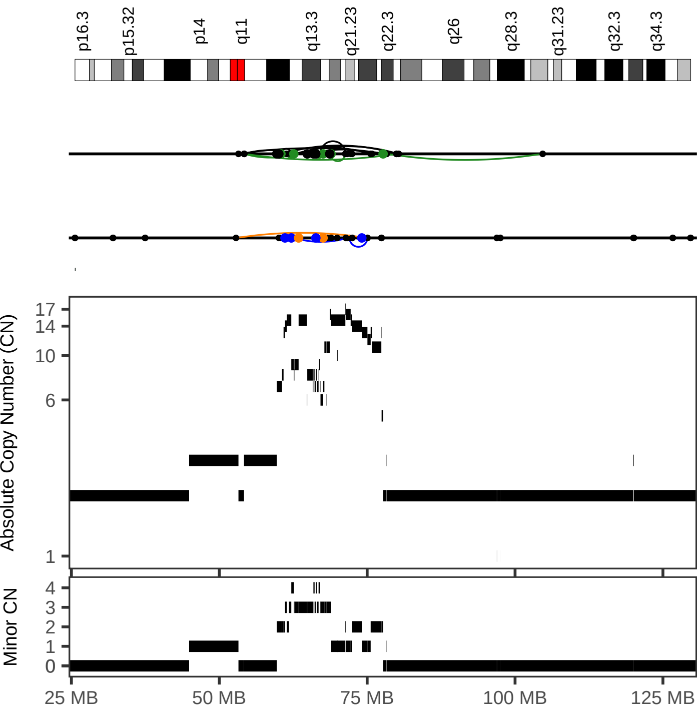

	AOCS−142
Cancer type	Ovary−AdenoCA
Position	4:52826977−104671727
Type	With other complex events
Interleaved intrachr. SVs	22
Total SVs (intrachr. + transl.)	41
SV types	DEL: 3; DUP: 3; h2hINV: 10; t2tINV: 6; TRA: 19
SVs in sample	587
Oscillating CN (2 and 3 states)	7, 8
CN segments	52
FDR fragment joints	0.48
FDR chr. breakp. enrich.	0.03
Linked to chrs	
Purity, ploidy	0.4, 3.64

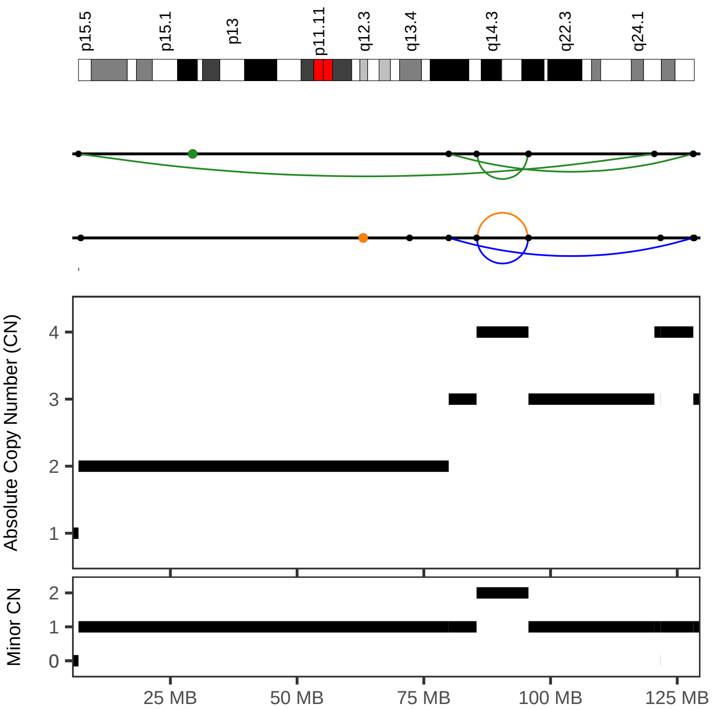

	AOCS−153
Cancer type	Ovary−AdenoCA
Position	11:6869185−128159617
Type	After polyploidization
Interleaved intrachr. SVs	3
Total SVs (intrachr. + transl.)	10
SV types	DEL: 1; DUP: 2; h2hINV: 0; t2tINV: 3; TRA: 4
SVs in sample	194
Oscillating CN (2 and 3 states)	7, 8
CN segments	8
FDR fragment joints	0.59
FDR chr. breakp. enrich.	0.1
Linked to chrs	1:78956017−180480315;3:73753832−116449819
Purity, ploidy	0.71, 1.89

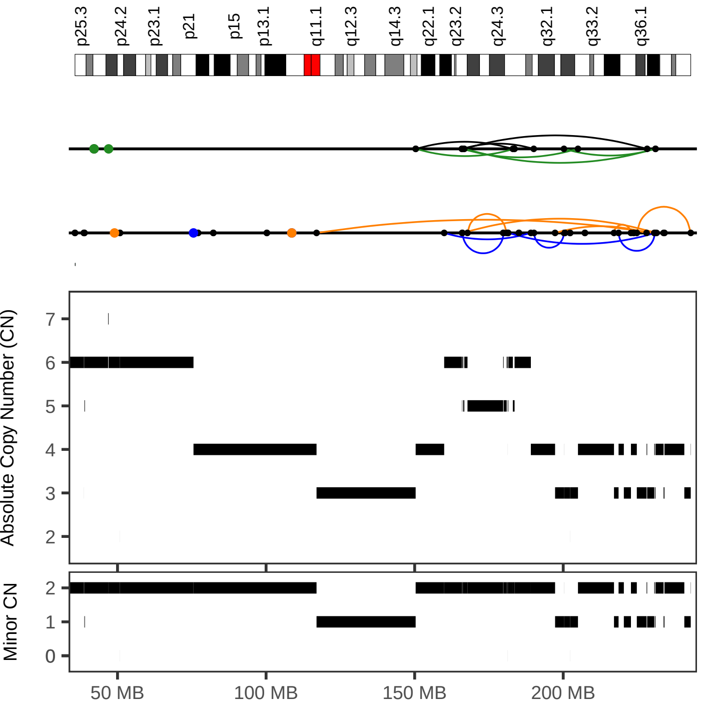

AOCS−155

Cancer type	Ovary−AdenoCA
Position	2:116979827−242917384
Type	With other complex events
Interleaved intrachr. SVs	19
Total SVs (intrachr. + transl.)	19
SV types	DEL: 6; DUP: 6; h2hINV: 3; t2tINV: 4; TRA: 0
SVs in sample	268
Oscillating CN (2 and 3 states)	16, 21
CN segments	40
FDR fragment joints	0.78
FDR chr. breakp. enrich.	0
Linked to chrs	
Purity, ploidy	0.81, 3.71

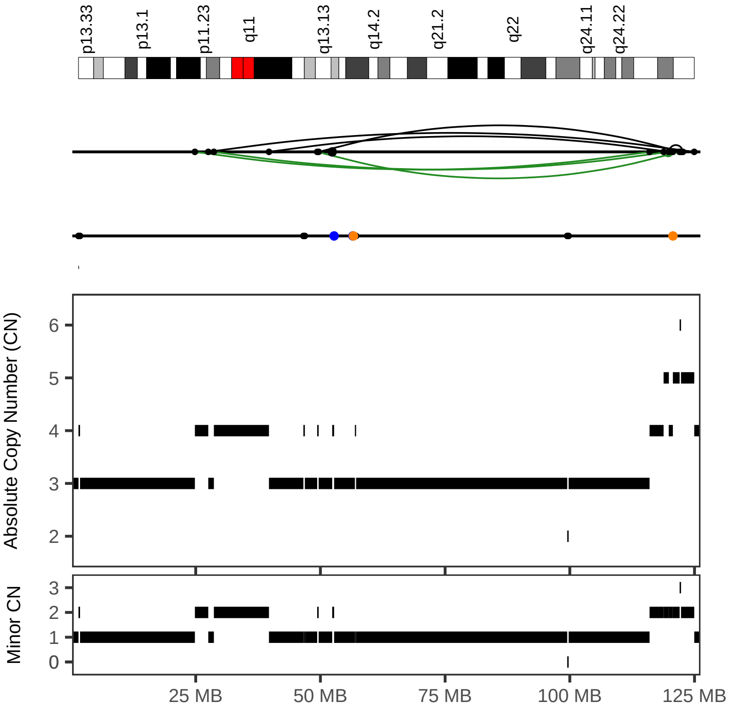

AOCS−159

Cancer type	Ovary−AdenoCA
Position	12:24836900−124945298
Type	With other complex events
Interleaved intrachr. SVs	8
Total SVs (intrachr. + transl.)	13
SV types	DEL: 0; DUP: 0; h2hINV: 4; t2tINV: 4; TRA: 5
SVs in sample	259
Oscillating CN (2 and 3 states)	12, 23
CN segments	23
FDR fragment joints	0.46
FDR chr. breakp. enrich.	0.4
Linked to chrs	7:66581838−110075762;
Purity, ploidy	0.65, 2.93

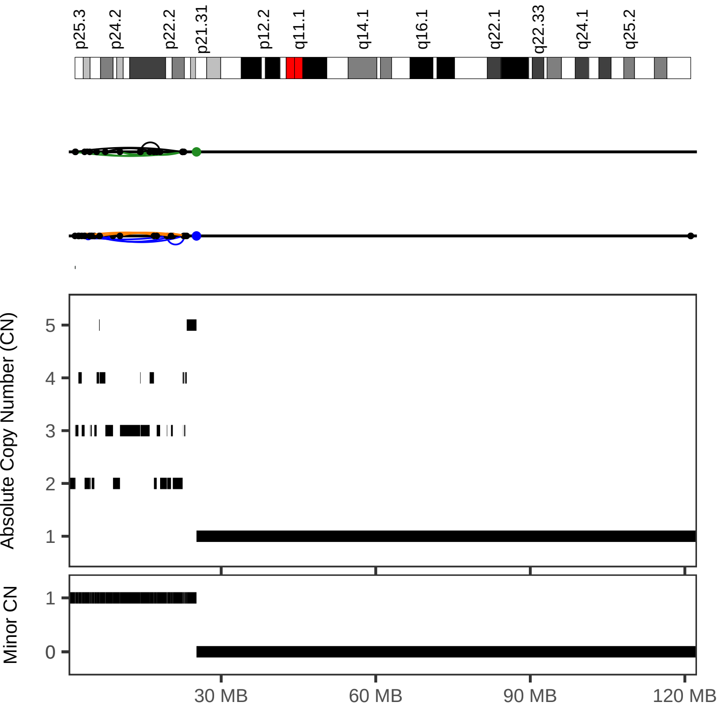

AOCS−160

Cancer type	Ovary−AdenoCA
Position	6:1616031−23308796
Type	With other complex events
Interleaved intrachr. SVs	18
Total SVs (intrachr. + transl.)	18
SV types	DEL: 4; DUP: 6; h2hINV: 3; t2tINV: 5; TRA: 0
SVs in sample	172
Oscillating CN (2 and 3 states)	9, 22
CN segments	37
FDR fragment joints	0.84
FDR chr. breakp. enrich.	0
Linked to chrs	
Purity, ploidy	0.85, 1.76

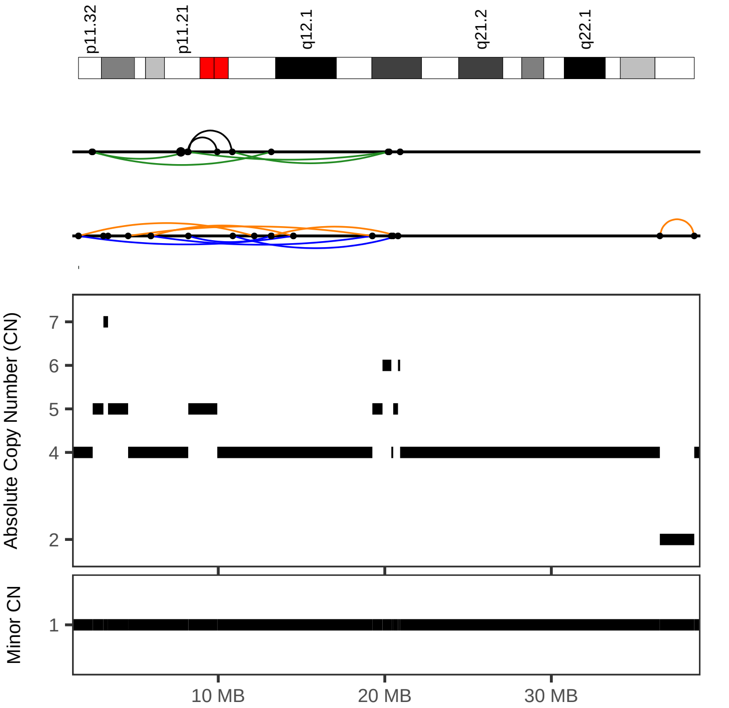

AOCS−162

Cancer type	Ovary−AdenoCA
Position	18:1605416−20794812
Type	With other complex events
Interleaved intrachr. SVs	14
Total SVs (intrachr. + transl.)	15
SV types	DEL: 4; DUP: 4; h2hINV: 2; t2tINV: 4; TRA: 1
SVs in sample	172
Oscillating CN (2 and 3 states)	7, 7
CN segments	14
FDR fragment joints	0.88
FDR chr. breakp. enrich.	0
Linked to chrs	
Purity, ploidy	0.79, 3.31

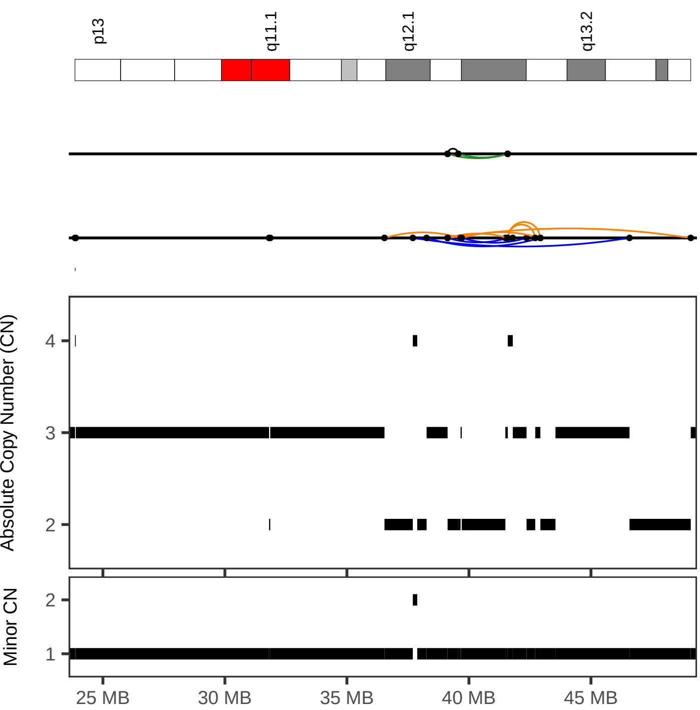

**AOCS−166**	
Cancer type	Ovary−AdenoCA
Position	22:36538809−49089280
Type	With other complex events
Interleaved intrachr. SVs	14
Total SVs (intrachr. + transl.)	14
SV types	DEL: 6; DUP: 5; h2hINV: 1; t2tINV: 2; TRA: 0
SVs in sample	170
Oscillating CN (2 and 3 states)	8, 16
CN segments	18
FDR fragment joints	0.59
FDR chr. breakp. enrich.	0
Linked to chrs	
Purity, ploidy	0.77, 3.46

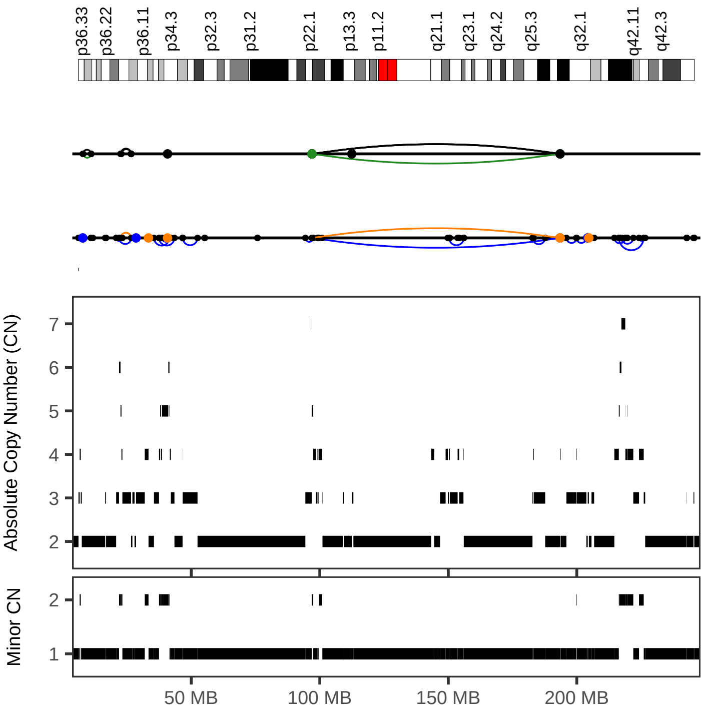

**AOCS−169**	
Cancer type	Ovary−AdenoCA
Position	1:94394953−193703467
Type	With other complex events
Interleaved intrachr. SVs	7
Total SVs (intrachr. + transl.)	20
SV types	DEL: 1; DUP: 2; h2hINV: 3; t2tINV: 1; TRA: 13
SVs in sample	413
Oscillating CN (2 and 3 states)	8, 10
CN segments	32
FDR fragment joints	0.74
FDR chr. breakp. enrich.	0
Linked to chrs	4:67409369−77723303;
Purity, ploidy	0.65, 2.07

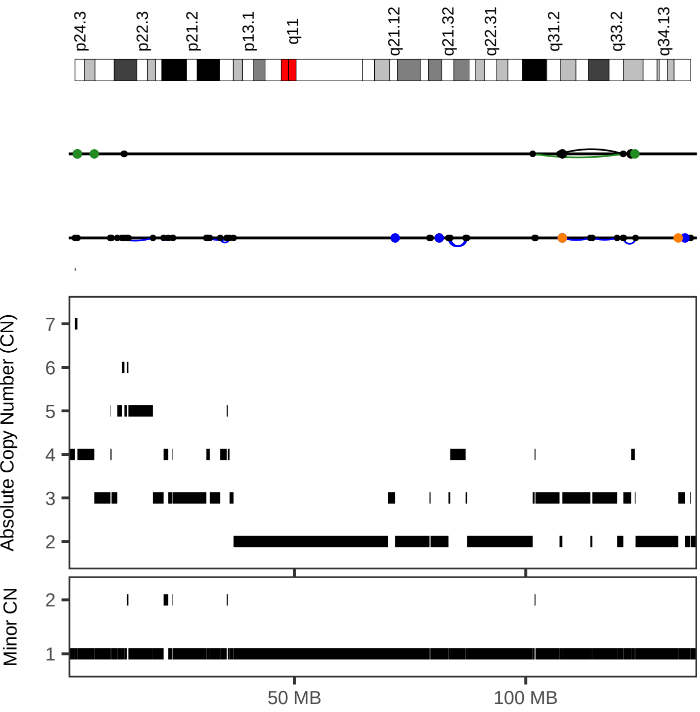

**AOCS−169**	
Cancer type	Ovary−AdenoCA
Position	9:12691740−121121973
Type	With other complex events
Interleaved intrachr. SVs	3
Total SVs (intrachr. + transl.)	13
SV types	DEL: 0; DUP: 2; h2hINV: 2; t2tINV: 2; TRA: 7
SVs in sample	413
Oscillating CN (2 and 3 states)	8, 30
CN segments	34
FDR fragment joints	0.64
FDR chr. breakp. enrich.	0
Linked to chrs	13:23398289−85684635;20:4534875−59811572
Purity, ploidy	0.65, 2.07

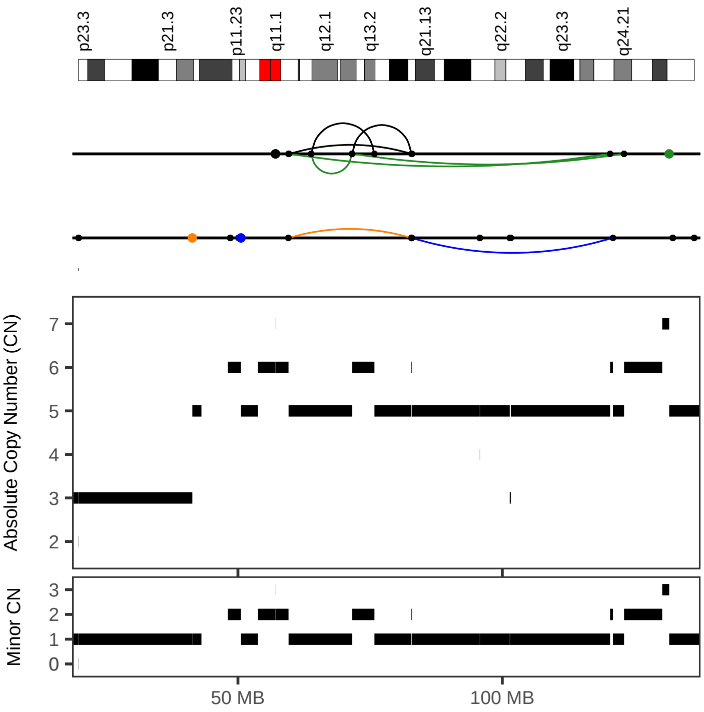

**0c1a2e7d−e7e4−481e−a012−ef214c444497**	
Cancer type	Ovary−AdenoCA
Position	8:59542153−123021272
Type	After polyploidization
Interleaved intrachr. SVs	8
Total SVs (intrachr. + transl.)	8
SV types	DEL: 1; DUP: 1; h2hINV: 3; t2tINV: 3; TRA: 0
SVs in sample	174
Oscillating CN (2 and 3 states)	11, 11
CN segments	18
FDR fragment joints	0.64
FDR chr. breakp. enrich.	0
Linked to chrs	
Purity, ploidy	0.9, 2.97

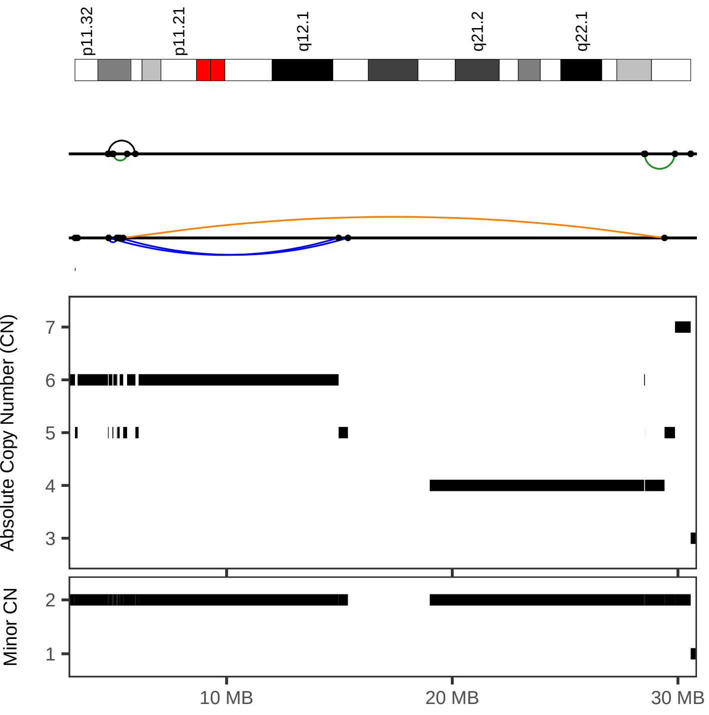

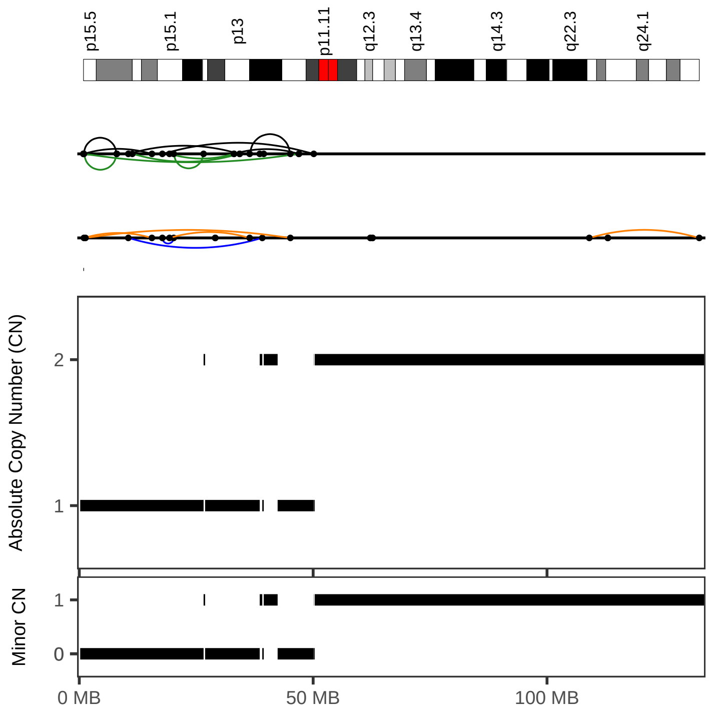

	0c1a2e7d−e7e4−481e−a012−ef214c444497
Cancer type	Ovary−AdenoCA
Position	18:4742587−29869159
Type	After polyploidization
Interleaved intrachr. SVs	8
Total SVs (intrachr. + transl.)	8
SV types	DEL: 1; DUP: 3; h2hINV: 2; t2tINV: 2; TRA: 0
SVs in sample	174
Oscillating CN (2 and 3 states)	15, 15
CN segments	21
FDR fragment joints	0.84
FDR chr. breakp. enrich.	0.02
Linked to chrs	
Purity, ploidy	0.9, 2.97

	30b8f4cd−9245−4496−a8a8−c3e59093bc0a
Cancer type	Ovary−AdenoCA
Position	11:898947−50134754
Type	Canonical without polyploidization
Interleaved intrachr. SVs	17
Total SVs (intrachr. + transl.)	17
SV types	DEL: 3; DUP: 2; h2hINV: 6; t2tINV: 6; TRA: 0
SVs in sample	206
Oscillating CN (2 and 3 states)	9, 9
CN segments	9
FDR fragment joints	0.59
FDR chr. breakp. enrich.	0
Linked to chrs	
Purity, ploidy	0.9, 1.74

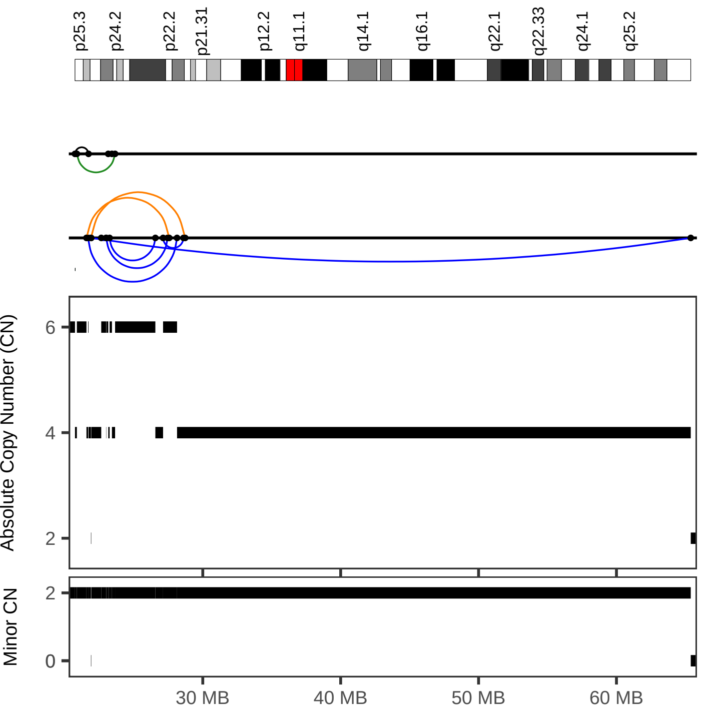

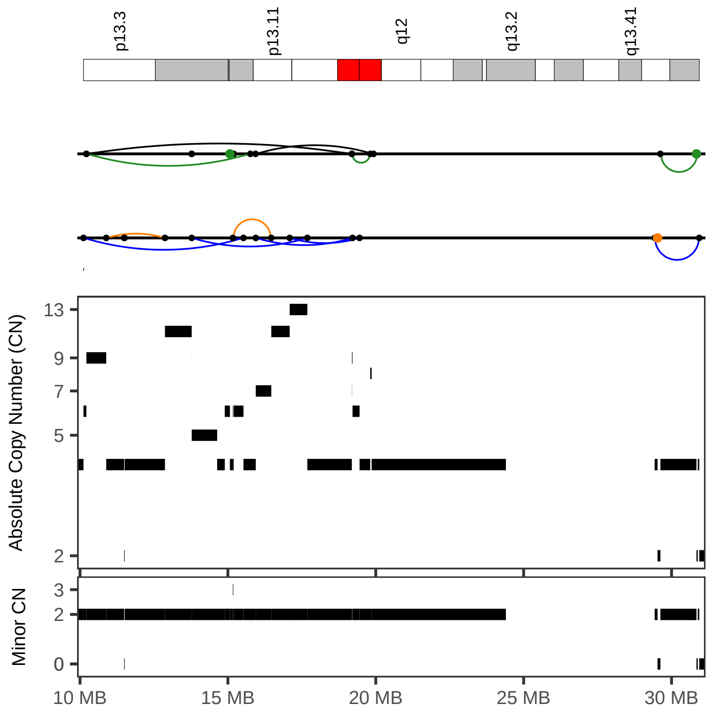

	4b930a10−4b12−4428−84f9−3255b4a3bc4f
Cancer type	Ovary−AdenoCA
Position	6:20732385−65386033
Type	Before polyploidization
Interleaved intrachr. SVs	10
Total SVs (intrachr. + transl.)	10
SV types	DEL: 2; DUP: 5; h2hINV: 2; t2tINV: 1; TRA: 0
SVs in sample	78
Oscillating CN (2 and 3 states)	11, 18
CN segments	18
FDR fragment joints	0.59
FDR chr. breakp. enrich.	0.03
Linked to chrs	
Purity, ploidy	0.78, 3.15

	4b930a10−4b12−4428−84f9−3255b4a3bc4f
Cancer type	Ovary−AdenoCA
Position	19:10117072−19925765
Type	With other complex events
Interleaved intrachr. SVs	10
Total SVs (intrachr. + transl.)	11
SV types	DEL: 1; DUP: 4; h2hINV: 2; t2tINV: 3; TRA: 1
SVs in sample	78
Oscillating CN (2 and 3 states)	7, 7
CN segments	26
FDR fragment joints	0.64
FDR chr. breakp. enrich.	0
Linked to chrs	
Purity, ploidy	0.78, 3.15

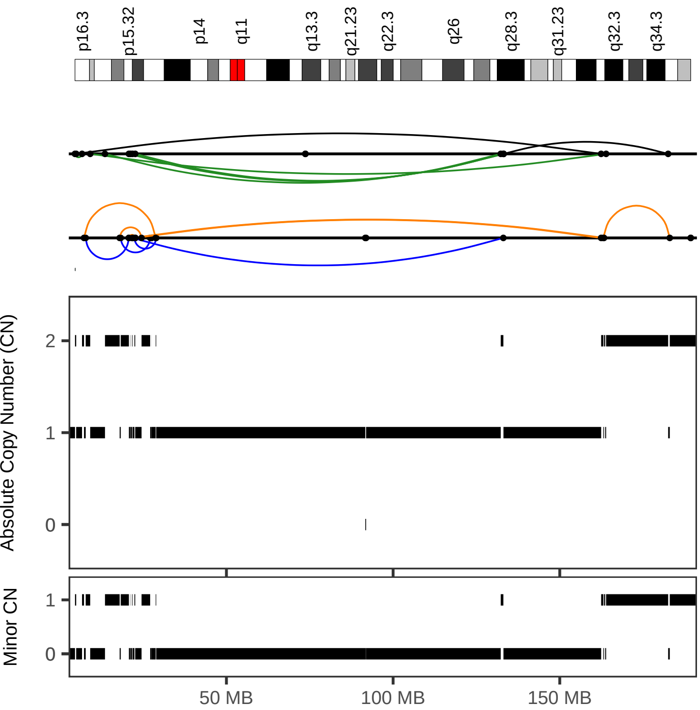

	**4c18d9cf−4af4−4a86−8b1c−f78795fbbd7e**
Cancer type	Ovary−AdenoCA
Position	4:4561511−183003967
Type	Canonical without polyploidization
Interleaved intrachr. SVs	19
Total SVs (intrachr. + transl.)	19
SV types	DEL: 6; DUP: 5; h2hINV: 3; t2tINV: 5; TRA: 0
SVs in sample	96
Oscillating CN (2 and 3 states)	22, 35
CN segments	35
FDR fragment joints	0.84
FDR chr. breakp. enrich.	0
Linked to chrs	
Purity, ploidy	0.83, 1.77

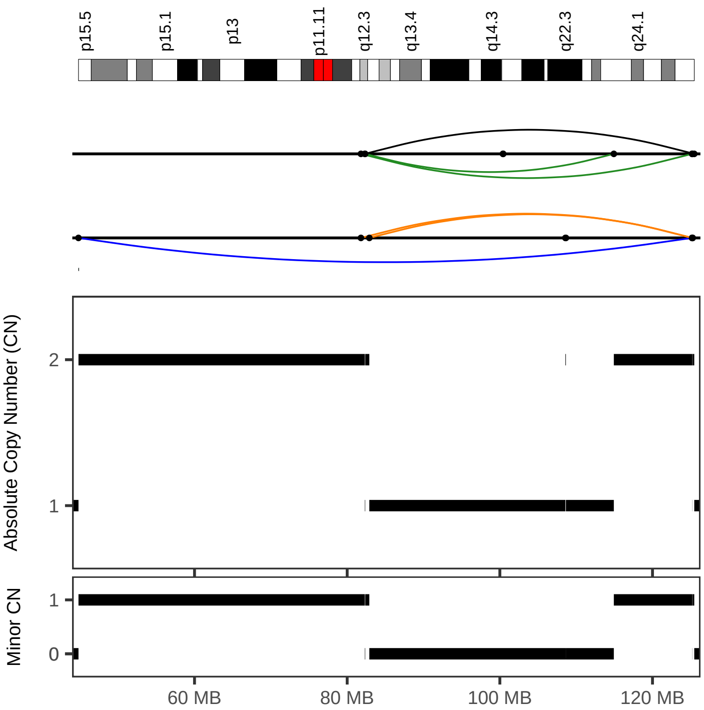

	**4c18d9cf−4af4−4a86−8b1c−f78795fbbd7e**
Cancer type	Ovary−AdenoCA
Position	11:44797680−125468262
Type	Canonical without polyploidization
Interleaved intrachr. SVs	7
Total SVs (intrachr. + transl.)	7
SV types	DEL: 2; DUP: 1; h2hINV: 2; t2tINV: 2; TRA: 0
SVs in sample	96
Oscillating CN (2 and 3 states)	12, 12
CN segments	12
FDR fragment joints	0.95
FDR chr. breakp. enrich.	0.06
Linked to chrs	
Purity, ploidy	0.83, 1.77

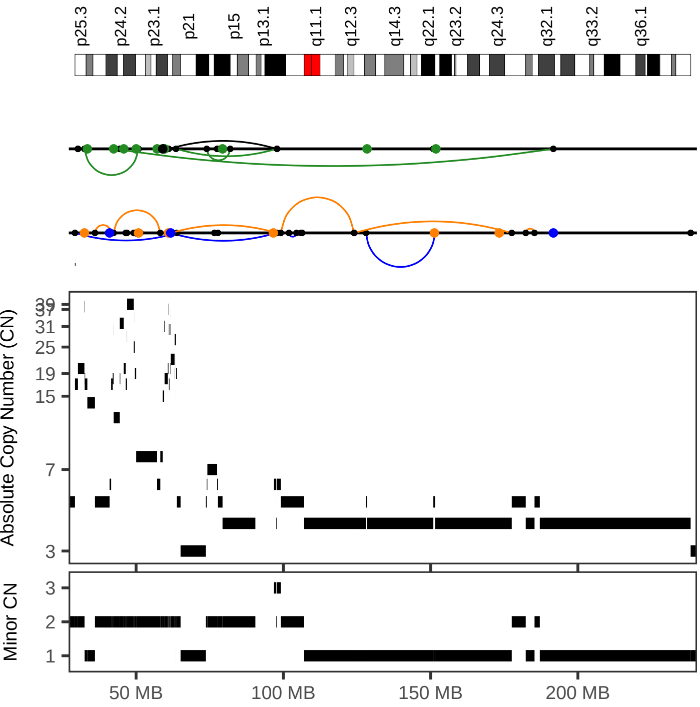

	**5c127332−5ca0−45f1−a5ac−4876ad94e491**
Cancer type	Ovary−AdenoCA
Position	2:29255147−191672689
Type	With other complex events
Interleaved intrachr. SVs	10
Total SVs (intrachr. + transl.)	30
SV types	DEL: 3; DUP: 3; h2hINV: 1; t2tINV: 3; TRA: 20
SVs in sample	318
Oscillating CN (2 and 3 states)	12, 15
CN segments	65
FDR fragment joints	0.83
FDR chr. breakp. enrich.	0
Linked to chrs	10:4028896−29263432;7:1928595−38075627
Purity, ploidy	0.8, 3.95

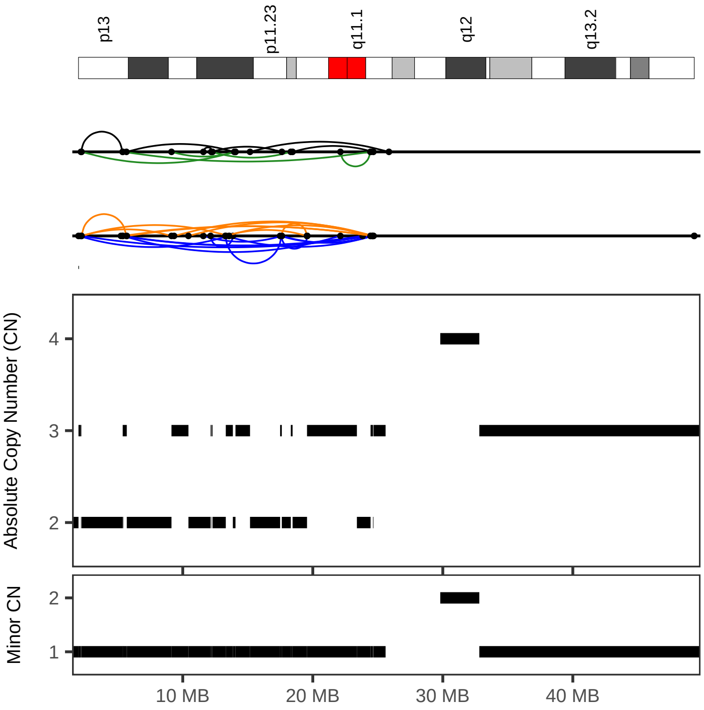

	**62379be5−13f0−474b−94d3−6f944ec4ee96**
Cancer type	Ovary−AdenoCA
Position	20:1979378−25856531
Type	Canonical without polyploidization
Interleaved intrachr. SVs	33
Total SVs (intrachr. + transl.)	33
SV types	DEL: 10; DUP: 12; h2hINV: 6; t2tINV: 5; TRA: 0
SVs in sample	216
Oscillating CN (2 and 3 states)	29, 30
CN segments	30
FDR fragment joints	0.59
FDR chr. breakp. enrich.	0
Linked to chrs	
Purity, ploidy	0.94, 2.68

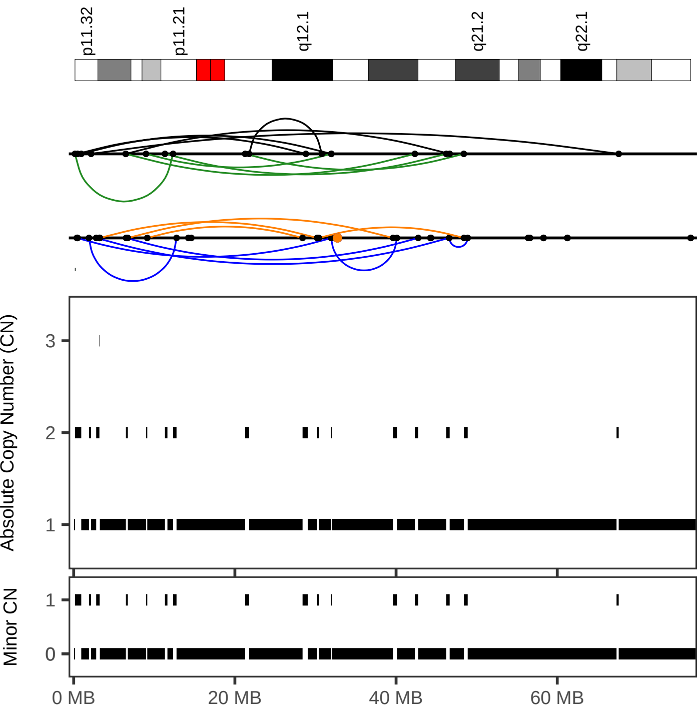

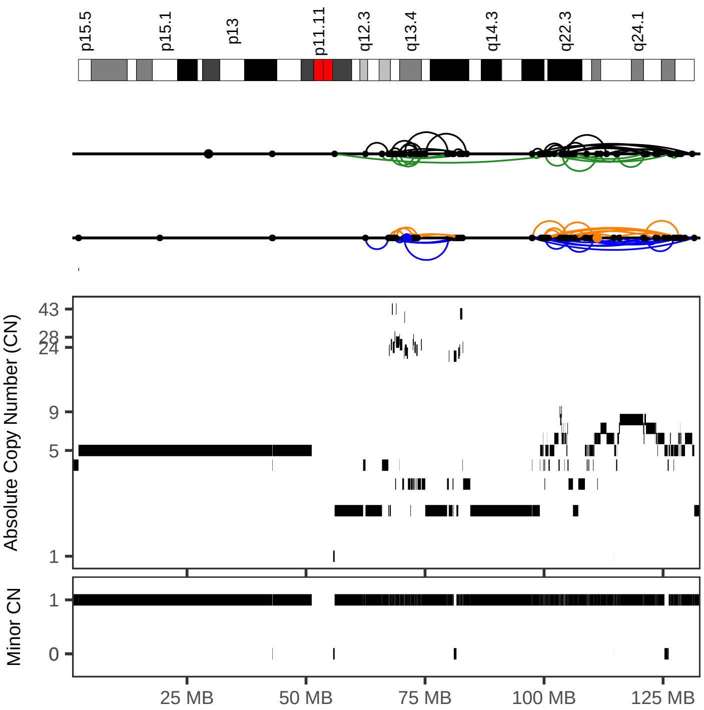

	700e91bb−d675−41b2−bbbd−935767c7b447
Cancer type	Ovary−AdenoCA
Position	18:158944−67628538
Type	Canonical without polyploidization
Interleaved intrachr. SVs	20
Total SVs (intrachr. + transl.)	22
SV types	DEL: 4; DUP: 6; h2hINV: 5; t2tINV: 5; TRA: 2
SVs in sample	686
Oscillating CN (2 and 3 states)	31, 33
CN segments	37
FDR fragment joints	0.96
FDR chr. breakp. enrich.	0.04
Linked to chrs	6:9405680−153296807;
Purity, ploidy	0.8, 1.88

	7ca97692−0bf5−4bbd−81ce−10a051d04bd5
Cancer type	Ovary−AdenoCA
Position	11:56003935−131544055
Type	With other complex events
Interleaved intrachr. SVs	63
Total SVs (intrachr. + transl.)	65
SV types	DEL: 16; DUP: 15; h2hINV: 16; t2tINV: 16; TRA: 2
SVs in sample	275
Oscillating CN (2 and 3 states)	11, 36
CN segments	152
FDR fragment joints	1
FDR chr. breakp. enrich.	0
Linked to chrs	
Purity, ploidy	0.96, 3.74

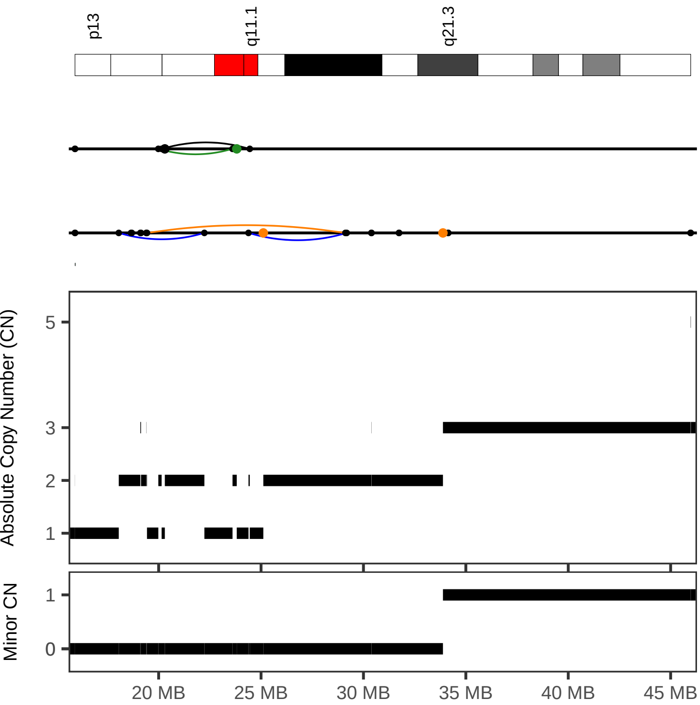

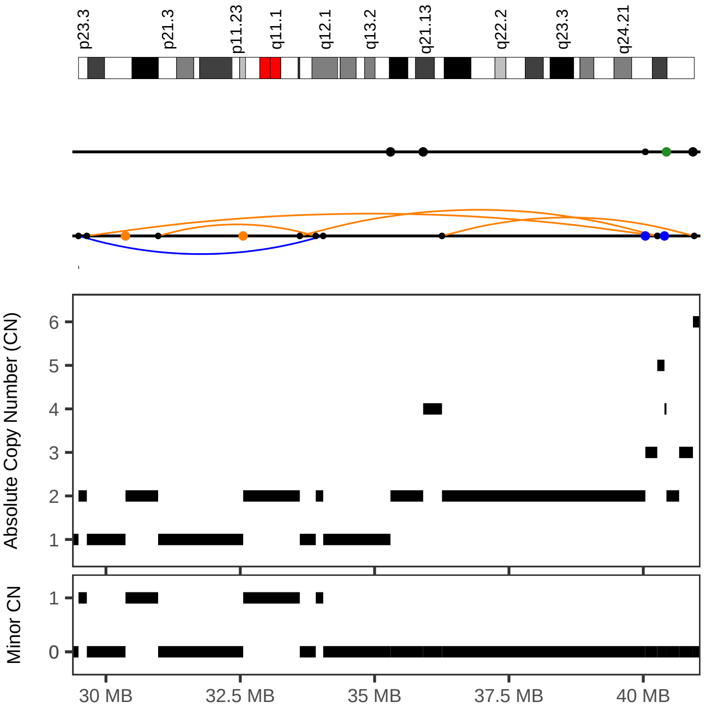

	8a6d2ce3−cc57−451b−9b07−8263782aa23f
Cancer type	Ovary−AdenoCA
Position	21:18052703−29183847
Type	Canonical without polyploidization
Interleaved intrachr. SVs	5
Total SVs (intrachr. + transl.)	8
SV types	DEL: 1; DUP: 2; h2hINV: 1; t2tINV: 1; TRA: 3
SVs in sample	704
Oscillating CN (2 and 3 states)	11, 15
CN segments	15
FDR fragment joints	0.92
FDR chr. breakp. enrich.	0.01
Linked to chrs	2:23974041−152146531;
Purity, ploidy	0.76, 3.3

	ba68f2cf−41fd−9655−1fac7681f588
Cancer type	Ovary−AdenoCA
Position	8:29488947−40948299
Type	With other complex events
Interleaved intrachr. SVs	5
Total SVs (intrachr. + transl.)	13
SV types	DEL: 4; DUP: 1; h2hINV: 0; t2tINV: 0; TRA: 8
SVs in sample	204
Oscillating CN (2 and 3 states)	9, 9
CN segments	17
FDR fragment joints	0.36
FDR chr. breakp. enrich.	0.23
Linked to chrs	
Purity, ploidy	0.89, 3.23

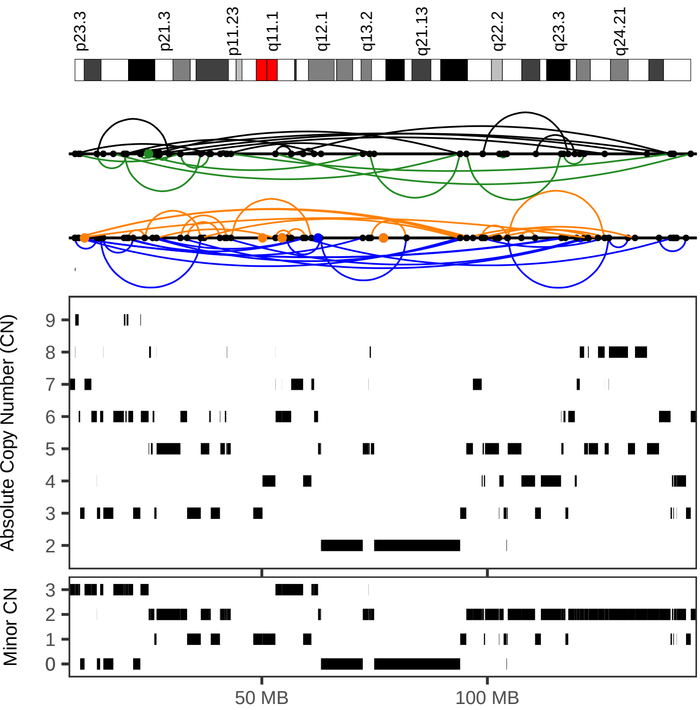

	**c435627c−159d−4a6d−a819−30abac24bf4d**
Cancer type	Ovary−AdenoCA
Position	8:8571303−145086772
Type	With other complex events
Interleaved intrachr. SVs	67
Total SVs (intrachr. + transl.)	77
SV types	DEL: 19; DUP: 18; h2hINV: 15; t2tINV: 15; TRA: 10
SVs in sample	283
Oscillating CN (2 and 3 states)	7, 14
CN segments	117
FDR fragment joints	0.9
FDR chr. breakp. enrich.	0
Linked to chrs	3:15395276−169948381;
Purity, ploidy	0.94, 3.57

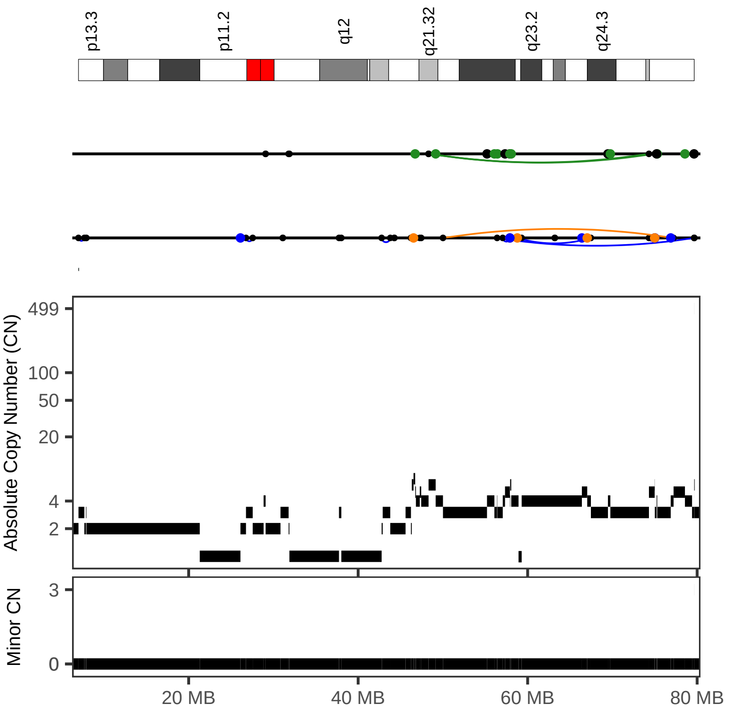

	**c9e28934−8379−4511−817c−d787f2c4ca3a**
Cancer type	Ovary−AdenoCA
Position	17:48309481−79654739
Type	With other complex events
Interleaved intrachr. SVs	5
Total SVs (intrachr. + transl.)	28
SV types	DEL: 1; DUP: 2; h2hINV: 0; t2tINV: 2; TRA: 23
SVs in sample	377
Oscillating CN (2 and 3 states)	7, 10
CN segments	30
FDR fragment joints	0.64
FDR chr. breakp. enrich.	0
Linked to chrs	1:22012754−22205189;16:288679−70722743 2:18960070−230123603;
Purity, ploidy	0.84, 2.56

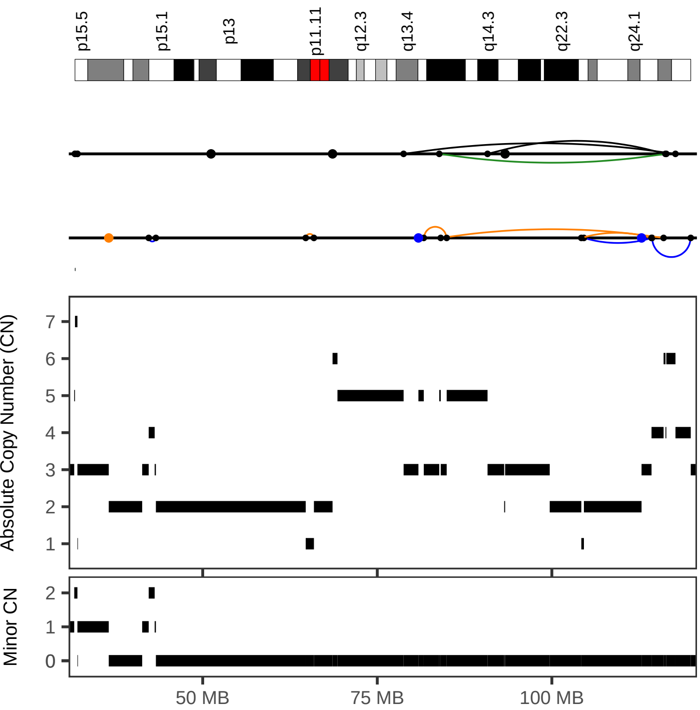

	**e9483296−cb91−497a−b955−39a3c3289dac**
Cancer type	Ovary−AdenoCA
Position	11:78769716−119886055
Type	With other complex events
Interleaved intrachr. SVs	6
Total SVs (intrachr. + transl.)	9
SV types	DEL: 2; DUP: 1; h2hINV: 2; t2tINV: 1; TRA: 3
SVs in sample	126
Oscillating CN (2 and 3 states)	7, 8
CN segments	19
FDR fragment joints	0.91
FDR chr. breakp. enrich.	0
Linked to chrs	6:69991793−73854949;
Purity, ploidy	0.92, 2.78

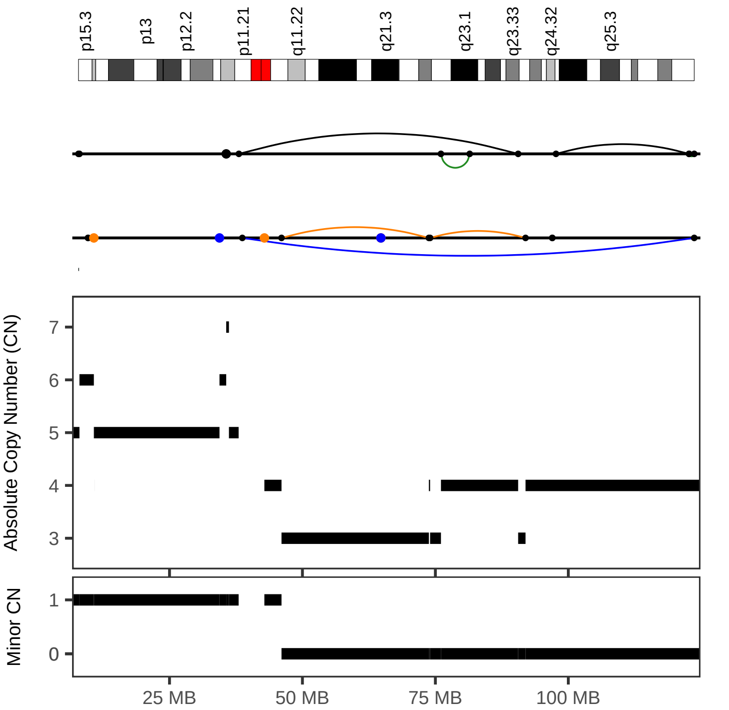

	**f0c353fd−947c−41e2−b643−3ecc0d69796c**
Cancer type	Ovary−AdenoCA
Position	10:38043794−123678267
Type	After polyploidization
Interleaved intrachr. SVs	4
Total SVs (intrachr. + transl.)	7
SV types	DEL: 1; DUP: 1; h2hINV: 1; t2tINV: 1; TRA: 3
SVs in sample	288
Oscillating CN (2 and 3 states)	7, 8
CN segments	8
FDR fragment joints	1
FDR chr. breakp. enrich.	0.5
Linked to chrs	7:64898151−152863433;
Purity, ploidy	0.91, 4.11

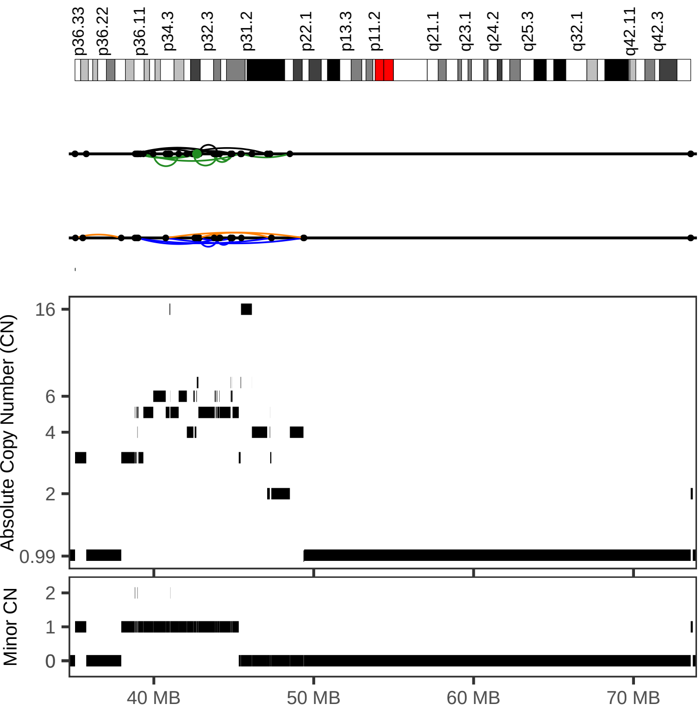

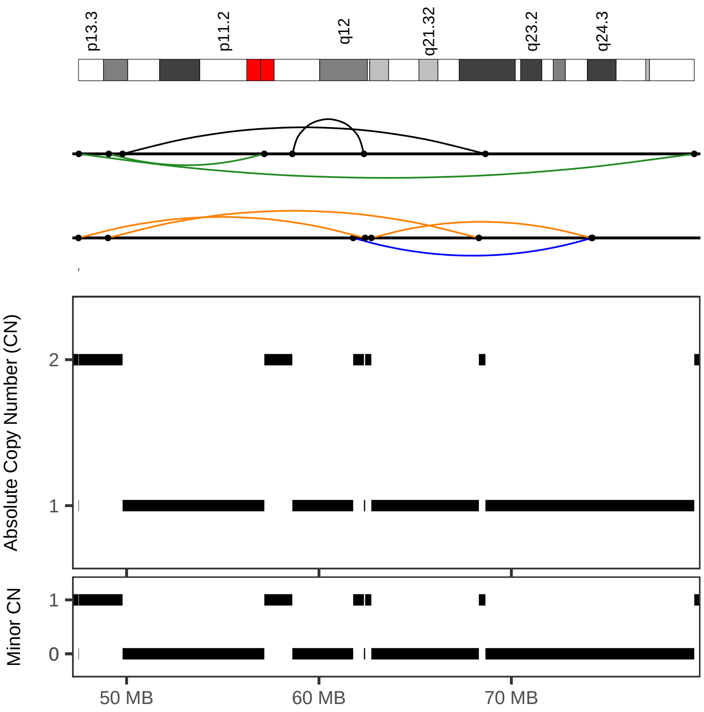

	**ICGC_0007**
Cancer type	Panc−AdenoCA
Position	1:38813433−49397759
Type	With other complex events
Interleaved intrachr. SVs	28
Total SVs (intrachr. + transl.)	29
SV types	DEL: 3; DUP: 8; h2hINV: 8;<br>t2tINV: 9; TRA: 1
SVs in sample	82
Oscillating CN (2 and 3 states)	7, 13
CN segments	45
FDR fragment joints	0.59
FDR chr. breakp. enrich.	0
Linked to chrs	
Purity, ploidy	0.47, 1.83

	**ICGC_0007**
Cancer type	Panc−AdenoCA
Position	17:47498985−79507305
Type	Canonical without polyploidization
Interleaved intrachr. SVs	8
Total SVs (intrachr. + transl.)	8
SV types	DEL: 3; DUP: 1; h2hINV: 2;<br>t2tINV: 2; TRA: 0
SVs in sample	82
Oscillating CN (2 and 3 states)	12, 12
CN segments	12
FDR fragment joints	0.84
FDR chr. breakp. enrich.	0.01
Linked to chrs	
Purity, ploidy	0.47, 1.83

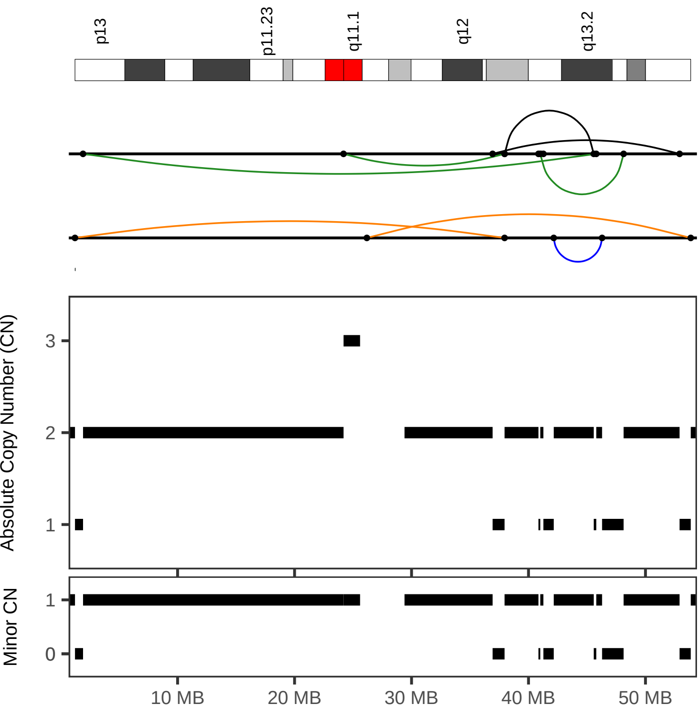

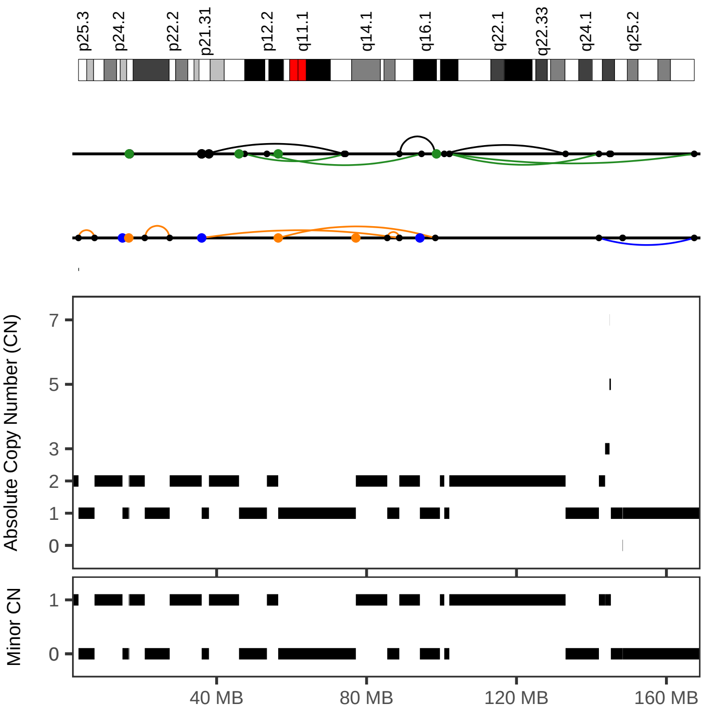

	**ICGC_0007**
Cancer type	Panc−AdenoCA
Position	20:1226919−53870564
Type	Canonical without polyploidization
Interleaved intrachr. SVs	9
Total SVs (intrachr. + transl.)	9
SV types	DEL: 2; DUP: 1; h2hINV: 3;<br>t2tINV: 3; TRA: 0
SVs in sample	82
Oscillating CN (2 and 3 states)	13, 16
CN segments	16
FDR fragment joints	0.83
FDR chr. breakp. enrich.	0
Linked to chrs	
Purity, ploidy	0.47, 1.83

	**ICGC_0020**
Cancer type	Panc−AdenoCA
Position	6:36021238−98348848
Type	Canonical without polyploidization
Interleaved intrachr. SVs	7
Total SVs (intrachr. + transl.)	13
SV types	DEL: 3; DUP: 0; h2hINV: 2;<br>t2tINV: 2; TRA: 6
SVs in sample	57
Oscillating CN (2 and 3 states)	9, 9
CN segments	9
FDR fragment joints	0.64
FDR chr. breakp. enrich.	0
Linked to chrs	11:2734690−58550647;
Purity, ploidy	0.54, 1.78

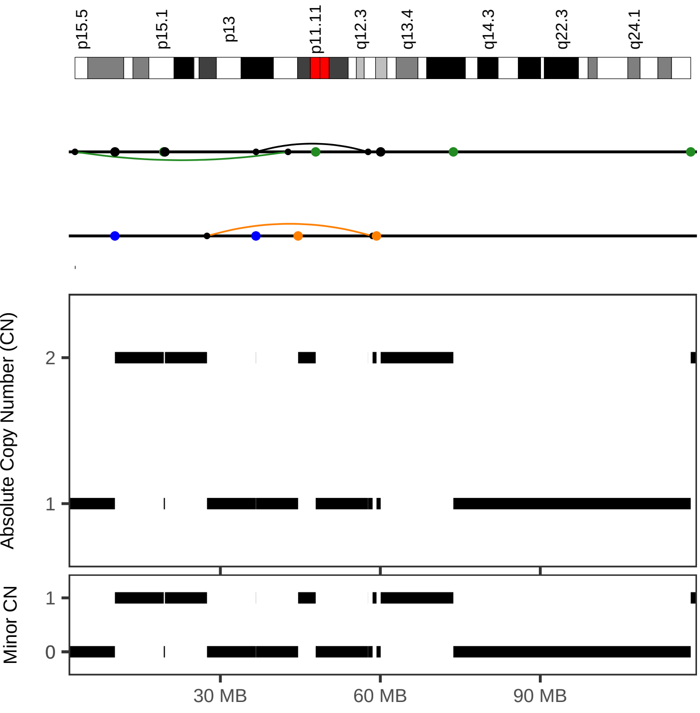

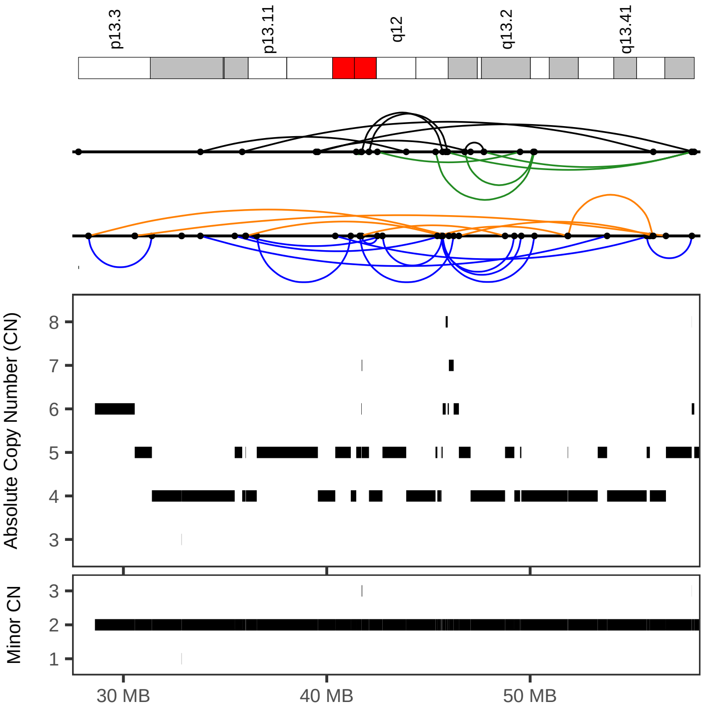

	**ICGC_0020**
Cancer type	Panc−AdenoCA
Position	11:2734690−58550648
Type	Canonical without polyploidization
Interleaved intrachr. SVs	3
Total SVs (intrachr. + transl.)	10
SV types	DEL: 1; DUP: 0; h2hINV: 1; t2tINV: 1; TRA: 7
SVs in sample	57
Oscillating CN (2 and 3 states)	12, 12
CN segments	12
FDR fragment joints	0.84
FDR chr. breakp. enrich.	0
Linked to chrs	
Purity, ploidy	0.54, 1.78

	**ICGC_0021**
Cancer type	Panc−AdenoCA
Position	19:28278241−58069967
Type	With other complex events
Interleaved intrachr. SVs	33
Total SVs (intrachr. + transl.)	33
SV types	DEL: 7; DUP: 13; h2hINV: 7; t2tINV: 6; TRA: 0
SVs in sample	162
Oscillating CN (2 and 3 states)	13, 17
CN segments	48
FDR fragment joints	0.59
FDR chr. breakp. enrich.	0
Linked to chrs	
Purity, ploidy	0.43, 2.88

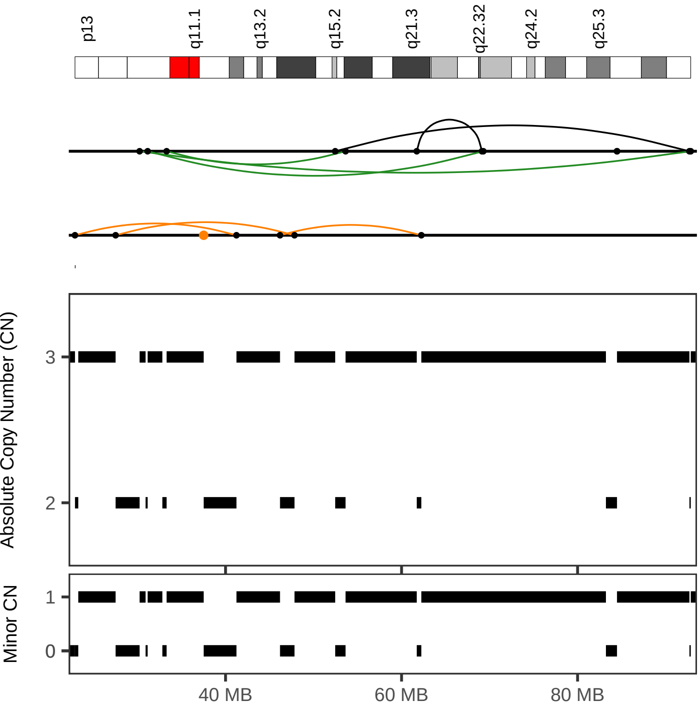

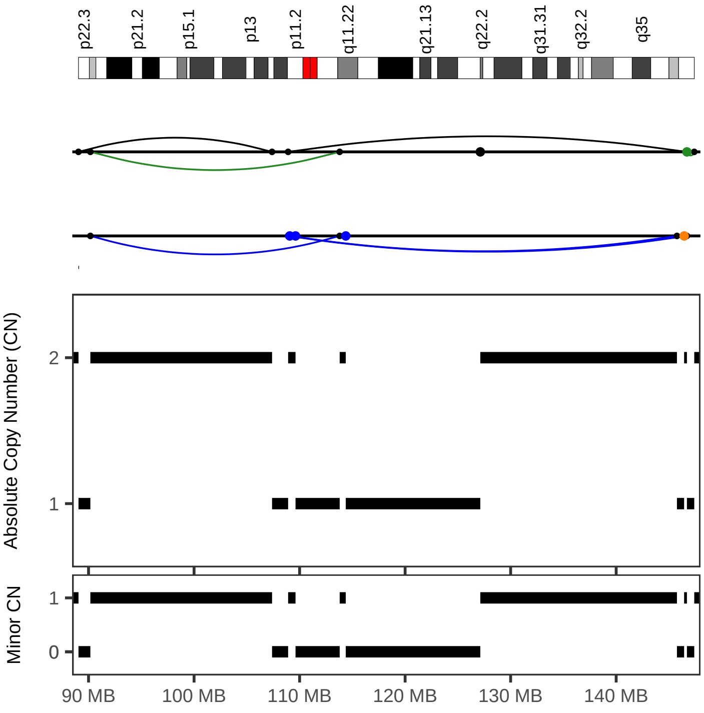

	**ICGC_0026**
Cancer type	Panc−AdenoCA
Position	15:22891529−92856416
Type	Canonical without polyploidization
Interleaved intrachr. SVs	8
Total SVs (intrachr. + transl.)	9
SV types	DEL: 3; DUP: 0; h2hINV: 2; t2tINV: 3; TRA: 1
SVs in sample	66
Oscillating CN (2 and 3 states)	20, 20
CN segments	20
FDR fragment joints	0.59
FDR chr. breakp. enrich.	0
Linked to chrs	
Purity, ploidy	0.62, 3.55

	**ICGC_0031**
Cancer type	Panc−AdenoCA
Position	7:89045625−146695947
Type	Canonical without polyploidization
Interleaved intrachr. SVs	6
Total SVs (intrachr. + transl.)	11
SV types	DEL: 0; DUP: 3; h2hINV: 2; t2tINV: 1; TRA: 5
SVs in sample	27
Oscillating CN (2 and 3 states)	10, 10
CN segments	10
FDR fragment joints	0.59
FDR chr. breakp. enrich.	0
Linked to chrs	3:20319589−174852805;
Purity, ploidy	0.42, 1.68

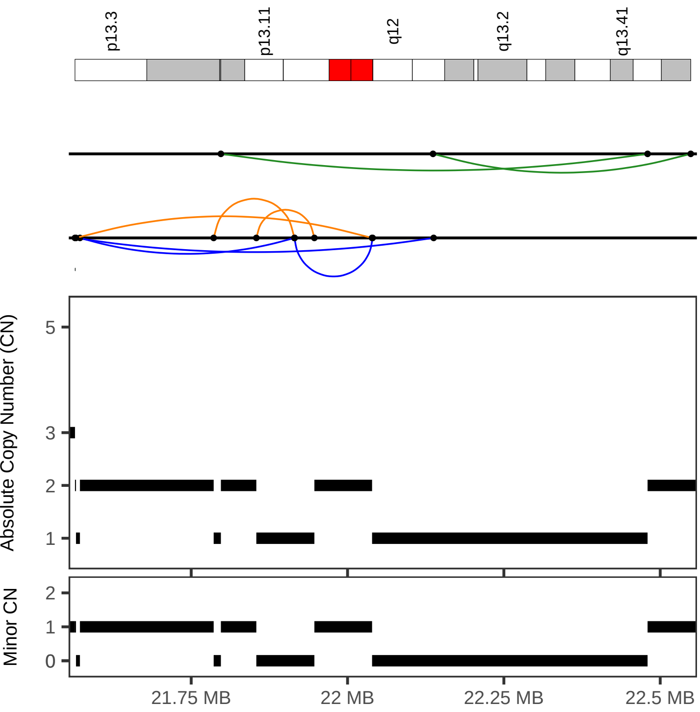

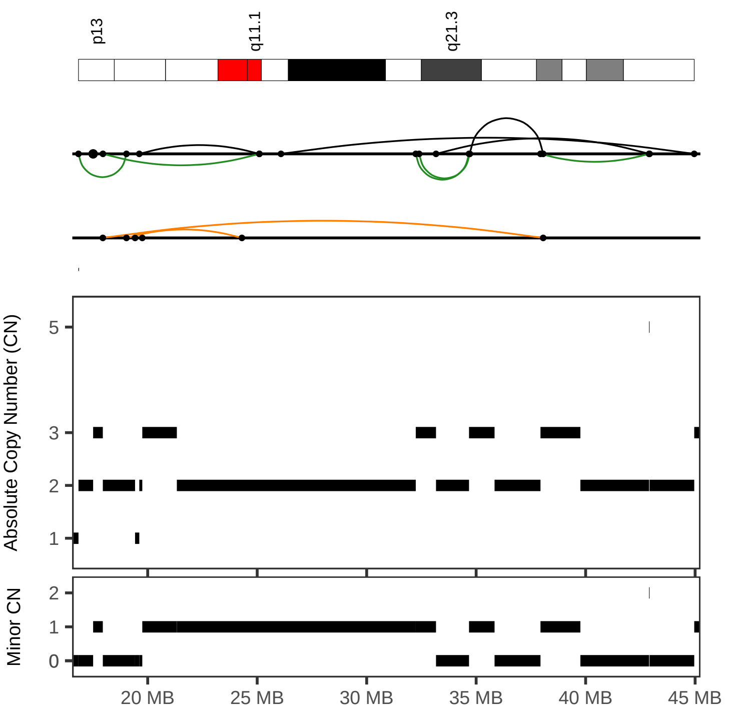

	ICGC_0037
Cancer type	Panc−AdenoCA
Position	19:21564139−22548813
Type	Canonical without polyploidization
Interleaved intrachr. SVs	8
Total SVs (intrachr. + transl.)	8
SV types	DEL: 3; DUP: 3; h2hINV: 0; t2tINV: 2; TRA: 0
SVs in sample	165
Oscillating CN (2 and 3 states)	9, 9
CN segments	9
FDR fragment joints	0.59
FDR chr. breakp. enrich.	0.01
Linked to chrs	
Purity, ploidy	0.48, 2.75

	ICGC_0052
Cancer type	Panc−AdenoCA
Position	21:16837999−44959353
Type	With other complex events
Interleaved intrachr. SVs	12
Total SVs (intrachr. + transl.)	13
SV types	DEL: 3; DUP: 0; h2hINV: 4; t2tINV: 5; TRA: 1
SVs in sample	83
Oscillating CN (2 and 3 states)	9, 16
CN segments	16
FDR fragment joints	0.59
FDR chr. breakp. enrich.	0
Linked to chrs	
Purity, ploidy	0.33, 2.89

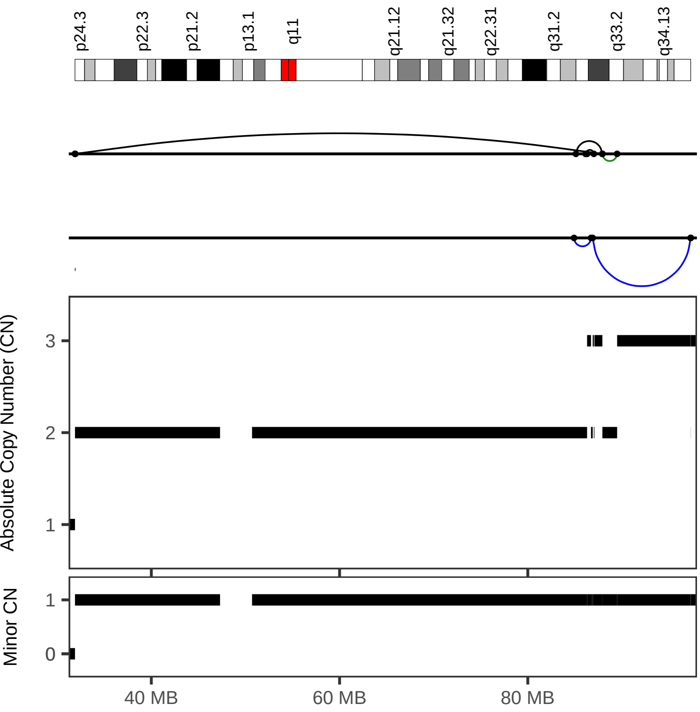

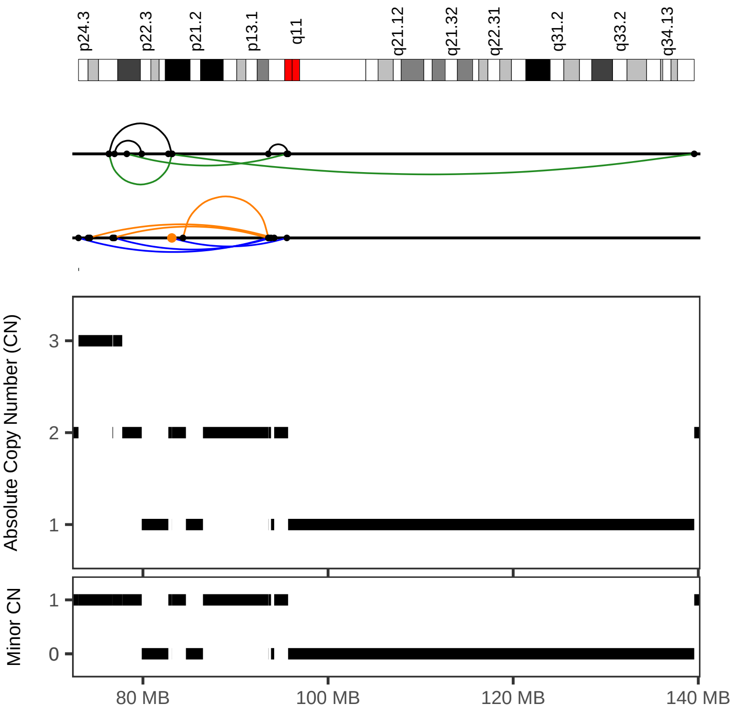

	ICGC_0055
Cancer type	Panc−AdenoCA
Position	9:31913973−97303304
Type	Canonical without polyploidization
Interleaved intrachr. SVs	7
Total SVs (intrachr. + transl.)	7
SV types	DEL: 0; DUP: 2; h2hINV: 3; t2tINV: 2; TRA: 0
SVs in sample	23
Oscillating CN (2 and 3 states)	9, 10
CN segments	10
FDR fragment joints	0.64
FDR chr. breakp. enrich.	0
Linked to chrs	
Purity, ploidy	0.51, 1.75

	ICGC_0067
Cancer type	Panc−AdenoCA
Position	9:73035221−139560353
Type	Canonical without polyploidization
Interleaved intrachr. SVs	12
Total SVs (intrachr. + transl.)	13
SV types	DEL: 3; DUP: 3; h2hINV: 3; t2tINV: 3; TRA: 1
SVs in sample	73
Oscillating CN (2 and 3 states)	13, 16
CN segments	16
FDR fragment joints	1
FDR chr. breakp. enrich.	0
Linked to chrs	
Purity, ploidy	0.32, 1.69

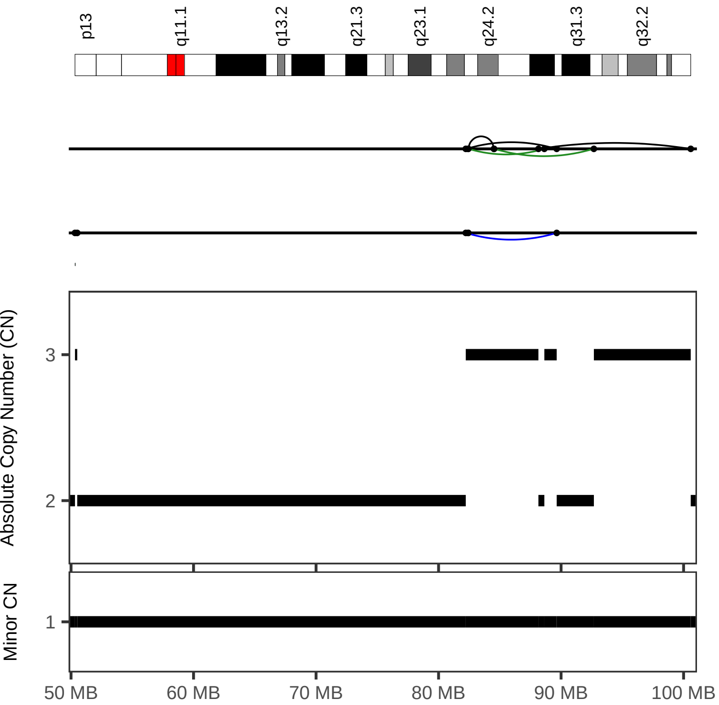

	ICGC_0067
Cancer type	Panc−AdenoCA
Position	14:82219284−100582142
Type	Canonical without polyploidization
Interleaved intrachr. SVs	6
Total SVs (intrachr. + transl.)	6
SV types	DEL: 0; DUP: 1; h2hINV: 3; t2tINV: 2; TRA: 0
SVs in sample	73
Oscillating CN (2 and 3 states)	7, 7
CN segments	7
FDR fragment joints	0.59
FDR chr. breakp. enrich.	0.01
Linked to chrs	
Purity, ploidy	0.32, 1.69

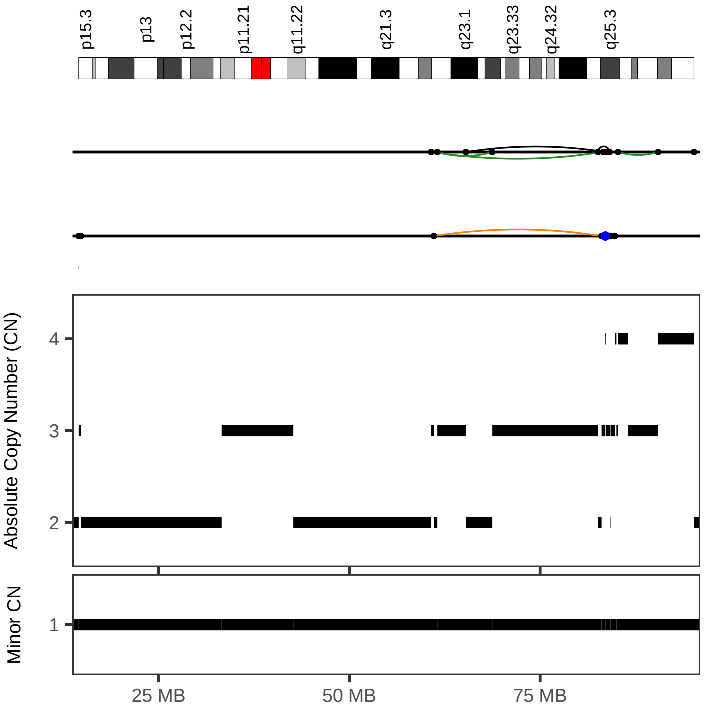

	ICGC_0087
Cancer type	Panc−AdenoCA
Position	10:60725577−84090610
Type	Canonical without polyploidization
Interleaved intrachr. SVs	6
Total SVs (intrachr. + transl.)	7
SV types	DEL: 2; DUP: 0; h2hINV: 2; t2tINV: 2; TRA: 1
SVs in sample	79
Oscillating CN (2 and 3 states)	7, 9
CN segments	9
FDR fragment joints	0.64
FDR chr. breakp. enrich.	0
Linked to chrs	
Purity, ploidy	0.29, 2.33

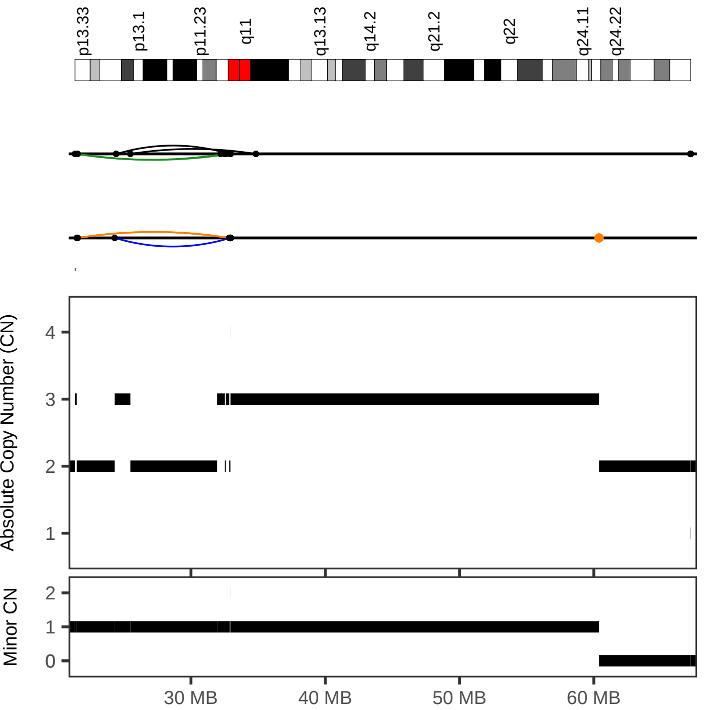

	ICGC_0088
Cancer type	Panc−AdenoCA
Position	12:21367968−34833918
Type	Canonical without polyploidization
Interleaved intrachr. SVs	9
Total SVs (intrachr. + transl.)	9
SV types	DEL: 3; DUP: 2; h2hINV: 2; t2tINV: 2; TRA: 0
SVs in sample	116
Oscillating CN (2 and 3 states)	9, 13
CN segments	13
FDR fragment joints	0.97
FDR chr. breakp. enrich.	0.02
Linked to chrs	
Purity, ploidy	0.51, 2.33

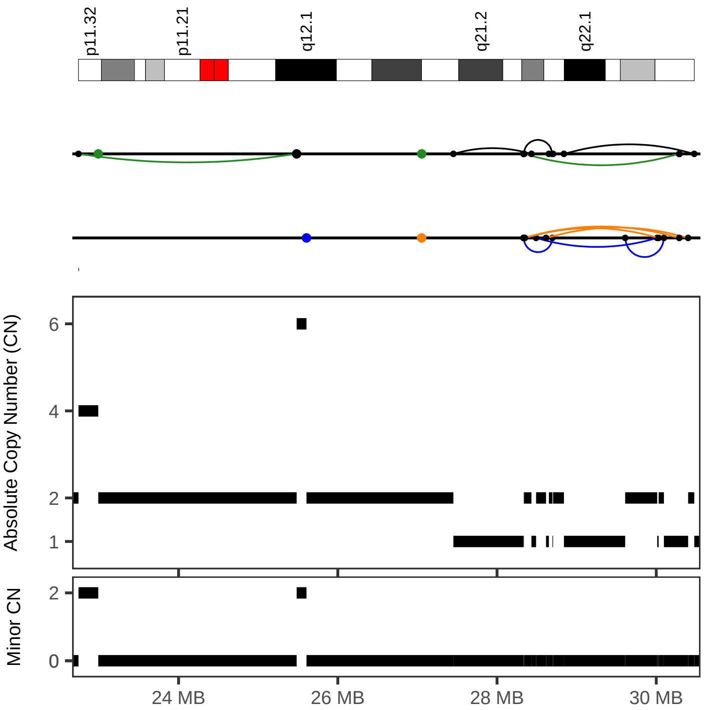

	ICGC_0088
Cancer type	Panc−AdenoCA
Position	18:27451678−30477084
Type	Canonical without polyploidization
Interleaved intrachr. SVs	11
Total SVs (intrachr. + transl.)	11
SV types	DEL: 3; DUP: 3; h2hINV: 3; t2tINV: 2; TRA: 0
SVs in sample	116
Oscillating CN (2 and 3 states)	17, 17
CN segments	17
FDR fragment joints	0.98
FDR chr. breakp. enrich.	0
Linked to chrs	
Purity, ploidy	0.51, 2.33

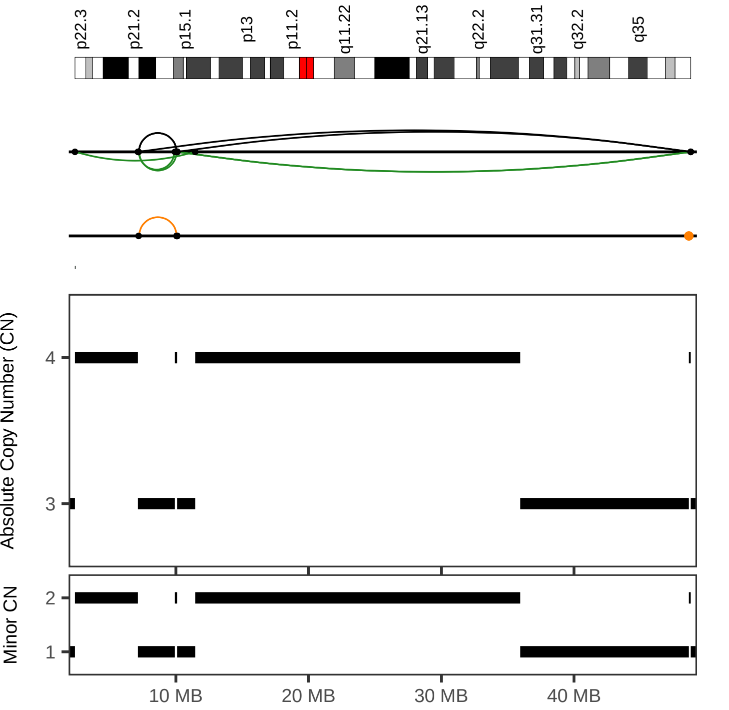

ICGC_0108	
Cancer type	Panc−AdenoCA
Position	7:2398212−48791277
Type	After polyploidization
Interleaved intrachr. SVs	11
Total SVs (intrachr. + transl.)	12
SV types	DEL: 1; DUP: 1; h2hINV: 4; t2tINV: 5; TRA: 1
SVs in sample	130
Oscillating CN (2 and 3 states)	8, 8
CN segments	8
FDR fragment joints	0.59
FDR chr. breakp. enrich.	0.03
Linked to chrs	
Purity, ploidy	0.31, 3.02

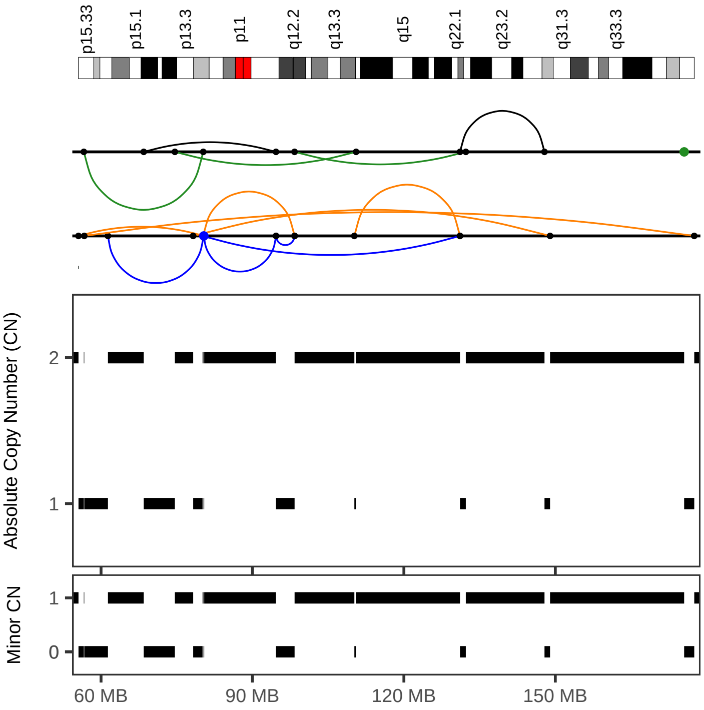

ICGC_0109	
Cancer type	Panc−AdenoCA
Position	5:55531264−177528041
Type	Canonical without polyploidization
Interleaved intrachr. SVs	14
Total SVs (intrachr. + transl.)	16
SV types	DEL: 5; DUP: 4; h2hINV: 2; t2tINV: 3; TRA: 2
SVs in sample	30
Oscillating CN (2 and 3 states)	22, 22
CN segments	22
FDR fragment joints	0.78
FDR chr. breakp. enrich.	0
Linked to chrs	
Purity, ploidy	0.48, 1.96

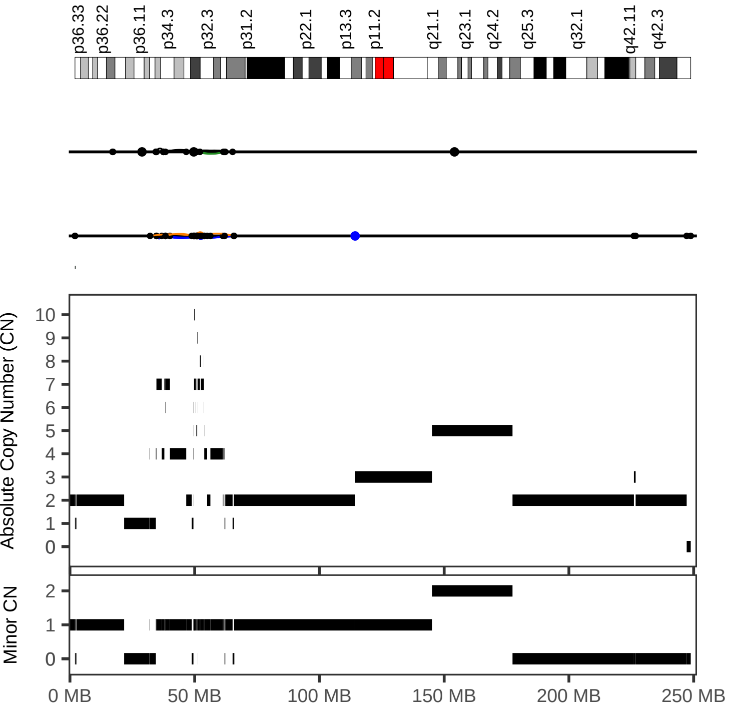

ICGC_0114	
Cancer type	Panc−AdenoCA
Position	1:32132328−65796925
Type	With other complex events
Interleaved intrachr. SVs	25
Total SVs (intrachr. + transl.)	26
SV types	DEL: 9; DUP: 8; h2hINV: 5; t2tINV: 3; TRA: 1
SVs in sample	195
Oscillating CN (2 and 3 states)	7, 7
CN segments	43
FDR fragment joints	0.59
FDR chr. breakp. enrich.	0
Linked to chrs	
Purity, ploidy	0.68, 2.73

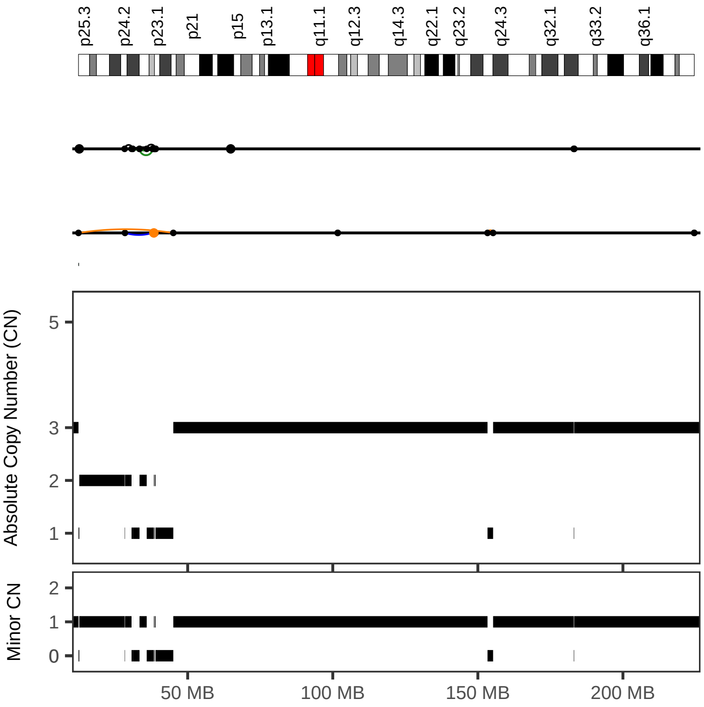

ICGC_0114	
Cancer type	Panc−AdenoCA
Position	2:28274298−38935751
Type	Canonical without polyploidization
Interleaved intrachr. SVs	12
Total SVs (intrachr. + transl.)	13
SV types	DEL: 0; DUP: 3; h2hINV: 4; t2tINV: 5; TRA: 1
SVs in sample	195
Oscillating CN (2 and 3 states)	11, 11
CN segments	11
FDR fragment joints	0.59
FDR chr. breakp. enrich.	0.17
Linked to chrs	
Purity, ploidy	0.68, 2.73

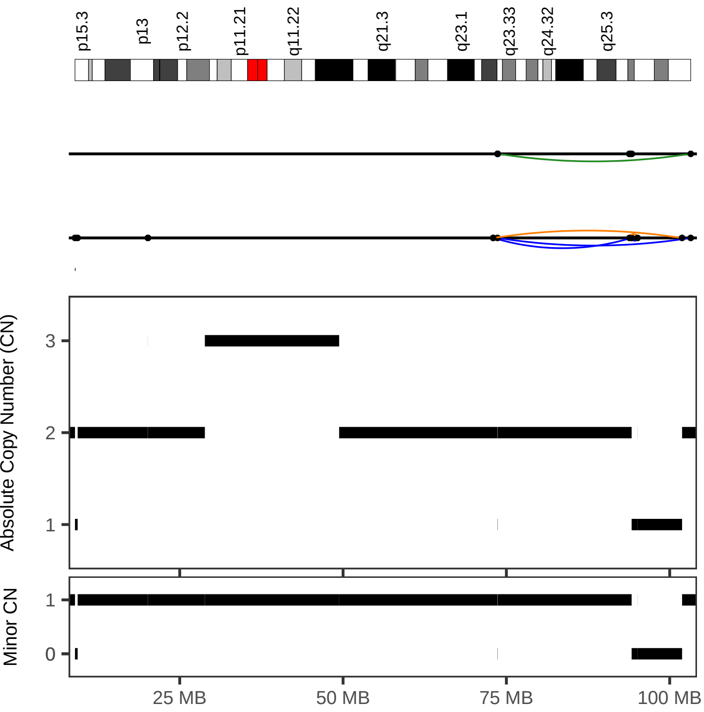

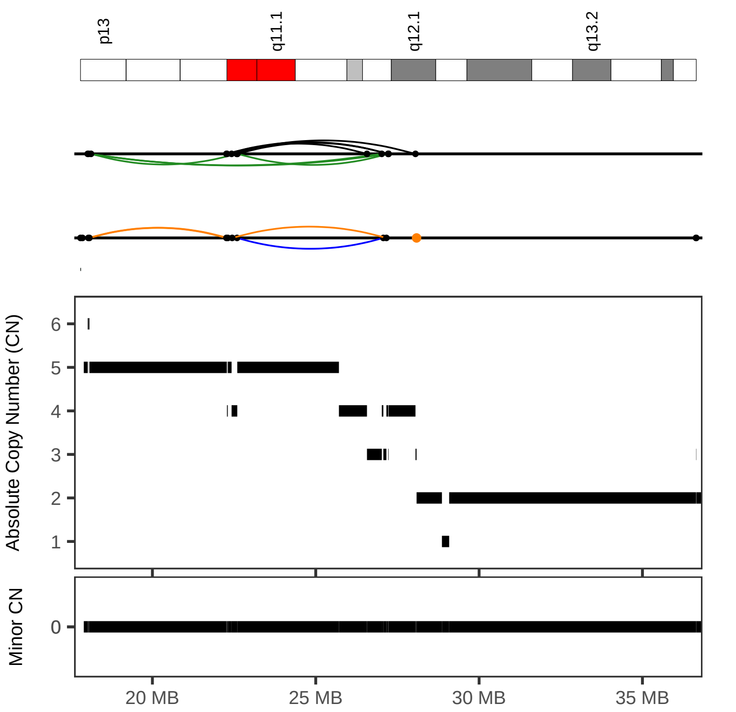

	ICGC_0114
Cancer type	Panc−AdenoCA
Position	10:72984995−103221106
Type	Canonical without polyploidization
Interleaved intrachr. SVs	7
Total SVs (intrachr. + transl.)	7
SV types	DEL: 2; DUP: 3; h2hINV: 1; t2tINV: 1; TRA: 0
SVs in sample	195
Oscillating CN (2 and 3 states)	7, 7
CN segments	7
FDR fragment joints	0.74
FDR chr. breakp. enrich.	0.55
Linked to chrs	
Purity, ploidy	0.68, 2.73

	ICGC_0134
Cancer type	Panc−AdenoCA
Position	22:18019664−28052500
Type	With other complex events
Interleaved intrachr. SVs	12
Total SVs (intrachr. + transl.)	12
SV types	DEL: 3; DUP: 1; h2hINV: 4; t2tINV: 4; TRA: 0
SVs in sample	142
Oscillating CN (2 and 3 states)	8, 18
CN segments	18
FDR fragment joints	0.64
FDR chr. breakp. enrich.	0
Linked to chrs	
Purity, ploidy	0.31, 2.71

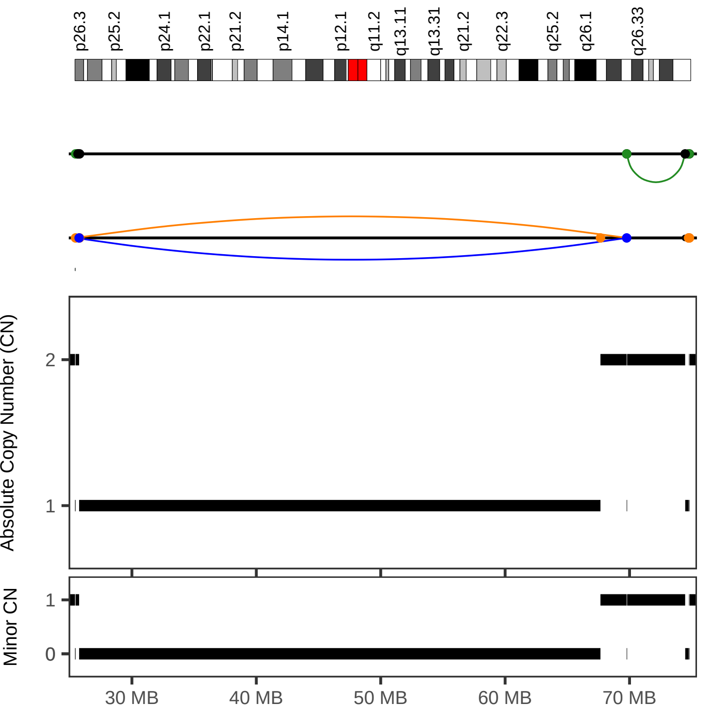

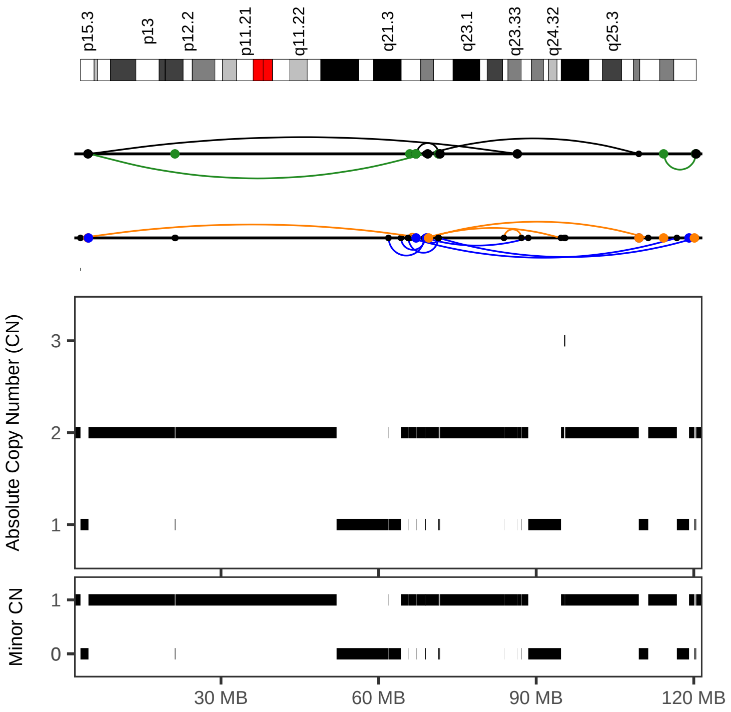

	ICGC_0146
Cancer type	Panc−AdenoCA
Position	3:25423800−74920530
Type	Canonical without polyploidization
Interleaved intrachr. SVs	3
Total SVs (intrachr. + transl.)	21
SV types	DEL: 2; DUP: 0; h2hINV: 0; t2tINV: 1; TRA: 18
SVs in sample	105
Oscillating CN (2 and 3 states)	12, 12
CN segments	12
FDR fragment joints	0.59
FDR chr. breakp. enrich.	0
Linked to chrs	5:13751277−82972678;10:3251990−120467282
Purity, ploidy	0.38, 1.76

	ICGC_0146
Cancer type	Panc−AdenoCA
Position	10:3251990−120467283
Type	Canonical without polyploidization
Interleaved intrachr. SVs	18
Total SVs (intrachr. + transl.)	44
SV types	DEL: 6; DUP: 7; h2hINV: 3; t2tINV: 2; TRA: 26
SVs in sample	105
Oscillating CN (2 and 3 states)	32, 42
CN segments	42
FDR fragment joints	0.59
FDR chr. breakp. enrich.	0
Linked to chrs	3:25423800−74920529;5:13751277−82972678
Purity, ploidy	0.38, 1.76

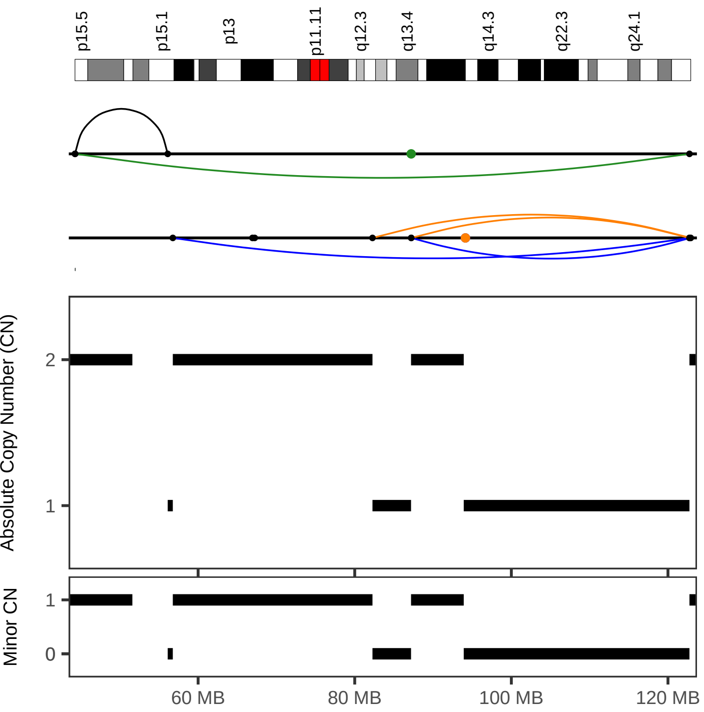

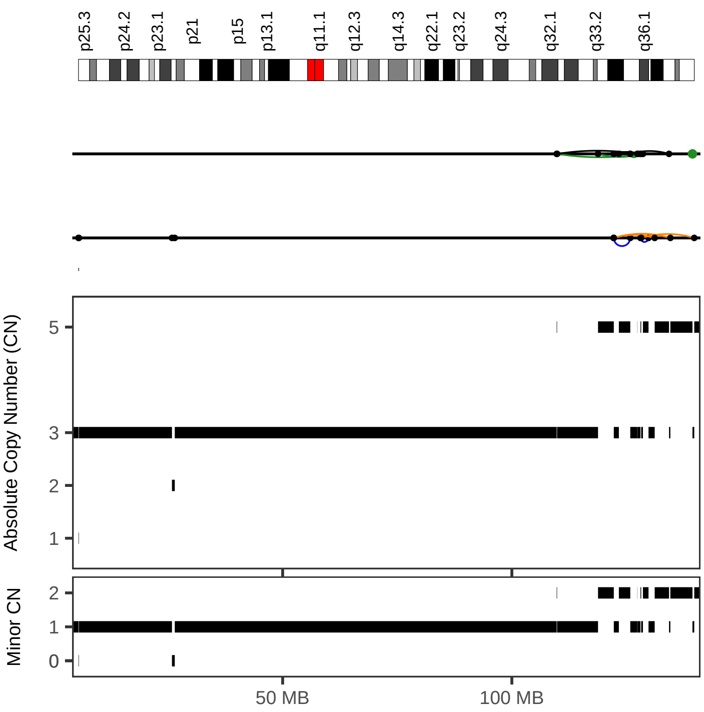

	ICGC_0146
Cancer type	Panc−AdenoCA
Position	11:44273470−122909222
Type	Canonical without polyploidization
Interleaved intrachr. SVs	6
Total SVs (intrachr. + transl.)	9
SV types	DEL: 2; DUP: 2; h2hINV: 1; t2tINV: 1; TRA: 3
SVs in sample	105
Oscillating CN (2 and 3 states)	7, 7
CN segments	7
FDR fragment joints	0.91
FDR chr. breakp. enrich.	0.05
Linked to chrs	5:13751277−82972678;
Purity, ploidy	0.38, 1.76

	ICGC_0199
Cancer type	Panc−AdenoCA
Position	2:109779279−139790794
Type	After polyploidization
Interleaved intrachr. SVs	12
Total SVs (intrachr. + transl.)	13
SV types	DEL: 3; DUP: 2; h2hINV: 3; t2tINV: 4; TRA: 1
SVs in sample	54
Oscillating CN (2 and 3 states)	17, 17
CN segments	17
FDR fragment joints	0.91
FDR chr. breakp. enrich.	0
Linked to chrs	
Purity, ploidy	0.22, 3.05

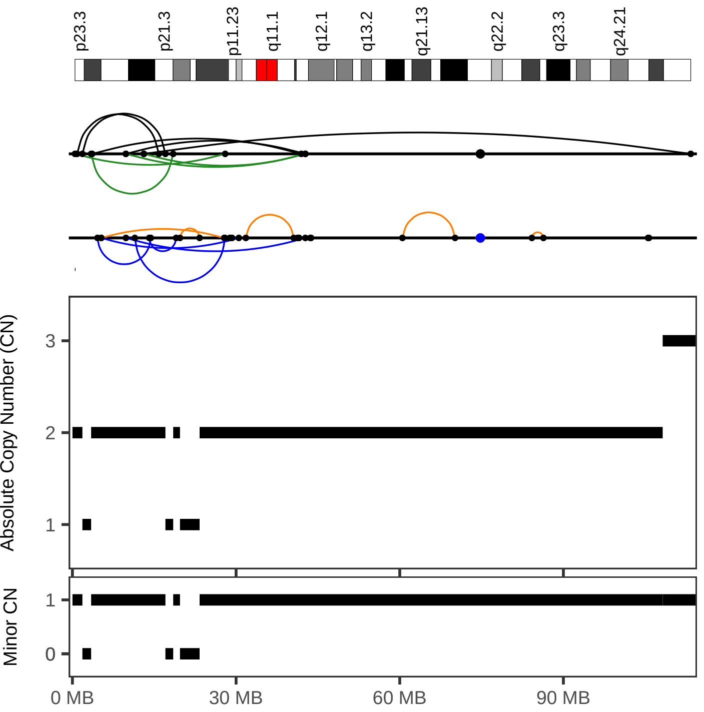

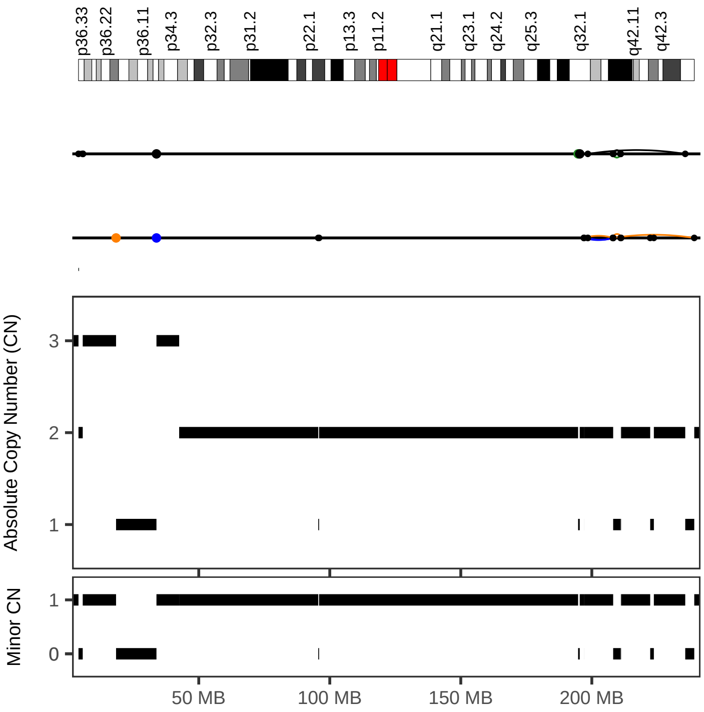

	ICGC_0205
Cancer type	Panc−AdenoCA
Position	8:466553−113346314
Type	Canonical without polyploidization
Interleaved intrachr. SVs	15
Total SVs (intrachr. + transl.)	18
SV types	DEL: 1; DUP: 5; h2hINV: 5; t2tINV: 4; TRA: 3
SVs in sample	127
Oscillating CN (2 and 3 states)	7, 8
CN segments	8
FDR fragment joints	0.62
FDR chr. breakp. enrich.	0
Linked to chrs	9:9187524−139636858;
Purity, ploidy	0.6, 1.83

	ICGC_0214
Cancer type	Panc−AdenoCA
Position	1:196999875−239097447
Type	Canonical without polyploidization
Interleaved intrachr. SVs	12
Total SVs (intrachr. + transl.)	12
SV types	DEL: 5; DUP: 2; h2hINV: 3; t2tINV: 2; TRA: 0
SVs in sample	254
Oscillating CN (2 and 3 states)	11, 11
CN segments	11
FDR fragment joints	0.64
FDR chr. breakp. enrich.	0.96
Linked to chrs	
Purity, ploidy	0.47, 1.82

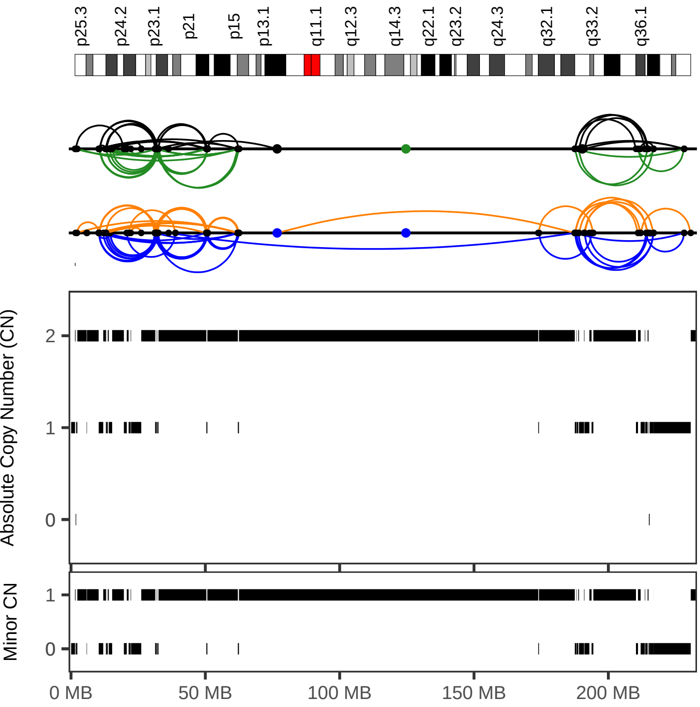

	**ICGC_0214**
Cancer type	Panc−AdenoCA
Position	2:1473520−230672823
Type	Canonical without polyploidization
Interleaved intrachr. SVs	142
Total SVs (intrachr. + transl.)	147
SV types	DEL: 30; DUP: 44; h2hINV: 35; t2tINV: 33; TRA: 5
SVs in sample	254
Oscillating CN (2 and 3 states)	61, 69
CN segments	69
FDR fragment joints	0.59
FDR chr. breakp. enrich.	0
Linked to chrs	4:77735561−162014257;X:98103871−154466372
Purity, ploidy	0.47, 1.82

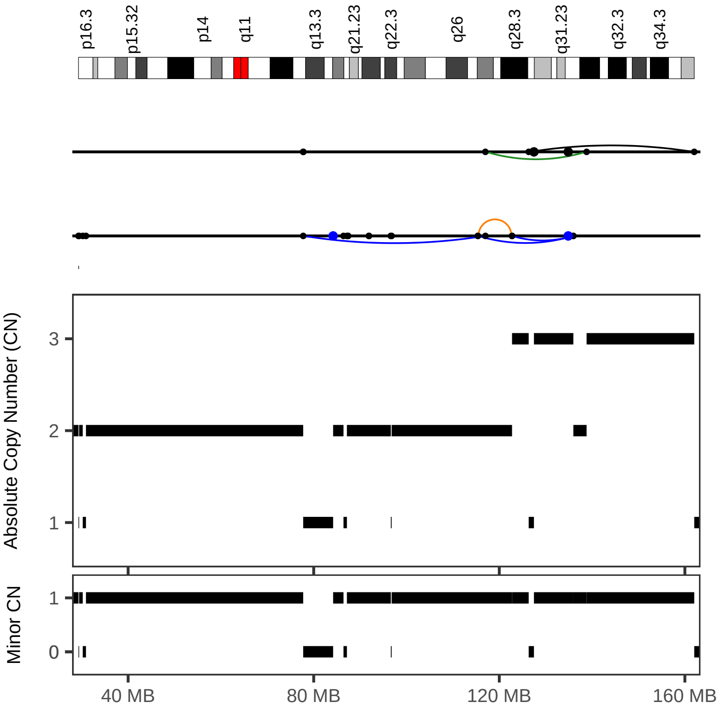

	**ICGC_0214**
Cancer type	Panc−AdenoCA
Position	4:77735561−162014258
Type	With other complex events
Interleaved intrachr. SVs	7
Total SVs (intrachr. + transl.)	11
SV types	DEL: 1; DUP: 3; h2hINV: 2; t2tINV: 1; TRA: 4
SVs in sample	254
Oscillating CN (2 and 3 states)	7, 7
CN segments	13
FDR fragment joints	0.74
FDR chr. breakp. enrich.	0.56
Linked to chrs	1:196999875−239097446;
Purity, ploidy	0.47, 1.82

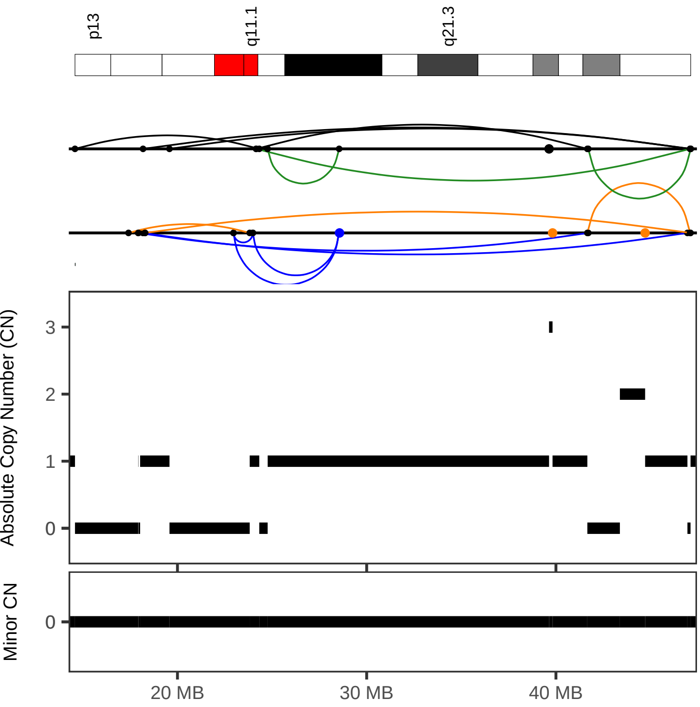

	**ICGC_0217**
Cancer type	Panc−AdenoCA
Position	21:14583619−47124626
Type	With other complex events
Interleaved intrachr. SVs	16
Total SVs (intrachr. + transl.)	20
SV types	DEL: 4; DUP: 5; h2hINV: 4; t2tINV: 3; TRA: 4
SVs in sample	99
Oscillating CN (2 and 3 states)	8, 8
CN segments	15
FDR fragment joints	0.94
FDR chr. breakp. enrich.	0
Linked to chrs	
Purity, ploidy	0.53, 1.92

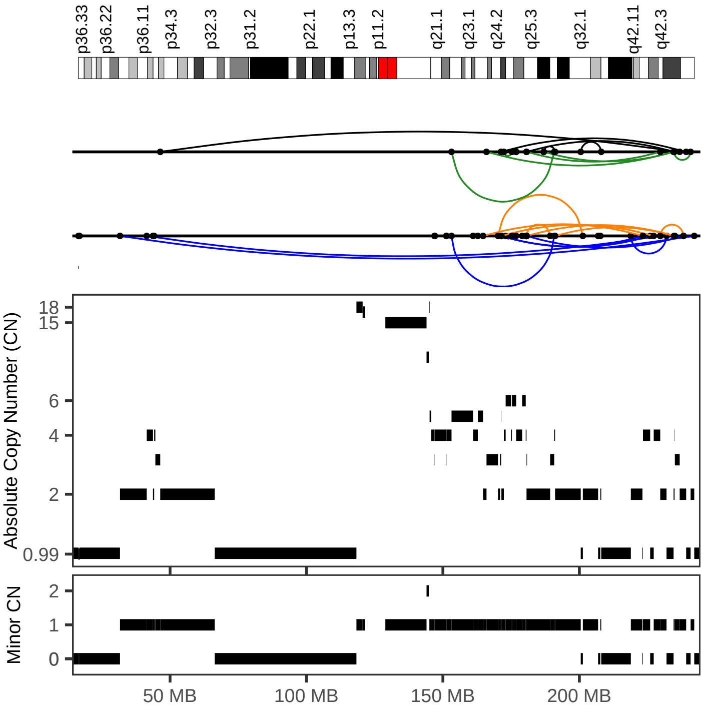

	**ICGC_0300**
Cancer type	Panc−AdenoCA
Position	1:41444007−240774472
Type	With other complex events
Interleaved intrachr. SVs	23
Total SVs (intrachr. + transl.)	23
SV types	DEL: 7; DUP: 6; h2hINV: 5; t2tINV: 5; TRA: 0
SVs in sample	68
Oscillating CN (2 and 3 states)	8, 13
CN segments	59
FDR fragment joints	0.94
FDR chr. breakp. enrich.	0
Linked to chrs	
Purity, ploidy	0.56, 2.02

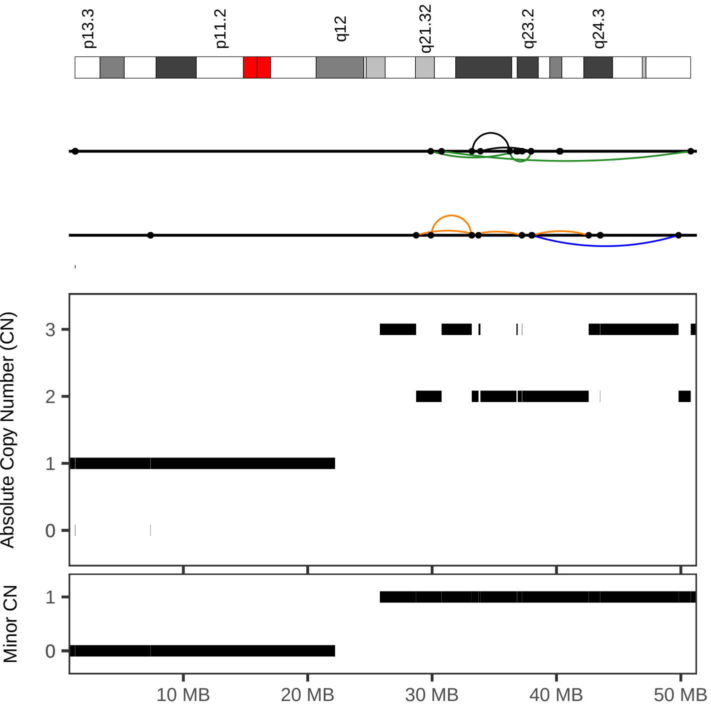

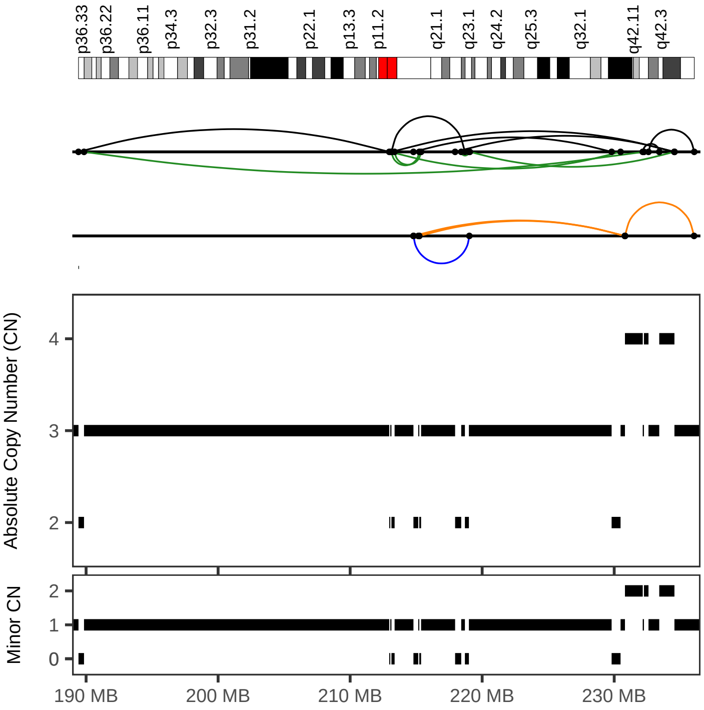

ICGC_0303

Cancer type	Panc−AdenoCA
Position	17:28715899−50792664
Type	Canonical without polyploidization
Interleaved intrachr. SVs	9
Total SVs (intrachr. + transl.)	9
SV types	DEL: 3; DUP: 0; h2hINV: 3; t2tINV: 3; TRA: 0
SVs in sample	132
Oscillating CN (2 and 3 states)	14, 14
CN segments	14
FDR fragment joints	0.59
FDR chr. breakp. enrich.	0
Linked to chrs	
Purity, ploidy	0.58, 1.9

ICGC_0313

Cancer type	Panc−AdenoCA
Position	1:189423365−236063825
Type	Canonical without polyploidization
Interleaved intrachr. SVs	18
Total SVs (intrachr. + transl.)	18
SV types	DEL: 3; DUP: 2; h2hINV: 7; t2tINV: 6; TRA: 0
SVs in sample	38
Oscillating CN (2 and 3 states)	16, 22
CN segments	22
FDR fragment joints	0.59
FDR chr. breakp. enrich.	0
Linked to chrs	
Purity, ploidy	0.28, 3.06

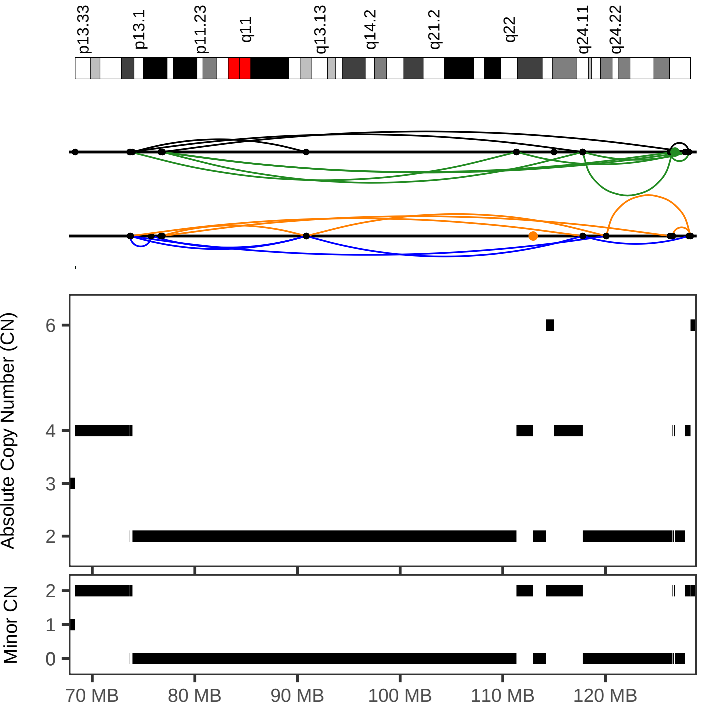

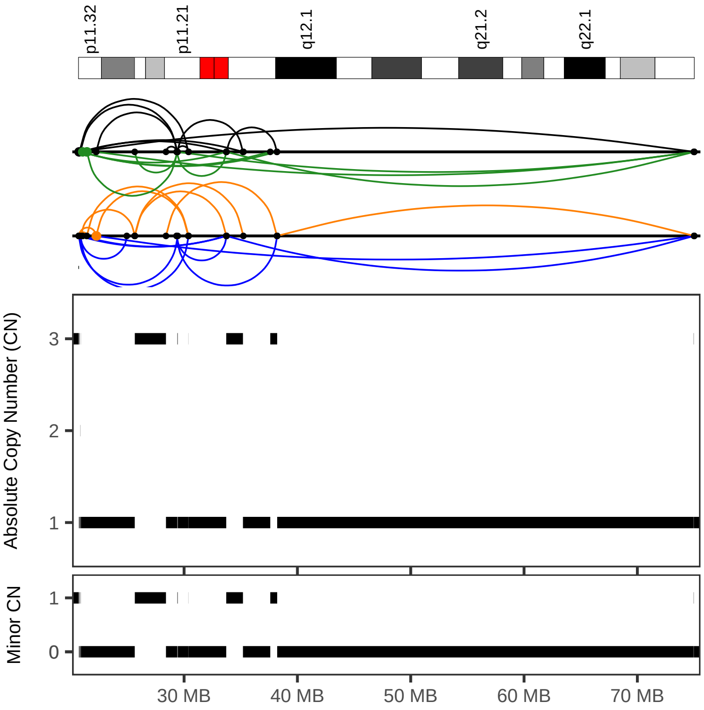

ICGC_0326

Cancer type	Panc−AdenoCA
Position	12:73647749−128290868
Type	With other complex events
Interleaved intrachr. SVs	24
Total SVs (intrachr. + transl.)	26
SV types	DEL: 6; DUP: 6; h2hINV: 4; t2tINV: 8; TRA: 2
SVs in sample	108
Oscillating CN (2 and 3 states)	7, 7
CN segments	14
FDR fragment joints	0.8
FDR chr. breakp. enrich.	0
Linked to chrs	
Purity, ploidy	0.44, 2.52

ICGC_0326

Cancer type	Panc−AdenoCA
Position	18:20681108−75019000
Type	Canonical without polyploidization
Interleaved intrachr. SVs	41
Total SVs (intrachr. + transl.)	47
SV types	DEL: 10; DUP: 9; h2hINV: 11; t2tINV: 11; TRA: 6
SVs in sample	108
Oscillating CN (2 and 3 states)	17, 24
CN segments	24
FDR fragment joints	0.98
FDR chr. breakp. enrich.	0
Linked to chrs	
Purity, ploidy	0.44, 2.52

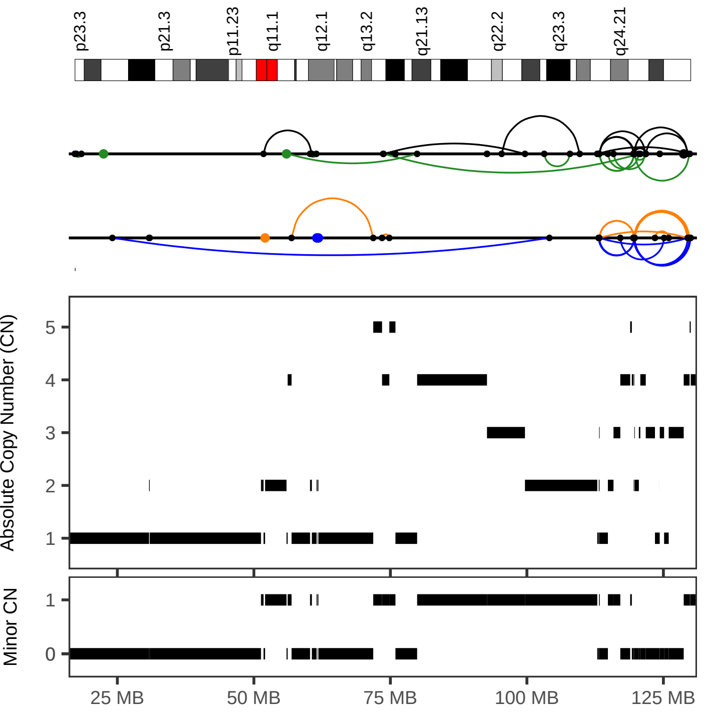

	ICGC_0392
Cancer type	Panc−AdenoCA
Position	8:24086553−130006493
Type	With other complex events
Interleaved intrachr. SVs	47
Total SVs (intrachr. + transl.)	52
SV types	DEL: 11; DUP: 11; h2hINV: 12; t2tINV: 13; TRA: 5
SVs in sample	205
Oscillating CN (2 and 3 states)	7, 15
CN segments	54
FDR fragment joints	0.98
FDR chr. breakp. enrich.	0
Linked to chrs	6:14848129−56658866;
Purity, ploidy	0.5, 1.82

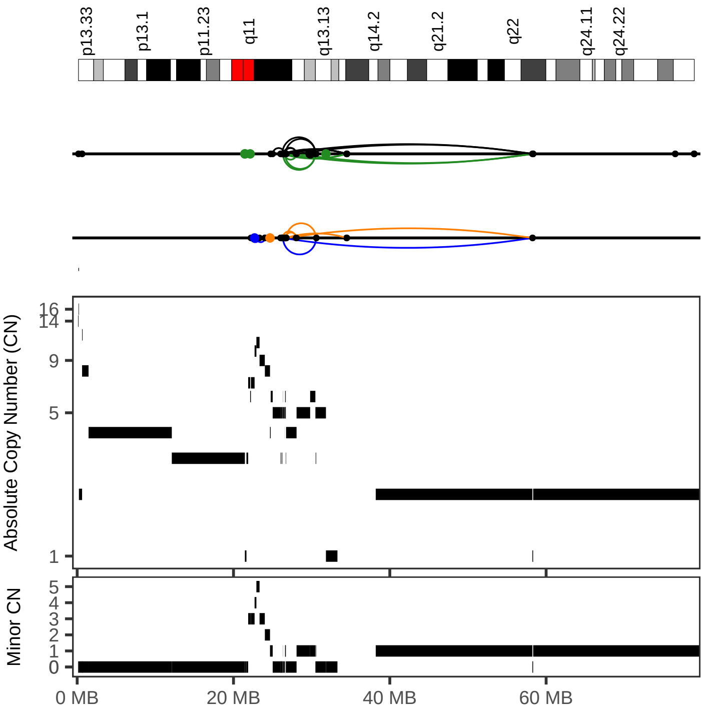

	ICGC_0392
Cancer type	Panc−AdenoCA
Position	12:25014211−58344707
Type	With other complex events
Interleaved intrachr. SVs	42
Total SVs (intrachr. + transl.)	44
SV types	DEL: 10; DUP: 6; h2hINV: 14; t2tINV: 12; TRA: 2
SVs in sample	205
Oscillating CN (2 and 3 states)	7, 17
CN segments	35
FDR fragment joints	0.59
FDR chr. breakp. enrich.	0
Linked to chrs	
Purity, ploidy	0.5, 1.82

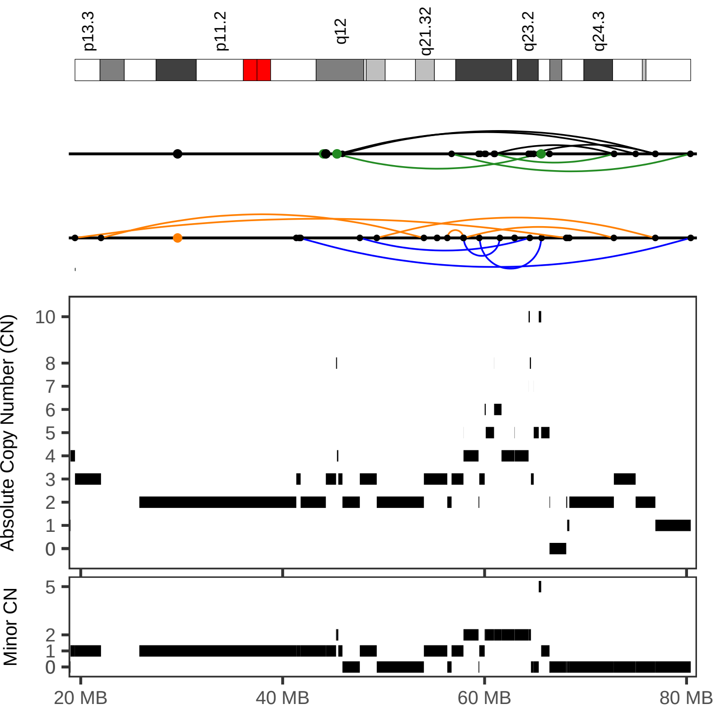

	ICGC_0392
Cancer type	Panc−AdenoCA
Position	17:19427565−80415097
Type	With other complex events
Interleaved intrachr. SVs	18
Total SVs (intrachr. + transl.)	25
SV types	DEL: 5; DUP: 5; h2hINV: 5; t2tINV: 3; TRA: 7
SVs in sample	205
Oscillating CN (2 and 3 states)	7, 7
CN segments	42
FDR fragment joints	0.91
FDR chr. breakp. enrich.	0
Linked to chrs	12:25014211−58344706;
Purity, ploidy	0.5, 1.82

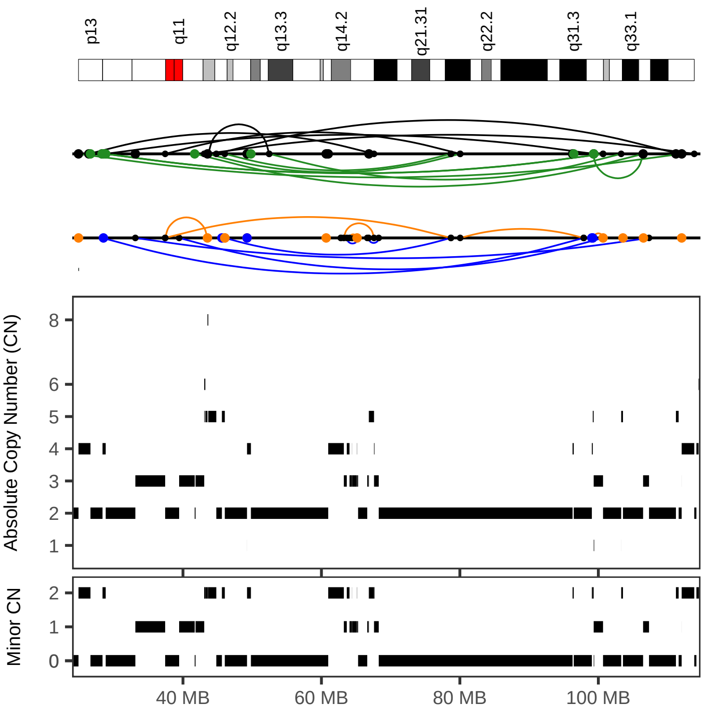

	ICGC_0393
Cancer type	Panc−AdenoCA
Position	13:24905703−113852941
Type	With other complex events
Interleaved intrachr. SVs	24
Total SVs (intrachr. + transl.)	63
SV types	DEL: 4; DUP: 6; h2hINV: 6; t2tINV: 8; TRA: 39
SVs in sample	158
Oscillating CN (2 and 3 states)	8, 10
CN segments	55
FDR fragment joints	0.8
FDR chr. breakp. enrich.	0
Linked to chrs	22:18789020−50349274;
Purity, ploidy	0.44, 2.08

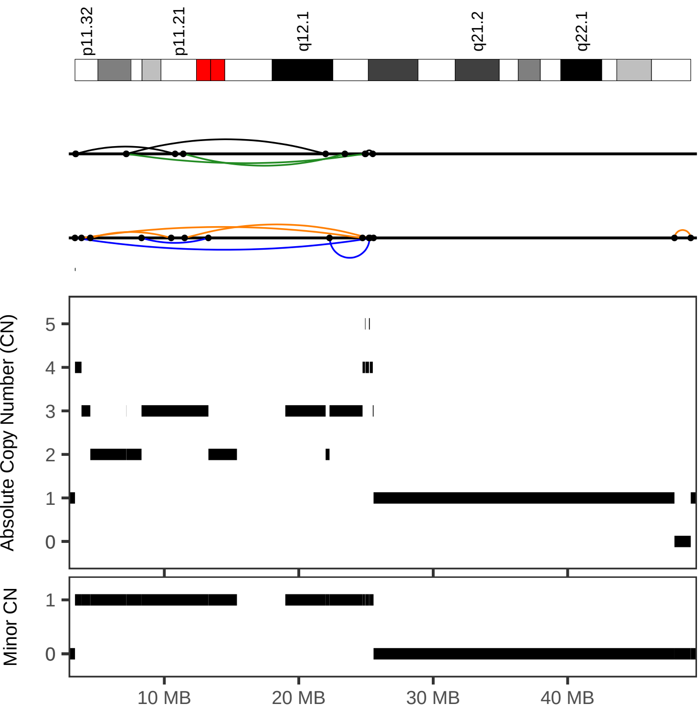

	ICGC_0412
Cancer type	Panc−AdenoCA
Position	18:3408603−25505804
Type	With other complex events
Interleaved intrachr. SVs	10
Total SVs (intrachr. + transl.)	10
SV types	DEL: 3; DUP: 2; h2hINV: 3; t2tINV: 2; TRA: 0
SVs in sample	34
Oscillating CN (2 and 3 states)	9, 16
CN segments	16
FDR fragment joints	0.96
FDR chr. breakp. enrich.	0
Linked to chrs	
Purity, ploidy	0.45, 1.85

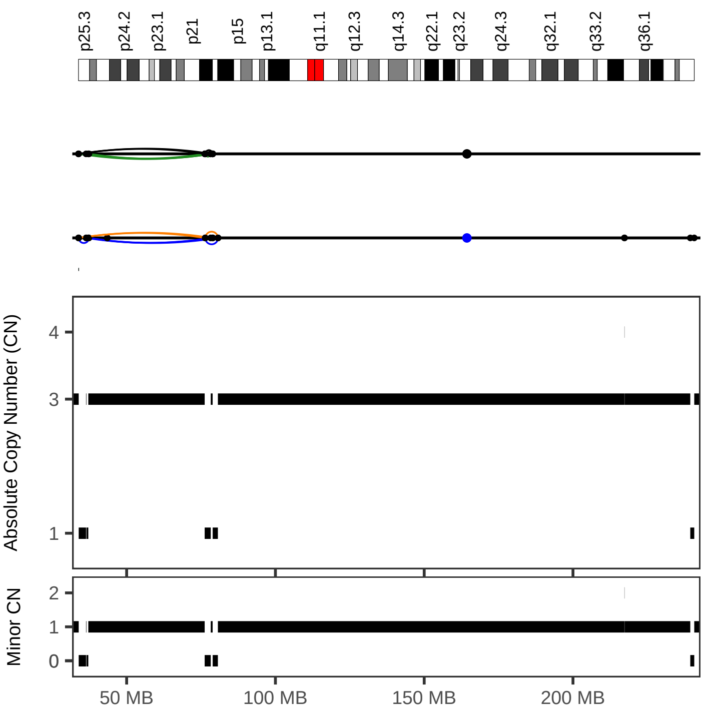

	ICGC_0419
Cancer type	Panc−AdenoCA
Position	2:33811228−80725292
Type	Canonical without polyploidization
Interleaved intrachr. SVs	19
Total SVs (intrachr. + transl.)	19
SV types	DEL: 4; DUP: 5; h2hINV: 5; t2tINV: 5; TRA: 0
SVs in sample	132
Oscillating CN (2 and 3 states)	11, 11
CN segments	11
FDR fragment joints	0.99
FDR chr. breakp. enrich.	0
Linked to chrs	
Purity, ploidy	0.33, 2.92

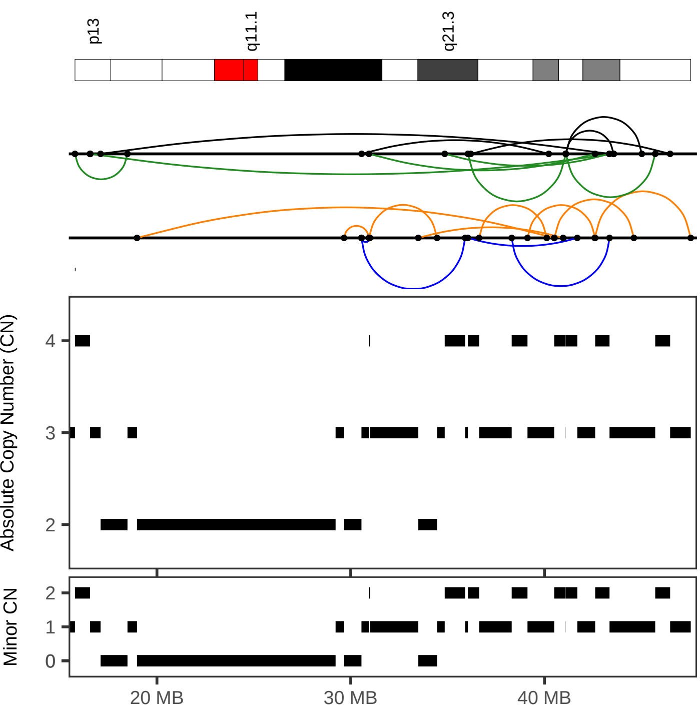

	ICGC_0419
Cancer type	Panc−AdenoCA
Position	21:15770299−47546356
Type	With other complex events
Interleaved intrachr. SVs	23
Total SVs (intrachr. + transl.)	23
SV types	DEL: 8; DUP: 4; h2hINV: 5; t2tINV: 6; TRA: 0
SVs in sample	132
Oscillating CN (2 and 3 states)	15, 26
CN segments	26
FDR fragment joints	0.75
FDR chr. breakp. enrich.	0
Linked to chrs	
Purity, ploidy	0.33, 2.92

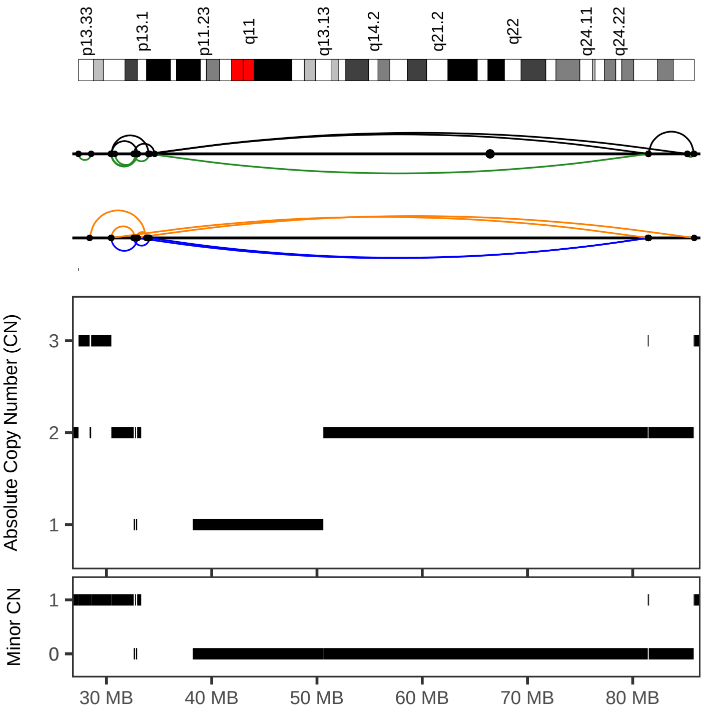

	ICGC_0420
Cancer type	Panc−AdenoCA
Position	12:27339007−85847160
Type	With other complex events
Interleaved intrachr. SVs	31
Total SVs (intrachr. + transl.)	32
SV types	DEL: 8; DUP: 7; h2hINV: 8; t2tINV: 8; TRA: 1
SVs in sample	90
Oscillating CN (2 and 3 states)	10, 19
CN segments	19
FDR fragment joints	1
FDR chr. breakp. enrich.	0
Linked to chrs	
Purity, ploidy	0.55, 1.66

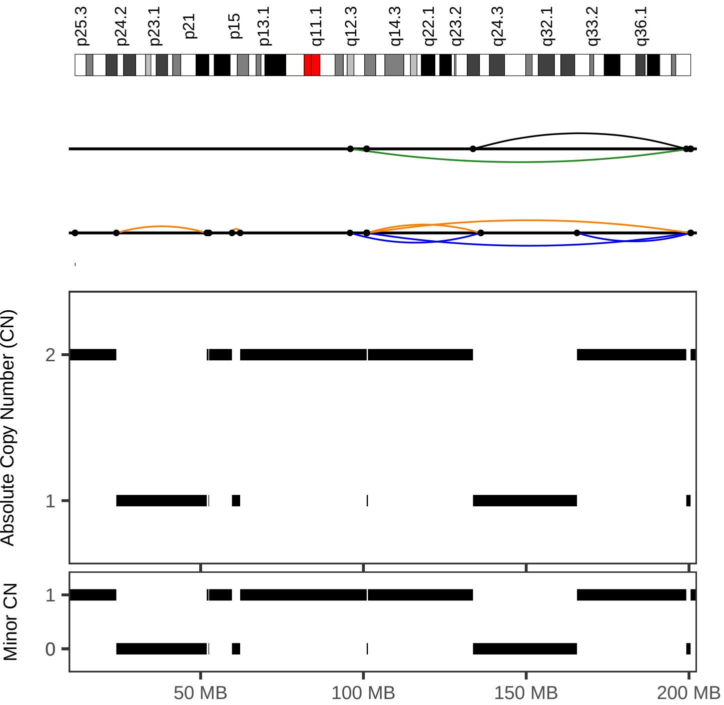

	ICGC_0486
Cancer type	Panc−AdenoCA
Position	2:95882672−200533578
Type	Canonical without polyploidization
Interleaved intrachr. SVs	8
Total SVs (intrachr. + transl.)	8
SV types	DEL: 3; DUP: 3; h2hINV: 1; t2tINV: 1; TRA: 0
SVs in sample	112
Oscillating CN (2 and 3 states)	9, 9
CN segments	9
FDR fragment joints	0.64
FDR chr. breakp. enrich.	0.13
Linked to chrs	
Purity, ploidy	0.75, 1.84

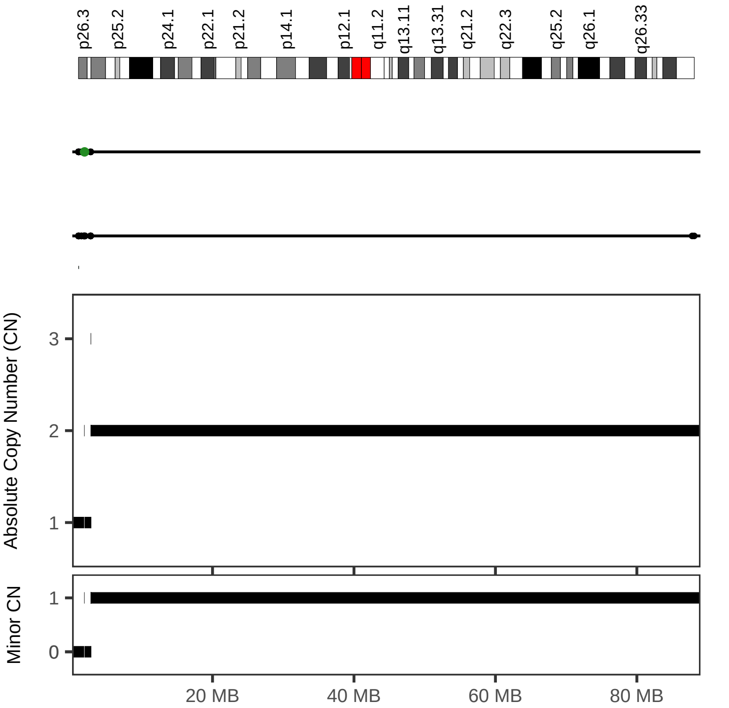

	ICGC_0502
Cancer type	Panc−AdenoCA
Position	3:1045935−2770954
Type	Canonical without polyploidization
Interleaved intrachr. SVs	38
Total SVs (intrachr. + transl.)	39
SV types	DEL: 9; DUP: 14; h2hINV: 6; t2tINV: 9; TRA: 1
SVs in sample	120
Oscillating CN (2 and 3 states)	9, 12
CN segments	12
FDR fragment joints	0.59
FDR chr. breakp. enrich.	0
Linked to chrs	
Purity, ploidy	0.53, 1.85

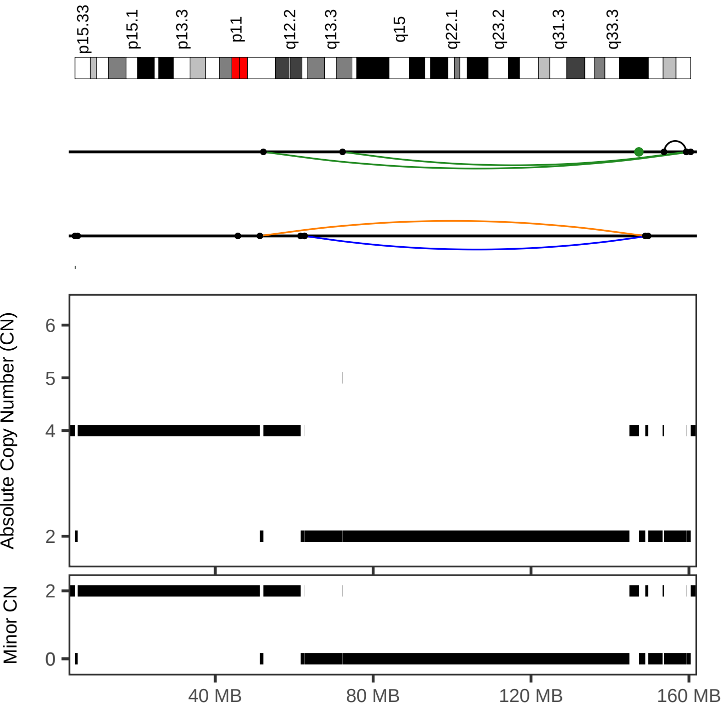

	ICGC_0521
Cancer type	Panc−AdenoCA
Position	5:51314841−160442765
Type	Before polyploidization
Interleaved intrachr. SVs	6
Total SVs (intrachr. + transl.)	7
SV types	DEL: 1; DUP: 2; h2hINV: 1; t2tINV: 2; TRA: 1
SVs in sample	107
Oscillating CN (2 and 3 states)	10, 16
CN segments	16
FDR fragment joints	0.91
FDR chr. breakp. enrich.	0.46
Linked to chrs	
Purity, ploidy	0.57, 3.43

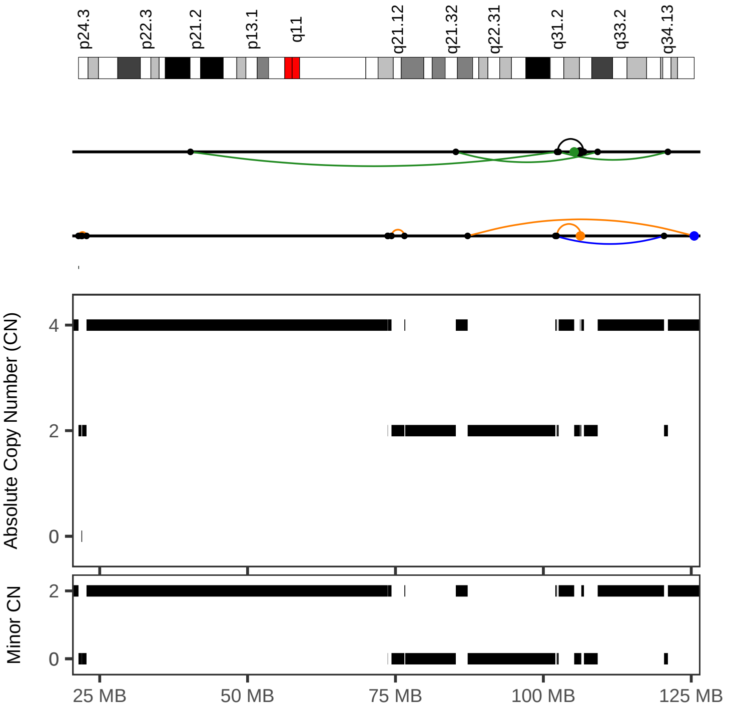

	ICGC_0521
Cancer type	Panc−AdenoCA
Position	9:40335567−125502754
Type	Before polyploidization
Interleaved intrachr. SVs	7
Total SVs (intrachr. + transl.)	11
SV types	DEL: 2; DUP: 1; h2hINV: 1; t2tINV: 3; TRA: 4
SVs in sample	107
Oscillating CN (2 and 3 states)	21, 21
CN segments	21
FDR fragment joints	0.74
FDR chr. breakp. enrich.	0
Linked to chrs	
Purity, ploidy	0.57, 3.43

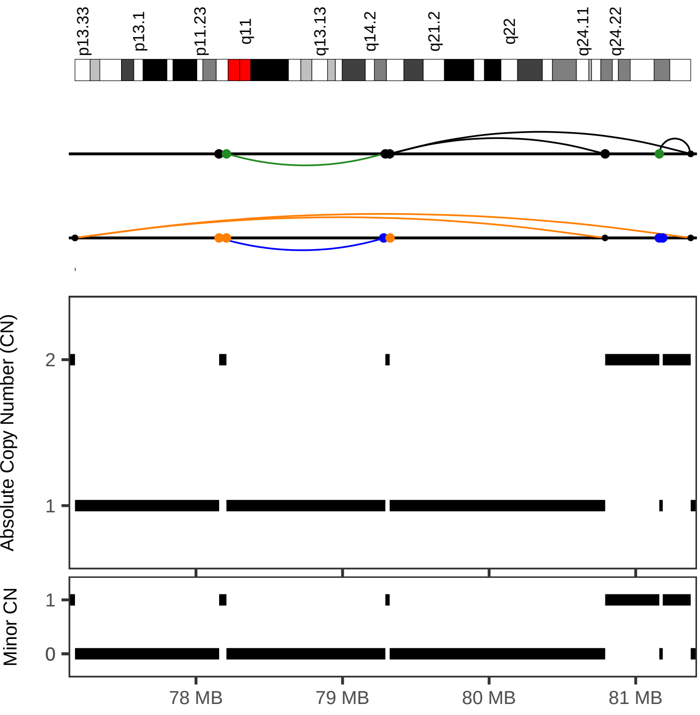

**ICGC_0526**	
Cancer type	Panc−AdenoCA
Position	12:77174515−81375488
Type	Canonical without polyploidization
Interleaved intrachr. SVs	4
Total SVs (intrachr. + transl.)	16
SV types	DEL: 2; DUP: 0; h2hINV: 2; t2tINV: 0; TRA: 12
SVs in sample	127
Oscillating CN (2 and 3 states)	10, 10
CN segments	10
FDR fragment joints	0.59
FDR chr. breakp. enrich.	0
Linked to chrs	13:42400145−51281145;
Purity, ploidy	0.54, 1.78

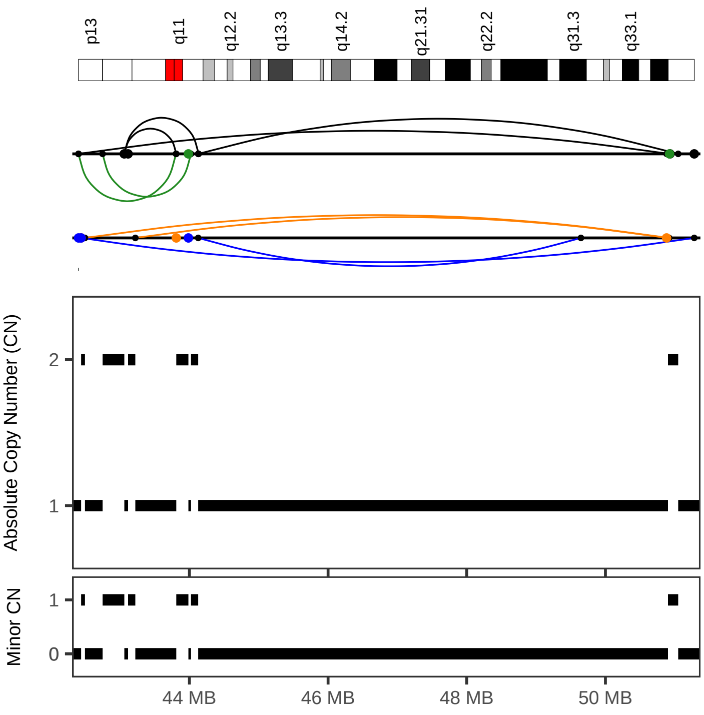

**ICGC_0526**	
Cancer type	Panc−AdenoCA
Position	13:42400145−51281146
Type	Canonical without polyploidization
Interleaved intrachr. SVs	11
Total SVs (intrachr. + transl.)	23
SV types	DEL: 2; DUP: 2; h2hINV: 5; t2tINV: 2; TRA: 12
SVs in sample	127
Oscillating CN (2 and 3 states)	13, 13
CN segments	13
FDR fragment joints	0.64
FDR chr. breakp. enrich.	0
Linked to chrs	12:77174515−81375487;
Purity, ploidy	0.54, 1.78

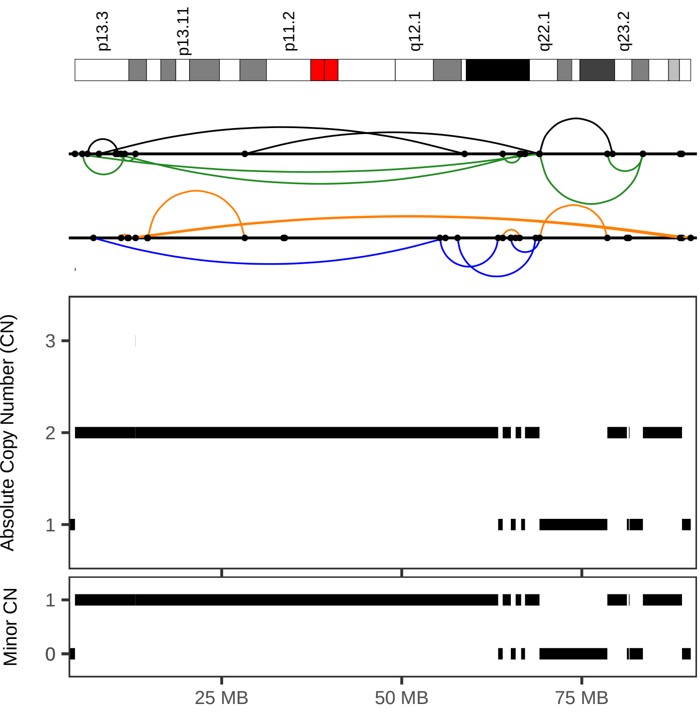

**ICGC_0526**	
Cancer type	Panc−AdenoCA
Position	16:4628924−90122371
Type	Canonical without polyploidization
Interleaved intrachr. SVs	21
Total SVs (intrachr. + transl.)	21
SV types	DEL: 5; DUP: 4; h2hINV: 5; t2tINV: 7; TRA: 0
SVs in sample	127
Oscillating CN (2 and 3 states)	14, 16
CN segments	16
FDR fragment joints	0.87
FDR chr. breakp. enrich.	0
Linked to chrs	
Purity, ploidy	0.54, 1.78

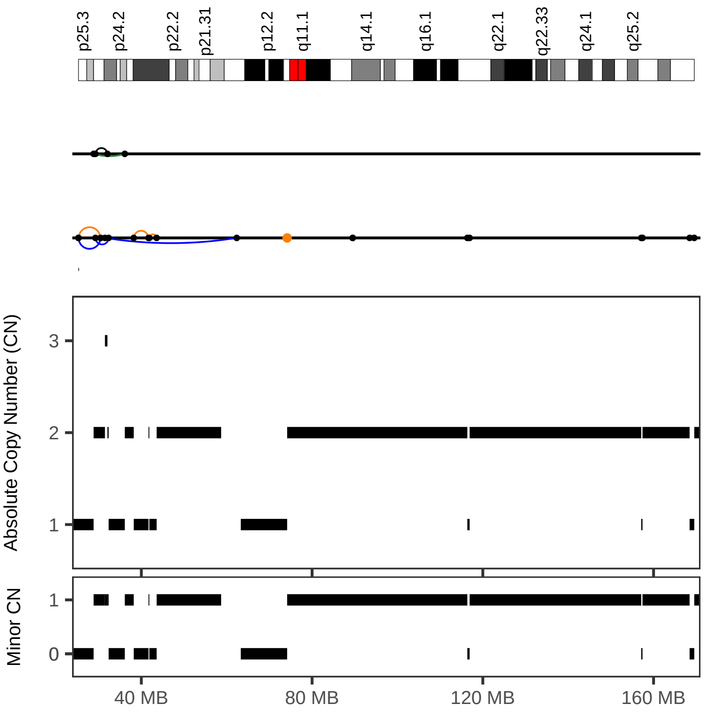

**ICGC_0535**	
Cancer type	Panc−AdenoCA
Position	6:25273614−62346185
Type	Canonical without polyploidization
Interleaved intrachr. SVs	6
Total SVs (intrachr. + transl.)	6
SV types	DEL: 1; DUP: 3; h2hINV: 1; t2tINV: 1; TRA: 0
SVs in sample	102
Oscillating CN (2 and 3 states)	8, 11
CN segments	11
FDR fragment joints	0.64
FDR chr. breakp. enrich.	0.05
Linked to chrs	
Purity, ploidy	0.46, 1.85

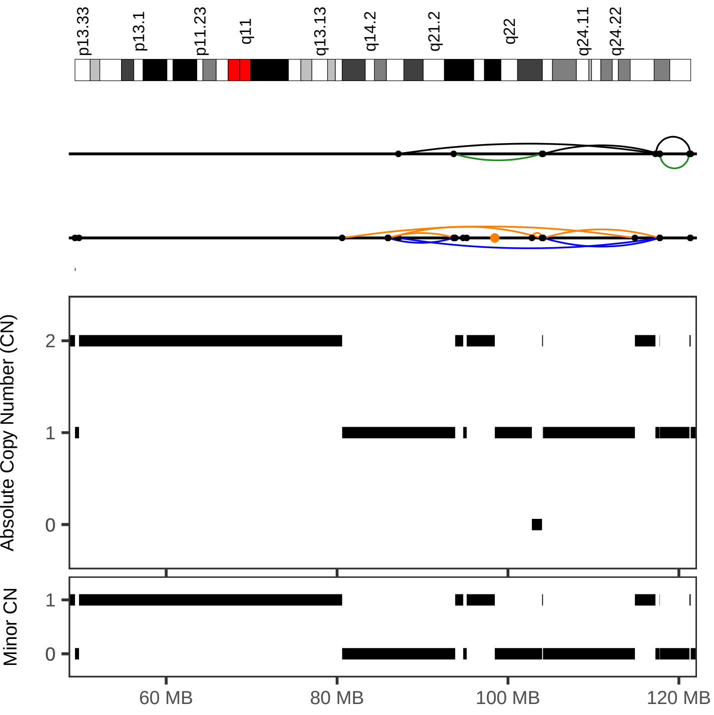

	ICGC_0535
Cancer type	Panc−AdenoCA
Position	12:80590486−121388006
Type	Canonical without polyploidization
Interleaved intrachr. SVs	13
Total SVs (intrachr. + transl.)	14
SV types	DEL: 5; DUP: 3; h2hINV: 3; t2tINV: 2; TRA: 1
SVs in sample	102
Oscillating CN (2 and 3 states)	11, 17
CN segments	17
FDR fragment joints	0.77
FDR chr. breakp. enrich.	0
Linked to chrs	
Purity, ploidy	0.46, 1.85

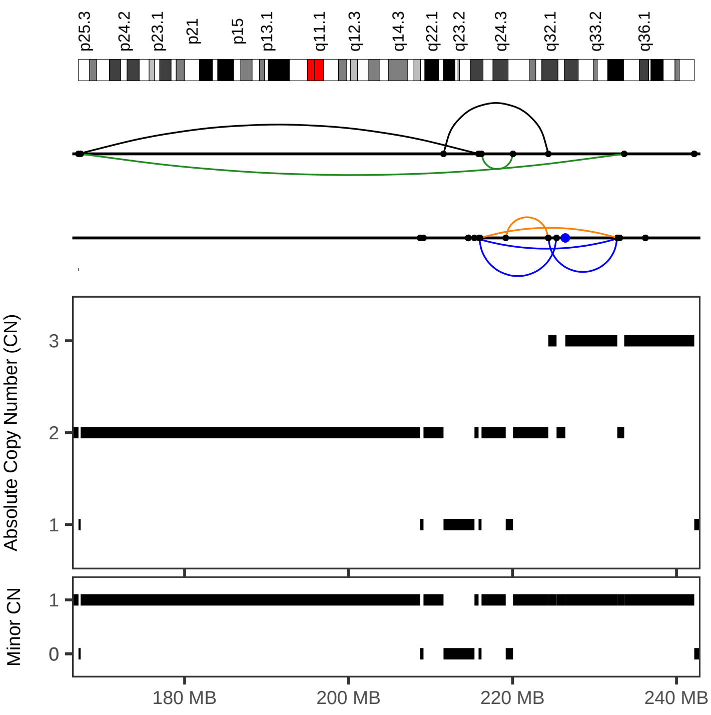

	ICGC_0536
Cancer type	Panc−AdenoCA
Position	2:167051773−233617455
Type	Canonical without polyploidization
Interleaved intrachr. SVs	9
Total SVs (intrachr. + transl.)	10
SV types	DEL: 2; DUP: 3; h2hINV: 2; t2tINV: 2; TRA: 1
SVs in sample	59
Oscillating CN (2 and 3 states)	10, 15
CN segments	15
FDR fragment joints	0.97
FDR chr. breakp. enrich.	0
Linked to chrs	
Purity, ploidy	0.38, 1.82

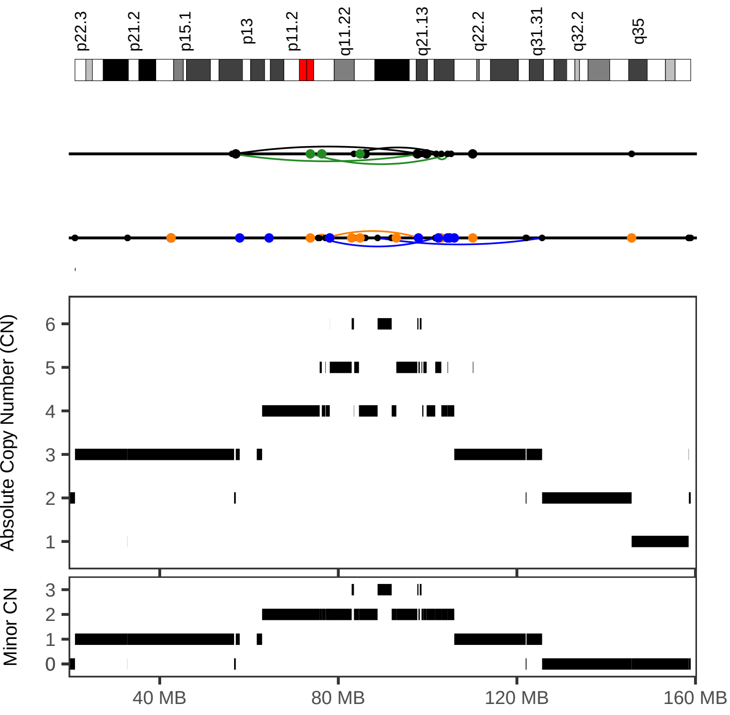

	PCSI_0015
Cancer type	Panc−AdenoCA
Position	7:56204033−125643006
Type	With other complex events
Interleaved intrachr. SVs	9
Total SVs (intrachr. + transl.)	32
SV types	DEL: 2; DUP: 2; h2hINV: 2; t2tINV: 3; TRA: 23
SVs in sample	541
Oscillating CN (2 and 3 states)	8, 10
CN segments	36
FDR fragment joints	0.97
FDR chr. breakp. enrich.	0
Linked to chrs	11:13107124−96905437;12:1762415−132632651
Purity, ploidy	0.94, 2.58

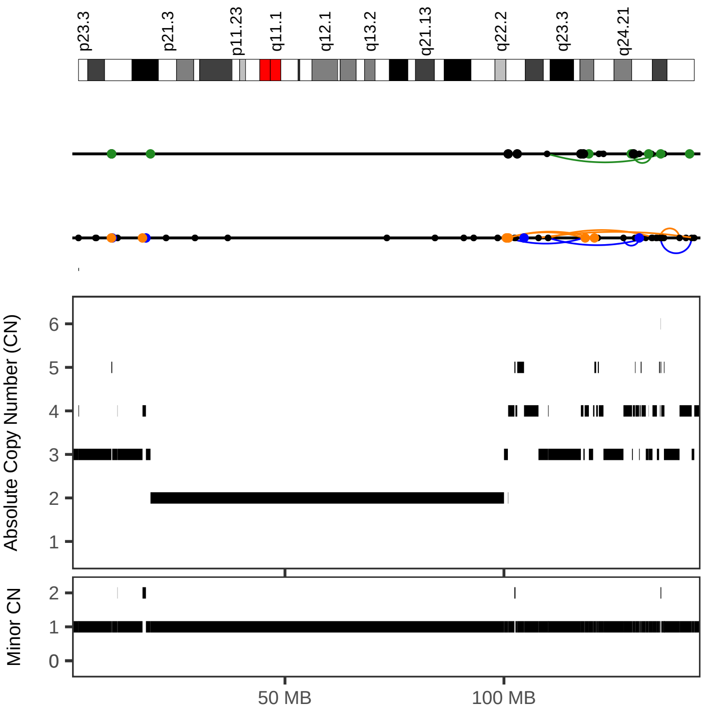

	PCSI_0015
Cancer type	Panc−AdenoCA
Position	8:100785898−143451880
Type	With other complex events
Interleaved intrachr. SVs	15
Total SVs (intrachr. + transl.)	30
SV types	DEL: 6; DUP: 5; h2hINV: 1; t2tINV: 3; TRA: 15
SVs in sample	541
Oscillating CN (2 and 3 states)	9, 34
CN segments	49
FDR fragment joints	0.59
FDR chr. breakp. enrich.	0
Linked to chrs	10:5398824−88241617;12:1762415−132632651 21:18184323−34748018;
Purity, ploidy	0.94, 2.58

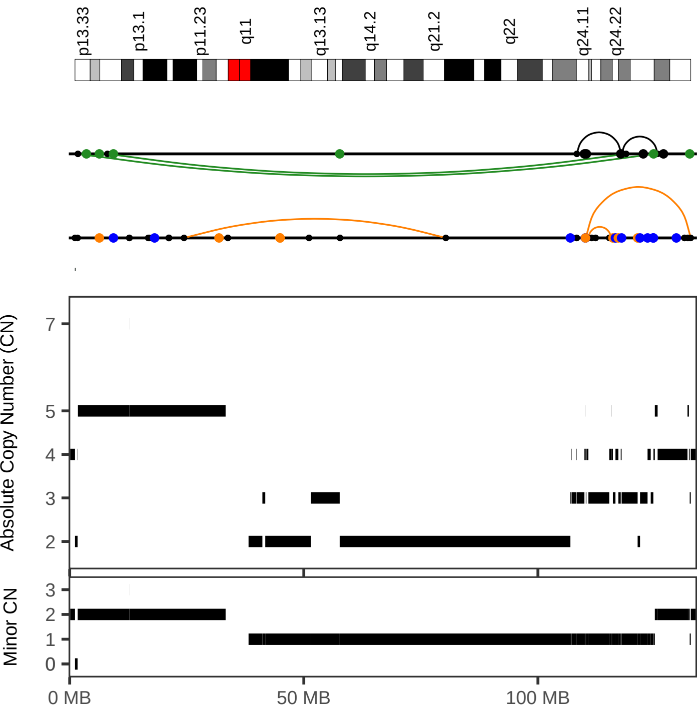

	**PCSI_0015**
Cancer type	Panc−AdenoCA
Position	12:1762415−132632652
Type	With other complex events
Interleaved intrachr. SVs	5
Total SVs (intrachr. + transl.)	36
SV types	DEL: 1; DUP: 0; h2hINV: 2;<br>t2tINV: 2; TRA: 31
SVs in sample	541
Oscillating CN (2 and 3 states)	8, 42
CN segments	44
FDR fragment joints	0.64
FDR chr. breakp. enrich.	0
Linked to chrs	1:3415964−191894580;2:154803413−189497987<br>7:56204033−125643005;8:100785898−143451879
Purity, ploidy	0.94, 2.58

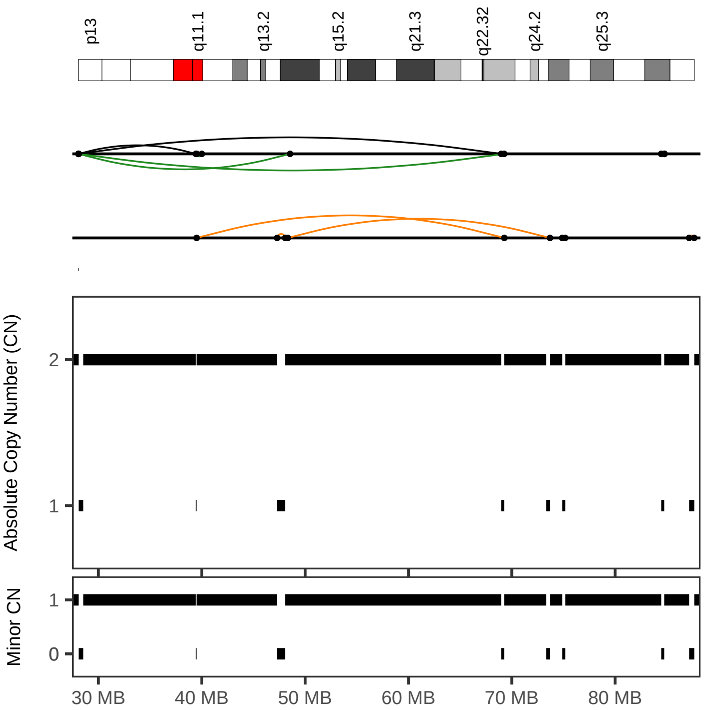

	**PCSI_0023**
Cancer type	Panc−AdenoCA
Position	15:28070426−73690087
Type	Canonical without polyploidization
Interleaved intrachr. SVs	6
Total SVs (intrachr. + transl.)	6
SV types	DEL: 2; DUP: 0; h2hINV: 2;<br>t2tINV: 2; TRA: 0
SVs in sample	114
Oscillating CN (2 and 3 states)	11, 11
CN segments	11
FDR fragment joints	0.64
FDR chr. breakp. enrich.	0
Linked to chrs	
Purity, ploidy	0.27, 1.96

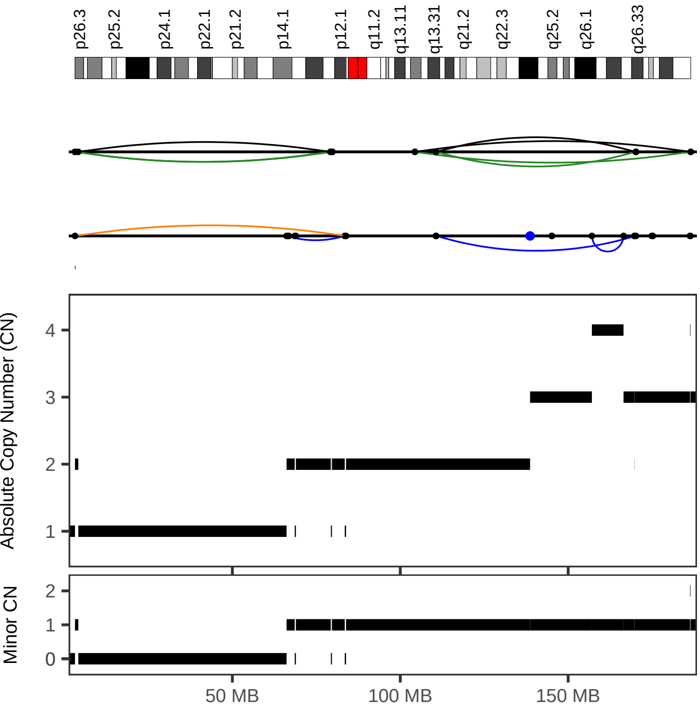

	**PCSI_0048**
Cancer type	Panc−AdenoCA
Position	3:3120897−83846277
Type	Canonical without polyploidization
Interleaved intrachr. SVs	6
Total SVs (intrachr. + transl.)	6
SV types	DEL: 1; DUP: 1; h2hINV: 2;<br>t2tINV: 2; TRA: 0
SVs in sample	119
Oscillating CN (2 and 3 states)	13, 13
CN segments	13
FDR fragment joints	0.91
FDR chr. breakp. enrich.	0
Linked to chrs	
Purity, ploidy	0.59, 1.95

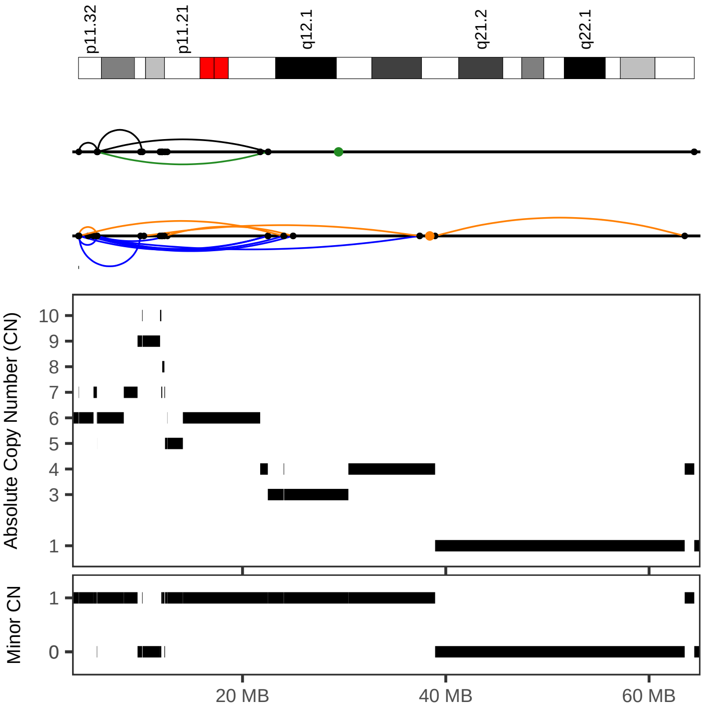

	**PCSI_0072**
Cancer type	Panc−AdenoCA
Position	18:3860804−37436169
Type	With other complex events
Interleaved intrachr. SVs	26
Total SVs (intrachr. + transl.)	27
SV types	DEL: 6; DUP: 9; h2hINV: 7;<br>t2tINV: 4; TRA: 1
SVs in sample	88
Oscillating CN (2 and 3 states)	7, 10
CN segments	26
FDR fragment joints	0.64
FDR chr. breakp. enrich.	0
Linked to chrs	
Purity, ploidy	0.45, 3.07

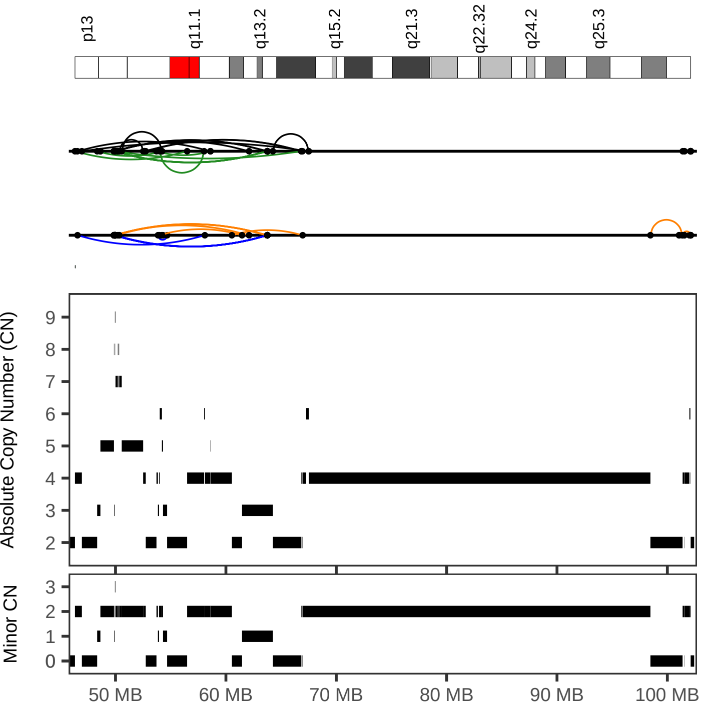

	**PCSI_0080**
Cancer type	Panc−AdenoCA
Position	15:46323229−67503083
Type	With other complex events
Interleaved intrachr. SVs	39
Total SVs (intrachr. + transl.)	39
SV types	DEL: 9; DUP: 11; h2hINV: 10; t2tINV: 9; TRA: 0
SVs in sample	138
Oscillating CN (2 and 3 states)	8, 8
CN segments	38
FDR fragment joints	0.98
FDR chr. breakp. enrich.	0
Linked to chrs	
Purity, ploidy	0.52, 3.16

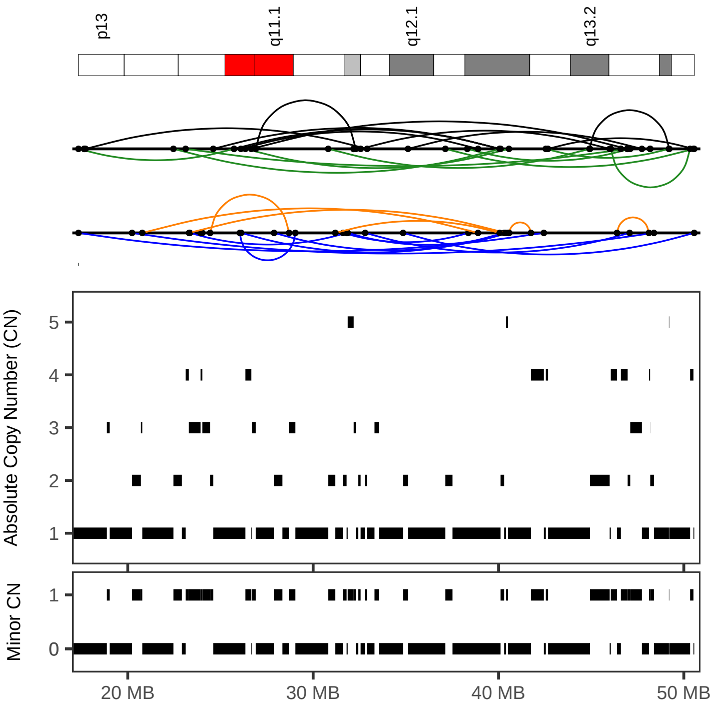

	**PCSI_0081**
Cancer type	Panc−AdenoCA
Position	22:17339868−50561700
Type	With other complex events
Interleaved intrachr. SVs	34
Total SVs (intrachr. + transl.)	34
SV types	DEL: 5; DUP: 10; h2hINV: 10; t2tINV: 9; TRA: 0
SVs in sample	195
Oscillating CN (2 and 3 states)	7, 14
CN segments	63
FDR fragment joints	0.64
FDR chr. breakp. enrich.	0
Linked to chrs	
Purity, ploidy	0.85, 1.65

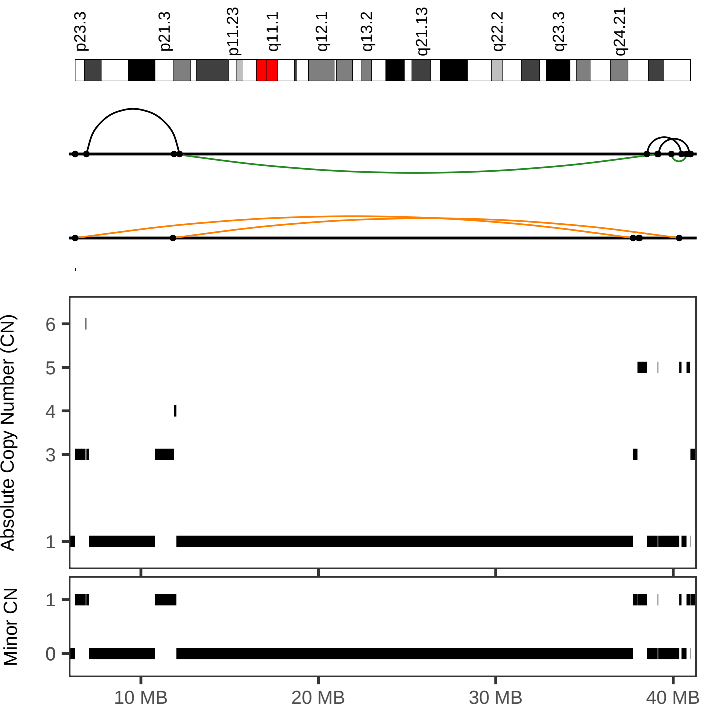

	**PCSI_0082**
Cancer type	Panc−AdenoCA
Position	8:6295682−40937004
Type	With other complex events
Interleaved intrachr. SVs	7
Total SVs (intrachr. + transl.)	7
SV types	DEL: 2; DUP: 0; h2hINV: 3; t2tINV: 2; TRA: 0
SVs in sample	160
Oscillating CN (2 and 3 states)	8, 9
CN segments	16
FDR fragment joints	0.64
FDR chr. breakp. enrich.	0.71
Linked to chrs	
Purity, ploidy	0.67, 2.94

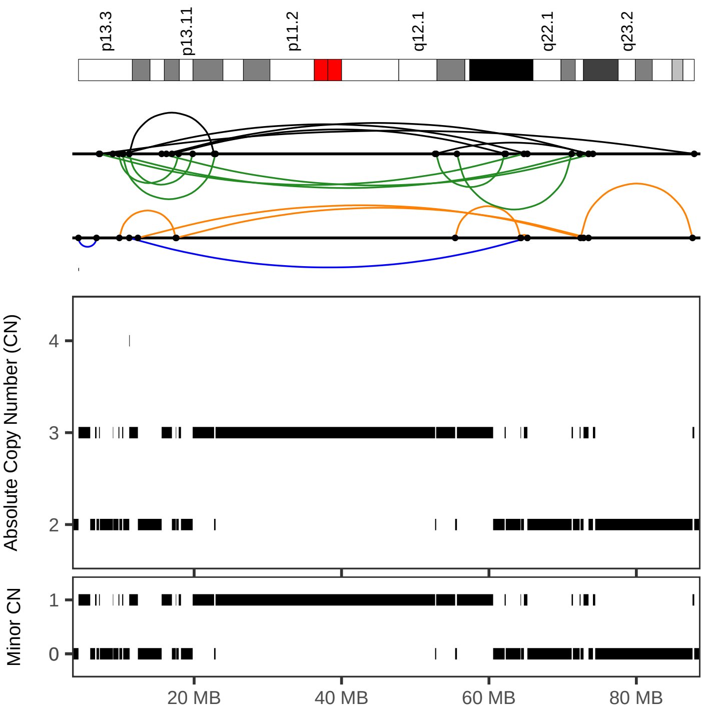

	**PCSI_0082**
Cancer type	Panc−AdenoCA
Position	16:7095949−87854086
Type	Canonical without polyploidization
Interleaved intrachr. SVs	21
Total SVs (intrachr. + transl.)	21
SV types	DEL: 6; DUP: 1; h2hINV: 6; t2tINV: 8; TRA: 0
SVs in sample	160
Oscillating CN (2 and 3 states)	32, 32
CN segments	41
FDR fragment joints	0.59
FDR chr. breakp. enrich.	0
Linked to chrs	
Purity, ploidy	0.67, 2.94

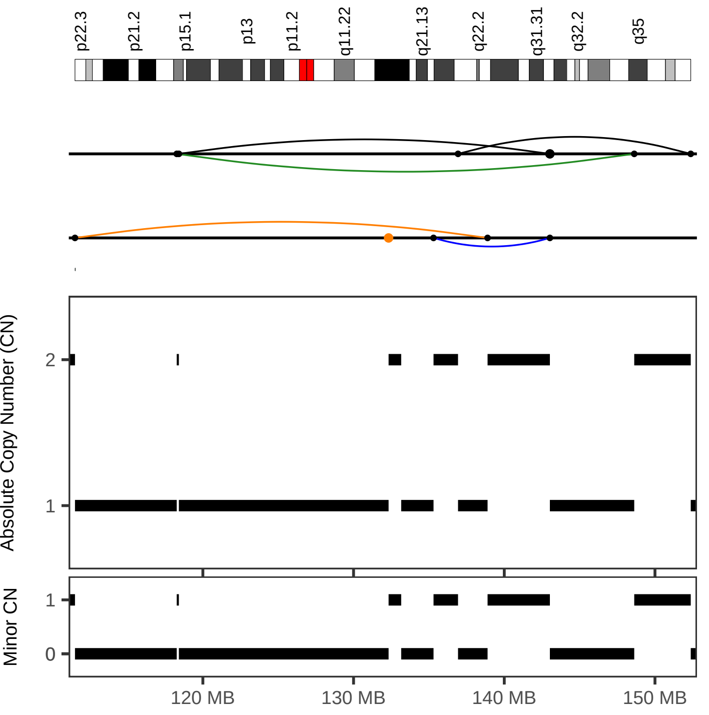

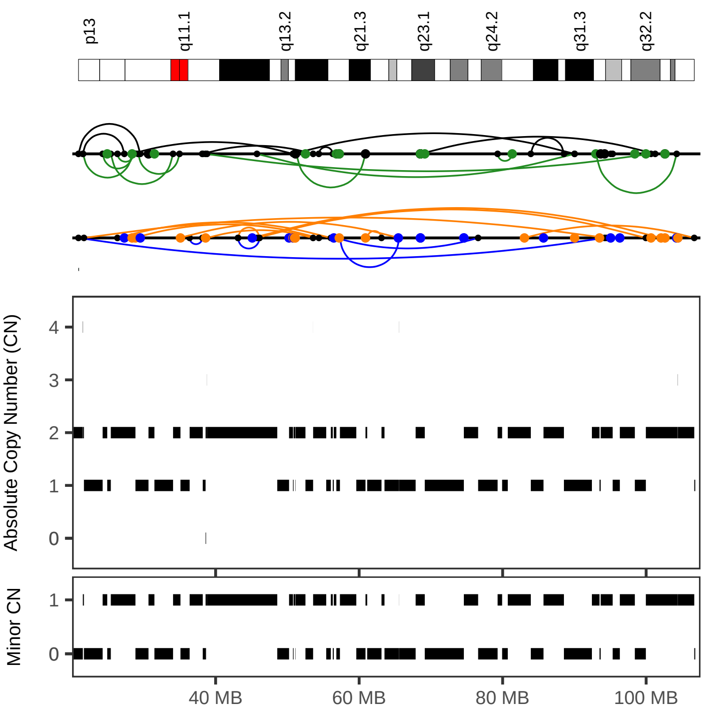

	PCSI_0101
Cancer type	Panc−AdenoCA
Position	7:111513717−152373524
Type	Canonical without polyploidization
Interleaved intrachr. SVs	5
Total SVs (intrachr. + transl.)	7
SV types	DEL: 1; DUP: 1; h2hINV: 2; t2tINV: 1; TRA: 2
SVs in sample	202
Oscillating CN (2 and 3 states)	11, 11
CN segments	11
FDR fragment joints	0.92
FDR chr. breakp. enrich.	0.52
Linked to chrs	14:20883702−106705000;
Purity, ploidy	0.21, 1.76

	PCSI_0101
Cancer type	Panc−AdenoCA
Position	14:20883702−106705001
Type	With other complex events
Interleaved intrachr. SVs	32
Total SVs (intrachr. + transl.)	88
SV types	DEL: 9; DUP: 5; h2hINV: 8; t2tINV: 10; TRA: 56
SVs in sample	202
Oscillating CN (2 and 3 states)	18, 34
CN segments	60
FDR fragment joints	0.7
FDR chr. breakp. enrich.	0
Linked to chrs	7:111513717−152373523;
Purity, ploidy	0.21, 1.76

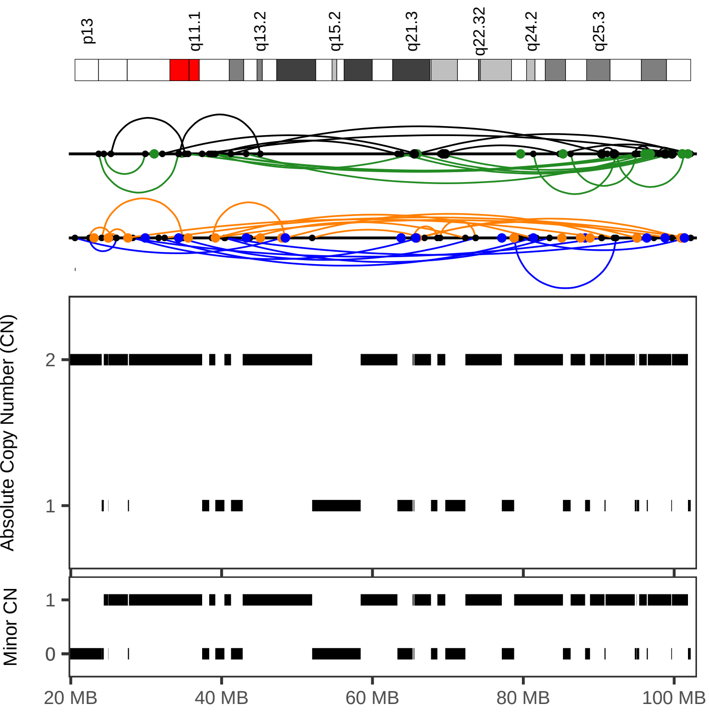

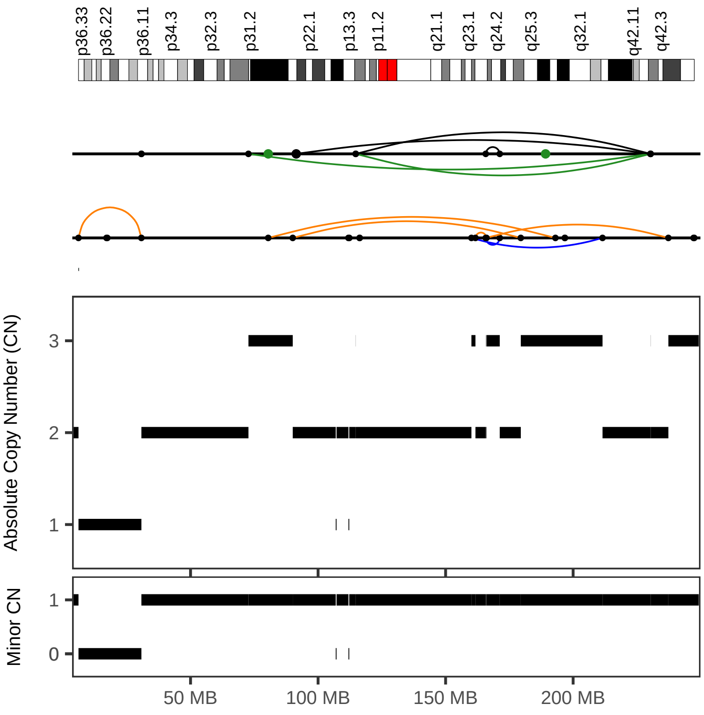

	PCSI_0101
Cancer type	Panc−AdenoCA
Position	15:20557095−102195722
Type	Canonical without polyploidization
Interleaved intrachr. SVs	53
Total SVs (intrachr. + transl.)	108
SV types	DEL: 16; DUP: 11; h2hINV: 12; t2tINV: 14; TRA: 55
SVs in sample	202
Oscillating CN (2 and 3 states)	41, 41
CN segments	41
FDR fragment joints	0.84
FDR chr. breakp. enrich.	0
Linked to chrs	14:20883702−106705000;
Purity, ploidy	0.21, 1.76

	PCSI_0104
Cancer type	Panc−AdenoCA
Position	1:72694825−237333439
Type	Canonical without polyploidization
Interleaved intrachr. SVs	11
Total SVs (intrachr. + transl.)	14
SV types	DEL: 4; DUP: 2; h2hINV: 3; t2tINV: 2; TRA: 3
SVs in sample	107
Oscillating CN (2 and 3 states)	14, 19
CN segments	19
FDR fragment joints	0.84
FDR chr. breakp. enrich.	0
Linked to chrs	
Purity, ploidy	0.85, 1.97

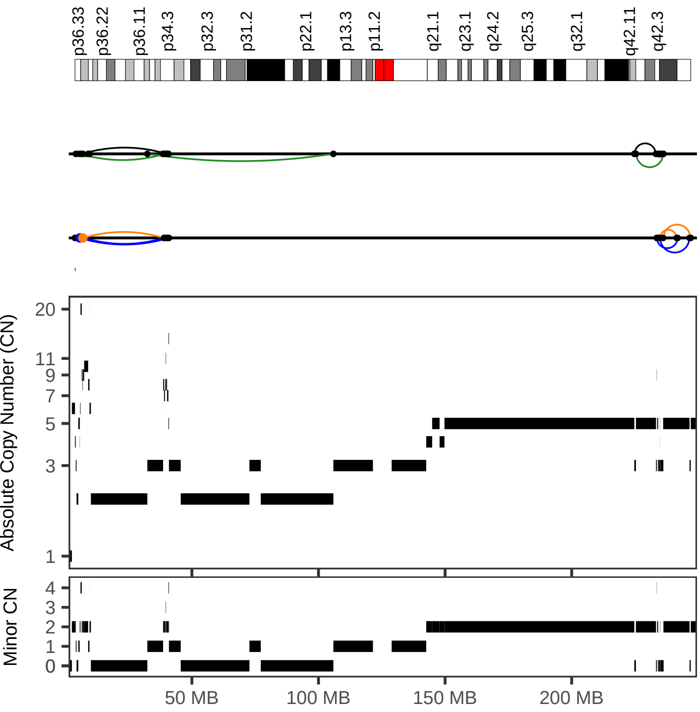

	**PCSI_0111**
Cancer type	Panc−AdenoCA
Position	1:224736774−247023067
Type	With other complex events
Interleaved intrachr. SVs	9
Total SVs (intrachr. + transl.)	9
SV types	DEL: 3; DUP: 2; h2hINV: 2; t2tINV: 2; TRA: 0
SVs in sample	260
Oscillating CN (2 and 3 states)	7, 8
CN segments	17
FDR fragment joints	0.97
FDR chr. breakp. enrich.	0.19
Linked to chrs	
Purity, ploidy	0.81, 3.09

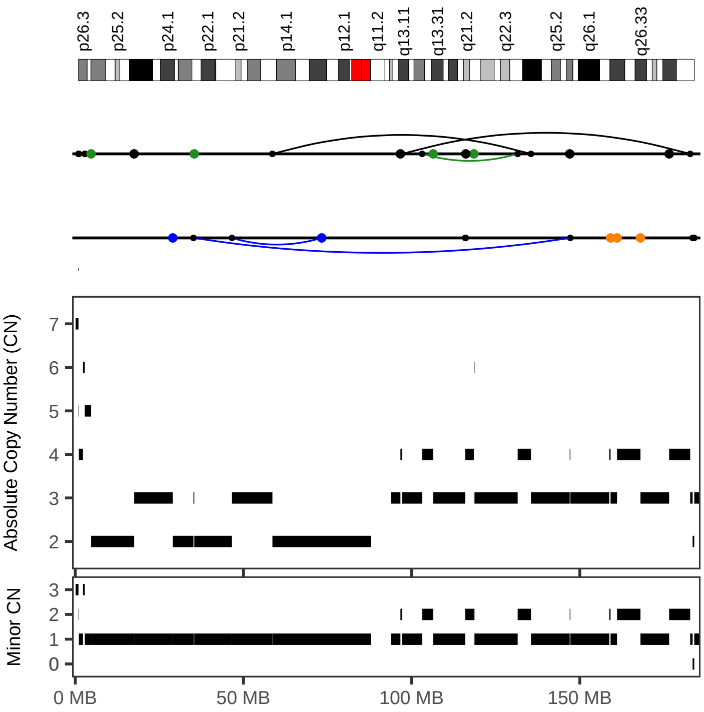

	**PCSI_0111**
Cancer type	Panc−AdenoCA
Position	3:35128015−182834759
Type	With other complex events
Interleaved intrachr. SVs	4
Total SVs (intrachr. + transl.)	15
SV types	DEL: 0; DUP: 2; h2hINV: 2; t2tINV: 0; TRA: 11
SVs in sample	260
Oscillating CN (2 and 3 states)	11, 23
CN segments	23
FDR fragment joints	0.59
FDR chr. breakp. enrich.	0.51
Linked to chrs	6:104571922−165438063;
Purity, ploidy	0.81, 3.09

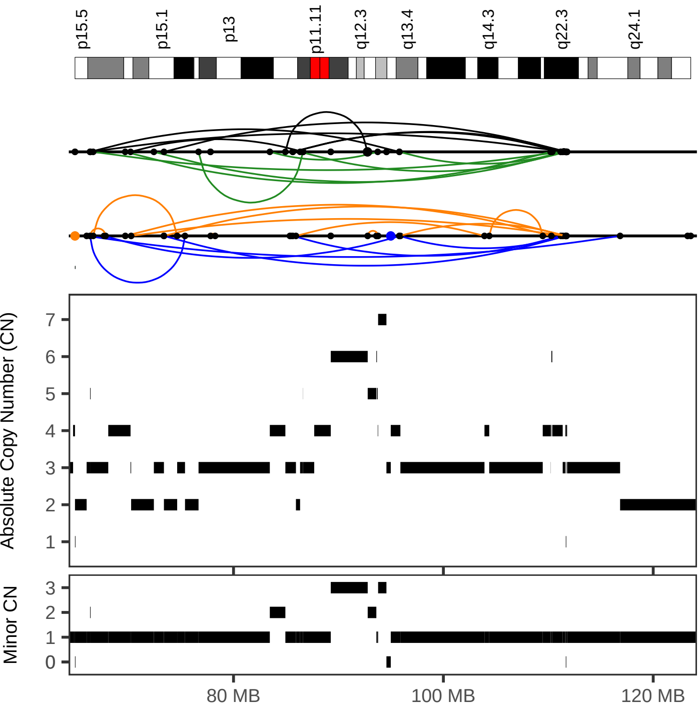

	**PCSI_0111**
Cancer type	Panc−AdenoCA
Position	11:66329047−111791324
Type	With other complex events
Interleaved intrachr. SVs	33
Total SVs (intrachr. + transl.)	35
SV types	DEL: 8; DUP: 6; h2hINV: 11; t2tINV: 8; TRA: 2
SVs in sample	260
Oscillating CN (2 and 3 states)	8, 13
CN segments	40
FDR fragment joints	0.75
FDR chr. breakp. enrich.	0
Linked to chrs	
Purity, ploidy	0.81, 3.09

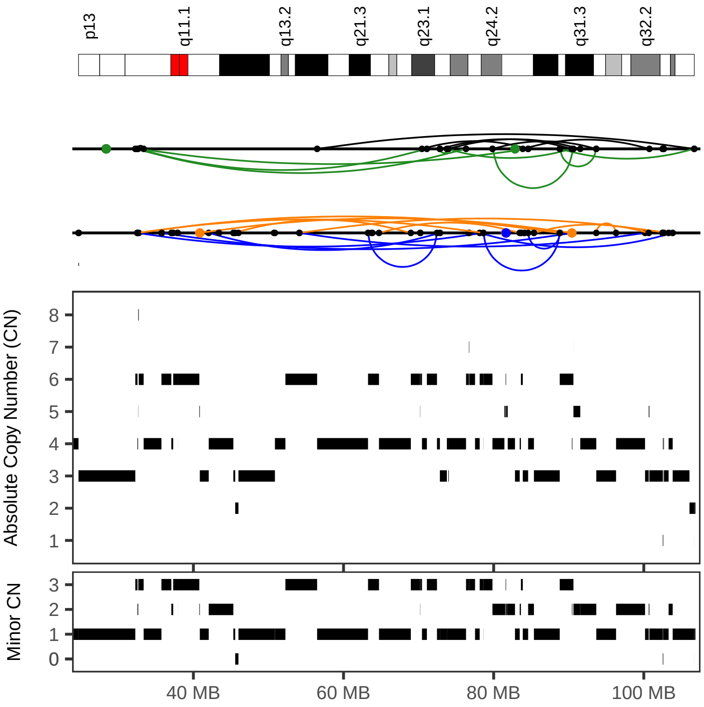

	**PCSI_0145**
Cancer type	Panc−AdenoCA
Position	14:32259593−106721663
Type	With other complex events
Interleaved intrachr. SVs	30
Total SVs (intrachr. + transl.)	35
SV types	DEL: 7; DUP: 8; h2hINV: 7; t2tINV: 8; TRA: 5
SVs in sample	550
Oscillating CN (2 and 3 states)	7, 11
CN segments	71
FDR fragment joints	1
FDR chr. breakp. enrich.	0
Linked to chrs	
Purity, ploidy	0.87, 2.72

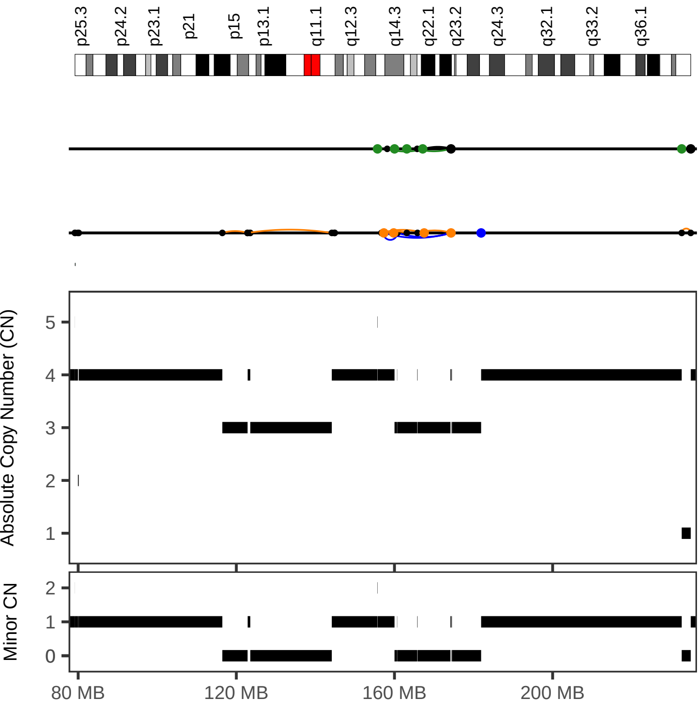

	**PCSI_0173**
Cancer type	Panc−AdenoCA
Position	2:156697897−174544813
Type	After polyploidization
Interleaved intrachr. SVs	9
Total SVs (intrachr. + transl.)	17
SV types	DEL: 3; DUP: 3; h2hINV: 1; t2tINV: 2; TRA: 8
SVs in sample	140
Oscillating CN (2 and 3 states)	10, 10
CN segments	10
FDR fragment joints	0.83
FDR chr. breakp. enrich.	0
Linked to chrs	
Purity, ploidy	0.42, 3.5

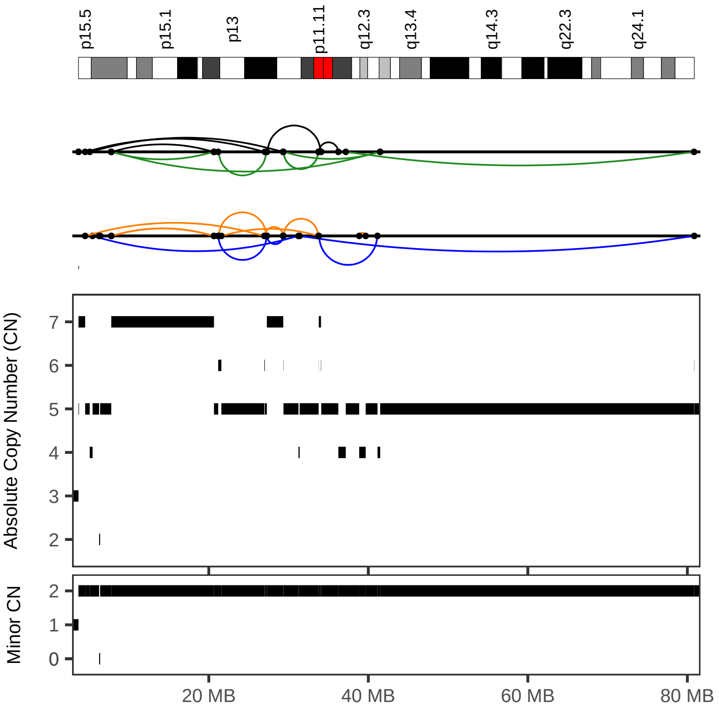

	**PCSI_0174**
Cancer type	Panc−AdenoCA
Position	11:4497415−80861714
Type	With other complex events
Interleaved intrachr. SVs	26
Total SVs (intrachr. + transl.)	26
SV types	DEL: 7; DUP: 6; h2hINV: 6; t2tINV: 7; TRA: 0
SVs in sample	110
Oscillating CN (2 and 3 states)	7, 17
CN segments	29
FDR fragment joints	0.99
FDR chr. breakp. enrich.	0
Linked to chrs	
Purity, ploidy	0.75, 3.5

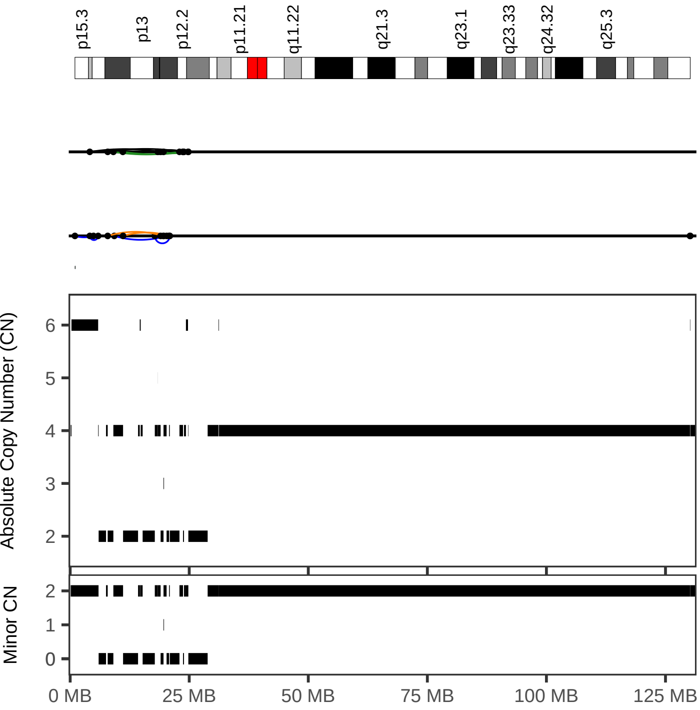

	**PCSI_0217**
Cancer type	Panc−AdenoCA
Position	10:997409−23898134
Type	With other complex events
Interleaved intrachr. SVs	14
Total SVs (intrachr. + transl.)	14
SV types	DEL: 3; DUP: 5; h2hINV: 3; t2tINV: 3; TRA: 0
SVs in sample	152
Oscillating CN (2 and 3 states)	7, 12
CN segments	23
FDR fragment joints	0.88
FDR chr. breakp. enrich.	0.02
Linked to chrs	
Purity, ploidy	0.77, 3.17

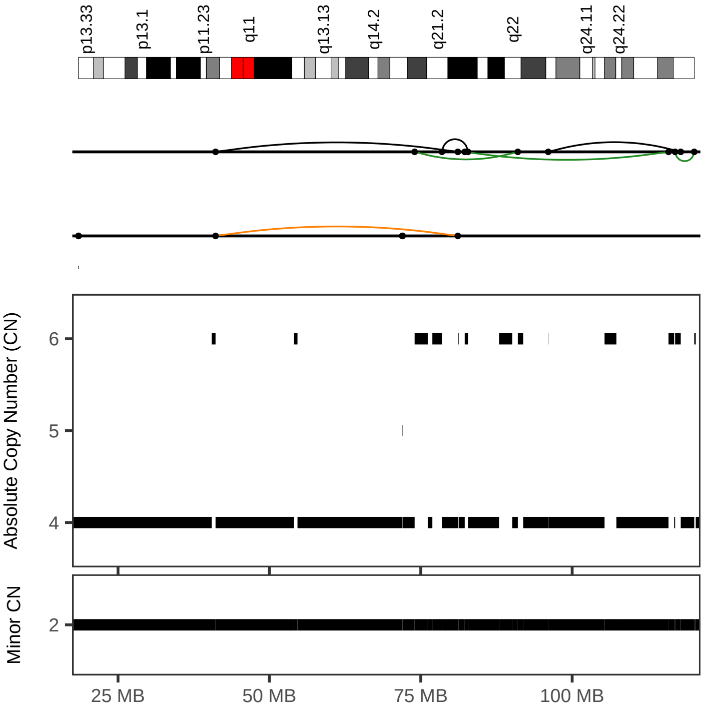

	**PCSI_0217**
Cancer type	Panc−AdenoCA
Position	12:41107420−120160634
Type	Before polyploidization
Interleaved intrachr. SVs	7
Total SVs (intrachr. + transl.)	7
SV types	DEL: 1; DUP: 0; h2hINV: 3; t2tINV: 3; TRA: 0
SVs in sample	152
Oscillating CN (2 and 3 states)	22, 27
CN segments	27
FDR fragment joints	0.59
FDR chr. breakp. enrich.	0.49
Linked to chrs	
Purity, ploidy	0.77, 3.17

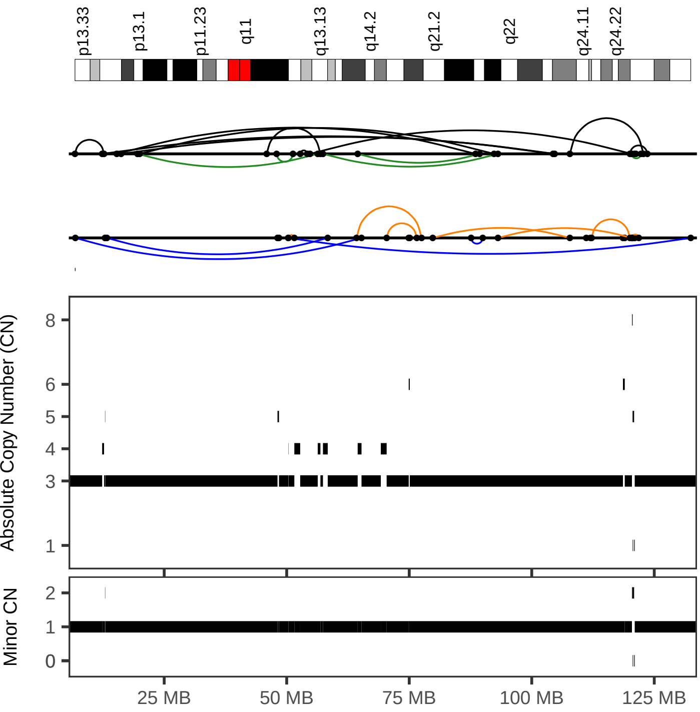

PCSI_0226	
Cancer type	Panc−AdenoCA
Position	12:6793815−132449370
Type	With other complex events
Interleaved intrachr. SVs	23
Total SVs (intrachr. + transl.)	23
SV types	DEL: 5; DUP: 4; h2hINV: 9; t2tINV: 5; TRA: 0
SVs in sample	144
Oscillating CN (2 and 3 states)	13, 19
CN segments	29
FDR fragment joints	0.64
FDR chr. breakp. enrich.	0
Linked to chrs	
Purity, ploidy	0.81, 2.95

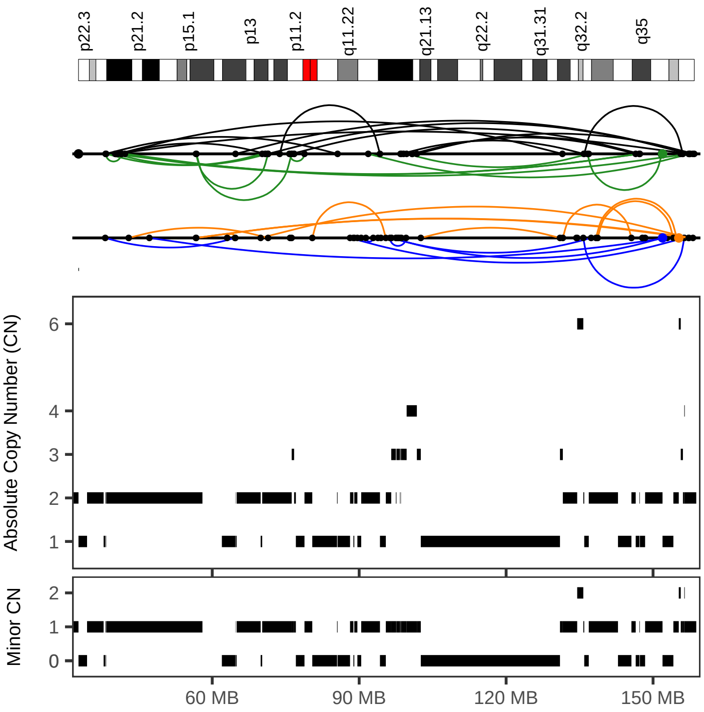

PCSI_0230	
Cancer type	Panc−AdenoCA
Position	7:38154775−158410033
Type	With other complex events
Interleaved intrachr. SVs	48
Total SVs (intrachr. + transl.)	52
SV types	DEL: 11; DUP: 9; h2hINV: 14; t2tINV: 14; TRA: 4
SVs in sample	235
Oscillating CN (2 and 3 states)	13, 32
CN segments	52
FDR fragment joints	0.76
FDR chr. breakp. enrich.	0
Linked to chrs	
Purity, ploidy	0.92, 1.83

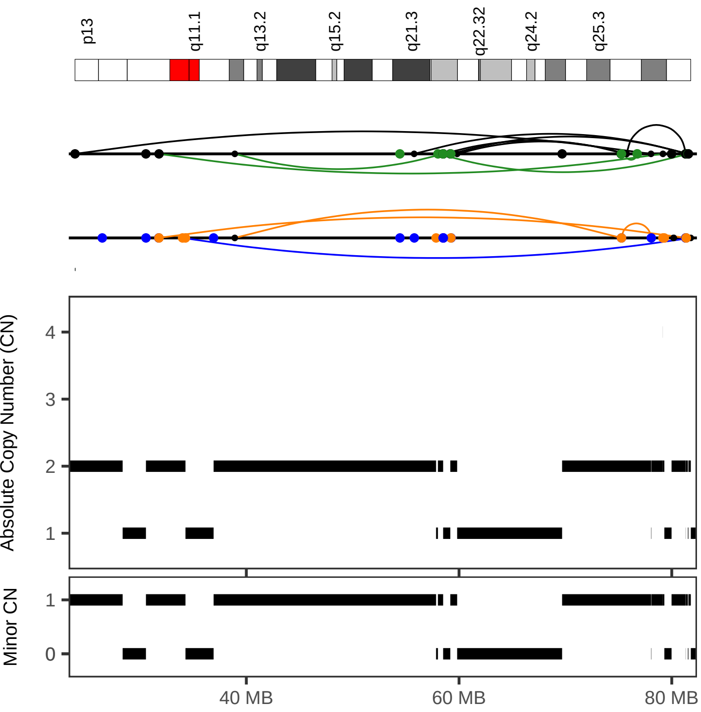

PCSI_0230	
Cancer type	Panc−AdenoCA
Position	15:23887647−81786260
Type	Canonical without polyploidization
Interleaved intrachr. SVs	15
Total SVs (intrachr. + transl.)	66
SV types	DEL: 3; DUP: 1; h2hINV: 6; t2tINV: 5; TRA: 51
SVs in sample	235
Oscillating CN (2 and 3 states)	13, 21
CN segments	21
FDR fragment joints	0.59
FDR chr. breakp. enrich.	0
Linked to chrs	6:8601646−159239116;8:2303876−24989800 21:24156719−42334956;
Purity, ploidy	0.92, 1.83

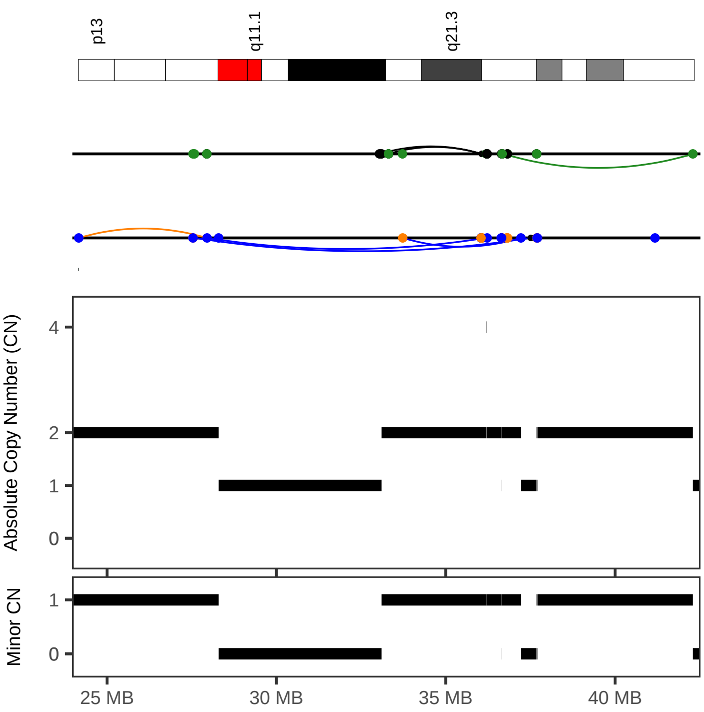

PCSI_0230	
Cancer type	Panc−AdenoCA
Position	21:24156719−42334957
Type	Canonical without polyploidization
Interleaved intrachr. SVs	7
Total SVs (intrachr. + transl.)	57
SV types	DEL: 1; DUP: 3; h2hINV: 2; t2tINV: 1; TRA: 50
SVs in sample	235
Oscillating CN (2 and 3 states)	8, 12
CN segments	12
FDR fragment joints	0.74
FDR chr. breakp. enrich.	0
Linked to chrs	8:2303876−24989800;
Purity, ploidy	0.92, 1.83

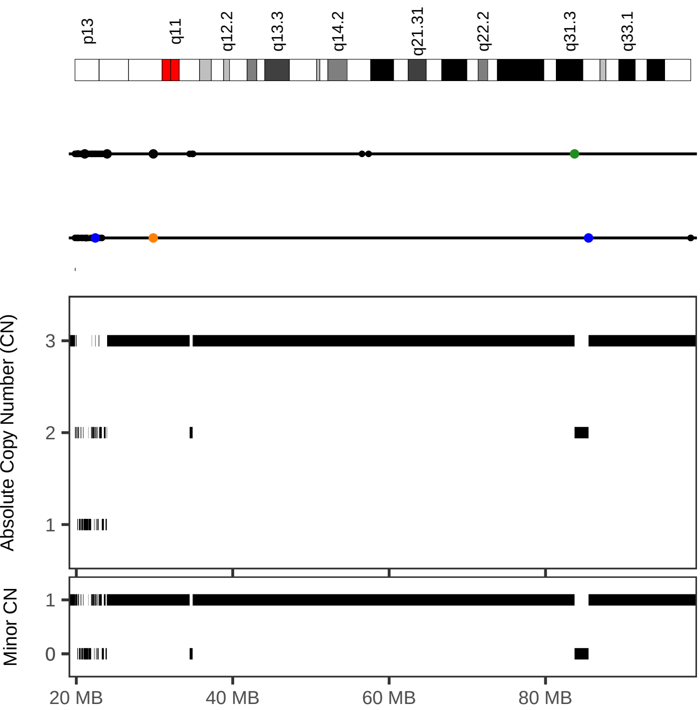

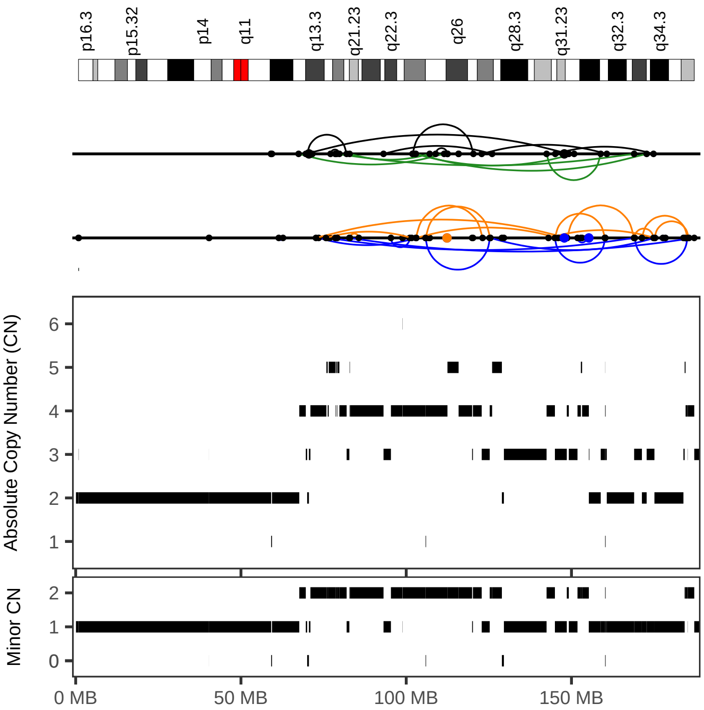

	**PCSI_0239**
Cancer type	Panc−AdenoCA
Position	13:19829855−23893773
Type	With other complex events
Interleaved intrachr. SVs	37
Total SVs (intrachr. + transl.)	39
SV types	DEL: 6; DUP: 10; h2hINV: 11; t2tINV: 10; TRA: 2
SVs in sample	111
Oscillating CN (2 and 3 states)	17, 45
CN segments	45
FDR fragment joints	0.74
FDR chr. breakp. enrich.	0
Linked to chrs	4:33126224−179897669;
Purity, ploidy	0.64, 1.95

	**PCSI_0261**
Cancer type	Panc−AdenoCA
Position	4:67443335−187140838
Type	With other complex events
Interleaved intrachr. SVs	41
Total SVs (intrachr. + transl.)	47
SV types	DEL: 12; DUP: 14; h2hINV: 9; t2tINV: 6; TRA: 6
SVs in sample	382
Oscillating CN (2 and 3 states)	9, 20
CN segments	58
FDR fragment joints	0.59
FDR chr. breakp. enrich.	0
Linked to chrs	9:9041105−26051500;
Purity, ploidy	0.55, 2.88

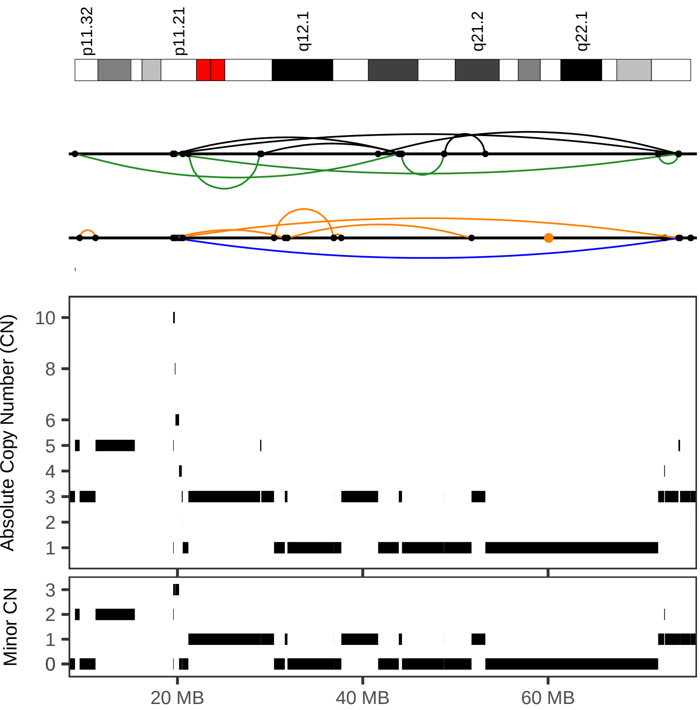

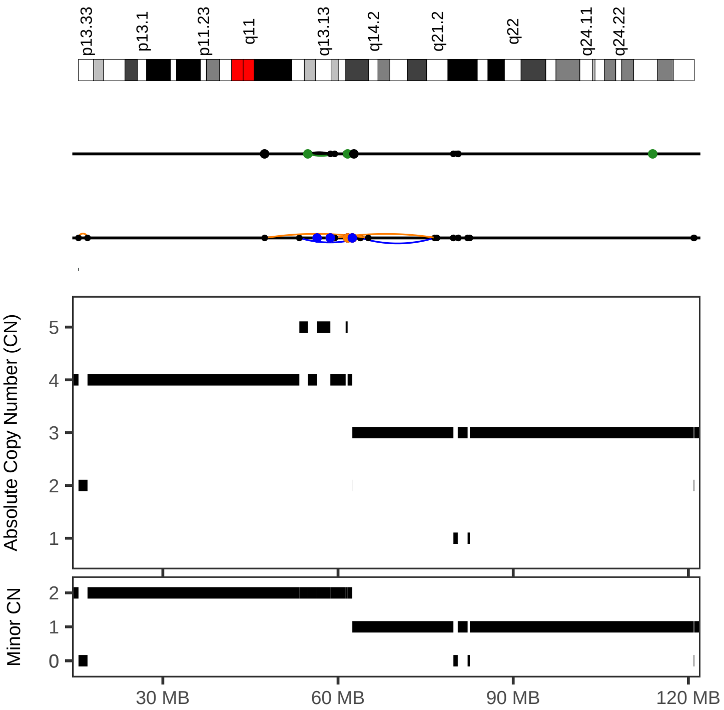

	**PCSI_0261**
Cancer type	Panc−AdenoCA
Position	18:8939813−74242436
Type	With other complex events
Interleaved intrachr. SVs	13
Total SVs (intrachr. + transl.)	15
SV types	DEL: 4; DUP: 1; h2hINV: 5; t2tINV: 3; TRA: 2
SVs in sample	382
Oscillating CN (2 and 3 states)	15, 18
CN segments	33
FDR fragment joints	0.64
FDR chr. breakp. enrich.	0
Linked to chrs	
Purity, ploidy	0.55, 2.88

	**PCSI_0274**
Cancer type	Panc−AdenoCA
Position	12:47428310−76942994
Type	After polyploidization
Interleaved intrachr. SVs	6
Total SVs (intrachr. + transl.)	19
SV types	DEL: 2; DUP: 3; h2hINV: 0; t2tINV: 1; TRA: 13
SVs in sample	202
Oscillating CN (2 and 3 states)	7, 10
CN segments	10
FDR fragment joints	0.59
FDR chr. breakp. enrich.	0
Linked to chrs	18:38904263−39580007;
Purity, ploidy	0.6, 3.17

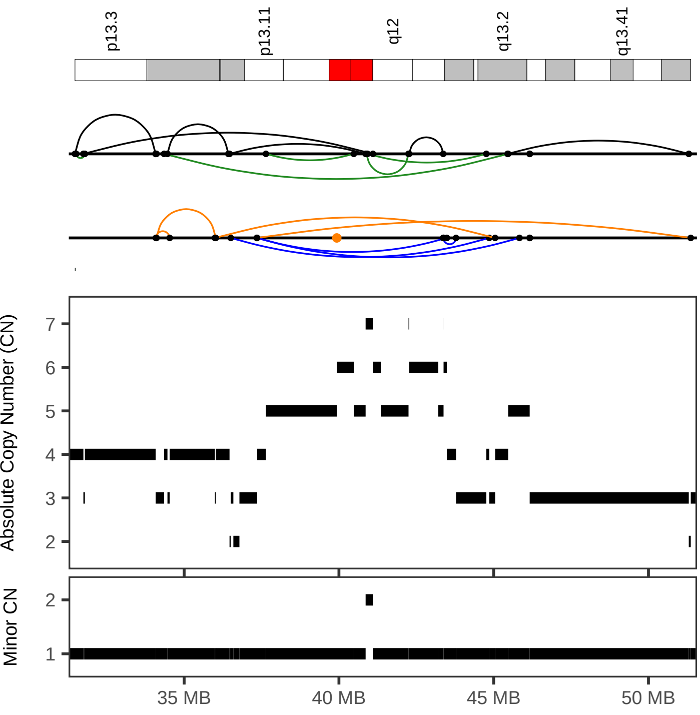

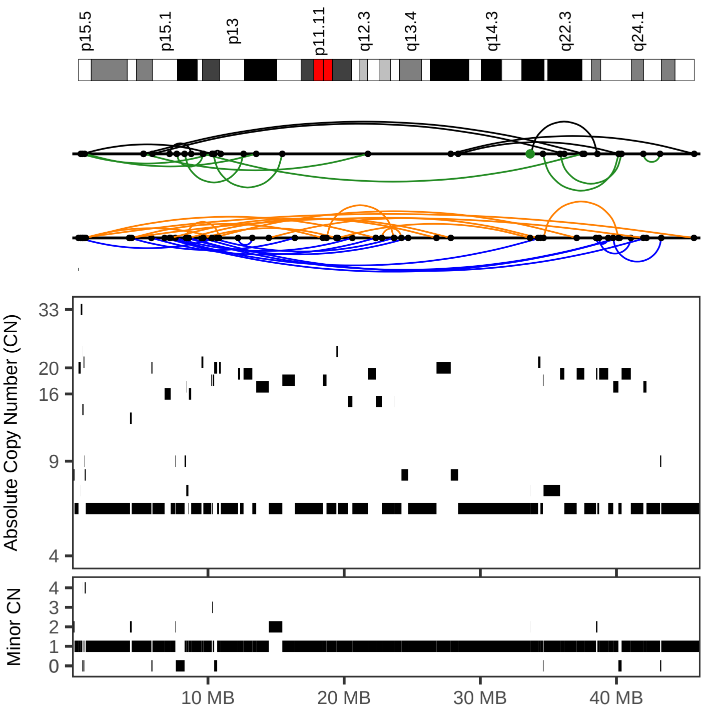

	**PCSI_0279**
Cancer type	Panc−AdenoCA
Position	19:31472618−51366174
Type	With other complex events
Interleaved intrachr. SVs	20
Total SVs (intrachr. + transl.)	21
SV types	DEL: 4; DUP: 5; h2hINV: 6; t2tINV: 5; TRA: 1
SVs in sample	57
Oscillating CN (2 and 3 states)	9, 13
CN segments	35
FDR fragment joints	0.96
FDR chr. breakp. enrich.	0
Linked to chrs	
Purity, ploidy	0.6, 2.03

	**PCSI_0280**
Cancer type	Panc−AdenoCA
Position	11:489225−45714066
Type	With other complex events
Interleaved intrachr. SVs	49
Total SVs (intrachr. + transl.)	50
SV types	DEL: 16; DUP: 15; h2hINV: 8; t2tINV: 10; TRA: 1
SVs in sample	96
Oscillating CN (2 and 3 states)	8, 14
CN segments	79
FDR fragment joints	0.59
FDR chr. breakp. enrich.	0
Linked to chrs	
Purity, ploidy	0.15, 4.76

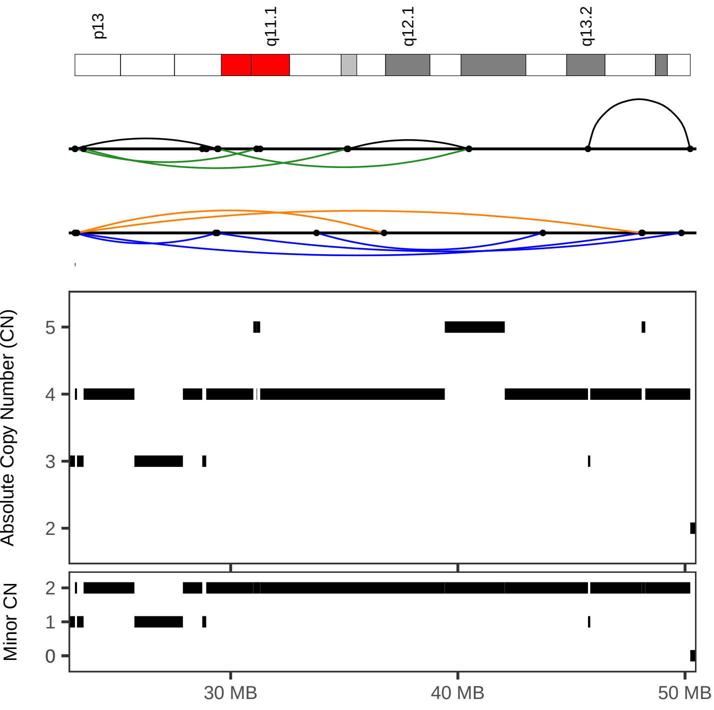

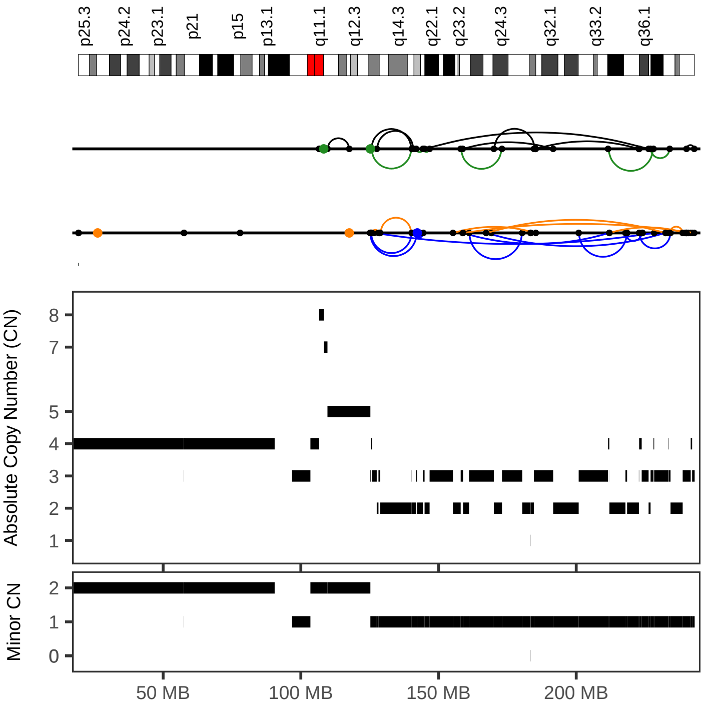

	**PCSI_0281**
Cancer type	Panc−AdenoCA
Position	22:23142389−50230074
Type	With other complex events
Interleaved intrachr. SVs	13
Total SVs (intrachr. + transl.)	13
SV types	DEL: 2; DUP: 4; h2hINV: 4; t2tINV: 3; TRA: 0
SVs in sample	85
Oscillating CN (2 and 3 states)	7, 17
CN segments	18
FDR fragment joints	0.88
FDR chr. breakp. enrich.	0
Linked to chrs	
Purity, ploidy	0.47, 3.9

	**PCSI_0284**
Cancer type	Panc−AdenoCA
Position	2:125112849−242849922
Type	With other complex events
Interleaved intrachr. SVs	30
Total SVs (intrachr. + transl.)	32
SV types	DEL: 6; DUP: 10; h2hINV: 7; t2tINV: 7; TRA: 2
SVs in sample	238
Oscillating CN (2 and 3 states)	18, 45
CN segments	46
FDR fragment joints	0.83
FDR chr. breakp. enrich.	0
Linked to chrs	
Purity, ploidy	0.56, 3.12

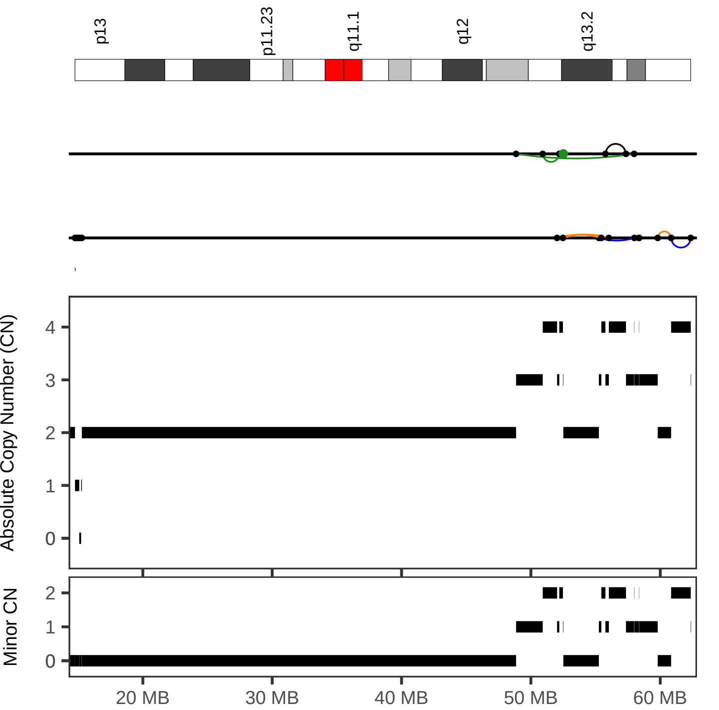

	**PCSI_0284**
Cancer type	Panc−AdenoCA
Position	20:48853377−57995207
Type	With other complex events
Interleaved intrachr. SVs	6
Total SVs (intrachr. + transl.)	7
SV types	DEL: 2; DUP: 1; h2hINV: 1;<br>t2tINV: 2; TRA: 1
SVs in sample	238
Oscillating CN (2 and 3 states)	7, 13
CN segments	13
FDR fragment joints	0.91
FDR chr. breakp. enrich.	0
Linked to chrs	
Purity, ploidy	0.56, 3.12

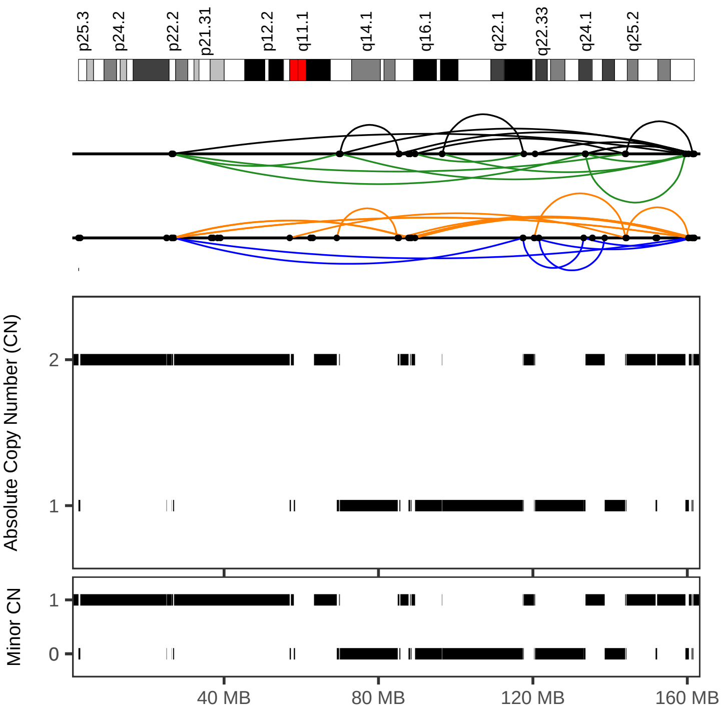

	**PCSI_0287**
Cancer type	Panc−AdenoCA
Position	6:26445451−161795623
Type	Canonical without polyploidization
Interleaved intrachr. SVs	41
Total SVs (intrachr. + transl.)	41
SV types	DEL: 12; DUP: 7; h2hINV: 12;<br>t2tINV: 10; TRA: 0
SVs in sample	152
Oscillating CN (2 and 3 states)	47, 47
CN segments	47
FDR fragment joints	0.73
FDR chr. breakp. enrich.	0
Linked to chrs	
Purity, ploidy	0.94, 1.94

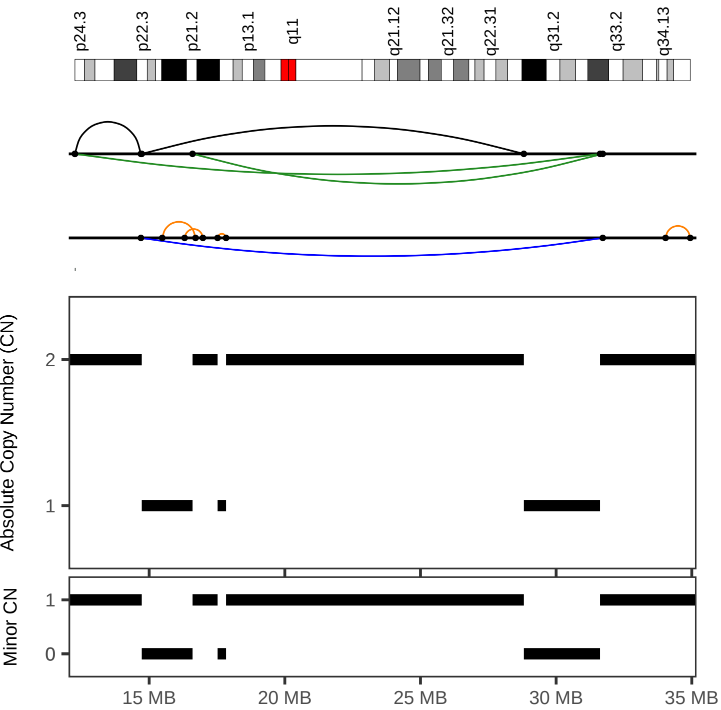

	**PCSI_0287**
Cancer type	Panc−AdenoCA
Position	9:12263449−31719162
Type	Canonical without polyploidization
Interleaved intrachr. SVs	7
Total SVs (intrachr. + transl.)	7
SV types	DEL: 2; DUP: 1; h2hINV: 2;<br>t2tINV: 2; TRA: 0
SVs in sample	152
Oscillating CN (2 and 3 states)	7, 7
CN segments	7
FDR fragment joints	0.95
FDR chr. breakp. enrich.	0.43
Linked to chrs	
Purity, ploidy	0.94, 1.94

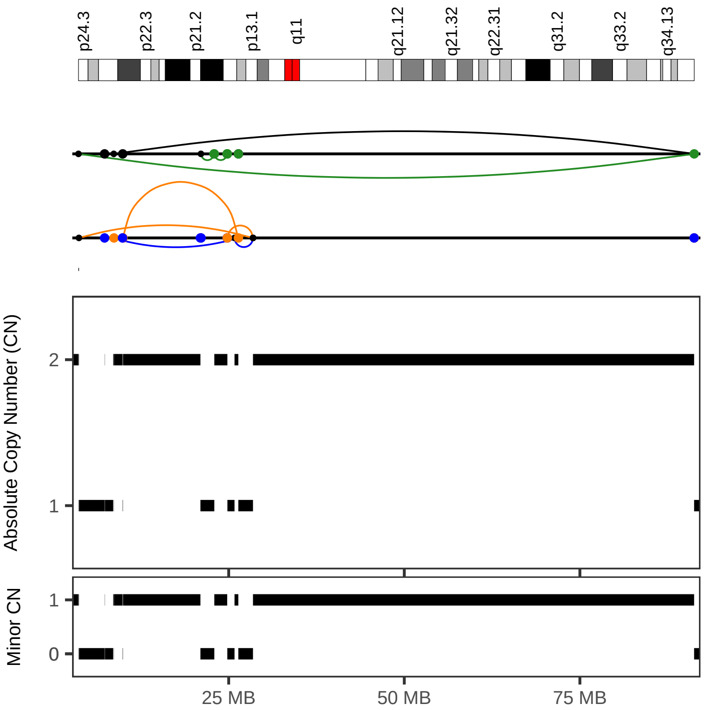

	**PCSI_0297**
Cancer type	Panc−AdenoCA
Position	9:3612495−91279620
Type	Canonical without polyploidization
Interleaved intrachr. SVs	7
Total SVs (intrachr. + transl.)	29
SV types	DEL: 3; DUP: 2; h2hINV: 1;<br>t2tINV: 1; TRA: 22
SVs in sample	181
Oscillating CN (2 and 3 states)	20, 20
CN segments	20
FDR fragment joints	0.74
FDR chr. breakp. enrich.	0
Linked to chrs	6:3725048−101996715;
Purity, ploidy	0.85, 1.89

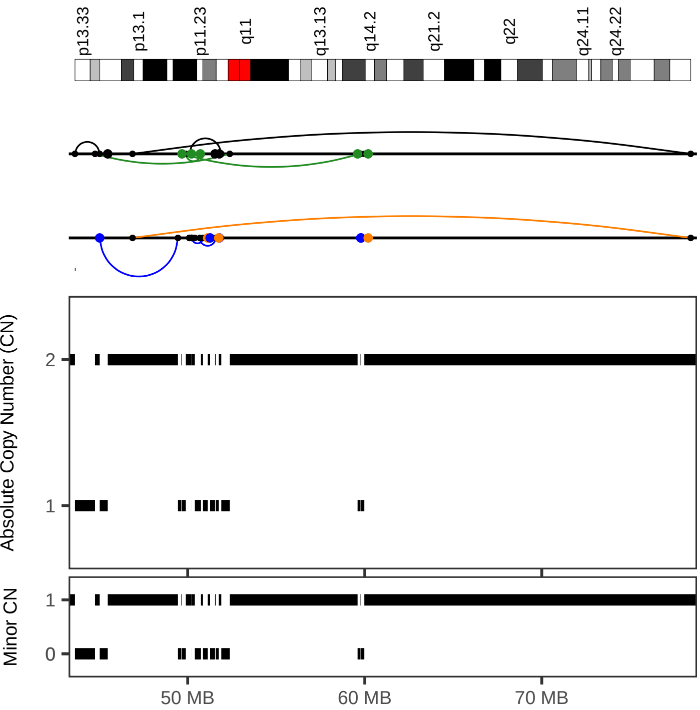

	**PCSI_0297**
Cancer type	Panc−AdenoCA
Position	12:49890146−51896557
Type	Canonical without polyploidization
Interleaved intrachr. SVs	8
Total SVs (intrachr. + transl.)	16
SV types	DEL: 0; DUP: 6; h2hINV: 1; t2tINV: 1; TRA: 8
SVs in sample	181
Oscillating CN (2 and 3 states)	12, 12
CN segments	12
FDR fragment joints	0.21
FDR chr. breakp. enrich.	0
Linked to chrs	
Purity, ploidy	0.85, 1.89

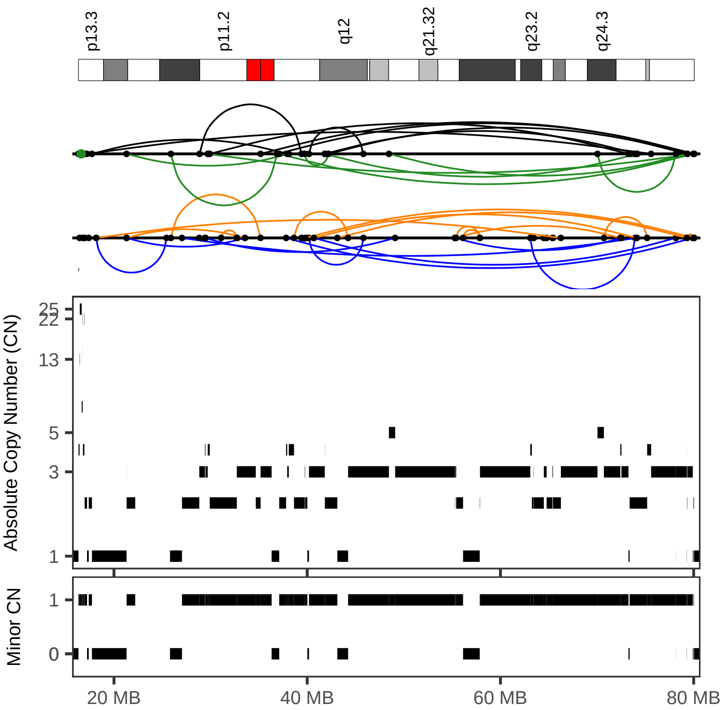

	**PCSI_0297**
Cancer type	Panc−AdenoCA
Position	17:16438476−80055892
Type	With other complex events
Interleaved intrachr. SVs	42
Total SVs (intrachr. + transl.)	43
SV types	DEL: 11; DUP: 12; h2hINV: 10; t2tINV: 9; TRA: 1
SVs in sample	181
Oscillating CN (2 and 3 states)	8, 10
CN segments	73
FDR fragment joints	0.94
FDR chr. breakp. enrich.	0
Linked to chrs	
Purity, ploidy	0.85, 1.89

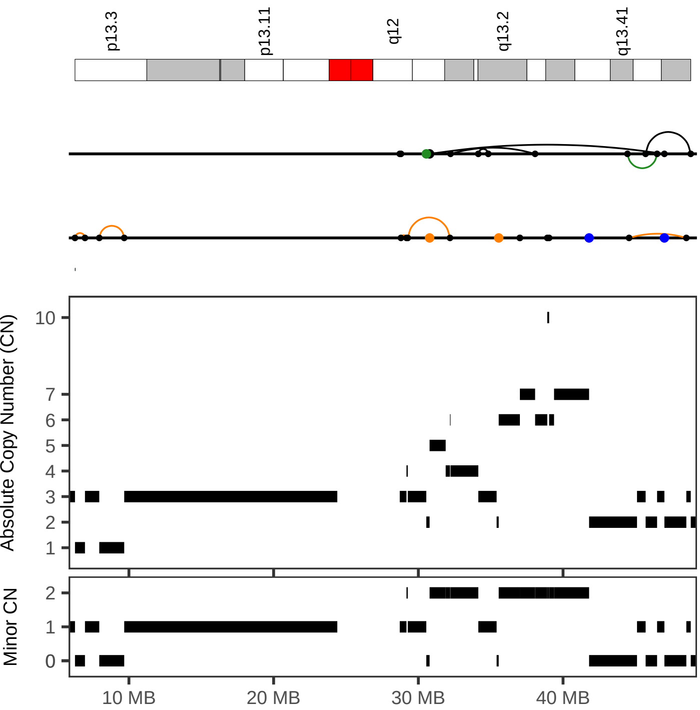

	**PCSI_0300**
Cancer type	Panc−AdenoCA
Position	19:29270995−48823894
Type	With other complex events
Interleaved intrachr. SVs	5
Total SVs (intrachr. + transl.)	11
SV types	DEL: 2; DUP: 0; h2hINV: 2; t2tINV: 1; TRA: 6
SVs in sample	208
Oscillating CN (2 and 3 states)	7, 7
CN segments	21
FDR fragment joints	0.64
FDR chr. breakp. enrich.	0
Linked to chrs	
Purity, ploidy	0.58, 2.82

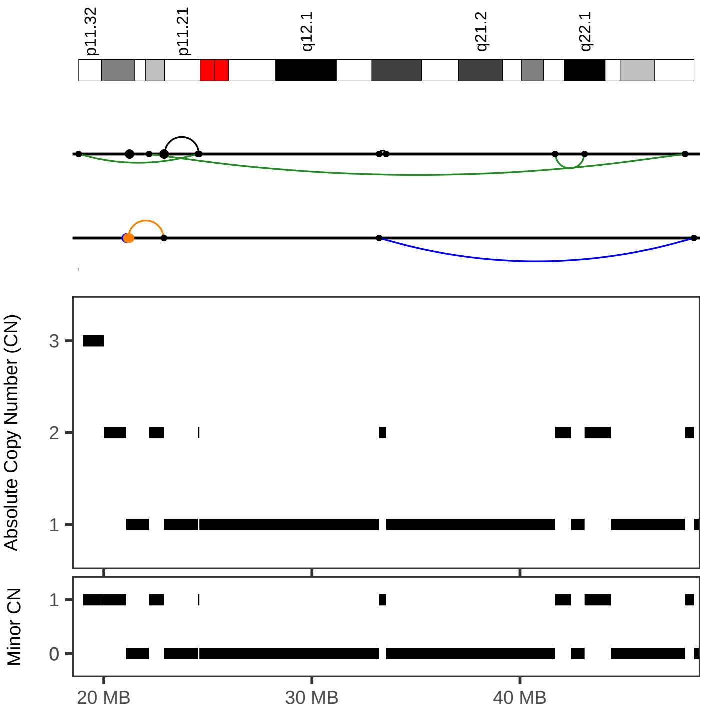

	**PCSI_0303**
Cancer type	Panc−AdenoCA
Position	18:18793459−48361192
Type	Canonical without polyploidization
Interleaved intrachr. SVs	5
Total SVs (intrachr. + transl.)	11
SV types	DEL: 1; DUP: 1; h2hINV: 1; t2tINV: 2; TRA: 6
SVs in sample	28
Oscillating CN (2 and 3 states)	14, 16
CN segments	16
FDR fragment joints	0.92
FDR chr. breakp. enrich.	0
Linked to chrs	
Purity, ploidy	0.52, 2.1

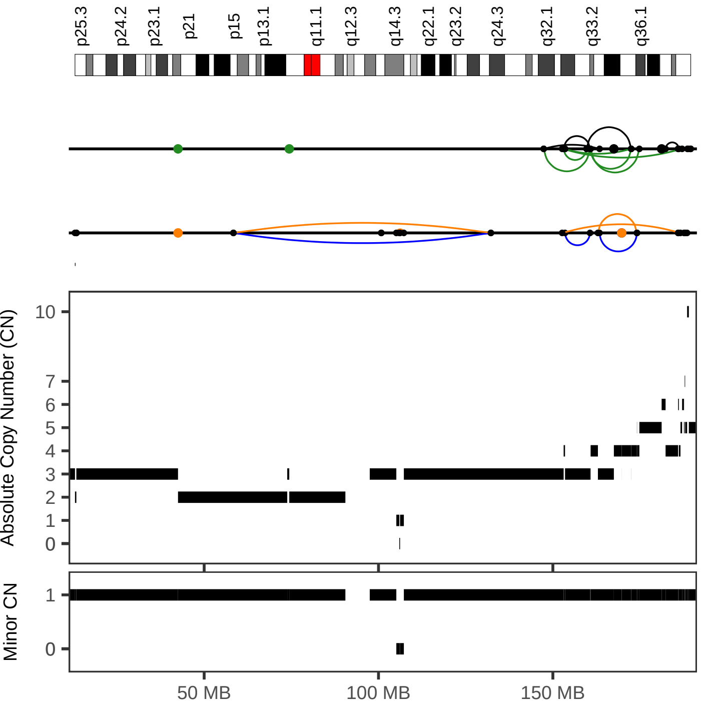

	PCSI_0305
Cancer type	Panc−AdenoCA
Position	2:147370933−187071951
Type	With other complex events
Interleaved intrachr. SVs	13
Total SVs (intrachr. + transl.)	17
SV types	DEL: 2; DUP: 2; h2hINV: 3; t2tINV: 6; TRA: 4
SVs in sample	166
Oscillating CN (2 and 3 states)	12, 16
CN segments	21
FDR fragment joints	0.59
FDR chr. breakp. enrich.	0
Linked to chrs	
Purity, ploidy	0.89, 3.08

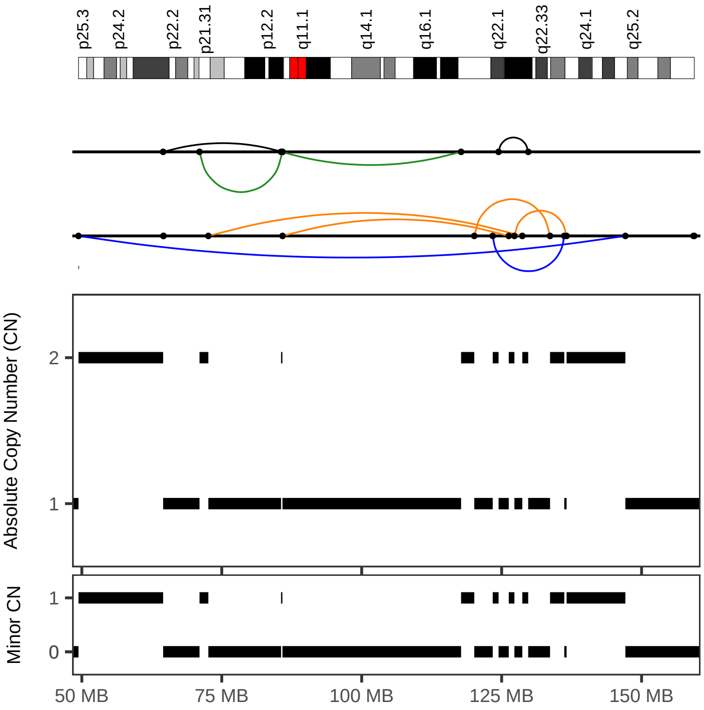

	PCSI_0307
Cancer type	Panc−AdenoCA
Position	6:64529563−136604930
Type	Canonical without polyploidization
Interleaved intrachr. SVs	9
Total SVs (intrachr. + transl.)	9
SV types	DEL: 4; DUP: 1; h2hINV: 2; t2tINV: 2; TRA: 0
SVs in sample	43
Oscillating CN (2 and 3 states)	18, 18
CN segments	18
FDR fragment joints	0.64
FDR chr. breakp. enrich.	0
Linked to chrs	
Purity, ploidy	0.81, 1.8

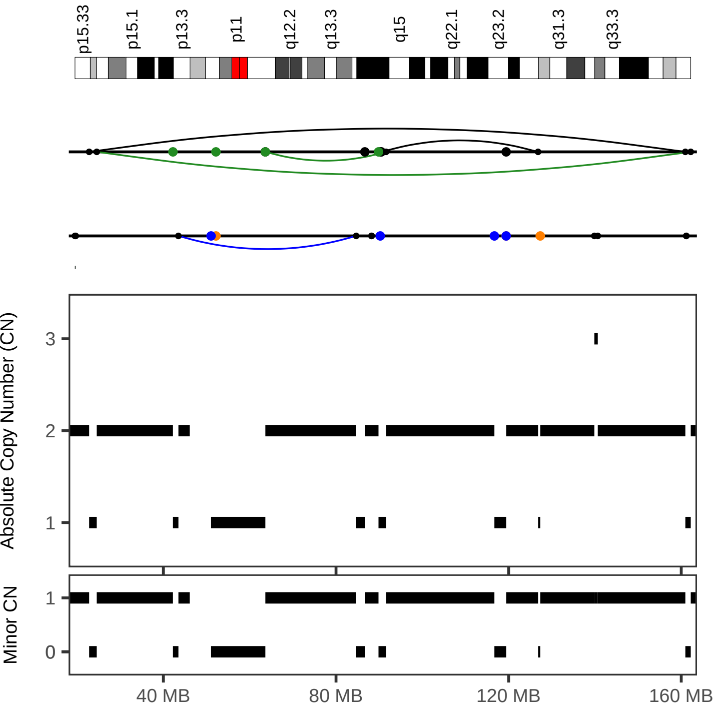

	PCSI_0324
Cancer type	Panc−AdenoCA
Position	5:43481679−126819152
Type	Canonical without polyploidization
Interleaved intrachr. SVs	3
Total SVs (intrachr. + transl.)	17
SV types	DEL: 0; DUP: 1; h2hINV: 1; t2tINV: 1; TRA: 14
SVs in sample	204
Oscillating CN (2 and 3 states)	10, 10
CN segments	10
FDR fragment joints	0.84
FDR chr. breakp. enrich.	0.01
Linked to chrs	
Purity, ploidy	0.56, 1.86

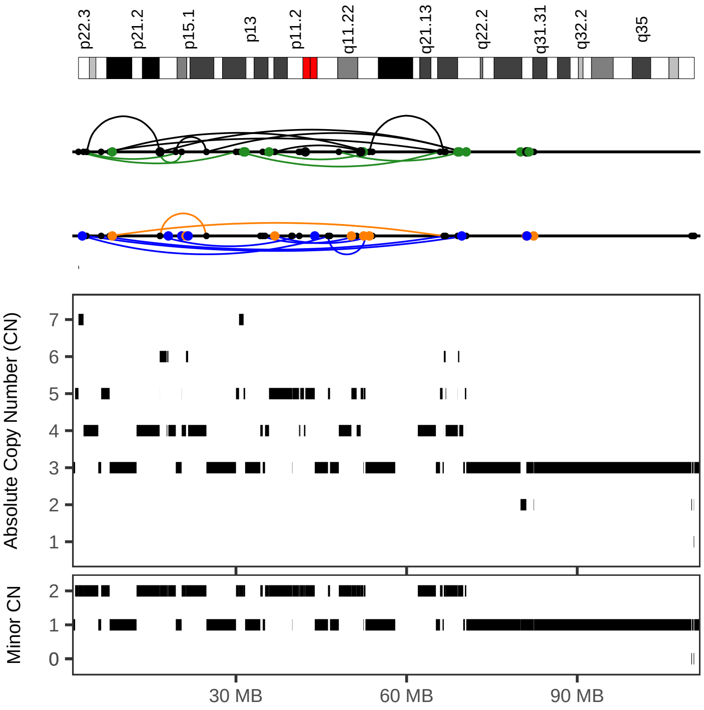

	PCSI_0325
Cancer type	Panc−AdenoCA
Position	7:2311735−70487292
Type	With other complex events
Interleaved intrachr. SVs	26
Total SVs (intrachr. + transl.)	49
SV types	DEL: 2; DUP: 7; h2hINV: 10; t2tINV: 7; TRA: 23
SVs in sample	207
Oscillating CN (2 and 3 states)	7, 14
CN segments	59
FDR fragment joints	0.59
FDR chr. breakp. enrich.	0
Linked to chrs	12:21197763−34726454;
Purity, ploidy	0.67, 2.85

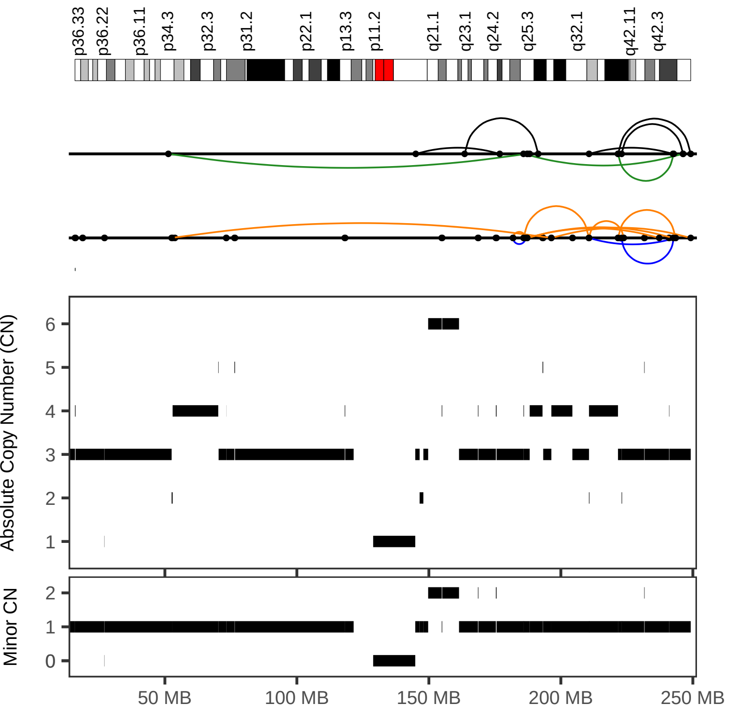

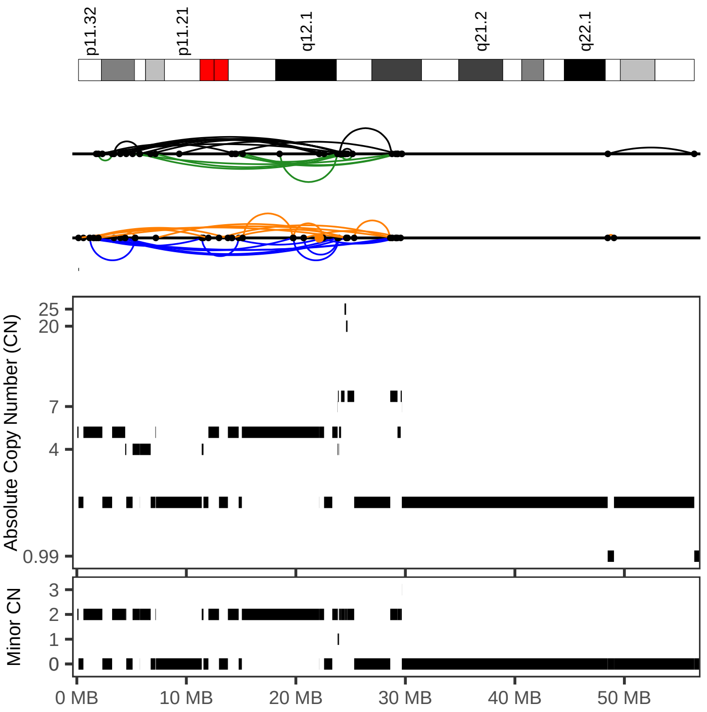

	**PCSI_0338**
Cancer type	Panc−AdenoCA
Position	1:51308307−249192722
Type	With other complex events
Interleaved intrachr. SVs	20
Total SVs (intrachr. + transl.)	20
SV types	DEL: 8; DUP: 4; h2hINV: 5; t2tINV: 3; TRA: 0
SVs in sample	225
Oscillating CN (2 and 3 states)	8, 9
CN segments	40
FDR fragment joints	0.63
FDR chr. breakp. enrich.	0
Linked to chrs	
Purity, ploidy	0.6, 2.02

	**PCSI_0340**
Cancer type	Panc−AdenoCA
Position	18:149060−29571306
Type	With other complex events
Interleaved intrachr. SVs	48
Total SVs (intrachr. + transl.)	49
SV types	DEL: 11; DUP: 13; h2hINV: 14; t2tINV: 10; TRA: 1
SVs in sample	237
Oscillating CN (2 and 3 states)	10, 19
CN segments	37
FDR fragment joints	0.88
FDR chr. breakp. enrich.	0
Linked to chrs	
Purity, ploidy	0.41, 3.29

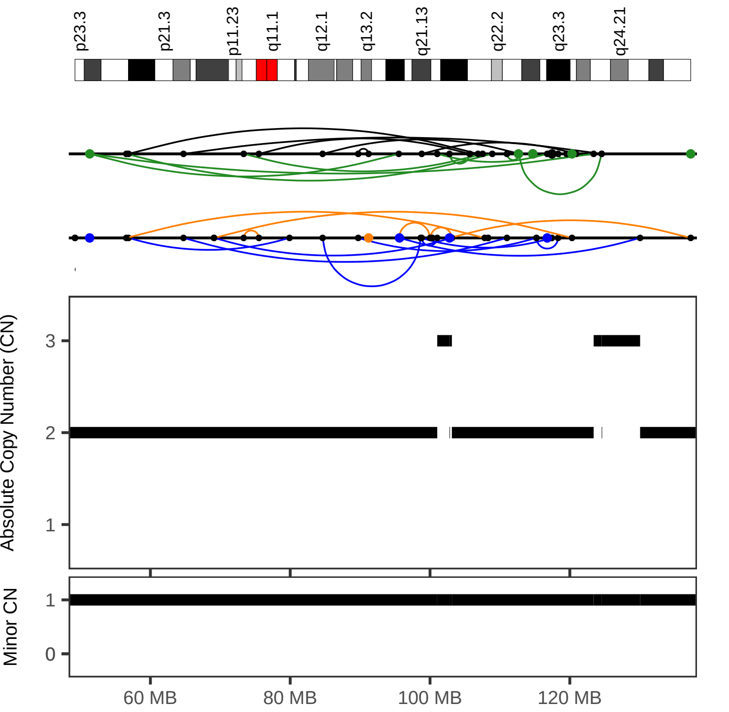

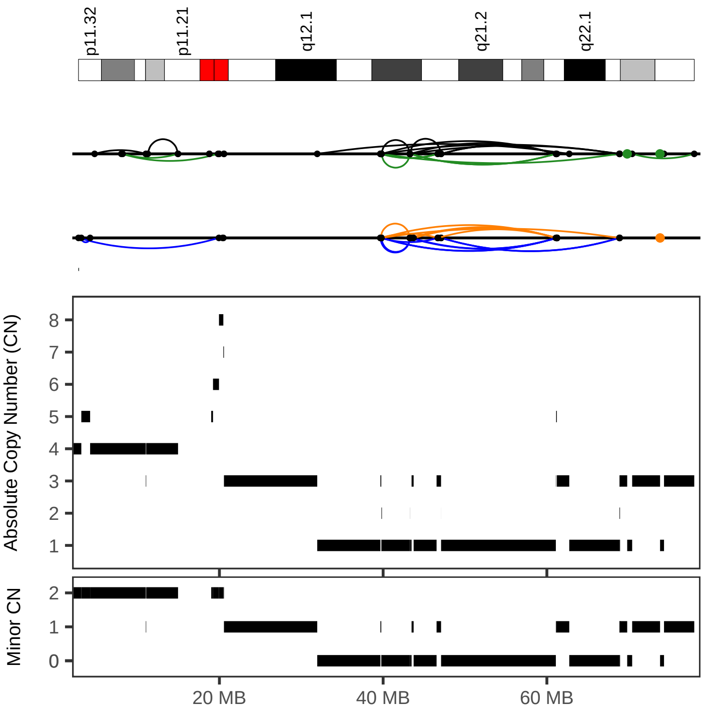

	**PCSI_0345**
Cancer type	Panc−AdenoCA
Position	8:51309933−137292850
Type	Canonical without polyploidization
Interleaved intrachr. SVs	29
Total SVs (intrachr. + transl.)	40
SV types	DEL: 6; DUP: 9; h2hINV: 6; t2tINV: 8; TRA: 11
SVs in sample	92
Oscillating CN (2 and 3 states)	9, 9
CN segments	9
FDR fragment joints	0.86
FDR chr. breakp. enrich.	0
Linked to chrs	19:21513068−58739451;
Purity, ploidy	0.53, 1.87

	**PCSI_0350**
Cancer type	Panc−AdenoCA
Position	18:31950397−68899945
Type	With other complex events
Interleaved intrachr. SVs	40
Total SVs (intrachr. + transl.)	40
SV types	DEL: 9; DUP: 12; h2hINV: 10; t2tINV: 9; TRA: 0
SVs in sample	100
Oscillating CN (2 and 3 states)	7, 13
CN segments	25
FDR fragment joints	0.92
FDR chr. breakp. enrich.	0
Linked to chrs	
Purity, ploidy	0.99, 3.25

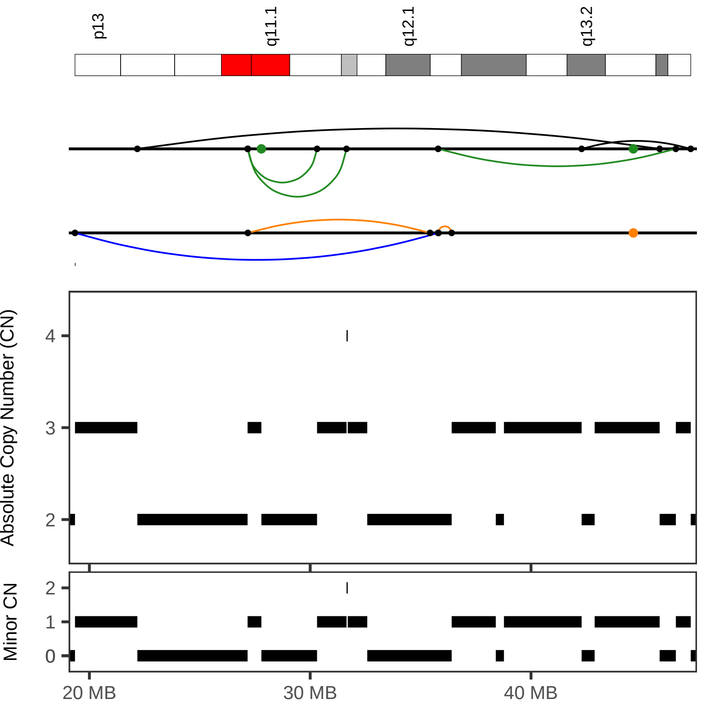

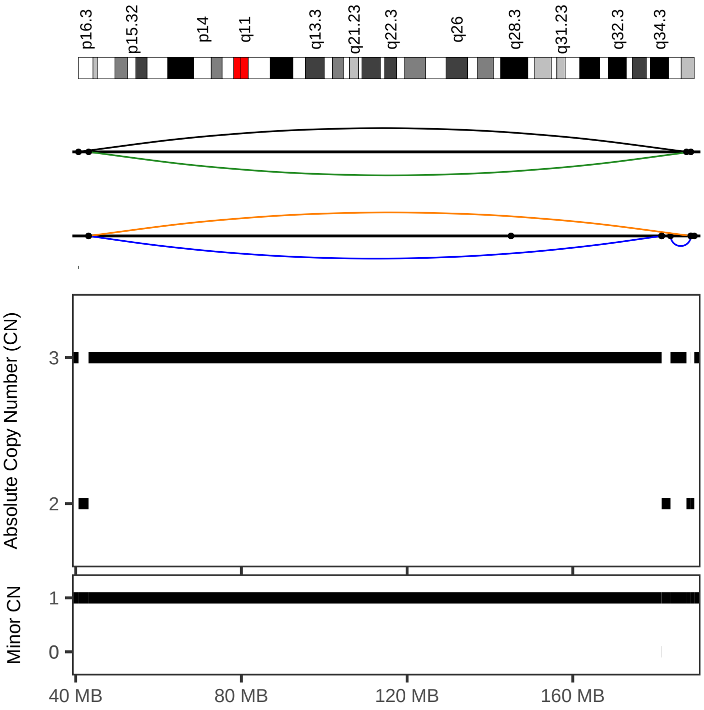

	**PCSI_0350**
Cancer type	Panc−AdenoCA
Position	22:19346879−47234372
Type	Canonical without polyploidization
Interleaved intrachr. SVs	5
Total SVs (intrachr. + transl.)	8
SV types	DEL: 1; DUP: 1; h2hINV: 2; t2tINV: 1; TRA: 3
SVs in sample	100
Oscillating CN (2 and 3 states)	10, 16
CN segments	16
FDR fragment joints	0.92
FDR chr. breakp. enrich.	0
Linked to chrs	
Purity, ploidy	0.99, 3.25

	**PCSI_0351**
Cancer type	Panc−AdenoCA
Position	4:40669996−189308624
Type	Canonical without polyploidization
Interleaved intrachr. SVs	7
Total SVs (intrachr. + transl.)	7
SV types	DEL: 3; DUP: 2; h2hINV: 1; t2tINV: 1; TRA: 0
SVs in sample	75
Oscillating CN (2 and 3 states)	12, 12
CN segments	12
FDR fragment joints	0.74
FDR chr. breakp. enrich.	0.12
Linked to chrs	
Purity, ploidy	0.98, 2.9

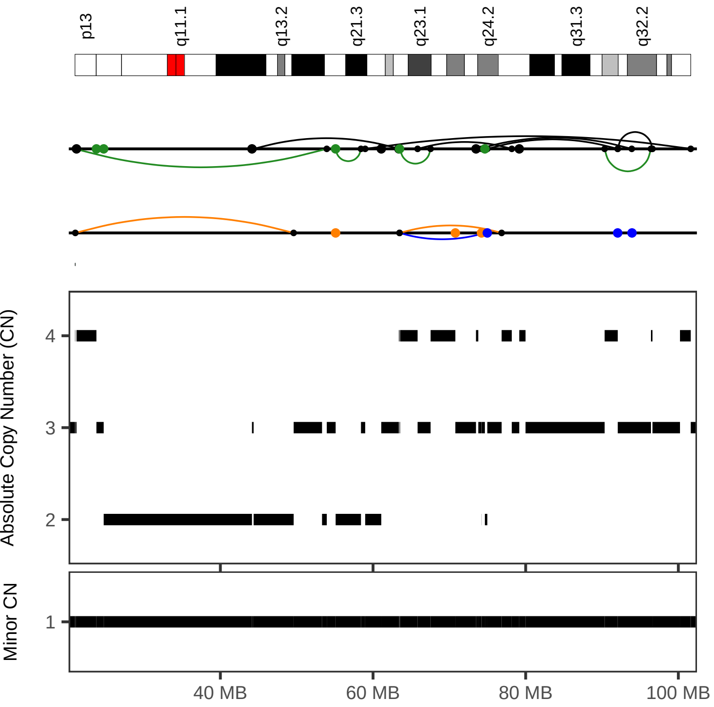

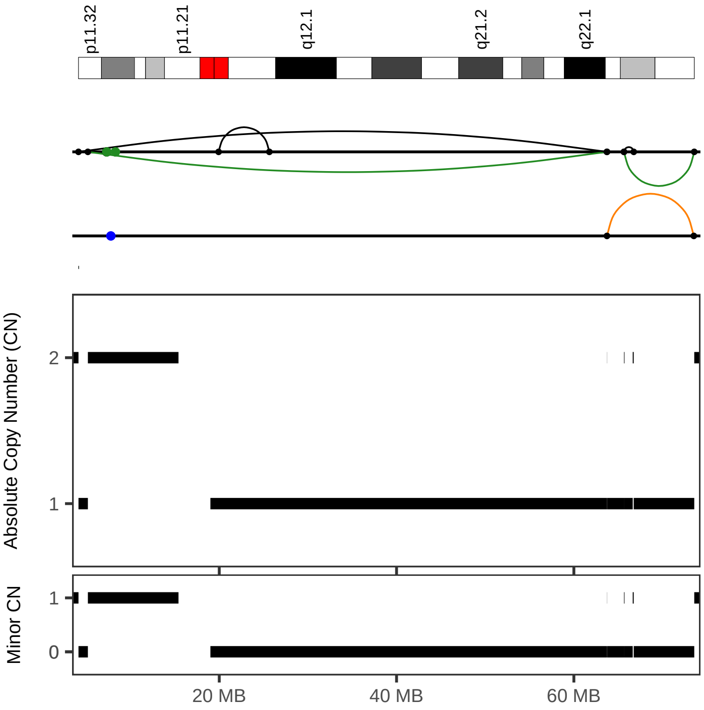

	**PCSI_0352**
Cancer type	Panc−AdenoCA
Position	14:20953269−101623829
Type	With other complex events
Interleaved intrachr. SVs	13
Total SVs (intrachr. + transl.)	30
SV types	DEL: 2; DUP: 1; h2hINV: 6; t2tINV: 4; TRA: 17
SVs in sample	72
Oscillating CN (2 and 3 states)	11, 36
CN segments	36
FDR fragment joints	0.59
FDR chr. breakp. enrich.	0
Linked to chrs	6:40037807−56846579;18:2451184−18686432
Purity, ploidy	NA, NA

	**PCSI_0354**
Cancer type	Panc−AdenoCA
Position	18:4121150−73576993
Type	Canonical without polyploidization
Interleaved intrachr. SVs	3
Total SVs (intrachr. + transl.)	7
SV types	DEL: 1; DUP: 0; h2hINV: 1; t2tINV: 1; TRA: 4
SVs in sample	73
Oscillating CN (2 and 3 states)	10, 10
CN segments	10
FDR fragment joints	0.84
FDR chr. breakp. enrich.	0
Linked to chrs	22:27238901−46022596;
Purity, ploidy	0.8, 1.97

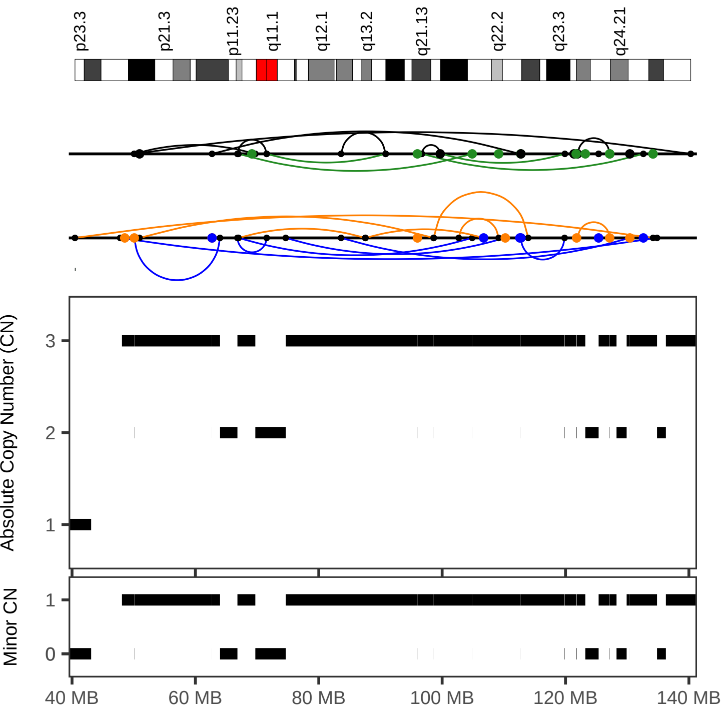

	PCSI_0356
Cancer type	Panc−AdenoCA
Position	8:40478806−140298863
Type	Canonical without polyploidization
Interleaved intrachr. SVs	23
Total SVs (intrachr. + transl.)	54
SV types	DEL: 6; DUP: 7; h2hINV: 6; t2tINV: 4; TRA: 31
SVs in sample	228
Oscillating CN (2 and 3 states)	31, 32
CN segments	32
FDR fragment joints	0.88
FDR chr. breakp. enrich.	0
Linked to chrs	17:22238087−79107549;
Purity, ploidy	0.77, 3.33

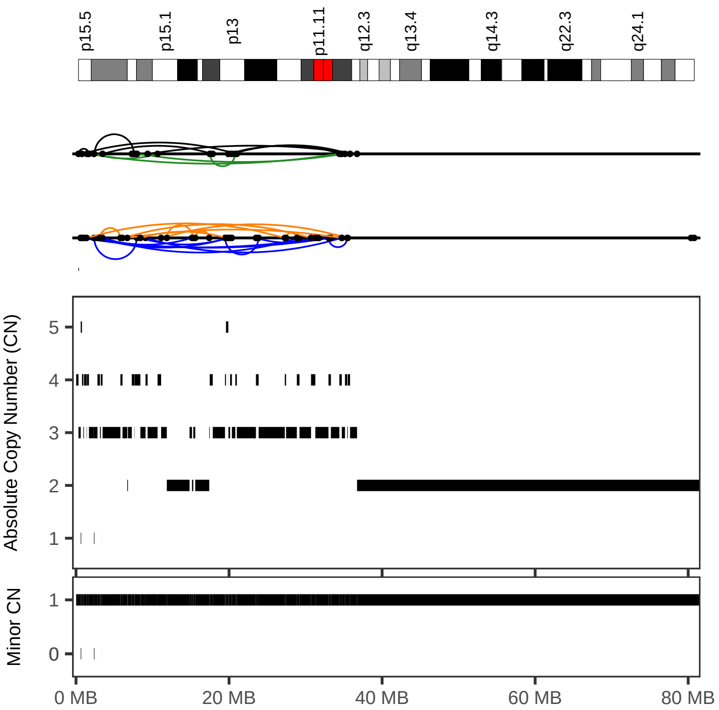

	PCSI_0356
Cancer type	Panc−AdenoCA
Position	11:323191−36726710
Type	With other complex events
Interleaved intrachr. SVs	34
Total SVs (intrachr. + transl.)	34
SV types	DEL: 8; DUP: 12; h2hINV: 9; t2tINV: 5; TRA: 0
SVs in sample	228
Oscillating CN (2 and 3 states)	24, 48
CN segments	63
FDR fragment joints	0.6
FDR chr. breakp. enrich.	0
Linked to chrs	
Purity, ploidy	0.77, 3.33

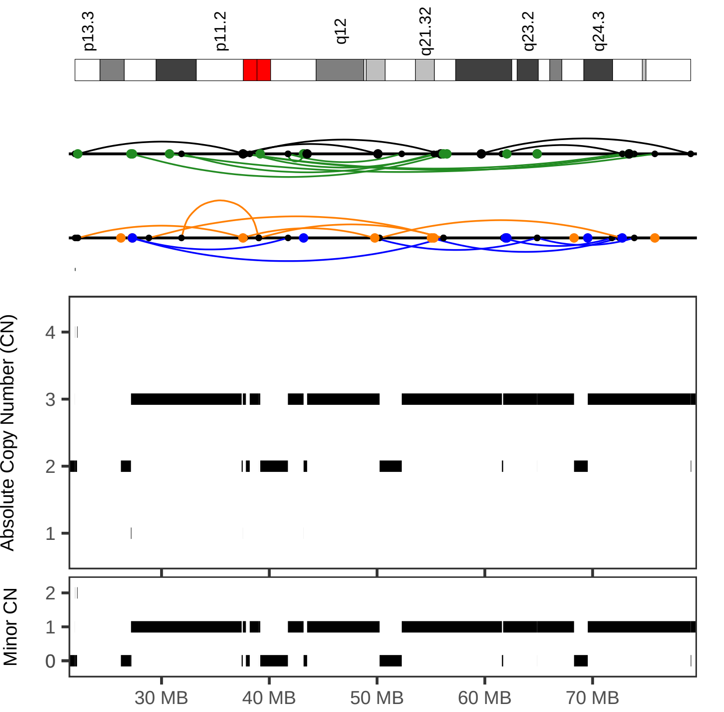

	PCSI_0356
Cancer type	Panc−AdenoCA
Position	17:22238087−79107550
Type	With other complex events
Interleaved intrachr. SVs	25
Total SVs (intrachr. + transl.)	58
SV types	DEL: 6; DUP: 6; h2hINV: 5; t2tINV: 8; TRA: 33
SVs in sample	228
Oscillating CN (2 and 3 states)	11, 14
CN segments	27
FDR fragment joints	0.9
FDR chr. breakp. enrich.	0
Linked to chrs	8:40478806−140298862;
Purity, ploidy	0.77, 3.33

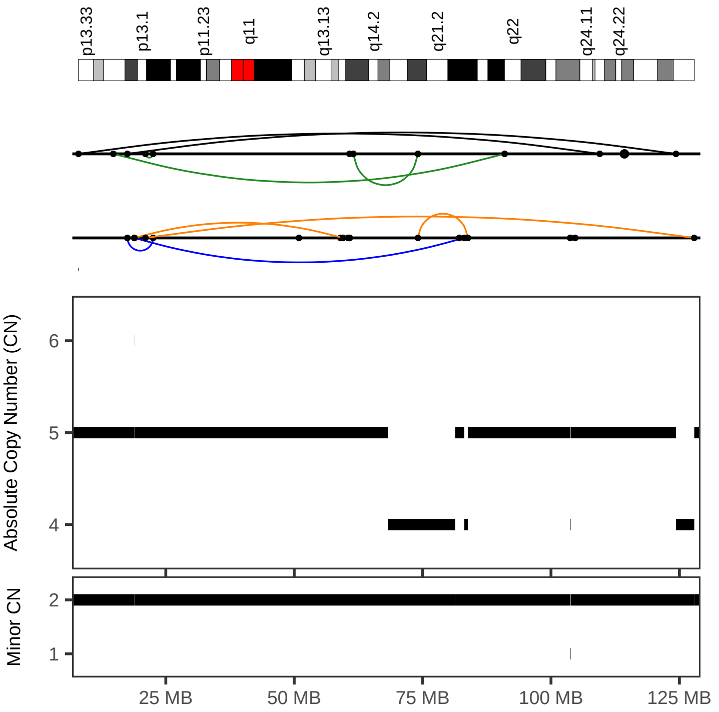

	PCSI_0392
Cancer type	Panc−AdenoCA
Position	12:7998293−127887019
Type	After polyploidization
Interleaved intrachr. SVs	9
Total SVs (intrachr. + transl.)	10
SV types	DEL: 4; DUP: 2; h2hINV: 2; t2tINV: 1; TRA: 1
SVs in sample	160
Oscillating CN (2 and 3 states)	9, 11
CN segments	11
FDR fragment joints	0.64
FDR chr. breakp. enrich.	0
Linked to chrs	
Purity, ploidy	0.99, 3.89

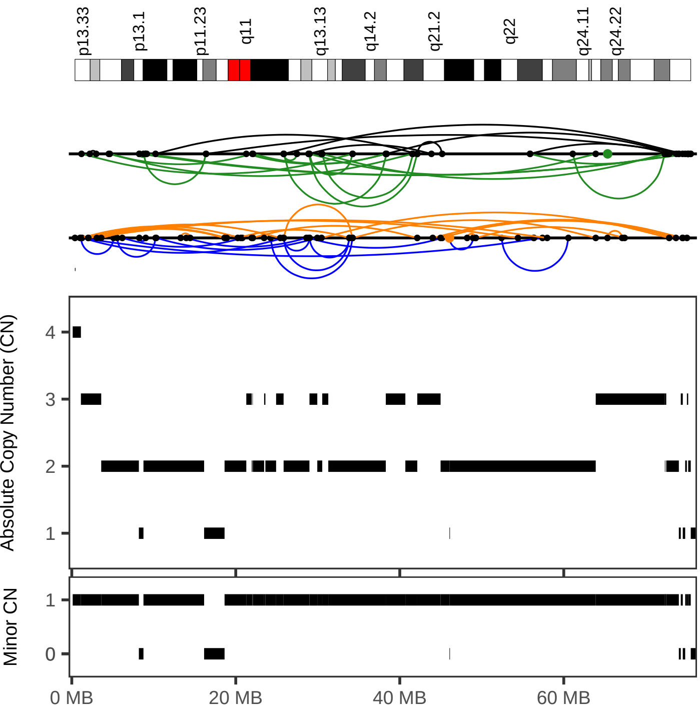

	PCSI_0403
Cancer type	Panc−AdenoCA
Position	12:388638−75487543
Type	With other complex events
Interleaved intrachr. SVs	59
Total SVs (intrachr. + transl.)	61
SV types	DEL: 17; DUP: 14; h2hINV: 9; t2tINV: 19; TRA: 2
SVs in sample	115
Oscillating CN (2 and 3 states)	21, 34
CN segments	41
FDR fragment joints	0.59
FDR chr. breakp. enrich.	0
Linked to chrs	
Purity, ploidy	0.89, 1.87

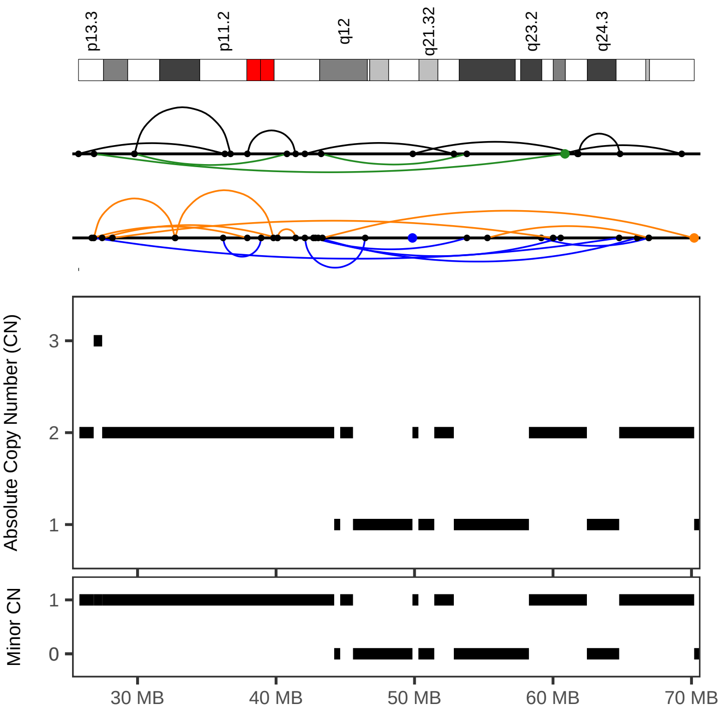

	PCSI_0403
Cancer type	Panc−AdenoCA
Position	17:25733074−70173103
Type	Canonical without polyploidization
Interleaved intrachr. SVs	25
Total SVs (intrachr. + transl.)	27
SV types	DEL: 8; DUP: 7; h2hINV: 7; t2tINV: 3; TRA: 2
SVs in sample	115
Oscillating CN (2 and 3 states)	11, 14
CN segments	14
FDR fragment joints	0.64
FDR chr. breakp. enrich.	0
Linked to chrs	
Purity, ploidy	0.89, 1.87

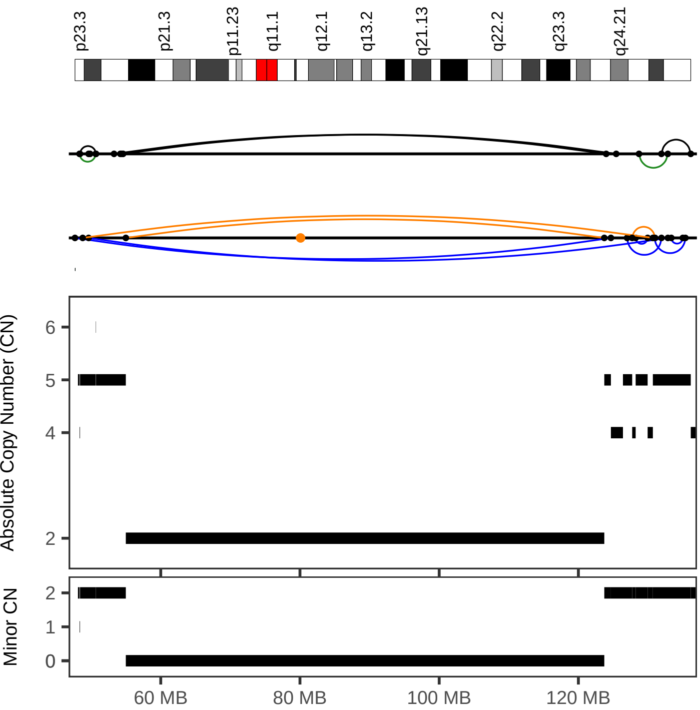

	PCSI_0451
Cancer type	Panc−AdenoCA
Position	8:47674760−136141807
Type	With other complex events
Interleaved intrachr. SVs	14
Total SVs (intrachr. + transl.)	15
SV types	DEL: 2; DUP: 5; h2hINV: 5; t2tINV: 2; TRA: 1
SVs in sample	56
Oscillating CN (2 and 3 states)	8, 10
CN segments	15
FDR fragment joints	0.64
FDR chr. breakp. enrich.	0
Linked to chrs	
Purity, ploidy	0.99, 1.86

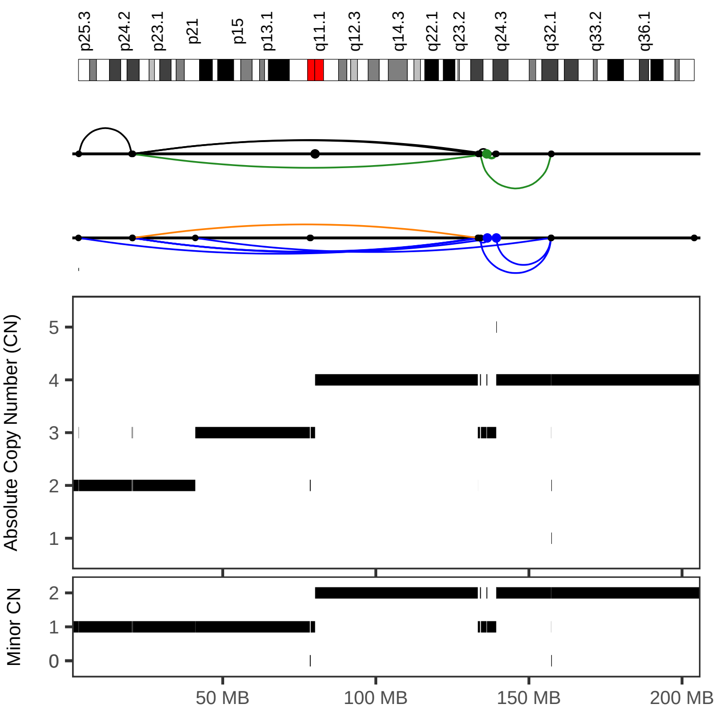

	PCSI_0458
Cancer type	Panc−AdenoCA
Position	2:2888790−157310482
Type	With other complex events
Interleaved intrachr. SVs	17
Total SVs (intrachr. + transl.)	21
SV types	DEL: 1; DUP: 8; h2hINV: 4; t2tINV: 4; TRA: 4
SVs in sample	83
Oscillating CN (2 and 3 states)	9, 23
CN segments	26
FDR fragment joints	0.52
FDR chr. breakp. enrich.	0
Linked to chrs	1:205733022−240669211;
Purity, ploidy	0.44, 2.75

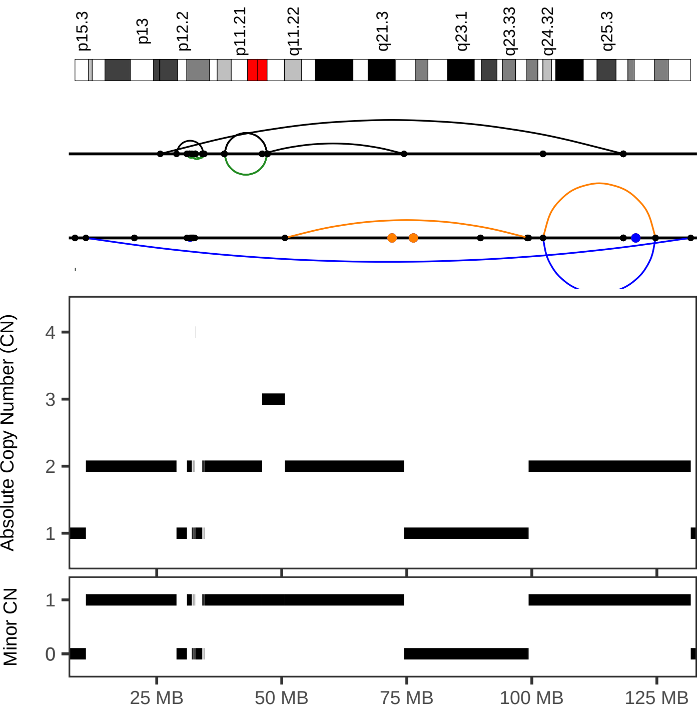

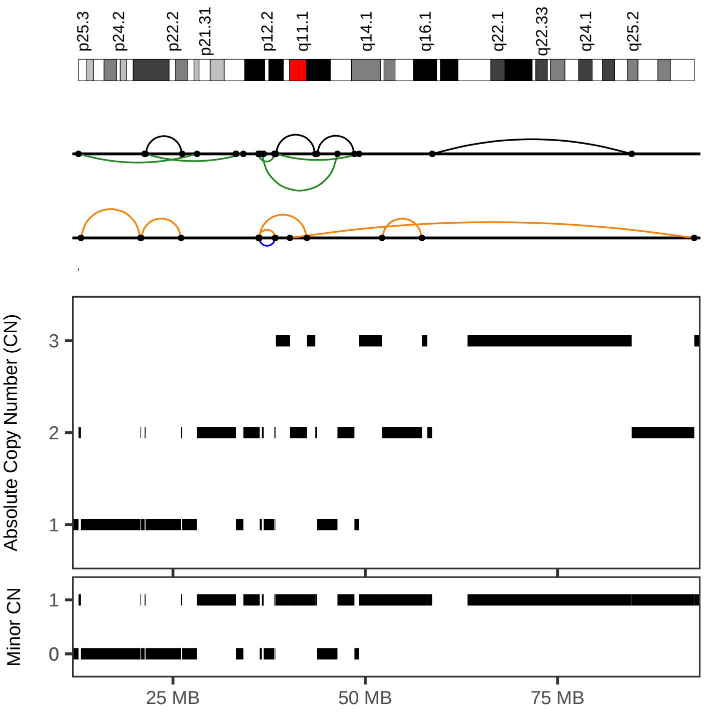

PCSI_0472

Cancer type	Panc−AdenoCA
Position	10:28947349−34511968
Type	Canonical without polyploidization
Interleaved intrachr. SVs	12
Total SVs (intrachr. + transl.)	12
SV types	DEL: 2; DUP: 3; h2hINV: 3; t2tINV: 4; TRA: 0
SVs in sample	238
Oscillating CN (2 and 3 states)	13, 18
CN segments	18
FDR fragment joints	0.91
FDR chr. breakp. enrich.	0
Linked to chrs	
Purity, ploidy	0.91, 1.74

PCSI_0473

Cancer type	Panc−AdenoCA
Position	6:36130410−92771456
Type	With other complex events
Interleaved intrachr. SVs	13
Total SVs (intrachr. + transl.)	13
SV types	DEL: 3; DUP: 2; h2hINV: 4; t2tINV: 4; TRA: 0
SVs in sample	61
Oscillating CN (2 and 3 states)	7, 11
CN segments	21
FDR fragment joints	0.88
FDR chr. breakp. enrich.	0
Linked to chrs	
Purity, ploidy	0.55, 1.87

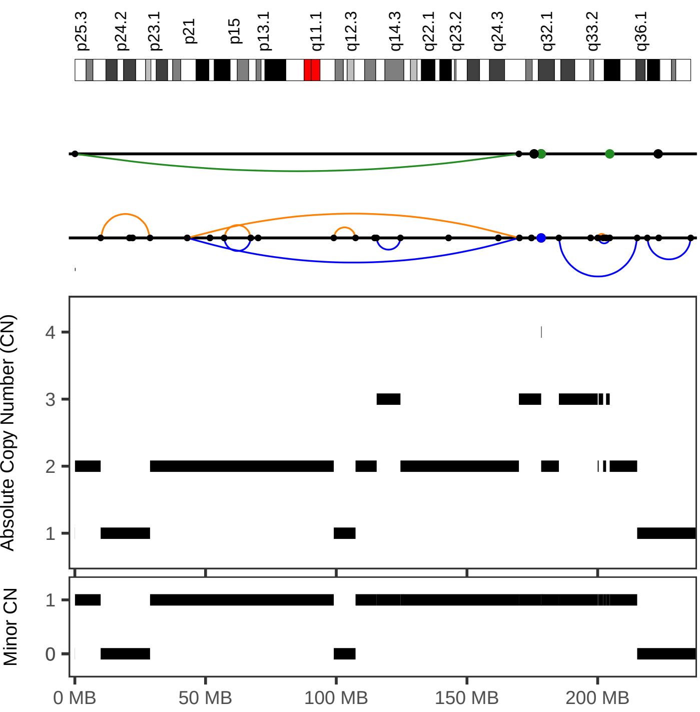

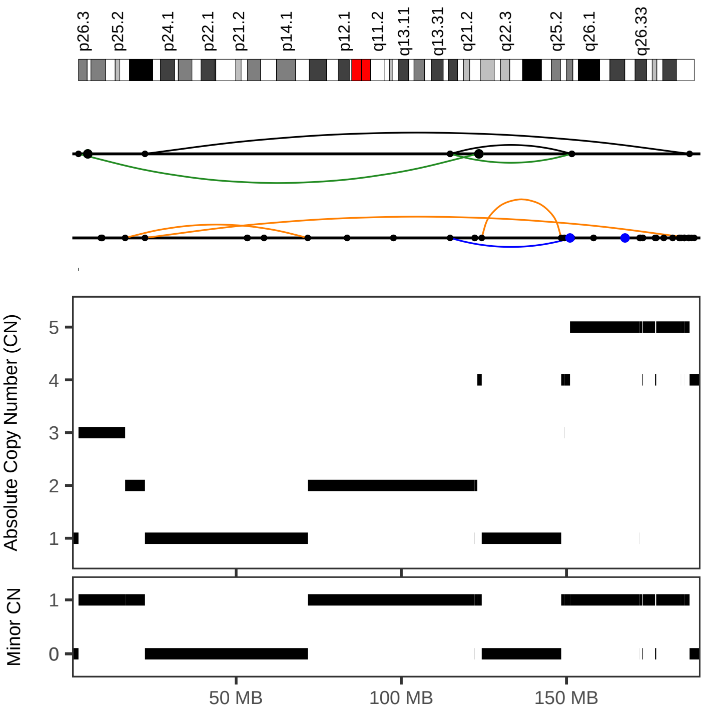

PCSI_0477

Cancer type	Panc−AdenoCA
Position	2:45422−204621489
Type	With other complex events
Interleaved intrachr. SVs	3
Total SVs (intrachr. + transl.)	14
SV types	DEL: 3; DUP: 2; h2hINV: 0; t2tINV: 1; TRA: 8
SVs in sample	323
Oscillating CN (2 and 3 states)	7, 9
CN segments	16
FDR fragment joints	0.59
FDR chr. breakp. enrich.	0.08
Linked to chrs	17:21709058−72004069;
Purity, ploidy	0.86, 1.86

PCSI_0477

Cancer type	Panc−AdenoCA
Position	3:2293198−187254007
Type	With other complex events
Interleaved intrachr. SVs	7
Total SVs (intrachr. + transl.)	13
SV types	DEL: 2; DUP: 1; h2hINV: 2; t2tINV: 2; TRA: 6
SVs in sample	323
Oscillating CN (2 and 3 states)	10, 10
CN segments	23
FDR fragment joints	0.95
FDR chr. breakp. enrich.	0.05
Linked to chrs	7:28529342−105022970;
Purity, ploidy	0.86, 1.86

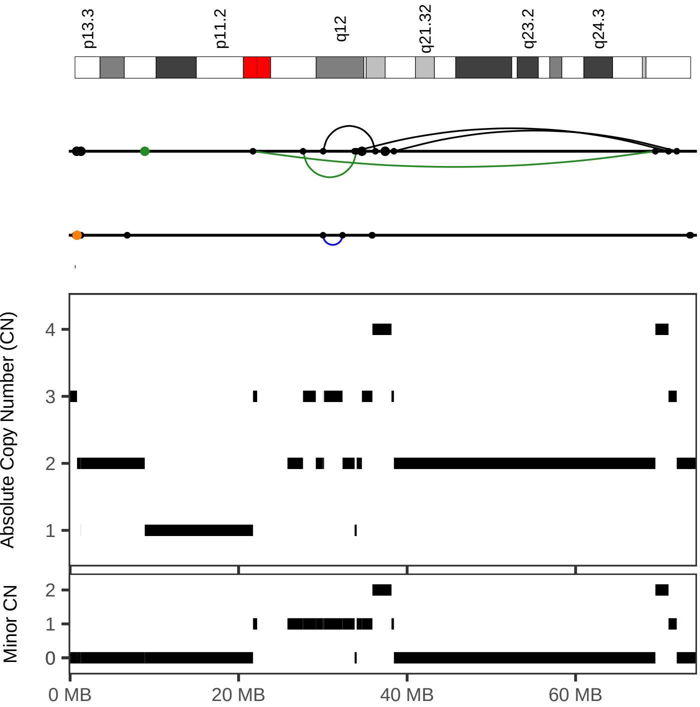

	PCSI_0477
Cancer type	Panc−AdenoCA
Position	17:21709058−72004070
Type	With other complex events
Interleaved intrachr. SVs	6
Total SVs (intrachr. + transl.)	9
SV types	DEL: 0; DUP: 1; h2hINV: 3; t2tINV: 2; TRA: 3
SVs in sample	323
Oscillating CN (2 and 3 states)	8, 14
CN segments	17
FDR fragment joints	0.59
FDR chr. breakp. enrich.	0
Linked to chrs	
Purity, ploidy	0.86, 1.86

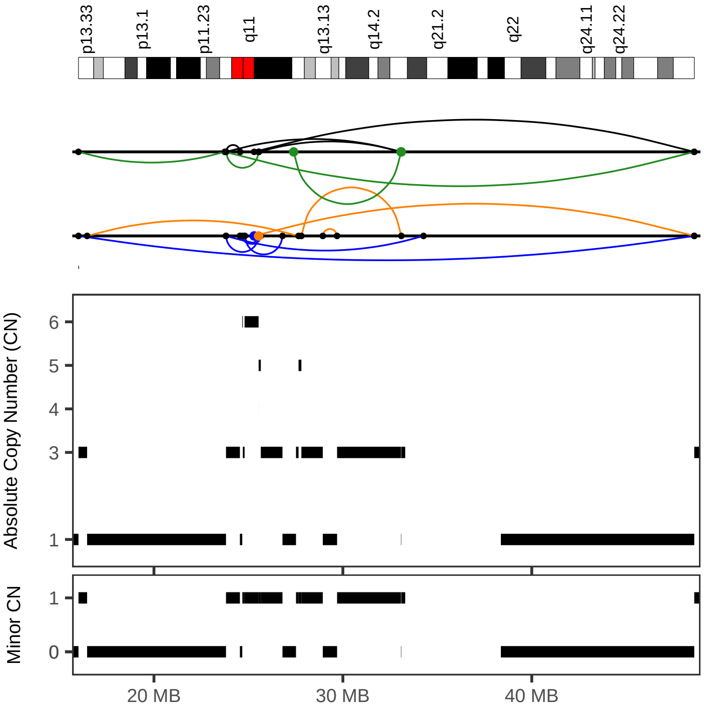

	PCSI_0527
Cancer type	Panc−AdenoCA
Position	12:16004740−48600593
Type	With other complex events
Interleaved intrachr. SVs	17
Total SVs (intrachr. + transl.)	22
SV types	DEL: 3; DUP: 6; h2hINV: 3; t2tINV: 5; TRA: 5
SVs in sample	53
Oscillating CN (2 and 3 states)	7, 7
CN segments	20
FDR fragment joints	0.74
FDR chr. breakp. enrich.	0
Linked to chrs	
Purity, ploidy	0.52, 1.9

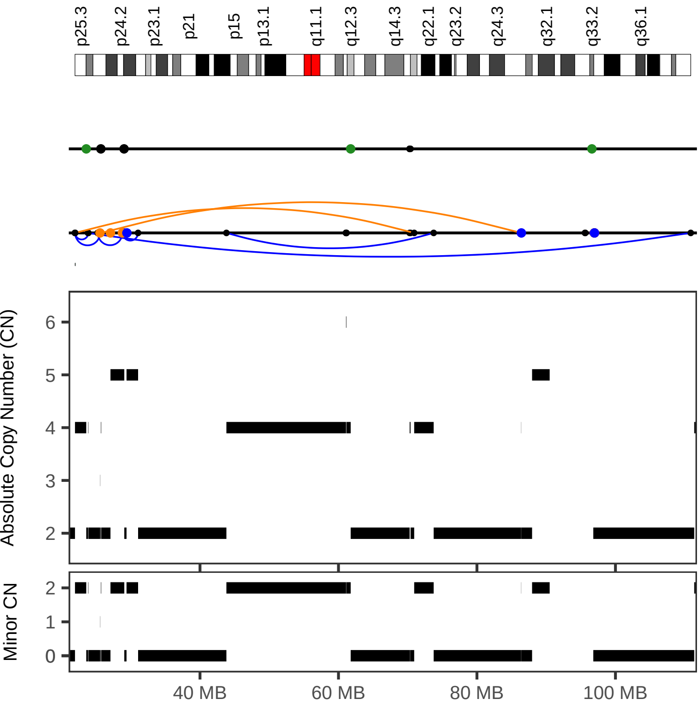

	PCSI_0572
Cancer type	Panc−AdenoCA
Position	2:21945480−110876001
Type	With other complex events
Interleaved intrachr. SVs	9
Total SVs (intrachr. + transl.)	24
SV types	DEL: 3; DUP: 6; h2hINV: 0; t2tINV: 0; TRA: 15
SVs in sample	86
Oscillating CN (2 and 3 states)	8, 13
CN segments	24
FDR fragment joints	0.21
FDR chr. breakp. enrich.	0
Linked to chrs	1:107128798−114787741;
Purity, ploidy	0.49, 2.71

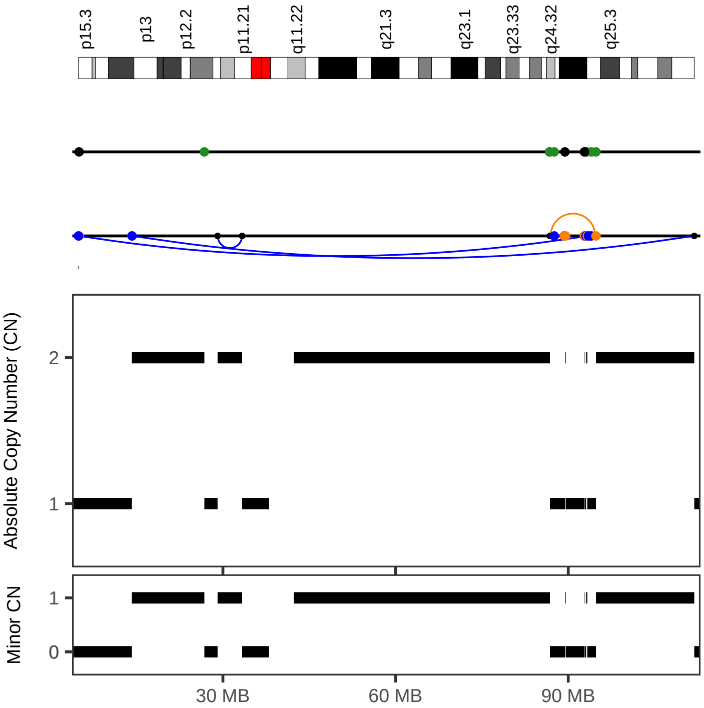

	ICGC_0431
Cancer type	Panc−Endocrine
Position	10:4904254−111891388
Type	Canonical without polyploidization
Interleaved intrachr. SVs	3
Total SVs (intrachr. + transl.)	32
SV types	DEL: 1; DUP: 2; h2hINV: 0; t2tINV: 0; TRA: 29
SVs in sample	68
Oscillating CN (2 and 3 states)	17, 17
CN segments	17
FDR fragment joints	0.59
FDR chr. breakp. enrich.	0
Linked to chrs	1:206839058−239207023;2:39442101−65926164
Purity, ploidy	0.89, 1.89

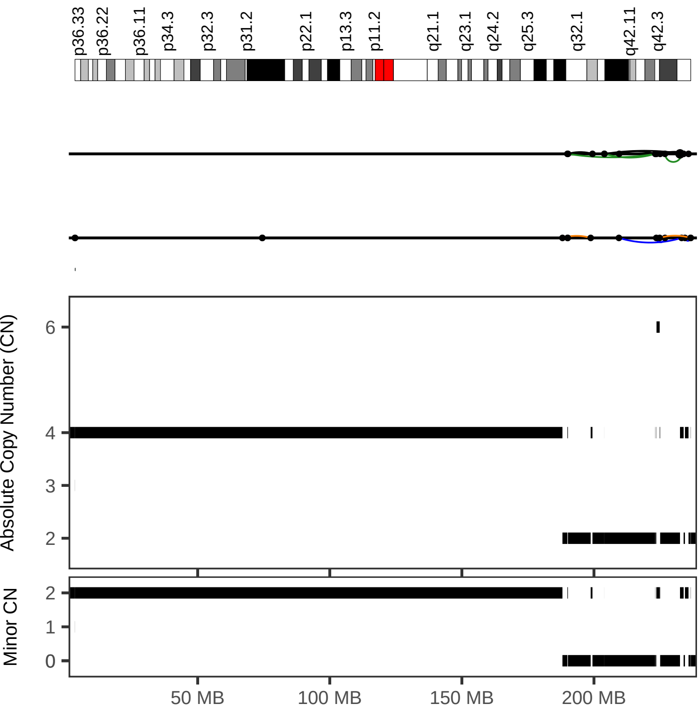

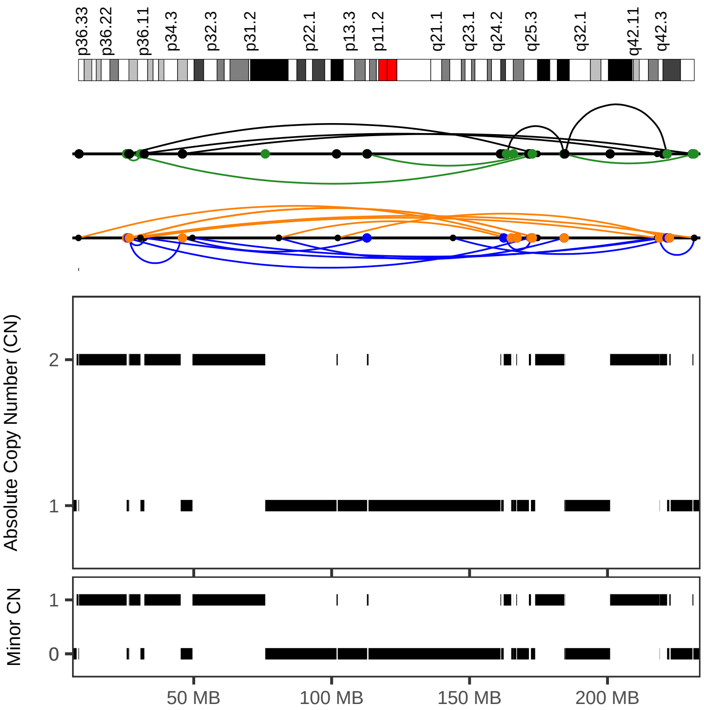

ICGC_0455

Cancer type	Panc−Endocrine
Position	1:188073080−236635572
Type	With other complex events
Interleaved intrachr. SVs	12
Total SVs (intrachr. + transl.)	13
SV types	DEL: 2; DUP: 3; h2hINV: 4; t2tINV: 3; TRA: 1
SVs in sample	72
Oscillating CN (2 and 3 states)	10, 19
CN segments	19
FDR fragment joints	0.91
FDR chr. breakp. enrich.	0
Linked to chrs	
Purity, ploidy	0.86, 2.78

ICGC_0498

Cancer type	Panc−Endocrine
Position	1:8214142−231450770
Type	Canonical without polyploidization
Interleaved intrachr. SVs	30
Total SVs (intrachr. + transl.)	96
SV types	DEL: 9; DUP: 11; h2hINV: 5; t2tINV: 5; TRA: 66
SVs in sample	148
Oscillating CN (2 and 3 states)	41, 41
CN segments	41
FDR fragment joints	0.59
FDR chr. breakp. enrich.	0
Linked to chrs	11:21255252−90295432;
Purity, ploidy	0.93, 1.64

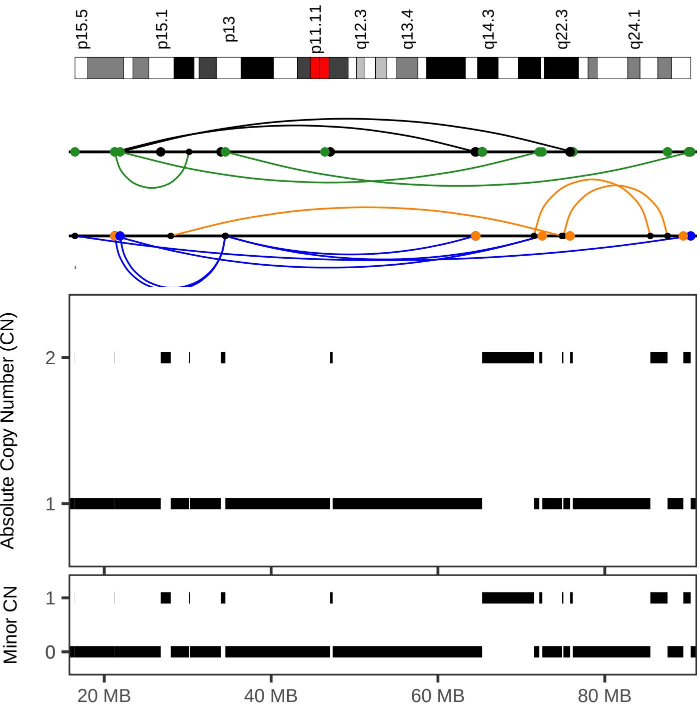

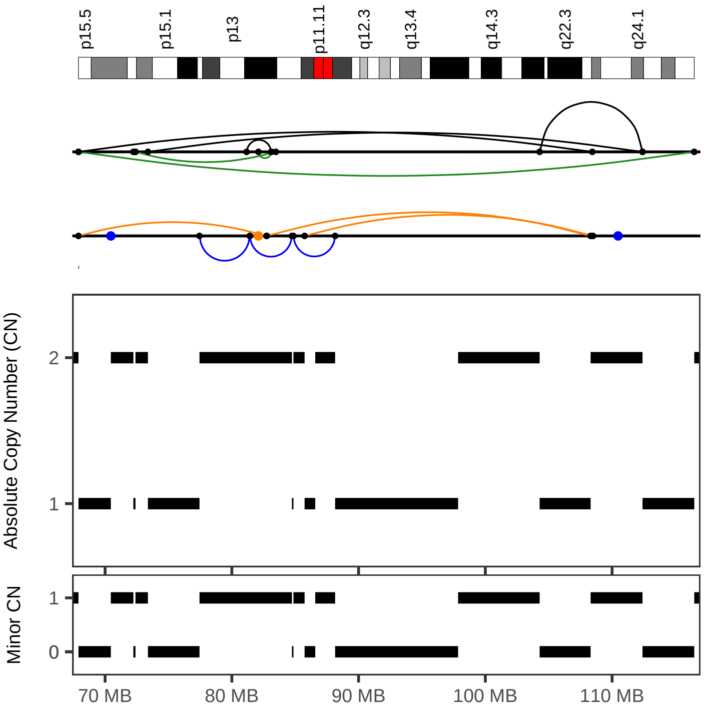

ICGC_0498

Cancer type	Panc−Endocrine
Position	11:21255252−90295433
Type	Canonical without polyploidization
Interleaved intrachr. SVs	12
Total SVs (intrachr. + transl.)	49
SV types	DEL: 3; DUP: 4; h2hINV: 2; t2tINV: 3; TRA: 37
SVs in sample	148
Oscillating CN (2 and 3 states)	26, 26
CN segments	26
FDR fragment joints	0.91
FDR chr. breakp. enrich.	0
Linked to chrs	1:8214142−231450769;
Purity, ploidy	0.93, 1.64

ITNET−1266

Cancer type	Panc−Endocrine
Position	11:67900950−116481177
Type	Canonical without polyploidization
Interleaved intrachr. SVs	14
Total SVs (intrachr. + transl.)	17
SV types	DEL: 3; DUP: 3; h2hINV: 5; t2tINV: 3; TRA: 3
SVs in sample	30
Oscillating CN (2 and 3 states)	17, 17
CN segments	17
FDR fragment joints	0.88
FDR chr. breakp. enrich.	0
Linked to chrs	17:47084966−57048643;
Purity, ploidy	0.95, 1.93

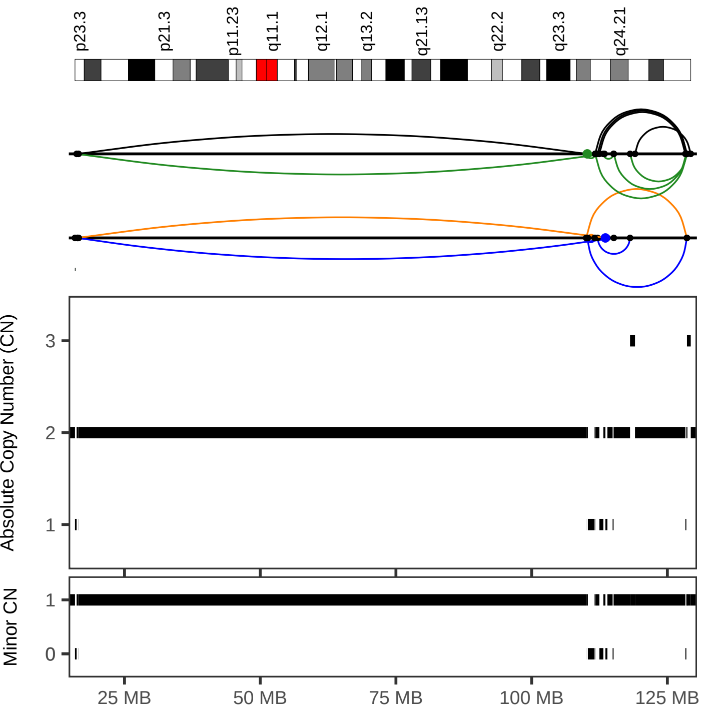

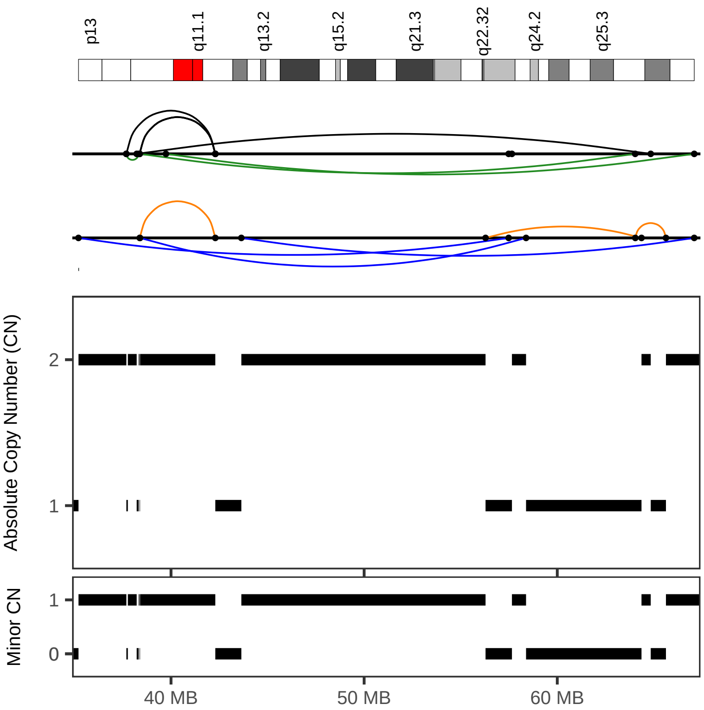

	ITNET−0087
Cancer type	Panc−Endocrine
Position	8:15876266−129305739
Type	Canonical without polyploidization
Interleaved intrachr. SVs	19
Total SVs (intrachr. + transl.)	21
SV types	DEL: 4; DUP: 4; h2hINV: 5; t2tINV: 6; TRA: 2
SVs in sample	28
Oscillating CN (2 and 3 states)	16, 16
CN segments	25
FDR fragment joints	0.92
FDR chr. breakp. enrich.	0
Linked to chrs	
Purity, ploidy	0.81, 2

	ITNET−0118
Cancer type	Panc−Endocrine
Position	15:35208509−67092577
Type	Canonical without polyploidization
Interleaved intrachr. SVs	14
Total SVs (intrachr. + transl.)	14
SV types	DEL: 3; DUP: 3; h2hINV: 4; t2tINV: 4; TRA: 0
SVs in sample	19
Oscillating CN (2 and 3 states)	15, 15
CN segments	15
FDR fragment joints	0.98
FDR chr. breakp. enrich.	0
Linked to chrs	
Purity, ploidy	0.56, 1.91

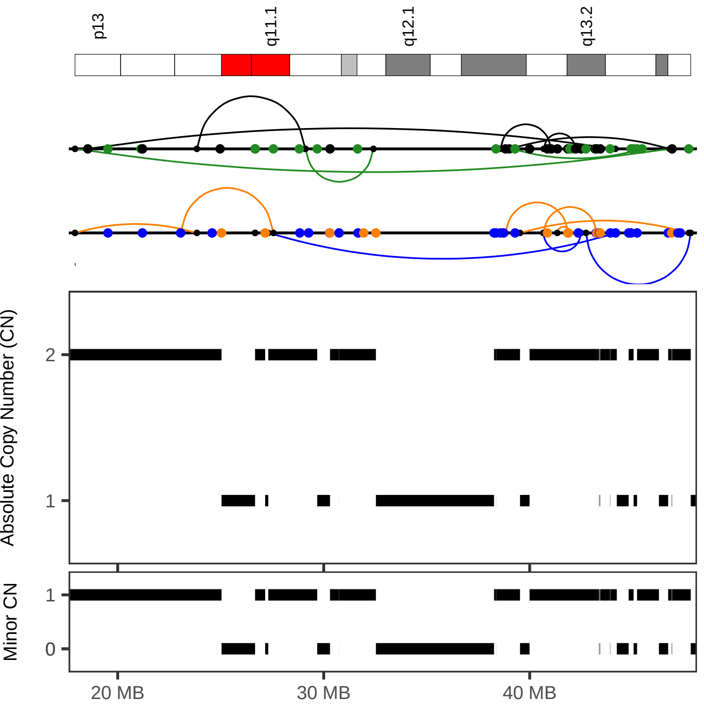

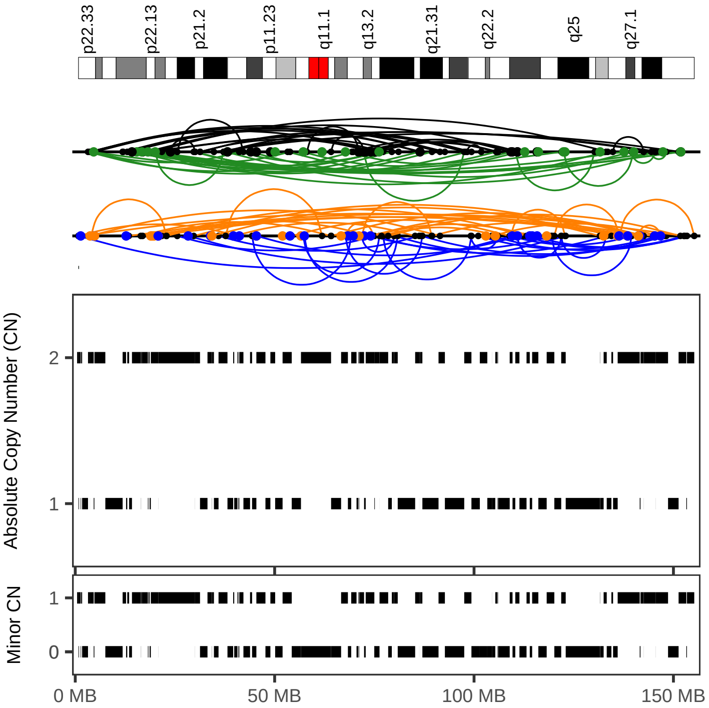

	ITNET−0152
Cancer type	Panc−Endocrine
Position	22:17924903−47818249
Type	Canonical without polyploidization
Interleaved intrachr. SVs	16
Total SVs (intrachr. + transl.)	99
SV types	DEL: 6; DUP: 3; h2hINV: 5; t2tINV: 2; TRA: 83
SVs in sample	227
Oscillating CN (2 and 3 states)	30, 30
CN segments	30
FDR fragment joints	0.64
FDR chr. breakp. enrich.	0
Linked to chrs	
Purity, ploidy	0.82, 1.96

	ITNET−0152
Cancer type	Panc−Endocrine
Position	X:808303−155214868
Type	Canonical without polyploidization
Interleaved intrachr. SVs	109
Total SVs (intrachr. + transl.)	196
SV types	DEL: 29; DUP: 26; h2hINV: 27; t2tINV: 27; TRA: 87
SVs in sample	227
Oscillating CN (2 and 3 states)	122, 122
CN segments	122
FDR fragment joints	0.99
FDR chr. breakp. enrich.	0
Linked to chrs	10:117460104−122847324;
Purity, ploidy	0.82, 1.96

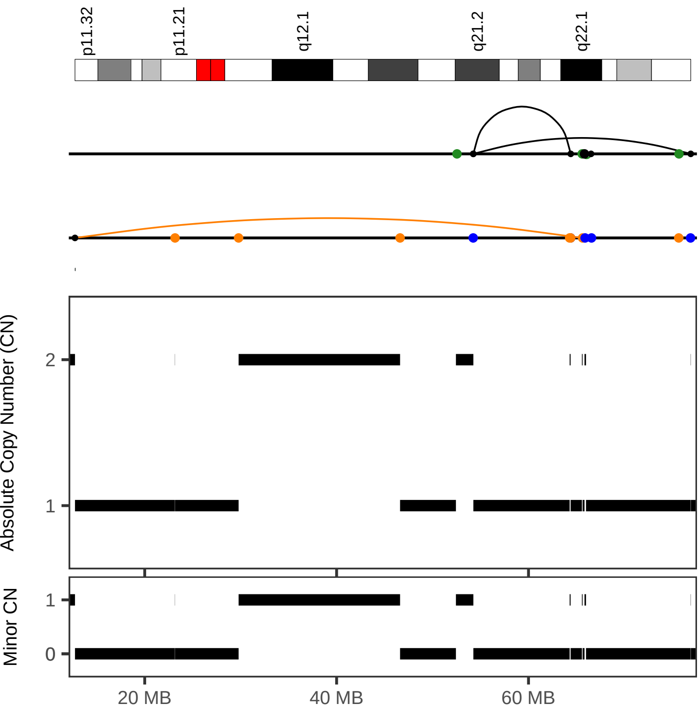

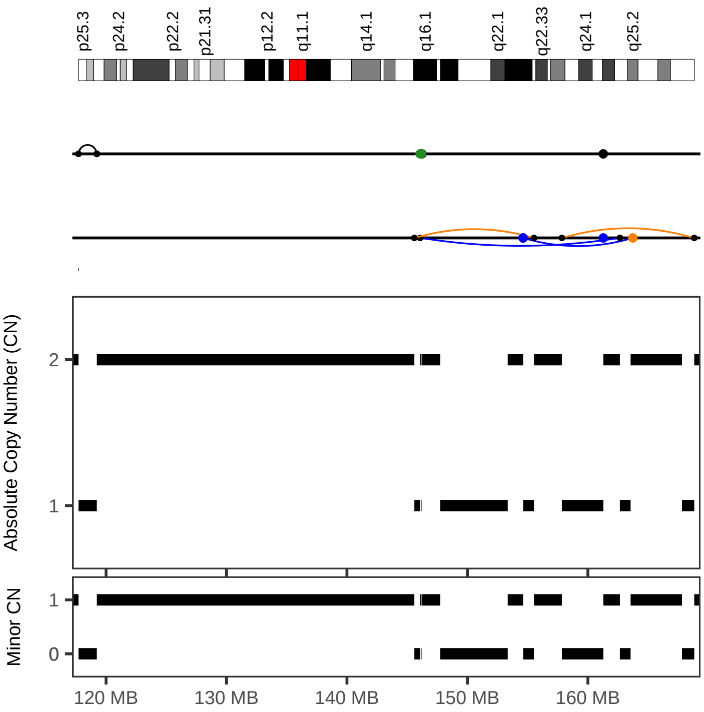

ITNET−0968

Cancer type	Panc−Endocrine
Position	18:12732238−76910328
Type	Canonical without polyploidization
Interleaved intrachr. SVs	3
Total SVs (intrachr. + transl.)	19
SV types	DEL: 1; DUP: 0; h2hINV: 1; t2tINV: 1; TRA: 16
SVs in sample	42
Oscillating CN (2 and 3 states)	15, 15
CN segments	15
FDR fragment joints	0.84
FDR chr. breakp. enrich.	0
Linked to chrs	1:95538888−234231402;
Purity, ploidy	0.85, 1.73

EOPC−011

Cancer type	Prost−AdenoCA
Position	6:145595779−168829760
Type	Canonical without polyploidization
Interleaved intrachr. SVs	4
Total SVs (intrachr. + transl.)	12
SV types	DEL: 2; DUP: 2; h2hINV: 0; t2tINV: 0; TRA: 8
SVs in sample	27
Oscillating CN (2 and 3 states)	14, 14
CN segments	14
FDR fragment joints	0.59
FDR chr. breakp. enrich.	0
Linked to chrs	3:36958690−96921576;10:21109565−51786602
Purity, ploidy	0.5, 1.89

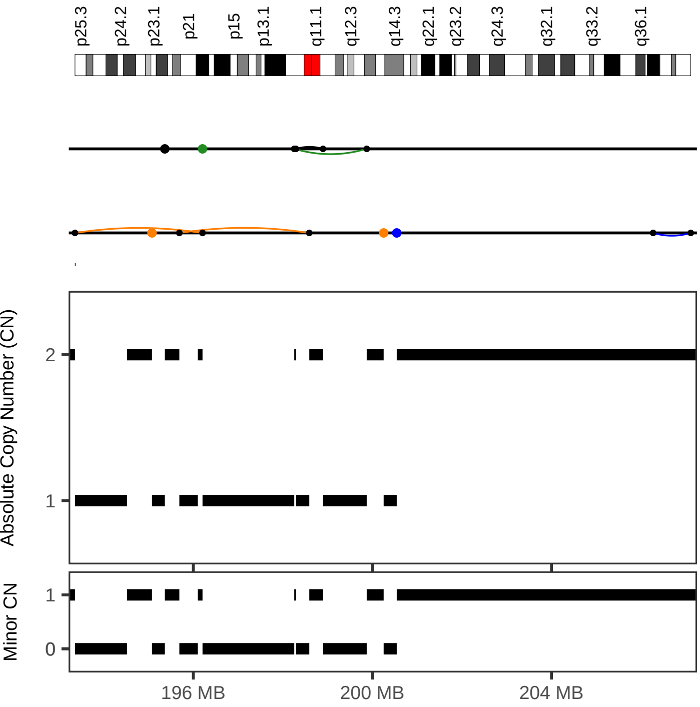

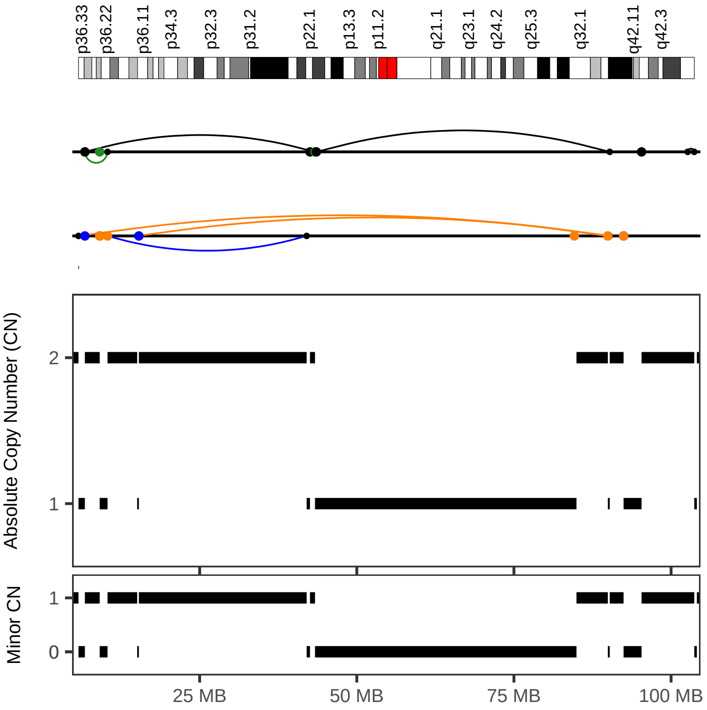

EOPC−018

Cancer type	Prost−AdenoCA
Position	2:193357450−199873274
Type	Canonical without polyploidization
Interleaved intrachr. SVs	4
Total SVs (intrachr. + transl.)	7
SV types	DEL: 2; DUP: 0; h2hINV: 1; t2tINV: 1; TRA: 3
SVs in sample	16
Oscillating CN (2 and 3 states)	12, 12
CN segments	12
FDR fragment joints	0.64
FDR chr. breakp. enrich.	0
Linked to chrs	
Purity, ploidy	0.43, 1.93

EOPC−021

Cancer type	Prost−AdenoCA
Position	1:5717619−90237612
Type	Canonical without polyploidization
Interleaved intrachr. SVs	5
Total SVs (intrachr. + transl.)	18
SV types	DEL: 2; DUP: 1; h2hINV: 2; t2tINV: 0; TRA: 13
SVs in sample	52
Oscillating CN (2 and 3 states)	12, 12
CN segments	12
FDR fragment joints	0.64
FDR chr. breakp. enrich.	0
Linked to chrs	2:55039051−149412024;
Purity, ploidy	0.28, 1.87

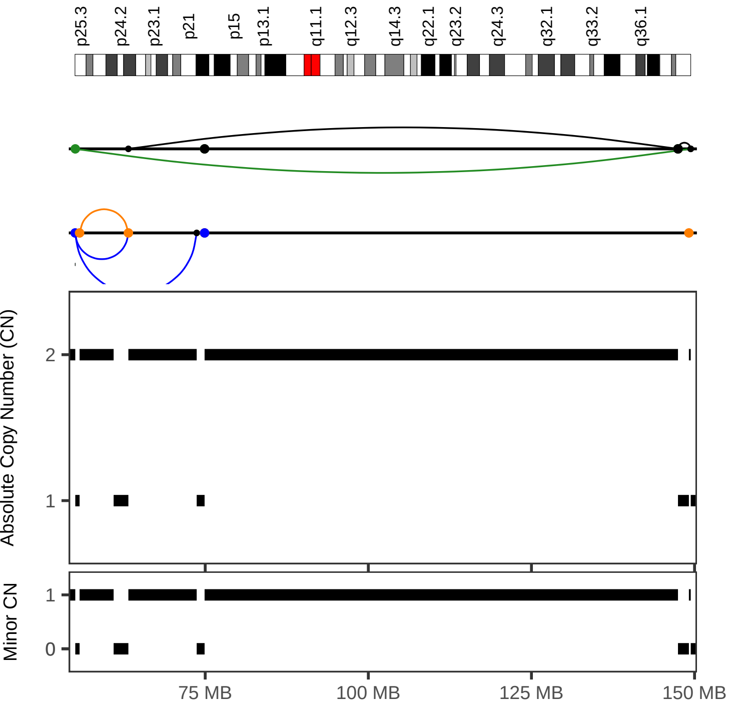

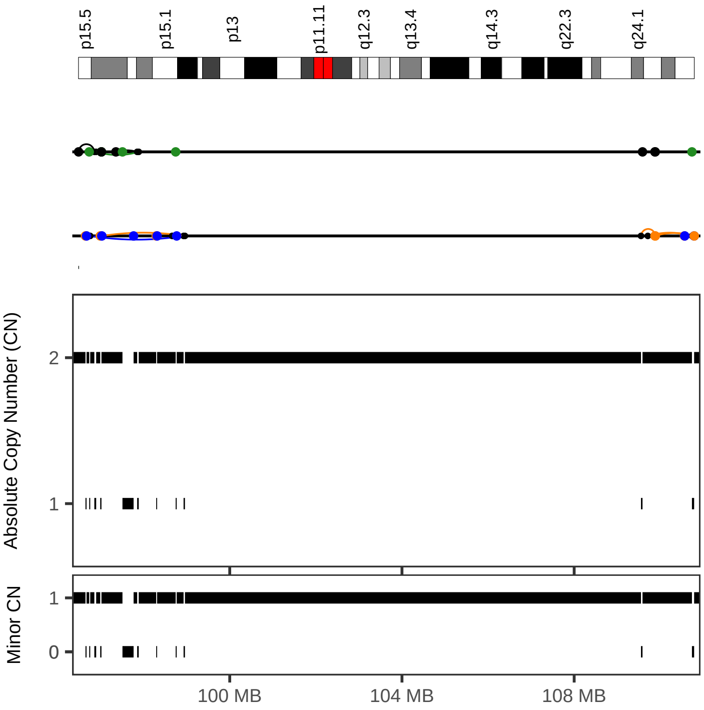

EOPC−021

Cancer type	Prost−AdenoCA
Position	2:55039051−149412025
Type	Canonical without polyploidization
Interleaved intrachr. SVs	3
Total SVs (intrachr. + transl.)	15
SV types	DEL: 0; DUP: 1; h2hINV: 1; t2tINV: 1; TRA: 12
SVs in sample	52
Oscillating CN (2 and 3 states)	10, 10
CN segments	10
FDR fragment joints	0.84
FDR chr. breakp. enrich.	0
Linked to chrs	1:5717619−90237611;
Purity, ploidy	0.28, 1.87

EOPC−029

Cancer type	Prost−AdenoCA
Position	11:96486263−98959029
Type	Canonical without polyploidization
Interleaved intrachr. SVs	4
Total SVs (intrachr. + transl.)	20
SV types	DEL: 1; DUP: 1; h2hINV: 1; t2tINV: 1; TRA: 16
SVs in sample	90
Oscillating CN (2 and 3 states)	19, 19
CN segments	19
FDR fragment joints	1
FDR chr. breakp. enrich.	0
Linked to chrs	
Purity, ploidy	0.42, 1.98

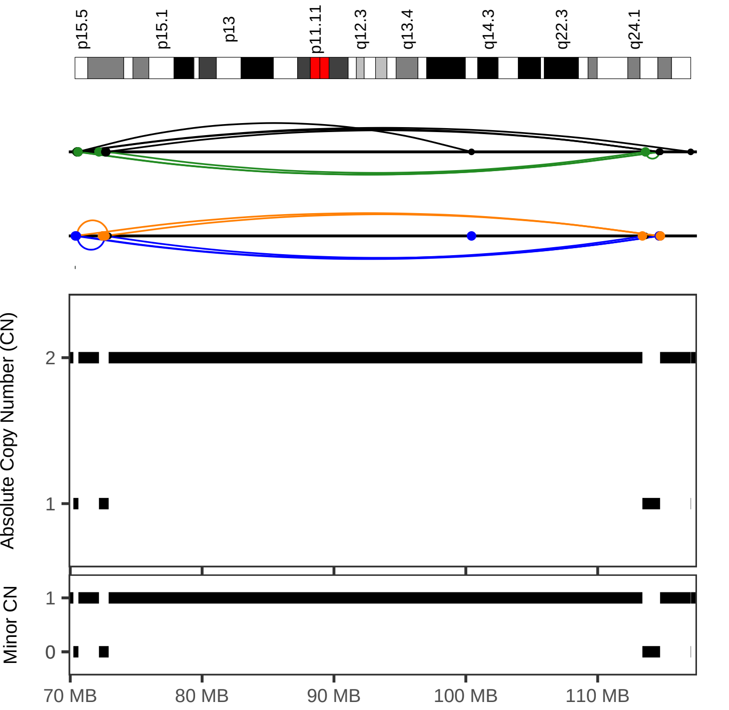

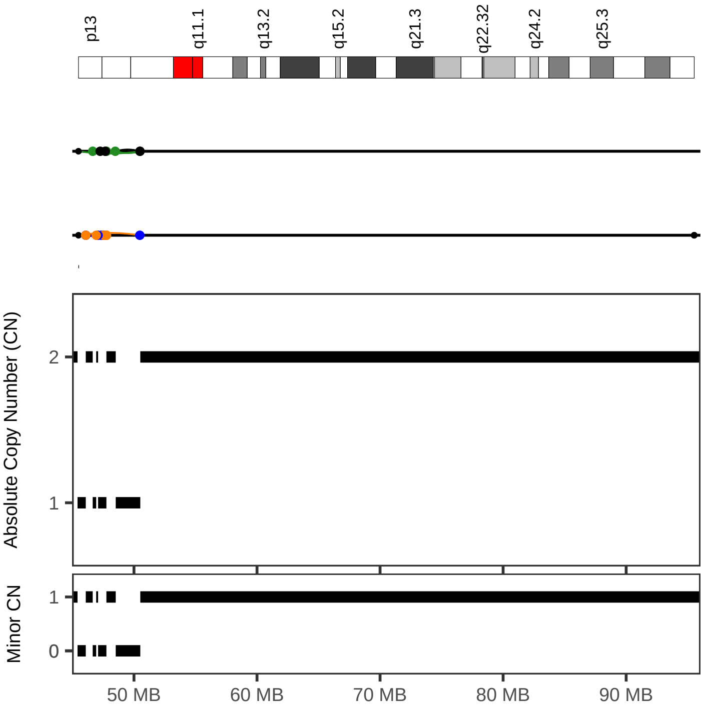

EOPC−030

Cancer type	Prost−AdenoCA
Position	11:70358348−117056058
Type	Canonical without polyploidization
Interleaved intrachr. SVs	21
Total SVs (intrachr. + transl.)	38
SV types	DEL: 6; DUP: 5; h2hINV: 5; t2tINV: 5; TRA: 17
SVs in sample	116
Oscillating CN (2 and 3 states)	7, 7
CN segments	7
FDR fragment joints	1
FDR chr. breakp. enrich.	0
Linked to chrs	15:45489848−50514323;
Purity, ploidy	0.67, 1.97

EOPC−030

Cancer type	Prost−AdenoCA
Position	15:45489848−50514324
Type	Canonical without polyploidization
Interleaved intrachr. SVs	4
Total SVs (intrachr. + transl.)	21
SV types	DEL: 1; DUP: 0; h2hINV: 1; t2tINV: 2; TRA: 17
SVs in sample	116
Oscillating CN (2 and 3 states)	8, 8
CN segments	8
FDR fragment joints	0.64
FDR chr. breakp. enrich.	0
Linked to chrs	
Purity, ploidy	0.67, 1.97

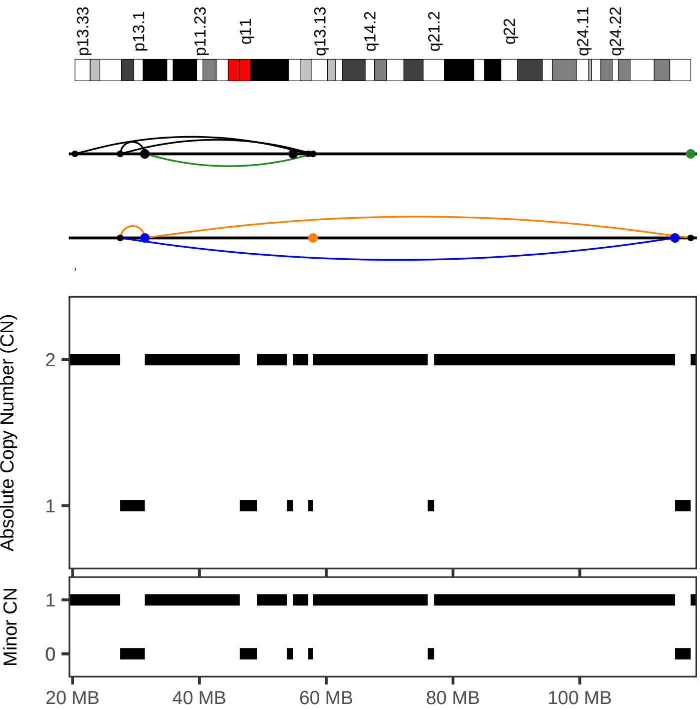

	EOPC−05
Cancer type	Prost−AdenoCA
Position	12:20367968−117498112
Type	Canonical without polyploidization
Interleaved intrachr. SVs	7
Total SVs (intrachr. + transl.)	16
SV types	DEL: 2; DUP: 1; h2hINV: 3; t2tINV: 1; TRA: 9
SVs in sample	35
Oscillating CN (2 and 3 states)	13, 13
CN segments	13
FDR fragment joints	0.74
FDR chr. breakp. enrich.	0
Linked to chrs	
Purity, ploidy	0.42, 1.87

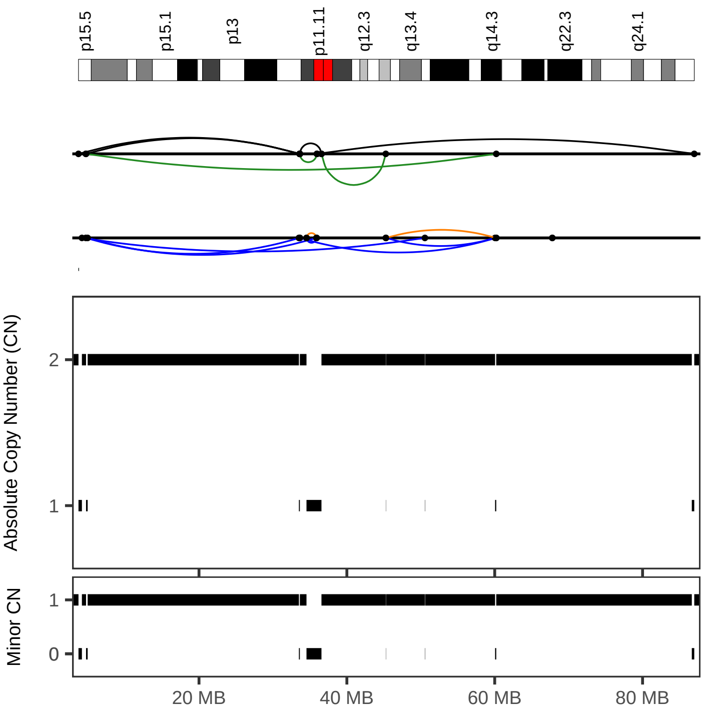

	EOPC−06
Cancer type	Prost−AdenoCA
Position	11:3698754−86989800
Type	Canonical without polyploidization
Interleaved intrachr. SVs	13
Total SVs (intrachr. + transl.)	13
SV types	DEL: 2; DUP: 5; h2hINV: 4; t2tINV: 2; TRA: 0
SVs in sample	62
Oscillating CN (2 and 3 states)	16, 16
CN segments	16
FDR fragment joints	0.64
FDR chr. breakp. enrich.	0
Linked to chrs	
Purity, ploidy	0.47, 1.82

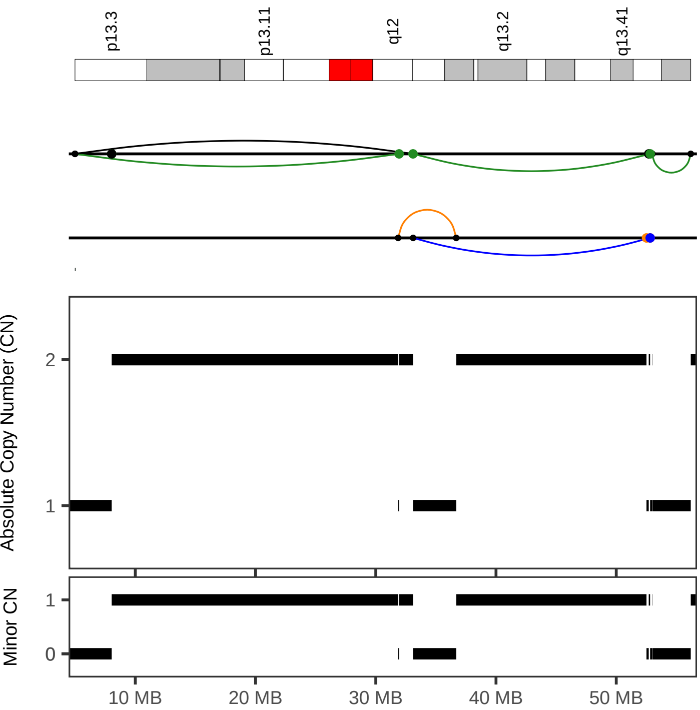

	EOPC−06
Cancer type	Prost−AdenoCA
Position	19:4983858−52796956
Type	Canonical without polyploidization
Interleaved intrachr. SVs	5
Total SVs (intrachr. + transl.)	11
SV types	DEL: 1; DUP: 1; h2hINV: 1; t2tINV: 2; TRA: 6
SVs in sample	62
Oscillating CN (2 and 3 states)	8, 8
CN segments	8
FDR fragment joints	0.92
FDR chr. breakp. enrich.	0
Linked to chrs	
Purity, ploidy	0.47, 1.82

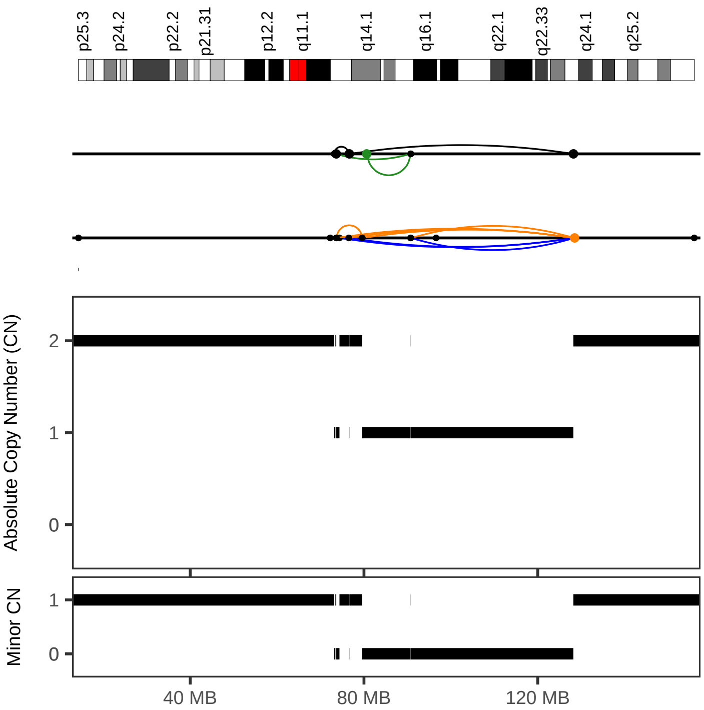

	CPCG0001
Cancer type	Prost−AdenoCA
Position	6:73106289−128515137
Type	Canonical without polyploidization
Interleaved intrachr. SVs	14
Total SVs (intrachr. + transl.)	19
SV types	DEL: 6; DUP: 3; h2hINV: 3; t2tINV: 2; TRA: 5
SVs in sample	183
Oscillating CN (2 and 3 states)	10, 10
CN segments	10
FDR fragment joints	0.64
FDR chr. breakp. enrich.	0
Linked to chrs	
Purity, ploidy	0.54, 1.9

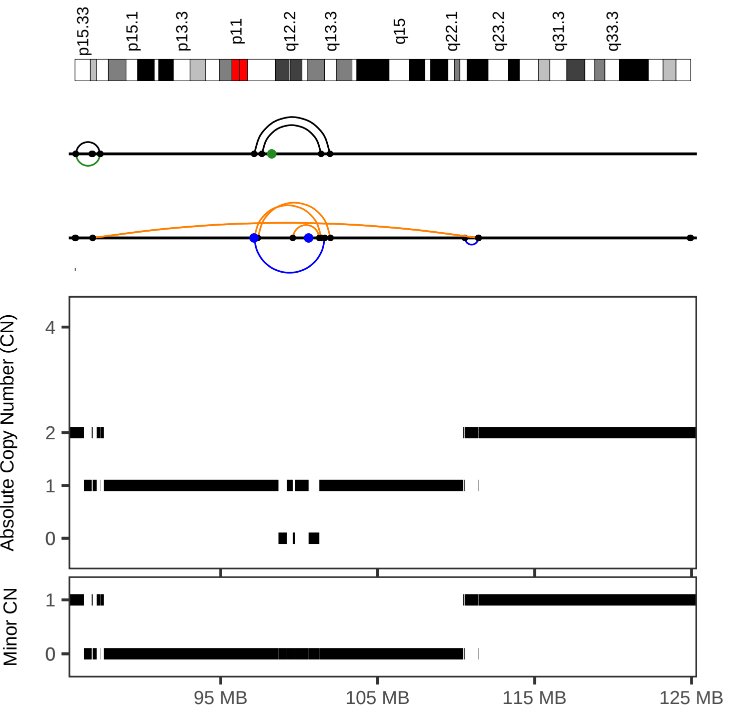

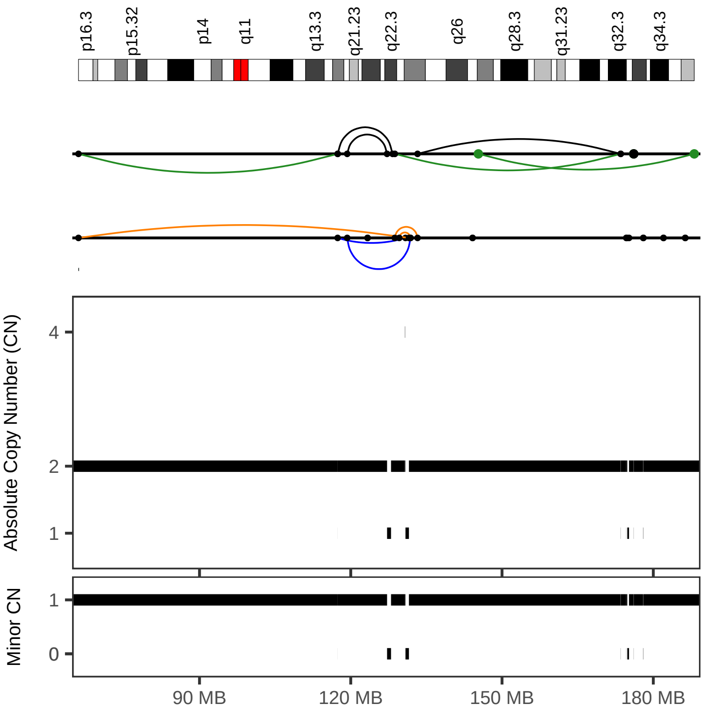

	CPCG0020
Cancer type	Prost−AdenoCA
Position	5:85713705−111436997
Type	With other complex events
Interleaved intrachr. SVs	7
Total SVs (intrachr. + transl.)	10
SV types	DEL: 3; DUP: 1; h2hINV: 1; t2tINV: 2; TRA: 3
SVs in sample	57
Oscillating CN (2 and 3 states)	8, 19
CN segments	19
FDR fragment joints	0.74
FDR chr. breakp. enrich.	0
Linked to chrs	
Purity, ploidy	0.48, 1.88

	CPCG0040
Cancer type	Prost−AdenoCA
Position	4:66000602−188117670
Type	Canonical without polyploidization
Interleaved intrachr. SVs	11
Total SVs (intrachr. + transl.)	15
SV types	DEL: 3; DUP: 2; h2hINV: 3; t2tINV: 3; TRA: 4
SVs in sample	86
Oscillating CN (2 and 3 states)	13, 19
CN segments	19
FDR fragment joints	0.98
FDR chr. breakp. enrich.	0
Linked to chrs	
Purity, ploidy	0.65, 1.87

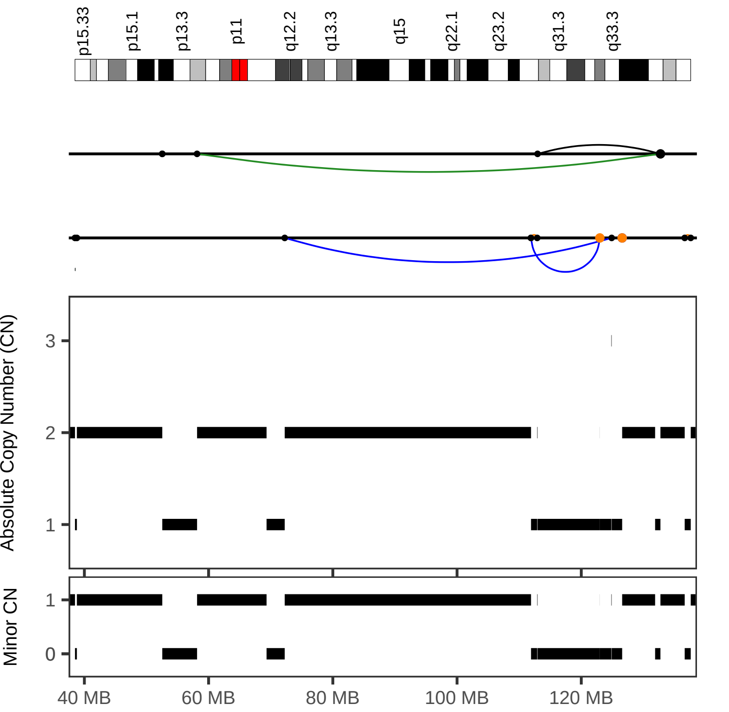

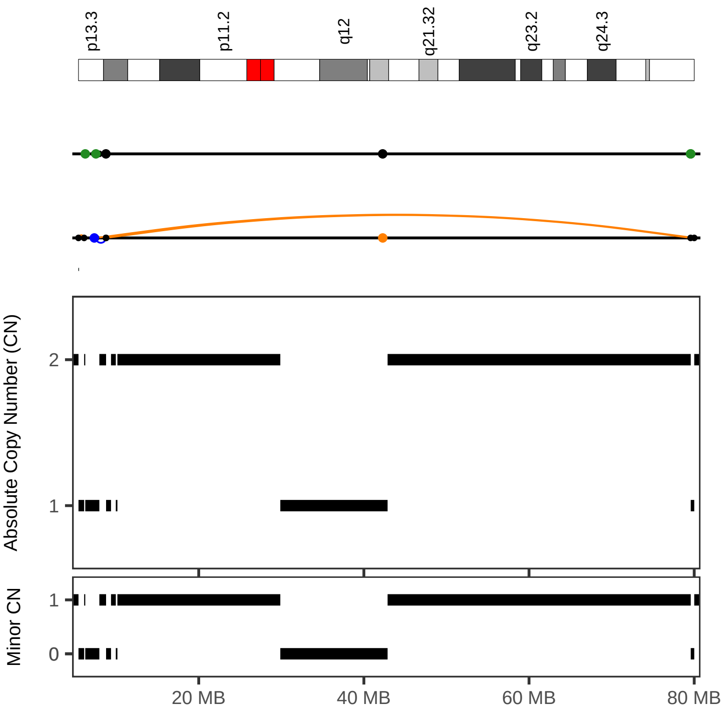

	CPCG0047
Cancer type	Prost−AdenoCA
Position	5:58141508−132747188
Type	Canonical without polyploidization
Interleaved intrachr. SVs	5
Total SVs (intrachr. + transl.)	8
SV types	DEL: 1; DUP: 2; h2hINV: 1; t2tINV: 1; TRA: 3
SVs in sample	74
Oscillating CN (2 and 3 states)	8, 13
CN segments	13
FDR fragment joints	0.92
FDR chr. breakp. enrich.	0.01
Linked to chrs	17:7344436−80003024;
Purity, ploidy	0.62, 1.91

	CPCG0047
Cancer type	Prost−AdenoCA
Position	17:7344436−80003025
Type	Canonical without polyploidization
Interleaved intrachr. SVs	3
Total SVs (intrachr. + transl.)	10
SV types	DEL: 1; DUP: 1; h2hINV: 0; t2tINV: 1; TRA: 7
SVs in sample	74
Oscillating CN (2 and 3 states)	10, 10
CN segments	10
FDR fragment joints	0.84
FDR chr. breakp. enrich.	0
Linked to chrs	5:58141508−132747187;
Purity, ploidy	0.62, 1.91

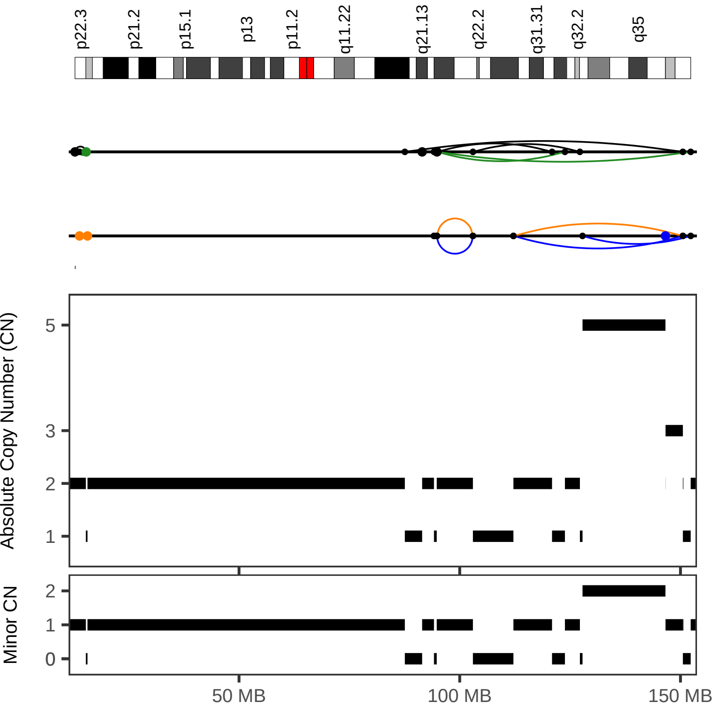

	**CPCG0078**
Cancer type	Prost−AdenoCA
Position	7:87564783−152327924
Type	Canonical without polyploidization
Interleaved intrachr. SVs	13
Total SVs (intrachr. + transl.)	17
SV types	DEL: 3; DUP: 4; h2hINV: 4; t2tINV: 2; TRA: 4
SVs in sample	75
Oscillating CN (2 and 3 states)	9, 9
CN segments	15
FDR fragment joints	0.88
FDR chr. breakp. enrich.	0
Linked to chrs	3:102500885−131983072;
Purity, ploidy	0.39, 2.01

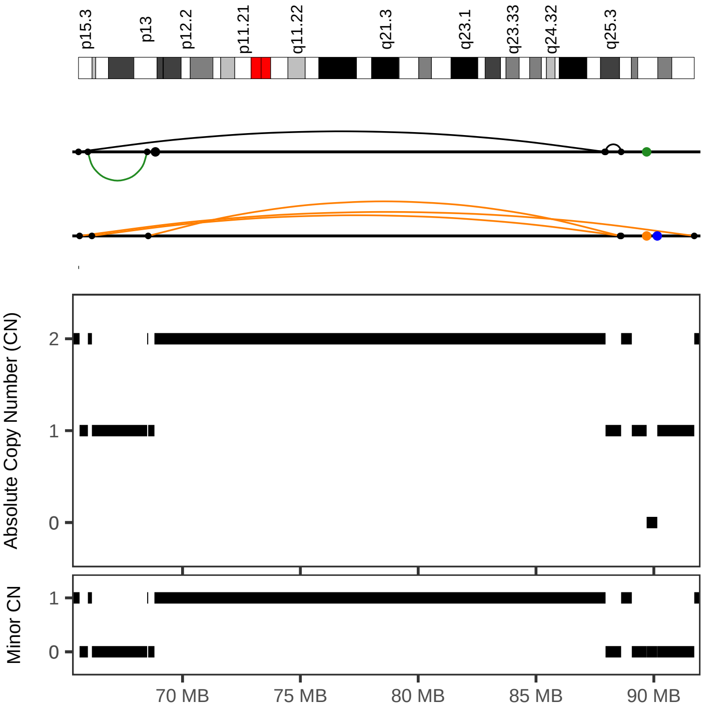

	**CPCG0087**
Cancer type	Prost−AdenoCA
Position	10:65582868−91714710
Type	Canonical without polyploidization
Interleaved intrachr. SVs	6
Total SVs (intrachr. + transl.)	10
SV types	DEL: 3; DUP: 0; h2hINV: 2; t2tINV: 1; TRA: 4
SVs in sample	96
Oscillating CN (2 and 3 states)	10, 13
CN segments	13
FDR fragment joints	0.59
FDR chr. breakp. enrich.	0
Linked to chrs	
Purity, ploidy	0.62, 1.86

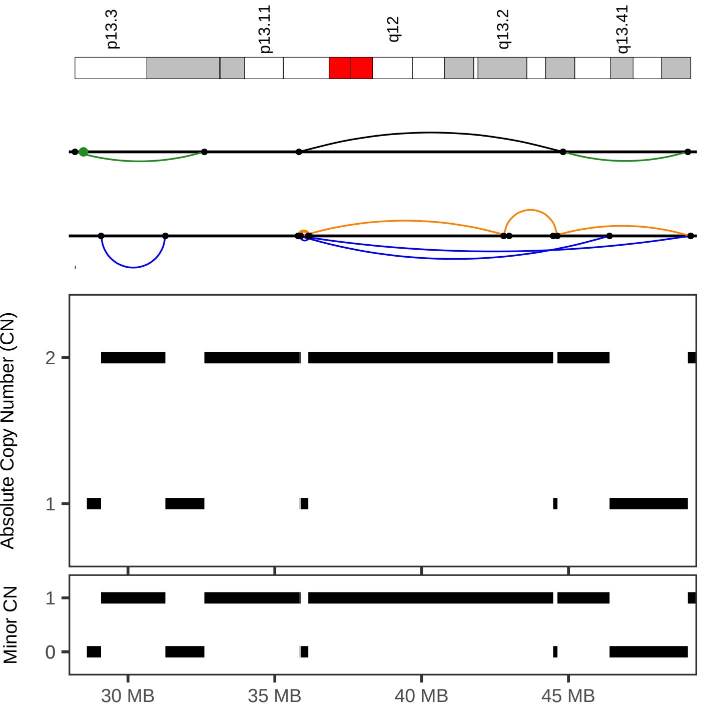

	**CPCG0095**
Cancer type	Prost−AdenoCA
Position	19:35792212−49163182
Type	Canonical without polyploidization
Interleaved intrachr. SVs	10
Total SVs (intrachr. + transl.)	10
SV types	DEL: 5; DUP: 3; h2hINV: 1; t2tINV: 1; TRA: 0
SVs in sample	66
Oscillating CN (2 and 3 states)	9, 9
CN segments	9
FDR fragment joints	0.59
FDR chr. breakp. enrich.	0
Linked to chrs	
Purity, ploidy	0.63, 1.95

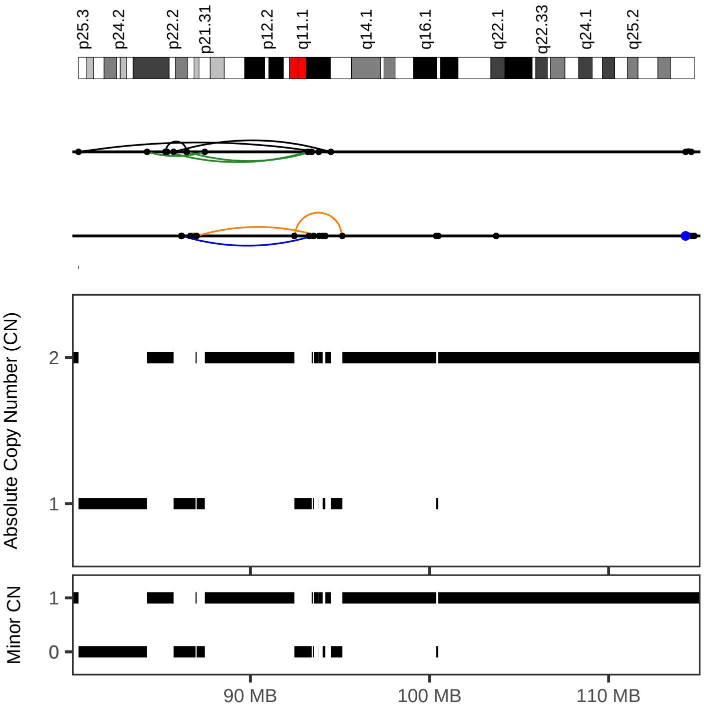

	**CPCG0098**
Cancer type	Prost−AdenoCA
Position	6:80395987−95133658
Type	Canonical without polyploidization
Interleaved intrachr. SVs	11
Total SVs (intrachr. + transl.)	11
SV types	DEL: 3; DUP: 2; h2hINV: 3; t2tINV: 3; TRA: 0
SVs in sample	100
Oscillating CN (2 and 3 states)	16, 16
CN segments	16
FDR fragment joints	0.98
FDR chr. breakp. enrich.	0
Linked to chrs	
Purity, ploidy	0.69, 1.93

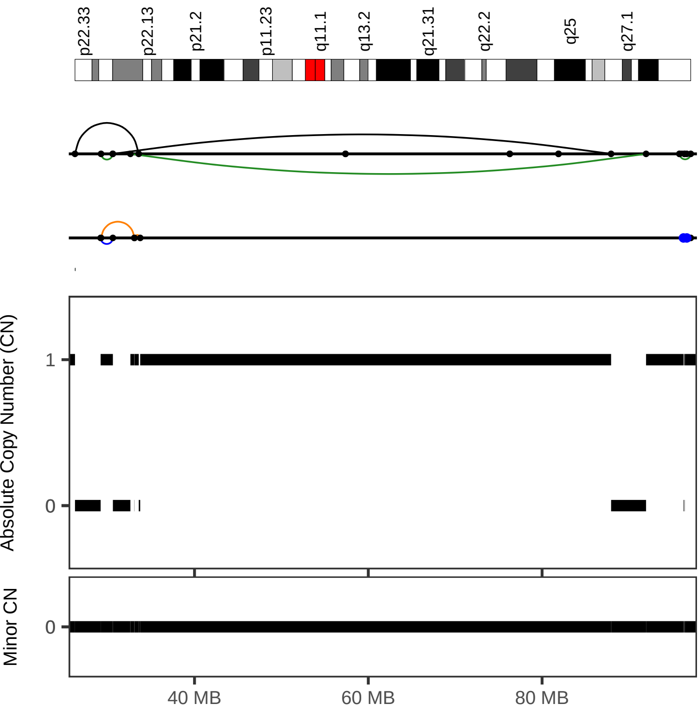

	CPCG0098
Cancer type	Prost−AdenoCA
Position	X:26243883−91967097
Type	Canonical without polyploidization
Interleaved intrachr. SVs	7
Total SVs (intrachr. + transl.)	7
SV types	DEL: 2; DUP: 1; h2hINV: 2; t2tINV: 2; TRA: 0
SVs in sample	100
Oscillating CN (2 and 3 states)	10, 10
CN segments	10
FDR fragment joints	0.95
FDR chr. breakp. enrich.	0
Linked to chrs	
Purity, ploidy	0.69, 1.93

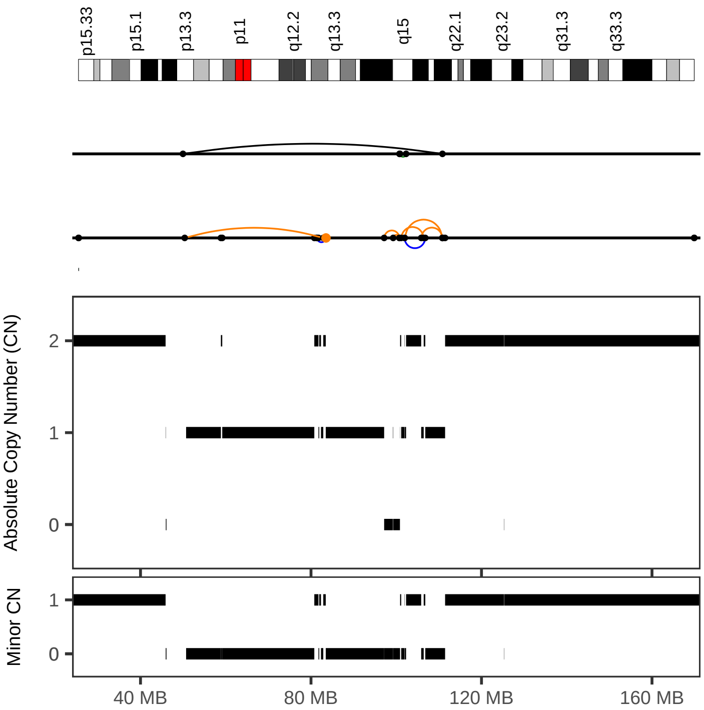

	CPCG0099
Cancer type	Prost−AdenoCA
Position	5:49962437−111461751
Type	With other complex events
Interleaved intrachr. SVs	8
Total SVs (intrachr. + transl.)	9
SV types	DEL: 4; DUP: 2; h2hINV: 1; t2tINV: 1; TRA: 1
SVs in sample	116
Oscillating CN (2 and 3 states)	10, 23
CN segments	23
FDR fragment joints	0.59
FDR chr. breakp. enrich.	0
Linked to chrs	
Purity, ploidy	0.62, 1.84

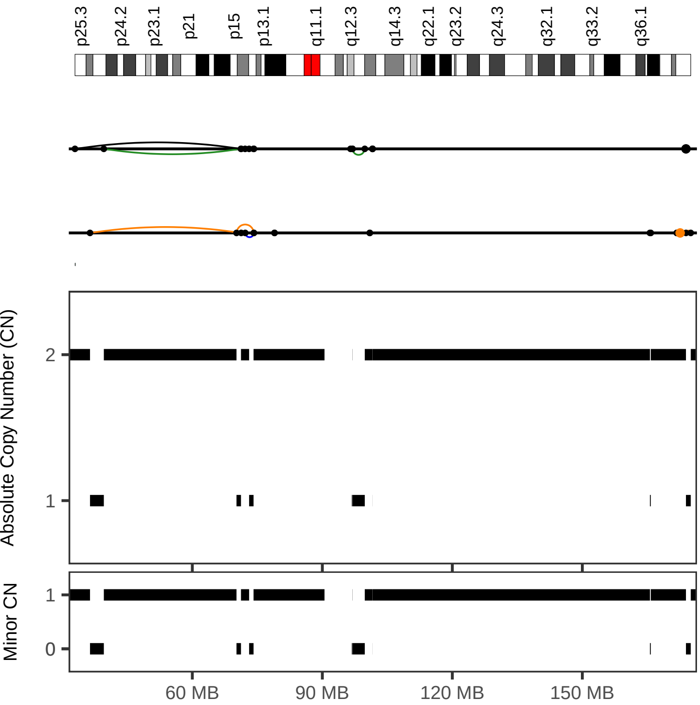

	CPCG0123
Cancer type	Prost−AdenoCA
Position	2:32918330−74219519
Type	Canonical without polyploidization
Interleaved intrachr. SVs	7
Total SVs (intrachr. + transl.)	7
SV types	DEL: 2; DUP: 1; h2hINV: 2; t2tINV: 2; TRA: 0
SVs in sample	144
Oscillating CN (2 and 3 states)	7, 7
CN segments	7
FDR fragment joints	0.95
FDR chr. breakp. enrich.	0.03
Linked to chrs	
Purity, ploidy	0.44, 1.9

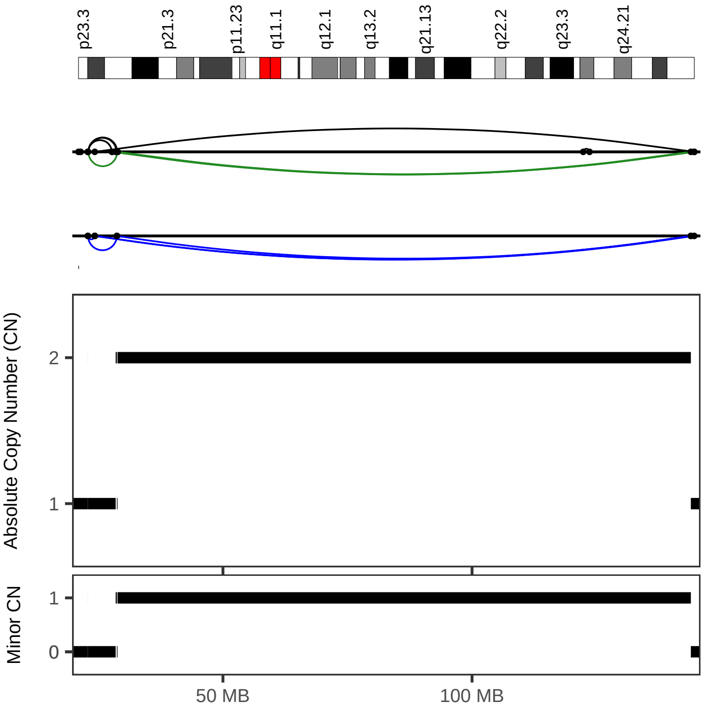

	CPCG0123
Cancer type	Prost−AdenoCA
Position	8:22894046−144595974
Type	Canonical without polyploidization
Interleaved intrachr. SVs	13
Total SVs (intrachr. + transl.)	13
SV types	DEL: 0; DUP: 5; h2hINV: 4; t2tINV: 4; TRA: 0
SVs in sample	144
Oscillating CN (2 and 3 states)	7, 7
CN segments	7
FDR fragment joints	0.59
FDR chr. breakp. enrich.	0.04
Linked to chrs	
Purity, ploidy	0.44, 1.9

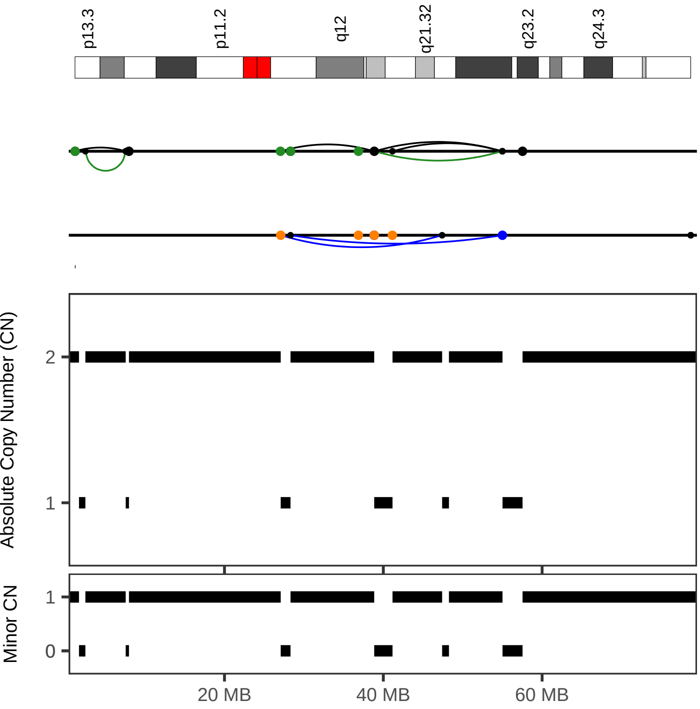

	**CPCG0123**
Cancer type	Prost−AdenoCA
Position	17:27075680−55017524
Type	Canonical without polyploidization
Interleaved intrachr. SVs	6
Total SVs (intrachr. + transl.)	17
SV types	DEL: 0; DUP: 2; h2hINV: 3; t2tINV: 1; TRA: 11
SVs in sample	144
Oscillating CN (2 and 3 states)	9, 9
CN segments	9
FDR fragment joints	0.59
FDR chr. breakp. enrich.	0
Linked to chrs	10:75239971−97413753;4:40113263−89954085
Purity, ploidy	0.44, 1.9

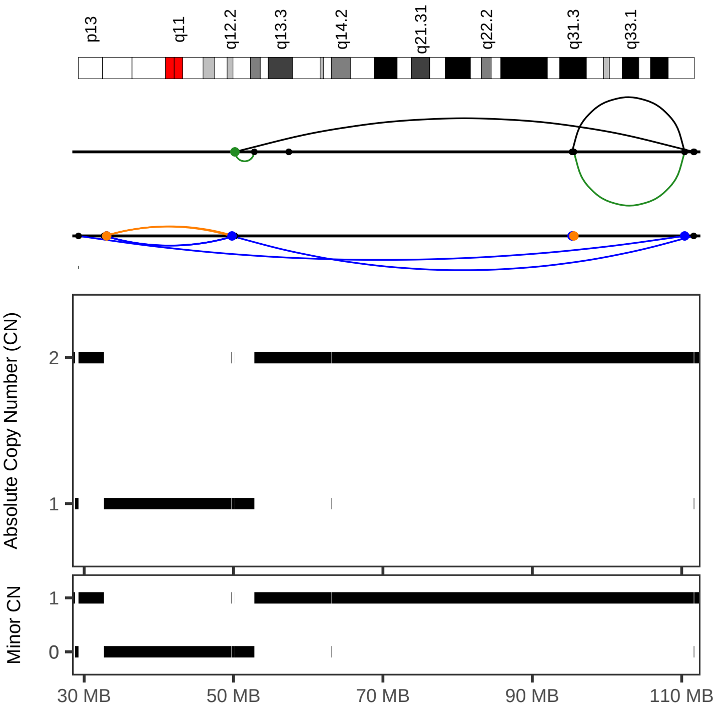

	**CPCG0124**
Cancer type	Prost−AdenoCA
Position	13:29235366−111679206
Type	Canonical without polyploidization
Interleaved intrachr. SVs	12
Total SVs (intrachr. + transl.)	19
SV types	DEL: 3; DUP: 4; h2hINV: 2; t2tINV: 3; TRA: 7
SVs in sample	121
Oscillating CN (2 and 3 states)	13, 13
CN segments	13
FDR fragment joints	0.91
FDR chr. breakp. enrich.	0
Linked to chrs	10:48369342−119949557;
Purity, ploidy	0.47, 1.87

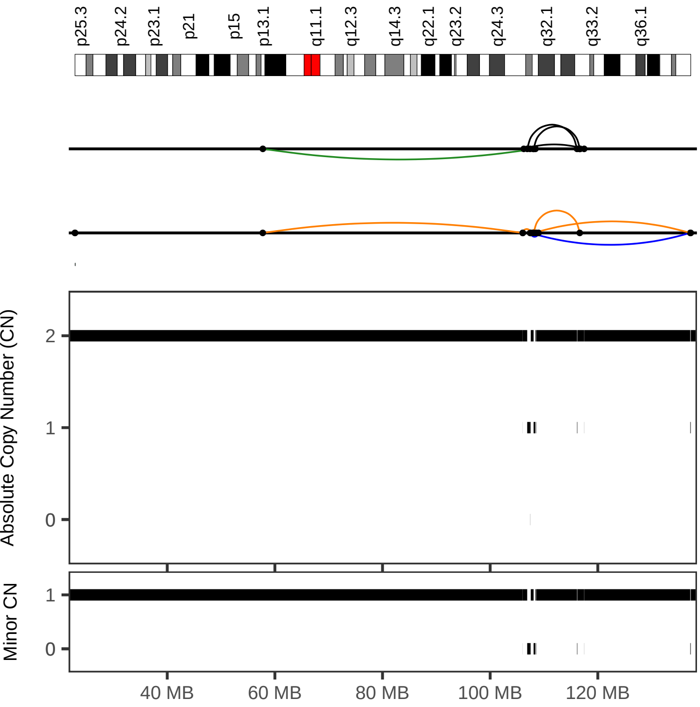

	**CPCG0128**
Cancer type	Prost−AdenoCA
Position	2:57746139−137270112
Type	Canonical without polyploidization
Interleaved intrachr. SVs	15
Total SVs (intrachr. + transl.)	15
SV types	DEL: 6; DUP: 2; h2hINV: 4; t2tINV: 3; TRA: 0
SVs in sample	84
Oscillating CN (2 and 3 states)	12, 19
CN segments	19
FDR fragment joints	0.64
FDR chr. breakp. enrich.	0
Linked to chrs	
Purity, ploidy	0.76, 1.9

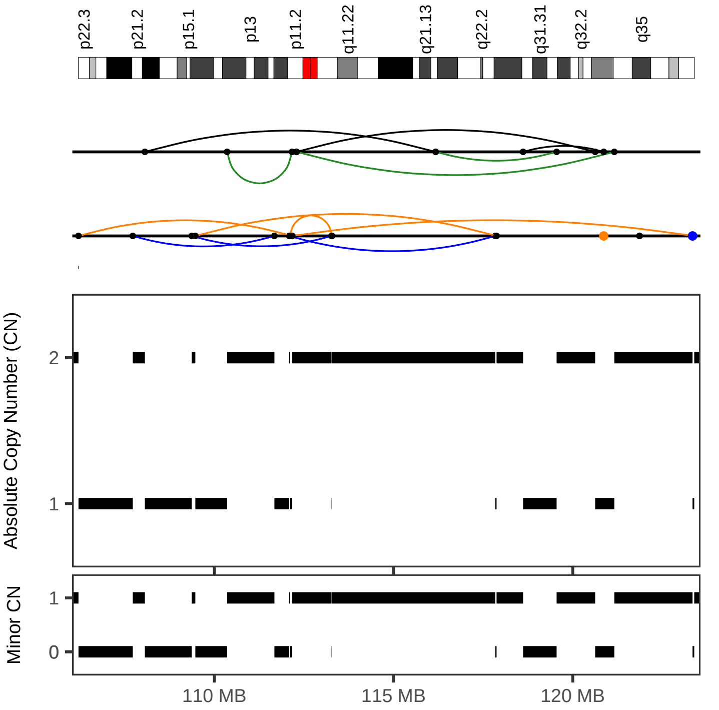

	**CPCG0128**
Cancer type	Prost−AdenoCA
Position	7:106208873−123387813
Type	Canonical without polyploidization
Interleaved intrachr. SVs	13
Total SVs (intrachr. + transl.)	15
SV types	DEL: 4; DUP: 3; h2hINV: 3; t2tINV: 3; TRA: 2
SVs in sample	84
Oscillating CN (2 and 3 states)	20, 20
CN segments	20
FDR fragment joints	0.98
FDR chr. breakp. enrich.	0
Linked to chrs	
Purity, ploidy	0.76, 1.9

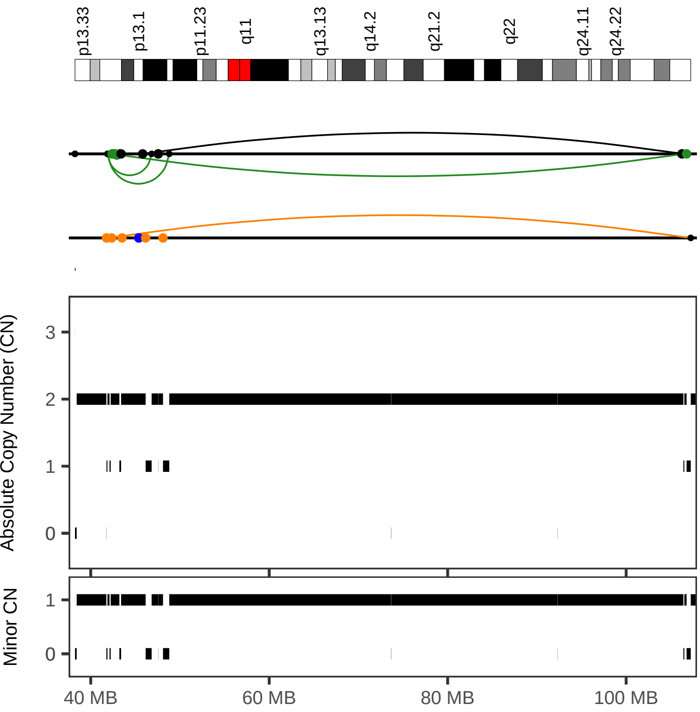

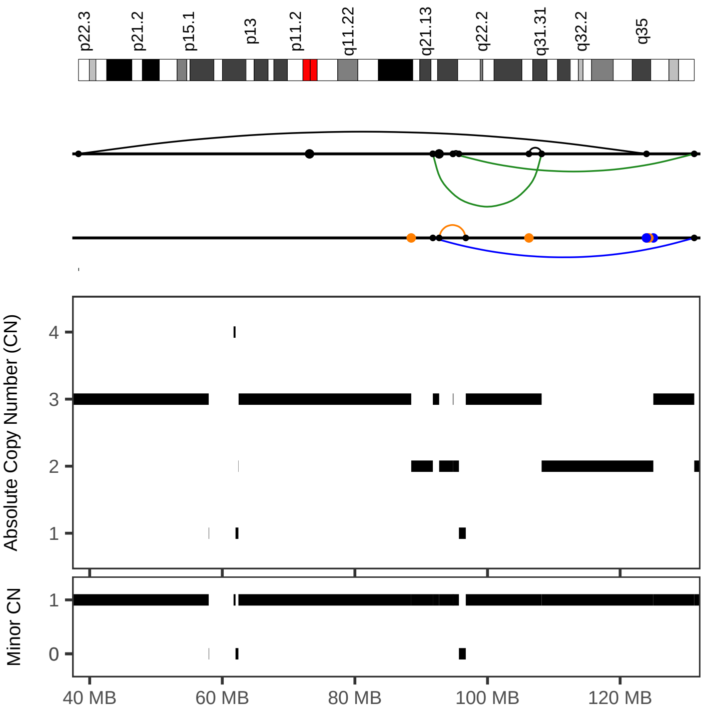

	**CPCG0154**
Cancer type	Prost−AdenoCA
Position	12:41871673−107239693
Type	With other complex events
Interleaved intrachr. SVs	7
Total SVs (intrachr. + transl.)	24
SV types	DEL: 2; DUP: 0; h2hINV: 1; t2tINV: 4; TRA: 17
SVs in sample	79
Oscillating CN (2 and 3 states)	11, 19
CN segments	19
FDR fragment joints	0.59
FDR chr. breakp. enrich.	0
Linked to chrs	
Purity, ploidy	0.4, 1.92

	**CPCG0158**
Cancer type	Prost−AdenoCA
Position	7:38307844−131180183
Type	With other complex events
Interleaved intrachr. SVs	5
Total SVs (intrachr. + transl.)	13
SV types	DEL: 1; DUP: 1; h2hINV: 1; t2tINV: 2; TRA: 8
SVs in sample	114
Oscillating CN (2 and 3 states)	7, 7
CN segments	16
FDR fragment joints	0.92
FDR chr. breakp. enrich.	0
Linked to chrs	
Purity, ploidy	0.56, 1.92

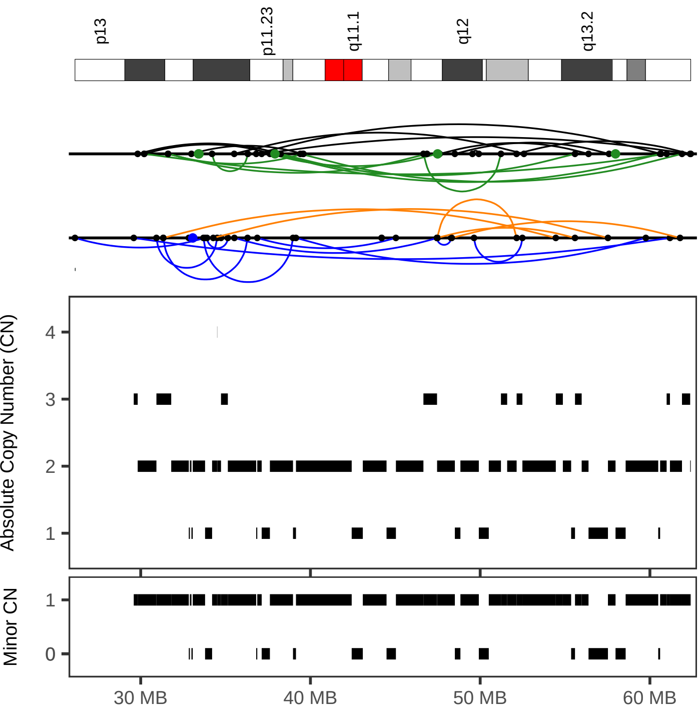

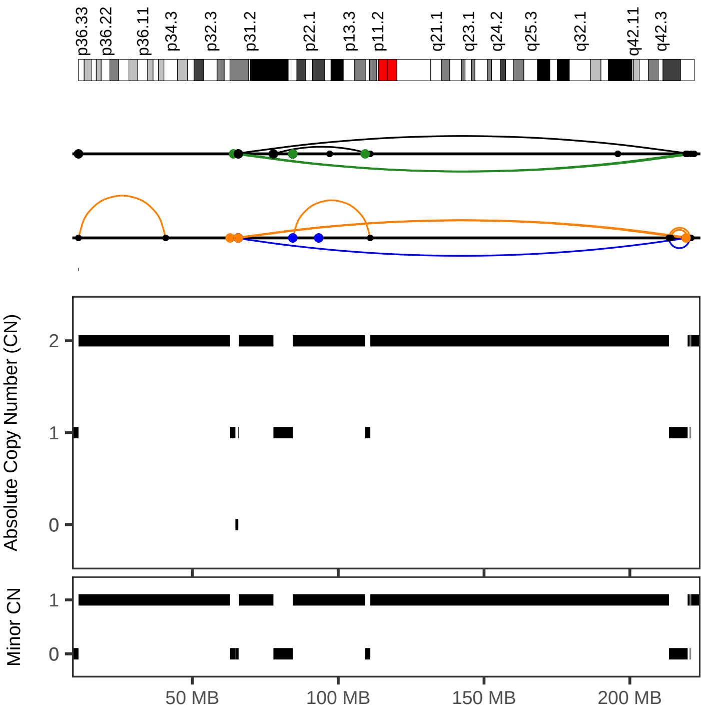

	**CPCG0158**
Cancer type	Prost−AdenoCA
Position	20:26127345−62406063
Type	With other complex events
Interleaved intrachr. SVs	35
Total SVs (intrachr. + transl.)	41
SV types	DEL: 5; DUP: 11; h2hINV: 10; t2tINV: 9; TRA: 6
SVs in sample	114
Oscillating CN (2 and 3 states)	11, 28
CN segments	52
FDR fragment joints	0.64
FDR chr. breakp. enrich.	0
Linked to chrs	
Purity, ploidy	0.56, 1.92

	**CPCG0166**
Cancer type	Prost−AdenoCA
Position	1:64280405−222074952
Type	Canonical without polyploidization
Interleaved intrachr. SVs	12
Total SVs (intrachr. + transl.)	27
SV types	DEL: 6; DUP: 2; h2hINV: 1; t2tINV: 3; TRA: 15
SVs in sample	126
Oscillating CN (2 and 3 states)	14, 16
CN segments	16
FDR fragment joints	0.59
FDR chr. breakp. enrich.	0
Linked to chrs	16:74306345−83503515;
Purity, ploidy	0.18, 1.86

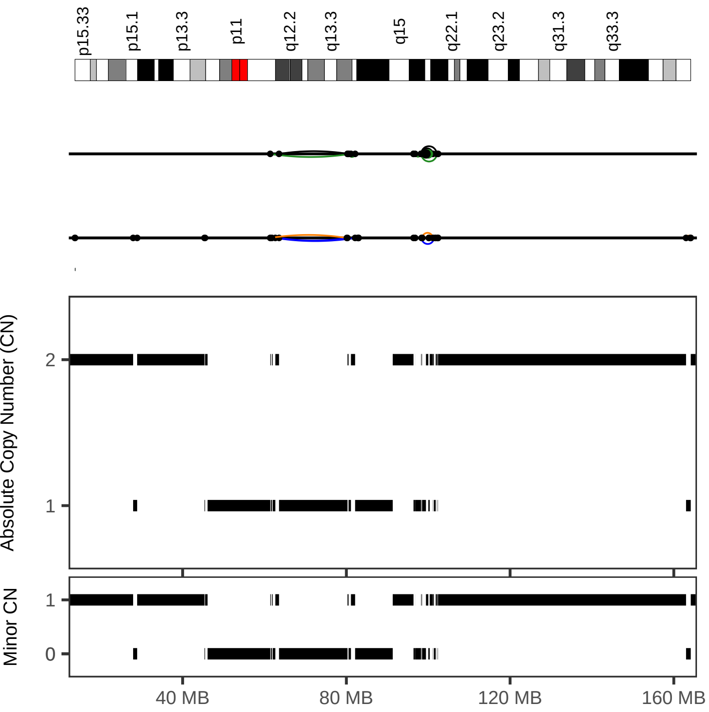

	CPCG0183
Cancer type	Prost−AdenoCA
Position	5:61391468−82150238
Type	Canonical without polyploidization
Interleaved intrachr. SVs	6
Total SVs (intrachr. + transl.)	6
SV types	DEL: 1; DUP: 2; h2hINV: 1; t2tINV: 2; TRA: 0
SVs in sample	124
Oscillating CN (2 and 3 states)	11, 11
CN segments	11
FDR fragment joints	0.91
FDR chr. breakp. enrich.	0
Linked to chrs	
Purity, ploidy	0.71, 1.88

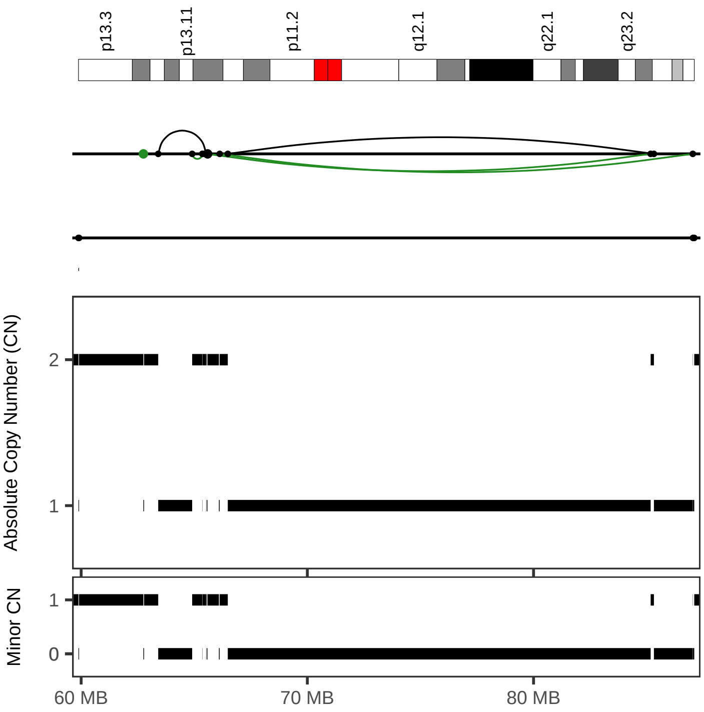

	CPCG0183
Cancer type	Prost−AdenoCA
Position	16:63407336−87039387
Type	Canonical without polyploidization
Interleaved intrachr. SVs	6
Total SVs (intrachr. + transl.)	7
SV types	DEL: 0; DUP: 0; h2hINV: 3; t2tINV: 3; TRA: 1
SVs in sample	124
Oscillating CN (2 and 3 states)	14, 14
CN segments	14
FDR fragment joints	0.48
FDR chr. breakp. enrich.	0.01
Linked to chrs	
Purity, ploidy	0.71, 1.88

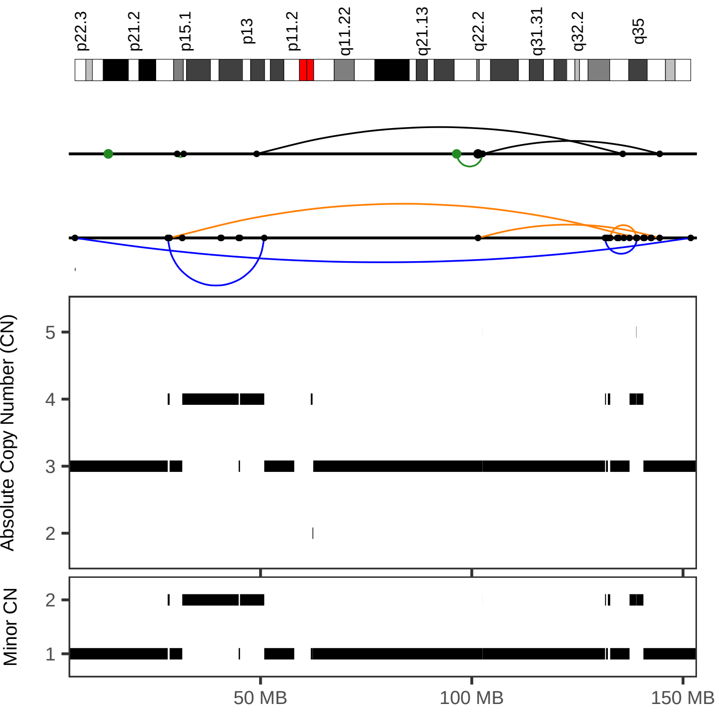

	CPCG0184
Cancer type	Prost−AdenoCA
Position	7:28022973−144475086
Type	With other complex events
Interleaved intrachr. SVs	8
Total SVs (intrachr. + transl.)	10
SV types	DEL: 3; DUP: 2; h2hINV: 2; t2tINV: 1; TRA: 2
SVs in sample	77
Oscillating CN (2 and 3 states)	7, 9
CN segments	19
FDR fragment joints	0.84
FDR chr. breakp. enrich.	0
Linked to chrs	
Purity, ploidy	0.57, 2.16

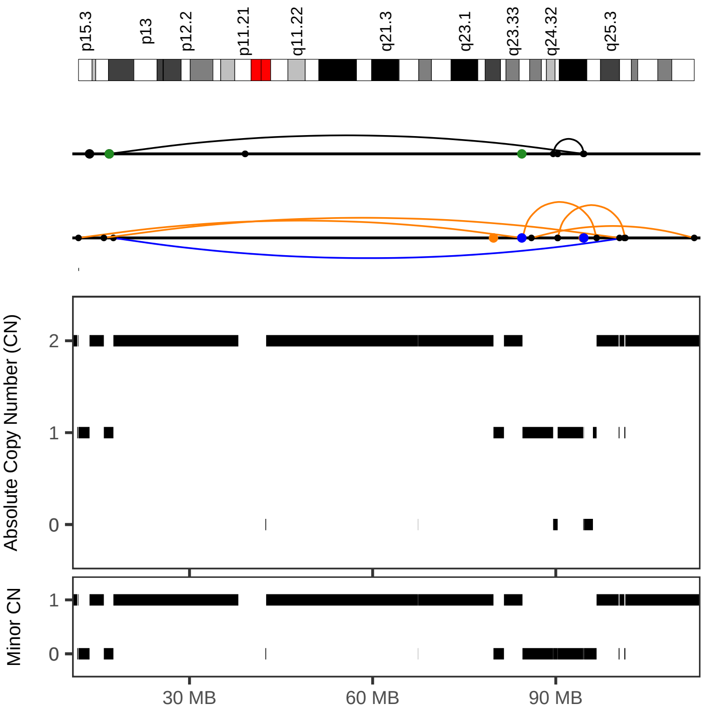

	CPCG0185
Cancer type	Prost−AdenoCA
Position	10:11812464−112681665
Type	With other complex events
Interleaved intrachr. SVs	8
Total SVs (intrachr. + transl.)	16
SV types	DEL: 5; DUP: 1; h2hINV: 2; t2tINV: 0; TRA: 8
SVs in sample	72
Oscillating CN (2 and 3 states)	7, 11
CN segments	22
FDR fragment joints	0.48
FDR chr. breakp. enrich.	0
Linked to chrs	
Purity, ploidy	0.56, 1.87

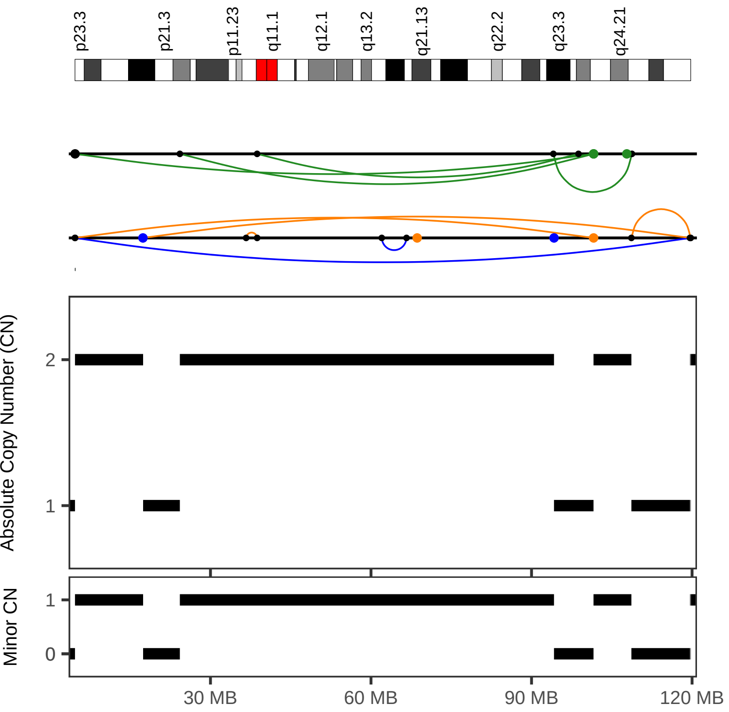

CPCG0191

Cancer type	Prost−AdenoCA
Position	8:4682515−119746552
Type	Canonical without polyploidization
Interleaved intrachr. SVs	9
Total SVs (intrachr. + transl.)	17
SV types	DEL: 4; DUP: 1; h2hINV: 0; t2tINV: 4; TRA: 8
SVs in sample	121
Oscillating CN (2 and 3 states)	9, 9
CN segments	9
FDR fragment joints	0.55
FDR chr. breakp. enrich.	0
Linked to chrs	12:51747202−124872407;
Purity, ploidy	0.55, 1.84

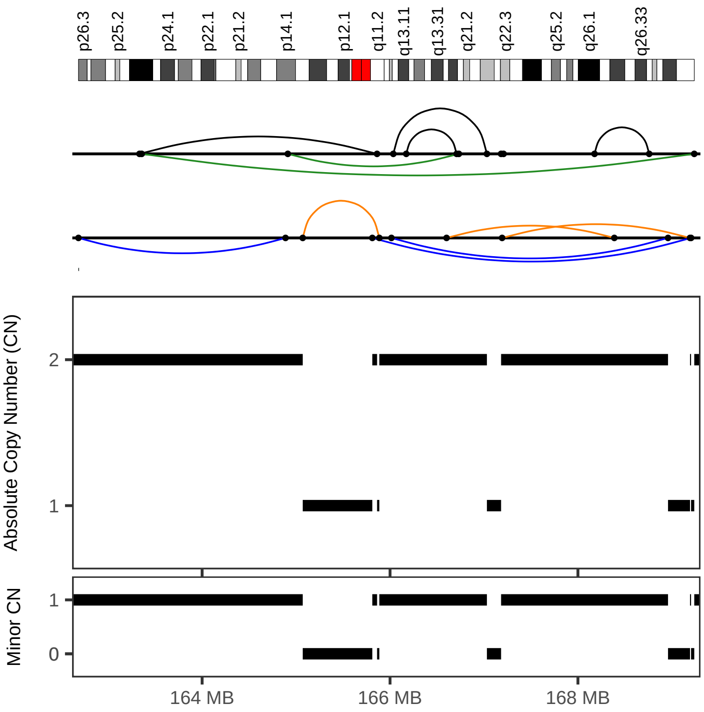

CPCG0199

Cancer type	Prost−AdenoCA
Position	3:162683867−169237872
Type	Canonical without polyploidization
Interleaved intrachr. SVs	13
Total SVs (intrachr. + transl.)	13
SV types	DEL: 3; DUP: 3; h2hINV: 4; t2tINV: 3; TRA: 0
SVs in sample	109
Oscillating CN (2 and 3 states)	11, 11
CN segments	11
FDR fragment joints	0.98
FDR chr. breakp. enrich.	0.18
Linked to chrs	
Purity, ploidy	0.28, 1.88

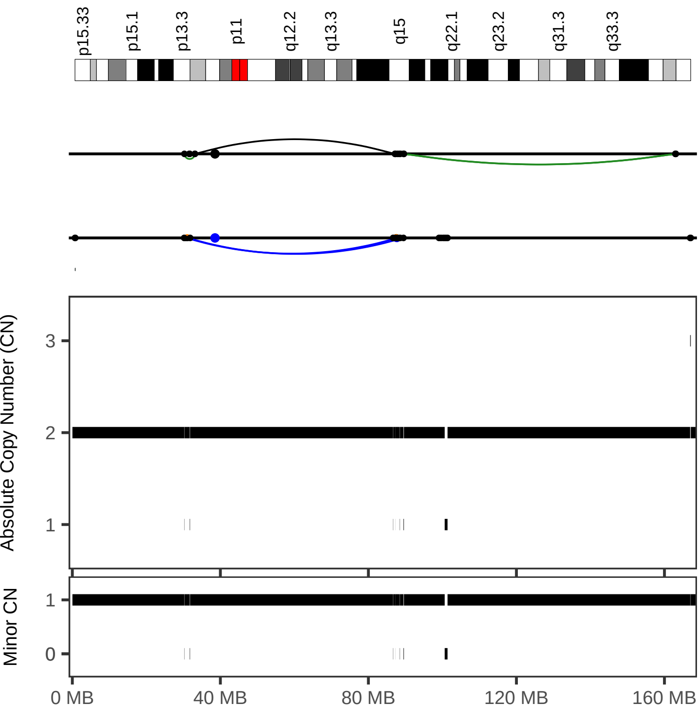

CPCG0199

Cancer type	Prost−AdenoCA
Position	5:30217746−163056821
Type	Canonical without polyploidization
Interleaved intrachr. SVs	14
Total SVs (intrachr. + transl.)	16
SV types	DEL: 3; DUP: 3; h2hINV: 3; t2tINV: 5; TRA: 2
SVs in sample	109
Oscillating CN (2 and 3 states)	16, 16
CN segments	16
FDR fragment joints	0.88
FDR chr. breakp. enrich.	0
Linked to chrs	
Purity, ploidy	0.28, 1.88

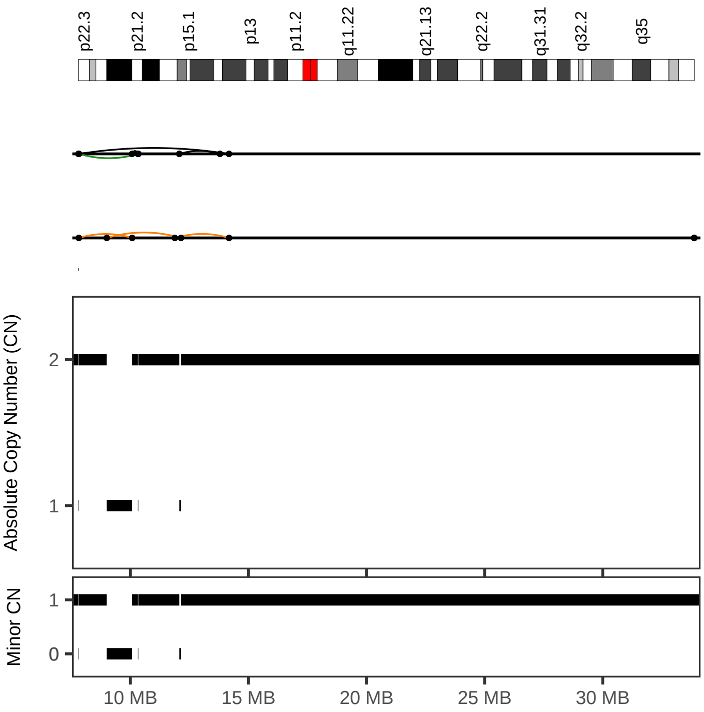

CPCG0199

Cancer type	Prost−AdenoCA
Position	7:7799682−14177776
Type	Canonical without polyploidization
Interleaved intrachr. SVs	7
Total SVs (intrachr. + transl.)	7
SV types	DEL: 3; DUP: 0; h2hINV: 3; t2tINV: 1; TRA: 0
SVs in sample	109
Oscillating CN (2 and 3 states)	8, 8
CN segments	8
FDR fragment joints	0.59
FDR chr. breakp. enrich.	0.64
Linked to chrs	
Purity, ploidy	0.28, 1.88

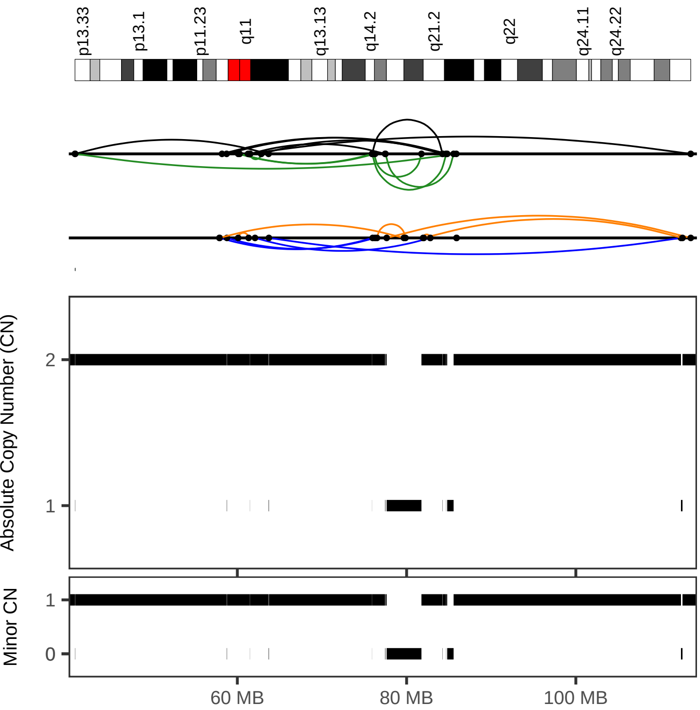

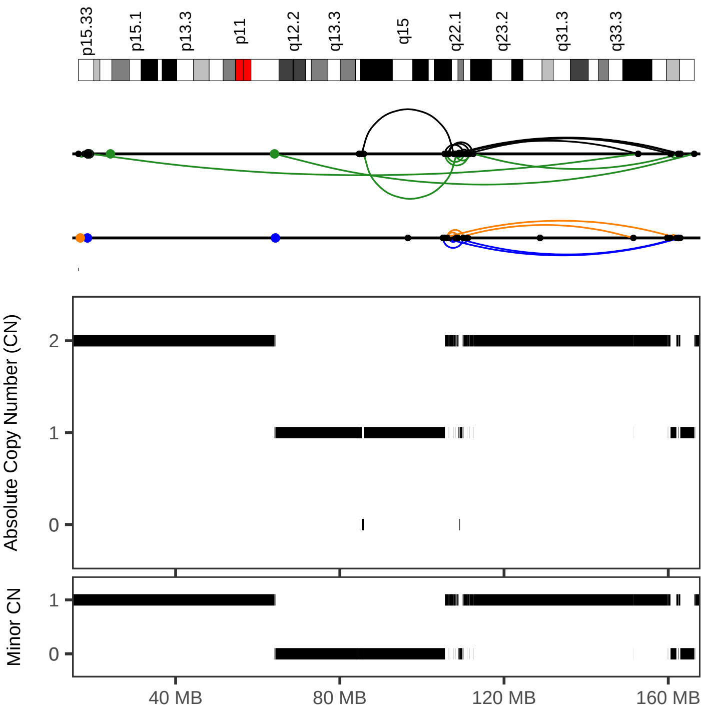

	CPCG0199
Cancer type	Prost−AdenoCA
Position	12:40811810−113578120
Type	Canonical without polyploidization
Interleaved intrachr. SVs	24
Total SVs (intrachr. + transl.)	24
SV types	DEL: 6; DUP: 4; h2hINV: 7; t2tINV: 7; TRA: 0
SVs in sample	109
Oscillating CN (2 and 3 states)	22, 22
CN segments	22
FDR fragment joints	0.84
FDR chr. breakp. enrich.	0
Linked to chrs	
Purity, ploidy	0.28, 1.88

	CPCG0201
Cancer type	Prost−AdenoCA
Position	5:19275862−166331721
Type	With other complex events
Interleaved intrachr. SVs	36
Total SVs (intrachr. + transl.)	39
SV types	DEL: 8; DUP: 5; h2hINV: 11; t2tINV: 12; TRA: 3
SVs in sample	350
Oscillating CN (2 and 3 states)	26, 29
CN segments	46
FDR fragment joints	0.59
FDR chr. breakp. enrich.	0
Linked to chrs	
Purity, ploidy	0.78, 1.83

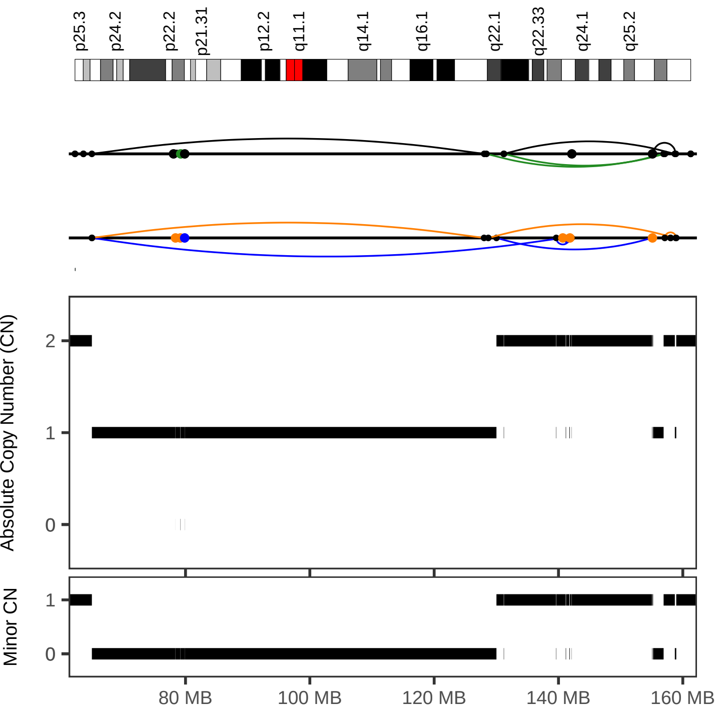

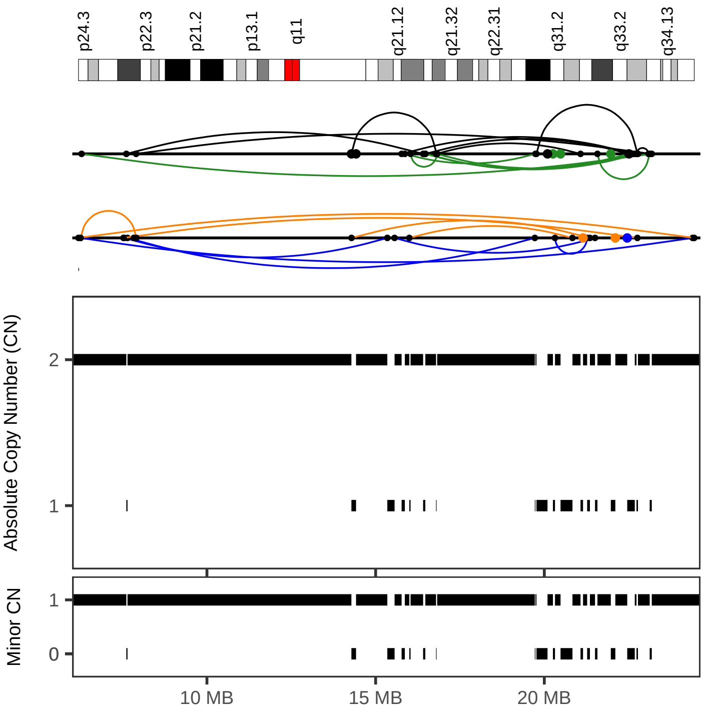

	CPCG0201
Cancer type	Prost−AdenoCA
Position	6:64935112−158946645
Type	Canonical without polyploidization
Interleaved intrachr. SVs	11
Total SVs (intrachr. + transl.)	22
SV types	DEL: 2; DUP: 4; h2hINV: 3; t2tINV: 2; TRA: 11
SVs in sample	350
Oscillating CN (2 and 3 states)	18, 24
CN segments	24
FDR fragment joints	0.84
FDR chr. breakp. enrich.	0.07
Linked to chrs	11:36858222−50765922;4:114833795−189168660
Purity, ploidy	0.78, 1.83

	CPCG0201
Cancer type	Prost−AdenoCA
Position	9:6256612−23184831
Type	Canonical without polyploidization
Interleaved intrachr. SVs	23
Total SVs (intrachr. + transl.)	33
SV types	DEL: 4; DUP: 4; h2hINV: 8; t2tINV: 7; TRA: 10
SVs in sample	350
Oscillating CN (2 and 3 states)	37, 37
CN segments	37
FDR fragment joints	0.64
FDR chr. breakp. enrich.	0
Linked to chrs	5:19275862−166331720;
Purity, ploidy	0.78, 1.83

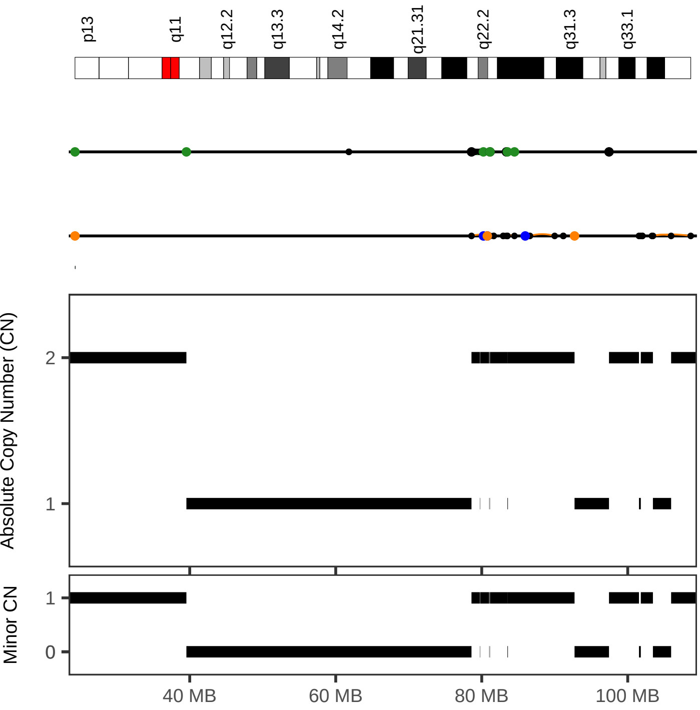

	CPCG0201
Cancer type	Prost−AdenoCA
Position	13:78597547−81366968
Type	Canonical without polyploidization
Interleaved intrachr. SVs	6
Total SVs (intrachr. + transl.)	13
SV types	DEL: 1; DUP: 0; h2hINV: 3; t2tINV: 2; TRA: 7
SVs in sample	350
Oscillating CN (2 and 3 states)	7, 7
CN segments	7
FDR fragment joints	0.59
FDR chr. breakp. enrich.	0
Linked to chrs	
Purity, ploidy	0.78, 1.83

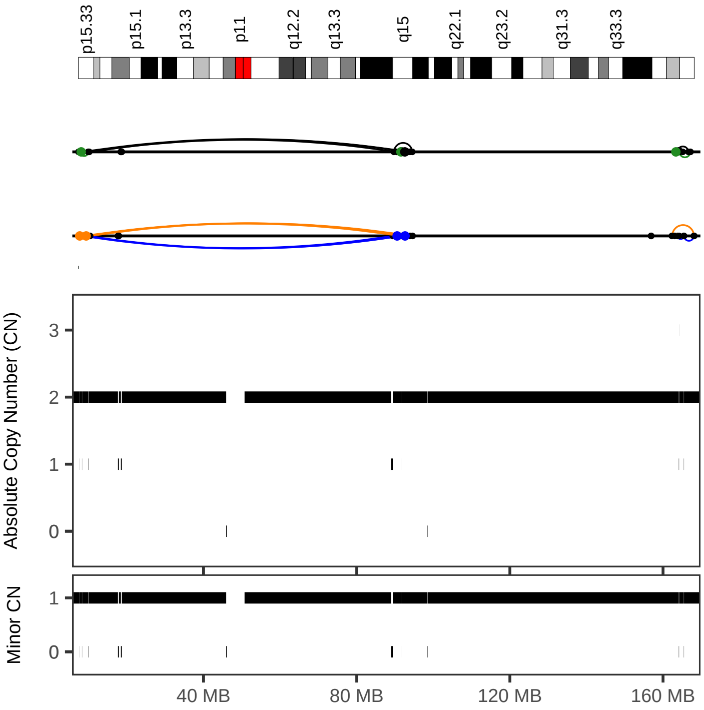

	CPCG0206
Cancer type	Prost−AdenoCA
Position	5:7327241−94584506
Type	Canonical without polyploidization
Interleaved intrachr. SVs	16
Total SVs (intrachr. + transl.)	23
SV types	DEL: 4; DUP: 4; h2hINV: 6; t2tINV: 2; TRA: 7
SVs in sample	173
Oscillating CN (2 and 3 states)	11, 17
CN segments	17
FDR fragment joints	0.64
FDR chr. breakp. enrich.	0
Linked to chrs	
Purity, ploidy	0.3, 1.94

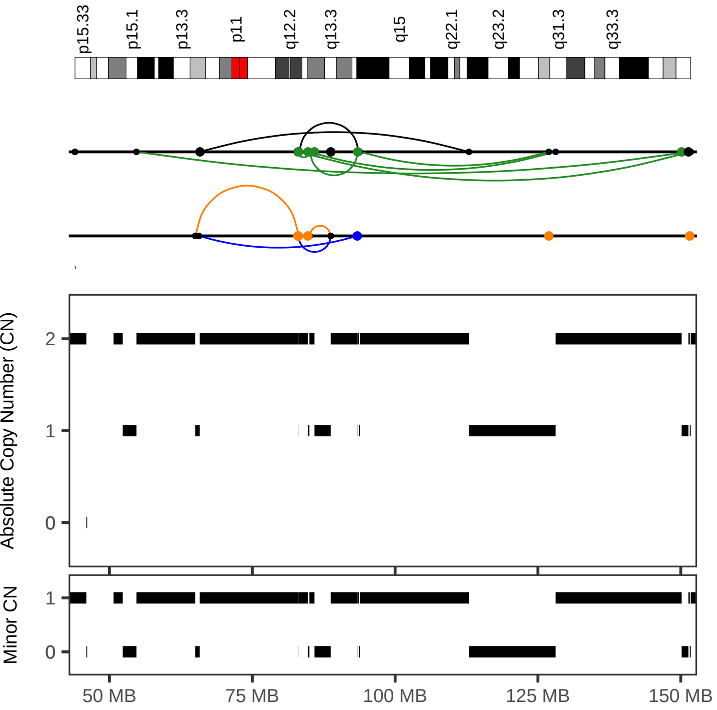

	CPCG0208
Cancer type	Prost−AdenoCA
Position	5:54711398−151747029
Type	Canonical without polyploidization
Interleaved intrachr. SVs	12
Total SVs (intrachr. + transl.)	28
SV types	DEL: 2; DUP: 2; h2hINV: 2; t2tINV: 6; TRA: 16
SVs in sample	90
Oscillating CN (2 and 3 states)	23, 23
CN segments	23
FDR fragment joints	0.59
FDR chr. breakp. enrich.	0
Linked to chrs	
Purity, ploidy	0.78, 1.91

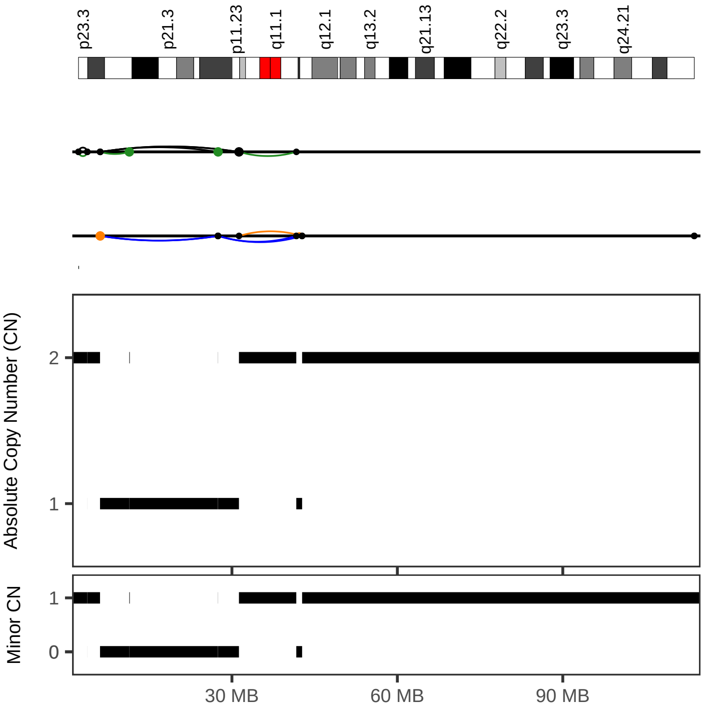

	CPCG0208
Cancer type	Prost−AdenoCA
Position	8:6075645−42724629
Type	Canonical without polyploidization
Interleaved intrachr. SVs	12
Total SVs (intrachr. + transl.)	16
SV types	DEL: 2; DUP: 4; h2hINV: 3; t2tINV: 3; TRA: 4
SVs in sample	90
Oscillating CN (2 and 3 states)	8, 8
CN segments	8
FDR fragment joints	0.91
FDR chr. breakp. enrich.	0
Linked to chrs	
Purity, ploidy	0.78, 1.91

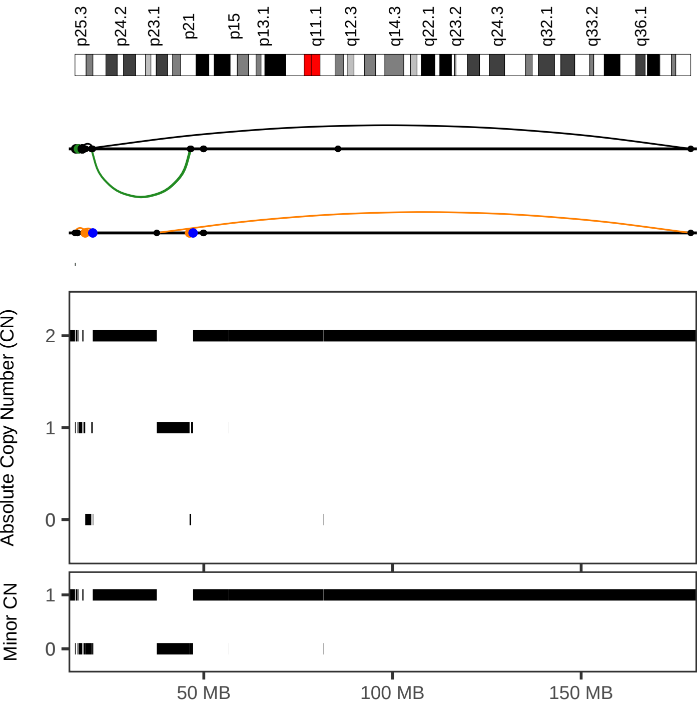

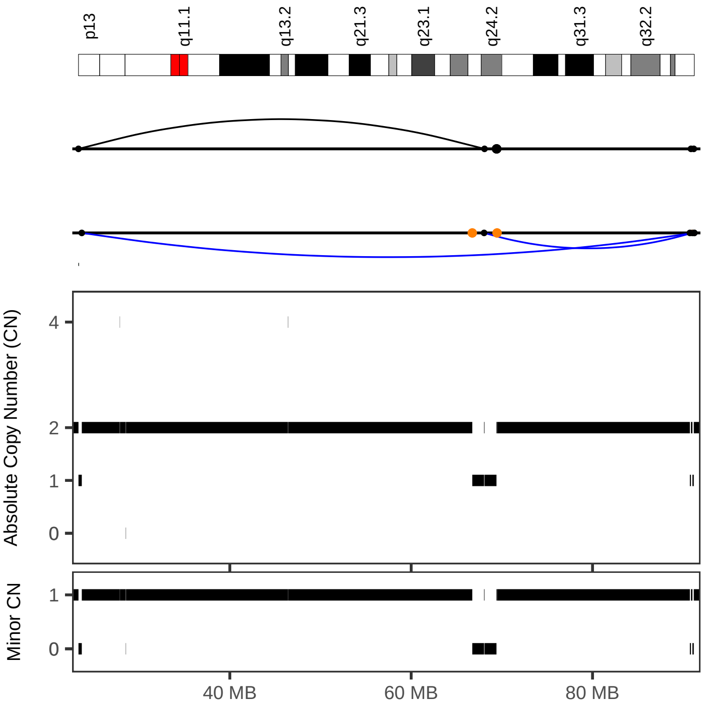

	**CPCG0211**
Cancer type	Prost−AdenoCA
Position	2:15850928−179041463
Type	With other complex events
Interleaved intrachr. SVs	7
Total SVs (intrachr. + transl.)	16
SV types	DEL: 3; DUP: 0; h2hINV: 2; t2tINV: 2; TRA: 9
SVs in sample	106
Oscillating CN (2 and 3 states)	7, 10
CN segments	20
FDR fragment joints	0.64
FDR chr. breakp. enrich.	0
Linked to chrs	3:37350014−175530480;
Purity, ploidy	0.48, 1.9

	**CPCG0211**
Cancer type	Prost−AdenoCA
Position	14:23310029−91217856
Type	Canonical without polyploidization
Interleaved intrachr. SVs	5
Total SVs (intrachr. + transl.)	9
SV types	DEL: 1; DUP: 2; h2hINV: 1; t2tINV: 1; TRA: 4
SVs in sample	106
Oscillating CN (2 and 3 states)	11, 13
CN segments	18
FDR fragment joints	0.92
FDR chr. breakp. enrich.	0.01
Linked to chrs	
Purity, ploidy	0.48, 1.9

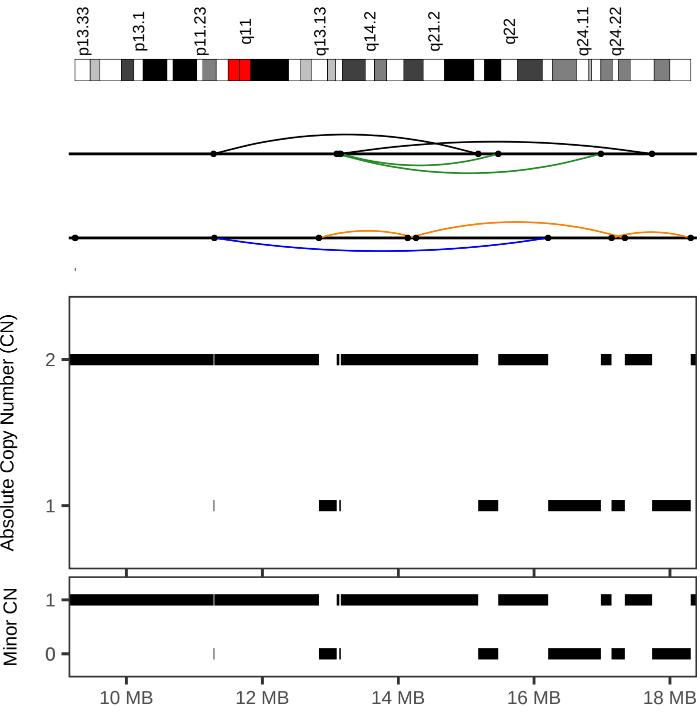

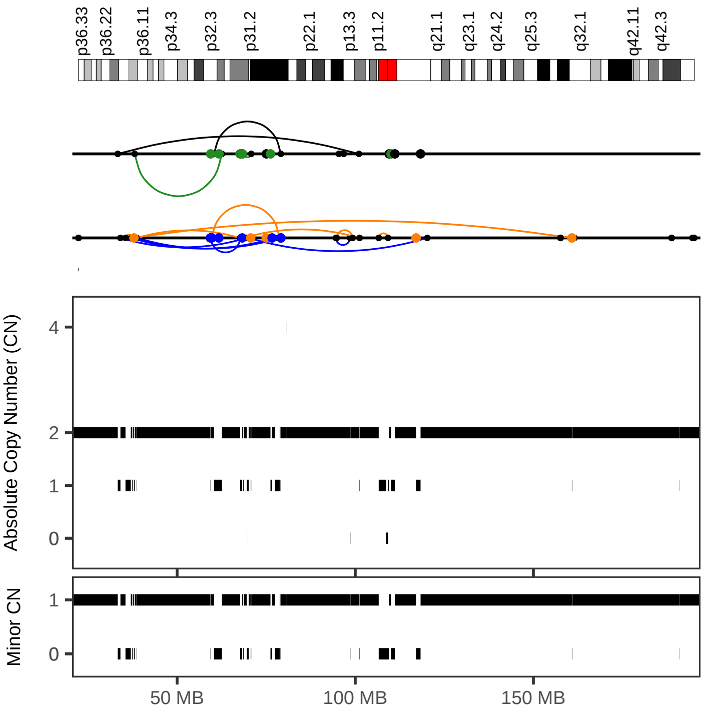

	**CPCG0217**
Cancer type	Prost−AdenoCA
Position	12:11281216−18305829
Type	Canonical without polyploidization
Interleaved intrachr. SVs	8
Total SVs (intrachr. + transl.)	8
SV types	DEL: 3; DUP: 1; h2hINV: 2; t2tINV: 2; TRA: 0
SVs in sample	30
Oscillating CN (2 and 3 states)	14, 14
CN segments	14
FDR fragment joints	0.84
FDR chr. breakp. enrich.	0
Linked to chrs	
Purity, ploidy	0.6, 1.92

	**CPCG0232**
Cancer type	Prost−AdenoCA
Position	1:33333284−160926638
Type	With other complex events
Interleaved intrachr. SVs	14
Total SVs (intrachr. + transl.)	38
SV types	DEL: 5; DUP: 5; h2hINV: 2; t2tINV: 2; TRA: 24
SVs in sample	235
Oscillating CN (2 and 3 states)	23, 34
CN segments	50
FDR fragment joints	0.64
FDR chr. breakp. enrich.	0
Linked to chrs	10:85447653−117550661;12:14965560−129555946
Purity, ploidy	0.54, 1.83

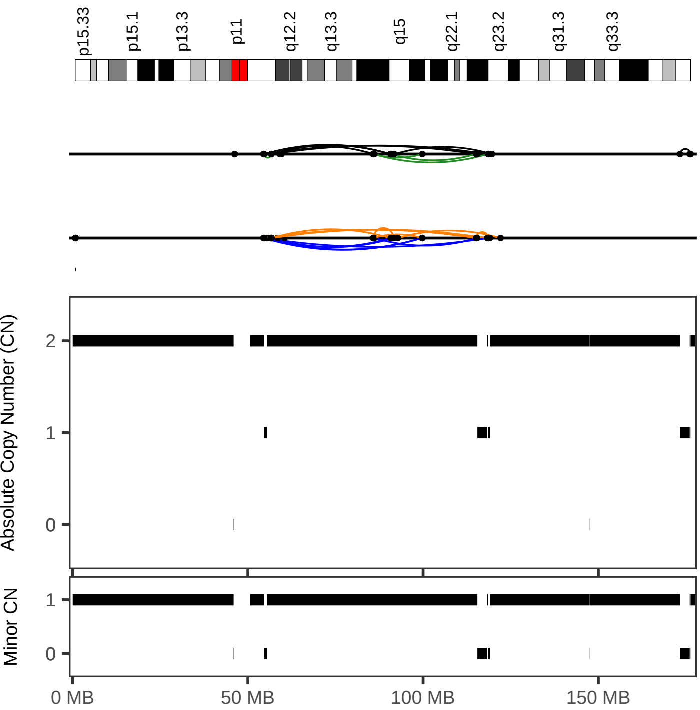

	CPCG0232
Cancer type	Prost−AdenoCA
Position	5:54443227−122107536
Type	Canonical without polyploidization
Interleaved intrachr. SVs	24
Total SVs (intrachr. + transl.)	24
SV types	DEL: 8; DUP: 6; h2hINV: 6; t2tINV: 4; TRA: 0
SVs in sample	235
Oscillating CN (2 and 3 states)	9, 9
CN segments	9
FDR fragment joints	0.8
FDR chr. breakp. enrich.	0
Linked to chrs	
Purity, ploidy	0.54, 1.83

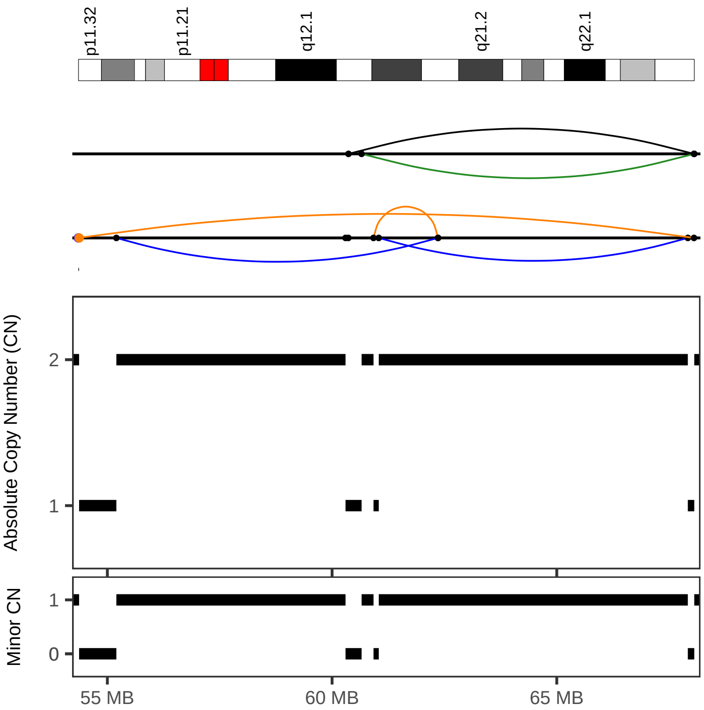

	CPCG0232
Cancer type	Prost−AdenoCA
Position	18:54357319−68058750
Type	Canonical without polyploidization
Interleaved intrachr. SVs	6
Total SVs (intrachr. + transl.)	8
SV types	DEL: 2; DUP: 2; h2hINV: 1; t2tINV: 1; TRA: 2
SVs in sample	235
Oscillating CN (2 and 3 states)	9, 9
CN segments	9
FDR fragment joints	0.91
FDR chr. breakp. enrich.	0.35
Linked to chrs	3:70605253−72494754;
Purity, ploidy	0.54, 1.83

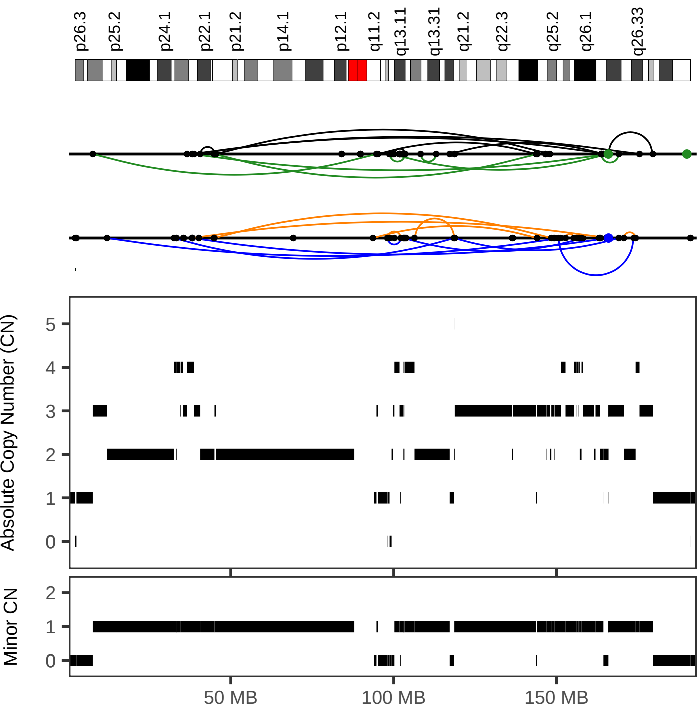

	CPCG0233
Cancer type	Prost−AdenoCA
Position	3:7634513−179524544
Type	With other complex events
Interleaved intrachr. SVs	39
Total SVs (intrachr. + transl.)	41
SV types	DEL: 8; DUP: 10; h2hINV: 11; t2tINV: 10; TRA: 2
SVs in sample	348
Oscillating CN (2 and 3 states)	8, 17
CN segments	92
FDR fragment joints	0.94
FDR chr. breakp. enrich.	0
Linked to chrs	8:91134186−115039928;
Purity, ploidy	0.59, 1.86

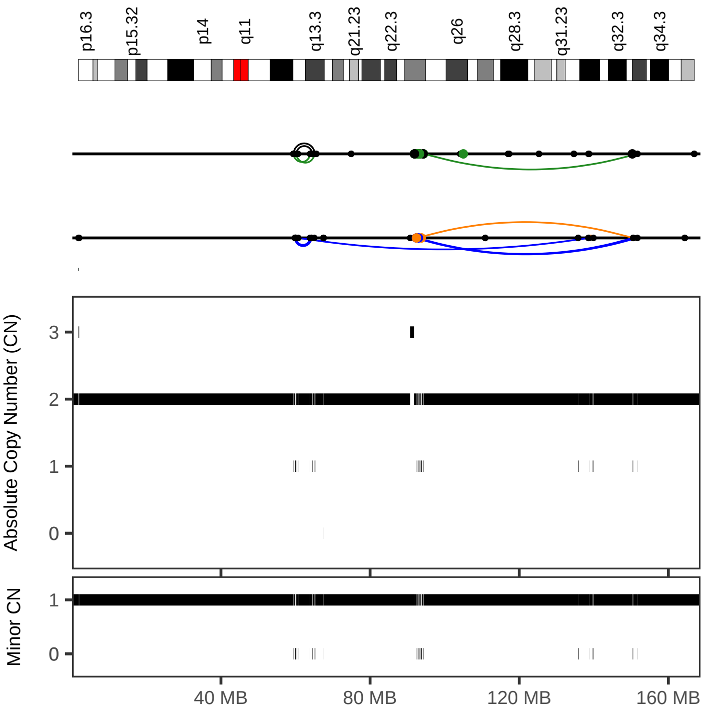

	CPCG0233
Cancer type	Prost−AdenoCA
Position	4:59417131−151727783
Type	With other complex events
Interleaved intrachr. SVs	18
Total SVs (intrachr. + transl.)	34
SV types	DEL: 2; DUP: 6; h2hINV: 4; t2tINV: 6; TRA: 16
SVs in sample	348
Oscillating CN (2 and 3 states)	29, 29
CN segments	50
FDR fragment joints	0.64
FDR chr. breakp. enrich.	0
Linked to chrs	11:31191605−104665238;
Purity, ploidy	0.59, 1.86

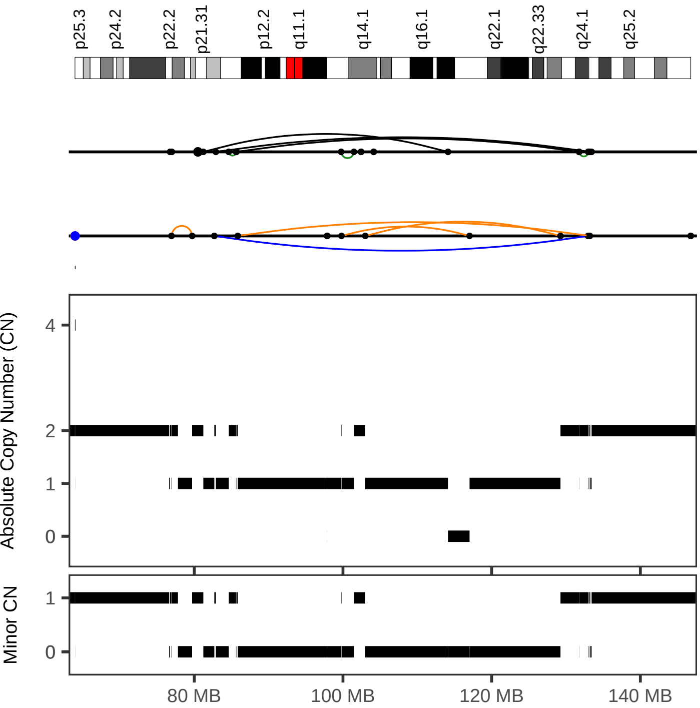

CPCG0233

Cancer type	Prost−AdenoCA
Position	6:81224216−133441817
Type	With other complex events
Interleaved intrachr. SVs	12
Total SVs (intrachr. + transl.)	12
SV types	DEL: 3; DUP: 1; h2hINV: 4; t2tINV: 4; TRA: 0
SVs in sample	348
Oscillating CN (2 and 3 states)	12, 26
CN segments	26
FDR fragment joints	0.64
FDR chr. breakp. enrich.	0.8
Linked to chrs	
Purity, ploidy	0.59, 1.86

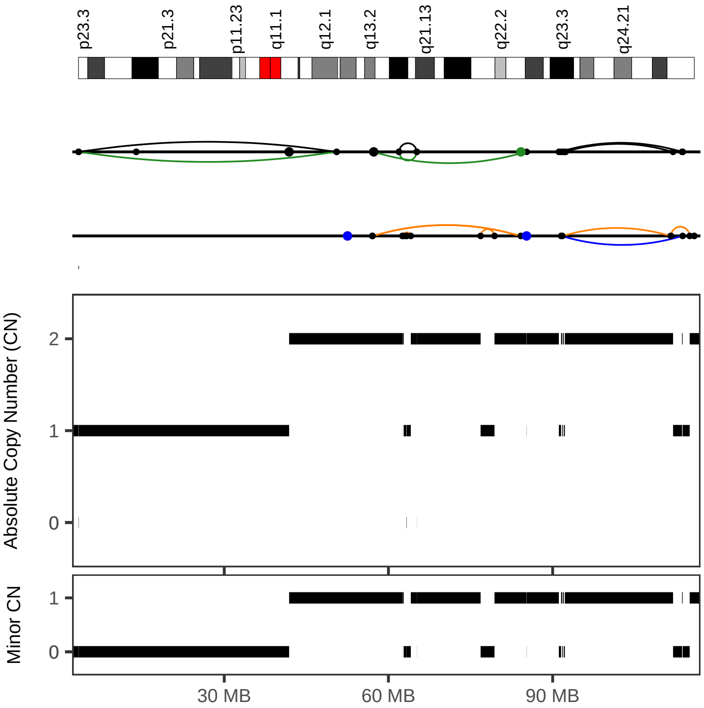

CPCG0233

Cancer type	Prost−AdenoCA
Position	8:91134186−115039929
Type	Canonical without polyploidization
Interleaved intrachr. SVs	8
Total SVs (intrachr. + transl.)	8
SV types	DEL: 2; DUP: 1; h2hINV: 3; t2tINV: 2; TRA: 0
SVs in sample	348
Oscillating CN (2 and 3 states)	10, 10
CN segments	10
FDR fragment joints	0.84
FDR chr. breakp. enrich.	0.08
Linked to chrs	
Purity, ploidy	0.59, 1.86

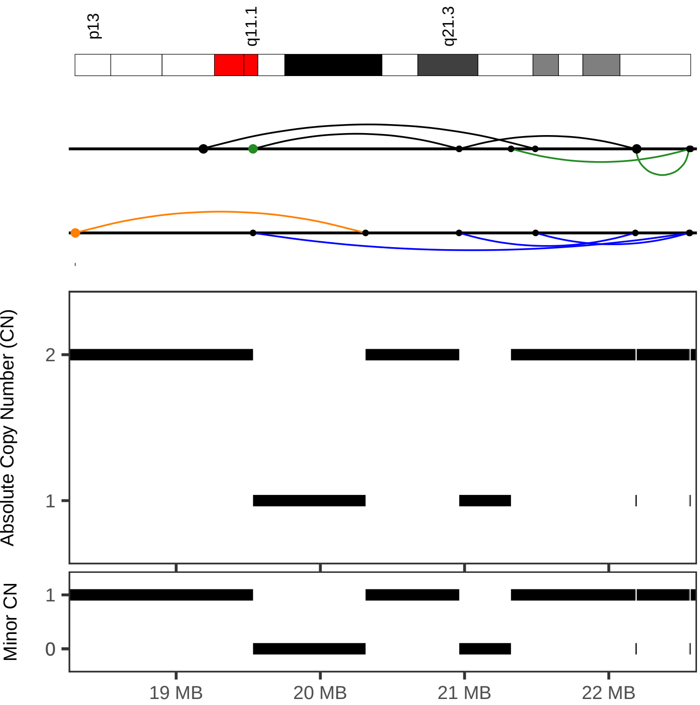

CPCG0233

Cancer type	Prost−AdenoCA
Position	21:18298400−22568498
Type	Canonical without polyploidization
Interleaved intrachr. SVs	9
Total SVs (intrachr. + transl.)	13
SV types	DEL: 1; DUP: 3; h2hINV: 3; t2tINV: 2; TRA: 4
SVs in sample	348
Oscillating CN (2 and 3 states)	11, 11
CN segments	11
FDR fragment joints	0.83
FDR chr. breakp. enrich.	0
Linked to chrs	
Purity, ploidy	0.59, 1.86

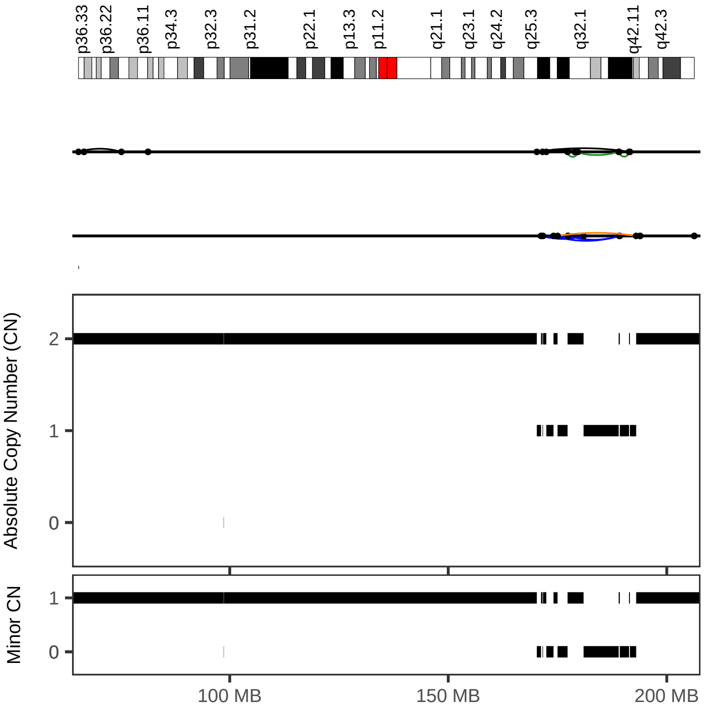

CPCG0236

Cancer type	Prost−AdenoCA
Position	1:170266116−192996844
Type	Canonical without polyploidization
Interleaved intrachr. SVs	11
Total SVs (intrachr. + transl.)	11
SV types	DEL: 1; DUP: 4; h2hINV: 3; t2tINV: 3; TRA: 0
SVs in sample	237
Oscillating CN (2 and 3 states)	14, 14
CN segments	14
FDR fragment joints	0.71
FDR chr. breakp. enrich.	0.84
Linked to chrs	
Purity, ploidy	0.48, 1.9

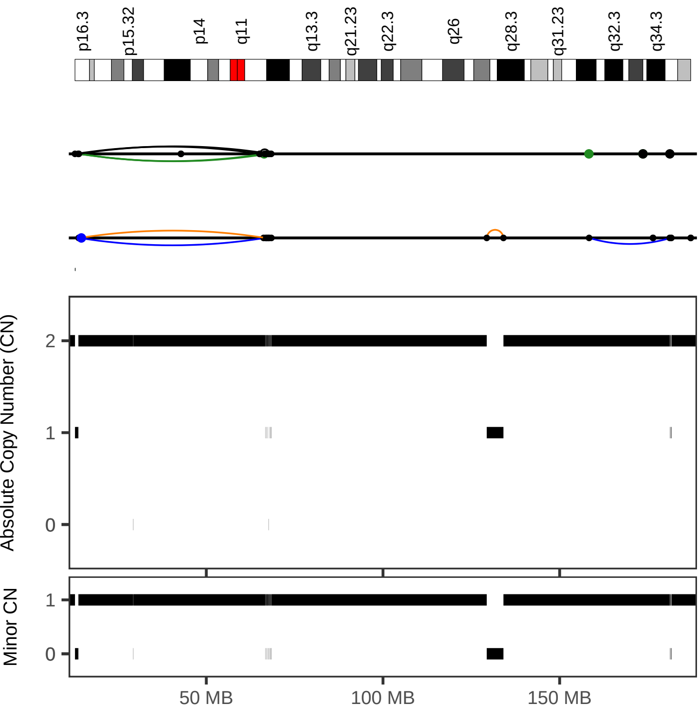

	CPCG0236
Cancer type	Prost−AdenoCA
Position	4:12833922−68421773
Type	With other complex events
Interleaved intrachr. SVs	15
Total SVs (intrachr. + transl.)	16
SV types	DEL: 3; DUP: 3; h2hINV: 4; t2tINV: 5; TRA: 1
SVs in sample	237
Oscillating CN (2 and 3 states)	7, 11
CN segments	19
FDR fragment joints	0.9
FDR chr. breakp. enrich.	0.06
Linked to chrs	
Purity, ploidy	0.48, 1.9

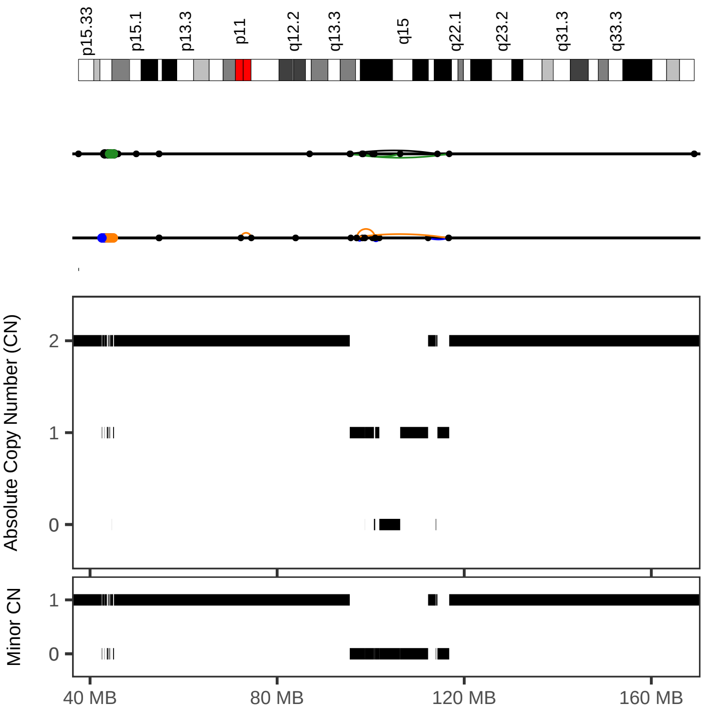

	CPCG0236
Cancer type	Prost−AdenoCA
Position	5:97010270−106309341
Type	Canonical without polyploidization
Interleaved intrachr. SVs	7
Total SVs (intrachr. + transl.)	7
SV types	DEL: 1; DUP: 2; h2hINV: 2; t2tINV: 2; TRA: 0
SVs in sample	237
Oscillating CN (2 and 3 states)	7, 7
CN segments	7
FDR fragment joints	0.95
FDR chr. breakp. enrich.	0
Linked to chrs	
Purity, ploidy	0.48, 1.9

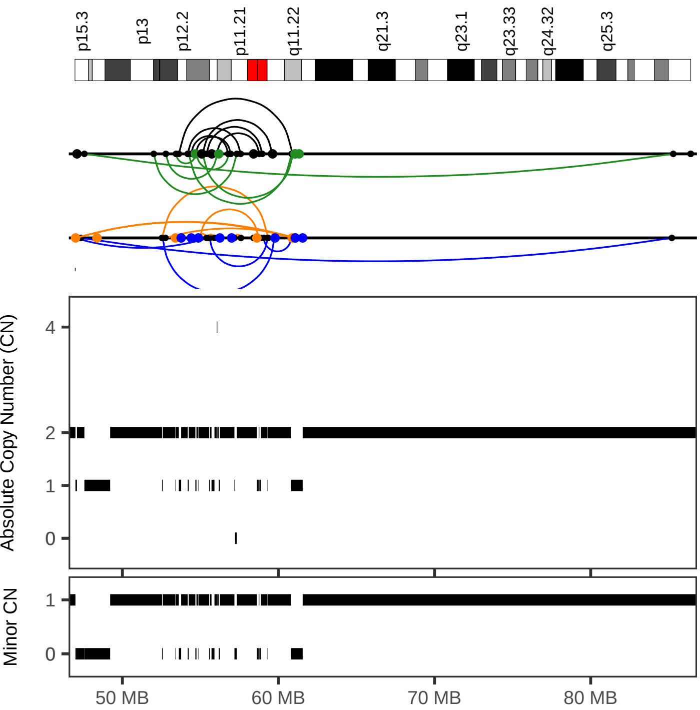

	CPCG0236
Cancer type	Prost−AdenoCA
Position	10:46962433−85287848
Type	With other complex events
Interleaved intrachr. SVs	28
Total SVs (intrachr. + transl.)	53
SV types	DEL: 9; DUP: 5; h2hINV: 7; t2tINV: 7; TRA: 25
SVs in sample	237
Oscillating CN (2 and 3 states)	21, 21
CN segments	36
FDR fragment joints	0.84
FDR chr. breakp. enrich.	0
Linked to chrs	11:39558829−123463707;5:97010270−106309340
Purity, ploidy	0.48, 1.9

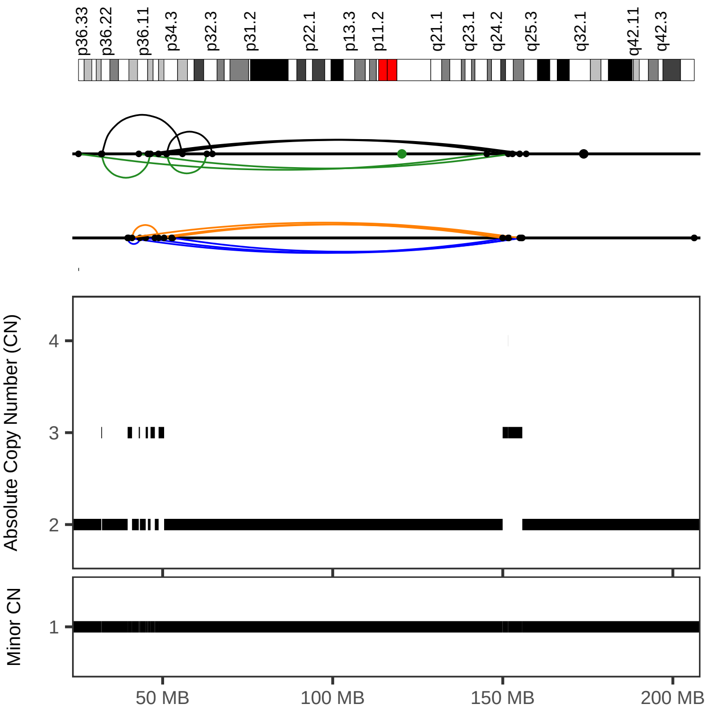

	CPCG0238
Cancer type	Prost−AdenoCA
Position	1:25248495−156885998
Type	Canonical without polyploidization
Interleaved intrachr. SVs	18
Total SVs (intrachr. + transl.)	19
SV types	DEL: 5; DUP: 4; h2hINV: 5; t2tINV: 4; TRA: 1
SVs in sample	132
Oscillating CN (2 and 3 states)	14, 17
CN segments	17
FDR fragment joints	0.98
FDR chr. breakp. enrich.	0
Linked to chrs	
Purity, ploidy	0.41, 1.91

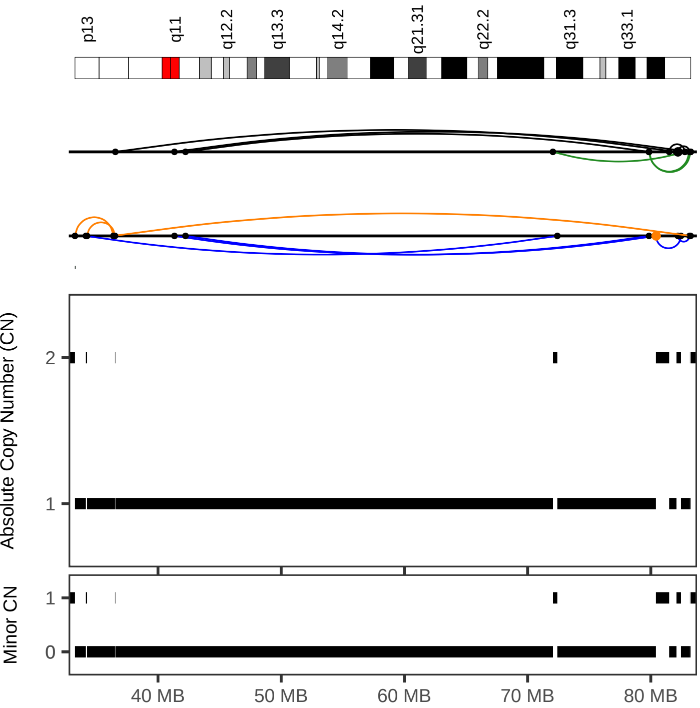

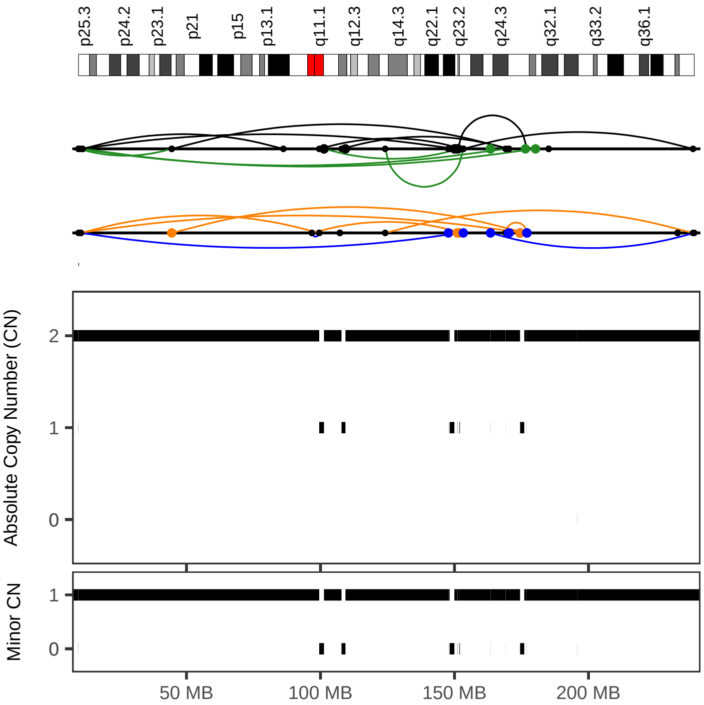

	**CPCG0238**
Cancer type	Prost−AdenoCA
Position	13:33264435−83241801
Type	Canonical without polyploidization
Interleaved intrachr. SVs	17
Total SVs (intrachr. + transl.)	19
SV types	DEL: 3; DUP: 5; h2hINV: 5; t2tINV: 4; TRA: 2
SVs in sample	132
Oscillating CN (2 and 3 states)	14, 14
CN segments	14
FDR fragment joints	0.92
FDR chr. breakp. enrich.	0
Linked to chrs	
Purity, ploidy	0.41, 1.91

	**CPCG0241**
Cancer type	Prost−AdenoCA
Position	2:9693687−239470346
Type	Canonical without polyploidization
Interleaved intrachr. SVs	21
Total SVs (intrachr. + transl.)	45
SV types	DEL: 6; DUP: 3; h2hINV: 7; t2tINV: 5; TRA: 24
SVs in sample	206
Oscillating CN (2 and 3 states)	23, 25
CN segments	25
FDR fragment joints	0.72
FDR chr. breakp. enrich.	0
Linked to chrs	3:53132905−194929595;
Purity, ploidy	0.42, 1.92

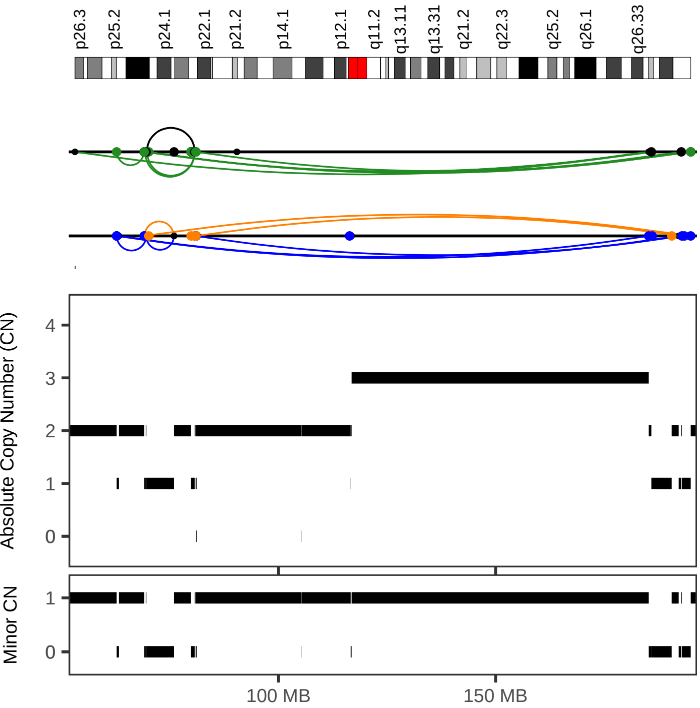

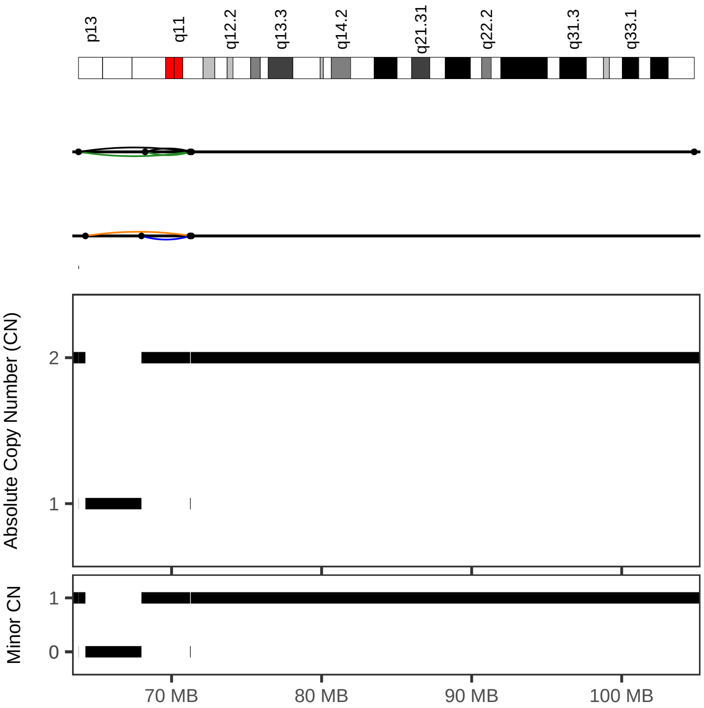

	**CPCG0241**
Cancer type	Prost−AdenoCA
Position	3:53132905−194929596
Type	With other complex events
Interleaved intrachr. SVs	24
Total SVs (intrachr. + transl.)	74
SV types	DEL: 4; DUP: 6; h2hINV: 5; t2tINV: 9; TRA: 50
SVs in sample	206
Oscillating CN (2 and 3 states)	15, 21
CN segments	29
FDR fragment joints	0.64
FDR chr. breakp. enrich.	0
Linked to chrs	18:6527402−73201775;2:9693687−239470345
Purity, ploidy	0.42, 1.92

	**CPCG0241**
Cancer type	Prost−AdenoCA
Position	13:63794744−71333917
Type	Canonical without polyploidization
Interleaved intrachr. SVs	7
Total SVs (intrachr. + transl.)	7
SV types	DEL: 2; DUP: 1; h2hINV: 2; t2tINV: 2; TRA: 0
SVs in sample	206
Oscillating CN (2 and 3 states)	8, 8
CN segments	8
FDR fragment joints	0.95
FDR chr. breakp. enrich.	0.9
Linked to chrs	
Purity, ploidy	0.42, 1.92

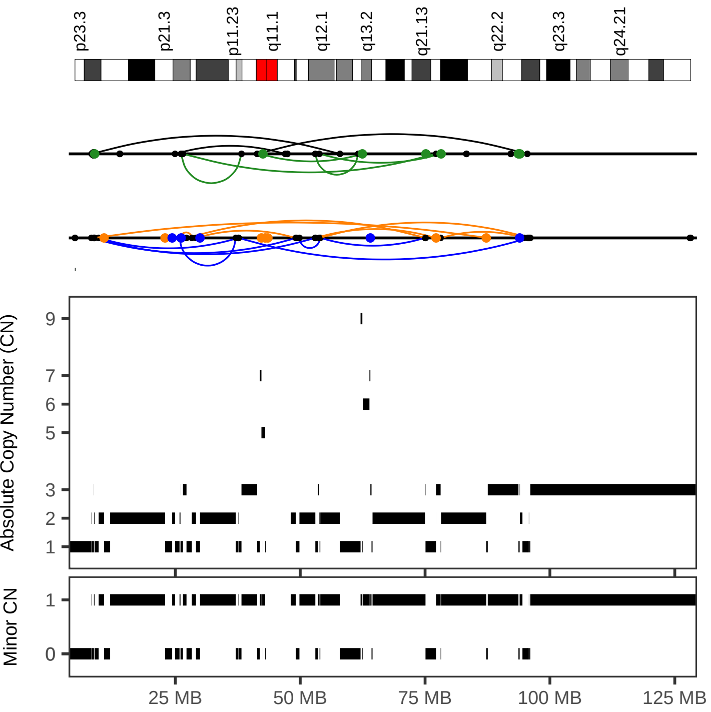

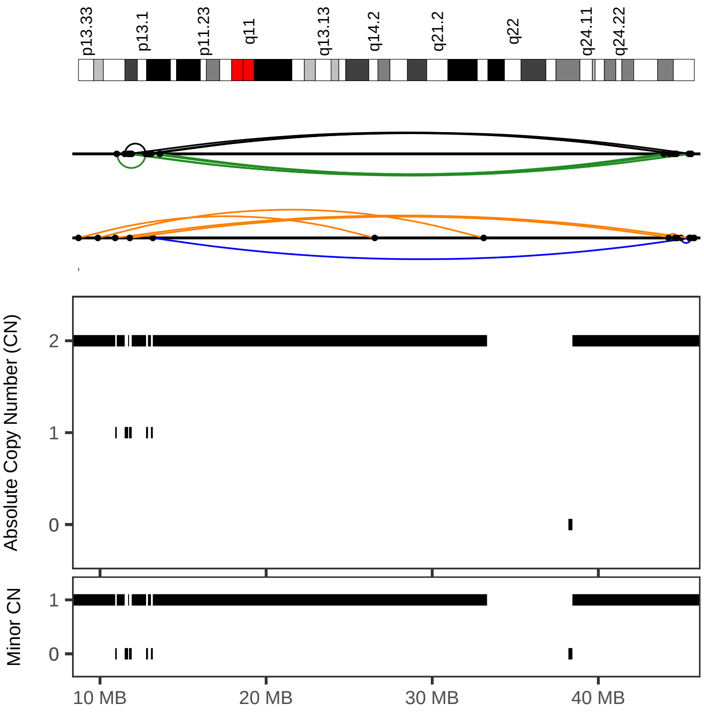

	**CPCG0242**
Cancer type	Prost−AdenoCA
Position	8:8188079−96079992
Type	With other complex events
Interleaved intrachr. SVs	23
Total SVs (intrachr. + transl.)	44
SV types	DEL: 7; DUP: 8; h2hINV: 3; t2tINV: 5; TRA: 21
SVs in sample	217
Oscillating CN (2 and 3 states)	11, 26
CN segments	66
FDR fragment joints	0.64
FDR chr. breakp. enrich.	0
Linked to chrs	19:14507324−55917568;
Purity, ploidy	0.65, 1.95

	**CPCG0246**
Cancer type	Prost−AdenoCA
Position	12:8702882−45773773
Type	Canonical without polyploidization
Interleaved intrachr. SVs	18
Total SVs (intrachr. + transl.)	18
SV types	DEL: 6; DUP: 2; h2hINV: 4; t2tINV: 6; TRA: 0
SVs in sample	25
Oscillating CN (2 and 3 states)	11, 13
CN segments	13
FDR fragment joints	0.64
FDR chr. breakp. enrich.	0
Linked to chrs	
Purity, ploidy	0.82, 1.93

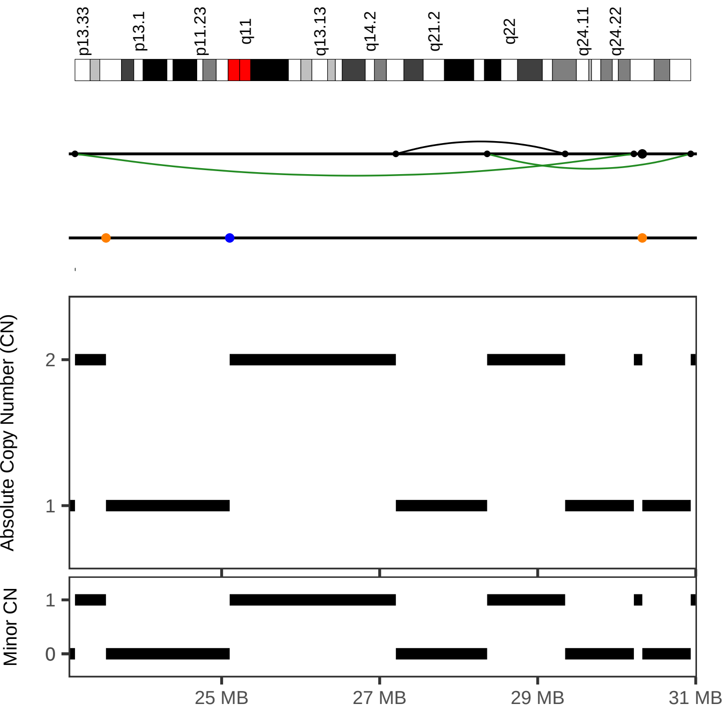

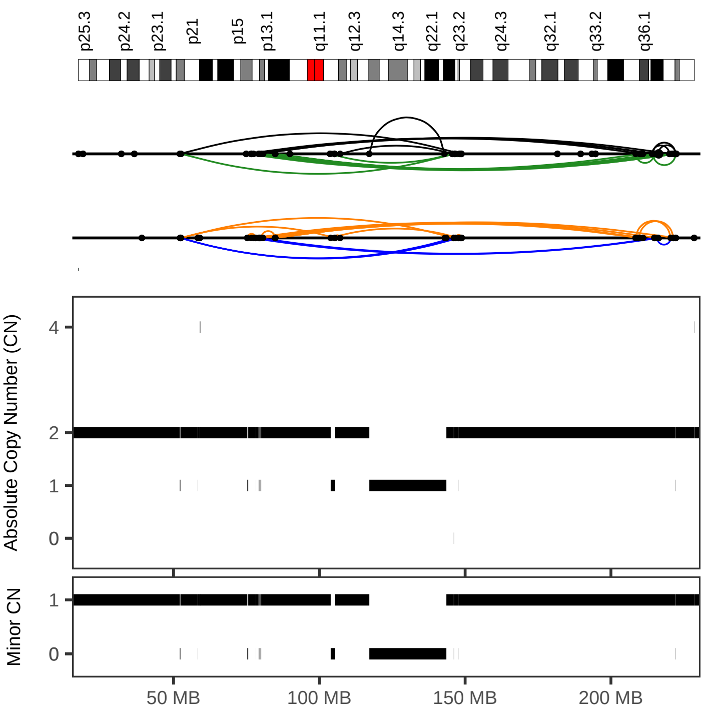

	**CPCG0248**
Cancer type	Prost−AdenoCA
Position	12:23143579−30936876
Type	Canonical without polyploidization
Interleaved intrachr. SVs	3
Total SVs (intrachr. + transl.)	7
SV types	DEL: 0; DUP: 0; h2hINV: 1; t2tINV: 2; TRA: 4
SVs in sample	110
Oscillating CN (2 and 3 states)	9, 9
CN segments	9
FDR fragment joints	0.59
FDR chr. breakp. enrich.	0.39
Linked to chrs	
Purity, ploidy	0.62, 1.89

	**CPCG0249**
Cancer type	Prost−AdenoCA
Position	2:52137503−222453077
Type	With other complex events
Interleaved intrachr. SVs	49
Total SVs (intrachr. + transl.)	50
SV types	DEL: 18; DUP: 5; h2hINV: 14; t2tINV: 12; TRA: 1
SVs in sample	458
Oscillating CN (2 and 3 states)	15, 21
CN segments	28
FDR fragment joints	0.48
FDR chr. breakp. enrich.	0
Linked to chrs	
Purity, ploidy	0.69, 1.87

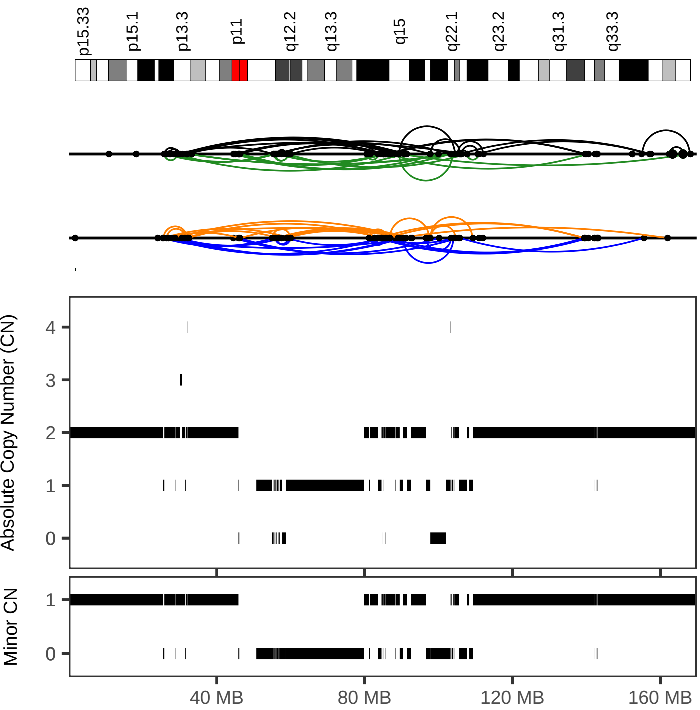

	CPCG0249
Cancer type	Prost−AdenoCA
Position	5:24023055−168167900
Type	With other complex events
Interleaved intrachr. SVs	99
Total SVs (intrachr. + transl.)	103
SV types	DEL: 22; DUP: 24; h2hINV: 29; t2tINV: 24; TRA: 4
SVs in sample	458
Oscillating CN (2 and 3 states)	20, 21
CN segments	78
FDR fragment joints	0.84
FDR chr. breakp. enrich.	0
Linked to chrs	8:12585867−142127031;
Purity, ploidy	0.69, 1.87

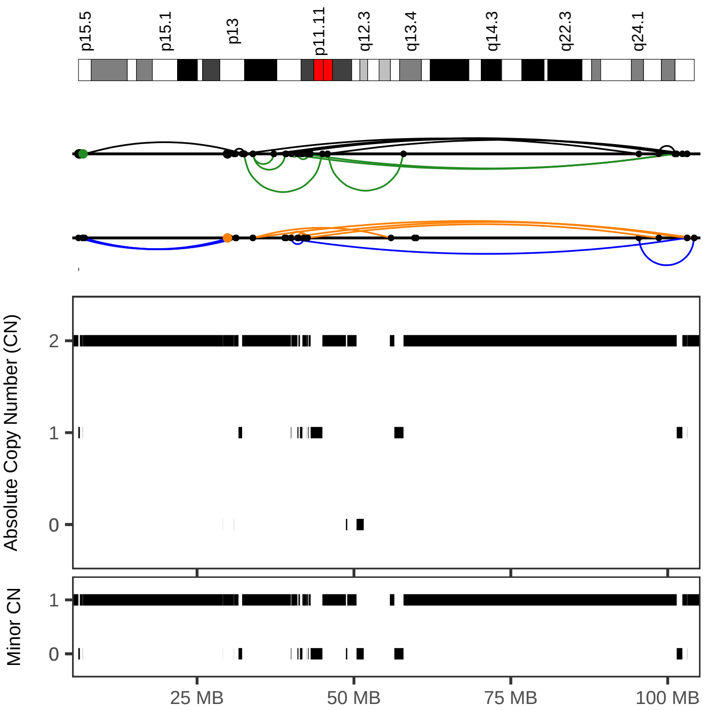

	CPCG0249
Cancer type	Prost−AdenoCA
Position	11:6102762−104228614
Type	With other complex events
Interleaved intrachr. SVs	33
Total SVs (intrachr. + transl.)	38
SV types	DEL: 7; DUP: 6; h2hINV: 9; t2tINV: 11; TRA: 5
SVs in sample	458
Oscillating CN (2 and 3 states)	15, 32
CN segments	32
FDR fragment joints	0.69
FDR chr. breakp. enrich.	0
Linked to chrs	13:82314051−113900733;
Purity, ploidy	0.69, 1.87

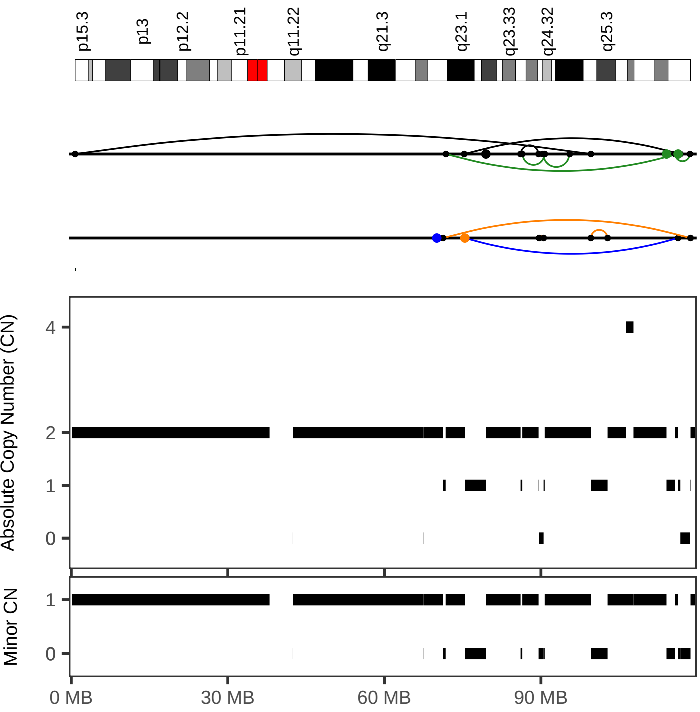

	CPCG0256
Cancer type	Prost−AdenoCA
Position	10:753210−118664596
Type	With other complex events
Interleaved intrachr. SVs	8
Total SVs (intrachr. + transl.)	13
SV types	DEL: 2; DUP: 2; h2hINV: 2; t2tINV: 2; TRA: 5
SVs in sample	64
Oscillating CN (2 and 3 states)	9, 15
CN segments	26
FDR fragment joints	1
FDR chr. breakp. enrich.	0
Linked to chrs	5:137274453−173139889;
Purity, ploidy	0.65, 1.91

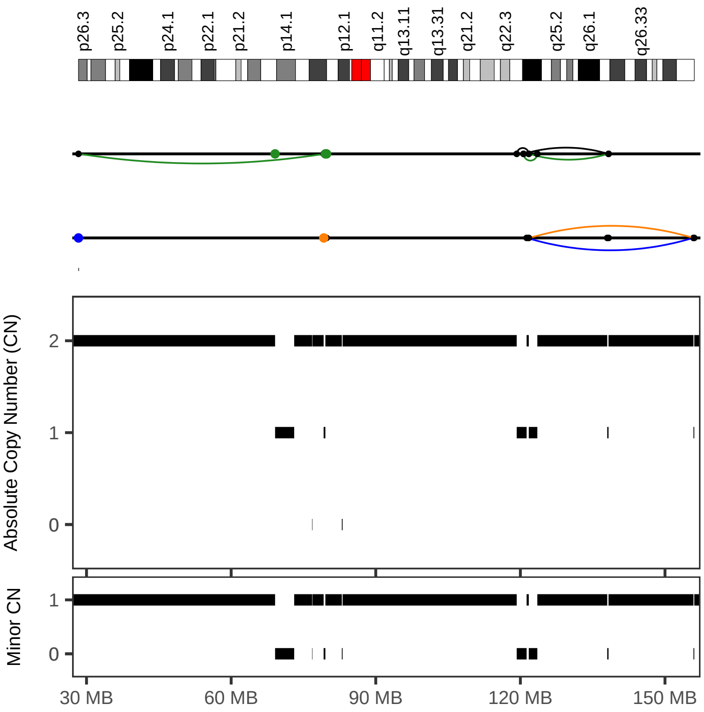

	CPCG0258
Cancer type	Prost−AdenoCA
Position	3:119241441−156069152
Type	Canonical without polyploidization
Interleaved intrachr. SVs	7
Total SVs (intrachr. + transl.)	7
SV types	DEL: 2; DUP: 1; h2hINV: 2; t2tINV: 2; TRA: 0
SVs in sample	45
Oscillating CN (2 and 3 states)	8, 8
CN segments	8
FDR fragment joints	0.95
FDR chr. breakp. enrich.	0
Linked to chrs	
Purity, ploidy	0.3, 1.92

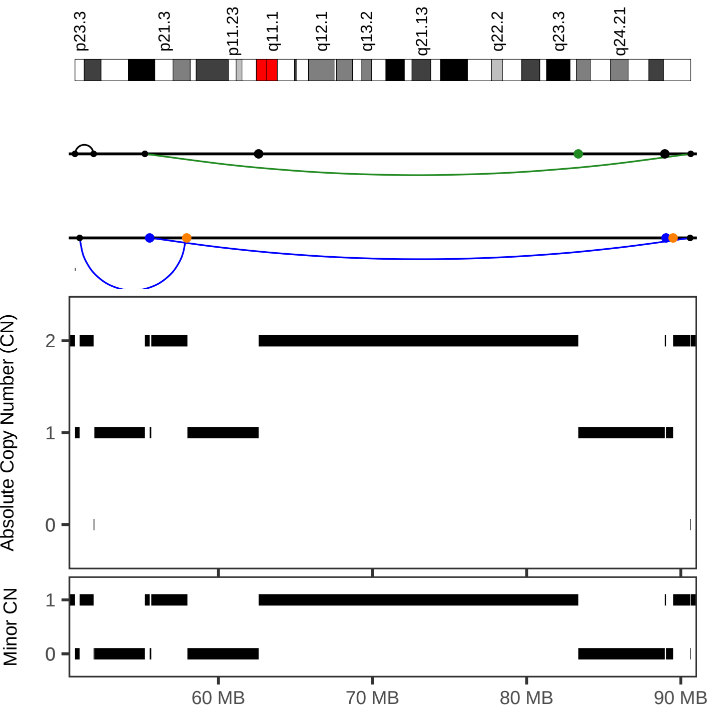

	**CPCG0263**
Cancer type	Prost−AdenoCA
Position	8:50690802−90644745
Type	Canonical without polyploidization
Interleaved intrachr. SVs	4
Total SVs (intrachr. + transl.)	11
SV types	DEL: 0; DUP: 2; h2hINV: 1;<br>t2tINV: 1; TRA: 7
SVs in sample	67
Oscillating CN (2 and 3 states)	10, 12
CN segments	15
FDR fragment joints	0.64
FDR chr. breakp. enrich.	0
Linked to chrs	11:121125846−122406012;
Purity, ploidy	0.39, 1.97

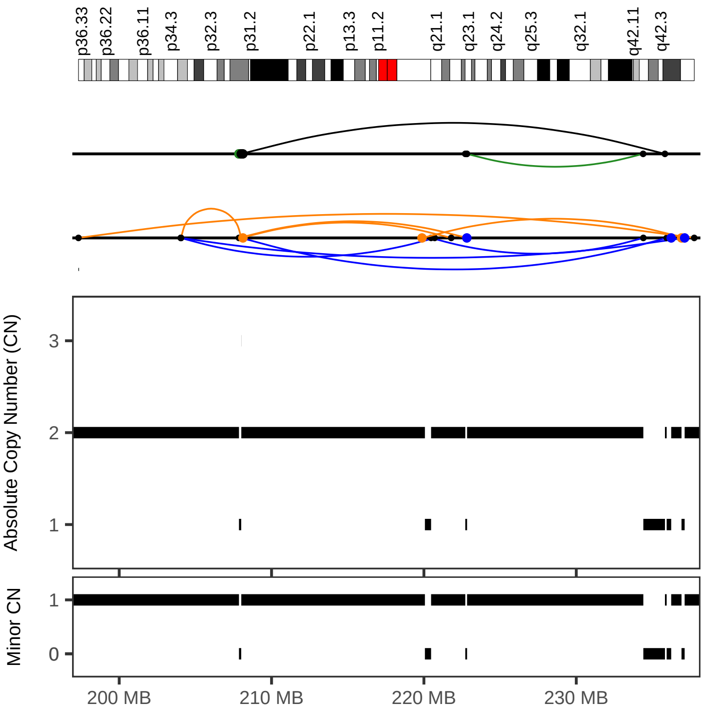

	**CPCG0267**
Cancer type	Prost−AdenoCA
Position	1:204042528−237113743
Type	Canonical without polyploidization
Interleaved intrachr. SVs	10
Total SVs (intrachr. + transl.)	20
SV types	DEL: 4; DUP: 4; h2hINV: 1;<br>t2tINV: 1; TRA: 10
SVs in sample	63
Oscillating CN (2 and 3 states)	11, 11
CN segments	14
FDR fragment joints	0.59
FDR chr. breakp. enrich.	0
Linked to chrs	3:10350196−84771996;
Purity, ploidy	0.91, 1.93

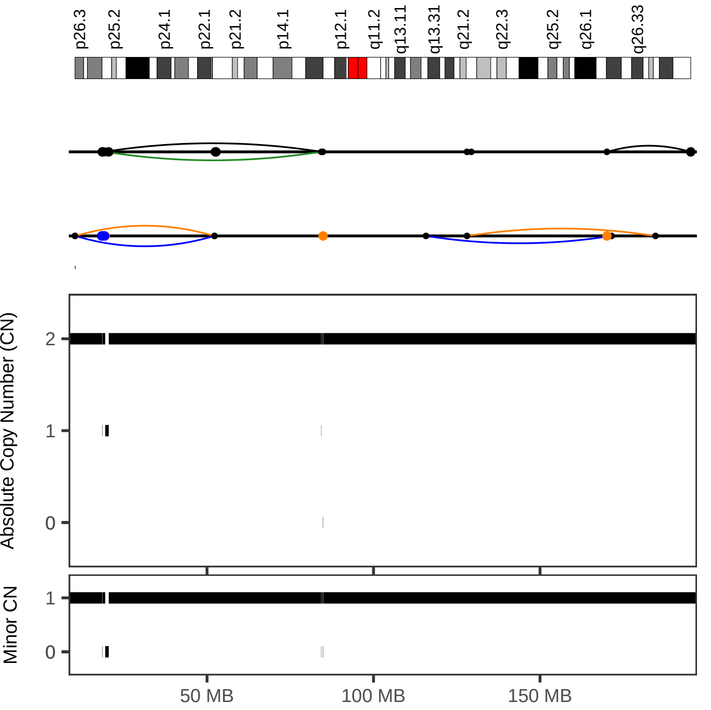

	**CPCG0267**
Cancer type	Prost−AdenoCA
Position	3:10350196−84771997
Type	Canonical without polyploidization
Interleaved intrachr. SVs	4
Total SVs (intrachr. + transl.)	15
SV types	DEL: 1; DUP: 1; h2hINV: 1;<br>t2tINV: 1; TRA: 11
SVs in sample	63
Oscillating CN (2 and 3 states)	7, 8
CN segments	8
FDR fragment joints	1
FDR chr. breakp. enrich.	0
Linked to chrs	1:204042528−237113742;
Purity, ploidy	0.91, 1.93

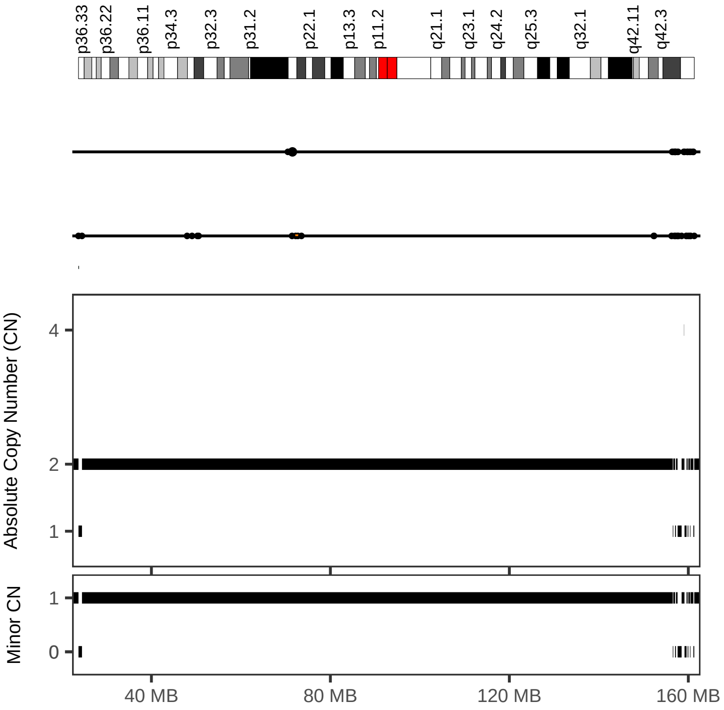

	**CPCG0268**
Cancer type	Prost−AdenoCA
Position	1:156391329−161122378
Type	With other complex events
Interleaved intrachr. SVs	18
Total SVs (intrachr. + transl.)	18
SV types	DEL: 4; DUP: 2; h2hINV: 6;<br>t2tINV: 6; TRA: 0
SVs in sample	135
Oscillating CN (2 and 3 states)	10, 20
CN segments	20
FDR fragment joints	0.64
FDR chr. breakp. enrich.	0
Linked to chrs	
Purity, ploidy	0.34, 1.89

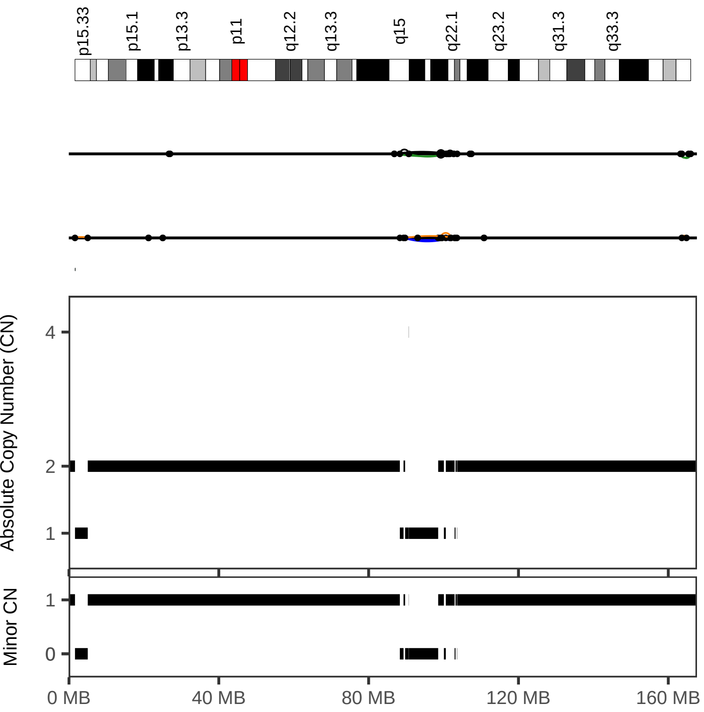

	**CPCG0268**
Cancer type	Prost−AdenoCA
Position	5:86850288−103636603
Type	Canonical without polyploidization
Interleaved intrachr. SVs	14
Total SVs (intrachr. + transl.)	15
SV types	DEL: 2; DUP: 4; h2hINV: 4; t2tINV: 4; TRA: 1
SVs in sample	135
Oscillating CN (2 and 3 states)	8, 13
CN segments	13
FDR fragment joints	0.88
FDR chr. breakp. enrich.	0
Linked to chrs	
Purity, ploidy	0.34, 1.89

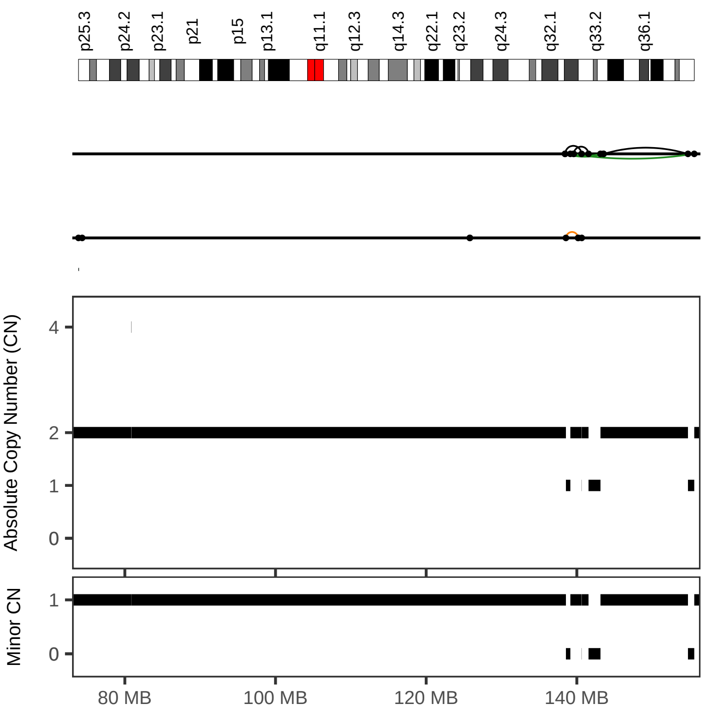

	**CPCG0324**
Cancer type	Prost−AdenoCA
Position	2:138429668−155586709
Type	Canonical without polyploidization
Interleaved intrachr. SVs	8
Total SVs (intrachr. + transl.)	8
SV types	DEL: 2; DUP: 0; h2hINV: 3; t2tINV: 3; TRA: 0
SVs in sample	81
Oscillating CN (2 and 3 states)	9, 9
CN segments	9
FDR fragment joints	0.59
FDR chr. breakp. enrich.	0.09
Linked to chrs	
Purity, ploidy	0.5, 1.87

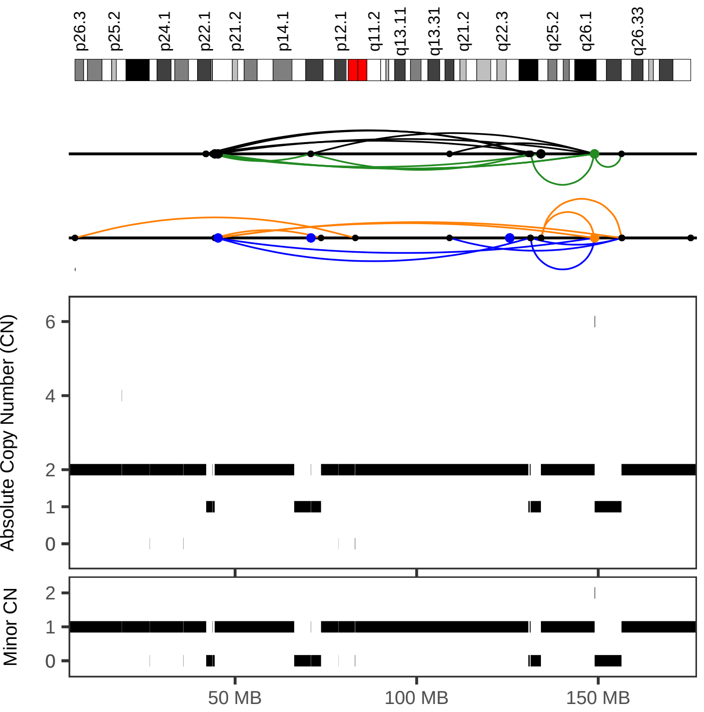

	**CPCG0331**
Cancer type	Prost−AdenoCA
Position	3:5905087−156509264
Type	With other complex events
Interleaved intrachr. SVs	25
Total SVs (intrachr. + transl.)	35
SV types	DEL: 6; DUP: 5; h2hINV: 6; t2tINV: 8; TRA: 10
SVs in sample	123
Oscillating CN (2 and 3 states)	9, 21
CN segments	27
FDR fragment joints	0.9
FDR chr. breakp. enrich.	0
Linked to chrs	2:71318777−235597577;
Purity, ploidy	0.41, 1.88

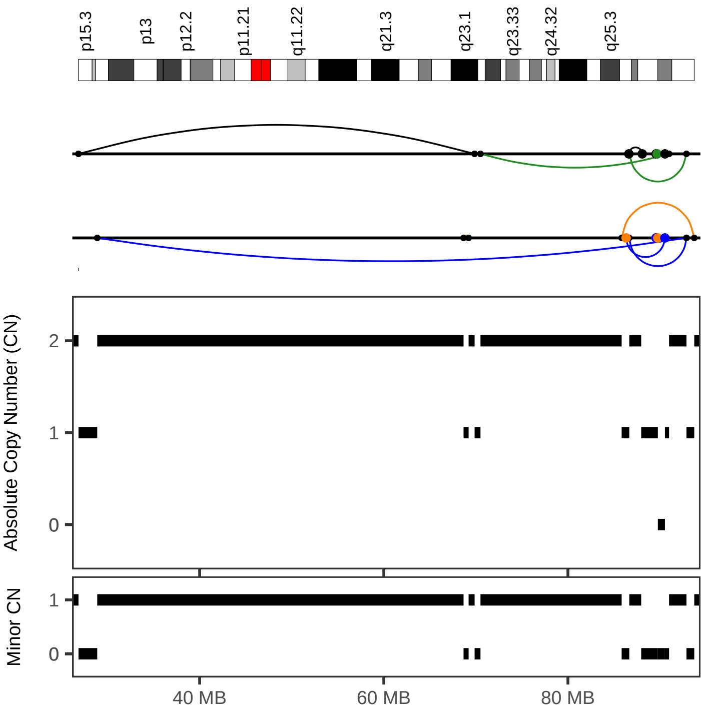

	**CPCG0331**
Cancer type	Prost−AdenoCA
Position	10:26837814−93718569
Type	Canonical without polyploidization
Interleaved intrachr. SVs	8
Total SVs (intrachr. + transl.)	18
SV types	DEL: 1; DUP: 3; h2hINV: 2; t2tINV: 2; TRA: 10
SVs in sample	123
Oscillating CN (2 and 3 states)	9, 14
CN segments	14
FDR fragment joints	0.84
FDR chr. breakp. enrich.	0
Linked to chrs	
Purity, ploidy	0.41, 1.88

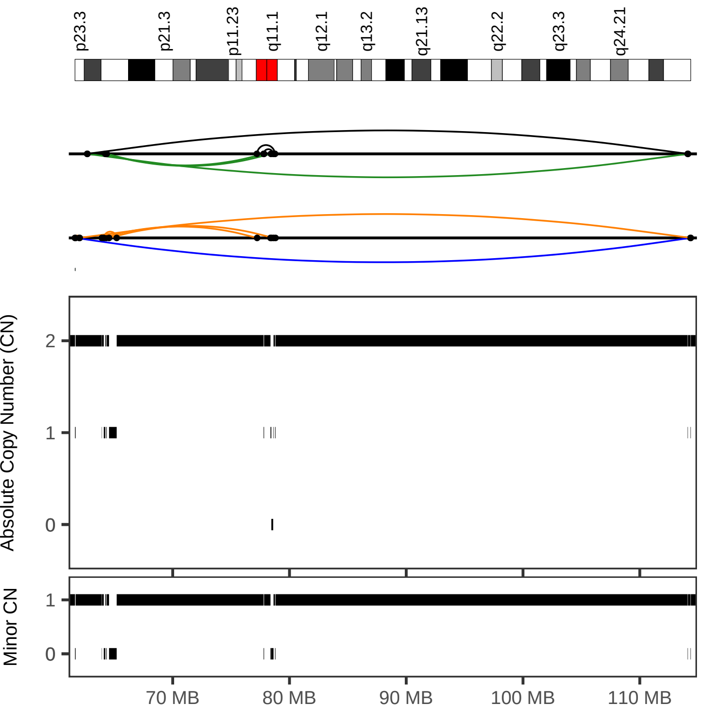

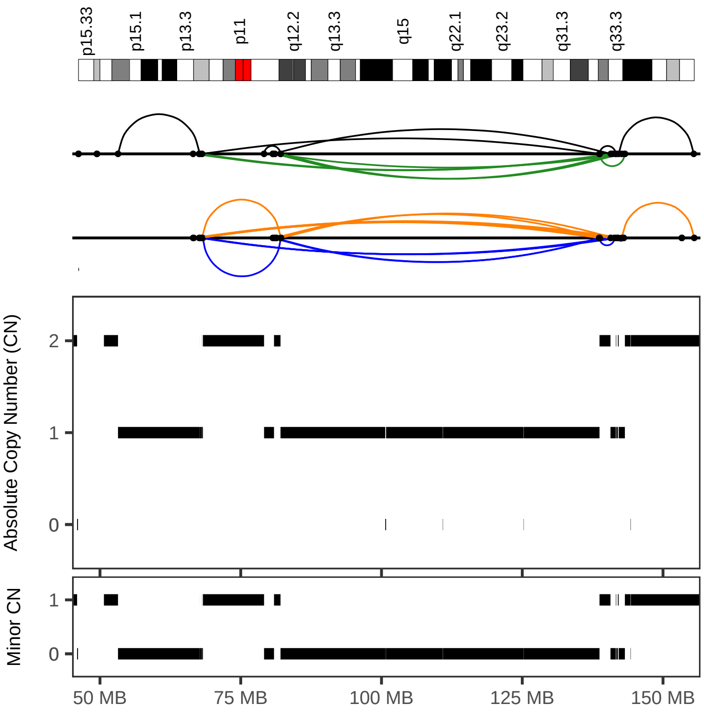

	**CPCG0336**
Cancer type	Prost−AdenoCA
Position	8:63930625−78804766
Type	Canonical without polyploidization
Interleaved intrachr. SVs	9
Total SVs (intrachr. + transl.)	9
SV types	DEL: 4; DUP: 1; h2hINV: 2; t2tINV: 2; TRA: 0
SVs in sample	68
Oscillating CN (2 and 3 states)	11, 16
CN segments	16
FDR fragment joints	0.64
FDR chr. breakp. enrich.	0
Linked to chrs	
Purity, ploidy	0.39, 1.93

	**CPCG0340**
Cancer type	Prost−AdenoCA
Position	5:53204904−155545927
Type	With other complex events
Interleaved intrachr. SVs	29
Total SVs (intrachr. + transl.)	29
SV types	DEL: 9; DUP: 8; h2hINV: 6; t2tINV: 6; TRA: 0
SVs in sample	139
Oscillating CN (2 and 3 states)	9, 22
CN segments	24
FDR fragment joints	0.86
FDR chr. breakp. enrich.	0
Linked to chrs	
Purity, ploidy	0.6, 1.9

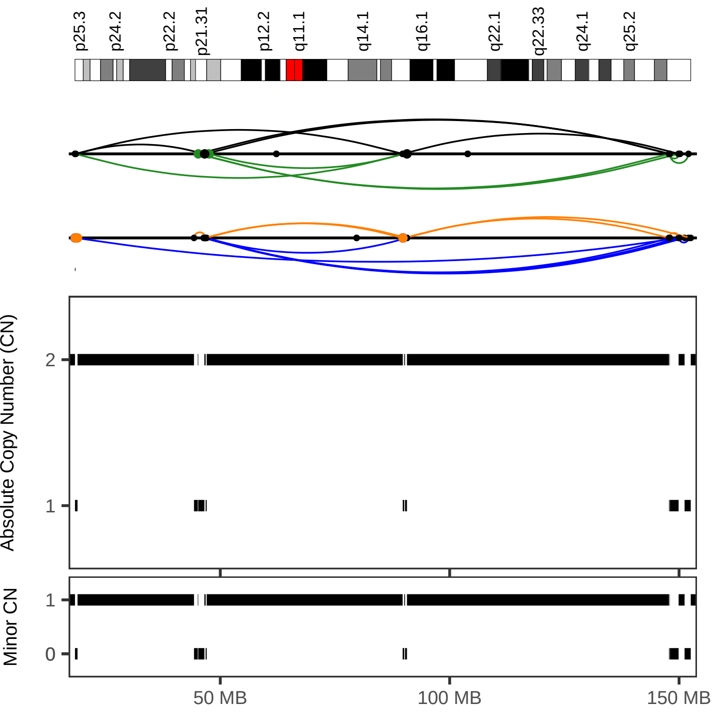

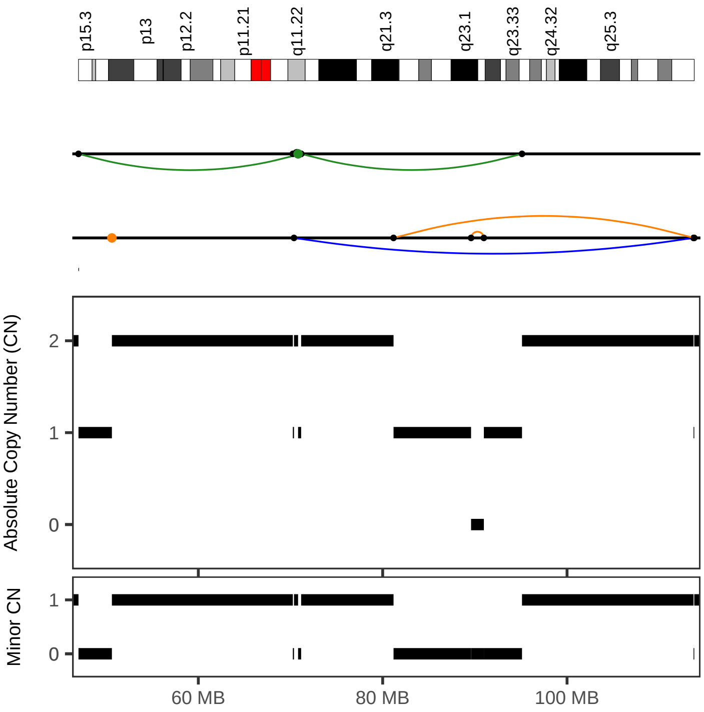

	**CPCG0340**
Cancer type	Prost−AdenoCA
Position	6:18322627−152548190
Type	Canonical without polyploidization
Interleaved intrachr. SVs	28
Total SVs (intrachr. + transl.)	39
SV types	DEL: 6; DUP: 9; h2hINV: 6; t2tINV: 7; TRA: 11
SVs in sample	139
Oscillating CN (2 and 3 states)	22, 22
CN segments	22
FDR fragment joints	0.88
FDR chr. breakp. enrich.	0
Linked to chrs	
Purity, ploidy	0.6, 1.9

	**CPCG0342**
Cancer type	Prost−AdenoCA
Position	10:46999321−113799131
Type	With other complex events
Interleaved intrachr. SVs	5
Total SVs (intrachr. + transl.)	7
SV types	DEL: 1; DUP: 1; h2hINV: 1; t2tINV: 2; TRA: 2
SVs in sample	72
Oscillating CN (2 and 3 states)	7, 12
CN segments	12
FDR fragment joints	0.92
FDR chr. breakp. enrich.	0.07
Linked to chrs	
Purity, ploidy	0.34, 1.92

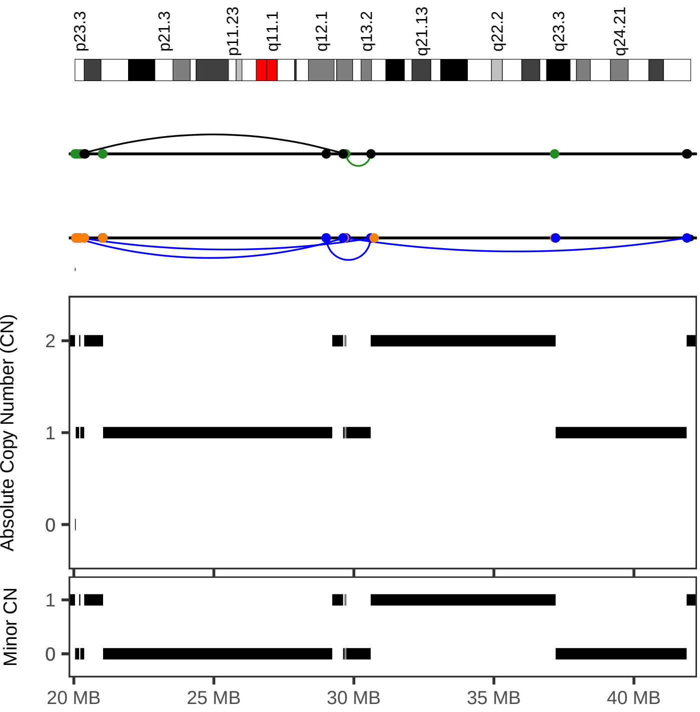

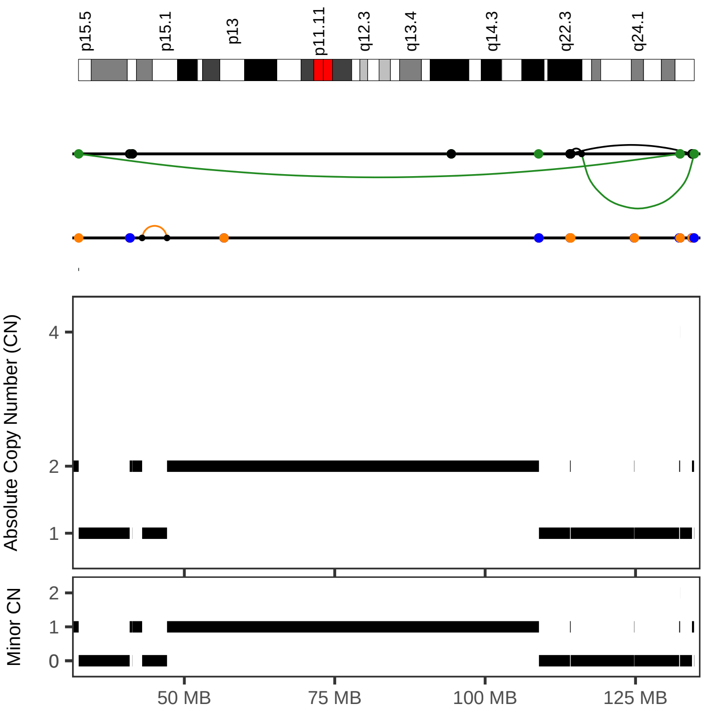

CPCG0350

Cancer type	Prost−AdenoCA
Position	8:20040649−42029377
Type	Canonical without polyploidization
Interleaved intrachr. SVs	8
Total SVs (intrachr. + transl.)	53
SV types	DEL: 0; DUP: 6; h2hINV: 1; t2tINV: 1; TRA: 45
SVs in sample	202
Oscillating CN (2 and 3 states)	14, 16
CN segments	16
FDR fragment joints	0.21
FDR chr. breakp. enrich.	0
Linked to chrs	18:4244316−74665299;
Purity, ploidy	0.74, 1.9

CPCG0350

Cancer type	Prost−AdenoCA
Position	11:32402370−134754988
Type	Canonical without polyploidization
Interleaved intrachr. SVs	4
Total SVs (intrachr. + transl.)	45
SV types	DEL: 0; DUP: 0; h2hINV: 2; t2tINV: 2; TRA: 41
SVs in sample	202
Oscillating CN (2 and 3 states)	14, 18
CN segments	18
FDR fragment joints	0.59
FDR chr. breakp. enrich.	0
Linked to chrs	8:20040649−42029376;18:4244316−74665299
Purity, ploidy	0.74, 1.9

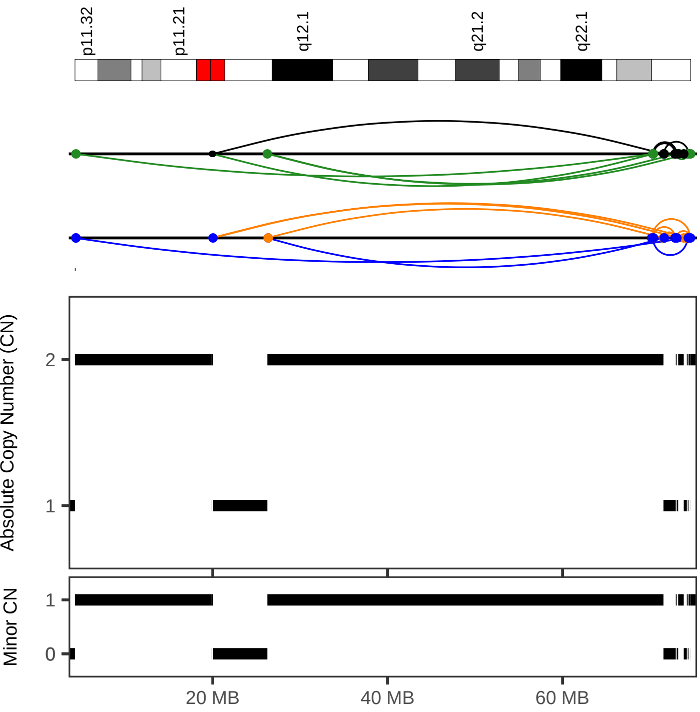

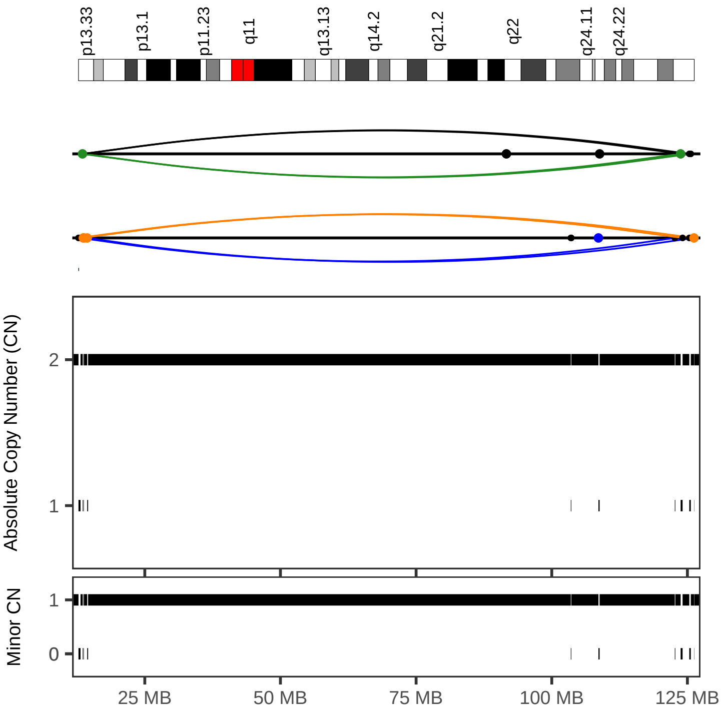

CPCG0350

Cancer type	Prost−AdenoCA
Position	18:4244316−74665300
Type	Canonical without polyploidization
Interleaved intrachr. SVs	22
Total SVs (intrachr. + transl.)	75
SV types	DEL: 7; DUP: 5; h2hINV: 5; t2tINV: 5; TRA: 53
SVs in sample	202
Oscillating CN (2 and 3 states)	19, 19
CN segments	19
FDR fragment joints	0.93
FDR chr. breakp. enrich.	0
Linked to chrs	8:20040649−42029376;
Purity, ploidy	0.74, 1.9

CPCG0354

Cancer type	Prost−AdenoCA
Position	12:12779174−126256048
Type	Canonical without polyploidization
Interleaved intrachr. SVs	9
Total SVs (intrachr. + transl.)	18
SV types	DEL: 3; DUP: 2; h2hINV: 2; t2tINV: 2; TRA: 9
SVs in sample	113
Oscillating CN (2 and 3 states)	19, 19
CN segments	19
FDR fragment joints	0.97
FDR chr. breakp. enrich.	0
Linked to chrs	16:29187104−85721630;
Purity, ploidy	0.97, 1.92

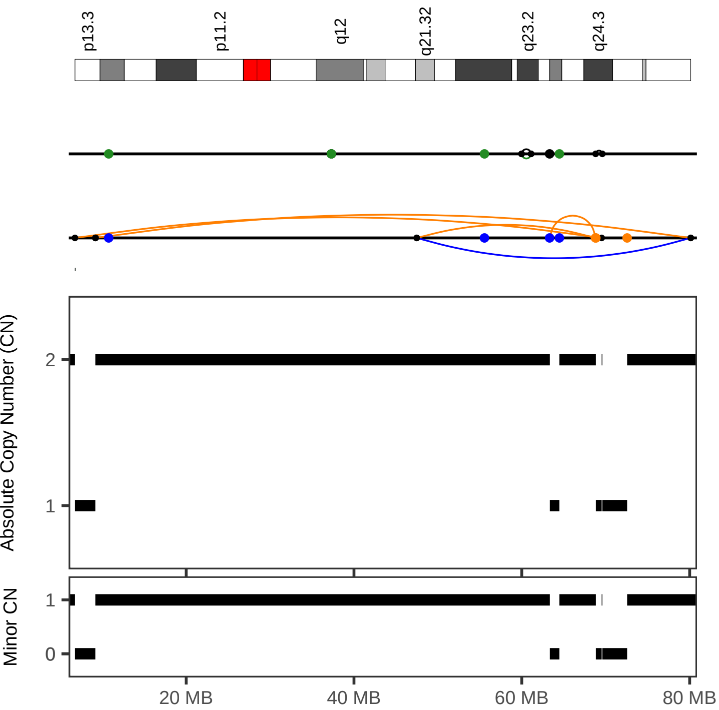

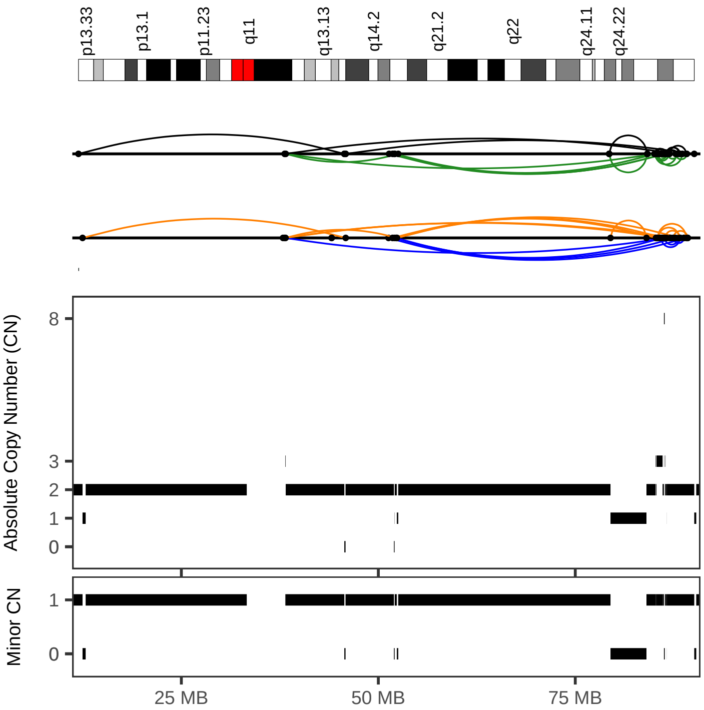

	**CPCG0354**
Cancer type	Prost−AdenoCA
Position	17:6755315−80126854
Type	Canonical without polyploidization
Interleaved intrachr. SVs	7
Total SVs (intrachr. + transl.)	20
SV types	DEL: 5; DUP: 1; h2hINV: 1; t2tINV: 0; TRA: 13
SVs in sample	113
Oscillating CN (2 and 3 states)	8, 8
CN segments	8
FDR fragment joints	0.38
FDR chr. breakp. enrich.	0
Linked to chrs	
Purity, ploidy	0.97, 1.92

	**CPCG0360**
Cancer type	Prost−AdenoCA
Position	12:11938733−90103272
Type	With other complex events
Interleaved intrachr. SVs	49
Total SVs (intrachr. + transl.)	49
SV types	DEL: 13; DUP: 9; h2hINV: 13; t2tINV: 14; TRA: 0
SVs in sample	171
Oscillating CN (2 and 3 states)	8, 14
CN segments	30
FDR fragment joints	0.83
FDR chr. breakp. enrich.	0
Linked to chrs	
Purity, ploidy	0.25, 1.86

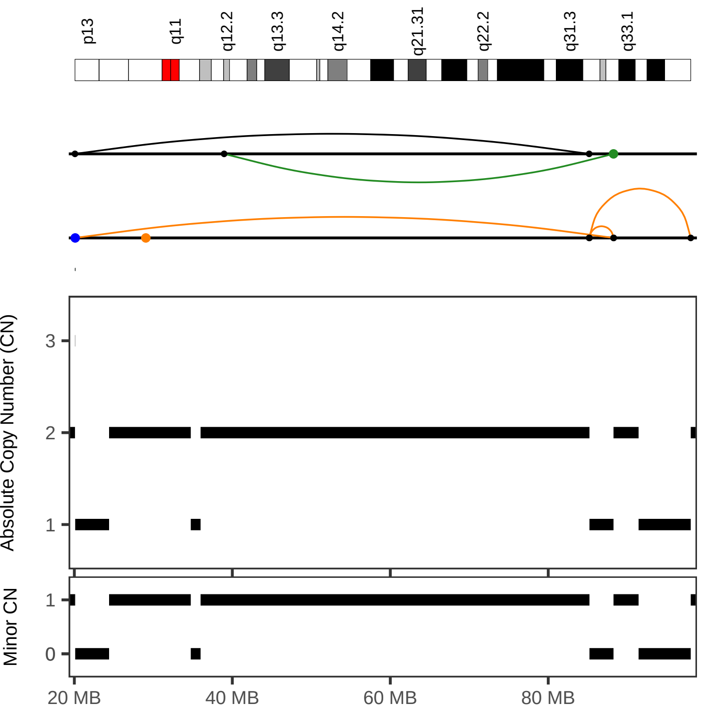

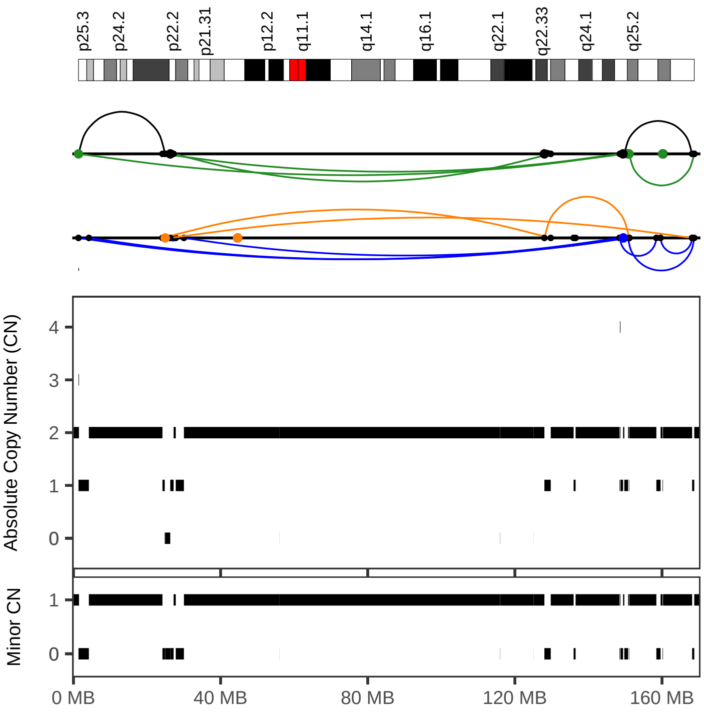

	**CPCG0362**
Cancer type	Prost−AdenoCA
Position	13:20078680−98039016
Type	Canonical without polyploidization
Interleaved intrachr. SVs	5
Total SVs (intrachr. + transl.)	8
SV types	DEL: 3; DUP: 0; h2hINV: 1; t2tINV: 1; TRA: 3
SVs in sample	48
Oscillating CN (2 and 3 states)	8, 9
CN segments	9
FDR fragment joints	0.59
FDR chr. breakp. enrich.	0
Linked to chrs	
Purity, ploidy	0.97, 1.93

	**CPCG0364**
Cancer type	Prost−AdenoCA
Position	6:1360399−168764390
Type	With other complex events
Interleaved intrachr. SVs	18
Total SVs (intrachr. + transl.)	31
SV types	DEL: 3; DUP: 7; h2hINV: 4; t2tINV: 4; TRA: 13
SVs in sample	78
Oscillating CN (2 and 3 states)	14, 14
CN segments	38
FDR fragment joints	0.64
FDR chr. breakp. enrich.	0
Linked to chrs	4:40146550−96108207;
Purity, ploidy	0.63, 1.91

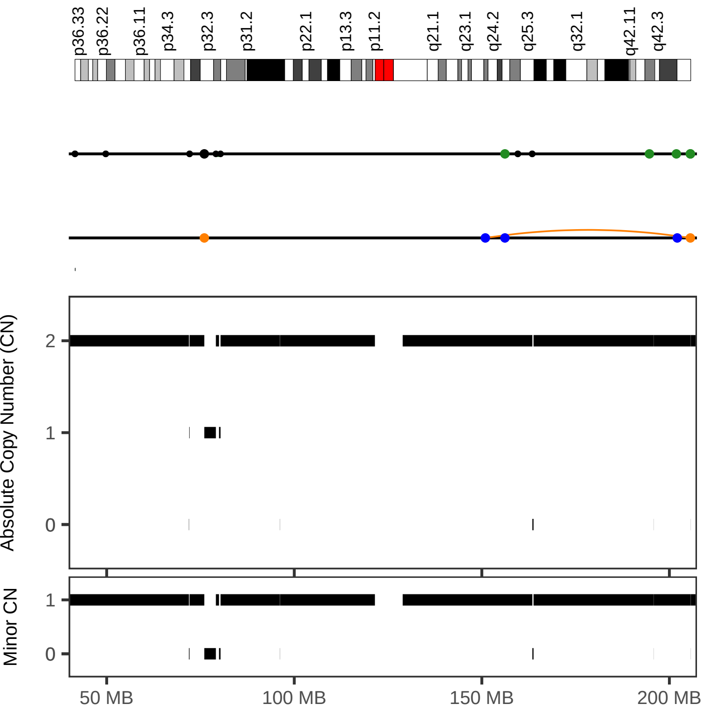

	CPCG0366
Cancer type	Prost−AdenoCA
Position	1:150909043−205693972
Type	Canonical without polyploidization
Interleaved intrachr. SVs	3
Total SVs (intrachr. + transl.)	12
SV types	DEL: 1; DUP: 1; h2hINV: 0; t2tINV: 1; TRA: 9
SVs in sample	213
Oscillating CN (2 and 3 states)	7, 7
CN segments	7
FDR fragment joints	0.84
FDR chr. breakp. enrich.	0.62
Linked to chrs	6:167735883−169038472;13:20995521−107382175
Purity, ploidy	0.55, 1.96

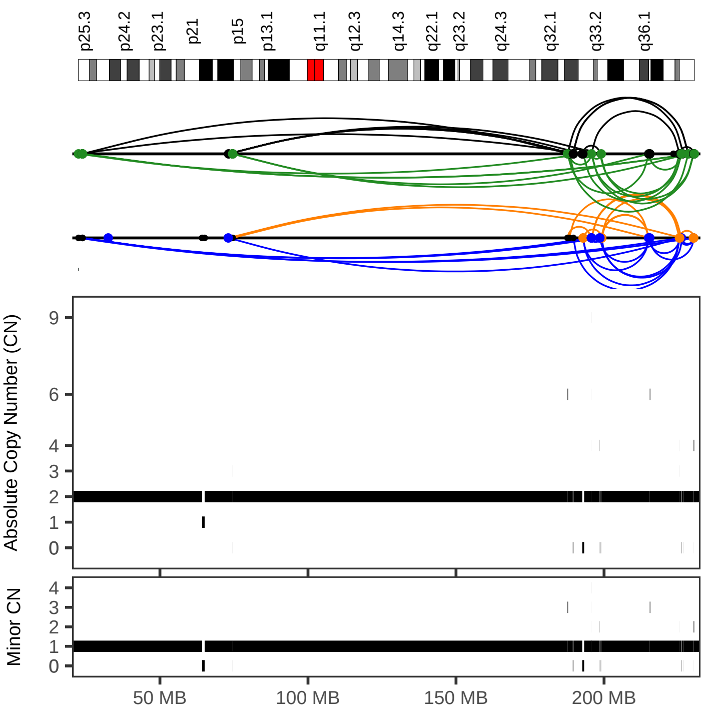

	CPCG0366
Cancer type	Prost−AdenoCA
Position	2:22493101−230456464
Type	With other complex events
Interleaved intrachr. SVs	62
Total SVs (intrachr. + transl.)	117
SV types	DEL: 12; DUP: 16; h2hINV: 16; t2tINV: 18; TRA: 55
SVs in sample	213
Oscillating CN (2 and 3 states)	7, 8
CN segments	44
FDR fragment joints	0.83
FDR chr. breakp. enrich.	0
Linked to chrs	6:167735883−169038472;
Purity, ploidy	0.55, 1.96

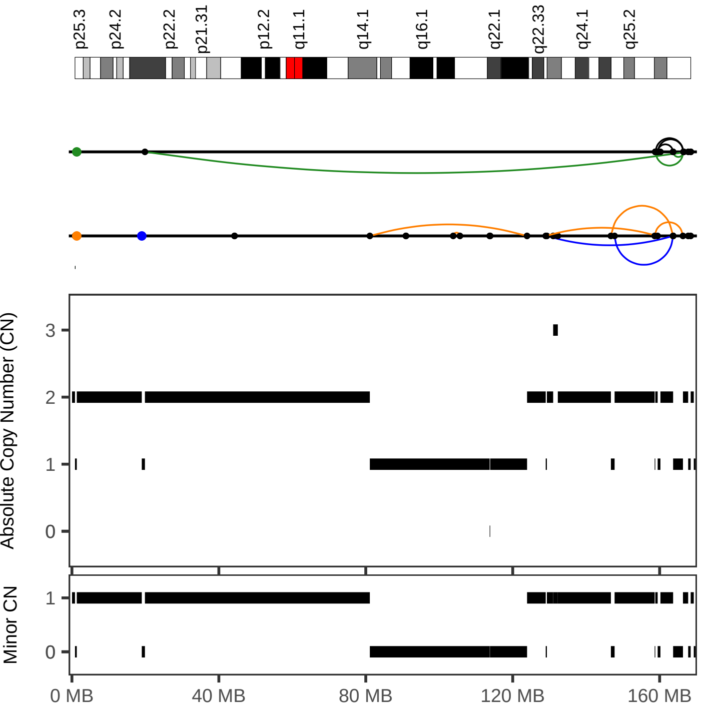

	CPCG0369
Cancer type	Prost−AdenoCA
Position	6:128987682−166522109
Type	Canonical without polyploidization
Interleaved intrachr. SVs	11
Total SVs (intrachr. + transl.)	11
SV types	DEL: 3; DUP: 2; h2hINV: 3; t2tINV: 3; TRA: 0
SVs in sample	131
Oscillating CN (2 and 3 states)	9, 12
CN segments	12
FDR fragment joints	0.98
FDR chr. breakp. enrich.	0
Linked to chrs	
Purity, ploidy	0.52, 1.89

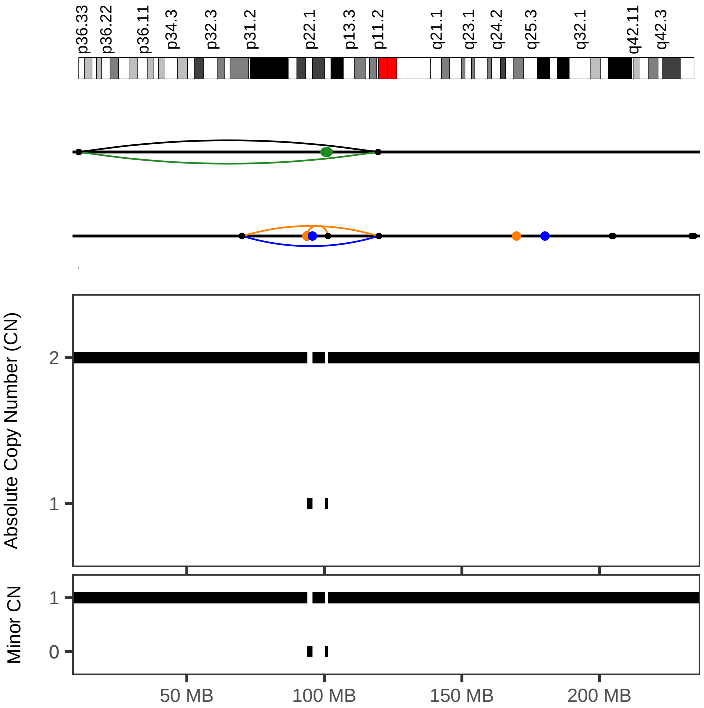

	CPCG0372
Cancer type	Prost−AdenoCA
Position	1:10695500−119824570
Type	Canonical without polyploidization
Interleaved intrachr. SVs	4
Total SVs (intrachr. + transl.)	10
SV types	DEL: 1; DUP: 1; h2hINV: 1; t2tINV: 1; TRA: 6
SVs in sample	86
Oscillating CN (2 and 3 states)	7, 7
CN segments	7
FDR fragment joints	1
FDR chr. breakp. enrich.	0.02
Linked to chrs	
Purity, ploidy	0.53, 1.91

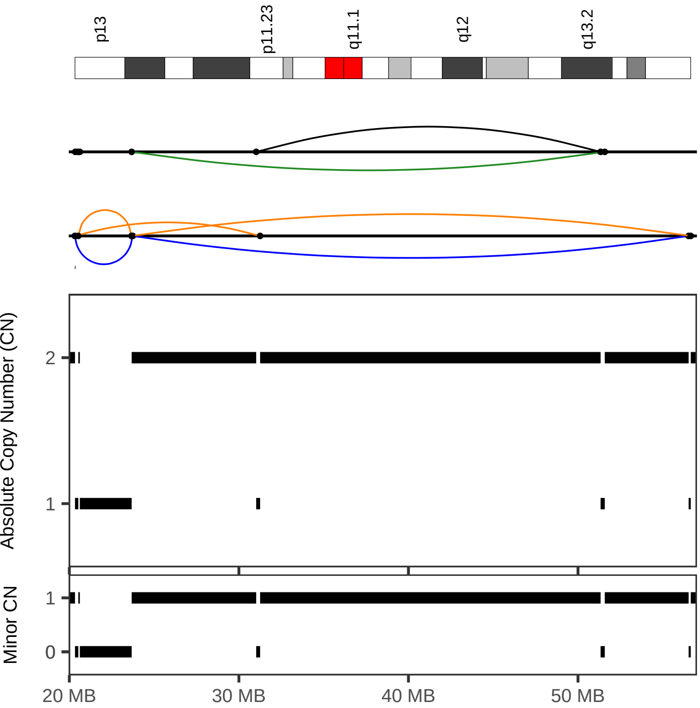

	CPCG0372
Cancer type	Prost−AdenoCA
Position	20:20333170−56648940
Type	Canonical without polyploidization
Interleaved intrachr. SVs	6
Total SVs (intrachr. + transl.)	6
SV types	DEL: 2; DUP: 2; h2hINV: 1; t2tINV: 1; TRA: 0
SVs in sample	86
Oscillating CN (2 and 3 states)	11, 11
CN segments	11
FDR fragment joints	0.91
FDR chr. breakp. enrich.	0
Linked to chrs	
Purity, ploidy	0.53, 1.91

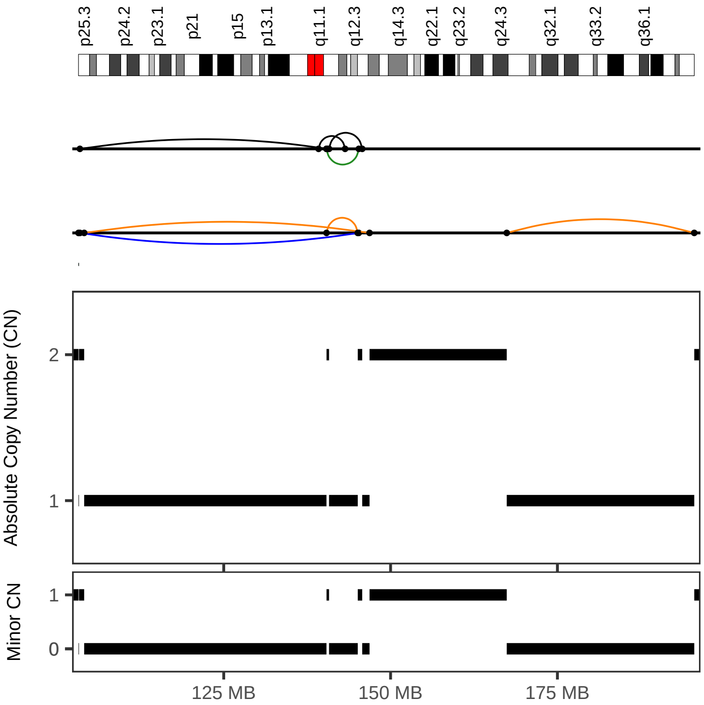

	CPCG0375
Cancer type	Prost−AdenoCA
Position	2:103449140−146851707
Type	Canonical without polyploidization
Interleaved intrachr. SVs	8
Total SVs (intrachr. + transl.)	8
SV types	DEL: 2; DUP: 1; h2hINV: 3; t2tINV: 2; TRA: 0
SVs in sample	88
Oscillating CN (2 and 3 states)	9, 9
CN segments	9
FDR fragment joints	0.84
FDR chr. breakp. enrich.	0.58
Linked to chrs	
Purity, ploidy	0.46, 1.87

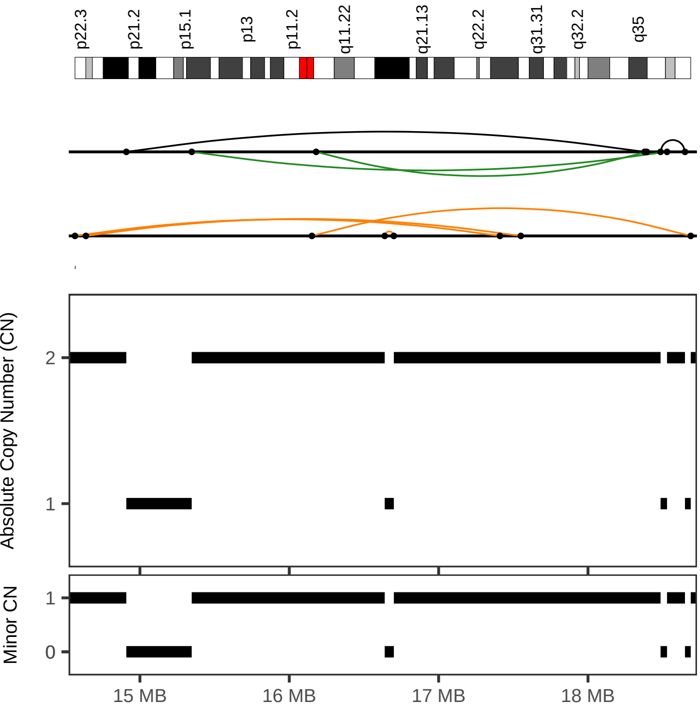

	CPCG0375
Cancer type	Prost−AdenoCA
Position	7:14565219−18687976
Type	Canonical without polyploidization
Interleaved intrachr. SVs	7
Total SVs (intrachr. + transl.)	7
SV types	DEL: 3; DUP: 0; h2hINV: 2; t2tINV: 2; TRA: 0
SVs in sample	88
Oscillating CN (2 and 3 states)	9, 9
CN segments	9
FDR fragment joints	0.64
FDR chr. breakp. enrich.	0.17
Linked to chrs	
Purity, ploidy	0.46, 1.87

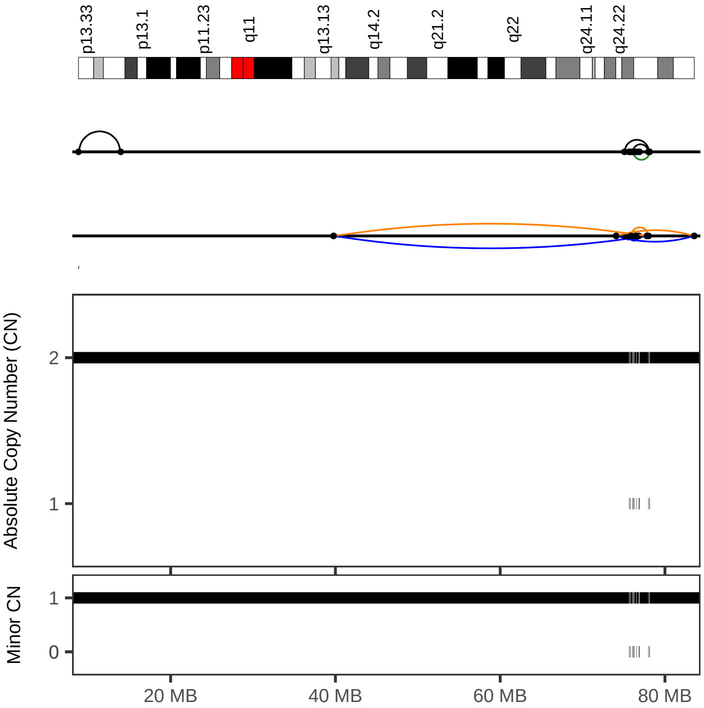

	CPCG0375
Cancer type	Prost−AdenoCA
Position	12:39777632−83554265
Type	Canonical without polyploidization
Interleaved intrachr. SVs	14
Total SVs (intrachr. + transl.)	14
SV types	DEL: 3; DUP: 5; h2hINV: 3; t2tINV: 3; TRA: 0
SVs in sample	88
Oscillating CN (2 and 3 states)	17, 17
CN segments	17
FDR fragment joints	0.88
FDR chr. breakp. enrich.	0
Linked to chrs	
Purity, ploidy	0.46, 1.87

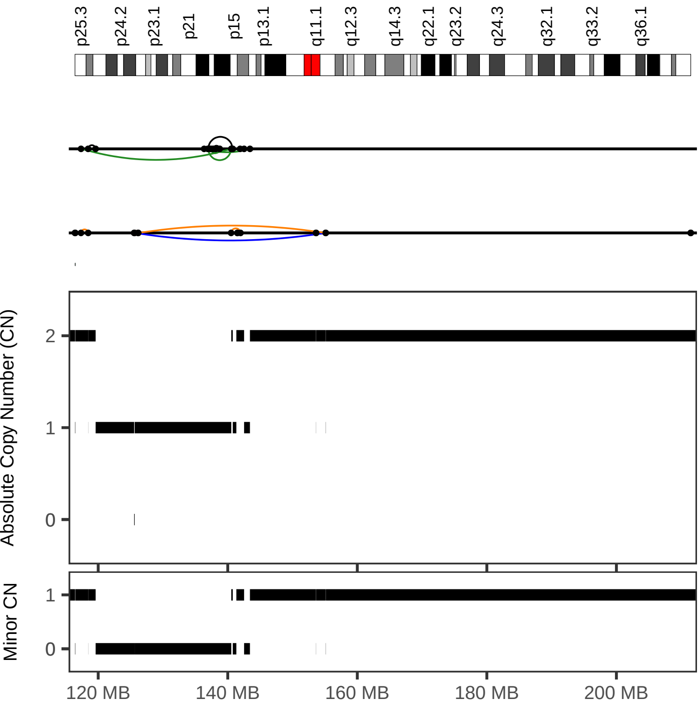

	**CPCG0388**
Cancer type	Prost−AdenoCA
Position	2:117339084−155136678
Type	Canonical without polyploidization
Interleaved intrachr. SVs	11
Total SVs (intrachr. + transl.)	11
SV types	DEL: 4; DUP: 1; h2hINV: 3; t2tINV: 3; TRA: 0
SVs in sample	212
Oscillating CN (2 and 3 states)	10, 15
CN segments	15
FDR fragment joints	0.71
FDR chr. breakp. enrich.	0.9
Linked to chrs	
Purity, ploidy	0.68, 1.85

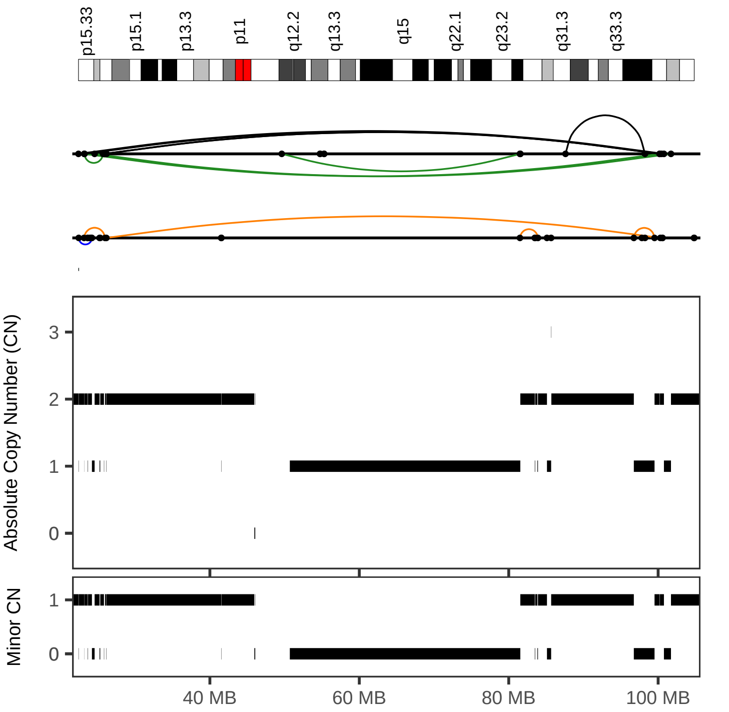

	**CPCG0388**
Cancer type	Prost−AdenoCA
Position	5:22412327−101716388
Type	With other complex events
Interleaved intrachr. SVs	11
Total SVs (intrachr. + transl.)	11
SV types	DEL: 2; DUP: 1; h2hINV: 4; t2tINV: 4; TRA: 0
SVs in sample	212
Oscillating CN (2 and 3 states)	16, 27
CN segments	32
FDR fragment joints	0.64
FDR chr. breakp. enrich.	0
Linked to chrs	
Purity, ploidy	0.68, 1.85

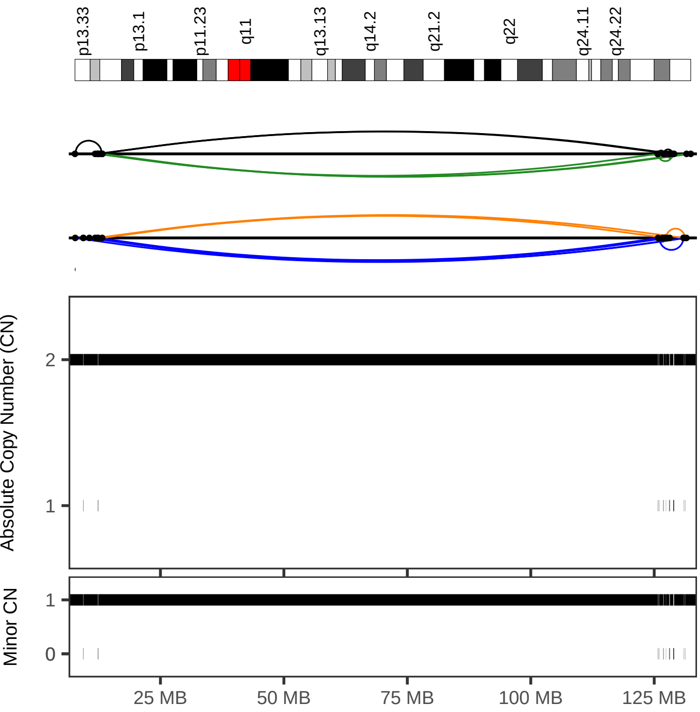

	**CPCG0388**
Cancer type	Prost−AdenoCA
Position	12:7695125−132397887
Type	Canonical without polyploidization
Interleaved intrachr. SVs	24
Total SVs (intrachr. + transl.)	24
SV types	DEL: 5; DUP: 7; h2hINV: 6; t2tINV: 6; TRA: 0
SVs in sample	212
Oscillating CN (2 and 3 states)	27, 27
CN segments	27
FDR fragment joints	0.97
FDR chr. breakp. enrich.	0
Linked to chrs	
Purity, ploidy	0.68, 1.85

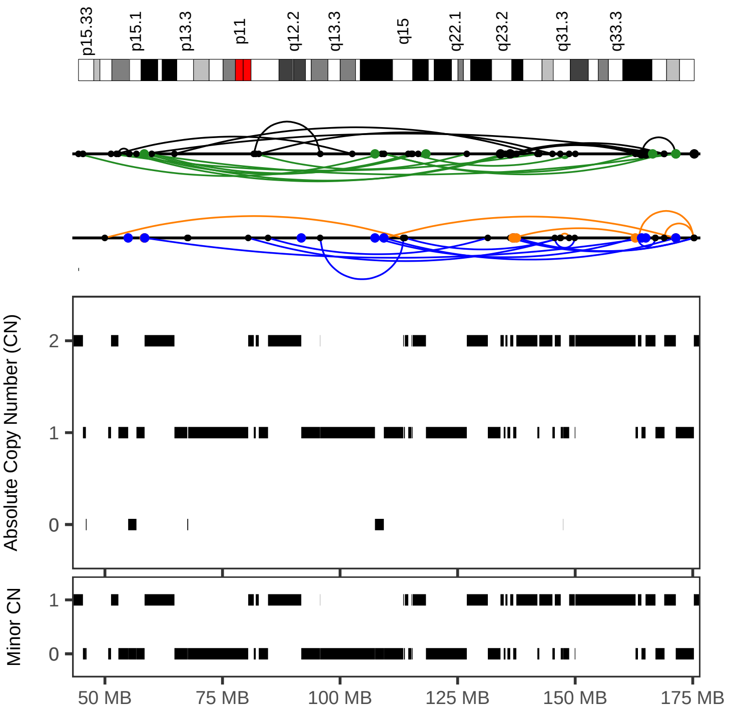

	**CPCG0392**
Cancer type	Prost−AdenoCA
Position	5:44374104−175313675
Type	With other complex events
Interleaved intrachr. SVs	43
Total SVs (intrachr. + transl.)	70
SV types	DEL: 6; DUP: 11; h2hINV: 13; t2tINV: 13; TRA: 27
SVs in sample	198
Oscillating CN (2 and 3 states)	25, 69
CN segments	69
FDR fragment joints	0.59
FDR chr. breakp. enrich.	0
Linked to chrs	12:62827736−124709303;3:94830204−149541269
Purity, ploidy	0.68, 1.85

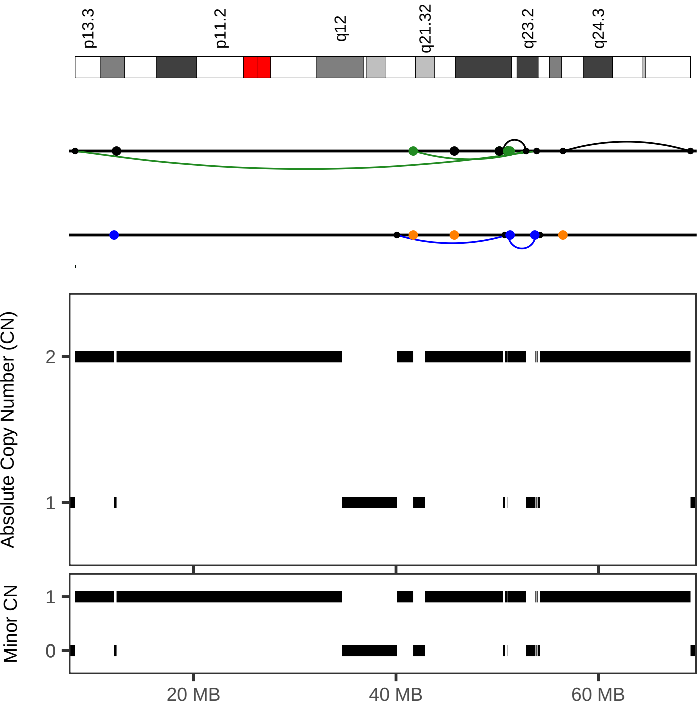

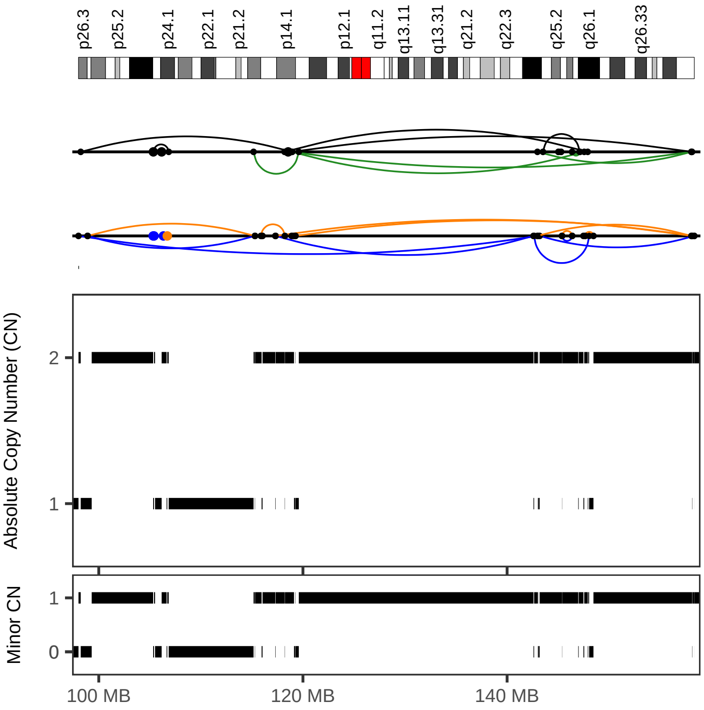

	CPCG0392
Cancer type	Prost−AdenoCA
Position	17:40076564−53795248
Type	Canonical without polyploidization
Interleaved intrachr. SVs	5
Total SVs (intrachr. + transl.)	16
SV types	DEL: 0; DUP: 3; h2hINV: 1; t2tINV: 1; TRA: 11
SVs in sample	198
Oscillating CN (2 and 3 states)	10, 10
CN segments	10
FDR fragment joints	0.59
FDR chr. breakp. enrich.	0
Linked to chrs	
Purity, ploidy	0.68, 1.85

	CPCG0404
Cancer type	Prost−AdenoCA
Position	3:98011541−158330216
Type	Canonical without polyploidization
Interleaved intrachr. SVs	21
Total SVs (intrachr. + transl.)	28
SV types	DEL: 8; DUP: 6; h2hINV: 3; t2tINV: 4; TRA: 7
SVs in sample	176
Oscillating CN (2 and 3 states)	43, 43
CN segments	43
FDR fragment joints	0.63
FDR chr. breakp. enrich.	0
Linked to chrs	8:131182988−138911084;
Purity, ploidy	0.65, 1.92

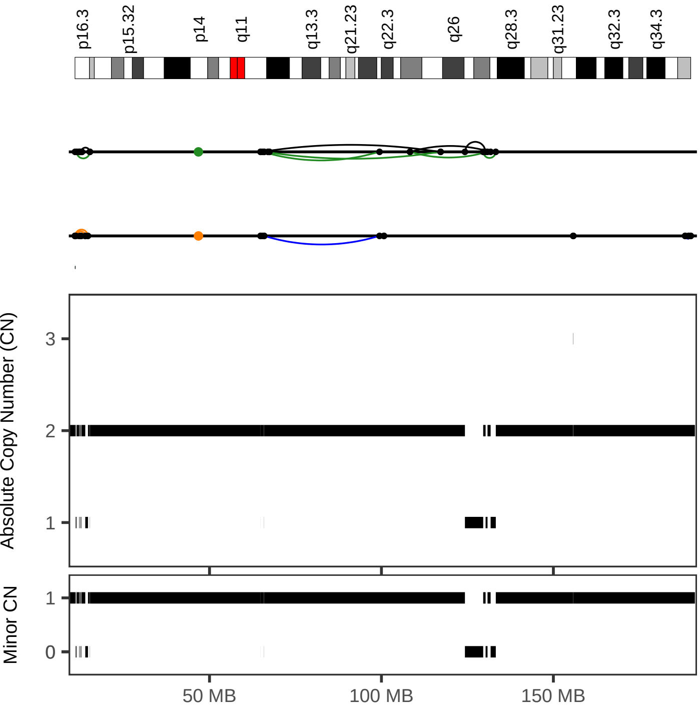

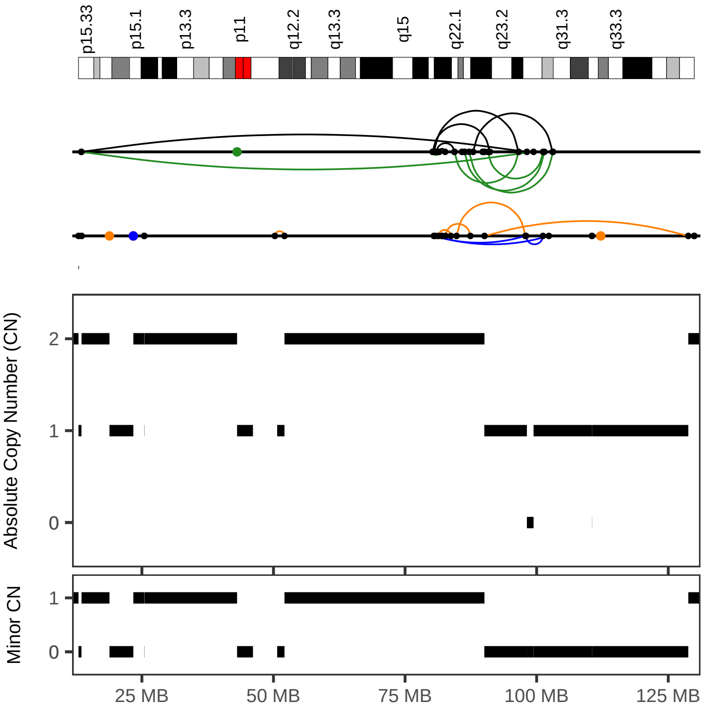

	CPCG0404
Cancer type	Prost−AdenoCA
Position	4:10877187−133253578
Type	Canonical without polyploidization
Interleaved intrachr. SVs	11
Total SVs (intrachr. + transl.)	24
SV types	DEL: 2; DUP: 5; h2hINV: 7; t2tINV: 8; TRA: 2
SVs in sample	176
Oscillating CN (2 and 3 states)	30, 30
CN segments	30
FDR fragment joints	0.59
FDR chr. breakp. enrich.	0
Linked to chrs	
Purity, ploidy	0.65, 1.92

	CPCG0404
Cancer type	Prost−AdenoCA
Position	5:12953930−128804942
Type	With other complex events
Interleaved intrachr. SVs	21
Total SVs (intrachr. + transl.)	27
SV types	DEL: 5; DUP: 3; h2hINV: 7; t2tINV: 6; TRA: 6
SVs in sample	176
Oscillating CN (2 and 3 states)	9, 14
CN segments	19
FDR fragment joints	0.72
FDR chr. breakp. enrich.	0
Linked to chrs	
Purity, ploidy	0.65, 1.92

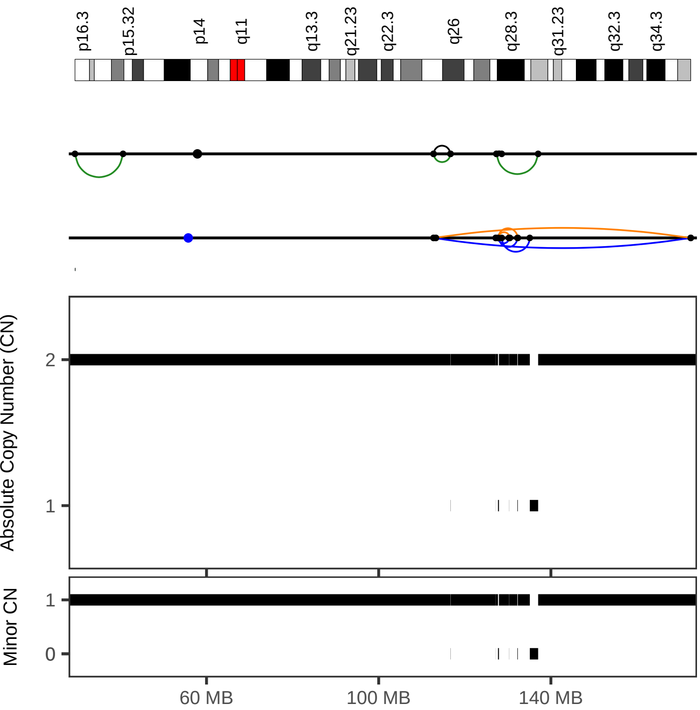

**CPCG0407**	
Cancer type	Prost−AdenoCA
Position	4:127348851−137036859
Type	Canonical without polyploidization
Interleaved intrachr. SVs	9
Total SVs (intrachr. + transl.)	9
SV types	DEL: 3; DUP: 4; h2hINV: 1; t2tINV: 1; TRA: 0
SVs in sample	72
Oscillating CN (2 and 3 states)	9, 9
CN segments	9
FDR fragment joints	0.59
FDR chr. breakp. enrich.	0
Linked to chrs	
Purity, ploidy	0.73, 1.89

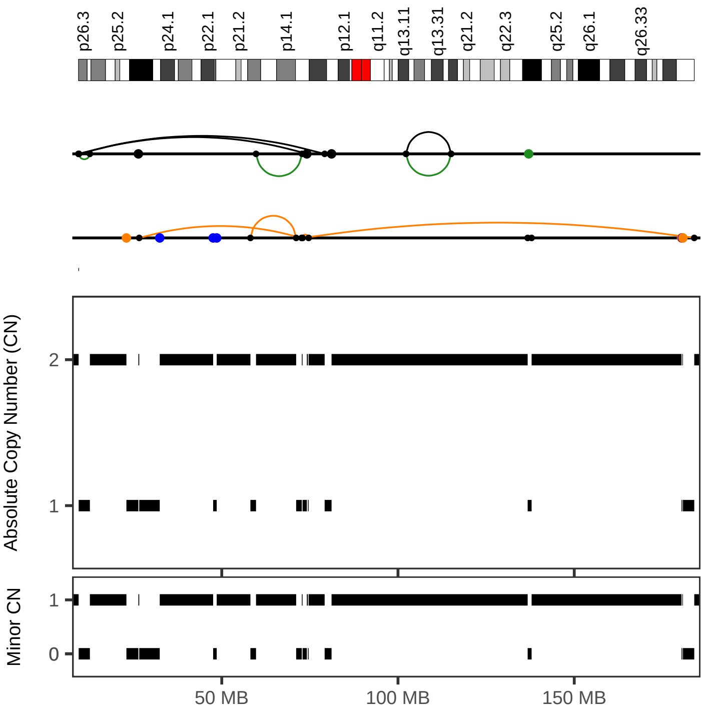

**CPCG0409**	
Cancer type	Prost−AdenoCA
Position	3:9353418−184040107
Type	Canonical without polyploidization
Interleaved intrachr. SVs	8
Total SVs (intrachr. + transl.)	19
SV types	DEL: 4; DUP: 0; h2hINV: 2; t2tINV: 2; TRA: 11
SVs in sample	106
Oscillating CN (2 and 3 states)	28, 28
CN segments	28
FDR fragment joints	0.59
FDR chr. breakp. enrich.	0
Linked to chrs	8:75912919−113963126;
Purity, ploidy	0.38, 1.91

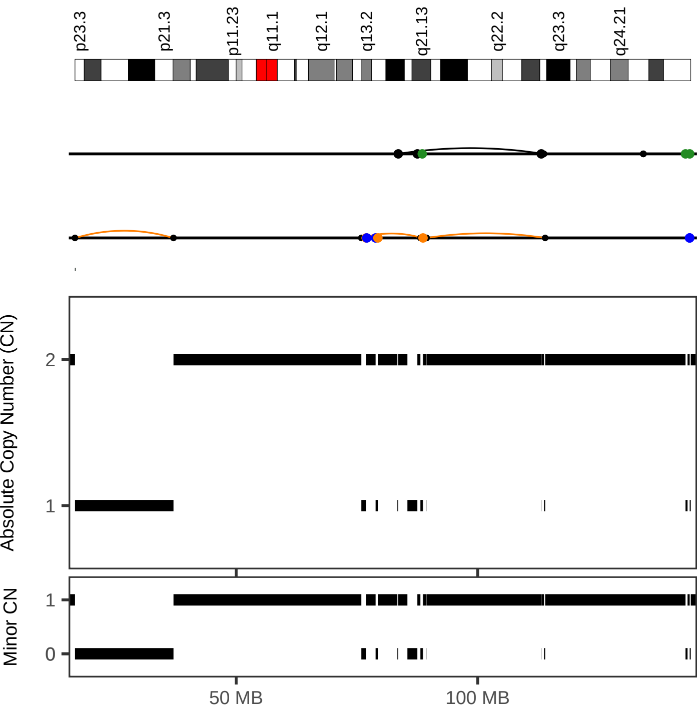

**CPCG0409**	
Cancer type	Prost−AdenoCA
Position	8:75912919−113963127
Type	Canonical without polyploidization
Interleaved intrachr. SVs	4
Total SVs (intrachr. + transl.)	13
SV types	DEL: 3; DUP: 0; h2hINV: 1; t2tINV: 0; TRA: 9
SVs in sample	106
Oscillating CN (2 and 3 states)	18, 18
CN segments	18
FDR fragment joints	0.48
FDR chr. breakp. enrich.	0
Linked to chrs	3:9353418−184040106;
Purity, ploidy	0.38, 1.91

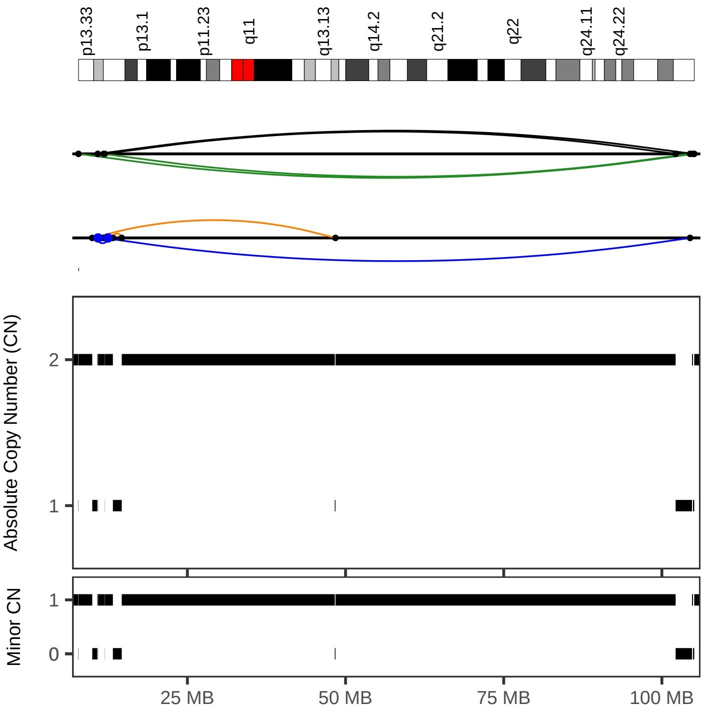

**CPCG0409**	
Cancer type	Prost−AdenoCA
Position	12:9962423−104916091
Type	Canonical without polyploidization
Interleaved intrachr. SVs	7
Total SVs (intrachr. + transl.)	9
SV types	DEL: 2; DUP: 2; h2hINV: 2; t2tINV: 1; TRA: 2
SVs in sample	106
Oscillating CN (2 and 3 states)	13, 13
CN segments	13
FDR fragment joints	0.95
FDR chr. breakp. enrich.	0.01
Linked to chrs	
Purity, ploidy	0.38, 1.91

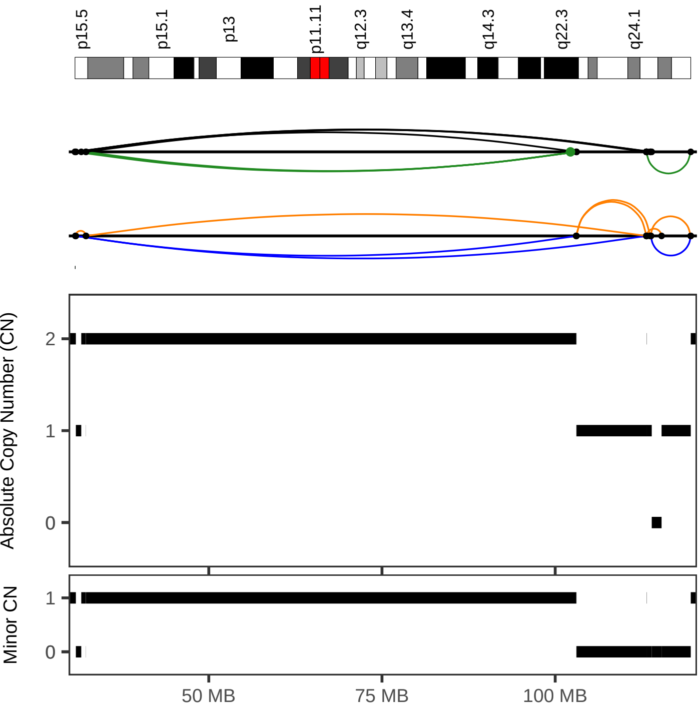

	CPCG0412
Cancer type	Prost−AdenoCA
Position	11:30685891−119565687
Type	Canonical without polyploidization
Interleaved intrachr. SVs	23
Total SVs (intrachr. + transl.)	24
SV types	DEL: 7; DUP: 3; h2hINV: 7; t2tINV: 6; TRA: 1
SVs in sample	55
Oscillating CN (2 and 3 states)	16, 19
CN segments	19
FDR fragment joints	0.67
FDR chr. breakp. enrich.	0
Linked to chrs	
Purity, ploidy	0.55, 1.8

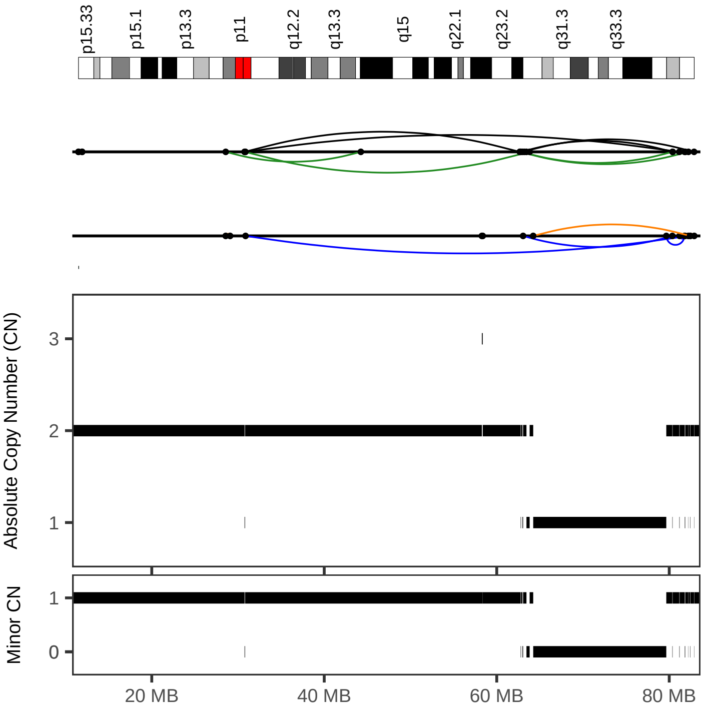

	CPCG0413
Cancer type	Prost−AdenoCA
Position	5:28572732−82901949
Type	Canonical without polyploidization
Interleaved intrachr. SVs	16
Total SVs (intrachr. + transl.)	16
SV types	DEL: 3; DUP: 4; h2hINV: 4; t2tINV: 5; TRA: 0
SVs in sample	55
Oscillating CN (2 and 3 states)	23, 27
CN segments	27
FDR fragment joints	0.94
FDR chr. breakp. enrich.	0
Linked to chrs	
Purity, ploidy	0.47, 1.9

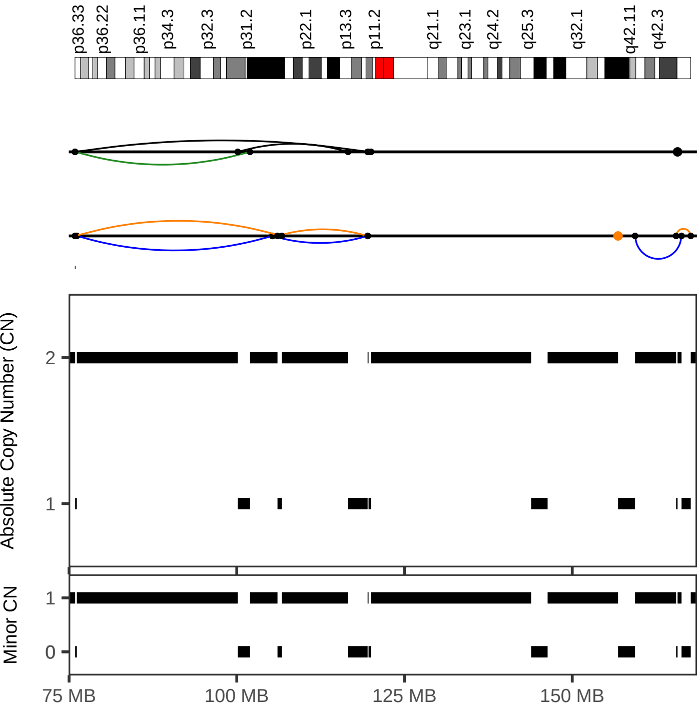

	0065_CRUK_PC_0065
Cancer type	Prost−AdenoCA
Position	1:75879779−120025710
Type	Canonical without polyploidization
Interleaved intrachr. SVs	8
Total SVs (intrachr. + transl.)	8
SV types	DEL: 2; DUP: 2; h2hINV: 2; t2tINV: 2; TRA: 0
SVs in sample	36
Oscillating CN (2 and 3 states)	11, 11
CN segments	11
FDR fragment joints	1
FDR chr. breakp. enrich.	0
Linked to chrs	
Purity, ploidy	0.56, 1.88

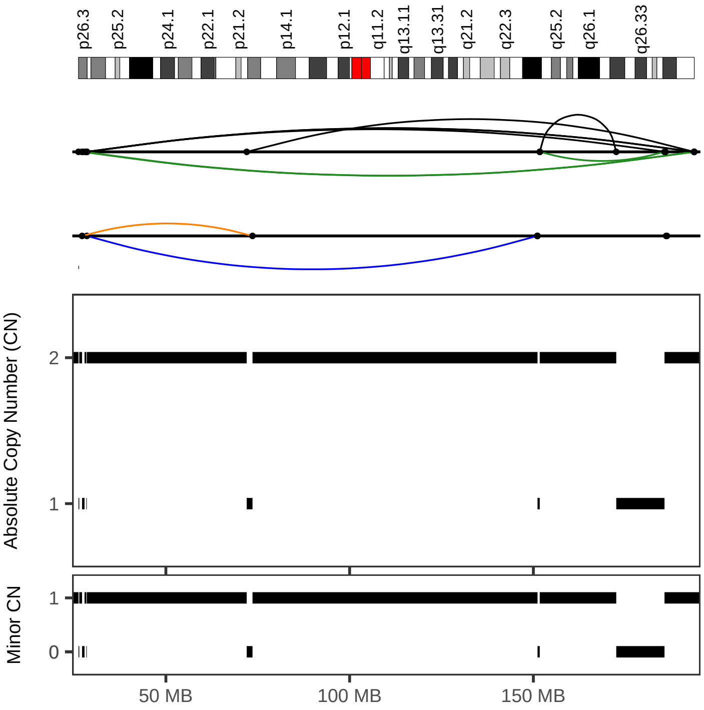

	0065_CRUK_PC_0065
Cancer type	Prost−AdenoCA
Position	3:27203160−193771532
Type	Canonical without polyploidization
Interleaved intrachr. SVs	7
Total SVs (intrachr. + transl.)	7
SV types	DEL: 1; DUP: 1; h2hINV: 4; t2tINV: 1; TRA: 0
SVs in sample	36
Oscillating CN (2 and 3 states)	10, 10
CN segments	10
FDR fragment joints	0.59
FDR chr. breakp. enrich.	0
Linked to chrs	
Purity, ploidy	0.56, 1.88

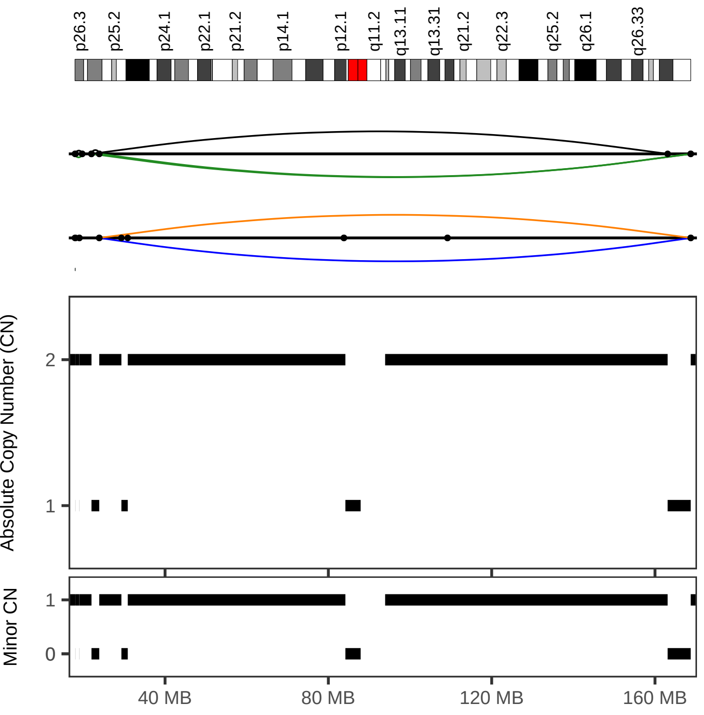

	**0067_CRUK_PC_0067**
Cancer type	Prost−AdenoCA
Position	3:21978772−168757004
Type	Canonical without polyploidization
Interleaved intrachr. SVs	6
Total SVs (intrachr. + transl.)	6
SV types	DEL: 1; DUP: 1; h2hINV: 2; t2tINV: 2; TRA: 0
SVs in sample	83
Oscillating CN (2 and 3 states)	9, 9
CN segments	9
FDR fragment joints	0.91
FDR chr. breakp. enrich.	0.04
Linked to chrs	
Purity, ploidy	0.8, 1.89

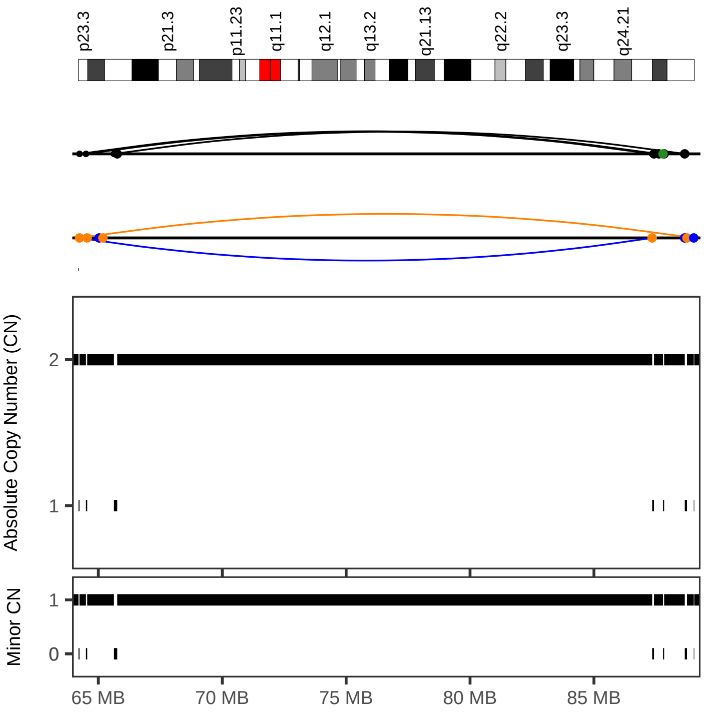

	**0067_CRUK_PC_0067**
Cancer type	Prost−AdenoCA
Position	8:64237001−88665512
Type	Canonical without polyploidization
Interleaved intrachr. SVs	4
Total SVs (intrachr. + transl.)	16
SV types	DEL: 0; DUP: 1; h2hINV: 3; t2tINV: 0; TRA: 12
SVs in sample	83
Oscillating CN (2 and 3 states)	11, 11
CN segments	11
FDR fragment joints	0.48
FDR chr. breakp. enrich.	0
Linked to chrs	13:67945892−69823504;
Purity, ploidy	0.8, 1.89

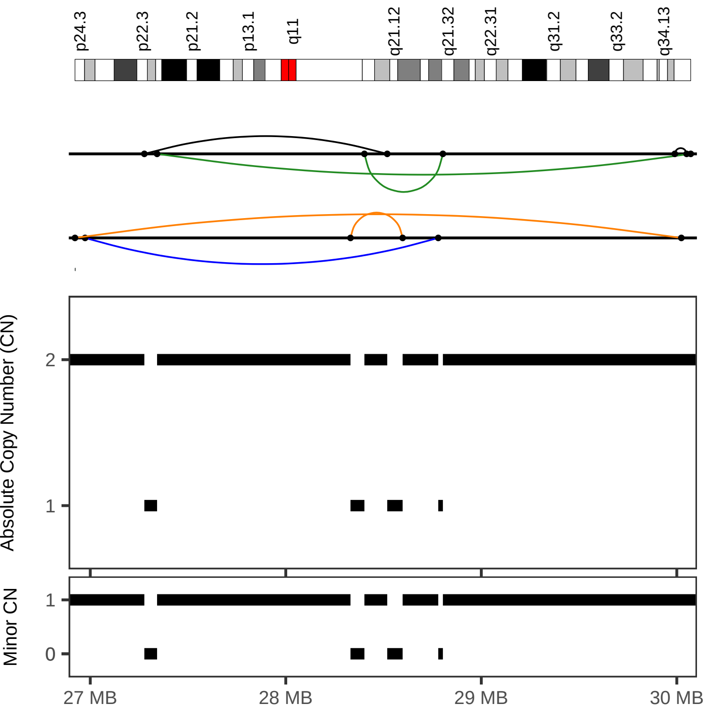

	**0067_CRUK_PC_0067**
Cancer type	Prost−AdenoCA
Position	9:26921758−30071295
Type	Canonical without polyploidization
Interleaved intrachr. SVs	7
Total SVs (intrachr. + transl.)	7
SV types	DEL: 2; DUP: 1; h2hINV: 2; t2tINV: 2; TRA: 0
SVs in sample	83
Oscillating CN (2 and 3 states)	9, 9
CN segments	9
FDR fragment joints	0.95
FDR chr. breakp. enrich.	0.15
Linked to chrs	
Purity, ploidy	0.8, 1.89

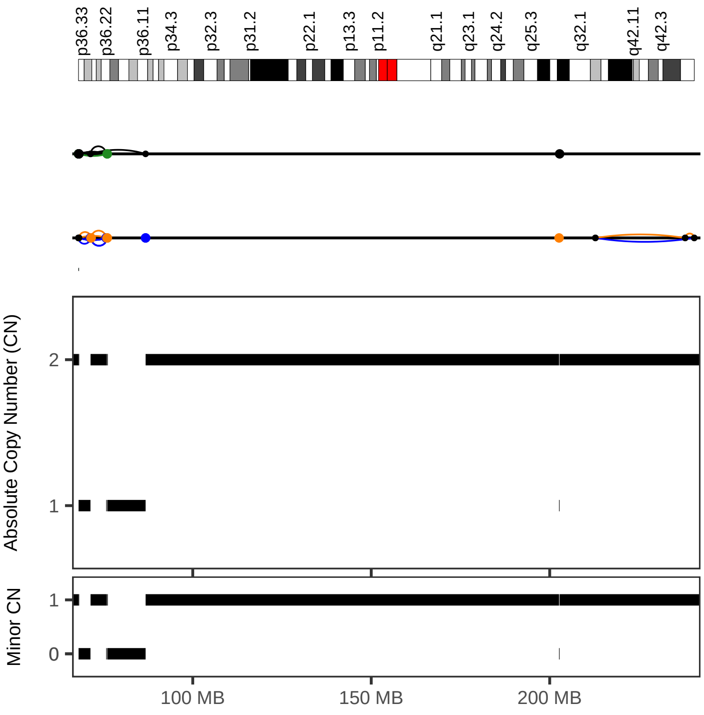

	**0072_CRUK_PC_0072**
Cancer type	Prost−AdenoCA
Position	1:67986816−86784290
Type	Canonical without polyploidization
Interleaved intrachr. SVs	19
Total SVs (intrachr. + transl.)	38
SV types	DEL: 5; DUP: 6; h2hINV: 4; t2tINV: 4; TRA: 19
SVs in sample	165
Oscillating CN (2 and 3 states)	20, 20
CN segments	20
FDR fragment joints	0.92
FDR chr. breakp. enrich.	0
Linked to chrs	
Purity, ploidy	0.59, 1.88

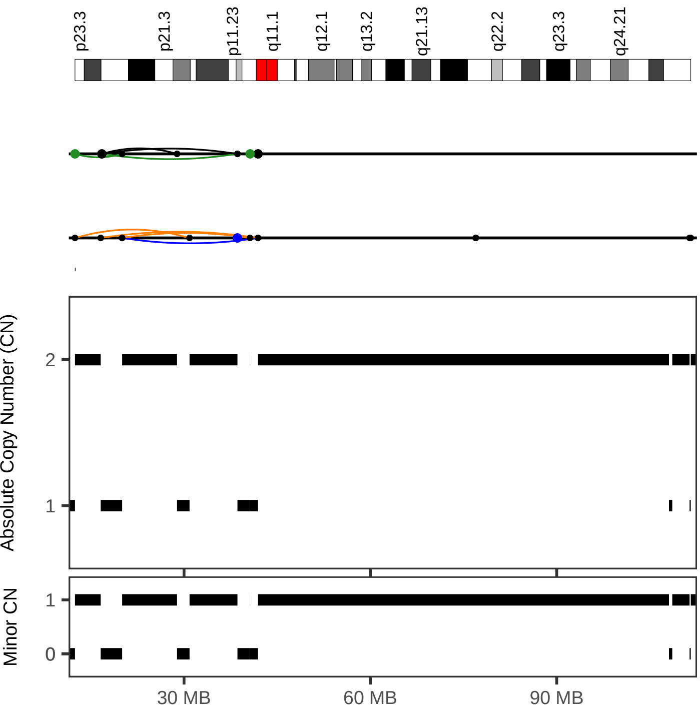

	**0072_CRUK_PC_0072**
Cancer type	Prost−AdenoCA
Position	8:12443102−41913983
Type	Canonical without polyploidization
Interleaved intrachr. SVs	8
Total SVs (intrachr. + transl.)	12
SV types	DEL: 3; DUP: 1; h2hINV: 2; t2tINV: 2; TRA: 4
SVs in sample	165
Oscillating CN (2 and 3 states)	10, 10
CN segments	10
FDR fragment joints	0.84
FDR chr. breakp. enrich.	0.11
Linked to chrs	13:34142528−82507263;
Purity, ploidy	0.59, 1.88

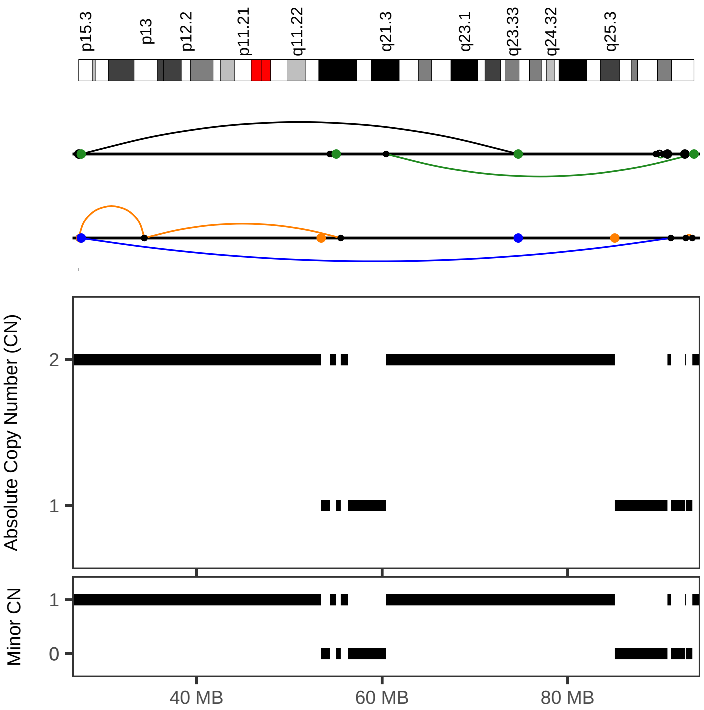

	**0072_CRUK_PC_0072**
Cancer type	Prost−AdenoCA
Position	10:27291487−93614346
Type	Canonical without polyploidization
Interleaved intrachr. SVs	5
Total SVs (intrachr. + transl.)	17
SV types	DEL: 2; DUP: 1; h2hINV: 1; t2tINV: 1; TRA: 12
SVs in sample	165
Oscillating CN (2 and 3 states)	13, 13
CN segments	13
FDR fragment joints	0.92
FDR chr. breakp. enrich.	0
Linked to chrs	
Purity, ploidy	0.59, 1.88

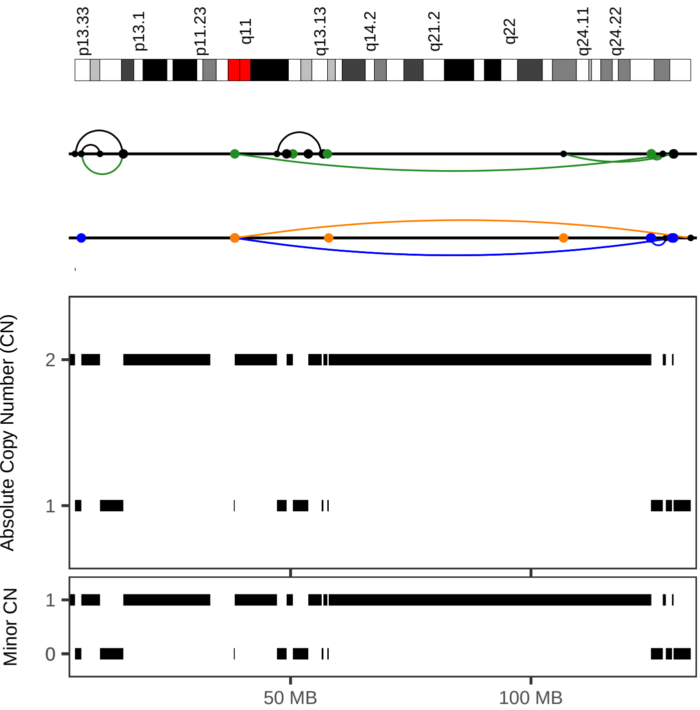

	**0072_CRUK_PC_0072**
Cancer type	Prost−AdenoCA
Position	12:38382189−133229840
Type	Canonical without polyploidization
Interleaved intrachr. SVs	4
Total SVs (intrachr. + transl.)	33
SV types	DEL: 1; DUP: 2; h2hINV: 0; t2tINV: 1; TRA: 29
SVs in sample	165
Oscillating CN (2 and 3 states)	17, 17
CN segments	17
FDR fragment joints	0.64
FDR chr. breakp. enrich.	0
Linked to chrs	1:67986816−86784289;4:3129136−148497920
Purity, ploidy	0.59, 1.88

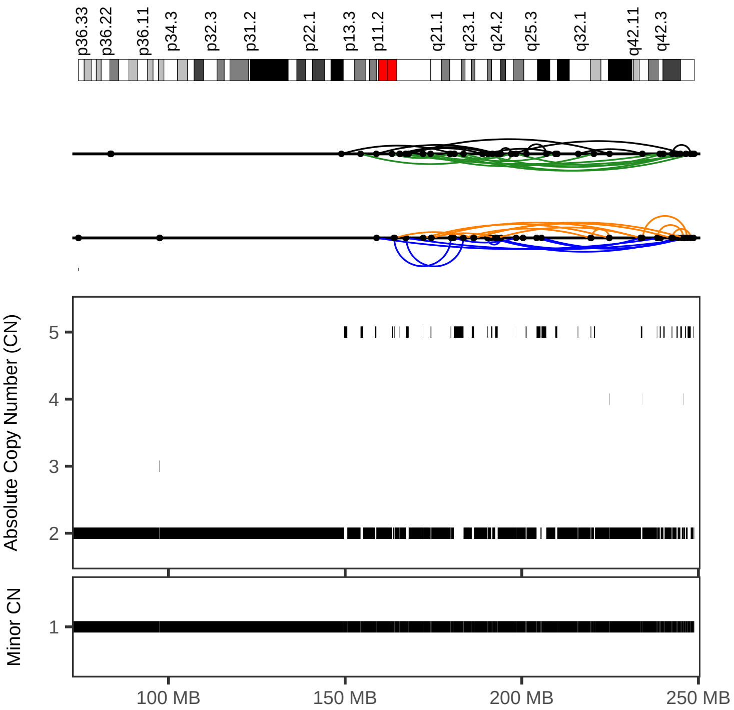

	**0077_CRUK_PC_0077**
Cancer type	Prost−AdenoCA
Position	1:148930966−248810132
Type	With other complex events
Interleaved intrachr. SVs	45
Total SVs (intrachr. + transl.)	45
SV types	DEL: 12; DUP: 10; h2hINV: 11; t2tINV: 12; TRA: 0
SVs in sample	198
Oscillating CN (2 and 3 states)	53, 56
CN segments	78
FDR fragment joints	0.98
FDR chr. breakp. enrich.	0
Linked to chrs	
Purity, ploidy	0.68, 1.89

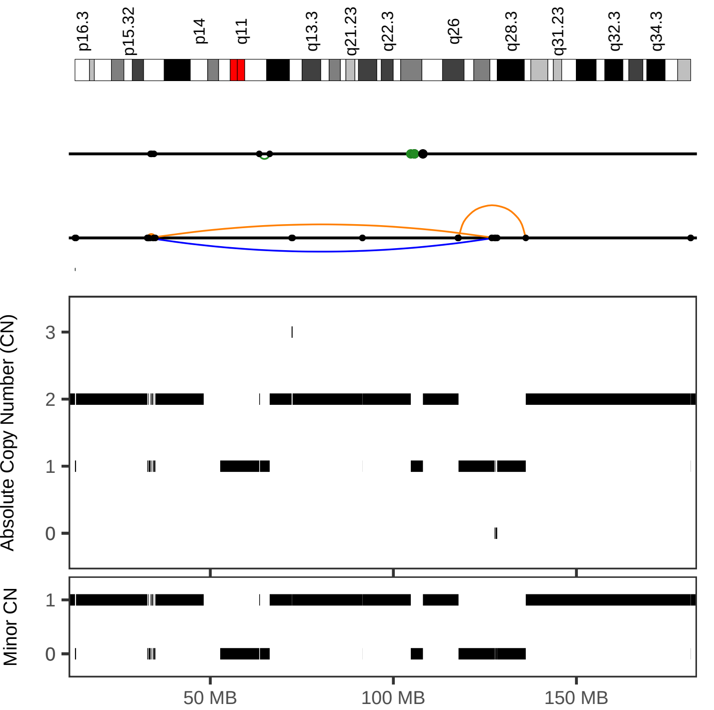

0077_CRUK_PC_0077	
Cancer type	Prost−AdenoCA
Position	4:32778170−136168076
Type	With other complex events
Interleaved intrachr. SVs	9
Total SVs (intrachr. + transl.)	13
SV types	DEL: 4; DUP: 3; h2hINV: 1; t2tINV: 1; TRA: 4
SVs in sample	198
Oscillating CN (2 and 3 states)	16, 28
CN segments	28
FDR fragment joints	0.59
FDR chr. breakp. enrich.	0.12
Linked to chrs	5:46053227−172902120;
Purity, ploidy	0.68, 1.89

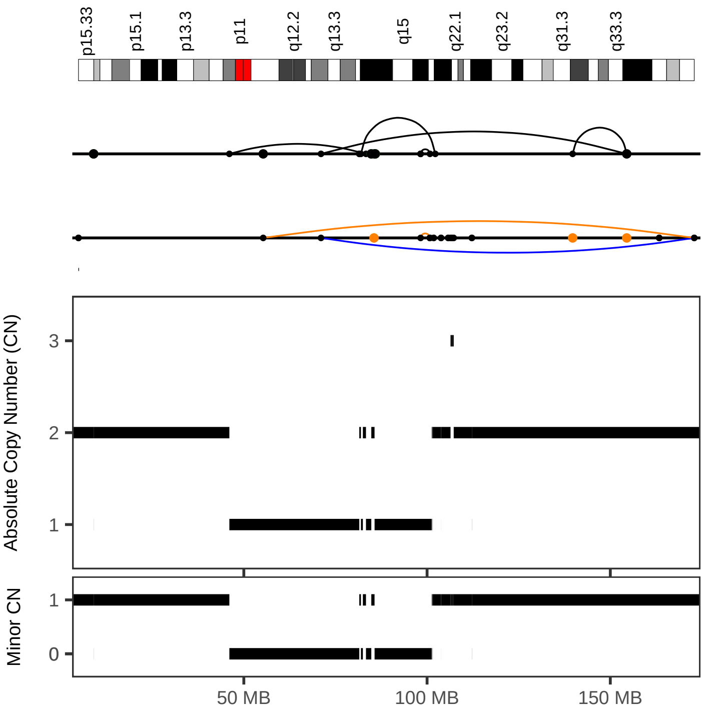

0077_CRUK_PC_0077	
Cancer type	Prost−AdenoCA
Position	5:46053227−172902121
Type	Canonical without polyploidization
Interleaved intrachr. SVs	5
Total SVs (intrachr. + transl.)	15
SV types	DEL: 1; DUP: 1; h2hINV: 3; t2tINV: 0; TRA: 10
SVs in sample	198
Oscillating CN (2 and 3 states)	16, 22
CN segments	22
FDR fragment joints	0.59
FDR chr. breakp. enrich.	0
Linked to chrs	2:34203351−199138380;4:32778170−136168075
Purity, ploidy	0.68, 1.89

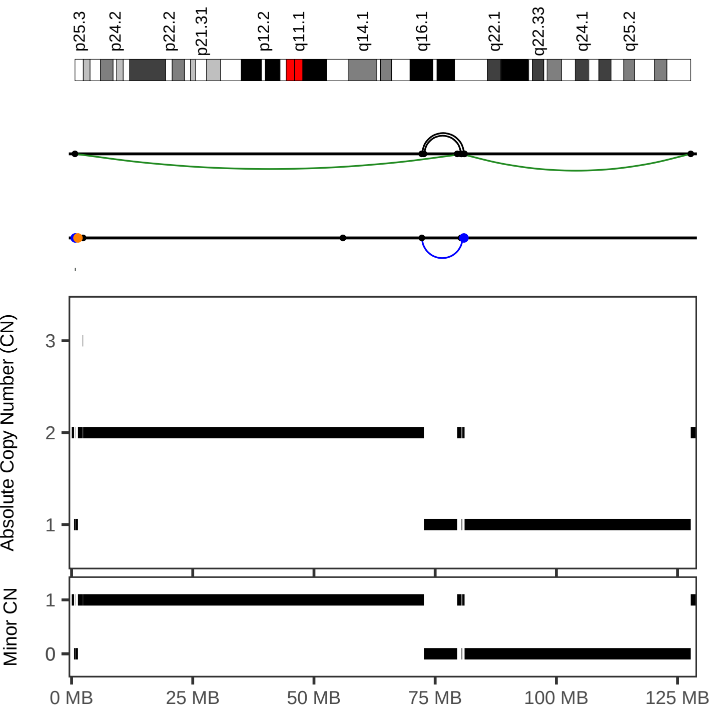

0077_CRUK_PC_0077	
Cancer type	Prost−AdenoCA
Position	6:697961−127720347
Type	Canonical without polyploidization
Interleaved intrachr. SVs	7
Total SVs (intrachr. + transl.)	10
SV types	DEL: 1; DUP: 1; h2hINV: 2; t2tINV: 3; TRA: 3
SVs in sample	198
Oscillating CN (2 and 3 states)	9, 13
CN segments	13
FDR fragment joints	0.74
FDR chr. breakp. enrich.	0.86
Linked to chrs	12:10457601−23828589;
Purity, ploidy	0.68, 1.89

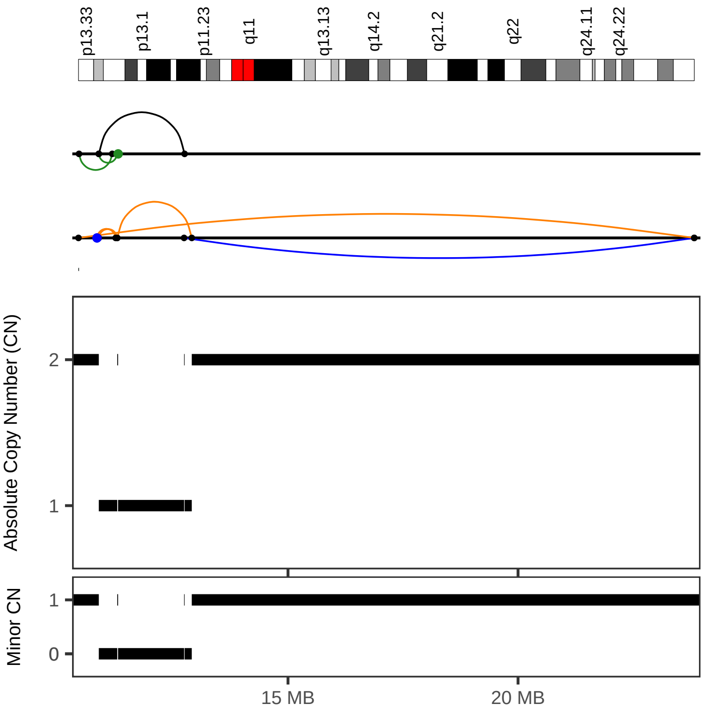

0077_CRUK_PC_0077	
Cancer type	Prost−AdenoCA
Position	12:10457601−23828590
Type	Canonical without polyploidization
Interleaved intrachr. SVs	7
Total SVs (intrachr. + transl.)	9
SV types	DEL: 3; DUP: 1; h2hINV: 1; t2tINV: 2; TRA: 2
SVs in sample	198
Oscillating CN (2 and 3 states)	7, 7
CN segments	7
FDR fragment joints	0.74
FDR chr. breakp. enrich.	0.57
Linked to chrs	6:697961−127720346;
Purity, ploidy	0.68, 1.89

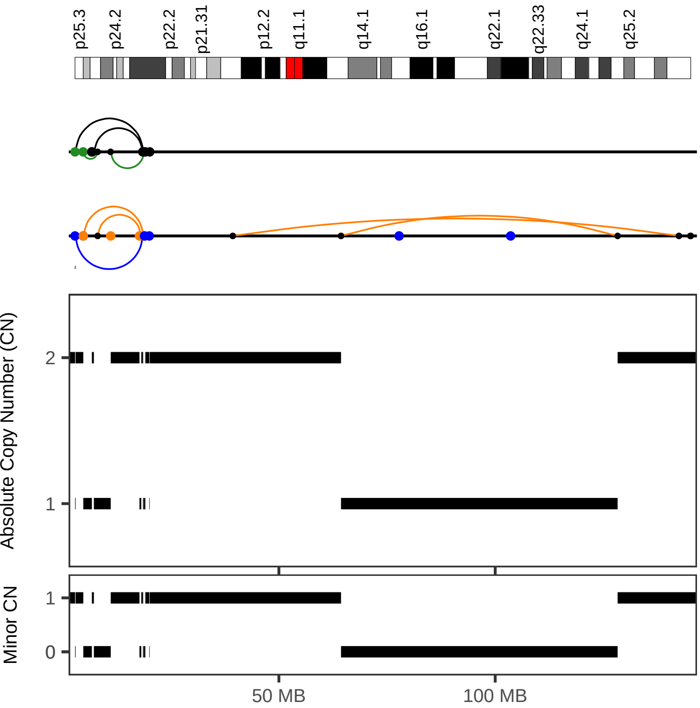

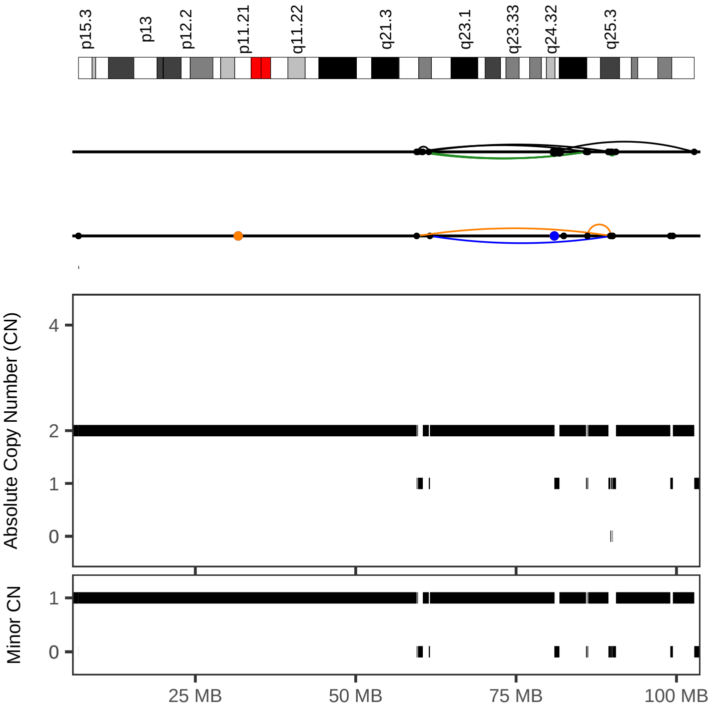

	**0082_CRUK_PC_0082**
Cancer type	Prost−AdenoCA
Position	6:2865588−18891521
Type	Canonical without polyploidization
Interleaved intrachr. SVs	7
Total SVs (intrachr. + transl.)	21
SV types	DEL: 2; DUP: 1; h2hINV: 2; t2tINV: 2; TRA: 14
SVs in sample	128
Oscillating CN (2 and 3 states)	14, 14
CN segments	14
FDR fragment joints	0.95
FDR chr. breakp. enrich.	0
Linked to chrs	10:59502181−102770425;
Purity, ploidy	0.57, 1.88

	**0082_CRUK_PC_0082**
Cancer type	Prost−AdenoCA
Position	10:59502181−102770426
Type	Canonical without polyploidization
Interleaved intrachr. SVs	17
Total SVs (intrachr. + transl.)	22
SV types	DEL: 3; DUP: 1; h2hINV: 7; t2tINV: 6; TRA: 5
SVs in sample	128
Oscillating CN (2 and 3 states)	16, 24
CN segments	24
FDR fragment joints	0.59
FDR chr. breakp. enrich.	0
Linked to chrs	
Purity, ploidy	0.57, 1.88

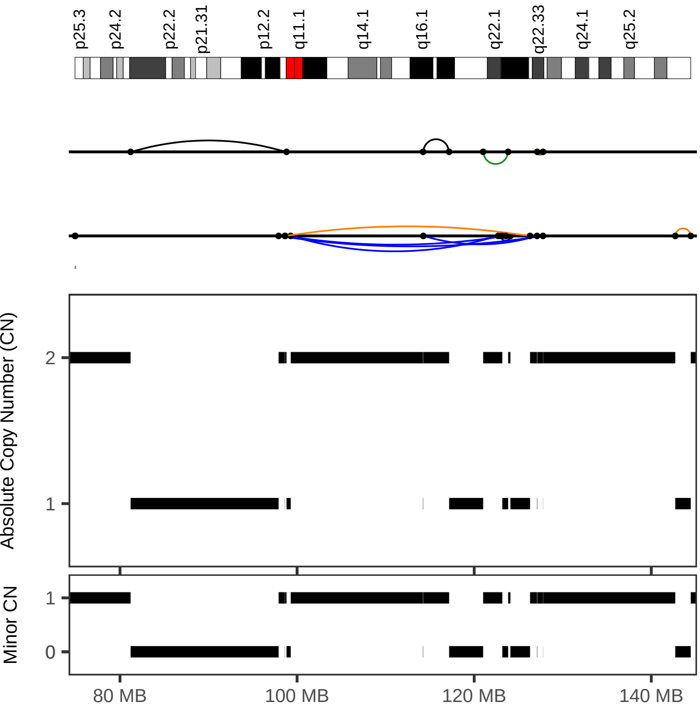

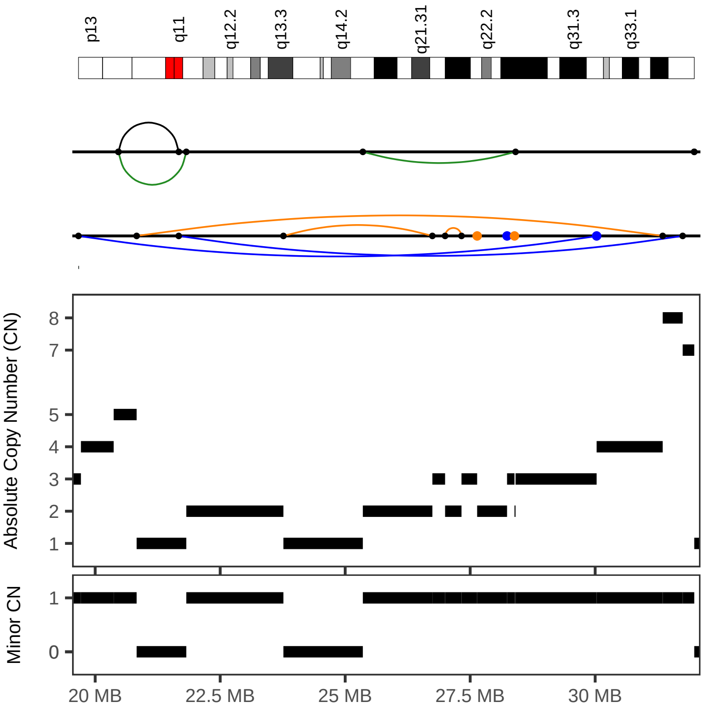

	**0089_CRUK_PC_0089**
Cancer type	Prost−AdenoCA
Position	6:81197888−127781306
Type	Canonical without polyploidization
Interleaved intrachr. SVs	10
Total SVs (intrachr. + transl.)	10
SV types	DEL: 1; DUP: 5; h2hINV: 2; t2tINV: 2; TRA: 0
SVs in sample	52
Oscillating CN (2 and 3 states)	18, 18
CN segments	18
FDR fragment joints	0.59
FDR chr. breakp. enrich.	0
Linked to chrs	
Purity, ploidy	0.74, 1.92

	**0089_CRUK_PC_0089**
Cancer type	Prost−AdenoCA
Position	13:19665581−31750172
Type	With other complex events
Interleaved intrachr. SVs	5
Total SVs (intrachr. + transl.)	9
SV types	DEL: 1; DUP: 2; h2hINV: 1; t2tINV: 1; TRA: 4
SVs in sample	52
Oscillating CN (2 and 3 states)	8, 12
CN segments	17
FDR fragment joints	0.92
FDR chr. breakp. enrich.	0
Linked to chrs	
Purity, ploidy	0.74, 1.92

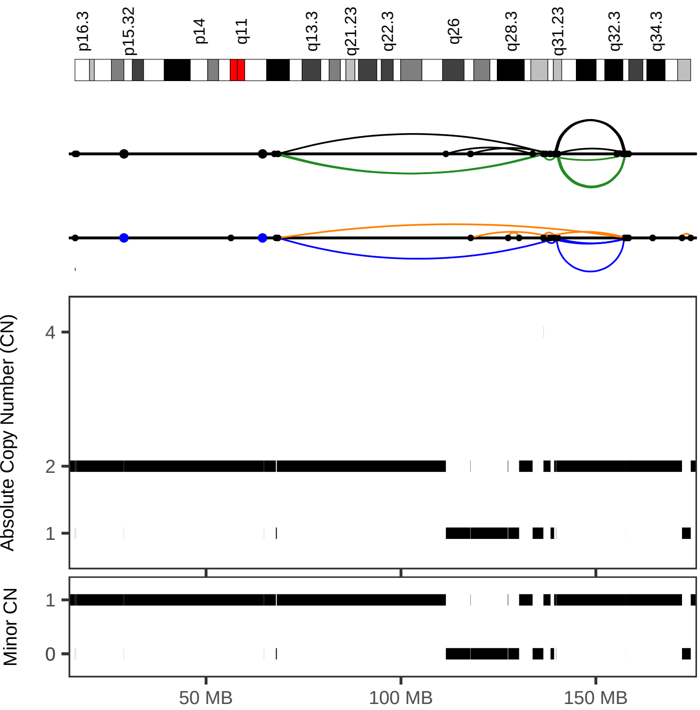

	**0090_CRUK_PC_0090**
Cancer type	Prost−AdenoCA
Position	4:67524856−158414913
Type	Canonical without polyploidization
Interleaved intrachr. SVs	25
Total SVs (intrachr. + transl.)	25
SV types	DEL: 5; DUP: 5; h2hINV: 7; t2tINV: 8; TRA: 0
SVs in sample	194
Oscillating CN (2 and 3 states)	14, 14
CN segments	22
FDR fragment joints	0.84
FDR chr. breakp. enrich.	0
Linked to chrs	
Purity, ploidy	0.21, 1.92

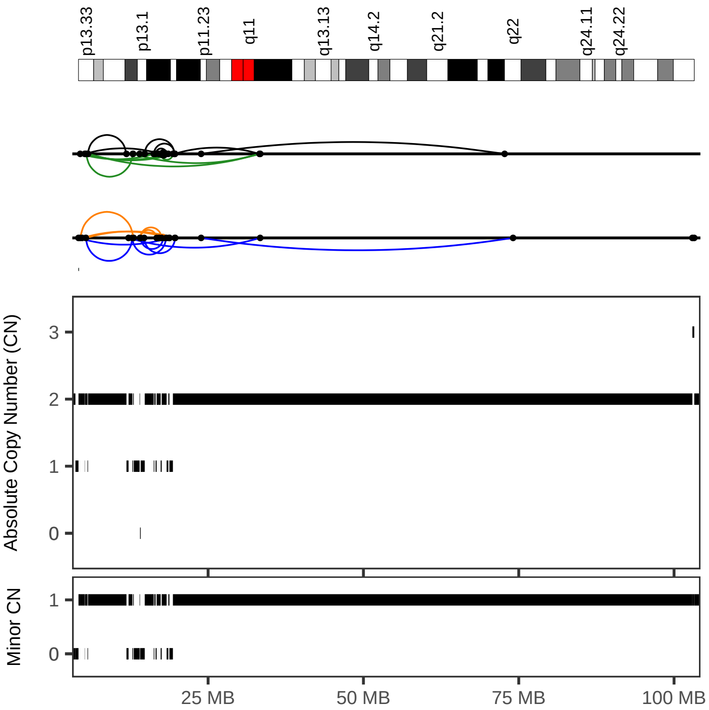

	**0090_CRUK_PC_0090**
Cancer type	Prost−AdenoCA
Position	12:4134323−74087961
Type	With other complex events
Interleaved intrachr. SVs	31
Total SVs (intrachr. + transl.)	32
SV types	DEL: 6; DUP: 9; h2hINV: 8; t2tINV: 8; TRA: 1
SVs in sample	194
Oscillating CN (2 and 3 states)	12, 13
CN segments	24
FDR fragment joints	0.92
FDR chr. breakp. enrich.	0
Linked to chrs	
Purity, ploidy	0.21, 1.92

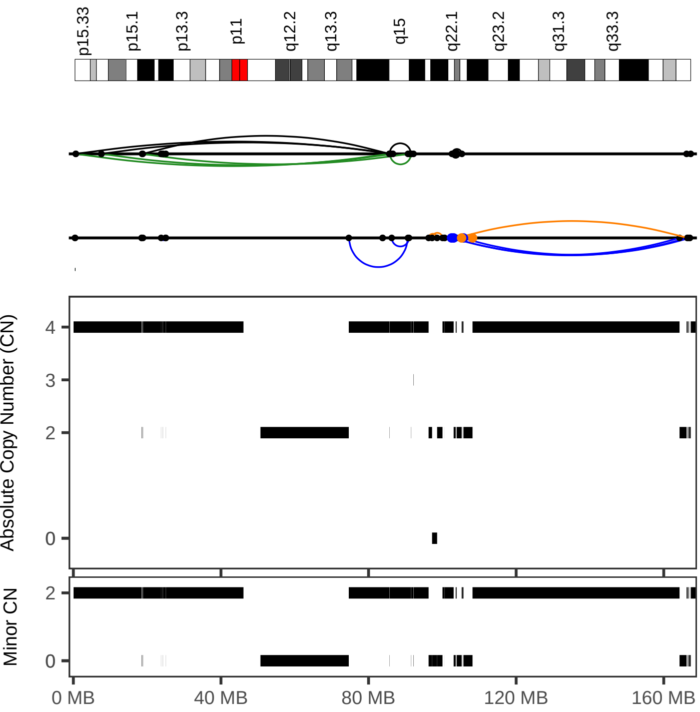

	**0091_CRUK_PC_0091**
Cancer type	Prost−AdenoCA
Position	5:654406−92191496
Type	Before polyploidization
Interleaved intrachr. SVs	12
Total SVs (intrachr. + transl.)	12
SV types	DEL: 1; DUP: 2; h2hINV: 4; t2tINV: 5; TRA: 0
SVs in sample	308
Oscillating CN (2 and 3 states)	21, 23
CN segments	23
FDR fragment joints	0.59
FDR chr. breakp. enrich.	0.01
Linked to chrs	
Purity, ploidy	0.86, 3.7

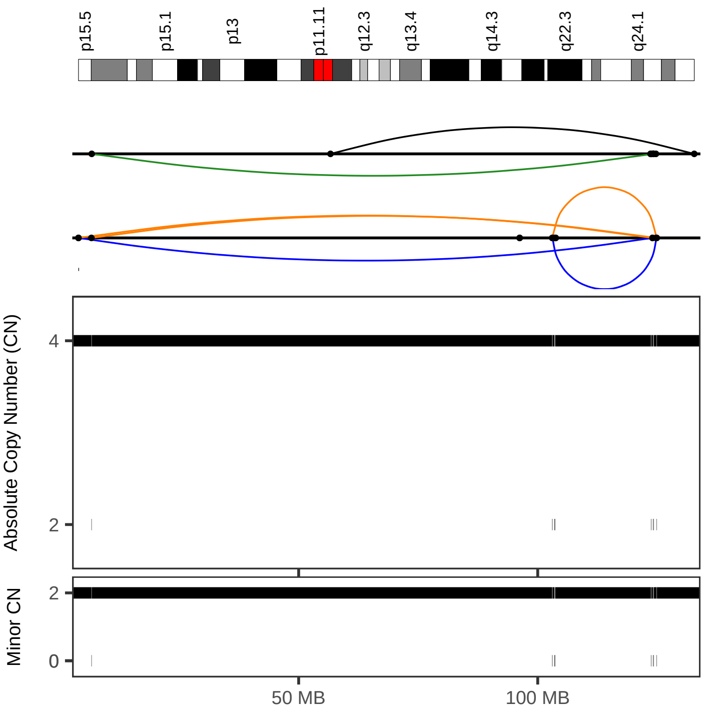

	**0091_CRUK_PC_0091**
Cancer type	Prost−AdenoCA
Position	11:3944706−132738959
Type	Before polyploidization
Interleaved intrachr. SVs	10
Total SVs (intrachr. + transl.)	10
SV types	DEL: 3; DUP: 2; h2hINV: 3; t2tINV: 2; TRA: 0
SVs in sample	308
Oscillating CN (2 and 3 states)	15, 15
CN segments	15
FDR fragment joints	0.96
FDR chr. breakp. enrich.	1
Linked to chrs	
Purity, ploidy	0.86, 3.7

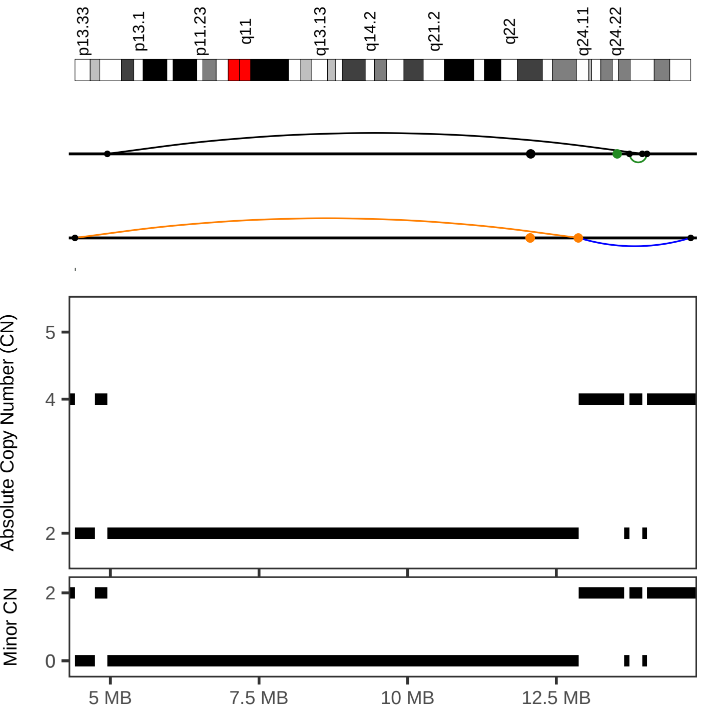

**0091_CRUK_PC_0091**	
Cancer type	Prost−AdenoCA
Position	12:4404224−14760891
Type	Before polyploidization
Interleaved intrachr. SVs	4
Total SVs (intrachr. + transl.)	9
SV types	DEL: 1; DUP: 1; h2hINV: 1; t2tINV: 1; TRA: 5
SVs in sample	308
Oscillating CN (2 and 3 states)	8, 8
CN segments	8
FDR fragment joints	1
FDR chr. breakp. enrich.	0.67
Linked to chrs	13:61651222−91504851;
Purity, ploidy	0.86, 3.7

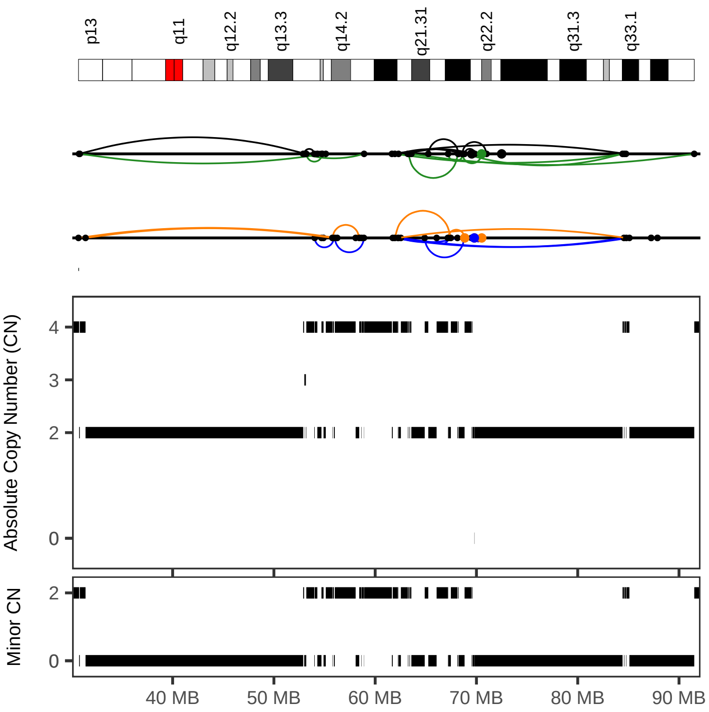

**0091_CRUK_PC_0091**	
Cancer type	Prost−AdenoCA
Position	13:61651222−91504852
Type	Before polyploidization
Interleaved intrachr. SVs	23
Total SVs (intrachr. + transl.)	30
SV types	DEL: 4; DUP: 6; h2hINV: 7; t2tINV: 6; TRA: 7
SVs in sample	308
Oscillating CN (2 and 3 states)	25, 34
CN segments	34
FDR fragment joints	0.88
FDR chr. breakp. enrich.	0
Linked to chrs	12:4404224−14760890;
Purity, ploidy	0.86, 3.7

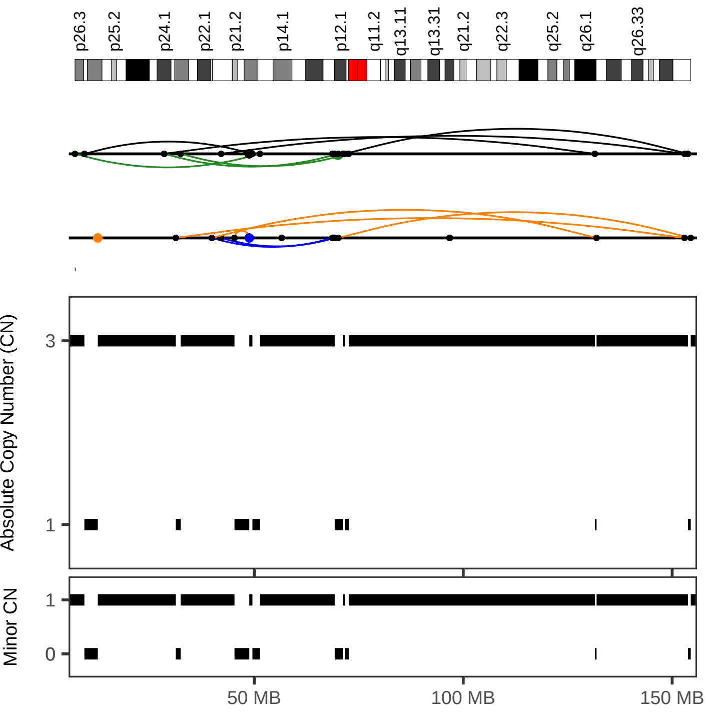

**A12−0020_CRUK_PC_0020**	
Cancer type	Prost−AdenoCA
Position	3:7099331−154439230
Type	Canonical without polyploidization
Interleaved intrachr. SVs	15
Total SVs (intrachr. + transl.)	18
SV types	DEL: 3; DUP: 3; h2hINV: 4; t2tINV: 5; TRA: 3
SVs in sample	189
Oscillating CN (2 and 3 states)	17, 17
CN segments	17
FDR fragment joints	0.9
FDR chr. breakp. enrich.	0.08
Linked to chrs	
Purity, ploidy	NA, NA

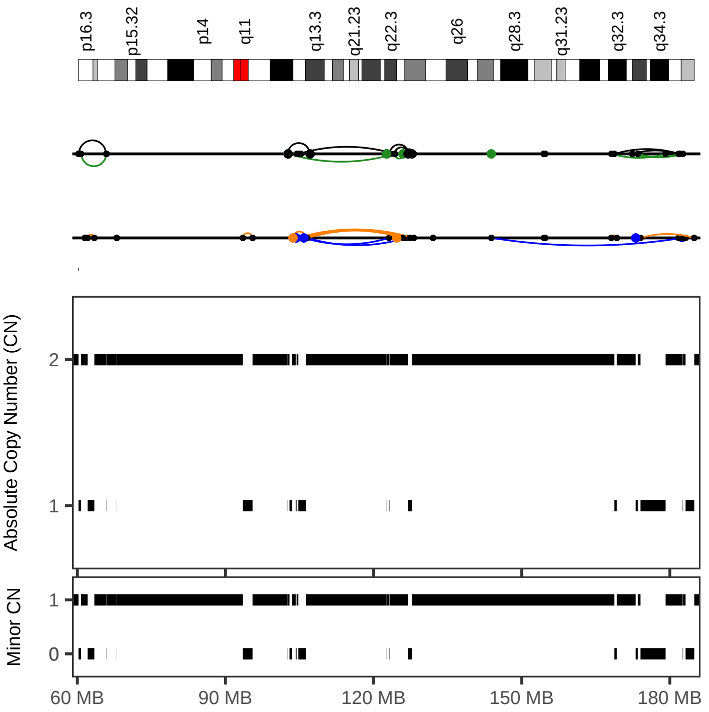

**A21−0096_CRUK_PC_0096**	
Cancer type	Prost−AdenoCA
Position	4:102585699−128146094
Type	Canonical without polyploidization
Interleaved intrachr. SVs	16
Total SVs (intrachr. + transl.)	28
SV types	DEL: 5; DUP: 2; h2hINV: 5; t2tINV: 4; TRA: 12
SVs in sample	496
Oscillating CN (2 and 3 states)	28, 28
CN segments	28
FDR fragment joints	0.76
FDR chr. breakp. enrich.	0.03
Linked to chrs	
Purity, ploidy	NA, NA

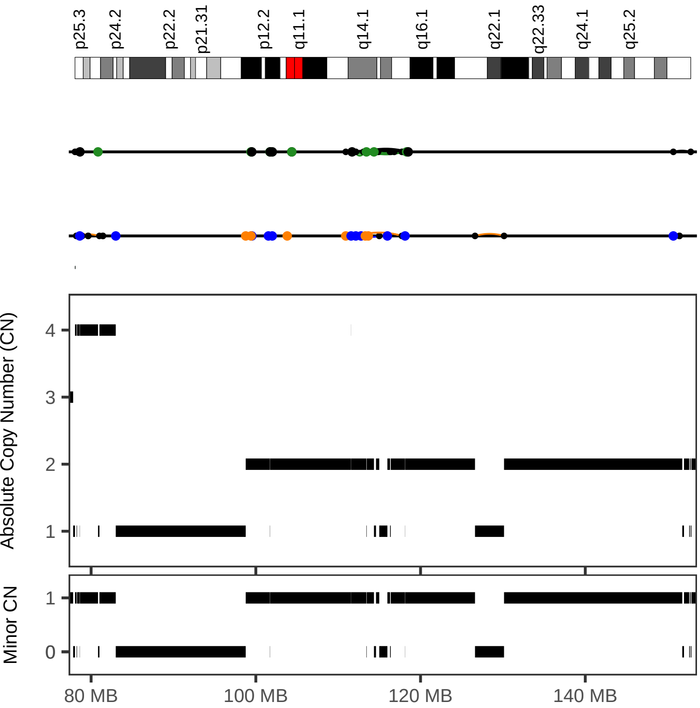

	A21−0096_CRUK_PC_0096
Cancer type	Prost−AdenoCA
Position	6:110920236−118495780
Type	Canonical without polyploidization
Interleaved intrachr. SVs	13
Total SVs (intrachr. + transl.)	24
SV types	DEL: 2; DUP: 1; h2hINV: 5; t2tINV: 5; TRA: 11
SVs in sample	496
Oscillating CN (2 and 3 states)	11, 13
CN segments	13
FDR fragment joints	0.59
FDR chr. breakp. enrich.	0
Linked to chrs	8:38045408−103847168;
Purity, ploidy	NA, NA

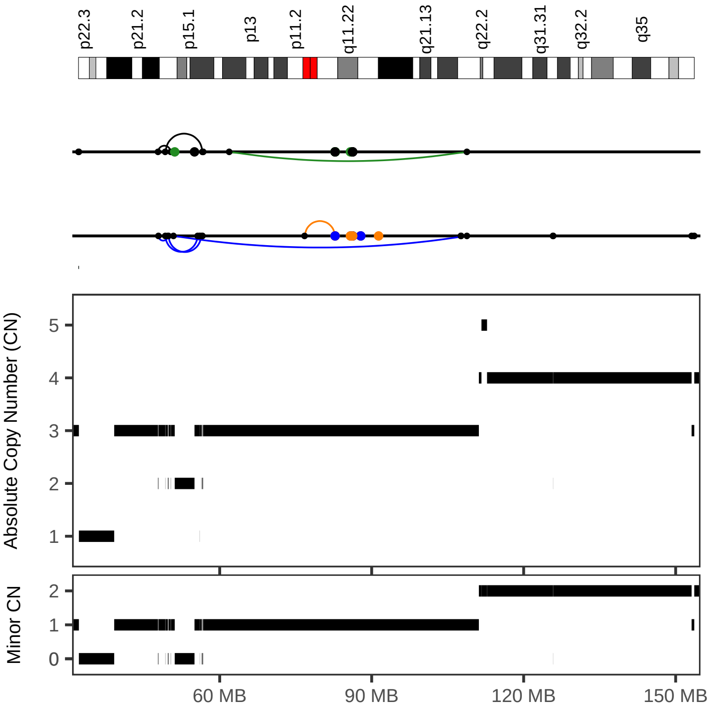

	A21−0096_CRUK_PC_0096
Cancer type	Prost−AdenoCA
Position	7:47819326−108795651
Type	Canonical without polyploidization
Interleaved intrachr. SVs	8
Total SVs (intrachr. + transl.)	35
SV types	DEL: 0; DUP: 4; h2hINV: 3; t2tINV: 1; TRA: 27
SVs in sample	496
Oscillating CN (2 and 3 states)	10, 16
CN segments	16
FDR fragment joints	0.59
FDR chr. breakp. enrich.	0.02
Linked to chrs	10:9280803−129008364;
Purity, ploidy	NA, NA

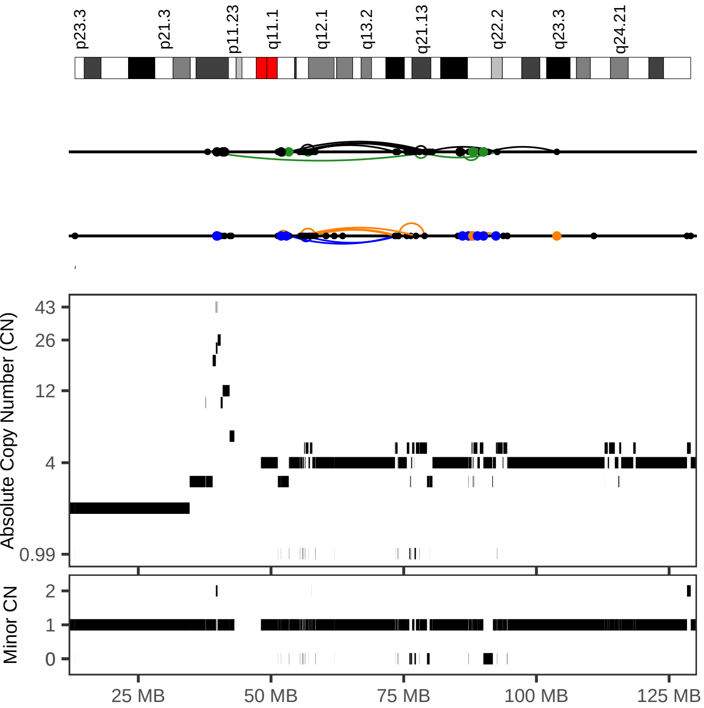

	A21−0096_CRUK_PC_0096
Cancer type	Prost−AdenoCA
Position	8:38045408−103847169
Type	With other complex events
Interleaved intrachr. SVs	27
Total SVs (intrachr. + transl.)	49
SV types	DEL: 6; DUP: 3; h2hINV: 9; t2tINV: 9; TRA: 22
SVs in sample	496
Oscillating CN (2 and 3 states)	14, 19
CN segments	87
FDR fragment joints	0.59
FDR chr. breakp. enrich.	0
Linked to chrs	6:110920236−118495779;
Purity, ploidy	NA, NA

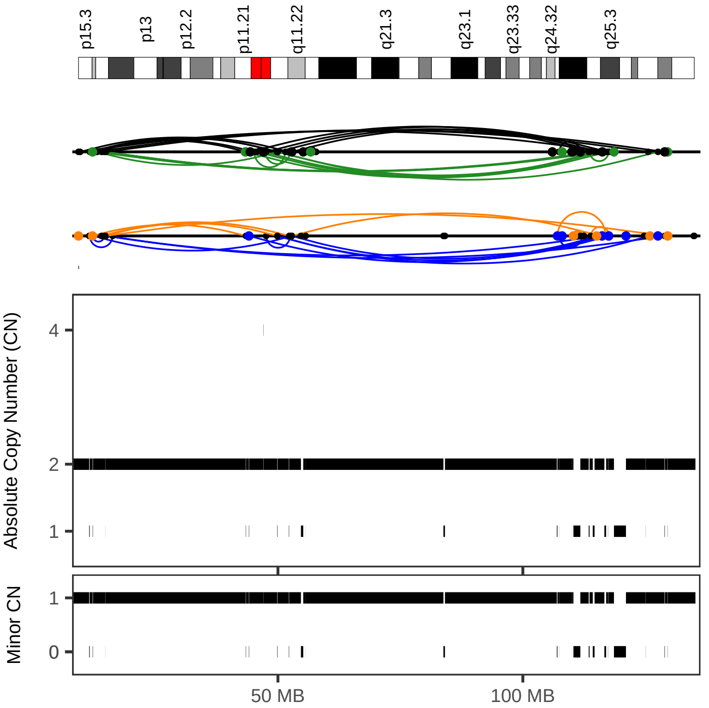

	A21−0096_CRUK_PC_0096
Cancer type	Prost−AdenoCA
Position	10:9280803−129008365
Type	Canonical without polyploidization
Interleaved intrachr. SVs	44
Total SVs (intrachr. + transl.)	74
SV types	DEL: 8; DUP: 10; h2hINV: 13; t2tINV: 13; TRA: 30
SVs in sample	496
Oscillating CN (2 and 3 states)	39, 55
CN segments	55
FDR fragment joints	0.73
FDR chr. breakp. enrich.	0
Linked to chrs	6:110920236−118495779;7:47819326−108795650
Purity, ploidy	NA, NA

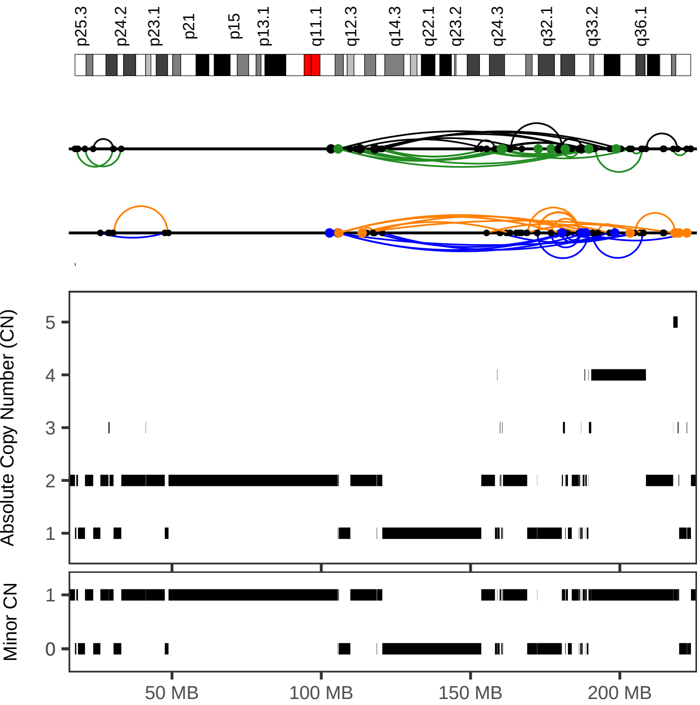

	**A24−0021_CRUK_PC_0021**
Cancer type	Prost−AdenoCA
Position	2:104733057−222529357
Type	With other complex events
Interleaved intrachr. SVs	67
Total SVs (intrachr. + transl.)	92
SV types	DEL: 16; DUP: 20; h2hINV: 12; t2tINV: 19; TRA: 25
SVs in sample	313
Oscillating CN (2 and 3 states)	10, 15
CN segments	57
FDR fragment joints	0.64
FDR chr. breakp. enrich.	0
Linked to chrs	1:39903182−157347896;16:1786815−72144385
Purity, ploidy	NA, NA

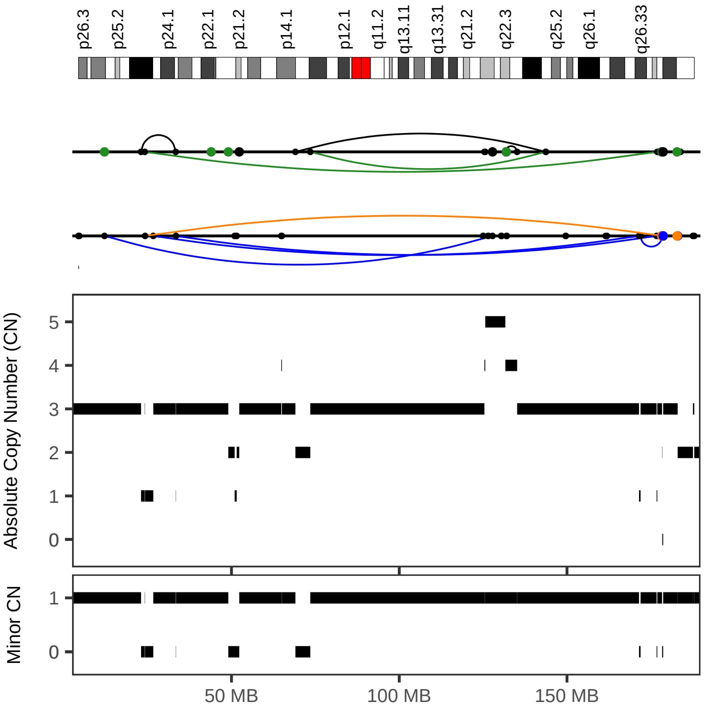

	**A24−0021_CRUK_PC_0021**
Cancer type	Prost−AdenoCA
Position	3:12152327−178682467
Type	With other complex events
Interleaved intrachr. SVs	11
Total SVs (intrachr. + transl.)	31
SV types	DEL: 1; DUP: 4; h2hINV: 3; t2tINV: 3; TRA: 20
SVs in sample	313
Oscillating CN (2 and 3 states)	7, 13
CN segments	26
FDR fragment joints	0.71
FDR chr. breakp. enrich.	0
Linked to chrs	11:14837827−64851797;6:78541847−151587658
Purity, ploidy	NA, NA

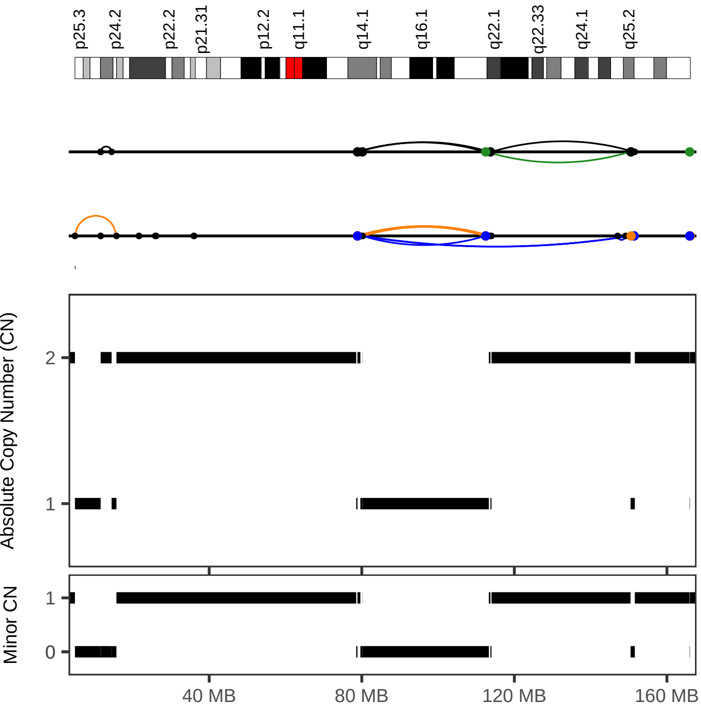

	**A24−0021_CRUK_PC_0021**
Cancer type	Prost−AdenoCA
Position	6:78541847−151587659
Type	Canonical without polyploidization
Interleaved intrachr. SVs	15
Total SVs (intrachr. + transl.)	29
SV types	DEL: 5; DUP: 6; h2hINV: 3; t2tINV: 1; TRA: 14
SVs in sample	313
Oscillating CN (2 and 3 states)	13, 13
CN segments	13
FDR fragment joints	0.59
FDR chr. breakp. enrich.	0
Linked to chrs	3:12152327−178682466;
Purity, ploidy	NA, NA

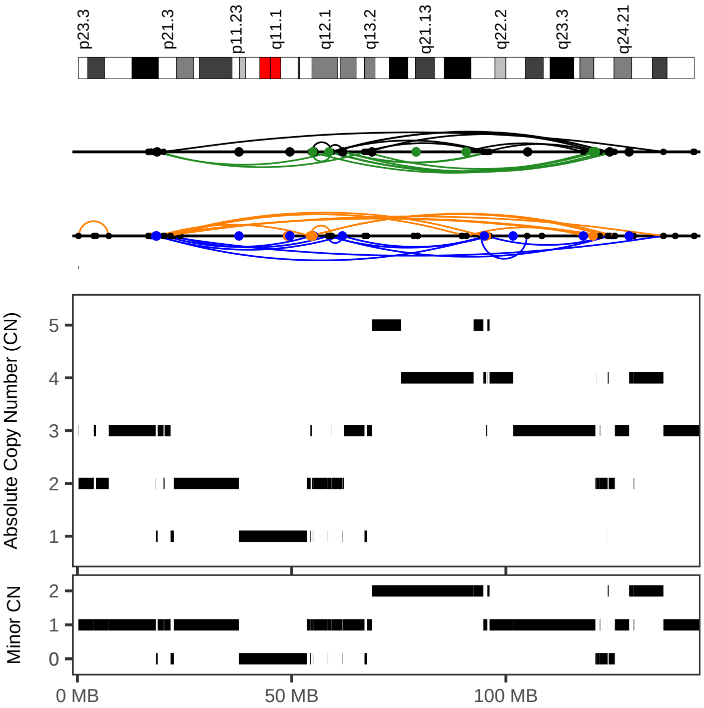

	**A34−0022_CRUK_PC_0022**
Cancer type	Prost−AdenoCA
Position	8:18259748−136762151
Type	With other complex events
Interleaved intrachr. SVs	51
Total SVs (intrachr. + transl.)	93
SV types	DEL: 13; DUP: 12; h2hINV: 13; t2tINV: 13; TRA: 42
SVs in sample	461
Oscillating CN (2 and 3 states)	11, 16
CN segments	67
FDR fragment joints	1
FDR chr. breakp. enrich.	0
Linked to chrs	13:75922651−79259285;2:31507439−200933012
Purity, ploidy	NA, NA

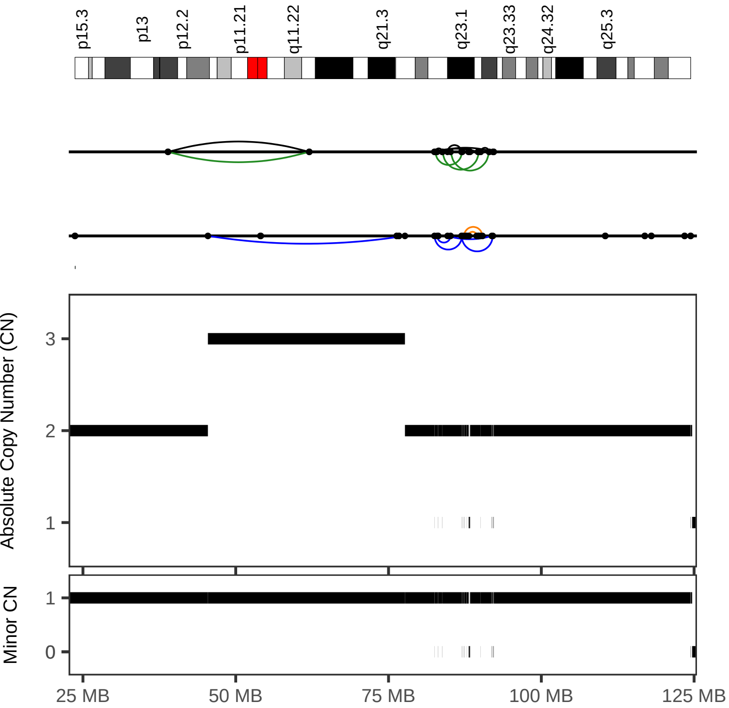

	A34−0022_CRUK_PC_0022
Cancer type	Prost−AdenoCA
Position	10:82501407−92221074
Type	Canonical without polyploidization
Interleaved intrachr. SVs	19
Total SVs (intrachr. + transl.)	19
SV types	DEL: 4; DUP: 5; h2hINV: 5; t2tINV: 5; TRA: 0
SVs in sample	461
Oscillating CN (2 and 3 states)	23, 23
CN segments	23
FDR fragment joints	0.99
FDR chr. breakp. enrich.	0.01
Linked to chrs	
Purity, ploidy	NA, NA

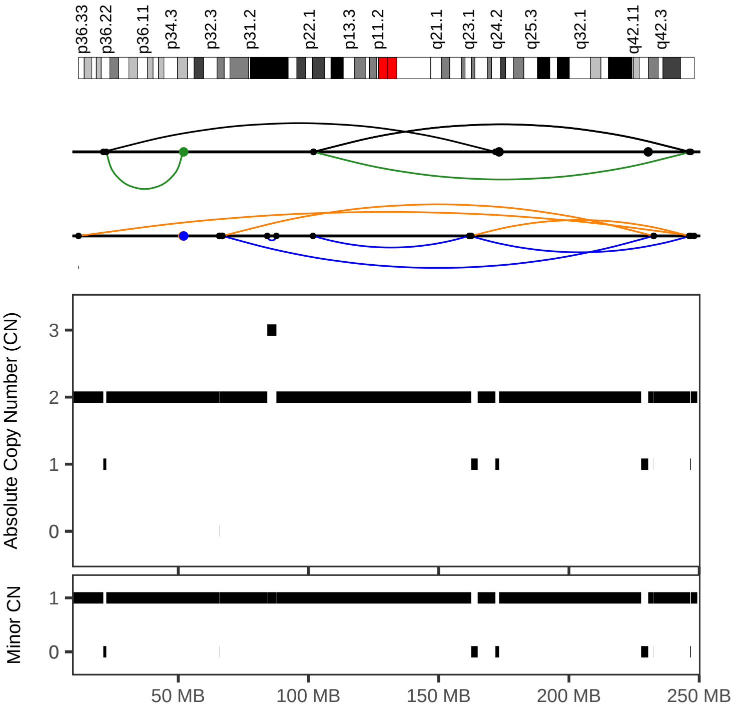

	3acd8328−76f7−4f43−9660−394b95748c3d
Cancer type	Prost−AdenoCA
Position	1:21235981−246814200
Type	Canonical without polyploidization
Interleaved intrachr. SVs	9
Total SVs (intrachr. + transl.)	14
SV types	DEL: 2; DUP: 3; h2hINV: 3; t2tINV: 1; TRA: 5
SVs in sample	88
Oscillating CN (2 and 3 states)	11, 11
CN segments	16
FDR fragment joints	0.83
FDR chr. breakp. enrich.	0
Linked to chrs	5:54730905−58095208;
Purity, ploidy	0.29, 1.87

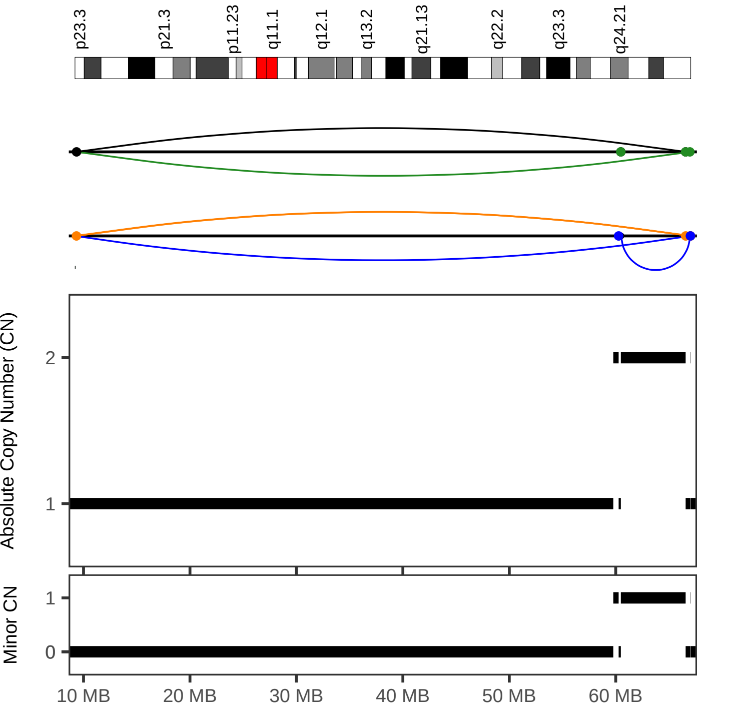

	3acd8328−76f7−4f43−9660−394b95748c3d
Cancer type	Prost−AdenoCA
Position	8:9194649−67046989
Type	Canonical without polyploidization
Interleaved intrachr. SVs	7
Total SVs (intrachr. + transl.)	20
SV types	DEL: 2; DUP: 3; h2hINV: 1; t2tINV: 1; TRA: 13
SVs in sample	88
Oscillating CN (2 and 3 states)	9, 9
CN segments	9
FDR fragment joints	0.74
FDR chr. breakp. enrich.	0
Linked to chrs	17:10827228−11022985;
Purity, ploidy	0.29, 1.87

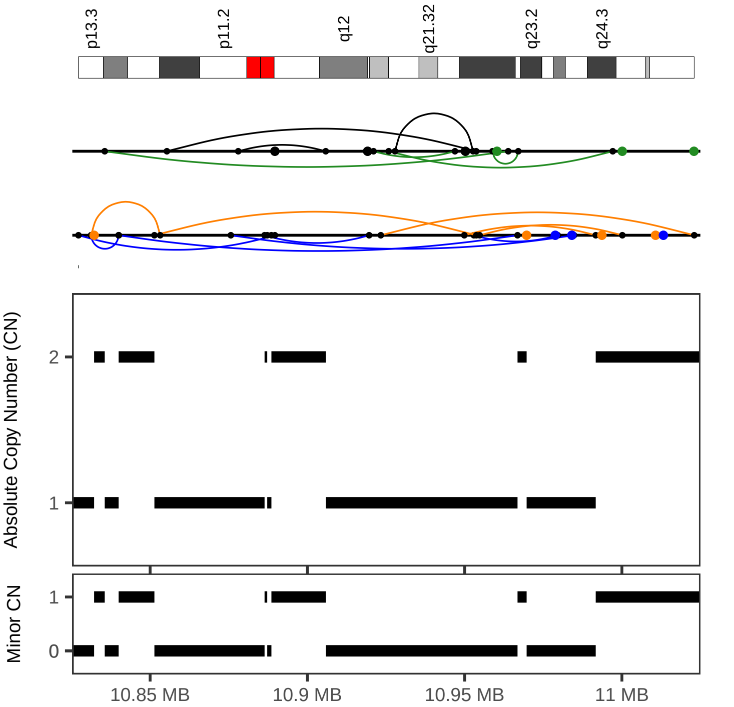

	3acd8328−76f7−4f43−9660−394b95748c3d
Cancer type	Prost−AdenoCA
Position	17:10827228−11022986
Type	Canonical without polyploidization
Interleaved intrachr. SVs	16
Total SVs (intrachr. + transl.)	29
SV types	DEL: 5; DUP: 5; h2hINV: 3; t2tINV: 3; TRA: 13
SVs in sample	88
Oscillating CN (2 and 3 states)	12, 12
CN segments	12
FDR fragment joints	0.84
FDR chr. breakp. enrich.	0
Linked to chrs	8:9194649−67046988;
Purity, ploidy	0.29, 1.87

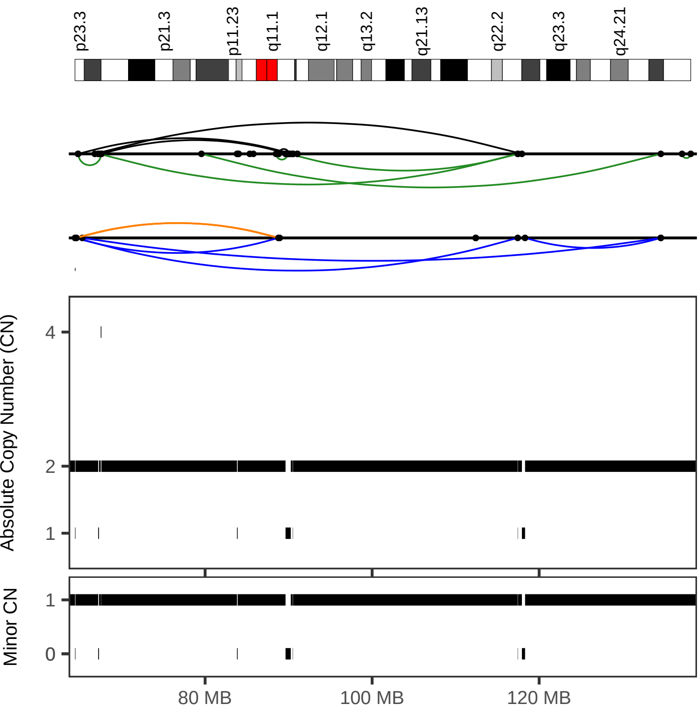

	4fe004e5−49f9−4336−a690−6ebad512c813
Cancer type	Prost−AdenoCA
Position	8:64418681−134574191
Type	Canonical without polyploidization
Interleaved intrachr. SVs	18
Total SVs (intrachr. + transl.)	18
SV types	DEL: 2; DUP: 3; h2hINV: 6; t2tINV: 7; TRA: 0
SVs in sample	126
Oscillating CN (2 and 3 states)	11, 16
CN segments	16
FDR fragment joints	0.59
FDR chr. breakp. enrich.	0
Linked to chrs	
Purity, ploidy	0.21, 1.89

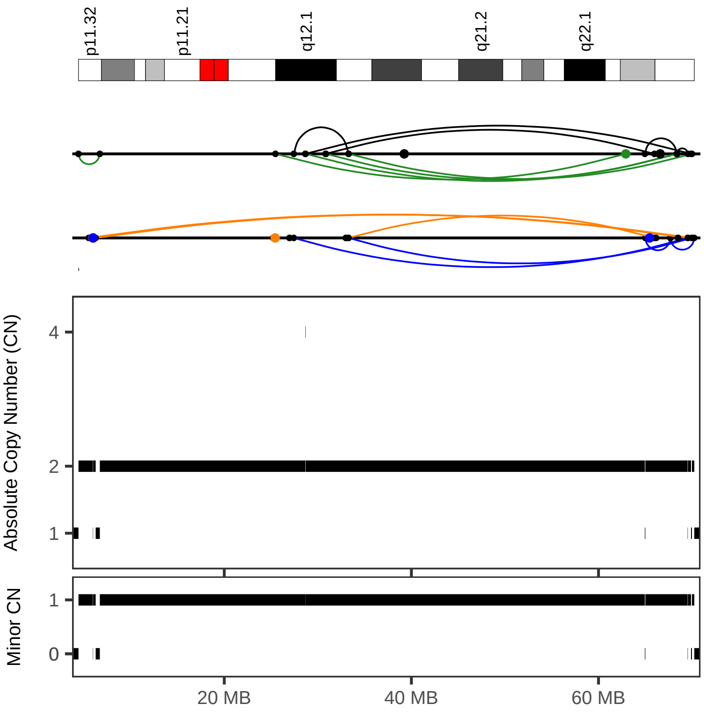

	4fe004e5−49f9−4336−a690−6ebad512c813
Cancer type	Prost−AdenoCA
Position	18:4417928−70232682
Type	With other complex events
Interleaved intrachr. SVs	17
Total SVs (intrachr. + transl.)	24
SV types	DEL: 3; DUP: 4; h2hINV: 5; t2tINV: 5; TRA: 7
SVs in sample	126
Oscillating CN (2 and 3 states)	8, 14
CN segments	14
FDR fragment joints	0.92
FDR chr. breakp. enrich.	0
Linked to chrs	
Purity, ploidy	0.21, 1.89

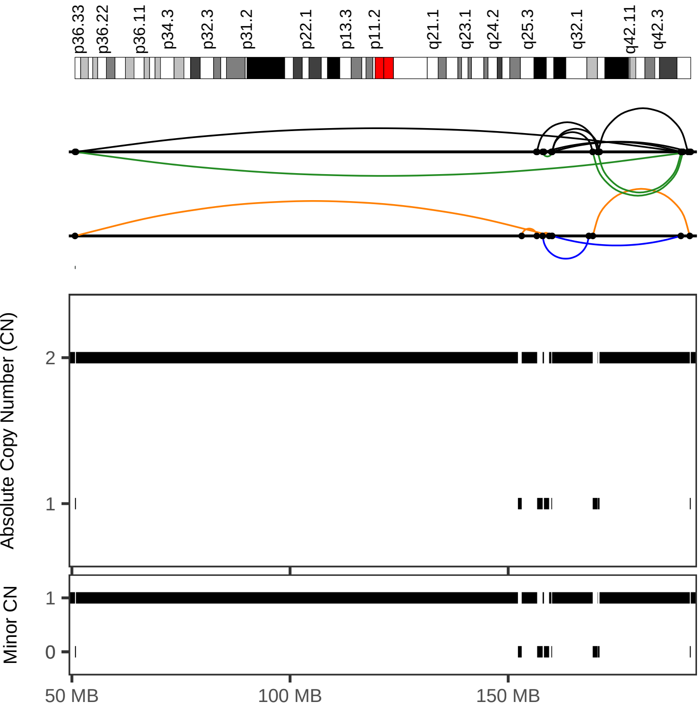

	5dd69c3e−9a3d−450c−9f10−10b4d6daeeba
Cancer type	Prost−AdenoCA
Position	1:50713589−191622424
Type	Canonical without polyploidization
Interleaved intrachr. SVs	19
Total SVs (intrachr. + transl.)	19
SV types	DEL: 4; DUP: 2; h2hINV: 9; t2tINV: 4; TRA: 0
SVs in sample	39
Oscillating CN (2 and 3 states)	15, 15
CN segments	15
FDR fragment joints	0.56
FDR chr. breakp. enrich.	0
Linked to chrs	
Purity, ploidy	0.79, 1.92

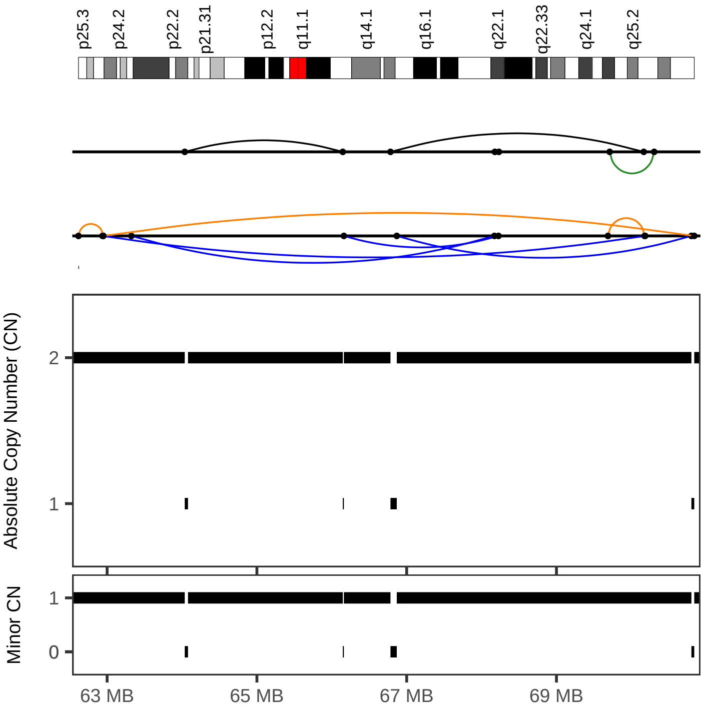

	5dd69c3e−9a3d−450c−9f10−10b4d6daeeba
Cancer type	Prost−AdenoCA
Position	6:62617511−70840186
Type	Canonical without polyploidization
Interleaved intrachr. SVs	10
Total SVs (intrachr. + transl.)	10
SV types	DEL: 3; DUP: 4; h2hINV: 1; t2tINV: 2; TRA: 0
SVs in sample	39
Oscillating CN (2 and 3 states)	9, 9
CN segments	9
FDR fragment joints	0.64
FDR chr. breakp. enrich.	0
Linked to chrs	
Purity, ploidy	0.79, 1.92

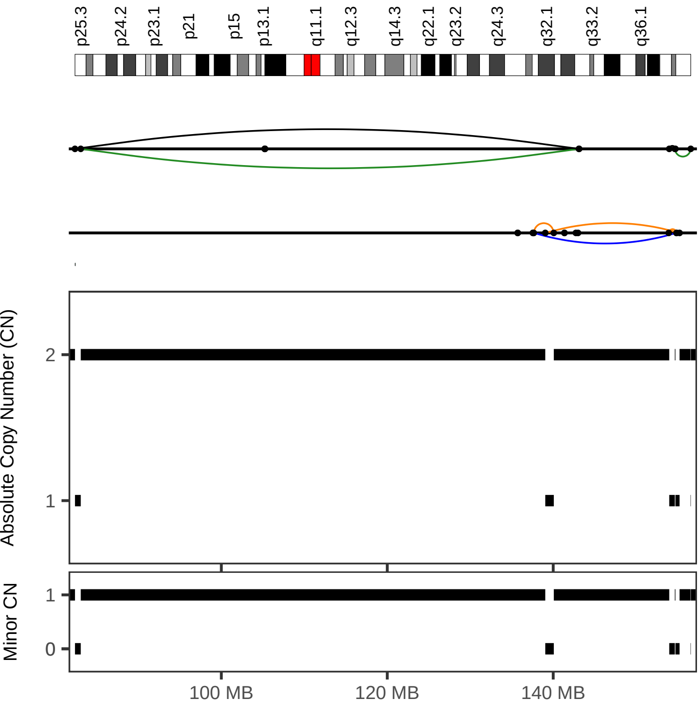

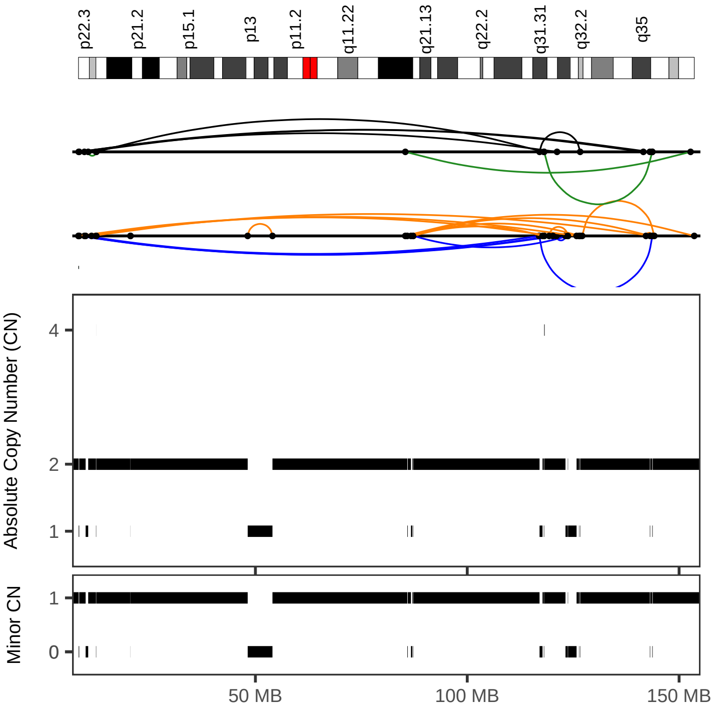

	73f875cb−bcfc−43ba−ab97−4e94caf836d4
Cancer type	Prost−AdenoCA
Position	2:82362285−156575950
Type	Canonical without polyploidization
Interleaved intrachr. SVs	8
Total SVs (intrachr. + transl.)	8
SV types	DEL: 3; DUP: 1; h2hINV: 2; t2tINV: 2; TRA: 0
SVs in sample	144
Oscillating CN (2 and 3 states)	10, 10
CN segments	10
FDR fragment joints	0.84
FDR chr. breakp. enrich.	0.77
Linked to chrs	
Purity, ploidy	0.29, 1.84

	73f875cb−bcfc−43ba−ab97−4e94caf836d4
Cancer type	Prost−AdenoCA
Position	7:8238638−153576066
Type	With other complex events
Interleaved intrachr. SVs	25
Total SVs (intrachr. + transl.)	25
SV types	DEL: 11; DUP: 6; h2hINV: 5; t2tINV: 3; TRA: 0
SVs in sample	144
Oscillating CN (2 and 3 states)	14, 14
CN segments	35
FDR fragment joints	0.58
FDR chr. breakp. enrich.	0
Linked to chrs	
Purity, ploidy	0.29, 1.84

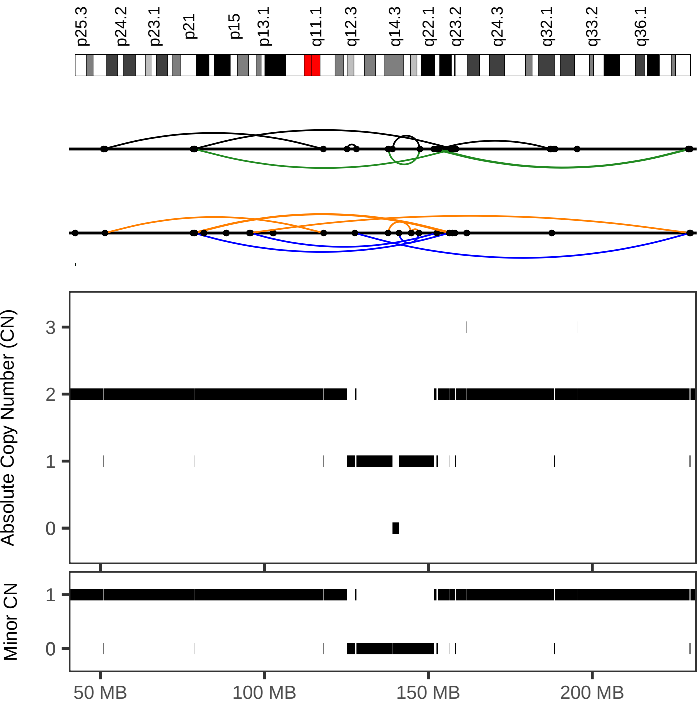

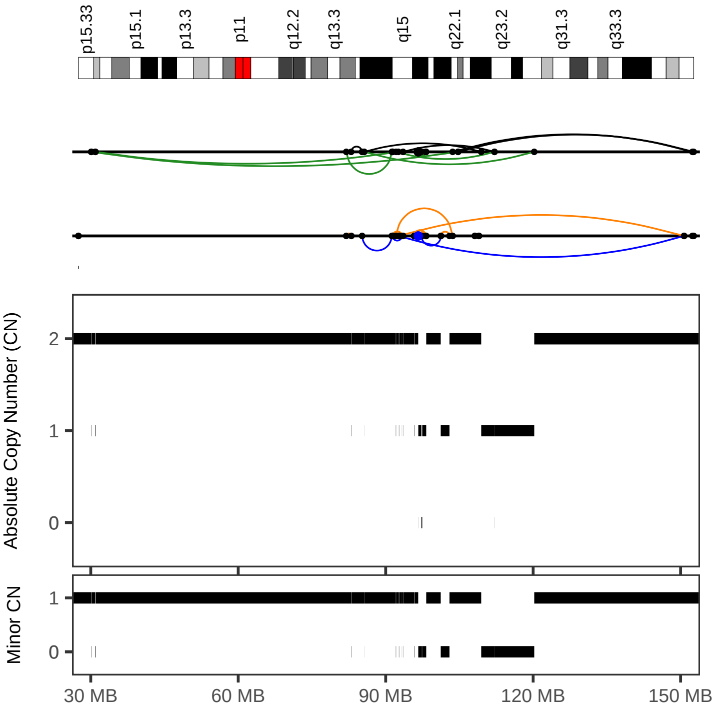

	c039014f−821c−43d6−9d3c−914f9c8d98fa
Cancer type	Prost−AdenoCA
Position	2:50901965−229972547
Type	With other complex events
Interleaved intrachr. SVs	24
Total SVs (intrachr. + transl.)	24
SV types	DEL: 6; DUP: 3; h2hINV: 7; t2tINV: 8; TRA: 0
SVs in sample	222
Oscillating CN (2 and 3 states)	15, 36
CN segments	36
FDR fragment joints	0.64
FDR chr. breakp. enrich.	0
Linked to chrs	
Purity, ploidy	0.85, 1.96

	c039014f−821c−43d6−9d3c−914f9c8d98fa
Cancer type	Prost−AdenoCA
Position	5:30074933−152675583
Type	Canonical without polyploidization
Interleaved intrachr. SVs	23
Total SVs (intrachr. + transl.)	25
SV types	DEL: 7; DUP: 3; h2hINV: 7; t2tINV: 6; TRA: 2
SVs in sample	222
Oscillating CN (2 and 3 states)	21, 32
CN segments	32
FDR fragment joints	0.67
FDR chr. breakp. enrich.	0
Linked to chrs	
Purity, ploidy	0.85, 1.96

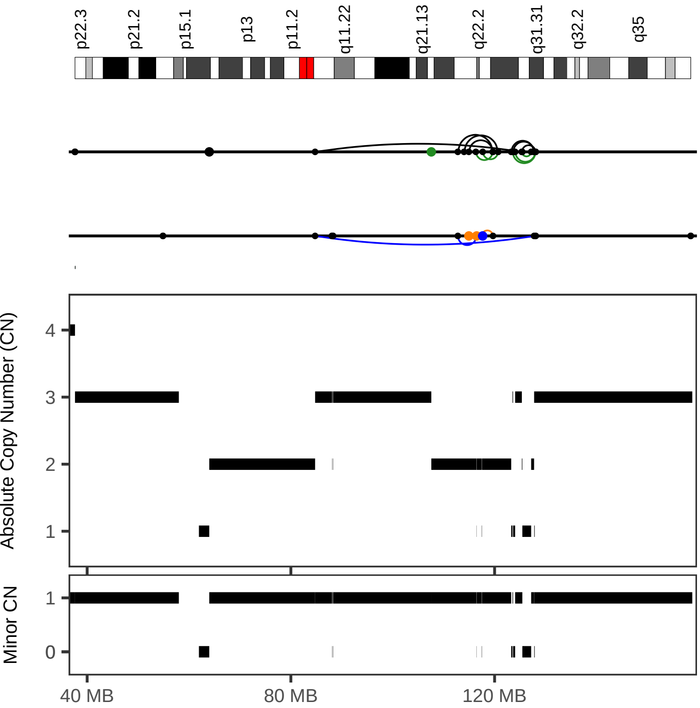

	c039014f−821c−43d6−9d3c−914f9c8d98fa
Cancer type	Prost−AdenoCA
Position	7:84753907−128099139
Type	With other complex events
Interleaved intrachr. SVs	10
Total SVs (intrachr. + transl.)	14
SV types	DEL: 0; DUP: 2; h2hINV: 4; t2tINV: 4; TRA: 4
SVs in sample	222
Oscillating CN (2 and 3 states)	7, 10
CN segments	21
FDR fragment joints	0.59
FDR chr. breakp. enrich.	0
Linked to chrs	11:29320290−106781286;
Purity, ploidy	0.85, 1.96

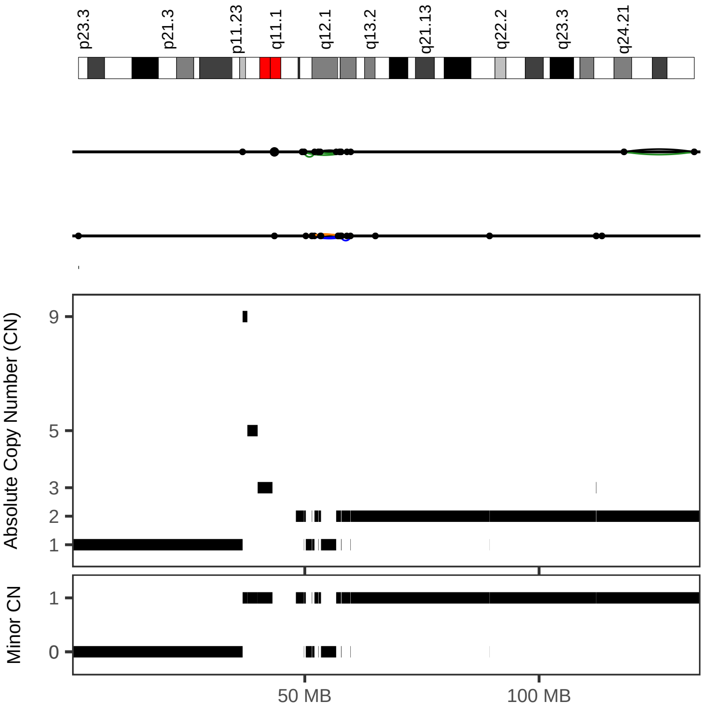

	c039014f−821c−43d6−9d3c−914f9c8d98fa
Cancer type	Prost−AdenoCA
Position	8:49431428−59807696
Type	Canonical without polyploidization
Interleaved intrachr. SVs	17
Total SVs (intrachr. + transl.)	17
SV types	DEL: 5; DUP: 4; h2hINV: 4; t2tINV: 4; TRA: 0
SVs in sample	222
Oscillating CN (2 and 3 states)	19, 19
CN segments	19
FDR fragment joints	0.99
FDR chr. breakp. enrich.	0
Linked to chrs	
Purity, ploidy	0.85, 1.96

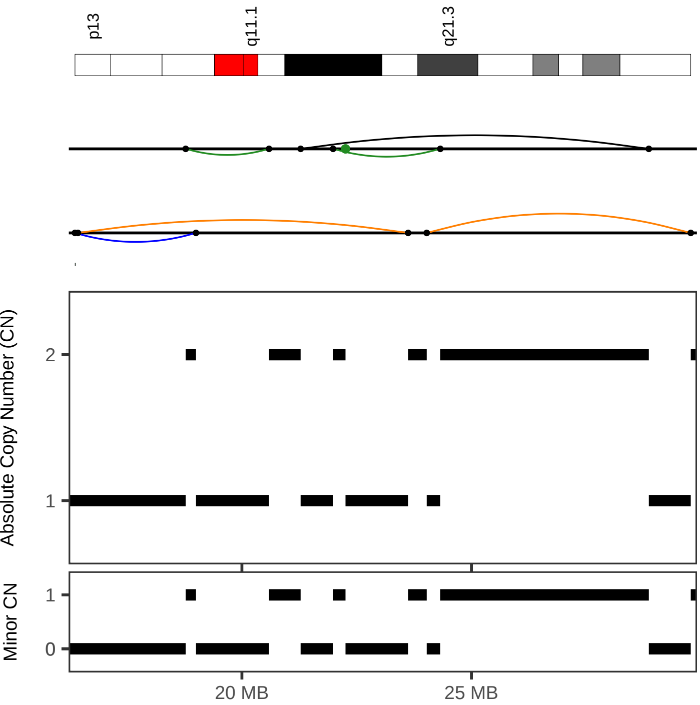

	c039014f−821c−43d6−9d3c−914f9c8d98fa
Cancer type	Prost−AdenoCA
Position	21:16364103−29777341
Type	Canonical without polyploidization
Interleaved intrachr. SVs	6
Total SVs (intrachr. + transl.)	7
SV types	DEL: 2; DUP: 1; h2hINV: 1; t2tINV: 2; TRA: 1
SVs in sample	222
Oscillating CN (2 and 3 states)	12, 12
CN segments	12
FDR fragment joints	0.91
FDR chr. breakp. enrich.	0.08
Linked to chrs	
Purity, ploidy	0.85, 1.96

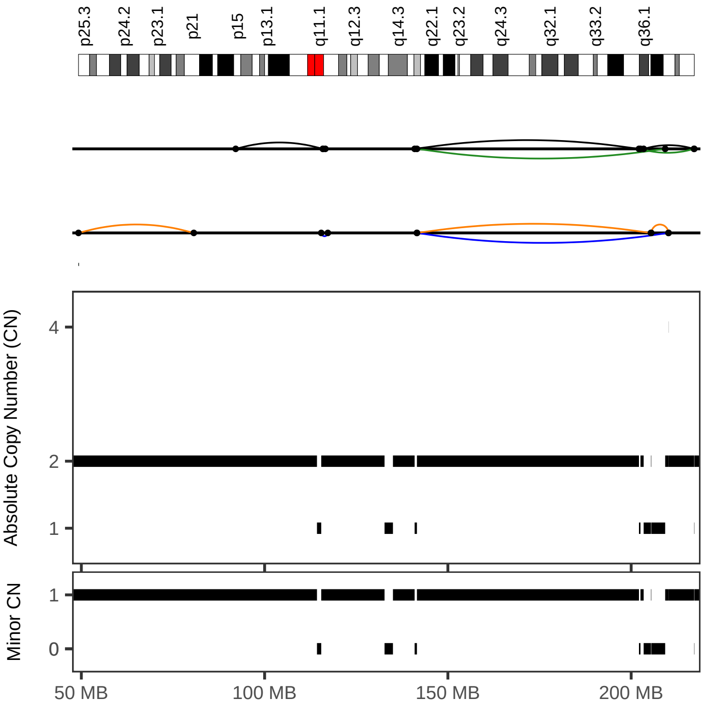

	da5b0169−e4f4−4674−b348−220af03ff4b5
Cancer type	Prost−AdenoCA
Position	2:140924944−217210101
Type	Canonical without polyploidization
Interleaved intrachr. SVs	7
Total SVs (intrachr. + transl.)	7
SV types	DEL: 2; DUP: 1; h2hINV: 2; t2tINV: 2; TRA: 0
SVs in sample	29
Oscillating CN (2 and 3 states)	8, 12
CN segments	12
FDR fragment joints	0.95
FDR chr. breakp. enrich.	0
Linked to chrs	
Purity, ploidy	0.19, 1.91

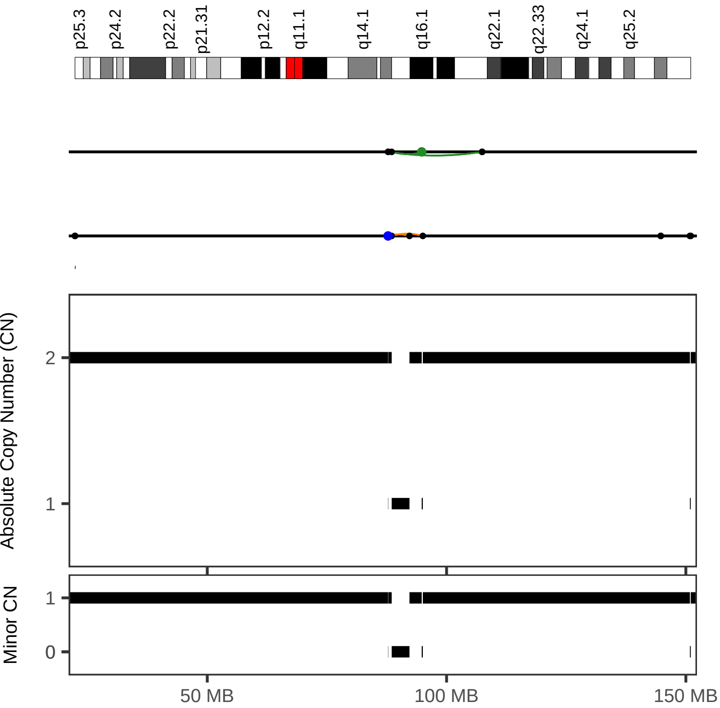

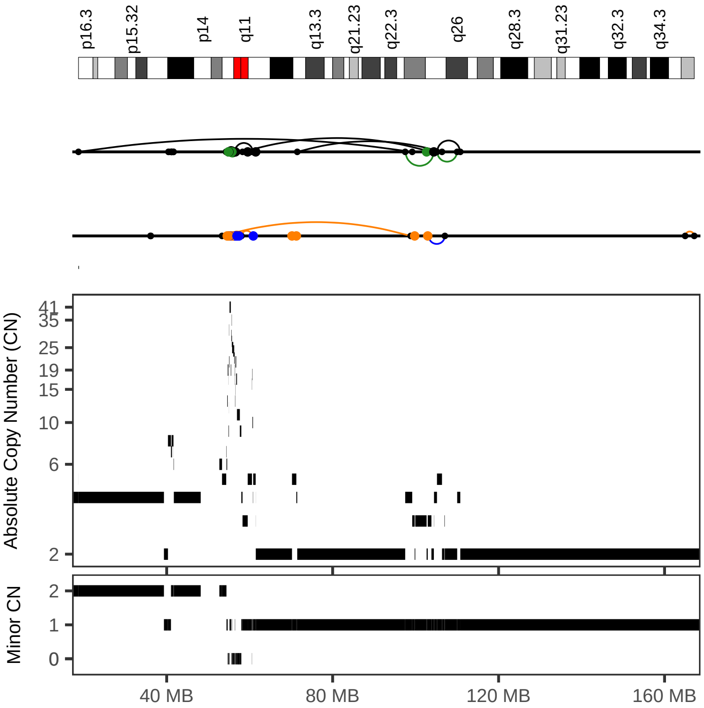

	f6381367−142c−45d0−92b3−c1727d1813ce
Cancer type	Prost−AdenoCA
Position	6:87766263−107431907
Type	Canonical without polyploidization
Interleaved intrachr. SVs	5
Total SVs (intrachr. + transl.)	7
SV types	DEL: 2; DUP: 0; h2hINV: 0; t2tINV: 3; TRA: 2
SVs in sample	133
Oscillating CN (2 and 3 states)	7, 7
CN segments	7
FDR fragment joints	0.59
FDR chr. breakp. enrich.	0.26
Linked to chrs	
Purity, ploidy	0.92, 1.9

	MELA−0001
Cancer type	Skin−Melanoma
Position	4:18741843−110768809
Type	With other complex events
Interleaved intrachr. SVs	16
Total SVs (intrachr. + transl.)	44
SV types	DEL: 4; DUP: 1; h2hINV: 6; t2tINV: 5; TRA: 28
SVs in sample	460
Oscillating CN (2 and 3 states)	7, 13
CN segments	68
FDR fragment joints	0.59
FDR chr. breakp. enrich.	0
Linked to chrs	
Purity, ploidy	0.75, 3.01

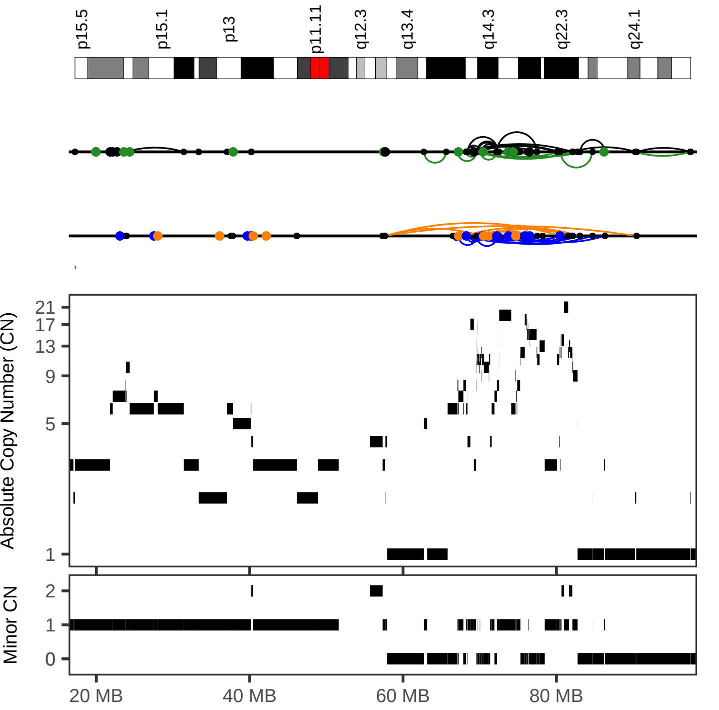

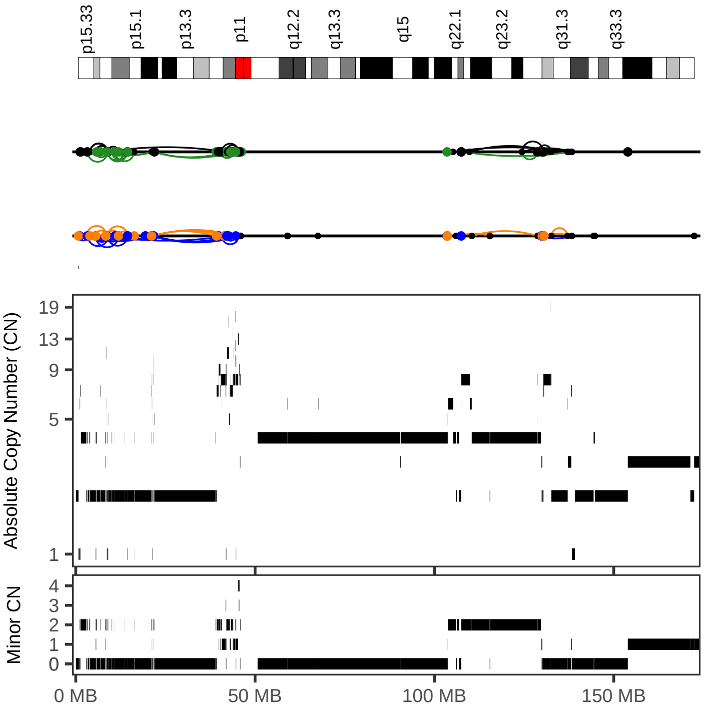

	MELA−0001
Cancer type	Skin−Melanoma
Position	11:57335469−97526275
Type	With other complex events
Interleaved intrachr. SVs	56
Total SVs (intrachr. + transl.)	79
SV types	DEL: 10; DUP: 15; h2hINV: 19; t2tINV: 12; TRA: 23
SVs in sample	460
Oscillating CN (2 and 3 states)	7, 9
CN segments	95
FDR fragment joints	0.59
FDR chr. breakp. enrich.	0
Linked to chrs	5:20025209−45767558;
Purity, ploidy	0.75, 3.01

	MELA−0002
Cancer type	Skin−Melanoma
Position	5:759044−45795365
Type	With other complex events
Interleaved intrachr. SVs	71
Total SVs (intrachr. + transl.)	135
SV types	DEL: 13; DUP: 26; h2hINV: 15; t2tINV: 17; TRA: 64
SVs in sample	748
Oscillating CN (2 and 3 states)	10, 12
CN segments	122
FDR fragment joints	0.57
FDR chr. breakp. enrich.	0
Linked to chrs	11:48612434−104669301;14:43855218−104600367
Purity, ploidy	0.54, 2.62

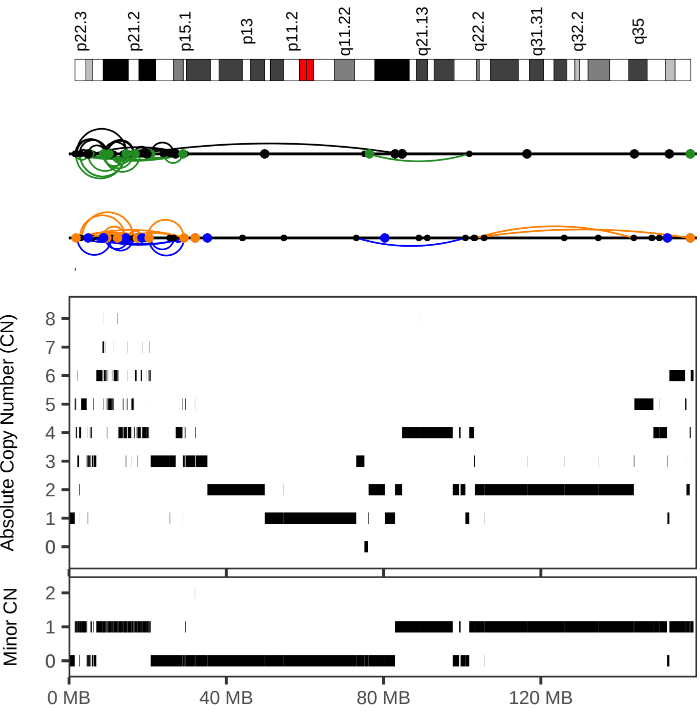

	**MELA−0002**
Cancer type	Skin−Melanoma
Position	7:1508469−101805272
Type	With other complex events
Interleaved intrachr. SVs	73
Total SVs (intrachr. + transl.)	113
SV types	DEL: 12; DUP: 15; h2hINV: 22;<br>t2tINV: 24; TRA: 40
SVs in sample	748
Oscillating CN (2 and 3 states)	10, 14
CN segments	137
FDR fragment joints	0.59
FDR chr. breakp. enrich.	0
Linked to chrs	11:48612434−104669301;2:11161529−144813239<br>22:17741148−36618399;3:69823911−79435314
Purity, ploidy	0.54, 2.62

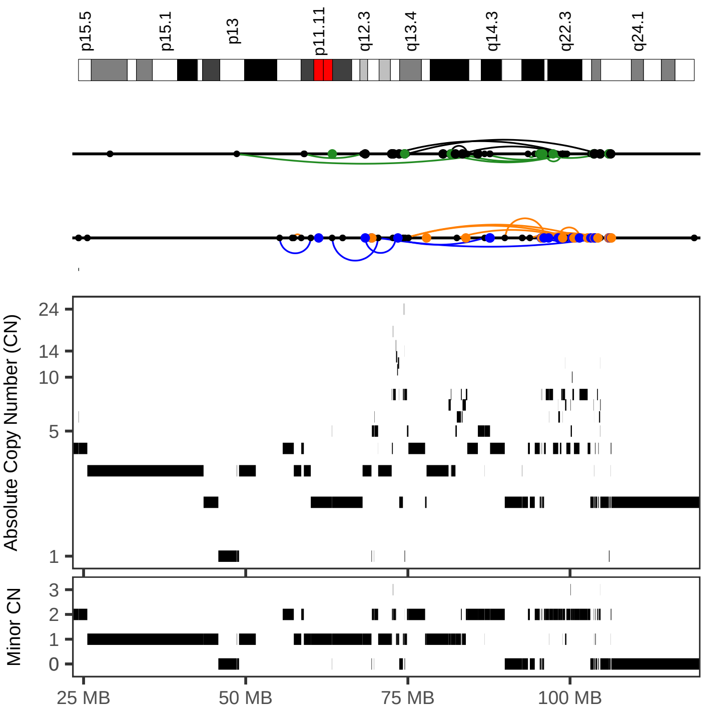

	**MELA−0002**
Cancer type	Skin−Melanoma
Position	11:48612434−104669302
Type	With other complex events
Interleaved intrachr. SVs	34
Total SVs (intrachr. + transl.)	83
SV types	DEL: 10; DUP: 7; h2hINV: 8;<br>t2tINV: 9; TRA: 49
SVs in sample	748
Oscillating CN (2 and 3 states)	7, 10
CN segments	113
FDR fragment joints	0.92
FDR chr. breakp. enrich.	0
Linked to chrs	5:759044−45795364;7:1508469−101805271
Purity, ploidy	0.54, 2.62

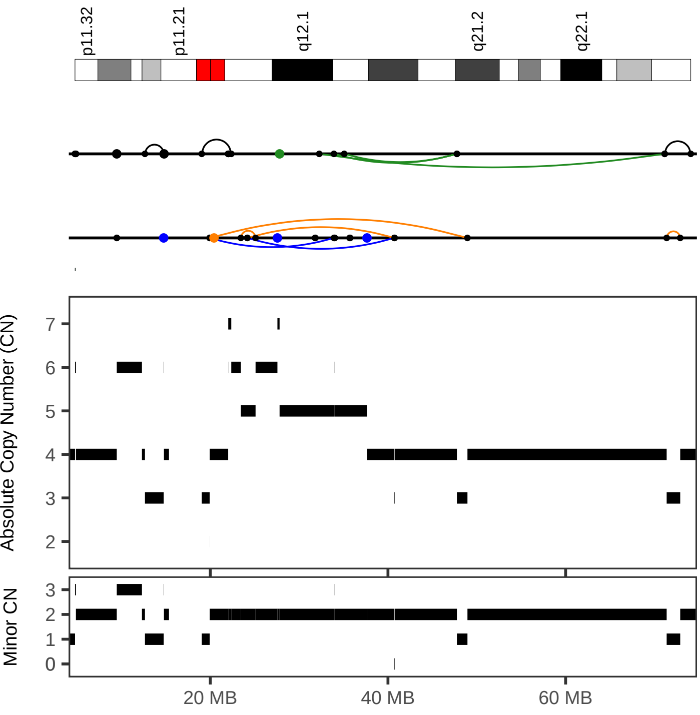

	**MELA−0002**
Cancer type	Skin−Melanoma
Position	18:19034698−74083616
Type	With other complex events
Interleaved intrachr. SVs	10
Total SVs (intrachr. + transl.)	14
SV types	DEL: 3; DUP: 2; h2hINV: 2;<br>t2tINV: 3; TRA: 4
SVs in sample	748
Oscillating CN (2 and 3 states)	7, 10
CN segments	21
FDR fragment joints	0.96
FDR chr. breakp. enrich.	0.29
Linked to chrs	
Purity, ploidy	0.54, 2.62

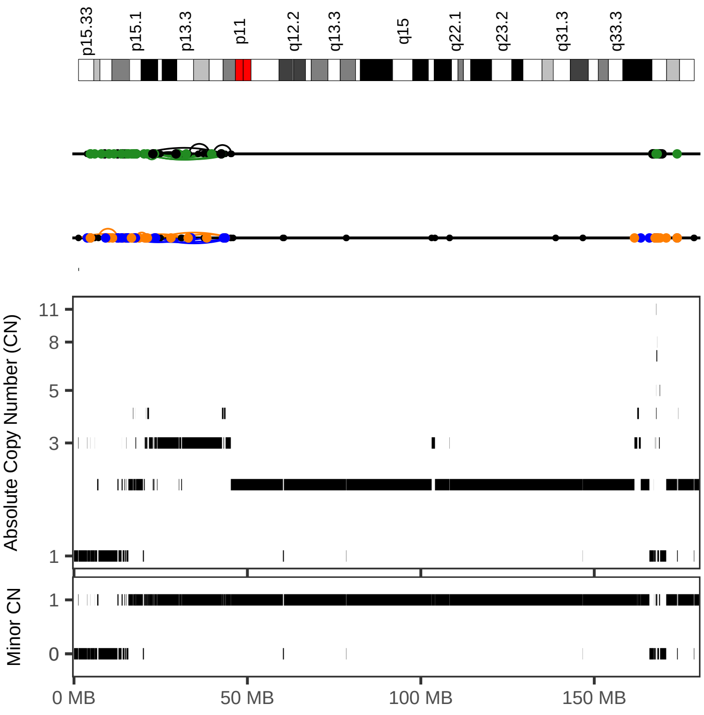

	**MELA−0003**
Cancer type	Skin−Melanoma
Position	5:1296136−45874996
Type	With other complex events
Interleaved intrachr. SVs	33
Total SVs (intrachr. + transl.)	109
SV types	DEL: 10; DUP: 7; h2hINV: 6;<br>t2tINV: 10; TRA: 76
SVs in sample	1187
Oscillating CN (2 and 3 states)	11, 27
CN segments	52
FDR fragment joints	0.75
FDR chr. breakp. enrich.	0
Linked to chrs	12:43076099−128657836;
Purity, ploidy	0.96, 1.84

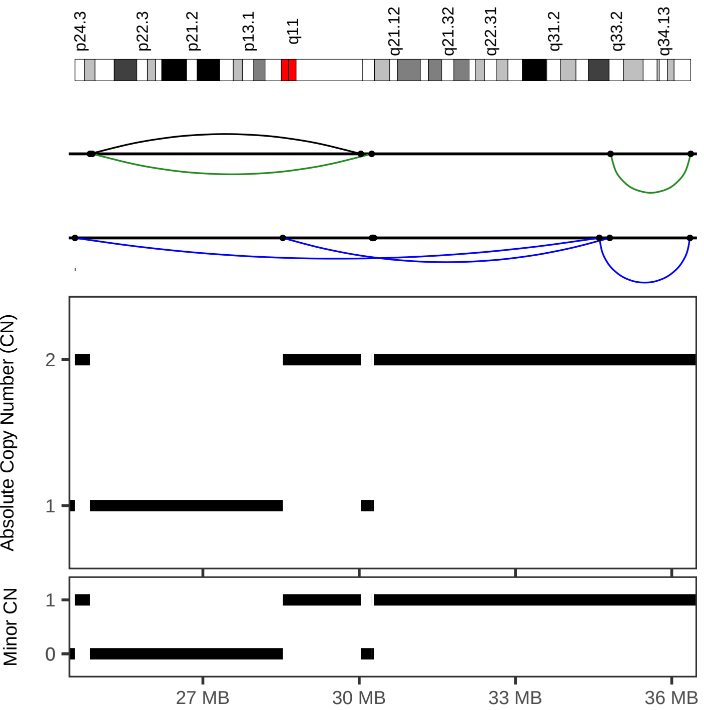

	MELA−0003
Cancer type	Skin−Melanoma
Position	9:24544471−36364603
Type	Canonical without polyploidization
Interleaved intrachr. SVs	6
Total SVs (intrachr. + transl.)	6
SV types	DEL: 0; DUP: 3; h2hINV: 1; t2tINV: 2; TRA: 0
SVs in sample	1187
Oscillating CN (2 and 3 states)	7, 7
CN segments	7
FDR fragment joints	0.59
FDR chr. breakp. enrich.	0
Linked to chrs	
Purity, ploidy	0.96, 1.84

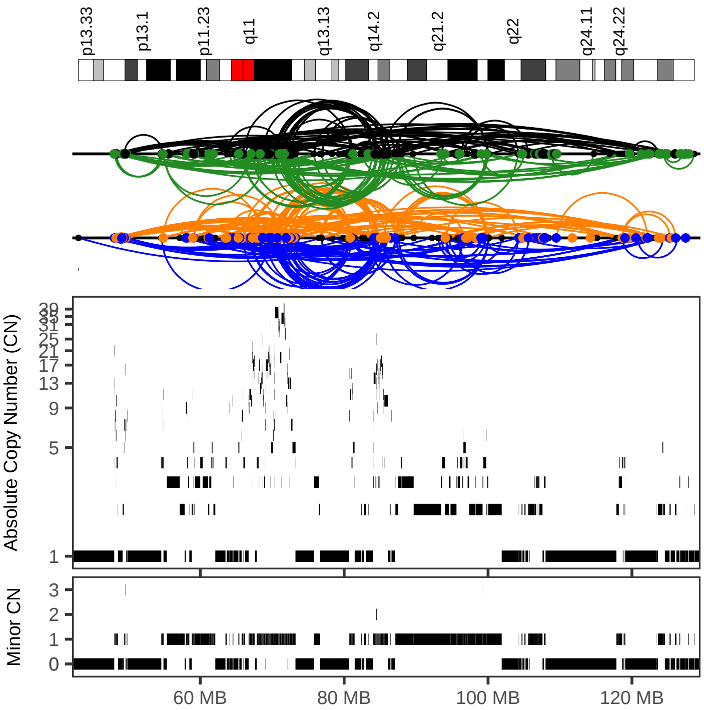

	MELA−0003
Cancer type	Skin−Melanoma
Position	12:43076099−128657837
Type	With other complex events
Interleaved intrachr. SVs	536
Total SVs (intrachr. + transl.)	881
SV types	DEL: 150; DUP: 120; h2hINV: 130; t2tINV: 136; TRA: 345
SVs in sample	1187
Oscillating CN (2 and 3 states)	11, 24
CN segments	377
FDR fragment joints	0.59
FDR chr. breakp. enrich.	0
Linked to chrs	1:15885881−58152235;4:41842565−58016946
Purity, ploidy	0.96, 1.84

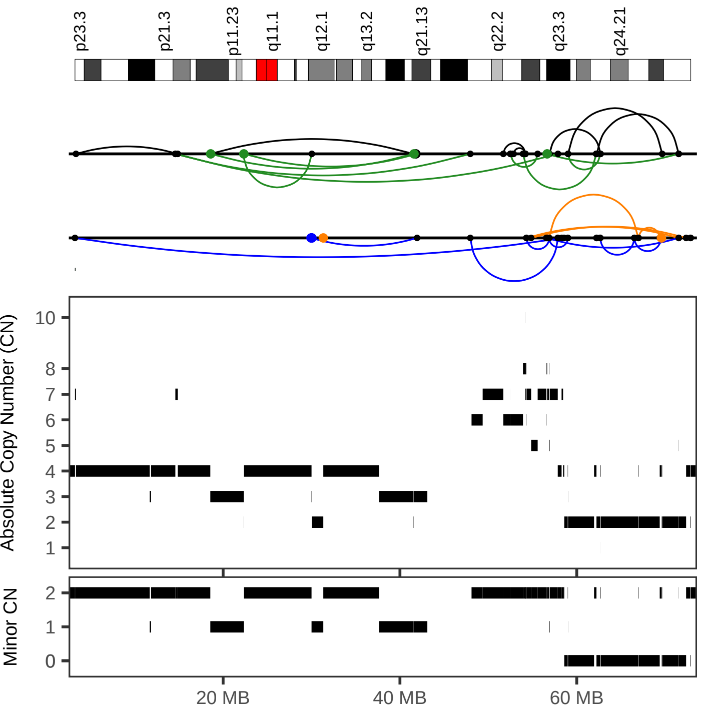

	MELA−0007
Cancer type	Skin−Melanoma
Position	8:3242350−72370952
Type	With other complex events
Interleaved intrachr. SVs	29
Total SVs (intrachr. + transl.)	39
SV types	DEL: 6; DUP: 8; h2hINV: 8; t2tINV: 7; TRA: 10
SVs in sample	377
Oscillating CN (2 and 3 states)	8, 19
CN segments	59
FDR fragment joints	0.96
FDR chr. breakp. enrich.	0
Linked to chrs	
Purity, ploidy	0.69, 3.61

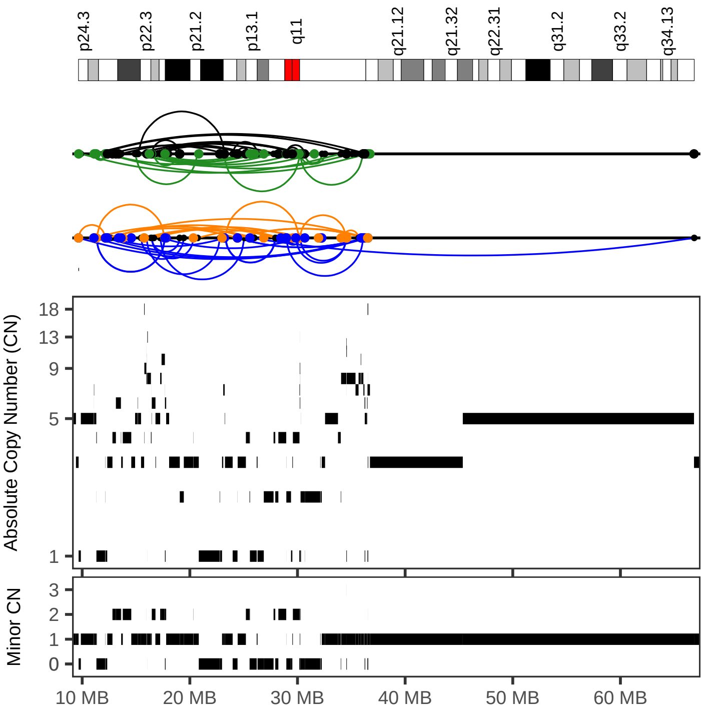

	MELA−0007
Cancer type	Skin−Melanoma
Position	9:9654286−66858139
Type	With other complex events
Interleaved intrachr. SVs	80
Total SVs (intrachr. + transl.)	202
SV types	DEL: 17; DUP: 25; h2hINV: 17; t2tINV: 21; TRA: 122
SVs in sample	377
Oscillating CN (2 and 3 states)	7, 10
CN segments	156
FDR fragment joints	0.64
FDR chr. breakp. enrich.	0
Linked to chrs	12:20581752−32372624;
Purity, ploidy	0.69, 3.61

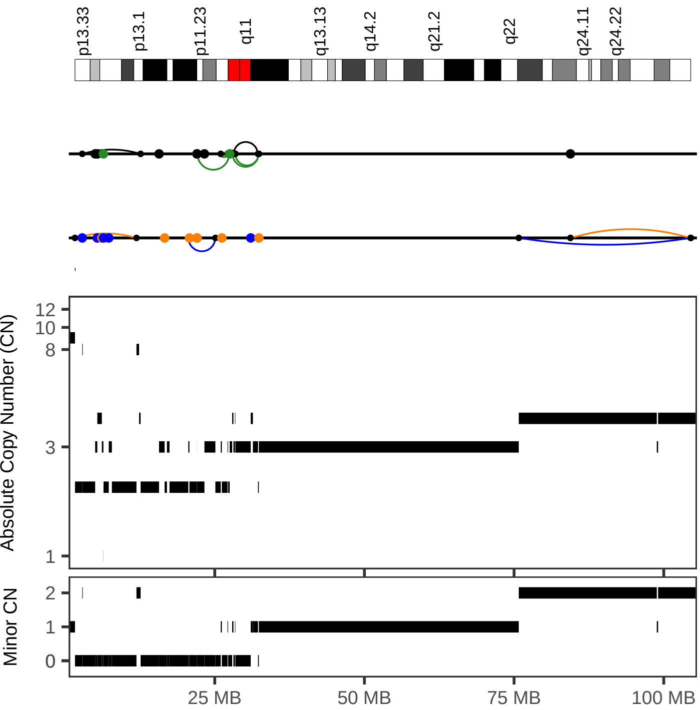

**MELA−0007**	
Cancer type	Skin−Melanoma
Position	12:20581752−32372625
Type	With other complex events
Interleaved intrachr. SVs	6
Total SVs (intrachr. + transl.)	17
SV types	DEL: 0; DUP: 1; h2hINV: 2; t2tINV: 3; TRA: 11
SVs in sample	377
Oscillating CN (2 and 3 states)	11, 21
CN segments	21
FDR fragment joints	0.59
FDR chr. breakp. enrich.	0
Linked to chrs	5:265764−28190998;9:9654286−66858138
Purity, ploidy	0.69, 3.61

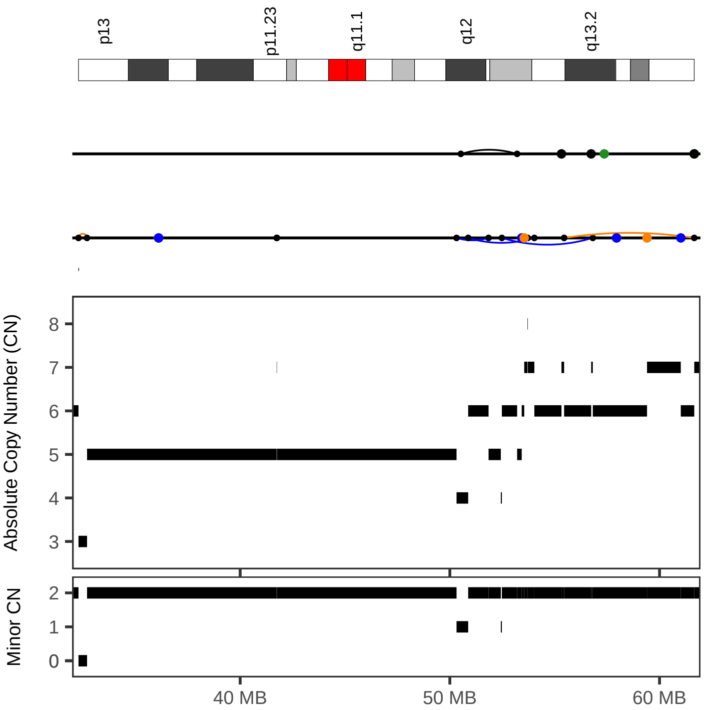

**MELA−0009**	
Cancer type	Skin−Melanoma
Position	20:50318897−61655152
Type	With other complex events
Interleaved intrachr. SVs	5
Total SVs (intrachr. + transl.)	17
SV types	DEL: 1; DUP: 3; h2hINV: 1; t2tINV: 0; TRA: 12
SVs in sample	351
Oscillating CN (2 and 3 states)	9, 14
CN segments	18
FDR fragment joints	0.59
FDR chr. breakp. enrich.	0
Linked to chrs	11:41485920−112489308;
Purity, ploidy	0.49, 3.33

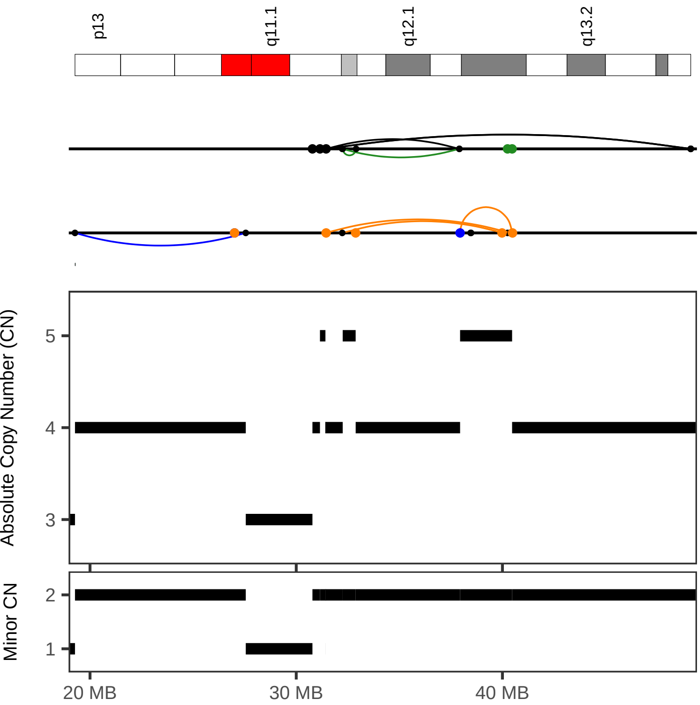

**MELA−0009**	
Cancer type	Skin−Melanoma
Position	22:31415162−49131333
Type	After polyploidization
Interleaved intrachr. SVs	9
Total SVs (intrachr. + transl.)	17
SV types	DEL: 3; DUP: 2; h2hINV: 3; t2tINV: 1; TRA: 8
SVs in sample	351
Oscillating CN (2 and 3 states)	7, 7
CN segments	7
FDR fragment joints	0.83
FDR chr. breakp. enrich.	0
Linked to chrs	3:19250926−195326588;
Purity, ploidy	0.49, 3.33

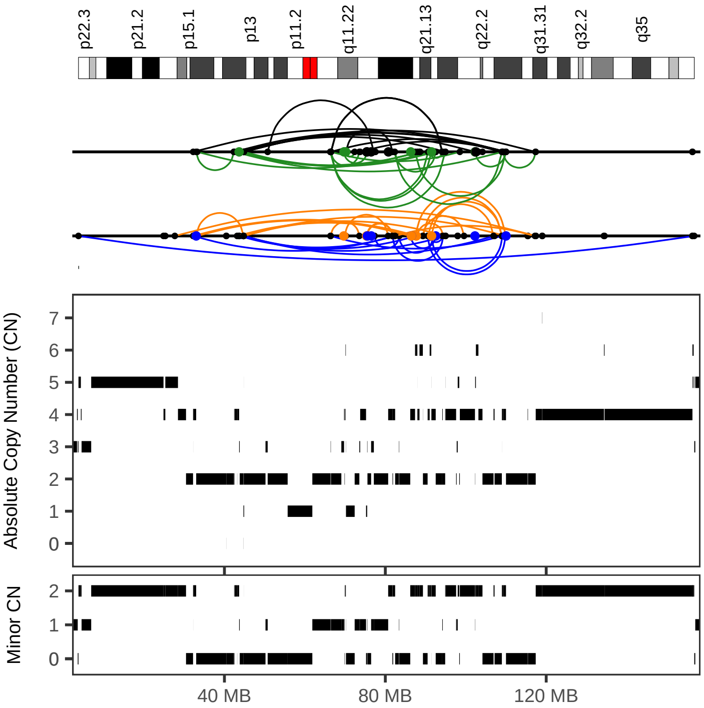

**MELA−0012**	
Cancer type	Skin−Melanoma
Position	7:32236661−117391259
Type	With other complex events
Interleaved intrachr. SVs	70
Total SVs (intrachr. + transl.)	96
SV types	DEL: 18; DUP: 15; h2hINV: 16; t2tINV: 21; TRA: 26
SVs in sample	508
Oscillating CN (2 and 3 states)	7, 15
CN segments	97
FDR fragment joints	0.83
FDR chr. breakp. enrich.	0
Linked to chrs	3:131435629−180741701;9:4860008−31734960
Purity, ploidy	0.79, 2.87

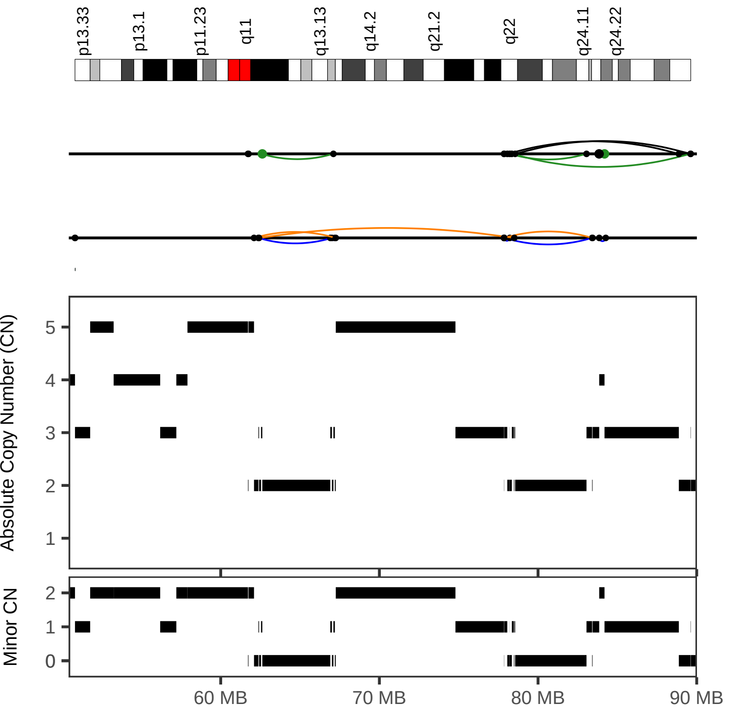

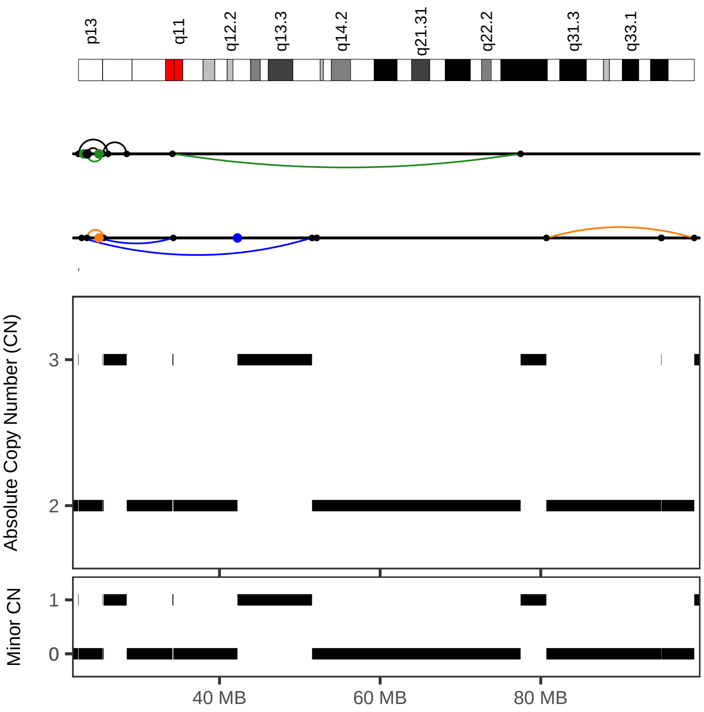

	**MELA−0012**
Cancer type	Skin−Melanoma
Position	12:62098962−89622897
Type	With other complex events
Interleaved intrachr. SVs	12
Total SVs (intrachr. + transl.)	15
SV types	DEL: 3; DUP: 4; h2hINV: 2; t2tINV: 3; TRA: 3
SVs in sample	508
Oscillating CN (2 and 3 states)	13, 18
CN segments	28
FDR fragment joints	0.91
FDR chr. breakp. enrich.	0.97
Linked to chrs	11:60665556−76908359;
Purity, ploidy	0.79, 2.87

	**MELA−0012**
Cancer type	Skin−Melanoma
Position	13:22438944−77491950
Type	Canonical without polyploidization
Interleaved intrachr. SVs	8
Total SVs (intrachr. + transl.)	14
SV types	DEL: 1; DUP: 2; h2hINV: 3; t2tINV: 2; TRA: 6
SVs in sample	508
Oscillating CN (2 and 3 states)	10, 10
CN segments	10
FDR fragment joints	0.84
FDR chr. breakp. enrich.	0.77
Linked to chrs	8:508734−74087760;
Purity, ploidy	0.79, 2.87

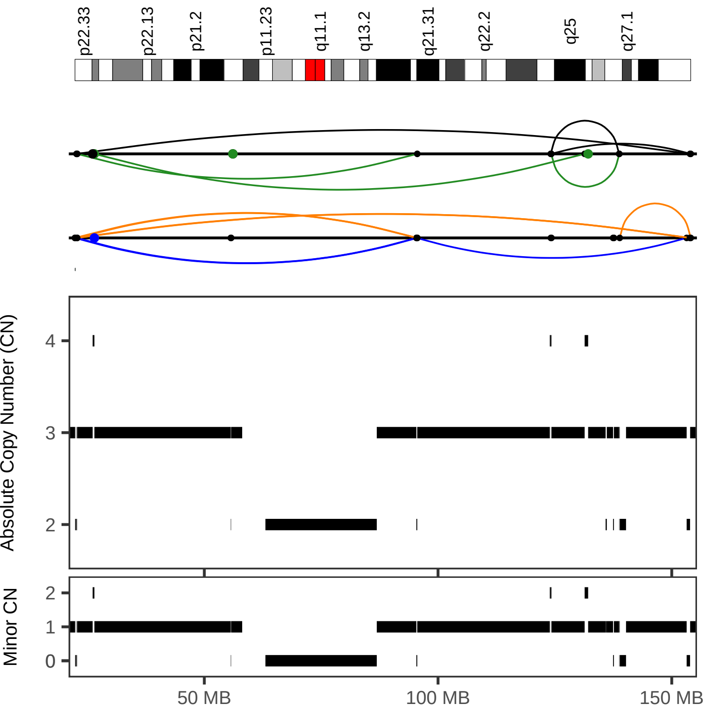

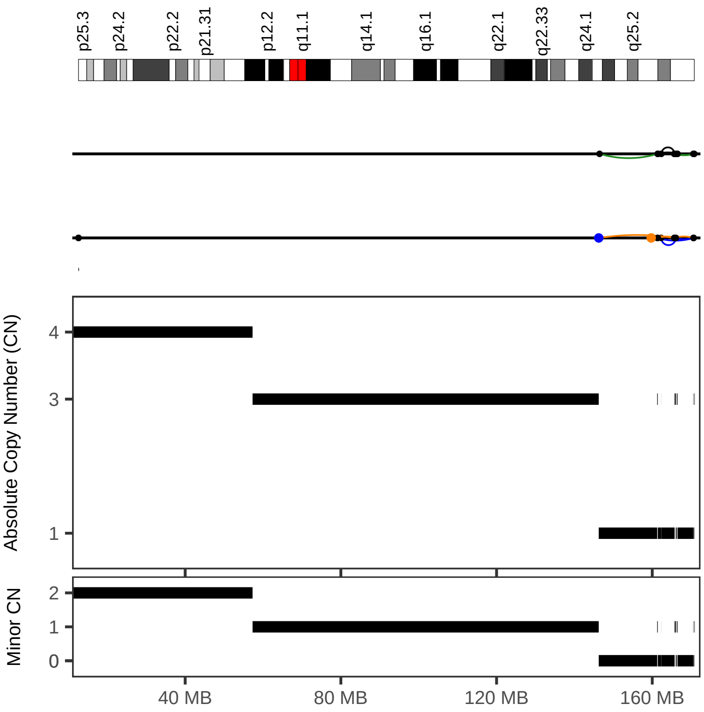

	**MELA−0012**
Cancer type	Skin−Melanoma
Position	X:22327741−154060135
Type	With other complex events
Interleaved intrachr. SVs	16
Total SVs (intrachr. + transl.)	22
SV types	DEL: 5; DUP: 4; h2hINV: 3; t2tINV: 4; TRA: 6
SVs in sample	508
Oscillating CN (2 and 3 states)	11, 34
CN segments	34
FDR fragment joints	0.94
FDR chr. breakp. enrich.	1
Linked to chrs	
Purity, ploidy	0.79, 2.87

	**MELA−0043**
Cancer type	Skin−Melanoma
Position	6:146452116−170788391
Type	Canonical without polyploidization
Interleaved intrachr. SVs	11
Total SVs (intrachr. + transl.)	12
SV types	DEL: 3; DUP: 3; h2hINV: 2; t2tINV: 3; TRA: 1
SVs in sample	106
Oscillating CN (2 and 3 states)	14, 14
CN segments	14
FDR fragment joints	0.98
FDR chr. breakp. enrich.	0.03
Linked to chrs	
Purity, ploidy	0.63, 3.54

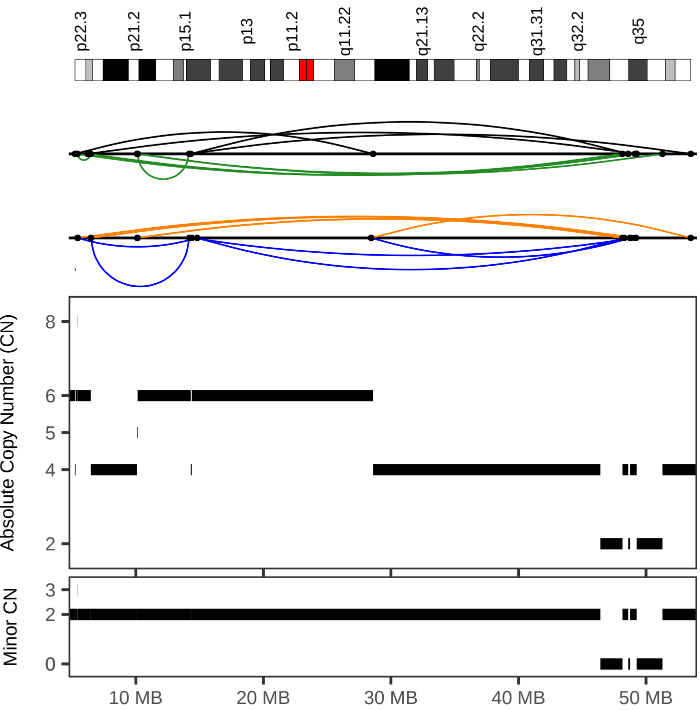

	**MELA−0043**
Cancer type	Skin−Melanoma
Position	7:5229877−53502420
Type	With other complex events
Interleaved intrachr. SVs	23
Total SVs (intrachr. + transl.)	23
SV types	DEL: 6; DUP: 5; h2hINV: 6; t2tINV: 6; TRA: 0
SVs in sample	106
Oscillating CN (2 and 3 states)	7, 10
CN segments	16
FDR fragment joints	1
FDR chr. breakp. enrich.	0
Linked to chrs	
Purity, ploidy	0.63, 3.54

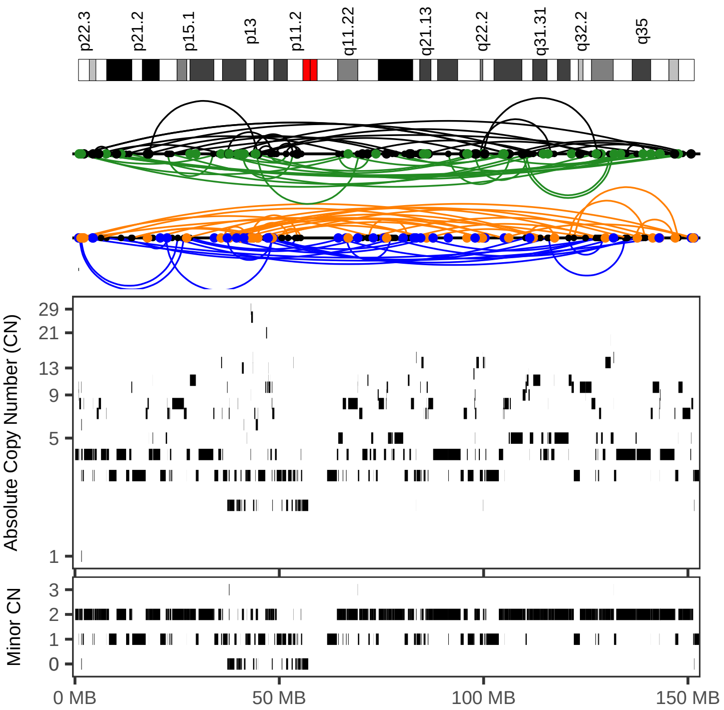

	**MELA−0048**
Cancer type	Skin−Melanoma
Position	7:858862−151538983
Type	With other complex events
Interleaved intrachr. SVs	121
Total SVs (intrachr. + transl.)	279
SV types	DEL: 34; DUP: 25; h2hINV: 29; t2tINV: 33; TRA: 158
SVs in sample	444
Oscillating CN (2 and 3 states)	8, 8
CN segments	316
FDR fragment joints	0.72
FDR chr. breakp. enrich.	0
Linked to chrs	
Purity, ploidy	0.9, 2.98

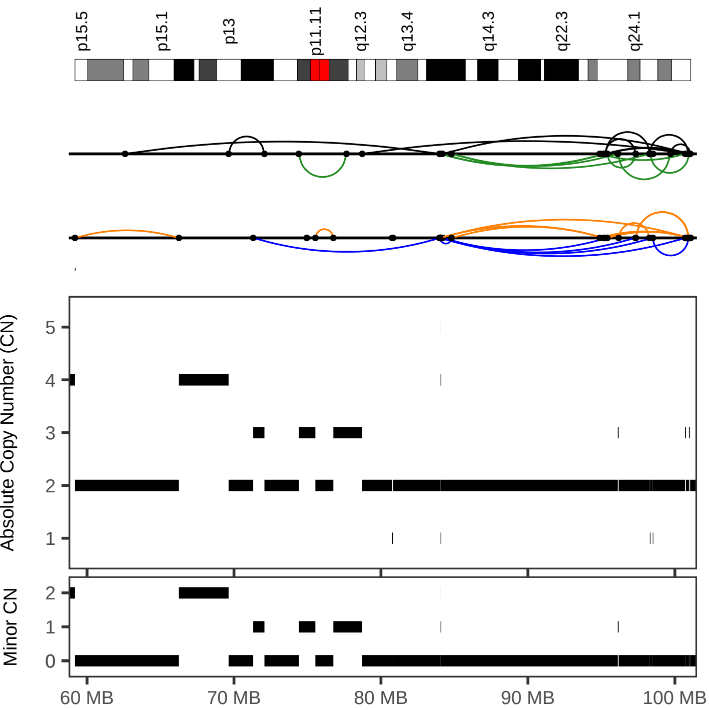

	**MELA−0052**
Cancer type	Skin−Melanoma
Position	11:59180890−101076970
Type	With other complex events
Interleaved intrachr. SVs	40
Total SVs (intrachr. + transl.)	40
SV types	DEL: 11; DUP: 9; h2hINV: 12; t2tINV: 8; TRA: 0
SVs in sample	91
Oscillating CN (2 and 3 states)	7, 9
CN segments	29
FDR fragment joints	0.84
FDR chr. breakp. enrich.	0
Linked to chrs	
Purity, ploidy	0.66, 3.22

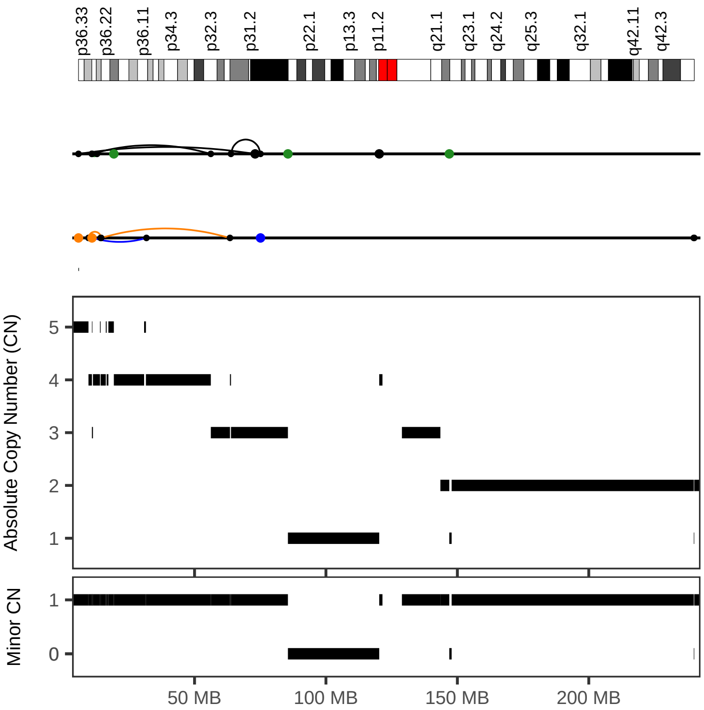

	**MELA−0055**
Cancer type	Skin−Melanoma
Position	1:9644166−63480813
Type	After polyploidization
Interleaved intrachr. SVs	5
Total SVs (intrachr. + transl.)	7
SV types	DEL: 2; DUP: 1; h2hINV: 1; t2tINV: 1; TRA: 2
SVs in sample	38
Oscillating CN (2 and 3 states)	9, 9
CN segments	14
FDR fragment joints	0.92
FDR chr. breakp. enrich.	0
Linked to chrs	
Purity, ploidy	0.87, 2.05

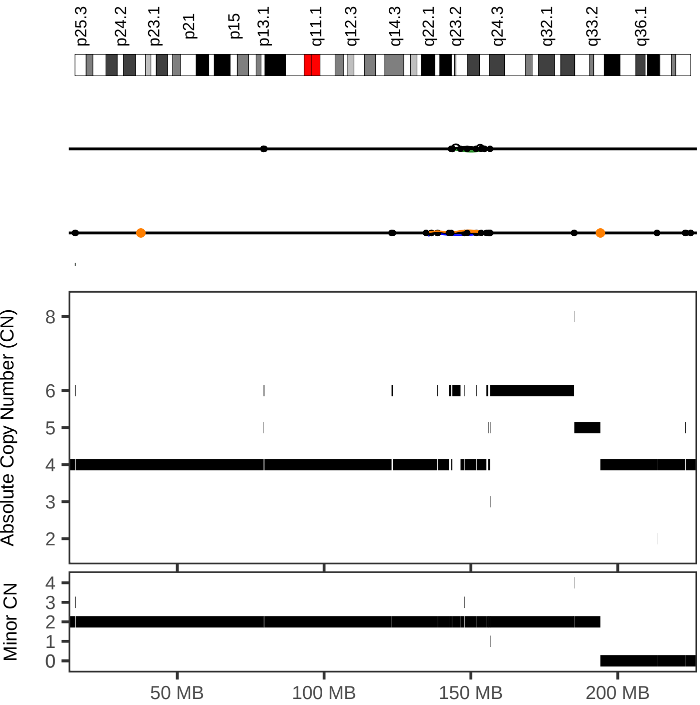

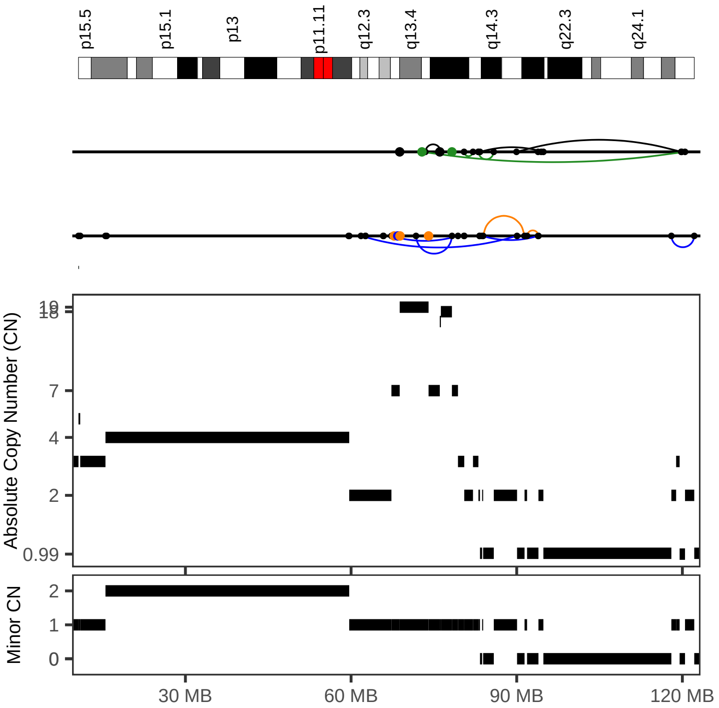

	MELA−0056
Cancer type	Skin−Melanoma
Position	2:134717568−156505138
Type	Before polyploidization
Interleaved intrachr. SVs	14
Total SVs (intrachr. + transl.)	14
SV types	DEL: 4; DUP: 4; h2hINV: 3; t2tINV: 3; TRA: 0
SVs in sample	224
Oscillating CN (2 and 3 states)	12, 13
CN segments	17
FDR fragment joints	0.98
FDR chr. breakp. enrich.	0.17
Linked to chrs	
Purity, ploidy	0.23, 4.05

	MELA−0056
Cancer type	Skin−Melanoma
Position	11:61794852−122148653
Type	With other complex events
Interleaved intrachr. SVs	12
Total SVs (intrachr. + transl.)	20
SV types	DEL: 2; DUP: 5; h2hINV: 2; t2tINV: 3; TRA: 8
SVs in sample	224
Oscillating CN (2 and 3 states)	11, 16
CN segments	25
FDR fragment joints	0.64
FDR chr. breakp. enrich.	0
Linked to chrs	
Purity, ploidy	0.23, 4.05

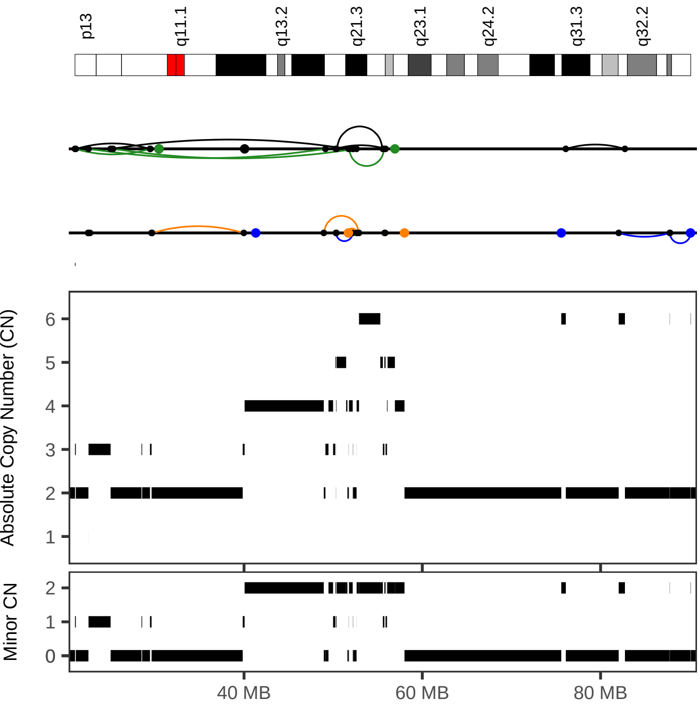

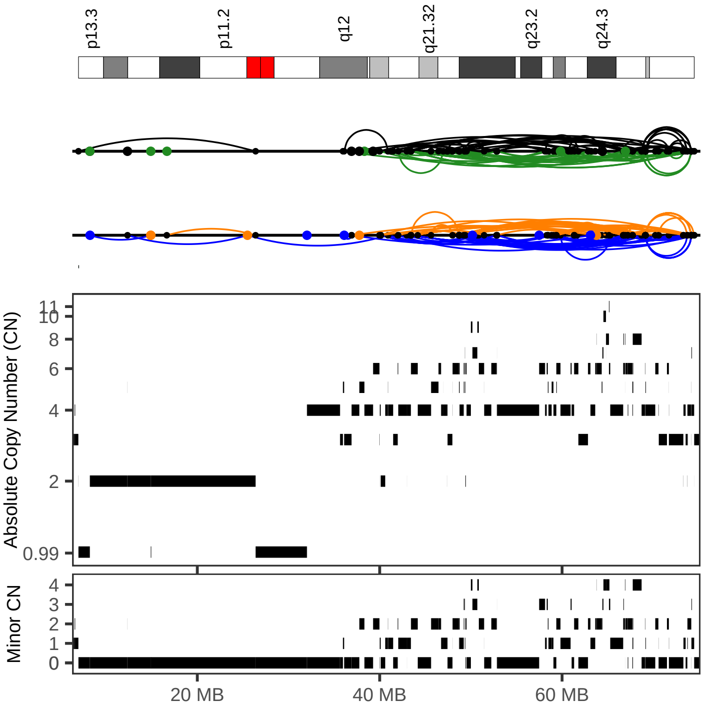

	MELA−0060
Cancer type	Skin−Melanoma
Position	14:21034990−55876063
Type	With other complex events
Interleaved intrachr. SVs	14
Total SVs (intrachr. + transl.)	19
SV types	DEL: 3; DUP: 1; h2hINV: 5; t2tINV: 5; TRA: 5
SVs in sample	213
Oscillating CN (2 and 3 states)	10, 10
CN segments	39
FDR fragment joints	0.59
FDR chr. breakp. enrich.	0
Linked to chrs	7:146640987−147701428;
Purity, ploidy	0.46, 3.71

	MELA−0066
Cancer type	Skin−Melanoma
Position	17:6966673−74491773
Type	With other complex events
Interleaved intrachr. SVs	211
Total SVs (intrachr. + transl.)	235
SV types	DEL: 54; DUP: 57; h2hINV: 48; t2tINV: 52; TRA: 24
SVs in sample	355
Oscillating CN (2 and 3 states)	7, 21
CN segments	160
FDR fragment joints	0.89
FDR chr. breakp. enrich.	0
Linked to chrs	1:160797429−216909158;
Purity, ploidy	0.42, 3.25

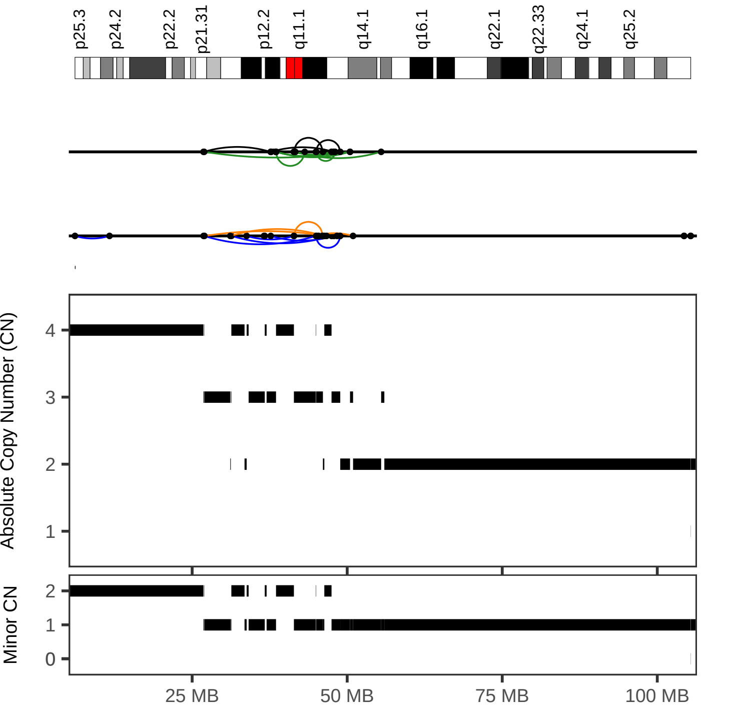

	MELA−0067
Cancer type	Skin−Melanoma
Position	6:26830084−55474034
Type	With other complex events
Interleaved intrachr. SVs	22
Total SVs (intrachr. + transl.)	22
SV types	DEL: 5; DUP: 5; h2hINV: 5; t2tINV: 7; TRA: 0
SVs in sample	676
Oscillating CN (2 and 3 states)	8, 11
CN segments	22
FDR fragment joints	0.93
FDR chr. breakp. enrich.	0.15
Linked to chrs	
Purity, ploidy	0.76, 2.03

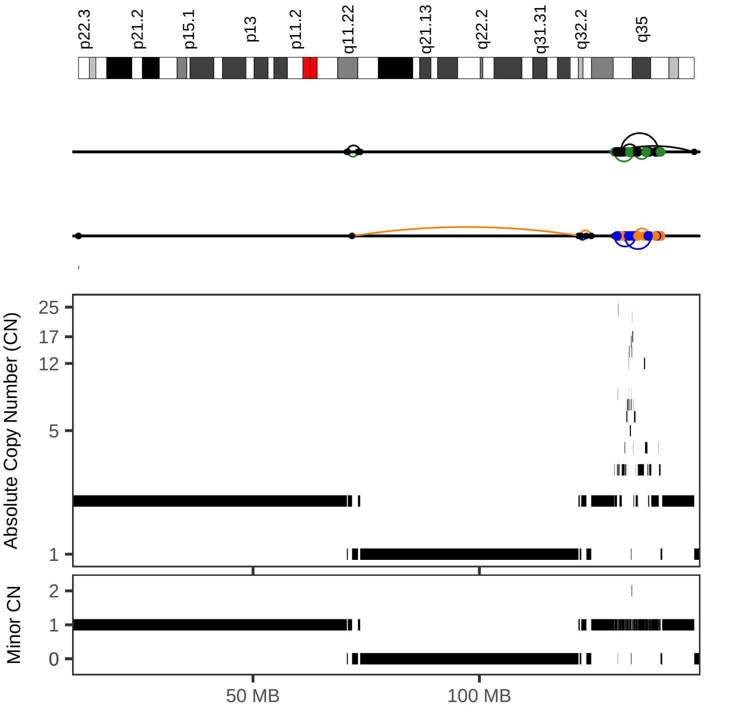

	MELA−0067
Cancer type	Skin−Melanoma
Position	7:129744428−147453672
Type	With other complex events
Interleaved intrachr. SVs	13
Total SVs (intrachr. + transl.)	149
SV types	DEL: 1; DUP: 5; h2hINV: 4; t2tINV: 3; TRA: 136
SVs in sample	676
Oscillating CN (2 and 3 states)	8, 10
CN segments	64
FDR fragment joints	0.64
FDR chr. breakp. enrich.	0
Linked to chrs	11:58806330−124114051;
Purity, ploidy	0.76, 2.03

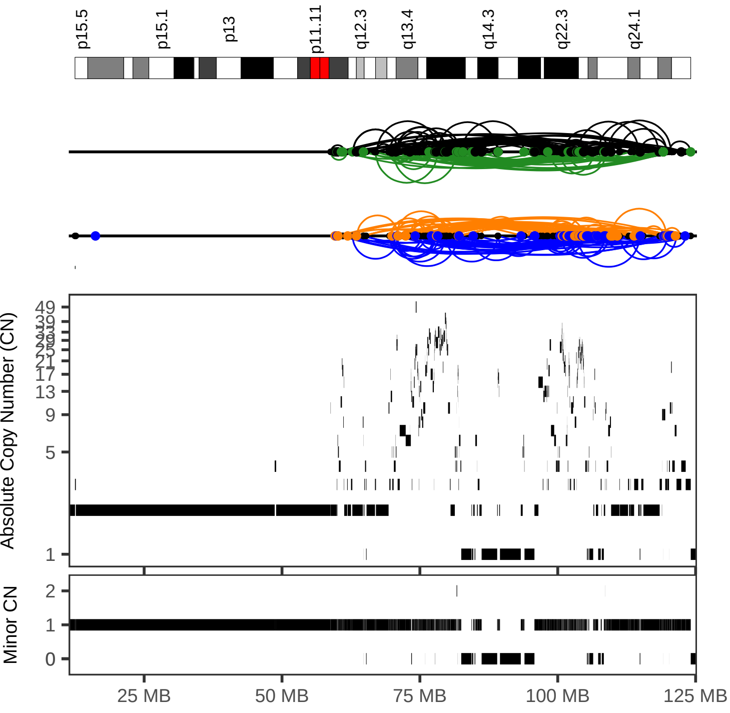

	MELA−0067
Cancer type	Skin−Melanoma
Position	11:58806330−124114052
Type	With other complex events
Interleaved intrachr. SVs	405
Total SVs (intrachr. + transl.)	547
SV types	DEL: 96; DUP: 109; h2hINV: 104; t2tINV: 96; TRA: 142
SVs in sample	676
Oscillating CN (2 and 3 states)	11, 11
CN segments	344
FDR fragment joints	0.83
FDR chr. breakp. enrich.	0
Linked to chrs	20:18728454−50145268;7:129744428−147453671
Purity, ploidy	0.76, 2.03

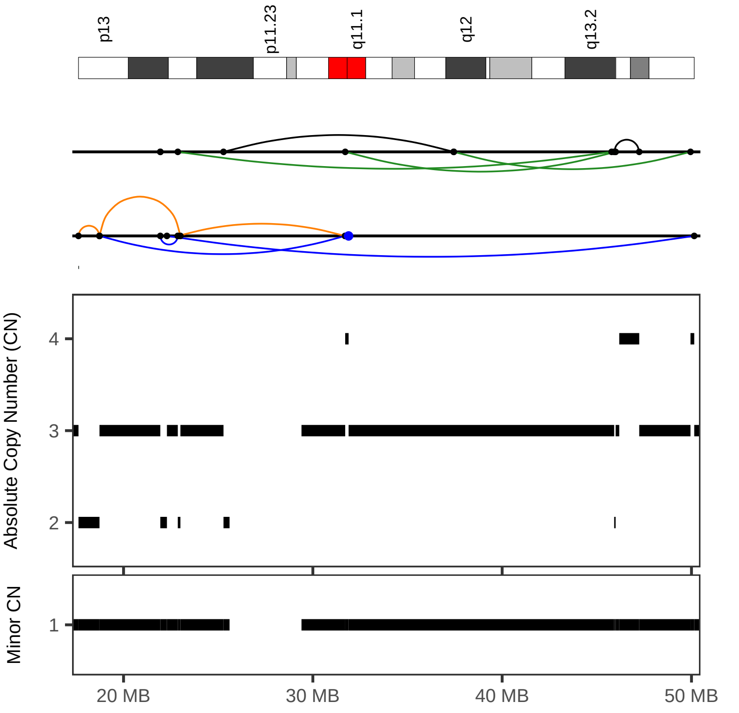

	MELA−0067
Cancer type	Skin−Melanoma
Position	20:18728454−50145269
Type	With other complex events
Interleaved intrachr. SVs	10
Total SVs (intrachr. + transl.)	11
SV types	DEL: 2; DUP: 3; h2hINV: 2; t2tINV: 3; TRA: 1
SVs in sample	676
Oscillating CN (2 and 3 states)	7, 15
CN segments	15
FDR fragment joints	0.96
FDR chr. breakp. enrich.	1
Linked to chrs	
Purity, ploidy	0.76, 2.03

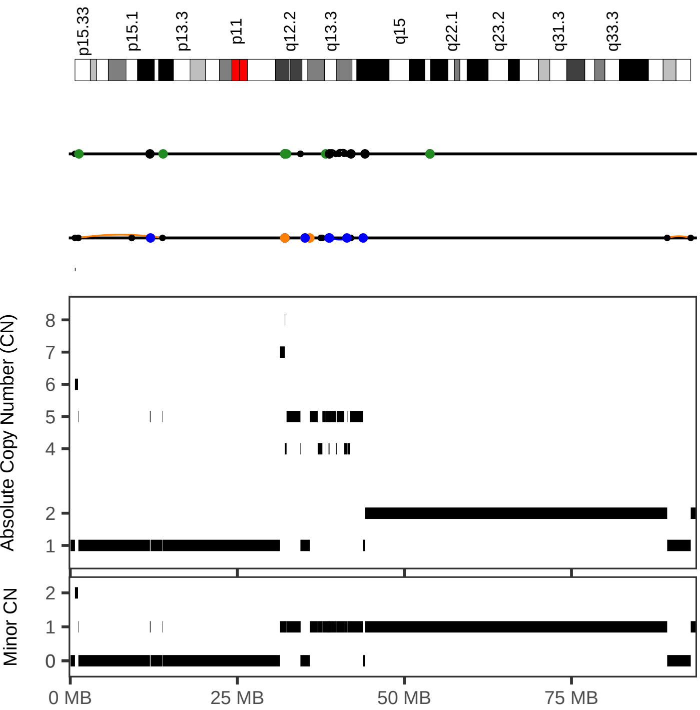

	MELA−0070
Cancer type	Skin−Melanoma
Position	5:703450−42018198
Type	With other complex events
Interleaved intrachr. SVs	3
Total SVs (intrachr. + transl.)	25
SV types	DEL: 1; DUP: 2; h2hINV: 1;<br>t2tINV: 2; TRA: 19
SVs in sample	450
Oscillating CN (2 and 3 states)	15, 15
CN segments	30
FDR fragment joints	0.91
FDR chr. breakp. enrich.	0.34
Linked to chrs	20:37323724−59336021;9:447092−9513408
Purity, ploidy	0.75, 2.64

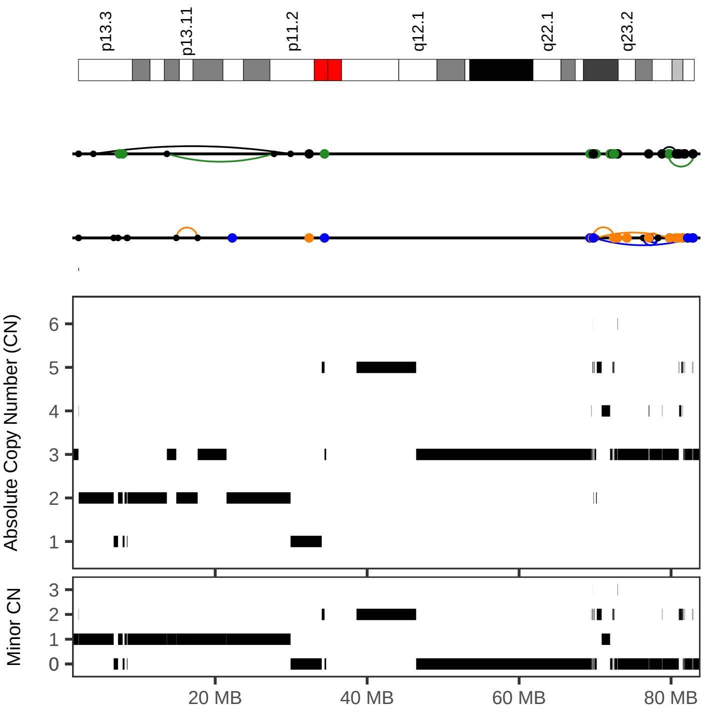

	MELA−0070
Cancer type	Skin−Melanoma
Position	16:69653875−83050346
Type	With other complex events
Interleaved intrachr. SVs	5
Total SVs (intrachr. + transl.)	52
SV types	DEL: 2; DUP: 1; h2hINV: 1;<br>t2tINV: 1; TRA: 47
SVs in sample	450
Oscillating CN (2 and 3 states)	9, 10
CN segments	34
FDR fragment joints	0.92
FDR chr. breakp. enrich.	0
Linked to chrs	17:38259169−65526579;
Purity, ploidy	0.75, 2.64

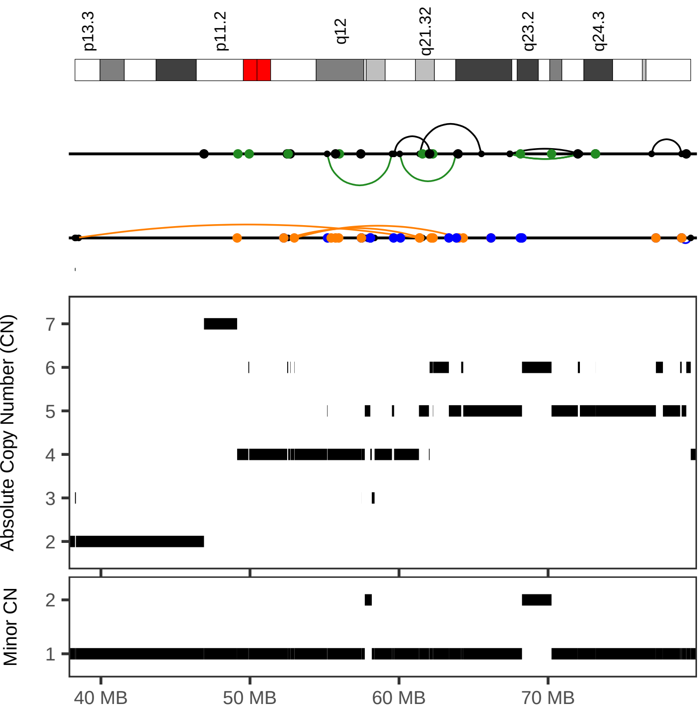

	MELA−0070
Cancer type	Skin−Melanoma
Position	17:38259169−65526580
Type	With other complex events
Interleaved intrachr. SVs	7
Total SVs (intrachr. + transl.)	44
SV types	DEL: 3; DUP: 1; h2hINV: 2;<br>t2tINV: 1; TRA: 37
SVs in sample	450
Oscillating CN (2 and 3 states)	9, 13
CN segments	30
FDR fragment joints	0.74
FDR chr. breakp. enrich.	0
Linked to chrs	12:55086091−64480689;16:69653875−83050345<br>9:447092−9513408;
Purity, ploidy	0.75, 2.64

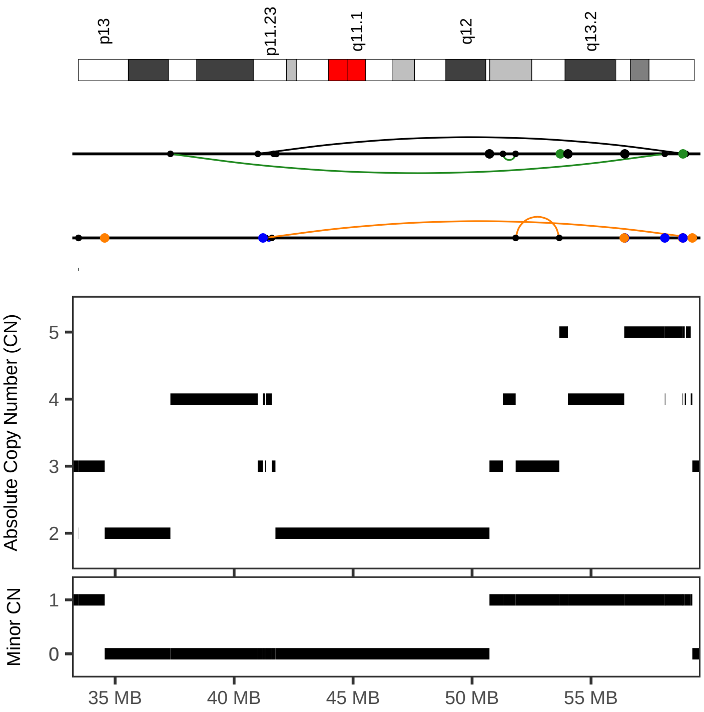

	MELA−0070
Cancer type	Skin−Melanoma
Position	20:37323724−59336022
Type	With other complex events
Interleaved intrachr. SVs	4
Total SVs (intrachr. + transl.)	16
SV types	DEL: 1; DUP: 0; h2hINV: 1;<br>t2tINV: 2; TRA: 12
SVs in sample	450
Oscillating CN (2 and 3 states)	10, 11
CN segments	21
FDR fragment joints	0.64
FDR chr. breakp. enrich.	0
Linked to chrs	14:22746323−50625573;5:703450−42018197<br>9:447092−9513408;
Purity, ploidy	0.75, 2.64

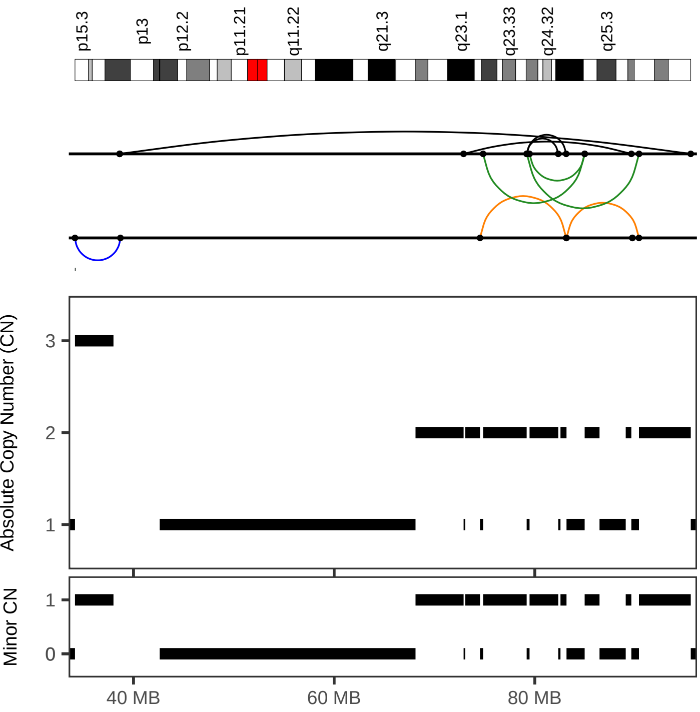

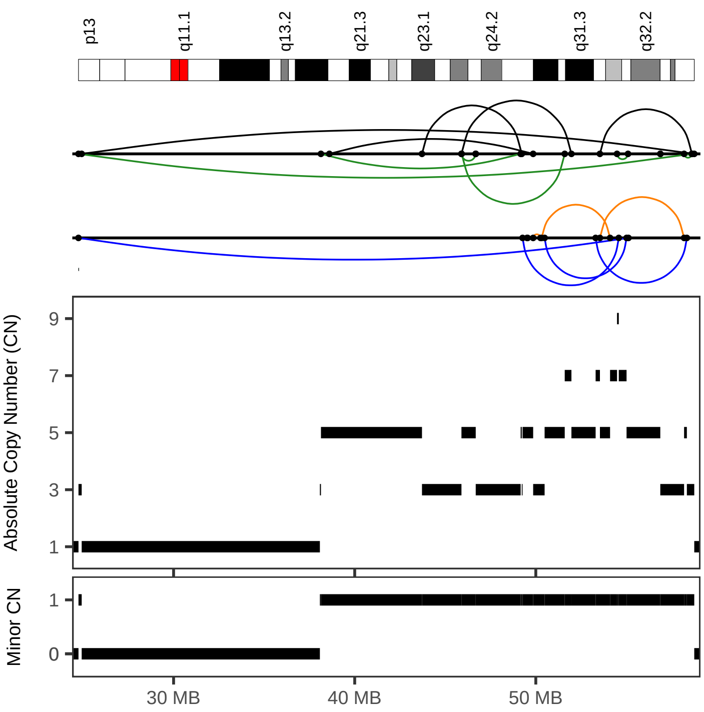

	**MELA−0075**
Cancer type	Skin−Melanoma
Position	10:72902980−90393121
Type	Canonical without polyploidization
Interleaved intrachr. SVs	8
Total SVs (intrachr. + transl.)	8
SV types	DEL: 2; DUP: 0; h2hINV: 3; t2tINV: 3; TRA: 0
SVs in sample	42
Oscillating CN (2 and 3 states)	14, 14
CN segments	14
FDR fragment joints	0.59
FDR chr. breakp. enrich.	0
Linked to chrs	
Purity, ploidy	0.88, 2.02

	**MELA−0076**
Cancer type	Skin−Melanoma
Position	14:24748061−58748685
Type	With other complex events
Interleaved intrachr. SVs	17
Total SVs (intrachr. + transl.)	17
SV types	DEL: 2; DUP: 4; h2hINV: 5; t2tINV: 6; TRA: 0
SVs in sample	59
Oscillating CN (2 and 3 states)	10, 24
CN segments	24
FDR fragment joints	0.64
FDR chr. breakp. enrich.	0
Linked to chrs	
Purity, ploidy	0.53, 1.84

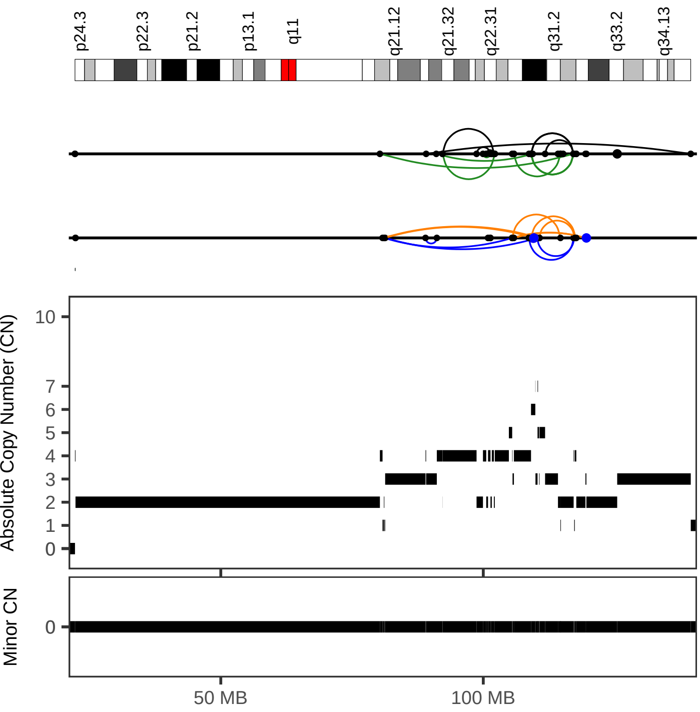

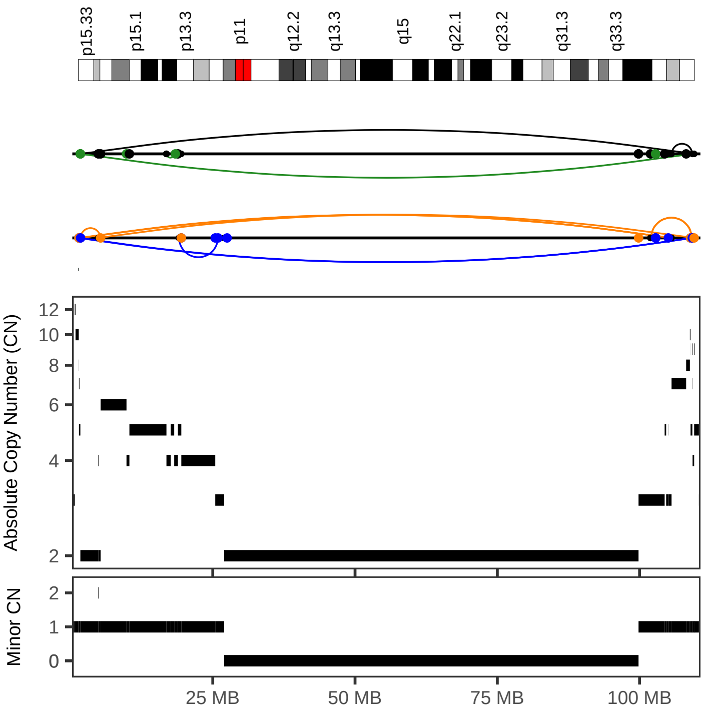

	**MELA−0160**
Cancer type	Skin−Melanoma
Position	9:80306032−139519485
Type	With other complex events
Interleaved intrachr. SVs	34
Total SVs (intrachr. + transl.)	38
SV types	DEL: 13; DUP: 7; h2hINV: 5; t2tINV: 9; TRA: 4
SVs in sample	96
Oscillating CN (2 and 3 states)	13, 15
CN segments	43
FDR fragment joints	0.59
FDR chr. breakp. enrich.	0
Linked to chrs	
Purity, ploidy	0.68, 3.2

	**MELA−0167**
Cancer type	Skin−Melanoma
Position	5:1403933−109603948
Type	With other complex events
Interleaved intrachr. SVs	14
Total SVs (intrachr. + transl.)	52
SV types	DEL: 6; DUP: 4; h2hINV: 2; t2tINV: 2; TRA: 38
SVs in sample	339
Oscillating CN (2 and 3 states)	7, 10
CN segments	31
FDR fragment joints	0.59
FDR chr. breakp. enrich.	0
Linked to chrs	12:45517029−71387081;7:29323351−150218312
Purity, ploidy	0.74, 2.9

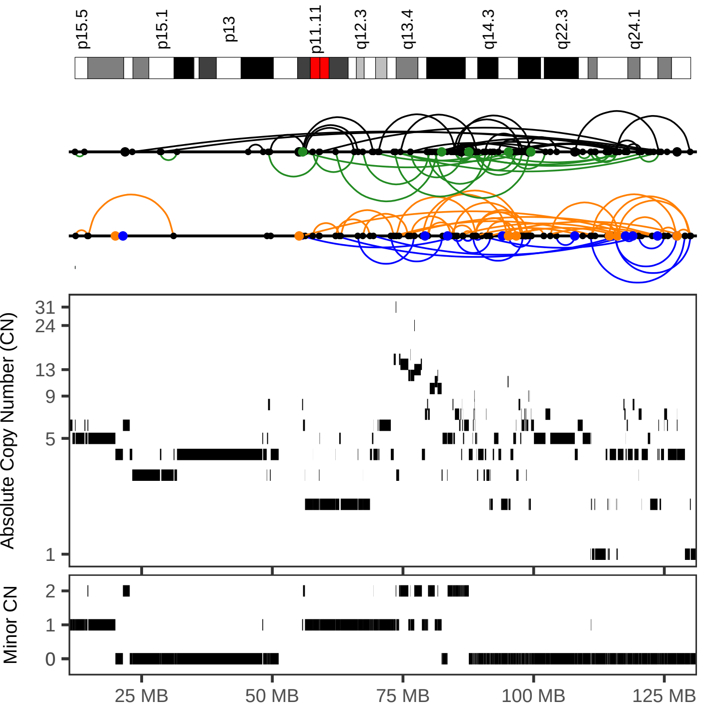

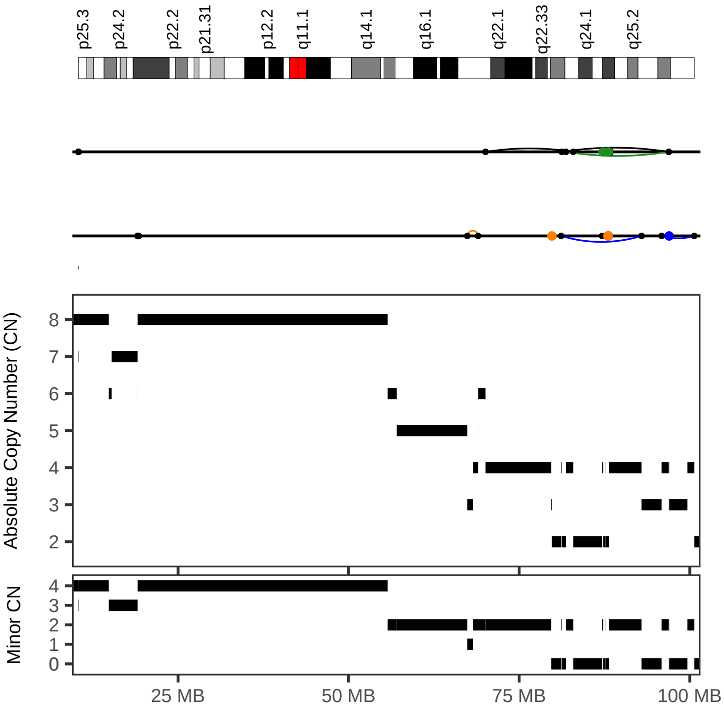

	**MELA−0167**
Cancer type	Skin−Melanoma
Position	11:14788852−130055978
Type	With other complex events
Interleaved intrachr. SVs	137
Total SVs (intrachr. + transl.)	167
SV types	DEL: 44; DUP: 25; h2hINV: 34; t2tINV: 34; TRA: 30
SVs in sample	339
Oscillating CN (2 and 3 states)	7, 12
CN segments	188
FDR fragment joints	0.59
FDR chr. breakp. enrich.	0
Linked to chrs	
Purity, ploidy	0.74, 2.9

	**MELA−0169**
Cancer type	Skin−Melanoma
Position	6:70072538−100661050
Type	With other complex events
Interleaved intrachr. SVs	5
Total SVs (intrachr. + transl.)	13
SV types	DEL: 0; DUP: 2; h2hINV: 2; t2tINV: 1; TRA: 8
SVs in sample	95
Oscillating CN (2 and 3 states)	10, 20
CN segments	20
FDR fragment joints	0.64
FDR chr. breakp. enrich.	0
Linked to chrs	
Purity, ploidy	0.78, 4

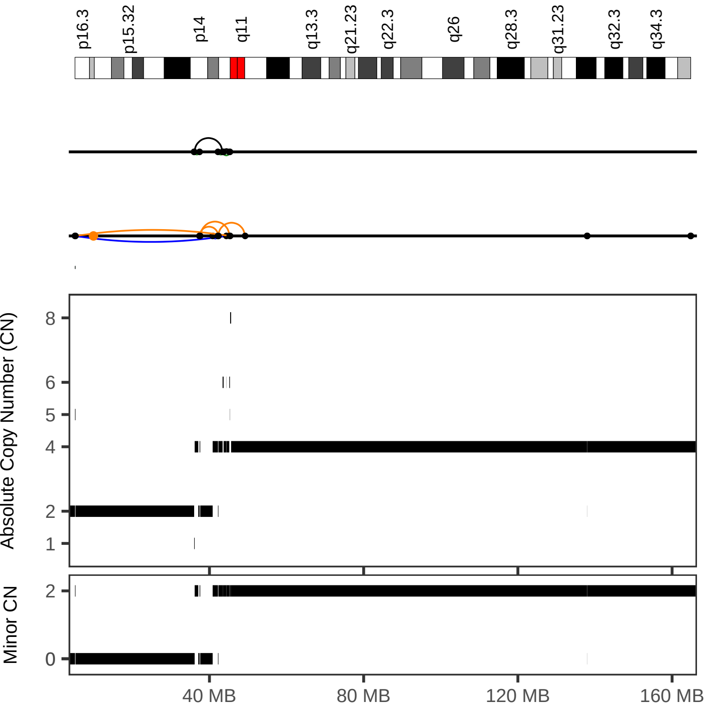

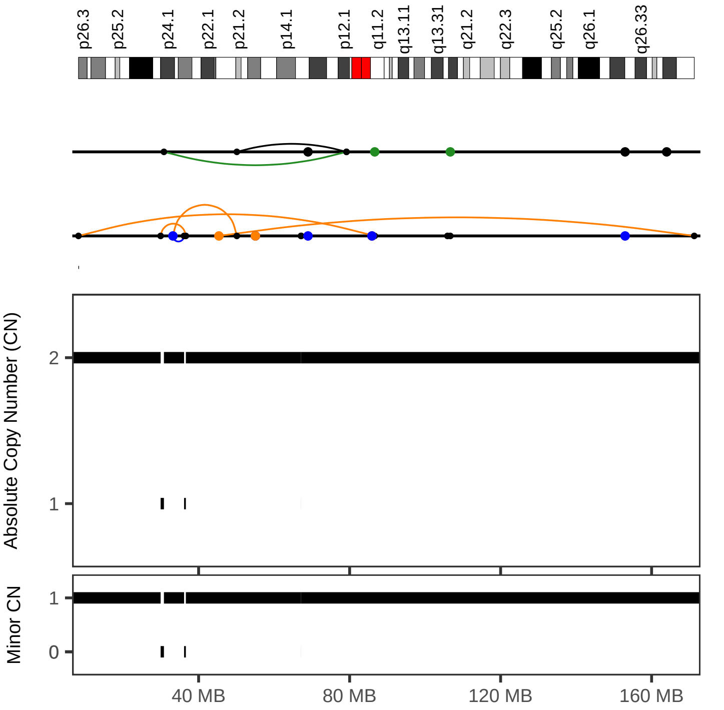

	**MELA−0170**
Cancer type	Skin−Melanoma
Position	4:5123431−49258772
Type	With other complex events
Interleaved intrachr. SVs	11
Total SVs (intrachr. + transl.)	12
SV types	DEL: 4; DUP: 2; h2hINV: 2; t2tINV: 3; TRA: 1
SVs in sample	83
Oscillating CN (2 and 3 states)	7, 8
CN segments	19
FDR fragment joints	0.84
FDR chr. breakp. enrich.	0.01
Linked to chrs	
Purity, ploidy	0.66, 3.36

	**MELA−0174**
Cancer type	Skin−Melanoma
Position	3:8169851−171292232
Type	Canonical without polyploidization
Interleaved intrachr. SVs	5
Total SVs (intrachr. + transl.)	18
SV types	DEL: 4; DUP: 0; h2hINV: 0; t2tINV: 1; TRA: 13
SVs in sample	249
Oscillating CN (2 and 3 states)	7, 7
CN segments	7
FDR fragment joints	0.36
FDR chr. breakp. enrich.	0.15
Linked to chrs	2:36739938−238611838;
Purity, ploidy	0.94, 2.15

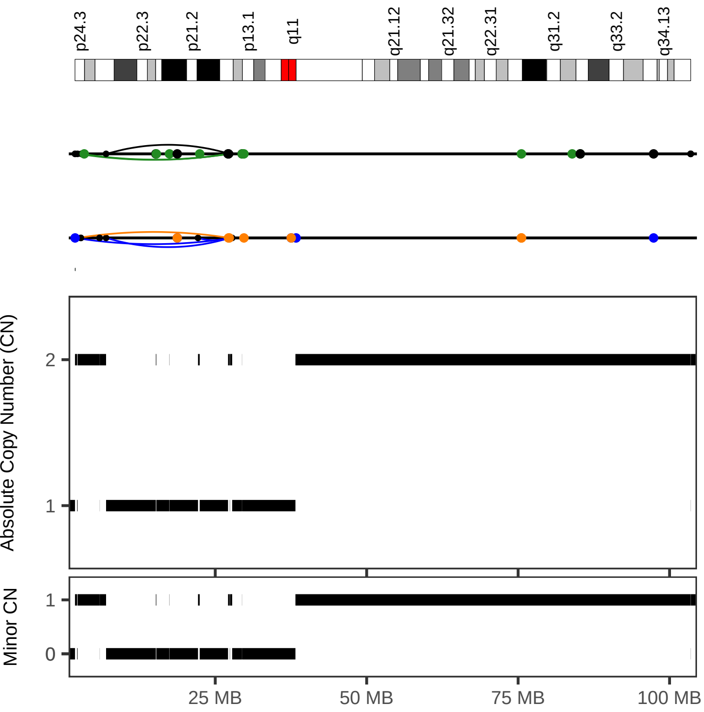

	MELA−0174
Cancer type	Skin−Melanoma
Position	9:2192834−27453601
Type	Canonical without polyploidization
Interleaved intrachr. SVs	6
Total SVs (intrachr. + transl.)	18
SV types	DEL: 1; DUP: 2; h2hINV: 1; t2tINV: 2; TRA: 12
SVs in sample	249
Oscillating CN (2 and 3 states)	14, 14
CN segments	14
FDR fragment joints	0.91
FDR chr. breakp. enrich.	0
Linked to chrs	5:120366559−126309299;
Purity, ploidy	0.94, 2.15

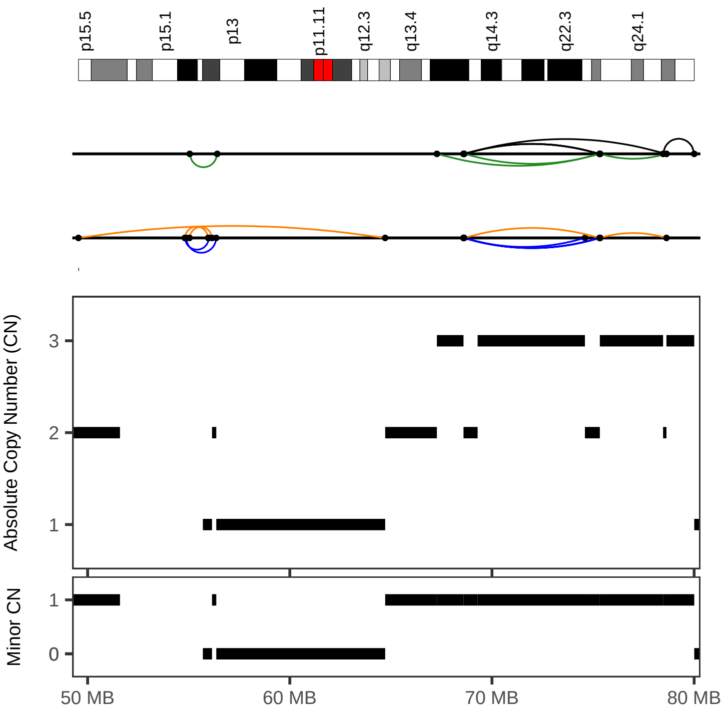

	MELA−0180
Cancer type	Skin−Melanoma
Position	11:67275408−80004995
Type	Canonical without polyploidization
Interleaved intrachr. SVs	16
Total SVs (intrachr. + transl.)	16
SV types	DEL: 2; DUP: 5; h2hINV: 6; t2tINV: 3; TRA: 0
SVs in sample	82
Oscillating CN (2 and 3 states)	7, 8
CN segments	8
FDR fragment joints	0.64
FDR chr. breakp. enrich.	0
Linked to chrs	
Purity, ploidy	0.39, 1.81

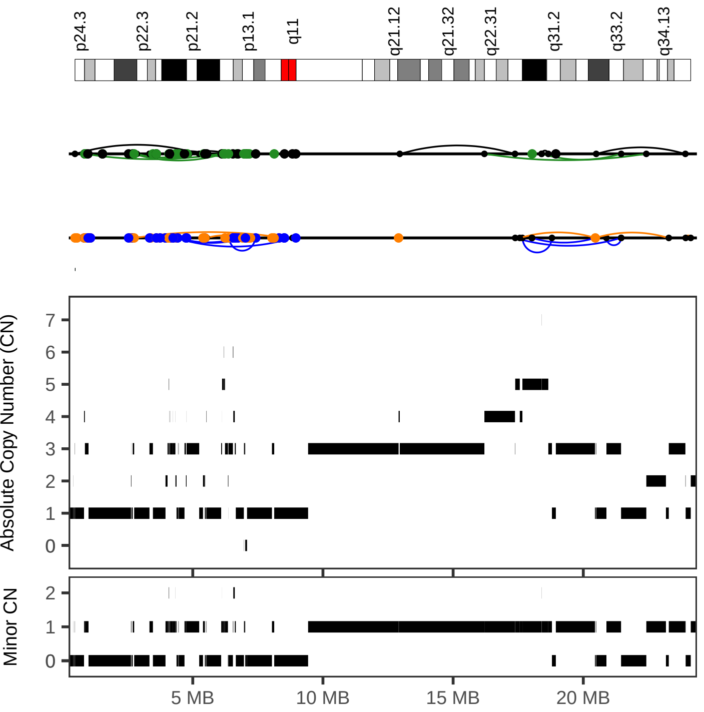

	MELA−0184
Cancer type	Skin−Melanoma
Position	9:474983−8830179
Type	With other complex events
Interleaved intrachr. SVs	13
Total SVs (intrachr. + transl.)	115
SV types	DEL: 2; DUP: 4; h2hINV: 3; t2tINV: 4; TRA: 102
SVs in sample	394
Oscillating CN (2 and 3 states)	8, 11
CN segments	62
FDR fragment joints	0.88
FDR chr. breakp. enrich.	0
Linked to chrs	17:47916−22130643;
Purity, ploidy	0.39, 3.23

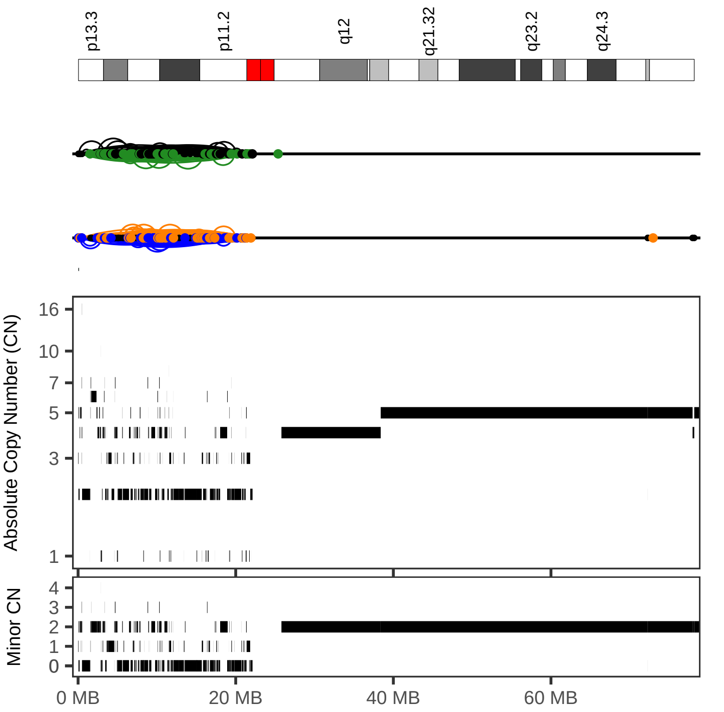

	MELA−0184
Cancer type	Skin−Melanoma
Position	17:47916−22130644
Type	With other complex events
Interleaved intrachr. SVs	190
Total SVs (intrachr. + transl.)	308
SV types	DEL: 49; DUP: 44; h2hINV: 49; t2tINV: 48; TRA: 118
SVs in sample	394
Oscillating CN (2 and 3 states)	8, 11
CN segments	251
FDR fragment joints	0.96
FDR chr. breakp. enrich.	0
Linked to chrs	
Purity, ploidy	0.39, 3.23

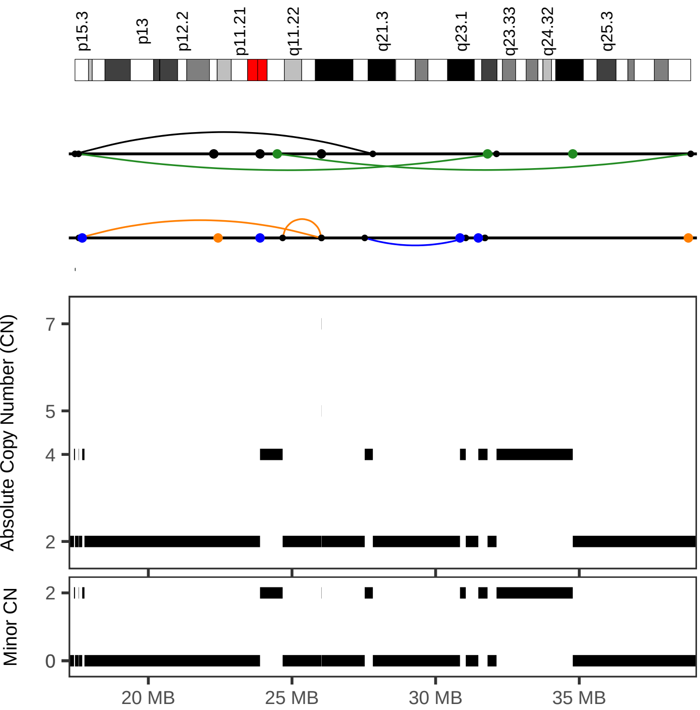

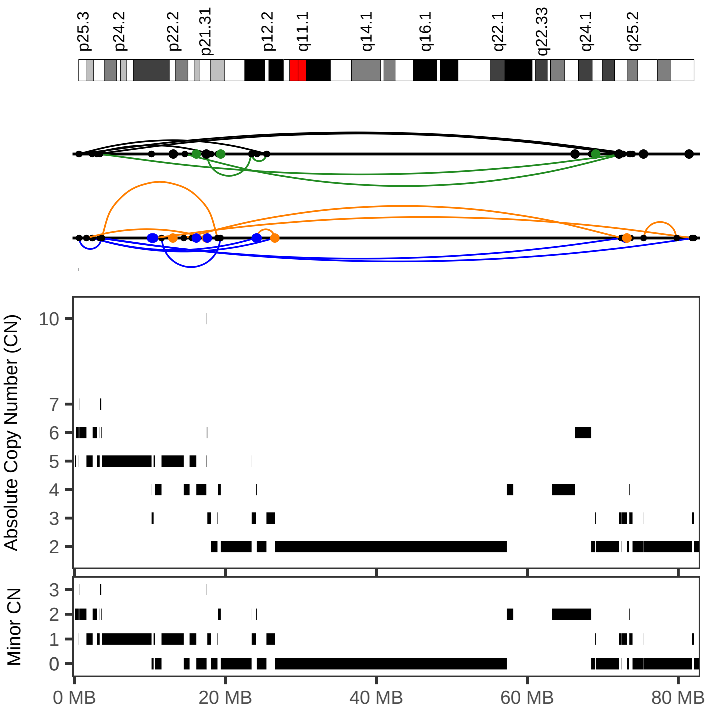

**MELA−0185**	
Cancer type	Skin−Melanoma
Position	10:17445989−38881097
Type	With other complex events
Interleaved intrachr. SVs	6
Total SVs (intrachr. + transl.)	18
SV types	DEL: 2; DUP: 1; h2hINV: 1; t2tINV: 2; TRA: 12
SVs in sample	60
Oscillating CN (2 and 3 states)	9, 11
CN segments	18
FDR fragment joints	0.91
FDR chr. breakp. enrich.	0
Linked to chrs	9:18382271−35495731;
Purity, ploidy	0.85, 3.46

**MELA−0193**	
Cancer type	Skin−Melanoma
Position	6:548107−82083993
Type	With other complex events
Interleaved intrachr. SVs	26
Total SVs (intrachr. + transl.)	46
SV types	DEL: 6; DUP: 9; h2hINV: 6; t2tINV: 5; TRA: 20
SVs in sample	264
Oscillating CN (2 and 3 states)	8, 17
CN segments	59
FDR fragment joints	0.79
FDR chr. breakp. enrich.	0
Linked to chrs	
Purity, ploidy	0.9, 4.07

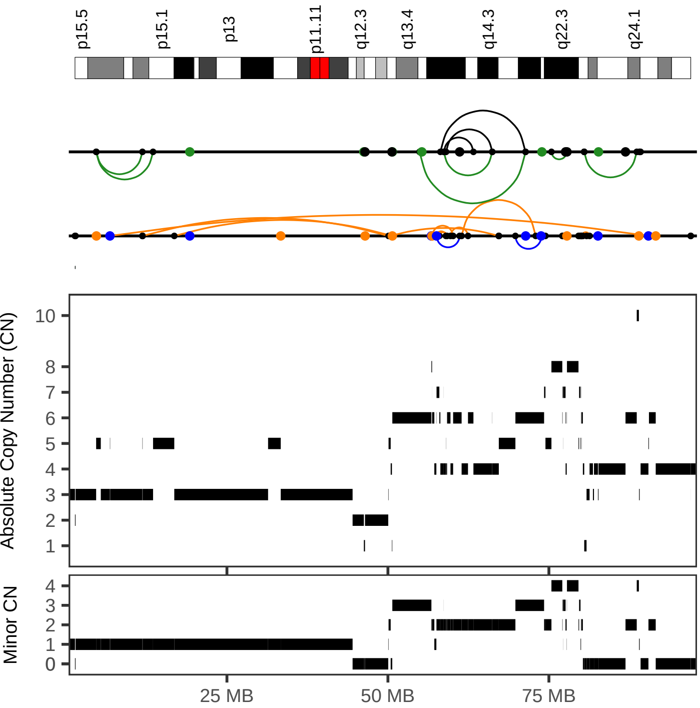

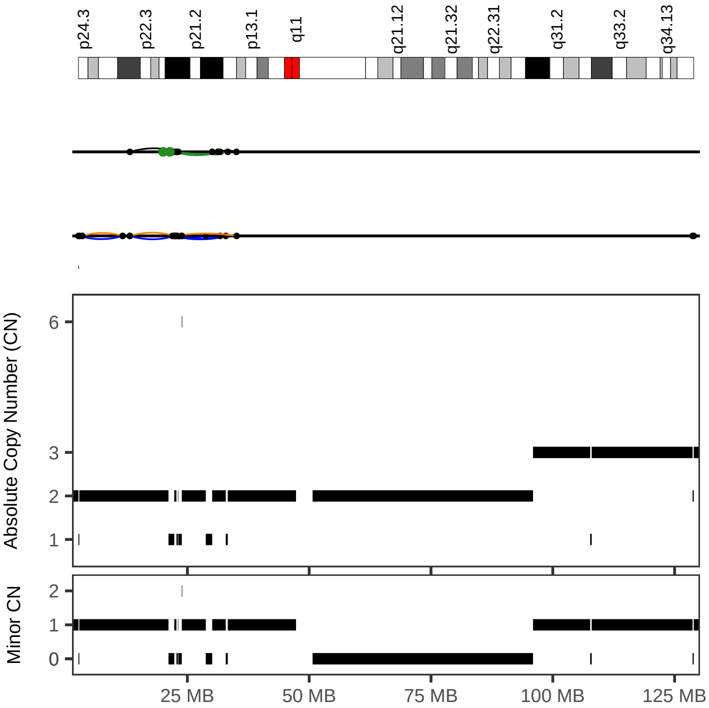

**MELA−0193**	
Cancer type	Skin−Melanoma
Position	11:4690426−90534996
Type	With other complex events
Interleaved intrachr. SVs	19
Total SVs (intrachr. + transl.)	46
SV types	DEL: 9; DUP: 3; h2hINV: 3; t2tINV: 4; TRA: 27
SVs in sample	264
Oscillating CN (2 and 3 states)	10, 12
CN segments	75
FDR fragment joints	0.59
FDR chr. breakp. enrich.	0
Linked to chrs	1:29465201−244012994;
Purity, ploidy	0.9, 4.07

**MELA−0200**	
Cancer type	Skin−Melanoma
Position	9:13186614−35103688
Type	With other complex events
Interleaved intrachr. SVs	19
Total SVs (intrachr. + transl.)	23
SV types	DEL: 4; DUP: 7; h2hINV: 4; t2tINV: 4; TRA: 4
SVs in sample	55
Oscillating CN (2 and 3 states)	8, 8
CN segments	14
FDR fragment joints	0.78
FDR chr. breakp. enrich.	0
Linked to chrs	
Purity, ploidy	0.26, 2.04

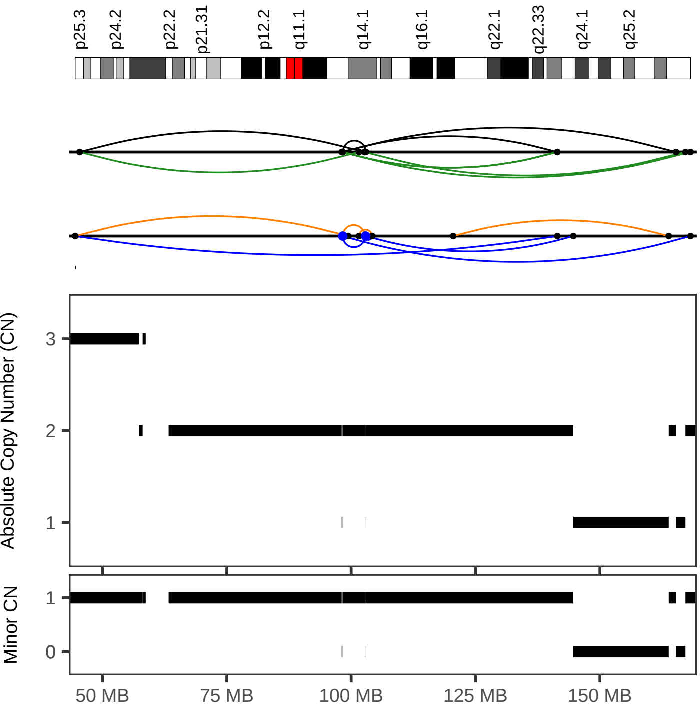

MELA−0203	
Cancer type	Skin−Melanoma
Position	6:44528437−168230895
Type	Canonical without polyploidization
Interleaved intrachr. SVs	21
Total SVs (intrachr. + transl.)	23
SV types	DEL: 5; DUP: 6; h2hINV: 5; t2tINV: 5; TRA: 2
SVs in sample	44
Oscillating CN (2 and 3 states)	11, 14
CN segments	14
FDR fragment joints	1
FDR chr. breakp. enrich.	0
Linked to chrs	
Purity, ploidy	0.98, 1.96

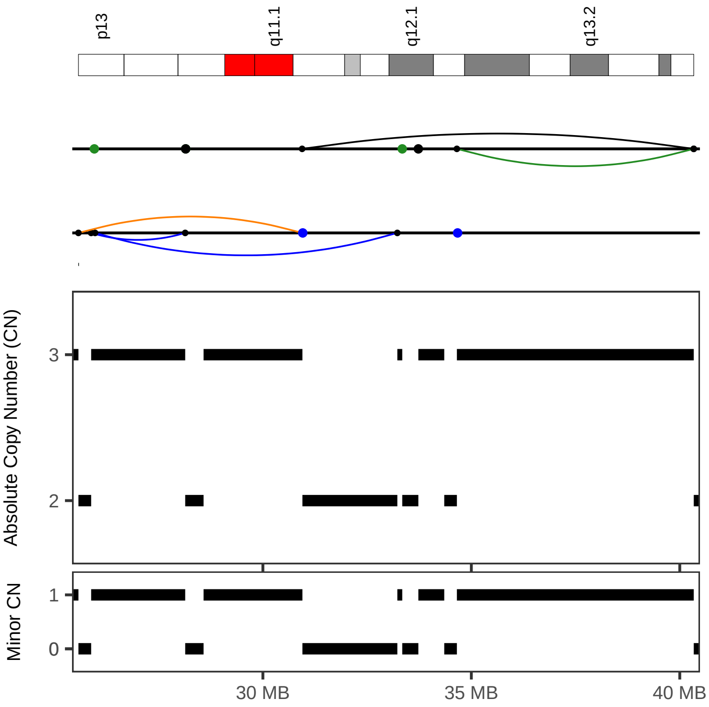

MELA−0223	
Cancer type	Skin−Melanoma
Position	22:25575062−40335606
Type	Canonical without polyploidization
Interleaved intrachr. SVs	5
Total SVs (intrachr. + transl.)	12
SV types	DEL: 1; DUP: 2; h2hINV: 1; t2tINV: 1; TRA: 7
SVs in sample	104
Oscillating CN (2 and 3 states)	11, 11
CN segments	11
FDR fragment joints	0.92
FDR chr. breakp. enrich.	0
Linked to chrs	
Purity, ploidy	0.87, 4.03

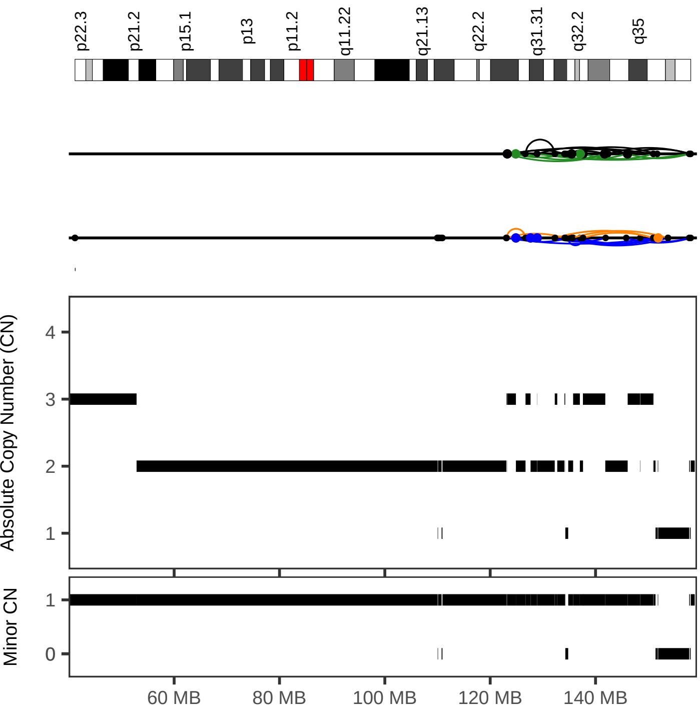

MELA−0226	
Cancer type	Skin−Melanoma
Position	7:123055563−158071677
Type	With other complex events
Interleaved intrachr. SVs	32
Total SVs (intrachr. + transl.)	43
SV types	DEL: 5; DUP: 10; h2hINV: 10; t2tINV: 7; TRA: 11
SVs in sample	152
Oscillating CN (2 and 3 states)	17, 42
CN segments	42
FDR fragment joints	0.64
FDR chr. breakp. enrich.	0
Linked to chrs	14:76917881−90636669;
Purity, ploidy	0.48, 1.86

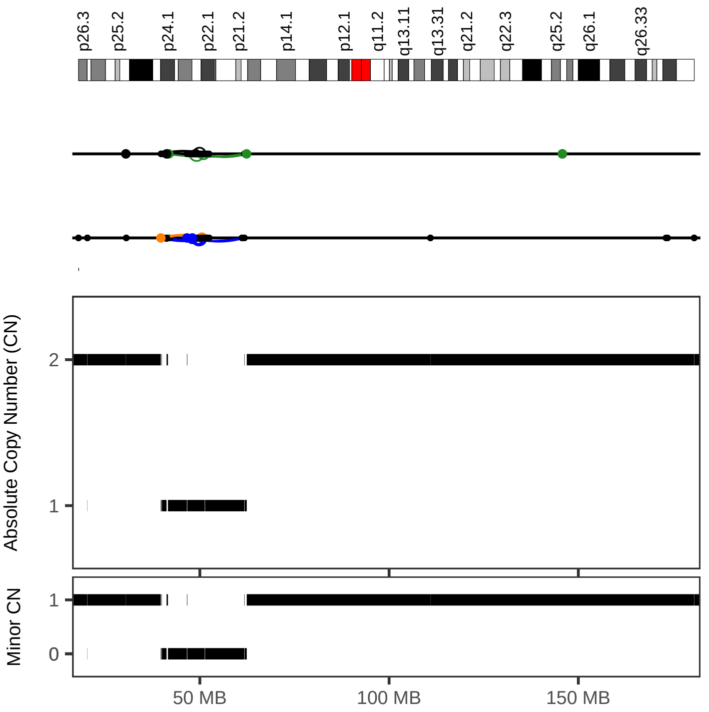

MELA−0229	
Cancer type	Skin−Melanoma
Position	3:39682784−62393971
Type	Canonical without polyploidization
Interleaved intrachr. SVs	41
Total SVs (intrachr. + transl.)	47
SV types	DEL: 10; DUP: 13; h2hINV: 11; t2tINV: 7; TRA: 6
SVs in sample	155
Oscillating CN (2 and 3 states)	7, 11
CN segments	11
FDR fragment joints	0.68
FDR chr. breakp. enrich.	0
Linked to chrs	
Purity, ploidy	0.31, 1.85

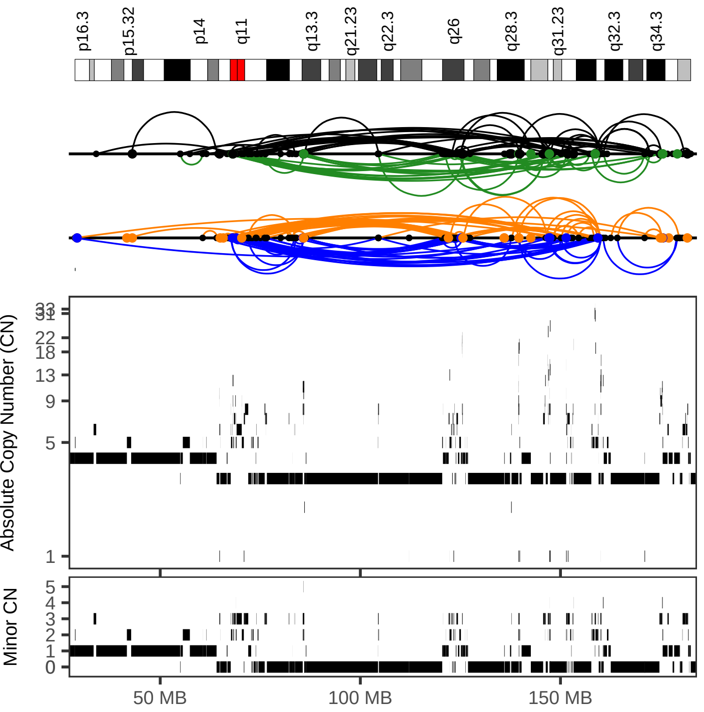

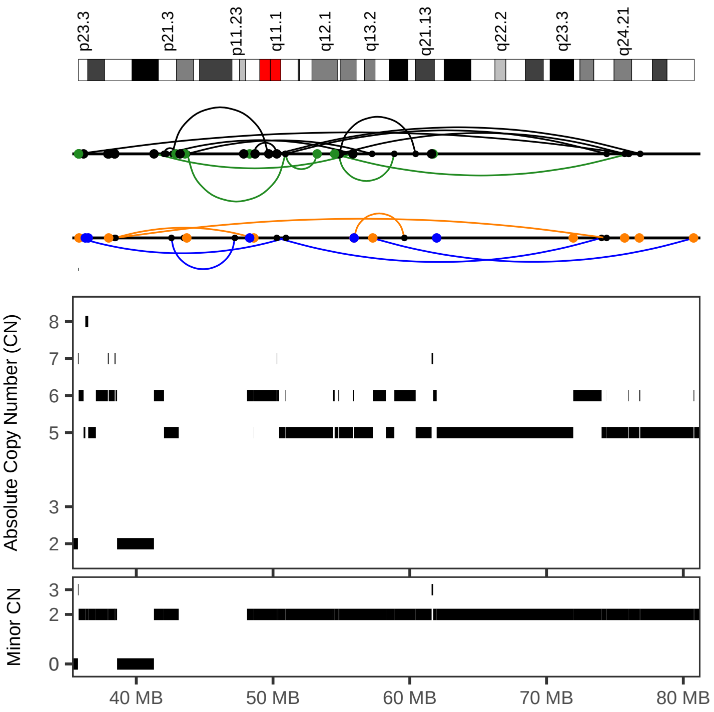

	MELA−0230
Cancer type	Skin−Melanoma
Position	4:28707038−182542170
Type	With other complex events
Interleaved intrachr. SVs	242
Total SVs (intrachr. + transl.)	316
SV types	DEL: 66; DUP: 56; h2hINV: 66; t2tINV: 54; TRA: 74
SVs in sample	441
Oscillating CN (2 and 3 states)	7, 13
CN segments	347
FDR fragment joints	0.64
FDR chr. breakp. enrich.	0
Linked to chrs	8:35775519−80798641;
Purity, ploidy	0.74, 3.69

	MELA−0230
Cancer type	Skin−Melanoma
Position	8:35775519−80798642
Type	With other complex events
Interleaved intrachr. SVs	23
Total SVs (intrachr. + transl.)	63
SV types	DEL: 3; DUP: 5; h2hINV: 10; t2tINV: 5; TRA: 40
SVs in sample	441
Oscillating CN (2 and 3 states)	14, 20
CN segments	46
FDR fragment joints	0.59
FDR chr. breakp. enrich.	0
Linked to chrs	4:28707038−182542169;
Purity, ploidy	0.74, 3.69

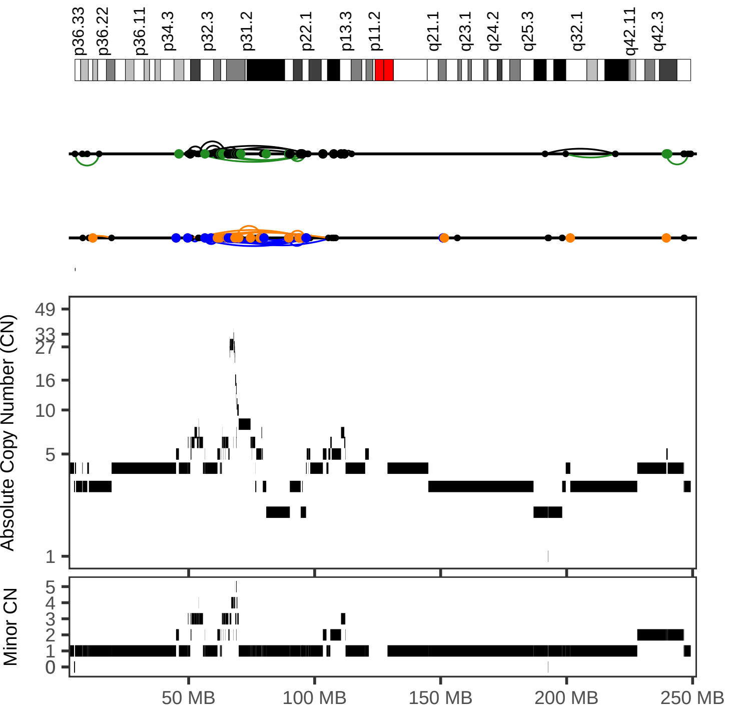

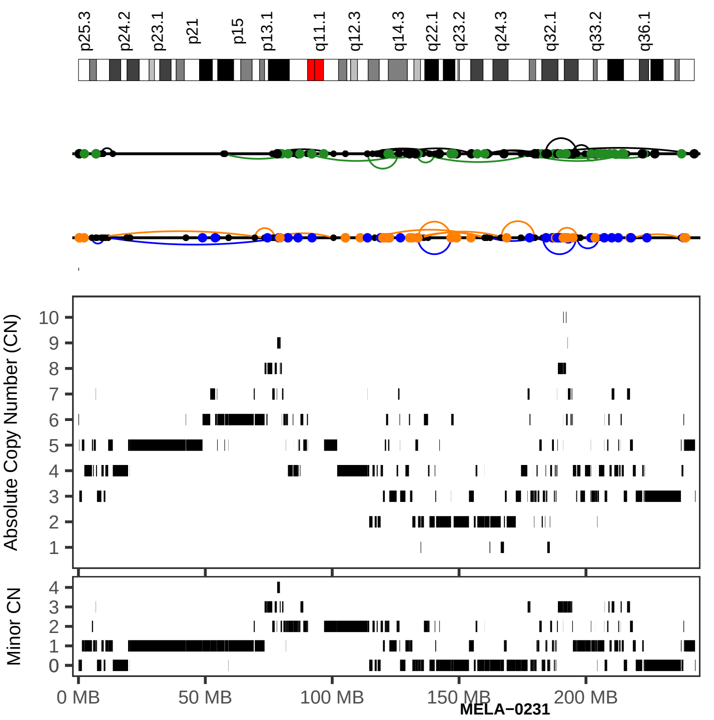

	MELA−0231
Cancer type	Skin−Melanoma
Position	1:49615754−106759581
Type	With other complex events
Interleaved intrachr. SVs	43
Total SVs (intrachr. + transl.)	100
SV types	DEL: 10; DUP: 13; h2hINV: 11; t2tINV: 9; TRA: 57
SVs in sample	1234
Oscillating CN (2 and 3 states)	10, 11
CN segments	77
FDR fragment joints	0.89
FDR chr. breakp. enrich.	0
Linked to chrs	11:85905386−127883070;12:4111597−34617369 2:5310579−242675571;20:5565486−62296241 5:4413255−157081451;6:76285221−169646111 7:712296−54531287;9:396152−20238661
Purity, ploidy	0.92, 4.03

	MELA−0231
Cancer type	Skin−Melanoma
Position	2:5310579−242675572
Type	With other complex events
Interleaved intrachr. SVs	57
Total SVs (intrachr. + transl.)	198
SV types	DEL: 16; DUP: 11; h2hINV: 13; t2tINV: 17; TRA: 141
SVs in sample	1234
Oscillating CN (2 and 3 states)	7, 19
CN segments	198
FDR fragment joints	0.74
FDR chr. breakp. enrich.	0
Linked to chrs	10:19180867−131722345;11:85905386−127883070 18:58999177−77021614;20:5565486−62296241 4:58934408−183626933;5:4413255−157081451 6:76285221−169646111;7:712296−54531287 8:9543398−106192555;9:396152−20238661
Purity, ploidy	0.92, 4.03

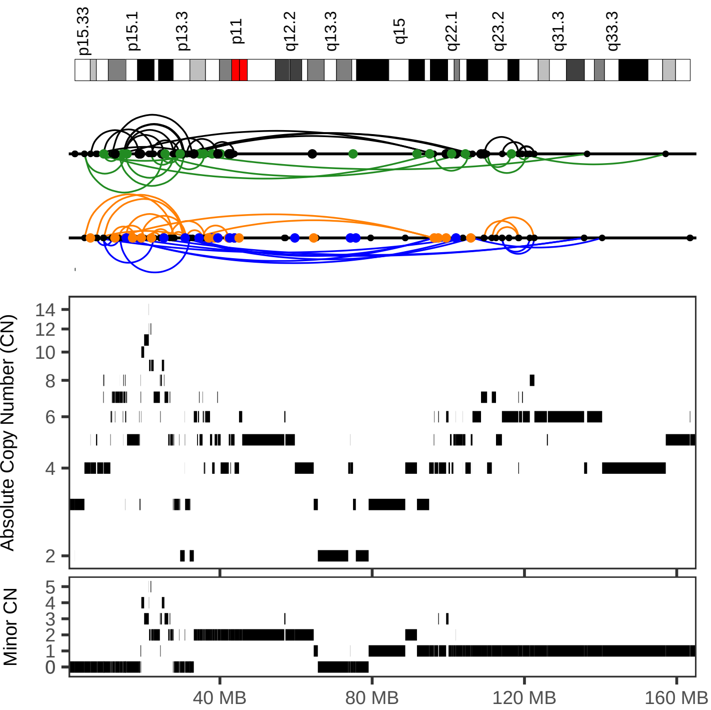

MELA−0231

Cancer type	Skin−Melanoma
Position	5:4413255−157081452
Type	With other complex events
Interleaved intrachr. SVs	80
Total SVs (intrachr. + transl.)	146
SV types	DEL: 20; DUP: 15; h2hINV: 21; t2tINV: 24; TRA: 66
SVs in sample	1234
Oscillating CN (2 and 3 states)	8, 12
CN segments	150
FDR fragment joints	0.64
FDR chr. breakp. enrich.	0
Linked to chrs	10:19180867−131722345;11:85905386−127883070 14:27456610−34055767;2:5310579−242675571 20:5565486−62296241;4:58934408−183626933 7:712296−54531287;8:9543398−106192555
Purity, ploidy	0.92, 4.03

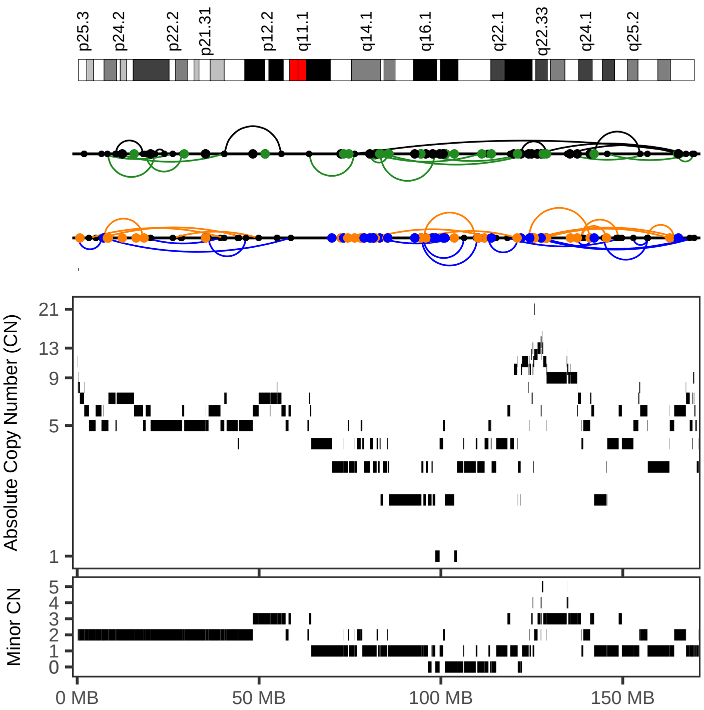

MELA−0231

Cancer type	Skin−Melanoma
Position	6:76285221−169646112
Type	With other complex events
Interleaved intrachr. SVs	37
Total SVs (intrachr. + transl.)	133
SV types	DEL: 10; DUP: 10; h2hINV: 6; t2tINV: 11; TRA: 96
SVs in sample	1234
Oscillating CN (2 and 3 states)	8, 9
CN segments	117
FDR fragment joints	0.74
FDR chr. breakp. enrich.	0
Linked to chrs	1:49615754−106759580;11:85905386−127883070 2:5310579−242675571;20:5565486−62296241 4:58934408−183626933;7:712296−54531287 8:9543398−106192555;
Purity, ploidy	0.92, 4.03

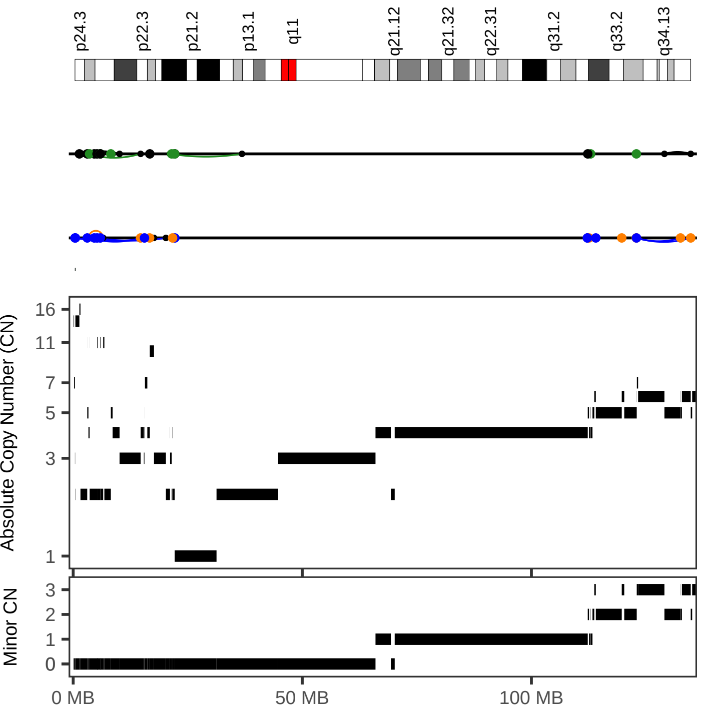

MELA−0231

Cancer type	Skin−Melanoma
Position	9:396152−20238662
Type	With other complex events
Interleaved intrachr. SVs	9
Total SVs (intrachr. + transl.)	27
SV types	DEL: 1; DUP: 4; h2hINV: 3; t2tINV: 1; TRA: 18
SVs in sample	1234
Oscillating CN (2 and 3 states)	8, 9
CN segments	29
FDR fragment joints	0.59
FDR chr. breakp. enrich.	0.78
Linked to chrs	1:49615754−106759580;11:85905386−127883070
Purity, ploidy	0.92, 4.03

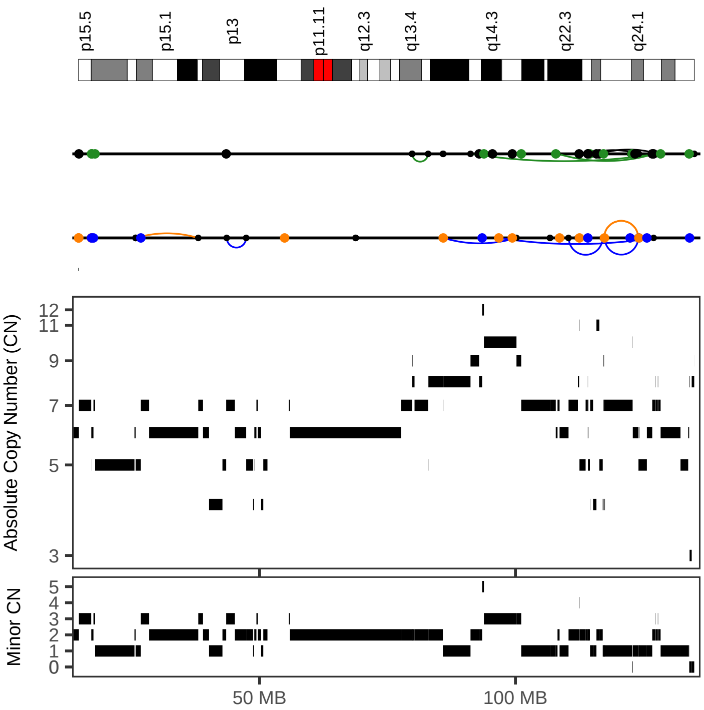

MELA−0231

Cancer type	Skin−Melanoma
Position	11:85905386−127883071
Type	With other complex events
Interleaved intrachr. SVs	7
Total SVs (intrachr. + transl.)	35
SV types	DEL: 0; DUP: 3; h2hINV: 2; t2tINV: 2; TRA: 28
SVs in sample	1234
Oscillating CN (2 and 3 states)	7, 11
CN segments	43
FDR fragment joints	0.64
FDR chr. breakp. enrich.	0.11
Linked to chrs	1:49615754−106759580;2:5310579−242675571 20:5565486−62296241;4:58934408−183626933 6:76285221−169646111;9:396152−20238661
Purity, ploidy	0.92, 4.03

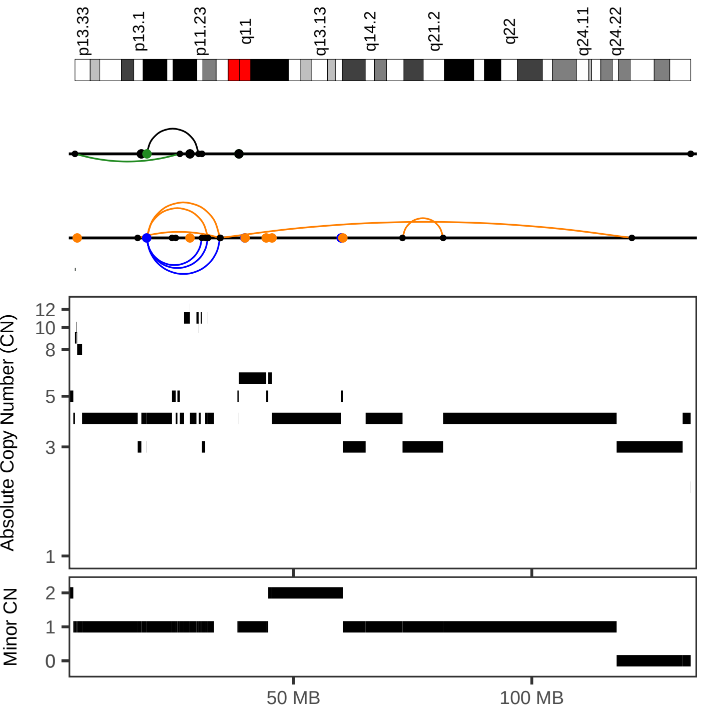

	**MELA−0231**
Cancer type	Skin−Melanoma
Position	12:4111597−34617370
Type	With other complex events
Interleaved intrachr. SVs	9
Total SVs (intrachr. + transl.)	15
SV types	DEL: 3; DUP: 3; h2hINV: 1;<br>t2tINV: 2; TRA: 6
SVs in sample	1234
Oscillating CN (2 and 3 states)	7, 7
CN segments	27
FDR fragment joints	0.83
FDR chr. breakp. enrich.	0
Linked to chrs	9:396152−20238661;
Purity, ploidy	0.92, 4.03

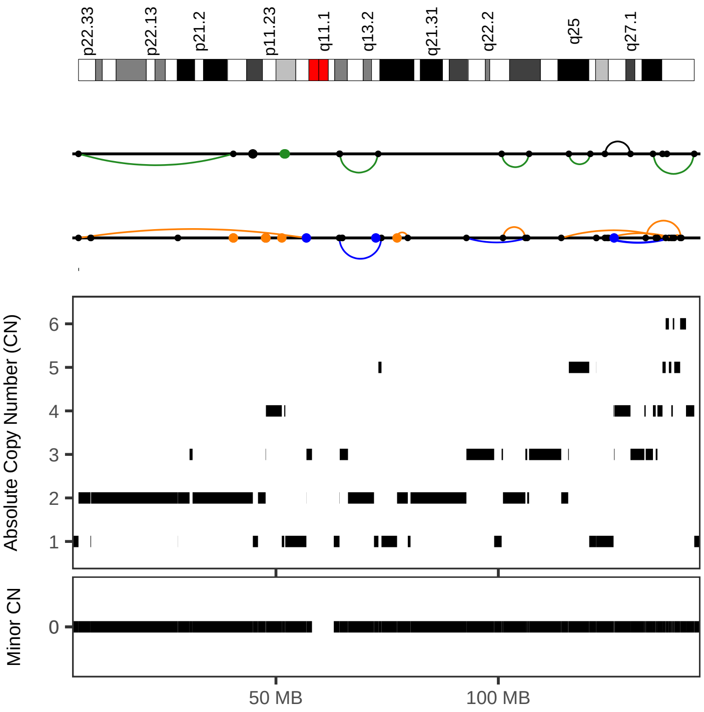

	**MELA−0231**
Cancer type	Skin−Melanoma
Position	X:114145722−144057315
Type	With other complex events
Interleaved intrachr. SVs	8
Total SVs (intrachr. + transl.)	9
SV types	DEL: 3; DUP: 2; h2hINV: 1;<br>t2tINV: 2; TRA: 1
SVs in sample	1234
Oscillating CN (2 and 3 states)	9, 14
CN segments	24
FDR fragment joints	0.84
FDR chr. breakp. enrich.	0.07
Linked to chrs	
Purity, ploidy	0.92, 4.03

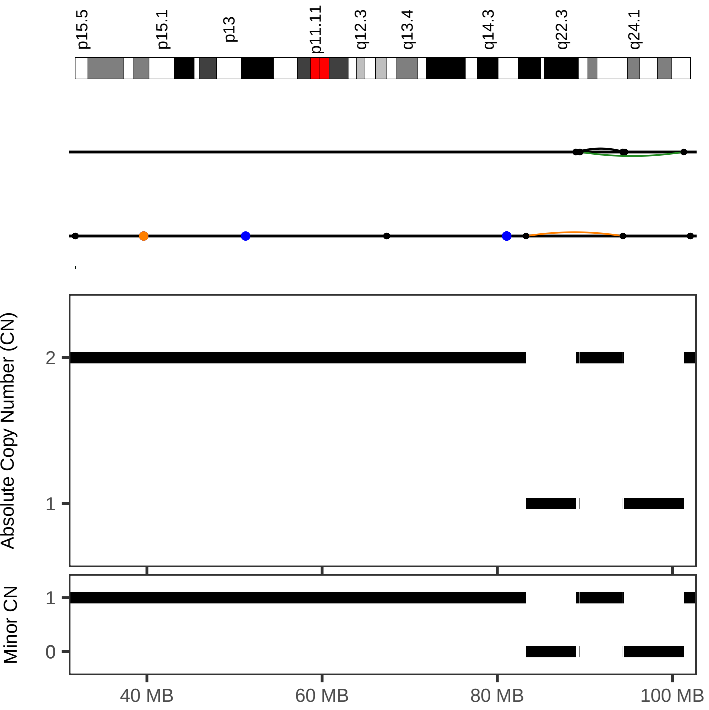

	**MELA−0234**
Cancer type	Skin−Melanoma
Position	11:83286872−101298726
Type	Canonical without polyploidization
Interleaved intrachr. SVs	6
Total SVs (intrachr. + transl.)	6
SV types	DEL: 1; DUP: 0; h2hINV: 3;<br>t2tINV: 2; TRA: 0
SVs in sample	128
Oscillating CN (2 and 3 states)	8, 8
CN segments	8
FDR fragment joints	0.59
FDR chr. breakp. enrich.	0
Linked to chrs	
Purity, ploidy	0.92, 1.98

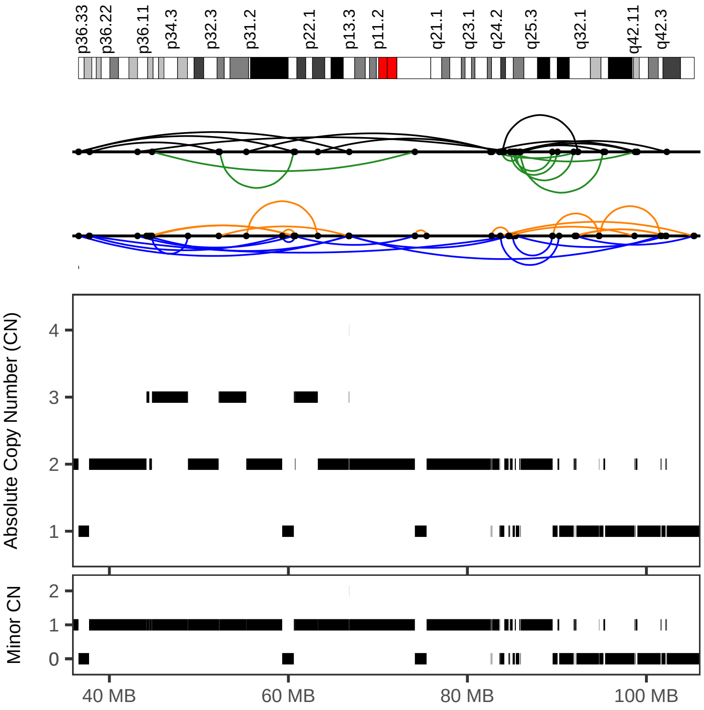

	**MELA−0239**
Cancer type	Skin−Melanoma
Position	1:36546861−105343304
Type	Canonical without polyploidization
Interleaved intrachr. SVs	48
Total SVs (intrachr. + transl.)	48
SV types	DEL: 13; DUP: 14; h2hINV: 11;<br>t2tINV: 10; TRA: 0
SVs in sample	93
Oscillating CN (2 and 3 states)	42, 42
CN segments	63
FDR fragment joints	0.88
FDR chr. breakp. enrich.	0
Linked to chrs	
Purity, ploidy	0.82, 2.22

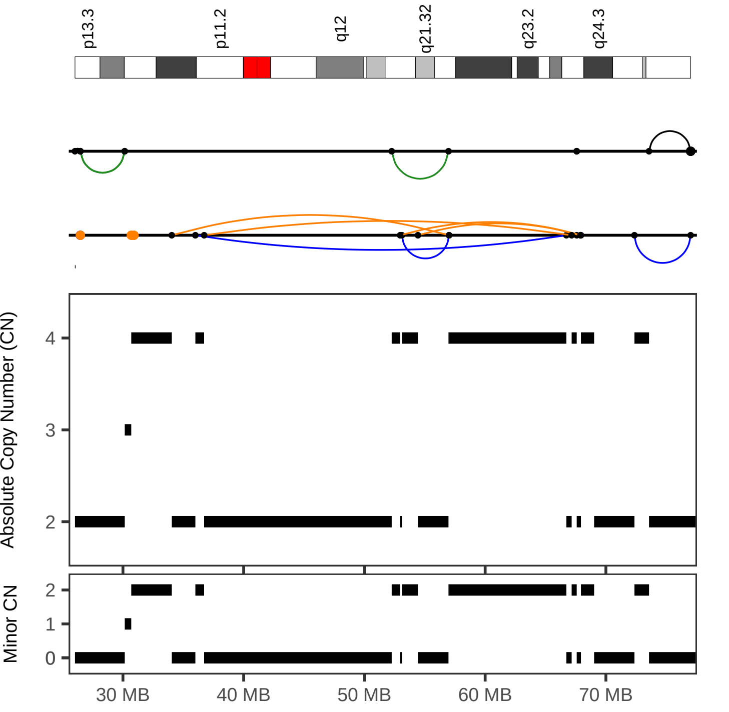

	01ad975d−c2ed−4e4d−bd3b−c9512fc9073c
Cancer type	Skin−Melanoma
Position	17:34042841−67932342
Type	Before polyploidization
Interleaved intrachr. SVs	8
Total SVs (intrachr. + transl.)	8
SV types	DEL: 4; DUP: 3; h2hINV: 0;<br>t2tINV: 1; TRA: 0
SVs in sample	50
Oscillating CN (2 and 3 states)	14, 14
CN segments	14
FDR fragment joints	0.59
FDR chr. breakp. enrich.	0
Linked to chrs	
Purity, ploidy	0.78, 2.85

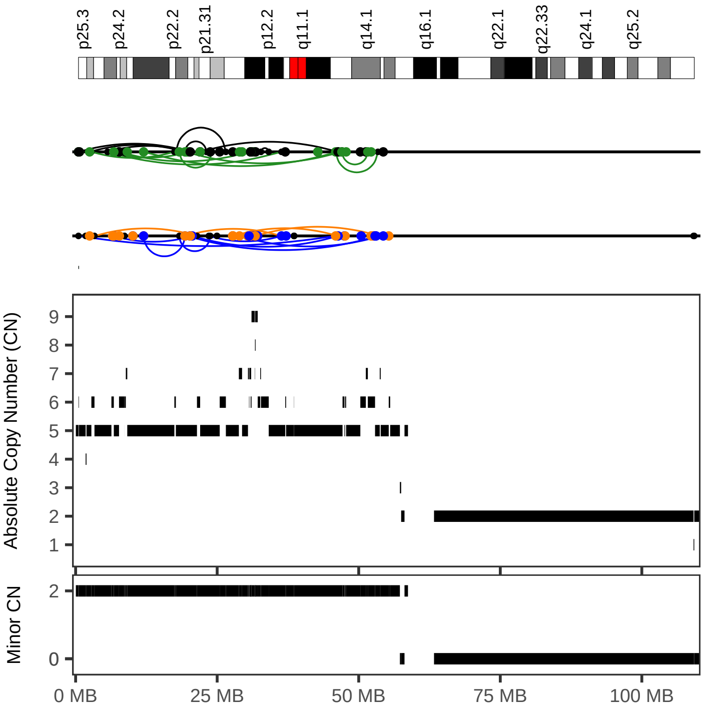

	1404791b−86bf−4dfd−85b4−c8ff356f109c
Cancer type	Skin−Melanoma
Position	6:503244−53879504
Type	With other complex events
Interleaved intrachr. SVs	30
Total SVs (intrachr. + transl.)	124
SV types	DEL: 4; DUP: 9; h2hINV: 7;<br>t2tINV: 10; TRA: 94
SVs in sample	573
Oscillating CN (2 and 3 states)	11, 29
CN segments	53
FDR fragment joints	0.63
FDR chr. breakp. enrich.	0
Linked to chrs	11:33008500−79611121;19:32205724−58908288<br>22:29353674−50585361;7:306183−65693058
Purity, ploidy	0.89, 2.68

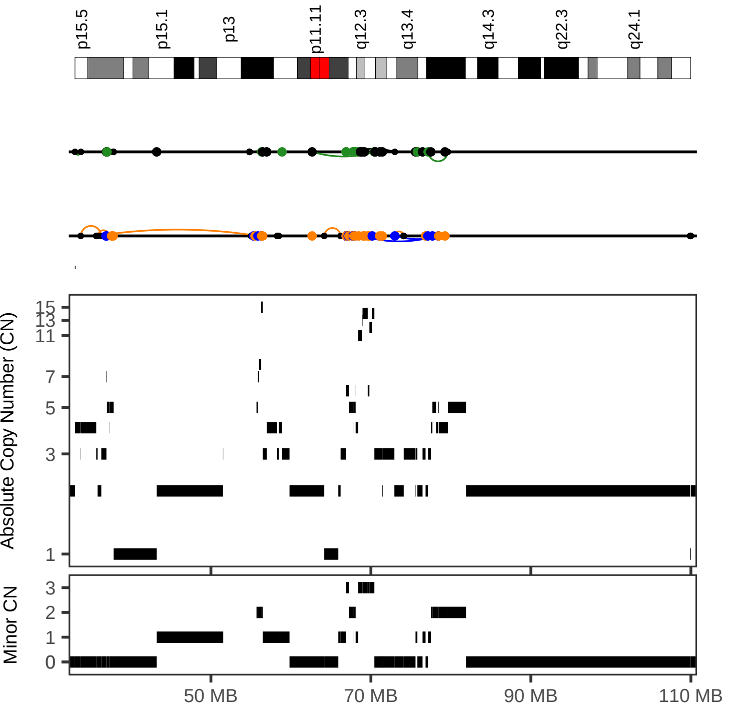

	1404791b−86bf−4dfd−85b4−c8ff356f109c
Cancer type	Skin−Melanoma
Position	11:33008500−79611122
Type	With other complex events
Interleaved intrachr. SVs	6
Total SVs (intrachr. + transl.)	89
SV types	DEL: 5; DUP: 2; h2hINV: 1;<br>t2tINV: 4; TRA: 77
SVs in sample	573
Oscillating CN (2 and 3 states)	13, 19
CN segments	58
FDR fragment joints	0.59
FDR chr. breakp. enrich.	0
Linked to chrs	19:32205724−58908288;6:503244−53879503<br>7:306183−65693058;
Purity, ploidy	0.89, 2.68

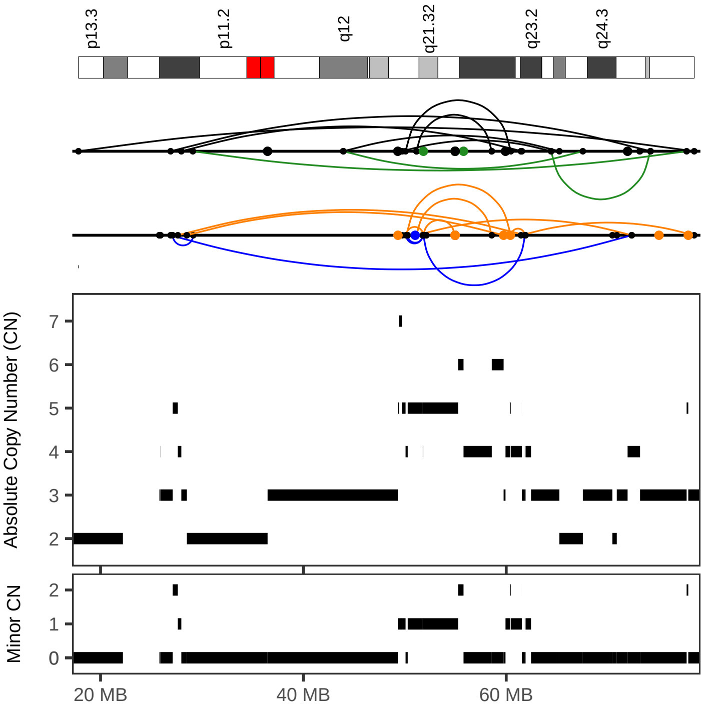

	1404791b−86bf−4dfd−85b4−c8ff356f109c
Cancer type	Skin−Melanoma
Position	17:26903274−78531573
Type	With other complex events
Interleaved intrachr. SVs	26
Total SVs (intrachr. + transl.)	45
SV types	DEL: 9; DUP: 6; h2hINV: 7;<br>t2tINV: 4; TRA: 19
SVs in sample	573
Oscillating CN (2 and 3 states)	7, 18
CN segments	37
FDR fragment joints	0.64
FDR chr. breakp. enrich.	0
Linked to chrs	
Purity, ploidy	0.89, 2.68

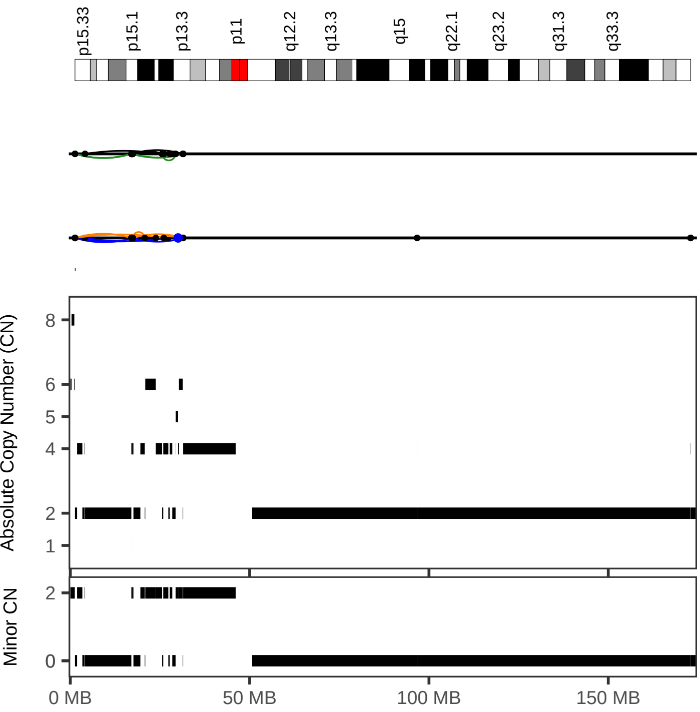

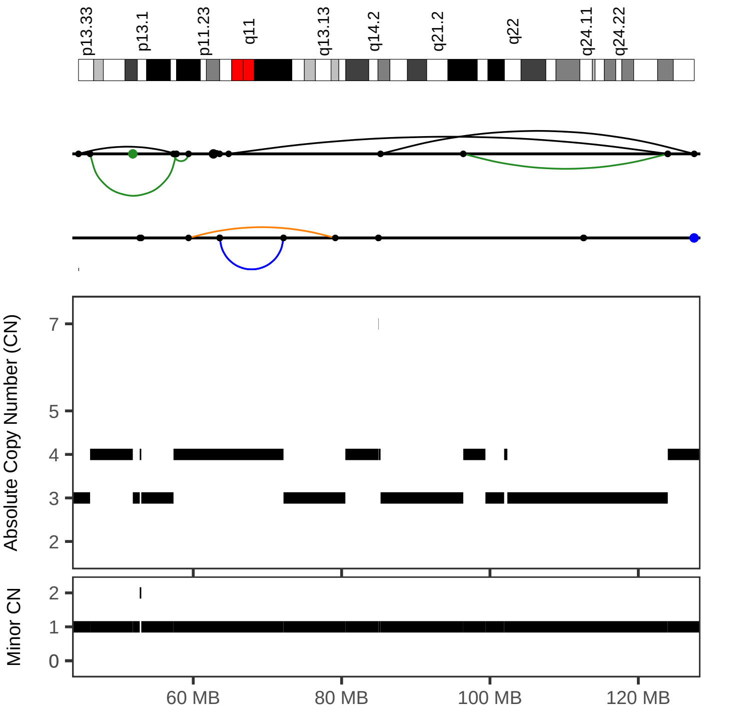

	210726bd−db19−48ae−9a3d−e6ac44480e85
Cancer type	Skin−Melanoma
Position	5:1296282−31464643
Type	With other complex events
Interleaved intrachr. SVs	24
Total SVs (intrachr. + transl.)	25
SV types	DEL: 6; DUP: 4; h2hINV: 7; t2tINV: 7; TRA: 1
SVs in sample	108
Oscillating CN (2 and 3 states)	7, 7
CN segments	24
FDR fragment joints	0.84
FDR chr. breakp. enrich.	0
Linked to chrs	
Purity, ploidy	0.66, 2.88

	210726bd−db19−48ae−9a3d−e6ac44480e85
Cancer type	Skin−Melanoma
Position	12:44527293−127533801
Type	With other complex events
Interleaved intrachr. SVs	10
Total SVs (intrachr. + transl.)	13
SV types	DEL: 1; DUP: 3; h2hINV: 3; t2tINV: 3; TRA: 3
SVs in sample	108
Oscillating CN (2 and 3 states)	8, 16
CN segments	16
FDR fragment joints	0.83
FDR chr. breakp. enrich.	0
Linked to chrs	
Purity, ploidy	0.66, 2.88

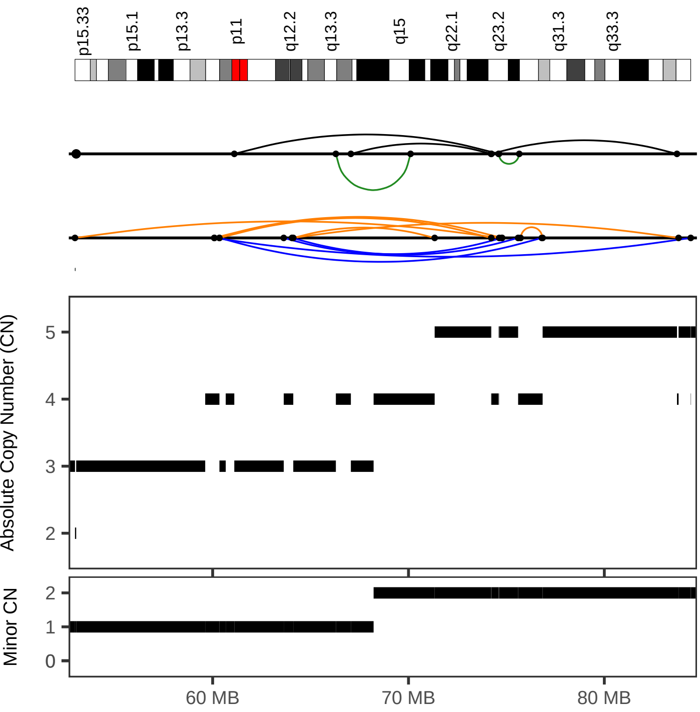

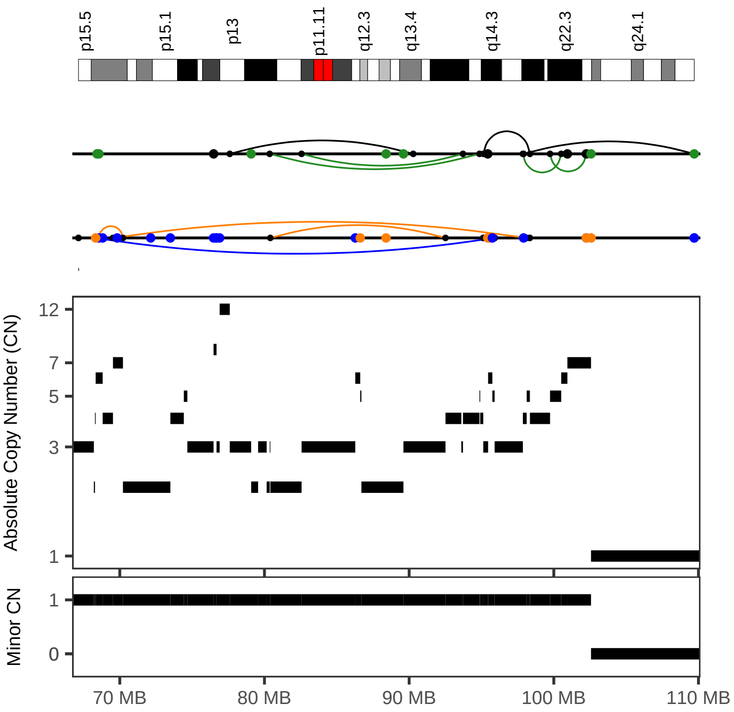

	3582fb74−f43d−4fcd−8f22−bb1506fcff1c
Cancer type	Skin−Melanoma
Position	5:52969272−84413143
Type	With other complex events
Interleaved intrachr. SVs	15
Total SVs (intrachr. + transl.)	16
SV types	DEL: 6; DUP: 4; h2hINV: 3; t2tINV: 2; TRA: 1
SVs in sample	179
Oscillating CN (2 and 3 states)	13, 23
CN segments	23
FDR fragment joints	0.64
FDR chr. breakp. enrich.	0
Linked to chrs	
Purity, ploidy	0.94, 2.36

	3582fb74−f43d−4fcd−8f22−bb1506fcff1c
Cancer type	Skin−Melanoma
Position	11:68283918−109705790
Type	With other complex events
Interleaved intrachr. SVs	7
Total SVs (intrachr. + transl.)	40
SV types	DEL: 2; DUP: 1; h2hINV: 2; t2tINV: 2; TRA: 33
SVs in sample	179
Oscillating CN (2 and 3 states)	7, 11
CN segments	40
FDR fragment joints	0.95
FDR chr. breakp. enrich.	0
Linked to chrs	
Purity, ploidy	0.94, 2.36

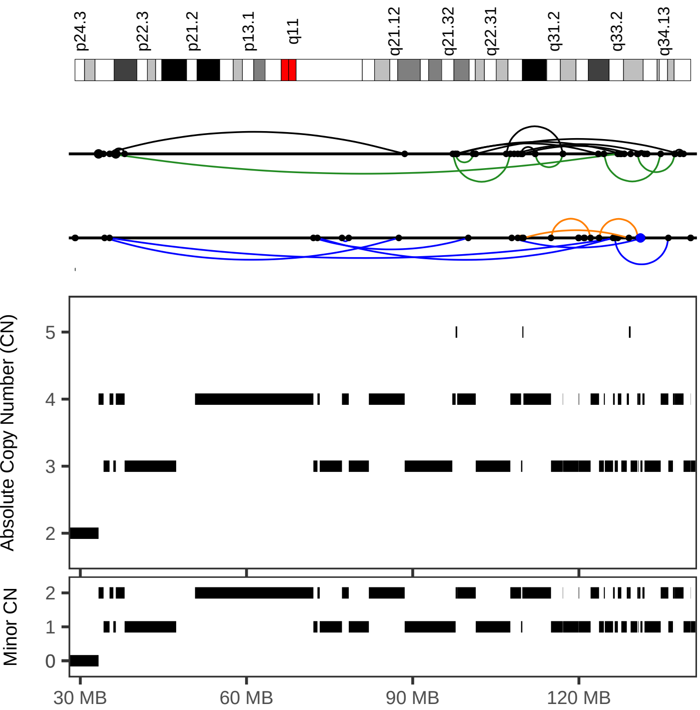

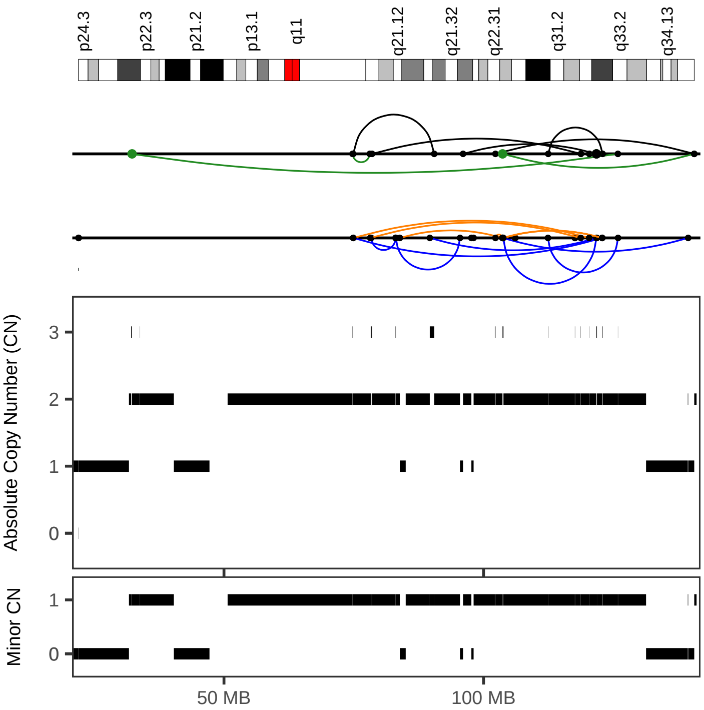

	3fea3f02−2db3−44e0−984e−c61130d92c9b
Cancer type	Skin−Melanoma
Position	9:34192125−138885872
Type	With other complex events
Interleaved intrachr. SVs	28
Total SVs (intrachr. + transl.)	30
SV types	DEL: 4; DUP: 6; h2hINV: 10;<br>t2tINV: 8; TRA: 2
SVs in sample	262
Oscillating CN (2 and 3 states)	15, 37
CN segments	50
FDR fragment joints	0.62
FDR chr. breakp. enrich.	0
Linked to chrs	
Purity, ploidy	0.74, 3.05

	40e17bf8−6c19−4e5e−b60d−ad54f504a970
Cancer type	Skin−Melanoma
Position	9:32139979−140579391
Type	With other complex events
Interleaved intrachr. SVs	21
Total SVs (intrachr. + transl.)	25
SV types	DEL: 5; DUP: 8; h2hINV: 5;<br>t2tINV: 3; TRA: 4
SVs in sample	140
Oscillating CN (2 and 3 states)	21, 46
CN segments	46
FDR fragment joints	0.64
FDR chr. breakp. enrich.	0
Linked to chrs	22:29683567−49998523;
Purity, ploidy	0.76, 1.94

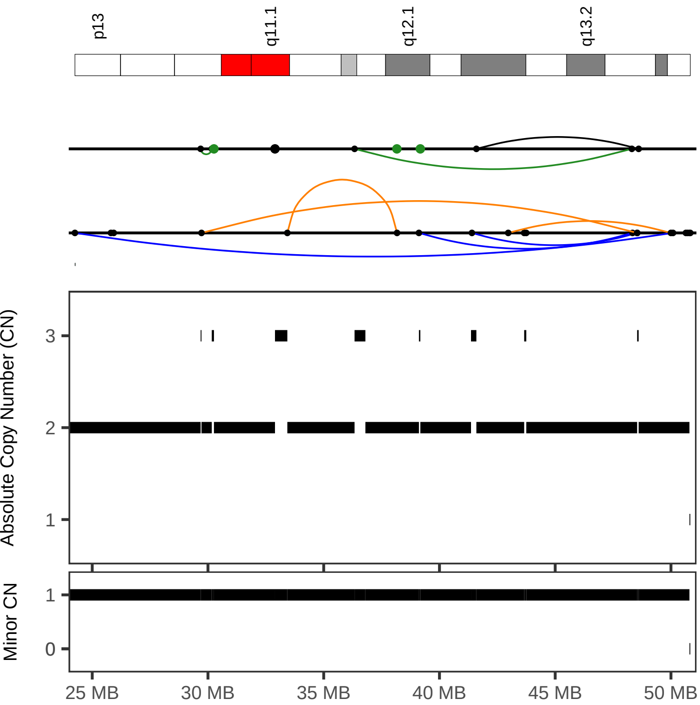

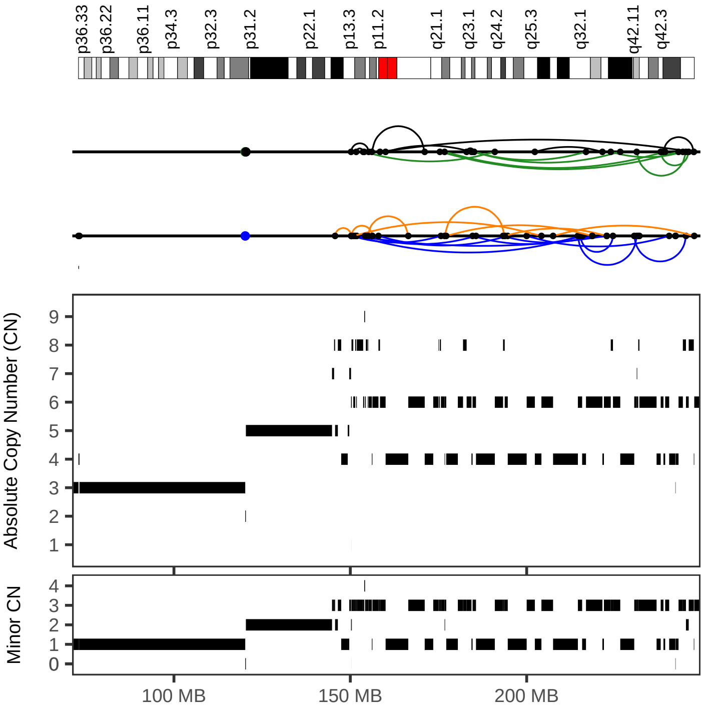

	40e17bf8−6c19−4e5e−b60d−ad54f504a970
Cancer type	Skin−Melanoma
Position	22:29683567−49998524
Type	Canonical without polyploidization
Interleaved intrachr. SVs	8
Total SVs (intrachr. + transl.)	12
SV types	DEL: 3; DUP: 2; h2hINV: 1;<br>t2tINV: 2; TRA: 4
SVs in sample	140
Oscillating CN (2 and 3 states)	16, 16
CN segments	16
FDR fragment joints	0.84
FDR chr. breakp. enrich.	0
Linked to chrs	9:32139979−140579390;
Purity, ploidy	0.76, 1.94

	5bc515c8−7727−4d69−98e9−31bbcb748550
Cancer type	Skin−Melanoma
Position	1:145684513−247510948
Type	With other complex events
Interleaved intrachr. SVs	36
Total SVs (intrachr. + transl.)	36
SV types	DEL: 9; DUP: 11; h2hINV: 8;<br>t2tINV: 8; TRA: 0
SVs in sample	178
Oscillating CN (2 and 3 states)	11, 43
CN segments	77
FDR fragment joints	0.91
FDR chr. breakp. enrich.	0
Linked to chrs	
Purity, ploidy	0.47, 3.2

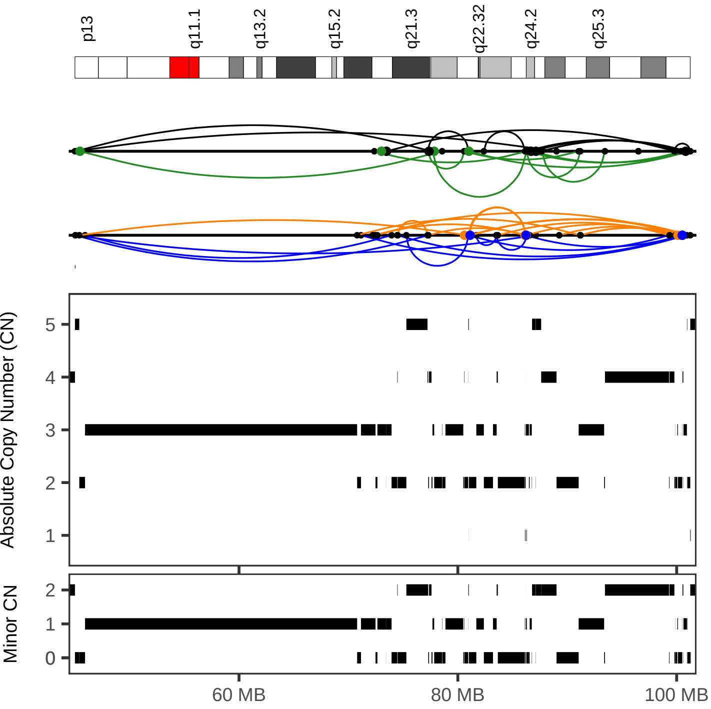

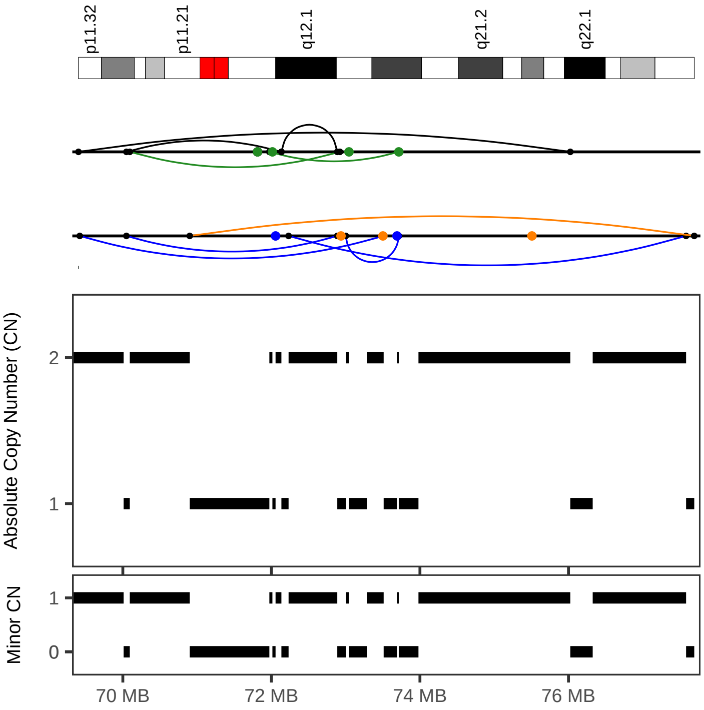

	**5bc515c8−7727−4d69−98e9−31bbcb748550**
Cancer type	Skin−Melanoma
Position	15:45003571−101242417
Type	With other complex events
Interleaved intrachr. SVs	50
Total SVs (intrachr. + transl.)	68
SV types	DEL: 19; DUP: 11; h2hINV: 10; t2tINV: 10; TRA: 18
SVs in sample	178
Oscillating CN (2 and 3 states)	9, 15
CN segments	74
FDR fragment joints	0.59
FDR chr. breakp. enrich.	0
Linked to chrs	1:145684513−247510947;
Purity, ploidy	0.47, 3.2

	**5f6df69c−4ebf−4811−8b28−ad393dfbe1a3**
Cancer type	Skin−Melanoma
Position	18:69402625−77693227
Type	Canonical without polyploidization
Interleaved intrachr. SVs	10
Total SVs (intrachr. + transl.)	20
SV types	DEL: 1; DUP: 4; h2hINV: 3; t2tINV: 2; TRA: 10
SVs in sample	160
Oscillating CN (2 and 3 states)	20, 20
CN segments	20
FDR fragment joints	0.64
FDR chr. breakp. enrich.	0
Linked to chrs	2:15930327−35023335;3:81208042−140376908 6:80713179−118504283;
Purity, ploidy	0.86, 1.94

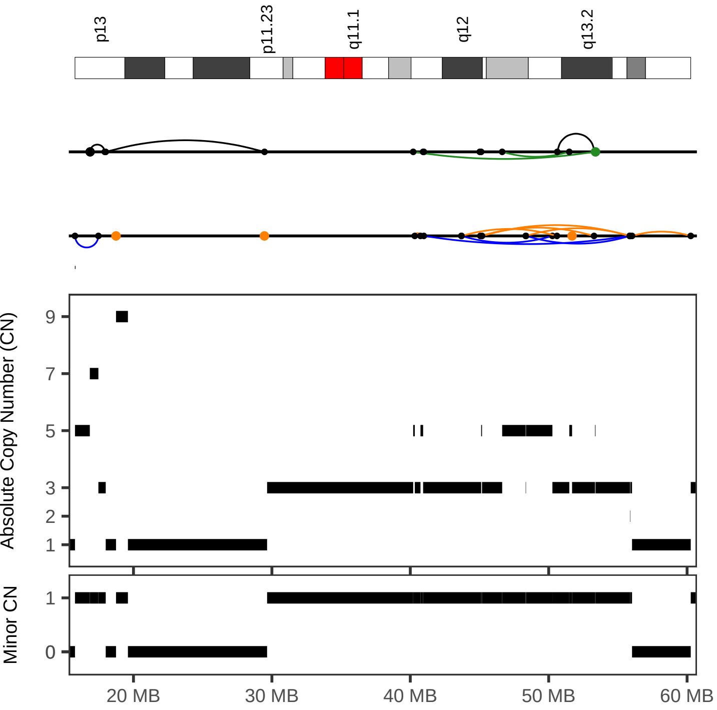

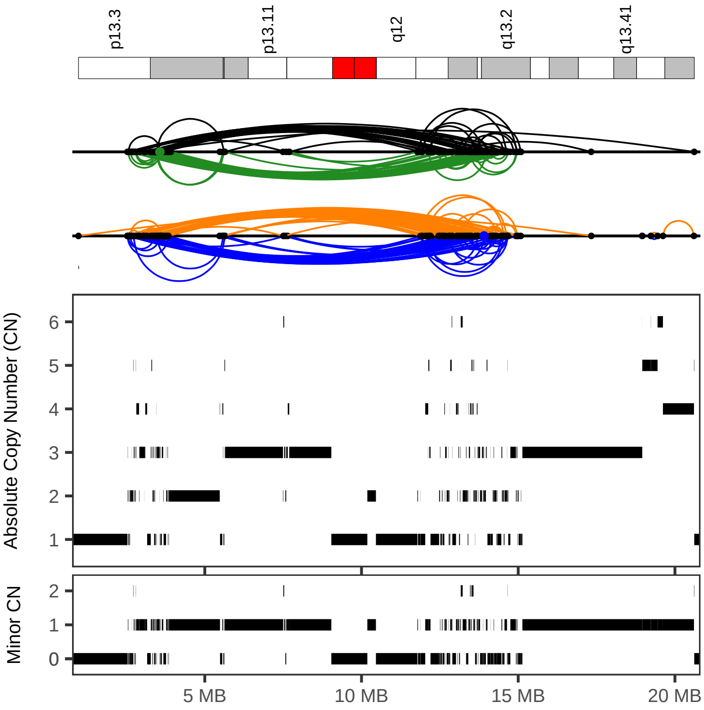

	**5f6df69c−4ebf−4811−8b28−ad393dfbe1a3**
Cancer type	Skin−Melanoma
Position	20:40210929−55897885
Type	After polyploidization
Interleaved intrachr. SVs	12
Total SVs (intrachr. + transl.)	14
SV types	DEL: 4; DUP: 3; h2hINV: 2; t2tINV: 3; TRA: 2
SVs in sample	160
Oscillating CN (2 and 3 states)	14, 16
CN segments	16
FDR fragment joints	0.91
FDR chr. breakp. enrich.	0
Linked to chrs	
Purity, ploidy	0.86, 1.94

	**68f75ab5−9d77−40c5−a8ce−f56016bbe9e4**
Cancer type	Skin−Melanoma
Position	19:969992−20616012
Type	With other complex events
Interleaved intrachr. SVs	269
Total SVs (intrachr. + transl.)	271
SV types	DEL: 79; DUP: 69; h2hINV: 58; t2tINV: 63; TRA: 2
SVs in sample	305
Oscillating CN (2 and 3 states)	11, 14
CN segments	211
FDR fragment joints	0.59
FDR chr. breakp. enrich.	0
Linked to chrs	
Purity, ploidy	0.56, 2.15

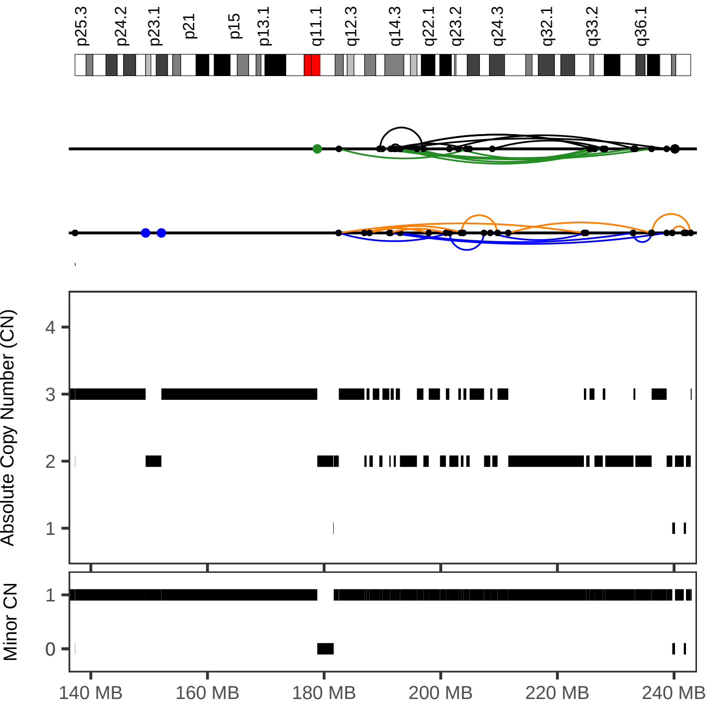

76cc1d42−acec−46b3−9663−5e4de2550353	
Cancer type	Skin−Melanoma
Position	2:182474699−242863436
Type	Canonical without polyploidization
Interleaved intrachr. SVs	27
Total SVs (intrachr. + transl.)	28
SV types	DEL: 7; DUP: 6; h2hINV: 8; t2tINV: 6; TRA: 1
SVs in sample	245
Oscillating CN (2 and 3 states)	42, 47
CN segments	47
FDR fragment joints	0.96
FDR chr. breakp. enrich.	0
Linked to chrs	
Purity, ploidy	0.83, 2.94

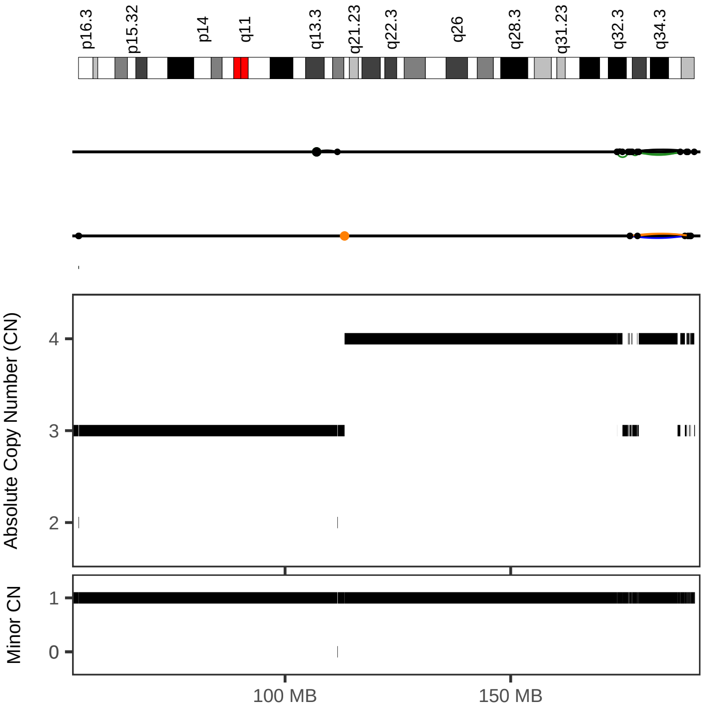

76cc1d42−acec−46b3−9663−5e4de2550353	
Cancer type	Skin−Melanoma
Position	4:176289985−190678345
Type	After polyploidization
Interleaved intrachr. SVs	9
Total SVs (intrachr. + transl.)	9
SV types	DEL: 2; DUP: 2; h2hINV: 2; t2tINV: 3; TRA: 0
SVs in sample	245
Oscillating CN (2 and 3 states)	18, 18
CN segments	18
FDR fragment joints	0.97
FDR chr. breakp. enrich.	1
Linked to chrs	
Purity, ploidy	0.83, 2.94

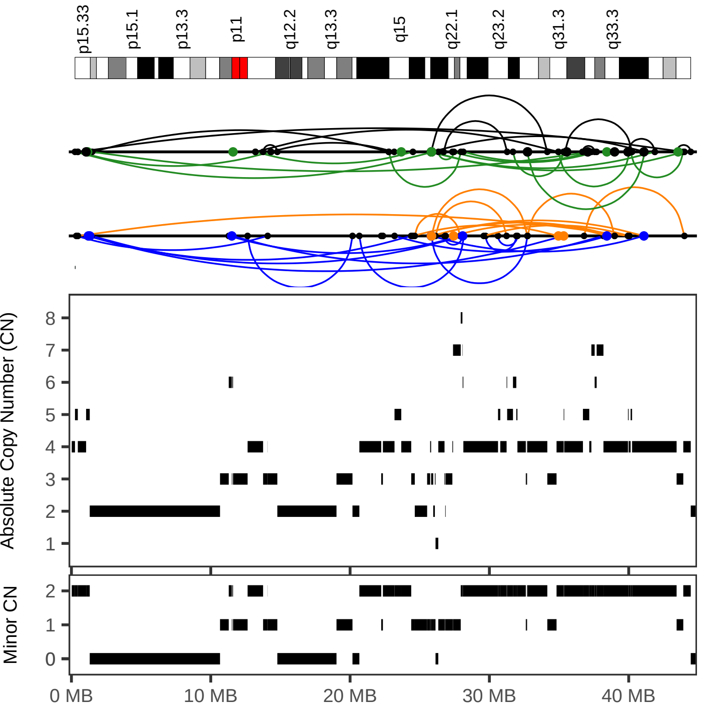

76cc1d42−acec−46b3−9663−5e4de2550353	
Cancer type	Skin−Melanoma
Position	5:253936−44452670
Type	With other complex events
Interleaved intrachr. SVs	52
Total SVs (intrachr. + transl.)	86
SV types	DEL: 11; DUP: 14; h2hINV: 13; t2tINV: 14; TRA: 34
SVs in sample	245
Oscillating CN (2 and 3 states)	7, 25
CN segments	67
FDR fragment joints	0.95
FDR chr. breakp. enrich.	0
Linked to chrs	16:63130093−68046856;
Purity, ploidy	0.83, 2.94

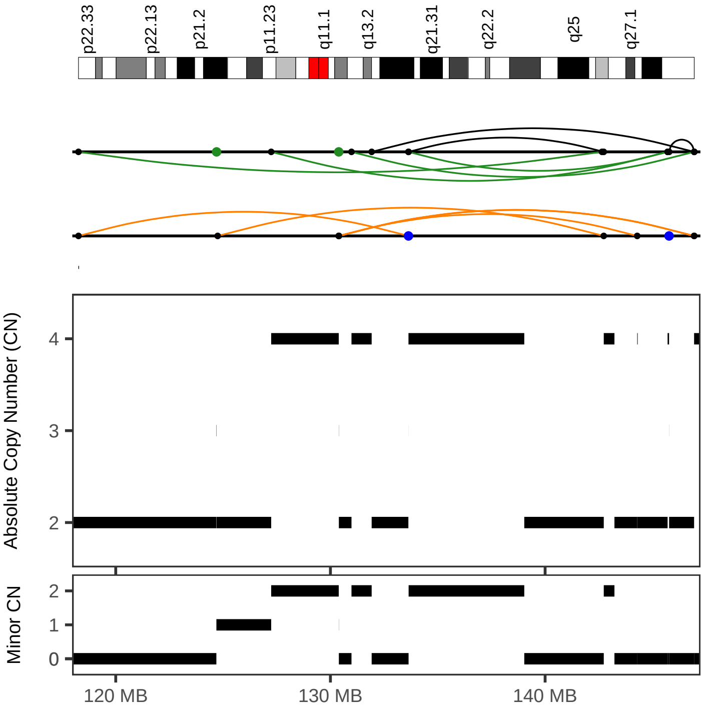

7b92fcd4−f9be−4b97−94a2−b2e8348aeaba	
Cancer type	Skin−Melanoma
Position	X:118264440−146947067
Type	With other complex events
Interleaved intrachr. SVs	12
Total SVs (intrachr. + transl.)	16
SV types	DEL: 5; DUP: 0; h2hINV: 3; t2tINV: 4; TRA: 4
SVs in sample	137
Oscillating CN (2 and 3 states)	7, 14
CN segments	20
FDR fragment joints	0.59
FDR chr. breakp. enrich.	0
Linked to chrs	
Purity, ploidy	0.67, 4.4

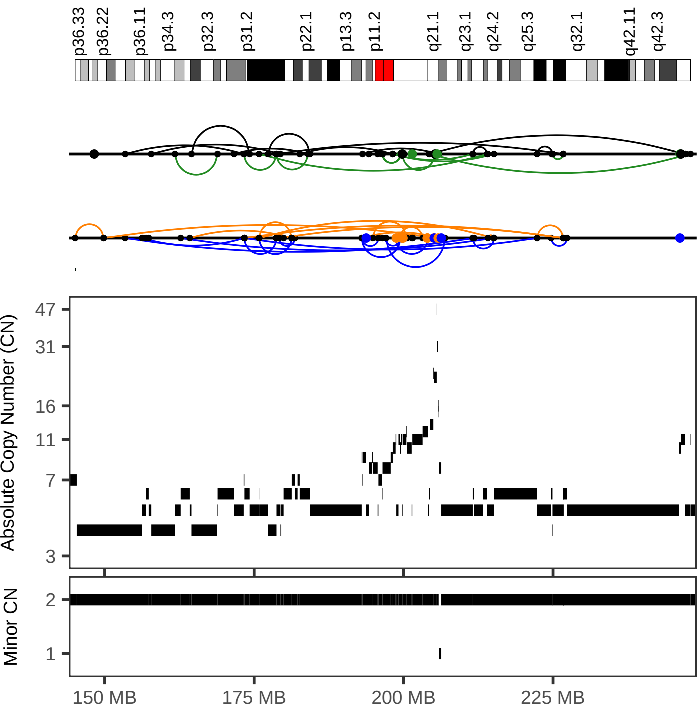

	7ce09a09−074e−4f38−bed7−98ab34347fbe
Cancer type	Skin−Melanoma
Position	1:149816333−248002432
Type	With other complex events
Interleaved intrachr. SVs	52
Total SVs (intrachr. + transl.)	70
SV types	DEL: 13; DUP: 15; h2hINV: 12; t2tINV: 12; TRA: 18
SVs in sample	201
Oscillating CN (2 and 3 states)	10, 19
CN segments	91
FDR fragment joints	0.95
FDR chr. breakp. enrich.	0
Linked to chrs	11:5892683−117914382;7:61559135−158613295
Purity, ploidy	0.88, 3.45

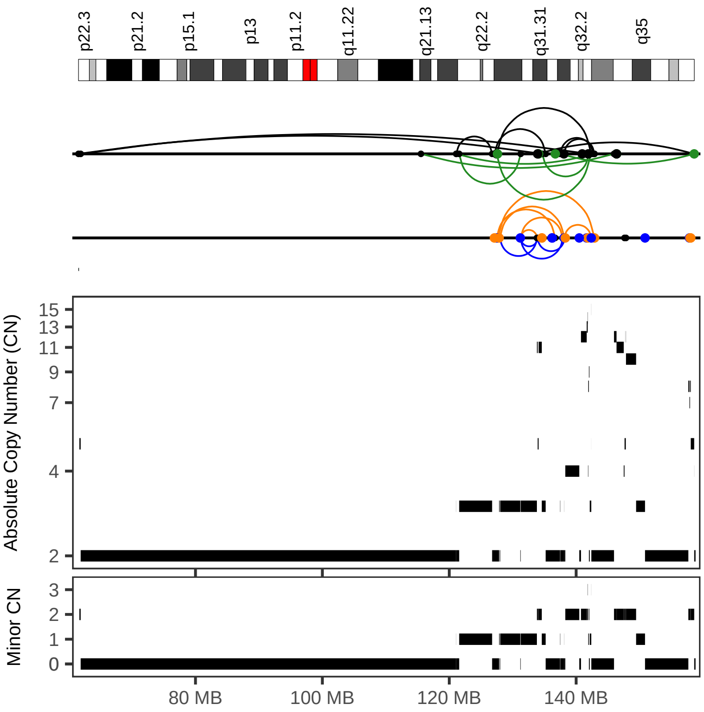

	7ce09a09−074e−4f38−bed7−98ab34347fbe
Cancer type	Skin−Melanoma
Position	7:61559135−158613296
Type	With other complex events
Interleaved intrachr. SVs	26
Total SVs (intrachr. + transl.)	57
SV types	DEL: 6; DUP: 4; h2hINV: 8; t2tINV: 8; TRA: 31
SVs in sample	201
Oscillating CN (2 and 3 states)	10, 11
CN segments	49
FDR fragment joints	0.71
FDR chr. breakp. enrich.	0
Linked to chrs	1:149816333−248002431;11:5892683−117914382 3:120763840−170508367;
Purity, ploidy	0.88, 3.45

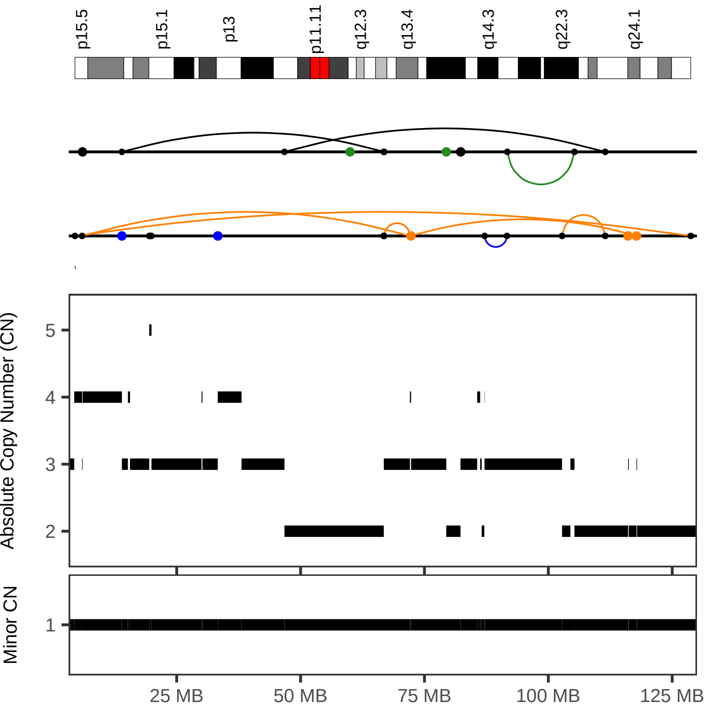

	7ce09a09−074e−4f38−bed7−98ab34347fbe
Cancer type	Skin−Melanoma
Position	11:5892683−117914383
Type	With other complex events
Interleaved intrachr. SVs	5
Total SVs (intrachr. + transl.)	15
SV types	DEL: 3; DUP: 0; h2hINV: 2; t2tINV: 0; TRA: 10
SVs in sample	201
Oscillating CN (2 and 3 states)	8, 24
CN segments	30
FDR fragment joints	0.59
FDR chr. breakp. enrich.	0
Linked to chrs	1:149816333−248002431;7:61559135−158613295
Purity, ploidy	0.88, 3.45

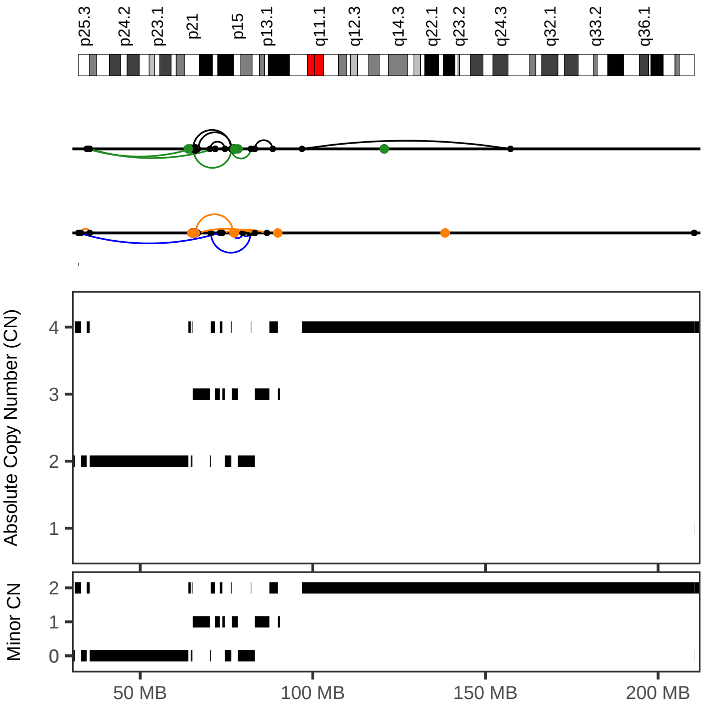

	800d3a17−269f−4d97−94ff−8585f20038d8
Cancer type	Skin−Melanoma
Position	2:32129440−88367600
Type	With other complex events
Interleaved intrachr. SVs	17
Total SVs (intrachr. + transl.)	34
SV types	DEL: 4; DUP: 4; h2hINV: 5; t2tINV: 4; TRA: 17
SVs in sample	337
Oscillating CN (2 and 3 states)	8, 14
CN segments	23
FDR fragment joints	0.99
FDR chr. breakp. enrich.	0.01
Linked to chrs	
Purity, ploidy	0.51, 3.31

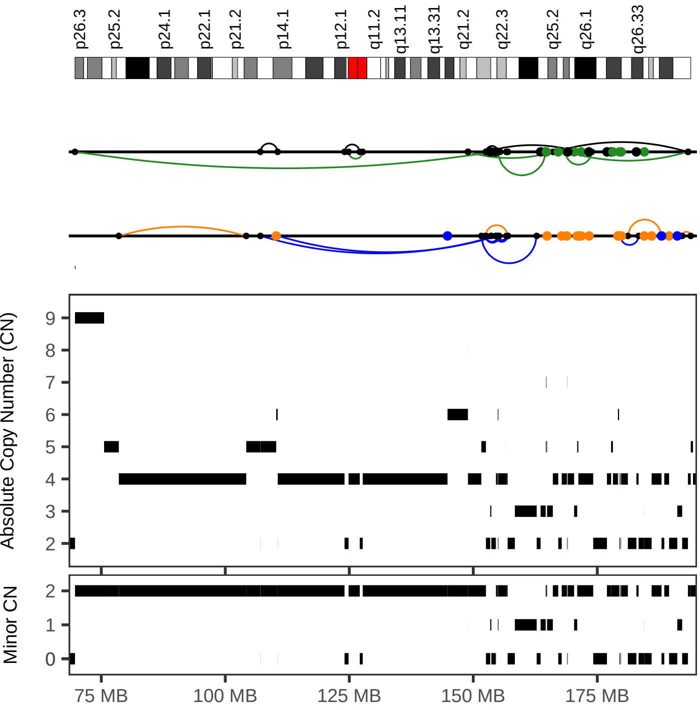

**800d3a17−269f−4d97−94ff−8585f20038d8**	
Cancer type	Skin−Melanoma
Position	3:69695493−193847204
Type	With other complex events
Interleaved intrachr. SVs	29
Total SVs (intrachr. + transl.)	67
SV types	DEL: 4; DUP: 10; h2hINV: 5; t2tINV: 10; TRA: 38
SVs in sample	337
Oscillating CN (2 and 3 states)	8, 17
CN segments	76
FDR fragment joints	0.59
FDR chr. breakp. enrich.	0
Linked to chrs	15:60170393−102151414;7:112828−25746189
Purity, ploidy	0.51, 3.31

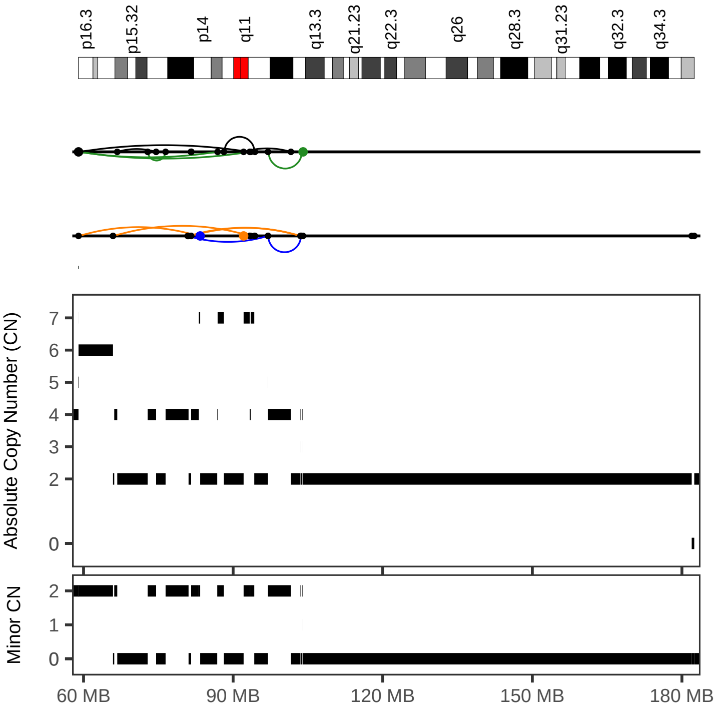

**800d3a17−269f−4d97−94ff−8585f20038d8**	
Cancer type	Skin−Melanoma
Position	4:58983388−104017952
Type	With other complex events
Interleaved intrachr. SVs	15
Total SVs (intrachr. + transl.)	18
SV types	DEL: 6; DUP: 2; h2hINV: 3; t2tINV: 4; TRA: 3
SVs in sample	337
Oscillating CN (2 and 3 states)	8, 9
CN segments	29
FDR fragment joints	0.64
FDR chr. breakp. enrich.	0.36
Linked to chrs	
Purity, ploidy	0.51, 3.31

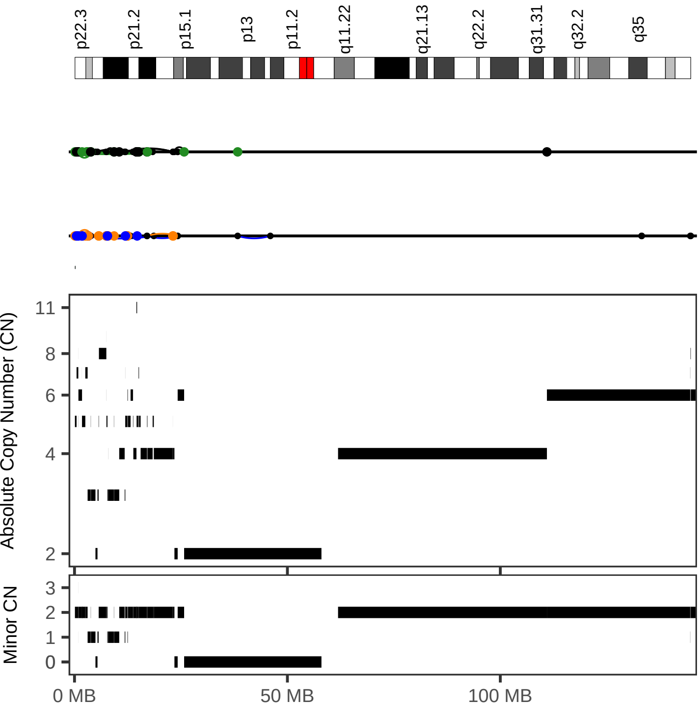

**800d3a17−269f−4d97−94ff−8585f20038d8**	
Cancer type	Skin−Melanoma
Position	7:112828−25746190
Type	With other complex events
Interleaved intrachr. SVs	15
Total SVs (intrachr. + transl.)	62
SV types	DEL: 3; DUP: 3; h2hINV: 5; t2tINV: 4; TRA: 47
SVs in sample	337
Oscillating CN (2 and 3 states)	8, 9
CN segments	56
FDR fragment joints	0.9
FDR chr. breakp. enrich.	0
Linked to chrs	15:60170393−102151414;3:69695493−193847203
Purity, ploidy	0.51, 3.31

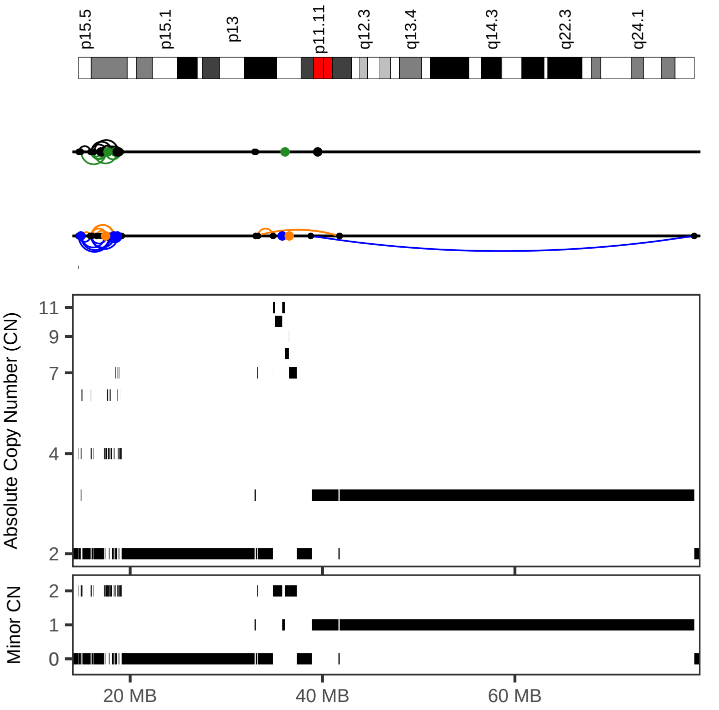

**800d3a17−269f−4d97−94ff−8585f20038d8**	
Cancer type	Skin−Melanoma
Position	11:14627440−19120746
Type	With other complex events
Interleaved intrachr. SVs	44
Total SVs (intrachr. + transl.)	53
SV types	DEL: 8; DUP: 14; h2hINV: 10; t2tINV: 12; TRA: 9
SVs in sample	337
Oscillating CN (2 and 3 states)	11, 29
CN segments	48
FDR fragment joints	0.69
FDR chr. breakp. enrich.	0
Linked to chrs	15:60170393−102151414;
Purity, ploidy	0.51, 3.31

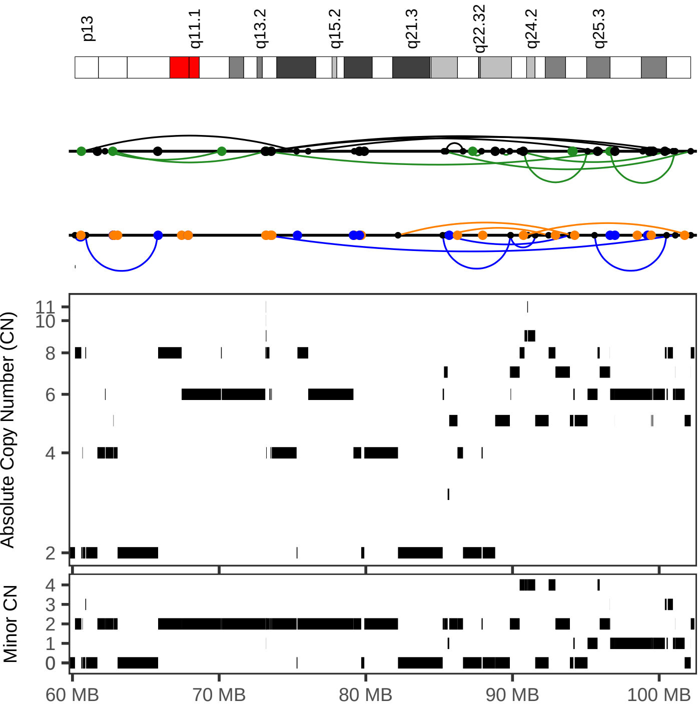

	800d3a17−269f−4d97−94ff−8585f20038d8
Cancer type	Skin−Melanoma
Position	15:60170393−102151415
Type	With other complex events
Interleaved intrachr. SVs	23
Total SVs (intrachr. + transl.)	81
SV types	DEL: 3; DUP: 7; h2hINV: 6; t2tINV: 7; TRA: 58
SVs in sample	337
Oscillating CN (2 and 3 states)	7, 7
CN segments	80
FDR fragment joints	0.67
FDR chr. breakp. enrich.	0
Linked to chrs	11:14627440−19120745;3:69695493−193847203 7:112828−25746189;
Purity, ploidy	0.51, 3.31

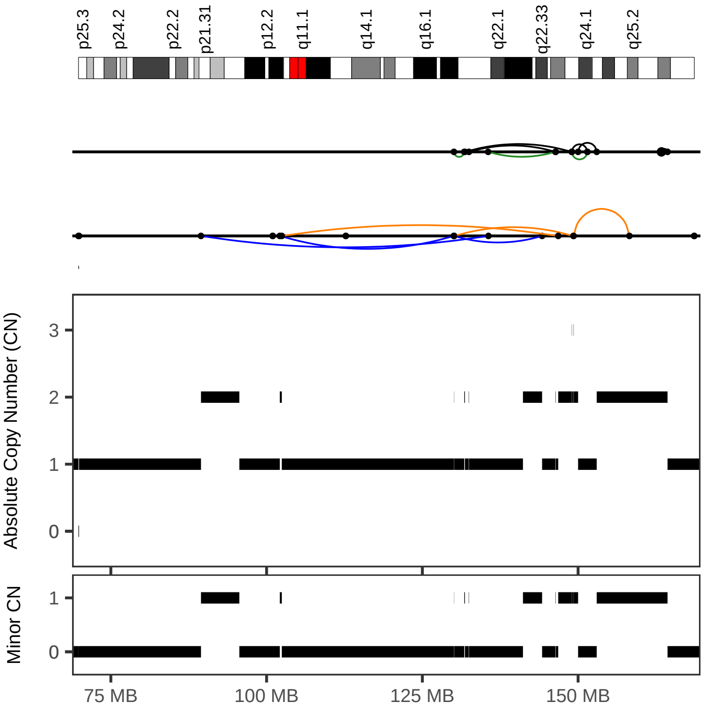

	81cedb9e−ee43−476b−9ed9−8d0fdb346065
Cancer type	Skin−Melanoma
Position	6:89466188−158235277
Type	Canonical without polyploidization
Interleaved intrachr. SVs	13
Total SVs (intrachr. + transl.)	13
SV types	DEL: 3; DUP: 3; h2hINV: 4; t2tINV: 3; TRA: 0
SVs in sample	57
Oscillating CN (2 and 3 states)	15, 21
CN segments	21
FDR fragment joints	0.98
FDR chr. breakp. enrich.	0
Linked to chrs	
Purity, ploidy	0.38, 1.88

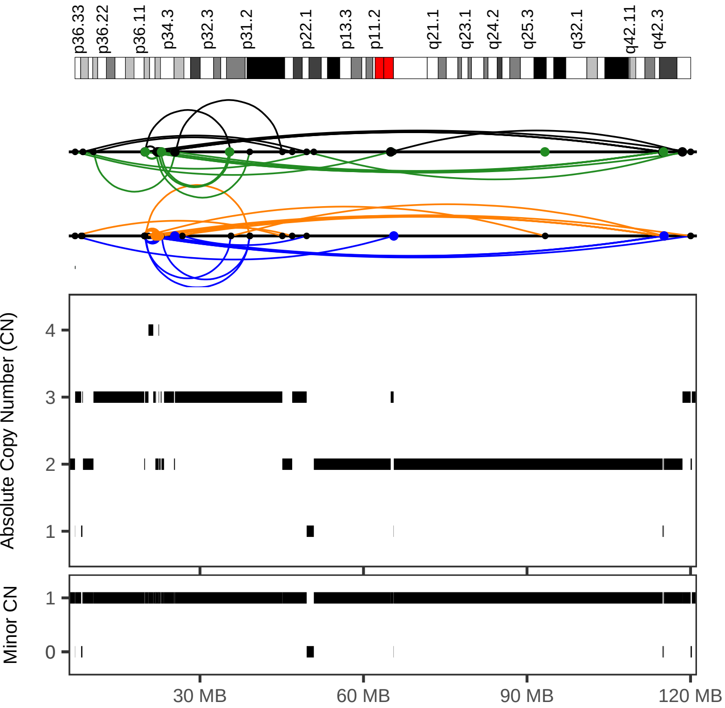

	85400bf1−23e0−4e0f−8df7−f0d108641aee
Cancer type	Skin−Melanoma
Position	1:7068840−120031293
Type	With other complex events
Interleaved intrachr. SVs	77
Total SVs (intrachr. + transl.)	102
SV types	DEL: 20; DUP: 21; h2hINV: 18; t2tINV: 18; TRA: 25
SVs in sample	219
Oscillating CN (2 and 3 states)	12, 19
CN segments	42
FDR fragment joints	0.97
FDR chr. breakp. enrich.	0
Linked to chrs	8:19835544−144243958;9:72055003−138463854
Purity, ploidy	0.91, 2.02

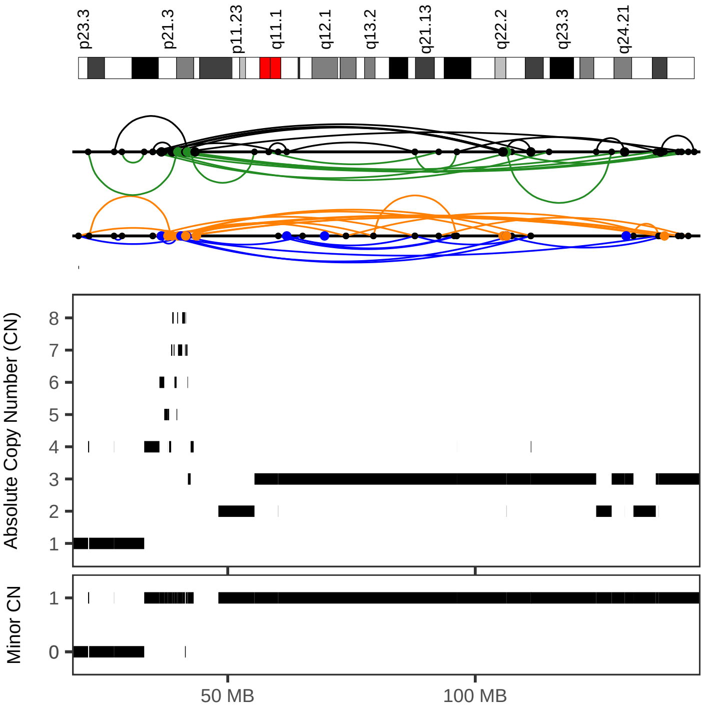

	85400bf1−23e0−4e0f−8df7−f0d108641aee
Cancer type	Skin−Melanoma
Position	8:19835544−144243959
Type	With other complex events
Interleaved intrachr. SVs	55
Total SVs (intrachr. + transl.)	90
SV types	DEL: 16; DUP: 12; h2hINV: 15; t2tINV: 12; TRA: 35
SVs in sample	219
Oscillating CN (2 and 3 states)	9, 18
CN segments	43
FDR fragment joints	0.86
FDR chr. breakp. enrich.	0
Linked to chrs	1:7068840−120031292;9:72055003−138463854
Purity, ploidy	0.91, 2.02

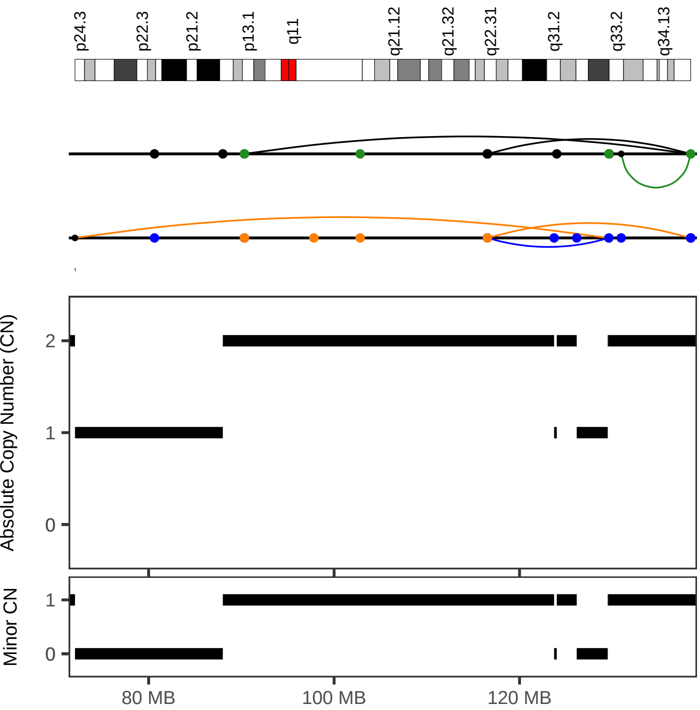

85400bf1−23e0−4e0f−8df7−f0d108641aee

Cancer type	Skin−Melanoma
Position	9:72055003−138463855
Type	Canonical without polyploidization
Interleaved intrachr. SVs	5
Total SVs (intrachr. + transl.)	38
SV types	DEL: 2; DUP: 1; h2hINV: 2; t2tINV: 0; TRA: 33
SVs in sample	219
Oscillating CN (2 and 3 states)	8, 8
CN segments	8
FDR fragment joints	0.64
FDR chr. breakp. enrich.	0
Linked to chrs	1:7068840−120031292;8:19835544−144243958
Purity, ploidy	0.91, 2.02

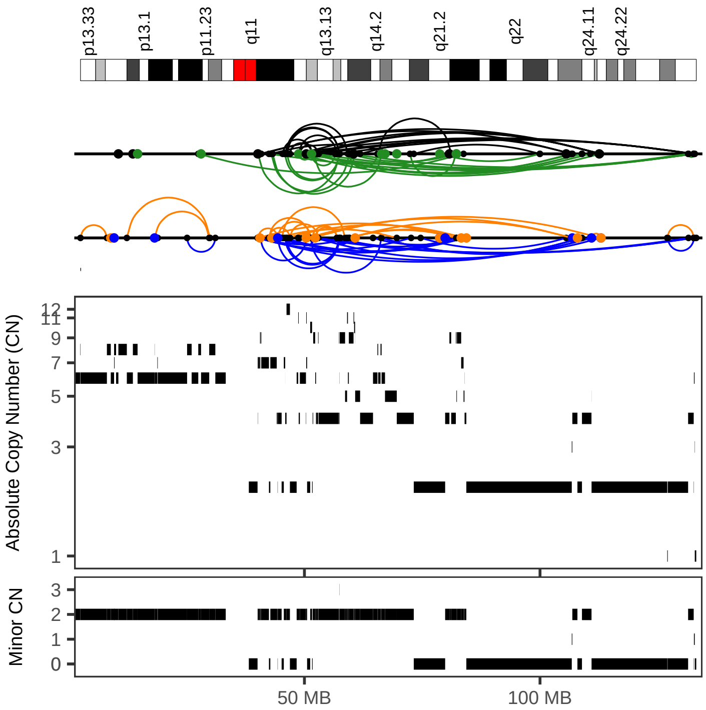

8c54fcfd−999f−43c5−b31f−26d006f5fff3

Cancer type	Skin−Melanoma
Position	12:12308231−133162422
Type	With other complex events
Interleaved intrachr. SVs	97
Total SVs (intrachr. + transl.)	134
SV types	DEL: 23; DUP: 27; h2hINV: 21; t2tINV: 26; TRA: 37
SVs in sample	351
Oscillating CN (2 and 3 states)	7, 13
CN segments	99
FDR fragment joints	0.86
FDR chr. breakp. enrich.	0
Linked to chrs	8:27003357−144300867;
Purity, ploidy	0.9, 3.17

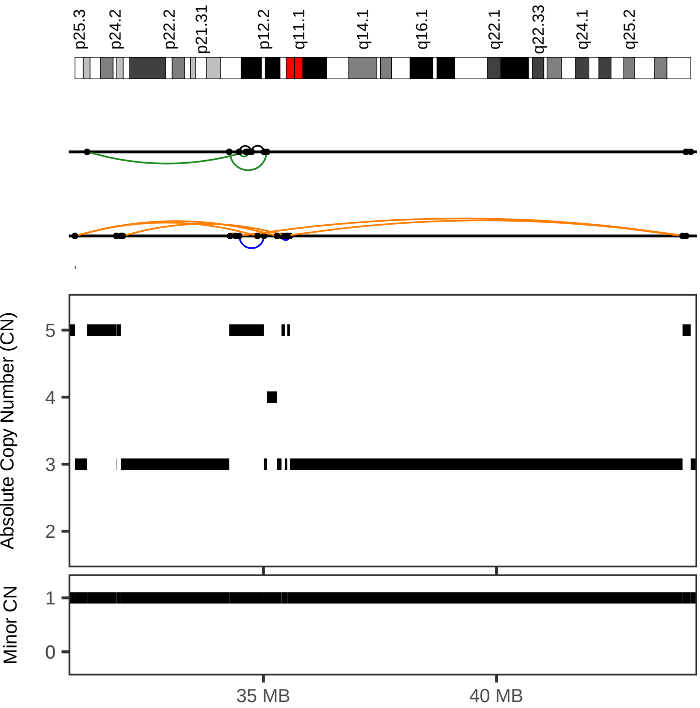

97d9dfc9−c937−48b4−b453−df081950419f

Cancer type	Skin−Melanoma
Position	6:30956994−44170027
Type	With other complex events
Interleaved intrachr. SVs	14
Total SVs (intrachr. + transl.)	14
SV types	DEL: 5; DUP: 2; h2hINV: 4; t2tINV: 3; TRA: 0
SVs in sample	38
Oscillating CN (2 and 3 states)	8, 16
CN segments	16
FDR fragment joints	0.78
FDR chr. breakp. enrich.	0
Linked to chrs	
Purity, ploidy	0.42, 1.95

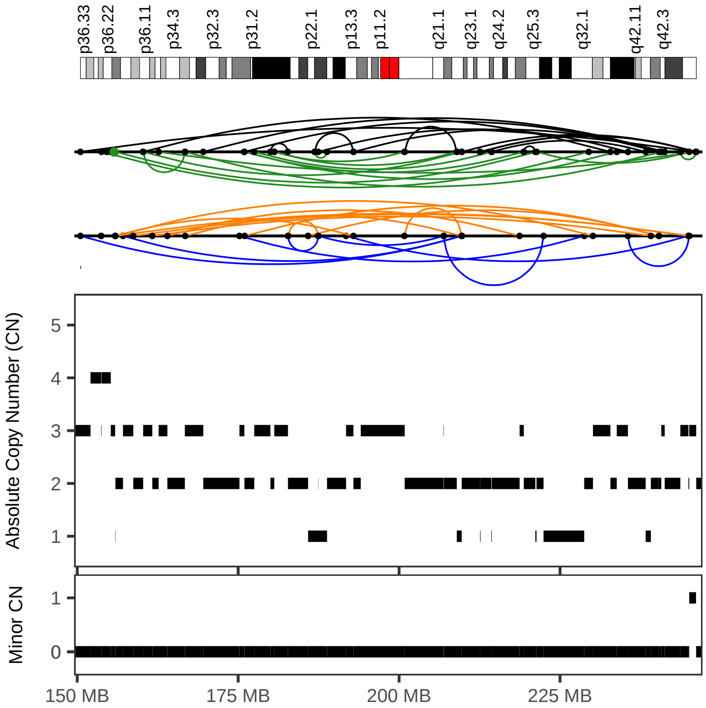

a01a350d−64be−4af3−91c3−7d681c3db11d

Cancer type	Skin−Melanoma
Position	1:150504464−246161534
Type	With other complex events
Interleaved intrachr. SVs	47
Total SVs (intrachr. + transl.)	48
SV types	DEL: 11; DUP: 8; h2hINV: 13; t2tINV: 15; TRA: 1
SVs in sample	78
Oscillating CN (2 and 3 states)	17, 60
CN segments	65
FDR fragment joints	0.64
FDR chr. breakp. enrich.	0
Linked to chrs	
Purity, ploidy	0.93, 1.81

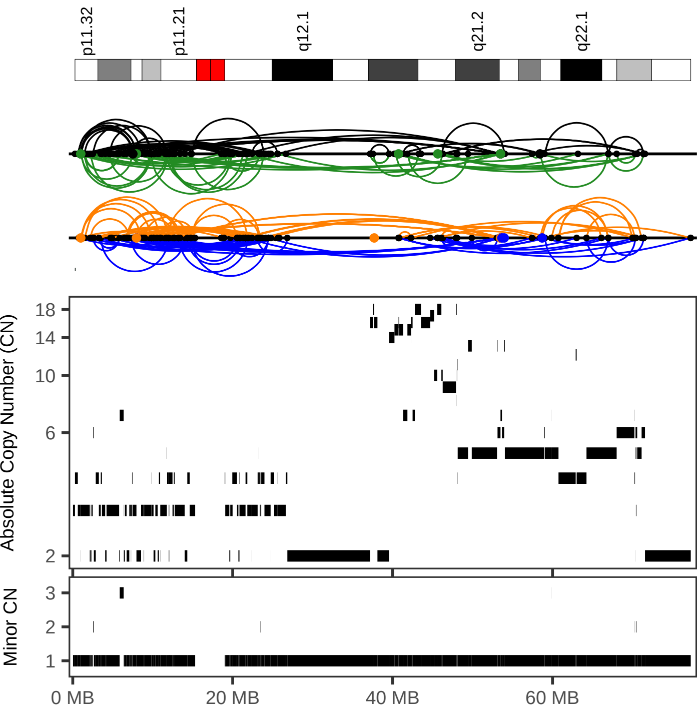

	b85b14d7−3b5a−4800−af12−622ec03b9fe5
Cancer type	Skin−Melanoma
Position	18:274892−77288441
Type	With other complex events
Interleaved intrachr. SVs	232
Total SVs (intrachr. + transl.)	254
SV types	DEL: 56; DUP: 58; h2hINV: 58; t2tINV: 60; TRA: 22
SVs in sample	633
Oscillating CN (2 and 3 states)	10, 20
CN segments	145
FDR fragment joints	1
FDR chr. breakp. enrich.	0
Linked to chrs	3:47423861−128885161;
Purity, ploidy	0.72, 3.36

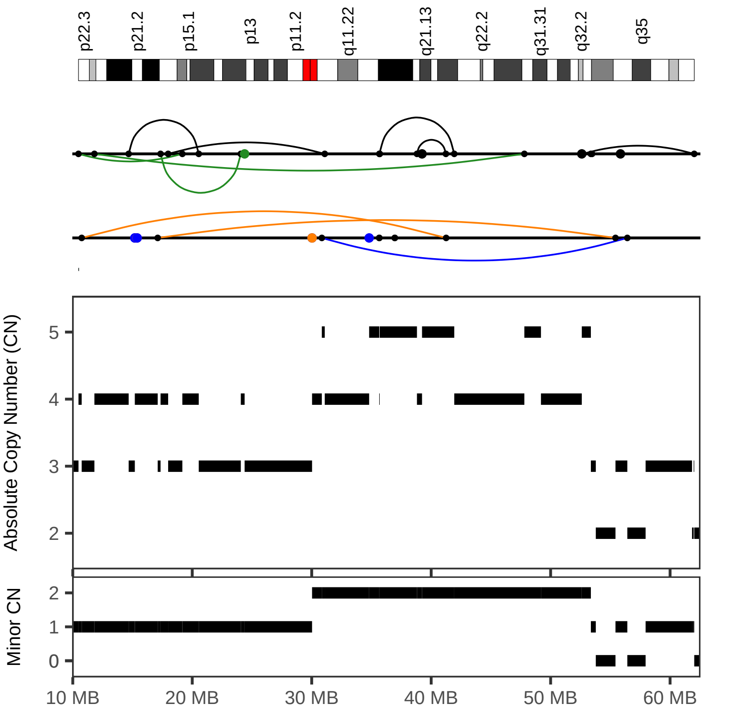

	be73fde2−d843−426b−9306−a02d3f2a754b
Cancer type	Skin−Melanoma
Position	7:10477885−62019633
Type	With other complex events
Interleaved intrachr. SVs	10
Total SVs (intrachr. + transl.)	21
SV types	DEL: 2; DUP: 1; h2hINV: 4; t2tINV: 3; TRA: 11
SVs in sample	50
Oscillating CN (2 and 3 states)	13, 13
CN segments	32
FDR fragment joints	0.64
FDR chr. breakp. enrich.	0
Linked to chrs	
Purity, ploidy	0.84, 2.36

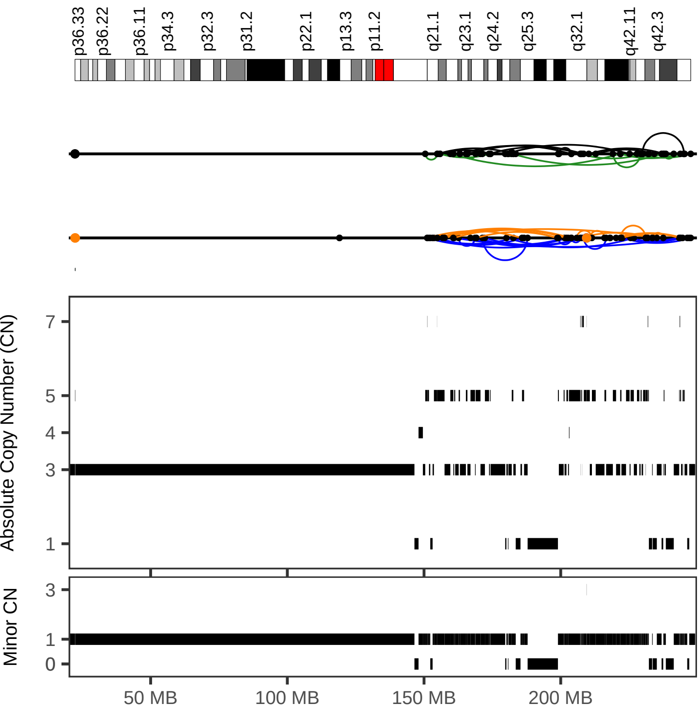

	c2948fe9−5798−43bc−a743−511e94e417f8
Cancer type	Skin−Melanoma
Position	1:150445691−247569477
Type	With other complex events
Interleaved intrachr. SVs	56
Total SVs (intrachr. + transl.)	58
SV types	DEL: 15; DUP: 16; h2hINV: 13; t2tINV: 12; TRA: 2
SVs in sample	236
Oscillating CN (2 and 3 states)	21, 37
CN segments	97
FDR fragment joints	0.91
FDR chr. breakp. enrich.	0
Linked to chrs	
Purity, ploidy	0.87, 3.29

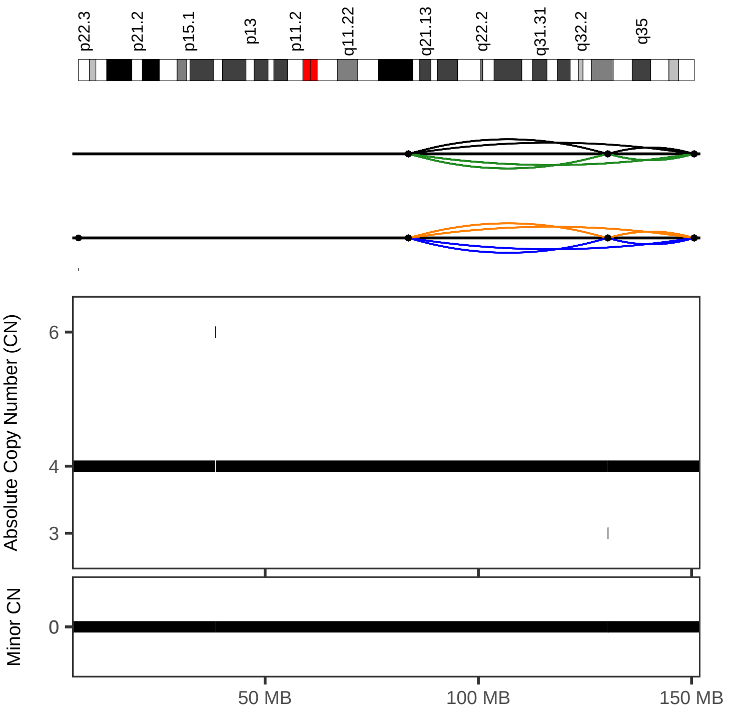

	c2948fe9−5798−43bc−a743−511e94e417f8
Cancer type	Skin−Melanoma
Position	7:83569717−150614810
Type	After polyploidization
Interleaved intrachr. SVs	57
Total SVs (intrachr. + transl.)	57
SV types	DEL: 14; DUP: 13; h2hINV: 11; t2tINV: 19; TRA: 0
SVs in sample	236
Oscillating CN (2 and 3 states)	9, 9
CN segments	9
FDR fragment joints	0.64
FDR chr. breakp. enrich.	0
Linked to chrs	
Purity, ploidy	0.87, 3.29

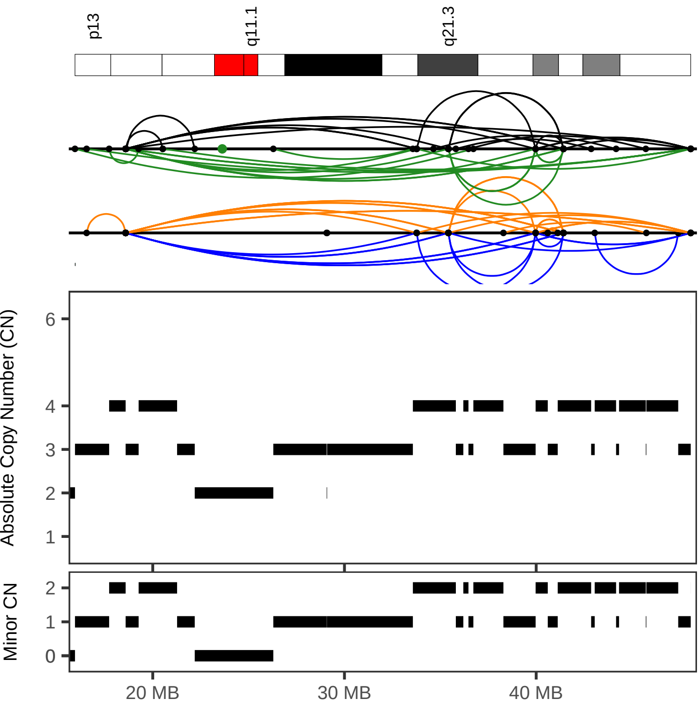

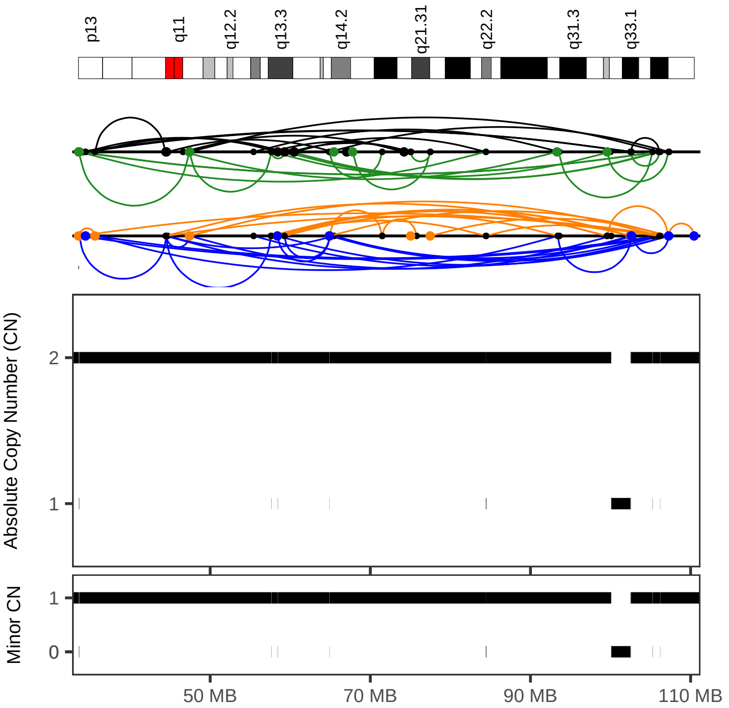

	d8eac750−9daf−4465−b672−9e31fd369057
Cancer type	Skin−Melanoma
Position	21:15944010−48061613
Type	After polyploidization
Interleaved intrachr. SVs	72
Total SVs (intrachr. + transl.)	73
SV types	DEL: 21; DUP: 15; h2hINV: 19; t2tINV: 17; TRA: 1
SVs in sample	266
Oscillating CN (2 and 3 states)	17, 26
CN segments	26
FDR fragment joints	0.84
FDR chr. breakp. enrich.	0
Linked to chrs	
Purity, ploidy	0.47, 3.89

	e36c9411−773b−4a8e−9b43−895b3cb044c7
Cancer type	Skin−Melanoma
Position	13:33553524−110451695
Type	Canonical without polyploidization
Interleaved intrachr. SVs	72
Total SVs (intrachr. + transl.)	105
SV types	DEL: 19; DUP: 19; h2hINV: 16; t2tINV: 18; TRA: 33
SVs in sample	167
Oscillating CN (2 and 3 states)	22, 22
CN segments	22
FDR fragment joints	0.97
FDR chr. breakp. enrich.	0
Linked to chrs	
Purity, ploidy	0.97, 1.8

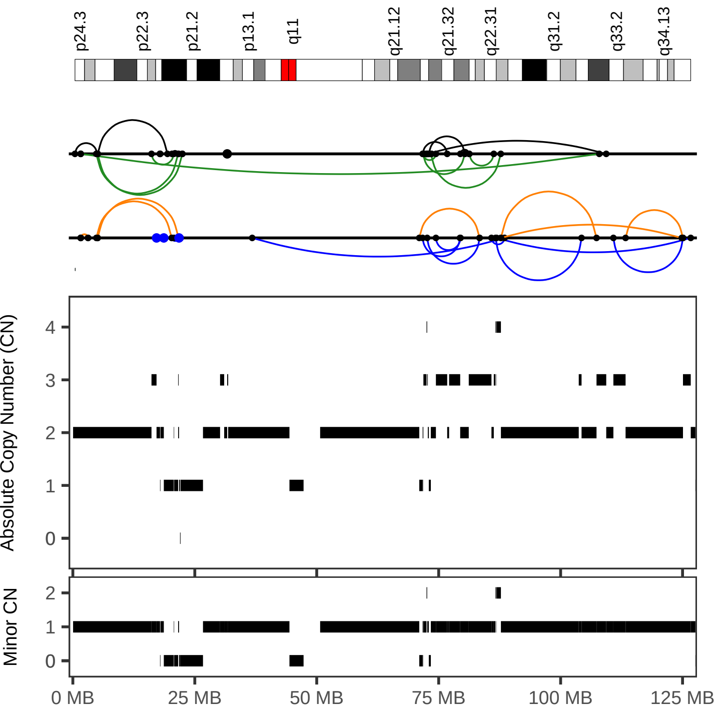

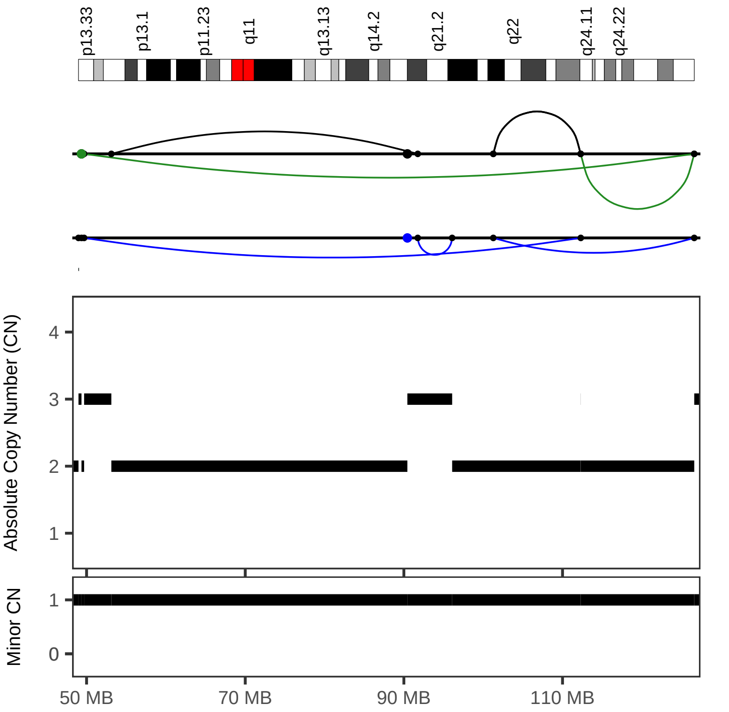

	12bfd446−2ee5−4664−a492−c023d134f60f
Cancer type	SoftTissue−Leiomyo
Position	9:434615−126676283
Type	With other complex events
Interleaved intrachr. SVs	37
Total SVs (intrachr. + transl.)	41
SV types	DEL: 8; DUP: 9; h2hINV: 10; t2tINV: 10; TRA: 4
SVs in sample	247
Oscillating CN (2 and 3 states)	9, 34
CN segments	54
FDR fragment joints	0.97
FDR chr. breakp. enrich.	0
Linked to chrs	10:11662562−50056639;
Purity, ploidy	0.88, 1.73

	12bfd446−2ee5−4664−a492−c023d134f60f
Cancer type	SoftTissue−Leiomyo
Position	12:49689929−126602595
Type	Canonical without polyploidization
Interleaved intrachr. SVs	5
Total SVs (intrachr. + transl.)	7
SV types	DEL: 0; DUP: 2; h2hINV: 1; t2tINV: 2; TRA: 2
SVs in sample	247
Oscillating CN (2 and 3 states)	7, 7
CN segments	7
FDR fragment joints	0.64
FDR chr. breakp. enrich.	0.54
Linked to chrs	17:37441745−62969497;
Purity, ploidy	0.88, 1.73

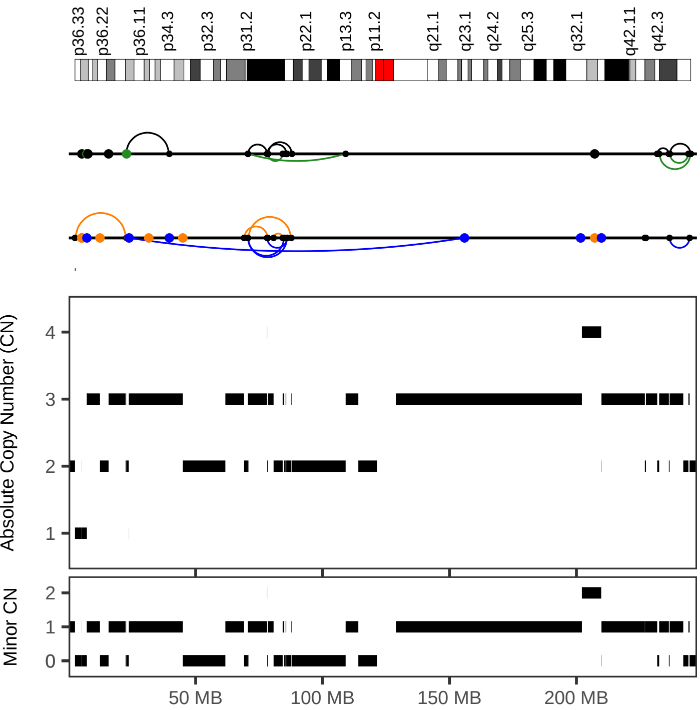

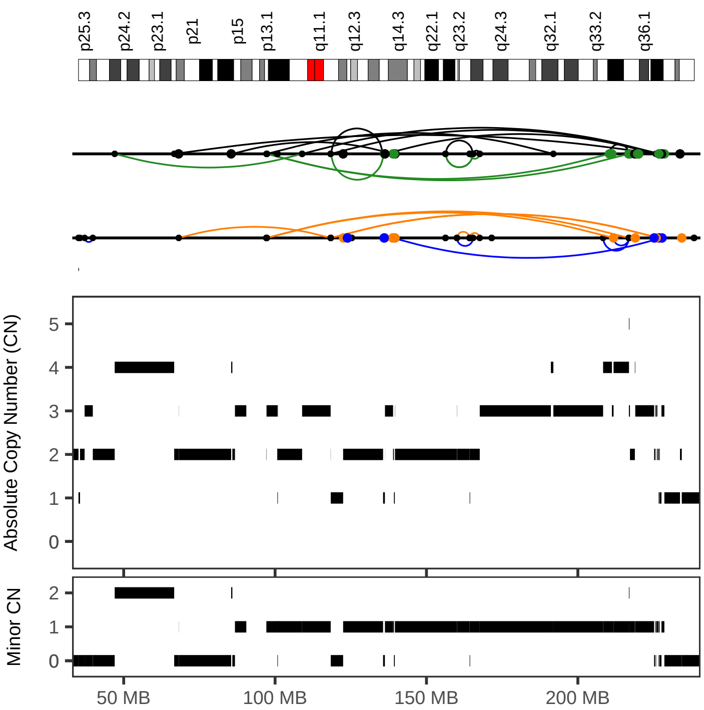

	**257dc623−41e1−4fa3−859c−c787db10bd60**
Cancer type	SoftTissue−Leiomyo
Position	1:69086265−109086561
Type	Canonical without polyploidization
Interleaved intrachr. SVs	13
Total SVs (intrachr. + transl.)	13
SV types	DEL: 2; DUP: 3; h2hINV: 4; t2tINV: 4; TRA: 0
SVs in sample	356
Oscillating CN (2 and 3 states)	16, 18
CN segments	21
FDR fragment joints	0.88
FDR chr. breakp. enrich.	0
Linked to chrs	
Purity, ploidy	0.62, 3.3

	**257dc623−41e1−4fa3−859c−c787db10bd60**
Cancer type	SoftTissue−Leiomyo
Position	2:47029935−227773557
Type	With other complex events
Interleaved intrachr. SVs	22
Total SVs (intrachr. + transl.)	63
SV types	DEL: 5; DUP: 3; h2hINV: 8; t2tINV: 6; TRA: 41
SVs in sample	356
Oscillating CN (2 and 3 states)	7, 13
CN segments	56
FDR fragment joints	0.64
FDR chr. breakp. enrich.	0
Linked to chrs	4:18377040−178289697;
Purity, ploidy	0.62, 3.3

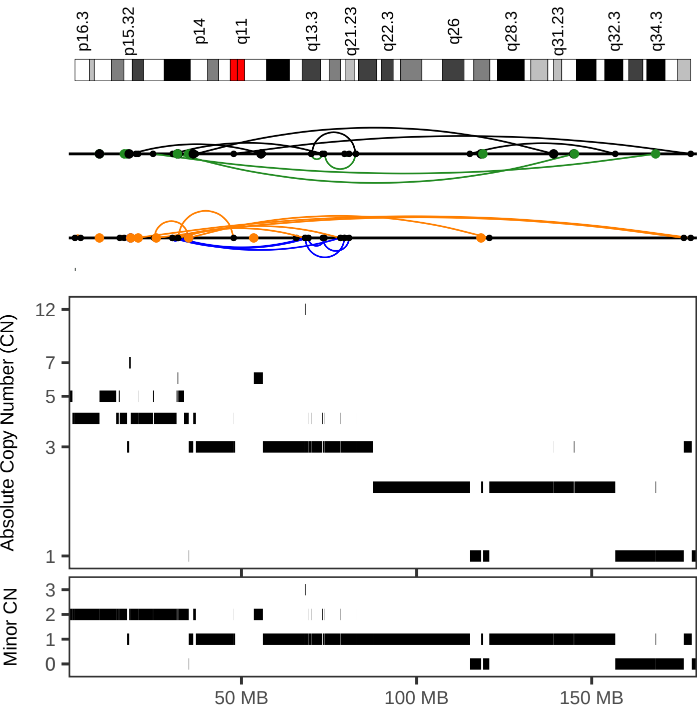

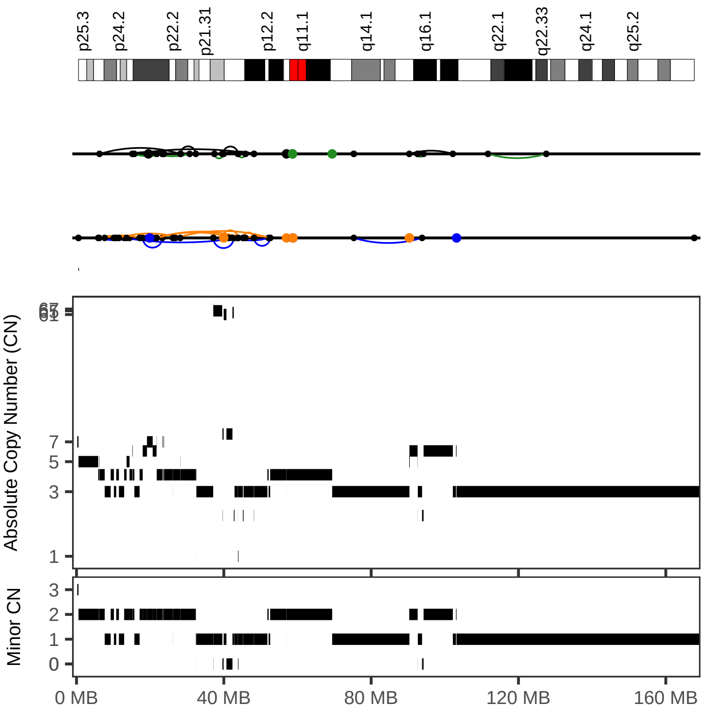

	**257dc623−41e1−4fa3−859c−c787db10bd60**
Cancer type	SoftTissue−Leiomyo
Position	4:18377040−178289698
Type	With other complex events
Interleaved intrachr. SVs	27
Total SVs (intrachr. + transl.)	45
SV types	DEL: 7; DUP: 8; h2hINV: 6; t2tINV: 6; TRA: 18
SVs in sample	356
Oscillating CN (2 and 3 states)	13, 25
CN segments	46
FDR fragment joints	0.96
FDR chr. breakp. enrich.	0
Linked to chrs	2:47029935−227773556;9:11837191−29559821
Purity, ploidy	0.62, 3.3

	**257dc623−41e1−4fa3−859c−c787db10bd60**
Cancer type	SoftTissue−Leiomyo
Position	6:5887196−52586986
Type	With other complex events
Interleaved intrachr. SVs	27
Total SVs (intrachr. + transl.)	30
SV types	DEL: 9; DUP: 6; h2hINV: 7; t2tINV: 5; TRA: 3
SVs in sample	356
Oscillating CN (2 and 3 states)	7, 11
CN segments	52
FDR fragment joints	0.81
FDR chr. breakp. enrich.	0
Linked to chrs	4:18377040−178289697;
Purity, ploidy	0.62, 3.3

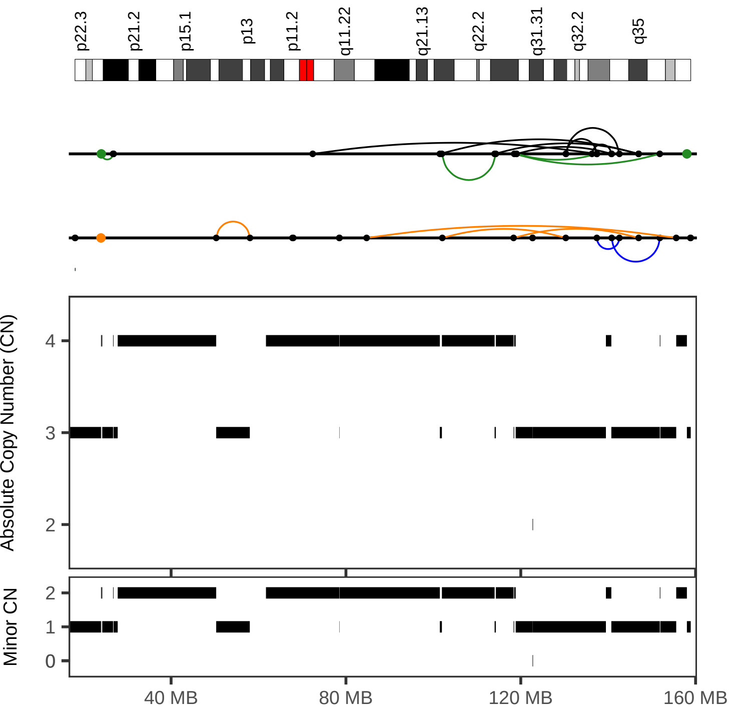

	257dc623−41e1−4fa3−859c−c787db10bd60
Cancer type	SoftTissue−Leiomyo
Position	7:72402131−155562912
Type	With other complex events
Interleaved intrachr. SVs	15
Total SVs (intrachr. + transl.)	15
SV types	DEL: 3; DUP: 2; h2hINV: 7; t2tINV: 3; TRA: 0
SVs in sample	356
Oscillating CN (2 and 3 states)	10, 17
CN segments	17
FDR fragment joints	0.59
FDR chr. breakp. enrich.	0.31
Linked to chrs	
Purity, ploidy	0.62, 3.3

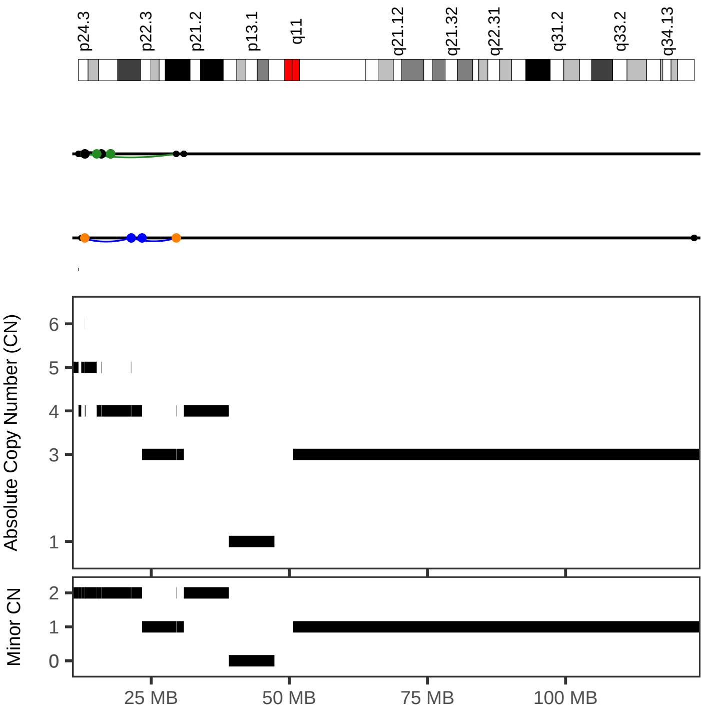

	257dc623−41e1−4fa3−859c−c787db10bd60
Cancer type	SoftTissue−Leiomyo
Position	9:11837191−29559822
Type	With other complex events
Interleaved intrachr. SVs	6
Total SVs (intrachr. + transl.)	16
SV types	DEL: 1; DUP: 2; h2hINV: 2; t2tINV: 1; TRA: 10
SVs in sample	356
Oscillating CN (2 and 3 states)	7, 9
CN segments	15
FDR fragment joints	0.91
FDR chr. breakp. enrich.	0.27
Linked to chrs	4:18377040−178289697;
Purity, ploidy	0.62, 3.3

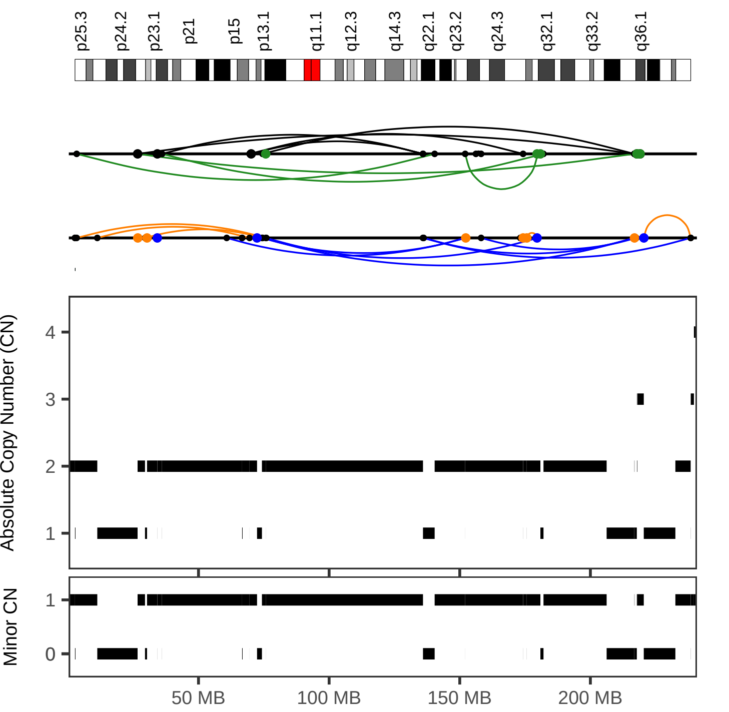

	5a64f7e9−3833−4a3c−962b−13eb3e305c69
Cancer type	SoftTissue−Leiomyo
Position	2:3363332−238409727
Type	Canonical without polyploidization
Interleaved intrachr. SVs	23
Total SVs (intrachr. + transl.)	43
SV types	DEL: 6; DUP: 7; h2hINV: 5; t2tINV: 5; TRA: 20
SVs in sample	339
Oscillating CN (2 and 3 states)	32, 39
CN segments	39
FDR fragment joints	0.94
FDR chr. breakp. enrich.	0
Linked to chrs	
Purity, ploidy	0.72, 1.65

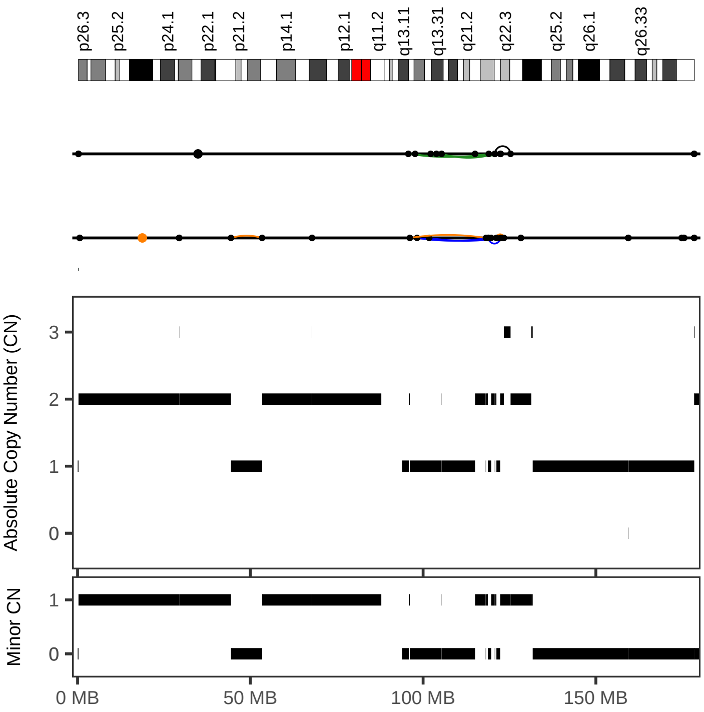

	5a64f7e9−3833−4a3c−962b−13eb3e305c69
Cancer type	SoftTissue−Leiomyo
Position	3:95733851−125309240
Type	Canonical without polyploidization
Interleaved intrachr. SVs	12
Total SVs (intrachr. + transl.)	12
SV types	DEL: 3; DUP: 3; h2hINV: 1; t2tINV: 5; TRA: 0
SVs in sample	339
Oscillating CN (2 and 3 states)	14, 16
CN segments	16
FDR fragment joints	0.64
FDR chr. breakp. enrich.	0.59
Linked to chrs	
Purity, ploidy	0.72, 1.65

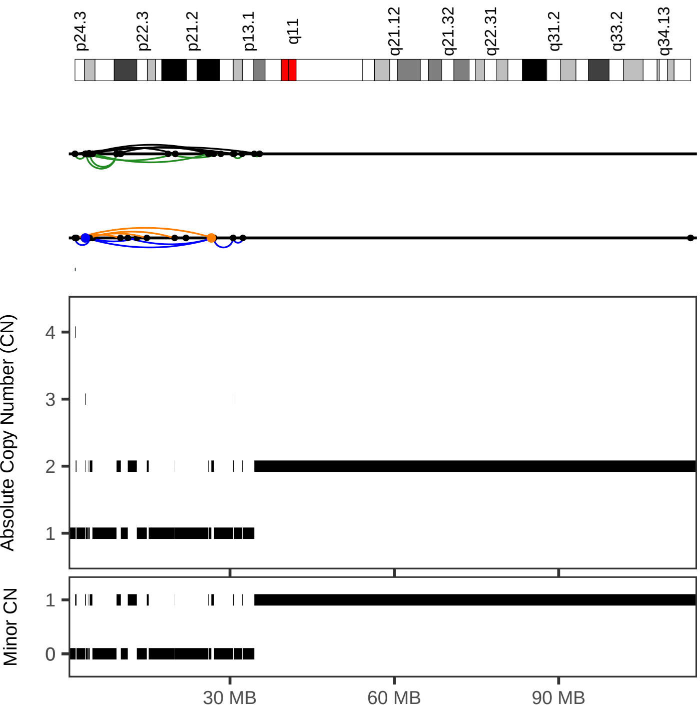

	5a64f7e9−3833−4a3c−962b−13eb3e305c69
Cancer type	SoftTissue−Leiomyo
Position	9:1710868−35411917
Type	Canonical without polyploidization
Interleaved intrachr. SVs	31
Total SVs (intrachr. + transl.)	33
SV types	DEL: 5; DUP: 8; h2hINV: 8; t2tINV: 10; TRA: 2
SVs in sample	339
Oscillating CN (2 and 3 states)	32, 34
CN segments	44
FDR fragment joints	0.73
FDR chr. breakp. enrich.	0
Linked to chrs	
Purity, ploidy	0.72, 1.65

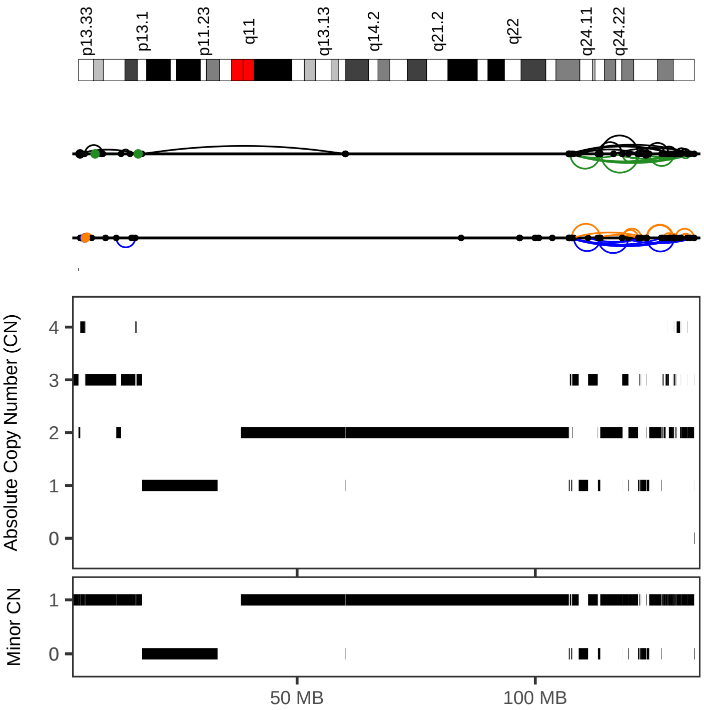

	5a64f7e9−3833−4a3c−962b−13eb3e305c69
Cancer type	SoftTissue−Leiomyo
Position	12:107038045−133376551
Type	With other complex events
Interleaved intrachr. SVs	80
Total SVs (intrachr. + transl.)	81
SV types	DEL: 22; DUP: 17; h2hINV: 21; t2tINV: 20; TRA: 1
SVs in sample	339
Oscillating CN (2 and 3 states)	10, 24
CN segments	87
FDR fragment joints	0.91
FDR chr. breakp. enrich.	0
Linked to chrs	
Purity, ploidy	0.72, 1.65

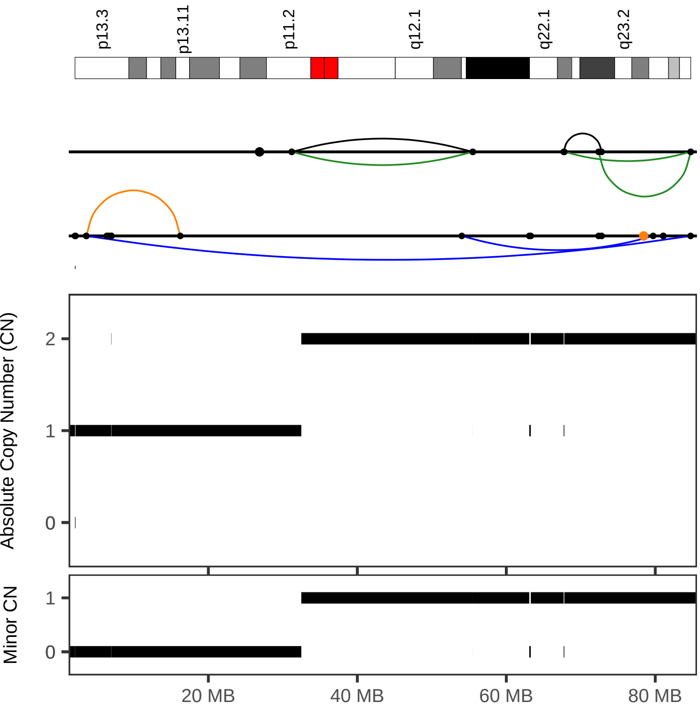

	5a64f7e9−3833−4a3c−962b−13eb3e305c69
Cancer type	SoftTissue−Leiomyo
Position	16:3616081−84764407
Type	Canonical without polyploidization
Interleaved intrachr. SVs	9
Total SVs (intrachr. + transl.)	11
SV types	DEL: 2; DUP: 2; h2hINV: 2; t2tINV: 3; TRA: 2
SVs in sample	339
Oscillating CN (2 and 3 states)	10, 10
CN segments	10
FDR fragment joints	0.97
FDR chr. breakp. enrich.	0.02
Linked to chrs	
Purity, ploidy	0.72, 1.65

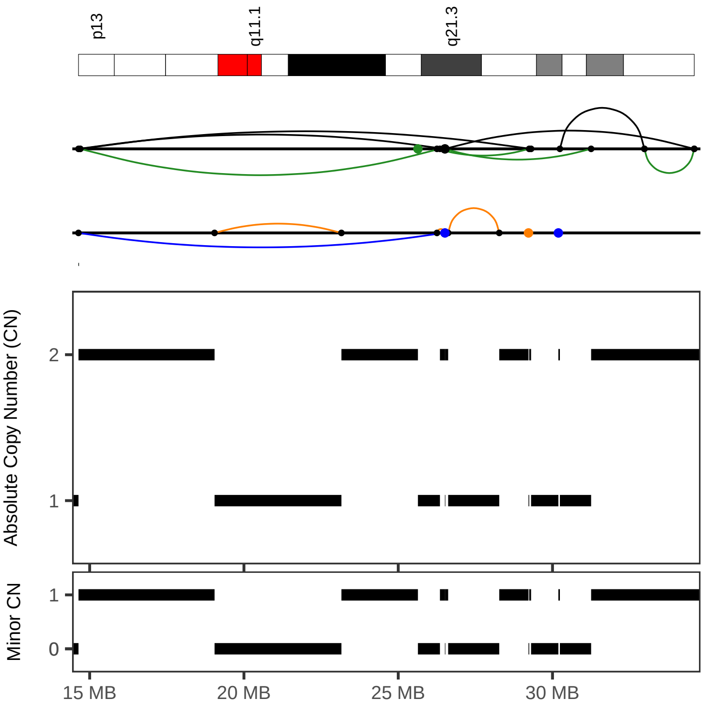

	7f9031da−124a−4a38−83e6−878a50e58c24
Cancer type	SoftTissue−Leiomyo
Position	21:14637168−34593655
Type	Canonical without polyploidization
Interleaved intrachr. SVs	9
Total SVs (intrachr. + transl.)	14
SV types	DEL: 1; DUP: 1; h2hINV: 4; t2tINV: 3; TRA: 5
SVs in sample	229
Oscillating CN (2 and 3 states)	16, 16
CN segments	16
FDR fragment joints	0.59
FDR chr. breakp. enrich.	0
Linked to chrs	17:28526654−65297775;
Purity, ploidy	0.86, 1.89

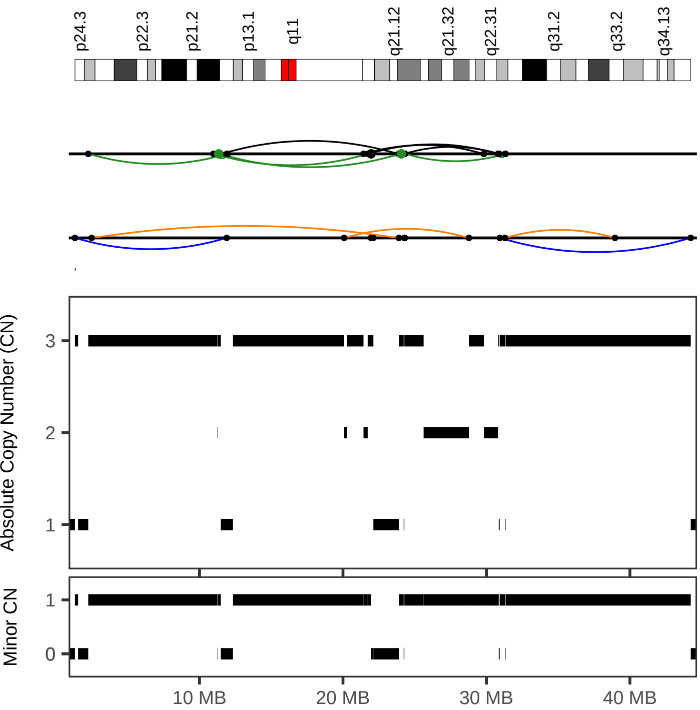

	**8654c2b5−27d7−43c5−9a39−7fdefd7a0324**
Cancer type	SoftTissue−Leiomyo
Position	9:1318657−44240245
Type	With other complex events
Interleaved intrachr. SVs	17
Total SVs (intrachr. + transl.)	20
SV types	DEL: 3; DUP: 3; h2hINV: 5;<br>t2tINV: 6; TRA: 3
SVs in sample	38
Oscillating CN (2 and 3 states)	9, 22
CN segments	29
FDR fragment joints	0.74
FDR chr. breakp. enrich.	0
Linked to chrs	3:12932880−76585300;
Purity, ploidy	0.94, 2.94

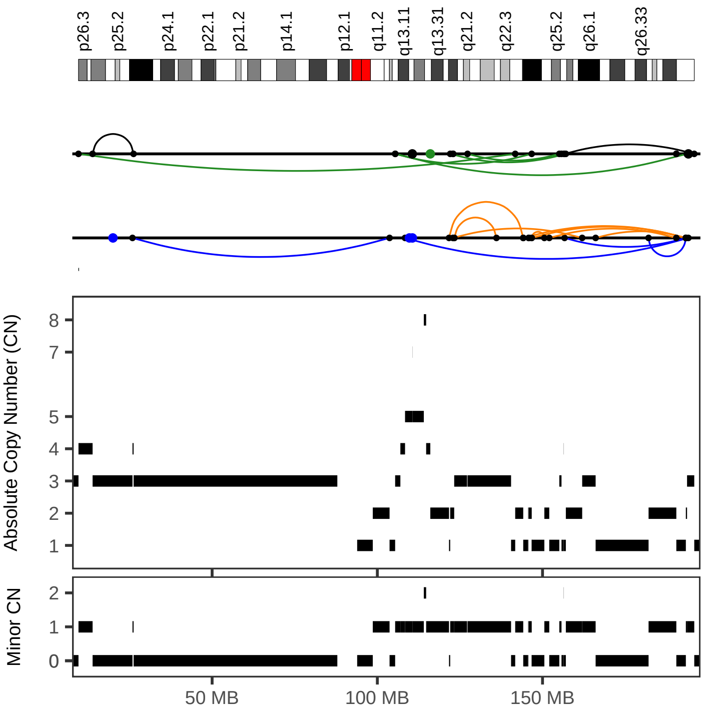

	**9c9b53e5−cdbe−4510−a839−4a3b5039681b**
Cancer type	SoftTissue−Leiomyo
Position	3:9549122−195919822
Type	With other complex events
Interleaved intrachr. SVs	18
Total SVs (intrachr. + transl.)	24
SV types	DEL: 8; DUP: 3; h2hINV: 1;<br>t2tINV: 6; TRA: 6
SVs in sample	240
Oscillating CN (2 and 3 states)	7, 15
CN segments	41
FDR fragment joints	0.48
FDR chr. breakp. enrich.	0.01
Linked to chrs	18:25133083−67867957;
Purity, ploidy	0.91, 2.73

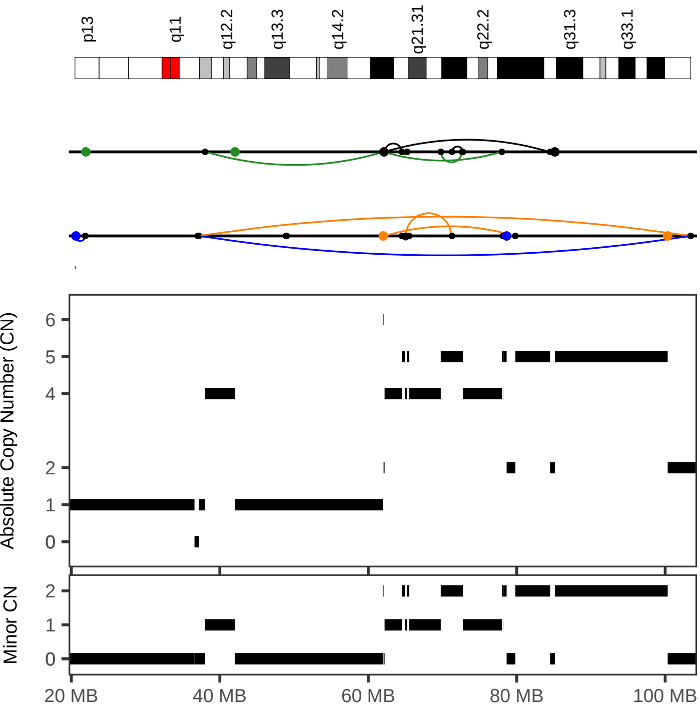

	**9c9b53e5−cdbe−4510−a839−4a3b5039681b**
Cancer type	SoftTissue−Leiomyo
Position	13:62209862−72754123
Type	After polyploidization
Interleaved intrachr. SVs	6
Total SVs (intrachr. + transl.)	6
SV types	DEL: 1; DUP: 1; h2hINV: 2;<br>t2tINV: 2; TRA: 0
SVs in sample	240
Oscillating CN (2 and 3 states)	7, 7
CN segments	7
FDR fragment joints	0.91
FDR chr. breakp. enrich.	0
Linked to chrs	
Purity, ploidy	0.91, 2.73

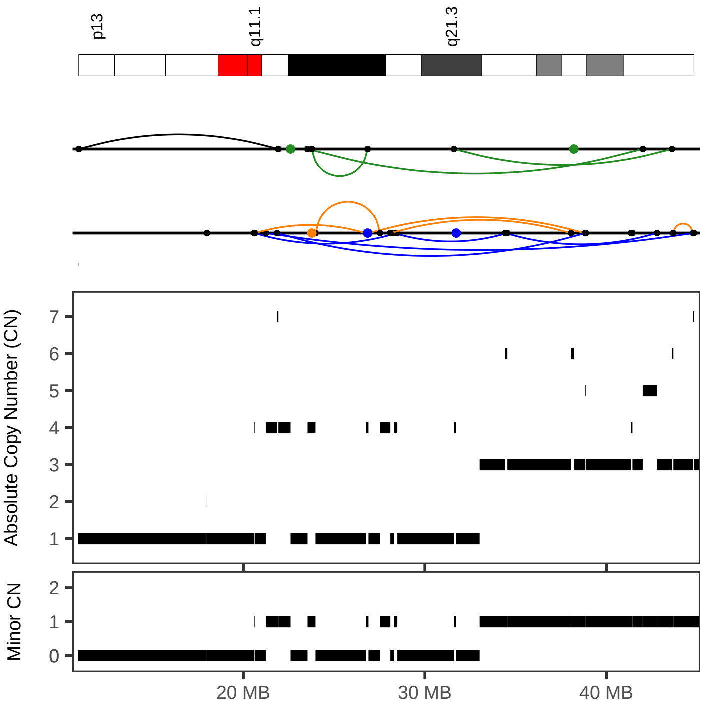

	**9c9b53e5−cdbe−4510−a839−4a3b5039681b**
Cancer type	SoftTissue−Leiomyo
Position	21:10931511−44826284
Type	With other complex events
Interleaved intrachr. SVs	13
Total SVs (intrachr. + transl.)	18
SV types	DEL: 4; DUP: 5; h2hINV: 1;<br>t2tINV: 3; TRA: 5
SVs in sample	240
Oscillating CN (2 and 3 states)	12, 15
CN segments	34
FDR fragment joints	0.64
FDR chr. breakp. enrich.	0
Linked to chrs	7:78585846−144766063;
Purity, ploidy	0.91, 2.73

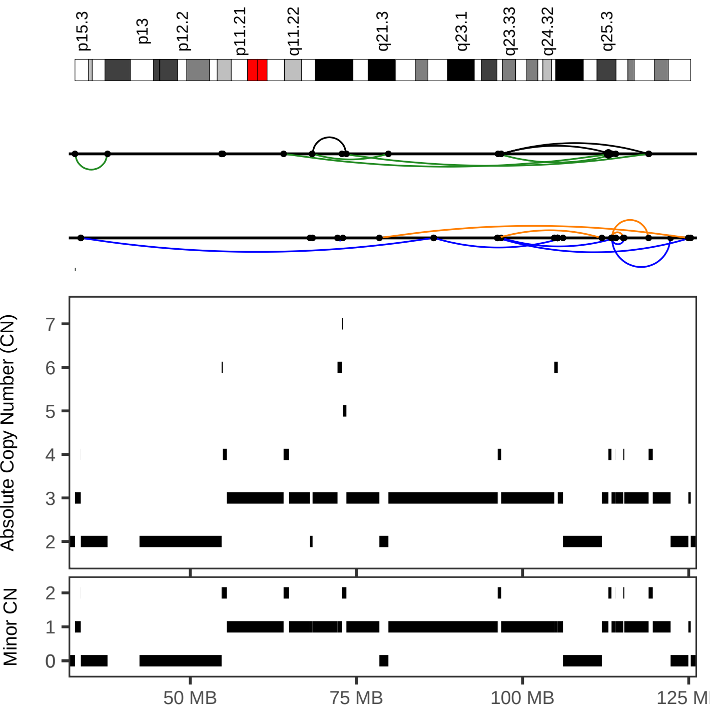

	c20682a1−f340−430d−99f9−077fe093ed19
Cancer type	SoftTissue−Leiomyo
Position	10:32651421−125302235
Type	With other complex events
Interleaved intrachr. SVs	21
Total SVs (intrachr. + transl.)	22
SV types	DEL: 6; DUP: 7; h2hINV: 3; t2tINV: 5; TRA: 1
SVs in sample	215
Oscillating CN (2 and 3 states)	11, 16
CN segments	44
FDR fragment joints	0.72
FDR chr. breakp. enrich.	0
Linked to chrs	
Purity, ploidy	0.82, 3.11

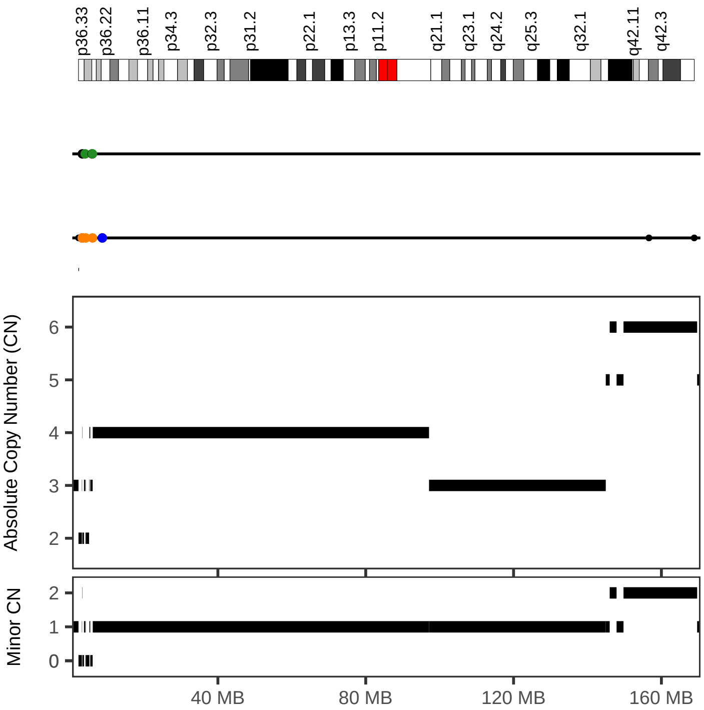

	ea3a524b−6ee3−4904−8924−29fcaa20985c
Cancer type	SoftTissue−Leiomyo
Position	1:2466876−6163124
Type	With other complex events
Interleaved intrachr. SVs	7
Total SVs (intrachr. + transl.)	16
SV types	DEL: 3; DUP: 2; h2hINV: 0; t2tINV: 2; TRA: 9
SVs in sample	318
Oscillating CN (2 and 3 states)	7, 14
CN segments	14
FDR fragment joints	0.64
FDR chr. breakp. enrich.	1
Linked to chrs	2:9263222−242771261;
Purity, ploidy	0.87, 3.16

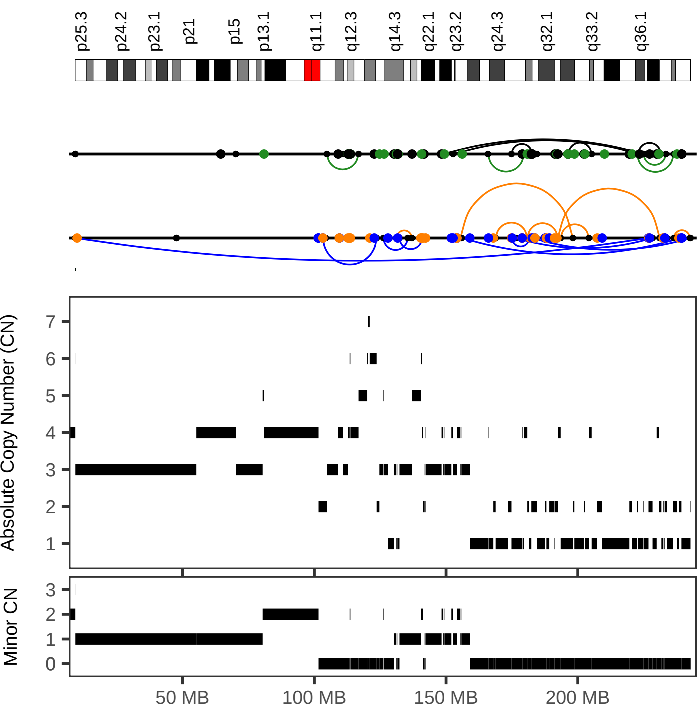

	ea3a524b−6ee3−4904−8924−29fcaa20985c
Cancer type	SoftTissue−Leiomyo
Position	2:9263222−242771262
Type	With other complex events
Interleaved intrachr. SVs	22
Total SVs (intrachr. + transl.)	126
SV types	DEL: 7; DUP: 6; h2hINV: 6; t2tINV: 3; TRA: 104
SVs in sample	318
Oscillating CN (2 and 3 states)	12, 19
CN segments	103
FDR fragment joints	0.73
FDR chr. breakp. enrich.	0
Linked to chrs	1:2466876−6163123;18:275901−68359362 21:14714130−47619465;
Purity, ploidy	0.87, 3.16

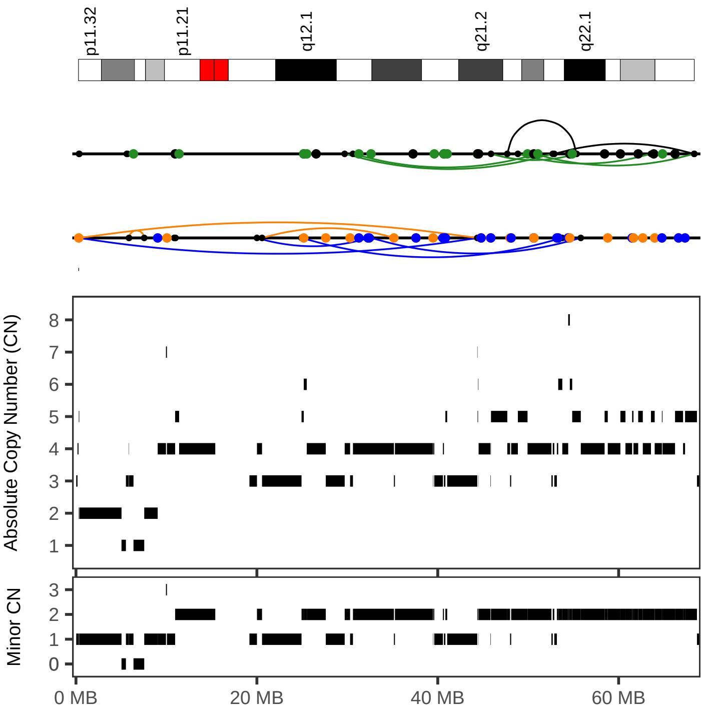

	ea3a524b−6ee3−4904−8924−29fcaa20985c
Cancer type	SoftTissue−Leiomyo
Position	18:275901−68359363
Type	With other complex events
Interleaved intrachr. SVs	13
Total SVs (intrachr. + transl.)	78
SV types	DEL: 2; DUP: 4; h2hINV: 2; t2tINV: 5; TRA: 65
SVs in sample	318
Oscillating CN (2 and 3 states)	17, 18
CN segments	72
FDR fragment joints	0.64
FDR chr. breakp. enrich.	0
Linked to chrs	2:9263222−242771261;21:14714130−47619465
Purity, ploidy	0.87, 3.16

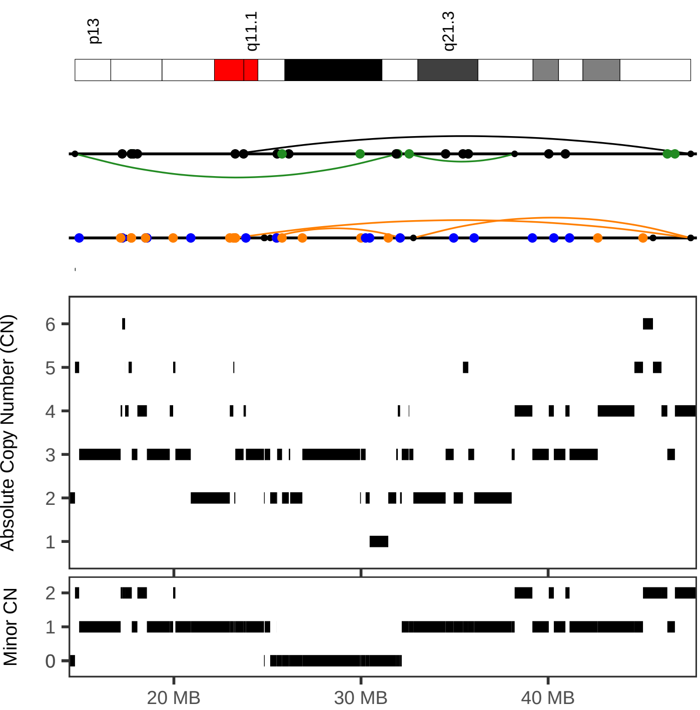

ea3a524b−6ee3−4904−8924−29fcaa20985c	
Cancer type	SoftTissue−Leiomyo
Position	21:14714130−47619466
Type	With other complex events
Interleaved intrachr. SVs	6
Total SVs (intrachr. + transl.)	61
SV types	DEL: 3; DUP: 0; h2hINV: 1; t2tINV: 2; TRA: 55
SVs in sample	318
Oscillating CN (2 and 3 states)	14, 23
CN segments	61
FDR fragment joints	0.59
FDR chr. breakp. enrich.	0
Linked to chrs	2:9263222−242771261;18:275901−68359362
Purity, ploidy	0.87, 3.16

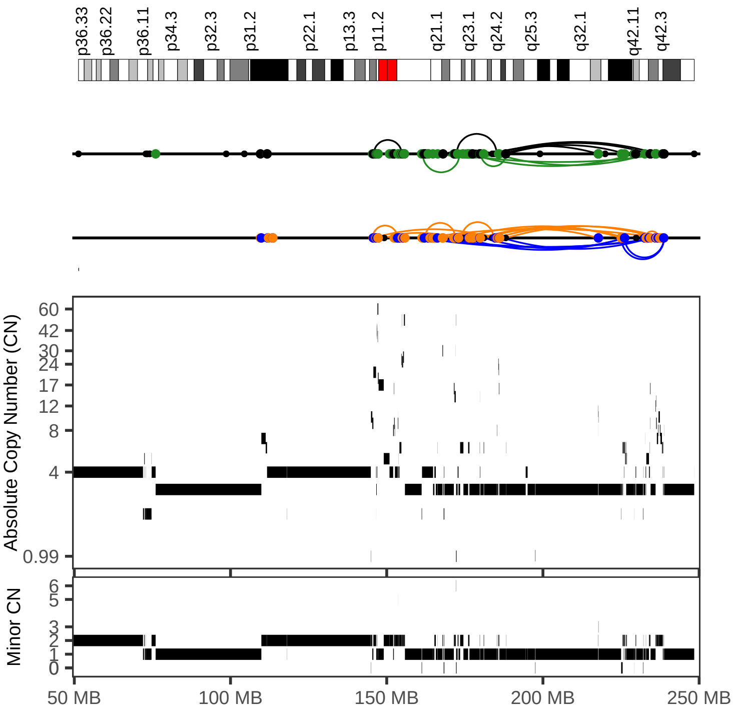

2905cbd1−719b−46d9−b8af−8fe4927bc473	
Cancer type	SoftTissue−Liposarc
Position	1:145389851−238867163
Type	With other complex events
Interleaved intrachr. SVs	45
Total SVs (intrachr. + transl.)	207
SV types	DEL: 18; DUP: 9; h2hINV: 10; t2tINV: 8; TRA: 162
SVs in sample	1958
Oscillating CN (2 and 3 states)	7, 7
CN segments	138
FDR fragment joints	0.57
FDR chr. breakp. enrich.	0
Linked to chrs	11:12674170−130091311;12:8404400−101121300 18:19701585−65299989;19:10607945−56113175 2:103504725−236615449;5:893523−179304806 7:159034−86160840;X:1766391−152187953
Purity, ploidy	0.92, 3.54

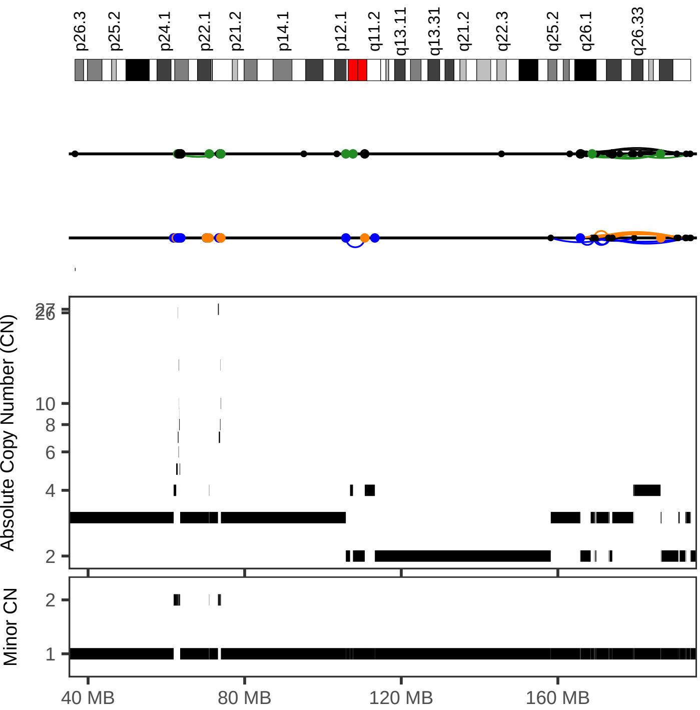

2905cbd1−719b−46d9−b8af−8fe4927bc473	
Cancer type	SoftTissue−Liposarc
Position	3:158182221−193942170
Type	With other complex events
Interleaved intrachr. SVs	25
Total SVs (intrachr. + transl.)	32
SV types	DEL: 7; DUP: 8; h2hINV: 4; t2tINV: 6; TRA: 7
SVs in sample	1958
Oscillating CN (2 and 3 states)	17, 17
CN segments	33
FDR fragment joints	0.78
FDR chr. breakp. enrich.	0
Linked to chrs	10:9380961−36837630;2:103504725−236615449
Purity, ploidy	0.92, 3.54

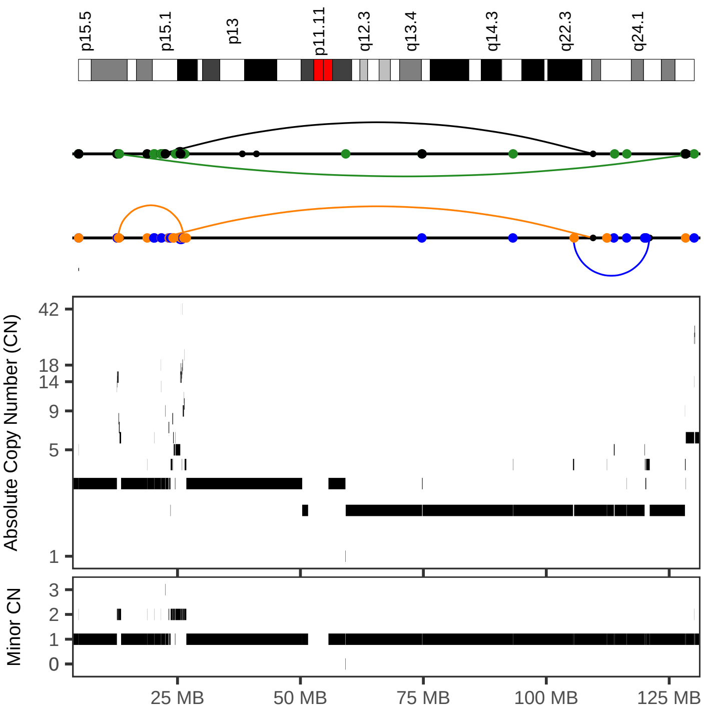

2905cbd1−719b−46d9−b8af−8fe4927bc473	
Cancer type	SoftTissue−Liposarc
Position	11:12674170−130091312
Type	With other complex events
Interleaved intrachr. SVs	8
Total SVs (intrachr. + transl.)	74
SV types	DEL: 2; DUP: 3; h2hINV: 2; t2tINV: 1; TRA: 66
SVs in sample	1958
Oscillating CN (2 and 3 states)	7, 9
CN segments	72
FDR fragment joints	0.84
FDR chr. breakp. enrich.	0.64
Linked to chrs	1:145389851−238867162;12:8404400−101121300 17:50057932−77131856;19:10607945−56113175 2:103504725−236615449;5:893523−179304806 7:159034−86160840;X:1766391−152187953
Purity, ploidy	0.92, 3.54

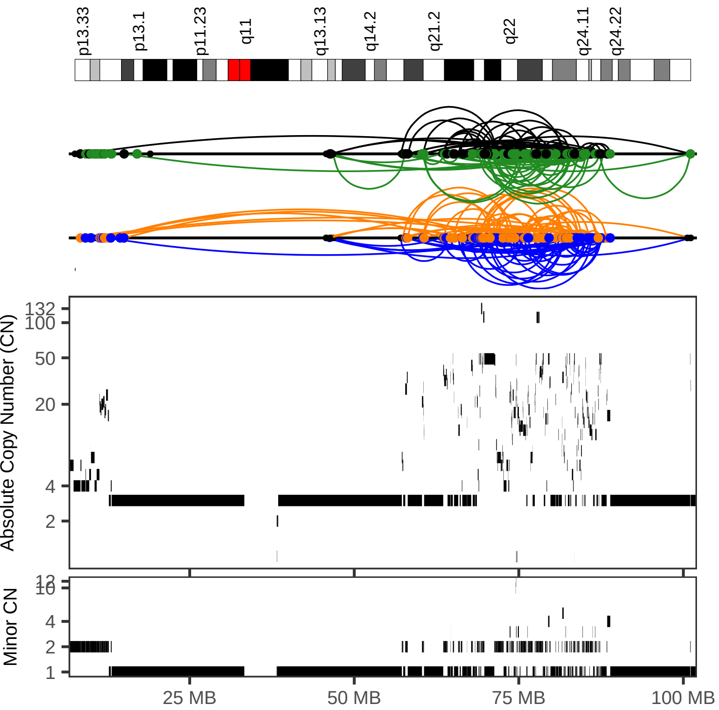

2905cbd1−719b−46d9−b8af−8fe4927bc473

Cancer type	SoftTissue−Liposarc
Position	12:8404400−101121301
Type	With other complex events
Interleaved intrachr. SVs	243
Total SVs (intrachr. + transl.)	725
SV types	DEL: 63; DUP: 64; h2hINV: 56;<br>t2tINV: 60; TRA: 482
SVs in sample	1958
Oscillating CN (2 and 3 states)	7, 9
CN segments	292
FDR fragment joints	0.92
FDR chr. breakp. enrich.	0
Linked to chrs	1:145389851−238867162;11:12674170−130091311<br>16:83655547−88637357;2:103504725−236615449<br>20:48743238−54339929;3:158182221−193942169<br>7:159034−86160840;9:7234461−14473861<br>X:1766391−152187953;
Purity, ploidy	0.92, 3.54

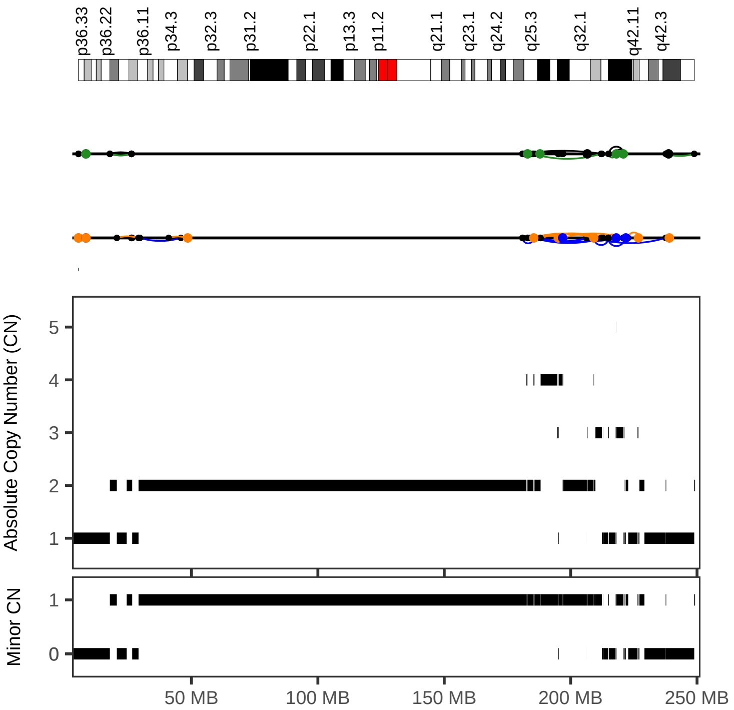

2cacd9d0−8414−4f17−8259−c9d838597f39

Cancer type	SoftTissue−Liposarc
Position	1:180979548−248890604
Type	With other complex events
Interleaved intrachr. SVs	32
Total SVs (intrachr. + transl.)	46
SV types	DEL: 9; DUP: 10; h2hINV: 6;<br>t2tINV: 7; TRA: 14
SVs in sample	531
Oscillating CN (2 and 3 states)	16, 22
CN segments	68
FDR fragment joints	0.82
FDR chr. breakp. enrich.	0
Linked to chrs	12:17335568−78414574;4:7659968−47714737
Purity, ploidy	0.88, 1.92

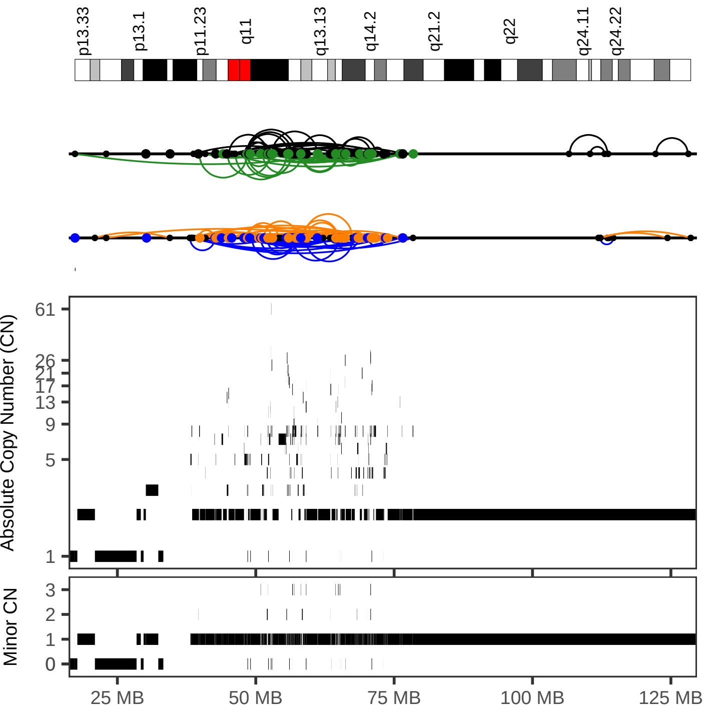

2cacd9d0−8414−4f17−8259−c9d838597f39

Cancer type	SoftTissue−Liposarc
Position	12:17335568−78414575
Type	With other complex events
Interleaved intrachr. SVs	119
Total SVs (intrachr. + transl.)	288
SV types	DEL: 31; DUP: 29; h2hINV: 28;<br>t2tINV: 31; TRA: 169
SVs in sample	531
Oscillating CN (2 and 3 states)	7, 7
CN segments	266
FDR fragment joints	0.98
FDR chr. breakp. enrich.	0
Linked to chrs	1:180979548−248890603;4:7659968−47714737
Purity, ploidy	0.88, 1.92

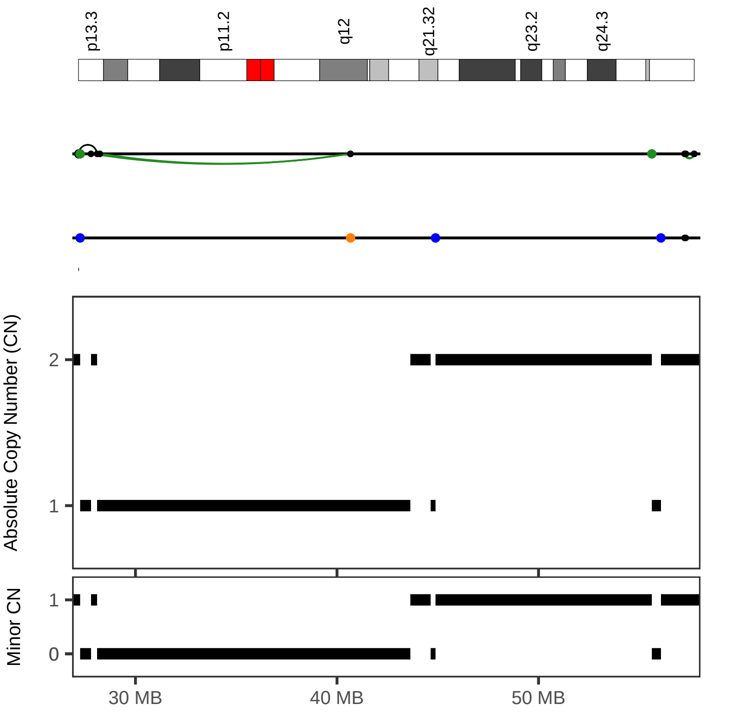

2cacd9d0−8414−4f17−8259−c9d838597f39

Cancer type	SoftTissue−Liposarc
Position	17:27172002−57726288
Type	Canonical without polyploidization
Interleaved intrachr. SVs	3
Total SVs (intrachr. + transl.)	13
SV types	DEL: 0; DUP: 1; h2hINV: 2;<br>t2tINV: 3; TRA: 7
SVs in sample	531
Oscillating CN (2 and 3 states)	9, 9
CN segments	9
FDR fragment joints	0.59
FDR chr. breakp. enrich.	1
Linked to chrs	4:7659968−47714737;
Purity, ploidy	0.88, 1.92

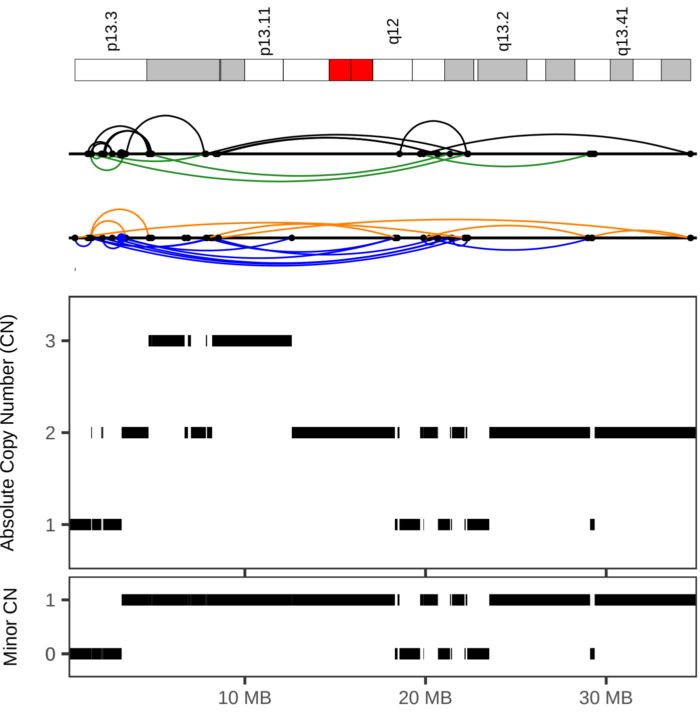

2cacd9d0−8414−4f17−8259−c9d838597f39	
Cancer type	SoftTissue−Liposarc
Position	19:596348−34678229
Type	With other complex events
Interleaved intrachr. SVs	43
Total SVs (intrachr. + transl.)	45
SV types	DEL: 8; DUP: 16; h2hINV: 11; t2tINV: 8; TRA: 2
SVs in sample	531
Oscillating CN (2 and 3 states)	23, 40
CN segments	40
FDR fragment joints	0.59
FDR chr. breakp. enrich.	0
Linked to chrs	
Purity, ploidy	0.88, 1.92

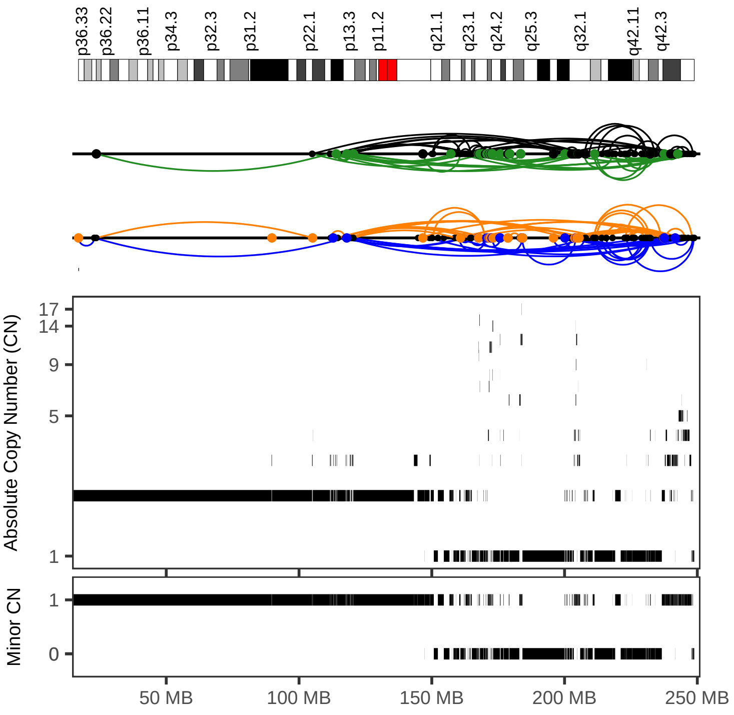

2ff6aede−7bcd−440b−99ab−7a023faf658e	
Cancer type	SoftTissue−Liposarc
Position	1:16936361−248844598
Type	With other complex events
Interleaved intrachr. SVs	163
Total SVs (intrachr. + transl.)	259
SV types	DEL: 38; DUP: 40; h2hINV: 44; t2tINV: 41; TRA: 96
SVs in sample	1218
Oscillating CN (2 and 3 states)	39, 74
CN segments	251
FDR fragment joints	0.95
FDR chr. breakp. enrich.	0
Linked to chrs	5:86635−168032759;
Purity, ploidy	0.89, 1.88

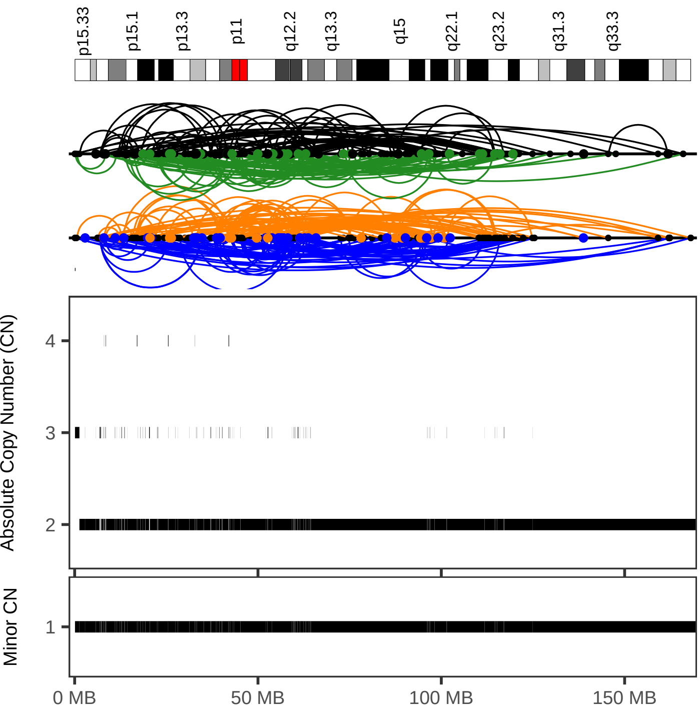

2ff6aede−7bcd−440b−99ab−7a023faf658e	
Cancer type	SoftTissue−Liposarc
Position	5:86635−168032760
Type	With other complex events
Interleaved intrachr. SVs	417
Total SVs (intrachr. + transl.)	528
SV types	DEL: 110; DUP: 102; h2hINV: 98; t2tINV: 107; TRA: 111
SVs in sample	1218
Oscillating CN (2 and 3 states)	88, 88
CN segments	181
FDR fragment joints	0.89
FDR chr. breakp. enrich.	0
Linked to chrs	
Purity, ploidy	0.89, 1.88

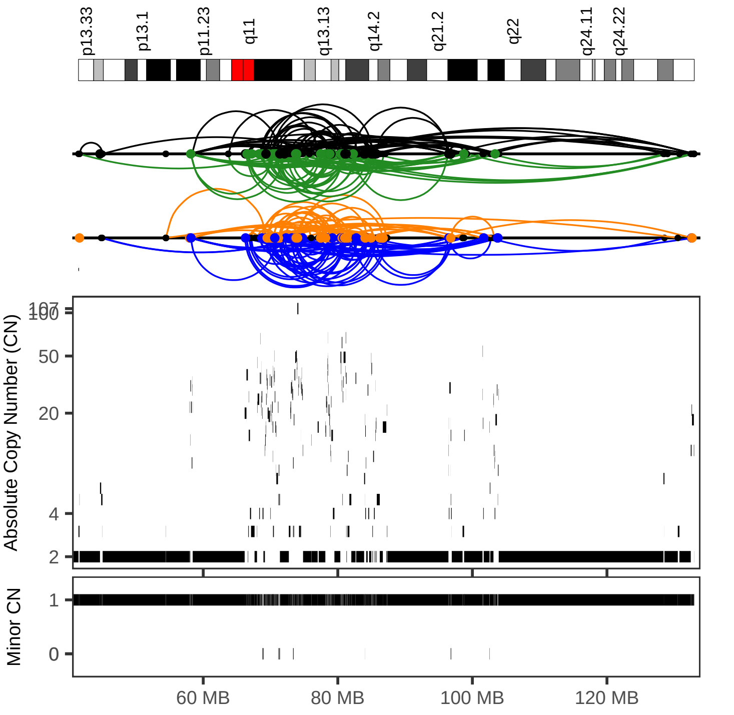

2ff6aede−7bcd−440b−99ab−7a023faf658e	
Cancer type	SoftTissue−Liposarc
Position	12:41458405−132968720
Type	With other complex events
Interleaved intrachr. SVs	294
Total SVs (intrachr. + transl.)	430
SV types	DEL: 62; DUP: 85; h2hINV: 76; t2tINV: 71; TRA: 136
SVs in sample	1218
Oscillating CN (2 and 3 states)	5, 7
CN segments	261
FDR fragment joints	0.59
FDR chr. breakp. enrich.	0
Linked to chrs	
Purity, ploidy	0.89, 1.88

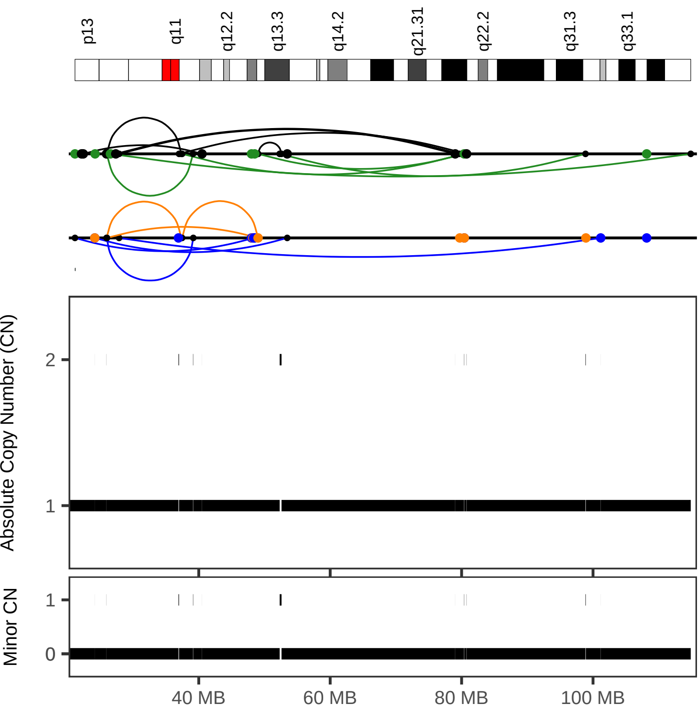

	2ff6aede−7bcd−440b−99ab−7a023faf658e
Cancer type	SoftTissue−Liposarc
Position	13:21166372−114867952
Type	Canonical without polyploidization
Interleaved intrachr. SVs	19
Total SVs (intrachr. + transl.)	49
SV types	DEL: 3; DUP: 5; h2hINV: 6; t2tINV: 5; TRA: 30
SVs in sample	1218
Oscillating CN (2 and 3 states)	25, 25
CN segments	25
FDR fragment joints	0.84
FDR chr. breakp. enrich.	0.51
Linked to chrs	5:86635−168032759;
Purity, ploidy	0.89, 1.88

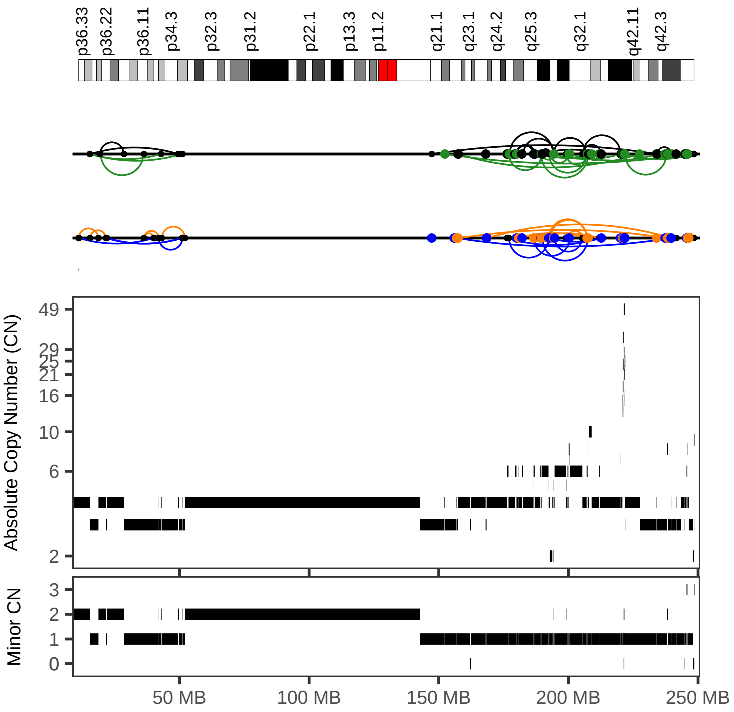

	3505f91d−bc66−4732−84b6−18c5b32ca6b8
Cancer type	SoftTissue−Liposarc
Position	1:147269177−248454297
Type	With other complex events
Interleaved intrachr. SVs	59
Total SVs (intrachr. + transl.)	204
SV types	DEL: 13; DUP: 13; h2hINV: 15; t2tINV: 18; TRA: 145
SVs in sample	1246
Oscillating CN (2 and 3 states)	17, 25
CN segments	175
FDR fragment joints	0.84
FDR chr. breakp. enrich.	0
Linked to chrs	12:4488859−131646236;2:2725830−47762583 4:64107549−135664103;5:111534825−180595775 9:107267770−135095318;
Purity, ploidy	0.61, 3.84

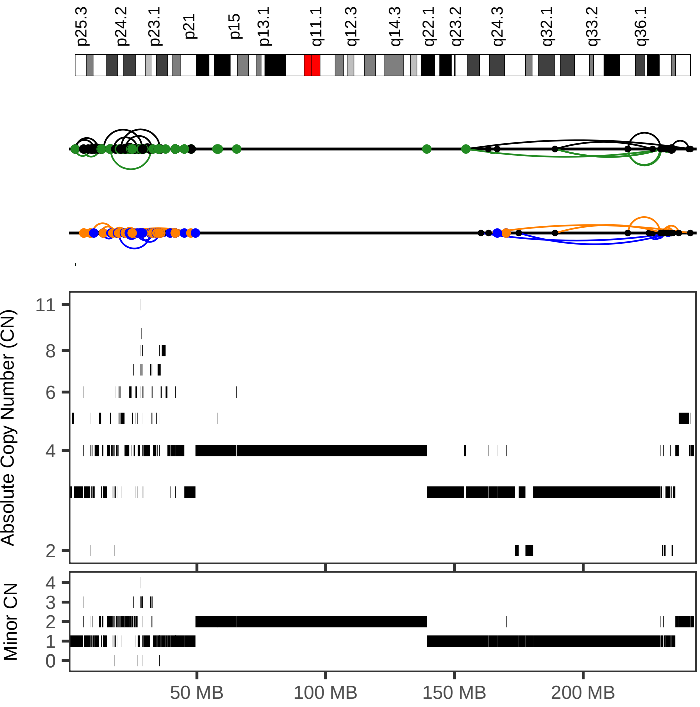

	3505f91d−bc66−4732−84b6−18c5b32ca6b8
Cancer type	SoftTissue−Liposarc
Position	2:2725830−47762584
Type	With other complex events
Interleaved intrachr. SVs	36
Total SVs (intrachr. + transl.)	155
SV types	DEL: 10; DUP: 9; h2hINV: 11; t2tINV: 6; TRA: 119
SVs in sample	1246
Oscillating CN (2 and 3 states)	8, 12
CN segments	122
FDR fragment joints	0.74
FDR chr. breakp. enrich.	0
Linked to chrs	1:147269177−248454296;12:4488859−131646236 5:111534825−180595775;X:77058127−154924952
Purity, ploidy	0.61, 3.84

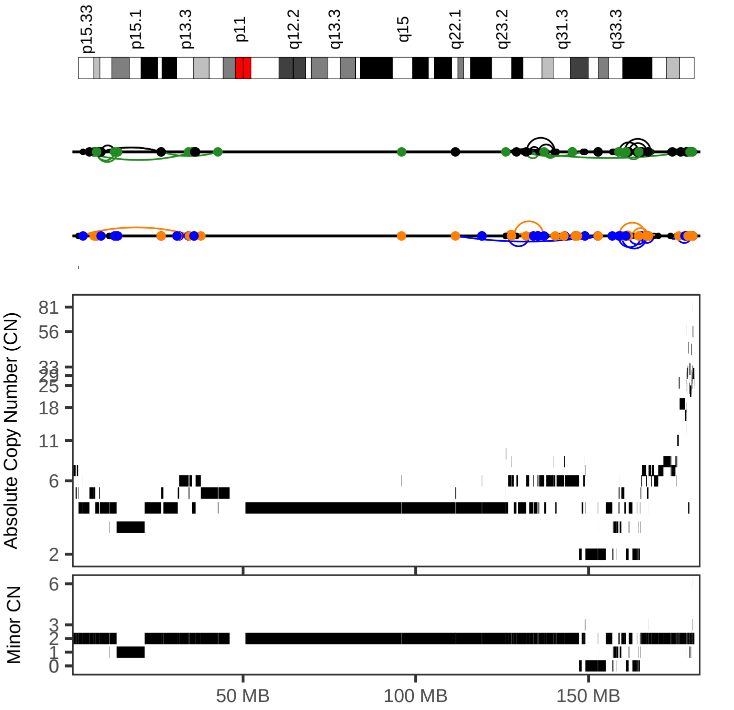

	3505f91d−bc66−4732−84b6−18c5b32ca6b8
Cancer type	SoftTissue−Liposarc
Position	5:111534825−180595776
Type	With other complex events
Interleaved intrachr. SVs	22
Total SVs (intrachr. + transl.)	127
SV types	DEL: 5; DUP: 4; h2hINV: 6; t2tINV: 7; TRA: 105
SVs in sample	1246
Oscillating CN (2 and 3 states)	15, 15
CN segments	135
FDR fragment joints	0.86
FDR chr. breakp. enrich.	0
Linked to chrs	1:147269177−248454296;12:4488859−131646236 13:20111633−31209009;X:77058127−154924952
Purity, ploidy	0.61, 3.84

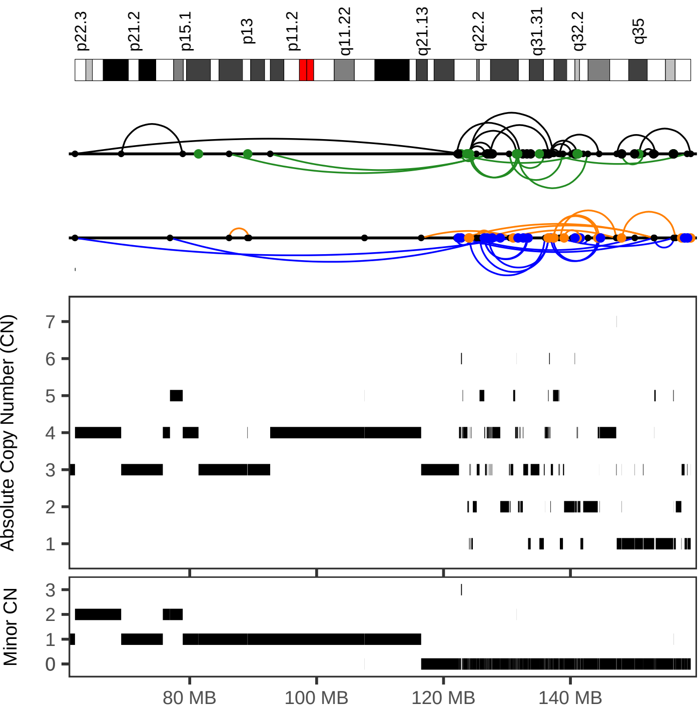

3505f91d−bc66−4732−84b6−18c5b32ca6b8	
Cancer type	SoftTissue−Liposarc
Position	7:61912885−158960074
Type	With other complex events
Interleaved intrachr. SVs	57
Total SVs (intrachr. + transl.)	126
SV types	DEL: 10; DUP: 19; h2hINV: 15; t2tINV: 13; TRA: 69
SVs in sample	1246
Oscillating CN (2 and 3 states)	15, 19
CN segments	126
FDR fragment joints	0.59
FDR chr. breakp. enrich.	0
Linked to chrs	
Purity, ploidy	0.61, 3.84

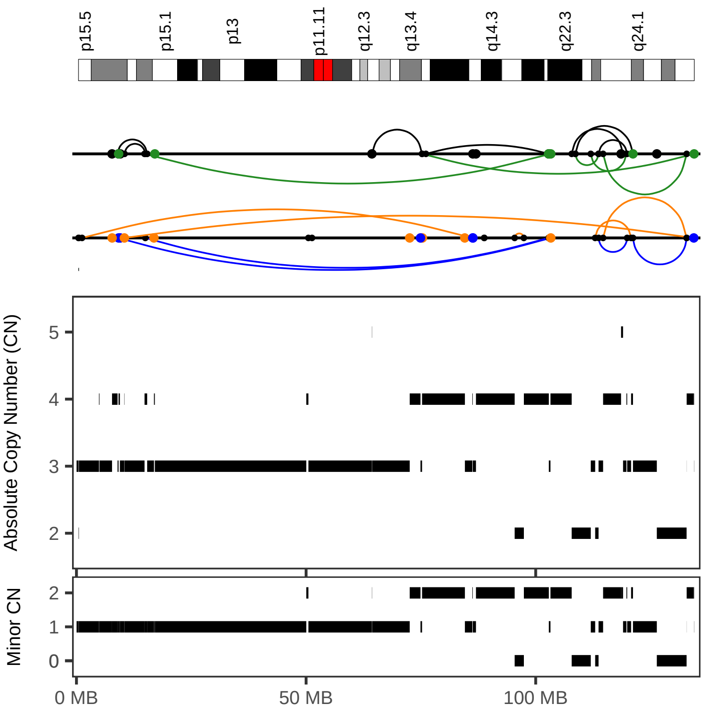

3505f91d−bc66−4732−84b6−18c5b32ca6b8	
Cancer type	SoftTissue−Liposarc
Position	11:1215903−134523511
Type	With other complex events
Interleaved intrachr. SVs	10
Total SVs (intrachr. + transl.)	49
SV types	DEL: 4; DUP: 5; h2hINV: 6; t2tINV: 5; TRA: 29
SVs in sample	1246
Oscillating CN (2 and 3 states)	15, 32
CN segments	45
FDR fragment joints	0.96
FDR chr. breakp. enrich.	0.91
Linked to chrs	1:147269177−248454296;12:4488859−131646236 6:57953521−119636360;
Purity, ploidy	0.61, 3.84

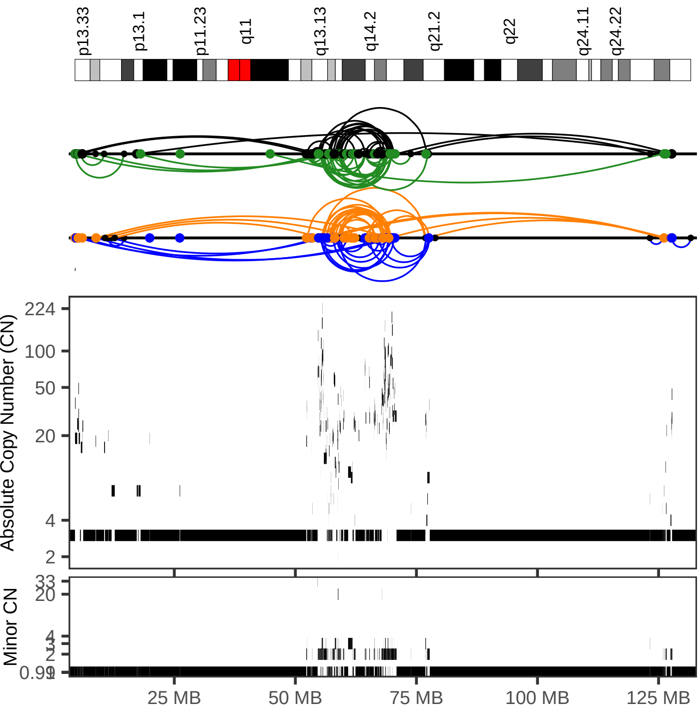

3505f91d−bc66−4732−84b6−18c5b32ca6b8	
Cancer type	SoftTissue−Liposarc
Position	12:4488859−131646237
Type	With other complex events
Interleaved intrachr. SVs	146
Total SVs (intrachr. + transl.)	391
SV types	DEL: 31; DUP: 35; h2hINV: 37; t2tINV: 43; TRA: 245
SVs in sample	1246
Oscillating CN (2 and 3 states)	7, 7
CN segments	384
FDR fragment joints	0.64
FDR chr. breakp. enrich.	0
Linked to chrs	1:147269177−248454296;13:20111633−31209009 2:2725830−47762583;4:64107549−135664103 5:111534825−180595775;9:107267770−135095318
Purity, ploidy	0.61, 3.84

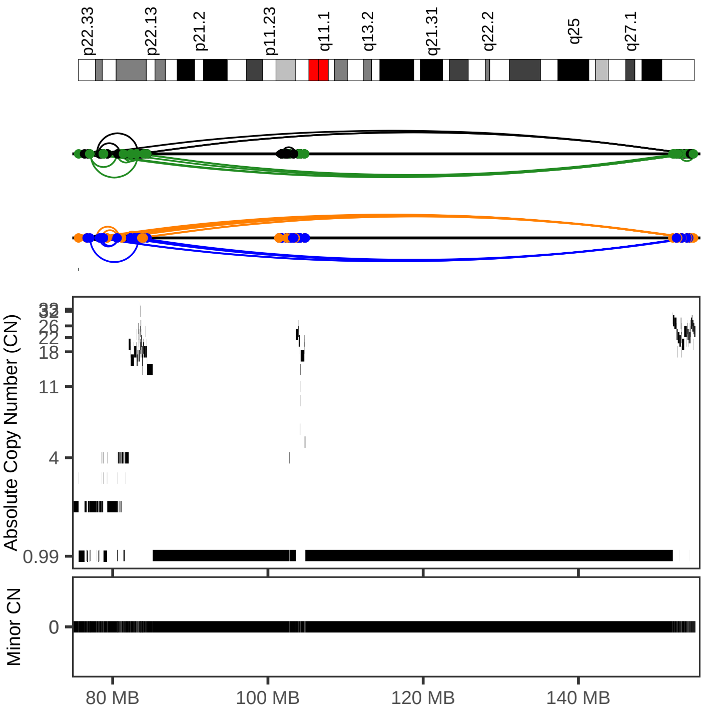

3505f91d−bc66−4732−84b6−18c5b32ca6b8	
Cancer type	SoftTissue−Liposarc
Position	X:77058127−154924953
Type	With other complex events
Interleaved intrachr. SVs	35
Total SVs (intrachr. + transl.)	205
SV types	DEL: 10; DUP: 9; h2hINV: 5; t2tINV: 11; TRA: 170
SVs in sample	1246
Oscillating CN (2 and 3 states)	7, 7
CN segments	147
FDR fragment joints	0.64
FDR chr. breakp. enrich.	0
Linked to chrs	1:147269177−248454296;12:4488859−131646236 13:20111633−31209009;2:2725830−47762583 4:64107549−135664103;
Purity, ploidy	0.61, 3.84

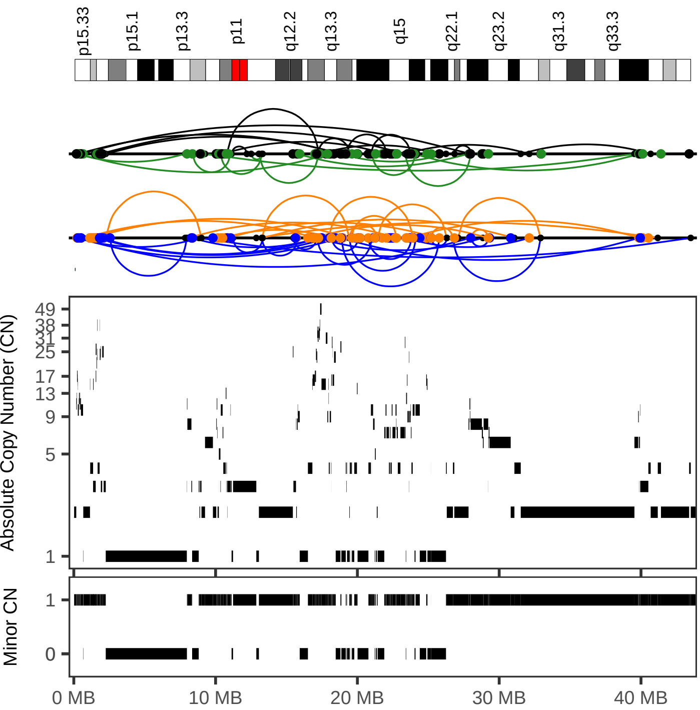

	6b189eff−b919−49d6−8775−dbf32c9ccbba
Cancer type	SoftTissue−Liposarc
Position	5:82214−43509706
Type	With other complex events
Interleaved intrachr. SVs	74
Total SVs (intrachr. + transl.)	315
SV types	DEL: 21; DUP: 23; h2hINV: 15; t2tINV: 15; TRA: 241
SVs in sample	890
Oscillating CN (2 and 3 states)	6, 8
CN segments	196
FDR fragment joints	0.64
FDR chr. breakp. enrich.	0
Linked to chrs	14:95901595−105399402;
Purity, ploidy	0.88, 2

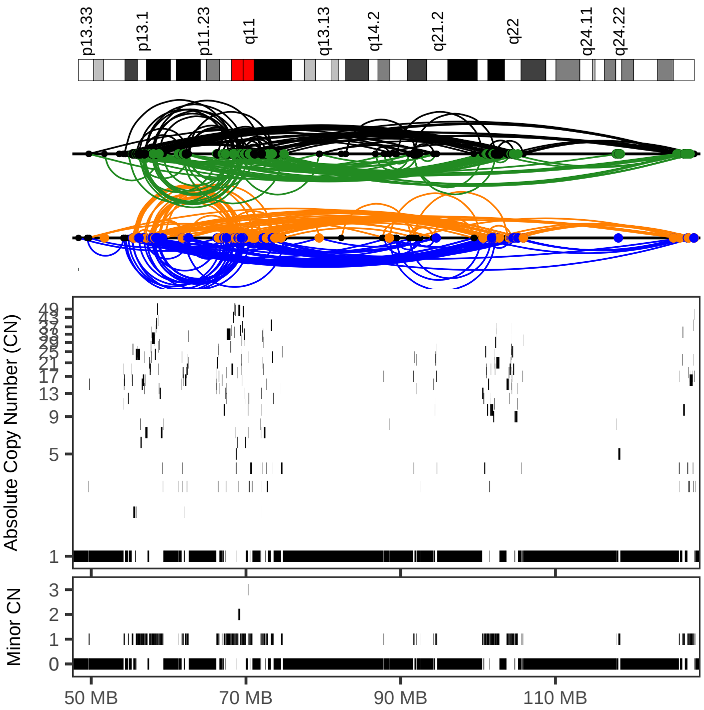

	6b189eff−b919−49d6−8775−dbf32c9ccbba
Cancer type	SoftTissue−Liposarc
Position	12:48350328−127948831
Type	With other complex events
Interleaved intrachr. SVs	400
Total SVs (intrachr. + transl.)	708
SV types	DEL: 102; DUP: 98; h2hINV: 95; t2tINV: 105; TRA: 308
SVs in sample	890
Oscillating CN (2 and 3 states)	4, 4
CN segments	291
FDR fragment joints	0.92
FDR chr. breakp. enrich.	0
Linked to chrs	5:82214−43509705;14:95901595−105399402
Purity, ploidy	0.88, 2

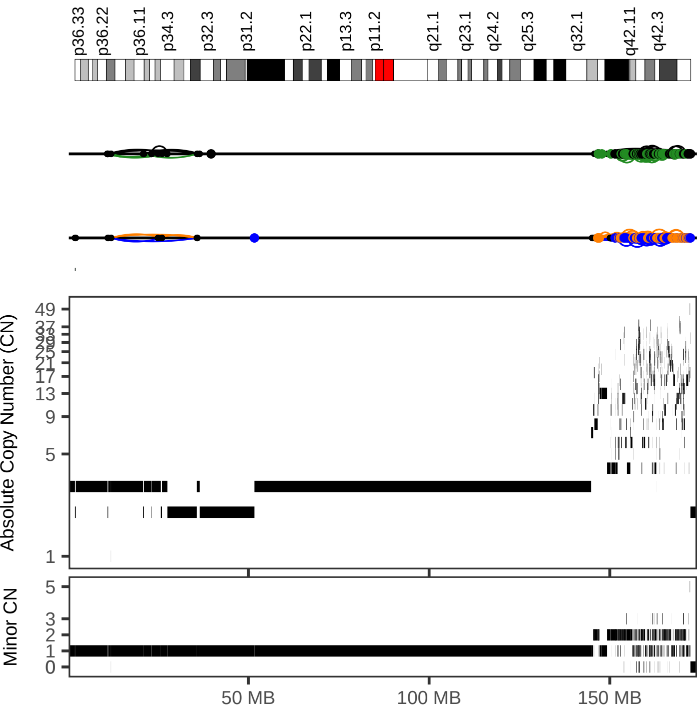

	73d2e8ef−4e10−416a−845b−f48374e48ecb
Cancer type	SoftTissue−Liposarc
Position	1:145112810−172403220
Type	With other complex events
Interleaved intrachr. SVs	256
Total SVs (intrachr. + transl.)	432
SV types	DEL: 73; DUP: 65; h2hINV: 56; t2tINV: 62; TRA: 176
SVs in sample	1088
Oscillating CN (2 and 3 states)	7, 7
CN segments	440
FDR fragment joints	0.64
FDR chr. breakp. enrich.	0
Linked to chrs	7:113482279−131109556;
Purity, ploidy	0.55, 3.11

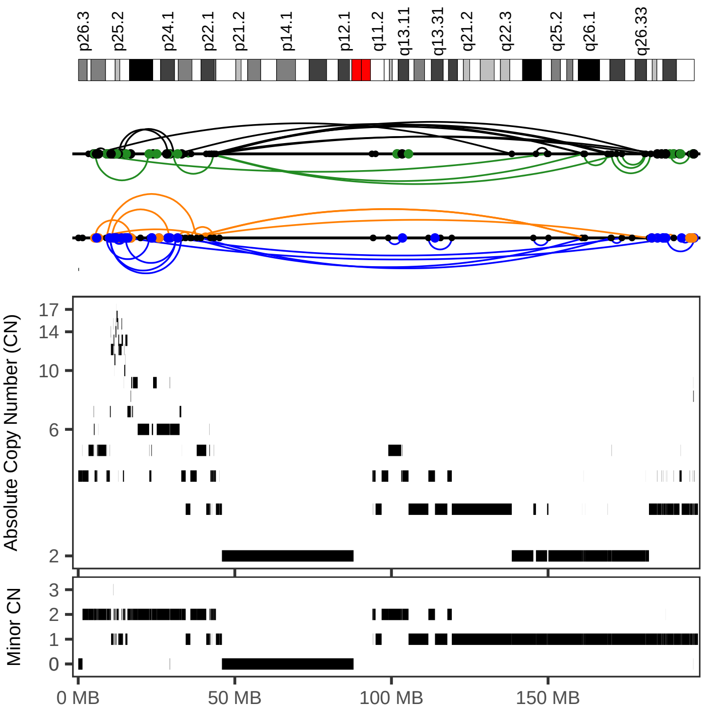

	73d2e8ef−4e10−416a−845b−f48374e48ecb
Cancer type	SoftTissue−Liposarc
Position	3:3279482−196670440
Type	With other complex events
Interleaved intrachr. SVs	58
Total SVs (intrachr. + transl.)	170
SV types	DEL: 16; DUP: 15; h2hINV: 14; t2tINV: 13; TRA: 112
SVs in sample	1088
Oscillating CN (2 and 3 states)	14, 24
CN segments	153
FDR fragment joints	0.97
FDR chr. breakp. enrich.	0
Linked to chrs	12:57518989−72095051;14:29873311−73266123 17:38121352−80696807;2:1619612−6515518 22:32381273−48585046;5:703397−41841708 8:13601211−30077762;
Purity, ploidy	0.55, 3.11

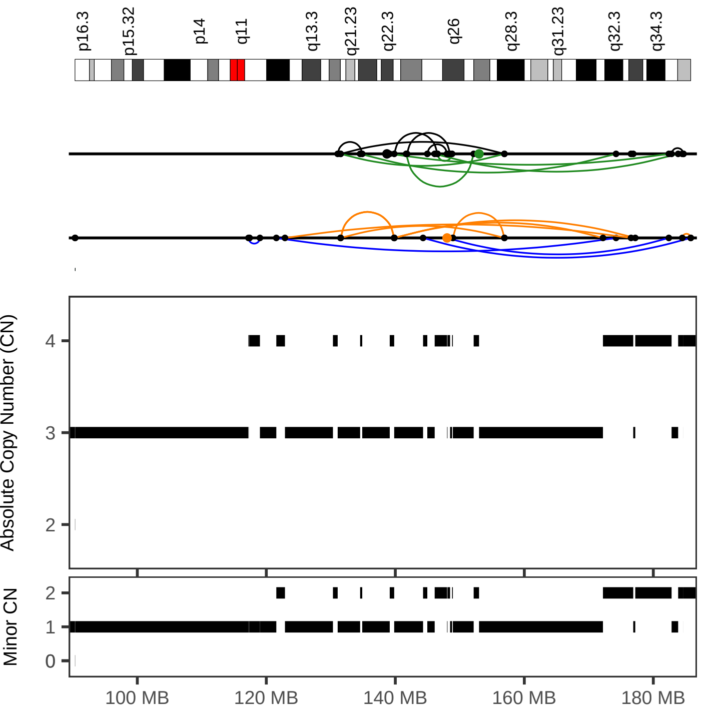

	73d2e8ef−4e10−416a−845b−f48374e48ecb
Cancer type	SoftTissue−Liposarc
Position	4:121551106−185803467
Type	After polyploidization
Interleaved intrachr. SVs	27
Total SVs (intrachr. + transl.)	30
SV types	DEL: 9; DUP: 3; h2hINV: 7; t2tINV: 8; TRA: 3
SVs in sample	1088
Oscillating CN (2 and 3 states)	25, 25
CN segments	25
FDR fragment joints	0.59
FDR chr. breakp. enrich.	0
Linked to chrs	
Purity, ploidy	0.55, 3.11

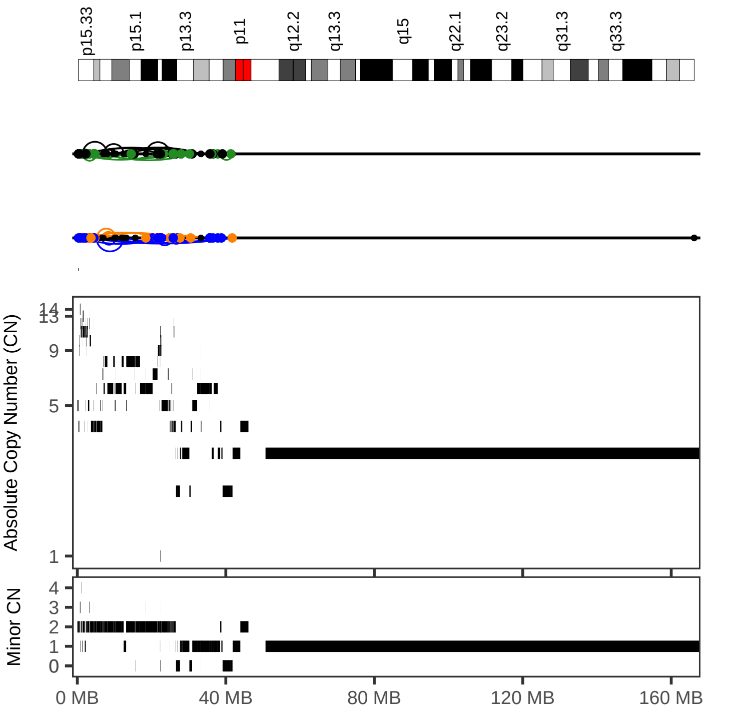

	73d2e8ef−4e10−416a−845b−f48374e48ecb
Cancer type	SoftTissue−Liposarc
Position	5:703397−41841709
Type	With other complex events
Interleaved intrachr. SVs	53
Total SVs (intrachr. + transl.)	170
SV types	DEL: 10; DUP: 12; h2hINV: 18; t2tINV: 13; TRA: 117
SVs in sample	1088
Oscillating CN (2 and 3 states)	9, 20
CN segments	158
FDR fragment joints	0.64
FDR chr. breakp. enrich.	0
Linked to chrs	12:57518989−72095051;17:38121352−80696807 22:32381273−48585046;3:3279482−196670439 8:13601211−30077762;
Purity, ploidy	0.55, 3.11

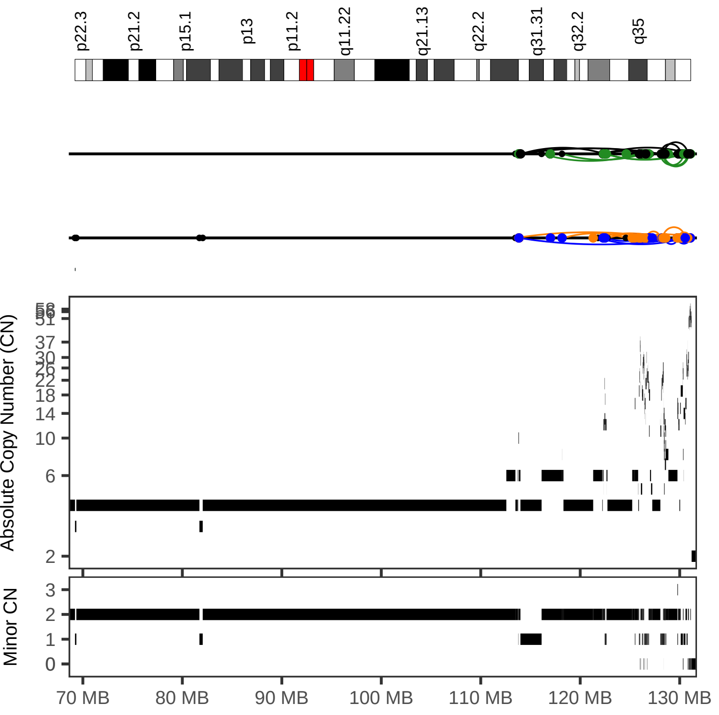

	73d2e8ef−4e10−416a−845b−f48374e48ecb
Cancer type	SoftTissue−Liposarc
Position	7:113482279−131109557
Type	With other complex events
Interleaved intrachr. SVs	47
Total SVs (intrachr. + transl.)	215
SV types	DEL: 12; DUP: 8; h2hINV: 14; t2tINV: 13; TRA: 168
SVs in sample	1088
Oscillating CN (2 and 3 states)	5, 6
CN segments	167
FDR fragment joints	0.7
FDR chr. breakp. enrich.	0
Linked to chrs	1:145112810−172403219;
Purity, ploidy	0.55, 3.11

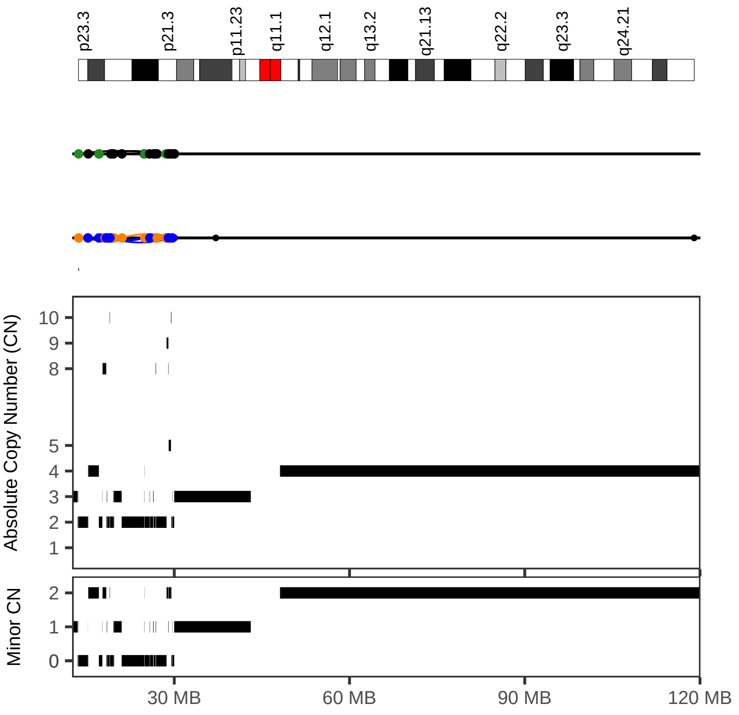

	73d2e8ef−4e10−416a−845b−f48374e48ecb
Cancer type	SoftTissue−Liposarc
Position	8:13601211−30077763
Type	With other complex events
Interleaved intrachr. SVs	7
Total SVs (intrachr. + transl.)	60
SV types	DEL: 1; DUP: 4; h2hINV: 2; t2tINV: 0; TRA: 53
SVs in sample	1088
Oscillating CN (2 and 3 states)	10, 10
CN segments	40
FDR fragment joints	0.59
FDR chr. breakp. enrich.	0.13
Linked to chrs	12:57518989−72095051;14:29873311−73266123 17:38121352−80696807;3:3279482−196670439 5:703397−41841708;
Purity, ploidy	0.55, 3.11

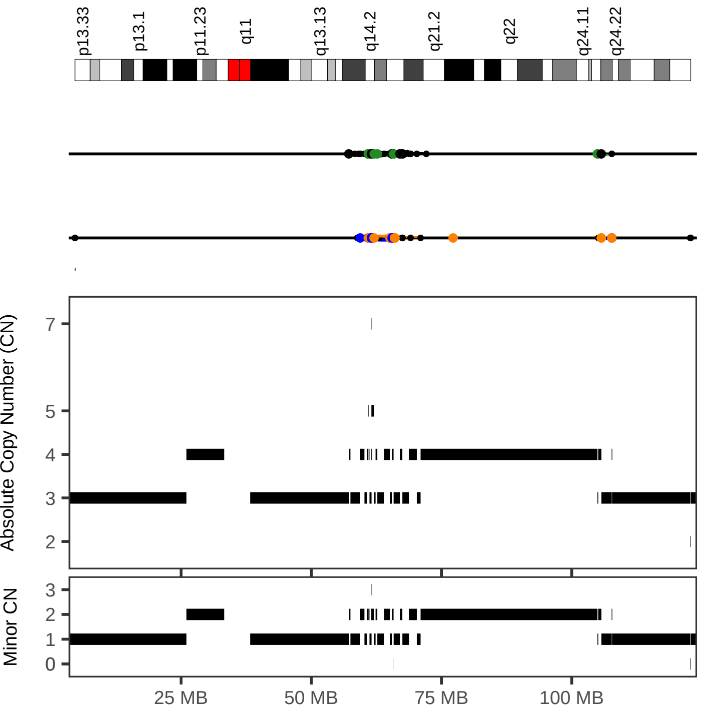

73d2e8ef−4e10−416a−845b−f48374e48ecb

Cancer type	SoftTissue−Liposarc
Position	12:57518989−72095052
Type	With other complex events
Interleaved intrachr. SVs	25
Total SVs (intrachr. + transl.)	74
SV types	DEL: 7; DUP: 6; h2hINV: 5; t2tINV: 7; TRA: 49
SVs in sample	1088
Oscillating CN (2 and 3 states)	19, 22
CN segments	37
FDR fragment joints	0.95
FDR chr. breakp. enrich.	0
Linked to chrs	17:38121352−80696807;22:32381273−48585046 3:3279482−196670439;5:703397−41841708 8:13601211−30077762;
Purity, ploidy	0.55, 3.11

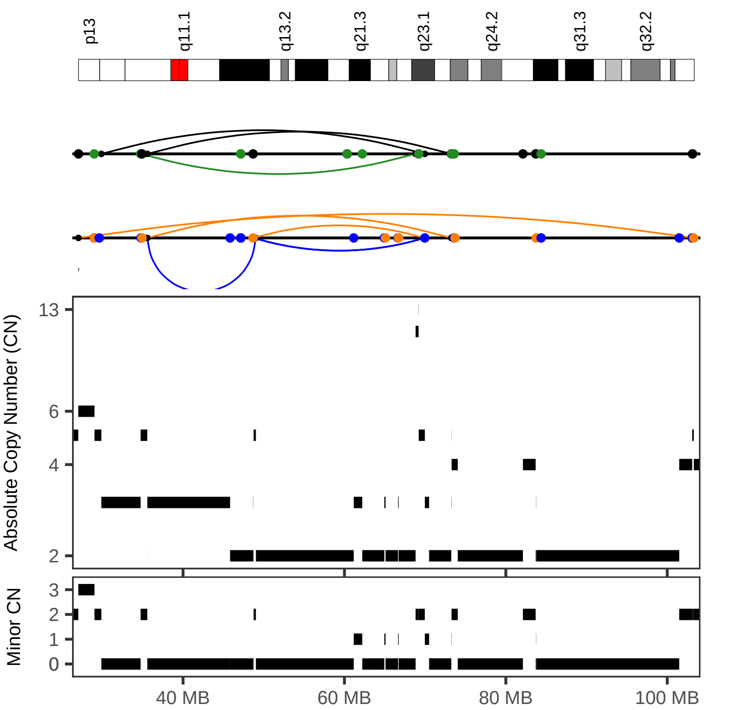

73d2e8ef−4e10−416a−845b−f48374e48ecb

Cancer type	SoftTissue−Liposarc
Position	14:29873311−73266124
Type	With other complex events
Interleaved intrachr. SVs	9
Total SVs (intrachr. + transl.)	32
SV types	DEL: 3; DUP: 3; h2hINV: 2; t2tINV: 1; TRA: 23
SVs in sample	1088
Oscillating CN (2 and 3 states)	7, 7
CN segments	25
FDR fragment joints	0.83
FDR chr. breakp. enrich.	0
Linked to chrs	3:3279482−196670439;5:703397−41841708 8:13601211−30077762;
Purity, ploidy	0.55, 3.11

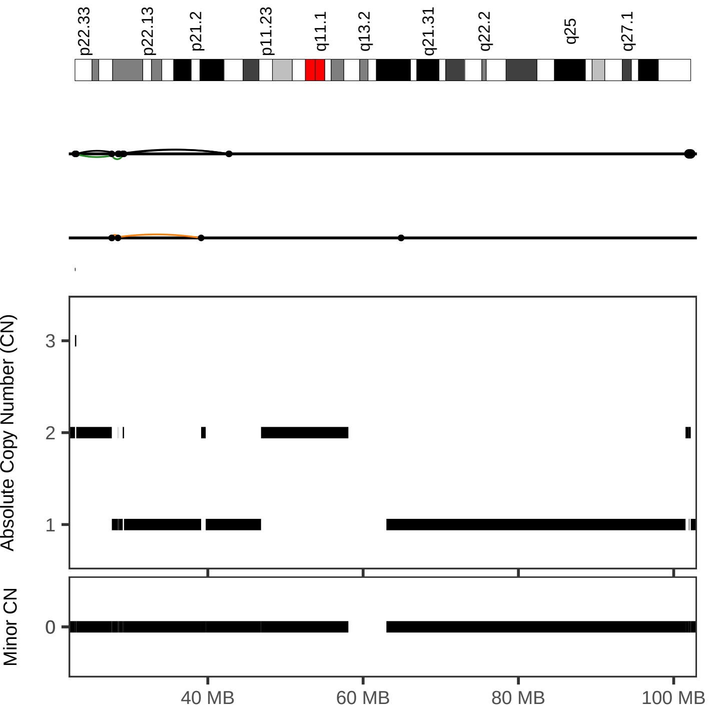

73d2e8ef−4e10−416a−845b−f48374e48ecb

Cancer type	SoftTissue−Liposarc
Position	X:22888722−42758155
Type	Canonical without polyploidization
Interleaved intrachr. SVs	7
Total SVs (intrachr. + transl.)	7
SV types	DEL: 2; DUP: 0; h2hINV: 3; t2tINV: 2; TRA: 0
SVs in sample	1088
Oscillating CN (2 and 3 states)	12, 13
CN segments	13
FDR fragment joints	0.64
FDR chr. breakp. enrich.	0
Linked to chrs	
Purity, ploidy	0.55, 3.11

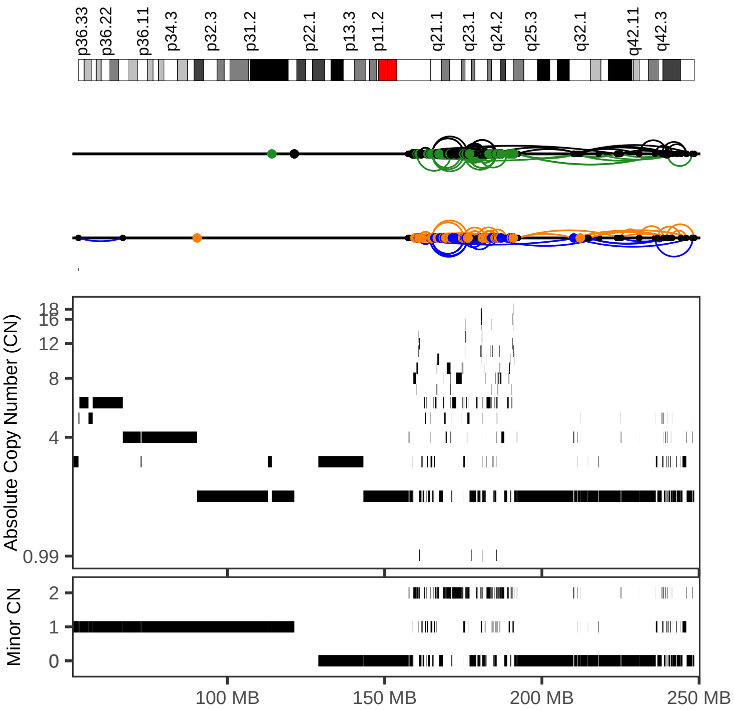

854e42bc−95b2−4b77−a4b2−ffbd6c28b281

Cancer type	SoftTissue−Liposarc
Position	1:157434186−248437131
Type	With other complex events
Interleaved intrachr. SVs	179
Total SVs (intrachr. + transl.)	598
SV types	DEL: 52; DUP: 35; h2hINV: 44; t2tINV: 48; TRA: 419
SVs in sample	2036
Oscillating CN (2 and 3 states)	9, 13
CN segments	240
FDR fragment joints	0.59
FDR chr. breakp. enrich.	0
Linked to chrs	10:3043555−124930156;11:20106252−97980851 12:27498321−130397264;13:56101373−108997400 16:261147−88704040;3:8155512−83721536 6:1095728−156611835;X:70422197−98898337
Purity, ploidy	0.84, 2.88

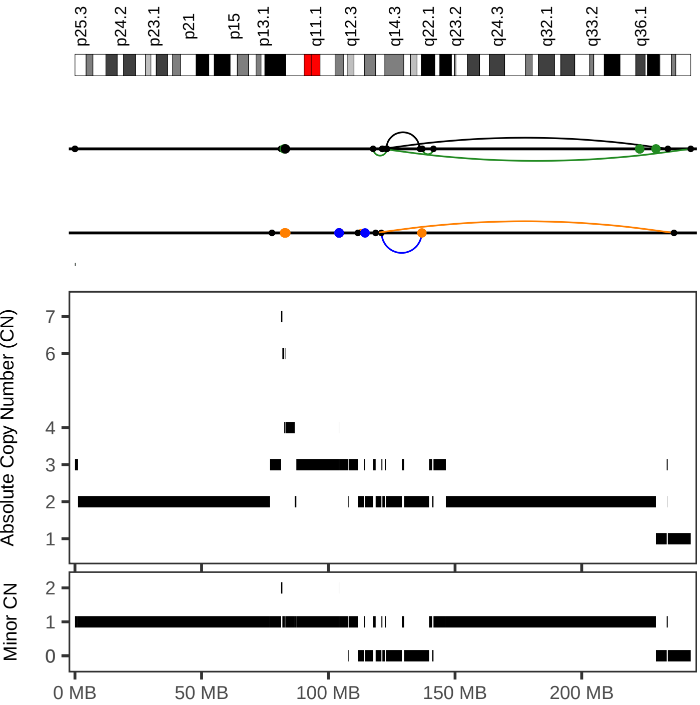

	854e42bc−95b2−4b77−a4b2−ffbd6c28b281
Cancer type	SoftTissue−Liposarc
Position	2:117676635−243016298
Type	Canonical without polyploidization
Interleaved intrachr. SVs	6
Total SVs (intrachr. + transl.)	10
SV types	DEL: 1; DUP: 1; h2hINV: 2; t2tINV: 2; TRA: 4
SVs in sample	2036
Oscillating CN (2 and 3 states)	14, 14
CN segments	18
FDR fragment joints	0.91
FDR chr. breakp. enrich.	0
Linked to chrs	8:80454829−117232183;
Purity, ploidy	0.84, 2.88

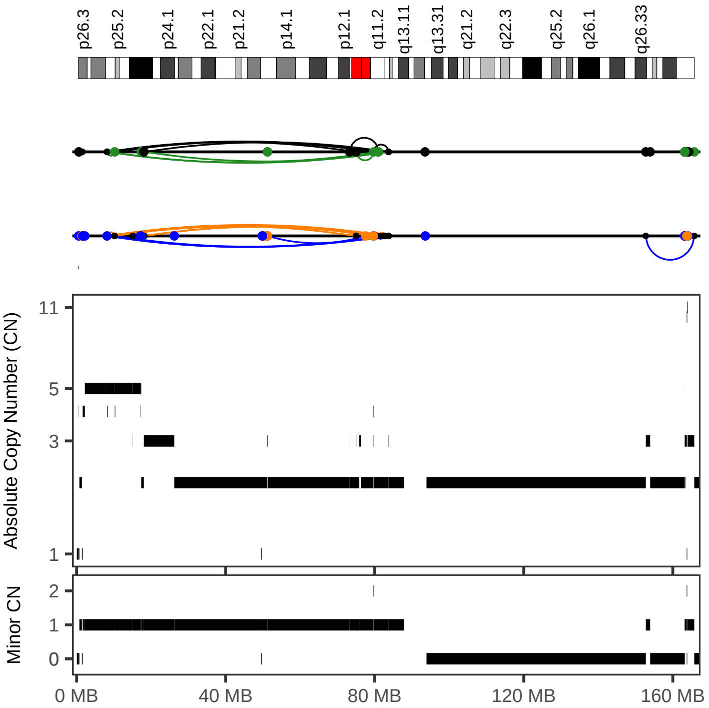

	854e42bc−95b2−4b77−a4b2−ffbd6c28b281
Cancer type	SoftTissue−Liposarc
Position	3:8155512−83721537
Type	With other complex events
Interleaved intrachr. SVs	29
Total SVs (intrachr. + transl.)	55
SV types	DEL: 8; DUP: 5; h2hINV: 8; t2tINV: 8; TRA: 26
SVs in sample	2036
Oscillating CN (2 and 3 states)	12, 12
CN segments	32
FDR fragment joints	0.86
FDR chr. breakp. enrich.	0.73
Linked to chrs	11:20106252−97980851;16:261147−88704040 6:1095728−156611835;X:70422197−98898337
Purity, ploidy	0.84, 2.88

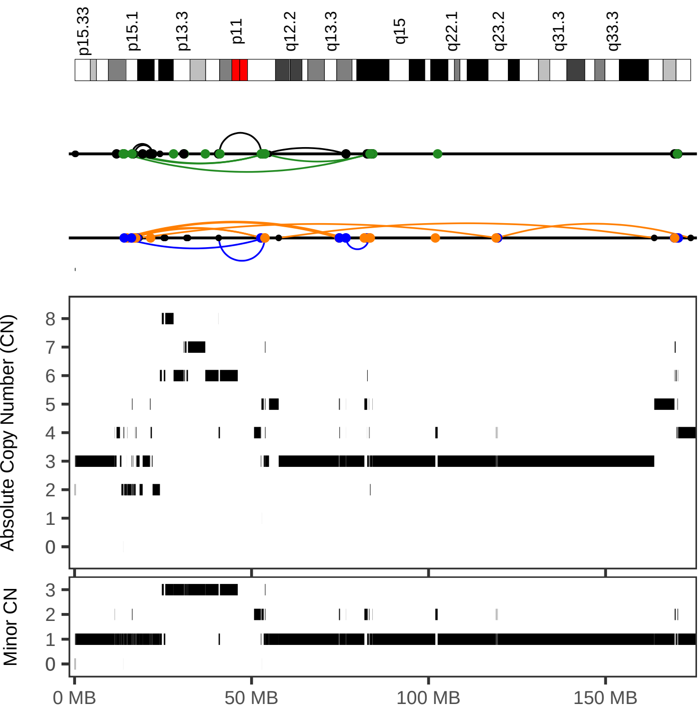

	854e42bc−95b2−4b77−a4b2−ffbd6c28b281
Cancer type	SoftTissue−Liposarc
Position	5:13726553−174060331
Type	With other complex events
Interleaved intrachr. SVs	25
Total SVs (intrachr. + transl.)	105
SV types	DEL: 8; DUP: 5; h2hINV: 4; t2tINV: 8; TRA: 80
SVs in sample	2036
Oscillating CN (2 and 3 states)	7, 12
CN segments	97
FDR fragment joints	0.64
FDR chr. breakp. enrich.	0.61
Linked to chrs	12:27498321−130397264;9:1604237−38289691
Purity, ploidy	0.84, 2.88

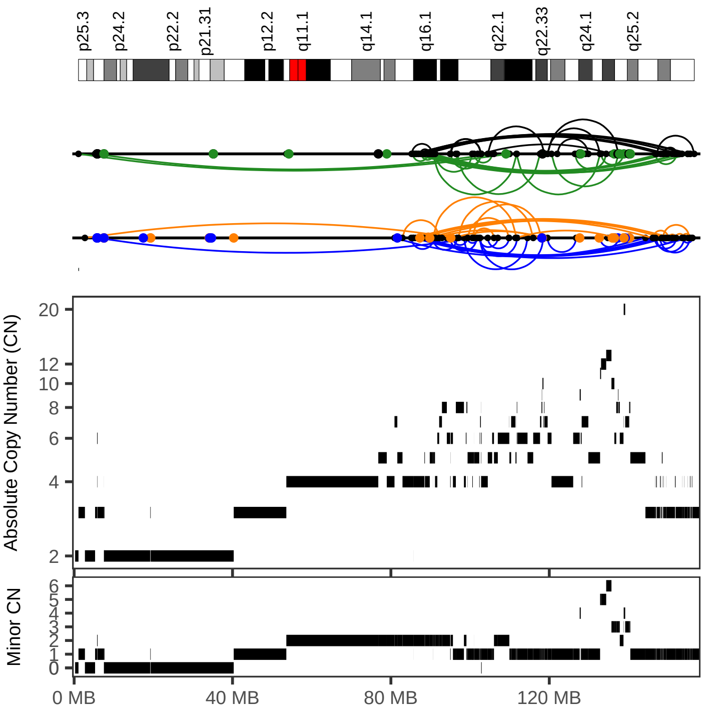

	854e42bc−95b2−4b77−a4b2−ffbd6c28b281
Cancer type	SoftTissue−Liposarc
Position	6:1095728−156611836
Type	With other complex events
Interleaved intrachr. SVs	93
Total SVs (intrachr. + transl.)	148
SV types	DEL: 23; DUP: 26; h2hINV: 19; t2tINV: 25; TRA: 55
SVs in sample	2036
Oscillating CN (2 and 3 states)	20, 29
CN segments	135
FDR fragment joints	0.82
FDR chr. breakp. enrich.	0.02
Linked to chrs	12:27498321−130397264;13:56101373−108997400 14:21880534−104480455;16:261147−88704040 3:8155512−83721536;X:70422197−98898337
Purity, ploidy	0.84, 2.88

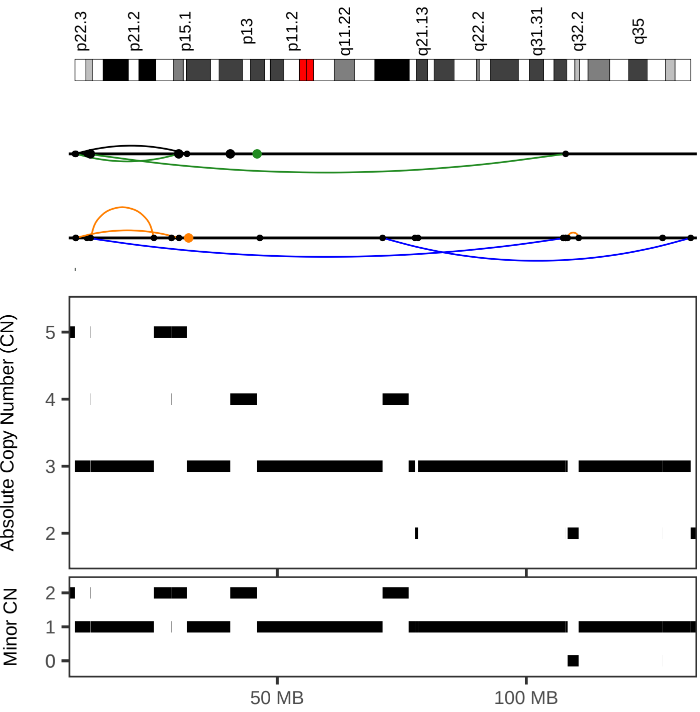

	854e42bc−95b2−4b77−a4b2−ffbd6c28b281
Cancer type	SoftTissue−Liposarc
Position	7:9379433−133023159
Type	With other complex events
Interleaved intrachr. SVs	7
Total SVs (intrachr. + transl.)	12
SV types	DEL: 1; DUP: 3; h2hINV: 1; t2tINV: 2; TRA: 5
SVs in sample	2036
Oscillating CN (2 and 3 states)	10, 11
CN segments	21
FDR fragment joints	0.74
FDR chr. breakp. enrich.	0
Linked to chrs	16:261147−88704040;9:1604237−38289691
Purity, ploidy	0.84, 2.88

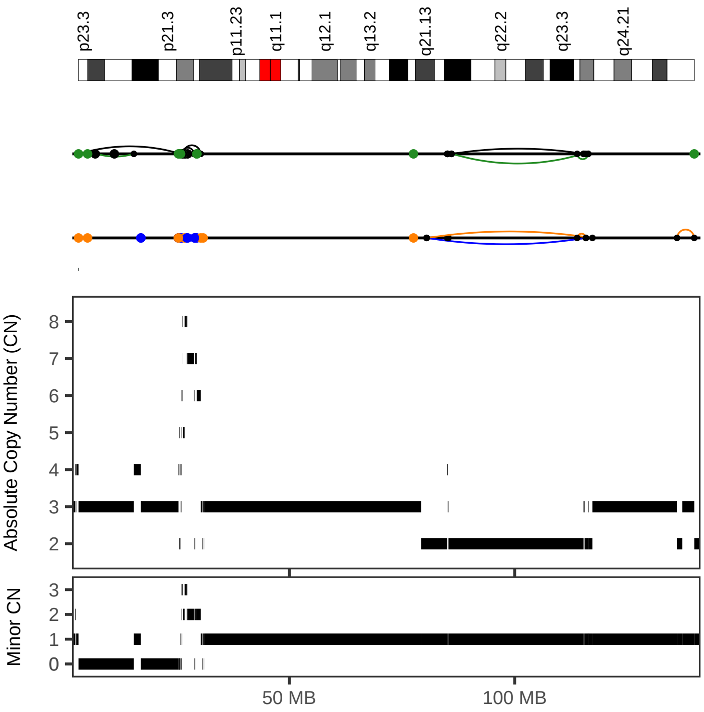

	854e42bc−95b2−4b77−a4b2−ffbd6c28b281
Cancer type	SoftTissue−Liposarc
Position	8:80454829−117232184
Type	Canonical without polyploidization
Interleaved intrachr. SVs	8
Total SVs (intrachr. + transl.)	8
SV types	DEL: 2; DUP: 1; h2hINV: 1; t2tINV: 4; TRA: 0
SVs in sample	2036
Oscillating CN (2 and 3 states)	7, 10
CN segments	10
FDR fragment joints	0.59
FDR chr. breakp. enrich.	0
Linked to chrs	
Purity, ploidy	0.84, 2.88

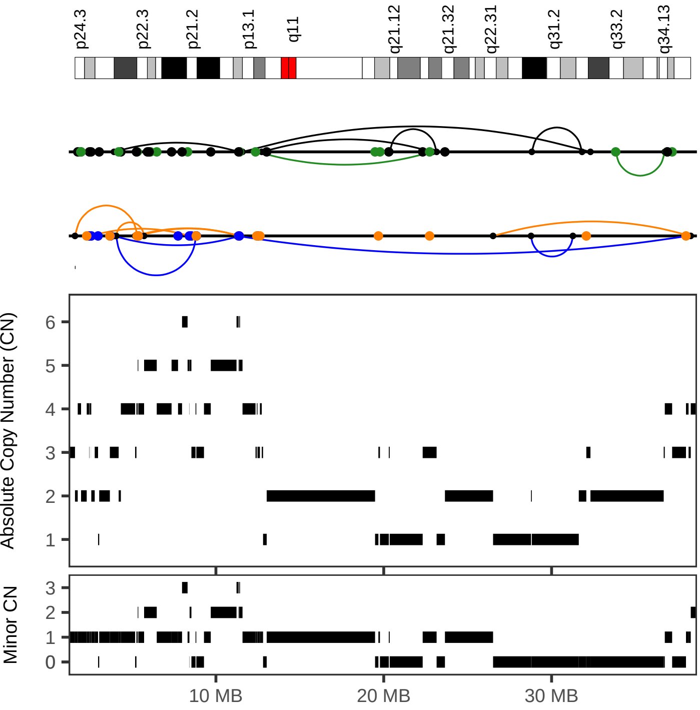

	854e42bc−95b2−4b77−a4b2−ffbd6c28b281
Cancer type	SoftTissue−Liposarc
Position	9:1604237−38289692
Type	With other complex events
Interleaved intrachr. SVs	12
Total SVs (intrachr. + transl.)	76
SV types	DEL: 6; DUP: 4; h2hINV: 2; t2tINV: 0; TRA: 64
SVs in sample	2036
Oscillating CN (2 and 3 states)	7, 15
CN segments	68
FDR fragment joints	0.48
FDR chr. breakp. enrich.	0.58
Linked to chrs	10:3043555−124930156;14:21880534−104480455 16:261147−88704040;19:5386099−49674391 5:13726553−174060330;7:9379433−133023158
Purity, ploidy	0.84, 2.88

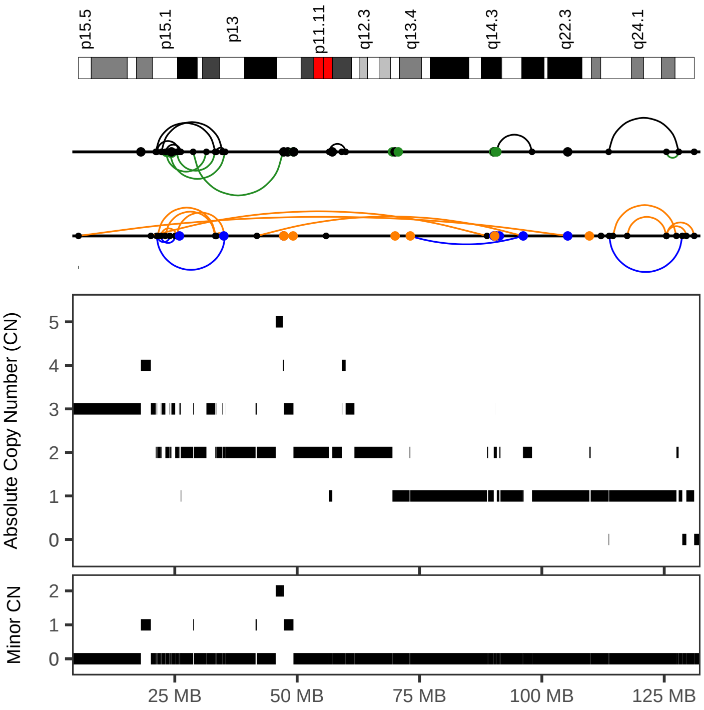

	854e42bc−95b2−4b77−a4b2−ffbd6c28b281
Cancer type	SoftTissue−Liposarc
Position	11:20106252−97980852
Type	With other complex events
Interleaved intrachr. SVs	26
Total SVs (intrachr. + transl.)	67
SV types	DEL: 7; DUP: 5; h2hINV: 7; t2tINV: 7; TRA: 41
SVs in sample	2036
Oscillating CN (2 and 3 states)	23, 39
CN segments	62
FDR fragment joints	0.95
FDR chr. breakp. enrich.	0.7
Linked to chrs	12:27498321−130397264;3:8155512−83721536 8:80454829−117232183;
Purity, ploidy	0.84, 2.88

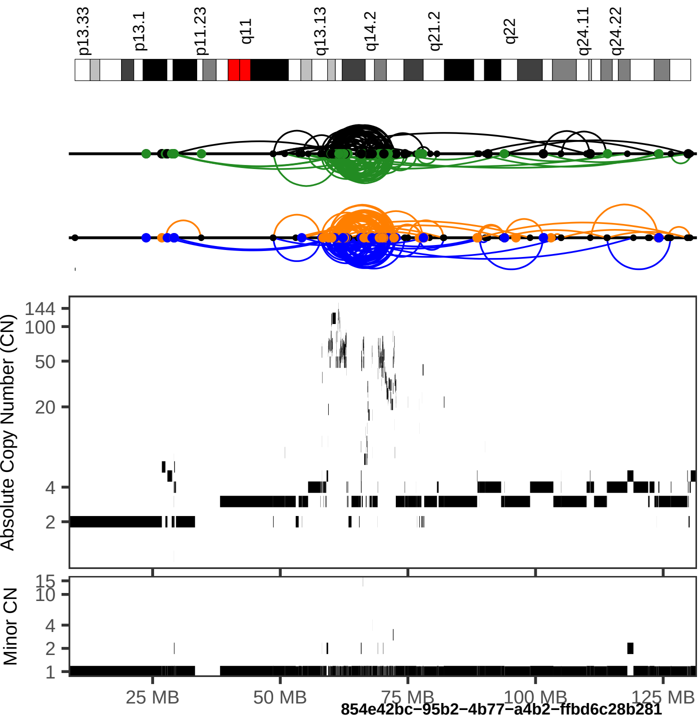

854e42bc−95b2−4b77−a4b2−ffbd6c28b281	
Cancer type	SoftTissue−Liposarc
Position	12:27498321−130397265
Type	With other complex events
Interleaved intrachr. SVs	409
Total SVs (intrachr. + transl.)	913
SV types	DEL: 97; DUP: 106; h2hINV: 101; t2tINV: 105; TRA: 504
SVs in sample	2036
Oscillating CN (2 and 3 states)	11, 14
CN segments	461
FDR fragment joints	0.94
FDR chr. breakp. enrich.	0
Linked to chrs	10:3043555−124930156;11:20106252−97980851 13:56101373−108997400;15:22917198−97545745 16:261147−88704040;20:37676020−59930521 3:8155512−83721536;5:13726553−174060330 6:1095728−156611835;8:80454829−117232183 X:70422197−98898337;
Purity, ploidy	0.84, 2.88

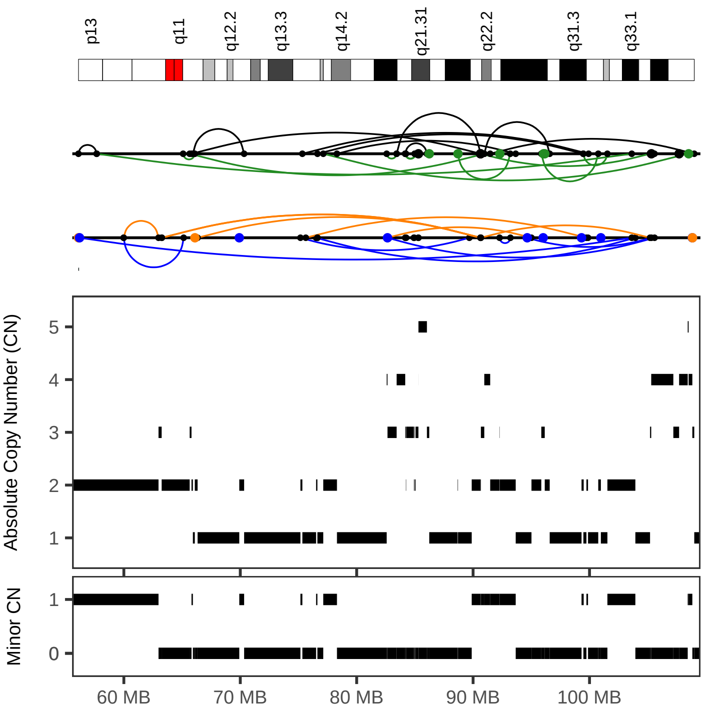

854e42bc−95b2−4b77−a4b2−ffbd6c28b281	
Cancer type	SoftTissue−Liposarc
Position	13:56101373−108997401
Type	With other complex events
Interleaved intrachr. SVs	33
Total SVs (intrachr. + transl.)	55
SV types	DEL: 7; DUP: 7; h2hINV: 9; t2tINV: 10; TRA: 22
SVs in sample	2036
Oscillating CN (2 and 3 states)	12, 28
CN segments	63
FDR fragment joints	0.89
FDR chr. breakp. enrich.	1
Linked to chrs	12:27498321−130397264;19:5386099−49674391 4:637238−140609576;6:1095728−156611835
Purity, ploidy	0.84, 2.88

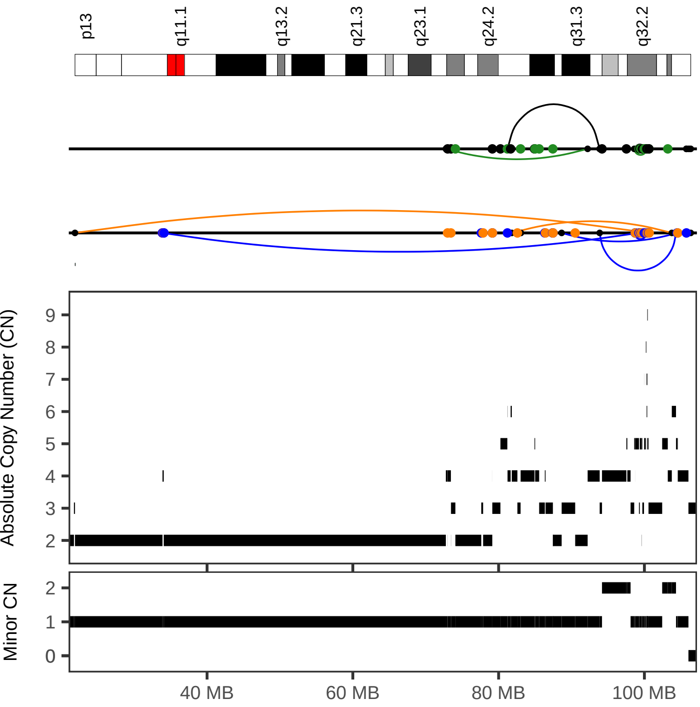

854e42bc−95b2−4b77−a4b2−ffbd6c28b281	
Cancer type	SoftTissue−Liposarc
Position	14:21880534−104480456
Type	With other complex events
Interleaved intrachr. SVs	20
Total SVs (intrachr. + transl.)	86
SV types	DEL: 4; DUP: 7; h2hINV: 5; t2tINV: 4; TRA: 66
SVs in sample	2036
Oscillating CN (2 and 3 states)	7, 19
CN segments	70
FDR fragment joints	0.83
FDR chr. breakp. enrich.	0
Linked to chrs	5:13726553−174060330;6:1095728−156611835 9:1604237−38289691;
Purity, ploidy	0.84, 2.88

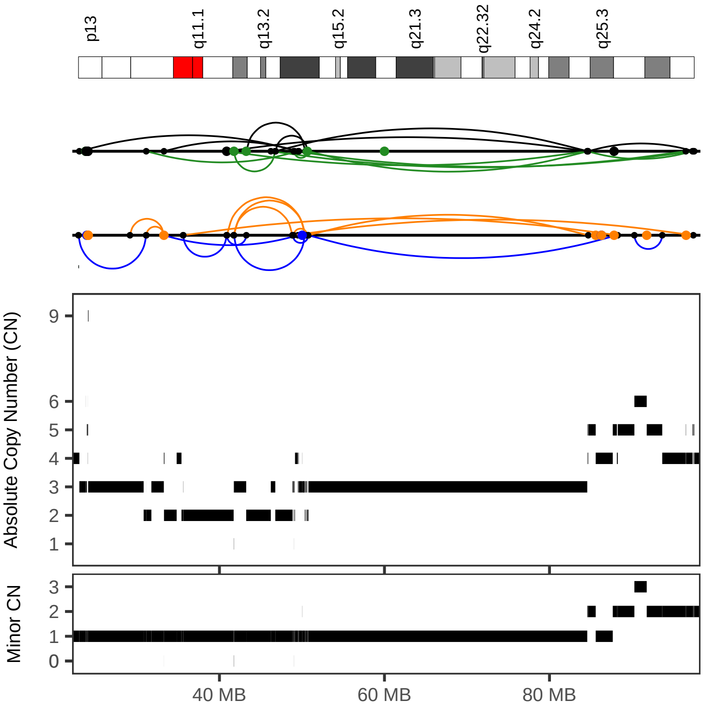

854e42bc−95b2−4b77−a4b2−ffbd6c28b281	
Cancer type	SoftTissue−Liposarc
Position	15:22917198−97545746
Type	With other complex events
Interleaved intrachr. SVs	35
Total SVs (intrachr. + transl.)	58
SV types	DEL: 10; DUP: 8; h2hINV: 9; t2tINV: 8; TRA: 23
SVs in sample	2036
Oscillating CN (2 and 3 states)	10, 14
CN segments	69
FDR fragment joints	0.97
FDR chr. breakp. enrich.	0.56
Linked to chrs	16:261147−88704040;19:5386099−49674391 X:70422197−98898337;
Purity, ploidy	0.84, 2.88

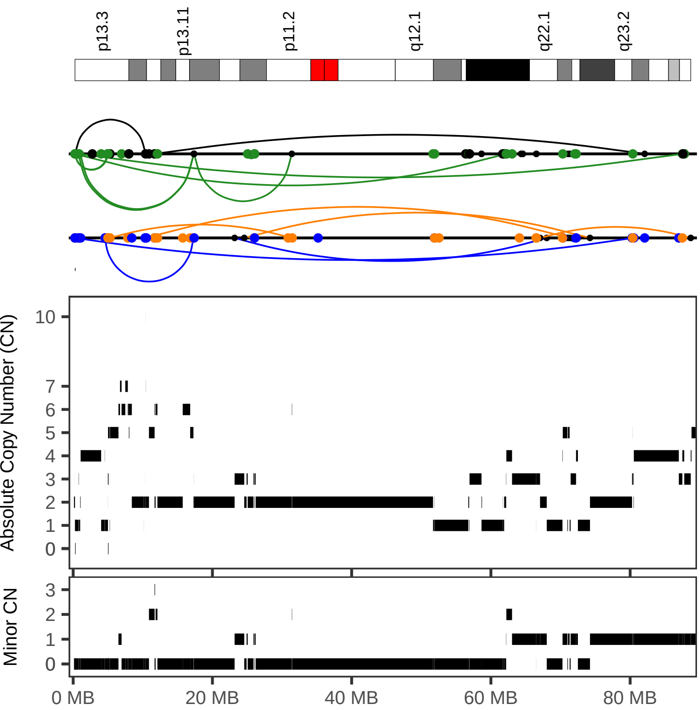

854e42bc−95b2−4b77−a4b2−ffbd6c28b281

Cancer type	SoftTissue−Liposarc
Position	16:261147−88704041
Type	With other complex events
Interleaved intrachr. SVs	19
Total SVs (intrachr. + transl.)	118
SV types	DEL: 6; DUP: 3; h2hINV: 3; t2tINV: 7; TRA: 99
SVs in sample	2036
Oscillating CN (2 and 3 states)	11, 12
CN segments	98
FDR fragment joints	0.64
FDR chr. breakp. enrich.	0
Linked to chrs	12:27498321−130397264;15:22917198−97545745 19:5386099−49674391;6:1095728−156611835 7:9379433−133023158;8:80454829−117232183 9:1604237−38289691;X:70422197−98898337
Purity, ploidy	0.84, 2.88

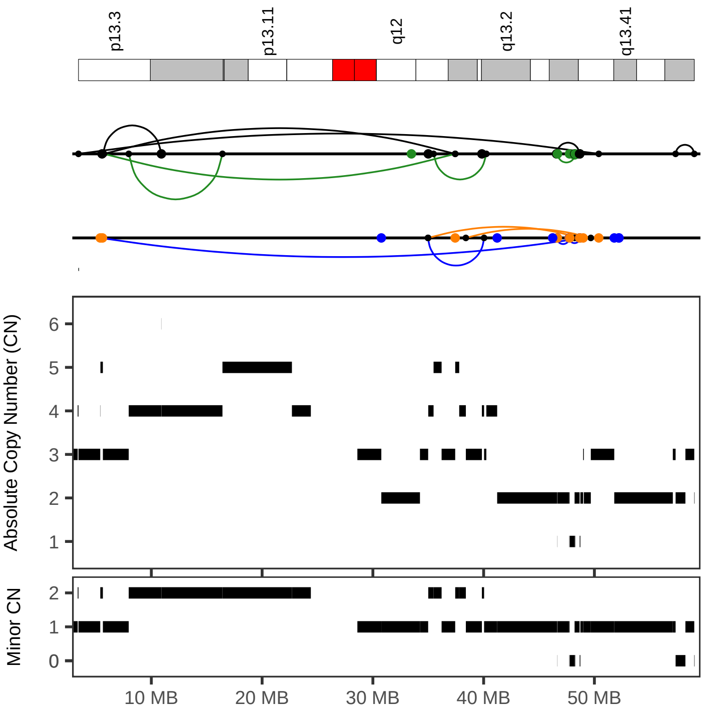

854e42bc−95b2−4b77−a4b2−ffbd6c28b281

Cancer type	SoftTissue−Liposarc
Position	19:5386099−49674392
Type	With other complex events
Interleaved intrachr. SVs	10
Total SVs (intrachr. + transl.)	41
SV types	DEL: 3; DUP: 2; h2hINV: 2; t2tINV: 3; TRA: 31
SVs in sample	2036
Oscillating CN (2 and 3 states)	19, 19
CN segments	46
FDR fragment joints	0.96
FDR chr. breakp. enrich.	0.03
Linked to chrs	10:3043555−124930156;15:22917198−97545745 16:261147−88704040;9:1604237−38289691
Purity, ploidy	0.84, 2.88

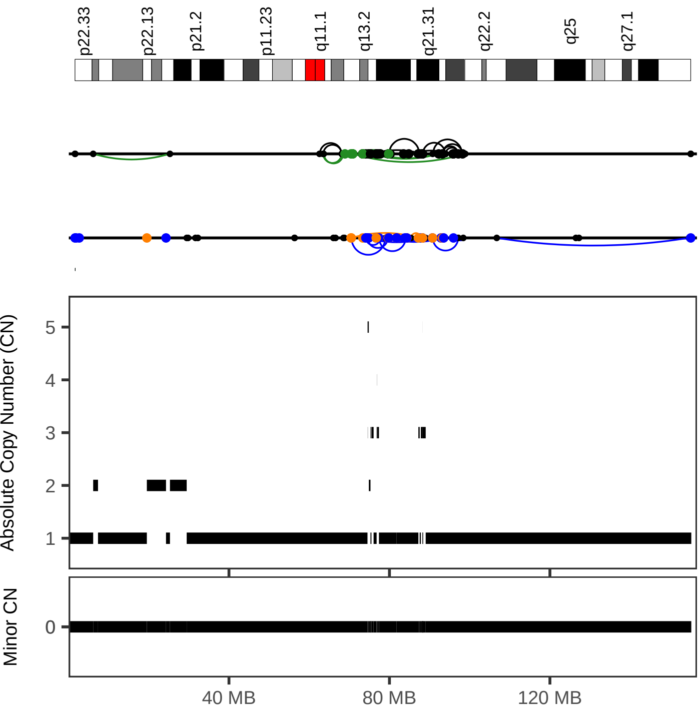

854e42bc−95b2−4b77−a4b2−ffbd6c28b281

Cancer type	SoftTissue−Liposarc
Position	X:70422197−98898338
Type	With other complex events
Interleaved intrachr. SVs	23
Total SVs (intrachr. + transl.)	94
SV types	DEL: 3; DUP: 8; h2hINV: 8; t2tINV: 4; TRA: 71
SVs in sample	2036
Oscillating CN (2 and 3 states)	7, 13
CN segments	28
FDR fragment joints	0.59
FDR chr. breakp. enrich.	0.2
Linked to chrs	12:27498321−130397264;15:22917198−97545745 16:261147−88704040;6:1095728−156611835 8:80454829−117232183;
Purity, ploidy	0.84, 2.88

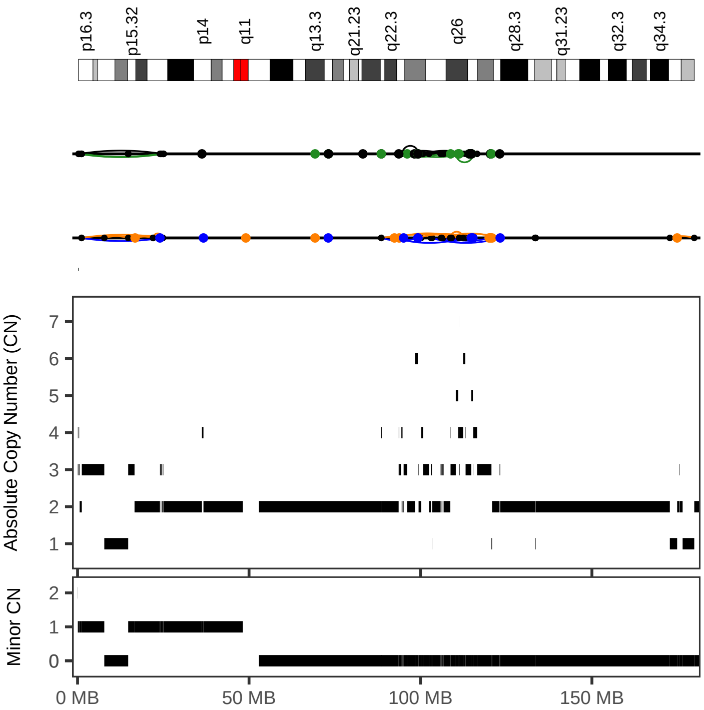

8f3f6f7c−4b50−467e−a6e8−d836735913f6

Cancer type	SoftTissue−Liposarc
Position	4:88570552−123136247
Type	With other complex events
Interleaved intrachr. SVs	24
Total SVs (intrachr. + transl.)	57
SV types	DEL: 6; DUP: 7; h2hINV: 5; t2tINV: 6; TRA: 33
SVs in sample	782
Oscillating CN (2 and 3 states)	9, 13
CN segments	46
FDR fragment joints	0.97
FDR chr. breakp. enrich.	0
Linked to chrs	12:1089811−133349178;6:9401122−151337808
Purity, ploidy	0.62, 2.12

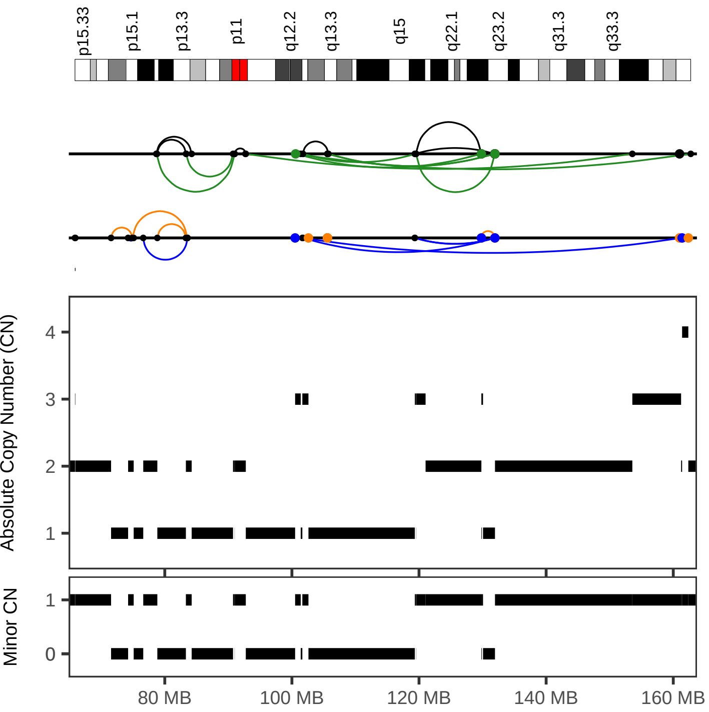

	8f3f6f7c−4b50−467e−a6e8−d836735913f6
Cancer type	SoftTissue−Liposarc
Position	5:71550993−162747485
Type	With other complex events
Interleaved intrachr. SVs	25
Total SVs (intrachr. + transl.)	44
SV types	DEL: 4; DUP: 6; h2hINV: 6; t2tINV: 9; TRA: 19
SVs in sample	782
Oscillating CN (2 and 3 states)	11, 19
CN segments	29
FDR fragment joints	0.64
FDR chr. breakp. enrich.	0.87
Linked to chrs	8:65806479−108515176;
Purity, ploidy	0.62, 2.12

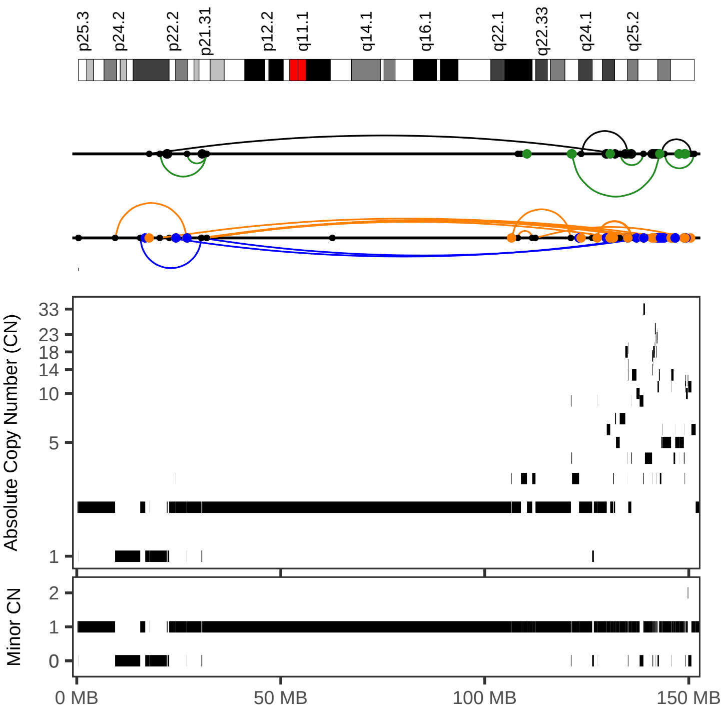

	8f3f6f7c−4b50−467e−a6e8−d836735913f6
Cancer type	SoftTissue−Liposarc
Position	6:9401122−151337809
Type	With other complex events
Interleaved intrachr. SVs	20
Total SVs (intrachr. + transl.)	91
SV types	DEL: 9; DUP: 3; h2hINV: 3; t2tINV: 5; TRA: 71
SVs in sample	782
Oscillating CN (2 and 3 states)	8, 20
CN segments	88
FDR fragment joints	0.59
FDR chr. breakp. enrich.	0
Linked to chrs	12:1089811−133349178;4:88570552−123136246
Purity, ploidy	0.62, 2.12

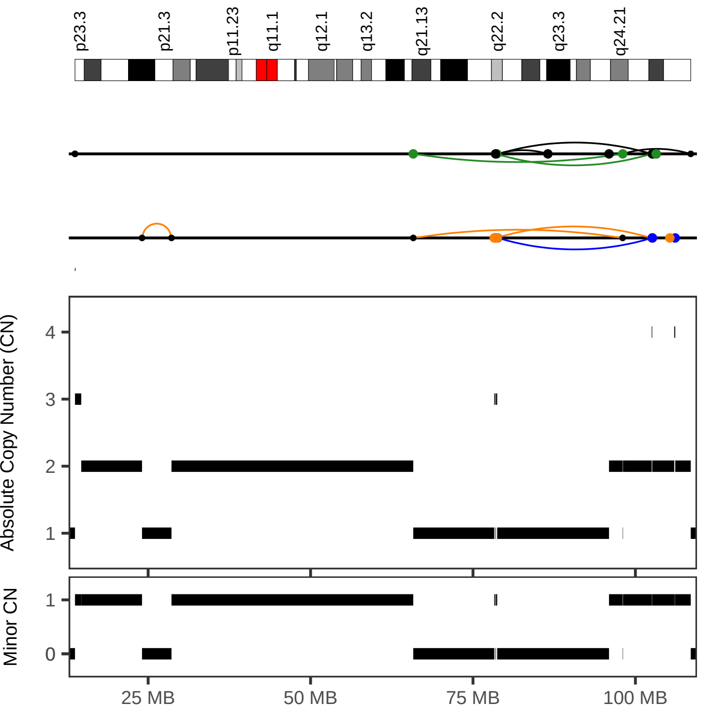

	8f3f6f7c−4b50−467e−a6e8−d836735913f6
Cancer type	SoftTissue−Liposarc
Position	8:65806479−108515177
Type	With other complex events
Interleaved intrachr. SVs	7
Total SVs (intrachr. + transl.)	29
SV types	DEL: 2; DUP: 1; h2hINV: 2; t2tINV: 2; TRA: 22
SVs in sample	782
Oscillating CN (2 and 3 states)	7, 9
CN segments	15
FDR fragment joints	0.95
FDR chr. breakp. enrich.	0.54
Linked to chrs	5:71550993−162747484;
Purity, ploidy	0.62, 2.12

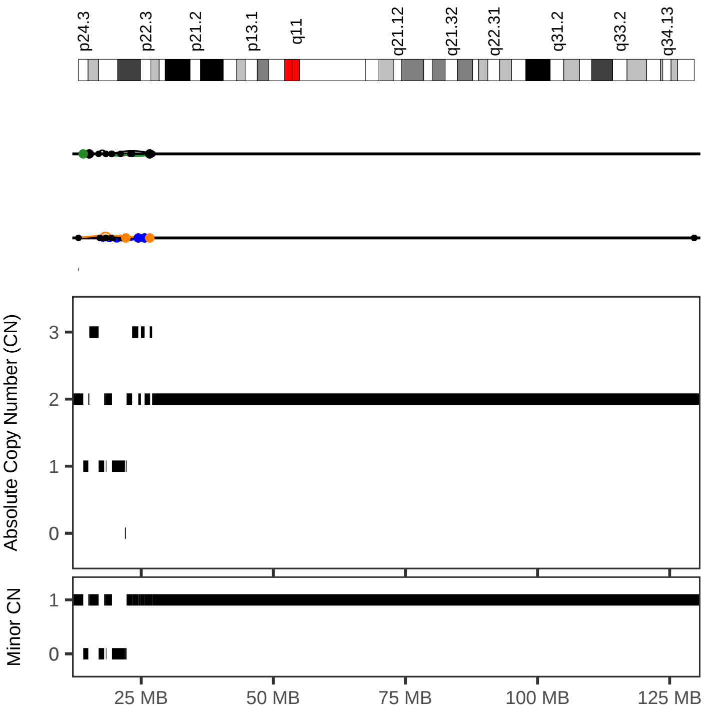

	8f3f6f7c−4b50−467e−a6e8−d836735913f6
Cancer type	SoftTissue−Liposarc
Position	9:13132039−27112660
Type	With other complex events
Interleaved intrachr. SVs	12
Total SVs (intrachr. + transl.)	19
SV types	DEL: 3; DUP: 4; h2hINV: 2; t2tINV: 3; TRA: 7
SVs in sample	782
Oscillating CN (2 and 3 states)	11, 18
CN segments	22
FDR fragment joints	0.91
FDR chr. breakp. enrich.	0.61
Linked to chrs	12:1089811−133349178;
Purity, ploidy	0.62, 2.12

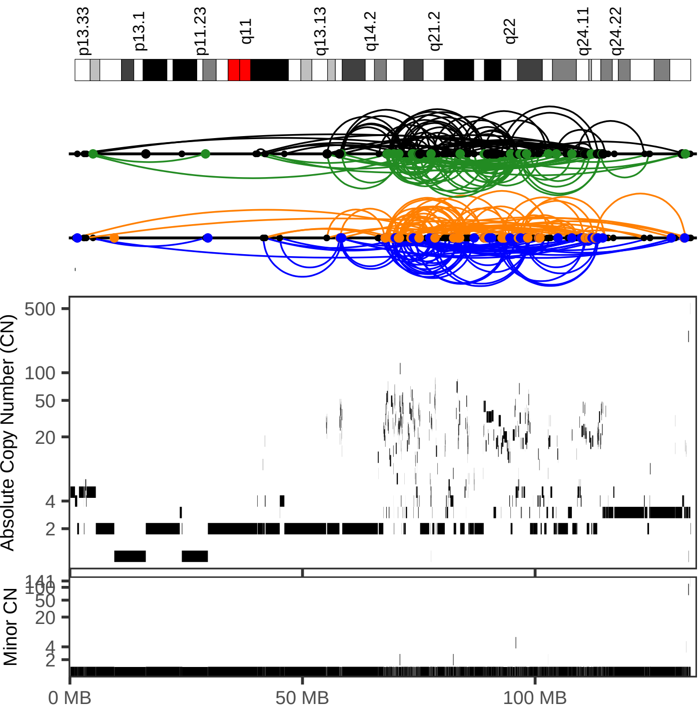

8f3f6f7c−4b50−467e−a6e8−d836735913f6

Cancer type	SoftTissue−Liposarc
Position	12:1089811−133349179
Type	With other complex events
Interleaved intrachr. SVs	334
Total SVs (intrachr. + transl.)	459
SV types	DEL: 84; DUP: 84; h2hINV: 85; t2tINV: 81; TRA: 125
SVs in sample	782
Oscillating CN (2 and 3 states)	6, 7
CN segments	580
FDR fragment joints	1
FDR chr. breakp. enrich.	0
Linked to chrs	11:14830528−20532173;6:9401122−151337808 9:13132039−27112659;
Purity, ploidy	0.62, 2.12

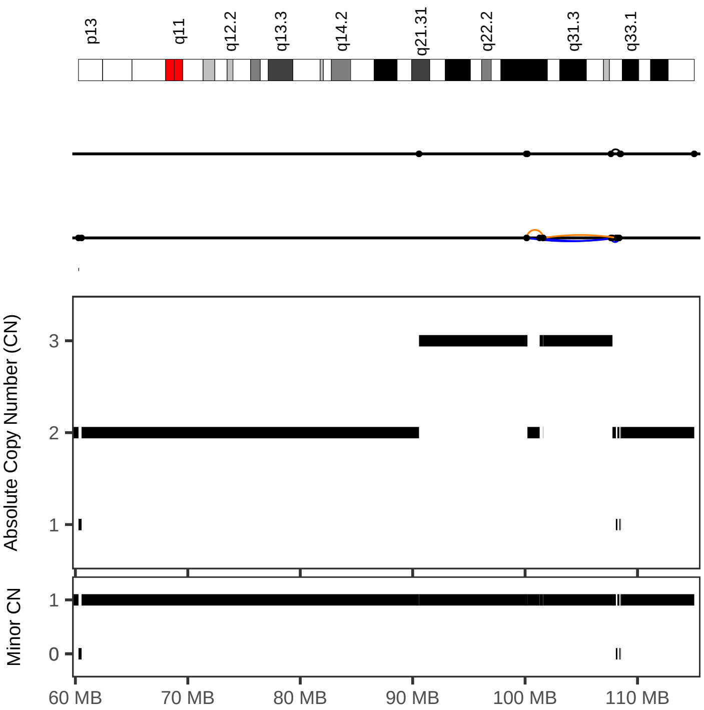

8f3f6f7c−4b50−467e−a6e8−d836735913f6

Cancer type	SoftTissue−Liposarc
Position	13:100098950−108510778
Type	With other complex events
Interleaved intrachr. SVs	8
Total SVs (intrachr. + transl.)	8
SV types	DEL: 2; DUP: 3; h2hINV: 2; t2tINV: 1; TRA: 0
SVs in sample	782
Oscillating CN (2 and 3 states)	7, 12
CN segments	12
FDR fragment joints	0.84
FDR chr. breakp. enrich.	0.01
Linked to chrs	
Purity, ploidy	0.62, 2.12

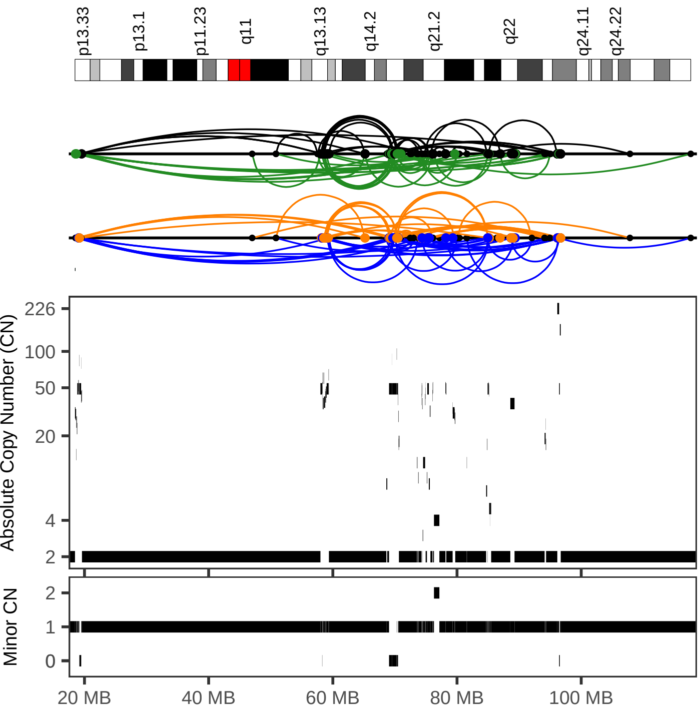

9517413b−b5b8−4130−8ba1−d86d44abe2ec

Cancer type	SoftTissue−Liposarc
Position	12:18468583−117642876
Type	With other complex events
Interleaved intrachr. SVs	167
Total SVs (intrachr. + transl.)	330
SV types	DEL: 39; DUP: 45; h2hINV: 36; t2tINV: 47; TRA: 163
SVs in sample	498
Oscillating CN (2 and 3 states)	4, 4
CN segments	101
FDR fragment joints	0.67
FDR chr. breakp. enrich.	0
Linked to chrs	
Purity, ploidy	0.6, 2.24

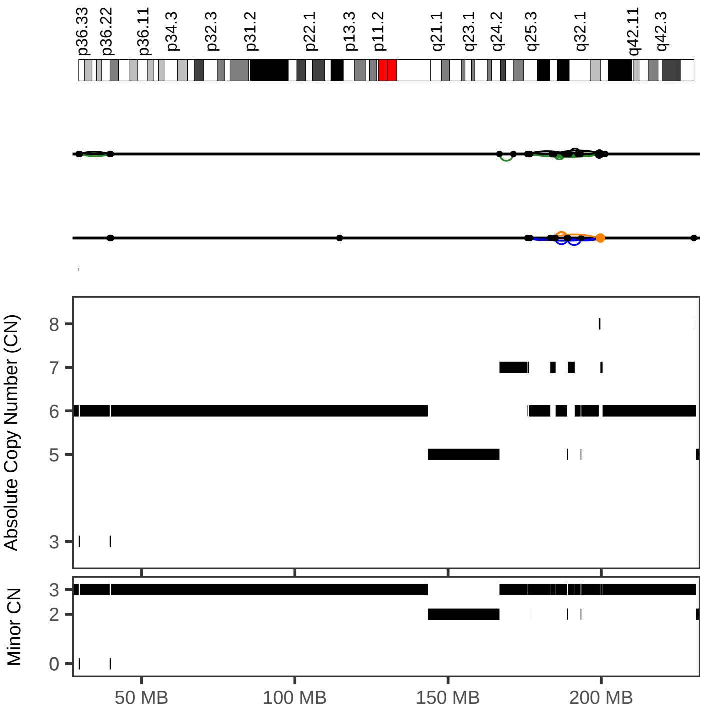

b5f27002−4277−418d−90b0−cc5a2742692e

Cancer type	SoftTissue−Liposarc
Position	1:175909202−201271527
Type	With other complex events
Interleaved intrachr. SVs	19
Total SVs (intrachr. + transl.)	21
SV types	DEL: 3; DUP: 5; h2hINV: 6; t2tINV: 5; TRA: 2
SVs in sample	895
Oscillating CN (2 and 3 states)	7, 7
CN segments	15
FDR fragment joints	0.84
FDR chr. breakp. enrich.	0
Linked to chrs	X:115283407−149711206;
Purity, ploidy	0.87, 6.21

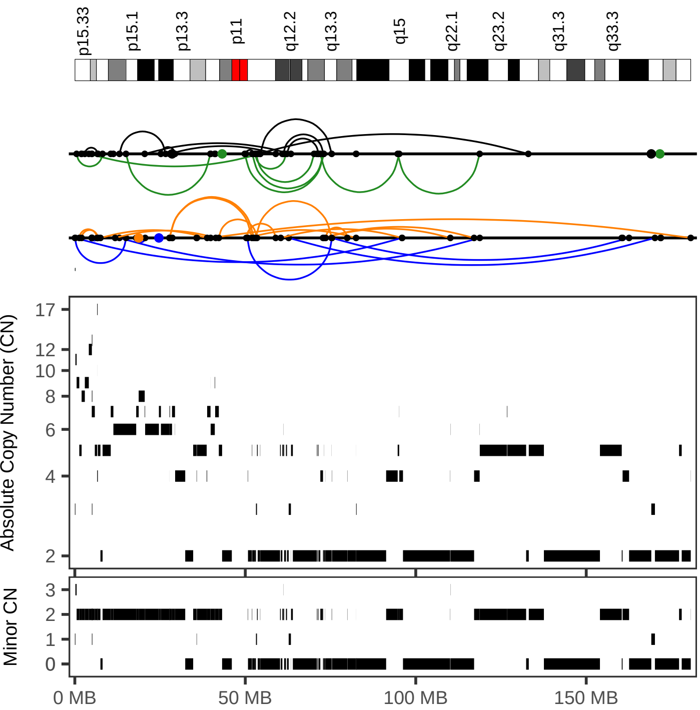

cd73a55f−03c1−4da0−9fa7−1ae4a263664e	
Cancer type	SoftTissue−Liposarc
Position	5:40979−180658104
Type	With other complex events
Interleaved intrachr. SVs	48
Total SVs (intrachr. + transl.)	54
SV types	DEL: 16; DUP: 9; h2hINV: 11; t2tINV: 12; TRA: 6
SVs in sample	595
Oscillating CN (2 and 3 states)	7, 21
CN segments	115
FDR fragment joints	0.64
FDR chr. breakp. enrich.	0
Linked to chrs	12:39672405−132182028;
Purity, ploidy	0.74, 3.24

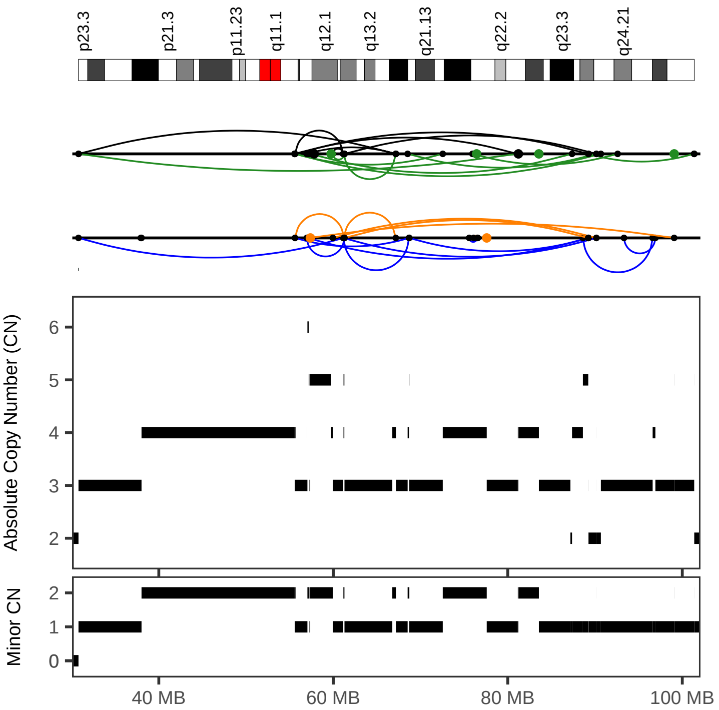

cd73a55f−03c1−4da0−9fa7−1ae4a263664e	
Cancer type	SoftTissue−Liposarc
Position	8:30791212−101358676
Type	With other complex events
Interleaved intrachr. SVs	35
Total SVs (intrachr. + transl.)	43
SV types	DEL: 6; DUP: 10; h2hINV: 8; t2tINV: 11; TRA: 8
SVs in sample	595
Oscillating CN (2 and 3 states)	7, 18
CN segments	49
FDR fragment joints	0.72
FDR chr. breakp. enrich.	0.01
Linked to chrs	12:39672405−132182028;
Purity, ploidy	0.74, 3.24

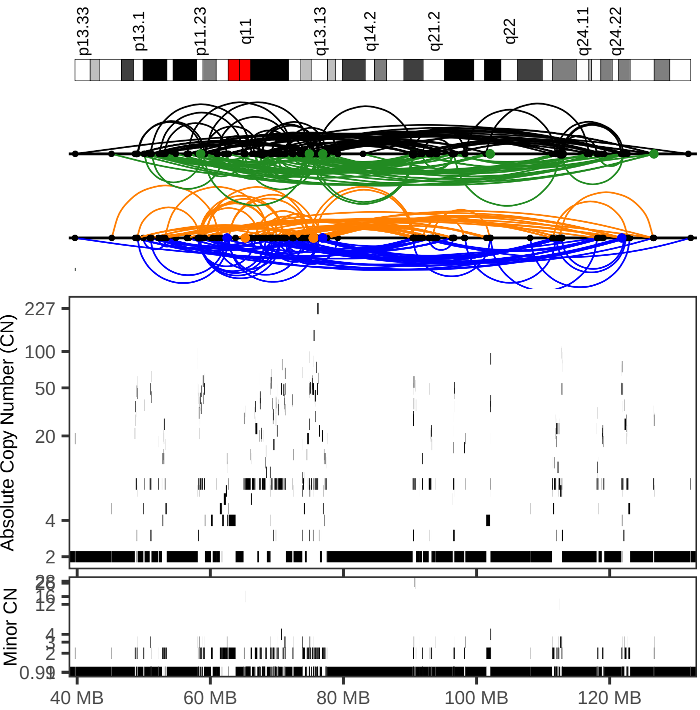

cd73a55f−03c1−4da0−9fa7−1ae4a263664e	
Cancer type	SoftTissue−Liposarc
Position	12:39672405−132182029
Type	With other complex events
Interleaved intrachr. SVs	376
Total SVs (intrachr. + transl.)	394
SV types	DEL: 90; DUP: 86; h2hINV: 99; t2tINV: 101; TRA: 18
SVs in sample	595
Oscillating CN (2 and 3 states)	8, 8
CN segments	516
FDR fragment joints	0.73
FDR chr. breakp. enrich.	0
Linked to chrs	5:40979−180658103;8:30791212−101358675 13:23586259−110740998;19:4529269−35019474
Purity, ploidy	0.74, 3.24

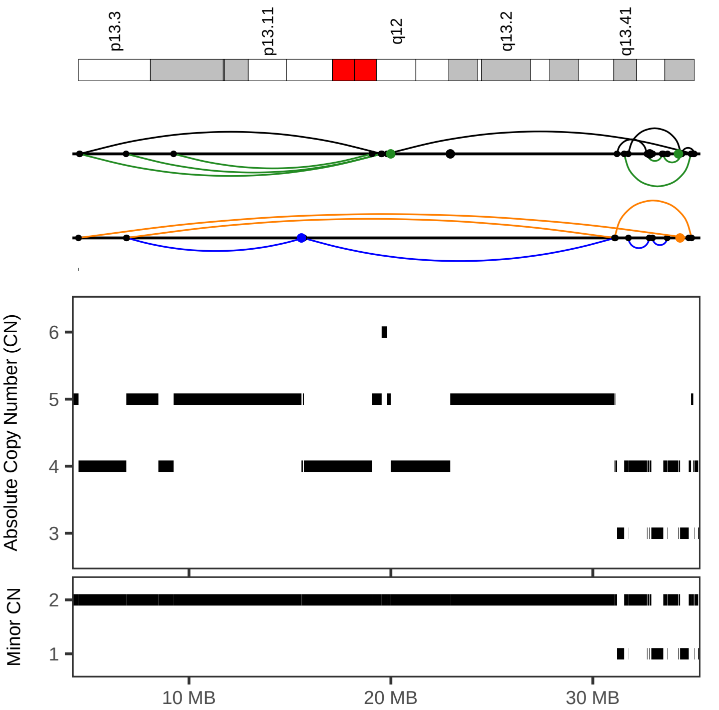

cd73a55f−03c1−4da0−9fa7−1ae4a263664e	
Cancer type	SoftTissue−Liposarc
Position	19:4529269−35019475
Type	With other complex events
Interleaved intrachr. SVs	21
Total SVs (intrachr. + transl.)	27
SV types	DEL: 3; DUP: 4; h2hINV: 6; t2tINV: 8; TRA: 6
SVs in sample	595
Oscillating CN (2 and 3 states)	17, 34
CN segments	34
FDR fragment joints	0.63
FDR chr. breakp. enrich.	0
Linked to chrs	3:116497225−116813983;12:39672405−132182028
Purity, ploidy	0.74, 3.24

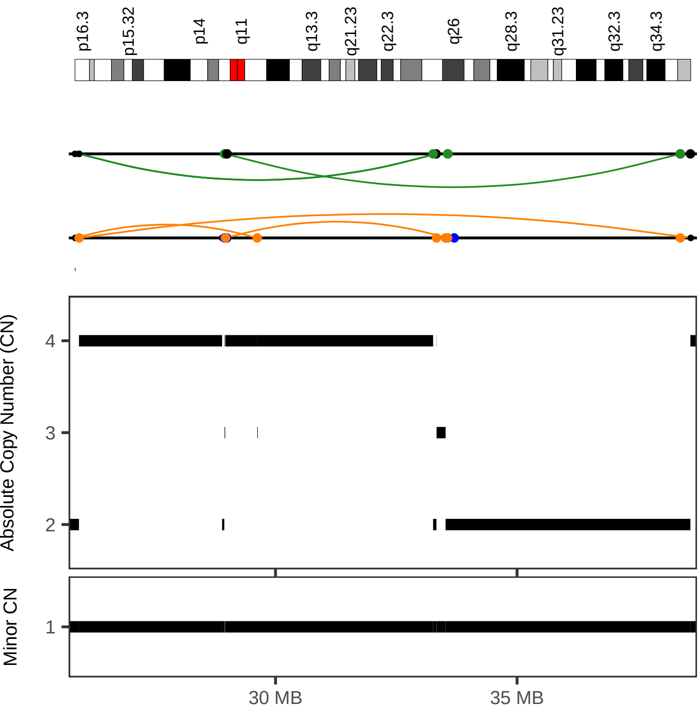

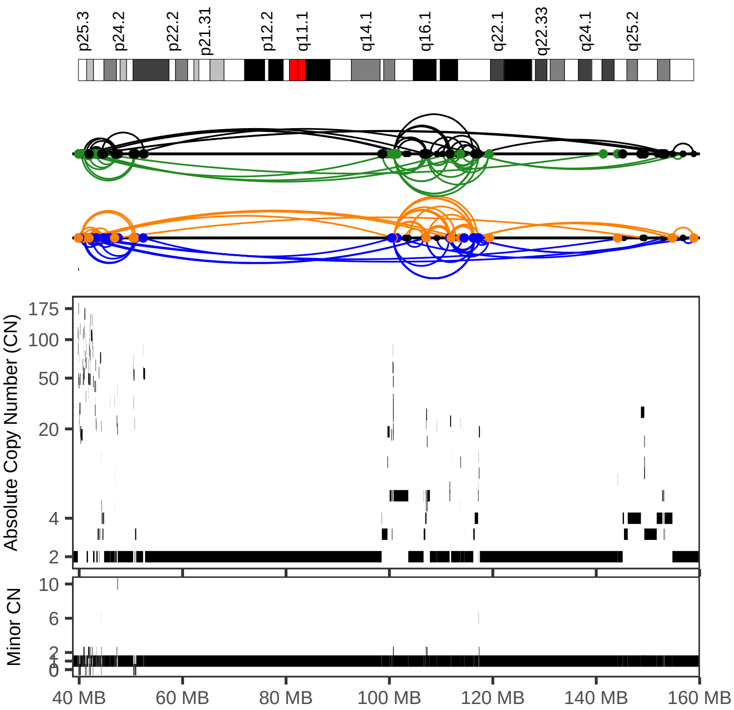

	e786de34−4c21−460f−89ab−008de4347049
Cancer type	SoftTissue−Liposarc
Position	4:25848307−38595195
Type	With other complex events
Interleaved intrachr. SVs	7
Total SVs (intrachr. + transl.)	31
SV types	DEL: 3; DUP: 0; h2hINV: 0; t2tINV: 4; TRA: 24
SVs in sample	872
Oscillating CN (2 and 3 states)	7, 13
CN segments	15
FDR fragment joints	0.48
FDR chr. breakp. enrich.	0.07
Linked to chrs	6:39847295−158859759;
Purity, ploidy	0.38, 2.37

	e786de34−4c21−460f−89ab−008de4347049
Cancer type	SoftTissue−Liposarc
Position	6:39847295−158859760
Type	With other complex events
Interleaved intrachr. SVs	197
Total SVs (intrachr. + transl.)	494
SV types	DEL: 55; DUP: 48; h2hINV: 48; t2tINV: 46; TRA: 297
SVs in sample	872
Oscillating CN (2 and 3 states)	5, 6
CN segments	213
FDR fragment joints	0.86
FDR chr. breakp. enrich.	0
Linked to chrs	4:25848307−38595194;
Purity, ploidy	0.38, 2.37

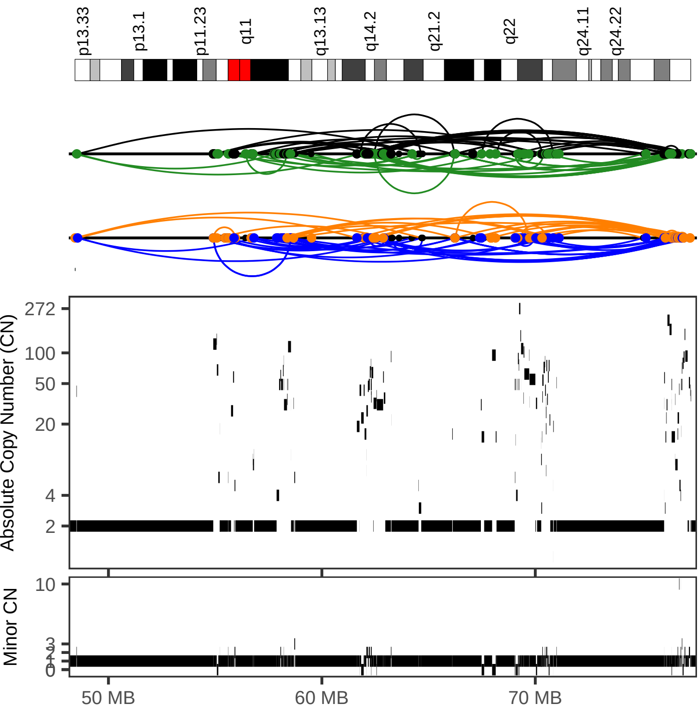

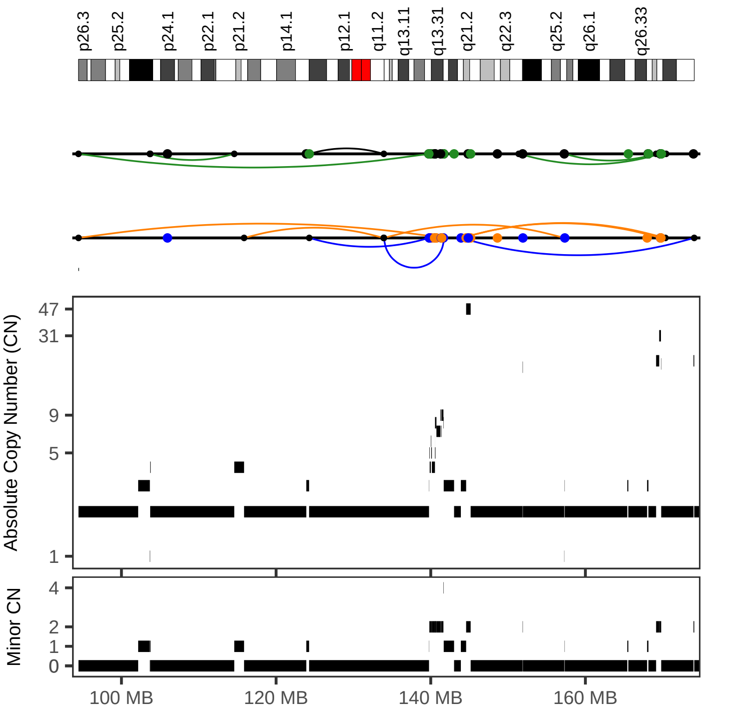

	e786de34−4c21−460f−89ab−008de4347049
Cancer type	SoftTissue−Liposarc
Position	12:48431354−77284404
Type	With other complex events
Interleaved intrachr. SVs	155
Total SVs (intrachr. + transl.)	448
SV types	DEL: 34; DUP: 42; h2hINV: 45; t2tINV: 34; TRA: 293
SVs in sample	872
Oscillating CN (2 and 3 states)	3, 4
CN segments	171
FDR fragment joints	0.64
FDR chr. breakp. enrich.	0
Linked to chrs	4:25848307−38595194;6:39847295−158859759
Purity, ploidy	0.38, 2.37

	e7a72f7e−77b1−4d95−8f37−39a6cffc35dc
Cancer type	SoftTissue−Liposarc
Position	3:94442780−174075990
Type	With other complex events
Interleaved intrachr. SVs	14
Total SVs (intrachr. + transl.)	69
SV types	DEL: 5; DUP: 3; h2hINV: 3; t2tINV: 3; TRA: 55
SVs in sample	696
Oscillating CN (2 and 3 states)	7, 13
CN segments	50
FDR fragment joints	0.88
FDR chr. breakp. enrich.	0.01
Linked to chrs	11:993833−37433475;5:1295334−20553338 6:42555034−48239598;
Purity, ploidy	0.62, 3.8

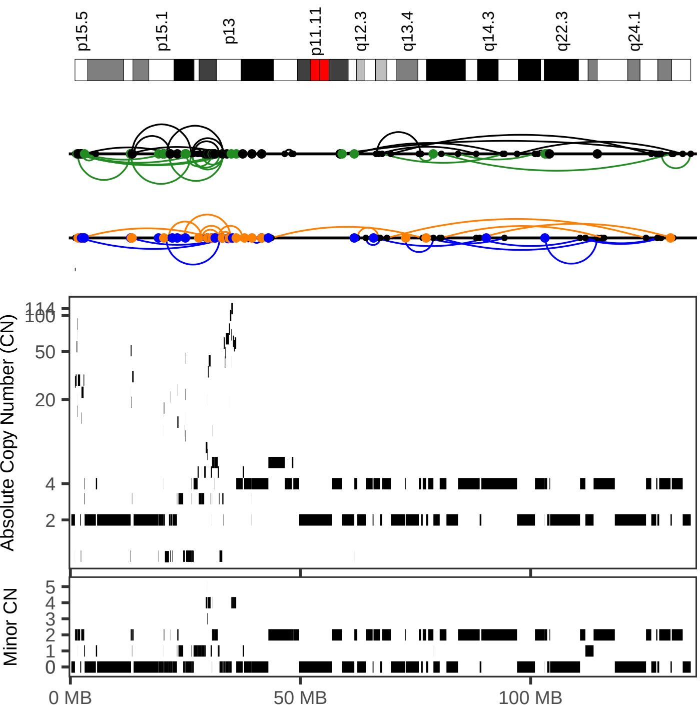

e7a72f7e−77b1−4d95−8f37−39a6cffc35dc

Cancer type	SoftTissue−Liposarc
Position	11:993833−37433476
Type	With other complex events
Interleaved intrachr. SVs	55
Total SVs (intrachr. + transl.)	138
SV types	DEL: 17; DUP: 6; h2hINV: 15;<br>t2tINV: 17; TRA: 83
SVs in sample	696
Oscillating CN (2 and 3 states)	8, 8
CN segments	103
FDR fragment joints	0.48
FDR chr. breakp. enrich.	0
Linked to chrs	3:94442780−174075989;5:1295334−20553338<br>6:42555034−48239598;
Purity, ploidy	0.62, 3.8

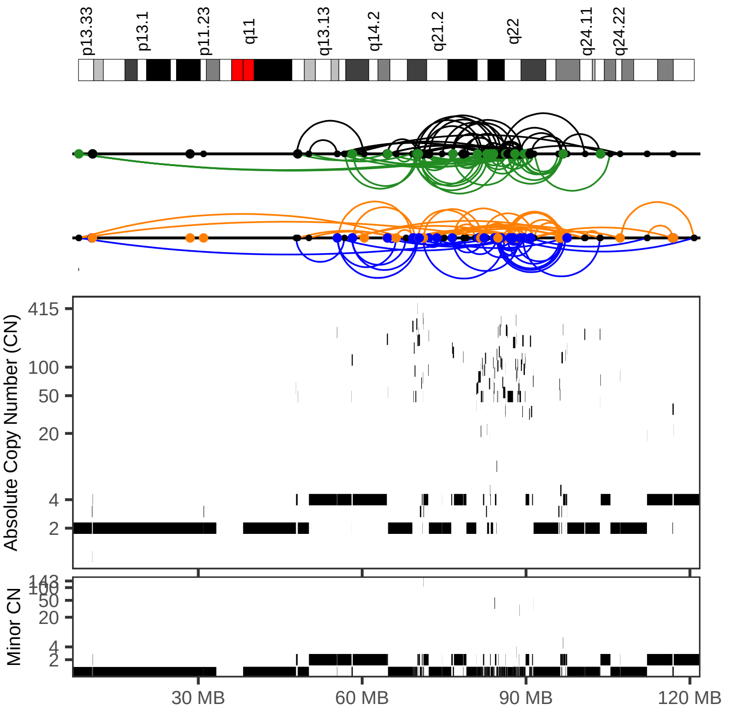

e7a72f7e−77b1−4d95−8f37−39a6cffc35dc

Cancer type	SoftTissue−Liposarc
Position	12:8061289−120789609
Type	With other complex events
Interleaved intrachr. SVs	195
Total SVs (intrachr. + transl.)	354
SV types	DEL: 46; DUP: 42; h2hINV: 49;<br>t2tINV: 58; TRA: 159
SVs in sample	696
Oscillating CN (2 and 3 states)	4, 8
CN segments	174
FDR fragment joints	0.62
FDR chr. breakp. enrich.	0
Linked to chrs	11:993833−37433475;21:35308379−35766982<br>3:94442780−174075989;6:42555034−48239598
Purity, ploidy	0.62, 3.8

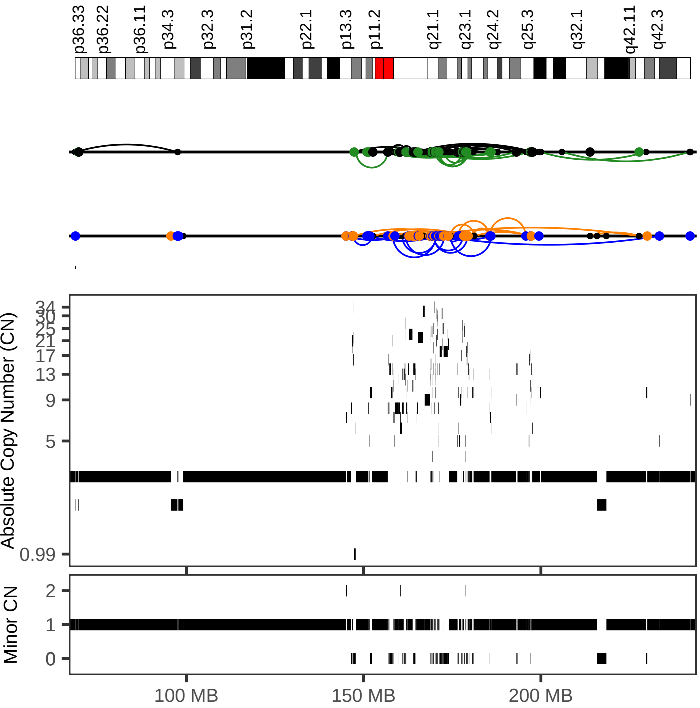

ea0e5ae7−1f15−4504−aea3−efd1f6733304

Cancer type	SoftTissue−Liposarc
Position	1:145025792−241989428
Type	With other complex events
Interleaved intrachr. SVs	66
Total SVs (intrachr. + transl.)	182
SV types	DEL: 14; DUP: 16; h2hINV: 16;<br>t2tINV: 20; TRA: 116
SVs in sample	595
Oscillating CN (2 and 3 states)	5, 6
CN segments	228
FDR fragment joints	0.84
FDR chr. breakp. enrich.	0
Linked to chrs	12:8047626−76351791;
Purity, ploidy	0.44, 3.26

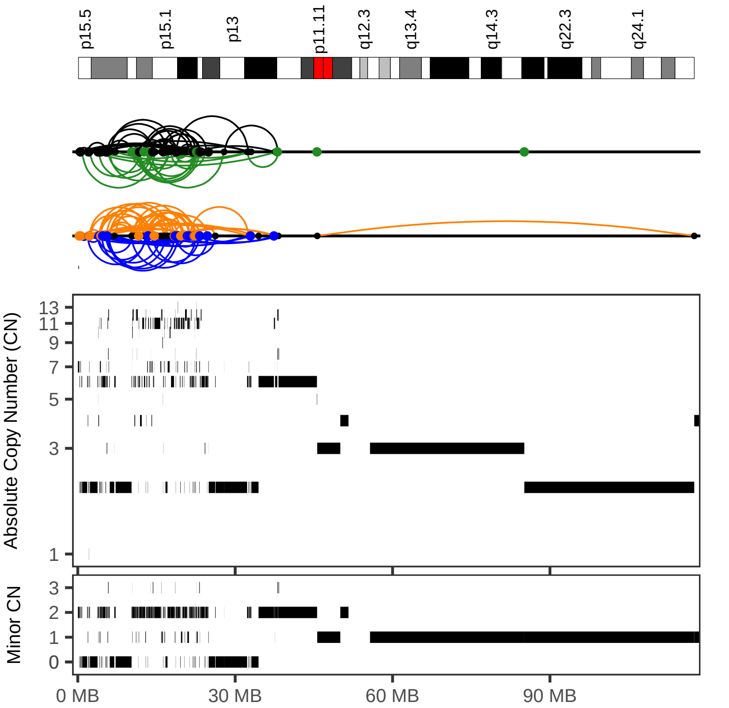

ea0e5ae7−1f15−4504−aea3−efd1f6733304

Cancer type	SoftTissue−Liposarc
Position	11:132789−38263176
Type	With other complex events
Interleaved intrachr. SVs	134
Total SVs (intrachr. + transl.)	184
SV types	DEL: 39; DUP: 33; h2hINV: 34;<br>t2tINV: 28; TRA: 50
SVs in sample	595
Oscillating CN (2 and 3 states)	8, 25
CN segments	268
FDR fragment joints	0.68
FDR chr. breakp. enrich.	0
Linked to chrs	
Purity, ploidy	0.44, 3.26

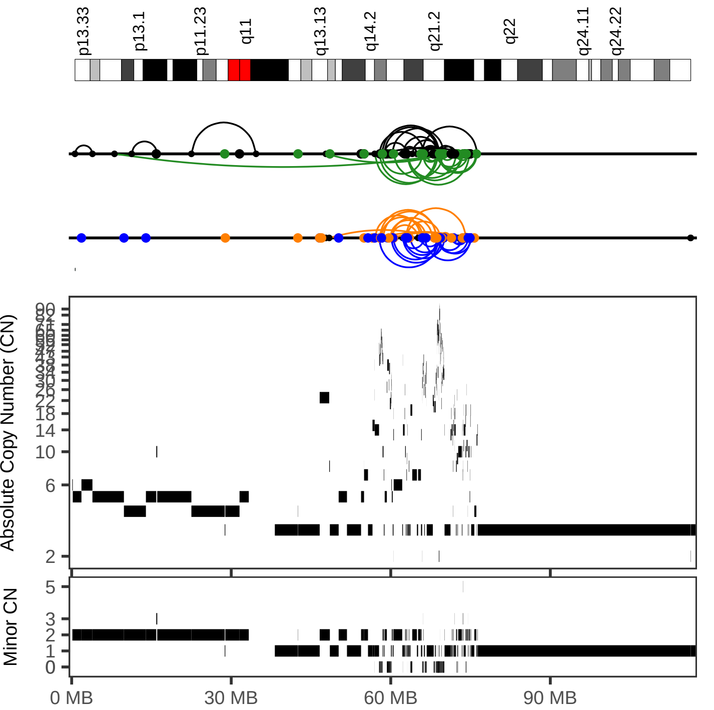

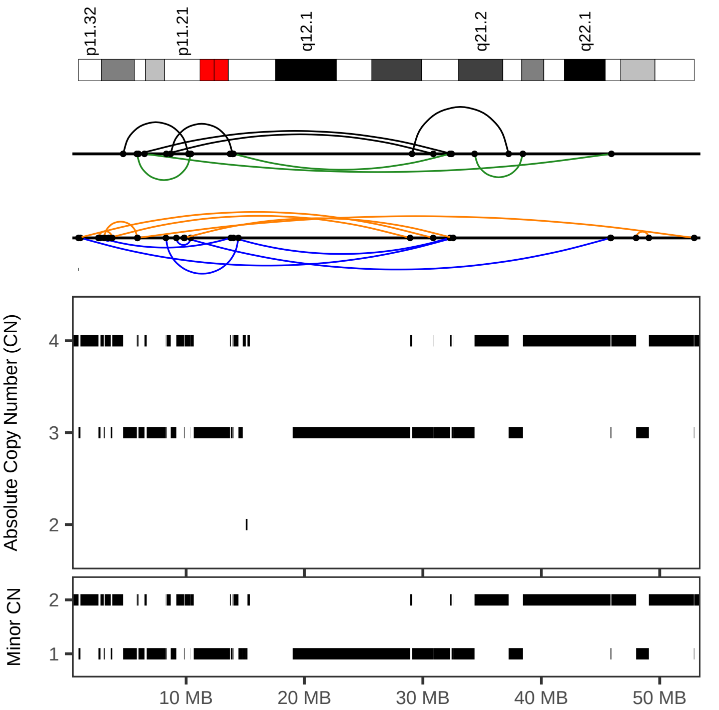

	ea0e5ae7−1f15−4504−aea3−efd1f6733304
Cancer type	SoftTissue−Liposarc
Position	12:8047626−76351792
Type	With other complex events
Interleaved intrachr. SVs	81
Total SVs (intrachr. + transl.)	202
SV types	DEL: 20; DUP: 21; h2hINV: 18; t2tINV: 22; TRA: 121
SVs in sample	595
Oscillating CN (2 and 3 states)	6, 7
CN segments	250
FDR fragment joints	0.95
FDR chr. breakp. enrich.	0
Linked to chrs	
Purity, ploidy	0.44, 3.26

	ea0e5ae7−1f15−4504−aea3−efd1f6733304
Cancer type	SoftTissue−Liposarc
Position	18:915255−52915207
Type	After polyploidization
Interleaved intrachr. SVs	23
Total SVs (intrachr. + transl.)	23
SV types	DEL: 6; DUP: 7; h2hINV: 5; t2tINV: 5; TRA: 0
SVs in sample	595
Oscillating CN (2 and 3 states)	32, 52
CN segments	52
FDR fragment joints	0.94
FDR chr. breakp. enrich.	0.07
Linked to chrs	
Purity, ploidy	0.44, 3.26

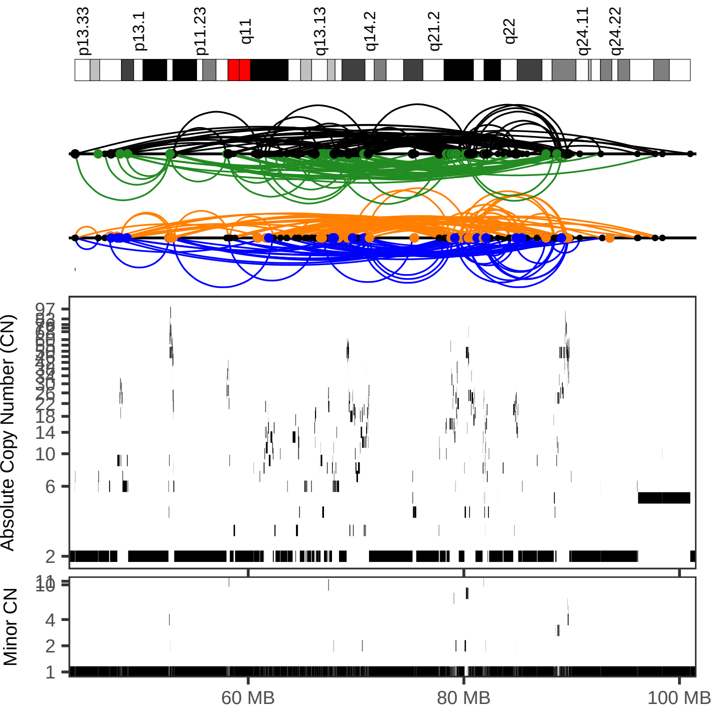

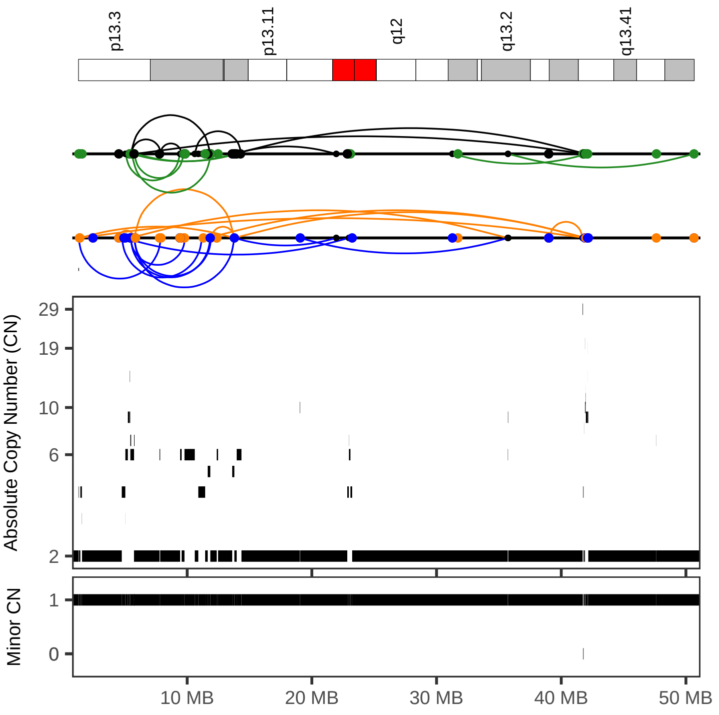

	f75d2589−fd7b−49a1−a1c8−0b4726b81f3c
Cancer type	SoftTissue−Liposarc
Position	12:43926078−100997188
Type	With other complex events
Interleaved intrachr. SVs	336
Total SVs (intrachr. + transl.)	484
SV types	DEL: 88; DUP: 80; h2hINV: 91; t2tINV: 77; TRA: 148
SVs in sample	562
Oscillating CN (2 and 3 states)	7, 8
CN segments	441
FDR fragment joints	0.75
FDR chr. breakp. enrich.	0
Linked to chrs	19:1283296−50665824;
Purity, ploidy	0.9, 2.01

	f75d2589−fd7b−49a1−a1c8−0b4726b81f3c
Cancer type	SoftTissue−Liposarc
Position	19:1283296−50665825
Type	With other complex events
Interleaved intrachr. SVs	37
Total SVs (intrachr. + transl.)	135
SV types	DEL: 9; DUP: 10; h2hINV: 8; t2tINV: 10; TRA: 98
SVs in sample	562
Oscillating CN (2 and 3 states)	7, 13
CN segments	65
FDR fragment joints	0.97
FDR chr. breakp. enrich.	0
Linked to chrs	12:43926078−100997187;
Purity, ploidy	0.9, 2.01

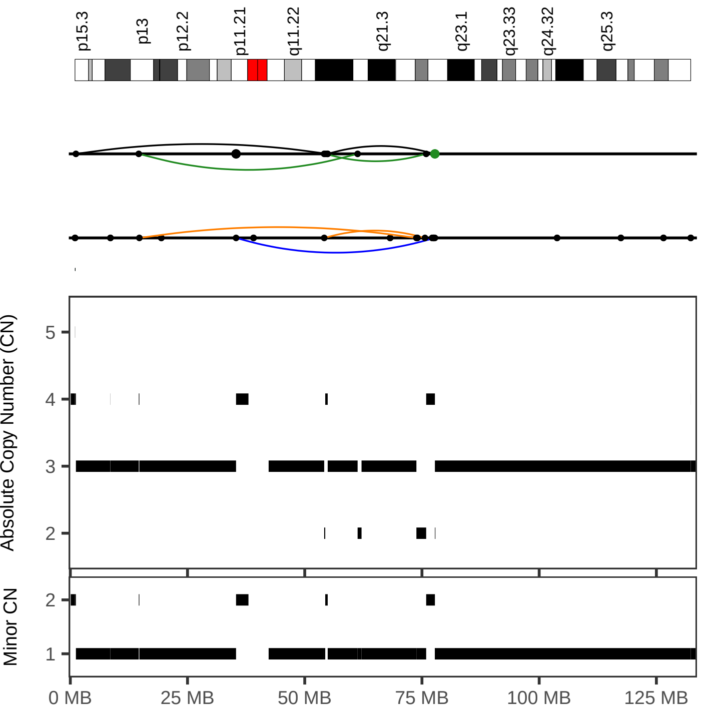

	CGP_donor_GC00015
Cancer type	Stomach−AdenoCA
Position	10:1196201−77760857
Type	With other complex events
Interleaved intrachr. SVs	8
Total SVs (intrachr. + transl.)	10
SV types	DEL: 2; DUP: 1; h2hINV: 2; t2tINV: 3; TRA: 2
SVs in sample	170
Oscillating CN (2 and 3 states)	7, 7
CN segments	16
FDR fragment joints	0.84
FDR chr. breakp. enrich.	0
Linked to chrs	
Purity, ploidy	0.61, 2.62

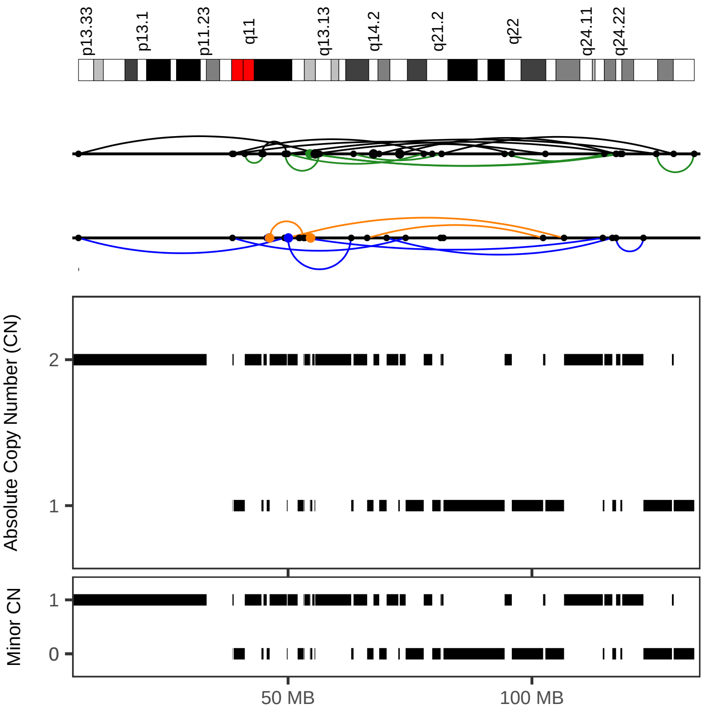

	CGP_donor_GC00017
Cancer type	Stomach−AdenoCA
Position	12:7028514−133278608
Type	Canonical without polyploidization
Interleaved intrachr. SVs	30
Total SVs (intrachr. + transl.)	39
SV types	DEL: 6; DUP: 7; h2hINV: 9; t2tINV: 8; TRA: 9
SVs in sample	115
Oscillating CN (2 and 3 states)	50, 50
CN segments	50
FDR fragment joints	0.91
FDR chr. breakp. enrich.	0
Linked to chrs	
Purity, ploidy	0.32, 2.17

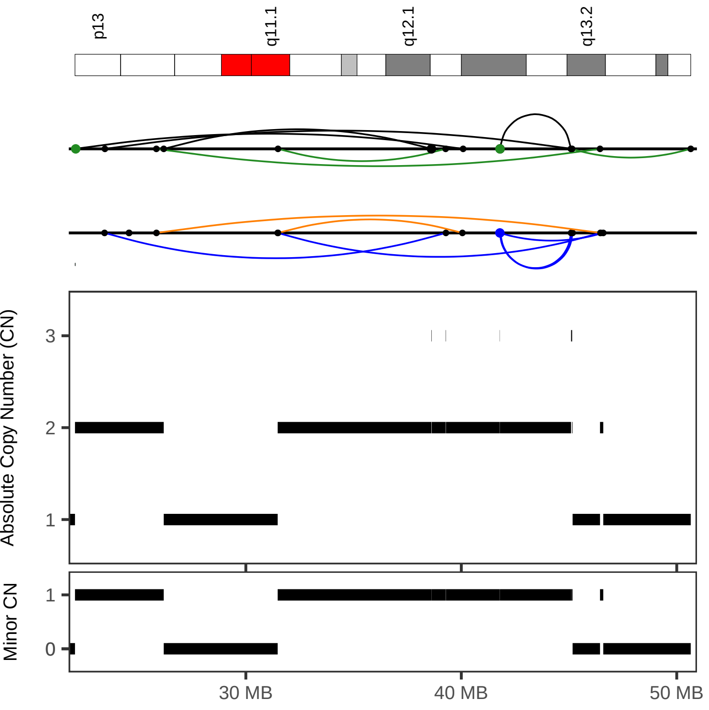

	CGP_donor_GC00017
Cancer type	Stomach−AdenoCA
Position	22:22068632−50649237
Type	Canonical without polyploidization
Interleaved intrachr. SVs	15
Total SVs (intrachr. + transl.)	20
SV types	DEL: 2; DUP: 5; h2hINV: 4; t2tINV: 4; TRA: 5
SVs in sample	115
Oscillating CN (2 and 3 states)	9, 14
CN segments	14
FDR fragment joints	0.82
FDR chr. breakp. enrich.	0
Linked to chrs	
Purity, ploidy	0.32, 2.17

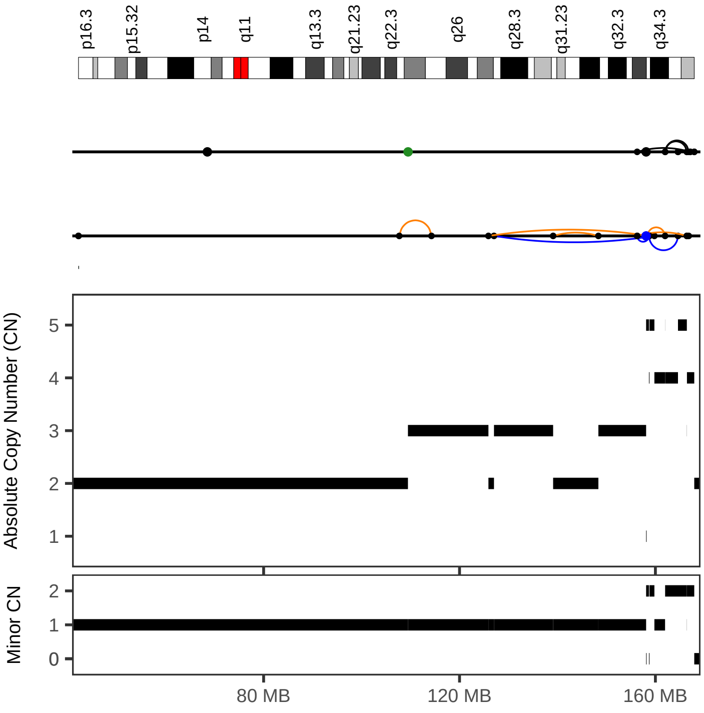

	CGP_donor_GC00029
Cancer type	Stomach−AdenoCA
Position	4:125902490−167111168
Type	With other complex events
Interleaved intrachr. SVs	11
Total SVs (intrachr. + transl.)	13
SV types	DEL: 4; DUP: 3; h2hINV: 3; t2tINV: 1; TRA: 2
SVs in sample	246
Oscillating CN (2 and 3 states)	7, 9
CN segments	14
FDR fragment joints	0.71
FDR chr. breakp. enrich.	0.37
Linked to chrs	6:8337578−165204117;
Purity, ploidy	0.53, 3.33

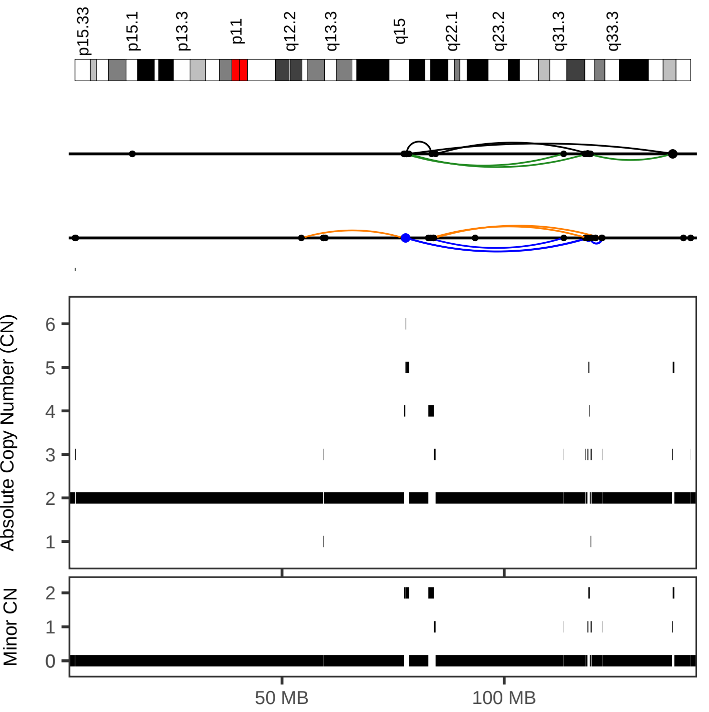

CGP_donor_GC00029	
Cancer type	Stomach−AdenoCA
Position	5:54358154−138271539
Type	With other complex events
Interleaved intrachr. SVs	19
Total SVs (intrachr. + transl.)	21
SV types	DEL: 3; DUP: 6; h2hINV: 6; t2tINV: 4; TRA: 2
SVs in sample	246
Oscillating CN (2 and 3 states)	7, 7
CN segments	29
FDR fragment joints	0.78
FDR chr. breakp. enrich.	0
Linked to chrs	8:112366160−131135554;
Purity, ploidy	0.53, 3.33

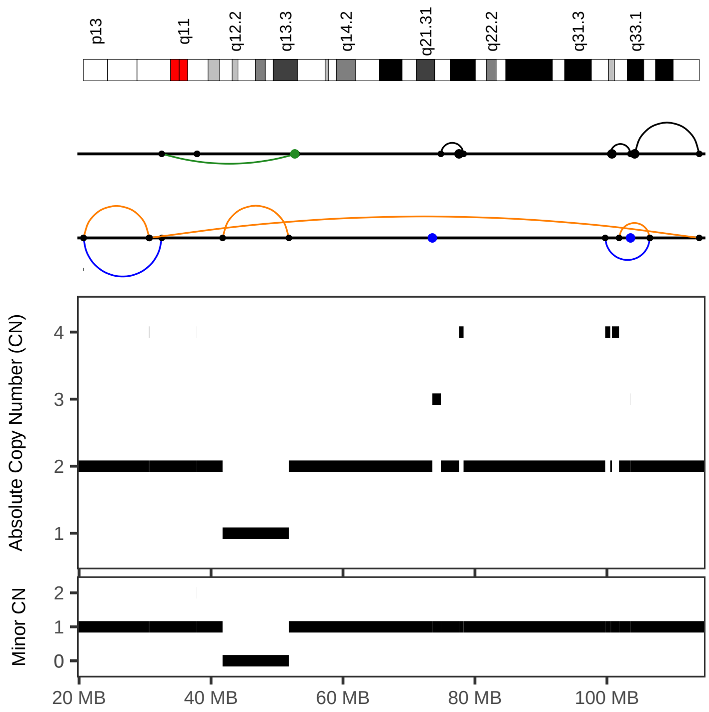

CGP_donor_GC00047	
Cancer type	Stomach−AdenoCA
Position	13:20671166−113982594
Type	With other complex events
Interleaved intrachr. SVs	7
Total SVs (intrachr. + transl.)	13
SV types	DEL: 2; DUP: 2; h2hINV: 2; t2tINV: 1; TRA: 6
SVs in sample	187
Oscillating CN (2 and 3 states)	7, 11
CN segments	17
FDR fragment joints	0.95
FDR chr. breakp. enrich.	0
Linked to chrs	
Purity, ploidy	0.38, 2.11

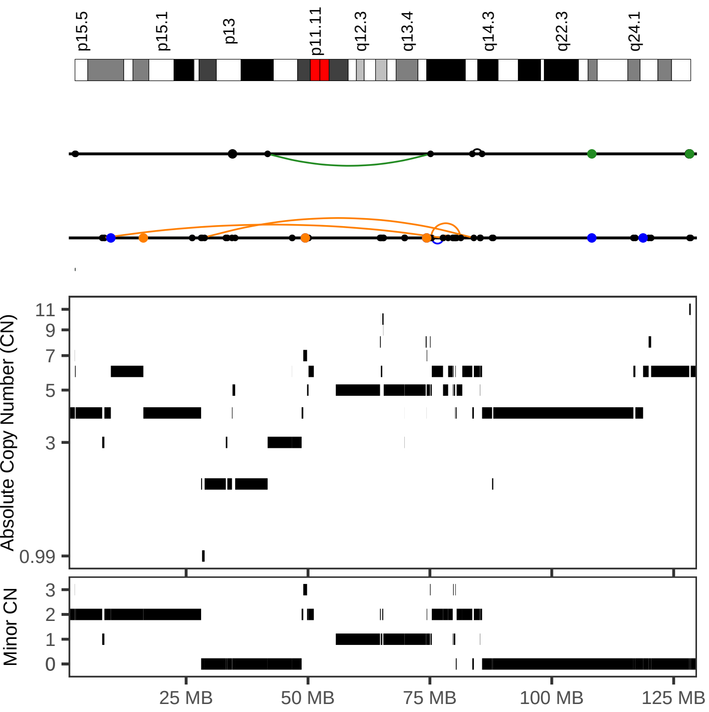

CGP_donor_GC00054	
Cancer type	Stomach−AdenoCA
Position	11:7783797−85700498
Type	With other complex events
Interleaved intrachr. SVs	9
Total SVs (intrachr. + transl.)	16
SV types	DEL: 5; DUP: 2; h2hINV: 1; t2tINV: 1; TRA: 7
SVs in sample	550
Oscillating CN (2 and 3 states)	7, 7
CN segments	52
FDR fragment joints	0.59
FDR chr. breakp. enrich.	0
Linked to chrs	
Purity, ploidy	0.34, 4.27

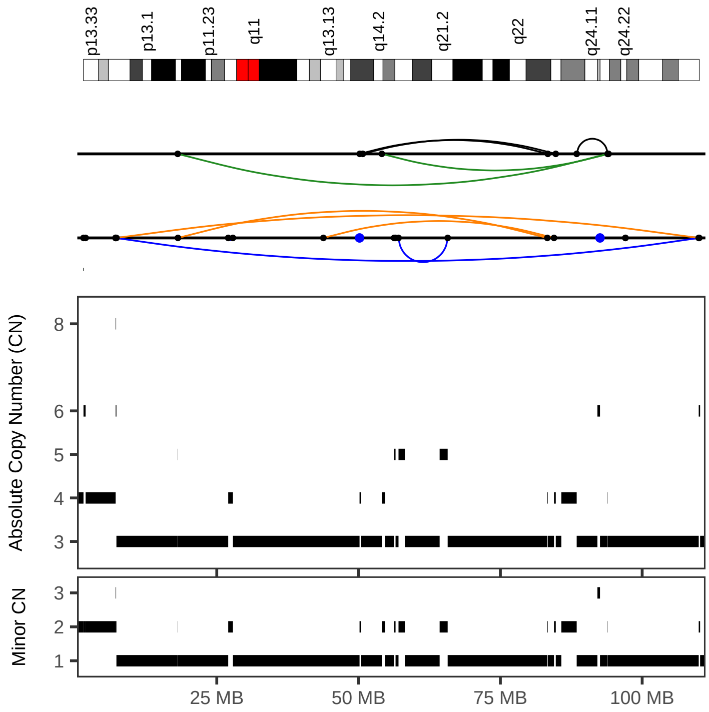

CGP_donor_GC00054	
Cancer type	Stomach−AdenoCA
Position	12:18101997−94069586
Type	With other complex events
Interleaved intrachr. SVs	8
Total SVs (intrachr. + transl.)	10
SV types	DEL: 2; DUP: 1; h2hINV: 3; t2tINV: 2; TRA: 2
SVs in sample	550
Oscillating CN (2 and 3 states)	7, 20
CN segments	24
FDR fragment joints	0.84
FDR chr. breakp. enrich.	0.41
Linked to chrs	
Purity, ploidy	0.34, 4.27

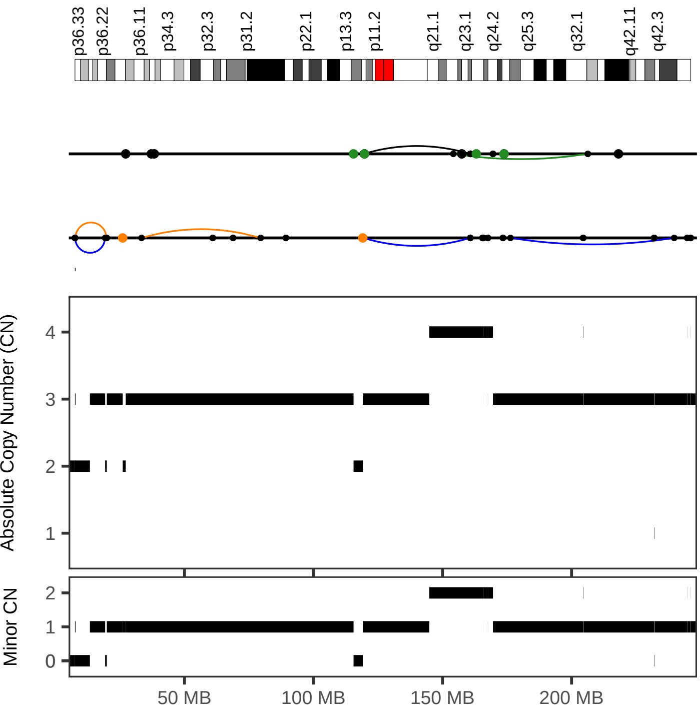

	647dc54f−a51b−40ea−9d97−c93598c2af71
Cancer type	Stomach−AdenoCA
Position	1:119235280−239786526
Type	After polyploidization
Interleaved intrachr. SVs	4
Total SVs (intrachr. + transl.)	11
SV types	DEL: 0; DUP: 2; h2hINV: 1; t2tINV: 1; TRA: 7
SVs in sample	294
Oscillating CN (2 and 3 states)	9, 11
CN segments	11
FDR fragment joints	0.64
FDR chr. breakp. enrich.	0
Linked to chrs	11:2708193−95973109;
Purity, ploidy	0.75, 2.88

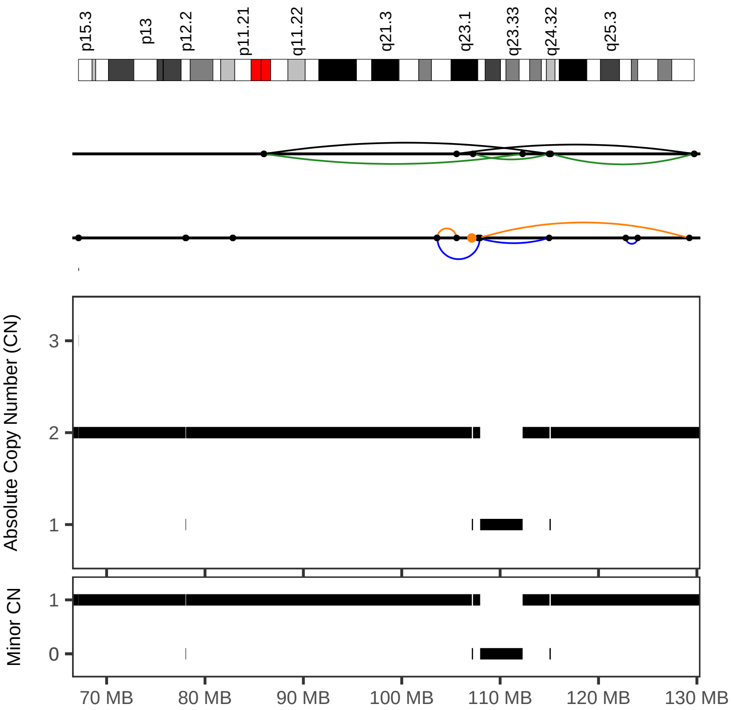

	87c217d4−66f4−46ca−8244−7856ce658fd3
Cancer type	Stomach−AdenoCA
Position	10:85982825−129728054
Type	Canonical without polyploidization
Interleaved intrachr. SVs	9
Total SVs (intrachr. + transl.)	10
SV types	DEL: 2; DUP: 2; h2hINV: 2; t2tINV: 3; TRA: 1
SVs in sample	85
Oscillating CN (2 and 3 states)	7, 7
CN segments	7
FDR fragment joints	0.97
FDR chr. breakp. enrich.	0
Linked to chrs	
Purity, ploidy	0.66, 1.71

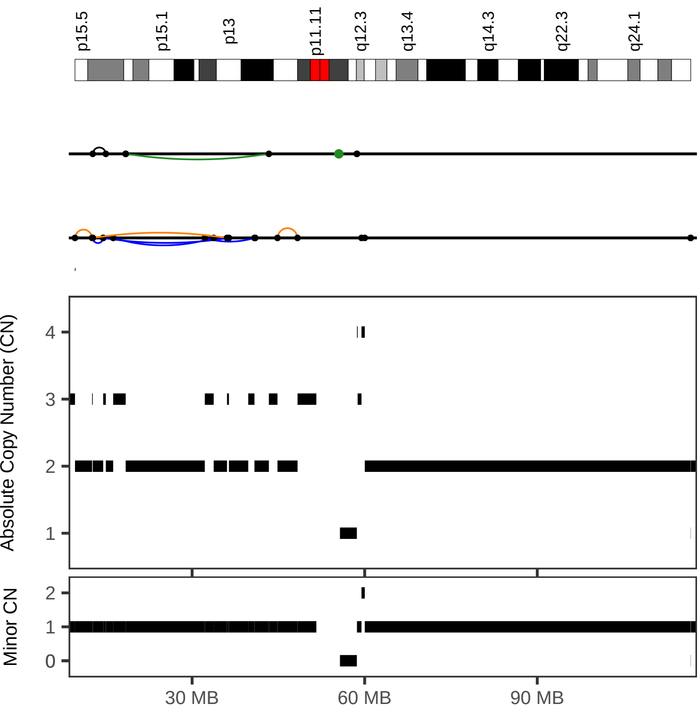

	8b5746f9−dbee−40bd−9141−1081960cf286
Cancer type	Stomach−AdenoCA
Position	11:12725196−43348618
Type	Canonical without polyploidization
Interleaved intrachr. SVs	8
Total SVs (intrachr. + transl.)	8
SV types	DEL: 1; DUP: 4; h2hINV: 2; t2tINV: 1; TRA: 0
SVs in sample	363
Oscillating CN (2 and 3 states)	13, 13
CN segments	13
FDR fragment joints	0.59
FDR chr. breakp. enrich.	1
Linked to chrs	
Purity, ploidy	0.27, 2.91

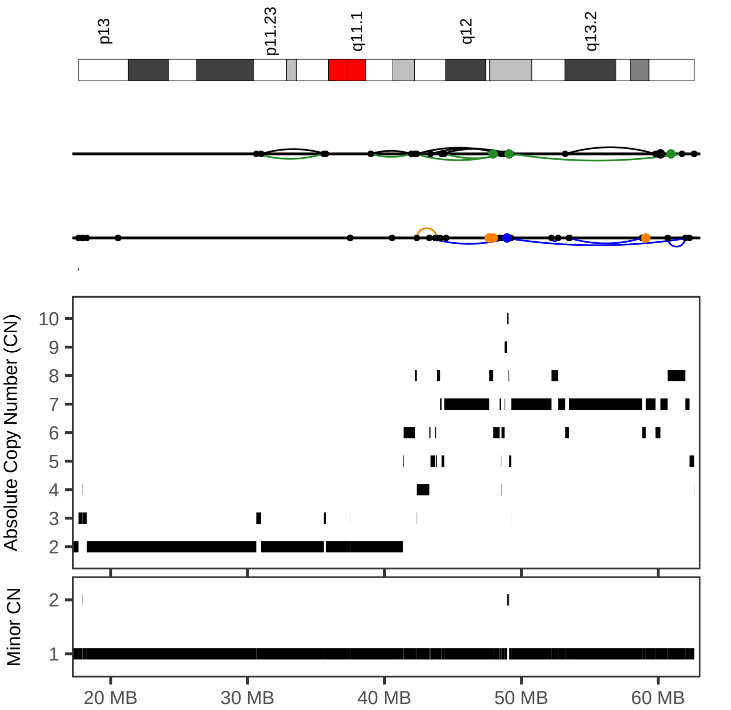

	8cb0144b−be6b−40a1−86a2−708f96d9b615
Cancer type	Stomach−AdenoCA
Position	20:42226186−62284371
Type	With other complex events
Interleaved intrachr. SVs	10
Total SVs (intrachr. + transl.)	18
SV types	DEL: 1; DUP: 4; h2hINV: 3; t2tINV: 2; TRA: 8
SVs in sample	507
Oscillating CN (2 and 3 states)	9, 9
CN segments	40
FDR fragment joints	0.64
FDR chr. breakp. enrich.	0
Linked to chrs	7:86655436−99825257;
Purity, ploidy	0.88, 1.89

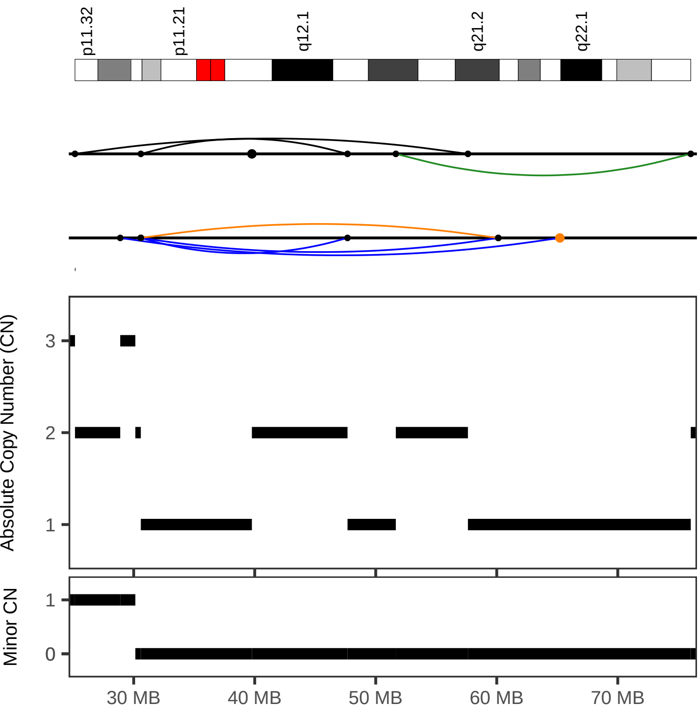

8d3e3d09−bc61−4d5e−b8d6−5aee6cb488ae	
Cancer type	Stomach−AdenoCA
Position	18:25148058−76030080
Type	Canonical without polyploidization
Interleaved intrachr. SVs	5
Total SVs (intrachr. + transl.)	7
SV types	DEL: 1; DUP: 2; h2hINV: 1; t2tINV: 1; TRA: 2
SVs in sample	42
Oscillating CN (2 and 3 states)	7, 9
CN segments	9
FDR fragment joints	0.92
FDR chr. breakp. enrich.	0
Linked to chrs	
Purity, ploidy	0.69, 1.97

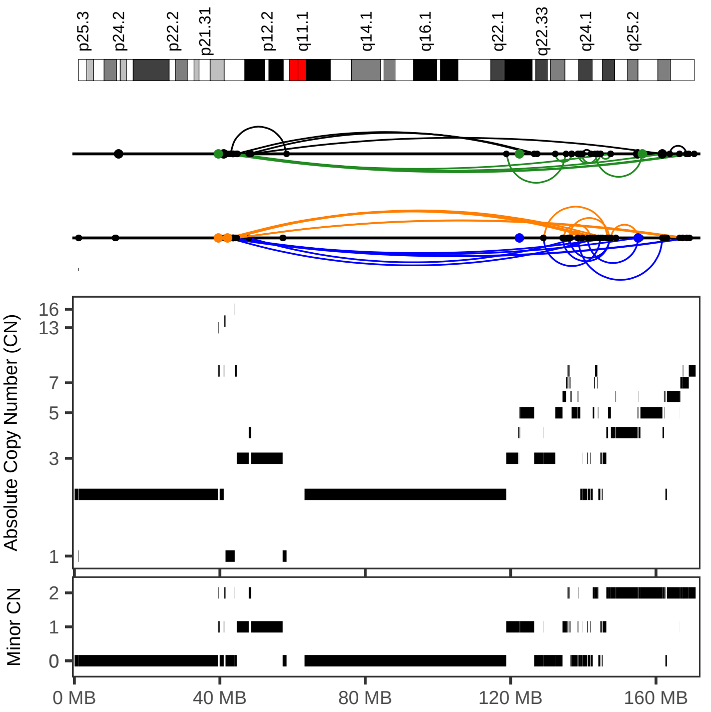

b8a1c8c7−3945−4d84−8f58−3a1a1eb10730	
Cancer type	Stomach−AdenoCA
Position	6:39514580−170510916
Type	With other complex events
Interleaved intrachr. SVs	56
Total SVs (intrachr. + transl.)	66
SV types	DEL: 17; DUP: 16; h2hINV: 10; t2tINV: 13; TRA: 10
SVs in sample	298
Oscillating CN (2 and 3 states)	7, 13
CN segments	80
FDR fragment joints	0.64
FDR chr. breakp. enrich.	0
Linked to chrs	9:74822475−138742365;
Purity, ploidy	0.49, 3.06

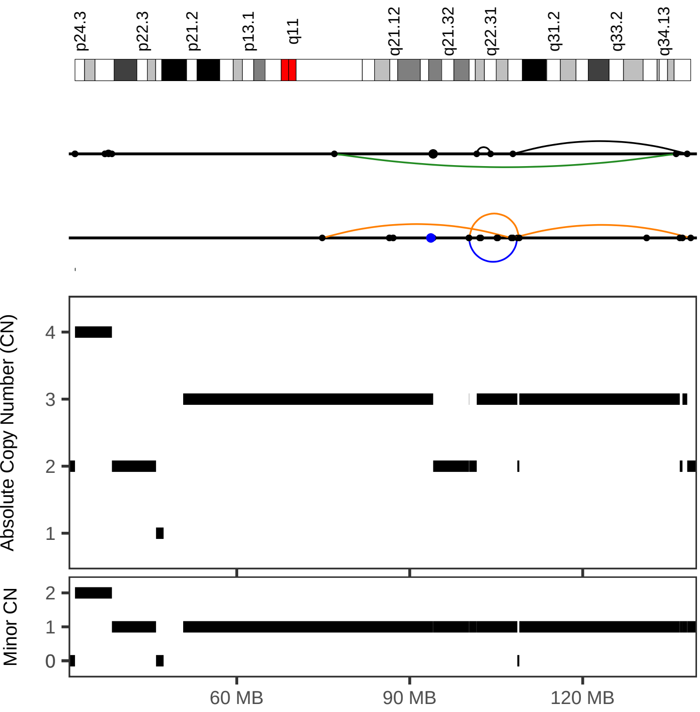

b8a1c8c7−3945−4d84−8f58−3a1a1eb10730	
Cancer type	Stomach−AdenoCA
Position	9:74822475−138742366
Type	Canonical without polyploidization
Interleaved intrachr. SVs	6
Total SVs (intrachr. + transl.)	8
SV types	DEL: 3; DUP: 1; h2hINV: 1; t2tINV: 1; TRA: 2
SVs in sample	298
Oscillating CN (2 and 3 states)	10, 10
CN segments	10
FDR fragment joints	0.64
FDR chr. breakp. enrich.	0.13
Linked to chrs	6:39514580−170510915;
Purity, ploidy	0.49, 3.06

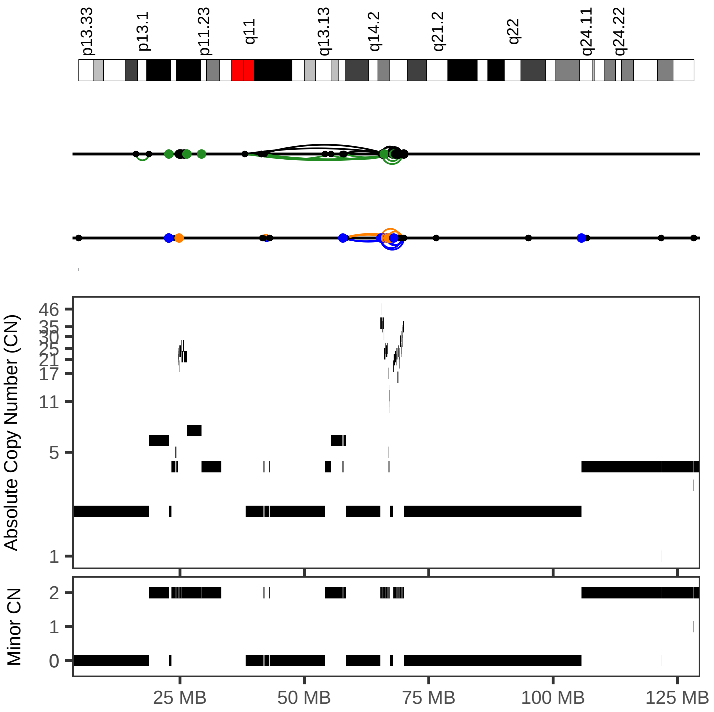

e37cae9e−0656−4b89−b326−739a5cc859e0	
Cancer type	Stomach−AdenoCA
Position	12:38025321−70035232
Type	With other complex events
Interleaved intrachr. SVs	49
Total SVs (intrachr. + transl.)	67
SV types	DEL: 7; DUP: 9; h2hINV: 16; t2tINV: 17; TRA: 18
SVs in sample	303
Oscillating CN (2 and 3 states)	7, 8
CN segments	61
FDR fragment joints	0.48
FDR chr. breakp. enrich.	0
Linked to chrs	
Purity, ploidy	0.74, 3.26

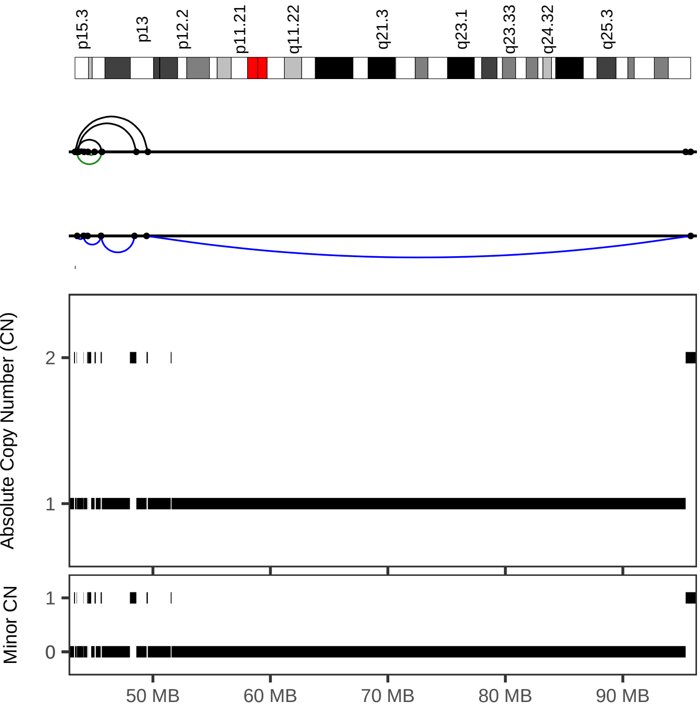

	085ffcdc−8600−42e2−852a−506465f716a2
Cancer type	Thy−AdenoCA
Position	10:43499619−48588565
Type	Canonical without polyploidization
Interleaved intrachr. SVs	8
Total SVs (intrachr. + transl.)	8
SV types	DEL: 0; DUP: 4; h2hINV: 3; t2tINV: 1; TRA: 0
SVs in sample	22
Oscillating CN (2 and 3 states)	16, 16
CN segments	16
FDR fragment joints	0.59
FDR chr. breakp. enrich.	0
Linked to chrs	
Purity, ploidy	0.75, 1.98

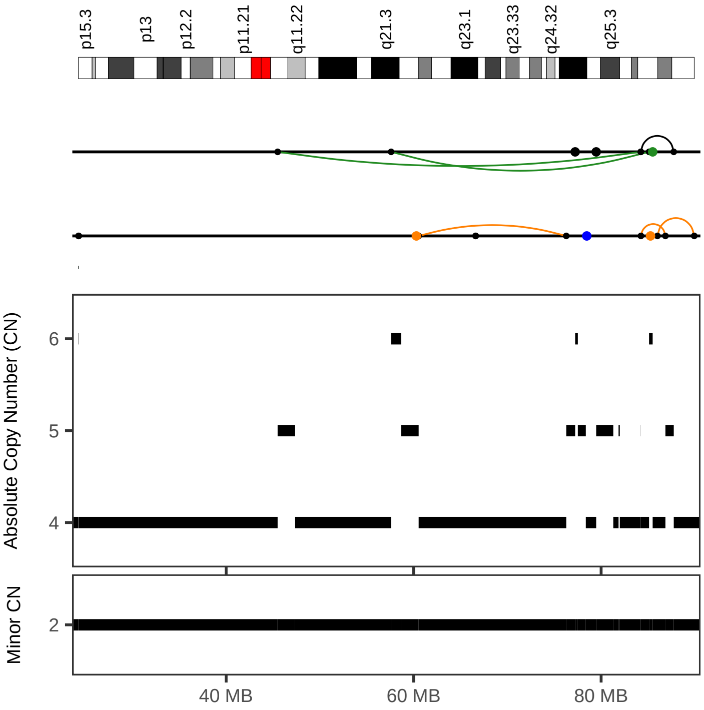

	24118b8f−59fe−482a−aed3−7b1bde1571b7
Cancer type	Uterus−AdenoCA
Position	10:45492675−89934330
Type	With other complex events
Interleaved intrachr. SVs	5
Total SVs (intrachr. + transl.)	11
SV types	DEL: 2; DUP: 0; h2hINV: 1; t2tINV: 2; TRA: 6
SVs in sample	152
Oscillating CN (2 and 3 states)	8, 12
CN segments	19
FDR fragment joints	0.64
FDR chr. breakp. enrich.	0.02
Linked to chrs	1:8226155−101008006;18:3979871−48656506
Purity, ploidy	0.65, 3.57

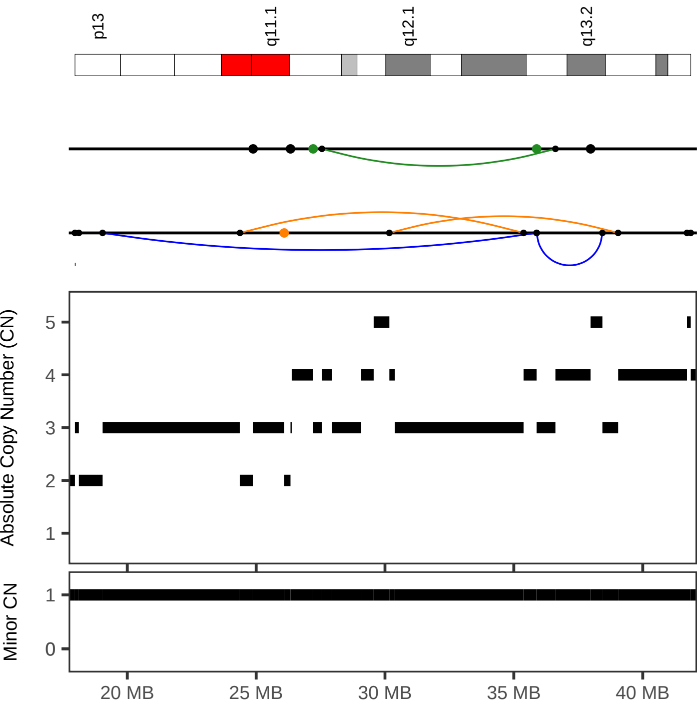

	8d9e4917−334b−4c76−aee1−1e22be772db0
Cancer type	Uterus−AdenoCA
Position	22:19039995−39045748
Type	With other complex events
Interleaved intrachr. SVs	5
Total SVs (intrachr. + transl.)	11
SV types	DEL: 2; DUP: 2; h2hINV: 0; t2tINV: 1; TRA: 6
SVs in sample	422
Oscillating CN (2 and 3 states)	8, 19
CN segments	21
FDR fragment joints	0.64
FDR chr. breakp. enrich.	0
Linked to chrs	
Purity, ploidy	0.46, 3.34

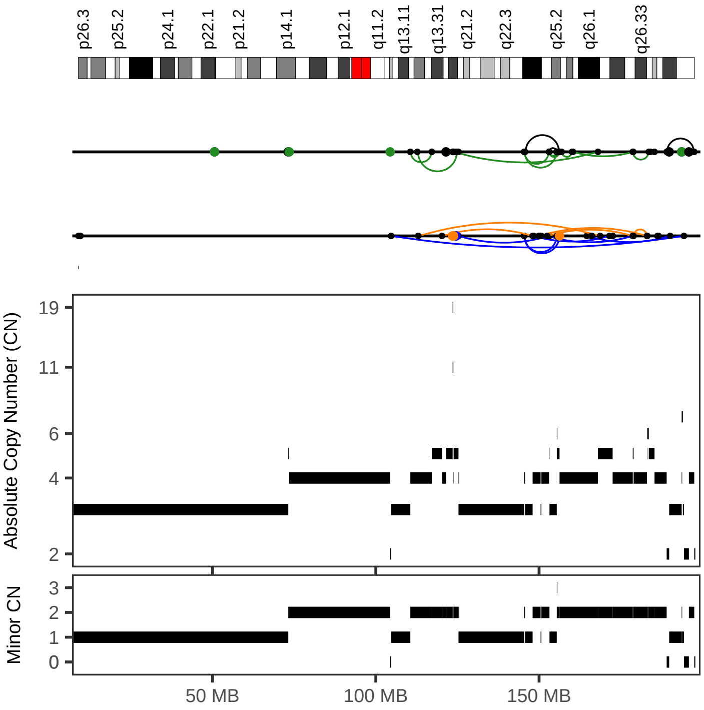

	944a102f−3475−45aa−9e30−4b92d1449f00
Cancer type	Uterus−AdenoCA
Position	3:104698386−197525118
Type	With other complex events
Interleaved intrachr. SVs	33
Total SVs (intrachr. + transl.)	43
SV types	DEL: 7; DUP: 9; h2hINV: 6; t2tINV: 11; TRA: 10
SVs in sample	189
Oscillating CN (2 and 3 states)	8, 14
CN segments	40
FDR fragment joints	0.69
FDR chr. breakp. enrich.	0
Linked to chrs	X:7578980−79744209;
Purity, ploidy	0.63, 3.6

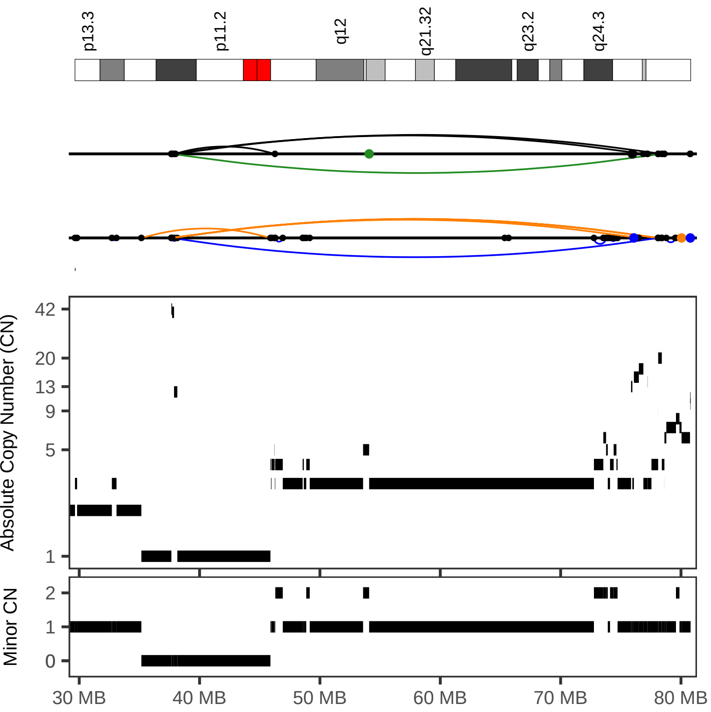

	af37d8f0−fea0−47f6−be0a−2fb55a5da5c4
Cancer type	Uterus−AdenoCA
Position	17:35162507−78407459
Type	With other complex events
Interleaved intrachr. SVs	13
Total SVs (intrachr. + transl.)	16
SV types	DEL: 5; DUP: 4; h2hINV: 3; t2tINV: 1; TRA: 3
SVs in sample	315
Oscillating CN (2 and 3 states)	8, 13
CN segments	39
FDR fragment joints	0.64
FDR chr. breakp. enrich.	0
Linked to chrs	
Purity, ploidy	0.89, 1.95

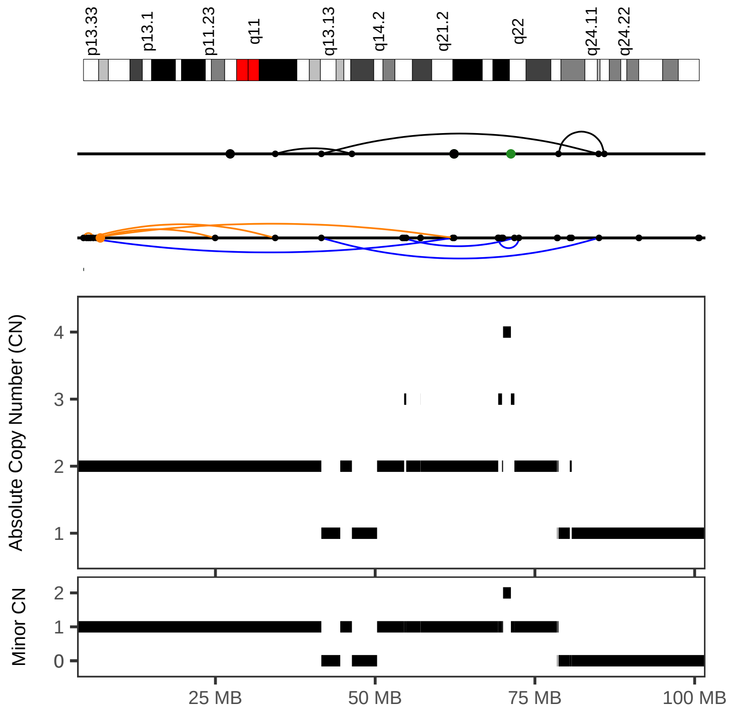

	b60f22ac−a659−4f33−b01d−820e86a9a5c9
Cancer type	Uterus−AdenoCA
Position	12:4347137−85863796
Type	With other complex events
Interleaved intrachr. SVs	12
Total SVs (intrachr. + transl.)	17
SV types	DEL: 4; DUP: 5; h2hINV: 3; t2tINV: 0; TRA: 5
SVs in sample	353
Oscillating CN (2 and 3 states)	7, 11
CN segments	19
FDR fragment joints	0.59
FDR chr. breakp. enrich.	0.09
Linked to chrs	
Purity, ploidy	0.84, 1.87

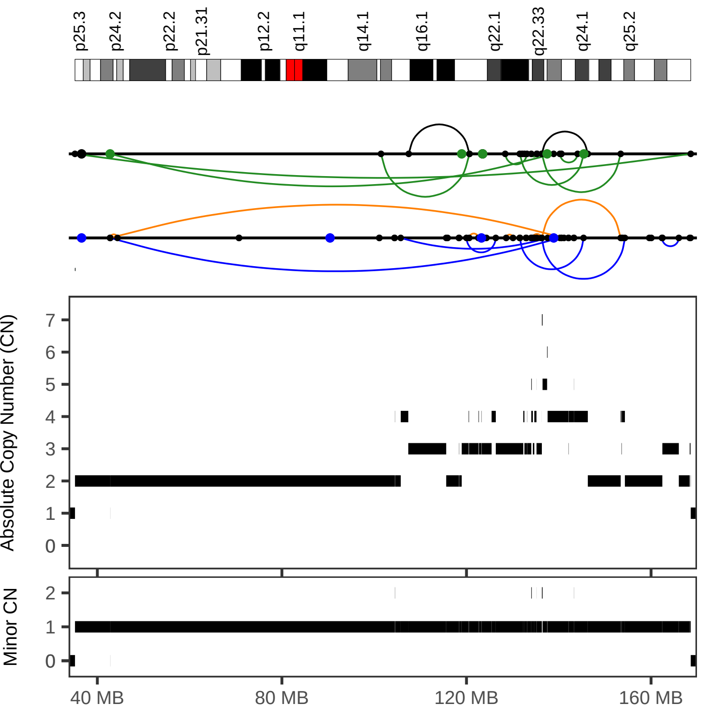

	bad34c92−1e64−4187−a0b4−061105fd13ba
Cancer type	Uterus−AdenoCA
Position	6:42773558−154331741
Type	With other complex events
Interleaved intrachr. SVs	16
Total SVs (intrachr. + transl.)	24
SV types	DEL: 3; DUP: 5; h2hINV: 2; t2tINV: 6; TRA: 8
SVs in sample	315
Oscillating CN (2 and 3 states)	13, 18
CN segments	42
FDR fragment joints	0.64
FDR chr. breakp. enrich.	0
Linked to chrs	1:1127335−152612347;
Purity, ploidy	0.78, 2.01

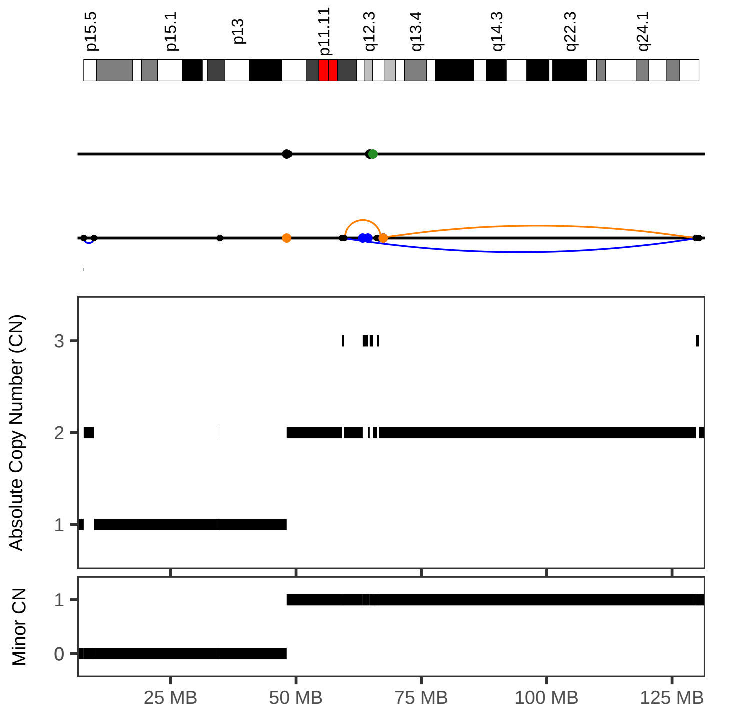

	c350b53b−3c27−4b9e−8b57−d7b6a1205a1f
Cancer type	Uterus−AdenoCA
Position	11:59618659−129738667
Type	Canonical without polyploidization
Interleaved intrachr. SVs	3
Total SVs (intrachr. + transl.)	9
SV types	DEL: 2; DUP: 1; h2hINV: 0; t2tINV: 0; TRA: 6
SVs in sample	278
Oscillating CN (2 and 3 states)	8, 8
CN segments	8
FDR fragment joints	0.59
FDR chr. breakp. enrich.	0.49
Linked to chrs	15:40483864−102277235;2:68246324−234694542
Purity, ploidy	0.93, 2.08
