# Supplementary material for: Comprehensive analysis of chromothripsis in 2,658 human cancers using whole-genome sequencing
Source: Nat Genet. 2020 Feb 5;52(3):331–41. doi: 10.1038/s41588-019-0576-7 (PMC7058534; doi:10.1038/s41588-019-0576-7)
Supplement: Supplementary file 7 — Low-confidence chromothripsis calls. [file 41588_2019_576_MOESM7_ESM.pdf]

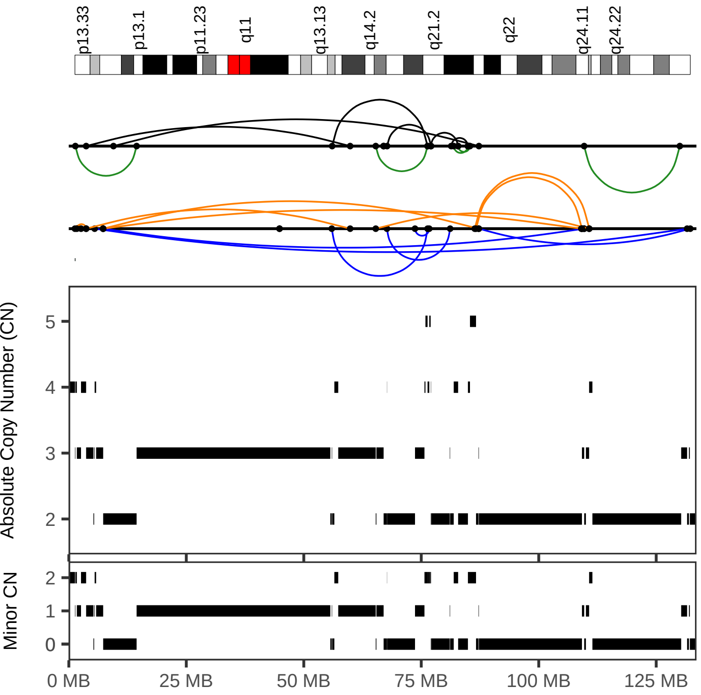

	**BTCA_donor_A096**
Cancer type	Biliary−AdenoCA
Position	12:1297595−132243589
Type	With other complex events
Interleaved intrachr. SVs	26
Total SVs (intrachr. + transl.)	26
SV types	DEL: 8; DUP: 7; h2hINV: 6; t2tINV: 5; TRA: 0
SVs in sample	292
Oscillating CN (2 and 3 states)	6, 14
CN segments	47
FDR fragment joints	0.9179372
FDR chr. breakp. enrich.	0
Linked to chrs	
Purity, ploidy	0.63, 2.77

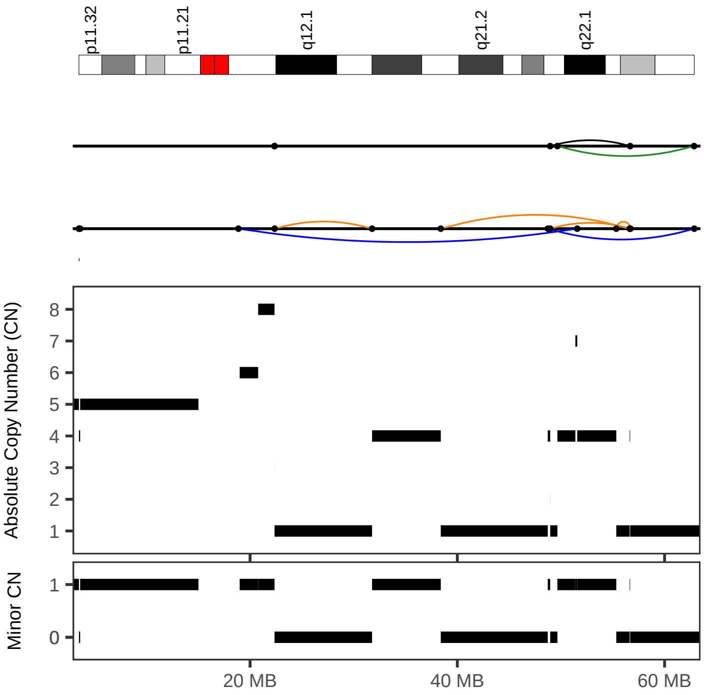

	**BTCA_donor_A096**
Cancer type	Biliary−AdenoCA
Position	18:18873445−62863653
Type	With other complex events
Interleaved intrachr. SVs	7
Total SVs (intrachr. + transl.)	7
SV types	DEL: 3; DUP: 2; h2hINV: 1; t2tINV: 1; TRA: 0
SVs in sample	292
Oscillating CN (2 and 3 states)	4, 4
CN segments	16
FDR fragment joints	0.7735152
FDR chr. breakp. enrich.	0.62
Linked to chrs	
Purity, ploidy	0.63, 2.77

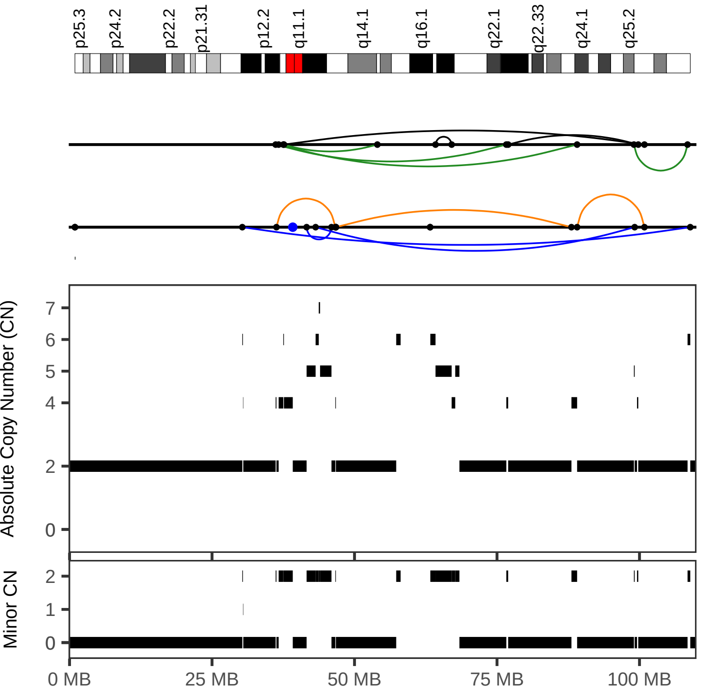

	**BTCA_donor_A153**
Cancer type	Biliary−AdenoCA
Position	6:36165437−108436463
Type	With other complex events
Interleaved intrachr. SVs	11
Total SVs (intrachr. + transl.)	12
SV types	DEL: 3; DUP: 2; h2hINV: 2; t2tINV: 4; TRA: 1
SVs in sample	116
Oscillating CN (2 and 3 states)	5, 10
CN segments	28
FDR fragment joints	0.9284301
FDR chr. breakp. enrich.	0
Linked to chrs	
Purity, ploidy	0.42, 3.59

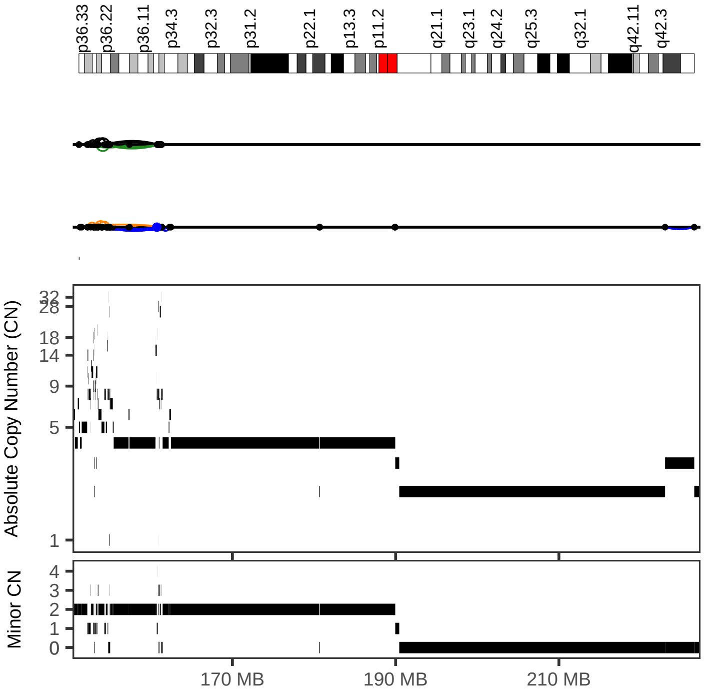

	**BTCA_donor_B070**
Cancer type	Biliary−AdenoCA
Position	1:152201706−162468614
Type	With other complex events
Interleaved intrachr. SVs	47
Total SVs (intrachr. + transl.)	48
SV types	DEL: 9; DUP: 14; h2hINV: 15; t2tINV: 9; TRA: 1
SVs in sample	166
Oscillating CN (2 and 3 states)	4, 5
CN segments	74
FDR fragment joints	0.615458
FDR chr. breakp. enrich.	0
Linked to chrs	
Purity, ploidy	0.56, 2.93

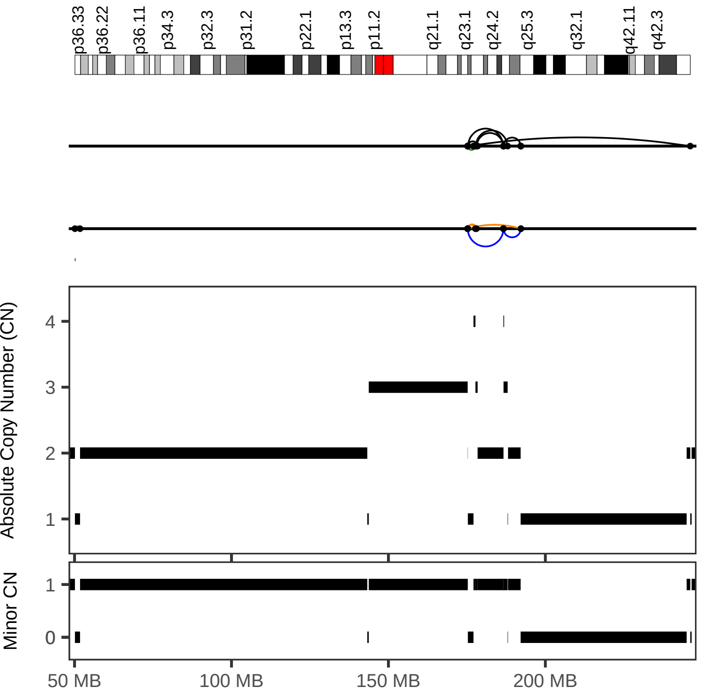

	**BTCA_donor_B083**
Cancer type	Biliary−AdenoCA
Position	1:175210783−246161909
Type	With other complex events
Interleaved intrachr. SVs	14
Total SVs (intrachr. + transl.)	14
SV types	DEL: 3; DUP: 2; h2hINV: 7; t2tINV: 2; TRA: 0
SVs in sample	112
Oscillating CN (2 and 3 states)	5, 6
CN segments	16
FDR fragment joints	0.615458
FDR chr. breakp. enrich.	0.04
Linked to chrs	
Purity, ploidy	0.45, 1.9

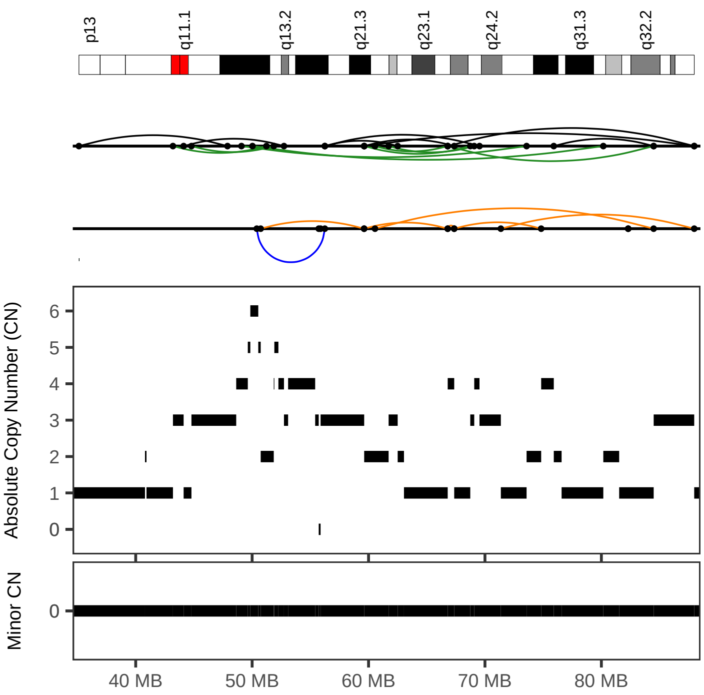

	**BTCA_donor_B083**
Cancer type	Biliary−AdenoCA
Position	14:35121332−87977558
Type	With other complex events
Interleaved intrachr. SVs	23
Total SVs (intrachr. + transl.)	23
SV types	DEL: 6; DUP: 1; h2hINV: 8; t2tINV: 8; TRA: 0
SVs in sample	112
Oscillating CN (2 and 3 states)	4, 9
CN segments	37
FDR fragment joints	0.615458
FDR chr. breakp. enrich.	0
Linked to chrs	
Purity, ploidy	0.45, 1.9

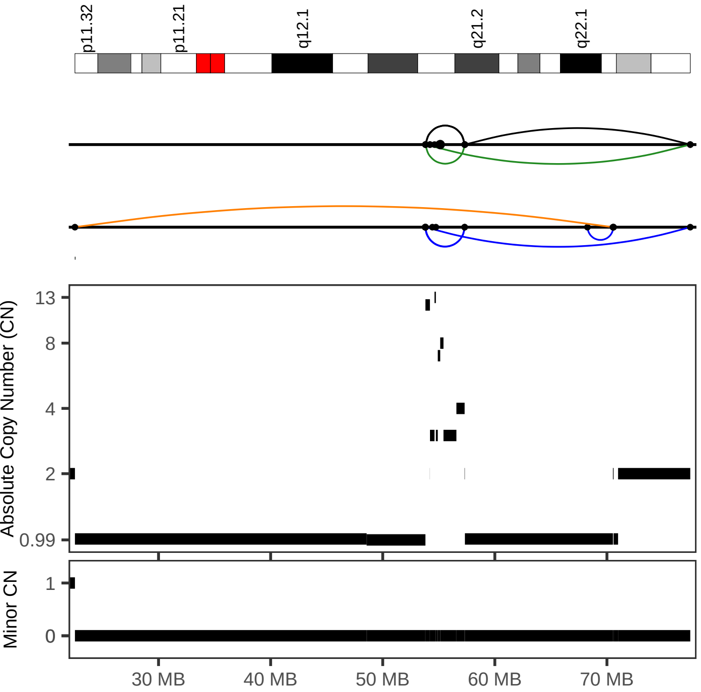

	**BTCA_donor_B083**
Cancer type	Biliary−AdenoCA
Position	18:22541678−77433482
Type	With other complex events
Interleaved intrachr. SVs	13
Total SVs (intrachr. + transl.)	14
SV types	DEL: 2; DUP: 6; h2hINV: 3; t2tINV: 2; TRA: 1
SVs in sample	112
Oscillating CN (2 and 3 states)	5, 5
CN segments	16
FDR fragment joints	0.6776251
FDR chr. breakp. enrich.	0
Linked to chrs	
Purity, ploidy	0.45, 1.9

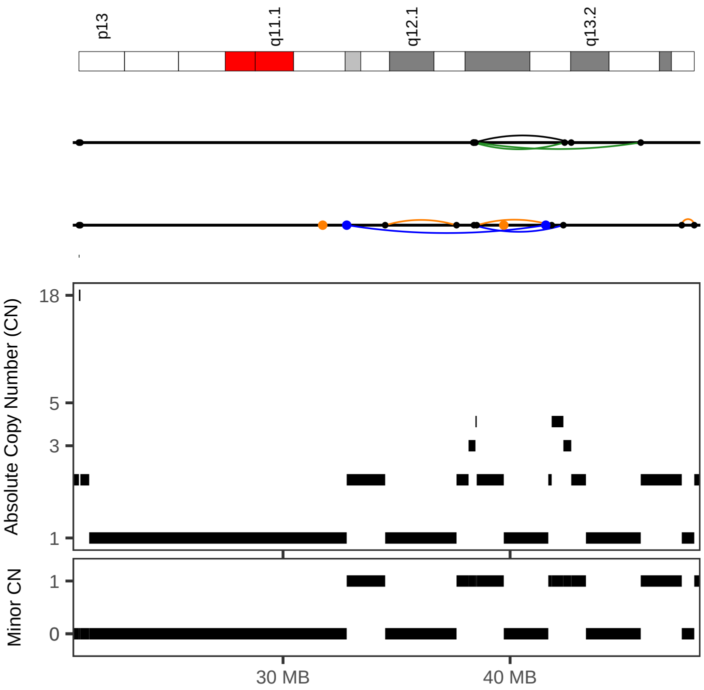

	**BTCA_donor_B083**
Cancer type	Biliary−AdenoCA
Position	22:32803334−45752862
Type	With other complex events
Interleaved intrachr. SVs	6
Total SVs (intrachr. + transl.)	9
SV types	DEL: 1; DUP: 2; h2hINV: 1; t2tINV: 2; TRA: 3
SVs in sample	112
Oscillating CN (2 and 3 states)	4, 6
CN segments	14
FDR fragment joints	0.6776251
FDR chr. breakp. enrich.	0
Linked to chrs	4:2189376−19547021;
Purity, ploidy	0.45, 1.9

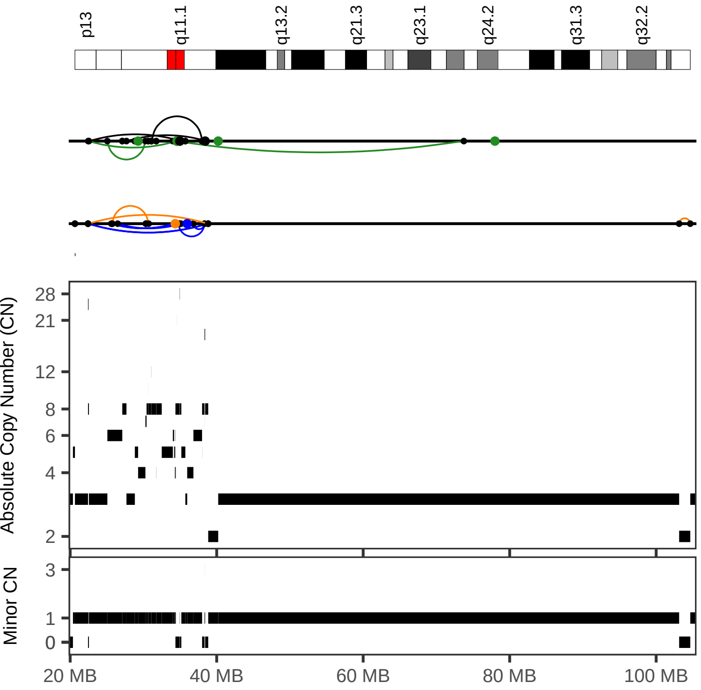

BTCA_donor_C080

Cancer type	Biliary−AdenoCA
Position	14:22429630−73744179
Type	With other complex events
Interleaved intrachr. SVs	17
Total SVs (intrachr. + transl.)	26
SV types	DEL: 3; DUP: 7; h2hINV: 3; t2tINV: 4; TRA: 9
SVs in sample	142
Oscillating CN (2 and 3 states)	4, 7
CN segments	40
FDR fragment joints	0.8172348
FDR chr. breakp. enrich.	0
Linked to chrs	
Purity, ploidy	0.6, 2.79

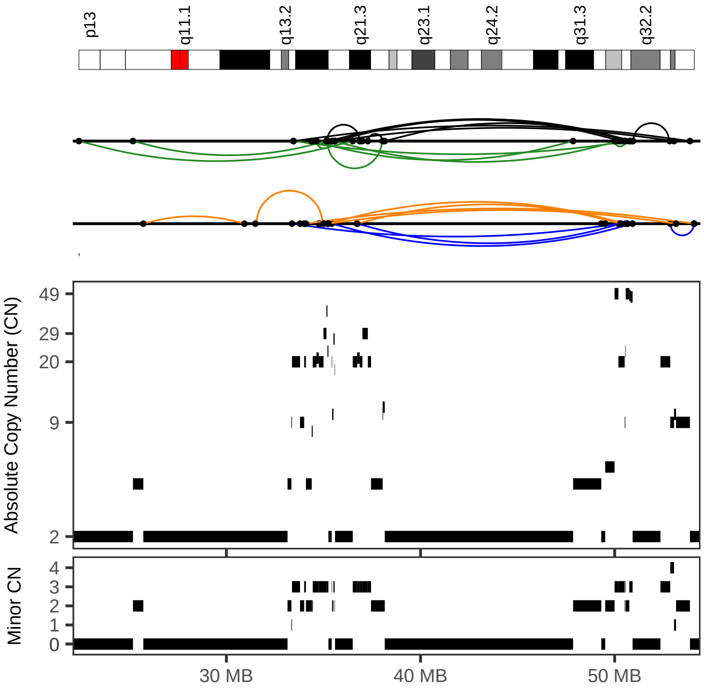

BTCA_donor_R149

Cancer type	Biliary−AdenoCA
Position	14:22402704−54099947
Type	With other complex events
Interleaved intrachr. SVs	29
Total SVs (intrachr. + transl.)	29
SV types	DEL: 5; DUP: 5; h2hINV: 10; t2tINV: 9; TRA: 0
SVs in sample	108
Oscillating CN (2 and 3 states)	4, 5
CN segments	47
FDR fragment joints	0.6482122
FDR chr. breakp. enrich.	0
Linked to chrs	
Purity, ploidy	0.34, 3.04

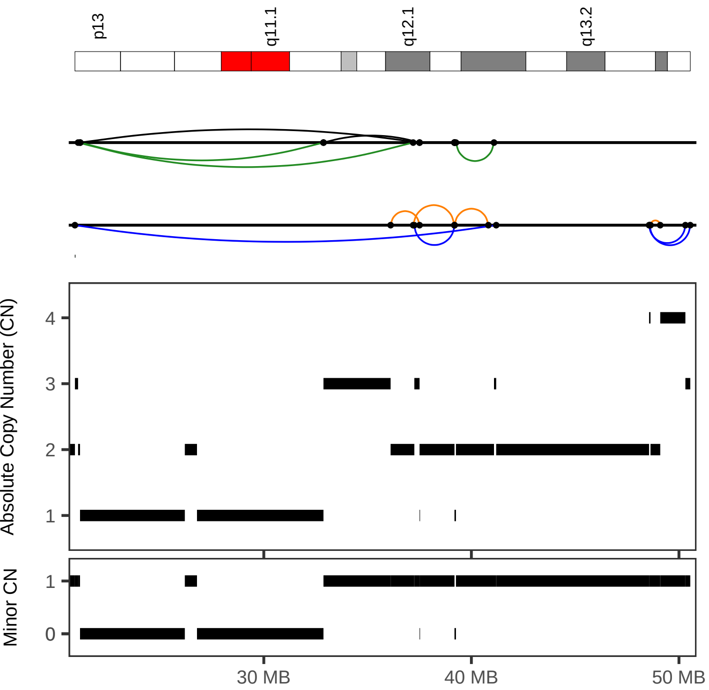

BTCA_donor_Y008

Cancer type	Biliary−AdenoCA
Position	22:21053371−41089205
Type	With other complex events
Interleaved intrachr. SVs	9
Total SVs (intrachr. + transl.)	9
SV types	DEL: 3; DUP: 1; h2hINV: 2; t2tINV: 3; TRA: 0
SVs in sample	48
Oscillating CN (2 and 3 states)	4, 11
CN segments	12
FDR fragment joints	0.854603
FDR chr. breakp. enrich.	0
Linked to chrs	
Purity, ploidy	0.48, 1.88

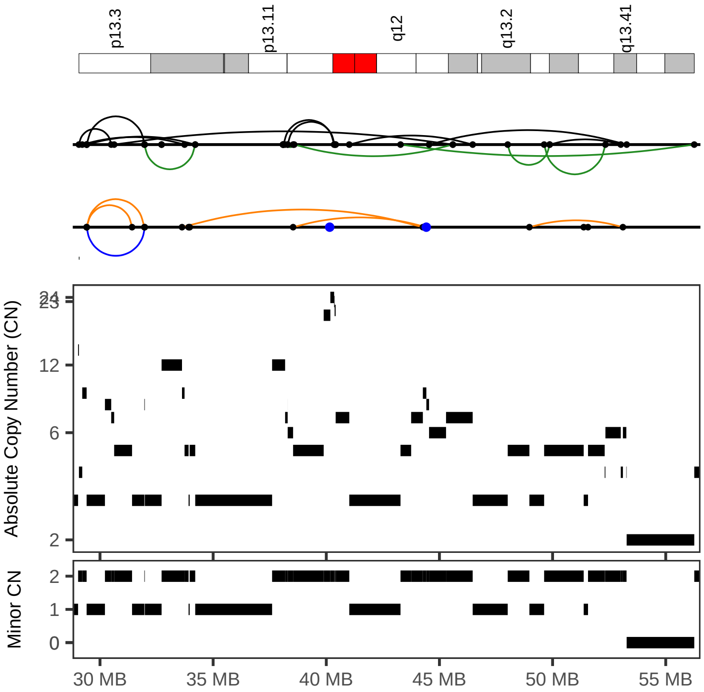

BTCA_donor_Y140

Cancer type	Biliary−AdenoCA
Position	19:29068313−56261033
Type	With other complex events
Interleaved intrachr. SVs	19
Total SVs (intrachr. + transl.)	21
SV types	DEL: 4; DUP: 1; h2hINV: 10; t2tINV: 4; TRA: 2
SVs in sample	96
Oscillating CN (2 and 3 states)	6, 7
CN segments	45
FDR fragment joints	0.5774122
FDR chr. breakp. enrich.	0
Linked to chrs	
Purity, ploidy	0.78, 3.66

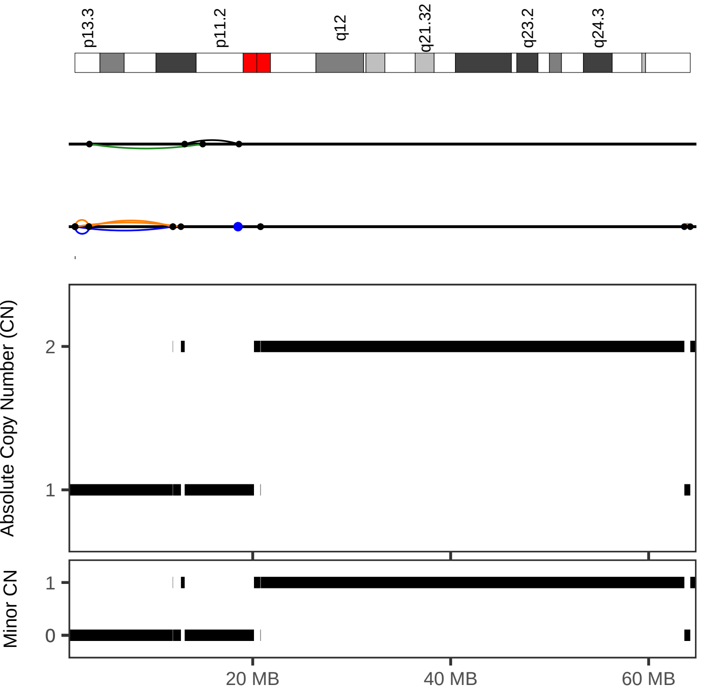

	RK137
Cancer type	Biliary−AdenoCA
Position	17:2039238−18611038
Type	Canonical without polyploidization
Interleaved intrachr. SVs	7
Total SVs (intrachr. + transl.)	8
SV types	DEL: 3; DUP: 2; h2hINV: 1; t2tINV: 1; TRA: 1
SVs in sample	43
Oscillating CN (2 and 3 states)	5, 5
CN segments	5
FDR fragment joints	0.6776251
FDR chr. breakp. enrich.	0
Linked to chrs	
Purity, ploidy	0.5, 2.3

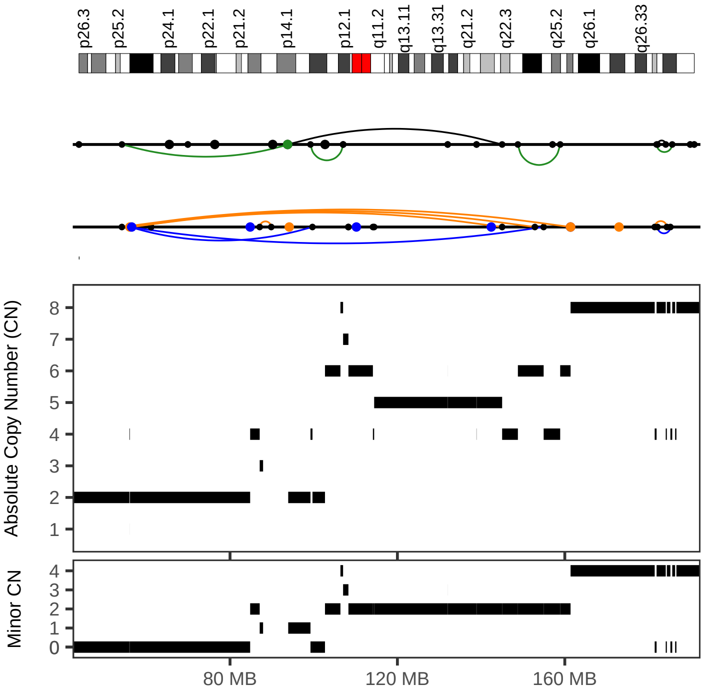

	RK208
Cancer type	Biliary−AdenoCA
Position	3:54159227−158898997
Type	With other complex events
Interleaved intrachr. SVs	9
Total SVs (intrachr. + transl.)	21
SV types	DEL: 2; DUP: 2; h2hINV: 2; t2tINV: 3; TRA: 12
SVs in sample	294
Oscillating CN (2 and 3 states)	4, 7
CN segments	23
FDR fragment joints	0.6610257
FDR chr. breakp. enrich.	0
Linked to chrs	
Purity, ploidy	0.2, 3.83

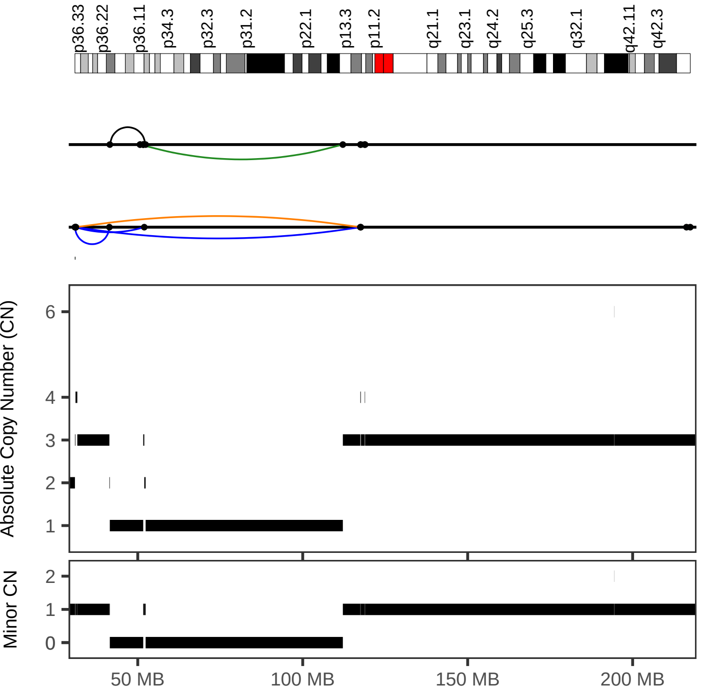

	RK279
Cancer type	Biliary−AdenoCA
Position	1:30955993−118910093
Type	With other complex events
Interleaved intrachr. SVs	8
Total SVs (intrachr. + transl.)	8
SV types	DEL: 1; DUP: 3; h2hINV: 2; t2tINV: 2; TRA: 0
SVs in sample	35
Oscillating CN (2 and 3 states)	5, 5
CN segments	15
FDR fragment joints	0.8653243
FDR chr. breakp. enrich.	0
Linked to chrs	
Purity, ploidy	0.35, 1.84

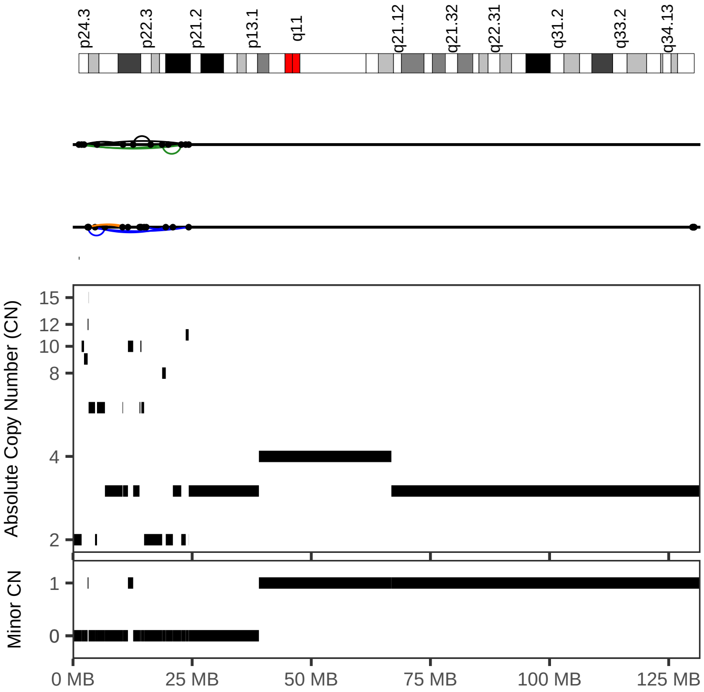

	RK298
Cancer type	Biliary−AdenoCA
Position	9:1239088−24282207
Type	With other complex events
Interleaved intrachr. SVs	12
Total SVs (intrachr. + transl.)	12
SV types	DEL: 2; DUP: 4; h2hINV: 3; t2tINV: 3; TRA: 0
SVs in sample	74
Oscillating CN (2 and 3 states)	4, 6
CN segments	24
FDR fragment joints	0.9284301
FDR chr. breakp. enrich.	0
Linked to chrs	
Purity, ploidy	0.28, 3.37

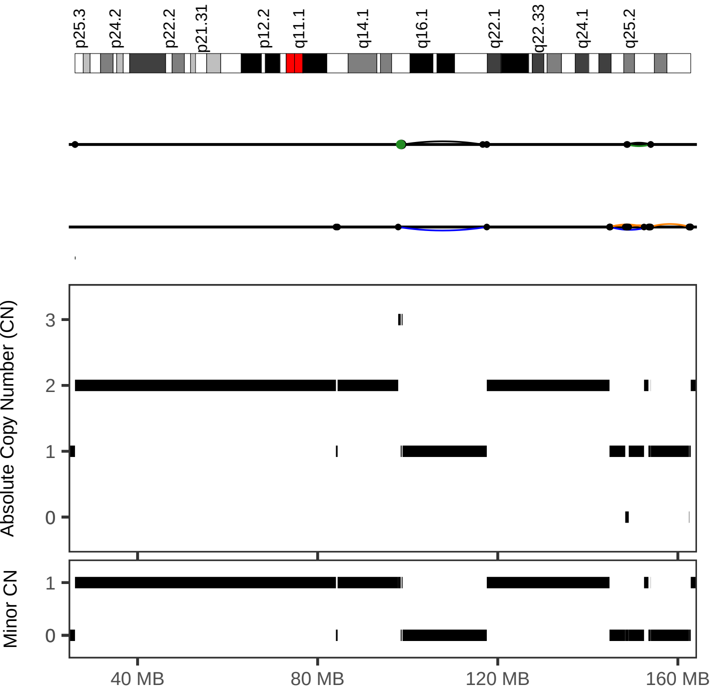

	RK307
Cancer type	Biliary−AdenoCA
Position	6:144819172−162778287
Type	With other complex events
Interleaved intrachr. SVs	8
Total SVs (intrachr. + transl.)	8
SV types	DEL: 5; DUP: 1; h2hINV: 1; t2tINV: 1; TRA: 0
SVs in sample	89
Oscillating CN (2 and 3 states)	5, 9
CN segments	9
FDR fragment joints	0.5435077
FDR chr. breakp. enrich.	0
Linked to chrs	
Purity, ploidy	0.37, 1.79

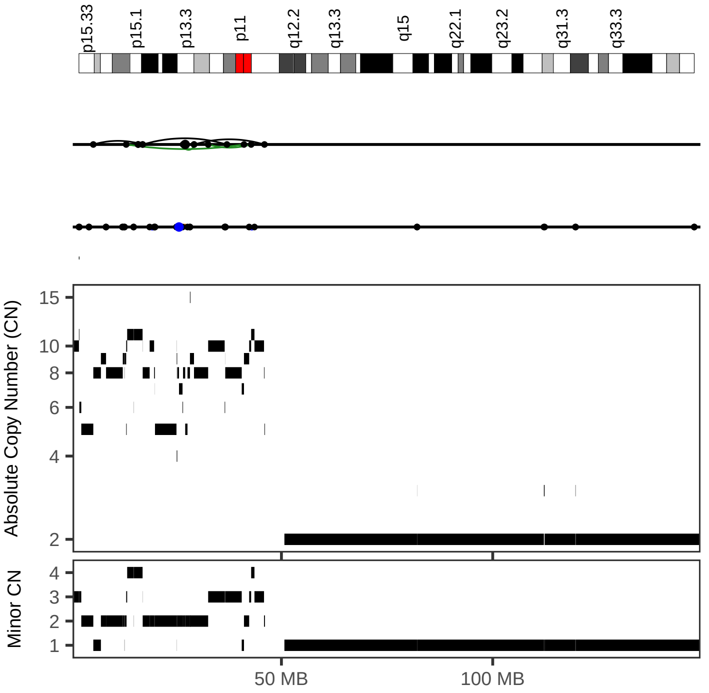

	24f21425−b001−4986−aedf−5b4dd851c6ad
Cancer type	Bladder−TCC
Position	5:5481718−45983726
Type	With other complex events
Interleaved intrachr. SVs	8
Total SVs (intrachr. + transl.)	10
SV types	DEL: 1; DUP: 1; h2hINV: 3; t2tINV: 3; TRA: 2
SVs in sample	271
Oscillating CN (2 and 3 states)	6, 9
CN segments	42
FDR fragment joints	0.6776251
FDR chr. breakp. enrich.	0
Linked to chrs	
Purity, ploidy	0.64, 3.43

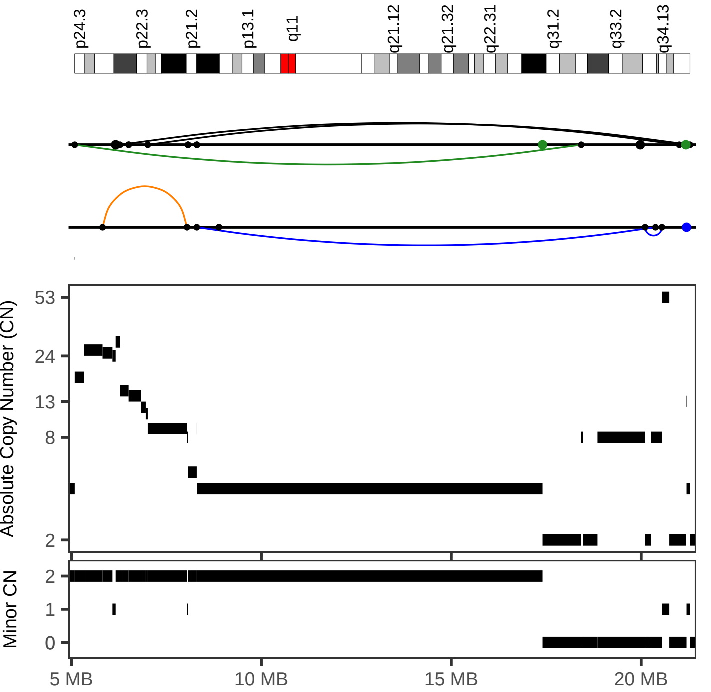

	24f21425−b001−4986−aedf−5b4dd851c6ad
Cancer type	Bladder−TCC
Position	9:5086998−21286063
Type	With other complex events
Interleaved intrachr. SVs	6
Total SVs (intrachr. + transl.)	11
SV types	DEL: 1; DUP: 2; h2hINV: 2; t2tINV: 1; TRA: 5
SVs in sample	271
Oscillating CN (2 and 3 states)	6, 6
CN segments	26
FDR fragment joints	0.7425546
FDR chr. breakp. enrich.	0.29
Linked to chrs	
Purity, ploidy	0.64, 3.43

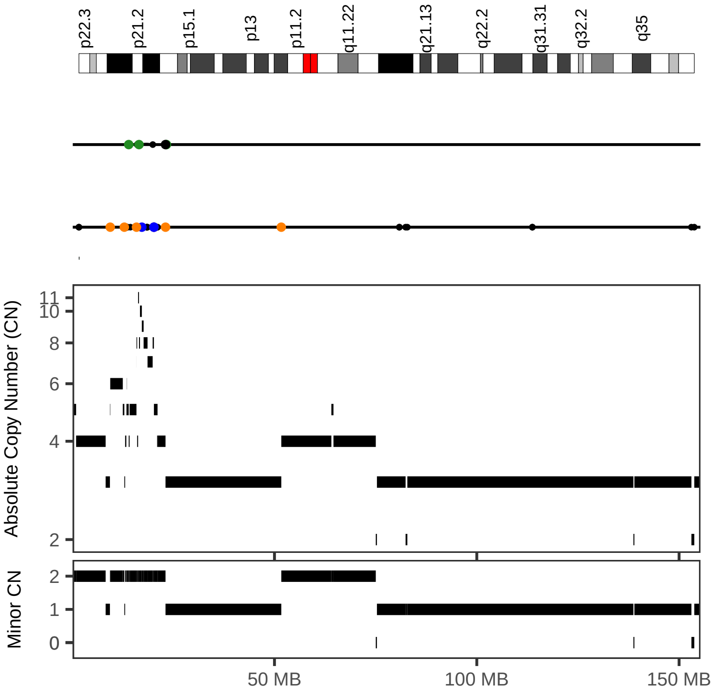

	3ed614e7−f356−4d87−985b−d3bbbae3bb40
Cancer type	Bladder−TCC
Position	7:12512048−21119497
Type	With other complex events
Interleaved intrachr. SVs	6
Total SVs (intrachr. + transl.)	14
SV types	DEL: 2; DUP: 3; h2hINV: 1; t2tINV: 0; TRA: 8
SVs in sample	449
Oscillating CN (2 and 3 states)	4, 7
CN segments	22
FDR fragment joints	0.5435077
FDR chr. breakp. enrich.	0.22
Linked to chrs	
Purity, ploidy	0.74, 3.29

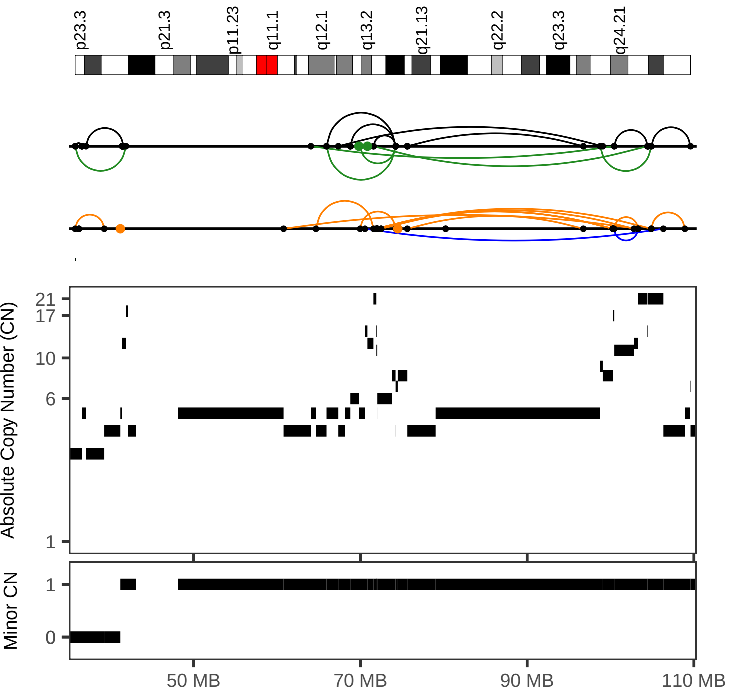

3ed614e7−f356−4d87−985b−d3bbbae3bb40	
Cancer type	Bladder−TCC
Position	8:60780756−109595289
Type	With other complex events
Interleaved intrachr. SVs	23
Total SVs (intrachr. + transl.)	26
SV types	DEL: 9; DUP: 2; h2hINV: 7; t2tINV: 5; TRA: 3
SVs in sample	449
Oscillating CN (2 and 3 states)	6, 6
CN segments	40
FDR fragment joints	0.5435077
FDR chr. breakp. enrich.	0
Linked to chrs	2:14647349−206284284;
Purity, ploidy	0.74, 3.29

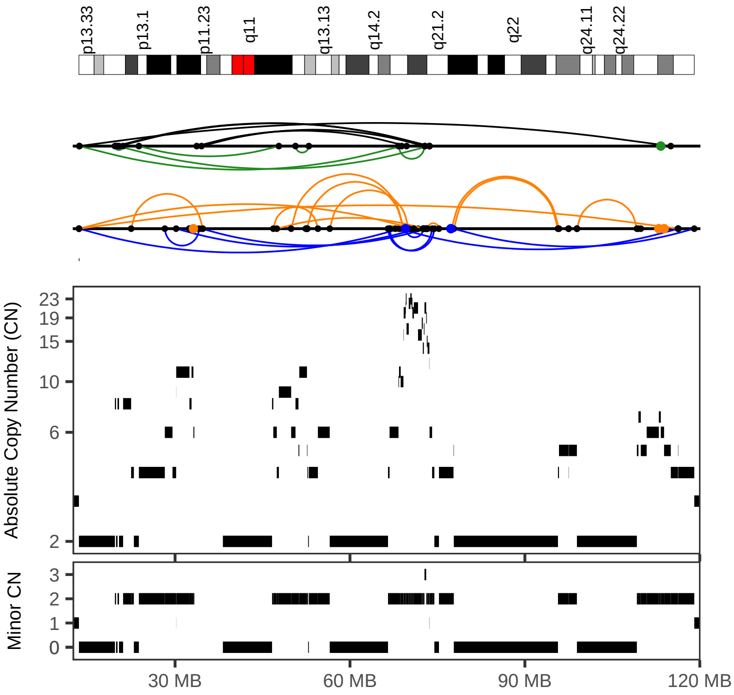

3ed614e7−f356−4d87−985b−d3bbbae3bb40	
Cancer type	Bladder−TCC
Position	12:13507658−119049122
Type	With other complex events
Interleaved intrachr. SVs	32
Total SVs (intrachr. + transl.)	38
SV types	DEL: 11; DUP: 10; h2hINV: 5; t2tINV: 6; TRA: 6
SVs in sample	449
Oscillating CN (2 and 3 states)	6, 9
CN segments	79
FDR fragment joints	0.615458
FDR chr. breakp. enrich.	0
Linked to chrs	
Purity, ploidy	0.74, 3.29

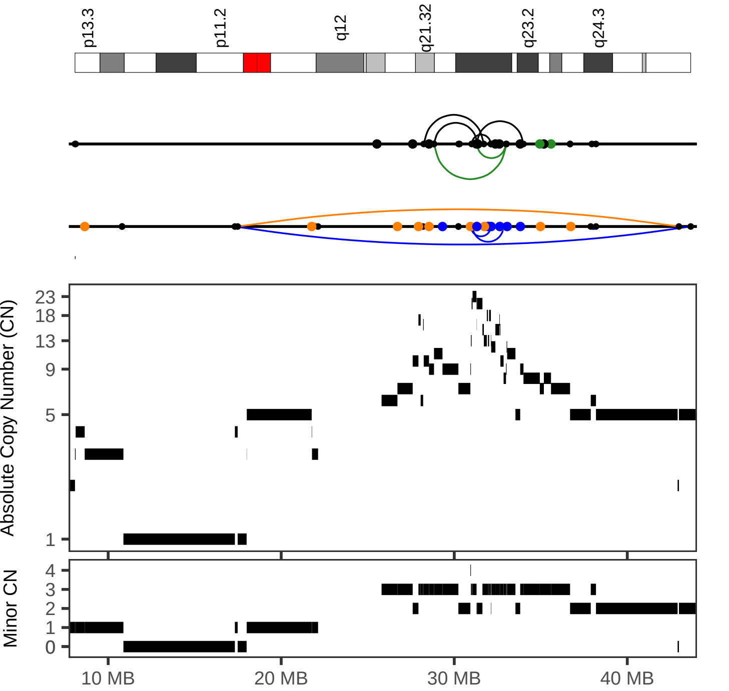

3ed614e7−f356−4d87−985b−d3bbbae3bb40	
Cancer type	Bladder−TCC
Position	17:28237017−34001517
Type	With other complex events
Interleaved intrachr. SVs	9
Total SVs (intrachr. + transl.)	28
SV types	DEL: 0; DUP: 2; h2hINV: 5; t2tINV: 2; TRA: 19
SVs in sample	449
Oscillating CN (2 and 3 states)	5, 5
CN segments	31
FDR fragment joints	0.6776251
FDR chr. breakp. enrich.	0
Linked to chrs	11:8965507−99216922;X:3858348−76848684
Purity, ploidy	0.74, 3.29

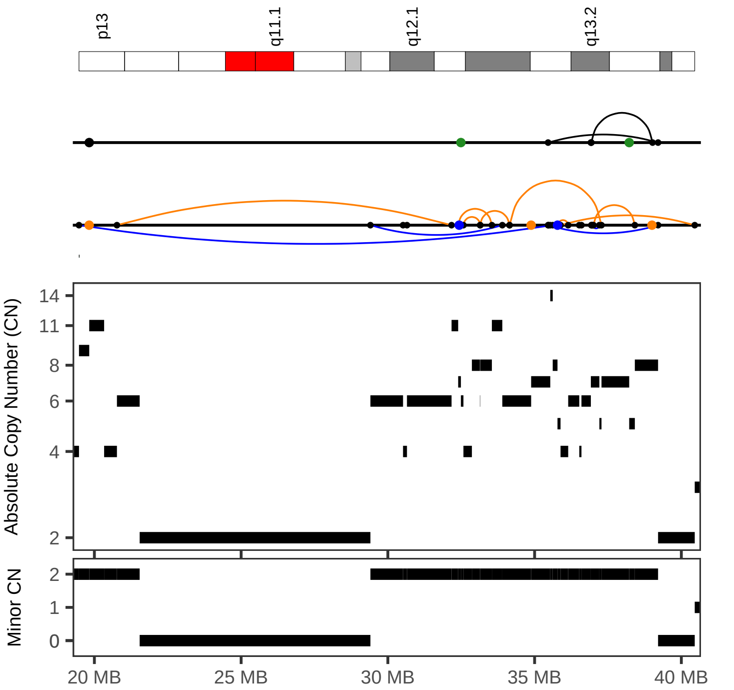

3ed614e7−f356−4d87−985b−d3bbbae3bb40	
Cancer type	Bladder−TCC
Position	22:19474849−40455649
Type	With other complex events
Interleaved intrachr. SVs	10
Total SVs (intrachr. + transl.)	18
SV types	DEL: 4; DUP: 3; h2hINV: 2; t2tINV: 1; TRA: 8
SVs in sample	449
Oscillating CN (2 and 3 states)	4, 6
CN segments	32
FDR fragment joints	0.641841
FDR chr. breakp. enrich.	0
Linked to chrs	12:13507658−119049121;17:28237017−34001516
Purity, ploidy	0.74, 3.29

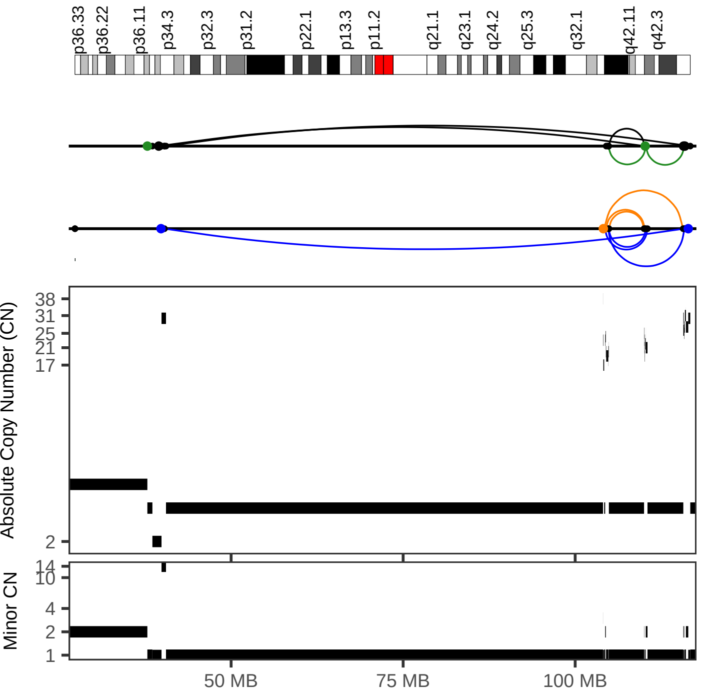

	5d54c742−5a8e−4c40−8d62−95e75e210ab8
Cancer type	Bladder−TCC
Position	1:39492660−116722898
Type	With other complex events
Interleaved intrachr. SVs	18
Total SVs (intrachr. + transl.)	24
SV types	DEL: 2; DUP: 4; h2hINV: 6; t2tINV: 6; TRA: 6
SVs in sample	175
Oscillating CN (2 and 3 states)	4, 5
CN segments	39
FDR fragment joints	0.6776251
FDR chr. breakp. enrich.	0
Linked to chrs	4:72403638−190385401;
Purity, ploidy	0.6, 3.23

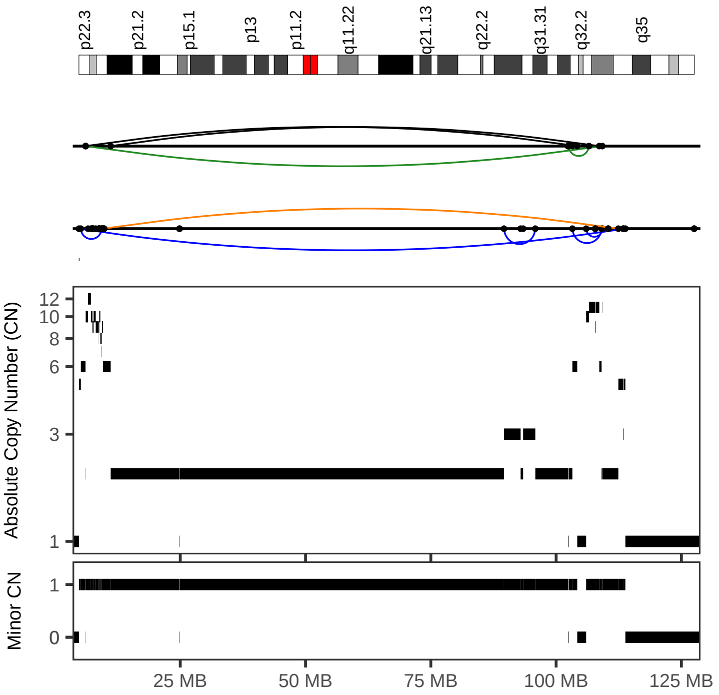

	8c1dd7f7−b74a−4fa2−b6a7−86f0348d2567
Cancer type	Bladder−TCC
Position	7:5163068−112416677
Type	With other complex events
Interleaved intrachr. SVs	10
Total SVs (intrachr. + transl.)	10
SV types	DEL: 2; DUP: 3; h2hINV: 3; t2tINV: 2; TRA: 0
SVs in sample	80
Oscillating CN (2 and 3 states)	5, 5
CN segments	35
FDR fragment joints	0.9625775
FDR chr. breakp. enrich.	0
Linked to chrs	
Purity, ploidy	0.84, 2.09

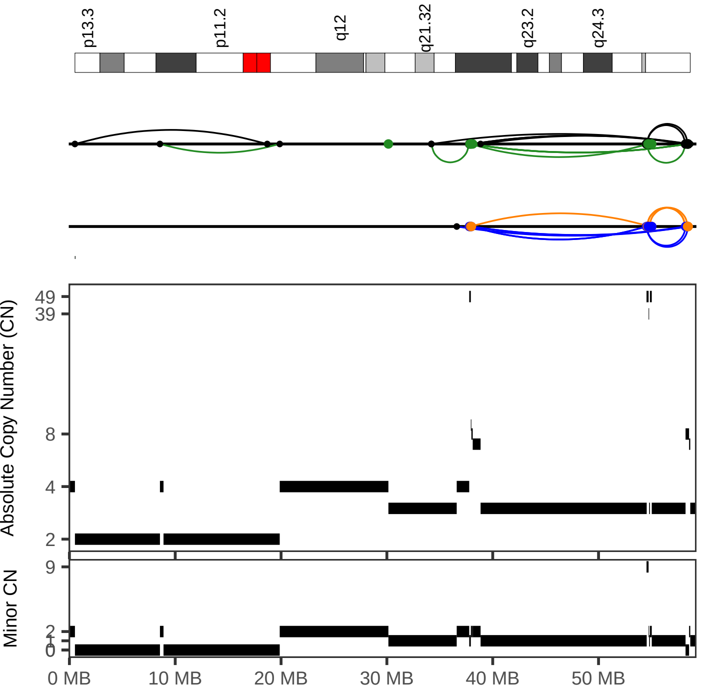

	904a8757−e0c5−41ef−b583−c8f170caaac1
Cancer type	Bladder−TCC
Position	17:34199415−58666962
Type	With other complex events
Interleaved intrachr. SVs	29
Total SVs (intrachr. + transl.)	74
SV types	DEL: 5; DUP: 9; h2hINV: 7; t2tINV: 8; TRA: 45
SVs in sample	274
Oscillating CN (2 and 3 states)	4, 4
CN segments	17
FDR fragment joints	0.9807783
FDR chr. breakp. enrich.	0
Linked to chrs	16:70993199−81194913;
Purity, ploidy	0.6, 2.92

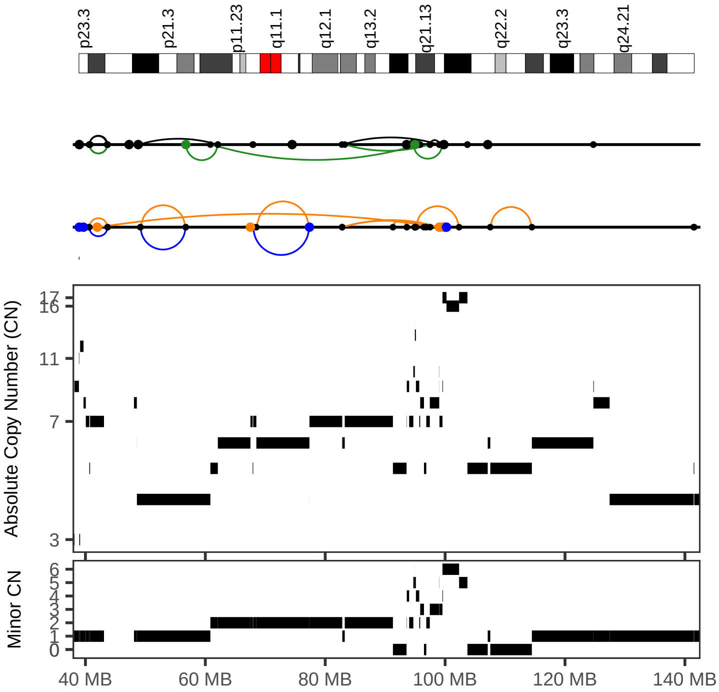

	9c274536−3ca1−4f0e−93a8−1688074d862f
Cancer type	Bladder−TCC
Position	8:40629167−102330843
Type	With other complex events
Interleaved intrachr. SVs	20
Total SVs (intrachr. + transl.)	34
SV types	DEL: 6; DUP: 2; h2hINV: 6; t2tINV: 6; TRA: 14
SVs in sample	270
Oscillating CN (2 and 3 states)	6, 8
CN segments	39
FDR fragment joints	0.6776251
FDR chr. breakp. enrich.	0
Linked to chrs	
Purity, ploidy	0.71, 3.43

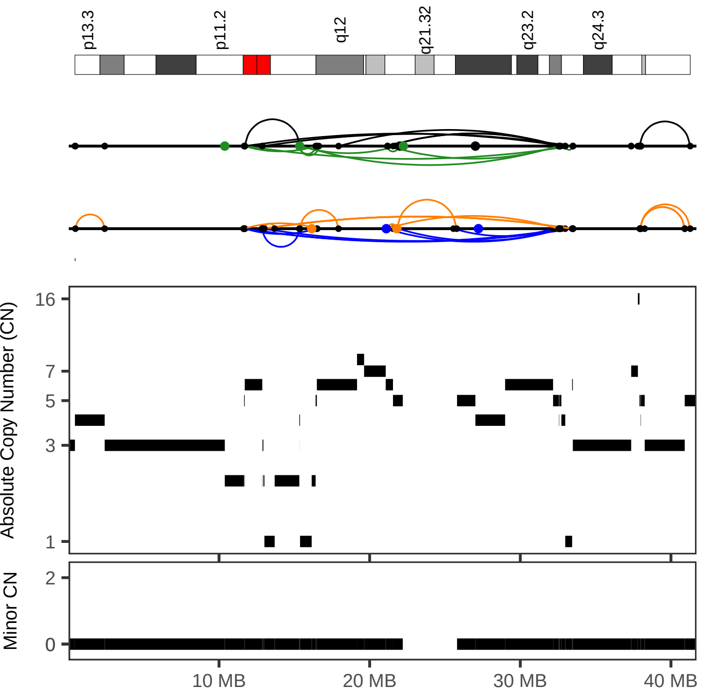

9c274536−3ca1−4f0e−93a8−1688074d862f	
Cancer type	Bladder−TCC
Position	17:11629450−33500976
Type	With other complex events
Interleaved intrachr. SVs	37
Total SVs (intrachr. + transl.)	45
SV types	DEL: 10; DUP: 9; h2hINV: 8; t2tINV: 10; TRA: 8
SVs in sample	270
Oscillating CN (2 and 3 states)	6, 7
CN segments	34
FDR fragment joints	0.9849105
FDR chr. breakp. enrich.	0
Linked to chrs	
Purity, ploidy	0.71, 3.43

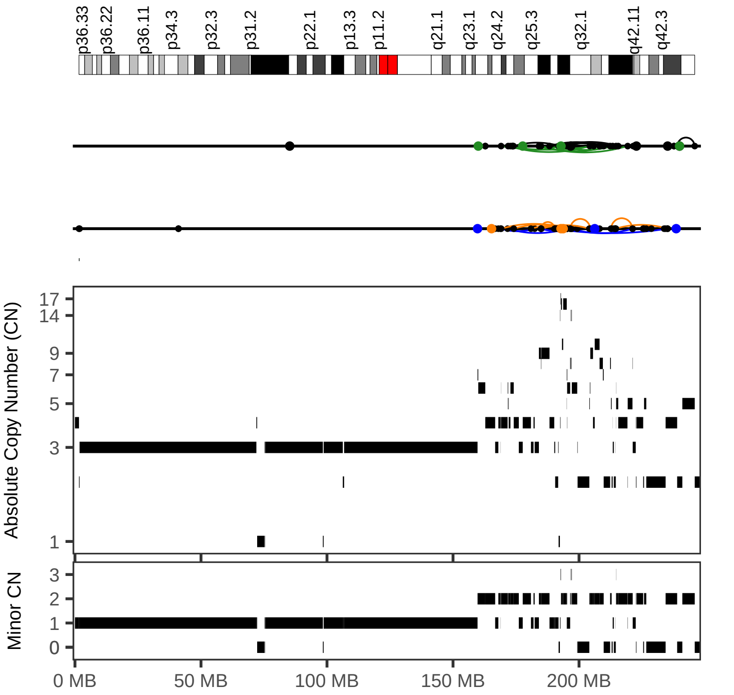

ca554128−da9d−4f37−9560−ca083509e01d	
Cancer type	Bladder−TCC
Position	1:168849602−235165090
Type	With other complex events
Interleaved intrachr. SVs	30
Total SVs (intrachr. + transl.)	38
SV types	DEL: 11; DUP: 7; h2hINV: 6; t2tINV: 6; TRA: 8
SVs in sample	231
Oscillating CN (2 and 3 states)	6, 8
CN segments	72
FDR fragment joints	0.7282798
FDR chr. breakp. enrich.	0
Linked to chrs	3:330518−149204905;
Purity, ploidy	0.79, 3.15

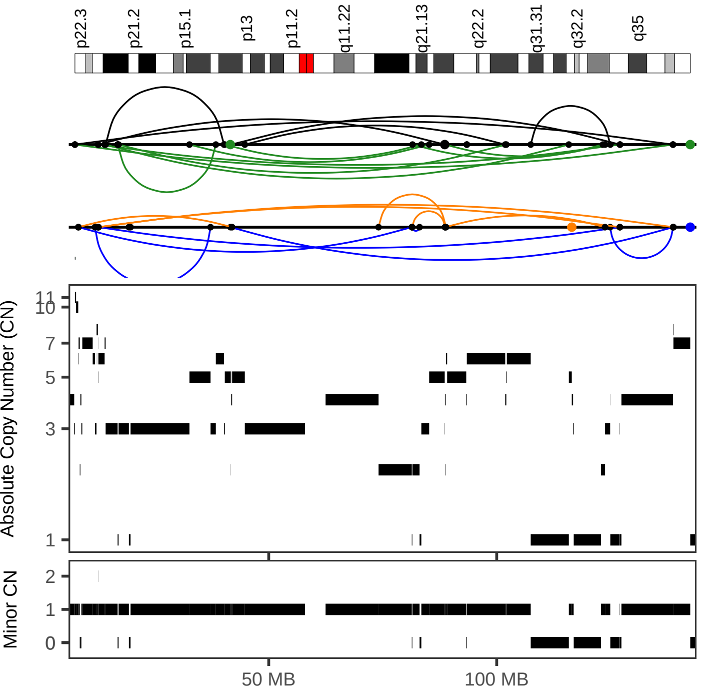

ca554128−da9d−4f37−9560−ca083509e01d	
Cancer type	Bladder−TCC
Position	7:7489162−138751893
Type	With other complex events
Interleaved intrachr. SVs	28
Total SVs (intrachr. + transl.)	31
SV types	DEL: 6; DUP: 6; h2hINV: 7; t2tINV: 9; TRA: 3
SVs in sample	231
Oscillating CN (2 and 3 states)	6, 8
CN segments	66
FDR fragment joints	0.8653243
FDR chr. breakp. enrich.	0
Linked to chrs	
Purity, ploidy	0.79, 3.15

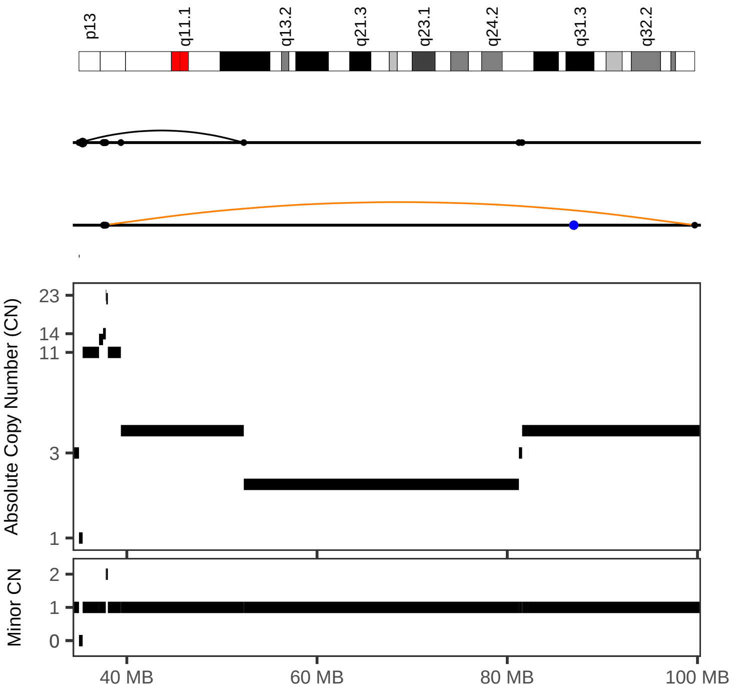

ca554128−da9d−4f37−9560−ca083509e01d	
Cancer type	Bladder−TCC
Position	14:34967294−99710526
Type	With other complex events
Interleaved intrachr. SVs	16
Total SVs (intrachr. + transl.)	20
SV types	DEL: 4; DUP: 4; h2hINV: 4; t2tINV: 4; TRA: 4
SVs in sample	231
Oscillating CN (2 and 3 states)	4, 4
CN segments	13
FDR fragment joints	0.9625775
FDR chr. breakp. enrich.	0
Linked to chrs	
Purity, ploidy	0.79, 3.15

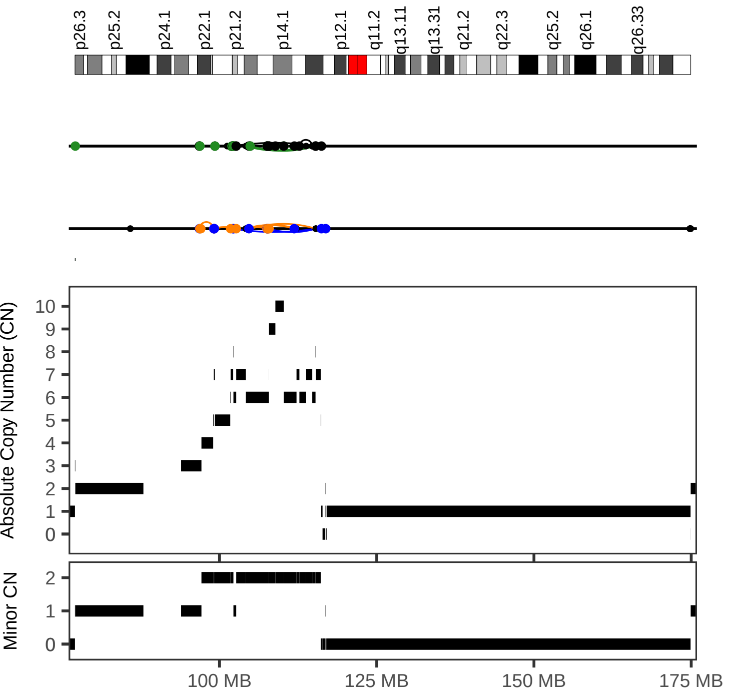

	**CGP_donor_1437423**
Cancer type	Bone−Benign
Position	3:99097210−115323895
Type	With other complex events
Interleaved intrachr. SVs	20
Total SVs (intrachr. + transl.)	62
SV types	DEL: 7; DUP: 5; h2hINV: 5; t2tINV: 3; TRA: 42
SVs in sample	145
Oscillating CN (2 and 3 states)	5, 8
CN segments	19
FDR fragment joints	0.7091283
FDR chr. breakp. enrich.	0
Linked to chrs	6:41870611−56460500;
Purity, ploidy	0.33, 2.04

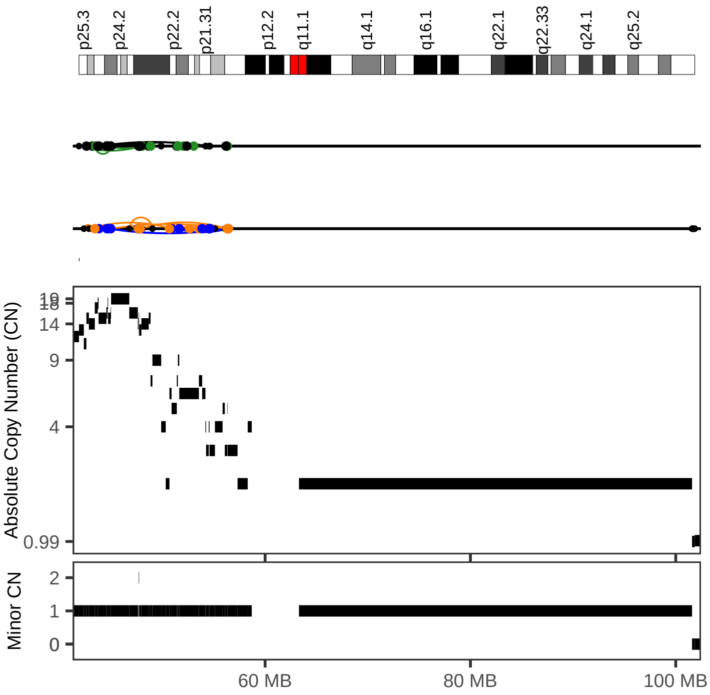

	**CGP_donor_1437423**
Cancer type	Bone−Benign
Position	6:41870611−56460501
Type	With other complex events
Interleaved intrachr. SVs	20
Total SVs (intrachr. + transl.)	70
SV types	DEL: 8; DUP: 3; h2hINV: 5; t2tINV: 4; TRA: 50
SVs in sample	145
Oscillating CN (2 and 3 states)	5, 6
CN segments	40
FDR fragment joints	0.8572806
FDR chr. breakp. enrich.	0
Linked to chrs	
Purity, ploidy	0.33, 2.04

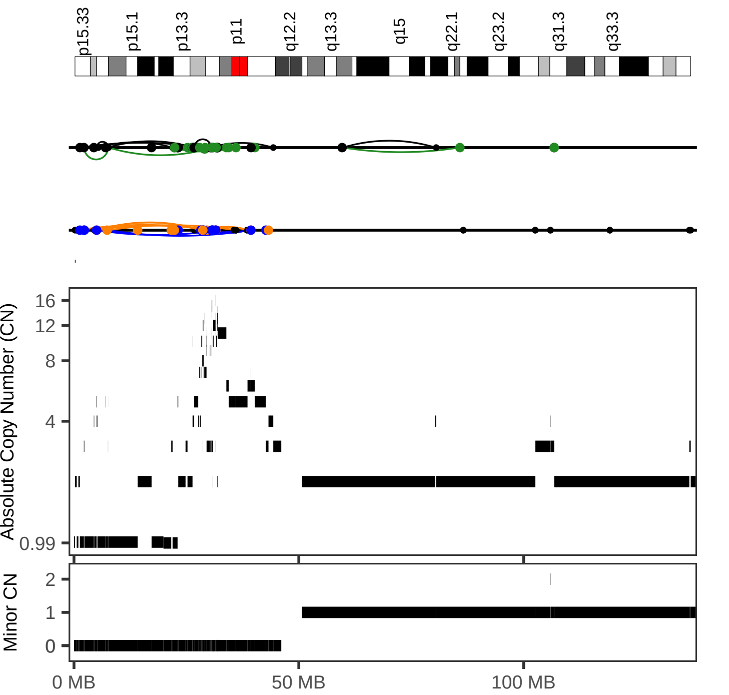

	**CGP_donor_1397077**
Cancer type	Bone−Osteosarc
Position	5:759551−44322947
Type	With other complex events
Interleaved intrachr. SVs	25
Total SVs (intrachr. + transl.)	90
SV types	DEL: 8; DUP: 6; h2hINV: 6; t2tINV: 5; TRA: 65
SVs in sample	379
Oscillating CN (2 and 3 states)	5, 8
CN segments	88
FDR fragment joints	0.935101
FDR chr. breakp. enrich.	0
Linked to chrs	18:26924845−64011243;6:88458092−155828130
Purity, ploidy	0.47, 2.53

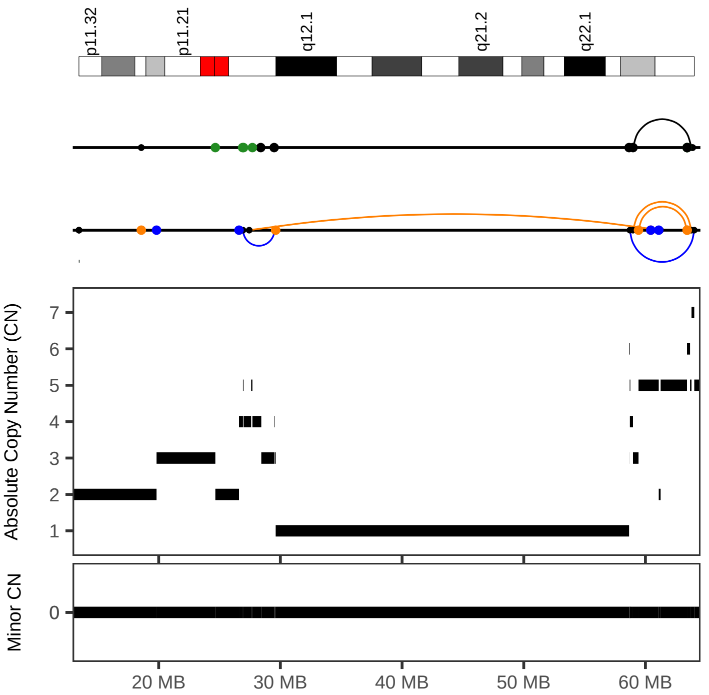

	**CGP_donor_1397077**
Cancer type	Bone−Osteosarc
Position	18:26924845−64011244
Type	With other complex events
Interleaved intrachr. SVs	6
Total SVs (intrachr. + transl.)	22
SV types	DEL: 3; DUP: 2; h2hINV: 1; t2tINV: 0; TRA: 16
SVs in sample	379
Oscillating CN (2 and 3 states)	4, 7
CN segments	21
FDR fragment joints	0.6776251
FDR chr. breakp. enrich.	0
Linked to chrs	5:759551−44322946;
Purity, ploidy	0.47, 2.53

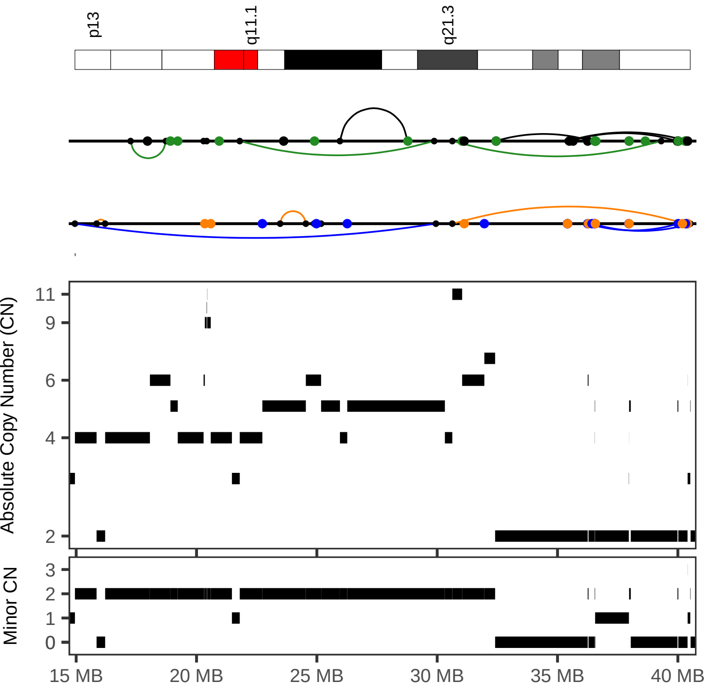

	**CGP_donor_1397077**
Cancer type	Bone−Osteosarc
Position	21:30627381−40515319
Type	With other complex events
Interleaved intrachr. SVs	9
Total SVs (intrachr. + transl.)	47
SV types	DEL: 2; DUP: 2; h2hINV: 4; t2tINV: 1; TRA: 38
SVs in sample	379
Oscillating CN (2 and 3 states)	4, 6
CN segments	21
FDR fragment joints	0.8864524
FDR chr. breakp. enrich.	0
Linked to chrs	5:759551−44322946;6:88458092−155828130
Purity, ploidy	0.47, 2.53

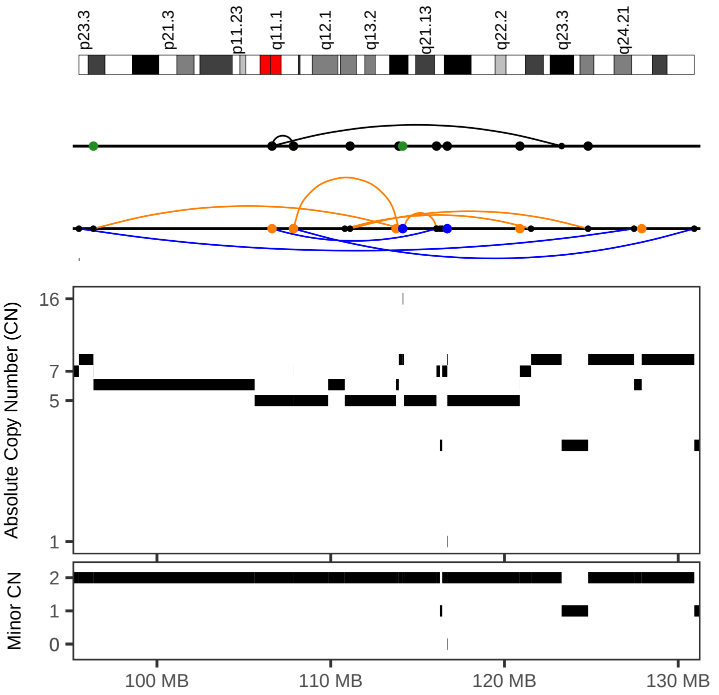

	**CGP_donor_1397083**
Cancer type	Bone−Osteosarc
Position	8:95509557−130921648
Type	With other complex events
Interleaved intrachr. SVs	9
Total SVs (intrachr. + transl.)	29
SV types	DEL: 4; DUP: 3; h2hINV: 2; t2tINV: 0; TRA: 20
SVs in sample	142
Oscillating CN (2 and 3 states)	4, 5
CN segments	28
FDR fragment joints	0.6776251
FDR chr. breakp. enrich.	0
Linked to chrs	13:101502303−104764633;
Purity, ploidy	0.57, 3.02

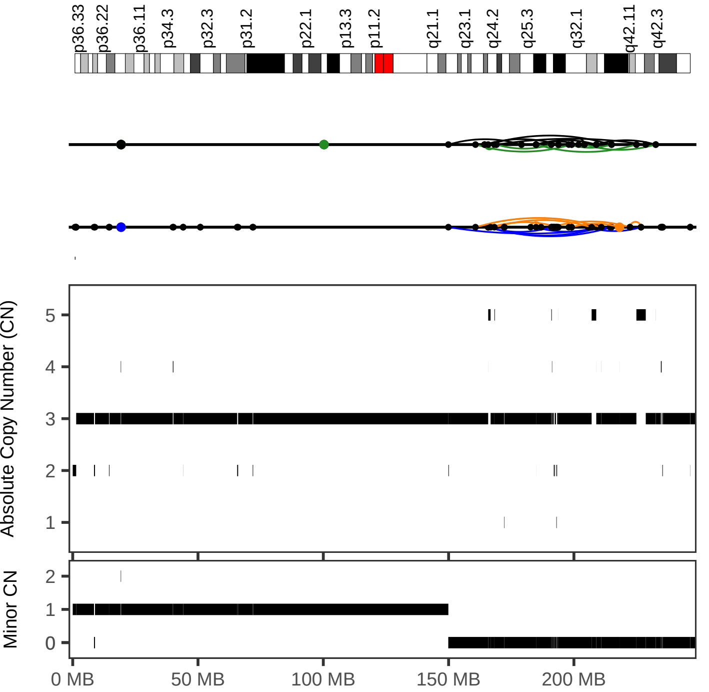

	**CGP_donor_1437403**
Cancer type	Bone−Osteosarc
Position	1:149900456−232658583
Type	With other complex events
Interleaved intrachr. SVs	28
Total SVs (intrachr. + transl.)	29
SV types	DEL: 8; DUP: 6; h2hINV: 8; t2tINV: 6; TRA: 1
SVs in sample	445
Oscillating CN (2 and 3 states)	6, 11
CN segments	36
FDR fragment joints	0.8824283
FDR chr. breakp. enrich.	0
Linked to chrs	
Purity, ploidy	0.8, 2.57

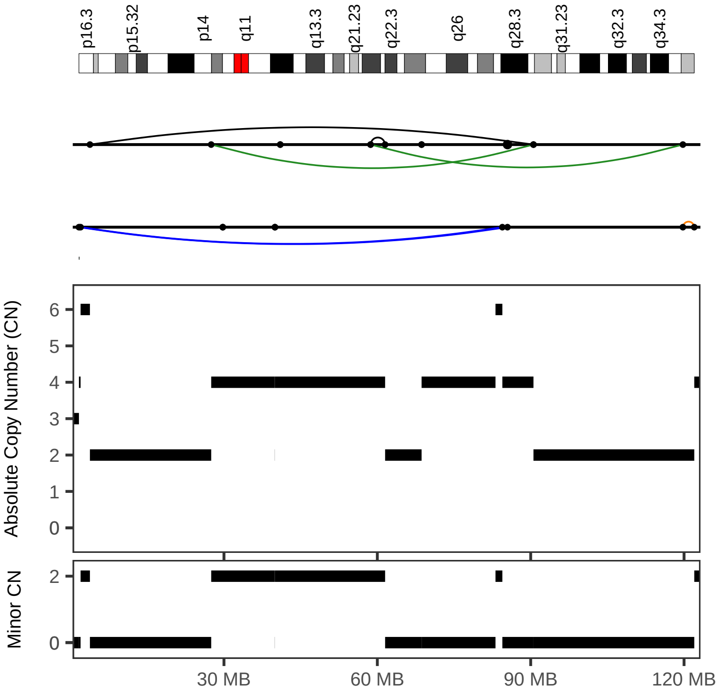

	**CGP_donor_1475256**
Cancer type	Bone−Osteosarc
Position	4:1628586−119770886
Type	With other complex events
Interleaved intrachr. SVs	6
Total SVs (intrachr. + transl.)	7
SV types	DEL: 0; DUP: 2; h2hINV: 2; t2tINV: 2; TRA: 1
SVs in sample	217
Oscillating CN (2 and 3 states)	6, 8
CN segments	11
FDR fragment joints	0.6776251
FDR chr. breakp. enrich.	0.81
Linked to chrs	
Purity, ploidy	0.79, 3.21

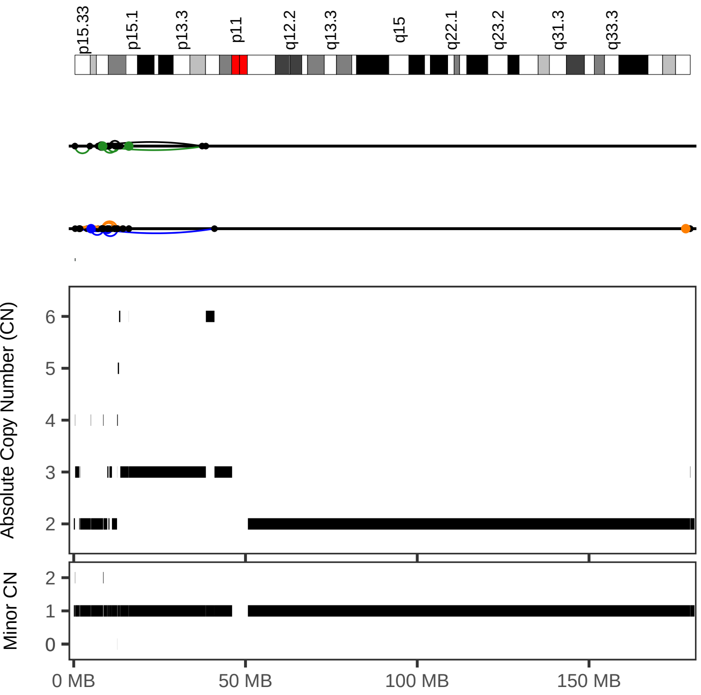

	CGP_donor_1490914
Cancer type	Bone−Osteosarc
Position	5:343444−40967400
Type	With other complex events
Interleaved intrachr. SVs	28
Total SVs (intrachr. + transl.)	32
SV types	DEL: 10; DUP: 8; h2hINV: 4; t2tINV: 6; TRA: 4
SVs in sample	462
Oscillating CN (2 and 3 states)	6, 15
CN segments	23
FDR fragment joints	0.8505431
FDR chr. breakp. enrich.	0.49
Linked to chrs	10:50348934−97731678;
Purity, ploidy	0.64, 2.03

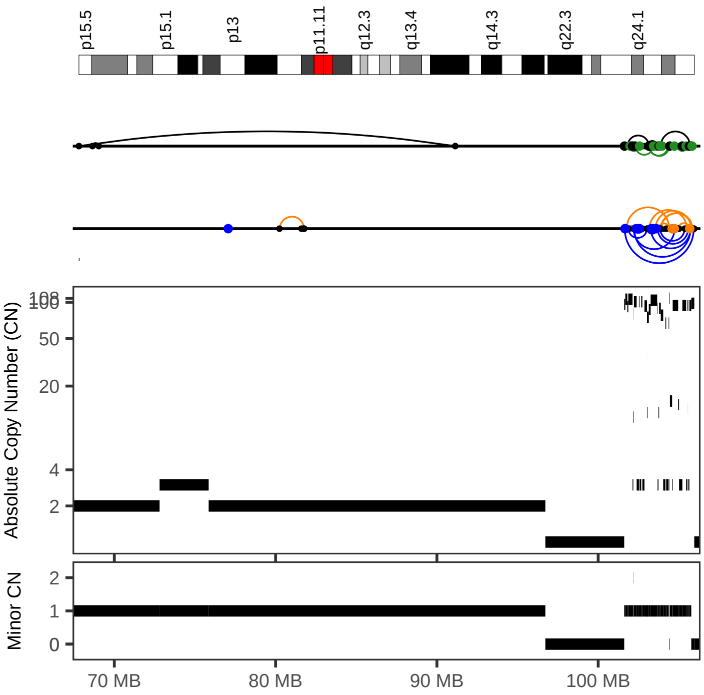

	CGP_donor_1490914
Cancer type	Bone−Osteosarc
Position	11:101720341−105872169
Type	With other complex events
Interleaved intrachr. SVs	35
Total SVs (intrachr. + transl.)	64
SV types	DEL: 11; DUP: 11; h2hINV: 5; t2tINV: 8; TRA: 29
SVs in sample	462
Oscillating CN (2 and 3 states)	6, 7
CN segments	43
FDR fragment joints	0.9840364
FDR chr. breakp. enrich.	0
Linked to chrs	6:76106264−166727719;
Purity, ploidy	0.64, 2.03

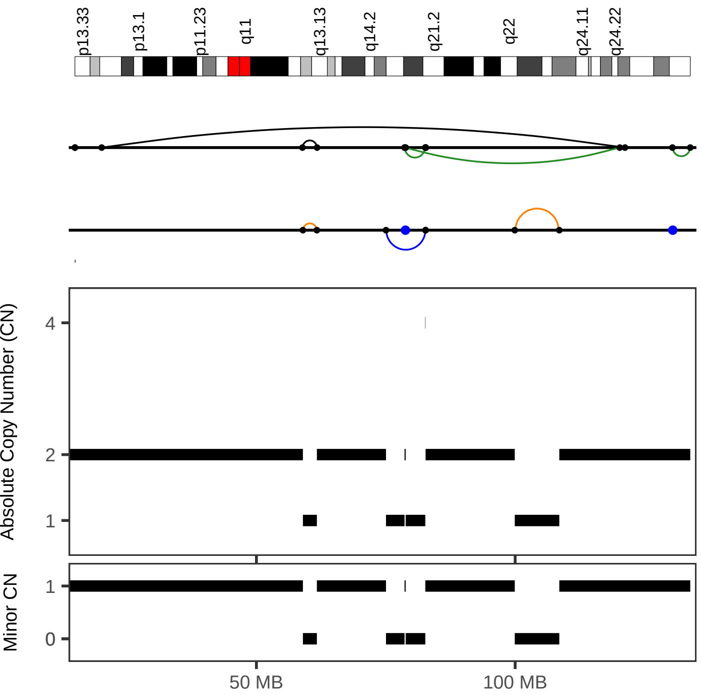

	CGP_donor_1490914
Cancer type	Bone−Osteosarc
Position	12:75035974−120211927
Type	With other complex events
Interleaved intrachr. SVs	6
Total SVs (intrachr. + transl.)	7
SV types	DEL: 1; DUP: 1; h2hINV: 2; t2tINV: 2; TRA: 1
SVs in sample	462
Oscillating CN (2 and 3 states)	4, 8
CN segments	8
FDR fragment joints	0.9599662
FDR chr. breakp. enrich.	0.51
Linked to chrs	
Purity, ploidy	0.64, 2.03

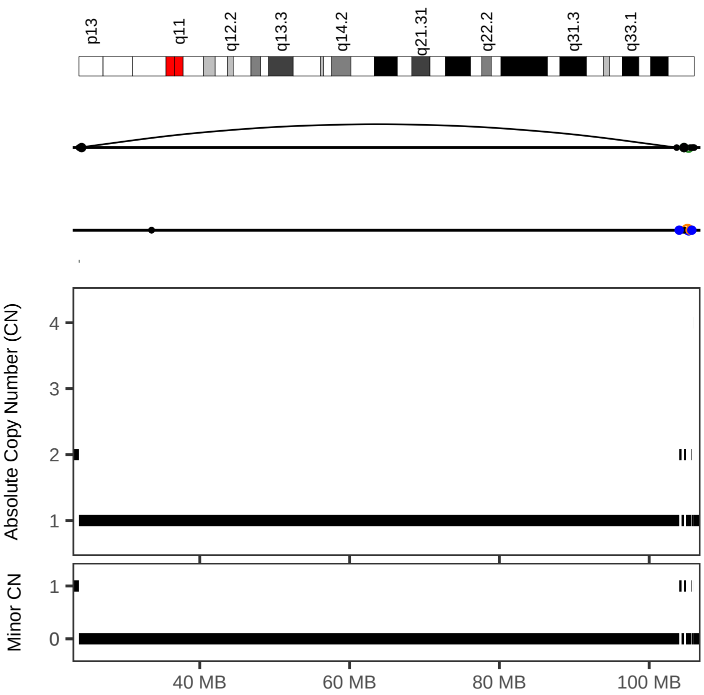

	CGP_donor_1490914
Cancer type	Bone−Osteosarc
Position	13:104316198−106010540
Type	Canonical without polyploidization
Interleaved intrachr. SVs	9
Total SVs (intrachr. + transl.)	13
SV types	DEL: 2; DUP: 4; h2hINV: 2; t2tINV: 1; TRA: 4
SVs in sample	462
Oscillating CN (2 and 3 states)	5, 7
CN segments	7
FDR fragment joints	0.7995907
FDR chr. breakp. enrich.	0.82
Linked to chrs	17:7531323−7839259;
Purity, ploidy	0.64, 2.03

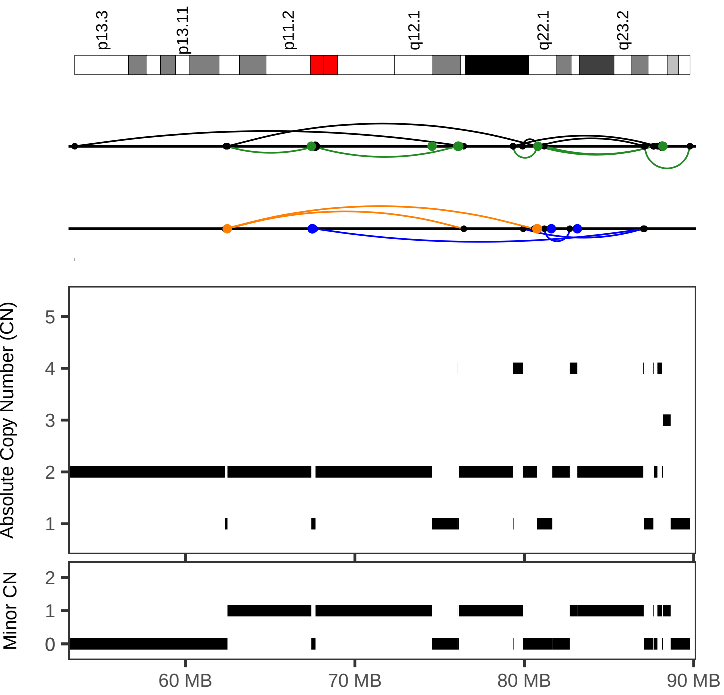

**CGP_donor_1490914**	
Cancer type	Bone−Osteosarc
Position	16:53455552−89780377
Type	With other complex events
Interleaved intrachr. SVs	15
Total SVs (intrachr. + transl.)	30
SV types	DEL: 2; DUP: 2; h2hINV: 5; t2tINV: 6; TRA: 15
SVs in sample	462
Oscillating CN (2 and 3 states)	6, 11
CN segments	26
FDR fragment joints	0.662962
FDR chr. breakp. enrich.	0
Linked to chrs	
Purity, ploidy	0.64, 2.03

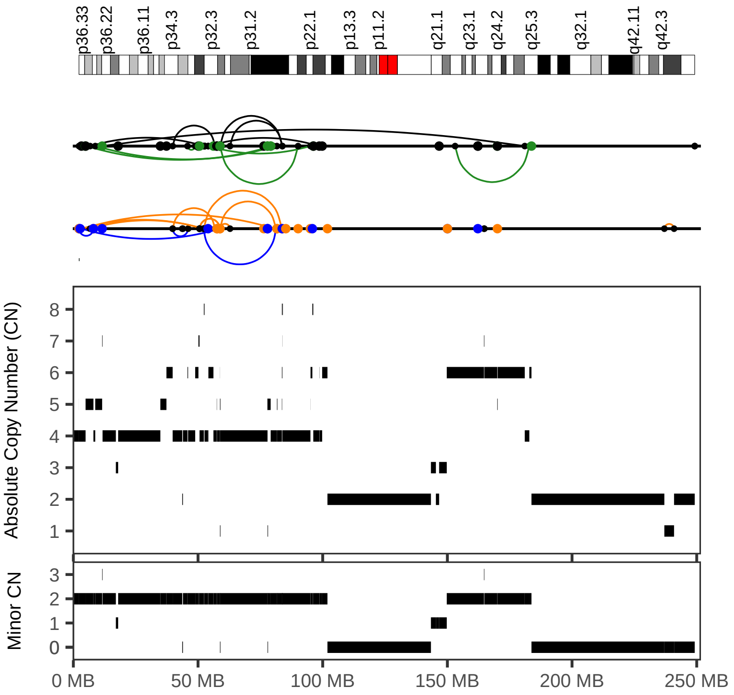

**CGP_donor_1528364**	
Cancer type	Bone−Osteosarc
Position	1:2249362−182869104
Type	With other complex events
Interleaved intrachr. SVs	29
Total SVs (intrachr. + transl.)	86
SV types	DEL: 10; DUP: 5; h2hINV: 7; t2tINV: 7; TRA: 57
SVs in sample	927
Oscillating CN (2 and 3 states)	6, 8
CN segments	64
FDR fragment joints	0.615458
FDR chr. breakp. enrich.	0.04
Linked to chrs	2:98265301−240710955;3:39705096−116872806 9:1299761−9380244;
Purity, ploidy	0.79, 3.29

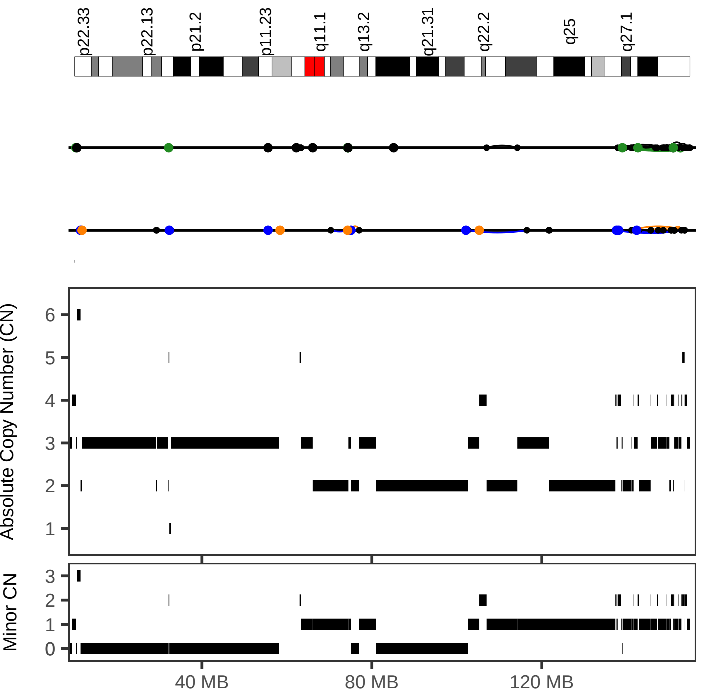

**CGP_donor_1528371**	
Cancer type	Bone−Osteosarc
Position	X:137305293−154877002
Type	With other complex events
Interleaved intrachr. SVs	23
Total SVs (intrachr. + transl.)	30
SV types	DEL: 5; DUP: 3; h2hINV: 6; t2tINV: 9; TRA: 7
SVs in sample	349
Oscillating CN (2 and 3 states)	6, 14
CN segments	34
FDR fragment joints	0.6776251
FDR chr. breakp. enrich.	0
Linked to chrs	
Purity, ploidy	0.33, 3.49

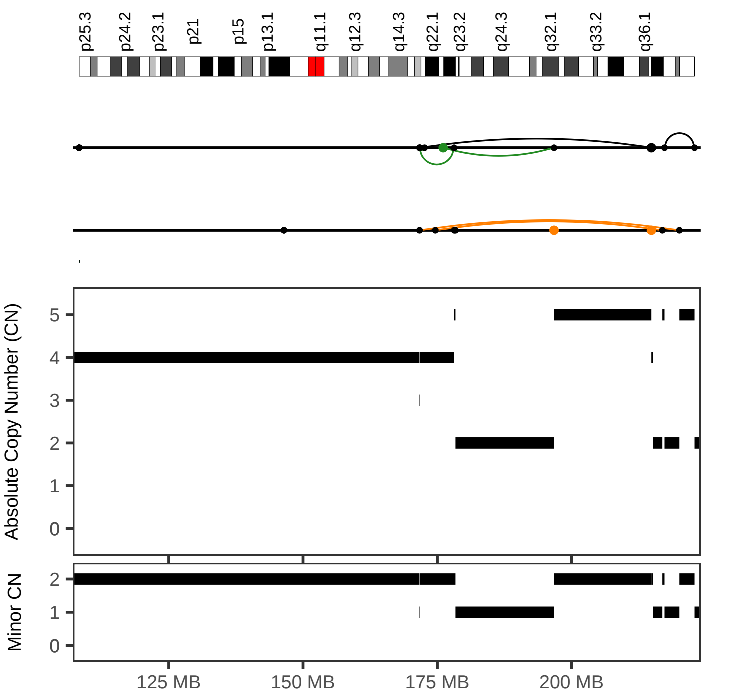

**CGP_donor_1528374**	
Cancer type	Bone−Osteosarc
Position	2:171676936−222890322
Type	With other complex events
Interleaved intrachr. SVs	7
Total SVs (intrachr. + transl.)	11
SV types	DEL: 2; DUP: 0; h2hINV: 2; t2tINV: 3; TRA: 4
SVs in sample	113
Oscillating CN (2 and 3 states)	5, 6
CN segments	11
FDR fragment joints	0.615458
FDR chr. breakp. enrich.	0
Linked to chrs	
Purity, ploidy	0.69, 2.64

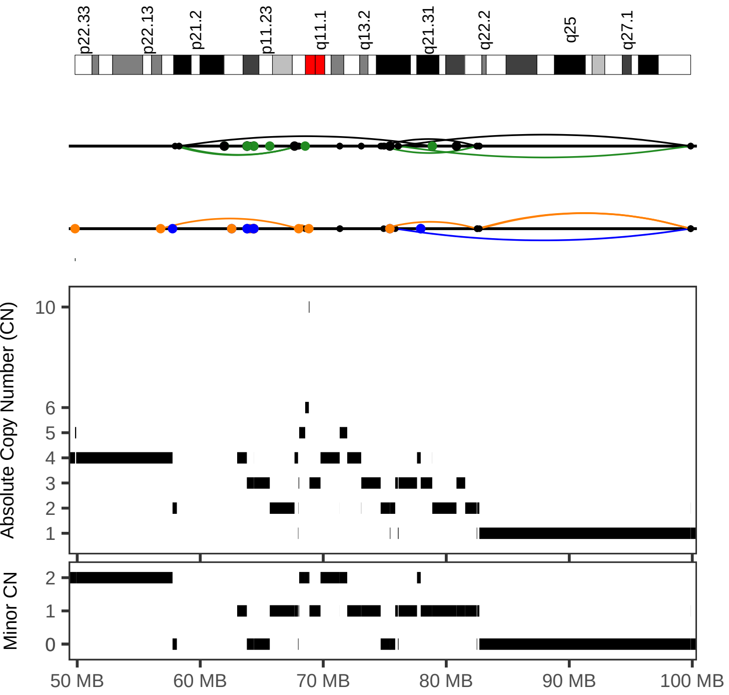

	**CGP_donor_1691133**
Cancer type	Bone−Osteosarc
Position	X:56780045−99873812
Type	With other complex events
Interleaved intrachr. SVs	14
Total SVs (intrachr. + transl.)	48
SV types	DEL: 6; DUP: 1; h2hINV: 3; t2tINV: 4; TRA: 34
SVs in sample	325
Oscillating CN (2 and 3 states)	6, 7
CN segments	38
FDR fragment joints	0.7908979
FDR chr. breakp. enrich.	0
Linked to chrs	19:1003699−31085706;6:32255732−170226667 8:54201065−139568173;
Purity, ploidy	0.68, 2.87

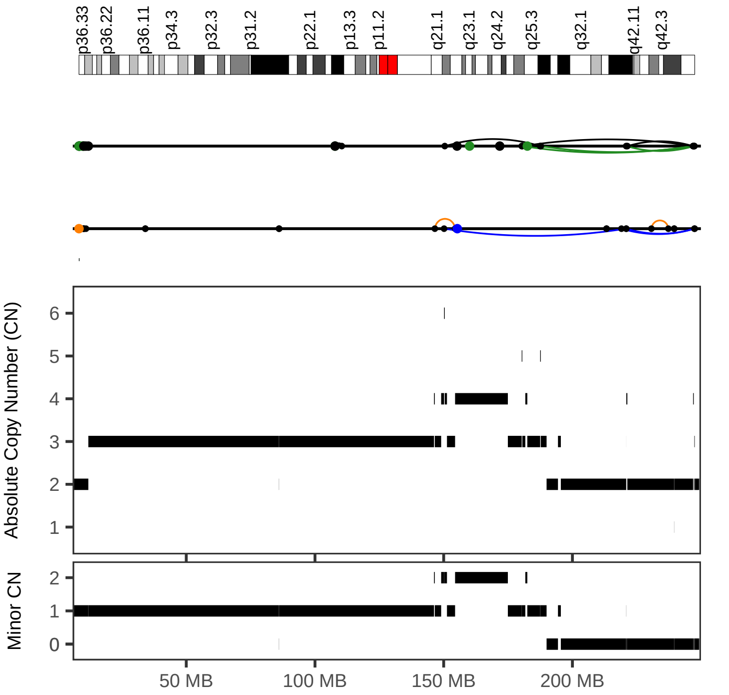

	**CGP_donor_1691207**
Cancer type	Bone−Osteosarc
Position	1:146556486−247517843
Type	With other complex events
Interleaved intrachr. SVs	10
Total SVs (intrachr. + transl.)	15
SV types	DEL: 1; DUP: 3; h2hINV: 3; t2tINV: 3; TRA: 5
SVs in sample	415
Oscillating CN (2 and 3 states)	6, 10
CN segments	26
FDR fragment joints	0.6471662
FDR chr. breakp. enrich.	0.57
Linked to chrs	2:5332159−125723075;7:3613250−157967148
Purity, ploidy	0.71, 2.58

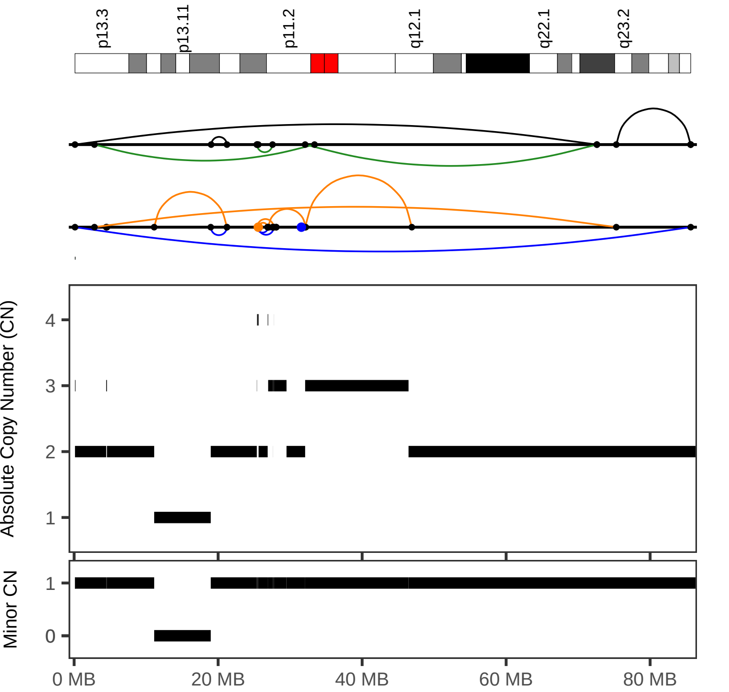

	**CGP_donor_1691207**
Cancer type	Bone−Osteosarc
Position	16:117927−85638502
Type	With other complex events
Interleaved intrachr. SVs	16
Total SVs (intrachr. + transl.)	18
SV types	DEL: 4; DUP: 5; h2hINV: 2; t2tINV: 5; TRA: 2
SVs in sample	415
Oscillating CN (2 and 3 states)	4, 11
CN segments	21
FDR fragment joints	0.6776251
FDR chr. breakp. enrich.	0
Linked to chrs	
Purity, ploidy	0.71, 2.58

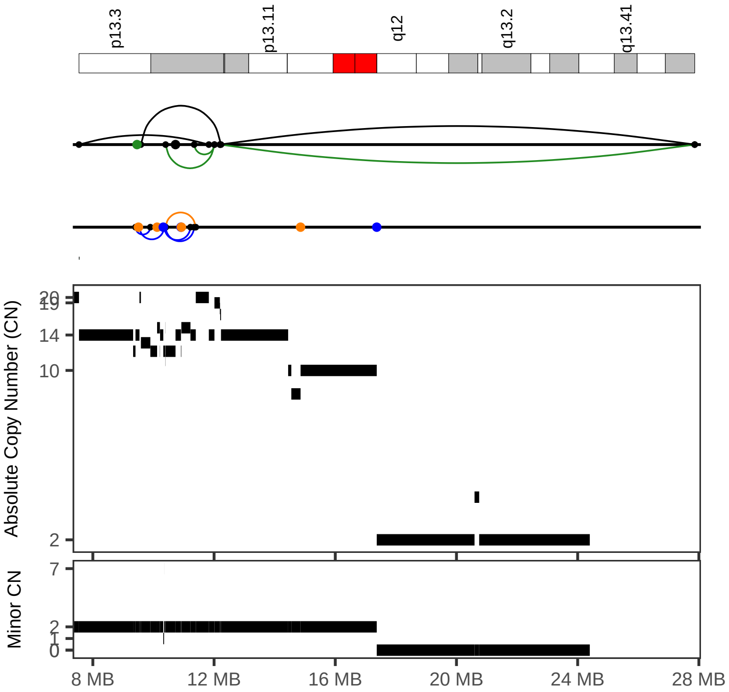

	**CGP_donor_1691210**
Cancer type	Bone−Osteosarc
Position	19:7542257−27863221
Type	With other complex events
Interleaved intrachr. SVs	11
Total SVs (intrachr. + transl.)	20
SV types	DEL: 1; DUP: 4; h2hINV: 3; t2tINV: 3; TRA: 9
SVs in sample	183
Oscillating CN (2 and 3 states)	5, 9
CN segments	32
FDR fragment joints	0.8572806
FDR chr. breakp. enrich.	0
Linked to chrs	17:25441812−34068071;
Purity, ploidy	0.85, 3.59

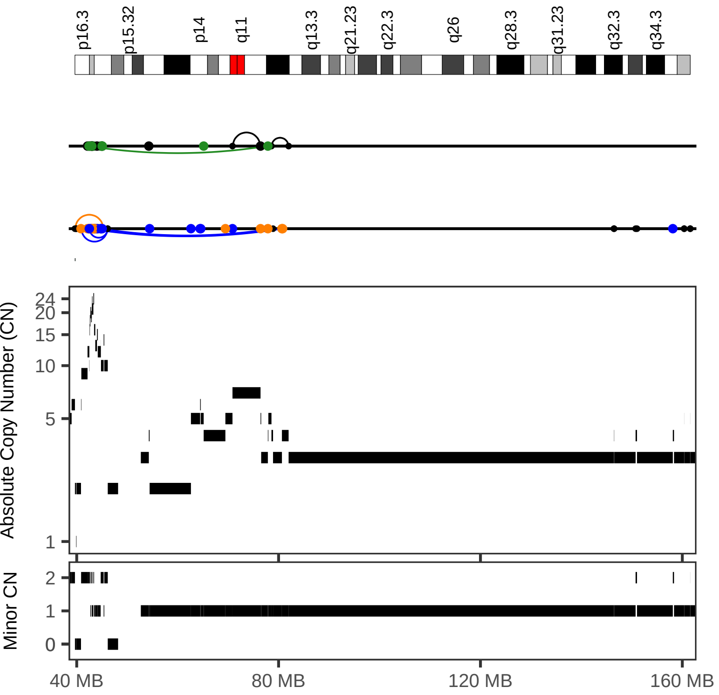

	**CGP_donor_1691211**
Cancer type	Bone−Osteosarc
Position	4:39643156−81985932
Type	With other complex events
Interleaved intrachr. SVs	8
Total SVs (intrachr. + transl.)	51
SV types	DEL: 1; DUP: 4; h2hINV: 1; t2tINV: 2; TRA: 43
SVs in sample	606
Oscillating CN (2 and 3 states)	4, 5
CN segments	43
FDR fragment joints	0.6776251
FDR chr. breakp. enrich.	0
Linked to chrs	16:6326850−23636185;20:14482537−55742990 5:27518365−44838184;9:72321762−137935608
Purity, ploidy	0.62, 3.16

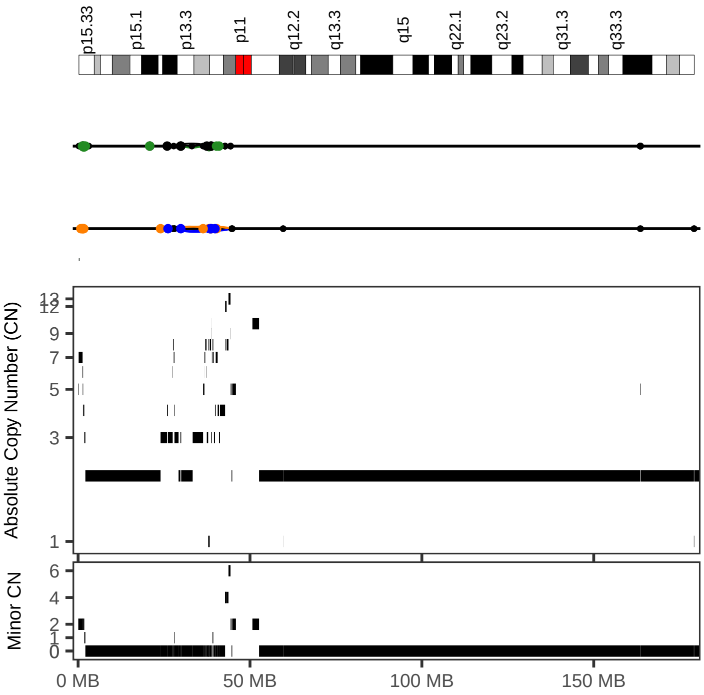

	**CGP_donor_1691211**
Cancer type	Bone−Osteosarc
Position	5:27518365−44838185
Type	With other complex events
Interleaved intrachr. SVs	15
Total SVs (intrachr. + transl.)	30
SV types	DEL: 4; DUP: 5; h2hINV: 4; t2tINV: 2; TRA: 15
SVs in sample	606
Oscillating CN (2 and 3 states)	5, 10
CN segments	43
FDR fragment joints	0.8572806
FDR chr. breakp. enrich.	0.01
Linked to chrs	9:72321762−137935608;
Purity, ploidy	0.62, 3.16

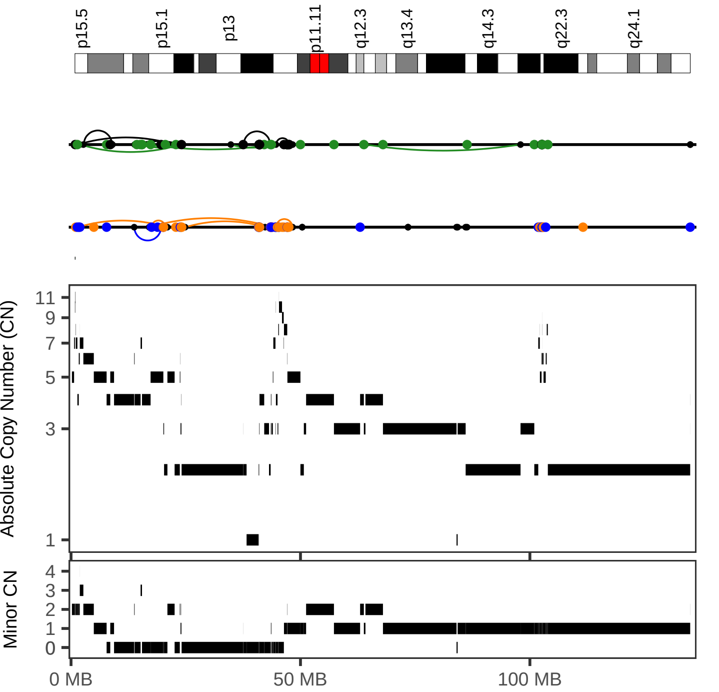

	**CGP_donor_1691211**
Cancer type	Bone−Osteosarc
Position	11:1368421−44085211
Type	With other complex events
Interleaved intrachr. SVs	15
Total SVs (intrachr. + transl.)	65
SV types	DEL: 6; DUP: 1; h2hINV: 3; t2tINV: 5; TRA: 50
SVs in sample	606
Oscillating CN (2 and 3 states)	4, 11
CN segments	43
FDR fragment joints	0.9794281
FDR chr. breakp. enrich.	0
Linked to chrs	1:20463312−245809115;16:6326850−23636185 2:9233757−238199399;9:72321762−137935608
Purity, ploidy	0.62, 3.16

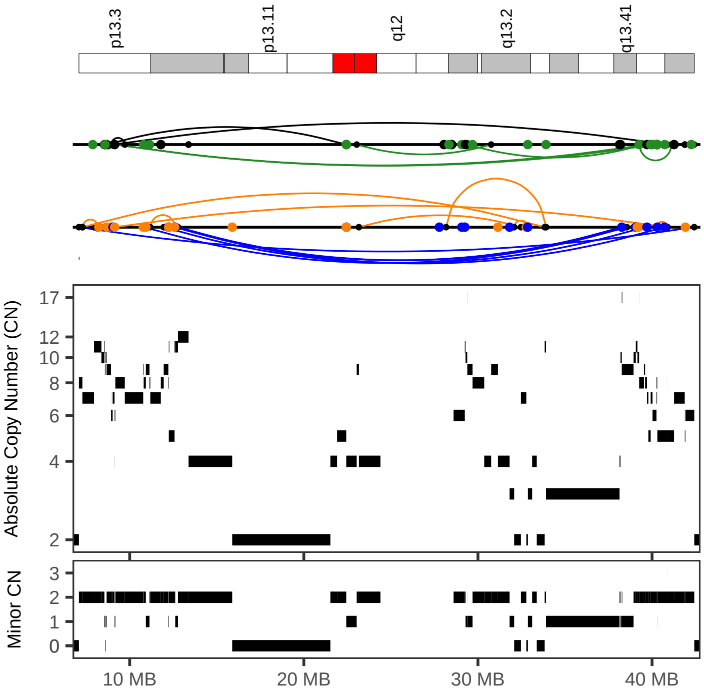

	**CGP_donor_1691211**
Cancer type	Bone−Osteosarc
Position	19:7281972−41134872
Type	With other complex events
Interleaved intrachr. SVs	23
Total SVs (intrachr. + transl.)	86
SV types	DEL: 7; DUP: 7; h2hINV: 3; t2tINV: 6; TRA: 63
SVs in sample	606
Oscillating CN (2 and 3 states)	4, 8
CN segments	77
FDR fragment joints	0.7829107
FDR chr. breakp. enrich.	0
Linked to chrs	16:6326850−23636185;17:996864−13569640 2:9233757−238199399;5:27518365−44838184 9:72321762−137935608;
Purity, ploidy	0.62, 3.16

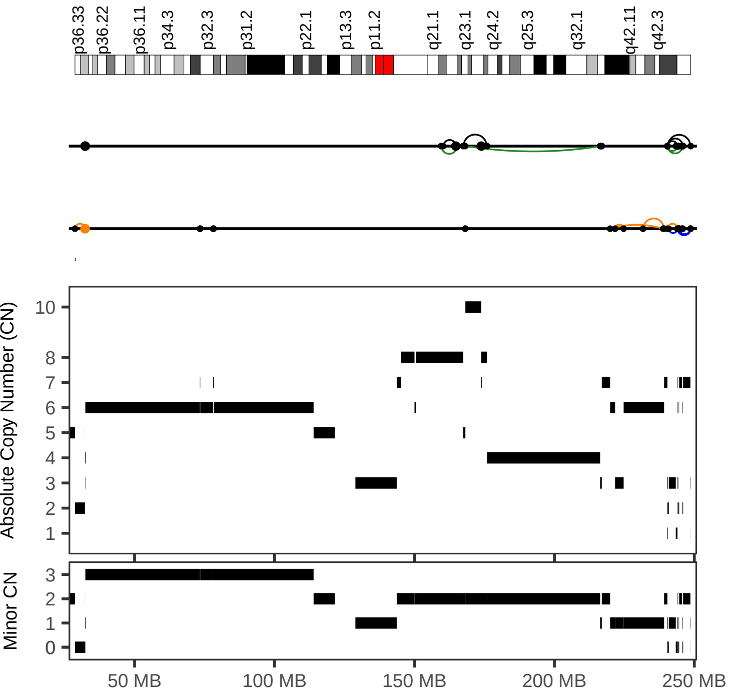

	**CGP_donor_1691212**
Cancer type	Bone−Osteosarc
Position	1:240384329−248714171
Type	With other complex events
Interleaved intrachr. SVs	19
Total SVs (intrachr. + transl.)	19
SV types	DEL: 1; DUP: 7; h2hINV: 7; t2tINV: 4; TRA: 0
SVs in sample	135
Oscillating CN (2 and 3 states)	6, 17
CN segments	23
FDR fragment joints	0.615458
FDR chr. breakp. enrich.	0
Linked to chrs	
Purity, ploidy	0.74, 4

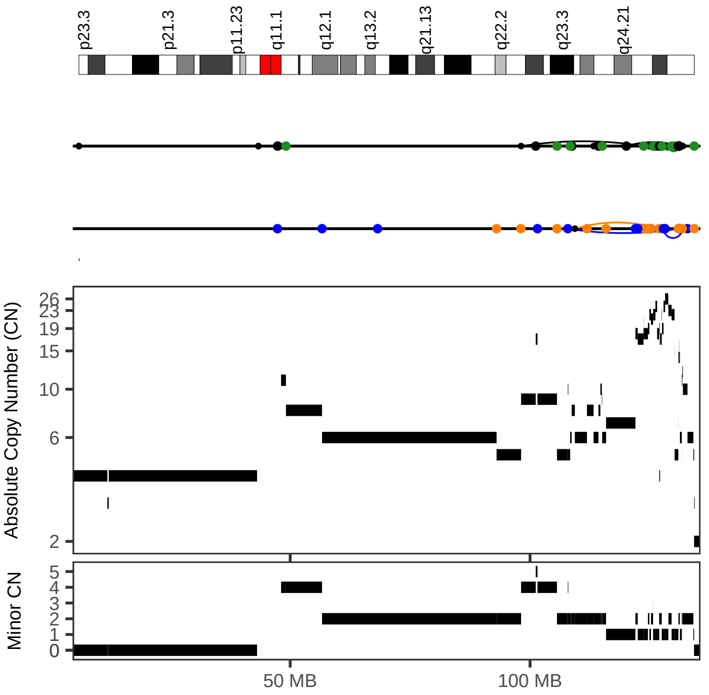

	**CGP_donor_1691213**
Cancer type	Bone−Osteosarc
Position	8:98131760−131862131
Type	With other complex events
Interleaved intrachr. SVs	10
Total SVs (intrachr. + transl.)	54
SV types	DEL: 1; DUP: 2; h2hINV: 3; t2tINV: 4; TRA: 44
SVs in sample	596
Oscillating CN (2 and 3 states)	6, 6
CN segments	53
FDR fragment joints	0.9284301
FDR chr. breakp. enrich.	0
Linked to chrs	12:38831735−119704558;
Purity, ploidy	0.87, 3.53

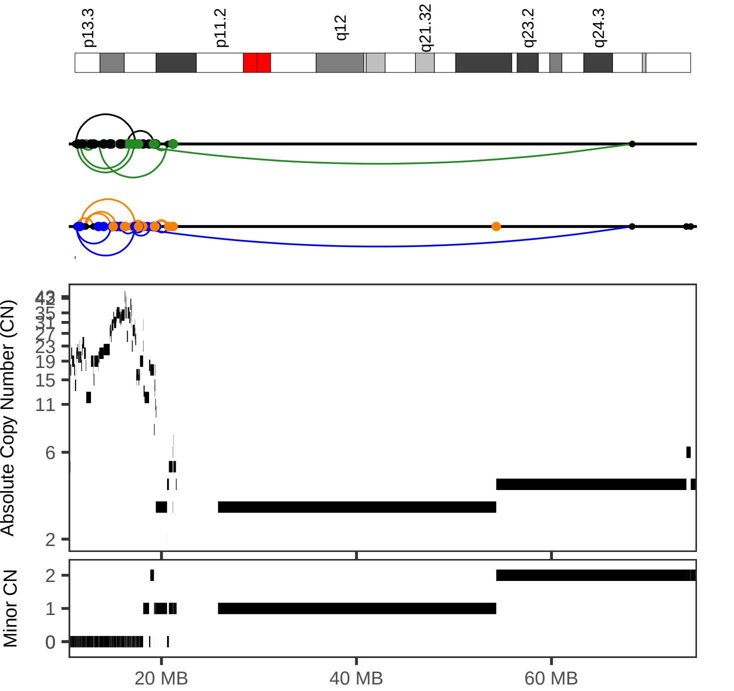

	**CGP_donor_1691213**
Cancer type	Bone−Osteosarc
Position	17:11128144−68285285
Type	With other complex events
Interleaved intrachr. SVs	31
Total SVs (intrachr. + transl.)	108
SV types	DEL: 11; DUP: 8; h2hINV: 5; t2tINV: 7; TRA: 77
SVs in sample	596
Oscillating CN (2 and 3 states)	4, 10
CN segments	98
FDR fragment joints	0.9685443
FDR chr. breakp. enrich.	0
Linked to chrs	10:104364960−104413206;19:7659219−23705866 8:98131760−131862130;
Purity, ploidy	0.87, 3.53

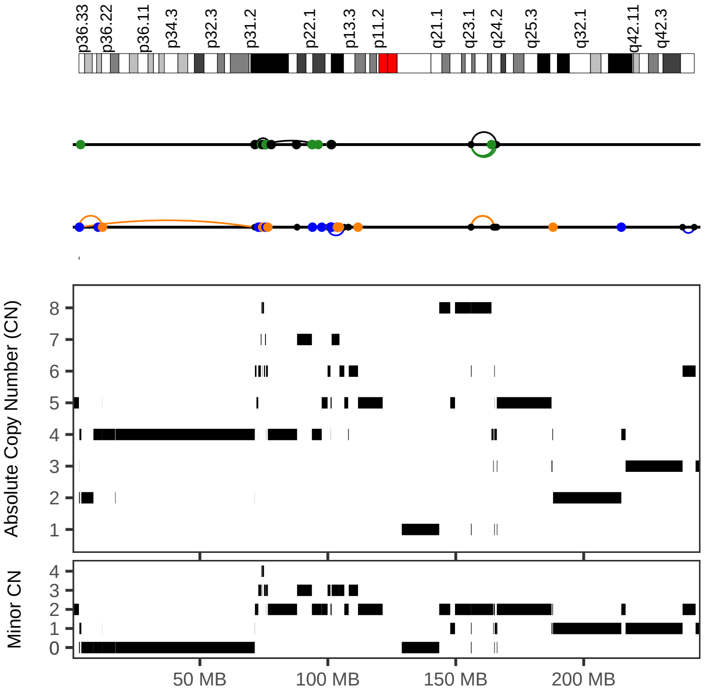

	**CGP_donor_1691214**
Cancer type	Bone−Osteosarc
Position	1:155935299−166050473
Type	With other complex events
Interleaved intrachr. SVs	14
Total SVs (intrachr. + transl.)	15
SV types	DEL: 7; DUP: 0; h2hINV: 2; t2tINV: 5; TRA: 1
SVs in sample	652
Oscillating CN (2 and 3 states)	6, 6
CN segments	19
FDR fragment joints	0.5435077
FDR chr. breakp. enrich.	0.16
Linked to chrs	
Purity, ploidy	0.49, 3.33

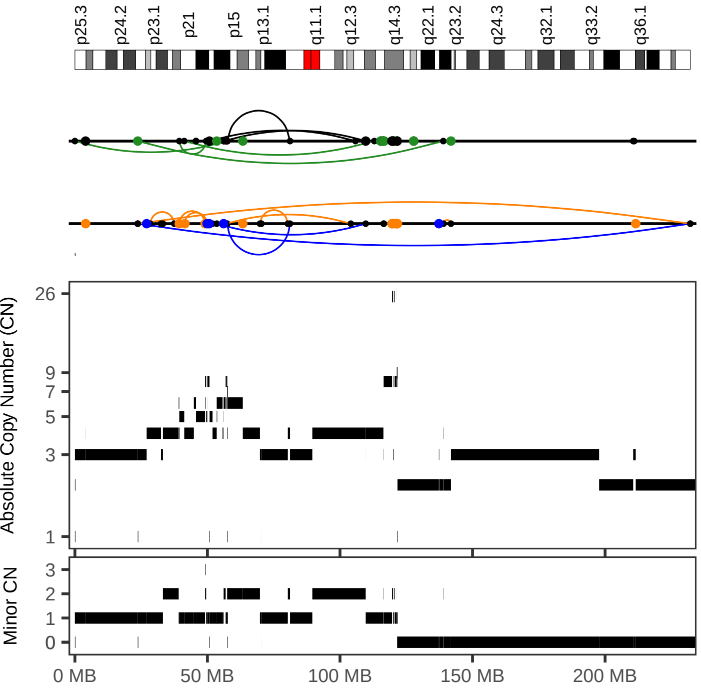

	CGP_donor_1691214
Cancer type	Bone−Osteosarc
Position	2:11400−232123609
Type	With other complex events
Interleaved intrachr. SVs	20
Total SVs (intrachr. + transl.)	60
SV types	DEL: 5; DUP: 5; h2hINV: 4; t2tINV: 6; TRA: 40
SVs in sample	652
Oscillating CN (2 and 3 states)	6, 9
CN segments	70
FDR fragment joints	0.6776251
FDR chr. breakp. enrich.	0.04
Linked to chrs	1:155935299−166050472;14:41964680−78837997 3:29117586−189128944;X:48348455−150018346
Purity, ploidy	0.49, 3.33

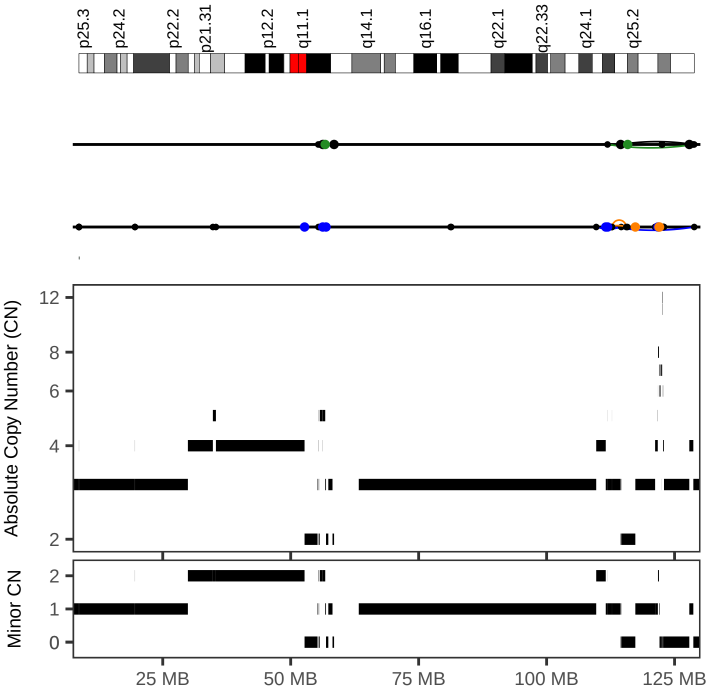

	CGP_donor_1691217
Cancer type	Bone−Osteosarc
Position	6:109694663−128848927
Type	With other complex events
Interleaved intrachr. SVs	6
Total SVs (intrachr. + transl.)	21
SV types	DEL: 2; DUP: 6; h2hINV: 2; t2tINV: 2; TRA: 9
SVs in sample	380
Oscillating CN (2 and 3 states)	5, 8
CN segments	26
FDR fragment joints	0.615458
FDR chr. breakp. enrich.	0.02
Linked to chrs	14:27063592−79165065;X:88486379−153095440
Purity, ploidy	0.53, 2.89

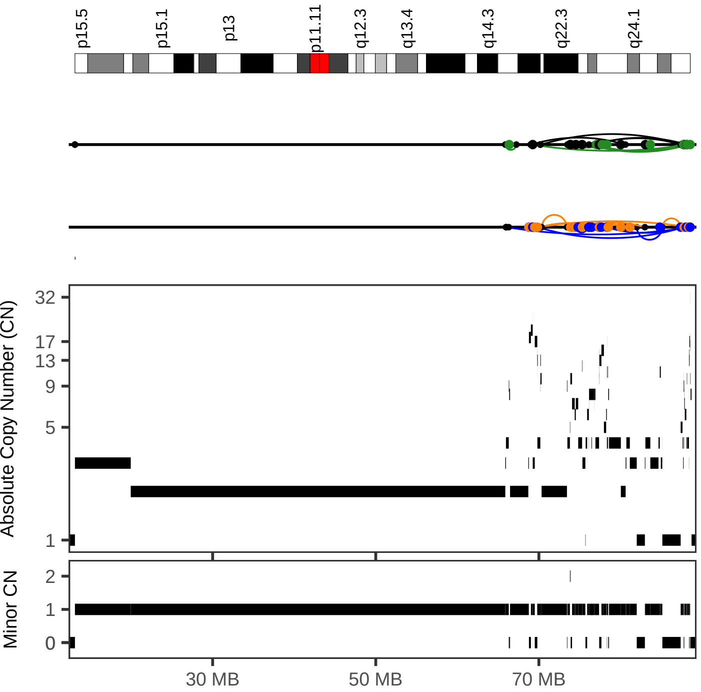

	CGP_donor_1232859
Cancer type	Breast−AdenoCA
Position	11:68987281−88452236
Type	With other complex events
Interleaved intrachr. SVs	24
Total SVs (intrachr. + transl.)	73
SV types	DEL: 7; DUP: 5; h2hINV: 7; t2tINV: 5; TRA: 49
SVs in sample	316
Oscillating CN (2 and 3 states)	4, 8
CN segments	82
FDR fragment joints	0.8653243
FDR chr. breakp. enrich.	0
Linked to chrs	6:56201574−162603779;20:33258582−62543744
Purity, ploidy	0.54, 2.17

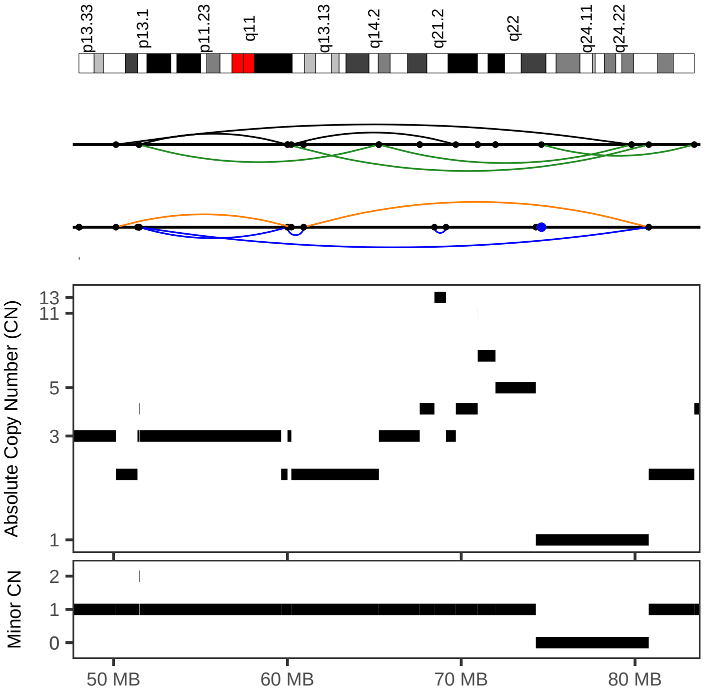

	CGP_donor_1232859
Cancer type	Breast−AdenoCA
Position	12:50134439−83407108
Type	With other complex events
Interleaved intrachr. SVs	14
Total SVs (intrachr. + transl.)	16
SV types	DEL: 3; DUP: 4; h2hINV: 3; t2tINV: 4; TRA: 2
SVs in sample	316
Oscillating CN (2 and 3 states)	5, 9
CN segments	18
FDR fragment joints	0.9501265
FDR chr. breakp. enrich.	0.09
Linked to chrs	
Purity, ploidy	0.54, 2.17

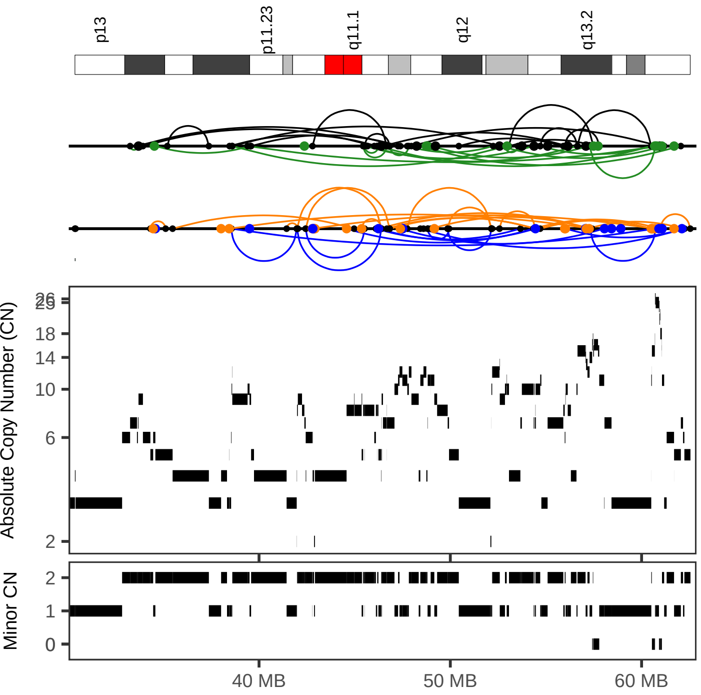

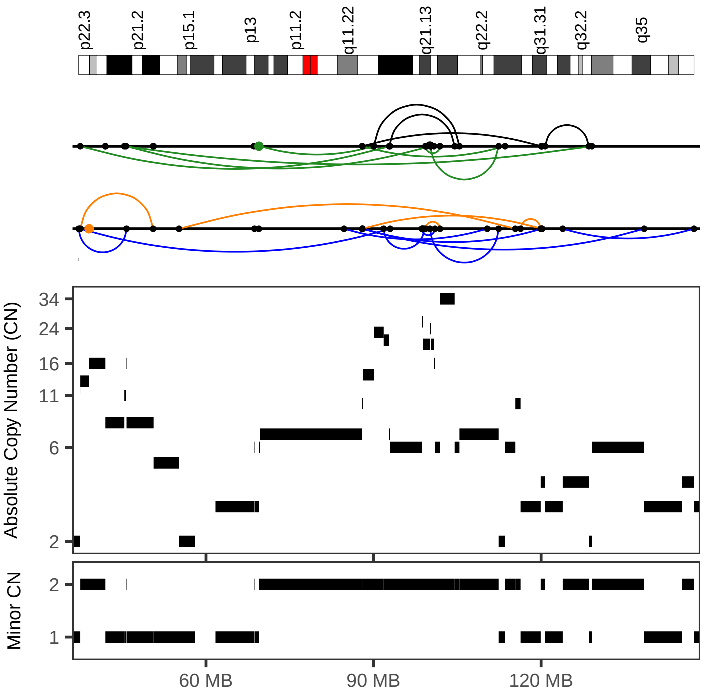

	**CGP_donor_1232859**
Cancer type	Breast−AdenoCA
Position	20:33258582−62543745
Type	With other complex events
Interleaved intrachr. SVs	71
Total SVs (intrachr. + transl.)	130
SV types	DEL: 21; DUP: 14; h2hINV: 18; t2tINV: 18; TRA: 59
SVs in sample	316
Oscillating CN (2 and 3 states)	5, 10
CN segments	145
FDR fragment joints	0.6776251
FDR chr. breakp. enrich.	0
Linked to chrs	6:56201574−162603779;12:50134439−83407107
Purity, ploidy	0.54, 2.17

	**CGP_donor_1234121**
Cancer type	Breast−AdenoCA
Position	7:37201530−147378535
Type	With other complex events
Interleaved intrachr. SVs	25
Total SVs (intrachr. + transl.)	27
SV types	DEL: 4; DUP: 9; h2hINV: 5; t2tINV: 7; TRA: 2
SVs in sample	149
Oscillating CN (2 and 3 states)	4, 7
CN segments	44
FDR fragment joints	0.8393975
FDR chr. breakp. enrich.	0
Linked to chrs	
Purity, ploidy	0.4, 3.56

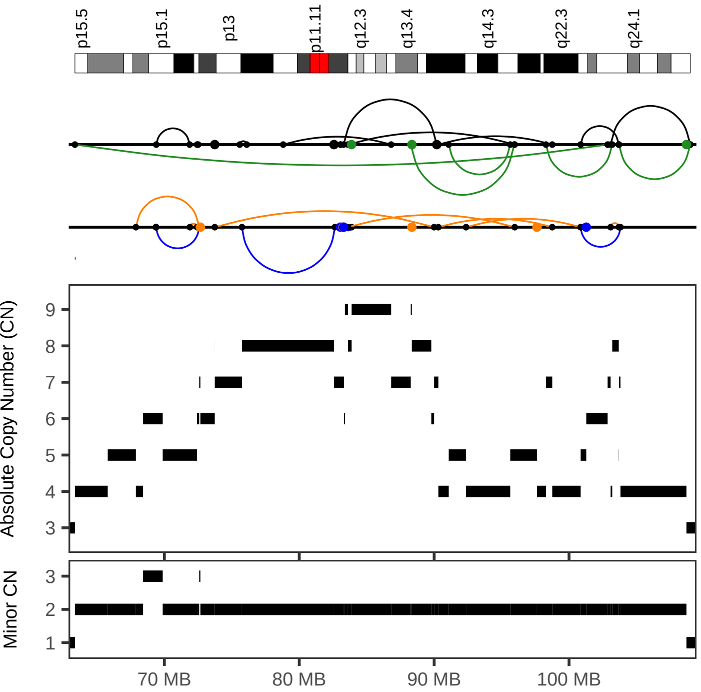

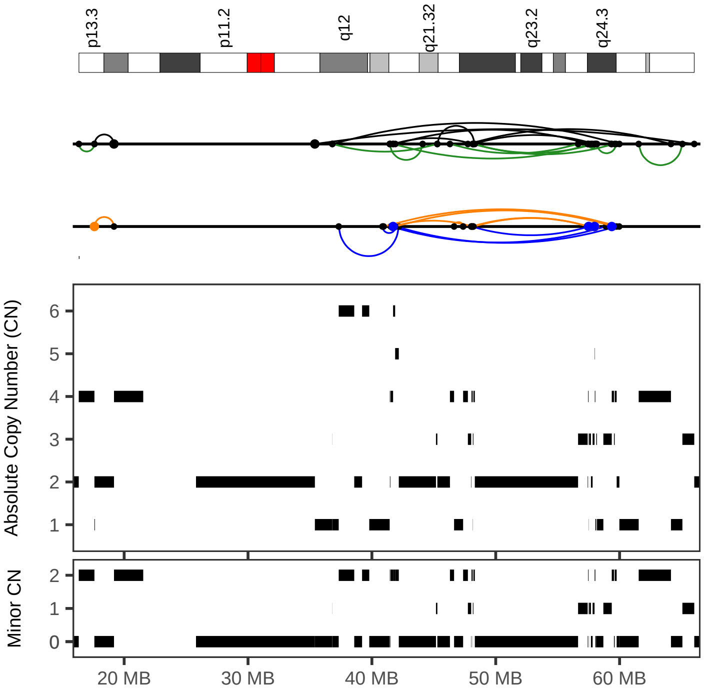

	**CGP_donor_1234121**
Cancer type	Breast−AdenoCA
Position	11:63368164−108986358
Type	With other complex events
Interleaved intrachr. SVs	20
Total SVs (intrachr. + transl.)	33
SV types	DEL: 5; DUP: 3; h2hINV: 7; t2tINV: 5; TRA: 13
SVs in sample	149
Oscillating CN (2 and 3 states)	5, 7
CN segments	37
FDR fragment joints	0.8653243
FDR chr. breakp. enrich.	0
Linked to chrs	
Purity, ploidy	0.4, 3.56

	**CGP_donor_1234124**
Cancer type	Breast−AdenoCA
Position	17:36804652−65070726
Type	With other complex events
Interleaved intrachr. SVs	26
Total SVs (intrachr. + transl.)	32
SV types	DEL: 5; DUP: 5; h2hINV: 7; t2tINV: 9; TRA: 6
SVs in sample	95
Oscillating CN (2 and 3 states)	5, 8
CN segments	49
FDR fragment joints	0.9501265
FDR chr. breakp. enrich.	0
Linked to chrs	
Purity, ploidy	0.59, 3

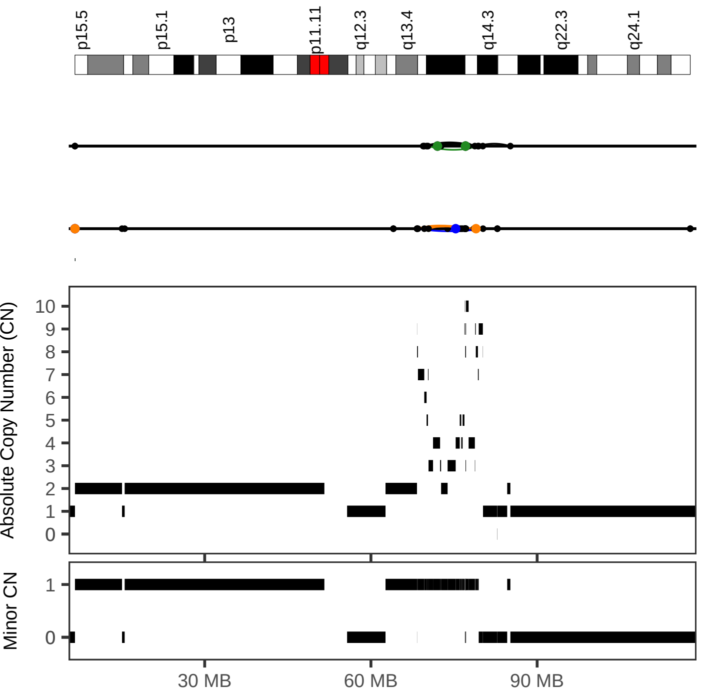

CGP_donor_1337214	
Cancer type	Breast−AdenoCA
Position	11:68314464−85167264
Type	With other complex events
Interleaved intrachr. SVs	12
Total SVs (intrachr. + transl.)	19
SV types	DEL: 4; DUP: 2; h2hINV: 5; t2tINV: 1; TRA: 7
SVs in sample	161
Oscillating CN (2 and 3 states)	4, 9
CN segments	36
FDR fragment joints	0.615458
FDR chr. breakp. enrich.	0
Linked to chrs	
Purity, ploidy	0.63, 1.91

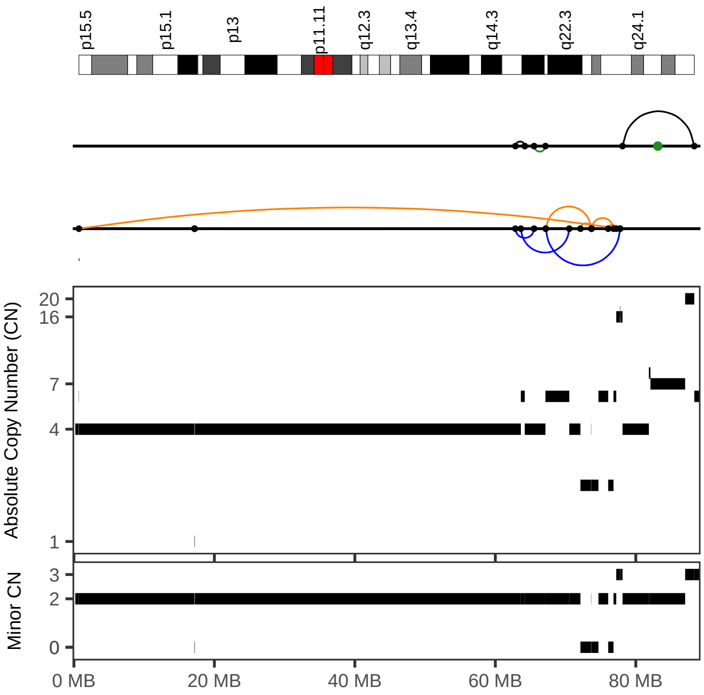

CGP_donor_1337217	
Cancer type	Breast−AdenoCA
Position	11:712704−77789504
Type	With other complex events
Interleaved intrachr. SVs	7
Total SVs (intrachr. + transl.)	7
SV types	DEL: 2; DUP: 3; h2hINV: 1; t2tINV: 1; TRA: 0
SVs in sample	143
Oscillating CN (2 and 3 states)	5, 7
CN segments	16
FDR fragment joints	0.7735152
FDR chr. breakp. enrich.	0.02
Linked to chrs	
Purity, ploidy	0.29, 3.57

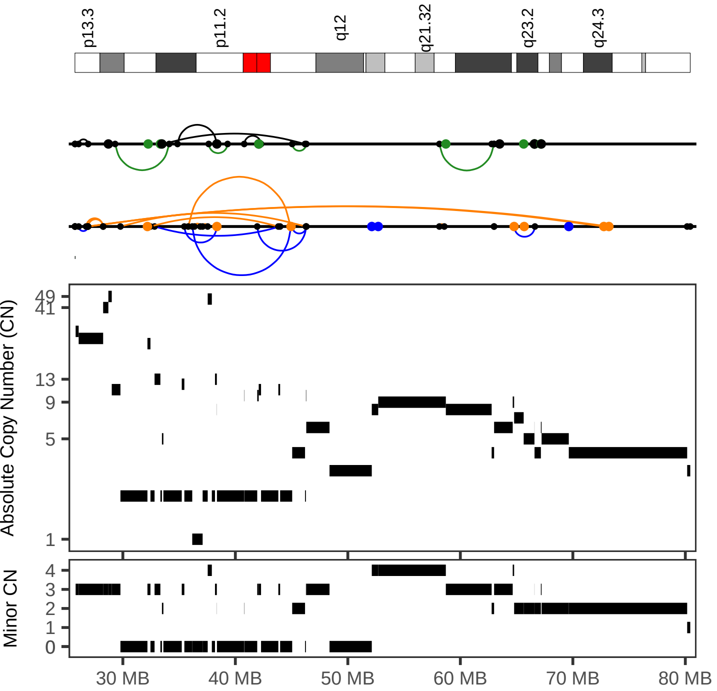

CGP_donor_1337217	
Cancer type	Breast−AdenoCA
Position	17:29313383−46309227
Type	With other complex events
Interleaved intrachr. SVs	14
Total SVs (intrachr. + transl.)	23
SV types	DEL: 3; DUP: 5; h2hINV: 3; t2tINV: 3; TRA: 9
SVs in sample	143
Oscillating CN (2 and 3 states)	4, 6
CN segments	29
FDR fragment joints	0.9990251
FDR chr. breakp. enrich.	0
Linked to chrs	
Purity, ploidy	0.29, 3.57

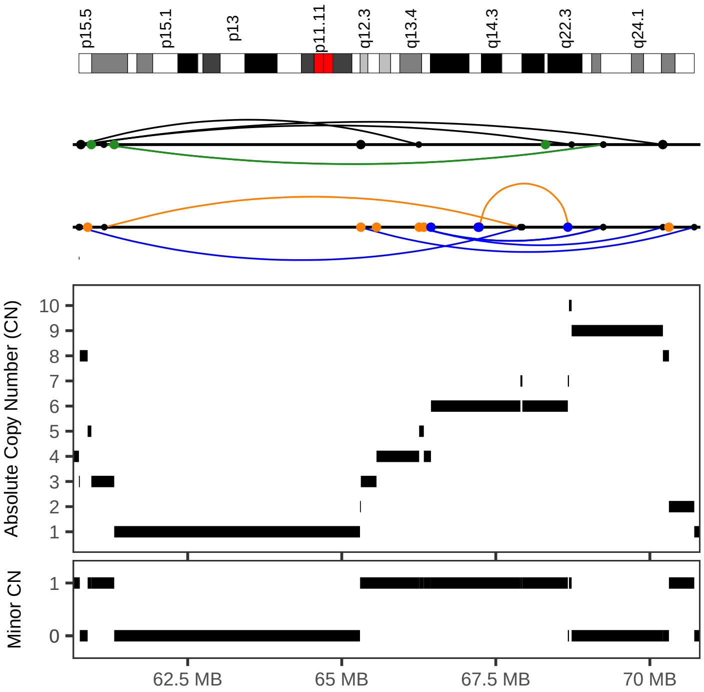

CGP_donor_1337220	
Cancer type	Breast−AdenoCA
Position	11:60734477−70720476
Type	With other complex events
Interleaved intrachr. SVs	13
Total SVs (intrachr. + transl.)	29
SV types	DEL: 3; DUP: 5; h2hINV: 3; t2tINV: 2; TRA: 16
SVs in sample	46
Oscillating CN (2 and 3 states)	4, 6
CN segments	19
FDR fragment joints	0.7359483
FDR chr. breakp. enrich.	0
Linked to chrs	
Purity, ploidy	0.51, 2.07

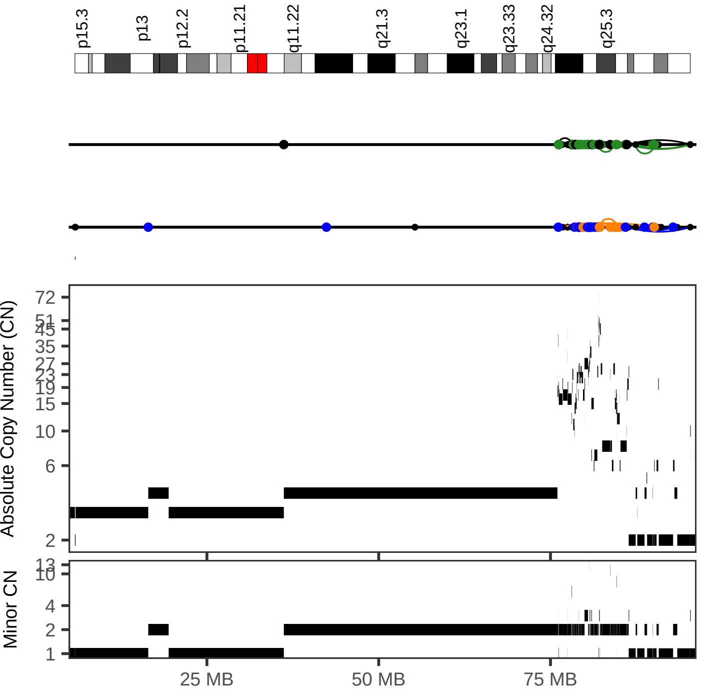

	**CGP_donor_1337223**
Cancer type	Breast−AdenoCA
Position	10:76016429−95343437
Type	With other complex events
Interleaved intrachr. SVs	27
Total SVs (intrachr. + transl.)	89
SV types	DEL: 8; DUP: 4; h2hINV: 9; t2tINV: 6; TRA: 62
SVs in sample	318
Oscillating CN (2 and 3 states)	4, 6
CN segments	107
FDR fragment joints	0.615458
FDR chr. breakp. enrich.	0
Linked to chrs	
Purity, ploidy	0.48, 3.45

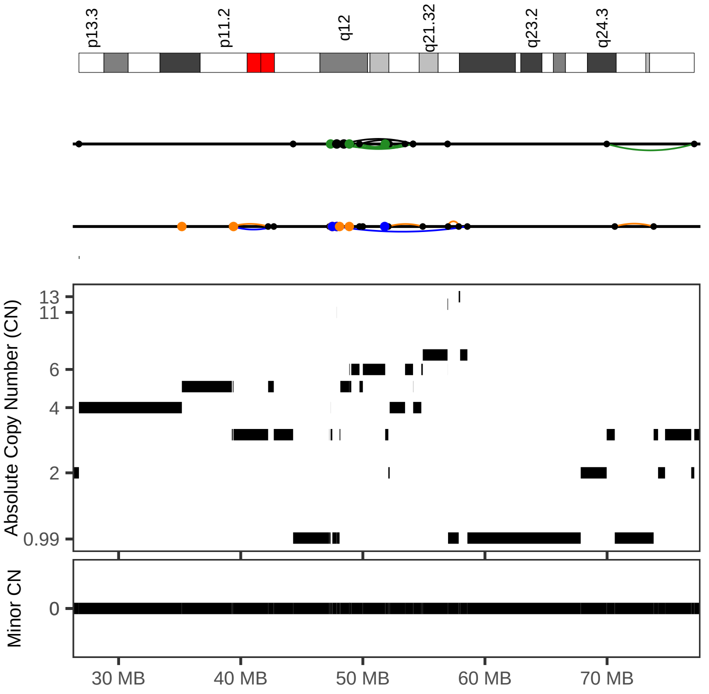

	**CGP_donor_1337223**
Cancer type	Breast−AdenoCA
Position	17:48154795−54905796
Type	With other complex events
Interleaved intrachr. SVs	8
Total SVs (intrachr. + transl.)	13
SV types	DEL: 2; DUP: 0; h2hINV: 2; t2tINV: 4; TRA: 5
SVs in sample	318
Oscillating CN (2 and 3 states)	6, 6
CN segments	14
FDR fragment joints	0.6776251
FDR chr. breakp. enrich.	0
Linked to chrs	
Purity, ploidy	0.48, 3.45

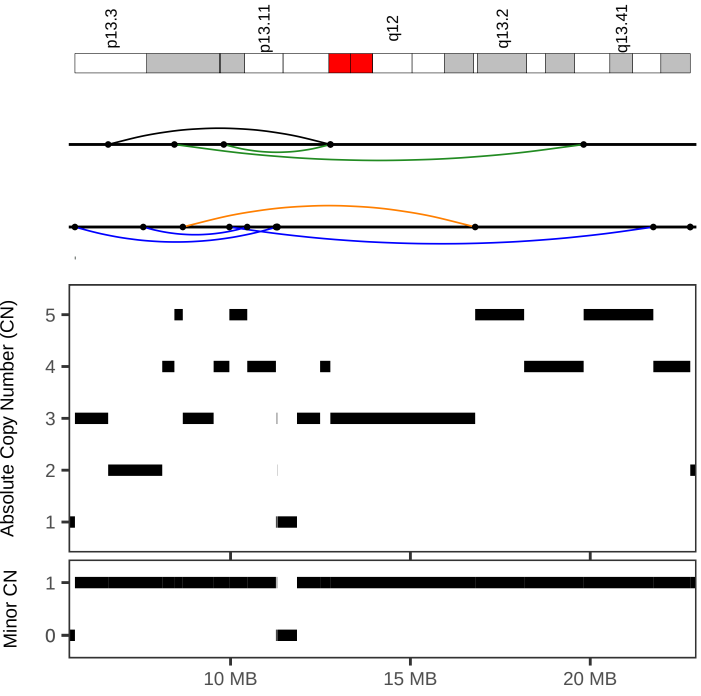

	**CGP_donor_1337225**
Cancer type	Breast−AdenoCA
Position	19:5673576−21753985
Type	With other complex events
Interleaved intrachr. SVs	10
Total SVs (intrachr. + transl.)	10
SV types	DEL: 2; DUP: 5; h2hINV: 1; t2tINV: 2; TRA: 0
SVs in sample	53
Oscillating CN (2 and 3 states)	4, 9
CN segments	22
FDR fragment joints	0.615458
FDR chr. breakp. enrich.	0
Linked to chrs	
Purity, ploidy	0.47, 2.32

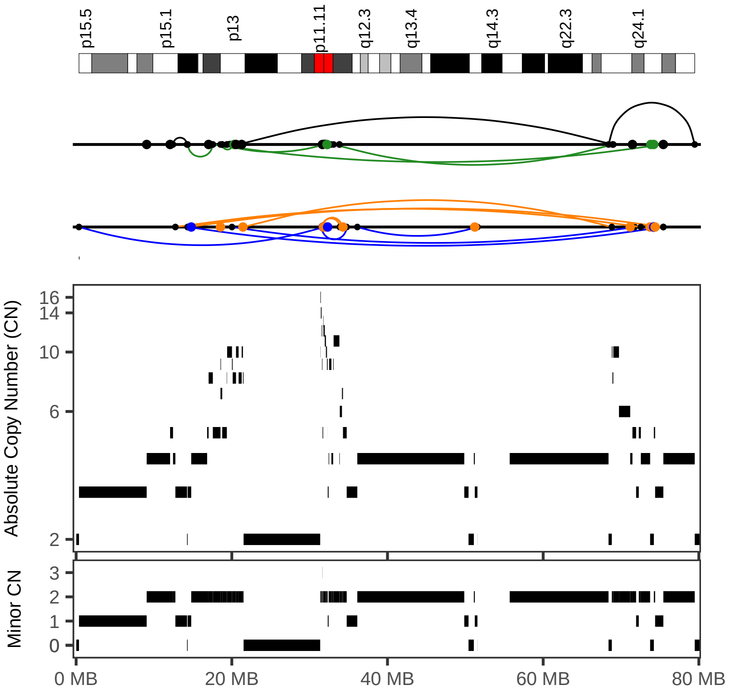

	**CGP_donor_1337226**
Cancer type	Breast−AdenoCA
Position	11:364948−79470446
Type	With other complex events
Interleaved intrachr. SVs	21
Total SVs (intrachr. + transl.)	48
SV types	DEL: 5; DUP: 4; h2hINV: 5; t2tINV: 7; TRA: 27
SVs in sample	473
Oscillating CN (2 and 3 states)	5, 6
CN segments	69
FDR fragment joints	0.6776251
FDR chr. breakp. enrich.	0
Linked to chrs	
Purity, ploidy	0.7, 3.48

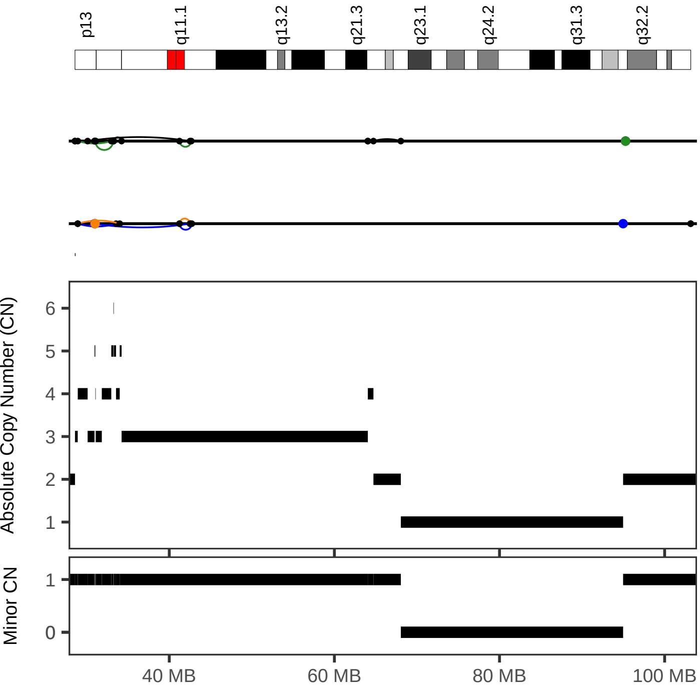

	**CGP_donor_1347742**
Cancer type	Breast−AdenoCA
Position	14:28909864−42732595
Type	With other complex events
Interleaved intrachr. SVs	13
Total SVs (intrachr. + transl.)	15
SV types	DEL: 2; DUP: 4; h2hINV: 3; t2tINV: 4; TRA: 2
SVs in sample	230
Oscillating CN (2 and 3 states)	4, 9
CN segments	16
FDR fragment joints	0.925252
FDR chr. breakp. enrich.	0
Linked to chrs	
Purity, ploidy	0.52, 2.12

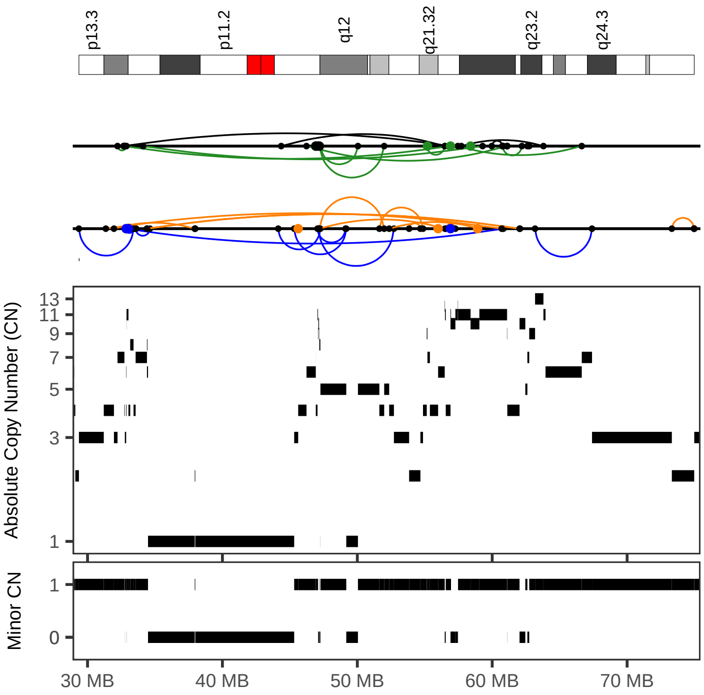

	**CGP_donor_1347742**
Cancer type	Breast−AdenoCA
Position	17:29361754−67397898
Type	With other complex events
Interleaved intrachr. SVs	35
Total SVs (intrachr. + transl.)	49
SV types	DEL: 11; DUP: 9; h2hINV: 6; t2tINV: 9; TRA: 14
SVs in sample	230
Oscillating CN (2 and 3 states)	4, 8
CN segments	68
FDR fragment joints	0.8653243
FDR chr. breakp. enrich.	0
Linked to chrs	3:25050231−162503836;15:66537420−102369379
Purity, ploidy	0.52, 2.12

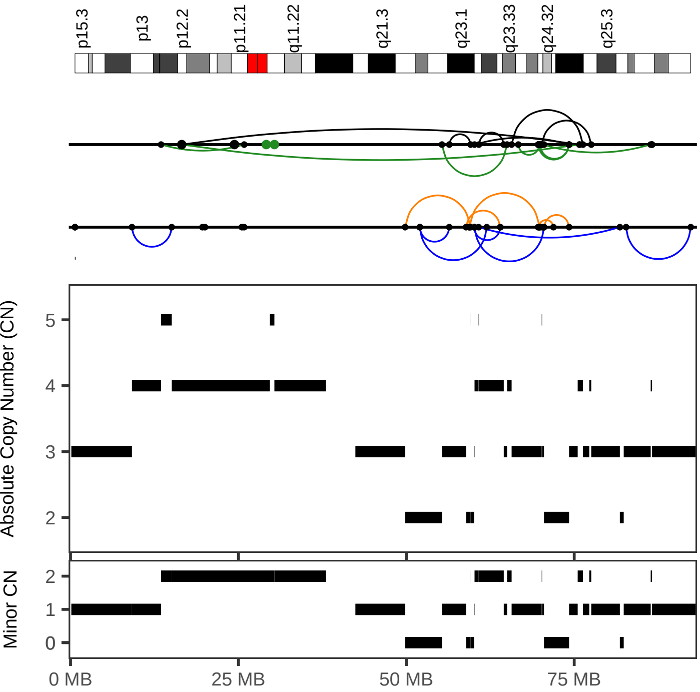

	**CGP_donor_1353427**
Cancer type	Breast−AdenoCA
Position	10:9142443−92352593
Type	With other complex events
Interleaved intrachr. SVs	30
Total SVs (intrachr. + transl.)	34
SV types	DEL: 6; DUP: 9; h2hINV: 7; t2tINV: 8; TRA: 4
SVs in sample	267
Oscillating CN (2 and 3 states)	5, 18
CN segments	30
FDR fragment joints	0.8653243
FDR chr. breakp. enrich.	0
Linked to chrs	6:100548524−101591933;
Purity, ploidy	0.29, 2.76

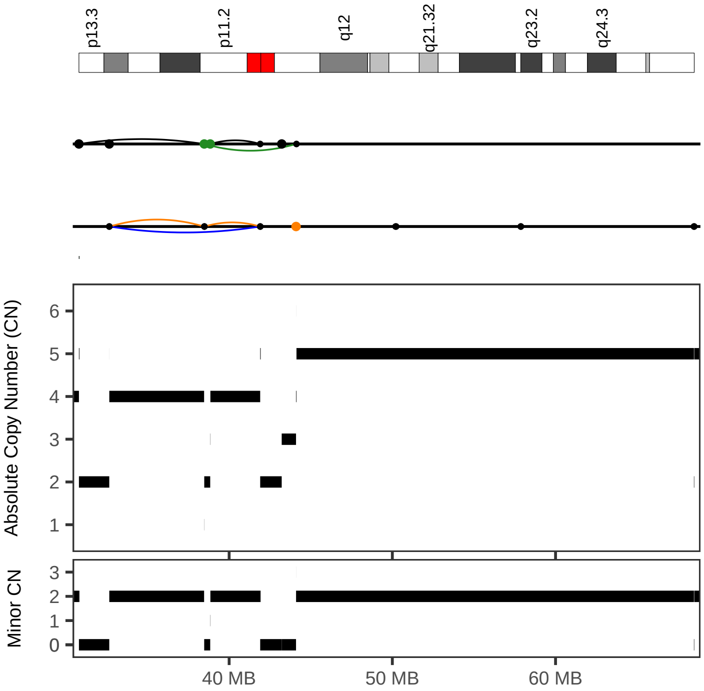

	**CGP_donor_1353427**
Cancer type	Breast−AdenoCA
Position	17:30795695−44125802
Type	With other complex events
Interleaved intrachr. SVs	6
Total SVs (intrachr. + transl.)	12
SV types	DEL: 2; DUP: 1; h2hINV: 1; t2tINV: 2; TRA: 6
SVs in sample	267
Oscillating CN (2 and 3 states)	4, 9
CN segments	16
FDR fragment joints	0.6776251
FDR chr. breakp. enrich.	0
Linked to chrs	
Purity, ploidy	0.29, 2.76

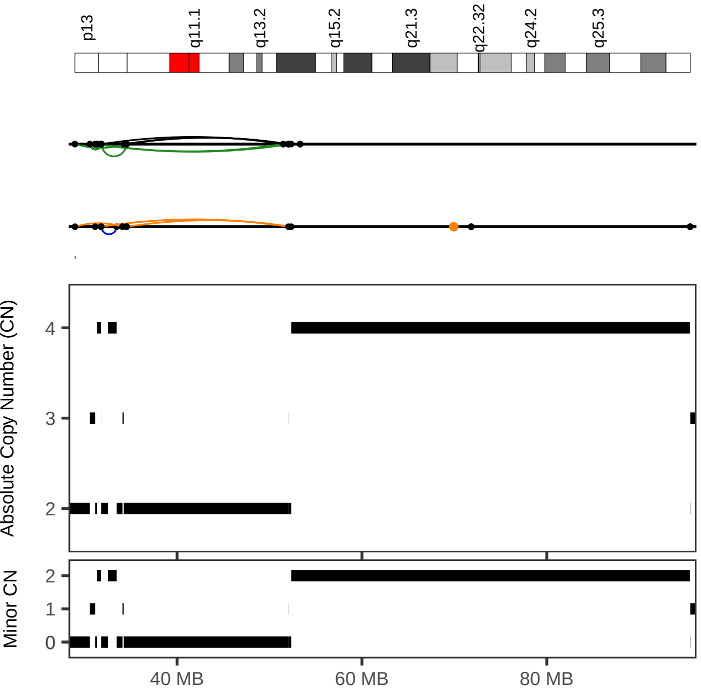

	**CGP_donor_1353434**
Cancer type	Breast−AdenoCA
Position	15:28941386−52345122
Type	With other complex events
Interleaved intrachr. SVs	16
Total SVs (intrachr. + transl.)	16
SV types	DEL: 5; DUP: 1; h2hINV: 4; t2tINV: 6; TRA: 0
SVs in sample	291
Oscillating CN (2 and 3 states)	5, 14
CN segments	14
FDR fragment joints	0.615458
FDR chr. breakp. enrich.	0
Linked to chrs	
Purity, ploidy	0.5, 2.94

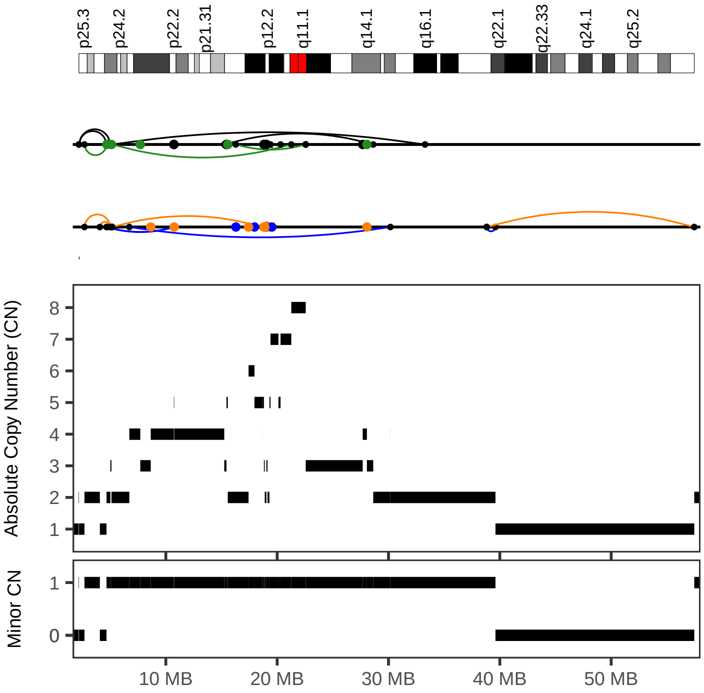

	**CGP_donor_1364028**
Cancer type	Breast−AdenoCA
Position	6:2192497−33282947
Type	With other complex events
Interleaved intrachr. SVs	14
Total SVs (intrachr. + transl.)	42
SV types	DEL: 3; DUP: 4; h2hINV: 4; t2tINV: 3; TRA: 28
SVs in sample	203
Oscillating CN (2 and 3 states)	4, 6
CN segments	33
FDR fragment joints	0.8536199
FDR chr. breakp. enrich.	0
Linked to chrs	14:84761805−88311295;20:13487327−62399710 9:18053450−78248432;
Purity, ploidy	0.68, 2.2

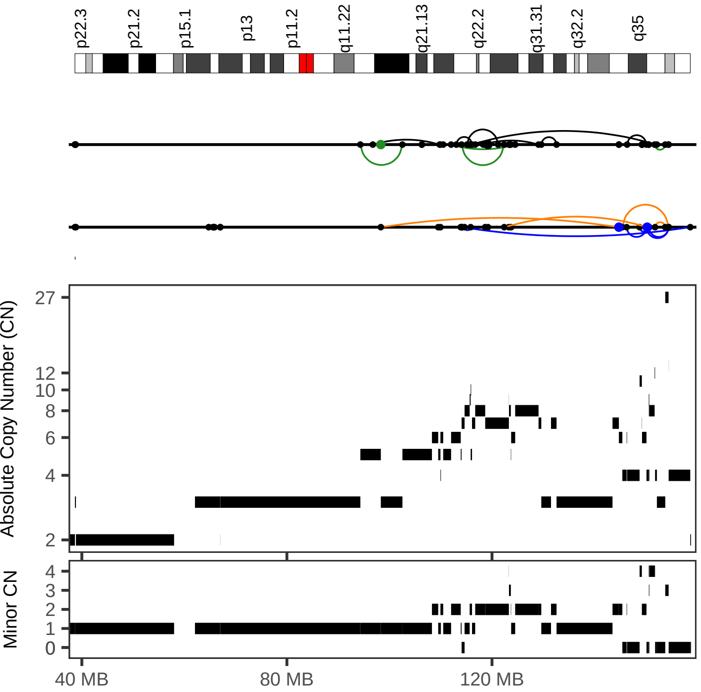

	**CGP_donor_1364033**
Cancer type	Breast−AdenoCA
Position	7:94323289−158670701
Type	With other complex events
Interleaved intrachr. SVs	25
Total SVs (intrachr. + transl.)	29
SV types	DEL: 7; DUP: 6; h2hINV: 6; t2tINV: 6; TRA: 4
SVs in sample	529
Oscillating CN (2 and 3 states)	5, 9
CN segments	50
FDR fragment joints	1
FDR chr. breakp. enrich.	0.01
Linked to chrs	16:54538858−83487716;20:23008810−61019045
Purity, ploidy	0.32, 3.04

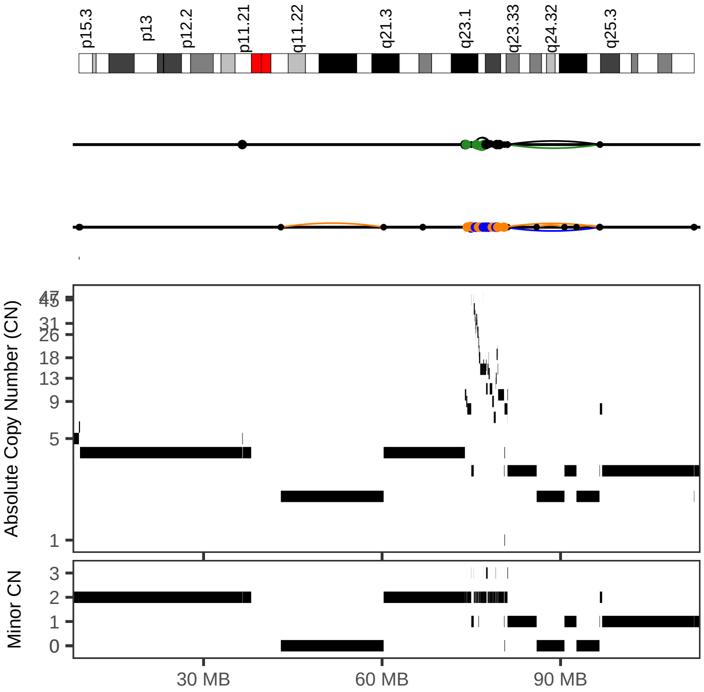

	**CGP_donor_1364033**
Cancer type	Breast−AdenoCA
Position	10:74137063−96621191
Type	With other complex events
Interleaved intrachr. SVs	16
Total SVs (intrachr. + transl.)	41
SV types	DEL: 4; DUP: 3; h2hINV: 6; t2tINV: 3; TRA: 25
SVs in sample	529
Oscillating CN (2 and 3 states)	5, 5
CN segments	56
FDR fragment joints	0.615458
FDR chr. breakp. enrich.	0
Linked to chrs	
Purity, ploidy	0.32, 3.04

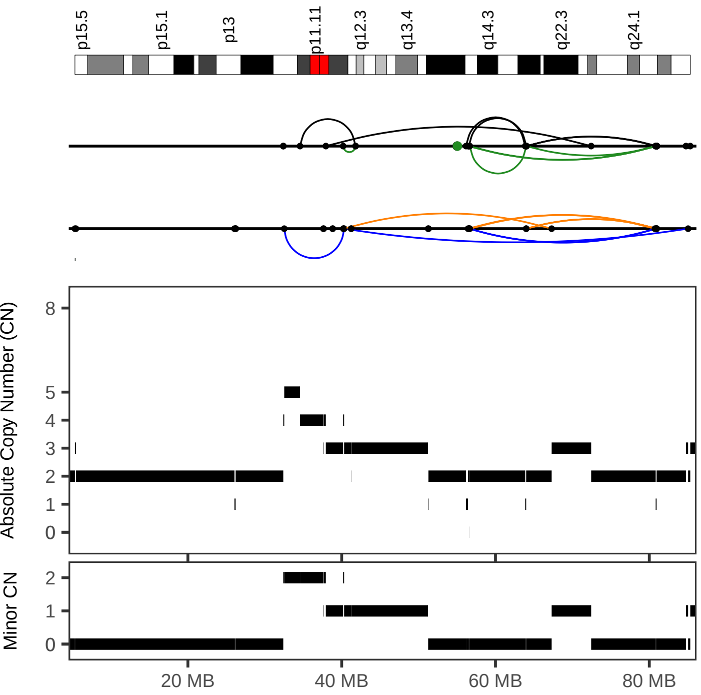

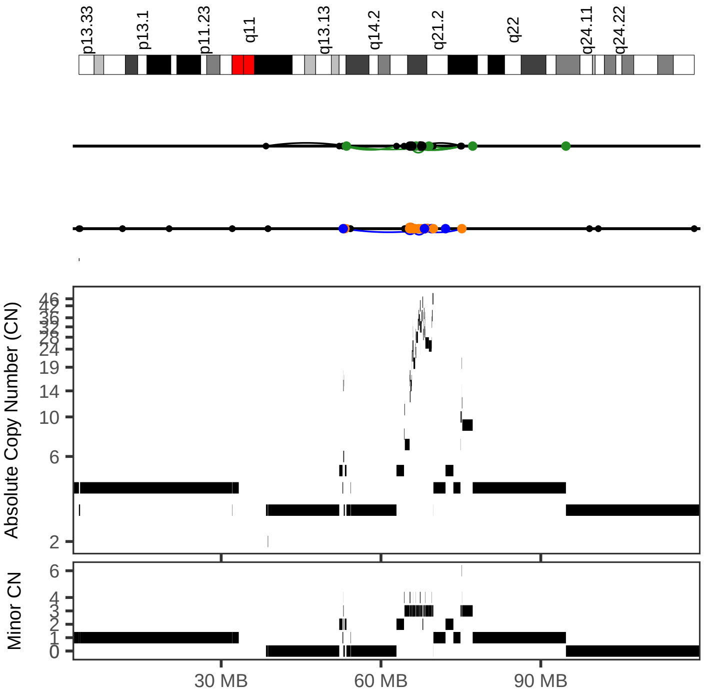

	**CGP_donor_1364033**
Cancer type	Breast−AdenoCA
Position	11:32528936−85324051
Type	With other complex events
Interleaved intrachr. SVs	26
Total SVs (intrachr. + transl.)	28
SV types	DEL: 5; DUP: 4; h2hINV: 8; t2tINV: 9; TRA: 2
SVs in sample	529
Oscillating CN (2 and 3 states)	6, 14
CN segments	25
FDR fragment joints	0.615458
FDR chr. breakp. enrich.	0.03
Linked to chrs	3:132552596−181241473;
Purity, ploidy	0.32, 3.04

	**CGP_donor_1364033**
Cancer type	Breast−AdenoCA
Position	12:38404016−75256147
Type	With other complex events
Interleaved intrachr. SVs	32
Total SVs (intrachr. + transl.)	58
SV types	DEL: 6; DUP: 7; h2hINV: 7; t2tINV: 12; TRA: 26
SVs in sample	529
Oscillating CN (2 and 3 states)	4, 5
CN segments	86
FDR fragment joints	0.6776251
FDR chr. breakp. enrich.	0
Linked to chrs	10:74137063−96621190;
Purity, ploidy	0.32, 3.04

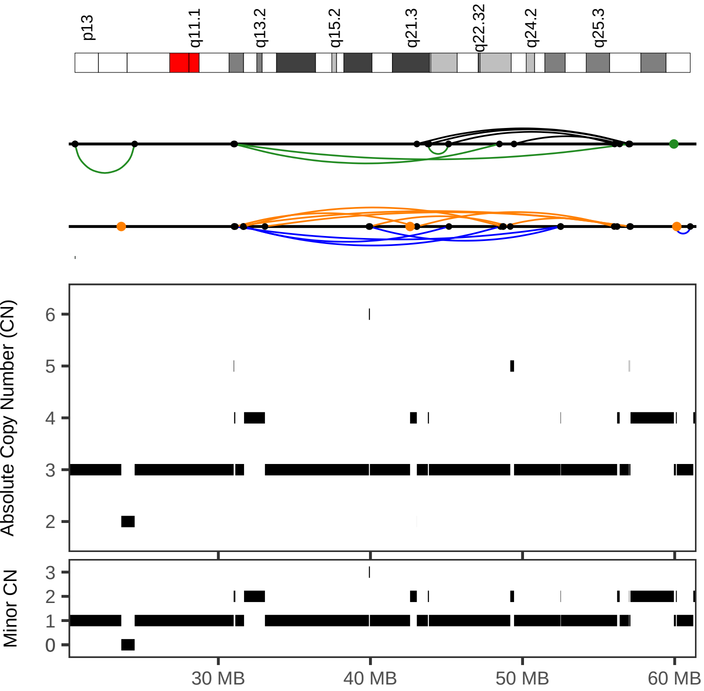

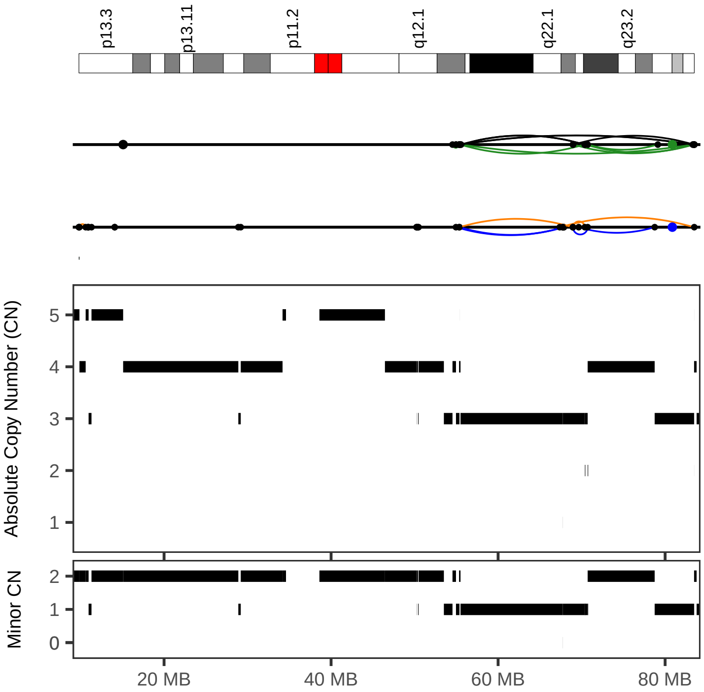

	**CGP_donor_1364033**
Cancer type	Breast−AdenoCA
Position	15:31008365−57097215
Type	With other complex events
Interleaved intrachr. SVs	18
Total SVs (intrachr. + transl.)	19
SV types	DEL: 7; DUP: 4; h2hINV: 4; t2tINV: 3; TRA: 1
SVs in sample	529
Oscillating CN (2 and 3 states)	5, 14
CN segments	23
FDR fragment joints	0.615458
FDR chr. breakp. enrich.	0.03
Linked to chrs	
Purity, ploidy	0.32, 3.04

	**CGP_donor_1364033**
Cancer type	Breast−AdenoCA
Position	16:54538858−83487717
Type	With other complex events
Interleaved intrachr. SVs	21
Total SVs (intrachr. + transl.)	23
SV types	DEL: 3; DUP: 4; h2hINV: 7; t2tINV: 7; TRA: 2
SVs in sample	529
Oscillating CN (2 and 3 states)	5, 11
CN segments	20
FDR fragment joints	0.6776251
FDR chr. breakp. enrich.	0
Linked to chrs	7:94323289−158670700;
Purity, ploidy	0.32, 3.04

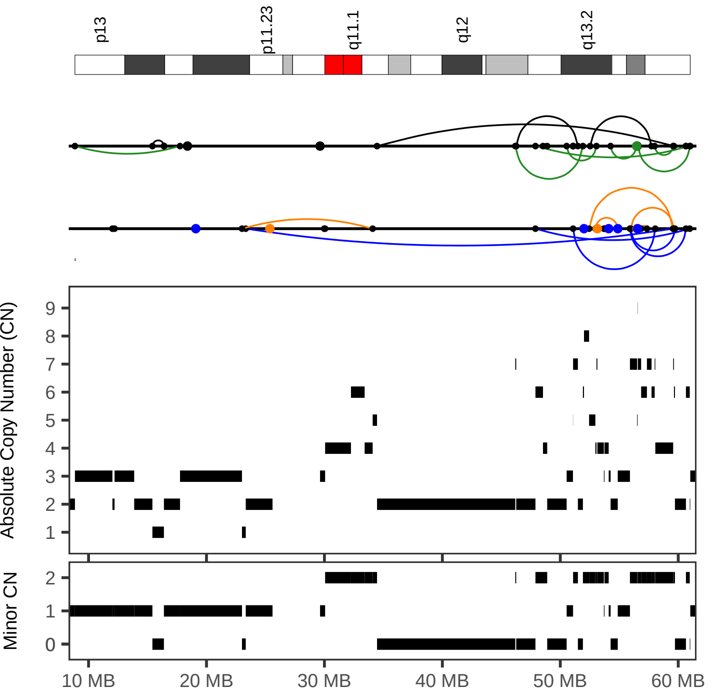

	**CGP_donor_1364033**
Cancer type	Breast−AdenoCA
Position	20:23008810−61019046
Type	With other complex events
Interleaved intrachr. SVs	19
Total SVs (intrachr. + transl.)	29
SV types	DEL: 4; DUP: 5; h2hINV: 3; t2tINV: 7; TRA: 10
SVs in sample	529
Oscillating CN (2 and 3 states)	5, 8
CN segments	44
FDR fragment joints	0.6776251
FDR chr. breakp. enrich.	0
Linked to chrs	17:15573740−80067669;5:154600947−177590688 6:57399664−170784375;
Purity, ploidy	0.32, 3.04

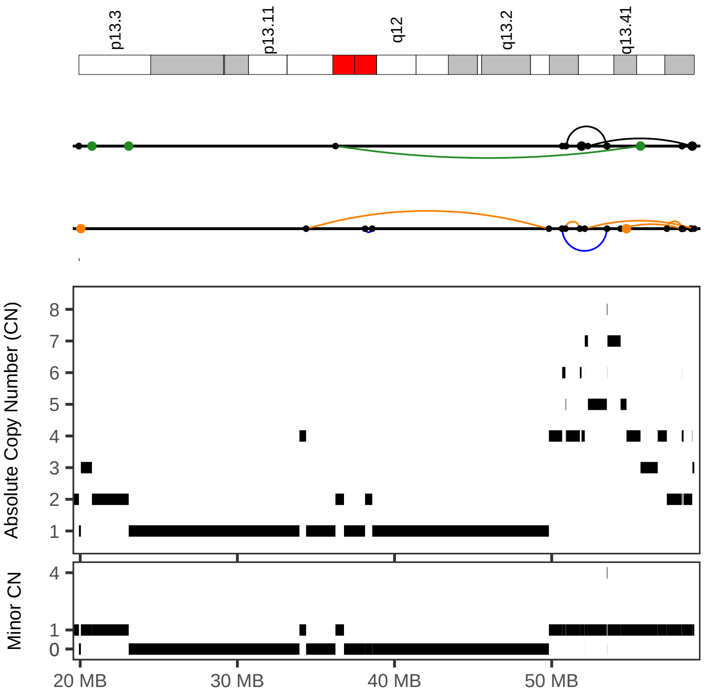

	**CGP_donor_1374616**
Cancer type	Breast−AdenoCA
Position	19:34372322−59071044
Type	With other complex events
Interleaved intrachr. SVs	13
Total SVs (intrachr. + transl.)	17
SV types	DEL: 5; DUP: 2; h2hINV: 2; t2tINV: 4; TRA: 4
SVs in sample	144
Oscillating CN (2 and 3 states)	5, 8
CN segments	31
FDR fragment joints	0.615458
FDR chr. breakp. enrich.	0
Linked to chrs	X:3867520−67168966;
Purity, ploidy	0.5, 1.95

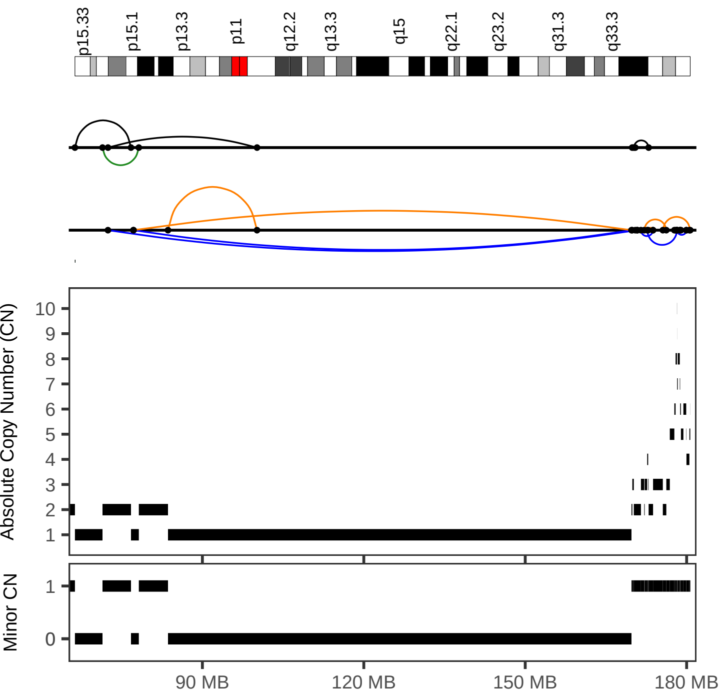

	**CGP_donor_1397086**
Cancer type	Breast−AdenoCA
Position	5:66307170−180674011
Type	With other complex events
Interleaved intrachr. SVs	17
Total SVs (intrachr. + transl.)	17
SV types	DEL: 5; DUP: 7; h2hINV: 3; t2tINV: 2; TRA: 0
SVs in sample	82
Oscillating CN (2 and 3 states)	6, 17
CN segments	32
FDR fragment joints	0.615458
FDR chr. breakp. enrich.	0
Linked to chrs	
Purity, ploidy	0.59, 1.98

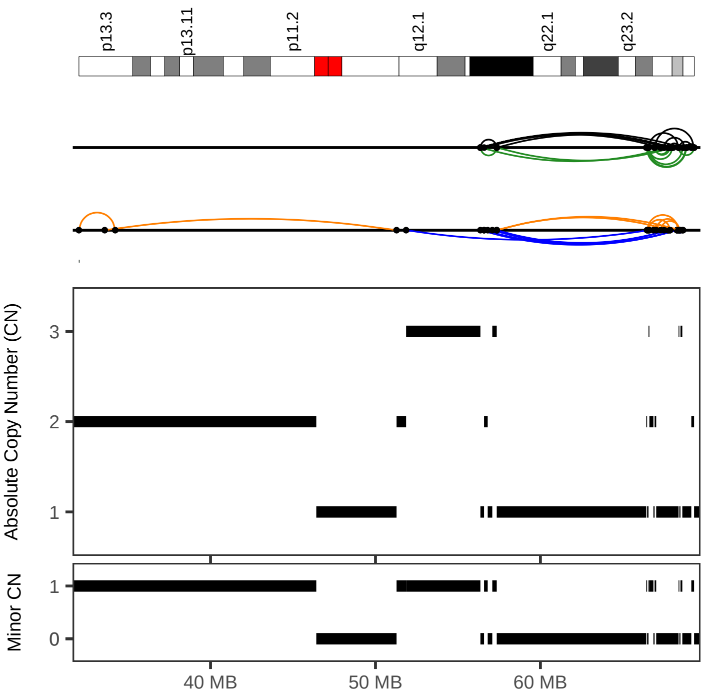

	**CGP_donor_1397086**
Cancer type	Breast−AdenoCA
Position	16:51856069−69324699
Type	With other complex events
Interleaved intrachr. SVs	39
Total SVs (intrachr. + transl.)	39
SV types	DEL: 8; DUP: 7; h2hINV: 14; t2tINV: 10; TRA: 0
SVs in sample	82
Oscillating CN (2 and 3 states)	5, 10
CN segments	19
FDR fragment joints	0.6278017
FDR chr. breakp. enrich.	0
Linked to chrs	
Purity, ploidy	0.59, 1.98

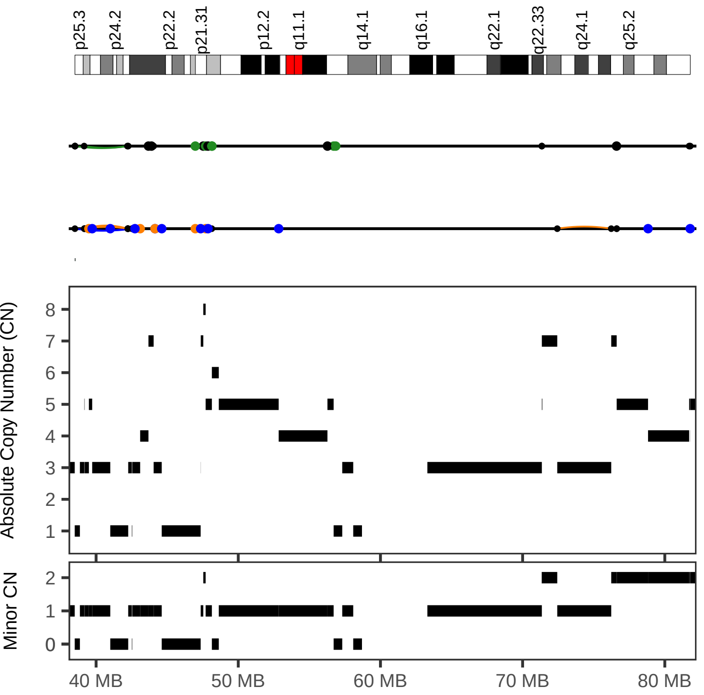

	**CGP_donor_1397260**
Cancer type	Breast−AdenoCA
Position	6:38510448−42541219
Type	With other complex events
Interleaved intrachr. SVs	7
Total SVs (intrachr. + transl.)	10
SV types	DEL: 3; DUP: 2; h2hINV: 0; t2tINV: 2; TRA: 3
SVs in sample	160
Oscillating CN (2 and 3 states)	5, 12
CN segments	12
FDR fragment joints	0.615458
FDR chr. breakp. enrich.	0
Linked to chrs	
Purity, ploidy	0.41, 3.03

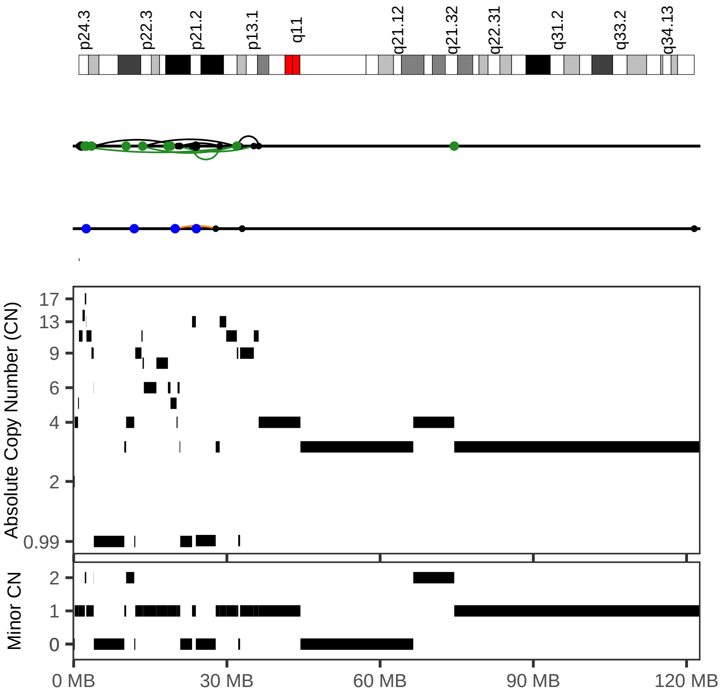

	**CGP_donor_1397262**
Cancer type	Breast−AdenoCA
Position	9:1026203−36225240
Type	With other complex events
Interleaved intrachr. SVs	9
Total SVs (intrachr. + transl.)	23
SV types	DEL: 1; DUP: 0; h2hINV: 4; t2tINV: 4; TRA: 14
SVs in sample	235
Oscillating CN (2 and 3 states)	4, 4
CN segments	32
FDR fragment joints	0.2592493
FDR chr. breakp. enrich.	0
Linked to chrs	
Purity, ploidy	0.19, 3.34

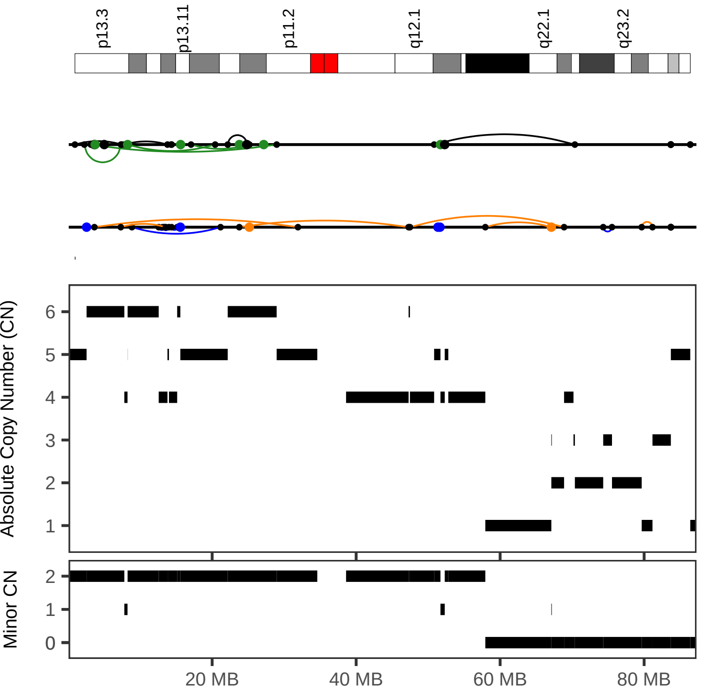

	**CGP_donor_1397262**
Cancer type	Breast−AdenoCA
Position	16:937916−47287050
Type	With other complex events
Interleaved intrachr. SVs	13
Total SVs (intrachr. + transl.)	24
SV types	DEL: 3; DUP: 3; h2hINV: 3; t2tINV: 4; TRA: 11
SVs in sample	235
Oscillating CN (2 and 3 states)	4, 9
CN segments	14
FDR fragment joints	0.7568568
FDR chr. breakp. enrich.	0
Linked to chrs	
Purity, ploidy	0.19, 3.34

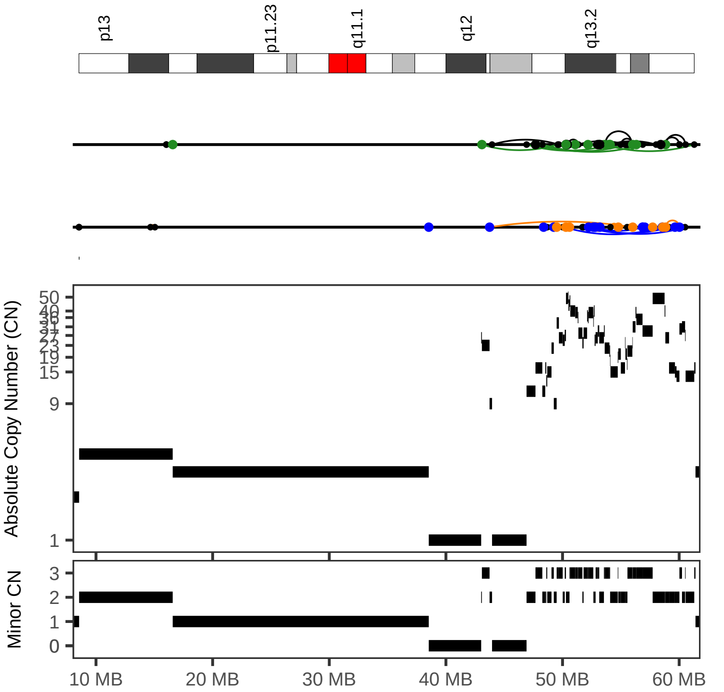

	**CGP_donor_1397262**
Cancer type	Breast−AdenoCA
Position	20:43024445−61292598
Type	With other complex events
Interleaved intrachr. SVs	25
Total SVs (intrachr. + transl.)	68
SV types	DEL: 5; DUP: 4; h2hINV: 8; t2tINV: 8; TRA: 43
SVs in sample	235
Oscillating CN (2 and 3 states)	4, 6
CN segments	64
FDR fragment joints	0.7345058
FDR chr. breakp. enrich.	0
Linked to chrs	
Purity, ploidy	0.19, 3.34

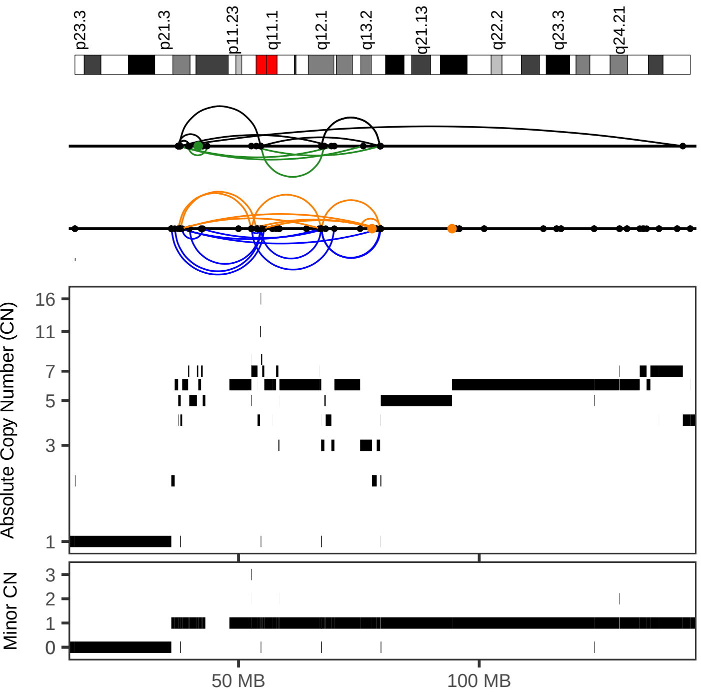

	CGP_donor_1397278
Cancer type	Breast−AdenoCA
Position	8:36028735−142305374
Type	With other complex events
Interleaved intrachr. SVs	40
Total SVs (intrachr. + transl.)	43
SV types	DEL: 9; DUP: 12; h2hINV: 10; t2tINV: 9; TRA: 3
SVs in sample	340
Oscillating CN (2 and 3 states)	6, 12
CN segments	72
FDR fragment joints	0.9599662
FDR chr. breakp. enrich.	0
Linked to chrs	
Purity, ploidy	0.42, 2.02

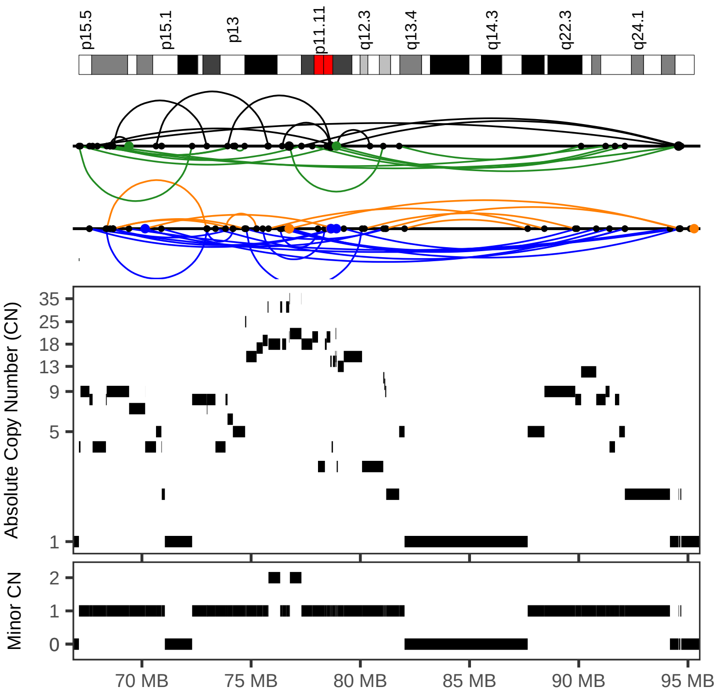

	CGP_donor_1425260
Cancer type	Breast−AdenoCA
Position	11:67115970−94692580
Type	With other complex events
Interleaved intrachr. SVs	52
Total SVs (intrachr. + transl.)	65
SV types	DEL: 9; DUP: 18; h2hINV: 11; t2tINV: 14; TRA: 13
SVs in sample	91
Oscillating CN (2 and 3 states)	6, 6
CN segments	72
FDR fragment joints	0.615458
FDR chr. breakp. enrich.	0
Linked to chrs	6:34298680−40481487;
Purity, ploidy	0.8, 2.01

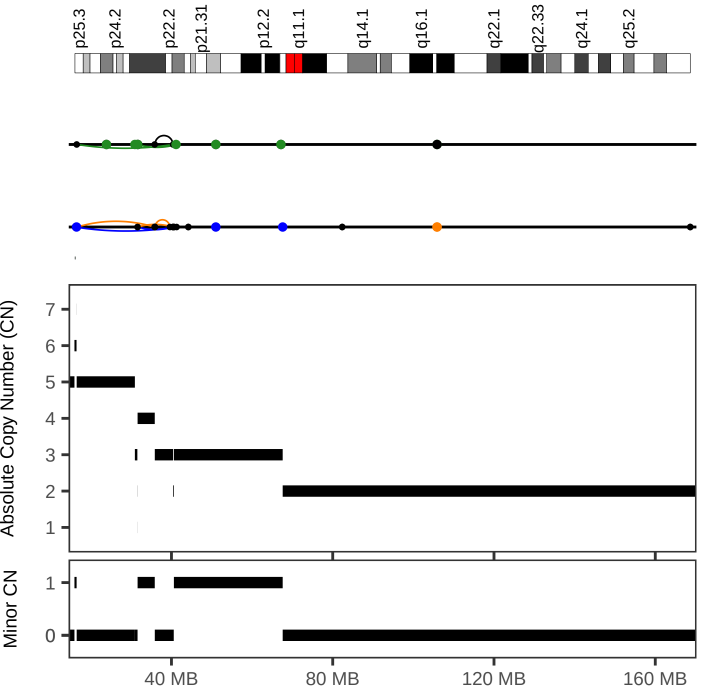

	CGP_donor_1475200
Cancer type	Breast−AdenoCA
Position	6:16058789−41282014
Type	With other complex events
Interleaved intrachr. SVs	7
Total SVs (intrachr. + transl.)	14
SV types	DEL: 2; DUP: 2; h2hINV: 1; t2tINV: 2; TRA: 7
SVs in sample	331
Oscillating CN (2 and 3 states)	5, 7
CN segments	17
FDR fragment joints	0.8066159
FDR chr. breakp. enrich.	0
Linked to chrs	
Purity, ploidy	0.6, 3.69

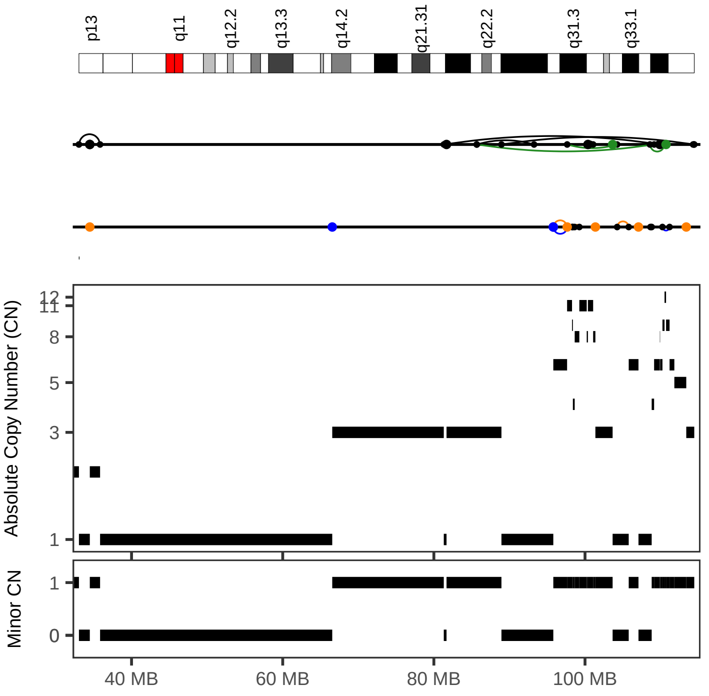

	CGP_donor_1475201
Cancer type	Breast−AdenoCA
Position	13:81313916−114454357
Type	With other complex events
Interleaved intrachr. SVs	6
Total SVs (intrachr. + transl.)	17
SV types	DEL: 0; DUP: 1; h2hINV: 3; t2tINV: 2; TRA: 11
SVs in sample	45
Oscillating CN (2 and 3 states)	5, 5
CN segments	26
FDR fragment joints	0.930656
FDR chr. breakp. enrich.	0
Linked to chrs	
Purity, ploidy	0.76, 2.06

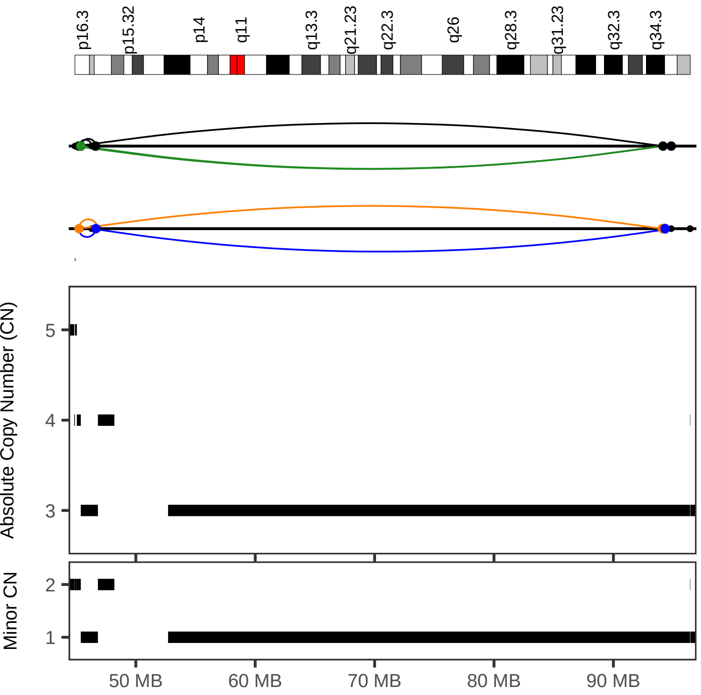

	CGP_donor_1475202
Cancer type	Breast−AdenoCA
Position	4:44897680−94849273
Type	After polyploidization
Interleaved intrachr. SVs	12
Total SVs (intrachr. + transl.)	28
SV types	DEL: 3; DUP: 2; h2hINV: 4; t2tINV: 3; TRA: 16
SVs in sample	217
Oscillating CN (2 and 3 states)	4, 5
CN segments	5
FDR fragment joints	0.8988396
FDR chr. breakp. enrich.	0
Linked to chrs	1:80341830−241765598;
Purity, ploidy	0.87, 3.64

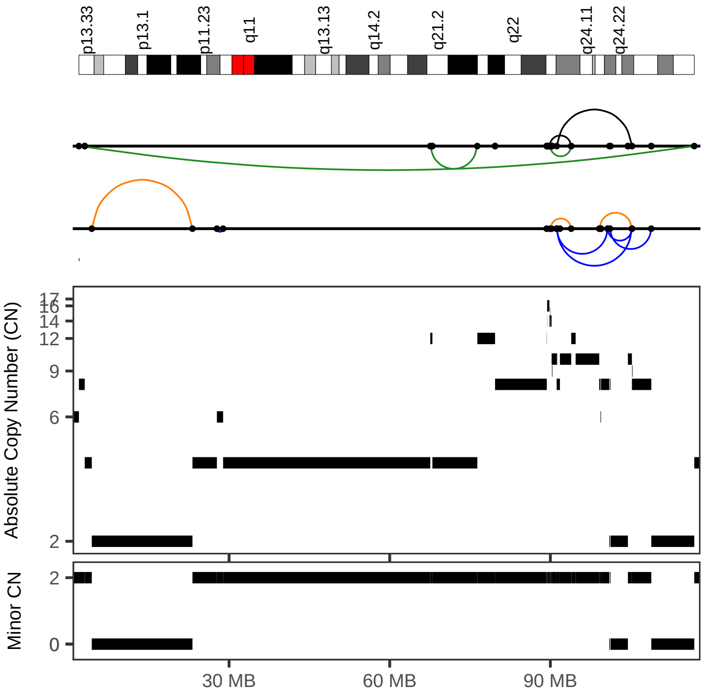

	CGP_donor_1475202
Cancer type	Breast−AdenoCA
Position	12:89302895−108838113
Type	With other complex events
Interleaved intrachr. SVs	17
Total SVs (intrachr. + transl.)	17
SV types	DEL: 3; DUP: 5; h2hINV: 6; t2tINV: 3; TRA: 0
SVs in sample	217
Oscillating CN (2 and 3 states)	5, 6
CN segments	29
FDR fragment joints	0.7735152
FDR chr. breakp. enrich.	0
Linked to chrs	
Purity, ploidy	0.87, 3.64

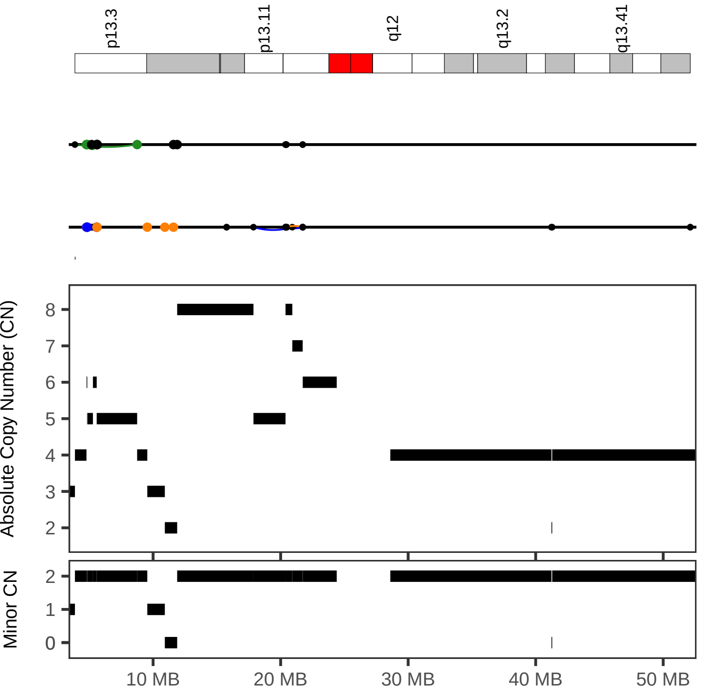

	CGP_donor_1475202
Cancer type	Breast−AdenoCA
Position	19:4796337−21735946
Type	With other complex events
Interleaved intrachr. SVs	7
Total SVs (intrachr. + transl.)	32
SV types	DEL: 3; DUP: 6; h2hINV: 3; t2tINV: 2; TRA: 18
SVs in sample	217
Oscillating CN (2 and 3 states)	6, 9
CN segments	16
FDR fragment joints	0.9224215
FDR chr. breakp. enrich.	0
Linked to chrs	3:4105086−194006756;
Purity, ploidy	0.87, 3.64

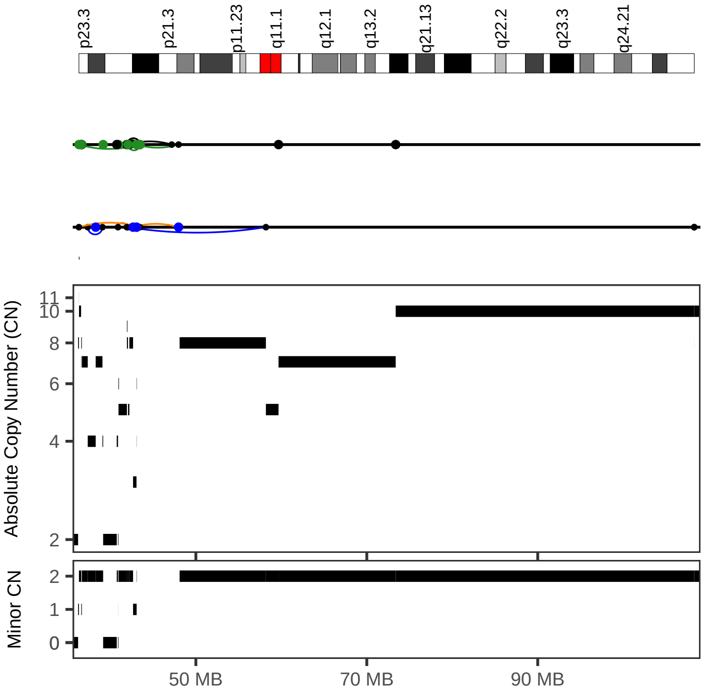

	CGP_donor_1503014
Cancer type	Breast−AdenoCA
Position	8:36316585−58193474
Type	With other complex events
Interleaved intrachr. SVs	9
Total SVs (intrachr. + transl.)	28
SV types	DEL: 2; DUP: 1; h2hINV: 3; t2tINV: 3; TRA: 19
SVs in sample	161
Oscillating CN (2 and 3 states)	5, 5
CN segments	24
FDR fragment joints	0.5435077
FDR chr. breakp. enrich.	0
Linked to chrs	11:57620337−81529410;
Purity, ploidy	0.48, 2.97

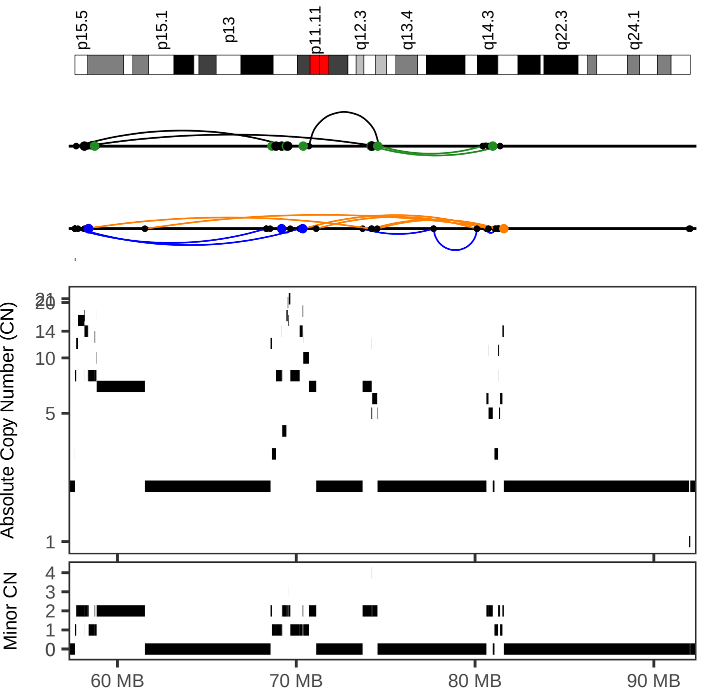

CGP_donor_1503014	
Cancer type	Breast−AdenoCA
Position	11:57620337−81529411
Type	With other complex events
Interleaved intrachr. SVs	20
Total SVs (intrachr. + transl.)	40
SV types	DEL: 7; DUP: 6; h2hINV: 4; t2tINV: 3; TRA: 20
SVs in sample	161
Oscillating CN (2 and 3 states)	5, 7
CN segments	53
FDR fragment joints	0.8135044
FDR chr. breakp. enrich.	0
Linked to chrs	8:36316585−58193473;
Purity, ploidy	0.48, 2.97

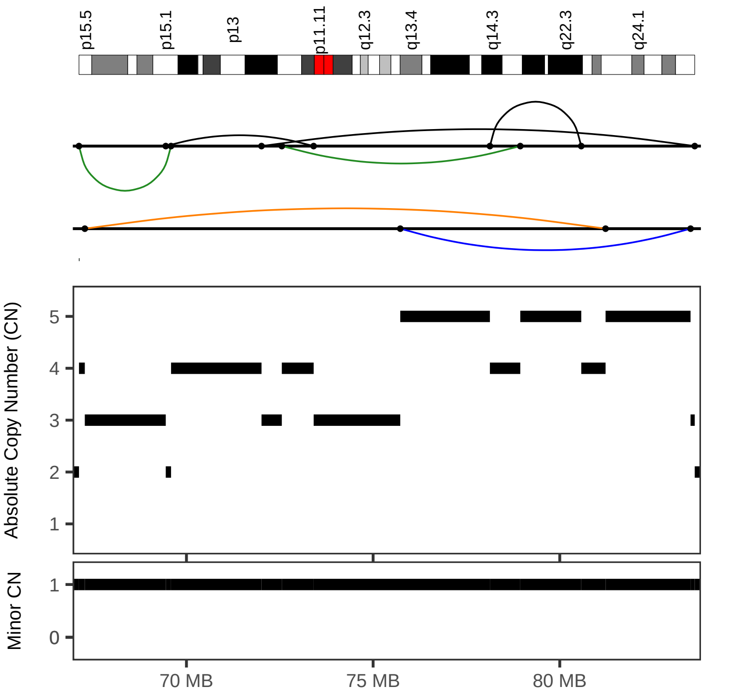

CGP_donor_1503016	
Cancer type	Breast−AdenoCA
Position	11:67121530−83614852
Type	With other complex events
Interleaved intrachr. SVs	7
Total SVs (intrachr. + transl.)	7
SV types	DEL: 1; DUP: 1; h2hINV: 3; t2tINV: 2; TRA: 0
SVs in sample	72
Oscillating CN (2 and 3 states)	5, 12
CN segments	14
FDR fragment joints	0.7735152
FDR chr. breakp. enrich.	0.08
Linked to chrs	
Purity, ploidy	0.54, 2.1

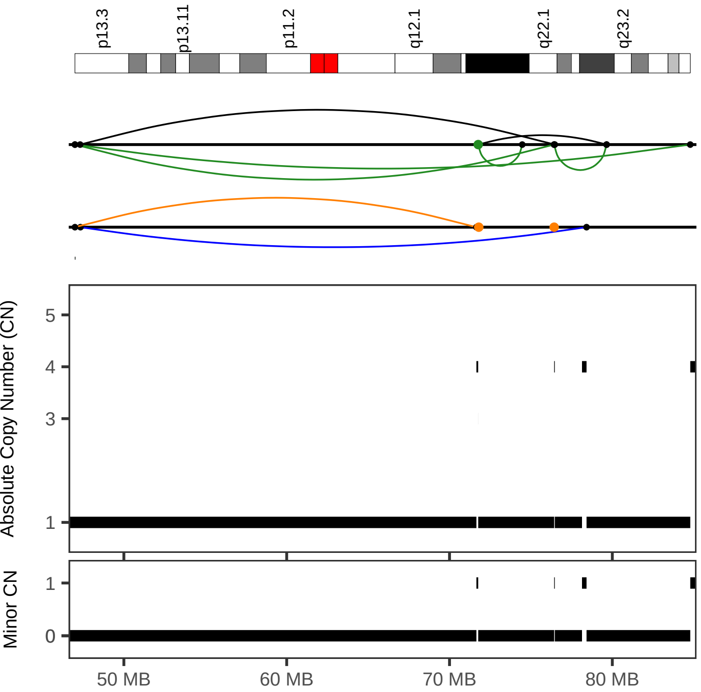

CGP_donor_1503021	
Cancer type	Breast−AdenoCA
Position	16:46993430−84786026
Type	Before polyploidization
Interleaved intrachr. SVs	8
Total SVs (intrachr. + transl.)	11
SV types	DEL: 1; DUP: 1; h2hINV: 2; t2tINV: 4; TRA: 3
SVs in sample	25
Oscillating CN (2 and 3 states)	6, 7
CN segments	9
FDR fragment joints	0.7425546
FDR chr. breakp. enrich.	0
Linked to chrs	
Purity, ploidy	0.67, 2.05

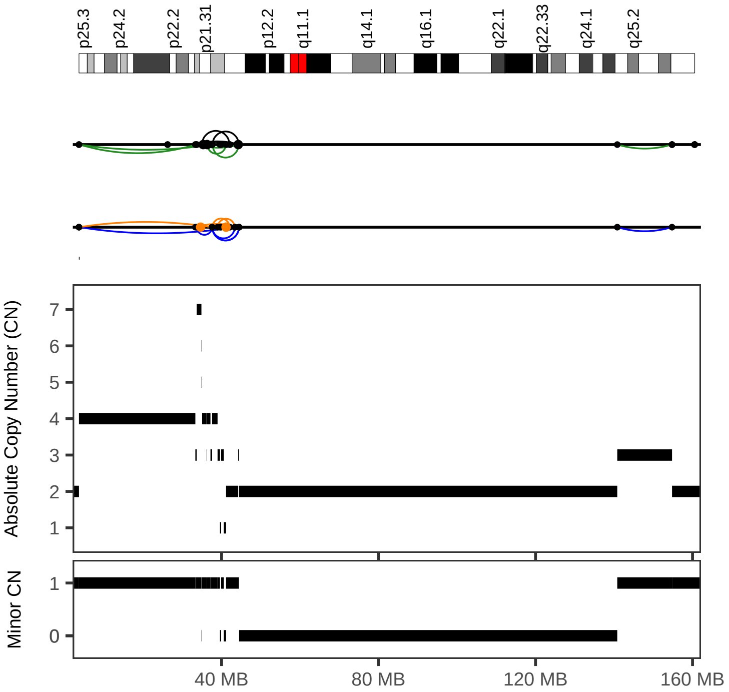

CGP_donor_1503150	
Cancer type	Breast−AdenoCA
Position	6:3658692−44465119
Type	With other complex events
Interleaved intrachr. SVs	21
Total SVs (intrachr. + transl.)	26
SV types	DEL: 4; DUP: 5; h2hINV: 6; t2tINV: 6; TRA: 5
SVs in sample	180
Oscillating CN (2 and 3 states)	6, 9
CN segments	18
FDR fragment joints	0.8172348
FDR chr. breakp. enrich.	0
Linked to chrs	3:175252560−194564989;
Purity, ploidy	0.82, 1.98

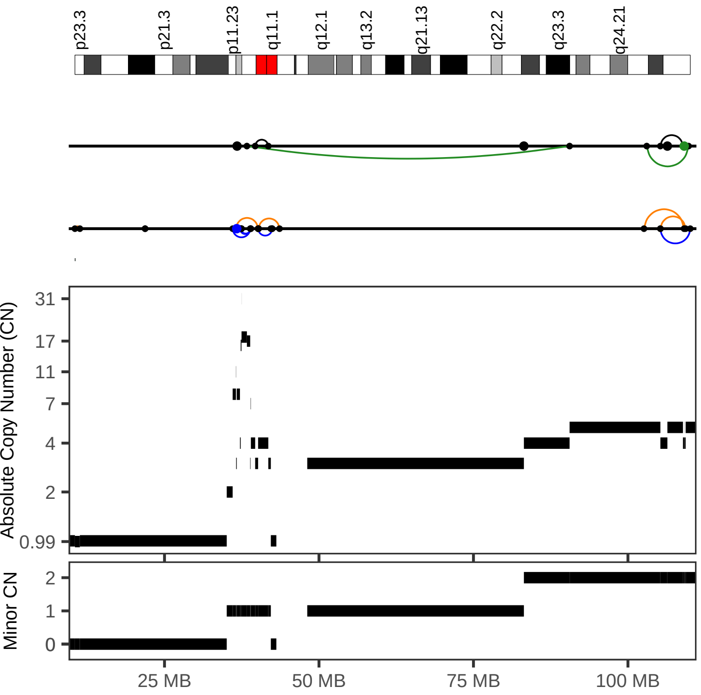

	CGP_donor_1503150
Cancer type	Breast−AdenoCA
Position	8:36031131−90557044
Type	With other complex events
Interleaved intrachr. SVs	8
Total SVs (intrachr. + transl.)	11
SV types	DEL: 2; DUP: 4; h2hINV: 1; t2tINV: 1; TRA: 3
SVs in sample	180
Oscillating CN (2 and 3 states)	4, 7
CN segments	19
FDR fragment joints	0.615458
FDR chr. breakp. enrich.	0
Linked to chrs	
Purity, ploidy	0.82, 1.98

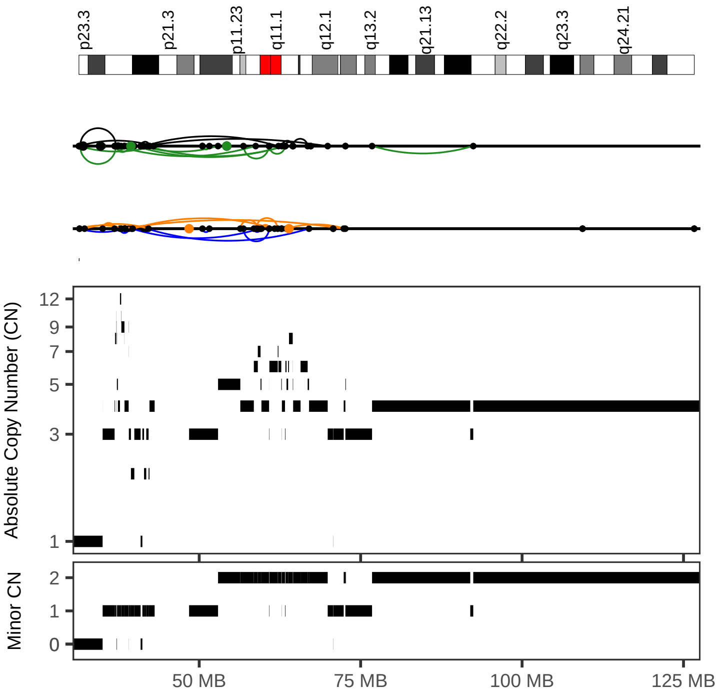

	CGP_donor_1114881
Cancer type	Breast−AdenoCA
Position	8:31373451−72632912
Type	With other complex events
Interleaved intrachr. SVs	39
Total SVs (intrachr. + transl.)	47
SV types	DEL: 9; DUP: 7; h2hINV: 11; t2tINV: 12; TRA: 8
SVs in sample	249
Oscillating CN (2 and 3 states)	4, 13
CN segments	60
FDR fragment joints	0.615458
FDR chr. breakp. enrich.	0
Linked to chrs	6:65175120−68525091;
Purity, ploidy	0.77, 1.96

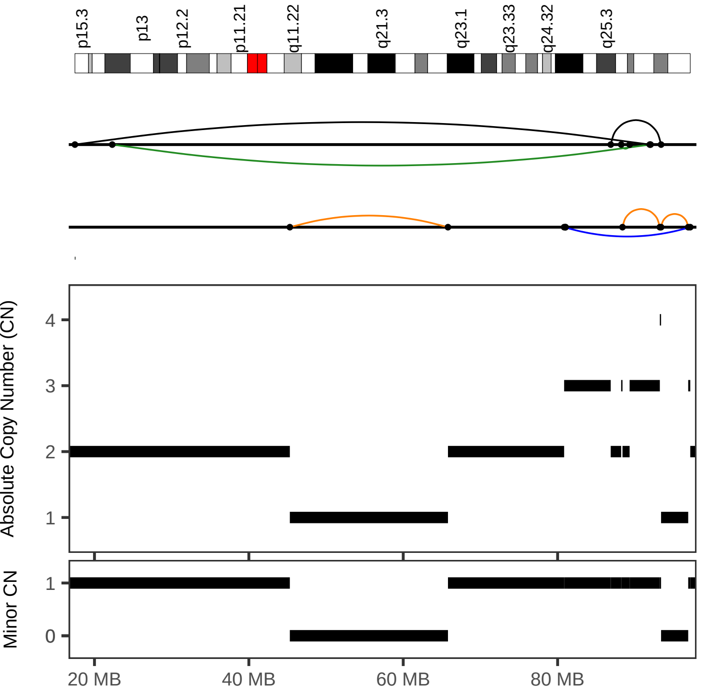

	CGP_donor_1114881
Cancer type	Breast−AdenoCA
Position	10:17464930−97184502
Type	With other complex events
Interleaved intrachr. SVs	6
Total SVs (intrachr. + transl.)	6
SV types	DEL: 1; DUP: 1; h2hINV: 2; t2tINV: 2; TRA: 0
SVs in sample	249
Oscillating CN (2 and 3 states)	6, 10
CN segments	12
FDR fragment joints	0.9284301
FDR chr. breakp. enrich.	0.77
Linked to chrs	
Purity, ploidy	0.77, 1.96

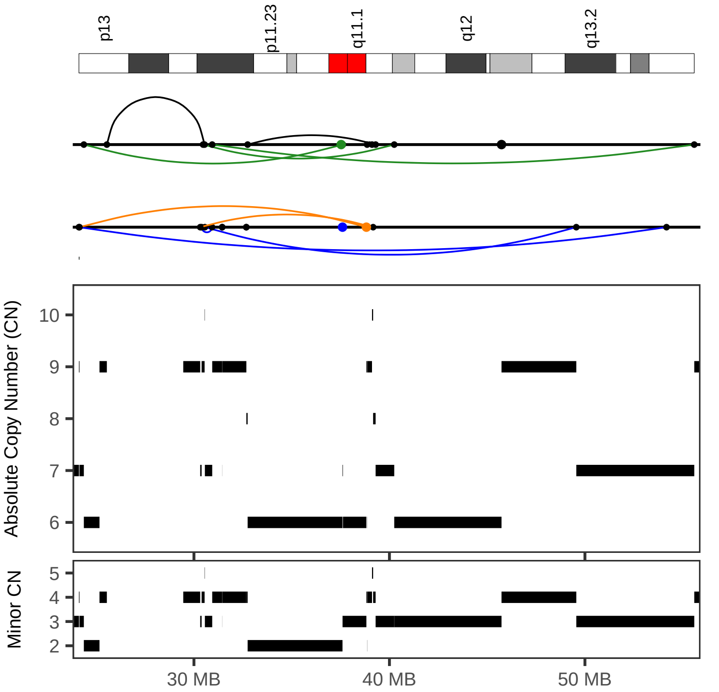

	CGP_donor_1187030
Cancer type	Breast−AdenoCA
Position	20:24116036−55592291
Type	With other complex events
Interleaved intrachr. SVs	11
Total SVs (intrachr. + transl.)	15
SV types	DEL: 2; DUP: 3; h2hINV: 3; t2tINV: 3; TRA: 4
SVs in sample	98
Oscillating CN (2 and 3 states)	4, 6
CN segments	26
FDR fragment joints	0.9905774
FDR chr. breakp. enrich.	0
Linked to chrs	
Purity, ploidy	0.54, 6.22

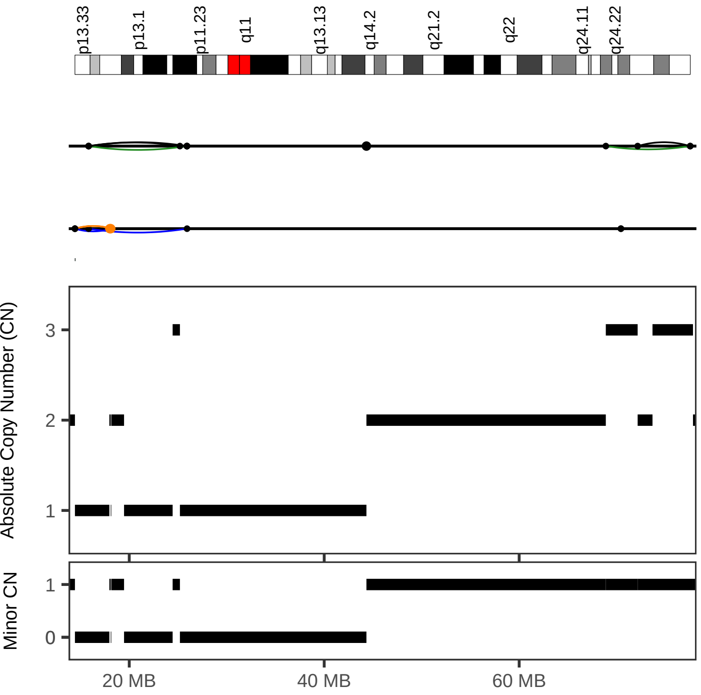

	**CGP_donor_1187033**
Cancer type	Breast−AdenoCA
Position	12:14425084−25928588
Type	Canonical without polyploidization
Interleaved intrachr. SVs	8
Total SVs (intrachr. + transl.)	10
SV types	DEL: 2; DUP: 2; h2hINV: 2; t2tINV: 2; TRA: 2
SVs in sample	228
Oscillating CN (2 and 3 states)	6, 8
CN segments	8
FDR fragment joints	0.8572806
FDR chr. breakp. enrich.	0.1
Linked to chrs	
Purity, ploidy	0.59, 1.81

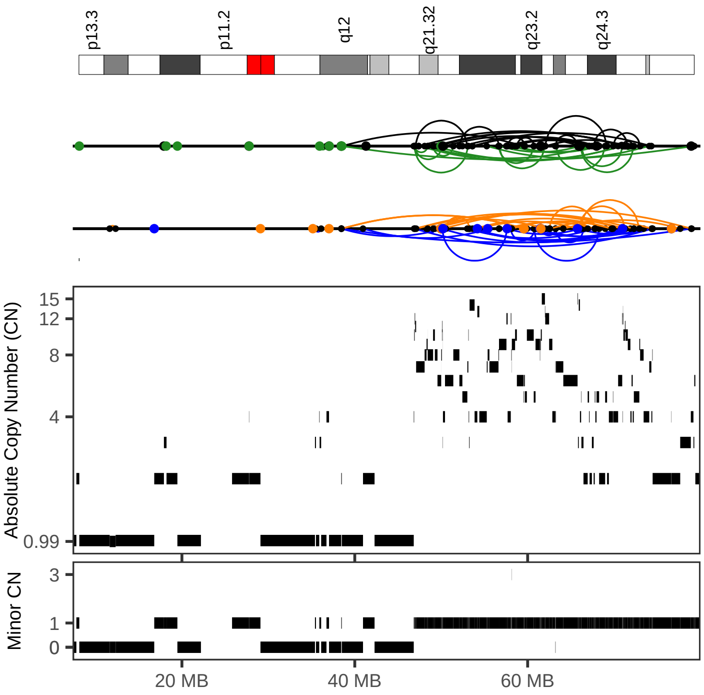

	**CGP_donor_1187033**
Cancer type	Breast−AdenoCA
Position	17:38442067−79276431
Type	With other complex events
Interleaved intrachr. SVs	84
Total SVs (intrachr. + transl.)	114
SV types	DEL: 20; DUP: 22; h2hINV: 23; t2tINV: 19; TRA: 30
SVs in sample	228
Oscillating CN (2 and 3 states)	5, 5
CN segments	116
FDR fragment joints	0.8784945
FDR chr. breakp. enrich.	0
Linked to chrs	
Purity, ploidy	0.59, 1.81

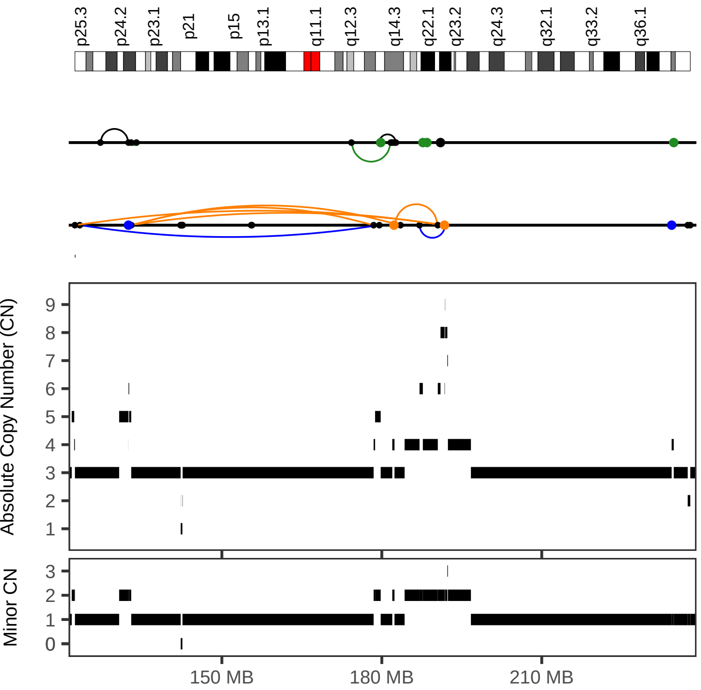

	**CGP_donor_1199104**
Cancer type	Breast−AdenoCA
Position	2:122442175−191880486
Type	With other complex events
Interleaved intrachr. SVs	10
Total SVs (intrachr. + transl.)	17
SV types	DEL: 5; DUP: 2; h2hINV: 1; t2tINV: 2; TRA: 7
SVs in sample	803
Oscillating CN (2 and 3 states)	6, 8
CN segments	26
FDR fragment joints	0.6776251
FDR chr. breakp. enrich.	0
Linked to chrs	4:53118926−163564695;8:13736456−132773371
Purity, ploidy	0.53, 3.83

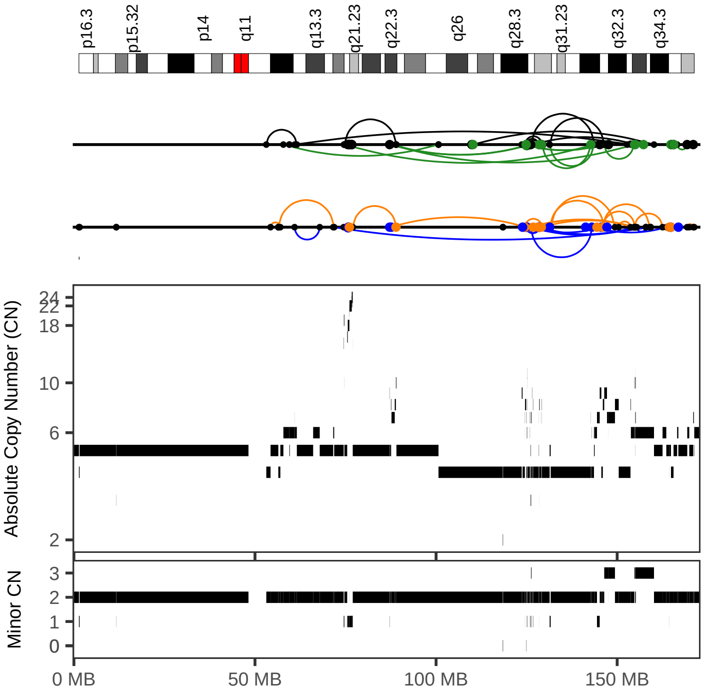

	**CGP_donor_1199104**
Cancer type	Breast−AdenoCA
Position	4:53118926−163564696
Type	With other complex events
Interleaved intrachr. SVs	52
Total SVs (intrachr. + transl.)	106
SV types	DEL: 14; DUP: 11; h2hINV: 15; t2tINV: 12; TRA: 54
SVs in sample	803
Oscillating CN (2 and 3 states)	6, 11
CN segments	115
FDR fragment joints	0.9922157
FDR chr. breakp. enrich.	0
Linked to chrs	5:77648424−175567949;8:13736456−132773371
Purity, ploidy	0.53, 3.83

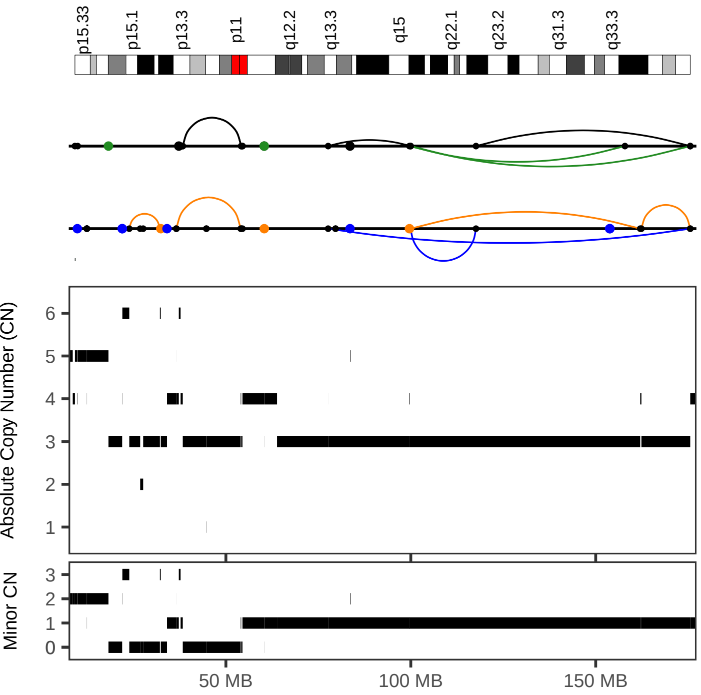

	CGP_donor_1199104
Cancer type	Breast−AdenoCA
Position	5:77648424−175567950
Type	After polyploidization
Interleaved intrachr. SVs	7
Total SVs (intrachr. + transl.)	12
SV types	DEL: 1; DUP: 2; h2hINV: 2; t2tINV: 2; TRA: 5
SVs in sample	803
Oscillating CN (2 and 3 states)	6, 9
CN segments	9
FDR fragment joints	0.6776251
FDR chr. breakp. enrich.	0.23
Linked to chrs	12:24474408−110217684;4:53118926−163564695
Purity, ploidy	0.53, 3.83

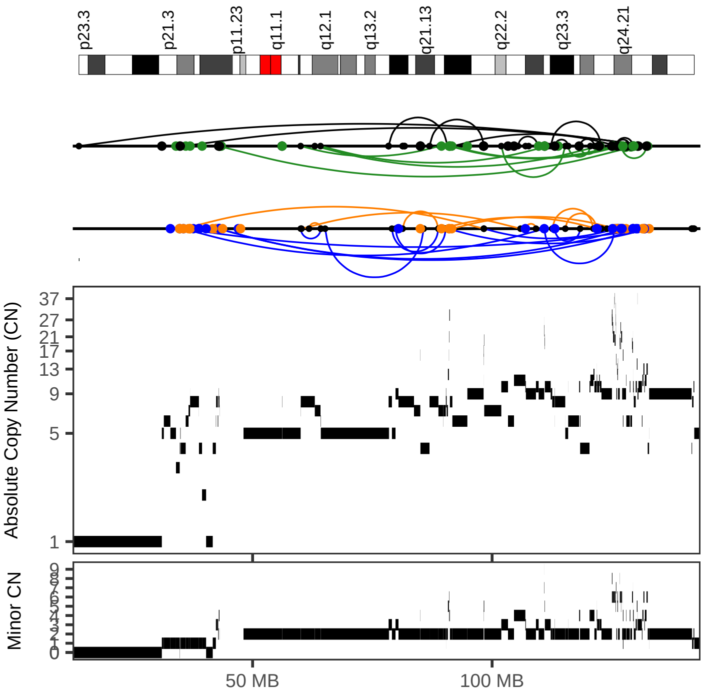

	CGP_donor_1199104
Cancer type	Breast−AdenoCA
Position	8:13736456−132773372
Type	With other complex events
Interleaved intrachr. SVs	50
Total SVs (intrachr. + transl.)	164
SV types	DEL: 12; DUP: 14; h2hINV: 11; t2tINV: 13; TRA: 114
SVs in sample	803
Oscillating CN (2 and 3 states)	6, 6
CN segments	155
FDR fragment joints	0.9339031
FDR chr. breakp. enrich.	0
Linked to chrs	11:66056632−80789771;12:24474408−110217684 2:122442175−191880485;4:53118926−163564695 5:77648424−175567949;7:75587775−134670400
Purity, ploidy	0.53, 3.83

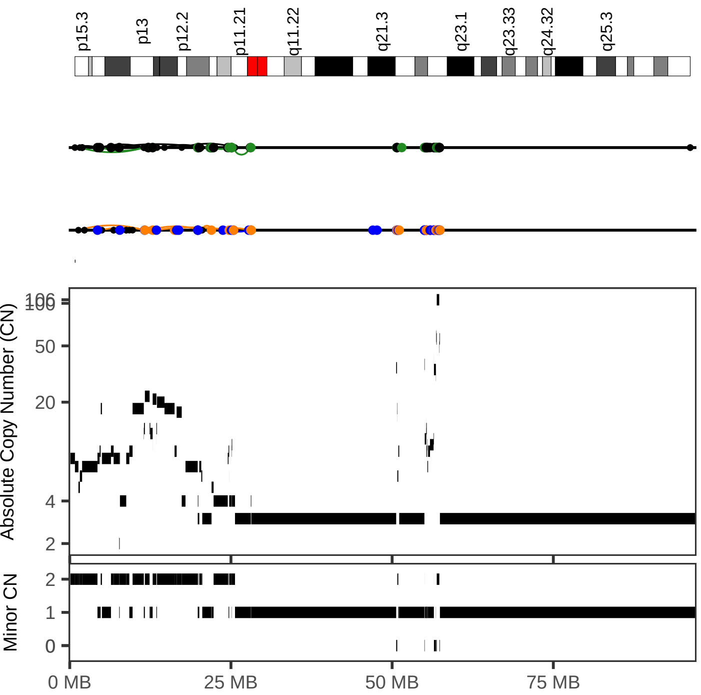

	CGP_donor_1199104
Cancer type	Breast−AdenoCA
Position	10:800474−27783066
Type	With other complex events
Interleaved intrachr. SVs	20
Total SVs (intrachr. + transl.)	65
SV types	DEL: 8; DUP: 1; h2hINV: 7; t2tINV: 4; TRA: 45
SVs in sample	803
Oscillating CN (2 and 3 states)	4, 6
CN segments	50
FDR fragment joints	0.9147564
FDR chr. breakp. enrich.	0
Linked to chrs	4:53118926−163564695;
Purity, ploidy	0.53, 3.83

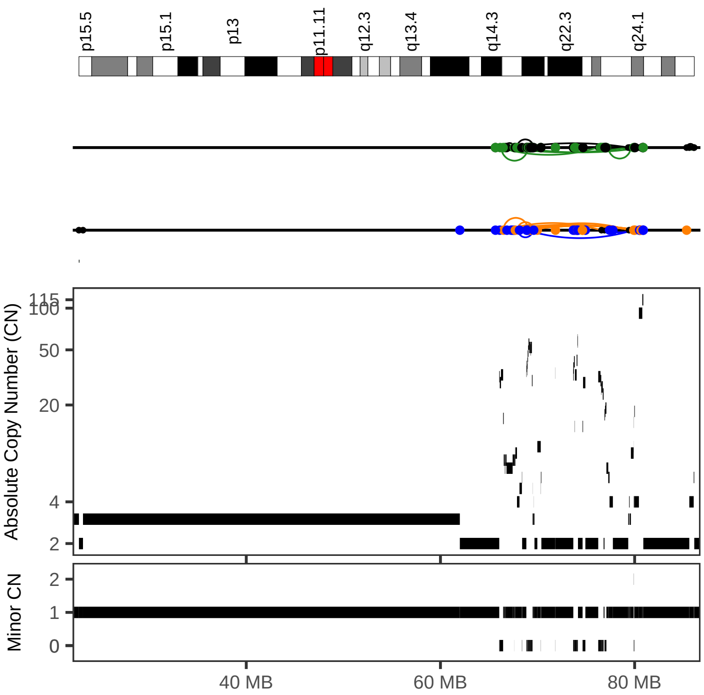

	CGP_donor_1199104
Cancer type	Breast−AdenoCA
Position	11:66056632−80789772
Type	With other complex events
Interleaved intrachr. SVs	21
Total SVs (intrachr. + transl.)	96
SV types	DEL: 9; DUP: 3; h2hINV: 3; t2tINV: 6; TRA: 75
SVs in sample	803
Oscillating CN (2 and 3 states)	5, 5
CN segments	75
FDR fragment joints	0.9343093
FDR chr. breakp. enrich.	0
Linked to chrs	12:24474408−110217684;8:13736456−132773371
Purity, ploidy	0.53, 3.83

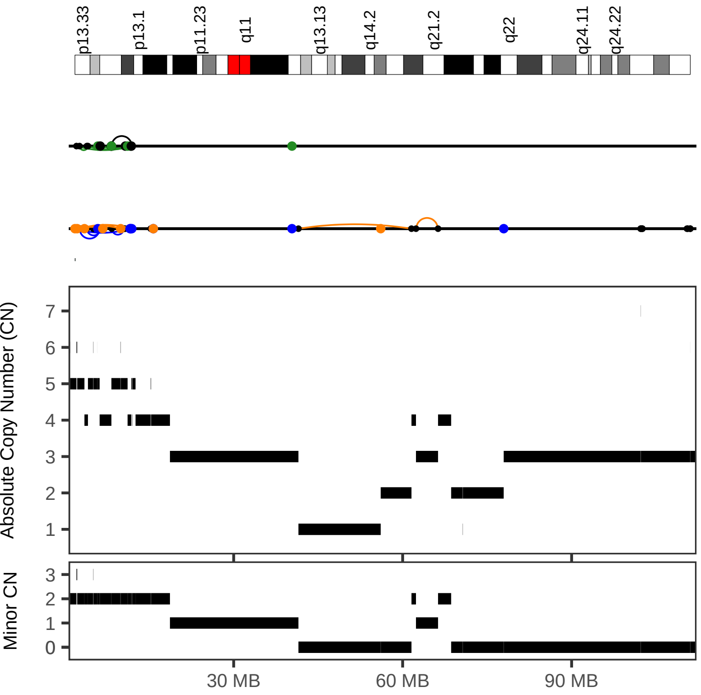

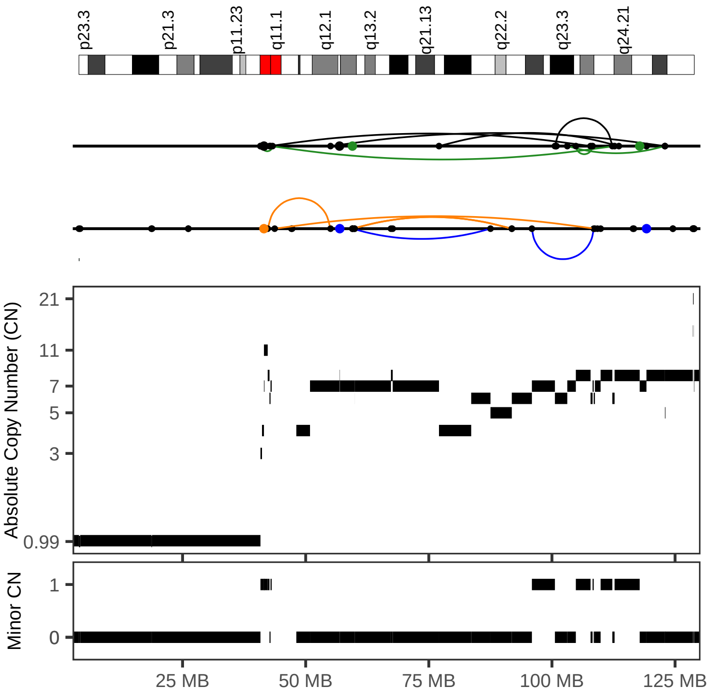

	**CGP_donor_1199129**
Cancer type	Breast−AdenoCA
Position	12:1812499−12002882
Type	With other complex events
Interleaved intrachr. SVs	13
Total SVs (intrachr. + transl.)	31
SV types	DEL: 3; DUP: 5; h2hINV: 1; t2tINV: 4; TRA: 18
SVs in sample	416
Oscillating CN (2 and 3 states)	5, 17
CN segments	17
FDR fragment joints	0.8168871
FDR chr. breakp. enrich.	0
Linked to chrs	
Purity, ploidy	0.56, 2.88

	**CGP_donor_1199131**
Cancer type	Breast−AdenoCA
Position	8:41127422−122943006
Type	With other complex events
Interleaved intrachr. SVs	14
Total SVs (intrachr. + transl.)	21
SV types	DEL: 4; DUP: 2; h2hINV: 4; t2tINV: 4; TRA: 7
SVs in sample	240
Oscillating CN (2 and 3 states)	4, 6
CN segments	33
FDR fragment joints	0.9462199
FDR chr. breakp. enrich.	0
Linked to chrs	
Purity, ploidy	0.48, 3.16

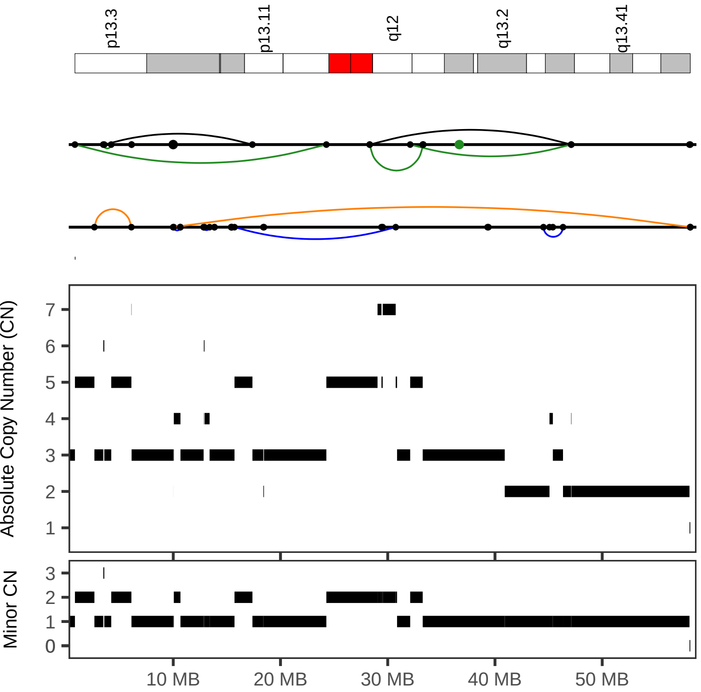

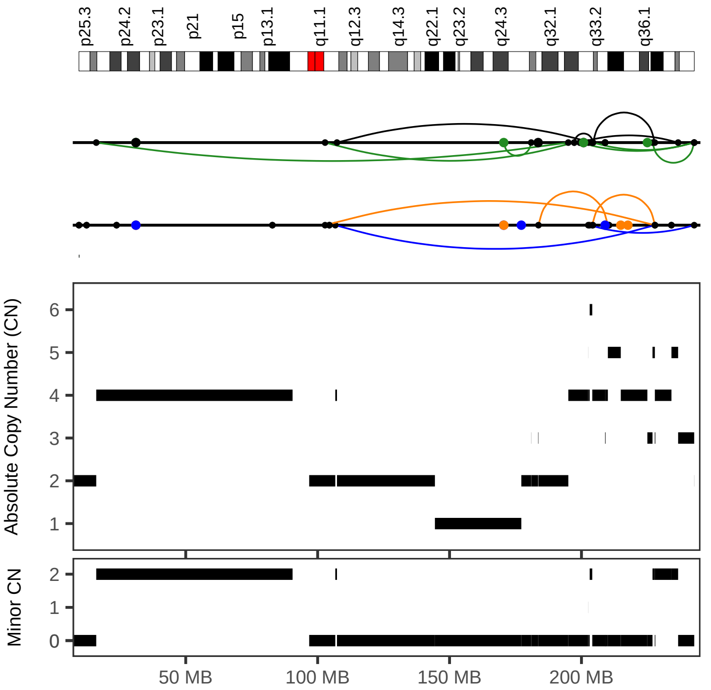

	**CGP_donor_1199131**
Cancer type	Breast−AdenoCA
Position	19:831618−58208210
Type	With other complex events
Interleaved intrachr. SVs	11
Total SVs (intrachr. + transl.)	13
SV types	DEL: 2; DUP: 1; h2hINV: 4; t2tINV: 4; TRA: 2
SVs in sample	240
Oscillating CN (2 and 3 states)	5, 5
CN segments	34
FDR fragment joints	0.615458
FDR chr. breakp. enrich.	0
Linked to chrs	
Purity, ploidy	0.48, 3.16

	**CGP_donor_1199138**
Cancer type	Breast−AdenoCA
Position	2:16077672−242757221
Type	With other complex events
Interleaved intrachr. SVs	16
Total SVs (intrachr. + transl.)	31
SV types	DEL: 4; DUP: 2; h2hINV: 4; t2tINV: 6; TRA: 15
SVs in sample	426
Oscillating CN (2 and 3 states)	5, 7
CN segments	30
FDR fragment joints	0.930656
FDR chr. breakp. enrich.	0.23
Linked to chrs	1:217357432−229030469;
Purity, ploidy	0.72, 3.18

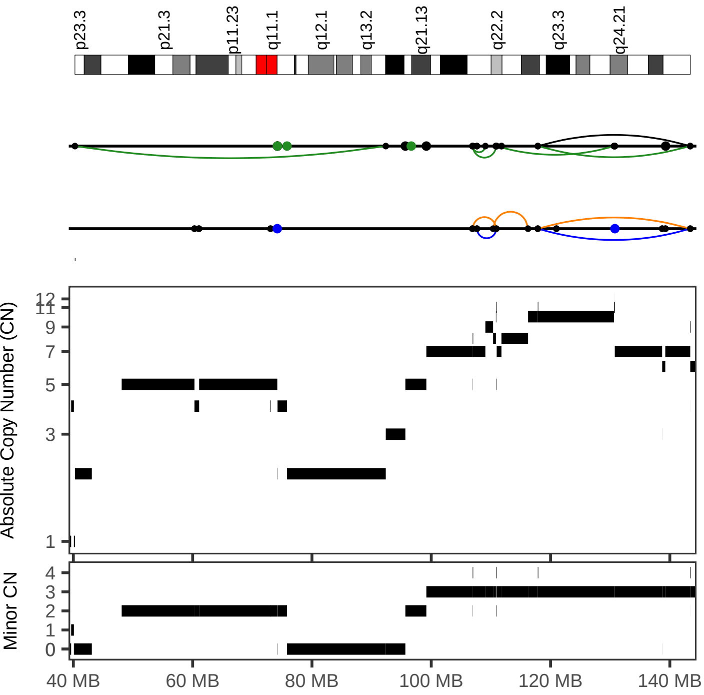

	**CGP_donor_1199138**
Cancer type	Breast−AdenoCA
Position	8:106917395−143420924
Type	With other complex events
Interleaved intrachr. SVs	15
Total SVs (intrachr. + transl.)	17
SV types	DEL: 4; DUP: 3; h2hINV: 4; t2tINV: 4; TRA: 2
SVs in sample	426
Oscillating CN (2 and 3 states)	4, 7
CN segments	24
FDR fragment joints	0.9934091
FDR chr. breakp. enrich.	0.01
Linked to chrs	
Purity, ploidy	0.72, 3.18

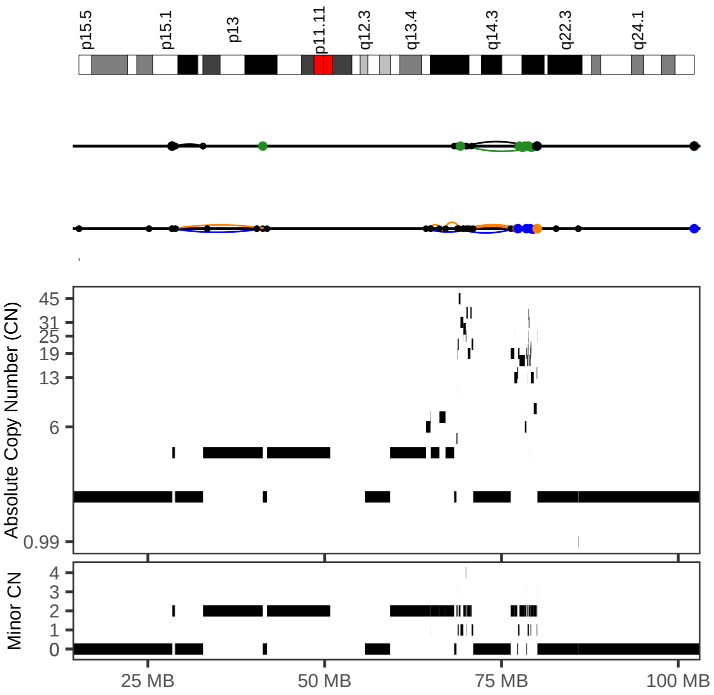

	**CGP_donor_1199138**
Cancer type	Breast−AdenoCA
Position	11:64326051−80060406
Type	With other complex events
Interleaved intrachr. SVs	23
Total SVs (intrachr. + transl.)	35
SV types	DEL: 6; DUP: 7; h2hINV: 3; t2tINV: 7; TRA: 12
SVs in sample	426
Oscillating CN (2 and 3 states)	5, 5
CN segments	56
FDR fragment joints	0.9757835
FDR chr. breakp. enrich.	0
Linked to chrs	
Purity, ploidy	0.72, 3.18

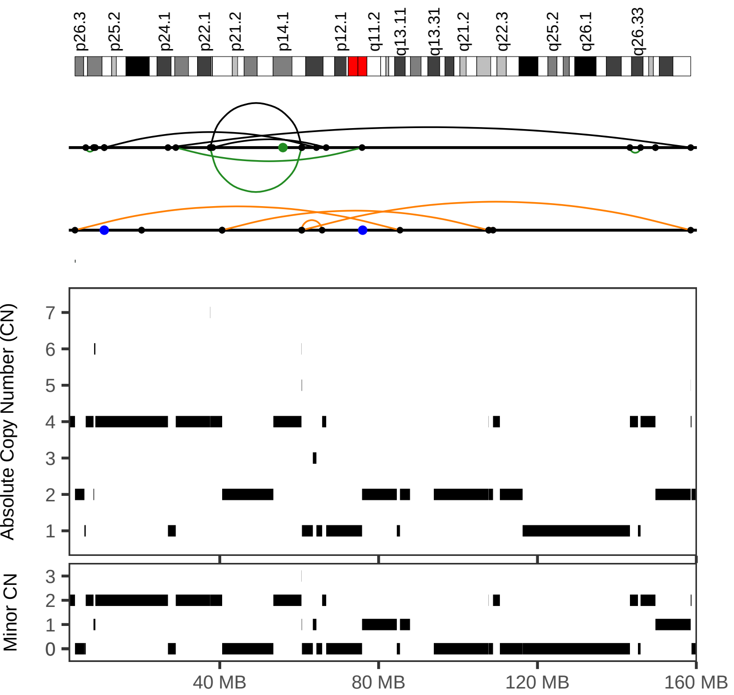

	**CGP_donor_1230722**
Cancer type	Breast−AdenoCA
Position	3:3536437−158590135
Type	With other complex events
Interleaved intrachr. SVs	10
Total SVs (intrachr. + transl.)	13
SV types	DEL: 4; DUP: 0; h2hINV: 4; t2tINV: 2; TRA: 3
SVs in sample	192
Oscillating CN (2 and 3 states)	5, 6
CN segments	34
FDR fragment joints	0.6776251
FDR chr. breakp. enrich.	0.15
Linked to chrs	
Purity, ploidy	0.27, 2.38

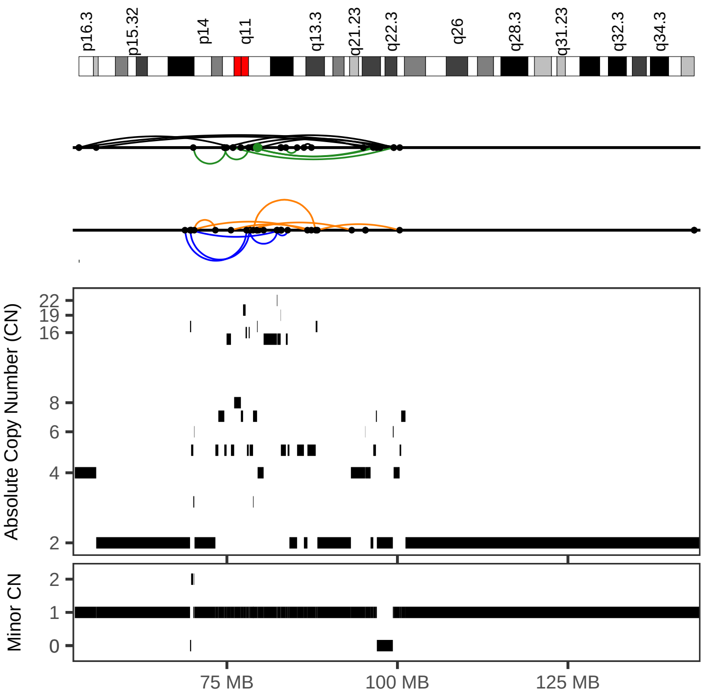

	**CGP_donor_1230722**
Cancer type	Breast−AdenoCA
Position	4:53307836−100323926
Type	With other complex events
Interleaved intrachr. SVs	27
Total SVs (intrachr. + transl.)	28
SV types	DEL: 7; DUP: 6; h2hINV: 7; t2tINV: 7; TRA: 1
SVs in sample	192
Oscillating CN (2 and 3 states)	5, 5
CN segments	46
FDR fragment joints	0.9794281
FDR chr. breakp. enrich.	0
Linked to chrs	
Purity, ploidy	0.27, 2.38

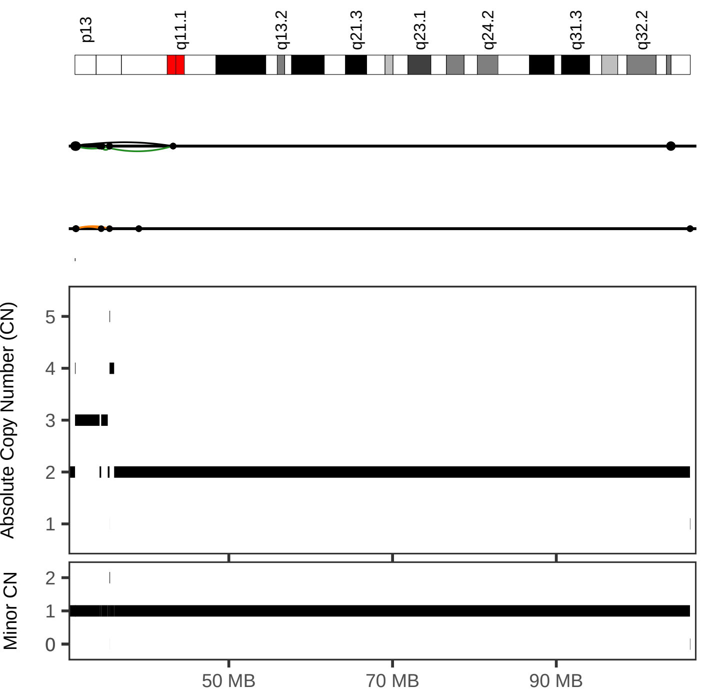

	**CGP_donor_1230722**
Cancer type	Breast−AdenoCA
Position	14:31249224−43197371
Type	With other complex events
Interleaved intrachr. SVs	9
Total SVs (intrachr. + transl.)	10
SV types	DEL: 3; DUP: 0; h2hINV: 2; t2tINV: 4; TRA: 1
SVs in sample	192
Oscillating CN (2 and 3 states)	4, 5
CN segments	8
FDR fragment joints	0.615458
FDR chr. breakp. enrich.	0
Linked to chrs	
Purity, ploidy	0.27, 2.38

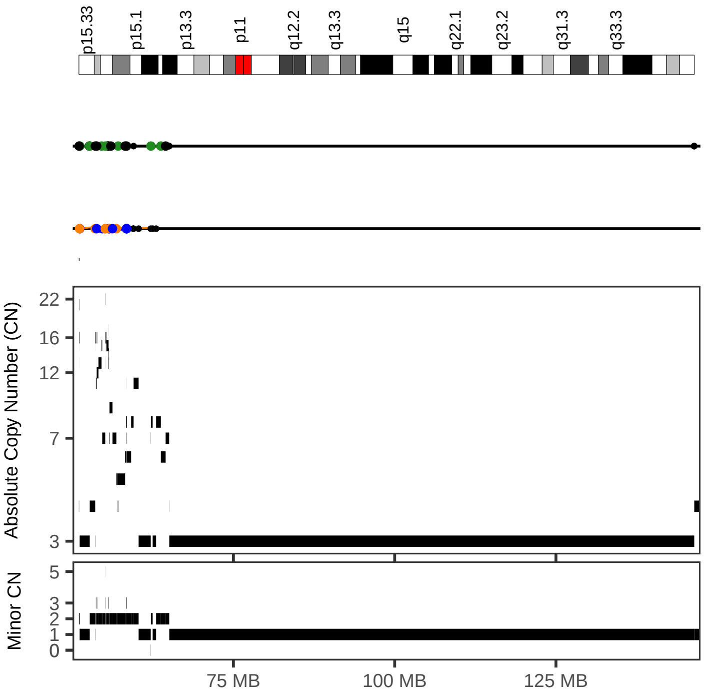

	**CGP_donor_1230728**
Cancer type	Breast−AdenoCA
Position	5:51047194−59523791
Type	With other complex events
Interleaved intrachr. SVs	7
Total SVs (intrachr. + transl.)	36
SV types	DEL: 1; DUP: 3; h2hINV: 0; t2tINV: 3; TRA: 29
SVs in sample	329
Oscillating CN (2 and 3 states)	4, 4
CN segments	41
FDR fragment joints	0.641841
FDR chr. breakp. enrich.	0
Linked to chrs	
Purity, ploidy	0.27, 3.5

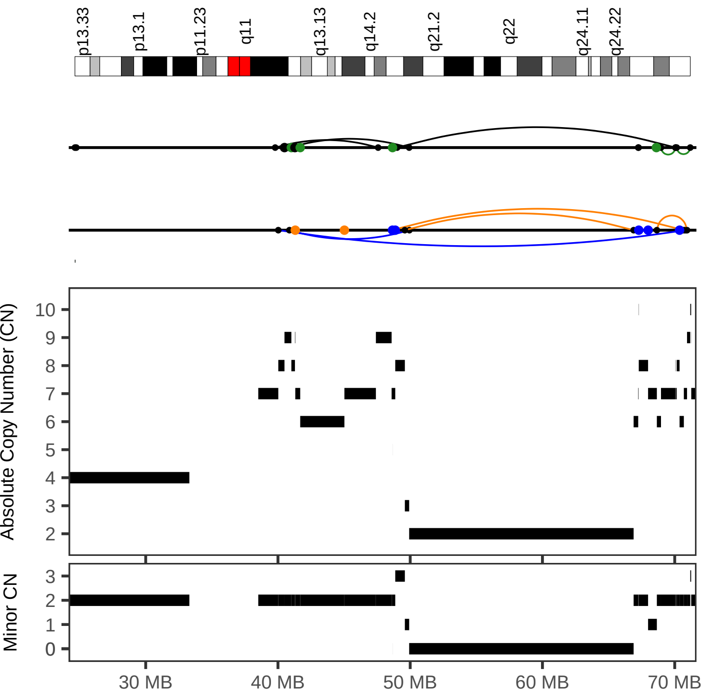

	**CGP_donor_1230728**
Cancer type	Breast−AdenoCA
Position	12:39789055−71175213
Type	With other complex events
Interleaved intrachr. SVs	9
Total SVs (intrachr. + transl.)	22
SV types	DEL: 3; DUP: 2; h2hINV: 3; t2tINV: 1; TRA: 13
SVs in sample	329
Oscillating CN (2 and 3 states)	4, 6
CN segments	29
FDR fragment joints	0.6776251
FDR chr. breakp. enrich.	0
Linked to chrs	
Purity, ploidy	0.27, 3.5

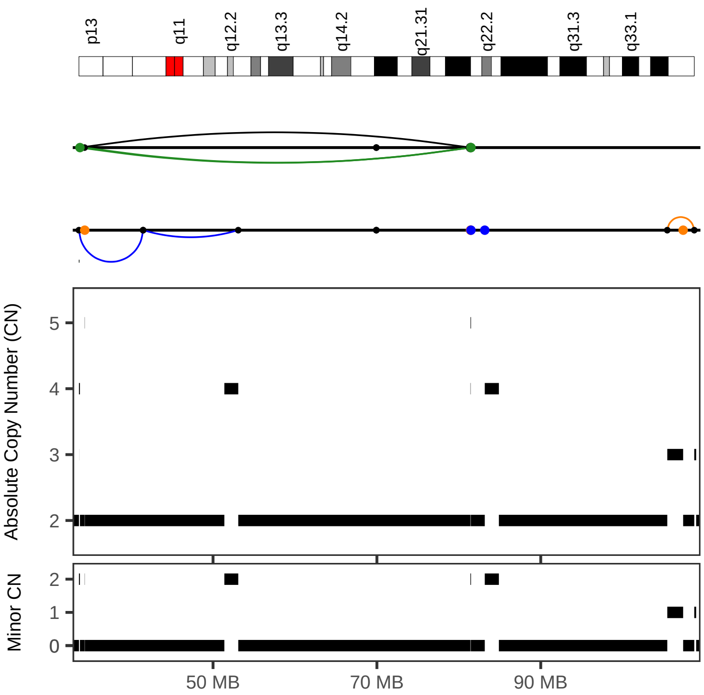

	**CGP_donor_1230728**
Cancer type	Breast−AdenoCA
Position	13:33627141−81483301
Type	With other complex events
Interleaved intrachr. SVs	6
Total SVs (intrachr. + transl.)	12
SV types	DEL: 0; DUP: 3; h2hINV: 1; t2tINV: 2; TRA: 6
SVs in sample	329
Oscillating CN (2 and 3 states)	4, 9
CN segments	13
FDR fragment joints	0.9284301
FDR chr. breakp. enrich.	0.04
Linked to chrs	16:2388599−19726670;
Purity, ploidy	0.27, 3.5

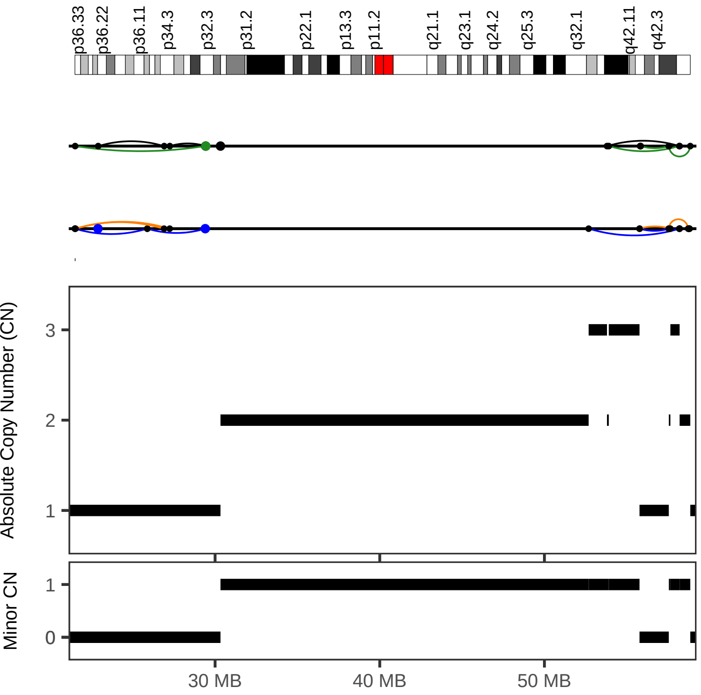

CGP_donor_1230729

Cancer type	Breast−AdenoCA
Position	1:52686225−58856084
Type	With other complex events
Interleaved intrachr. SVs	10
Total SVs (intrachr. + transl.)	10
SV types	DEL: 3; DUP: 2; h2hINV: 2; t2tINV: 3; TRA: 0
SVs in sample	107
Oscillating CN (2 and 3 states)	4, 7
CN segments	10
FDR fragment joints	0.9625775
FDR chr. breakp. enrich.	0
Linked to chrs	
Purity, ploidy	0.52, 1.7

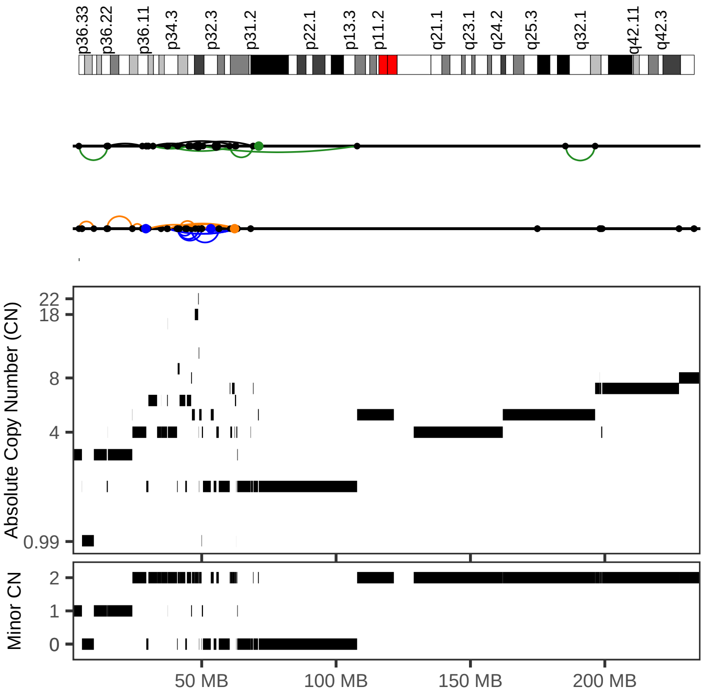

CGP_donor_1230778

Cancer type	Breast−AdenoCA
Position	1:30154115−107830373
Type	With other complex events
Interleaved intrachr. SVs	26
Total SVs (intrachr. + transl.)	31
SV types	DEL: 8; DUP: 9; h2hINV: 4; t2tINV: 5; TRA: 5
SVs in sample	182
Oscillating CN (2 and 3 states)	5, 10
CN segments	44
FDR fragment joints	0.7013708
FDR chr. breakp. enrich.	0
Linked to chrs	
Purity, ploidy	0.24, 3.39

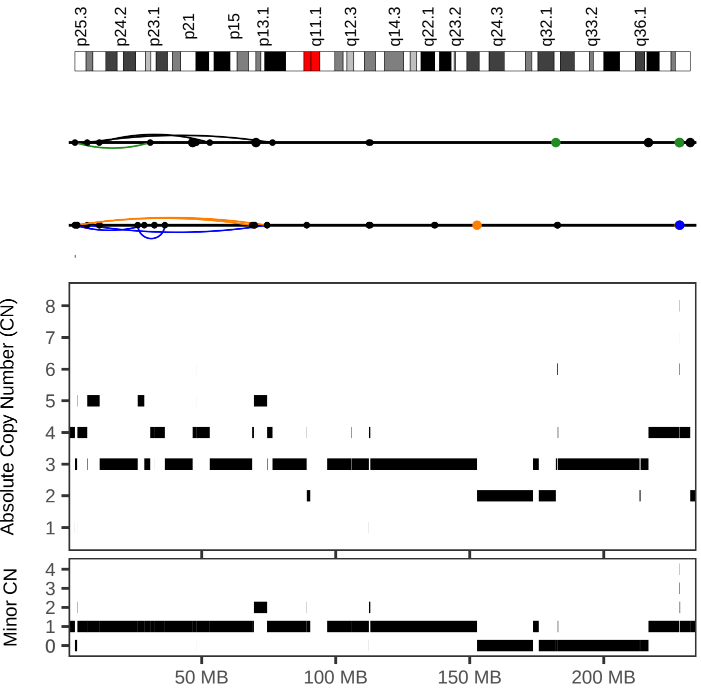

CGP_donor_1230785

Cancer type	Breast−AdenoCA
Position	2:2703128−76418672
Type	With other complex events
Interleaved intrachr. SVs	9
Total SVs (intrachr. + transl.)	11
SV types	DEL: 2; DUP: 4; h2hINV: 2; t2tINV: 1; TRA: 2
SVs in sample	502
Oscillating CN (2 and 3 states)	6, 8
CN segments	25
FDR fragment joints	0.8653243
FDR chr. breakp. enrich.	0.63
Linked to chrs	
Purity, ploidy	0.35, 2.71

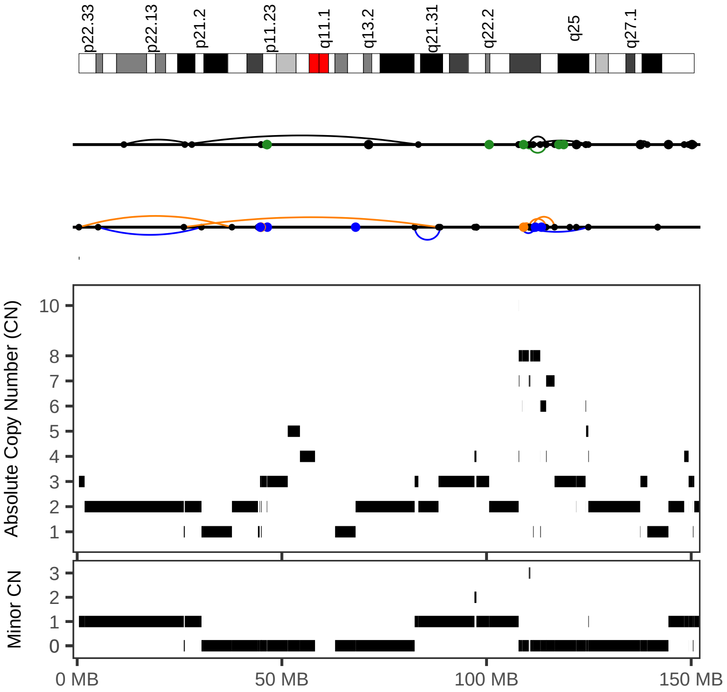

CGP_donor_1230785

Cancer type	Breast−AdenoCA
Position	X:108712192−124865542
Type	With other complex events
Interleaved intrachr. SVs	12
Total SVs (intrachr. + transl.)	19
SV types	DEL: 5; DUP: 3; h2hINV: 2; t2tINV: 2; TRA: 7
SVs in sample	502
Oscillating CN (2 and 3 states)	5, 6
CN segments	23
FDR fragment joints	0.8653243
FDR chr. breakp. enrich.	0
Linked to chrs	
Purity, ploidy	0.35, 2.71

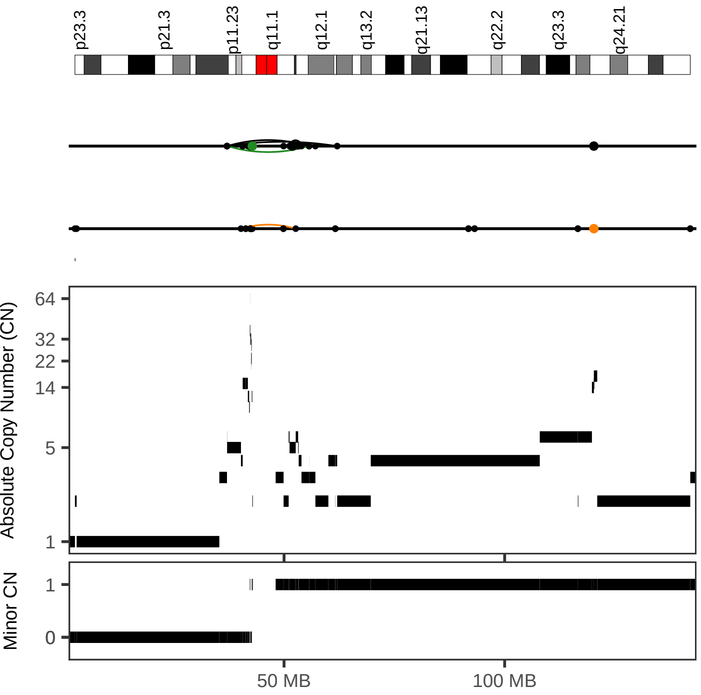

	CGP_donor_1310131
Cancer type	Breast−AdenoCA
Position	8:37059484−62040279
Type	With other complex events
Interleaved intrachr. SVs	6
Total SVs (intrachr. + transl.)	9
SV types	DEL: 1; DUP: 0; h2hINV: 4; t2tINV: 1; TRA: 3
SVs in sample	256
Oscillating CN (2 and 3 states)	5, 9
CN segments	35
FDR fragment joints	0.5435077
FDR chr. breakp. enrich.	0
Linked to chrs	
Purity, ploidy	0.22, 2.91

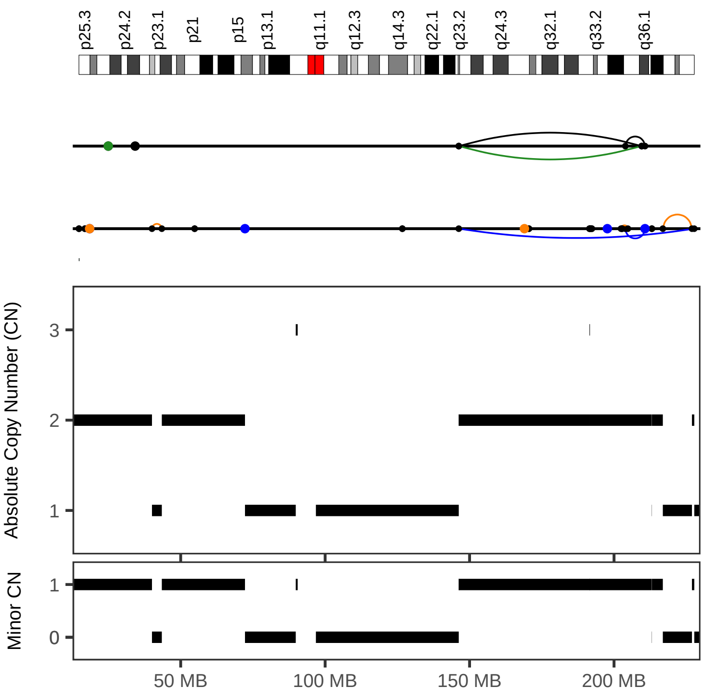

	CGP_donor_1347720
Cancer type	Breast−AdenoCA
Position	2:146257863−227776482
Type	Canonical without polyploidization
Interleaved intrachr. SVs	6
Total SVs (intrachr. + transl.)	9
SV types	DEL: 1; DUP: 2; h2hINV: 2; t2tINV: 1; TRA: 3
SVs in sample	173
Oscillating CN (2 and 3 states)	6, 6
CN segments	9
FDR fragment joints	0.6776251
FDR chr. breakp. enrich.	0.01
Linked to chrs	
Purity, ploidy	0.37, 1.59

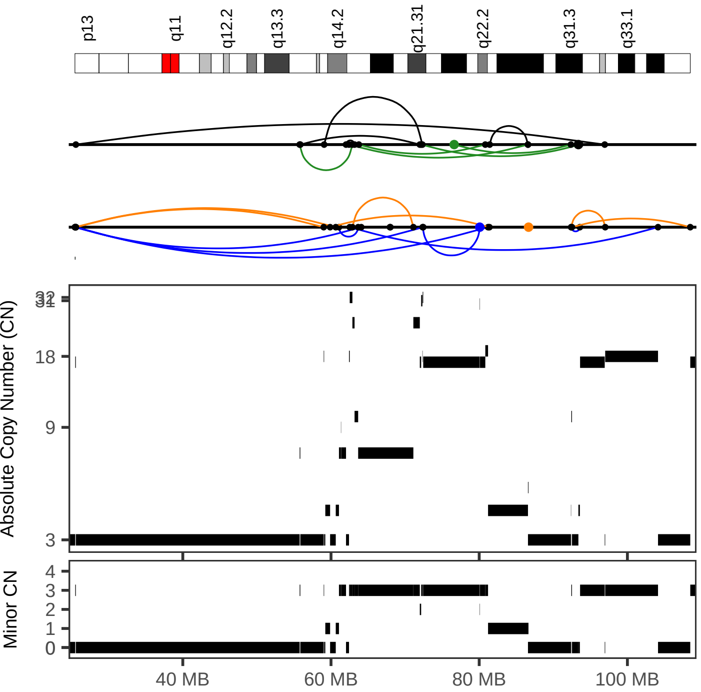

	CGP_donor_1347737
Cancer type	Breast−AdenoCA
Position	13:25444057−108478748
Type	With other complex events
Interleaved intrachr. SVs	22
Total SVs (intrachr. + transl.)	26
SV types	DEL: 6; DUP: 7; h2hINV: 4; t2tINV: 5; TRA: 4
SVs in sample	245
Oscillating CN (2 and 3 states)	4, 6
CN segments	41
FDR fragment joints	0.9179372
FDR chr. breakp. enrich.	0
Linked to chrs	8:54710029−141665224;
Purity, ploidy	0.59, 5.63

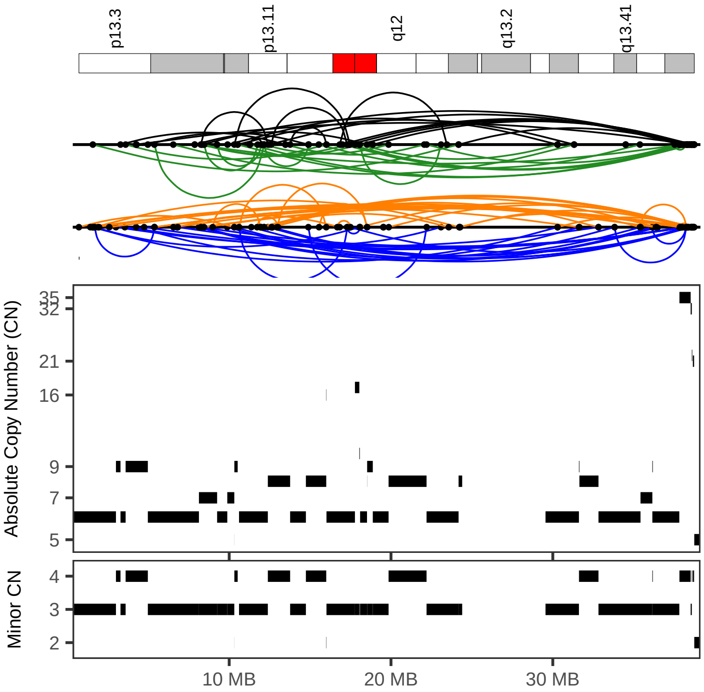

	CGP_donor_1347737
Cancer type	Breast−AdenoCA
Position	19:712477−38656509
Type	With other complex events
Interleaved intrachr. SVs	102
Total SVs (intrachr. + transl.)	102
SV types	DEL: 31; DUP: 27; h2hINV: 21; t2tINV: 23; TRA: 0
SVs in sample	245
Oscillating CN (2 and 3 states)	5, 7
CN segments	36
FDR fragment joints	0.6776251
FDR chr. breakp. enrich.	0
Linked to chrs	
Purity, ploidy	0.59, 5.63

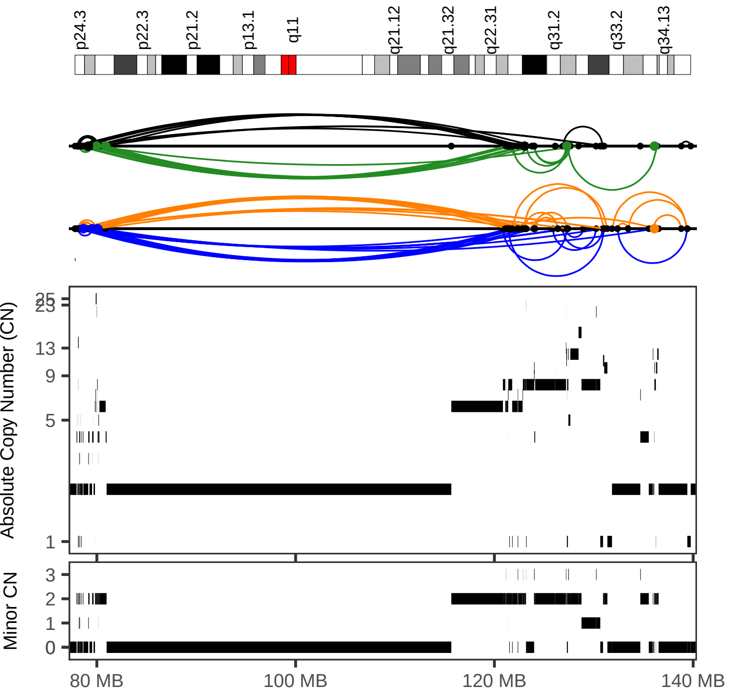

CGP_donor_1353431	
Cancer type	Breast−AdenoCA
Position	9:77800927−139754035
Type	With other complex events
Interleaved intrachr. SVs	103
Total SVs (intrachr. + transl.)	113
SV types	DEL: 33; DUP: 28; h2hINV: 21; t2tINV: 21; TRA: 10
SVs in sample	276
Oscillating CN (2 and 3 states)	5, 8
CN segments	130
FDR fragment joints	0.6661212
FDR chr. breakp. enrich.	0
Linked to chrs	11:65669076−121347314;17:27282661−72008911
Purity, ploidy	0.51, 3.26

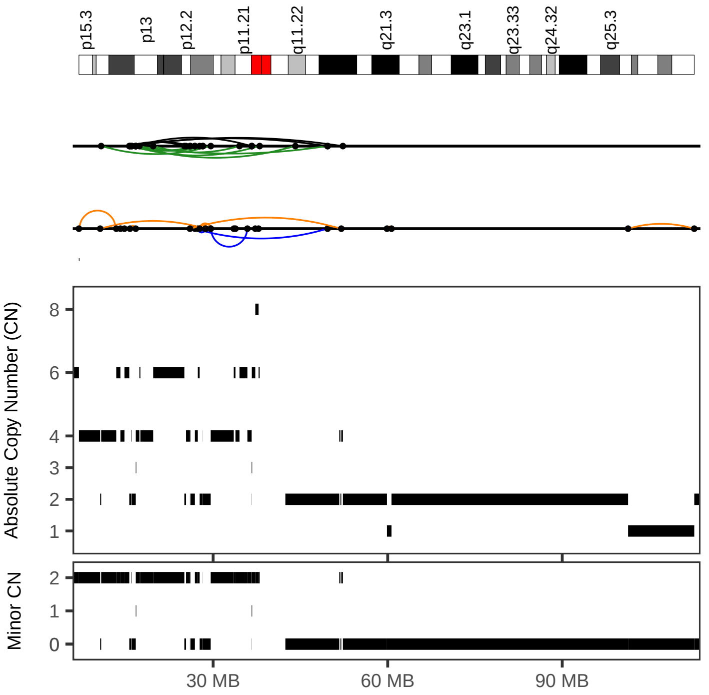

CGP_donor_1353431	
Cancer type	Breast−AdenoCA
Position	10:6930033−52299947
Type	With other complex events
Interleaved intrachr. SVs	23
Total SVs (intrachr. + transl.)	23
SV types	DEL: 5; DUP: 3; h2hINV: 6; t2tINV: 9; TRA: 0
SVs in sample	276
Oscillating CN (2 and 3 states)	6, 10
CN segments	40
FDR fragment joints	0.615458
FDR chr. breakp. enrich.	0
Linked to chrs	
Purity, ploidy	0.51, 3.26

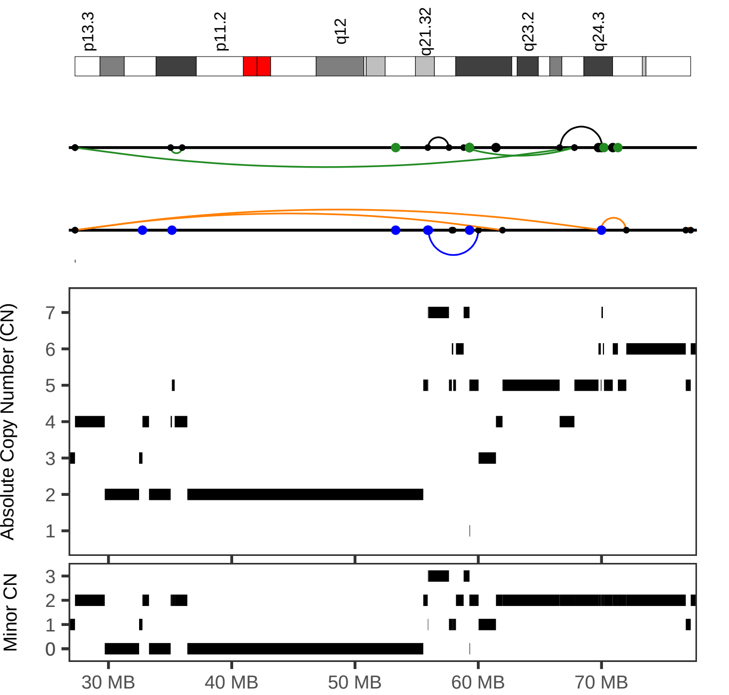

CGP_donor_1353431	
Cancer type	Breast−AdenoCA
Position	17:27282661−72008912
Type	With other complex events
Interleaved intrachr. SVs	8
Total SVs (intrachr. + transl.)	25
SV types	DEL: 3; DUP: 1; h2hINV: 2; t2tINV: 2; TRA: 17
SVs in sample	276
Oscillating CN (2 and 3 states)	5, 8
CN segments	34
FDR fragment joints	0.6776251
FDR chr. breakp. enrich.	0
Linked to chrs	9:77800927−139754034;
Purity, ploidy	0.51, 3.26

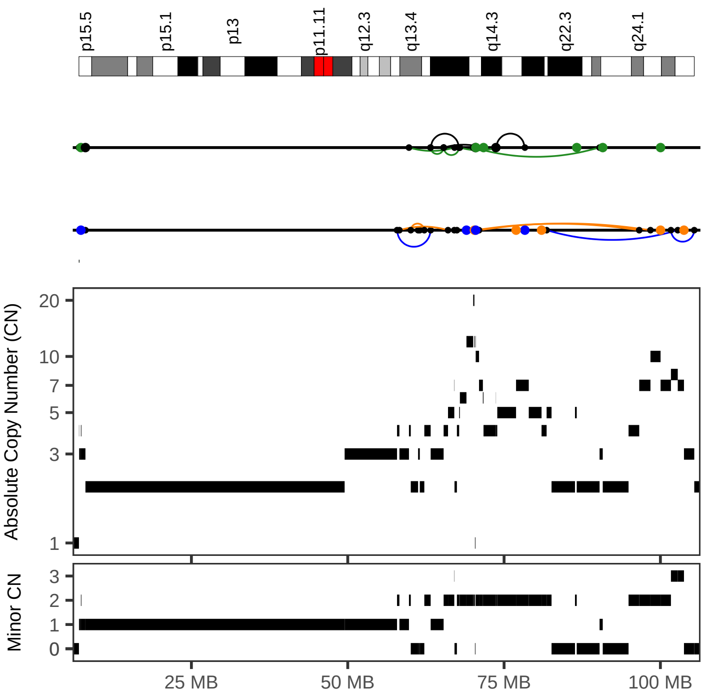

CGP_donor_1353432	
Cancer type	Breast−AdenoCA
Position	11:57883853−105414478
Type	With other complex events
Interleaved intrachr. SVs	15
Total SVs (intrachr. + transl.)	30
SV types	DEL: 4; DUP: 3; h2hINV: 3; t2tINV: 5; TRA: 15
SVs in sample	185
Oscillating CN (2 and 3 states)	4, 6
CN segments	45
FDR fragment joints	0.9284301
FDR chr. breakp. enrich.	0
Linked to chrs	
Purity, ploidy	0.89, 3.18

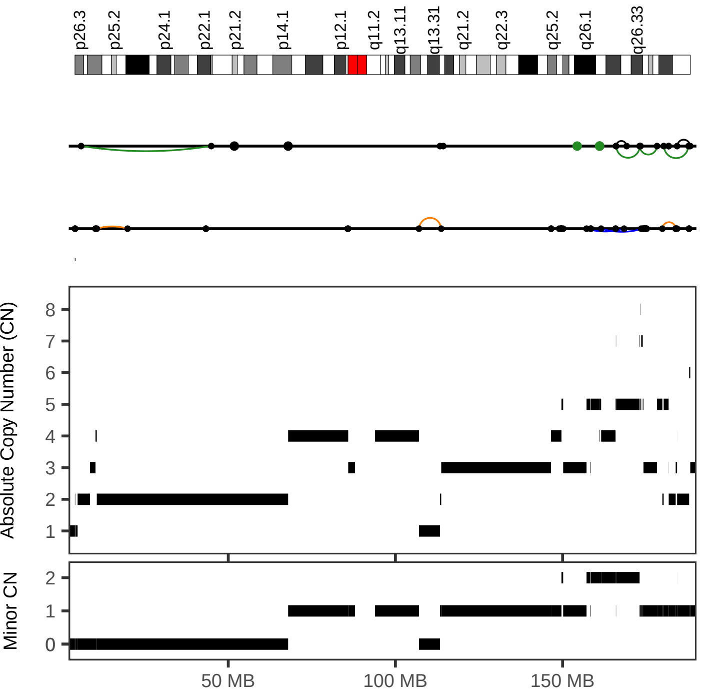

	CGP_donor_1363963
Cancer type	Breast−AdenoCA
Position	3:157158956−178255600
Type	With other complex events
Interleaved intrachr. SVs	6
Total SVs (intrachr. + transl.)	8
SV types	DEL: 0; DUP: 3; h2hINV: 1; t2tINV: 2; TRA: 2
SVs in sample	324
Oscillating CN (2 and 3 states)	5, 7
CN segments	16
FDR fragment joints	0.615458
FDR chr. breakp. enrich.	0.05
Linked to chrs	
Purity, ploidy	0.18, 2.98

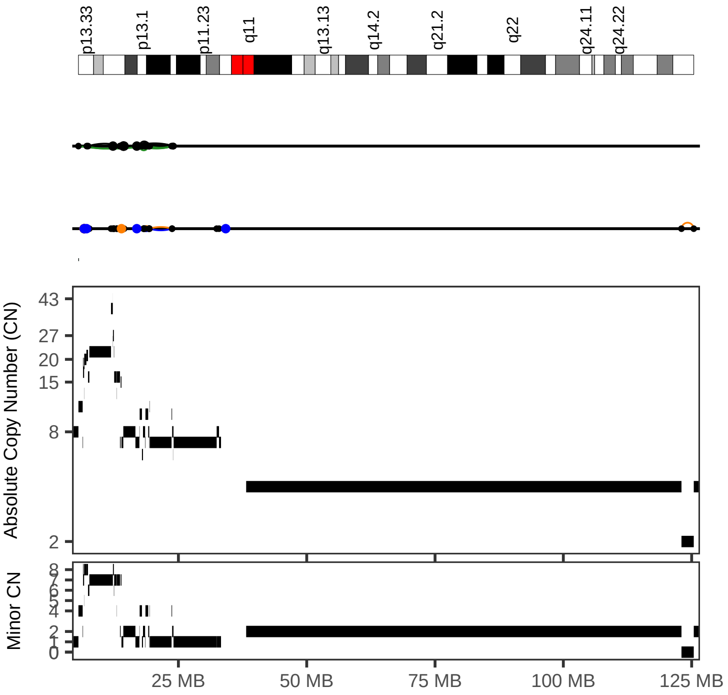

	CGP_donor_1456607
Cancer type	Breast−AdenoCA
Position	12:5519258−24071782
Type	With other complex events
Interleaved intrachr. SVs	15
Total SVs (intrachr. + transl.)	27
SV types	DEL: 3; DUP: 2; h2hINV: 4; t2tINV: 6; TRA: 12
SVs in sample	172
Oscillating CN (2 and 3 states)	4, 5
CN segments	39
FDR fragment joints	0.615458
FDR chr. breakp. enrich.	0
Linked to chrs	
Purity, ploidy	0.57, 3.21

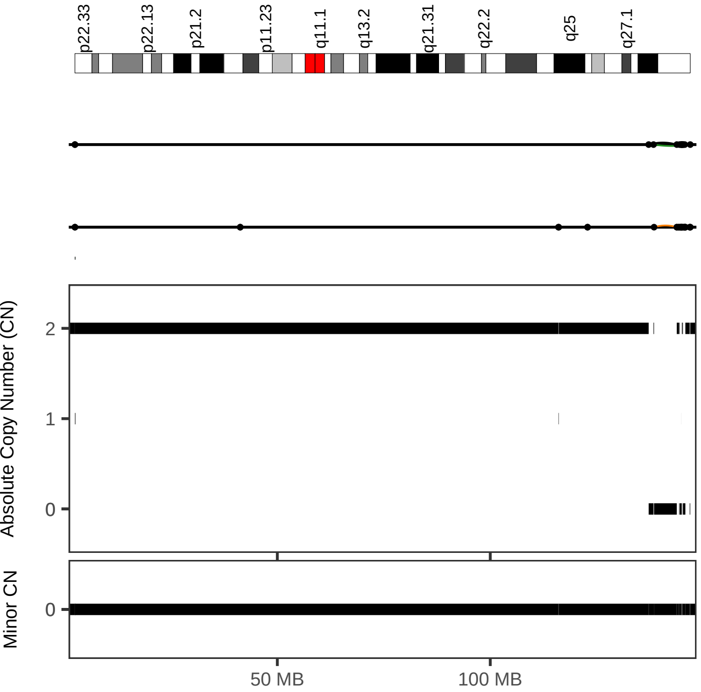

	CGP_donor_1456607
Cancer type	Breast−AdenoCA
Position	X:144433858−145805852
Type	Canonical without polyploidization
Interleaved intrachr. SVs	9
Total SVs (intrachr. + transl.)	9
SV types	DEL: 3; DUP: 2; h2hINV: 2; t2tINV: 2; TRA: 0
SVs in sample	172
Oscillating CN (2 and 3 states)	6, 8
CN segments	8
FDR fragment joints	0.9723381
FDR chr. breakp. enrich.	0
Linked to chrs	
Purity, ploidy	0.57, 3.21

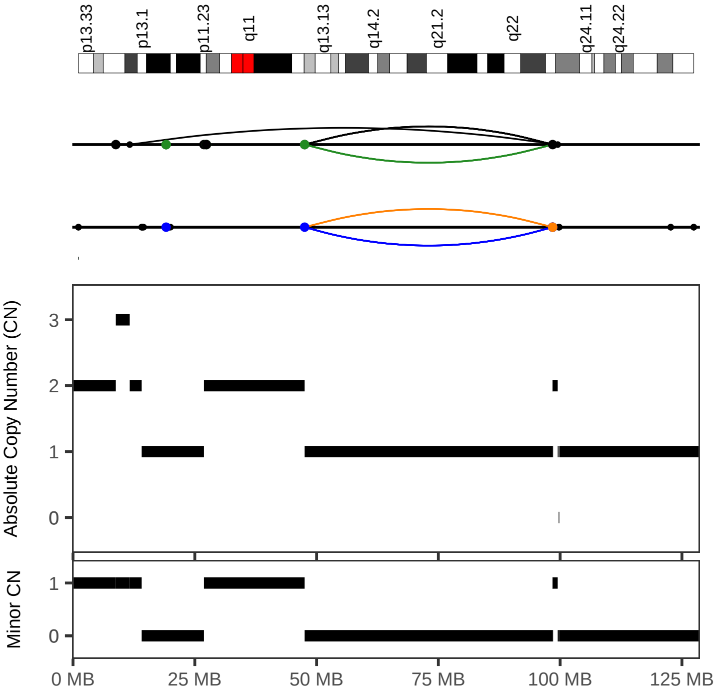

	CGP_donor_1472394
Cancer type	Breast−AdenoCA
Position	12:47550216−98458711
Type	Canonical without polyploidization
Interleaved intrachr. SVs	13
Total SVs (intrachr. + transl.)	21
SV types	DEL: 2; DUP: 4; h2hINV: 4; t2tINV: 3; TRA: 8
SVs in sample	366
Oscillating CN (2 and 3 states)	5, 5
CN segments	5
FDR fragment joints	0.8882853
FDR chr. breakp. enrich.	0
Linked to chrs	11:22556697−111968888;
Purity, ploidy	0.7, 1.76

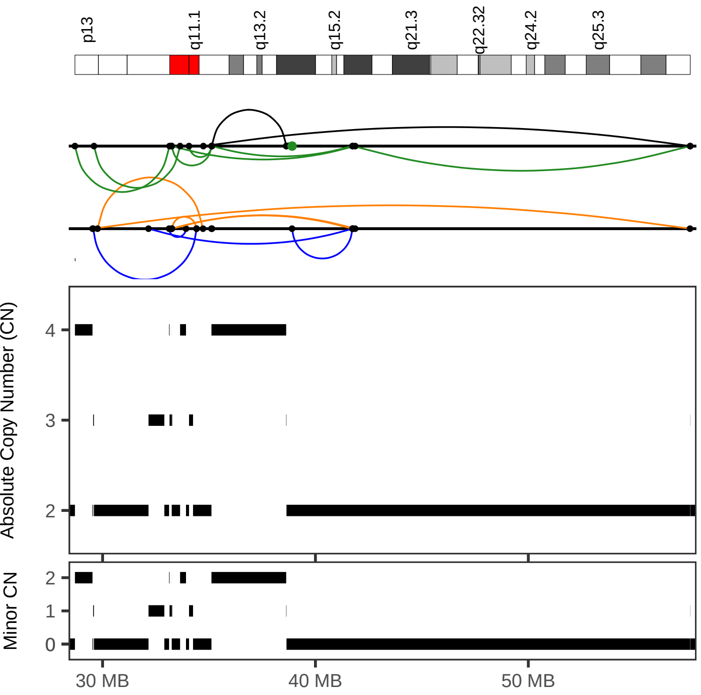

	**CGP_donor_1472395**
Cancer type	Breast−AdenoCA
Position	15:28701023−57607104
Type	With other complex events
Interleaved intrachr. SVs	20
Total SVs (intrachr. + transl.)	21
SV types	DEL: 5; DUP: 5; h2hINV: 3; t2tINV: 7; TRA: 1
SVs in sample	132
Oscillating CN (2 and 3 states)	5, 10
CN segments	20
FDR fragment joints	0.8882853
FDR chr. breakp. enrich.	0
Linked to chrs	
Purity, ploidy	0.5, 3.23

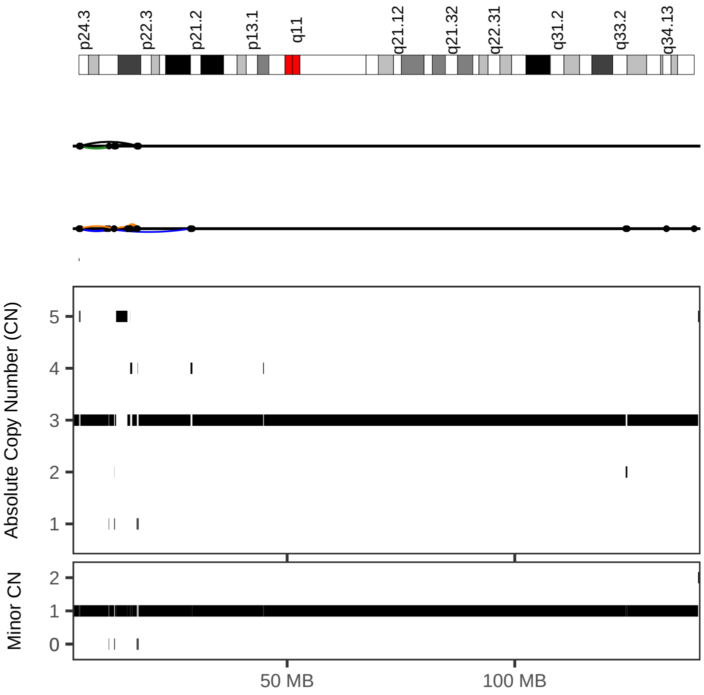

	**CGP_donor_1606179**
Cancer type	Breast−AdenoCA
Position	9:4256540−29177994
Type	With other complex events
Interleaved intrachr. SVs	12
Total SVs (intrachr. + transl.)	12
SV types	DEL: 3; DUP: 3; h2hINV: 3; t2tINV: 3; TRA: 0
SVs in sample	372
Oscillating CN (2 and 3 states)	4, 7
CN segments	22
FDR fragment joints	1
FDR chr. breakp. enrich.	0.28
Linked to chrs	
Purity, ploidy	0.52, 2.83

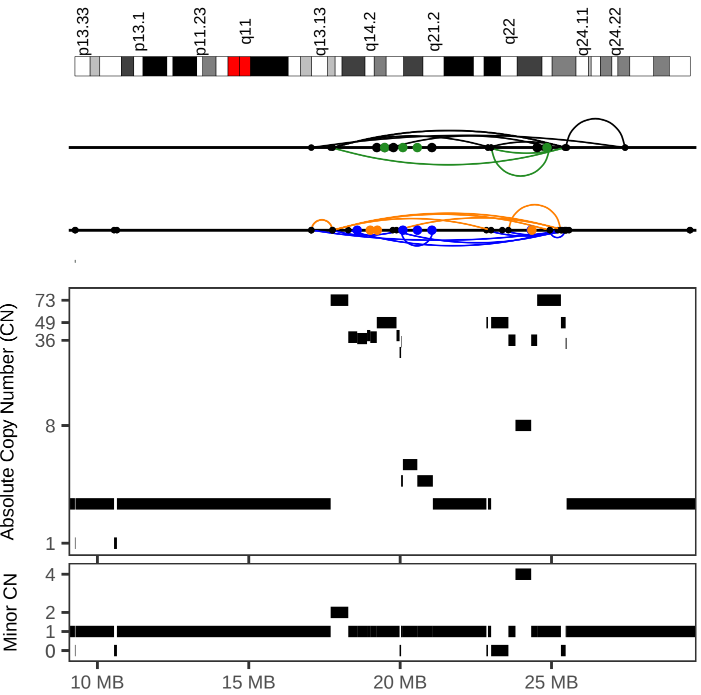

	**CGP_donor_1606179**
Cancer type	Breast−AdenoCA
Position	12:17064462−27430583
Type	With other complex events
Interleaved intrachr. SVs	25
Total SVs (intrachr. + transl.)	42
SV types	DEL: 8; DUP: 7; h2hINV: 7; t2tINV: 3; TRA: 17
SVs in sample	372
Oscillating CN (2 and 3 states)	4, 4
CN segments	24
FDR fragment joints	0.8988396
FDR chr. breakp. enrich.	0
Linked to chrs	6:947294−169801582;
Purity, ploidy	0.52, 2.83

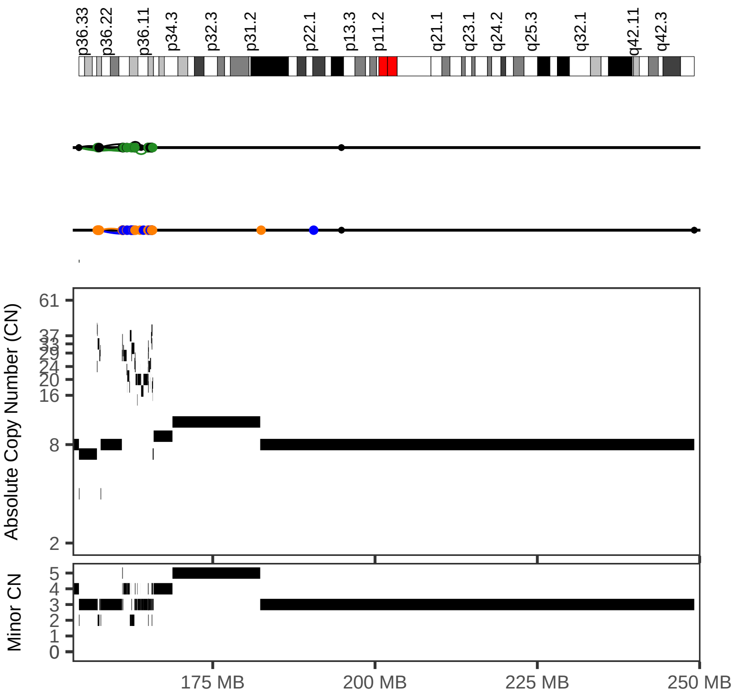

	**CGP_donor_1654385**
Cancer type	Breast−AdenoCA
Position	1:154387036−165898515
Type	With other complex events
Interleaved intrachr. SVs	26
Total SVs (intrachr. + transl.)	111
SV types	DEL: 6; DUP: 6; h2hINV: 7; t2tINV: 7; TRA: 85
SVs in sample	372
Oscillating CN (2 and 3 states)	4, 7
CN segments	63
FDR fragment joints	0.6776251
FDR chr. breakp. enrich.	0
Linked to chrs	21:10701912−45821257;
Purity, ploidy	0.72, 4.42

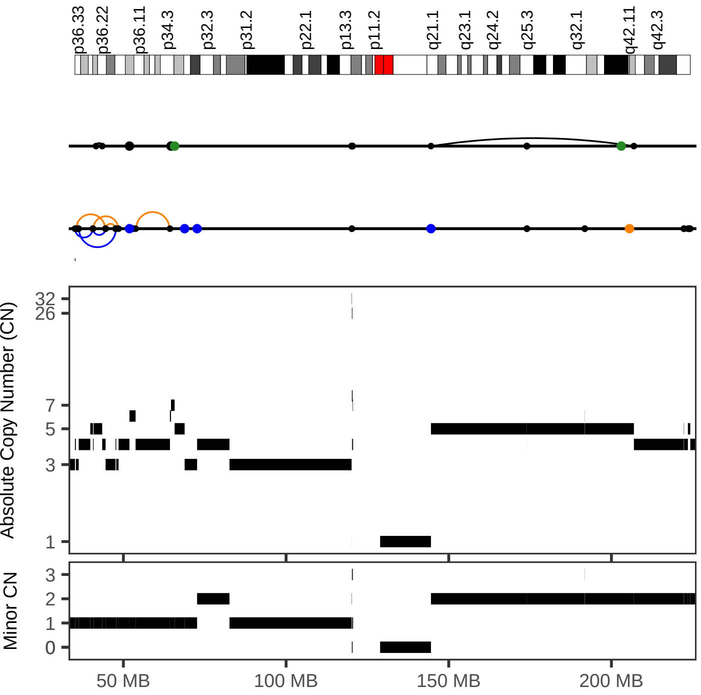

	CGP_donor_1701345
Cancer type	Breast−AdenoCA
Position	1:35101528−48512693
Type	With other complex events
Interleaved intrachr. SVs	6
Total SVs (intrachr. + transl.)	6
SV types	DEL: 3; DUP: 3; h2hINV: 0; t2tINV: 0; TRA: 0
SVs in sample	368
Oscillating CN (2 and 3 states)	5, 11
CN segments	11
FDR fragment joints	0.5435077
FDR chr. breakp. enrich.	0.69
Linked to chrs	
Purity, ploidy	0.43, 2.84

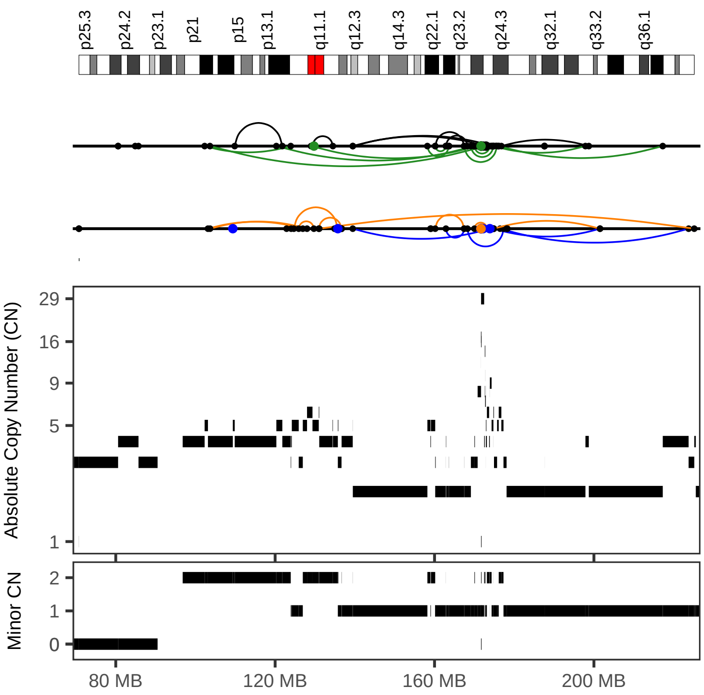

	CGP_donor_1701345
Cancer type	Breast−AdenoCA
Position	2:102310161−225180735
Type	With other complex events
Interleaved intrachr. SVs	37
Total SVs (intrachr. + transl.)	55
SV types	DEL: 11; DUP: 6; h2hINV: 9; t2tINV: 11; TRA: 18
SVs in sample	368
Oscillating CN (2 and 3 states)	6, 12
CN segments	78
FDR fragment joints	0.9723381
FDR chr. breakp. enrich.	0
Linked to chrs	1:35101528−48512692;10:290666−86528974
Purity, ploidy	0.43, 2.84

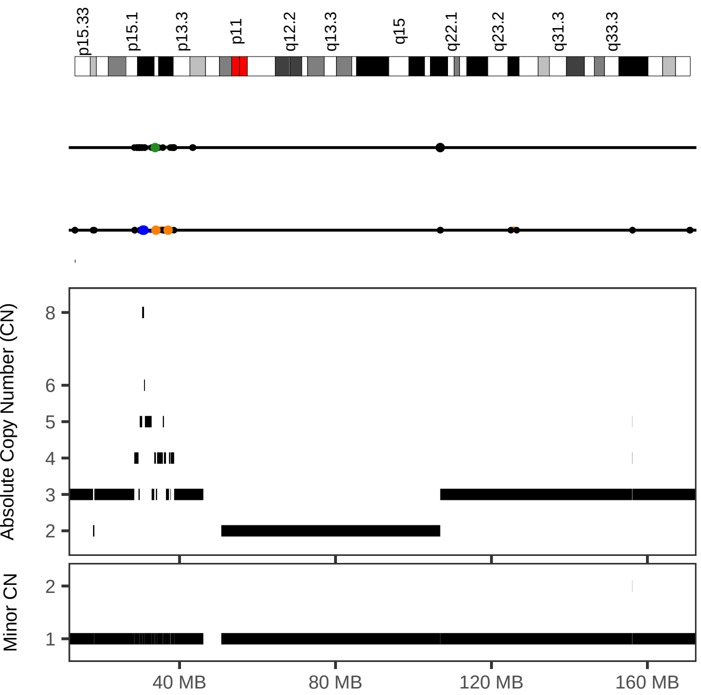

	CGP_donor_1701345
Cancer type	Breast−AdenoCA
Position	5:28386068−38607621
Type	With other complex events
Interleaved intrachr. SVs	11
Total SVs (intrachr. + transl.)	16
SV types	DEL: 2; DUP: 2; h2hINV: 4; t2tINV: 3; TRA: 5
SVs in sample	368
Oscillating CN (2 and 3 states)	6, 6
CN segments	17
FDR fragment joints	0.9501265
FDR chr. breakp. enrich.	0.42
Linked to chrs	
Purity, ploidy	0.43, 2.84

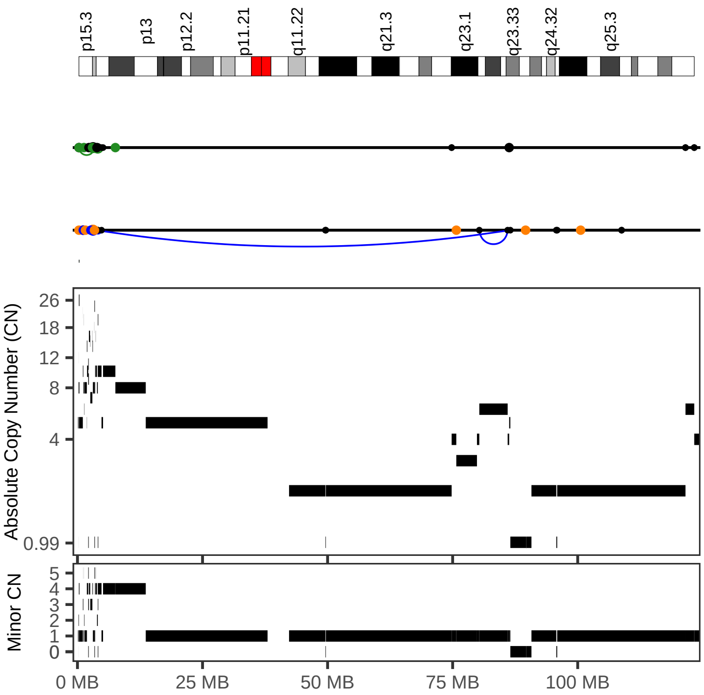

	CGP_donor_1701345
Cancer type	Breast−AdenoCA
Position	10:290666−86528975
Type	With other complex events
Interleaved intrachr. SVs	18
Total SVs (intrachr. + transl.)	34
SV types	DEL: 4; DUP: 6; h2hINV: 3; t2tINV: 5; TRA: 16
SVs in sample	368
Oscillating CN (2 and 3 states)	5, 7
CN segments	52
FDR fragment joints	0.9717738
FDR chr. breakp. enrich.	0
Linked to chrs	2:102310161−225180734;
Purity, ploidy	0.43, 2.84

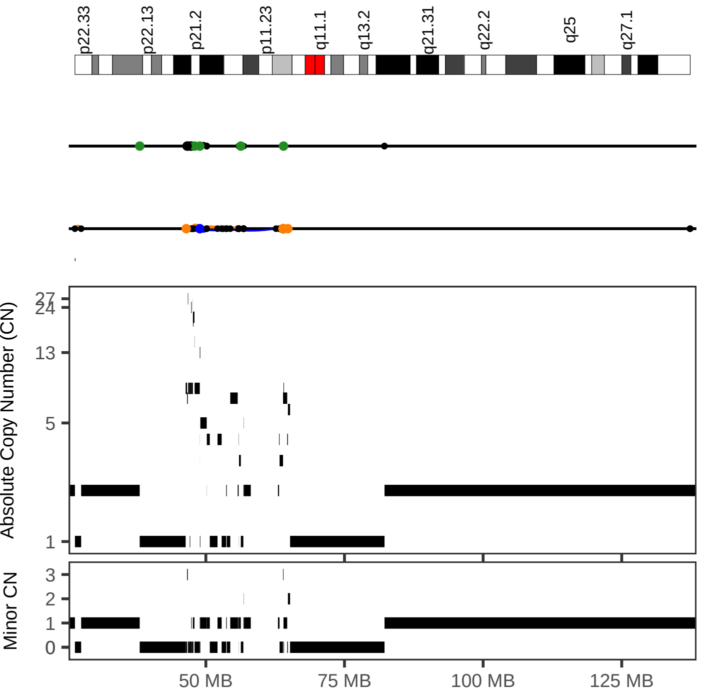

CGP_donor_1701345

Cancer type	Breast−AdenoCA
Position	X:47359209−62646302
Type	With other complex events
Interleaved intrachr. SVs	14
Total SVs (intrachr. + transl.)	19
SV types	DEL: 7; DUP: 5; h2hINV: 2; t2tINV: 0; TRA: 5
SVs in sample	368
Oscillating CN (2 and 3 states)	4, 8
CN segments	33
FDR fragment joints	0.615458
FDR chr. breakp. enrich.	0
Linked to chrs	10:290666−86528974;2:102310161−225180734
Purity, ploidy	0.43, 2.84

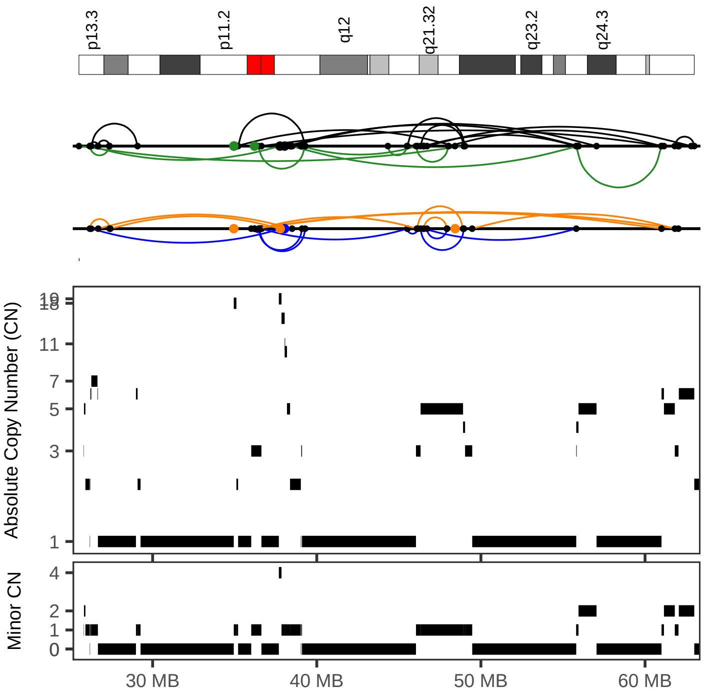

084c6aec−94f7−4090−8f3f−59fa9e89721a

Cancer type	Breast−AdenoCA
Position	17:25508004−62998961
Type	With other complex events
Interleaved intrachr. SVs	40
Total SVs (intrachr. + transl.)	49
SV types	DEL: 9; DUP: 8; h2hINV: 14; t2tINV: 9; TRA: 9
SVs in sample	257
Oscillating CN (2 and 3 states)	4, 5
CN segments	41
FDR fragment joints	0.6776251
FDR chr. breakp. enrich.	0
Linked to chrs	
Purity, ploidy	0.88, 1.98

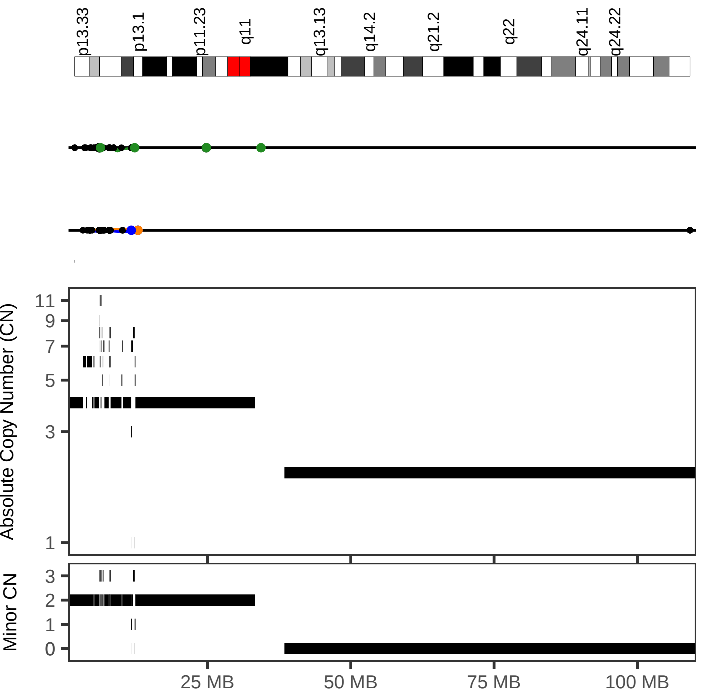

08da7c4c−3067−4bcf−9d7a−78566df72e69

Cancer type	Breast−AdenoCA
Position	12:3255392−12445504
Type	With other complex events
Interleaved intrachr. SVs	37
Total SVs (intrachr. + transl.)	42
SV types	DEL: 11; DUP: 9; h2hINV: 8; t2tINV: 9; TRA: 5
SVs in sample	151
Oscillating CN (2 and 3 states)	6, 9
CN segments	56
FDR fragment joints	1
FDR chr. breakp. enrich.	0
Linked to chrs	1:1177876−186668387;
Purity, ploidy	0.63, 3.03

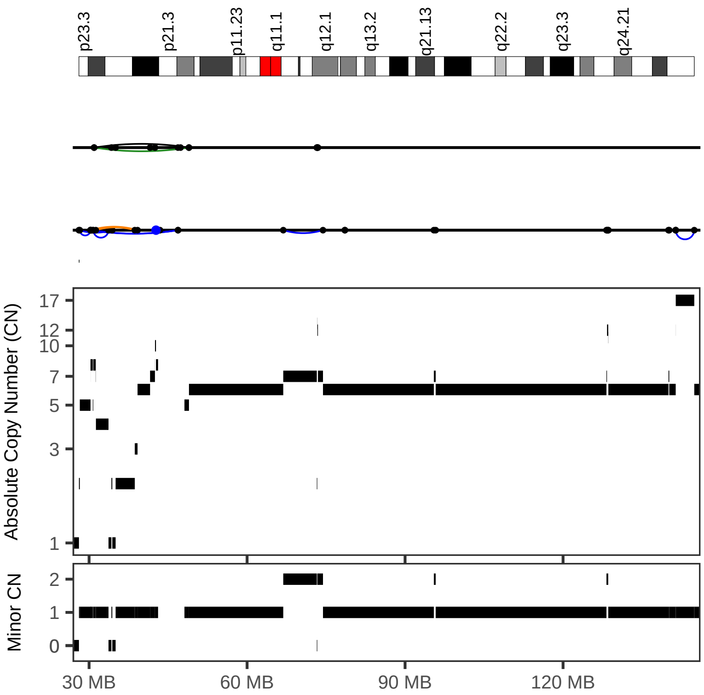

0a2a3529−f645−4967−9a58−89ee20b8bb62

Cancer type	Breast−AdenoCA
Position	8:28068350−48961471
Type	With other complex events
Interleaved intrachr. SVs	12
Total SVs (intrachr. + transl.)	13
SV types	DEL: 2; DUP: 6; h2hINV: 1; t2tINV: 3; TRA: 1
SVs in sample	190
Oscillating CN (2 and 3 states)	4, 7
CN segments	21
FDR fragment joints	0.5435077
FDR chr. breakp. enrich.	0
Linked to chrs	
Purity, ploidy	0.66, 3.57

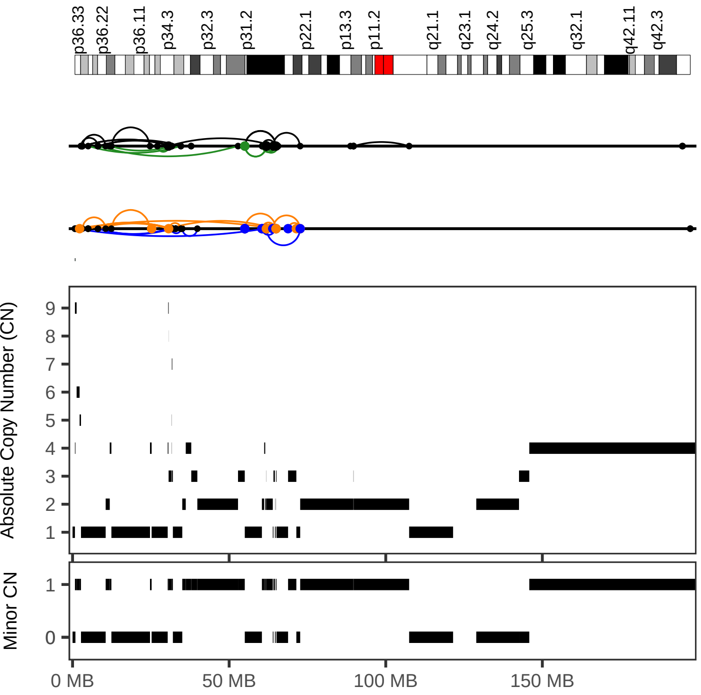

	0bc5744c−5fa3−45bb−87d0−70a02068b392
Cancer type	Breast−AdenoCA
Position	1:755344−72678036
Type	With other complex events
Interleaved intrachr. SVs	50
Total SVs (intrachr. + transl.)	85
SV types	DEL: 18; DUP: 7; h2hINV: 14; t2tINV: 11; TRA: 35
SVs in sample	487
Oscillating CN (2 and 3 states)	5, 7
CN segments	43
FDR fragment joints	0.615458
FDR chr. breakp. enrich.	0
Linked to chrs	14:39530849−45584133;
Purity, ploidy	0.43, 2.16

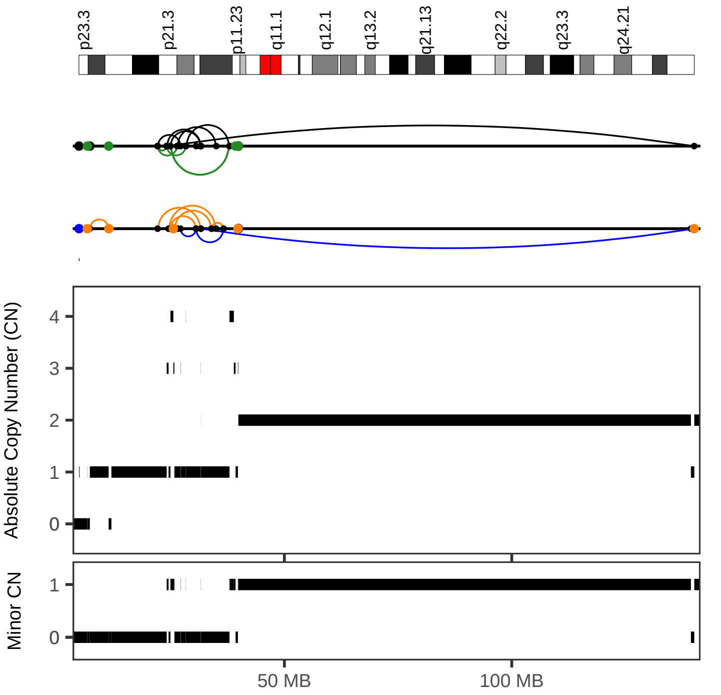

	0bc5744c−5fa3−45bb−87d0−70a02068b392
Cancer type	Breast−AdenoCA
Position	8:22116077−140133049
Type	With other complex events
Interleaved intrachr. SVs	22
Total SVs (intrachr. + transl.)	37
SV types	DEL: 7; DUP: 3; h2hINV: 6; t2tINV: 6; TRA: 15
SVs in sample	487
Oscillating CN (2 and 3 states)	4, 5
CN segments	21
FDR fragment joints	0.8653243
FDR chr. breakp. enrich.	0
Linked to chrs	1:755344−72678035;14:39530849−45584133
Purity, ploidy	0.43, 2.16

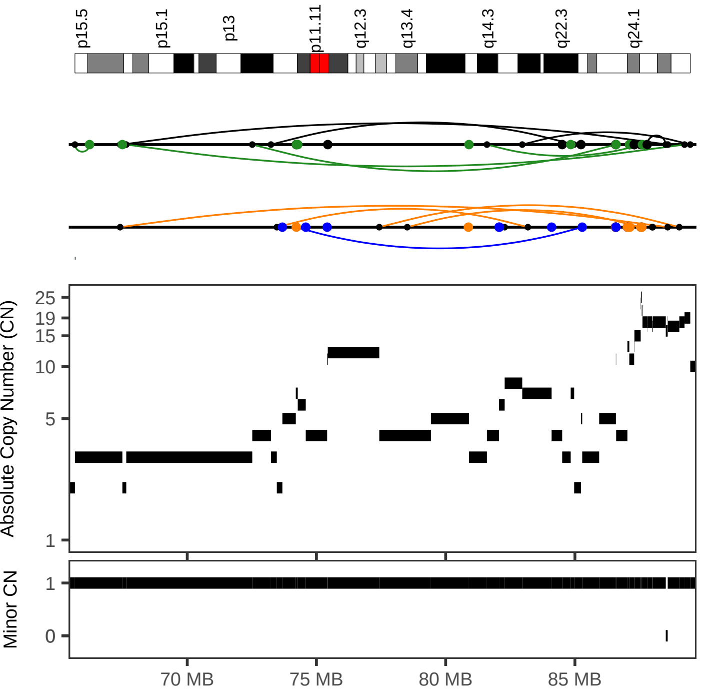

	0bc5744c−5fa3−45bb−87d0−70a02068b392
Cancer type	Breast−AdenoCA
Position	11:67403366−89463830
Type	With other complex events
Interleaved intrachr. SVs	12
Total SVs (intrachr. + transl.)	56
SV types	DEL: 4; DUP: 1; h2hINV: 3; t2tINV: 4; TRA: 44
SVs in sample	487
Oscillating CN (2 and 3 states)	5, 9
CN segments	47
FDR fragment joints	0.8653243
FDR chr. breakp. enrich.	0
Linked to chrs	
Purity, ploidy	0.43, 2.16

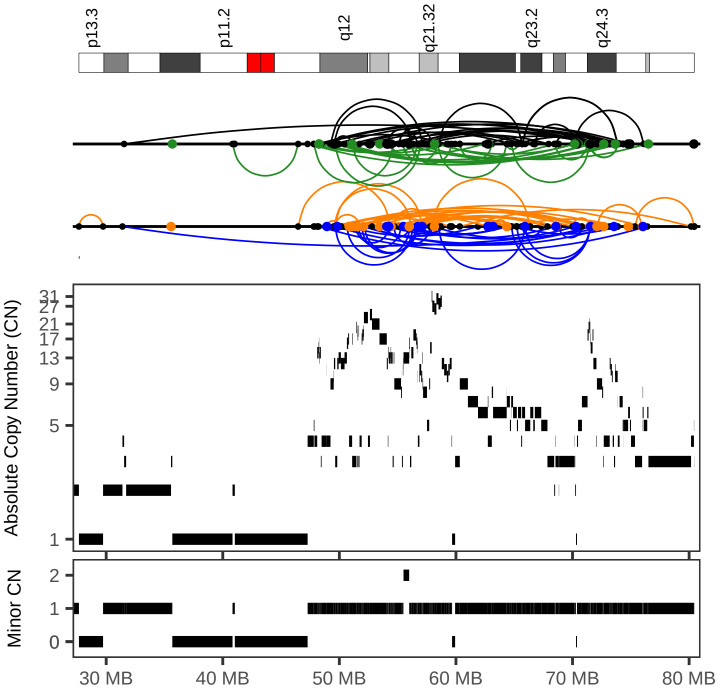

	0bc5744c−5fa3−45bb−87d0−70a02068b392
Cancer type	Breast−AdenoCA
Position	17:31395557−80441361
Type	With other complex events
Interleaved intrachr. SVs	137
Total SVs (intrachr. + transl.)	206
SV types	DEL: 37; DUP: 36; h2hINV: 31; t2tINV: 33; TRA: 69
SVs in sample	487
Oscillating CN (2 and 3 states)	6, 14
CN segments	179
FDR fragment joints	0.7425546
FDR chr. breakp. enrich.	0
Linked to chrs	11:67403366−89463829;14:39530849−45584133
Purity, ploidy	0.43, 2.16

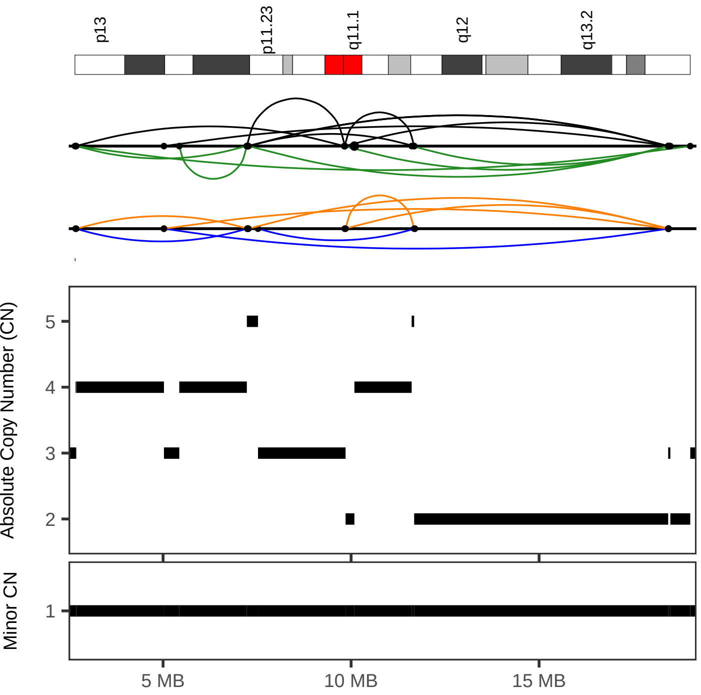

	0bc5744c−5fa3−45bb−87d0−70a02068b392
Cancer type	Breast−AdenoCA
Position	20:2672158−18490675
Type	With other complex events
Interleaved intrachr. SVs	22
Total SVs (intrachr. + transl.)	23
SV types	DEL: 6; DUP: 3; h2hINV: 8; t2tINV: 5; TRA: 1
SVs in sample	487
Oscillating CN (2 and 3 states)	5, 6
CN segments	13
FDR fragment joints	0.6776251
FDR chr. breakp. enrich.	0
Linked to chrs	
Purity, ploidy	0.43, 2.16

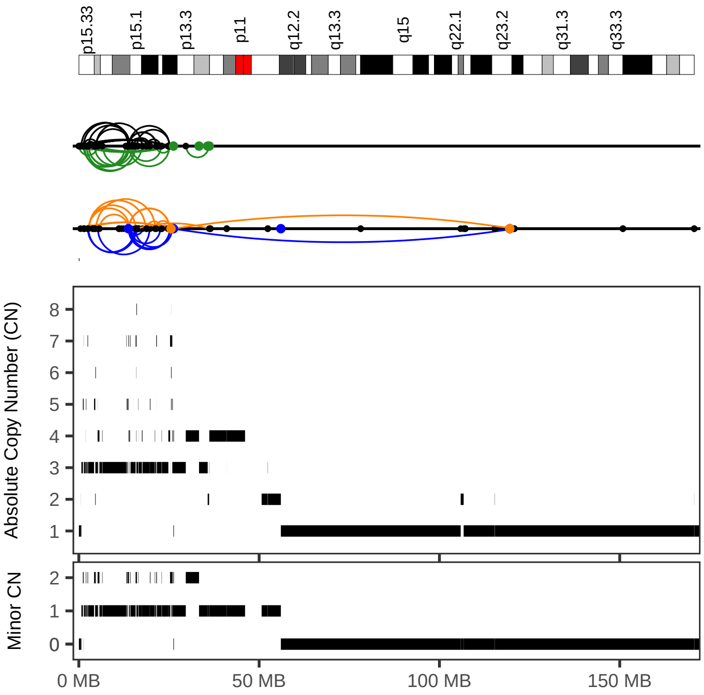

	0dca98b0−f43e−45b6−9a02−00092c78678c
Cancer type	Breast−AdenoCA
Position	5:24520−120779185
Type	With other complex events
Interleaved intrachr. SVs	70
Total SVs (intrachr. + transl.)	81
SV types	DEL: 17; DUP: 12; h2hINV: 19; t2tINV: 22; TRA: 11
SVs in sample	552
Oscillating CN (2 and 3 states)	6, 15
CN segments	97
FDR fragment joints	0.8086009
FDR chr. breakp. enrich.	0
Linked to chrs	10:14391438−105152621;6:53464511−167310325 X:96927939−148453405;
Purity, ploidy	0.76, 2.13

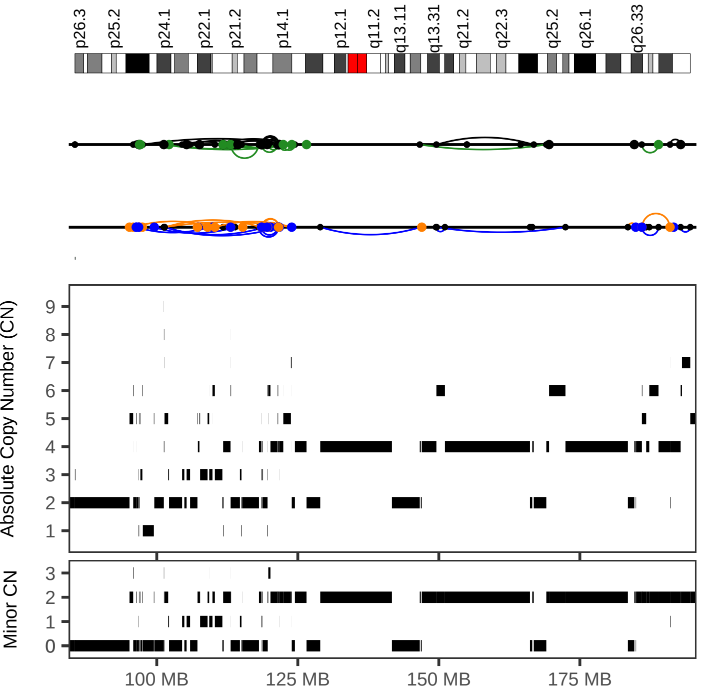

	1174f6e4−ffbe−4e59−a000−8d861c968369
Cancer type	Breast−AdenoCA
Position	3:95813661−124485880
Type	With other complex events
Interleaved intrachr. SVs	46
Total SVs (intrachr. + transl.)	111
SV types	DEL: 13; DUP: 9; h2hINV: 13; t2tINV: 11; TRA: 65
SVs in sample	357
Oscillating CN (2 and 3 states)	6, 10
CN segments	94
FDR fragment joints	0.7814391
FDR chr. breakp. enrich.	0
Linked to chrs	
Purity, ploidy	0.49, 3.26

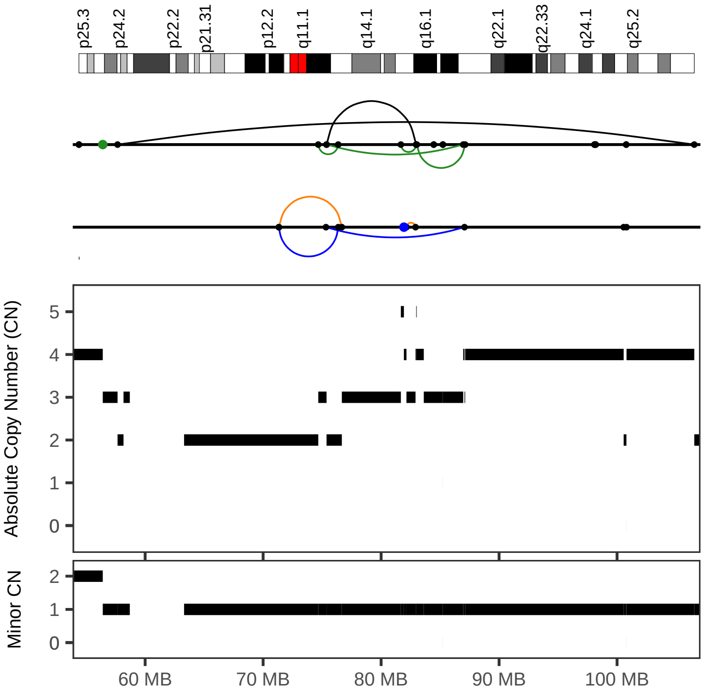

	1174f6e4−ffbe−4e59−a000−8d861c968369
Cancer type	Breast−AdenoCA
Position	6:71335682−87123000
Type	With other complex events
Interleaved intrachr. SVs	8
Total SVs (intrachr. + transl.)	9
SV types	DEL: 1; DUP: 2; h2hINV: 1; t2tINV: 4; TRA: 1
SVs in sample	357
Oscillating CN (2 and 3 states)	4, 7
CN segments	16
FDR fragment joints	0.615458
FDR chr. breakp. enrich.	1
Linked to chrs	
Purity, ploidy	0.49, 3.26

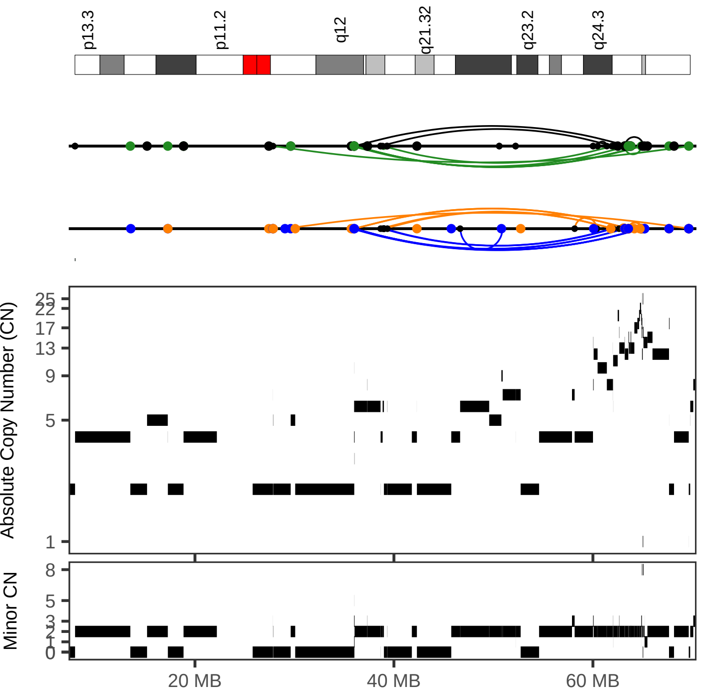

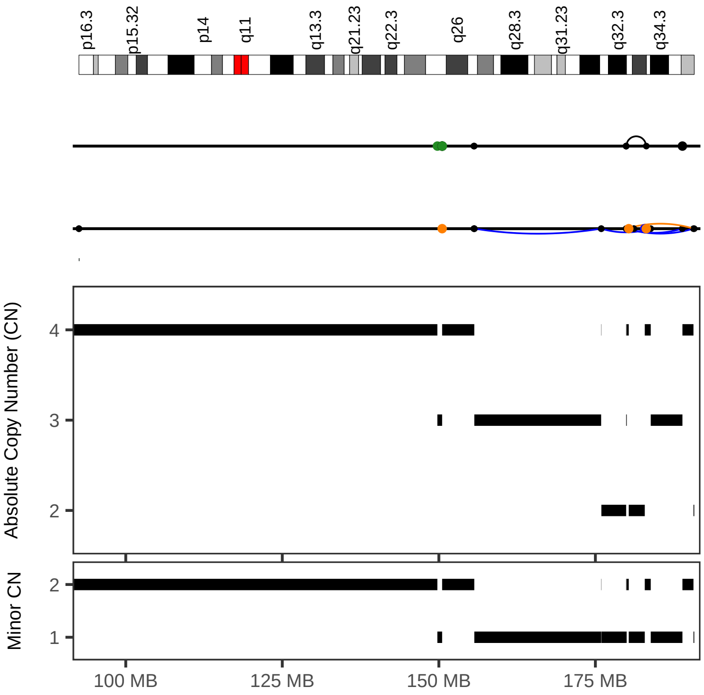

	1174f6e4−ffbe−4e59−a000−8d861c968369
Cancer type	Breast−AdenoCA
Position	17:35714118−65191896
Type	With other complex events
Interleaved intrachr. SVs	21
Total SVs (intrachr. + transl.)	71
SV types	DEL: 5; DUP: 7; h2hINV: 4; t2tINV: 5; TRA: 50
SVs in sample	357
Oscillating CN (2 and 3 states)	6, 9
CN segments	81
FDR fragment joints	0.615458
FDR chr. breakp. enrich.	0
Linked to chrs	3:95813661−124485879;
Purity, ploidy	0.49, 3.26

	1557183c−aedd−4df6−b98a−3654667f7b69
Cancer type	Breast−AdenoCA
Position	4:155589998−190789670
Type	With other complex events
Interleaved intrachr. SVs	9
Total SVs (intrachr. + transl.)	13
SV types	DEL: 1; DUP: 6; h2hINV: 2; t2tINV: 0; TRA: 4
SVs in sample	435
Oscillating CN (2 and 3 states)	5, 14
CN segments	14
FDR fragment joints	0.5435077
FDR chr. breakp. enrich.	0.44
Linked to chrs	17:29145267−69232448;
Purity, ploidy	0.6, 3.86

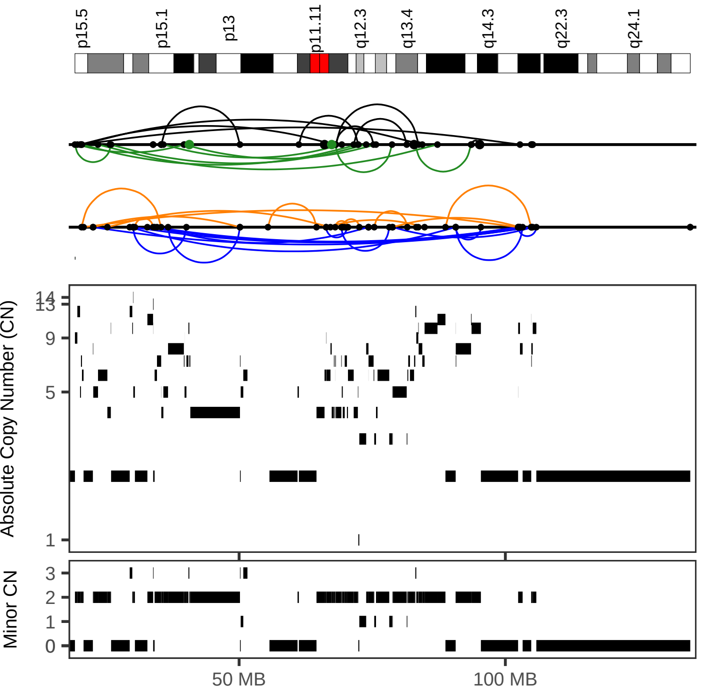

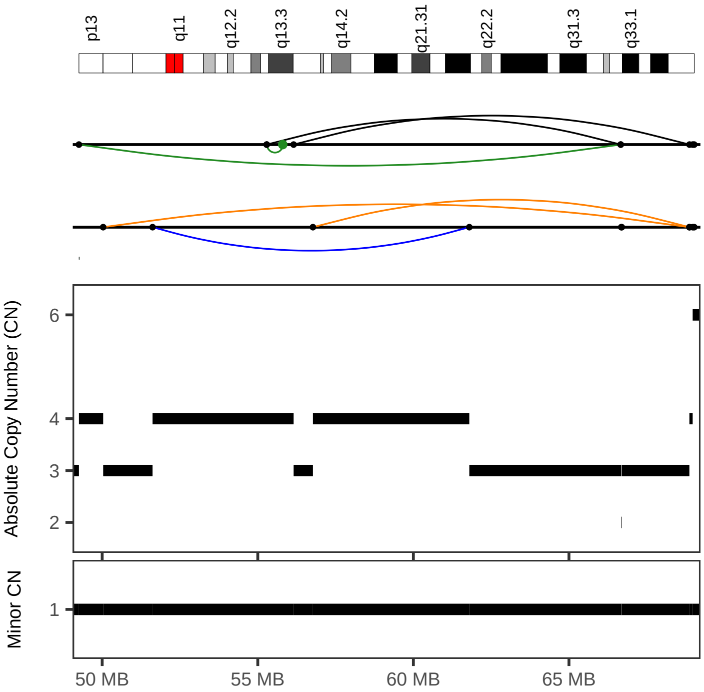

	1557183c−aedd−4df6−b98a−3654667f7b69
Cancer type	Breast−AdenoCA
Position	11:19167099−105816357
Type	With other complex events
Interleaved intrachr. SVs	46
Total SVs (intrachr. + transl.)	51
SV types	DEL: 13; DUP: 14; h2hINV: 9; t2tINV: 10; TRA: 5
SVs in sample	435
Oscillating CN (2 and 3 states)	5, 9
CN segments	103
FDR fragment joints	0.9681319
FDR chr. breakp. enrich.	0
Linked to chrs	
Purity, ploidy	0.6, 3.86

	1557183c−aedd−4df6−b98a−3654667f7b69
Cancer type	Breast−AdenoCA
Position	13:49252644−68873475
Type	After polyploidization
Interleaved intrachr. SVs	6
Total SVs (intrachr. + transl.)	7
SV types	DEL: 2; DUP: 1; h2hINV: 2; t2tINV: 1; TRA: 1
SVs in sample	435
Oscillating CN (2 and 3 states)	6, 9
CN segments	9
FDR fragment joints	0.7735152
FDR chr. breakp. enrich.	0.61
Linked to chrs	
Purity, ploidy	0.6, 3.86

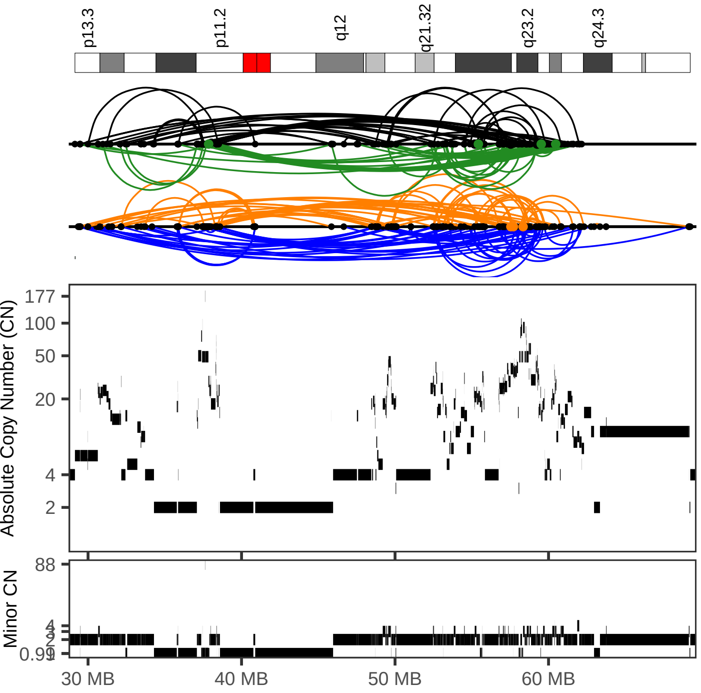

	1557183c−aedd−4df6−b98a−3654667f7b69
Cancer type	Breast−AdenoCA
Position	17:29145267−69232449
Type	With other complex events
Interleaved intrachr. SVs	255
Total SVs (intrachr. + transl.)	265
SV types	DEL: 71; DUP: 67; h2hINV: 59; t2tINV: 58; TRA: 10
SVs in sample	435
Oscillating CN (2 and 3 states)	5, 7
CN segments	324
FDR fragment joints	0.7933887
FDR chr. breakp. enrich.	0
Linked to chrs	
Purity, ploidy	0.6, 3.86

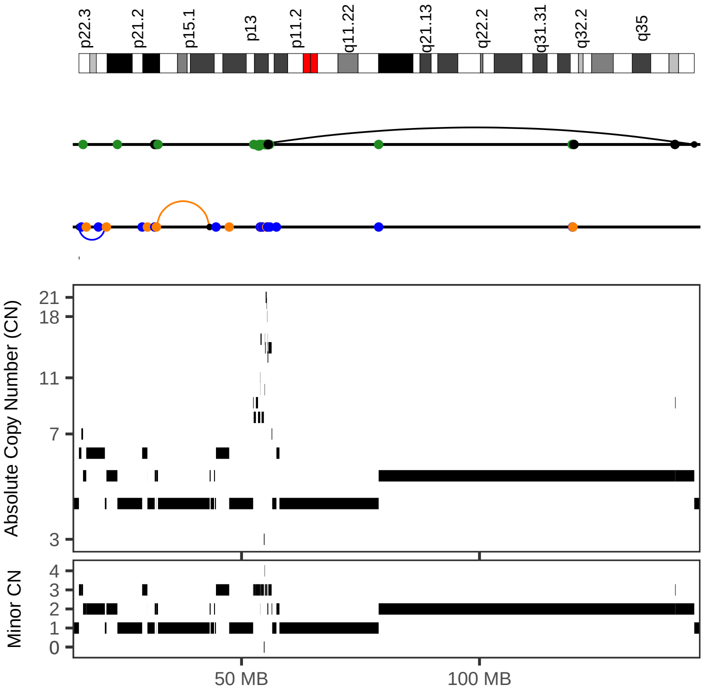

	233b02f3−c4f0−4a67−9db5−e68d5cdaccb6
Cancer type	Breast−AdenoCA
Position	7:52434693−145134157
Type	With other complex events
Interleaved intrachr. SVs	7
Total SVs (intrachr. + transl.)	35
SV types	DEL: 1; DUP: 2; h2hINV: 1; t2tINV: 3; TRA: 28
SVs in sample	618
Oscillating CN (2 and 3 states)	4, 5
CN segments	31
FDR fragment joints	0.7526697
FDR chr. breakp. enrich.	0
Linked to chrs	8:63956033−143798716;X:3683303−142419843
Purity, ploidy	0.7, 5.48

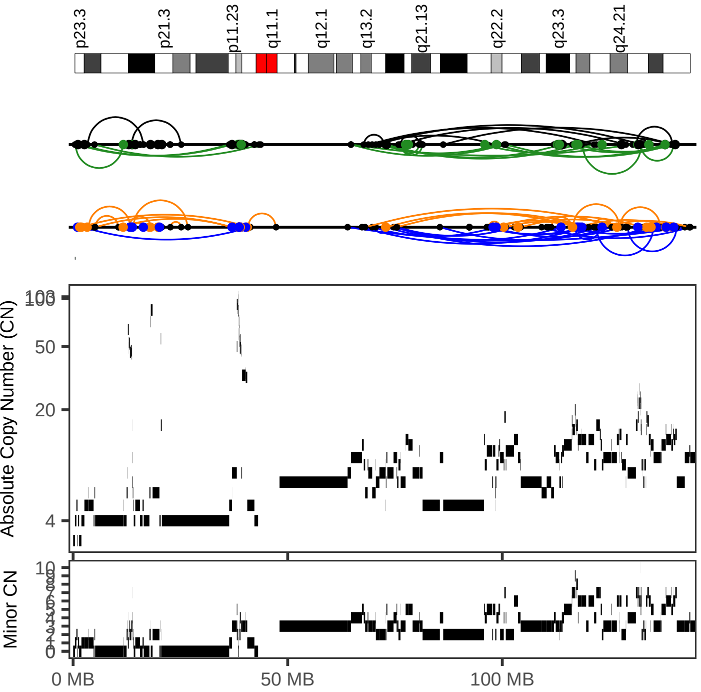

	233b02f3−c4f0−4a67−9db5−e68d5cdaccb6
Cancer type	Breast−AdenoCA
Position	8:63956033−143798717
Type	With other complex events
Interleaved intrachr. SVs	63
Total SVs (intrachr. + transl.)	108
SV types	DEL: 15; DUP: 20; h2hINV: 12; t2tINV: 16; TRA: 45
SVs in sample	618
Oscillating CN (2 and 3 states)	5, 13
CN segments	150
FDR fragment joints	0.7137217
FDR chr. breakp. enrich.	0
Linked to chrs	17:29900854−72983270;X:3683303−142419843
Purity, ploidy	0.7, 5.48

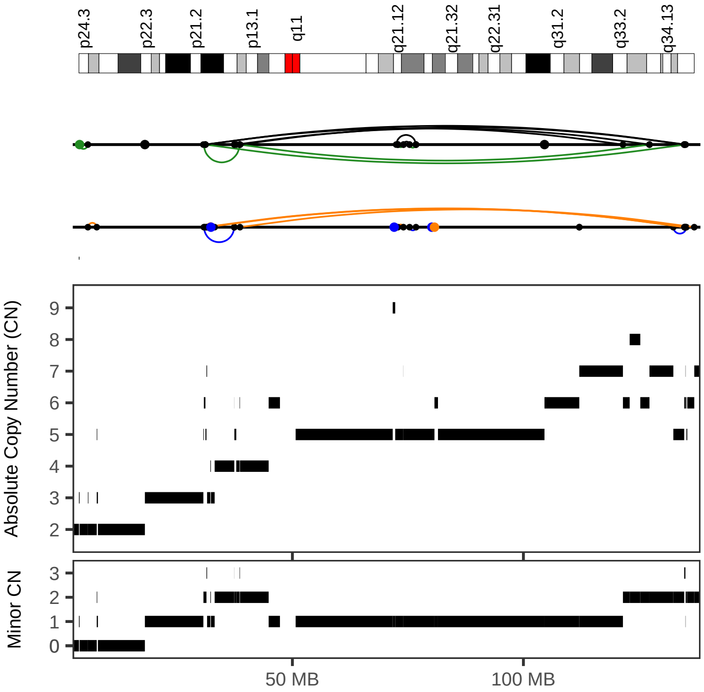

	233b02f3−c4f0−4a67−9db5−e68d5cdaccb6
Cancer type	Breast−AdenoCA
Position	9:30724383−136978634
Type	With other complex events
Interleaved intrachr. SVs	16
Total SVs (intrachr. + transl.)	21
SV types	DEL: 5; DUP: 3; h2hINV: 4; t2tINV: 4; TRA: 5
SVs in sample	618
Oscillating CN (2 and 3 states)	4, 8
CN segments	34
FDR fragment joints	0.9462199
FDR chr. breakp. enrich.	0.09
Linked to chrs	
Purity, ploidy	0.7, 5.48

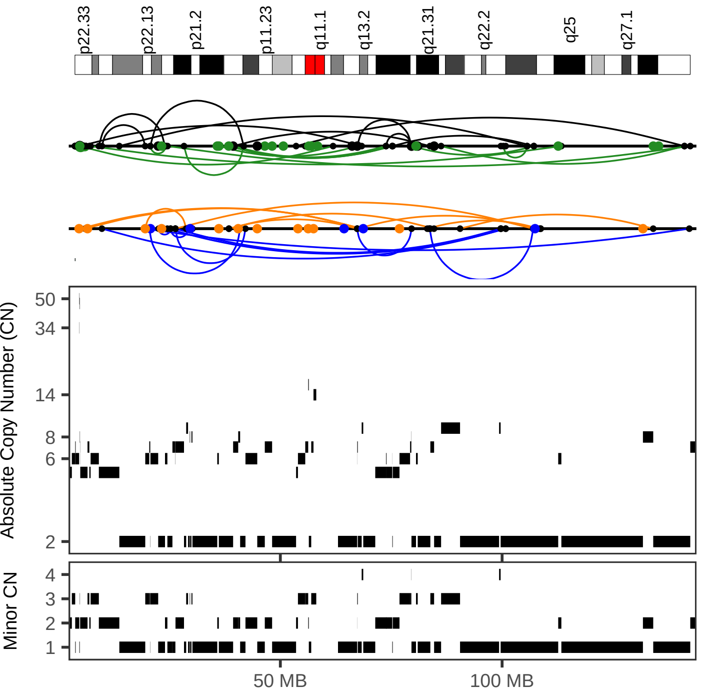

233b02f3−c4f0−4a67−9db5−e68d5cdaccb6

Cancer type	Breast−AdenoCA
Position	X:3683303−142419844
Type	With other complex events
Interleaved intrachr. SVs	53
Total SVs (intrachr. + transl.)	94
SV types	DEL: 10; DUP: 13; h2hINV: 14; t2tINV: 16; TRA: 41
SVs in sample	618
Oscillating CN (2 and 3 states)	5, 7
CN segments	77
FDR fragment joints	0.6714613
FDR chr. breakp. enrich.	0
Linked to chrs	17:29900854−72983270;18:1797061−70607437 8:63956033−143798716;
Purity, ploidy	0.7, 5.48

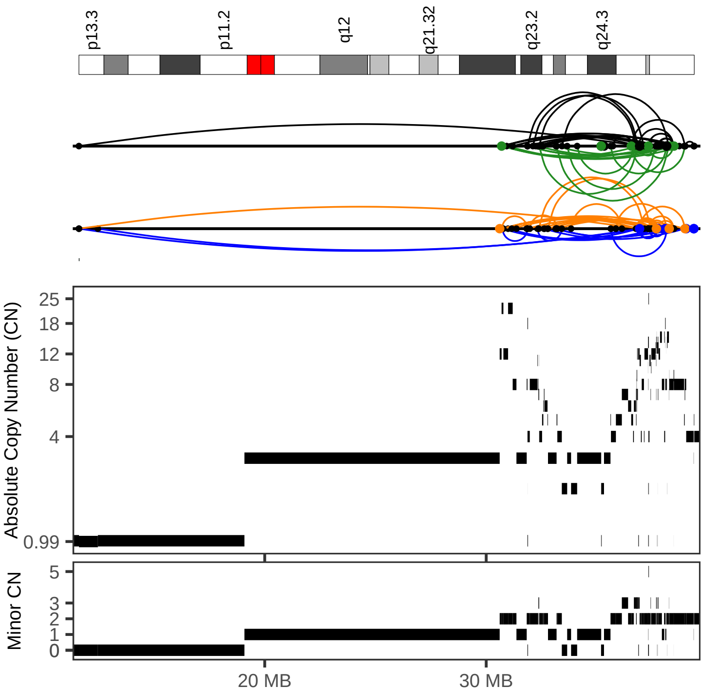

2779fa01−ac93−4e80−a997−3385f72172c3

Cancer type	Breast−AdenoCA
Position	17:11610139−39394274
Type	With other complex events
Interleaved intrachr. SVs	103
Total SVs (intrachr. + transl.)	127
SV types	DEL: 35; DUP: 20; h2hINV: 27; t2tINV: 21; TRA: 24
SVs in sample	190
Oscillating CN (2 and 3 states)	5, 9
CN segments	128
FDR fragment joints	0.615458
FDR chr. breakp. enrich.	0
Linked to chrs	
Purity, ploidy	0.85, 2.06

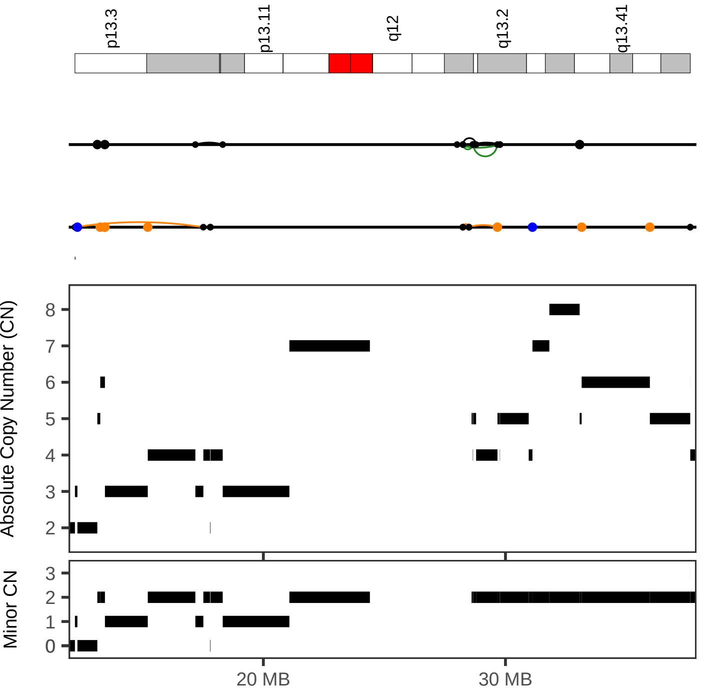

38a8b734−9acc−42f9−b5b7−e51b0dfc6504

Cancer type	Breast−AdenoCA
Position	19:28234779−29767781
Type	After polyploidization
Interleaved intrachr. SVs	6
Total SVs (intrachr. + transl.)	7
SV types	DEL: 2; DUP: 0; h2hINV: 2; t2tINV: 2; TRA: 1
SVs in sample	54
Oscillating CN (2 and 3 states)	6, 7
CN segments	7
FDR fragment joints	0.6776251
FDR chr. breakp. enrich.	0
Linked to chrs	
Purity, ploidy	0.73, 3.36

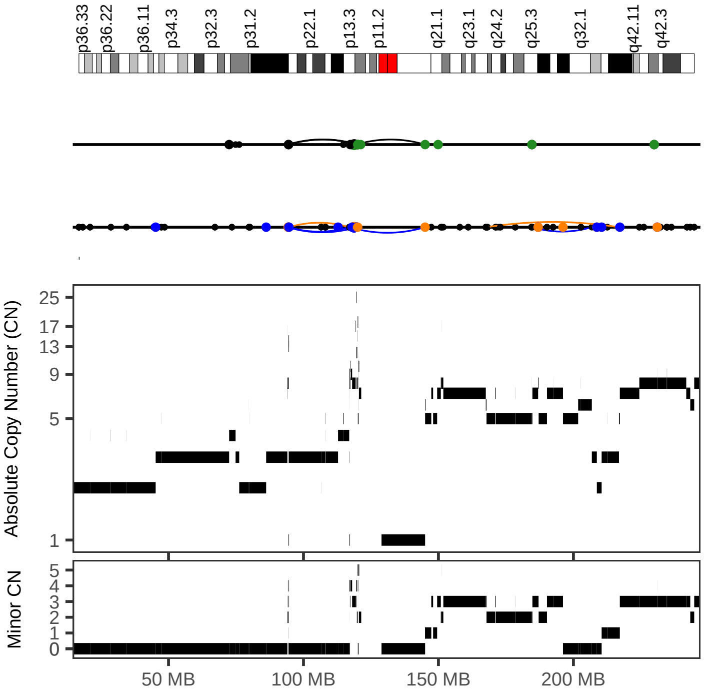

441919eb−8c9f−478c−bda3−8de4f8295e8a

Cancer type	Breast−AdenoCA
Position	1:94026241−145056864
Type	With other complex events
Interleaved intrachr. SVs	18
Total SVs (intrachr. + transl.)	37
SV types	DEL: 4; DUP: 6; h2hINV: 5; t2tINV: 3; TRA: 19
SVs in sample	744
Oscillating CN (2 and 3 states)	4, 6
CN segments	51
FDR fragment joints	0.818888
FDR chr. breakp. enrich.	0
Linked to chrs	17:31874561−36100025;8:1680713−143764778
Purity, ploidy	0.5, 3.35

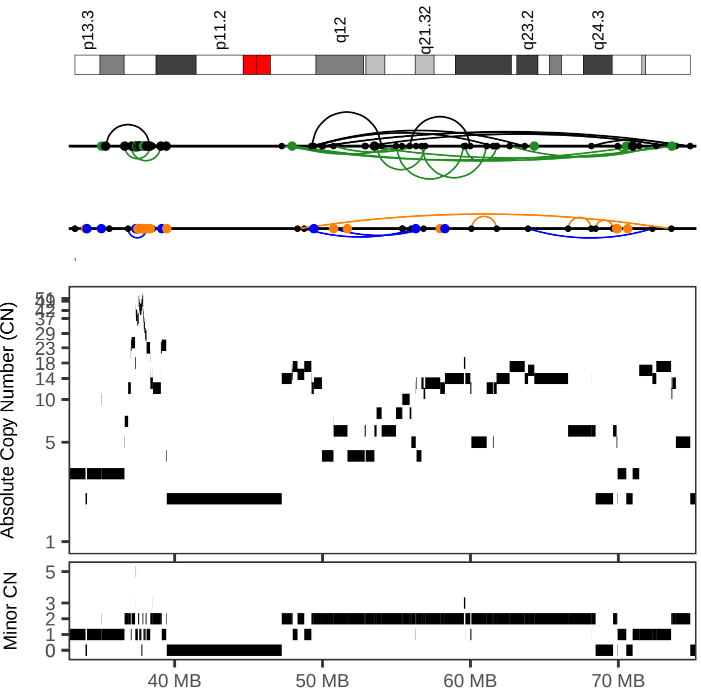

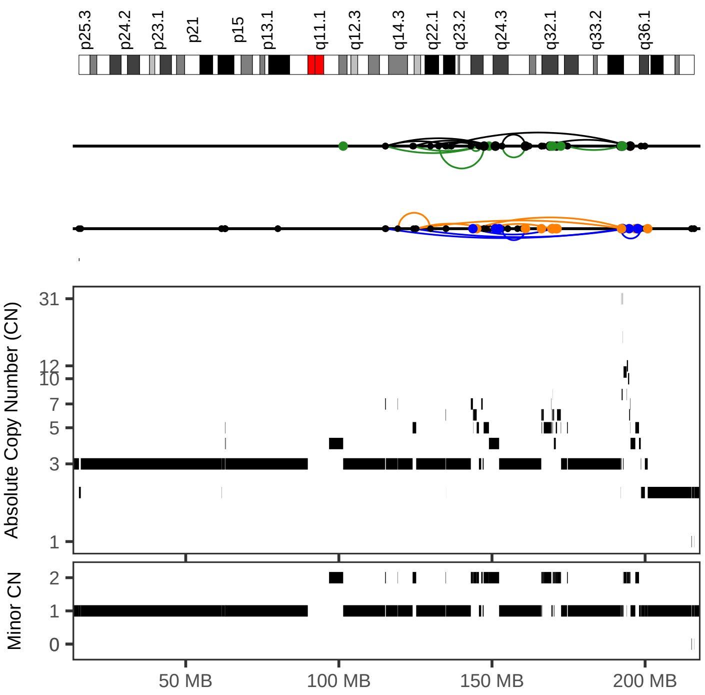

	44bec761−b603−49c0−8634−f6bfe0319bb1
Cancer type	Breast−AdenoCA
Position	17:47241742−74862785
Type	With other complex events
Interleaved intrachr. SVs	26
Total SVs (intrachr. + transl.)	41
SV types	DEL: 2; DUP: 4; h2hINV: 9; t2tINV: 11; TRA: 15
SVs in sample	320
Oscillating CN (2 and 3 states)	6, 7
CN segments	71
FDR fragment joints	0.615458
FDR chr. breakp. enrich.	0
Linked to chrs	22:18266499−42732086;3:79667212−191335907
Purity, ploidy	0.59, 3.37

	4da999a0−ef41−4a0b−b1d1−446b39cc855a
Cancer type	Breast−AdenoCA
Position	2:115111736−198568025
Type	With other complex events
Interleaved intrachr. SVs	25
Total SVs (intrachr. + transl.)	63
SV types	DEL: 6; DUP: 6; h2hINV: 8; t2tINV: 5; TRA: 38
SVs in sample	423
Oscillating CN (2 and 3 states)	5, 9
CN segments	63
FDR fragment joints	0.9936601
FDR chr. breakp. enrich.	0
Linked to chrs	15:56597551−99238230;17:2048893−25902796 8:35905735−108901611;
Purity, ploidy	0.77, 3.16

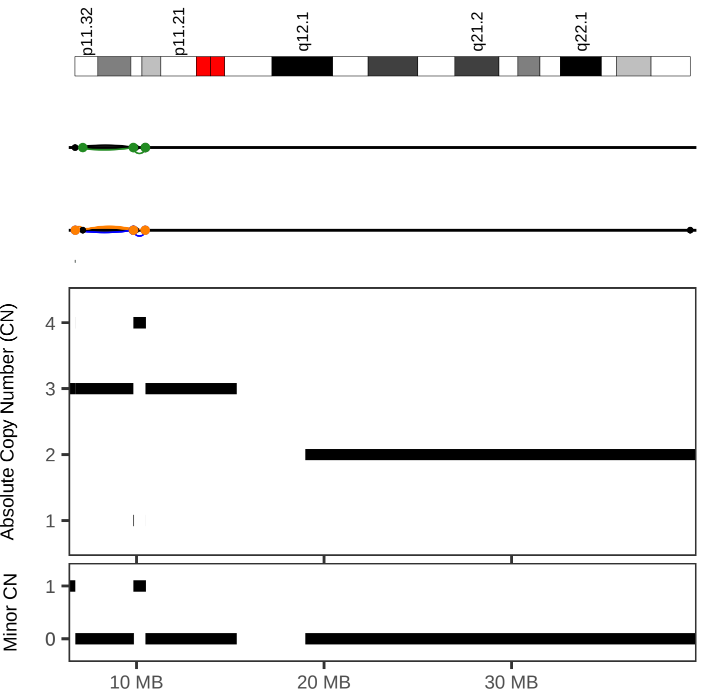

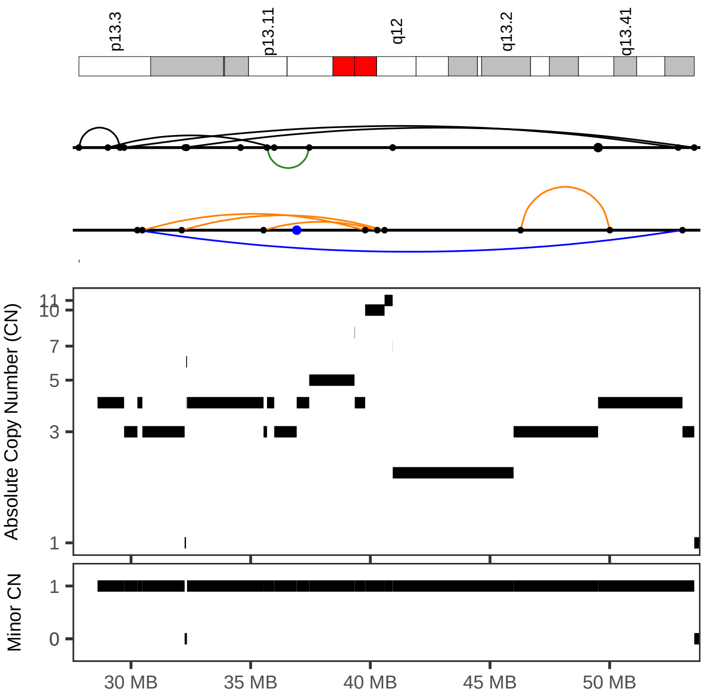

	4da999a0−ef41−4a0b−b1d1−446b39cc855a
Cancer type	Breast−AdenoCA
Position	18:6714837−10477636
Type	With other complex events
Interleaved intrachr. SVs	12
Total SVs (intrachr. + transl.)	26
SV types	DEL: 5; DUP: 3; h2hINV: 2; t2tINV: 2; TRA: 14
SVs in sample	423
Oscillating CN (2 and 3 states)	4, 5
CN segments	11
FDR fragment joints	0.6338796
FDR chr. breakp. enrich.	0
Linked to chrs	17:2048893−25902796;8:35905735−108901611
Purity, ploidy	0.77, 3.16

	53886143−c1c6−40e9−88e6−e4e5e0271fc8
Cancer type	Breast−AdenoCA
Position	19:27822952−53535690
Type	With other complex events
Interleaved intrachr. SVs	9
Total SVs (intrachr. + transl.)	11
SV types	DEL: 3; DUP: 1; h2hINV: 4; t2tINV: 1; TRA: 2
SVs in sample	113
Oscillating CN (2 and 3 states)	5, 7
CN segments	24
FDR fragment joints	0.8653243
FDR chr. breakp. enrich.	0
Linked to chrs	
Purity, ploidy	0.54, 2.98

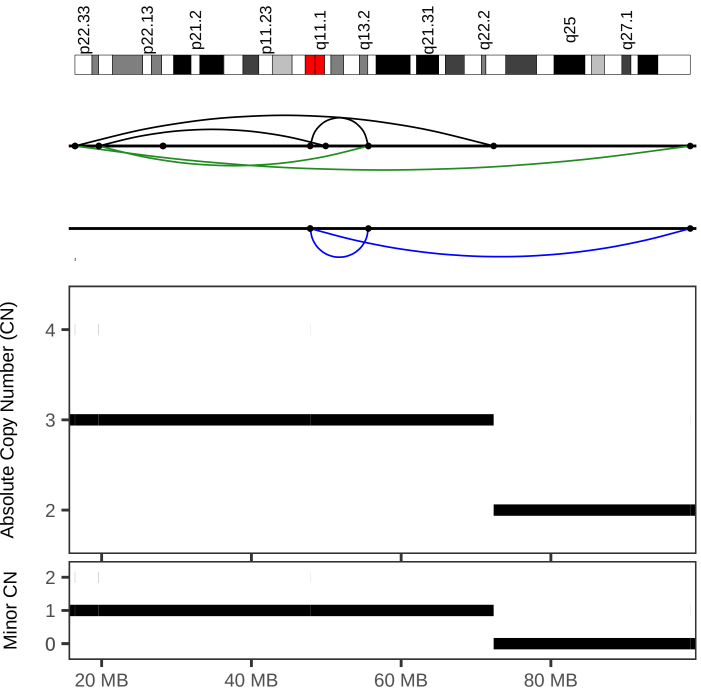

	53886143−c1c6−40e9−88e6−e4e5e0271fc8
Cancer type	Breast−AdenoCA
Position	X:16428213−98616120
Type	After polyploidization
Interleaved intrachr. SVs	7
Total SVs (intrachr. + transl.)	7
SV types	DEL: 0; DUP: 2; h2hINV: 3; t2tINV: 2; TRA: 0
SVs in sample	113
Oscillating CN (2 and 3 states)	6, 9
CN segments	9
FDR fragment joints	0.6776251
FDR chr. breakp. enrich.	0.53
Linked to chrs	
Purity, ploidy	0.54, 2.98

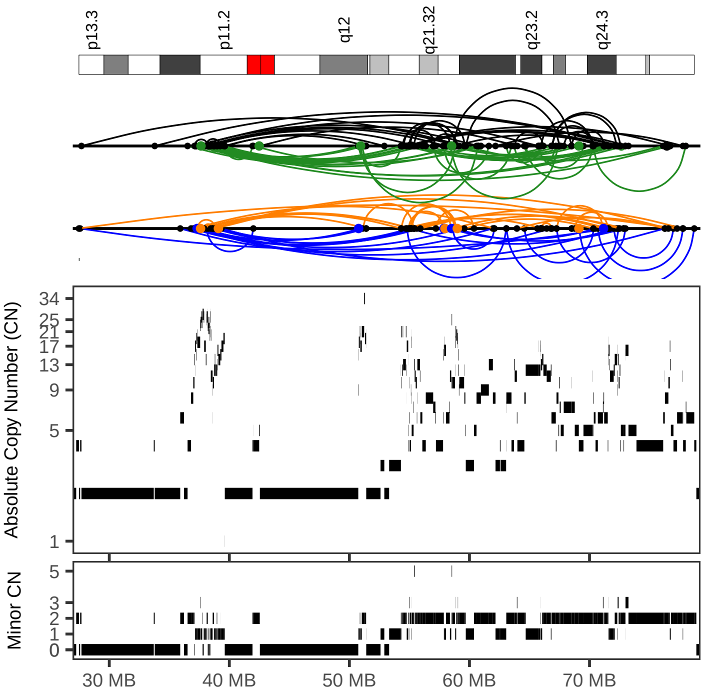

	5580b21a−2cdb−4777−ad79−6e06654144f5
Cancer type	Breast−AdenoCA
Position	17:27472457−78723957
Type	With other complex events
Interleaved intrachr. SVs	141
Total SVs (intrachr. + transl.)	158
SV types	DEL: 29; DUP: 28; h2hINV: 42; t2tINV: 42; TRA: 17
SVs in sample	252
Oscillating CN (2 and 3 states)	5, 8
CN segments	232
FDR fragment joints	0.615458
FDR chr. breakp. enrich.	0
Linked to chrs	
Purity, ploidy	0.55, 3.56

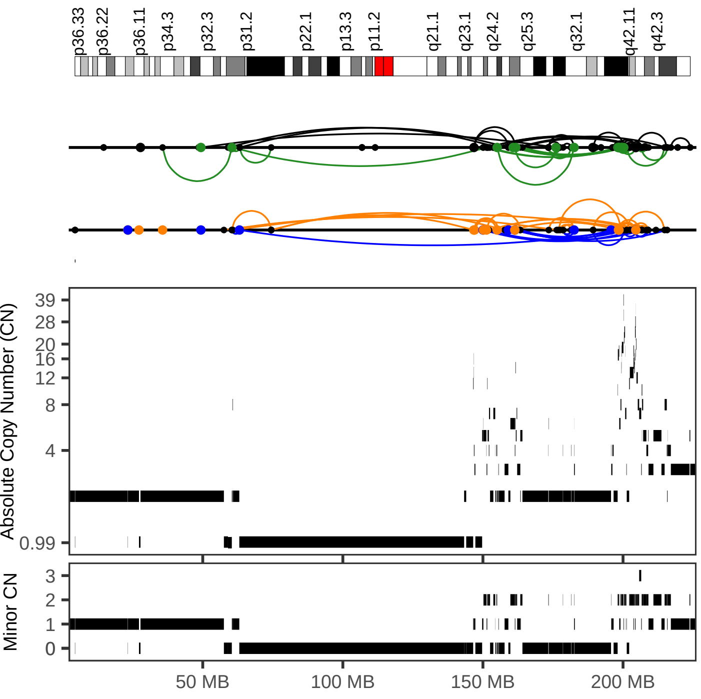

	566792ae−f853−4a47−856d−f02cdcfcb18a
Cancer type	Breast−AdenoCA
Position	1:35669387−215888510
Type	With other complex events
Interleaved intrachr. SVs	91
Total SVs (intrachr. + transl.)	130
SV types	DEL: 25; DUP: 18; h2hINV: 25; t2tINV: 23; TRA: 39
SVs in sample	579
Oscillating CN (2 and 3 states)	6, 10
CN segments	117
FDR fragment joints	0.615458
FDR chr. breakp. enrich.	0
Linked to chrs	10:109264246−123915302;13:20809449−38507870 17:25885913−74155396;
Purity, ploidy	0.72, 2.01

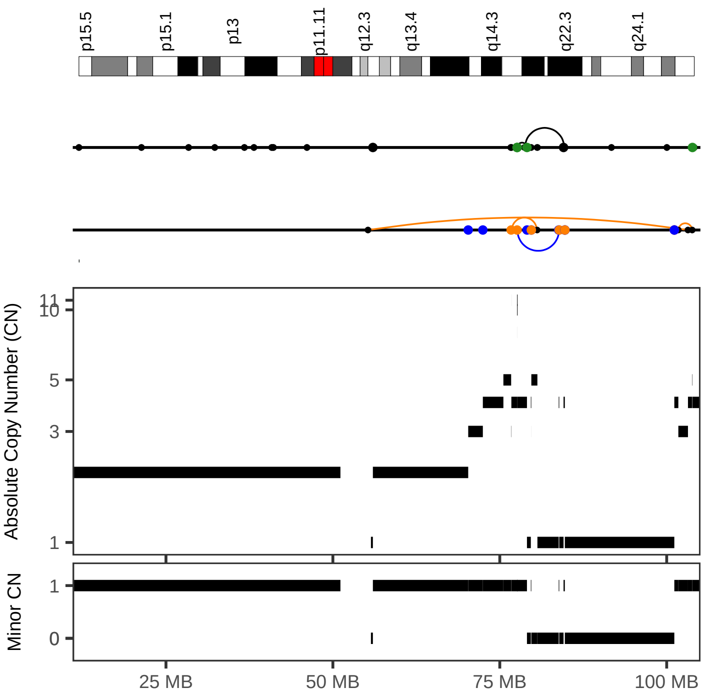

	566792ae−f853−4a47−856d−f02cdcfcb18a
Cancer type	Breast−AdenoCA
Position	11:76728327−84744117
Type	With other complex events
Interleaved intrachr. SVs	6
Total SVs (intrachr. + transl.)	16
SV types	DEL: 1; DUP: 1; h2hINV: 3; t2tINV: 1; TRA: 10
SVs in sample	579
Oscillating CN (2 and 3 states)	5, 8
CN segments	18
FDR fragment joints	0.6776251
FDR chr. breakp. enrich.	0.01
Linked to chrs	22:22420637−33518316;
Purity, ploidy	0.72, 2.01

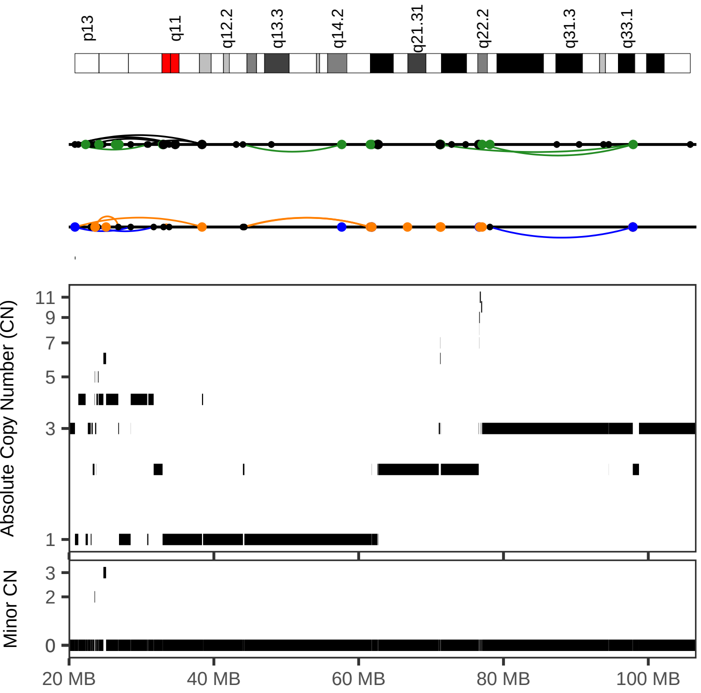

	566792ae−f853−4a47−856d−f02cdcfcb18a
Cancer type	Breast−AdenoCA
Position	13:20809449−38507871
Type	With other complex events
Interleaved intrachr. SVs	15
Total SVs (intrachr. + transl.)	28
SV types	DEL: 3; DUP: 2; h2hINV: 6; t2tINV: 4; TRA: 13
SVs in sample	579
Oscillating CN (2 and 3 states)	6, 11
CN segments	32
FDR fragment joints	0.615458
FDR chr. breakp. enrich.	0
Linked to chrs	17:25885913−74155396;
Purity, ploidy	0.72, 2.01

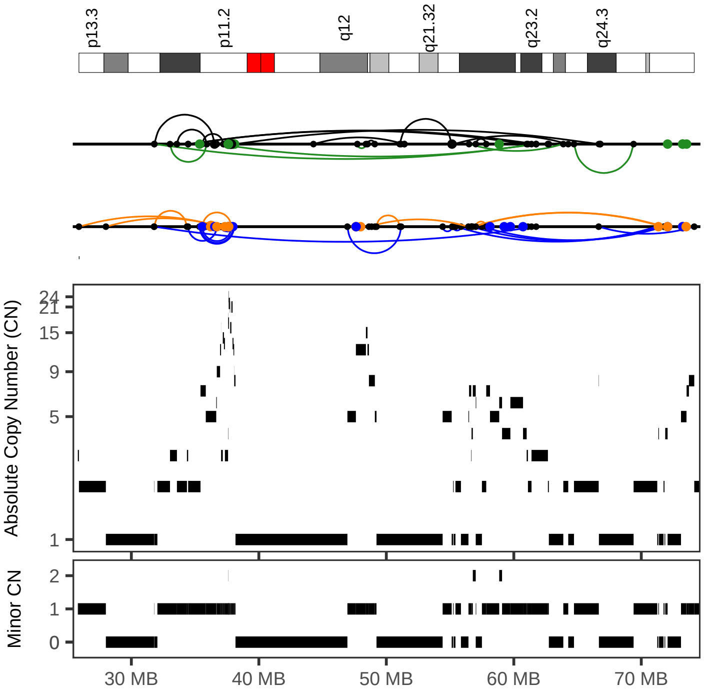

	566792ae−f853−4a47−856d−f02cdcfcb18a
Cancer type	Breast−AdenoCA
Position	17:25885913−74155397
Type	With other complex events
Interleaved intrachr. SVs	43
Total SVs (intrachr. + transl.)	90
SV types	DEL: 10; DUP: 15; h2hINV: 12; t2tINV: 6; TRA: 47
SVs in sample	579
Oscillating CN (2 and 3 states)	5, 9
CN segments	84
FDR fragment joints	0.615458
FDR chr. breakp. enrich.	0
Linked to chrs	1:35669387−215888509;10:109264246−123915302 13:20809449−38507870;
Purity, ploidy	0.72, 2.01

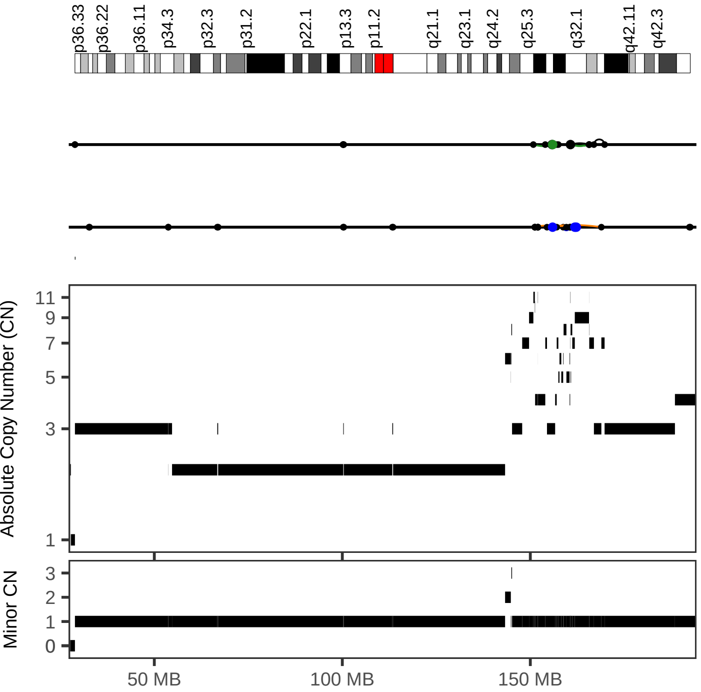

	5dd423e8−feaa−4568−a750−500948c41d6c
Cancer type	Breast−AdenoCA
Position	1:150805216−169752869
Type	With other complex events
Interleaved intrachr. SVs	16
Total SVs (intrachr. + transl.)	22
SV types	DEL: 4; DUP: 2; h2hINV: 5; t2tINV: 5; TRA: 6
SVs in sample	255
Oscillating CN (2 and 3 states)	4, 6
CN segments	33
FDR fragment joints	0.8874881
FDR chr. breakp. enrich.	0
Linked to chrs	
Purity, ploidy	0.8, 2.15

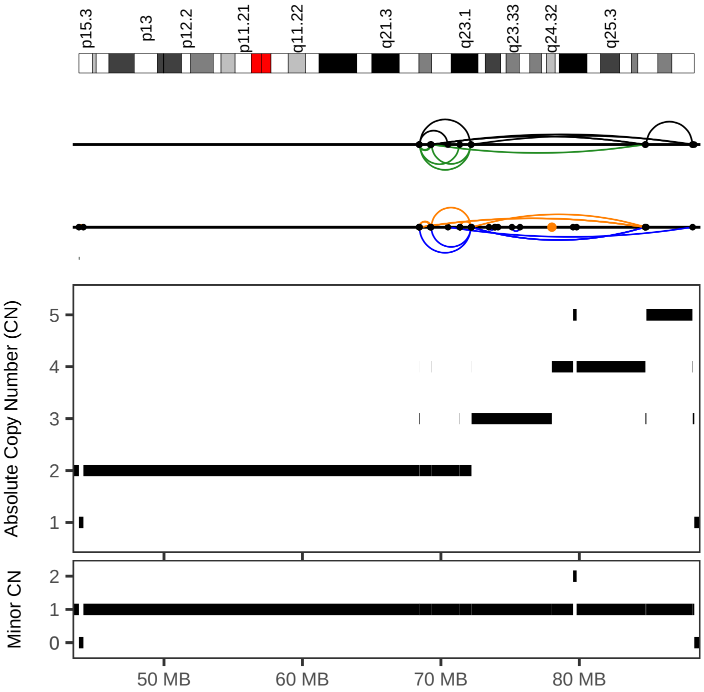

	5dd423e8−feaa−4568−a750−500948c41d6c
Cancer type	Breast−AdenoCA
Position	10:68422126−88297594
Type	With other complex events
Interleaved intrachr. SVs	27
Total SVs (intrachr. + transl.)	28
SV types	DEL: 8; DUP: 5; h2hINV: 7; t2tINV: 7; TRA: 1
SVs in sample	255
Oscillating CN (2 and 3 states)	4, 11
CN segments	25
FDR fragment joints	0.8653243
FDR chr. breakp. enrich.	0
Linked to chrs	
Purity, ploidy	0.8, 2.15

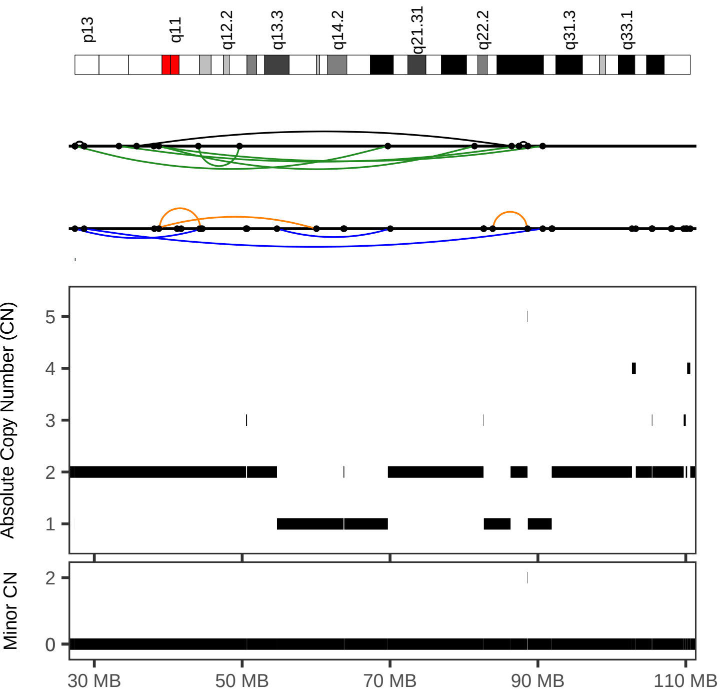

	5ed024e8−d05e−4c65−9441−eda9930ccc82
Cancer type	Breast−AdenoCA
Position	13:27374782−90659951
Type	With other complex events
Interleaved intrachr. SVs	15
Total SVs (intrachr. + transl.)	15
SV types	DEL: 3; DUP: 3; h2hINV: 4; t2tINV: 5; TRA: 0
SVs in sample	475
Oscillating CN (2 and 3 states)	5, 5
CN segments	14
FDR fragment joints	0.925252
FDR chr. breakp. enrich.	0.04
Linked to chrs	
Purity, ploidy	0.38, 2.04

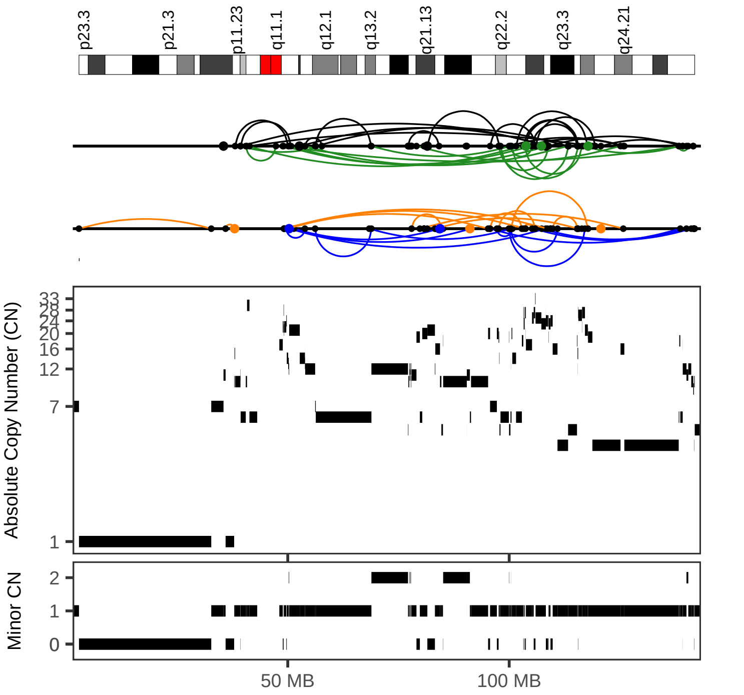

	60df7543−6da5−4c75−943b−5800c1e08234
Cancer type	Breast−AdenoCA
Position	8:38109487−141109862
Type	With other complex events
Interleaved intrachr. SVs	66
Total SVs (intrachr. + transl.)	77
SV types	DEL: 11; DUP: 15; h2hINV: 22; t2tINV: 18; TRA: 11
SVs in sample	156
Oscillating CN (2 and 3 states)	4, 5
CN segments	106
FDR fragment joints	0.615458
FDR chr. breakp. enrich.	0
Linked to chrs	
Purity, ploidy	0.39, 3.73

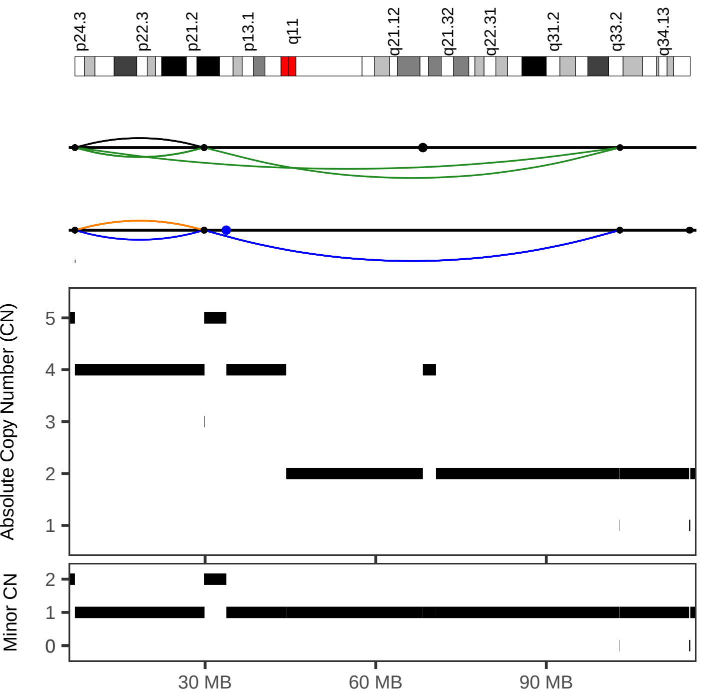

	60df7543−6da5−4c75−943b−5800c1e08234
Cancer type	Breast−AdenoCA
Position	9:7101017−102960994
Type	With other complex events
Interleaved intrachr. SVs	18
Total SVs (intrachr. + transl.)	20
SV types	DEL: 4; DUP: 6; h2hINV: 3; t2tINV: 5; TRA: 2
SVs in sample	156
Oscillating CN (2 and 3 states)	4, 8
CN segments	10
FDR fragment joints	0.6776251
FDR chr. breakp. enrich.	0
Linked to chrs	
Purity, ploidy	0.39, 3.73

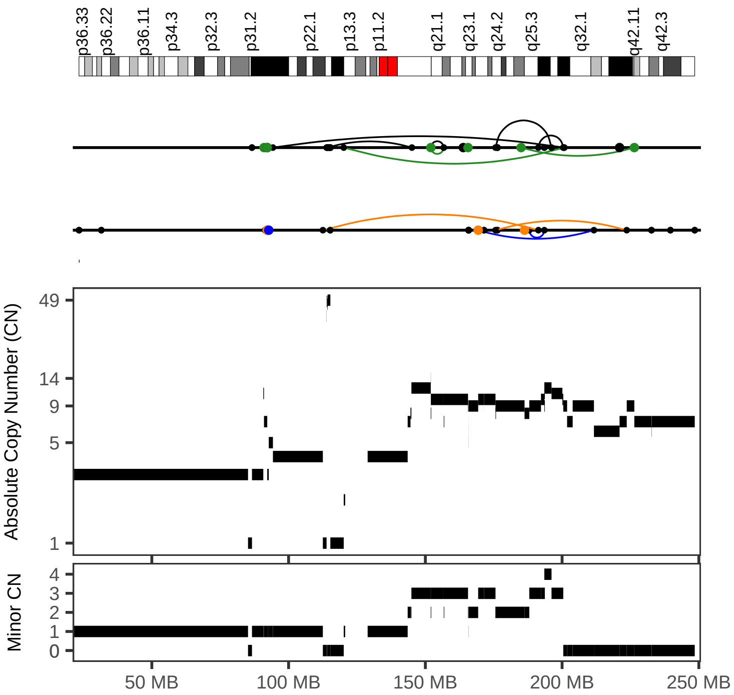

	6fa2a667−9c36−4526−8a58−1975e863a806
Cancer type	Breast−AdenoCA
Position	1:94274301−226426398
Type	With other complex events
Interleaved intrachr. SVs	10
Total SVs (intrachr. + transl.)	18
SV types	DEL: 2; DUP: 2; h2hINV: 4; t2tINV: 2; TRA: 8
SVs in sample	259
Oscillating CN (2 and 3 states)	4, 6
CN segments	42
FDR fragment joints	0.615458
FDR chr. breakp. enrich.	0
Linked to chrs	
Purity, ploidy	0.88, 3.98

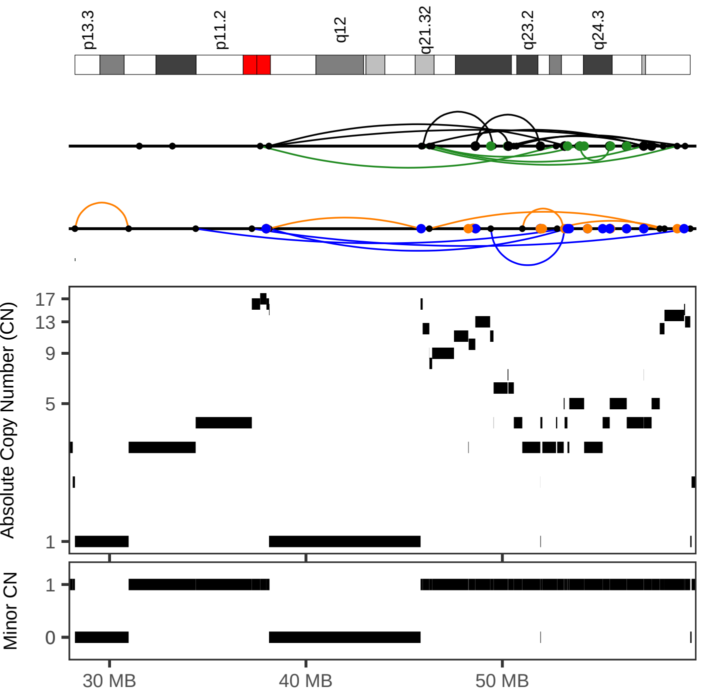

733a2a0f−b37a−4b81−b49e−3c0f30d1eb37

Cancer type	Breast−AdenoCA
Position	17:34384789−59566711
Type	With other complex events
Interleaved intrachr. SVs	21
Total SVs (intrachr. + transl.)	70
SV types	DEL: 4; DUP: 4; h2hINV: 8; t2tINV: 5; TRA: 49
SVs in sample	403
Oscillating CN (2 and 3 states)	5, 7
CN segments	47
FDR fragment joints	0.7592608
FDR chr. breakp. enrich.	0
Linked to chrs	1:145117775−243098982;8:31896789−139505358
Purity, ploidy	0.6, 1.93

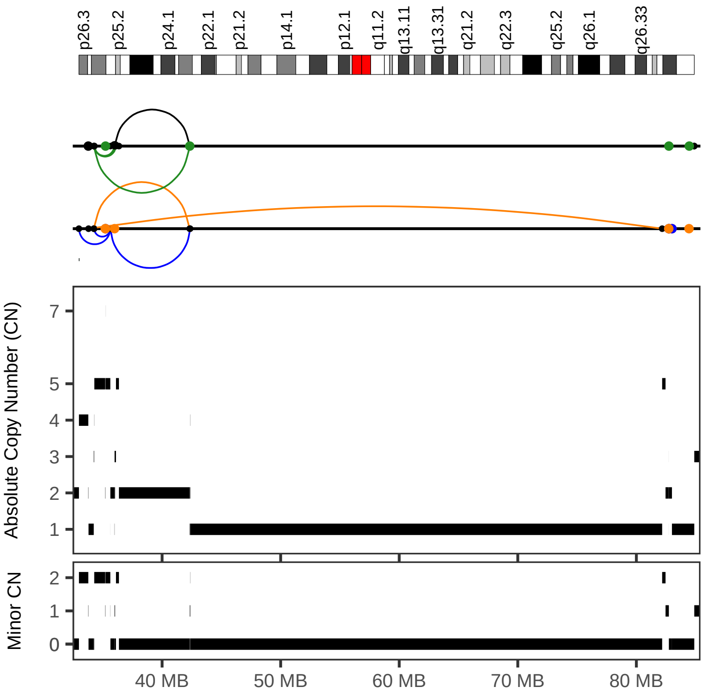

75113445−d2d6−44a0−866c−c9175e6d214b

Cancer type	Breast−AdenoCA
Position	3:32984474−82175838
Type	With other complex events
Interleaved intrachr. SVs	11
Total SVs (intrachr. + transl.)	18
SV types	DEL: 2; DUP: 4; h2hINV: 2; t2tINV: 3; TRA: 7
SVs in sample	212
Oscillating CN (2 and 3 states)	5, 6
CN segments	23
FDR fragment joints	0.9875525
FDR chr. breakp. enrich.	0
Linked to chrs	10:54407919−63446306;
Purity, ploidy	0.76, 2.35

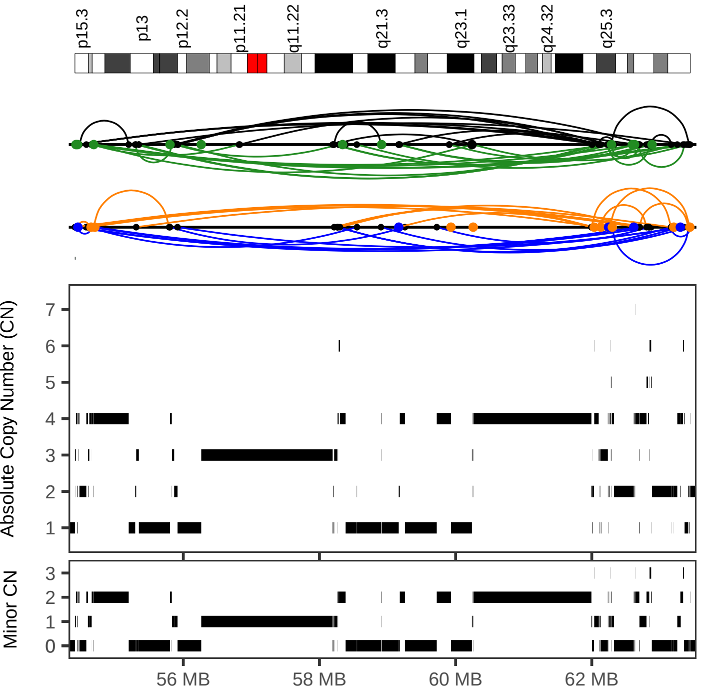

75113445−d2d6−44a0−866c−c9175e6d214b

Cancer type	Breast−AdenoCA
Position	10:54407919−63446307
Type	With other complex events
Interleaved intrachr. SVs	74
Total SVs (intrachr. + transl.)	102
SV types	DEL: 15; DUP: 15; h2hINV: 19; t2tINV: 25; TRA: 28
SVs in sample	212
Oscillating CN (2 and 3 states)	5, 10
CN segments	109
FDR fragment joints	0.6776251
FDR chr. breakp. enrich.	0
Linked to chrs	3:32984474−82175837;
Purity, ploidy	0.76, 2.35

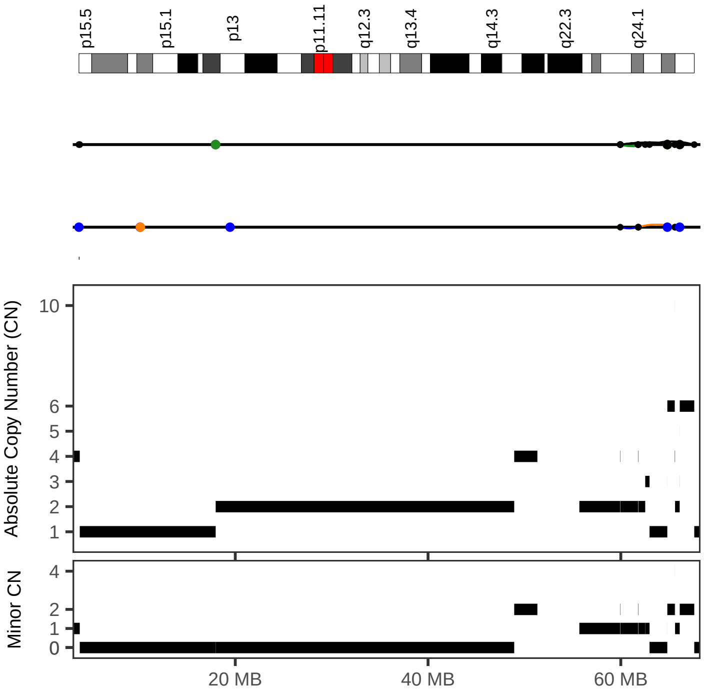

75113445−d2d6−44a0−866c−c9175e6d214b

Cancer type	Breast−AdenoCA
Position	11:59935523−67621236
Type	With other complex events
Interleaved intrachr. SVs	10
Total SVs (intrachr. + transl.)	17
SV types	DEL: 2; DUP: 1; h2hINV: 4; t2tINV: 3; TRA: 7
SVs in sample	212
Oscillating CN (2 and 3 states)	5, 8
CN segments	17
FDR fragment joints	0.615458
FDR chr. breakp. enrich.	0
Linked to chrs	
Purity, ploidy	0.76, 2.35

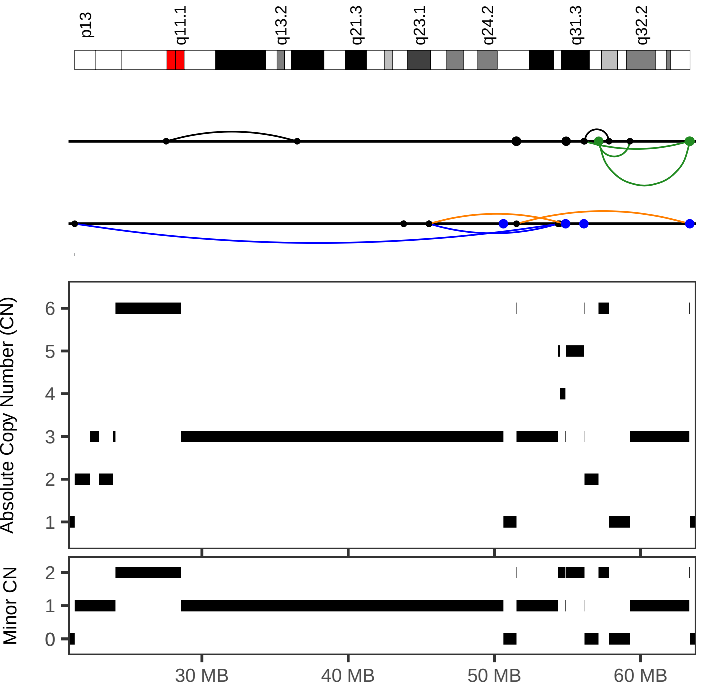

	**75113445−d2d6−44a0−866c−c9175e6d214b**
Cancer type	Breast−AdenoCA
Position	14:21299867−63371689
Type	With other complex events
Interleaved intrachr. SVs	8
Total SVs (intrachr. + transl.)	18
SV types	DEL: 2; DUP: 2; h2hINV: 1; t2tINV: 3; TRA: 10
SVs in sample	212
Oscillating CN (2 and 3 states)	4, 7
CN segments	22
FDR fragment joints	0.6776251
FDR chr. breakp. enrich.	0
Linked to chrs	11:59935523−67621235;
Purity, ploidy	0.76, 2.35

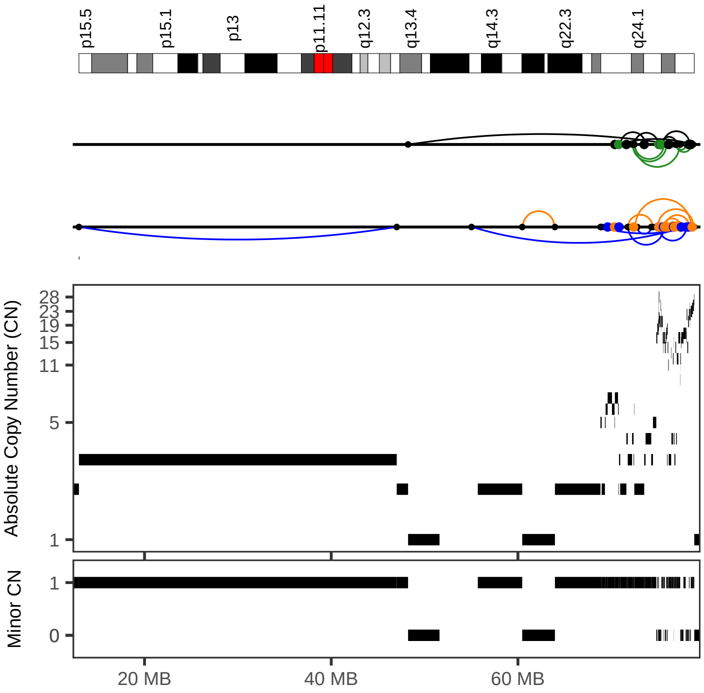

	**784de7ac−8424−42eb−83d4−a1bebaa42b97**
Cancer type	Breast−AdenoCA
Position	11:48231454−78887152
Type	With other complex events
Interleaved intrachr. SVs	34
Total SVs (intrachr. + transl.)	69
SV types	DEL: 10; DUP: 8; h2hINV: 6; t2tINV: 10; TRA: 35
SVs in sample	106
Oscillating CN (2 and 3 states)	4, 6
CN segments	86
FDR fragment joints	0.615458
FDR chr. breakp. enrich.	0
Linked to chrs	
Purity, ploidy	0.6, 2.04

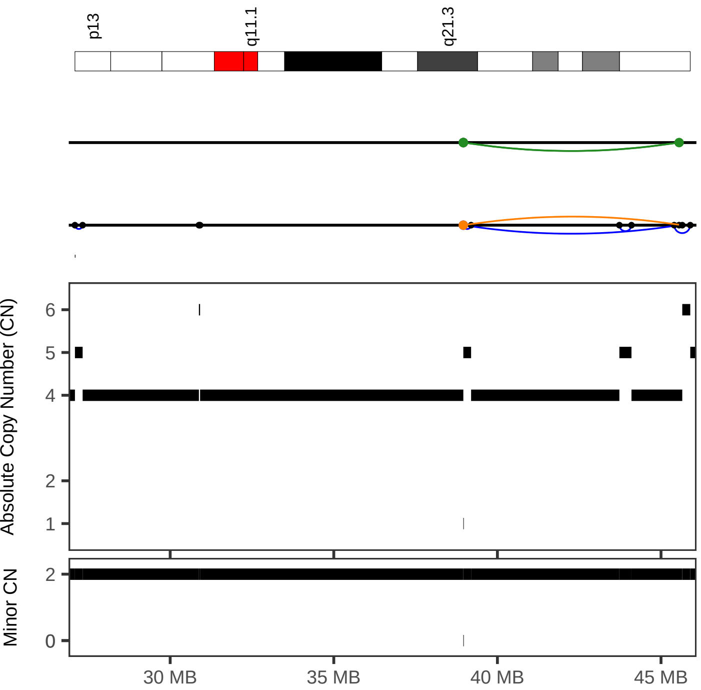

	**8785012f−f73e−4d68−87cf−1d804af32782**
Cancer type	Breast−AdenoCA
Position	21:38958060−45893770
Type	With other complex events
Interleaved intrachr. SVs	6
Total SVs (intrachr. + transl.)	12
SV types	DEL: 1; DUP: 3; h2hINV: 0; t2tINV: 2; TRA: 6
SVs in sample	530
Oscillating CN (2 and 3 states)	4, 6
CN segments	8
FDR fragment joints	0.9284301
FDR chr. breakp. enrich.	0.01
Linked to chrs	10:949660−110004863;
Purity, ploidy	0.56, 2.92

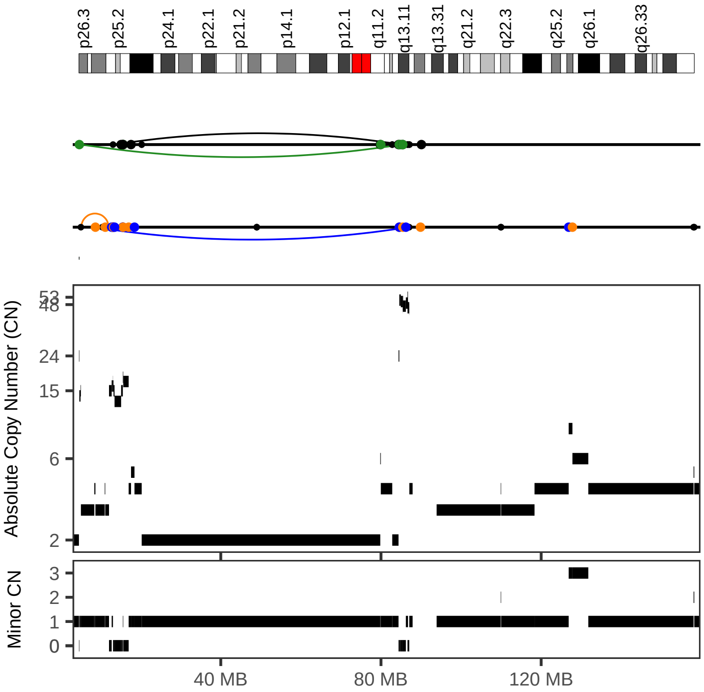

	**8cf54607−01ce−42b0−9bd9−8627edd9f3b7**
Cancer type	Breast−AdenoCA
Position	3:4979331−87053735
Type	With other complex events
Interleaved intrachr. SVs	8
Total SVs (intrachr. + transl.)	36
SV types	DEL: 1; DUP: 2; h2hINV: 2; t2tINV: 3; TRA: 28
SVs in sample	500
Oscillating CN (2 and 3 states)	5, 5
CN segments	35
FDR fragment joints	0.8653243
FDR chr. breakp. enrich.	0.02
Linked to chrs	10:37398211−116249332;4:39957449−163913724 6:66019356−135278032;
Purity, ploidy	0.55, 3.76

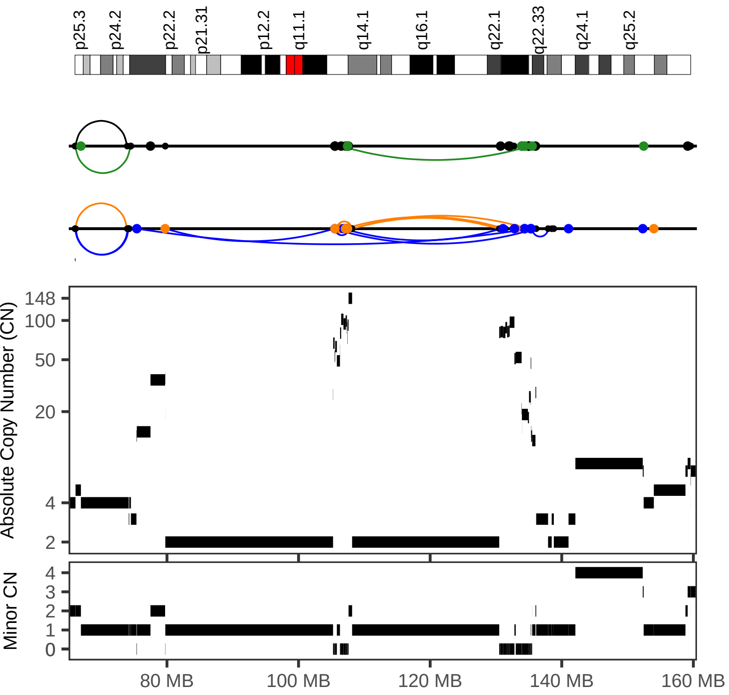

8cf54607−01ce−42b0−9bd9−8627edd9f3b7	
Cancer type	Breast−AdenoCA
Position	6:66019356−135278033
Type	With other complex events
Interleaved intrachr. SVs	6
Total SVs (intrachr. + transl.)	50
SV types	DEL: 5; DUP: 5; h2hINV: 1; t2tINV: 1; TRA: 38
SVs in sample	500
Oscillating CN (2 and 3 states)	6, 6
CN segments	57
FDR fragment joints	0.6776251
FDR chr. breakp. enrich.	0
Linked to chrs	12:2943203−96943877;13:22850041−105164538 3:4979331−87053734;
Purity, ploidy	0.55, 3.76

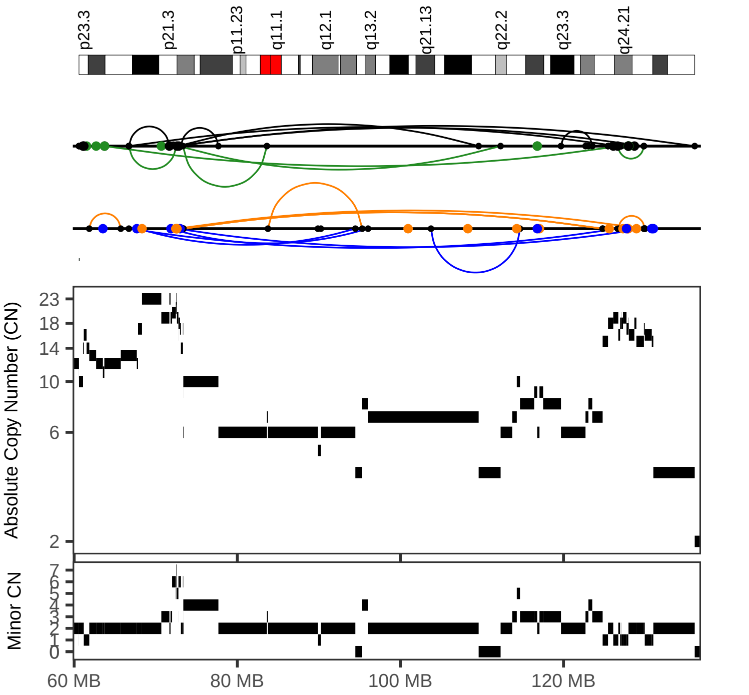

8cf54607−01ce−42b0−9bd9−8627edd9f3b7	
Cancer type	Breast−AdenoCA
Position	8:61853482−136089944
Type	With other complex events
Interleaved intrachr. SVs	26
Total SVs (intrachr. + transl.)	63
SV types	DEL: 7; DUP: 7; h2hINV: 7; t2tINV: 5; TRA: 37
SVs in sample	500
Oscillating CN (2 and 3 states)	4, 7
CN segments	65
FDR fragment joints	0.7735152
FDR chr. breakp. enrich.	0
Linked to chrs	10:37398211−116249332;15:32410181−71959925 3:4979331−87053734;4:39957449−163913724 6:66019356−135278032;
Purity, ploidy	0.55, 3.76

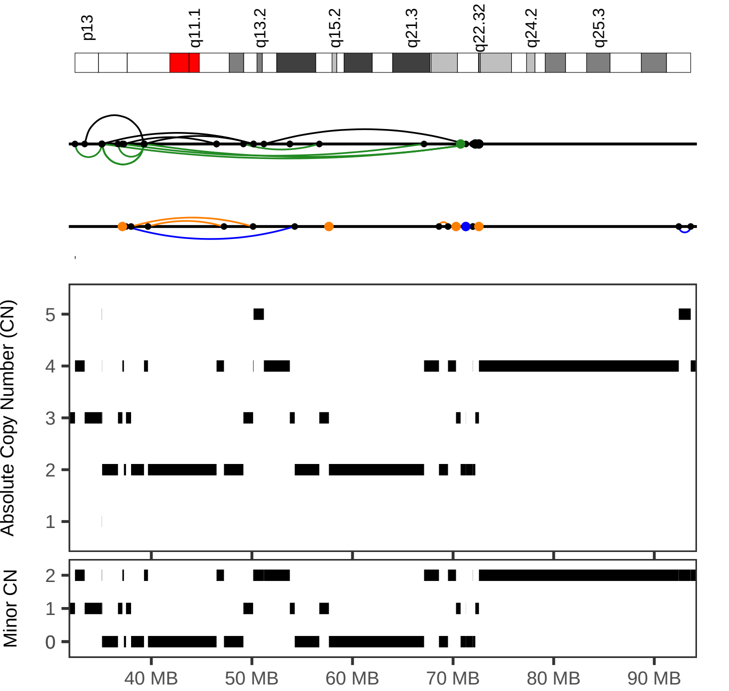

8cf54607−01ce−42b0−9bd9−8627edd9f3b7	
Cancer type	Breast−AdenoCA
Position	15:32410181−71959926
Type	With other complex events
Interleaved intrachr. SVs	17
Total SVs (intrachr. + transl.)	22
SV types	DEL: 2; DUP: 1; h2hINV: 5; t2tINV: 9; TRA: 5
SVs in sample	500
Oscillating CN (2 and 3 states)	5, 10
CN segments	34
FDR fragment joints	0.615458
FDR chr. breakp. enrich.	0
Linked to chrs	
Purity, ploidy	0.55, 3.76

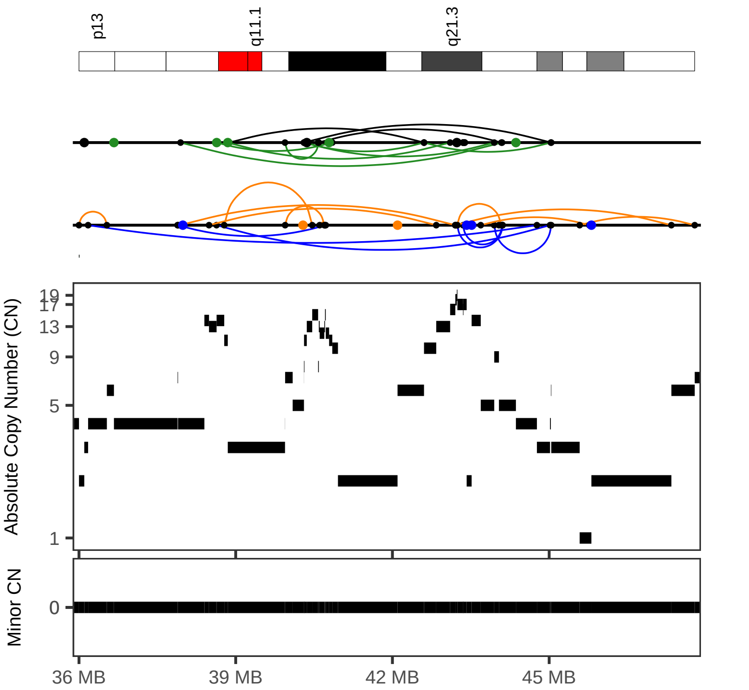

8cf54607−01ce−42b0−9bd9−8627edd9f3b7	
Cancer type	Breast−AdenoCA
Position	21:35999819−47786448
Type	With other complex events
Interleaved intrachr. SVs	29
Total SVs (intrachr. + transl.)	43
SV types	DEL: 11; DUP: 7; h2hINV: 3; t2tINV: 8; TRA: 14
SVs in sample	500
Oscillating CN (2 and 3 states)	5, 5
CN segments	54
FDR fragment joints	0.8609429
FDR chr. breakp. enrich.	0
Linked to chrs	20:11131846−59577546;
Purity, ploidy	0.55, 3.76

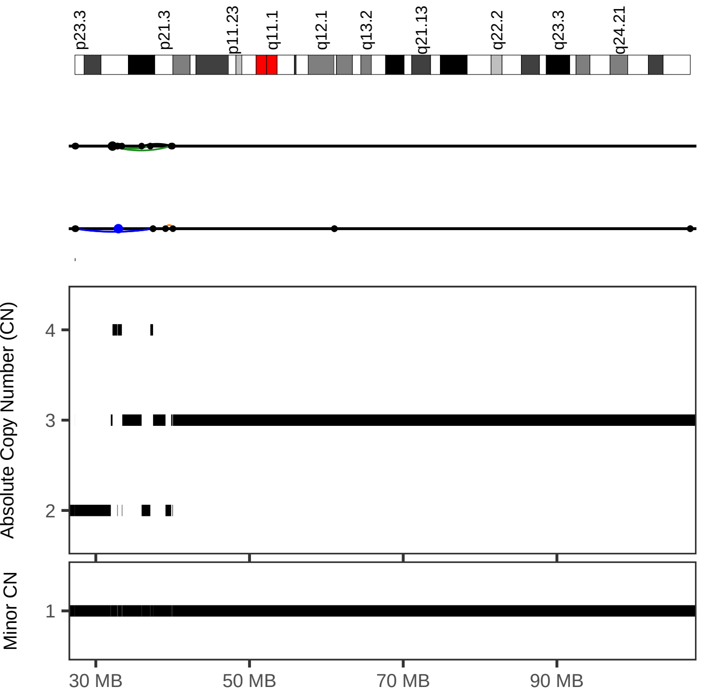

	9435447e−d65f−408b−863b−6576b1d652dd
Cancer type	Breast−AdenoCA
Position	8:27280903−40031251
Type	With other complex events
Interleaved intrachr. SVs	8
Total SVs (intrachr. + transl.)	10
SV types	DEL: 1; DUP: 2; h2hINV: 3; t2tINV: 2; TRA: 2
SVs in sample	137
Oscillating CN (2 and 3 states)	5, 9
CN segments	15
FDR fragment joints	0.8572806
FDR chr. breakp. enrich.	0.22
Linked to chrs	6:11657826−66411416;
Purity, ploidy	0.63, 3.27

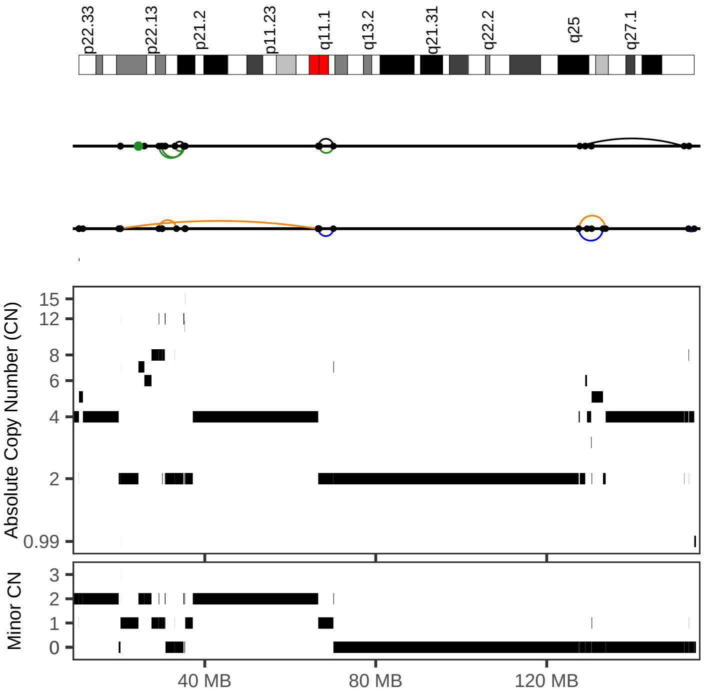

	993103b1−e5a1−4c33−8629−be53ebc41d64
Cancer type	Breast−AdenoCA
Position	X:29228730−35455689
Type	With other complex events
Interleaved intrachr. SVs	7
Total SVs (intrachr. + transl.)	7
SV types	DEL: 2; DUP: 0; h2hINV: 2; t2tINV: 3; TRA: 0
SVs in sample	202
Oscillating CN (2 and 3 states)	4, 10
CN segments	15
FDR fragment joints	0.6776251
FDR chr. breakp. enrich.	0
Linked to chrs	
Purity, ploidy	0.41, 3.34

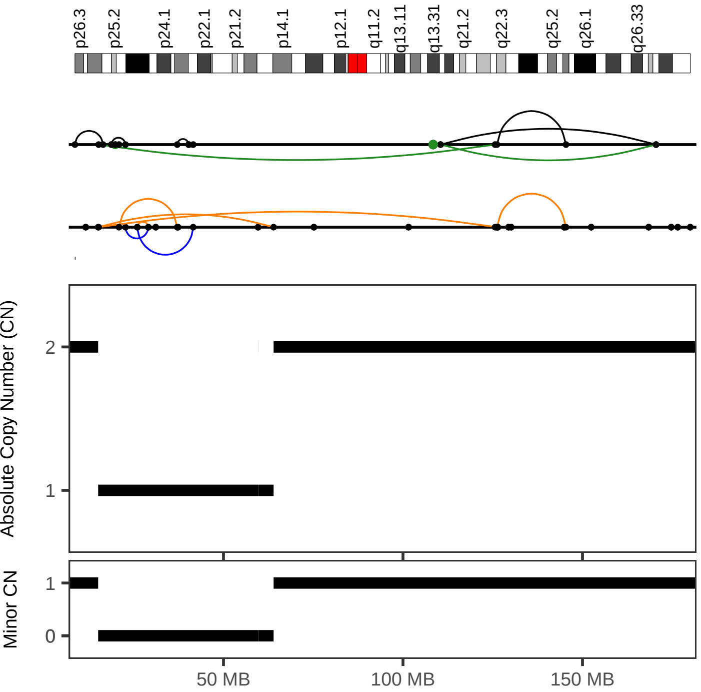

	9938ce5c−e74e−446e−a932−f096f85cc3b1
Cancer type	Breast−AdenoCA
Position	3:8619684−170470349
Type	Canonical without polyploidization
Interleaved intrachr. SVs	9
Total SVs (intrachr. + transl.)	11
SV types	DEL: 4; DUP: 0; h2hINV: 3; t2tINV: 2; TRA: 2
SVs in sample	361
Oscillating CN (2 and 3 states)	5, 5
CN segments	5
FDR fragment joints	0.615458
FDR chr. breakp. enrich.	0.16
Linked to chrs	
Purity, ploidy	0.5, 1.84

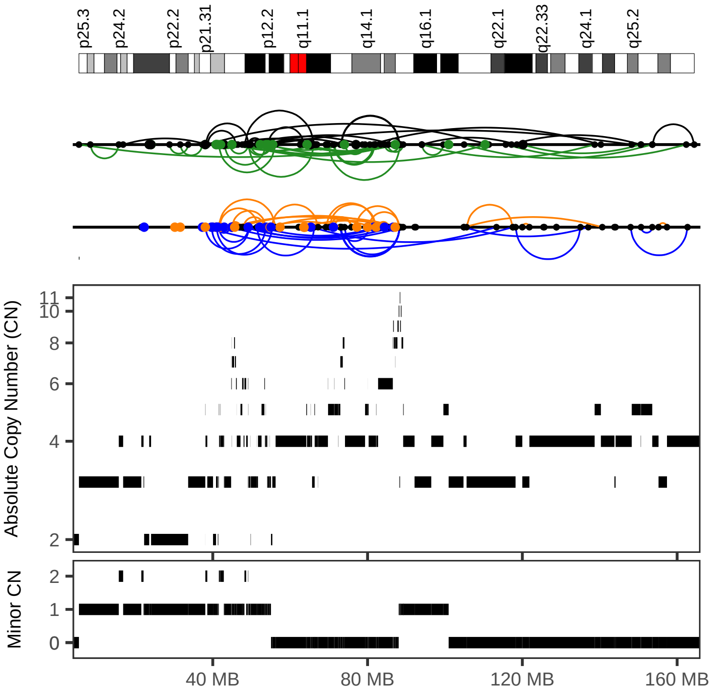

	9fefbe7c−f66a−4940−843e−285cb7b392c1
Cancer type	Breast−AdenoCA
Position	6:5413201−164420806
Type	With other complex events
Interleaved intrachr. SVs	102
Total SVs (intrachr. + transl.)	163
SV types	DEL: 23; DUP: 27; h2hINV: 23; t2tINV: 29; TRA: 61
SVs in sample	740
Oscillating CN (2 and 3 states)	6, 16
CN segments	129
FDR fragment joints	0.8517294
FDR chr. breakp. enrich.	0
Linked to chrs	1:31254630−243789936;11:77294848−126487191 7:63694811−105774033;
Purity, ploidy	0.83, 3.26

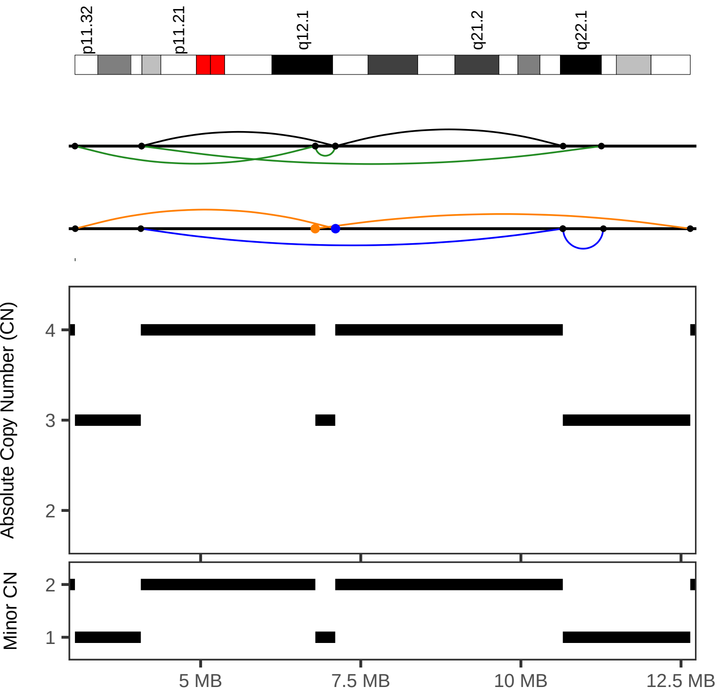

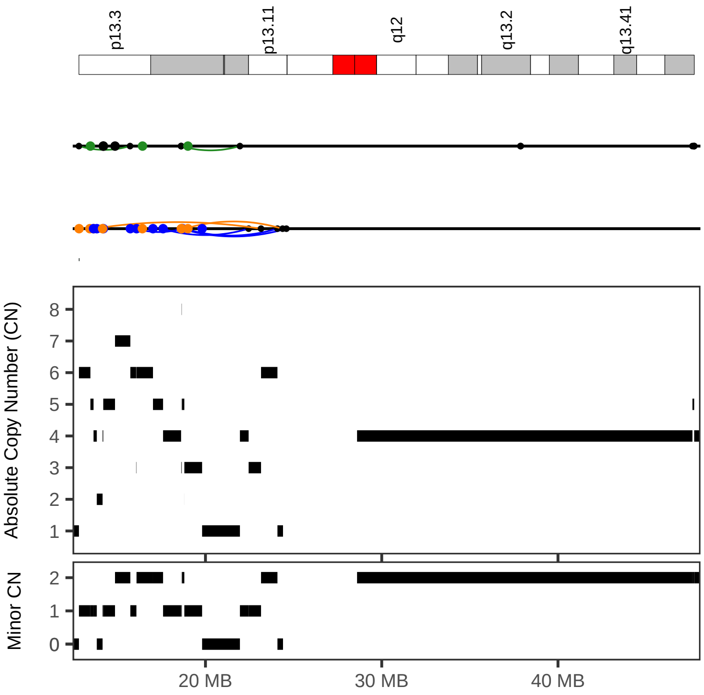

	9fefbe7c−f66a−4940−843e−285cb7b392c1
Cancer type	Breast−AdenoCA
Position	18:3037071−12644210
Type	After polyploidization
Interleaved intrachr. SVs	9
Total SVs (intrachr. + transl.)	11
SV types	DEL: 2; DUP: 2; h2hINV: 2; t2tINV: 3; TRA: 2
SVs in sample	740
Oscillating CN (2 and 3 states)	6, 6
CN segments	6
FDR fragment joints	0.9804396
FDR chr. breakp. enrich.	0.33
Linked to chrs	
Purity, ploidy	0.83, 3.26

	9fefbe7c−f66a−4940−843e−285cb7b392c1
Cancer type	Breast−AdenoCA
Position	19:12822132−24591286
Type	With other complex events
Interleaved intrachr. SVs	8
Total SVs (intrachr. + transl.)	31
SV types	DEL: 2; DUP: 4; h2hINV: 0; t2tINV: 2; TRA: 23
SVs in sample	740
Oscillating CN (2 and 3 states)	4, 6
CN segments	27
FDR fragment joints	0.5257473
FDR chr. breakp. enrich.	0
Linked to chrs	1:31254630−243789936;
Purity, ploidy	0.83, 3.26

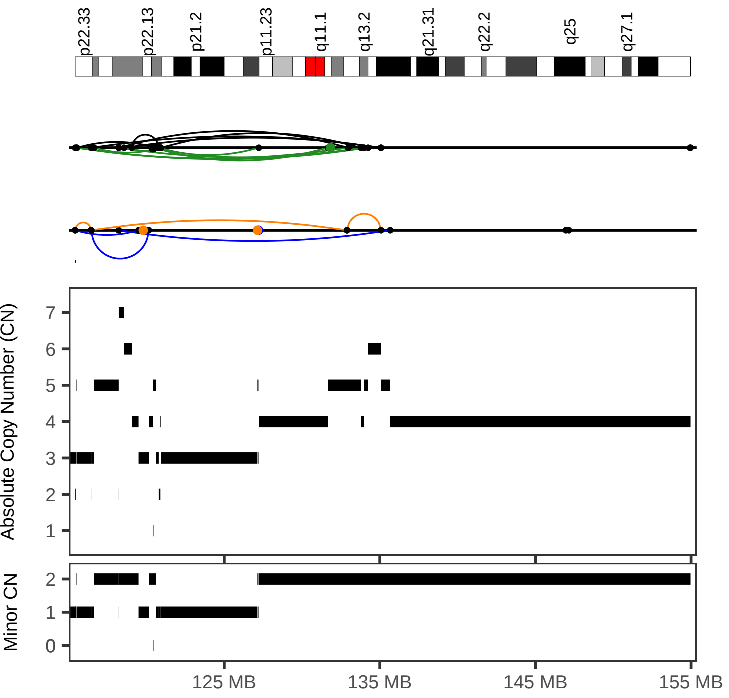

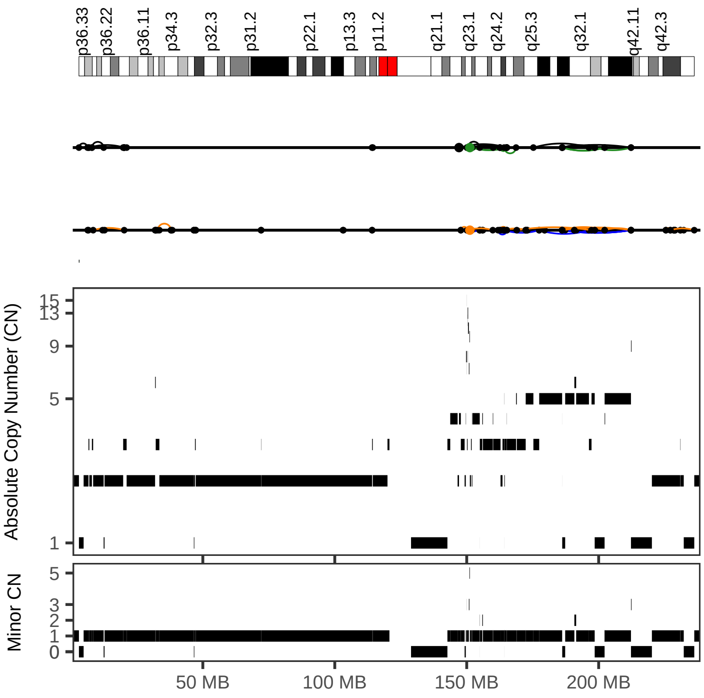

	9fefbe7c−f66a−4940−843e−285cb7b392c1
Cancer type	Breast−AdenoCA
Position	X:115424165−135666891
Type	With other complex events
Interleaved intrachr. SVs	19
Total SVs (intrachr. + transl.)	24
SV types	DEL: 3; DUP: 3; h2hINV: 7; t2tINV: 6; TRA: 5
SVs in sample	740
Oscillating CN (2 and 3 states)	4, 7
CN segments	29
FDR fragment joints	0.7568568
FDR chr. breakp. enrich.	0.25
Linked to chrs	
Purity, ploidy	0.83, 3.26

	a824b3bd−34d5−4cc1−a92f−f9d6ac0f1814
Cancer type	Breast−AdenoCA
Position	1:147408680−212272247
Type	With other complex events
Interleaved intrachr. SVs	37
Total SVs (intrachr. + transl.)	39
SV types	DEL: 9; DUP: 9; h2hINV: 10; t2tINV: 9; TRA: 2
SVs in sample	463
Oscillating CN (2 and 3 states)	6, 9
CN segments	62
FDR fragment joints	0.9800011
FDR chr. breakp. enrich.	0
Linked to chrs	3:29682227−151361517;
Purity, ploidy	0.51, 2.1

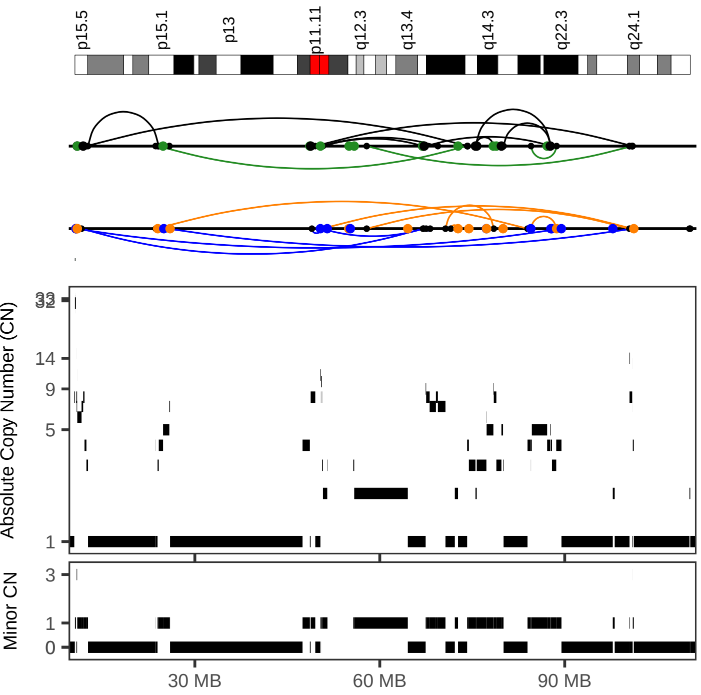

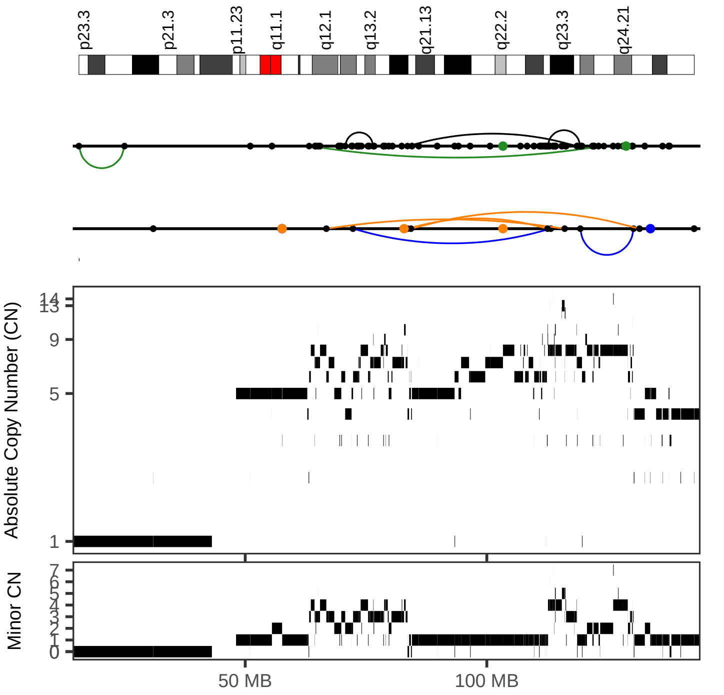

a824b3bd−34d5−4cc1−a92f−f9d6ac0f1814	
Cancer type	Breast−AdenoCA
Position	11:10547187−101046437
Type	With other complex events
Interleaved intrachr. SVs	25
Total SVs (intrachr. + transl.)	70
SV types	DEL: 6; DUP: 5; h2hINV: 9; t2tINV: 5; TRA: 45
SVs in sample	463
Oscillating CN (2 and 3 states)	5, 15
CN segments	78
FDR fragment joints	0.8066159
FDR chr. breakp. enrich.	0
Linked to chrs	2:26459899−233537708;5:108659861−118357838
Purity, ploidy	0.51, 2.1

a9b7d7fe−be31−4f71−afee−c1bfdf511888	
Cancer type	Breast−AdenoCA
Position	8:63227110−131599267
Type	With other complex events
Interleaved intrachr. SVs	10
Total SVs (intrachr. + transl.)	14
SV types	DEL: 3; DUP: 2; h2hINV: 3; t2tINV: 2; TRA: 4
SVs in sample	460
Oscillating CN (2 and 3 states)	6, 8
CN segments	173
FDR fragment joints	0.6776251
FDR chr. breakp. enrich.	0
Linked to chrs	
Purity, ploidy	0.47, 2.76

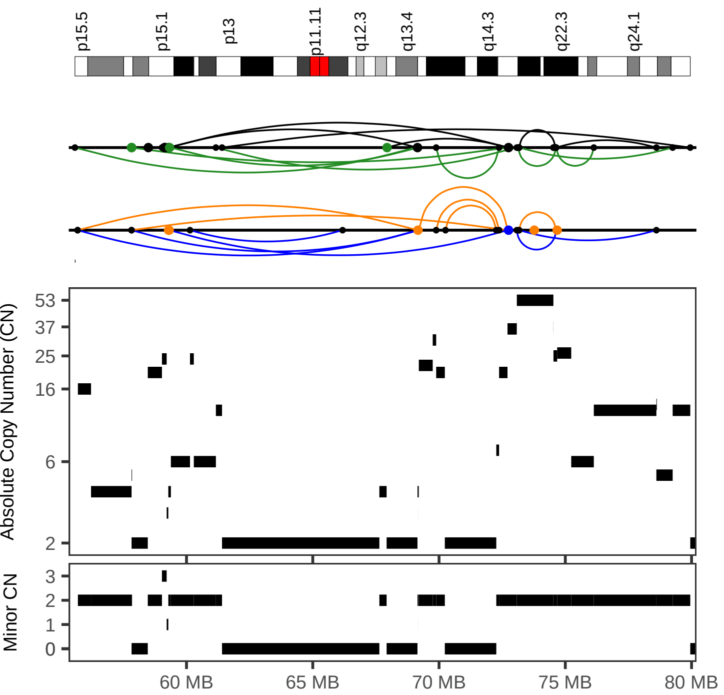

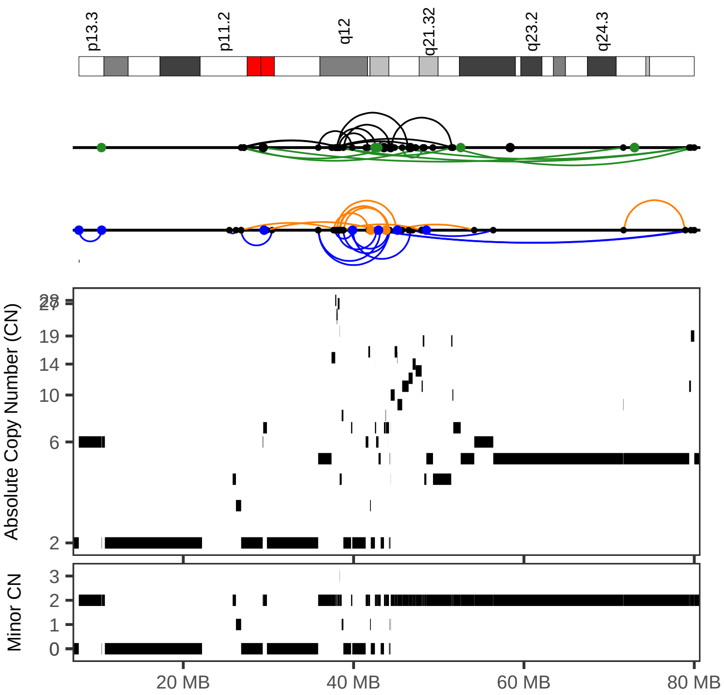

b43e41af−1d82−4b5f−b8f1−add0510e6b86	
Cancer type	Breast−AdenoCA
Position	11:55581976−79948001
Type	With other complex events
Interleaved intrachr. SVs	15
Total SVs (intrachr. + transl.)	31
SV types	DEL: 3; DUP: 4; h2hINV: 4; t2tINV: 4; TRA: 16
SVs in sample	296
Oscillating CN (2 and 3 states)	4, 6
CN segments	37
FDR fragment joints	0.7622832
FDR chr. breakp. enrich.	0
Linked to chrs	22:24093006−30283045;
Purity, ploidy	0.72, 4.04

b43e41af−1d82−4b5f−b8f1−add0510e6b86	
Cancer type	Breast−AdenoCA
Position	17:26793465−79998471
Type	With other complex events
Interleaved intrachr. SVs	49
Total SVs (intrachr. + transl.)	66
SV types	DEL: 13; DUP: 12; h2hINV: 13; t2tINV: 11; TRA: 17
SVs in sample	296
Oscillating CN (2 and 3 states)	4, 7
CN segments	64
FDR fragment joints	0.992996
FDR chr. breakp. enrich.	0
Linked to chrs	19:48961354−58347530;
Purity, ploidy	0.72, 4.04

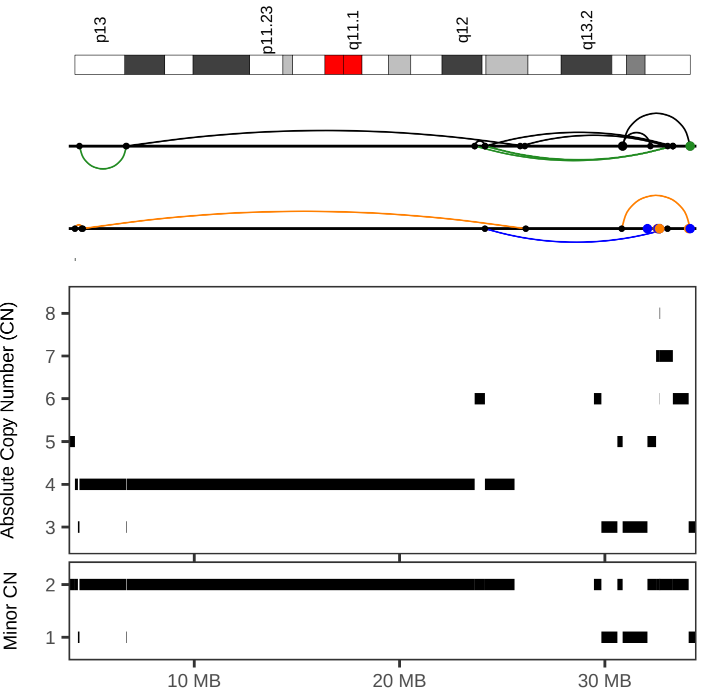

	**b43e41af−1d82−4b5f−b8f1−add0510e6b86**
Cancer type	Breast−AdenoCA
Position	20:4186490−34159090
Type	With other complex events
Interleaved intrachr. SVs	14
Total SVs (intrachr. + transl.)	28
SV types	DEL: 3; DUP: 1; h2hINV: 6; t2tINV: 4; TRA: 14
SVs in sample	296
Oscillating CN (2 and 3 states)	5, 8
CN segments	19
FDR fragment joints	0.9794281
FDR chr. breakp. enrich.	0
Linked to chrs	22:24093006−30283045;
Purity, ploidy	0.72, 4.04

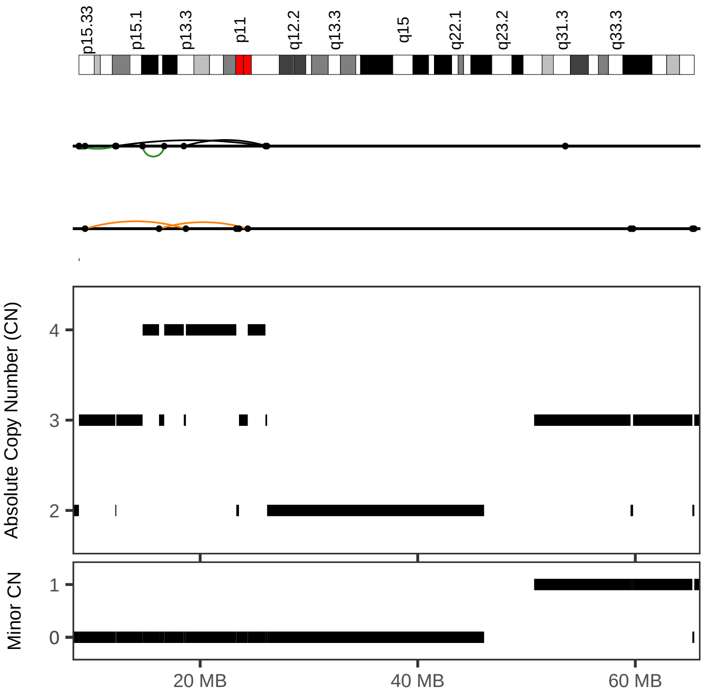

	**b97bf89a−7a85−4eef−ae7e−f787aead1f0a**
Cancer type	Breast−AdenoCA
Position	5:8856070−26153658
Type	With other complex events
Interleaved intrachr. SVs	6
Total SVs (intrachr. + transl.)	6
SV types	DEL: 2; DUP: 0; h2hINV: 2; t2tINV: 2; TRA: 0
SVs in sample	194
Oscillating CN (2 and 3 states)	6, 8
CN segments	13
FDR fragment joints	0.6776251
FDR chr. breakp. enrich.	0.78
Linked to chrs	
Purity, ploidy	0.47, 3.8

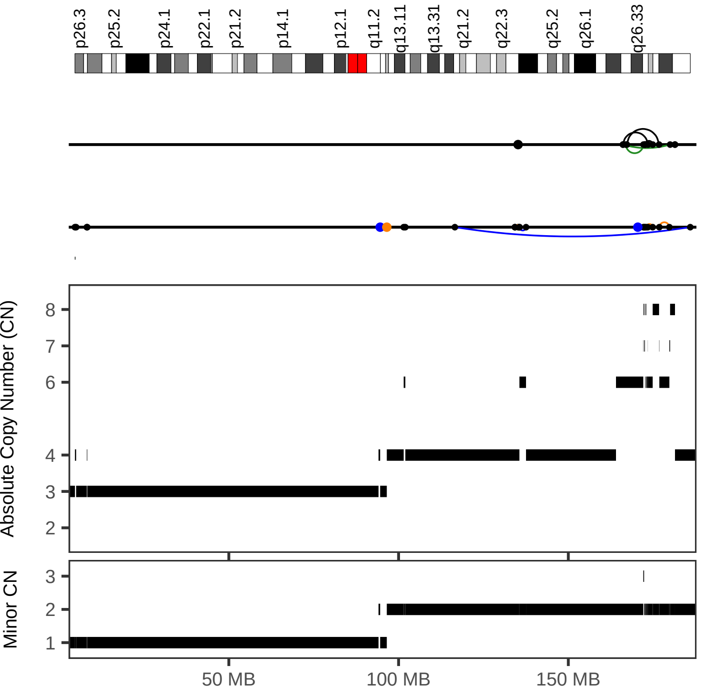

	**c0892598−1f7b−4f23−9cd8−731f797753d5**
Cancer type	Breast−AdenoCA
Position	3:166105643−176738060
Type	With other complex events
Interleaved intrachr. SVs	8
Total SVs (intrachr. + transl.)	9
SV types	DEL: 2; DUP: 1; h2hINV: 3; t2tINV: 2; TRA: 1
SVs in sample	358
Oscillating CN (2 and 3 states)	5, 8
CN segments	13
FDR fragment joints	0.9723381
FDR chr. breakp. enrich.	1
Linked to chrs	
Purity, ploidy	0.61, 3.55

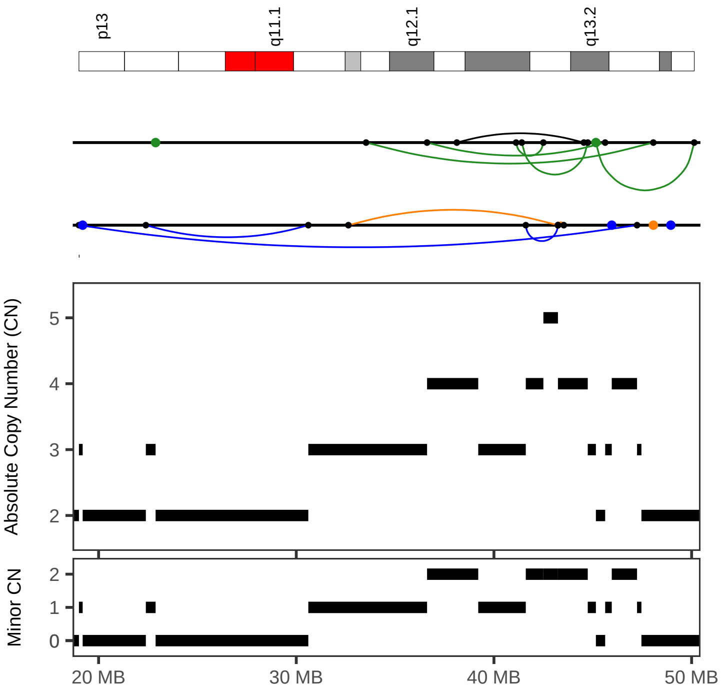

	**c0892598−1f7b−4f23−9cd8−731f797753d5**
Cancer type	Breast−AdenoCA
Position	22:19004440−50133204
Type	With other complex events
Interleaved intrachr. SVs	10
Total SVs (intrachr. + transl.)	16
SV types	DEL: 2; DUP: 2; h2hINV: 1; t2tINV: 5; TRA: 6
SVs in sample	358
Oscillating CN (2 and 3 states)	5, 16
CN segments	16
FDR fragment joints	0.8653243
FDR chr. breakp. enrich.	0
Linked to chrs	1:150239597−180719222;3:166105643−176738059
Purity, ploidy	0.61, 3.55

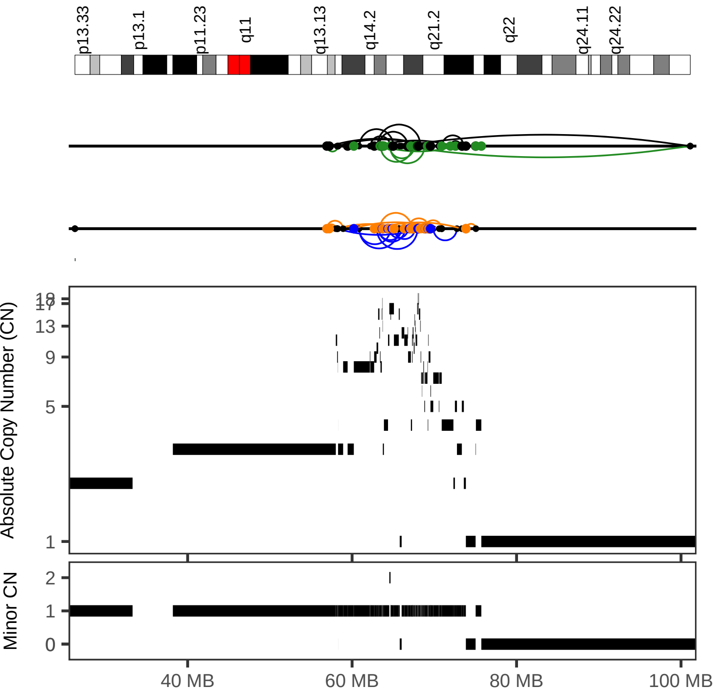

c364e81c−eb1e−4870−ab37−9c661f5f2e3d	
Cancer type	Breast−AdenoCA
Position	12:56913152−101125708
Type	With other complex events
Interleaved intrachr. SVs	50
Total SVs (intrachr. + transl.)	124
SV types	DEL: 10; DUP: 13; h2hINV: 13; t2tINV: 14; TRA: 74
SVs in sample	397
Oscillating CN (2 and 3 states)	5, 8
CN segments	81
FDR fragment joints	0.854603
FDR chr. breakp. enrich.	0
Linked to chrs	16:5415371−33325042;20:24138933−61546428
Purity, ploidy	0.41, 2.02

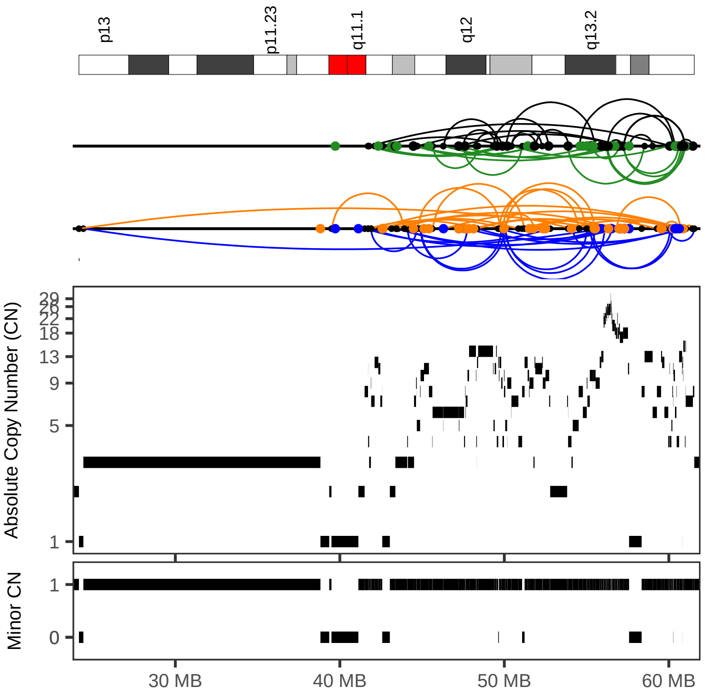

c364e81c−eb1e−4870−ab37−9c661f5f2e3d	
Cancer type	Breast−AdenoCA
Position	20:24138933−61546429
Type	With other complex events
Interleaved intrachr. SVs	87
Total SVs (intrachr. + transl.)	196
SV types	DEL: 25; DUP: 20; h2hINV: 23; t2tINV: 19; TRA: 109
SVs in sample	397
Oscillating CN (2 and 3 states)	5, 7
CN segments	148
FDR fragment joints	0.615458
FDR chr. breakp. enrich.	0
Linked to chrs	12:56913152−101125707;16:5415371−33325042
Purity, ploidy	0.41, 2.02

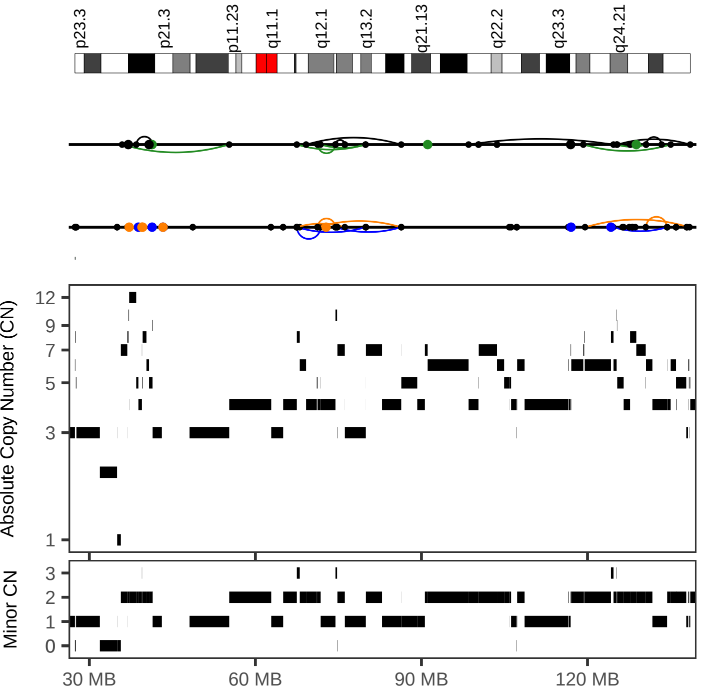

d3d545b3−457f−4389−821f−704cb24aff7f	
Cancer type	Breast−AdenoCA
Position	8:67453419−86357134
Type	With other complex events
Interleaved intrachr. SVs	14
Total SVs (intrachr. + transl.)	15
SV types	DEL: 3; DUP: 5; h2hINV: 3; t2tINV: 3; TRA: 1
SVs in sample	709
Oscillating CN (2 and 3 states)	5, 6
CN segments	20
FDR fragment joints	0.925252
FDR chr. breakp. enrich.	0
Linked to chrs	
Purity, ploidy	0.76, 3.03

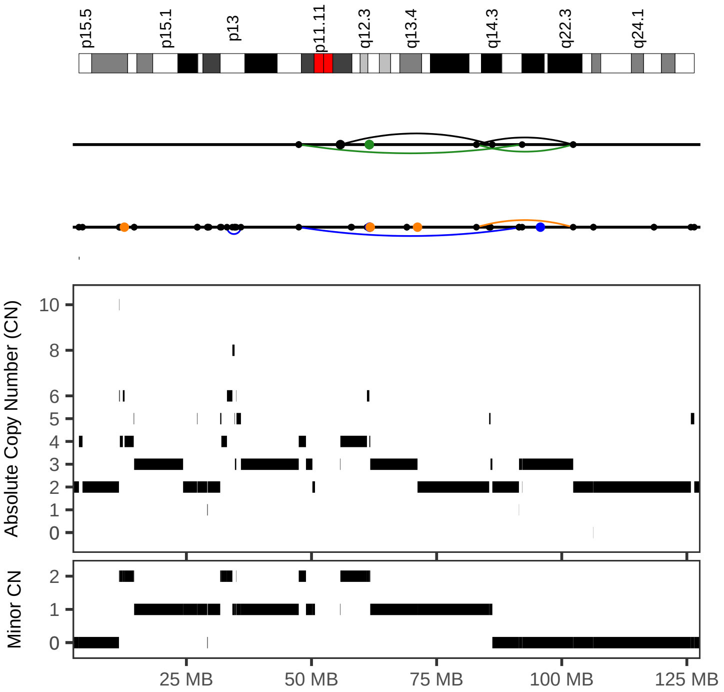

d3d545b3−457f−4389−821f−704cb24aff7f	
Cancer type	Breast−AdenoCA
Position	11:47447817−102287472
Type	With other complex events
Interleaved intrachr. SVs	6
Total SVs (intrachr. + transl.)	15
SV types	DEL: 1; DUP: 1; h2hINV: 2; t2tINV: 2; TRA: 9
SVs in sample	709
Oscillating CN (2 and 3 states)	4, 6
CN segments	18
FDR fragment joints	0.9905774
FDR chr. breakp. enrich.	0.44
Linked to chrs	
Purity, ploidy	0.76, 3.03

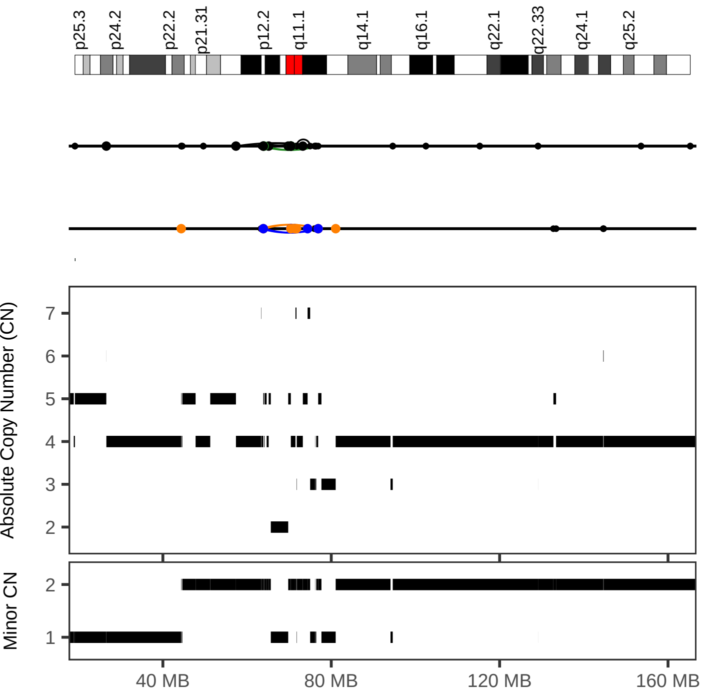

	d67cd793−2931−429a−9084−2f3c4c8be7ad
Cancer type	Breast−AdenoCA
Position	6:57423175−76896760
Type	With other complex events
Interleaved intrachr. SVs	13
Total SVs (intrachr. + transl.)	35
SV types	DEL: 2; DUP: 4; h2hINV: 3; t2tINV: 4; TRA: 22
SVs in sample	427
Oscillating CN (2 and 3 states)	6, 12
CN segments	21
FDR fragment joints	0.615458
FDR chr. breakp. enrich.	0
Linked to chrs	14:20136324−55556439;
Purity, ploidy	0.54, 3.31

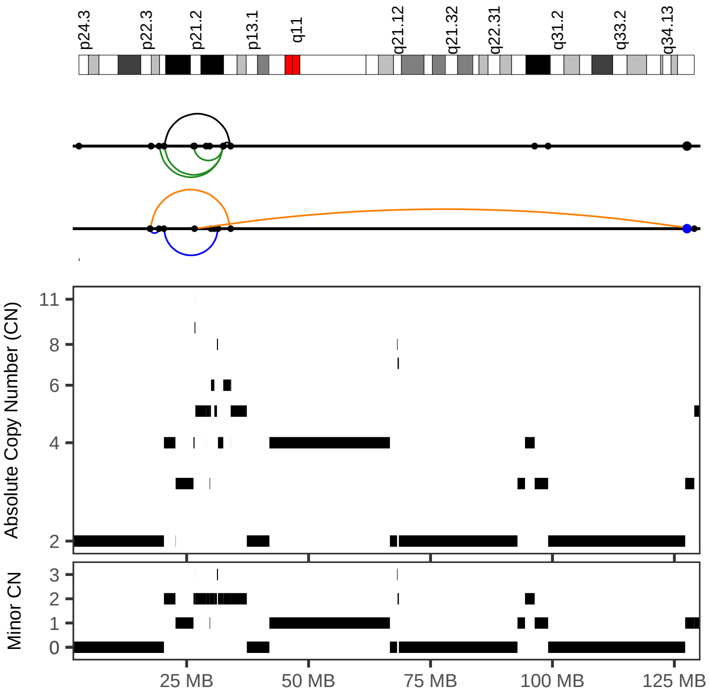

	d67cd793−2931−429a−9084−2f3c4c8be7ad
Cancer type	Breast−AdenoCA
Position	9:17497087−129076121
Type	With other complex events
Interleaved intrachr. SVs	10
Total SVs (intrachr. + transl.)	12
SV types	DEL: 2; DUP: 2; h2hINV: 2; t2tINV: 4; TRA: 2
SVs in sample	427
Oscillating CN (2 and 3 states)	4, 5
CN segments	32
FDR fragment joints	0.6776251
FDR chr. breakp. enrich.	0.76
Linked to chrs	12:33908610−132258020;
Purity, ploidy	0.54, 3.31

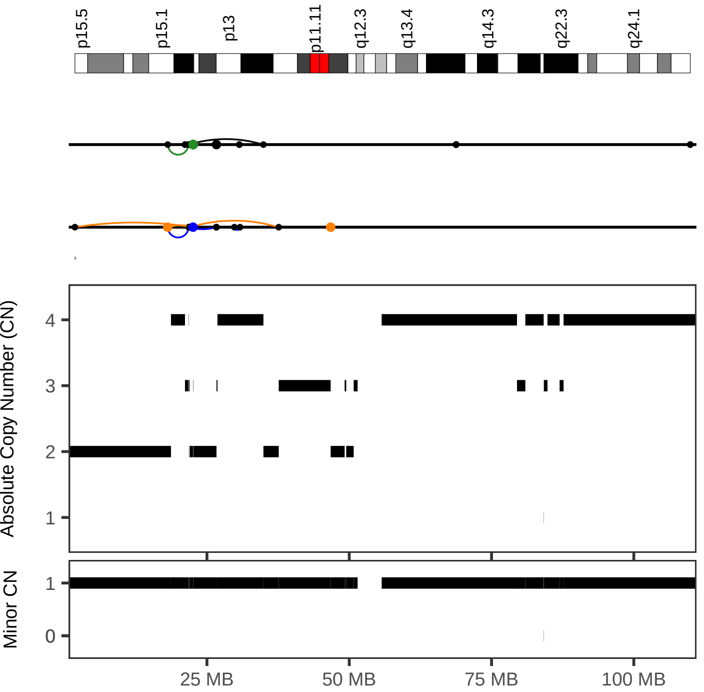

	d67cd793−2931−429a−9084−2f3c4c8be7ad
Cancer type	Breast−AdenoCA
Position	11:1800449−37610887
Type	With other complex events
Interleaved intrachr. SVs	7
Total SVs (intrachr. + transl.)	14
SV types	DEL: 3; DUP: 2; h2hINV: 1; t2tINV: 1; TRA: 7
SVs in sample	427
Oscillating CN (2 and 3 states)	5, 5
CN segments	13
FDR fragment joints	0.9794281
FDR chr. breakp. enrich.	0.79
Linked to chrs	
Purity, ploidy	0.54, 3.31

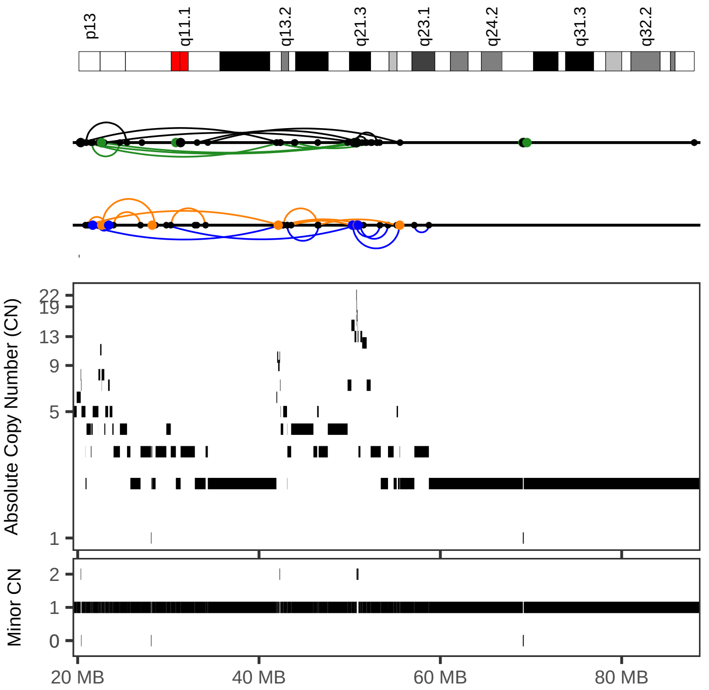

	d67cd793−2931−429a−9084−2f3c4c8be7ad
Cancer type	Breast−AdenoCA
Position	14:20136324−55556440
Type	With other complex events
Interleaved intrachr. SVs	34
Total SVs (intrachr. + transl.)	55
SV types	DEL: 9; DUP: 7; h2hINV: 9; t2tINV: 9; TRA: 21
SVs in sample	427
Oscillating CN (2 and 3 states)	6, 8
CN segments	76
FDR fragment joints	0.930656
FDR chr. breakp. enrich.	0
Linked to chrs	6:57423175−76896759;
Purity, ploidy	0.54, 3.31

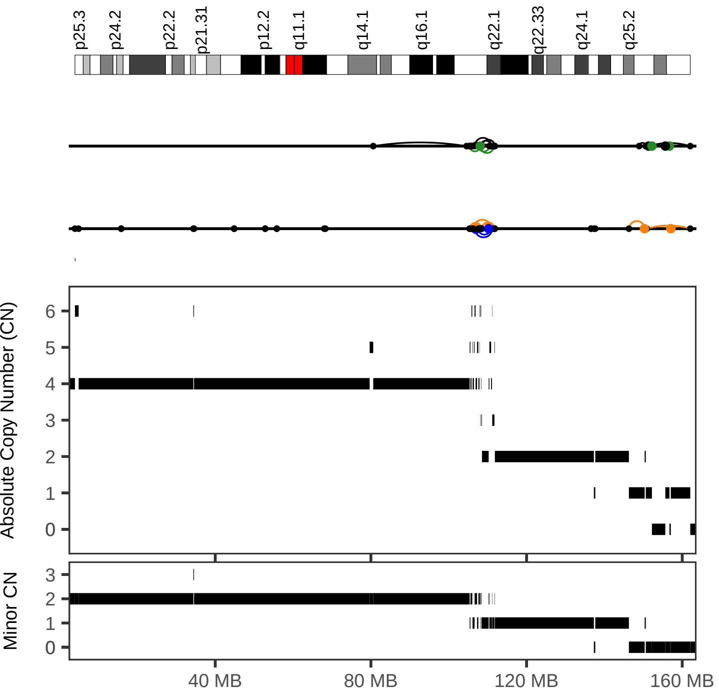

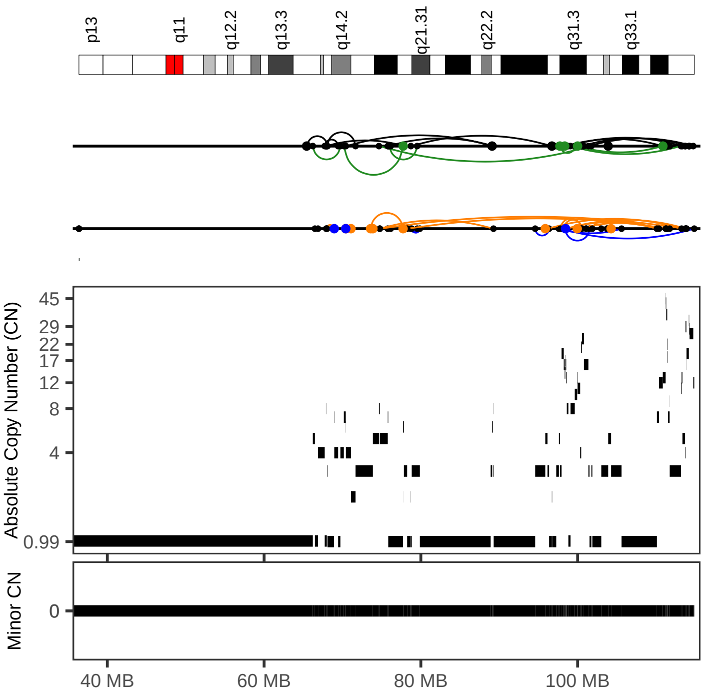

	d6f7afc0−1558−43ad−acb1−2b5311ed2264
Cancer type	Breast−AdenoCA
Position	6:105302877−111874190
Type	With other complex events
Interleaved intrachr. SVs	27
Total SVs (intrachr. + transl.)	29
SV types	DEL: 9; DUP: 8; h2hINV: 6; t2tINV: 4; TRA: 2
SVs in sample	304
Oscillating CN (2 and 3 states)	4, 9
CN segments	35
FDR fragment joints	0.6776251
FDR chr. breakp. enrich.	0
Linked to chrs	
Purity, ploidy	0.27, 2.34

	d6f7afc0−1558−43ad−acb1−2b5311ed2264
Cancer type	Breast−AdenoCA
Position	13:65488647−114881095
Type	With other complex events
Interleaved intrachr. SVs	52
Total SVs (intrachr. + transl.)	71
SV types	DEL: 13; DUP: 9; h2hINV: 17; t2tINV: 13; TRA: 19
SVs in sample	304
Oscillating CN (2 and 3 states)	5, 8
CN segments	93
FDR fragment joints	0.615458
FDR chr. breakp. enrich.	0
Linked to chrs	12:66502753−130003738;
Purity, ploidy	0.27, 2.34

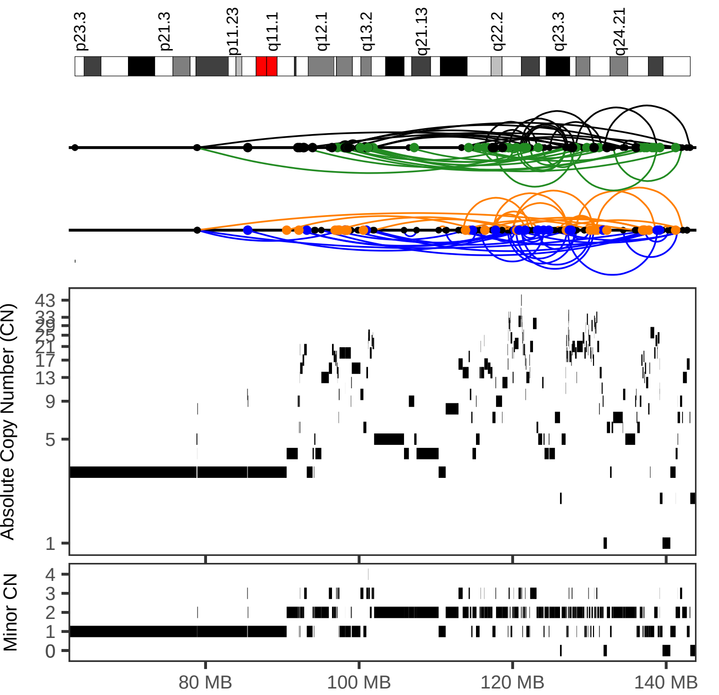

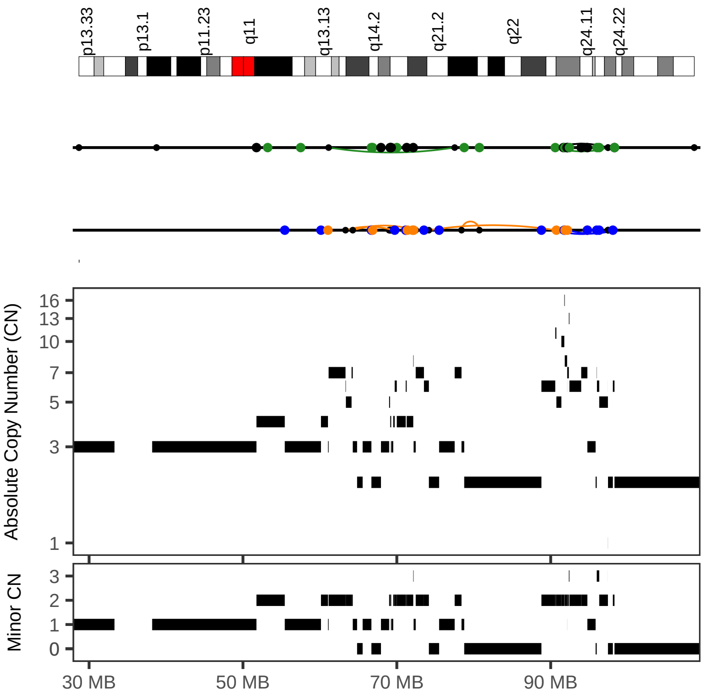

	d8d7a6c2−6427−4f47−968c−6c3affba4617
Cancer type	Breast−AdenoCA
Position	8:78814327−143134735
Type	With other complex events
Interleaved intrachr. SVs	116
Total SVs (intrachr. + transl.)	220
SV types	DEL: 30; DUP: 32; h2hINV: 29; t2tINV: 25; TRA: 104
SVs in sample	453
Oscillating CN (2 and 3 states)	4, 7
CN segments	214
FDR fragment joints	0.8274637
FDR chr. breakp. enrich.	0
Linked to chrs	12:61134794−98290461;
Purity, ploidy	0.62, 3.59

	d8d7a6c2−6427−4f47−968c−6c3affba4617
Cancer type	Breast−AdenoCA
Position	12:61134794−98290462
Type	With other complex events
Interleaved intrachr. SVs	8
Total SVs (intrachr. + transl.)	48
SV types	DEL: 2; DUP: 2; h2hINV: 2; t2tINV: 2; TRA: 40
SVs in sample	453
Oscillating CN (2 and 3 states)	5, 6
CN segments	48
FDR fragment joints	0.9501265
FDR chr. breakp. enrich.	0
Linked to chrs	
Purity, ploidy	0.62, 3.59

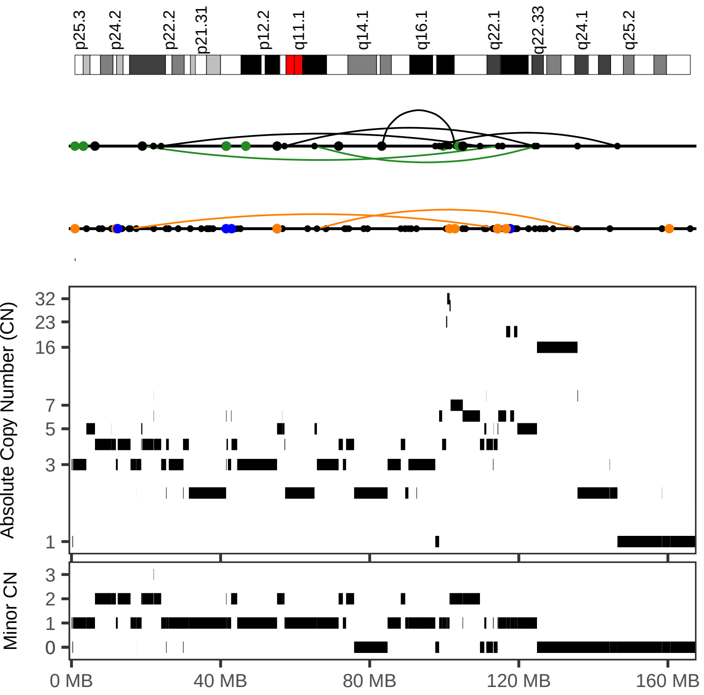

	d9dc3b59−613d−469e−8b4f−6c5a557eb26a
Cancer type	Breast−AdenoCA
Position	6:15838701−146459167
Type	With other complex events
Interleaved intrachr. SVs	8
Total SVs (intrachr. + transl.)	28
SV types	DEL: 2; DUP: 0; h2hINV: 4; t2tINV: 2; TRA: 20
SVs in sample	1222
Oscillating CN (2 and 3 states)	4, 9
CN segments	70
FDR fragment joints	0.8653243
FDR chr. breakp. enrich.	0.01
Linked to chrs	17:2481365−78698345;
Purity, ploidy	0.41, 2.07

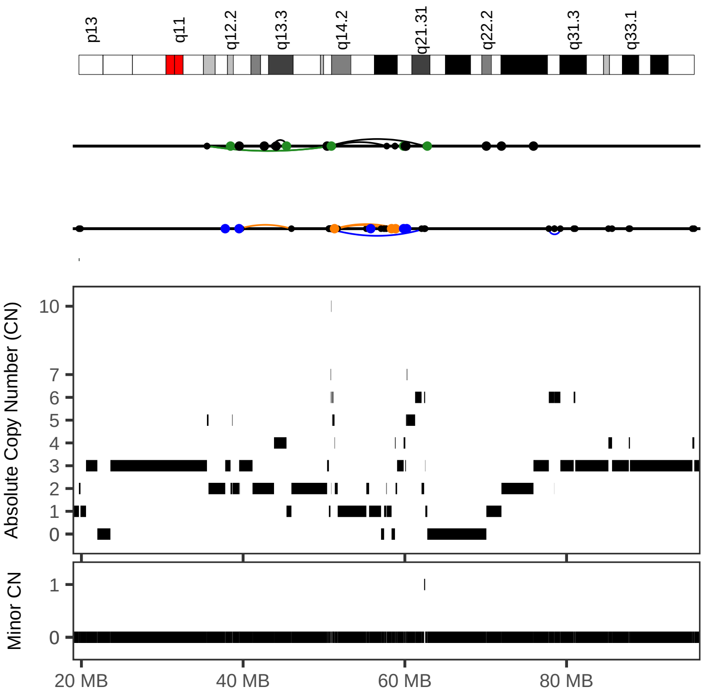

	e4fc0909−f284−4471−866d−d8967b6adcbc
Cancer type	Breast−AdenoCA
Position	13:35528163−62533552
Type	With other complex events
Interleaved intrachr. SVs	9
Total SVs (intrachr. + transl.)	27
SV types	DEL: 1; DUP: 4; h2hINV: 2; t2tINV: 2; TRA: 18
SVs in sample	630
Oscillating CN (2 and 3 states)	6, 10
CN segments	48
FDR fragment joints	0.6776251
FDR chr. breakp. enrich.	0
Linked to chrs	6:93474674−108070112;
Purity, ploidy	0.49, 2.63

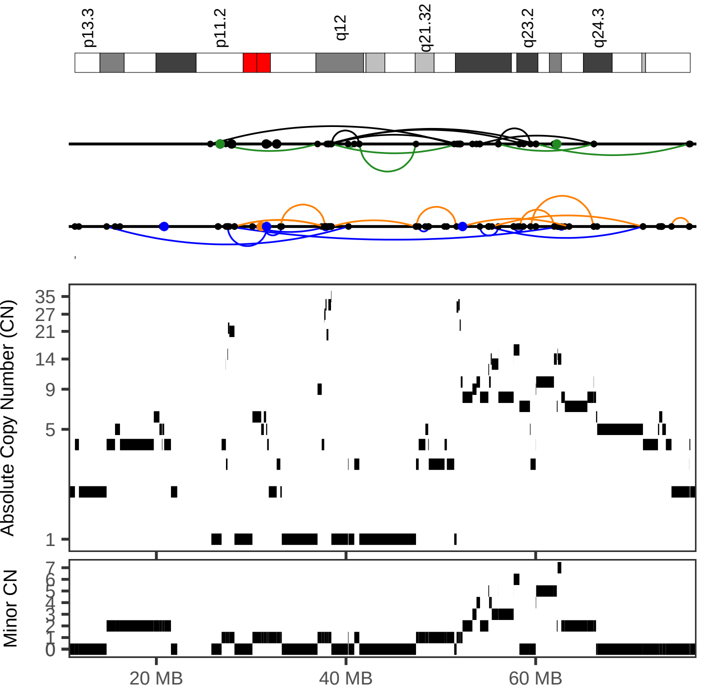

	e4fc0909−f284−4471−866d−d8967b6adcbc
Cancer type	Breast−AdenoCA
Position	17:14753198−76302171
Type	With other complex events
Interleaved intrachr. SVs	44
Total SVs (intrachr. + transl.)	54
SV types	DEL: 11; DUP: 17; h2hINV: 9; t2tINV: 7; TRA: 10
SVs in sample	630
Oscillating CN (2 and 3 states)	6, 7
CN segments	91
FDR fragment joints	0.615458
FDR chr. breakp. enrich.	0
Linked to chrs	20:34495099−60210988;7:10063721−147714910
Purity, ploidy	0.49, 2.63

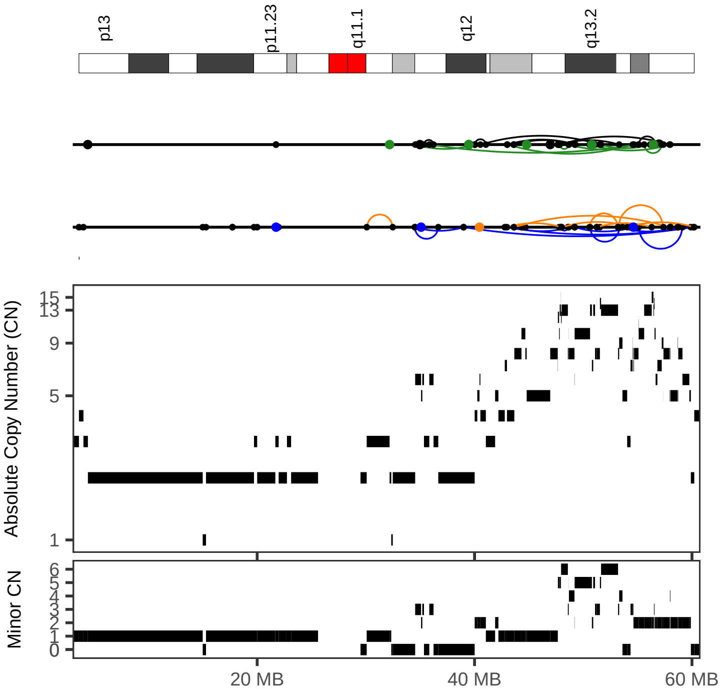

	e4fc0909−f284−4471−866d−d8967b6adcbc
Cancer type	Breast−AdenoCA
Position	20:34495099−60210989
Type	With other complex events
Interleaved intrachr. SVs	39
Total SVs (intrachr. + transl.)	49
SV types	DEL: 10; DUP: 12; h2hINV: 9; t2tINV: 8; TRA: 10
SVs in sample	630
Oscillating CN (2 and 3 states)	5, 10
CN segments	75
FDR fragment joints	0.9410753
FDR chr. breakp. enrich.	0
Linked to chrs	17:14753198−76302170;
Purity, ploidy	0.49, 2.63

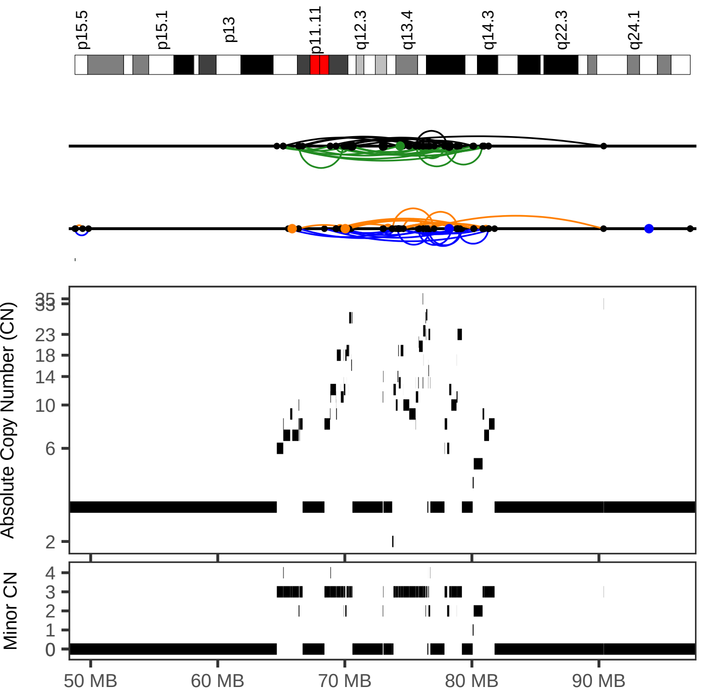

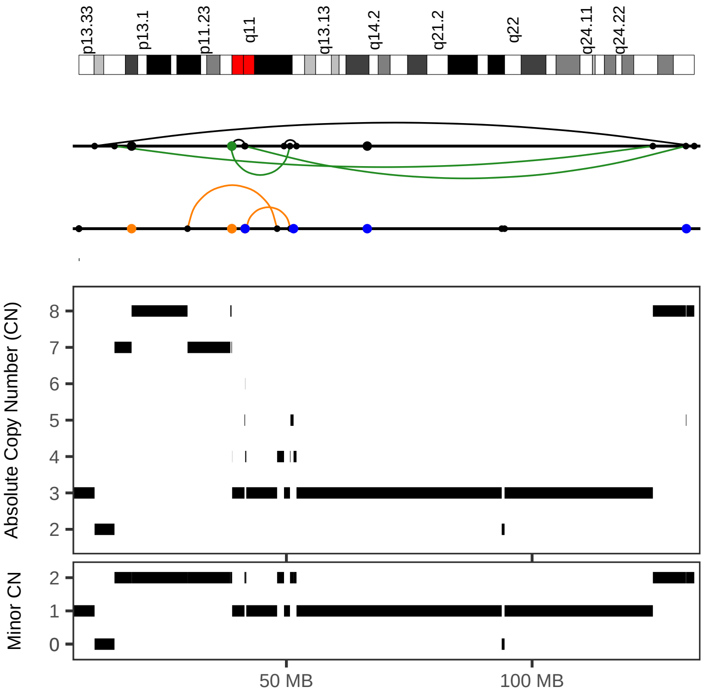

	f0787165−6f58−4d67−b510−928eea2c4882
Cancer type	Breast−AdenoCA
Position	11:64649854−90375457
Type	With other complex events
Interleaved intrachr. SVs	70
Total SVs (intrachr. + transl.)	78
SV types	DEL: 17; DUP: 19; h2hINV: 13; t2tINV: 21; TRA: 8
SVs in sample	421
Oscillating CN (2 and 3 states)	4, 8
CN segments	90
FDR fragment joints	0.615458
FDR chr. breakp. enrich.	0
Linked to chrs	
Purity, ploidy	0.69, 3.99

	f45210d3−9e66−4f5e−bef1−5ee5547cc893
Cancer type	Breast−AdenoCA
Position	12:15014157−131294963
Type	With other complex events
Interleaved intrachr. SVs	8
Total SVs (intrachr. + transl.)	19
SV types	DEL: 2; DUP: 0; h2hINV: 3; t2tINV: 3; TRA: 11
SVs in sample	179
Oscillating CN (2 and 3 states)	6, 10
CN segments	22
FDR fragment joints	0.8082862
FDR chr. breakp. enrich.	0
Linked to chrs	
Purity, ploidy	0.84, 3.82

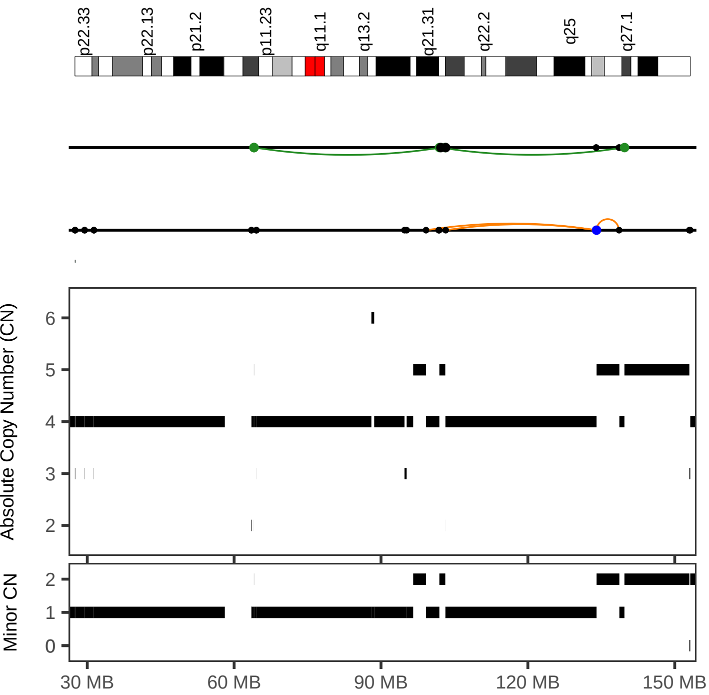

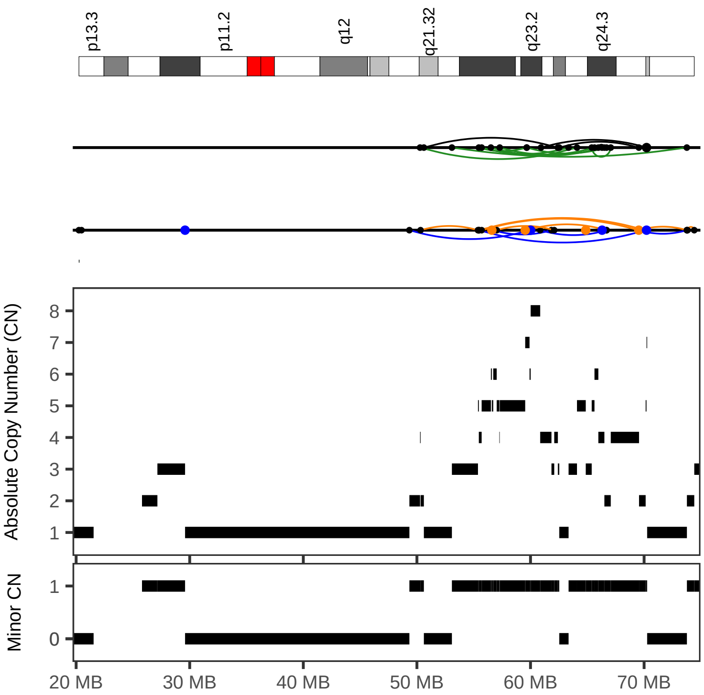

	f45210d3−9e66−4f5e−bef1−5ee5547cc893
Cancer type	Breast−AdenoCA
Position	X:64048649−139724918
Type	With other complex events
Interleaved intrachr. SVs	6
Total SVs (intrachr. + transl.)	12
SV types	DEL: 3; DUP: 0; h2hINV: 1; t2tINV: 2; TRA: 6
SVs in sample	179
Oscillating CN (2 and 3 states)	6, 12
CN segments	19
FDR fragment joints	0.6776251
FDR chr. breakp. enrich.	0
Linked to chrs	
Purity, ploidy	0.84, 3.82

	f51af6db−2655−47fb−9ffc−83a503a728ea
Cancer type	Breast−AdenoCA
Position	17:49337625−73767807
Type	With other complex events
Interleaved intrachr. SVs	23
Total SVs (intrachr. + transl.)	31
SV types	DEL: 6; DUP: 5; h2hINV: 4; t2tINV: 8; TRA: 8
SVs in sample	49
Oscillating CN (2 and 3 states)	5, 5
CN segments	35
FDR fragment joints	0.6776251
FDR chr. breakp. enrich.	0
Linked to chrs	
Purity, ploidy	0.87, 2.04

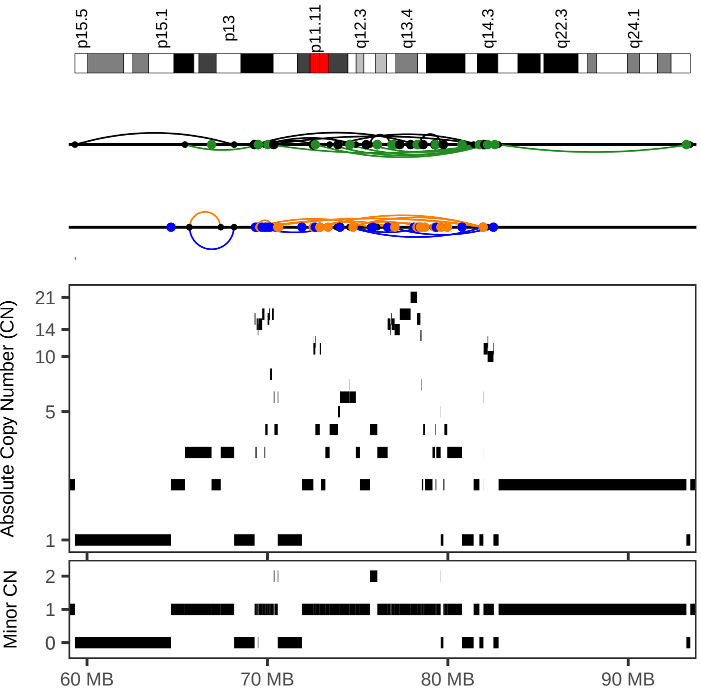

	f6e1ec78−5ad9−4879−9b7e−262d17b166ad
Cancer type	Breast−AdenoCA
Position	11:59328137−82524424
Type	With other complex events
Interleaved intrachr. SVs	32
Total SVs (intrachr. + transl.)	108
SV types	DEL: 12; DUP: 6; h2hINV: 8; t2tINV: 6; TRA: 76
SVs in sample	320
Oscillating CN (2 and 3 states)	4, 5
CN segments	77
FDR fragment joints	0.8298498
FDR chr. breakp. enrich.	0
Linked to chrs	3:20060433−168725244;
Purity, ploidy	0.53, 2.23

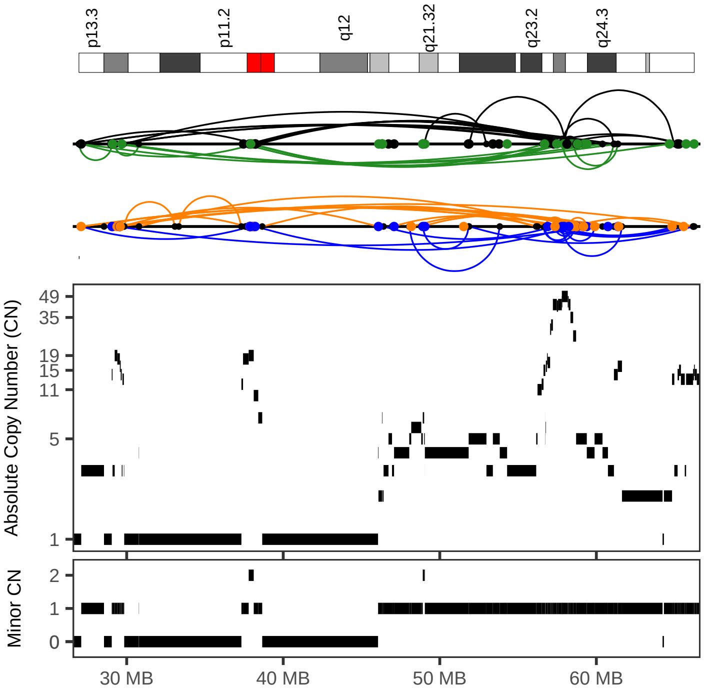

	f6e1ec78−5ad9−4879−9b7e−262d17b166ad
Cancer type	Breast−AdenoCA
Position	17:26949478−66253676
Type	With other complex events
Interleaved intrachr. SVs	77
Total SVs (intrachr. + transl.)	161
SV types	DEL: 19; DUP: 20; h2hINV: 21; t2tINV: 17; TRA: 84
SVs in sample	320
Oscillating CN (2 and 3 states)	4, 5
CN segments	81
FDR fragment joints	0.988335
FDR chr. breakp. enrich.	0
Linked to chrs	3:20060433−168725244;
Purity, ploidy	0.53, 2.23

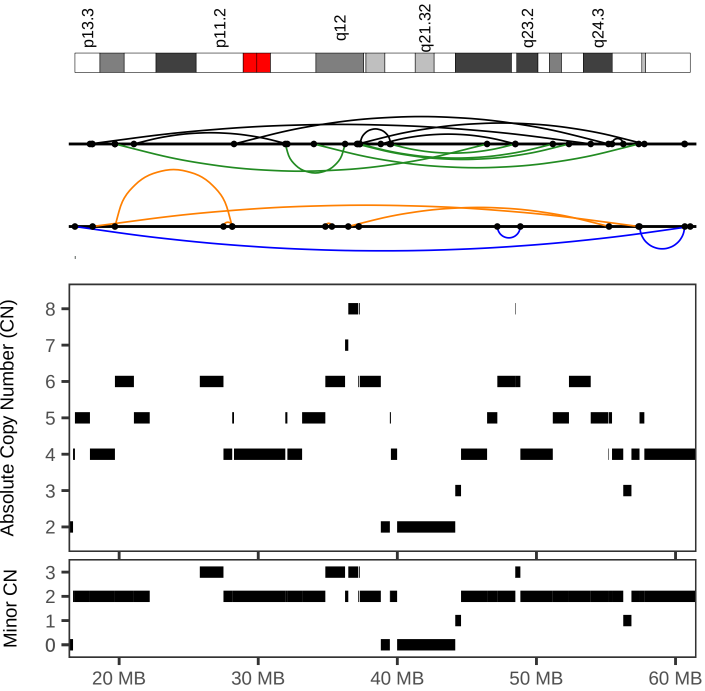

	CGP_donor_1451426
Cancer type	Breast−DCIS
Position	17:17901920−60667439
Type	With other complex events
Interleaved intrachr. SVs	19
Total SVs (intrachr. + transl.)	19
SV types	DEL: 4; DUP: 2; h2hINV: 6; t2tINV: 7; TRA: 0
SVs in sample	52
Oscillating CN (2 and 3 states)	6, 11
CN segments	37
FDR fragment joints	0.615458
FDR chr. breakp. enrich.	0
Linked to chrs	
Purity, ploidy	0.42, 3.49

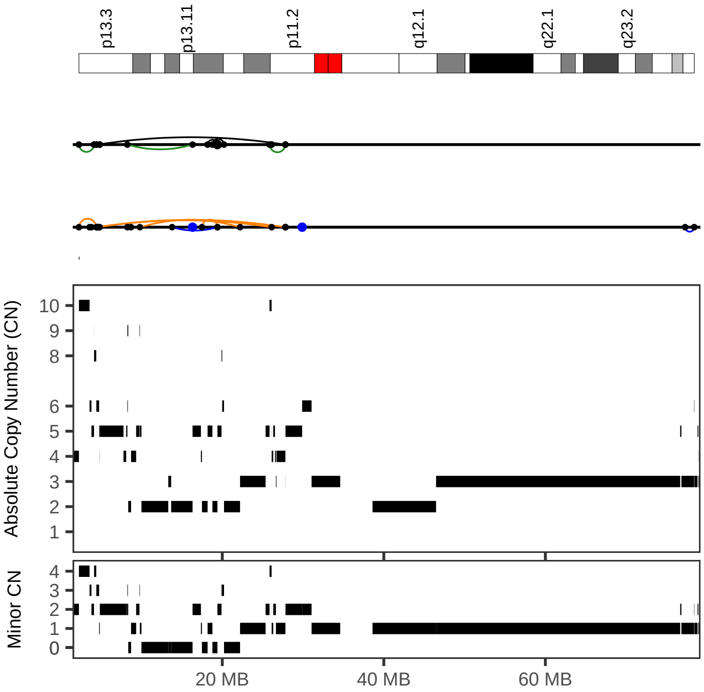

	CGP_donor_1114930
Cancer type	Breast−DCIS
Position	16:8249679−22198632
Type	With other complex events
Interleaved intrachr. SVs	7
Total SVs (intrachr. + transl.)	9
SV types	DEL: 3; DUP: 1; h2hINV: 2; t2tINV: 1; TRA: 2
SVs in sample	33
Oscillating CN (2 and 3 states)	4, 6
CN segments	20
FDR fragment joints	0.9723381
FDR chr. breakp. enrich.	0
Linked to chrs	
Purity, ploidy	0.56, 3.33

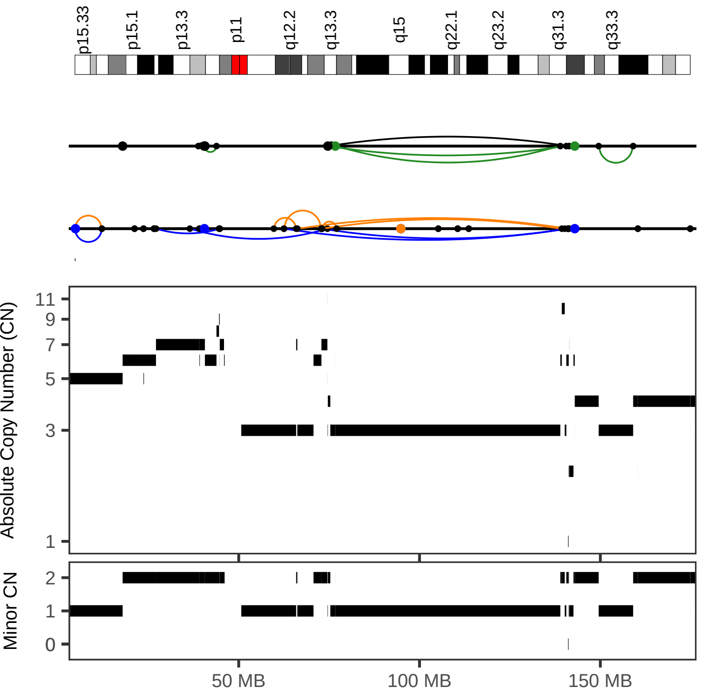

CGP_donor_1234120	
Cancer type	Breast−LobularCA
Position	5:27117220−142566044
Type	With other complex events
Interleaved intrachr. SVs	15
Total SVs (intrachr. + transl.)	23
SV types	DEL: 7; DUP: 4; h2hINV: 2; t2tINV: 2; TRA: 8
SVs in sample	344
Oscillating CN (2 and 3 states)	4, 7
CN segments	32
FDR fragment joints	0.7861024
FDR chr. breakp. enrich.	0
Linked to chrs	11:62942614−98446661;
Purity, ploidy	0.39, 2.94

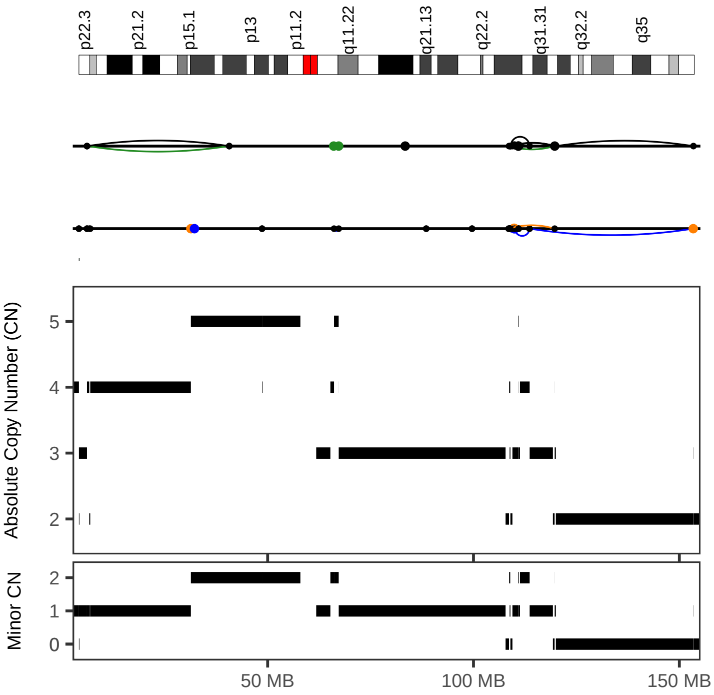

CGP_donor_1234120	
Cancer type	Breast−LobularCA
Position	7:108549083−153423652
Type	With other complex events
Interleaved intrachr. SVs	20
Total SVs (intrachr. + transl.)	24
SV types	DEL: 3; DUP: 8; h2hINV: 6; t2tINV: 3; TRA: 4
SVs in sample	344
Oscillating CN (2 and 3 states)	5, 7
CN segments	22
FDR fragment joints	0.8298498
FDR chr. breakp. enrich.	0
Linked to chrs	
Purity, ploidy	0.39, 2.94

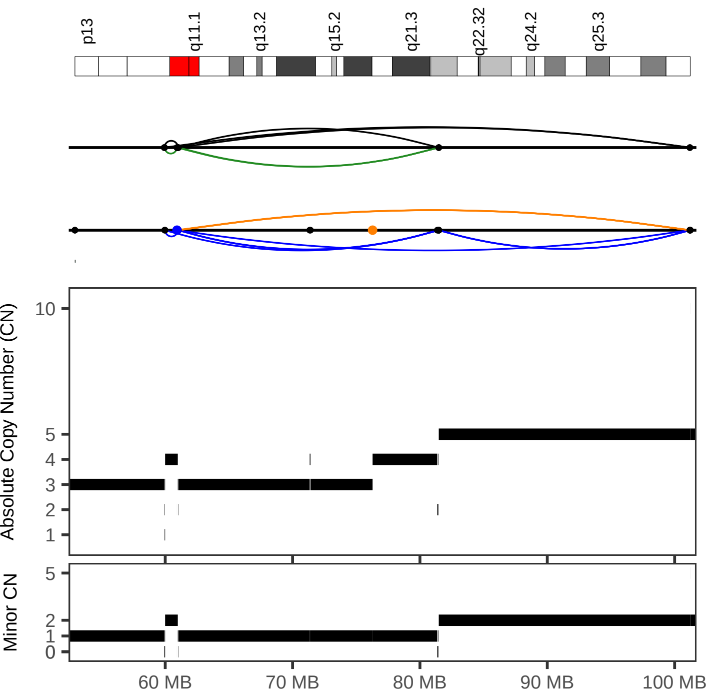

CGP_donor_1234120	
Cancer type	Breast−LobularCA
Position	15:59922904−101226389
Type	With other complex events
Interleaved intrachr. SVs	18
Total SVs (intrachr. + transl.)	20
SV types	DEL: 4; DUP: 7; h2hINV: 4; t2tINV: 3; TRA: 2
SVs in sample	344
Oscillating CN (2 and 3 states)	4, 6
CN segments	18
FDR fragment joints	0.662962
FDR chr. breakp. enrich.	0
Linked to chrs	
Purity, ploidy	0.39, 2.94

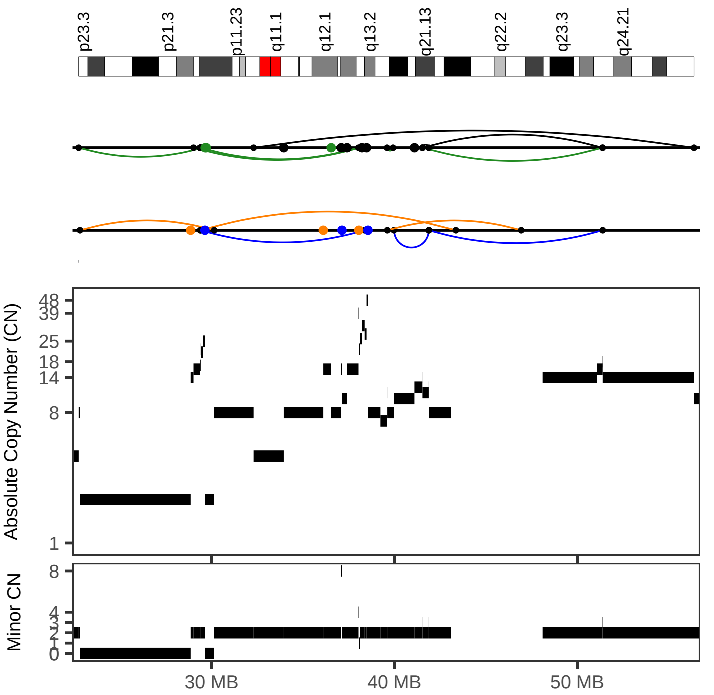

CGP_donor_1353429	
Cancer type	Breast−LobularCA
Position	8:22728682−56379069
Type	With other complex events
Interleaved intrachr. SVs	16
Total SVs (intrachr. + transl.)	31
SV types	DEL: 3; DUP: 3; h2hINV: 4; t2tINV: 6; TRA: 15
SVs in sample	83
Oscillating CN (2 and 3 states)	4, 5
CN segments	45
FDR fragment joints	0.615458
FDR chr. breakp. enrich.	0
Linked to chrs	
Purity, ploidy	0.27, 4.13

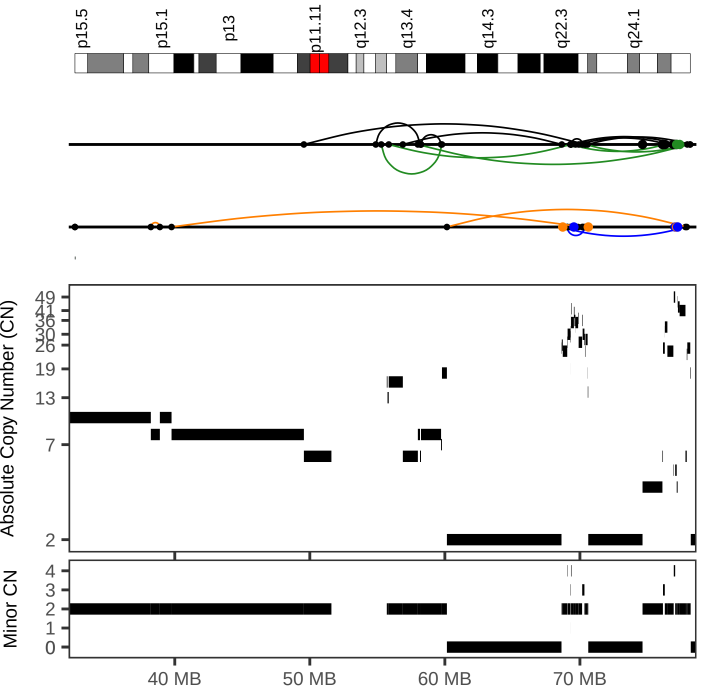

CGP_donor_1353429	
Cancer type	Breast−LobularCA
Position	11:39761387−78170654
Type	With other complex events
Interleaved intrachr. SVs	21
Total SVs (intrachr. + transl.)	36
SV types	DEL: 3; DUP: 5; h2hINV: 7; t2tINV: 6; TRA: 15
SVs in sample	83
Oscillating CN (2 and 3 states)	4, 5
CN segments	55
FDR fragment joints	0.641841
FDR chr. breakp. enrich.	0
Linked to chrs	
Purity, ploidy	0.27, 4.13

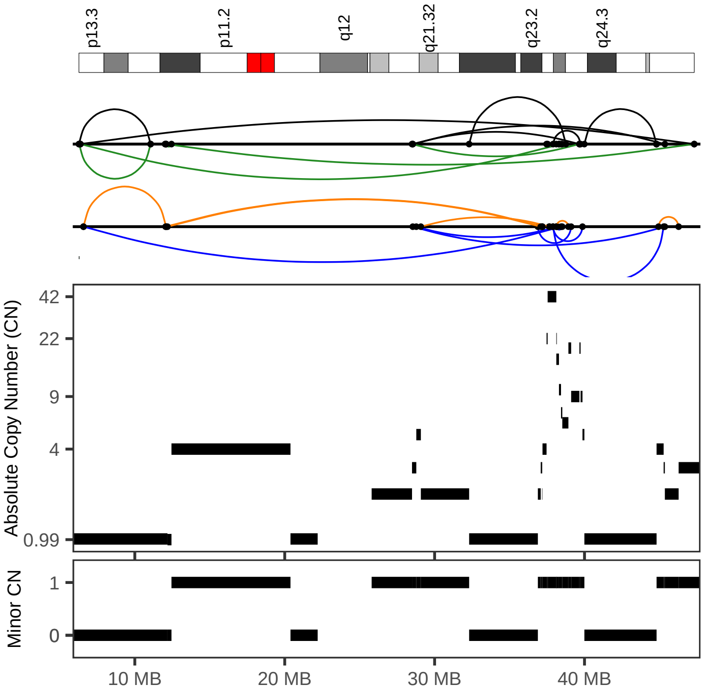

CGP_donor_1230724	
Cancer type	Breast−LobularCA
Position	17:6272054−47320751
Type	With other complex events
Interleaved intrachr. SVs	28
Total SVs (intrachr. + transl.)	28
SV types	DEL: 6; DUP: 8; h2hINV: 9; t2tINV: 5; TRA: 0
SVs in sample	33
Oscillating CN (2 and 3 states)	4, 5
CN segments	30
FDR fragment joints	0.8066159
FDR chr. breakp. enrich.	0
Linked to chrs	
Purity, ploidy	0.53, 2

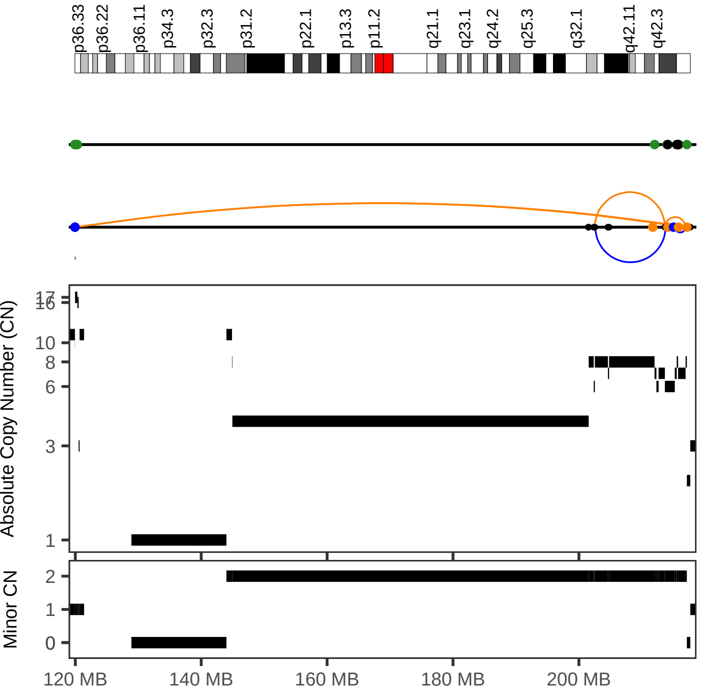

2d29a4ac−98e7−4663−9dd6−5681bc32ac2e	
Cancer type	Breast−LobularCA
Position	1:202357683−216952427
Type	With other complex events
Interleaved intrachr. SVs	8
Total SVs (intrachr. + transl.)	18
SV types	DEL: 3; DUP: 5; h2hINV: 0; t2tINV: 0; TRA: 10
SVs in sample	105
Oscillating CN (2 and 3 states)	5, 11
CN segments	12
FDR fragment joints	0.6776251
FDR chr. breakp. enrich.	0
Linked to chrs	11:68233404−80019930;
Purity, ploidy	0.5, 2.06

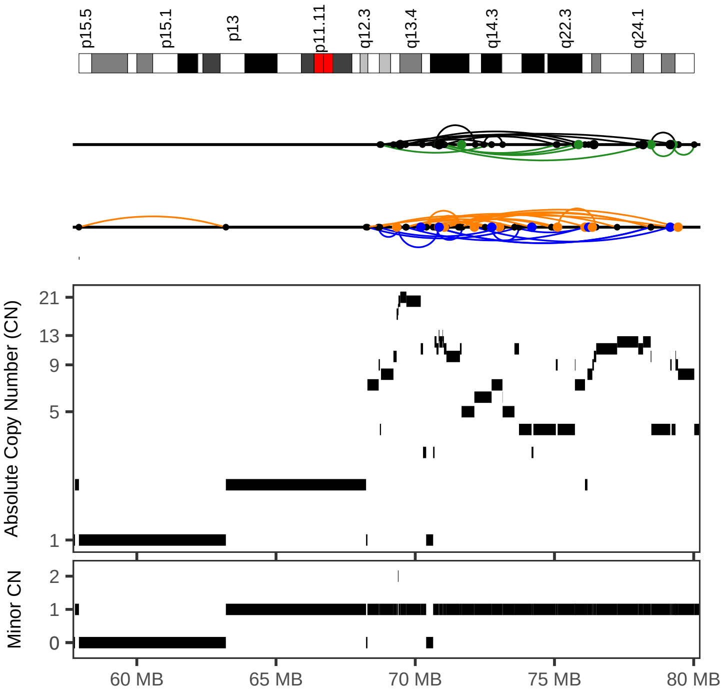

2d29a4ac−98e7−4663−9dd6−5681bc32ac2e	
Cancer type	Breast−LobularCA
Position	11:68233404−80019931
Type	With other complex events
Interleaved intrachr. SVs	48
Total SVs (intrachr. + transl.)	74
SV types	DEL: 17; DUP: 13; h2hINV: 10; t2tINV: 8; TRA: 26
SVs in sample	105
Oscillating CN (2 and 3 states)	5, 10
CN segments	57
FDR fragment joints	0.615458
FDR chr. breakp. enrich.	0
Linked to chrs	
Purity, ploidy	0.5, 2.06

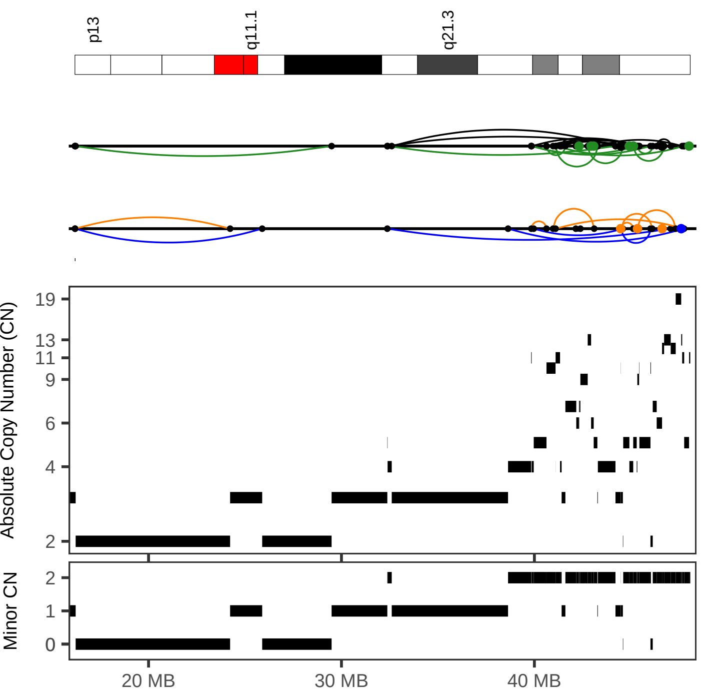

	ef4cbd38−bc79−4d60−a715−647edd2ebe9e
Cancer type	Breast−LobularCA
Position	21:32373739−47753333
Type	With other complex events
Interleaved intrachr. SVs	33
Total SVs (intrachr. + transl.)	44
SV types	DEL: 7; DUP: 5; h2hINV: 11; t2tINV: 10; TRA: 11
SVs in sample	134
Oscillating CN (2 and 3 states)	5, 8
CN segments	45
FDR fragment joints	0.615458
FDR chr. breakp. enrich.	0
Linked to chrs	8:42881572−135695929;13:19943737−57122358
Purity, ploidy	0.77, 3.15

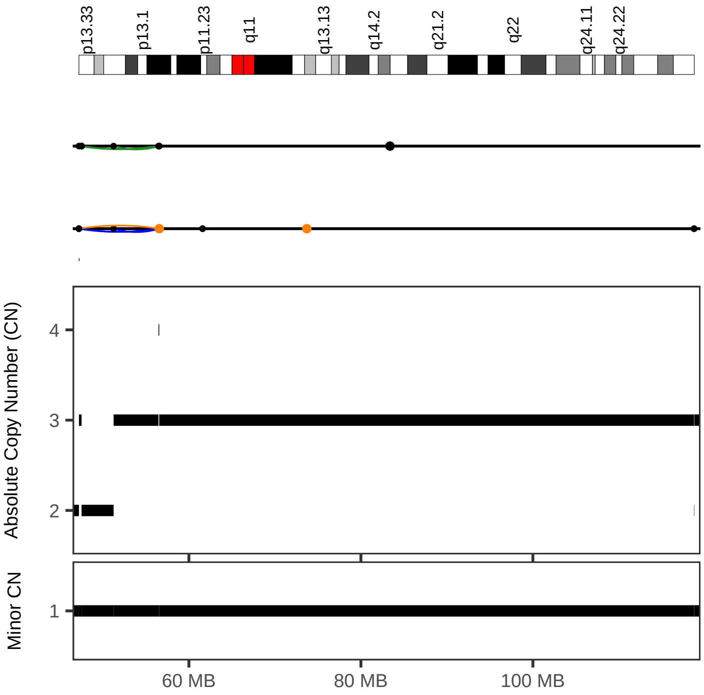

	00bca18c−b3d4−45a3−8f19−034cc40449a4
Cancer type	Cervix−SCC
Position	12:47208673−56550744
Type	Canonical without polyploidization
Interleaved intrachr. SVs	7
Total SVs (intrachr. + transl.)	7
SV types	DEL: 1; DUP: 3; h2hINV: 0; t2tINV: 3; TRA: 0
SVs in sample	168
Oscillating CN (2 and 3 states)	4, 6
CN segments	6
FDR fragment joints	0.615458
FDR chr. breakp. enrich.	0.07
Linked to chrs	
Purity, ploidy	0.76, 2.78

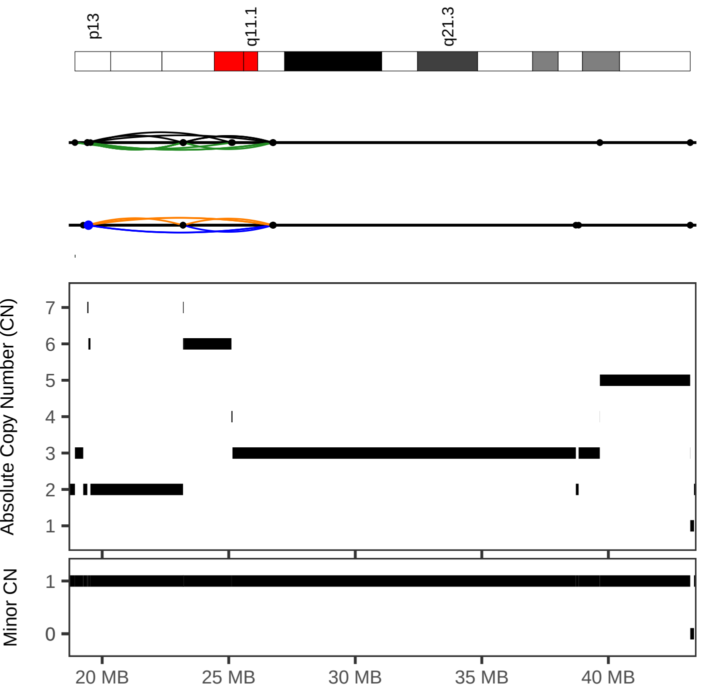

	8a4da4f0−30f2−497e−8a7c−988fd6b813cb
Cancer type	Cervix−SCC
Position	21:18922646−26760076
Type	With other complex events
Interleaved intrachr. SVs	19
Total SVs (intrachr. + transl.)	20
SV types	DEL: 4; DUP: 3; h2hINV: 5; t2tINV: 7; TRA: 1
SVs in sample	117
Oscillating CN (2 and 3 states)	4, 5
CN segments	11
FDR fragment joints	0.8572806
FDR chr. breakp. enrich.	0
Linked to chrs	
Purity, ploidy	0.79, 1.97

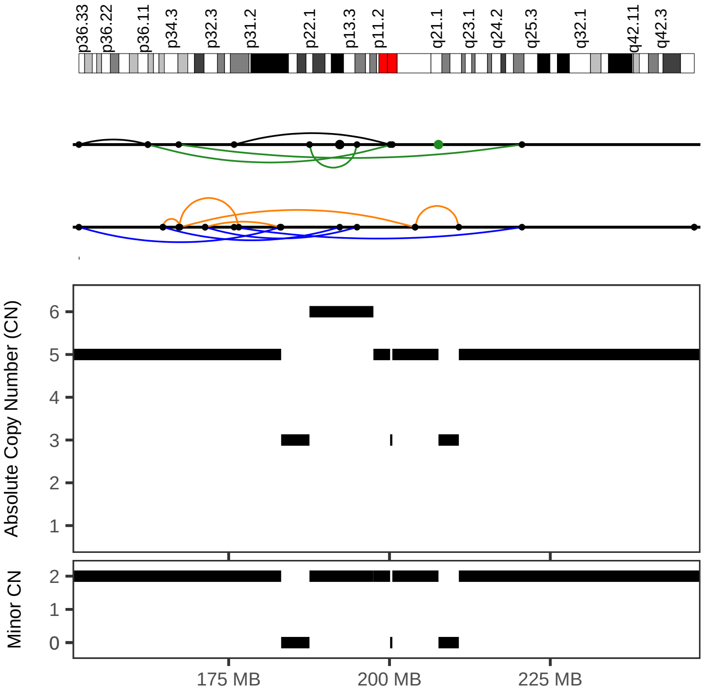

	eb59cf69−1997−41b9−bf69−69ad7da292a1
Cancer type	Cervix−SCC
Position	1:151719688−220597001
Type	After polyploidization
Interleaved intrachr. SVs	14
Total SVs (intrachr. + transl.)	16
SV types	DEL: 5; DUP: 4; h2hINV: 2; t2tINV: 3; TRA: 2
SVs in sample	63
Oscillating CN (2 and 3 states)	5, 5
CN segments	8
FDR fragment joints	0.9501265
FDR chr. breakp. enrich.	0
Linked to chrs	
Purity, ploidy	0.4, 3.41

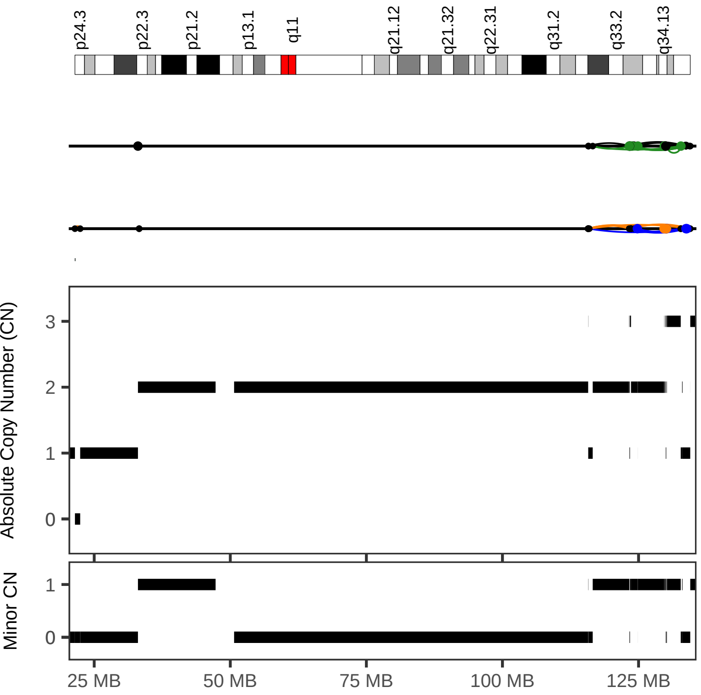

	01a92062−967a−4900−8dc7−a5ecd3b3f8e2
Cancer type	CNS−GBM
Position	9:115736690−134498722
Type	With other complex events
Interleaved intrachr. SVs	27
Total SVs (intrachr. + transl.)	44
SV types	DEL: 7; DUP: 4; h2hINV: 6; t2tINV: 10; TRA: 17
SVs in sample	98
Oscillating CN (2 and 3 states)	5, 9
CN segments	27
FDR fragment joints	0.8874881
FDR chr. breakp. enrich.	0
Linked to chrs	
Purity, ploidy	0.2, 2.05

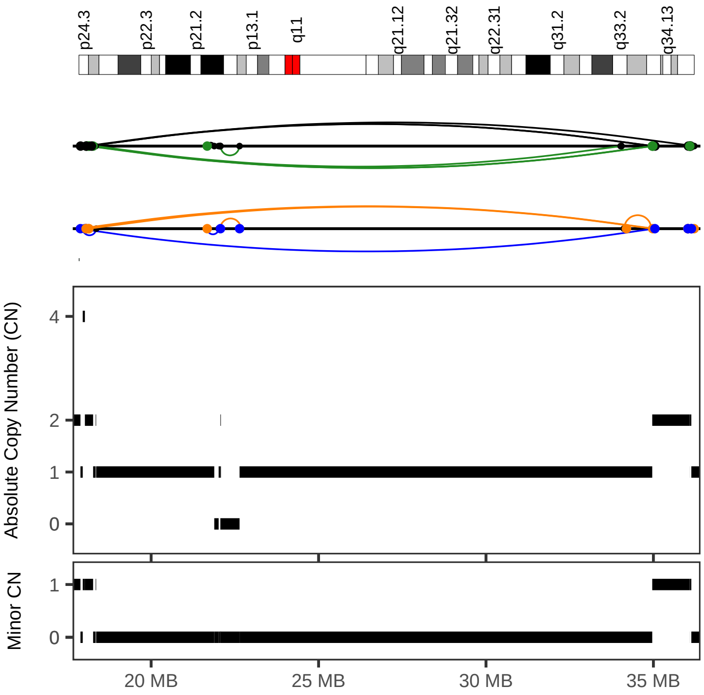

	0798dbe2−1914−427c−a2fe−2a865d0d6eda
Cancer type	CNS−GBM
Position	9:17843117−36219343
Type	With other complex events
Interleaved intrachr. SVs	12
Total SVs (intrachr. + transl.)	51
SV types	DEL: 2; DUP: 2; h2hINV: 5; t2tINV: 3; TRA: 39
SVs in sample	209
Oscillating CN (2 and 3 states)	5, 9
CN segments	19
FDR fragment joints	0.9284301
FDR chr. breakp. enrich.	0
Linked to chrs	1:8762210−205453074;10:5388962−135052040
Purity, ploidy	0.8, 2.05

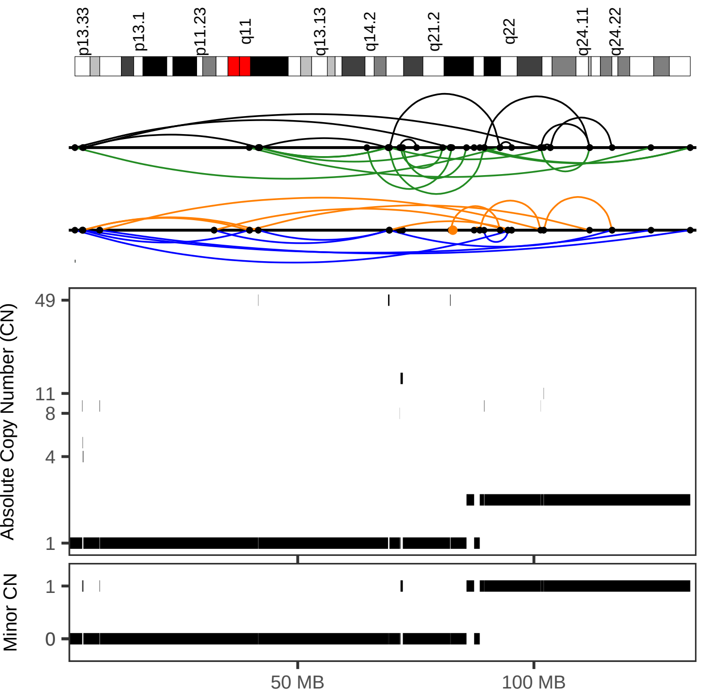

	2c5fa2d4−8e35−42e4−8bca−9fb3371a19c8
Cancer type	CNS−GBM
Position	12:2835782−133092445
Type	With other complex events
Interleaved intrachr. SVs	44
Total SVs (intrachr. + transl.)	45
SV types	DEL: 10; DUP: 9; h2hINV: 12; t2tINV: 13; TRA: 1
SVs in sample	77
Oscillating CN (2 and 3 states)	5, 5
CN segments	28
FDR fragment joints	0.9161301
FDR chr. breakp. enrich.	0
Linked to chrs	
Purity, ploidy	0.87, 1.89

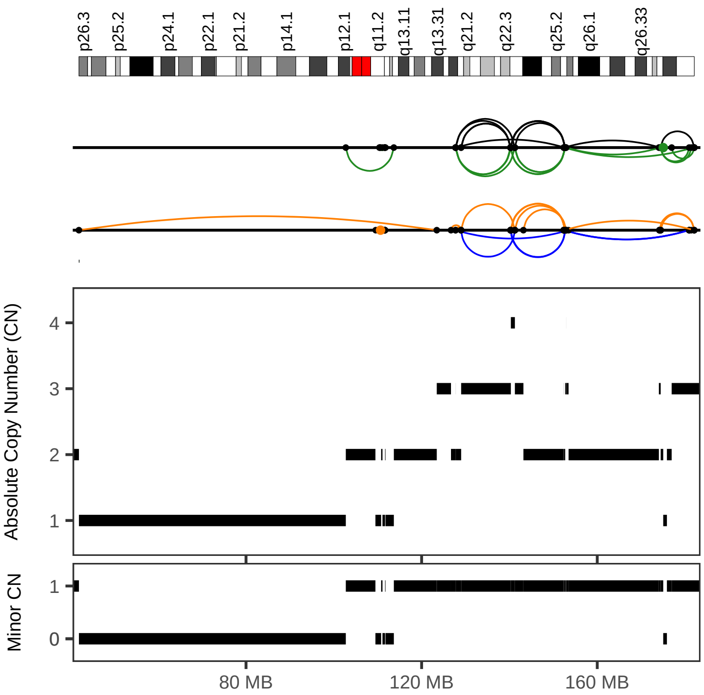

	386b629e−fab1−4033−b088−45d6eeb4a13e
Cancer type	CNS−GBM
Position	3:126688674−182097458
Type	With other complex events
Interleaved intrachr. SVs	54
Total SVs (intrachr. + transl.)	55
SV types	DEL: 14; DUP: 11; h2hINV: 15; t2tINV: 14; TRA: 1
SVs in sample	114
Oscillating CN (2 and 3 states)	5, 18
CN segments	18
FDR fragment joints	0.8833361
FDR chr. breakp. enrich.	0
Linked to chrs	
Purity, ploidy	0.82, 1.88

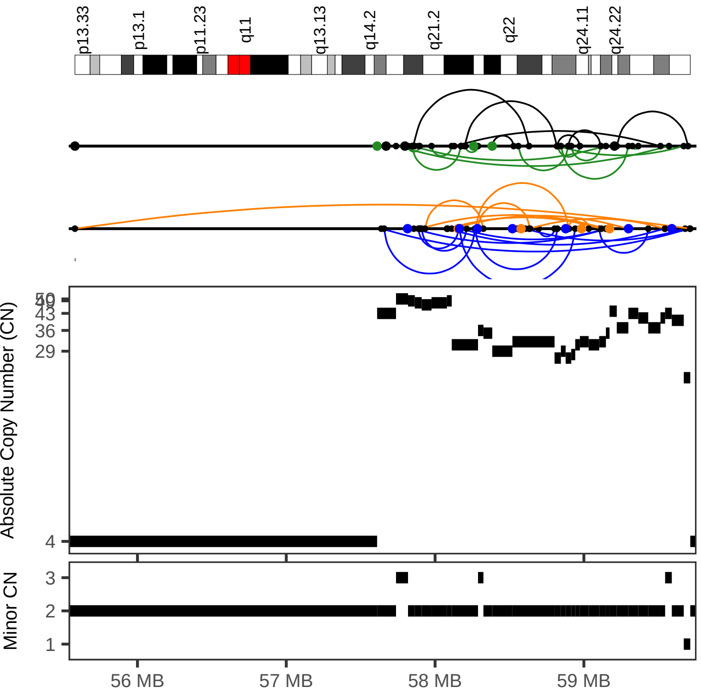

	521ea765−1bd1−423d−a75d−091243df37a9
Cancer type	CNS−GBM
Position	12:57657463−59700261
Type	With other complex events
Interleaved intrachr. SVs	36
Total SVs (intrachr. + transl.)	52
SV types	DEL: 8; DUP: 10; h2hINV: 7; t2tINV: 11; TRA: 16
SVs in sample	90
Oscillating CN (2 and 3 states)	4, 6
CN segments	30
FDR fragment joints	0.6776251
FDR chr. breakp. enrich.	0
Linked to chrs	
Purity, ploidy	0.84, 4

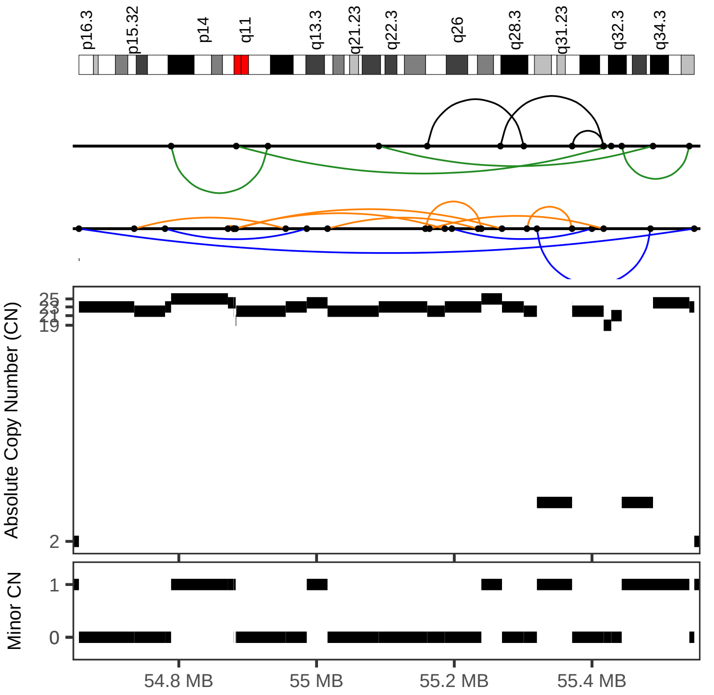

	5c984433−33cf−42fc−b3ba−511efcdcab19
Cancer type	CNS−GBM
Position	4:54735188−55541337
Type	With other complex events
Interleaved intrachr. SVs	17
Total SVs (intrachr. + transl.)	17
SV types	DEL: 7; DUP: 3; h2hINV: 3; t2tINV: 4; TRA: 0
SVs in sample	107
Oscillating CN (2 and 3 states)	4, 7
CN segments	25
FDR fragment joints	0.6776251
FDR chr. breakp. enrich.	0
Linked to chrs	
Purity, ploidy	0.73, 2

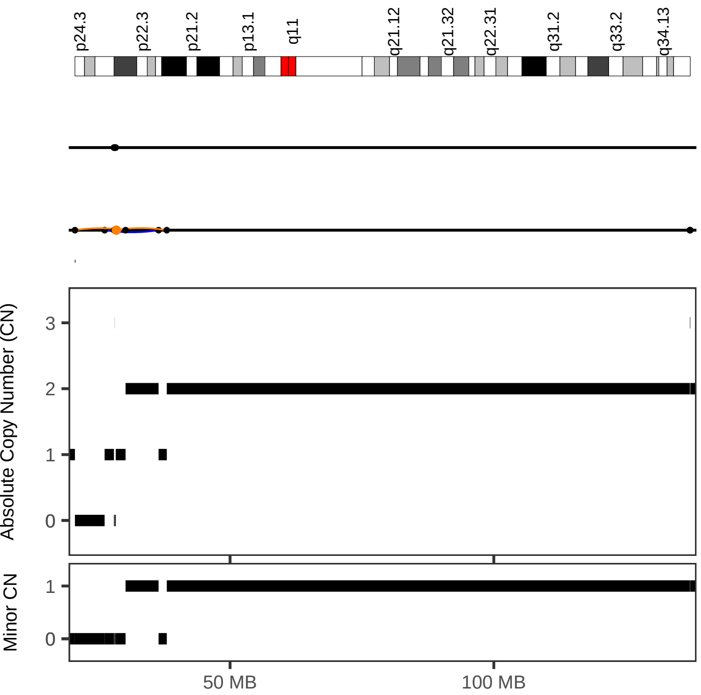

	5c984433−33cf−42fc−b3ba−511efcdcab19
Cancer type	CNS−GBM
Position	9:20593576−38007522
Type	With other complex events
Interleaved intrachr. SVs	6
Total SVs (intrachr. + transl.)	7
SV types	DEL: 2; DUP: 2; h2hINV: 1; t2tINV: 1; TRA: 1
SVs in sample	107
Oscillating CN (2 and 3 states)	5, 11
CN segments	11
FDR fragment joints	0.7735152
FDR chr. breakp. enrich.	0.21
Linked to chrs	
Purity, ploidy	0.73, 2

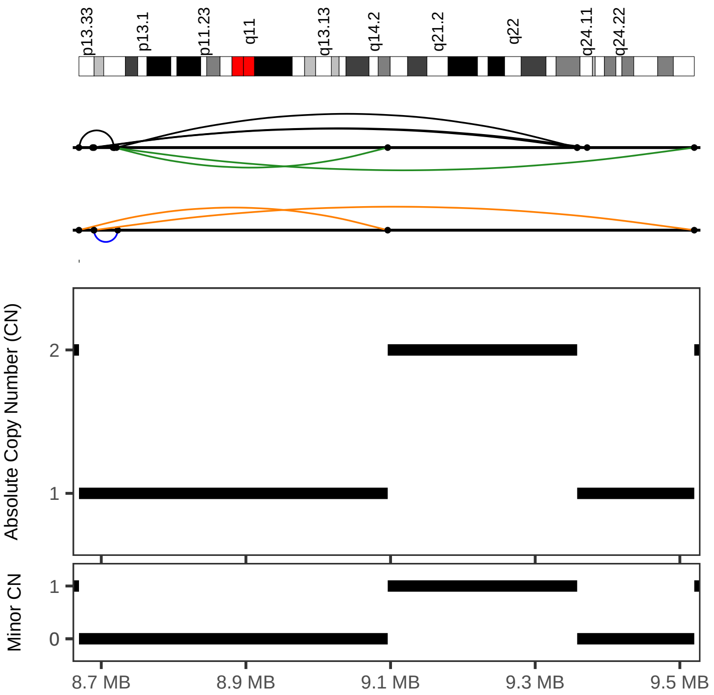

	737b35e1−d668−4fce−9b6e−76946c7952b6
Cancer type	CNS−GBM
Position	12:8669087−9519921
Type	Canonical without polyploidization
Interleaved intrachr. SVs	9
Total SVs (intrachr. + transl.)	9
SV types	DEL: 2; DUP: 1; h2hINV: 4; t2tINV: 2; TRA: 0
SVs in sample	173
Oscillating CN (2 and 3 states)	5, 5
CN segments	5
FDR fragment joints	0.6776251
FDR chr. breakp. enrich.	0.9
Linked to chrs	
Purity, ploidy	0.93, 1.97

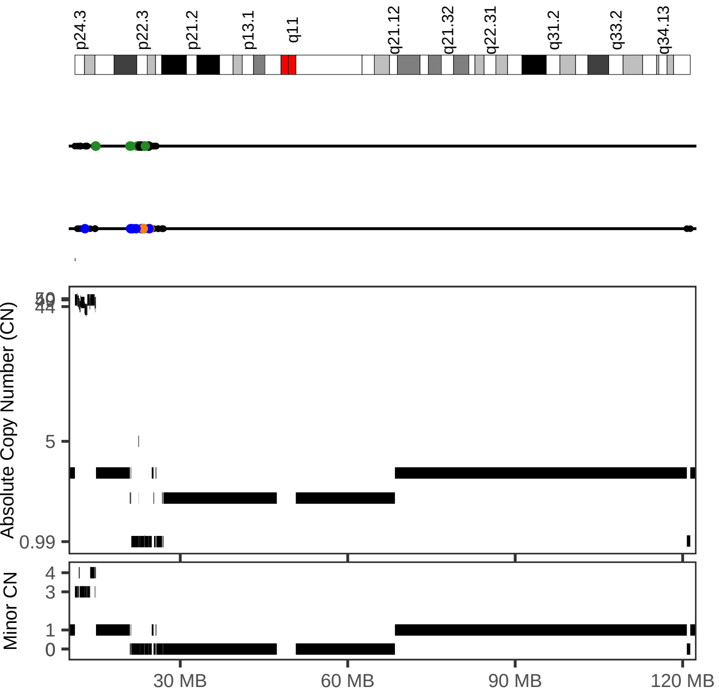

74139255−a635−4c87−814d−3dd04ed630a8	
Cancer type	CNS−GBM
Position	9:21130542−26979040
Type	With other complex events
Interleaved intrachr. SVs	17
Total SVs (intrachr. + transl.)	35
SV types	DEL: 4; DUP: 3; h2hINV: 6; t2tINV: 4; TRA: 18
SVs in sample	291
Oscillating CN (2 and 3 states)	5, 9
CN segments	21
FDR fragment joints	0.8653243
FDR chr. breakp. enrich.	0
Linked to chrs	15:34114739−74116043;
Purity, ploidy	0.38, 3.59

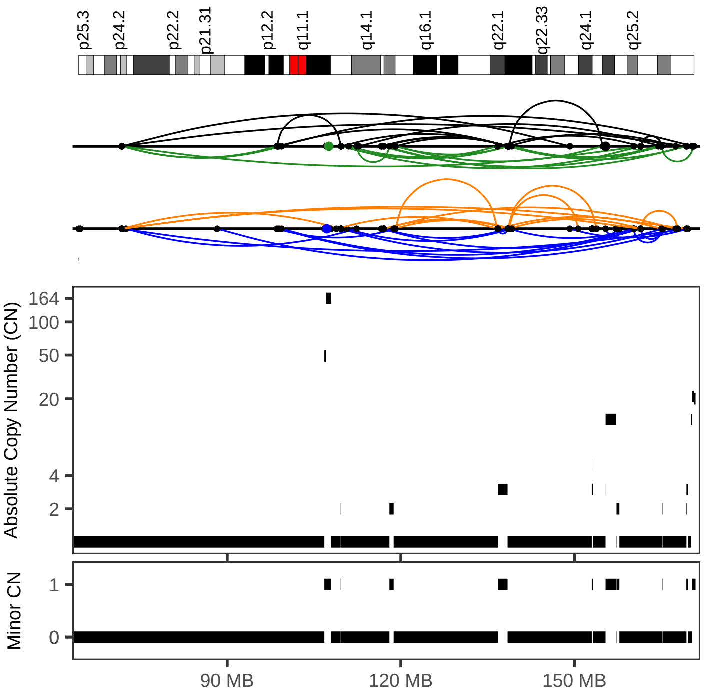

93ed7a2b−b0cb−4a84−871f−5c34a0b6a640	
Cancer type	CNS−GBM
Position	6:71789357−170660594
Type	With other complex events
Interleaved intrachr. SVs	63
Total SVs (intrachr. + transl.)	66
SV types	DEL: 13; DUP: 17; h2hINV: 15; t2tINV: 18; TRA: 3
SVs in sample	175
Oscillating CN (2 and 3 states)	6, 8
CN segments	27
FDR fragment joints	0.845361
FDR chr. breakp. enrich.	0
Linked to chrs	12:44665062−82704890;
Purity, ploidy	0.92, 1.83

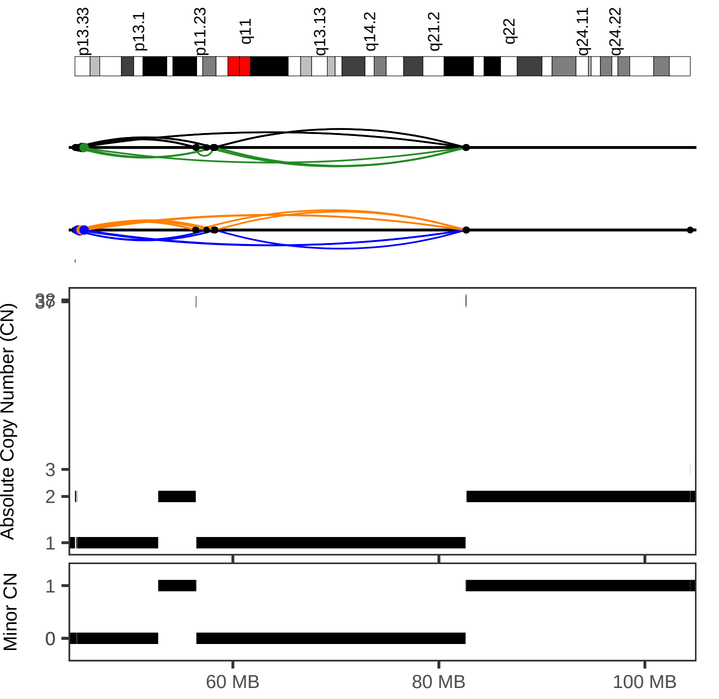

93ed7a2b−b0cb−4a84−871f−5c34a0b6a640	
Cancer type	CNS−GBM
Position	12:44665062−82704891
Type	With other complex events
Interleaved intrachr. SVs	47
Total SVs (intrachr. + transl.)	52
SV types	DEL: 12; DUP: 12; h2hINV: 13; t2tINV: 10; TRA: 5
SVs in sample	175
Oscillating CN (2 and 3 states)	6, 6
CN segments	12
FDR fragment joints	0.958094
FDR chr. breakp. enrich.	0
Linked to chrs	6:71789357−170660593;
Purity, ploidy	0.92, 1.83

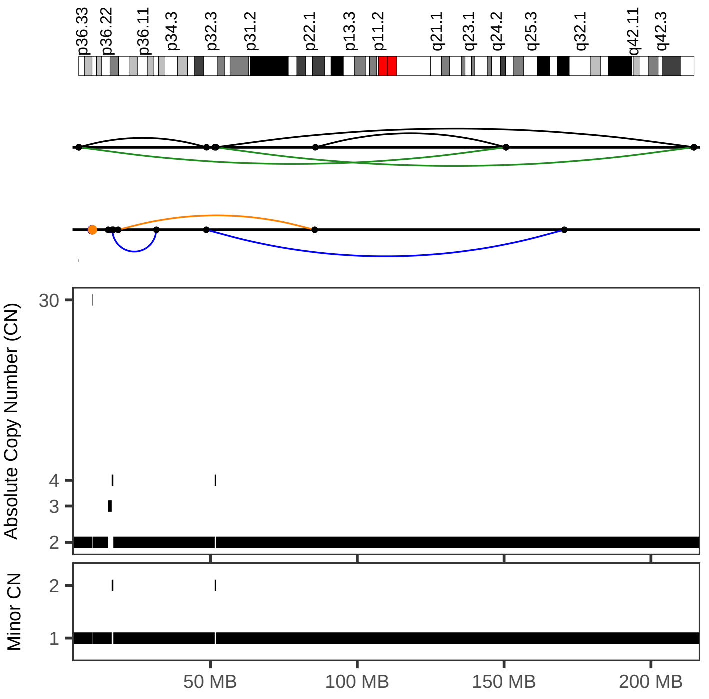

a2ac9937−f351−4d78−9261−264bf6c21e0c	
Cancer type	CNS−GBM
Position	1:5166554−214663754
Type	With other complex events
Interleaved intrachr. SVs	9
Total SVs (intrachr. + transl.)	11
SV types	DEL: 1; DUP: 3; h2hINV: 3; t2tINV: 2; TRA: 2
SVs in sample	102
Oscillating CN (2 and 3 states)	4, 5
CN segments	8
FDR fragment joints	0.8653243
FDR chr. breakp. enrich.	0.51
Linked to chrs	
Purity, ploidy	0.75, 1.94

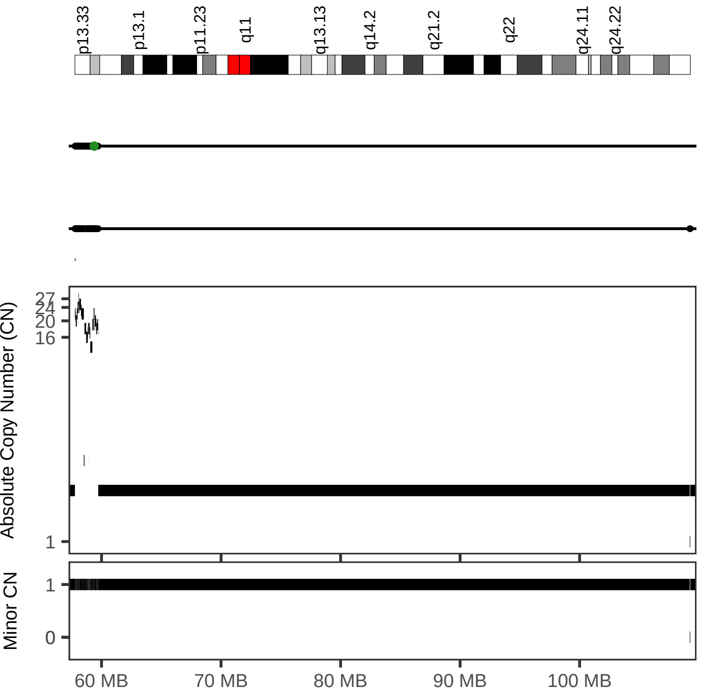

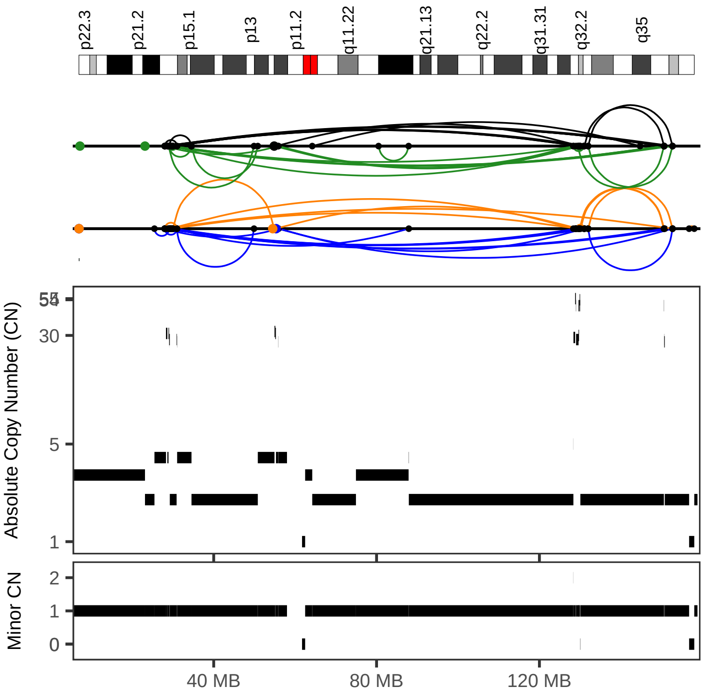

	b37d944c−c65c−46d6−bb8b−3eb37cb85b68
Cancer type	CNS−GBM
Position	12:57781868−59706359
Type	With other complex events
Interleaved intrachr. SVs	81
Total SVs (intrachr. + transl.)	82
SV types	DEL: 22; DUP: 14; h2hINV: 23; t2tINV: 22; TRA: 1
SVs in sample	130
Oscillating CN (2 and 3 states)	4, 5
CN segments	29
FDR fragment joints	0.6444888
FDR chr. breakp. enrich.	0
Linked to chrs	
Purity, ploidy	0.89, 1.95

	be3a7ef3−34ed−40e1−9d9c−187940596b26
Cancer type	CNS−GBM
Position	7:25442783−152694109
Type	With other complex events
Interleaved intrachr. SVs	60
Total SVs (intrachr. + transl.)	63
SV types	DEL: 13; DUP: 16; h2hINV: 15; t2tINV: 16; TRA: 3
SVs in sample	93
Oscillating CN (2 and 3 states)	4, 5
CN segments	39
FDR fragment joints	0.9599662
FDR chr. breakp. enrich.	0
Linked to chrs	
Purity, ploidy	0.71, 1.97

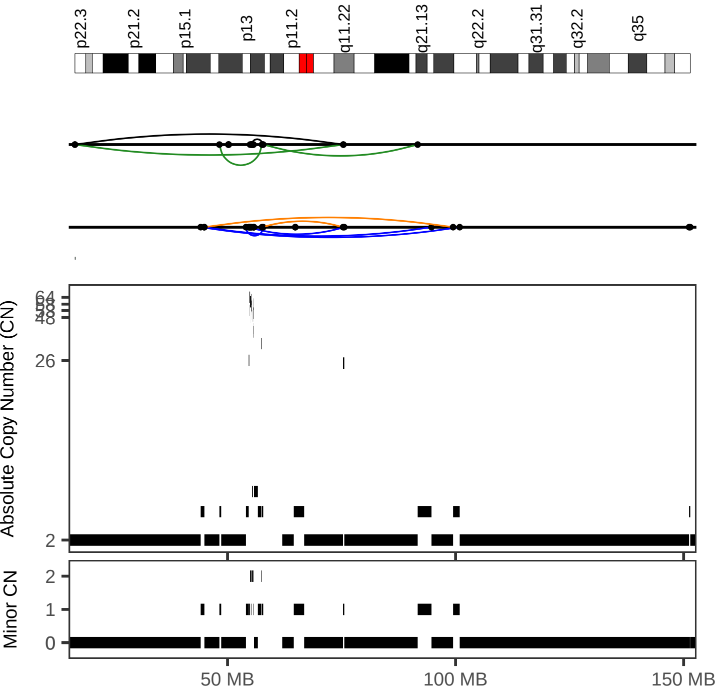

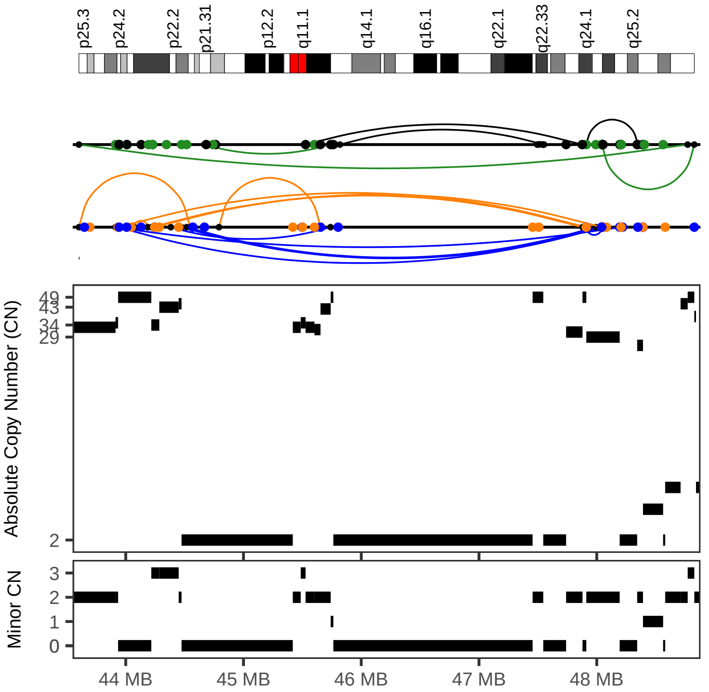

	bf339349−062f−4ea9−a0b2−d87d3a21099e
Cancer type	CNS−GBM
Position	7:16533917−100901571
Type	With other complex events
Interleaved intrachr. SVs	19
Total SVs (intrachr. + transl.)	19
SV types	DEL: 3; DUP: 7; h2hINV: 4; t2tINV: 5; TRA: 0
SVs in sample	338
Oscillating CN (2 and 3 states)	6, 6
CN segments	36
FDR fragment joints	0.7158792
FDR chr. breakp. enrich.	0.46
Linked to chrs	
Purity, ploidy	0.83, 1.97

	c931f3bd−74c5−4ebd−bc0f−c7c6becd25ab
Cancer type	CNS−GBM
Position	6:43602359−48828217
Type	With other complex events
Interleaved intrachr. SVs	16
Total SVs (intrachr. + transl.)	106
SV types	DEL: 5; DUP: 6; h2hINV: 2; t2tINV: 3; TRA: 90
SVs in sample	241
Oscillating CN (2 and 3 states)	4, 4
CN segments	26
FDR fragment joints	0.7498275
FDR chr. breakp. enrich.	0
Linked to chrs	
Purity, ploidy	0.7, 4.05

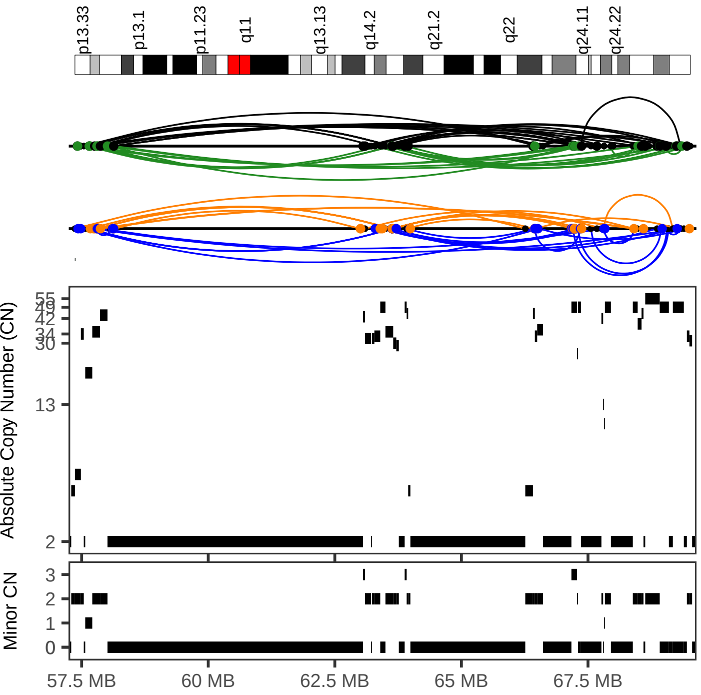

	c931f3bd−74c5−4ebd−bc0f−c7c6becd25ab
Cancer type	CNS−GBM
Position	12:57367262−69519356
Type	With other complex events
Interleaved intrachr. SVs	79
Total SVs (intrachr. + transl.)	181
SV types	DEL: 16; DUP: 20; h2hINV: 24; t2tINV: 19; TRA: 102
SVs in sample	241
Oscillating CN (2 and 3 states)	4, 5
CN segments	46
FDR fragment joints	1
FDR chr. breakp. enrich.	0
Linked to chrs	
Purity, ploidy	0.7, 4.05

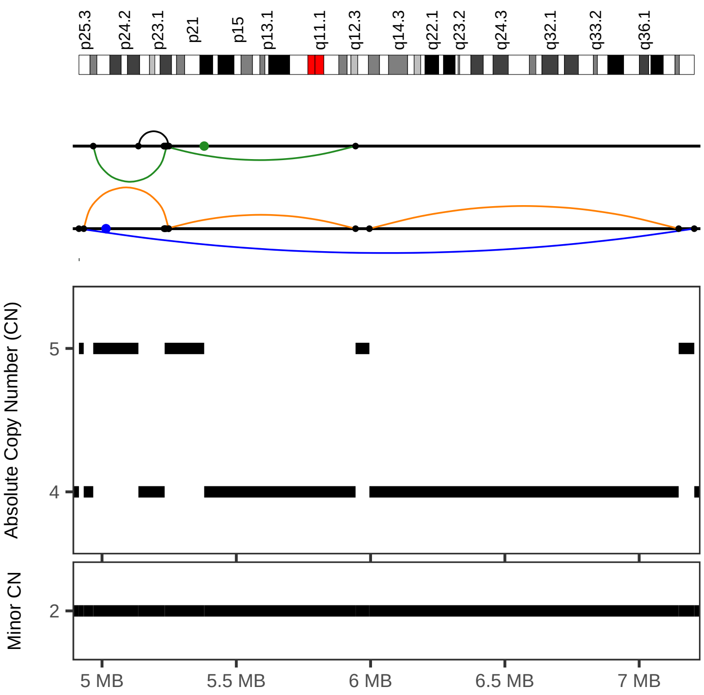

	de7b7cac−f094−4d59−8651−e991e34ea093
Cancer type	CNS−GBM
Position	2:4931914−5944046
Type	After polyploidization
Interleaved intrachr. SVs	6
Total SVs (intrachr. + transl.)	8
SV types	DEL: 2; DUP: 1; h2hINV: 1; t2tINV: 2; TRA: 2
SVs in sample	105
Oscillating CN (2 and 3 states)	6, 6
CN segments	6
FDR fragment joints	1
FDR chr. breakp. enrich.	0.36
Linked to chrs	
Purity, ploidy	0.56, 3.59

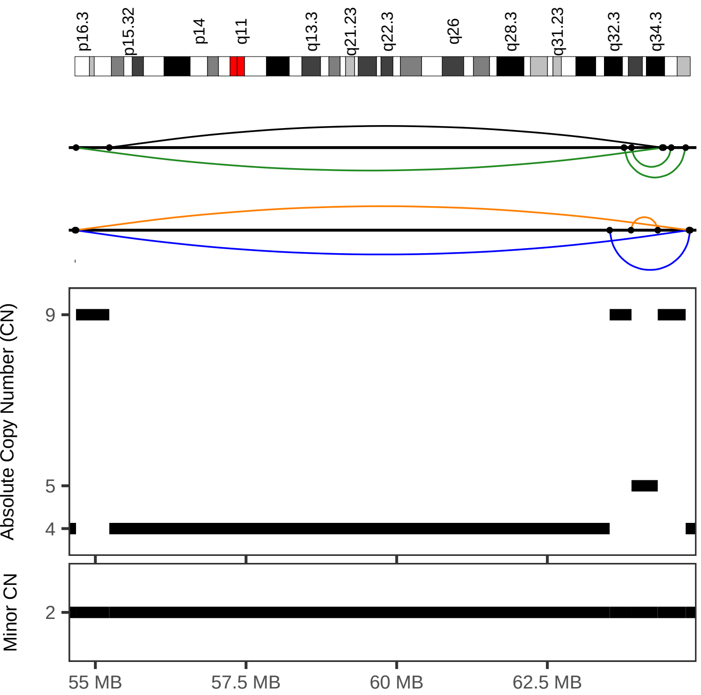

	de7b7cac−f094−4d59−8651−e991e34ea093
Cancer type	CNS−GBM
Position	4:54660832−64870856
Type	With other complex events
Interleaved intrachr. SVs	8
Total SVs (intrachr. + transl.)	8
SV types	DEL: 2; DUP: 2; h2hINV: 1; t2tINV: 3; TRA: 0
SVs in sample	105
Oscillating CN (2 and 3 states)	4, 7
CN segments	7
FDR fragment joints	0.8653243
FDR chr. breakp. enrich.	0.89
Linked to chrs	
Purity, ploidy	0.56, 3.59

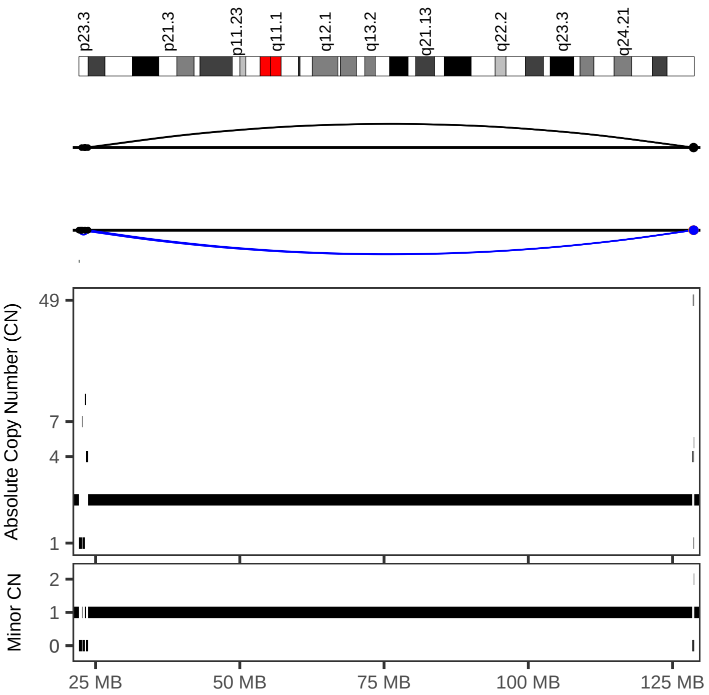

	ICGC_MB131
Cancer type	CNS−Medullo
Position	8:22113923−128760777
Type	With other complex events
Interleaved intrachr. SVs	13
Total SVs (intrachr. + transl.)	19
SV types	DEL: 1; DUP: 6; h2hINV: 4; t2tINV: 2; TRA: 6
SVs in sample	39
Oscillating CN (2 and 3 states)	5, 6
CN segments	25
FDR fragment joints	0.615458
FDR chr. breakp. enrich.	0
Linked to chrs	3:38417904−38895250;
Purity, ploidy	0.96, 2.05

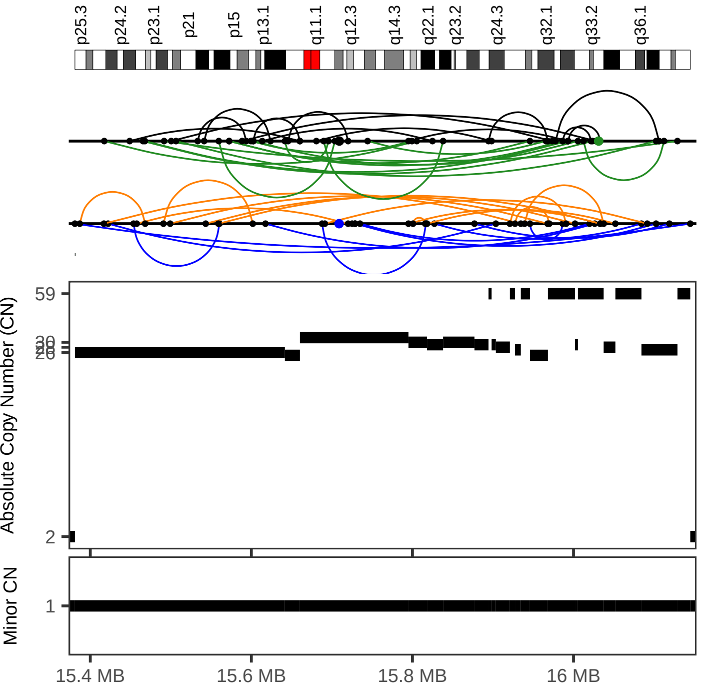

	ICGC_MB177
Cancer type	CNS−Medullo
Position	2:15386910−16129075
Type	With other complex events
Interleaved intrachr. SVs	51
Total SVs (intrachr. + transl.)	54
SV types	DEL: 14; DUP: 9; h2hINV: 14; t2tINV: 14; TRA: 3
SVs in sample	59
Oscillating CN (2 and 3 states)	4, 7
CN segments	21
FDR fragment joints	0.8481558
FDR chr. breakp. enrich.	0
Linked to chrs	
Purity, ploidy	0.99, 1.97

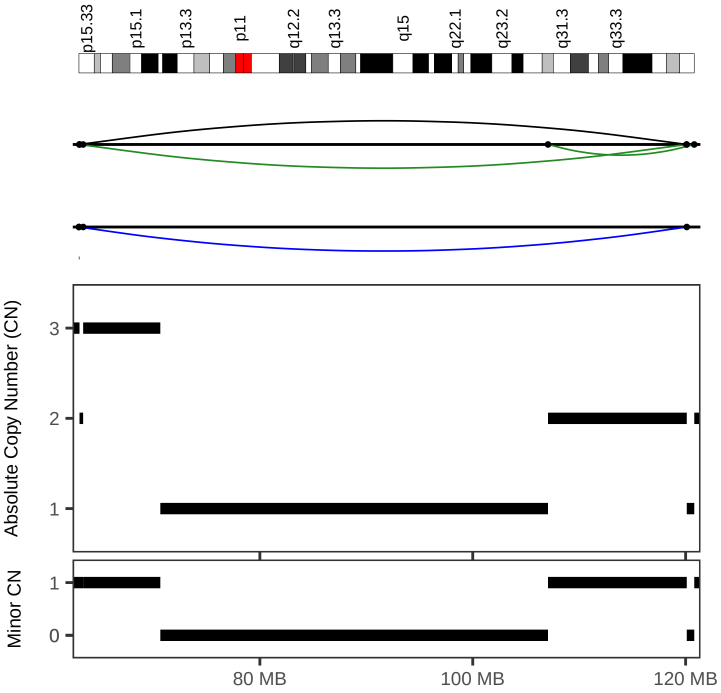

	ICGC_MB181
Cancer type	CNS−Medullo
Position	5:63000287−120810285
Type	With other complex events
Interleaved intrachr. SVs	6
Total SVs (intrachr. + transl.)	6
SV types	DEL: 1; DUP: 1; h2hINV: 2; t2tINV: 2; TRA: 0
SVs in sample	6
Oscillating CN (2 and 3 states)	4, 7
CN segments	7
FDR fragment joints	0.9284301
FDR chr. breakp. enrich.	0
Linked to chrs	
Purity, ploidy	0.97, 1.99

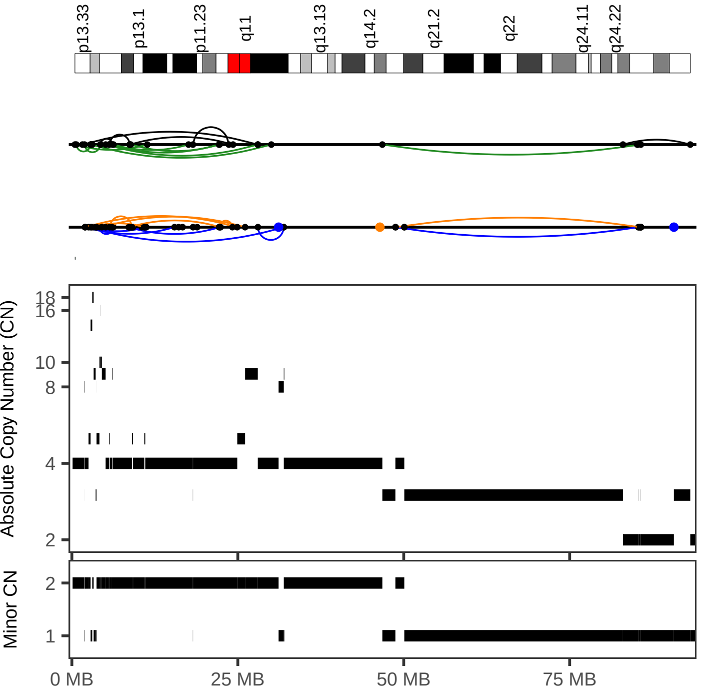

	ICGC_MB34
Cancer type	CNS−Medullo
Position	12:461707−31932605
Type	With other complex events
Interleaved intrachr. SVs	30
Total SVs (intrachr. + transl.)	31
SV types	DEL: 7; DUP: 9; h2hINV: 6; t2tINV: 8; TRA: 1
SVs in sample	242
Oscillating CN (2 and 3 states)	5, 10
CN segments	32
FDR fragment joints	0.8653243
FDR chr. breakp. enrich.	0
Linked to chrs	
Purity, ploidy	0.97, 3.62

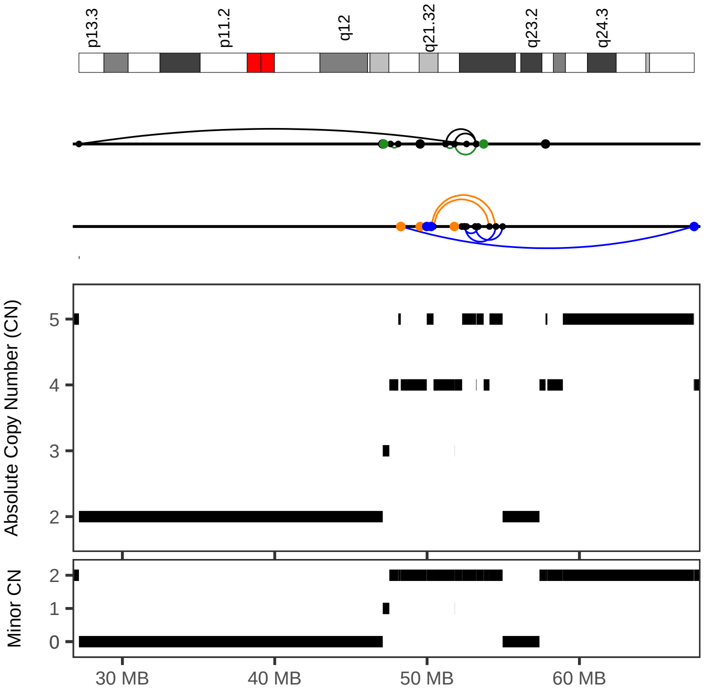

	ICGC_MB34
Cancer type	CNS−Medullo
Position	17:27146708−67502119
Type	With other complex events
Interleaved intrachr. SVs	12
Total SVs (intrachr. + transl.)	23
SV types	DEL: 4; DUP: 4; h2hINV: 3; t2tINV: 1; TRA: 11
SVs in sample	242
Oscillating CN (2 and 3 states)	6, 15
CN segments	20
FDR fragment joints	0.7861024
FDR chr. breakp. enrich.	0
Linked to chrs	3:80545898−151249120;
Purity, ploidy	0.97, 3.62

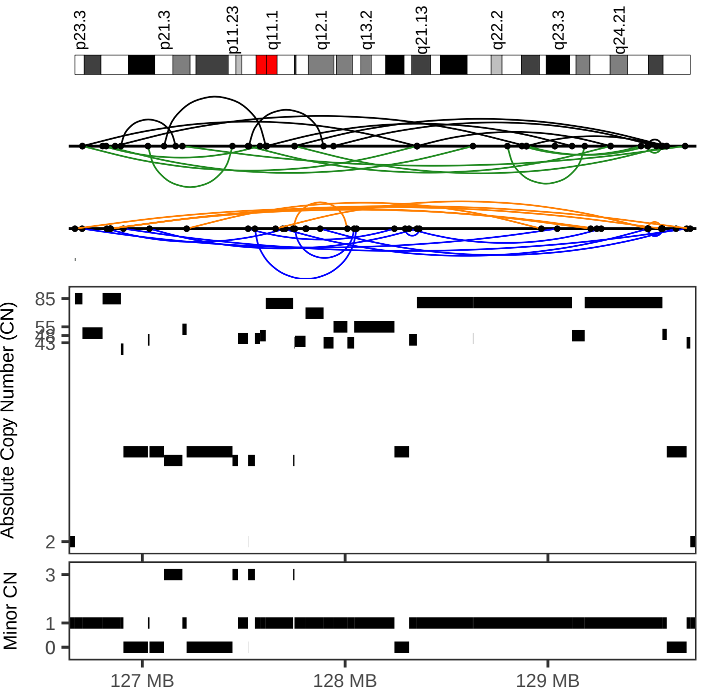

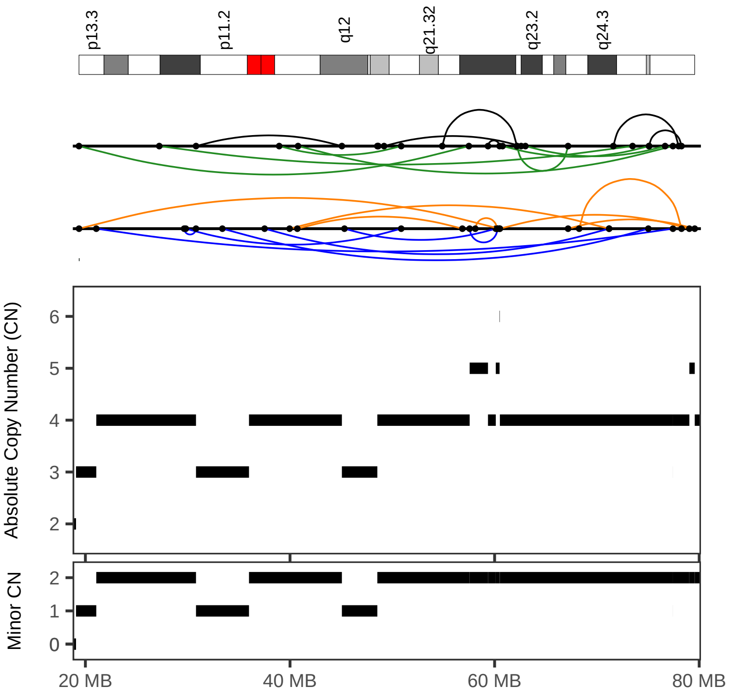

	ICGC_MB50
Cancer type	CNS−Medullo
Position	8:126667508−129701837
Type	With other complex events
Interleaved intrachr. SVs	43
Total SVs (intrachr. + transl.)	43
SV types	DEL: 8; DUP: 13; h2hINV: 11; t2tINV: 11; TRA: 0
SVs in sample	60
Oscillating CN (2 and 3 states)	4, 4
CN segments	36
FDR fragment joints	0.8609429
FDR chr. breakp. enrich.	0
Linked to chrs	
Purity, ploidy	0.95, 2.31

	ICGC_MB91
Cancer type	CNS−Medullo
Position	17:19371748−79563637
Type	With other complex events
Interleaved intrachr. SVs	27
Total SVs (intrachr. + transl.)	27
SV types	DEL: 7; DUP: 7; h2hINV: 6; t2tINV: 7; TRA: 0
SVs in sample	34
Oscillating CN (2 and 3 states)	6, 10
CN segments	15
FDR fragment joints	1
FDR chr. breakp. enrich.	0
Linked to chrs	
Purity, ploidy	0.86, 1.99

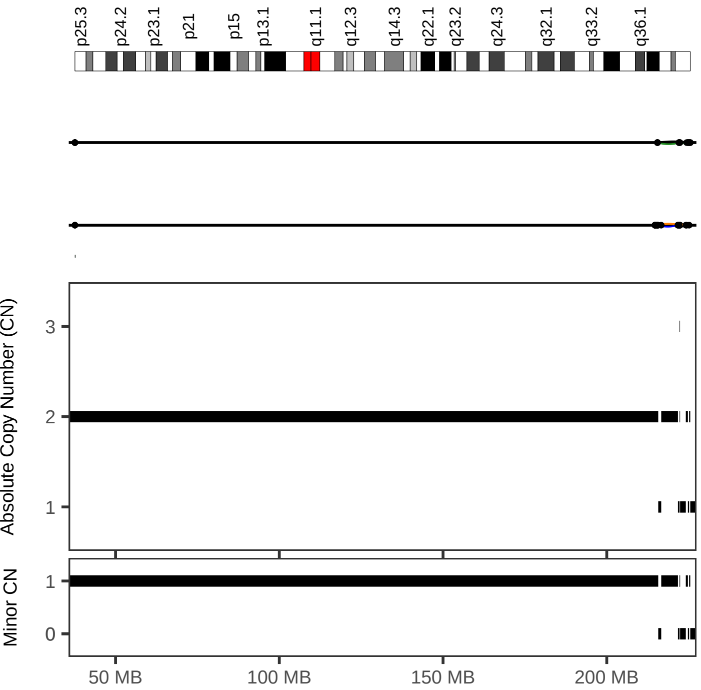

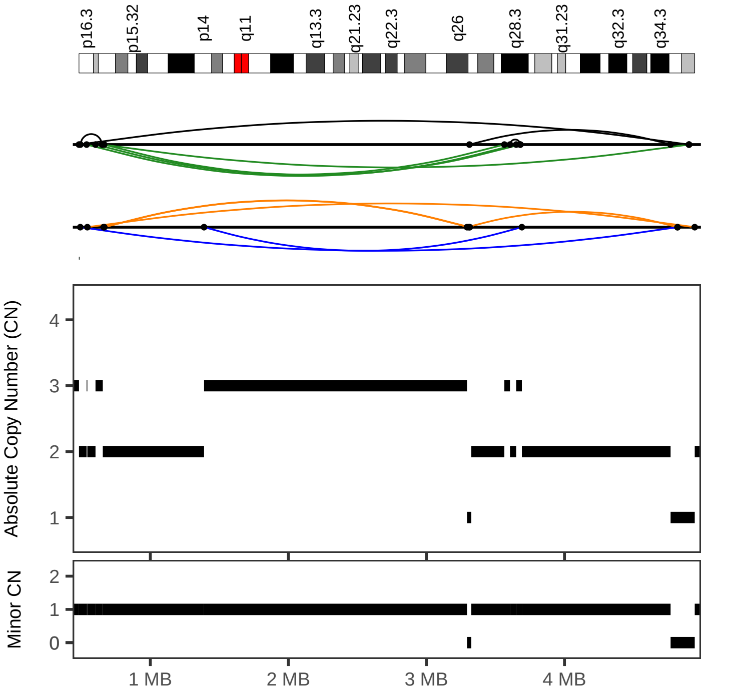

	6a0bcf0c−fa4c−4119−99d2−f722b781d20f
Cancer type	CNS−Oligo
Position	2:214706062−225494723
Type	With other complex events
Interleaved intrachr. SVs	16
Total SVs (intrachr. + transl.)	16
SV types	DEL: 4; DUP: 4; h2hINV: 5; t2tINV: 3; TRA: 0
SVs in sample	45
Oscillating CN (2 and 3 states)	6, 6
CN segments	11
FDR fragment joints	0.9501265
FDR chr. breakp. enrich.	0
Linked to chrs	
Purity, ploidy	0.21, 1.99

	70939245−41e0−4845−a473−5fef719b9828
Cancer type	CNS−Oligo
Position	4:483333−4943380
Type	With other complex events
Interleaved intrachr. SVs	14
Total SVs (intrachr. + transl.)	14
SV types	DEL: 4; DUP: 2; h2hINV: 4; t2tINV: 4; TRA: 0
SVs in sample	62
Oscillating CN (2 and 3 states)	6, 8
CN segments	14
FDR fragment joints	0.8988396
FDR chr. breakp. enrich.	0
Linked to chrs	
Purity, ploidy	0.8, 2.06

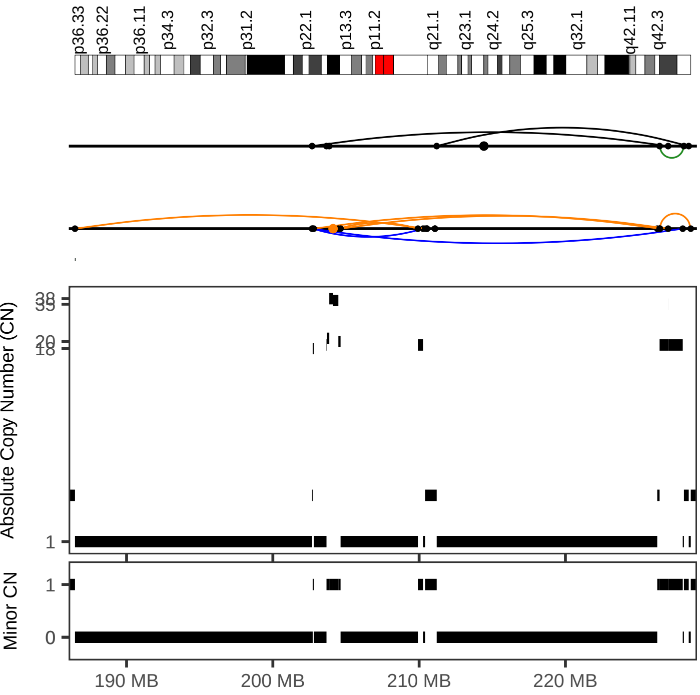

	f86fa219−34e9−4812−8edf−2b66d7219779
Cancer type	CNS−Oligo
Position	1:186471142−228569911
Type	With other complex events
Interleaved intrachr. SVs	11
Total SVs (intrachr. + transl.)	13
SV types	DEL: 5; DUP: 3; h2hINV: 2; t2tINV: 1; TRA: 2
SVs in sample	22
Oscillating CN (2 and 3 states)	4, 4
CN segments	22
FDR fragment joints	0.615458
FDR chr. breakp. enrich.	0
Linked to chrs	
Purity, ploidy	0.8, 2.01

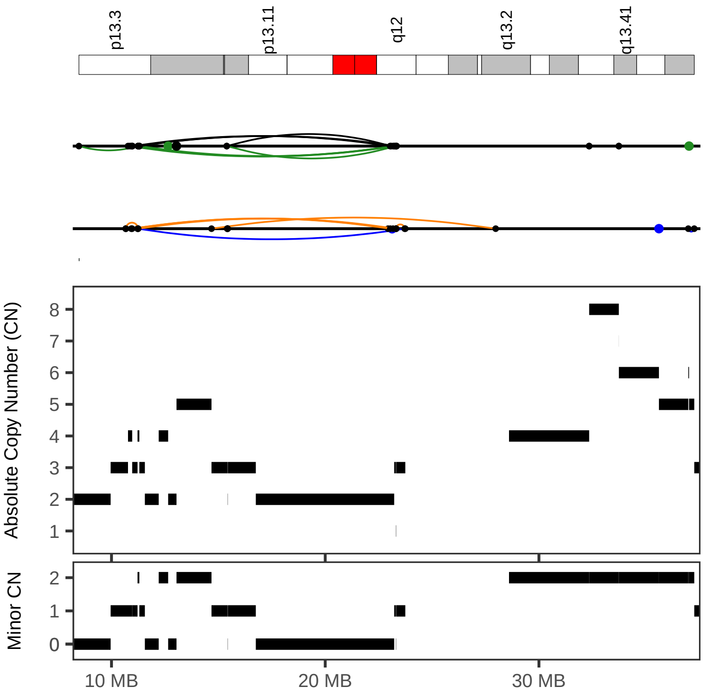

	5116e3b4−2bac−40f5−8046−b9c1783faaa5
Cancer type	ColoRect−AdenoCA
Position	19:8475496−27976857
Type	With other complex events
Interleaved intrachr. SVs	19
Total SVs (intrachr. + transl.)	21
SV types	DEL: 6; DUP: 2; h2hINV: 5; t2tINV: 6; TRA: 2
SVs in sample	322
Oscillating CN (2 and 3 states)	5, 7
CN segments	18
FDR fragment joints	0.6610257
FDR chr. breakp. enrich.	0
Linked to chrs	
Purity, ploidy	0.5, 3.09

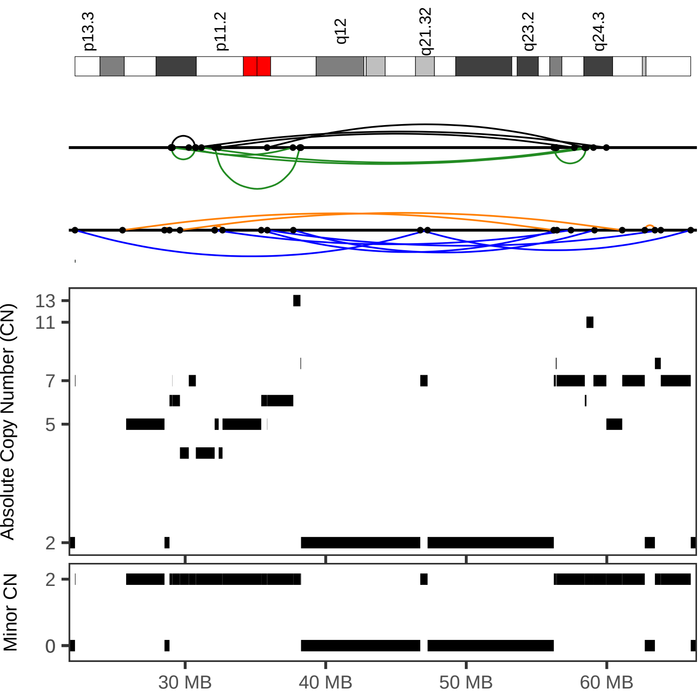

	6fa41234−9077−4d05−a295−6820f3bedf5b
Cancer type	ColoRect−AdenoCA
Position	17:22158710−65973469
Type	With other complex events
Interleaved intrachr. SVs	21
Total SVs (intrachr. + transl.)	21
SV types	DEL: 3; DUP: 6; h2hINV: 5; t2tINV: 7; TRA: 0
SVs in sample	74
Oscillating CN (2 and 3 states)	4, 5
CN segments	32
FDR fragment joints	0.7568568
FDR chr. breakp. enrich.	0
Linked to chrs	
Purity, ploidy	0.8, 2.22

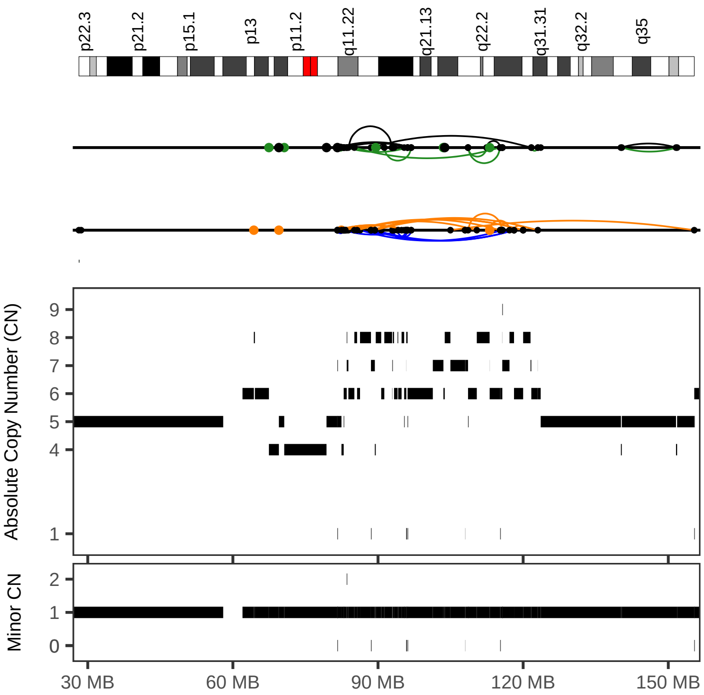

	d8e9ea52−ff28−4626−baa3−71350fd53ddf
Cancer type	ColoRect−AdenoCA
Position	7:81604311−155367371
Type	With other complex events
Interleaved intrachr. SVs	53
Total SVs (intrachr. + transl.)	60
SV types	DEL: 21; DUP: 11; h2hINV: 12; t2tINV: 9; TRA: 7
SVs in sample	181
Oscillating CN (2 and 3 states)	5, 9
CN segments	71
FDR fragment joints	0.5435077
FDR chr. breakp. enrich.	0
Linked to chrs	
Purity, ploidy	0.5, 3.72

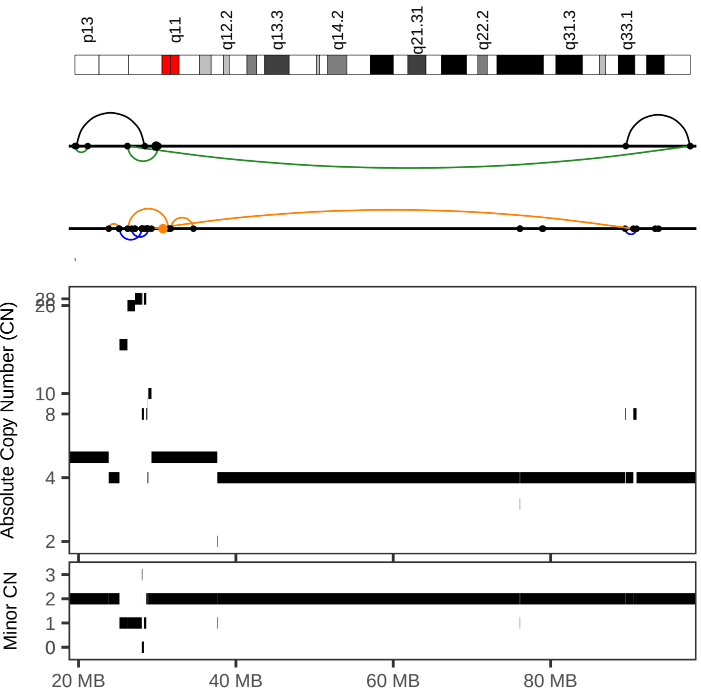

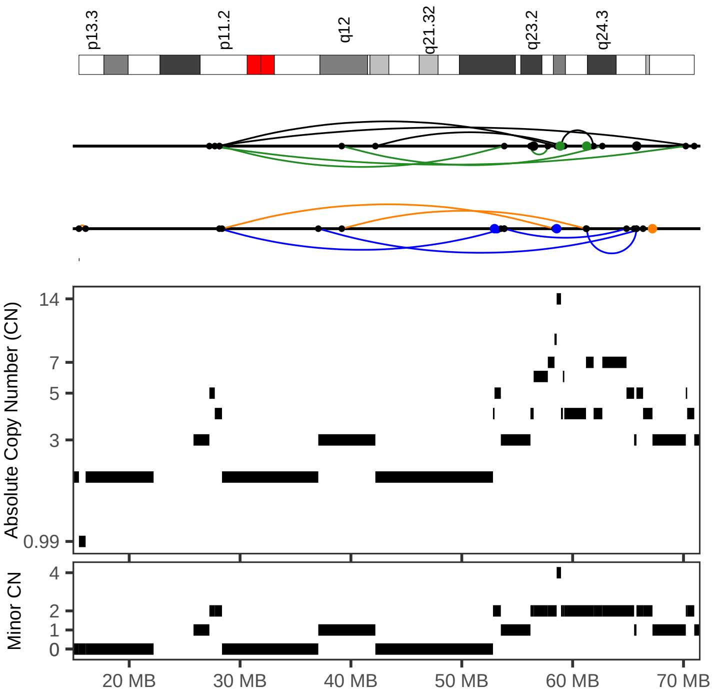

	d8e9ea52−ff28−4626−baa3−71350fd53ddf
Cancer type	ColoRect−AdenoCA
Position	13:19541475−97747877
Type	With other complex events
Interleaved intrachr. SVs	13
Total SVs (intrachr. + transl.)	15
SV types	DEL: 2; DUP: 6; h2hINV: 2; t2tINV: 3; TRA: 2
SVs in sample	181
Oscillating CN (2 and 3 states)	5, 5
CN segments	22
FDR fragment joints	0.6776251
FDR chr. breakp. enrich.	0
Linked to chrs	
Purity, ploidy	0.5, 3.72

	335d0351−3740−4847−9ff4−be2f078c70f7
Cancer type	ColoRect−AdenoCA
Position	17:28133401−66354370
Type	With other complex events
Interleaved intrachr. SVs	11
Total SVs (intrachr. + transl.)	17
SV types	DEL: 2; DUP: 4; h2hINV: 3; t2tINV: 2; TRA: 6
SVs in sample	72
Oscillating CN (2 and 3 states)	4, 7
CN segments	22
FDR fragment joints	0.6776251
FDR chr. breakp. enrich.	0
Linked to chrs	
Purity, ploidy	0.62, 3.26

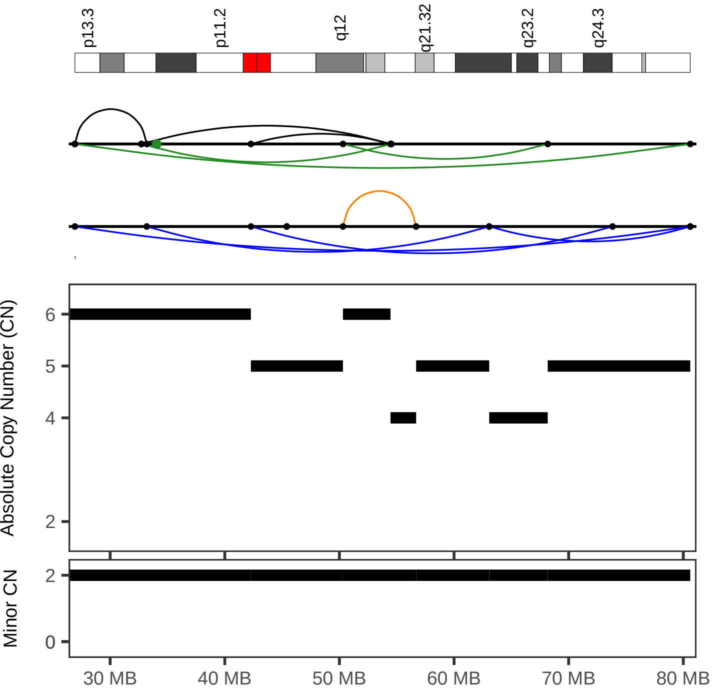

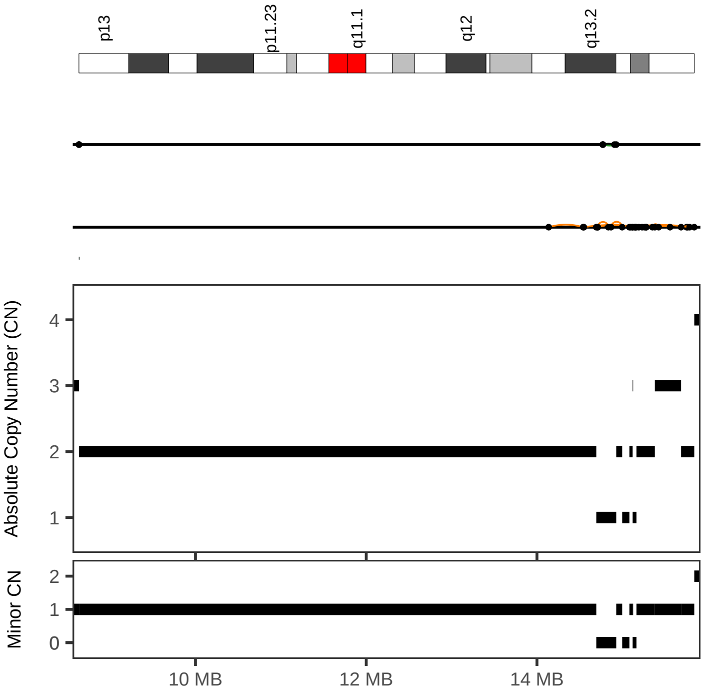

	8e00e7e7−ffaf−44f0−91a7−172671f18e08
Cancer type	ColoRect−AdenoCA
Position	17:26926287−80606072
Type	With other complex events
Interleaved intrachr. SVs	11
Total SVs (intrachr. + transl.)	13
SV types	DEL: 1; DUP: 4; h2hINV: 3; t2tINV: 3; TRA: 2
SVs in sample	126
Oscillating CN (2 and 3 states)	4, 7
CN segments	7
FDR fragment joints	0.6776251
FDR chr. breakp. enrich.	0
Linked to chrs	18:20312665−35616751;
Purity, ploidy	0.69, 3.49

	9af35239−ffb9−4d4f−889e−f743da271eef
Cancer type	ColoRect−AdenoCA
Position	20:14694871−15096752
Type	Canonical without polyploidization
Interleaved intrachr. SVs	6
Total SVs (intrachr. + transl.)	6
SV types	DEL: 4; DUP: 0; h2hINV: 1; t2tINV: 1; TRA: 0
SVs in sample	89
Oscillating CN (2 and 3 states)	4, 4
CN segments	4
FDR fragment joints	0.5435077
FDR chr. breakp. enrich.	0
Linked to chrs	
Purity, ploidy	0.64, 2.52

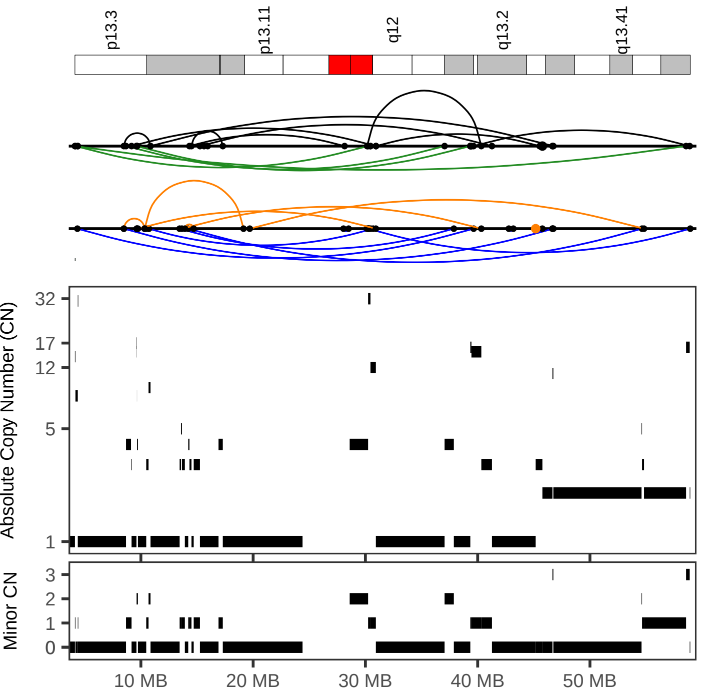

	**OCCAMS−AH−021**
Cancer type	Eso−AdenoCA
Position	19:4125375−58935360
Type	With other complex events
Interleaved intrachr. SVs	31
Total SVs (intrachr. + transl.)	33
SV types	DEL: 6; DUP: 10; h2hINV: 10; t2tINV: 5; TRA: 2
SVs in sample	262
Oscillating CN (2 and 3 states)	4, 7
CN segments	45
FDR fragment joints	0.780201
FDR chr. breakp. enrich.	0
Linked to chrs	
Purity, ploidy	0.26, 2.05

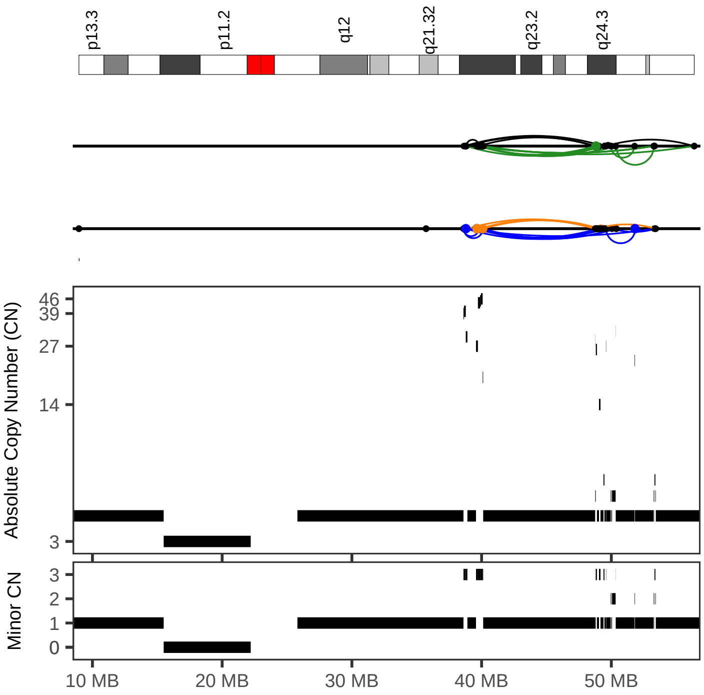

	**OCCAMS−AH−036**
Cancer type	Eso−AdenoCA
Position	17:38600780−56393627
Type	With other complex events
Interleaved intrachr. SVs	40
Total SVs (intrachr. + transl.)	46
SV types	DEL: 9; DUP: 11; h2hINV: 9; t2tINV: 11; TRA: 6
SVs in sample	149
Oscillating CN (2 and 3 states)	4, 5
CN segments	33
FDR fragment joints	0.9152324
FDR chr. breakp. enrich.	0
Linked to chrs	
Purity, ploidy	0.65, 4.49

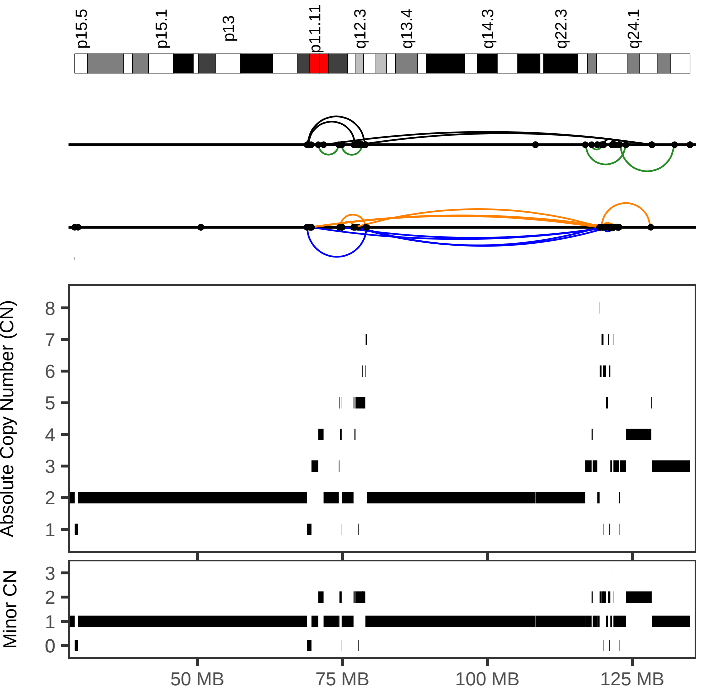

	**OCCAMS−AH−039**
Cancer type	Eso−AdenoCA
Position	11:68863900−132284467
Type	With other complex events
Interleaved intrachr. SVs	32
Total SVs (intrachr. + transl.)	32
SV types	DEL: 13; DUP: 7; h2hINV: 6; t2tINV: 6; TRA: 0
SVs in sample	146
Oscillating CN (2 and 3 states)	4, 6
CN segments	54
FDR fragment joints	0.615458
FDR chr. breakp. enrich.	0
Linked to chrs	
Purity, ploidy	0.38, 1.99

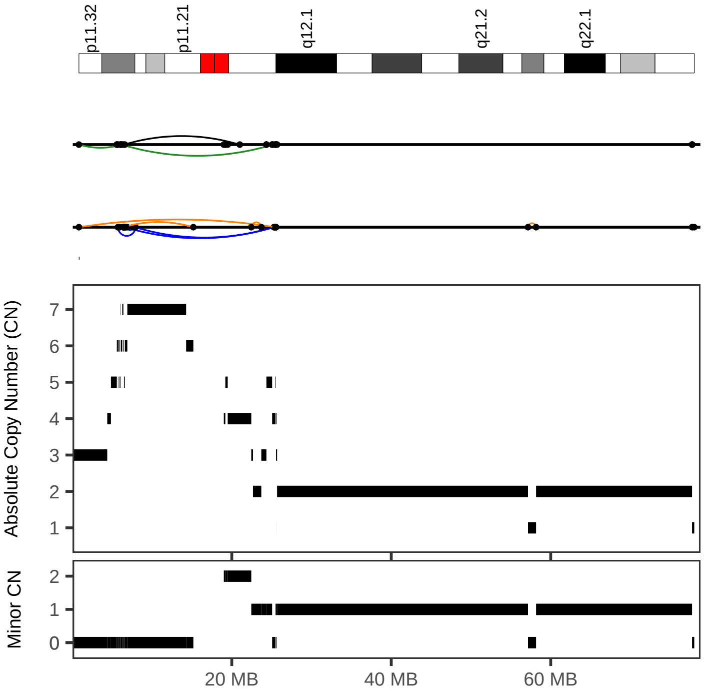

	**OCCAMS−AH−042**
Cancer type	Eso−AdenoCA
Position	18:827310−25488324
Type	With other complex events
Interleaved intrachr. SVs	12
Total SVs (intrachr. + transl.)	12
SV types	DEL: 3; DUP: 4; h2hINV: 2; t2tINV: 3; TRA: 0
SVs in sample	85
Oscillating CN (2 and 3 states)	6, 18
CN segments	27
FDR fragment joints	0.9284301
FDR chr. breakp. enrich.	0
Linked to chrs	
Purity, ploidy	0.48, 3.19

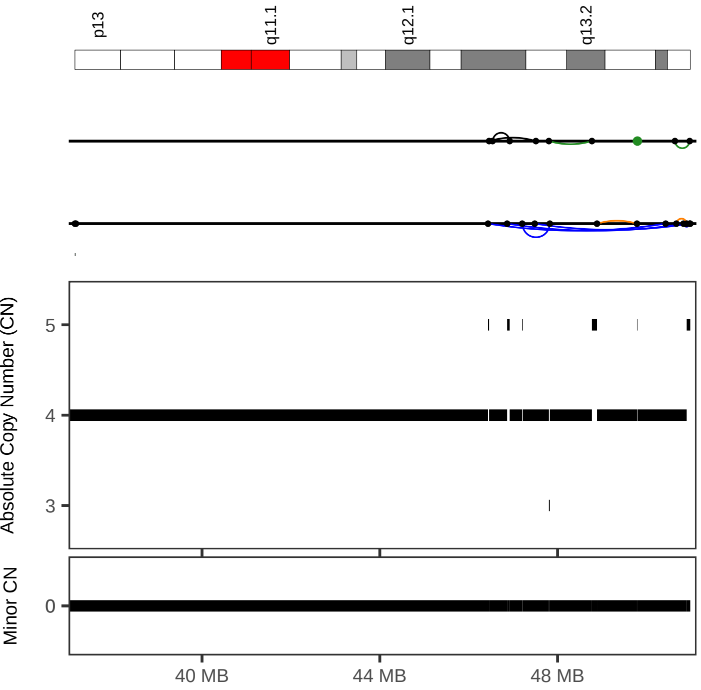

	**OCCAMS−AH−047**
Cancer type	Eso−AdenoCA
Position	22:46434583−50985112
Type	With other complex events
Interleaved intrachr. SVs	10
Total SVs (intrachr. + transl.)	11
SV types	DEL: 1; DUP: 5; h2hINV: 2; t2tINV: 2; TRA: 1
SVs in sample	211
Oscillating CN (2 and 3 states)	6, 13
CN segments	13
FDR fragment joints	0.615458
FDR chr. breakp. enrich.	0
Linked to chrs	
Purity, ploidy	0.31, 4.28

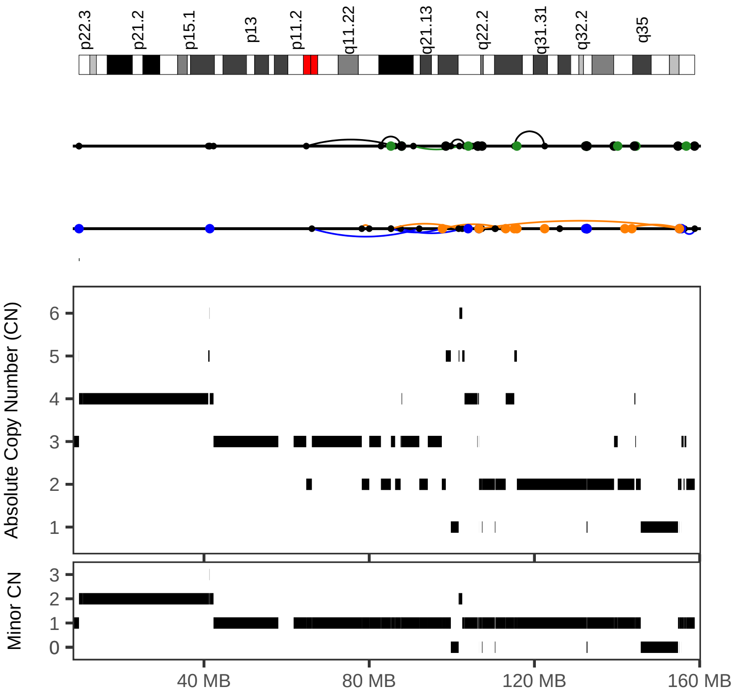

	**OCCAMS−AH−064**
Cancer type	Eso−AdenoCA
Position	7:64763385−155596659
Type	With other complex events
Interleaved intrachr. SVs	11
Total SVs (intrachr. + transl.)	49
SV types	DEL: 3; DUP: 4; h2hINV: 3; t2tINV: 1; TRA: 38
SVs in sample	448
Oscillating CN (2 and 3 states)	6, 9
CN segments	48
FDR fragment joints	0.8653243
FDR chr. breakp. enrich.	0
Linked to chrs	3:1926043−196423190;
Purity, ploidy	0.65, 2.65

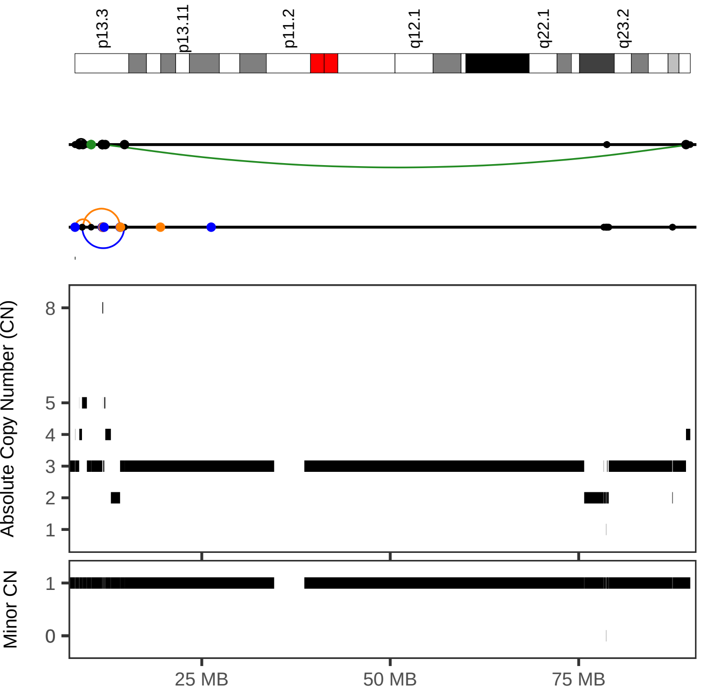

	**OCCAMS−AH−064**
Cancer type	Eso−AdenoCA
Position	16:8170195−89798592
Type	With other complex events
Interleaved intrachr. SVs	7
Total SVs (intrachr. + transl.)	29
SV types	DEL: 2; DUP: 1; h2hINV: 1; t2tINV: 3; TRA: 22
SVs in sample	448
Oscillating CN (2 and 3 states)	5, 8
CN segments	35
FDR fragment joints	0.5680234
FDR chr. breakp. enrich.	0
Linked to chrs	3:1926043−196423190;7:64763385−155596658
Purity, ploidy	0.65, 2.65

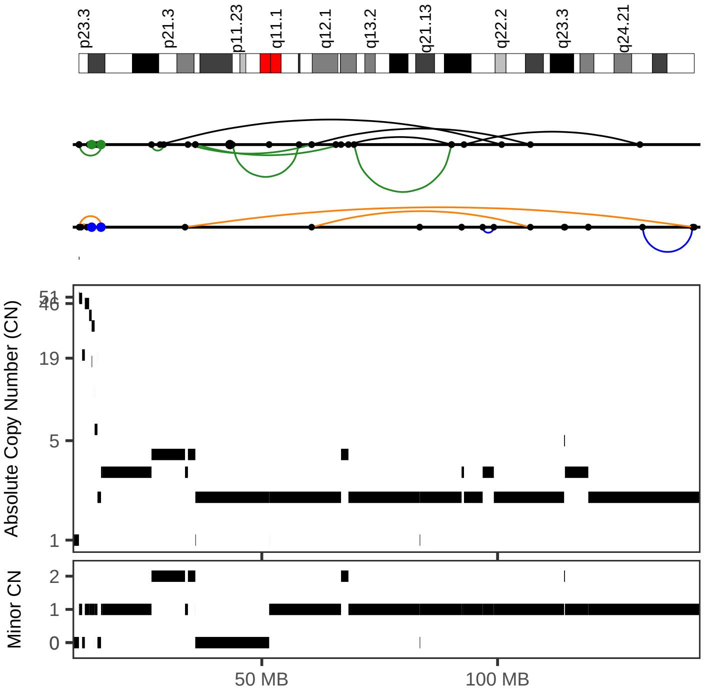

	**OCCAMS−AH−071**
Cancer type	Eso−AdenoCA
Position	8:26605057−141712244
Type	With other complex events
Interleaved intrachr. SVs	8
Total SVs (intrachr. + transl.)	9
SV types	DEL: 2; DUP: 0; h2hINV: 3; t2tINV: 3; TRA: 1
SVs in sample	134
Oscillating CN (2 and 3 states)	5, 10
CN segments	19
FDR fragment joints	0.615458
FDR chr. breakp. enrich.	0
Linked to chrs	
Purity, ploidy	0.39, 2.25

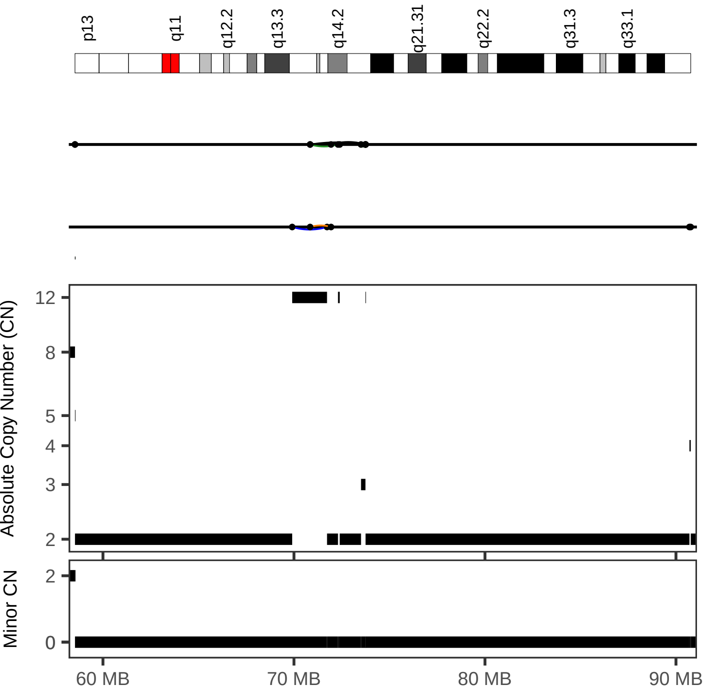

OCCAMS−AH−077

Cancer type	Eso−AdenoCA
Position	13:69907738−73751361
Type	With other complex events
Interleaved intrachr. SVs	7
Total SVs (intrachr. + transl.)	7
SV types	DEL: 1; DUP: 1; h2hINV: 3; t2tINV: 2; TRA: 0
SVs in sample	68
Oscillating CN (2 and 3 states)	4, 5
CN segments	9
FDR fragment joints	0.7735152
FDR chr. breakp. enrich.	0
Linked to chrs	
Purity, ploidy	0.54, 4.09

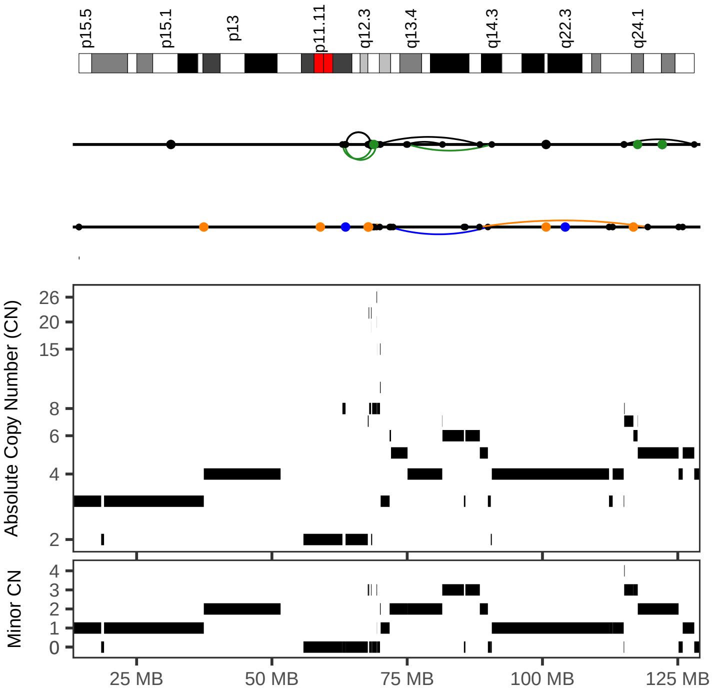

OCCAMS−AH−096

Cancer type	Eso−AdenoCA
Position	11:63032046−128045151
Type	With other complex events
Interleaved intrachr. SVs	12
Total SVs (intrachr. + transl.)	24
SV types	DEL: 2; DUP: 2; h2hINV: 4; t2tINV: 4; TRA: 12
SVs in sample	326
Oscillating CN (2 and 3 states)	4, 6
CN segments	39
FDR fragment joints	0.8653243
FDR chr. breakp. enrich.	0
Linked to chrs	
Purity, ploidy	0.26, 2.74

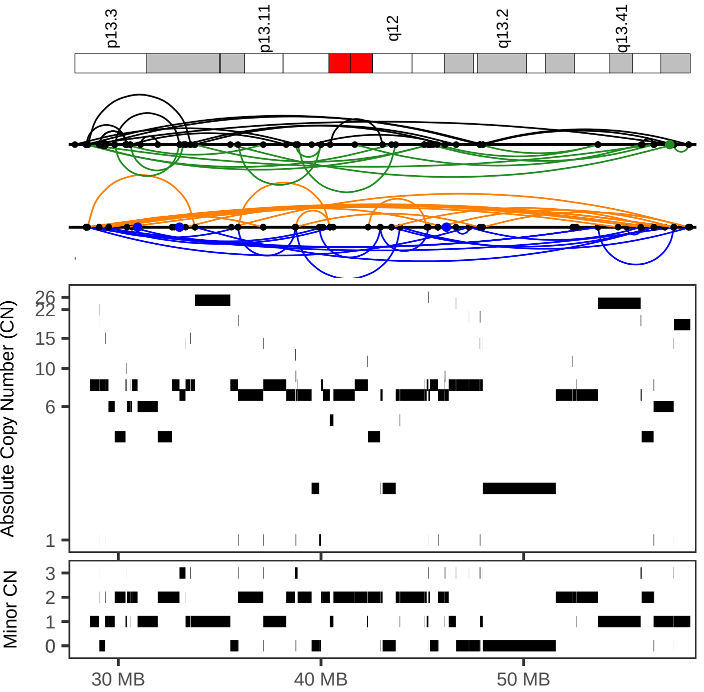

OCCAMS−AH−096

Cancer type	Eso−AdenoCA
Position	19:27855418−58222100
Type	With other complex events
Interleaved intrachr. SVs	70
Total SVs (intrachr. + transl.)	75
SV types	DEL: 16; DUP: 18; h2hINV: 18; t2tINV: 18; TRA: 5
SVs in sample	326
Oscillating CN (2 and 3 states)	6, 7
CN segments	98
FDR fragment joints	0.9284301
FDR chr. breakp. enrich.	0
Linked to chrs	22:16884779−49630407;
Purity, ploidy	0.26, 2.74

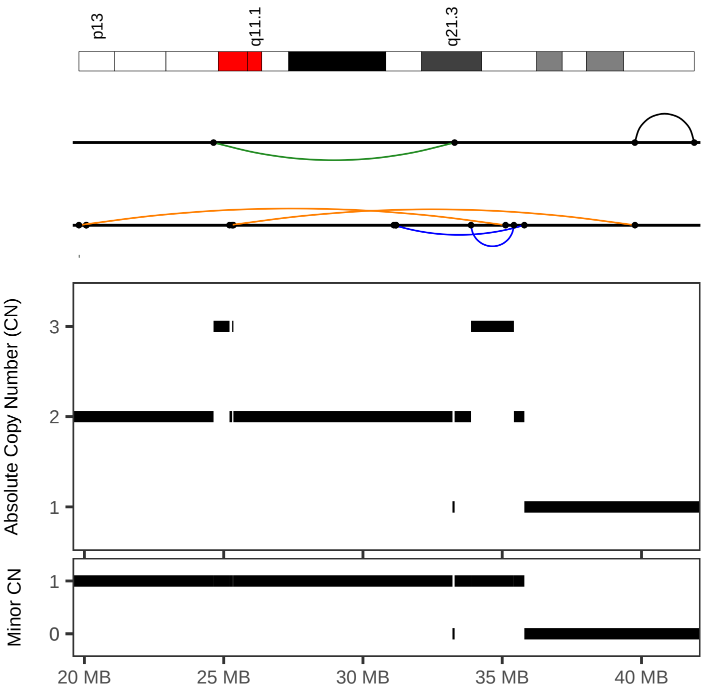

OCCAMS−AH−108

Cancer type	Eso−AdenoCA
Position	21:19802561−41893624
Type	With other complex events
Interleaved intrachr. SVs	6
Total SVs (intrachr. + transl.)	6
SV types	DEL: 2; DUP: 2; h2hINV: 1; t2tINV: 1; TRA: 0
SVs in sample	162
Oscillating CN (2 and 3 states)	5, 10
CN segments	10
FDR fragment joints	0.9284301
FDR chr. breakp. enrich.	0
Linked to chrs	
Purity, ploidy	0.47, 2.83

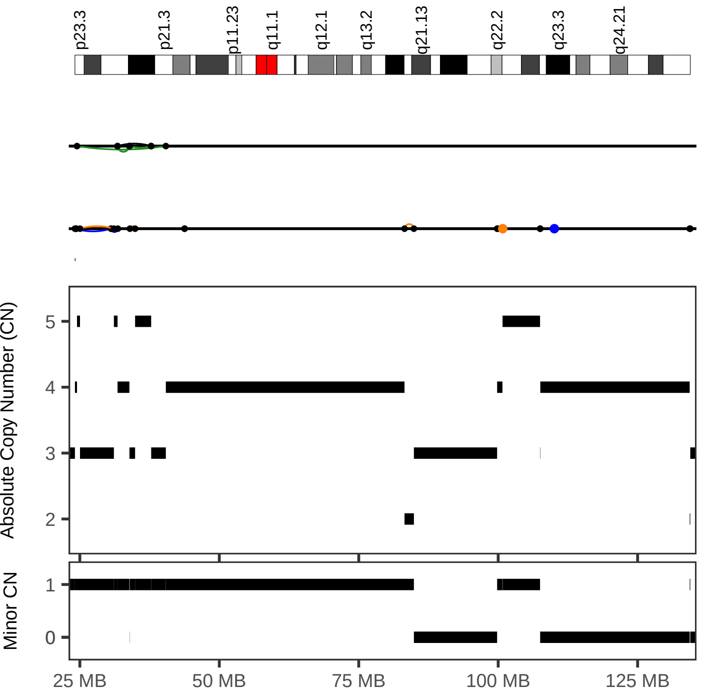

	**OCCAMS−AH−127**
Cancer type	Eso−AdenoCA
Position	8:24116542−40417339
Type	With other complex events
Interleaved intrachr. SVs	6
Total SVs (intrachr. + transl.)	6
SV types	DEL: 1; DUP: 2; h2hINV: 1; t2tINV: 2; TRA: 0
SVs in sample	148
Oscillating CN (2 and 3 states)	4, 7
CN segments	11
FDR fragment joints	0.9284301
FDR chr. breakp. enrich.	0.01
Linked to chrs	
Purity, ploidy	0.36, 3.12

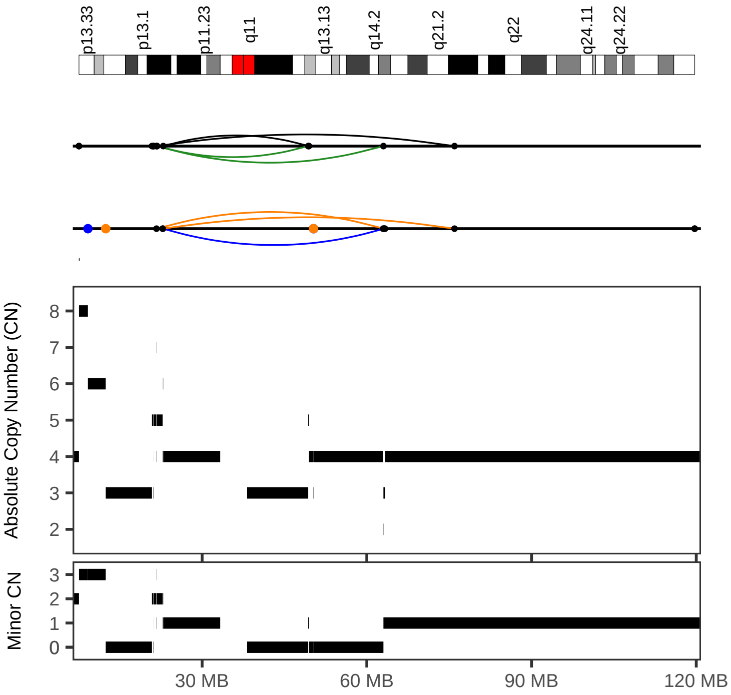

	**OCCAMS−AH−127**
Cancer type	Eso−AdenoCA
Position	12:21670505−75969433
Type	With other complex events
Interleaved intrachr. SVs	8
Total SVs (intrachr. + transl.)	10
SV types	DEL: 3; DUP: 1; h2hINV: 2; t2tINV: 2; TRA: 2
SVs in sample	148
Oscillating CN (2 and 3 states)	4, 6
CN segments	15
FDR fragment joints	0.8572806
FDR chr. breakp. enrich.	0.01
Linked to chrs	2:5457516−205931055;
Purity, ploidy	0.36, 3.12

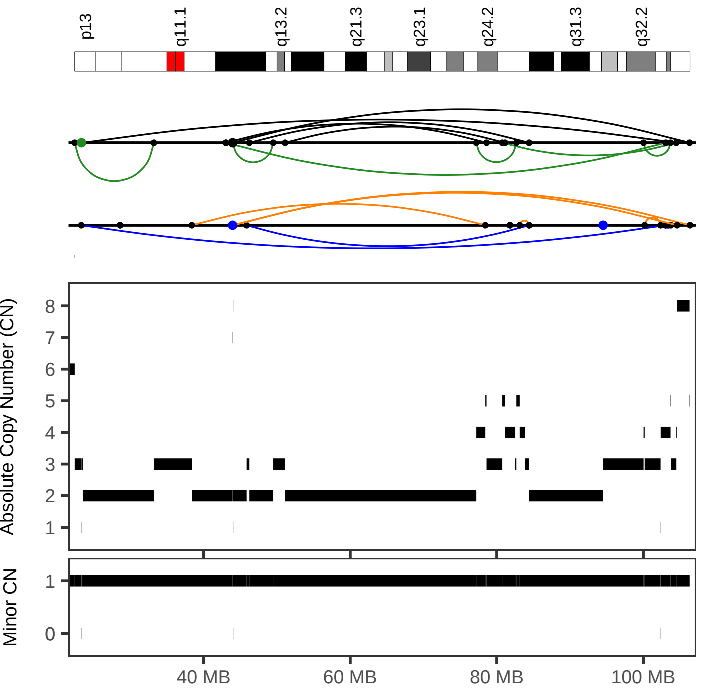

	**OCCAMS−AH−131**
Cancer type	Eso−AdenoCA
Position	14:22399406−106374496
Type	With other complex events
Interleaved intrachr. SVs	17
Total SVs (intrachr. + transl.)	21
SV types	DEL: 3; DUP: 3; h2hINV: 5; t2tINV: 6; TRA: 4
SVs in sample	266
Oscillating CN (2 and 3 states)	5, 6
CN segments	39
FDR fragment joints	0.7568568
FDR chr. breakp. enrich.	0
Linked to chrs	8:74740233−78745926;
Purity, ploidy	0.53, 2.63

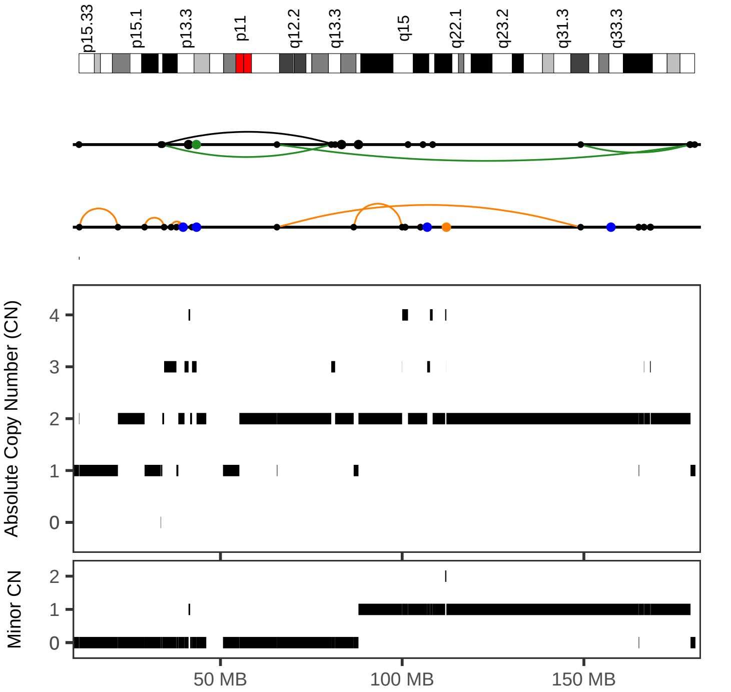

	**OCCAMS−AH−135**
Cancer type	Eso−AdenoCA
Position	5:29118627−180500252
Type	With other complex events
Interleaved intrachr. SVs	7
Total SVs (intrachr. + transl.)	16
SV types	DEL: 2; DUP: 0; h2hINV: 2; t2tINV: 3; TRA: 9
SVs in sample	298
Oscillating CN (2 and 3 states)	5, 13
CN segments	37
FDR fragment joints	0.7908979
FDR chr. breakp. enrich.	0.01
Linked to chrs	6:39890387−56314679;
Purity, ploidy	0.55, 2.85

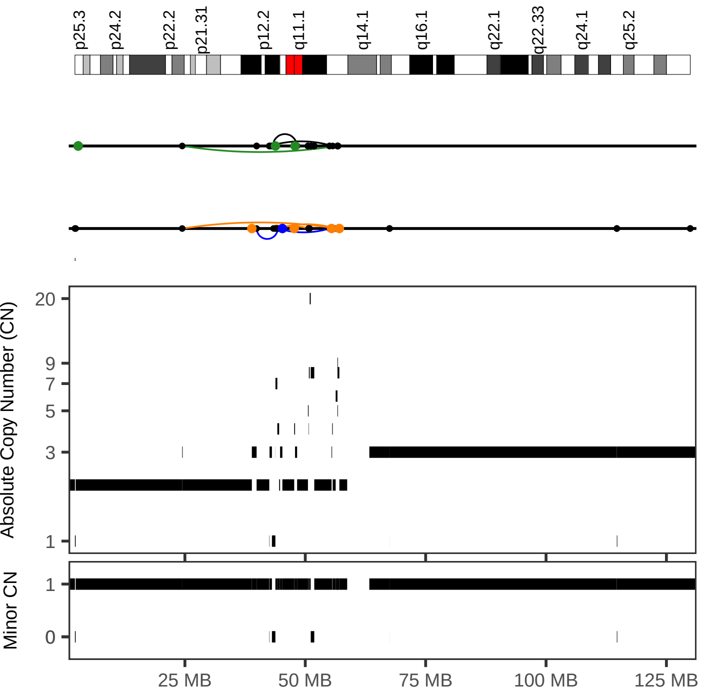

	OCCAMS−AH−135
Cancer type	Eso−AdenoCA
Position	6:39890387−56314680
Type	With other complex events
Interleaved intrachr. SVs	9
Total SVs (intrachr. + transl.)	14
SV types	DEL: 4; DUP: 2; h2hINV: 2; t2tINV: 1; TRA: 5
SVs in sample	298
Oscillating CN (2 and 3 states)	5, 7
CN segments	27
FDR fragment joints	0.615458
FDR chr. breakp. enrich.	0
Linked to chrs	5:29118627−180500251;
Purity, ploidy	0.55, 2.85

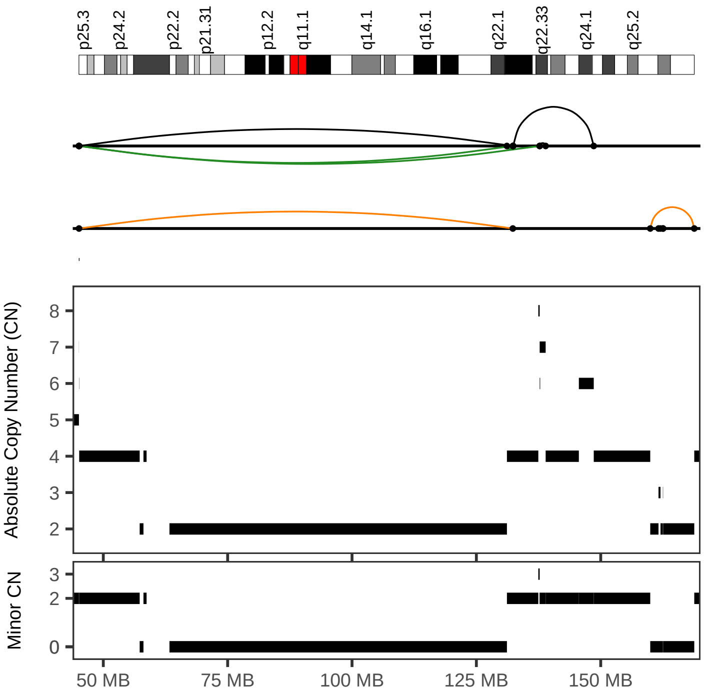

	OCCAMS−AH−136
Cancer type	Eso−AdenoCA
Position	6:45096268−148600879
Type	With other complex events
Interleaved intrachr. SVs	6
Total SVs (intrachr. + transl.)	6
SV types	DEL: 1; DUP: 0; h2hINV: 2; t2tINV: 3; TRA: 0
SVs in sample	67
Oscillating CN (2 and 3 states)	5, 5
CN segments	14
FDR fragment joints	0.615458
FDR chr. breakp. enrich.	0
Linked to chrs	
Purity, ploidy	0.25, 3.12

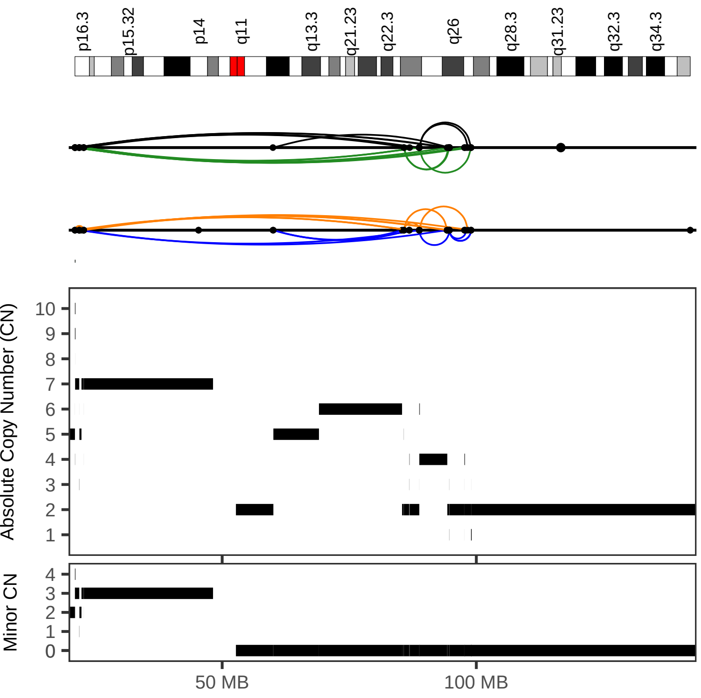

	OCCAMS−AH−146
Cancer type	Eso−AdenoCA
Position	4:21026782−98969606
Type	With other complex events
Interleaved intrachr. SVs	41
Total SVs (intrachr. + transl.)	41
SV types	DEL: 9; DUP: 10; h2hINV: 10; t2tINV: 12; TRA: 0
SVs in sample	195
Oscillating CN (2 and 3 states)	4, 7
CN segments	40
FDR fragment joints	0.9577901
FDR chr. breakp. enrich.	0
Linked to chrs	
Purity, ploidy	0.4, 4.65

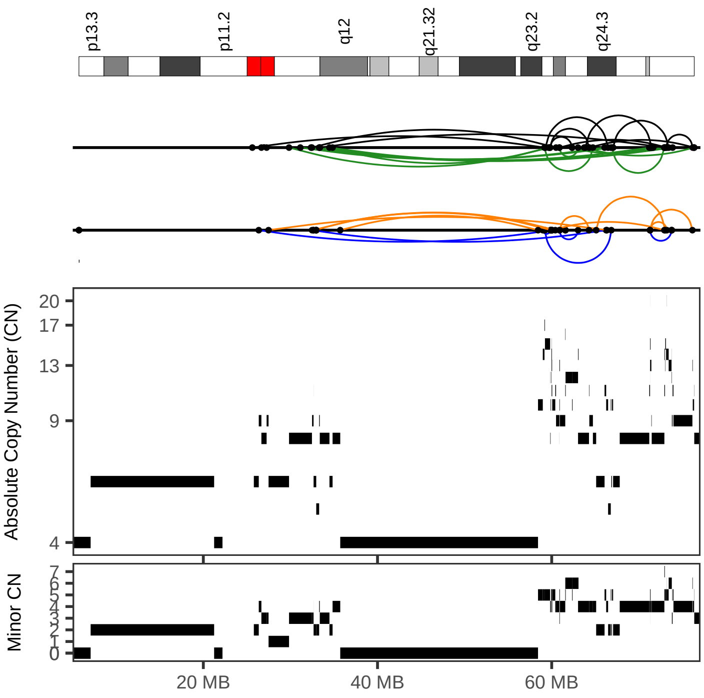

	OCCAMS−AH−146
Cancer type	Eso−AdenoCA
Position	17:25626424−76366954
Type	With other complex events
Interleaved intrachr. SVs	42
Total SVs (intrachr. + transl.)	42
SV types	DEL: 8; DUP: 10; h2hINV: 12; t2tINV: 12; TRA: 0
SVs in sample	195
Oscillating CN (2 and 3 states)	4, 6
CN segments	80
FDR fragment joints	0.8653243
FDR chr. breakp. enrich.	0
Linked to chrs	
Purity, ploidy	0.4, 4.65

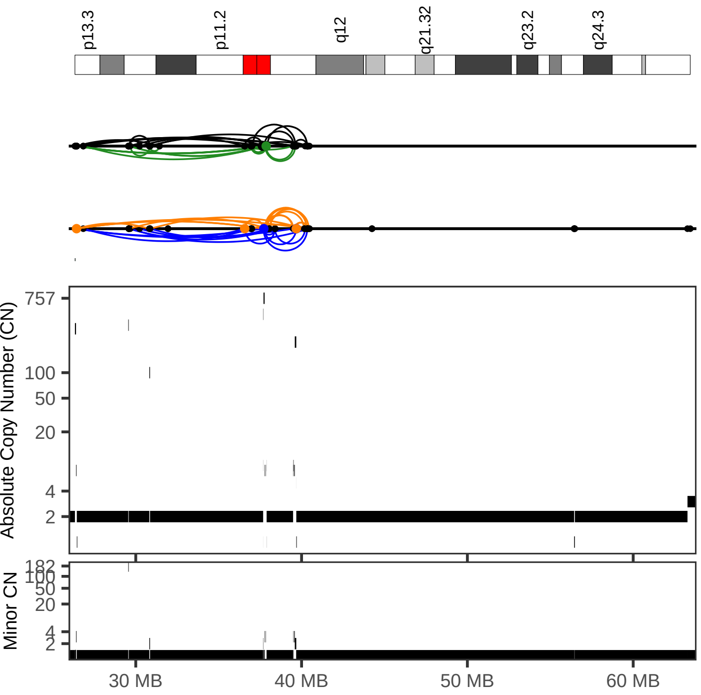

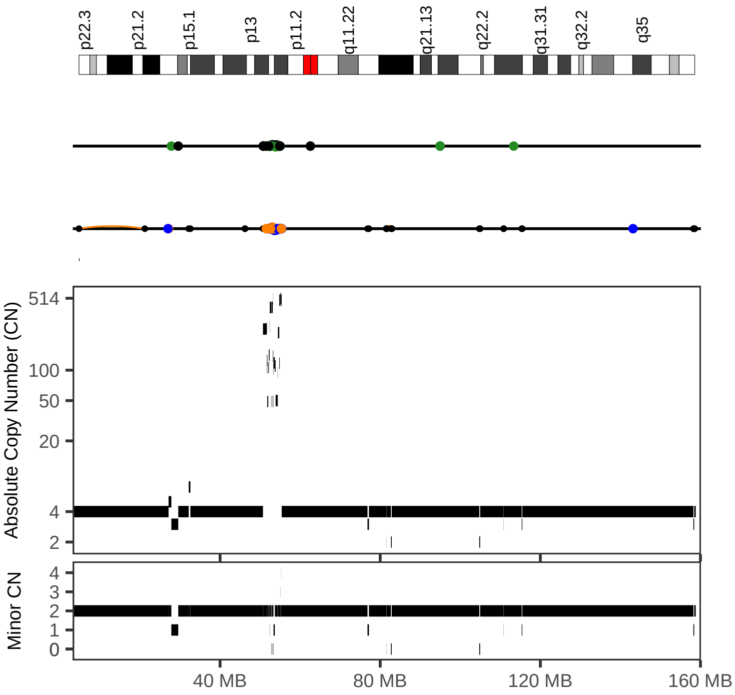

	**OCCAMS−AH−167**
Cancer type	Eso−AdenoCA
Position	17:26331304−40471971
Type	With other complex events
Interleaved intrachr. SVs	73
Total SVs (intrachr. + transl.)	82
SV types	DEL: 18; DUP: 17; h2hINV: 19; t2tINV: 19; TRA: 9
SVs in sample	307
Oscillating CN (2 and 3 states)	5, 6
CN segments	28
FDR fragment joints	1
FDR chr. breakp. enrich.	0
Linked to chrs	
Purity, ploidy	0.28, 2.52

	**OCCAMS−AH−174**
Cancer type	Eso−AdenoCA
Position	7:50712649−55386047
Type	With other complex events
Interleaved intrachr. SVs	50
Total SVs (intrachr. + transl.)	99
SV types	DEL: 16; DUP: 11; h2hINV: 12; t2tINV: 11; TRA: 49
SVs in sample	572
Oscillating CN (2 and 3 states)	4, 5
CN segments	28
FDR fragment joints	0.615458
FDR chr. breakp. enrich.	0
Linked to chrs	2:32595317−181665721;9:73771445−82788409
Purity, ploidy	0.29, 4.26

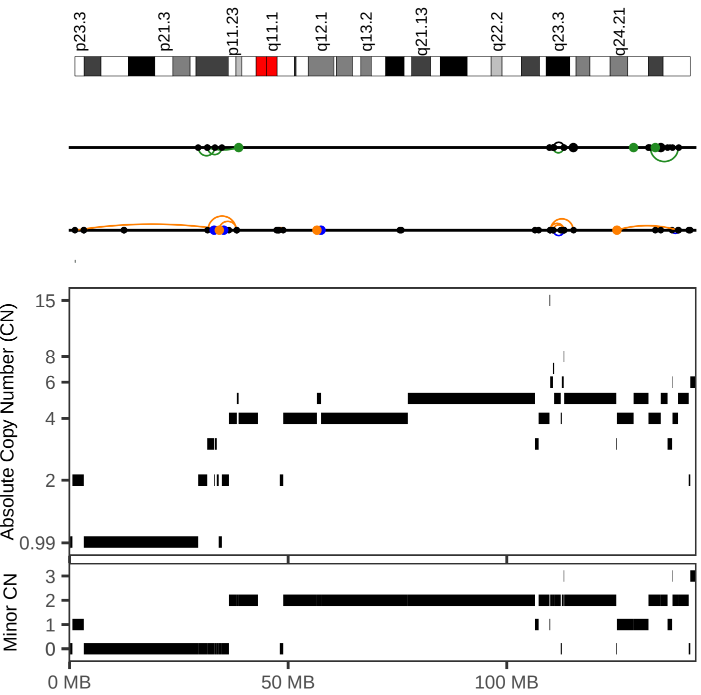

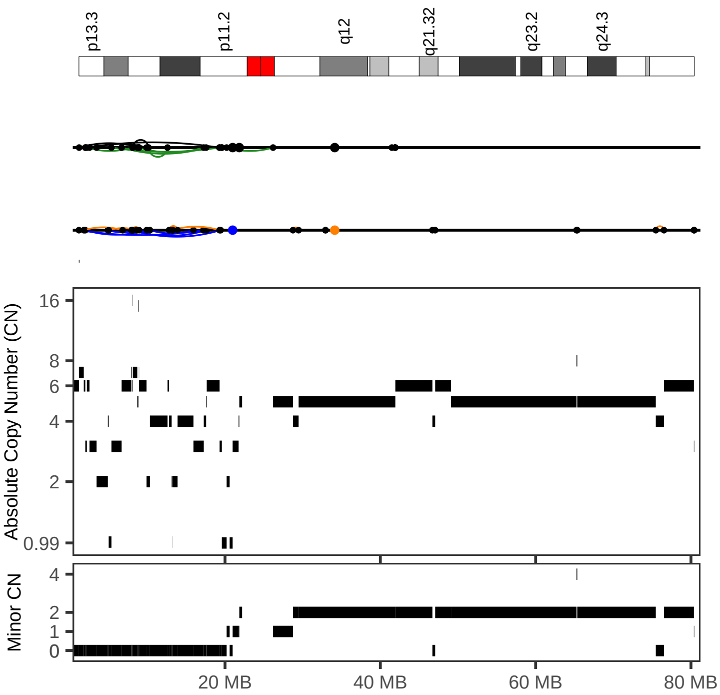

	**OCCAMS−AH−174**
Cancer type	Eso−AdenoCA
Position	8:1230941−38668853
Type	With other complex events
Interleaved intrachr. SVs	7
Total SVs (intrachr. + transl.)	10
SV types	DEL: 4; DUP: 0; h2hINV: 0; t2tINV: 3; TRA: 3
SVs in sample	572
Oscillating CN (2 and 3 states)	5, 9
CN segments	12
FDR fragment joints	0.615458
FDR chr. breakp. enrich.	0.02
Linked to chrs	
Purity, ploidy	0.29, 4.26

	**OCCAMS−AH−174**
Cancer type	Eso−AdenoCA
Position	17:1185559−19439852
Type	With other complex events
Interleaved intrachr. SVs	22
Total SVs (intrachr. + transl.)	22
SV types	DEL: 5; DUP: 6; h2hINV: 6; t2tINV: 5; TRA: 0
SVs in sample	572
Oscillating CN (2 and 3 states)	4, 5
CN segments	31
FDR fragment joints	0.992996
FDR chr. breakp. enrich.	0
Linked to chrs	
Purity, ploidy	0.29, 4.26

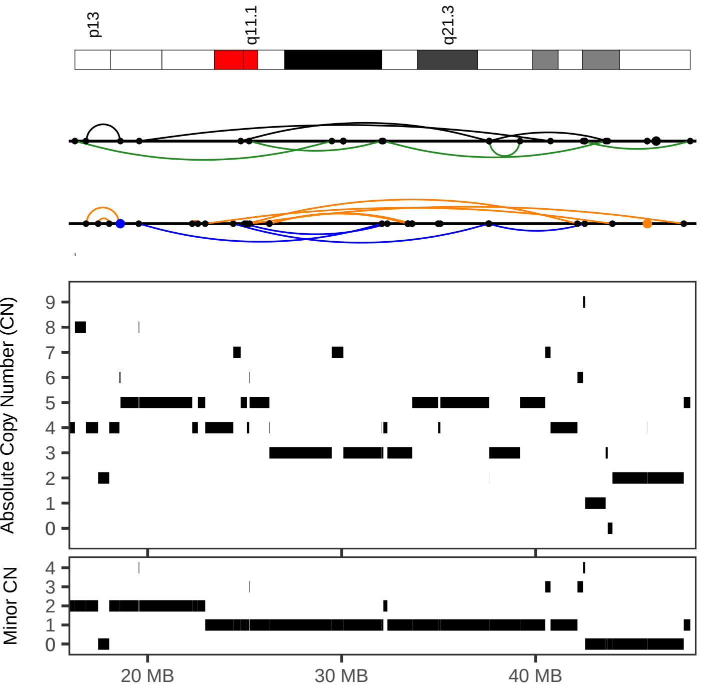

	**OCCAMS−AH−174**
Cancer type	Eso−AdenoCA
Position	21:16251242−47972973
Type	With other complex events
Interleaved intrachr. SVs	18
Total SVs (intrachr. + transl.)	21
SV types	DEL: 6; DUP: 4; h2hINV: 3; t2tINV: 5; TRA: 3
SVs in sample	572
Oscillating CN (2 and 3 states)	5, 8
CN segments	41
FDR fragment joints	0.7568568
FDR chr. breakp. enrich.	0
Linked to chrs	
Purity, ploidy	0.29, 4.26

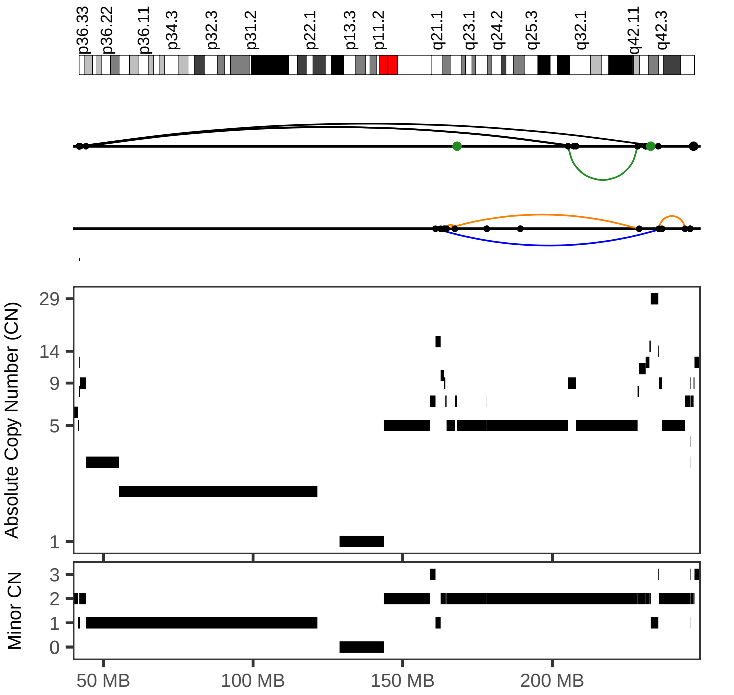

	**OCCAMS−AH−182**
Cancer type	Eso−AdenoCA
Position	1:41909912−244361254
Type	With other complex events
Interleaved intrachr. SVs	7
Total SVs (intrachr. + transl.)	9
SV types	DEL: 2; DUP: 1; h2hINV: 3; t2tINV: 1; TRA: 2
SVs in sample	223
Oscillating CN (2 and 3 states)	6, 6
CN segments	28
FDR fragment joints	0.615458
FDR chr. breakp. enrich.	0.9
Linked to chrs	
Purity, ploidy	0.17, 3.08

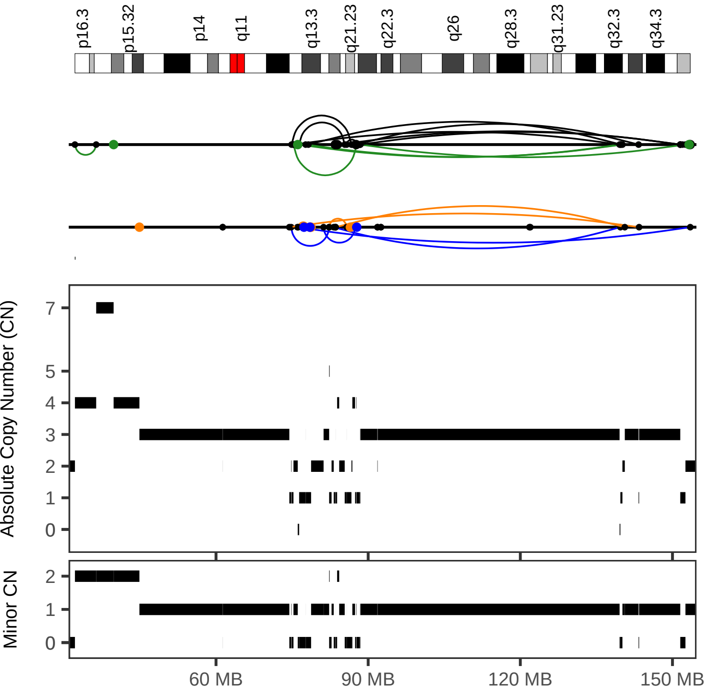

	**OCCAMS−AH−182**
Cancer type	Eso−AdenoCA
Position	4:74456231−153507115
Type	With other complex events
Interleaved intrachr. SVs	23
Total SVs (intrachr. + transl.)	35
SV types	DEL: 4; DUP: 5; h2hINV: 8; t2tINV: 6; TRA: 12
SVs in sample	223
Oscillating CN (2 and 3 states)	4, 10
CN segments	41
FDR fragment joints	0.9176394
FDR chr. breakp. enrich.	0
Linked to chrs	
Purity, ploidy	0.17, 3.08

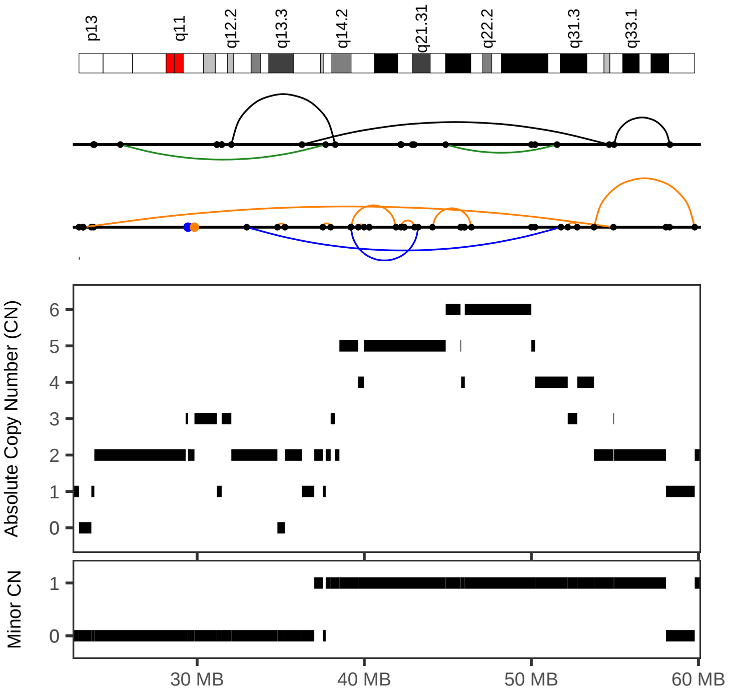

	**OCCAMS−GS−002**
Cancer type	Eso−AdenoCA
Position	13:23188644−59775992
Type	With other complex events
Interleaved intrachr. SVs	7
Total SVs (intrachr. + transl.)	9
SV types	DEL: 3; DUP: 1; h2hINV: 2; t2tINV: 1; TRA: 2
SVs in sample	134
Oscillating CN (2 and 3 states)	5, 13
CN segments	33
FDR fragment joints	0.6776251
FDR chr. breakp. enrich.	0
Linked to chrs	
Purity, ploidy	0.69, 1.97

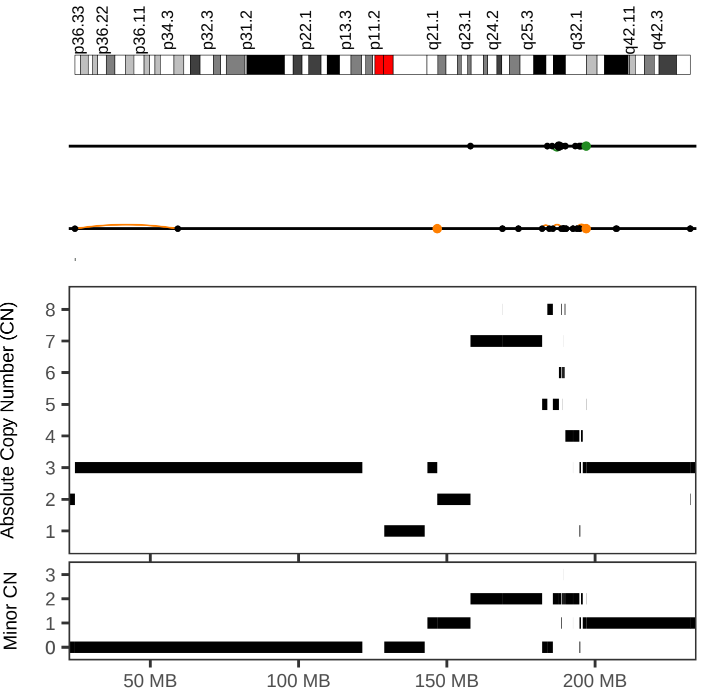

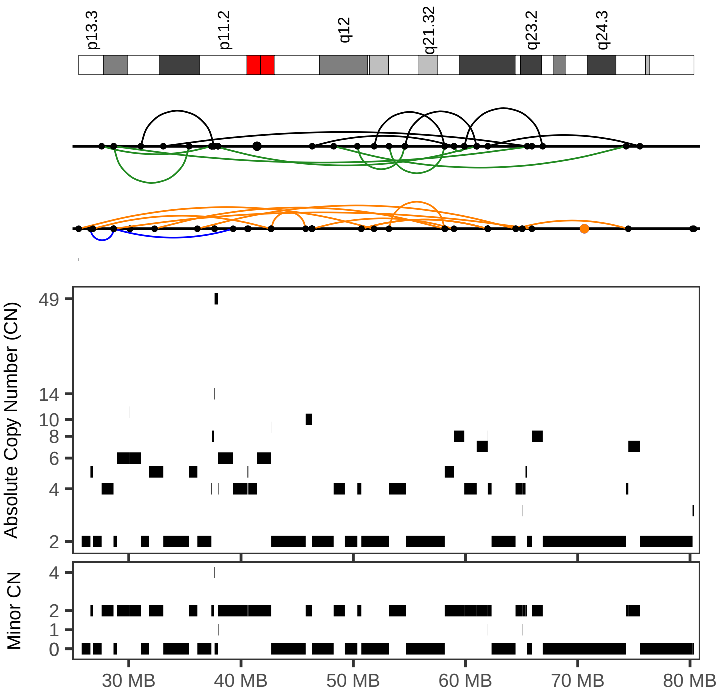

OCCAMS−PS−013

Cancer type	Eso−AdenoCA
Position	1:193309911−196936704
Type	With other complex events
Interleaved intrachr. SVs	7
Total SVs (intrachr. + transl.)	7
SV types	DEL: 3; DUP: 1; h2hINV: 1; t2tINV: 2; TRA: 0
SVs in sample	162
Oscillating CN (2 and 3 states)	4, 5
CN segments	8
FDR fragment joints	0.7735152
FDR chr. breakp. enrich.	0
Linked to chrs	
Purity, ploidy	0.41, 2.62

OCCAMS−QE−095

Cancer type	Eso−AdenoCA
Position	17:25534782−75535929
Type	With other complex events
Interleaved intrachr. SVs	29
Total SVs (intrachr. + transl.)	32
SV types	DEL: 11; DUP: 3; h2hINV: 7; t2tINV: 8; TRA: 3
SVs in sample	247
Oscillating CN (2 and 3 states)	6, 7
CN segments	56
FDR fragment joints	0.615458
FDR chr. breakp. enrich.	0
Linked to chrs	2:47217884−215282384;
Purity, ploidy	0.61, 2.48

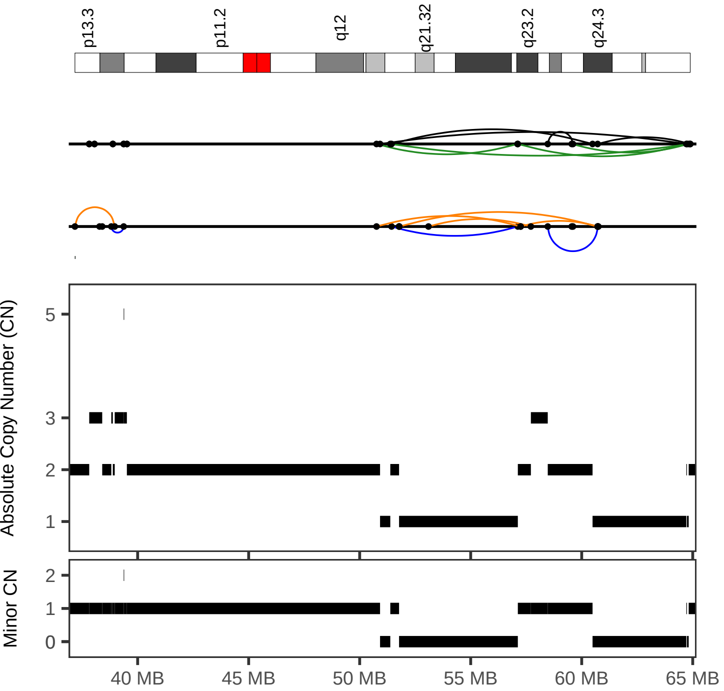

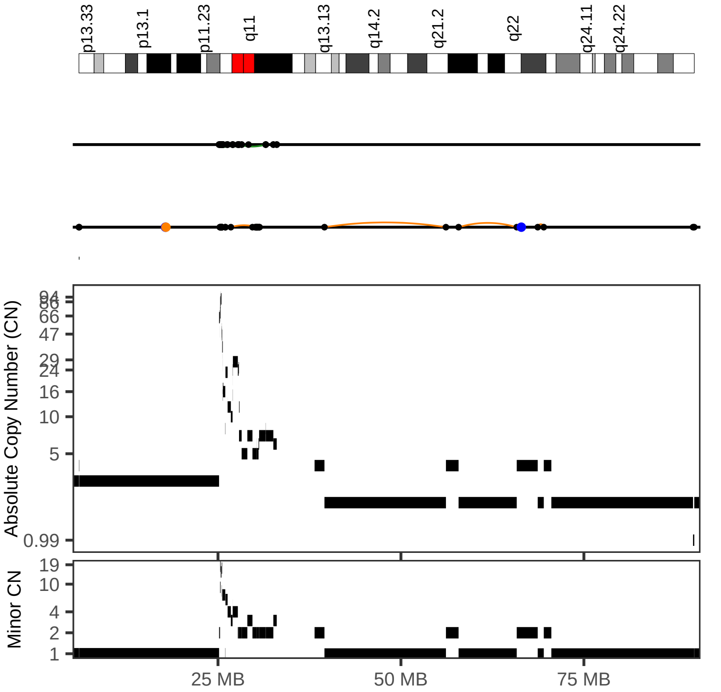

OCCAMS−RS−006

Cancer type	Eso−AdenoCA
Position	17:50758967−64888510
Type	With other complex events
Interleaved intrachr. SVs	16
Total SVs (intrachr. + transl.)	16
SV types	DEL: 5; DUP: 3; h2hINV: 4; t2tINV: 4; TRA: 0
SVs in sample	198
Oscillating CN (2 and 3 states)	5, 11
CN segments	11
FDR fragment joints	0.9501265
FDR chr. breakp. enrich.	0
Linked to chrs	
Purity, ploidy	0.49, 1.67

OCCAMS−RS−007

Cancer type	Eso−AdenoCA
Position	12:25246063−31515072
Type	With other complex events
Interleaved intrachr. SVs	11
Total SVs (intrachr. + transl.)	11
SV types	DEL: 6; DUP: 1; h2hINV: 0; t2tINV: 4; TRA: 0
SVs in sample	242
Oscillating CN (2 and 3 states)	4, 4
CN segments	41
FDR fragment joints	0.4888368
FDR chr. breakp. enrich.	0
Linked to chrs	
Purity, ploidy	0.48, 2.58

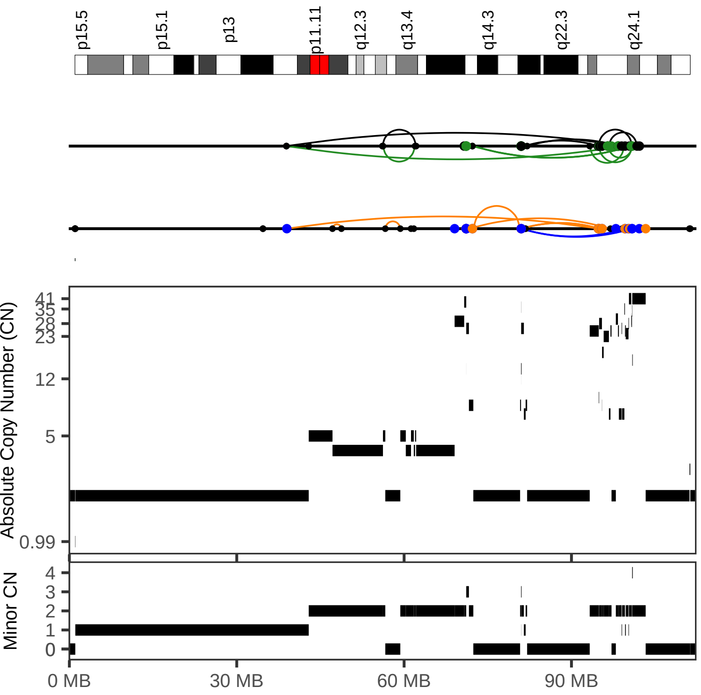

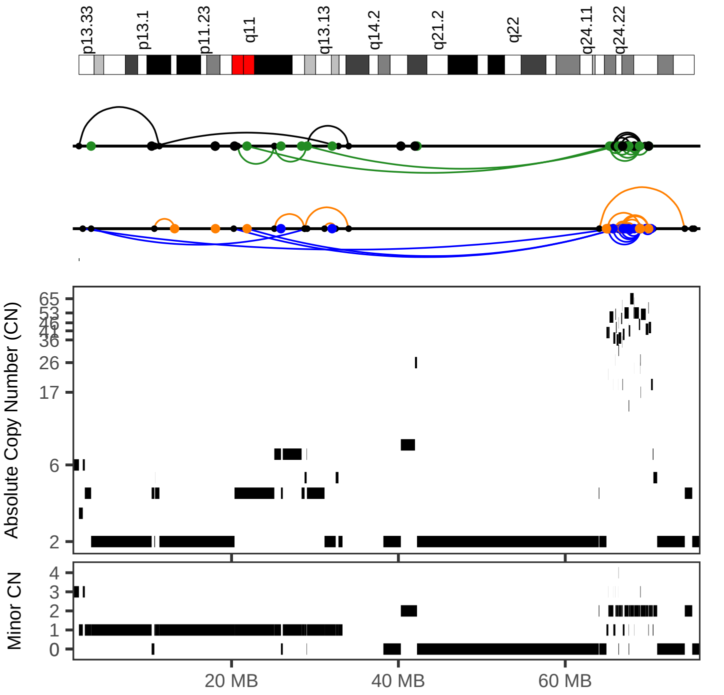

	OCCAMS−RS−008
Cancer type	Eso−AdenoCA
Position	11:38894113−101762285
Type	With other complex events
Interleaved intrachr. SVs	21
Total SVs (intrachr. + transl.)	80
SV types	DEL: 5; DUP: 4; h2hINV: 6; t2tINV: 6; TRA: 59
SVs in sample	303
Oscillating CN (2 and 3 states)	6, 11
CN segments	51
FDR fragment joints	0.6776251
FDR chr. breakp. enrich.	0
Linked to chrs	12:1702270−74329203;2:43419451−218499372
Purity, ploidy	0.19, 2.9

	OCCAMS−RS−008
Cancer type	Eso−AdenoCA
Position	12:1702270−74329204
Type	With other complex events
Interleaved intrachr. SVs	57
Total SVs (intrachr. + transl.)	131
SV types	DEL: 17; DUP: 12; h2hINV: 12; t2tINV: 16; TRA: 74
SVs in sample	303
Oscillating CN (2 and 3 states)	5, 5
CN segments	69
FDR fragment joints	0.964753
FDR chr. breakp. enrich.	0
Linked to chrs	11:38894113−101762284;2:43419451−218499372
Purity, ploidy	0.19, 2.9

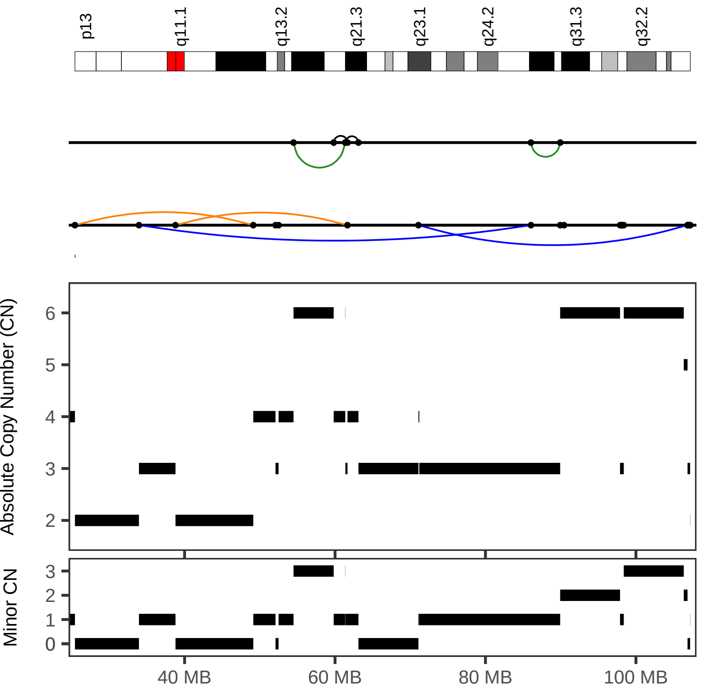

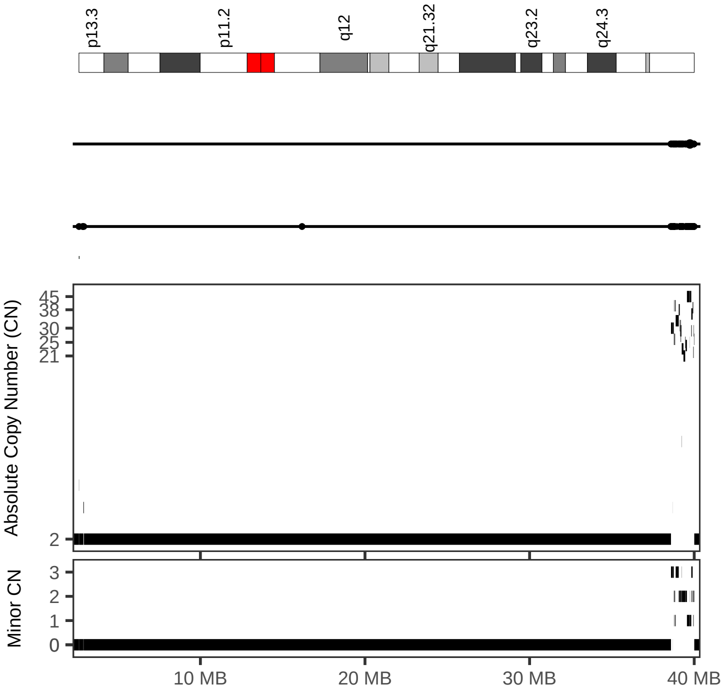

	OCCAMS−RS−031
Cancer type	Eso−AdenoCA
Position	14:25421773−106863854
Type	With other complex events
Interleaved intrachr. SVs	9
Total SVs (intrachr. + transl.)	9
SV types	DEL: 3; DUP: 2; h2hINV: 2; t2tINV: 2; TRA: 0
SVs in sample	156
Oscillating CN (2 and 3 states)	5, 6
CN segments	19
FDR fragment joints	0.9723381
FDR chr. breakp. enrich.	0
Linked to chrs	
Purity, ploidy	0.4, 3.7

	OCCAMS−RS−036
Cancer type	Eso−AdenoCA
Position	17:38588621−39990043
Type	With other complex events
Interleaved intrachr. SVs	78
Total SVs (intrachr. + transl.)	79
SV types	DEL: 18; DUP: 18; h2hINV: 19; t2tINV: 23; TRA: 1
SVs in sample	332
Oscillating CN (2 and 3 states)	4, 7
CN segments	30
FDR fragment joints	0.8497879
FDR chr. breakp. enrich.	0
Linked to chrs	
Purity, ploidy	0.73, 2.22

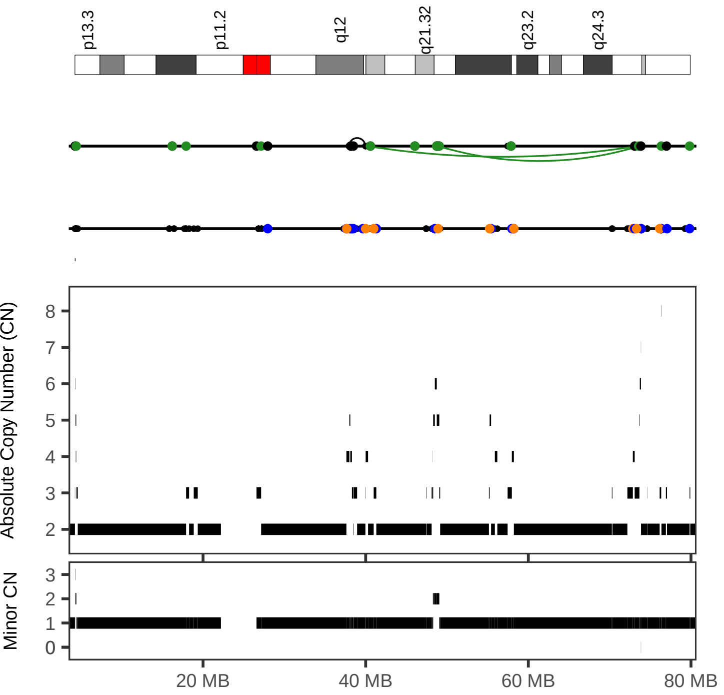

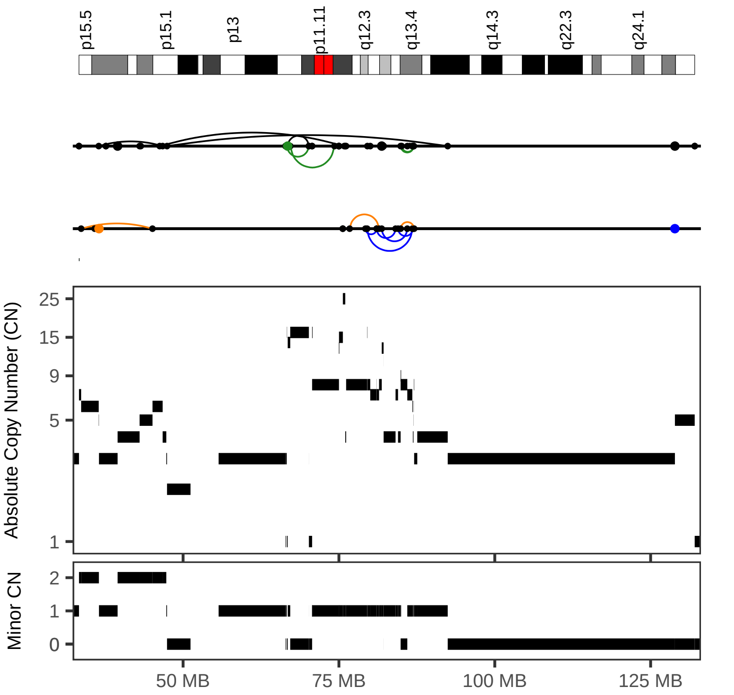

OCCAMS−RS−047

Cancer type	Eso−AdenoCA
Position	17:37640778−73863270
Type	With other complex events
Interleaved intrachr. SVs	9
Total SVs (intrachr. + transl.)	48
SV types	DEL: 0; DUP: 6; h2hINV: 1; t2tINV: 2; TRA: 39
SVs in sample	894
Oscillating CN (2 and 3 states)	6, 8
CN segments	39
FDR fragment joints	0.615458
FDR chr. breakp. enrich.	0
Linked to chrs	1:23826407−235519805;11:66812374−111289395 12:56993350−123682991;2:43059538−85800998 5:32316033−176769547;6:41252622−127972763
Purity, ploidy	0.48, 2.04

OCCAMS−SH−003

Cancer type	Eso−AdenoCA
Position	11:76719544−87065454
Type	With other complex events
Interleaved intrachr. SVs	11
Total SVs (intrachr. + transl.)	12
SV types	DEL: 2; DUP: 5; h2hINV: 1; t2tINV: 3; TRA: 1
SVs in sample	250
Oscillating CN (2 and 3 states)	5, 5
CN segments	21
FDR fragment joints	0.615458
FDR chr. breakp. enrich.	0
Linked to chrs	
Purity, ploidy	0.53, 3.64

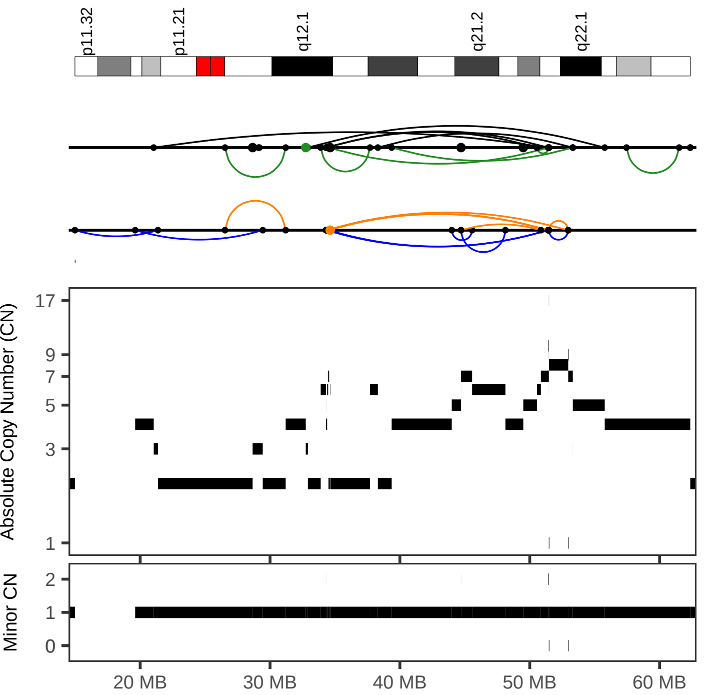

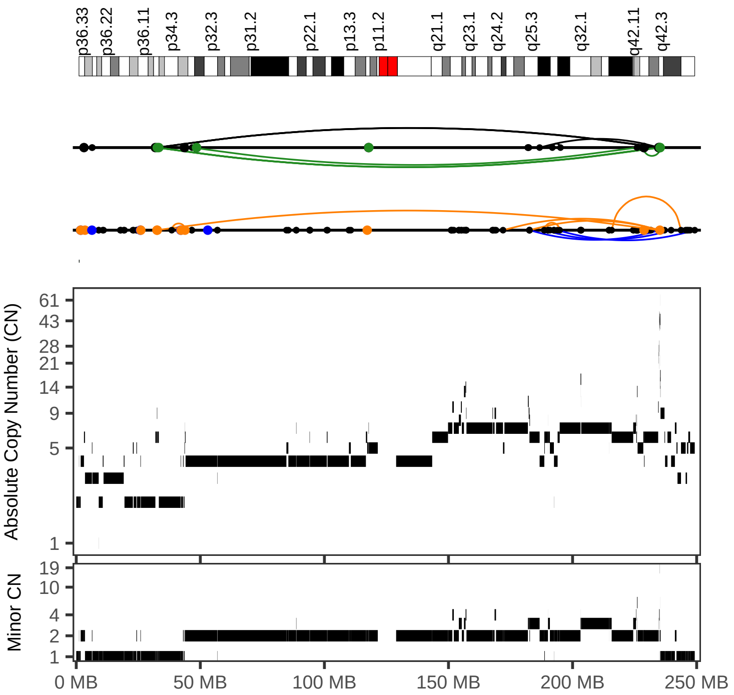

OCCAMS−SH−003

Cancer type	Eso−AdenoCA
Position	18:14973143−55776093
Type	With other complex events
Interleaved intrachr. SVs	23
Total SVs (intrachr. + transl.)	29
SV types	DEL: 6; DUP: 7; h2hINV: 5; t2tINV: 5; TRA: 6
SVs in sample	250
Oscillating CN (2 and 3 states)	5, 9
CN segments	46
FDR fragment joints	0.930656
FDR chr. breakp. enrich.	0
Linked to chrs	
Purity, ploidy	0.53, 3.64

OCCAMS−SH−024

Cancer type	Eso−AdenoCA
Position	1:32513613−249217731
Type	With other complex events
Interleaved intrachr. SVs	30
Total SVs (intrachr. + transl.)	52
SV types	DEL: 6; DUP: 12; h2hINV: 6; t2tINV: 6; TRA: 22
SVs in sample	656
Oscillating CN (2 and 3 states)	6, 13
CN segments	114
FDR fragment joints	0.9961719
FDR chr. breakp. enrich.	0
Linked to chrs	3:101679749−195089022;
Purity, ploidy	0.25, 4.5

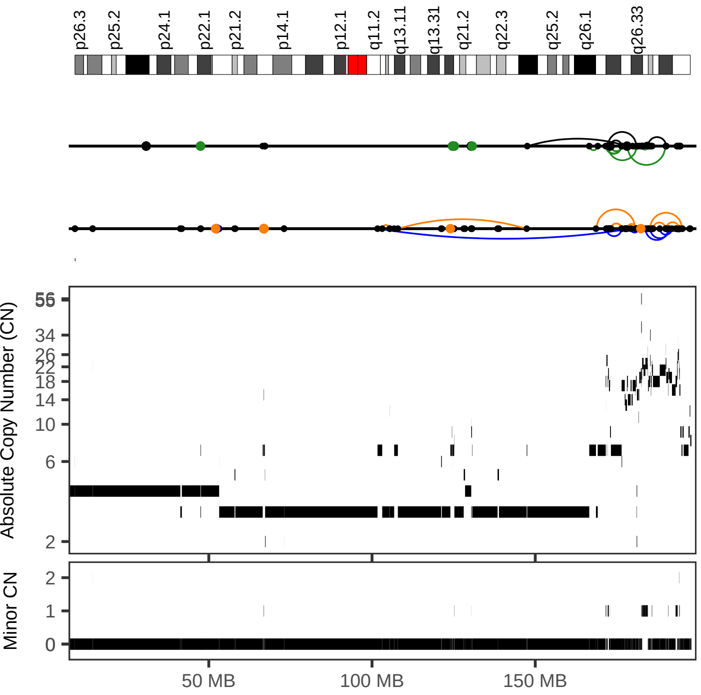

	**OCCAMS−SH−024**
Cancer type	Eso−AdenoCA
Position	3:101679749−195089023
Type	With other complex events
Interleaved intrachr. SVs	40
Total SVs (intrachr. + transl.)	49
SV types	DEL: 9; DUP: 14; h2hINV: 6; t2tINV: 11; TRA: 9
SVs in sample	656
Oscillating CN (2 and 3 states)	6, 6
CN segments	116
FDR fragment joints	0.7124738
FDR chr. breakp. enrich.	0
Linked to chrs	
Purity, ploidy	0.25, 4.5

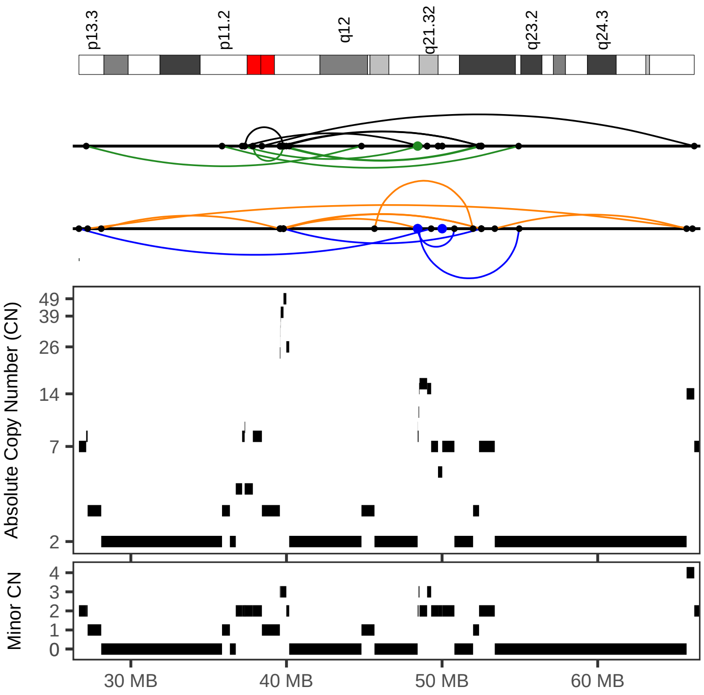

	**OCCAMS−SH−038**
Cancer type	Eso−AdenoCA
Position	17:26663234−66204181
Type	With other complex events
Interleaved intrachr. SVs	24
Total SVs (intrachr. + transl.)	28
SV types	DEL: 7; DUP: 4; h2hINV: 5; t2tINV: 8; TRA: 4
SVs in sample	146
Oscillating CN (2 and 3 states)	4, 4
CN segments	38
FDR fragment joints	0.9794281
FDR chr. breakp. enrich.	0
Linked to chrs	4:13404057−187928989;
Purity, ploidy	0.25, 2.35

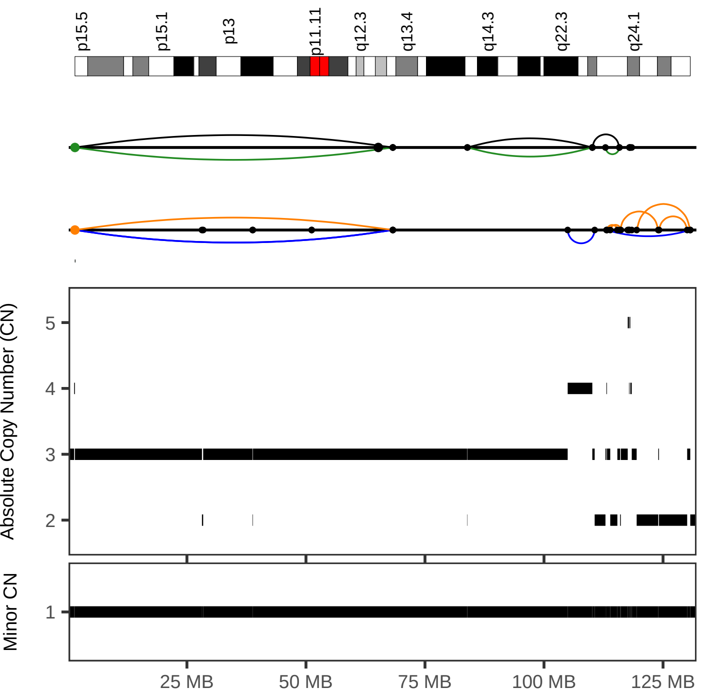

	**OCCAMS−SH−051**
Cancer type	Eso−AdenoCA
Position	11:83876112−130719233
Type	With other complex events
Interleaved intrachr. SVs	10
Total SVs (intrachr. + transl.)	10
SV types	DEL: 4; DUP: 2; h2hINV: 2; t2tINV: 2; TRA: 0
SVs in sample	450
Oscillating CN (2 and 3 states)	6, 12
CN segments	23
FDR fragment joints	0.8572806
FDR chr. breakp. enrich.	0.3
Linked to chrs	
Purity, ploidy	0.69, 2.44

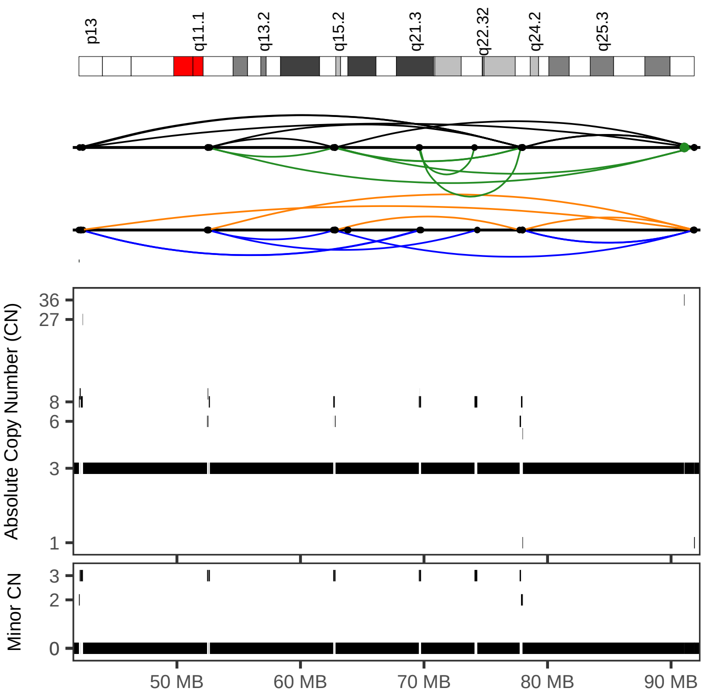

	**OCCAMS−SH−071**
Cancer type	Eso−AdenoCA
Position	15:42066409−91876190
Type	With other complex events
Interleaved intrachr. SVs	29
Total SVs (intrachr. + transl.)	32
SV types	DEL: 4; DUP: 10; h2hINV: 8; t2tINV: 7; TRA: 3
SVs in sample	284
Oscillating CN (2 and 3 states)	5, 5
CN segments	35
FDR fragment joints	0.615458
FDR chr. breakp. enrich.	0
Linked to chrs	
Purity, ploidy	0.42, 3.56

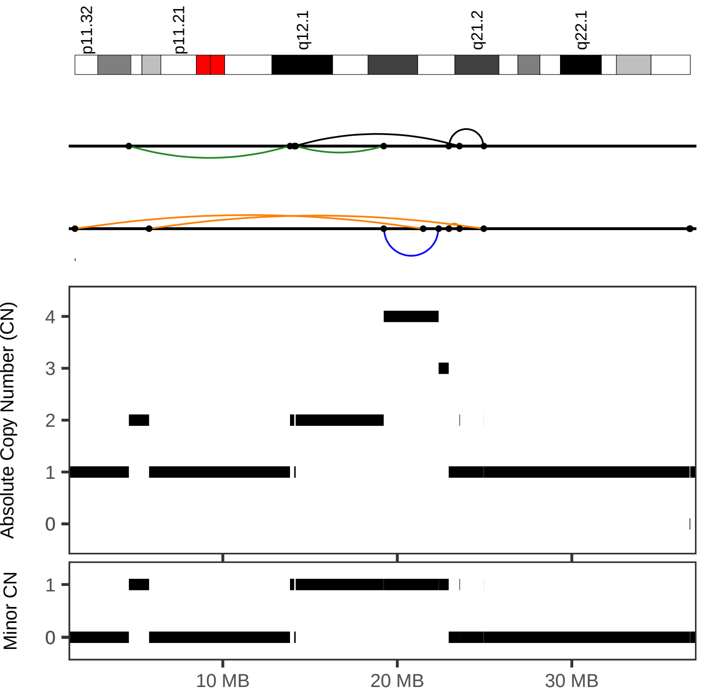

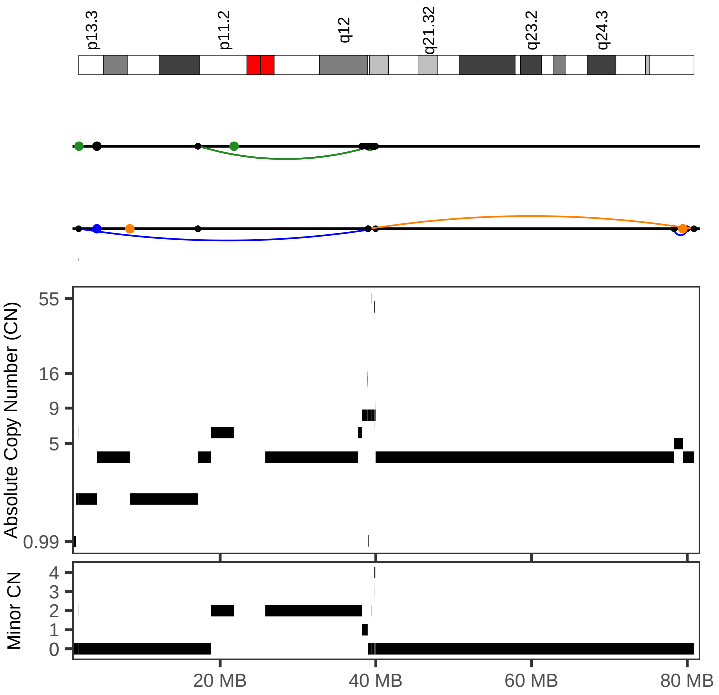

OCCAMS−ST−020	
Cancer type	Eso−AdenoCA
Position	18:1524644−24959091
Type	With other complex events
Interleaved intrachr. SVs	6
Total SVs (intrachr. + transl.)	6
SV types	DEL: 2; DUP: 1; h2hINV: 2; t2tINV: 1; TRA: 0
SVs in sample	165
Oscillating CN (2 and 3 states)	6, 6
CN segments	13
FDR fragment joints	0.9284301
FDR chr. breakp. enrich.	0.15
Linked to chrs	
Purity, ploidy	0.43, 1.76

OCCAMS−ST−029	
Cancer type	Eso−AdenoCA
Position	17:1828973−80871688
Type	With other complex events
Interleaved intrachr. SVs	9
Total SVs (intrachr. + transl.)	15
SV types	DEL: 2; DUP: 1; h2hINV: 2; t2tINV: 4; TRA: 6
SVs in sample	286
Oscillating CN (2 and 3 states)	4, 5
CN segments	29
FDR fragment joints	0.8462769
FDR chr. breakp. enrich.	0
Linked to chrs	
Purity, ploidy	0.35, 3.04

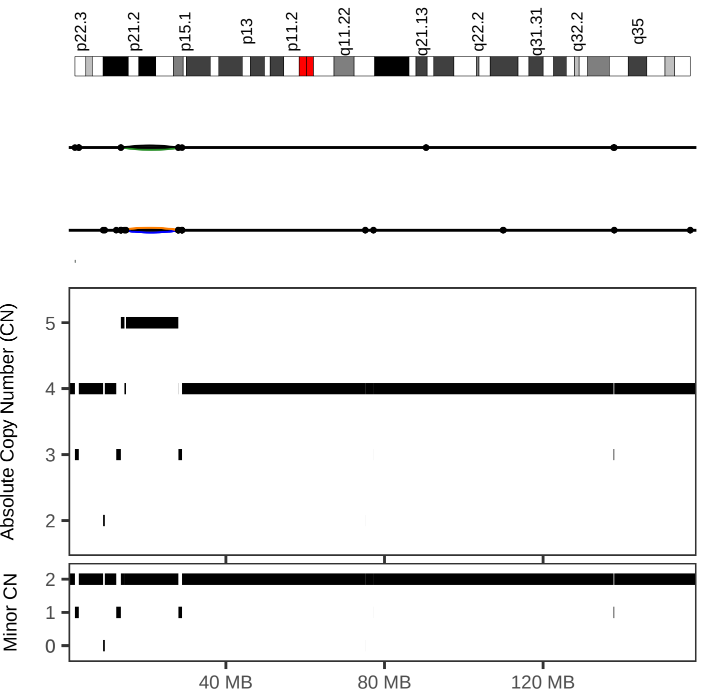

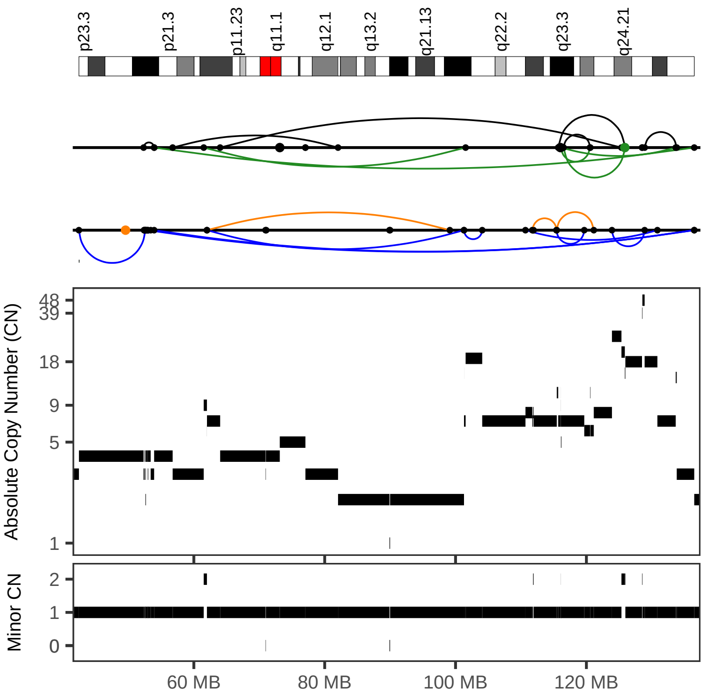

OCCAMS−ST−037	
Cancer type	Eso−AdenoCA
Position	7:12338056−28936629
Type	With other complex events
Interleaved intrachr. SVs	10
Total SVs (intrachr. + transl.)	10
SV types	DEL: 4; DUP: 3; h2hINV: 1; t2tINV: 2; TRA: 0
SVs in sample	438
Oscillating CN (2 and 3 states)	4, 5
CN segments	7
FDR fragment joints	0.6776251
FDR chr. breakp. enrich.	1
Linked to chrs	
Purity, ploidy	0.35, 3.47

OCCAMS−ST−037	
Cancer type	Eso−AdenoCA
Position	8:56764256−133807235
Type	With other complex events
Interleaved intrachr. SVs	17
Total SVs (intrachr. + transl.)	20
SV types	DEL: 3; DUP: 5; h2hINV: 5; t2tINV: 4; TRA: 3
SVs in sample	438
Oscillating CN (2 and 3 states)	5, 5
CN segments	40
FDR fragment joints	0.8572806
FDR chr. breakp. enrich.	0.01
Linked to chrs	
Purity, ploidy	0.35, 3.47

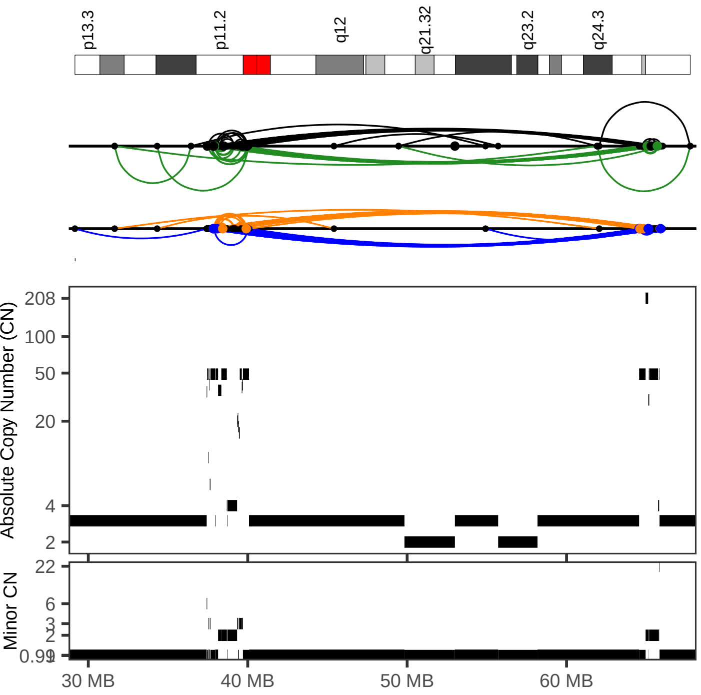

	OCCAMS−ST−037
Cancer type	Eso−AdenoCA
Position	17:29160620−67758256
Type	With other complex events
Interleaved intrachr. SVs	186
Total SVs (intrachr. + transl.)	215
SV types	DEL: 39; DUP: 51; h2hINV: 48; t2tINV: 48; TRA: 29
SVs in sample	438
Oscillating CN (2 and 3 states)	5, 5
CN segments	36
FDR fragment joints	0.7170622
FDR chr. breakp. enrich.	0
Linked to chrs	
Purity, ploidy	0.35, 3.47

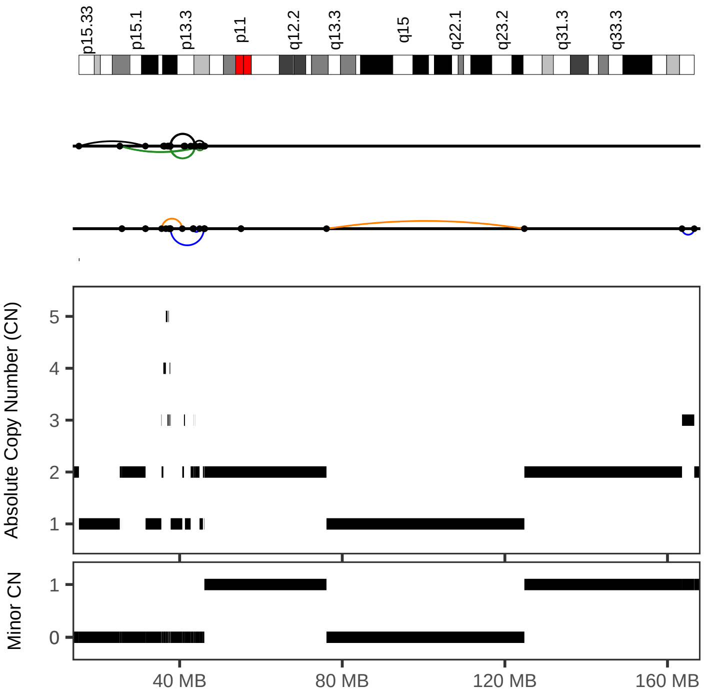

	OCCAMS−ST−041
Cancer type	Eso−AdenoCA
Position	5:15221088−46212518
Type	With other complex events
Interleaved intrachr. SVs	15
Total SVs (intrachr. + transl.)	15
SV types	DEL: 1; DUP: 4; h2hINV: 5; t2tINV: 5; TRA: 0
SVs in sample	163
Oscillating CN (2 and 3 states)	5, 8
CN segments	27
FDR fragment joints	0.6471662
FDR chr. breakp. enrich.	0
Linked to chrs	
Purity, ploidy	0.52, 2.4

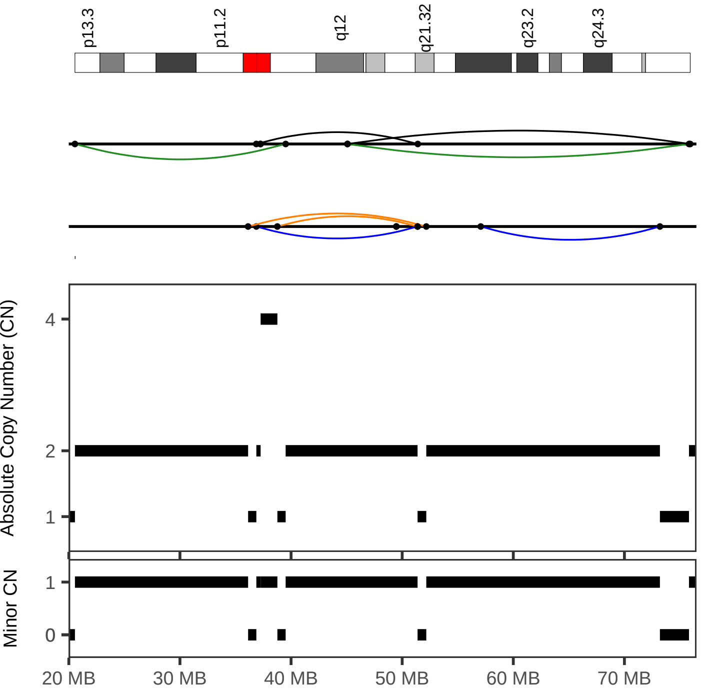

	OCCAMS−WG−001
Cancer type	Eso−AdenoCA
Position	17:20549700−75923156
Type	Canonical without polyploidization
Interleaved intrachr. SVs	7
Total SVs (intrachr. + transl.)	7
SV types	DEL: 2; DUP: 1; h2hINV: 2; t2tINV: 2; TRA: 0
SVs in sample	151
Oscillating CN (2 and 3 states)	6, 6
CN segments	10
FDR fragment joints	0.9599662
FDR chr. breakp. enrich.	0.02
Linked to chrs	
Purity, ploidy	0.53, 1.91

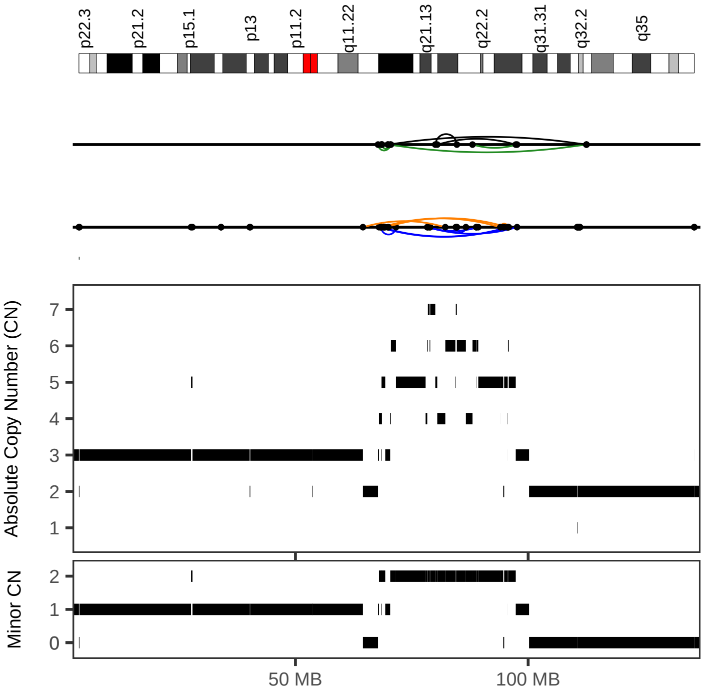

	OCCAMS−WG−005
Cancer type	Eso−AdenoCA
Position	7:64499272−112538689
Type	With other complex events
Interleaved intrachr. SVs	19
Total SVs (intrachr. + transl.)	19
SV types	DEL: 5; DUP: 6; h2hINV: 4; t2tINV: 4; TRA: 0
SVs in sample	277
Oscillating CN (2 and 3 states)	4, 6
CN segments	40
FDR fragment joints	0.9348624
FDR chr. breakp. enrich.	0
Linked to chrs	
Purity, ploidy	0.38, 2.47

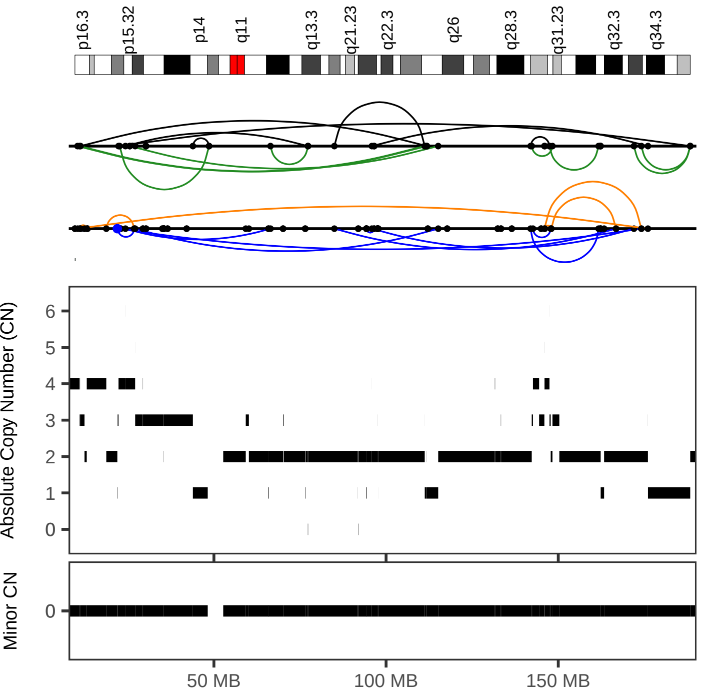

	OCCAMS−WG−008
Cancer type	Eso−AdenoCA
Position	4:10386453−188335991
Type	With other complex events
Interleaved intrachr. SVs	35
Total SVs (intrachr. + transl.)	36
SV types	DEL: 6; DUP: 11; h2hINV: 8; t2tINV: 10; TRA: 1
SVs in sample	357
Oscillating CN (2 and 3 states)	4, 11
CN segments	67
FDR fragment joints	0.6776251
FDR chr. breakp. enrich.	0
Linked to chrs	
Purity, ploidy	0.4, 2.6

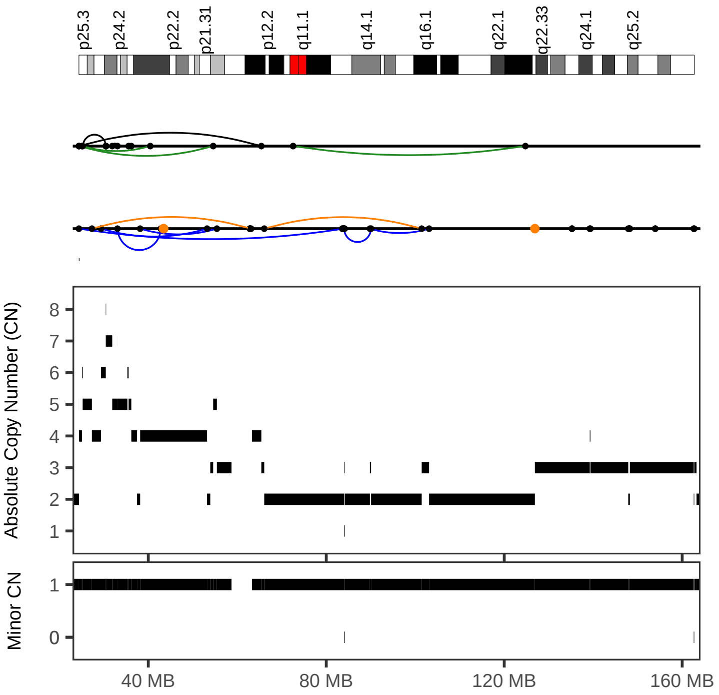

	OCCAMS−WG−008
Cancer type	Eso−AdenoCA
Position	6:24415766−65407598
Type	With other complex events
Interleaved intrachr. SVs	10
Total SVs (intrachr. + transl.)	12
SV types	DEL: 1; DUP: 4; h2hINV: 3; t2tINV: 2; TRA: 2
SVs in sample	357
Oscillating CN (2 and 3 states)	4, 6
CN segments	23
FDR fragment joints	0.9284301
FDR chr. breakp. enrich.	0.21
Linked to chrs	
Purity, ploidy	0.4, 2.6

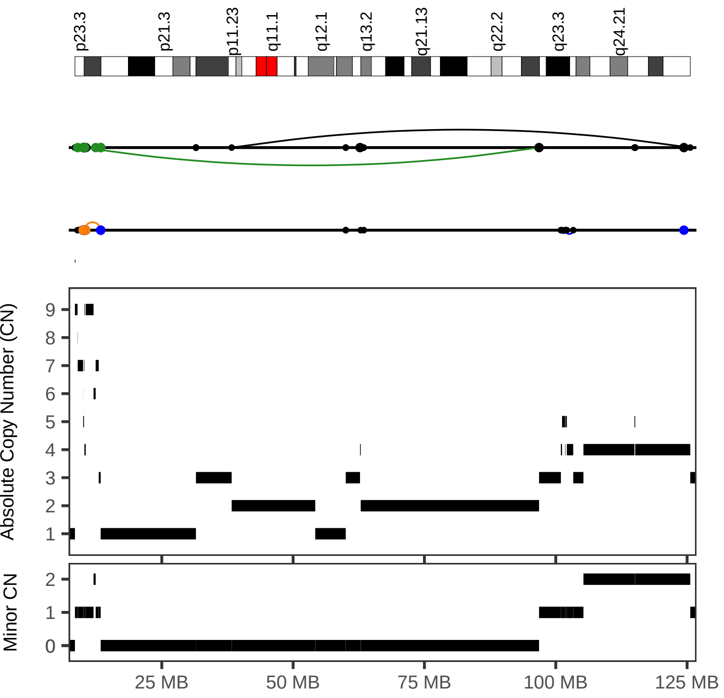

	OCCAMS−ZZ−004
Cancer type	Eso−AdenoCA
Position	8:8960637−125604409
Type	With other complex events
Interleaved intrachr. SVs	7
Total SVs (intrachr. + transl.)	47
SV types	DEL: 3; DUP: 1; h2hINV: 1; t2tINV: 2; TRA: 40
SVs in sample	323
Oscillating CN (2 and 3 states)	5, 12
CN segments	31
FDR fragment joints	0.6776251
FDR chr. breakp. enrich.	0
Linked to chrs	
Purity, ploidy	0.56, 2.11

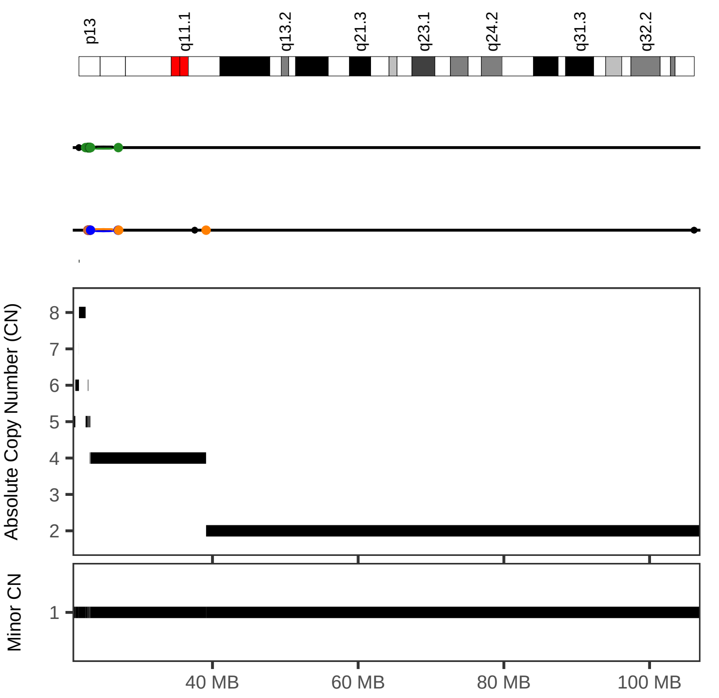

	OCCAMS−ZZ−004
Cancer type	Eso−AdenoCA
Position	14:22899463−27108658
Type	After polyploidization
Interleaved intrachr. SVs	38
Total SVs (intrachr. + transl.)	70
SV types	DEL: 8; DUP: 6; h2hINV: 12; t2tINV: 12; TRA: 32
SVs in sample	323
Oscillating CN (2 and 3 states)	6, 7
CN segments	7
FDR fragment joints	0.615458
FDR chr. breakp. enrich.	0
Linked to chrs	8:8960637−125604408;
Purity, ploidy	0.56, 2.11

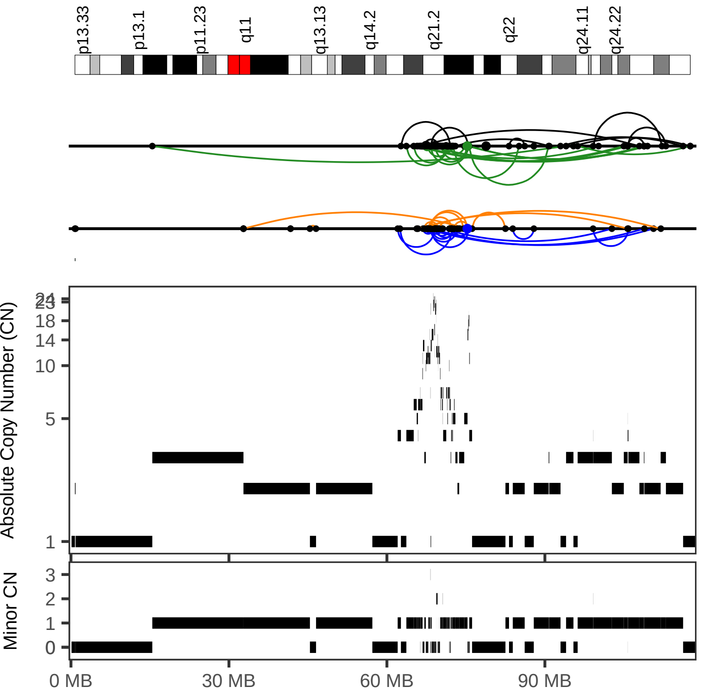

	**OCCAMS−ZZ−009**
Cancer type	Eso−AdenoCA
Position	12:15448042−117609293
Type	With other complex events
Interleaved intrachr. SVs	82
Total SVs (intrachr. + transl.)	90
SV types	DEL: 13; DUP: 19; h2hINV: 22; t2tINV: 28; TRA: 8
SVs in sample	353
Oscillating CN (2 and 3 states)	6, 10
CN segments	110
FDR fragment joints	0.5435077
FDR chr. breakp. enrich.	0
Linked to chrs	1:2485334−247277614;
Purity, ploidy	0.44, 2.04

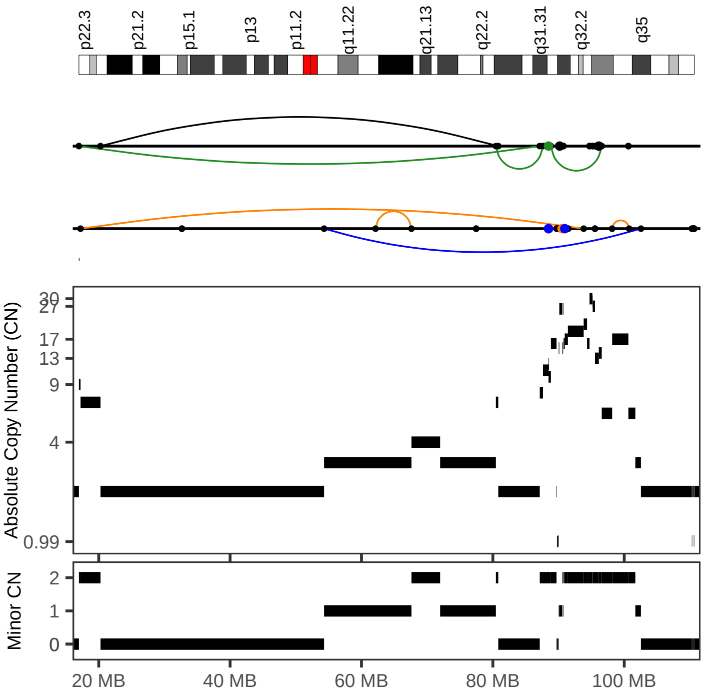

	**OCCAMS−ZZ−016**
Cancer type	Eso−AdenoCA
Position	7:16983311−102542434
Type	With other complex events
Interleaved intrachr. SVs	6
Total SVs (intrachr. + transl.)	13
SV types	DEL: 1; DUP: 1; h2hINV: 1; t2tINV: 3; TRA: 7
SVs in sample	286
Oscillating CN (2 and 3 states)	6, 6
CN segments	38
FDR fragment joints	0.7995907
FDR chr. breakp. enrich.	0
Linked to chrs	
Purity, ploidy	0.5, 2.45

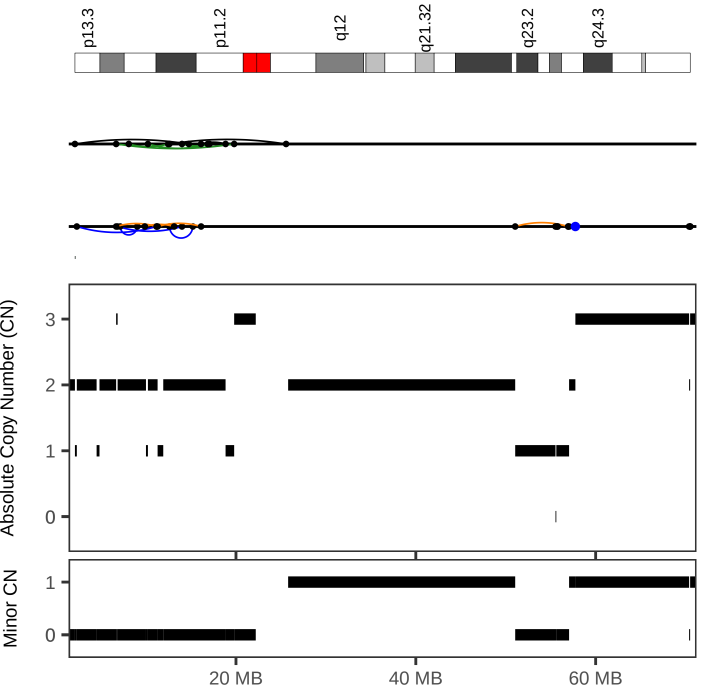

	**OCCAMS−ZZ−016**
Cancer type	Eso−AdenoCA
Position	17:2088600−25571414
Type	With other complex events
Interleaved intrachr. SVs	14
Total SVs (intrachr. + transl.)	14
SV types	DEL: 3; DUP: 4; h2hINV: 3; t2tINV: 4; TRA: 0
SVs in sample	286
Oscillating CN (2 and 3 states)	6, 8
CN segments	13
FDR fragment joints	0.9794281
FDR chr. breakp. enrich.	0
Linked to chrs	
Purity, ploidy	0.5, 2.45

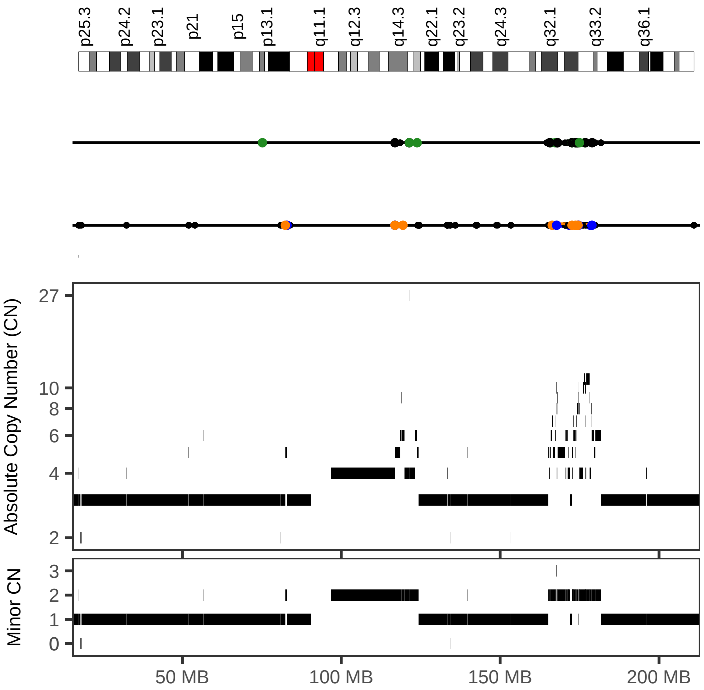

	**3e5f451a−5882−4914−ae1a−95c898c2bcca**
Cancer type	Head−SCC
Position	2:164623105−179954501
Type	With other complex events
Interleaved intrachr. SVs	30
Total SVs (intrachr. + transl.)	52
SV types	DEL: 6; DUP: 10; h2hINV: 6; t2tINV: 8; TRA: 22
SVs in sample	362
Oscillating CN (2 and 3 states)	5, 15
CN segments	56
FDR fragment joints	0.6776251
FDR chr. breakp. enrich.	0
Linked to chrs	17:37011270−62375855;
Purity, ploidy	0.58, 3.09

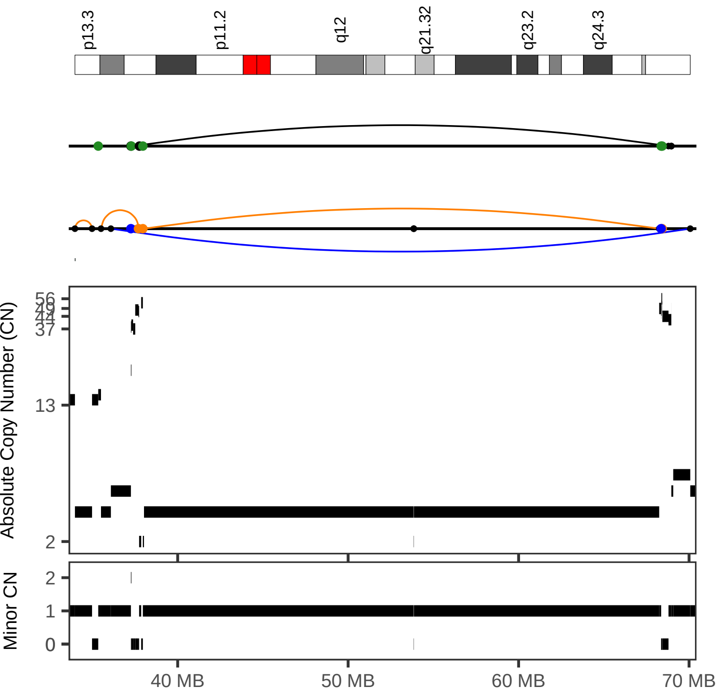

	64bb5550−2735−4401−a0db−58ec1020a32d
Cancer type	Head−SCC
Position	17:35502073−70069761
Type	With other complex events
Interleaved intrachr. SVs	6
Total SVs (intrachr. + transl.)	17
SV types	DEL: 2; DUP: 3; h2hINV: 1; t2tINV: 0; TRA: 11
SVs in sample	179
Oscillating CN (2 and 3 states)	4, 4
CN segments	26
FDR fragment joints	0.7735152
FDR chr. breakp. enrich.	0
Linked to chrs	
Purity, ploidy	0.36, 3.11

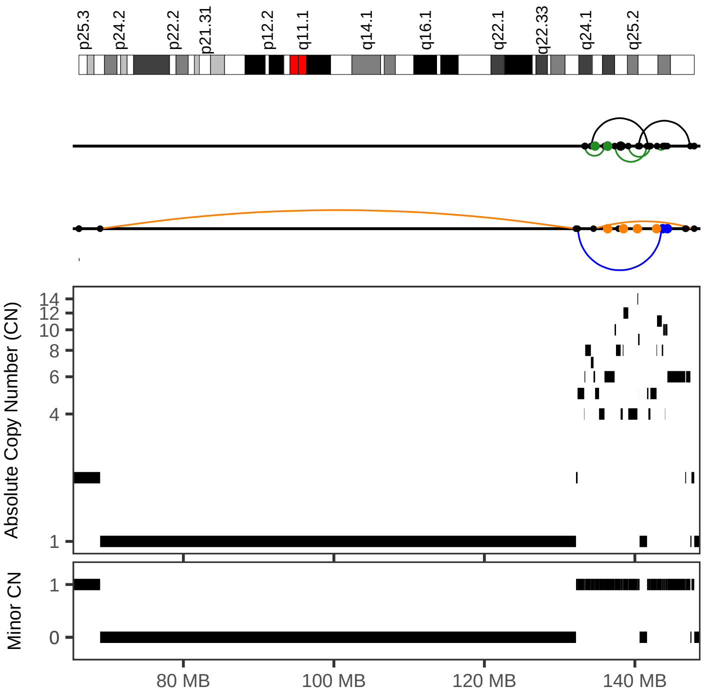

	7de19081−d5fd−468c−ad0d−f6e3e8b2ad70
Cancer type	Head−SCC
Position	6:132384731−147888916
Type	With other complex events
Interleaved intrachr. SVs	8
Total SVs (intrachr. + transl.)	18
SV types	DEL: 1; DUP: 1; h2hINV: 2; t2tINV: 4; TRA: 10
SVs in sample	140
Oscillating CN (2 and 3 states)	4, 5
CN segments	36
FDR fragment joints	0.8653243
FDR chr. breakp. enrich.	0
Linked to chrs	
Purity, ploidy	0.83, 1.95

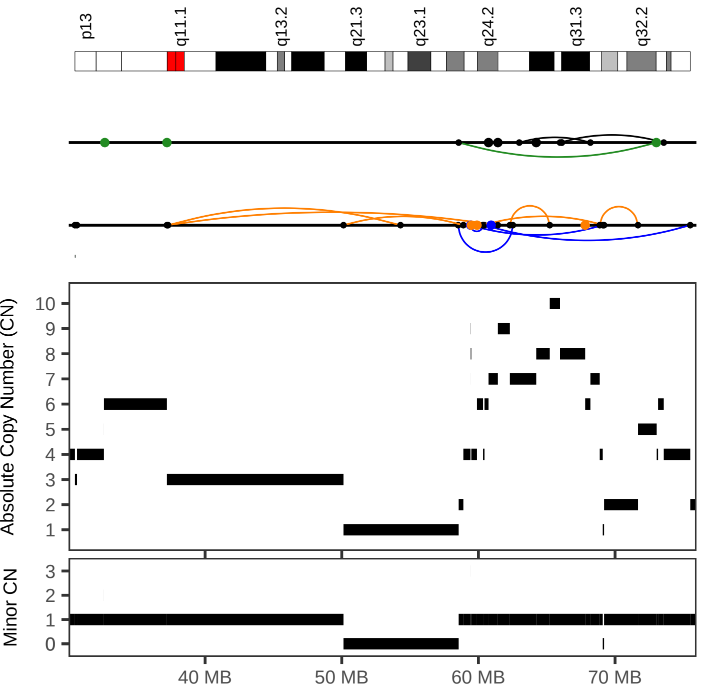

	7de19081−d5fd−468c−ad0d−f6e3e8b2ad70
Cancer type	Head−SCC
Position	14:37204606−75501405
Type	With other complex events
Interleaved intrachr. SVs	13
Total SVs (intrachr. + transl.)	22
SV types	DEL: 6; DUP: 4; h2hINV: 2; t2tINV: 1; TRA: 9
SVs in sample	140
Oscillating CN (2 and 3 states)	4, 4
CN segments	27
FDR fragment joints	0.615458
FDR chr. breakp. enrich.	0
Linked to chrs	
Purity, ploidy	0.83, 1.95

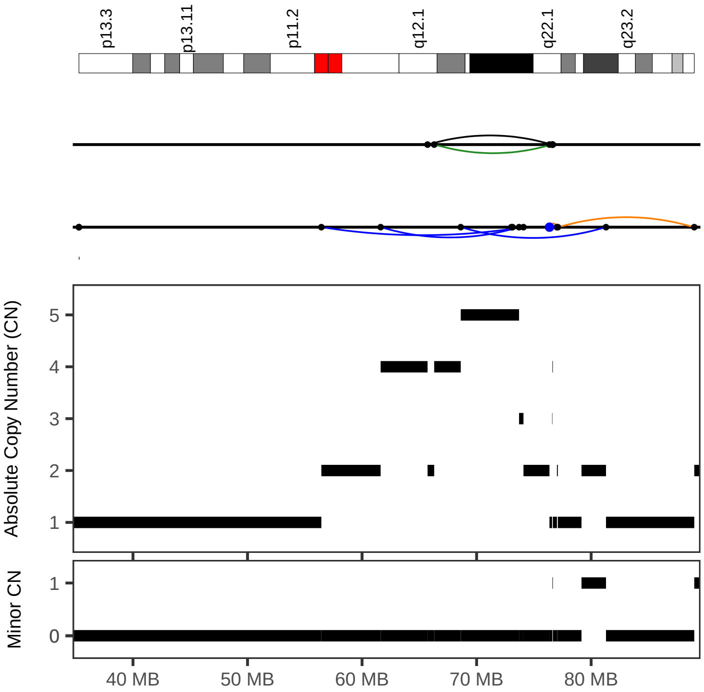

	7de19081−d5fd−468c−ad0d−f6e3e8b2ad70
Cancer type	Head−SCC
Position	16:56444067−89001559
Type	With other complex events
Interleaved intrachr. SVs	8
Total SVs (intrachr. + transl.)	9
SV types	DEL: 2; DUP: 3; h2hINV: 2; t2tINV: 1; TRA: 1
SVs in sample	140
Oscillating CN (2 and 3 states)	6, 8
CN segments	16
FDR fragment joints	0.6776251
FDR chr. breakp. enrich.	0
Linked to chrs	
Purity, ploidy	0.83, 1.95

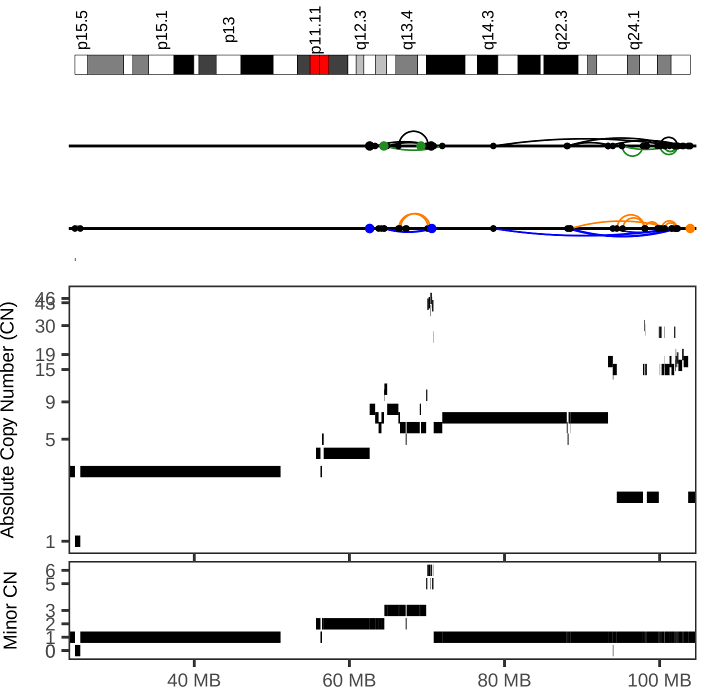

	8fc1f1be−d2d5−4b3a−9973−f4d964018beb
Cancer type	Head−SCC
Position	11:78556791−103094093
Type	With other complex events
Interleaved intrachr. SVs	24
Total SVs (intrachr. + transl.)	24
SV types	DEL: 5; DUP: 7; h2hINV: 6; t2tINV: 6; TRA: 0
SVs in sample	216
Oscillating CN (2 and 3 states)	5, 6
CN segments	34
FDR fragment joints	0.9723381
FDR chr. breakp. enrich.	0
Linked to chrs	
Purity, ploidy	0.71, 2.99

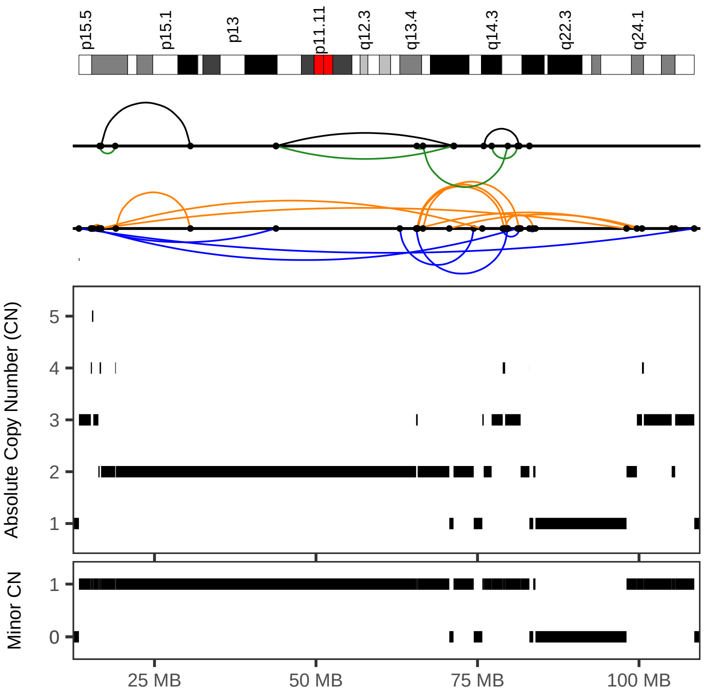

	90a8d2cb−1f31−45f9−8575−eed3cb9a7798
Cancer type	Head−SCC
Position	11:15147379−100462923
Type	With other complex events
Interleaved intrachr. SVs	24
Total SVs (intrachr. + transl.)	24
SV types	DEL: 9; DUP: 7; h2hINV: 3; t2tINV: 5; TRA: 0
SVs in sample	67
Oscillating CN (2 and 3 states)	5, 8
CN segments	26
FDR fragment joints	0.615458
FDR chr. breakp. enrich.	0
Linked to chrs	
Purity, ploidy	0.61, 1.9

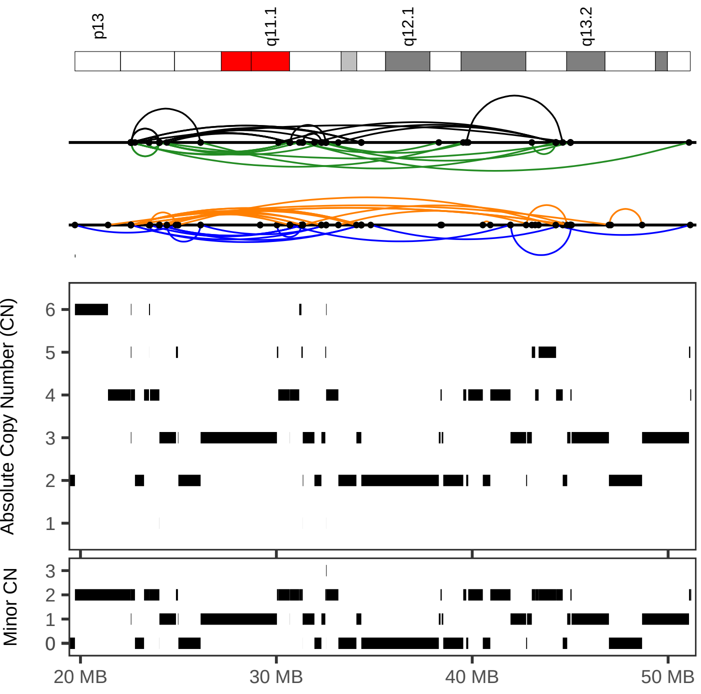

	ad6c9d09−2c03−4786−a72d−dd2aa5f603d4
Cancer type	Head−SCC
Position	22:19715951−51129703
Type	With other complex events
Interleaved intrachr. SVs	68
Total SVs (intrachr. + transl.)	68
SV types	DEL: 18; DUP: 15; h2hINV: 19; t2tINV: 16; TRA: 0
SVs in sample	360
Oscillating CN (2 and 3 states)	6, 8
CN segments	61
FDR fragment joints	0.9332614
FDR chr. breakp. enrich.	0
Linked to chrs	
Purity, ploidy	0.65, 3.12

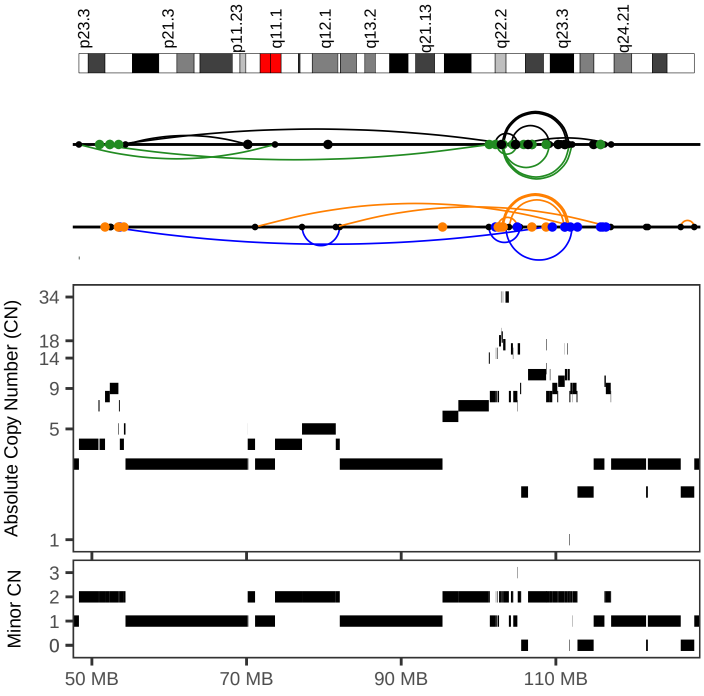

	70e306ee−9584−49d6−81a1−a49d837ab6aa
Cancer type	Kidney−ChRCC
Position	8:48323075−117143870
Type	With other complex events
Interleaved intrachr. SVs	26
Total SVs (intrachr. + transl.)	74
SV types	DEL: 7; DUP: 5; h2hINV: 8; t2tINV: 6; TRA: 48
SVs in sample	305
Oscillating CN (2 and 3 states)	5, 8
CN segments	70
FDR fragment joints	0.9807783
FDR chr. breakp. enrich.	0
Linked to chrs	15:40179423−68153781;3:27225774−76470708
Purity, ploidy	0.89, 3.06

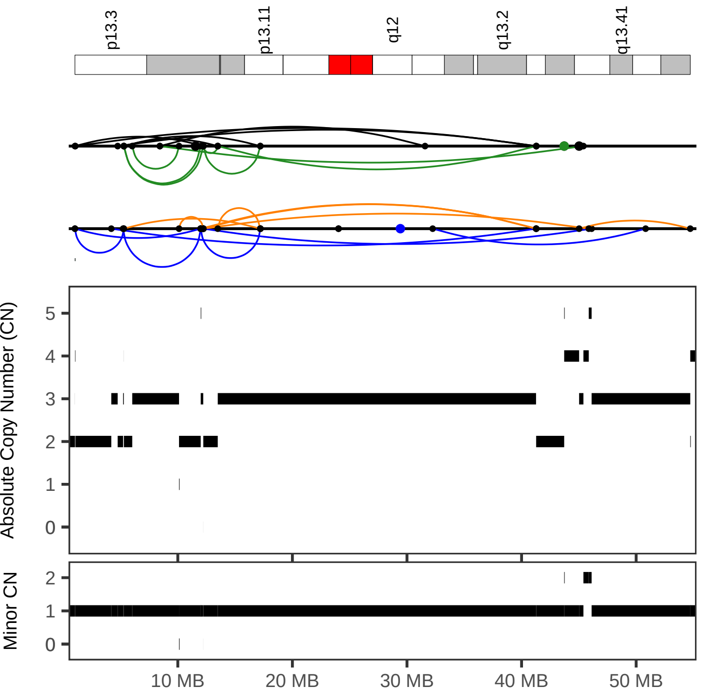

	**b809c2f2−185c−496a−a1d4−589294443a1d**
Cancer type	Kidney−RCC
Position	19:1021778−54719797
Type	With other complex events
Interleaved intrachr. SVs	28
Total SVs (intrachr. + transl.)	33
SV types	DEL: 7; DUP: 8; h2hINV: 6; t2tINV: 7; TRA: 5
SVs in sample	64
Oscillating CN (2 and 3 states)	5, 9
CN segments	30
FDR fragment joints	0.9067785
FDR chr. breakp. enrich.	0
Linked to chrs	
Purity, ploidy	0.78, 3.63

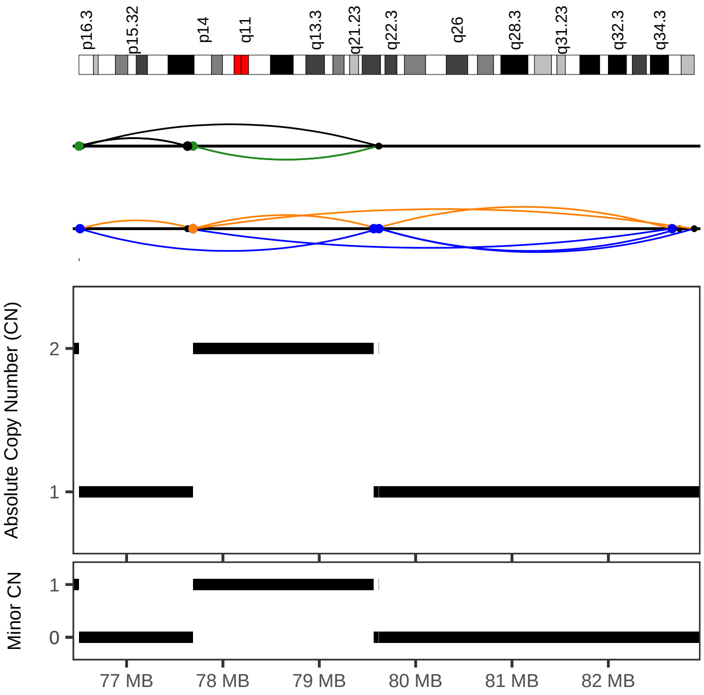

	**d80f601b−1064−4ff0−9c51−7f6afb22d1f5**
Cancer type	Kidney−RCC
Position	4:76505435−82891389
Type	Canonical without polyploidization
Interleaved intrachr. SVs	17
Total SVs (intrachr. + transl.)	27
SV types	DEL: 5; DUP: 5; h2hINV: 3; t2tINV: 4; TRA: 10
SVs in sample	61
Oscillating CN (2 and 3 states)	6, 6
CN segments	6
FDR fragment joints	0.6776251
FDR chr. breakp. enrich.	0
Linked to chrs	
Purity, ploidy	0.84, 1.98

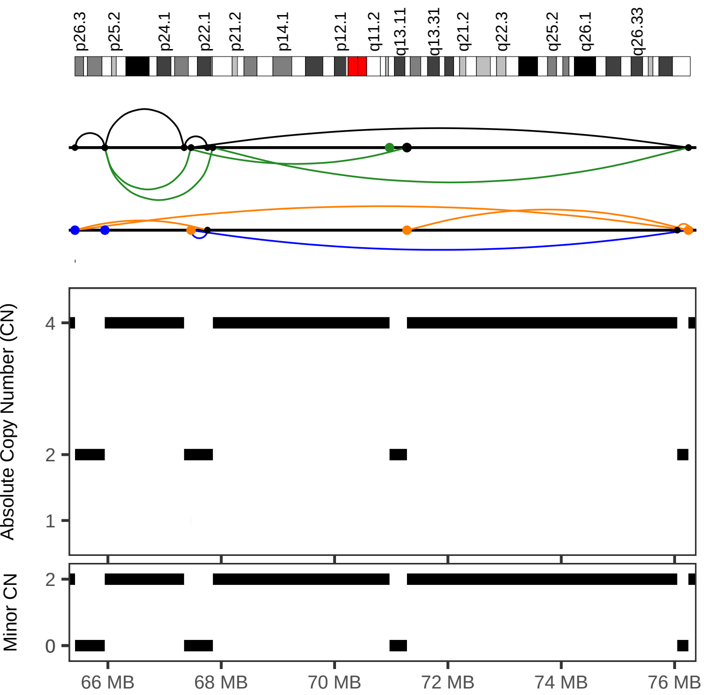

	**C0066**
Cancer type	Kidney−RCC
Position	3:65418245−76275813
Type	With other complex events
Interleaved intrachr. SVs	14
Total SVs (intrachr. + transl.)	22
SV types	DEL: 4; DUP: 2; h2hINV: 4; t2tINV: 4; TRA: 8
SVs in sample	25
Oscillating CN (2 and 3 states)	6, 11
CN segments	11
FDR fragment joints	0.8874881
FDR chr. breakp. enrich.	0
Linked to chrs	
Purity, ploidy	0.41, 3.57

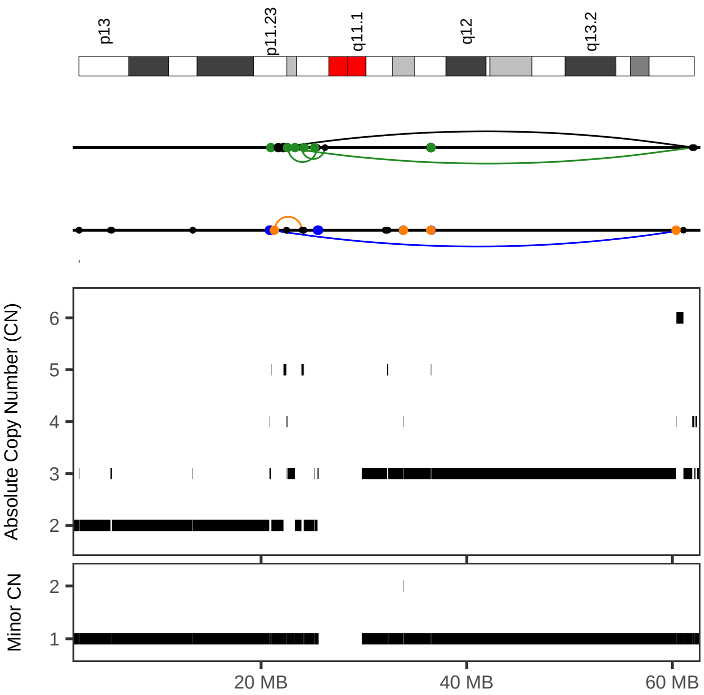

	**1987b453−f97d−45c7−9c89−b9a33313d645**
Cancer type	Liver−HCC
Position	20:20959449−62129528
Type	With other complex events
Interleaved intrachr. SVs	8
Total SVs (intrachr. + transl.)	25
SV types	DEL: 1; DUP: 2; h2hINV: 1; t2tINV: 4; TRA: 17
SVs in sample	524
Oscillating CN (2 and 3 states)	6, 8
CN segments	28
FDR fragment joints	0.9599662
FDR chr. breakp. enrich.	0
Linked to chrs	1:9273332−226103049;16:25250822−30860383 4:122039841−145149273;6:5868963−162834995
Purity, ploidy	0.74, 1.99

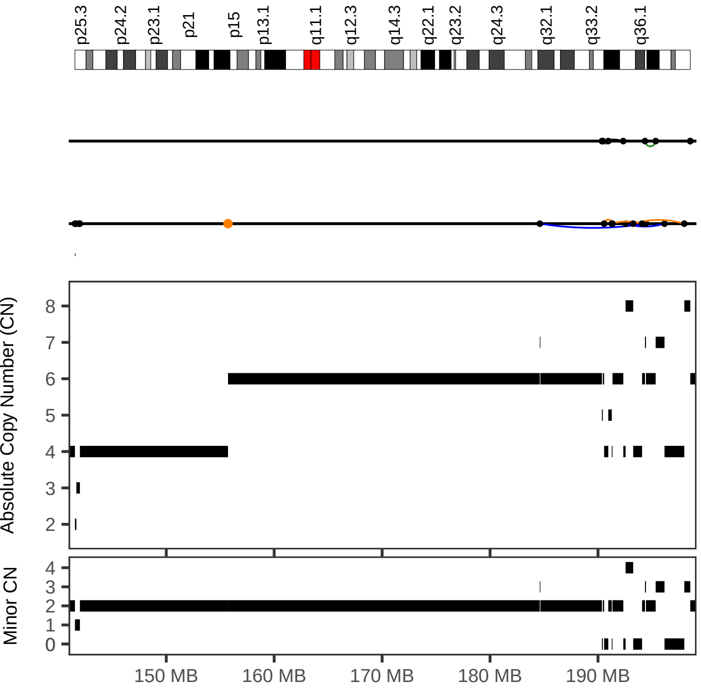

	2854ffe8−1f77−497a−bf49−11e0189fee35
Cancer type	Liver−HCC
Position	2:184611141−197996965
Type	With other complex events
Interleaved intrachr. SVs	8
Total SVs (intrachr. + transl.)	8
SV types	DEL: 3; DUP: 2; h2hINV: 1;<br>t2tINV: 2; TRA: 0
SVs in sample	43
Oscillating CN (2 and 3 states)	4, 8
CN segments	17
FDR fragment joints	0.8653243
FDR chr. breakp. enrich.	0
Linked to chrs	
Purity, ploidy	0.69, 3.74

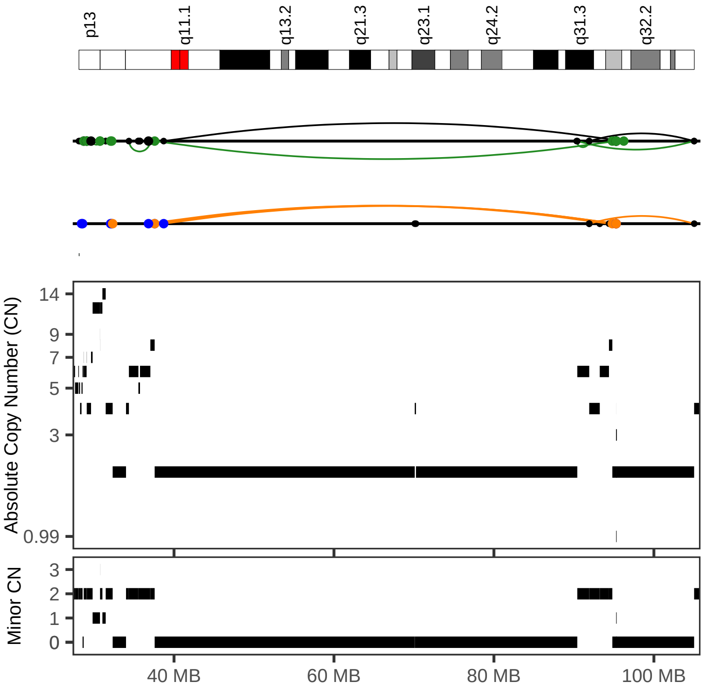

	3acf7438−fd65−442c−8a30−68b6714537f3
Cancer type	Liver−HCC
Position	14:34361058−105047156
Type	With other complex events
Interleaved intrachr. SVs	10
Total SVs (intrachr. + transl.)	31
SV types	DEL: 4; DUP: 0; h2hINV: 2;<br>t2tINV: 4; TRA: 21
SVs in sample	222
Oscillating CN (2 and 3 states)	4, 5
CN segments	21
FDR fragment joints	0.6776251
FDR chr. breakp. enrich.	0
Linked to chrs	5:141997−63388892;
Purity, ploidy	0.36, 3.55

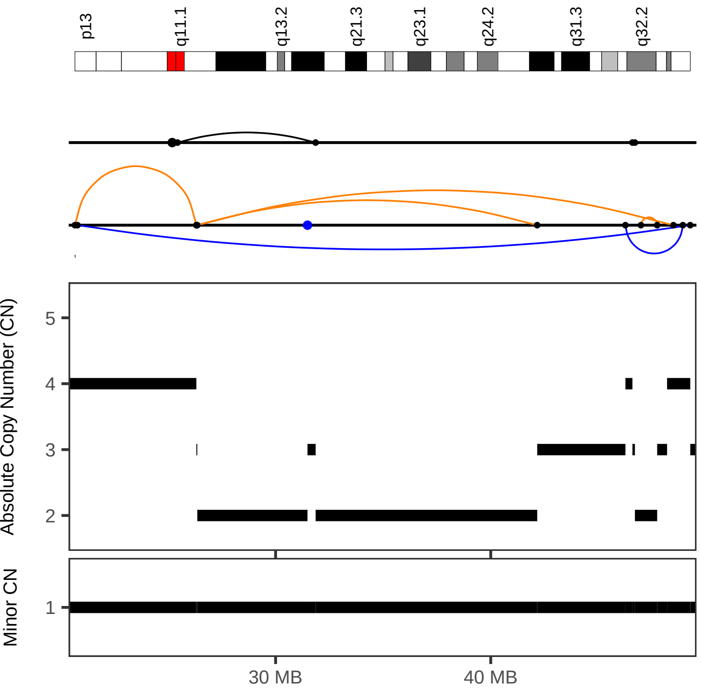

	52292ffc−0902−4d97−b461−20723987a177
Cancer type	Liver−HCC
Position	14:20673089−49273351
Type	With other complex events
Interleaved intrachr. SVs	6
Total SVs (intrachr. + transl.)	8
SV types	DEL: 3; DUP: 2; h2hINV: 1;<br>t2tINV: 0; TRA: 2
SVs in sample	129
Oscillating CN (2 and 3 states)	5, 12
CN segments	12
FDR fragment joints	0.6776251
FDR chr. breakp. enrich.	0.01
Linked to chrs	
Purity, ploidy	0.79, 3.28

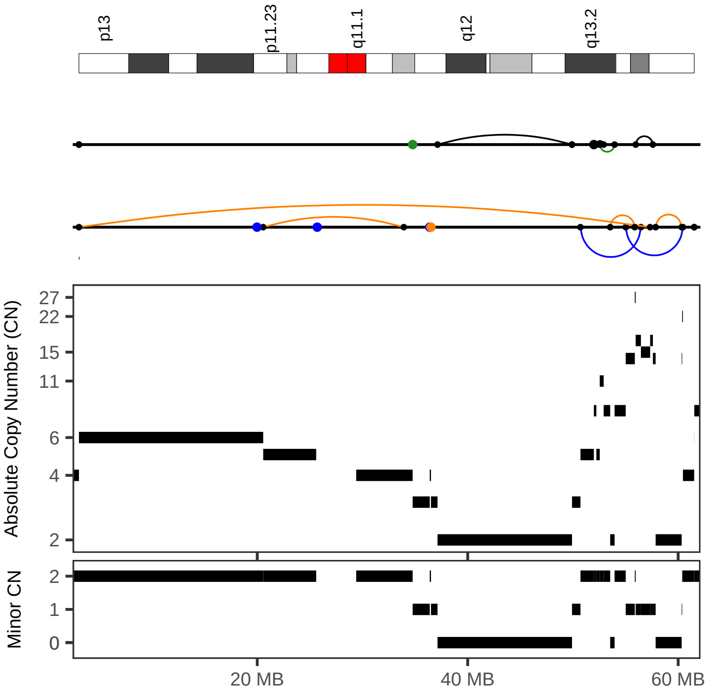

	56a82a56−0241−4d3d−9de2−696b0c36df91
Cancer type	Liver−HCC
Position	20:3053950−60453503
Type	With other complex events
Interleaved intrachr. SVs	7
Total SVs (intrachr. + transl.)	13
SV types	DEL: 2; DUP: 2; h2hINV: 2;<br>t2tINV: 1; TRA: 6
SVs in sample	109
Oscillating CN (2 and 3 states)	4, 5
CN segments	25
FDR fragment joints	0.7995907
FDR chr. breakp. enrich.	0
Linked to chrs	
Purity, ploidy	0.88, 3.52

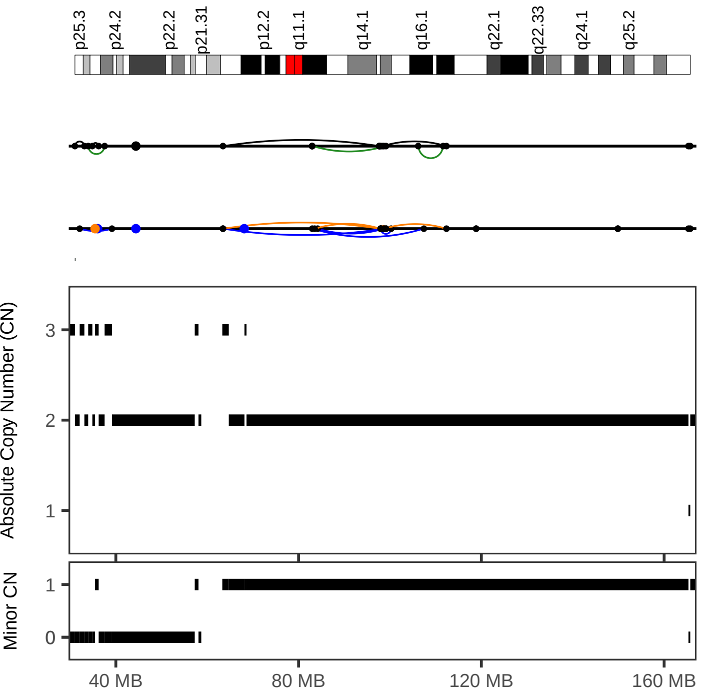

	614c0ecf−6a84−4c86−8706−79be5bb00991
Cancer type	Liver−HCC
Position	6:63448509−112361461
Type	Canonical without polyploidization
Interleaved intrachr. SVs	16
Total SVs (intrachr. + transl.)	17
SV types	DEL: 4; DUP: 5; h2hINV: 4; t2tINV: 3; TRA: 1
SVs in sample	171
Oscillating CN (2 and 3 states)	4, 4
CN segments	4
FDR fragment joints	0.8653243
FDR chr. breakp. enrich.	0
Linked to chrs	
Purity, ploidy	0.82, 2.44

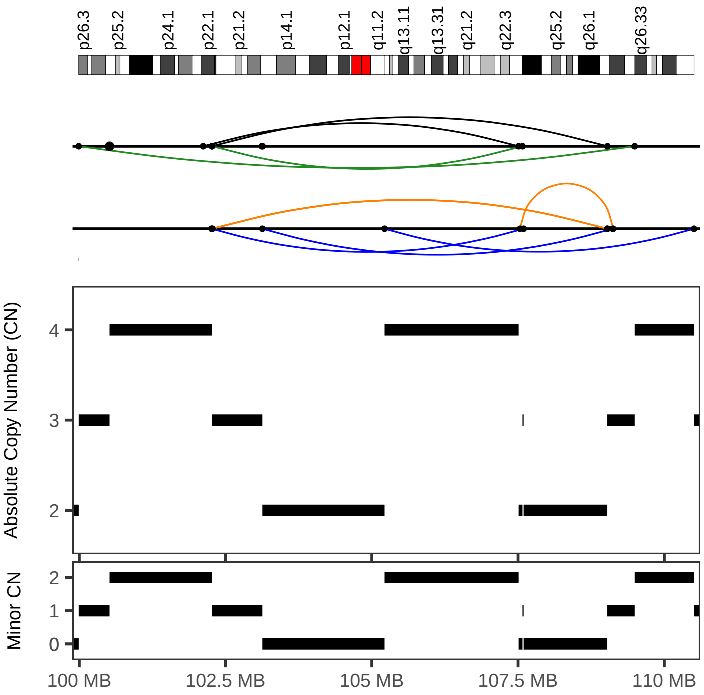

	65e430e1−0c9a−4045−83a9−653b7cff811d
Cancer type	Liver−HCC
Position	3:99990176−110508418
Type	With other complex events
Interleaved intrachr. SVs	11
Total SVs (intrachr. + transl.)	12
SV types	DEL: 3; DUP: 3; h2hINV: 3; t2tINV: 2; TRA: 1
SVs in sample	54
Oscillating CN (2 and 3 states)	4, 7
CN segments	11
FDR fragment joints	1
FDR chr. breakp. enrich.	0
Linked to chrs	
Purity, ploidy	0.64, 2.32

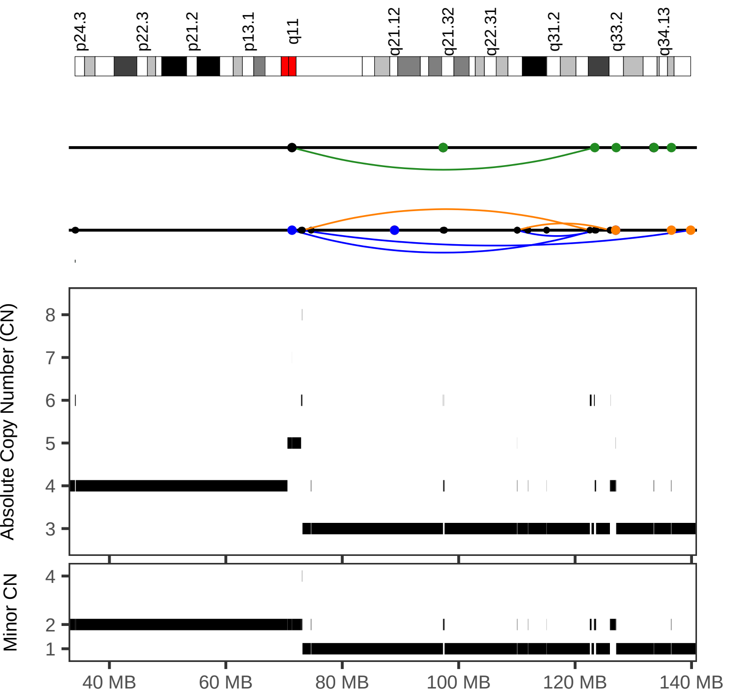

	6dcfc418−ab28−4365−95ea−c1ae254f2341
Cancer type	Liver−HCC
Position	9:71361828−139860149
Type	With other complex events
Interleaved intrachr. SVs	6
Total SVs (intrachr. + transl.)	20
SV types	DEL: 2; DUP: 3; h2hINV: 0; t2tINV: 1; TRA: 14
SVs in sample	572
Oscillating CN (2 and 3 states)	6, 6
CN segments	36
FDR fragment joints	0.8572806
FDR chr. breakp. enrich.	0.11
Linked to chrs	14:70988852−75703456;19:1843528−50016660
Purity, ploidy	0.85, 3.57

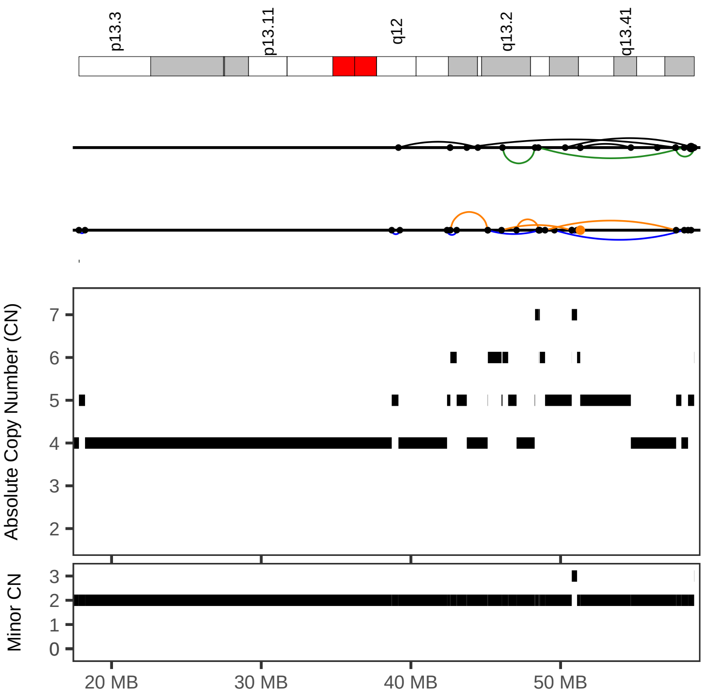

	8afa3140−dcbd−44c0−ab13−4ada6a0444d4
Cancer type	Liver−HCC
Position	19:38719064−58932021
Type	With other complex events
Interleaved intrachr. SVs	14
Total SVs (intrachr. + transl.)	16
SV types	DEL: 4; DUP: 4; h2hINV: 3; t2tINV: 3; TRA: 2
SVs in sample	301
Oscillating CN (2 and 3 states)	5, 14
CN segments	27
FDR fragment joints	0.9501265
FDR chr. breakp. enrich.	0
Linked to chrs	
Purity, ploidy	0.84, 3.54

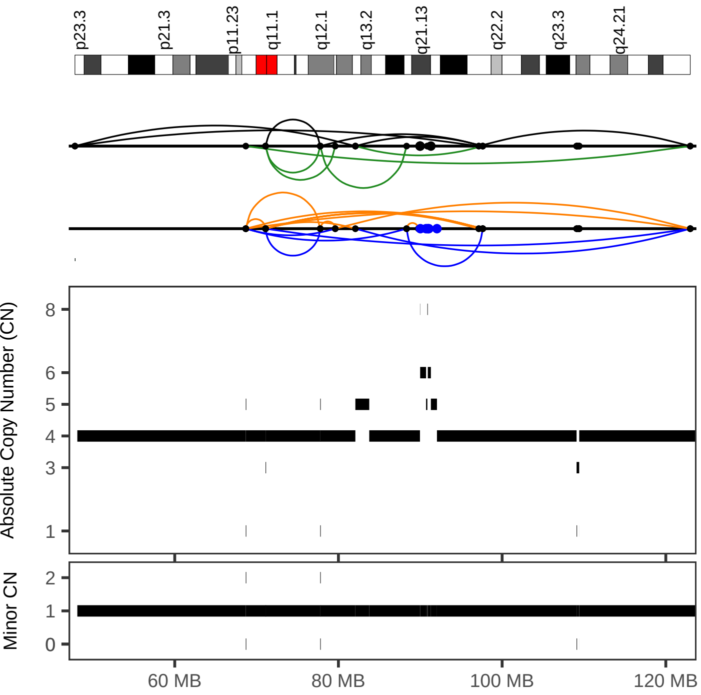

	b865dec4−f051−4fbe−9405−f832ff2010d7
Cancer type	Liver−HCC
Position	8:47804145−122986495
Type	With other complex events
Interleaved intrachr. SVs	27
Total SVs (intrachr. + transl.)	35
SV types	DEL: 10; DUP: 6; h2hINV: 6;<br>t2tINV: 5; TRA: 8
SVs in sample	245
Oscillating CN (2 and 3 states)	4, 6
CN segments	25
FDR fragment joints	0.7526697
FDR chr. breakp. enrich.	0
Linked to chrs	11:49377920−77893091;
Purity, ploidy	0.78, 3.47

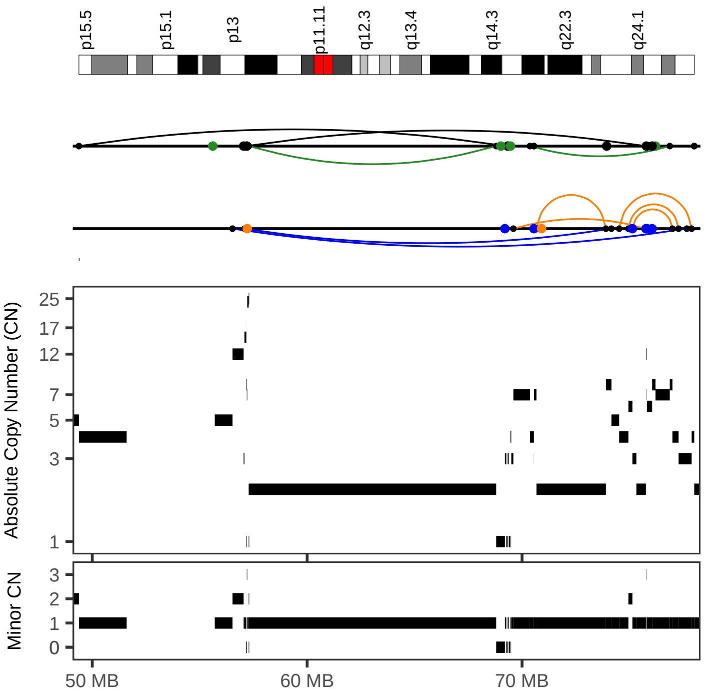

	b865dec4−f051−4fbe−9405−f832ff2010d7
Cancer type	Liver−HCC
Position	11:49377920−77893092
Type	With other complex events
Interleaved intrachr. SVs	9
Total SVs (intrachr. + transl.)	27
SV types	DEL: 4; DUP: 2; h2hINV: 2;<br>t2tINV: 1; TRA: 18
SVs in sample	245
Oscillating CN (2 and 3 states)	5, 9
CN segments	42
FDR fragment joints	0.9625775
FDR chr. breakp. enrich.	0
Linked to chrs	1:77623355−98337818;
Purity, ploidy	0.78, 3.47

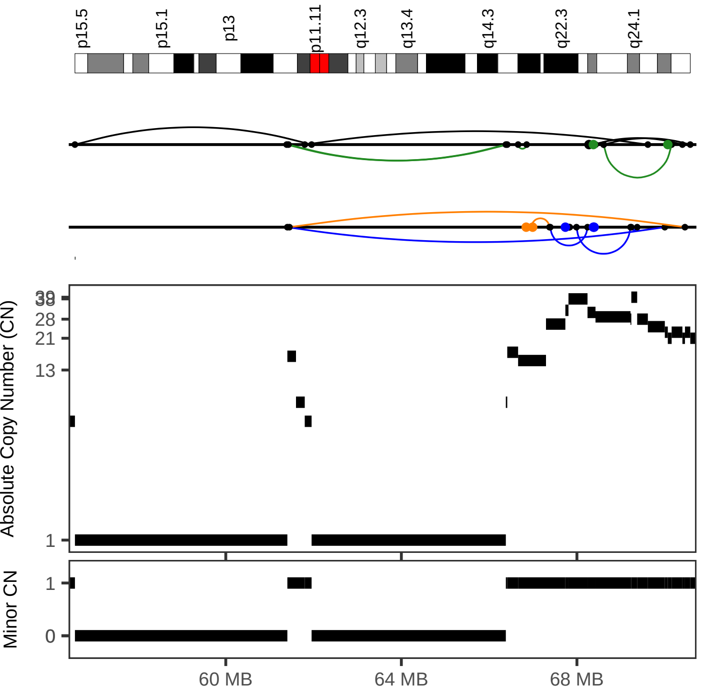

	c0bf9278−9cbb−4361−b4d8−ab172b67e276
Cancer type	Liver−HCC
Position	11:56563250−70581761
Type	With other complex events
Interleaved intrachr. SVs	11
Total SVs (intrachr. + transl.)	19
SV types	DEL: 1; DUP: 3; h2hINV: 4;<br>t2tINV: 3; TRA: 8
SVs in sample	49
Oscillating CN (2 and 3 states)	6, 6
CN segments	23
FDR fragment joints	0.8082862
FDR chr. breakp. enrich.	0
Linked to chrs	
Purity, ploidy	0.4, 2.18

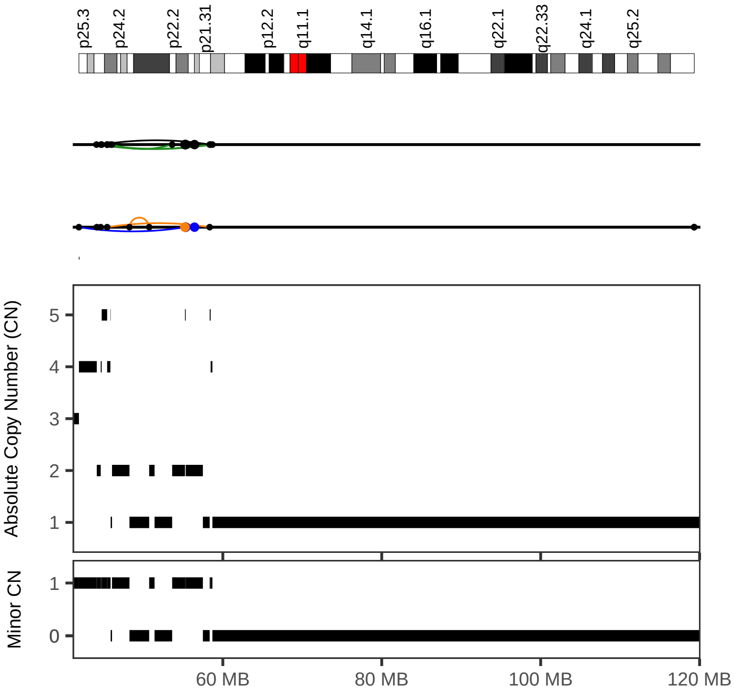

	c95ed407−bcc2−44aa−bc59−bf7549f2d665
Cancer type	Liver−HCC
Position	6:41892254−58688812
Type	With other complex events
Interleaved intrachr. SVs	6
Total SVs (intrachr. + transl.)	18
SV types	DEL: 1; DUP: 1; h2hINV: 1;<br>t2tINV: 3; TRA: 12
SVs in sample	81
Oscillating CN (2 and 3 states)	6, 12
CN segments	18
FDR fragment joints	0.615458
FDR chr. breakp. enrich.	0
Linked to chrs	
Purity, ploidy	0.82, 1.97

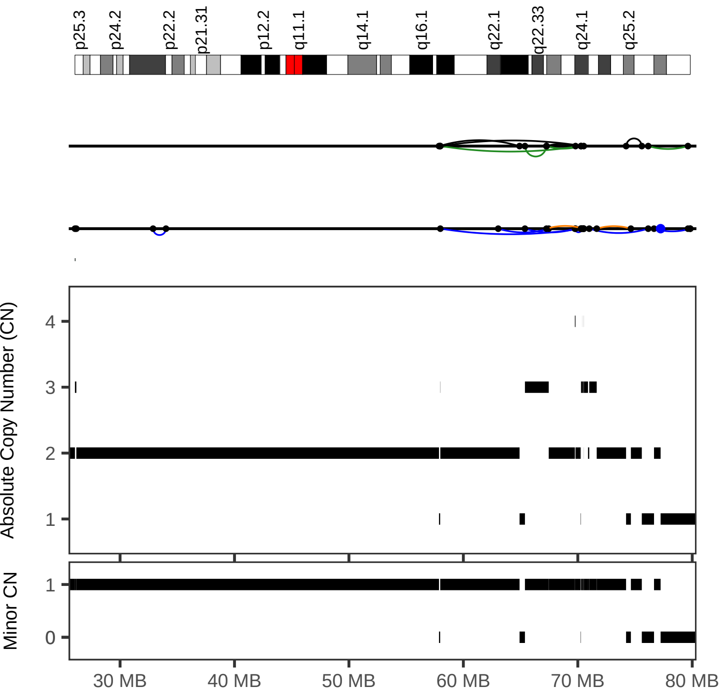

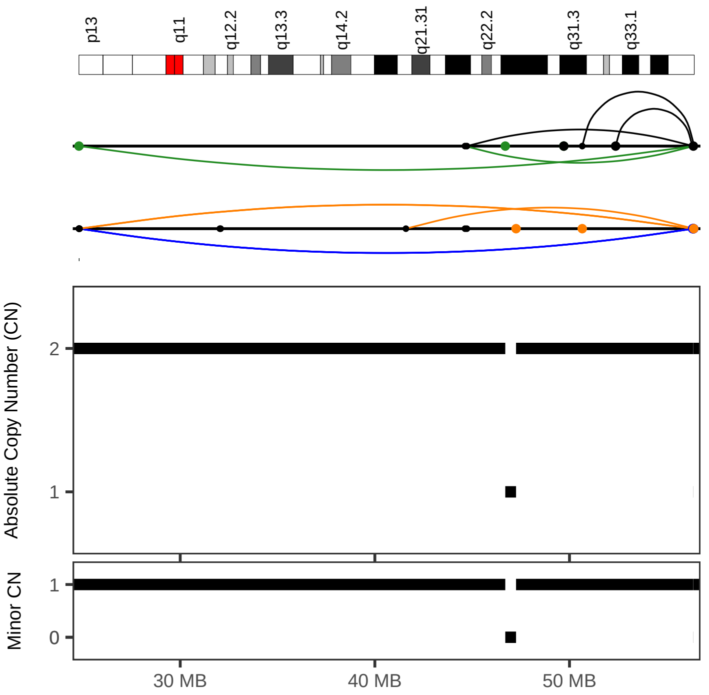

	ca8afb2c−be3d−41e8−8dbc−19fe7ab6154b
Cancer type	Liver−HCC
Position	6:57869537−70524555
Type	With other complex events
Interleaved intrachr. SVs	12
Total SVs (intrachr. + transl.)	12
SV types	DEL: 2; DUP: 5; h2hINV: 3; t2tINV: 2; TRA: 0
SVs in sample	125
Oscillating CN (2 and 3 states)	4, 6
CN segments	17
FDR fragment joints	0.6776251
FDR chr. breakp. enrich.	0
Linked to chrs	
Purity, ploidy	0.57, 1.9

	d680df09−368e−42b5−b540−45c41ed31042
Cancer type	Liver−HCC
Position	13:24796654−56409610
Type	Canonical without polyploidization
Interleaved intrachr. SVs	12
Total SVs (intrachr. + transl.)	29
SV types	DEL: 3; DUP: 4; h2hINV: 3; t2tINV: 2; TRA: 17
SVs in sample	82
Oscillating CN (2 and 3 states)	5, 5
CN segments	5
FDR fragment joints	0.7359483
FDR chr. breakp. enrich.	0
Linked to chrs	
Purity, ploidy	0.64, 1.94

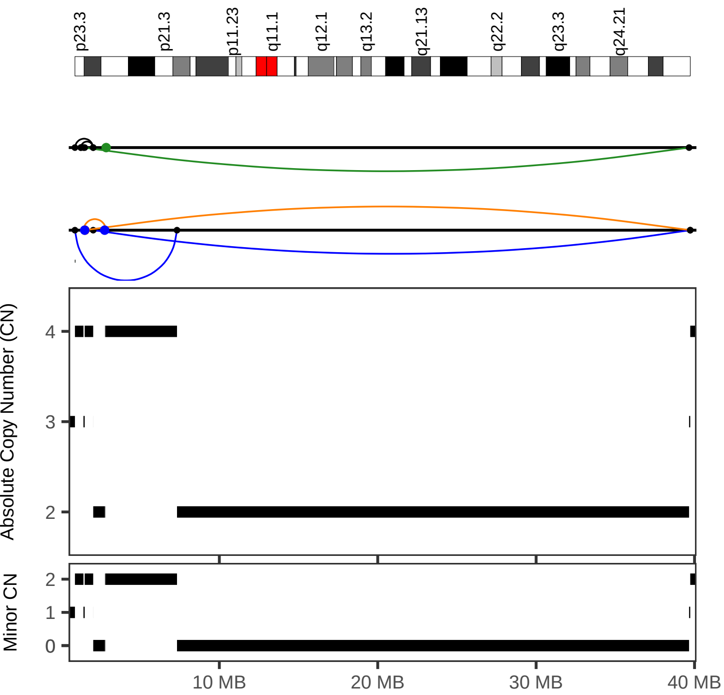

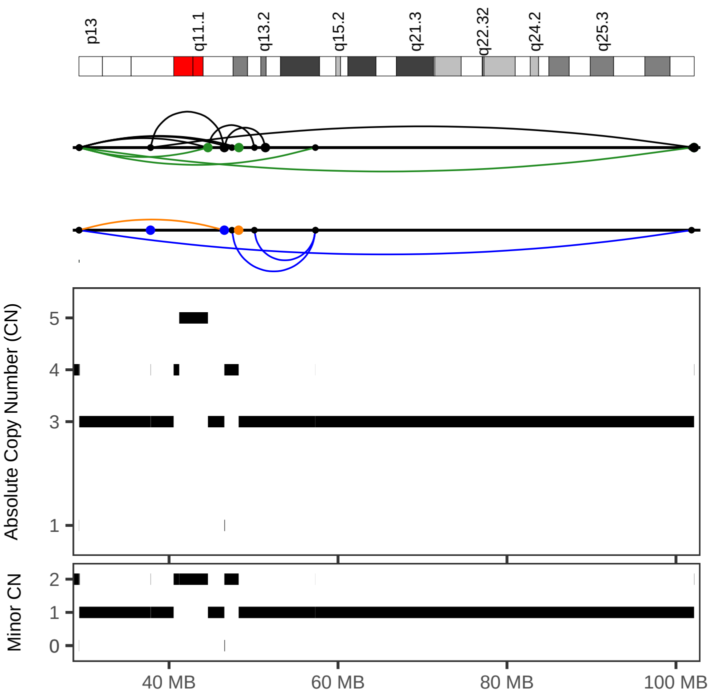

	dfa15fbe−bb14−40ce−afc6−70694112e3e2
Cancer type	Liver−HCC
Position	8:882836−39730717
Type	With other complex events
Interleaved intrachr. SVs	8
Total SVs (intrachr. + transl.)	12
SV types	DEL: 2; DUP: 2; h2hINV: 2; t2tINV: 2; TRA: 4
SVs in sample	27
Oscillating CN (2 and 3 states)	5, 7
CN segments	12
FDR fragment joints	0.9284301
FDR chr. breakp. enrich.	0
Linked to chrs	3:20901418−127446356;
Purity, ploidy	0.73, 4.19

	HX12
Cancer type	Liver−HCC
Position	15:29329993−102165441
Type	With other complex events
Interleaved intrachr. SVs	14
Total SVs (intrachr. + transl.)	23
SV types	DEL: 1; DUP: 3; h2hINV: 7; t2tINV: 3; TRA: 9
SVs in sample	66
Oscillating CN (2 and 3 states)	5, 9
CN segments	17
FDR fragment joints	0.615458
FDR chr. breakp. enrich.	0
Linked to chrs	
Purity, ploidy	0.65, 3.6

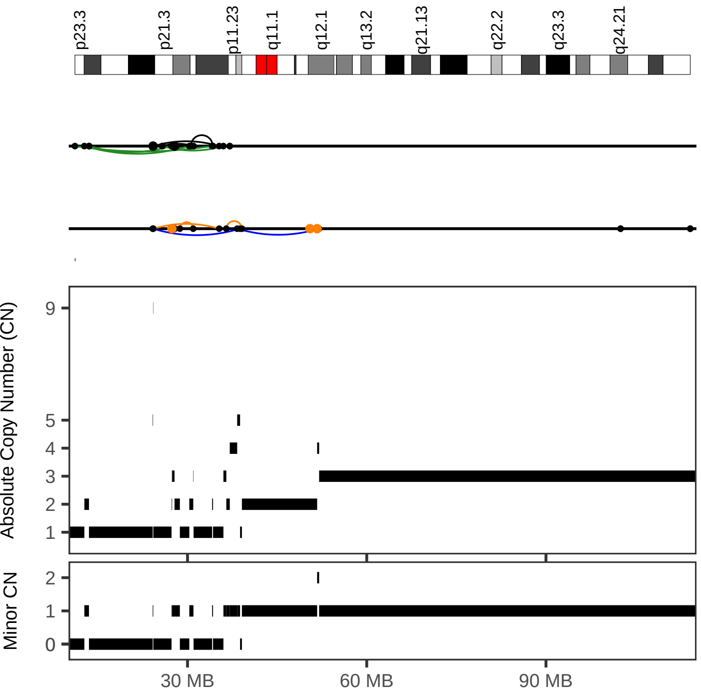

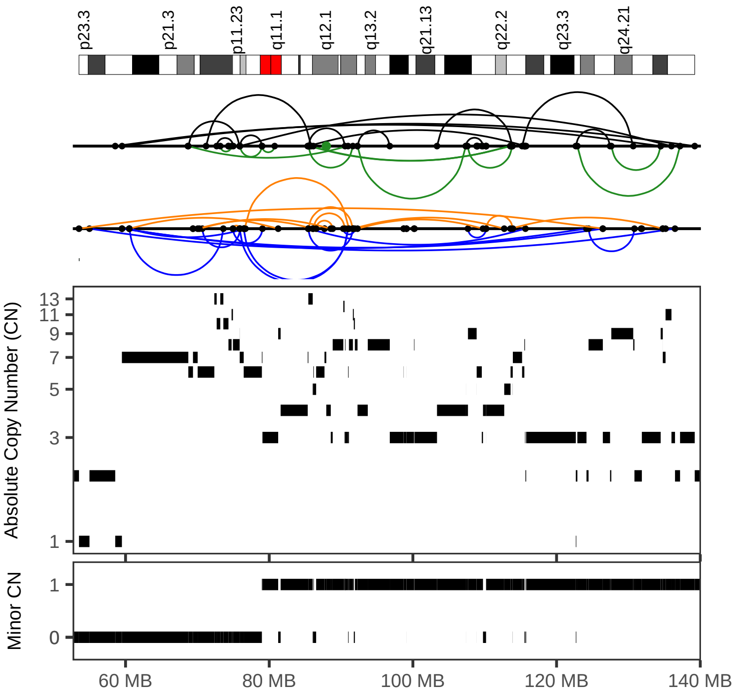

	HX14
Cancer type	Liver−HCC
Position	8:11152586−52033115
Type	With other complex events
Interleaved intrachr. SVs	12
Total SVs (intrachr. + transl.)	17
SV types	DEL: 3; DUP: 2; h2hINV: 3; t2tINV: 4; TRA: 5
SVs in sample	70
Oscillating CN (2 and 3 states)	5, 8
CN segments	25
FDR fragment joints	0.615458
FDR chr. breakp. enrich.	0
Linked to chrs	
Purity, ploidy	0.86, 1.84

	HX21
Cancer type	Liver−HCC
Position	8:53518261−139231447
Type	With other complex events
Interleaved intrachr. SVs	52
Total SVs (intrachr. + transl.)	53
SV types	DEL: 13; DUP: 14; h2hINV: 14; t2tINV: 11; TRA: 1
SVs in sample	133
Oscillating CN (2 and 3 states)	5, 12
CN segments	89
FDR fragment joints	0.9299383
FDR chr. breakp. enrich.	0
Linked to chrs	
Purity, ploidy	0.61, 1.81

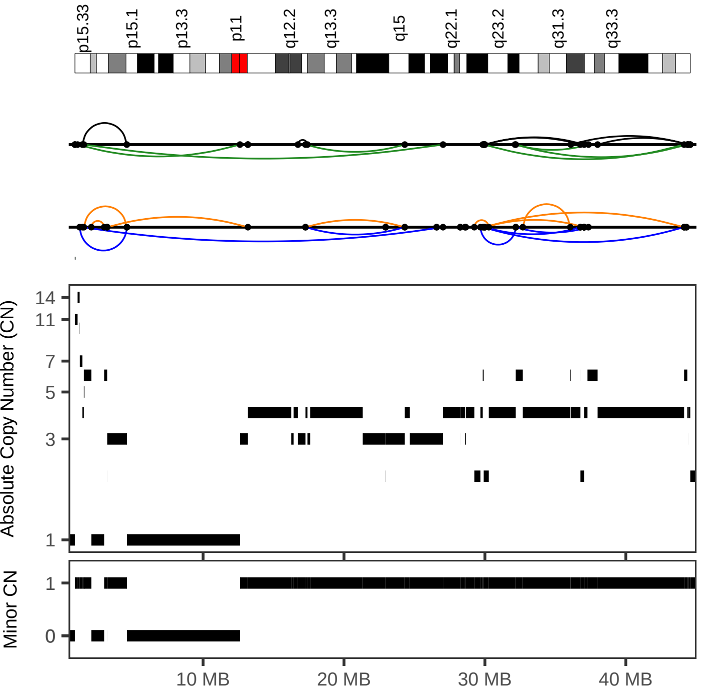

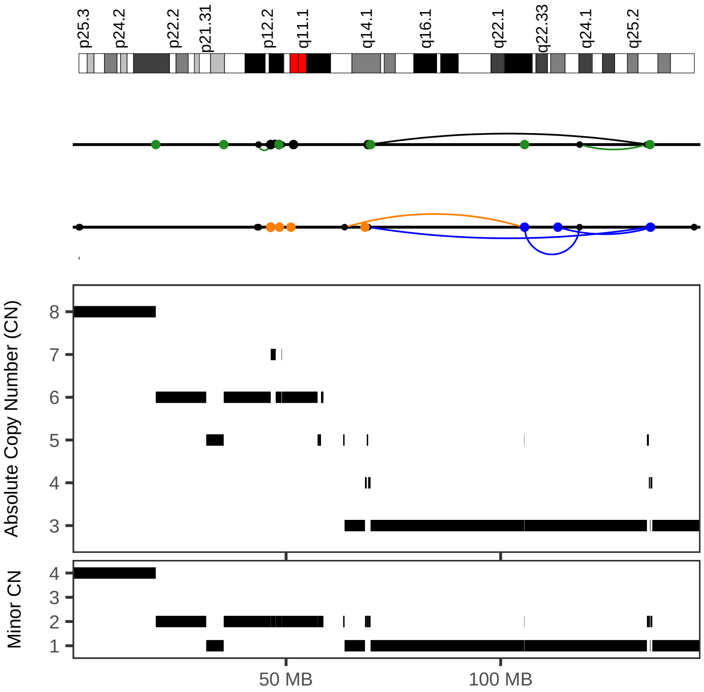

	HX25
Cancer type	Liver−HCC
Position	5:29249343−44428121
Type	With other complex events
Interleaved intrachr. SVs	15
Total SVs (intrachr. + transl.)	15
SV types	DEL: 3; DUP: 4; h2hINV: 4; t2tINV: 4; TRA: 0
SVs in sample	111
Oscillating CN (2 and 3 states)	6, 8
CN segments	18
FDR fragment joints	0.9905774
FDR chr. breakp. enrich.	0
Linked to chrs	
Purity, ploidy	0.77, 1.73

	HX28
Cancer type	Liver−HCC
Position	6:63623695−135334641
Type	With other complex events
Interleaved intrachr. SVs	6
Total SVs (intrachr. + transl.)	14
SV types	DEL: 1; DUP: 3; h2hINV: 1; t2tINV: 1; TRA: 8
SVs in sample	160
Oscillating CN (2 and 3 states)	4, 5
CN segments	12
FDR fragment joints	0.615458
FDR chr. breakp. enrich.	0
Linked to chrs	
Purity, ploidy	0.65, 3.46

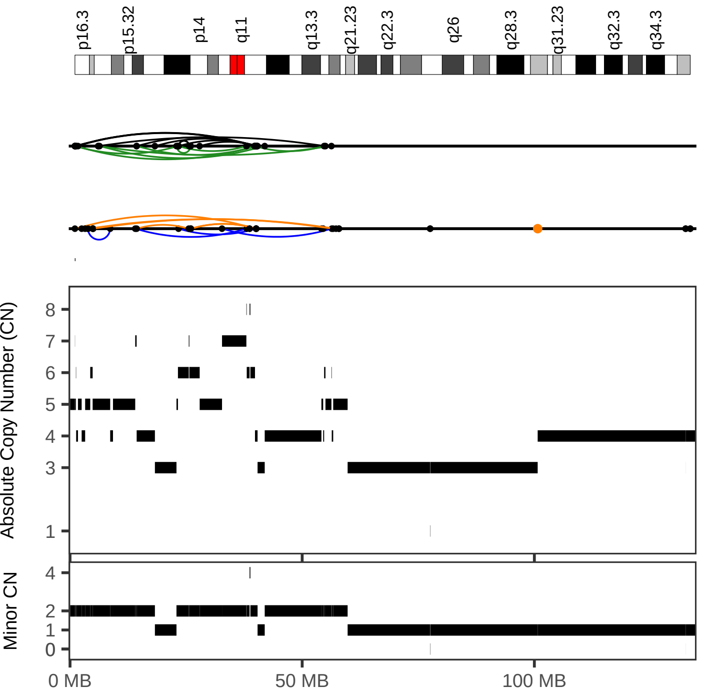

	HX35
Cancer type	Liver−HCC
Position	4:1056612−57295598
Type	With other complex events
Interleaved intrachr. SVs	29
Total SVs (intrachr. + transl.)	29
SV types	DEL: 6; DUP: 4; h2hINV: 10; t2tINV: 9; TRA: 0
SVs in sample	135
Oscillating CN (2 and 3 states)	4, 8
CN segments	36
FDR fragment joints	0.615458
FDR chr. breakp. enrich.	0
Linked to chrs	
Purity, ploidy	0.75, 3.67

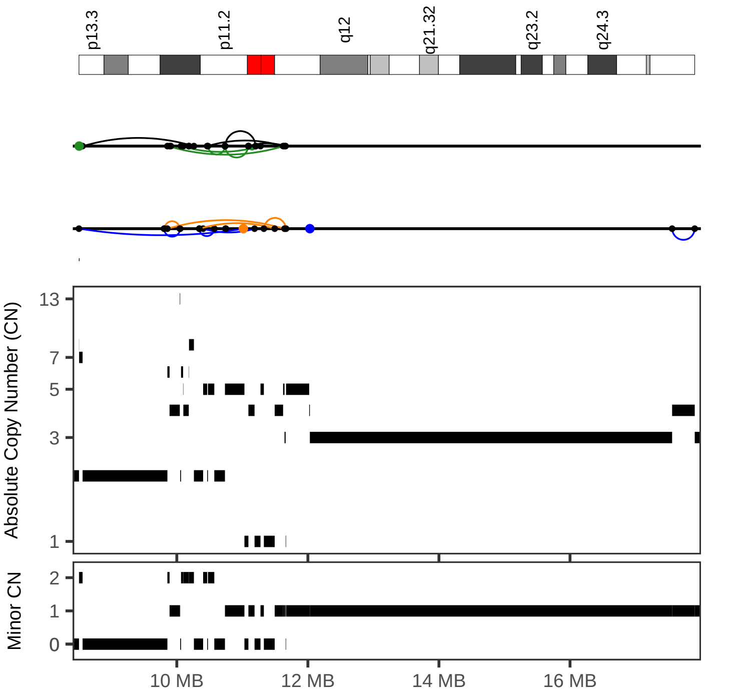

	HX35
Cancer type	Liver−HCC
Position	17:8507299−11666681
Type	With other complex events
Interleaved intrachr. SVs	17
Total SVs (intrachr. + transl.)	19
SV types	DEL: 4; DUP: 4; h2hINV: 4; t2tINV: 5; TRA: 2
SVs in sample	135
Oscillating CN (2 and 3 states)	6, 12
CN segments	28
FDR fragment joints	0.9958447
FDR chr. breakp. enrich.	0
Linked to chrs	
Purity, ploidy	0.75, 3.67

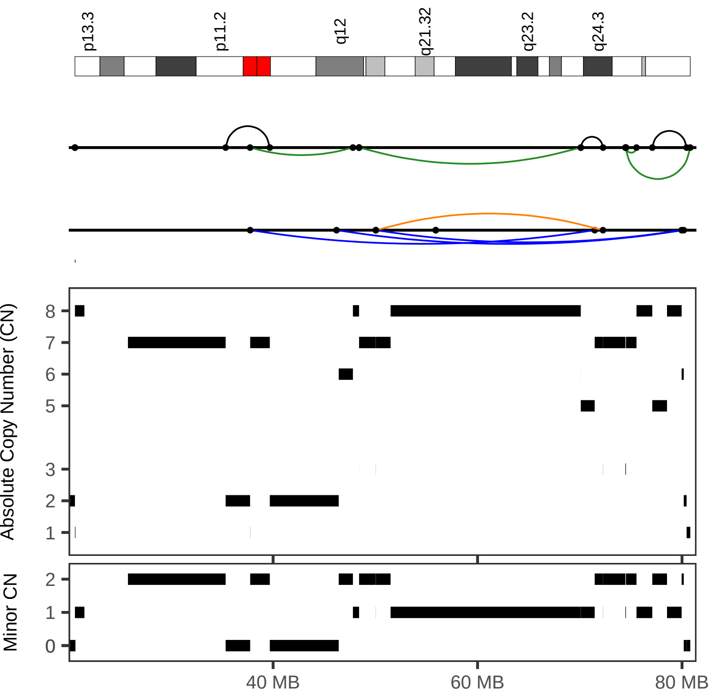

	RK003
Cancer type	Liver−HCC
Position	17:35358262−80803775
Type	With other complex events
Interleaved intrachr. SVs	10
Total SVs (intrachr. + transl.)	10
SV types	DEL: 1; DUP: 3; h2hINV: 3; t2tINV: 3; TRA: 0
SVs in sample	51
Oscillating CN (2 and 3 states)	5, 5
CN segments	24
FDR fragment joints	0.8572806
FDR chr. breakp. enrich.	0
Linked to chrs	
Purity, ploidy	0.54, 3.3

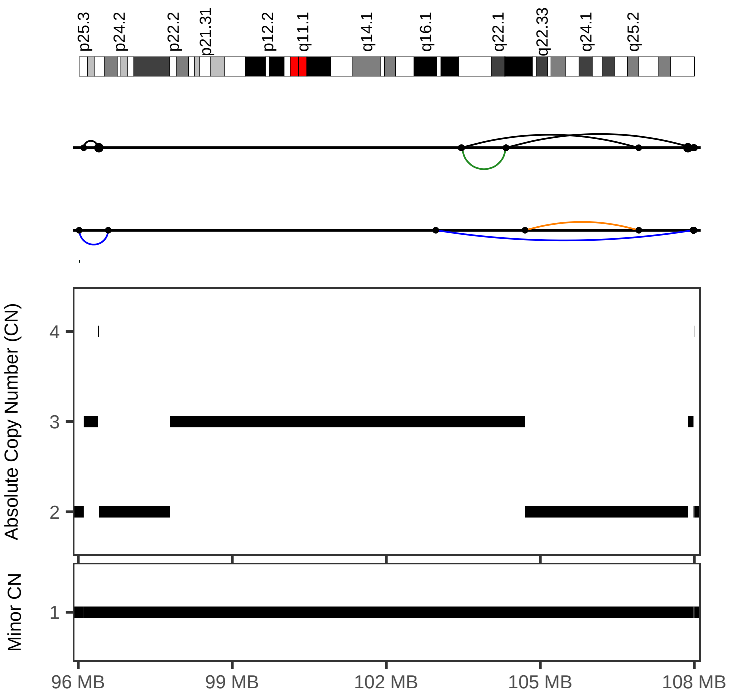

	RK038
Cancer type	Liver−HCC
Position	6:102965111−108005363
Type	Canonical without polyploidization
Interleaved intrachr. SVs	10
Total SVs (intrachr. + transl.)	11
SV types	DEL: 2; DUP: 3; h2hINV: 3; t2tINV: 2; TRA: 1
SVs in sample	99
Oscillating CN (2 and 3 states)	6, 8
CN segments	8
FDR fragment joints	0.9804396
FDR chr. breakp. enrich.	0
Linked to chrs	
Purity, ploidy	0.69, 1.92

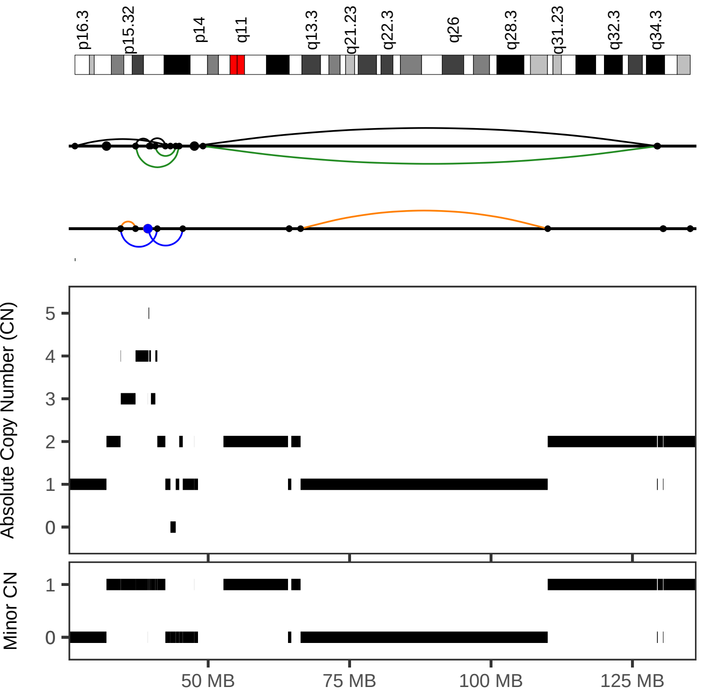

	RK042
Cancer type	Liver−HCC
Position	4:26444895−45511514
Type	With other complex events
Interleaved intrachr. SVs	7
Total SVs (intrachr. + transl.)	10
SV types	DEL: 0; DUP: 2; h2hINV: 3; t2tINV: 2; TRA: 3
SVs in sample	93
Oscillating CN (2 and 3 states)	5, 9
CN segments	17
FDR fragment joints	0.8572806
FDR chr. breakp. enrich.	0
Linked to chrs	
Purity, ploidy	0.88, 1.81

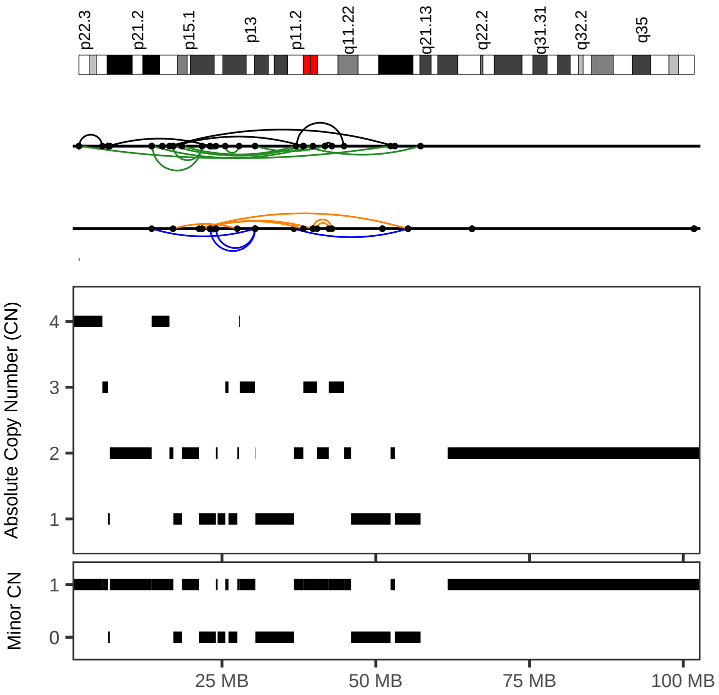

	RK061
Cancer type	Liver−HCC
Position	7:1738702−57285072
Type	With other complex events
Interleaved intrachr. SVs	28
Total SVs (intrachr. + transl.)	28
SV types	DEL: 7; DUP: 5; h2hINV: 6; t2tINV: 10; TRA: 0
SVs in sample	229
Oscillating CN (2 and 3 states)	6, 9
CN segments	28
FDR fragment joints	0.6776251
FDR chr. breakp. enrich.	0
Linked to chrs	
Purity, ploidy	0.82, 1.85

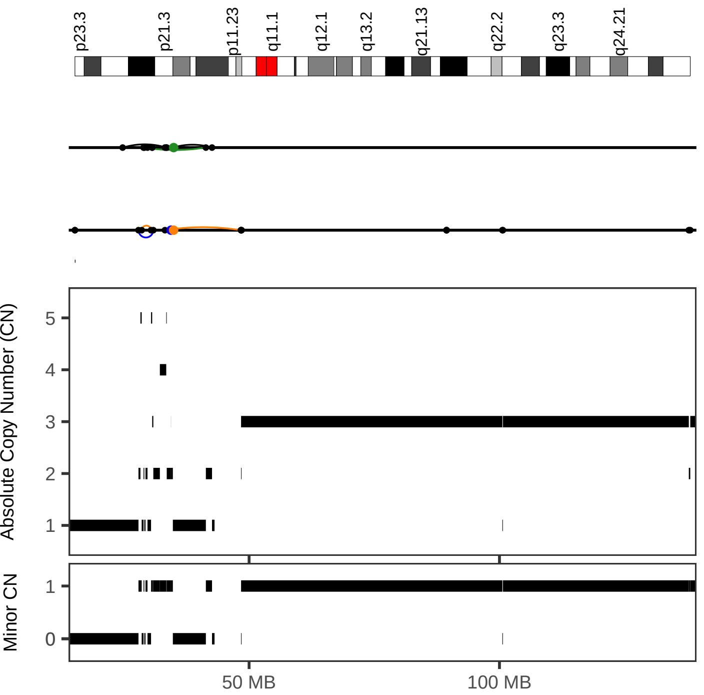

	RK061
Cancer type	Liver−HCC
Position	8:24743909−48399104
Type	With other complex events
Interleaved intrachr. SVs	7
Total SVs (intrachr. + transl.)	10
SV types	DEL: 2; DUP: 1; h2hINV: 2; t2tINV: 2; TRA: 3
SVs in sample	229
Oscillating CN (2 and 3 states)	5, 9
CN segments	20
FDR fragment joints	0.9625775
FDR chr. breakp. enrich.	0.02
Linked to chrs	
Purity, ploidy	0.82, 1.85

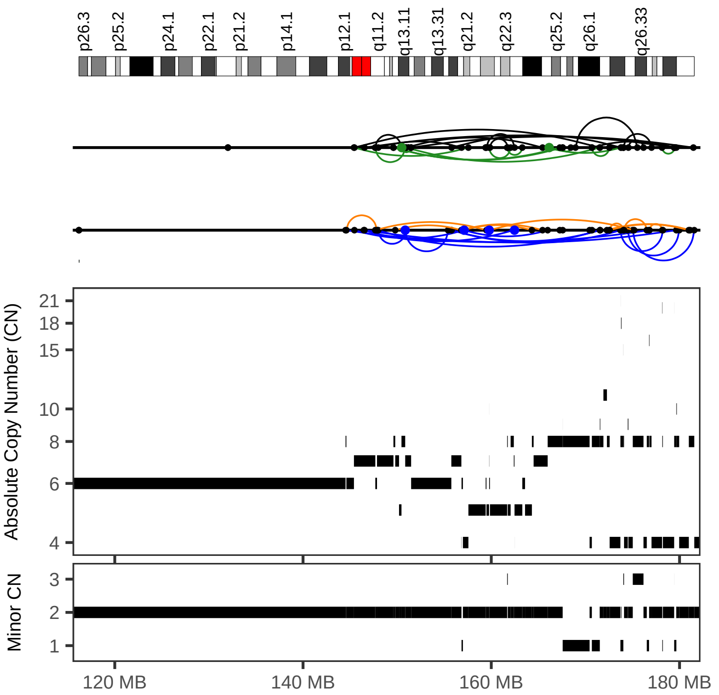

	RK072
Cancer type	Liver−HCC
Position	3:144516195−181561816
Type	With other complex events
Interleaved intrachr. SVs	52
Total SVs (intrachr. + transl.)	58
SV types	DEL: 13; DUP: 13; h2hINV: 14; t2tINV: 12; TRA: 6
SVs in sample	282
Oscillating CN (2 and 3 states)	4, 10
CN segments	67
FDR fragment joints	0.8644076
FDR chr. breakp. enrich.	0
Linked to chrs	
Purity, ploidy	0.7, 4.49

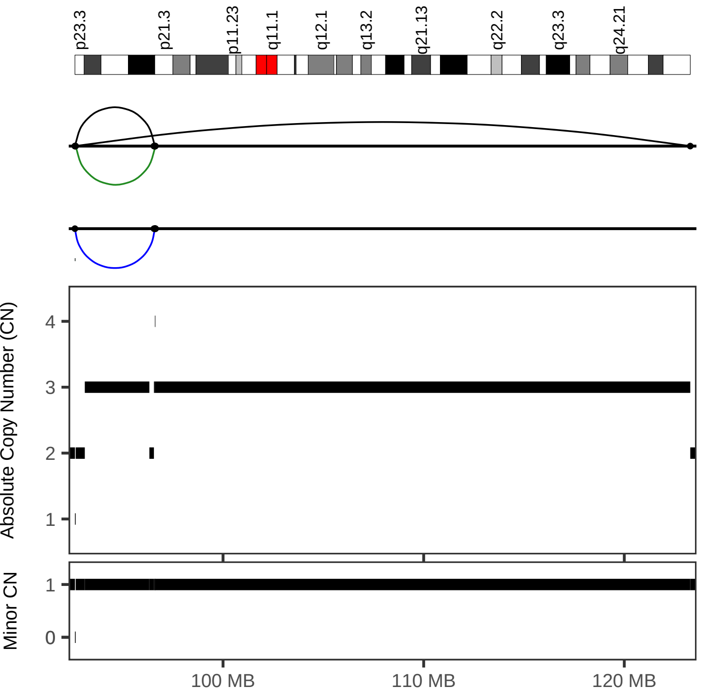

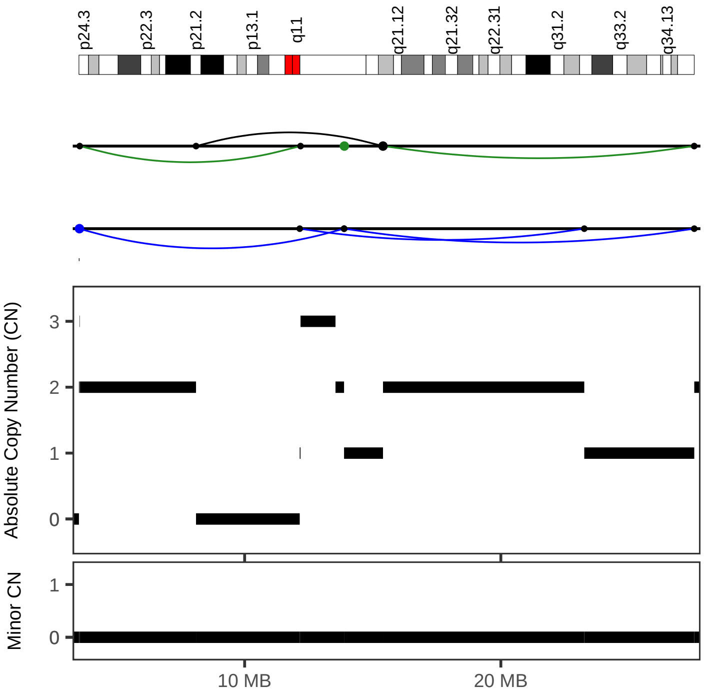

	**RK079**
Cancer type	Liver−HCC
Position	8:92616813−123287245
Type	With other complex events
Interleaved intrachr. SVs	7
Total SVs (intrachr. + transl.)	7
SV types	DEL: 1; DUP: 2; h2hINV: 2; t2tINV: 2; TRA: 0
SVs in sample	80
Oscillating CN (2 and 3 states)	4, 9
CN segments	9
FDR fragment joints	0.9599662
FDR chr. breakp. enrich.	0.01
Linked to chrs	
Purity, ploidy	0.76, 1.92

	**RK079**
Cancer type	Liver−HCC
Position	9:3532306−27548765
Type	With other complex events
Interleaved intrachr. SVs	6
Total SVs (intrachr. + transl.)	9
SV types	DEL: 0; DUP: 3; h2hINV: 1; t2tINV: 2; TRA: 3
SVs in sample	80
Oscillating CN (2 and 3 states)	5, 5
CN segments	11
FDR fragment joints	0.615458
FDR chr. breakp. enrich.	0
Linked to chrs	
Purity, ploidy	0.76, 1.92

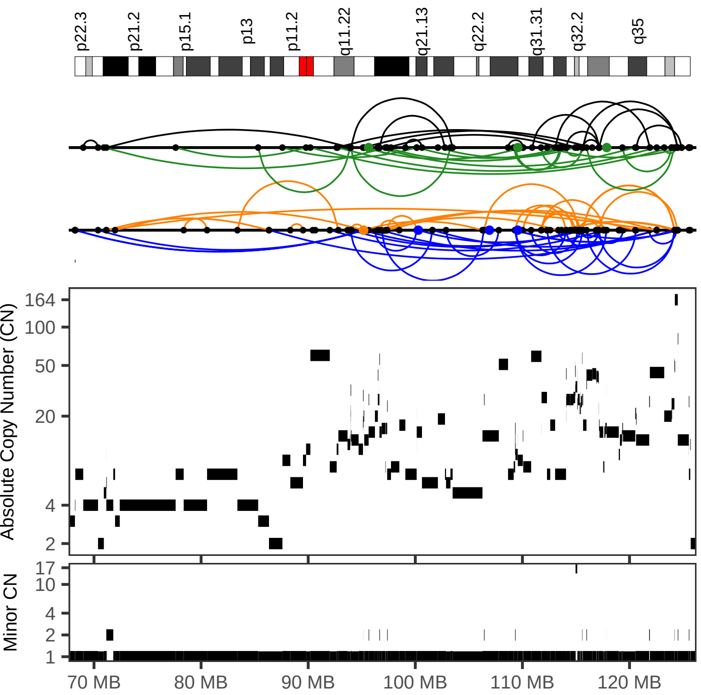

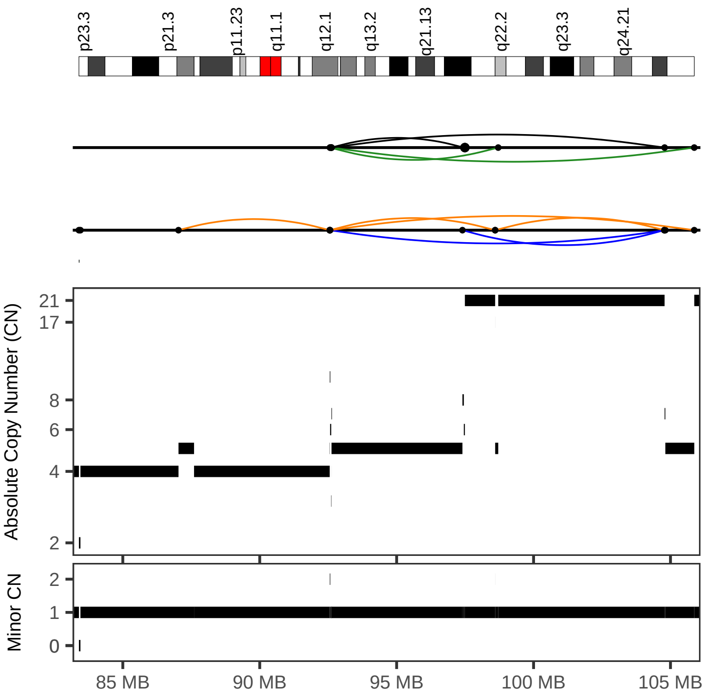

	**RK085**
Cancer type	Liver−HCC
Position	7:68220087−124871888
Type	With other complex events
Interleaved intrachr. SVs	98
Total SVs (intrachr. + transl.)	106
SV types	DEL: 29; DUP: 32; h2hINV: 16; t2tINV: 21; TRA: 8
SVs in sample	208
Oscillating CN (2 and 3 states)	5, 7
CN segments	147
FDR fragment joints	0.5435077
FDR chr. breakp. enrich.	0
Linked to chrs	
Purity, ploidy	0.49, 3.12

	**RK085**
Cancer type	Liver−HCC
Position	8:87033701−105868006
Type	With other complex events
Interleaved intrachr. SVs	11
Total SVs (intrachr. + transl.)	13
SV types	DEL: 4; DUP: 2; h2hINV: 3; t2tINV: 2; TRA: 2
SVs in sample	208
Oscillating CN (2 and 3 states)	4, 5
CN segments	20
FDR fragment joints	0.9000538
FDR chr. breakp. enrich.	0.51
Linked to chrs	
Purity, ploidy	0.49, 3.12

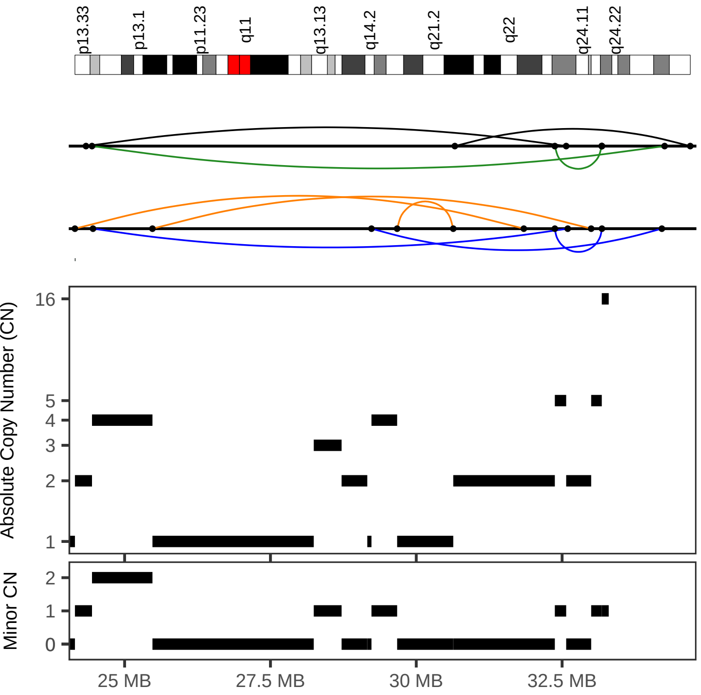

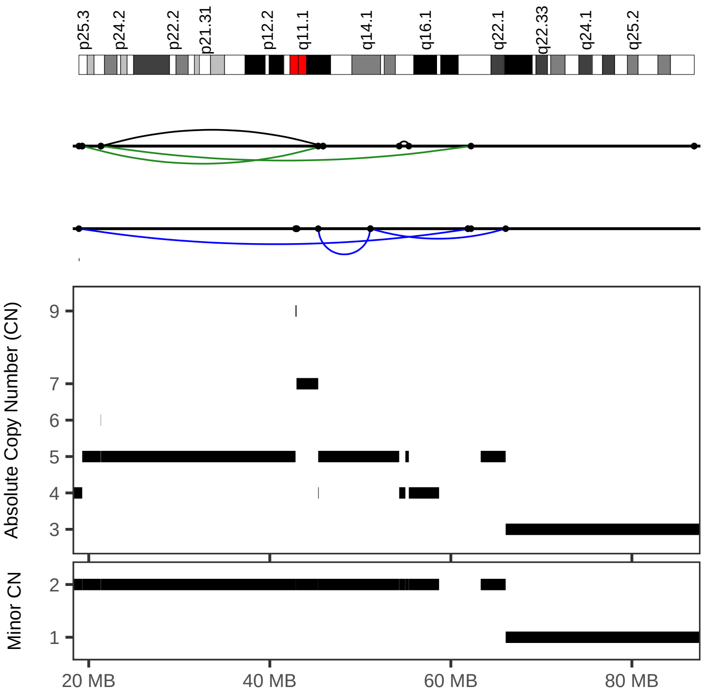

	**RK087**
Cancer type	Liver−HCC
Position	12:24148141−34697150
Type	With other complex events
Interleaved intrachr. SVs	9
Total SVs (intrachr. + transl.)	9
SV types	DEL: 2; DUP: 3; h2hINV: 2; t2tINV: 2; TRA: 0
SVs in sample	62
Oscillating CN (2 and 3 states)	4, 5
CN segments	14
FDR fragment joints	0.9723381
FDR chr. breakp. enrich.	0
Linked to chrs	
Purity, ploidy	0.26, 1.9

	**RK088**
Cancer type	Liver−HCC
Position	6:18913889−66058087
Type	With other complex events
Interleaved intrachr. SVs	7
Total SVs (intrachr. + transl.)	7
SV types	DEL: 0; DUP: 3; h2hINV: 2; t2tINV: 2; TRA: 0
SVs in sample	75
Oscillating CN (2 and 3 states)	6, 7
CN segments	13
FDR fragment joints	0.6776251
FDR chr. breakp. enrich.	0
Linked to chrs	
Purity, ploidy	0.78, 3.47

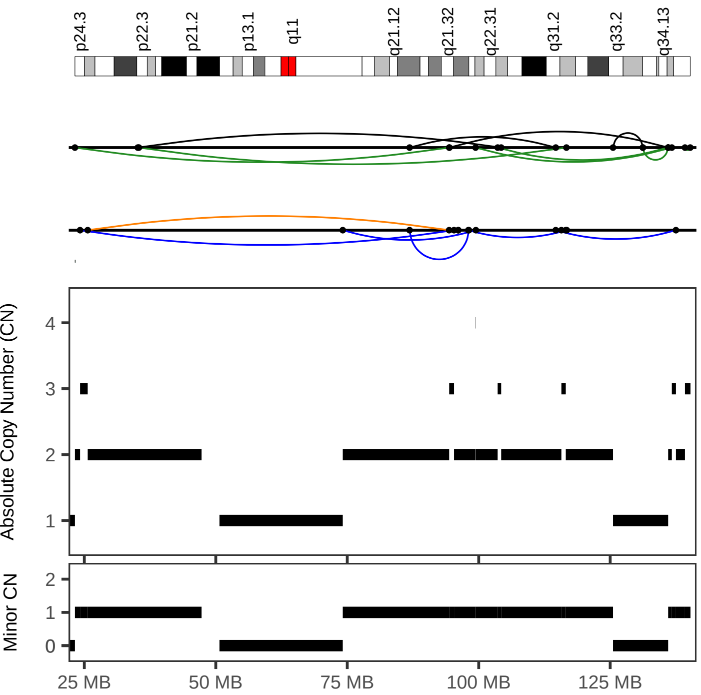

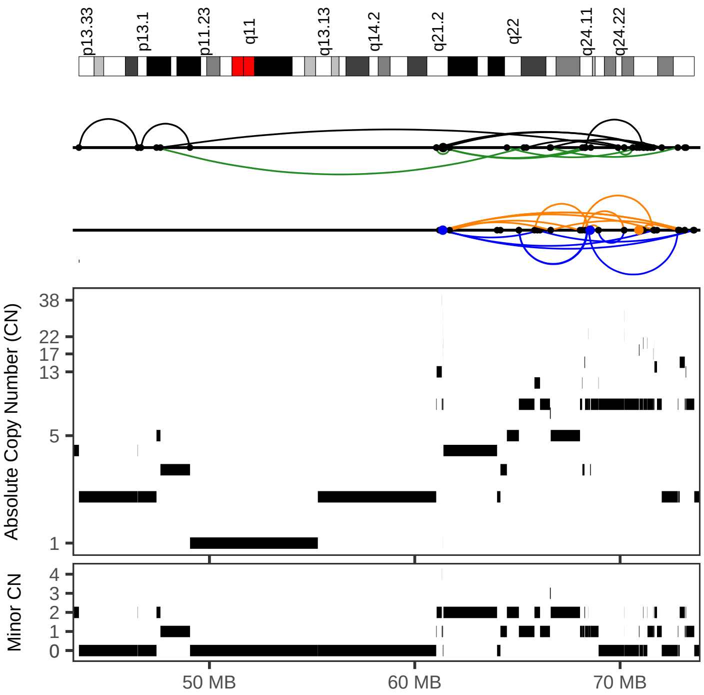

	**RK089**
Cancer type	Liver−HCC
Position	9:23213827−137506288
Type	With other complex events
Interleaved intrachr. SVs	13
Total SVs (intrachr. + transl.)	13
SV types	DEL: 1; DUP: 5; h2hINV: 3; t2tINV: 4; TRA: 0
SVs in sample	57
Oscillating CN (2 and 3 states)	5, 9
CN segments	17
FDR fragment joints	0.6776251
FDR chr. breakp. enrich.	0
Linked to chrs	
Purity, ploidy	0.63, 1.65

	**RK091**
Cancer type	Liver−HCC
Position	12:46659315−73611218
Type	With other complex events
Interleaved intrachr. SVs	39
Total SVs (intrachr. + transl.)	43
SV types	DEL: 12; DUP: 9; h2hINV: 9; t2tINV: 9; TRA: 4
SVs in sample	427
Oscillating CN (2 and 3 states)	5, 6
CN segments	61
FDR fragment joints	0.9077541
FDR chr. breakp. enrich.	0
Linked to chrs	
Purity, ploidy	0.74, 1.97

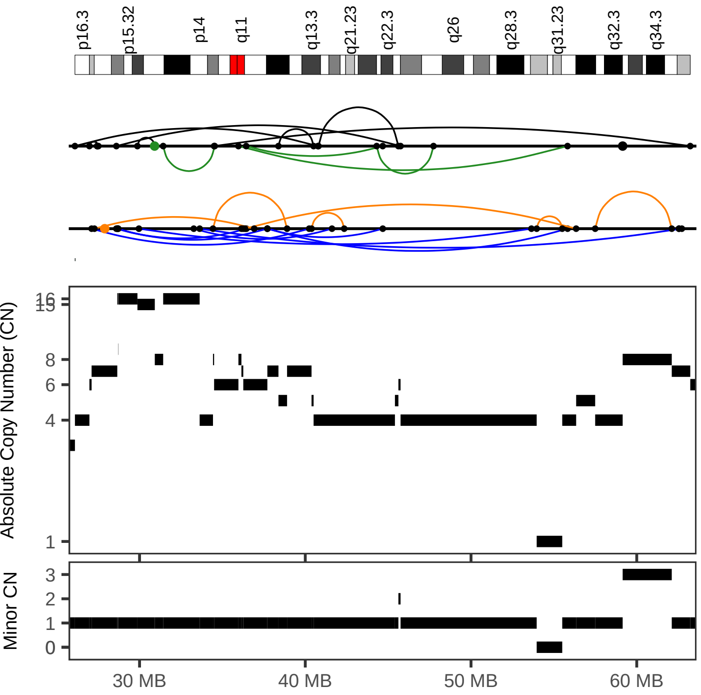

	**RK092**
Cancer type	Liver−HCC
Position	4:26100061−63228862
Type	With other complex events
Interleaved intrachr. SVs	23
Total SVs (intrachr. + transl.)	26
SV types	DEL: 4; DUP: 8; h2hINV: 7; t2tINV: 4; TRA: 3
SVs in sample	89
Oscillating CN (2 and 3 states)	4, 4
CN segments	30
FDR fragment joints	0.8172348
FDR chr. breakp. enrich.	0
Linked to chrs	
Purity, ploidy	0.55, 1.93

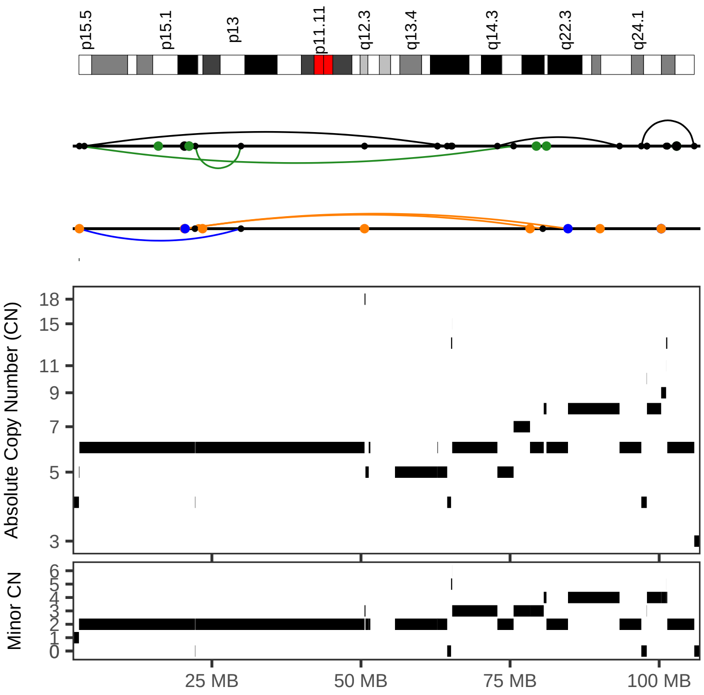

	**RK098**
Cancer type	Liver−HCC
Position	11:2705633−93356459
Type	With other complex events
Interleaved intrachr. SVs	8
Total SVs (intrachr. + transl.)	22
SV types	DEL: 3; DUP: 1; h2hINV: 2; t2tINV: 2; TRA: 14
SVs in sample	145
Oscillating CN (2 and 3 states)	5, 8
CN segments	21
FDR fragment joints	0.615458
FDR chr. breakp. enrich.	0
Linked to chrs	
Purity, ploidy	0.38, 3.78

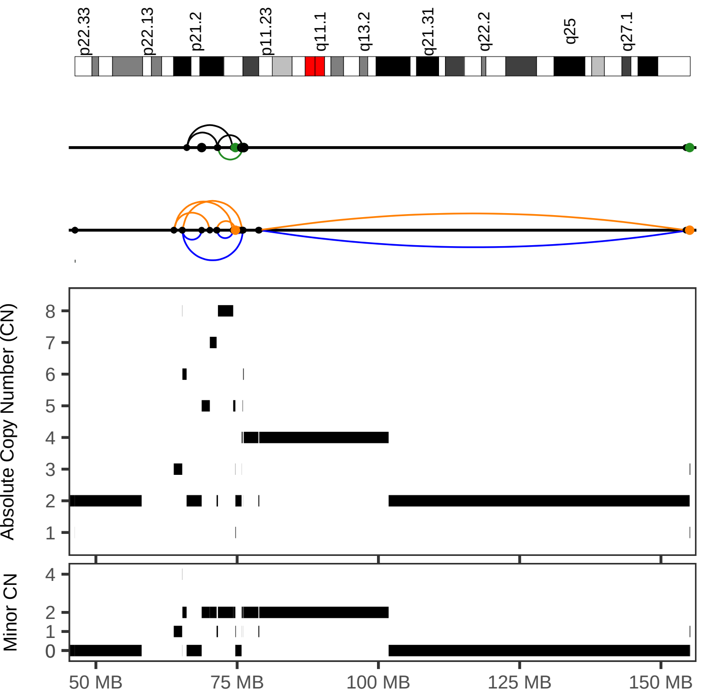

	**RK098**
Cancer type	Liver−HCC
Position	X:63781853−76077025
Type	With other complex events
Interleaved intrachr. SVs	15
Total SVs (intrachr. + transl.)	19
SV types	DEL: 6; DUP: 4; h2hINV: 3; t2tINV: 2; TRA: 4
SVs in sample	145
Oscillating CN (2 and 3 states)	5, 6
CN segments	24
FDR fragment joints	0.8082862
FDR chr. breakp. enrich.	0
Linked to chrs	
Purity, ploidy	0.38, 3.78

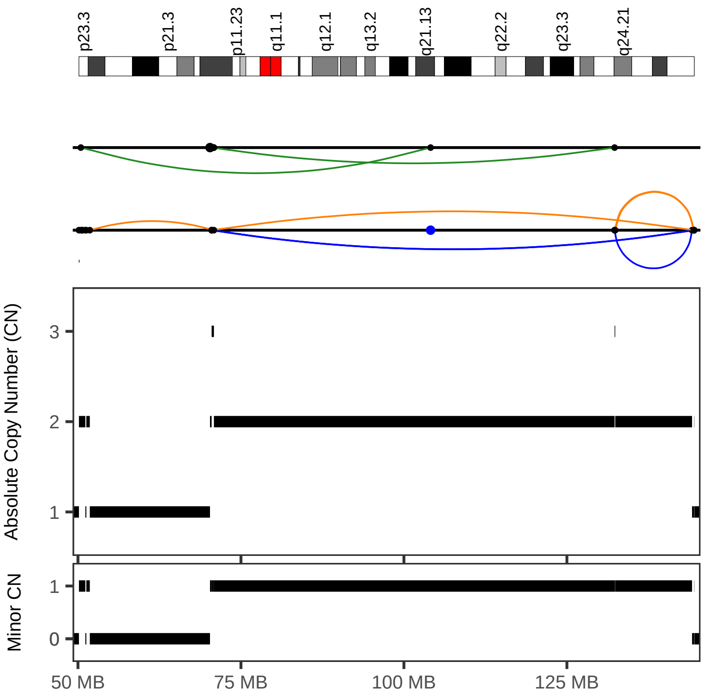

	**RK099**
Cancer type	Liver−HCC
Position	8:50144650−144536434
Type	With other complex events
Interleaved intrachr. SVs	7
Total SVs (intrachr. + transl.)	9
SV types	DEL: 2; DUP: 3; h2hINV: 0; t2tINV: 2; TRA: 2
SVs in sample	56
Oscillating CN (2 and 3 states)	5, 12
CN segments	12
FDR fragment joints	0.615458
FDR chr. breakp. enrich.	0
Linked to chrs	
Purity, ploidy	0.56, 1.7

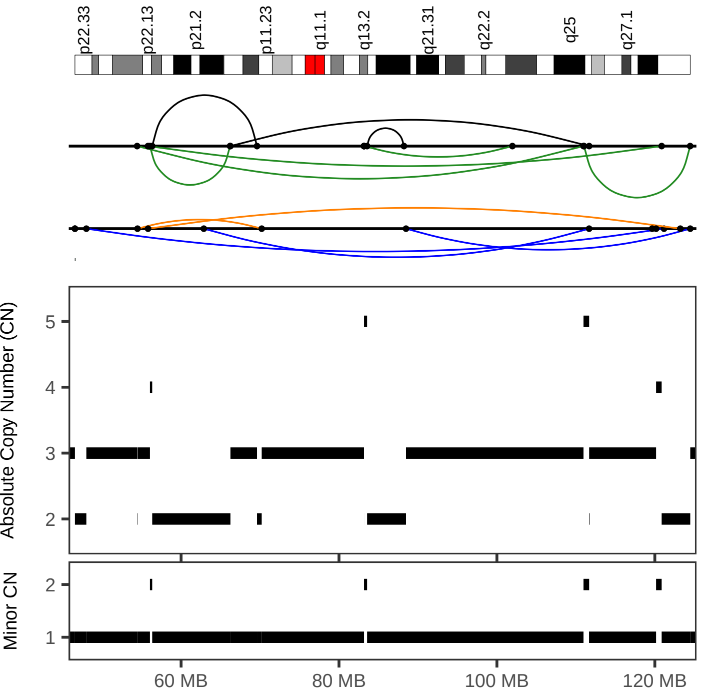

	**RK100**
Cancer type	Liver−HCC
Position	X:48002589−124488576
Type	With other complex events
Interleaved intrachr. SVs	12
Total SVs (intrachr. + transl.)	12
SV types	DEL: 2; DUP: 3; h2hINV: 2; t2tINV: 5; TRA: 0
SVs in sample	104
Oscillating CN (2 and 3 states)	5, 7
CN segments	18
FDR fragment joints	0.6776251
FDR chr. breakp. enrich.	0
Linked to chrs	
Purity, ploidy	0.56, 3.33

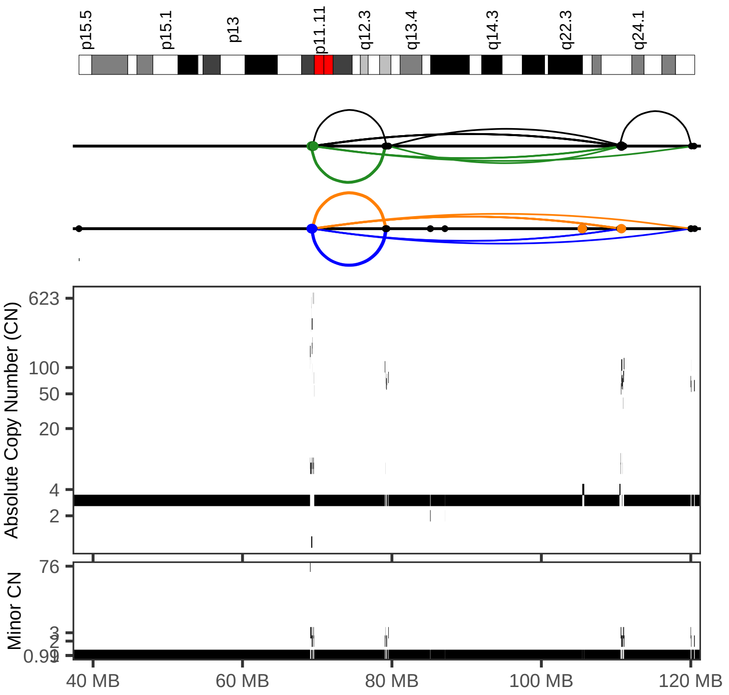

	**RK101**
Cancer type	Liver−HCC
Position	11:69040190−120528008
Type	With other complex events
Interleaved intrachr. SVs	59
Total SVs (intrachr. + transl.)	73
SV types	DEL: 18; DUP: 15; h2hINV: 14; t2tINV: 12; TRA: 14
SVs in sample	218
Oscillating CN (2 and 3 states)	5, 7
CN segments	92
FDR fragment joints	0.8246614
FDR chr. breakp. enrich.	0
Linked to chrs	9:4642656−38474065;
Purity, ploidy	0.81, 3.69

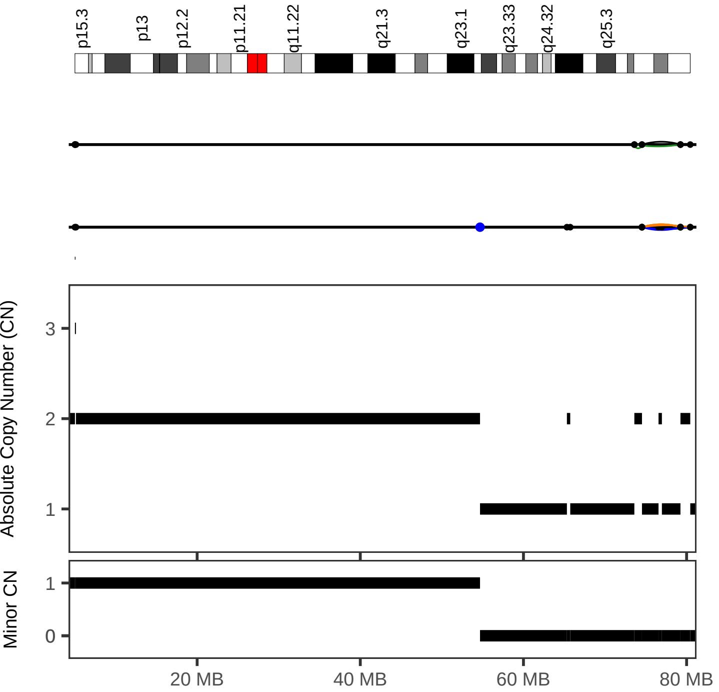

	**RK111**
Cancer type	Liver−HCC
Position	10:73592301−80444902
Type	Canonical without polyploidization
Interleaved intrachr. SVs	10
Total SVs (intrachr. + transl.)	10
SV types	DEL: 4; DUP: 2; h2hINV: 2; t2tINV: 2; TRA: 0
SVs in sample	176
Oscillating CN (2 and 3 states)	6, 6
CN segments	6
FDR fragment joints	0.8572806
FDR chr. breakp. enrich.	0.05
Linked to chrs	
Purity, ploidy	0.9, 1.83

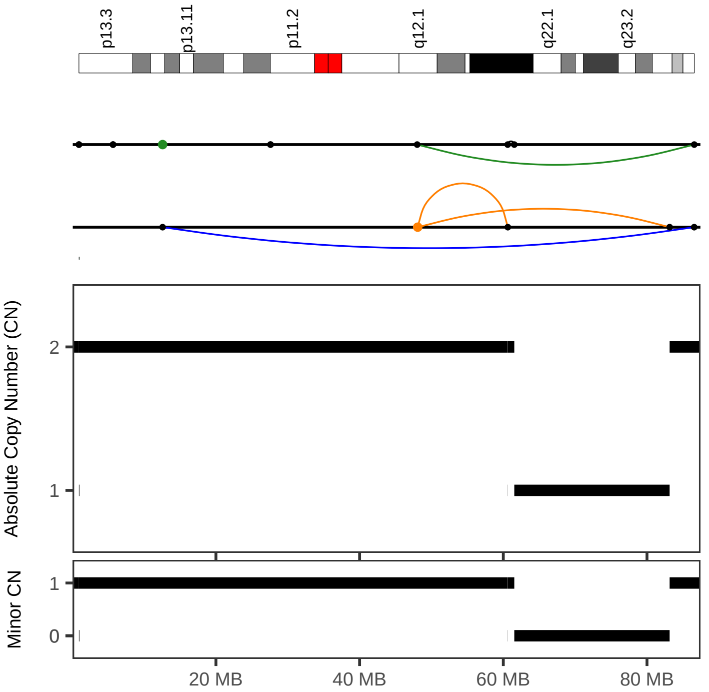

	**RK117**
Cancer type	Liver−HCC
Position	16:12581311−86577989
Type	Canonical without polyploidization
Interleaved intrachr. SVs	6
Total SVs (intrachr. + transl.)	8
SV types	DEL: 3; DUP: 1; h2hINV: 1; t2tINV: 1; TRA: 2
SVs in sample	130
Oscillating CN (2 and 3 states)	5, 5
CN segments	5
FDR fragment joints	0.615458
FDR chr. breakp. enrich.	0.01
Linked to chrs	13:29029306−51769998;
Purity, ploidy	0.73, 1.94

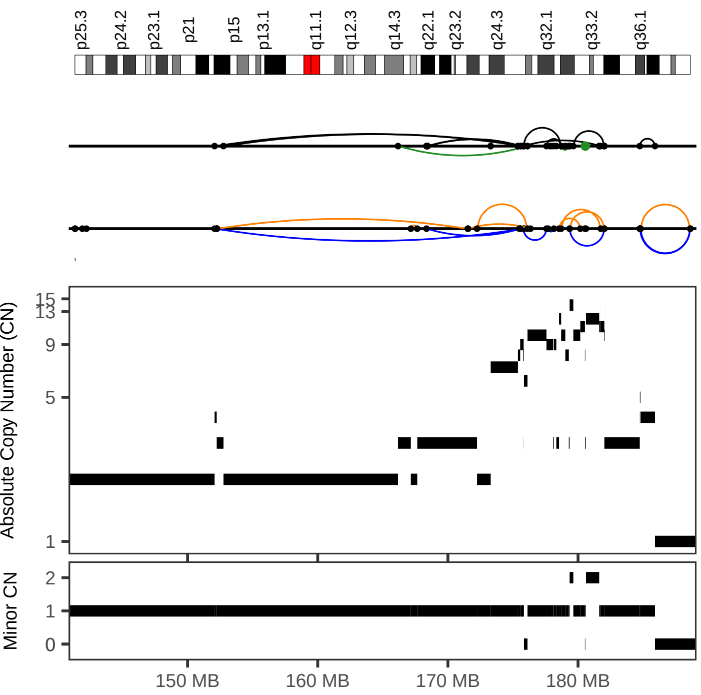

	RK119
Cancer type	Liver−HCC
Position	2:152075233−182020726
Type	With other complex events
Interleaved intrachr. SVs	21
Total SVs (intrachr. + transl.)	22
SV types	DEL: 6; DUP: 4; h2hINV: 8; t2tINV: 3; TRA: 1
SVs in sample	110
Oscillating CN (2 and 3 states)	6, 7
CN segments	37
FDR fragment joints	0.615458
FDR chr. breakp. enrich.	0
Linked to chrs	
Purity, ploidy	0.7, 2.09

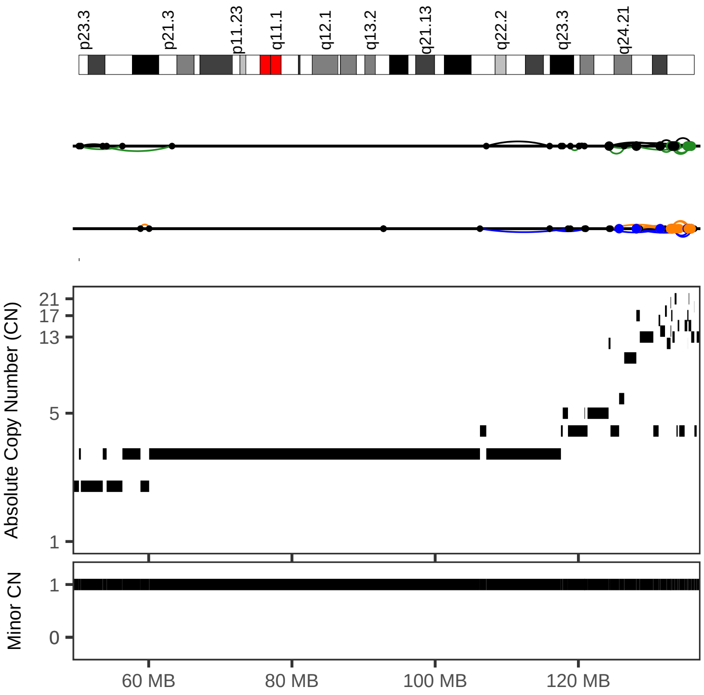

	RK135
Cancer type	Liver−HCC
Position	8:124217126−136178634
Type	With other complex events
Interleaved intrachr. SVs	27
Total SVs (intrachr. + transl.)	78
SV types	DEL: 7; DUP: 8; h2hINV: 5; t2tINV: 7; TRA: 51
SVs in sample	192
Oscillating CN (2 and 3 states)	4, 6
CN segments	29
FDR fragment joints	0.7510435
FDR chr. breakp. enrich.	0
Linked to chrs	
Purity, ploidy	0.7, 1.9

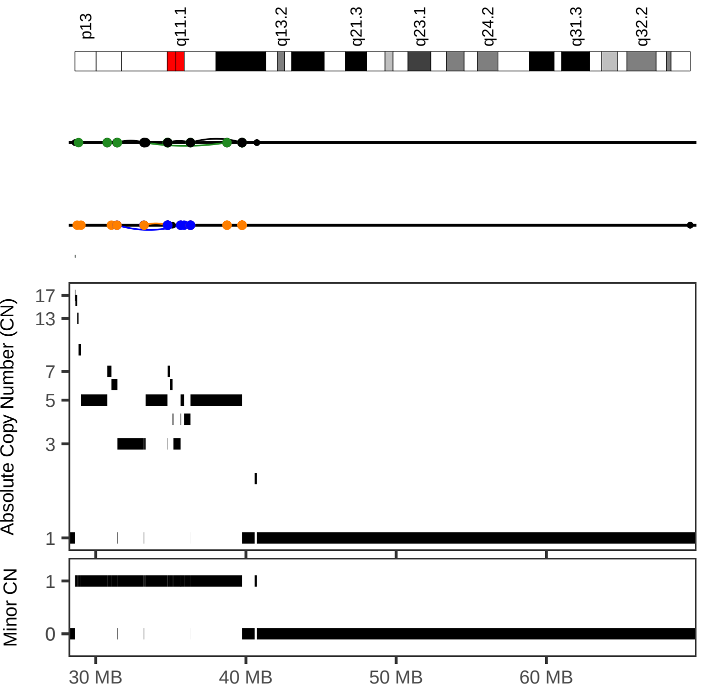

	RK135
Cancer type	Liver−HCC
Position	14:31416617−39741028
Type	With other complex events
Interleaved intrachr. SVs	6
Total SVs (intrachr. + transl.)	45
SV types	DEL: 1; DUP: 1; h2hINV: 3; t2tINV: 1; TRA: 39
SVs in sample	192
Oscillating CN (2 and 3 states)	4, 6
CN segments	16
FDR fragment joints	0.793803
FDR chr. breakp. enrich.	0
Linked to chrs	
Purity, ploidy	0.7, 1.9

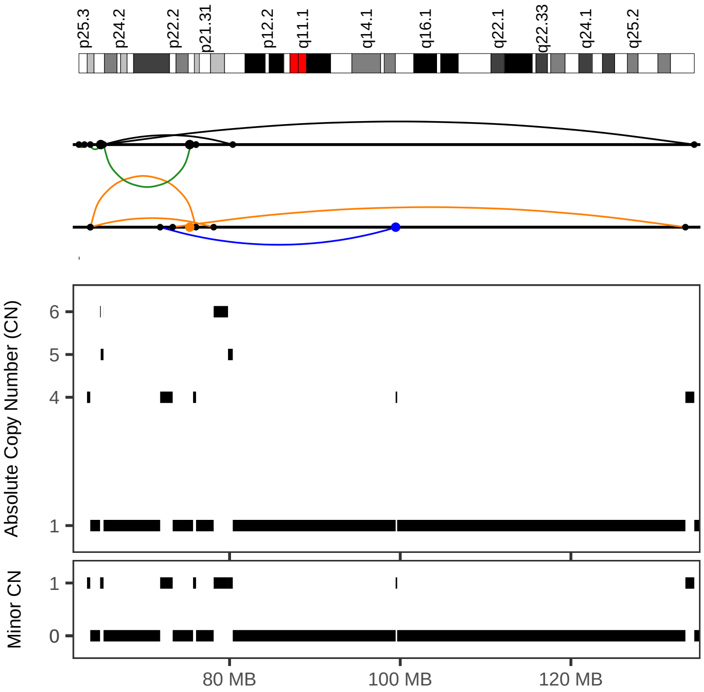

	RK141
Cancer type	Liver−HCC
Position	6:63658682−134453042
Type	With other complex events
Interleaved intrachr. SVs	9
Total SVs (intrachr. + transl.)	13
SV types	DEL: 3; DUP: 1; h2hINV: 3; t2tINV: 2; TRA: 4
SVs in sample	30
Oscillating CN (2 and 3 states)	5, 7
CN segments	18
FDR fragment joints	0.9000538
FDR chr. breakp. enrich.	0
Linked to chrs	
Purity, ploidy	0.7, 2.08

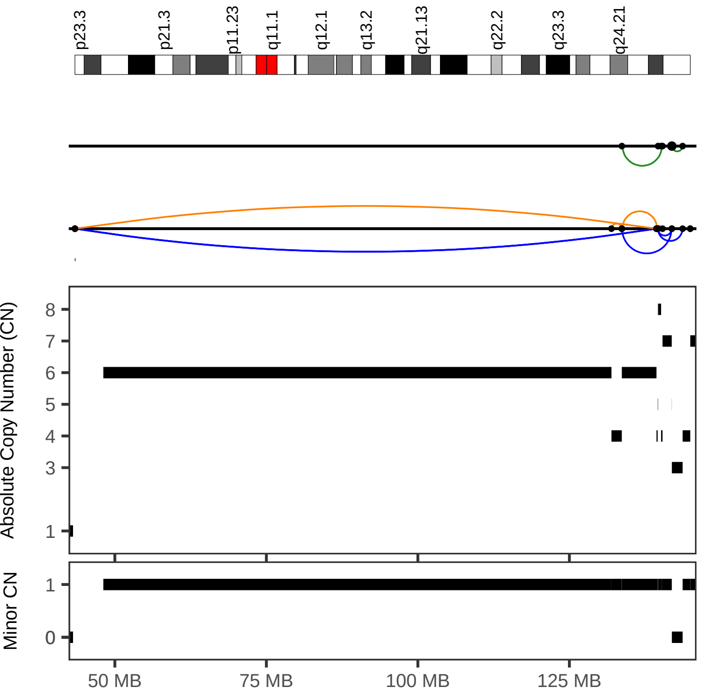

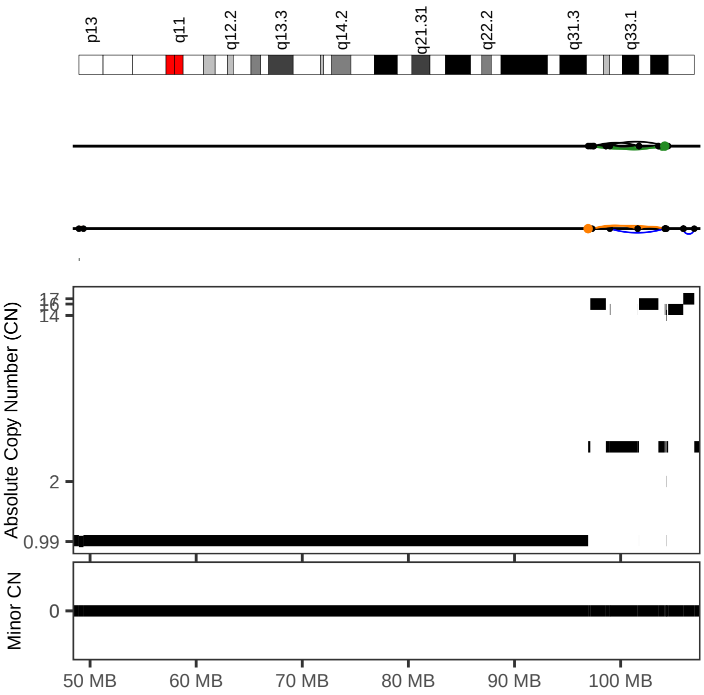

	**RK152**
Cancer type	Liver−HCC
Position	8:43410563−143686658
Type	With other complex events
Interleaved intrachr. SVs	11
Total SVs (intrachr. + transl.)	12
SV types	DEL: 4; DUP: 5; h2hINV: 0; t2tINV: 2; TRA: 1
SVs in sample	53
Oscillating CN (2 and 3 states)	6, 7
CN segments	15
FDR fragment joints	0.615458
FDR chr. breakp. enrich.	0
Linked to chrs	
Purity, ploidy	0.35, 2.24

	**RK166**
Cancer type	Liver−HCC
Position	13:96936658−104475344
Type	With other complex events
Interleaved intrachr. SVs	15
Total SVs (intrachr. + transl.)	17
SV types	DEL: 4; DUP: 1; h2hINV: 3; t2tINV: 7; TRA: 2
SVs in sample	74
Oscillating CN (2 and 3 states)	5, 7
CN segments	18
FDR fragment joints	0.615458
FDR chr. breakp. enrich.	0
Linked to chrs	
Purity, ploidy	0.48, 1.74

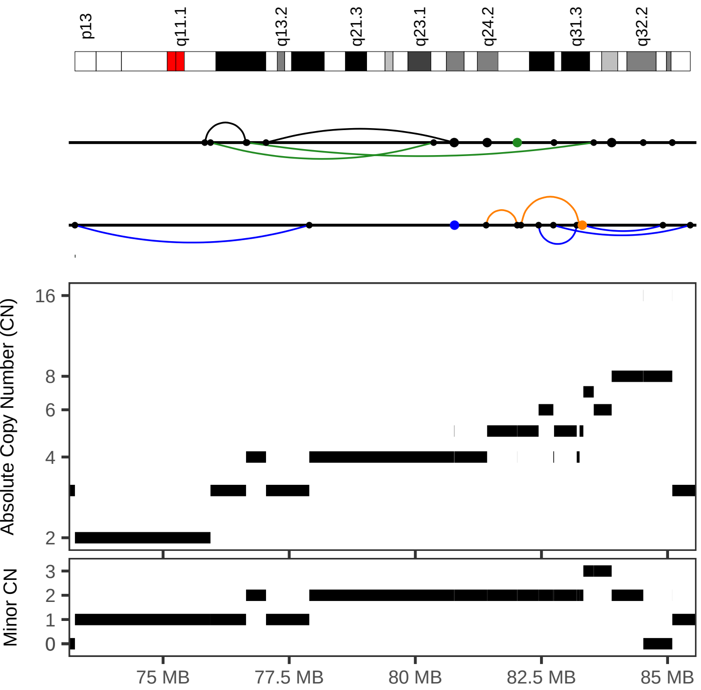

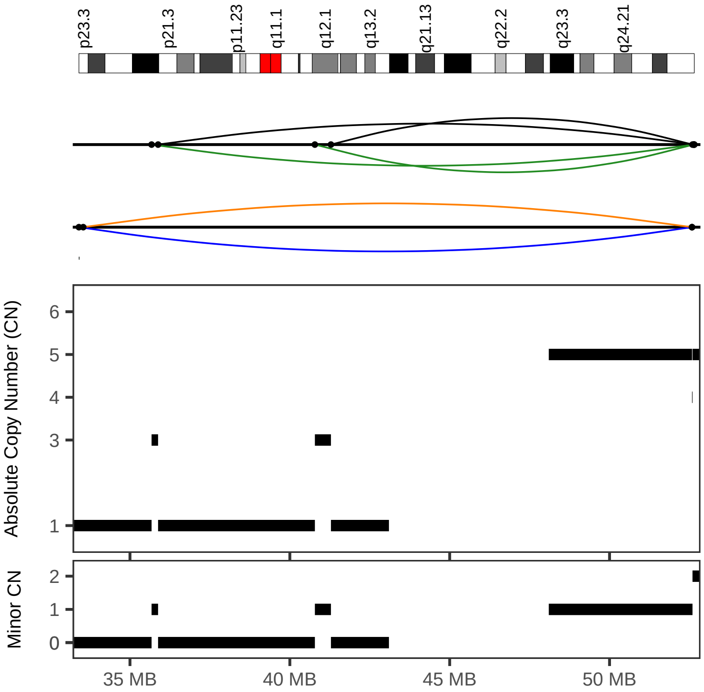

	**RK169**
Cancer type	Liver−HCC
Position	14:73252280−85449372
Type	With other complex events
Interleaved intrachr. SVs	9
Total SVs (intrachr. + transl.)	15
SV types	DEL: 1; DUP: 4; h2hINV: 2; t2tINV: 2; TRA: 6
SVs in sample	75
Oscillating CN (2 and 3 states)	6, 6
CN segments	22
FDR fragment joints	0.7257048
FDR chr. breakp. enrich.	0
Linked to chrs	
Purity, ploidy	0.24, 3.37

	**RK171**
Cancer type	Liver−HCC
Position	8:33403096−52649600
Type	Canonical without polyploidization
Interleaved intrachr. SVs	6
Total SVs (intrachr. + transl.)	6
SV types	DEL: 1; DUP: 1; h2hINV: 2; t2tINV: 2; TRA: 0
SVs in sample	11
Oscillating CN (2 and 3 states)	5, 6
CN segments	8
FDR fragment joints	0.9284301
FDR chr. breakp. enrich.	0
Linked to chrs	
Purity, ploidy	0.39, 2.23

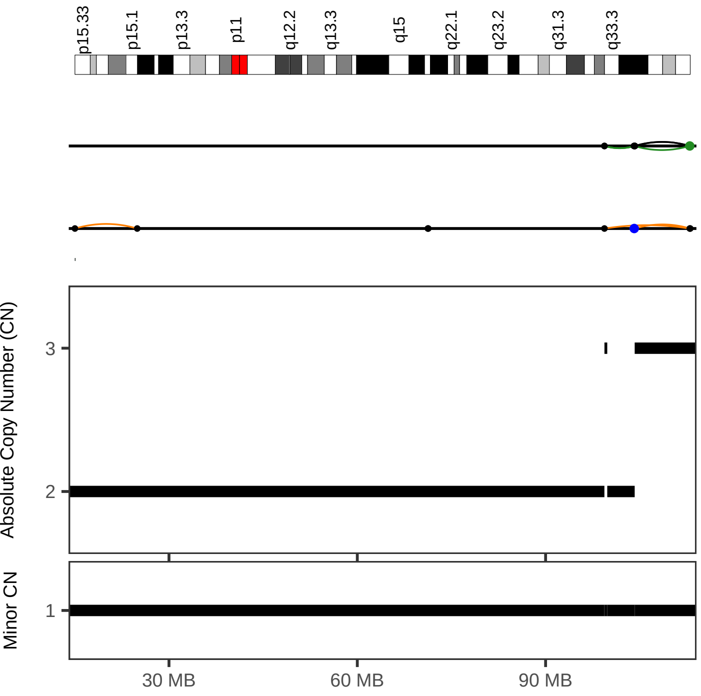

	**RK176**
Cancer type	Liver−HCC
Position	5:99356960−113044705
Type	Canonical without polyploidization
Interleaved intrachr. SVs	6
Total SVs (intrachr. + transl.)	8
SV types	DEL: 2; DUP: 0; h2hINV: 1; t2tINV: 3; TRA: 2
SVs in sample	94
Oscillating CN (2 and 3 states)	5, 5
CN segments	5
FDR fragment joints	0.8653243
FDR chr. breakp. enrich.	0.16
Linked to chrs	
Purity, ploidy	0.62, 2.03

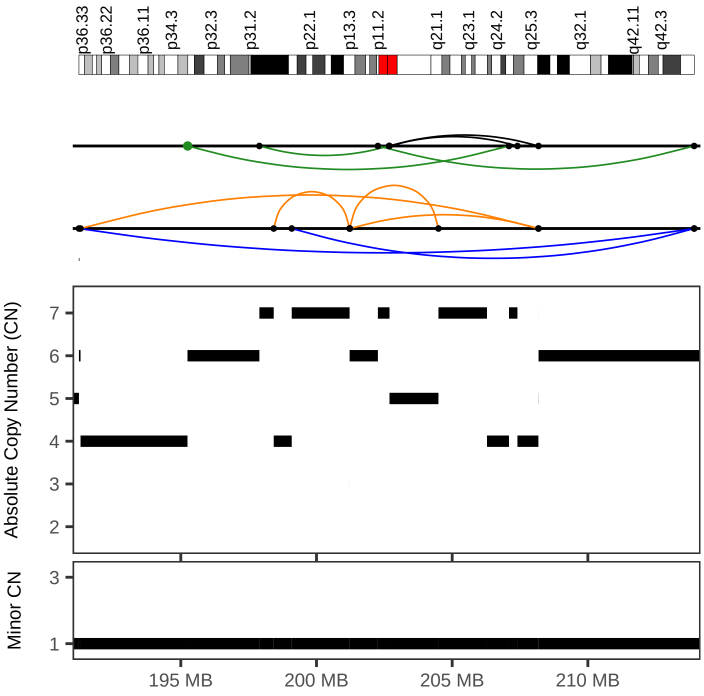

	**RK178**
Cancer type	Liver−HCC
Position	1:191304671−213916961
Type	With other complex events
Interleaved intrachr. SVs	10
Total SVs (intrachr. + transl.)	11
SV types	DEL: 4; DUP: 1; h2hINV: 2; t2tINV: 3; TRA: 1
SVs in sample	63
Oscillating CN (2 and 3 states)	4, 7
CN segments	16
FDR fragment joints	0.7425546
FDR chr. breakp. enrich.	0.02
Linked to chrs	
Purity, ploidy	0.47, 2.18

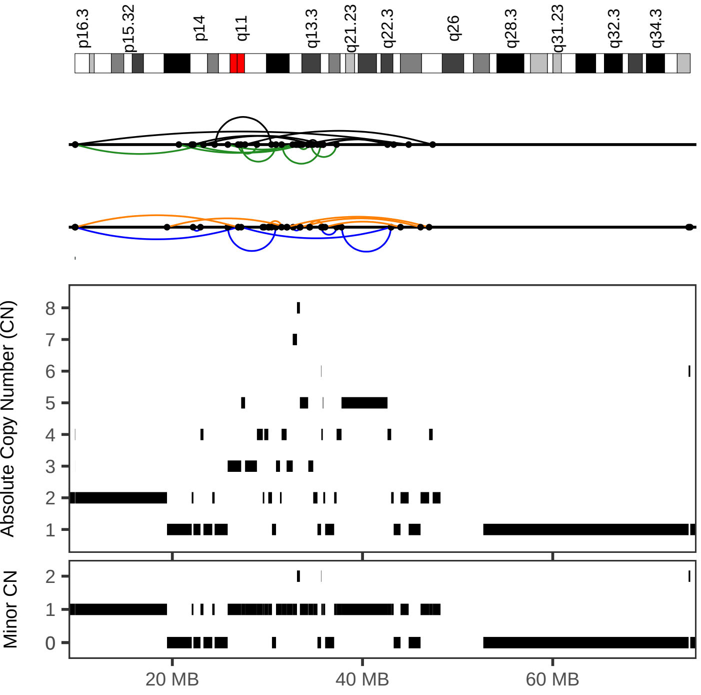

	**RK191**
Cancer type	Liver−HCC
Position	4:9755982−47376821
Type	With other complex events
Interleaved intrachr. SVs	34
Total SVs (intrachr. + transl.)	34
SV types	DEL: 9; DUP: 7; h2hINV: 9; t2tINV: 9; TRA: 0
SVs in sample	64
Oscillating CN (2 and 3 states)	5, 13
CN segments	44
FDR fragment joints	0.9717738
FDR chr. breakp. enrich.	0
Linked to chrs	
Purity, ploidy	0.41, 1.87

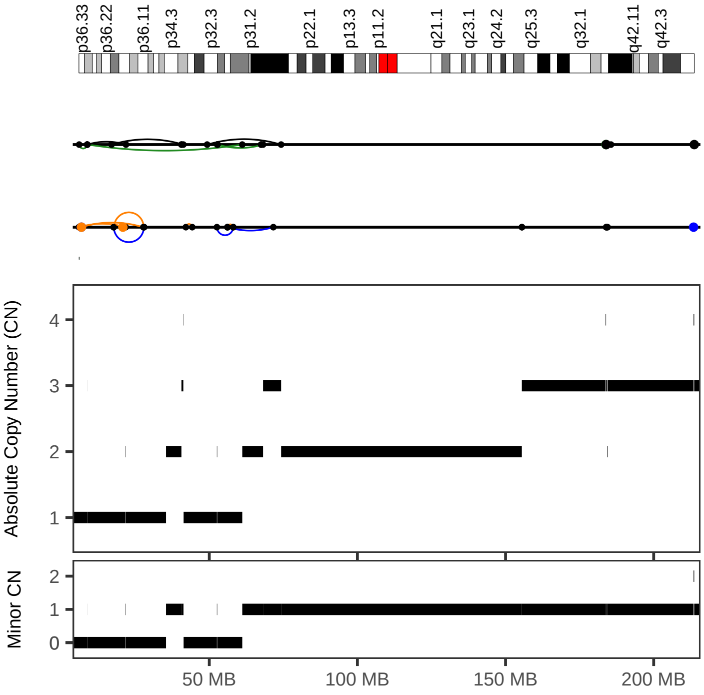

	**RK195**
Cancer type	Liver−HCC
Position	1:5994422−74247978
Type	With other complex events
Interleaved intrachr. SVs	14
Total SVs (intrachr. + transl.)	17
SV types	DEL: 4; DUP: 3; h2hINV: 4; t2tINV: 3; TRA: 3
SVs in sample	121
Oscillating CN (2 and 3 states)	4, 12
CN segments	14
FDR fragment joints	0.8653243
FDR chr. breakp. enrich.	0
Linked to chrs	
Purity, ploidy	0.8, 2.02

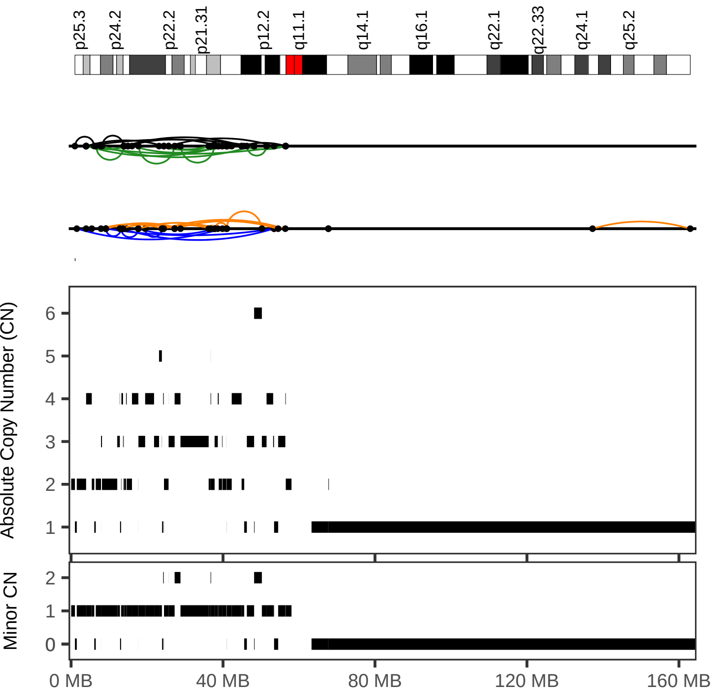

	**RK195**
Cancer type	Liver−HCC
Position	6:1017490−56519171
Type	With other complex events
Interleaved intrachr. SVs	44
Total SVs (intrachr. + transl.)	44
SV types	DEL: 11; DUP: 9; h2hINV: 10; t2tINV: 14; TRA: 0
SVs in sample	121
Oscillating CN (2 and 3 states)	5, 8
CN segments	63
FDR fragment joints	0.845361
FDR chr. breakp. enrich.	0
Linked to chrs	
Purity, ploidy	0.8, 2.02

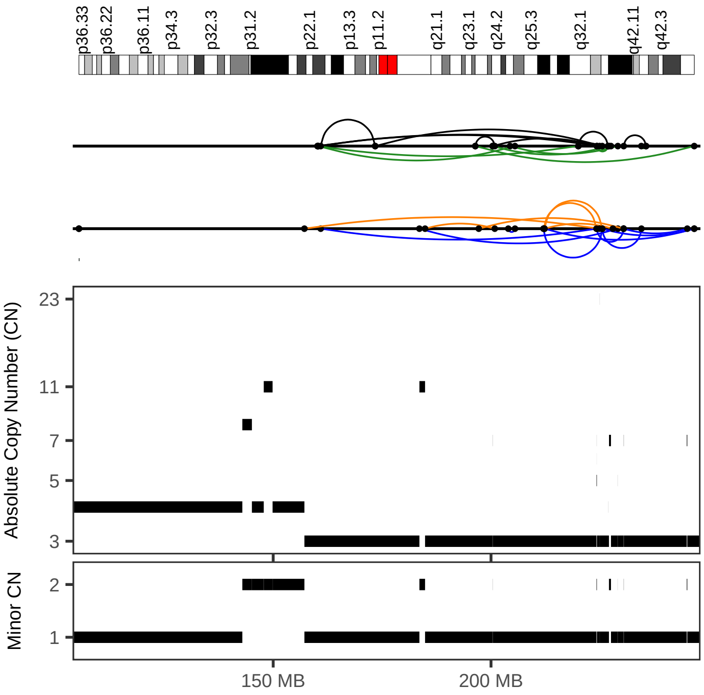

	**RK200**
Cancer type	Liver−HCC
Position	1:157162614−246658065
Type	With other complex events
Interleaved intrachr. SVs	29
Total SVs (intrachr. + transl.)	29
SV types	DEL: 6; DUP: 9; h2hINV: 7; t2tINV: 7; TRA: 0
SVs in sample	79
Oscillating CN (2 and 3 states)	5, 9
CN segments	21
FDR fragment joints	0.930656
FDR chr. breakp. enrich.	0
Linked to chrs	
Purity, ploidy	0.57, 3.64

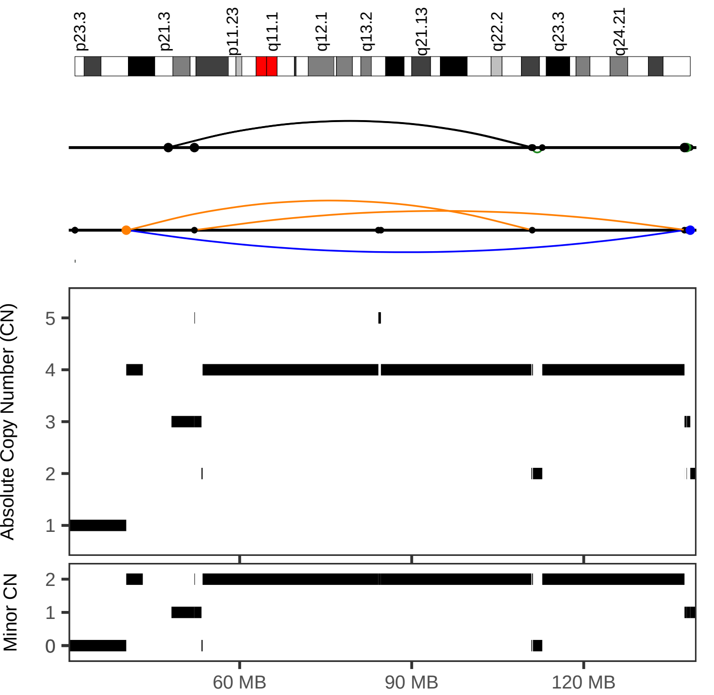

	**RK201**
Cancer type	Liver−HCC
Position	8:40469509−138579299
Type	With other complex events
Interleaved intrachr. SVs	7
Total SVs (intrachr. + transl.)	12
SV types	DEL: 2; DUP: 1; h2hINV: 2; t2tINV: 2; TRA: 5
SVs in sample	65
Oscillating CN (2 and 3 states)	6, 10
CN segments	17
FDR fragment joints	0.6776251
FDR chr. breakp. enrich.	0
Linked to chrs	6:57987979−165869685;
Purity, ploidy	0.29, 2.15

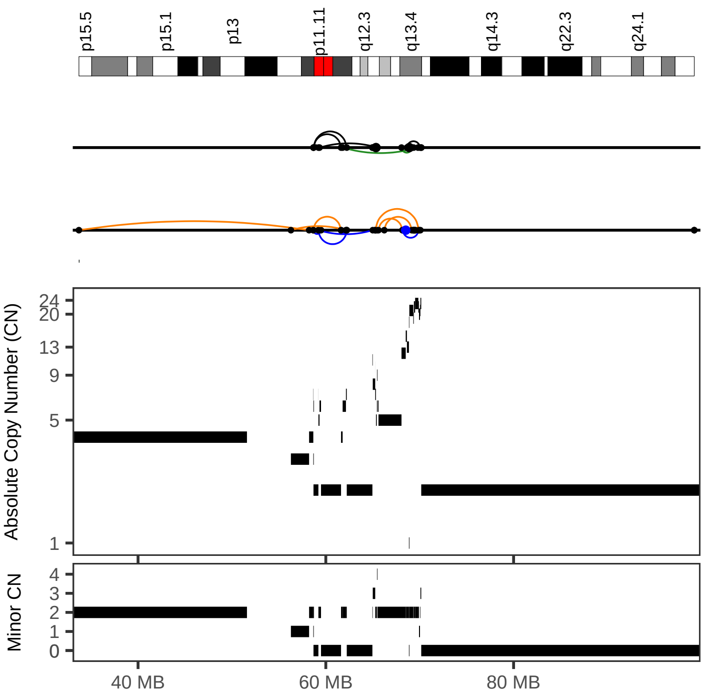

	**RK207**
Cancer type	Liver−HCC
Position	11:33703119−70073261
Type	With other complex events
Interleaved intrachr. SVs	23
Total SVs (intrachr. + transl.)	26
SV types	DEL: 9; DUP: 6; h2hINV: 5; t2tINV: 3; TRA: 3
SVs in sample	122
Oscillating CN (2 and 3 states)	4, 6
CN segments	40
FDR fragment joints	0.7510435
FDR chr. breakp. enrich.	0
Linked to chrs	
Purity, ploidy	0.38, 3.72

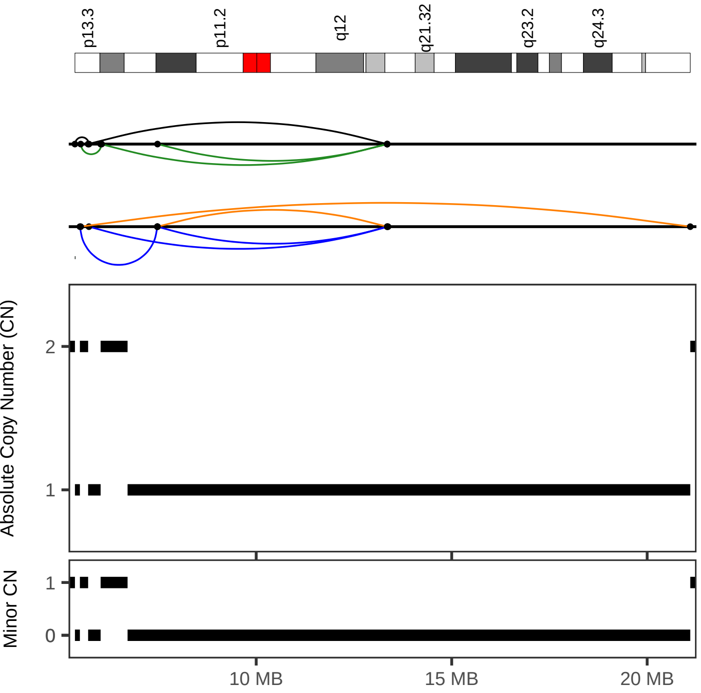

	**RK214**
Cancer type	Liver−HCC
Position	17:5363418−21101598
Type	Canonical without polyploidization
Interleaved intrachr. SVs	10
Total SVs (intrachr. + transl.)	10
SV types	DEL: 2; DUP: 3; h2hINV: 2; t2tINV: 3; TRA: 0
SVs in sample	43
Oscillating CN (2 and 3 states)	6, 6
CN segments	6
FDR fragment joints	0.9625775
FDR chr. breakp. enrich.	0
Linked to chrs	
Purity, ploidy	0.93, 1.96

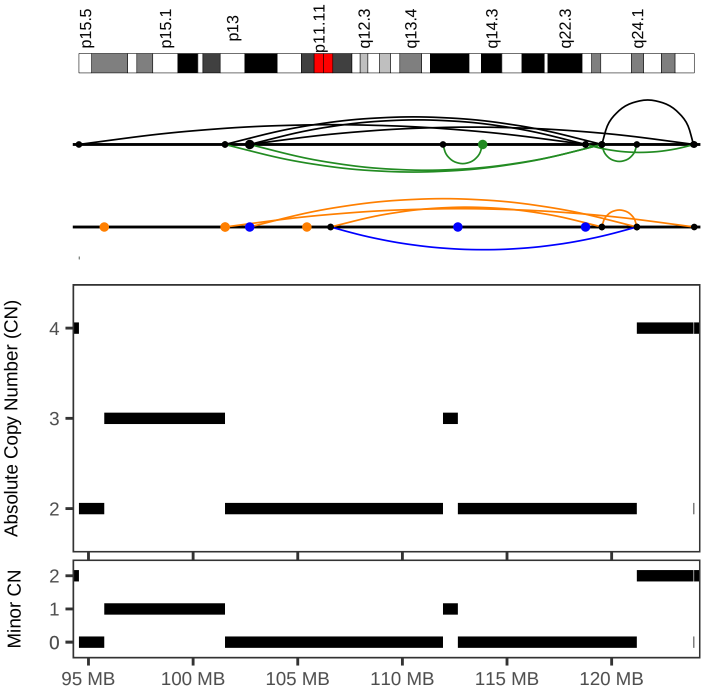

	**RK216**
Cancer type	Liver−HCC
Position	11:94543805−123946518
Type	Before polyploidization
Interleaved intrachr. SVs	14
Total SVs (intrachr. + transl.)	23
SV types	DEL: 4; DUP: 1; h2hINV: 5; t2tINV: 4; TRA: 9
SVs in sample	38
Oscillating CN (2 and 3 states)	6, 10
CN segments	10
FDR fragment joints	0.7861024
FDR chr. breakp. enrich.	0
Linked to chrs	
Purity, ploidy	0.84, 3.4

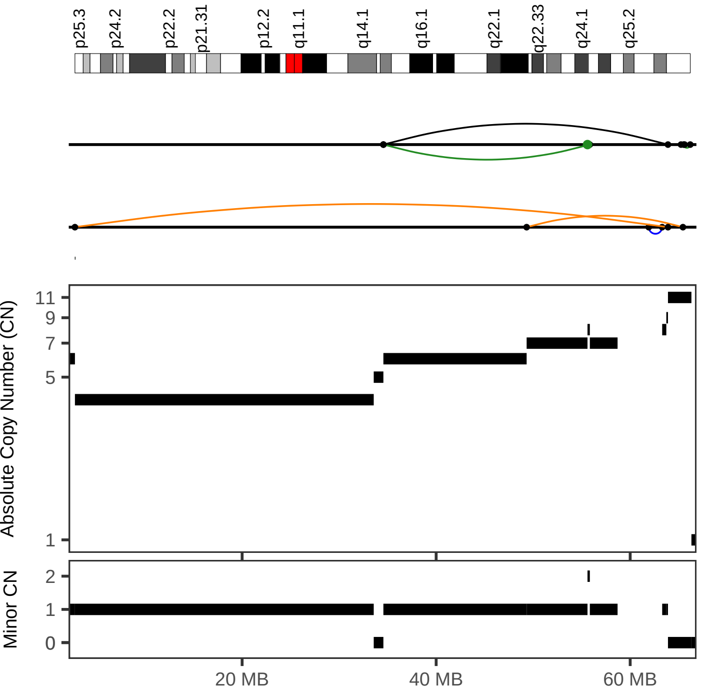

	**RK217**
Cancer type	Liver−HCC
Position	6:2765669−66192793
Type	With other complex events
Interleaved intrachr. SVs	6
Total SVs (intrachr. + transl.)	7
SV types	DEL: 2; DUP: 0; h2hINV: 2; t2tINV: 2; TRA: 1
SVs in sample	44
Oscillating CN (2 and 3 states)	4, 8
CN segments	9
FDR fragment joints	0.6776251
FDR chr. breakp. enrich.	0.01
Linked to chrs	
Purity, ploidy	0.46, 1.7

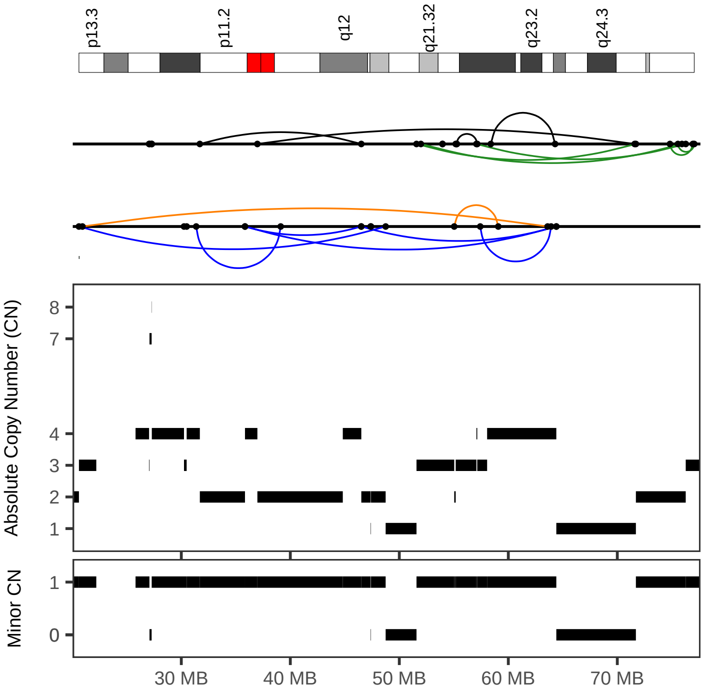

	**RK232**
Cancer type	Liver−HCC
Position	17:20606173−77059952
Type	With other complex events
Interleaved intrachr. SVs	18
Total SVs (intrachr. + transl.)	18
SV types	DEL: 2; DUP: 6; h2hINV: 4; t2tINV: 6; TRA: 0
SVs in sample	56
Oscillating CN (2 and 3 states)	6, 9
CN segments	25
FDR fragment joints	0.6776251
FDR chr. breakp. enrich.	0
Linked to chrs	
Purity, ploidy	0.61, 2.06

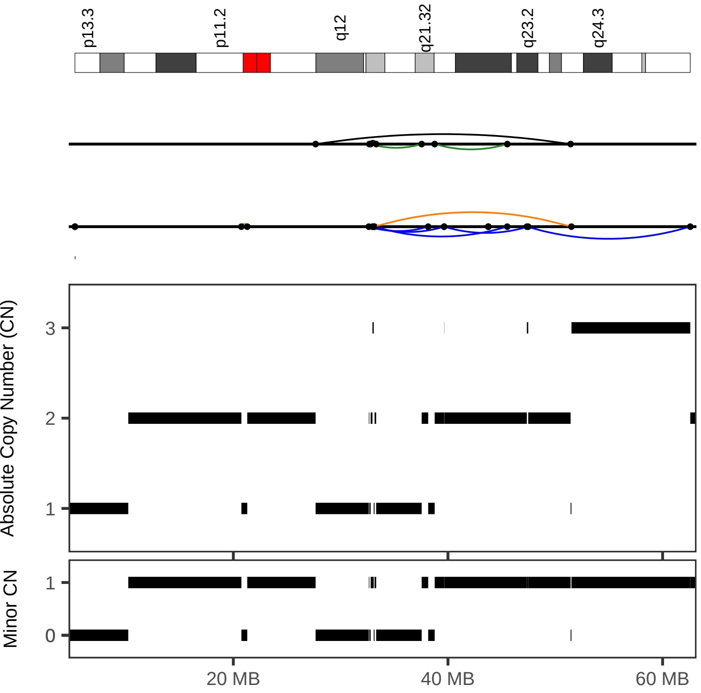

RK256	
Cancer type	Liver−HCC
Position	17:27669465−62576041
Type	With other complex events
Interleaved intrachr. SVs	10
Total SVs (intrachr. + transl.)	10
SV types	DEL: 1; DUP: 5; h2hINV: 2; t2tINV: 2; TRA: 0
SVs in sample	91
Oscillating CN (2 and 3 states)	6, 8
CN segments	18
FDR fragment joints	0.615458
FDR chr. breakp. enrich.	0
Linked to chrs	
Purity, ploidy	0.89, 1.86

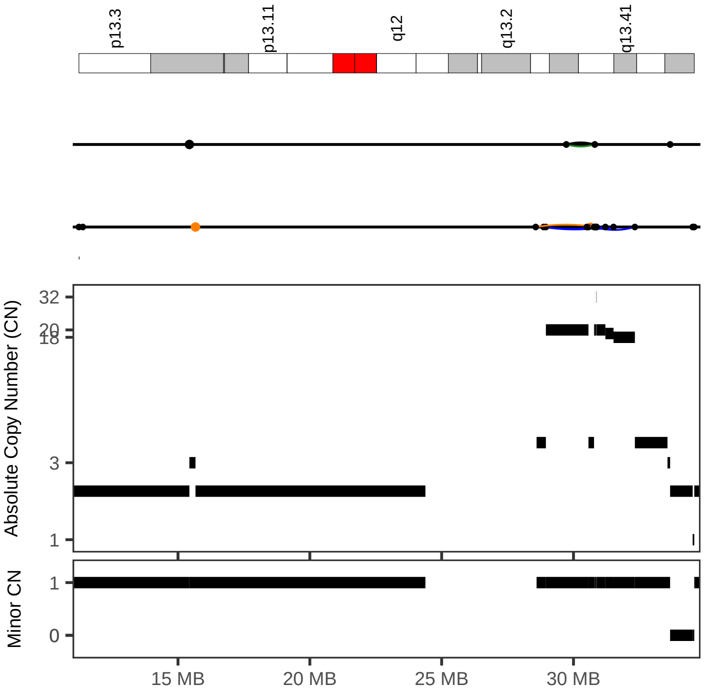

RK256	
Cancer type	Liver−HCC
Position	19:28565715−32327841
Type	With other complex events
Interleaved intrachr. SVs	8
Total SVs (intrachr. + transl.)	8
SV types	DEL: 2; DUP: 4; h2hINV: 1; t2tINV: 1; TRA: 0
SVs in sample	91
Oscillating CN (2 and 3 states)	5, 5
CN segments	12
FDR fragment joints	0.615458
FDR chr. breakp. enrich.	0
Linked to chrs	
Purity, ploidy	0.89, 1.86

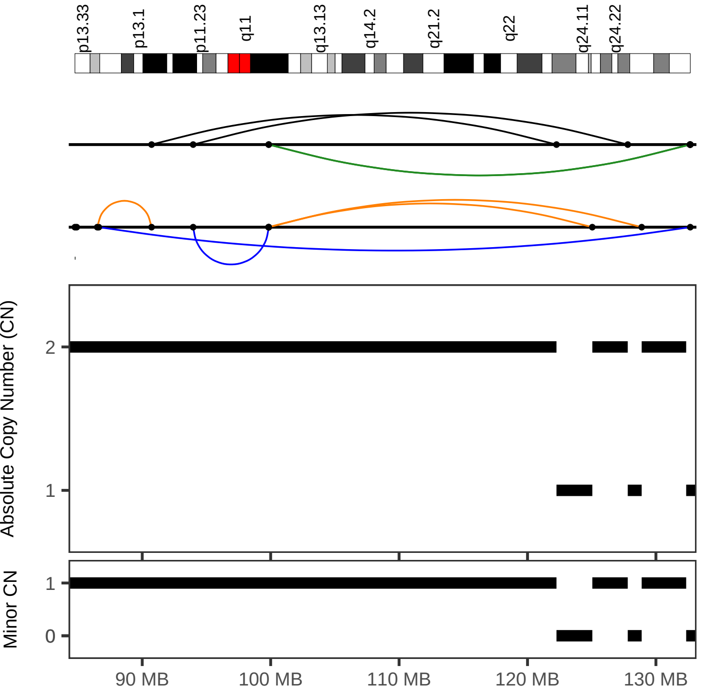

RK275	
Cancer type	Liver−HCC
Position	12:86498848−132672926
Type	Canonical without polyploidization
Interleaved intrachr. SVs	10
Total SVs (intrachr. + transl.)	10
SV types	DEL: 3; DUP: 2; h2hINV: 3; t2tINV: 2; TRA: 0
SVs in sample	69
Oscillating CN (2 and 3 states)	6, 6
CN segments	6
FDR fragment joints	0.9625775
FDR chr. breakp. enrich.	0
Linked to chrs	
Purity, ploidy	0.64, 1.96

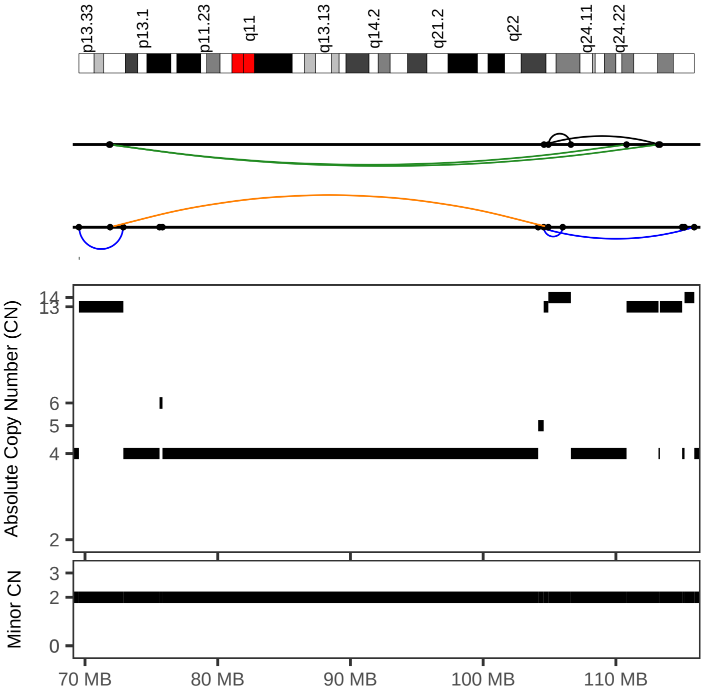

RK280	
Cancer type	Liver−HCC
Position	12:69541748−115904220
Type	With other complex events
Interleaved intrachr. SVs	8
Total SVs (intrachr. + transl.)	8
SV types	DEL: 1; DUP: 3; h2hINV: 2; t2tINV: 2; TRA: 0
SVs in sample	111
Oscillating CN (2 and 3 states)	5, 8
CN segments	14
FDR fragment joints	0.8653243
FDR chr. breakp. enrich.	0.03
Linked to chrs	
Purity, ploidy	0.87, 3.75

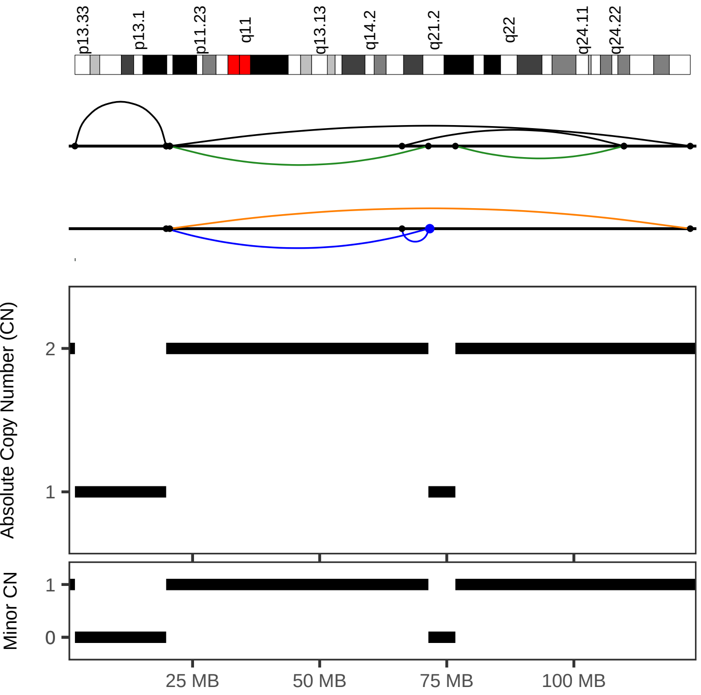

	**RK282**
Cancer type	Liver−HCC
Position	12:1853016−122894996
Type	Canonical without polyploidization
Interleaved intrachr. SVs	9
Total SVs (intrachr. + transl.)	10
SV types	DEL: 1; DUP: 2; h2hINV: 3; t2tINV: 3; TRA: 1
SVs in sample	40
Oscillating CN (2 and 3 states)	4, 4
CN segments	4
FDR fragment joints	0.8572806
FDR chr. breakp. enrich.	0
Linked to chrs	
Purity, ploidy	0.93, 2.24

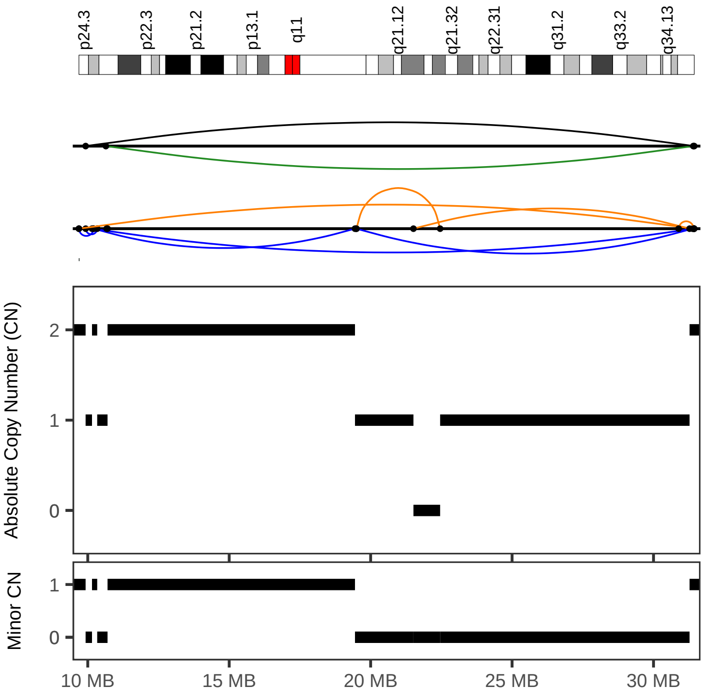

	**RK297**
Cancer type	Liver−HCC
Position	9:9687693−31438879
Type	Canonical without polyploidization
Interleaved intrachr. SVs	11
Total SVs (intrachr. + transl.)	11
SV types	DEL: 4; DUP: 5; h2hINV: 1; t2tINV: 1; TRA: 0
SVs in sample	34
Oscillating CN (2 and 3 states)	6, 9
CN segments	9
FDR fragment joints	0.615458
FDR chr. breakp. enrich.	0
Linked to chrs	
Purity, ploidy	0.33, 1.95

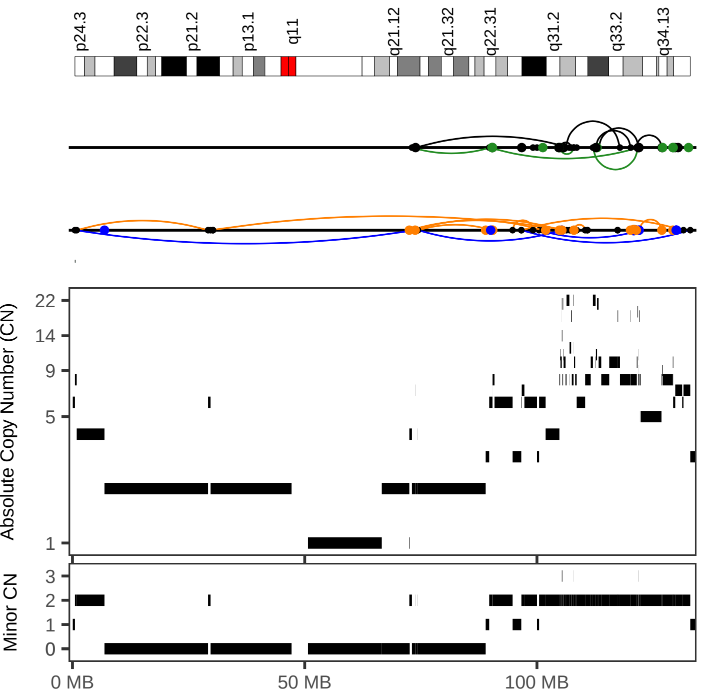

	**0d66bf6c−eed0−4726−bd5b−3bf6d610b4e0**
Cancer type	Lung−AdenoCA
Position	9:524316−133004751
Type	With other complex events
Interleaved intrachr. SVs	30
Total SVs (intrachr. + transl.)	74
SV types	DEL: 10; DUP: 7; h2hINV: 9; t2tINV: 4; TRA: 44
SVs in sample	414
Oscillating CN (2 and 3 states)	6, 8
CN segments	90
FDR fragment joints	0.7745035
FDR chr. breakp. enrich.	0
Linked to chrs	12:61686111−128942892;6:26318534−27718360 8:89155199−132576363;X:1621688−36004056
Purity, ploidy	0.48, 3.84

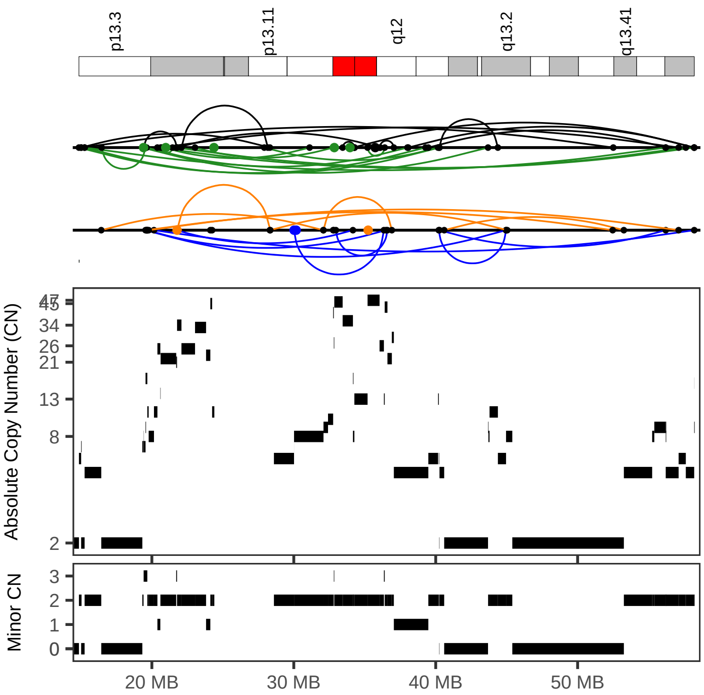

	**0d66bf6c−eed0−4726−bd5b−3bf6d610b4e0**
Cancer type	Lung−AdenoCA
Position	19:14859949−58216996
Type	With other complex events
Interleaved intrachr. SVs	41
Total SVs (intrachr. + transl.)	51
SV types	DEL: 7; DUP: 9; h2hINV: 12; t2tINV: 13; TRA: 10
SVs in sample	414
Oscillating CN (2 and 3 states)	4, 5
CN segments	64
FDR fragment joints	0.6434443
FDR chr. breakp. enrich.	0
Linked to chrs	12:61686111−128942892;X:1621688−36004056
Purity, ploidy	0.48, 3.84

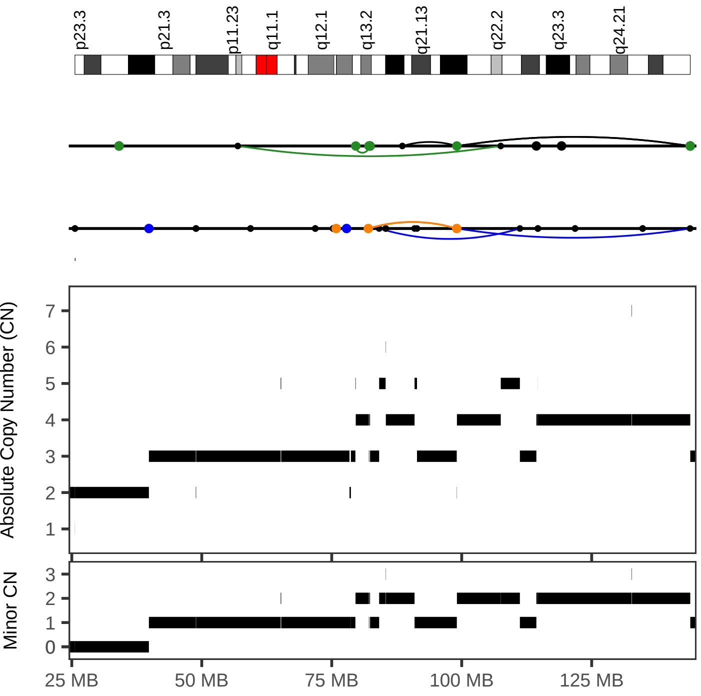

	0df573ee−28f0−4244−b434−09e6ca59fbf0
Cancer type	Lung−AdenoCA
Position	8:56923598−143961644
Type	With other complex events
Interleaved intrachr. SVs	9
Total SVs (intrachr. + transl.)	22
SV types	DEL: 2; DUP: 2; h2hINV: 3; t2tINV: 2; TRA: 13
SVs in sample	350
Oscillating CN (2 and 3 states)	4, 5
CN segments	25
FDR fragment joints	0.6776251
FDR chr. breakp. enrich.	0
Linked to chrs	20:15841444−61654373;
Purity, ploidy	0.42, 2.67

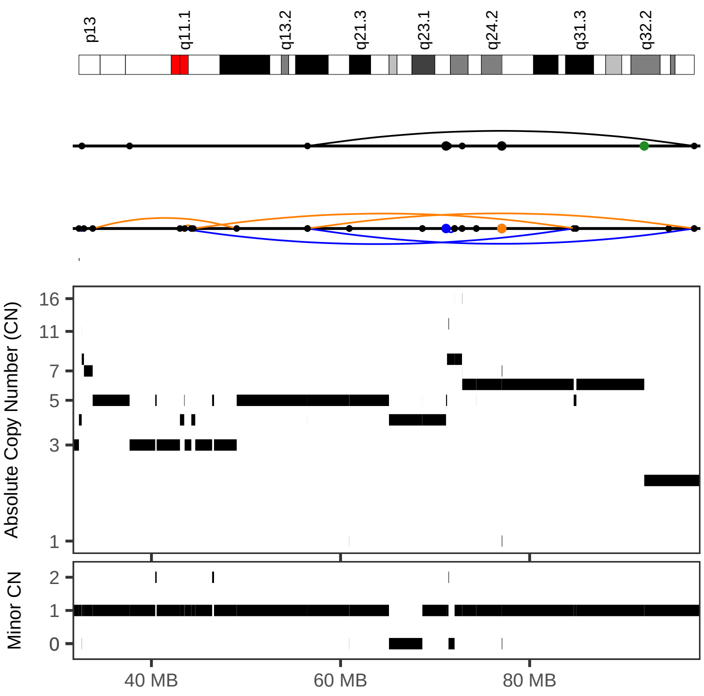

	0df573ee−28f0−4244−b434−09e6ca59fbf0
Cancer type	Lung−AdenoCA
Position	14:33787461−97407133
Type	With other complex events
Interleaved intrachr. SVs	6
Total SVs (intrachr. + transl.)	12
SV types	DEL: 3; DUP: 2; h2hINV: 1; t2tINV: 0; TRA: 6
SVs in sample	350
Oscillating CN (2 and 3 states)	5, 10
CN segments	36
FDR fragment joints	0.8298498
FDR chr. breakp. enrich.	0
Linked to chrs	13:30424355−100177239;
Purity, ploidy	0.42, 2.67

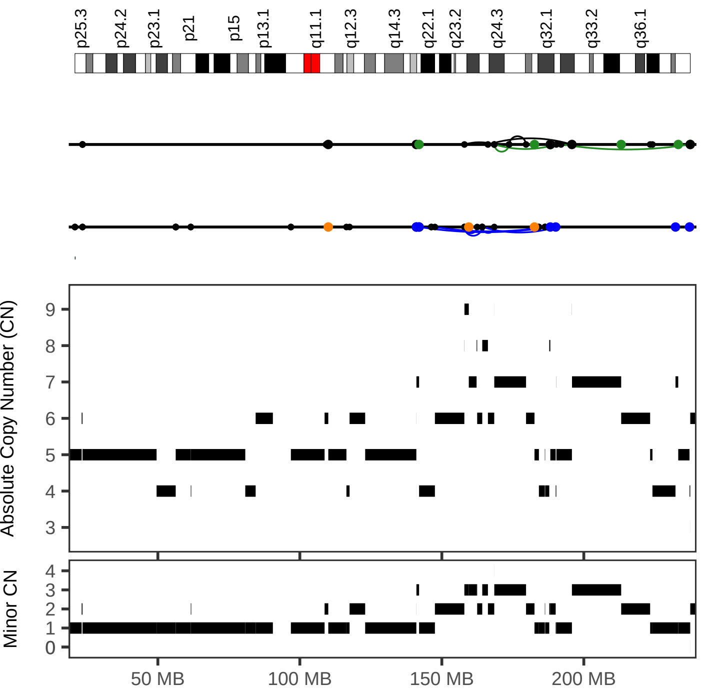

	31c96e35−5e2f−429c−b12a−7bc5a497a300
Cancer type	Lung−AdenoCA
Position	2:141983404−237479460
Type	With other complex events
Interleaved intrachr. SVs	11
Total SVs (intrachr. + transl.)	31
SV types	DEL: 0; DUP: 6; h2hINV: 2; t2tINV: 3; TRA: 20
SVs in sample	276
Oscillating CN (2 and 3 states)	4, 8
CN segments	34
FDR fragment joints	0.5435077
FDR chr. breakp. enrich.	0
Linked to chrs	1:14760541−105675265;
Purity, ploidy	0.49, 4.79

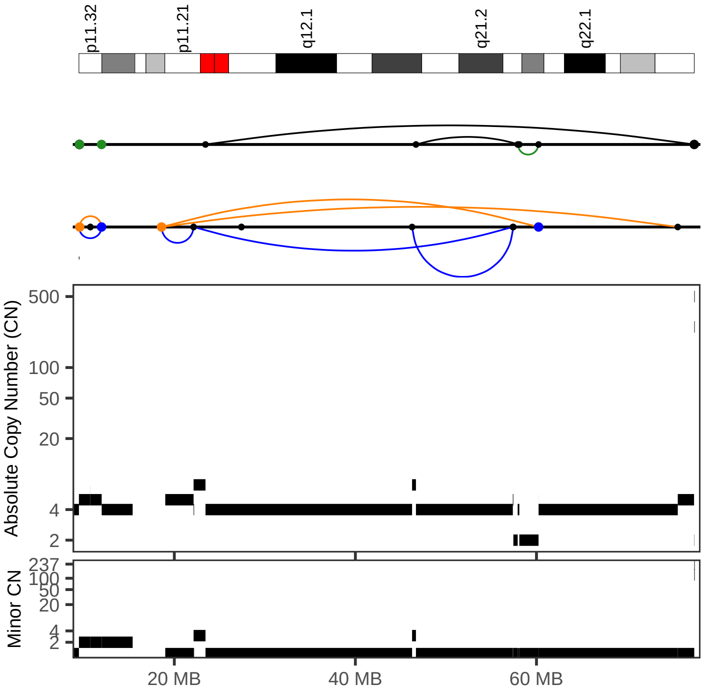

	31c96e35−5e2f−429c−b12a−7bc5a497a300
Cancer type	Lung−AdenoCA
Position	18:18573755−77402934
Type	With other complex events
Interleaved intrachr. SVs	8
Total SVs (intrachr. + transl.)	10
SV types	DEL: 2; DUP: 3; h2hINV: 2; t2tINV: 1; TRA: 2
SVs in sample	276
Oscillating CN (2 and 3 states)	5, 9
CN segments	15
FDR fragment joints	0.6776251
FDR chr. breakp. enrich.	0
Linked to chrs	
Purity, ploidy	0.49, 4.79

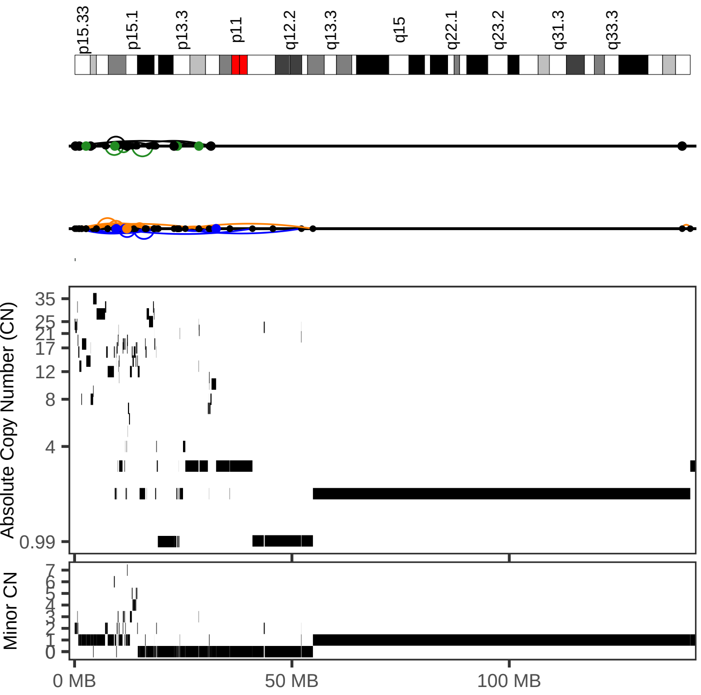

35cb7841−9b09−465a−90c5−e3b8a9faad49	
Cancer type	Lung−AdenoCA
Position	5:78381−54831894
Type	With other complex events
Interleaved intrachr. SVs	57
Total SVs (intrachr. + transl.)	71
SV types	DEL: 16; DUP: 18; h2hINV: 13; t2tINV: 10; TRA: 14
SVs in sample	208
Oscillating CN (2 and 3 states)	5, 10
CN segments	109
FDR fragment joints	0.9037055
FDR chr. breakp. enrich.	0
Linked to chrs	
Purity, ploidy	0.26, 2.72

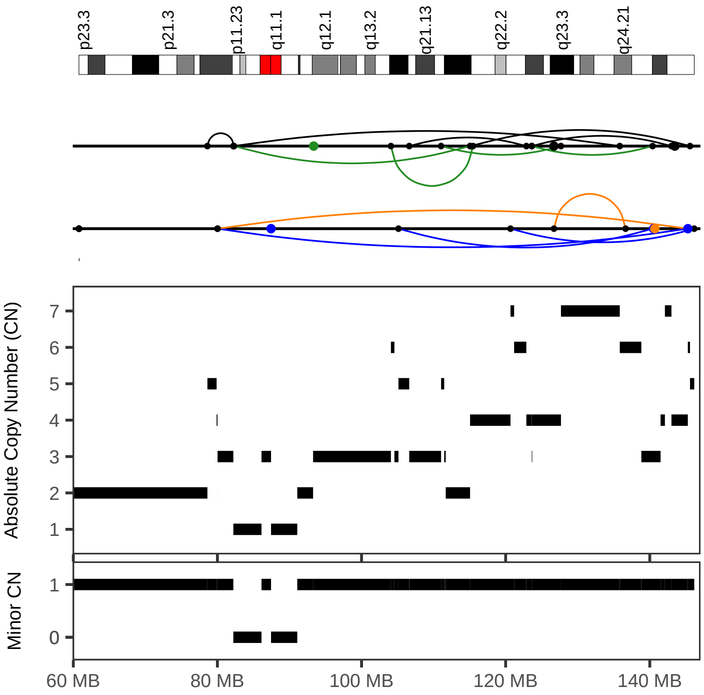

61c655ec−52b5−453f−a6cc−b2aba445b027	
Cancer type	Lung−AdenoCA
Position	8:78610917−146172492
Type	With other complex events
Interleaved intrachr. SVs	14
Total SVs (intrachr. + transl.)	21
SV types	DEL: 2; DUP: 3; h2hINV: 5; t2tINV: 4; TRA: 7
SVs in sample	44
Oscillating CN (2 and 3 states)	5, 7
CN segments	30
FDR fragment joints	0.8419568
FDR chr. breakp. enrich.	0
Linked to chrs	
Purity, ploidy	0.72, 2.02

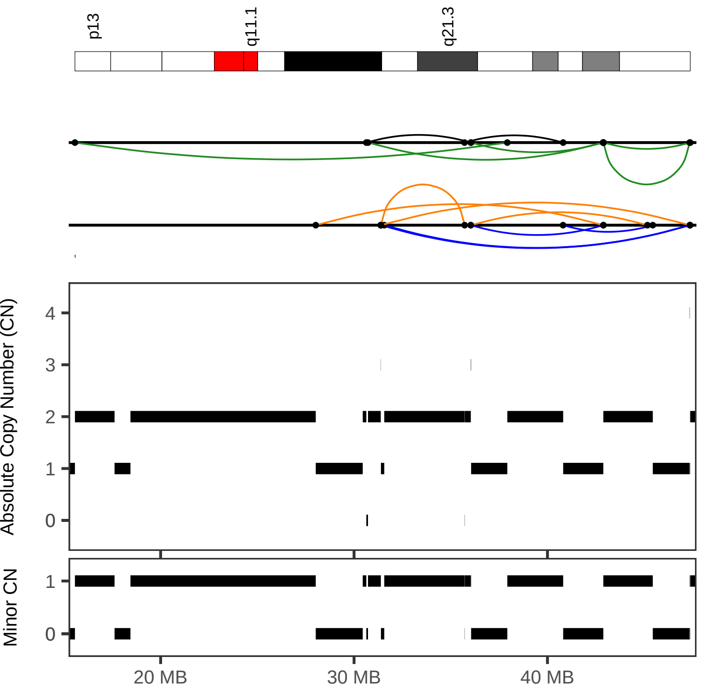

6dfd47d2−831a−4386−9051−f78199a16bb5	
Cancer type	Lung−AdenoCA
Position	21:15568990−47382700
Type	With other complex events
Interleaved intrachr. SVs	15
Total SVs (intrachr. + transl.)	15
SV types	DEL: 4; DUP: 4; h2hINV: 2; t2tINV: 5; TRA: 0
SVs in sample	113
Oscillating CN (2 and 3 states)	5, 7
CN segments	23
FDR fragment joints	0.8462769
FDR chr. breakp. enrich.	0
Linked to chrs	
Purity, ploidy	0.86, 1.97

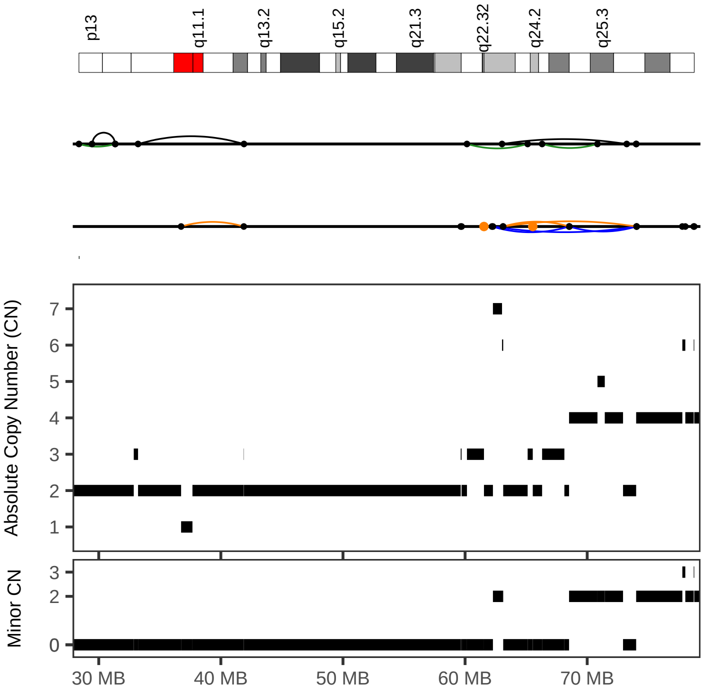

762dea8a−5b41−4058−979a−b7876ed13d7e	
Cancer type	Lung−AdenoCA
Position	15:60153021−74051084
Type	With other complex events
Interleaved intrachr. SVs	8
Total SVs (intrachr. + transl.)	10
SV types	DEL: 2; DUP: 3; h2hINV: 1; t2tINV: 2; TRA: 2
SVs in sample	74
Oscillating CN (2 and 3 states)	5, 6
CN segments	14
FDR fragment joints	0.6776251
FDR chr. breakp. enrich.	0
Linked to chrs	
Purity, ploidy	0.62, 2.94

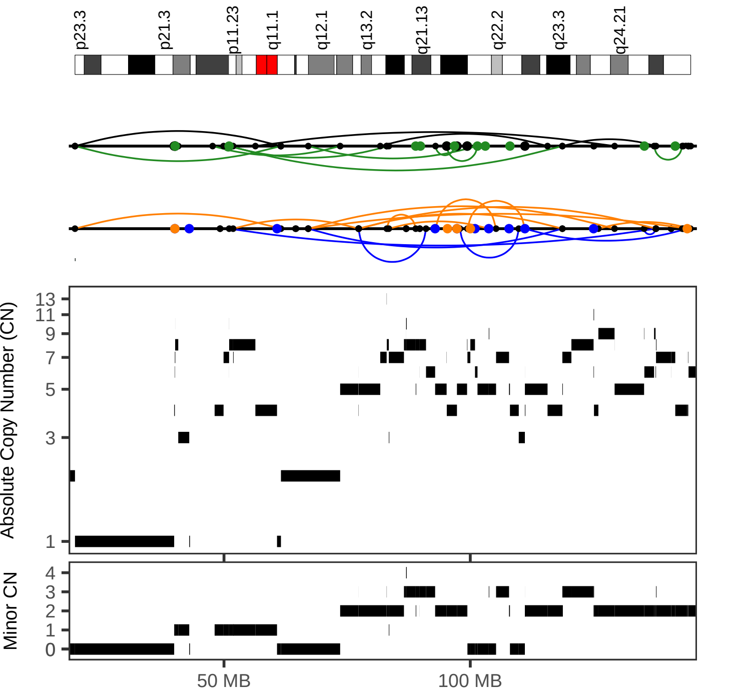

	97831d28−ab41−4c18−bfc2−c4c6bc757d13
Cancer type	Lung−AdenoCA
Position	8:19746812−144761257
Type	With other complex events
Interleaved intrachr. SVs	34
Total SVs (intrachr. + transl.)	68
SV types	DEL: 12; DUP: 6; h2hINV: 5; t2tINV: 11; TRA: 34
SVs in sample	229
Oscillating CN (2 and 3 states)	5, 8
CN segments	79
FDR fragment joints	1
FDR chr. breakp. enrich.	0
Linked to chrs	
Purity, ploidy	0.82, 3.49

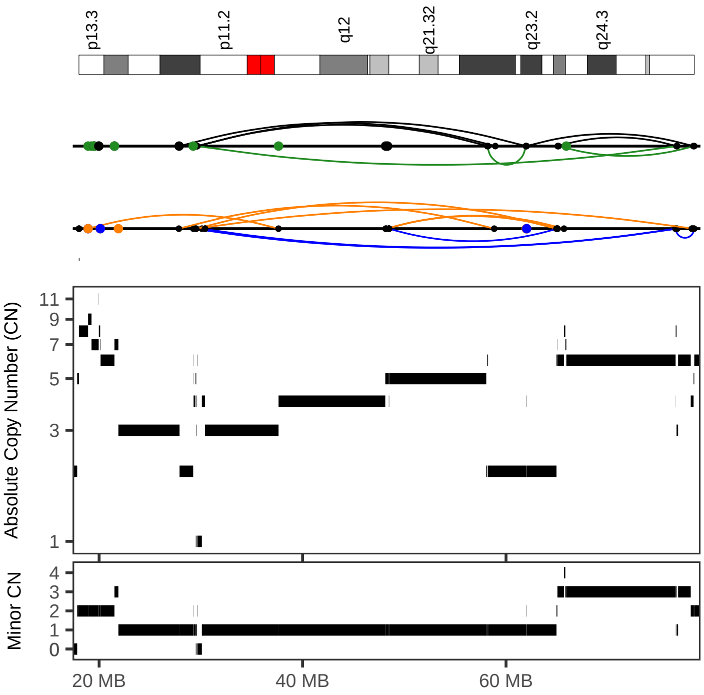

	97831d28−ab41−4c18−bfc2−c4c6bc757d13
Cancer type	Lung−AdenoCA
Position	17:18917497−78501499
Type	With other complex events
Interleaved intrachr. SVs	23
Total SVs (intrachr. + transl.)	45
SV types	DEL: 6; DUP: 4; h2hINV: 6; t2tINV: 7; TRA: 22
SVs in sample	229
Oscillating CN (2 and 3 states)	4, 9
CN segments	46
FDR fragment joints	0.6776251
FDR chr. breakp. enrich.	0
Linked to chrs	8:19746812−144761256;
Purity, ploidy	0.82, 3.49

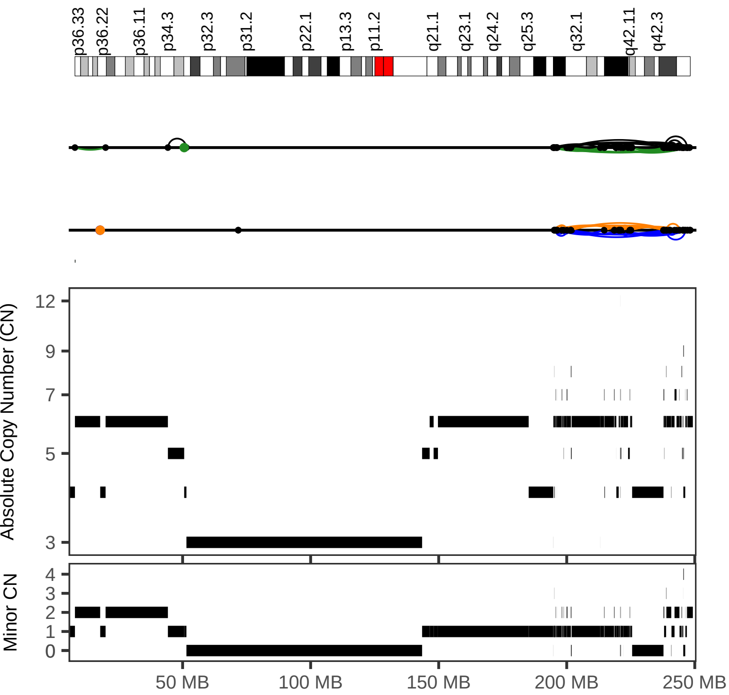

	9b132e4f−7e35−4cc5−8711−43ad62b906d0
Cancer type	Lung−AdenoCA
Position	1:194754133−247990684
Type	With other complex events
Interleaved intrachr. SVs	73
Total SVs (intrachr. + transl.)	73
SV types	DEL: 21; DUP: 18; h2hINV: 18; t2tINV: 16; TRA: 0
SVs in sample	209
Oscillating CN (2 and 3 states)	5, 9
CN segments	79
FDR fragment joints	0.9284301
FDR chr. breakp. enrich.	0
Linked to chrs	
Purity, ploidy	0.9, 4.33

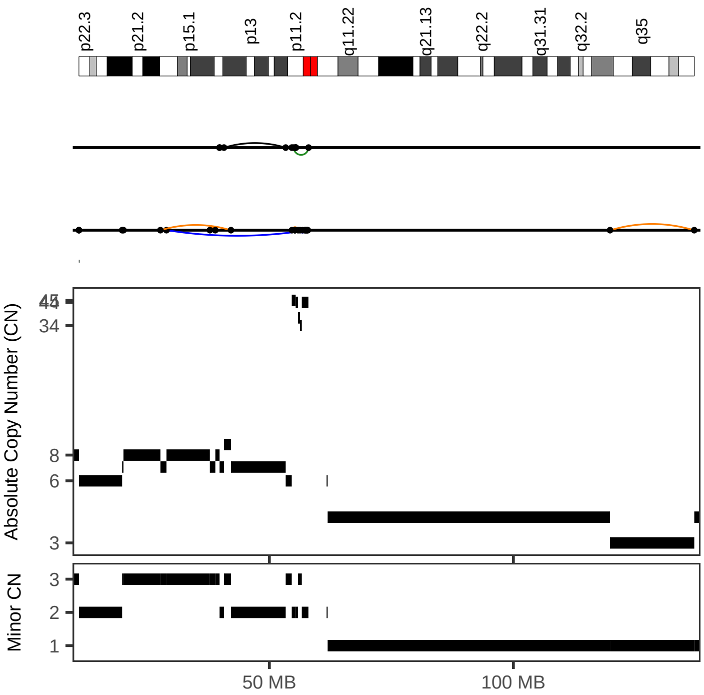

	9b132e4f−7e35−4cc5−8711−43ad62b906d0
Cancer type	Lung−AdenoCA
Position	7:27678717−58038295
Type	With other complex events
Interleaved intrachr. SVs	6
Total SVs (intrachr. + transl.)	6
SV types	DEL: 2; DUP: 1; h2hINV: 1; t2tINV: 2; TRA: 0
SVs in sample	209
Oscillating CN (2 and 3 states)	5, 5
CN segments	14
FDR fragment joints	0.9284301
FDR chr. breakp. enrich.	0.37
Linked to chrs	
Purity, ploidy	0.9, 4.33

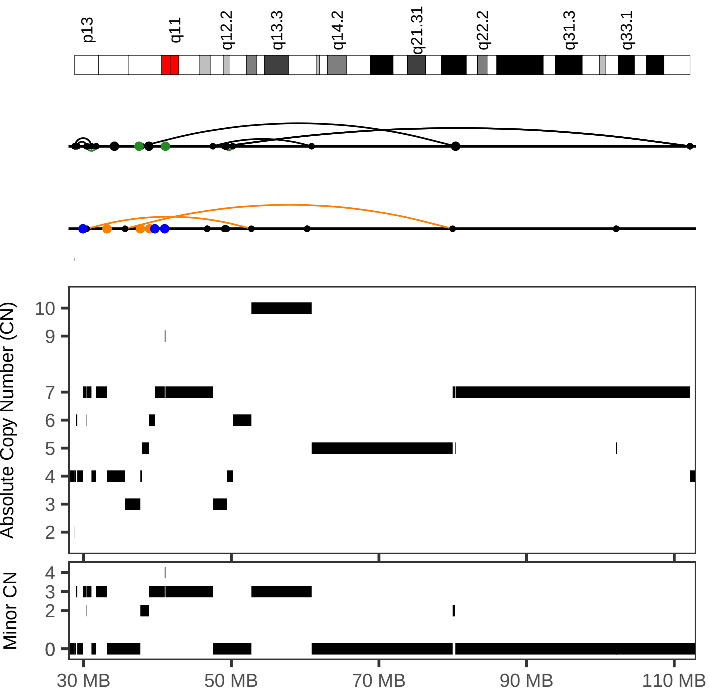

	9b132e4f−7e35−4cc5−8711−43ad62b906d0
Cancer type	Lung−AdenoCA
Position	13:28780200−112141515
Type	With other complex events
Interleaved intrachr. SVs	21
Total SVs (intrachr. + transl.)	32
SV types	DEL: 5; DUP: 2; h2hINV: 8; t2tINV: 6; TRA: 11
SVs in sample	209
Oscillating CN (2 and 3 states)	6, 7
CN segments	31
FDR fragment joints	0.7379502
FDR chr. breakp. enrich.	0
Linked to chrs	
Purity, ploidy	0.9, 4.33

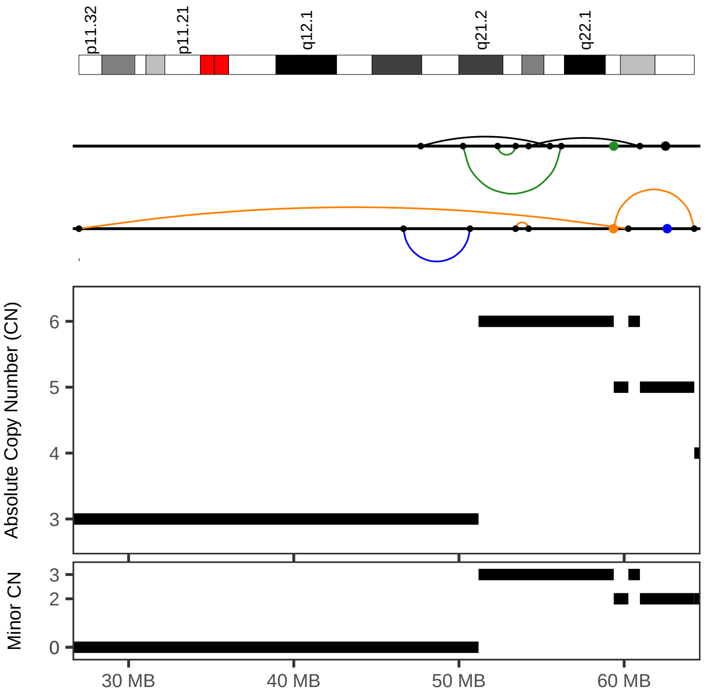

	9b132e4f−7e35−4cc5−8711−43ad62b906d0
Cancer type	Lung−AdenoCA
Position	18:26992235−64244136
Type	After polyploidization
Interleaved intrachr. SVs	6
Total SVs (intrachr. + transl.)	10
SV types	DEL: 2; DUP: 1; h2hINV: 2; t2tINV: 1; TRA: 4
SVs in sample	209
Oscillating CN (2 and 3 states)	4, 6
CN segments	6
FDR fragment joints	0.9625775
FDR chr. breakp. enrich.	0.06
Linked to chrs	13:28780200−112141514;
Purity, ploidy	0.9, 4.33

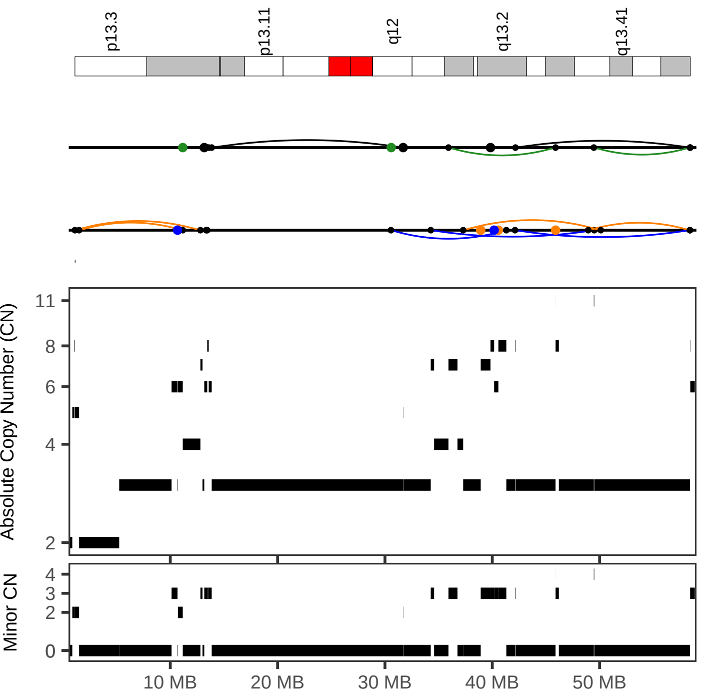

	9b132e4f−7e35−4cc5−8711−43ad62b906d0
Cancer type	Lung−AdenoCA
Position	19:13858390−58445196
Type	With other complex events
Interleaved intrachr. SVs	9
Total SVs (intrachr. + transl.)	16
SV types	DEL: 2; DUP: 3; h2hINV: 2; t2tINV: 2; TRA: 7
SVs in sample	209
Oscillating CN (2 and 3 states)	4, 7
CN segments	22
FDR fragment joints	0.9501265
FDR chr. breakp. enrich.	0
Linked to chrs	13:28780200−112141514;
Purity, ploidy	0.9, 4.33

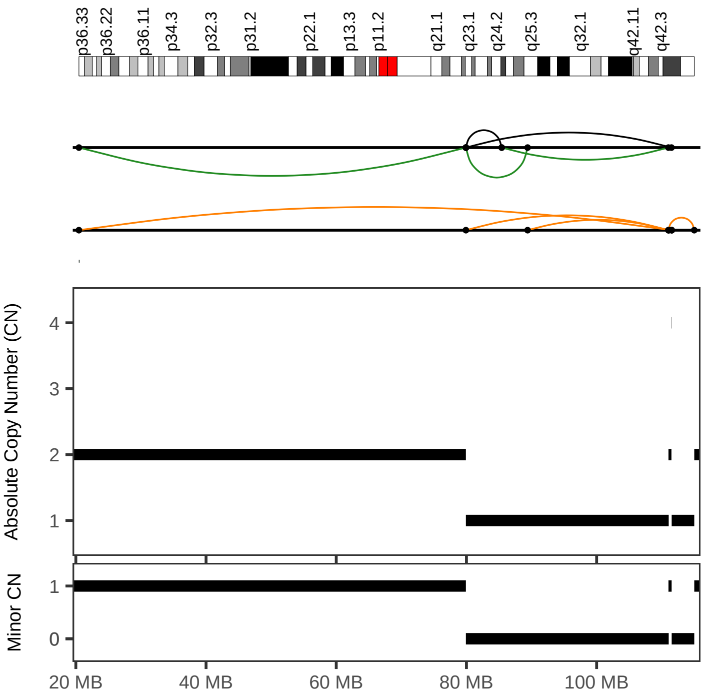

	a8d6694c−a213−4544−ac0b−63bce16d8f4e
Cancer type	Lung−AdenoCA
Position	1:20472132−114994801
Type	Canonical without polyploidization
Interleaved intrachr. SVs	8
Total SVs (intrachr. + transl.)	8
SV types	DEL: 3; DUP: 0; h2hINV: 2; t2tINV: 3; TRA: 0
SVs in sample	21
Oscillating CN (2 and 3 states)	5, 5
CN segments	8
FDR fragment joints	0.615458
FDR chr. breakp. enrich.	0
Linked to chrs	
Purity, ploidy	0.62, 2.3

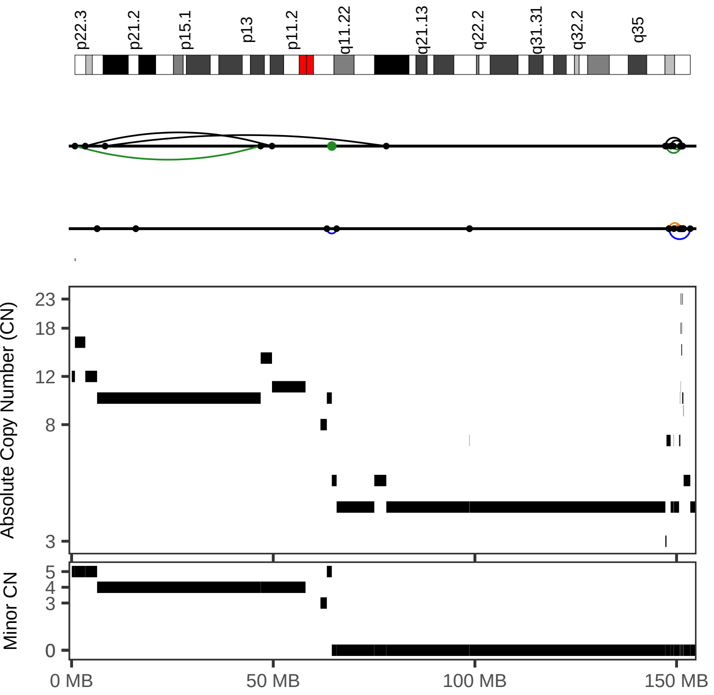

	bf15f7ad−9d92−473b−91d1−f24aa373ab97
Cancer type	Lung−AdenoCA
Position	7:147214684−153396315
Type	With other complex events
Interleaved intrachr. SVs	11
Total SVs (intrachr. + transl.)	11
SV types	DEL: 2; DUP: 4; h2hINV: 3; t2tINV: 2; TRA: 0
SVs in sample	108
Oscillating CN (2 and 3 states)	5, 7
CN segments	19
FDR fragment joints	0.8653243
FDR chr. breakp. enrich.	0
Linked to chrs	
Purity, ploidy	0.4, 3.48

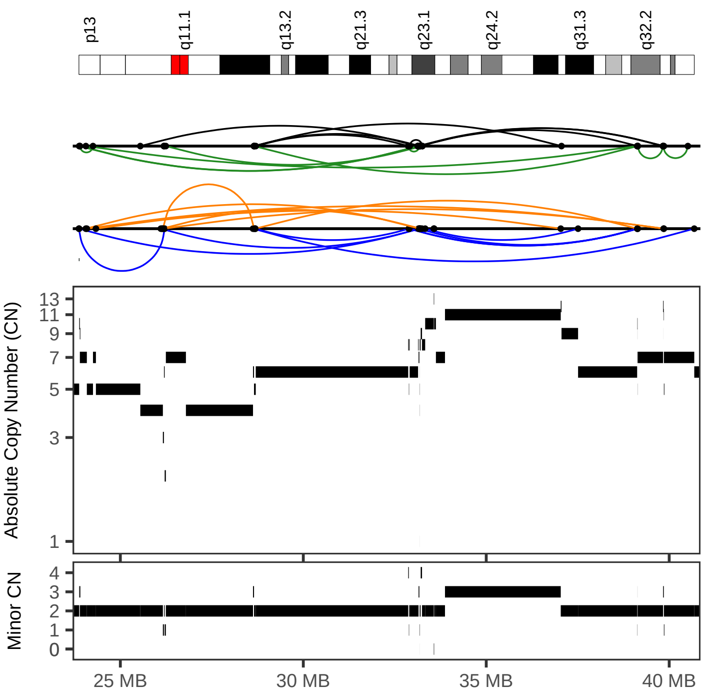

	bf15f7ad−9d92−473b−91d1−f24aa373ab97
Cancer type	Lung−AdenoCA
Position	14:23868021−40684899
Type	With other complex events
Interleaved intrachr. SVs	35
Total SVs (intrachr. + transl.)	35
SV types	DEL: 8; DUP: 9; h2hINV: 8; t2tINV: 10; TRA: 0
SVs in sample	108
Oscillating CN (2 and 3 states)	4, 8
CN segments	54
FDR fragment joints	0.9757835
FDR chr. breakp. enrich.	0
Linked to chrs	
Purity, ploidy	0.4, 3.48

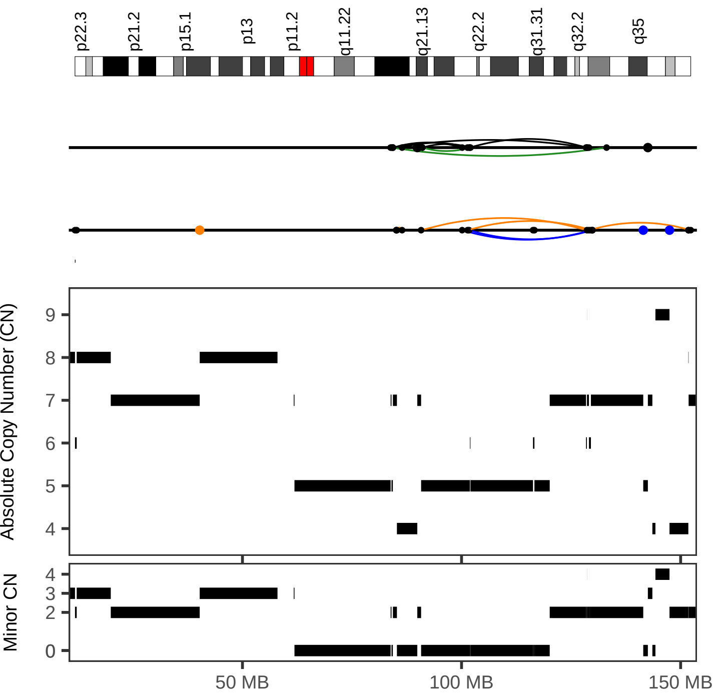

	cc4bd56a−25c5−4c48−b583−ac3aeb778ca6
Cancer type	Lung−AdenoCA
Position	7:83811028−152303288
Type	With other complex events
Interleaved intrachr. SVs	14
Total SVs (intrachr. + transl.)	18
SV types	DEL: 3; DUP: 2; h2hINV: 5; t2tINV: 4; TRA: 4
SVs in sample	237
Oscillating CN (2 and 3 states)	5, 15
CN segments	27
FDR fragment joints	0.8653243
FDR chr. breakp. enrich.	0
Linked to chrs	
Purity, ploidy	0.5, 5.16

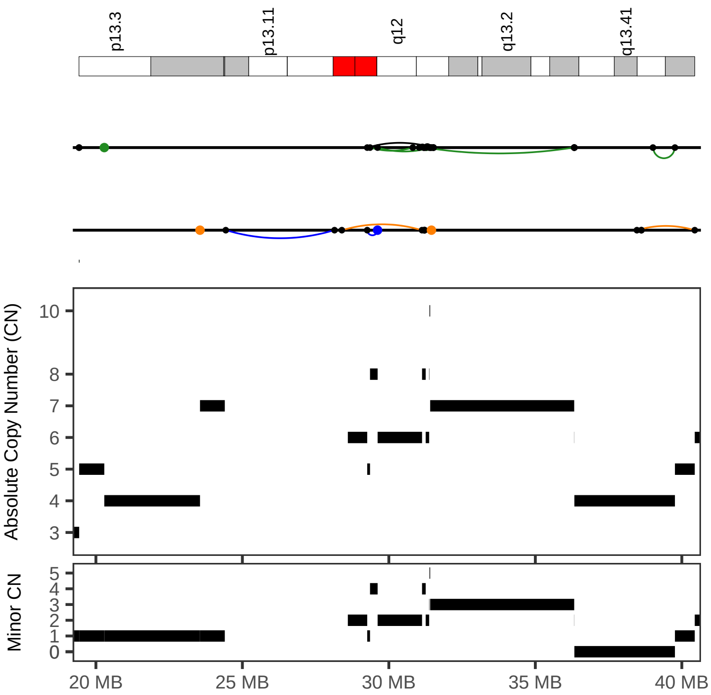

	cc4bd56a−25c5−4c48−b583−ac3aeb778ca6
Cancer type	Lung−AdenoCA
Position	19:28395079−36324916
Type	With other complex events
Interleaved intrachr. SVs	10
Total SVs (intrachr. + transl.)	12
SV types	DEL: 1; DUP: 1; h2hINV: 4; t2tINV: 4; TRA: 2
SVs in sample	237
Oscillating CN (2 and 3 states)	5, 6
CN segments	11
FDR fragment joints	0.8298498
FDR chr. breakp. enrich.	0
Linked to chrs	
Purity, ploidy	0.5, 5.16

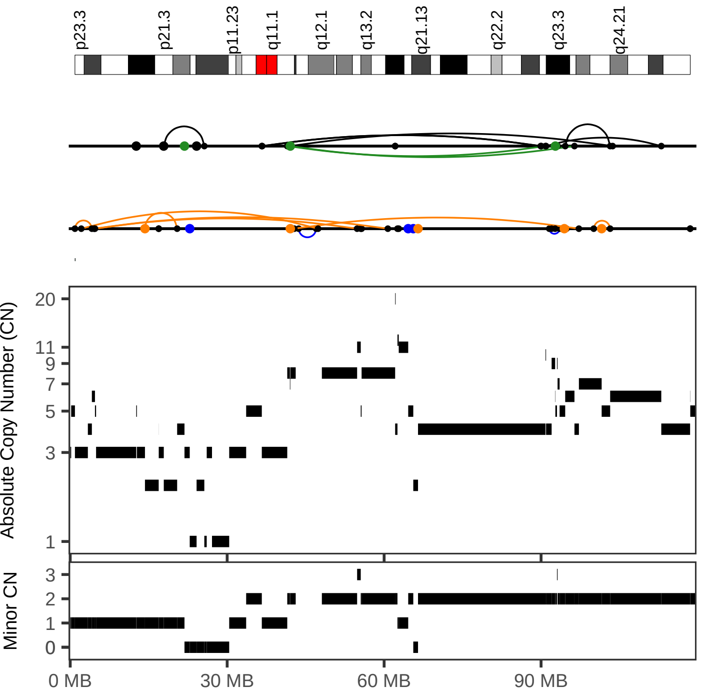

	cd0dc947−b708−464e−beb7−954c9d4583e7
Cancer type	Lung−AdenoCA
Position	8:898539−112976203
Type	With other complex events
Interleaved intrachr. SVs	14
Total SVs (intrachr. + transl.)	28
SV types	DEL: 6; DUP: 1; h2hINV: 5; t2tINV: 2; TRA: 14
SVs in sample	73
Oscillating CN (2 and 3 states)	4, 8
CN segments	49
FDR fragment joints	0.615458
FDR chr. breakp. enrich.	0
Linked to chrs	3:111543−188368918;
Purity, ploidy	0.36, 3.47

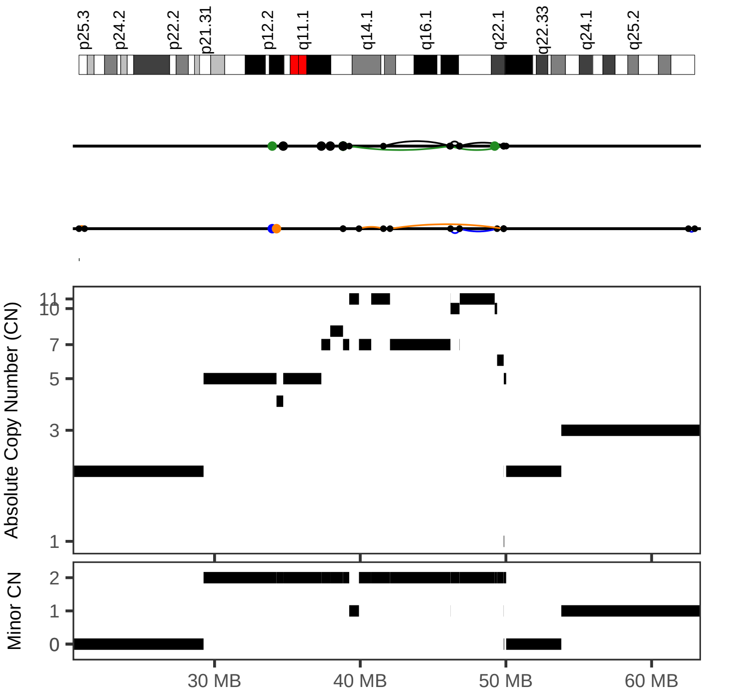

	d5326429−9805−47f9−97b0−fbda658e3f01
Cancer type	Lung−AdenoCA
Position	6:39244076−50016258
Type	With other complex events
Interleaved intrachr. SVs	10
Total SVs (intrachr. + transl.)	11
SV types	DEL: 2; DUP: 2; h2hINV: 3; t2tINV: 3; TRA: 1
SVs in sample	209
Oscillating CN (2 and 3 states)	5, 5
CN segments	14
FDR fragment joints	0.8653243
FDR chr. breakp. enrich.	0
Linked to chrs	
Purity, ploidy	0.63, 2.78

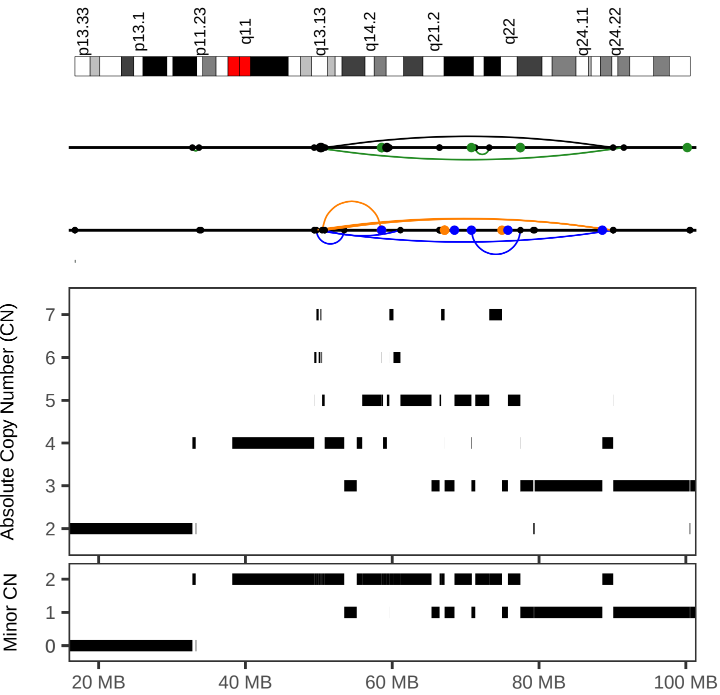

	d5326429−9805−47f9−97b0−fbda658e3f01
Cancer type	Lung−AdenoCA
Position	12:49376535−90124478
Type	With other complex events
Interleaved intrachr. SVs	7
Total SVs (intrachr. + transl.)	26
SV types	DEL: 3; DUP: 3; h2hINV: 1; t2tINV: 0; TRA: 19
SVs in sample	209
Oscillating CN (2 and 3 states)	5, 20
CN segments	41
FDR fragment joints	0.7510435
FDR chr. breakp. enrich.	0
Linked to chrs	
Purity, ploidy	0.63, 2.78

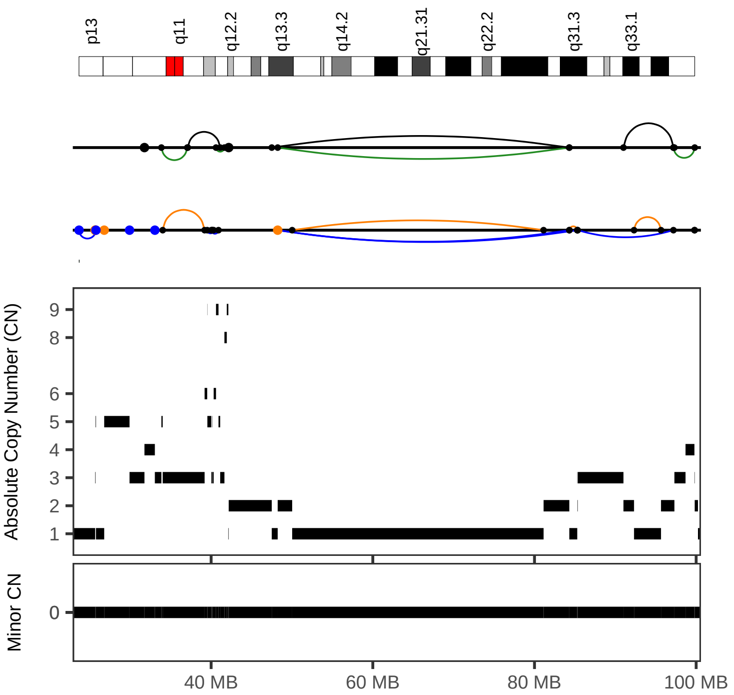

	d5326429−9805−47f9−97b0−fbda658e3f01
Cancer type	Lung−AdenoCA
Position	13:33846175−41641608
Type	With other complex events
Interleaved intrachr. SVs	6
Total SVs (intrachr. + transl.)	6
SV types	DEL: 1; DUP: 2; h2hINV: 1; t2tINV: 2; TRA: 0
SVs in sample	209
Oscillating CN (2 and 3 states)	4, 5
CN segments	13
FDR fragment joints	0.9284301
FDR chr. breakp. enrich.	0
Linked to chrs	
Purity, ploidy	0.63, 2.78

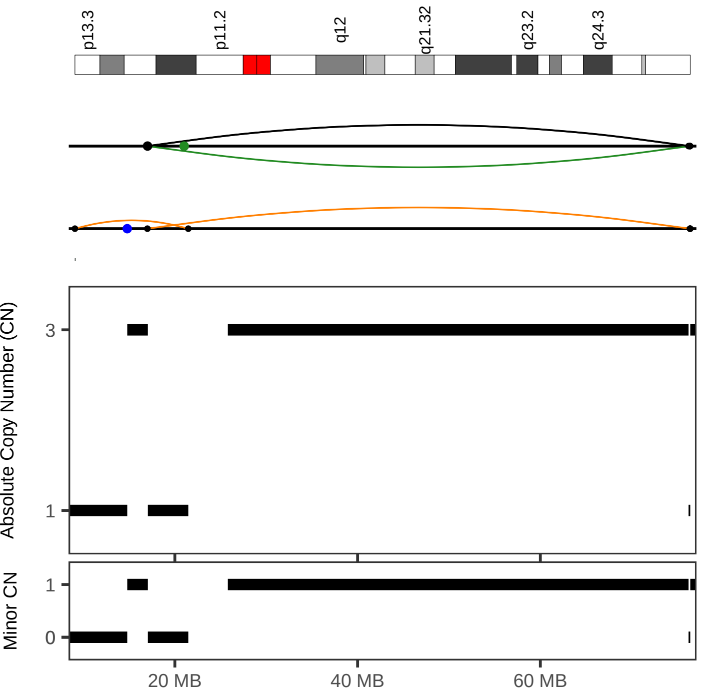

	dbd5b0de−94c9−45dd−afb3−6820a7ecaca2
Cancer type	Lung−AdenoCA
Position	17:9065515−76403933
Type	Canonical without polyploidization
Interleaved intrachr. SVs	6
Total SVs (intrachr. + transl.)	10
SV types	DEL: 3; DUP: 0; h2hINV: 2; t2tINV: 1; TRA: 4
SVs in sample	40
Oscillating CN (2 and 3 states)	6, 6
CN segments	6
FDR fragment joints	0.8572806
FDR chr. breakp. enrich.	0
Linked to chrs	6:3073059−49773929;
Purity, ploidy	0.72, 2.22

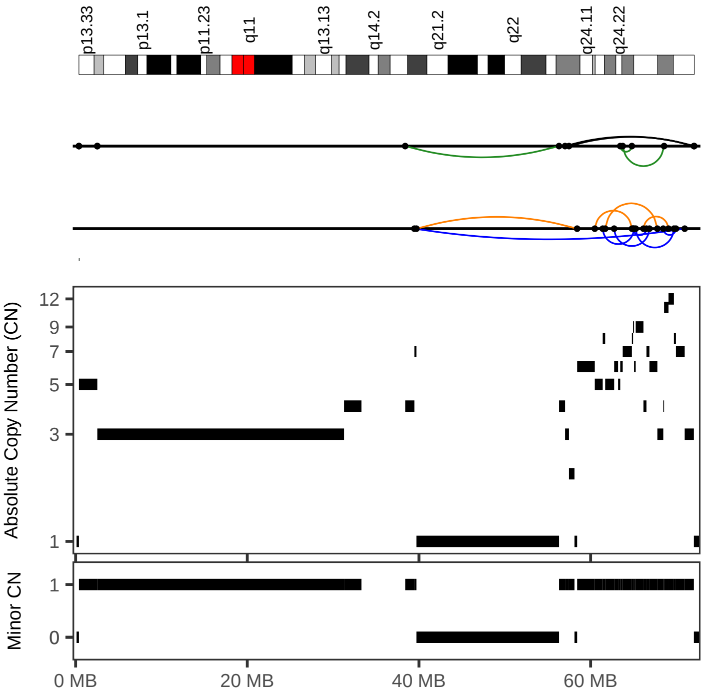

	f063ddbb−1668−40df−9ec2−b0a23ca2c389
Cancer type	Lung−AdenoCA
Position	12:60485092−69941157
Type	With other complex events
Interleaved intrachr. SVs	10
Total SVs (intrachr. + transl.)	10
SV types	DEL: 3; DUP: 5; h2hINV: 0; t2tINV: 2; TRA: 0
SVs in sample	20
Oscillating CN (2 and 3 states)	4, 14
CN segments	20
FDR fragment joints	0.615458
FDR chr. breakp. enrich.	0
Linked to chrs	
Purity, ploidy	0.42, 2.05

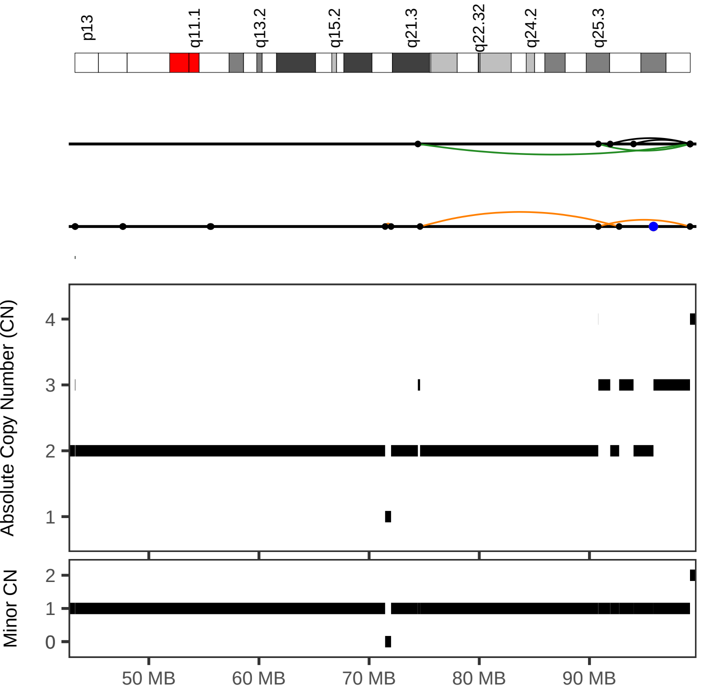

	037c57d1−b4a5−45dc−bda4−0550461d321b
Cancer type	Lung−SCC
Position	15:74425114−99150104
Type	With other complex events
Interleaved intrachr. SVs	6
Total SVs (intrachr. + transl.)	8
SV types	DEL: 2; DUP: 0; h2hINV: 2; t2tINV: 2; TRA: 2
SVs in sample	307
Oscillating CN (2 and 3 states)	5, 5
CN segments	9
FDR fragment joints	0.8653243
FDR chr. breakp. enrich.	0.08
Linked to chrs	
Purity, ploidy	0.35, 2.51

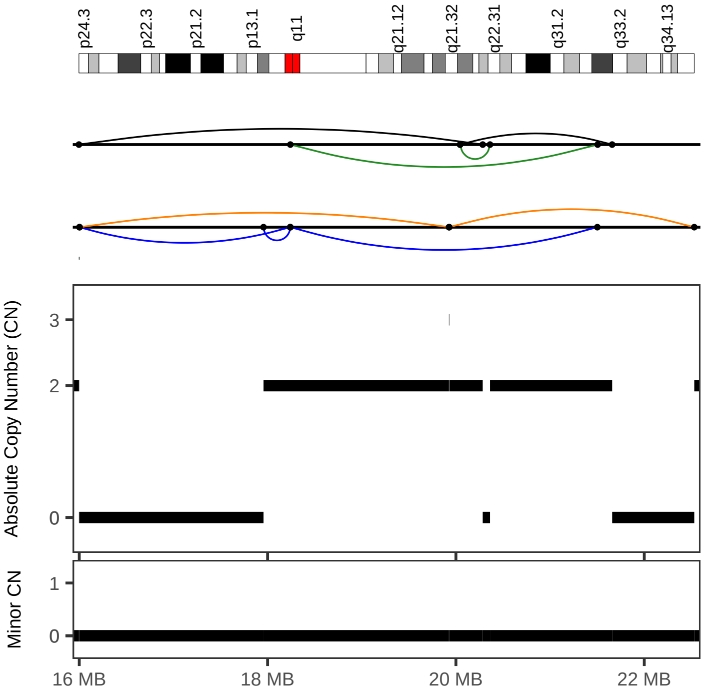

	0398eae1−7216−4595−80a5−6b117d96e070
Cancer type	Lung−SCC
Position	9:15996503−22530833
Type	With other complex events
Interleaved intrachr. SVs	9
Total SVs (intrachr. + transl.)	9
SV types	DEL: 2; DUP: 3; h2hINV: 2; t2tINV: 2; TRA: 0
SVs in sample	209
Oscillating CN (2 and 3 states)	5, 10
CN segments	10
FDR fragment joints	0.9723381
FDR chr. breakp. enrich.	0.9
Linked to chrs	
Purity, ploidy	0.57, 3.08

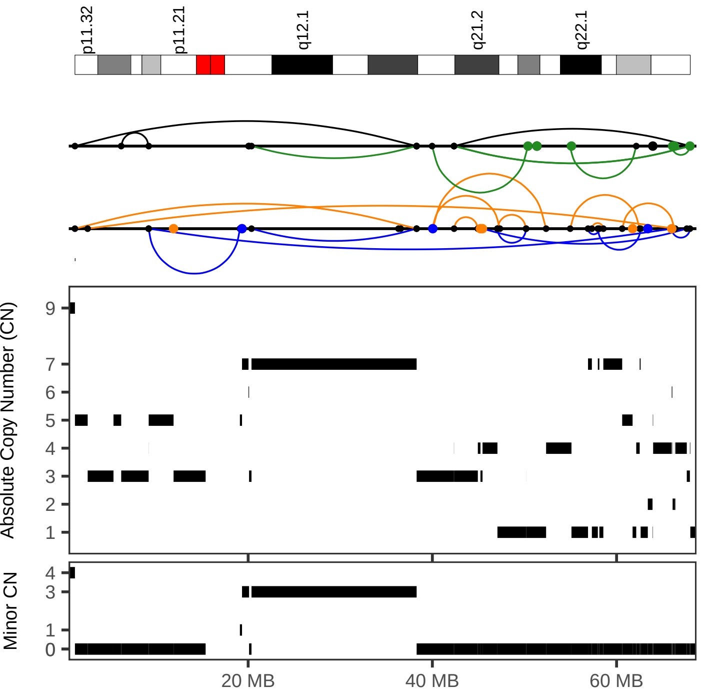

	0398eae1−7216−4595−80a5−6b117d96e070
Cancer type	Lung−SCC
Position	18:1190790−67995083
Type	With other complex events
Interleaved intrachr. SVs	25
Total SVs (intrachr. + transl.)	41
SV types	DEL: 8; DUP: 8; h2hINV: 3; t2tINV: 6; TRA: 16
SVs in sample	209
Oscillating CN (2 and 3 states)	6, 7
CN segments	46
FDR fragment joints	0.8053298
FDR chr. breakp. enrich.	0
Linked to chrs	
Purity, ploidy	0.57, 3.08

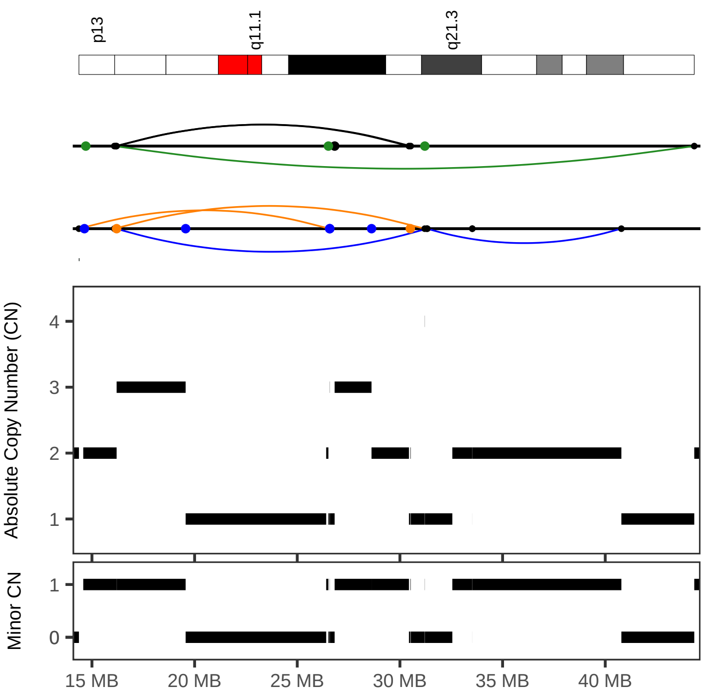

	0398eae1−7216−4595−80a5−6b117d96e070
Cancer type	Lung−SCC
Position	21:14366223−44334417
Type	With other complex events
Interleaved intrachr. SVs	7
Total SVs (intrachr. + transl.)	18
SV types	DEL: 2; DUP: 2; h2hINV: 2; t2tINV: 1; TRA: 11
SVs in sample	209
Oscillating CN (2 and 3 states)	6, 10
CN segments	20
FDR fragment joints	0.641841
FDR chr. breakp. enrich.	0
Linked to chrs	
Purity, ploidy	0.57, 3.08

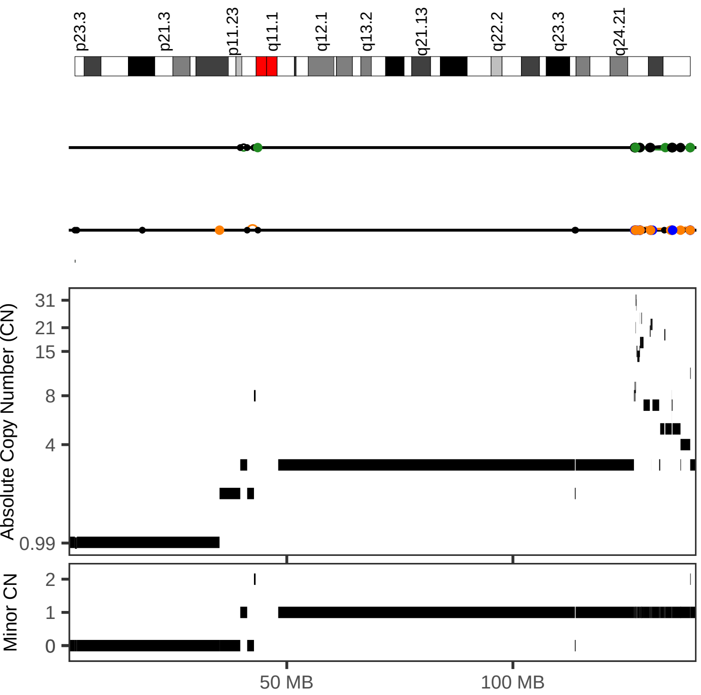

	1ee543d5−b8c0−4f79−8373−6bb6319f2ee2
Cancer type	Lung−SCC
Position	8:127118996−135131269
Type	With other complex events
Interleaved intrachr. SVs	6
Total SVs (intrachr. + transl.)	30
SV types	DEL: 4; DUP: 0; h2hINV: 1; t2tINV: 1; TRA: 24
SVs in sample	287
Oscillating CN (2 and 3 states)	4, 5
CN segments	23
FDR fragment joints	0.6776251
FDR chr. breakp. enrich.	0
Linked to chrs	5:8531769−45801498;
Purity, ploidy	0.4, 2.43

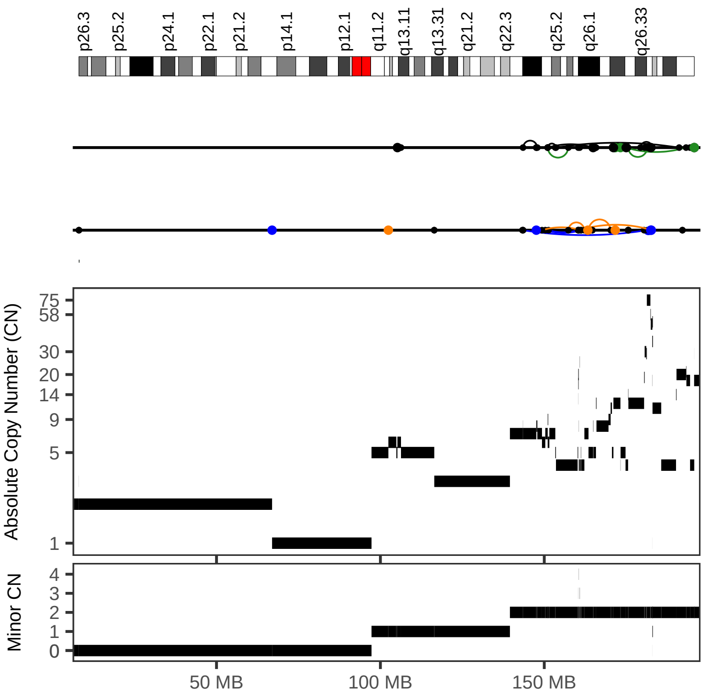

	1f11de87−a5fe−49fd−80c1−e279b2bc69de
Cancer type	Lung−SCC
Position	3:143326907−193269619
Type	With other complex events
Interleaved intrachr. SVs	17
Total SVs (intrachr. + transl.)	28
SV types	DEL: 4; DUP: 5; h2hINV: 5; t2tINV: 3; TRA: 11
SVs in sample	165
Oscillating CN (2 and 3 states)	5, 5
CN segments	53
FDR fragment joints	0.9794281
FDR chr. breakp. enrich.	0
Linked to chrs	
Purity, ploidy	0.63, 3.26

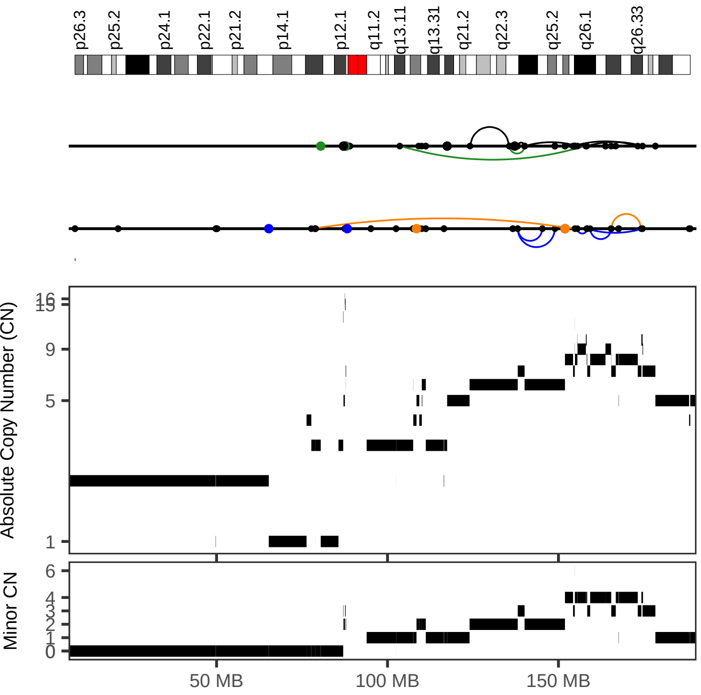

	**1f6b2aca−7357−40d1−ba7a−99227d9900a2**
Cancer type	Lung−SCC
Position	3:77709025−174601176
Type	With other complex events
Interleaved intrachr. SVs	16
Total SVs (intrachr. + transl.)	26
SV types	DEL: 2; DUP: 5; h2hINV: 5; t2tINV: 4; TRA: 10
SVs in sample	602
Oscillating CN (2 and 3 states)	5, 9
CN segments	49
FDR fragment joints	0.8172348
FDR chr. breakp. enrich.	0.1
Linked to chrs	21:14740825−17506647;4:2720902−165169008
Purity, ploidy	0.86, 2.97

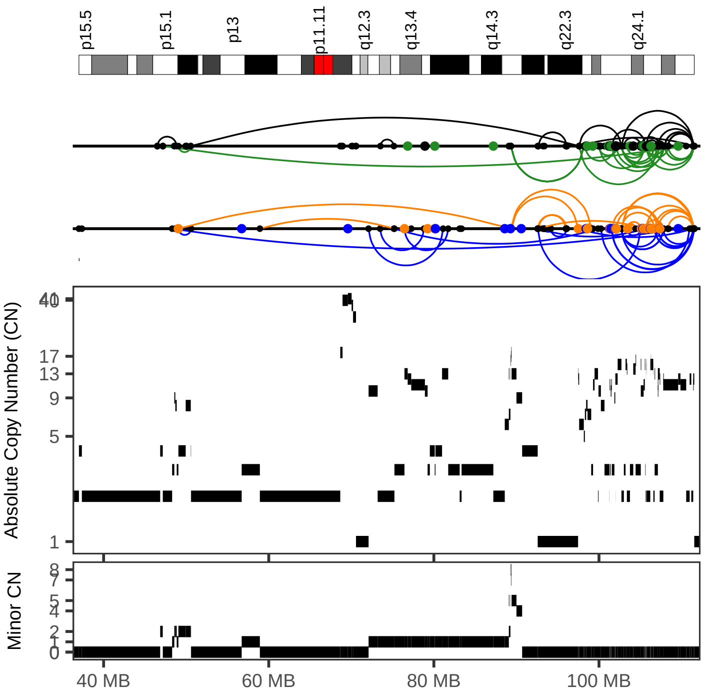

	**1f6b2aca−7357−40d1−ba7a−99227d9900a2**
Cancer type	Lung−SCC
Position	11:46508302−111537992
Type	With other complex events
Interleaved intrachr. SVs	112
Total SVs (intrachr. + transl.)	195
SV types	DEL: 27; DUP: 31; h2hINV: 26; t2tINV: 28; TRA: 83
SVs in sample	602
Oscillating CN (2 and 3 states)	5, 8
CN segments	129
FDR fragment joints	0.9723381
FDR chr. breakp. enrich.	0
Linked to chrs	12:4696888−43872964;18:21140095−63388412 4:2720902−165169008;6:47045197−150310816
Purity, ploidy	0.86, 2.97

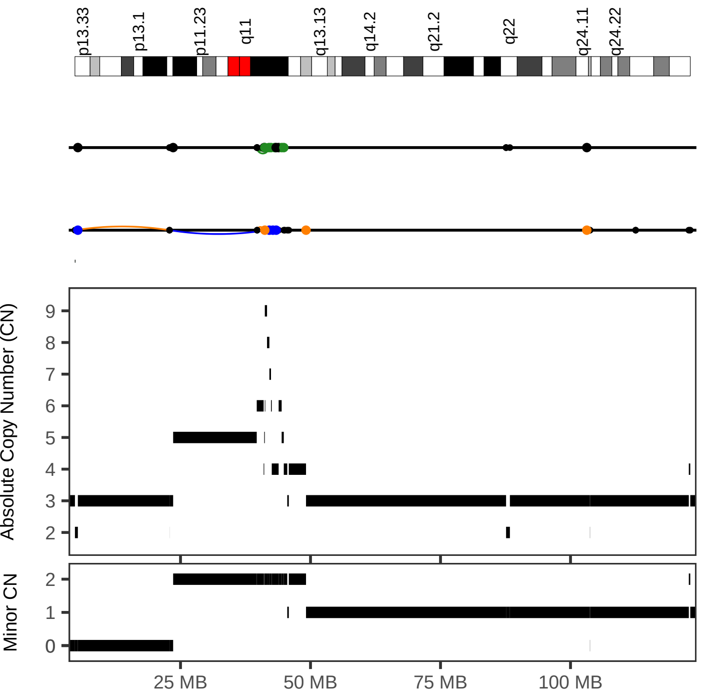

	**1f6b2aca−7357−40d1−ba7a−99227d9900a2**
Cancer type	Lung−SCC
Position	12:4696888−43872965
Type	With other complex events
Interleaved intrachr. SVs	7
Total SVs (intrachr. + transl.)	20
SV types	DEL: 3; DUP: 1; h2hINV: 0; t2tINV: 3; TRA: 13
SVs in sample	602
Oscillating CN (2 and 3 states)	4, 5
CN segments	14
FDR fragment joints	0.8572806
FDR chr. breakp. enrich.	0.35
Linked to chrs	11:46508302−111537991;18:21140095−63388412
Purity, ploidy	0.86, 2.97

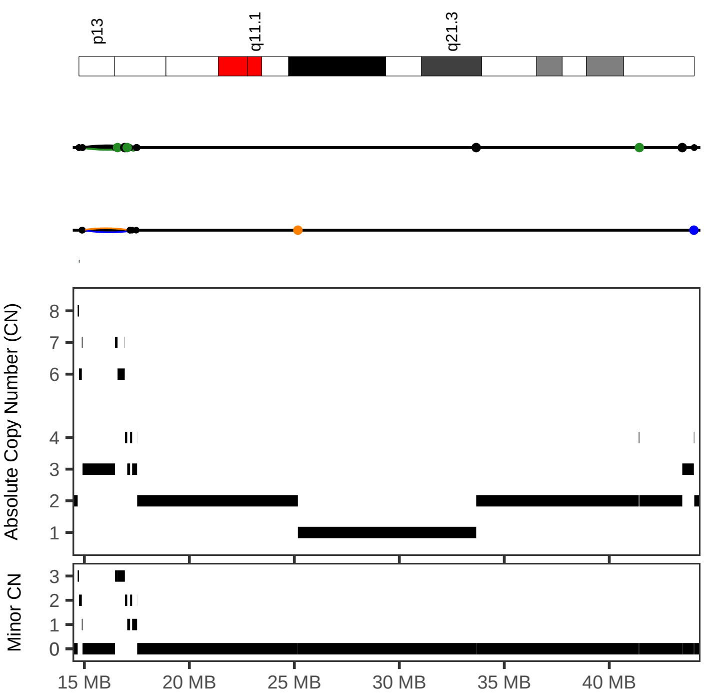

	**1f6b2aca−7357−40d1−ba7a−99227d9900a2**
Cancer type	Lung−SCC
Position	21:14740825−17506648
Type	With other complex events
Interleaved intrachr. SVs	10
Total SVs (intrachr. + transl.)	13
SV types	DEL: 2; DUP: 1; h2hINV: 3; t2tINV: 4; TRA: 3
SVs in sample	602
Oscillating CN (2 and 3 states)	5, 6
CN segments	12
FDR fragment joints	0.615458
FDR chr. breakp. enrich.	0
Linked to chrs	
Purity, ploidy	0.86, 2.97

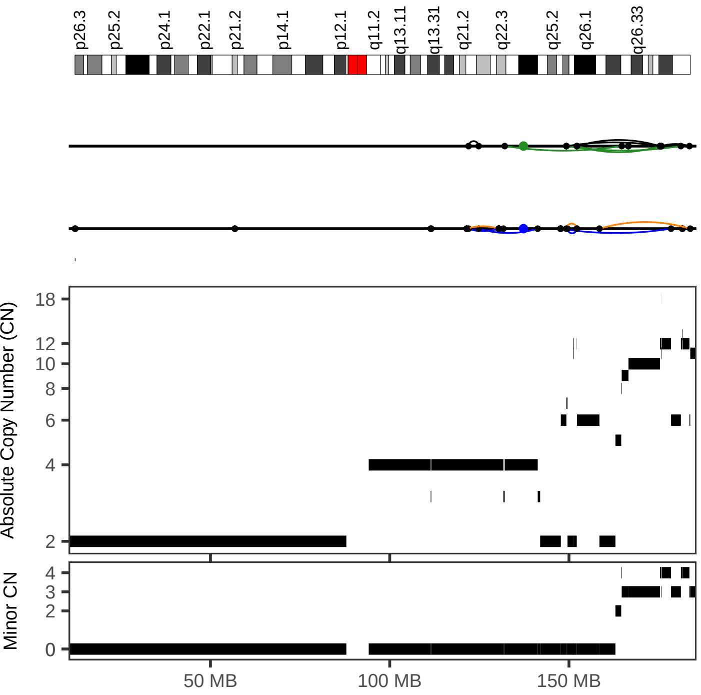

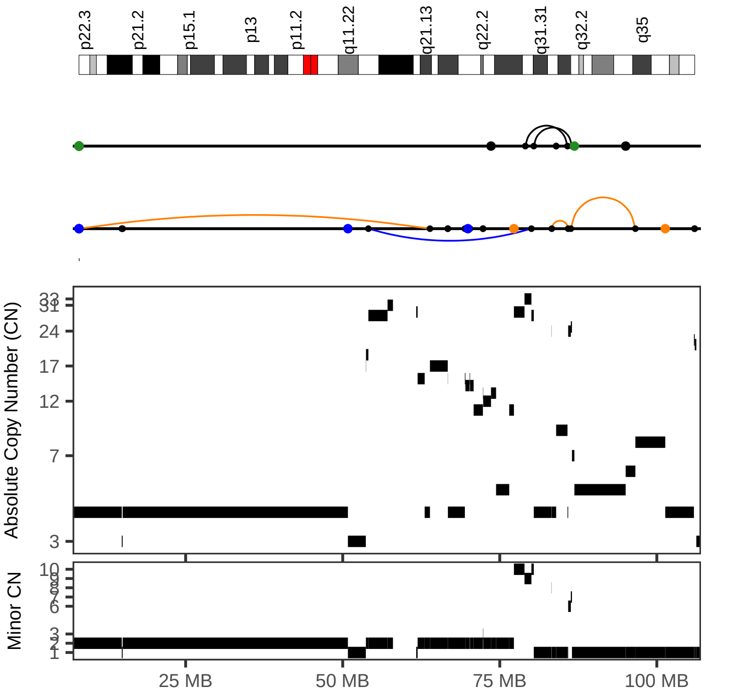

	3666bc65−8e40−409e−9a1f−41583dd6d978
Cancer type	Lung−SCC
Position	3:121469766−183835935
Type	With other complex events
Interleaved intrachr. SVs	16
Total SVs (intrachr. + transl.)	18
SV types	DEL: 3; DUP: 5; h2hINV: 4; t2tINV: 4; TRA: 2
SVs in sample	154
Oscillating CN (2 and 3 states)	4, 4
CN segments	28
FDR fragment joints	0.8653243
FDR chr. breakp. enrich.	0
Linked to chrs	
Purity, ploidy	0.64, 3.53

	3666bc65−8e40−409e−9a1f−41583dd6d978
Cancer type	Lung−SCC
Position	7:8021176−96585488
Type	With other complex events
Interleaved intrachr. SVs	6
Total SVs (intrachr. + transl.)	20
SV types	DEL: 3; DUP: 1; h2hINV: 2; t2tINV: 0; TRA: 14
SVs in sample	154
Oscillating CN (2 and 3 states)	4, 5
CN segments	38
FDR fragment joints	0.8572806
FDR chr. breakp. enrich.	0
Linked to chrs	5:1891003−59309258;
Purity, ploidy	0.64, 3.53

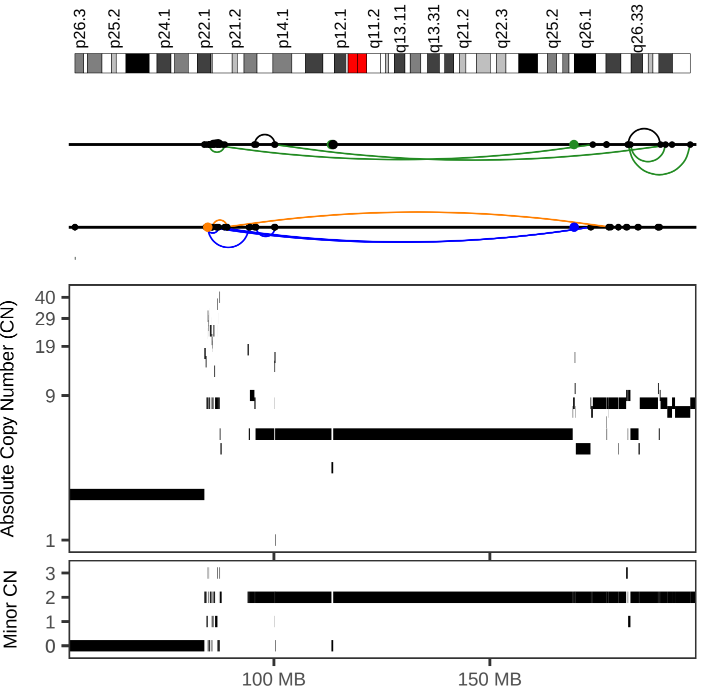

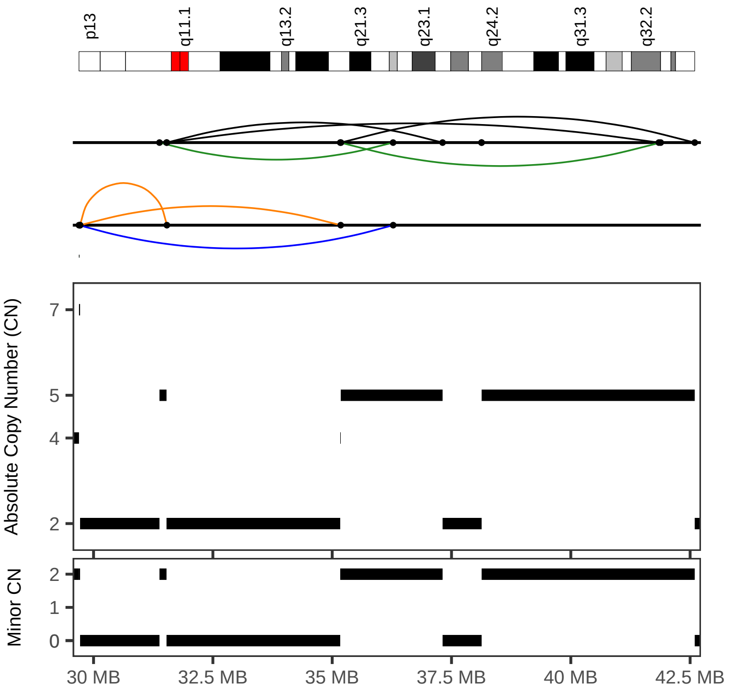

	376dfd27−68e8−4a1a−9c4f−5064279b2a9e
Cancer type	Lung−SCC
Position	3:84207634−196397331
Type	With other complex events
Interleaved intrachr. SVs	30
Total SVs (intrachr. + transl.)	35
SV types	DEL: 6; DUP: 10; h2hINV: 8; t2tINV: 6; TRA: 5
SVs in sample	234
Oscillating CN (2 and 3 states)	5, 5
CN segments	76
FDR fragment joints	0.8007013
FDR chr. breakp. enrich.	0
Linked to chrs	
Purity, ploidy	0.44, 3.03

	376dfd27−68e8−4a1a−9c4f−5064279b2a9e
Cancer type	Lung−SCC
Position	14:29694281−42596449
Type	With other complex events
Interleaved intrachr. SVs	8
Total SVs (intrachr. + transl.)	8
SV types	DEL: 2; DUP: 1; h2hINV: 3; t2tINV: 2; TRA: 0
SVs in sample	234
Oscillating CN (2 and 3 states)	4, 5
CN segments	9
FDR fragment joints	0.8653243
FDR chr. breakp. enrich.	0.7
Linked to chrs	
Purity, ploidy	0.44, 3.03

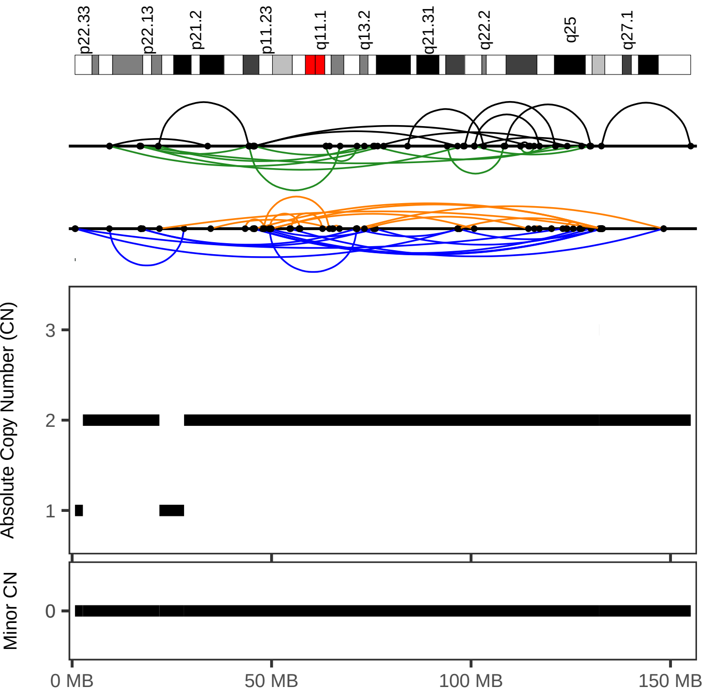

	376dfd27−68e8−4a1a−9c4f−5064279b2a9e
Cancer type	Lung−SCC
Position	X:714035−155073186
Type	Canonical without polyploidization
Interleaved intrachr. SVs	55
Total SVs (intrachr. + transl.)	55
SV types	DEL: 13; DUP: 16; h2hINV: 14; t2tINV: 12; TRA: 0
SVs in sample	234
Oscillating CN (2 and 3 states)	4, 6
CN segments	6
FDR fragment joints	0.930656
FDR chr. breakp. enrich.	0
Linked to chrs	
Purity, ploidy	0.44, 3.03

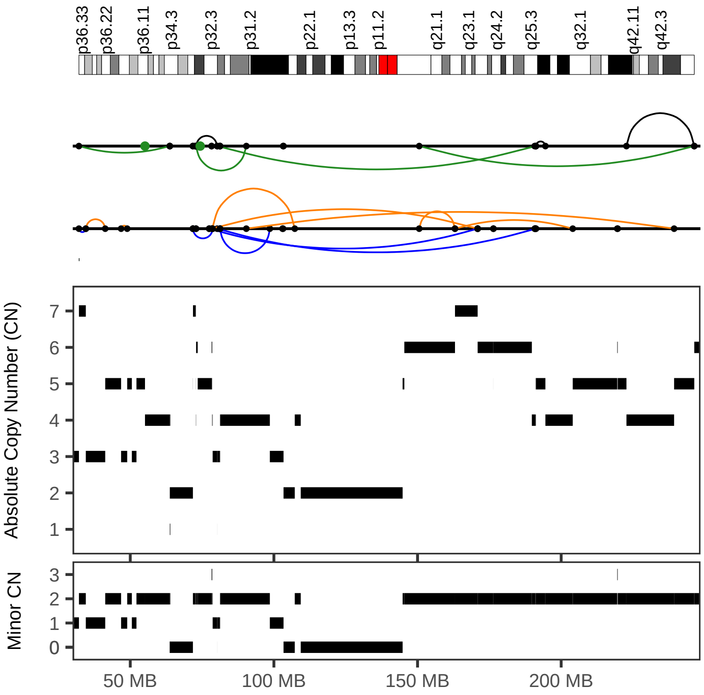

	395babb3−3f5d−4e71−a675−af4443f23028
Cancer type	Lung−SCC
Position	1:71829305−246323132
Type	With other complex events
Interleaved intrachr. SVs	19
Total SVs (intrachr. + transl.)	20
SV types	DEL: 7; DUP: 4; h2hINV: 4; t2tINV: 4; TRA: 1
SVs in sample	113
Oscillating CN (2 and 3 states)	6, 11
CN segments	38
FDR fragment joints	0.8572806
FDR chr. breakp. enrich.	0
Linked to chrs	
Purity, ploidy	0.38, 3.13

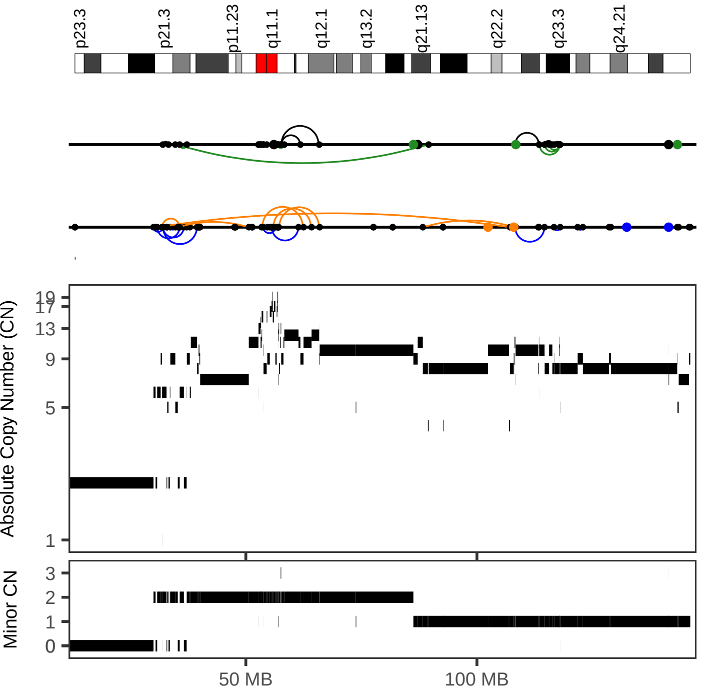

	43abe847−4ba7−466e−8283−5d7b80b999a7
Cancer type	Lung−SCC
Position	8:30016639−108126673
Type	With other complex events
Interleaved intrachr. SVs	13
Total SVs (intrachr. + transl.)	31
SV types	DEL: 8; DUP: 9; h2hINV: 4; t2tINV: 5; TRA: 5
SVs in sample	500
Oscillating CN (2 and 3 states)	5, 7
CN segments	82
FDR fragment joints	0.7013708
FDR chr. breakp. enrich.	0
Linked to chrs	
Purity, ploidy	0.69, 3.74

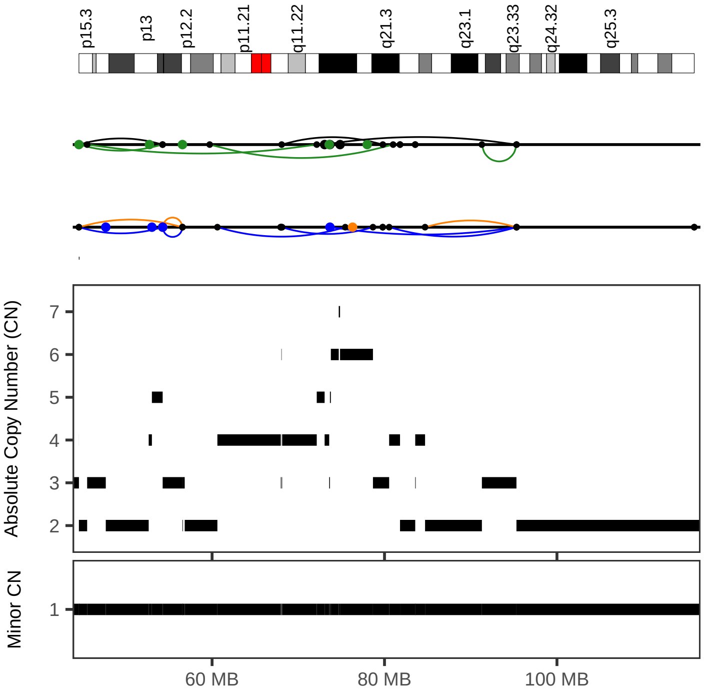

	43abe847−4ba7−466e−8283−5d7b80b999a7
Cancer type	Lung−SCC
Position	10:44557512−95329436
Type	With other complex events
Interleaved intrachr. SVs	17
Total SVs (intrachr. + transl.)	31
SV types	DEL: 4; DUP: 6; h2hINV: 3; t2tINV: 4; TRA: 14
SVs in sample	500
Oscillating CN (2 and 3 states)	4, 8
CN segments	34
FDR fragment joints	0.8653243
FDR chr. breakp. enrich.	0.01
Linked to chrs	
Purity, ploidy	0.69, 3.74

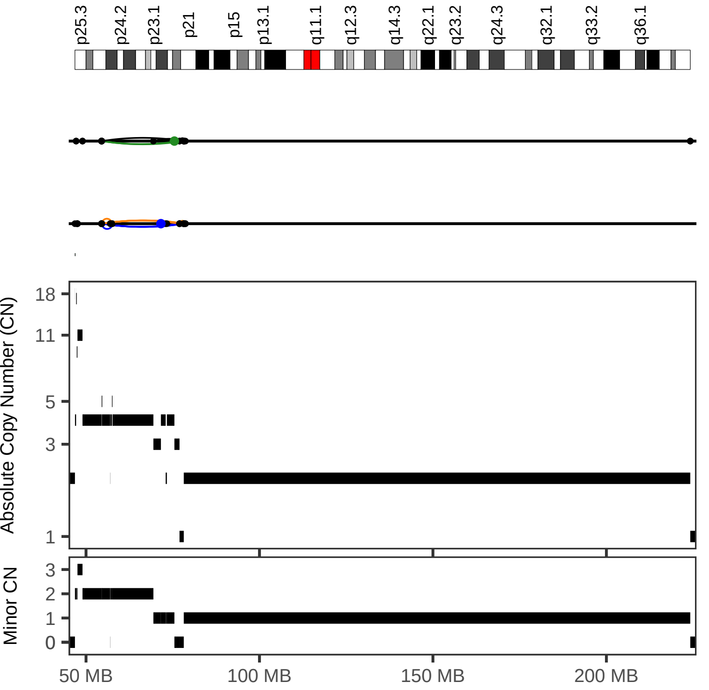

	4ca13c92−84b4−4edf−842a−b20b7e713415
Cancer type	Lung−SCC
Position	2:54467990−78652935
Type	With other complex events
Interleaved intrachr. SVs	17
Total SVs (intrachr. + transl.)	19
SV types	DEL: 5; DUP: 5; h2hINV: 4; t2tINV: 3; TRA: 2
SVs in sample	103
Oscillating CN (2 and 3 states)	5, 6
CN segments	16
FDR fragment joints	0.8653243
FDR chr. breakp. enrich.	0
Linked to chrs	
Purity, ploidy	0.4, 1.72

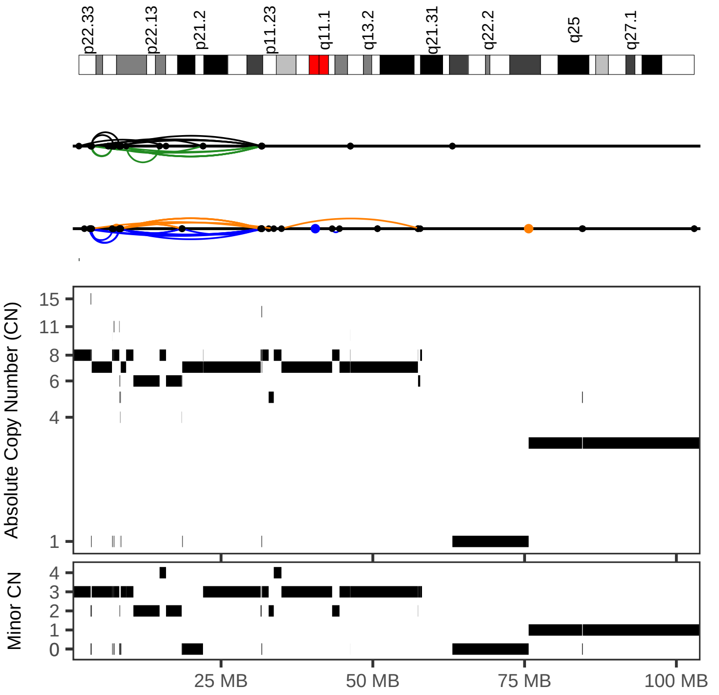

	585e6487−b0a3−4828−8a06−46bee01dff74
Cancer type	Lung−SCC
Position	X:1585455−33684999
Type	With other complex events
Interleaved intrachr. SVs	45
Total SVs (intrachr. + transl.)	45
SV types	DEL: 9; DUP: 13; h2hINV: 11; t2tINV: 12; TRA: 0
SVs in sample	129
Oscillating CN (2 and 3 states)	4, 5
CN segments	41
FDR fragment joints	0.9161301
FDR chr. breakp. enrich.	0
Linked to chrs	
Purity, ploidy	0.64, 3.46

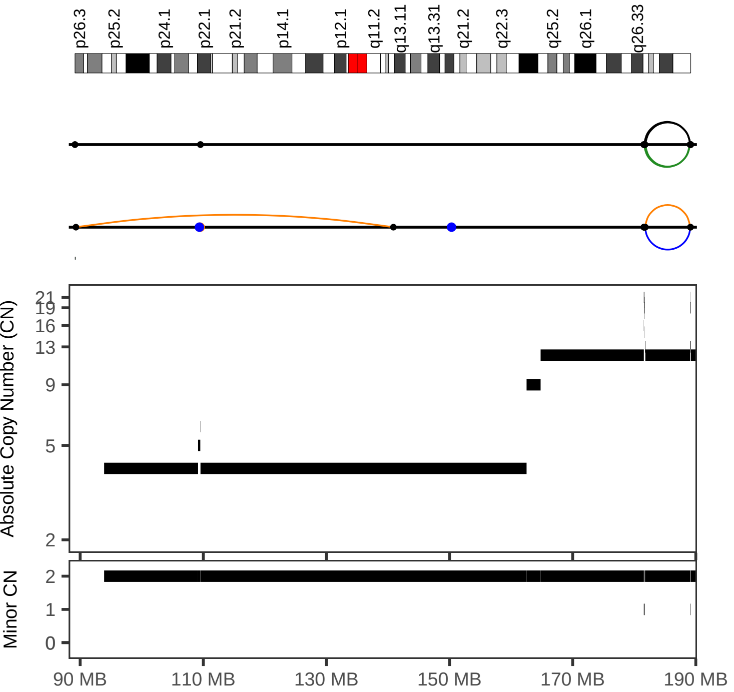

	6fd72426−f6c8−47ca−a500−d5d3600b9b15
Cancer type	Lung−SCC
Position	3:181546045−189173080
Type	With other complex events
Interleaved intrachr. SVs	13
Total SVs (intrachr. + transl.)	13
SV types	DEL: 1; DUP: 5; h2hINV: 5; t2tINV: 2; TRA: 0
SVs in sample	130
Oscillating CN (2 and 3 states)	4, 8
CN segments	18
FDR fragment joints	0.615458
FDR chr. breakp. enrich.	0
Linked to chrs	
Purity, ploidy	0.8, 3.37

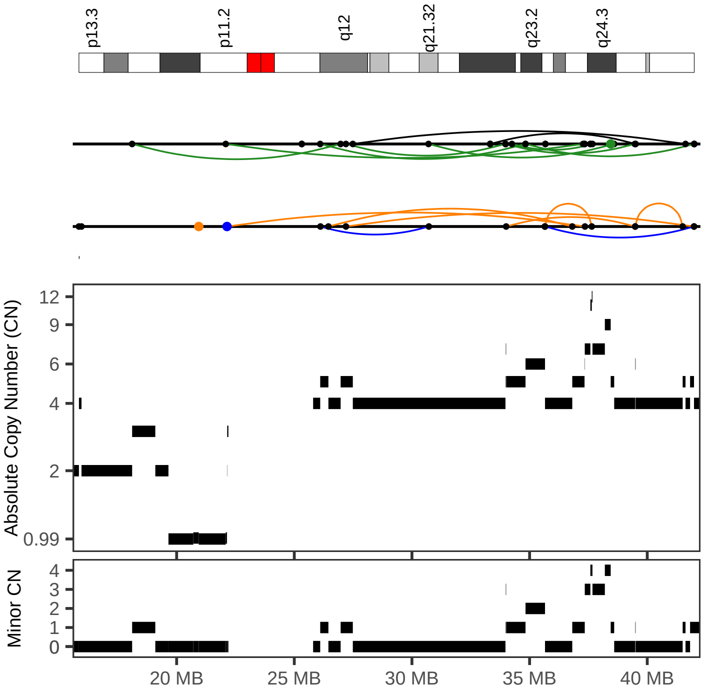

	6fd72426−f6c8−47ca−a500−d5d3600b9b15
Cancer type	Lung−SCC
Position	17:18108890−41996624
Type	With other complex events
Interleaved intrachr. SVs	18
Total SVs (intrachr. + transl.)	21
SV types	DEL: 6; DUP: 2; h2hINV: 2; t2tINV: 8; TRA: 3
SVs in sample	130
Oscillating CN (2 and 3 states)	6, 12
CN segments	33
FDR fragment joints	0.615458
FDR chr. breakp. enrich.	0
Linked to chrs	
Purity, ploidy	0.8, 3.37

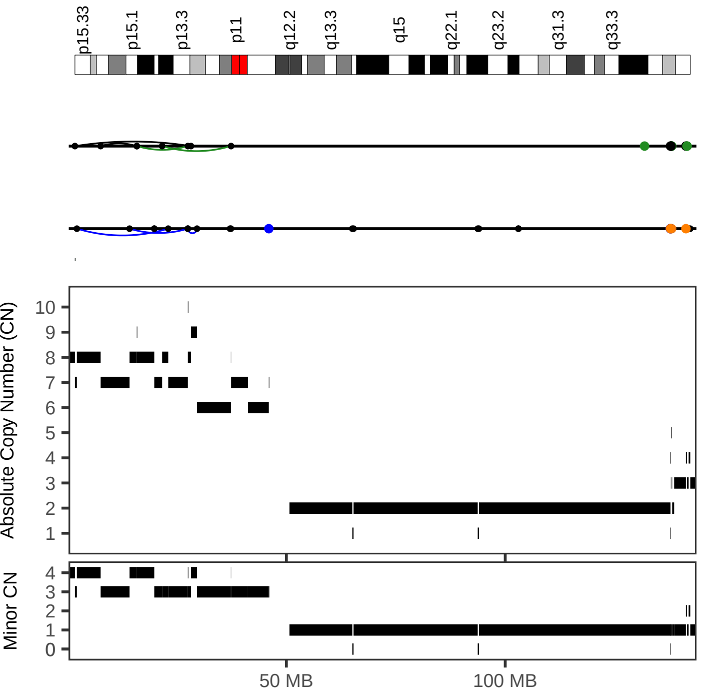

	7d905f8f−3967−4ea8−96c8−17b1b03fbec3
Cancer type	Lung−SCC
Position	5:1687513−37366455
Type	With other complex events
Interleaved intrachr. SVs	7
Total SVs (intrachr. + transl.)	7
SV types	DEL: 0; DUP: 3; h2hINV: 2; t2tINV: 2; TRA: 0
SVs in sample	385
Oscillating CN (2 and 3 states)	4, 4
CN segments	15
FDR fragment joints	0.6776251
FDR chr. breakp. enrich.	0.05
Linked to chrs	
Purity, ploidy	0.57, 2.6

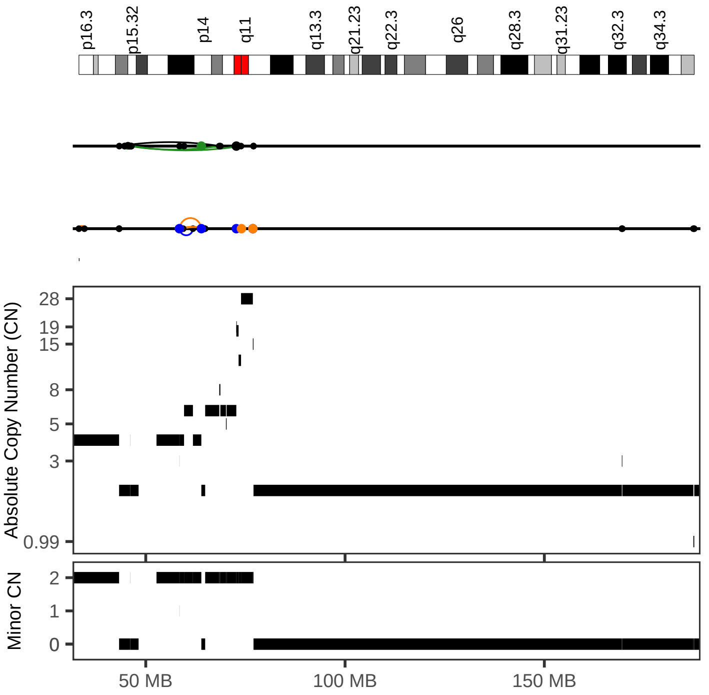

	9293e197−e38a−4e19−a7d0−1b45d1ad48bd
Cancer type	Lung−SCC
Position	4:43364661−73902886
Type	With other complex events
Interleaved intrachr. SVs	6
Total SVs (intrachr. + transl.)	12
SV types	DEL: 0; DUP: 0; h2hINV: 3; t2tINV: 3; TRA: 6
SVs in sample	187
Oscillating CN (2 and 3 states)	4, 5
CN segments	18
FDR fragment joints	0.6776251
FDR chr. breakp. enrich.	0.01
Linked to chrs	
Purity, ploidy	0.53, 3.3

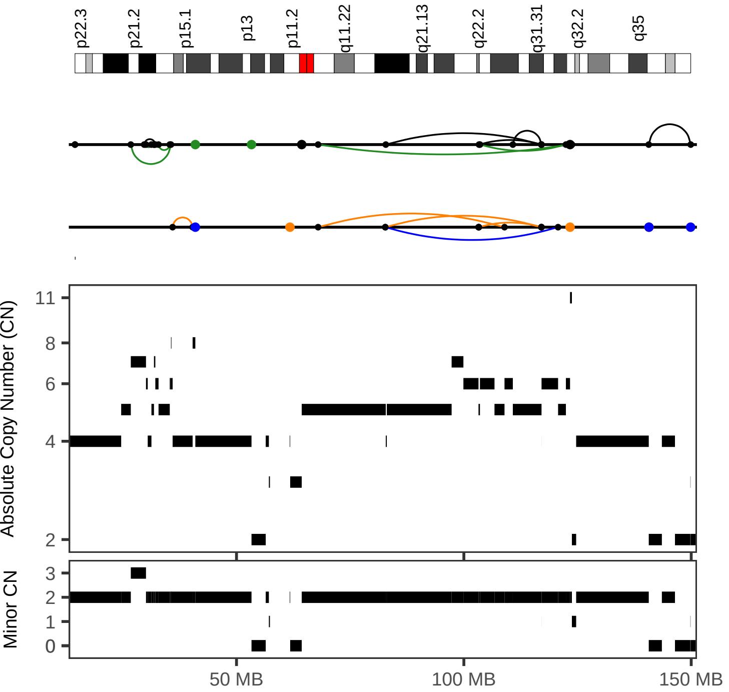

	9293e197−e38a−4e19−a7d0−1b45d1ad48bd
Cancer type	Lung−SCC
Position	7:67945786−122441992
Type	With other complex events
Interleaved intrachr. SVs	10
Total SVs (intrachr. + transl.)	10
SV types	DEL: 4; DUP: 2; h2hINV: 3; t2tINV: 1; TRA: 0
SVs in sample	187
Oscillating CN (2 and 3 states)	6, 12
CN segments	15
FDR fragment joints	0.6776251
FDR chr. breakp. enrich.	0
Linked to chrs	
Purity, ploidy	0.53, 3.3

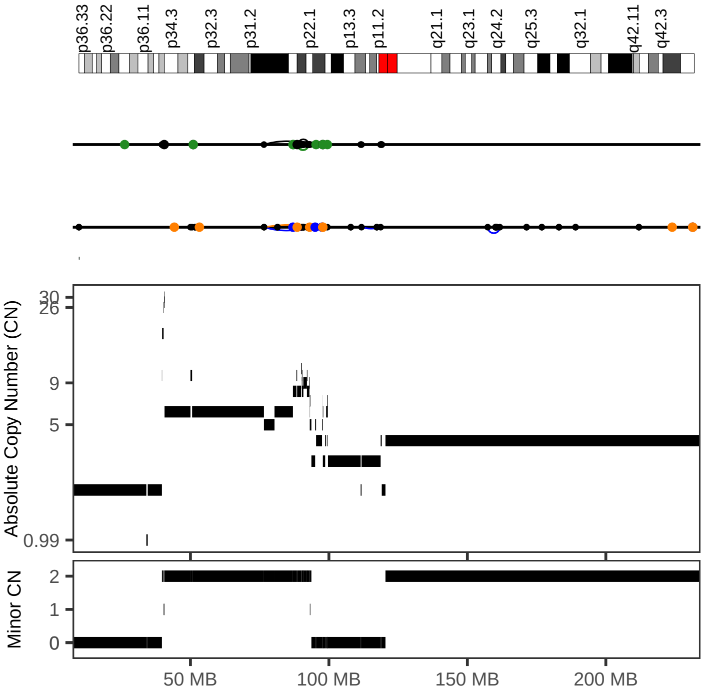

	9af6ed4e−8cdc−4f49−84e9−ba1053b5b3ca
Cancer type	Lung−SCC
Position	1:76535798−99663065
Type	With other complex events
Interleaved intrachr. SVs	47
Total SVs (intrachr. + transl.)	61
SV types	DEL: 8; DUP: 11; h2hINV: 16; t2tINV: 12; TRA: 14
SVs in sample	428
Oscillating CN (2 and 3 states)	5, 5
CN segments	41
FDR fragment joints	0.615458
FDR chr. breakp. enrich.	0
Linked to chrs	2:54782646−232522085;5:30463771−37215412
Purity, ploidy	0.82, 3.26

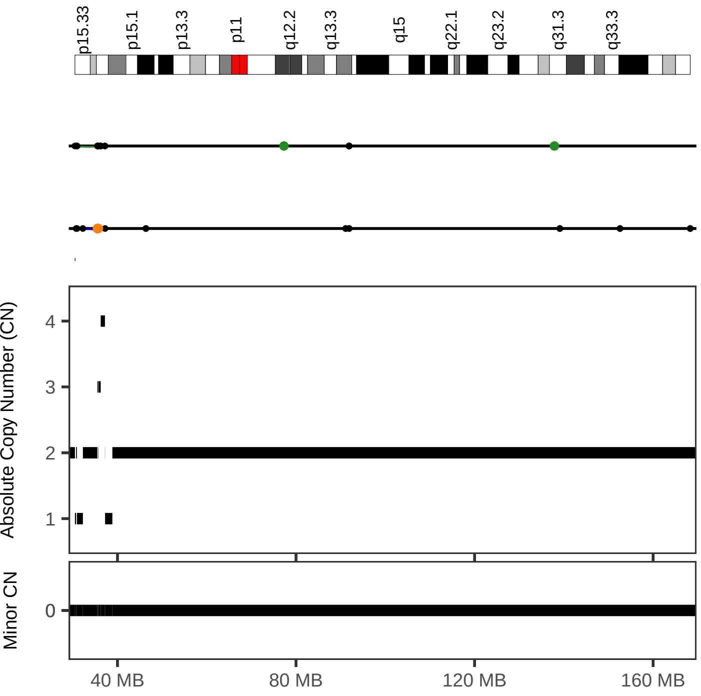

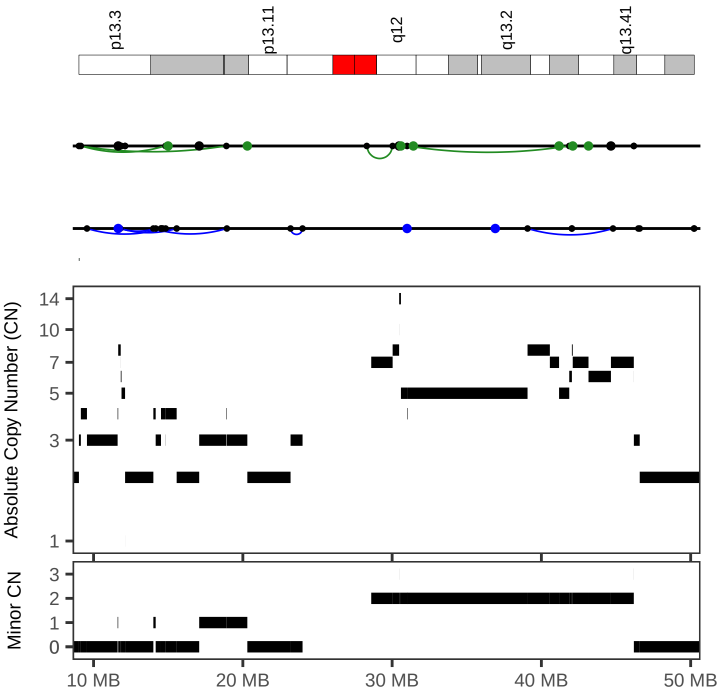

	9af6ed4e−8cdc−4f49−84e9−ba1053b5b3ca
Cancer type	Lung−SCC
Position	5:30463771−37215413
Type	With other complex events
Interleaved intrachr. SVs	6
Total SVs (intrachr. + transl.)	9
SV types	DEL: 0; DUP: 2; h2hINV: 2; t2tINV: 2; TRA: 3
SVs in sample	428
Oscillating CN (2 and 3 states)	4, 8
CN segments	10
FDR fragment joints	0.9723381
FDR chr. breakp. enrich.	0.78
Linked to chrs	1:76535798−99663064;
Purity, ploidy	0.82, 3.26

	aecf85cc−058c−46f3−9cdc−3573ac3b8438
Cancer type	Lung−SCC
Position	19:9018583−18930769
Type	With other complex events
Interleaved intrachr. SVs	9
Total SVs (intrachr. + transl.)	13
SV types	DEL: 0; DUP: 5; h2hINV: 1; t2tINV: 3; TRA: 4
SVs in sample	232
Oscillating CN (2 and 3 states)	5, 8
CN segments	19
FDR fragment joints	0.5435077
FDR chr. breakp. enrich.	0
Linked to chrs	2:24457554−239354640;
Purity, ploidy	0.56, 3.19

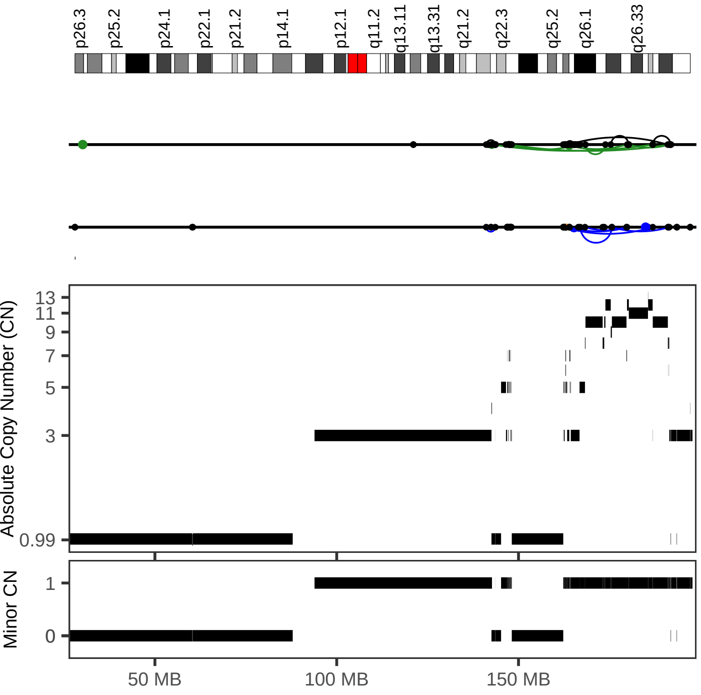

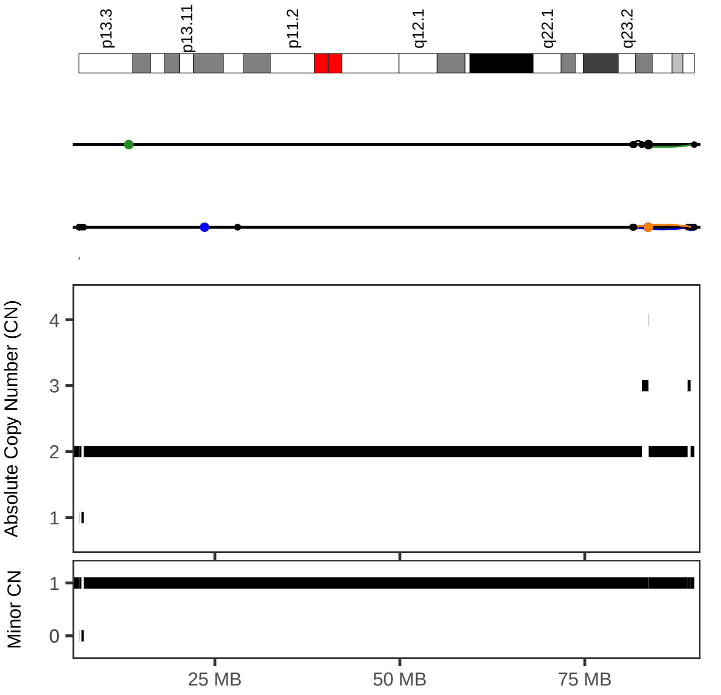

	c2598334−f866−4cc3−93ec−2d2e8b85d319
Cancer type	Lung−SCC
Position	3:141119758−191946026
Type	With other complex events
Interleaved intrachr. SVs	43
Total SVs (intrachr. + transl.)	44
SV types	DEL: 3; DUP: 14; h2hINV: 14; t2tINV: 12; TRA: 1
SVs in sample	165
Oscillating CN (2 and 3 states)	5, 21
CN segments	61
FDR fragment joints	0.5076332
FDR chr. breakp. enrich.	0
Linked to chrs	
Purity, ploidy	0.72, 1.86

	c2598334−f866−4cc3−93ec−2d2e8b85d319
Cancer type	Lung−SCC
Position	16:81457961−89793192
Type	Canonical without polyploidization
Interleaved intrachr. SVs	9
Total SVs (intrachr. + transl.)	13
SV types	DEL: 3; DUP: 4; h2hINV: 0; t2tINV: 2; TRA: 4
SVs in sample	165
Oscillating CN (2 and 3 states)	5, 7
CN segments	8
FDR fragment joints	0.615458
FDR chr. breakp. enrich.	0
Linked to chrs	
Purity, ploidy	0.72, 1.86

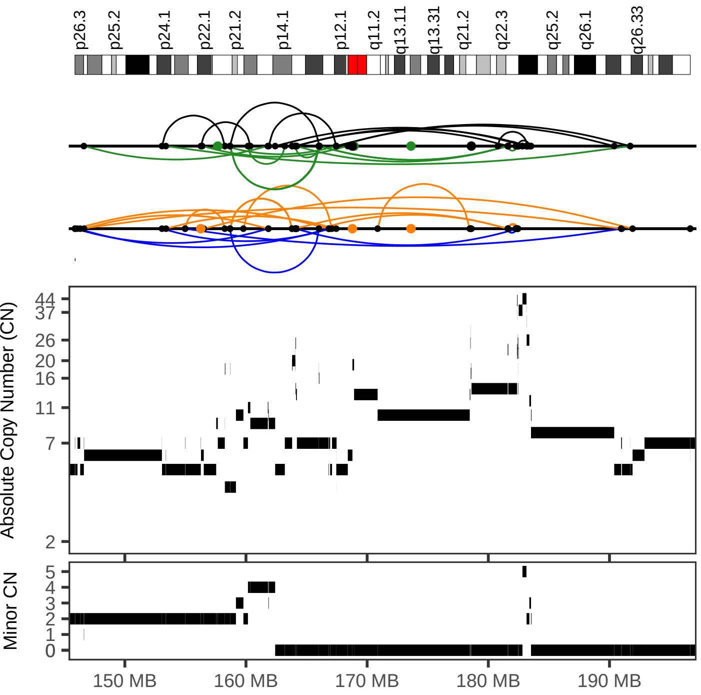

	ce8612ab−3149−4a6a−b424−29c0c21c9b8b
Cancer type	Lung−SCC
Position	3:145882159−191906843
Type	With other complex events
Interleaved intrachr. SVs	65
Total SVs (intrachr. + transl.)	73
SV types	DEL: 16; DUP: 10; h2hINV: 23; t2tINV: 16; TRA: 8
SVs in sample	213
Oscillating CN (2 and 3 states)	5, 14
CN segments	88
FDR fragment joints	0.5435077
FDR chr. breakp. enrich.	0
Linked to chrs	
Purity, ploidy	0.59, 3.12

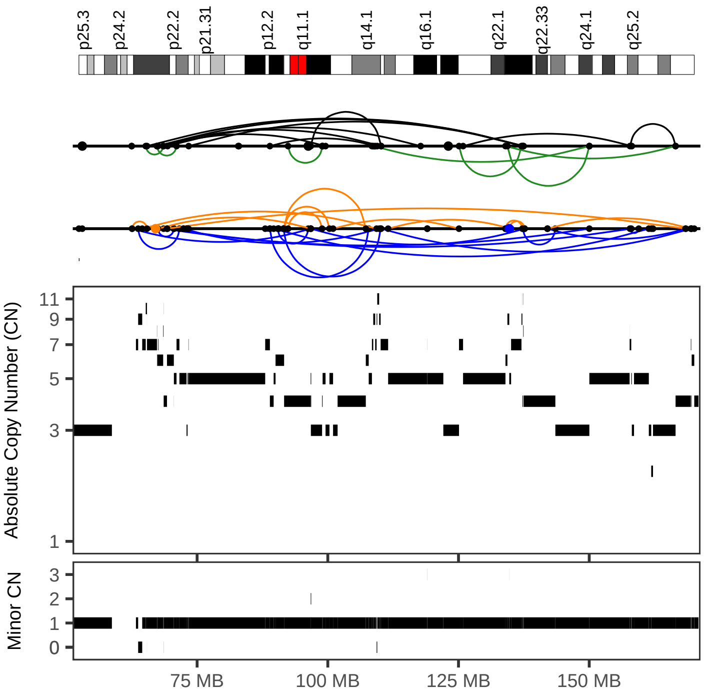

	e6b72c24−1607−43b9−8b8a−7bf83eea5895
Cancer type	Lung−SCC
Position	6:62569792−169514744
Type	With other complex events
Interleaved intrachr. SVs	48
Total SVs (intrachr. + transl.)	52
SV types	DEL: 12; DUP: 17; h2hINV: 10; t2tINV: 9; TRA: 4
SVs in sample	265
Oscillating CN (2 and 3 states)	4, 8
CN segments	78
FDR fragment joints	0.6338796
FDR chr. breakp. enrich.	0
Linked to chrs	
Purity, ploidy	0.53, 3.12

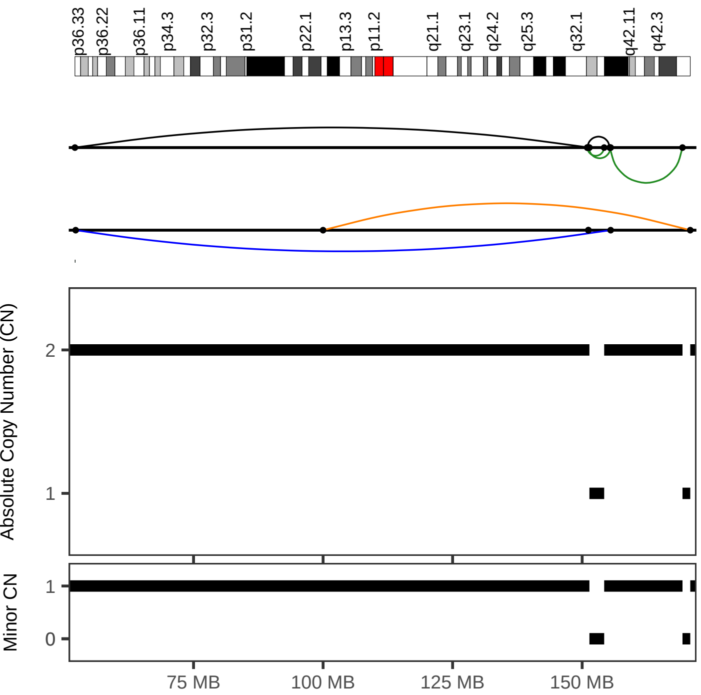

	a8e2df1e−4042−42af−9231−3a00e83489f0
Cancer type	Lymph−BNHL
Position	1:52105012−170864080
Type	Canonical without polyploidization
Interleaved intrachr. SVs	7
Total SVs (intrachr. + transl.)	7
SV types	DEL: 1; DUP: 1; h2hINV: 2; t2tINV: 3; TRA: 0
SVs in sample	112
Oscillating CN (2 and 3 states)	5, 5
CN segments	5
FDR fragment joints	0.7735152
FDR chr. breakp. enrich.	1
Linked to chrs	
Purity, ploidy	0.88, 1.98

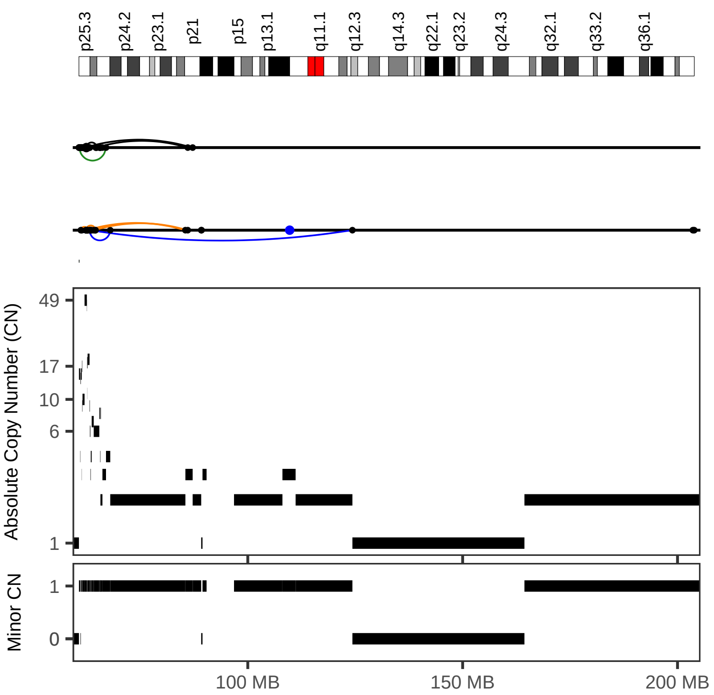

	a8e2df1e−4042−42af−9231−3a00e83489f0
Cancer type	Lymph−BNHL
Position	2:60753889−124345172
Type	With other complex events
Interleaved intrachr. SVs	29
Total SVs (intrachr. + transl.)	31
SV types	DEL: 8; DUP: 5; h2hINV: 7; t2tINV: 9; TRA: 2
SVs in sample	112
Oscillating CN (2 and 3 states)	4, 5
CN segments	34
FDR fragment joints	0.8653243
FDR chr. breakp. enrich.	0
Linked to chrs	
Purity, ploidy	0.88, 1.98

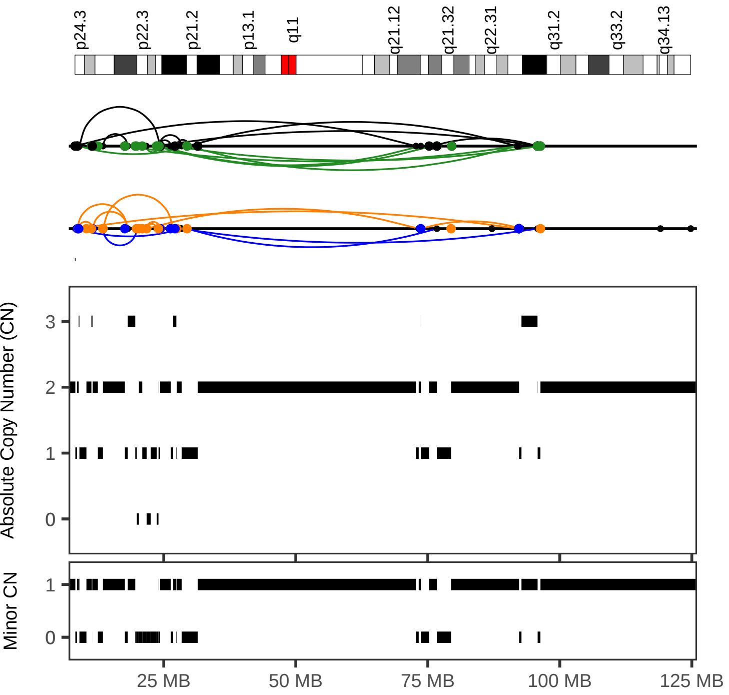

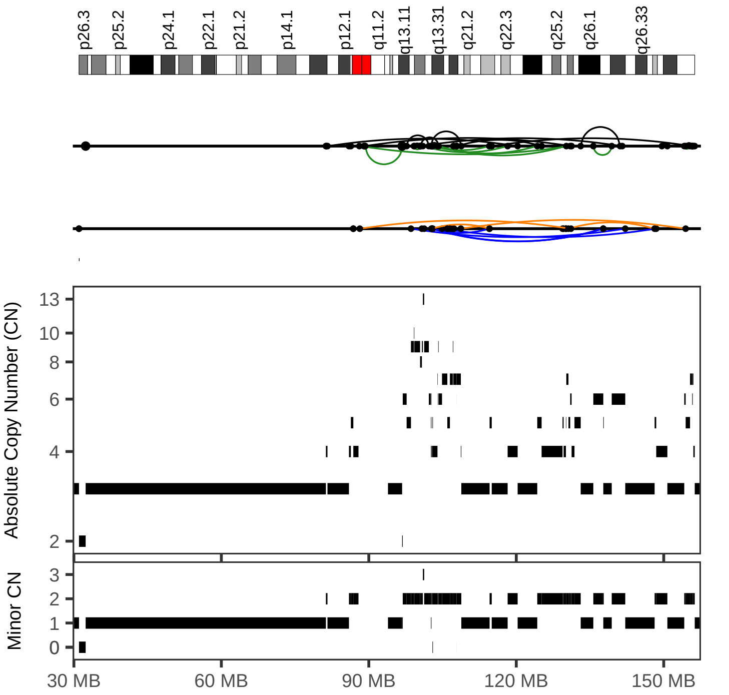

f0a326d2−1f3e−4a5d−bca8−32aaccc52338

Cancer type	Lymph−BNHL
Position	9:8189397−96357054
Type	With other complex events
Interleaved intrachr. SVs	30
Total SVs (intrachr. + transl.)	89
SV types	DEL: 10; DUP: 4; h2hINV: 9; t2tINV: 7; TRA: 59
SVs in sample	194
Oscillating CN (2 and 3 states)	6, 12
CN segments	49
FDR fragment joints	0.615458
FDR chr. breakp. enrich.	0
Linked to chrs	18:24492930−66759171;
Purity, ploidy	0.8, 2.27

4101316

Cancer type	Lymph−BNHL
Position	3:81591651−155801731
Type	With other complex events
Interleaved intrachr. SVs	32
Total SVs (intrachr. + transl.)	33
SV types	DEL: 4; DUP: 6; h2hINV: 13; t2tINV: 9; TRA: 1
SVs in sample	69
Oscillating CN (2 and 3 states)	5, 11
CN segments	64
FDR fragment joints	0.565038
FDR chr. breakp. enrich.	0
Linked to chrs	
Purity, ploidy	0.18, 2.41

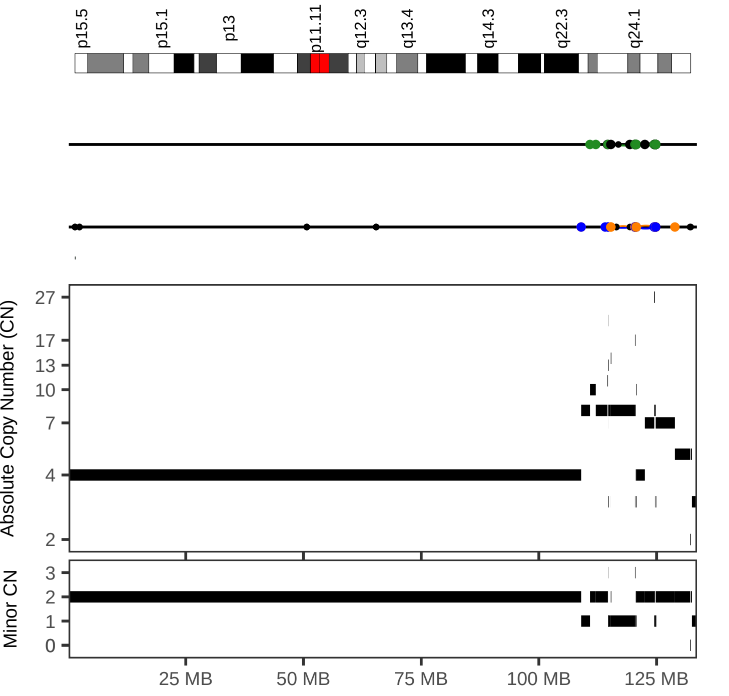

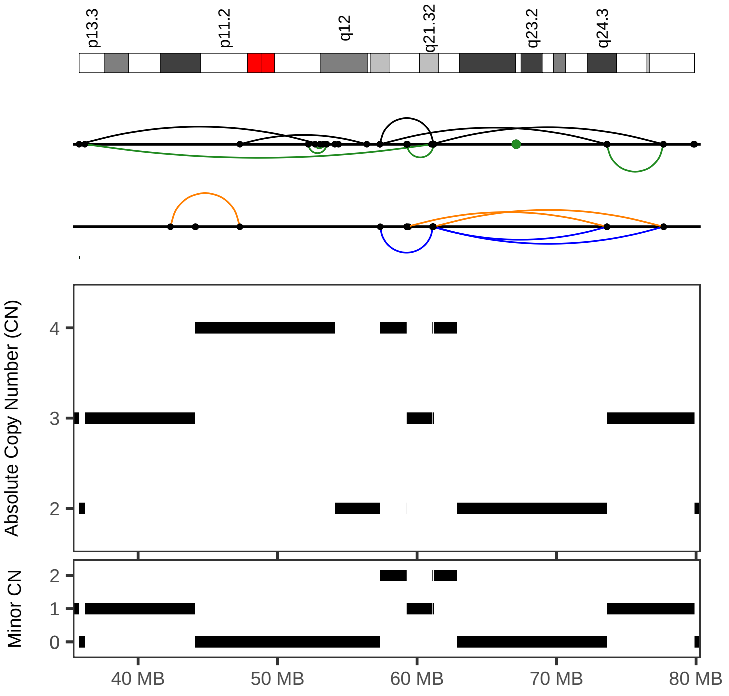

4111337

Cancer type	Lymph−BNHL
Position	11:114660441−124818719
Type	With other complex events
Interleaved intrachr. SVs	18
Total SVs (intrachr. + transl.)	69
SV types	DEL: 3; DUP: 6; h2hINV: 3; t2tINV: 6; TRA: 51
SVs in sample	226
Oscillating CN (2 and 3 states)	4, 5
CN segments	27
FDR fragment joints	0.6776251
FDR chr. breakp. enrich.	0
Linked to chrs	
Purity, ploidy	0.62, 4.03

4115001

Cancer type	Lymph−BNHL
Position	17:35776511−77685244
Type	With other complex events
Interleaved intrachr. SVs	19
Total SVs (intrachr. + transl.)	21
SV types	DEL: 3; DUP: 5; h2hINV: 5; t2tINV: 6; TRA: 2
SVs in sample	61
Oscillating CN (2 and 3 states)	4, 8
CN segments	15
FDR fragment joints	0.7568568
FDR chr. breakp. enrich.	0
Linked to chrs	
Purity, ploidy	0.46, 3.56

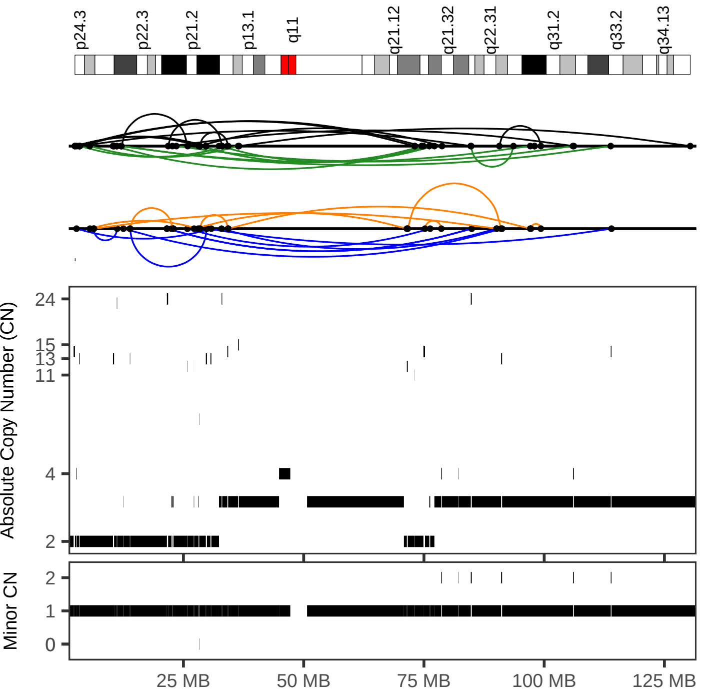

	4120193
Cancer type	Lymph−BNHL
Position	9:2435903−130371802
Type	With other complex events
Interleaved intrachr. SVs	40
Total SVs (intrachr. + transl.)	40
SV types	DEL: 8; DUP: 10; h2hINV: 12; t2tINV: 10; TRA: 0
SVs in sample	101
Oscillating CN (2 and 3 states)	5, 7
CN segments	64
FDR fragment joints	0.9109641
FDR chr. breakp. enrich.	0
Linked to chrs	
Purity, ploidy	0.47, 2.12

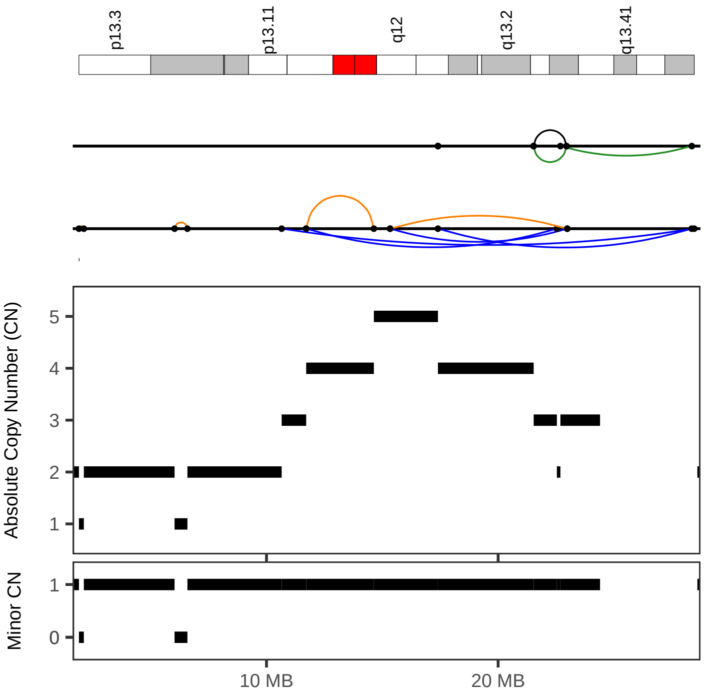

	4120193
Cancer type	Lymph−BNHL
Position	19:11710100−28359979
Type	With other complex events
Interleaved intrachr. SVs	8
Total SVs (intrachr. + transl.)	8
SV types	DEL: 2; DUP: 3; h2hINV: 1; t2tINV: 2; TRA: 0
SVs in sample	101
Oscillating CN (2 and 3 states)	4, 8
CN segments	8
FDR fragment joints	0.8653243
FDR chr. breakp. enrich.	0
Linked to chrs	
Purity, ploidy	0.47, 2.12

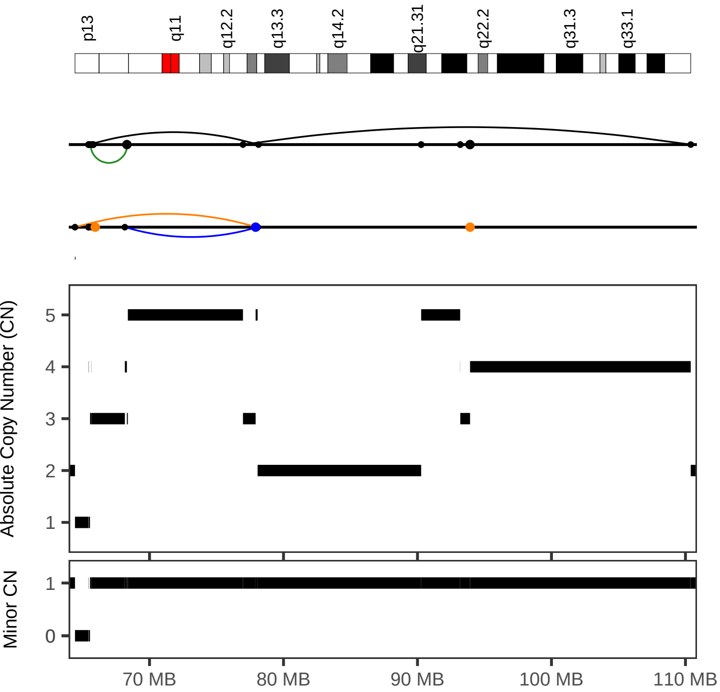

	4133511
Cancer type	Lymph−BNHL
Position	13:64436002−110393802
Type	With other complex events
Interleaved intrachr. SVs	8
Total SVs (intrachr. + transl.)	13
SV types	DEL: 2; DUP: 1; h2hINV: 3; t2tINV: 2; TRA: 5
SVs in sample	33
Oscillating CN (2 and 3 states)	5, 10
CN segments	19
FDR fragment joints	0.9000538
FDR chr. breakp. enrich.	0
Linked to chrs	
Purity, ploidy	0.81, 1.95

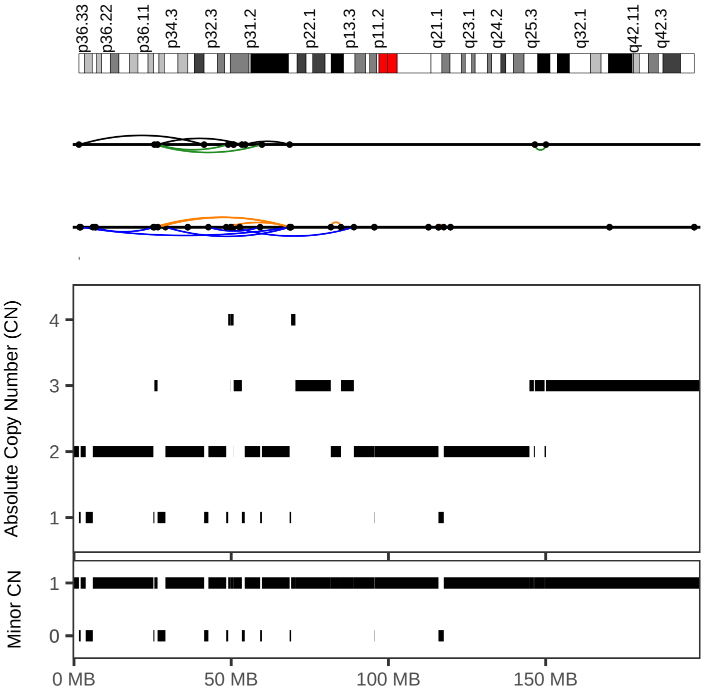

	4135099
Cancer type	Lymph−BNHL
Position	1:1563247−89014486
Type	With other complex events
Interleaved intrachr. SVs	13
Total SVs (intrachr. + transl.)	13
SV types	DEL: 3; DUP: 5; h2hINV: 3; t2tINV: 2; TRA: 0
SVs in sample	297
Oscillating CN (2 and 3 states)	5, 13
CN segments	28
FDR fragment joints	0.7995907
FDR chr. breakp. enrich.	0.37
Linked to chrs	
Purity, ploidy	0.79, 1.91

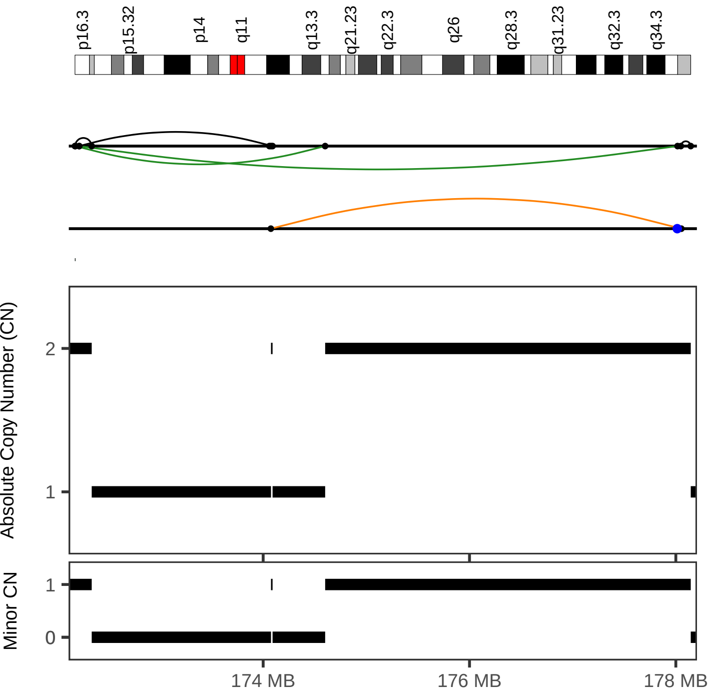

	4139696
Cancer type	Lymph−BNHL
Position	4:172175721−178144474
Type	Canonical without polyploidization
Interleaved intrachr. SVs	7
Total SVs (intrachr. + transl.)	8
SV types	DEL: 1; DUP: 0; h2hINV: 3; t2tINV: 3; TRA: 1
SVs in sample	33
Oscillating CN (2 and 3 states)	6, 6
CN segments	6
FDR fragment joints	0.6776251
FDR chr. breakp. enrich.	0.01
Linked to chrs	
Purity, ploidy	0.44, 2.02

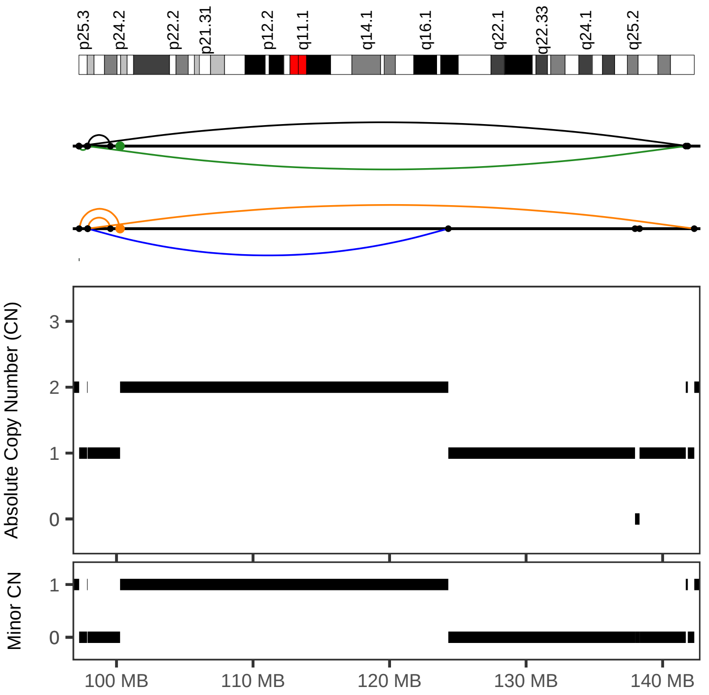

	4147081
Cancer type	Lymph−BNHL
Position	6:97247266−142316157
Type	With other complex events
Interleaved intrachr. SVs	7
Total SVs (intrachr. + transl.)	9
SV types	DEL: 2; DUP: 1; h2hINV: 2; t2tINV: 2; TRA: 2
SVs in sample	107
Oscillating CN (2 and 3 states)	6, 11
CN segments	11
FDR fragment joints	0.854603
FDR chr. breakp. enrich.	0.03
Linked to chrs	5:149829718−164575330;
Purity, ploidy	0.78, 1.94

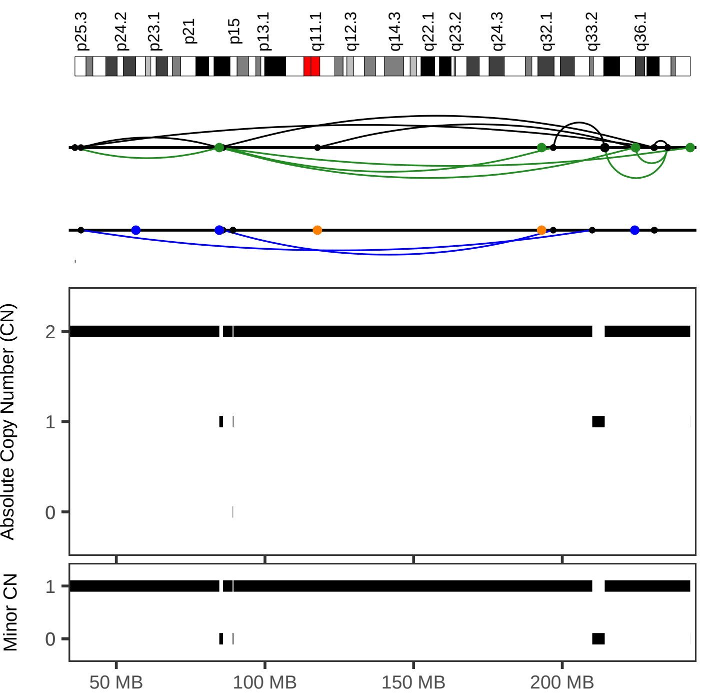

	4157186
Cancer type	Lymph−BNHL
Position	2:36073033−243043088
Type	With other complex events
Interleaved intrachr. SVs	15
Total SVs (intrachr. + transl.)	28
SV types	DEL: 0; DUP: 3; h2hINV: 6; t2tINV: 6; TRA: 13
SVs in sample	46
Oscillating CN (2 and 3 states)	5, 10
CN segments	10
FDR fragment joints	0.615458
FDR chr. breakp. enrich.	0
Linked to chrs	
Purity, ploidy	0.75, 2.04

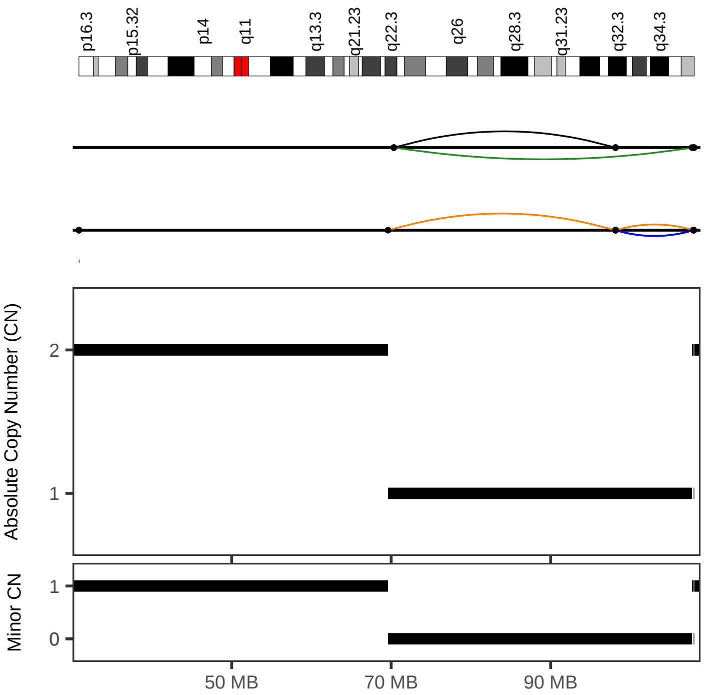

	4157186
Cancer type	Lymph−BNHL
Position	4:69602537−108006799
Type	Canonical without polyploidization
Interleaved intrachr. SVs	8
Total SVs (intrachr. + transl.)	8
SV types	DEL: 2; DUP: 2; h2hINV: 2; t2tINV: 2; TRA: 0
SVs in sample	46
Oscillating CN (2 and 3 states)	4, 4
CN segments	4
FDR fragment joints	1
FDR chr. breakp. enrich.	0.01
Linked to chrs	
Purity, ploidy	0.75, 2.04

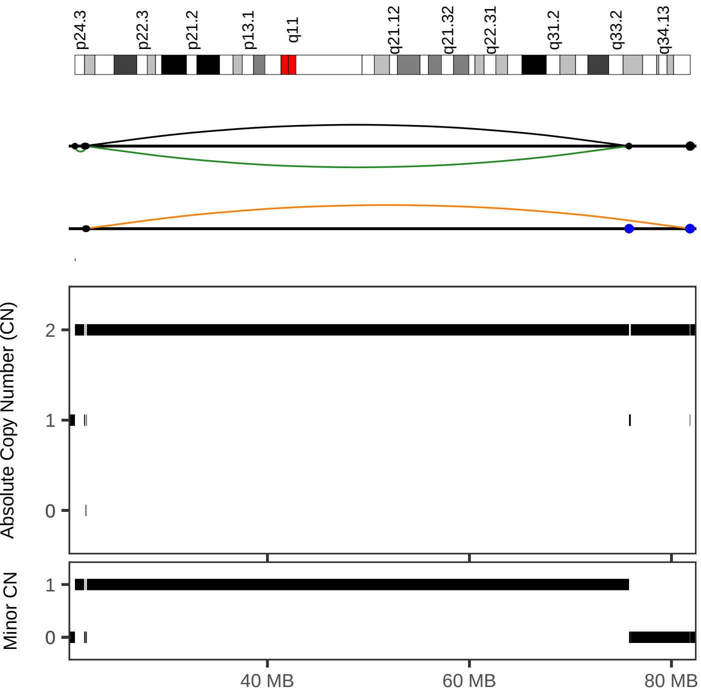

	**4158726**
Cancer type	Lymph−BNHL
Position	9:20938863−81861602
Type	With other complex events
Interleaved intrachr. SVs	6
Total SVs (intrachr. + transl.)	11
SV types	DEL: 3; DUP: 0; h2hINV: 1; t2tINV: 2; TRA: 5
SVs in sample	47
Oscillating CN (2 and 3 states)	6, 11
CN segments	11
FDR fragment joints	0.7425546
FDR chr. breakp. enrich.	0
Linked to chrs	18:49801362−72732255;
Purity, ploidy	0.45, 1.93

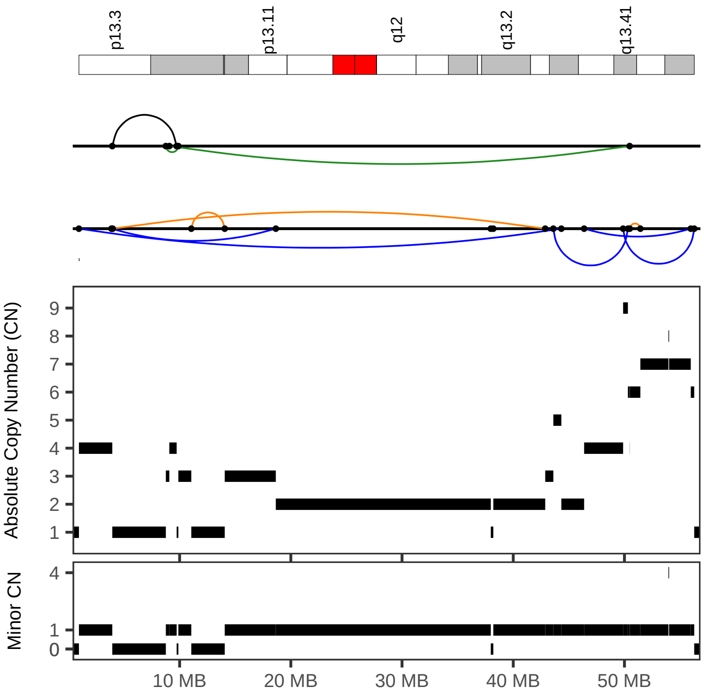

	**4160100**
Cancer type	Lymph−BNHL
Position	19:945638−56277099
Type	With other complex events
Interleaved intrachr. SVs	9
Total SVs (intrachr. + transl.)	9
SV types	DEL: 1; DUP: 5; h2hINV: 1; t2tINV: 2; TRA: 0
SVs in sample	60
Oscillating CN (2 and 3 states)	4, 5
CN segments	24
FDR fragment joints	0.615458
FDR chr. breakp. enrich.	0
Linked to chrs	
Purity, ploidy	0.76, 2.08

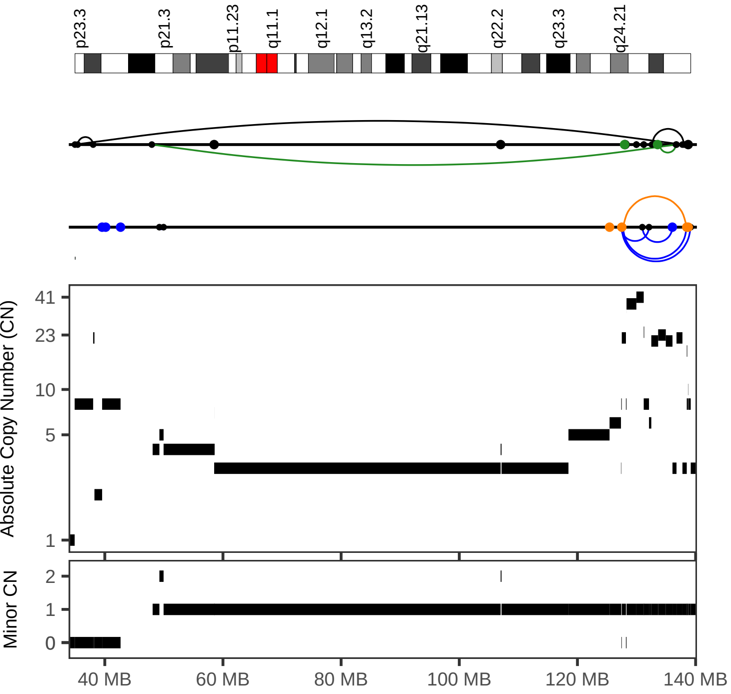

	**AOCS−060**
Cancer type	Ovary−AdenoCA
Position	8:34955549−139173410
Type	With other complex events
Interleaved intrachr. SVs	9
Total SVs (intrachr. + transl.)	23
SV types	DEL: 1; DUP: 4; h2hINV: 2; t2tINV: 2; TRA: 14
SVs in sample	197
Oscillating CN (2 and 3 states)	4, 6
CN segments	36
FDR fragment joints	0.7861024
FDR chr. breakp. enrich.	0
Linked to chrs	19:10867230−47792769;3:721690−195014230
Purity, ploidy	0.68, 2.95

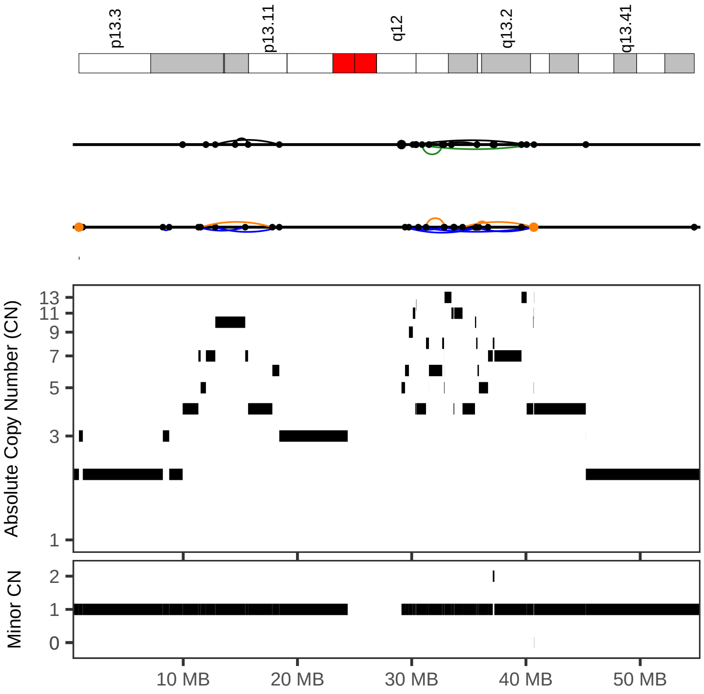

	**AOCS−061**
Cancer type	Ovary−AdenoCA
Position	19:29415891−40712326
Type	With other complex events
Interleaved intrachr. SVs	13
Total SVs (intrachr. + transl.)	14
SV types	DEL: 3; DUP: 6; h2hINV: 2; t2tINV: 2; TRA: 1
SVs in sample	177
Oscillating CN (2 and 3 states)	4, 5
CN segments	34
FDR fragment joints	0.615458
FDR chr. breakp. enrich.	0
Linked to chrs	
Purity, ploidy	0.88, 3.14

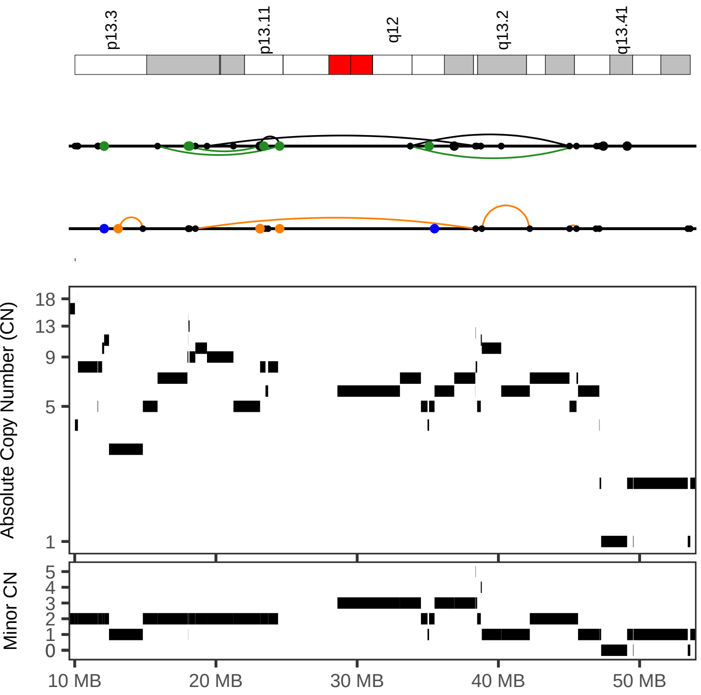

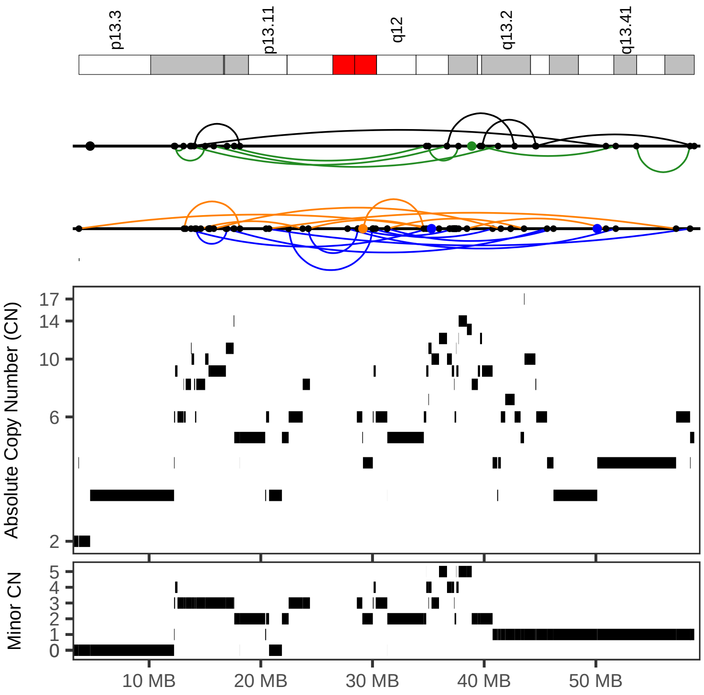

	**AOCS−080**
Cancer type	Ovary−AdenoCA
Position	19:15866356−45529483
Type	With other complex events
Interleaved intrachr. SVs	10
Total SVs (intrachr. + transl.)	21
SV types	DEL: 3; DUP: 0; h2hINV: 3; t2tINV: 4; TRA: 11
SVs in sample	207
Oscillating CN (2 and 3 states)	4, 8
CN segments	30
FDR fragment joints	0.615458
FDR chr. breakp. enrich.	0
Linked to chrs	20:15018409−22766306;
Purity, ploidy	0.92, 3.05

	**AOCS−083**
Cancer type	Ovary−AdenoCA
Position	19:3716326−58806899
Type	With other complex events
Interleaved intrachr. SVs	37
Total SVs (intrachr. + transl.)	42
SV types	DEL: 13; DUP: 11; h2hINV: 5; t2tINV: 8; TRA: 5
SVs in sample	242
Oscillating CN (2 and 3 states)	4, 6
CN segments	75
FDR fragment joints	0.615458
FDR chr. breakp. enrich.	0
Linked to chrs	16:4385507−32390908;
Purity, ploidy	0.56, 4.18

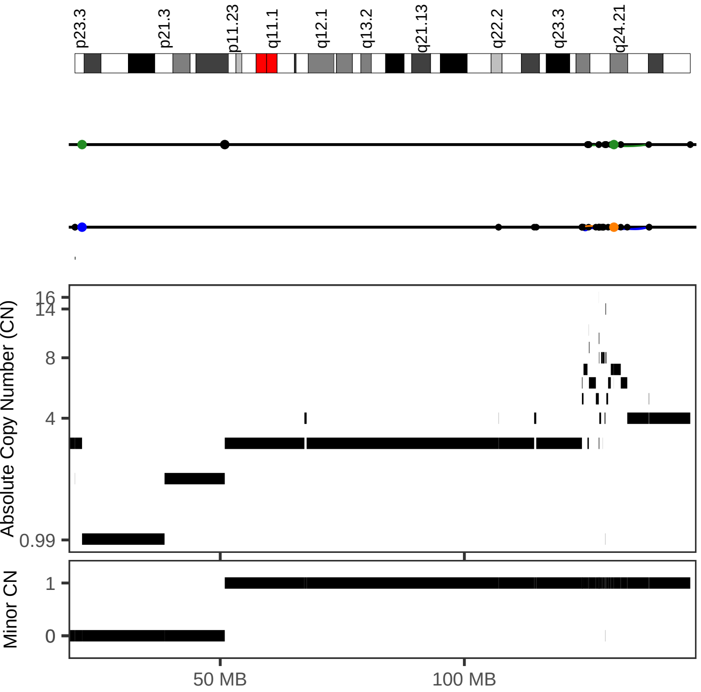

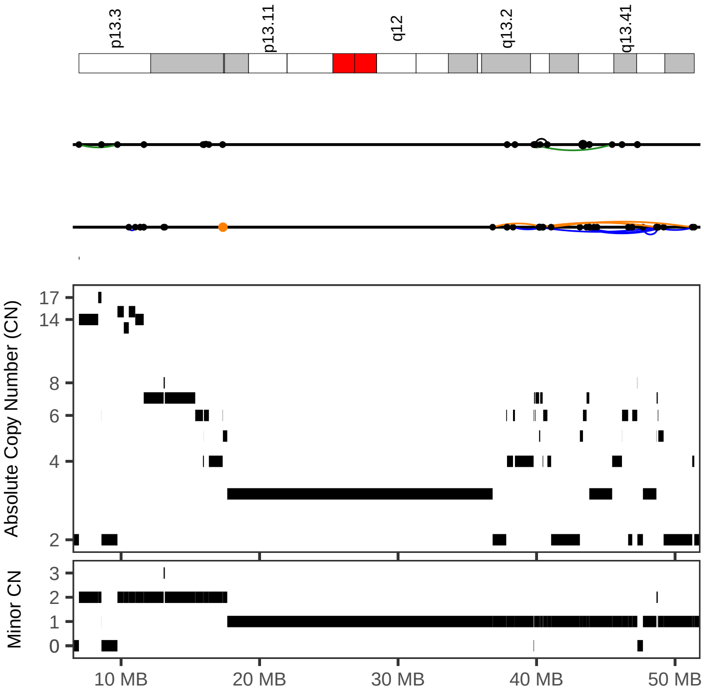

	**AOCS−084**
Cancer type	Ovary−AdenoCA
Position	8:124079382−137843454
Type	With other complex events
Interleaved intrachr. SVs	11
Total SVs (intrachr. + transl.)	13
SV types	DEL: 2; DUP: 5; h2hINV: 1; t2tINV: 3; TRA: 2
SVs in sample	146
Oscillating CN (2 and 3 states)	5, 7
CN segments	32
FDR fragment joints	0.7995907
FDR chr. breakp. enrich.	0
Linked to chrs	1:37638433−46640508;
Purity, ploidy	0.64, 3.09

	**AOCS−090**
Cancer type	Ovary−AdenoCA
Position	19:36827818−51385886
Type	With other complex events
Interleaved intrachr. SVs	14
Total SVs (intrachr. + transl.)	15
SV types	DEL: 5; DUP: 6; h2hINV: 1; t2tINV: 2; TRA: 1
SVs in sample	334
Oscillating CN (2 and 3 states)	5, 9
CN segments	34
FDR fragment joints	0.615458
FDR chr. breakp. enrich.	0
Linked to chrs	
Purity, ploidy	0.78, 2.65

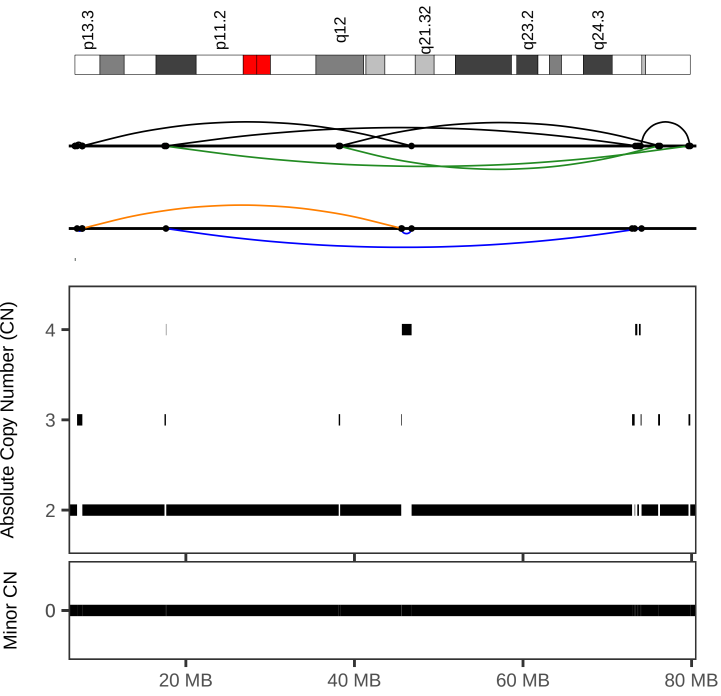

AOCS−096

Cancer type	Ovary−AdenoCA
Position	17:6837884−79840758
Type	With other complex events
Interleaved intrachr. SVs	14
Total SVs (intrachr. + transl.)	14
SV types	DEL: 1; DUP: 3; h2hINV: 5; t2tINV: 5; TRA: 0
SVs in sample	197
Oscillating CN (2 and 3 states)	6, 9
CN segments	22
FDR fragment joints	0.615458
FDR chr. breakp. enrich.	0
Linked to chrs	
Purity, ploidy	0.57, 3.38

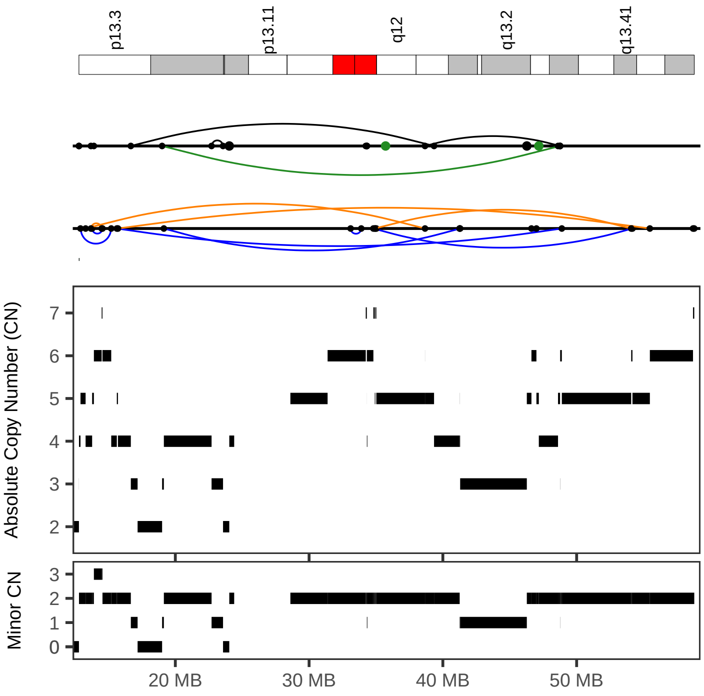

AOCS−096

Cancer type	Ovary−AdenoCA
Position	19:12910667−55473944
Type	With other complex events
Interleaved intrachr. SVs	10
Total SVs (intrachr. + transl.)	14
SV types	DEL: 3; DUP: 4; h2hINV: 2; t2tINV: 1; TRA: 4
SVs in sample	197
Oscillating CN (2 and 3 states)	5, 9
CN segments	43
FDR fragment joints	0.9794281
FDR chr. breakp. enrich.	0
Linked to chrs	3:967694−180645312;5:129143431−180006097
Purity, ploidy	0.57, 3.38

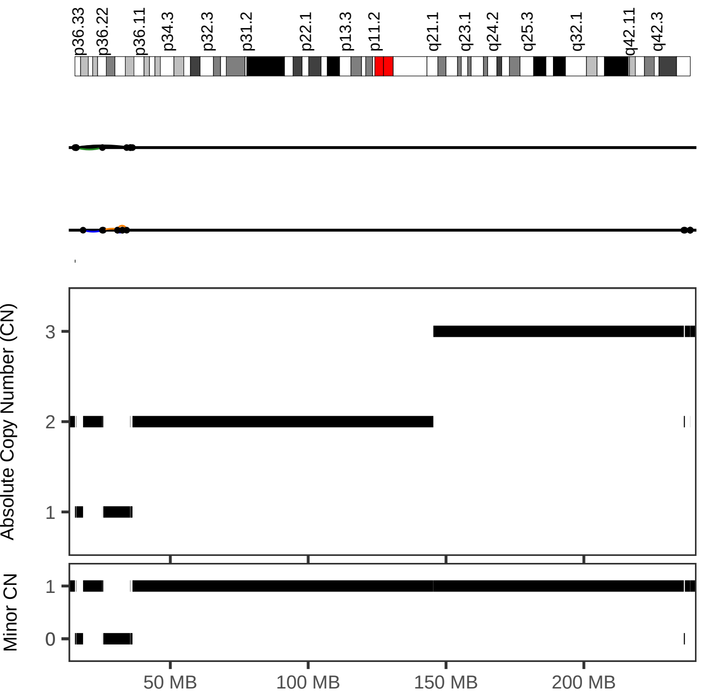

AOCS−097

Cancer type	Ovary−AdenoCA
Position	1:15828920−34169833
Type	Canonical without polyploidization
Interleaved intrachr. SVs	6
Total SVs (intrachr. + transl.)	6
SV types	DEL: 2; DUP: 2; h2hINV: 1; t2tINV: 1; TRA: 0
SVs in sample	51
Oscillating CN (2 and 3 states)	6, 6
CN segments	6
FDR fragment joints	0.9284301
FDR chr. breakp. enrich.	0.01
Linked to chrs	
Purity, ploidy	0.94, 1.81

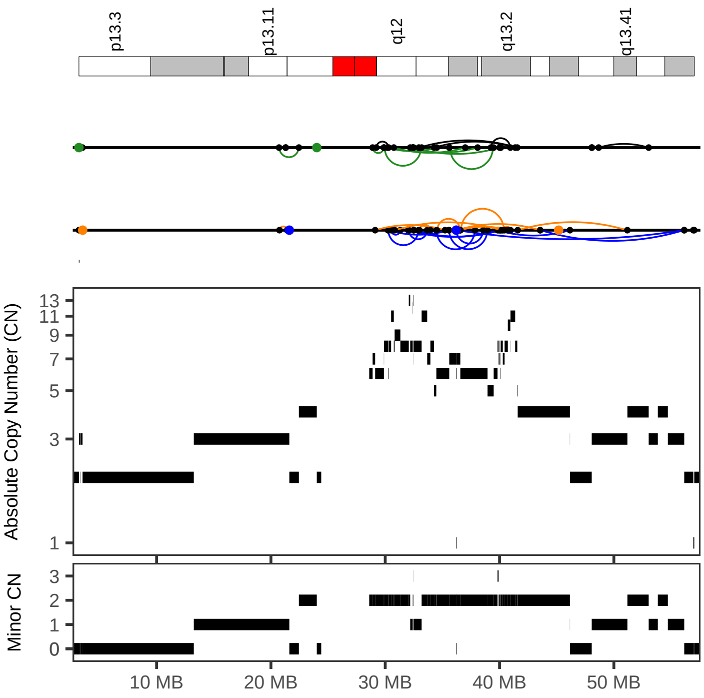

AOCS−111

Cancer type	Ovary−AdenoCA
Position	19:28894477−56155202
Type	With other complex events
Interleaved intrachr. SVs	36
Total SVs (intrachr. + transl.)	39
SV types	DEL: 9; DUP: 13; h2hINV: 6; t2tINV: 8; TRA: 3
SVs in sample	106
Oscillating CN (2 and 3 states)	5, 10
CN segments	54
FDR fragment joints	0.615458
FDR chr. breakp. enrich.	0
Linked to chrs	21:17237944−40394459;
Purity, ploidy	0.62, 3.09

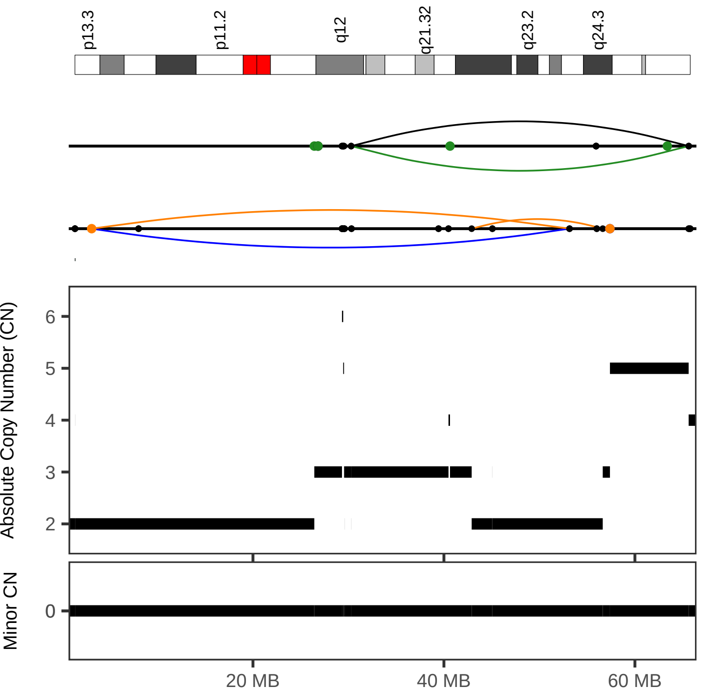

	AOCS−120
Cancer type	Ovary−AdenoCA
Position	17:3078252−65644573
Type	With other complex events
Interleaved intrachr. SVs	6
Total SVs (intrachr. + transl.)	20
SV types	DEL: 3; DUP: 4; h2hINV: 3; t2tINV: 2; TRA: 8
SVs in sample	790
Oscillating CN (2 and 3 states)	5, 11
CN segments	17
FDR fragment joints	0.7735152
FDR chr. breakp. enrich.	0.07
Linked to chrs	15:40235127−98220741;
Purity, ploidy	0.84, 2.89

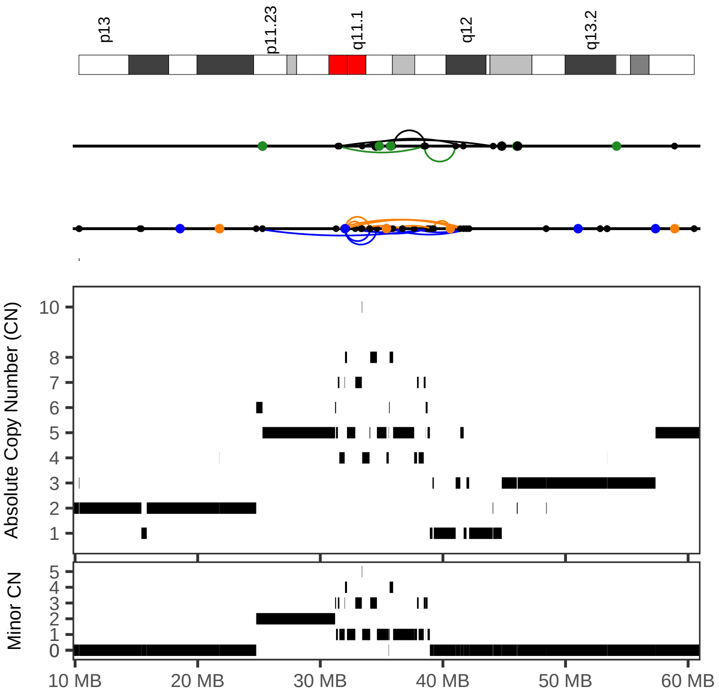

	AOCS−120
Cancer type	Ovary−AdenoCA
Position	20:24772924−44101646
Type	With other complex events
Interleaved intrachr. SVs	18
Total SVs (intrachr. + transl.)	27
SV types	DEL: 6; DUP: 7; h2hINV: 3; t2tINV: 2; TRA: 9
SVs in sample	790
Oscillating CN (2 and 3 states)	4, 7
CN segments	37
FDR fragment joints	0.6776251
FDR chr. breakp. enrich.	0
Linked to chrs	9:16411704−136399897;X:6640984−97824094
Purity, ploidy	0.84, 2.89

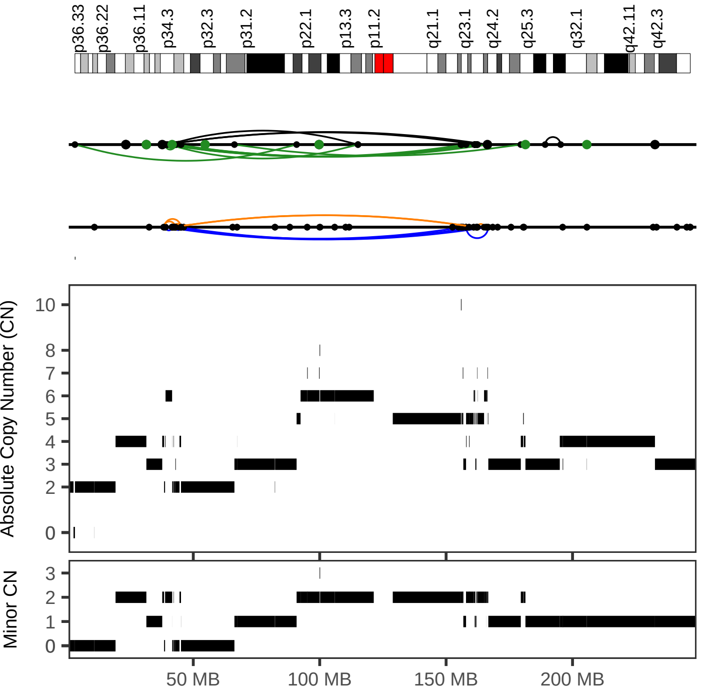

	AOCS−128
Cancer type	Ovary−AdenoCA
Position	1:3190607−179536707
Type	With other complex events
Interleaved intrachr. SVs	37
Total SVs (intrachr. + transl.)	47
SV types	DEL: 9; DUP: 9; h2hINV: 10; t2tINV: 9; TRA: 10
SVs in sample	496
Oscillating CN (2 and 3 states)	6, 13
CN segments	64
FDR fragment joints	0.6776251
FDR chr. breakp. enrich.	0
Linked to chrs	2:19515679−227555411;
Purity, ploidy	0.37, 3.11

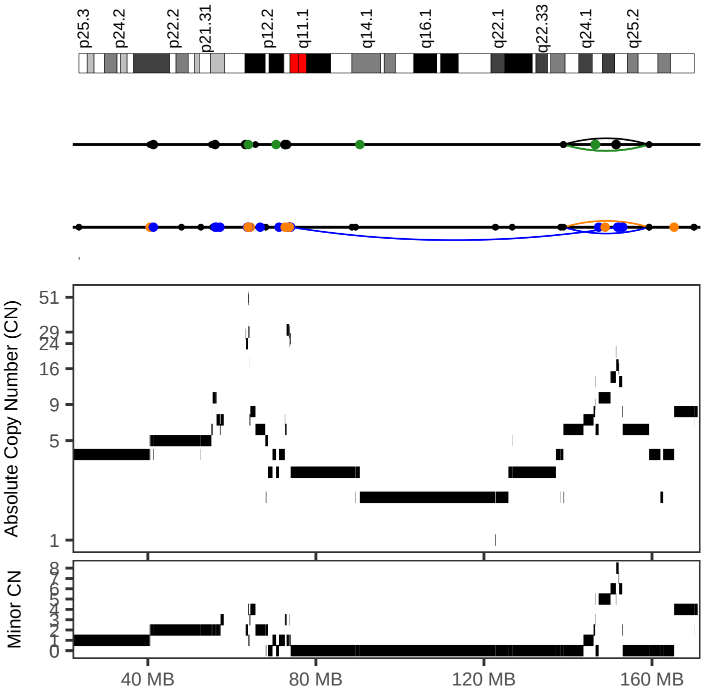

	AOCS−142
Cancer type	Ovary−AdenoCA
Position	6:73883485−159307243
Type	With other complex events
Interleaved intrachr. SVs	13
Total SVs (intrachr. + transl.)	26
SV types	DEL: 1; DUP: 2; h2hINV: 2; t2tINV: 8; TRA: 13
SVs in sample	587
Oscillating CN (2 and 3 states)	4, 6
CN segments	30
FDR fragment joints	0.615458
FDR chr. breakp. enrich.	0
Linked to chrs	
Purity, ploidy	0.4, 3.64

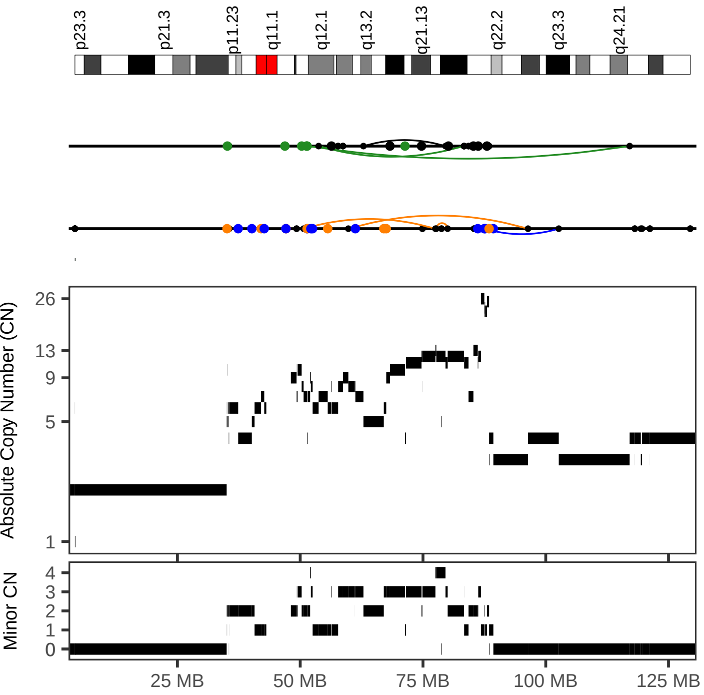

	**AOCS−142**
Cancer type	Ovary−AdenoCA
Position	8:35024834−35538986
Type	With other complex events
Interleaved intrachr. SVs	10
Total SVs (intrachr. + transl.)	12
SV types	DEL: 5; DUP: 1; h2hINV: 2; t2tINV: 2; TRA: 2
SVs in sample	587
Oscillating CN (2 and 3 states)	4, 11
CN segments	17
FDR fragment joints	0.615458
FDR chr. breakp. enrich.	0
Linked to chrs	1:14110378−72283224;
Purity, ploidy	0.4, 3.64

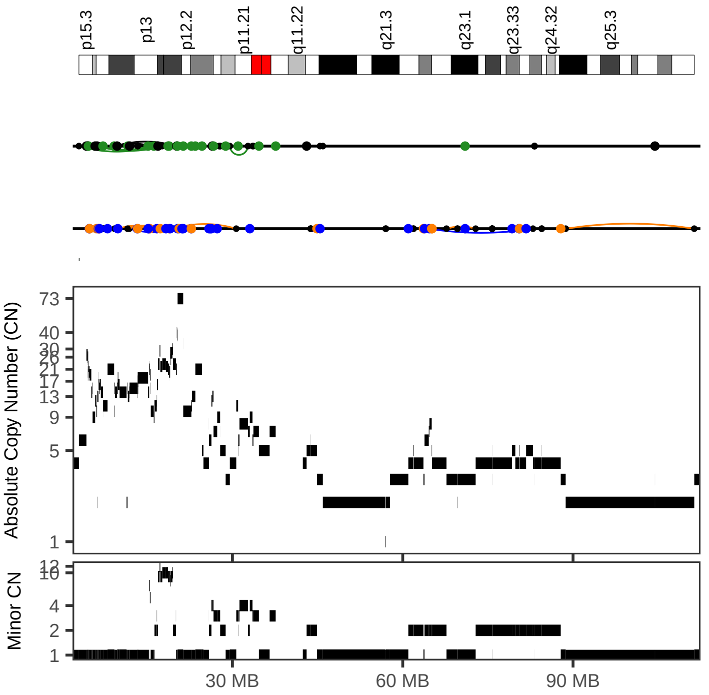

	**AOCS−142**
Cancer type	Ovary−AdenoCA
Position	10:2950088−32730004
Type	With other complex events
Interleaved intrachr. SVs	16
Total SVs (intrachr. + transl.)	70
SV types	DEL: 7; DUP: 2; h2hINV: 3; t2tINV: 4; TRA: 54
SVs in sample	587
Oscillating CN (2 and 3 states)	4, 5
CN segments	96
FDR fragment joints	0.6776251
FDR chr. breakp. enrich.	0
Linked to chrs	1:14110378−72283224;5:5590317−141885071 7:978895−144549791;
Purity, ploidy	0.4, 3.64

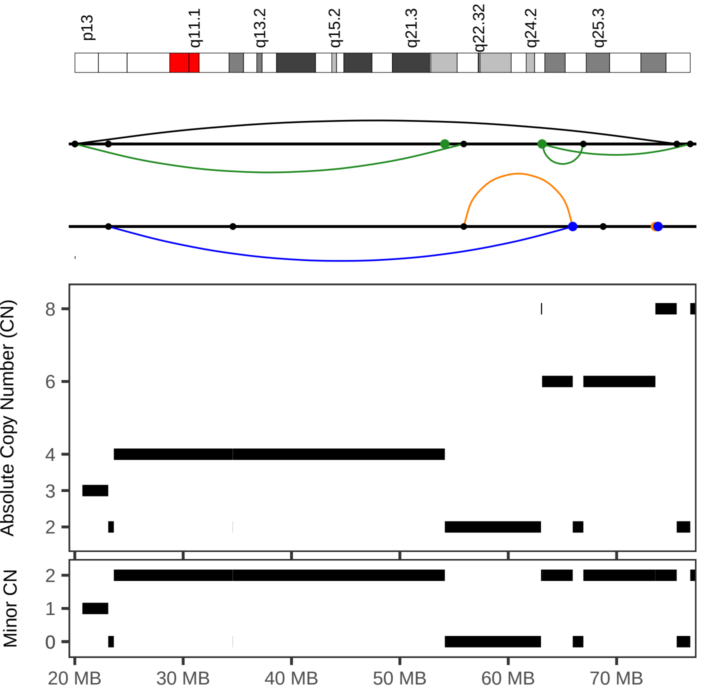

	**AOCS−155**
Cancer type	Ovary−AdenoCA
Position	15:20012055−76793985
Type	With other complex events
Interleaved intrachr. SVs	7
Total SVs (intrachr. + transl.)	13
SV types	DEL: 1; DUP: 1; h2hINV: 2; t2tINV: 3; TRA: 6
SVs in sample	268
Oscillating CN (2 and 3 states)	5, 7
CN segments	14
FDR fragment joints	0.9865804
FDR chr. breakp. enrich.	0.01
Linked to chrs	
Purity, ploidy	0.81, 3.71

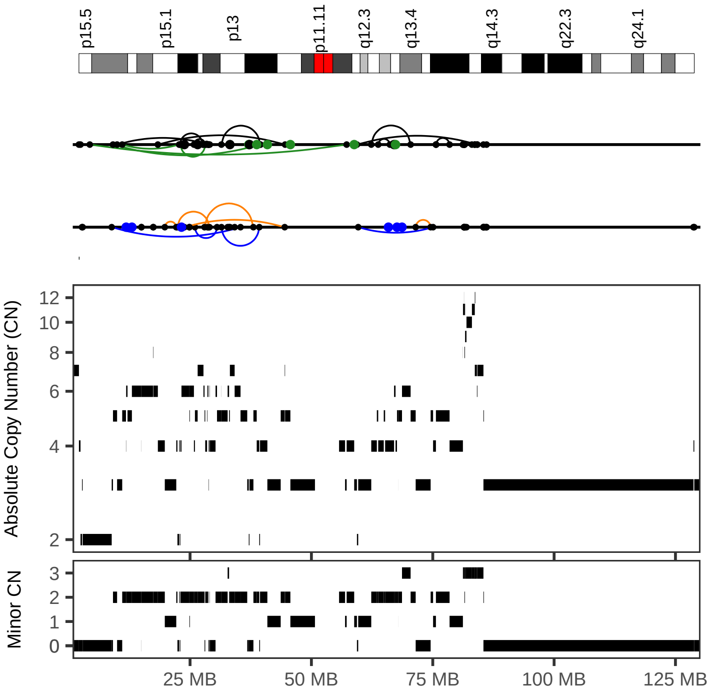

	**AOCS−159**
Cancer type	Ovary−AdenoCA
Position	11:8857856−44579253
Type	With other complex events
Interleaved intrachr. SVs	17
Total SVs (intrachr. + transl.)	27
SV types	DEL: 3; DUP: 4; h2hINV: 6; t2tINV: 4; TRA: 10
SVs in sample	259
Oscillating CN (2 and 3 states)	6, 11
CN segments	55
FDR fragment joints	0.615458
FDR chr. breakp. enrich.	0
Linked to chrs	18:132344−49228551;
Purity, ploidy	0.65, 2.93

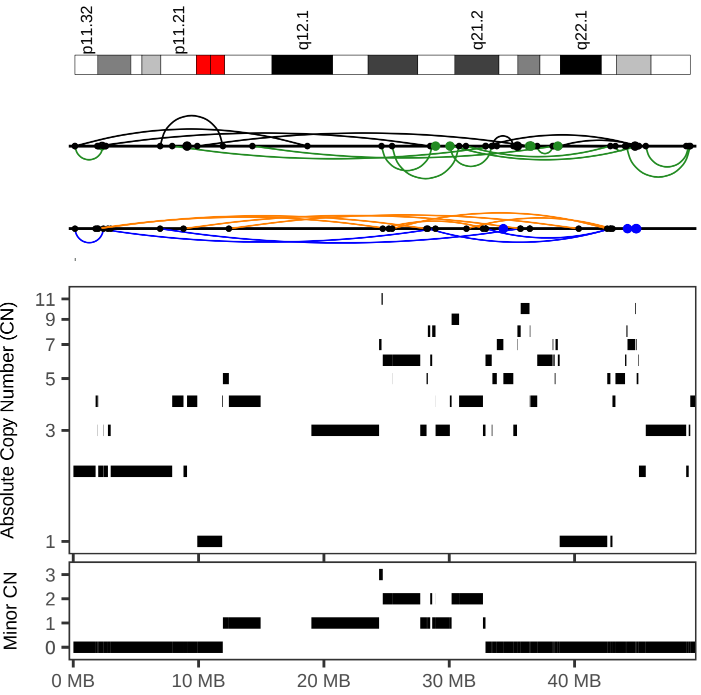

AOCS−159	
Cancer type	Ovary−AdenoCA
Position	18:132344−49228552
Type	With other complex events
Interleaved intrachr. SVs	30
Total SVs (intrachr. + transl.)	43
SV types	DEL: 7; DUP: 5; h2hINV: 8; t2tINV: 10; TRA: 13
SVs in sample	259
Oscillating CN (2 and 3 states)	5, 14
CN segments	69
FDR fragment joints	0.615458
FDR chr. breakp. enrich.	0
Linked to chrs	
Purity, ploidy	0.65, 2.93

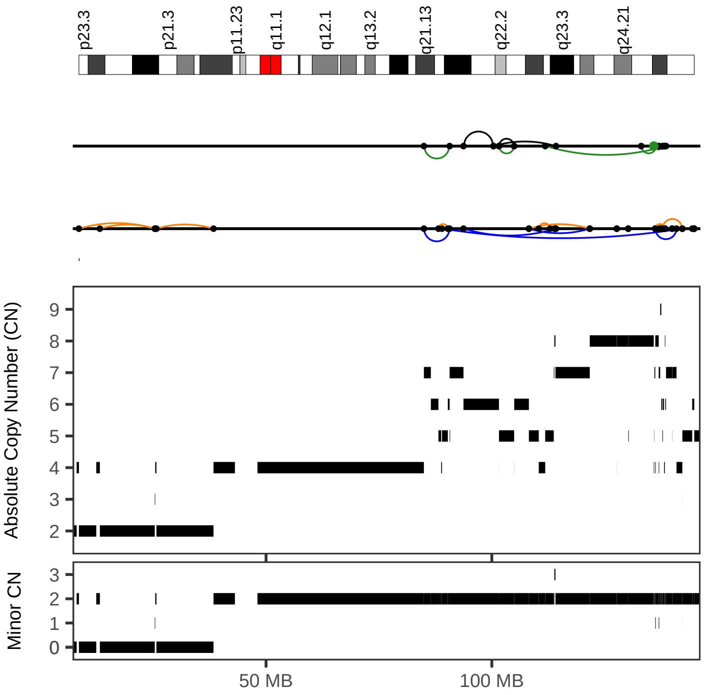

AOCS−162	
Cancer type	Ovary−AdenoCA
Position	8:84960470−142202378
Type	With other complex events
Interleaved intrachr. SVs	20
Total SVs (intrachr. + transl.)	21
SV types	DEL: 5; DUP: 7; h2hINV: 3; t2tINV: 5; TRA: 1
SVs in sample	172
Oscillating CN (2 and 3 states)	4, 6
CN segments	45
FDR fragment joints	0.8882853
FDR chr. breakp. enrich.	0
Linked to chrs	
Purity, ploidy	0.79, 3.31

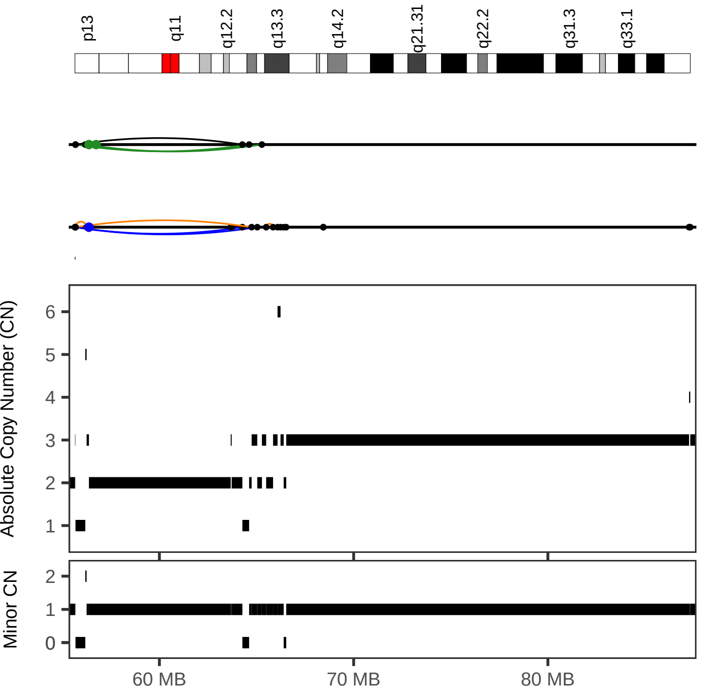

AOCS−163	
Cancer type	Ovary−AdenoCA
Position	13:55650980−65277402
Type	With other complex events
Interleaved intrachr. SVs	7
Total SVs (intrachr. + transl.)	11
SV types	DEL: 2; DUP: 2; h2hINV: 1; t2tINV: 2; TRA: 4
SVs in sample	152
Oscillating CN (2 and 3 states)	4, 9
CN segments	13
FDR fragment joints	0.9804396
FDR chr. breakp. enrich.	0
Linked to chrs	18:11220437−65546089;
Purity, ploidy	0.81, 3.24

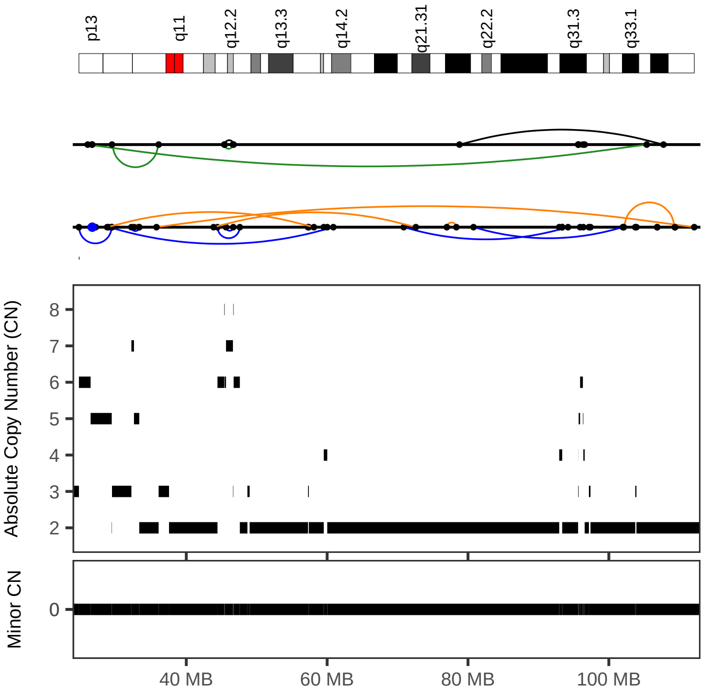

098acb76−0bf5−44e5−bcae−f919cf5fa5e5	
Cancer type	Ovary−AdenoCA
Position	13:24753200−112130103
Type	With other complex events
Interleaved intrachr. SVs	12
Total SVs (intrachr. + transl.)	13
SV types	DEL: 4; DUP: 5; h2hINV: 1; t2tINV: 2; TRA: 1
SVs in sample	718
Oscillating CN (2 and 3 states)	6, 12
CN segments	36
FDR fragment joints	0.615458
FDR chr. breakp. enrich.	0.2
Linked to chrs	
Purity, ploidy	0.82, 3.31

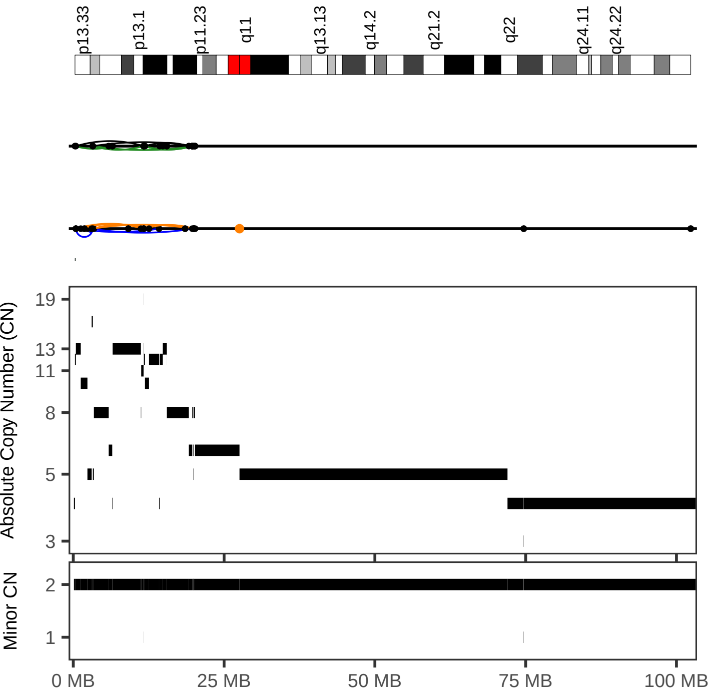

	15556b28−c6bd−455a−87b6−4b7b3e33d0e4
Cancer type	Ovary−AdenoCA
Position	12:280423−20208244
Type	With other complex events
Interleaved intrachr. SVs	20
Total SVs (intrachr. + transl.)	20
SV types	DEL: 6; DUP: 5; h2hINV: 4; t2tINV: 5; TRA: 0
SVs in sample	154
Oscillating CN (2 and 3 states)	4, 4
CN segments	29
FDR fragment joints	0.9625775
FDR chr. breakp. enrich.	0
Linked to chrs	
Purity, ploidy	0.84, 3.91

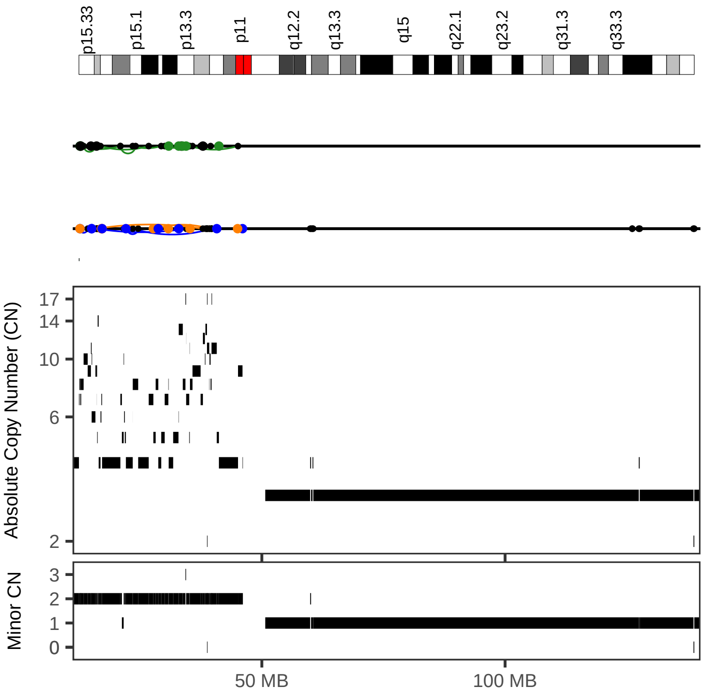

	21f6bf91−dfc6−4a59−8b76−88a0f95c7b47
Cancer type	Ovary−AdenoCA
Position	5:12399998−45103030
Type	With other complex events
Interleaved intrachr. SVs	18
Total SVs (intrachr. + transl.)	46
SV types	DEL: 4; DUP: 6; h2hINV: 1; t2tINV: 7; TRA: 28
SVs in sample	204
Oscillating CN (2 and 3 states)	6, 6
CN segments	64
FDR fragment joints	0.7198692
FDR chr. breakp. enrich.	0
Linked to chrs	
Purity, ploidy	0.9, 2.96

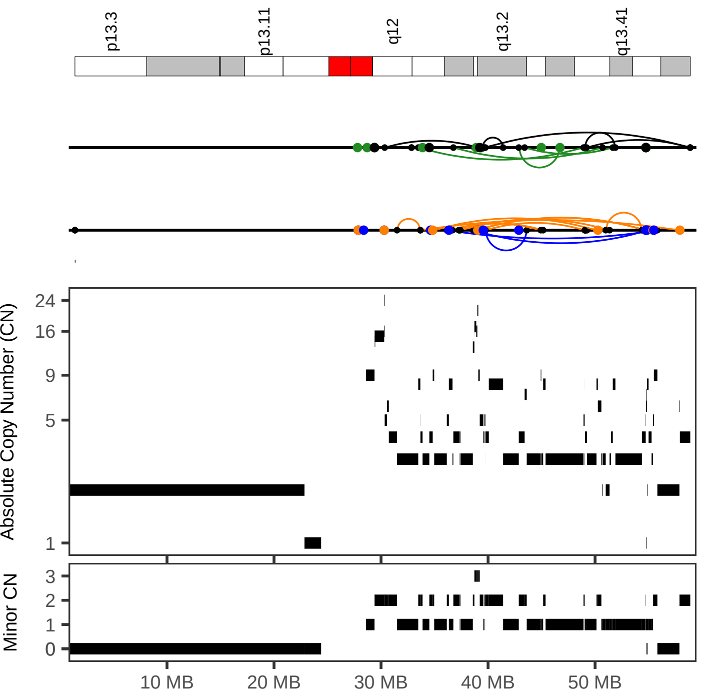

	21f6bf91−dfc6−4a59−8b76−88a0f95c7b47
Cancer type	Ovary−AdenoCA
Position	19:30340862−58891648
Type	With other complex events
Interleaved intrachr. SVs	24
Total SVs (intrachr. + transl.)	47
SV types	DEL: 10; DUP: 4; h2hINV: 5; t2tINV: 5; TRA: 23
SVs in sample	204
Oscillating CN (2 and 3 states)	5, 6
CN segments	68
FDR fragment joints	0.6933701
FDR chr. breakp. enrich.	0
Linked to chrs	1:8799389−247051700;4:42264826−44424729 5:12399998−45103029;
Purity, ploidy	0.9, 2.96

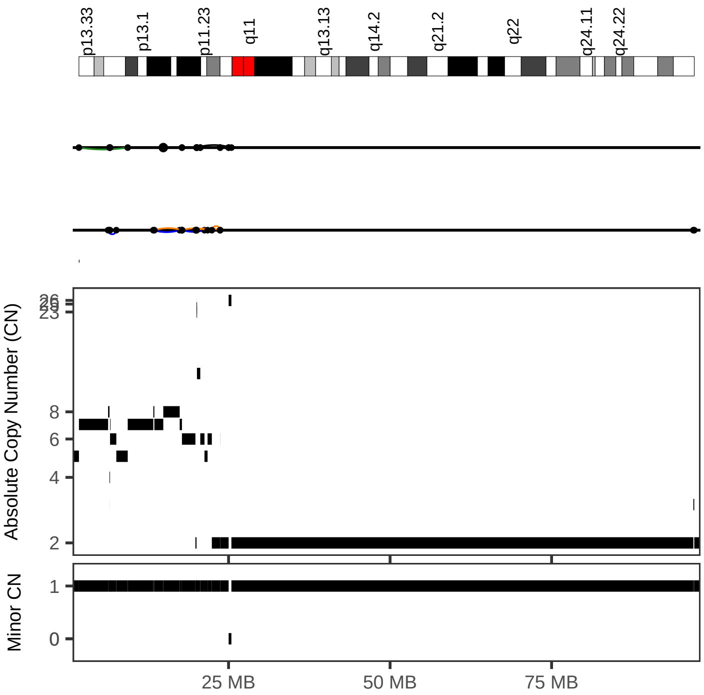

	58d34254−4f5b−40a4−9e9f−7160062fb2a4
Cancer type	Ovary−AdenoCA
Position	12:17804209−25436246
Type	With other complex events
Interleaved intrachr. SVs	6
Total SVs (intrachr. + transl.)	6
SV types	DEL: 3; DUP: 2; h2hINV: 1; t2tINV: 0; TRA: 0
SVs in sample	113
Oscillating CN (2 and 3 states)	4, 4
CN segments	15
FDR fragment joints	0.615458
FDR chr. breakp. enrich.	0
Linked to chrs	
Purity, ploidy	0.93, 1.9

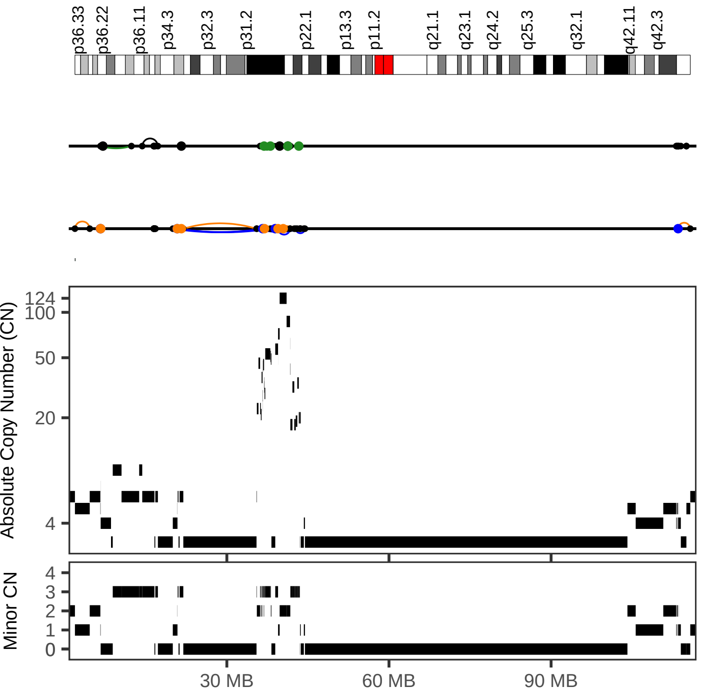

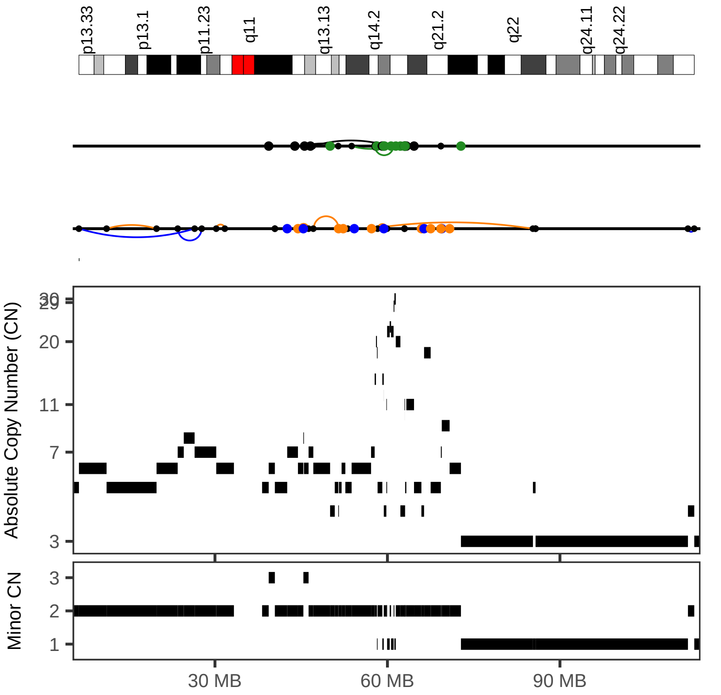

	5c127332−5ca0−45f1−a5ac−4876ad94e491
Cancer type	Ovary−AdenoCA
Position	1:19995601−44453593
Type	With other complex events
Interleaved intrachr. SVs	14
Total SVs (intrachr. + transl.)	29
SV types	DEL: 4; DUP: 7; h2hINV: 1; t2tINV: 2; TRA: 15
SVs in sample	318
Oscillating CN (2 and 3 states)	5, 8
CN segments	44
FDR fragment joints	0.6482122
FDR chr. breakp. enrich.	0
Linked to chrs	5:37201411−40485616;
Purity, ploidy	0.8, 3.95

	5c127332−5ca0−45f1−a5ac−4876ad94e491
Cancer type	Ovary−AdenoCA
Position	12:44542331−85299341
Type	With other complex events
Interleaved intrachr. SVs	7
Total SVs (intrachr. + transl.)	36
SV types	DEL: 3; DUP: 0; h2hINV: 2; t2tINV: 2; TRA: 29
SVs in sample	318
Oscillating CN (2 and 3 states)	4, 5
CN segments	44
FDR fragment joints	0.641841
FDR chr. breakp. enrich.	0
Linked to chrs	10:4028896−29263432;2:29255147−191672688 5:37201411−40485616;7:1928595−38075627
Purity, ploidy	0.8, 3.95

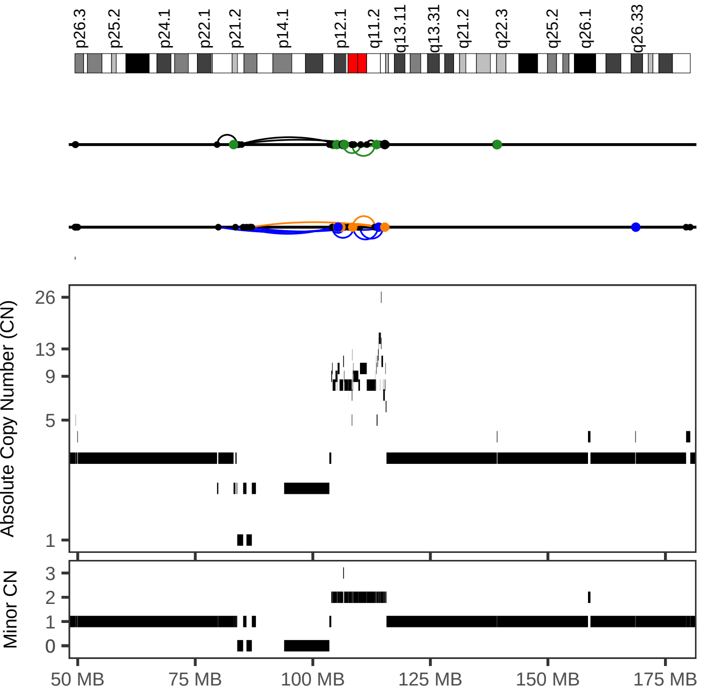

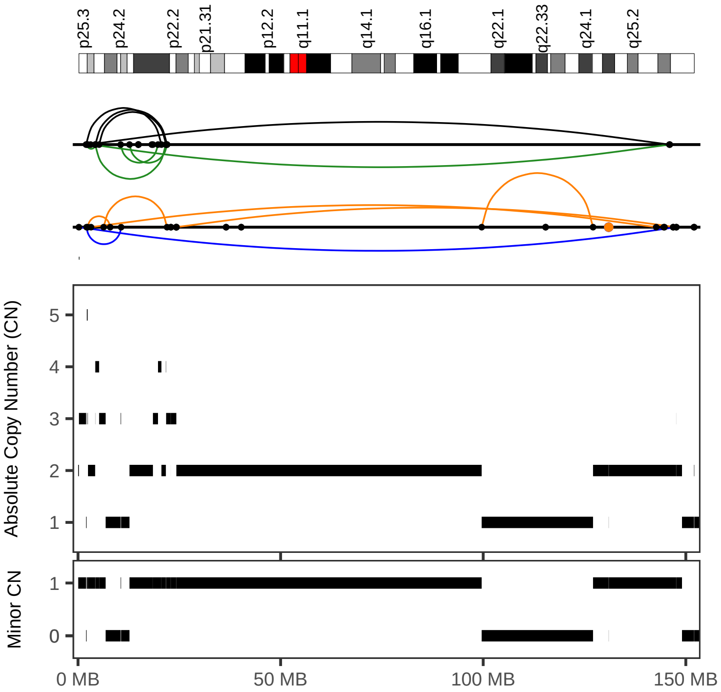

	62379be5−13f0−474b−94d3−6f944ec4ee96
Cancer type	Ovary−AdenoCA
Position	3:79636893−115666941
Type	With other complex events
Interleaved intrachr. SVs	36
Total SVs (intrachr. + transl.)	48
SV types	DEL: 9; DUP: 12; h2hINV: 7; t2tINV: 8; TRA: 12
SVs in sample	216
Oscillating CN (2 and 3 states)	5, 7
CN segments	57
FDR fragment joints	0.9031749
FDR chr. breakp. enrich.	0
Linked to chrs	7:17472055−156811082;
Purity, ploidy	0.94, 2.68

	6d10d4ee−6331−4bba−93bc−a7b64cc0b22a
Cancer type	Ovary−AdenoCA
Position	6:1989523−147659776
Type	With other complex events
Interleaved intrachr. SVs	15
Total SVs (intrachr. + transl.)	18
SV types	DEL: 4; DUP: 2; h2hINV: 4; t2tINV: 5; TRA: 3
SVs in sample	239
Oscillating CN (2 and 3 states)	6, 6
CN segments	28
FDR fragment joints	0.9875525
FDR chr. breakp. enrich.	0
Linked to chrs	
Purity, ploidy	0.87, 1.84

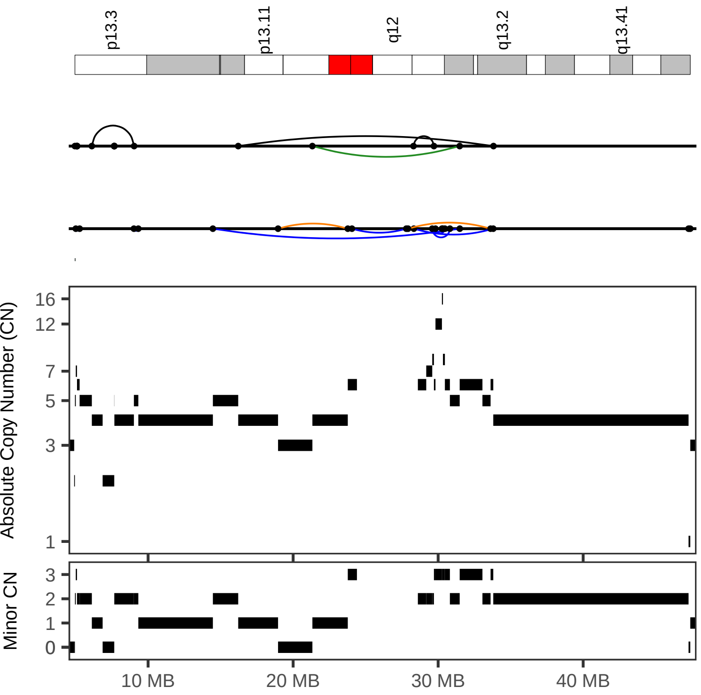

	90f4b65c−cfd4−4066−a5b9−842885c172e2
Cancer type	Ovary−AdenoCA
Position	19:14465820−33816769
Type	With other complex events
Interleaved intrachr. SVs	8
Total SVs (intrachr. + transl.)	8
SV types	DEL: 2; DUP: 3; h2hINV: 2; t2tINV: 1; TRA: 0
SVs in sample	163
Oscillating CN (2 and 3 states)	5, 6
CN segments	18
FDR fragment joints	0.8653243
FDR chr. breakp. enrich.	0
Linked to chrs	
Purity, ploidy	0.85, 3.35

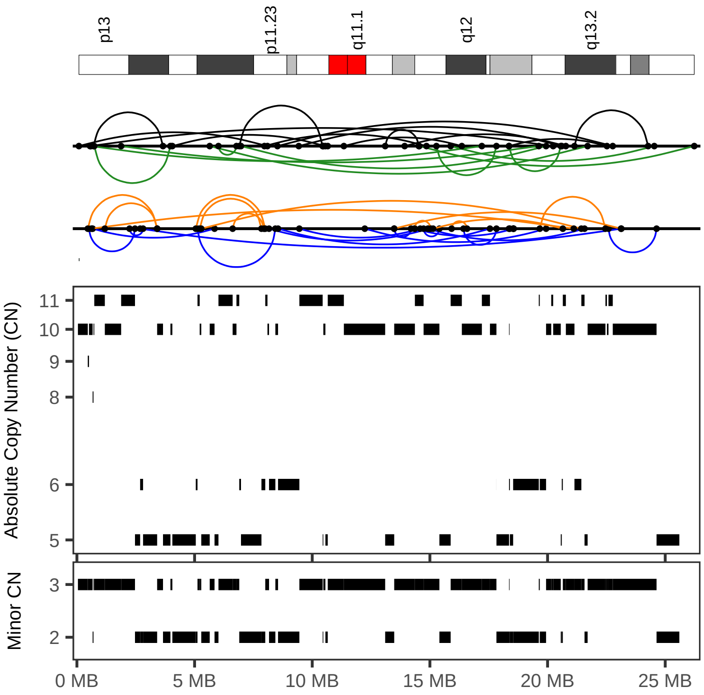

	90f4b65c−cfd4−4066−a5b9−842885c172e2
Cancer type	Ovary−AdenoCA
Position	20:85330−26233761
Type	With other complex events
Interleaved intrachr. SVs	52
Total SVs (intrachr. + transl.)	52
SV types	DEL: 12; DUP: 16; h2hINV: 13; t2tINV: 11; TRA: 0
SVs in sample	163
Oscillating CN (2 and 3 states)	5, 9
CN segments	72
FDR fragment joints	0.8653243
FDR chr. breakp. enrich.	0
Linked to chrs	
Purity, ploidy	0.85, 3.35

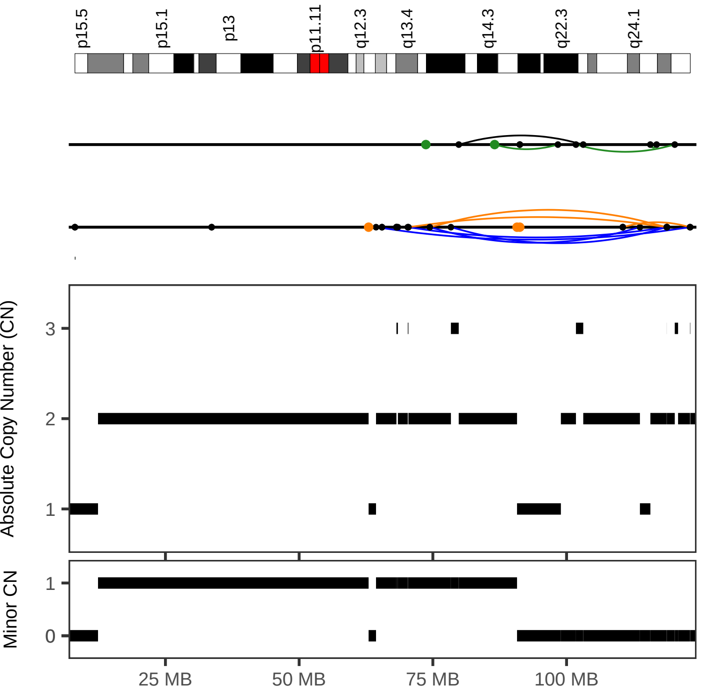

	ee0a4a13−613e−4c5d−96c3−8083a013702d
Cancer type	Ovary−AdenoCA
Position	11:70316616−123074109
Type	With other complex events
Interleaved intrachr. SVs	8
Total SVs (intrachr. + transl.)	12
SV types	DEL: 3; DUP: 3; h2hINV: 1; t2tINV: 1; TRA: 4
SVs in sample	173
Oscillating CN (2 and 3 states)	6, 15
CN segments	15
FDR fragment joints	0.6776251
FDR chr. breakp. enrich.	0
Linked to chrs	
Purity, ploidy	0.55, 1.67

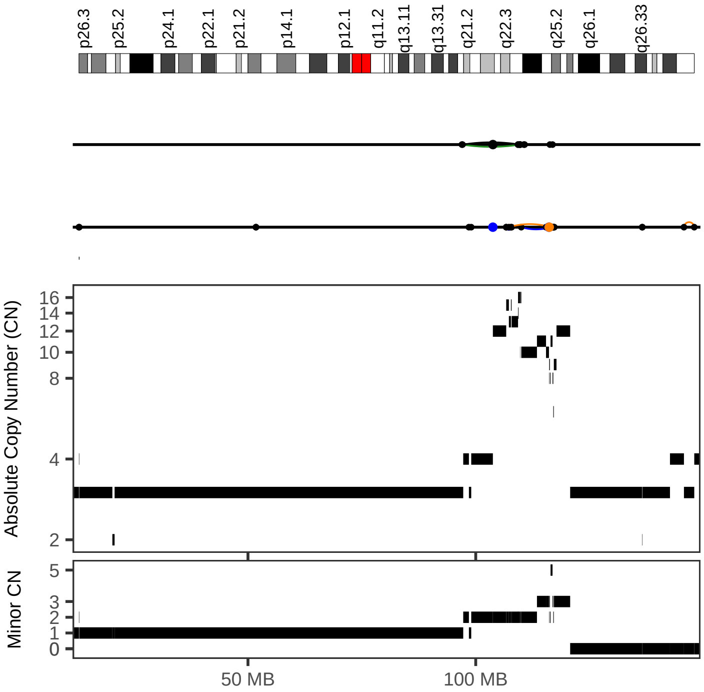

	ff530f28−0ec0−4494−bb54−44bb055bae1c
Cancer type	Ovary−AdenoCA
Position	3:96961383−117251520
Type	With other complex events
Interleaved intrachr. SVs	10
Total SVs (intrachr. + transl.)	14
SV types	DEL: 4; DUP: 3; h2hINV: 2; t2tINV: 1; TRA: 4
SVs in sample	170
Oscillating CN (2 and 3 states)	4, 6
CN segments	23
FDR fragment joints	0.6776251
FDR chr. breakp. enrich.	0.01
Linked to chrs	4:20228916−83508024;
Purity, ploidy	0.7, 3.15

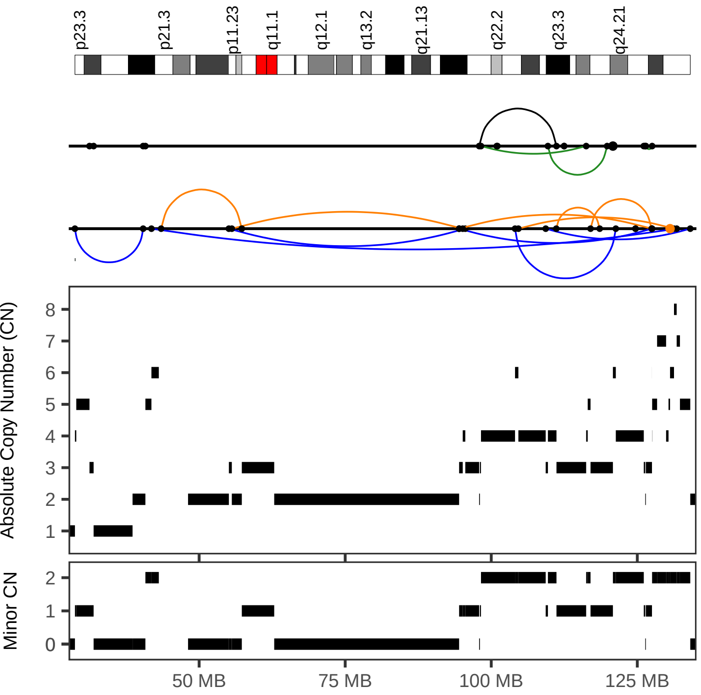

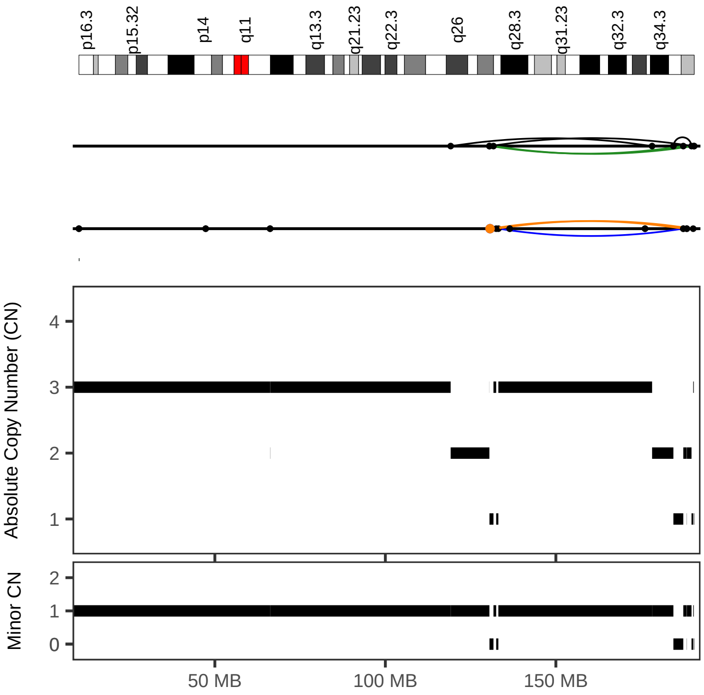

	**ff530f28−0ec0−4494−bb54−44bb055bae1c**
Cancer type	Ovary−AdenoCA
Position	8:41830249−134064686
Type	With other complex events
Interleaved intrachr. SVs	15
Total SVs (intrachr. + transl.)	17
SV types	DEL: 6; DUP: 5; h2hINV: 1; t2tINV: 3; TRA: 2
SVs in sample	170
Oscillating CN (2 and 3 states)	6, 6
CN segments	36
FDR fragment joints	0.615458
FDR chr. breakp. enrich.	0
Linked to chrs	
Purity, ploidy	0.7, 3.15

	**ICGC_0006**
Cancer type	Panc−AdenoCA
Position	4:119147689−190577193
Type	With other complex events
Interleaved intrachr. SVs	8
Total SVs (intrachr. + transl.)	10
SV types	DEL: 2; DUP: 1; h2hINV: 3; t2tINV: 2; TRA: 2
SVs in sample	258
Oscillating CN (2 and 3 states)	6, 8
CN segments	14
FDR fragment joints	0.9625775
FDR chr. breakp. enrich.	1
Linked to chrs	2:7594273−219080787;
Purity, ploidy	0.41, 3.23

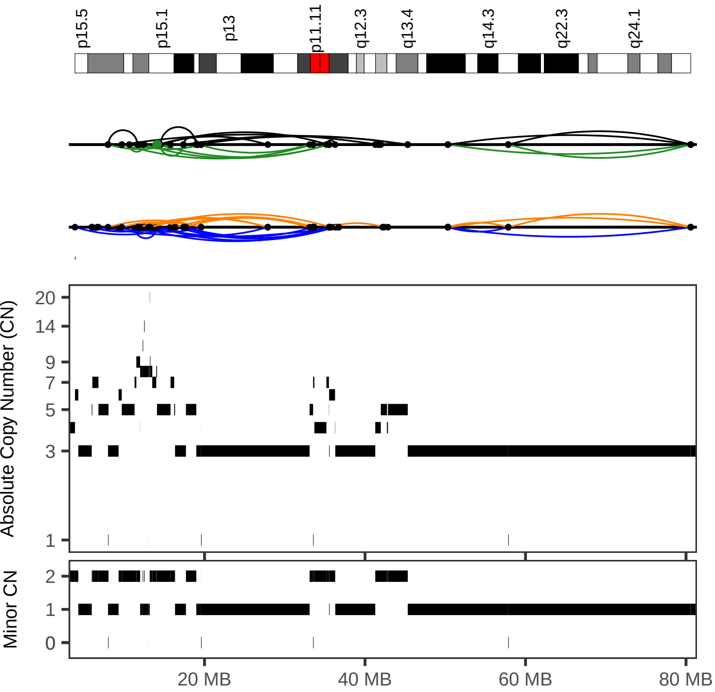

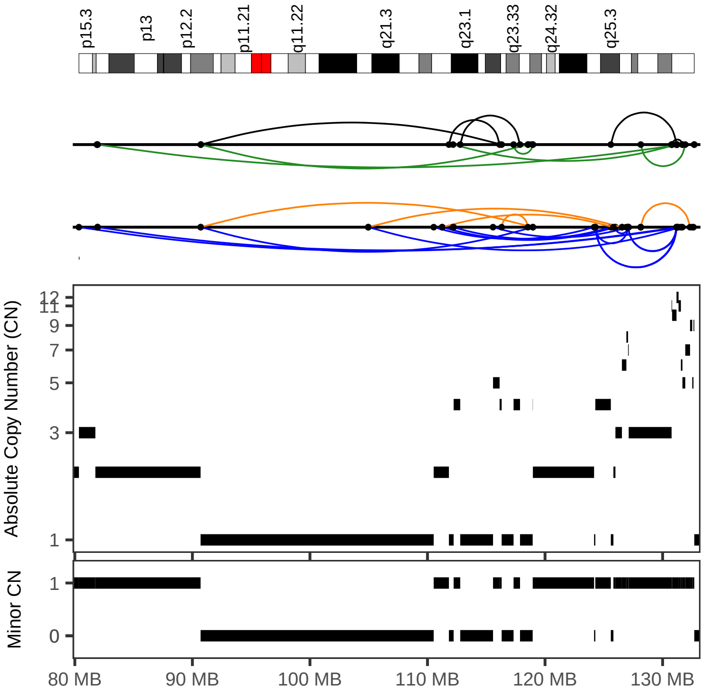

	**ICGC_0006**
Cancer type	Panc−AdenoCA
Position	11:3857736−45323041
Type	With other complex events
Interleaved intrachr. SVs	46
Total SVs (intrachr. + transl.)	47
SV types	DEL: 10; DUP: 17; h2hINV: 9; t2tINV: 10; TRA: 1
SVs in sample	258
Oscillating CN (2 and 3 states)	4, 5
CN segments	49
FDR fragment joints	0.615458
FDR chr. breakp. enrich.	0
Linked to chrs	
Purity, ploidy	0.41, 3.23

	**ICGC_0009**
Cancer type	Panc−AdenoCA
Position	10:80347920−132334500
Type	With other complex events
Interleaved intrachr. SVs	31
Total SVs (intrachr. + transl.)	31
SV types	DEL: 5; DUP: 14; h2hINV: 7; t2tINV: 5; TRA: 0
SVs in sample	41
Oscillating CN (2 and 3 states)	5, 6
CN segments	31
FDR fragment joints	0.5435077
FDR chr. breakp. enrich.	0
Linked to chrs	
Purity, ploidy	0.7, 1.86

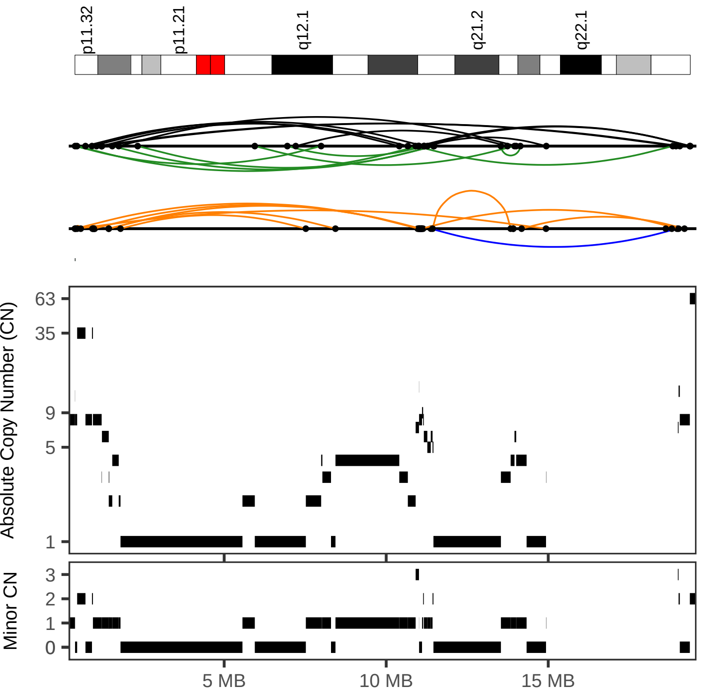

	ICGC_0021
Cancer type	Panc−AdenoCA
Position	18:394262−19382550
Type	With other complex events
Interleaved intrachr. SVs	33
Total SVs (intrachr. + transl.)	33
SV types	DEL: 11; DUP: 2; h2hINV: 11; t2tINV: 9; TRA: 0
SVs in sample	162
Oscillating CN (2 and 3 states)	5, 8
CN segments	42
FDR fragment joints	0.5435077
FDR chr. breakp. enrich.	0
Linked to chrs	
Purity, ploidy	0.43, 2.88

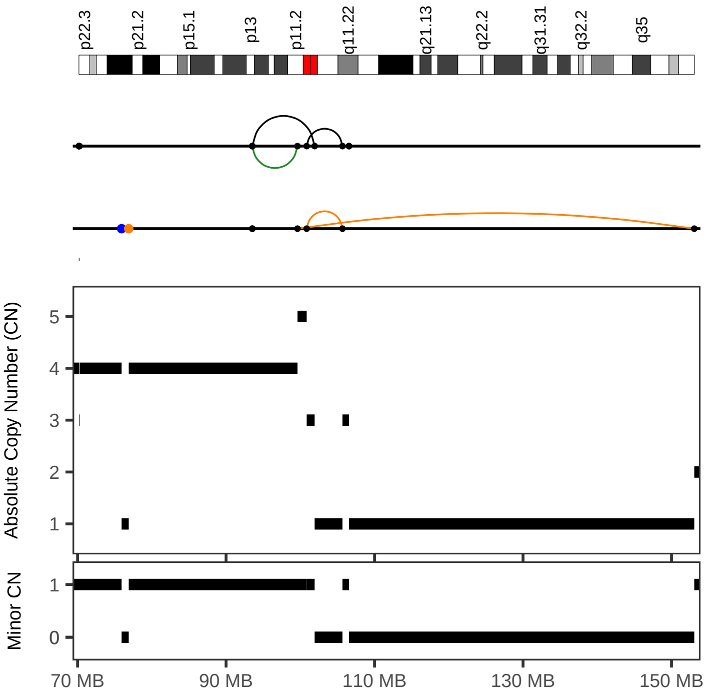

	ICGC_0025
Cancer type	Panc−AdenoCA
Position	7:93519827−153063819
Type	With other complex events
Interleaved intrachr. SVs	6
Total SVs (intrachr. + transl.)	6
SV types	DEL: 3; DUP: 0; h2hINV: 2; t2tINV: 1; TRA: 0
SVs in sample	118
Oscillating CN (2 and 3 states)	4, 5
CN segments	7
FDR fragment joints	0.615458
FDR chr. breakp. enrich.	0.2
Linked to chrs	
Purity, ploidy	0.51, 1.65

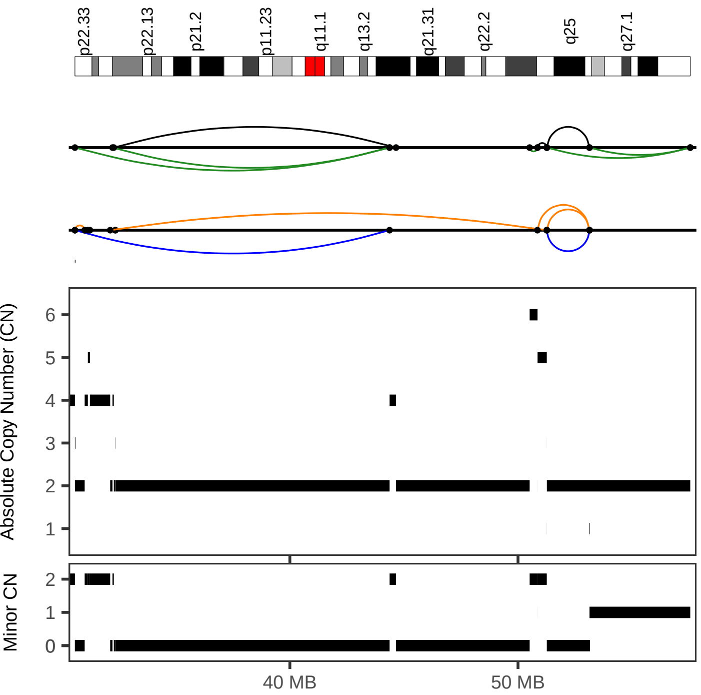

	ICGC_0026
Cancer type	Panc−AdenoCA
Position	X:30577518−57551527
Type	With other complex events
Interleaved intrachr. SVs	14
Total SVs (intrachr. + transl.)	14
SV types	DEL: 4; DUP: 2; h2hINV: 3; t2tINV: 5; TRA: 0
SVs in sample	66
Oscillating CN (2 and 3 states)	4, 8
CN segments	22
FDR fragment joints	0.8066159
FDR chr. breakp. enrich.	0
Linked to chrs	
Purity, ploidy	0.62, 3.55

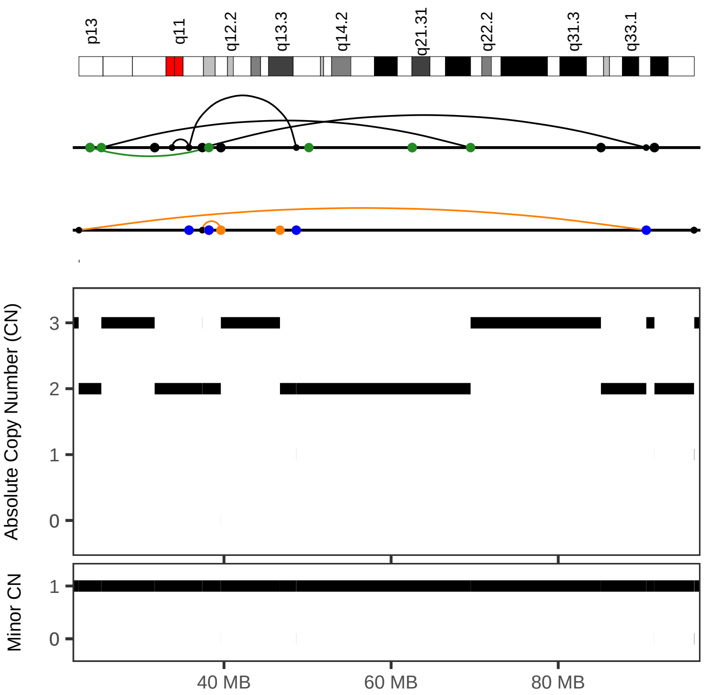

	ICGC_0037
Cancer type	Panc−AdenoCA
Position	13:22638525−90523945
Type	With other complex events
Interleaved intrachr. SVs	7
Total SVs (intrachr. + transl.)	31
SV types	DEL: 2; DUP: 0; h2hINV: 4; t2tINV: 1; TRA: 24
SVs in sample	165
Oscillating CN (2 and 3 states)	6, 13
CN segments	13
FDR fragment joints	0.615458
FDR chr. breakp. enrich.	0
Linked to chrs	
Purity, ploidy	0.48, 2.75

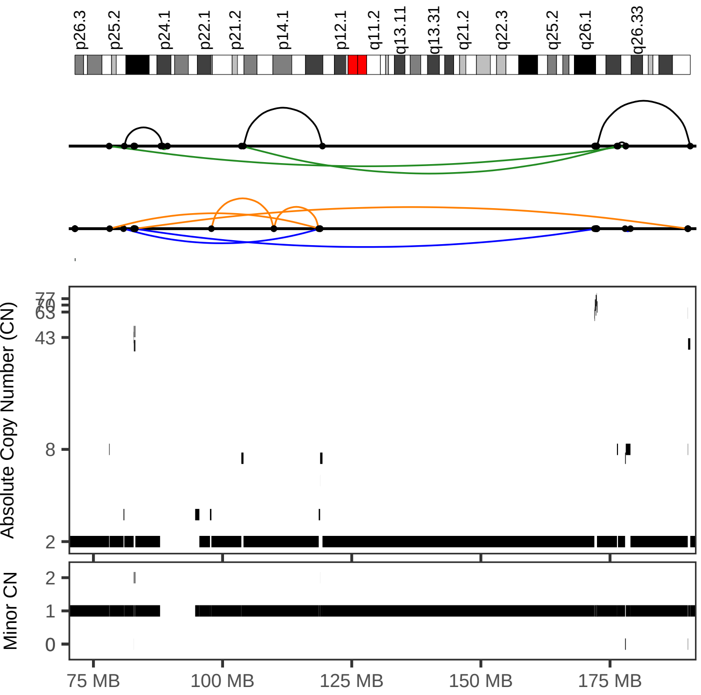

	ICGC_0059
Cancer type	Panc−AdenoCA
Position	3:78038205−190530121
Type	With other complex events
Interleaved intrachr. SVs	13
Total SVs (intrachr. + transl.)	13
SV types	DEL: 3; DUP: 3; h2hINV: 4; t2tINV: 3; TRA: 0
SVs in sample	68
Oscillating CN (2 and 3 states)	5, 5
CN segments	44
FDR fragment joints	0.9865804
FDR chr. breakp. enrich.	0
Linked to chrs	
Purity, ploidy	0.44, 3.41

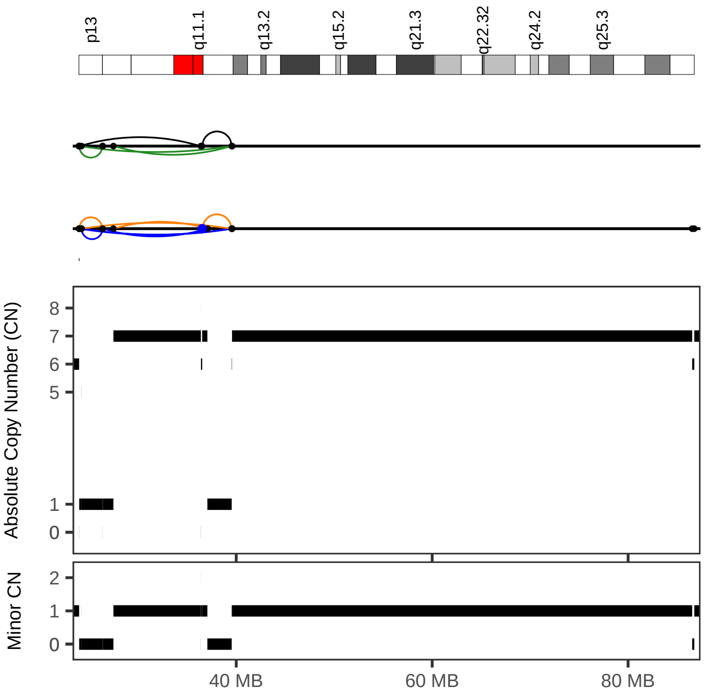

	ICGC_0066
Cancer type	Panc−AdenoCA
Position	15:23941952−39581480
Type	With other complex events
Interleaved intrachr. SVs	16
Total SVs (intrachr. + transl.)	17
SV types	DEL: 5; DUP: 4; h2hINV: 4; t2tINV: 3; TRA: 1
SVs in sample	66
Oscillating CN (2 and 3 states)	4, 7
CN segments	17
FDR fragment joints	0.930656
FDR chr. breakp. enrich.	0
Linked to chrs	
Purity, ploidy	0.42, 3.85

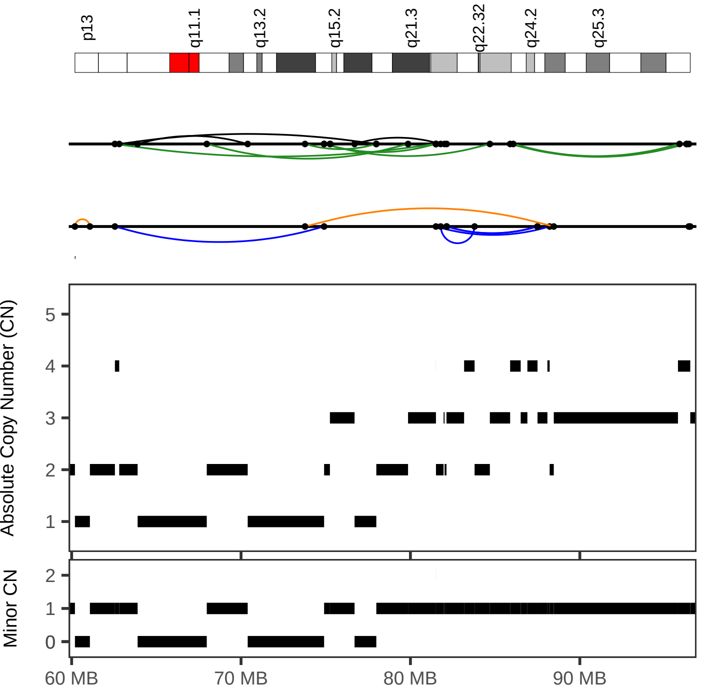

	ICGC_0069
Cancer type	Panc−AdenoCA
Position	15:62553528−96290031
Type	With other complex events
Interleaved intrachr. SVs	17
Total SVs (intrachr. + transl.)	17
SV types	DEL: 1; DUP: 5; h2hINV: 4; t2tINV: 7; TRA: 0
SVs in sample	73
Oscillating CN (2 and 3 states)	6, 8
CN segments	26
FDR fragment joints	0.615458
FDR chr. breakp. enrich.	0
Linked to chrs	
Purity, ploidy	0.58, 1.81

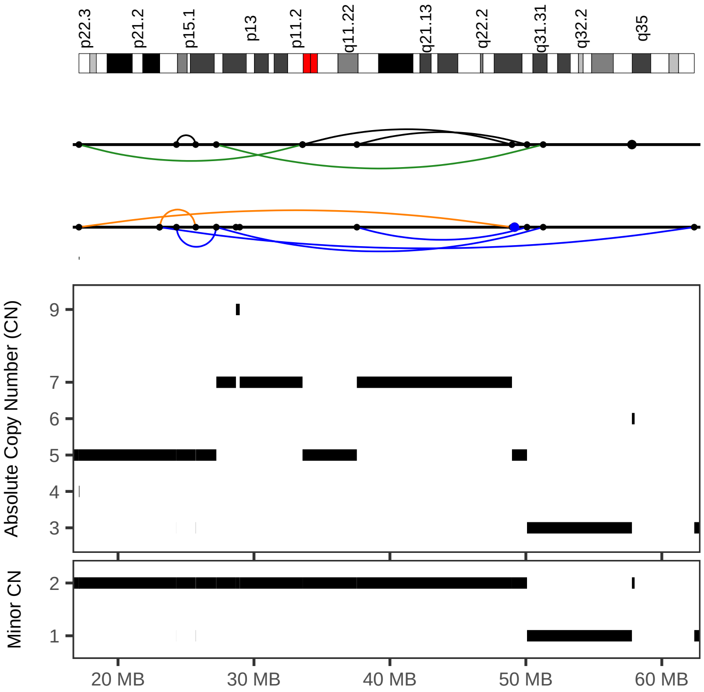

	ICGC_0087
Cancer type	Panc−AdenoCA
Position	7:17124522−62387137
Type	With other complex events
Interleaved intrachr. SVs	11
Total SVs (intrachr. + transl.)	14
SV types	DEL: 2; DUP: 4; h2hINV: 3; t2tINV: 2; TRA: 3
SVs in sample	79
Oscillating CN (2 and 3 states)	5, 12
CN segments	15
FDR fragment joints	0.8988396
FDR chr. breakp. enrich.	0
Linked to chrs	
Purity, ploidy	0.29, 2.33

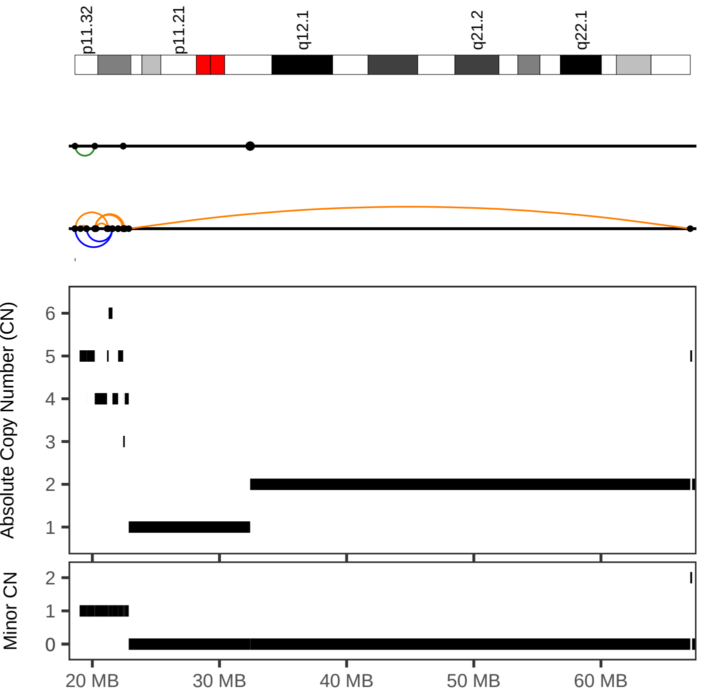

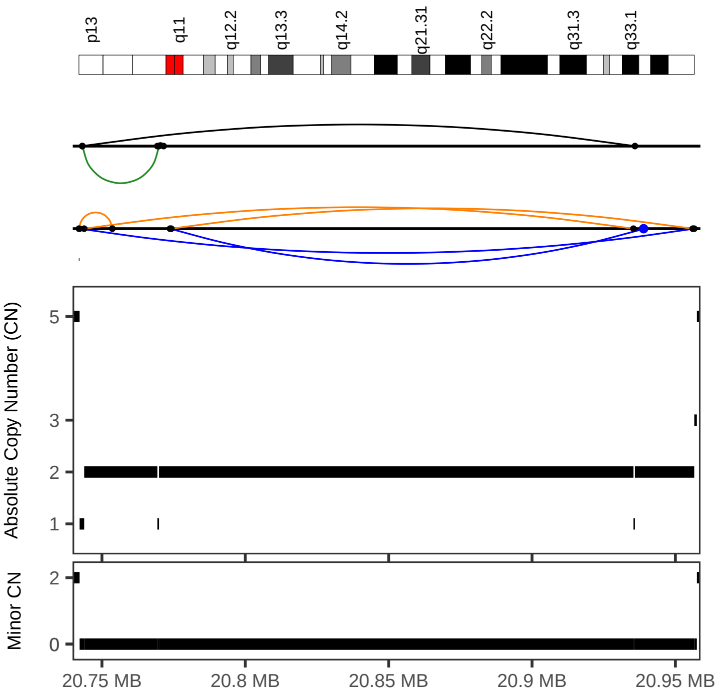

	ICGC_0087
Cancer type	Panc−AdenoCA
Position	18:18629528−22549984
Type	With other complex events
Interleaved intrachr. SVs	8
Total SVs (intrachr. + transl.)	8
SV types	DEL: 4; DUP: 2; h2hINV: 1; t2tINV: 1; TRA: 0
SVs in sample	79
Oscillating CN (2 and 3 states)	5, 5
CN segments	12
FDR fragment joints	0.615458
FDR chr. breakp. enrich.	0
Linked to chrs	
Purity, ploidy	0.29, 2.33

	ICGC_0103
Cancer type	Panc−AdenoCA
Position	13:20741975−20956570
Type	Canonical without polyploidization
Interleaved intrachr. SVs	7
Total SVs (intrachr. + transl.)	8
SV types	DEL: 3; DUP: 2; h2hINV: 1; t2tINV: 1; TRA: 1
SVs in sample	28
Oscillating CN (2 and 3 states)	6, 7
CN segments	8
FDR fragment joints	0.6776251
FDR chr. breakp. enrich.	0
Linked to chrs	
Purity, ploidy	0.32, 3.16

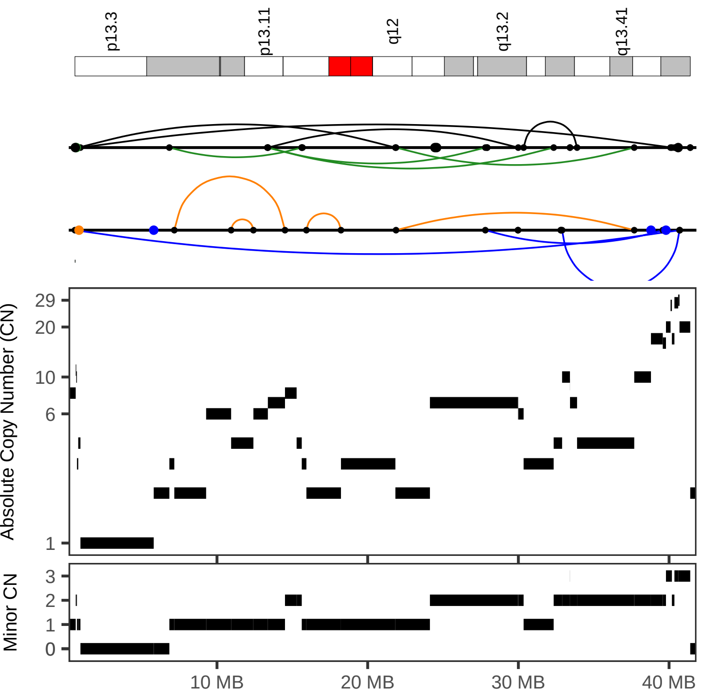

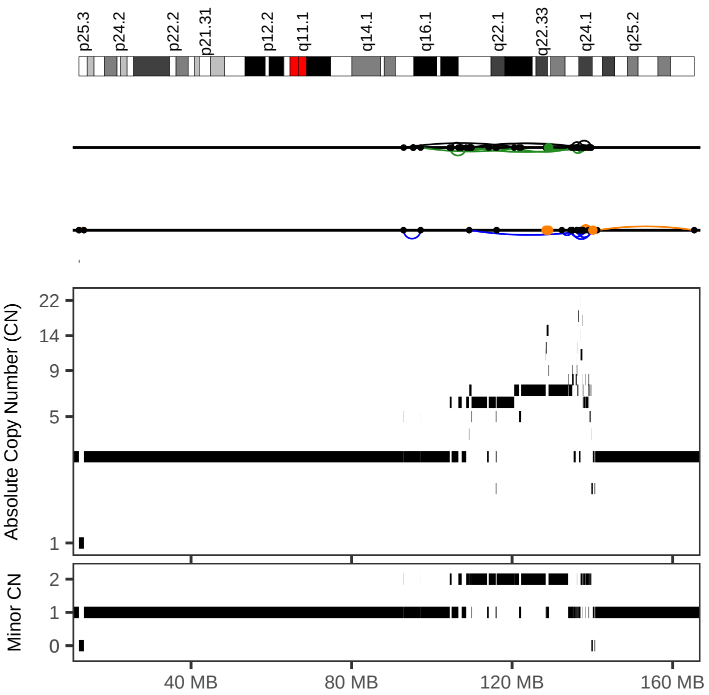

	ICGC_0108
Cancer type	Panc−AdenoCA
Position	19:793019−40693235
Type	With other complex events
Interleaved intrachr. SVs	14
Total SVs (intrachr. + transl.)	21
SV types	DEL: 2; DUP: 2; h2hINV: 5; t2tINV: 5; TRA: 7
SVs in sample	130
Oscillating CN (2 and 3 states)	4, 4
CN segments	34
FDR fragment joints	0.6776251
FDR chr. breakp. enrich.	0
Linked to chrs	
Purity, ploidy	0.31, 3.02

	ICGC_0114
Cancer type	Panc−AdenoCA
Position	6:92940256−139746601
Type	With other complex events
Interleaved intrachr. SVs	33
Total SVs (intrachr. + transl.)	36
SV types	DEL: 7; DUP: 7; h2hINV: 9; t2tINV: 10; TRA: 3
SVs in sample	195
Oscillating CN (2 and 3 states)	6, 14
CN segments	66
FDR fragment joints	0.9284301
FDR chr. breakp. enrich.	0
Linked to chrs	
Purity, ploidy	0.68, 2.73

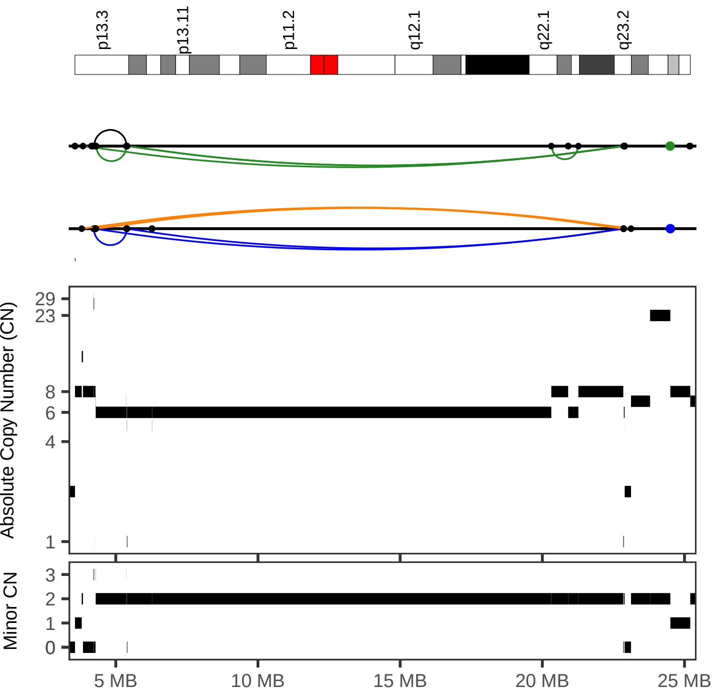

ICGC_0115	
Cancer type	Panc−AdenoCA
Position	16:3802028−23118217
Type	With other complex events
Interleaved intrachr. SVs	13
Total SVs (intrachr. + transl.)	13
SV types	DEL: 3; DUP: 3; h2hINV: 2; t2tINV: 5; TRA: 0
SVs in sample	104
Oscillating CN (2 and 3 states)	4, 5
CN segments	26
FDR fragment joints	0.7995907
FDR chr. breakp. enrich.	0
Linked to chrs	
Purity, ploidy	0.39, 3.3

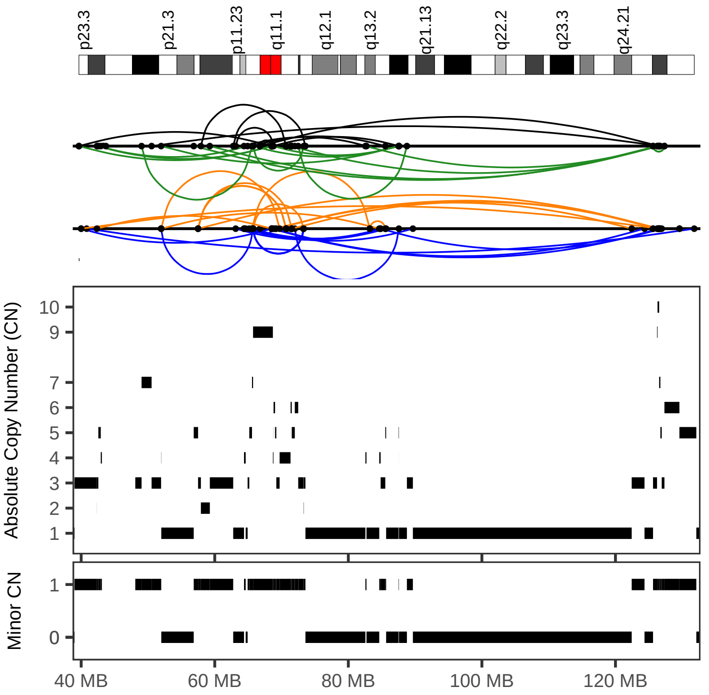

ICGC_0149	
Cancer type	Panc−AdenoCA
Position	8:39662139−131797224
Type	With other complex events
Interleaved intrachr. SVs	63
Total SVs (intrachr. + transl.)	63
SV types	DEL: 16; DUP: 18; h2hINV: 11; t2tINV: 18; TRA: 0
SVs in sample	238
Oscillating CN (2 and 3 states)	6, 7
CN segments	59
FDR fragment joints	0.6776251
FDR chr. breakp. enrich.	0
Linked to chrs	
Purity, ploidy	0.56, 1.87

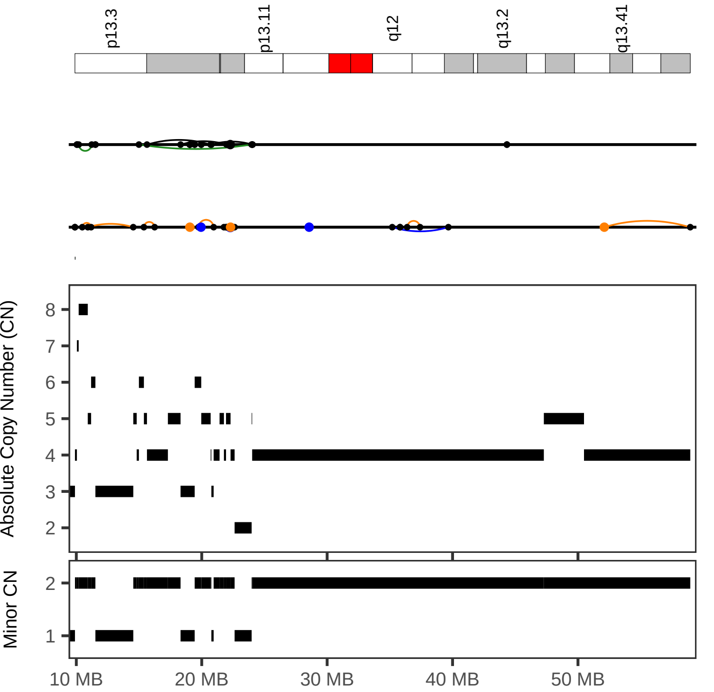

ICGC_0150	
Cancer type	Panc−AdenoCA
Position	19:14991068−24021988
Type	With other complex events
Interleaved intrachr. SVs	8
Total SVs (intrachr. + transl.)	12
SV types	DEL: 3; DUP: 1; h2hINV: 3; t2tINV: 1; TRA: 4
SVs in sample	115
Oscillating CN (2 and 3 states)	5, 10
CN segments	17
FDR fragment joints	0.6776251
FDR chr. breakp. enrich.	0
Linked to chrs	
Purity, ploidy	0.65, 3.71

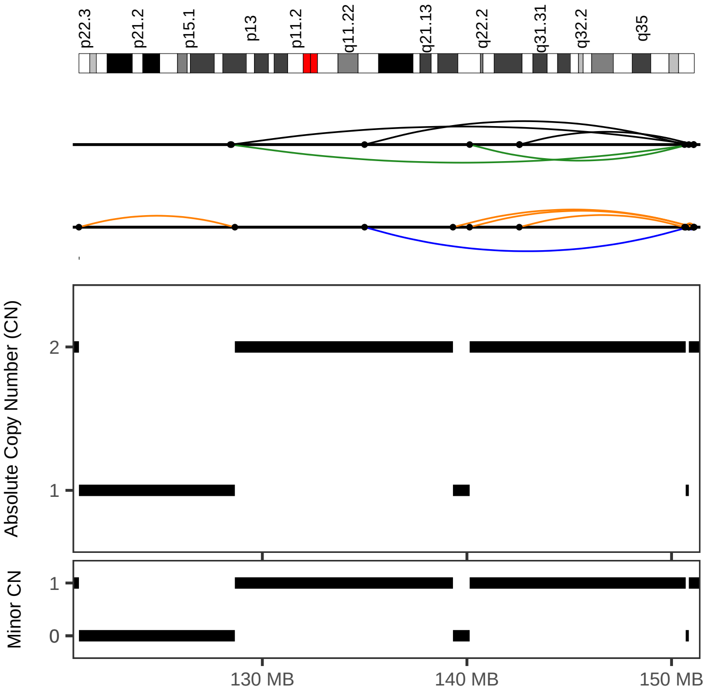

ICGC_0207	
Cancer type	Panc−AdenoCA
Position	7:121034912−151110416
Type	Canonical without polyploidization
Interleaved intrachr. SVs	12
Total SVs (intrachr. + transl.)	12
SV types	DEL: 5; DUP: 2; h2hINV: 3; t2tINV: 2; TRA: 0
SVs in sample	27
Oscillating CN (2 and 3 states)	6, 6
CN segments	6
FDR fragment joints	0.6776251
FDR chr. breakp. enrich.	0
Linked to chrs	
Purity, ploidy	0.61, 1.91

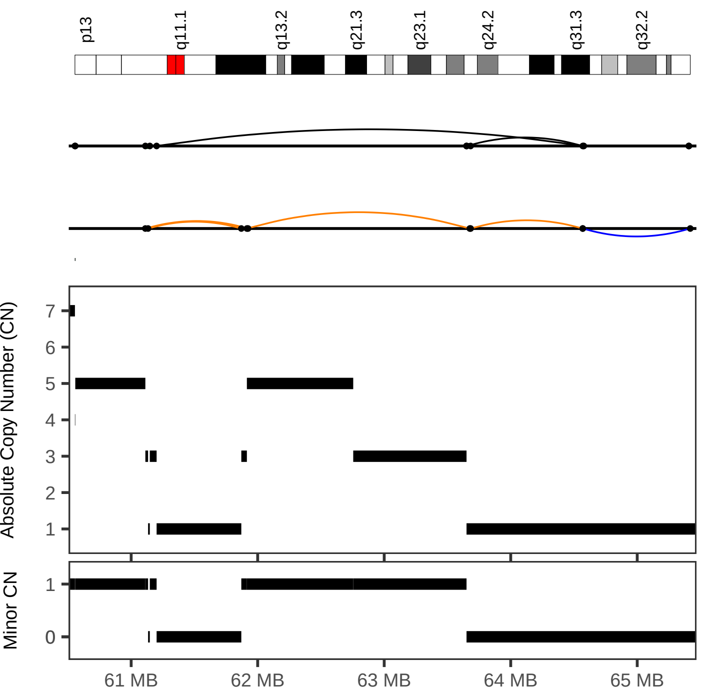

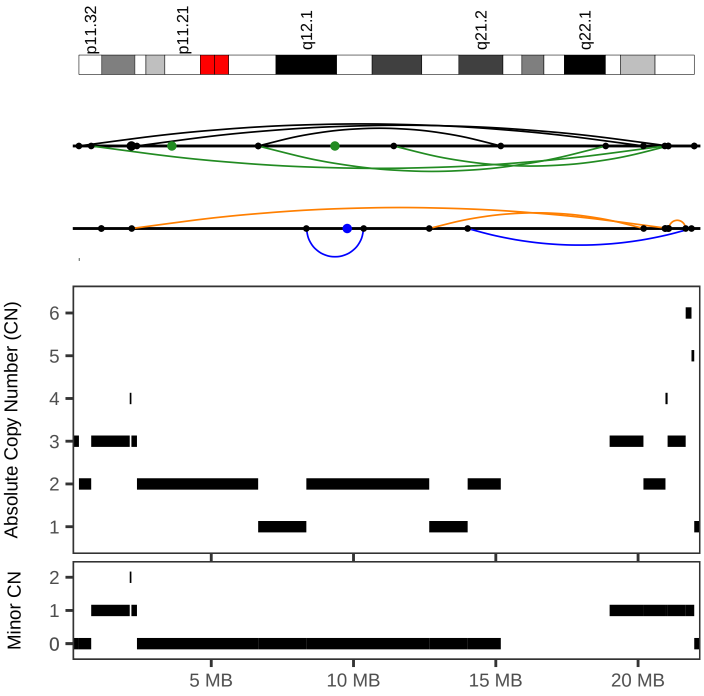

	**ICGC_0224**
Cancer type	Panc−AdenoCA
Position	14:61112011−65419128
Type	With other complex events
Interleaved intrachr. SVs	9
Total SVs (intrachr. + transl.)	9
SV types	DEL: 4; DUP: 1; h2hINV: 3; t2tINV: 1; TRA: 0
SVs in sample	76
Oscillating CN (2 and 3 states)	5, 9
CN segments	9
FDR fragment joints	0.615458
FDR chr. breakp. enrich.	0
Linked to chrs	
Purity, ploidy	0.77, 1.96

	**ICGC_0296**
Cancer type	Panc−AdenoCA
Position	18:350045−21877313
Type	With other complex events
Interleaved intrachr. SVs	9
Total SVs (intrachr. + transl.)	13
SV types	DEL: 2; DUP: 1; h2hINV: 3; t2tINV: 3; TRA: 4
SVs in sample	115
Oscillating CN (2 and 3 states)	5, 11
CN segments	15
FDR fragment joints	0.6776251
FDR chr. breakp. enrich.	0
Linked to chrs	
Purity, ploidy	0.54, 1.83

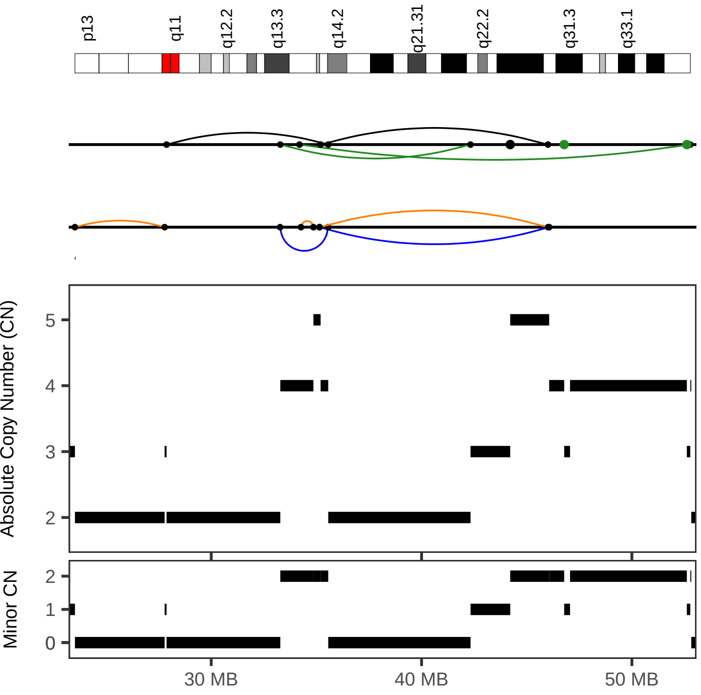

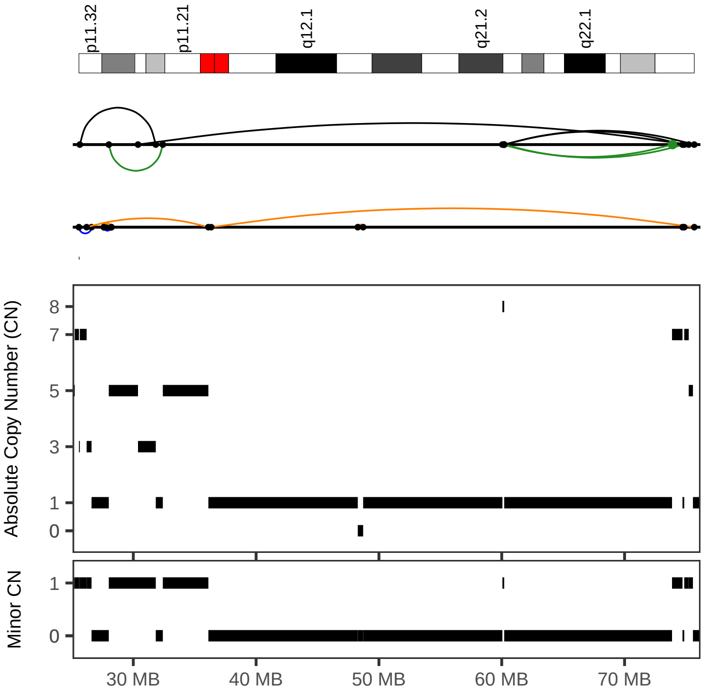

	**ICGC_0300**
Cancer type	Panc−AdenoCA
Position	13:27875998−52774140
Type	With other complex events
Interleaved intrachr. SVs	7
Total SVs (intrachr. + transl.)	10
SV types	DEL: 1; DUP: 2; h2hINV: 2; t2tINV: 2; TRA: 3
SVs in sample	68
Oscillating CN (2 and 3 states)	5, 5
CN segments	12
FDR fragment joints	0.6776251
FDR chr. breakp. enrich.	0
Linked to chrs	9:20973200−32637210;
Purity, ploidy	0.56, 2.02

	**ICGC_0301**
Cancer type	Panc−AdenoCA
Position	18:25572442−75675714
Type	With other complex events
Interleaved intrachr. SVs	15
Total SVs (intrachr. + transl.)	16
SV types	DEL: 4; DUP: 4; h2hINV: 4; t2tINV: 3; TRA: 1
SVs in sample	27
Oscillating CN (2 and 3 states)	4, 4
CN segments	18
FDR fragment joints	0.9501265
FDR chr. breakp. enrich.	0
Linked to chrs	
Purity, ploidy	0.61, 1.78

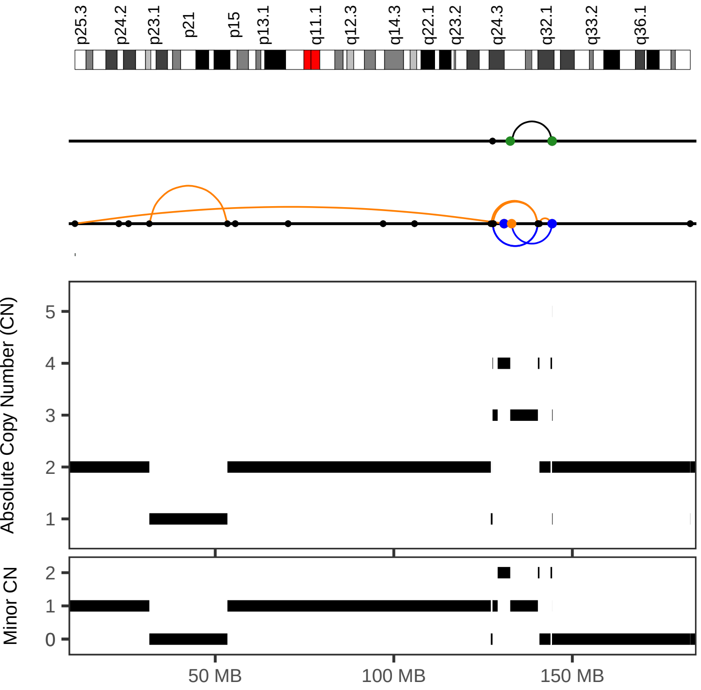

	**ICGC_0303**
Cancer type	Panc−AdenoCA
Position	2:10700671−144337304
Type	With other complex events
Interleaved intrachr. SVs	7
Total SVs (intrachr. + transl.)	14
SV types	DEL: 3; DUP: 3; h2hINV: 1; t2tINV: 0; TRA: 7
SVs in sample	132
Oscillating CN (2 and 3 states)	4, 6
CN segments	18
FDR fragment joints	0.615458
FDR chr. breakp. enrich.	0
Linked to chrs	1:109159140−230863765;
Purity, ploidy	0.58, 1.9

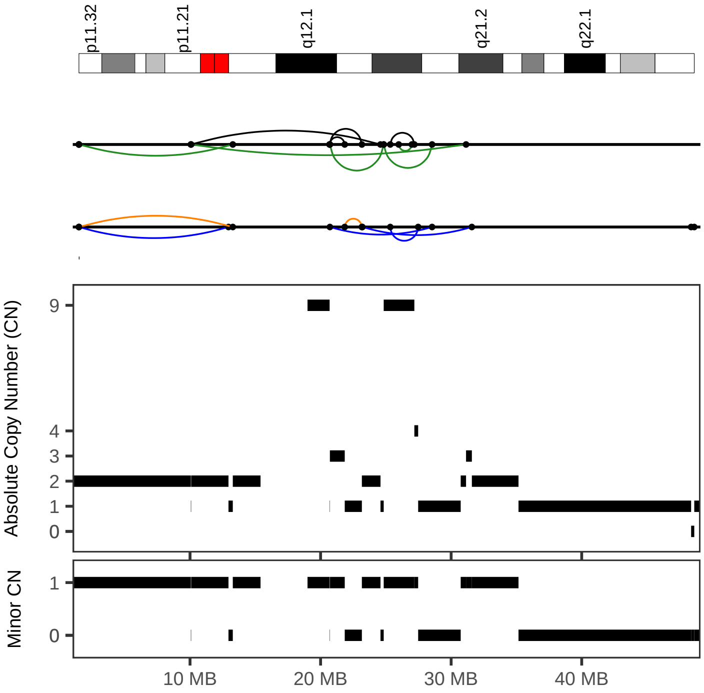

	**ICGC_0304**
Cancer type	Panc−AdenoCA
Position	18:1490133−31586695
Type	With other complex events
Interleaved intrachr. SVs	12
Total SVs (intrachr. + transl.)	12
SV types	DEL: 2; DUP: 3; h2hINV: 3; t2tINV: 4; TRA: 0
SVs in sample	55
Oscillating CN (2 and 3 states)	5, 5
CN segments	17
FDR fragment joints	0.9284301
FDR chr. breakp. enrich.	0
Linked to chrs	
Purity, ploidy	0.67, 1.96

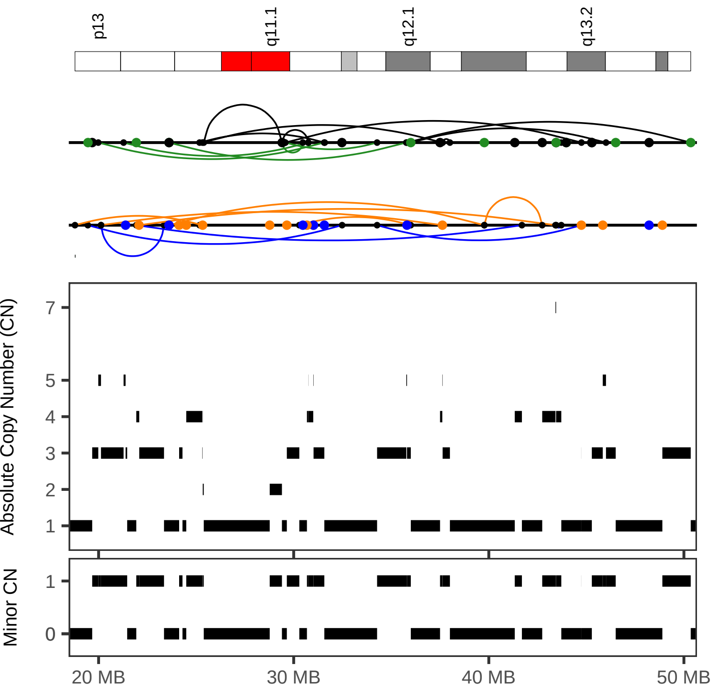

	**ICGC_0393**
Cancer type	Panc−AdenoCA
Position	22:18789020−50349275
Type	With other complex events
Interleaved intrachr. SVs	22
Total SVs (intrachr. + transl.)	62
SV types	DEL: 6; DUP: 5; h2hINV: 6; t2tINV: 5; TRA: 40
SVs in sample	158
Oscillating CN (2 and 3 states)	5, 7
CN segments	51
FDR fragment joints	0.8249489
FDR chr. breakp. enrich.	0
Linked to chrs	13:24905703−113852940;
Purity, ploidy	0.44, 2.08

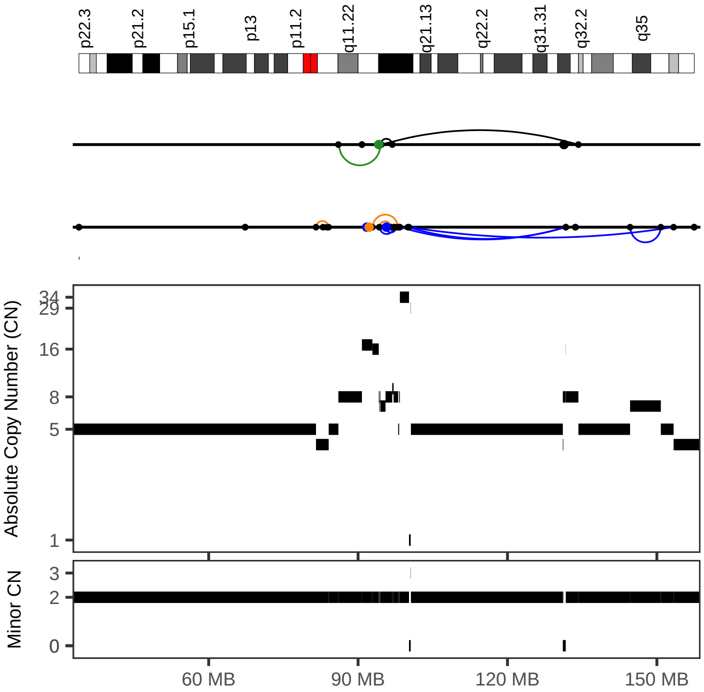

	**ICGC_0395**
Cancer type	Panc−AdenoCA
Position	7:86041901−153362571
Type	With other complex events
Interleaved intrachr. SVs	11
Total SVs (intrachr. + transl.)	16
SV types	DEL: 2; DUP: 6; h2hINV: 2; t2tINV: 1; TRA: 5
SVs in sample	87
Oscillating CN (2 and 3 states)	5, 5
CN segments	24
FDR fragment joints	0.615458
FDR chr. breakp. enrich.	0
Linked to chrs	
Purity, ploidy	0.43, 3.29

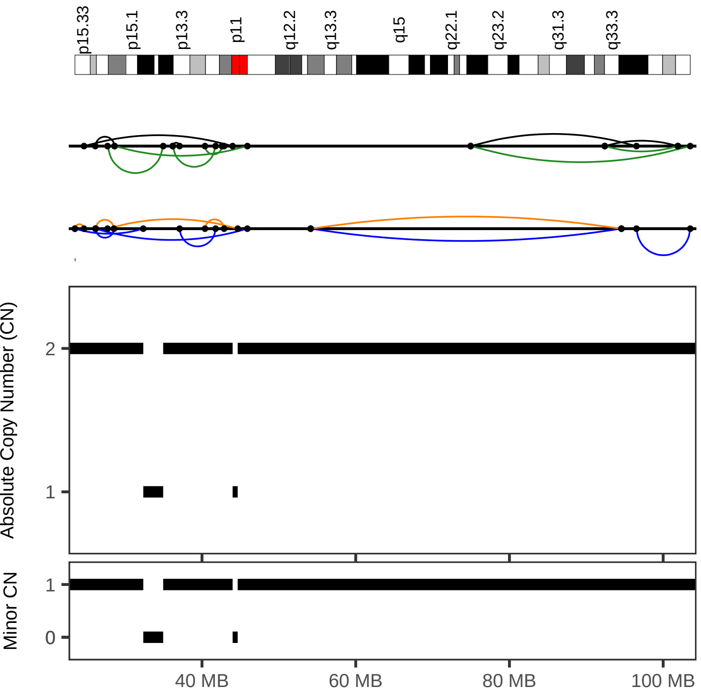

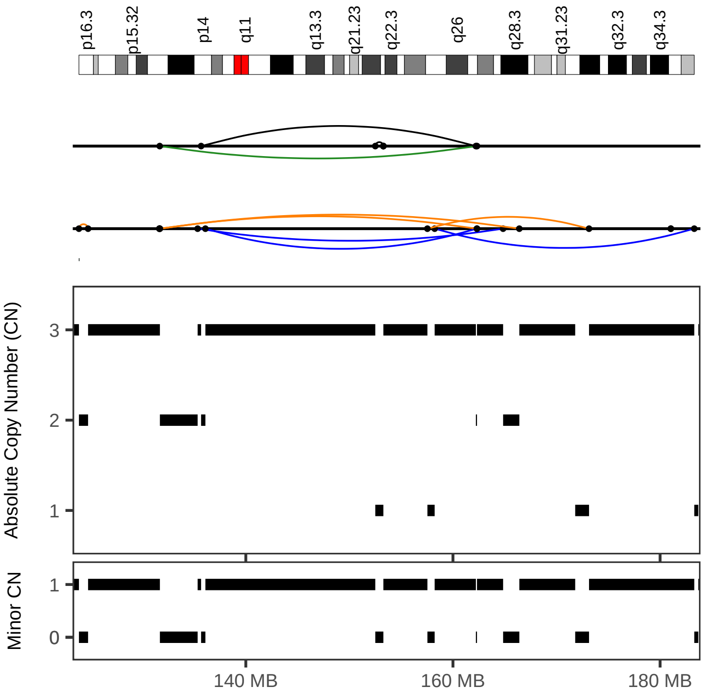

ICGC_0502

Cancer type	Panc−AdenoCA
Position	5:23468779−45905623
Type	Canonical without polyploidization
Interleaved intrachr. SVs	10
Total SVs (intrachr. + transl.)	10
SV types	DEL: 3; DUP: 3; h2hINV: 2; t2tINV: 2; TRA: 0
SVs in sample	120
Oscillating CN (2 and 3 states)	5, 5
CN segments	5
FDR fragment joints	0.9625775
FDR chr. breakp. enrich.	0
Linked to chrs	
Purity, ploidy	0.53, 1.85

ICGC_0521

Cancer type	Panc−AdenoCA
Position	4:131685289−183295953
Type	With other complex events
Interleaved intrachr. SVs	8
Total SVs (intrachr. + transl.)	8
SV types	DEL: 3; DUP: 3; h2hINV: 1; t2tINV: 1; TRA: 0
SVs in sample	107
Oscillating CN (2 and 3 states)	5, 16
CN segments	16
FDR fragment joints	0.6776251
FDR chr. breakp. enrich.	0.12
Linked to chrs	
Purity, ploidy	0.57, 3.43

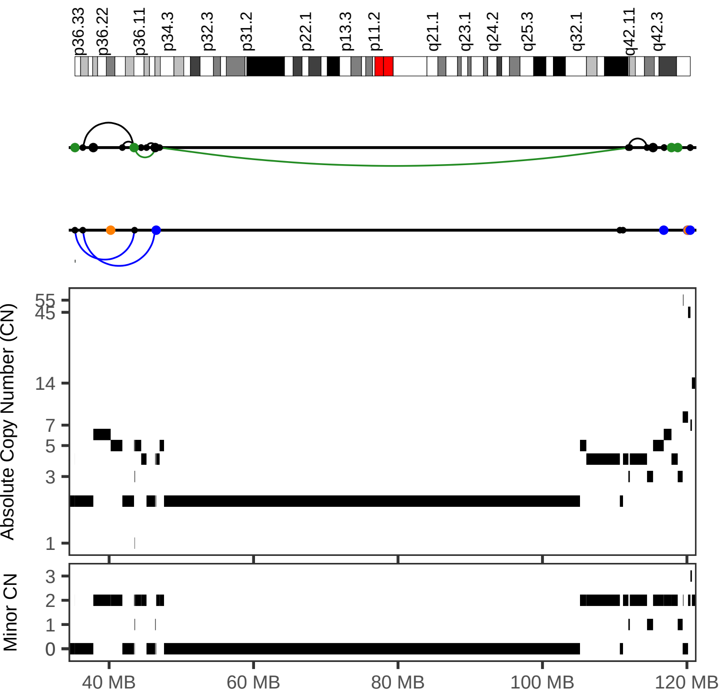

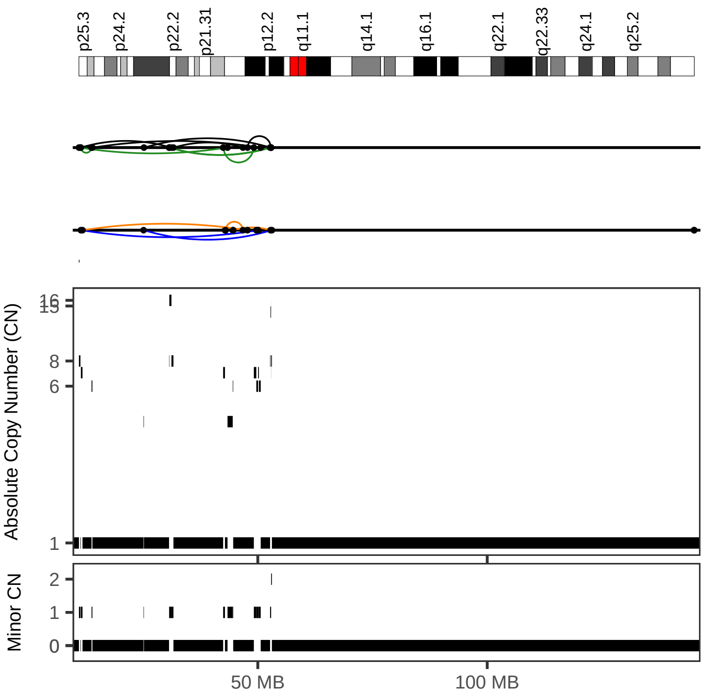

ICGC_0522

Cancer type	Panc−AdenoCA
Position	1:35264067−46448813
Type	With other complex events
Interleaved intrachr. SVs	7
Total SVs (intrachr. + transl.)	12
SV types	DEL: 0; DUP: 2; h2hINV: 3; t2tINV: 2; TRA: 5
SVs in sample	80
Oscillating CN (2 and 3 states)	4, 6
CN segments	13
FDR fragment joints	0.615458
FDR chr. breakp. enrich.	0
Linked to chrs	8:12160765−82189365;
Purity, ploidy	0.4, 3.42

ICGC_0526

Cancer type	Panc−AdenoCA
Position	6:11005830−53074096
Type	With other complex events
Interleaved intrachr. SVs	16
Total SVs (intrachr. + transl.)	16
SV types	DEL: 3; DUP: 3; h2hINV: 5; t2tINV: 5; TRA: 0
SVs in sample	127
Oscillating CN (2 and 3 states)	4, 6
CN segments	28
FDR fragment joints	0.8653243
FDR chr. breakp. enrich.	0.01
Linked to chrs	
Purity, ploidy	0.54, 1.78

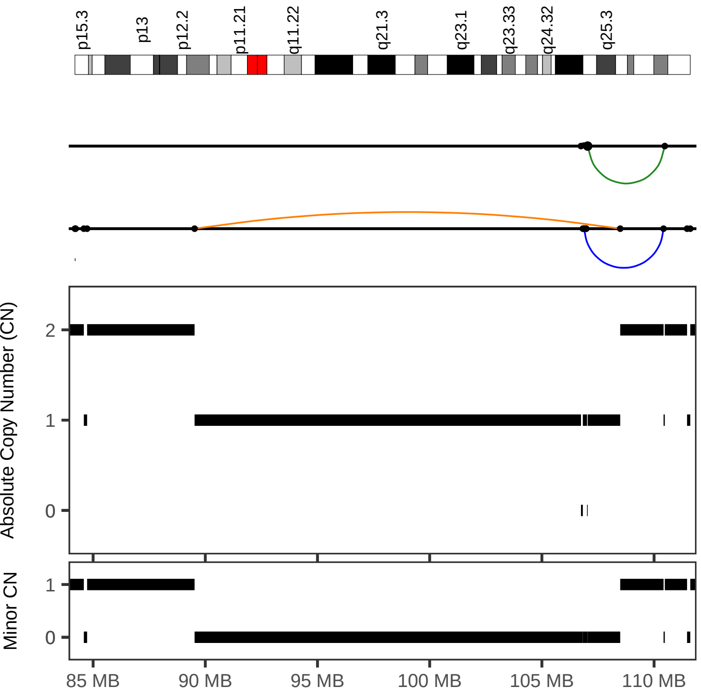

	ICGC_0535
Cancer type	Panc−AdenoCA
Position	10:89522467−110477428
Type	Canonical without polyploidization
Interleaved intrachr. SVs	6
Total SVs (intrachr. + transl.)	7
SV types	DEL: 2; DUP: 2; h2hINV: 1; t2tINV: 1; TRA: 1
SVs in sample	102
Oscillating CN (2 and 3 states)	5, 8
CN segments	8
FDR fragment joints	0.9599662
FDR chr. breakp. enrich.	0.1
Linked to chrs	
Purity, ploidy	0.46, 1.85

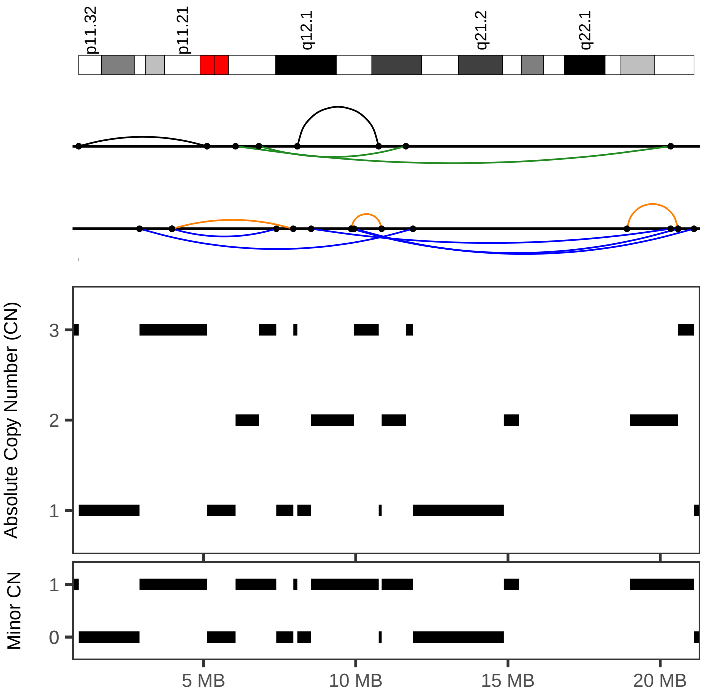

	ICGC_0535
Cancer type	Panc−AdenoCA
Position	18:896558−21110916
Type	With other complex events
Interleaved intrachr. SVs	12
Total SVs (intrachr. + transl.)	12
SV types	DEL: 3; DUP: 5; h2hINV: 2; t2tINV: 2; TRA: 0
SVs in sample	102
Oscillating CN (2 and 3 states)	4, 7
CN segments	18
FDR fragment joints	0.6776251
FDR chr. breakp. enrich.	0
Linked to chrs	
Purity, ploidy	0.46, 1.85

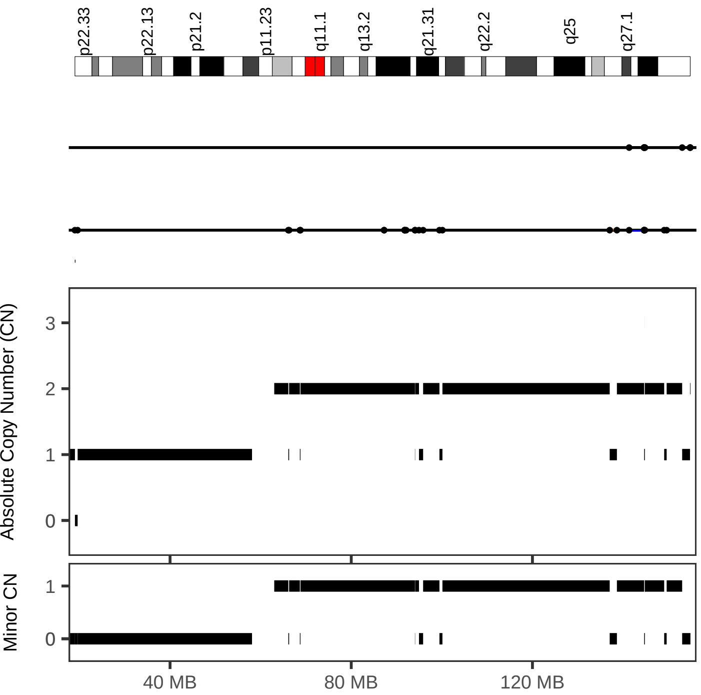

	ICGC_0535
Cancer type	Panc−AdenoCA
Position	X:141345603−144825631
Type	Canonical without polyploidization
Interleaved intrachr. SVs	6
Total SVs (intrachr. + transl.)	6
SV types	DEL: 1; DUP: 2; h2hINV: 1; t2tINV: 2; TRA: 0
SVs in sample	102
Oscillating CN (2 and 3 states)	5, 7
CN segments	7
FDR fragment joints	0.9284301
FDR chr. breakp. enrich.	0
Linked to chrs	
Purity, ploidy	0.46, 1.85

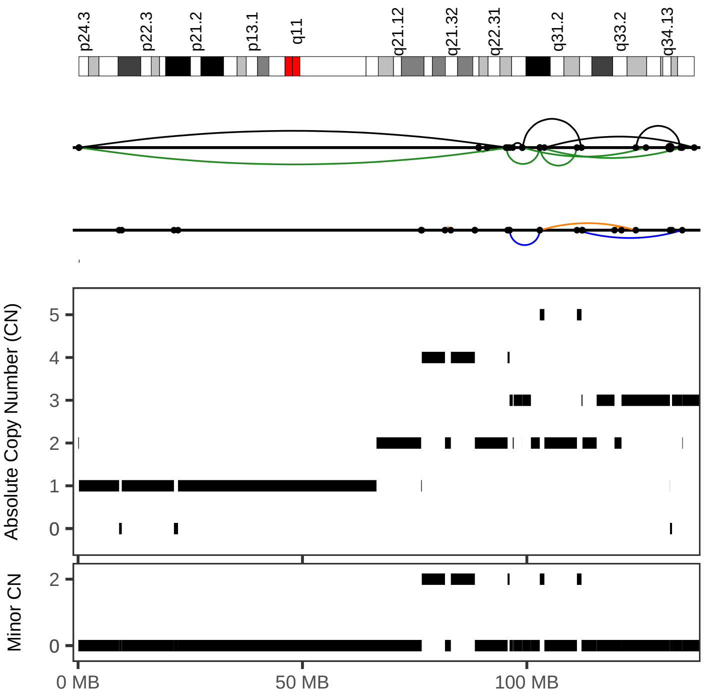

	ICGC_0543
Cancer type	Panc−AdenoCA
Position	9:187480−137300565
Type	With other complex events
Interleaved intrachr. SVs	14
Total SVs (intrachr. + transl.)	16
SV types	DEL: 2; DUP: 2; h2hINV: 5; t2tINV: 5; TRA: 2
SVs in sample	161
Oscillating CN (2 and 3 states)	6, 10
CN segments	32
FDR fragment joints	0.615458
FDR chr. breakp. enrich.	0
Linked to chrs	7:28956141−31357723;
Purity, ploidy	0.53, 1.89

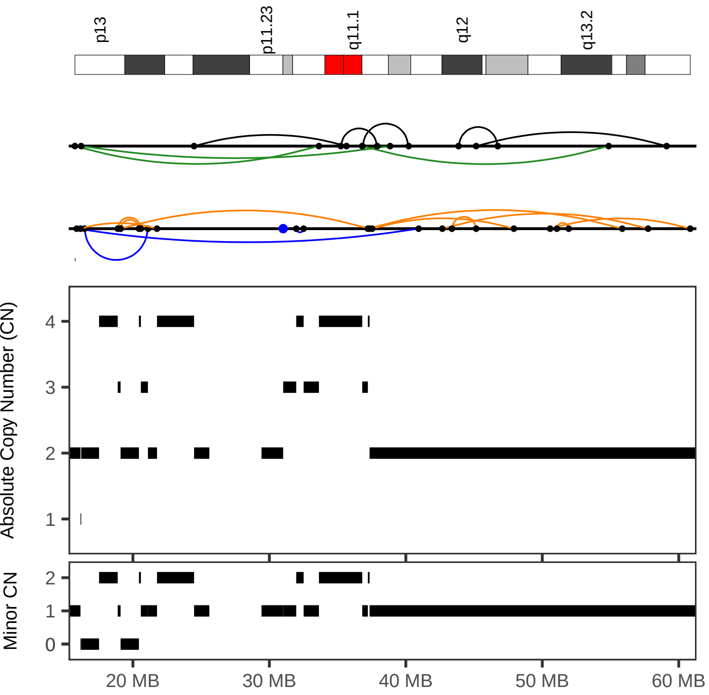

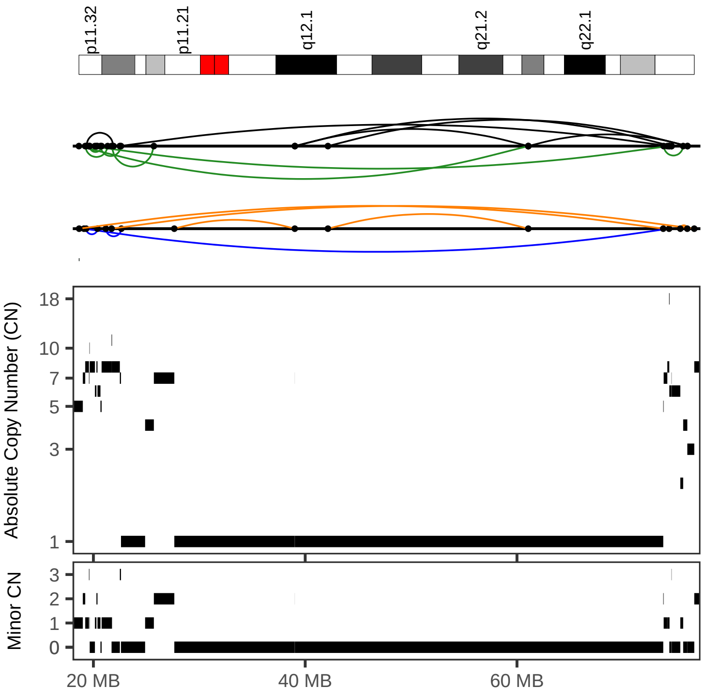

	**ICGC_0543**
Cancer type	Panc−AdenoCA
Position	20:15750921−60842706
Type	With other complex events
Interleaved intrachr. SVs	18
Total SVs (intrachr. + transl.)	19
SV types	DEL: 8; DUP: 2; h2hINV: 5; t2tINV: 3; TRA: 1
SVs in sample	161
Oscillating CN (2 and 3 states)	6, 7
CN segments	19
FDR fragment joints	0.615458
FDR chr. breakp. enrich.	0
Linked to chrs	
Purity, ploidy	0.53, 1.89

	**PCSI_0001**
Cancer type	Panc−AdenoCA
Position	18:18631561−76740272
Type	With other complex events
Interleaved intrachr. SVs	22
Total SVs (intrachr. + transl.)	22
SV types	DEL: 4; DUP: 3; h2hINV: 6; t2tINV: 9; TRA: 0
SVs in sample	37
Oscillating CN (2 and 3 states)	4, 5
CN segments	32
FDR fragment joints	0.615458
FDR chr. breakp. enrich.	0
Linked to chrs	
Purity, ploidy	0.15, 1.99

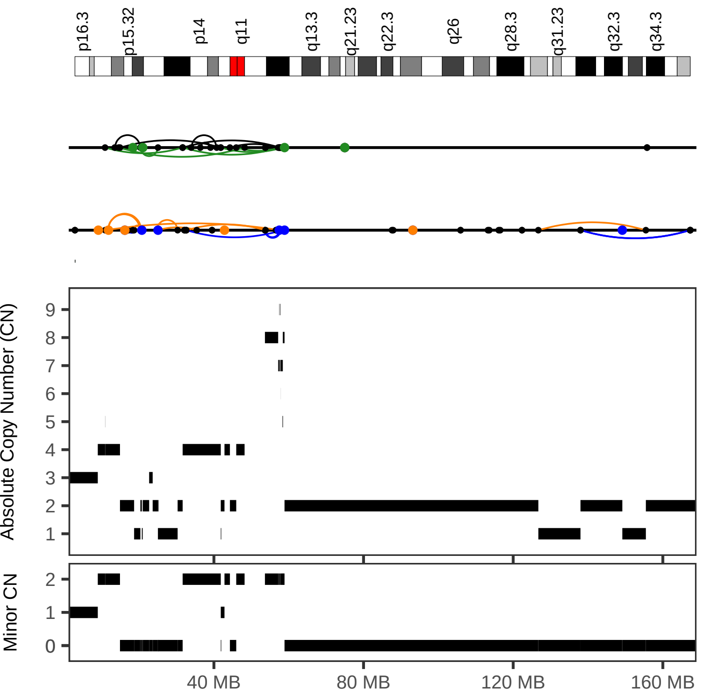

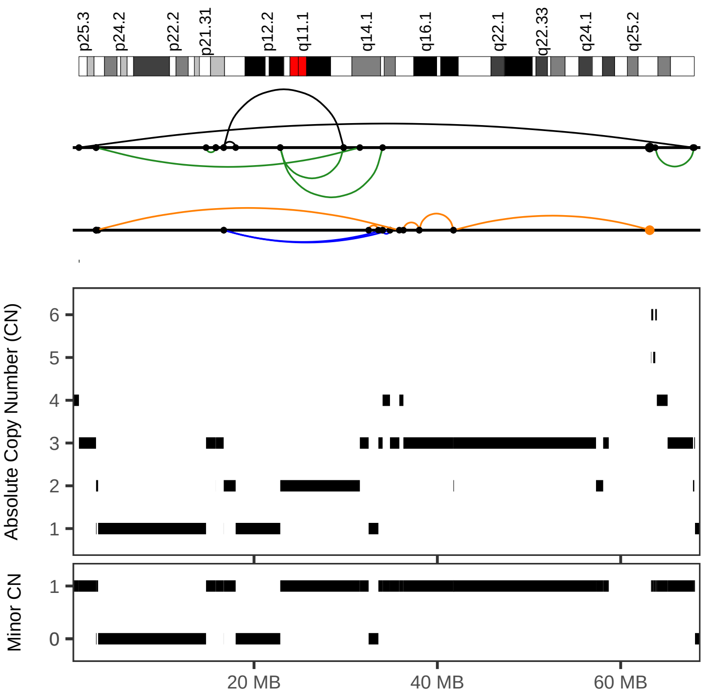

	**PCSI_0015**
Cancer type	Panc−AdenoCA
Position	4:10893126−58422207
Type	With other complex events
Interleaved intrachr. SVs	26
Total SVs (intrachr. + transl.)	35
SV types	DEL: 9; DUP: 5; h2hINV: 6; t2tINV: 6; TRA: 9
SVs in sample	541
Oscillating CN (2 and 3 states)	5, 7
CN segments	29
FDR fragment joints	0.6776251
FDR chr. breakp. enrich.	0
Linked to chrs	8:100785898−143451879;
Purity, ploidy	0.94, 2.58

	**PCSI_0044**
Cancer type	Panc−AdenoCA
Position	6:2763282−35849850
Type	With other complex events
Interleaved intrachr. SVs	10
Total SVs (intrachr. + transl.)	10
SV types	DEL: 1; DUP: 4; h2hINV: 2; t2tINV: 3; TRA: 0
SVs in sample	98
Oscillating CN (2 and 3 states)	5, 8
CN segments	18
FDR fragment joints	0.6776251
FDR chr. breakp. enrich.	0
Linked to chrs	
Purity, ploidy	0.68, 1.87

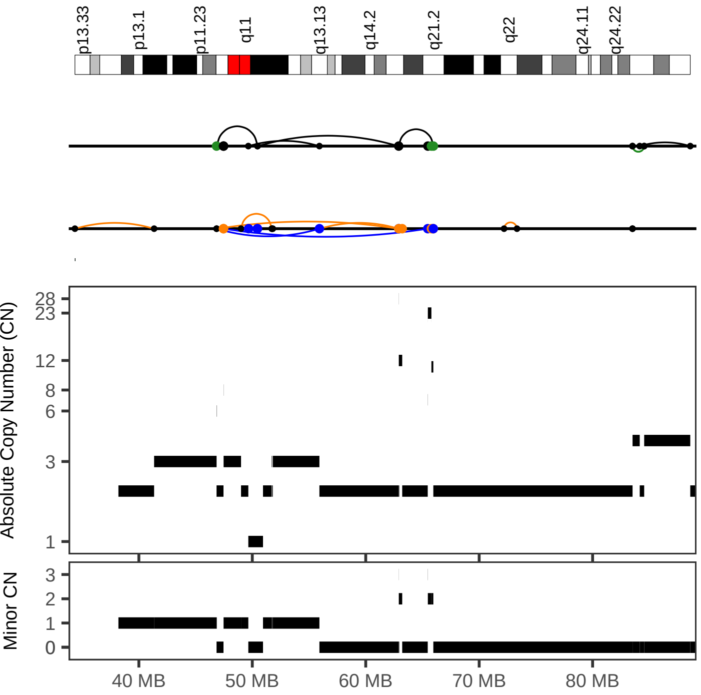

	PCSI_0044
Cancer type	Panc−AdenoCA
Position	12:46852610−65968411
Type	With other complex events
Interleaved intrachr. SVs	9
Total SVs (intrachr. + transl.)	28
SV types	DEL: 3; DUP: 2; h2hINV: 4; t2tINV: 0; TRA: 19
SVs in sample	98
Oscillating CN (2 and 3 states)	5, 8
CN segments	22
FDR fragment joints	0.6776251
FDR chr. breakp. enrich.	0
Linked to chrs	18:42542834−54212828;
Purity, ploidy	0.68, 1.87

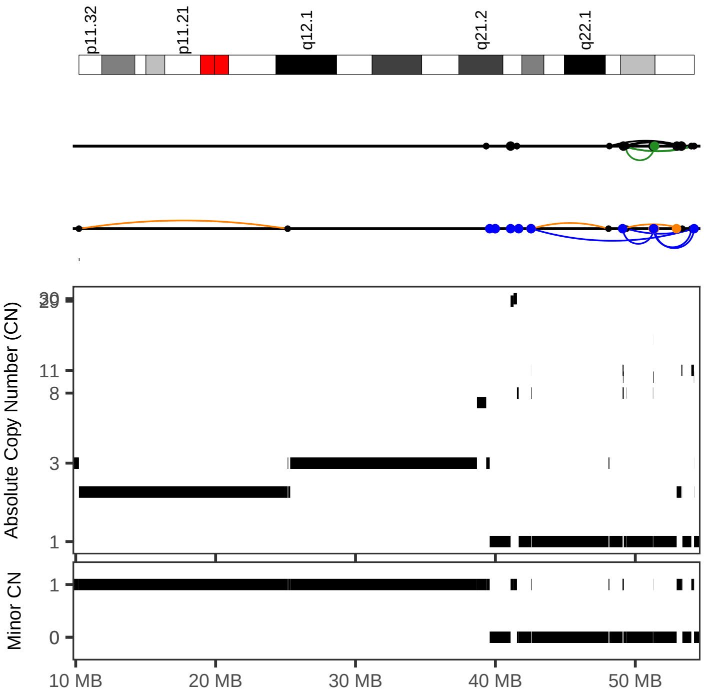

	PCSI_0044
Cancer type	Panc−AdenoCA
Position	18:42542834−54212829
Type	With other complex events
Interleaved intrachr. SVs	15
Total SVs (intrachr. + transl.)	28
SV types	DEL: 1; DUP: 5; h2hINV: 5; t2tINV: 4; TRA: 13
SVs in sample	98
Oscillating CN (2 and 3 states)	6, 10
CN segments	30
FDR fragment joints	0.6776251
FDR chr. breakp. enrich.	0
Linked to chrs	12:46852610−65968410;
Purity, ploidy	0.68, 1.87

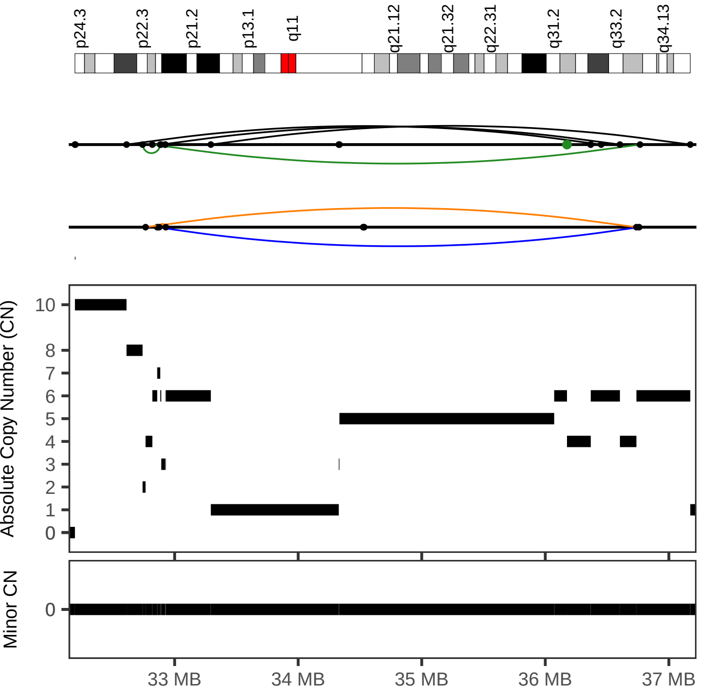

	PCSI_0077
Cancer type	Panc−AdenoCA
Position	9:32610603−37173324
Type	With other complex events
Interleaved intrachr. SVs	8
Total SVs (intrachr. + transl.)	10
SV types	DEL: 2; DUP: 1; h2hINV: 3; t2tINV: 2; TRA: 2
SVs in sample	74
Oscillating CN (2 and 3 states)	5, 6
CN segments	19
FDR fragment joints	0.615458
FDR chr. breakp. enrich.	0
Linked to chrs	
Purity, ploidy	0.56, 1.81

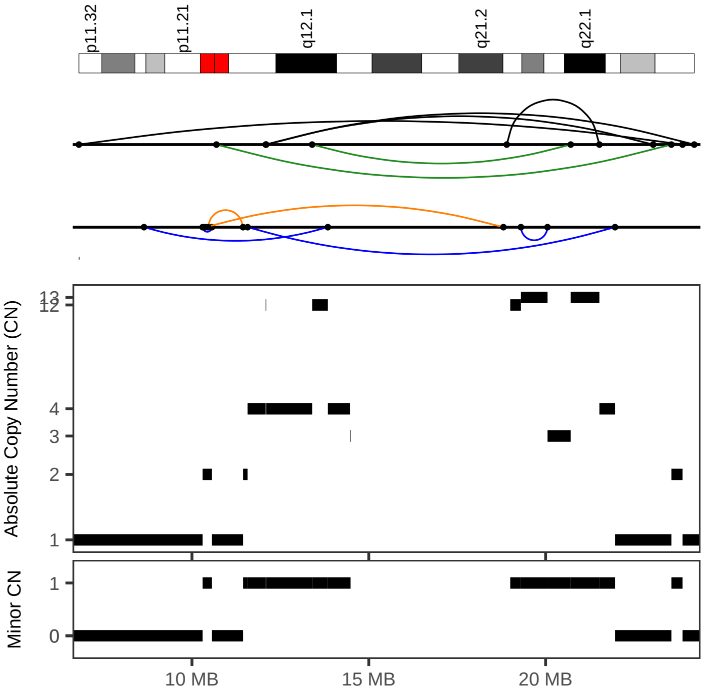

	PCSI_0077
Cancer type	Panc−AdenoCA
Position	18:6803844−24203012
Type	With other complex events
Interleaved intrachr. SVs	11
Total SVs (intrachr. + transl.)	11
SV types	DEL: 2; DUP: 3; h2hINV: 4; t2tINV: 2; TRA: 0
SVs in sample	74
Oscillating CN (2 and 3 states)	5, 6
CN segments	18
FDR fragment joints	0.8653243
FDR chr. breakp. enrich.	0
Linked to chrs	
Purity, ploidy	0.56, 1.81

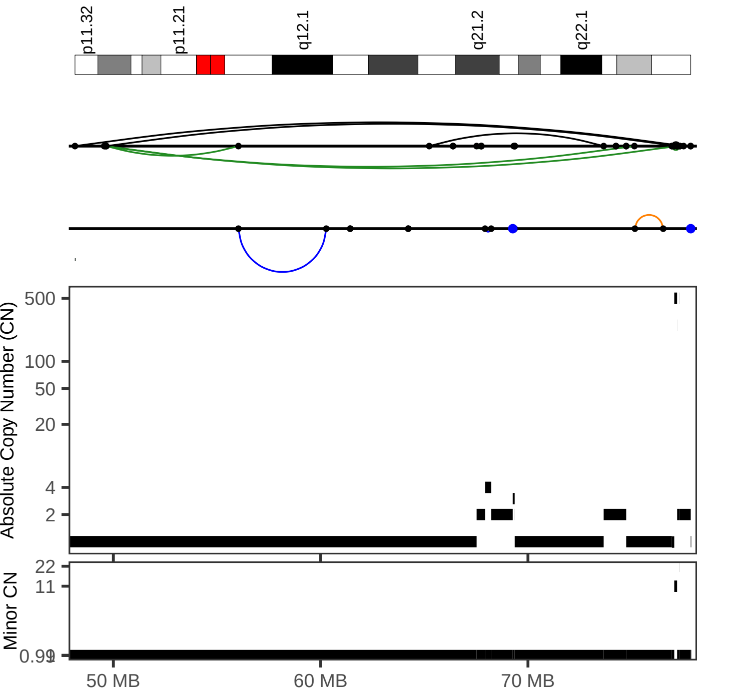

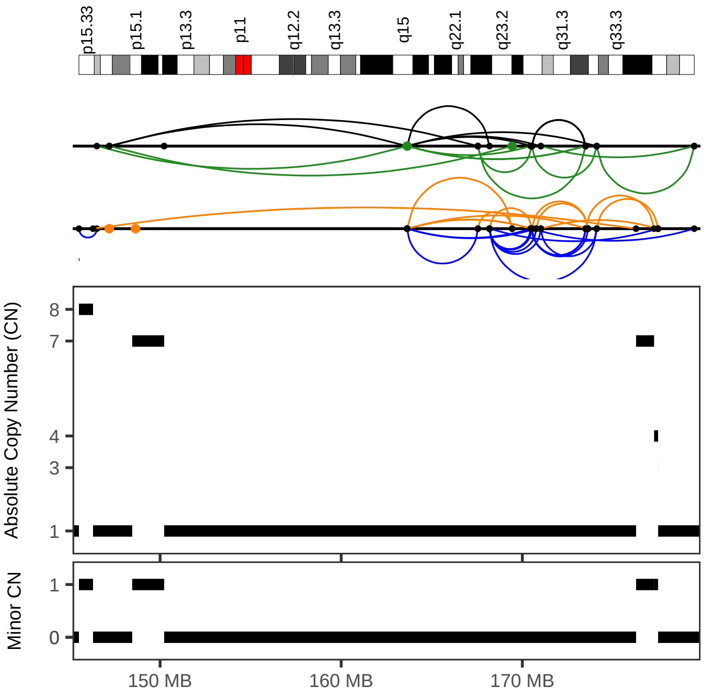

	PCSI_0078
Cancer type	Panc−AdenoCA
Position	18:49556708−77332270
Type	With other complex events
Interleaved intrachr. SVs	7
Total SVs (intrachr. + transl.)	8
SV types	DEL: 0; DUP: 1; h2hINV: 2; t2tINV: 4; TRA: 1
SVs in sample	101
Oscillating CN (2 and 3 states)	4, 7
CN segments	15
FDR fragment joints	0.615458
FDR chr. breakp. enrich.	0
Linked to chrs	
Purity, ploidy	0.7, 2.14

	PCSI_0081
Cancer type	Panc−AdenoCA
Position	5:145519578−179491764
Type	With other complex events
Interleaved intrachr. SVs	50
Total SVs (intrachr. + transl.)	54
SV types	DEL: 14; DUP: 17; h2hINV: 9; t2tINV: 10; TRA: 4
SVs in sample	195
Oscillating CN (2 and 3 states)	4, 5
CN segments	8
FDR fragment joints	0.6776251
FDR chr. breakp. enrich.	0
Linked to chrs	19:33279297−57149842;
Purity, ploidy	0.85, 1.65

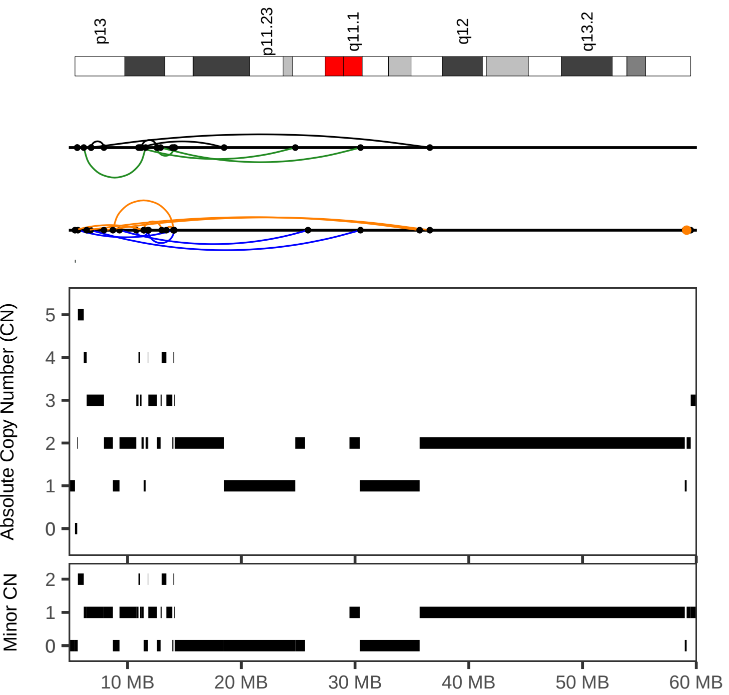

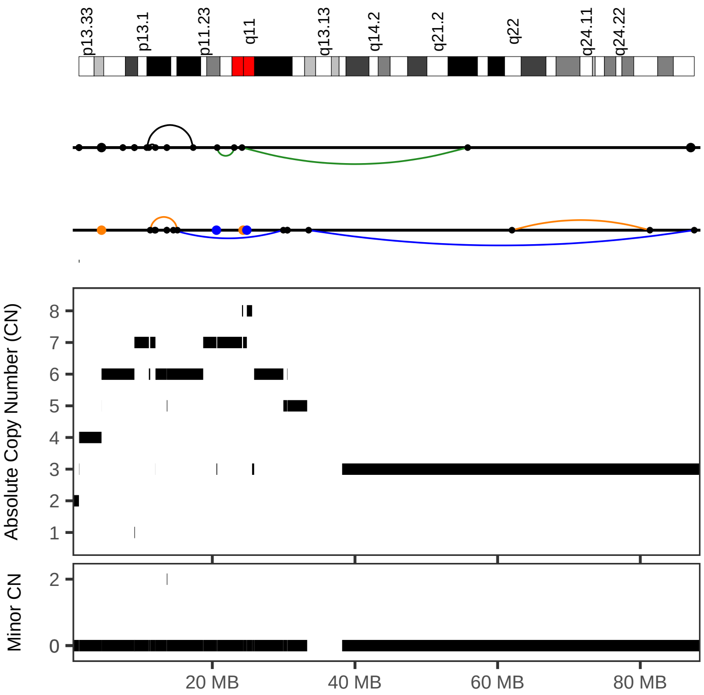

	PCSI_0081
Cancer type	Panc−AdenoCA
Position	20:5377429−36576271
Type	With other complex events
Interleaved intrachr. SVs	20
Total SVs (intrachr. + transl.)	20
SV types	DEL: 6; DUP: 5; h2hINV: 5; t2tINV: 4; TRA: 0
SVs in sample	195
Oscillating CN (2 and 3 states)	5, 13
CN segments	31
FDR fragment joints	0.9625775
FDR chr. breakp. enrich.	0
Linked to chrs	
Purity, ploidy	0.85, 1.65

	PCSI_0082
Cancer type	Panc−AdenoCA
Position	12:10809610−87566605
Type	With other complex events
Interleaved intrachr. SVs	7
Total SVs (intrachr. + transl.)	11
SV types	DEL: 2; DUP: 2; h2hINV: 2; t2tINV: 1; TRA: 4
SVs in sample	160
Oscillating CN (2 and 3 states)	4, 6
CN segments	19
FDR fragment joints	0.8653243
FDR chr. breakp. enrich.	0
Linked to chrs	
Purity, ploidy	0.67, 2.94

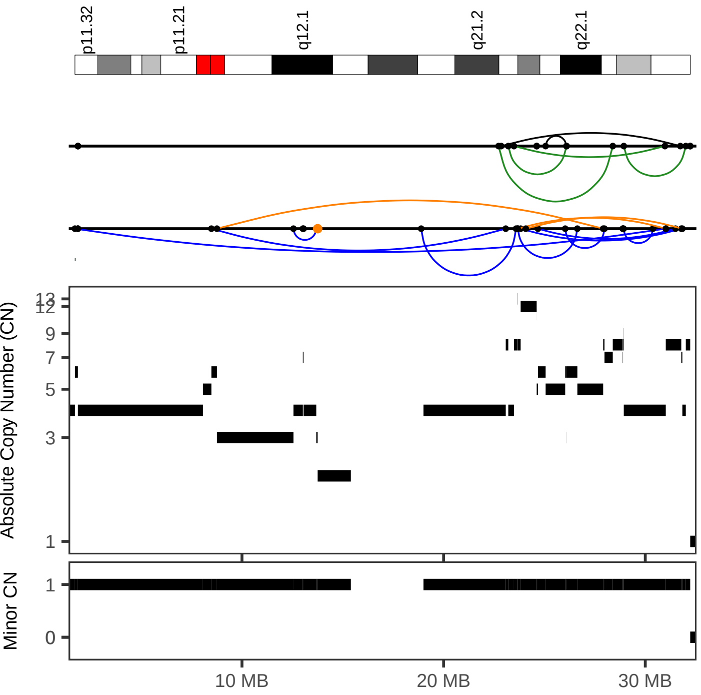

PCSI_0082	
Cancer type	Panc−AdenoCA
Position	18:1716536−32009794
Type	With other complex events
Interleaved intrachr. SVs	17
Total SVs (intrachr. + transl.)	18
SV types	DEL: 3; DUP: 8; h2hINV: 2; t2tINV: 4; TRA: 1
SVs in sample	160
Oscillating CN (2 and 3 states)	5, 13
CN segments	36
FDR fragment joints	0.615458
FDR chr. breakp. enrich.	0
Linked to chrs	
Purity, ploidy	0.67, 2.94

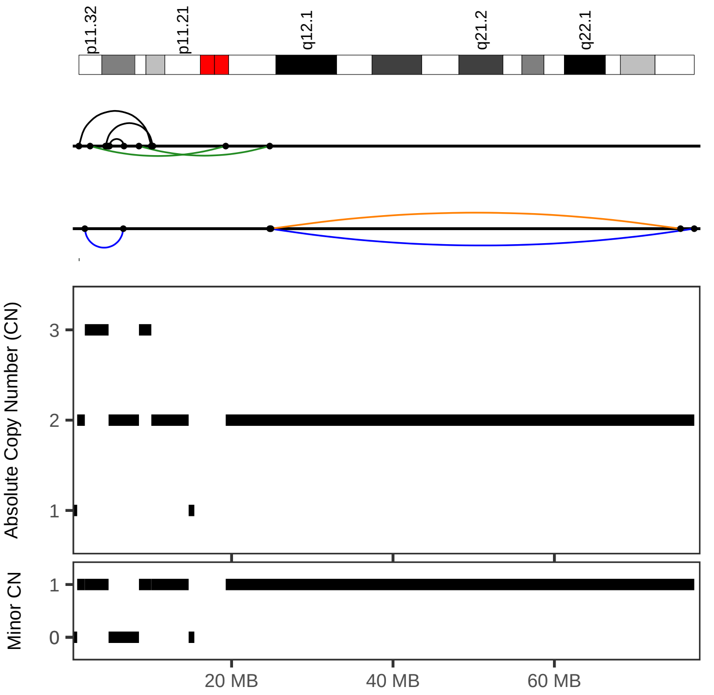

PCSI_0086	
Cancer type	Panc−AdenoCA
Position	18:1082109−77329138
Type	Canonical without polyploidization
Interleaved intrachr. SVs	7
Total SVs (intrachr. + transl.)	7
SV types	DEL: 0; DUP: 2; h2hINV: 3; t2tINV: 2; TRA: 0
SVs in sample	23
Oscillating CN (2 and 3 states)	5, 7
CN segments	7
FDR fragment joints	0.6776251
FDR chr. breakp. enrich.	0
Linked to chrs	
Purity, ploidy	0.55, 1.88

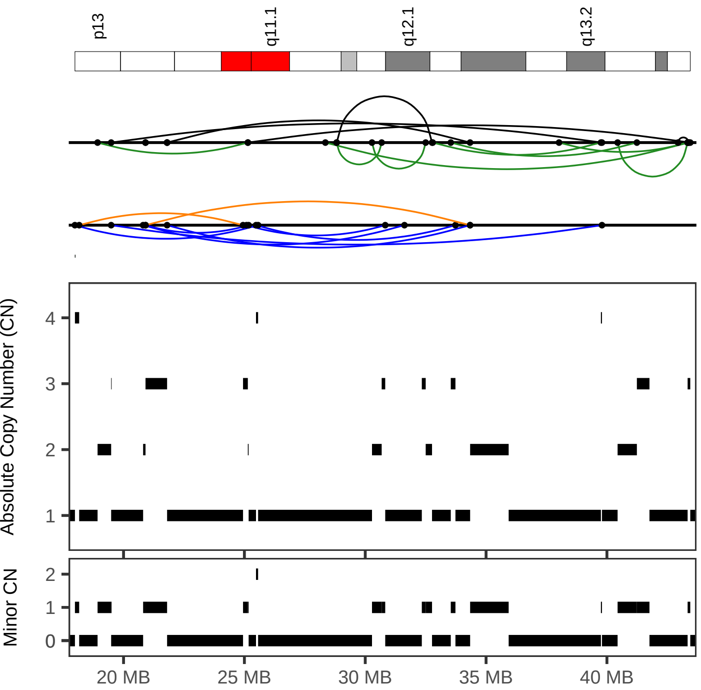

PCSI_0101	
Cancer type	Panc−AdenoCA
Position	22:17987673−43447488
Type	With other complex events
Interleaved intrachr. SVs	22
Total SVs (intrachr. + transl.)	22
SV types	DEL: 2; DUP: 7; h2hINV: 5; t2tINV: 8; TRA: 0
SVs in sample	202
Oscillating CN (2 and 3 states)	4, 7
CN segments	30
FDR fragment joints	0.615458
FDR chr. breakp. enrich.	0
Linked to chrs	
Purity, ploidy	0.21, 1.76

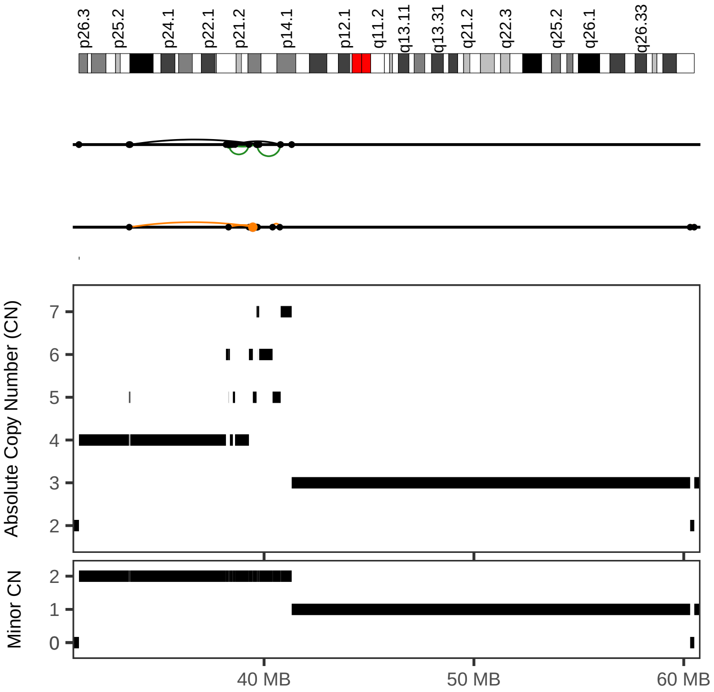

PCSI_0105	
Cancer type	Panc−AdenoCA
Position	3:33556195−40791777
Type	With other complex events
Interleaved intrachr. SVs	11
Total SVs (intrachr. + transl.)	12
SV types	DEL: 2; DUP: 1; h2hINV: 3; t2tINV: 5; TRA: 1
SVs in sample	91
Oscillating CN (2 and 3 states)	4, 16
CN segments	18
FDR fragment joints	0.6776251
FDR chr. breakp. enrich.	0
Linked to chrs	
Purity, ploidy	0.56, 3.69

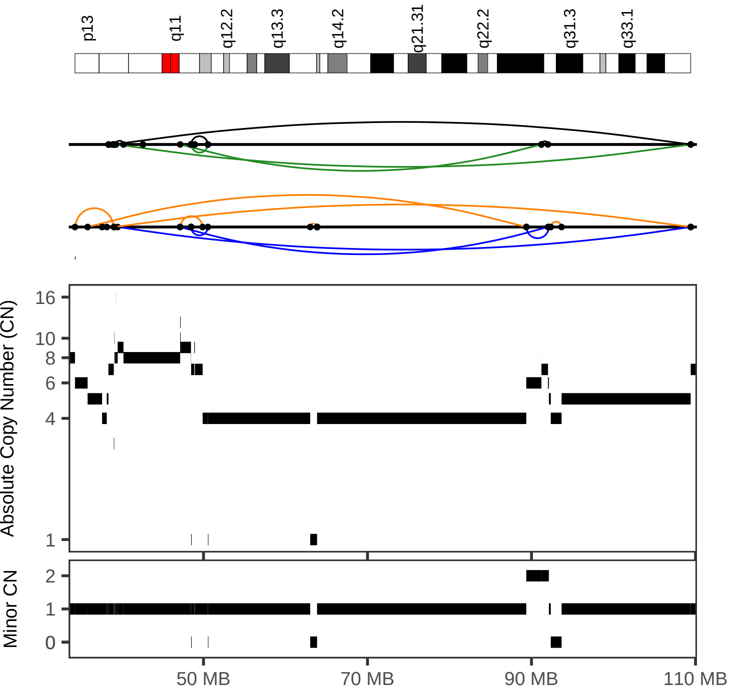

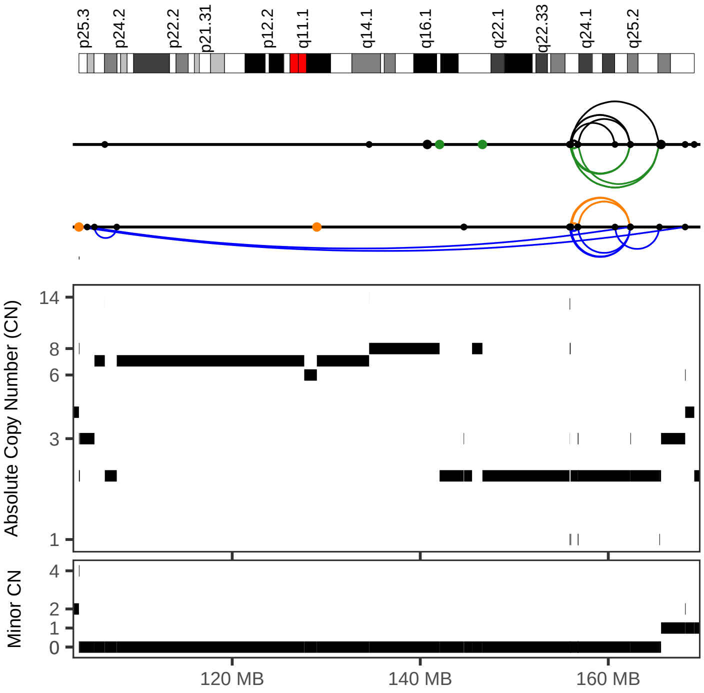

	**PCSI_0105**
Cancer type	Panc−AdenoCA
Position	13:34326449−109417492
Type	With other complex events
Interleaved intrachr. SVs	12
Total SVs (intrachr. + transl.)	12
SV types	DEL: 3; DUP: 3; h2hINV: 2; t2tINV: 4; TRA: 0
SVs in sample	91
Oscillating CN (2 and 3 states)	5, 5
CN segments	34
FDR fragment joints	0.9284301
FDR chr. breakp. enrich.	0
Linked to chrs	
Purity, ploidy	0.56, 3.69

	**PCSI_0111**
Cancer type	Panc−AdenoCA
Position	6:104571922−165438064
Type	With other complex events
Interleaved intrachr. SVs	42
Total SVs (intrachr. + transl.)	46
SV types	DEL: 10; DUP: 10; h2hINV: 11; t2tINV: 11; TRA: 4
SVs in sample	260
Oscillating CN (2 and 3 states)	6, 11
CN segments	37
FDR fragment joints	0.9599662
FDR chr. breakp. enrich.	0
Linked to chrs	3:35128015−182834758;
Purity, ploidy	0.81, 3.09

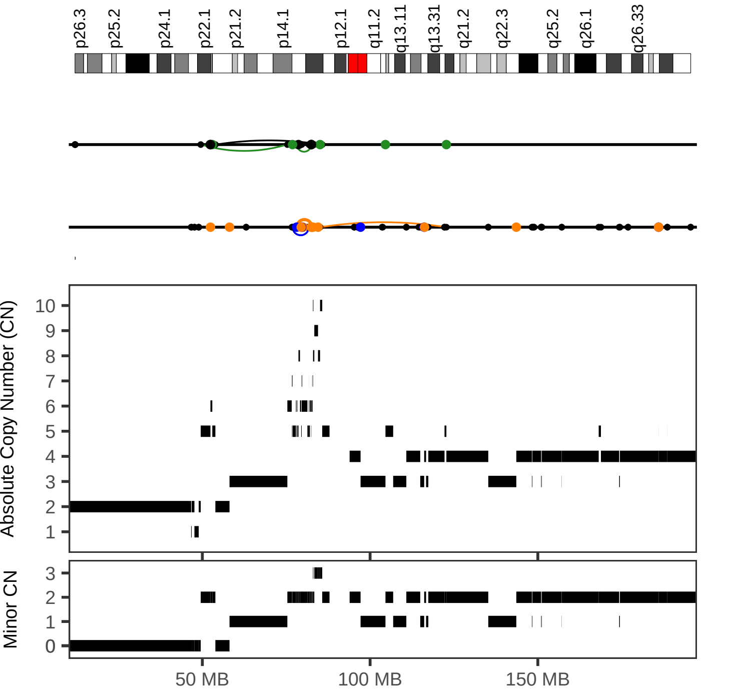

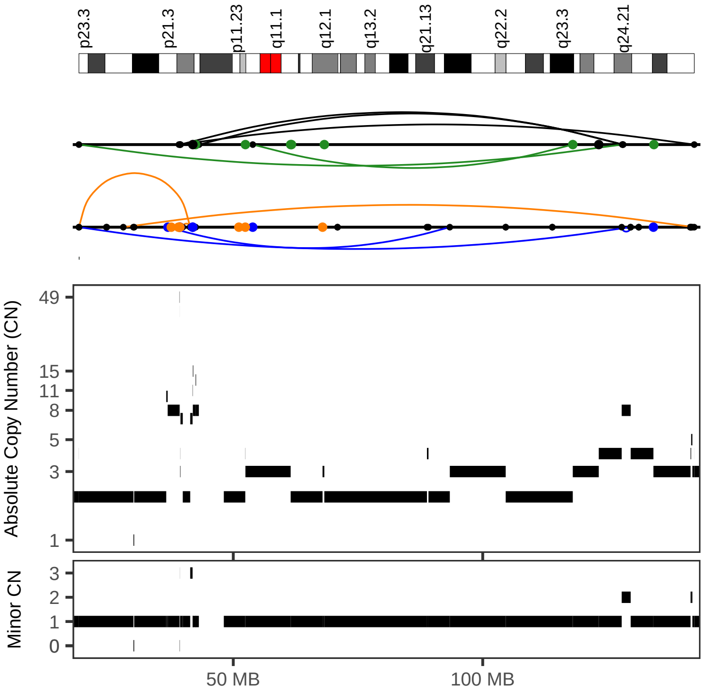

	**PCSI_0145**
Cancer type	Panc−AdenoCA
Position	3:76628774−82950282
Type	With other complex events
Interleaved intrachr. SVs	11
Total SVs (intrachr. + transl.)	24
SV types	DEL: 5; DUP: 2; h2hINV: 0; t2tINV: 4; TRA: 13
SVs in sample	550
Oscillating CN (2 and 3 states)	5, 8
CN segments	23
FDR fragment joints	0.5435077
FDR chr. breakp. enrich.	0
Linked to chrs	15:29942435−85558504;
Purity, ploidy	0.87, 2.72

	**PCSI_0145**
Cancer type	Panc−AdenoCA
Position	8:19058905−142399781
Type	With other complex events
Interleaved intrachr. SVs	11
Total SVs (intrachr. + transl.)	35
SV types	DEL: 2; DUP: 3; h2hINV: 3; t2tINV: 3; TRA: 24
SVs in sample	550
Oscillating CN (2 and 3 states)	4, 9
CN segments	41
FDR fragment joints	0.6776251
FDR chr. breakp. enrich.	0
Linked to chrs	14:32259593−106721662;7:3292583−143127799
Purity, ploidy	0.87, 2.72

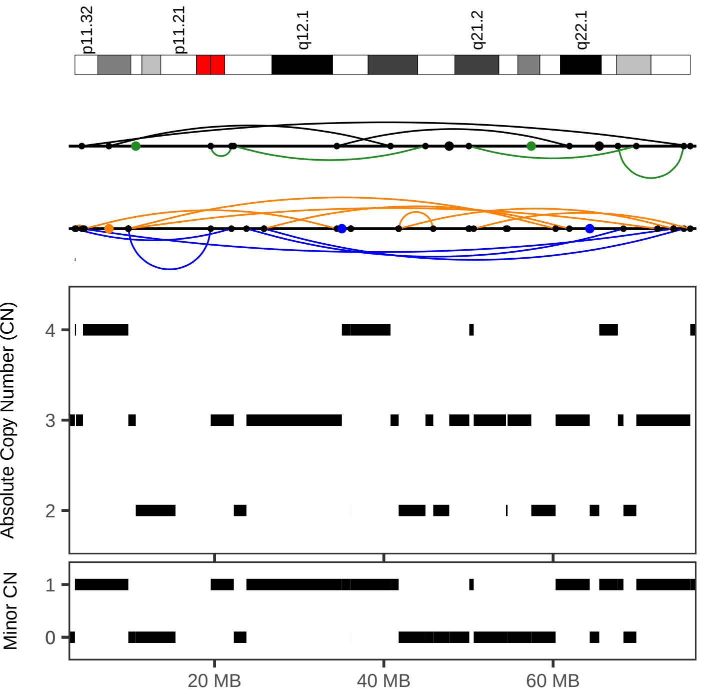

	PCSI_0145
Cancer type	Panc−AdenoCA
Position	18:3500844−76201208
Type	With other complex events
Interleaved intrachr. SVs	21
Total SVs (intrachr. + transl.)	28
SV types	DEL: 8; DUP: 6; h2hINV: 3; t2tINV: 4; TRA: 7
SVs in sample	550
Oscillating CN (2 and 3 states)	6, 13
CN segments	28
FDR fragment joints	0.8066159
FDR chr. breakp. enrich.	0
Linked to chrs	13:62732753−113705989;
Purity, ploidy	0.87, 2.72

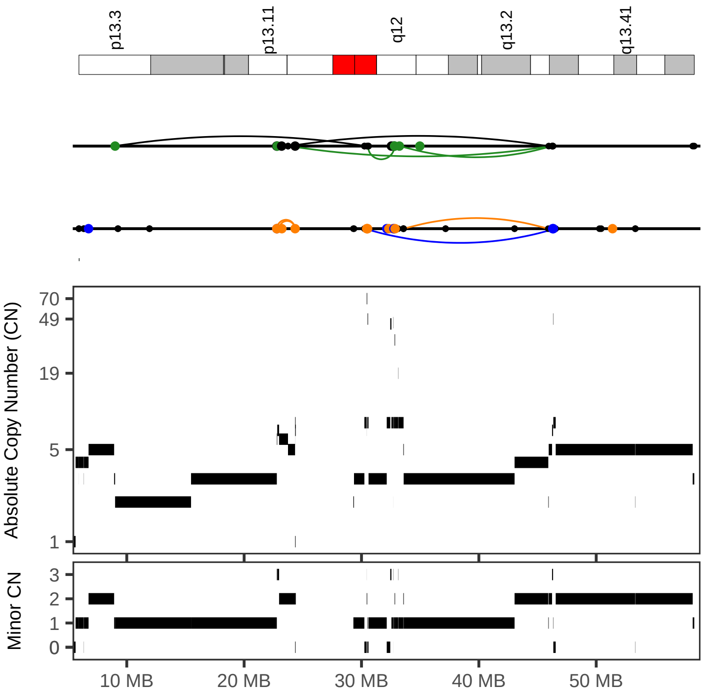

	PCSI_0145
Cancer type	Panc−AdenoCA
Position	19:8923906−46548614
Type	With other complex events
Interleaved intrachr. SVs	7
Total SVs (intrachr. + transl.)	33
SV types	DEL: 2; DUP: 1; h2hINV: 2; t2tINV: 2; TRA: 26
SVs in sample	550
Oscillating CN (2 and 3 states)	4, 6
CN segments	41
FDR fragment joints	0.6776251
FDR chr. breakp. enrich.	0
Linked to chrs	14:32259593−106721662;3:76628774−82950281
Purity, ploidy	0.87, 2.72

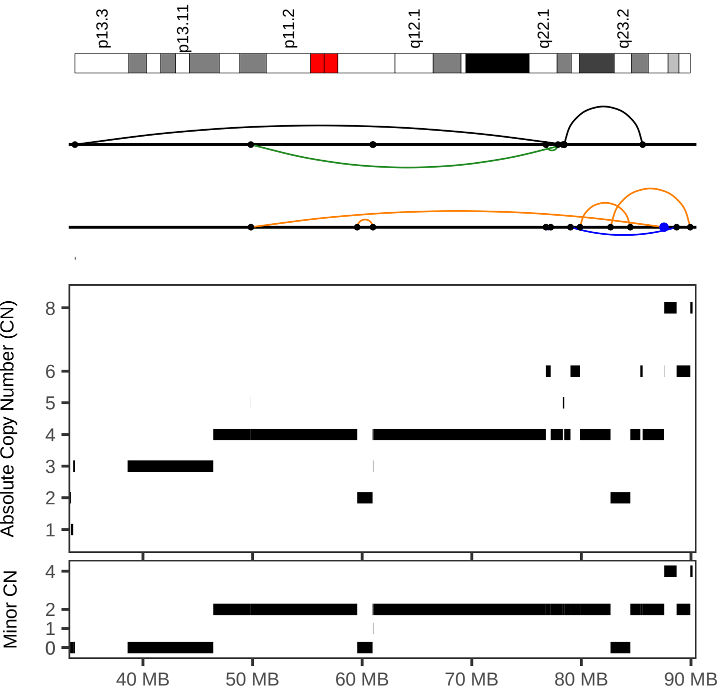

	PCSI_0162
Cancer type	Panc−AdenoCA
Position	16:33791525−89932613
Type	With other complex events
Interleaved intrachr. SVs	8
Total SVs (intrachr. + transl.)	9
SV types	DEL: 3; DUP: 1; h2hINV: 2; t2tINV: 2; TRA: 1
SVs in sample	66
Oscillating CN (2 and 3 states)	4, 7
CN segments	23
FDR fragment joints	0.9723381
FDR chr. breakp. enrich.	0
Linked to chrs	
Purity, ploidy	0.72, 2.93

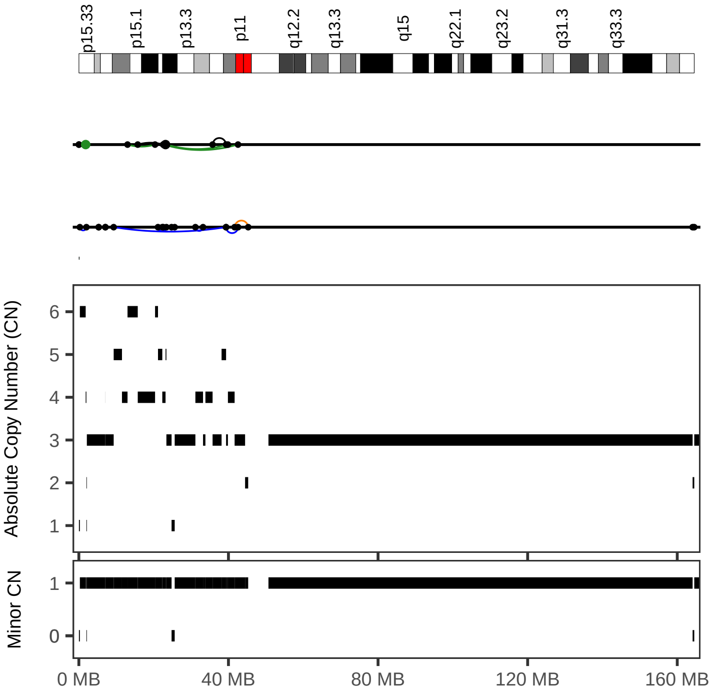

	PCSI_0173
Cancer type	Panc−AdenoCA
Position	5:9311584−45281354
Type	With other complex events
Interleaved intrachr. SVs	10
Total SVs (intrachr. + transl.)	11
SV types	DEL: 2; DUP: 3; h2hINV: 2; t2tINV: 3; TRA: 1
SVs in sample	140
Oscillating CN (2 and 3 states)	5, 9
CN segments	21
FDR fragment joints	0.8653243
FDR chr. breakp. enrich.	0.01
Linked to chrs	
Purity, ploidy	0.42, 3.5

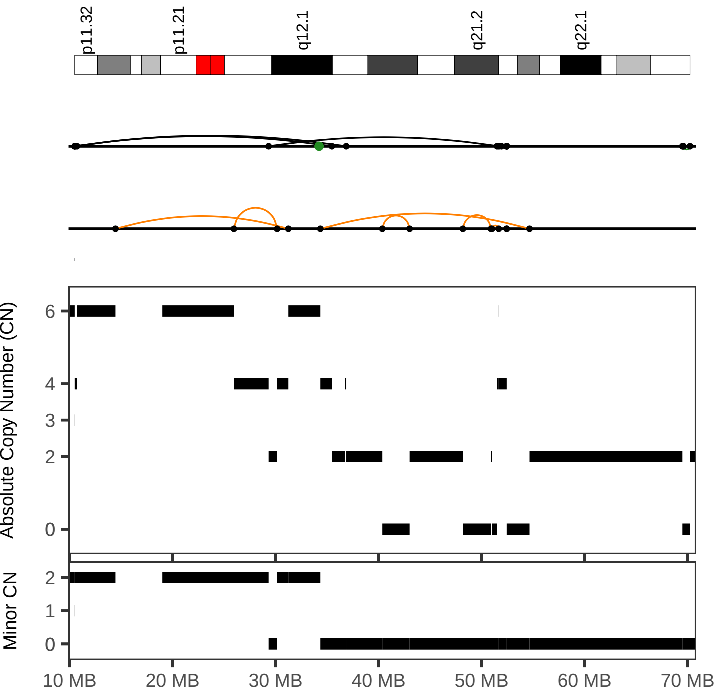

	**PCSI_0173**
Cancer type	Panc−AdenoCA
Position	18:10487843−54651877
Type	With other complex events
Interleaved intrachr. SVs	9
Total SVs (intrachr. + transl.)	10
SV types	DEL: 4; DUP: 0; h2hINV: 3; t2tINV: 2; TRA: 1
SVs in sample	140
Oscillating CN (2 and 3 states)	6, 7
CN segments	22
FDR fragment joints	0.615458
FDR chr. breakp. enrich.	0
Linked to chrs	
Purity, ploidy	0.42, 3.5

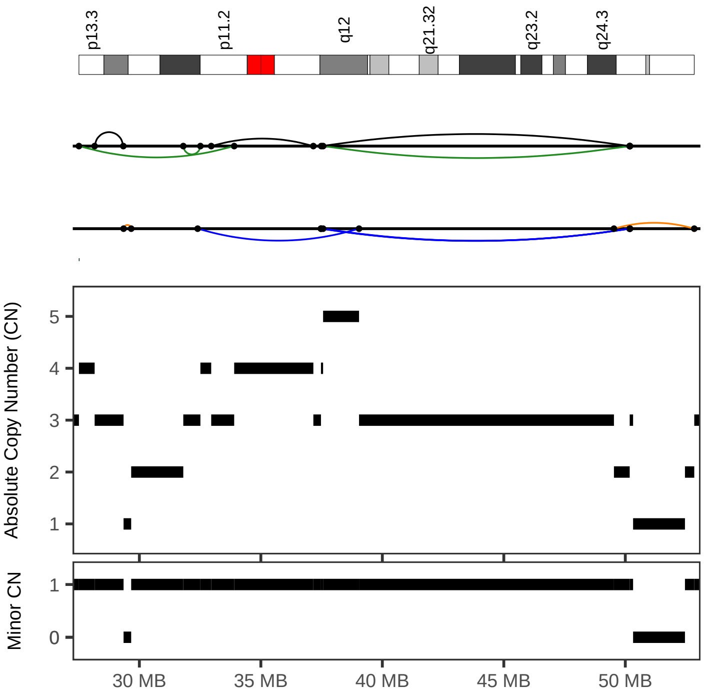

	**PCSI_0208**
Cancer type	Panc−AdenoCA
Position	17:27512548−52837653
Type	With other complex events
Interleaved intrachr. SVs	10
Total SVs (intrachr. + transl.)	10
SV types	DEL: 1; DUP: 4; h2hINV: 2; t2tINV: 3; TRA: 0
SVs in sample	27
Oscillating CN (2 and 3 states)	6, 6
CN segments	17
FDR fragment joints	0.6776251
FDR chr. breakp. enrich.	0
Linked to chrs	
Purity, ploidy	0.95, 1.91

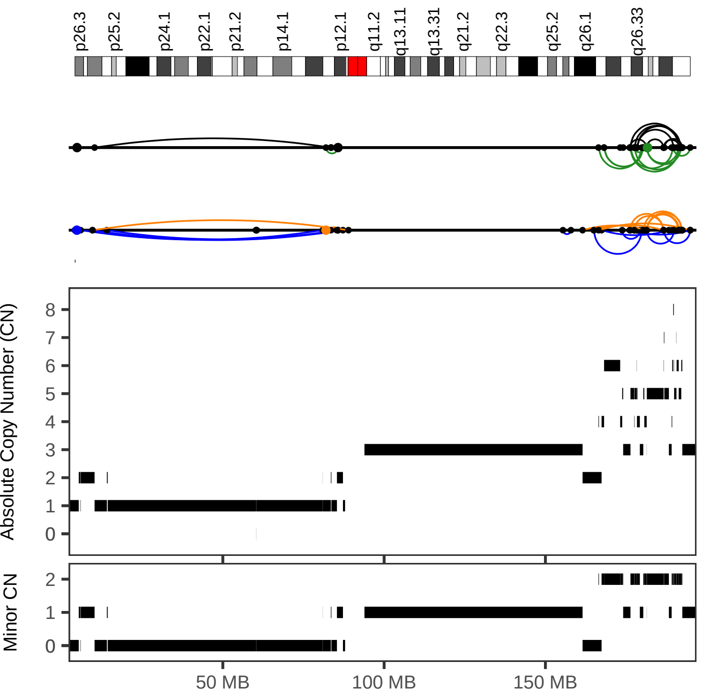

	**PCSI_0210**
Cancer type	Panc−AdenoCA
Position	3:161515687−194895663
Type	With other complex events
Interleaved intrachr. SVs	45
Total SVs (intrachr. + transl.)	47
SV types	DEL: 12; DUP: 12; h2hINV: 9; t2tINV: 12; TRA: 2
SVs in sample	194
Oscillating CN (2 and 3 states)	4, 8
CN segments	35
FDR fragment joints	0.9625775
FDR chr. breakp. enrich.	0
Linked to chrs	
Purity, ploidy	0.86, 2

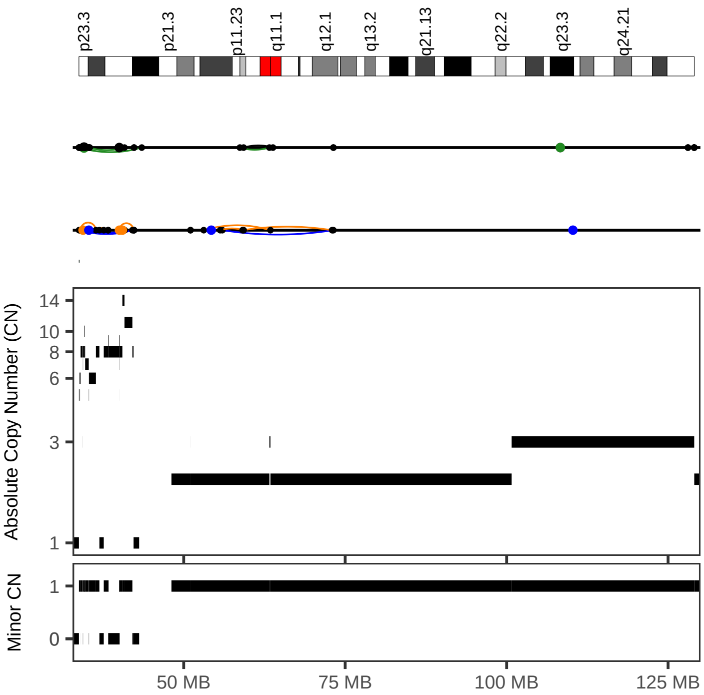

	**PCSI_0210**
Cancer type	Panc−AdenoCA
Position	8:33791127−43503910
Type	With other complex events
Interleaved intrachr. SVs	15
Total SVs (intrachr. + transl.)	21
SV types	DEL: 4; DUP: 4; h2hINV: 3; t2tINV: 4; TRA: 6
SVs in sample	194
Oscillating CN (2 and 3 states)	4, 6
CN segments	30
FDR fragment joints	0.7568568
FDR chr. breakp. enrich.	0
Linked to chrs	
Purity, ploidy	0.86, 2

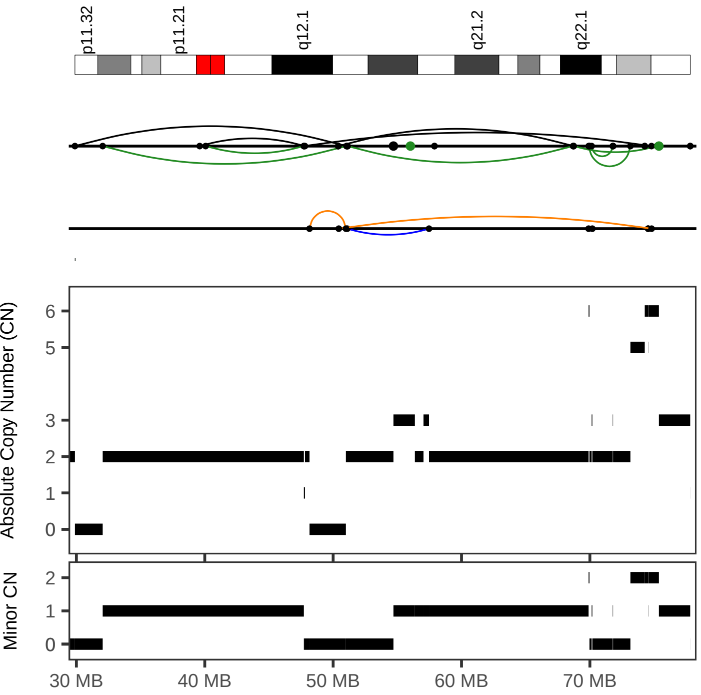

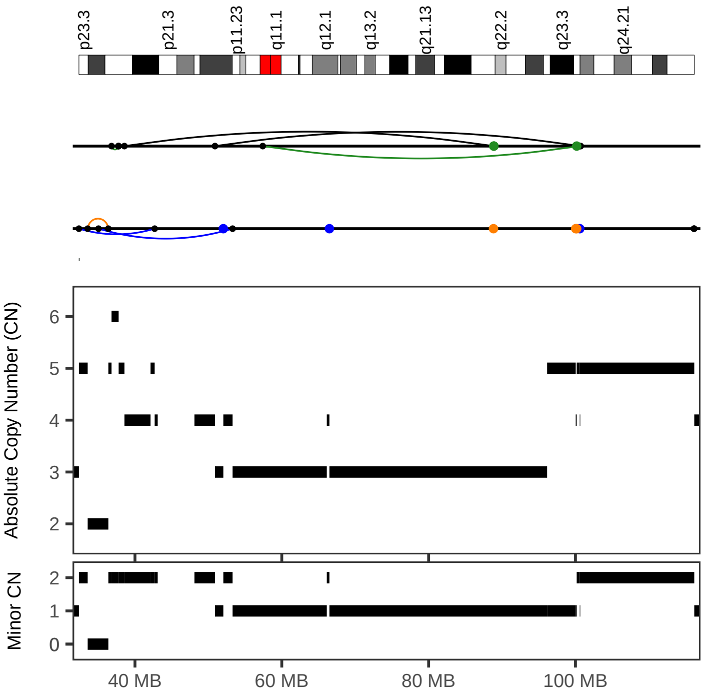

	PCSI_0230
Cancer type	Panc−AdenoCA
Position	18:29885709−75219927
Type	With other complex events
Interleaved intrachr. SVs	8
Total SVs (intrachr. + transl.)	10
SV types	DEL: 2; DUP: 0; h2hINV: 4; t2tINV: 2; TRA: 2
SVs in sample	235
Oscillating CN (2 and 3 states)	5, 13
CN segments	20
FDR fragment joints	0.615458
FDR chr. breakp. enrich.	0
Linked to chrs	
Purity, ploidy	0.92, 1.83

	PCSI_0233
Cancer type	Panc−AdenoCA
Position	8:32367518−100716200
Type	With other complex events
Interleaved intrachr. SVs	6
Total SVs (intrachr. + transl.)	15
SV types	DEL: 1; DUP: 2; h2hINV: 2; t2tINV: 1; TRA: 9
SVs in sample	71
Oscillating CN (2 and 3 states)	6, 15
CN segments	19
FDR fragment joints	0.6471662
FDR chr. breakp. enrich.	0
Linked to chrs	
Purity, ploidy	0.26, 2.92

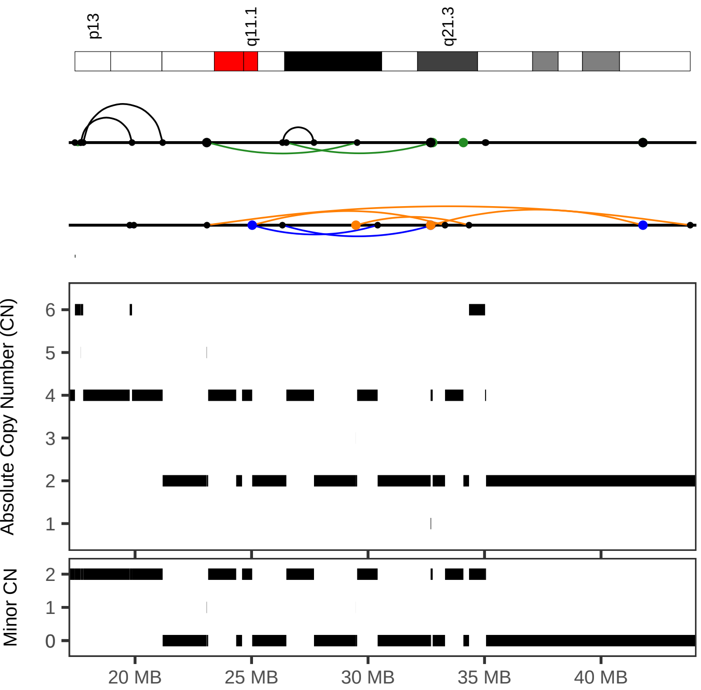

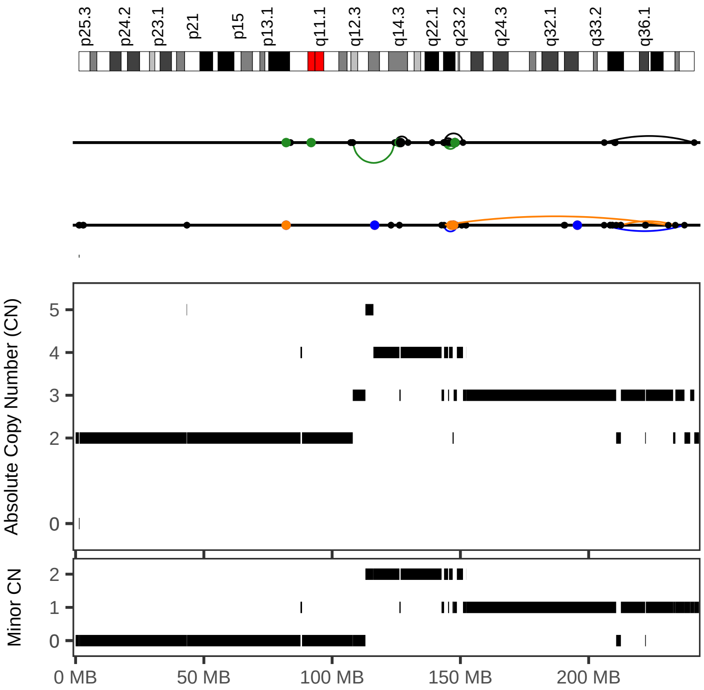

	PCSI_0240
Cancer type	Panc−AdenoCA
Position	21:23137397−41798616
Type	With other complex events
Interleaved intrachr. SVs	8
Total SVs (intrachr. + transl.)	17
SV types	DEL: 3; DUP: 2; h2hINV: 1; t2tINV: 2; TRA: 9
SVs in sample	65
Oscillating CN (2 and 3 states)	6, 10
CN segments	18
FDR fragment joints	0.930656
FDR chr. breakp. enrich.	0
Linked to chrs	
Purity, ploidy	0.65, 3.53

	PCSI_0261
Cancer type	Panc−AdenoCA
Position	2:143484174−151012579
Type	With other complex events
Interleaved intrachr. SVs	7
Total SVs (intrachr. + transl.)	13
SV types	DEL: 0; DUP: 1; h2hINV: 3; t2tINV: 3; TRA: 6
SVs in sample	382
Oscillating CN (2 and 3 states)	4, 8
CN segments	10
FDR fragment joints	0.9000538
FDR chr. breakp. enrich.	0.03
Linked to chrs	
Purity, ploidy	0.55, 2.88

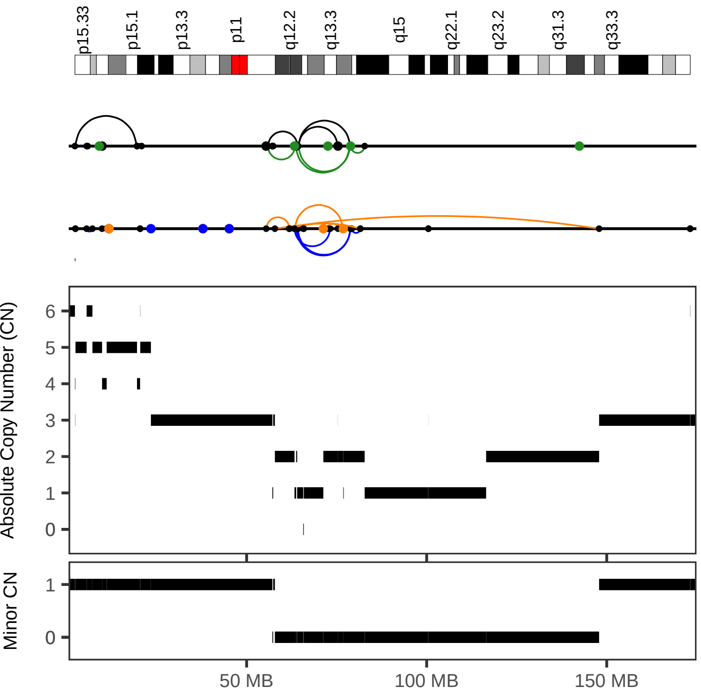

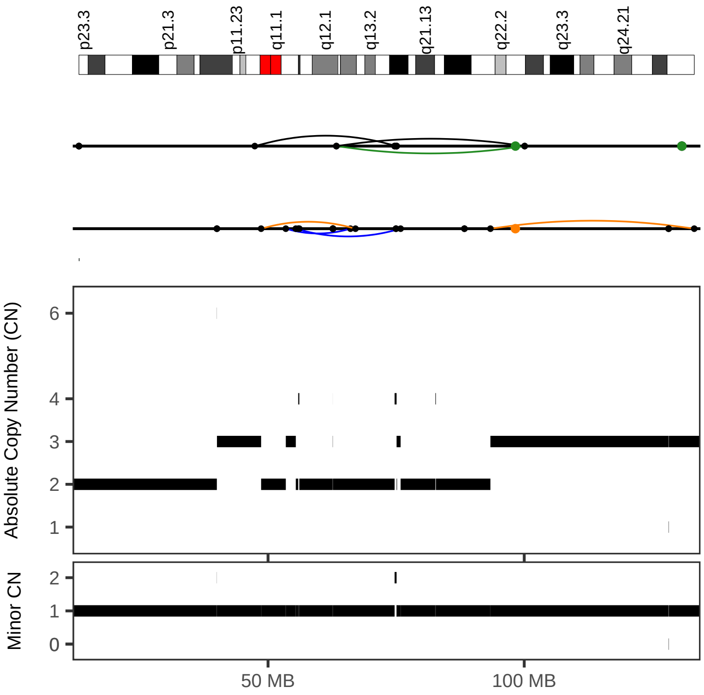

	**PCSI_0261**
Cancer type	Panc−AdenoCA
Position	5:55427909−147893239
Type	With other complex events
Interleaved intrachr. SVs	18
Total SVs (intrachr. + transl.)	26
SV types	DEL: 6; DUP: 4; h2hINV: 4; t2tINV: 4; TRA: 8
SVs in sample	382
Oscillating CN (2 and 3 states)	4, 13
CN segments	19
FDR fragment joints	0.7510435
FDR chr. breakp. enrich.	0
Linked to chrs	
Purity, ploidy	0.55, 2.88

	**PCSI_0261**
Cancer type	Panc−AdenoCA
Position	8:47403900−133181064
Type	With other complex events
Interleaved intrachr. SVs	7
Total SVs (intrachr. + transl.)	11
SV types	DEL: 2; DUP: 2; h2hINV: 2; t2tINV: 1; TRA: 4
SVs in sample	382
Oscillating CN (2 and 3 states)	4, 11
CN segments	18
FDR fragment joints	0.8653243
FDR chr. breakp. enrich.	0.66
Linked to chrs	12:34059230−90708383;
Purity, ploidy	0.55, 2.88

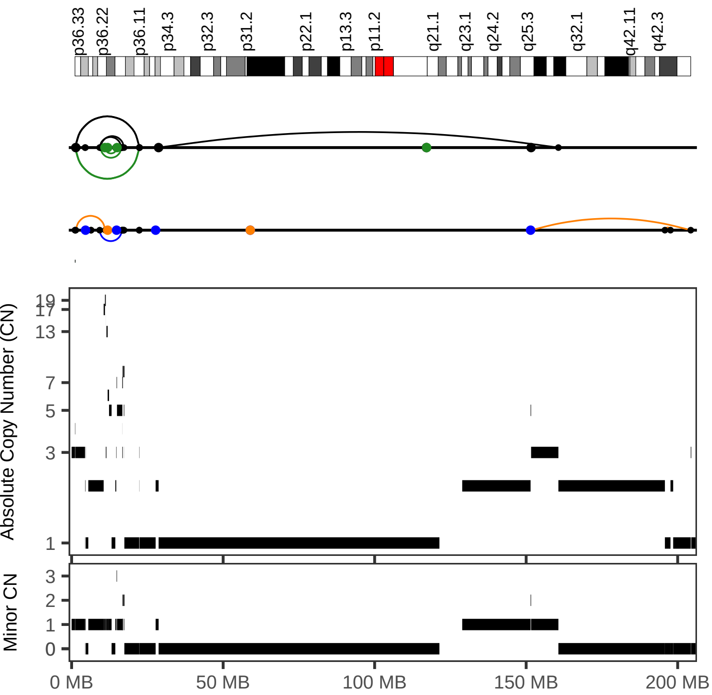

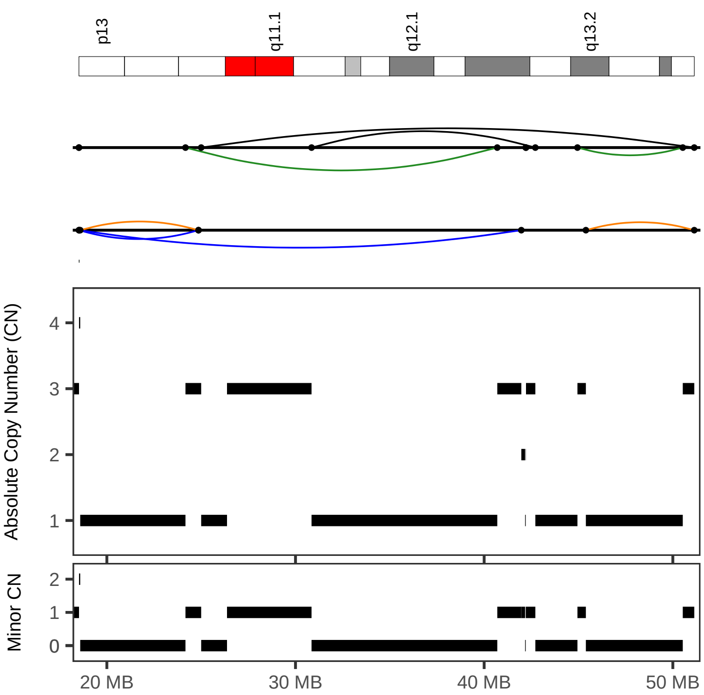

	**PCSI_0268**
Cancer type	Panc−AdenoCA
Position	1:1109651−22405832
Type	With other complex events
Interleaved intrachr. SVs	7
Total SVs (intrachr. + transl.)	14
SV types	DEL: 0; DUP: 2; h2hINV: 3; t2tINV: 2; TRA: 7
SVs in sample	104
Oscillating CN (2 and 3 states)	4, 5
CN segments	34
FDR fragment joints	0.615458
FDR chr. breakp. enrich.	0
Linked to chrs	
Purity, ploidy	0.67, 2.21

	**PCSI_0279**
Cancer type	Panc−AdenoCA
Position	22:18521810−51136116
Type	With other complex events
Interleaved intrachr. SVs	8
Total SVs (intrachr. + transl.)	8
SV types	DEL: 2; DUP: 2; h2hINV: 2; t2tINV: 2; TRA: 0
SVs in sample	57
Oscillating CN (2 and 3 states)	6, 8
CN segments	16
FDR fragment joints	1
FDR chr. breakp. enrich.	0
Linked to chrs	
Purity, ploidy	0.6, 2.03

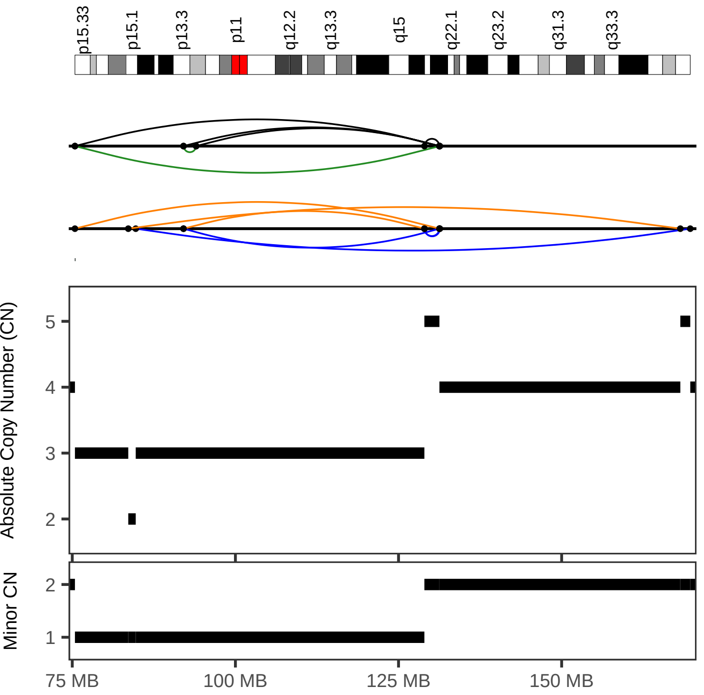

PCSI_0281	
Cancer type	Panc−AdenoCA
Position	5:75405316−169709323
Type	With other complex events
Interleaved intrachr. SVs	13
Total SVs (intrachr. + transl.)	13
SV types	DEL: 3; DUP: 4; h2hINV: 4; t2tINV: 2; TRA: 0
SVs in sample	85
Oscillating CN (2 and 3 states)	4, 5
CN segments	8
FDR fragment joints	0.9000538
FDR chr. breakp. enrich.	0.01
Linked to chrs	
Purity, ploidy	0.47, 3.9

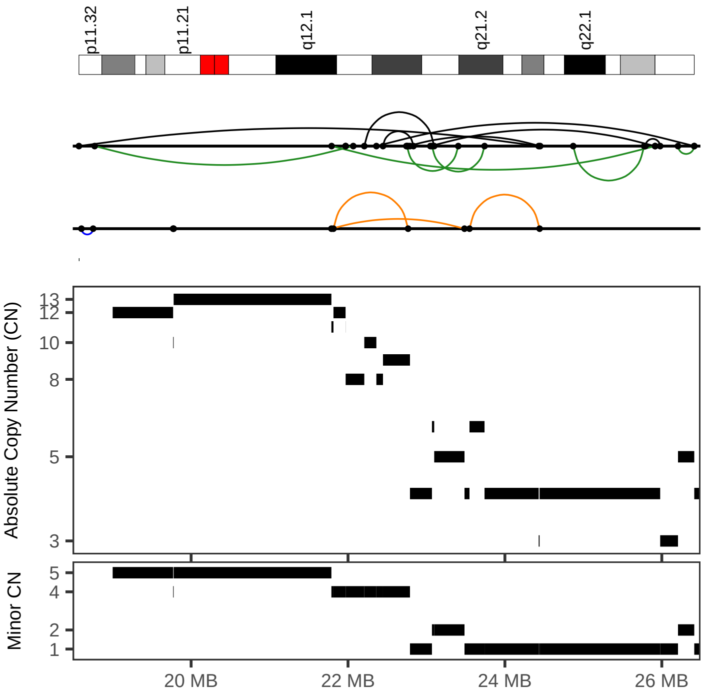

PCSI_0281	
Cancer type	Panc−AdenoCA
Position	18:18569763−26413726
Type	With other complex events
Interleaved intrachr. SVs	16
Total SVs (intrachr. + transl.)	16
SV types	DEL: 3; DUP: 0; h2hINV: 8; t2tINV: 5; TRA: 0
SVs in sample	85
Oscillating CN (2 and 3 states)	4, 6
CN segments	22
FDR fragment joints	0.4637733
FDR chr. breakp. enrich.	0
Linked to chrs	
Purity, ploidy	0.47, 3.9

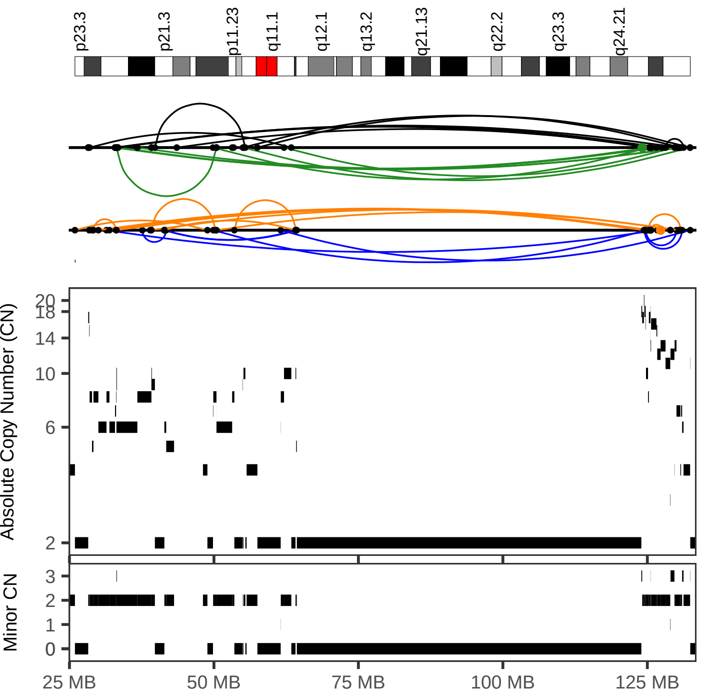

PCSI_0284	
Cancer type	Panc−AdenoCA
Position	8:25945162−132424548
Type	With other complex events
Interleaved intrachr. SVs	40
Total SVs (intrachr. + transl.)	42
SV types	DEL: 14; DUP: 8; h2hINV: 10; t2tINV: 8; TRA: 2
SVs in sample	238
Oscillating CN (2 and 3 states)	4, 7
CN segments	68
FDR fragment joints	0.615458
FDR chr. breakp. enrich.	0
Linked to chrs	
Purity, ploidy	0.56, 3.12

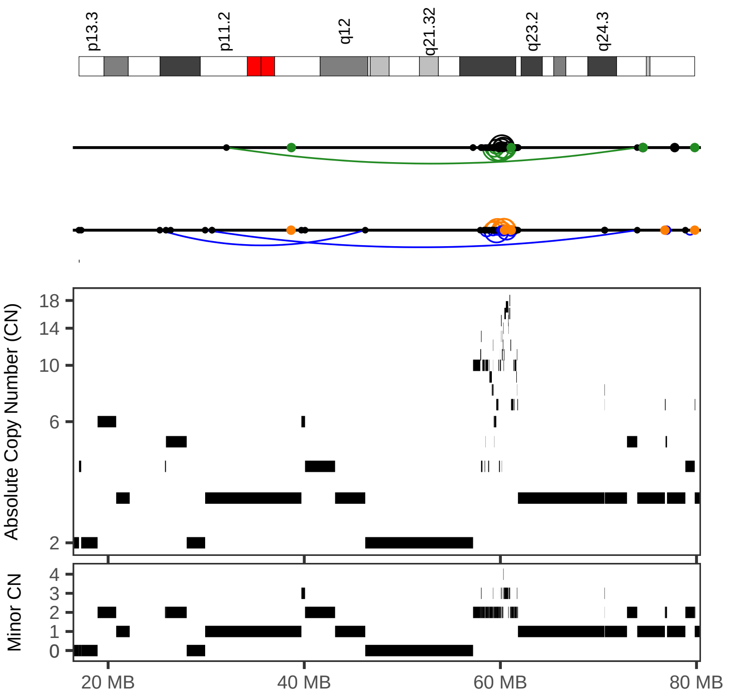

PCSI_0285	
Cancer type	Panc−AdenoCA
Position	17:57949419−61791871
Type	With other complex events
Interleaved intrachr. SVs	63
Total SVs (intrachr. + transl.)	70
SV types	DEL: 21; DUP: 14; h2hINV: 13; t2tINV: 15; TRA: 7
SVs in sample	446
Oscillating CN (2 and 3 states)	4, 8
CN segments	50
FDR fragment joints	0.6776251
FDR chr. breakp. enrich.	0
Linked to chrs	4:117110284−186019797;
Purity, ploidy	0.56, 3.15

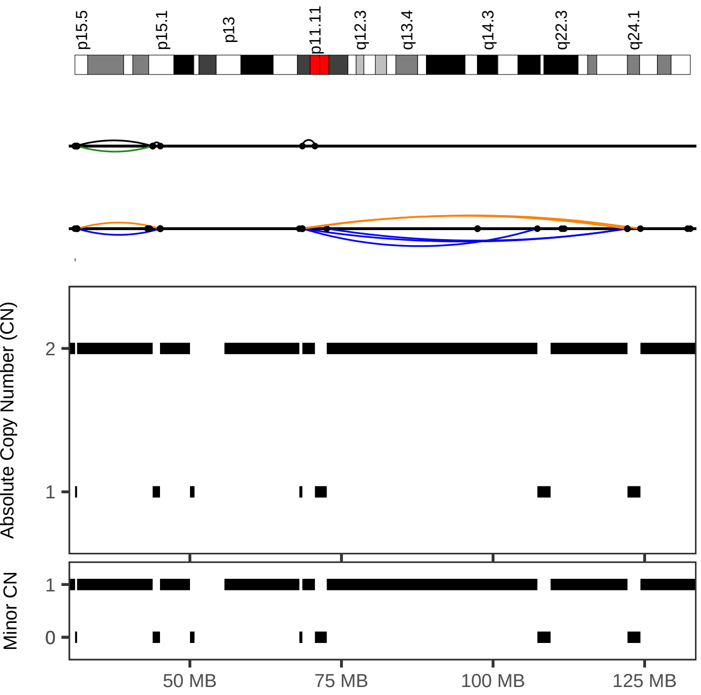

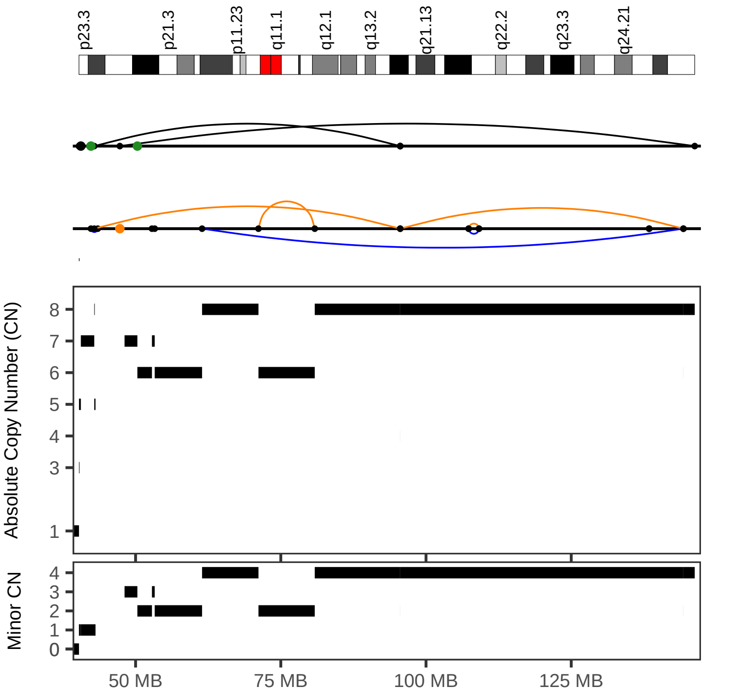

	**PCSI_0287**
Cancer type	Panc−AdenoCA
Position	11:31044158−45136475
Type	Canonical without polyploidization
Interleaved intrachr. SVs	7
Total SVs (intrachr. + transl.)	7
SV types	DEL: 2; DUP: 1; h2hINV: 2; t2tINV: 2; TRA: 0
SVs in sample	152
Oscillating CN (2 and 3 states)	5, 5
CN segments	5
FDR fragment joints	0.9599662
FDR chr. breakp. enrich.	0
Linked to chrs	
Purity, ploidy	0.94, 1.94

	**PCSI_0290**
Cancer type	Panc−AdenoCA
Position	8:42316395−146260778
Type	With other complex events
Interleaved intrachr. SVs	6
Total SVs (intrachr. + transl.)	8
SV types	DEL: 2; DUP: 2; h2hINV: 2; t2tINV: 0; TRA: 2
SVs in sample	137
Oscillating CN (2 and 3 states)	4, 9
CN segments	14
FDR fragment joints	0.615458
FDR chr. breakp. enrich.	0
Linked to chrs	11:55509544−78804415;
Purity, ploidy	0.72, 3.1

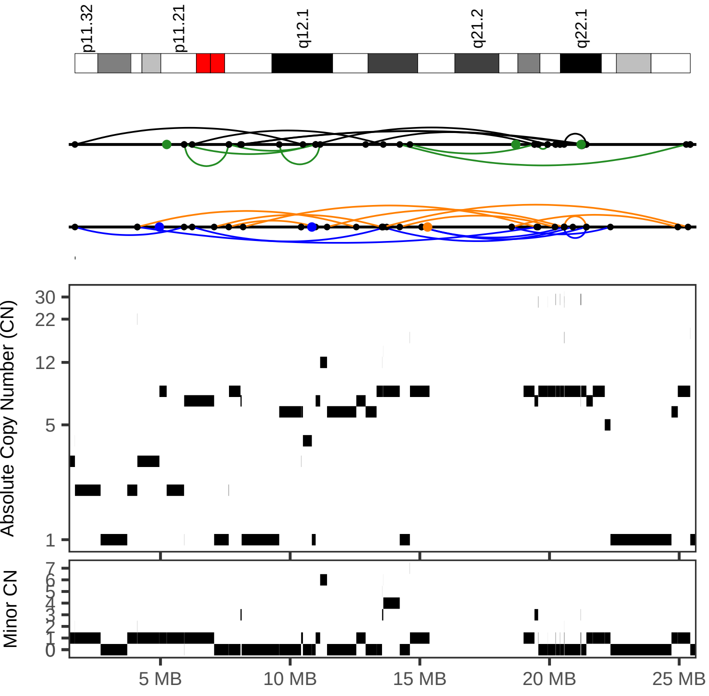

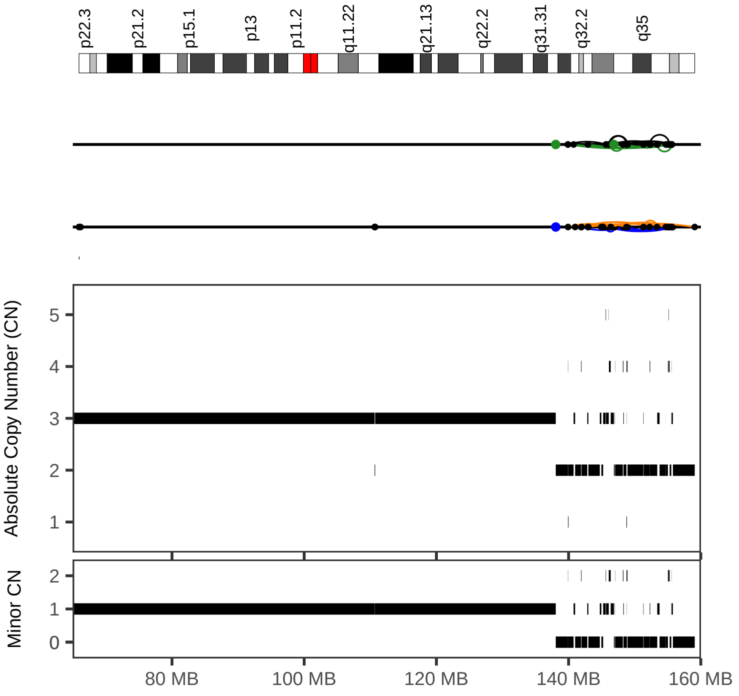

	**PCSI_0290**
Cancer type	Panc−AdenoCA
Position	18:1700778−25341902
Type	With other complex events
Interleaved intrachr. SVs	32
Total SVs (intrachr. + transl.)	39
SV types	DEL: 9; DUP: 8; h2hINV: 8; t2tINV: 7; TRA: 7
SVs in sample	137
Oscillating CN (2 and 3 states)	5, 10
CN segments	56
FDR fragment joints	0.952954
FDR chr. breakp. enrich.	0
Linked to chrs	
Purity, ploidy	0.72, 3.1

	**PCSI_0300**
Cancer type	Panc−AdenoCA
Position	7:139885932−159081404
Type	With other complex events
Interleaved intrachr. SVs	35
Total SVs (intrachr. + transl.)	36
SV types	DEL: 9; DUP: 9; h2hINV: 9; t2tINV: 8; TRA: 1
SVs in sample	208
Oscillating CN (2 and 3 states)	6, 13
CN segments	46
FDR fragment joints	0.9875525
FDR chr. breakp. enrich.	0
Linked to chrs	
Purity, ploidy	0.58, 2.82

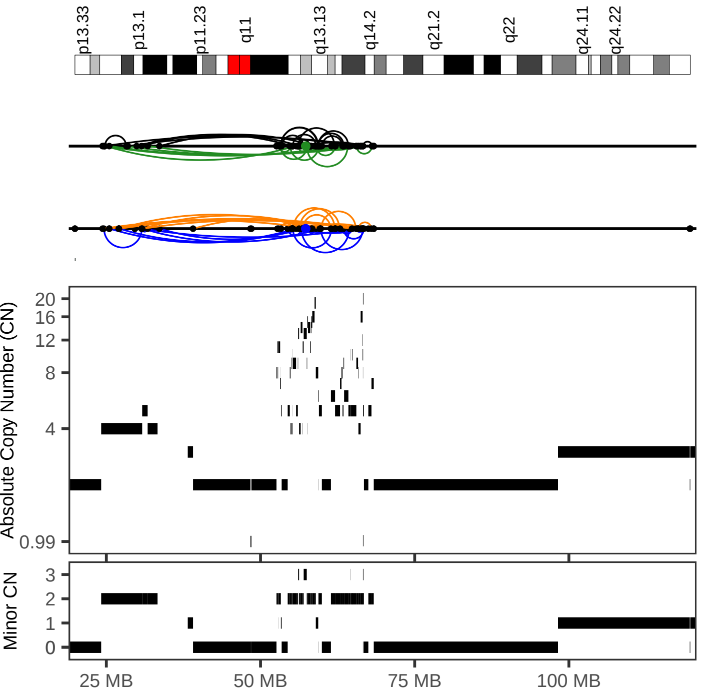

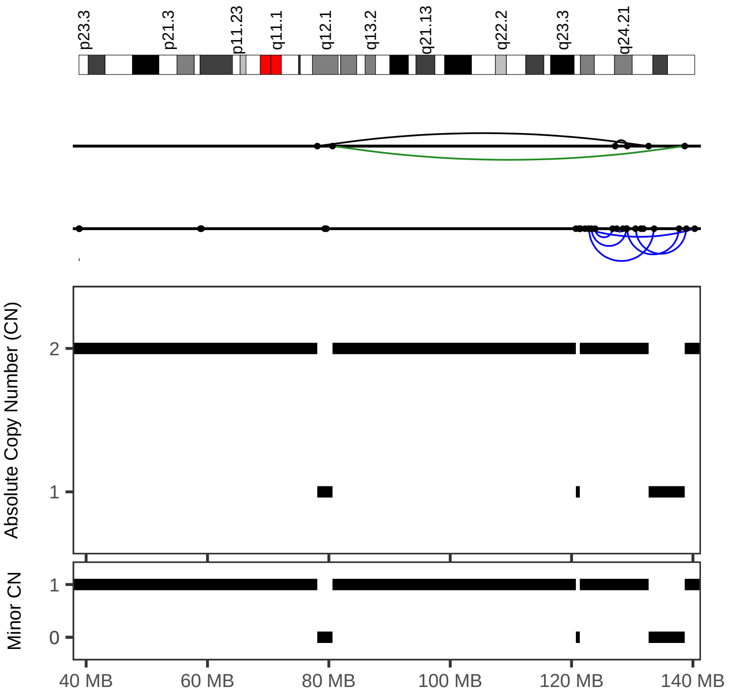

	**PCSI_0300**
Cancer type	Panc−AdenoCA
Position	12:24383751−68089214
Type	With other complex events
Interleaved intrachr. SVs	76
Total SVs (intrachr. + transl.)	78
SV types	DEL: 17; DUP: 21; h2hINV: 22; t2tINV: 16; TRA: 2
SVs in sample	208
Oscillating CN (2 and 3 states)	6, 7
CN segments	72
FDR fragment joints	0.7027324
FDR chr. breakp. enrich.	0
Linked to chrs	
Purity, ploidy	0.58, 2.82

	**PCSI_0324**
Cancer type	Panc−AdenoCA
Position	8:78094013−140299410
Type	Canonical without polyploidization
Interleaved intrachr. SVs	8
Total SVs (intrachr. + transl.)	8
SV types	DEL: 0; DUP: 5; h2hINV: 2; t2tINV: 1; TRA: 0
SVs in sample	204
Oscillating CN (2 and 3 states)	6, 6
CN segments	6
FDR fragment joints	0.5435077
FDR chr. breakp. enrich.	0.23
Linked to chrs	
Purity, ploidy	0.56, 1.86

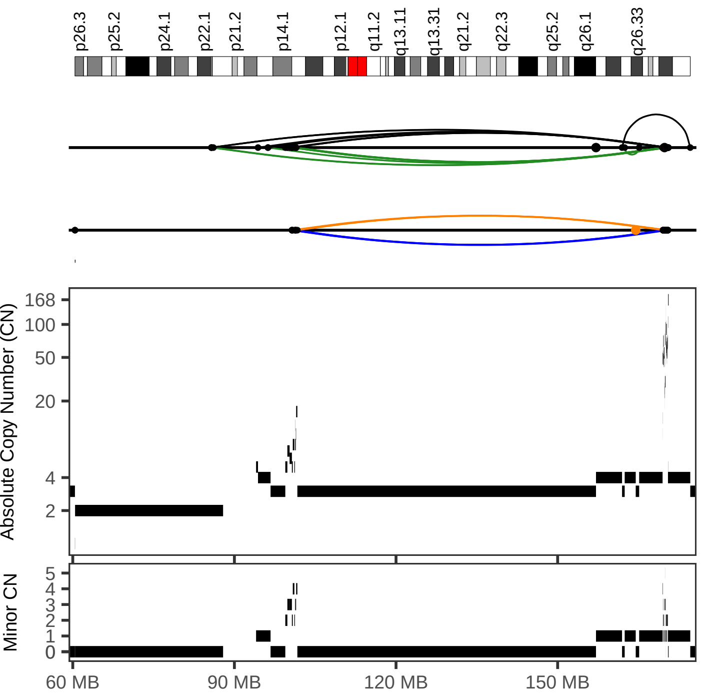

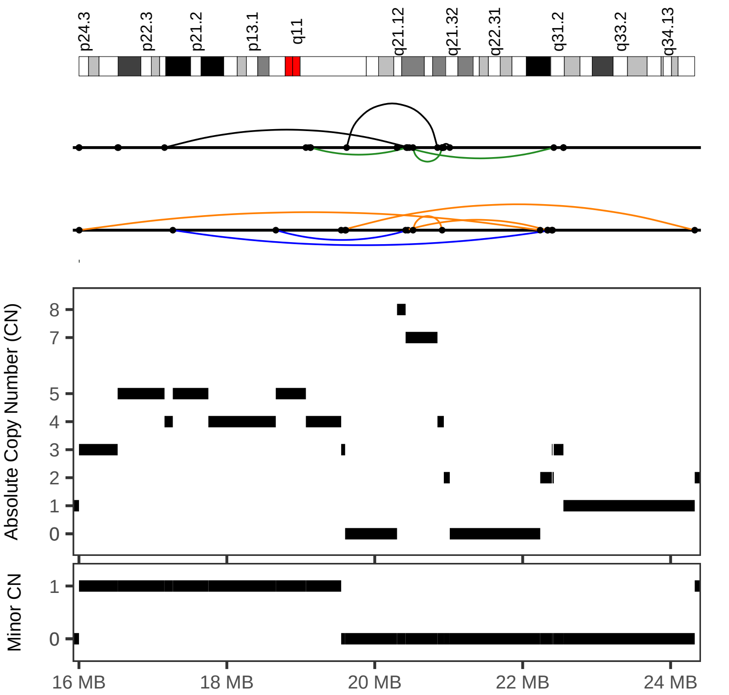

	**PCSI_0325**
Cancer type	Panc−AdenoCA
Position	3:85734262−174611972
Type	With other complex events
Interleaved intrachr. SVs	56
Total SVs (intrachr. + transl.)	59
SV types	DEL: 15; DUP: 13; h2hINV: 16; t2tINV: 12; TRA: 3
SVs in sample	207
Oscillating CN (2 and 3 states)	6, 6
CN segments	85
FDR fragment joints	0.9589247
FDR chr. breakp. enrich.	0
Linked to chrs	
Purity, ploidy	0.67, 2.85

	**PCSI_0337**
Cancer type	Panc−AdenoCA
Position	9:16003998−24326188
Type	With other complex events
Interleaved intrachr. SVs	13
Total SVs (intrachr. + transl.)	13
SV types	DEL: 4; DUP: 2; h2hINV: 4; t2tINV: 3; TRA: 0
SVs in sample	54
Oscillating CN (2 and 3 states)	6, 7
CN segments	20
FDR fragment joints	0.9000538
FDR chr. breakp. enrich.	0
Linked to chrs	
Purity, ploidy	0.63, 2.05

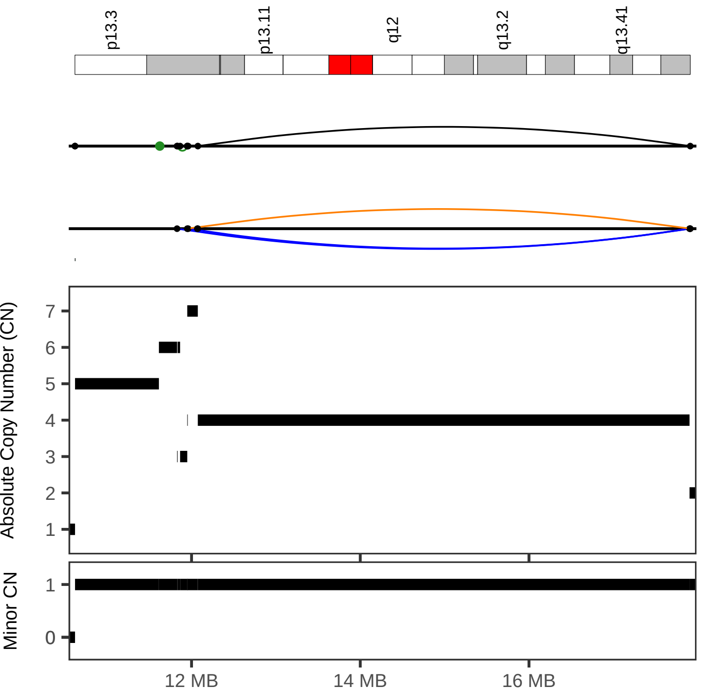

	PCSI_0337
Cancer type	Panc−AdenoCA
Position	19:11827395−17911949
Type	With other complex events
Interleaved intrachr. SVs	7
Total SVs (intrachr. + transl.)	7
SV types	DEL: 1; DUP: 2; h2hINV: 2; t2tINV: 2; TRA: 0
SVs in sample	54
Oscillating CN (2 and 3 states)	4, 5
CN segments	8
FDR fragment joints	0.9599662
FDR chr. breakp. enrich.	0
Linked to chrs	
Purity, ploidy	0.63, 2.05

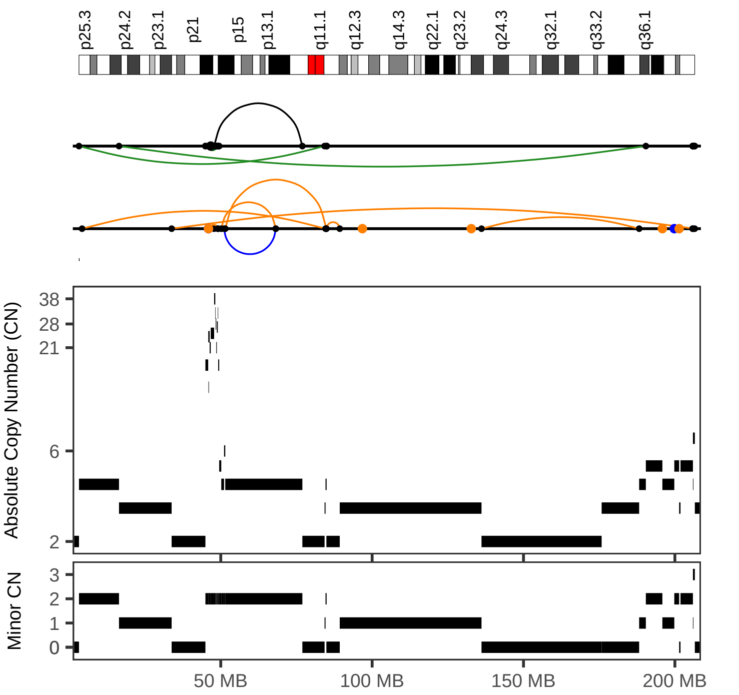

	PCSI_0340
Cancer type	Panc−AdenoCA
Position	2:3167627−206554663
Type	With other complex events
Interleaved intrachr. SVs	14
Total SVs (intrachr. + transl.)	22
SV types	DEL: 6; DUP: 2; h2hINV: 3; t2tINV: 3; TRA: 8
SVs in sample	237
Oscillating CN (2 and 3 states)	5, 7
CN segments	38
FDR fragment joints	0.5435077
FDR chr. breakp. enrich.	0.06
Linked to chrs	
Purity, ploidy	0.41, 3.29

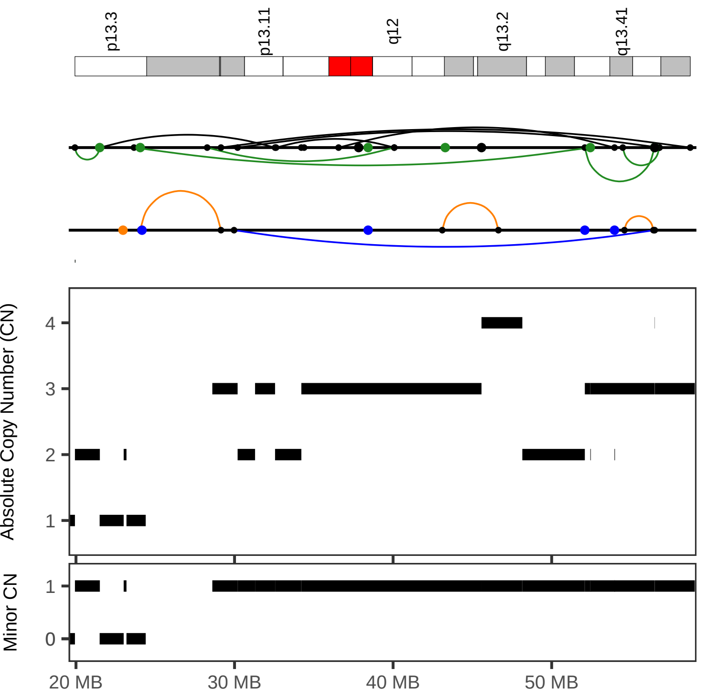

	PCSI_0345
Cancer type	Panc−AdenoCA
Position	19:21513068−58739452
Type	With other complex events
Interleaved intrachr. SVs	11
Total SVs (intrachr. + transl.)	24
SV types	DEL: 1; DUP: 1; h2hINV: 5; t2tINV: 4; TRA: 13
SVs in sample	92
Oscillating CN (2 and 3 states)	6, 8
CN segments	17
FDR fragment joints	0.615458
FDR chr. breakp. enrich.	0
Linked to chrs	
Purity, ploidy	0.53, 1.87

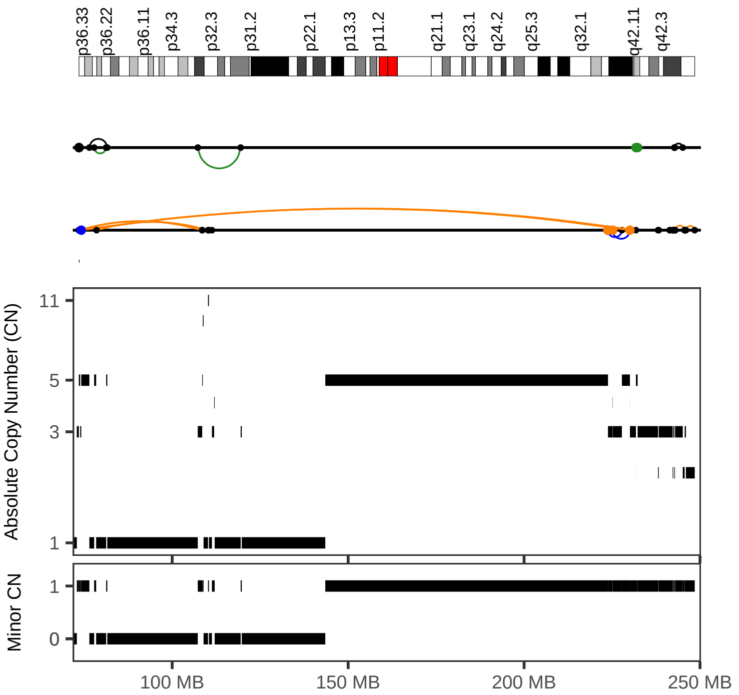

	PCSI_0347
Cancer type	Panc−AdenoCA
Position	1:73507136−231782377
Type	With other complex events
Interleaved intrachr. SVs	12
Total SVs (intrachr. + transl.)	19
SV types	DEL: 5; DUP: 4; h2hINV: 1; t2tINV: 2; TRA: 7
SVs in sample	63
Oscillating CN (2 and 3 states)	6, 9
CN segments	26
FDR fragment joints	0.615458
FDR chr. breakp. enrich.	0
Linked to chrs	
Purity, ploidy	NA, NA

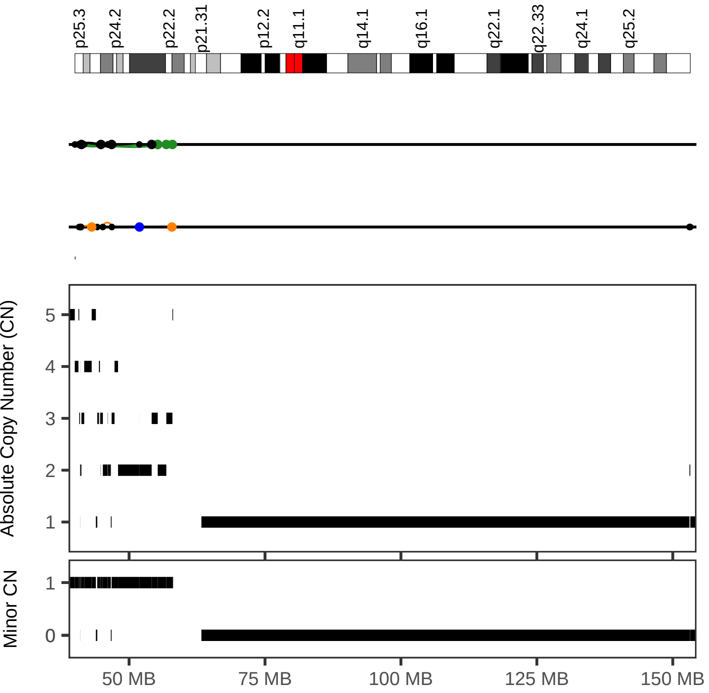

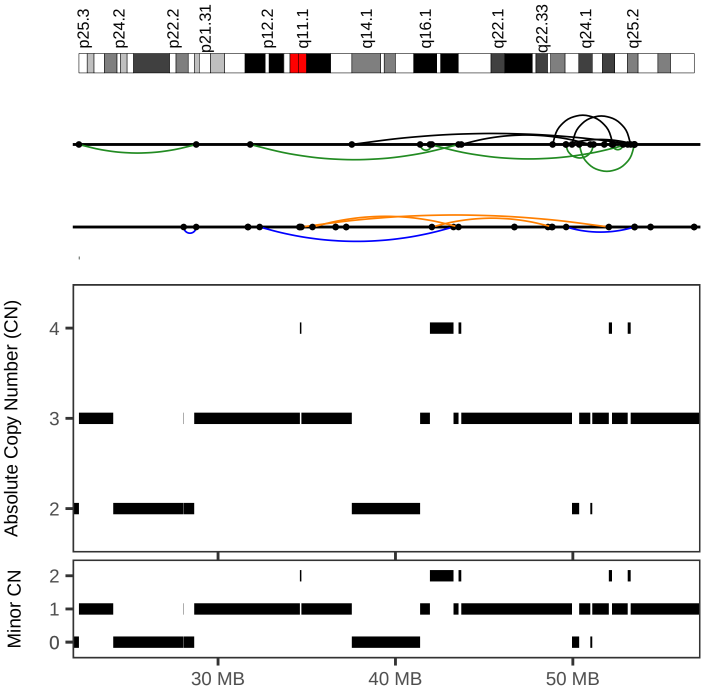

	**PCSI_0352**
Cancer type	Panc−AdenoCA
Position	6:40037807−56846580
Type	With other complex events
Interleaved intrachr. SVs	6
Total SVs (intrachr. + transl.)	14
SV types	DEL: 2; DUP: 0; h2hINV: 1; t2tINV: 3; TRA: 8
SVs in sample	72
Oscillating CN (2 and 3 states)	6, 12
CN segments	29
FDR fragment joints	0.615458
FDR chr. breakp. enrich.	0
Linked to chrs	14:20953269−101623828;
Purity, ploidy	NA, NA

	**PCSI_0353**
Cancer type	Panc−AdenoCA
Position	6:31811163−53484589
Type	With other complex events
Interleaved intrachr. SVs	17
Total SVs (intrachr. + transl.)	17
SV types	DEL: 3; DUP: 2; h2hINV: 6; t2tINV: 6; TRA: 0
SVs in sample	105
Oscillating CN (2 and 3 states)	5, 17
CN segments	17
FDR fragment joints	0.615458
FDR chr. breakp. enrich.	0
Linked to chrs	
Purity, ploidy	0.95, 3.75

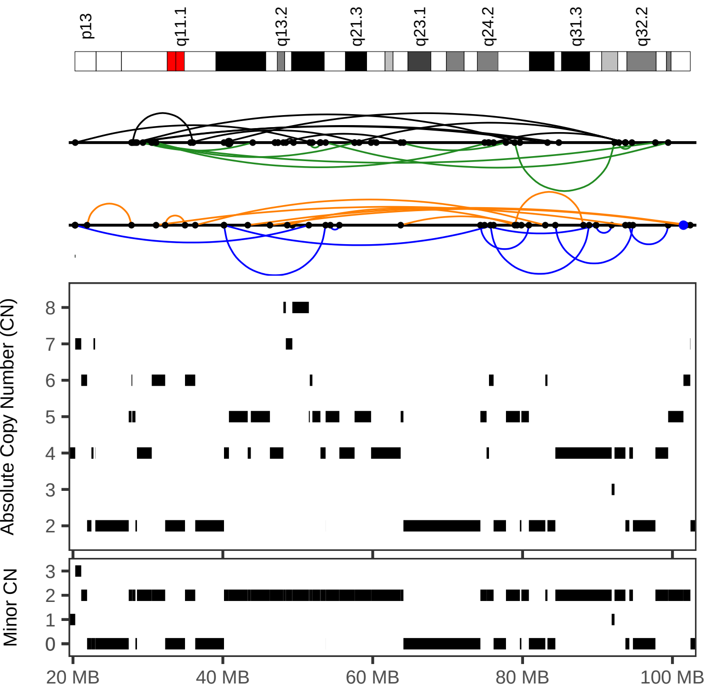

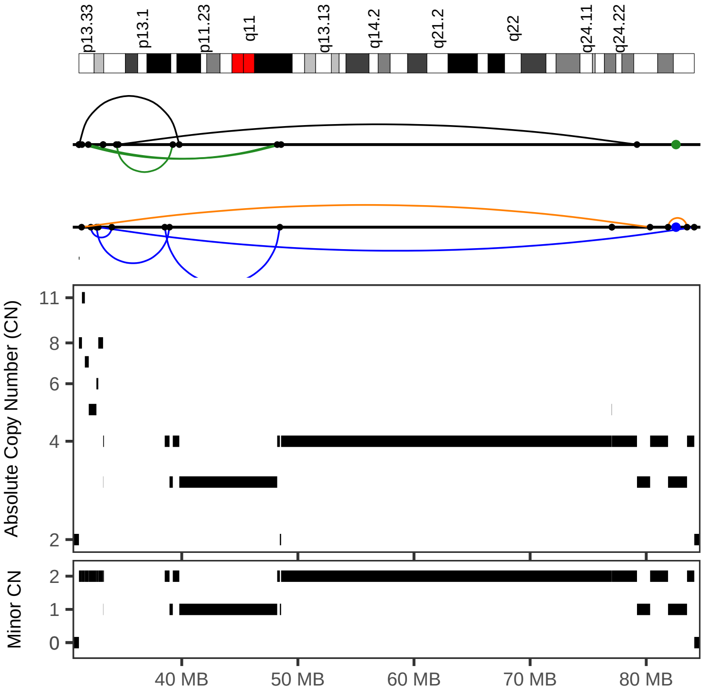

	**PCSI_0353**
Cancer type	Panc−AdenoCA
Position	14:20259866−102375473
Type	With other complex events
Interleaved intrachr. SVs	38
Total SVs (intrachr. + transl.)	40
SV types	DEL: 8; DUP: 9; h2hINV: 12; t2tINV: 9; TRA: 2
SVs in sample	105
Oscillating CN (2 and 3 states)	5, 9
CN segments	58
FDR fragment joints	0.9109641
FDR chr. breakp. enrich.	0
Linked to chrs	
Purity, ploidy	0.95, 3.75

	**PCSI_0356**
Cancer type	Panc−AdenoCA
Position	12:31165322−84134078
Type	With other complex events
Interleaved intrachr. SVs	10
Total SVs (intrachr. + transl.)	12
SV types	DEL: 1; DUP: 4; h2hINV: 2; t2tINV: 3; TRA: 2
SVs in sample	228
Oscillating CN (2 and 3 states)	5, 9
CN segments	22
FDR fragment joints	0.6776251
FDR chr. breakp. enrich.	0.07
Linked to chrs	
Purity, ploidy	0.77, 3.33

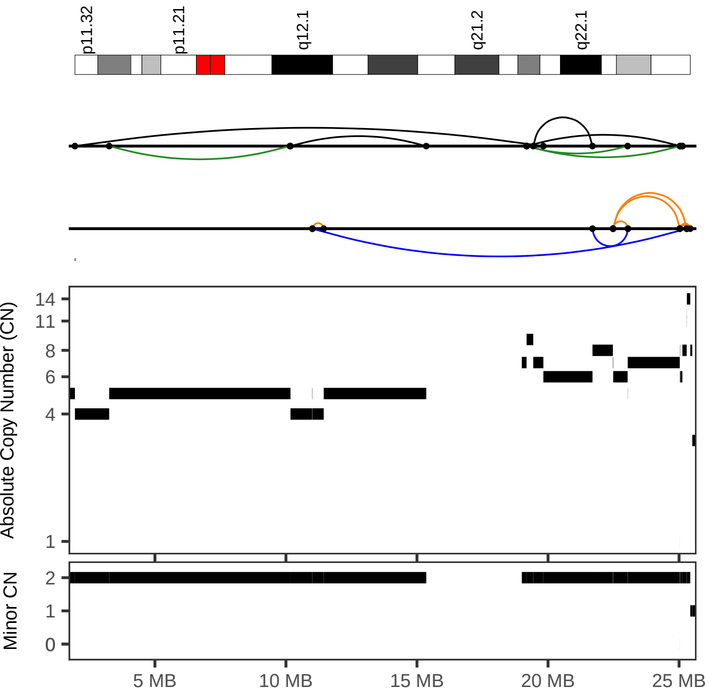

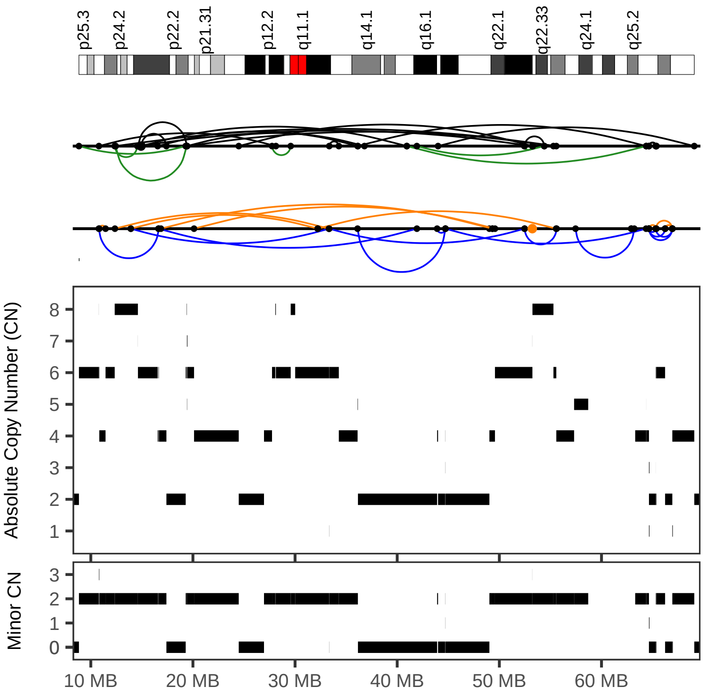

	PCSI_0356
Cancer type	Panc−AdenoCA
Position	18:1947225−25427315
Type	With other complex events
Interleaved intrachr. SVs	13
Total SVs (intrachr. + transl.)	13
SV types	DEL: 5; DUP: 2; h2hINV: 4; t2tINV: 2; TRA: 0
SVs in sample	228
Oscillating CN (2 and 3 states)	6, 6
CN segments	26
FDR fragment joints	0.6776251
FDR chr. breakp. enrich.	0
Linked to chrs	
Purity, ploidy	0.77, 3.33

	PCSI_0392
Cancer type	Panc−AdenoCA
Position	6:8838119−69082829
Type	With other complex events
Interleaved intrachr. SVs	34
Total SVs (intrachr. + transl.)	36
SV types	DEL: 5; DUP: 9; h2hINV: 13; t2tINV: 7; TRA: 2
SVs in sample	160
Oscillating CN (2 and 3 states)	5, 6
CN segments	57
FDR fragment joints	0.641841
FDR chr. breakp. enrich.	0
Linked to chrs	
Purity, ploidy	0.99, 3.89

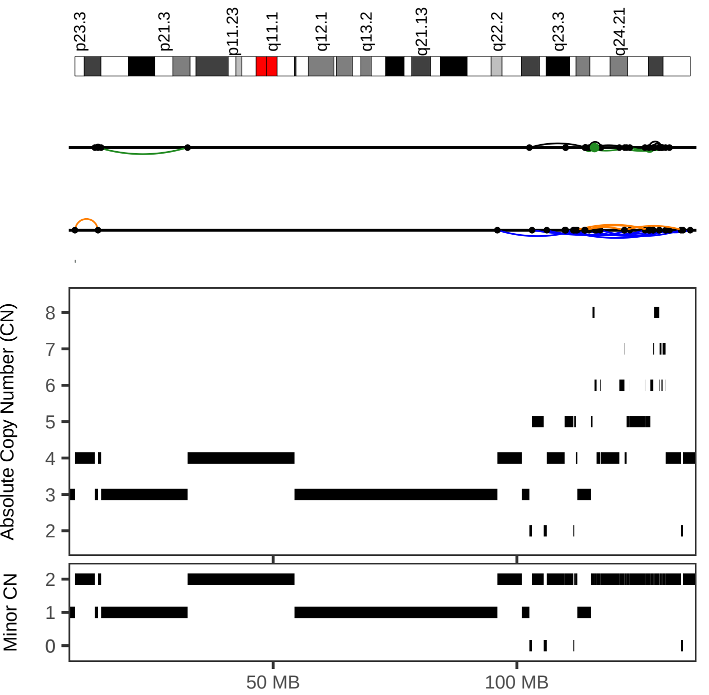

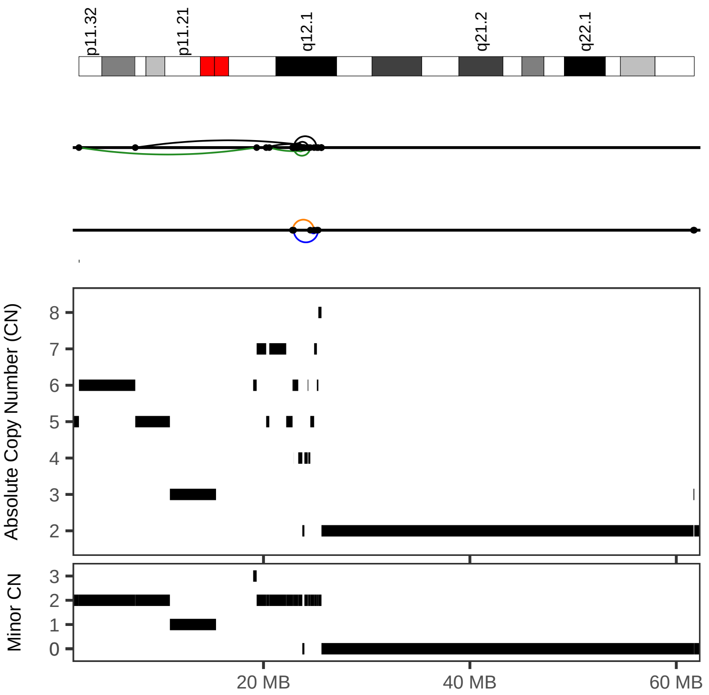

	PCSI_0392
Cancer type	Panc−AdenoCA
Position	8:95993359−135574872
Type	With other complex events
Interleaved intrachr. SVs	27
Total SVs (intrachr. + transl.)	28
SV types	DEL: 5; DUP: 11; h2hINV: 5; t2tINV: 6; TRA: 1
SVs in sample	160
Oscillating CN (2 and 3 states)	6, 17
CN segments	37
FDR fragment joints	0.615458
FDR chr. breakp. enrich.	0
Linked to chrs	
Purity, ploidy	0.99, 3.89

	PCSI_0392
Cancer type	Panc−AdenoCA
Position	18:20260488−25314382
Type	With other complex events
Interleaved intrachr. SVs	13
Total SVs (intrachr. + transl.)	13
SV types	DEL: 1; DUP: 3; h2hINV: 5; t2tINV: 4; TRA: 0
SVs in sample	160
Oscillating CN (2 and 3 states)	4, 7
CN segments	15
FDR fragment joints	0.6776251
FDR chr. breakp. enrich.	0
Linked to chrs	
Purity, ploidy	0.99, 3.89

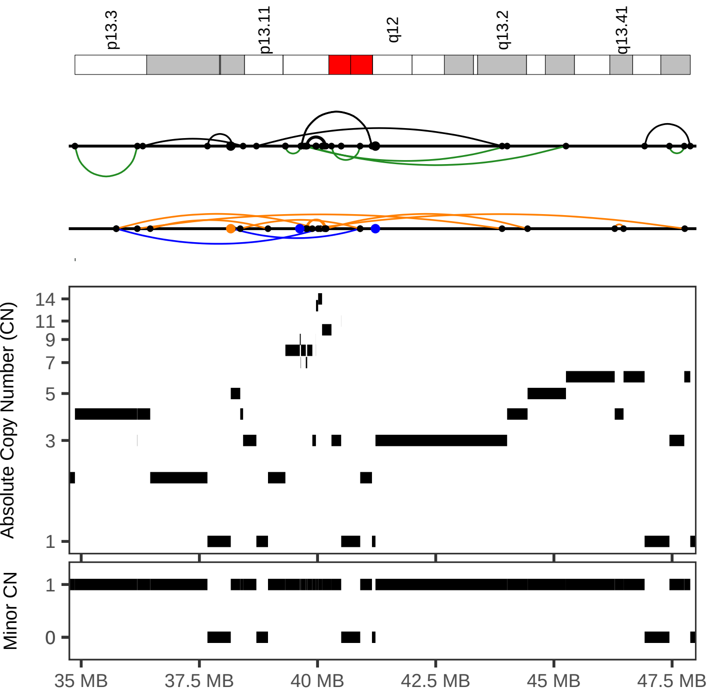

	**PCSI_0450**
Cancer type	Panc−AdenoCA
Position	19:34867315−47884220
Type	With other complex events
Interleaved intrachr. SVs	20
Total SVs (intrachr. + transl.)	25
SV types	DEL: 8; DUP: 2; h2hINV: 7; t2tINV: 3; TRA: 5
SVs in sample	80
Oscillating CN (2 and 3 states)	4, 6
CN segments	37
FDR fragment joints	0.6776251
FDR chr. breakp. enrich.	0
Linked to chrs	
Purity, ploidy	0.95, 1.78

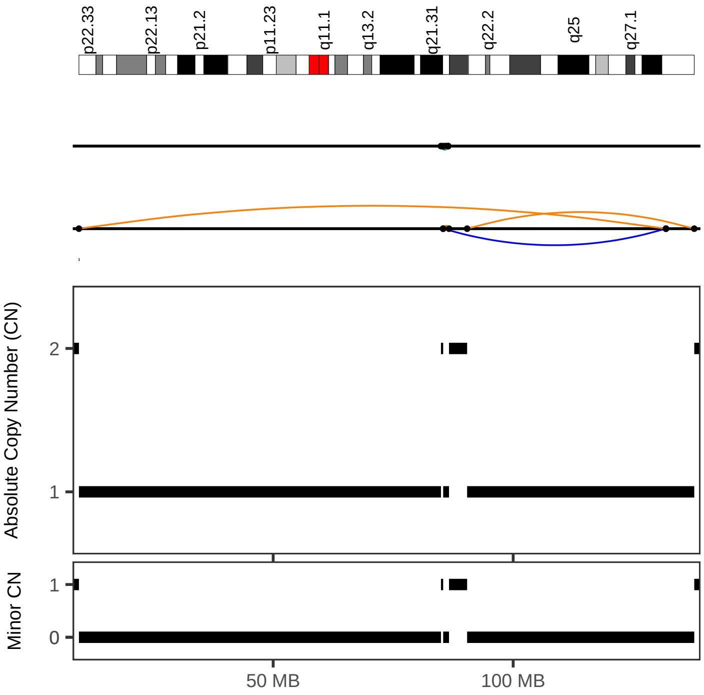

	**PCSI_0450**
Cancer type	Panc−AdenoCA
Position	X:9534069−137725602
Type	Canonical without polyploidization
Interleaved intrachr. SVs	6
Total SVs (intrachr. + transl.)	6
SV types	DEL: 3; DUP: 1; h2hINV: 1; t2tINV: 1; TRA: 0
SVs in sample	80
Oscillating CN (2 and 3 states)	6, 6
CN segments	6
FDR fragment joints	0.6776251
FDR chr. breakp. enrich.	0.55
Linked to chrs	
Purity, ploidy	0.95, 1.78

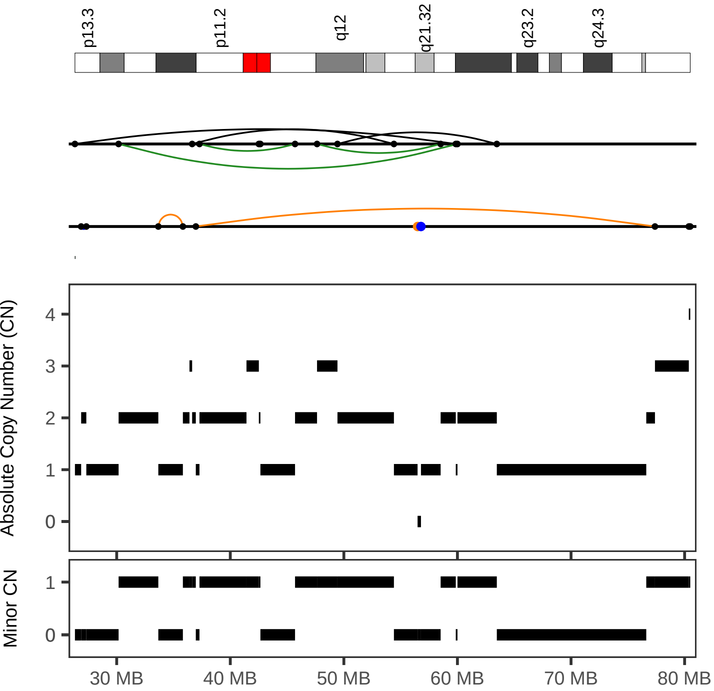

	**PCSI_0453**
Cancer type	Panc−AdenoCA
Position	17:26321720−77389422
Type	With other complex events
Interleaved intrachr. SVs	6
Total SVs (intrachr. + transl.)	8
SV types	DEL: 1; DUP: 0; h2hINV: 3; t2tINV: 2; TRA: 2
SVs in sample	102
Oscillating CN (2 and 3 states)	6, 25
CN segments	25
FDR fragment joints	0.8653243
FDR chr. breakp. enrich.	0
Linked to chrs	5:11299550−143890321;
Purity, ploidy	0.99, 1.96

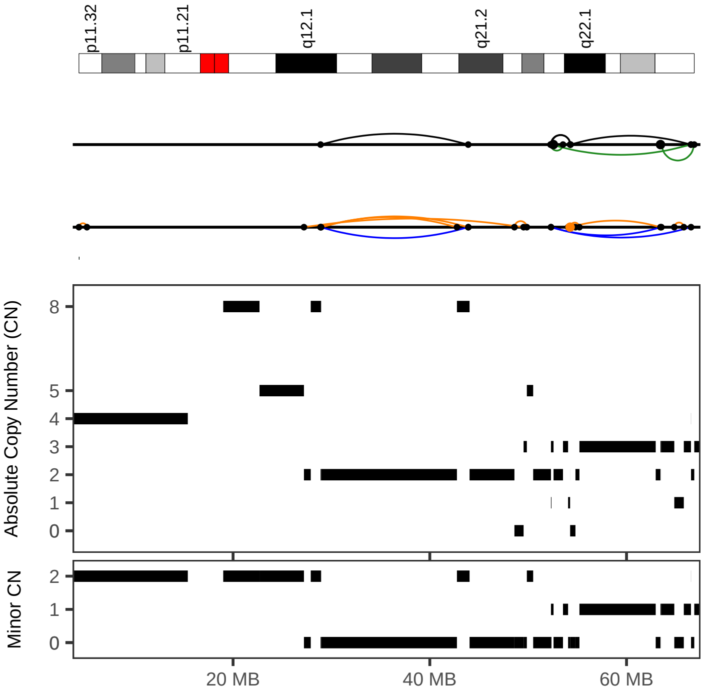

	**PCSI_0456**
Cancer type	Panc−AdenoCA
Position	18:52279996−66883860
Type	With other complex events
Interleaved intrachr. SVs	12
Total SVs (intrachr. + transl.)	15
SV types	DEL: 3; DUP: 2; h2hINV: 4; t2tINV: 3; TRA: 3
SVs in sample	99
Oscillating CN (2 and 3 states)	6, 11
CN segments	21
FDR fragment joints	0.8462769
FDR chr. breakp. enrich.	0
Linked to chrs	
Purity, ploidy	0.8, 3.6

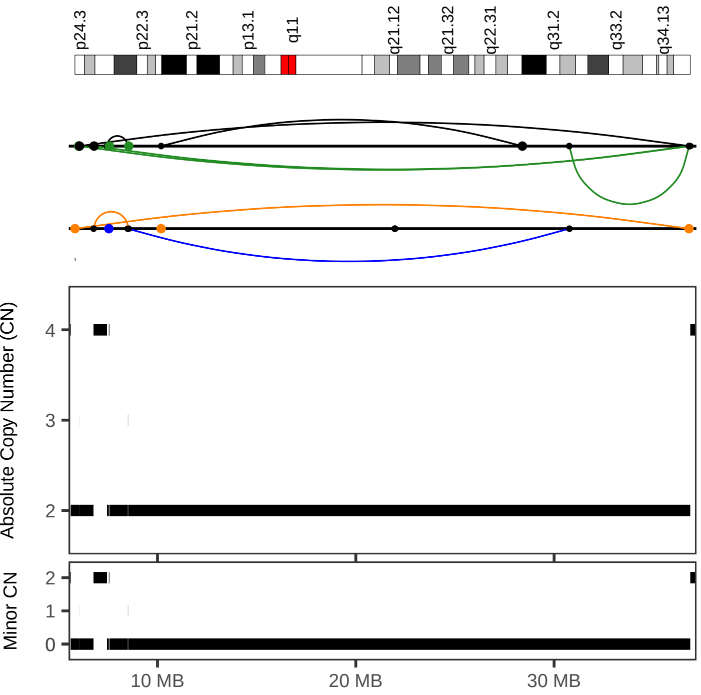

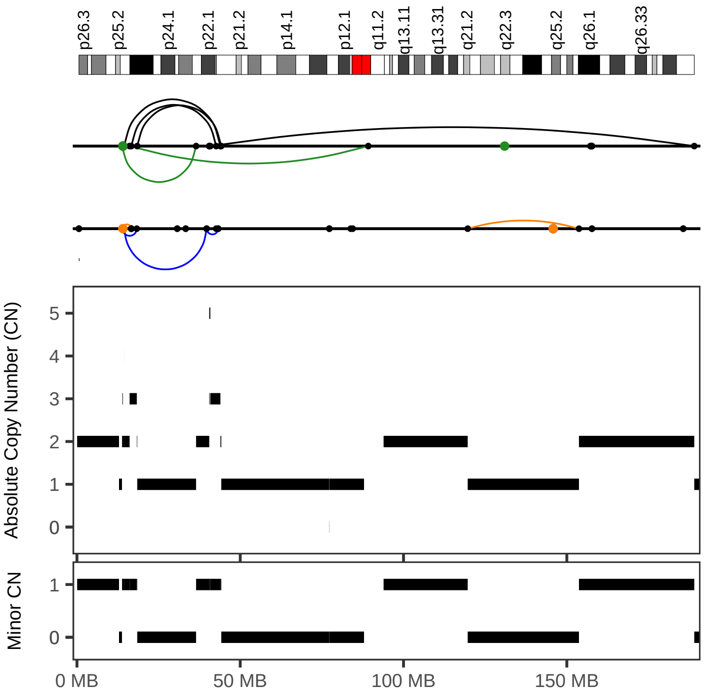

PCSI_0468	
Cancer type	Panc−AdenoCA
Position	9:5835505−36865594
Type	With other complex events
Interleaved intrachr. SVs	8
Total SVs (intrachr. + transl.)	19
SV types	DEL: 2; DUP: 1; h2hINV: 2; t2tINV: 3; TRA: 11
SVs in sample	105
Oscillating CN (2 and 3 states)	5, 12
CN segments	12
FDR fragment joints	0.6776251
FDR chr. breakp. enrich.	0
Linked to chrs	
Purity, ploidy	0.79, 3.59

PCSI_0472	
Cancer type	Panc−AdenoCA
Position	3:13788797−189021715
Type	With other complex events
Interleaved intrachr. SVs	12
Total SVs (intrachr. + transl.)	18
SV types	DEL: 2; DUP: 4; h2hINV: 4; t2tINV: 2; TRA: 6
SVs in sample	238
Oscillating CN (2 and 3 states)	5, 9
CN segments	20
FDR fragment joints	0.8653243
FDR chr. breakp. enrich.	0.01
Linked to chrs	10:28947349−34511967;
Purity, ploidy	0.91, 1.74

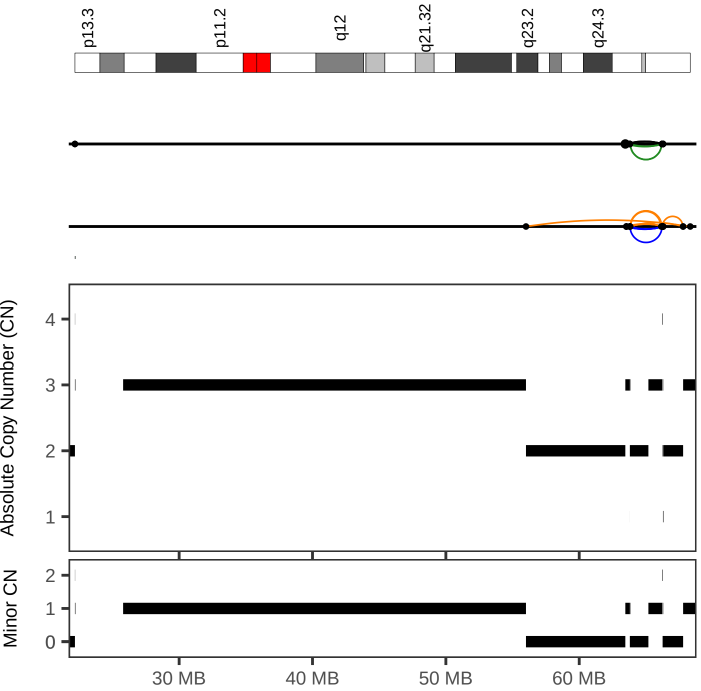

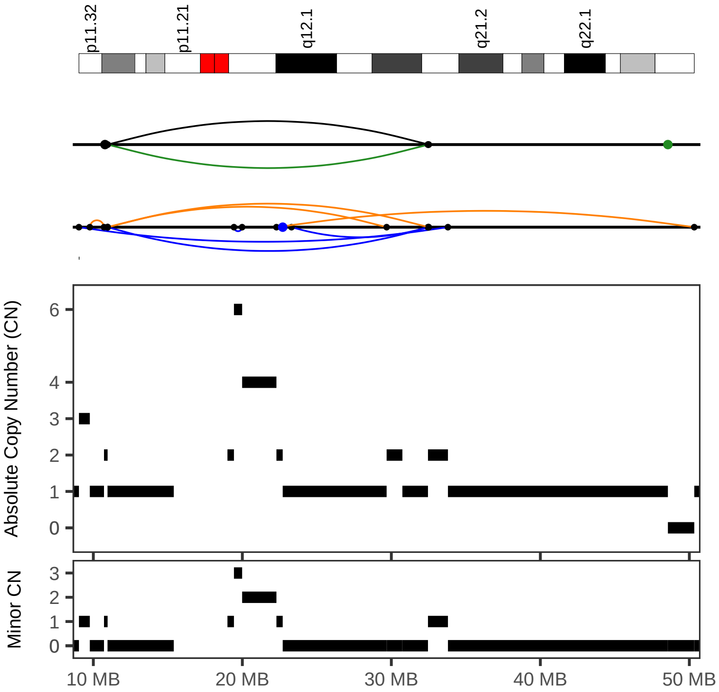

PCSI_0528	
Cancer type	Panc−AdenoCA
Position	17:63528018−67790466
Type	With other complex events
Interleaved intrachr. SVs	24
Total SVs (intrachr. + transl.)	24
SV types	DEL: 9; DUP: 5; h2hINV: 5; t2tINV: 5; TRA: 0
SVs in sample	76
Oscillating CN (2 and 3 states)	4, 7
CN segments	14
FDR fragment joints	0.6776251
FDR chr. breakp. enrich.	0
Linked to chrs	
Purity, ploidy	0.52, 3.06

PCSI_0531	
Cancer type	Panc−AdenoCA
Position	18:9032446−50330464
Type	With other complex events
Interleaved intrachr. SVs	8
Total SVs (intrachr. + transl.)	11
SV types	DEL: 3; DUP: 3; h2hINV: 1; t2tINV: 1; TRA: 3
SVs in sample	96
Oscillating CN (2 and 3 states)	6, 8
CN segments	17
FDR fragment joints	0.8653243
FDR chr. breakp. enrich.	0
Linked to chrs	
Purity, ploidy	0.31, 1.86

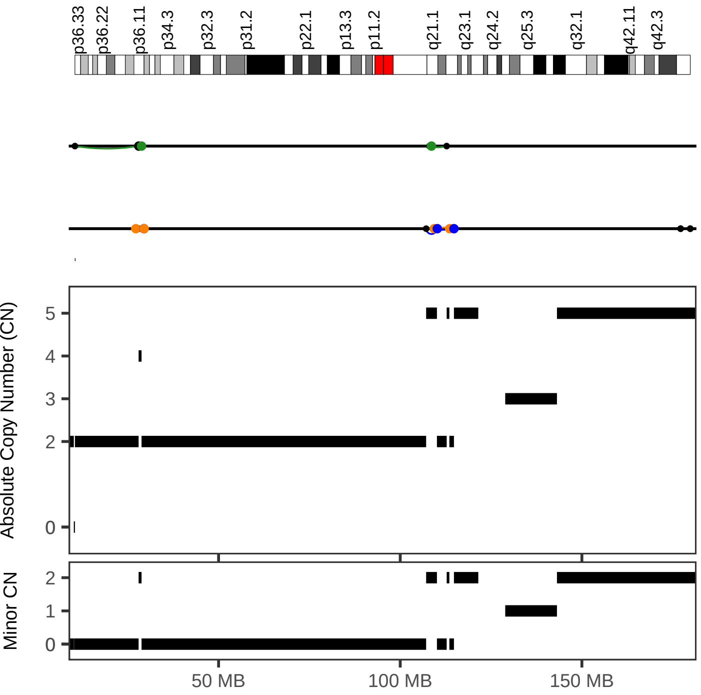

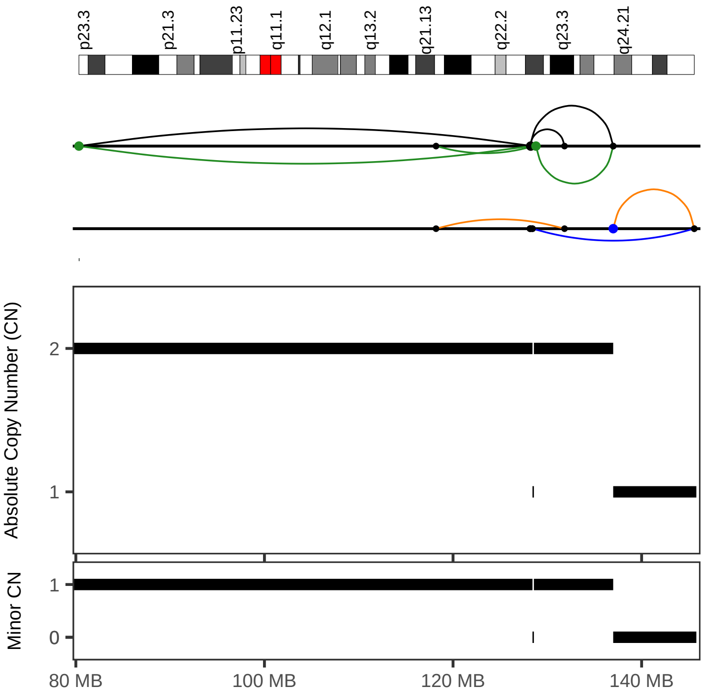

	PCSI_0572
Cancer type	Panc−AdenoCA
Position	1:107128798−114787742
Type	With other complex events
Interleaved intrachr. SVs	6
Total SVs (intrachr. + transl.)	10
SV types	DEL: 2; DUP: 3; h2hINV: 0; t2tINV: 1; TRA: 4
SVs in sample	86
Oscillating CN (2 and 3 states)	5, 5
CN segments	5
FDR fragment joints	0.615458
FDR chr. breakp. enrich.	0
Linked to chrs	2:21945480−110876000;
Purity, ploidy	0.49, 2.71

	ITNET−0134
Cancer type	Panc−Endocrine
Position	8:80326174−145581514
Type	Canonical without polyploidization
Interleaved intrachr. SVs	10
Total SVs (intrachr. + transl.)	14
SV types	DEL: 1; DUP: 3; h2hINV: 3; t2tINV: 3; TRA: 4
SVs in sample	55
Oscillating CN (2 and 3 states)	4, 4
CN segments	4
FDR fragment joints	0.6776251
FDR chr. breakp. enrich.	0
Linked to chrs	
Purity, ploidy	0.61, 2.02

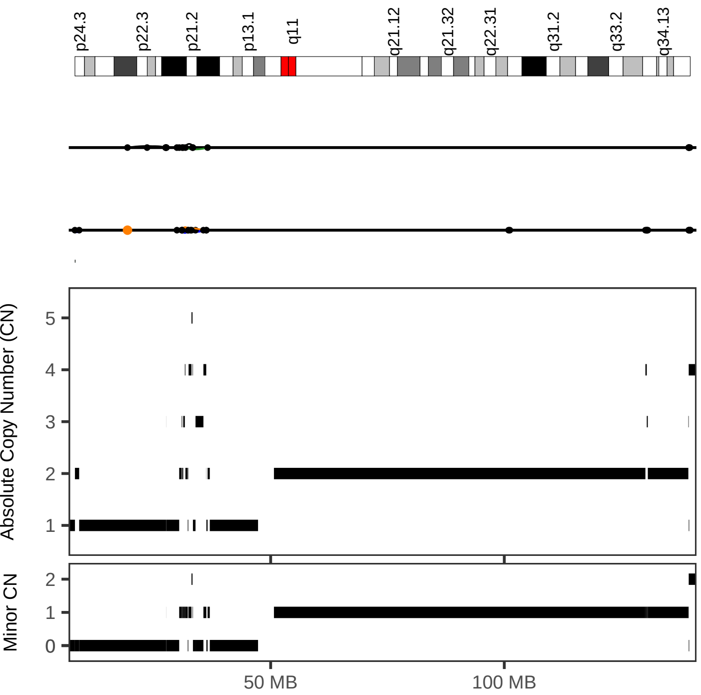

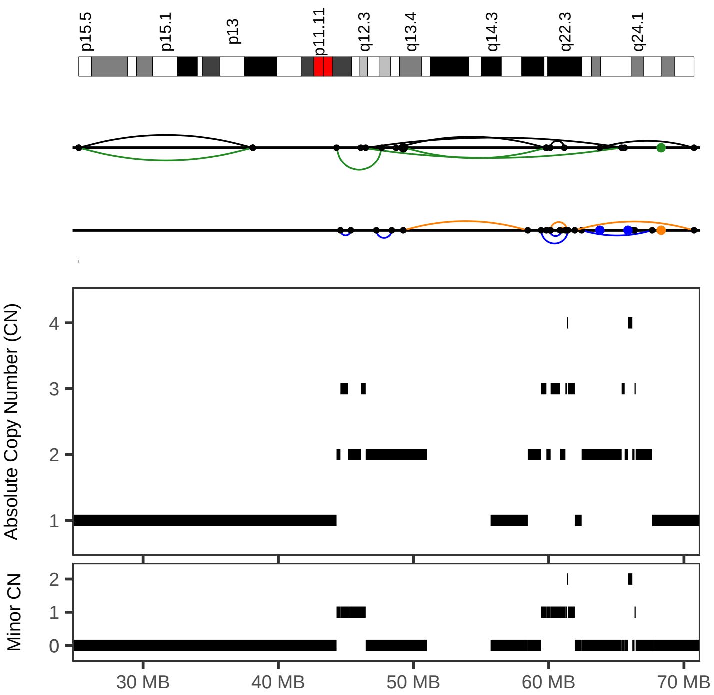

	ITNET−1047
Cancer type	Panc−Endocrine
Position	9:29933578−36500038
Type	With other complex events
Interleaved intrachr. SVs	9
Total SVs (intrachr. + transl.)	9
SV types	DEL: 2; DUP: 3; h2hINV: 2; t2tINV: 2; TRA: 0
SVs in sample	245
Oscillating CN (2 and 3 states)	4, 7
CN segments	18
FDR fragment joints	0.9723381
FDR chr. breakp. enrich.	0
Linked to chrs	
Purity, ploidy	0.9, 1.64

	ITNET−1047
Cancer type	Panc−Endocrine
Position	11:48710192−61425002
Type	Canonical without polyploidization
Interleaved intrachr. SVs	9
Total SVs (intrachr. + transl.)	10
SV types	DEL: 4; DUP: 2; h2hINV: 2; t2tINV: 1; TRA: 1
SVs in sample	245
Oscillating CN (2 and 3 states)	6, 10
CN segments	10
FDR fragment joints	0.8572806
FDR chr. breakp. enrich.	0
Linked to chrs	
Purity, ploidy	0.9, 1.64

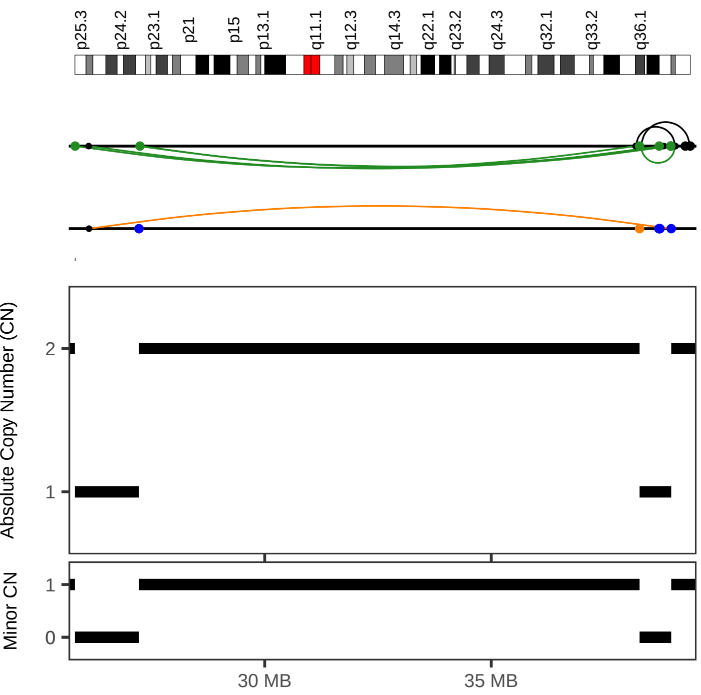

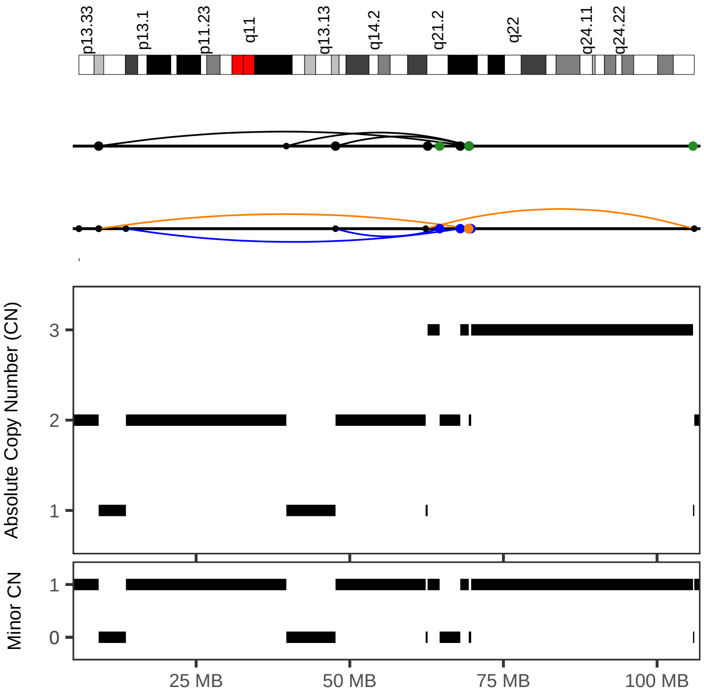

EOPC−022

Cancer type	Prost−AdenoCA
Position	2:25811734−39390809
Type	Canonical without polyploidization
Interleaved intrachr. SVs	8
Total SVs (intrachr. + transl.)	23
SV types	DEL: 1; DUP: 0; h2hINV: 2; t2tINV: 5; TRA: 15
SVs in sample	46
Oscillating CN (2 and 3 states)	4, 4
CN segments	4
FDR fragment joints	0.6776251
FDR chr. breakp. enrich.	0
Linked to chrs	1:100218185−116714121;
Purity, ploidy	0.34, 1.9

EOPC−030

Cancer type	Prost−AdenoCA
Position	12:9146108−106049040
Type	With other complex events
Interleaved intrachr. SVs	7
Total SVs (intrachr. + transl.)	21
SV types	DEL: 2; DUP: 2; h2hINV: 3; t2tINV: 0; TRA: 14
SVs in sample	116
Oscillating CN (2 and 3 states)	5, 12
CN segments	12
FDR fragment joints	0.7568568
FDR chr. breakp. enrich.	0
Linked to chrs	
Purity, ploidy	0.67, 1.97

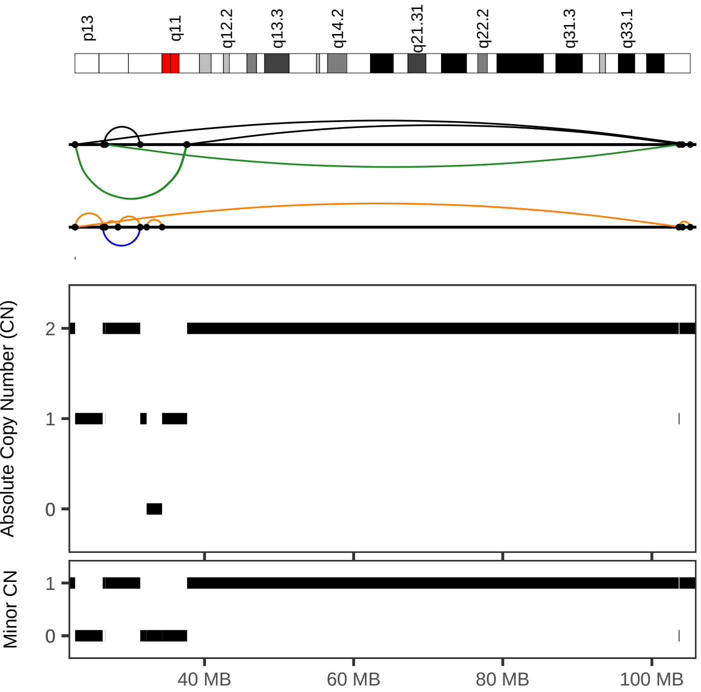

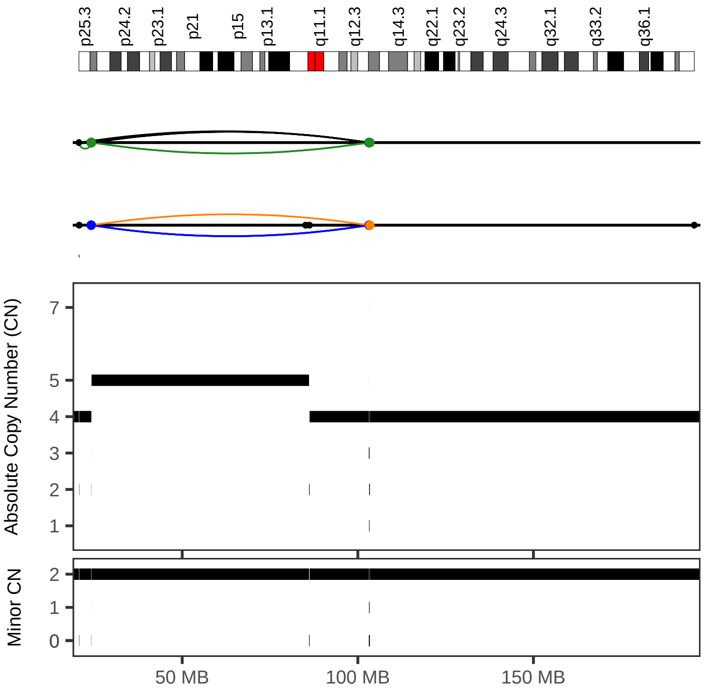

EOPC−030

Cancer type	Prost−AdenoCA
Position	13:22615390−105145326
Type	With other complex events
Interleaved intrachr. SVs	12
Total SVs (intrachr. + transl.)	12
SV types	DEL: 5; DUP: 1; h2hINV: 3; t2tINV: 3; TRA: 0
SVs in sample	116
Oscillating CN (2 and 3 states)	6, 11
CN segments	11
FDR fragment joints	0.6776251
FDR chr. breakp. enrich.	0
Linked to chrs	
Purity, ploidy	0.67, 1.97

EOPC−041

Cancer type	Prost−AdenoCA
Position	2:20591505−103430435
Type	With other complex events
Interleaved intrachr. SVs	16
Total SVs (intrachr. + transl.)	36
SV types	DEL: 4; DUP: 4; h2hINV: 4; t2tINV: 4; TRA: 20
SVs in sample	131
Oscillating CN (2 and 3 states)	5, 9
CN segments	25
FDR fragment joints	0.9284301
FDR chr. breakp. enrich.	0
Linked to chrs	19:42614927−44488344;
Purity, ploidy	0.44, 3.28

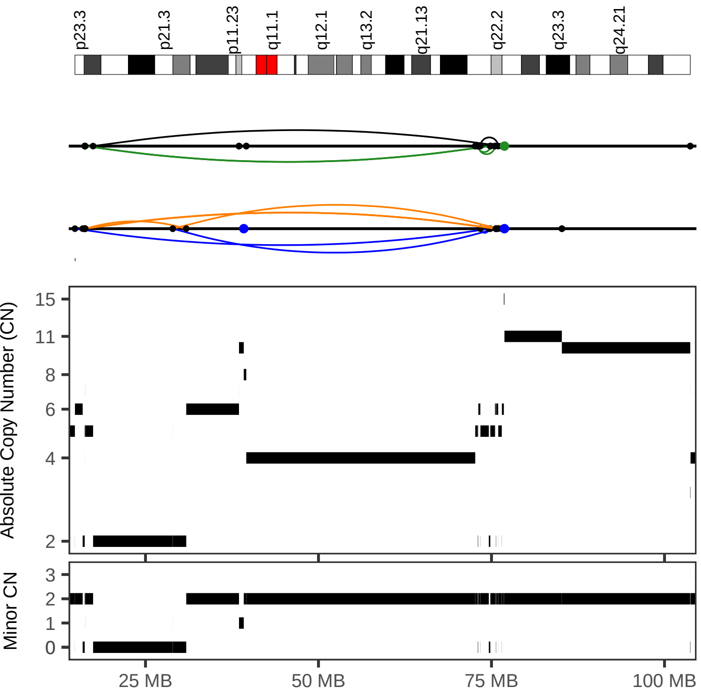

	EOPC−041
Cancer type	Prost−AdenoCA
Position	8:14804569−76501181
Type	With other complex events
Interleaved intrachr. SVs	14
Total SVs (intrachr. + transl.)	15
SV types	DEL: 5; DUP: 2; h2hINV: 3; t2tINV: 4; TRA: 1
SVs in sample	131
Oscillating CN (2 and 3 states)	4, 9
CN segments	33
FDR fragment joints	0.925252
FDR chr. breakp. enrich.	0
Linked to chrs	
Purity, ploidy	0.44, 3.28

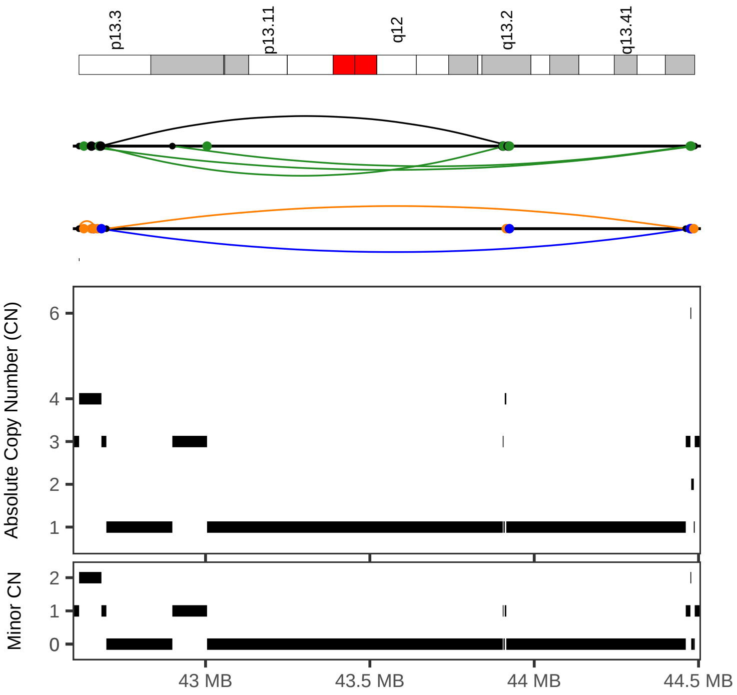

	EOPC−041
Cancer type	Prost−AdenoCA
Position	19:42614927−44488345
Type	With other complex events
Interleaved intrachr. SVs	8
Total SVs (intrachr. + transl.)	30
SV types	DEL: 2; DUP: 1; h2hINV: 2; t2tINV: 3; TRA: 22
SVs in sample	131
Oscillating CN (2 and 3 states)	6, 9
CN segments	15
FDR fragment joints	0.9284301
FDR chr. breakp. enrich.	0
Linked to chrs	2:20591505−103430434;
Purity, ploidy	0.44, 3.28

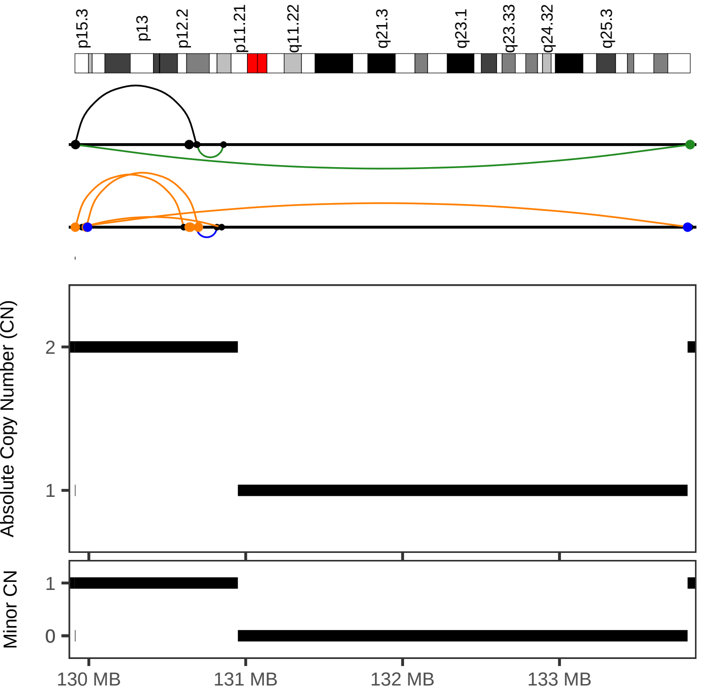

	EOPC−049
Cancer type	Prost−AdenoCA
Position	10:129911442−133833453
Type	Canonical without polyploidization
Interleaved intrachr. SVs	8
Total SVs (intrachr. + transl.)	19
SV types	DEL: 4; DUP: 1; h2hINV: 1; t2tINV: 2; TRA: 11
SVs in sample	50
Oscillating CN (2 and 3 states)	4, 4
CN segments	4
FDR fragment joints	0.615458
FDR chr. breakp. enrich.	0
Linked to chrs	
Purity, ploidy	0.68, 1.9

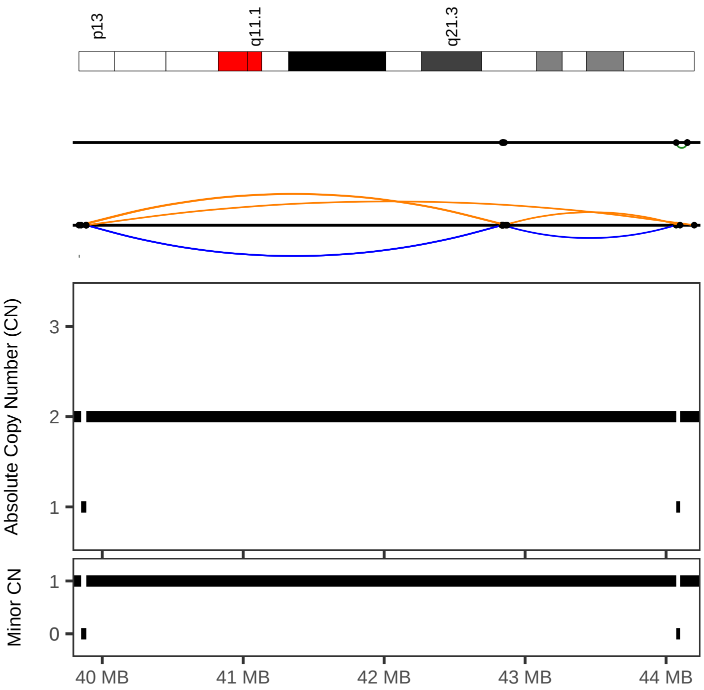

	CPCG0047
Cancer type	Prost−AdenoCA
Position	21:39834144−44199722
Type	Canonical without polyploidization
Interleaved intrachr. SVs	9
Total SVs (intrachr. + transl.)	9
SV types	DEL: 4; DUP: 3; h2hINV: 1; t2tINV: 1; TRA: 0
SVs in sample	74
Oscillating CN (2 and 3 states)	5, 5
CN segments	5
FDR fragment joints	0.615458
FDR chr. breakp. enrich.	0
Linked to chrs	
Purity, ploidy	0.62, 1.91

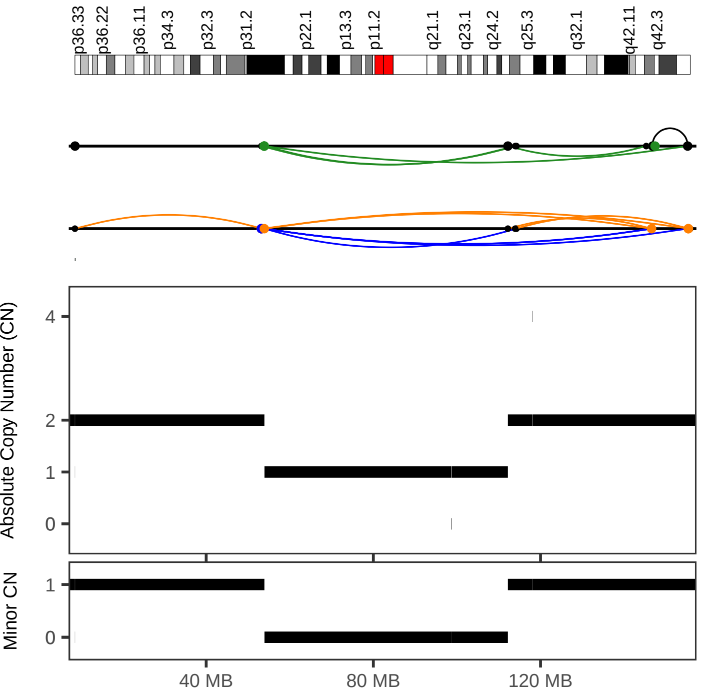

CPCG0094	
Cancer type	Prost−AdenoCA
Position	1:8589683−155821955
Type	With other complex events
Interleaved intrachr. SVs	15
Total SVs (intrachr. + transl.)	33
SV types	DEL: 5; DUP: 4; h2hINV: 2; t2tINV: 4; TRA: 18
SVs in sample	75
Oscillating CN (2 and 3 states)	5, 8
CN segments	10
FDR fragment joints	0.8378534
FDR chr. breakp. enrich.	0
Linked to chrs	2:13930709−194034057;
Purity, ploidy	0.46, 1.79

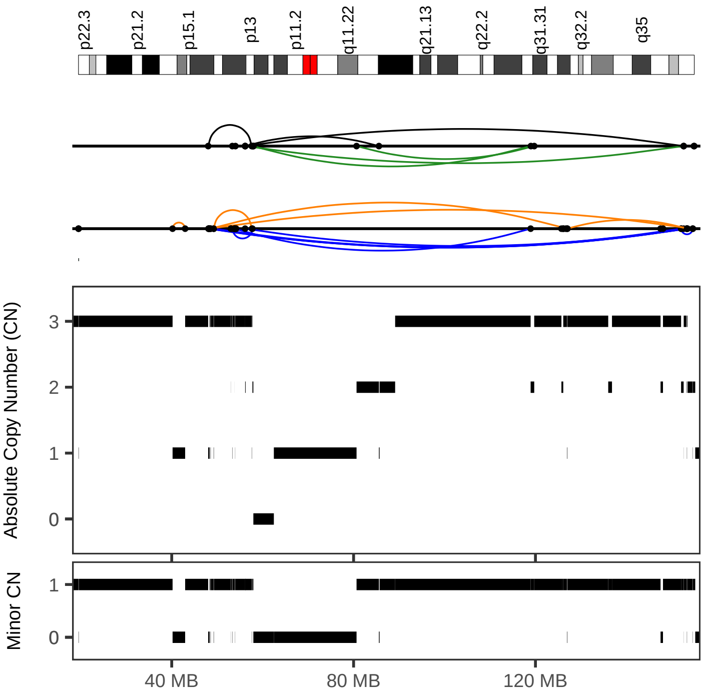

CPCG0099	
Cancer type	Prost−AdenoCA
Position	7:48017345−154624761
Type	With other complex events
Interleaved intrachr. SVs	20
Total SVs (intrachr. + transl.)	20
SV types	DEL: 5; DUP: 8; h2hINV: 4; t2tINV: 3; TRA: 0
SVs in sample	116
Oscillating CN (2 and 3 states)	6, 21
CN segments	46
FDR fragment joints	0.662962
FDR chr. breakp. enrich.	0
Linked to chrs	
Purity, ploidy	0.62, 1.84

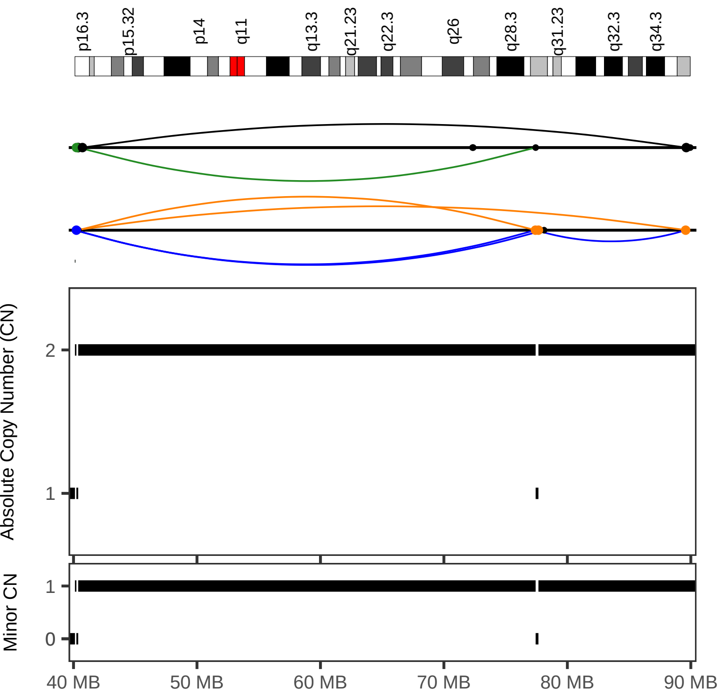

CPCG0123	
Cancer type	Prost−AdenoCA
Position	4:40113263−89954086
Type	Canonical without polyploidization
Interleaved intrachr. SVs	9
Total SVs (intrachr. + transl.)	18
SV types	DEL: 2; DUP: 4; h2hINV: 2; t2tINV: 1; TRA: 9
SVs in sample	144
Oscillating CN (2 and 3 states)	5, 5
CN segments	5
FDR fragment joints	0.9875525
FDR chr. breakp. enrich.	0
Linked to chrs	
Purity, ploidy	0.44, 1.9

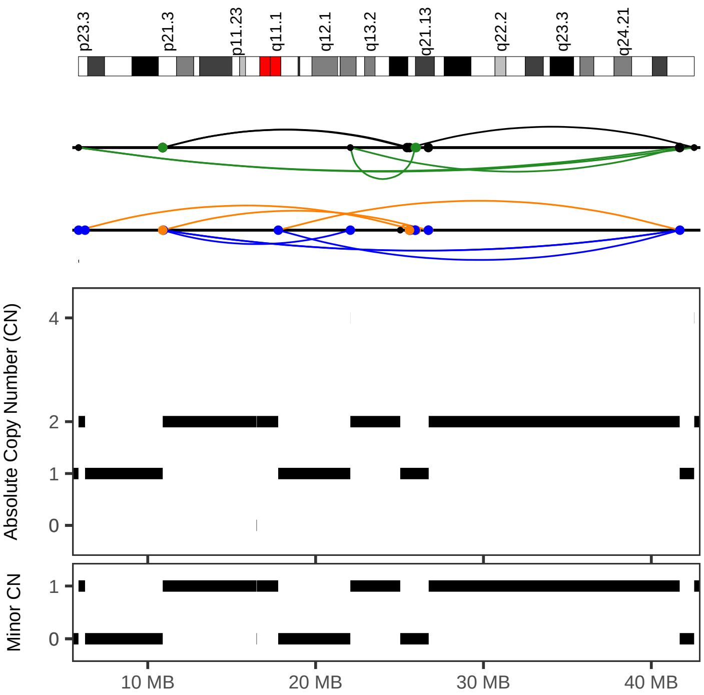

CPCG0124	
Cancer type	Prost−AdenoCA
Position	8:5872316−42557666
Type	With other complex events
Interleaved intrachr. SVs	15
Total SVs (intrachr. + transl.)	46
SV types	DEL: 4; DUP: 4; h2hINV: 3; t2tINV: 4; TRA: 31
SVs in sample	121
Oscillating CN (2 and 3 states)	4, 7
CN segments	14
FDR fragment joints	0.6776251
FDR chr. breakp. enrich.	0
Linked to chrs	
Purity, ploidy	0.47, 1.87

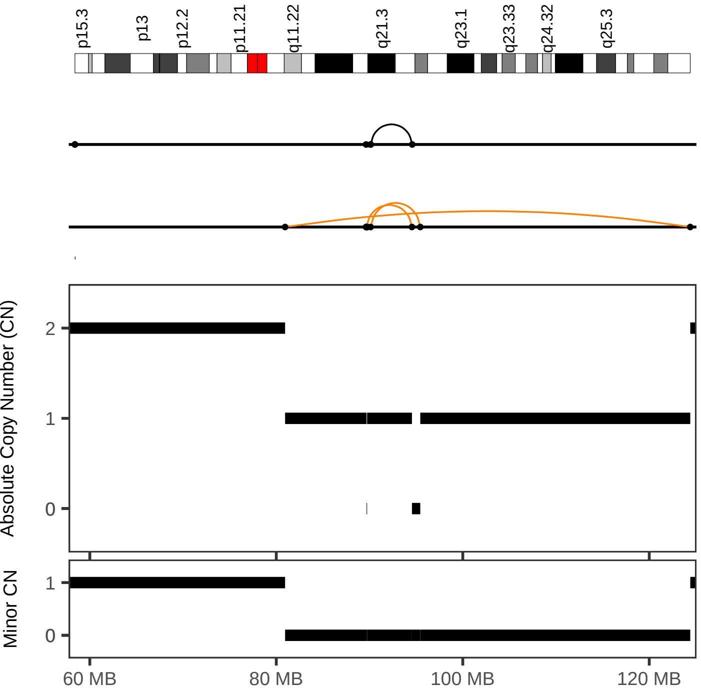

	**CPCG0166**
Cancer type	Prost−AdenoCA
Position	10:89626569−95441303
Type	Canonical without polyploidization
Interleaved intrachr. SVs	6
Total SVs (intrachr. + transl.)	6
SV types	DEL: 3; DUP: 1; h2hINV: 1; t2tINV: 1; TRA: 0
SVs in sample	126
Oscillating CN (2 and 3 states)	5, 5
CN segments	5
FDR fragment joints	0.6776251
FDR chr. breakp. enrich.	0.63
Linked to chrs	
Purity, ploidy	0.18, 1.86

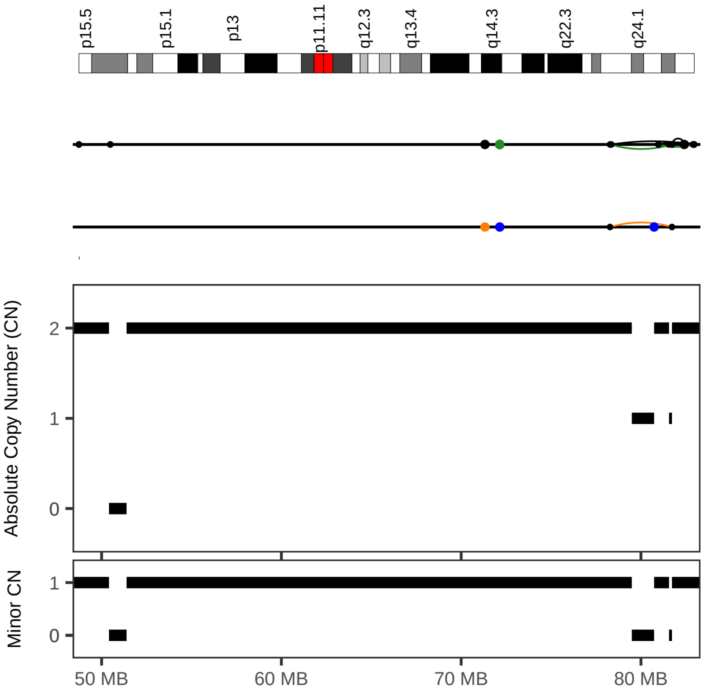

	**CPCG0189**
Cancer type	Prost−AdenoCA
Position	11:78278095−82964421
Type	Canonical without polyploidization
Interleaved intrachr. SVs	6
Total SVs (intrachr. + transl.)	8
SV types	DEL: 1; DUP: 0; h2hINV: 3; t2tINV: 2; TRA: 2
SVs in sample	92
Oscillating CN (2 and 3 states)	5, 5
CN segments	5
FDR fragment joints	0.6776251
FDR chr. breakp. enrich.	0
Linked to chrs	
Purity, ploidy	0.61, 1.89

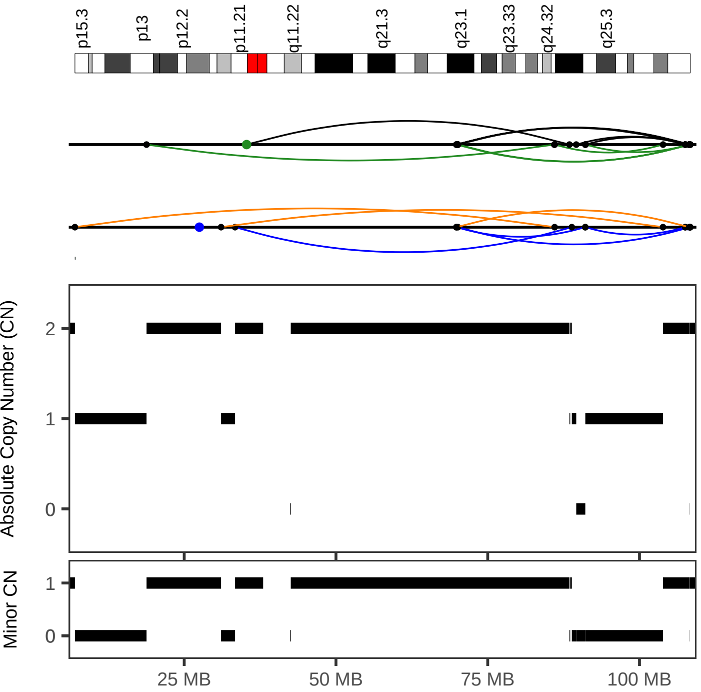

	**CPCG0191**
Cancer type	Prost−AdenoCA
Position	10:6994207−108361744
Type	With other complex events
Interleaved intrachr. SVs	23
Total SVs (intrachr. + transl.)	25
SV types	DEL: 3; DUP: 5; h2hINV: 7; t2tINV: 8; TRA: 2
SVs in sample	121
Oscillating CN (2 and 3 states)	4, 9
CN segments	14
FDR fragment joints	0.6776251
FDR chr. breakp. enrich.	0
Linked to chrs	
Purity, ploidy	0.55, 1.84

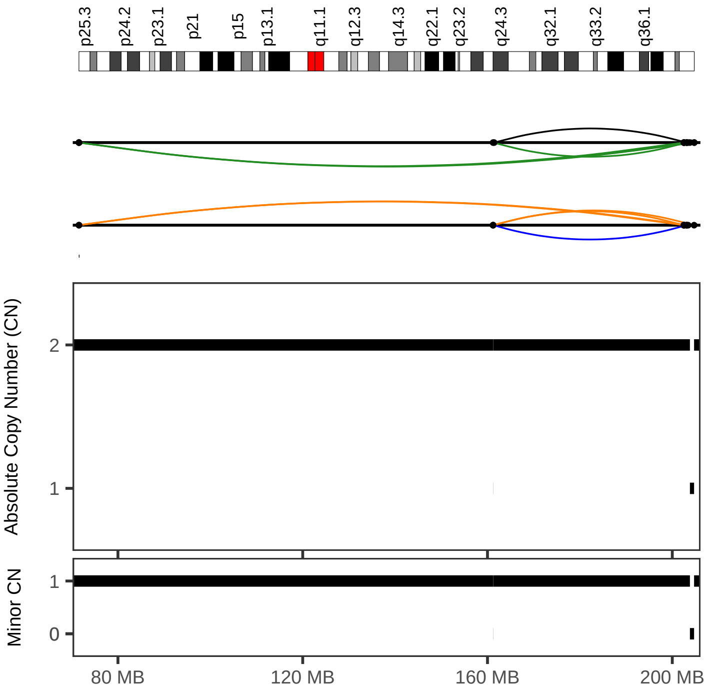

	**CPCG0196**
Cancer type	Prost−AdenoCA
Position	2:71553010−204756130
Type	Canonical without polyploidization
Interleaved intrachr. SVs	15
Total SVs (intrachr. + transl.)	15
SV types	DEL: 5; DUP: 2; h2hINV: 4; t2tINV: 4; TRA: 0
SVs in sample	116
Oscillating CN (2 and 3 states)	5, 5
CN segments	5
FDR fragment joints	0.8462769
FDR chr. breakp. enrich.	0.1
Linked to chrs	
Purity, ploidy	0.7, 1.91

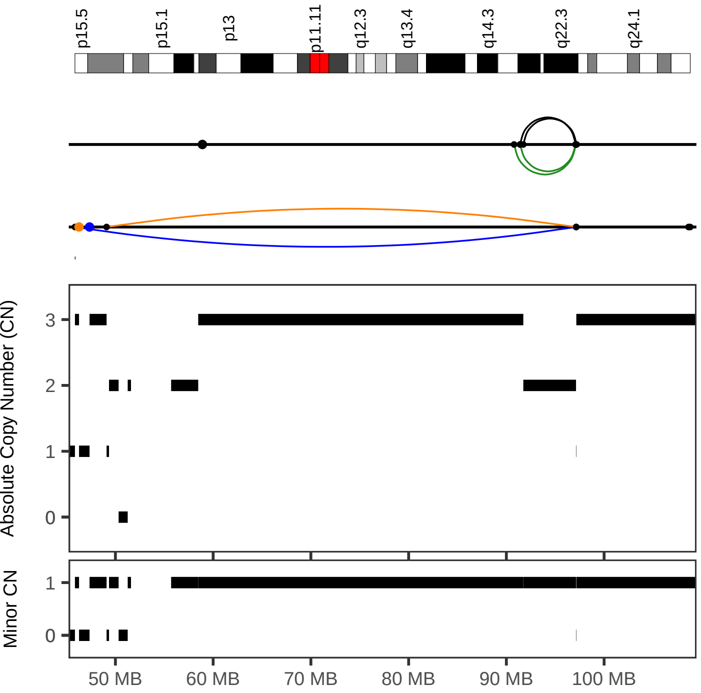

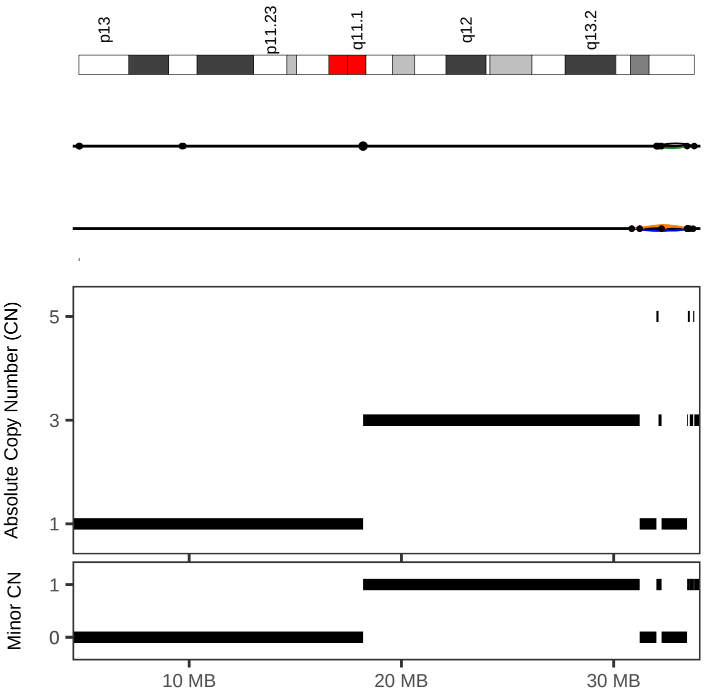

CPCG0206

Cancer type	Prost−AdenoCA
Position	11:45858528−97193270
Type	With other complex events
Interleaved intrachr. SVs	6
Total SVs (intrachr. + transl.)	9
SV types	DEL: 1; DUP: 1; h2hINV: 2; t2tINV: 2; TRA: 3
SVs in sample	173
Oscillating CN (2 and 3 states)	4, 7
CN segments	12
FDR fragment joints	0.9723381
FDR chr. breakp. enrich.	0.26
Linked to chrs	
Purity, ploidy	0.3, 1.94

CPCG0236

Cancer type	Prost−AdenoCA
Position	20:30850339−33795463
Type	With other complex events
Interleaved intrachr. SVs	15
Total SVs (intrachr. + transl.)	15
SV types	DEL: 7; DUP: 4; h2hINV: 2; t2tINV: 2; TRA: 0
SVs in sample	237
Oscillating CN (2 and 3 states)	5, 5
CN segments	10
FDR fragment joints	0.615458
FDR chr. breakp. enrich.	0
Linked to chrs	
Purity, ploidy	0.48, 1.9

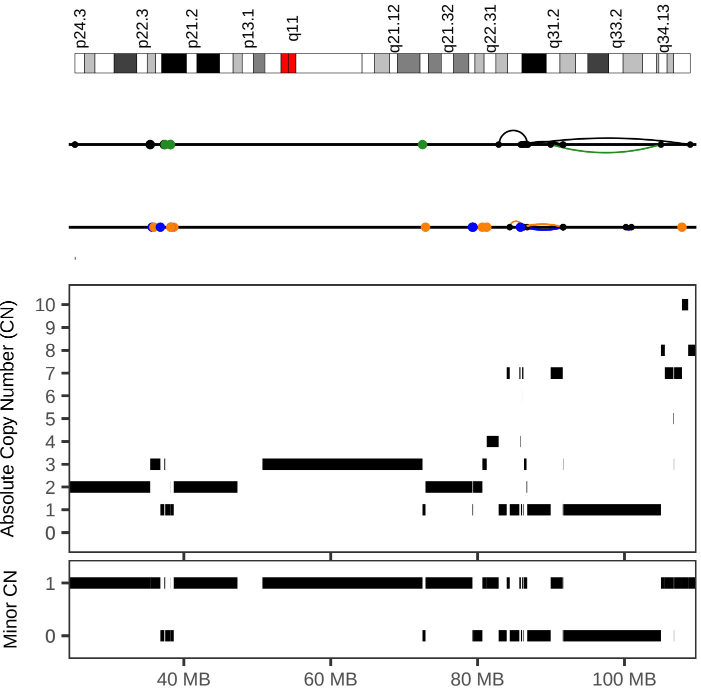

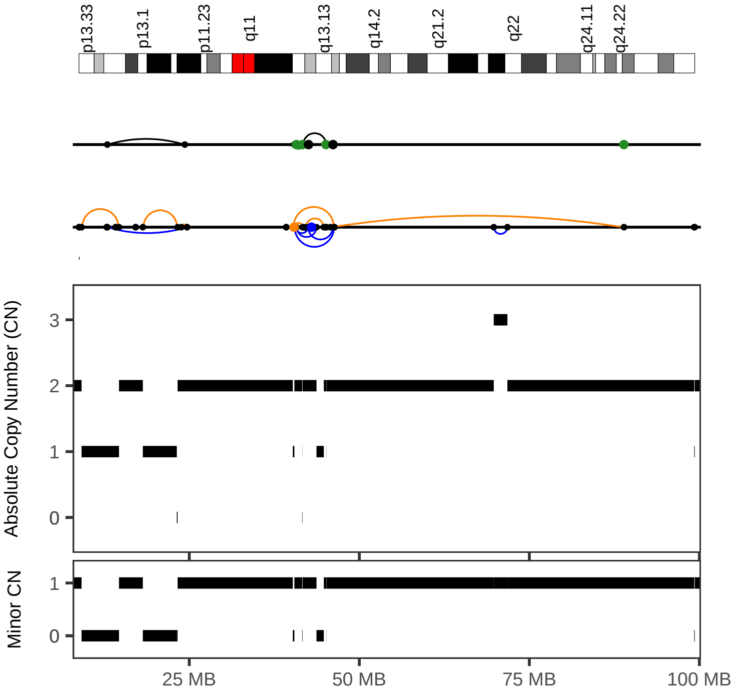

CPCG0242

Cancer type	Prost−AdenoCA
Position	9:82867617−108956321
Type	With other complex events
Interleaved intrachr. SVs	15
Total SVs (intrachr. + transl.)	17
SV types	DEL: 3; DUP: 2; h2hINV: 6; t2tINV: 4; TRA: 2
SVs in sample	217
Oscillating CN (2 and 3 states)	4, 4
CN segments	23
FDR fragment joints	0.8653243
FDR chr. breakp. enrich.	0
Linked to chrs	8:8188079−96079991;
Purity, ploidy	0.65, 1.95

CPCG0249

Cancer type	Prost−AdenoCA
Position	12:40224539−88916203
Type	With other complex events
Interleaved intrachr. SVs	12
Total SVs (intrachr. + transl.)	20
SV types	DEL: 5; DUP: 5; h2hINV: 1; t2tINV: 1; TRA: 8
SVs in sample	458
Oscillating CN (2 and 3 states)	6, 6
CN segments	11
FDR fragment joints	0.8572806
FDR chr. breakp. enrich.	0.1
Linked to chrs	
Purity, ploidy	0.69, 1.87

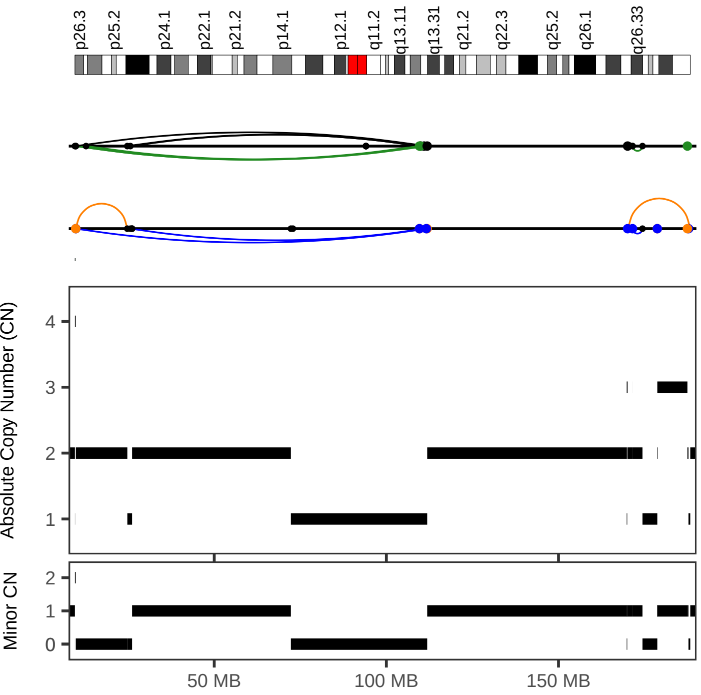

CPCG0251	
Cancer type	Prost−AdenoCA
Position	3:9531696−111833851
Type	Canonical without polyploidization
Interleaved intrachr. SVs	10
Total SVs (intrachr. + transl.)	20
SV types	DEL: 1; DUP: 2; h2hINV: 3; t2tINV: 4; TRA: 10
SVs in sample	67
Oscillating CN (2 and 3 states)	5, 6
CN segments	6
FDR fragment joints	0.9625775
FDR chr. breakp. enrich.	0
Linked to chrs	
Purity, ploidy	0.24, 2.2

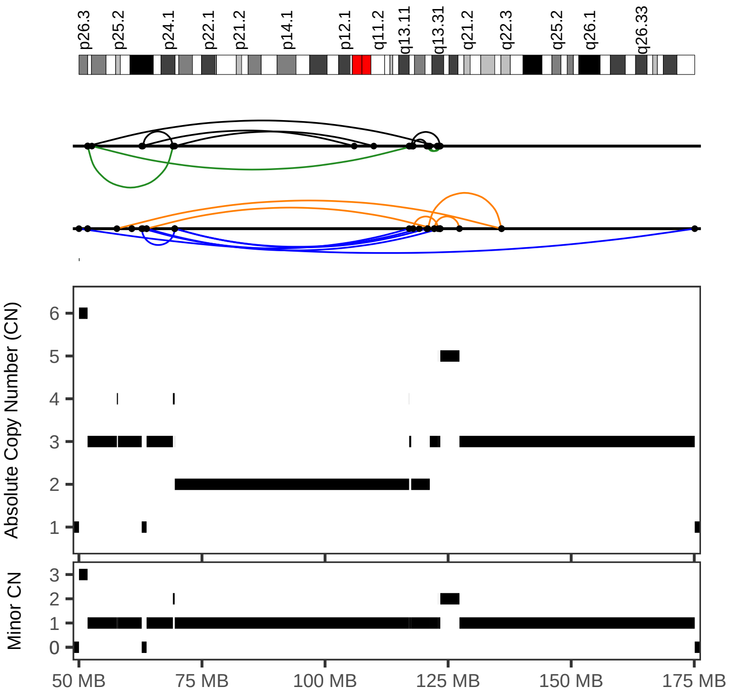

CPCG0263	
Cancer type	Prost−AdenoCA
Position	3:51760828−135855244
Type	With other complex events
Interleaved intrachr. SVs	21
Total SVs (intrachr. + transl.)	21
SV types	DEL: 6; DUP: 5; h2hINV: 6; t2tINV: 4; TRA: 0
SVs in sample	67
Oscillating CN (2 and 3 states)	5, 7
CN segments	17
FDR fragment joints	0.9462199
FDR chr. breakp. enrich.	0
Linked to chrs	
Purity, ploidy	0.39, 1.97

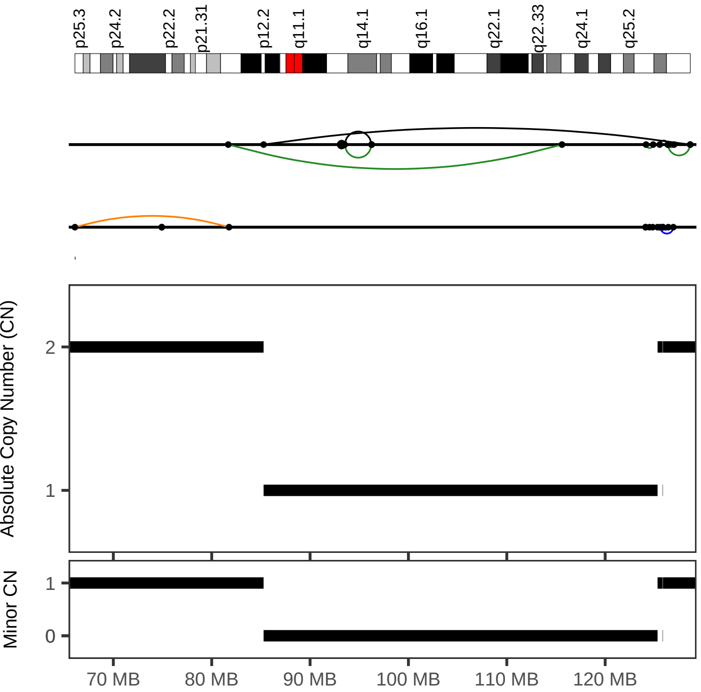

CPCG0268	
Cancer type	Prost−AdenoCA
Position	6:66095219−128649048
Type	Canonical without polyploidization
Interleaved intrachr. SVs	9
Total SVs (intrachr. + transl.)	10
SV types	DEL: 1; DUP: 3; h2hINV: 2; t2tINV: 3; TRA: 1
SVs in sample	135
Oscillating CN (2 and 3 states)	5, 5
CN segments	5
FDR fragment joints	0.6776251
FDR chr. breakp. enrich.	0.02
Linked to chrs	
Purity, ploidy	0.34, 1.89

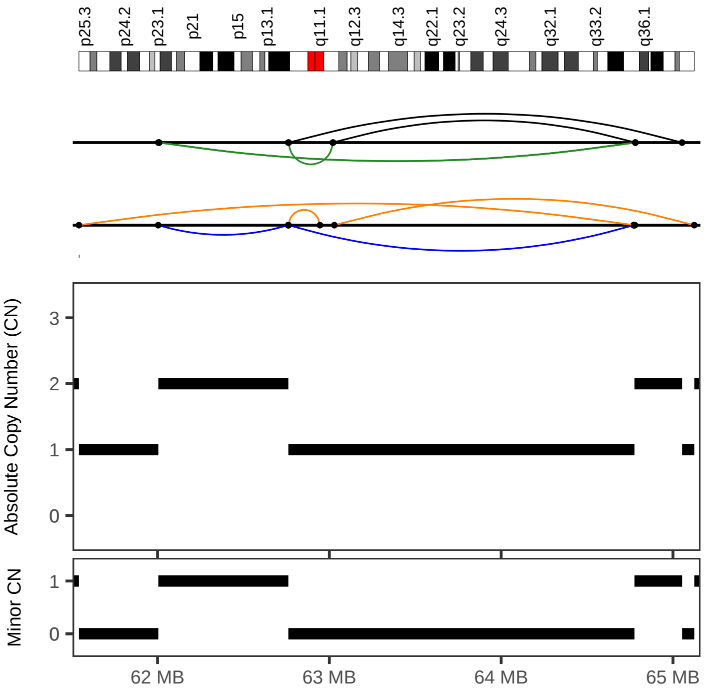

CPCG0342	
Cancer type	Prost−AdenoCA
Position	2:61542346−65123885
Type	Canonical without polyploidization
Interleaved intrachr. SVs	10
Total SVs (intrachr. + transl.)	10
SV types	DEL: 3; DUP: 2; h2hINV: 2; t2tINV: 3; TRA: 0
SVs in sample	72
Oscillating CN (2 and 3 states)	6, 6
CN segments	6
FDR fragment joints	0.9625775
FDR chr. breakp. enrich.	0.01
Linked to chrs	
Purity, ploidy	0.34, 1.92

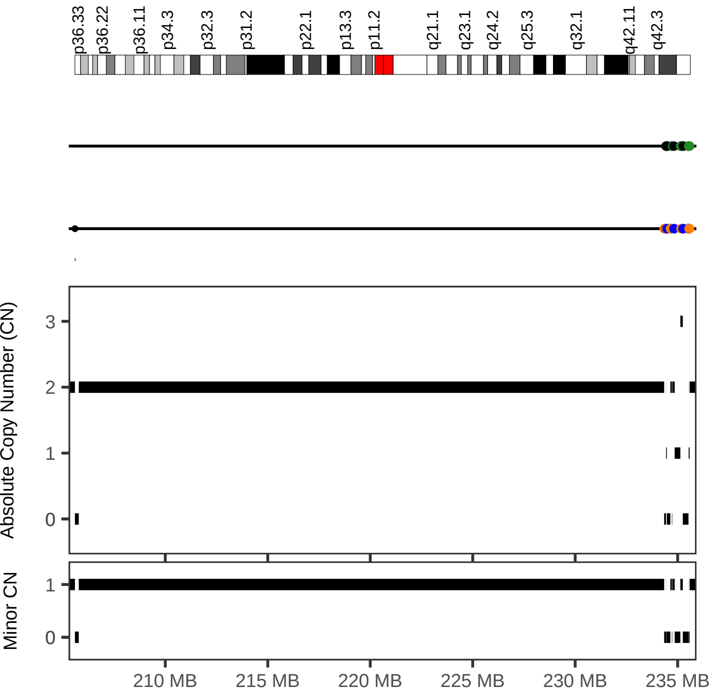

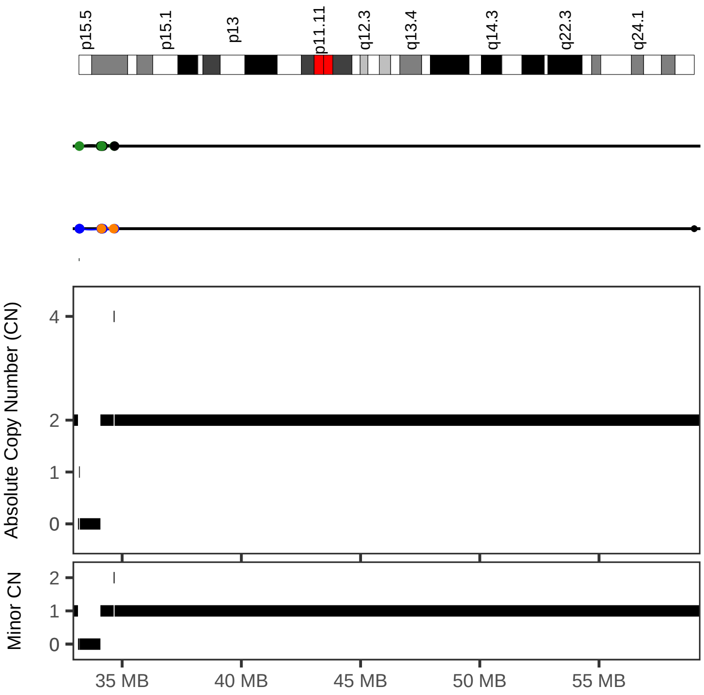

	CPCG0357
Cancer type	Prost−AdenoCA
Position	1:234433222−235613195
Type	With other complex events
Interleaved intrachr. SVs	33
Total SVs (intrachr. + transl.)	71
SV types	DEL: 11; DUP: 11; h2hINV: 7; t2tINV: 4; TRA: 38
SVs in sample	162
Oscillating CN (2 and 3 states)	4, 8
CN segments	13
FDR fragment joints	0.615458
FDR chr. breakp. enrich.	0
Linked to chrs	11:33186637−34681925;
Purity, ploidy	0.55, 1.92

	CPCG0357
Cancer type	Prost−AdenoCA
Position	11:33186637−34681926
Type	With other complex events
Interleaved intrachr. SVs	15
Total SVs (intrachr. + transl.)	44
SV types	DEL: 4; DUP: 3; h2hINV: 5; t2tINV: 3; TRA: 29
SVs in sample	162
Oscillating CN (2 and 3 states)	4, 5
CN segments	7
FDR fragment joints	0.6372084
FDR chr. breakp. enrich.	0
Linked to chrs	
Purity, ploidy	0.55, 1.92

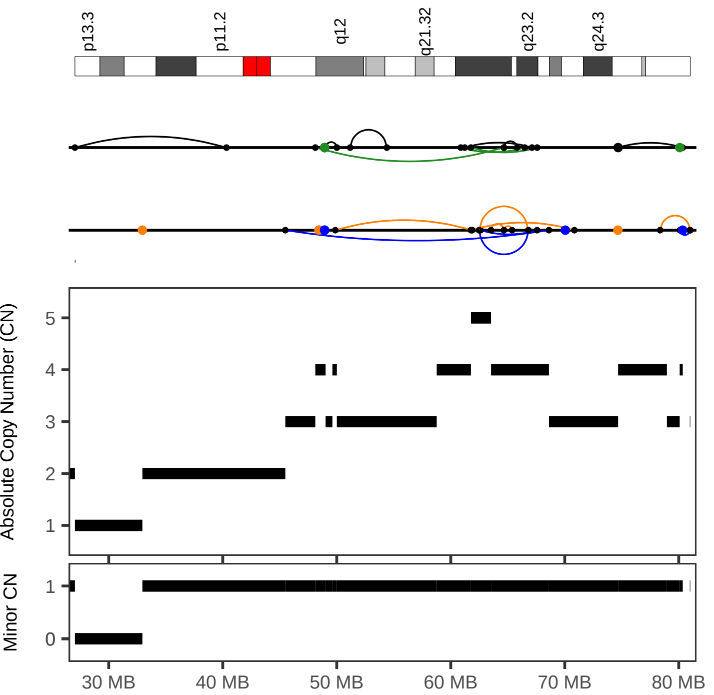

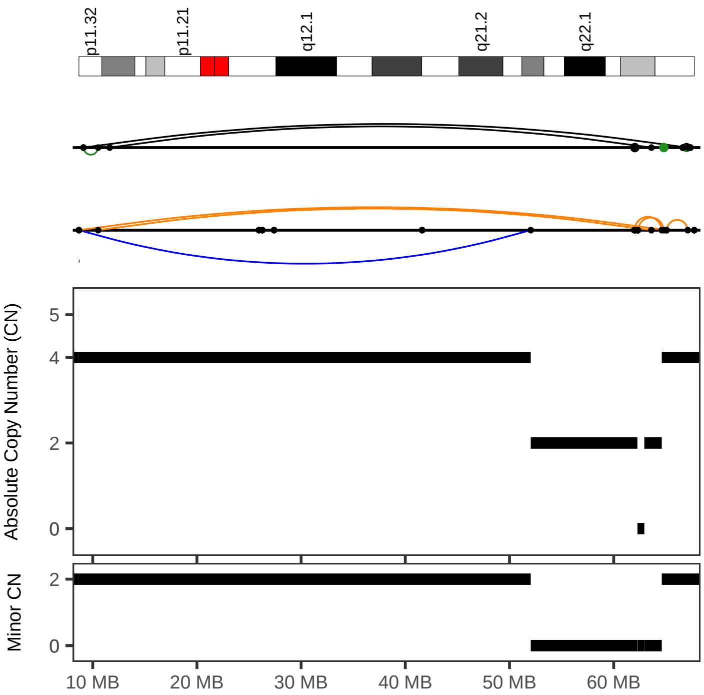

	CPCG0379
Cancer type	Prost−AdenoCA
Position	17:45491837−70847983
Type	After polyploidization
Interleaved intrachr. SVs	13
Total SVs (intrachr. + transl.)	20
SV types	DEL: 4; DUP: 3; h2hINV: 3; t2tINV: 3; TRA: 7
SVs in sample	107
Oscillating CN (2 and 3 states)	6, 9
CN segments	9
FDR fragment joints	0.8572806
FDR chr. breakp. enrich.	0
Linked to chrs	
Purity, ploidy	0.71, 1.98

	0091_CRUK_PC_0091
Cancer type	Prost−AdenoCA
Position	18:8673012−67370977
Type	Canonical without polyploidization
Interleaved intrachr. SVs	11
Total SVs (intrachr. + transl.)	13
SV types	DEL: 5; DUP: 1; h2hINV: 3; t2tINV: 2; TRA: 2
SVs in sample	308
Oscillating CN (2 and 3 states)	5, 7
CN segments	8
FDR fragment joints	0.6776251
FDR chr. breakp. enrich.	0
Linked to chrs	3:69780654−86522448;
Purity, ploidy	0.86, 3.7

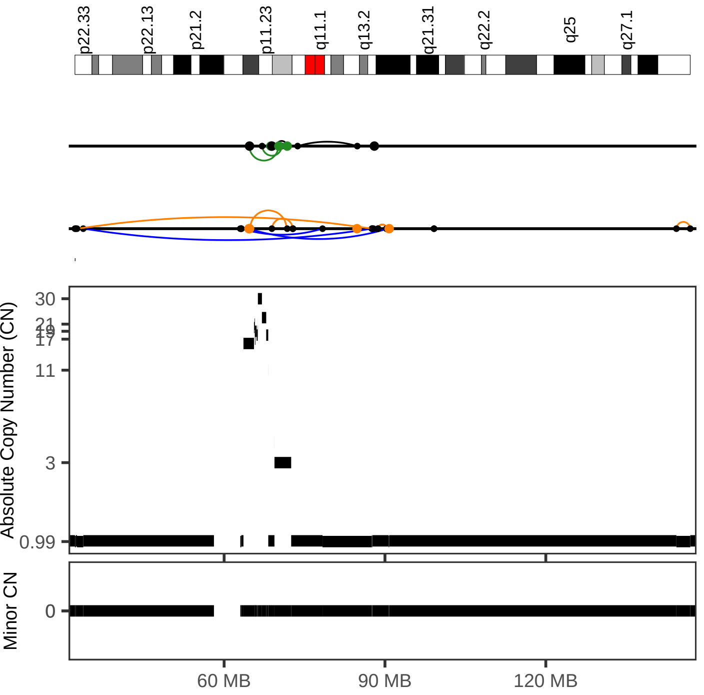

A21−0096_CRUK_PC_0096	
Cancer type	Prost−AdenoCA
Position	X:32559602−90575521
Type	With other complex events
Interleaved intrachr. SVs	7
Total SVs (intrachr. + transl.)	16
SV types	DEL: 2; DUP: 4; h2hINV: 1; t2tINV: 0; TRA: 9
SVs in sample	496
Oscillating CN (2 and 3 states)	5, 7
CN segments	24
FDR fragment joints	0.9501265
FDR chr. breakp. enrich.	0.55
Linked to chrs	
Purity, ploidy	NA, NA

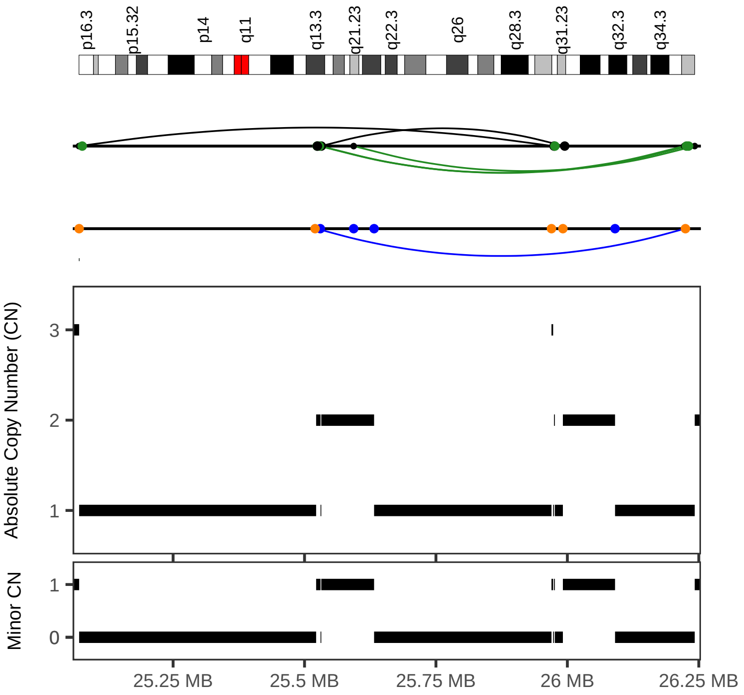

A22−0016_CRUK_PC_0016	
Cancer type	Prost−AdenoCA
Position	4:25070963−26242242
Type	With other complex events
Interleaved intrachr. SVs	6
Total SVs (intrachr. + transl.)	25
SV types	DEL: 0; DUP: 1; h2hINV: 2; t2tINV: 3; TRA: 19
SVs in sample	156
Oscillating CN (2 and 3 states)	6, 13
CN segments	13
FDR fragment joints	0.8653243
FDR chr. breakp. enrich.	0
Linked to chrs	2:210205164−210324505;5:54361714−175053850
Purity, ploidy	NA, NA

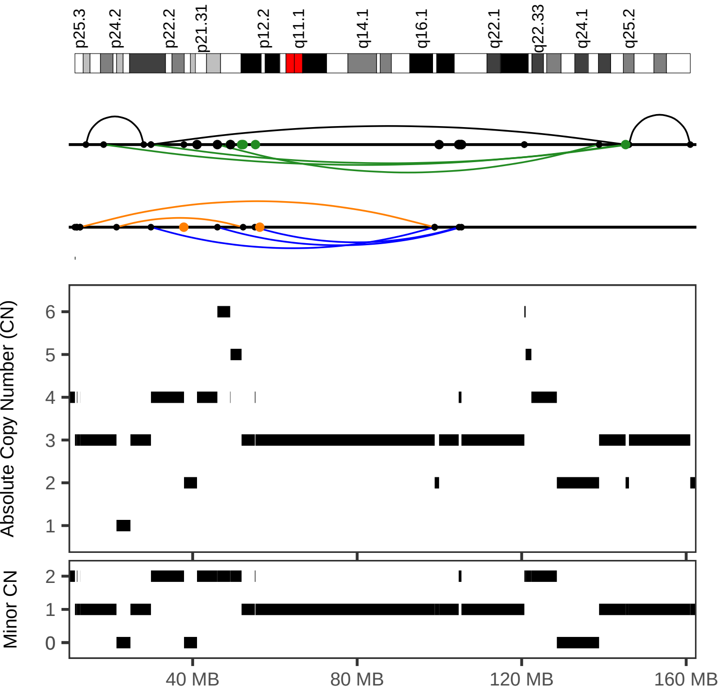

A22−0016_CRUK_PC_0016	
Cancer type	Prost−AdenoCA
Position	6:12626345−160987932
Type	With other complex events
Interleaved intrachr. SVs	11
Total SVs (intrachr. + transl.)	26
SV types	DEL: 2; DUP: 3; h2hINV: 3; t2tINV: 3; TRA: 15
SVs in sample	156
Oscillating CN (2 and 3 states)	5, 7
CN segments	24
FDR fragment joints	0.5435077
FDR chr. breakp. enrich.	0
Linked to chrs	12:32463480−122655729;
Purity, ploidy	NA, NA

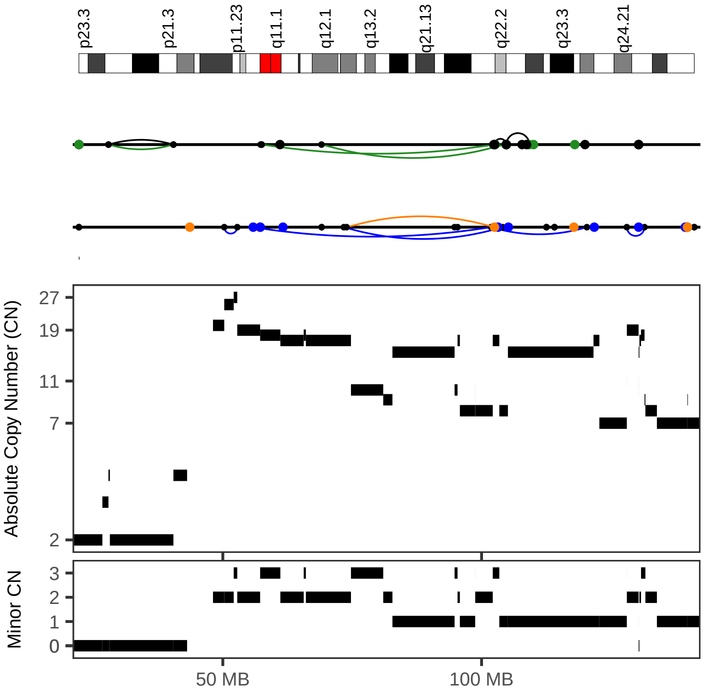

A29−0017_CRUK_PC_0017	
Cancer type	Prost−AdenoCA
Position	8:55705727−120347671
Type	With other complex events
Interleaved intrachr. SVs	10
Total SVs (intrachr. + transl.)	33
SV types	DEL: 1; DUP: 3; h2hINV: 3; t2tINV: 3; TRA: 23
SVs in sample	322
Oscillating CN (2 and 3 states)	4, 5
CN segments	17
FDR fragment joints	0.615458
FDR chr. breakp. enrich.	0
Linked to chrs	5:13735145−179718725;
Purity, ploidy	NA, NA

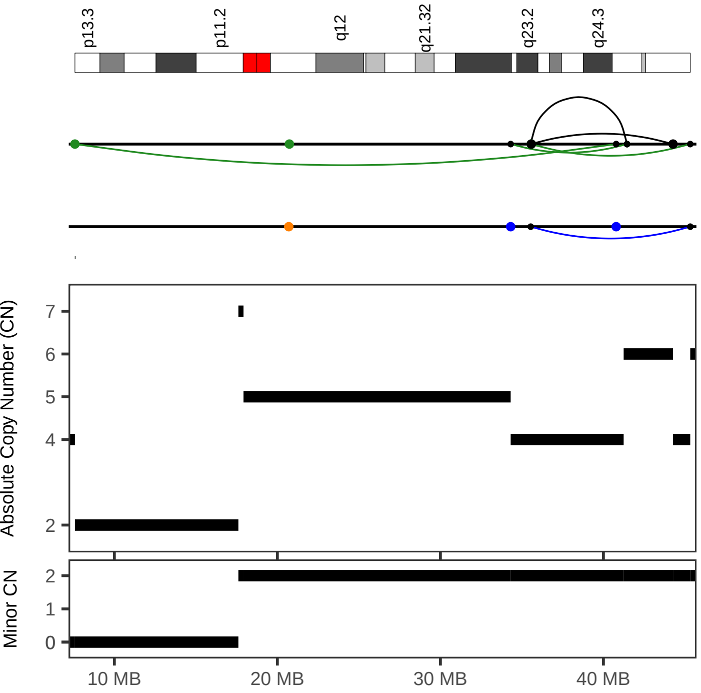

	**A29−0017_CRUK_PC_0017**
Cancer type	Prost−AdenoCA
Position	17:7580636−45324930
Type	With other complex events
Interleaved intrachr. SVs	6
Total SVs (intrachr. + transl.)	14
SV types	DEL: 0; DUP: 1; h2hINV: 2; t2tINV: 3; TRA: 8
SVs in sample	322
Oscillating CN (2 and 3 states)	4, 5
CN segments	8
FDR fragment joints	0.615458
FDR chr. breakp. enrich.	0.13
Linked to chrs	
Purity, ploidy	NA, NA

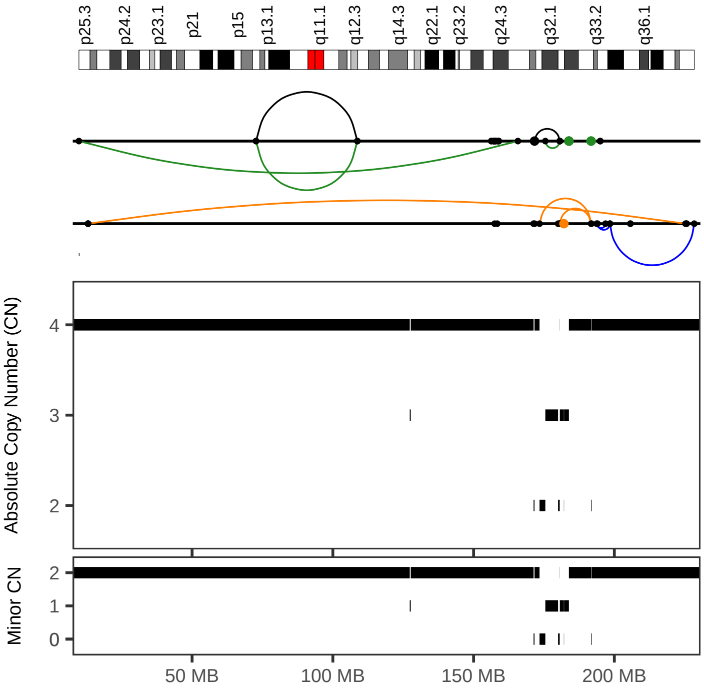

	**A32−0019_CRUK_PC_0019**
Cancer type	Prost−AdenoCA
Position	2:171308068−191866282
Type	With other complex events
Interleaved intrachr. SVs	6
Total SVs (intrachr. + transl.)	12
SV types	DEL: 4; DUP: 0; h2hINV: 1; t2tINV: 1; TRA: 6
SVs in sample	192
Oscillating CN (2 and 3 states)	5, 7
CN segments	14
FDR fragment joints	0.615458
FDR chr. breakp. enrich.	0
Linked to chrs	3:32985863−156750376;
Purity, ploidy	NA, NA

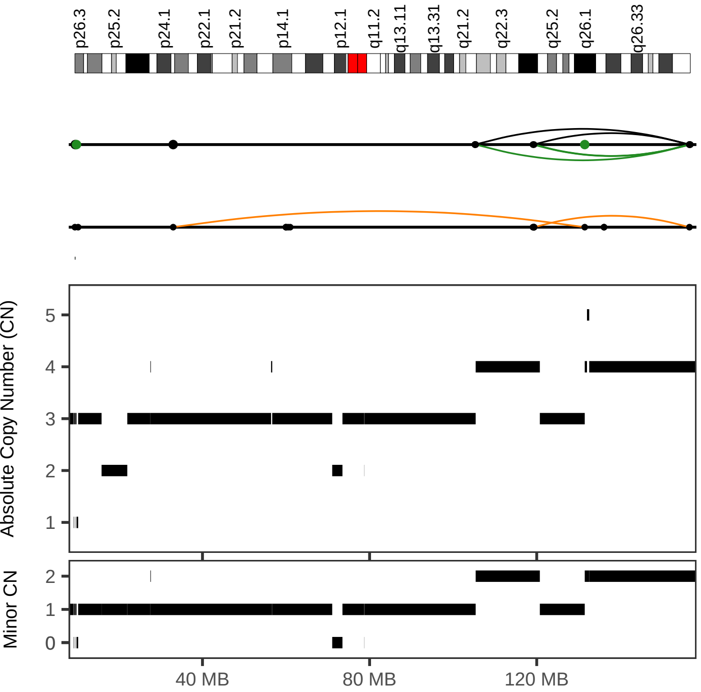

	**A32−0019_CRUK_PC_0019**
Cancer type	Prost−AdenoCA
Position	3:32985863−156750377
Type	With other complex events
Interleaved intrachr. SVs	10
Total SVs (intrachr. + transl.)	12
SV types	DEL: 3; DUP: 0; h2hINV: 4; t2tINV: 3; TRA: 2
SVs in sample	192
Oscillating CN (2 and 3 states)	5, 12
CN segments	12
FDR fragment joints	0.615458
FDR chr. breakp. enrich.	0.03
Linked to chrs	2:171308068−191866281;
Purity, ploidy	NA, NA

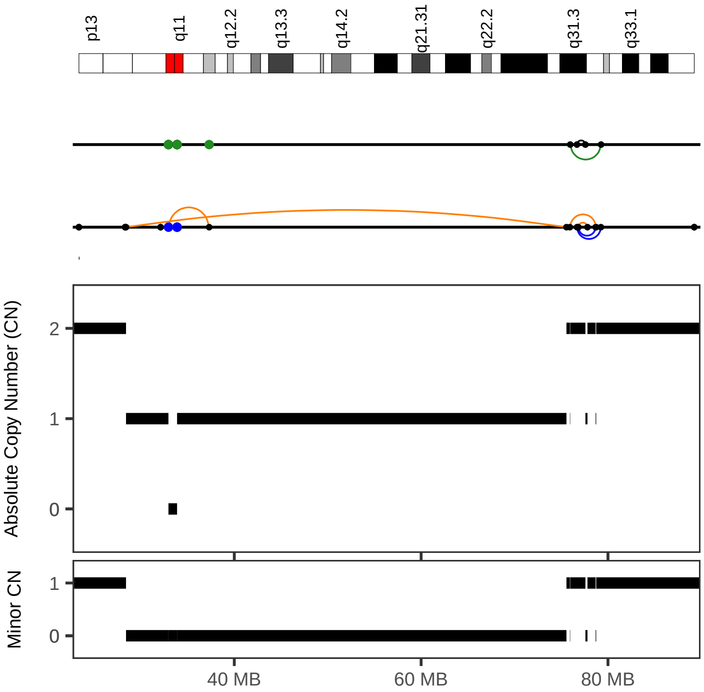

	**A34−0022_CRUK_PC_0022**
Cancer type	Prost−AdenoCA
Position	13:75922651−79259286
Type	Canonical without polyploidization
Interleaved intrachr. SVs	6
Total SVs (intrachr. + transl.)	6
SV types	DEL: 2; DUP: 2; h2hINV: 1; t2tINV: 1; TRA: 0
SVs in sample	461
Oscillating CN (2 and 3 states)	6, 6
CN segments	6
FDR fragment joints	0.9284301
FDR chr. breakp. enrich.	0.17
Linked to chrs	
Purity, ploidy	NA, NA

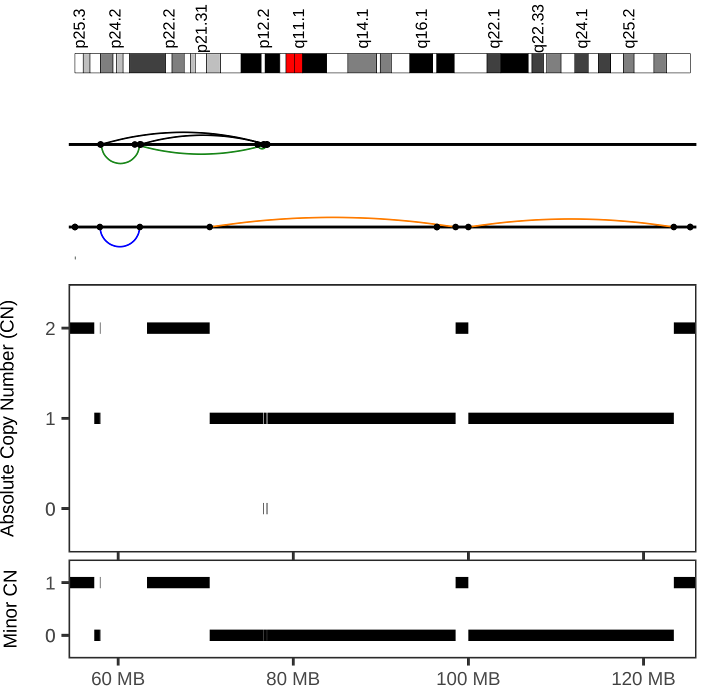

	**c039014f−821c−43d6−9d3c−914f9c8d98fa**
Cancer type	Prost−AdenoCA
Position	6:57910476−98537020
Type	With other complex events
Interleaved intrachr. SVs	10
Total SVs (intrachr. + transl.)	10
SV types	DEL: 1; DUP: 1; h2hINV: 3; t2tINV: 5; TRA: 0
SVs in sample	222
Oscillating CN (2 and 3 states)	5, 9
CN segments	9
FDR fragment joints	0.615458
FDR chr. breakp. enrich.	0.93
Linked to chrs	
Purity, ploidy	0.85, 1.96

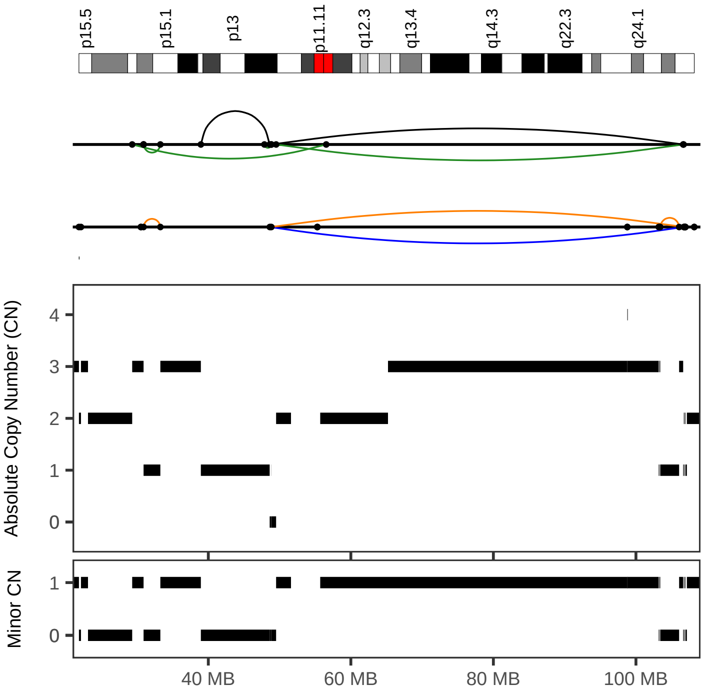

	**c039014f−821c−43d6−9d3c−914f9c8d98fa**
Cancer type	Prost−AdenoCA
Position	11:29320290−106781287
Type	With other complex events
Interleaved intrachr. SVs	7
Total SVs (intrachr. + transl.)	7
SV types	DEL: 1; DUP: 1; h2hINV: 2; t2tINV: 3; TRA: 0
SVs in sample	222
Oscillating CN (2 and 3 states)	6, 9
CN segments	20
FDR fragment joints	0.7735152
FDR chr. breakp. enrich.	0
Linked to chrs	
Purity, ploidy	0.85, 1.96

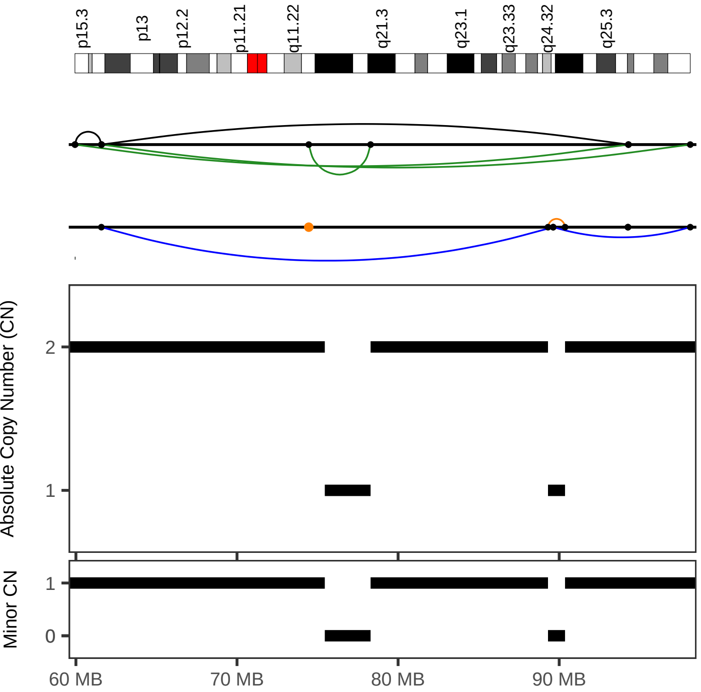

	**f6381367−142c−45d0−92b3−c1727d1813ce**
Cancer type	Prost−AdenoCA
Position	10:59938517−98135181
Type	Canonical without polyploidization
Interleaved intrachr. SVs	7
Total SVs (intrachr. + transl.)	8
SV types	DEL: 1; DUP: 2; h2hINV: 2; t2tINV: 2; TRA: 1
SVs in sample	133
Oscillating CN (2 and 3 states)	5, 5
CN segments	5
FDR fragment joints	1
FDR chr. breakp. enrich.	0.03
Linked to chrs	
Purity, ploidy	0.92, 1.9

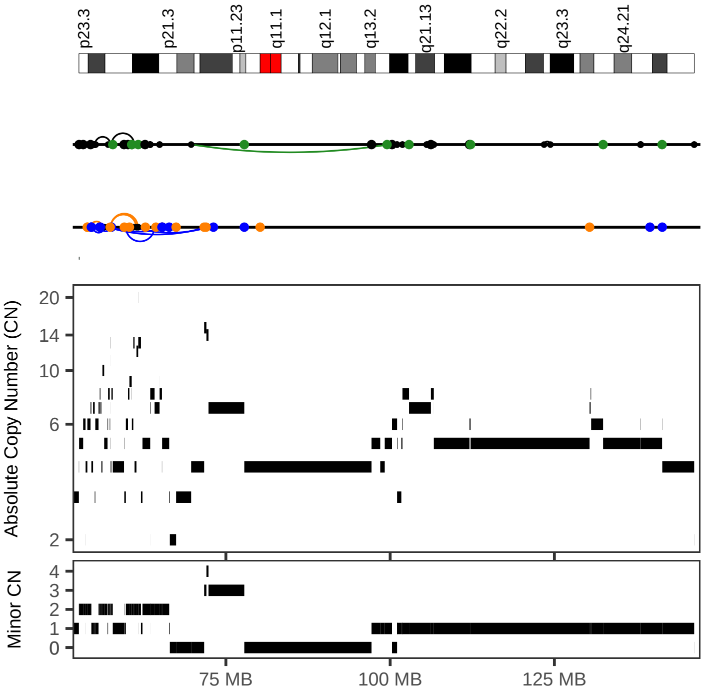

	**MELA−0001**
Cancer type	Skin−Melanoma
Position	8:54422125−99862970
Type	With other complex events
Interleaved intrachr. SVs	14
Total SVs (intrachr. + transl.)	45
SV types	DEL: 4; DUP: 6; h2hINV: 2; t2tINV: 2; TRA: 31
SVs in sample	460
Oscillating CN (2 and 3 states)	4, 7
CN segments	57
FDR fragment joints	0.5435077
FDR chr. breakp. enrich.	0
Linked to chrs	11:57335469−97526274;4:18741843−110768808
Purity, ploidy	0.75, 3.01

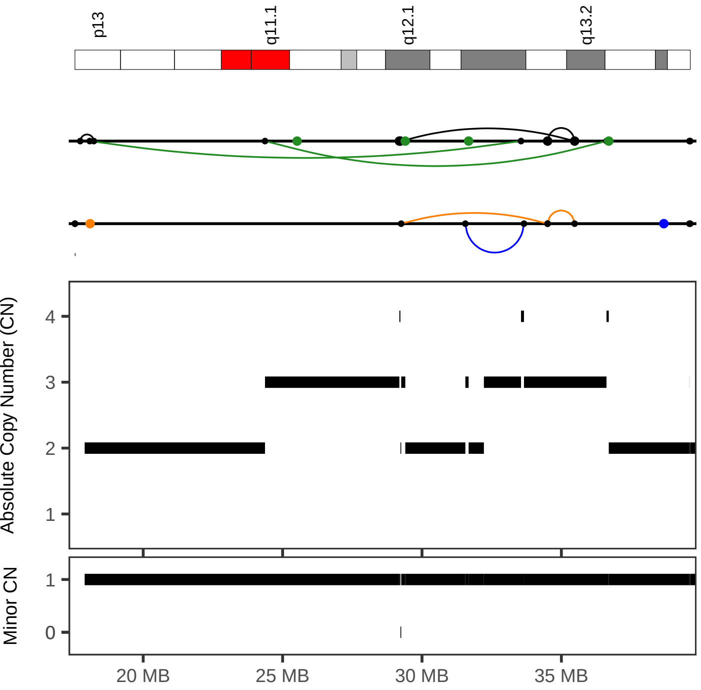

	MELA−0002
Cancer type	Skin−Melanoma
Position	22:17741148−36618400
Type	With other complex events
Interleaved intrachr. SVs	6
Total SVs (intrachr. + transl.)	14
SV types	DEL: 1; DUP: 1; h2hINV: 2; t2tINV: 2; TRA: 8
SVs in sample	748
Oscillating CN (2 and 3 states)	6, 8
CN segments	13
FDR fragment joints	0.615458
FDR chr. breakp. enrich.	0
Linked to chrs	2:11161529−144813239;7:1508469−101805271
Purity, ploidy	0.54, 2.62

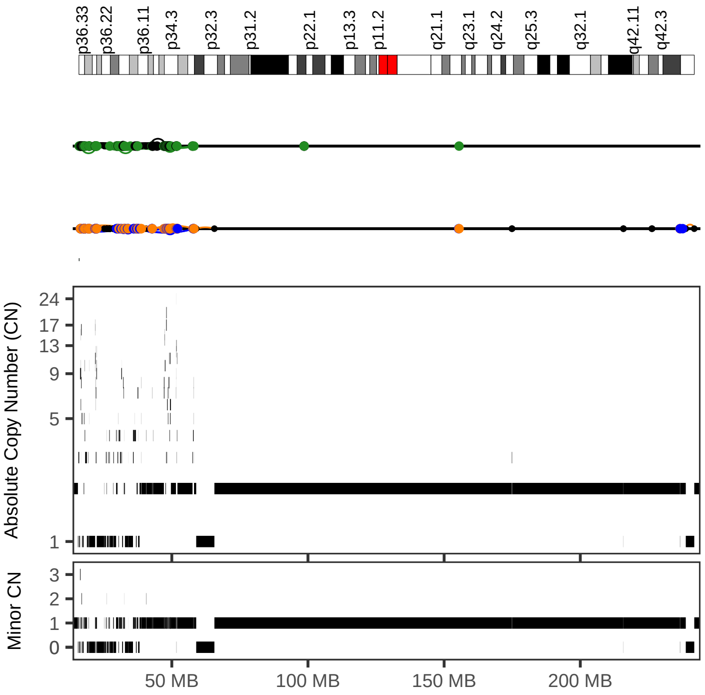

	MELA−0003
Cancer type	Skin−Melanoma
Position	1:15885881−58152236
Type	With other complex events
Interleaved intrachr. SVs	71
Total SVs (intrachr. + transl.)	308
SV types	DEL: 20; DUP: 20; h2hINV: 15; t2tINV: 16; TRA: 237
SVs in sample	1187
Oscillating CN (2 and 3 states)	6, 6
CN segments	160
FDR fragment joints	0.5558459
FDR chr. breakp. enrich.	0
Linked to chrs	12:43076099−128657836;5:1296136−45874995
Purity, ploidy	0.96, 1.84

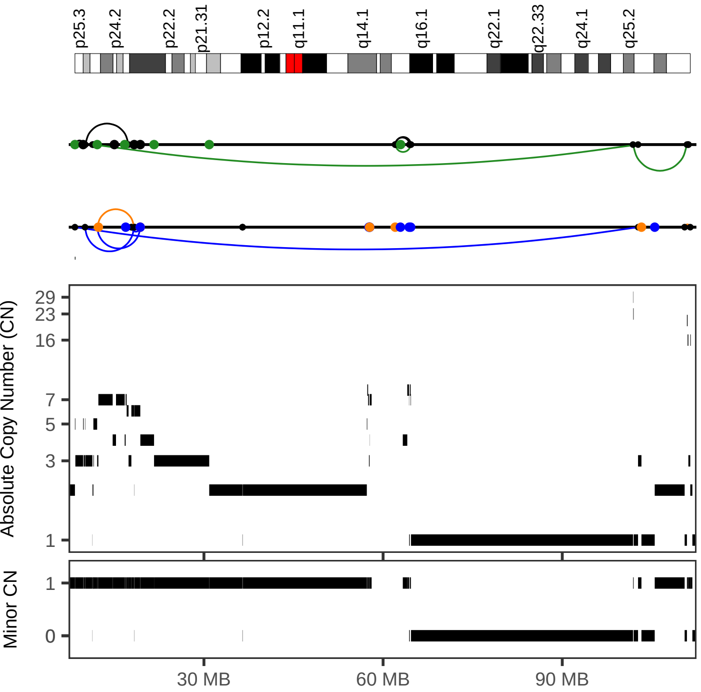

	MELA−0005
Cancer type	Skin−Melanoma
Position	6:8393693−111434013
Type	With other complex events
Interleaved intrachr. SVs	11
Total SVs (intrachr. + transl.)	35
SV types	DEL: 2; DUP: 5; h2hINV: 2; t2tINV: 2; TRA: 24
SVs in sample	220
Oscillating CN (2 and 3 states)	6, 7
CN segments	53
FDR fragment joints	0.615458
FDR chr. breakp. enrich.	0
Linked to chrs	5:1027171−19705106;
Purity, ploidy	0.79, 1.98

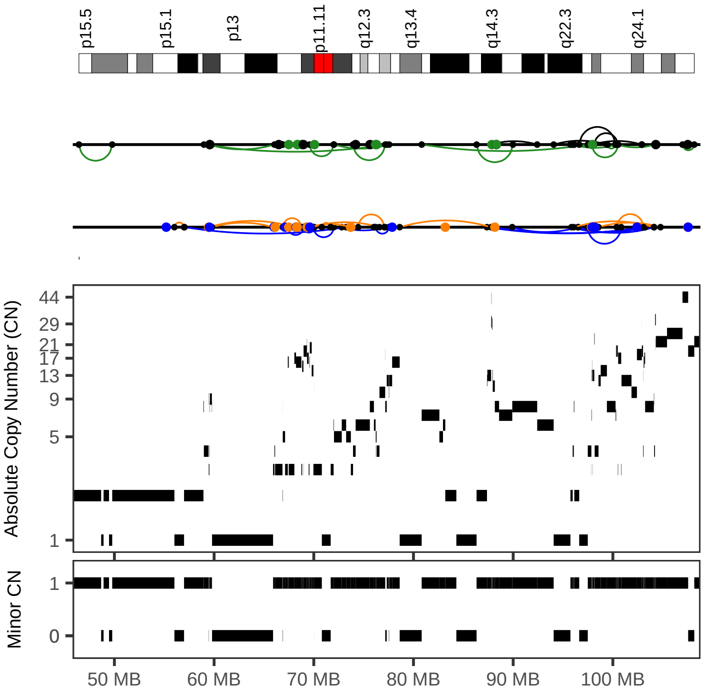

	MELA−0005
Cancer type	Skin−Melanoma
Position	11:78619623−104782805
Type	With other complex events
Interleaved intrachr. SVs	22
Total SVs (intrachr. + transl.)	34
SV types	DEL: 4; DUP: 6; h2hINV: 6; t2tINV: 6; TRA: 12
SVs in sample	220
Oscillating CN (2 and 3 states)	4, 5
CN segments	60
FDR fragment joints	0.9332614
FDR chr. breakp. enrich.	0
Linked to chrs	5:1027171−19705106;6:8393693−111434012
Purity, ploidy	0.79, 1.98

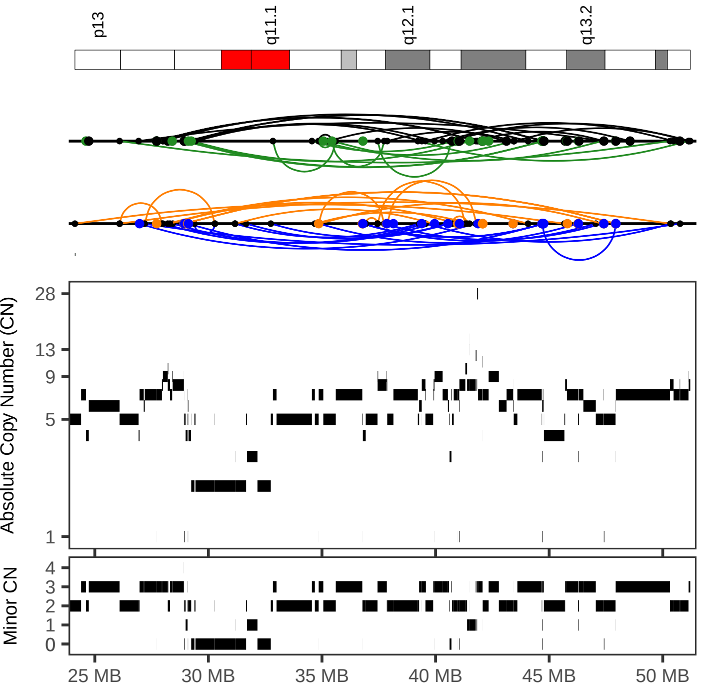

MELA−0007	
Cancer type	Skin−Melanoma
Position	22:24125398−51210651
Type	With other complex events
Interleaved intrachr. SVs	67
Total SVs (intrachr. + transl.)	171
SV types	DEL: 19; DUP: 17; h2hINV: 18; t2tINV: 13; TRA: 104
SVs in sample	377
Oscillating CN (2 and 3 states)	6, 9
CN segments	150
FDR fragment joints	1
FDR chr. breakp. enrich.	0
Linked to chrs	
Purity, ploidy	0.69, 3.61

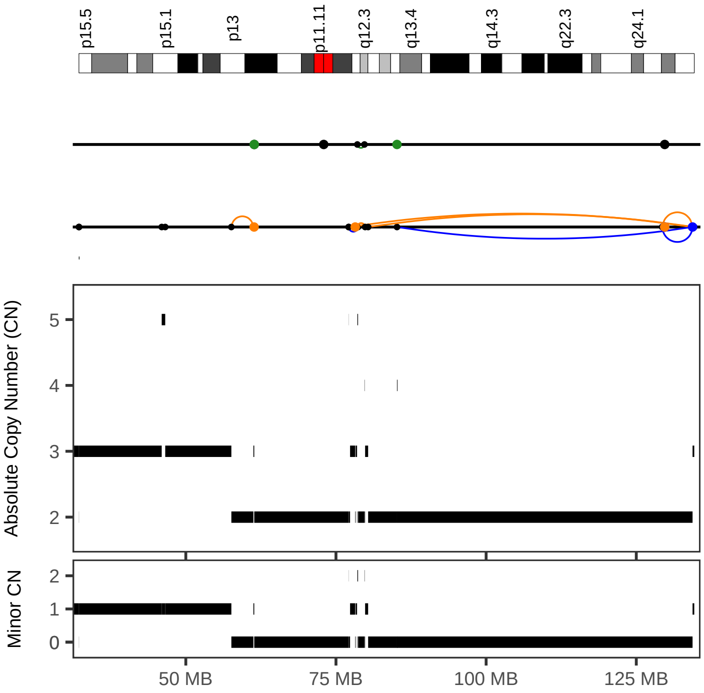

MELA−0008	
Cancer type	Skin−Melanoma
Position	11:77104049−134650672
Type	With other complex events
Interleaved intrachr. SVs	8
Total SVs (intrachr. + transl.)	14
SV types	DEL: 4; DUP: 3; h2hINV: 0; t2tINV: 1; TRA: 6
SVs in sample	142
Oscillating CN (2 and 3 states)	5, 10
CN segments	15
FDR fragment joints	0.615458
FDR chr. breakp. enrich.	0
Linked to chrs	22:41378272−41557630;
Purity, ploidy	0.79, 3.81

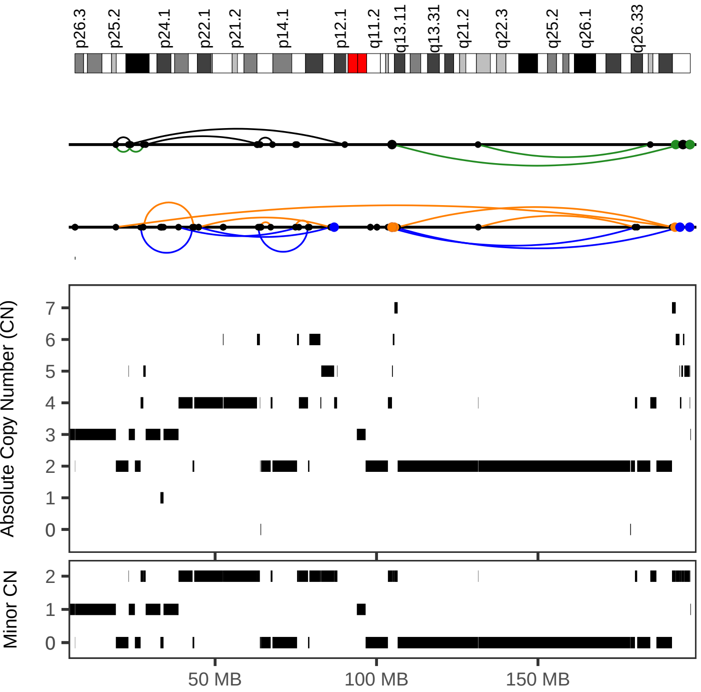

MELA−0009	
Cancer type	Skin−Melanoma
Position	3:19250926−195326589
Type	With other complex events
Interleaved intrachr. SVs	23
Total SVs (intrachr. + transl.)	33
SV types	DEL: 7; DUP: 6; h2hINV: 5; t2tINV: 5; TRA: 10
SVs in sample	351
Oscillating CN (2 and 3 states)	5, 12
CN segments	53
FDR fragment joints	0.7286575
FDR chr. breakp. enrich.	0
Linked to chrs	22:31415162−49131332;
Purity, ploidy	0.49, 3.33

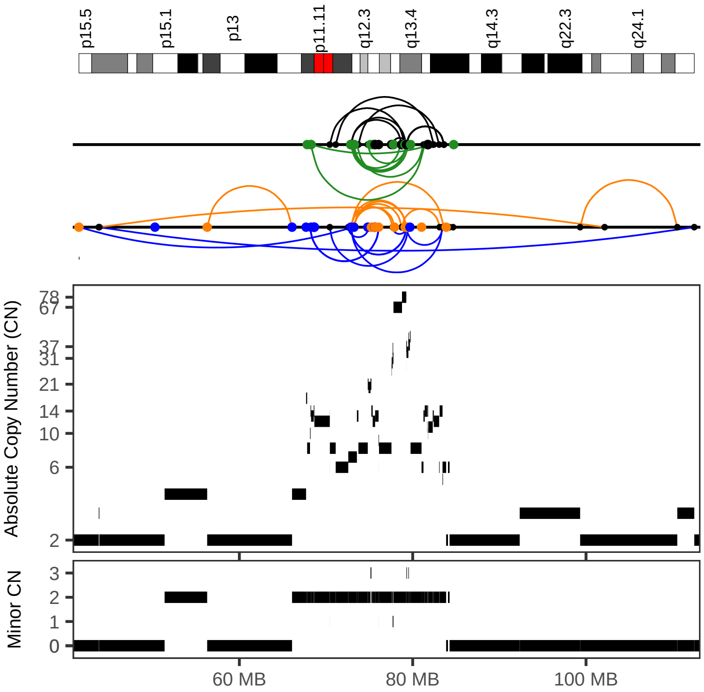

MELA−0009	
Cancer type	Skin−Melanoma
Position	11:41485920−112489309
Type	With other complex events
Interleaved intrachr. SVs	40
Total SVs (intrachr. + transl.)	94
SV types	DEL: 10; DUP: 11; h2hINV: 10; t2tINV: 9; TRA: 54
SVs in sample	351
Oscillating CN (2 and 3 states)	5, 8
CN segments	69
FDR fragment joints	0.7392975
FDR chr. breakp. enrich.	0
Linked to chrs	20:50318897−61655151;
Purity, ploidy	0.49, 3.33

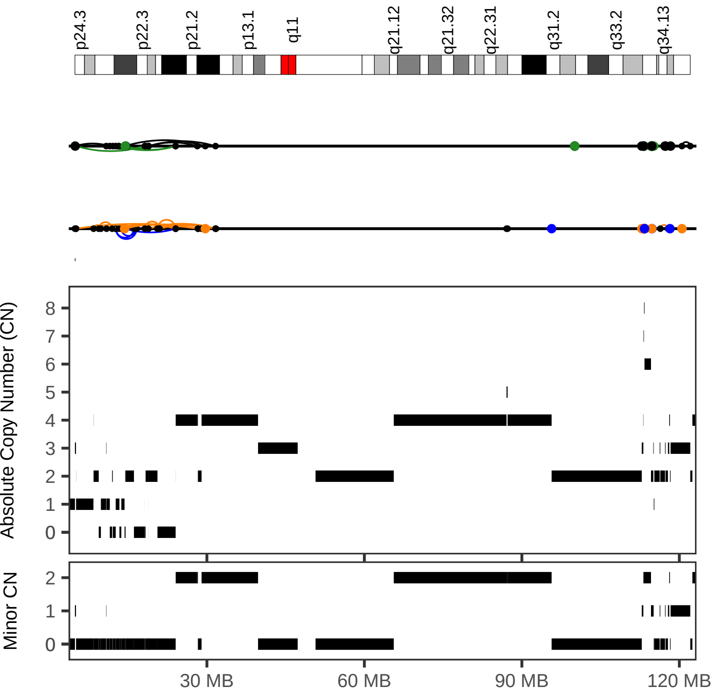

	**MELA−0012**
Cancer type	Skin−Melanoma
Position	9:4860008−31734961
Type	With other complex events
Interleaved intrachr. SVs	27
Total SVs (intrachr. + transl.)	32
SV types	DEL: 11; DUP: 5; h2hINV: 5; t2tINV: 6; TRA: 5
SVs in sample	508
Oscillating CN (2 and 3 states)	6, 21
CN segments	34
FDR fragment joints	0.615458
FDR chr. breakp. enrich.	0
Linked to chrs	7:32236661−117391258;
Purity, ploidy	0.79, 2.87

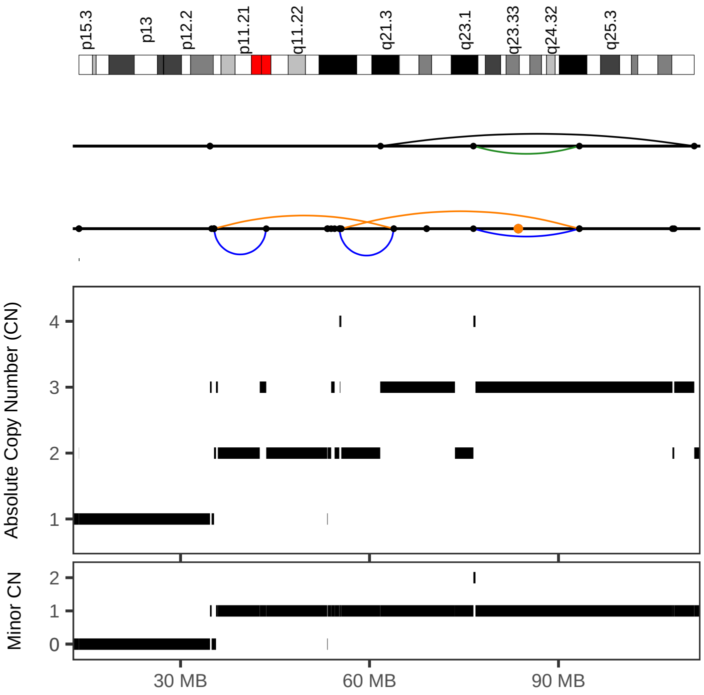

	**MELA−0012**
Cancer type	Skin−Melanoma
Position	10:34937946−111551297
Type	With other complex events
Interleaved intrachr. SVs	8
Total SVs (intrachr. + transl.)	11
SV types	DEL: 3; DUP: 3; h2hINV: 1; t2tINV: 1; TRA: 3
SVs in sample	508
Oscillating CN (2 and 3 states)	5, 11
CN segments	21
FDR fragment joints	0.615458
FDR chr. breakp. enrich.	0.53
Linked to chrs	
Purity, ploidy	0.79, 2.87

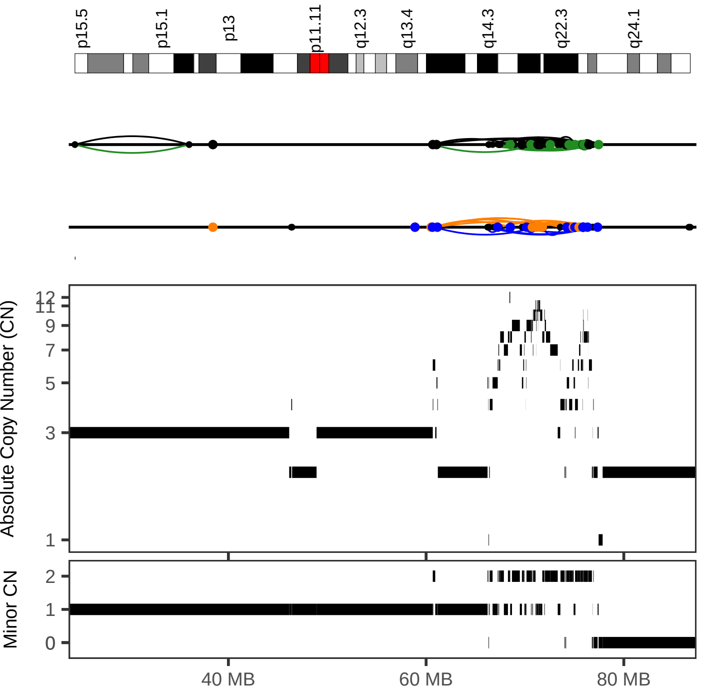

	**MELA−0012**
Cancer type	Skin−Melanoma
Position	11:60665556−76908360
Type	With other complex events
Interleaved intrachr. SVs	47
Total SVs (intrachr. + transl.)	80
SV types	DEL: 12; DUP: 10; h2hINV: 14; t2tINV: 11; TRA: 33
SVs in sample	508
Oscillating CN (2 and 3 states)	5, 9
CN segments	96
FDR fragment joints	0.9783158
FDR chr. breakp. enrich.	0
Linked to chrs	12:62098962−89622896;3:131435629−180741701 6:32272825−91026452;9:4860008−31734960
Purity, ploidy	0.79, 2.87

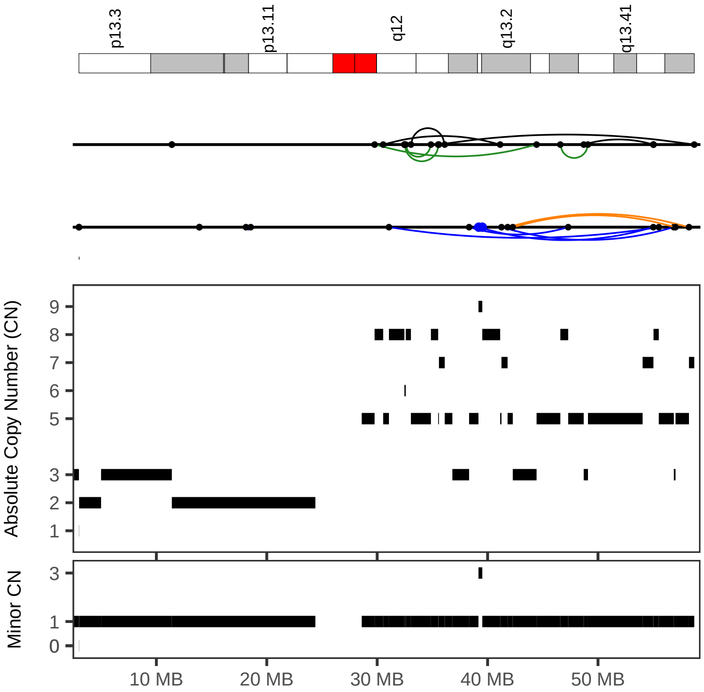

	**MELA−0015**
Cancer type	Skin−Melanoma
Position	19:29765034−58719123
Type	With other complex events
Interleaved intrachr. SVs	15
Total SVs (intrachr. + transl.)	17
SV types	DEL: 2; DUP: 4; h2hINV: 4; t2tINV: 5; TRA: 2
SVs in sample	201
Oscillating CN (2 and 3 states)	4, 5
CN segments	31
FDR fragment joints	0.6776251
FDR chr. breakp. enrich.	0
Linked to chrs	
Purity, ploidy	0.55, 3.39

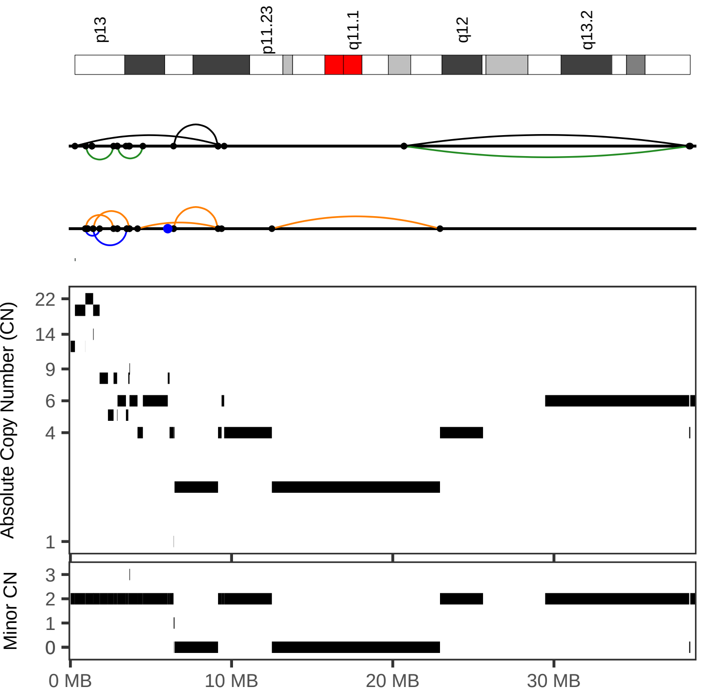

MELA−0043

Cancer type	Skin−Melanoma
Position	20:924675−9380462
Type	With other complex events
Interleaved intrachr. SVs	11
Total SVs (intrachr. + transl.)	12
SV types	DEL: 3; DUP: 4; h2hINV: 1; t2tINV: 3; TRA: 1
SVs in sample	106
Oscillating CN (2 and 3 states)	4, 7
CN segments	22
FDR fragment joints	0.6776251
FDR chr. breakp. enrich.	0
Linked to chrs	
Purity, ploidy	0.63, 3.54

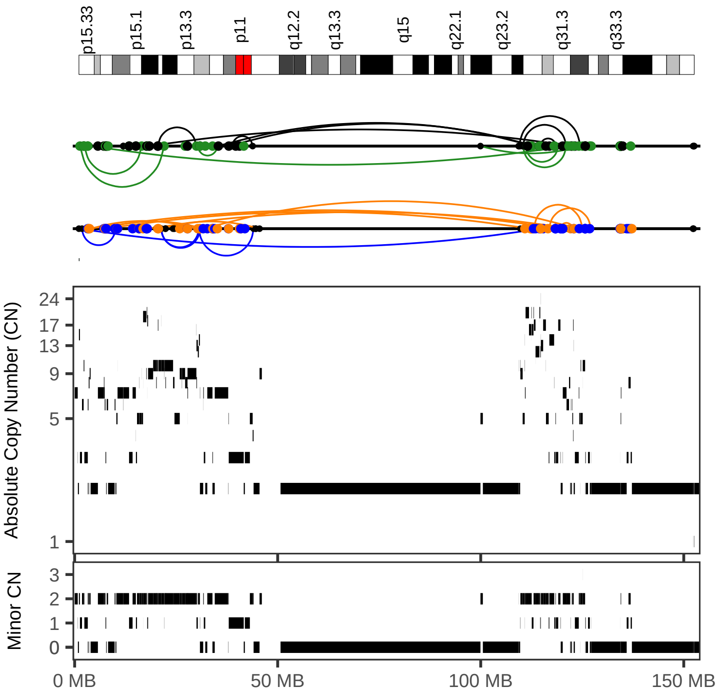

MELA−0048

Cancer type	Skin−Melanoma
Position	5:1054293−127185143
Type	With other complex events
Interleaved intrachr. SVs	32
Total SVs (intrachr. + transl.)	163
SV types	DEL: 11; DUP: 6; h2hINV: 7; t2tINV: 8; TRA: 131
SVs in sample	444
Oscillating CN (2 and 3 states)	6, 7
CN segments	161
FDR fragment joints	0.6942198
FDR chr. breakp. enrich.	0
Linked to chrs	2:35447197−238700316;7:858862−151538982
Purity, ploidy	0.9, 2.98

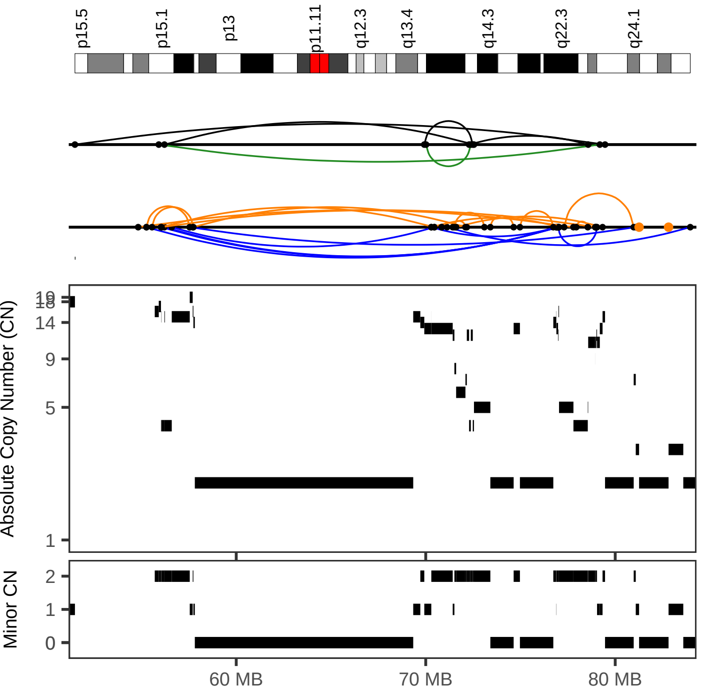

MELA−0050

Cancer type	Skin−Melanoma
Position	11:51487991−83958719
Type	With other complex events
Interleaved intrachr. SVs	26
Total SVs (intrachr. + transl.)	28
SV types	DEL: 11; DUP: 9; h2hINV: 4; t2tINV: 2; TRA: 2
SVs in sample	94
Oscillating CN (2 and 3 states)	6, 6
CN segments	52
FDR fragment joints	0.332153
FDR chr. breakp. enrich.	0
Linked to chrs	
Purity, ploidy	0.82, 2.77

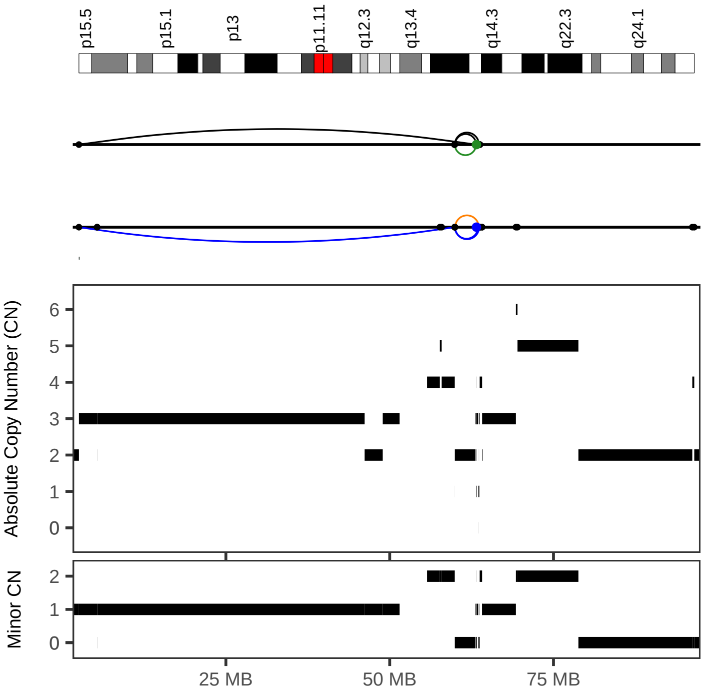

MELA−0060

Cancer type	Skin−Melanoma
Position	11:2569509−63788627
Type	With other complex events
Interleaved intrachr. SVs	20
Total SVs (intrachr. + transl.)	22
SV types	DEL: 4; DUP: 5; h2hINV: 5; t2tINV: 6; TRA: 2
SVs in sample	213
Oscillating CN (2 and 3 states)	5, 11
CN segments	31
FDR fragment joints	0.9419607
FDR chr. breakp. enrich.	0
Linked to chrs	
Purity, ploidy	0.46, 3.71

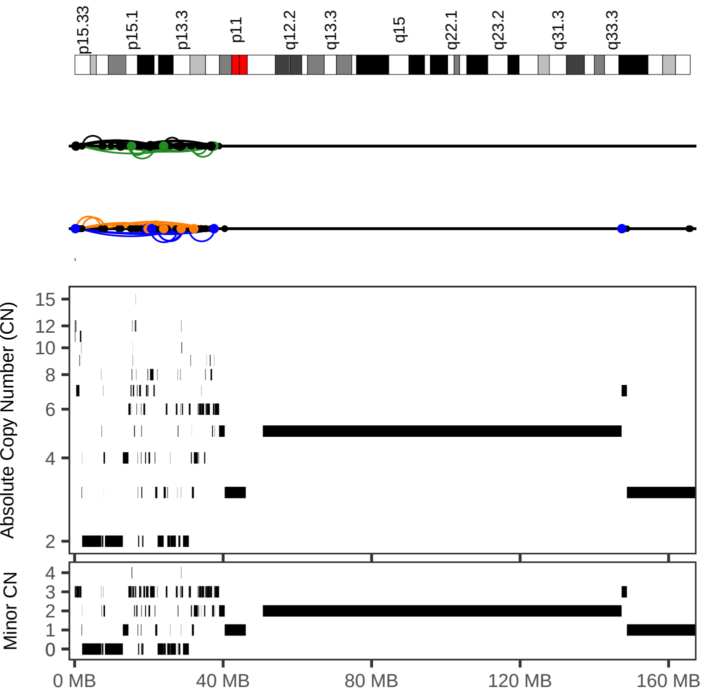

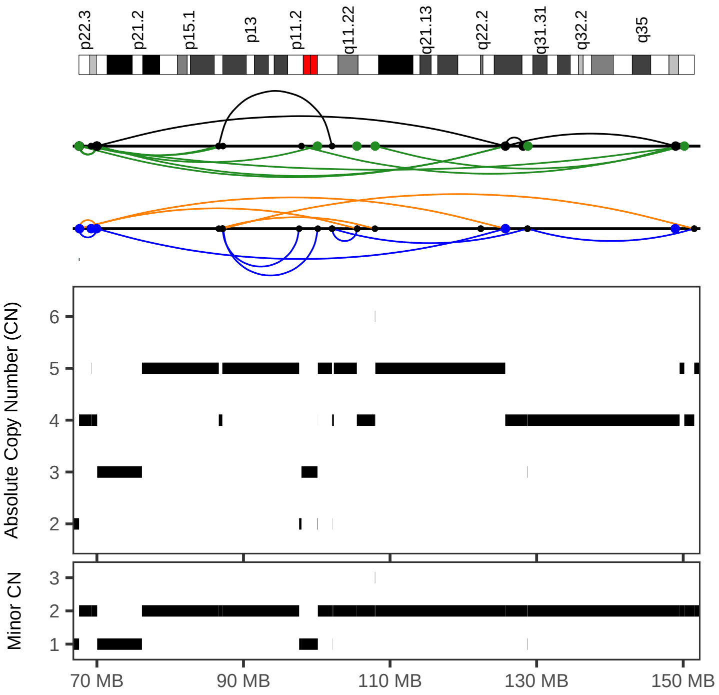

	**MELA−0064**
Cancer type	Skin−Melanoma
Position	5:75710−38913517
Type	With other complex events
Interleaved intrachr. SVs	79
Total SVs (intrachr. + transl.)	104
SV types	DEL: 22; DUP: 14; h2hINV: 21; t2tINV: 22; TRA: 25
SVs in sample	380
Oscillating CN (2 and 3 states)	5, 8
CN segments	105
FDR fragment joints	0.7995907
FDR chr. breakp. enrich.	0
Linked to chrs	
Purity, ploidy	0.66, 3.77

	**MELA−0064**
Cancer type	Skin−Melanoma
Position	7:67546621−151505707
Type	With other complex events
Interleaved intrachr. SVs	29
Total SVs (intrachr. + transl.)	49
SV types	DEL: 5; DUP: 8; h2hINV: 4; t2tINV: 12; TRA: 20
SVs in sample	380
Oscillating CN (2 and 3 states)	4, 7
CN segments	29
FDR fragment joints	0.5435077
FDR chr. breakp. enrich.	0
Linked to chrs	
Purity, ploidy	0.66, 3.77

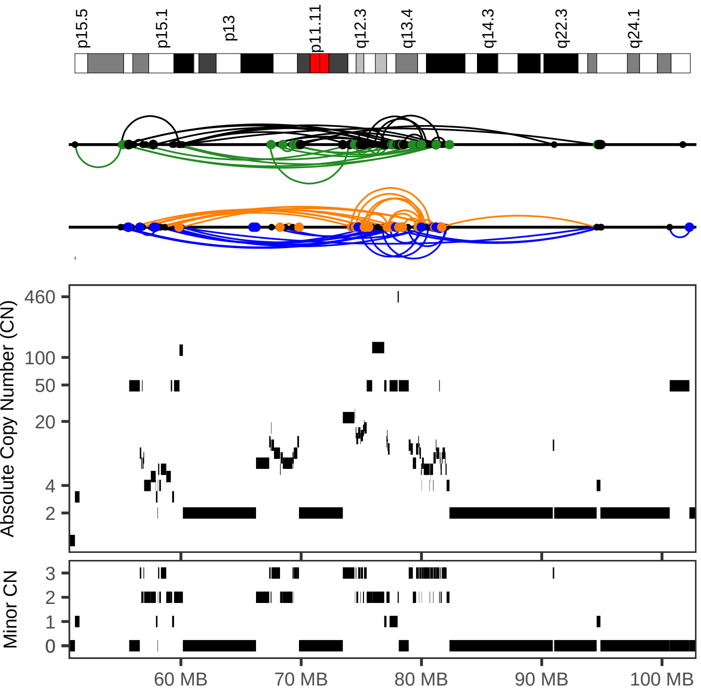

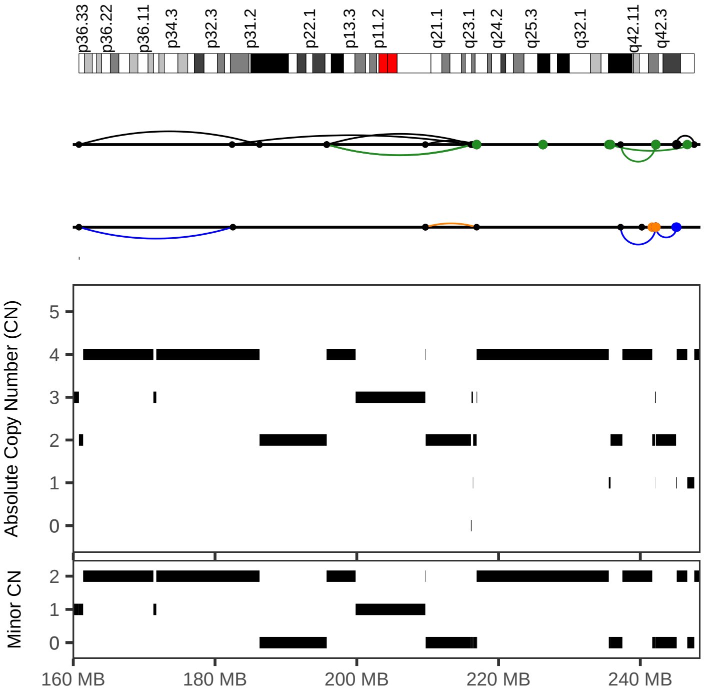

	**MELA−0064**
Cancer type	Skin−Melanoma
Position	11:51188282−94965346
Type	With other complex events
Interleaved intrachr. SVs	90
Total SVs (intrachr. + transl.)	178
SV types	DEL: 25; DUP: 23; h2hINV: 23; t2tINV: 19; TRA: 88
SVs in sample	380
Oscillating CN (2 and 3 states)	4, 7
CN segments	94
FDR fragment joints	0.7789125
FDR chr. breakp. enrich.	0
Linked to chrs	16:6114212−66283259;5:75710−38913516
Purity, ploidy	0.66, 3.77

	**MELA−0066**
Cancer type	Skin−Melanoma
Position	1:160797429−216909159
Type	With other complex events
Interleaved intrachr. SVs	9
Total SVs (intrachr. + transl.)	11
SV types	DEL: 1; DUP: 2; h2hINV: 4; t2tINV: 2; TRA: 2
SVs in sample	355
Oscillating CN (2 and 3 states)	5, 11
CN segments	17
FDR fragment joints	0.615458
FDR chr. breakp. enrich.	0.03
Linked to chrs	
Purity, ploidy	0.42, 3.25

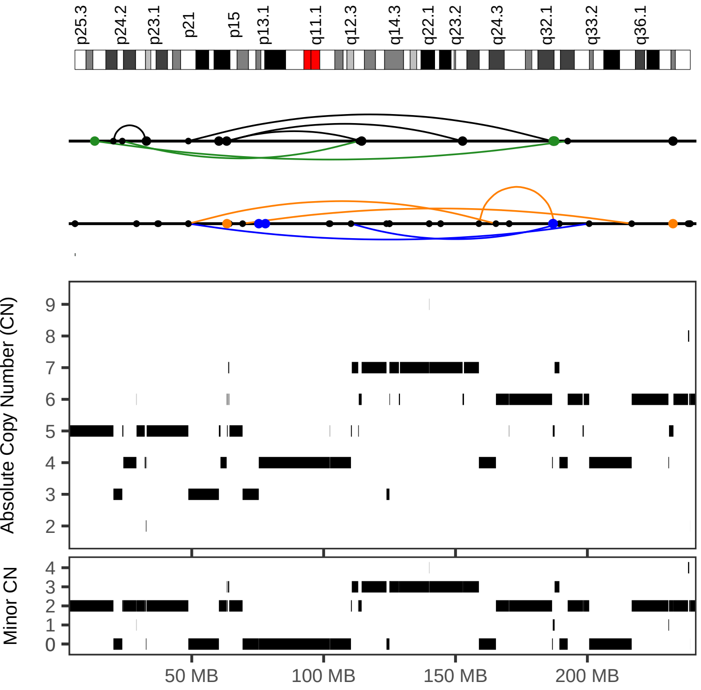

	MELA−0069
Cancer type	Skin−Melanoma
Position	2:13198317−216809264
Type	With other complex events
Interleaved intrachr. SVs	11
Total SVs (intrachr. + transl.)	26
SV types	DEL: 3; DUP: 2; h2hINV: 4; t2tINV: 2; TRA: 15
SVs in sample	401
Oscillating CN (2 and 3 states)	5, 8
CN segments	51
FDR fragment joints	0.7510435
FDR chr. breakp. enrich.	0.27
Linked to chrs	6:3865883−64390629;9:1637851−118991851
Purity, ploidy	0.97, 3.9

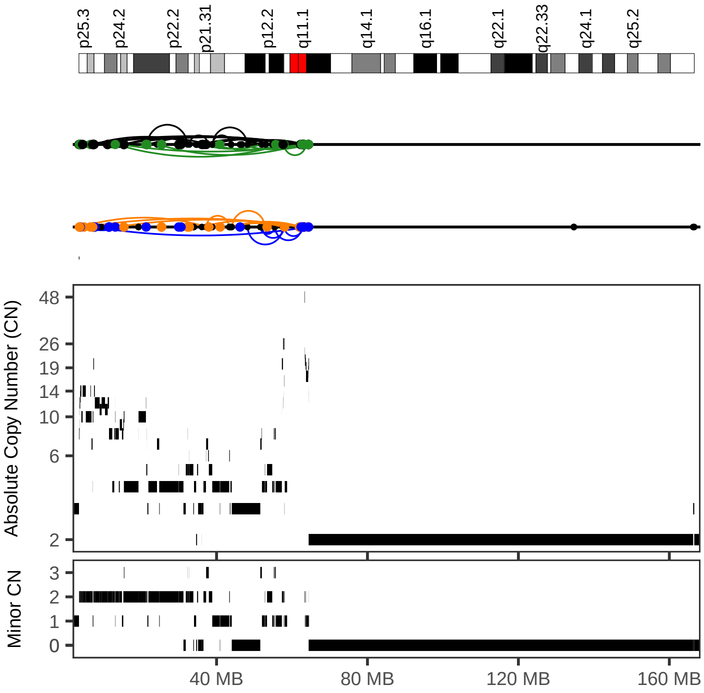

	MELA−0069
Cancer type	Skin−Melanoma
Position	6:3865883−64390630
Type	With other complex events
Interleaved intrachr. SVs	49
Total SVs (intrachr. + transl.)	129
SV types	DEL: 11; DUP: 9; h2hINV: 15; t2tINV: 14; TRA: 80
SVs in sample	401
Oscillating CN (2 and 3 states)	6, 7
CN segments	106
FDR fragment joints	0.615458
FDR chr. breakp. enrich.	0
Linked to chrs	14:77426835−91651121;2:13198317−216809263
Purity, ploidy	0.97, 3.9

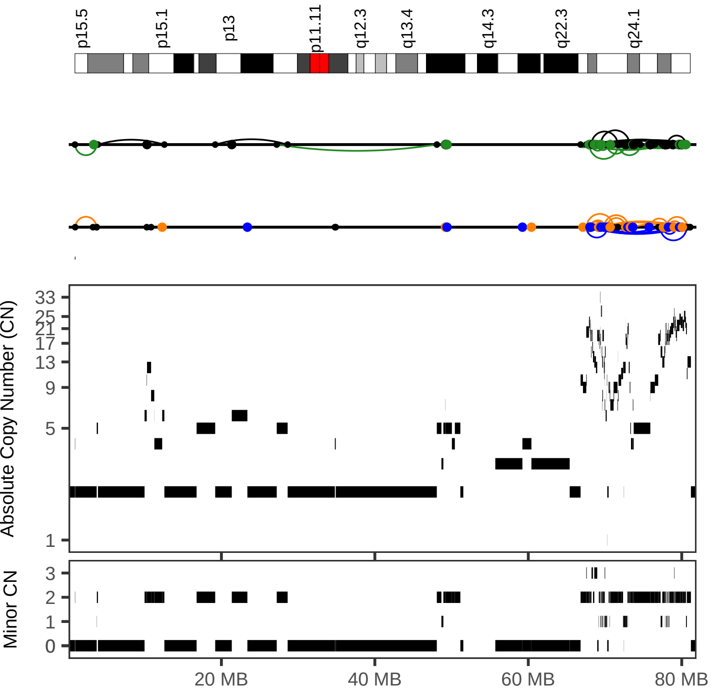

	MELA−0069
Cancer type	Skin−Melanoma
Position	11:66817953−80753867
Type	With other complex events
Interleaved intrachr. SVs	64
Total SVs (intrachr. + transl.)	134
SV types	DEL: 20; DUP: 14; h2hINV: 16; t2tINV: 14; TRA: 70
SVs in sample	401
Oscillating CN (2 and 3 states)	4, 7
CN segments	122
FDR fragment joints	0.8690452
FDR chr. breakp. enrich.	0
Linked to chrs	6:3865883−64390629;9:1637851−118991851
Purity, ploidy	0.97, 3.9

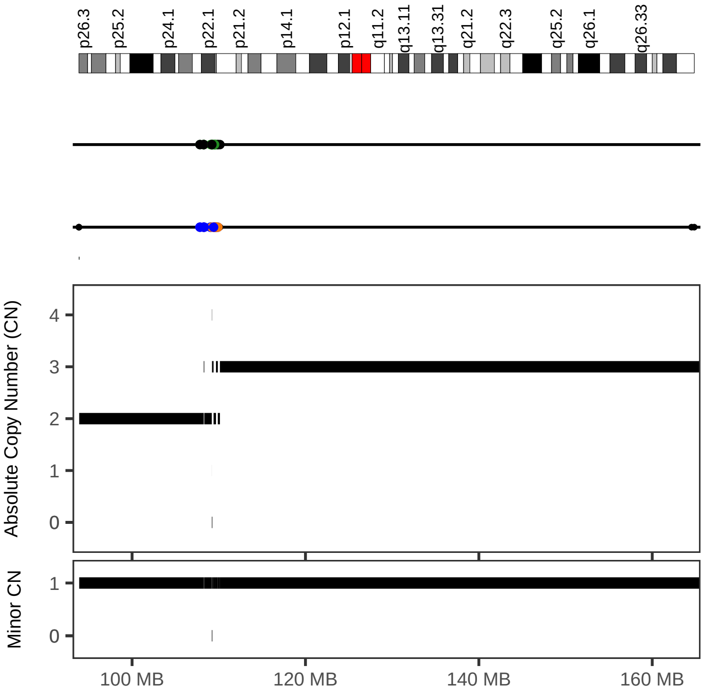

	MELA−0070
Cancer type	Skin−Melanoma
Position	3:107828103−110133076
Type	With other complex events
Interleaved intrachr. SVs	20
Total SVs (intrachr. + transl.)	65
SV types	DEL: 5; DUP: 7; h2hINV: 3; t2tINV: 5; TRA: 45
SVs in sample	450
Oscillating CN (2 and 3 states)	5, 7
CN segments	13
FDR fragment joints	0.7333688
FDR chr. breakp. enrich.	0
Linked to chrs	12:55086091−64480689;
Purity, ploidy	0.75, 2.64

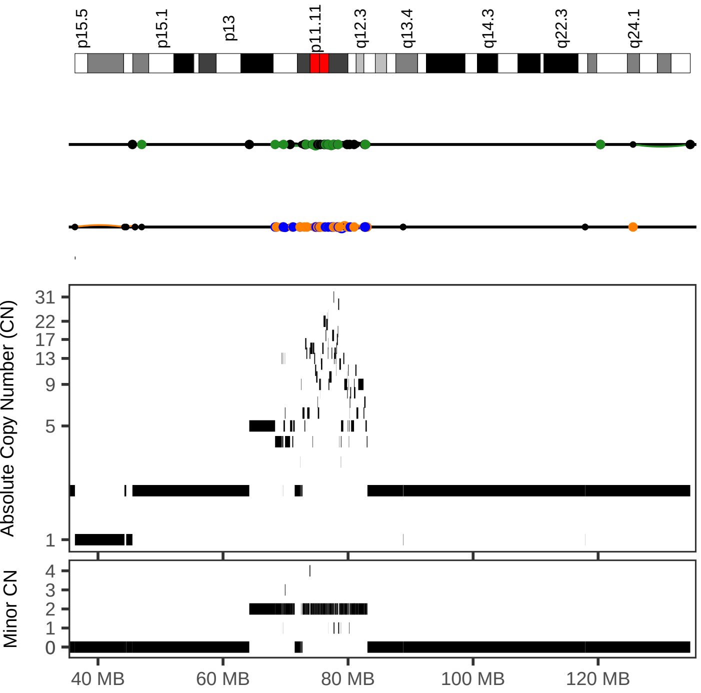

**MELA−0070**	
Cancer type	Skin−Melanoma
Position	11:69251922−83096232
Type	With other complex events
Interleaved intrachr. SVs	33
Total SVs (intrachr. + transl.)	107
SV types	DEL: 10; DUP: 7; h2hINV: 9; t2tINV: 7; TRA: 74
SVs in sample	450
Oscillating CN (2 and 3 states)	4, 7
CN segments	92
FDR fragment joints	0.7559325
FDR chr. breakp. enrich.	0
Linked to chrs	13:26276510−102632900;16:69653875−83050345 5:703450−42018197;9:447092−9513408
Purity, ploidy	0.75, 2.64

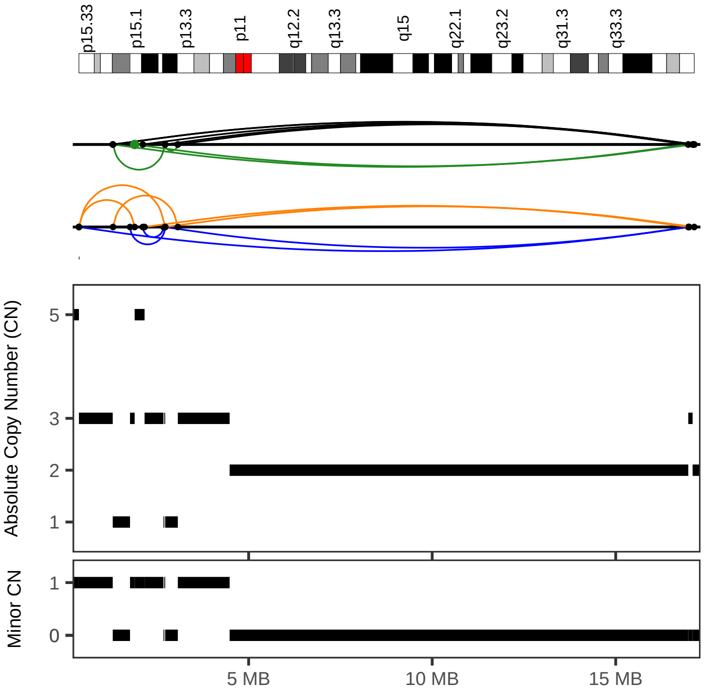

**MELA−0160**	
Cancer type	Skin−Melanoma
Position	5:376141−17139643
Type	With other complex events
Interleaved intrachr. SVs	18
Total SVs (intrachr. + transl.)	19
SV types	DEL: 5; DUP: 4; h2hINV: 5; t2tINV: 4; TRA: 1
SVs in sample	96
Oscillating CN (2 and 3 states)	5, 10
CN segments	13
FDR fragment joints	0.9348624
FDR chr. breakp. enrich.	0
Linked to chrs	
Purity, ploidy	0.68, 3.2

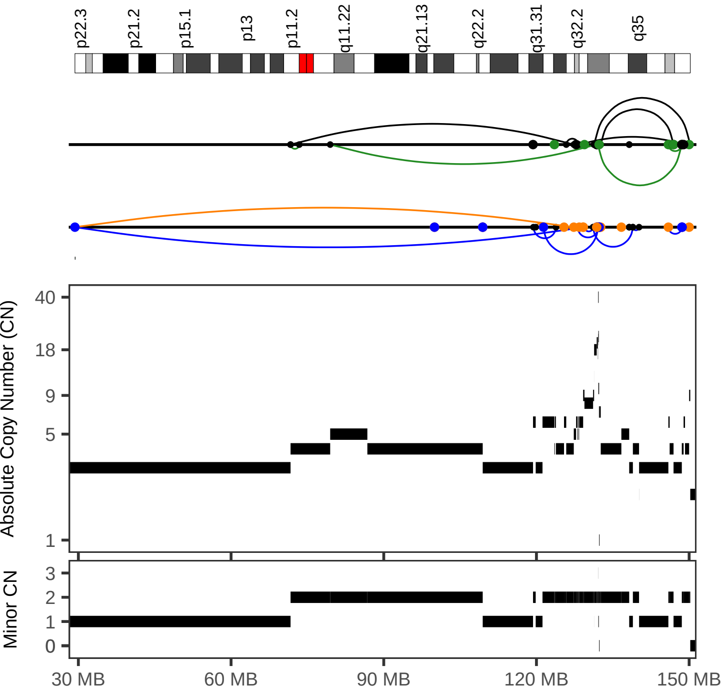

**MELA−0167**	
Cancer type	Skin−Melanoma
Position	7:29323351−150218313
Type	With other complex events
Interleaved intrachr. SVs	20
Total SVs (intrachr. + transl.)	64
SV types	DEL: 1; DUP: 9; h2hINV: 6; t2tINV: 4; TRA: 44
SVs in sample	339
Oscillating CN (2 and 3 states)	6, 10
CN segments	47
FDR fragment joints	0.615458
FDR chr. breakp. enrich.	0
Linked to chrs	
Purity, ploidy	0.74, 2.9

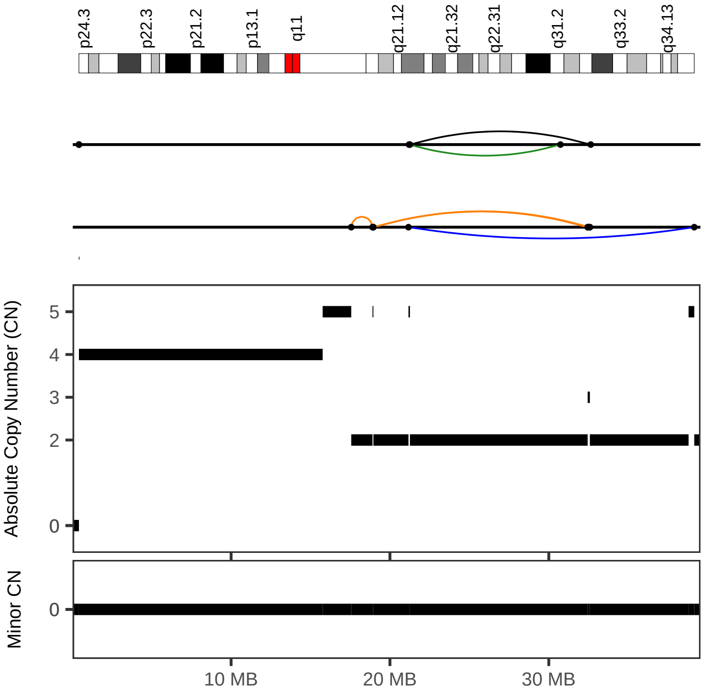

**MELA−0167**	
Cancer type	Skin−Melanoma
Position	9:18954759−39157408
Type	With other complex events
Interleaved intrachr. SVs	6
Total SVs (intrachr. + transl.)	6
SV types	DEL: 2; DUP: 2; h2hINV: 1; t2tINV: 1; TRA: 0
SVs in sample	339
Oscillating CN (2 and 3 states)	4, 8
CN segments	8
FDR fragment joints	0.9284301
FDR chr. breakp. enrich.	0.37
Linked to chrs	
Purity, ploidy	0.74, 2.9

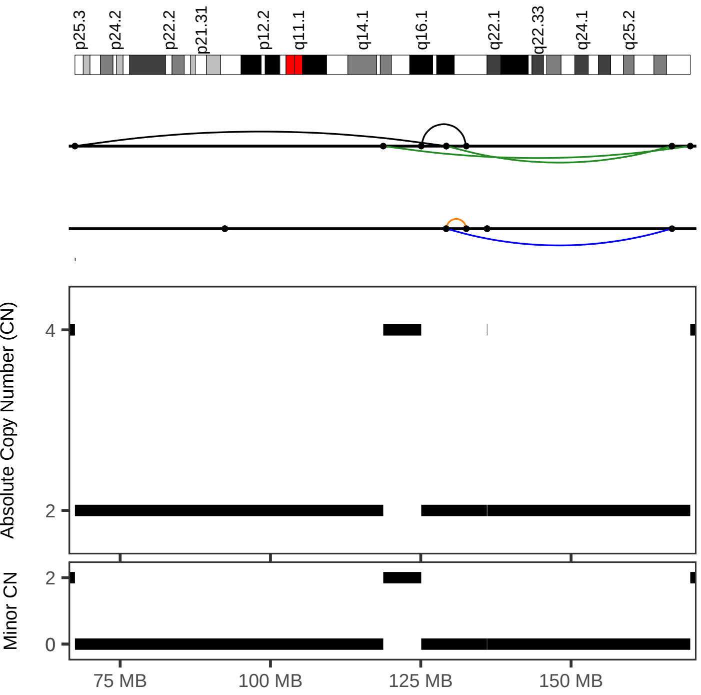

	MELA−0168
Cancer type	Skin−Melanoma
Position	6:67475142−169821533
Type	Before polyploidization
Interleaved intrachr. SVs	6
Total SVs (intrachr. + transl.)	6
SV types	DEL: 1; DUP: 1; h2hINV: 2; t2tINV: 2; TRA: 0
SVs in sample	57
Oscillating CN (2 and 3 states)	6, 6
CN segments	6
FDR fragment joints	0.9284301
FDR chr. breakp. enrich.	0.08
Linked to chrs	
Purity, ploidy	0.72, 3.64

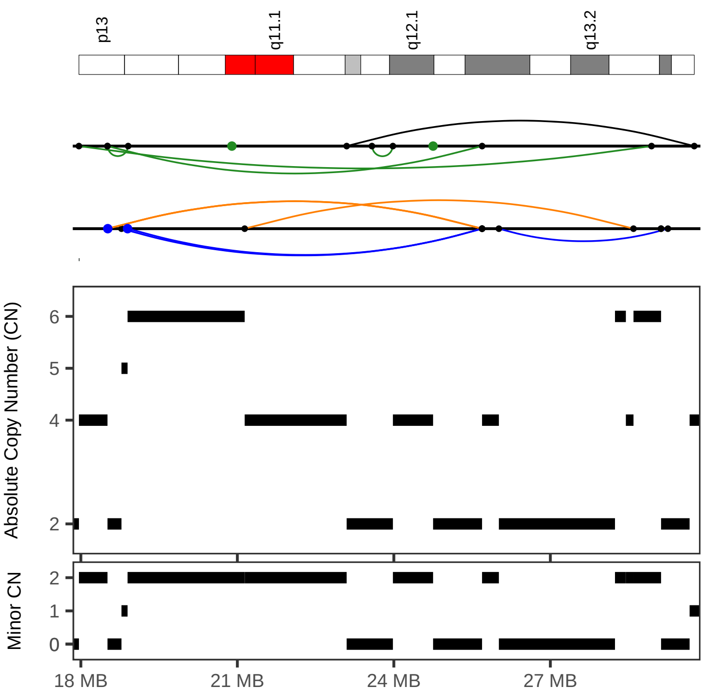

	MELA−0168
Cancer type	Skin−Melanoma
Position	22:17962914−29759005
Type	With other complex events
Interleaved intrachr. SVs	10
Total SVs (intrachr. + transl.)	14
SV types	DEL: 3; DUP: 3; h2hINV: 1; t2tINV: 3; TRA: 4
SVs in sample	57
Oscillating CN (2 and 3 states)	6, 11
CN segments	15
FDR fragment joints	0.8988396
FDR chr. breakp. enrich.	0
Linked to chrs	
Purity, ploidy	0.72, 3.64

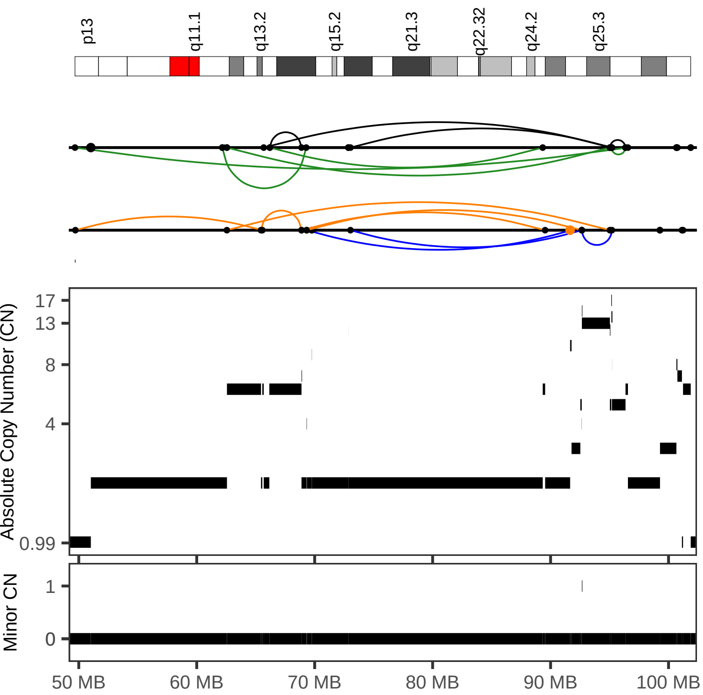

	MELA−0173
Cancer type	Skin−Melanoma
Position	15:49713725−96355825
Type	With other complex events
Interleaved intrachr. SVs	18
Total SVs (intrachr. + transl.)	21
SV types	DEL: 5; DUP: 3; h2hINV: 5; t2tINV: 5; TRA: 3
SVs in sample	90
Oscillating CN (2 and 3 states)	6, 8
CN segments	31
FDR fragment joints	0.7568568
FDR chr. breakp. enrich.	0
Linked to chrs	
Purity, ploidy	0.25, 3.01

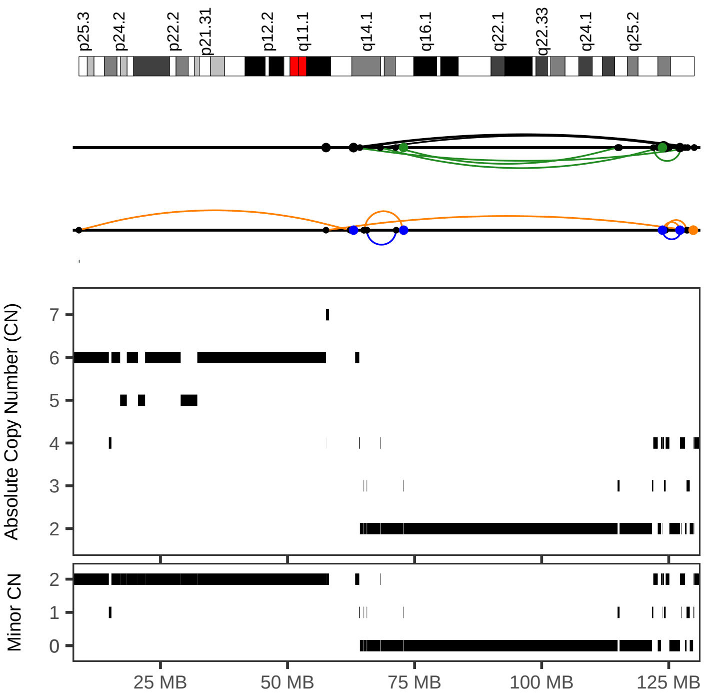

	MELA−0187
Cancer type	Skin−Melanoma
Position	6:64985024−128497164
Type	With other complex events
Interleaved intrachr. SVs	12
Total SVs (intrachr. + transl.)	20
SV types	DEL: 3; DUP: 3; h2hINV: 2; t2tINV: 4; TRA: 8
SVs in sample	313
Oscillating CN (2 and 3 states)	6, 14
CN segments	24
FDR fragment joints	0.8572806
FDR chr. breakp. enrich.	0
Linked to chrs	11:54893769−126272082;
Purity, ploidy	0.75, 3.27

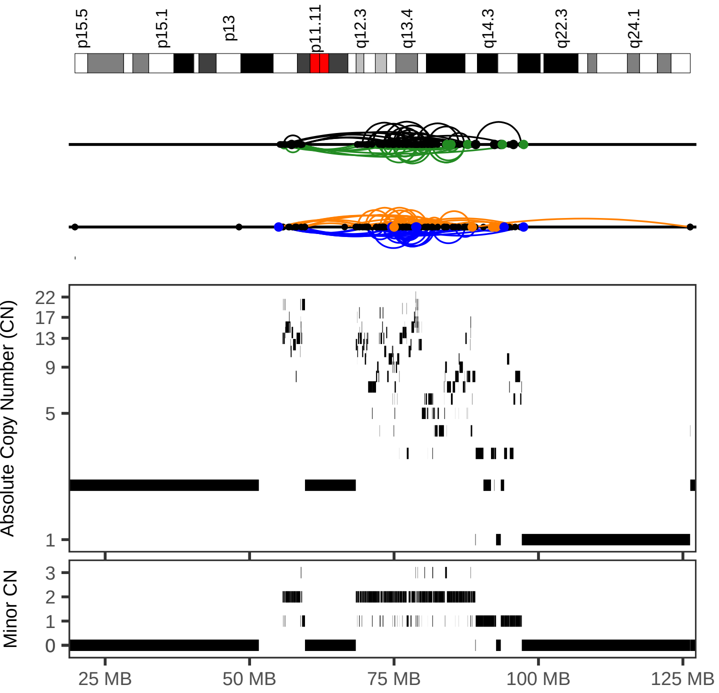

	MELA−0187
Cancer type	Skin−Melanoma
Position	11:54893769−126272083
Type	With other complex events
Interleaved intrachr. SVs	198
Total SVs (intrachr. + transl.)	218
SV types	DEL: 51; DUP: 52; h2hINV: 50; t2tINV: 45; TRA: 20
SVs in sample	313
Oscillating CN (2 and 3 states)	6, 12
CN segments	179
FDR fragment joints	0.930656
FDR chr. breakp. enrich.	0
Linked to chrs	
Purity, ploidy	0.75, 3.27

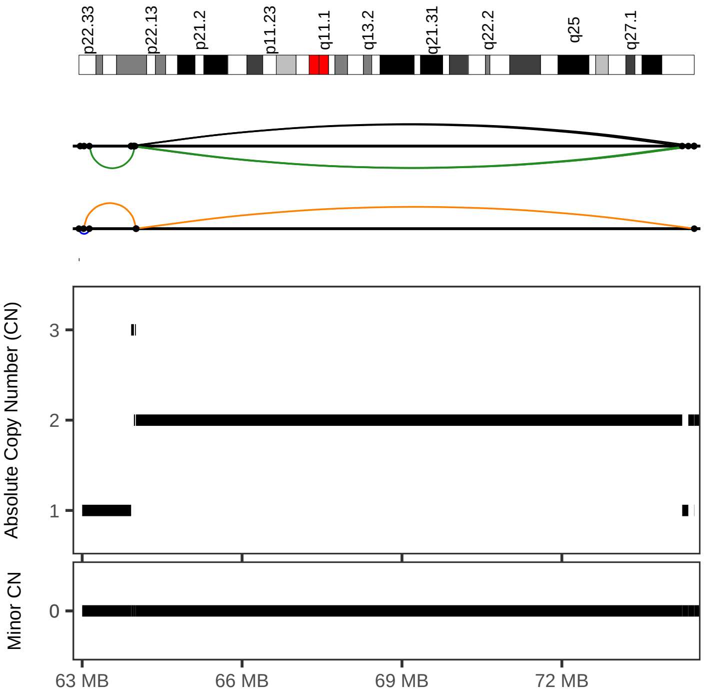

	MELA−0192
Cancer type	Skin−Melanoma
Position	X:62938682−74484110
Type	With other complex events
Interleaved intrachr. SVs	10
Total SVs (intrachr. + transl.)	10
SV types	DEL: 2; DUP: 1; h2hINV: 2; t2tINV: 5; TRA: 0
SVs in sample	55
Oscillating CN (2 and 3 states)	5, 5
CN segments	11
FDR fragment joints	0.615458
FDR chr. breakp. enrich.	0
Linked to chrs	
Purity, ploidy	0.75, 3.28

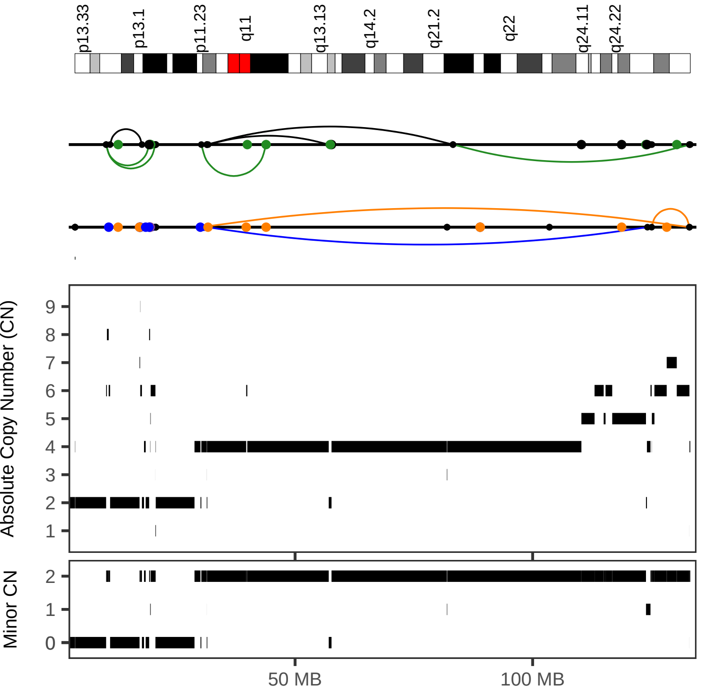

	MELA−0193
Cancer type	Skin−Melanoma
Position	12:30161467−133173511
Type	With other complex events
Interleaved intrachr. SVs	11
Total SVs (intrachr. + transl.)	28
SV types	DEL: 2; DUP: 3; h2hINV: 2; t2tINV: 4; TRA: 17
SVs in sample	264
Oscillating CN (2 and 3 states)	5, 6
CN segments	31
FDR fragment joints	0.6776251
FDR chr. breakp. enrich.	0
Linked to chrs	11:4690426−90534995;19:5218983−35407312 3:37537435−116274322;
Purity, ploidy	0.9, 4.07

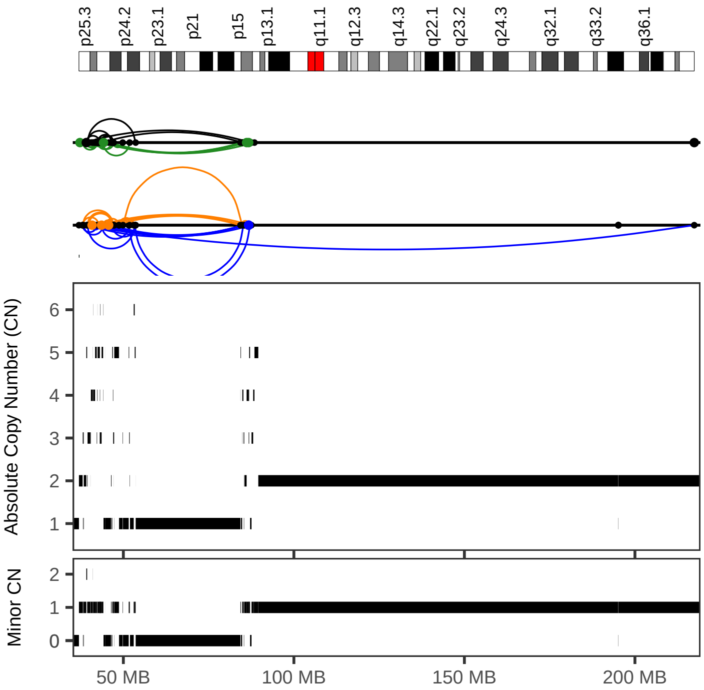

	MELA−0196
Cancer type	Skin−Melanoma
Position	2:38033476−88438303
Type	With other complex events
Interleaved intrachr. SVs	59
Total SVs (intrachr. + transl.)	68
SV types	DEL: 15; DUP: 17; h2hINV: 11; t2tINV: 16; TRA: 9
SVs in sample	101
Oscillating CN (2 and 3 states)	6, 12
CN segments	84
FDR fragment joints	0.905477
FDR chr. breakp. enrich.	0
Linked to chrs	
Purity, ploidy	0.73, 1.88

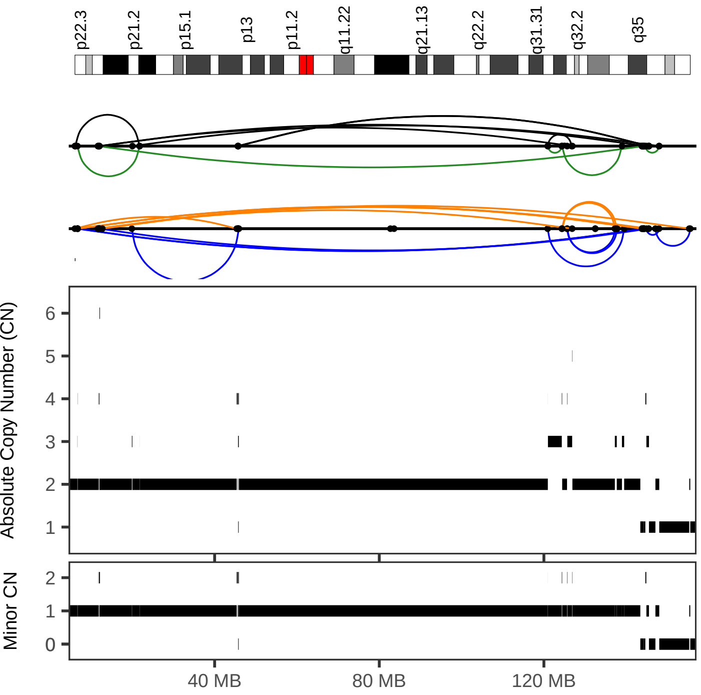

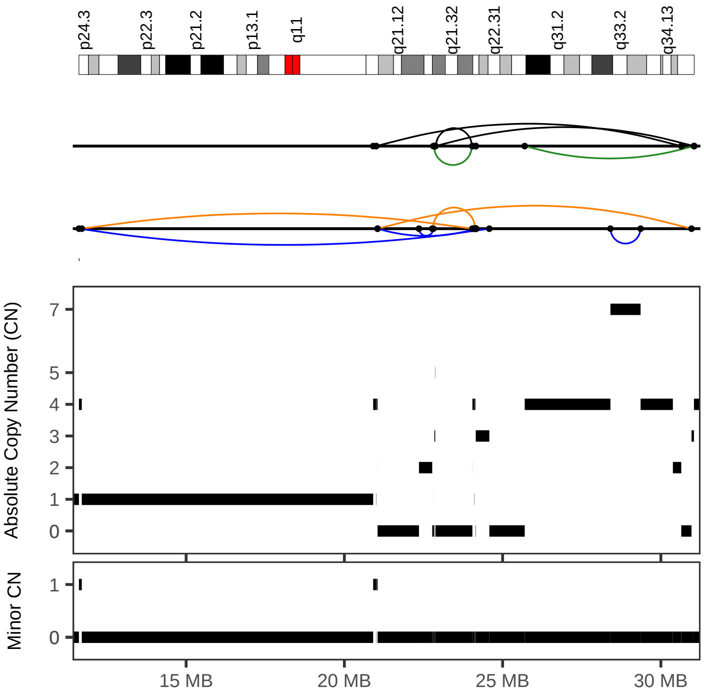

MELA−0202

Cancer type	Skin−Melanoma
Position	7:6051022−155559216
Type	With other complex events
Interleaved intrachr. SVs	42
Total SVs (intrachr. + transl.)	42
SV types	DEL: 10; DUP: 12; h2hINV: 11; t2tINV: 9; TRA: 0
SVs in sample	103
Oscillating CN (2 and 3 states)	5, 9
CN segments	44
FDR fragment joints	0.9550868
FDR chr. breakp. enrich.	0
Linked to chrs	
Purity, ploidy	0.73, 1.84

MELA−0205

Cancer type	Skin−Melanoma
Position	9:11612380−31055136
Type	With other complex events
Interleaved intrachr. SVs	14
Total SVs (intrachr. + transl.)	14
SV types	DEL: 4; DUP: 3; h2hINV: 3; t2tINV: 4; TRA: 0
SVs in sample	33
Oscillating CN (2 and 3 states)	5, 8
CN segments	28
FDR fragment joints	0.9794281
FDR chr. breakp. enrich.	0
Linked to chrs	
Purity, ploidy	0.24, 1.97

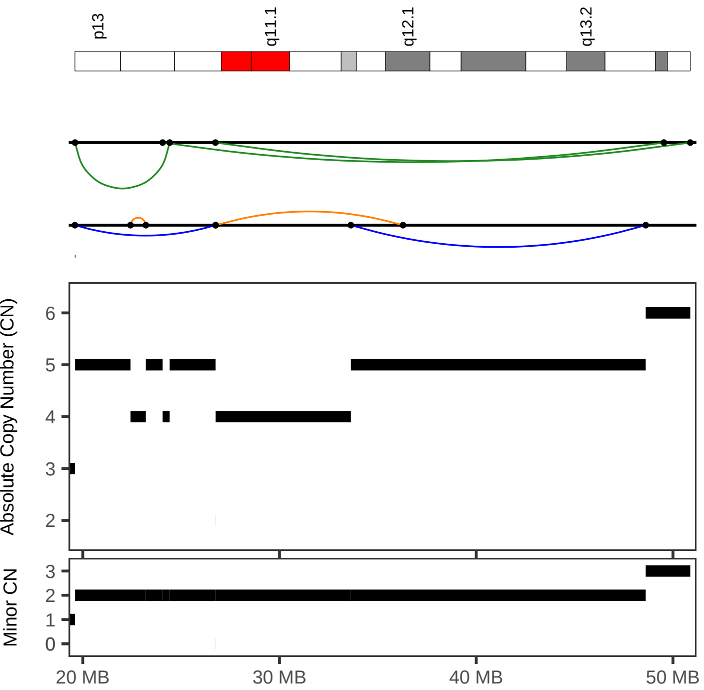

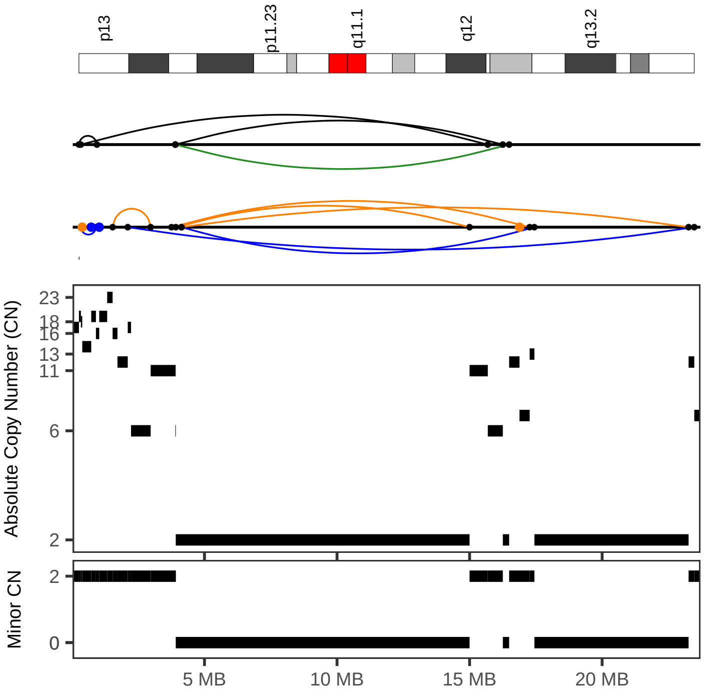

MELA−0213

Cancer type	Skin−Melanoma
Position	22:19600873−50879547
Type	With other complex events
Interleaved intrachr. SVs	6
Total SVs (intrachr. + transl.)	6
SV types	DEL: 1; DUP: 2; h2hINV: 0; t2tINV: 3; TRA: 0
SVs in sample	34
Oscillating CN (2 and 3 states)	5, 6
CN segments	10
FDR fragment joints	0.615458
FDR chr. breakp. enrich.	0
Linked to chrs	
Purity, ploidy	0.15, 3.66

MELA−0223

Cancer type	Skin−Melanoma
Position	20:264096−23474579
Type	With other complex events
Interleaved intrachr. SVs	10
Total SVs (intrachr. + transl.)	14
SV types	DEL: 4; DUP: 2; h2hINV: 3; t2tINV: 1; TRA: 4
SVs in sample	104
Oscillating CN (2 and 3 states)	4, 4
CN segments	24
FDR fragment joints	0.615458
FDR chr. breakp. enrich.	0
Linked to chrs	
Purity, ploidy	0.87, 4.03

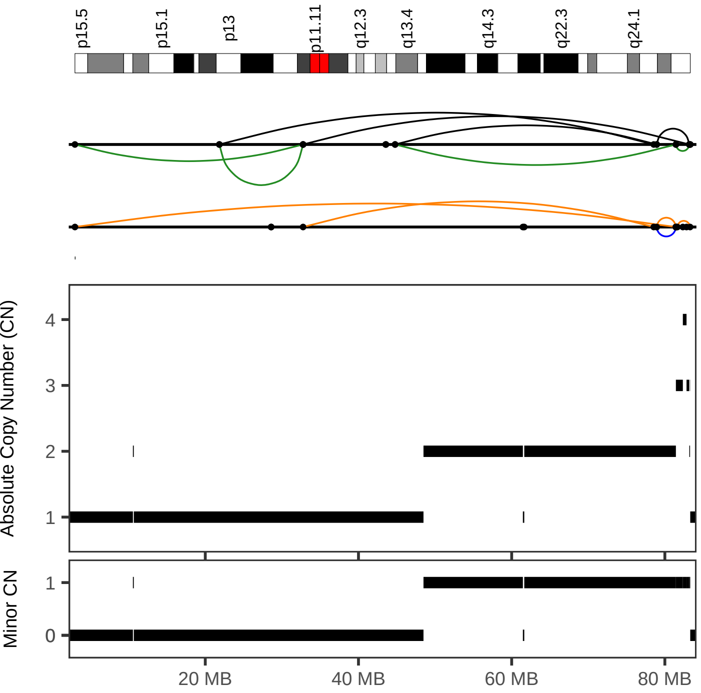

	**MELA−0226**
Cancer type	Skin−Melanoma
Position	11:2979632−83289483
Type	With other complex events
Interleaved intrachr. SVs	13
Total SVs (intrachr. + transl.)	13
SV types	DEL: 4; DUP: 1; h2hINV: 4; t2tINV: 4; TRA: 0
SVs in sample	152
Oscillating CN (2 and 3 states)	6, 11
CN segments	11
FDR fragment joints	0.6776251
FDR chr. breakp. enrich.	0
Linked to chrs	
Purity, ploidy	0.48, 1.86

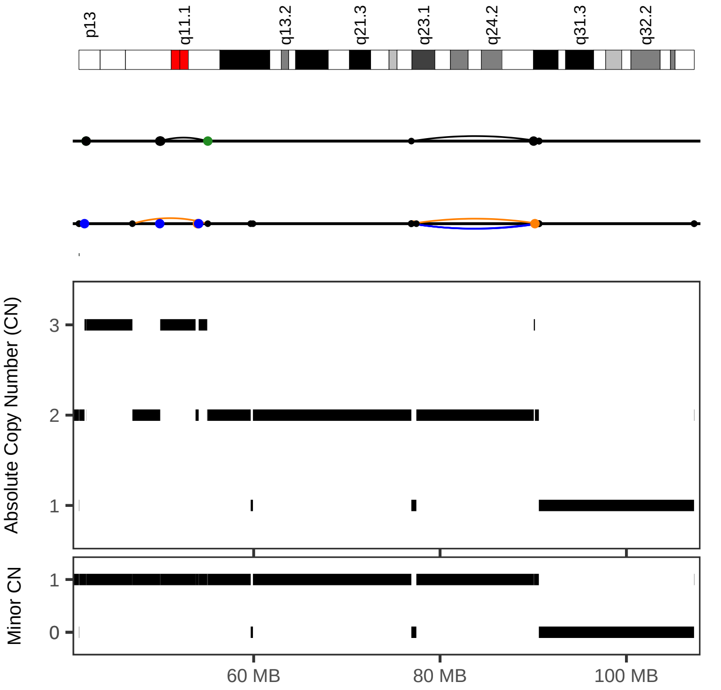

	**MELA−0226**
Cancer type	Skin−Melanoma
Position	14:76917881−90636670
Type	Canonical without polyploidization
Interleaved intrachr. SVs	11
Total SVs (intrachr. + transl.)	13
SV types	DEL: 3; DUP: 4; h2hINV: 1; t2tINV: 3; TRA: 2
SVs in sample	152
Oscillating CN (2 and 3 states)	5, 8
CN segments	8
FDR fragment joints	0.6776251
FDR chr. breakp. enrich.	0
Linked to chrs	7:123055563−158071676;
Purity, ploidy	0.48, 1.86

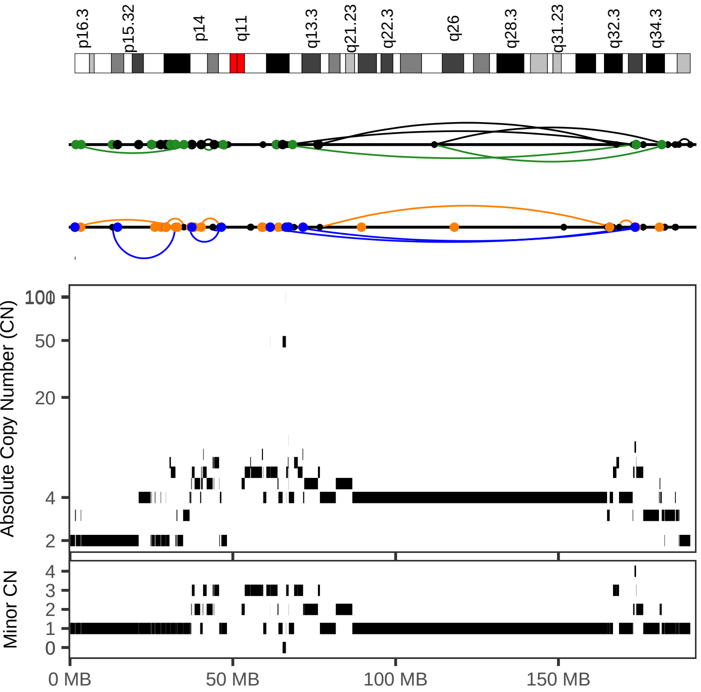

	**MELA−0231**
Cancer type	Skin−Melanoma
Position	4:58934408−183626934
Type	With other complex events
Interleaved intrachr. SVs	10
Total SVs (intrachr. + transl.)	38
SV types	DEL: 2; DUP: 2; h2hINV: 4; t2tINV: 2; TRA: 28
SVs in sample	1234
Oscillating CN (2 and 3 states)	5, 10
CN segments	47
FDR fragment joints	0.615458
FDR chr. breakp. enrich.	0.03
Linked to chrs	10:19180867−131722345;18:58999177−77021614 2:5310579−242675571;7:712296−54531287
Purity, ploidy	0.92, 4.03

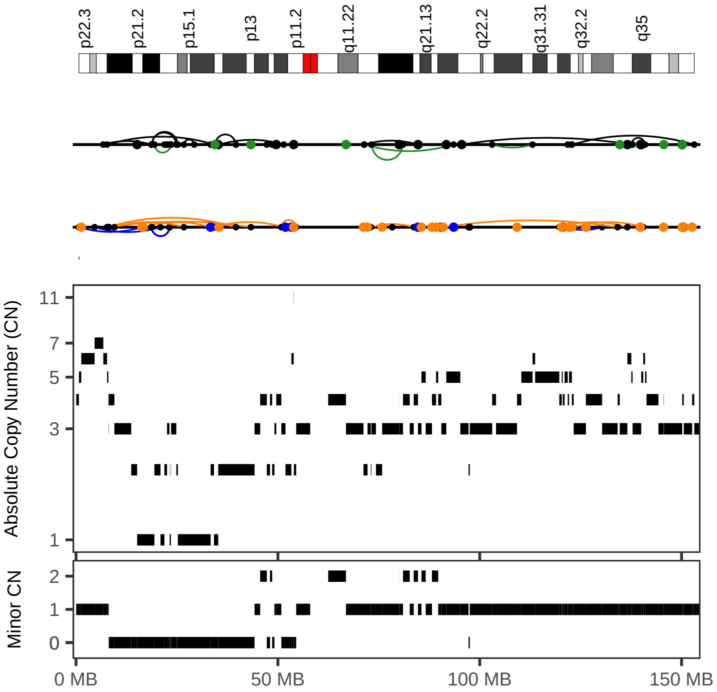

	**MELA−0231**
Cancer type	Skin−Melanoma
Position	7:712296−54531288
Type	With other complex events
Interleaved intrachr. SVs	23
Total SVs (intrachr. + transl.)	38
SV types	DEL: 8; DUP: 5; h2hINV: 6; t2tINV: 4; TRA: 15
SVs in sample	1234
Oscillating CN (2 and 3 states)	5, 26
CN segments	37
FDR fragment joints	0.7282798
FDR chr. breakp. enrich.	0
Linked to chrs	1:49615754−106759580;12:4111597−34617369 14:27456610−34055767;5:4413255−157081451
Purity, ploidy	0.92, 4.03

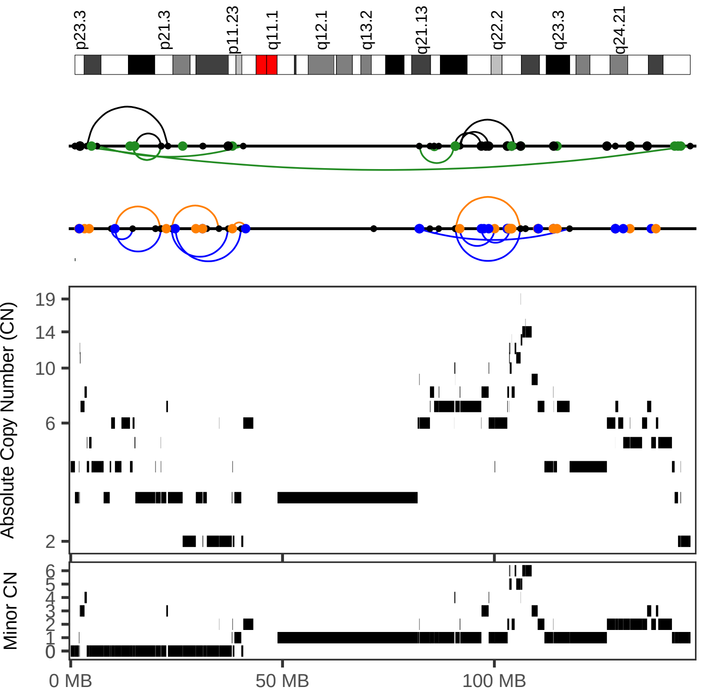

MELA−0231

Cancer type	Skin−Melanoma
Position	8:9543398−106192556
Type	With other complex events
Interleaved intrachr. SVs	7
Total SVs (intrachr. + transl.)	54
SV types	DEL: 2; DUP: 6; h2hINV: 4; t2tINV: 2; TRA: 40
SVs in sample	1234
Oscillating CN (2 and 3 states)	6, 8
CN segments	61
FDR fragment joints	0.615458
FDR chr. breakp. enrich.	0
Linked to chrs	12:4111597−34617369;15:27228030−90601941 18:58999177−77021614;2:5310579−242675571 6:76285221−169646111;
Purity, ploidy	0.92, 4.03

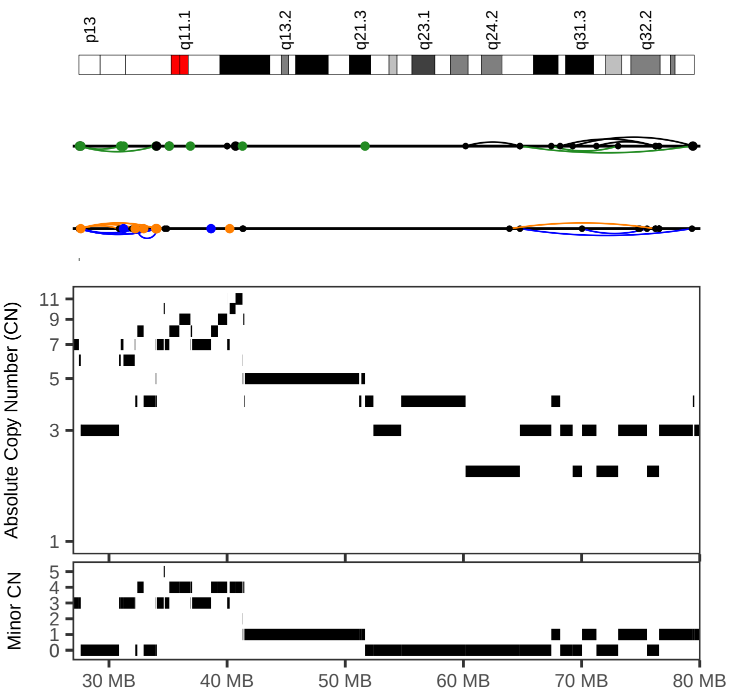

MELA−0231

Cancer type	Skin−Melanoma
Position	14:27456610−34055768
Type	With other complex events
Interleaved intrachr. SVs	17
Total SVs (intrachr. + transl.)	33
SV types	DEL: 5; DUP: 9; h2hINV: 1; t2tINV: 2; TRA: 16
SVs in sample	1234
Oscillating CN (2 and 3 states)	5, 5
CN segments	16
FDR fragment joints	0.6735663
FDR chr. breakp. enrich.	0
Linked to chrs	2:5310579−242675571;
Purity, ploidy	0.92, 4.03

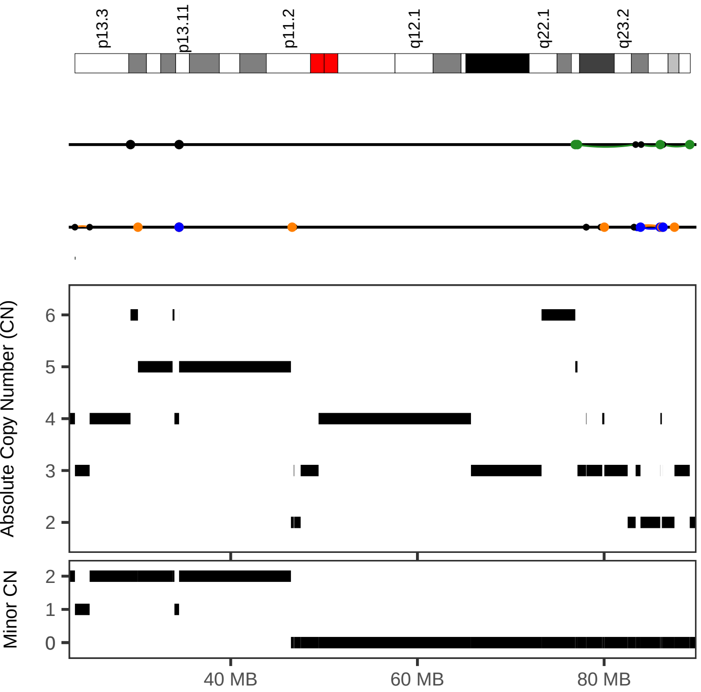

MELA−0231

Cancer type	Skin−Melanoma
Position	16:76956420−89223828
Type	With other complex events
Interleaved intrachr. SVs	9
Total SVs (intrachr. + transl.)	18
SV types	DEL: 2; DUP: 4; h2hINV: 0; t2tINV: 3; TRA: 9
SVs in sample	1234
Oscillating CN (2 and 3 states)	5, 9
CN segments	18
FDR fragment joints	0.6776251
FDR chr. breakp. enrich.	1
Linked to chrs	11:85905386−127883070;X:114145722−144057314
Purity, ploidy	0.92, 4.03

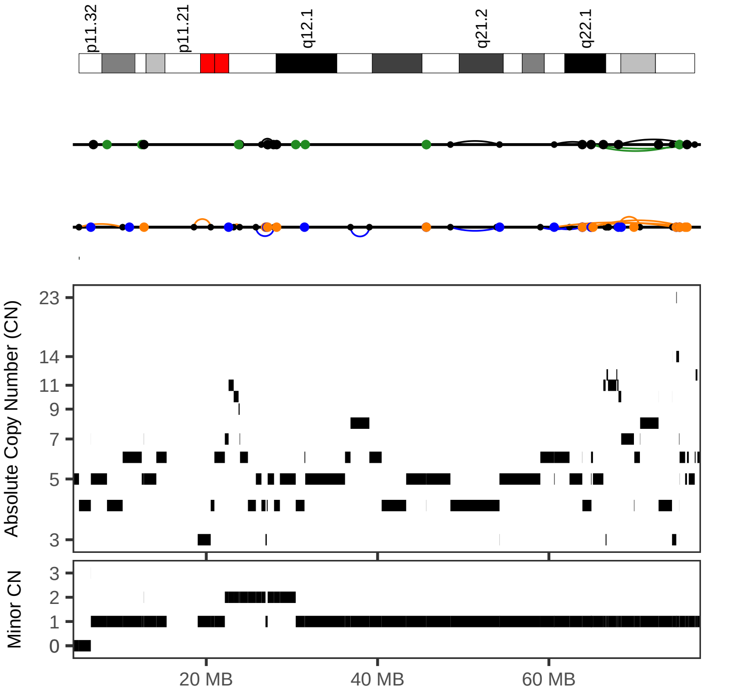

MELA−0231

Cancer type	Skin−Melanoma
Position	18:58999177−77021615
Type	With other complex events
Interleaved intrachr. SVs	10
Total SVs (intrachr. + transl.)	35
SV types	DEL: 4; DUP: 2; h2hINV: 2; t2tINV: 2; TRA: 25
SVs in sample	1234
Oscillating CN (2 and 3 states)	6, 10
CN segments	39
FDR fragment joints	0.615458
FDR chr. breakp. enrich.	0
Linked to chrs	6:76285221−169646111;
Purity, ploidy	0.92, 4.03

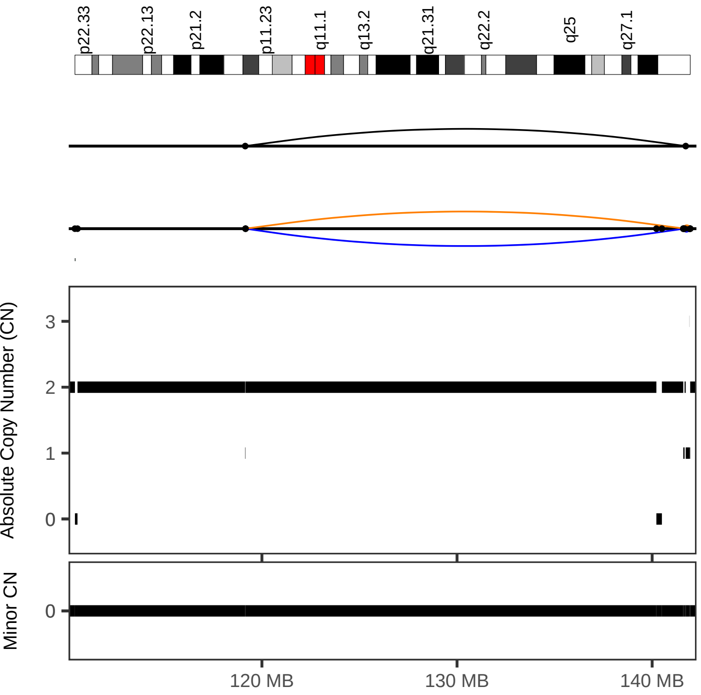

	**MELA−0256**
Cancer type	Skin−Melanoma
Position	X:119143192−141952863
Type	With other complex events
Interleaved intrachr. SVs	6
Total SVs (intrachr. + transl.)	6
SV types	DEL: 2; DUP: 3; h2hINV: 1; t2tINV: 0; TRA: 0
SVs in sample	92
Oscillating CN (2 and 3 states)	4, 10
CN segments	10
FDR fragment joints	0.615458
FDR chr. breakp. enrich.	0.34
Linked to chrs	
Purity, ploidy	0.7, 3.38

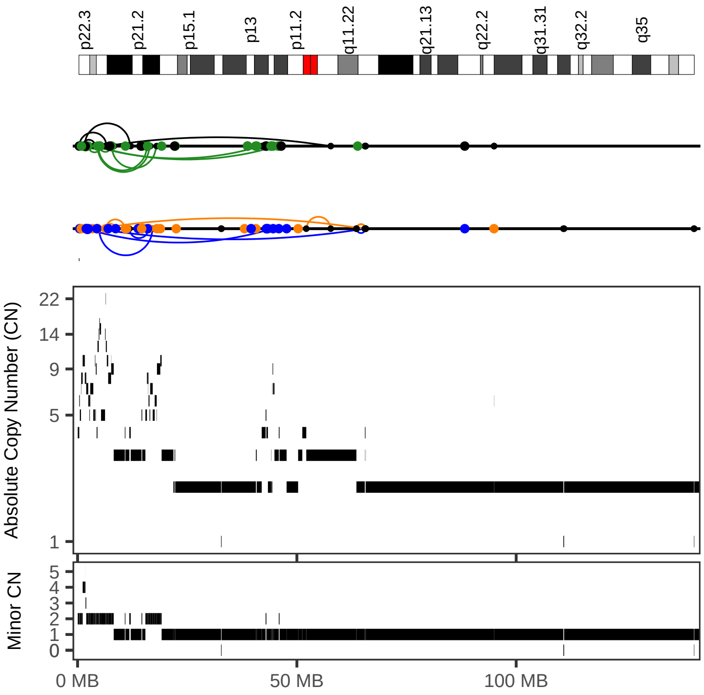

	**1404791b−86bf−4dfd−85b4−c8ff356f109c**
Cancer type	Skin−Melanoma
Position	7:306183−65693059
Type	With other complex events
Interleaved intrachr. SVs	31
Total SVs (intrachr. + transl.)	98
SV types	DEL: 7; DUP: 7; h2hINV: 7; t2tINV: 10; TRA: 67
SVs in sample	573
Oscillating CN (2 and 3 states)	6, 10
CN segments	89
FDR fragment joints	0.8988396
FDR chr. breakp. enrich.	0
Linked to chrs	11:33008500−79611121;19:32205724−58908288 22:29353674−50585361;6:503244−53879503 8:118357384−142919751;
Purity, ploidy	0.89, 2.68

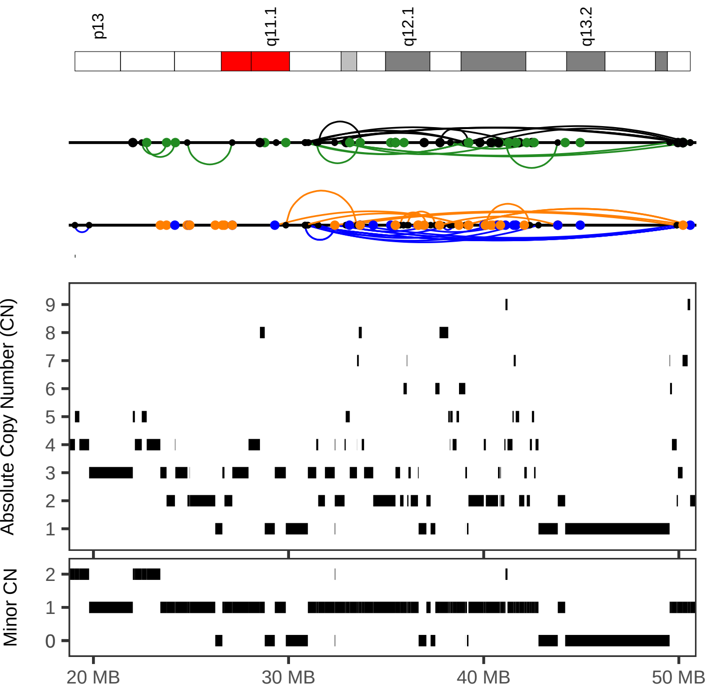

	**1404791b−86bf−4dfd−85b4−c8ff356f109c**
Cancer type	Skin−Melanoma
Position	22:29353674−50585362
Type	With other complex events
Interleaved intrachr. SVs	45
Total SVs (intrachr. + transl.)	127
SV types	DEL: 12; DUP: 15; h2hINV: 10; t2tINV: 8; TRA: 82
SVs in sample	573
Oscillating CN (2 and 3 states)	5, 8
CN segments	73
FDR fragment joints	0.9625775
FDR chr. breakp. enrich.	0
Linked to chrs	1:1375298−247642164;11:33008500−79611121 19:32205724−58908288;6:503244−53879503 7:306183−65693058;
Purity, ploidy	0.89, 2.68

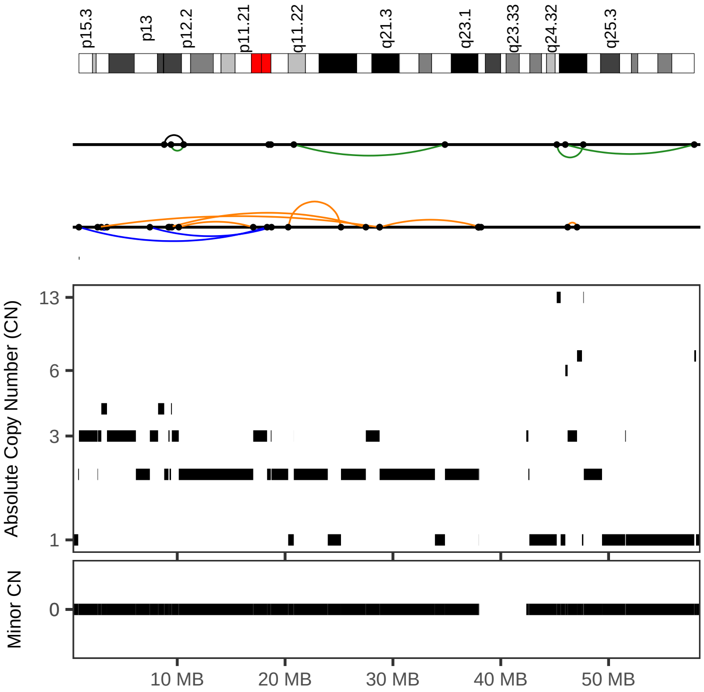

	**238111f9−a08b−49b4−9035−cc43026330e7**
Cancer type	Skin−Melanoma
Position	10:881509−38175147
Type	With other complex events
Interleaved intrachr. SVs	11
Total SVs (intrachr. + transl.)	11
SV types	DEL: 5; DUP: 3; h2hINV: 1; t2tINV: 2; TRA: 0
SVs in sample	87
Oscillating CN (2 and 3 states)	6, 11
CN segments	30
FDR fragment joints	0.615458
FDR chr. breakp. enrich.	0
Linked to chrs	
Purity, ploidy	0.89, 2.79

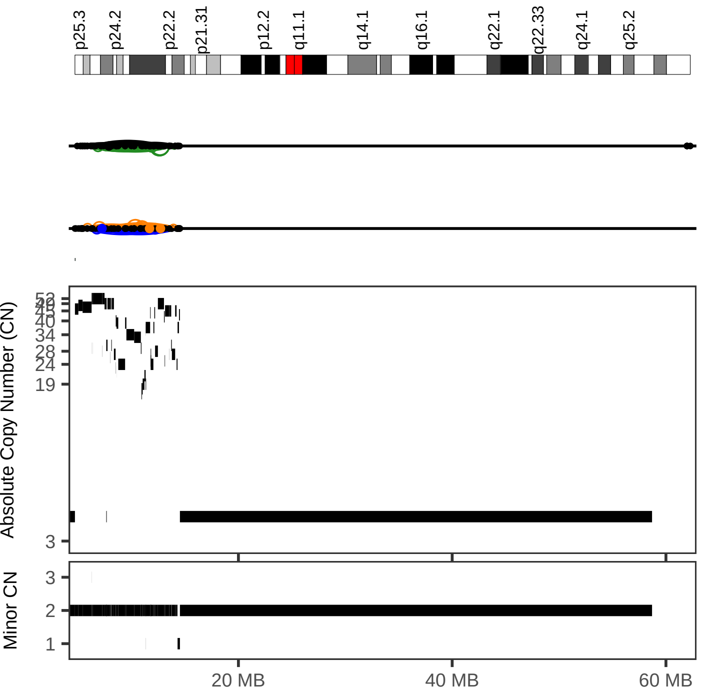

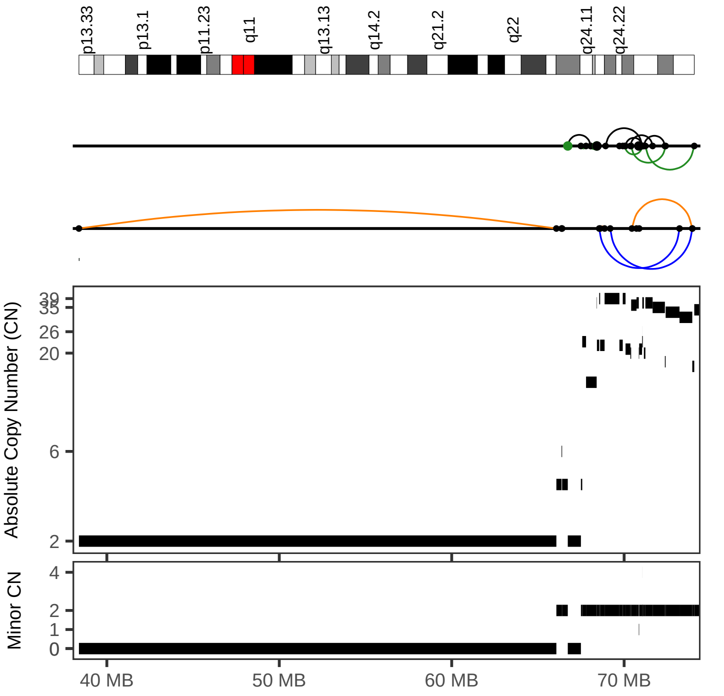

	296f44f9−3ecb−4b40−ae51−c3a06e3fbe00
Cancer type	Skin−Melanoma
Position	6:4799433−14498344
Type	With other complex events
Interleaved intrachr. SVs	79
Total SVs (intrachr. + transl.)	83
SV types	DEL: 22; DUP: 22; h2hINV: 17; t2tINV: 18; TRA: 4
SVs in sample	170
Oscillating CN (2 and 3 states)	4, 8
CN segments	57
FDR fragment joints	0.7735152
FDR chr. breakp. enrich.	0
Linked to chrs	
Purity, ploidy	0.86, 3.45

	296f44f9−3ecb−4b40−ae51−c3a06e3fbe00
Cancer type	Skin−Melanoma
Position	12:68553802−74066796
Type	With other complex events
Interleaved intrachr. SVs	10
Total SVs (intrachr. + transl.)	11
SV types	DEL: 1; DUP: 2; h2hINV: 4; t2tINV: 3; TRA: 1
SVs in sample	170
Oscillating CN (2 and 3 states)	5, 5
CN segments	24
FDR fragment joints	0.6776251
FDR chr. breakp. enrich.	0
Linked to chrs	
Purity, ploidy	0.86, 3.45

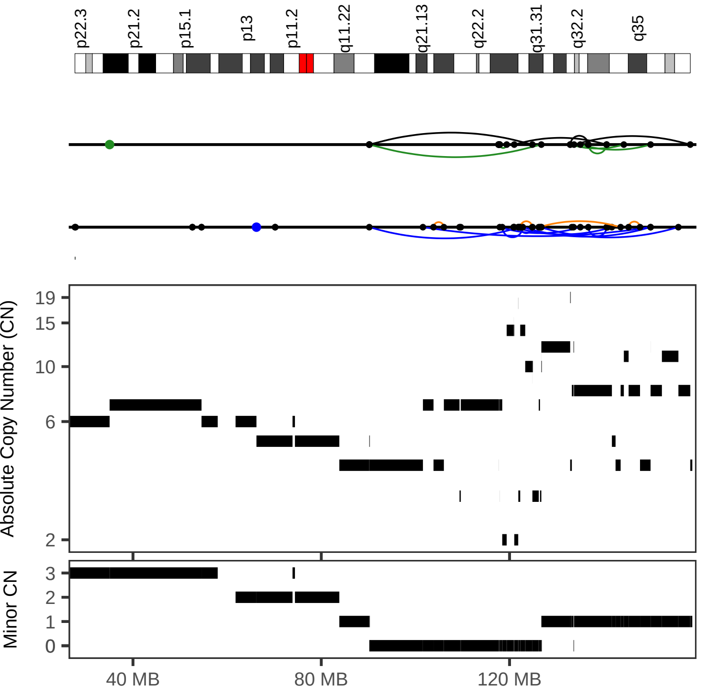

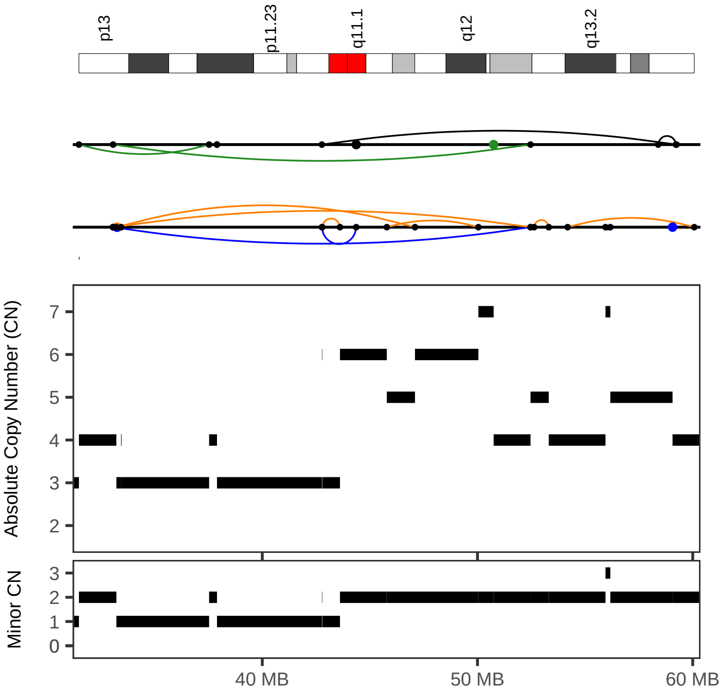

	3fea3f02−2db3−44e0−984e−c61130d92c9b
Cancer type	Skin−Melanoma
Position	7:90201391−158391119
Type	With other complex events
Interleaved intrachr. SVs	21
Total SVs (intrachr. + transl.)	21
SV types	DEL: 2; DUP: 9; h2hINV: 5; t2tINV: 5; TRA: 0
SVs in sample	262
Oscillating CN (2 and 3 states)	4, 11
CN segments	45
FDR fragment joints	0.615458
FDR chr. breakp. enrich.	0
Linked to chrs	
Purity, ploidy	0.74, 3.05

	3fea3f02−2db3−44e0−984e−c61130d92c9b
Cancer type	Skin−Melanoma
Position	20:31479759−60071503
Type	With other complex events
Interleaved intrachr. SVs	10
Total SVs (intrachr. + transl.)	13
SV types	DEL: 5; DUP: 2; h2hINV: 1; t2tINV: 2; TRA: 3
SVs in sample	262
Oscillating CN (2 and 3 states)	6, 16
CN segments	18
FDR fragment joints	0.7995907
FDR chr. breakp. enrich.	0
Linked to chrs	
Purity, ploidy	0.74, 3.05

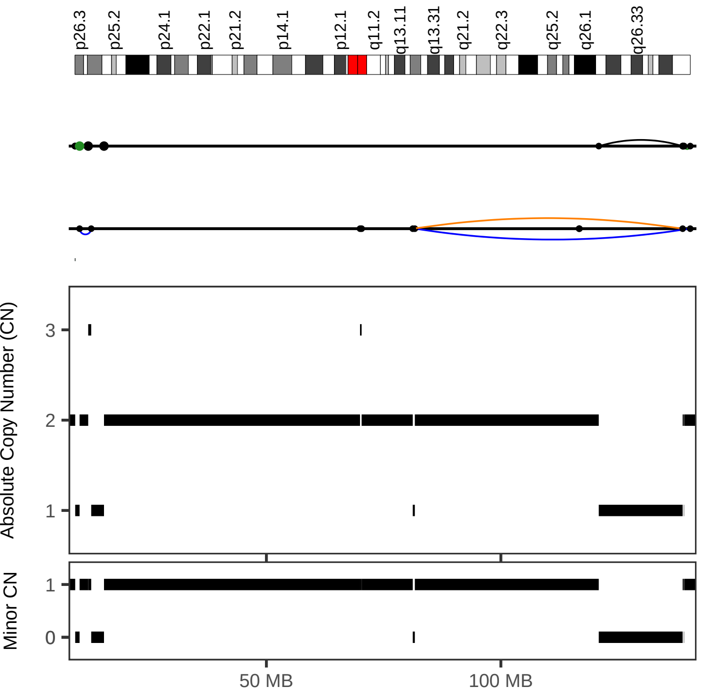

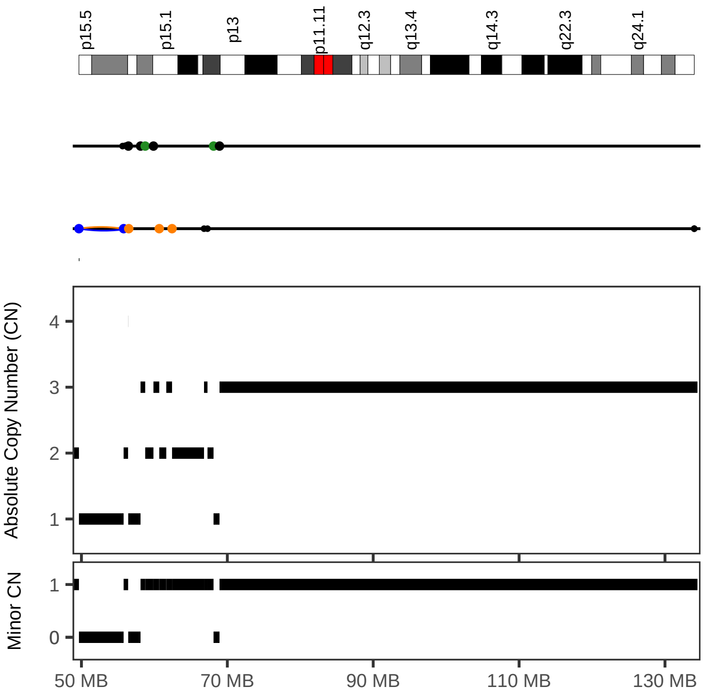

	5f6df69c−4ebf−4811−8b28−ad393dfbe1a3
Cancer type	Skin−Melanoma
Position	3:81208042−140376909
Type	Canonical without polyploidization
Interleaved intrachr. SVs	6
Total SVs (intrachr. + transl.)	6
SV types	DEL: 1; DUP: 1; h2hINV: 2; t2tINV: 2; TRA: 0
SVs in sample	160
Oscillating CN (2 and 3 states)	6, 6
CN segments	6
FDR fragment joints	0.9284301
FDR chr. breakp. enrich.	0.03
Linked to chrs	
Purity, ploidy	0.86, 1.94

	76cc1d42−acec−46b3−9663−5e4de2550353
Cancer type	Skin−Melanoma
Position	11:49654668−56498706
Type	Before polyploidization
Interleaved intrachr. SVs	6
Total SVs (intrachr. + transl.)	10
SV types	DEL: 1; DUP: 2; h2hINV: 2; t2tINV: 1; TRA: 4
SVs in sample	245
Oscillating CN (2 and 3 states)	5, 7
CN segments	7
FDR fragment joints	0.8572806
FDR chr. breakp. enrich.	0.01
Linked to chrs	
Purity, ploidy	0.83, 2.94

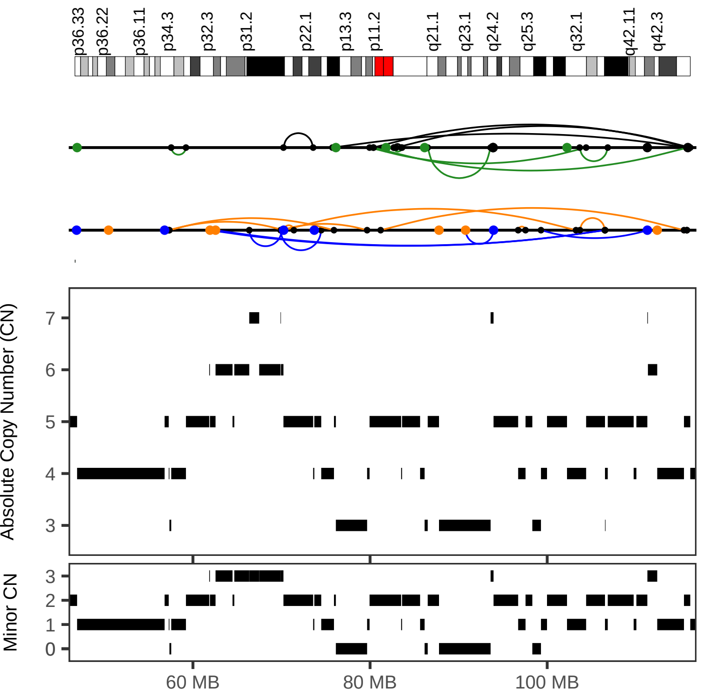

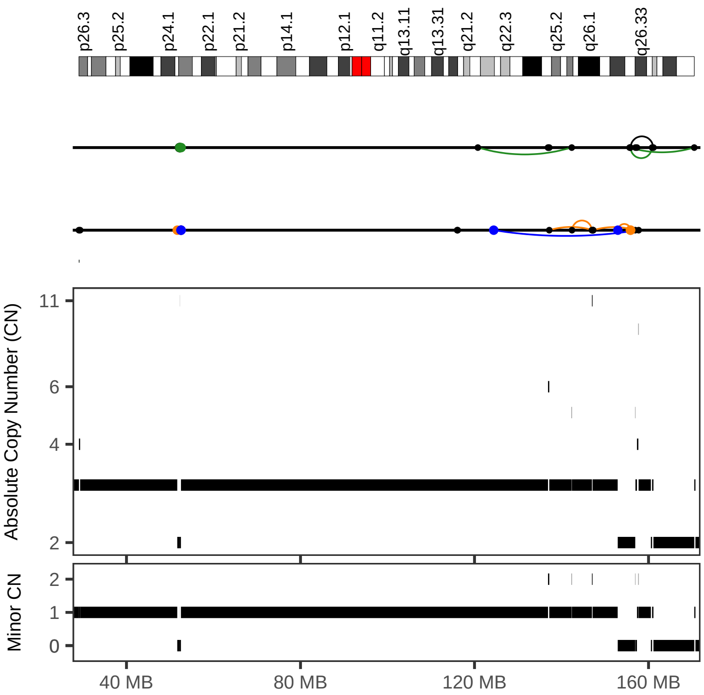

	7b92fcd4−f9be−4b97−94a2−b2e8348aeaba
Cancer type	Skin−Melanoma
Position	1:57268038−116153671
Type	With other complex events
Interleaved intrachr. SVs	21
Total SVs (intrachr. + transl.)	38
SV types	DEL: 6; DUP: 6; h2hINV: 5; t2tINV: 4; TRA: 17
SVs in sample	137
Oscillating CN (2 and 3 states)	6, 18
CN segments	46
FDR fragment joints	0.9460923
FDR chr. breakp. enrich.	0
Linked to chrs	
Purity, ploidy	0.67, 4.4

	7ce09a09−074e−4f38−bed7−98ab34347fbe
Cancer type	Skin−Melanoma
Position	3:120763840−170508368
Type	With other complex events
Interleaved intrachr. SVs	8
Total SVs (intrachr. + transl.)	12
SV types	DEL: 3; DUP: 1; h2hINV: 1; t2tINV: 3; TRA: 4
SVs in sample	201
Oscillating CN (2 and 3 states)	5, 5
CN segments	19
FDR fragment joints	0.6776251
FDR chr. breakp. enrich.	0
Linked to chrs	
Purity, ploidy	0.88, 3.45

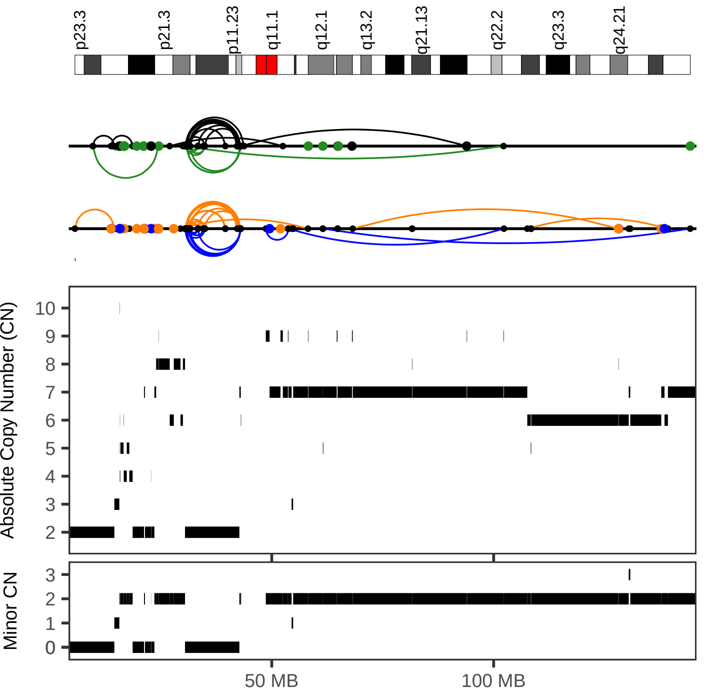

	8c54fcfd−999f−43c5−b31f−26d006f5fff3
Cancer type	Skin−Melanoma
Position	8:27003357−144300868
Type	With other complex events
Interleaved intrachr. SVs	70
Total SVs (intrachr. + transl.)	84
SV types	DEL: 23; DUP: 15; h2hINV: 18; t2tINV: 14; TRA: 14
SVs in sample	351
Oscillating CN (2 and 3 states)	6, 11
CN segments	41
FDR fragment joints	0.626975
FDR chr. breakp. enrich.	0
Linked to chrs	
Purity, ploidy	0.9, 3.17

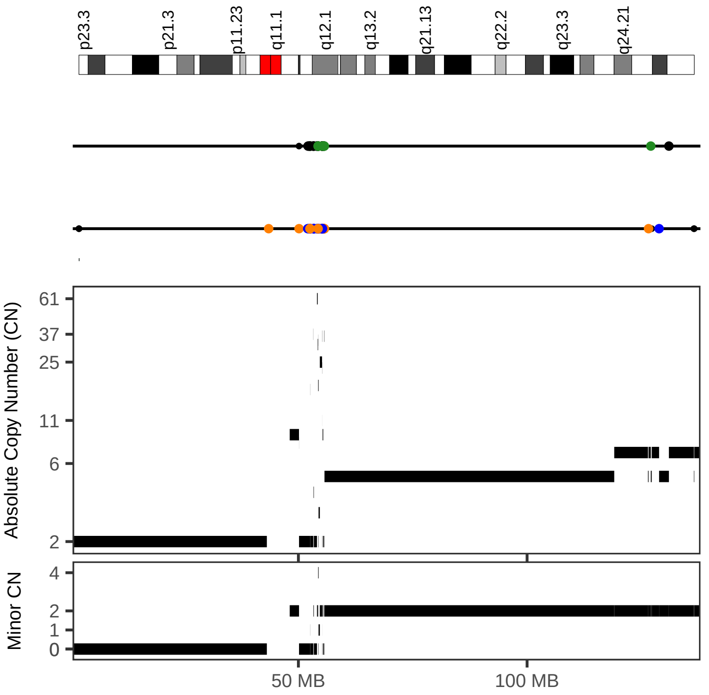

	b85b14d7−3b5a−4800−af12−622ec03b9fe5
Cancer type	Skin−Melanoma
Position	8:50126618−55588669
Type	With other complex events
Interleaved intrachr. SVs	8
Total SVs (intrachr. + transl.)	82
SV types	DEL: 1; DUP: 1; h2hINV: 5; t2tINV: 1; TRA: 74
SVs in sample	633
Oscillating CN (2 and 3 states)	4, 5
CN segments	23
FDR fragment joints	0.6776251
FDR chr. breakp. enrich.	0
Linked to chrs	11:68369991−101011601;
Purity, ploidy	0.72, 3.36

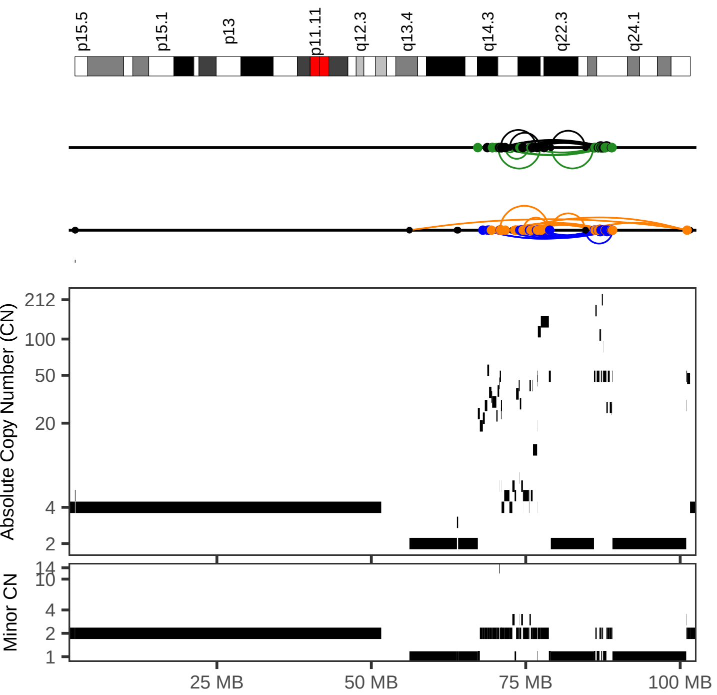

	b85b14d7−3b5a−4800−af12−622ec03b9fe5
Cancer type	Skin−Melanoma
Position	11:68369991−101011602
Type	With other complex events
Interleaved intrachr. SVs	67
Total SVs (intrachr. + transl.)	198
SV types	DEL: 15; DUP: 18; h2hINV: 20; t2tINV: 14; TRA: 131
SVs in sample	633
Oscillating CN (2 and 3 states)	6, 6
CN segments	63
FDR fragment joints	0.7549835
FDR chr. breakp. enrich.	0
Linked to chrs	
Purity, ploidy	0.72, 3.36

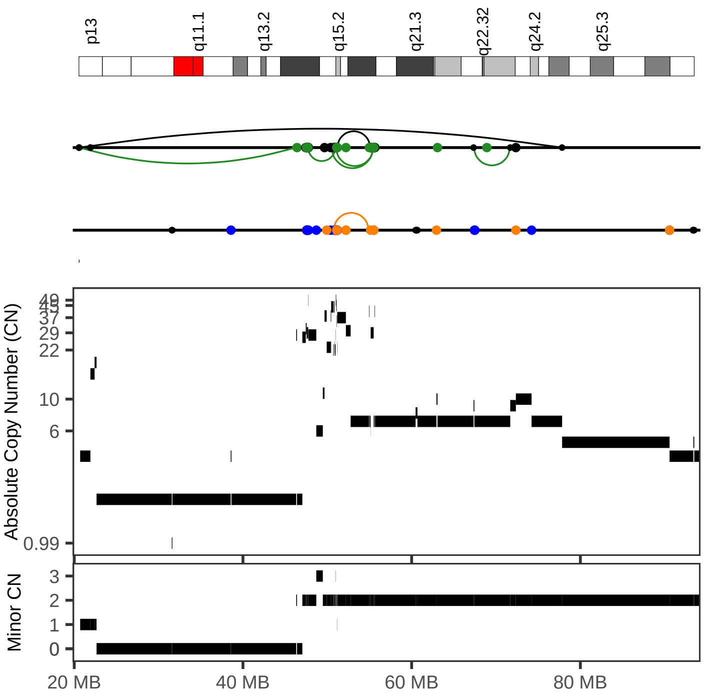

	b85b14d7−3b5a−4800−af12−622ec03b9fe5
Cancer type	Skin−Melanoma
Position	15:47687942−55499374
Type	With other complex events
Interleaved intrachr. SVs	8
Total SVs (intrachr. + transl.)	70
SV types	DEL: 2; DUP: 2; h2hINV: 1; t2tINV: 3; TRA: 62
SVs in sample	633
Oscillating CN (2 and 3 states)	4, 5
CN segments	28
FDR fragment joints	0.9483317
FDR chr. breakp. enrich.	0
Linked to chrs	11:68369991−101011601;
Purity, ploidy	0.72, 3.36

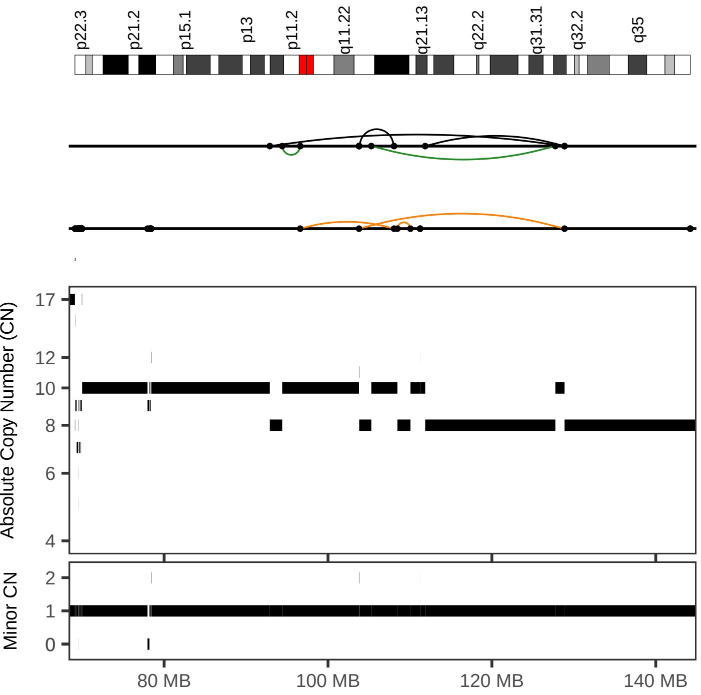

	d8eac750−9daf−4465−b672−9e31fd369057
Cancer type	Skin−Melanoma
Position	7:92899327−128870216
Type	With other complex events
Interleaved intrachr. SVs	7
Total SVs (intrachr. + transl.)	7
SV types	DEL: 2; DUP: 0; h2hINV: 3; t2tINV: 2; TRA: 0
SVs in sample	266
Oscillating CN (2 and 3 states)	4, 9
CN segments	12
FDR fragment joints	0.6776251
FDR chr. breakp. enrich.	0.03
Linked to chrs	
Purity, ploidy	0.47, 3.89

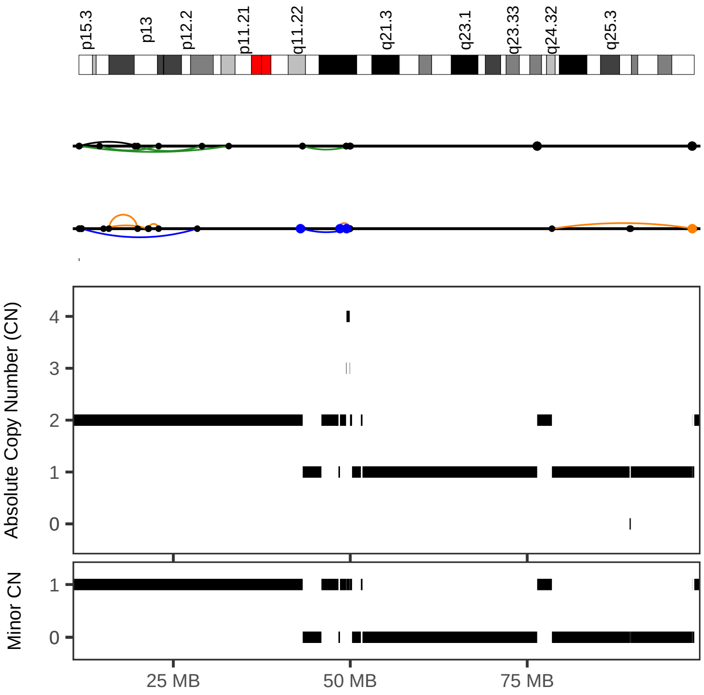

	12bfd446−2ee5−4664−a492−c023d134f60f
Cancer type	SoftTissue−Leiomyo
Position	10:11662562−50056640
Type	With other complex events
Interleaved intrachr. SVs	9
Total SVs (intrachr. + transl.)	21
SV types	DEL: 4; DUP: 5; h2hINV: 4; t2tINV: 5; TRA: 3
SVs in sample	247
Oscillating CN (2 and 3 states)	5, 5
CN segments	12
FDR fragment joints	0.6776251
FDR chr. breakp. enrich.	0
Linked to chrs	9:434615−126676282;
Purity, ploidy	0.88, 1.73

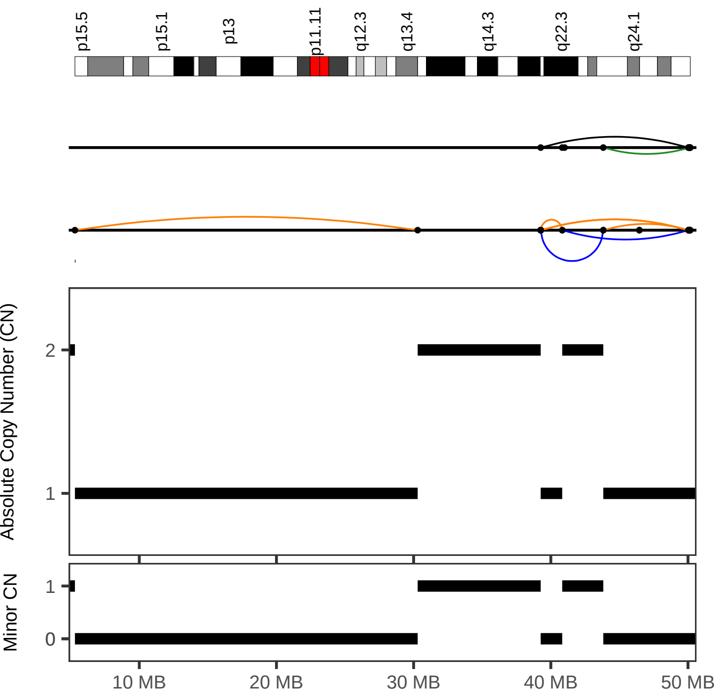

	12bfd446−2ee5−4664−a492−c023d134f60f
Cancer type	SoftTissue−Leiomyo
Position	11:39261849−50164804
Type	Canonical without polyploidization
Interleaved intrachr. SVs	14
Total SVs (intrachr. + transl.)	14
SV types	DEL: 6; DUP: 2; h2hINV: 2; t2tINV: 4; TRA: 0
SVs in sample	247
Oscillating CN (2 and 3 states)	4, 4
CN segments	4
FDR fragment joints	0.615458
FDR chr. breakp. enrich.	0.26
Linked to chrs	
Purity, ploidy	0.88, 1.73

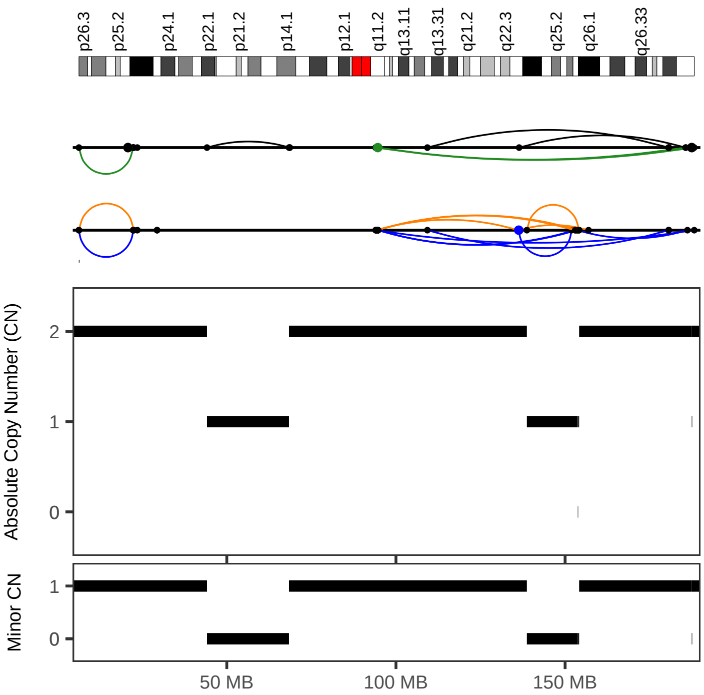

	7f9031da−124a−4a38−83e6−878a50e58c24
Cancer type	SoftTissue−Leiomyo
Position	3:94022172−188180159
Type	With other complex events
Interleaved intrachr. SVs	25
Total SVs (intrachr. + transl.)	29
SV types	DEL: 9; DUP: 7; h2hINV: 4; t2tINV: 5; TRA: 4
SVs in sample	229
Oscillating CN (2 and 3 states)	5, 9
CN segments	9
FDR fragment joints	0.8824283
FDR chr. breakp. enrich.	0
Linked to chrs	18:4330785−61517857;X:8748611−107791712
Purity, ploidy	0.86, 1.89

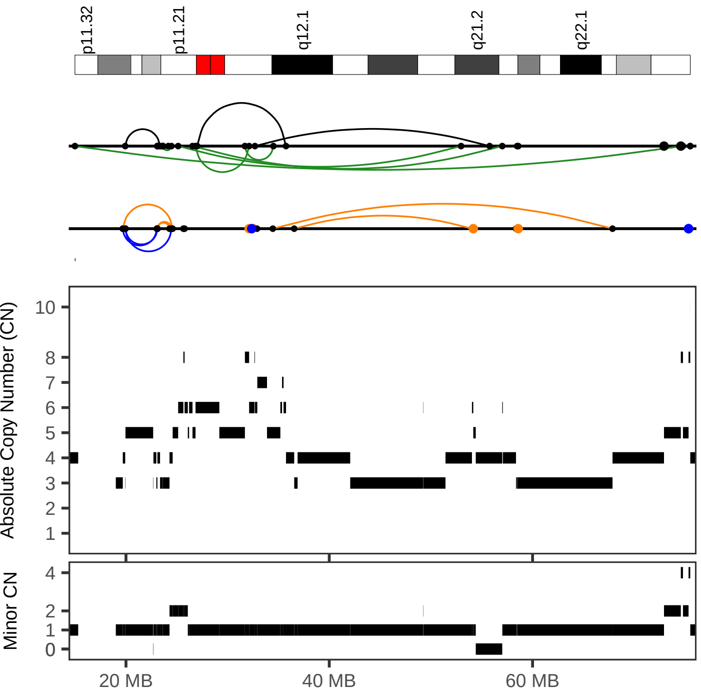

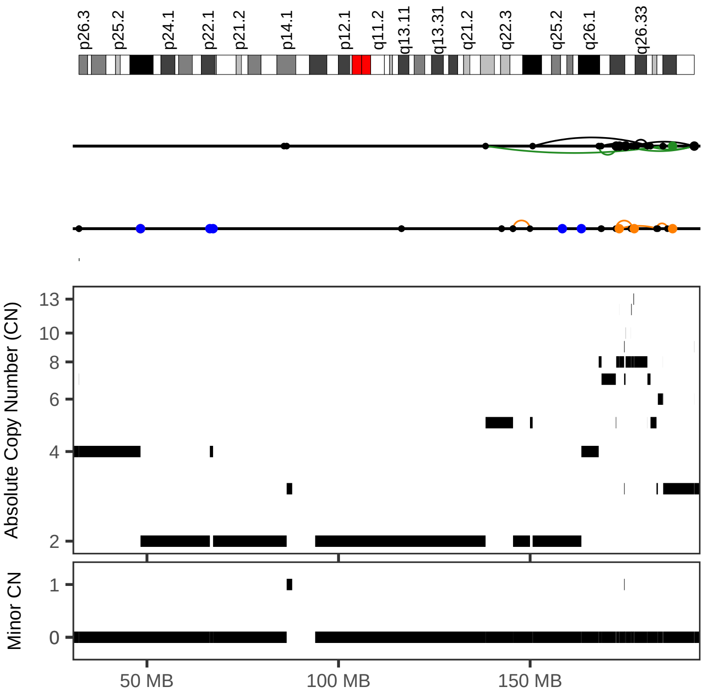

	9c9b53e5−cdbe−4510−a839−4a3b5039681b
Cancer type	SoftTissue−Leiomyo
Position	18:25133083−67867958
Type	With other complex events
Interleaved intrachr. SVs	9
Total SVs (intrachr. + transl.)	13
SV types	DEL: 3; DUP: 0; h2hINV: 2; t2tINV: 4; TRA: 4
SVs in sample	240
Oscillating CN (2 and 3 states)	6, 8
CN segments	32
FDR fragment joints	0.615458
FDR chr. breakp. enrich.	0
Linked to chrs	
Purity, ploidy	0.91, 2.73

	c20682a1−f340−430d−99f9−077fe093ed19
Cancer type	SoftTissue−Leiomyo
Position	3:138369667−192817448
Type	With other complex events
Interleaved intrachr. SVs	15
Total SVs (intrachr. + transl.)	25
SV types	DEL: 3; DUP: 1; h2hINV: 6; t2tINV: 5; TRA: 10
SVs in sample	215
Oscillating CN (2 and 3 states)	5, 7
CN segments	34
FDR fragment joints	0.615458
FDR chr. breakp. enrich.	0
Linked to chrs	6:18987971−150829167;
Purity, ploidy	0.82, 3.11

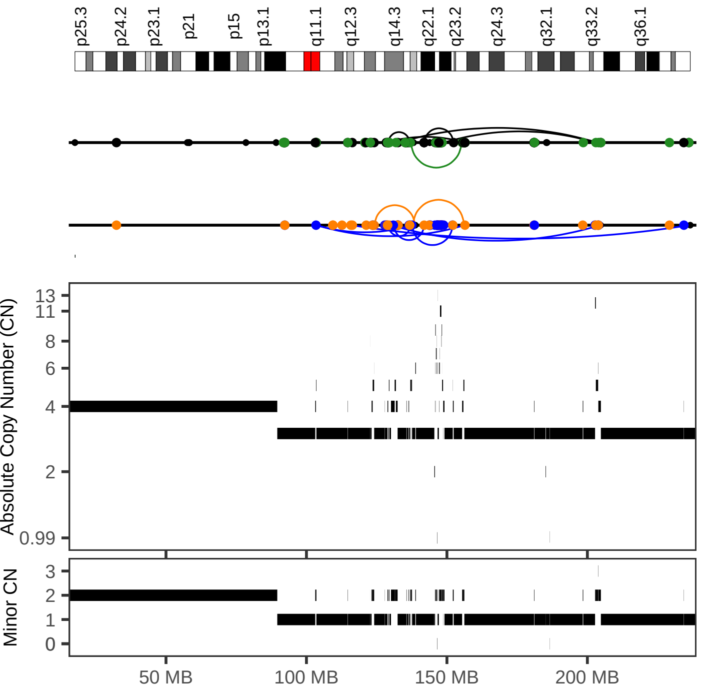

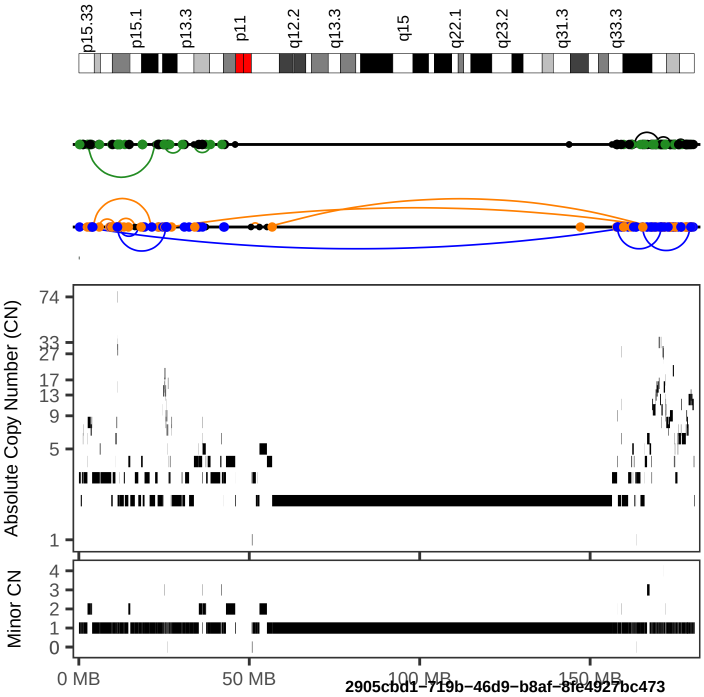

	2905cbd1−719b−46d9−b8af−8fe4927bc473
Cancer type	SoftTissue−Liposarc
Position	2:103504725−236615450
Type	With other complex events
Interleaved intrachr. SVs	15
Total SVs (intrachr. + transl.)	155
SV types	DEL: 2; DUP: 7; h2hINV: 5; t2tINV: 1; TRA: 140
SVs in sample	1958
Oscillating CN (2 and 3 states)	6, 12
CN segments	82
FDR fragment joints	0.9804396
FDR chr. breakp. enrich.	0.23
Linked to chrs	1:145389851−238867162;11:12674170−130091311 12:8404400−101121300;17:50057932−77131856 19:10607945−56113175;20:48743238−54339929 5:893523−179304806;7:159034−86160840 X:1766391−152187953;
Purity, ploidy	0.92, 3.54

	2905cbd1−719b−46d9−b8af−8fe4927bc473
Cancer type	SoftTissue−Liposarc
Position	5:893523−179304807
Type	With other complex events
Interleaved intrachr. SVs	19
Total SVs (intrachr. + transl.)	274
SV types	DEL: 5; DUP: 5; h2hINV: 4; t2tINV: 5; TRA: 255
SVs in sample	1958
Oscillating CN (2 and 3 states)	5, 15
CN segments	189
FDR fragment joints	0.9034513
FDR chr. breakp. enrich.	0
Linked to chrs	1:145389851−238867162;12:8404400−101121300 17:50057932−77131856;19:10607945−56113175 2:103504725−236615449;20:48743238−54339929 3:158182221−193942169;7:159034−86160840 9:7234461−14473861;X:1766391−152187953
Purity, ploidy	0.92, 3.54

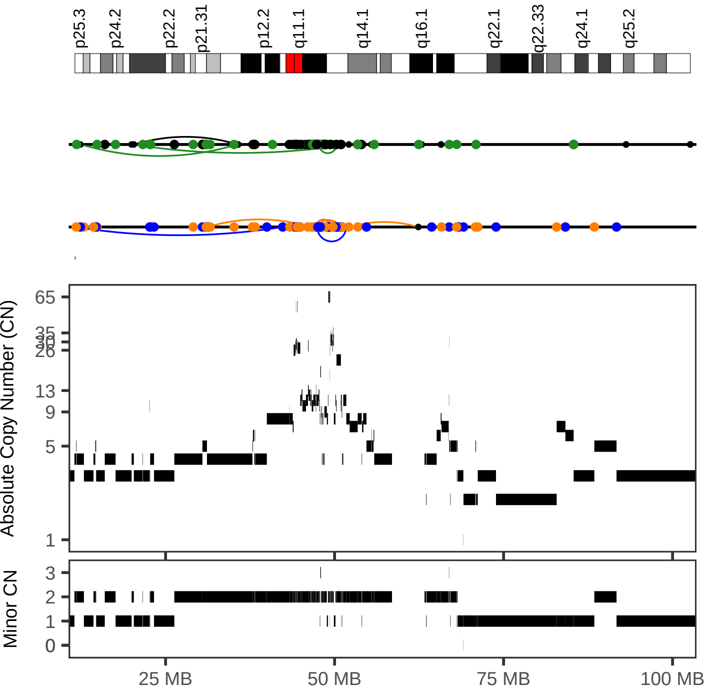

2905cbd1−719b−46d9−b8af−8fe4927bc473

Cancer type	SoftTissue−Liposarc
Position	6:47184887−51731046
Type	With other complex events
Interleaved intrachr. SVs	11
Total SVs (intrachr. + transl.)	60
SV types	DEL: 6; DUP: 2; h2hINV: 2; t2tINV: 1; TRA: 49
SVs in sample	1958
Oscillating CN (2 and 3 states)	4, 5
CN segments	42
FDR fragment joints	0.7993446
FDR chr. breakp. enrich.	0
Linked to chrs	1:145389851−238867162;11:12674170−130091311 12:8404400−101121300;17:50057932−77131856 2:103504725−236615449;7:159034−86160840 X:1766391−152187953;
Purity, ploidy	0.92, 3.54

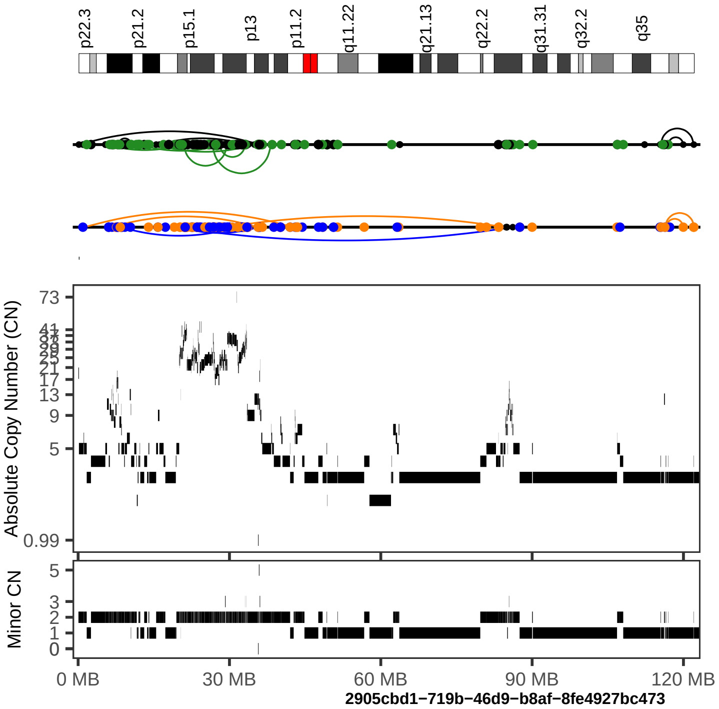

2905cbd1−719b−46d9−b8af−8fe4927bc473

Cancer type	SoftTissue−Liposarc
Position	7:159034−86160841
Type	With other complex events
Interleaved intrachr. SVs	22
Total SVs (intrachr. + transl.)	251
SV types	DEL: 5; DUP: 4; h2hINV: 5; t2tINV: 8; TRA: 229
SVs in sample	1958
Oscillating CN (2 and 3 states)	5, 8
CN segments	250
FDR fragment joints	0.8883474
FDR chr. breakp. enrich.	0
Linked to chrs	1:145389851−238867162;11:12674170−130091311 12:8404400−101121300;17:50057932−77131856 19:10607945−56113175;2:103504725−236615449 3:158182221−193942169;9:7234461−14473861 X:1766391−152187953;
Purity, ploidy	0.92, 3.54

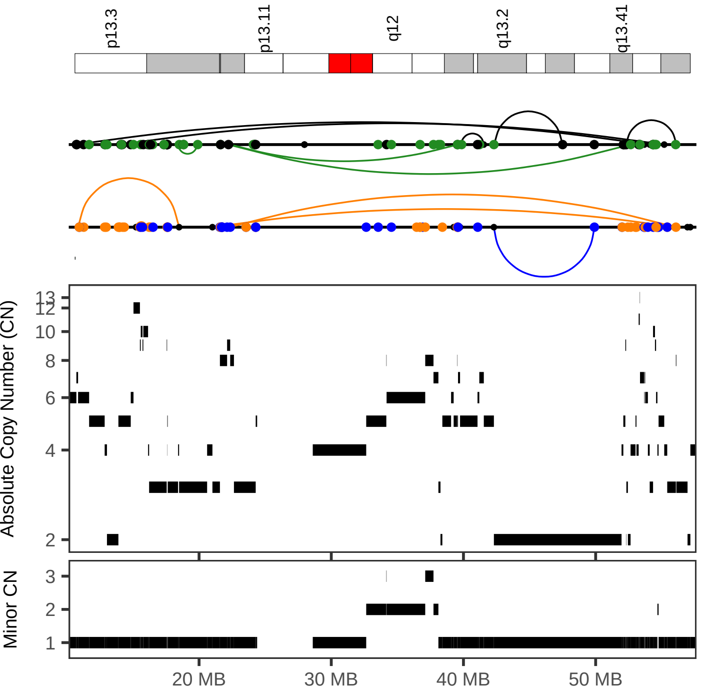

2905cbd1−719b−46d9−b8af−8fe4927bc473

Cancer type	SoftTissue−Liposarc
Position	19:10607945−56113176
Type	With other complex events
Interleaved intrachr. SVs	8
Total SVs (intrachr. + transl.)	140
SV types	DEL: 3; DUP: 0; h2hINV: 3; t2tINV: 2; TRA: 132
SVs in sample	1958
Oscillating CN (2 and 3 states)	5, 7
CN segments	73
FDR fragment joints	0.8653243
FDR chr. breakp. enrich.	0
Linked to chrs	1:145389851−238867162;10:9380961−36837630 11:12674170−130091311;12:8404400−101121300 17:50057932−77131856;2:103504725−236615449 5:893523−179304806;7:159034−86160840 X:1766391−152187953;
Purity, ploidy	0.92, 3.54

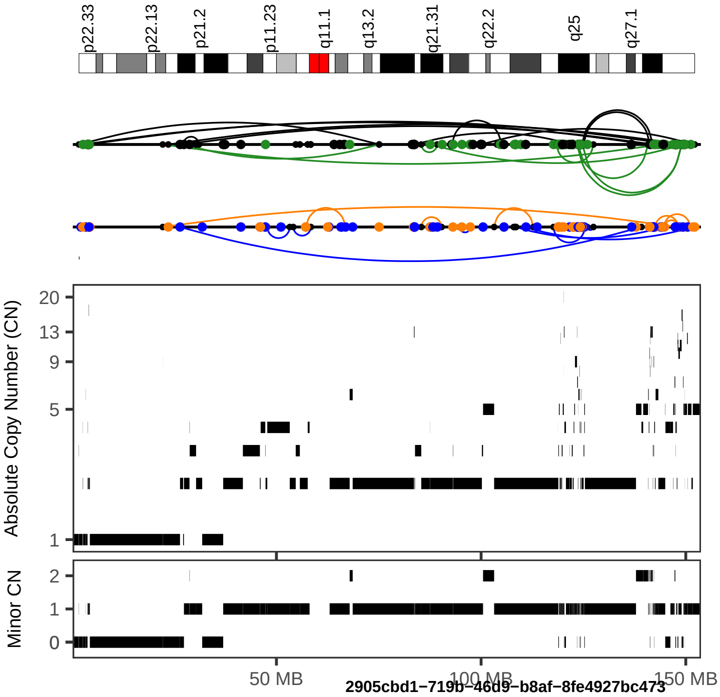

2905cbd1−719b−46d9−b8af−8fe4927bc473

Cancer type	SoftTissue−Liposarc
Position	X:1766391−152187954
Type	With other complex events
Interleaved intrachr. SVs	32
Total SVs (intrachr. + transl.)	230
SV types	DEL: 6; DUP: 6; h2hINV: 11; t2tINV: 9; TRA: 198
SVs in sample	1958
Oscillating CN (2 and 3 states)	5, 8
CN segments	145
FDR fragment joints	0.9728377
FDR chr. breakp. enrich.	0
Linked to chrs	1:145389851−238867162;10:9380961−36837630 11:12674170−130091311;12:8404400−101121300 17:50057932−77131856;19:10607945−56113175 2:103504725−236615449;20:48743238−54339929 5:893523−179304806;7:159034−86160840 9:7234461−14473861;
Purity, ploidy	0.92, 3.54

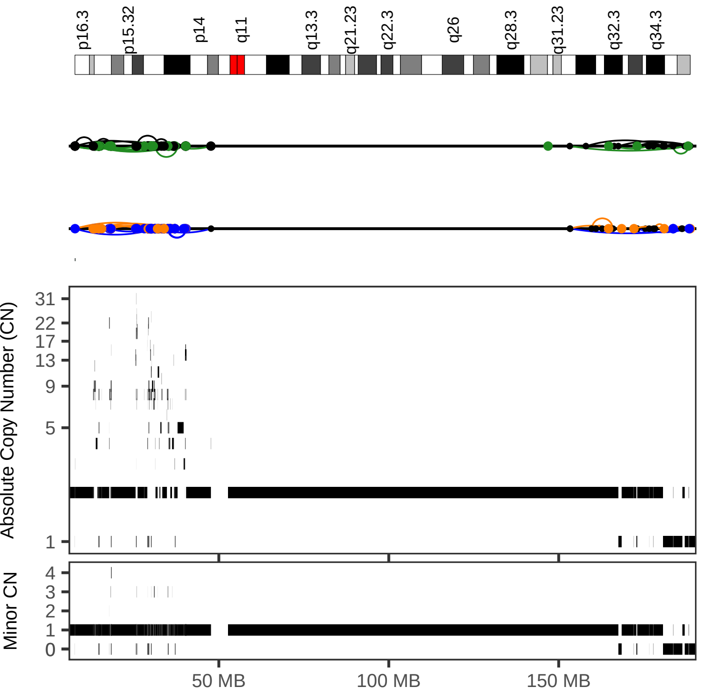

2cacd9d0−8414−4f17−8259−c9d838597f39

Cancer type	SoftTissue−Liposarc
Position	4:7659968−47714738
Type	With other complex events
Interleaved intrachr. SVs	34
Total SVs (intrachr. + transl.)	199
SV types	DEL: 8; DUP: 7; h2hINV: 9; t2tINV: 10; TRA: 165
SVs in sample	531
Oscillating CN (2 and 3 states)	5, 7
CN segments	164
FDR fragment joints	0.8548712
FDR chr. breakp. enrich.	0
Linked to chrs	1:180979548−248890603;12:17335568−78414574 17:27172002−57726287;
Purity, ploidy	0.88, 1.92

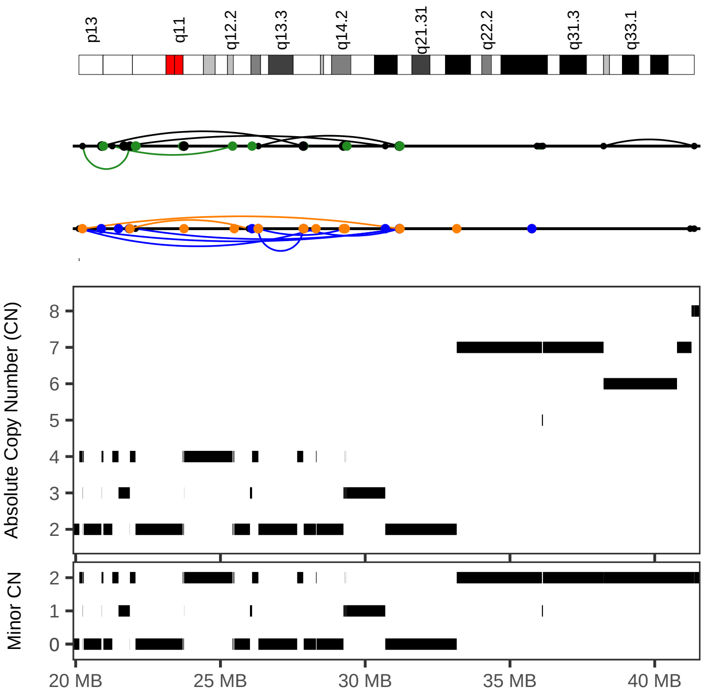

3505f91d−bc66−4732−84b6−18c5b32ca6b8

Cancer type	SoftTissue−Liposarc
Position	13:20111633−31209010
Type	With other complex events
Interleaved intrachr. SVs	14
Total SVs (intrachr. + transl.)	65
SV types	DEL: 3; DUP: 6; h2hINV: 3; t2tINV: 2; TRA: 51
SVs in sample	1246
Oscillating CN (2 and 3 states)	6, 9
CN segments	35
FDR fragment joints	0.6776251
FDR chr. breakp. enrich.	0
Linked to chrs	12:4488859−131646236;5:111534825−180595775 X:77058127−154924952;
Purity, ploidy	0.61, 3.84

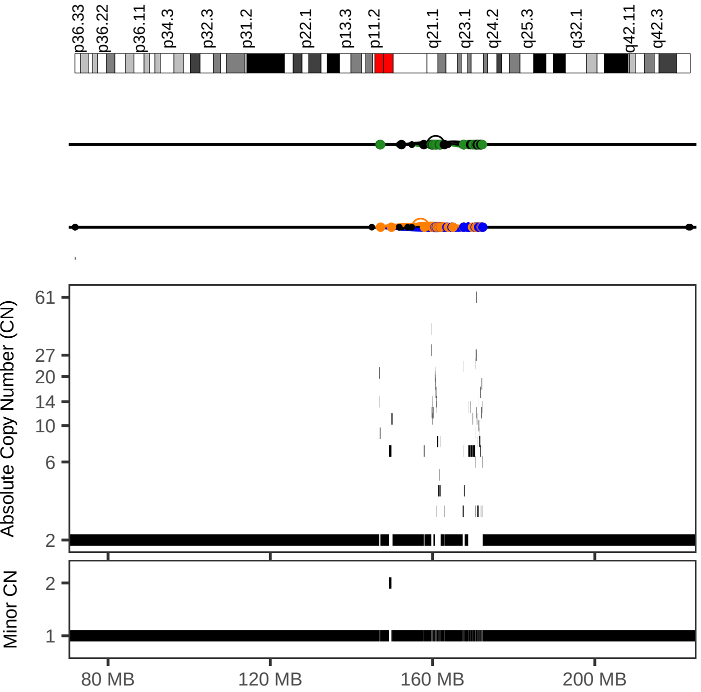

3608bfbb−2a92−4ec2−a330−bf4790a8b65c

Cancer type	SoftTissue−Liposarc
Position	1:145028117−172009311
Type	With other complex events
Interleaved intrachr. SVs	22
Total SVs (intrachr. + transl.)	127
SV types	DEL: 7; DUP: 6; h2hINV: 5; t2tINV: 4; TRA: 105
SVs in sample	448
Oscillating CN (2 and 3 states)	5, 8
CN segments	67
FDR fragment joints	0.6776251
FDR chr. breakp. enrich.	0
Linked to chrs	
Purity, ploidy	0.91, 2.17

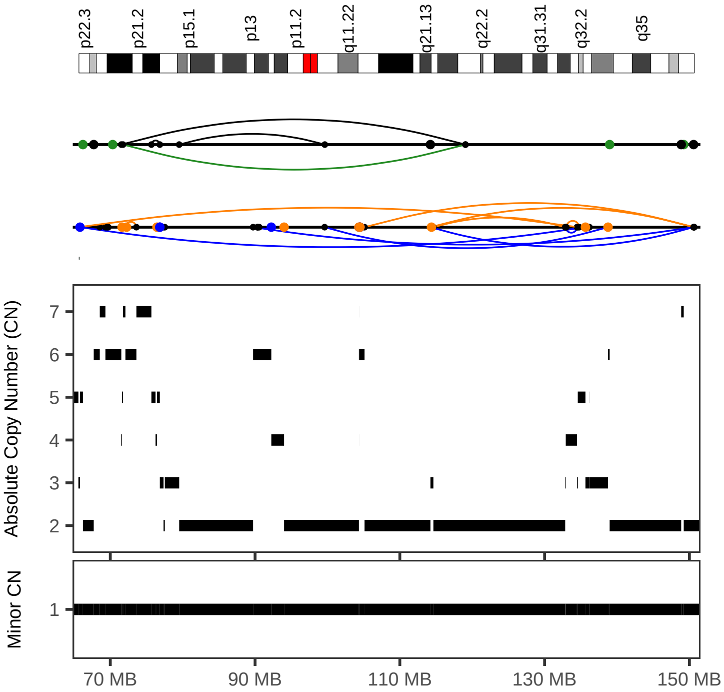

3608bfbb−2a92−4ec2−a330−bf4790a8b65c

Cancer type	SoftTissue−Liposarc
Position	7:65666837−150670275
Type	With other complex events
Interleaved intrachr. SVs	14
Total SVs (intrachr. + transl.)	38
SV types	DEL: 6; DUP: 5; h2hINV: 2; t2tINV: 1; TRA: 24
SVs in sample	448
Oscillating CN (2 and 3 states)	5, 11
CN segments	39
FDR fragment joints	0.4101979
FDR chr. breakp. enrich.	0
Linked to chrs	X:16247627−46643678;
Purity, ploidy	0.91, 2.17

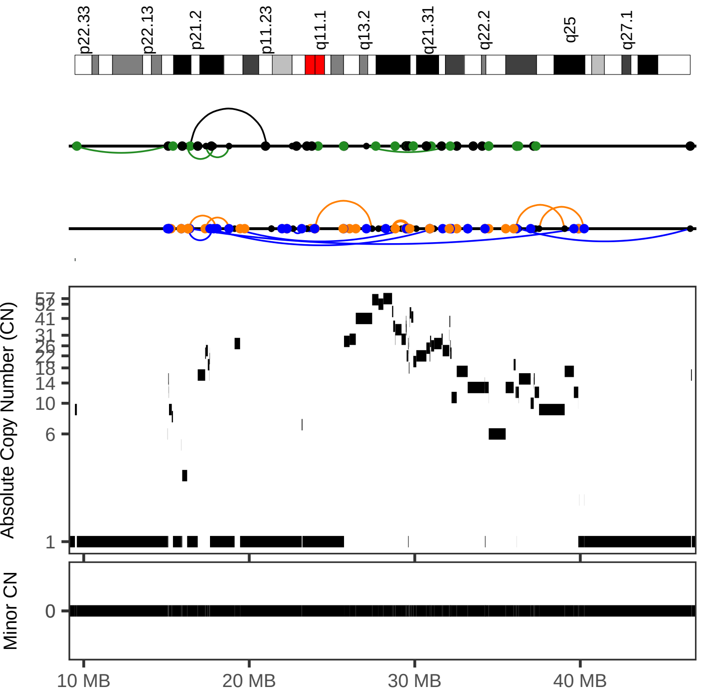

	**3608bfbb−2a92−4ec2−a330−bf4790a8b65c**
Cancer type	SoftTissue−Liposarc
Position	X:16247627−46643679
Type	With other complex events
Interleaved intrachr. SVs	20
Total SVs (intrachr. + transl.)	108
SV types	DEL: 10; DUP: 6; h2hINV: 1; t2tINV: 3; TRA: 88
SVs in sample	448
Oscillating CN (2 and 3 states)	5, 5
CN segments	85
FDR fragment joints	0.5435077
FDR chr. breakp. enrich.	0
Linked to chrs	1:145028117−172009310;7:65666837−150670274
Purity, ploidy	0.91, 2.17

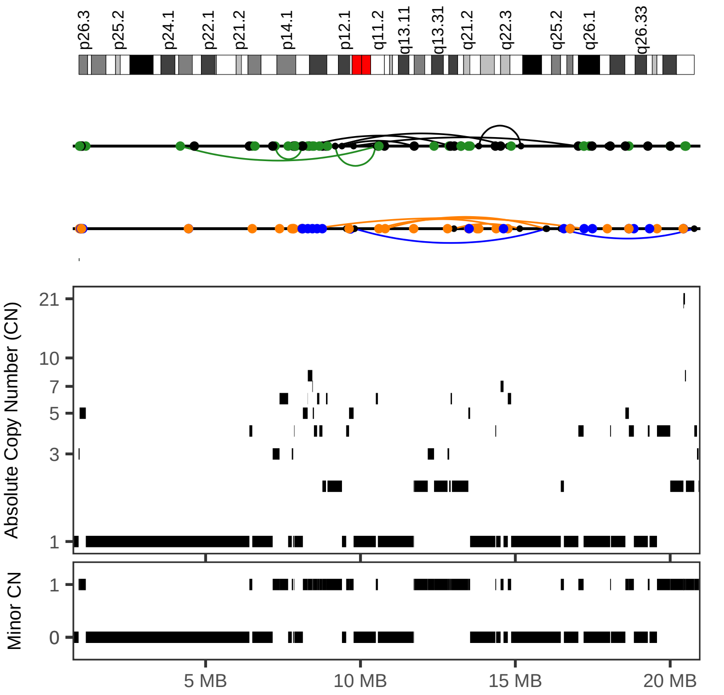

	**39f68467−3d37−4b19−aa43−ed7b120c619d**
Cancer type	SoftTissue−Liposarc
Position	3:4159559−20777597
Type	With other complex events
Interleaved intrachr. SVs	14
Total SVs (intrachr. + transl.)	107
SV types	DEL: 4; DUP: 3; h2hINV: 5; t2tINV: 2; TRA: 93
SVs in sample	1635
Oscillating CN (2 and 3 states)	5, 7
CN segments	61
FDR fragment joints	0.615458
FDR chr. breakp. enrich.	0.03
Linked to chrs	22:23956086−48888565;
Purity, ploidy	0.4, 2.04

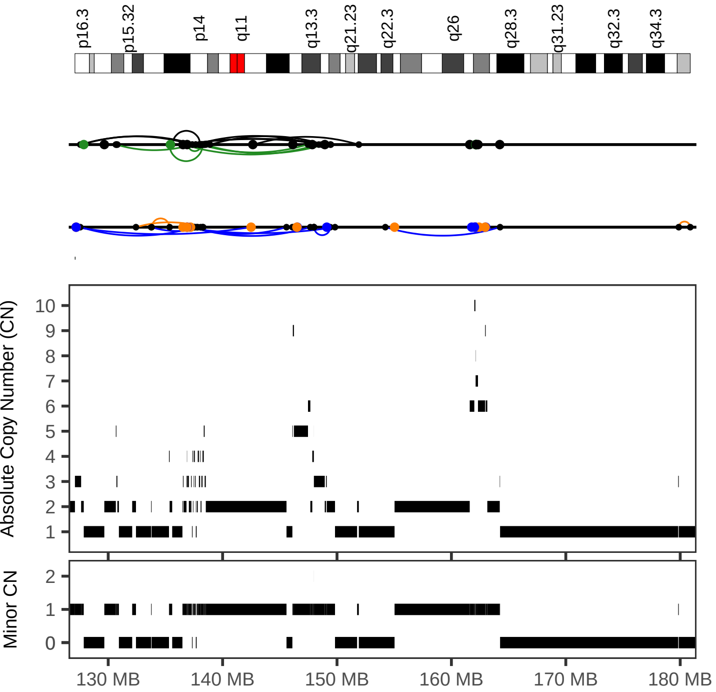

	**39f68467−3d37−4b19−aa43−ed7b120c619d**
Cancer type	SoftTissue−Liposarc
Position	4:127090720−151907883
Type	With other complex events
Interleaved intrachr. SVs	30
Total SVs (intrachr. + transl.)	49
SV types	DEL: 4; DUP: 8; h2hINV: 11; t2tINV: 7; TRA: 19
SVs in sample	1635
Oscillating CN (2 and 3 states)	6, 15
CN segments	60
FDR fragment joints	0.9284301
FDR chr. breakp. enrich.	0.12
Linked to chrs	20:33082736−57423557;
Purity, ploidy	0.4, 2.04

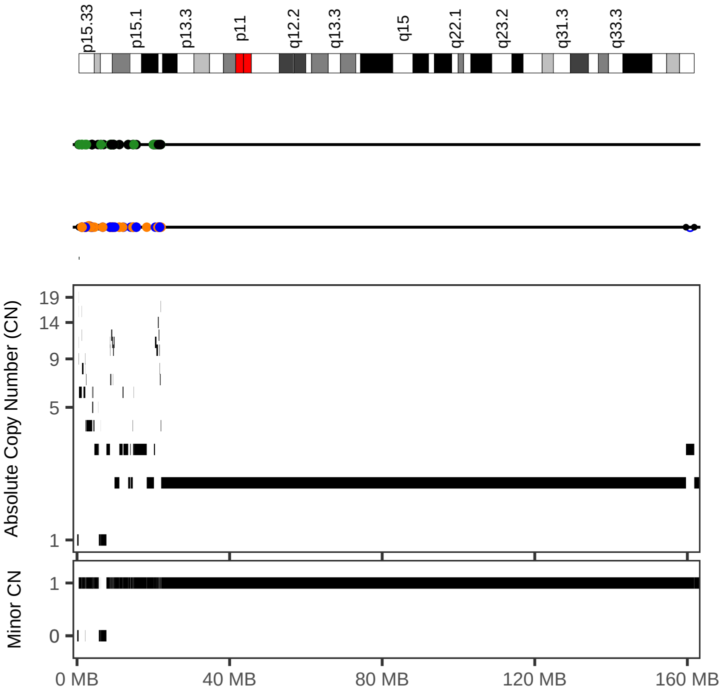

	**39f68467−3d37−4b19−aa43−ed7b120c619d**
Cancer type	SoftTissue−Liposarc
Position	5:492905−14929135
Type	With other complex events
Interleaved intrachr. SVs	16
Total SVs (intrachr. + transl.)	100
SV types	DEL: 9; DUP: 4; h2hINV: 3; t2tINV: 0; TRA: 84
SVs in sample	1635
Oscillating CN (2 and 3 states)	4, 6
CN segments	43
FDR fragment joints	0.5435077
FDR chr. breakp. enrich.	0
Linked to chrs	20:33082736−57423557;3:4159559−20777596 9:103346393−140789887;
Purity, ploidy	0.4, 2.04

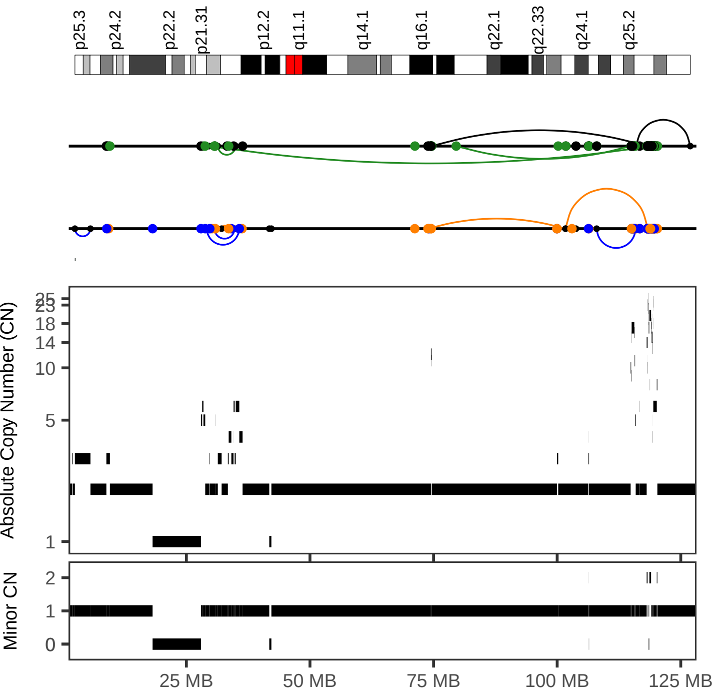

	39f68467−3d37−4b19−aa43−ed7b120c619d
Cancer type	SoftTissue−Liposarc
Position	6:29105248−126918492
Type	With other complex events
Interleaved intrachr. SVs	12
Total SVs (intrachr. + transl.)	206
SV types	DEL: 3; DUP: 3; h2hINV: 3; t2tINV: 3; TRA: 194
SVs in sample	1635
Oscillating CN (2 and 3 states)	4, 11
CN segments	64
FDR fragment joints	0.8474427
FDR chr. breakp. enrich.	0
Linked to chrs	20:33082736−57423557;3:4159559−20777596 9:103346393−140789887;
Purity, ploidy	0.4, 2.04

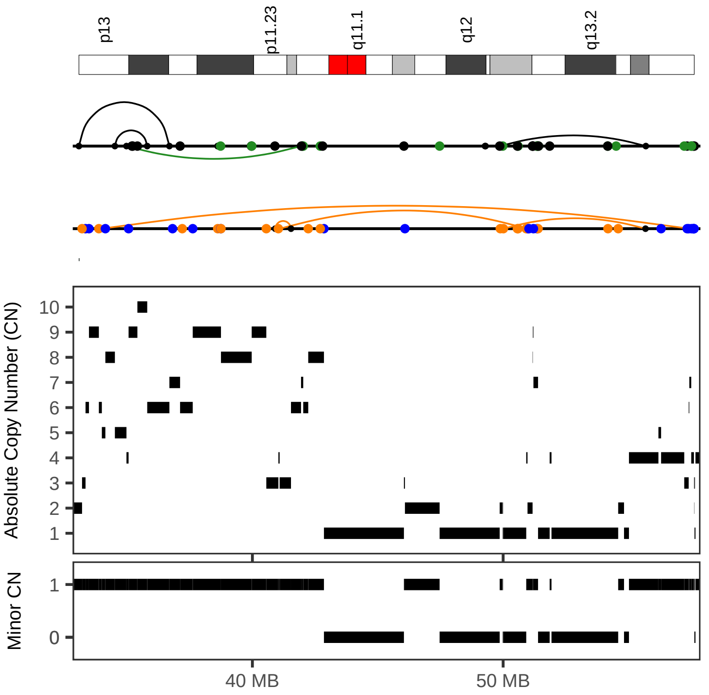

	39f68467−3d37−4b19−aa43−ed7b120c619d
Cancer type	SoftTissue−Liposarc
Position	20:33082736−57423558
Type	With other complex events
Interleaved intrachr. SVs	8
Total SVs (intrachr. + transl.)	73
SV types	DEL: 4; DUP: 0; h2hINV: 3; t2tINV: 1; TRA: 65
SVs in sample	1635
Oscillating CN (2 and 3 states)	6, 8
CN segments	48
FDR fragment joints	0.8653243
FDR chr. breakp. enrich.	0
Linked to chrs	3:4159559−20777596;4:127090720−151907882
Purity, ploidy	0.4, 2.04

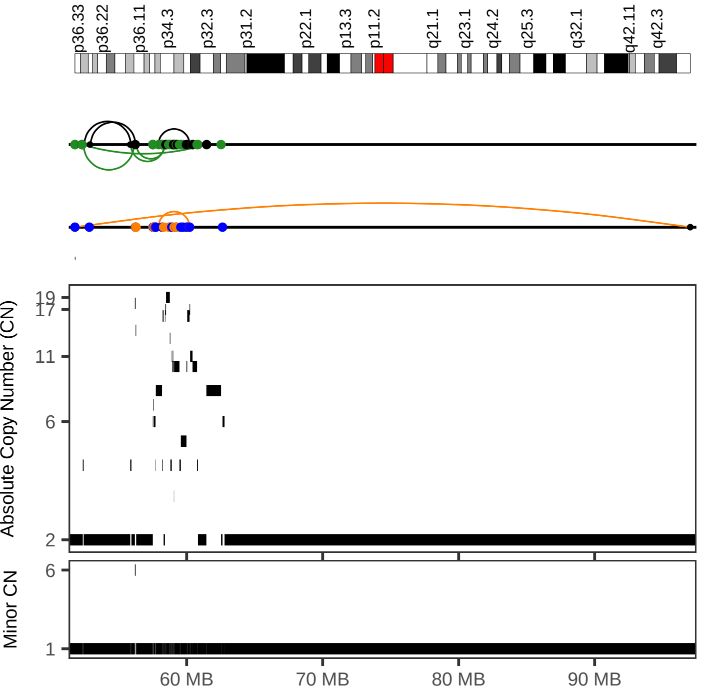

	6b189eff−b919−49d6−8775−dbf32c9ccbba
Cancer type	SoftTissue−Liposarc
Position	1:52359894−60254337
Type	With other complex events
Interleaved intrachr. SVs	7
Total SVs (intrachr. + transl.)	54
SV types	DEL: 1; DUP: 0; h2hINV: 3; t2tINV: 3; TRA: 47
SVs in sample	890
Oscillating CN (2 and 3 states)	4, 4
CN segments	33
FDR fragment joints	1
FDR chr. breakp. enrich.	0.7
Linked to chrs	5:82214−43509705;14:95901595−105399402
Purity, ploidy	0.88, 2

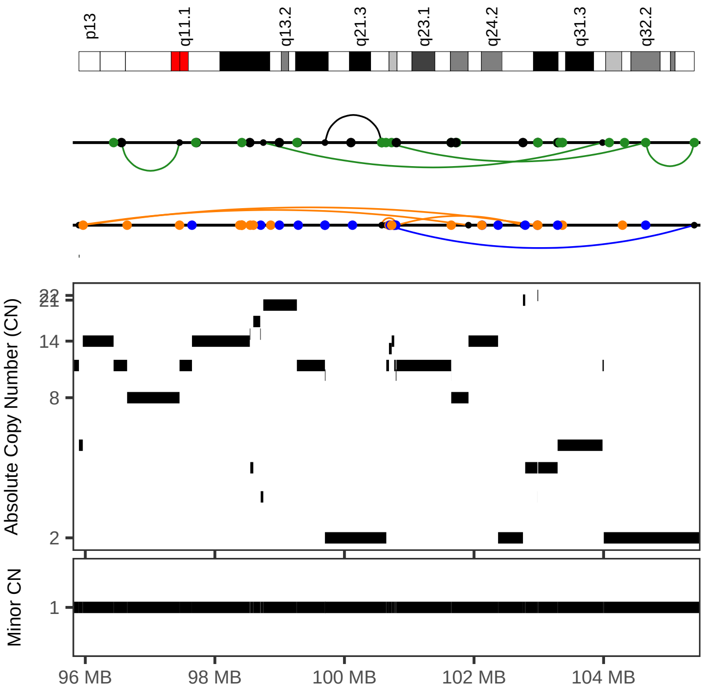

	6b189eff−b919−49d6−8775−dbf32c9ccbba
Cancer type	SoftTissue−Liposarc
Position	14:95901595−105399403
Type	With other complex events
Interleaved intrachr. SVs	10
Total SVs (intrachr. + transl.)	79
SV types	DEL: 4; DUP: 1; h2hINV: 1; t2tINV: 4; TRA: 69
SVs in sample	890
Oscillating CN (2 and 3 states)	5, 6
CN segments	34
FDR fragment joints	0.7590432
FDR chr. breakp. enrich.	0
Linked to chrs	5:82214−43509705;
Purity, ploidy	0.88, 2

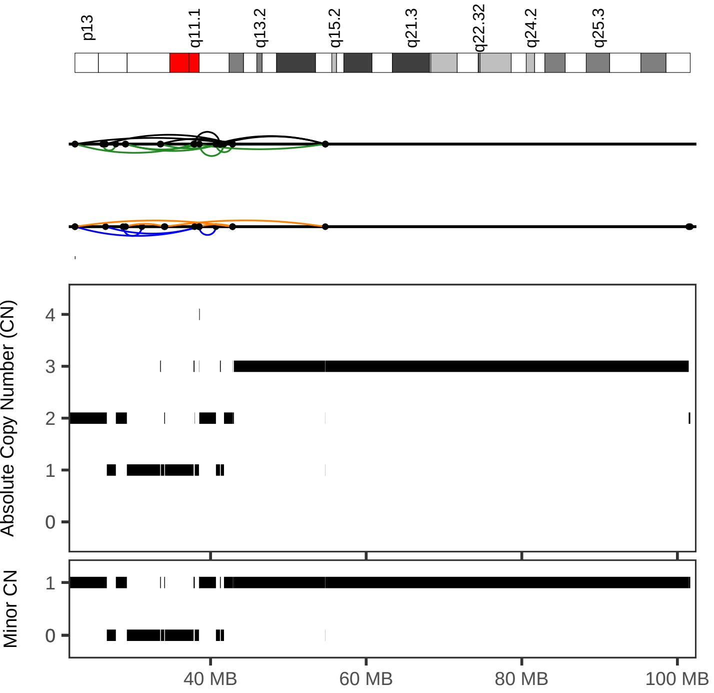

	**6b189eff−b919−49d6−8775−dbf32c9ccbba**
Cancer type	SoftTissue−Liposarc
Position	15:22563932−54759738
Type	With other complex events
Interleaved intrachr. SVs	23
Total SVs (intrachr. + transl.)	23
SV types	DEL: 5; DUP: 4; h2hINV: 6; t2tINV: 8; TRA: 0
SVs in sample	890
Oscillating CN (2 and 3 states)	4, 10
CN segments	26
FDR fragment joints	0.7861024
FDR chr. breakp. enrich.	1
Linked to chrs	
Purity, ploidy	0.88, 2

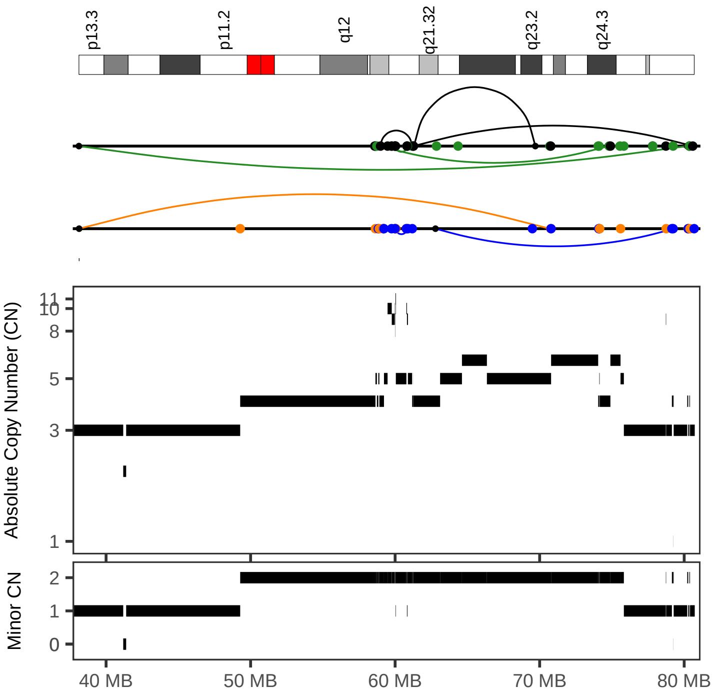

	**73d2e8ef−4e10−416a−845b−f48374e48ecb**
Cancer type	SoftTissue−Liposarc
Position	17:38121352−80696808
Type	With other complex events
Interleaved intrachr. SVs	7
Total SVs (intrachr. + transl.)	71
SV types	DEL: 1; DUP: 2; h2hINV: 2; t2tINV: 2; TRA: 64
SVs in sample	1088
Oscillating CN (2 and 3 states)	6, 11
CN segments	51
FDR fragment joints	0.930656
FDR chr. breakp. enrich.	0
Linked to chrs	12:57518989−72095051;22:32381273−48585046 3:3279482−196670439;5:703397−41841708 8:13601211−30077762;
Purity, ploidy	0.55, 3.11

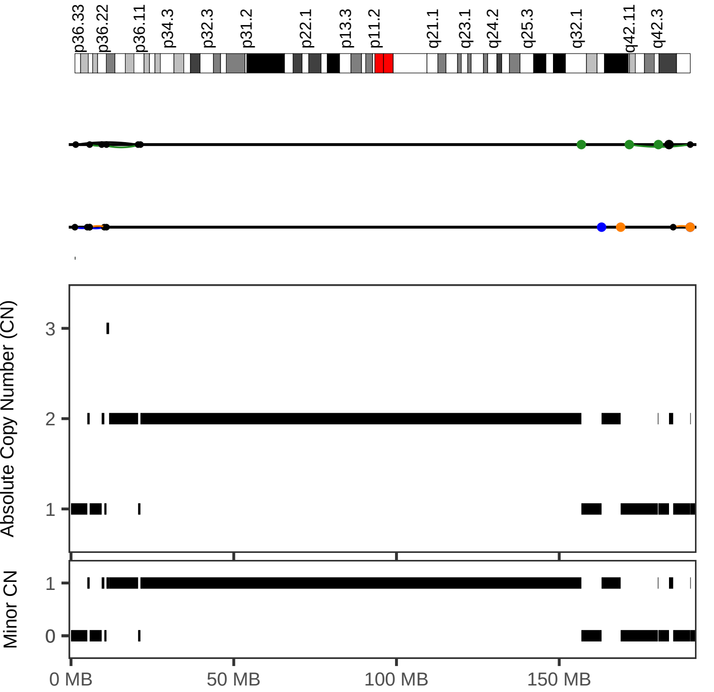

	**8f3f6f7c−4b50−467e−a6e8−d836735913f6**
Cancer type	SoftTissue−Liposarc
Position	1:1201892−21330306
Type	With other complex events
Interleaved intrachr. SVs	6
Total SVs (intrachr. + transl.)	6
SV types	DEL: 1; DUP: 2; h2hINV: 1; t2tINV: 2; TRA: 0
SVs in sample	782
Oscillating CN (2 and 3 states)	5, 7
CN segments	9
FDR fragment joints	0.9284301
FDR chr. breakp. enrich.	0
Linked to chrs	
Purity, ploidy	0.62, 2.12

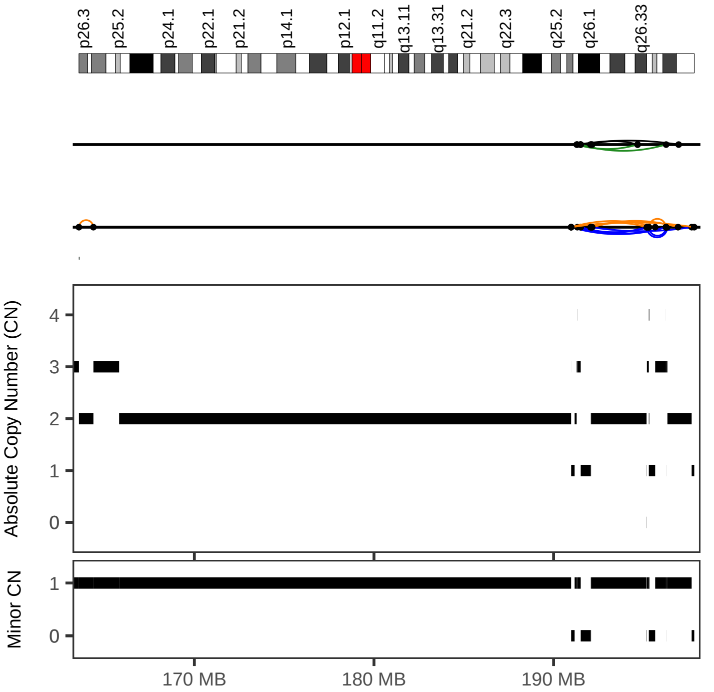

	**8f3f6f7c−4b50−467e−a6e8−d836735913f6**
Cancer type	SoftTissue−Liposarc
Position	3:190977465−197689209
Type	With other complex events
Interleaved intrachr. SVs	17
Total SVs (intrachr. + transl.)	17
SV types	DEL: 4; DUP: 8; h2hINV: 2; t2tINV: 3; TRA: 0
SVs in sample	782
Oscillating CN (2 and 3 states)	4, 7
CN segments	23
FDR fragment joints	0.615458
FDR chr. breakp. enrich.	0
Linked to chrs	
Purity, ploidy	0.62, 2.12

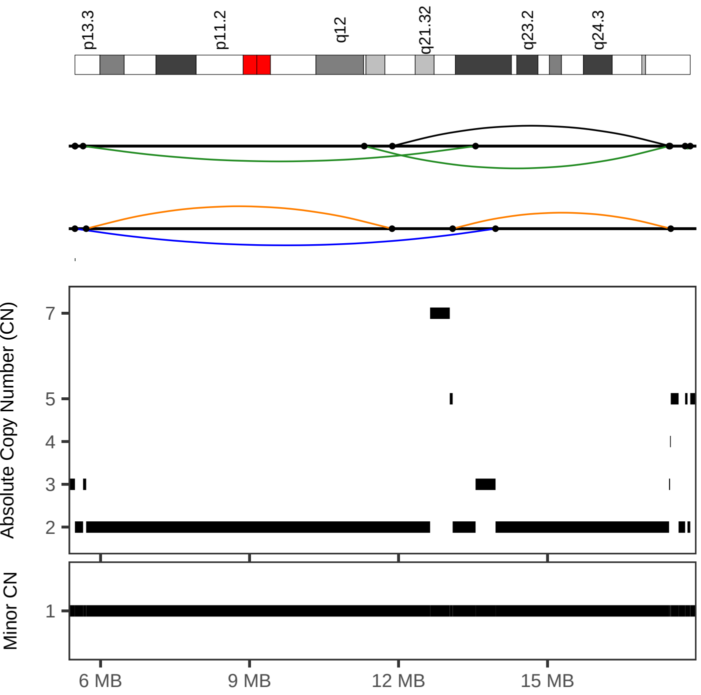

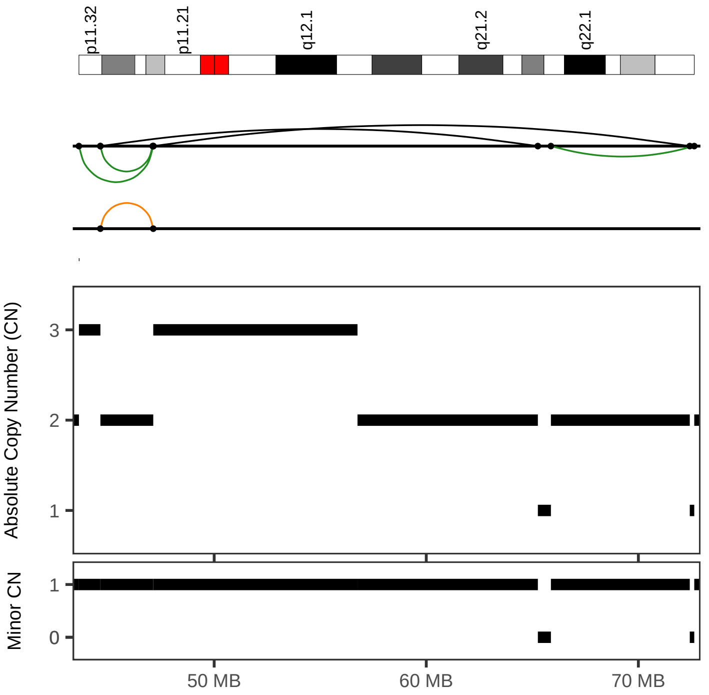

	**8f3f6f7c−4b50−467e−a6e8−d836735913f6**
Cancer type	SoftTissue−Liposarc
Position	17:5483205−17481065
Type	With other complex events
Interleaved intrachr. SVs	6
Total SVs (intrachr. + transl.)	6
SV types	DEL: 2; DUP: 1; h2hINV: 1; t2tINV: 2; TRA: 0
SVs in sample	782
Oscillating CN (2 and 3 states)	4, 7
CN segments	11
FDR fragment joints	0.9284301
FDR chr. breakp. enrich.	0.22
Linked to chrs	
Purity, ploidy	0.62, 2.12

	**8f3f6f7c−4b50−467e−a6e8−d836735913f6**
Cancer type	SoftTissue−Liposarc
Position	18:43622824−72635271
Type	Canonical without polyploidization
Interleaved intrachr. SVs	6
Total SVs (intrachr. + transl.)	6
SV types	DEL: 1; DUP: 0; h2hINV: 2; t2tINV: 3; TRA: 0
SVs in sample	782
Oscillating CN (2 and 3 states)	5, 8
CN segments	8
FDR fragment joints	0.615458
FDR chr. breakp. enrich.	0
Linked to chrs	
Purity, ploidy	0.62, 2.12

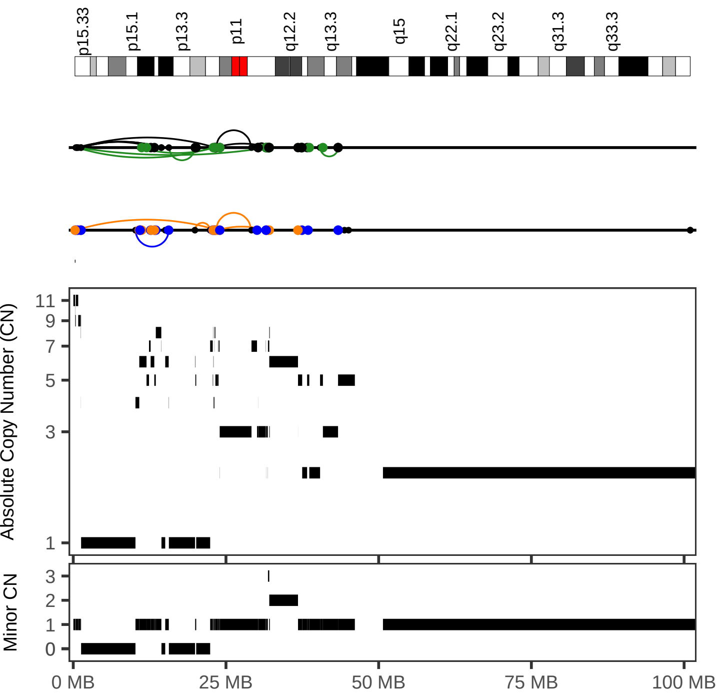

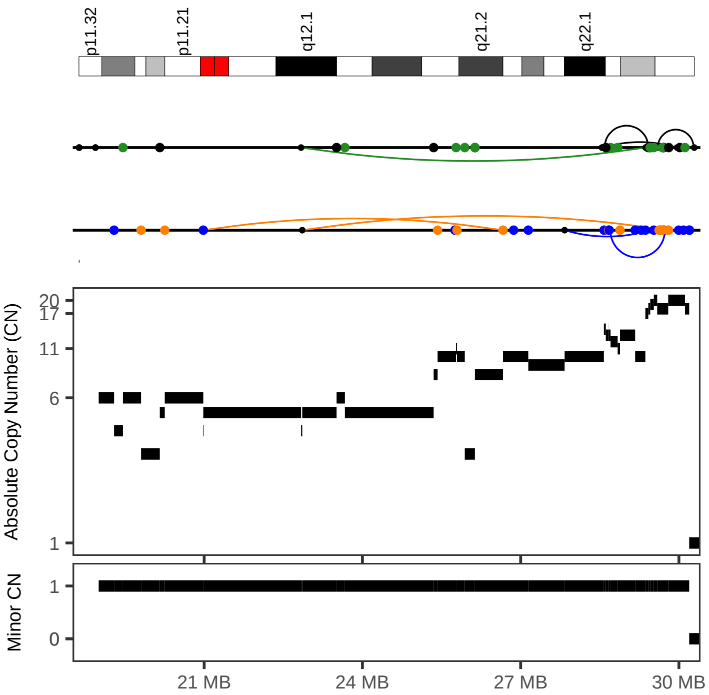

	**9517413b−b5b8−4130−8ba1−d86d44abe2ec**
Cancer type	SoftTissue−Liposarc
Position	5:274679−32089188
Type	With other complex events
Interleaved intrachr. SVs	19
Total SVs (intrachr. + transl.)	73
SV types	DEL: 4; DUP: 3; h2hINV: 7; t2tINV: 5; TRA: 54
SVs in sample	498
Oscillating CN (2 and 3 states)	5, 5
CN segments	50
FDR fragment joints	0.982453
FDR chr. breakp. enrich.	0
Linked to chrs	
Purity, ploidy	0.6, 2.24

	**9517413b−b5b8−4130−8ba1−d86d44abe2ec**
Cancer type	SoftTissue−Liposarc
Position	18:20983183−30290774
Type	With other complex events
Interleaved intrachr. SVs	9
Total SVs (intrachr. + transl.)	55
SV types	DEL: 2; DUP: 2; h2hINV: 3; t2tINV: 2; TRA: 46
SVs in sample	498
Oscillating CN (2 and 3 states)	4, 6
CN segments	30
FDR fragment joints	0.7257048
FDR chr. breakp. enrich.	0
Linked to chrs	5:274679−32089187;
Purity, ploidy	0.6, 2.24

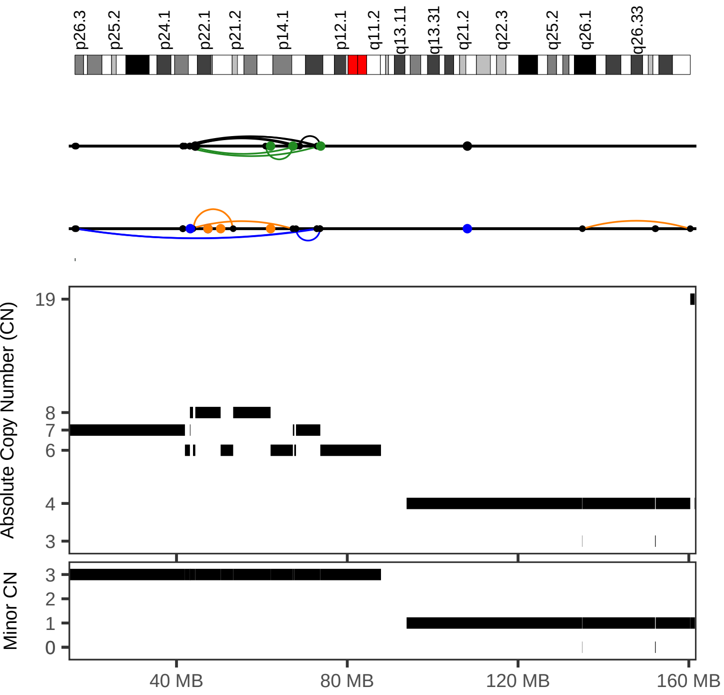

	**b5f27002−4277−418d−90b0−cc5a2742692e**
Cancer type	SoftTissue−Liposarc
Position	3:16208743−73683673
Type	With other complex events
Interleaved intrachr. SVs	16
Total SVs (intrachr. + transl.)	24
SV types	DEL: 2; DUP: 4; h2hINV: 6; t2tINV: 4; TRA: 8
SVs in sample	895
Oscillating CN (2 and 3 states)	6, 11
CN segments	13
FDR fragment joints	0.8653243
FDR chr. breakp. enrich.	0.22
Linked to chrs	9:1073973−27003043;
Purity, ploidy	0.87, 6.21

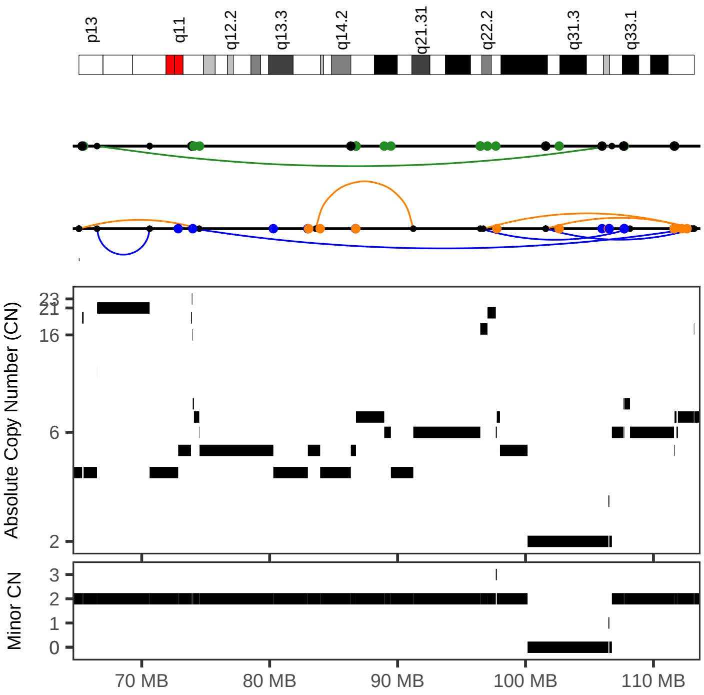

	**b5f27002−4277−418d−90b0−cc5a2742692e**
Cancer type	SoftTissue−Liposarc
Position	13:65093605−113188518
Type	With other complex events
Interleaved intrachr. SVs	7
Total SVs (intrachr. + transl.)	54
SV types	DEL: 3; DUP: 3; h2hINV: 0; t2tINV: 1; TRA: 47
SVs in sample	895
Oscillating CN (2 and 3 states)	5, 6
CN segments	41
FDR fragment joints	1
FDR chr. breakp. enrich.	0
Linked to chrs	1:175909202−201271526;15:77063545−87618665 3:16208743−73683672;6:121478880−150364307 7:2825814−148640815;X:115283407−149711206
Purity, ploidy	0.87, 6.21

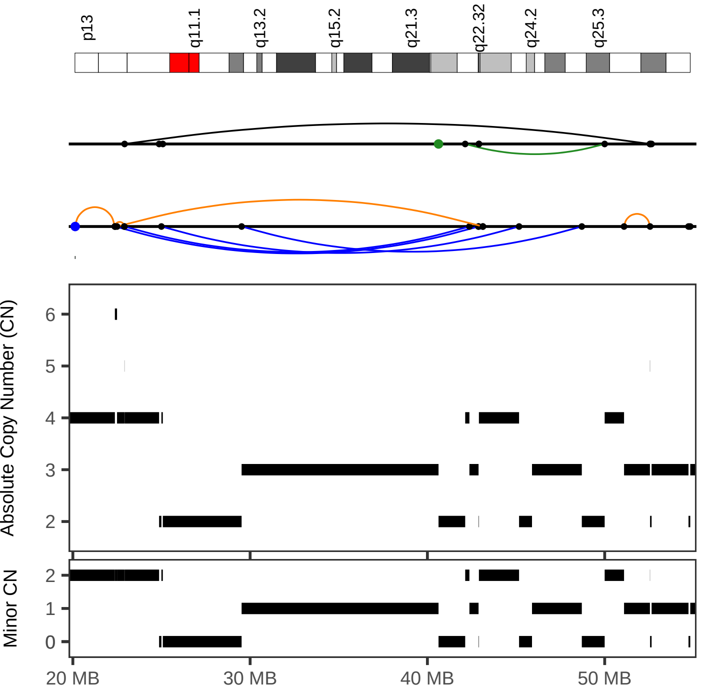

	**cd73a55f−03c1−4da0−9fa7−1ae4a263664e**
Cancer type	SoftTissue−Liposarc
Position	15:22370209−52646685
Type	With other complex events
Interleaved intrachr. SVs	12
Total SVs (intrachr. + transl.)	13
SV types	DEL: 3; DUP: 5; h2hINV: 3; t2tINV: 1; TRA: 1
SVs in sample	595
Oscillating CN (2 and 3 states)	4, 10
CN segments	21
FDR fragment joints	0.6776251
FDR chr. breakp. enrich.	0.5
Linked to chrs	
Purity, ploidy	0.74, 3.24

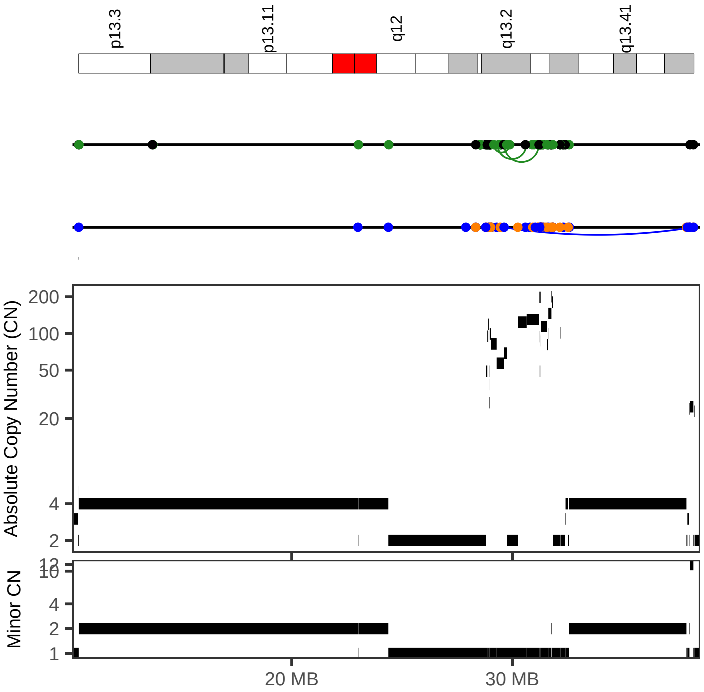

	**e7a72f7e−77b1−4d95−8f37−39a6cffc35dc**
Cancer type	SoftTissue−Liposarc
Position	19:29150130−38233328
Type	With other complex events
Interleaved intrachr. SVs	6
Total SVs (intrachr. + transl.)	79
SV types	DEL: 1; DUP: 2; h2hINV: 0; t2tINV: 3; TRA: 73
SVs in sample	696
Oscillating CN (2 and 3 states)	4, 7
CN segments	35
FDR fragment joints	0.930656
FDR chr. breakp. enrich.	0
Linked to chrs	11:993833−37433475;
Purity, ploidy	0.62, 3.8

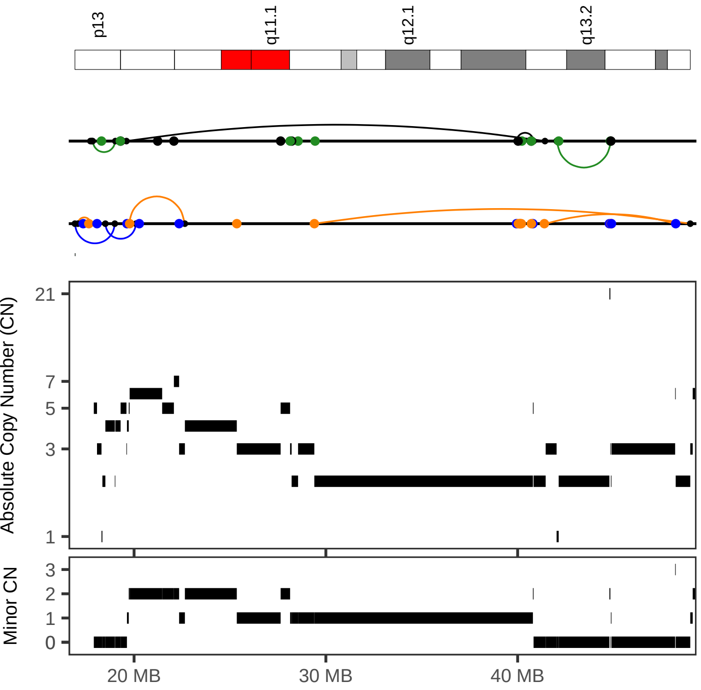

	**e7a72f7e−77b1−4d95−8f37−39a6cffc35dc**
Cancer type	SoftTissue−Liposarc
Position	22:16915315−49000417
Type	With other complex events
Interleaved intrachr. SVs	6
Total SVs (intrachr. + transl.)	44
SV types	DEL: 2; DUP: 2; h2hINV: 1; t2tINV: 1; TRA: 38
SVs in sample	696
Oscillating CN (2 and 3 states)	4, 5
CN segments	35
FDR fragment joints	0.8874881
FDR chr. breakp. enrich.	0
Linked to chrs	11:993833−37433475;5:1295334−20553338 6:42555034−48239598;
Purity, ploidy	0.62, 3.8

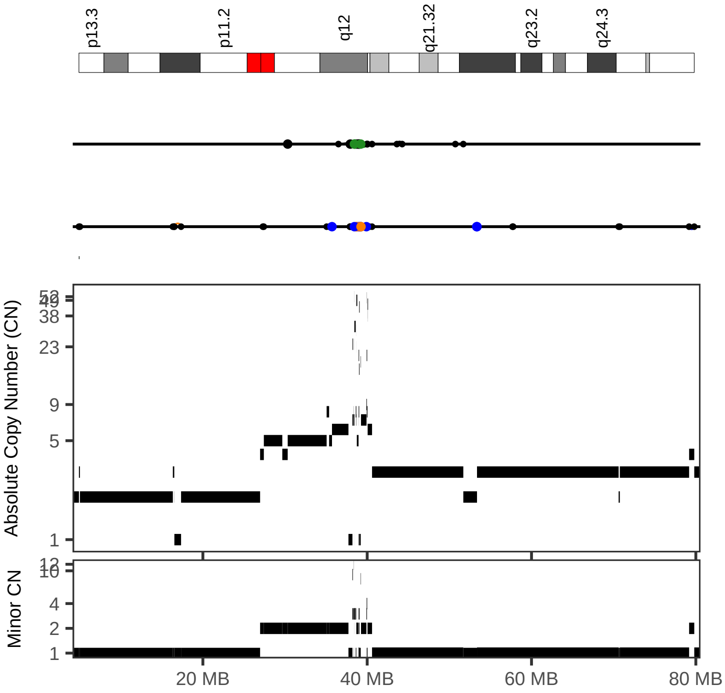

	**CGP_donor_GC00002**
Cancer type	Stomach−AdenoCA
Position	17:38202359−40046302
Type	With other complex events
Interleaved intrachr. SVs	42
Total SVs (intrachr. + transl.)	57
SV types	DEL: 13; DUP: 11; h2hINV: 6; t2tINV: 12; TRA: 15
SVs in sample	442
Oscillating CN (2 and 3 states)	5, 6
CN segments	54
FDR fragment joints	0.615458
FDR chr. breakp. enrich.	0
Linked to chrs	
Purity, ploidy	0.48, 3.17

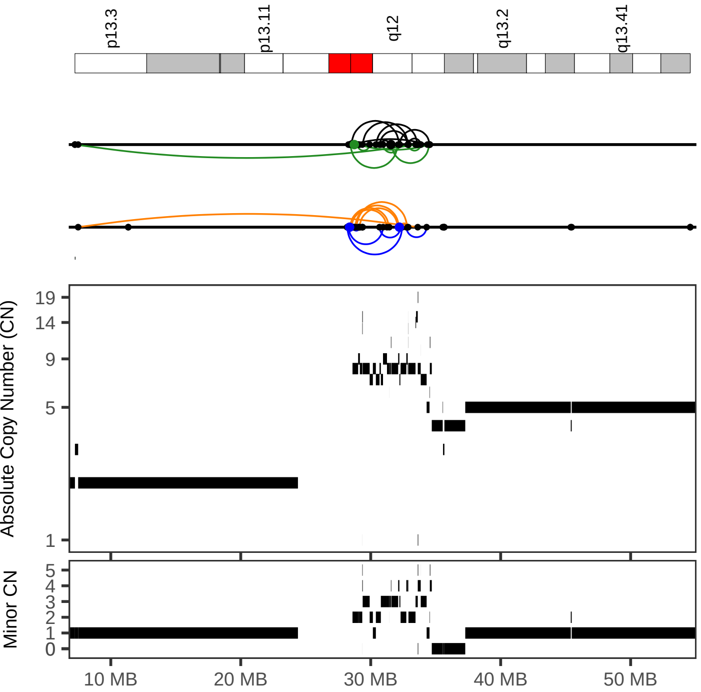

	**CGP_donor_GC00002**
Cancer type	Stomach−AdenoCA
Position	19:7239458−34549451
Type	With other complex events
Interleaved intrachr. SVs	38
Total SVs (intrachr. + transl.)	42
SV types	DEL: 11; DUP: 7; h2hINV: 11; t2tINV: 9; TRA: 4
SVs in sample	442
Oscillating CN (2 and 3 states)	6, 6
CN segments	42
FDR fragment joints	0.9550868
FDR chr. breakp. enrich.	0
Linked to chrs	
Purity, ploidy	0.48, 3.17

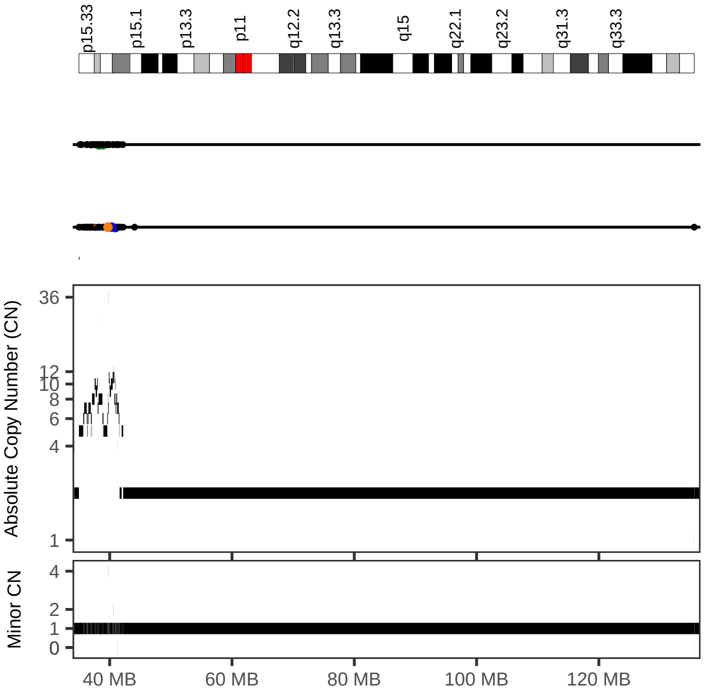

	**CGP_donor_GC00008**
Cancer type	Stomach−AdenoCA
Position	5:35107612−41952066
Type	With other complex events
Interleaved intrachr. SVs	43
Total SVs (intrachr. + transl.)	46
SV types	DEL: 9; DUP: 13; h2hINV: 10; t2tINV: 11; TRA: 3
SVs in sample	307
Oscillating CN (2 and 3 states)	5, 9
CN segments	48
FDR fragment joints	0.7964069
FDR chr. breakp. enrich.	0
Linked to chrs	
Purity, ploidy	0.93, 2.06

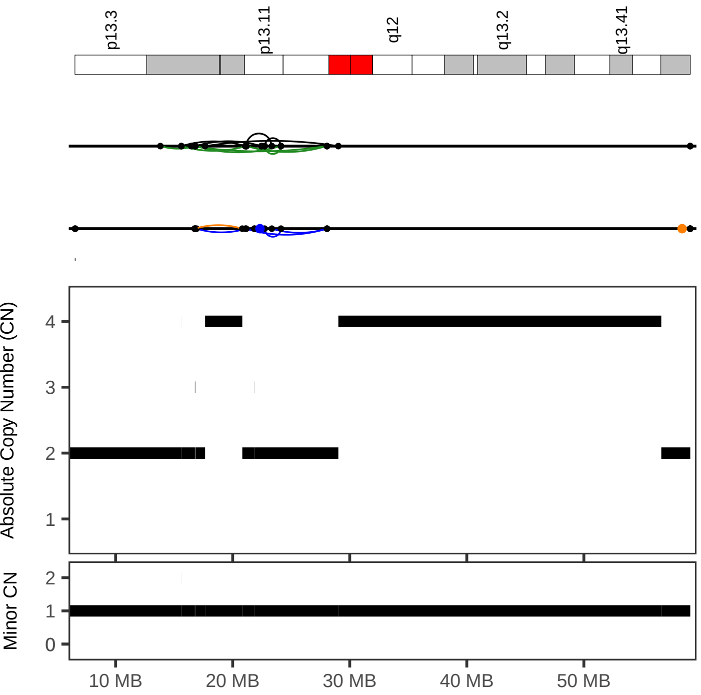

	**CGP_donor_GC00008**
Cancer type	Stomach−AdenoCA
Position	19:13824051−29025405
Type	With other complex events
Interleaved intrachr. SVs	19
Total SVs (intrachr. + transl.)	20
SV types	DEL: 1; DUP: 5; h2hINV: 7; t2tINV: 6; TRA: 1
SVs in sample	307
Oscillating CN (2 and 3 states)	5, 12
CN segments	12
FDR fragment joints	0.615458
FDR chr. breakp. enrich.	0
Linked to chrs	
Purity, ploidy	0.93, 2.06

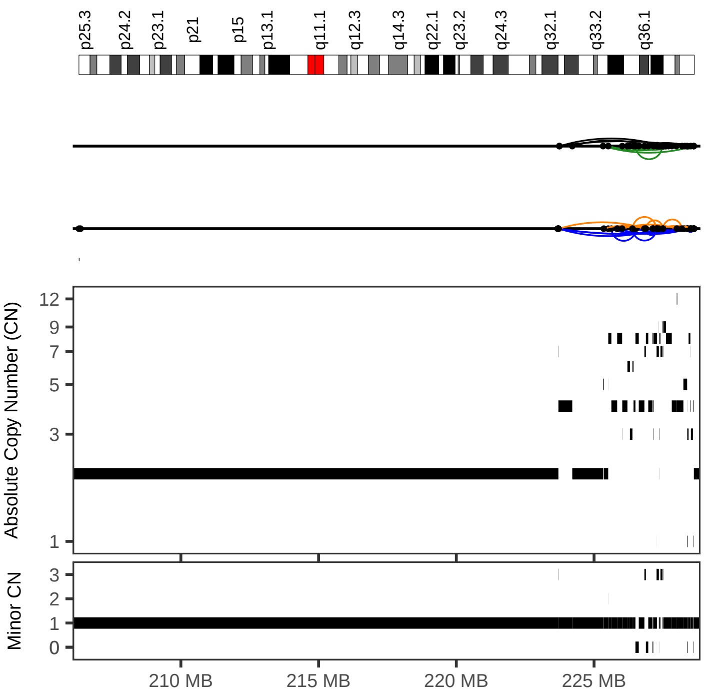

	**CGP_donor_GC00013**
Cancer type	Stomach−AdenoCA
Position	2:223705573−228623701
Type	With other complex events
Interleaved intrachr. SVs	40
Total SVs (intrachr. + transl.)	40
SV types	DEL: 11; DUP: 12; h2hINV: 7; t2tINV: 10; TRA: 0
SVs in sample	95
Oscillating CN (2 and 3 states)	4, 6
CN segments	52
FDR fragment joints	0.8135044
FDR chr. breakp. enrich.	0
Linked to chrs	
Purity, ploidy	0.92, 1.97

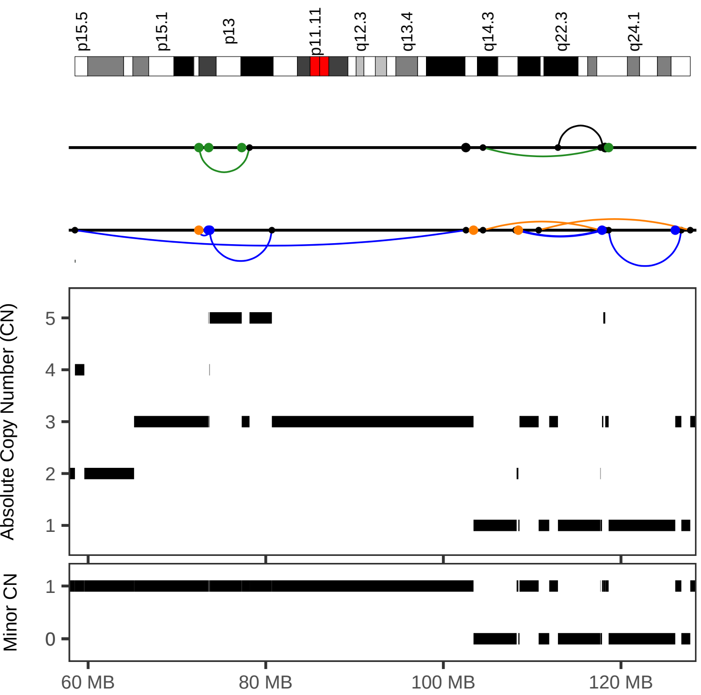

	**CGP_donor_GC00018**
Cancer type	Stomach−AdenoCA
Position	11:104455729−127800985
Type	With other complex events
Interleaved intrachr. SVs	8
Total SVs (intrachr. + transl.)	14
SV types	DEL: 2; DUP: 3; h2hINV: 2; t2tINV: 1; TRA: 6
SVs in sample	85
Oscillating CN (2 and 3 states)	5, 10
CN segments	16
FDR fragment joints	0.8066159
FDR chr. breakp. enrich.	0
Linked to chrs	20:35666161−59823117;
Purity, ploidy	0.25, 1.85

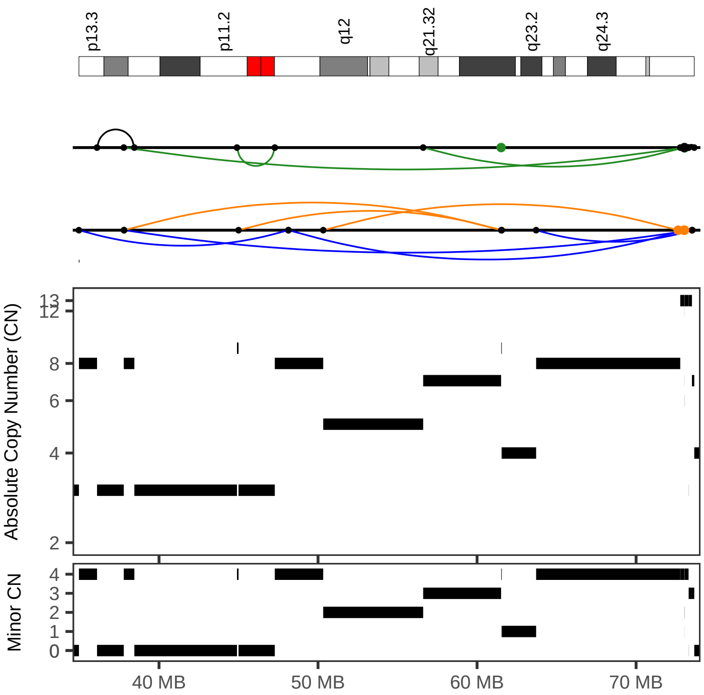

	**CGP_donor_GC00019**
Cancer type	Stomach−AdenoCA
Position	17:34964923−73655919
Type	With other complex events
Interleaved intrachr. SVs	13
Total SVs (intrachr. + transl.)	18
SV types	DEL: 3; DUP: 4; h2hINV: 2; t2tINV: 4; TRA: 5
SVs in sample	101
Oscillating CN (2 and 3 states)	4, 7
CN segments	22
FDR fragment joints	0.8653243
FDR chr. breakp. enrich.	0
Linked to chrs	
Purity, ploidy	0.13, 3.34

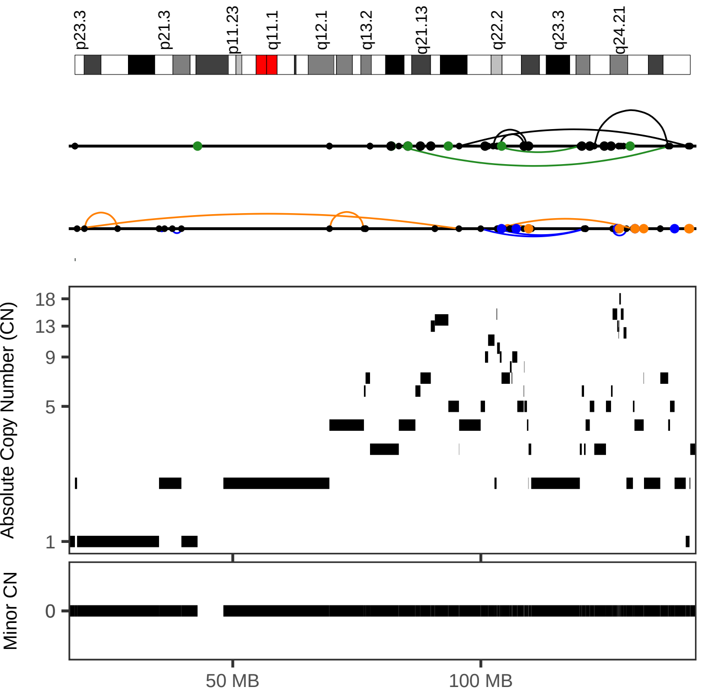

	**CGP_donor_GC00020**
Cancer type	Stomach−AdenoCA
Position	8:99969120−137759332
Type	With other complex events
Interleaved intrachr. SVs	6
Total SVs (intrachr. + transl.)	23
SV types	DEL: 1; DUP: 2; h2hINV: 2; t2tINV: 1; TRA: 17
SVs in sample	403
Oscillating CN (2 and 3 states)	4, 7
CN segments	41
FDR fragment joints	0.7861024
FDR chr. breakp. enrich.	0
Linked to chrs	1:22345572−221724578;
Purity, ploidy	0.44, 3.17

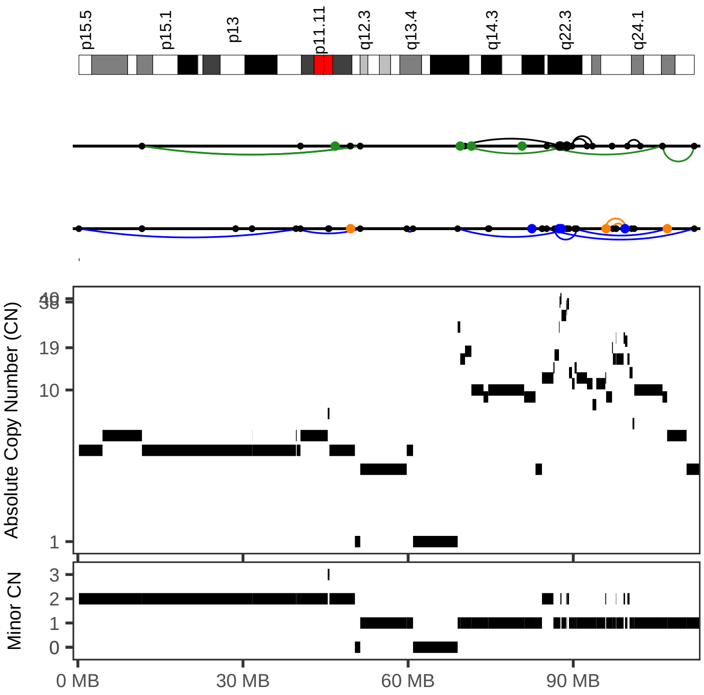

	**CGP_donor_GC00020**
Cancer type	Stomach−AdenoCA
Position	11:69004148−111983118
Type	With other complex events
Interleaved intrachr. SVs	11
Total SVs (intrachr. + transl.)	24
SV types	DEL: 0; DUP: 4; h2hINV: 3; t2tINV: 4; TRA: 13
SVs in sample	403
Oscillating CN (2 and 3 states)	4, 5
CN segments	40
FDR fragment joints	0.615458
FDR chr. breakp. enrich.	0
Linked to chrs	
Purity, ploidy	0.44, 3.17

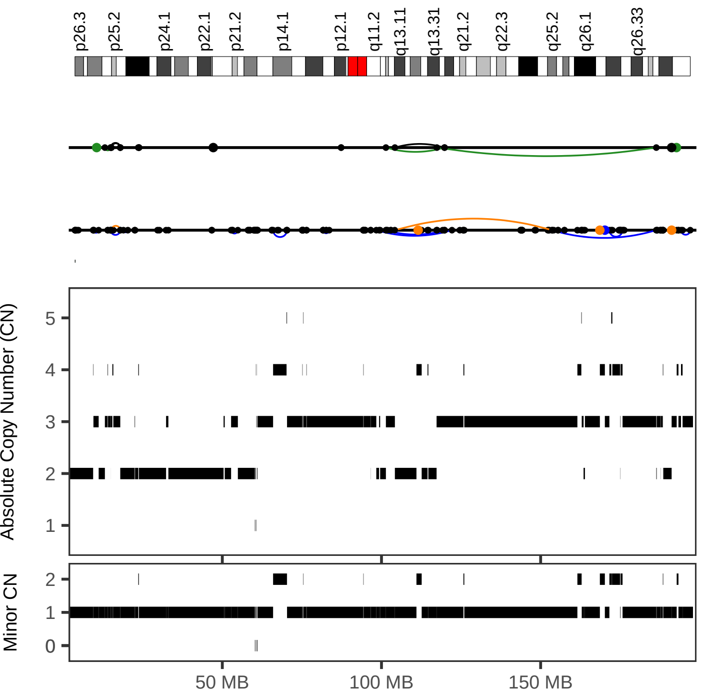

	**CGP_donor_GC00033**
Cancer type	Stomach−AdenoCA
Position	3:98369145−186328430
Type	With other complex events
Interleaved intrachr. SVs	10
Total SVs (intrachr. + transl.)	13
SV types	DEL: 1; DUP: 6; h2hINV: 1; t2tINV: 2; TRA: 3
SVs in sample	925
Oscillating CN (2 and 3 states)	5, 14
CN segments	28
FDR fragment joints	0.5435077
FDR chr. breakp. enrich.	0
Linked to chrs	
Purity, ploidy	0.49, 2.4

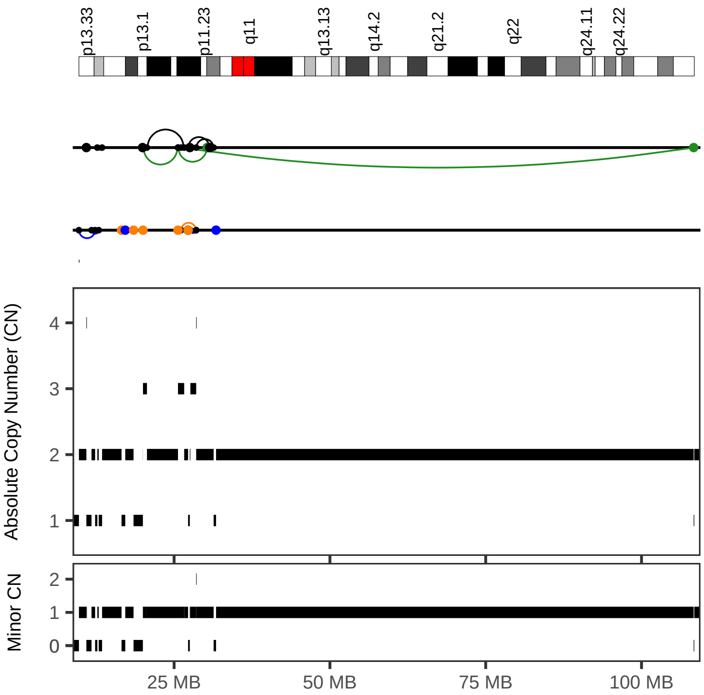

	**CGP_donor_GC00037**
Cancer type	Stomach−AdenoCA
Position	12:20006408−108461993
Type	With other complex events
Interleaved intrachr. SVs	7
Total SVs (intrachr. + transl.)	17
SV types	DEL: 1; DUP: 0; h2hINV: 3; t2tINV: 3; TRA: 10
SVs in sample	58
Oscillating CN (2 and 3 states)	5, 8
CN segments	13
FDR fragment joints	0.6776251
FDR chr. breakp. enrich.	0
Linked to chrs	
Purity, ploidy	0.72, 1.65

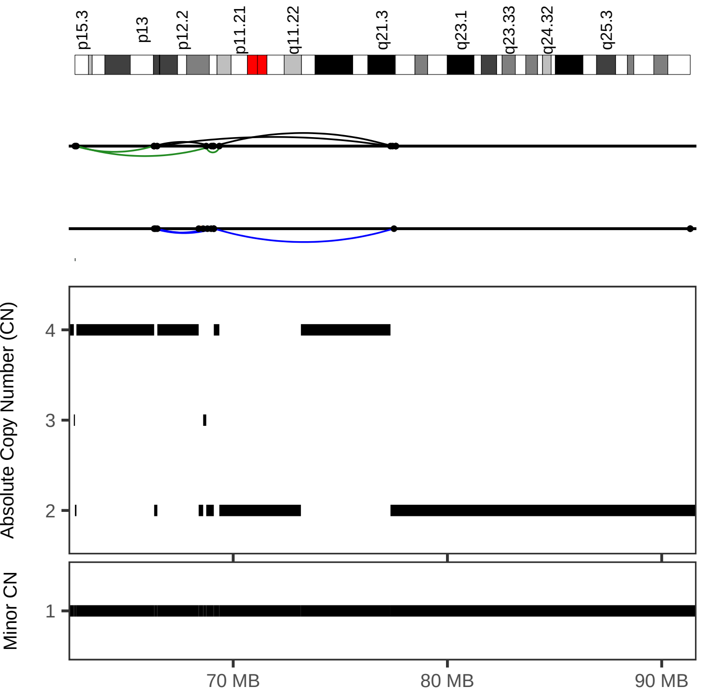

	**CGP_donor_GC00046**
Cancer type	Stomach−AdenoCA
Position	10:62613110−77504895
Type	With other complex events
Interleaved intrachr. SVs	10
Total SVs (intrachr. + transl.)	10
SV types	DEL: 0; DUP: 4; h2hINV: 3; t2tINV: 3; TRA: 0
SVs in sample	76
Oscillating CN (2 and 3 states)	5, 11
CN segments	11
FDR fragment joints	0.615458
FDR chr. breakp. enrich.	0
Linked to chrs	
Purity, ploidy	0.92, 1.94

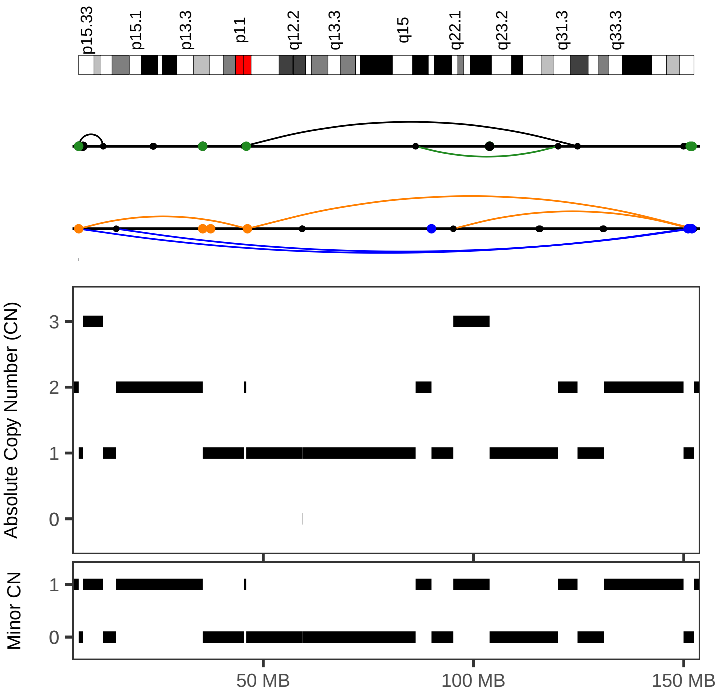

	**CGP_donor_GC00047**
Cancer type	Stomach−AdenoCA
Position	5:6115950−152429287
Type	With other complex events
Interleaved intrachr. SVs	10
Total SVs (intrachr. + transl.)	29
SV types	DEL: 3; DUP: 2; h2hINV: 2; t2tINV: 3; TRA: 19
SVs in sample	187
Oscillating CN (2 and 3 states)	6, 10
CN segments	19
FDR fragment joints	0.7359483
FDR chr. breakp. enrich.	0
Linked to chrs	1:26335498−206855060;
Purity, ploidy	0.38, 2.11

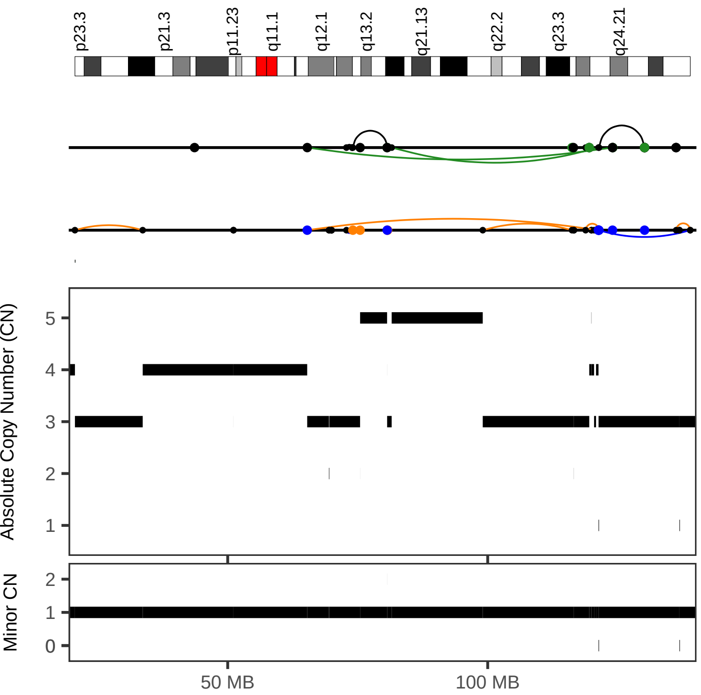

	**CGP_donor_GC00047**
Cancer type	Stomach−AdenoCA
Position	8:65287254−138950505
Type	With other complex events
Interleaved intrachr. SVs	7
Total SVs (intrachr. + transl.)	36
SV types	DEL: 2; DUP: 1; h2hINV: 1; t2tINV: 3; TRA: 29
SVs in sample	187
Oscillating CN (2 and 3 states)	4, 9
CN segments	24
FDR fragment joints	0.5435077
FDR chr. breakp. enrich.	0
Linked to chrs	1:26335498−206855060;5:6115950−152429286
Purity, ploidy	0.38, 2.11

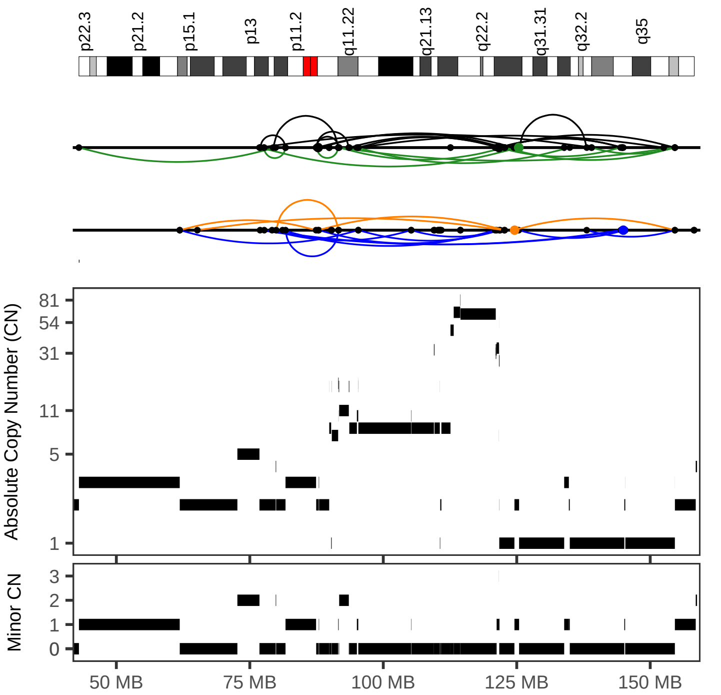

	**CGP_donor_GC00052**
Cancer type	Stomach−AdenoCA
Position	7:42989699−154627590
Type	With other complex events
Interleaved intrachr. SVs	48
Total SVs (intrachr. + transl.)	54
SV types	DEL: 5; DUP: 12; h2hINV: 16; t2tINV: 15; TRA: 6
SVs in sample	309
Oscillating CN (2 and 3 states)	6, 6
CN segments	62
FDR fragment joints	0.5435077
FDR chr. breakp. enrich.	0
Linked to chrs	4:20858263−181547734;
Purity, ploidy	0.44, 2.18

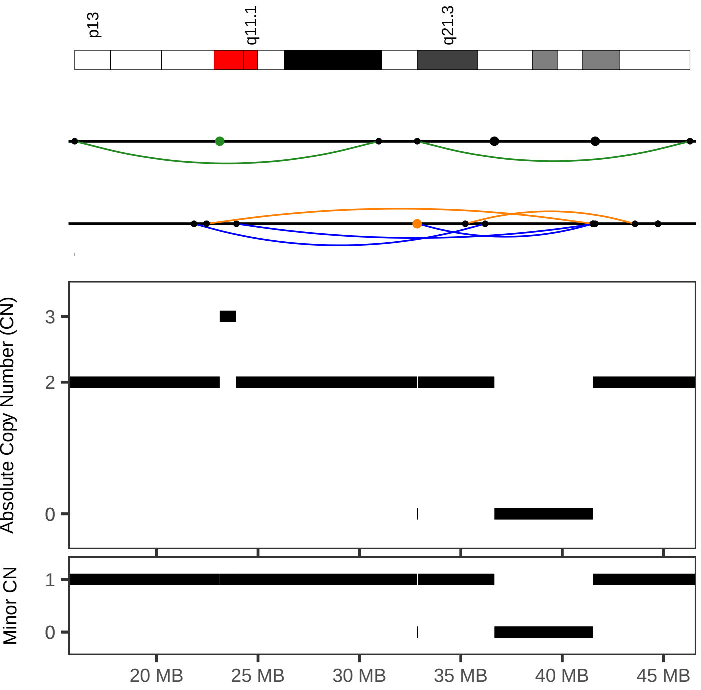

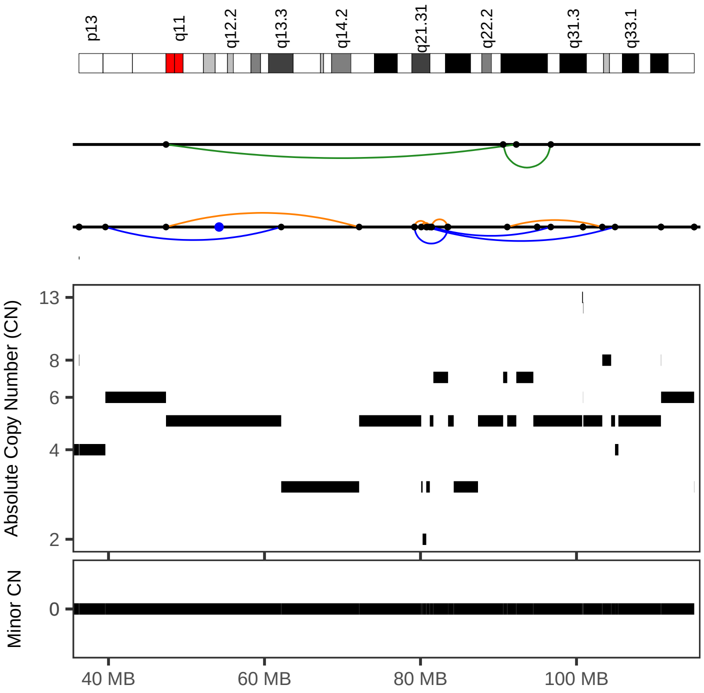

	**CGP_donor_GC00052**
Cancer type	Stomach−AdenoCA
Position	21:15959382−46300731
Type	Canonical without polyploidization
Interleaved intrachr. SVs	7
Total SVs (intrachr. + transl.)	11
SV types	DEL: 2; DUP: 3; h2hINV: 0; t2tINV: 2; TRA: 4
SVs in sample	309
Oscillating CN (2 and 3 states)	5, 7
CN segments	7
FDR fragment joints	0.7425546
FDR chr. breakp. enrich.	0
Linked to chrs	
Purity, ploidy	0.44, 2.18

	**CGP_donor_GC00053**
Cancer type	Stomach−AdenoCA
Position	13:39597021−104940825
Type	With other complex events
Interleaved intrachr. SVs	11
Total SVs (intrachr. + transl.)	12
SV types	DEL: 5; DUP: 4; h2hINV: 0; t2tINV: 2; TRA: 1
SVs in sample	317
Oscillating CN (2 and 3 states)	5, 10
CN segments	23
FDR fragment joints	0.5435077
FDR chr. breakp. enrich.	0.2
Linked to chrs	
Purity, ploidy	0.35, 4.48

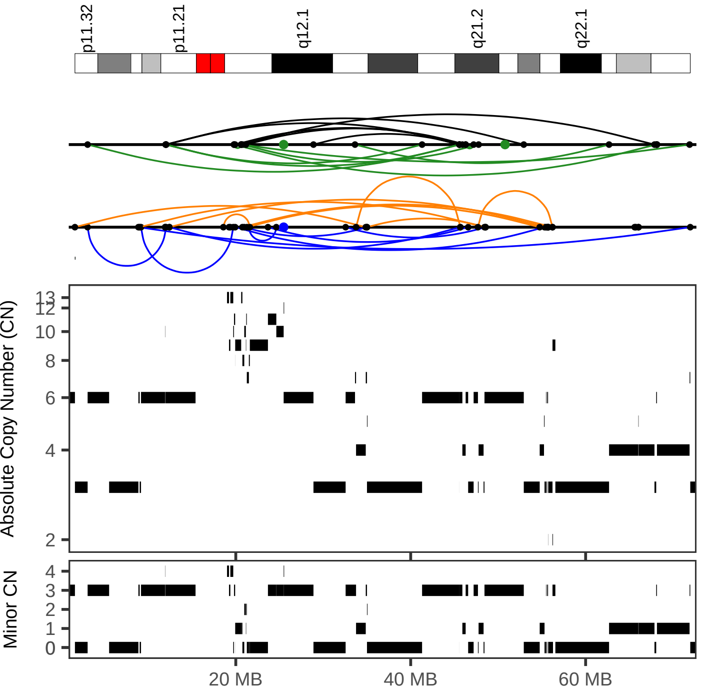

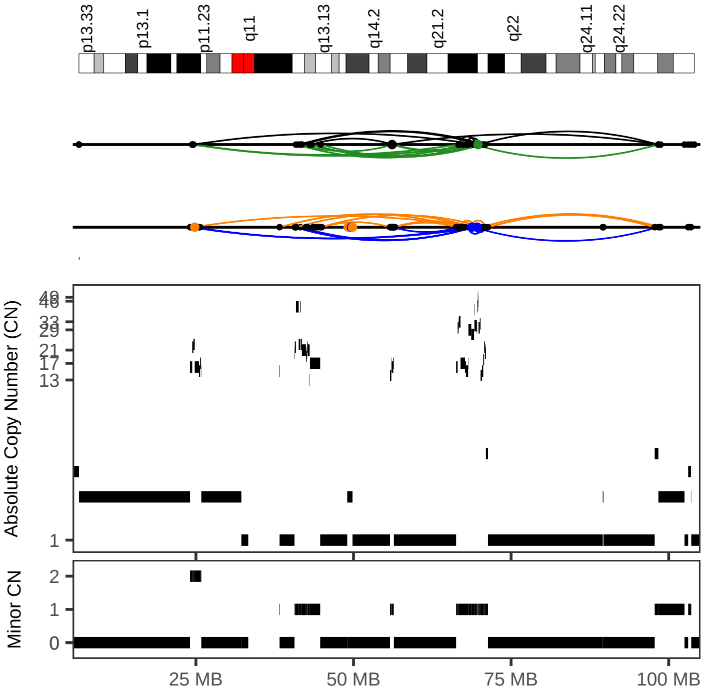

	**CGP_donor_GC00053**
Cancer type	Stomach−AdenoCA
Position	18:1608546−71962999
Type	With other complex events
Interleaved intrachr. SVs	33
Total SVs (intrachr. + transl.)	36
SV types	DEL: 9; DUP: 9; h2hINV: 6; t2tINV: 9; TRA: 3
SVs in sample	317
Oscillating CN (2 and 3 states)	6, 10
CN segments	66
FDR fragment joints	0.9875525
FDR chr. breakp. enrich.	0
Linked to chrs	
Purity, ploidy	0.35, 4.48

	**0aecac64−5982−4d76−8f31−958f6a00951d**
Cancer type	Stomach−AdenoCA
Position	12:24064615−98708265
Type	With other complex events
Interleaved intrachr. SVs	48
Total SVs (intrachr. + transl.)	56
SV types	DEL: 10; DUP: 16; h2hINV: 10; t2tINV: 12; TRA: 8
SVs in sample	332
Oscillating CN (2 and 3 states)	4, 4
CN segments	66
FDR fragment joints	0.6776251
FDR chr. breakp. enrich.	0
Linked to chrs	17:48341217−72770354;
Purity, ploidy	0.44, 1.75

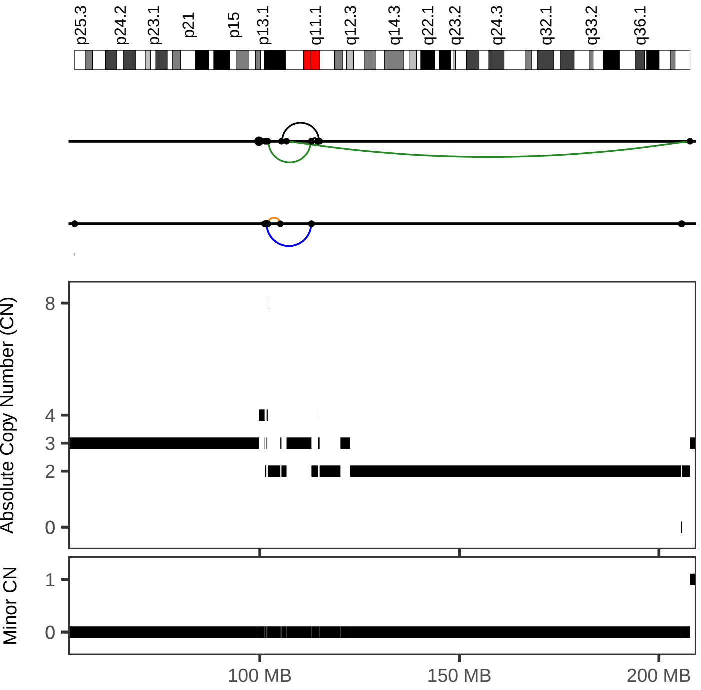

	0b4e8d73−459d−4930−9d1a−abb8d53e1844
Cancer type	Stomach−AdenoCA
Position	2:101189993−207792558
Type	With other complex events
Interleaved intrachr. SVs	9
Total SVs (intrachr. + transl.)	9
SV types	DEL: 2; DUP: 3; h2hINV: 2; t2tINV: 2; TRA: 0
SVs in sample	208
Oscillating CN (2 and 3 states)	5, 7
CN segments	19
FDR fragment joints	0.9723381
FDR chr. breakp. enrich.	0.83
Linked to chrs	
Purity, ploidy	0.64, 2.72

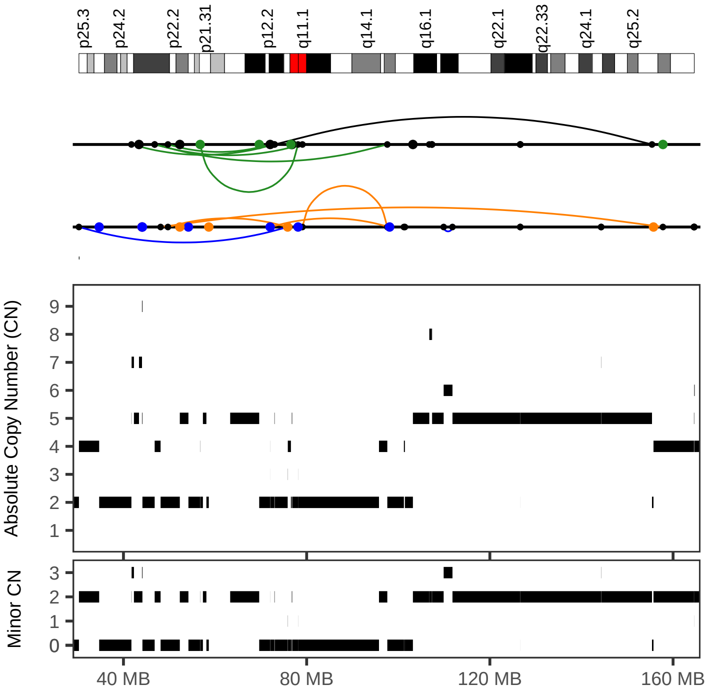

	1ca3c5e0−32b0−4467−8ec0−ca212e35d2b3
Cancer type	Stomach−AdenoCA
Position	6:30271712−157786893
Type	With other complex events
Interleaved intrachr. SVs	11
Total SVs (intrachr. + transl.)	33
SV types	DEL: 4; DUP: 1; h2hINV: 1; t2tINV: 5; TRA: 22
SVs in sample	313
Oscillating CN (2 and 3 states)	5, 12
CN segments	48
FDR fragment joints	0.8653243
FDR chr. breakp. enrich.	0
Linked to chrs	11:17850371−130169014;13:27261037−79173978
Purity, ploidy	0.3, 3.61

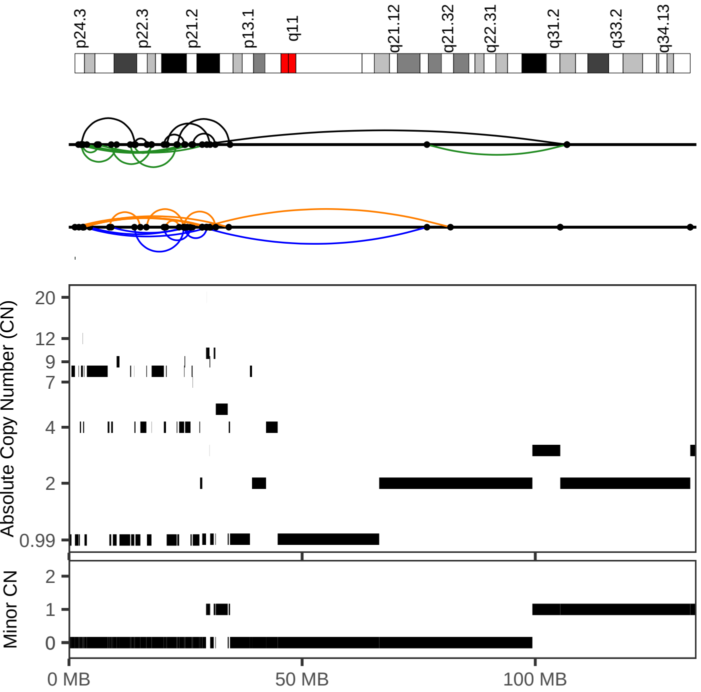

	283776cd−32e8−4a79−bed1−1411c9d3a9e0
Cancer type	Stomach−AdenoCA
Position	9:1273053−106799638
Type	With other complex events
Interleaved intrachr. SVs	35
Total SVs (intrachr. + transl.)	35
SV types	DEL: 10; DUP: 8; h2hINV: 7; t2tINV: 10; TRA: 0
SVs in sample	99
Oscillating CN (2 and 3 states)	4, 8
CN segments	63
FDR fragment joints	0.9176394
FDR chr. breakp. enrich.	0
Linked to chrs	
Purity, ploidy	0.9, 2.47

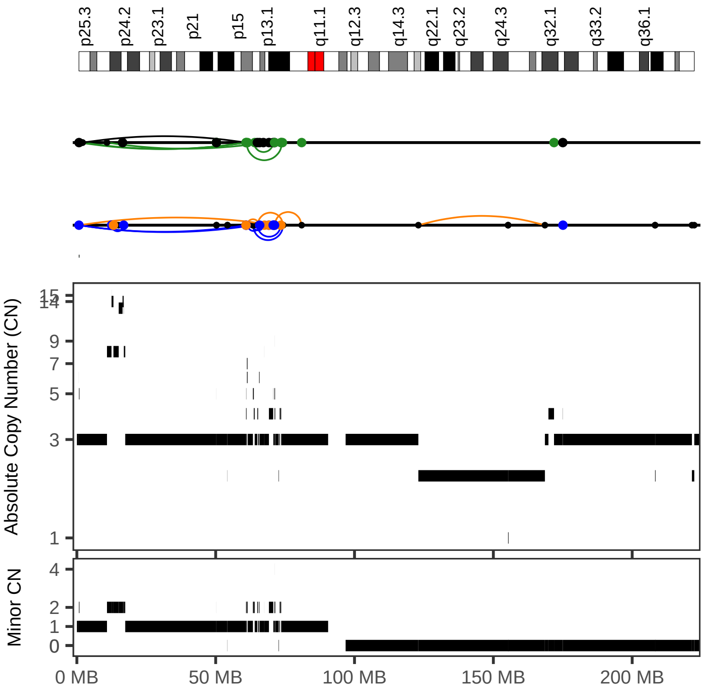

	2d291fa3−f0fd−4bc1−91a6−783863714190
Cancer type	Stomach−AdenoCA
Position	2:744061−80962231
Type	With other complex events
Interleaved intrachr. SVs	20
Total SVs (intrachr. + transl.)	57
SV types	DEL: 5; DUP: 7; h2hINV: 2; t2tINV: 6; TRA: 37
SVs in sample	408
Oscillating CN (2 and 3 states)	5, 9
CN segments	52
FDR fragment joints	0.6776251
FDR chr. breakp. enrich.	0
Linked to chrs	12:7030656−104650533;4:1493041−19429269
Purity, ploidy	0.62, 3.54

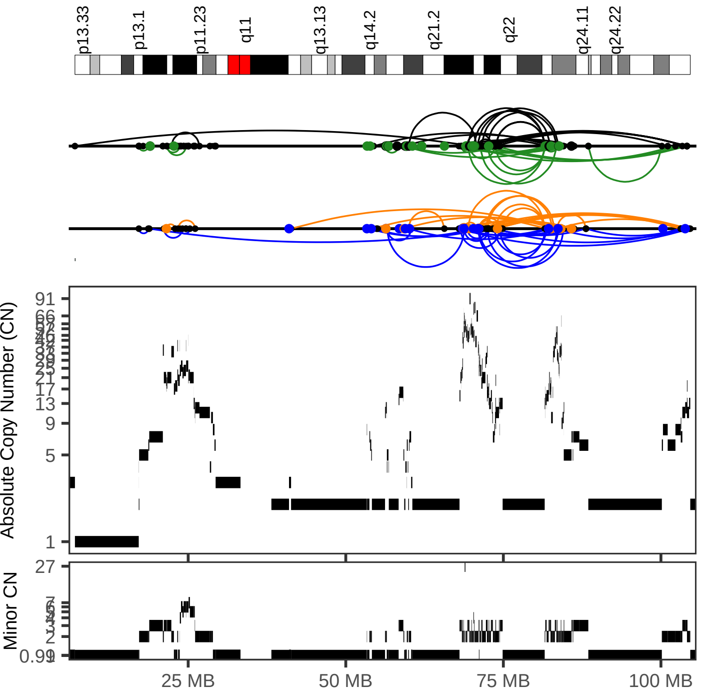

	2d291fa3−f0fd−4bc1−91a6−783863714190
Cancer type	Stomach−AdenoCA
Position	12:7030656−104650534
Type	With other complex events
Interleaved intrachr. SVs	103
Total SVs (intrachr. + transl.)	169
SV types	DEL: 27; DUP: 25; h2hINV: 24; t2tINV: 27; TRA: 66
SVs in sample	408
Oscillating CN (2 and 3 states)	6, 8
CN segments	243
FDR fragment joints	0.8572806
FDR chr. breakp. enrich.	0
Linked to chrs	10:83825888−84983844;2:744061−80962230
Purity, ploidy	0.62, 3.54

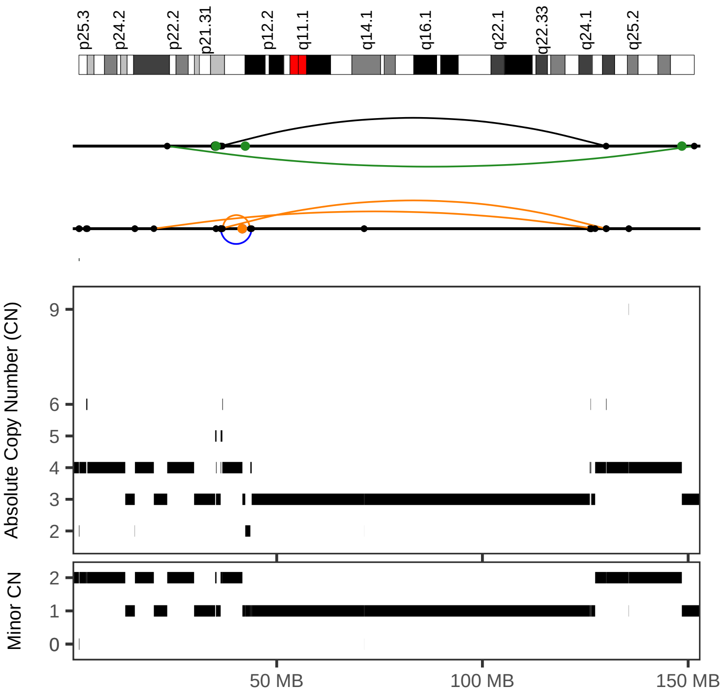

	337819d2−5281−4b41−9583−a476924bc837
Cancer type	Stomach−AdenoCA
Position	6:20138021−151484461
Type	With other complex events
Interleaved intrachr. SVs	7
Total SVs (intrachr. + transl.)	14
SV types	DEL: 2; DUP: 3; h2hINV: 1; t2tINV: 1; TRA: 7
SVs in sample	210
Oscillating CN (2 and 3 states)	4, 8
CN segments	28
FDR fragment joints	0.8988396
FDR chr. breakp. enrich.	0
Linked to chrs	14:56203717−106008232;2:63345548−242350180
Purity, ploidy	0.32, 2.97

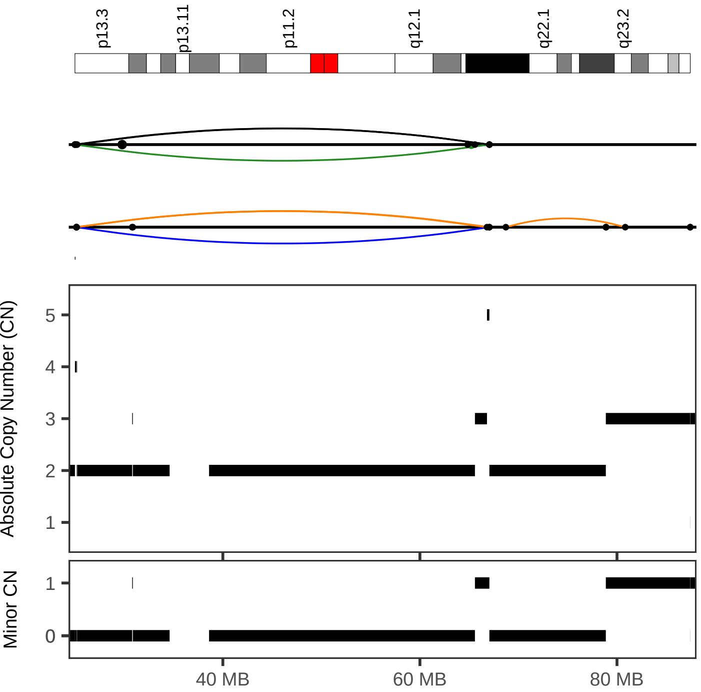

	337819d2−5281−4b41−9583−a476924bc837
Cancer type	Stomach−AdenoCA
Position	16:24987472−67049646
Type	With other complex events
Interleaved intrachr. SVs	8
Total SVs (intrachr. + transl.)	9
SV types	DEL: 3; DUP: 1; h2hINV: 2; t2tINV: 2; TRA: 1
SVs in sample	210
Oscillating CN (2 and 3 states)	5, 9
CN segments	11
FDR fragment joints	0.854603
FDR chr. breakp. enrich.	0.01
Linked to chrs	
Purity, ploidy	0.32, 2.97

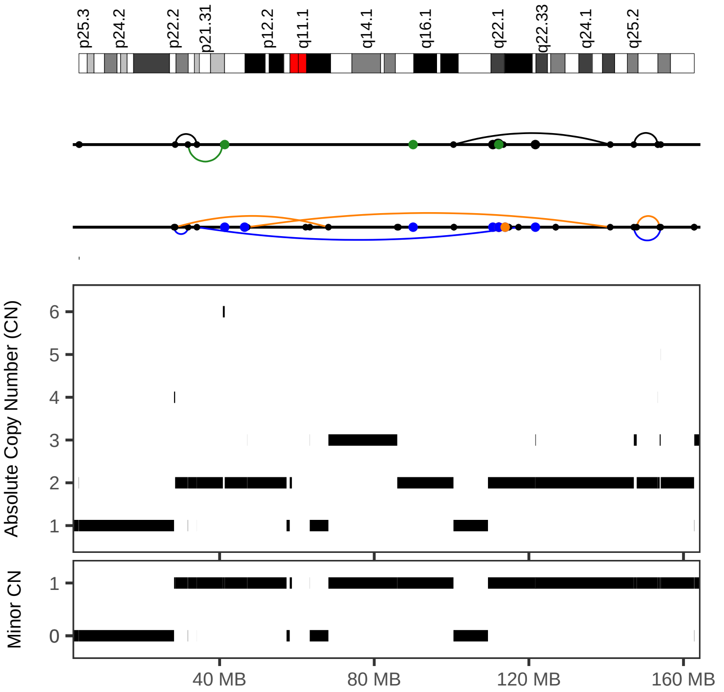

	53086e20−a727−40d6−b42d−8030109b5130
Cancer type	Stomach−AdenoCA
Position	6:28215328−141062702
Type	With other complex events
Interleaved intrachr. SVs	7
Total SVs (intrachr. + transl.)	24
SV types	DEL: 2; DUP: 2; h2hINV: 2; t2tINV: 1; TRA: 17
SVs in sample	246
Oscillating CN (2 and 3 states)	5, 6
CN segments	20
FDR fragment joints	0.5435077
FDR chr. breakp. enrich.	0
Linked to chrs	
Purity, ploidy	0.83, 1.96

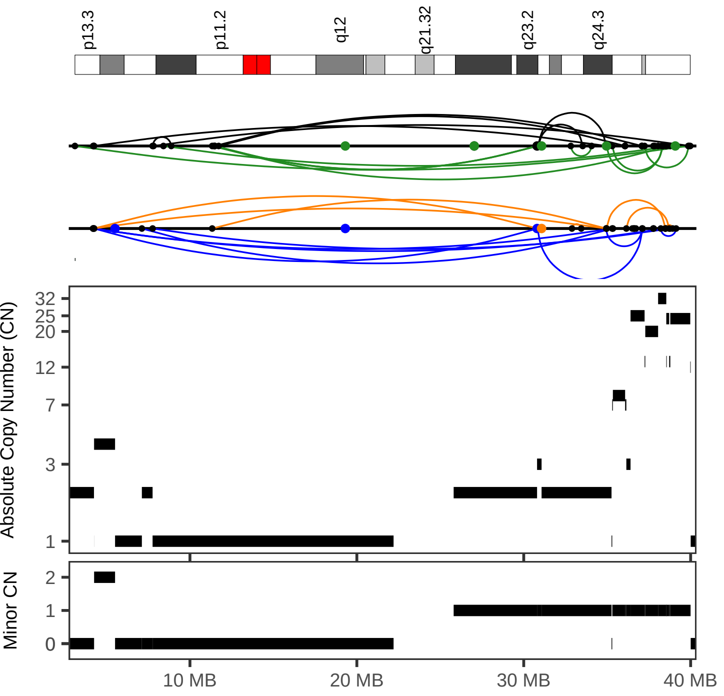

	53086e20−a727−40d6−b42d−8030109b5130
Cancer type	Stomach−AdenoCA
Position	17:3107404−39977217
Type	With other complex events
Interleaved intrachr. SVs	32
Total SVs (intrachr. + transl.)	43
SV types	DEL: 6; DUP: 8; h2hINV: 6; t2tINV: 12; TRA: 11
SVs in sample	246
Oscillating CN (2 and 3 states)	4, 10
CN segments	24
FDR fragment joints	0.6776251
FDR chr. breakp. enrich.	0
Linked to chrs	6:28215328−141062701;
Purity, ploidy	0.83, 1.96

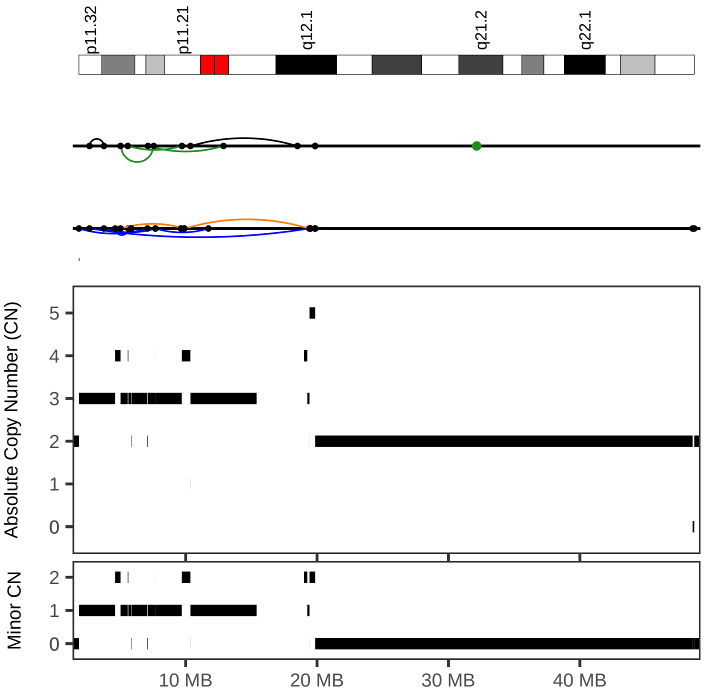

	647dc54f−a51b−40ea−9d97−c93598c2af71
Cancer type	Stomach−AdenoCA
Position	18:1875598−19427543
Type	With other complex events
Interleaved intrachr. SVs	12
Total SVs (intrachr. + transl.)	12
SV types	DEL: 2; DUP: 5; h2hINV: 2; t2tINV: 3; TRA: 0
SVs in sample	294
Oscillating CN (2 and 3 states)	6, 15
CN segments	20
FDR fragment joints	0.6776251
FDR chr. breakp. enrich.	0
Linked to chrs	
Purity, ploidy	0.75, 2.88

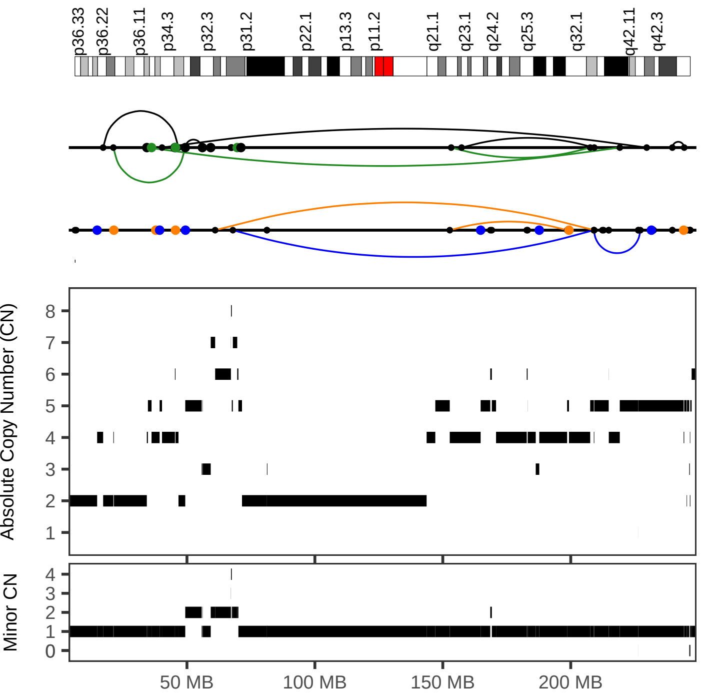

	8b5746f9−dbee−40bd−9141−1081960cf286
Cancer type	Stomach−AdenoCA
Position	1:17246972−229690987
Type	With other complex events
Interleaved intrachr. SVs	10
Total SVs (intrachr. + transl.)	27
SV types	DEL: 2; DUP: 2; h2hINV: 3; t2tINV: 3; TRA: 17
SVs in sample	363
Oscillating CN (2 and 3 states)	6, 11
CN segments	48
FDR fragment joints	0.8653243
FDR chr. breakp. enrich.	0
Linked to chrs	
Purity, ploidy	0.27, 2.91

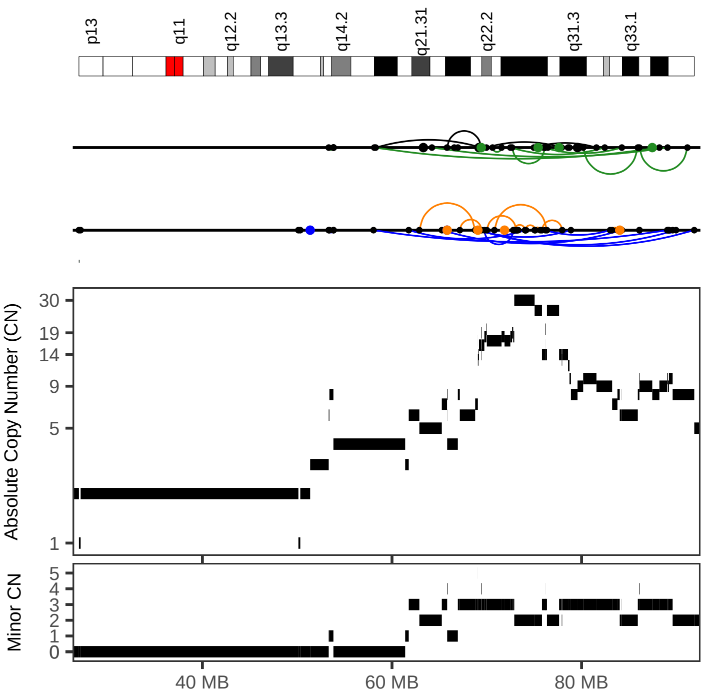

	8b5746f9−dbee−40bd−9141−1081960cf286
Cancer type	Stomach−AdenoCA
Position	13:58047889−91889039
Type	With other complex events
Interleaved intrachr. SVs	30
Total SVs (intrachr. + transl.)	42
SV types	DEL: 8; DUP: 9; h2hINV: 4; t2tINV: 9; TRA: 12
SVs in sample	363
Oscillating CN (2 and 3 states)	5, 7
CN segments	57
FDR fragment joints	0.8653243
FDR chr. breakp. enrich.	0
Linked to chrs	
Purity, ploidy	0.27, 2.91

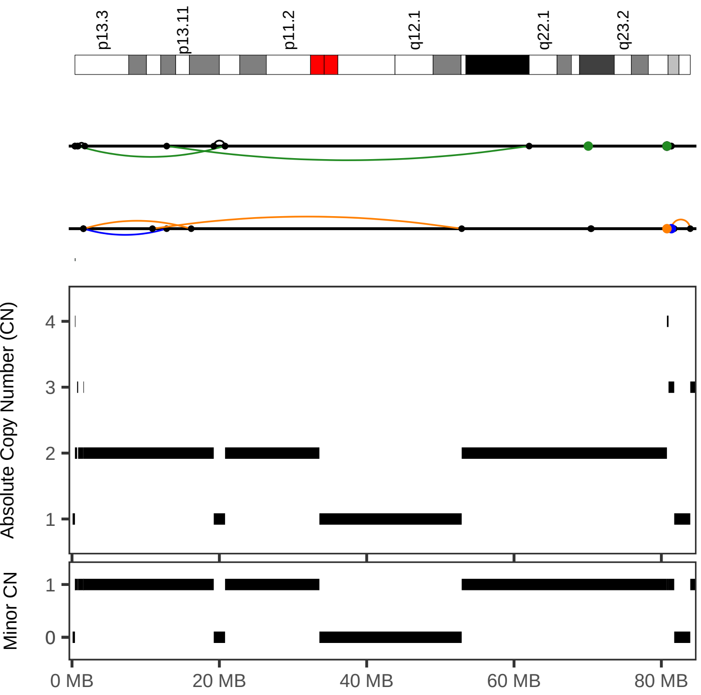

	8b5746f9−dbee−40bd−9141−1081960cf286
Cancer type	Stomach−AdenoCA
Position	16:693417−62053802
Type	Canonical without polyploidization
Interleaved intrachr. SVs	6
Total SVs (intrachr. + transl.)	6
SV types	DEL: 2; DUP: 1; h2hINV: 1; t2tINV: 2; TRA: 0
SVs in sample	363
Oscillating CN (2 and 3 states)	5, 8
CN segments	8
FDR fragment joints	0.9284301
FDR chr. breakp. enrich.	0.02
Linked to chrs	
Purity, ploidy	0.27, 2.91

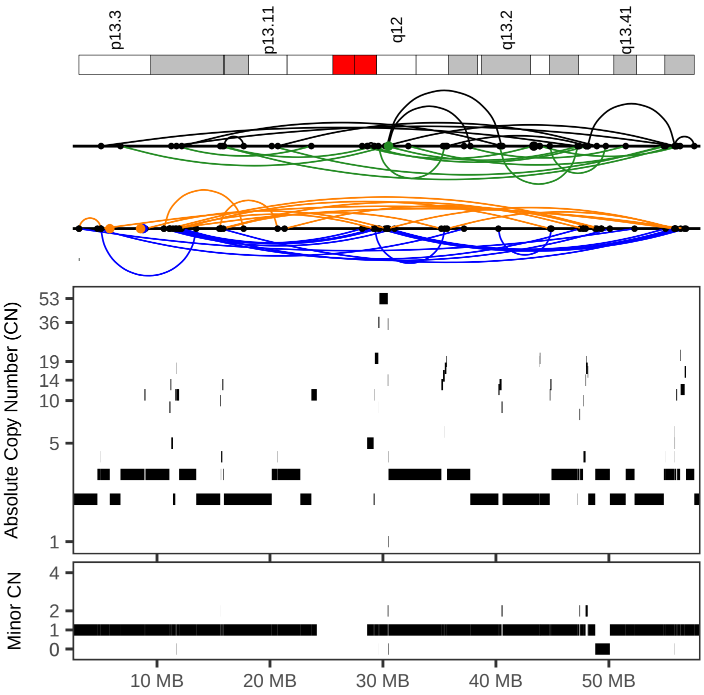

	8b5746f9−dbee−40bd−9141−1081960cf286
Cancer type	Stomach−AdenoCA
Position	19:3088176−57557538
Type	With other complex events
Interleaved intrachr. SVs	58
Total SVs (intrachr. + transl.)	63
SV types	DEL: 14; DUP: 17; h2hINV: 12; t2tINV: 15; TRA: 5
SVs in sample	363
Oscillating CN (2 and 3 states)	6, 9
CN segments	90
FDR fragment joints	0.9087805
FDR chr. breakp. enrich.	0
Linked to chrs	17:1358751−56764163;
Purity, ploidy	0.27, 2.91

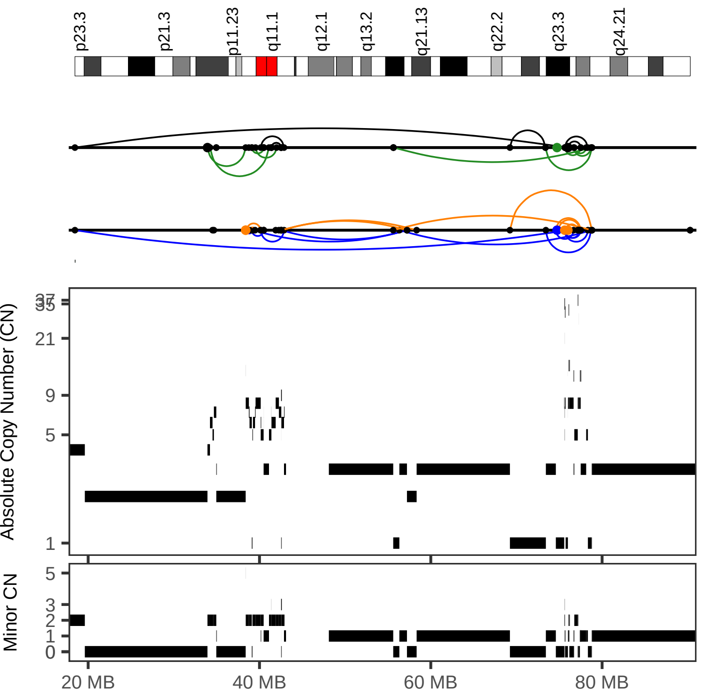

	c991697f−77f9−412a−9bd1−0e47f7387cb2
Cancer type	Stomach−AdenoCA
Position	8:18453850−78826170
Type	With other complex events
Interleaved intrachr. SVs	43
Total SVs (intrachr. + transl.)	51
SV types	DEL: 11; DUP: 13; h2hINV: 10; t2tINV: 9; TRA: 8
SVs in sample	187
Oscillating CN (2 and 3 states)	5, 8
CN segments	79
FDR fragment joints	0.8653243
FDR chr. breakp. enrich.	0
Linked to chrs	
Purity, ploidy	0.56, 3.09

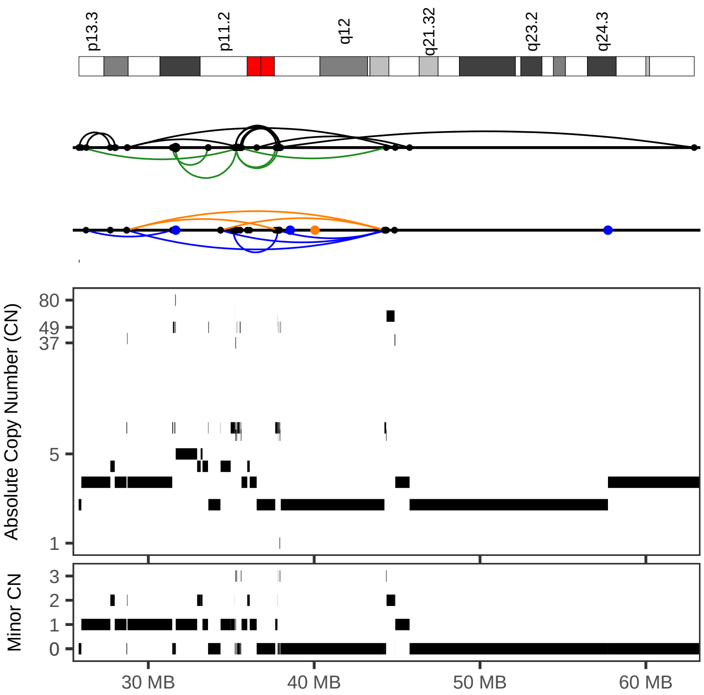

	c991697f−77f9−412a−9bd1−0e47f7387cb2
Cancer type	Stomach−AdenoCA
Position	17:25815792−62916421
Type	With other complex events
Interleaved intrachr. SVs	35
Total SVs (intrachr. + transl.)	40
SV types	DEL: 4; DUP: 7; h2hINV: 16; t2tINV: 8; TRA: 5
SVs in sample	187
Oscillating CN (2 and 3 states)	5, 9
CN segments	70
FDR fragment joints	0.5435077
FDR chr. breakp. enrich.	0
Linked to chrs	
Purity, ploidy	0.56, 3.09

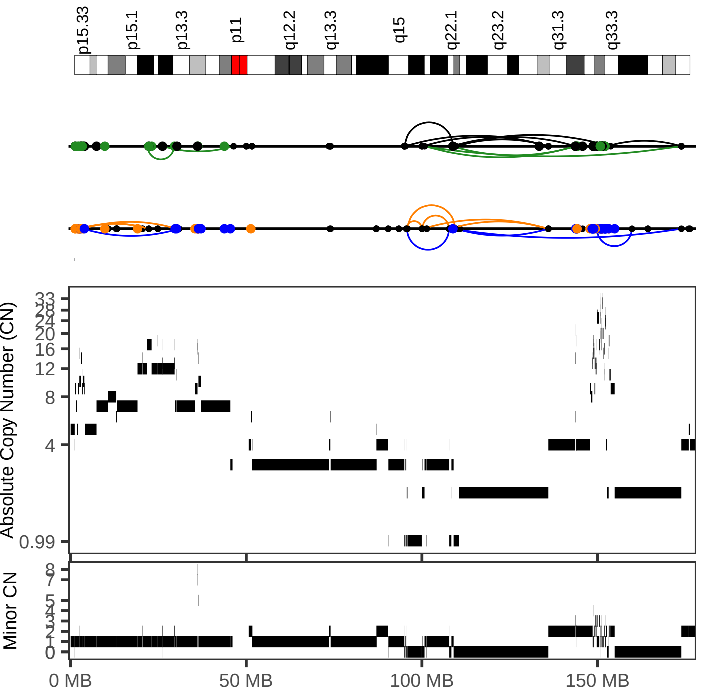

	**cb622cd6−776d−4681−8a67−4addd61c66b8**
Cancer type	Stomach−AdenoCA
Position	5:94993806−173860097
Type	With other complex events
Interleaved intrachr. SVs	29
Total SVs (intrachr. + transl.)	98
SV types	DEL: 6; DUP: 7; h2hINV: 11; t2tINV: 5; TRA: 69
SVs in sample	1322
Oscillating CN (2 and 3 states)	4, 5
CN segments	94
FDR fragment joints	0.5435077
FDR chr. breakp. enrich.	0
Linked to chrs	17:2599904−79841998;3:12028186−62799588
Purity, ploidy	0.46, 4.99

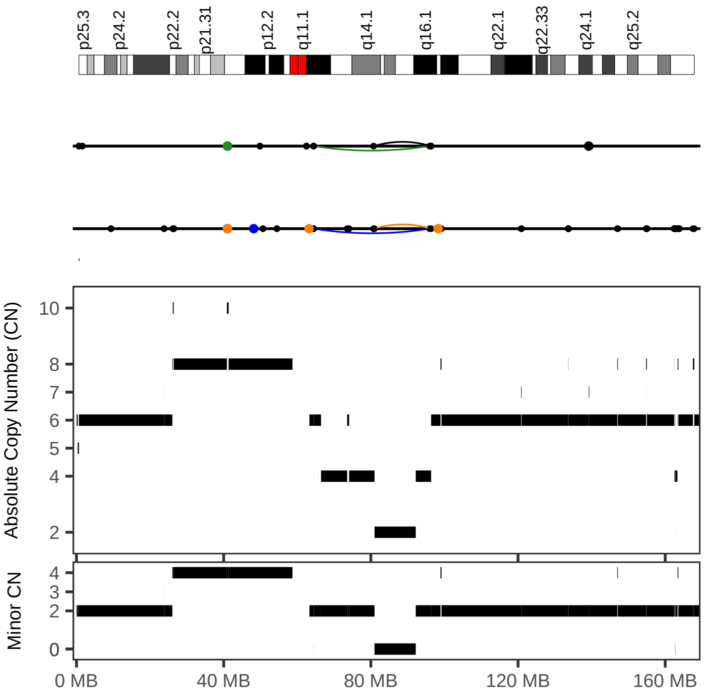

	**cb622cd6−776d−4681−8a67−4addd61c66b8**
Cancer type	Stomach−AdenoCA
Position	6:64442061−96377194
Type	Before polyploidization
Interleaved intrachr. SVs	6
Total SVs (intrachr. + transl.)	6
SV types	DEL: 1; DUP: 3; h2hINV: 1; t2tINV: 1; TRA: 0
SVs in sample	1322
Oscillating CN (2 and 3 states)	5, 8
CN segments	8
FDR fragment joints	0.6776251
FDR chr. breakp. enrich.	0
Linked to chrs	
Purity, ploidy	0.46, 4.99

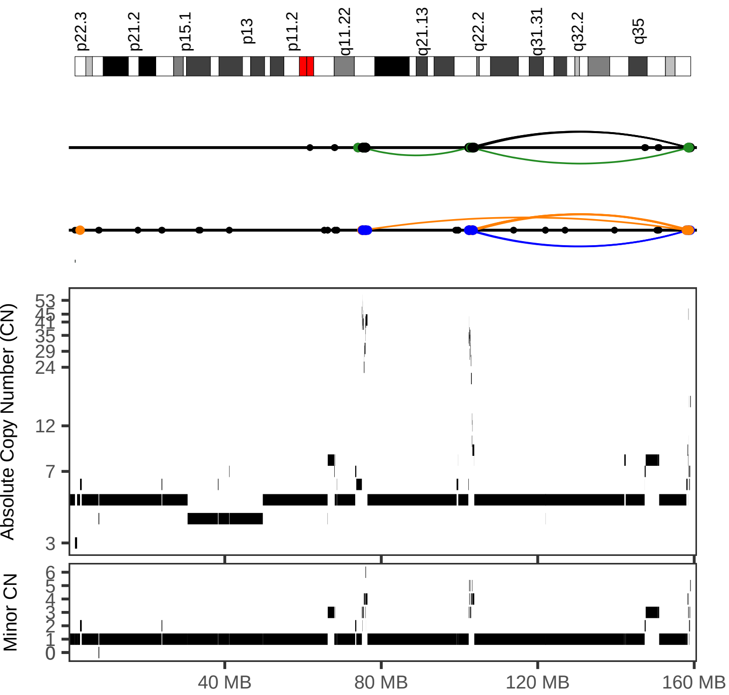

	**cb622cd6−776d−4681−8a67−4addd61c66b8**
Cancer type	Stomach−AdenoCA
Position	7:75111917−159113377
Type	With other complex events
Interleaved intrachr. SVs	14
Total SVs (intrachr. + transl.)	84
SV types	DEL: 5; DUP: 2; h2hINV: 4; t2tINV: 3; TRA: 70
SVs in sample	1322
Oscillating CN (2 and 3 states)	6, 6
CN segments	71
FDR fragment joints	0.8773673
FDR chr. breakp. enrich.	0
Linked to chrs	3:12028186−62799588;
Purity, ploidy	0.46, 4.99

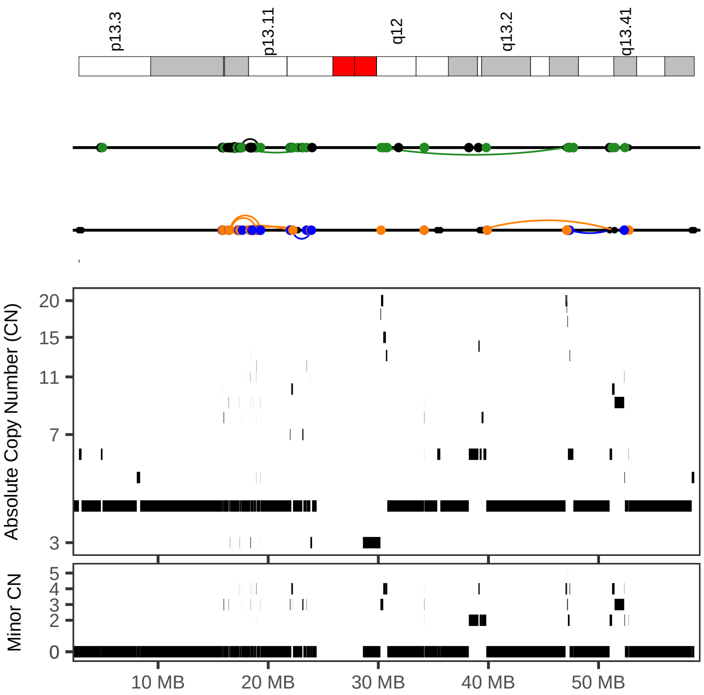

	**cb622cd6−776d−4681−8a67−4addd61c66b8**
Cancer type	Stomach−AdenoCA
Position	19:16006717−23829529
Type	With other complex events
Interleaved intrachr. SVs	11
Total SVs (intrachr. + transl.)	99
SV types	DEL: 5; DUP: 1; h2hINV: 2; t2tINV: 3; TRA: 88
SVs in sample	1322
Oscillating CN (2 and 3 states)	4, 7
CN segments	44
FDR fragment joints	0.9717738
FDR chr. breakp. enrich.	0
Linked to chrs	17:2599904−79841998;3:12028186−62799588
Purity, ploidy	0.46, 4.99

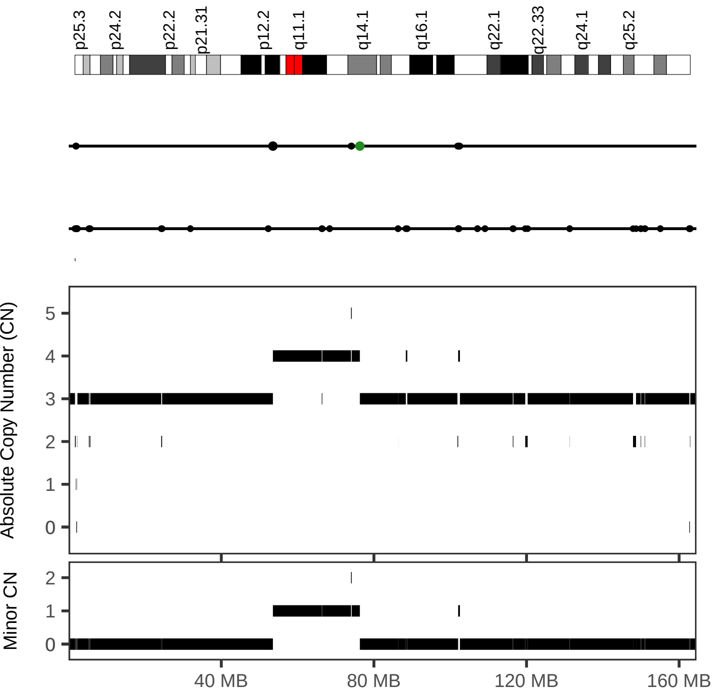

	e2aaabd9−f4ba−4763−a28a−c14d46a4bc5d
Cancer type	Stomach−AdenoCA
Position	6:1632693−2284818
Type	With other complex events
Interleaved intrachr. SVs	8
Total SVs (intrachr. + transl.)	8
SV types	DEL: 6; DUP: 0; h2hINV: 1; t2tINV: 1; TRA: 0
SVs in sample	565
Oscillating CN (2 and 3 states)	5, 7
CN segments	11
FDR fragment joints	0.2796253
FDR chr. breakp. enrich.	0.72
Linked to chrs	
Purity, ploidy	0.73, 2.22

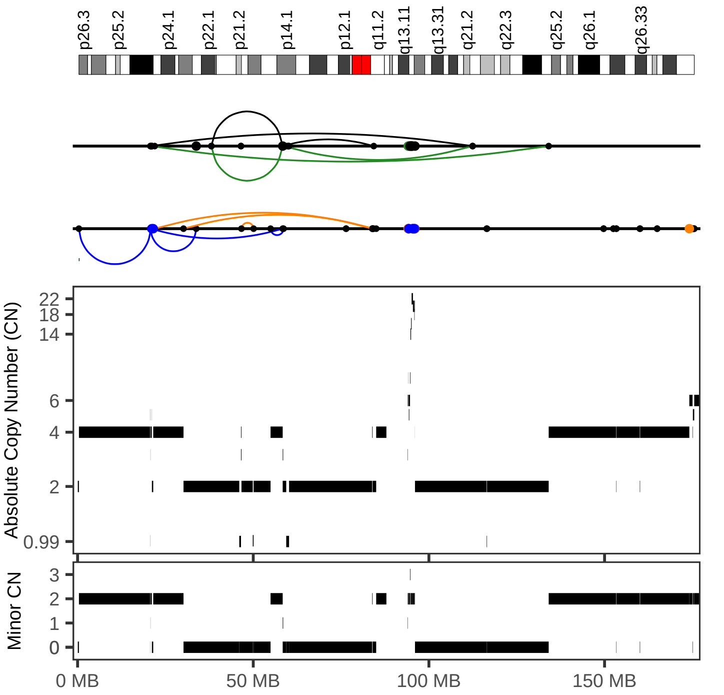

	e37cae9e−0656−4b89−b326−739a5cc859e0
Cancer type	Stomach−AdenoCA
Position	3:374952−134097822
Type	With other complex events
Interleaved intrachr. SVs	17
Total SVs (intrachr. + transl.)	39
SV types	DEL: 4; DUP: 5; h2hINV: 4; t2tINV: 4; TRA: 22
SVs in sample	303
Oscillating CN (2 and 3 states)	4, 7
CN segments	42
FDR fragment joints	0.615458
FDR chr. breakp. enrich.	0
Linked to chrs	12:38025321−70035231;7:5548287−146623411
Purity, ploidy	0.74, 3.26

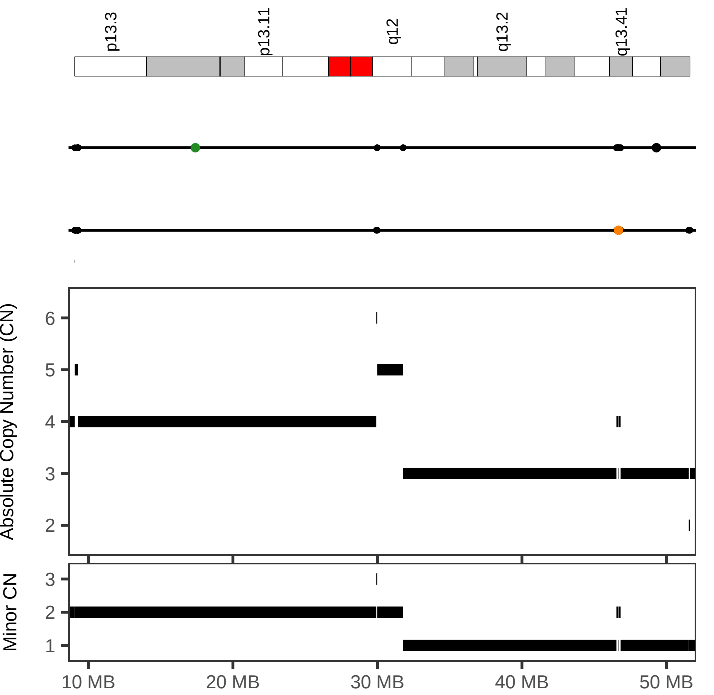

	2b76cc9c−c379−43e4−9c3f−56197a3353be
Cancer type	Uterus−AdenoCA
Position	19:46536637−46823329
Type	After polyploidization
Interleaved intrachr. SVs	9
Total SVs (intrachr. + transl.)	10
SV types	DEL: 2; DUP: 2; h2hINV: 4; t2tINV: 1; TRA: 1
SVs in sample	345
Oscillating CN (2 and 3 states)	5, 5
CN segments	5
FDR fragment joints	0.6776251
FDR chr. breakp. enrich.	0
Linked to chrs	
Purity, ploidy	0.62, 3.14

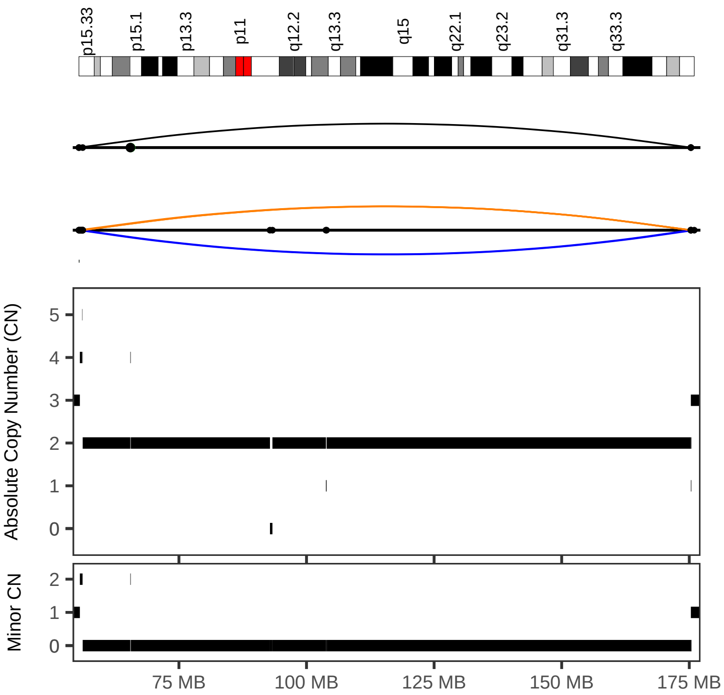

	2b865f8c−539f−4cb2−8f08−41709e9511e5
Cancer type	Uterus−AdenoCA
Position	5:55406655−175308957
Type	With other complex events
Interleaved intrachr. SVs	9
Total SVs (intrachr. + transl.)	11
SV types	DEL: 4; DUP: 3; h2hINV: 2; t2tINV: 0; TRA: 2
SVs in sample	221
Oscillating CN (2 and 3 states)	5, 7
CN segments	13
FDR fragment joints	0.7425546
FDR chr. breakp. enrich.	0.51
Linked to chrs	
Purity, ploidy	0.7, 3.19

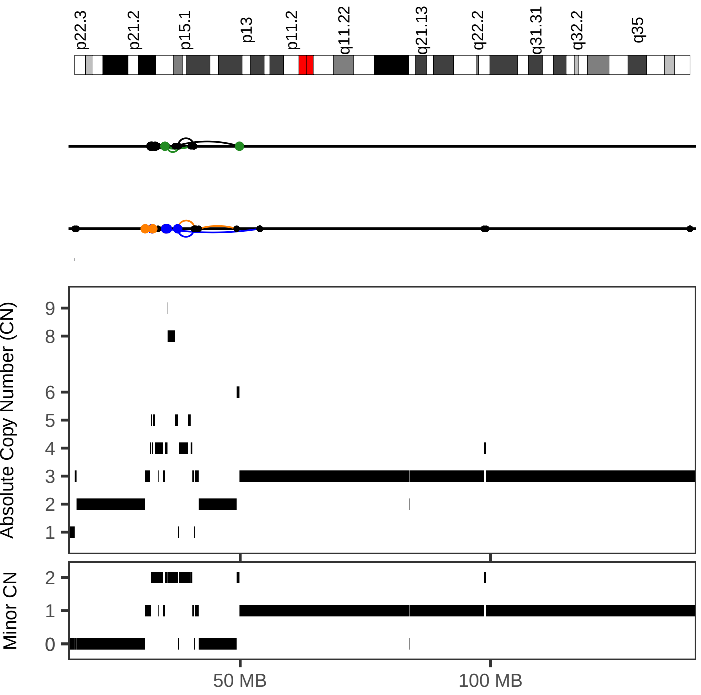

	5ffa2ec6−2d94−4b09−8fb9−3591cf38fa4f
Cancer type	Uterus−AdenoCA
Position	7:33023335−53917686
Type	With other complex events
Interleaved intrachr. SVs	13
Total SVs (intrachr. + transl.)	20
SV types	DEL: 3; DUP: 3; h2hINV: 2; t2tINV: 5; TRA: 7
SVs in sample	137
Oscillating CN (2 and 3 states)	5, 5
CN segments	24
FDR fragment joints	0.9109641
FDR chr. breakp. enrich.	0
Linked to chrs	
Purity, ploidy	0.79, 3.24

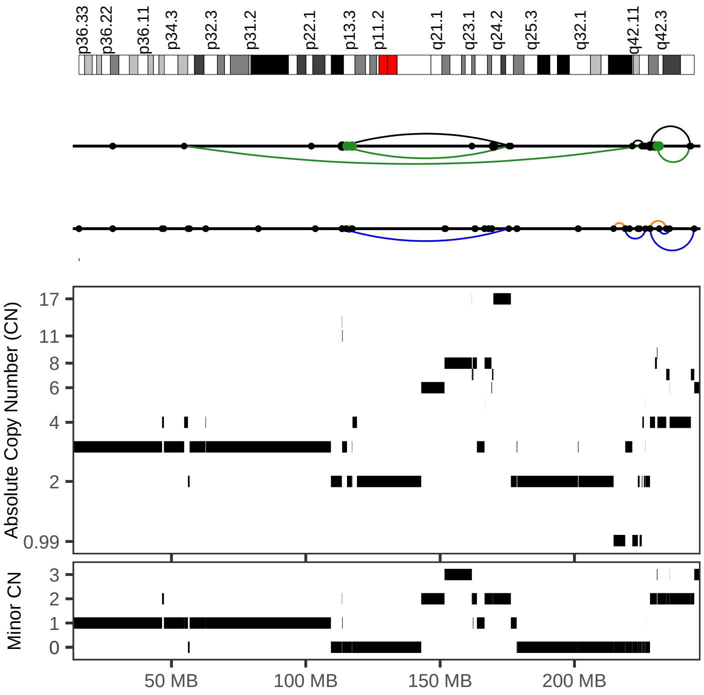

	748e38b1−2ead−4a0a−8881−d640617b856b
Cancer type	Uterus−AdenoCA
Position	1:54723498−244586486
Type	With other complex events
Interleaved intrachr. SVs	11
Total SVs (intrachr. + transl.)	19
SV types	DEL: 2; DUP: 3; h2hINV: 3; t2tINV: 3; TRA: 8
SVs in sample	409
Oscillating CN (2 and 3 states)	5, 6
CN segments	54
FDR fragment joints	0.615458
FDR chr. breakp. enrich.	0.07
Linked to chrs	
Purity, ploidy	0.38, 3.43

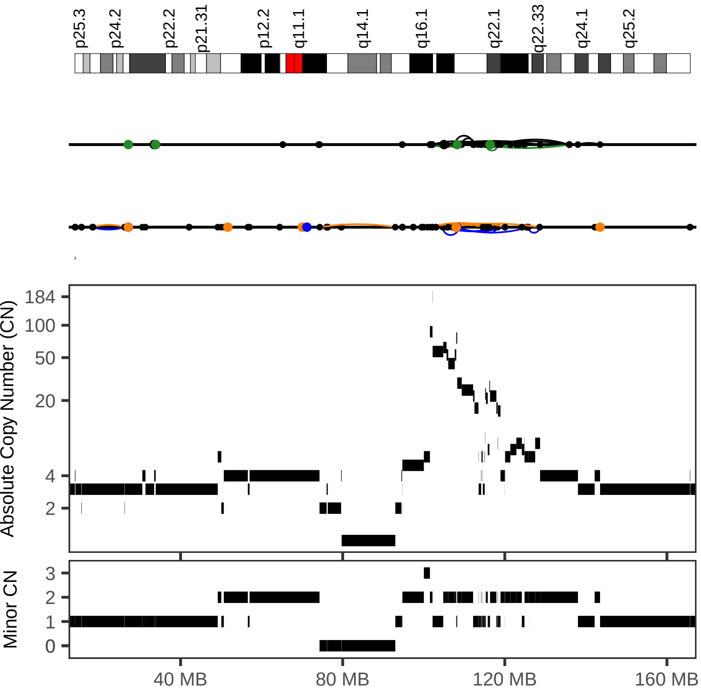

	748e38b1−2ead−4a0a−8881−d640617b856b
Cancer type	Uterus−AdenoCA
Position	6:101518828−135926443
Type	With other complex events
Interleaved intrachr. SVs	29
Total SVs (intrachr. + transl.)	35
SV types	DEL: 5; DUP: 8; h2hINV: 8; t2tINV: 8; TRA: 6
SVs in sample	409
Oscillating CN (2 and 3 states)	4, 5
CN segments	41
FDR fragment joints	0.9425269
FDR chr. breakp. enrich.	0
Linked to chrs	17:6570159−79796837;
Purity, ploidy	0.38, 3.43

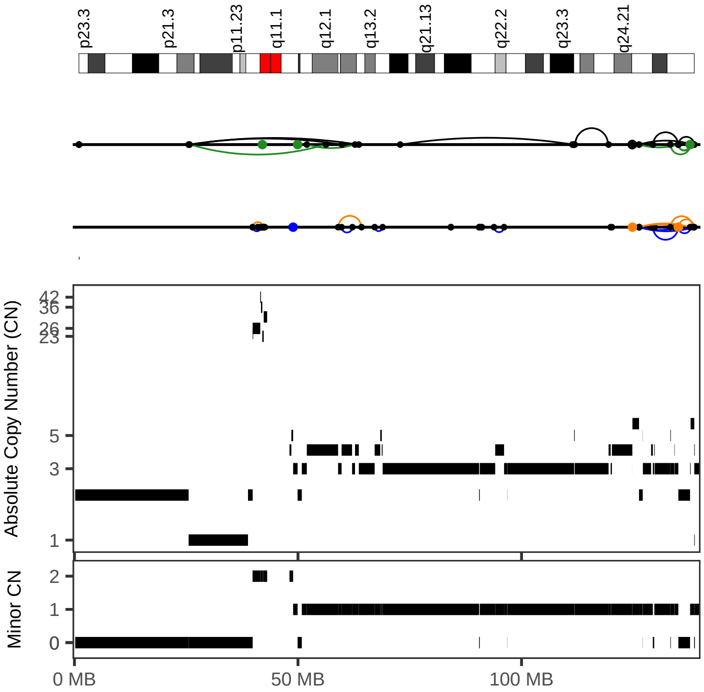

	748e38b1−2ead−4a0a−8881−d640617b856b
Cancer type	Uterus−AdenoCA
Position	8:126280423−138629562
Type	With other complex events
Interleaved intrachr. SVs	17
Total SVs (intrachr. + transl.)	19
SV types	DEL: 4; DUP: 5; h2hINV: 3; t2tINV: 5; TRA: 2
SVs in sample	409
Oscillating CN (2 and 3 states)	5, 11
CN segments	16
FDR fragment joints	0.9958447
FDR chr. breakp. enrich.	0
Linked to chrs	
Purity, ploidy	0.38, 3.43

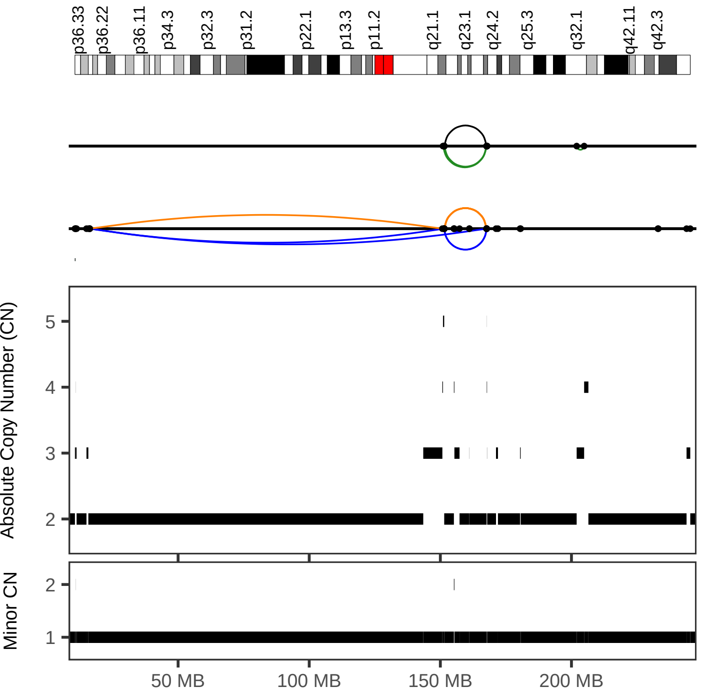

	af37d8f0−fea0−47f6−be0a−2fb55a5da5c4
Cancer type	Uterus−AdenoCA
Position	1:16306658−167711700
Type	With other complex events
Interleaved intrachr. SVs	15
Total SVs (intrachr. + transl.)	15
SV types	DEL: 4; DUP: 4; h2hINV: 4; t2tINV: 3; TRA: 0
SVs in sample	315
Oscillating CN (2 and 3 states)	5, 6
CN segments	15
FDR fragment joints	0.9905774
FDR chr. breakp. enrich.	0.54
Linked to chrs	
Purity, ploidy	0.89, 1.95

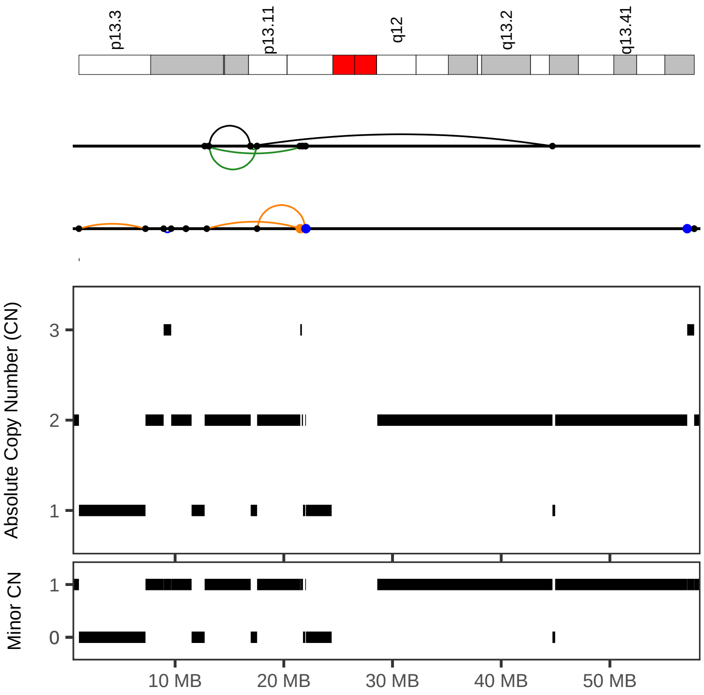

	b60f22ac−a659−4f33−b01d−820e86a9a5c9
Cancer type	Uterus−AdenoCA
Position	19:12718468−44715346
Type	Canonical without polyploidization
Interleaved intrachr. SVs	9
Total SVs (intrachr. + transl.)	11
SV types	DEL: 2; DUP: 0; h2hINV: 4; t2tINV: 3; TRA: 2
SVs in sample	353
Oscillating CN (2 and 3 states)	6, 10
CN segments	10
FDR fragment joints	0.7425546
FDR chr. breakp. enrich.	0.02
Linked to chrs	
Purity, ploidy	0.84, 1.87

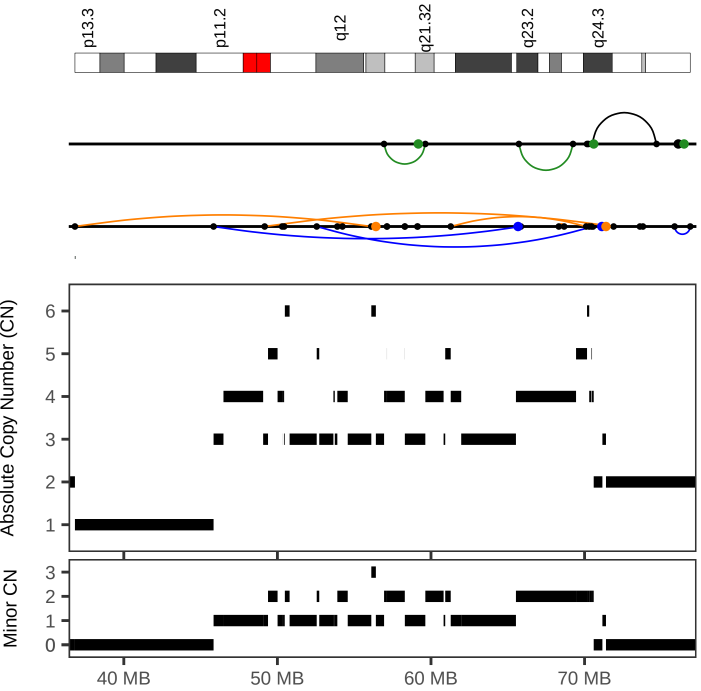

	bad34c92−1e64−4187−a0b4−061105fd13ba
Cancer type	Uterus−AdenoCA
Position	17:36816085−74685847
Type	With other complex events
Interleaved intrachr. SVs	7
Total SVs (intrachr. + transl.)	13
SV types	DEL: 3; DUP: 2; h2hINV: 1; t2tINV: 1; TRA: 6
SVs in sample	315
Oscillating CN (2 and 3 states)	5, 9
CN segments	38
FDR fragment joints	0.6776251
FDR chr. breakp. enrich.	0
Linked to chrs	
Purity, ploidy	0.78, 2.01

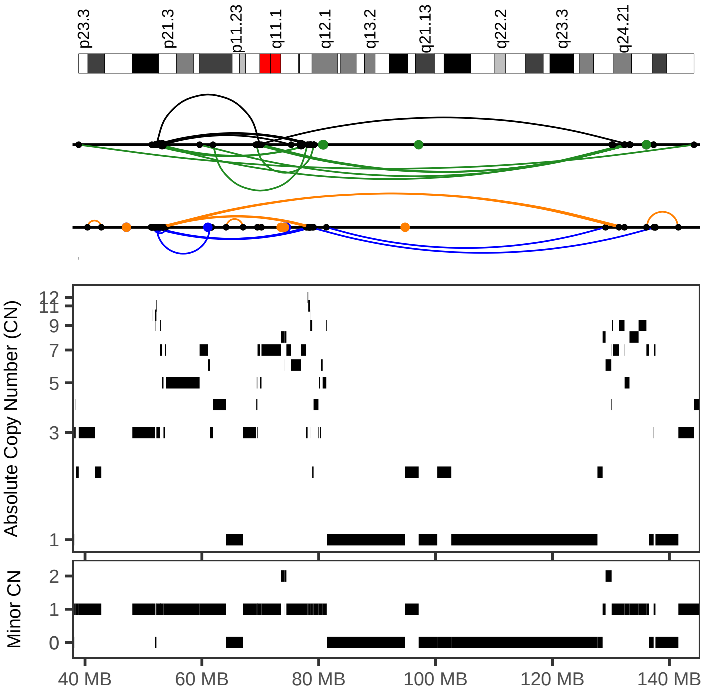

	c88e3901−f3ee−436a−ba80−143c8eab7d69
Cancer type	Uterus−AdenoCA
Position	8:51305779−141554762
Type	With other complex events
Interleaved intrachr. SVs	31
Total SVs (intrachr. + transl.)	45
SV types	DEL: 6; DUP: 8; h2hINV: 8; t2tINV: 9; TRA: 14
SVs in sample	382
Oscillating CN (2 and 3 states)	6, 7
CN segments	78
FDR fragment joints	0.9161301
FDR chr. breakp. enrich.	0
Linked to chrs	16:46736458−70526638;7:44704759−148485338
Purity, ploidy	0.96, 1.98
