# Supplementary material for: Comprehensive analysis of chromothripsis in 2,658 human cancers using whole-genome sequencing
Source: Nat Genet. 2020 Feb 5;52(3):331–41. doi: 10.1038/s41588-019-0576-7 (PMC7058534; doi:10.1038/s41588-019-0576-7)
Supplement: Supplementary file 8 — Regions displaying CN oscillations not classified as chromothripsis. These include: (1) CN oscillating profiles characterized by clusters of tandem duplications or deletions, (2) candidate chromothripsis cases satisfying the statistical criteria but considered false positives by visual inspection, and (3) chromosomes displaying at least 7 CN oscillations with few or no SVs mapped. [file 41588_2019_576_MOESM8_ESM.pdf]

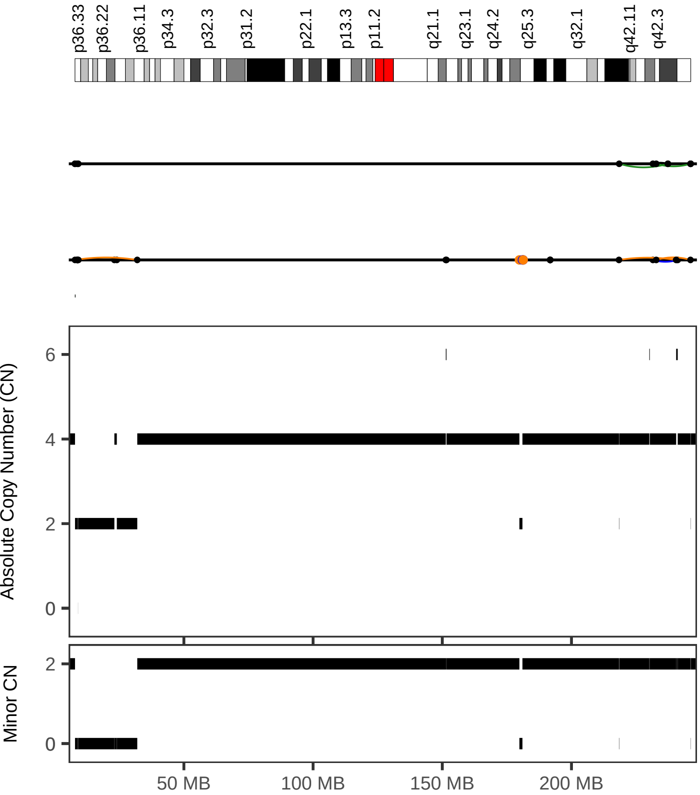

BTCA_donor_A153

Cancer type	Biliary−AdenoCA
Position	1:218376406−246159643
Interleaved intrachr. SVs	7
Total SVs (intrachr. + transl.)	7
SV types	DEL: 2; DUP: 1; h2hINV: 2; t2tINV: 2; TRA: 0
SVs in sample	116
Oscillating CN (2 and 3 states)	5, 8
CN segments	8
FDR fragment joints	0.9599662
FDR chr. breakp. enrich.	0
Linked to chrs	
Purity, ploidy	0.42, 3.59

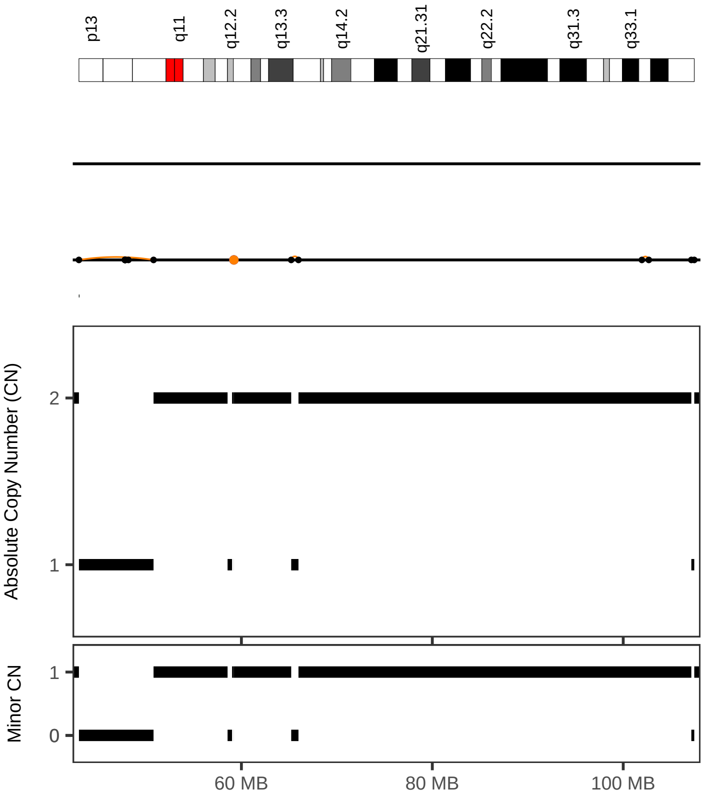

096b4f32−10c1−4737−a0dd−cae04c54ee33

Cancer type	Bladder−TCC
Position	13:42975600−107442606
Interleaved intrachr. SVs	1
Total SVs (intrachr. + transl.)	8
SV types	DEL: 6; DUP: 0; h2hINV: 0; t2tINV: 0; TRA: 2
SVs in sample	136
Oscillating CN (2 and 3 states)	10, 10
CN segments	10
FDR fragment joints	0.05741733
FDR chr. breakp. enrich.	0.17
Linked to chrs	
Purity, ploidy	0.74, 1.77

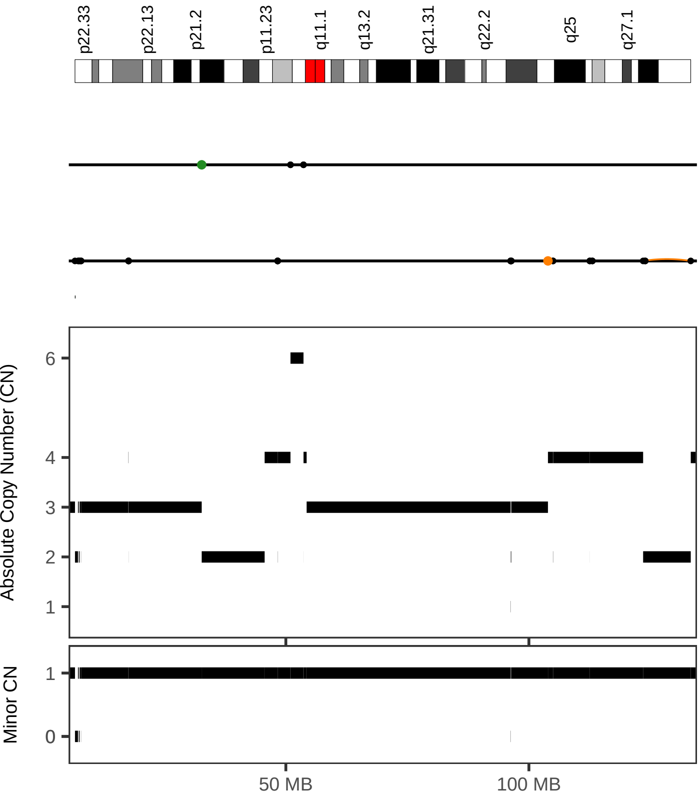

24f21425−b001−4986−aedf−5b4dd851c6ad

Cancer type	Bladder−TCC
Position	X:6615453−133296546
Interleaved intrachr. SVs	1
Total SVs (intrachr. + transl.)	13
SV types	DEL: 8; DUP: 3; h2hINV: 0; t2tINV: 0; TRA: 2
SVs in sample	271
Oscillating CN (2 and 3 states)	7, 9
CN segments	30
FDR fragment joints	0.1013295
FDR chr. breakp. enrich.	0.26
Linked to chrs	
Purity, ploidy	0.64, 3.43

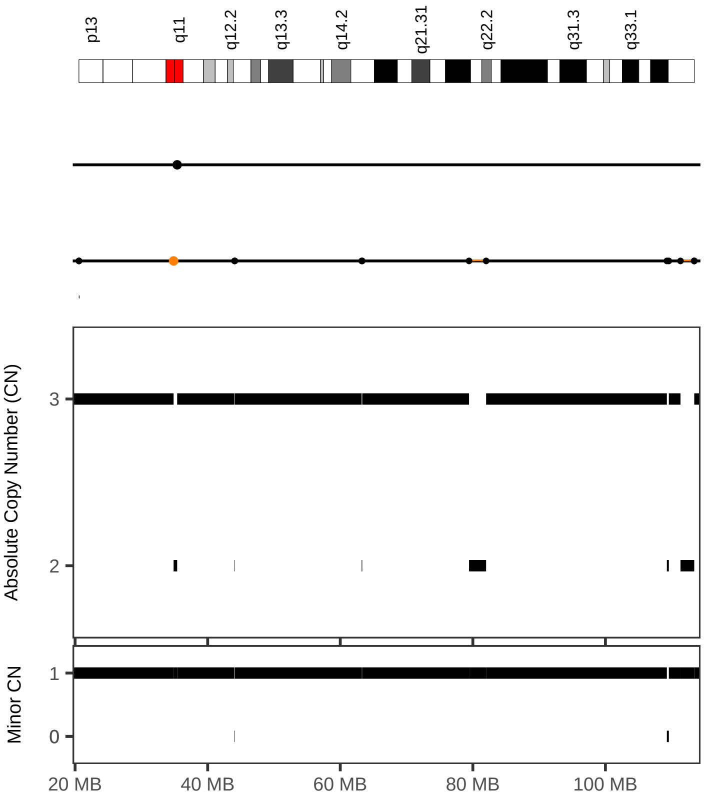

419deaac−ea45−4bdd−9fa0−b5cd8429b44f

Cancer type	Bladder−TCC
Position	13:20578507−113387449
Interleaved intrachr. SVs	1
Total SVs (intrachr. + transl.)	8
SV types	DEL: 6; DUP: 0; h2hINV: 0; t2tINV: 0; TRA: 2
SVs in sample	62
Oscillating CN (2 and 3 states)	12, 12
CN segments	12
FDR fragment joints	0.05741733
FDR chr. breakp. enrich.	0
Linked to chrs	
Purity, ploidy	0.66, 1.96

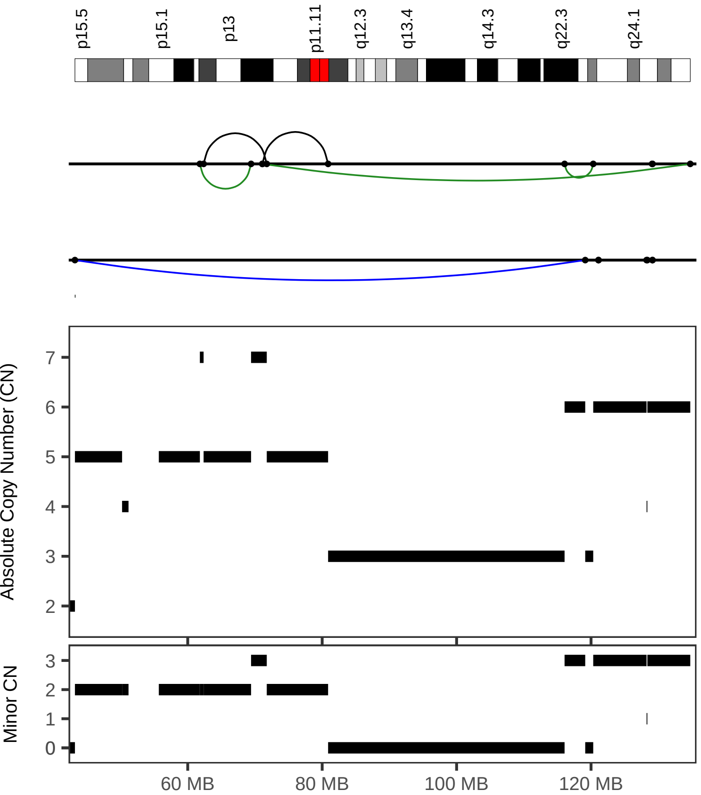

556fcbc8−172a−4af1−8822−ae036e8d68e8

Cancer type	Bladder−TCC
Position	11:43215596−134760626
Interleaved intrachr. SVs	6
Total SVs (intrachr. + transl.)	6
SV types	DEL: 0; DUP: 1; h2hINV: 2; t2tINV: 3; TRA: 0
SVs in sample	207
Oscillating CN (2 and 3 states)	5, 7
CN segments	13
FDR fragment joints	0.615458
FDR chr. breakp. enrich.	1
Linked to chrs	
Purity, ploidy	0.58, 3.53

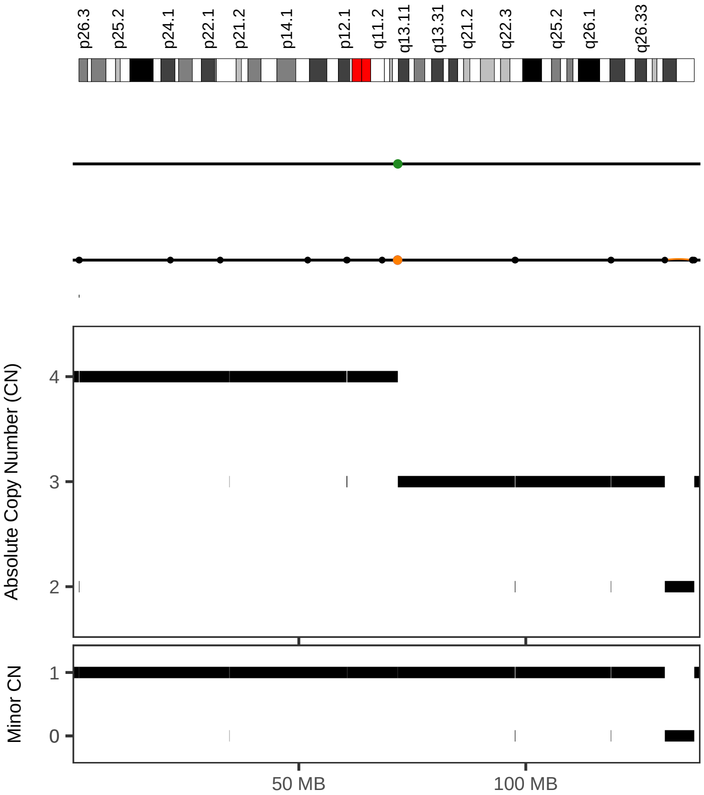

904a8757−e0c5−41ef−b583−c8f170caaac1

Cancer type	Bladder−TCC
Position	3:1555799−137115914
Interleaved intrachr. SVs	1
Total SVs (intrachr. + transl.)	10
SV types	DEL: 7; DUP: 0; h2hINV: 0; t2tINV: 0; TRA: 3
SVs in sample	274
Oscillating CN (2 and 3 states)	7, 7
CN segments	13
FDR fragment joints	0.0726553
FDR chr. breakp. enrich.	0.36
Linked to chrs	
Purity, ploidy	0.6, 2.92

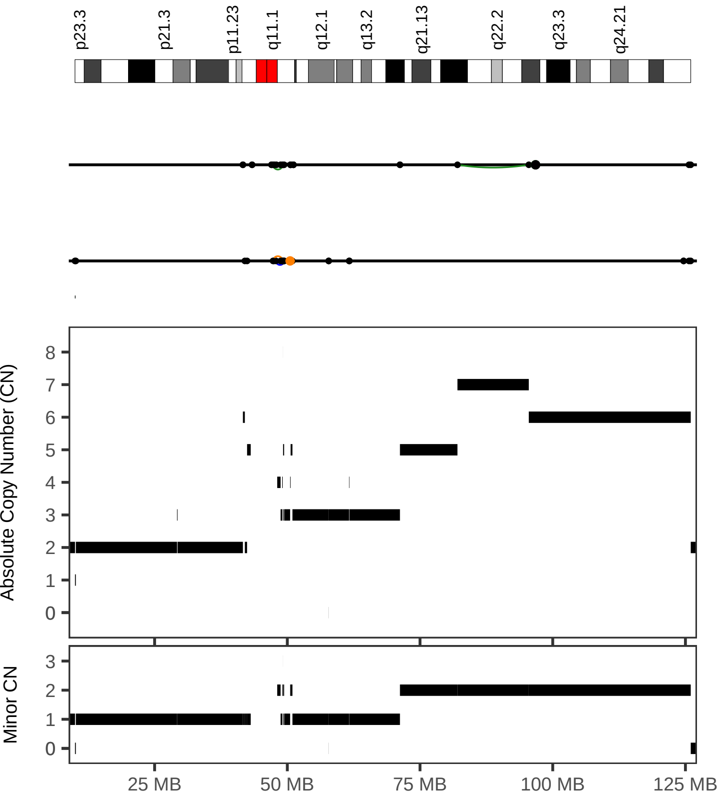

cb664eef−be6f−44ef−aa90−6f6da0e6440a

Cancer type	Bladder−TCC
Position	8:47026265−51150625
Interleaved intrachr. SVs	15
Total SVs (intrachr. + transl.)	16
SV types	DEL: 3; DUP: 3; h2hINV: 4; t2tINV: 5; TRA: 1
SVs in sample	180
Oscillating CN (2 and 3 states)	4, 4
CN segments	12
FDR fragment joints	0.9501265
FDR chr. breakp. enrich.	0
Linked to chrs	
Purity, ploidy	0.51, 3.57

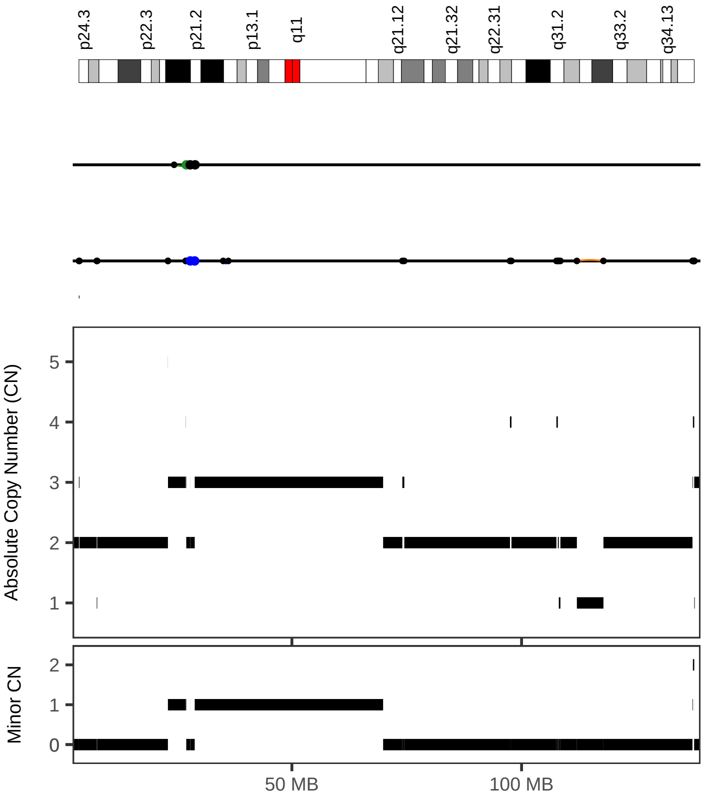

CGP_donor_1437403

Cancer type	Bone−Osteosarc
Position	9:3641509−137589235
Interleaved intrachr. SVs	1
Total SVs (intrachr. + transl.)	18
SV types	DEL: 3; DUP: 9; h2hINV: 0; t2tINV: 1; TRA: 5
SVs in sample	445
Oscillating CN (2 and 3 states)	8, 10
CN segments	28
FDR fragment joints	0.1845613
FDR chr. breakp. enrich.	0.84
Linked to chrs	
Purity, ploidy	0.8, 2.57

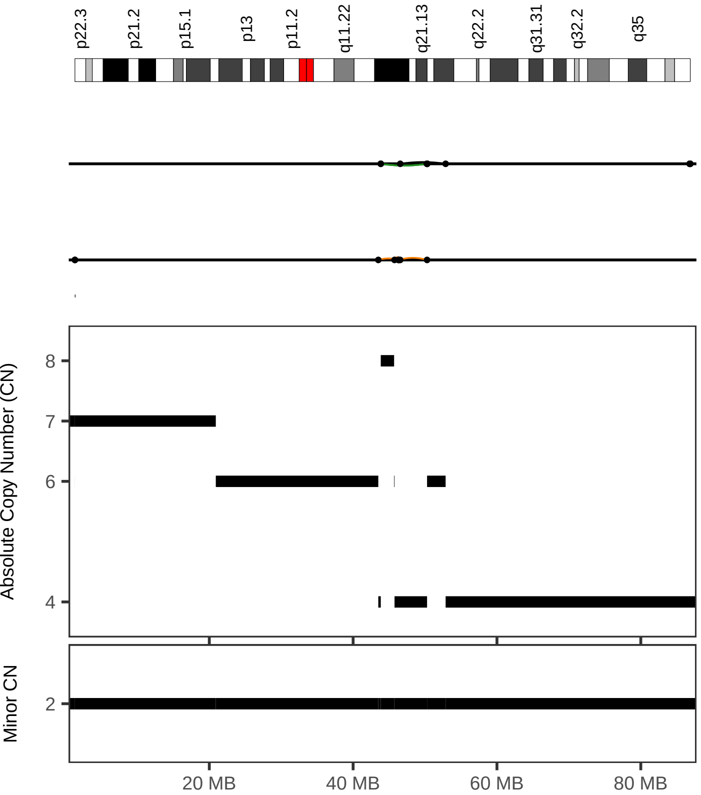

CGP_donor_1475256

Cancer type	Bone−Osteosarc
Position	7:43498754−52862136
Interleaved intrachr. SVs	6
Total SVs (intrachr. + transl.)	6
SV types	DEL: 3; DUP: 0; h2hINV: 1; t2tINV: 2; TRA: 0
SVs in sample	217
Oscillating CN (2 and 3 states)	4, 4
CN segments	6
FDR fragment joints	0.615458
FDR chr. breakp. enrich.	1
Linked to chrs	
Purity, ploidy	0.79, 3.21

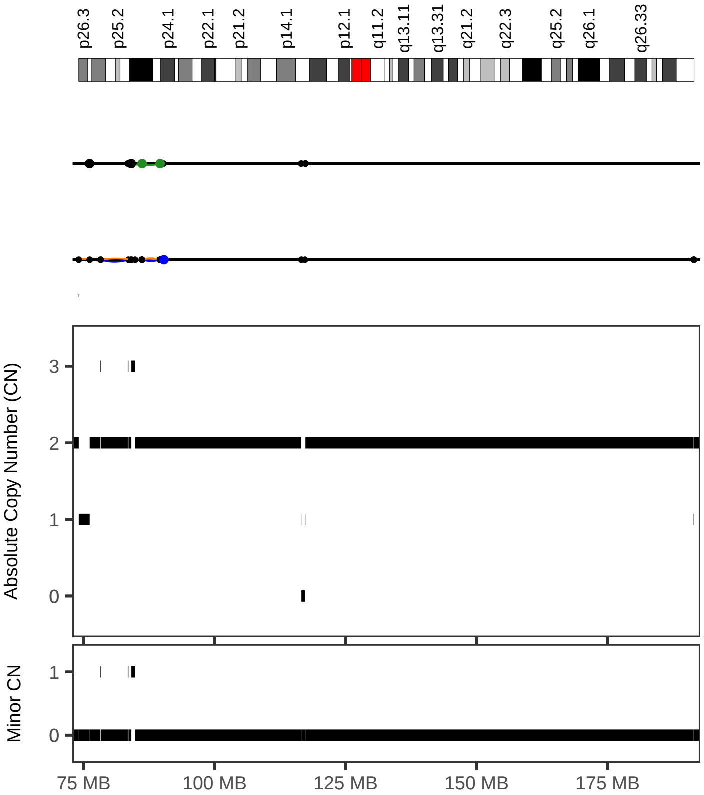

CGP_donor_1691147

Cancer type	Bone−Osteosarc
Position	3:78199279−90320009
Interleaved intrachr. SVs	5
Total SVs (intrachr. + transl.)	9
SV types	DEL: 3; DUP: 1; h2hINV: 0; t2tINV: 1; TRA: 4
SVs in sample	279
Oscillating CN (2 and 3 states)	8, 8
CN segments	8
FDR fragment joints	0.9723381
FDR chr. breakp. enrich.	0.9
Linked to chrs	18:2636202−13923207;
Purity, ploidy	0.72, 2.91

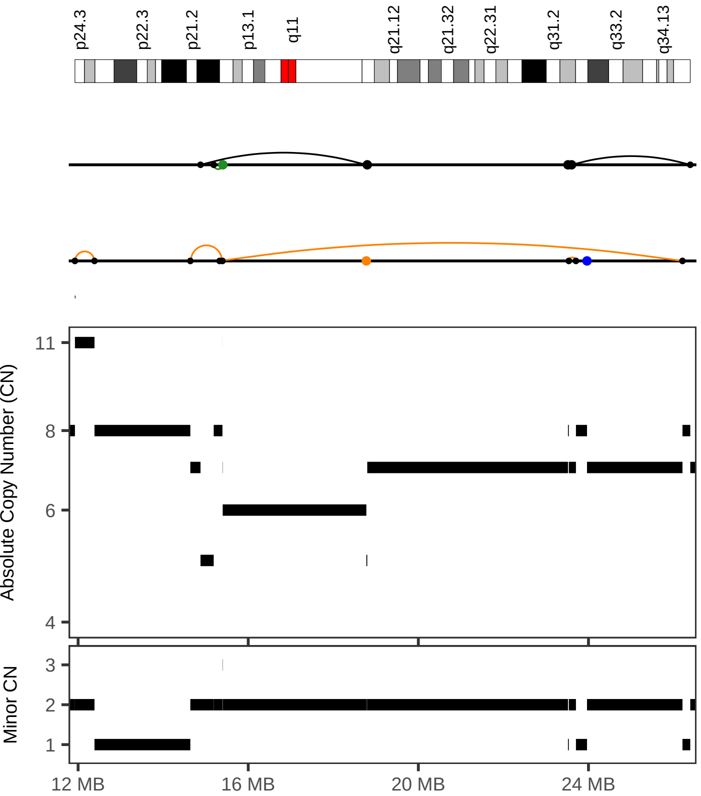

CGP_donor_1691212

Cancer type	Bone−Osteosarc
Position	9:14638768−26392657
Interleaved intrachr. SVs	6
Total SVs (intrachr. + transl.)	12
SV types	DEL: 3; DUP: 0; h2hINV: 2; t2tINV: 1; TRA: 6
SVs in sample	135
Oscillating CN (2 and 3 states)	7, 7
CN segments	14
FDR fragment joints	0.6776251
FDR chr. breakp. enrich.	0
Linked to chrs	21:25493177−41098739;
Purity, ploidy	0.74, 4

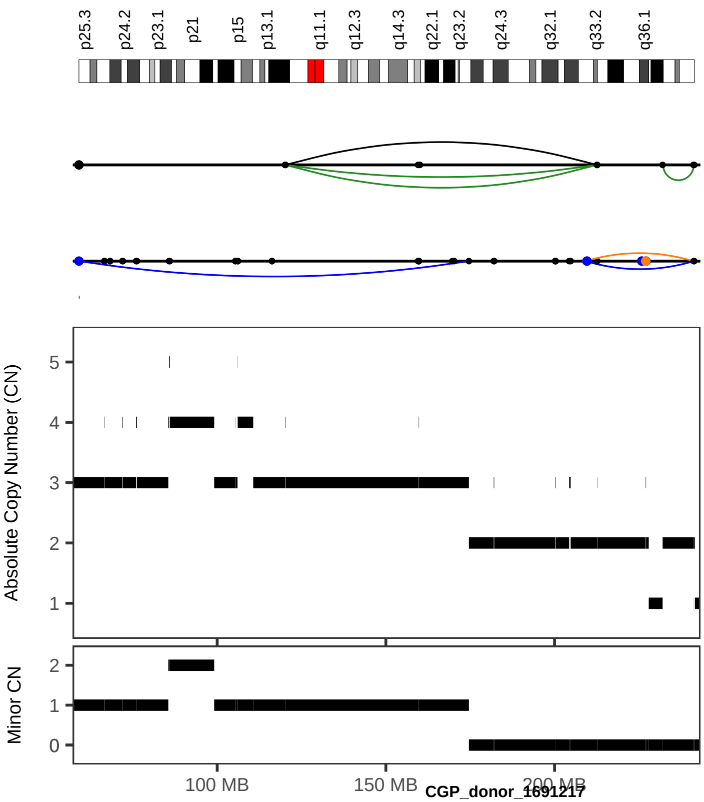

CGP_donor_1691217

Cancer type	Bone−Osteosarc
Position	2:59017074−241406602
Interleaved intrachr. SVs	9
Total SVs (intrachr. + transl.)	17
SV types	DEL: 1; DUP: 3; h2hINV: 2; t2tINV: 3; TRA: 8
SVs in sample	380
Oscillating CN (2 and 3 states)	14, 40
CN segments	40
FDR fragment joints	0.615458
FDR chr. breakp. enrich.	0.9
Linked to chrs	11:819052−86320877;19:43092652−56632953
Purity, ploidy	0.53, 2.89

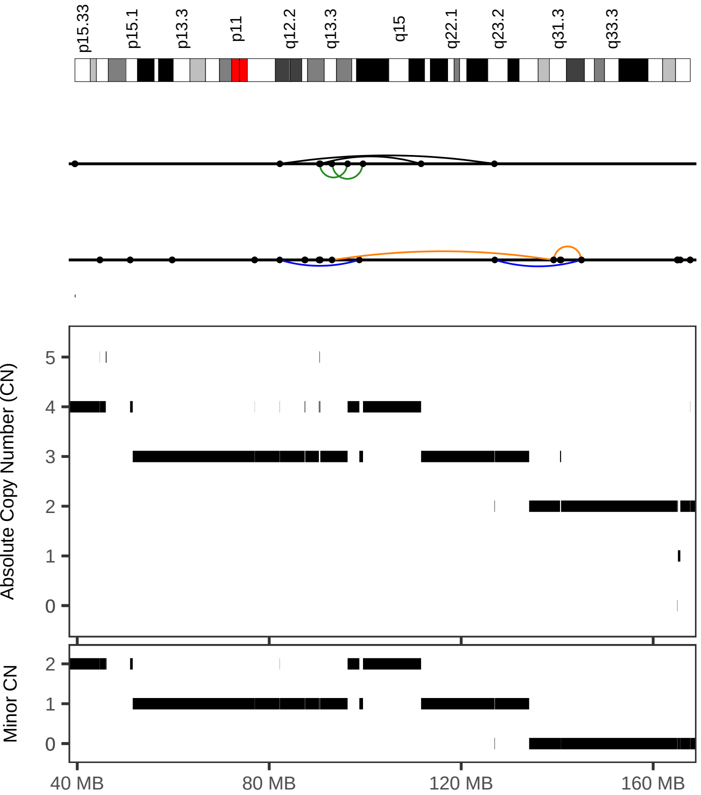

CGP_donor_1691217

Cancer type	Bone−Osteosarc
Position	5:82186991−145068339
Interleaved intrachr. SVs	8
Total SVs (intrachr. + transl.)	8
SV types	DEL: 1; DUP: 3; h2hINV: 2; t2tINV: 2; TRA: 0
SVs in sample	380
Oscillating CN (2 and 3 states)	6, 17
CN segments	17
FDR fragment joints	0.8653243
FDR chr. breakp. enrich.	0.87
Linked to chrs	
Purity, ploidy	0.53, 2.89

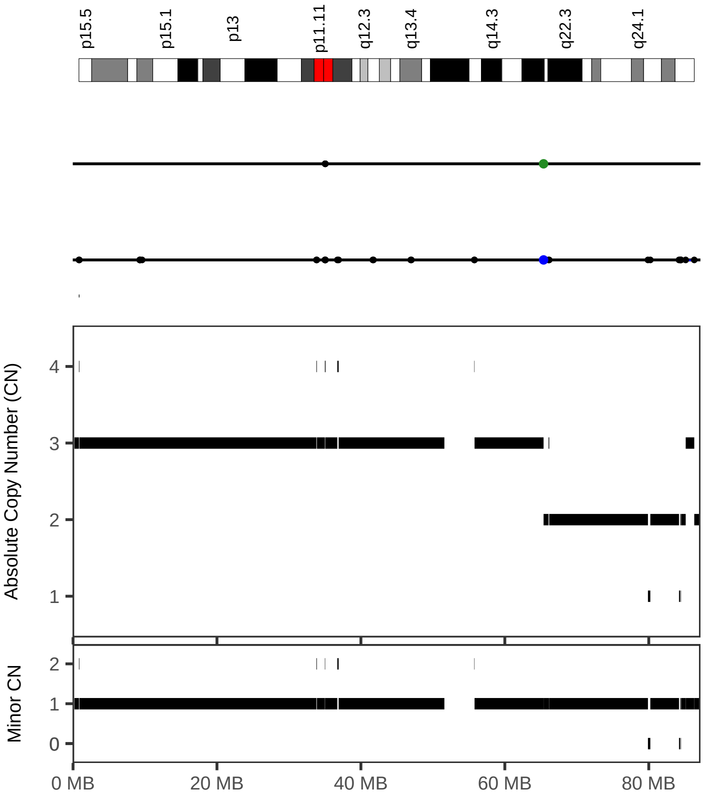

CGP_donor_1691217

Cancer type	Bone−Osteosarc
Position	11:819052−86320878
Interleaved intrachr. SVs	1
Total SVs (intrachr. + transl.)	16
SV types	DEL: 3; DUP: 11; h2hINV: 0; t2tINV: 0; TRA: 2
SVs in sample	380
Oscillating CN (2 and 3 states)	14, 25
CN segments	25
FDR fragment joints	0.01147689
FDR chr. breakp. enrich.	0.9
Linked to chrs	2:59017074−241406601;
Purity, ploidy	0.53, 2.89

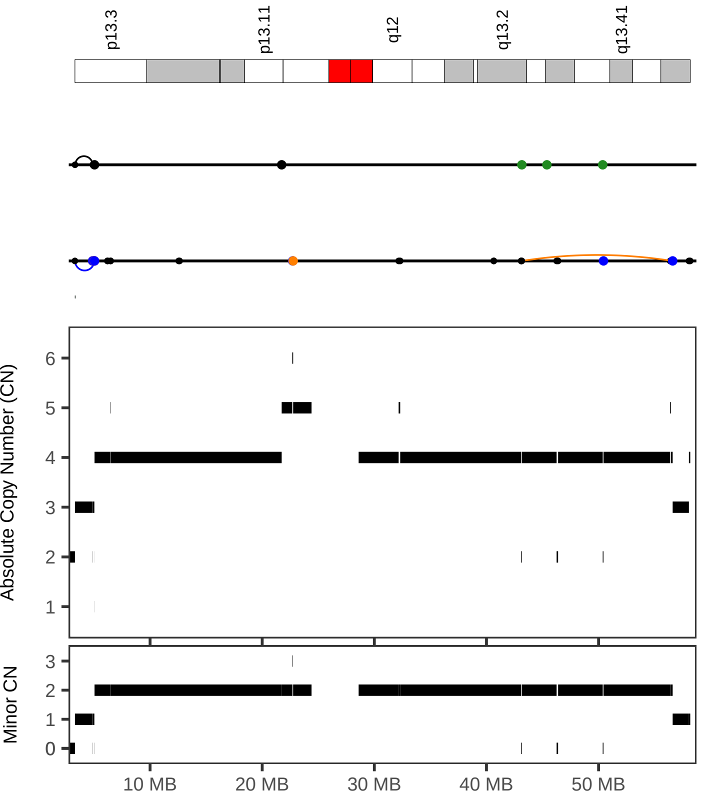

CGP_donor_1691217

Cancer type	Bone−Osteosarc
Position	19:43092652−56632954
Interleaved intrachr. SVs	3
Total SVs (intrachr. + transl.)	8
SV types	DEL: 2; DUP: 0; h2hINV: 0; t2tINV: 1; TRA: 5
SVs in sample	380
Oscillating CN (2 and 3 states)	7, 7
CN segments	10
FDR fragment joints	0.8653243
FDR chr. breakp. enrich.	0
Linked to chrs	17:461115−73256982;
Purity, ploidy	0.53, 2.89

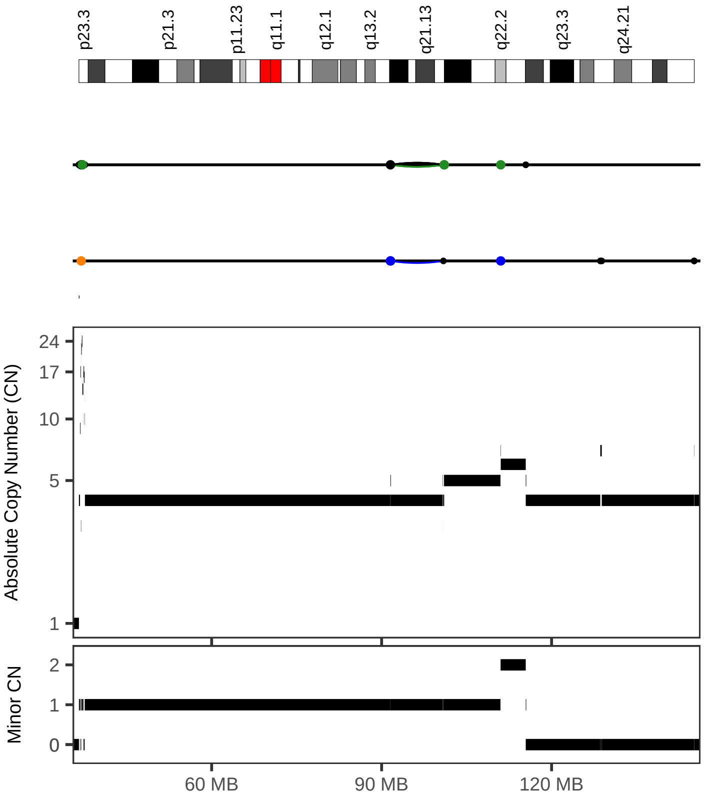

CGP_donor_1232859

Cancer type	Breast−AdenoCA
Position	8:91550520−101047671
Interleaved intrachr. SVs	8
Total SVs (intrachr. + transl.)	11
SV types	DEL: 0; DUP: 1; h2hINV: 3; t2tINV: 4; TRA: 3
SVs in sample	316
Oscillating CN (2 and 3 states)	7, 12
CN segments	12
FDR fragment joints	0.615458
FDR chr. breakp. enrich.	0
Linked to chrs	
Purity, ploidy	0.54, 2.17

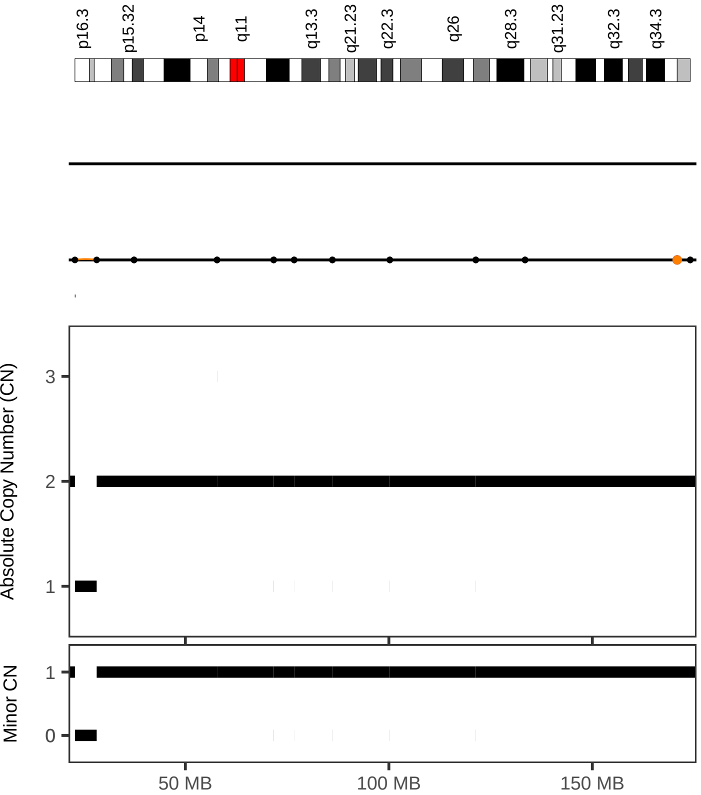

CGP_donor_1333048

Cancer type	Breast−AdenoCA
Position	4:22856554−121362620
Interleaved intrachr. SVs	1
Total SVs (intrachr. + transl.)	6
SV types	DEL: 6; DUP: 0; h2hINV: 0; t2tINV: 0; TRA: 0
SVs in sample	178
Oscillating CN (2 and 3 states)	11, 14
CN segments	14
FDR fragment joints	0.04139017
FDR chr. breakp. enrich.	1
Linked to chrs	
Purity, ploidy	0.54, 2.02

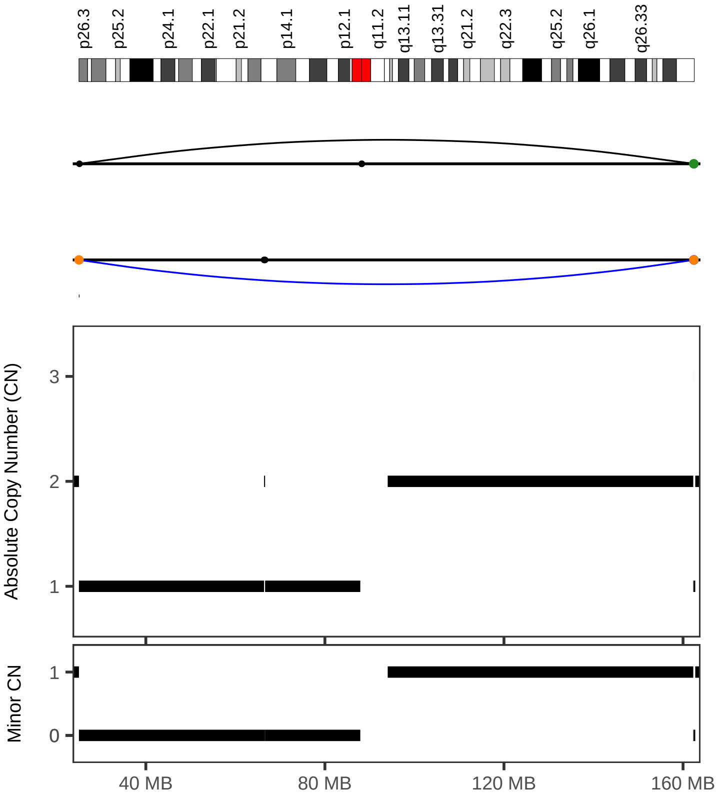

CGP_donor_1347742

Cancer type	Breast−AdenoCA
Position	3:25050231−162503837
Interleaved intrachr. SVs	7
Total SVs (intrachr. + transl.)	13
SV types	DEL: 3; DUP: 3; h2hINV: 1; t2tINV: 0; TRA: 6
SVs in sample	230
Oscillating CN (2 and 3 states)	9, 11
CN segments	11
FDR fragment joints	0.615458
FDR chr. breakp. enrich.	1
Linked to chrs	8:31533581−34500723;
Purity, ploidy	0.52, 2.12

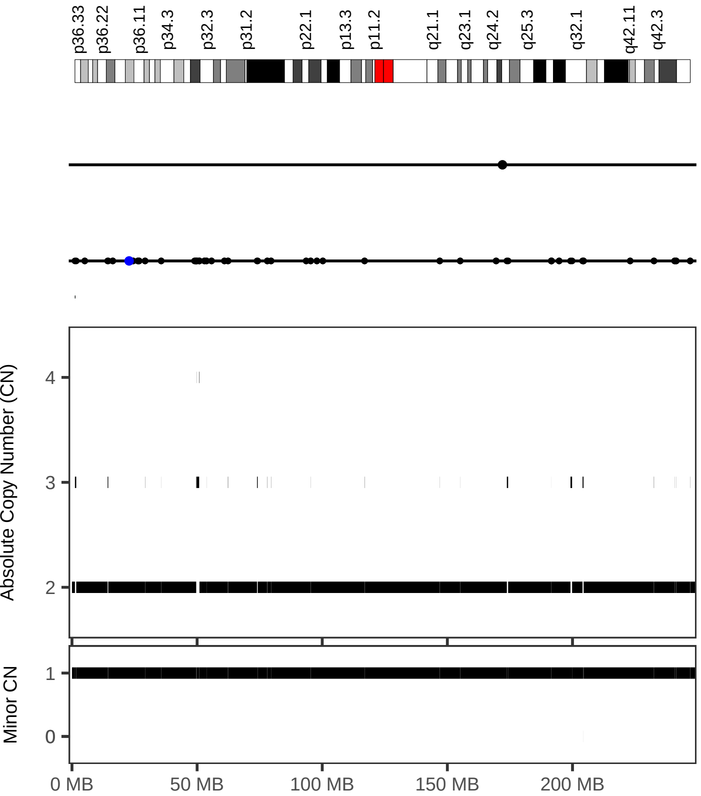

CGP_donor_1347751

Cancer type	Breast−AdenoCA
Position	1:1174539−247054567
Interleaved intrachr. SVs	1
Total SVs (intrachr. + transl.)	37
SV types	DEL: 2; DUP: 33; h2hINV: 0; t2tINV: 0; TRA: 2
SVs in sample	828
Oscillating CN (2 and 3 states)	38, 50
CN segments	50
FDR fragment joints	1.195208e−14
FDR chr. breakp. enrich.	0.03
Linked to chrs	
Purity, ploidy	0.9, 1.96

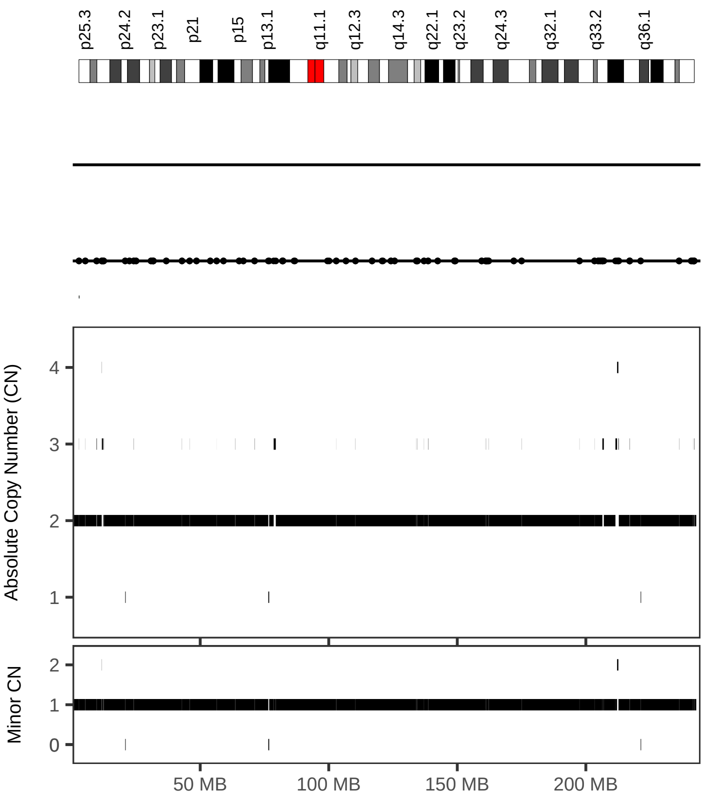

CGP_donor_1347751

Cancer type	Breast−AdenoCA
Position	2:11684173−217099835
Interleaved intrachr. SVs	2
Total SVs (intrachr. + transl.)	6
SV types	DEL: 0; DUP: 6; h2hINV: 0; t2tINV: 0; TRA: 0
SVs in sample	828
Oscillating CN (2 and 3 states)	30, 56
CN segments	56
FDR fragment joints	0.04139017
FDR chr. breakp. enrich.	0.9
Linked to chrs	
Purity, ploidy	0.9, 1.96

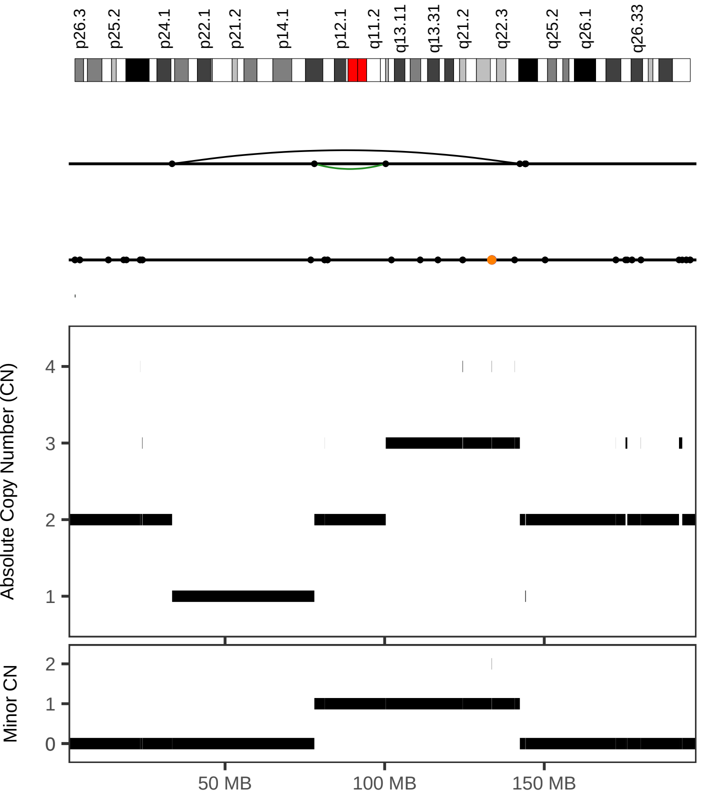

CGP_donor_1347751

Cancer type	Breast−AdenoCA
Position	3:2964392−195733210
Interleaved intrachr. SVs	1
Total SVs (intrachr. + transl.)	21
SV types	DEL: 1; DUP: 15; h2hINV: 1; t2tINV: 2; TRA: 2
SVs in sample	828
Oscillating CN (2 and 3 states)	9, 25
CN segments	27
FDR fragment joints	0.0009494935
FDR chr. breakp. enrich.	0
Linked to chrs	
Purity, ploidy	0.9, 1.96

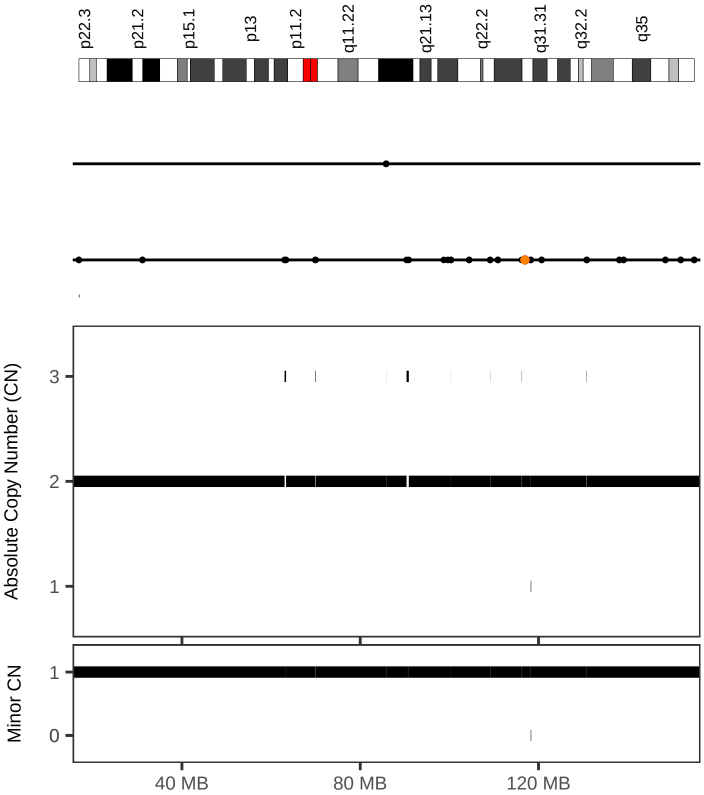

CGP_donor_1347751

Cancer type	Breast−AdenoCA
Position	7:63018209−154939154
Interleaved intrachr. SVs	1
Total SVs (intrachr. + transl.)	16
SV types	DEL: 0; DUP: 13; h2hINV: 0; t2tINV: 1; TRA: 2
SVs in sample	828
Oscillating CN (2 and 3 states)	16, 20
CN segments	20
FDR fragment joints	0.0002315962
FDR chr. breakp. enrich.	0
Linked to chrs	
Purity, ploidy	0.9, 1.96

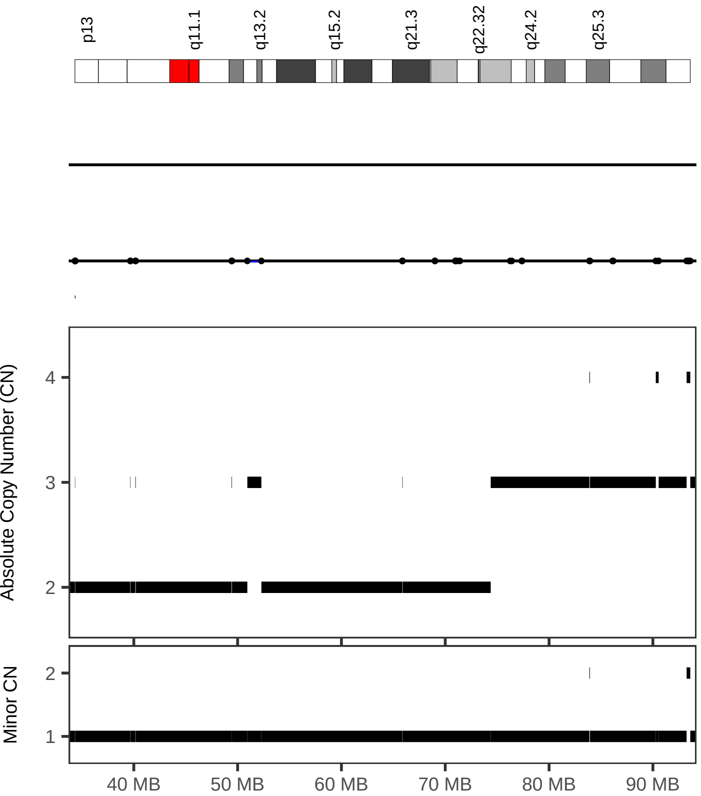

CGP_donor_1347751

Cancer type	Breast−AdenoCA
Position	15:34326314−93601501
Interleaved intrachr. SVs	1
Total SVs (intrachr. + transl.)	14
SV types	DEL: 0; DUP: 14; h2hINV: 0; t2tINV: 0; TRA: 0
SVs in sample	828
Oscillating CN (2 and 3 states)	13, 19
CN segments	19
FDR fragment joints	8.073633e−06
FDR chr. breakp. enrich.	0.48
Linked to chrs	
Purity, ploidy	0.9, 1.96

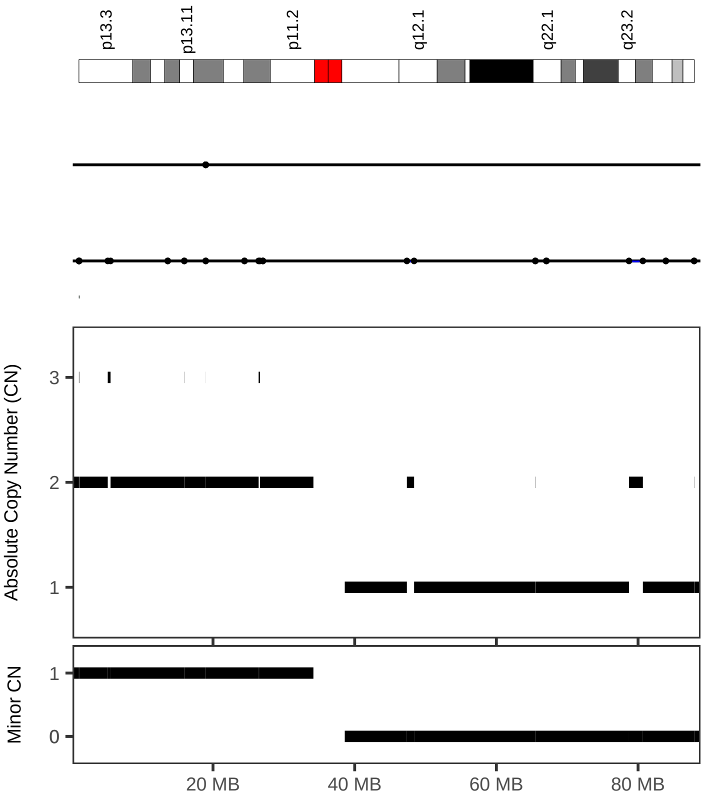

CGP_donor_1347751

Cancer type	Breast−AdenoCA
Position	16:1073804−87928916
Interleaved intrachr. SVs	1
Total SVs (intrachr. + transl.)	10
SV types	DEL: 0; DUP: 9; h2hINV: 1; t2tINV: 0; TRA: 0
SVs in sample	828
Oscillating CN (2 and 3 states)	10, 19
CN segments	19
FDR fragment joints	0.01020027
FDR chr. breakp. enrich.	0.69
Linked to chrs	
Purity, ploidy	0.9, 1.96

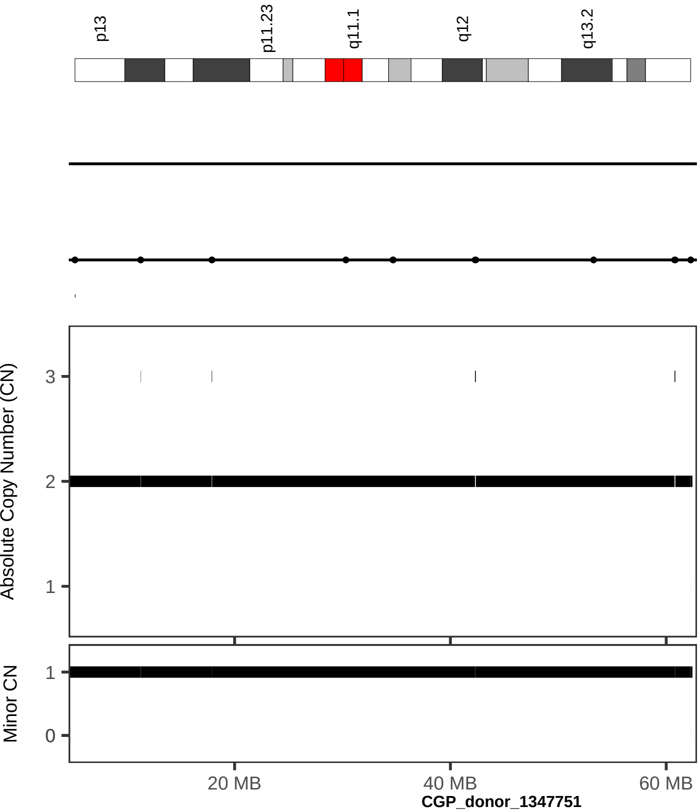

CGP_donor_1347751

Cancer type	Breast−AdenoCA
Position	20:11282201−60847010
Interleaved intrachr. SVs	1
Total SVs (intrachr. + transl.)	5
SV types	DEL: 0; DUP: 5; h2hINV: 0; t2tINV: 0; TRA: 0
SVs in sample	828
Oscillating CN (2 and 3 states)	8, 8
CN segments	8
FDR fragment joints	0.1013295
FDR chr. breakp. enrich.	0.11
Linked to chrs	
Purity, ploidy	0.9, 1.96

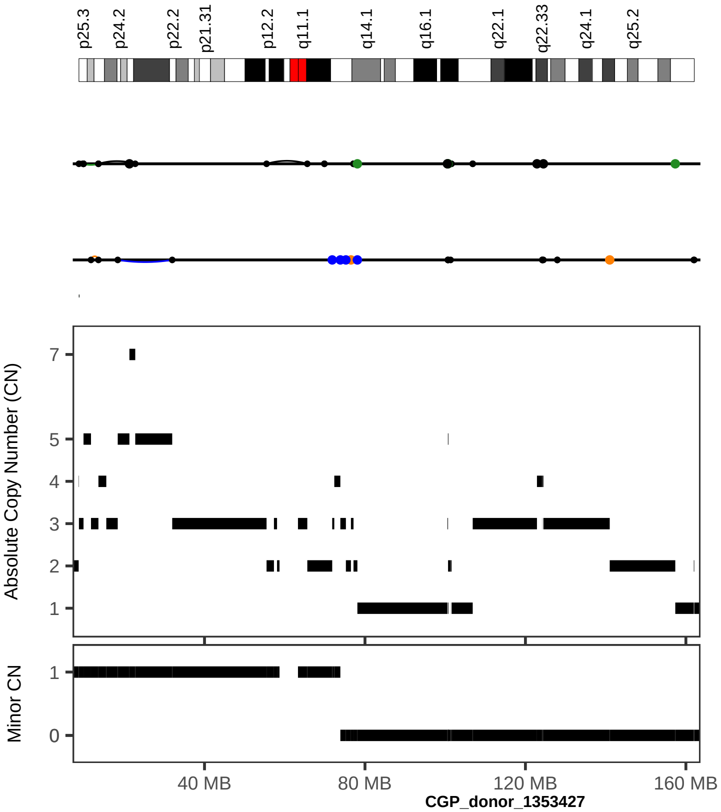

CGP_donor_1353427

Cancer type	Breast−AdenoCA
Position	6:100548524−101591934
Interleaved intrachr. SVs	3
Total SVs (intrachr. + transl.)	9
SV types	DEL: 0; DUP: 2; h2hINV: 2; t2tINV: 2; TRA: 3
SVs in sample	267
Oscillating CN (2 and 3 states)	7, 7
CN segments	11
FDR fragment joints	0.615458
FDR chr. breakp. enrich.	0
Linked to chrs	
Purity, ploidy	0.29, 2.76

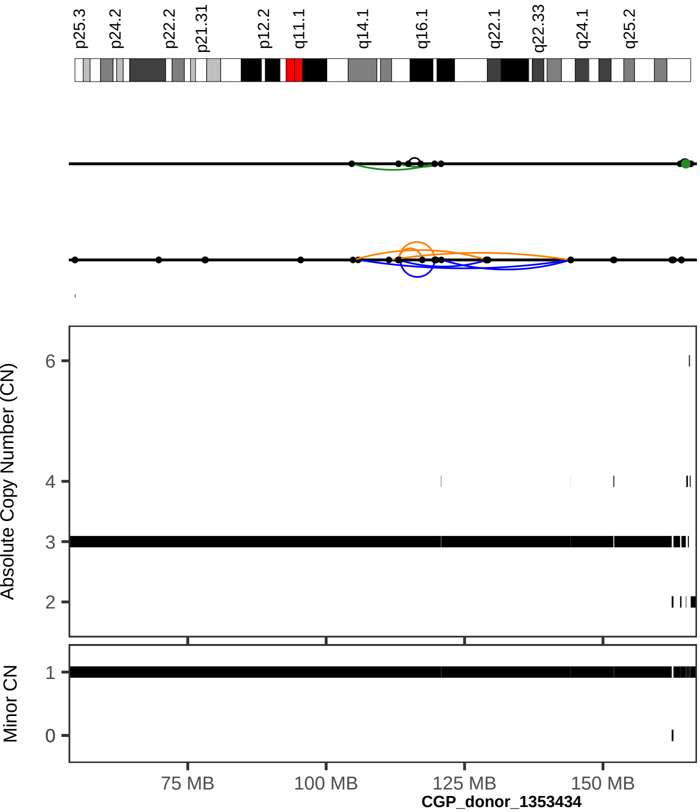

CGP_donor_1353434

Cancer type	Breast−AdenoCA
Position	6:104620681−144195202
Interleaved intrachr. SVs	10
Total SVs (intrachr. + transl.)	10
SV types	DEL: 4; DUP: 4; h2hINV: 0; t2tINV: 2; TRA: 0
SVs in sample	291
Oscillating CN (2 and 3 states)	5, 5
CN segments	5
FDR fragment joints	0.615458
FDR chr. breakp. enrich.	0.01
Linked to chrs	
Purity, ploidy	0.5, 2.94

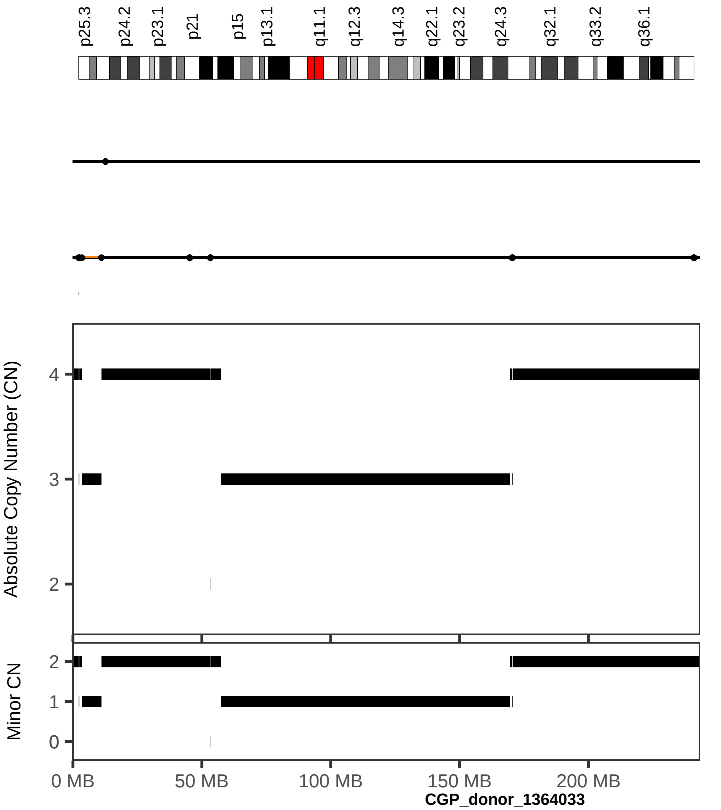

CGP_donor_1364033

Cancer type	Breast−AdenoCA
Position	2:2226914−240877127
Interleaved intrachr. SVs	1
Total SVs (intrachr. + transl.)	5
SV types	DEL: 5; DUP: 0; h2hINV: 0; t2tINV: 0; TRA: 0
SVs in sample	529
Oscillating CN (2 and 3 states)	7, 12
CN segments	12
FDR fragment joints	0.1013295
FDR chr. breakp. enrich.	0
Linked to chrs	
Purity, ploidy	0.32, 3.04

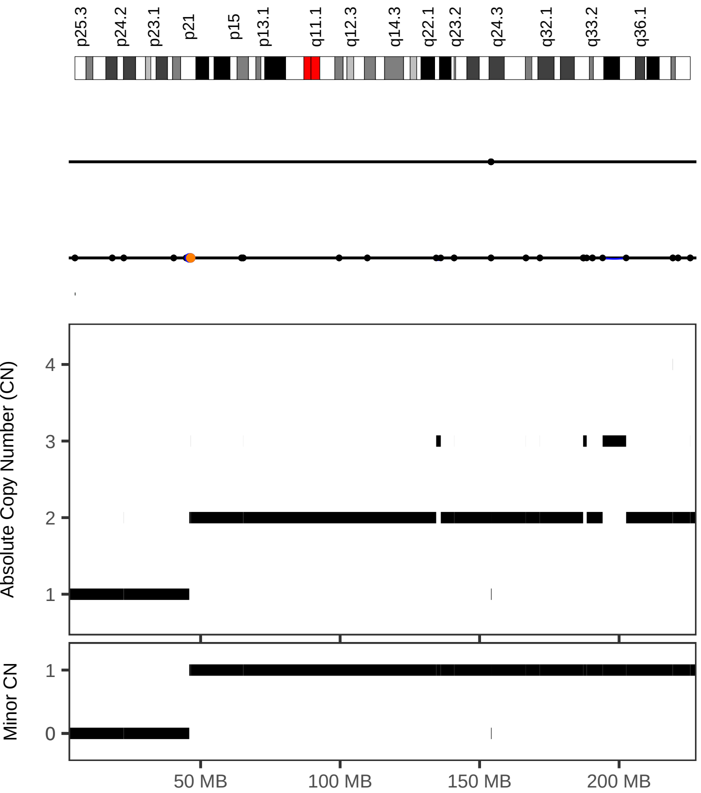

CGP_donor_1397278

Cancer type	Breast−AdenoCA
Position	2:22430768−219287015
Interleaved intrachr. SVs	1
Total SVs (intrachr. + transl.)	12
SV types	DEL: 0; DUP: 9; h2hINV: 0; t2tINV: 0; TRA: 3
SVs in sample	340
Oscillating CN (2 and 3 states)	9, 21
CN segments	23
FDR fragment joints	0.001274711
FDR chr. breakp. enrich.	0.89
Linked to chrs	
Purity, ploidy	0.42, 2.02

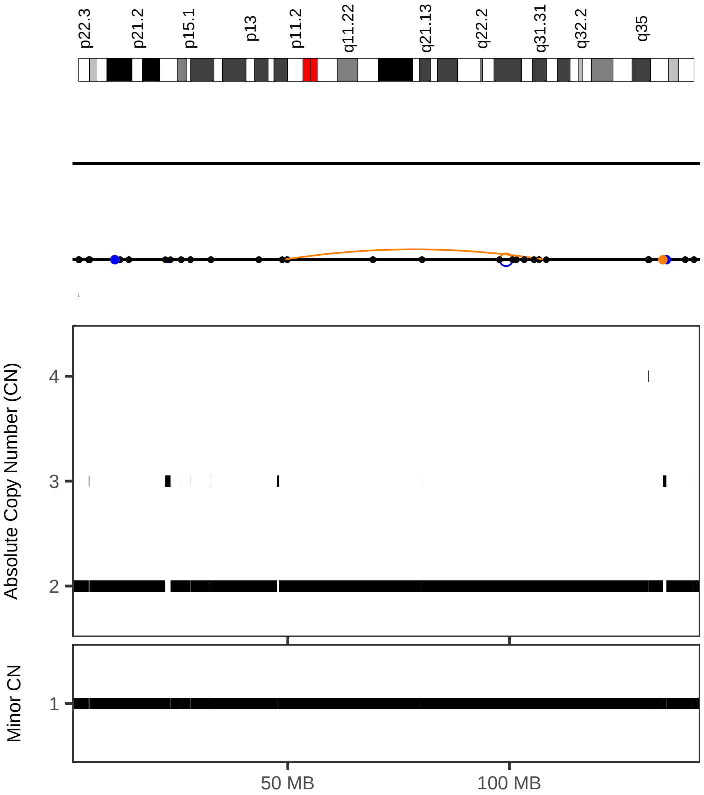

CGP_donor_1397278

Cancer type	Breast−AdenoCA
Position	7:5041125−108355553
Interleaved intrachr. SVs	1
Total SVs (intrachr. + transl.)	10
SV types	DEL: 2; DUP: 6; h2hINV: 0; t2tINV: 0; TRA: 2
SVs in sample	340
Oscillating CN (2 and 3 states)	16, 16
CN segments	16
FDR fragment joints	0.1790694
FDR chr. breakp. enrich.	0.07
Linked to chrs	
Purity, ploidy	0.42, 2.02

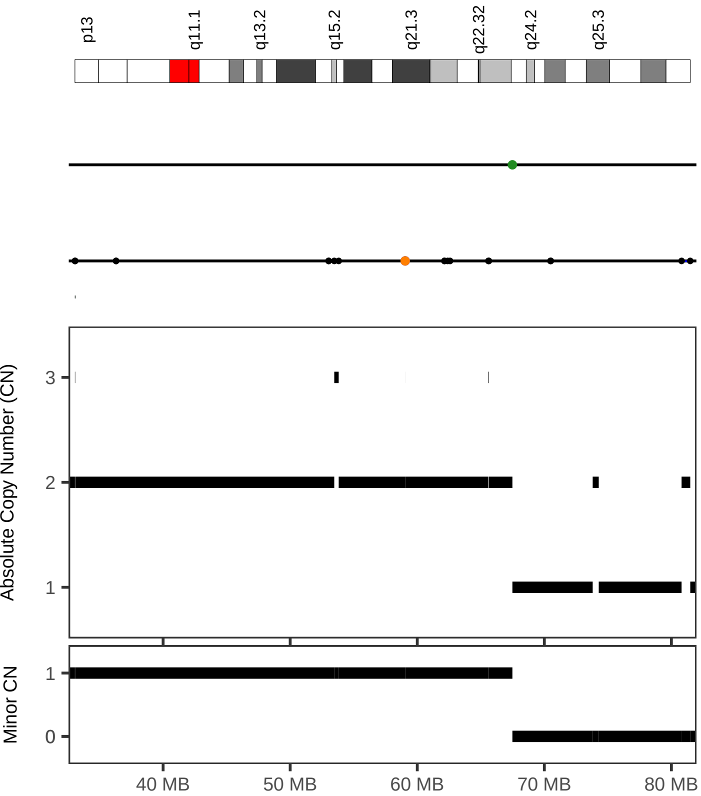

CGP_donor_1397278

Cancer type	Breast−AdenoCA
Position	15:33060156−81482186
Interleaved intrachr. SVs	1
Total SVs (intrachr. + transl.)	9
SV types	DEL: 0; DUP: 6; h2hINV: 0; t2tINV: 0; TRA: 3
SVs in sample	340
Oscillating CN (2 and 3 states)	8, 13
CN segments	13
FDR fragment joints	0.1488029
FDR chr. breakp. enrich.	0.33
Linked to chrs	
Purity, ploidy	0.42, 2.02

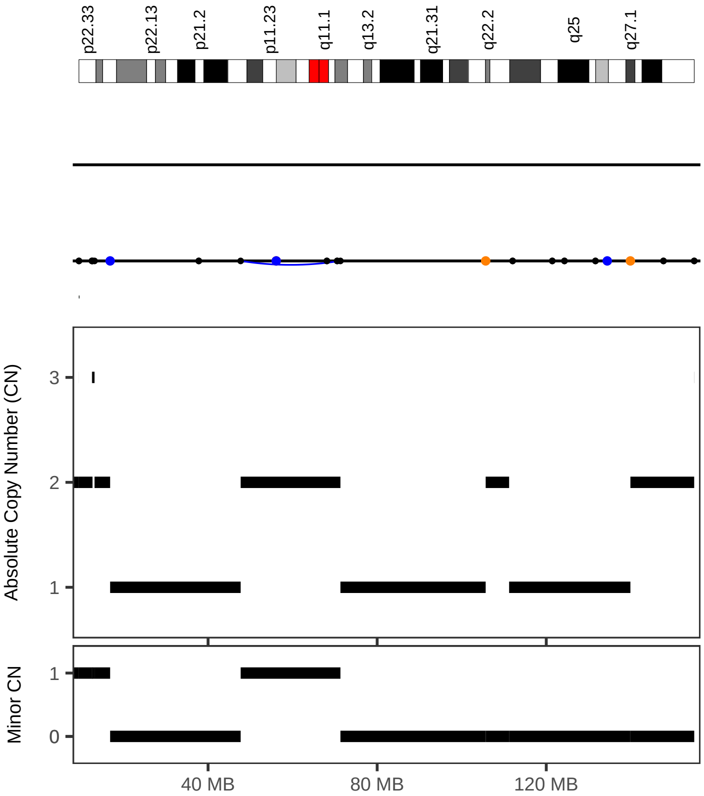

CGP_donor_1397278

Cancer type	Breast−AdenoCA
Position	X:12671854−155005826
Interleaved intrachr. SVs	1
Total SVs (intrachr. + transl.)	12
SV types	DEL: 0; DUP: 6; h2hINV: 0; t2tINV: 0; TRA: 6
SVs in sample	340
Oscillating CN (2 and 3 states)	7, 9
CN segments	9
FDR fragment joints	0.04139017
FDR chr. breakp. enrich.	0.83
Linked to chrs	7:5041125−108355552;
Purity, ploidy	0.42, 2.02

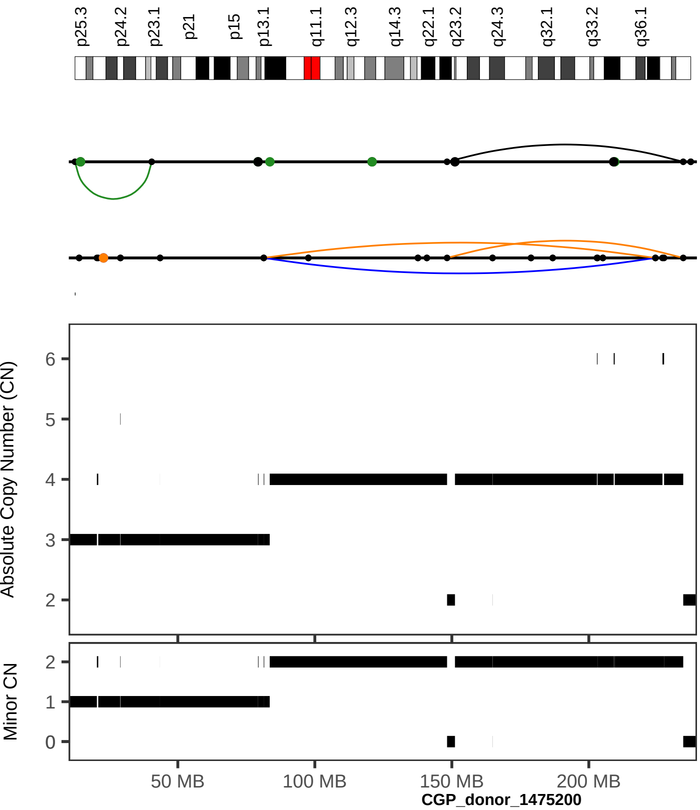

CGP_donor_1475200

Cancer type	Breast−AdenoCA
Position	2:81350895−234457223
Interleaved intrachr. SVs	4
Total SVs (intrachr. + transl.)	10
SV types	DEL: 2; DUP: 1; h2hINV: 1; t2tINV: 0; TRA: 6
SVs in sample	331
Oscillating CN (2 and 3 states)	7, 12
CN segments	14
FDR fragment joints	0.6776251
FDR chr. breakp. enrich.	0.47
Linked to chrs	
Purity, ploidy	0.6, 3.69

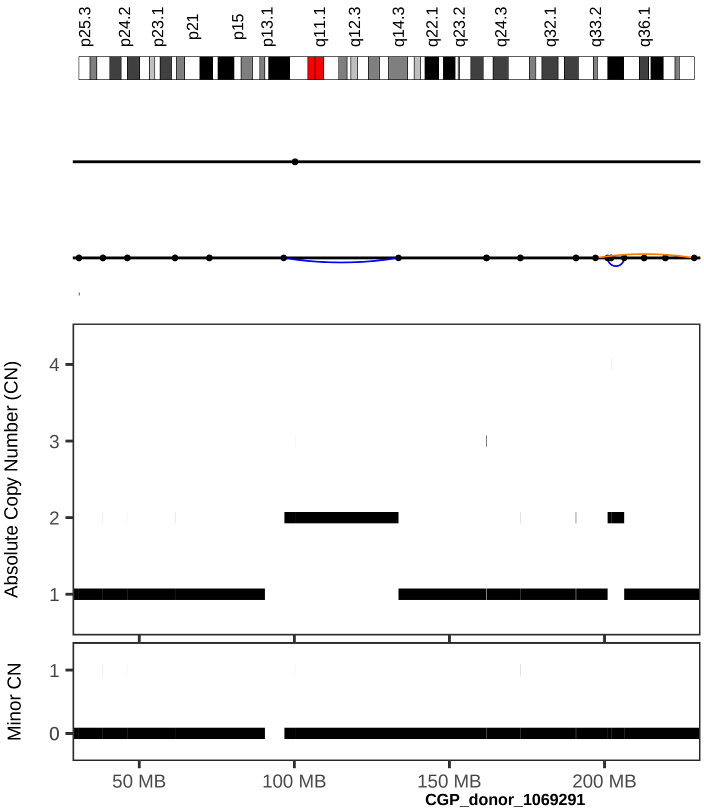

CGP_donor_1069291

Cancer type	Breast−AdenoCA
Position	2:30546371−228922869
Interleaved intrachr. SVs	1
Total SVs (intrachr. + transl.)	11
SV types	DEL: 1; DUP: 10; h2hINV: 0; t2tINV: 0; TRA: 0
SVs in sample	277
Oscillating CN (2 and 3 states)	9, 9
CN segments	22
FDR fragment joints	0.003601907
FDR chr. breakp. enrich.	0.32
Linked to chrs	
Purity, ploidy	0.46, 1.81

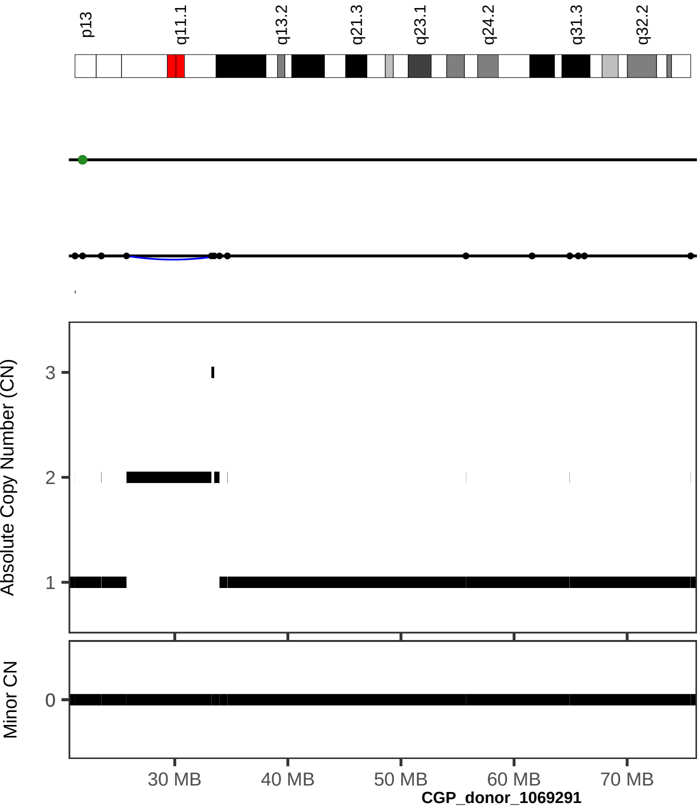

CGP_donor_1069291

Cancer type	Breast−AdenoCA
Position	14:23497943−75621029
Interleaved intrachr. SVs	1
Total SVs (intrachr. + transl.)	8
SV types	DEL: 0; DUP: 8; h2hINV: 0; t2tINV: 0; TRA: 0
SVs in sample	277
Oscillating CN (2 and 3 states)	10, 14
CN segments	14
FDR fragment joints	0.006736868
FDR chr. breakp. enrich.	0.23
Linked to chrs	
Purity, ploidy	0.46, 1.81

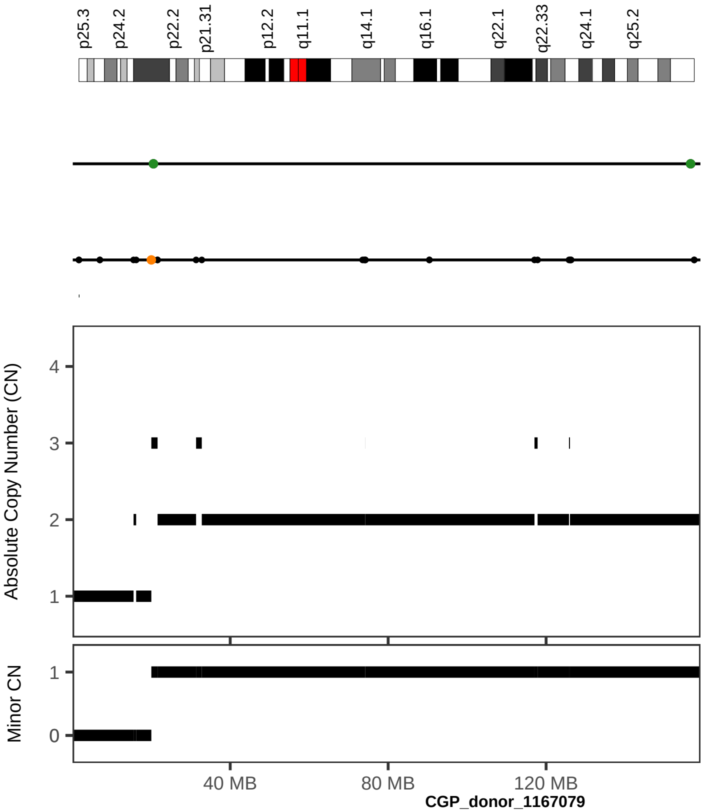

CGP_donor_1167079

Cancer type	Breast−AdenoCA
Position	6:6984465−157517645
Interleaved intrachr. SVs	1
Total SVs (intrachr. + transl.)	15
SV types	DEL: 0; DUP: 10; h2hINV: 0; t2tINV: 0; TRA: 5
SVs in sample	258
Oscillating CN (2 and 3 states)	10, 13
CN segments	13
FDR fragment joints	0.129004
FDR chr. breakp. enrich.	0.61
Linked to chrs	
Purity, ploidy	0.51, 1.83

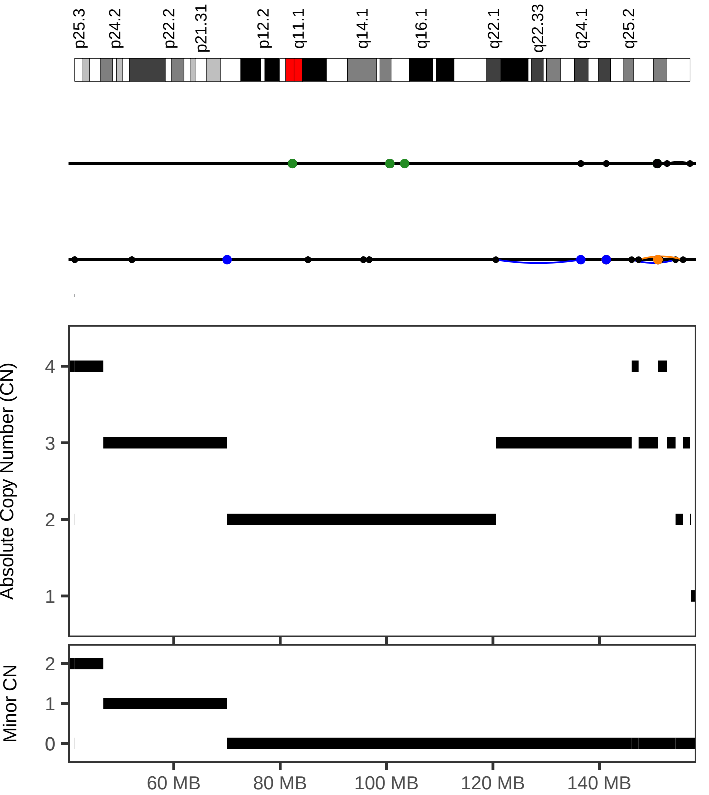

CGP_donor_1199138

Cancer type	Breast−AdenoCA
Position	6:146079326−157043927
Interleaved intrachr. SVs	3
Total SVs (intrachr. + transl.)	8
SV types	DEL: 1; DUP: 1; h2hINV: 1; t2tINV: 0; TRA: 5
SVs in sample	426
Oscillating CN (2 and 3 states)	8, 11
CN segments	11
FDR fragment joints	0.8653243
FDR chr. breakp. enrich.	0.5
Linked to chrs	
Purity, ploidy	0.72, 3.18

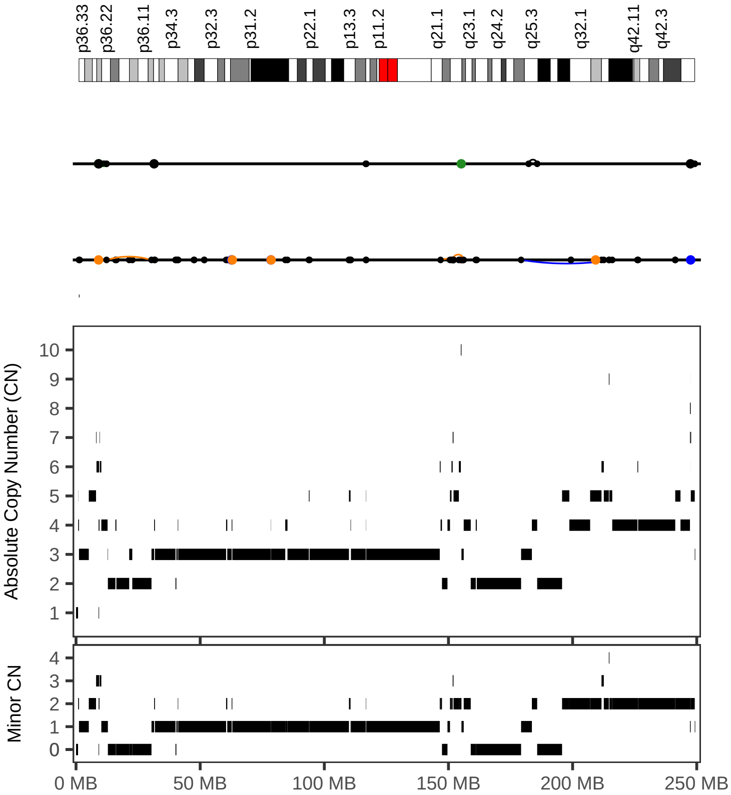

CGP_donor_1230785

Cancer type	Breast−AdenoCA
Position	1:10186027−116894133
Interleaved intrachr. SVs	3
Total SVs (intrachr. + transl.)	11
SV types	DEL: 1; DUP: 1; h2hINV: 2; t2tINV: 2; TRA: 5
SVs in sample	502
Oscillating CN (2 and 3 states)	11, 15
CN segments	32
FDR fragment joints	0.9804396
FDR chr. breakp. enrich.	0.08
Linked to chrs	
Purity, ploidy	0.35, 2.71

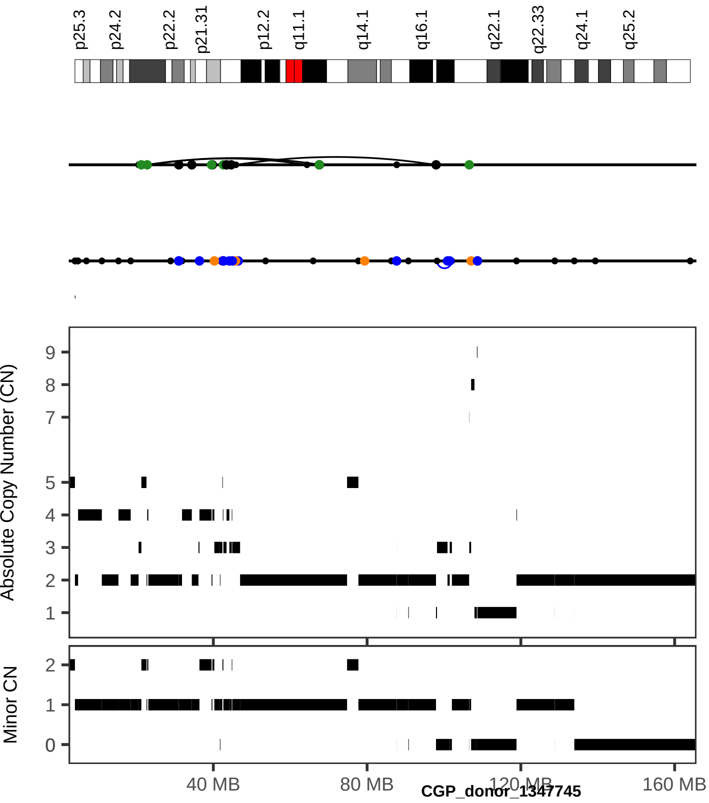

CGP_donor_1347745

Cancer type	Breast−AdenoCA
Position	6:22611379−97921915
Interleaved intrachr. SVs	3
Total SVs (intrachr. + transl.)	27
SV types	DEL: 0; DUP: 0; h2hINV: 3; t2tINV: 0; TRA: 24
SVs in sample	551
Oscillating CN (2 and 3 states)	7, 8
CN segments	30
FDR fragment joints	0.8393975
FDR chr. breakp. enrich.	0
Linked to chrs	1:35695011−87315086;8:2126333−142042838
Purity, ploidy	0.24, 2.41

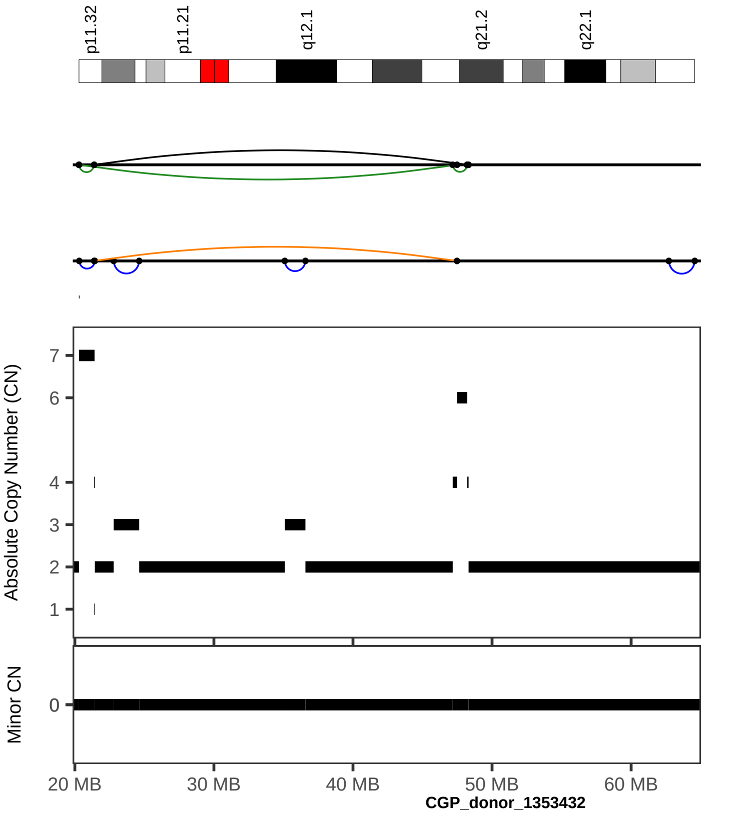

CGP_donor_1353432

Cancer type	Breast−AdenoCA
Position	18:20297595−48213819
Interleaved intrachr. SVs	6
Total SVs (intrachr. + transl.)	6
SV types	DEL: 1; DUP: 1; h2hINV: 1; t2tINV: 3; TRA: 0
SVs in sample	185
Oscillating CN (2 and 3 states)	5, 7
CN segments	12
FDR fragment joints	0.6776251
FDR chr. breakp. enrich.	0.13
Linked to chrs	
Purity, ploidy	0.89, 3.18

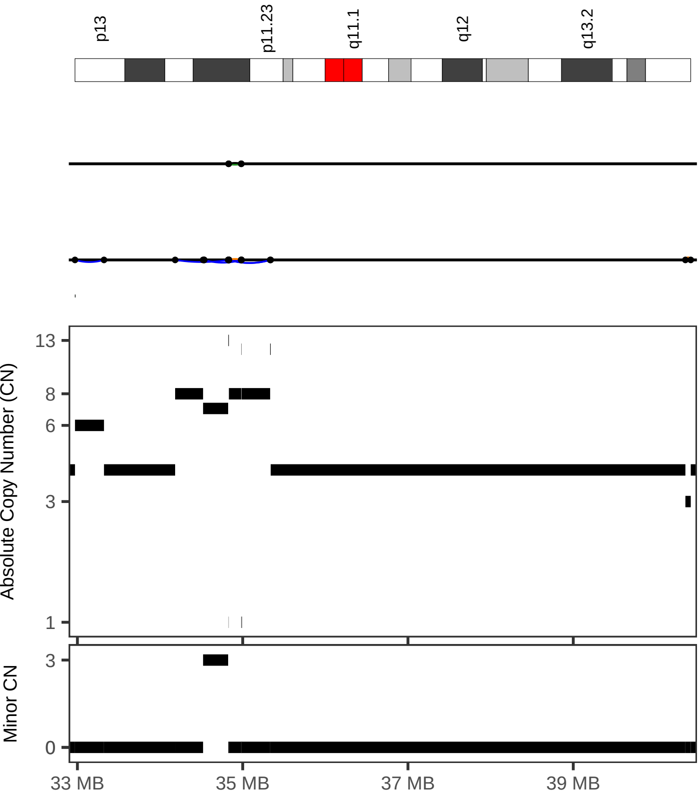

CGP_donor_1353432

Cancer type	Breast−AdenoCA
Position	20:34182540−35338513
Interleaved intrachr. SVs	8
Total SVs (intrachr. + transl.)	8
SV types	DEL: 1; DUP: 5; h2hINV: 1; t2tINV: 1; TRA: 0
SVs in sample	185
Oscillating CN (2 and 3 states)	4, 5
CN segments	13
FDR fragment joints	0.5435077
FDR chr. breakp. enrich.	0
Linked to chrs	
Purity, ploidy	0.89, 3.18

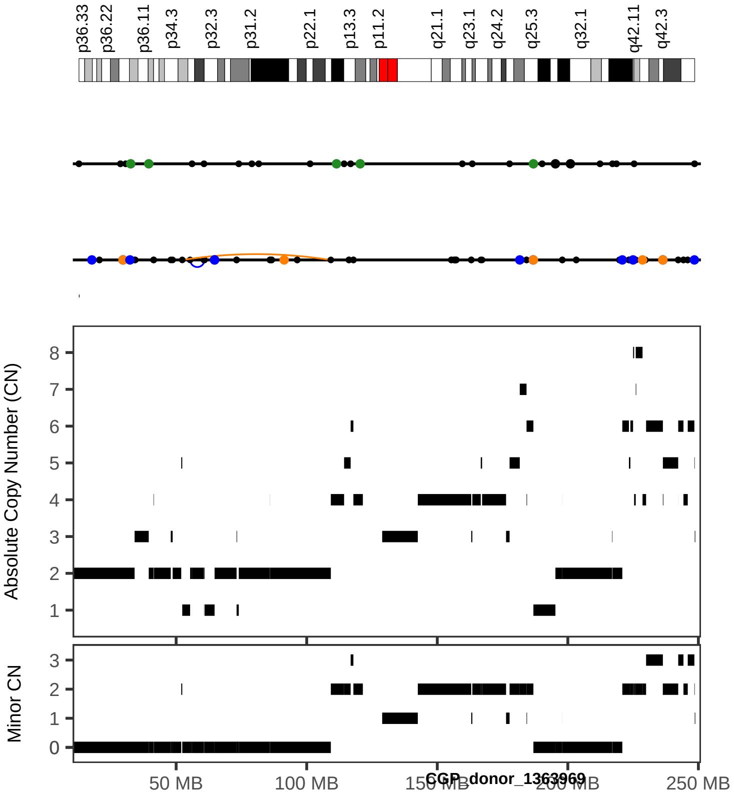

CGP_donor_1363969

Cancer type	Breast−AdenoCA
Position	1:20593050−248597403
Interleaved intrachr. SVs	1
Total SVs (intrachr. + transl.)	39
SV types	DEL: 2; DUP: 12; h2hINV: 2; t2tINV: 1; TRA: 22
SVs in sample	680
Oscillating CN (2 and 3 states)	8, 9
CN segments	59
FDR fragment joints	0.01578149
FDR chr. breakp. enrich.	0.02
Linked to chrs	10:15925151−65636394;15:49453754−101270858 2:24204828−232125013;4:46814412−190722754
[illegible]	[illegible]

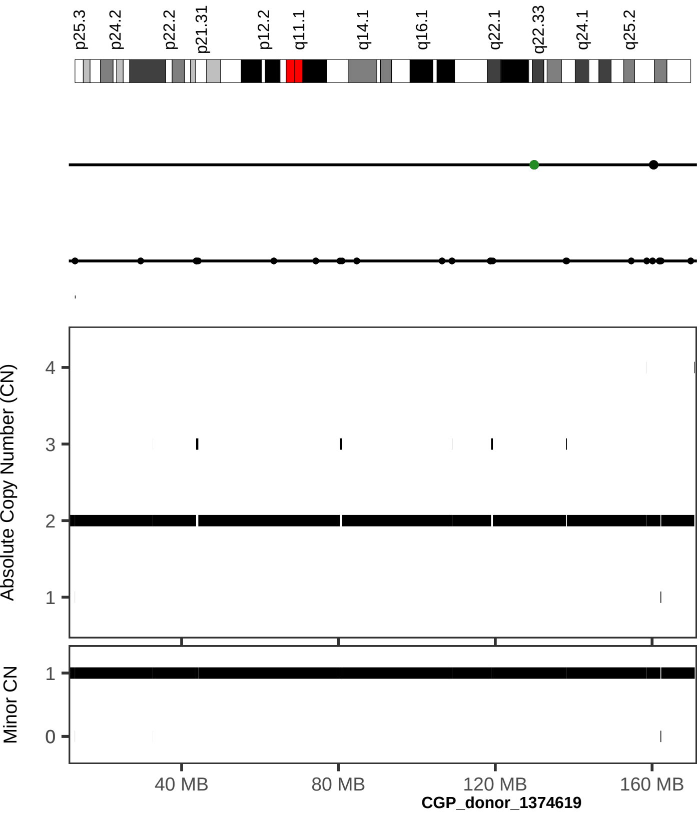

CGP_donor_1374619

Cancer type	Breast−AdenoCA
Position	6:12789227−162322086
Interleaved intrachr. SVs	1
Total SVs (intrachr. + transl.)	15
SV types	DEL: 2; DUP: 10; h2hINV: 0; t2tINV: 0; TRA: 3
SVs in sample	273
Oscillating CN (2 and 3 states)	13, 15
CN segments	18
FDR fragment joints	0.129004
FDR chr. breakp. enrich.	0.37
Linked to chrs	8:19513801−142877711;
Purity, ploidy	0.38, 1.62

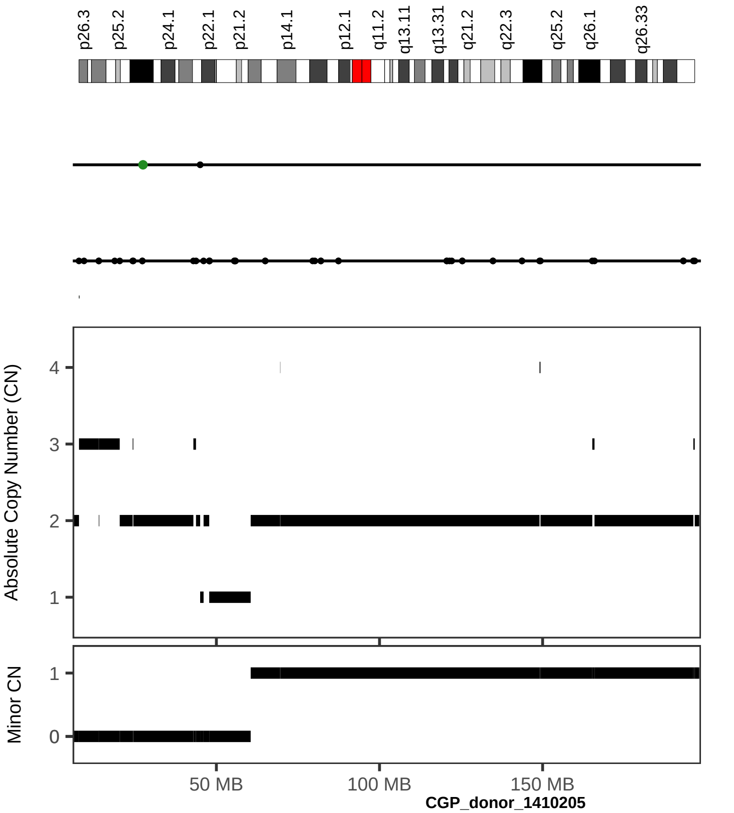

CGP_donor_1410205

Cancer type	Breast−AdenoCA
Position	3:7876202−196628059
Interleaved intrachr. SVs	1
Total SVs (intrachr. + transl.)	15
SV types	DEL: 1; DUP: 13; h2hINV: 0; t2tINV: 0; TRA: 1
SVs in sample	341
Oscillating CN (2 and 3 states)	8, 9
CN segments	20
FDR fragment joints	0.0006748813
FDR chr. breakp. enrich.	0.76
Linked to chrs	
Purity, ploidy	0.35, 1.83

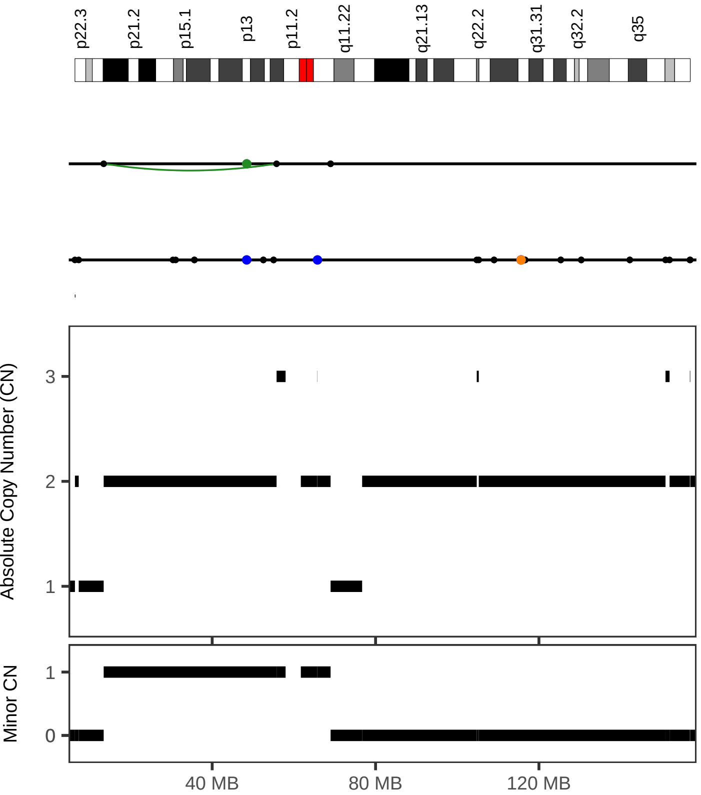

CGP_donor_1410205

Cancer type	Breast−AdenoCA
Position	7:6389159−157049440
Interleaved intrachr. SVs	1
Total SVs (intrachr. + transl.)	16
SV types	DEL: 0; DUP: 10; h2hINV: 0; t2tINV: 1; TRA: 5
SVs in sample	341
Oscillating CN (2 and 3 states)	7, 15
CN segments	15
FDR fragment joints	0.002298705
FDR chr. breakp. enrich.	0.84
Linked to chrs	
Purity, ploidy	0.35, 1.83

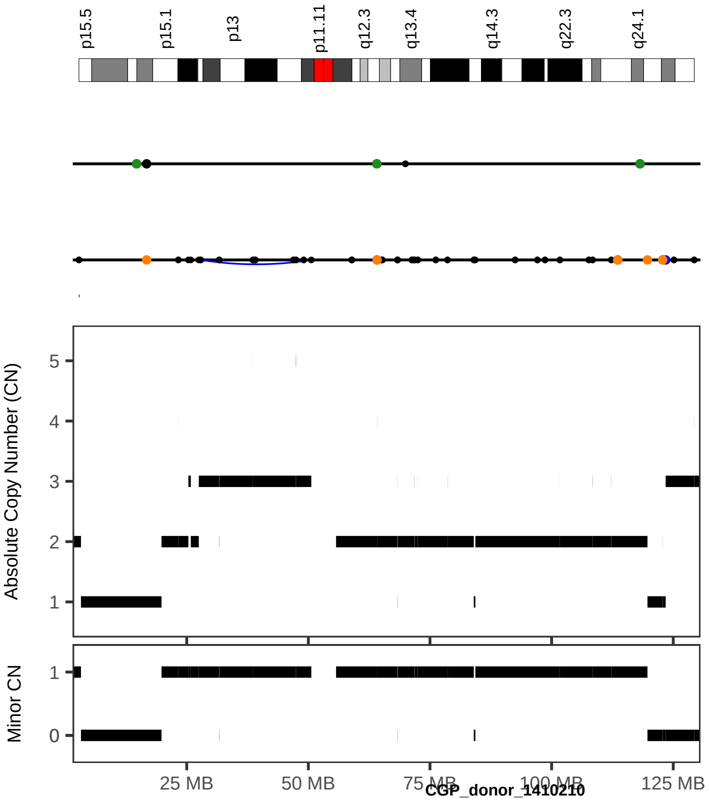

CGP_donor_1410210

Cancer type	Breast−AdenoCA
Position	11:2846968−112273249
Interleaved intrachr. SVs	1
Total SVs (intrachr. + transl.)	28
SV types	DEL: 4; DUP: 15; h2hINV: 0; t2tINV: 1; TRA: 8
SVs in sample	543
Oscillating CN (2 and 3 states)	9, 21
CN segments	37
FDR fragment joints	0.06904132
FDR chr. breakp. enrich.	0
Linked to chrs	4:74048710−156409342;8:27500339−142493804 X:2605722−149833496;
Purity, ploidy	0.66, 1.56

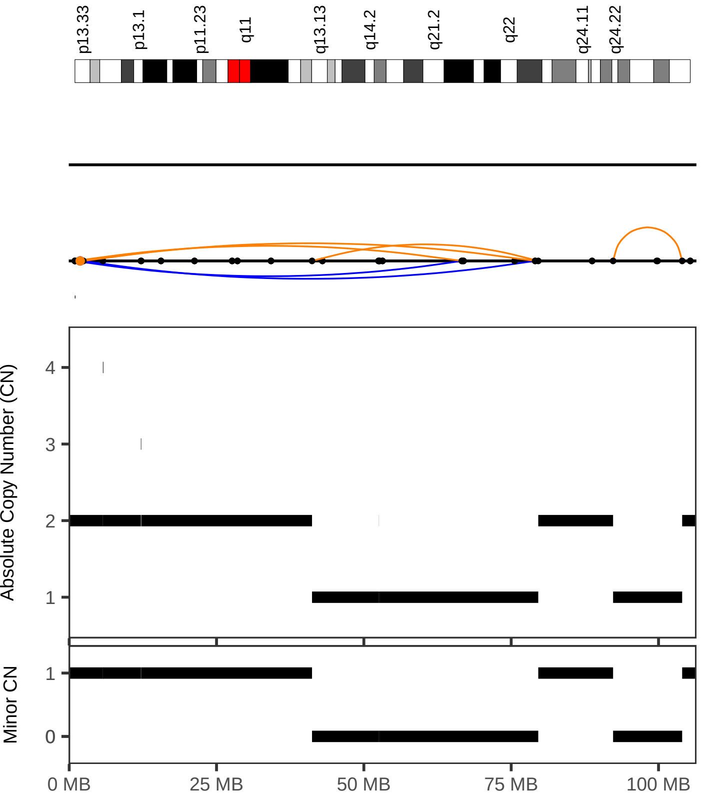

CGP_donor_1410210

Cancer type	Breast−AdenoCA
Position	12:972252−79594700
Interleaved intrachr. SVs	5
Total SVs (intrachr. + transl.)	7
SV types	DEL: 3; DUP: 2; h2hINV: 0; t2tINV: 0; TRA: 2
SVs in sample	543
Oscillating CN (2 and 3 states)	7, 7
CN segments	11
FDR fragment joints	0.5435077
FDR chr. breakp. enrich.	0.98
Linked to chrs	
Purity, ploidy	0.66, 1.56

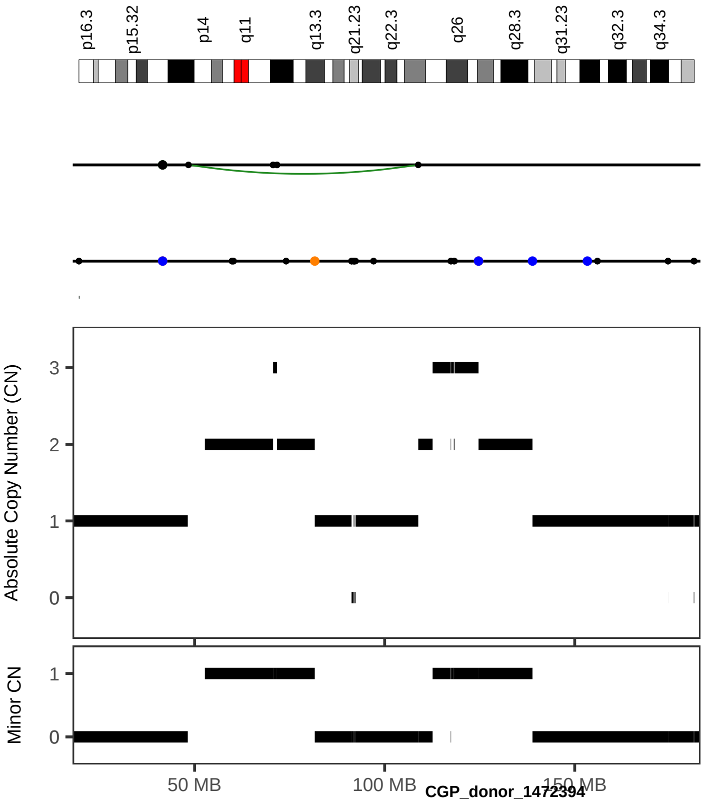

CGP_donor_1472394

Cancer type	Breast−AdenoCA
Position	4:19561889−181450628
Interleaved intrachr. SVs	1
Total SVs (intrachr. + transl.)	20
SV types	DEL: 10; DUP: 0; h2hINV: 0; t2tINV: 1; TRA: 9
SVs in sample	366
Oscillating CN (2 and 3 states)	7, 23
CN segments	23
FDR fragment joints	0.04139017
FDR chr. breakp. enrich.	0.92
Linked to chrs	13:36695786−97964893;3:128599014−194875397
Purity, ploidy	0.7, 1.76

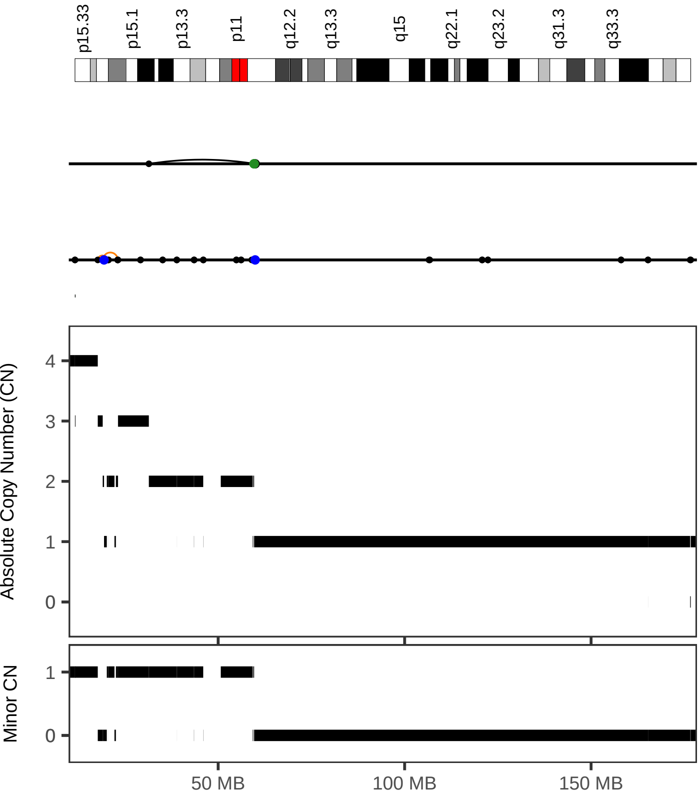

CGP_donor_1472394

Cancer type	Breast−AdenoCA
Position	5:31416901−59923452
Interleaved intrachr. SVs	4
Total SVs (intrachr. + transl.)	8
SV types	DEL: 1; DUP: 0; h2hINV: 2; t2tINV: 1; TRA: 4
SVs in sample	366
Oscillating CN (2 and 3 states)	10, 10
CN segments	10
FDR fragment joints	0.615458
FDR chr. breakp. enrich.	0.32
Linked to chrs	12:47550216−98458710;
Purity, ploidy	0.7, 1.76

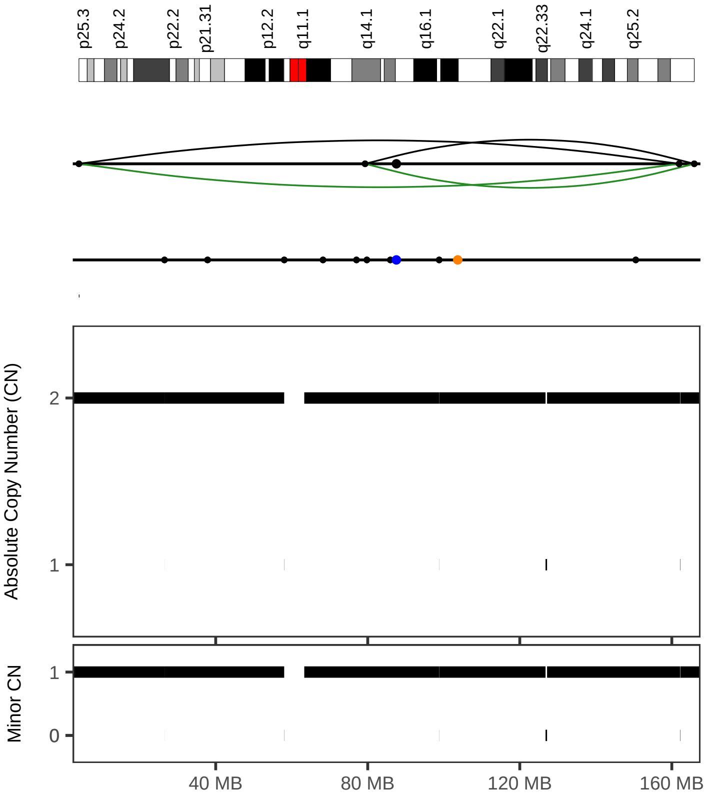

CGP_donor_1472394

Cancer type	Breast−AdenoCA
Position	6:4012667−165902670
Interleaved intrachr. SVs	4
Total SVs (intrachr. + transl.)	7
SV types	DEL: 0; DUP: 0; h2hINV: 2; t2tINV: 2; TRA: 3
SVs in sample	366
Oscillating CN (2 and 3 states)	11, 11
CN segments	11
FDR fragment joints	0.7735152
FDR chr. breakp. enrich.	0.46
Linked to chrs	14:38341626−81002028;
Purity, ploidy	0.7, 1.76

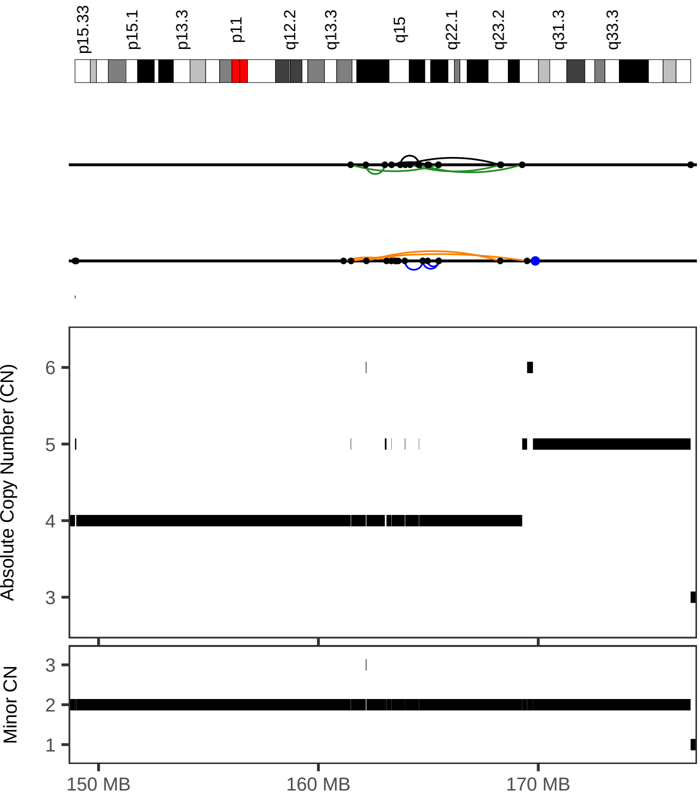

CGP_donor_1472395

Cancer type	Breast−AdenoCA
Position	5:161143076−169493045
Interleaved intrachr. SVs	14
Total SVs (intrachr. + transl.)	14
SV types	DEL: 4; DUP: 3; h2hINV: 3; t2tINV: 4; TRA: 0
SVs in sample	132
Oscillating CN (2 and 3 states)	10, 14
CN segments	15
FDR fragment joints	0.9794281
FDR chr. breakp. enrich.	0.01
Linked to chrs	
Purity, ploidy	0.5, 3.23

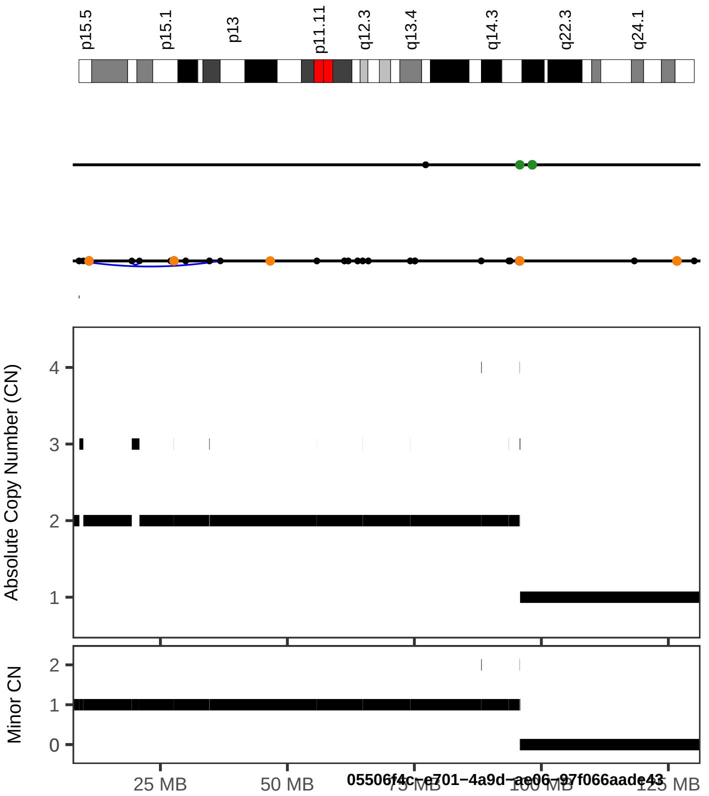

05506f4c−e701−4a9d−ae06−97f066aade43

Cancer type	Breast−AdenoCA
Position	11:8956771−130101571
Interleaved intrachr. SVs	1
Total SVs (intrachr. + transl.)	22
SV types	DEL: 0; DUP: 9; h2hINV: 0; t2tINV: 0; TRA: 13
SVs in sample	742
Oscillating CN (2 and 3 states)	15, 15
CN segments	24
FDR fragment joints	0.07914058
FDR chr. breakp. enrich.	0.95
Linked to chrs	13:26931051−109631660;15:59419044−77860746 20:11724482−45813710;7:12804785−147710049 9:1011854−128924972;

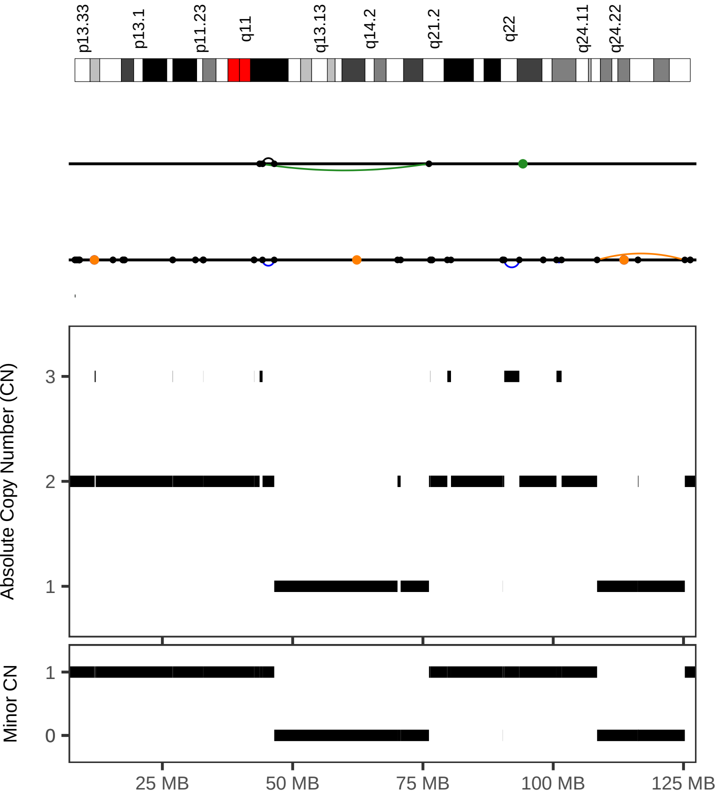

05506f4c−e701−4a9d−ae06−97f066aade43

Cancer type	Breast−AdenoCA
Position	12:8551694−125253036
Interleaved intrachr. SVs	1
Total SVs (intrachr. + transl.)	24
SV types	DEL: 3; DUP: 13; h2hINV: 1; t2tINV: 1; TRA: 6
SVs in sample	742
Oscillating CN (2 and 3 states)	13, 31
CN segments	31
FDR fragment joints	0.02295299
FDR chr. breakp. enrich.	0.87
Linked to chrs	7:12804785−147710049;
Purity, ploidy	0.66, 1.78

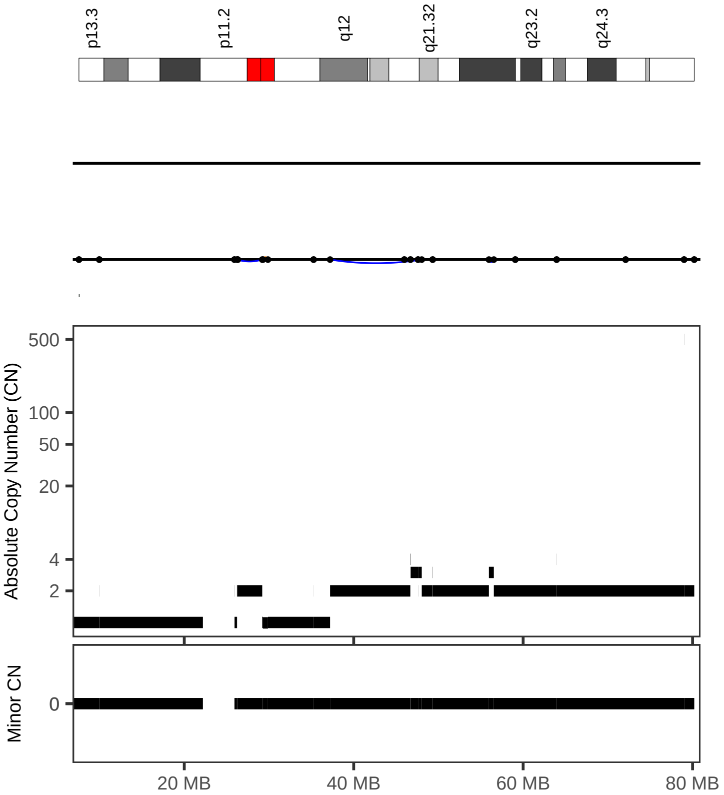

05506f4c−e701−4a9d−ae06−97f066aade43

Cancer type	Breast−AdenoCA
Position	17:7564505−56539923
Interleaved intrachr. SVs	1
Total SVs (intrachr. + transl.)	9
SV types	DEL: 2; DUP: 7; h2hINV: 0; t2tINV: 0; TRA: 0
SVs in sample	742
Oscillating CN (2 and 3 states)	9, 14
CN segments	23
FDR fragment joints	0.1178349
FDR chr. breakp. enrich.	0.91
Linked to chrs	
Purity, ploidy	0.66, 1.78

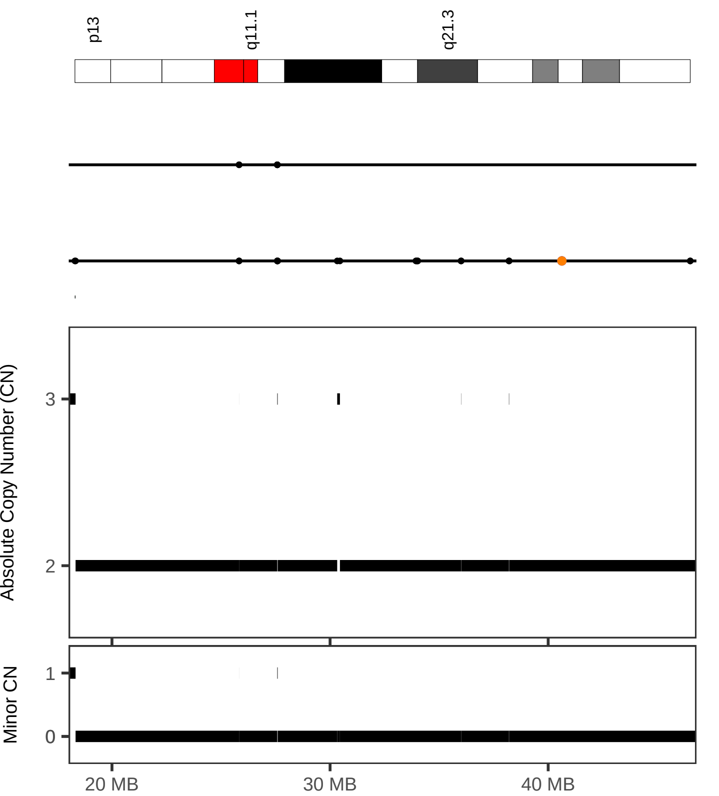

05506f4c−e701−4a9d−ae06−97f066aade43

Cancer type	Breast−AdenoCA
Position	21:18301501−38211721
Interleaved intrachr. SVs	1
Total SVs (intrachr. + transl.)	5
SV types	DEL: 0; DUP: 5; h2hINV: 0; t2tINV: 0; TRA: 0
SVs in sample	742
Oscillating CN (2 and 3 states)	12, 12
CN segments	12
FDR fragment joints	0.1013295
FDR chr. breakp. enrich.	0.19
Linked to chrs	
Purity, ploidy	0.66, 1.78

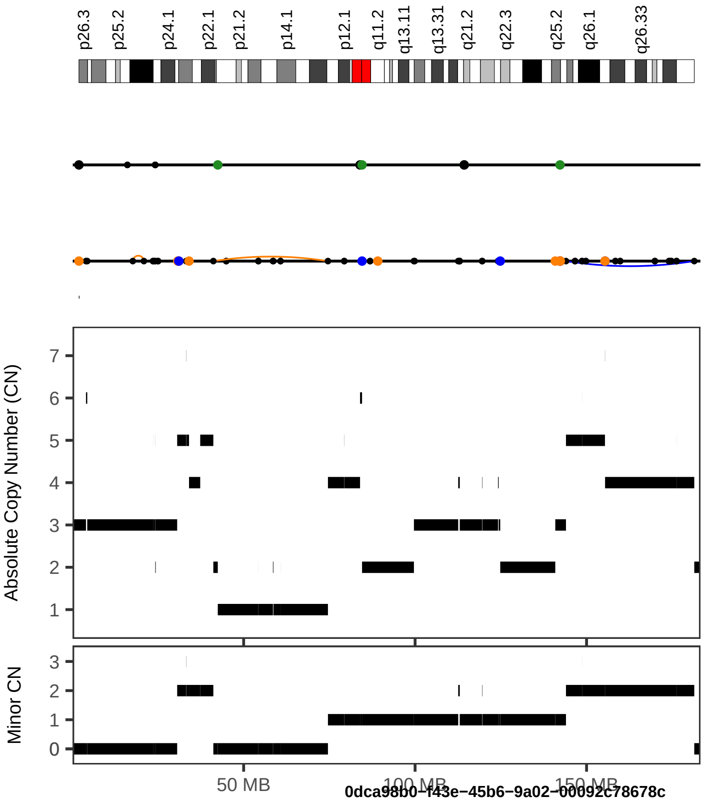

0dca98b0−f43e−45b6−9a02−00092c78678c

Cancer type	Breast−AdenoCA
Position	3:4014626−181407638
Interleaved intrachr. SVs	1
Total SVs (intrachr. + transl.)	38
SV types	DEL: 2; DUP: 14; h2hINV: 1; t2tINV: 0; TRA: 21
SVs in sample	552
Oscillating CN (2 and 3 states)	8, 8
CN segments	43
FDR fragment joints	0.02438658
FDR chr. breakp. enrich.	0.01
Linked to chrs	10:14391438−105152621;17:17662500−59054234
Purity, ploidy	0.76, 2.13

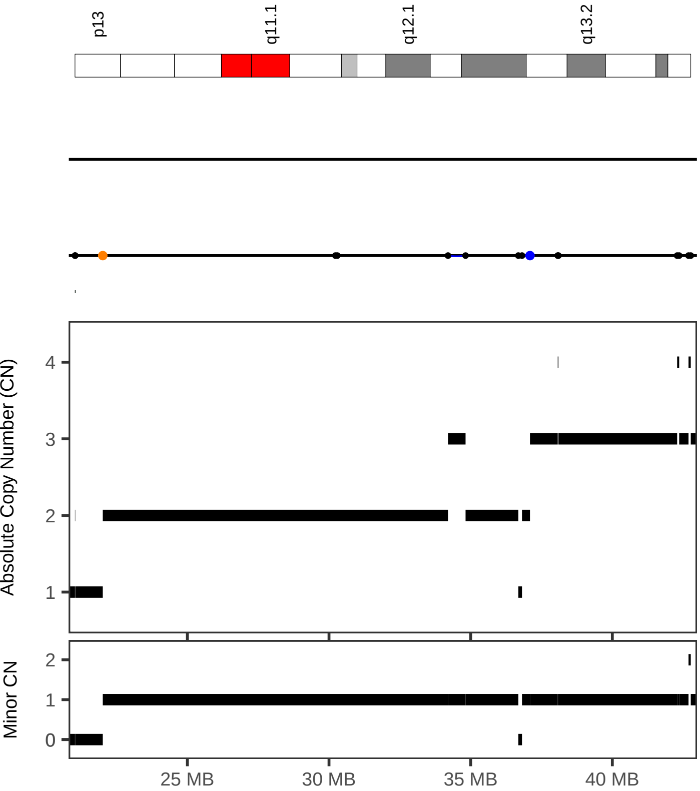

0dca98b0−f43e−45b6−9a02−00092c78678c

Cancer type	Breast−AdenoCA
Position	22:30225731−42768139
Interleaved intrachr. SVs	1
Total SVs (intrachr. + transl.)	7
SV types	DEL: 1; DUP: 5; h2hINV: 0; t2tINV: 0; TRA: 1
SVs in sample	552
Oscillating CN (2 and 3 states)	7, 12
CN segments	12
FDR fragment joints	0.1278363
FDR chr. breakp. enrich.	0.15
Linked to chrs	
Purity, ploidy	0.76, 2.13

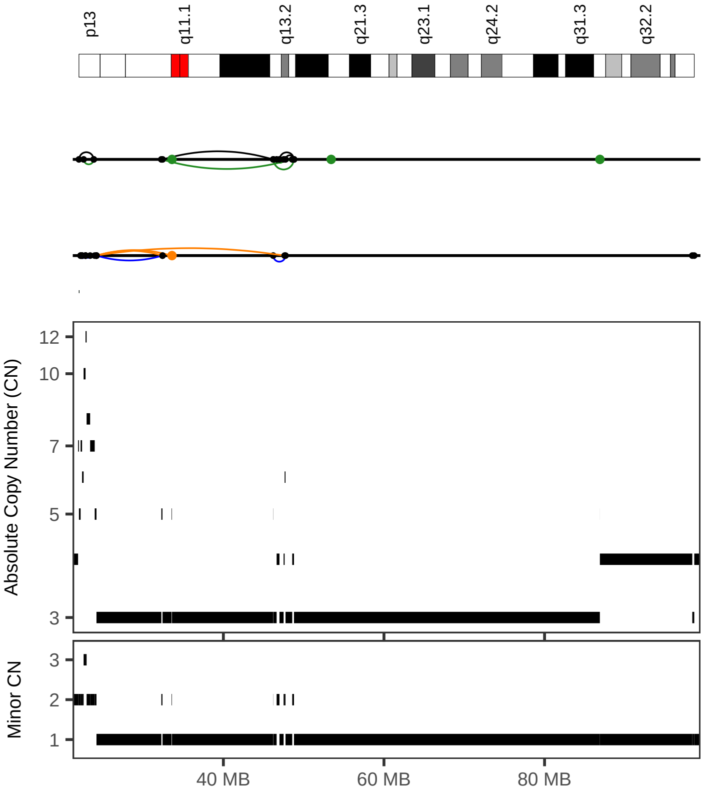

1557183c−aedd−4df6−b98a−3654667f7b69

Cancer type	Breast−AdenoCA
Position	14:23971383−48794055
Interleaved intrachr. SVs	11
Total SVs (intrachr. + transl.)	13
SV types	DEL: 3; DUP: 2; h2hINV: 3; t2tINV: 3; TRA: 2
SVs in sample	435
Oscillating CN (2 and 3 states)	10, 13
CN segments	17
FDR fragment joints	0.9000538
FDR chr. breakp. enrich.	0.07
Linked to chrs	
Purity, ploidy	0.6, 3.86

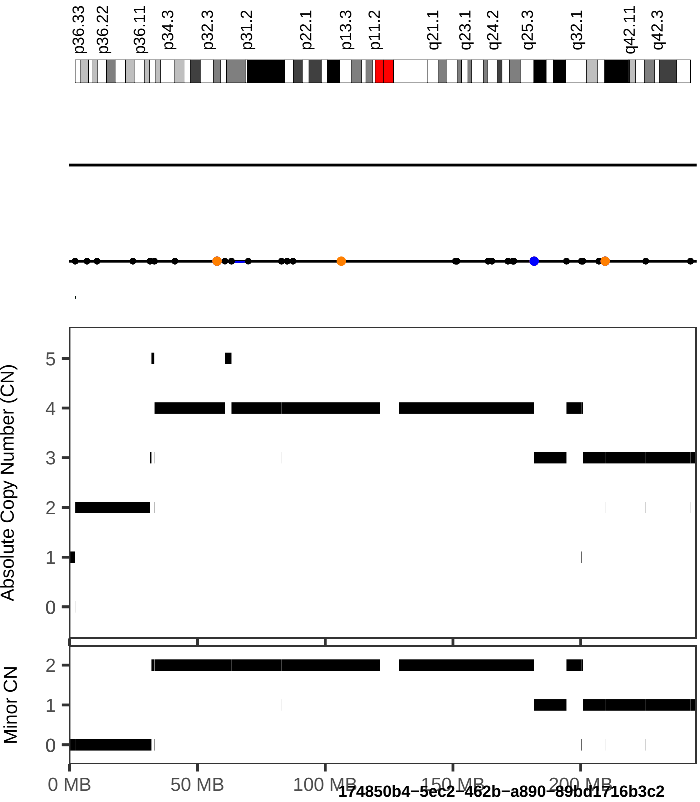

174850b4−5ec2−462b−a890−89bd1716b3c2

Cancer type	Breast−AdenoCA
Position	1:2158692−242947867
Interleaved intrachr. SVs	1
Total SVs (intrachr. + transl.)	17
SV types	DEL: 9; DUP: 1; h2hINV: 0; t2tINV: 0; TRA: 7
SVs in sample	456
Oscillating CN (2 and 3 states)	8, 11
CN segments	29
FDR fragment joints	0.002665934
FDR chr. breakp. enrich.	0.9
Linked to chrs	2:25587490−225396472;4:1872945−179290052
Purity, ploidy	0.47, 3.02

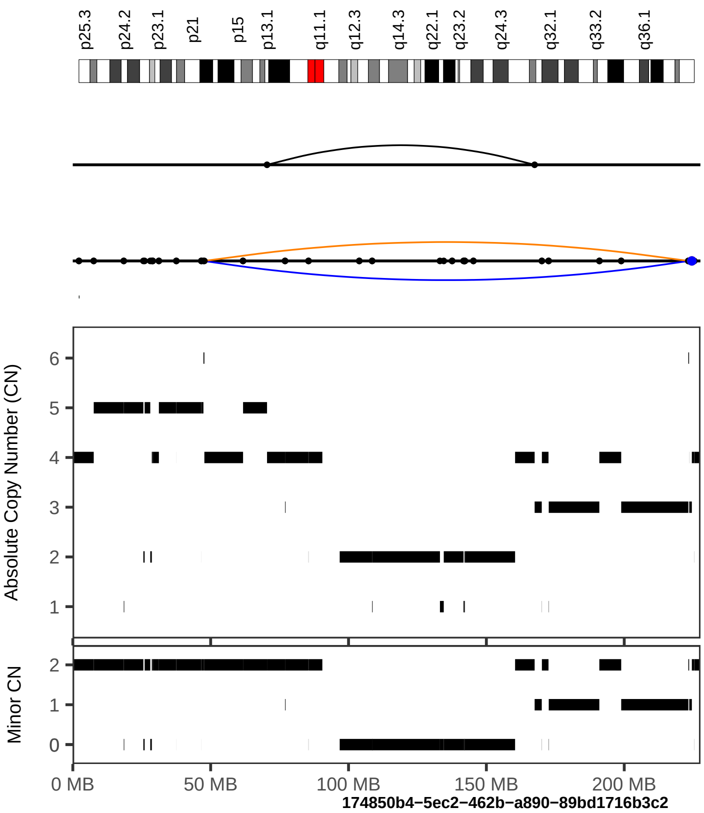

174850b4−5ec2−462b−a890−89bd1716b3c2

Cancer type	Breast−AdenoCA
Position	2:25587490−225396473
Interleaved intrachr. SVs	1
Total SVs (intrachr. + transl.)	20
SV types	DEL: 15; DUP: 2; h2hINV: 1; t2tINV: 0; TRA: 2
SVs in sample	456
Oscillating CN (2 and 3 states)	11, 11
CN segments	46
FDR fragment joints	0.0002466223
FDR chr. breakp. enrich.	0.88
Linked to chrs	1:2158692−242947866;
Purity, ploidy	0.47, 3.02

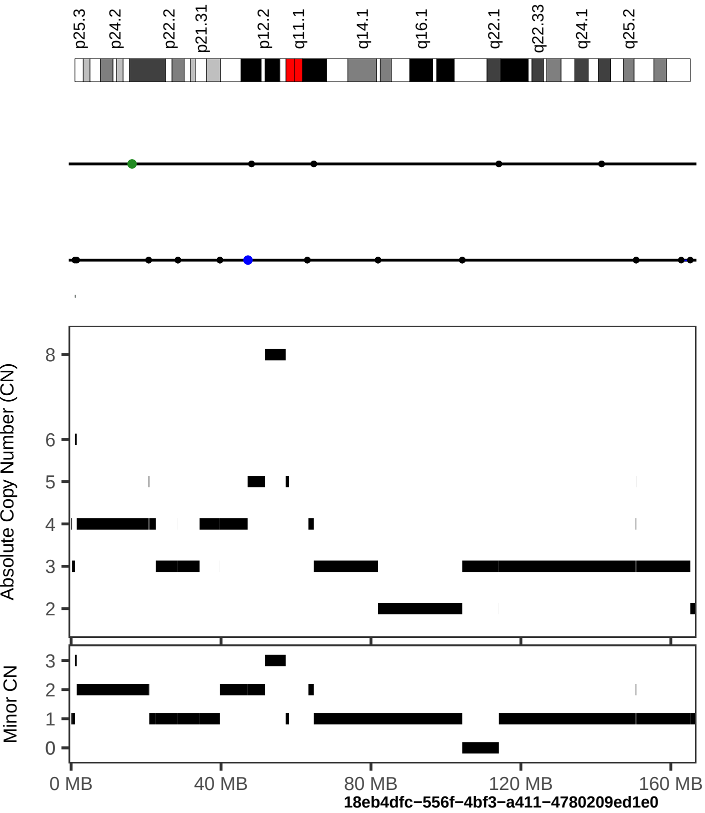

Cancer type	Breast−AdenoCA
Position	6:1006535−165186446
Interleaved intrachr. SVs	1
Total SVs (intrachr. + transl.)	7
SV types	DEL: 0; DUP: 5; h2hINV: 0; t2tINV: 0; TRA: 2
SVs in sample	280
Oscillating CN (2 and 3 states)	7, 10
CN segments	23
FDR fragment joints	0.1278363
FDR chr. breakp. enrich.	0.95
Linked to chrs	
Purity, ploidy	0.42, 2.75

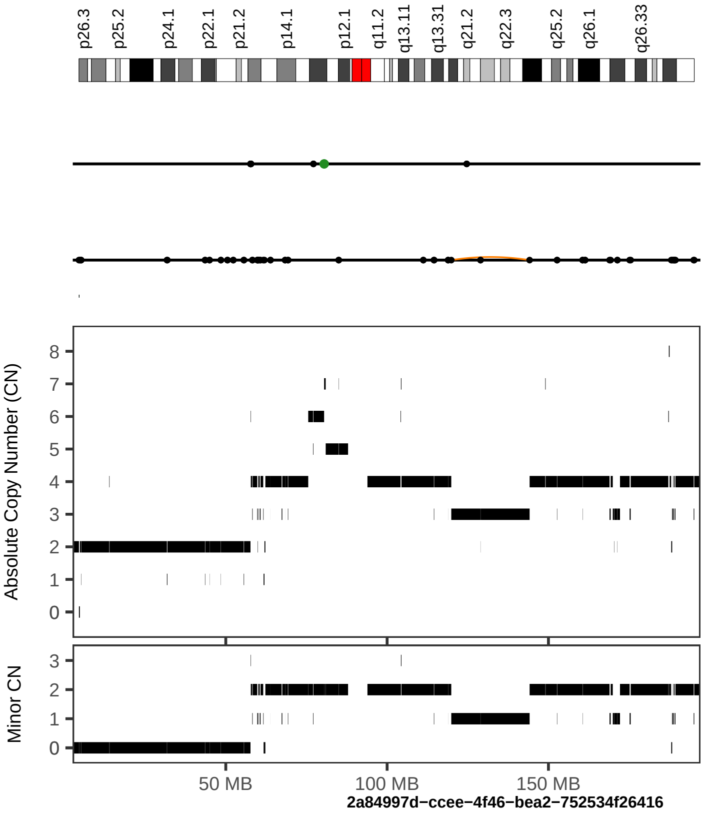

Cancer type	Breast−AdenoCA
Position	3:4487143−195190136
Interleaved intrachr. SVs	1
Total SVs (intrachr. + transl.)	34
SV types	DEL: 28; DUP: 1; h2hINV: 2; t2tINV: 2; TRA: 1
SVs in sample	596
Oscillating CN (2 and 3 states)	11, 15
CN segments	89
FDR fragment joints	4.073413e−09
FDR chr. breakp. enrich.	1
Linked to chrs	
Purity, ploidy	0.46, 2.83

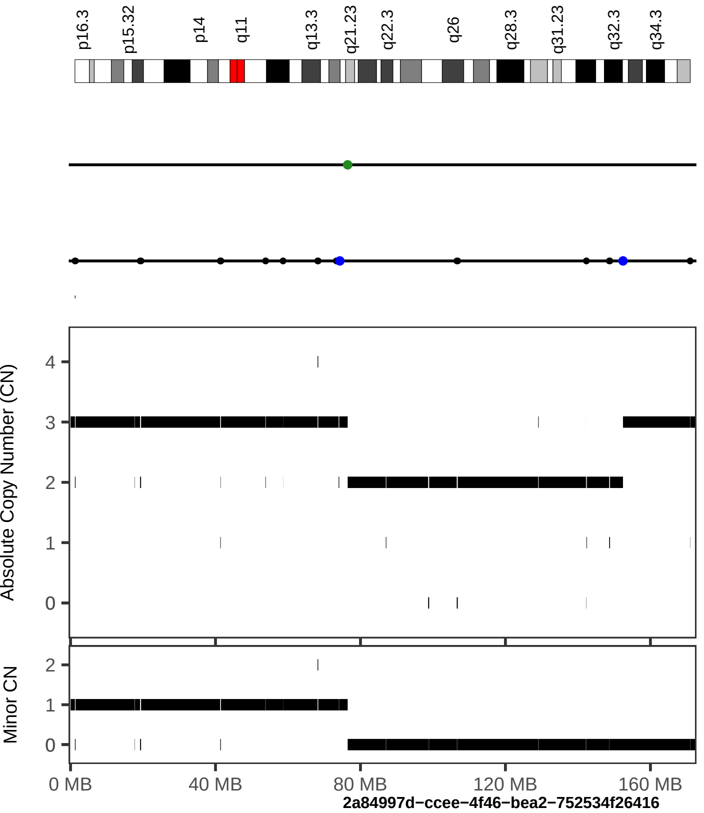

Cancer type	Breast−AdenoCA
Position	4:1211134−170990779
Interleaved intrachr. SVs	1
Total SVs (intrachr. + transl.)	14
SV types	DEL: 10; DUP: 1; h2hINV: 0; t2tINV: 0; TRA: 3
SVs in sample	596
Oscillating CN (2 and 3 states)	7, 21
CN segments	37
FDR fragment joints	0.05370273
FDR chr. breakp. enrich.	0
Linked to chrs	X:2221350−153108114;
Purity, ploidy	0.46, 2.83

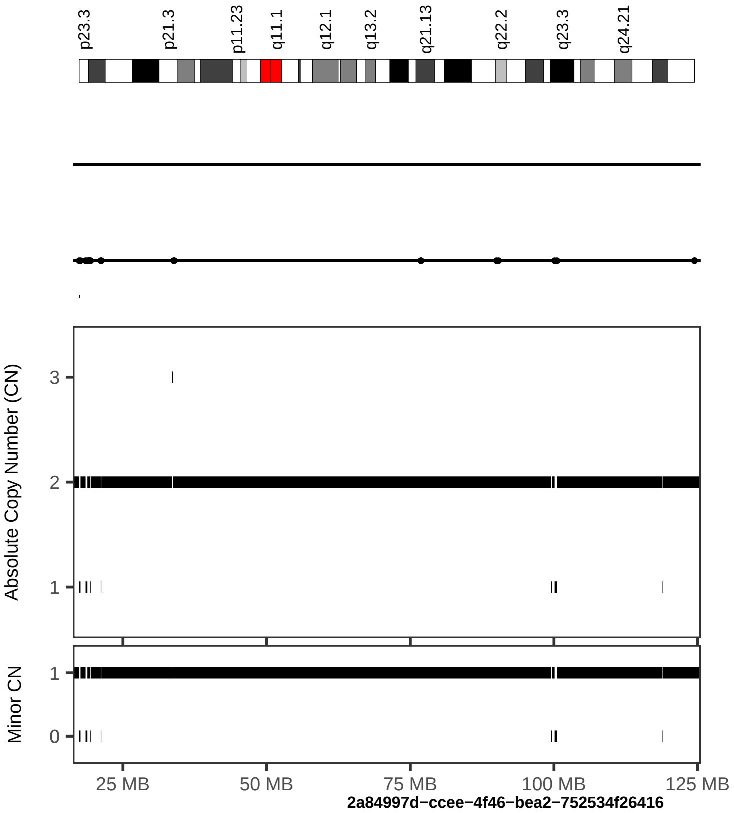

Cancer type	Breast−AdenoCA
Position	8:17394507−100542792
Interleaved intrachr. SVs	1
Total SVs (intrachr. + transl.)	7
SV types	DEL: 7; DUP: 0; h2hINV: 0; t2tINV: 0; TRA: 0
SVs in sample	596
Oscillating CN (2 and 3 states)	10, 16
CN segments	16
FDR fragment joints	0.01743054
FDR chr. breakp. enrich.	0
Linked to chrs	
Purity, ploidy	0.46, 2.83

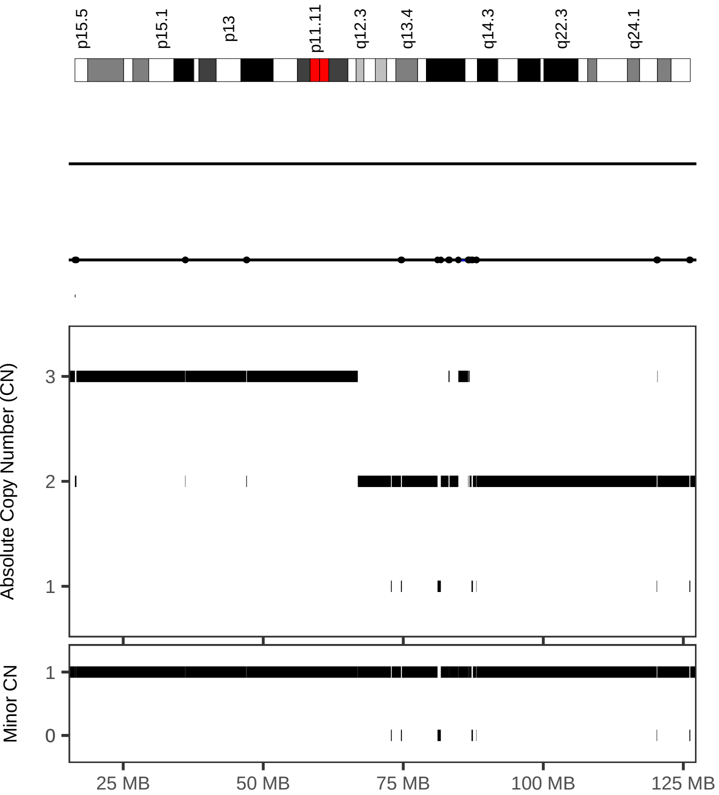

2a84997d−ccee−4f46−bea2−752534f26416

Cancer type	Breast−AdenoCA
Position	11:16375267−126235971
Interleaved intrachr. SVs	1
Total SVs (intrachr. + transl.)	13
SV types	DEL: 10; DUP: 3; h2hINV: 0; t2tINV: 0; TRA: 0
SVs in sample	596
Oscillating CN (2 and 3 states)	7, 29
CN segments	29
FDR fragment joints	0.02132783
FDR chr. breakp. enrich.	0.03
Linked to chrs	
Purity, ploidy	0.46, 2.83

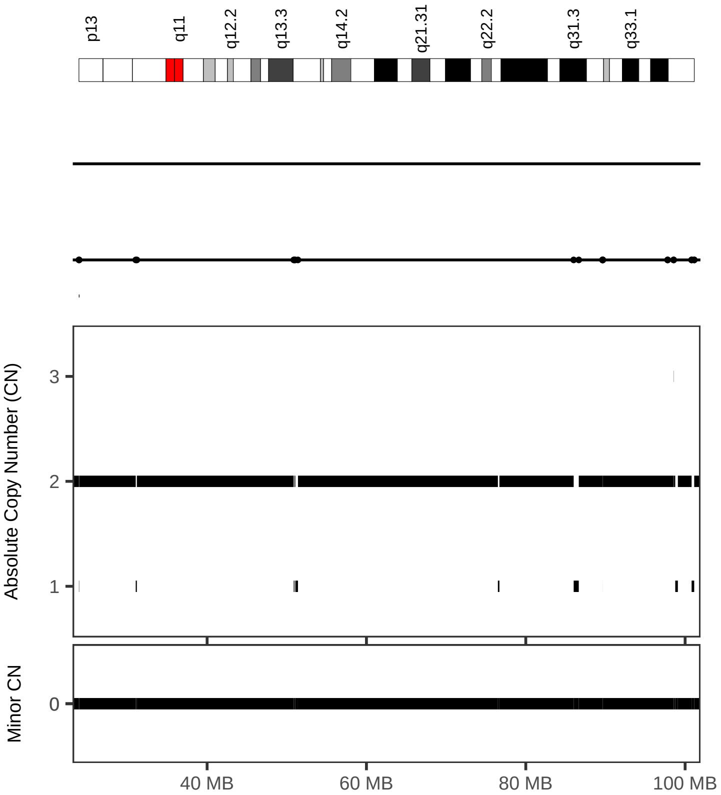

2a84997d−ccee−4f46−bea2−752534f26416

Cancer type	Breast−AdenoCA
Position	13:23906490−101146717
Interleaved intrachr. SVs	1
Total SVs (intrachr. + transl.)	10
SV types	DEL: 9; DUP: 1; h2hINV: 0; t2tINV: 0; TRA: 0
SVs in sample	596
Oscillating CN (2 and 3 states)	14, 20
CN segments	20
FDR fragment joints	0.01020027
FDR chr. breakp. enrich.	0.12
Linked to chrs	
Purity, ploidy	0.46, 2.83

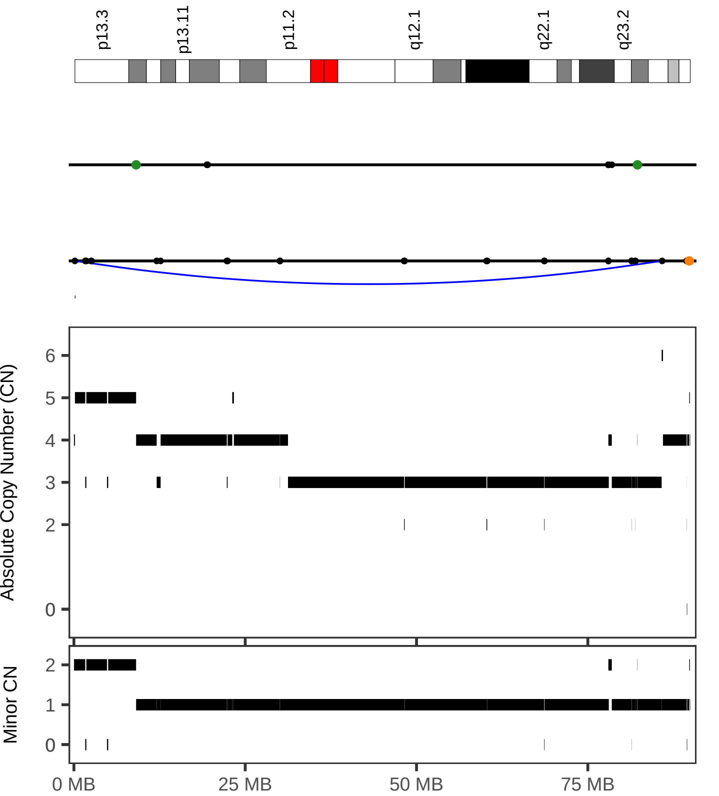

2a84997d−ccee−4f46−bea2−752534f26416

Cancer type	Breast−AdenoCA
Position	16:148109−89946753
Interleaved intrachr. SVs	1
Total SVs (intrachr. + transl.)	18
SV types	DEL: 12; DUP: 1; h2hINV: 2; t2tINV: 0; TRA: 3
SVs in sample	596
Oscillating CN (2 and 3 states)	7, 28
CN segments	39
FDR fragment joints	0.009092919
FDR chr. breakp. enrich.	0.43
Linked to chrs	
Purity, ploidy	0.46, 2.83

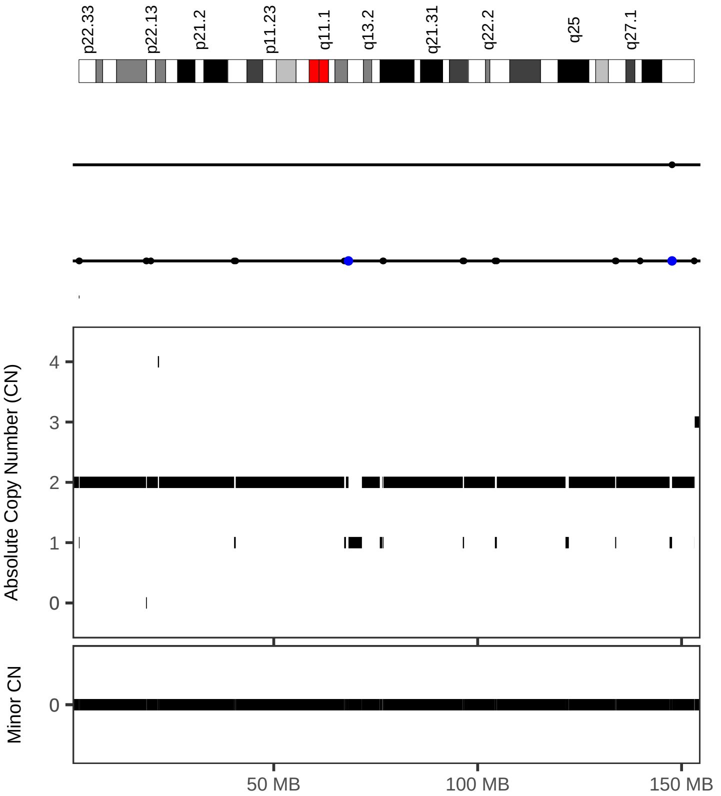

2a84997d−ccee−4f46−bea2−752534f26416

Cancer type	Breast−AdenoCA
Position	X:2221350−153108115
Interleaved intrachr. SVs	1
Total SVs (intrachr. + transl.)	11
SV types	DEL: 9; DUP: 0; h2hINV: 0; t2tINV: 0; TRA: 2
SVs in sample	596
Oscillating CN (2 and 3 states)	23, 23
CN segments	28
FDR fragment joints	0.02438658
FDR chr. breakp. enrich.	0.02
Linked to chrs	4:1211134−170990778;
Purity, ploidy	0.46, 2.83

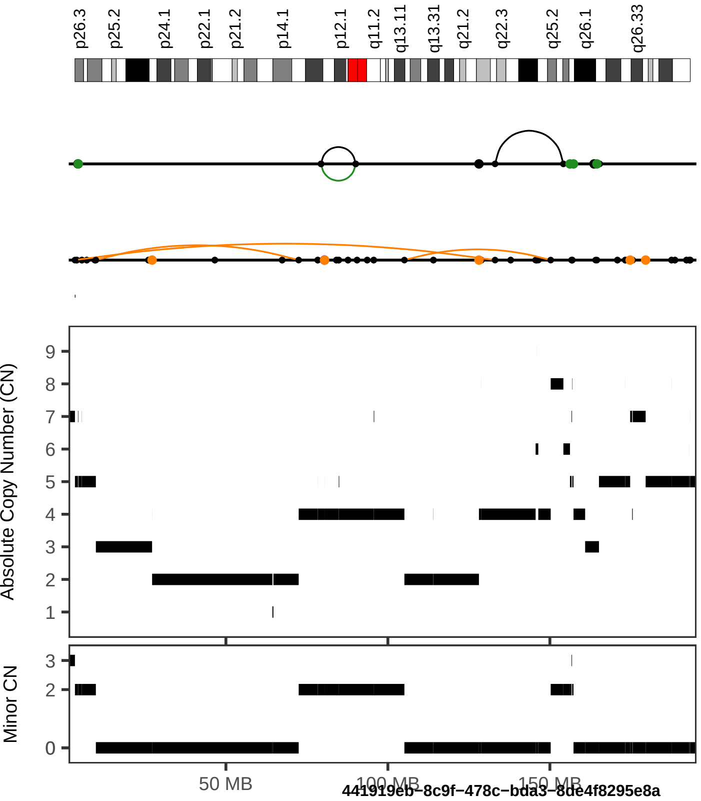

441919eb-8c9f-478c-bda3-8de4f8295e8a

Cancer type	Breast−AdenoCA
Position	3:3385898−154169466
Interleaved intrachr. SVs	3
Total SVs (intrachr. + transl.)	10
SV types	DEL: 2; DUP: 0; h2hINV: 1; t2tINV: 0; TRA: 7
SVs in sample	744
Oscillating CN (2 and 3 states)	7, 9
CN segments	31
FDR fragment joints	0.615458
FDR chr. breakp. enrich.	1
Linked to chrs	4:10183−123261412;7:1396546−142881742
Purity, ploidy	0.5, 3.35

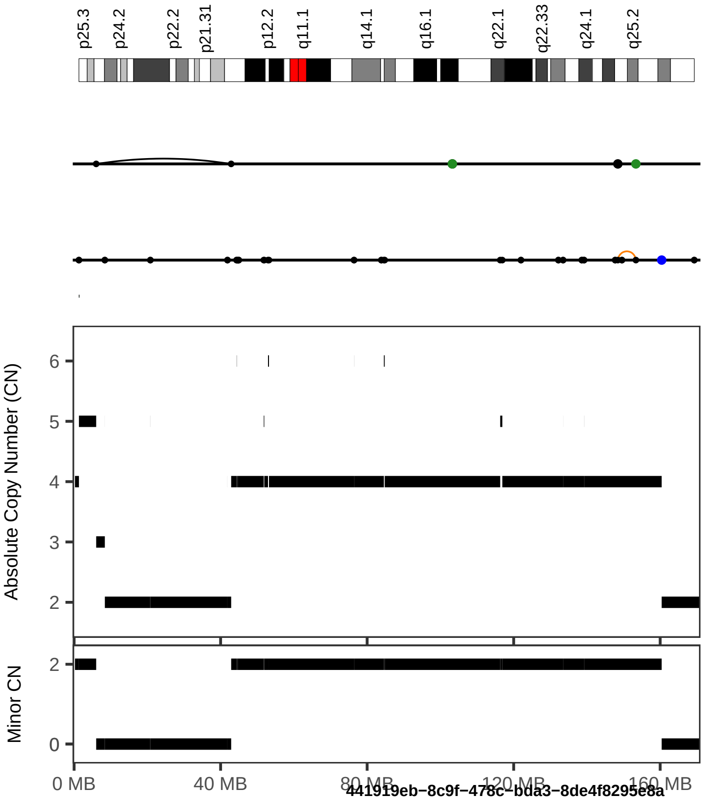

441919eb-8c9f-478c-bda3-8de4f8295e8a

Cancer type	Breast−AdenoCA
Position	6:6025768−153367099
Interleaved intrachr. SVs	1
Total SVs (intrachr. + transl.)	17
SV types	DEL: 1; DUP: 12; h2hINV: 1; t2tINV: 0; TRA: 3
SVs in sample	744
Oscillating CN (2 and 3 states)	7, 19
CN segments	26
FDR fragment joints	0.03488837
FDR chr. breakp. enrich.	0.04
Linked to chrs	2:18348976−72718038;7:1396546−142881742
Purity, ploidy	0.5, 3.35

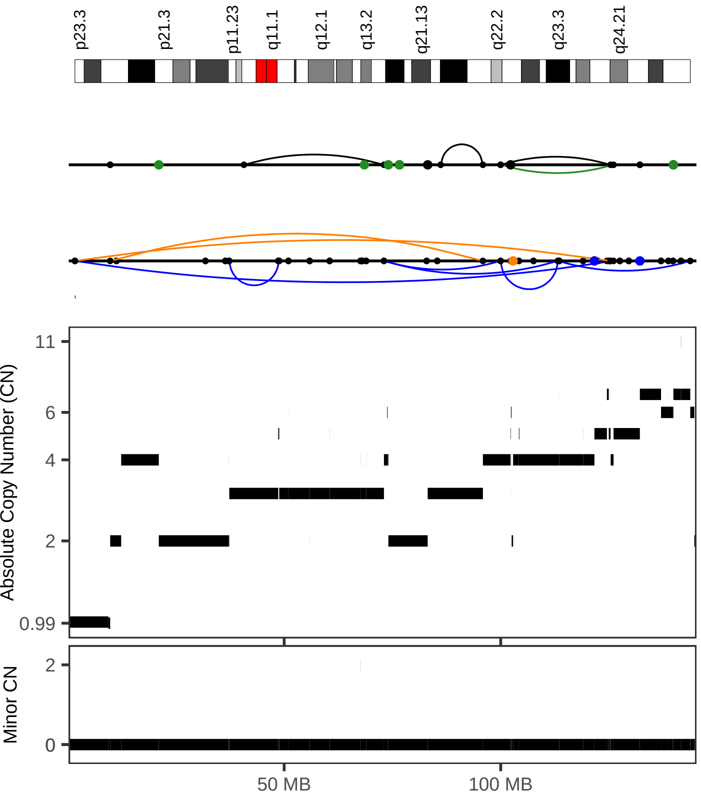

441919eb-8c9f-478c-bda3-8de4f8295e8a

Cancer type	Breast−AdenoCA
Position	8:1680713−143764779
Interleaved intrachr. SVs	9
Total SVs (intrachr. + transl.)	22
SV types	DEL: 2; DUP: 5; h2hINV: 1; t2tINV: 1; TRA: 13
SVs in sample	744
Oscillating CN (2 and 3 states)	7, 8
CN segments	56
FDR fragment joints	0.8874881
FDR chr. breakp. enrich.	0.02
Linked to chrs	17:31874561−36100025;
Purity, ploidy	0.5, 3.35

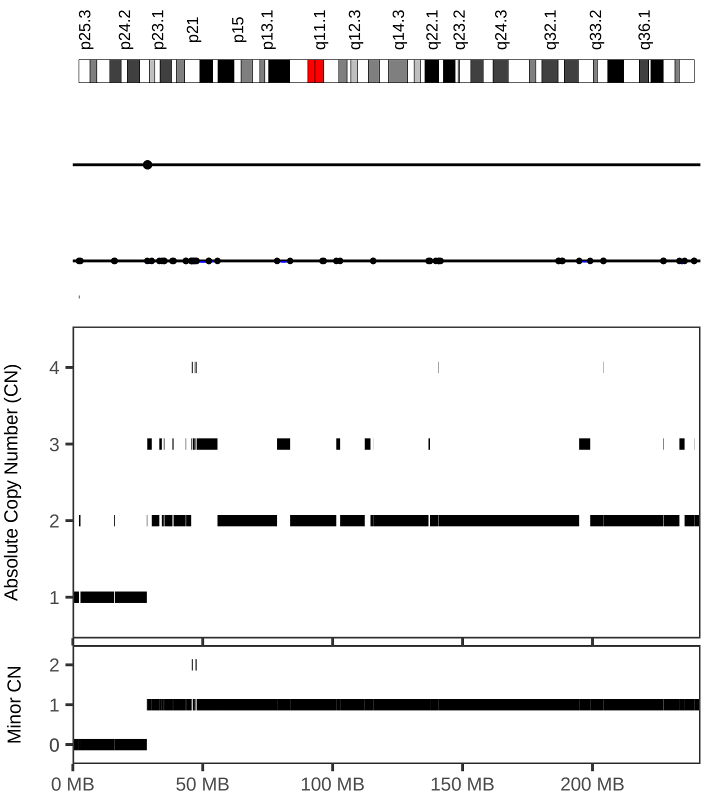

5ed024e8-d05e-4c65-9441-eda9930ccc82

Cancer type	Breast−AdenoCA
Position	2:2340260−239159179
Interleaved intrachr. SVs	1
Total SVs (intrachr. + transl.)	27
SV types	DEL: 0; DUP: 26; h2hINV: 0; t2tINV: 0; TRA: 1
SVs in sample	475
Oscillating CN (2 and 3 states)	14, 24
CN segments	47
FDR fragment joints	7.32348e−12
FDR chr. breakp. enrich.	0.3
Linked to chrs	
Purity, ploidy	0.38, 2.04

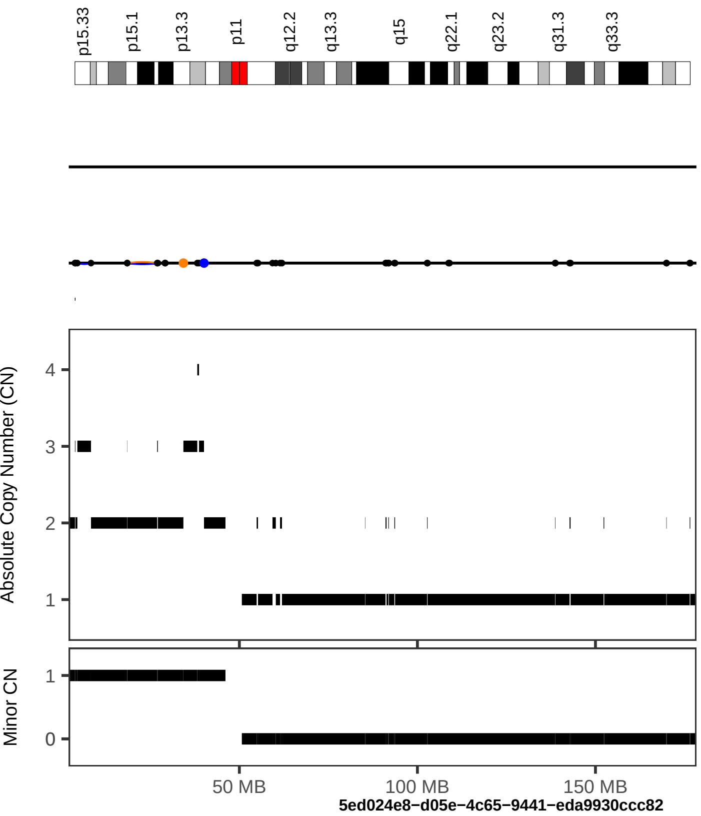

Cancer type	Breast−AdenoCA
Position	5:3827801−176614919
Interleaved intrachr. SVs	1
Total SVs (intrachr. + transl.)	20
SV types	DEL: 1; DUP: 17; h2hINV: 0; t2tINV: 0; TRA: 2
SVs in sample	475
Oscillating CN (2 and 3 states)	28, 39
CN segments	39
FDR fragment joints	1.804575e−06
FDR chr. breakp. enrich.	0.21
Linked to chrs	
Purity, ploidy	0.38, 2.04

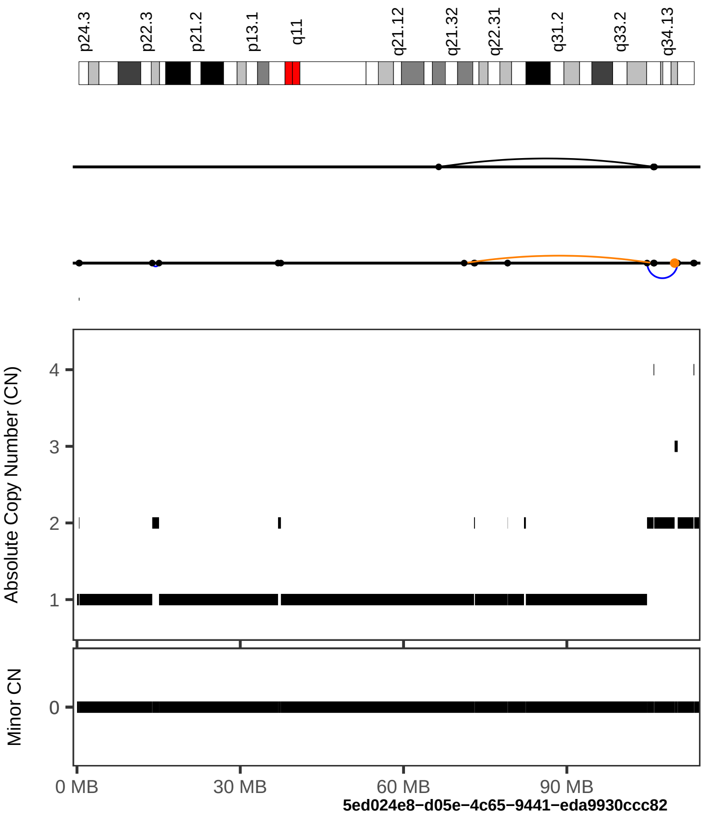

Cancer type	Breast−AdenoCA
Position	9:66454886−110368911
Interleaved intrachr. SVs	6
Total SVs (intrachr. + transl.)	7
SV types	DEL: 2; DUP: 1; h2hINV: 2; t2tINV: 1; TRA: 1
SVs in sample	475
Oscillating CN (2 and 3 states)	8, 8
CN segments	14
FDR fragment joints	0.7735152
FDR chr. breakp. enrich.	0.47
Linked to chrs	
Purity, ploidy	0.38, 2.04

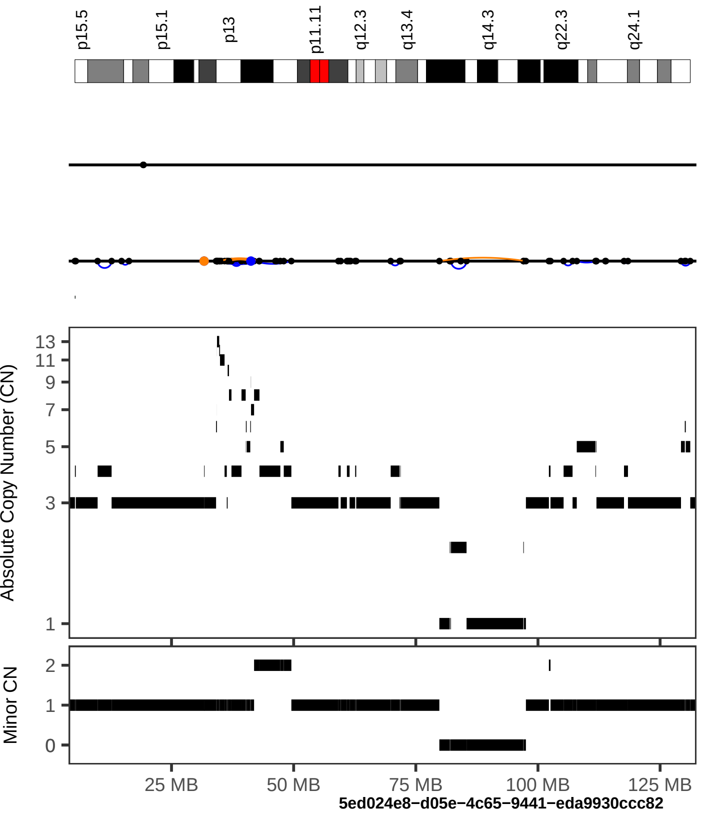

Cancer type	Breast−AdenoCA
Position	11:34123374−49526843
Interleaved intrachr. SVs	7
Total SVs (intrachr. + transl.)	8
SV types	DEL: 2; DUP: 5; h2hINV: 0; t2tINV: 0; TRA: 1
SVs in sample	475
Oscillating CN (2 and 3 states)	4, 5
CN segments	23
FDR fragment joints	0.2070134
FDR chr. breakp. enrich.	0
Linked to chrs	
Purity, ploidy	0.38, 2.04

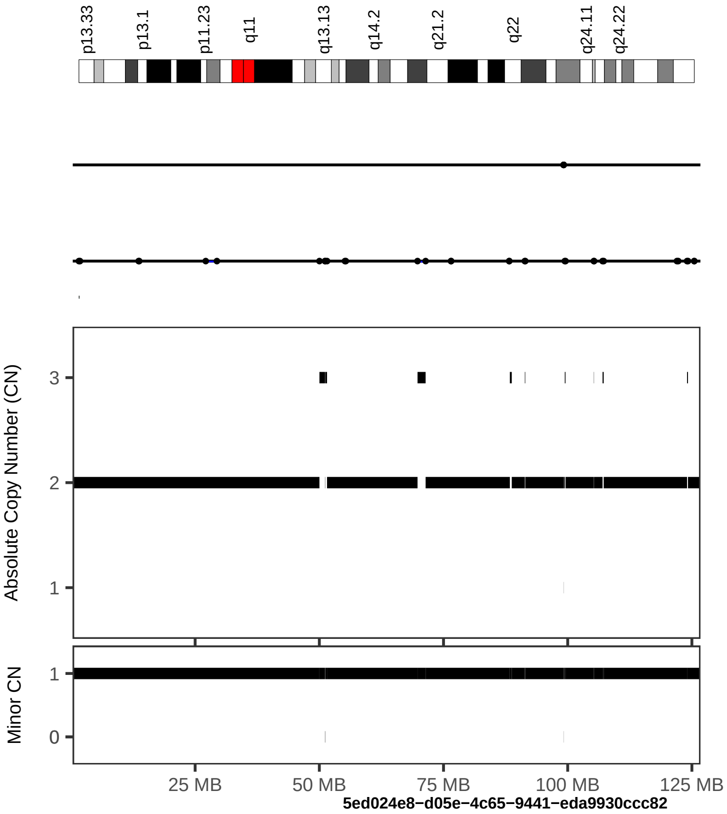

Cancer type	Breast−AdenoCA
Position	12:1602975−124219176
Interleaved intrachr. SVs	1
Total SVs (intrachr. + transl.)	15
SV types	DEL: 2; DUP: 12; h2hINV: 1; t2tINV: 0; TRA: 0
SVs in sample	475
Oscillating CN (2 and 3 states)	11, 21
CN segments	21
FDR fragment joints	0.005467199
FDR chr. breakp. enrich.	0.69
Linked to chrs	
Purity, ploidy	0.38, 2.04

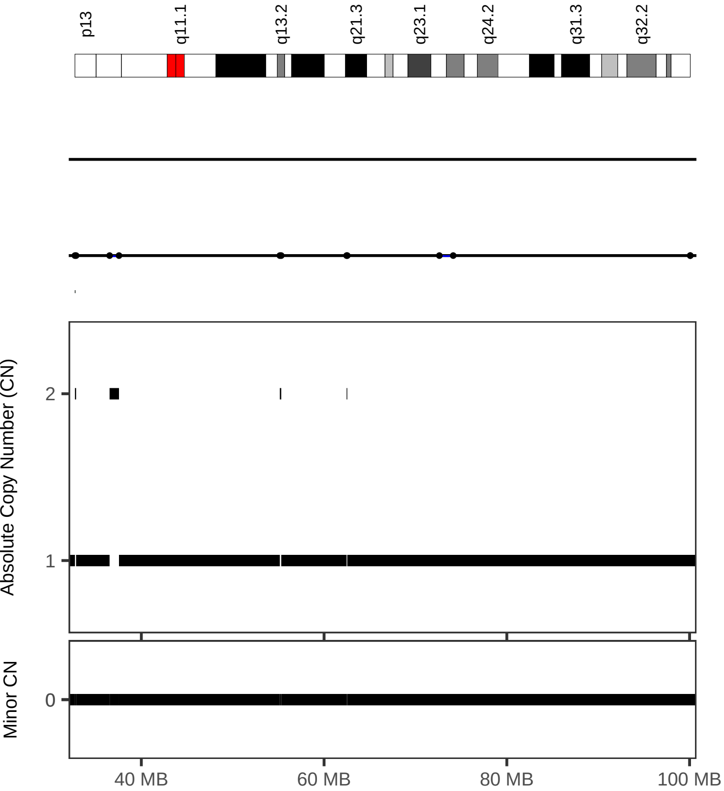

5ed024e8−d05e−4c65−9441−eda9930ccc82

Cancer type	Breast−AdenoCA
Position	14:32732923−74134194
Interleaved intrachr. SVs	1
Total SVs (intrachr. + transl.)	5
SV types	DEL: 0; DUP: 5; h2hINV: 0; t2tINV: 0; TRA: 0
SVs in sample	475
Oscillating CN (2 and 3 states)	8, 8
CN segments	8
FDR fragment joints	0.1013295
FDR chr. breakp. enrich.	0.07
Linked to chrs	
Purity, ploidy	0.38, 2.04

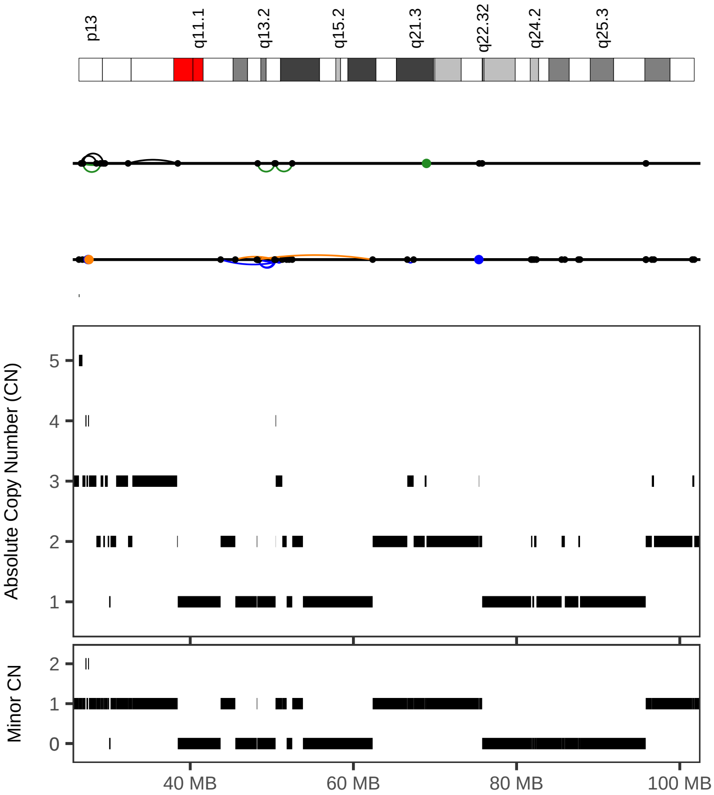

5ed024e8−d05e−4c65−9441−eda9930ccc82

Cancer type	Breast−AdenoCA
Position	15:43723632−62360621
Interleaved intrachr. SVs	11
Total SVs (intrachr. + transl.)	11
SV types	DEL: 2; DUP: 7; h2hINV: 0; t2tINV: 2; TRA: 0
SVs in sample	475
Oscillating CN (2 and 3 states)	5, 5
CN segments	12
FDR fragment joints	0.4101979
FDR chr. breakp. enrich.	0
Linked to chrs	
Purity, ploidy	0.38, 2.04

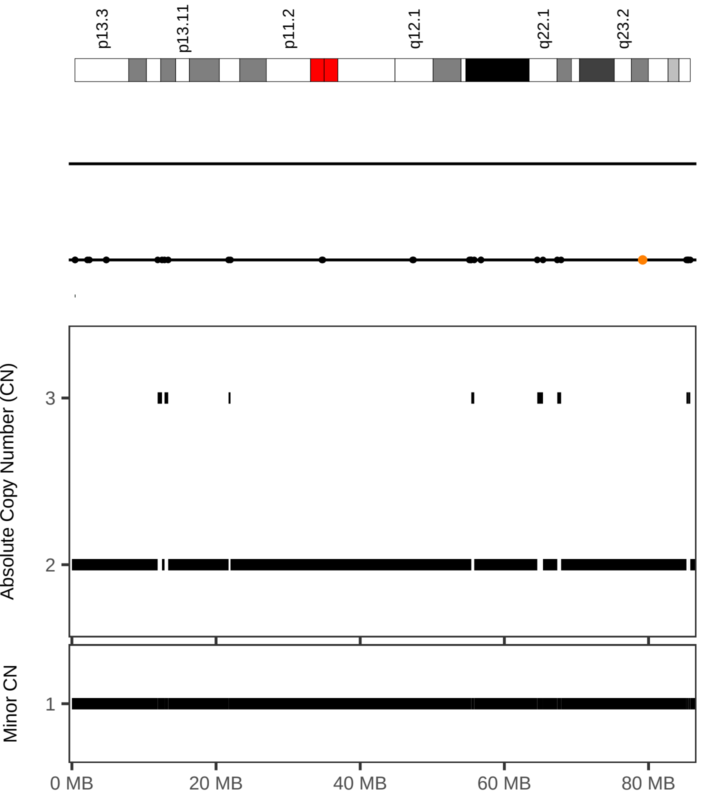

5ed024e8−d05e−4c65−9441−eda9930ccc82

Cancer type	Breast−AdenoCA
Position	16:421761−85786009
Interleaved intrachr. SVs	1
Total SVs (intrachr. + transl.)	15
SV types	DEL: 0; DUP: 14; h2hINV: 0; t2tINV: 0; TRA: 1
SVs in sample	475
Oscillating CN (2 and 3 states)	17, 17
CN segments	17
FDR fragment joints	4.035888e−05
FDR chr. breakp. enrich.	0.57
Linked to chrs	
Purity, ploidy	0.38, 2.04

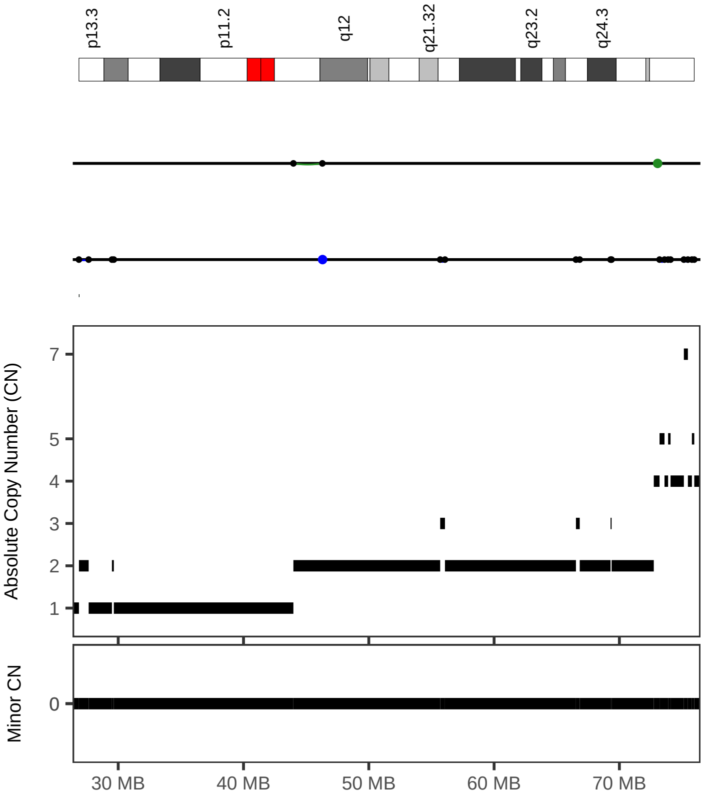

5ed024e8−d05e−4c65−9441−eda9930ccc82

Cancer type	Breast−AdenoCA
Position	17:26864514−75974296
Interleaved intrachr. SVs	1
Total SVs (intrachr. + transl.)	12
SV types	DEL: 0; DUP: 9; h2hINV: 0; t2tINV: 1; TRA: 2
SVs in sample	475
Oscillating CN (2 and 3 states)	7, 11
CN segments	20
FDR fragment joints	0.01333161
FDR chr. breakp. enrich.	1
Linked to chrs	
Purity, ploidy	0.38, 2.04

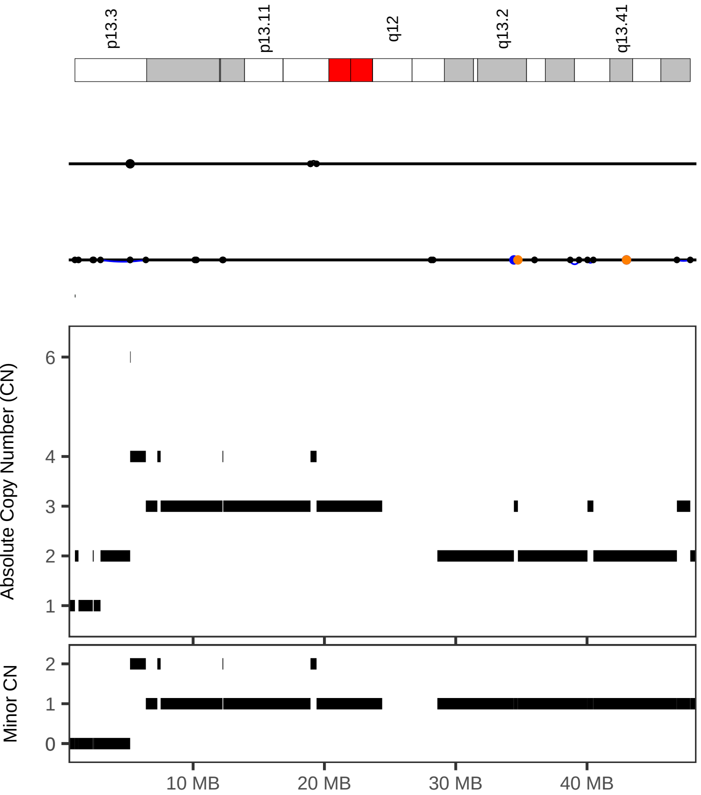

5ed024e8−d05e−4c65−9441−eda9930ccc82

Cancer type	Breast−AdenoCA
Position	19:998026−47861482
Interleaved intrachr. SVs	1
Total SVs (intrachr. + transl.)	15
SV types	DEL: 0; DUP: 9; h2hINV: 1; t2tINV: 0; TRA: 5
SVs in sample	475
Oscillating CN (2 and 3 states)	8, 15
CN segments	21
FDR fragment joints	0.0366383
FDR chr. breakp. enrich.	0.05
Linked to chrs	
Purity, ploidy	0.38, 2.04

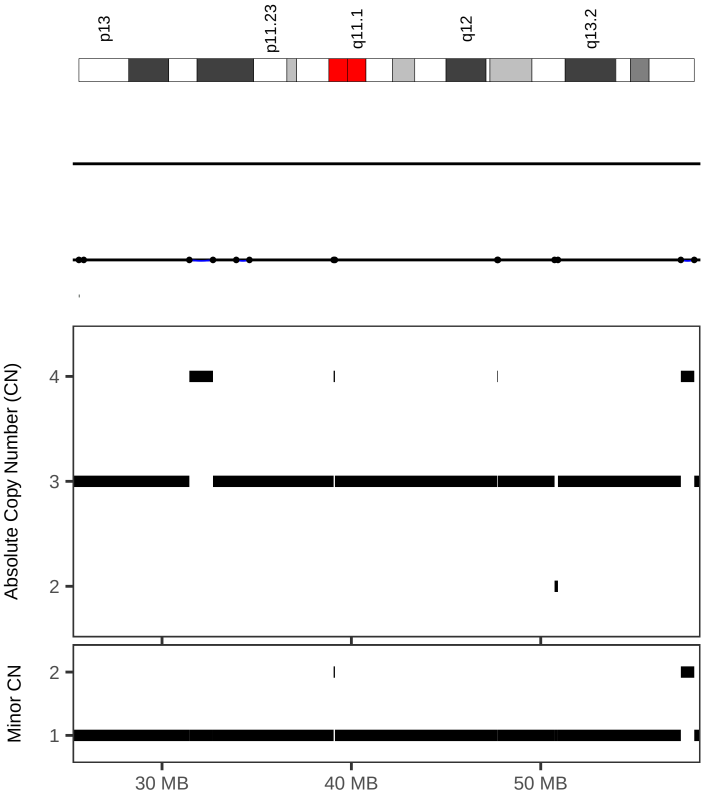

5ed024e8−d05e−4c65−9441−eda9930ccc82

Cancer type	Breast−AdenoCA
Position	20:25612239−58105683
Interleaved intrachr. SVs	1
Total SVs (intrachr. + transl.)	7
SV types	DEL: 1; DUP: 6; h2hINV: 0; t2tINV: 0; TRA: 0
SVs in sample	475
Oscillating CN (2 and 3 states)	7, 11
CN segments	11
FDR fragment joints	0.1278363
FDR chr. breakp. enrich.	0.68
Linked to chrs	
Purity, ploidy	0.38, 2.04

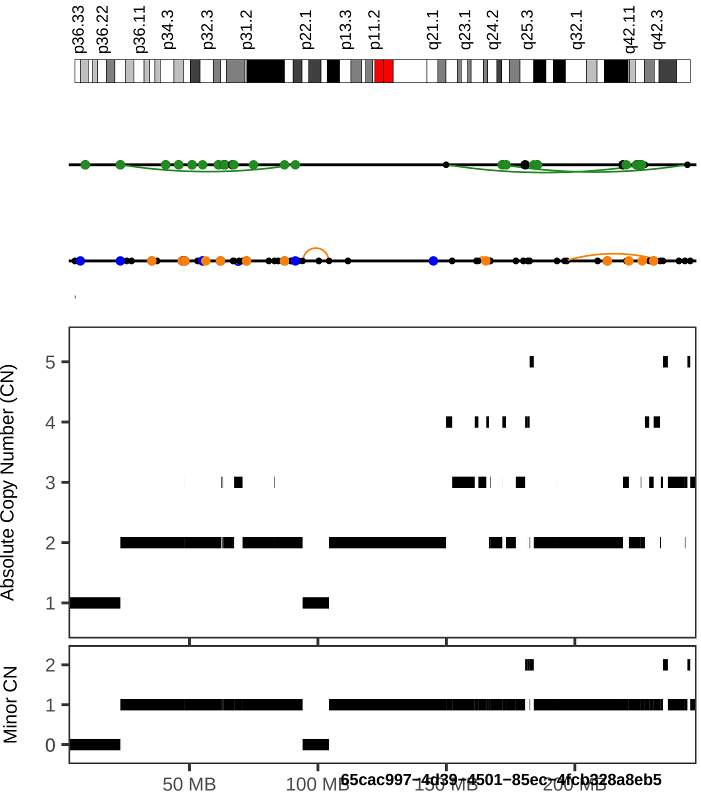

65cac997−4d39−4501−85ec−4fcb328a8eb5

Cancer type	Breast−AdenoCA
Position	1:149881190−243814704
Interleaved intrachr. SVs	3
Total SVs (intrachr. + transl.)	26
SV types	DEL: 1; DUP: 0; h2hINV: 0; t2tINV: 2; TRA: 23
SVs in sample	909
Oscillating CN (2 and 3 states)	7, 12
CN segments	36
FDR fragment joints	0.615458
FDR chr. breakp. enrich.	0
Linked to chrs	13:20691358−100667836;4:36731786−170830419 7:95697844−156942102;9:31378526−139868175 X:9604512−99744773;

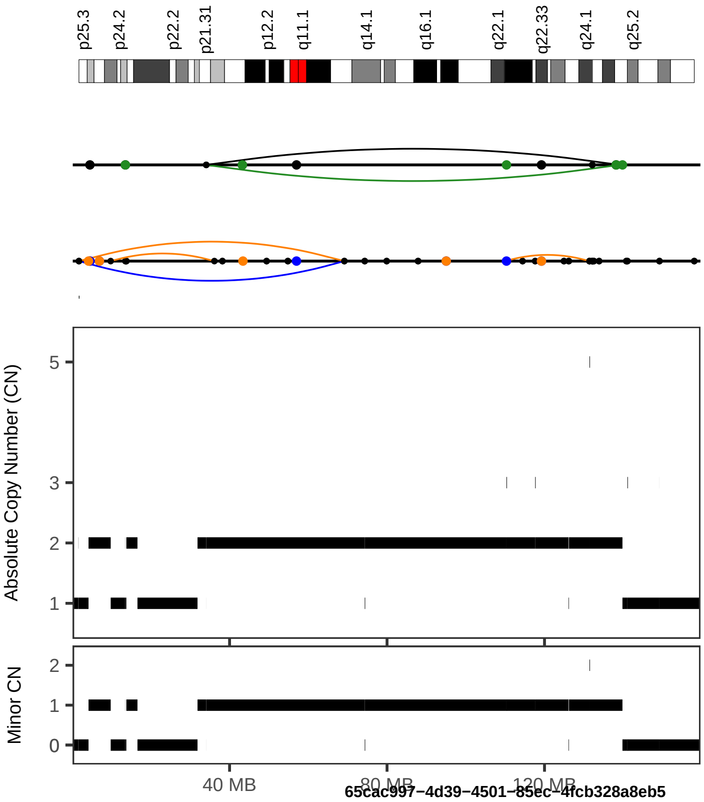

65cac997−4d39−4501−85ec−4fcb328a8eb5

Cancer type	Breast−AdenoCA
Position	6:1730813−139089485
Interleaved intrachr. SVs	5
Total SVs (intrachr. + transl.)	27
SV types	DEL: 2; DUP: 1; h2hINV: 1; t2tINV: 1; TRA: 22
SVs in sample	909
Oscillating CN (2 and 3 states)	13, 19
CN segments	21
FDR fragment joints	0.9284301
FDR chr. breakp. enrich.	0.76
Linked to chrs	1:149881190−243814703;15:38084470−80918007 8:22900664−128813813;
Purity, ploidy	0.66, 1.85

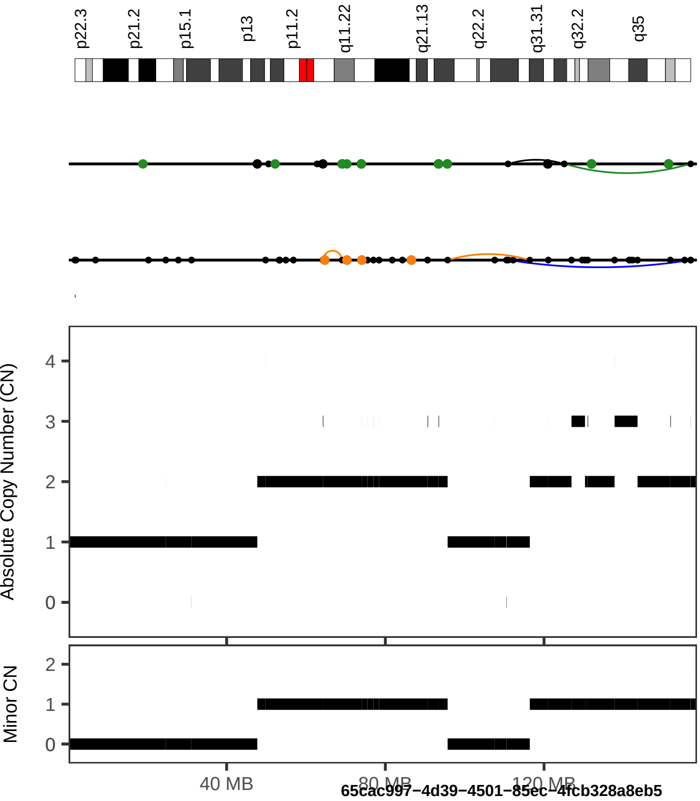

65cac997-4d39-4501-85ec-4fcb328a8eb5

Cancer type	Breast−AdenoCA
Position	7:95697844−156942103
Interleaved intrachr. SVs	4
Total SVs (intrachr. + transl.)	11
SV types	DEL: 1; DUP: 1; h2hINV: 1; t2tINV: 1; TRA: 7
SVs in sample	909
Oscillating CN (2 and 3 states)	7, 10
CN segments	18
FDR fragment joints	0.615458
FDR chr. breakp. enrich.	0.01
Linked to chrs	1:149881190−243814703;15:38084470−80918007
Purity, ploidy	0.66, 1.85

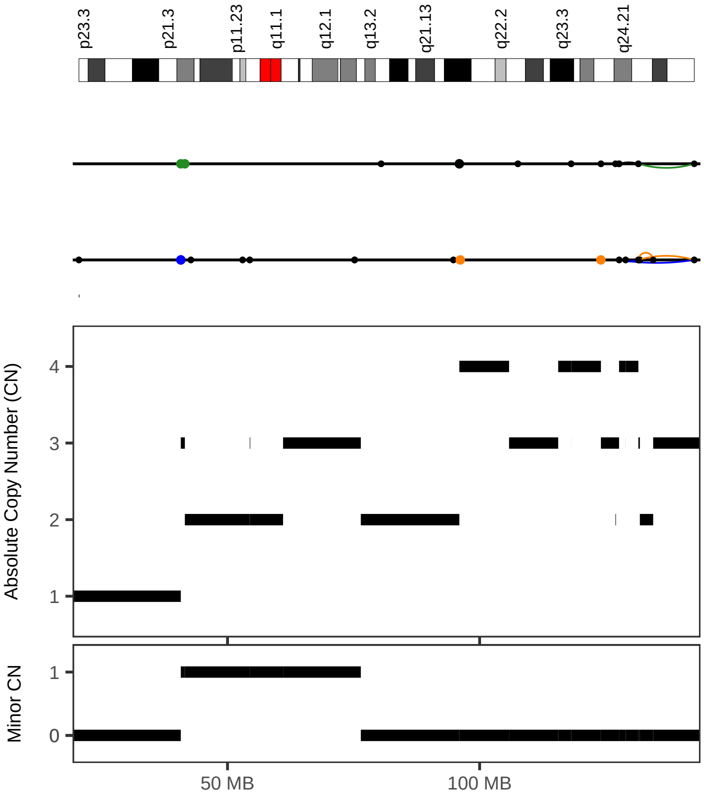

6956473b-92e5-4069-a26b-b48e280e76f2

Cancer type	Breast−AdenoCA
Position	8:127655333−142568575
Interleaved intrachr. SVs	6
Total SVs (intrachr. + transl.)	6
SV types	DEL: 1; DUP: 2; h2hINV: 1; t2tINV: 2; TRA: 0
SVs in sample	227
Oscillating CN (2 and 3 states)	4, 6
CN segments	6
FDR fragment joints	0.9284301
FDR chr. breakp. enrich.	0
Linked to chrs	
Purity, ploidy	0.56, 1.61

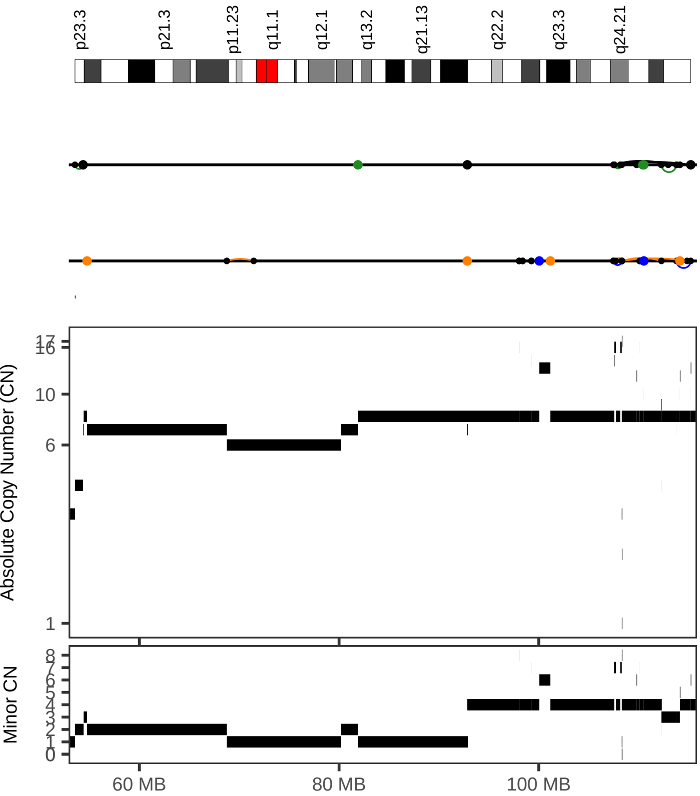

7861a5f2-8910-4bcb-9c34-f79c5acd6e21

Cancer type	Breast−AdenoCA
Position	8:107480527−115223161
Interleaved intrachr. SVs	13
Total SVs (intrachr. + transl.)	18
SV types	DEL: 4; DUP: 2; h2hINV: 4; t2tINV: 3; TRA: 5
SVs in sample	172
Oscillating CN (2 and 3 states)	4, 7
CN segments	32
FDR fragment joints	0.8653243
FDR chr. breakp. enrich.	0
Linked to chrs	
Purity, ploidy	0.54, 3.35

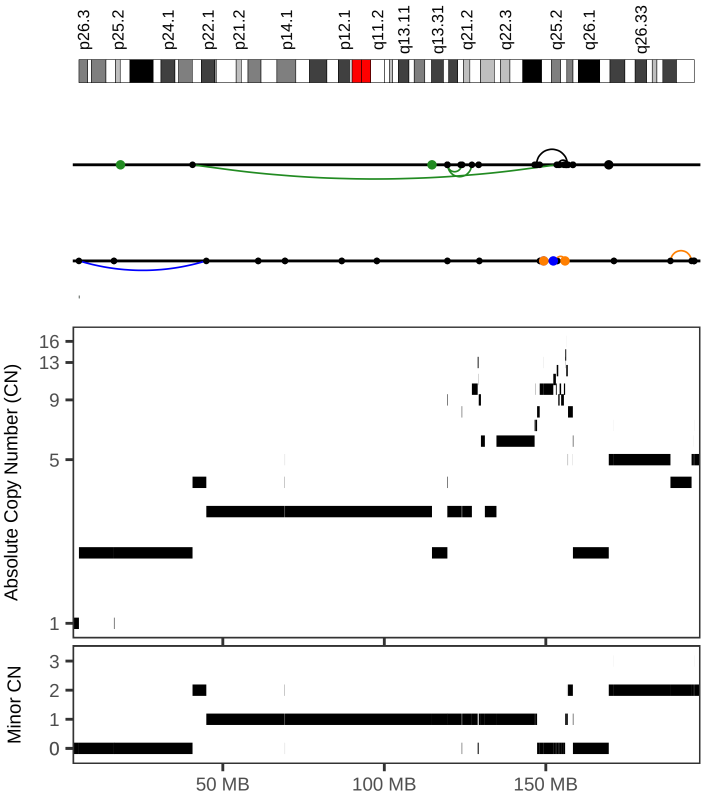

786e8dbe-442e-4551-87b3-b4c333b04dd4

Cancer type	Breast−AdenoCA
Position	3:5449076−156876693
Interleaved intrachr. SVs	6
Total SVs (intrachr. + transl.)	12
SV types	DEL: 1; DUP: 1; h2hINV: 2; t2tINV: 2; TRA: 6
SVs in sample	222
Oscillating CN (2 and 3 states)	4, 6
CN segments	44
FDR fragment joints	0.9284301
FDR chr. breakp. enrich.	0
Linked to chrs	
Purity, ploidy	0.61, 2.97

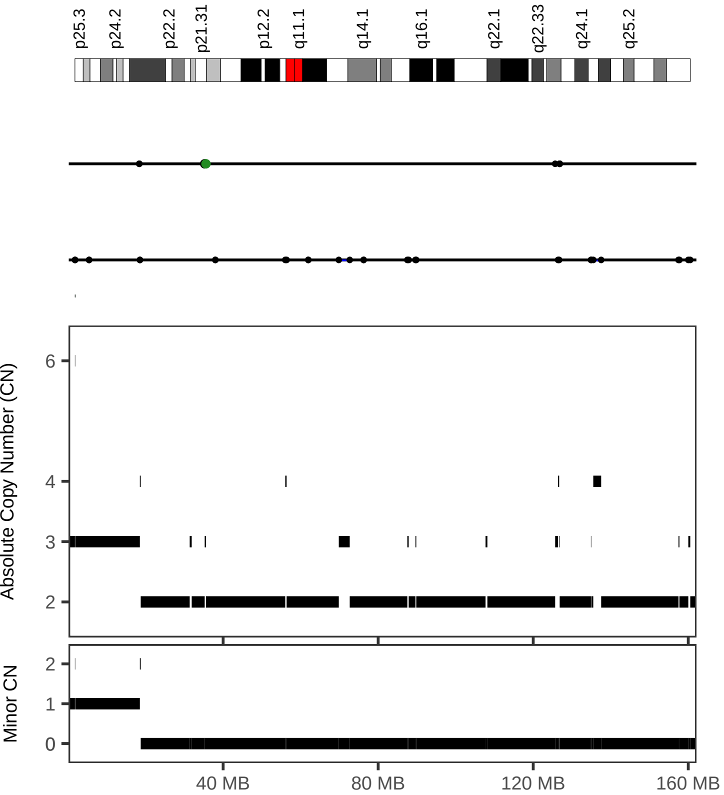

8785012f−f73e−4d68−87cf−1d804af32782

Cancer type	Breast−AdenoCA
Position	6:1759680−160510927
Interleaved intrachr. SVs	1
Total SVs (intrachr. + transl.)	16
SV types	DEL: 1; DUP: 11; h2hINV: 0; t2tINV: 2; TRA: 2
SVs in sample	530
Oscillating CN (2 and 3 states)	10, 18
CN segments	30
FDR fragment joints	0.05741733
FDR chr. breakp. enrich.	0.11
Linked to chrs	11:9158465−9546360;
Purity, ploidy	0.56, 2.92

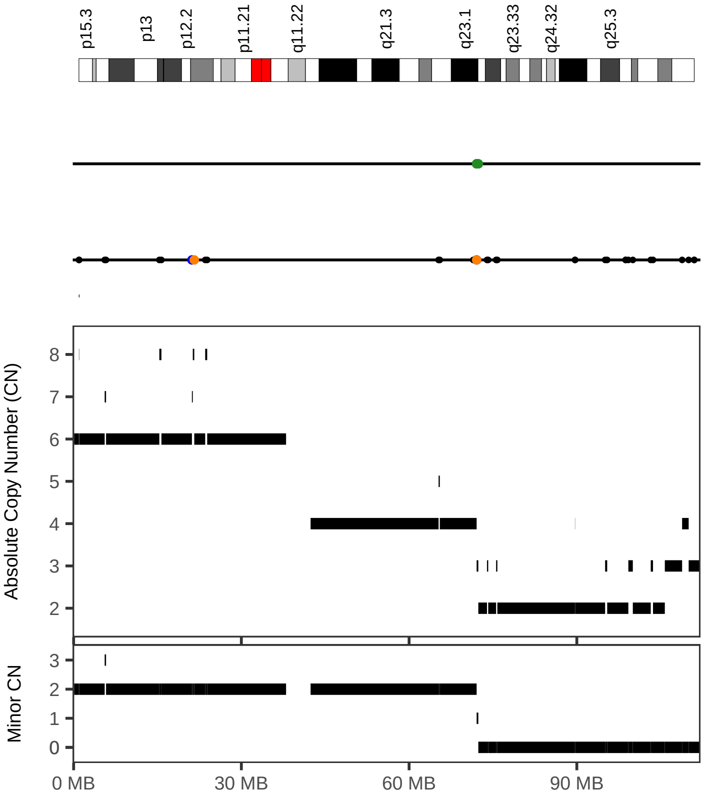

8785012f−f73e−4d68−87cf−1d804af32782

Cancer type	Breast−AdenoCA
Position	10:949660−110004864
Interleaved intrachr. SVs	1
Total SVs (intrachr. + transl.)	23
SV types	DEL: 1; DUP: 14; h2hINV: 0; t2tINV: 0; TRA: 8
SVs in sample	530
Oscillating CN (2 and 3 states)	8, 15
CN segments	31
FDR fragment joints	0.005291502
FDR chr. breakp. enrich.	0.97
Linked to chrs	
Purity, ploidy	0.56, 2.92

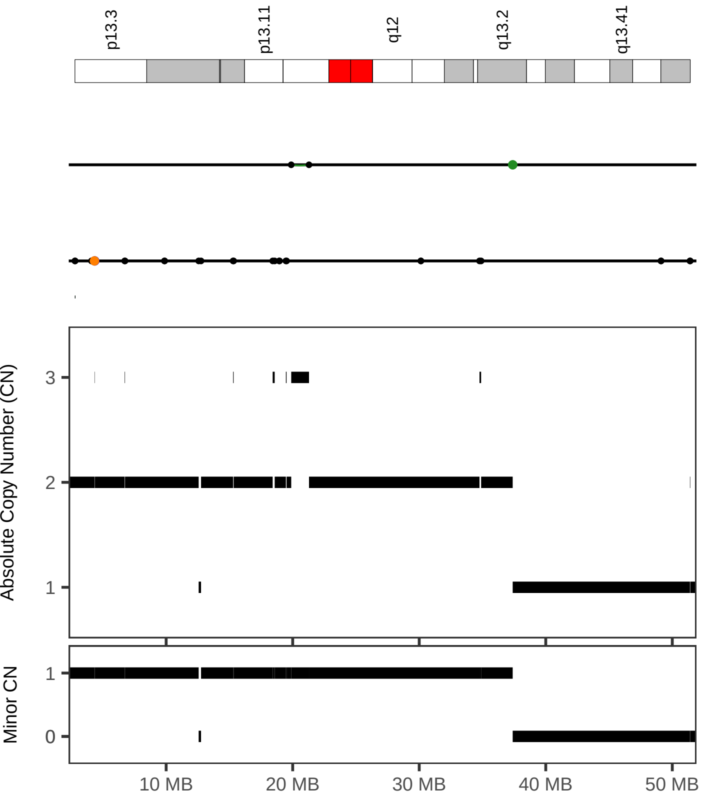

88db1340−e4bf−451a−87c0−6e9168296f5e

Cancer type	Breast−AdenoCA
Position	19:2788499−51420171
Interleaved intrachr. SVs	1
Total SVs (intrachr. + transl.)	15
SV types	DEL: 1; DUP: 10; h2hINV: 0; t2tINV: 1; TRA: 3
SVs in sample	415
Oscillating CN (2 and 3 states)	11, 20
CN segments	20
FDR fragment joints	0.0366383
FDR chr. breakp. enrich.	0.01
Linked to chrs	17:3491150−76396527;
Purity, ploidy	0.53, 1.49

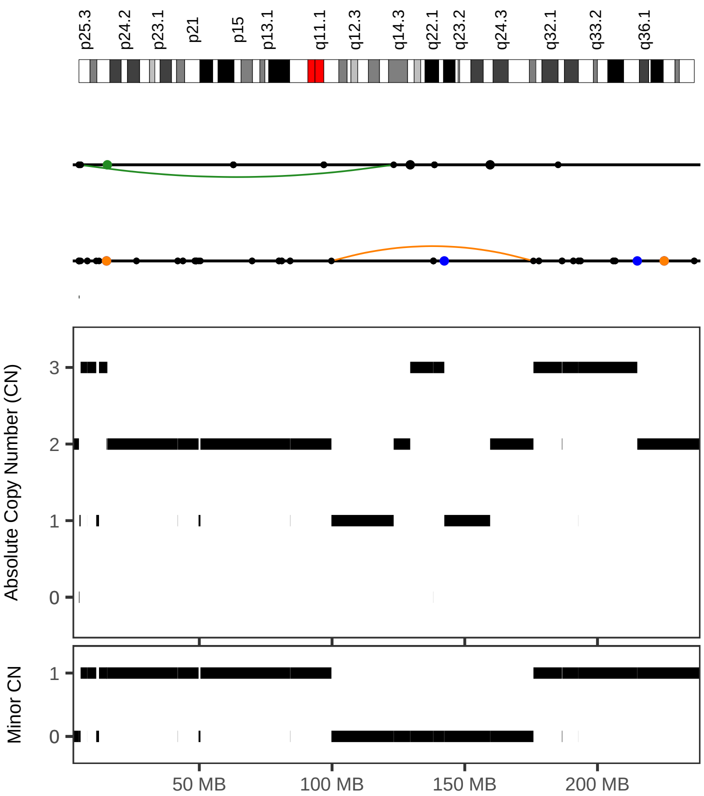

8bd68696−5eeb−46d7−9dc6−c39c849029a4

Cancer type	Breast−AdenoCA
Position	2:4653440−175863031
Interleaved intrachr. SVs	5
Total SVs (intrachr. + transl.)	11
SV types	DEL: 2; DUP: 0; h2hINV: 1; t2tINV: 2; TRA: 6
SVs in sample	343
Oscillating CN (2 and 3 states)	9, 15
CN segments	24
FDR fragment joints	0.8653243
FDR chr. breakp. enrich.	0.01
Linked to chrs	
Purity, ploidy	0.75, 1.87

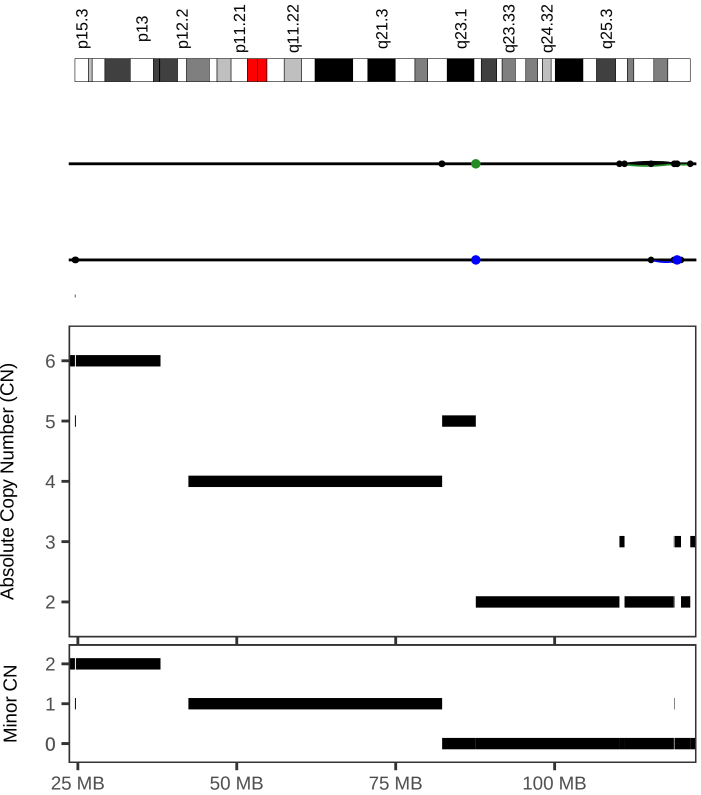

993103b1−e5a1−4c33−8629−be53ebc41d64

Cancer type	Breast−AdenoCA
Position	10:110197032−121335390
Interleaved intrachr. SVs	6
Total SVs (intrachr. + transl.)	7
SV types	DEL: 0; DUP: 2; h2hINV: 2; t2tINV: 2; TRA: 1
SVs in sample	202
Oscillating CN (2 and 3 states)	7, 7
CN segments	7
FDR fragment joints	0.6776251
FDR chr. breakp. enrich.	0.75
Linked to chrs	
Purity, ploidy	0.41, 3.34

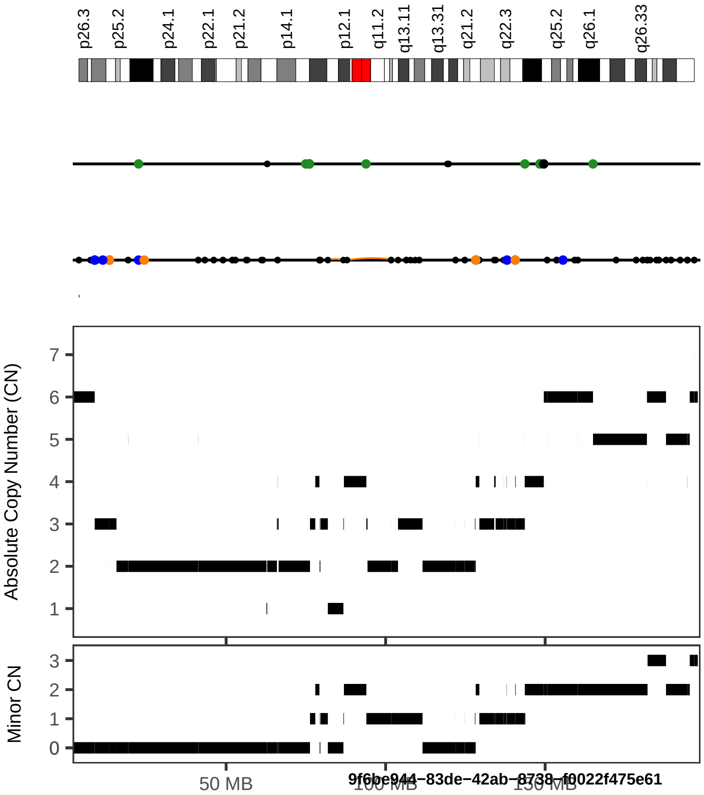

9f6be944−83de−42ab−8738−f0022f475e61

Cancer type	Breast−AdenoCA
Position	3:9745722−194626444
Interleaved intrachr. SVs	1
Total SVs (intrachr. + transl.)	46
SV types	DEL: 6; DUP: 16; h2hINV: 2; t2tINV: 0; TRA: 22
SVs in sample	864
Oscillating CN (2 and 3 states)	12, 14
CN segments	60
FDR fragment joints	0.04139017
FDR chr. breakp. enrich.	0.19
Linked to chrs	10:81006691−123761330;19:2570979−50976538 4:6056130−187601904;5:10244050−179437054 6:6002977−168886718;
Purity, ploidy	[illegible]

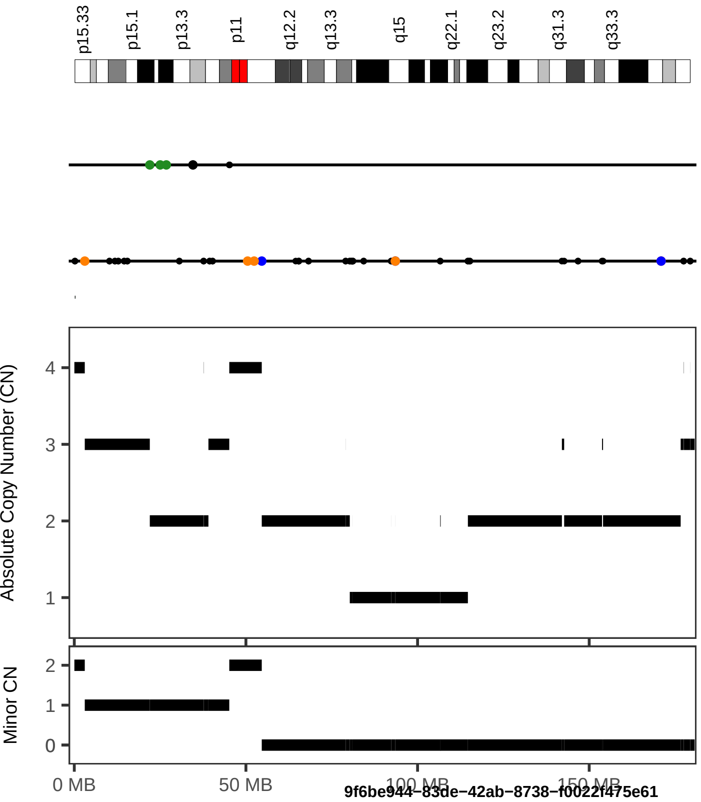

9f6be944−83de−42ab−8738−f0022f475e61

Cancer type	Breast−AdenoCA
Position	5:10244050−179437055
Interleaved intrachr. SVs	1
Total SVs (intrachr. + transl.)	27
SV types	DEL: 1; DUP: 14; h2hINV: 0; t2tINV: 1; TRA: 11
SVs in sample	864
Oscillating CN (2 and 3 states)	11, 11
CN segments	28
FDR fragment joints	0.01686763
FDR chr. breakp. enrich.	0.37
Linked to chrs	3:9745722−194626443;8:6488297−144963440
Purity, ploidy	0.6, 3.26

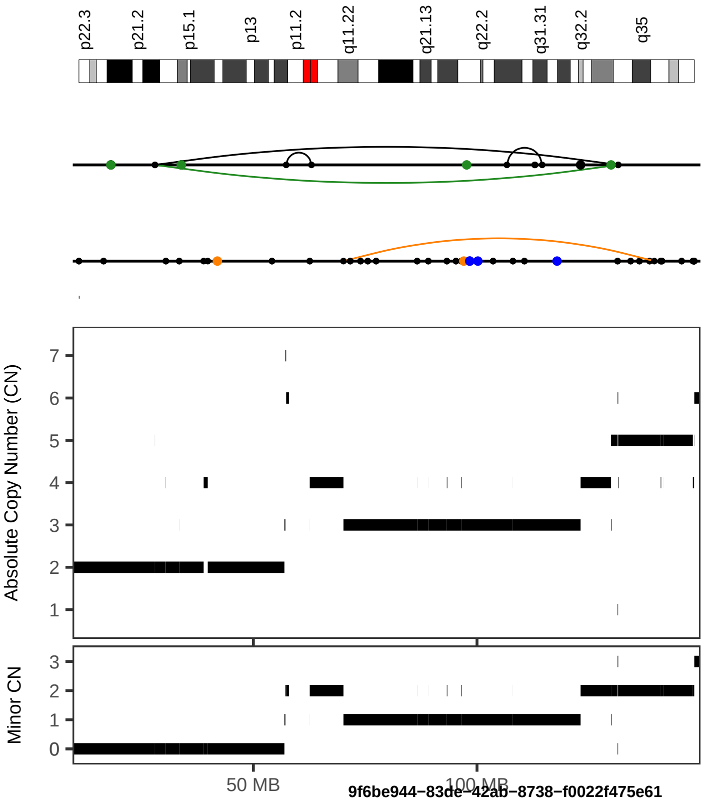

9f6be944−83de−42ab−8738−f0022f475e61

Cancer type	Breast−AdenoCA
Position	7:27995787−139646245
Interleaved intrachr. SVs	3
Total SVs (intrachr. + transl.)	14
SV types	DEL: 1; DUP: 0; h2hINV: 1; t2tINV: 1; TRA: 11
SVs in sample	864
Oscillating CN (2 and 3 states)	15, 16
CN segments	31
FDR fragment joints	0.9794281
FDR chr. breakp. enrich.	0.35
Linked to chrs	13:83228651−83363454;X:64211866−154270744
Purity, ploidy	0.6, 3.26

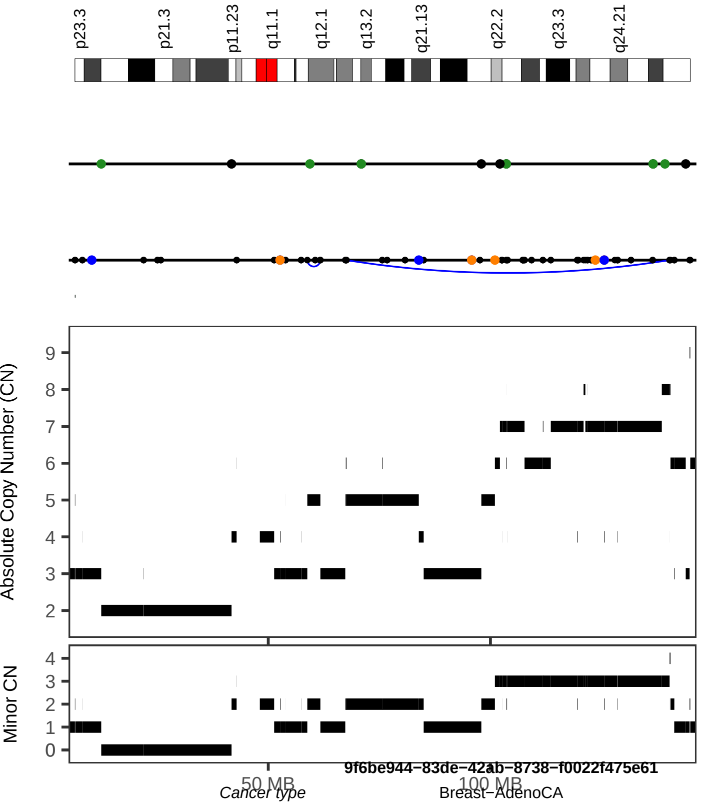

9f6be944−83de−42ab−8738−f0022f475e61

Cancer type	Breast−AdenoCA
Position	8:6488297−144963441
Interleaved intrachr. SVs	1
Total SVs (intrachr. + transl.)	39
SV types	DEL: 1; DUP: 15; h2hINV: 0; t2tINV: 0; TRA: 23
SVs in sample	864
Oscillating CN (2 and 3 states)	7, 10
CN segments	61
FDR fragment joints	0.1241446
FDR chr. breakp. enrich.	0
Linked to chrs	1:2210884−224017647;12:672188−132675127 5:10244050−179437054;6:6002977−168886718 7:27995787−139646244;X:64211866−154270744

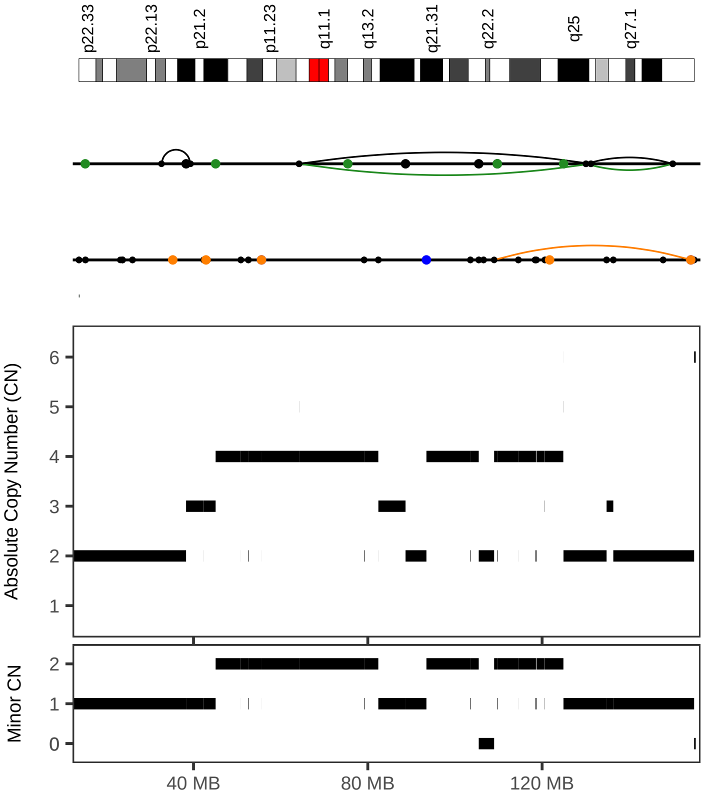

9f6be944−83de−42ab−8738−f0022f475e61

Cancer type	Breast−AdenoCA
Position	X:64211866−154270745
Interleaved intrachr. SVs	5
Total SVs (intrachr. + transl.)	15
SV types	DEL: 1; DUP: 0; h2hINV: 2; t2tINV: 2; TRA: 10
SVs in sample	864
Oscillating CN (2 and 3 states)	14, 23
CN segments	28
FDR fragment joints	0.7257048
FDR chr. breakp. enrich.	0.41
Linked to chrs	12:672188−132675127;
Purity, ploidy	0.6, 3.26

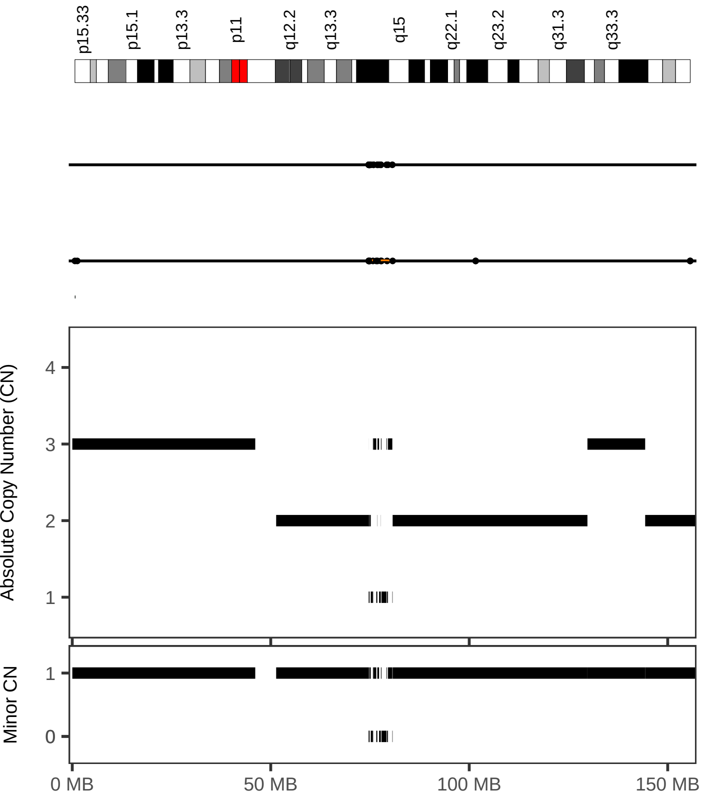

9fefbe7c−f66a−4940−843e−285cb7b392c1

Cancer type	Breast−AdenoCA
Position	5:74688832−80707030
Interleaved intrachr. SVs	15
Total SVs (intrachr. + transl.)	15
SV types	DEL: 2; DUP: 3; h2hINV: 5; t2tINV: 5; TRA: 0
SVs in sample	740
Oscillating CN (2 and 3 states)	6, 11
CN segments	25
FDR fragment joints	0.7257048
FDR chr. breakp. enrich.	0
Linked to chrs	
Purity, ploidy	0.83, 3.26

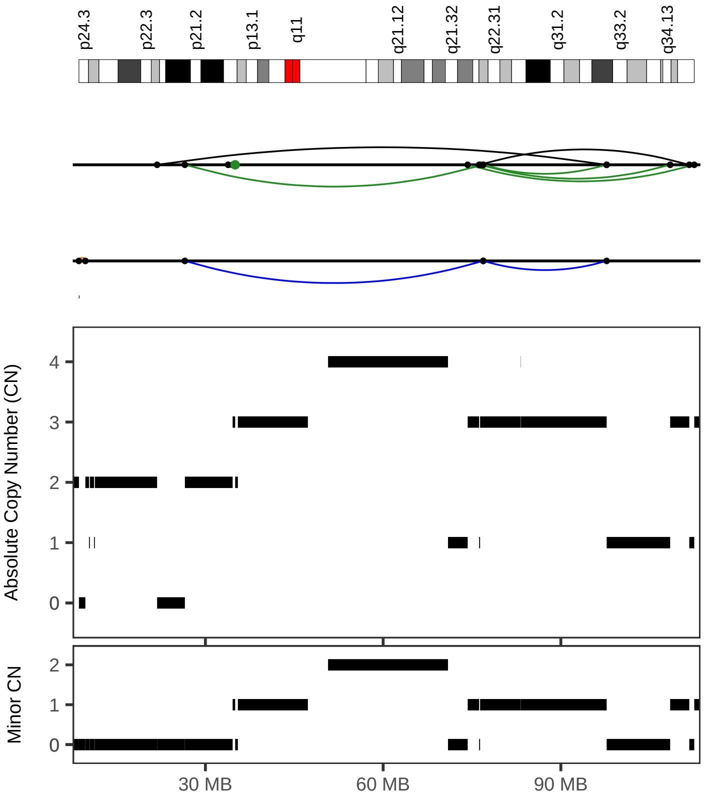

9fefbe7c−f66a−4940−843e−285cb7b392c1

Cancer type	Breast−AdenoCA
Position	9:21844042−112515969
Interleaved intrachr. SVs	8
Total SVs (intrachr. + transl.)	9
SV types	DEL: 0; DUP: 2; h2hINV: 2; t2tINV: 4; TRA: 1
SVs in sample	740
Oscillating CN (2 and 3 states)	5, 10
CN segments	16
FDR fragment joints	0.615458
FDR chr. breakp. enrich.	0
Linked to chrs	
Purity, ploidy	0.83, 3.26

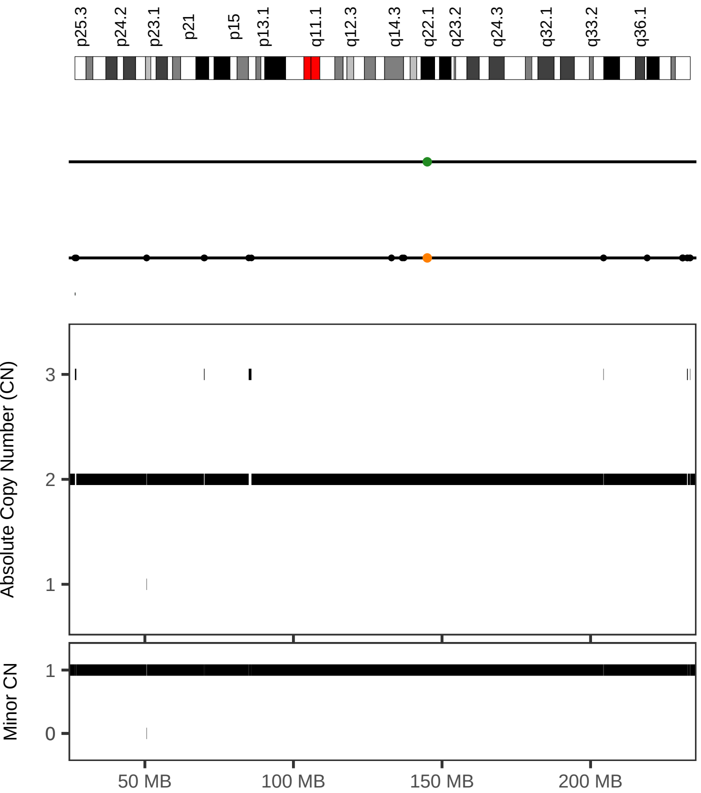

a824b3bd−34d5−4cc1−a92f−f9d6ac0f1814

Cancer type	Breast−AdenoCA
Position	2:26459899−233537709
Interleaved intrachr. SVs	1
Total SVs (intrachr. + transl.)	14
SV types	DEL: 2; DUP: 10; h2hINV: 0; t2tINV: 0; TRA: 2
SVs in sample	463
Oscillating CN (2 and 3 states)	11, 14
CN segments	14
FDR fragment joints	0.05370273
FDR chr. breakp. enrich.	0
Linked to chrs	11:10547187−101046436;
Purity, ploidy	0.51, 2.1

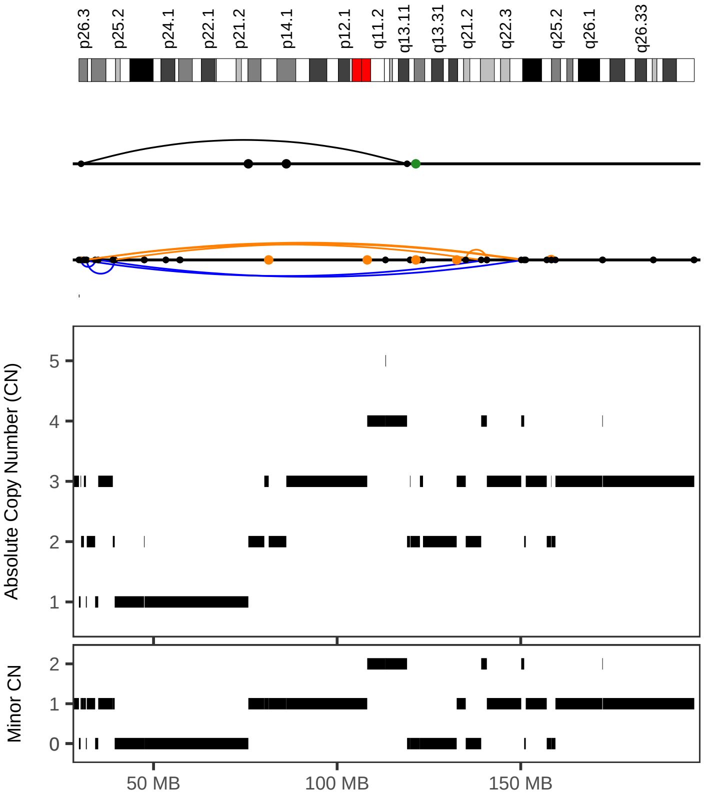

a824b3bd−34d5−4cc1−a92f−f9d6ac0f1814

Cancer type	Breast−AdenoCA
Position	3:29682227−151361518
Interleaved intrachr. SVs	10
Total SVs (intrachr. + transl.)	17
SV types	DEL: 5; DUP: 4; h2hINV: 1; t2tINV: 0; TRA: 7
SVs in sample	463
Oscillating CN (2 and 3 states)	7, 13
CN segments	31
FDR fragment joints	0.5435077
FDR chr. breakp. enrich.	0.71
Linked to chrs	1:147408680−212272246;
Purity, ploidy	0.51, 2.1

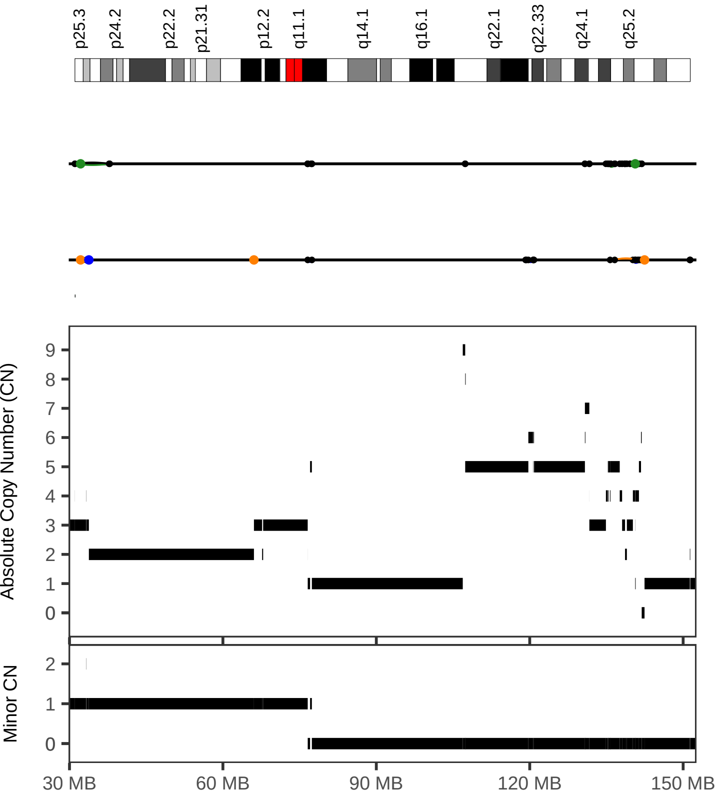

a824b3bd−34d5−4cc1−a92f−f9d6ac0f1814

Cancer type	Breast−AdenoCA
Position	6:134899220−141764105
Interleaved intrachr. SVs	13
Total SVs (intrachr. + transl.)	14
SV types	DEL: 2; DUP: 1; h2hINV: 4; t2tINV: 6; TRA: 1
SVs in sample	463
Oscillating CN (2 and 3 states)	7, 17
CN segments	17
FDR fragment joints	0.615458
FDR chr. breakp. enrich.	0.22
Linked to chrs	
Purity, ploidy	0.51, 2.1

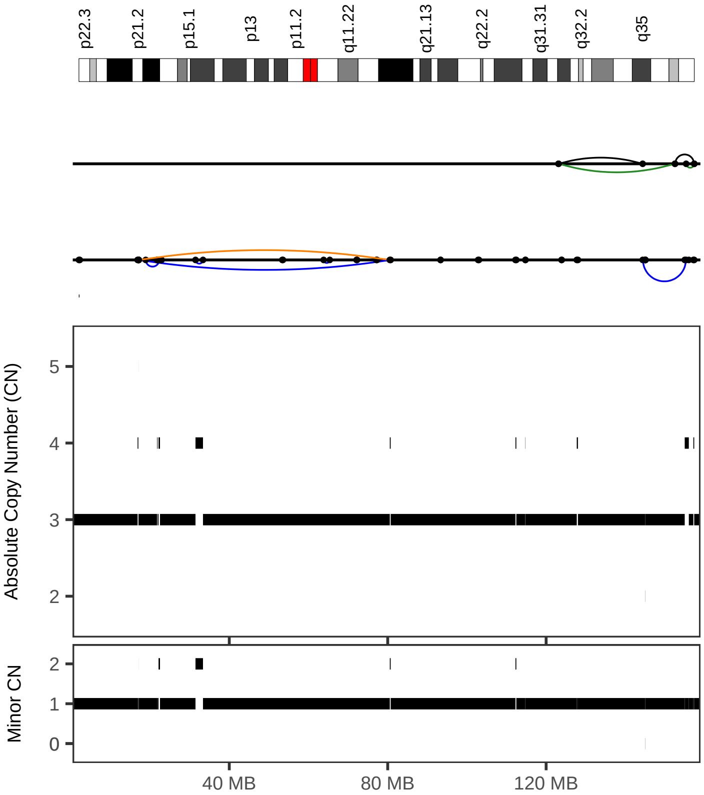

a824b3bd−34d5−4cc1−a92f−f9d6ac0f1814

Cancer type	Breast−AdenoCA
Position	7:123121252−157394758
Interleaved intrachr. SVs	6
Total SVs (intrachr. + transl.)	6
SV types	DEL: 0; DUP: 2; h2hINV: 2; t2tINV: 2; TRA: 0
SVs in sample	463
Oscillating CN (2 and 3 states)	5, 9
CN segments	9
FDR fragment joints	0.6776251
FDR chr. breakp. enrich.	0.66
Linked to chrs	
Purity, ploidy	0.51, 2.1

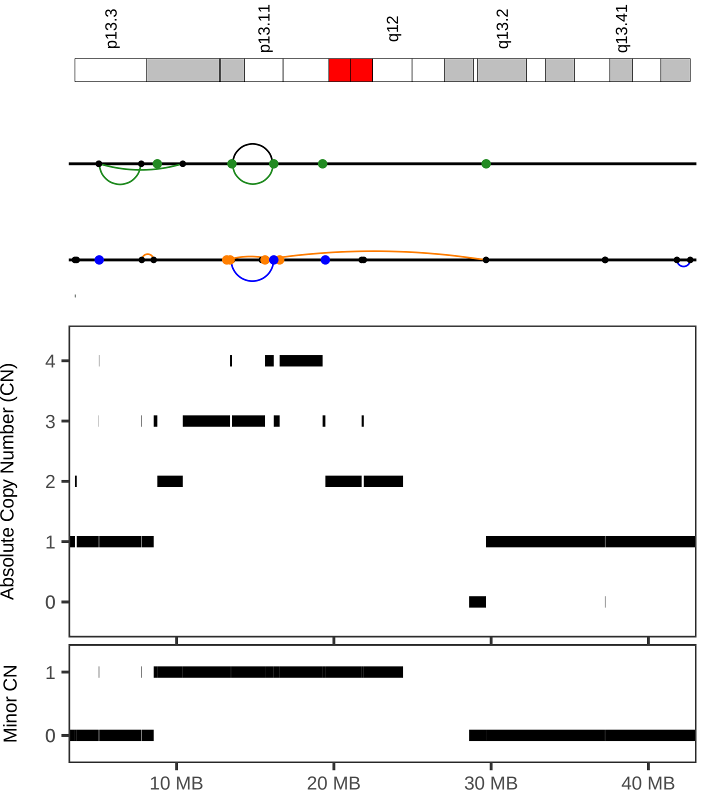

a824b3bd−34d5−4cc1−a92f−f9d6ac0f1814

Cancer type	Breast−AdenoCA
Position	19:13207846−29674953
Interleaved intrachr. SVs	5
Total SVs (intrachr. + transl.)	13
SV types	DEL: 2; DUP: 1; h2hINV: 1; t2tINV: 1; TRA: 8
SVs in sample	463
Oscillating CN (2 and 3 states)	7, 7
CN segments	12
FDR fragment joints	0.6776251
FDR chr. breakp. enrich.	0
Linked to chrs	11:10547187−101046436;
Purity, ploidy	0.51, 2.1

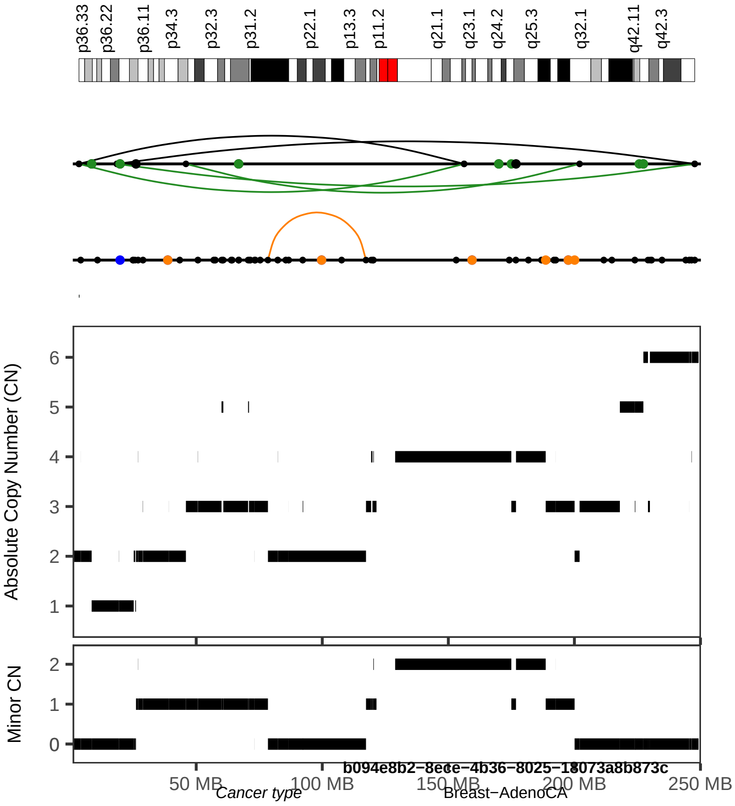

b094e8b2−8ece−4b36−8025−18073a8b873c

Cancer type	Breast−AdenoCA
Position	1:3498809−247725333
Interleaved intrachr. SVs	6
Total SVs (intrachr. + transl.)	31
SV types	DEL: 0; DUP: 1; h2hINV: 2; t2tINV: 3; TRA: 25
SVs in sample	763
Oscillating CN (2 and 3 states)	11, 11
CN segments	54
FDR fragment joints	0.7622832
FDR chr. breakp. enrich.	0.02
Linked to chrs	10:84390295−134360314;12:62348764−100607993 18:701058−54131138;2:9334743−209562257 20:4240904−56044212;3:4164393−197488085

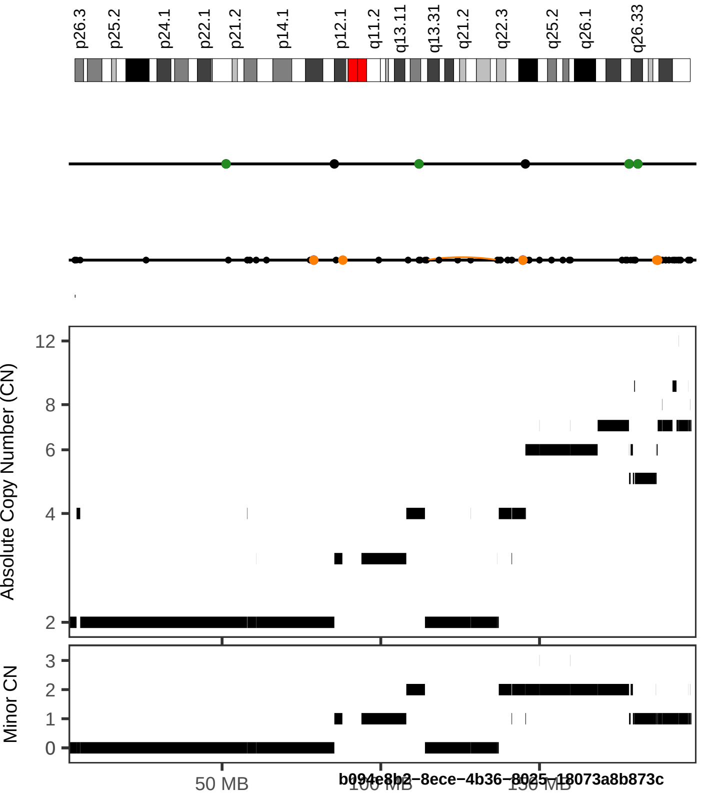

b094e8b2−8ece−4b36−8025−18073a8b873c

Cancer type	Breast−AdenoCA
Position	3:4164393−197488086
Interleaved intrachr. SVs	1
Total SVs (intrachr. + transl.)	45
SV types	DEL: 2; DUP: 24; h2hINV: 0; t2tINV: 0; TRA: 19
SVs in sample	763
Oscillating CN (2 and 3 states)	7, 17
CN segments	47
FDR fragment joints	0.0009227593
FDR chr. breakp. enrich.	0.16
Linked to chrs	1:3498809−247725332;12:62348764−100607993 2:9334743−209562257;4:20141203−160134616 7:31636190−151854818;

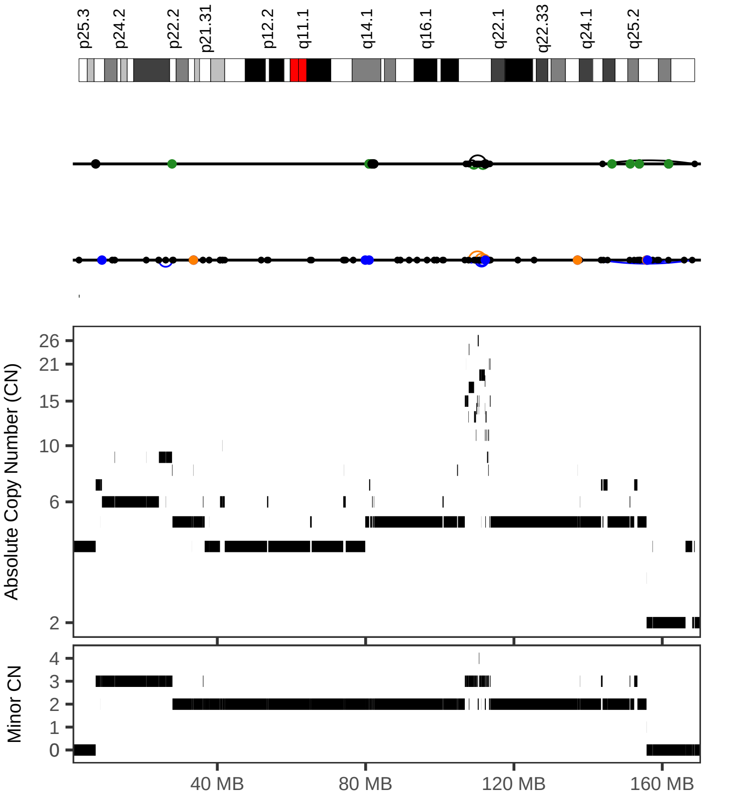

b094e8b2−8ece−4b36−8025−18073a8b873c

Cancer type	Breast−AdenoCA
Position	6:107054725−113512450
Interleaved intrachr. SVs	22
Total SVs (intrachr. + transl.)	24
SV types	DEL: 5; DUP: 6; h2hINV: 3; t2tINV: 8; TRA: 2
SVs in sample	763
Oscillating CN (2 and 3 states)	4, 4
CN segments	32
FDR fragment joints	0.615458
FDR chr. breakp. enrich.	0
Linked to chrs	
Purity, ploidy	0.82, 2.81

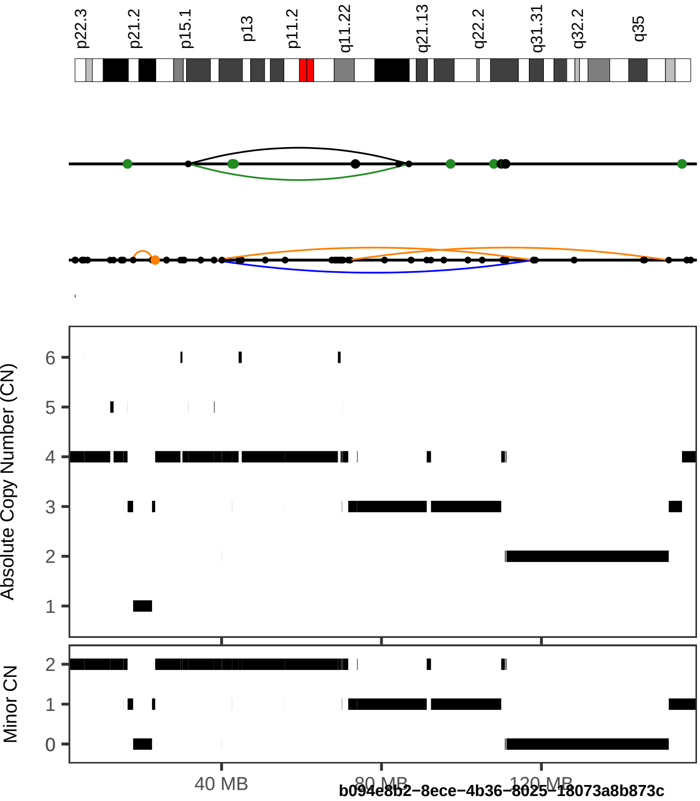

b094e8b2−8ece−4b36−8025−18073a8b873c

Cancer type	Breast−AdenoCA
Position	7:31636190−151854819
Interleaved intrachr. SVs	5
Total SVs (intrachr. + transl.)	18
SV types	DEL: 2; DUP: 1; h2hINV: 1; t2tINV: 1; TRA: 13
SVs in sample	763
Oscillating CN (2 and 3 states)	7, 11
CN segments	28
FDR fragment joints	0.4989394
FDR chr. breakp. enrich.	0
Linked to chrs	12:62348764−100607993;15:84737922−102193576
Purity, ploidy	0.82, 2.81

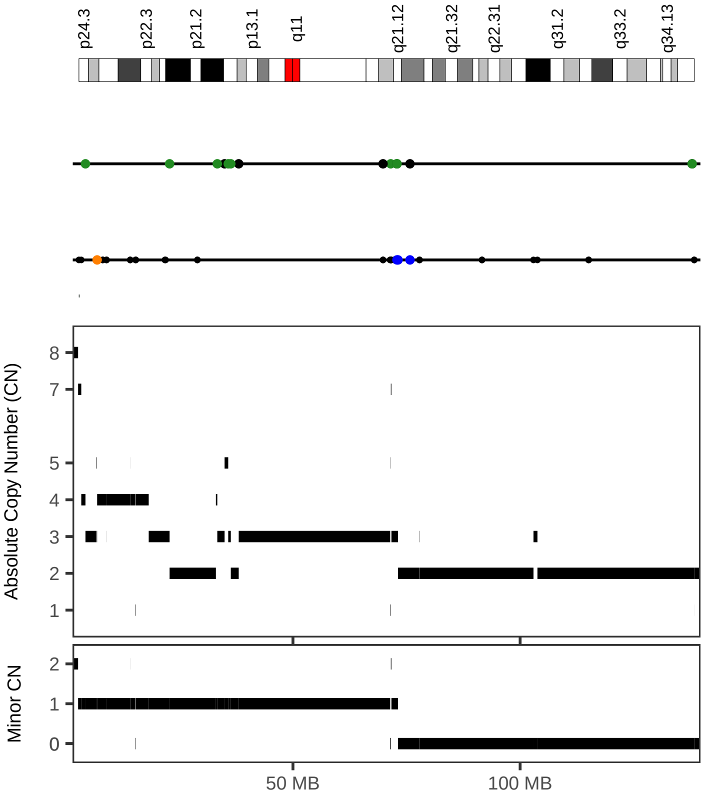

b094e8b2−8ece−4b36−8025−18073a8b873c

Cancer type	Breast−AdenoCA
Position	9:71374858−72890109
Interleaved intrachr. SVs	7
Total SVs (intrachr. + transl.)	12
SV types	DEL: 3; DUP: 1; h2hINV: 2; t2tINV: 1; TRA: 5
SVs in sample	763
Oscillating CN (2 and 3 states)	4, 5
CN segments	6
FDR fragment joints	0.6776251
FDR chr. breakp. enrich.	0.08
Linked to chrs	
Purity, ploidy	0.82, 2.81

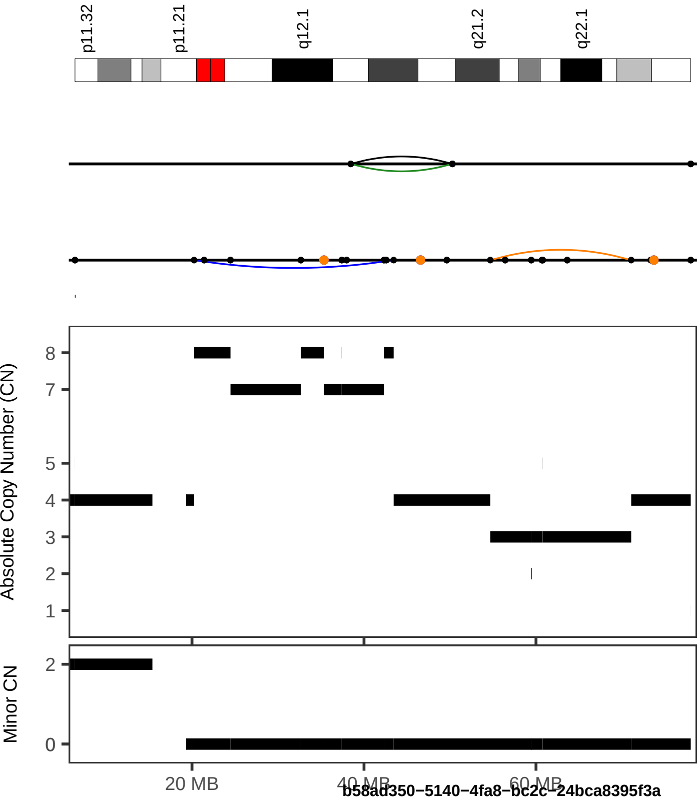

b58ad350−5140−4fa8−bc2c−24bca8395f3a

Cancer type	Breast−AdenoCA
Position	18:20248444−50278950
Interleaved intrachr. SVs	3
Total SVs (intrachr. + transl.)	7
SV types	DEL: 0; DUP: 1; h2hINV: 1; t2tINV: 1; TRA: 4
SVs in sample	518
Oscillating CN (2 and 3 states)	7, 8
CN segments	8
FDR fragment joints	0.7735152
FDR chr. breakp. enrich.	0
Linked to chrs	13:37442397−93491061;15:33755967−94821621
Purity, ploidy	0.65, 3.24

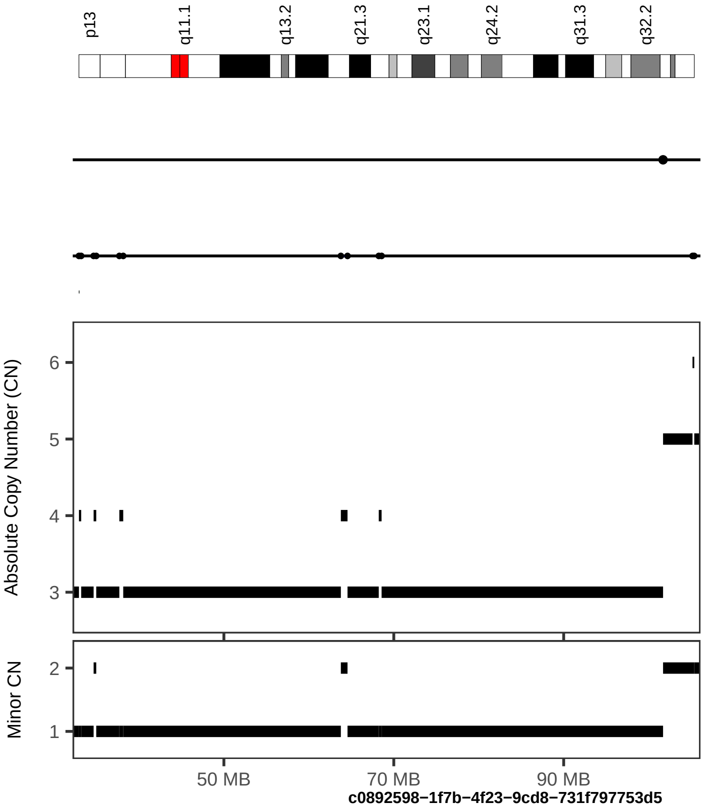

c0892598−1f7b−4f23−9cd8−731f797753d5

Cancer type	Breast−AdenoCA
Position	14:32936197−105369365
Interleaved intrachr. SVs	1
Total SVs (intrachr. + transl.)	7
SV types	DEL: 0; DUP: 6; h2hINV: 0; t2tINV: 0; TRA: 1
SVs in sample	358
Oscillating CN (2 and 3 states)	10, 11
CN segments	13
FDR fragment joints	0.1278363
FDR chr. breakp. enrich.	0.46
Linked to chrs	
Purity, ploidy	0.61, 3.55

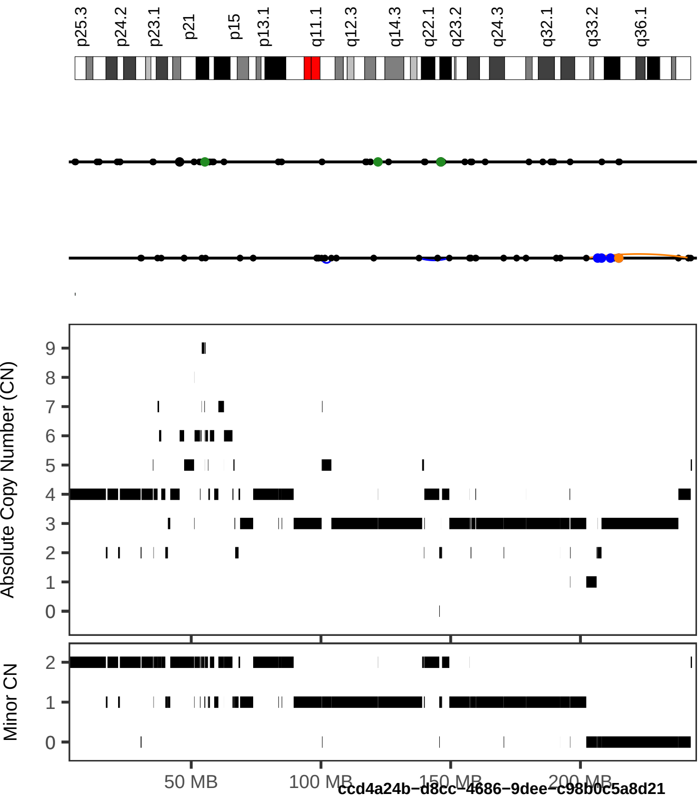

ccd4a24b−d8cc−4686−9dee−c98b0c5a8d21

Cancer type	Breast−AdenoCA
Position	2:30492069−242512963
Interleaved intrachr. SVs	1
Total SVs (intrachr. + transl.)	26
SV types	DEL: 2; DUP: 14; h2hINV: 0; t2tINV: 0; TRA: 10
SVs in sample	936
Oscillating CN (2 and 3 states)	8, 11
CN segments	86
FDR fragment joints	0.002298705
FDR chr. breakp. enrich.	0.69
Linked to chrs	4:19803347−158797787;7:7932046−156250630
Purity, ploidy	0.89, 3.26

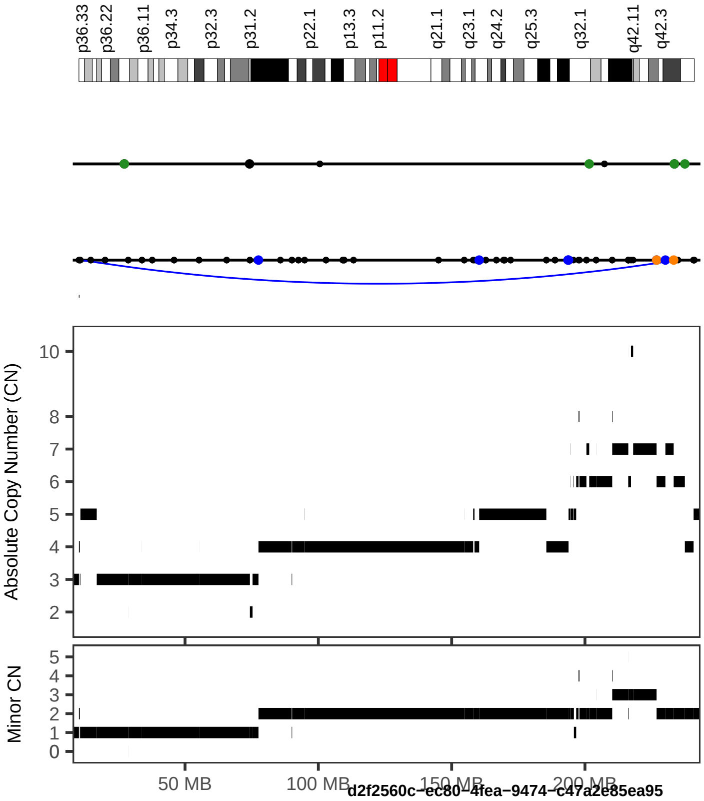

d2f2560c−ec80−4fea−9474−c47a2e85ea95

Cancer type	Breast−AdenoCA
Position	1:10197061−240751611
Interleaved intrachr. SVs	1
Total SVs (intrachr. + transl.)	36
SV types	DEL: 6; DUP: 14; h2hINV: 0; t2tINV: 1; TRA: 15
SVs in sample	546
Oscillating CN (2 and 3 states)	10, 10
CN segments	48
FDR fragment joints	0.03325273
FDR chr. breakp. enrich.	0.06
Linked to chrs	3:4264202−194052996;7:26783402−109202113
Purity, ploidy	0.67, 3.45

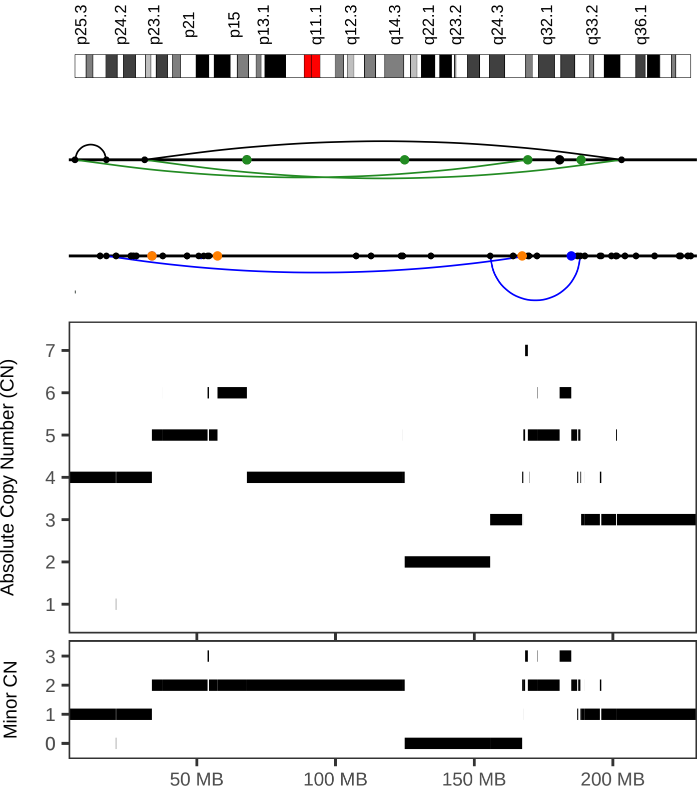

d2f2560c−ec80−4fea−9474−c47a2e85ea95

Cancer type	Breast−AdenoCA
Position	2:6059598−203121904
Interleaved intrachr. SVs	6
Total SVs (intrachr. + transl.)	17
SV types	DEL: 0; DUP: 2; h2hINV: 2; t2tINV: 2; TRA: 11
SVs in sample	546
Oscillating CN (2 and 3 states)	6, 9
CN segments	35
FDR fragment joints	0.8653243
FDR chr. breakp. enrich.	0.69
Linked to chrs	
Purity, ploidy	0.67, 3.45

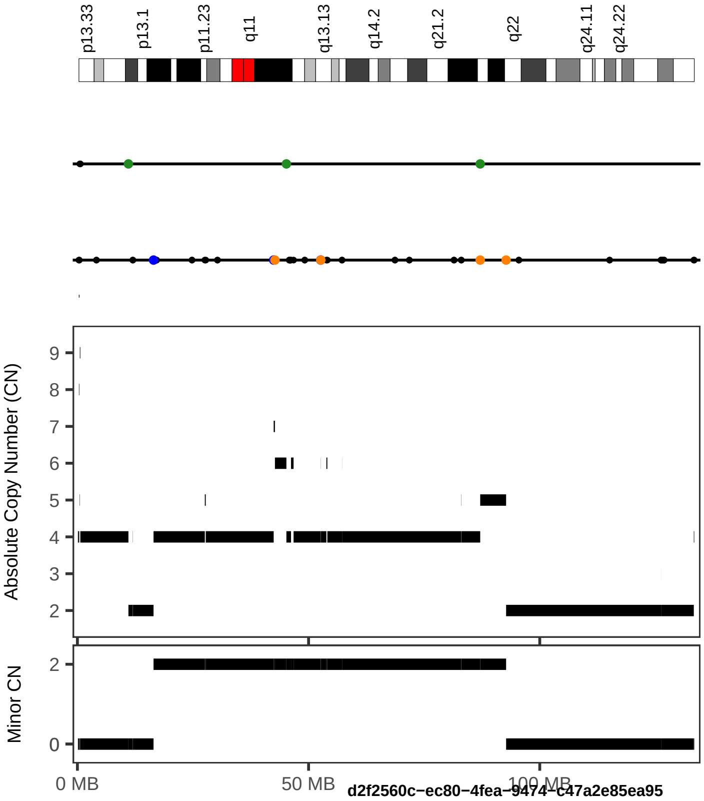

d2f2560c−ec80−4fea−9474−c47a2e85ea95

Cancer type	Breast−AdenoCA
Position	12:329064−133417882
Interleaved intrachr. SVs	1
Total SVs (intrachr. + transl.)	21
SV types	DEL: 1; DUP: 9; h2hINV: 1; t2tINV: 0; TRA: 10
SVs in sample	546
Oscillating CN (2 and 3 states)	10, 10
CN segments	29
FDR fragment joints	0.1278363
FDR chr. breakp. enrich.	0.2
Linked to chrs	20:30253204−59314862;8:6739069−135344821
Purity, ploidy	0.67, 3.45

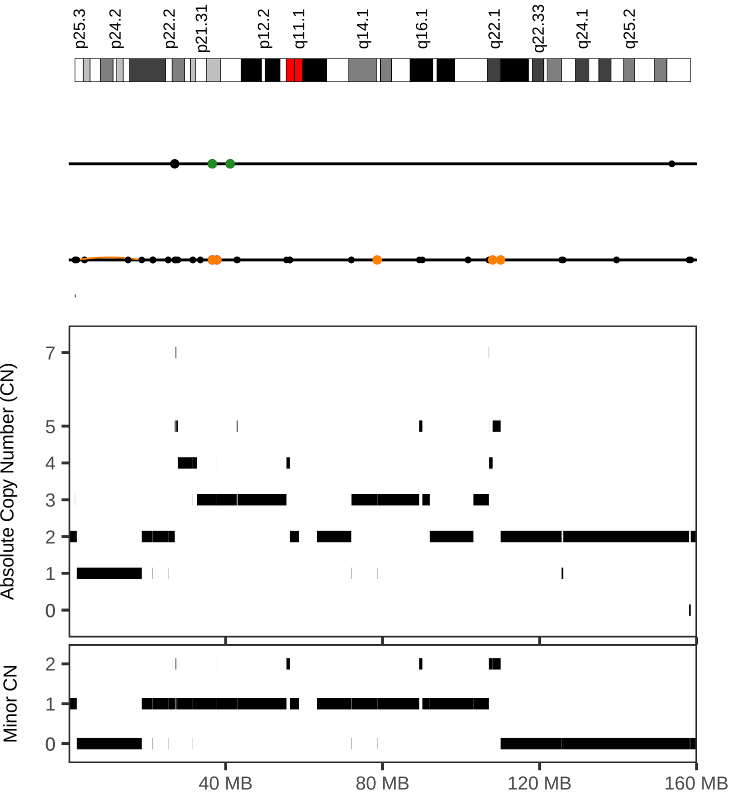

d3d545b3−457f−4389−821f−704cb24aff7f

Cancer type	Breast−AdenoCA
Position	6:1587687−158509485
Interleaved intrachr. SVs	1
Total SVs (intrachr. + transl.)	28
SV types	DEL: 9; DUP: 7; h2hINV: 0; t2tINV: 0; TRA: 12
SVs in sample	709
Oscillating CN (2 and 3 states)	7, 10
CN segments	40
FDR fragment joints	0.1635872
FDR chr. breakp. enrich.	0.68
Linked to chrs	1:8159480−247557942;
Purity, ploidy	0.76, 3.03

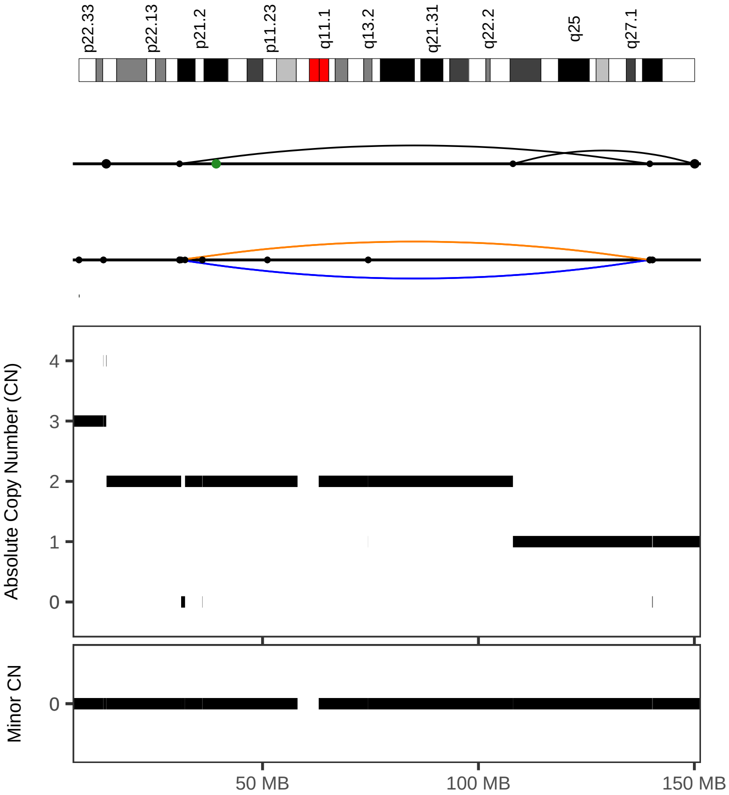

d3d545b3−457f−4389−821f−704cb24aff7f

Cancer type	Breast−AdenoCA
Position	X:30768634−150086913
Interleaved intrachr. SVs	6
Total SVs (intrachr. + transl.)	8
SV types	DEL: 2; DUP: 2; h2hINV: 2; t2tINV: 0; TRA: 2
SVs in sample	709
Oscillating CN (2 and 3 states)	5, 6
CN segments	11
FDR fragment joints	0.8653243
FDR chr. breakp. enrich.	0
Linked to chrs	
Purity, ploidy	0.76, 3.03

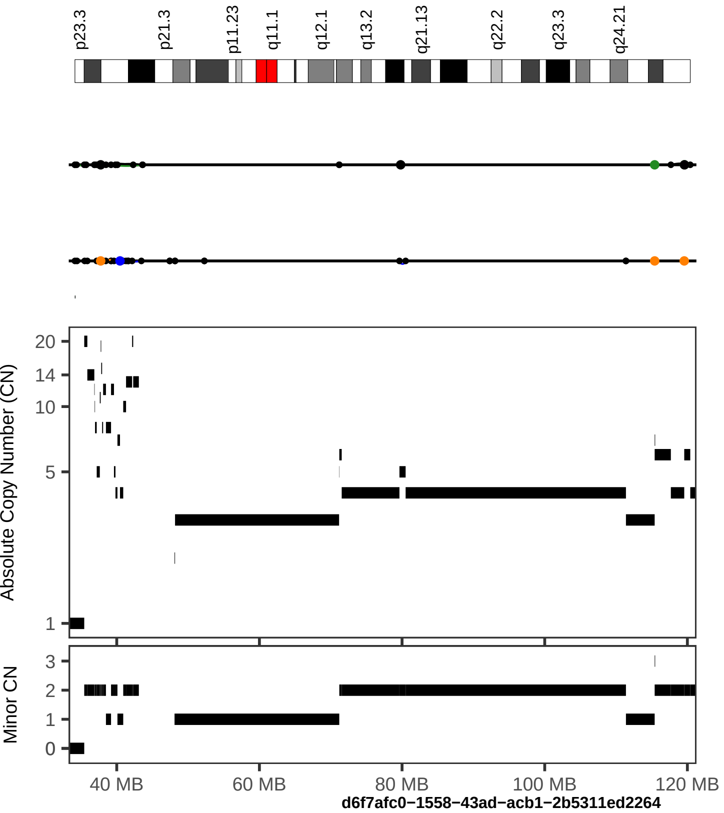

d6f7afc0−1558−43ad−acb1−2b5311ed2264

Cancer type	Breast−AdenoCA
Position	8:37191389−43639589
Interleaved intrachr. SVs	11
Total SVs (intrachr. + transl.)	14
SV types	DEL: 4; DUP: 2; h2hINV: 2; t2tINV: 3; TRA: 3
SVs in sample	304
Oscillating CN (2 and 3 states)	4, 6
CN segments	17
FDR fragment joints	0.8066159
FDR chr. breakp. enrich.	0
Linked to chrs	12:66502753−130003738;
Purity, ploidy	0.27, 2.34

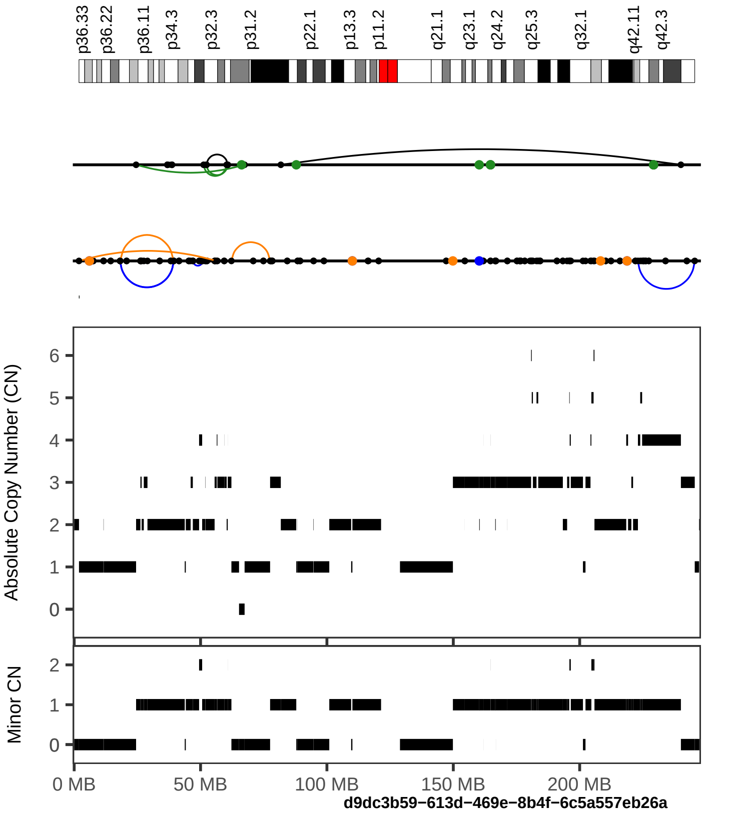

d9dc3b59−613d−469e−8b4f−6c5a557eb26a

Cancer type	Breast−AdenoCA
Position	1:1898876−77522550
Interleaved intrachr. SVs	10
Total SVs (intrachr. + transl.)	13
SV types	DEL: 3; DUP: 3; h2hINV: 1; t2tINV: 3; TRA: 3
SVs in sample	1222
Oscillating CN (2 and 3 states)	5, 12
CN segments	28
FDR fragment joints	0.9000538
FDR chr. breakp. enrich.	1
Linked to chrs	
Purity, ploidy	0.41, 2.07

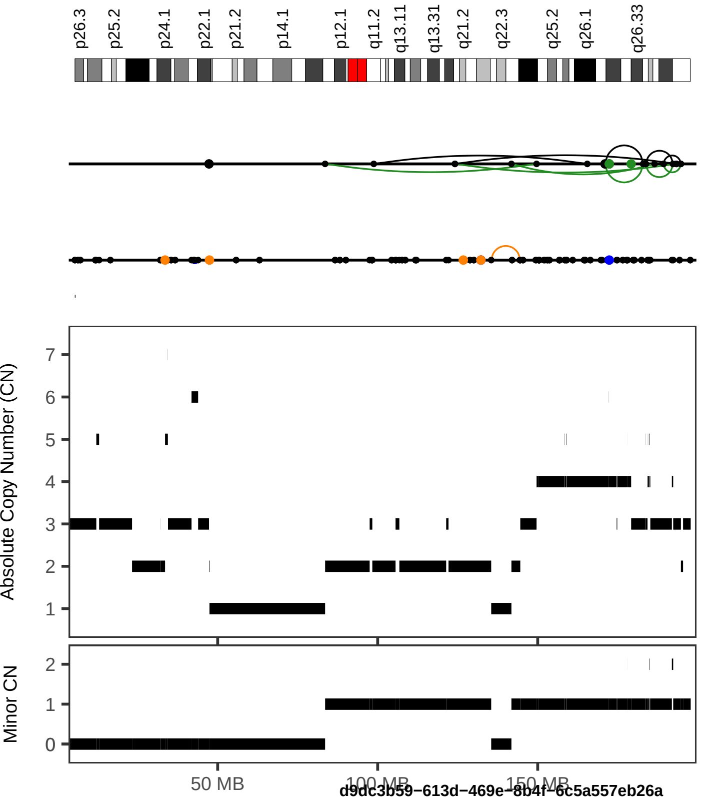

d9dc3b59−613d−469e−8b4f−6c5a557eb26a

Cancer type	Breast−AdenoCA
Position	3:83571470−194744514
Interleaved intrachr. SVs	11
Total SVs (intrachr. + transl.)	19
SV types	DEL: 1; DUP: 1; h2hINV: 4; t2tINV: 5; TRA: 8
SVs in sample	1222
Oscillating CN (2 and 3 states)	7, 11
CN segments	34
FDR fragment joints	0.6776251
FDR chr. breakp. enrich.	0.83
Linked to chrs	11:1073009−116625594;9:100142774−123611054
Purity, ploidy	0.41, 2.07

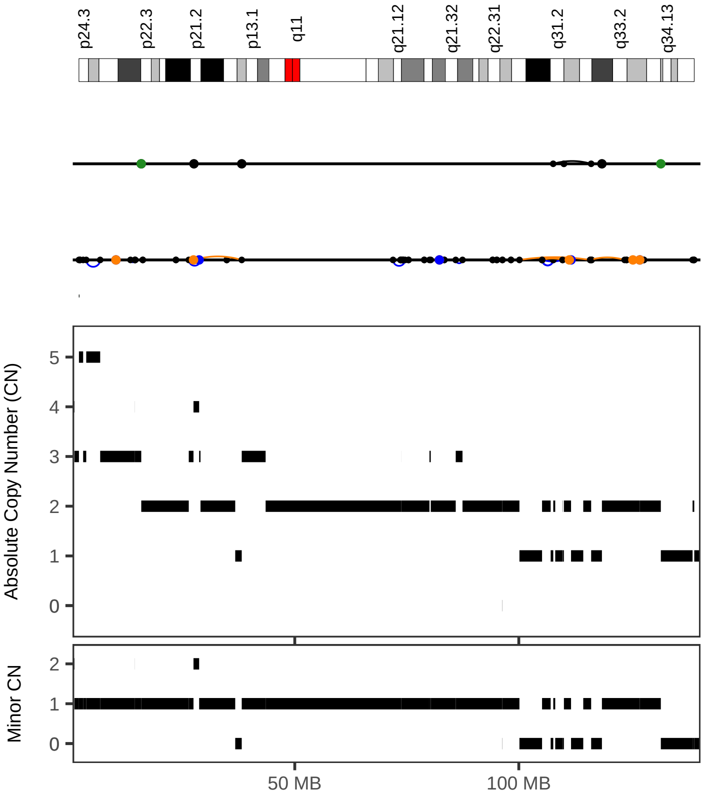

d9dc3b59−613d−469e−8b4f−6c5a557eb26a

Cancer type	Breast−AdenoCA
Position	9:100142774−123611055
Interleaved intrachr. SVs	7
Total SVs (intrachr. + transl.)	10
SV types	DEL: 3; DUP: 3; h2hINV: 1; t2tINV: 0; TRA: 3
SVs in sample	1222
Oscillating CN (2 and 3 states)	12, 12
CN segments	12
FDR fragment joints	0.615458
FDR chr. breakp. enrich.	0.12
Linked to chrs	
Purity, ploidy	0.41, 2.07

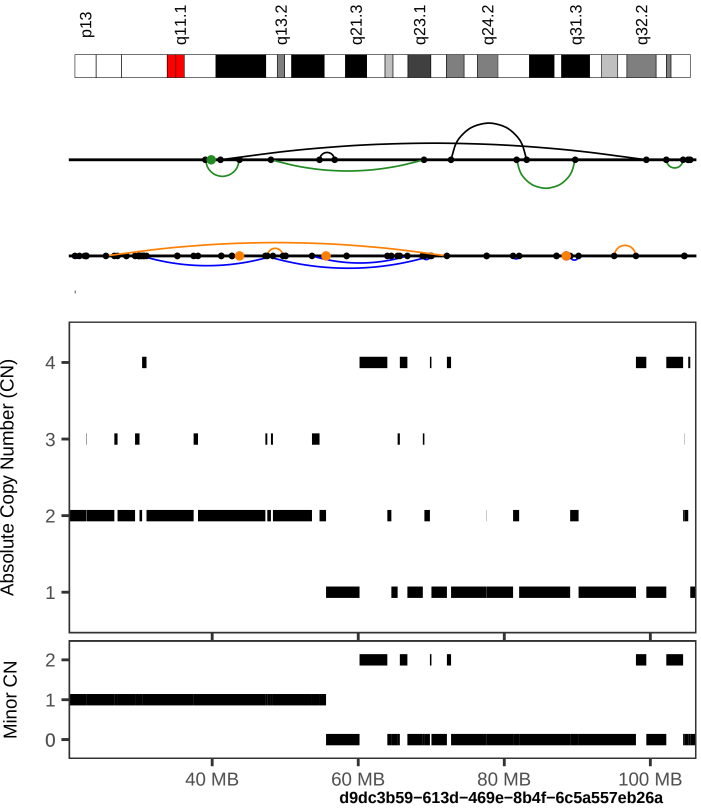

d9dc3b59−613d−469e−8b4f−6c5a557eb26a

Cancer type	Breast−AdenoCA
Position	14:25445910−99447397
Interleaved intrachr. SVs	9
Total SVs (intrachr. + transl.)	14
SV types	DEL: 2; DUP: 4; h2hINV: 1; t2tINV: 2; TRA: 5
SVs in sample	1222
Oscillating CN (2 and 3 states)	9, 15
CN segments	36
FDR fragment joints	0.6776251
FDR chr. breakp. enrich.	0.62
Linked to chrs	
Purity, ploidy	0.41, 2.07

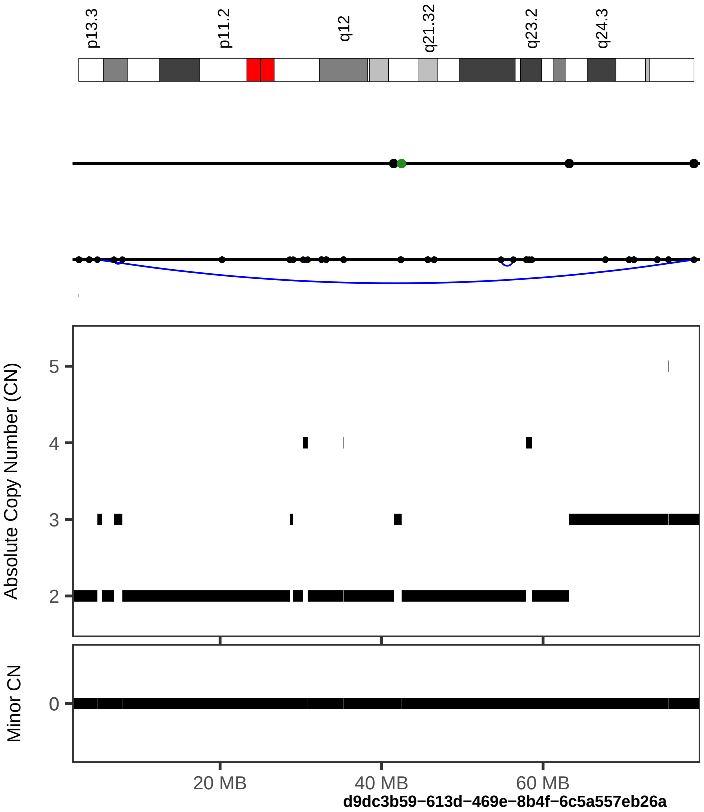

d9dc3b59−613d−469e−8b4f−6c5a557eb26a

Cancer type	Breast−AdenoCA
Position	17:2481365−78698346
Interleaved intrachr. SVs	1
Total SVs (intrachr. + transl.)	20
SV types	DEL: 0; DUP: 16; h2hINV: 0; t2tINV: 0; TRA: 4
SVs in sample	1222
Oscillating CN (2 and 3 states)	7, 16
CN segments	20
FDR fragment joints	0.0002466223
FDR chr. breakp. enrich.	0.4
Linked to chrs	
Purity, ploidy	0.41, 2.07

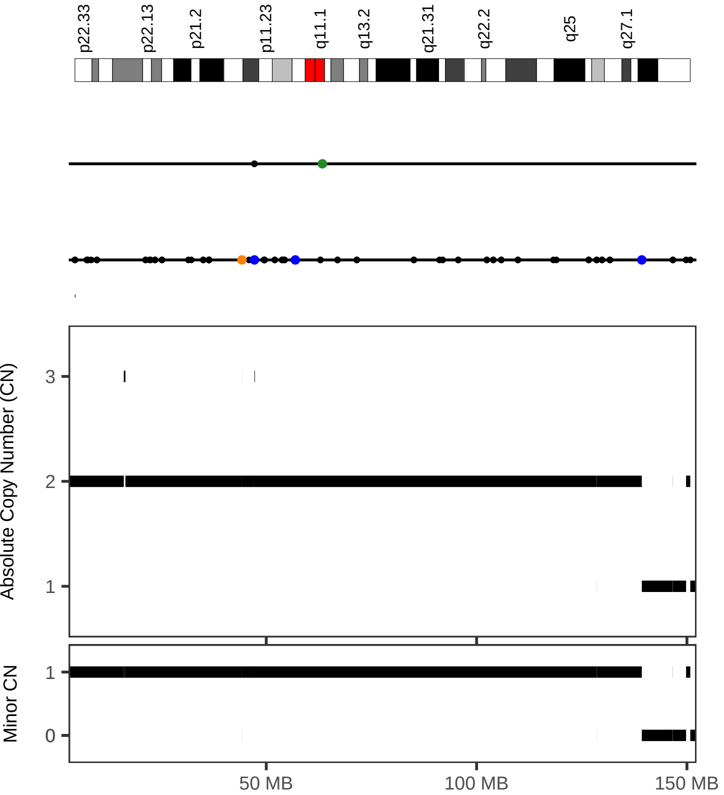

d9dc3b59−613d−469e−8b4f−6c5a557eb26a

Cancer type	Breast−AdenoCA
Position	X:7323507−150791833
Interleaved intrachr. SVs	1
Total SVs (intrachr. + transl.)	16
SV types	DEL: 2; DUP: 9; h2hINV: 0; t2tINV: 0; TRA: 5
SVs in sample	1222
Oscillating CN (2 and 3 states)	8, 14
CN segments	14
FDR fragment joints	0.01147689
FDR chr. breakp. enrich.	0.06
Linked to chrs	
Purity, ploidy	0.41, 2.07

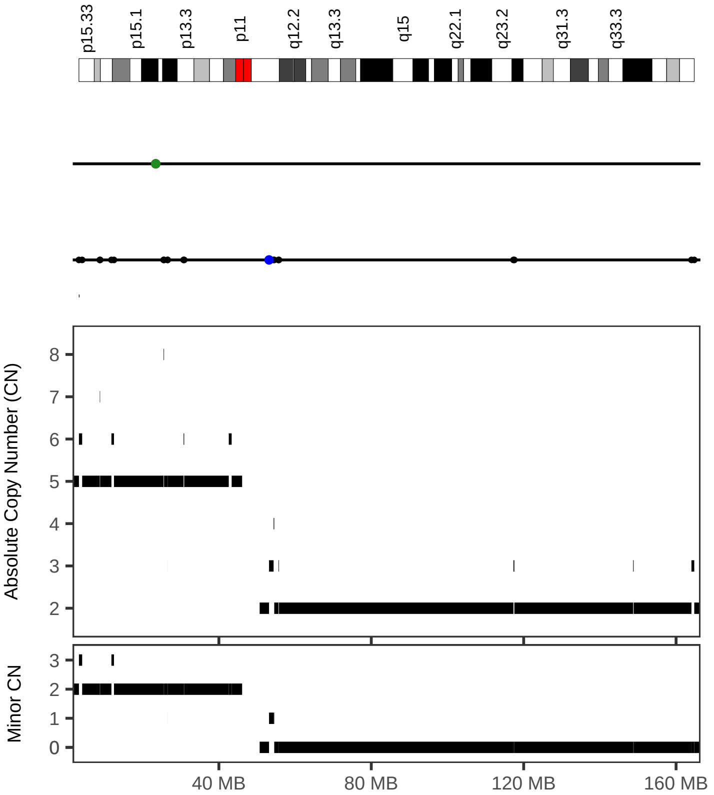

e4fc0909−f284−4471−866d−d8967b6adcbc

Cancer type	Breast−AdenoCA
Position	5:3262330−164768438
Interleaved intrachr. SVs	1
Total SVs (intrachr. + transl.)	12
SV types	DEL: 0; DUP: 9; h2hINV: 0; t2tINV: 0; TRA: 3
SVs in sample	630
Oscillating CN (2 and 3 states)	9, 11
CN segments	26
FDR fragment joints	0.01333161
FDR chr. breakp. enrich.	0
Linked to chrs	7:10063721−147714910;
Purity, ploidy	0.49, 2.63

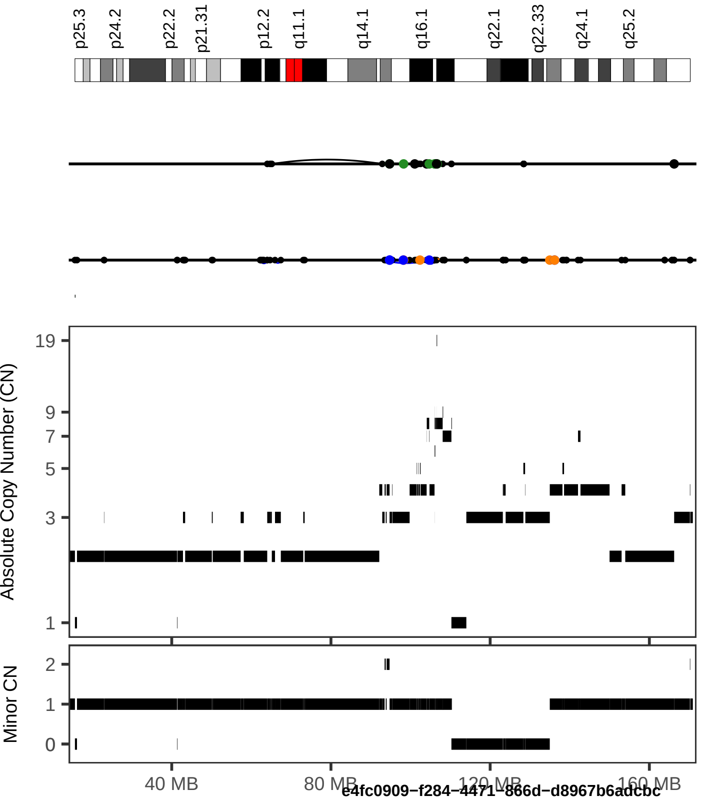

e4fc0909−f284−4471−866d−d8967b6adcbc

Cancer type	Breast−AdenoCA
Position	6:93474674−108070113
Interleaved intrachr. SVs	6
Total SVs (intrachr. + transl.)	23
SV types	DEL: 2; DUP: 2; h2hINV: 2; t2tINV: 0; TRA: 17
SVs in sample	630
Oscillating CN (2 and 3 states)	7, 14
CN segments	27
FDR fragment joints	0.7861024
FDR chr. breakp. enrich.	0
Linked to chrs	13:35528163−62533551;7:10063721−147714910
Purity, ploidy	0.49, 2.63

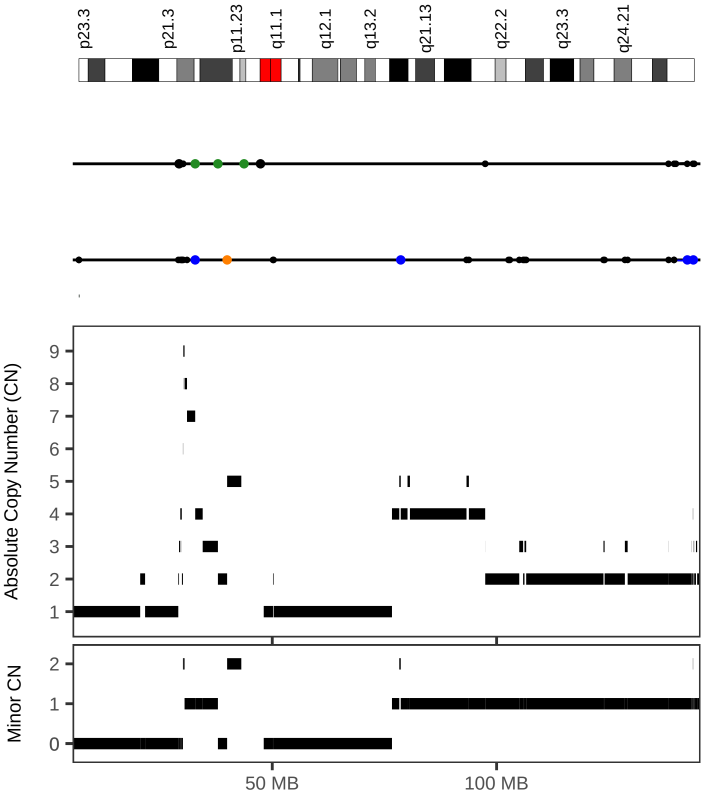

e4fc0909−f284−4471−866d−d8967b6adcbc

Cancer type	Breast−AdenoCA
Position	8:138302264−144043894
Interleaved intrachr. SVs	6
Total SVs (intrachr. + transl.)	8
SV types	DEL: 1; DUP: 2; h2hINV: 1; t2tINV: 2; TRA: 2
SVs in sample	630
Oscillating CN (2 and 3 states)	4, 10
CN segments	10
FDR fragment joints	0.615458
FDR chr. breakp. enrich.	0.9
Linked to chrs	
Purity, ploidy	0.49, 2.63

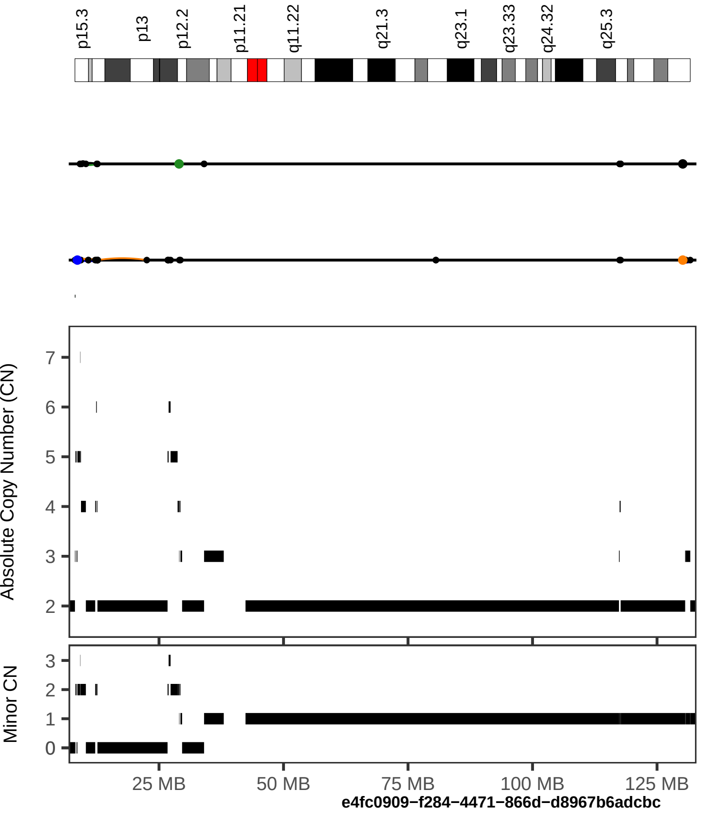

Cancer type	Breast−AdenoCA
Position	10:8113663−12635794
Interleaved intrachr. SVs	7
Total SVs (intrachr. + transl.)	8
SV types	DEL: 2; DUP: 2; h2hINV: 2; t2tINV: 1; TRA: 1
SVs in sample	630
Oscillating CN (2 and 3 states)	4, 6
CN segments	12
FDR fragment joints	0.8653243
FDR chr. breakp. enrich.	0.75
Linked to chrs	
Purity, ploidy	0.49, 2.63

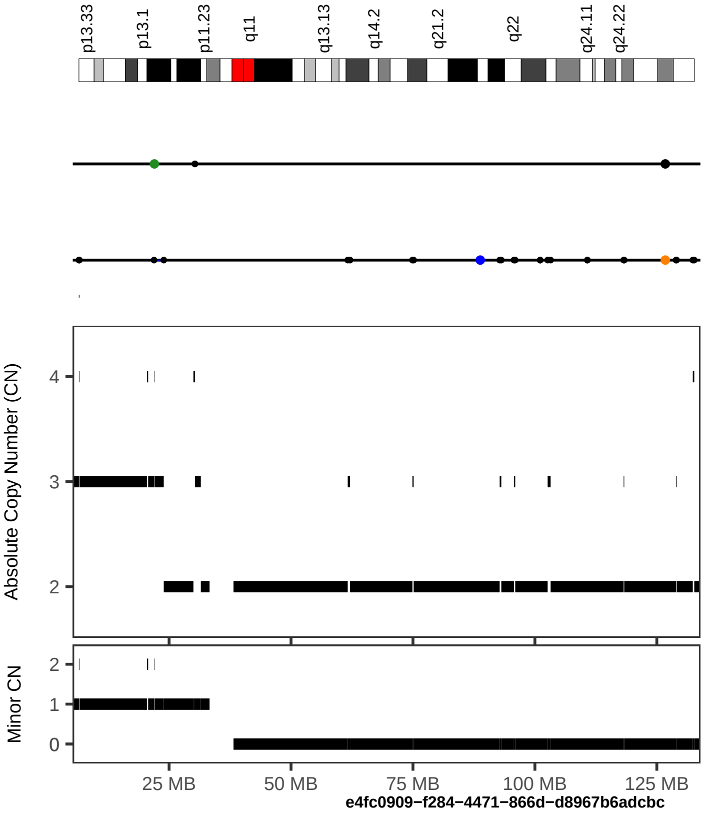

Cancer type	Breast−AdenoCA
Position	12:6511073−132672448
Interleaved intrachr. SVs	1
Total SVs (intrachr. + transl.)	15
SV types	DEL: 1; DUP: 10; h2hINV: 0; t2tINV: 0; TRA: 4
SVs in sample	630
Oscillating CN (2 and 3 states)	15, 19
CN segments	27
FDR fragment joints	0.0366383
FDR chr. breakp. enrich.	0.07
Linked to chrs	
Purity, ploidy	0.49, 2.63

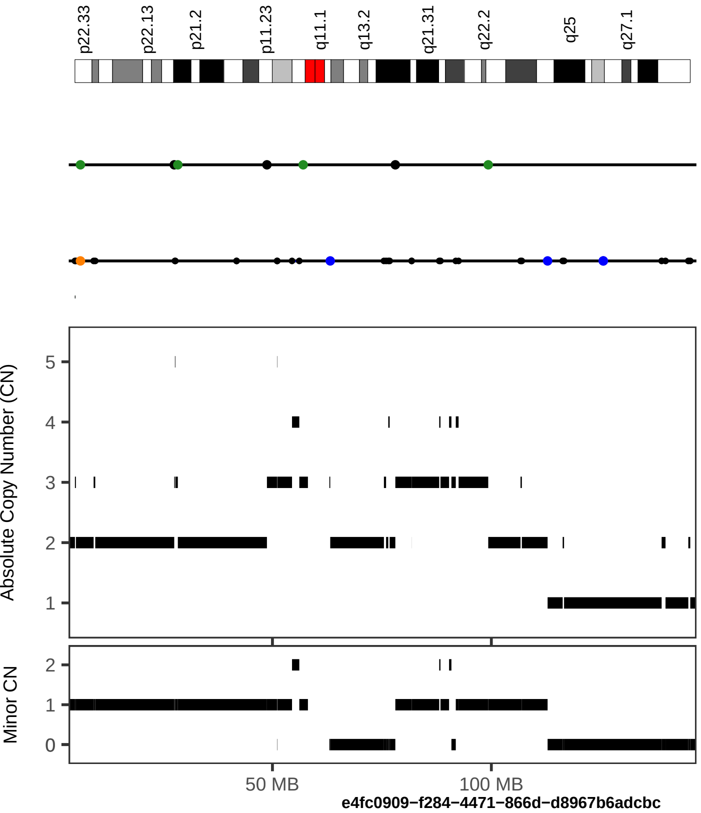

Cancer type	Breast−AdenoCA
Position	X:4881574−145429683
Interleaved intrachr. SVs	1
Total SVs (intrachr. + transl.)	25
SV types	DEL: 0; DUP: 13; h2hINV: 0; t2tINV: 0; TRA: 12
SVs in sample	630
Oscillating CN (2 and 3 states)	8, 14
CN segments	38
FDR fragment joints	0.004164391
FDR chr. breakp. enrich.	0.71
Linked to chrs	
Purity, ploidy	0.49, 2.63

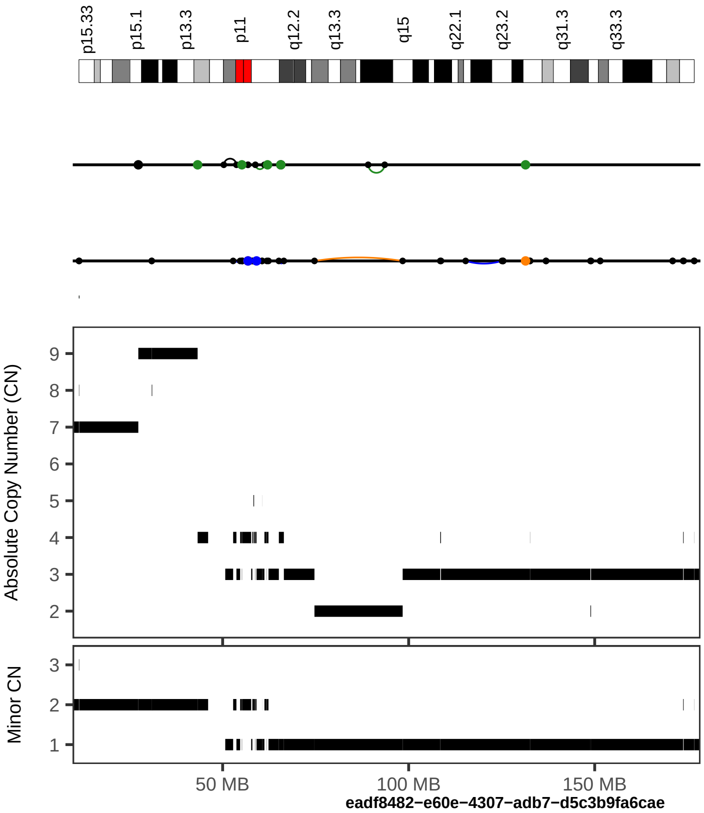

Cancer type	Breast−AdenoCA
Position	5:50309848−62318656
Interleaved intrachr. SVs	7
Total SVs (intrachr. + transl.)	12
SV types	DEL: 1; DUP: 5; h2hINV: 1; t2tINV: 0; TRA: 5
SVs in sample	662
Oscillating CN (2 and 3 states)	9, 10
CN segments	20
FDR fragment joints	0.445216
FDR chr. breakp. enrich.	0.84
Linked to chrs	
Purity, ploidy	0.65, 4.04

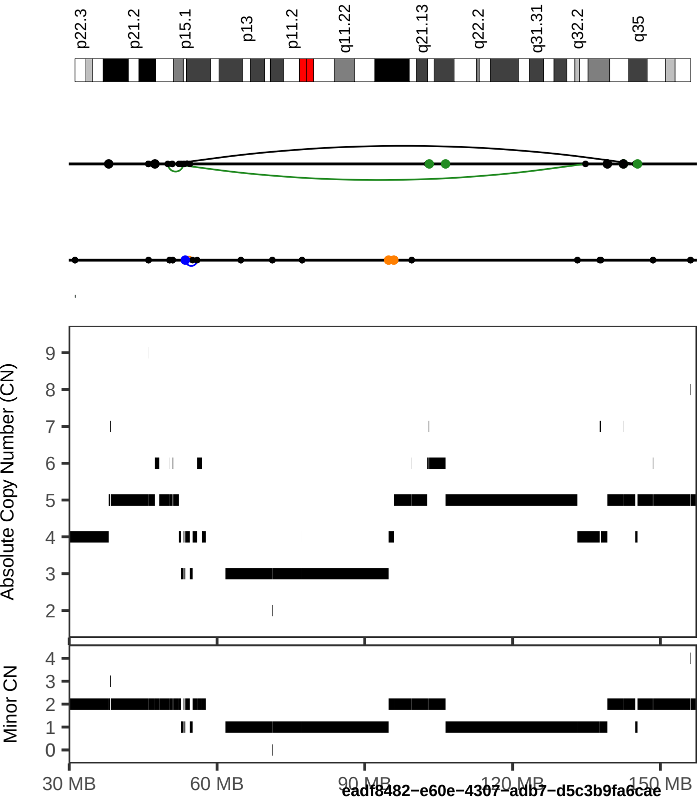

eadf8482−e60e−4307−adb7−d5c3b9fa6cae

Cancer type	Breast−AdenoCA
Position	7:49989231−144882103
Interleaved intrachr. SVs	3
Total SVs (intrachr. + transl.)	14
SV types	DEL: 1; DUP: 1; h2hINV: 2; t2tINV: 2; TRA: 8
SVs in sample	662
Oscillating CN (2 and 3 states)	7, 18
CN segments	36
FDR fragment joints	0.8066159
FDR chr. breakp. enrich.	0.91
Linked to chrs	15:39425396−101227080;9:67337170−114567752
Purity, ploidy	0.65, 4.04

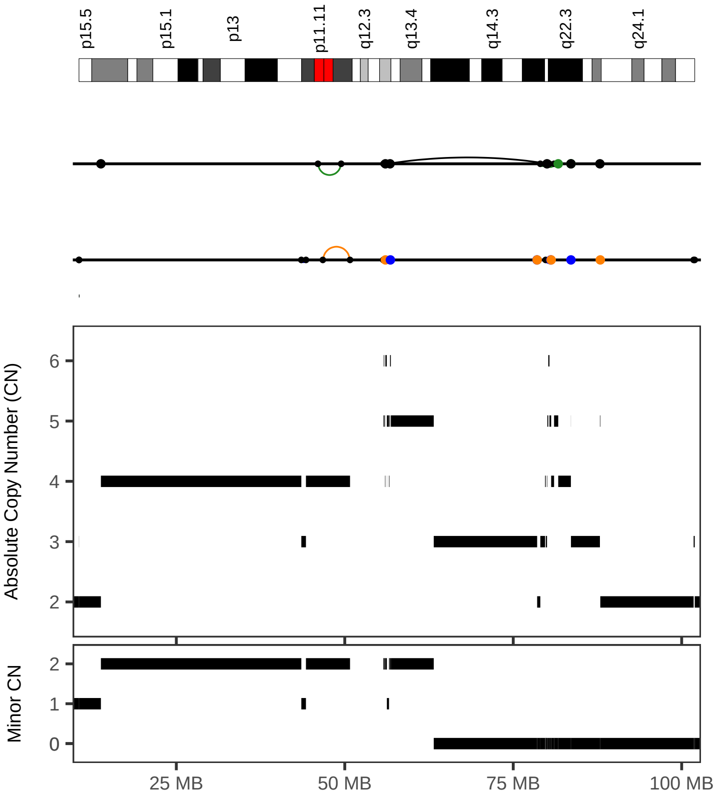

eadf8482−e60e−4307−adb7−d5c3b9fa6cae

Cancer type	Breast−AdenoCA
Position	11:55709941−81062381
Interleaved intrachr. SVs	13
Total SVs (intrachr. + transl.)	22
SV types	DEL: 2; DUP: 4; h2hINV: 3; t2tINV: 4; TRA: 9
SVs in sample	662
Oscillating CN (2 and 3 states)	8, 15
CN segments	28
FDR fragment joints	0.6776251
FDR chr. breakp. enrich.	0.12
Linked to chrs	3:143115106−168498932;
Purity, ploidy	0.65, 4.04

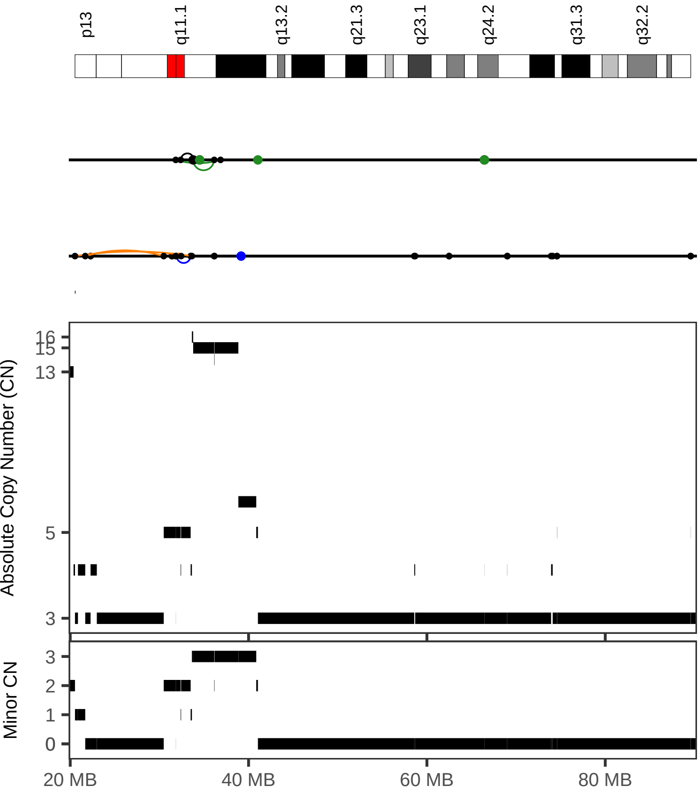

eadf8482−e60e−4307−adb7−d5c3b9fa6cae

Cancer type	Breast−AdenoCA
Position	14:20532445−36857210
Interleaved intrachr. SVs	7
Total SVs (intrachr. + transl.)	10
SV types	DEL: 3; DUP: 1; h2hINV: 1; t2tINV: 2; TRA: 3
SVs in sample	662
Oscillating CN (2 and 3 states)	5, 11
CN segments	15
FDR fragment joints	0.6776251
FDR chr. breakp. enrich.	0.69
Linked to chrs	3:143115106−168498932;
Purity, ploidy	0.65, 4.04

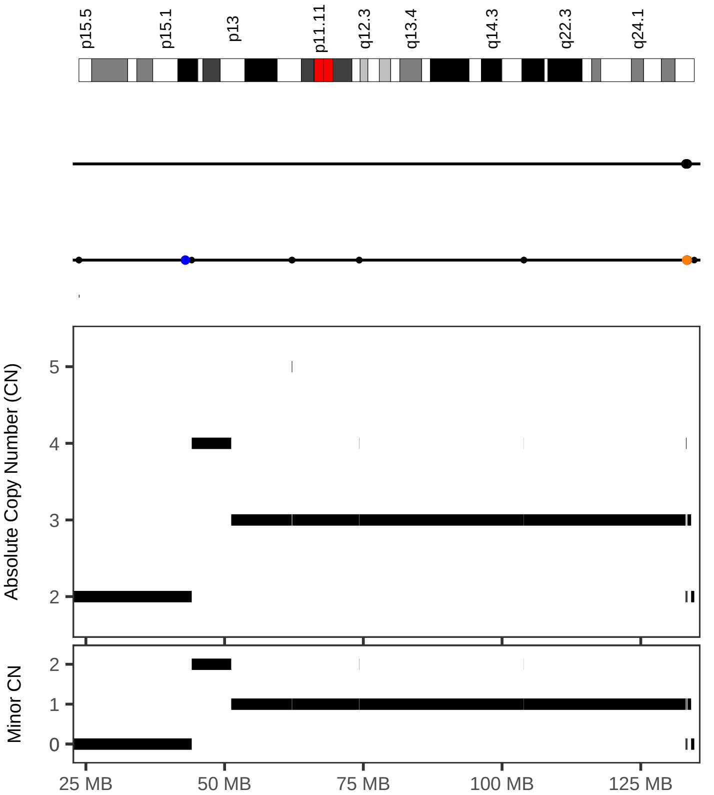

c13215d9−26a8−4c8b−b4fe−5da6aa3222af

Cancer type	Cervix−SCC
Position	11:133072701−134636862
Interleaved intrachr. SVs	9
Total SVs (intrachr. + transl.)	17
SV types	DEL: 4; DUP: 1; h2hINV: 3; t2tINV: 1; TRA: 8
SVs in sample	178
Oscillating CN (2 and 3 states)	12, 12
CN segments	14
FDR fragment joints	0.615458
FDR chr. breakp. enrich.	0
Linked to chrs	1:14796503−15137438;
Purity, ploidy	0.68, 3.49

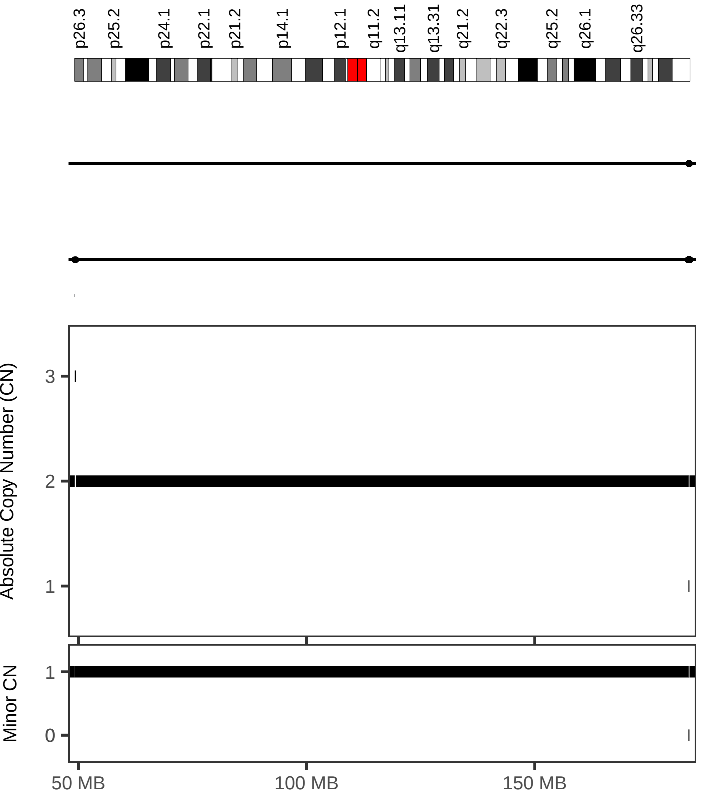

0798dbe2−1914−427c−a2fe−2a865d0d6eda

Cancer type	CNS−GBM
Position	3:183615477−183945373
Interleaved intrachr. SVs	12
Total SVs (intrachr. + transl.)	12
SV types	DEL: 3; DUP: 4; h2hINV: 2; t2tINV: 3; TRA: 0
SVs in sample	209
Oscillating CN (2 and 3 states)	12, 12
CN segments	12
FDR fragment joints	0.9284301
FDR chr. breakp. enrich.	0.74
Linked to chrs	
Purity, ploidy	0.8, 2.05

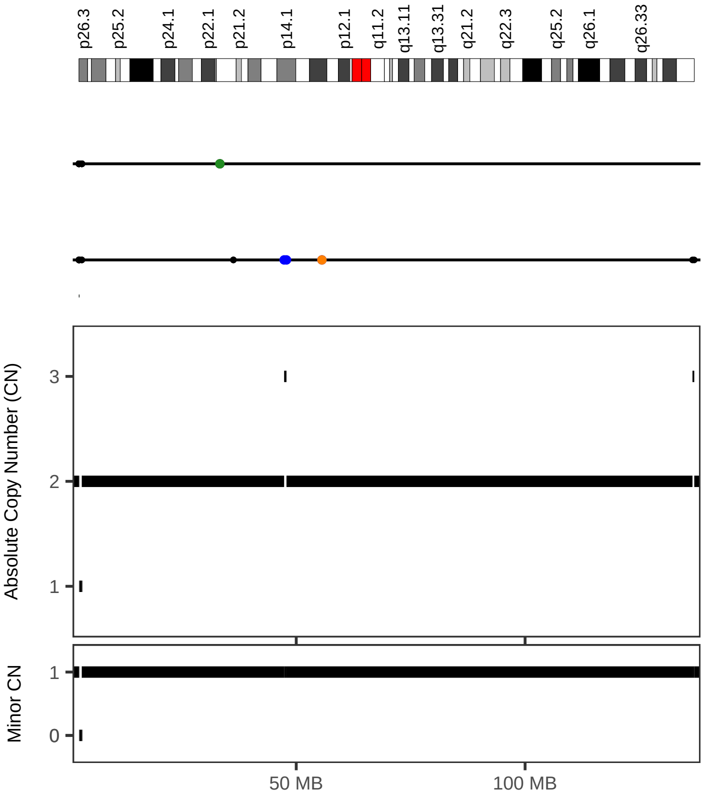

8da3103e−3e6c−4176−a583−d5fe5e60601e

Cancer type	CNS−GBM
Position	3:2515461−3160126
Interleaved intrachr. SVs	12
Total SVs (intrachr. + transl.)	12
SV types	DEL: 2; DUP: 3; h2hINV: 4; t2tINV: 3; TRA: 0
SVs in sample	131
Oscillating CN (2 and 3 states)	7, 7
CN segments	7
FDR fragment joints	0.9284301
FDR chr. breakp. enrich.	0
Linked to chrs	
Purity, ploidy	0.59, 2.03

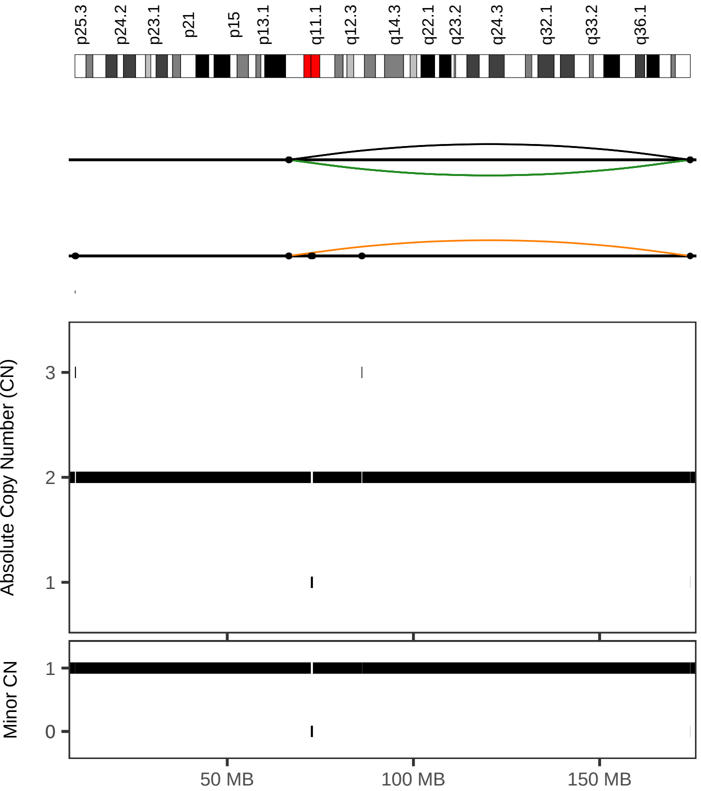

b1c4579c−72fa−4974−830c−b6a81ace45f4

Cancer type	CNS−GBM
Position	2:66469477−174340940
Interleaved intrachr. SVs	8
Total SVs (intrachr. + transl.)	8
SV types	DEL: 1; DUP: 1; h2hINV: 2; t2tINV: 4; TRA: 0
SVs in sample	87
Oscillating CN (2 and 3 states)	5, 9
CN segments	9
FDR fragment joints	0.615458
FDR chr. breakp. enrich.	0.03
Linked to chrs	
Purity, ploidy	0.79, 2.14

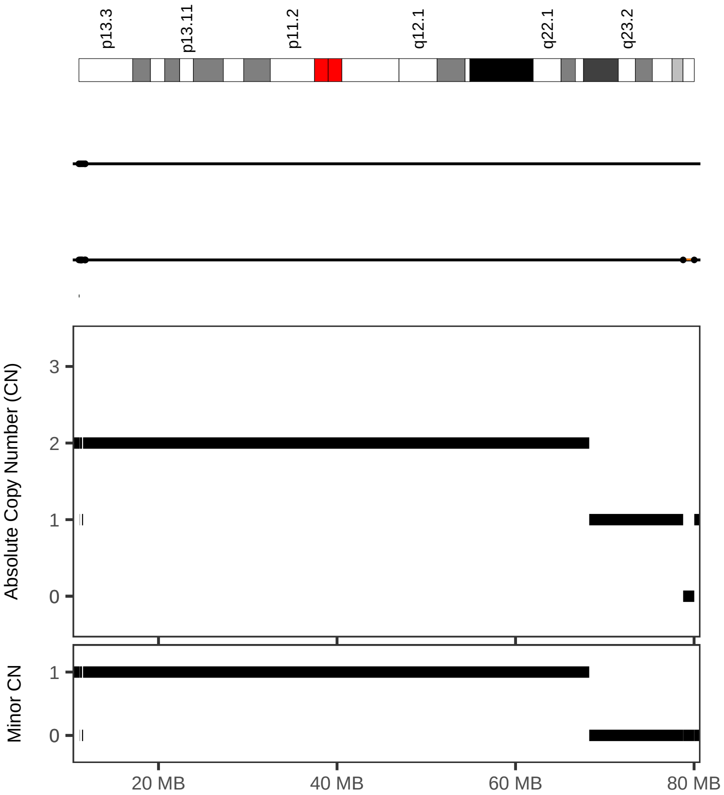

c184c3ca−7ad3−4202−b108−cb9fd5f5d947

Cancer type	CNS−GBM
Position	16:11082483−11804564
Interleaved intrachr. SVs	9
Total SVs (intrachr. + transl.)	9
SV types	DEL: 2; DUP: 3; h2hINV: 2; t2tINV: 2; TRA: 0
SVs in sample	263
Oscillating CN (2 and 3 states)	5, 5
CN segments	5
FDR fragment joints	0.9723381
FDR chr. breakp. enrich.	0.49
Linked to chrs	
Purity, ploidy	0.91, 2.1

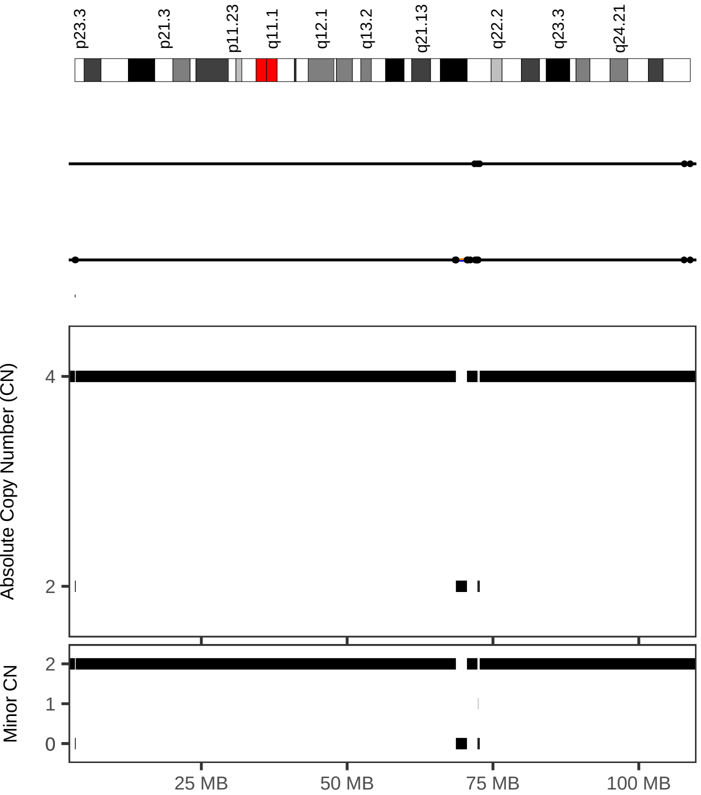

de7b7cac−f094−4d59−8651−e991e34ea093

Cancer type	CNS−GBM
Position	8:68506883−72724157
Interleaved intrachr. SVs	11
Total SVs (intrachr. + transl.)	11
SV types	DEL: 4; DUP: 5; h2hINV: 1; t2tINV: 1; TRA: 0
SVs in sample	105
Oscillating CN (2 and 3 states)	7, 7
CN segments	7
FDR fragment joints	0.615458
FDR chr. breakp. enrich.	0
Linked to chrs	
Purity, ploidy	0.56, 3.59

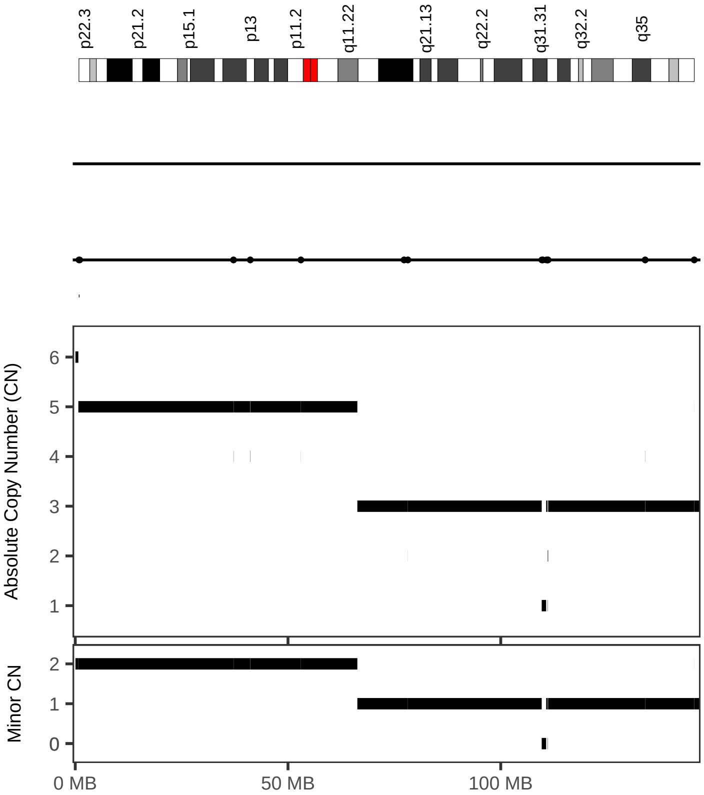

5116e3b4−2bac−40f5−8046−b9c1783faaa5

Cancer type	ColoRect−AdenoCA
Position	7:862136−133957567
Interleaved intrachr. SVs	1
Total SVs (intrachr. + transl.)	9
SV types	DEL: 8; DUP: 1; h2hINV: 0; t2tINV: 0; TRA: 0
SVs in sample	322
Oscillating CN (2 and 3 states)	7, 8
CN segments	18
FDR fragment joints	0.02438658
FDR chr. breakp. enrich.	0.63
Linked to chrs	
Purity, ploidy	0.5, 3.09

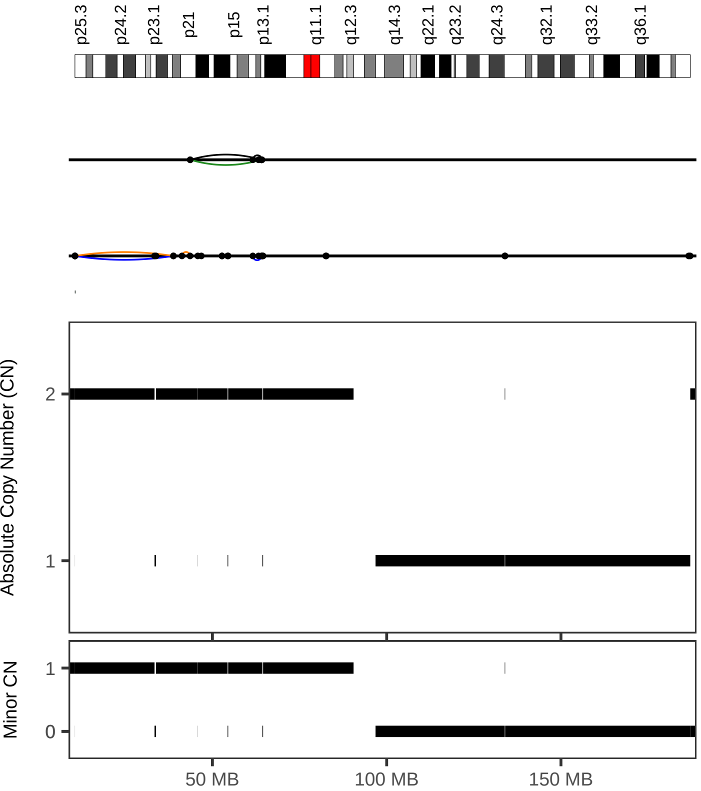

7d8eab0a−e6c8−4449−9ebf−50c41db94a06

Cancer type	ColoRect−AdenoCA
Position	2:43615678−64237004
Interleaved intrachr. SVs	6
Total SVs (intrachr. + transl.)	6
SV types	DEL: 1; DUP: 1; h2hINV: 2; t2tINV: 2; TRA: 0
SVs in sample	170
Oscillating CN (2 and 3 states)	5, 5
CN segments	5
FDR fragment joints	0.9284301
FDR chr. breakp. enrich.	0.58
Linked to chrs	
Purity, ploidy	0.7, 2.02

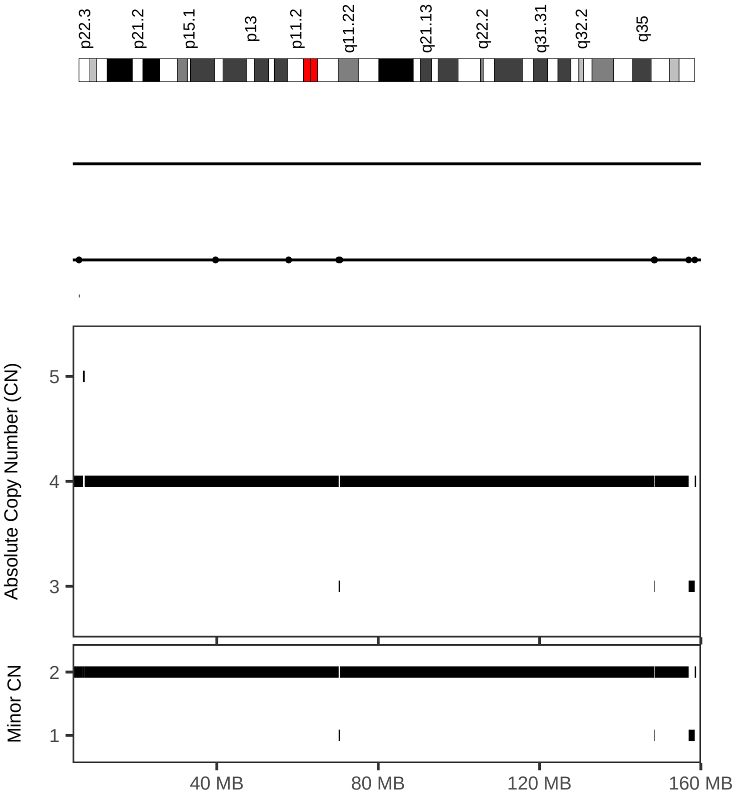

a512701f−88bb−4b05−8c94−fd285b9dd13e

Cancer type	ColoRect−AdenoCA
Position	7:39647779−158482174
Interleaved intrachr. SVs	1
Total SVs (intrachr. + transl.)	6
SV types	DEL: 6; DUP: 0; h2hINV: 0; t2tINV: 0; TRA: 0
SVs in sample	134
Oscillating CN (2 and 3 states)	7, 7
CN segments	7
FDR fragment joints	0.04139017
FDR chr. breakp. enrich.	1
Linked to chrs	
Purity, ploidy	0.8, 2.77

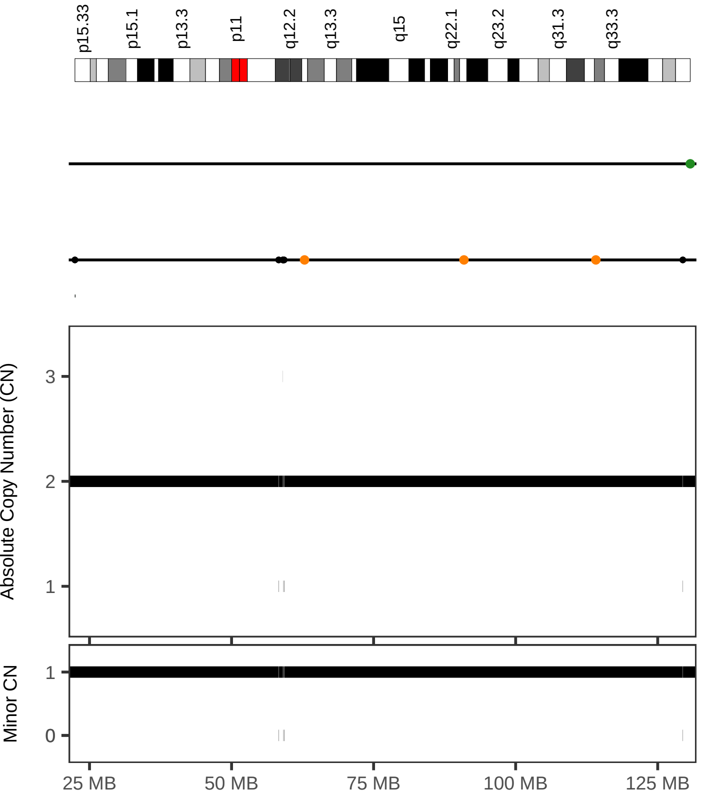

bfb07784−693b−4c25−874e−4ad6e04a5d46

Cancer type	ColoRect−AdenoCA
Position	5:58280145−129435613
Interleaved intrachr. SVs	1
Total SVs (intrachr. + transl.)	7
SV types	DEL: 4; DUP: 0; h2hINV: 0; t2tINV: 0; TRA: 3
SVs in sample	154
Oscillating CN (2 and 3 states)	7, 10
CN segments	10
FDR fragment joints	0.01743054
FDR chr. breakp. enrich.	0.4
Linked to chrs	1:4906911−246471749;
Purity, ploidy	0.58, 2.21

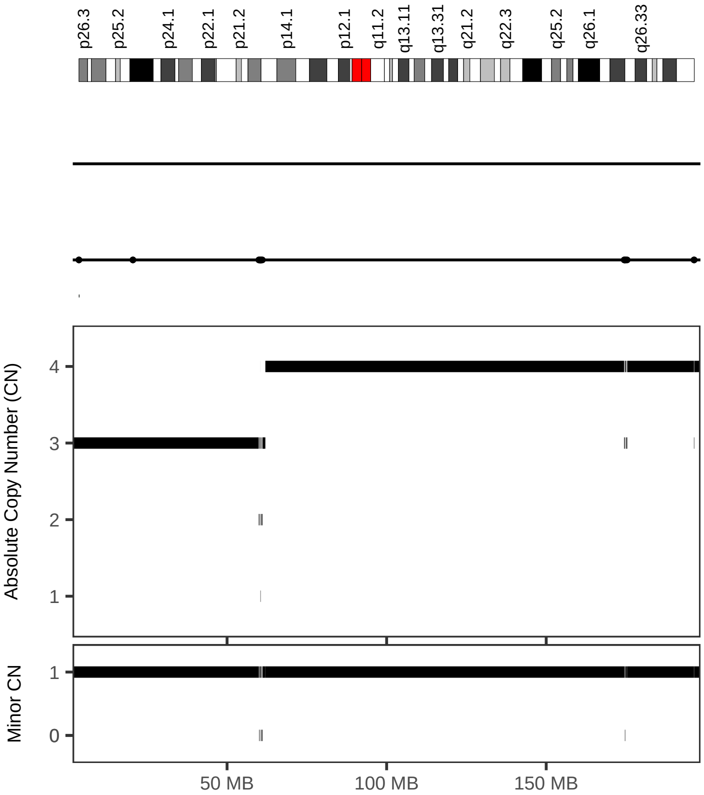

fafd4576−0709−49e1−926f−ad1ee09907ec

Cancer type	ColoRect−AdenoCA
Position	3:20473702−196392506
Interleaved intrachr. SVs	1
Total SVs (intrachr. + transl.)	11
SV types	DEL: 9; DUP: 2; h2hINV: 0; t2tINV: 0; TRA: 0
SVs in sample	146
Oscillating CN (2 and 3 states)	10, 22
CN segments	22
FDR fragment joints	0.02438658
FDR chr. breakp. enrich.	0.76
Linked to chrs	
Purity, ploidy	0.88, 3.93

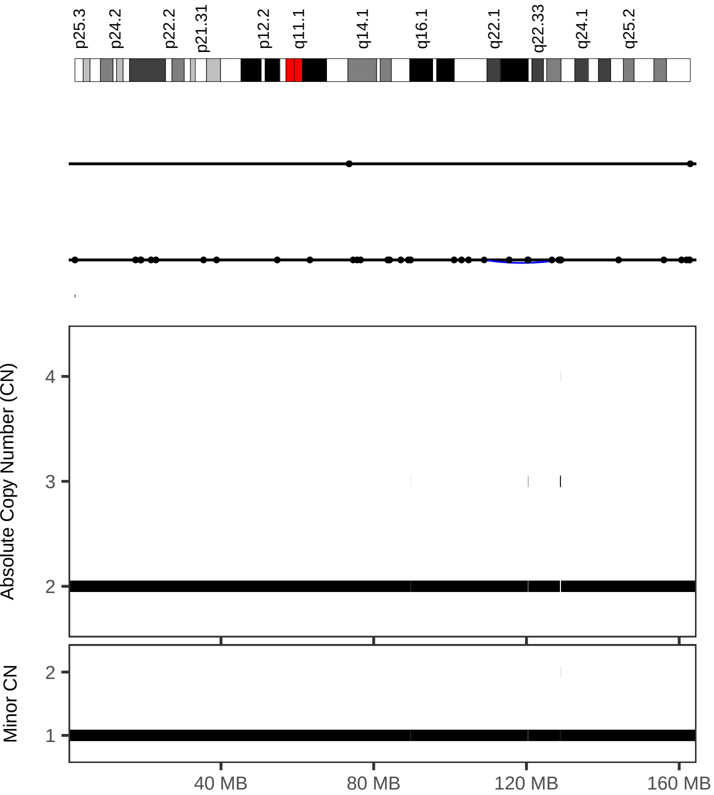

8feb0e8f−d09b−437d−8651−0cdecfe776bf

Cancer type	ColoRect−AdenoCA
Position	6:1750031−162731368
Interleaved intrachr. SVs	1
Total SVs (intrachr. + transl.)	24
SV types	DEL: 3; DUP: 20; h2hINV: 1; t2tINV: 0; TRA: 0
SVs in sample	603
Oscillating CN (2 and 3 states)	8, 8
CN segments	10
FDR fragment joints	3.049114e−06
FDR chr. breakp. enrich.	0.82
Linked to chrs	
Purity, ploidy	0.75, 1.99

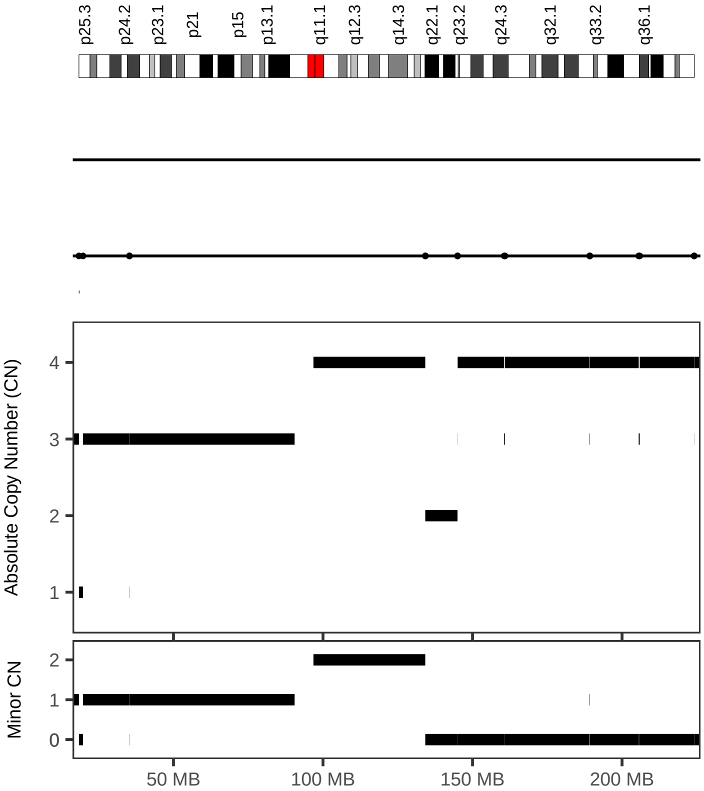

e2d395f8−bf13−4fe4−8031−683b0eedd875

Cancer type	ColoRect−AdenoCA
Position	2:18369748−224149589
Interleaved intrachr. SVs	1
Total SVs (intrachr. + transl.)	8
SV types	DEL: 7; DUP: 1; h2hINV: 0; t2tINV: 0; TRA: 0
SVs in sample	160
Oscillating CN (2 and 3 states)	10, 10
CN segments	16
FDR fragment joints	0.05741733
FDR chr. breakp. enrich.	0.35
Linked to chrs	
Purity, ploidy	0.54, 3.12

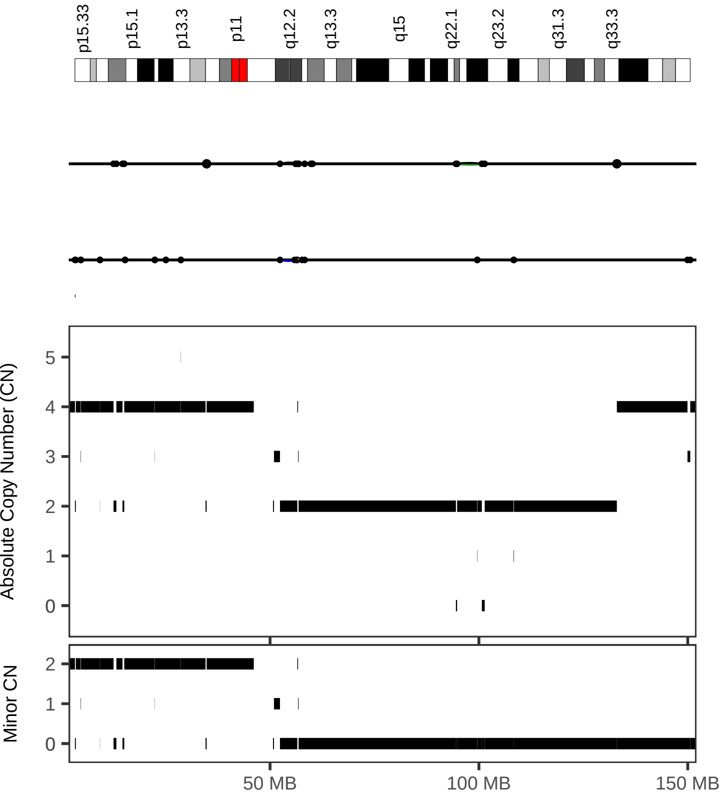

e2d395f8−bf13−4fe4−8031−683b0eedd875

Cancer type	ColoRect−AdenoCA
Position	5:52372824−60181728
Interleaved intrachr. SVs	8
Total SVs (intrachr. + transl.)	8
SV types	DEL: 1; DUP: 2; h2hINV: 2; t2tINV: 3; TRA: 0
SVs in sample	160
Oscillating CN (2 and 3 states)	4, 4
CN segments	6
FDR fragment joints	0.8653243
FDR chr. breakp. enrich.	0
Linked to chrs	
Purity, ploidy	0.54, 3.12

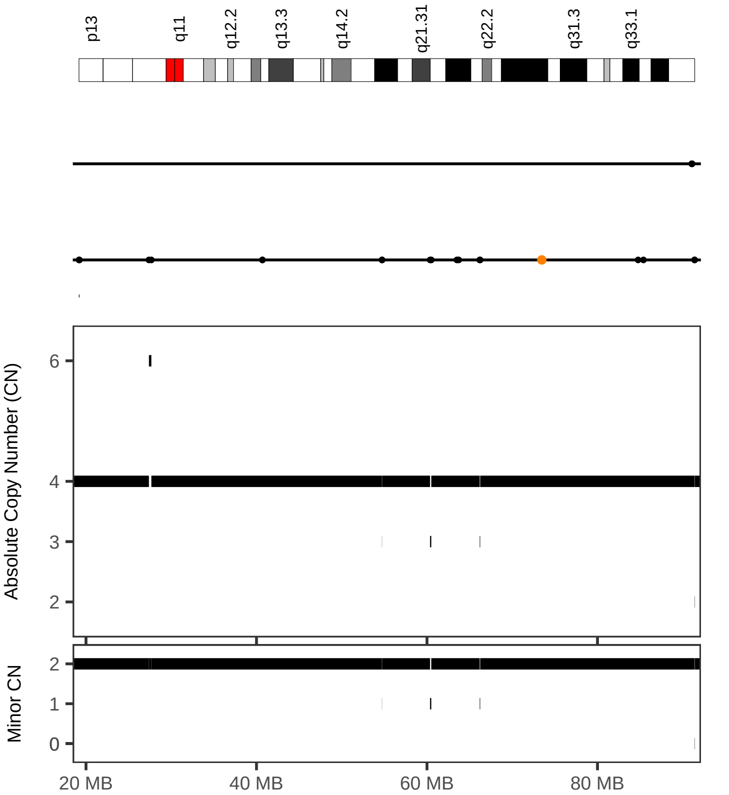

e2d395f8−bf13−4fe4−8031−683b0eedd875

Cancer type	ColoRect−AdenoCA
Position	13:19176863−91407141
Interleaved intrachr. SVs	1
Total SVs (intrachr. + transl.)	11
SV types	DEL: 8; DUP: 1; h2hINV: 0; t2tINV: 1; TRA: 1
SVs in sample	160
Oscillating CN (2 and 3 states)	7, 9
CN segments	11
FDR fragment joints	0.03230998
FDR chr. breakp. enrich.	0.06
Linked to chrs	
Purity, ploidy	0.54, 3.12

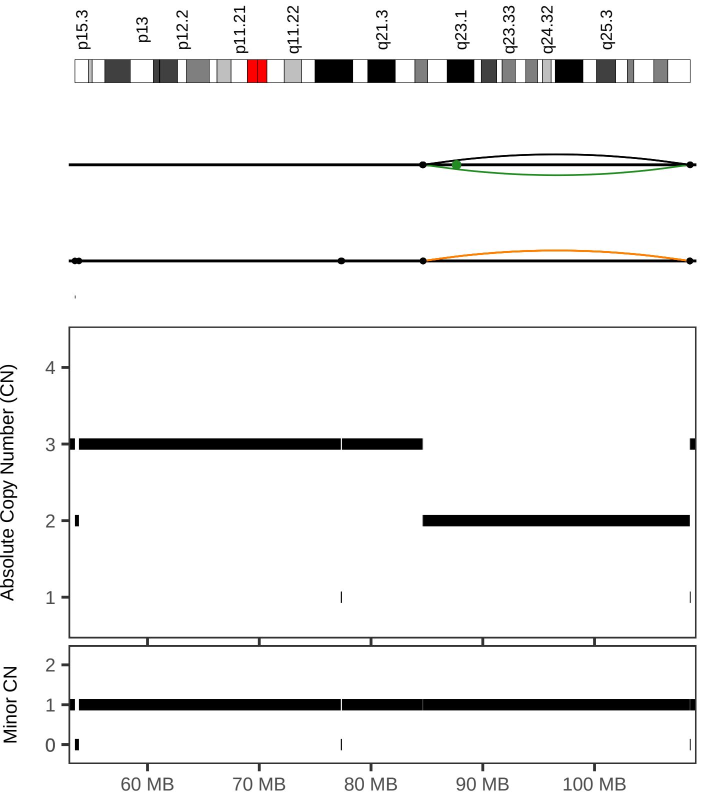

OCCAMS−AH−014

Cancer type	Eso−AdenoCA
Position	10:84597156−108564886
Interleaved intrachr. SVs	8
Total SVs (intrachr. + transl.)	10
SV types	DEL: 4; DUP: 0; h2hINV: 2; t2tINV: 2; TRA: 2
SVs in sample	299
Oscillating CN (2 and 3 states)	4, 5
CN segments	7
FDR fragment joints	0.615458
FDR chr. breakp. enrich.	1
Linked to chrs	
Purity, ploidy	0.49, 2.86

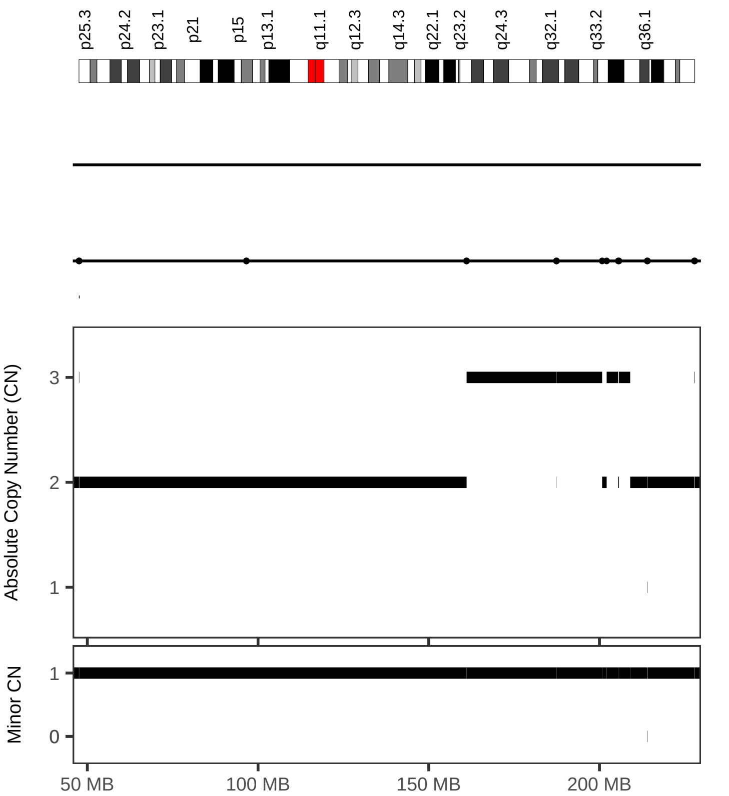

OCCAMS−AH−039

Cancer type	Eso−AdenoCA
Position	2:47547253−227912729
Interleaved intrachr. SVs	1
Total SVs (intrachr. + transl.)	7
SV types	DEL: 6; DUP: 1; h2hINV: 0; t2tINV: 0; TRA: 0
SVs in sample	146
Oscillating CN (2 and 3 states)	10, 14
CN segments	14
FDR fragment joints	0.1278363
FDR chr. breakp. enrich.	0.47
Linked to chrs	
Purity, ploidy	0.38, 1.99

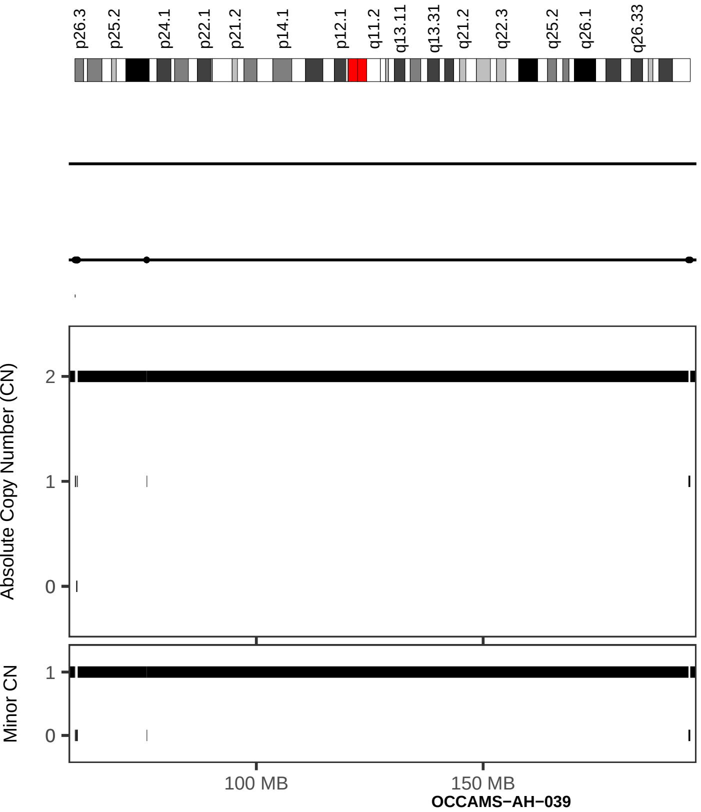

OCCAMS−AH−039

Cancer type	Eso−AdenoCA
Position	3:60184941−60611121
Interleaved intrachr. SVs	5
Total SVs (intrachr. + transl.)	5
SV types	DEL: 5; DUP: 0; h2hINV: 0; t2tINV: 0; TRA: 0
SVs in sample	146
Oscillating CN (2 and 3 states)	7, 8
CN segments	8
FDR fragment joints	0.1013295
FDR chr. breakp. enrich.	1
Linked to chrs	
Purity, ploidy	0.38, 1.99

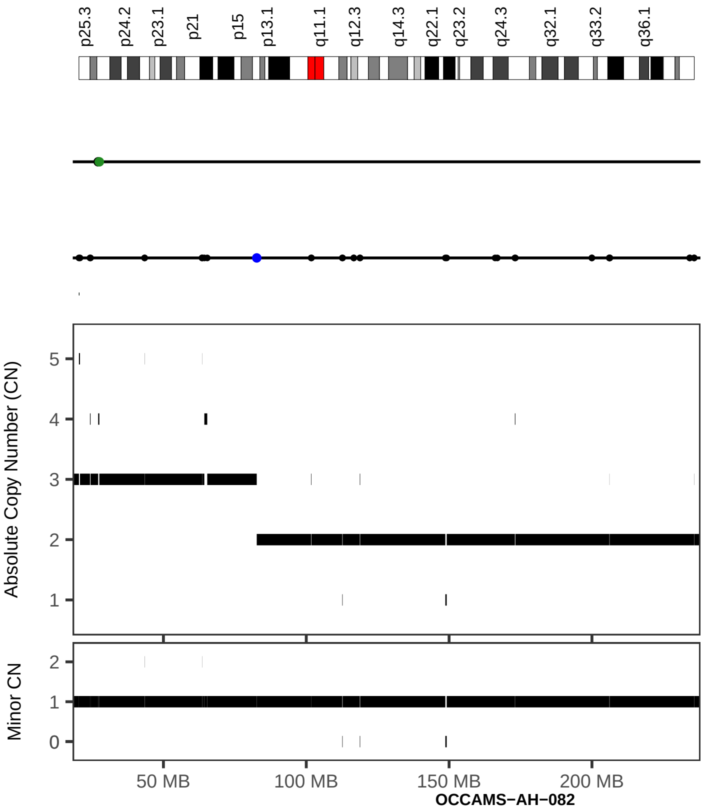

OCCAMS−AH−082

Cancer type	Eso−AdenoCA
Position	2:20418187−235815453
Interleaved intrachr. SVs	1
Total SVs (intrachr. + transl.)	18
SV types	DEL: 2; DUP: 13; h2hINV: 0; t2tINV: 0; TRA: 3
SVs in sample	433
Oscillating CN (2 and 3 states)	7, 12
CN segments	29
FDR fragment joints	0.002321411
FDR chr. breakp. enrich.	0.02
Linked to chrs	
Purity, ploidy	0.18, 2.48

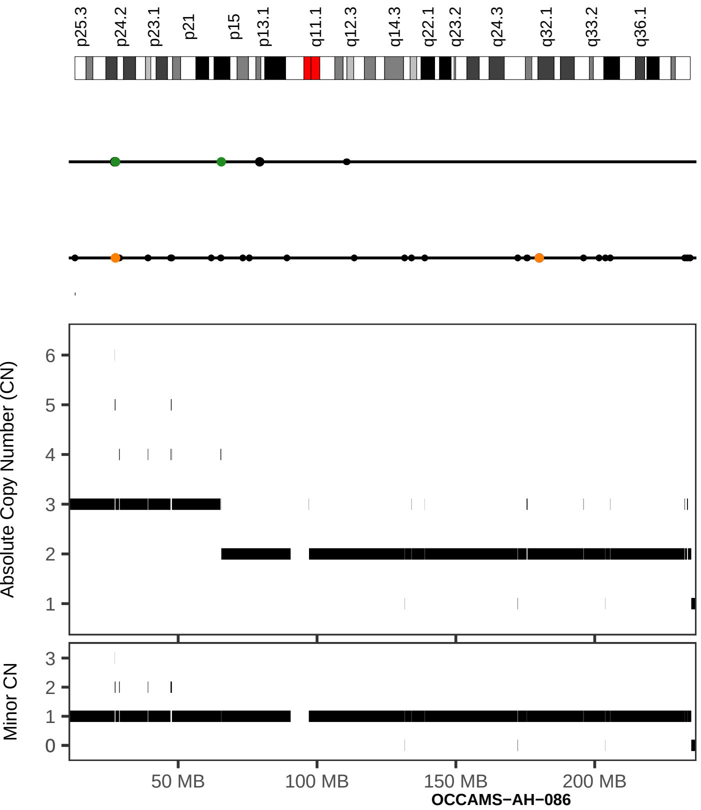

OCCAMS−AH−086

Cancer type	Eso−AdenoCA
Position	2:12813030−234404523
Interleaved intrachr. SVs	1
Total SVs (intrachr. + transl.)	34
SV types	DEL: 4; DUP: 18; h2hINV: 0; t2tINV: 1; TRA: 11
SVs in sample	619
Oscillating CN (2 and 3 states)	7, 34
CN segments	37
FDR fragment joints	0.04139017
FDR chr. breakp. enrich.	0.32
Linked to chrs	3:11605138−66344249;
Purity, ploidy	0.9, 2.18

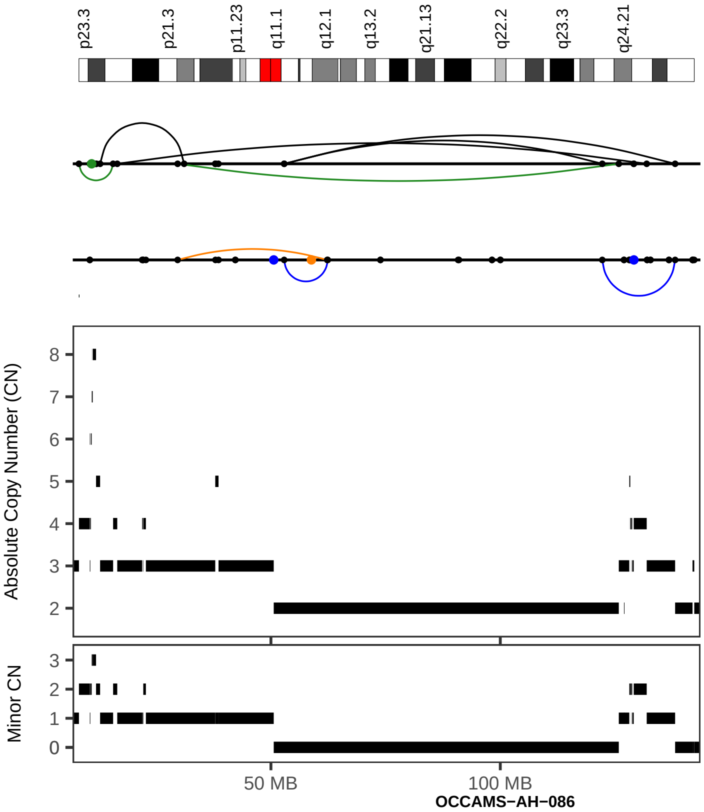

OCCAMS−AH−086

Cancer type	Eso−AdenoCA
Position	8:8145650−138110281
Interleaved intrachr. SVs	9
Total SVs (intrachr. + transl.)	13
SV types	DEL: 1; DUP: 2; h2hINV: 4; t2tINV: 2; TRA: 4
SVs in sample	619
Oscillating CN (2 and 3 states)	7, 14
CN segments	32
FDR fragment joints	0.6776251
FDR chr. breakp. enrich.	0.38
Linked to chrs	10:43601978−134914514;
Purity, ploidy	0.9, 2.18

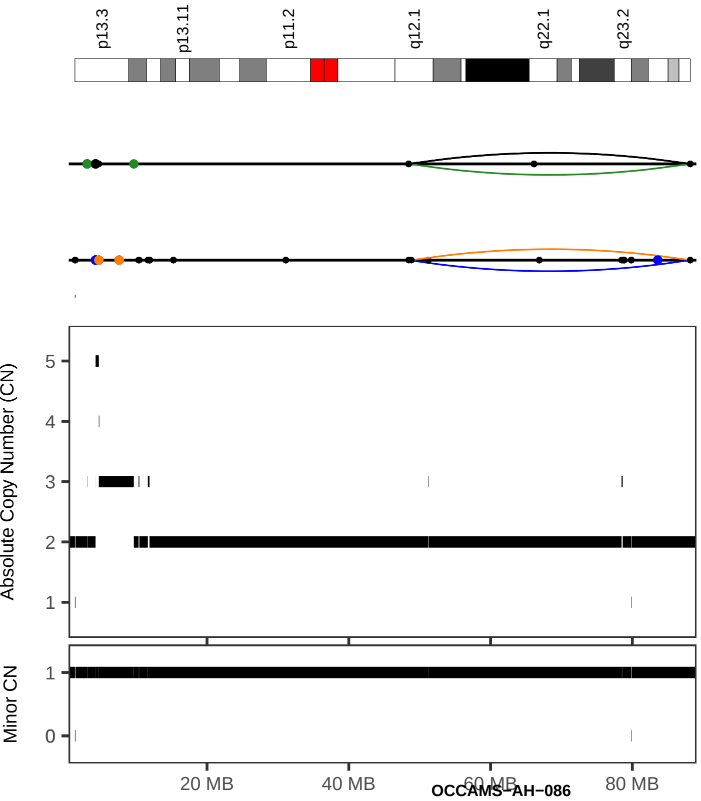

OCCAMS−AH−086

Cancer type	Eso−AdenoCA
Position	16:4274640−88167527
Interleaved intrachr. SVs	4
Total SVs (intrachr. + transl.)	25
SV types	DEL: 1; DUP: 2; h2hINV: 4; t2tINV: 1; TRA: 17
SVs in sample	619
Oscillating CN (2 and 3 states)	10, 14
CN segments	16
FDR fragment joints	0.6776251
FDR chr. breakp. enrich.	0
Linked to chrs	3:11605138−66344249;4:8173076−187978632
Purity, ploidy	0.9, 2.18

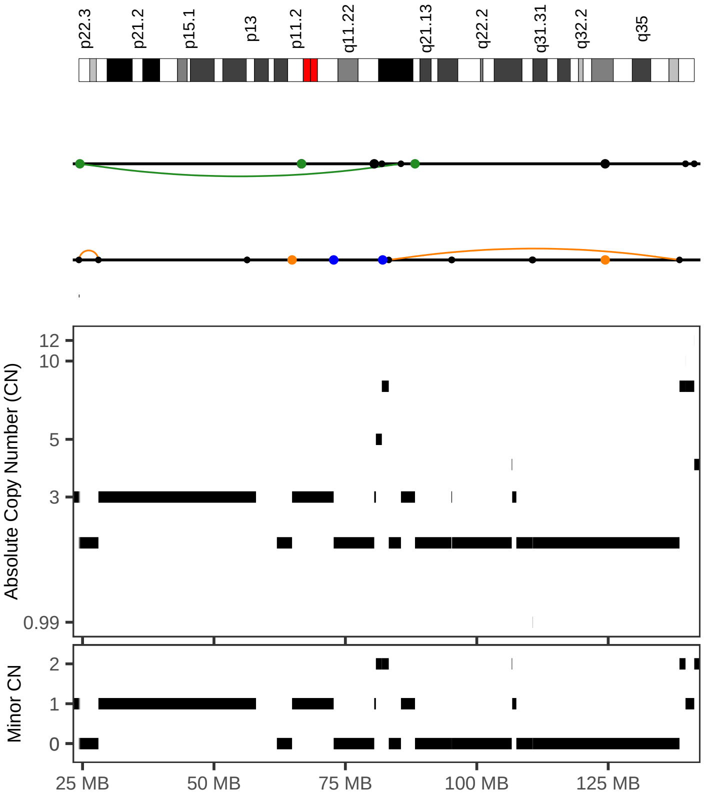

OCCAMS−AH−135

Cancer type	Eso−AdenoCA
Position	7:24306872−138562308
Interleaved intrachr. SVs	3
Total SVs (intrachr. + transl.)	13
SV types	DEL: 2; DUP: 0; h2hINV: 0; t2tINV: 1; TRA: 10
SVs in sample	298
Oscillating CN (2 and 3 states)	8, 9
CN segments	21
FDR fragment joints	0.9000538
FDR chr. breakp. enrich.	0.31
Linked to chrs	6:39890387−56314679;
Purity, ploidy	0.55, 2.85

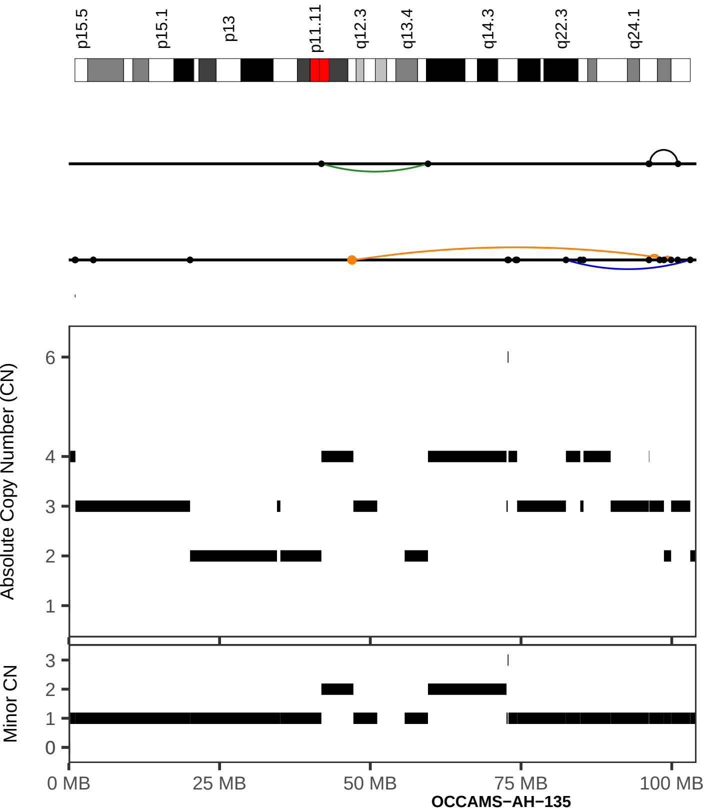

OCCAMS−AH−135

Cancer type	Eso−AdenoCA
Position	11:41887275−103067905
Interleaved intrachr. SVs	6
Total SVs (intrachr. + transl.)	7
SV types	DEL: 2; DUP: 1; h2hINV: 1; t2tINV: 2; TRA: 1
SVs in sample	298
Oscillating CN (2 and 3 states)	10, 10
CN segments	19
FDR fragment joints	0.7735152
FDR chr. breakp. enrich.	1
Linked to chrs	
Purity, ploidy	0.55, 2.85

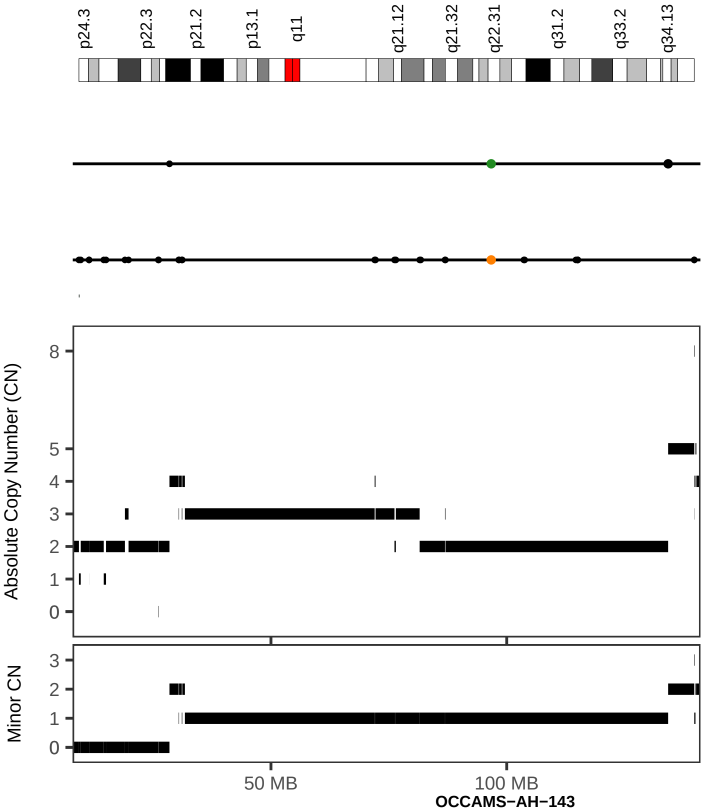

OCCAMS−AH−143

Cancer type	Eso−AdenoCA
Position	9:9270942−115069668
Interleaved intrachr. SVs	1
Total SVs (intrachr. + transl.)	15
SV types	DEL: 10; DUP: 3; h2hINV: 0; t2tINV: 0; TRA: 2
SVs in sample	596
Oscillating CN (2 and 3 states)	8, 13
CN segments	23
FDR fragment joints	0.02438658
FDR chr. breakp. enrich.	0.76
Linked to chrs	
Purity, ploidy	0.46, 2.6

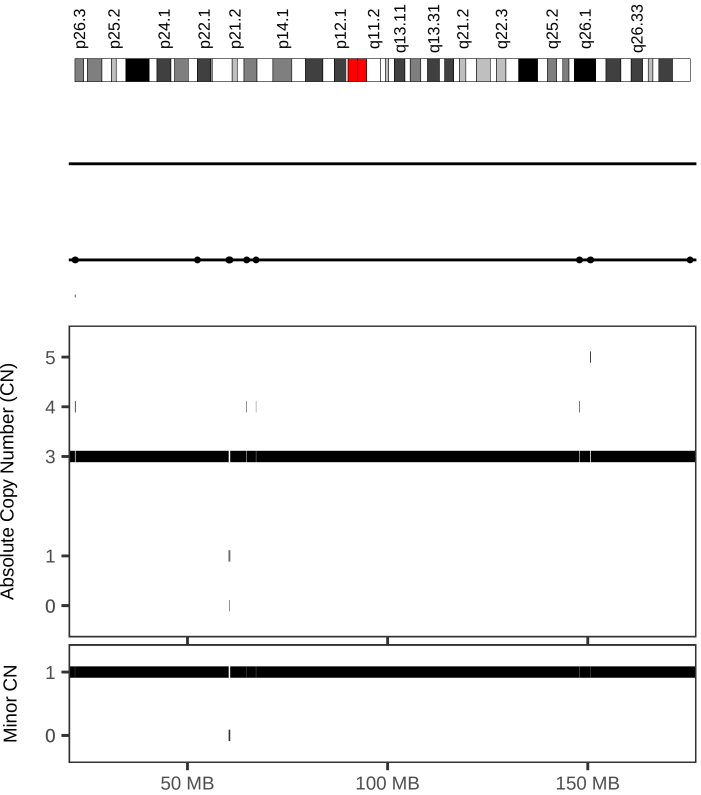

OCCAMS−AH−160

Cancer type	Eso−AdenoCA
Position	3:21878297−175600377
Interleaved intrachr. SVs	1
Total SVs (intrachr. + transl.)	9
SV types	DEL: 2; DUP: 7; h2hINV: 0; t2tINV: 0; TRA: 0
SVs in sample	102
Oscillating CN (2 and 3 states)	7, 12
CN segments	14
FDR fragment joints	0.1178349
FDR chr. breakp. enrich.	0.48
Linked to chrs	
Purity, ploidy	0.69, 2.68

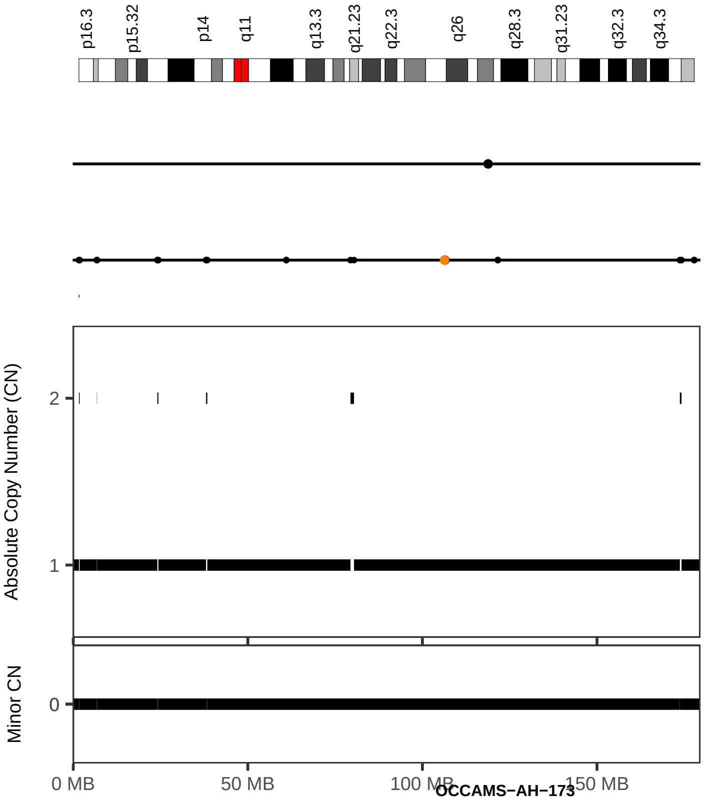

OCCAMS−AH−173

Cancer type	Eso−AdenoCA
Position	4:1618263−177859827
Interleaved intrachr. SVs	1
Total SVs (intrachr. + transl.)	13
SV types	DEL: 0; DUP: 9; h2hINV: 0; t2tINV: 0; TRA: 4
SVs in sample	410
Oscillating CN (2 and 3 states)	12, 12
CN segments	12
FDR fragment joints	0.03961725
FDR chr. breakp. enrich.	0.03
Linked to chrs	11:17492995−133541100;16:2300666−78688404
Purity, ploidy	0.6, 1.64

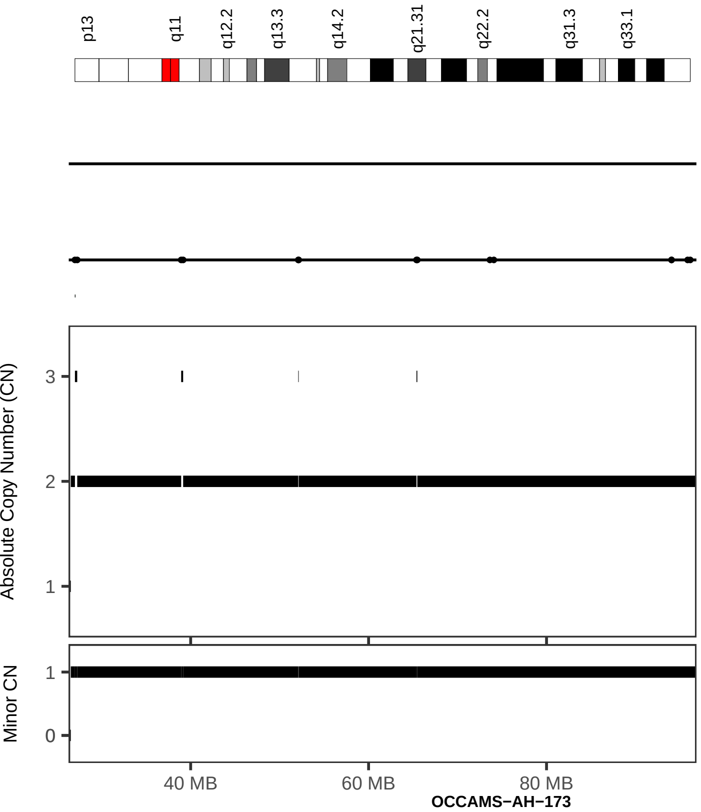

OCCAMS−AH−173

Cancer type	Eso−AdenoCA
Position	13:26991542−96155452
Interleaved intrachr. SVs	1
Total SVs (intrachr. + transl.)	6
SV types	DEL: 0; DUP: 6; h2hINV: 0; t2tINV: 0; TRA: 0
SVs in sample	410
Oscillating CN (2 and 3 states)	8, 8
CN segments	8
FDR fragment joints	0.04139017
FDR chr. breakp. enrich.	0.18
Linked to chrs	
Purity, ploidy	0.6, 1.64

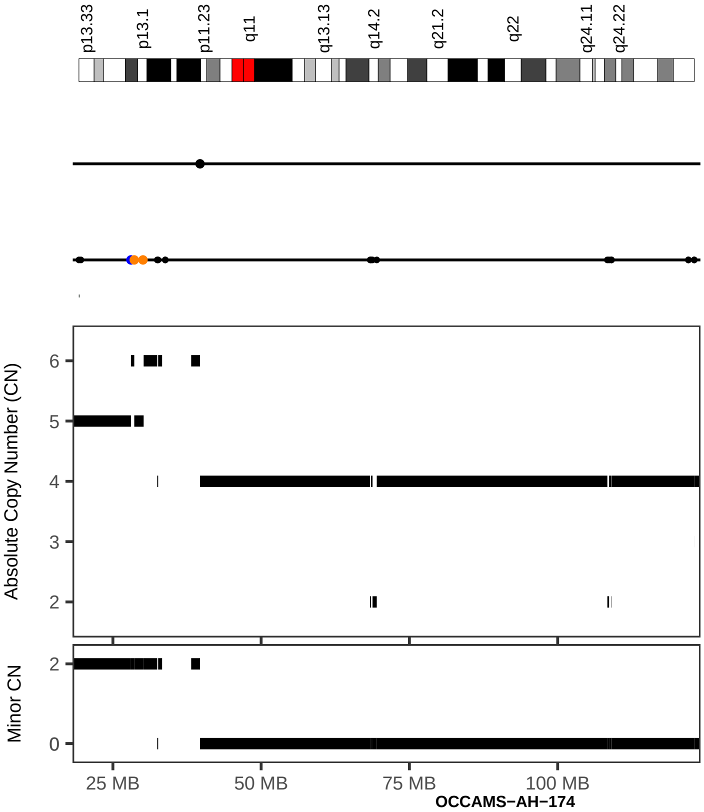

OCCAMS−AH−174

Cancer type	Eso−AdenoCA
Position	12:19271132−109088102
Interleaved intrachr. SVs	1
Total SVs (intrachr. + transl.)	11
SV types	DEL: 6; DUP: 1; h2hINV: 0; t2tINV: 0; TRA: 4
SVs in sample	572
Oscillating CN (2 and 3 states)	9, 9
CN segments	16
FDR fragment joints	0.129004
FDR chr. breakp. enrich.	0.05
Linked to chrs	
Purity, ploidy	0.29, 4.26

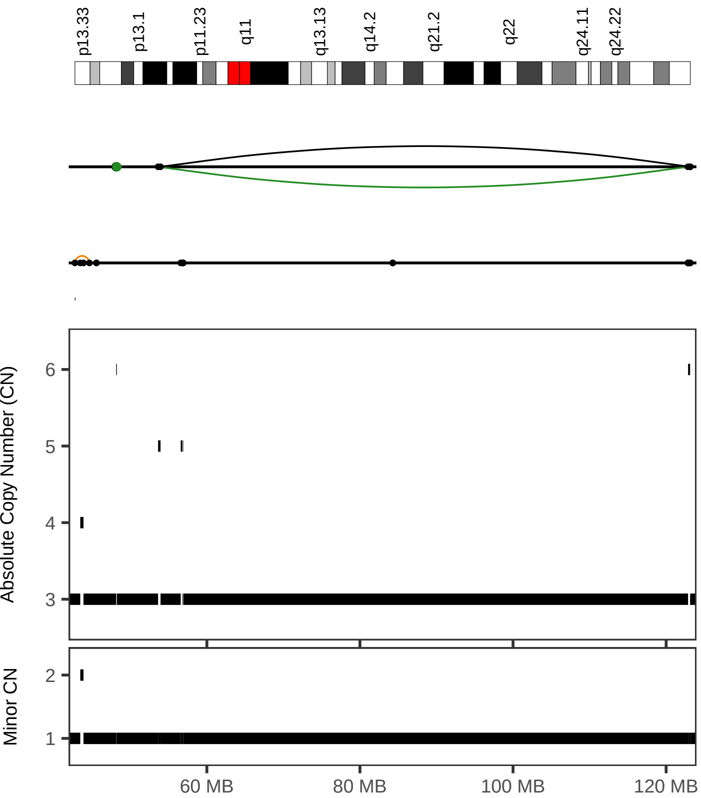

OCCAMS−AH−197

Cancer type	Eso−AdenoCA
Position	12:53626633−123151239
Interleaved intrachr. SVs	6
Total SVs (intrachr. + transl.)	6
SV types	DEL: 1; DUP: 2; h2hINV: 2; t2tINV: 1; TRA: 0
SVs in sample	269
Oscillating CN (2 and 3 states)	6, 8
CN segments	8
FDR fragment joints	0.9284301
FDR chr. breakp. enrich.	0.87
Linked to chrs	
Purity, ploidy	0.3, 3.09

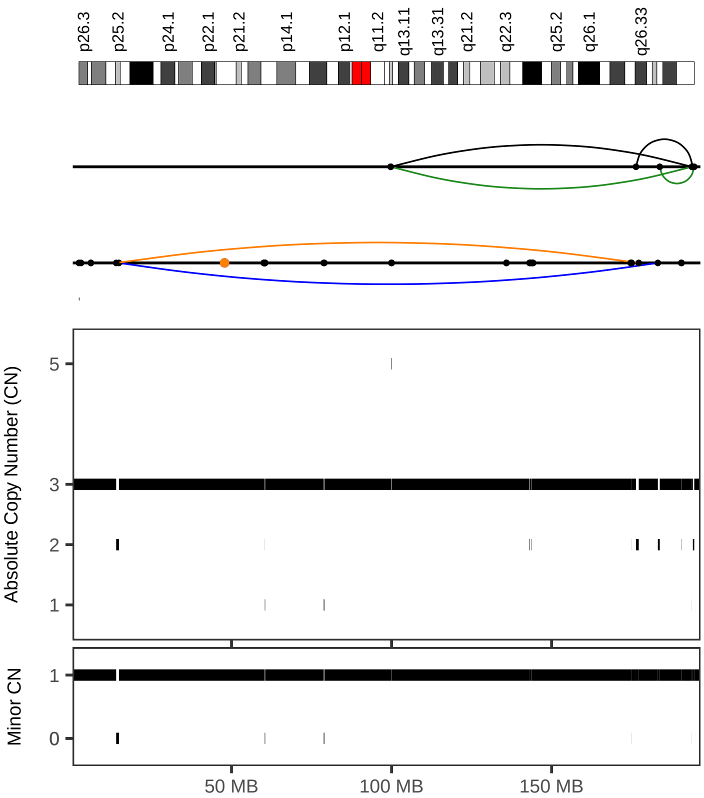

OCCAMS−AH−213

Cancer type	Eso−AdenoCA
Position	3:13968605−194565147
Interleaved intrachr. SVs	6
Total SVs (intrachr. + transl.)	8
SV types	DEL: 1; DUP: 1; h2hINV: 2; t2tINV: 2; TRA: 2
SVs in sample	283
Oscillating CN (2 and 3 states)	13, 17
CN segments	28
FDR fragment joints	1
FDR chr. breakp. enrich.	0.67
Linked to chrs	7:48065329−141802390;
Purity, ploidy	0.57, 2.68

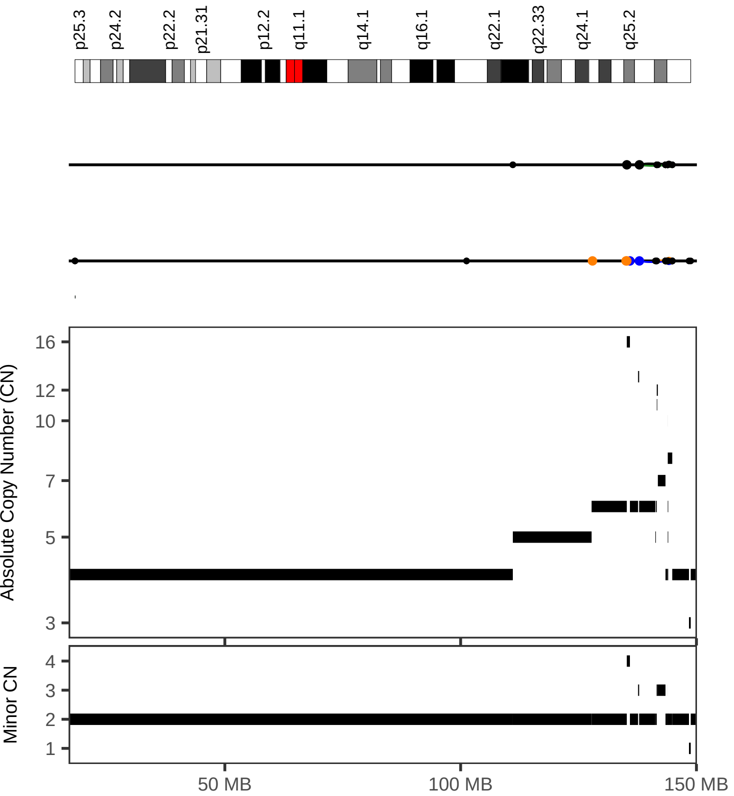

OCCAMS−ED−007

Cancer type	Eso−AdenoCA
Position	6:137614668−144874378
Interleaved intrachr. SVs	24
Total SVs (intrachr. + transl.)	26
SV types	DEL: 8; DUP: 7; h2hINV: 5; t2tINV: 4; TRA: 2
SVs in sample	163
Oscillating CN (2 and 3 states)	4, 6
CN segments	16
FDR fragment joints	0.8172348
FDR chr. breakp. enrich.	0
Linked to chrs	
Purity, ploidy	0.49, 4.07

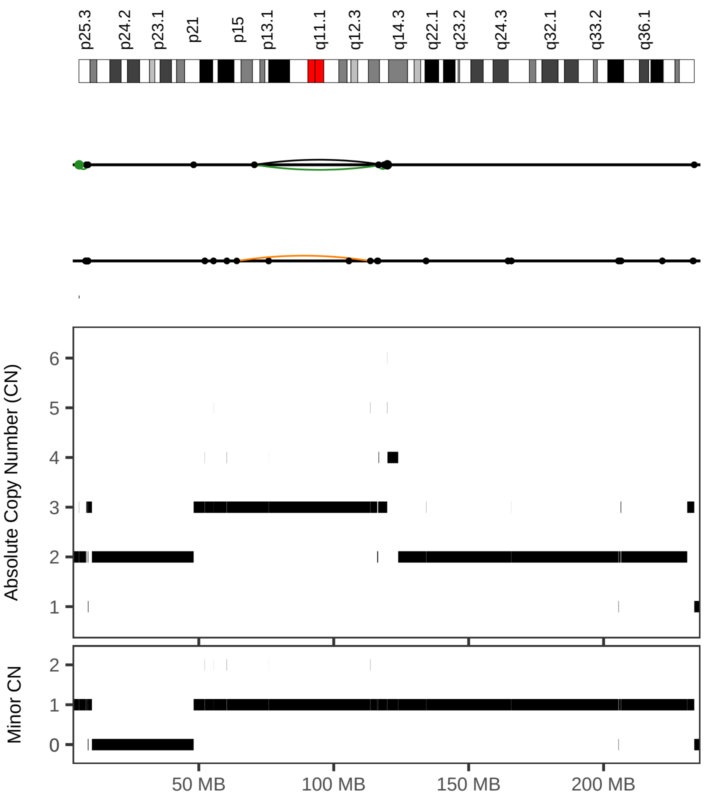

OCCAMS−PS−012

Cancer type	Eso−AdenoCA
Position	2:5579139−8930469
Interleaved intrachr. SVs	13
Total SVs (intrachr. + transl.)	14
SV types	DEL: 5; DUP: 5; h2hINV: 1; t2tINV: 2; TRA: 1
SVs in sample	165
Oscillating CN (2 and 3 states)	7, 11
CN segments	11
FDR fragment joints	0.6776251
FDR chr. breakp. enrich.	0
Linked to chrs	
Purity, ploidy	0.44, 2.14

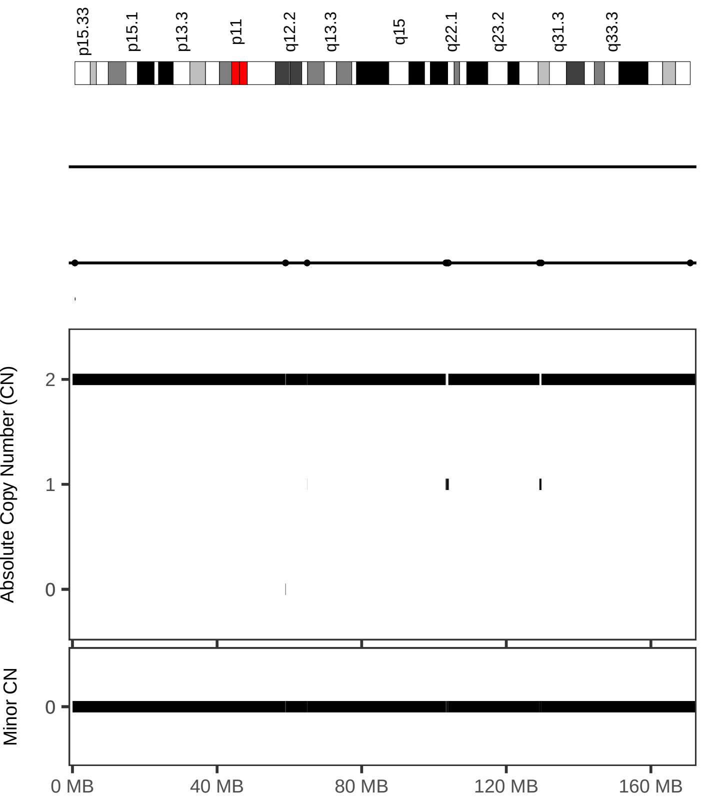

OCCAMS−QE−095

Cancer type	Eso−AdenoCA
Position	5:58889907−129722541
Interleaved intrachr. SVs	1
Total SVs (intrachr. + transl.)	5
SV types	DEL: 5; DUP: 0; h2hINV: 0; t2tINV: 0; TRA: 0
SVs in sample	247
Oscillating CN (2 and 3 states)	11, 12
CN segments	12
FDR fragment joints	0.1013295
FDR chr. breakp. enrich.	0.41
Linked to chrs	
Purity, ploidy	0.61, 2.48

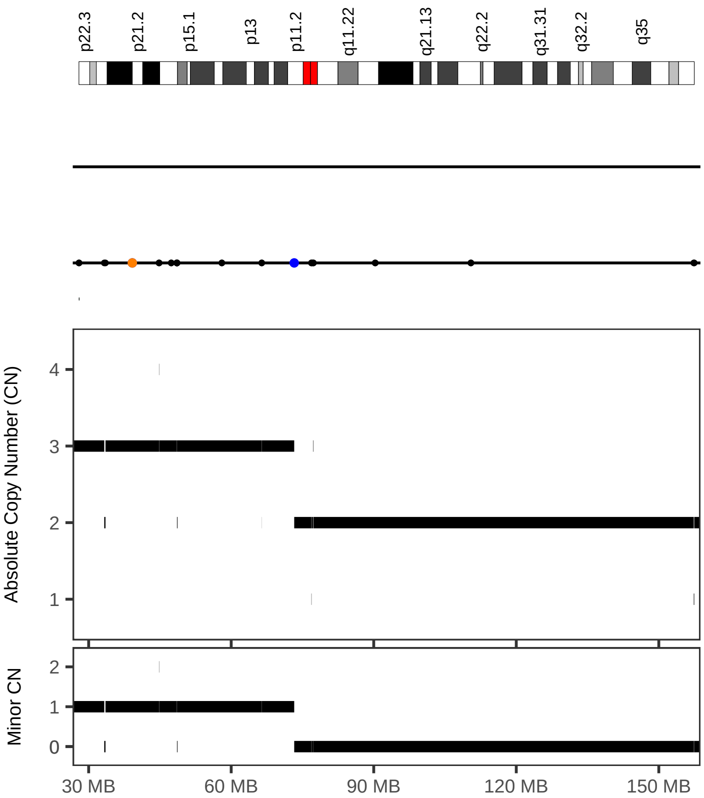

OCCAMS−QE−095

Cancer type	Eso−AdenoCA
Position	7:27948258−157460631
Interleaved intrachr. SVs	1
Total SVs (intrachr. + transl.)	13
SV types	DEL: 8; DUP: 2; h2hINV: 0; t2tINV: 0; TRA: 3
SVs in sample	247
Oscillating CN (2 and 3 states)	8, 18
CN segments	18
FDR fragment joints	0.06137436
FDR chr. breakp. enrich.	0.57
Linked to chrs	
Purity, ploidy	0.61, 2.48

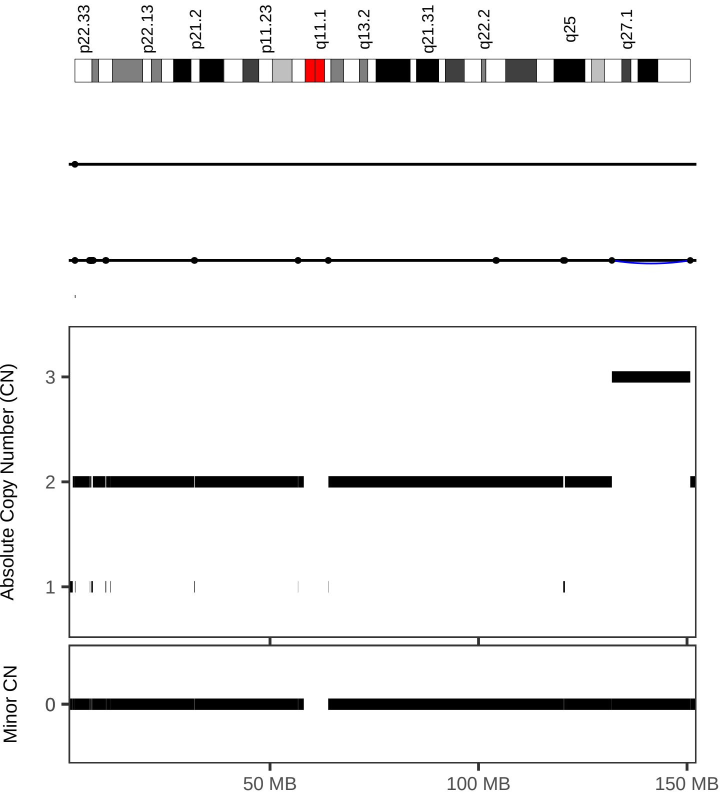

OCCAMS−QE−095

Cancer type	Eso−AdenoCA
Position	X:6665084−150768368
Interleaved intrachr. SVs	1
Total SVs (intrachr. + transl.)	10
SV types	DEL: 9; DUP: 1; h2hINV: 0; t2tINV: 0; TRA: 0
SVs in sample	247
Oscillating CN (2 and 3 states)	18, 20
CN segments	20
FDR fragment joints	0.01020027
FDR chr. breakp. enrich.	0.71
Linked to chrs	
Purity, ploidy	0.61, 2.48

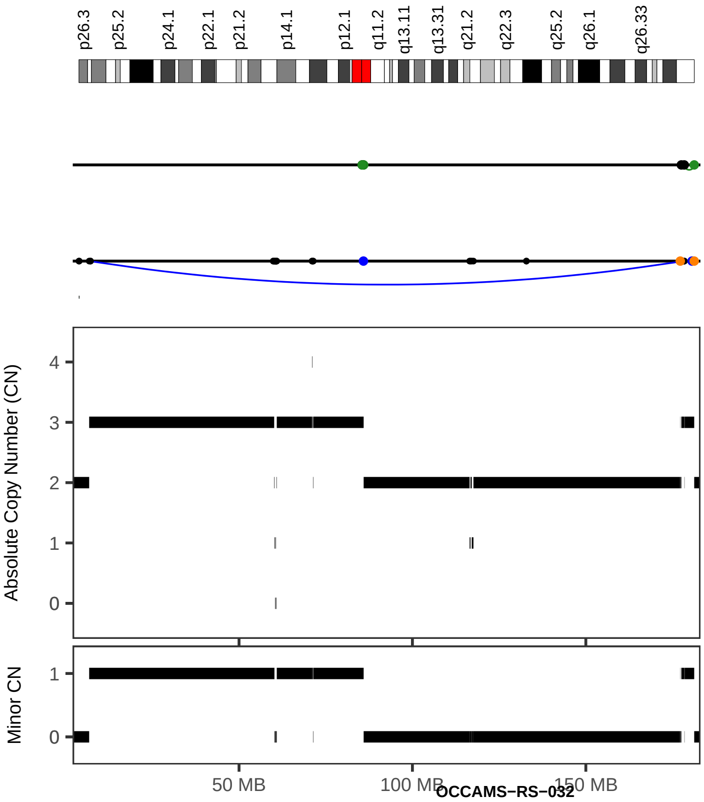

OCCAMS−RS−032

Cancer type	Eso−AdenoCA
Position	3:6793668−181259419
Interleaved intrachr. SVs	4
Total SVs (intrachr. + transl.)	19
SV types	DEL: 4; DUP: 3; h2hINV: 0; t2tINV: 1; TRA: 11
SVs in sample	159
Oscillating CN (2 and 3 states)	7, 17
CN segments	25
FDR fragment joints	0.9348624
FDR chr. breakp. enrich.	0
Linked to chrs	1:53896920−220849323;9:15830088−125695293
Purity, ploidy	0.61, 1.96

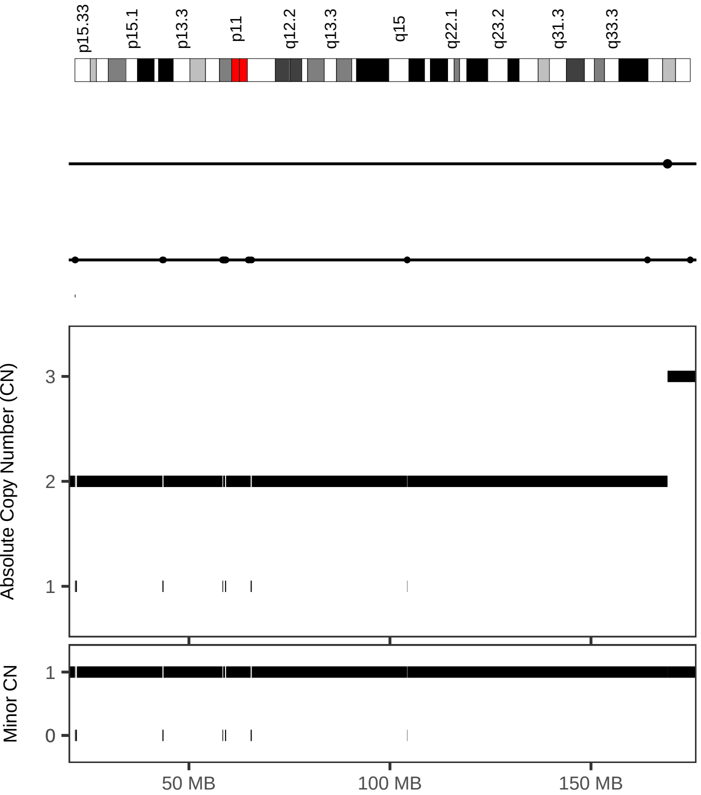

OCCAMS−RS−032

Cancer type	Eso−AdenoCA
Position	5:21645067−104326690
Interleaved intrachr. SVs	1
Total SVs (intrachr. + transl.)	7
SV types	DEL: 7; DUP: 0; h2hINV: 0; t2tINV: 0; TRA: 0
SVs in sample	159
Oscillating CN (2 and 3 states)	14, 14
CN segments	14
FDR fragment joints	0.01743054
FDR chr. breakp. enrich.	0.75
Linked to chrs	
Purity, ploidy	0.61, 1.96

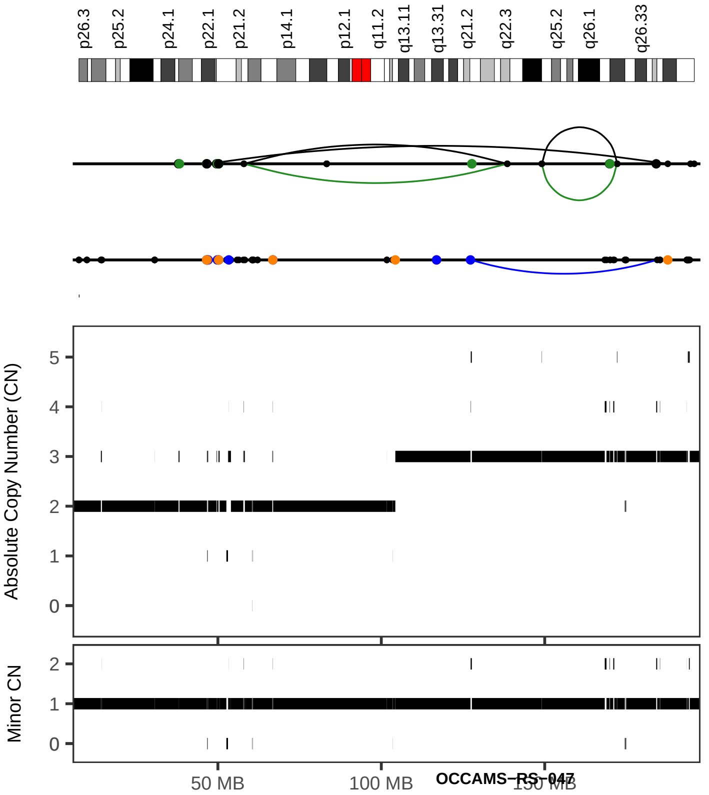

OCCAMS−RS−047

Cancer type	Eso−AdenoCA
Position	3:57769950−184424438
Interleaved intrachr. SVs	4
Total SVs (intrachr. + transl.)	15
SV types	DEL: 0; DUP: 2; h2hINV: 1; t2tINV: 1; TRA: 11
SVs in sample	894
Oscillating CN (2 and 3 states)	7, 23
CN segments	39
FDR fragment joints	0.925252
FDR chr. breakp. enrich.	0.2
Linked to chrs	1:23826407−235519805;12:56993350−123682991 17:37640778−73863269;4:6718600−185350689
Purity, ploidy	[illegible]

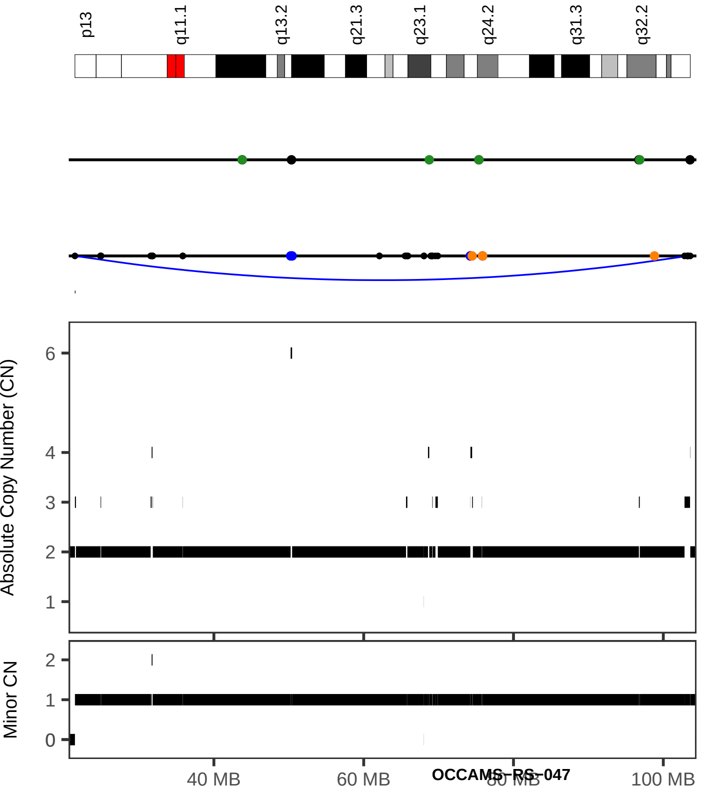

OCCAMS−RS−047

Cancer type	Eso−AdenoCA
Position	14:21444972−103597907
Interleaved intrachr. SVs	3
Total SVs (intrachr. + transl.)	20
SV types	DEL: 0; DUP: 3; h2hINV: 0; t2tINV: 0; TRA: 17
SVs in sample	894
Oscillating CN (2 and 3 states)	9, 17
CN segments	35
FDR fragment joints	0.8572806
FDR chr. breakp. enrich.	0.2
Linked to chrs	11:66812374−111289395;6:41252622−127972763 7:2419985−151823992;8:28471814−42053634 9:9115867−9827915;

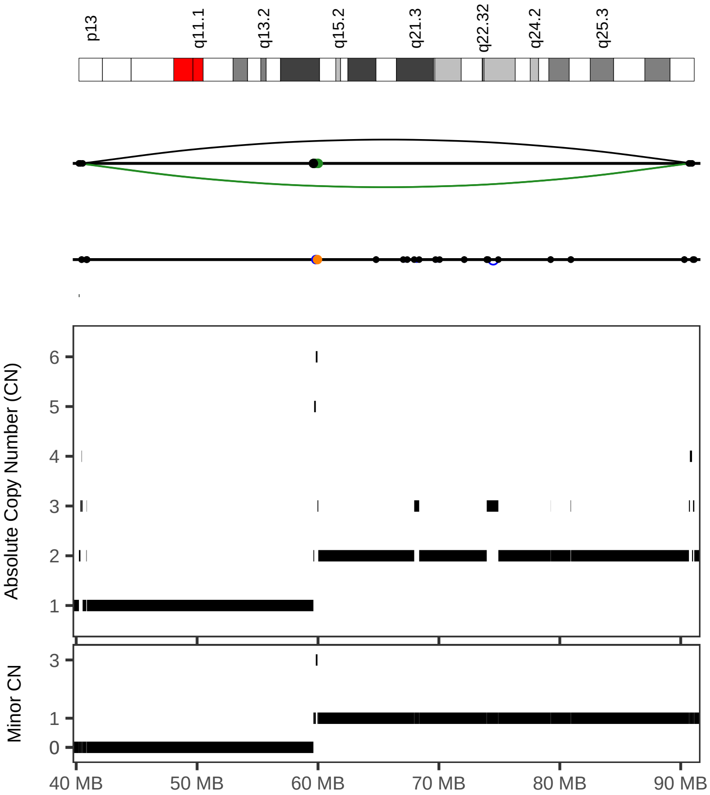

OCCAMS−RS−047

Cancer type	Eso−AdenoCA
Position	15:40224068−90935523
Interleaved intrachr. SVs	3
Total SVs (intrachr. + transl.)	11
SV types	DEL: 0; DUP: 0; h2hINV: 1; t2tINV: 2; TRA: 8
SVs in sample	894
Oscillating CN (2 and 3 states)	11, 11
CN segments	24
FDR fragment joints	0.615458
FDR chr. breakp. enrich.	0.72
Linked to chrs	18:19478394−72412982;
Purity, ploidy	0.48, 2.04

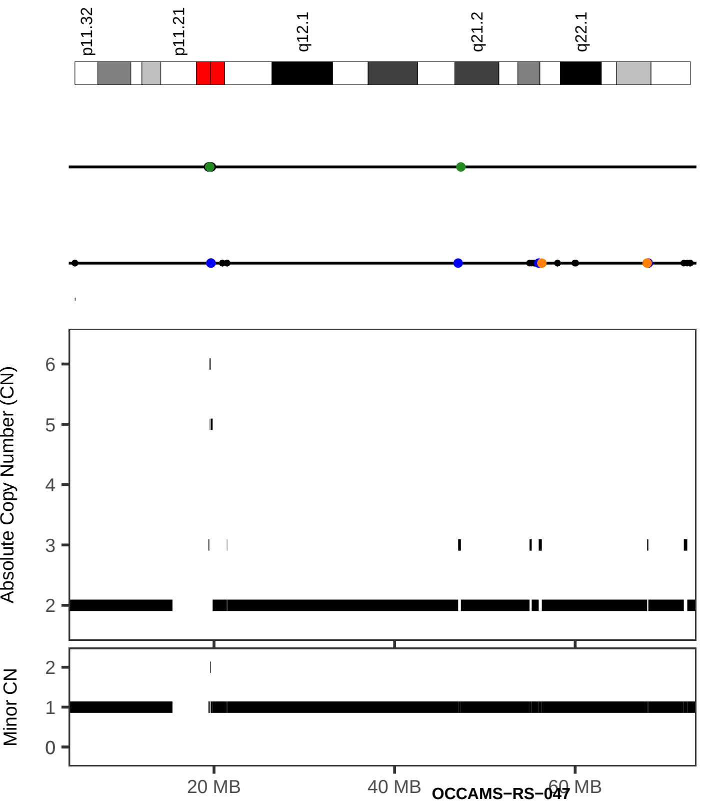

OCCAMS−RS−047

Cancer type	Eso−AdenoCA
Position	18:19478394−72412983
Interleaved intrachr. SVs	1
Total SVs (intrachr. + transl.)	21
SV types	DEL: 1; DUP: 9; h2hINV: 0; t2tINV: 0; TRA: 11
SVs in sample	894
Oscillating CN (2 and 3 states)	13, 14
CN segments	17
FDR fragment joints	0.02295299
FDR chr. breakp. enrich.	0.55
Linked to chrs	15:40224068−90935522;2:43059538−85800998 X:7262027−96789436;
Purity, ploidy	0.48, 2.04

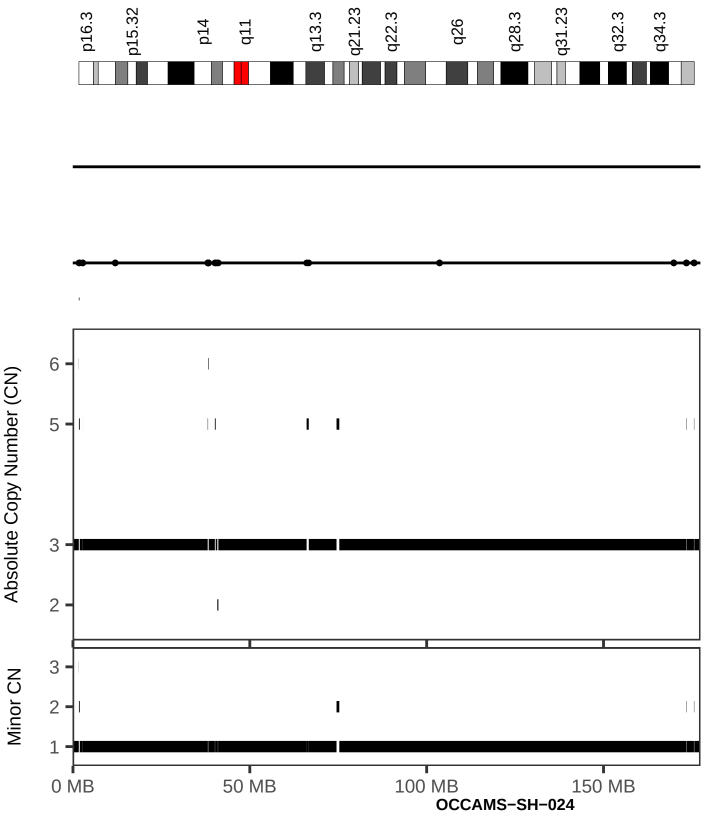

OCCAMS−SH−024

Cancer type	Eso−AdenoCA
Position	4:1687132−175651479
Interleaved intrachr. SVs	1
Total SVs (intrachr. + transl.)	10
SV types	DEL: 3; DUP: 7; h2hINV: 0; t2tINV: 0; TRA: 0
SVs in sample	656
Oscillating CN (2 and 3 states)	9, 9
CN segments	22
FDR fragment joints	0.1790694
FDR chr. breakp. enrich.	0
Linked to chrs	
Purity, ploidy	0.25, 4.5

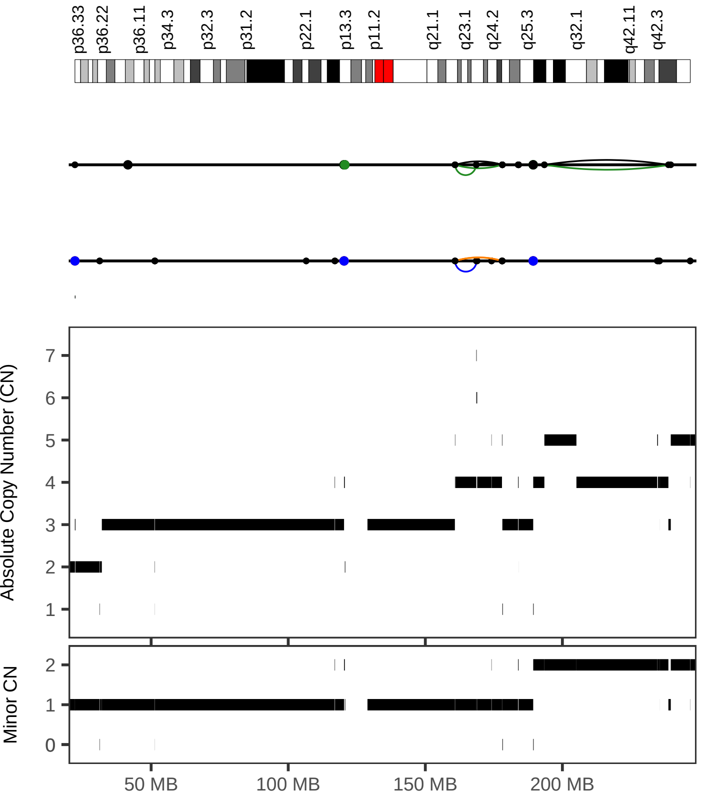

OCCAMS−SH−051

Cancer type	Eso−AdenoCA
Position	1:160780664−178102081
Interleaved intrachr. SVs	10
Total SVs (intrachr. + transl.)	10
SV types	DEL: 3; DUP: 3; h2hINV: 2; t2tINV: 2; TRA: 0
SVs in sample	450
Oscillating CN (2 and 3 states)	4, 6
CN segments	13
FDR fragment joints	0.9625775
FDR chr. breakp. enrich.	0.17
Linked to chrs	
Purity, ploidy	0.69, 2.44

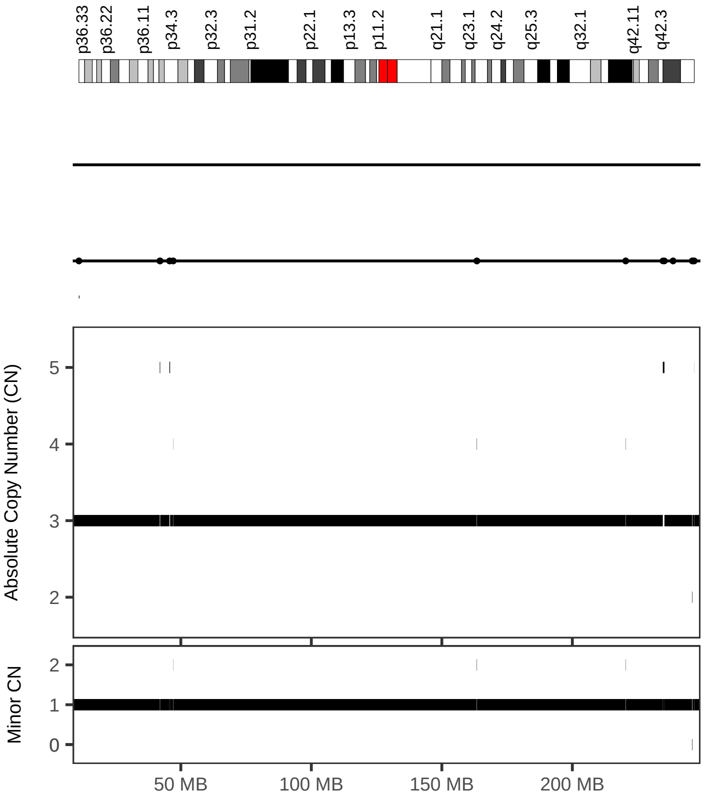

OCCAMS−ST−033

Cancer type	Eso−AdenoCA
Position	1:10953691−246705970
Interleaved intrachr. SVs	1
Total SVs (intrachr. + transl.)	9
SV types	DEL: 1; DUP: 8; h2hINV: 0; t2tINV: 0; TRA: 0
SVs in sample	131
Oscillating CN (2 and 3 states)	7, 13
CN segments	17
FDR fragment joints	0.02438658
FDR chr. breakp. enrich.	1
Linked to chrs	
Purity, ploidy	0.3, 2.82

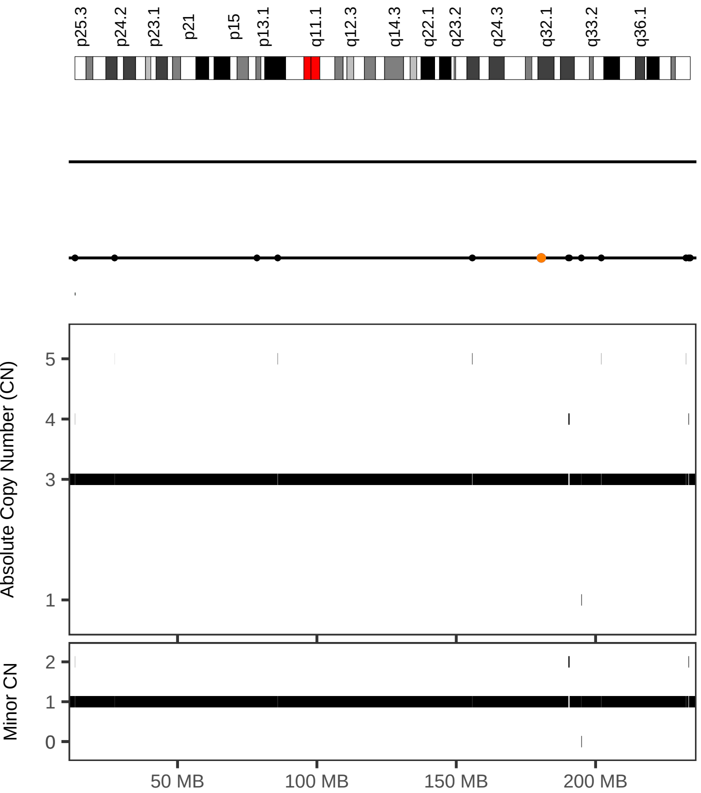

OCCAMS−ST−033

Cancer type	Eso−AdenoCA
Position	2:13178329−233510064
Interleaved intrachr. SVs	1
Total SVs (intrachr. + transl.)	10
SV types	DEL: 0; DUP: 8; h2hINV: 0; t2tINV: 0; TRA: 2
SVs in sample	131
Oscillating CN (2 and 3 states)	7, 10
CN segments	18
FDR fragment joints	0.01020027
FDR chr. breakp. enrich.	0.76
Linked to chrs	
Purity, ploidy	0.3, 2.82

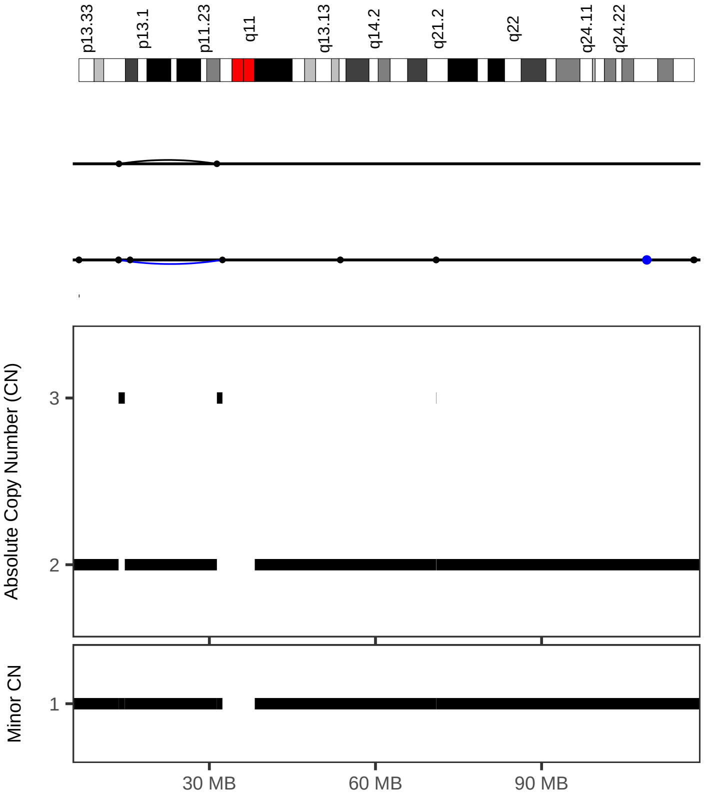

OCCAMS−ST−035

Cancer type	Eso−AdenoCA
Position	12:6443759−117567030
Interleaved intrachr. SVs	1
Total SVs (intrachr. + transl.)	8
SV types	DEL: 0; DUP: 6; h2hINV: 1; t2tINV: 0; TRA: 1
SVs in sample	137
Oscillating CN (2 and 3 states)	7, 7
CN segments	7
FDR fragment joints	0.05741733
FDR chr. breakp. enrich.	0.36
Linked to chrs	
Purity, ploidy	0.96, 1.93

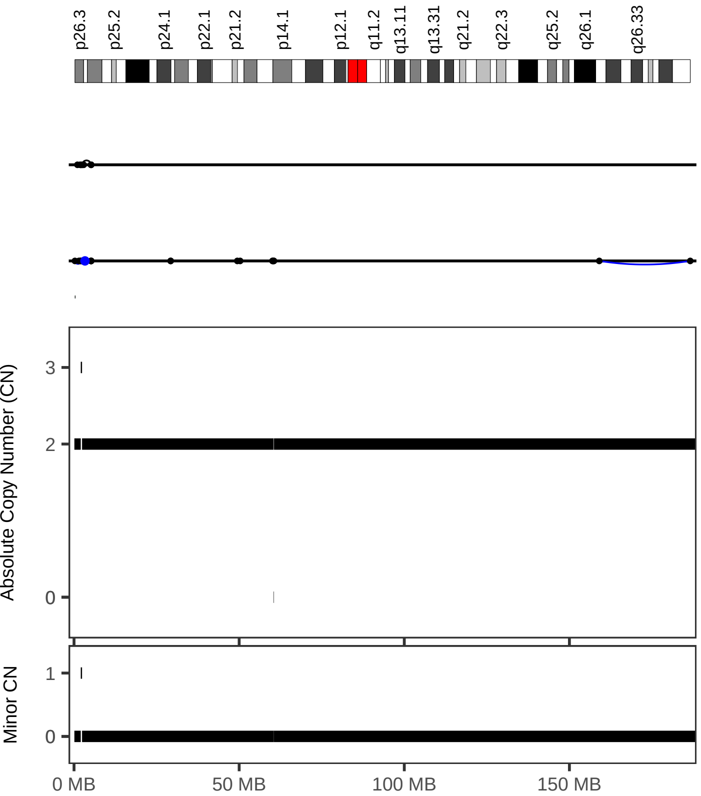

OCCAMS−WG−001

Cancer type	Eso−AdenoCA
Position	3:269704−5196742
Interleaved intrachr. SVs	8
Total SVs (intrachr. + transl.)	9
SV types	DEL: 0; DUP: 4; h2hINV: 1; t2tINV: 3; TRA: 1
SVs in sample	151
Oscillating CN (2 and 3 states)	5, 5
CN segments	5
FDR fragment joints	0.5435077
FDR chr. breakp. enrich.	0.17
Linked to chrs	
Purity, ploidy	0.53, 1.91

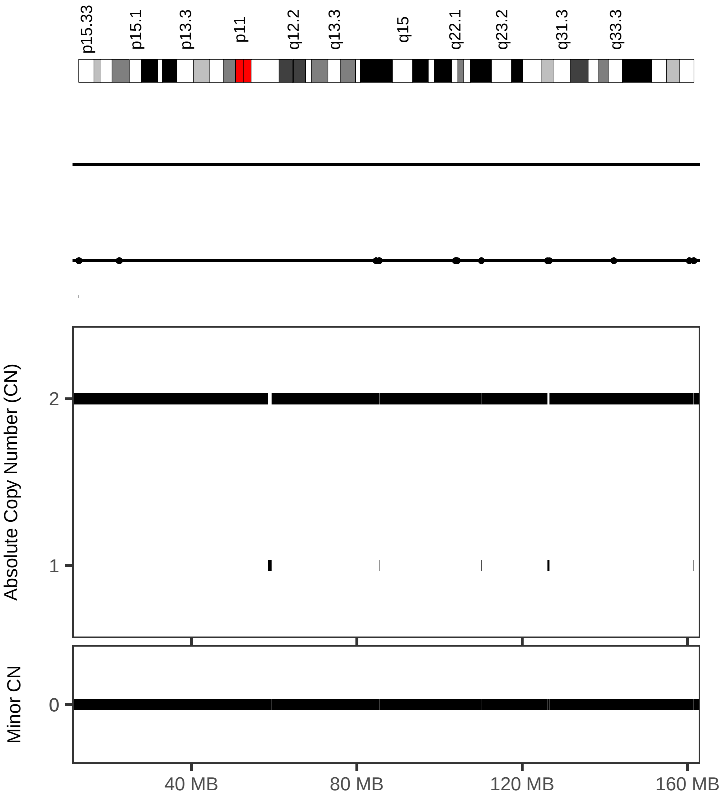

OCCAMS−WG−005

Cancer type	Eso−AdenoCA
Position	5:12714338−161549623
Interleaved intrachr. SVs	1
Total SVs (intrachr. + transl.)	7
SV types	DEL: 7; DUP: 0; h2hINV: 0; t2tINV: 0; TRA: 0
SVs in sample	277
Oscillating CN (2 and 3 states)	11, 11
CN segments	11
FDR fragment joints	0.01743054
FDR chr. breakp. enrich.	0.33
Linked to chrs	
Purity, ploidy	0.38, 2.47

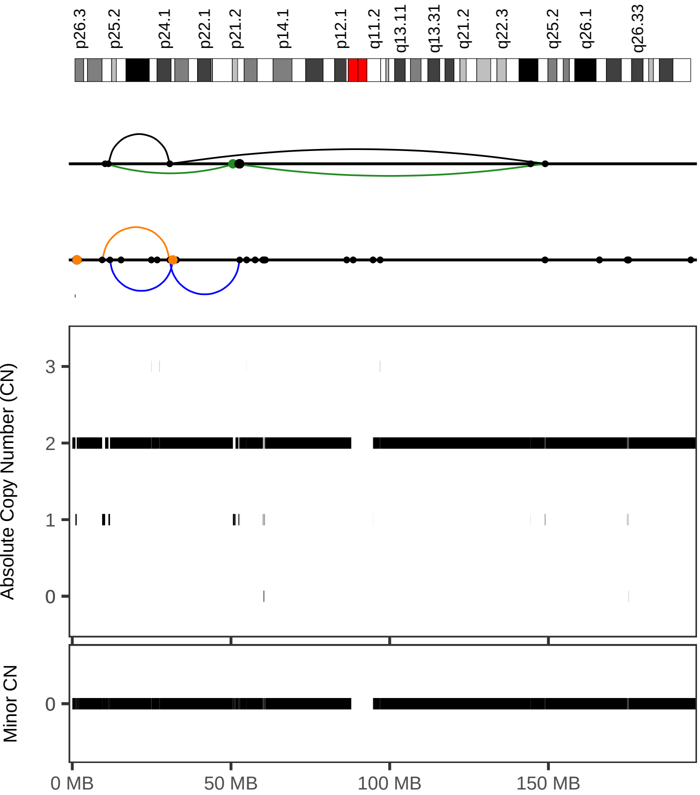

OCCAMS−WG−008

Cancer type	Eso−AdenoCA
Position	3:9406366−148985299
Interleaved intrachr. SVs	9
Total SVs (intrachr. + transl.)	13
SV types	DEL: 2; DUP: 2; h2hINV: 3; t2tINV: 2; TRA: 4
SVs in sample	357
Oscillating CN (2 and 3 states)	9, 36
CN segments	36
FDR fragment joints	0.9000538
FDR chr. breakp. enrich.	0
Linked to chrs	
Purity, ploidy	0.4, 2.6

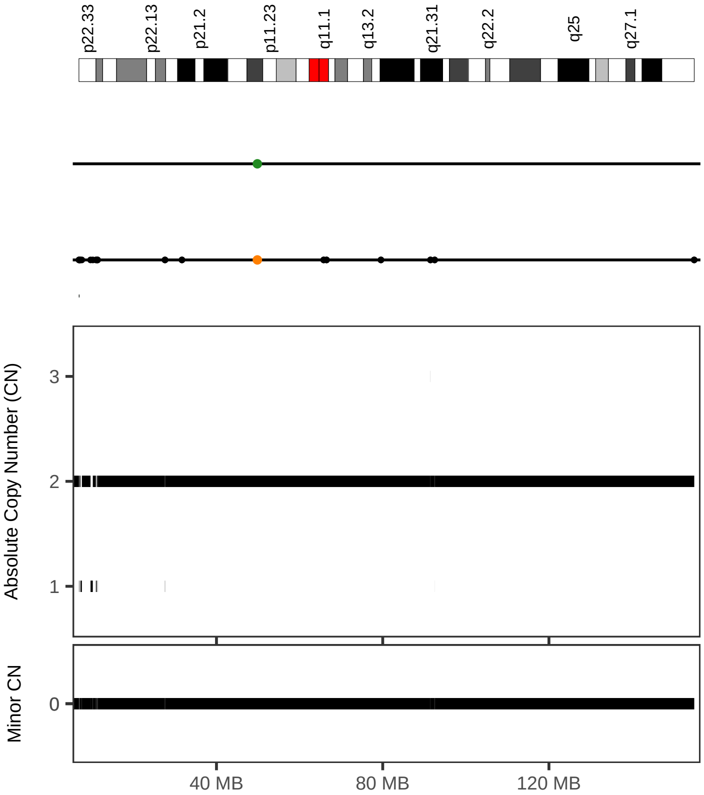

OCCAMS−WG−008

Cancer type	Eso−AdenoCA
Position	X:6894656−154973534
Interleaved intrachr. SVs	1
Total SVs (intrachr. + transl.)	11
SV types	DEL: 7; DUP: 2; h2hINV: 0; t2tINV: 0; TRA: 2
SVs in sample	357
Oscillating CN (2 and 3 states)	14, 18
CN segments	18
FDR fragment joints	0.129004
FDR chr. breakp. enrich.	0.9
Linked to chrs	13:52214952−85046098;
Purity, ploidy	0.4, 2.6

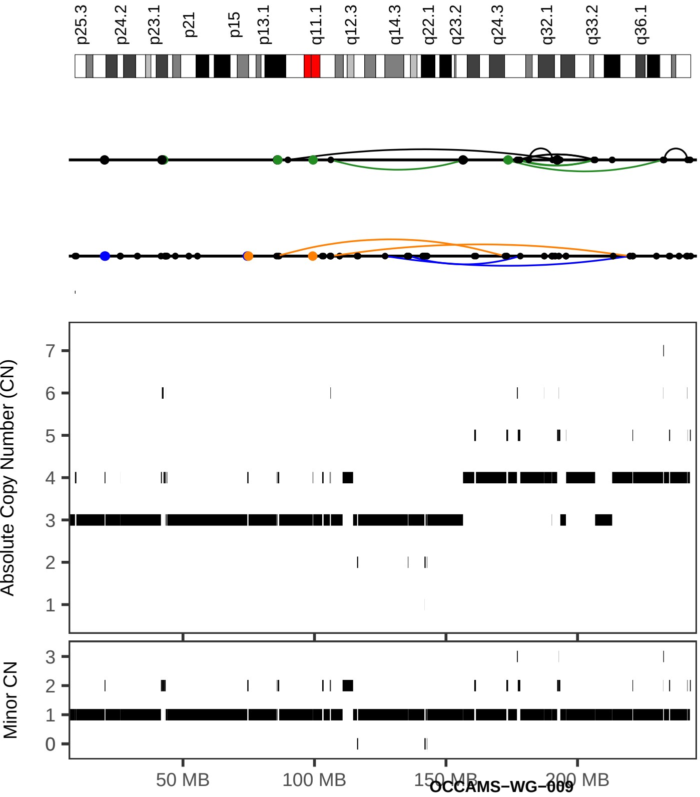

OCCAMS−WG−009

Cancer type	Eso−AdenoCA
Position	2:85478953−232558623
Interleaved intrachr. SVs	14
Total SVs (intrachr. + transl.)	22
SV types	DEL: 2; DUP: 4; h2hINV: 3; t2tINV: 5; TRA: 8
SVs in sample	490
Oscillating CN (2 and 3 states)	11, 19
CN segments	51
FDR fragment joints	0.615458
FDR chr. breakp. enrich.	0
Linked to chrs	11:7867256−37730506;12:1575828−123594121
Purity, ploidy	0.25, 2.63

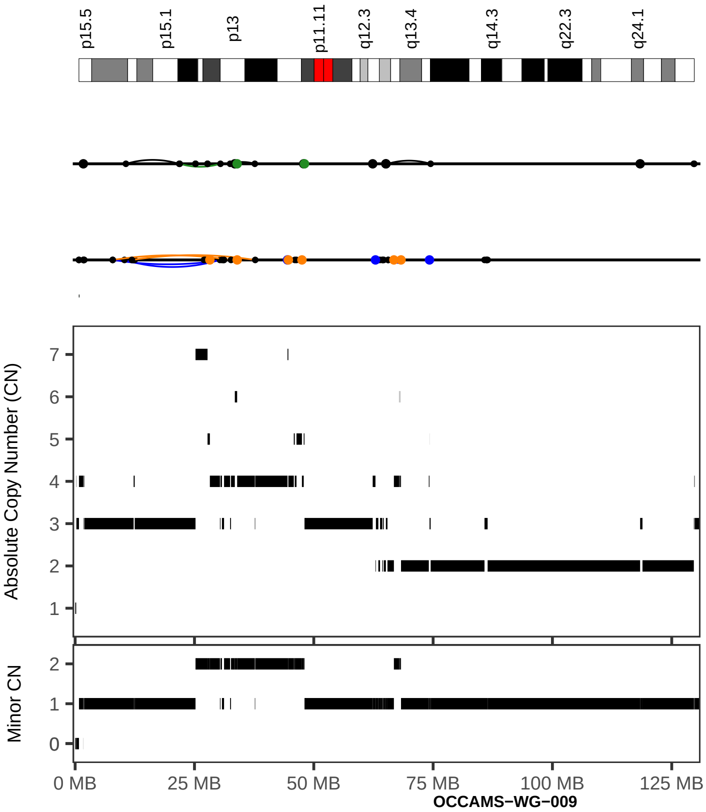

OCCAMS−WG−009

Cancer type	Eso−AdenoCA
Position	11:7867256−37730507
Interleaved intrachr. SVs	8
Total SVs (intrachr. + transl.)	12
SV types	DEL: 3; DUP: 2; h2hINV: 2; t2tINV: 1; TRA: 4
SVs in sample	490
Oscillating CN (2 and 3 states)	7, 11
CN segments	16
FDR fragment joints	0.6776251
FDR chr. breakp. enrich.	0
Linked to chrs	
Purity, ploidy	0.25, 2.63

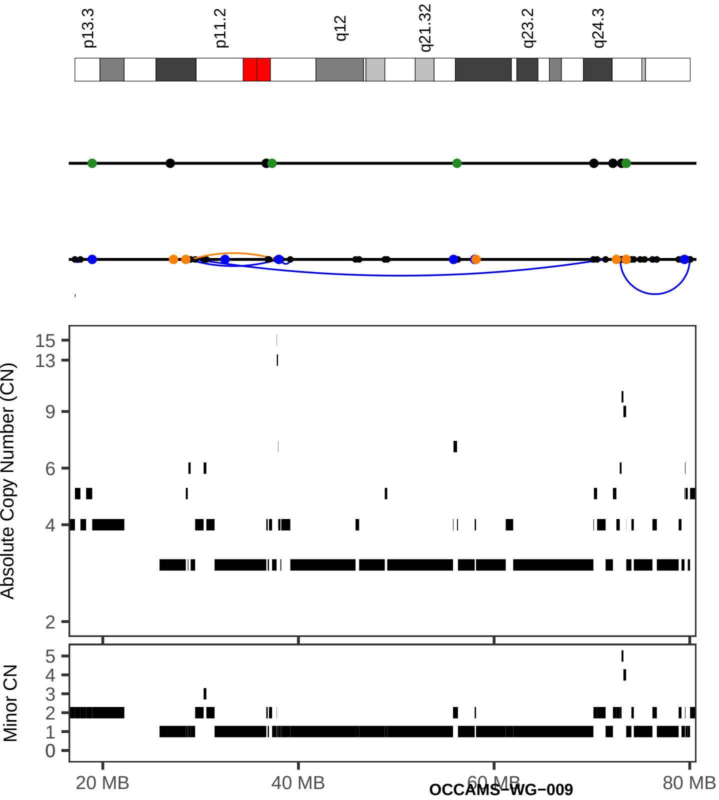

OCCAMS−WG−009

Cancer type	Eso−AdenoCA
Position	17:28749738−71397421
Interleaved intrachr. SVs	5
Total SVs (intrachr. + transl.)	14
SV types	DEL: 1; DUP: 4; h2hINV: 0; t2tINV: 0; TRA: 9
SVs in sample	490
Oscillating CN (2 and 3 states)	7, 10
CN segments	33
FDR fragment joints	0.5435077
FDR chr. breakp. enrich.	0
Linked to chrs	10:4579401−102025656;12:1575828−123594121
Purity, ploidy	0.25, 2.63

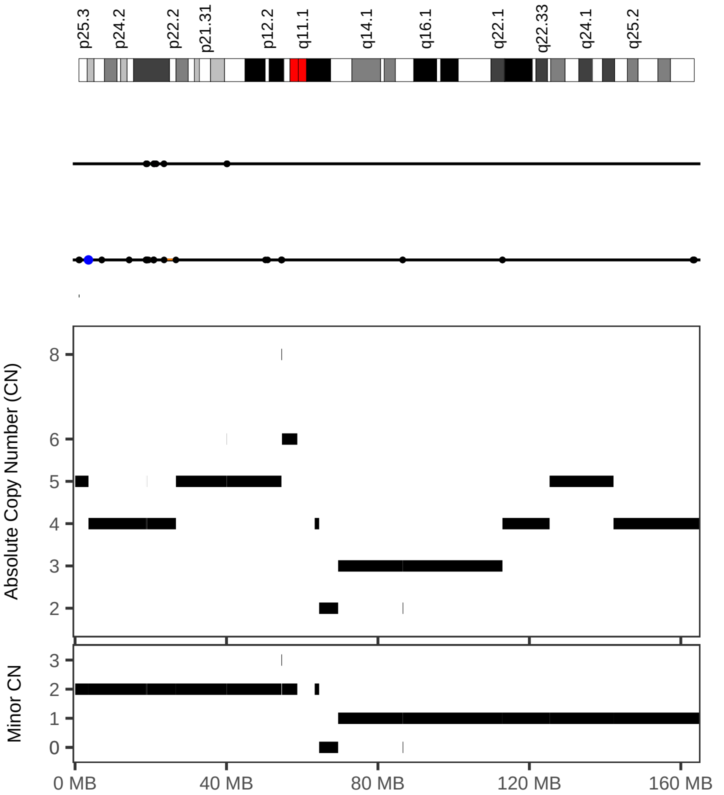

OCCAMS−WG−019

Cancer type	Eso−AdenoCA
Position	6:18740622−21421268
Interleaved intrachr. SVs	8
Total SVs (intrachr. + transl.)	8
SV types	DEL: 2; DUP: 2; h2hINV: 2; t2tINV: 2; TRA: 0
SVs in sample	263
Oscillating CN (2 and 3 states)	5, 5
CN segments	5
FDR fragment joints	1
FDR chr. breakp. enrich.	0.13
Linked to chrs	
Purity, ploidy	0.25, 3.97

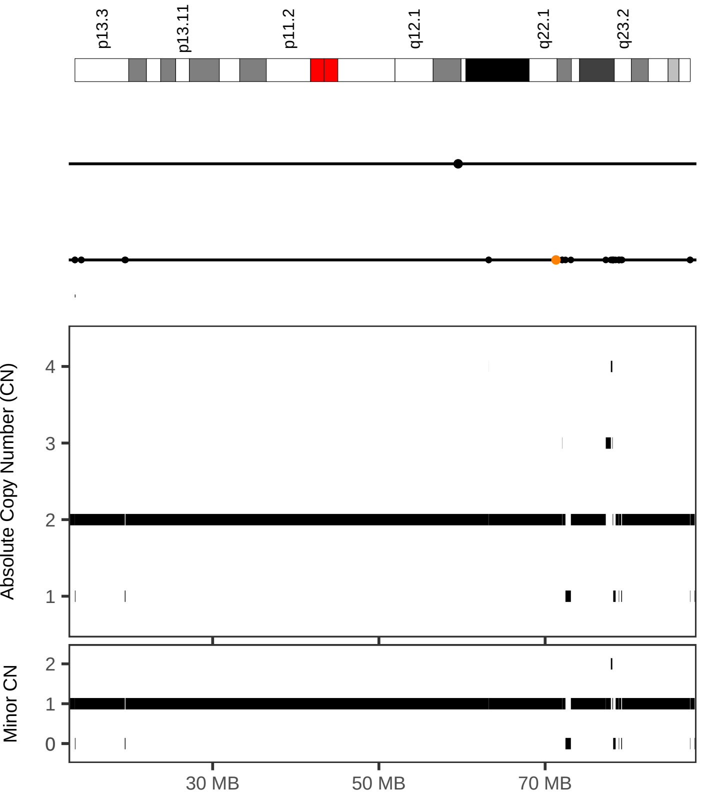

OCCAMS−ZZ−009

Cancer type	Eso−AdenoCA
Position	16:14176787−87458987
Interleaved intrachr. SVs	1
Total SVs (intrachr. + transl.)	14
SV types	DEL: 8; DUP: 4; h2hINV: 0; t2tINV: 0; TRA: 2
SVs in sample	353
Oscillating CN (2 and 3 states)	13, 21
CN segments	25
FDR fragment joints	0.1321718
FDR chr. breakp. enrich.	0.15
Linked to chrs	
Purity, ploidy	0.44, 2.04

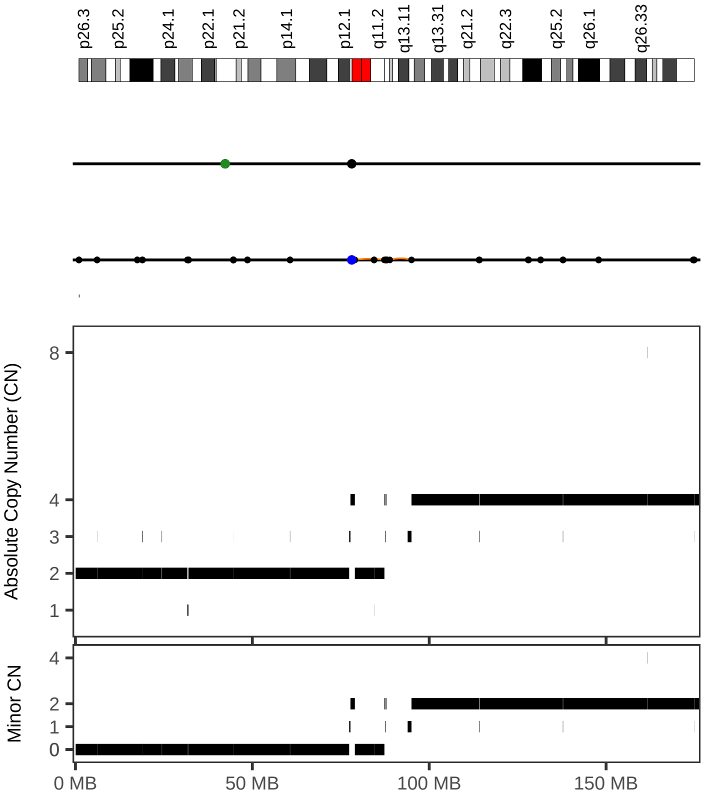

3e5f451a−5882−4914−ae1a−95c898c2bcca

Cancer type	Head−SCC
Position	3:980214−174918572
Interleaved intrachr. SVs	1
Total SVs (intrachr. + transl.)	25
SV types	DEL: 15; DUP: 4; h2hINV: 1; t2tINV: 0; TRA: 5
SVs in sample	362
Oscillating CN (2 and 3 states)	9, 17
CN segments	33
FDR fragment joints	0.01743054
FDR chr. breakp. enrich.	0.71
Linked to chrs	
Purity, ploidy	0.58, 3.09

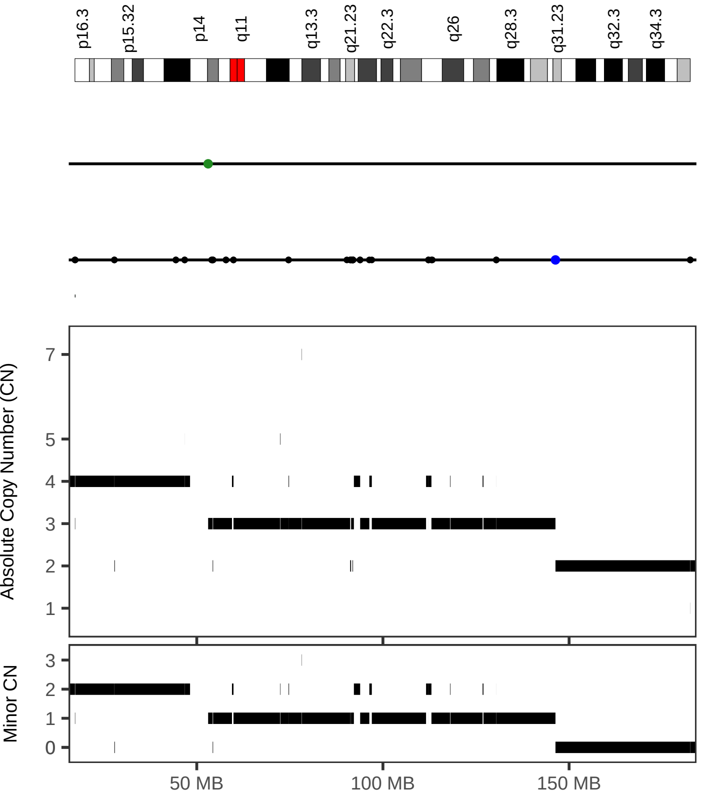

	3e5f451a−5882−4914−ae1a−95c898c2bcca
Cancer type	Head−SCC
Position	4:17276274−182542010
Interleaved intrachr. SVs	1
Total SVs (intrachr. + transl.)	14
SV types	DEL: 9; DUP: 3; h2hINV: 0; t2tINV: 0; TRA: 2
SVs in sample	362
Oscillating CN (2 and 3 states)	15, 22
CN segments	38
FDR fragment joints	0.1321718
FDR chr. breakp. enrich.	0.92
Linked to chrs	
Purity, ploidy	0.58, 3.09

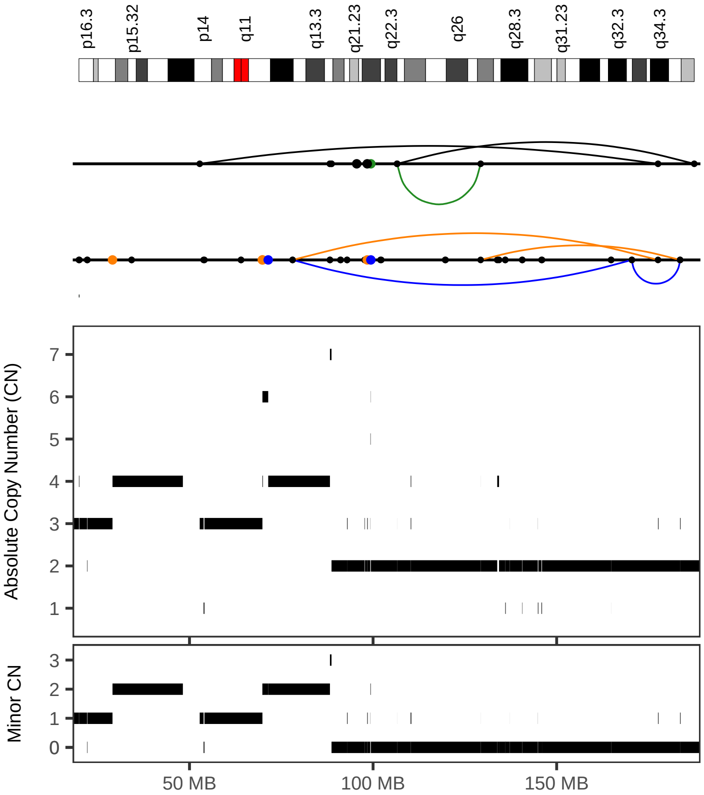

	ad6c9d09−2c03−4786−a72d−dd2aa5f603d4
Cancer type	Head−SCC
Position	4:52783484−187472712
Interleaved intrachr. SVs	7
Total SVs (intrachr. + transl.)	15
SV types	DEL: 2; DUP: 2; h2hINV: 2; t2tINV: 1; TRA: 8
SVs in sample	360
Oscillating CN (2 and 3 states)	8, 13
CN segments	44
FDR fragment joints	0.925252
FDR chr. breakp. enrich.	0.1
Linked to chrs	
Purity, ploidy	0.65, 3.12

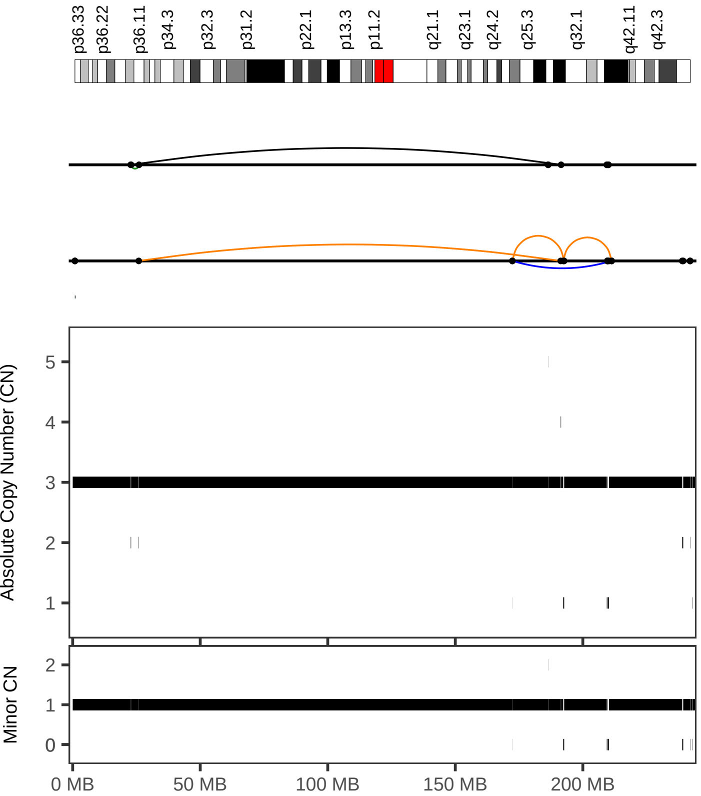

	c1f50a22−38df−41cc−a1f4−f7985504a7ac
Cancer type	Head−SCC
Position	1:22744462−211294614
Interleaved intrachr. SVs	6
Total SVs (intrachr. + transl.)	6
SV types	DEL: 3; DUP: 1; h2hINV: 1; t2tINV: 1; TRA: 0
SVs in sample	61
Oscillating CN (2 and 3 states)	11, 11
CN segments	20
FDR fragment joints	0.6776251
FDR chr. breakp. enrich.	0
Linked to chrs	
Purity, ploidy	0.7, 3.36

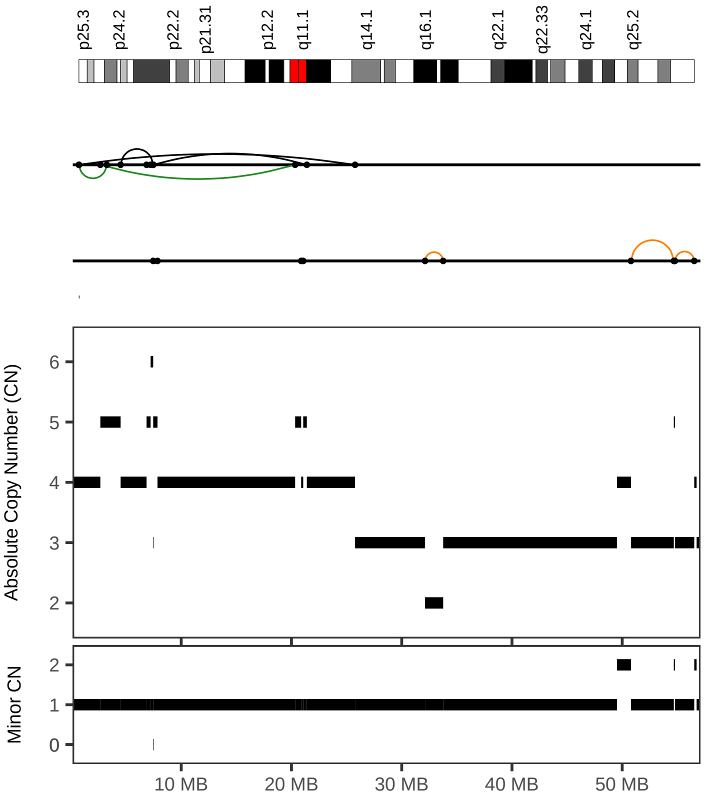

	cb99fb14−641c−4fb3−bf1d−17efb8cd982a
Cancer type	Head−SCC
Position	6:720079−25769089
Interleaved intrachr. SVs	6
Total SVs (intrachr. + transl.)	6
SV types	DEL: 0; DUP: 1; h2hINV: 3; t2tINV: 2; TRA: 0
SVs in sample	96
Oscillating CN (2 and 3 states)	6, 7
CN segments	13
FDR fragment joints	0.615458
FDR chr. breakp. enrich.	0.06
Linked to chrs	
Purity, ploidy	0.52, 2.94

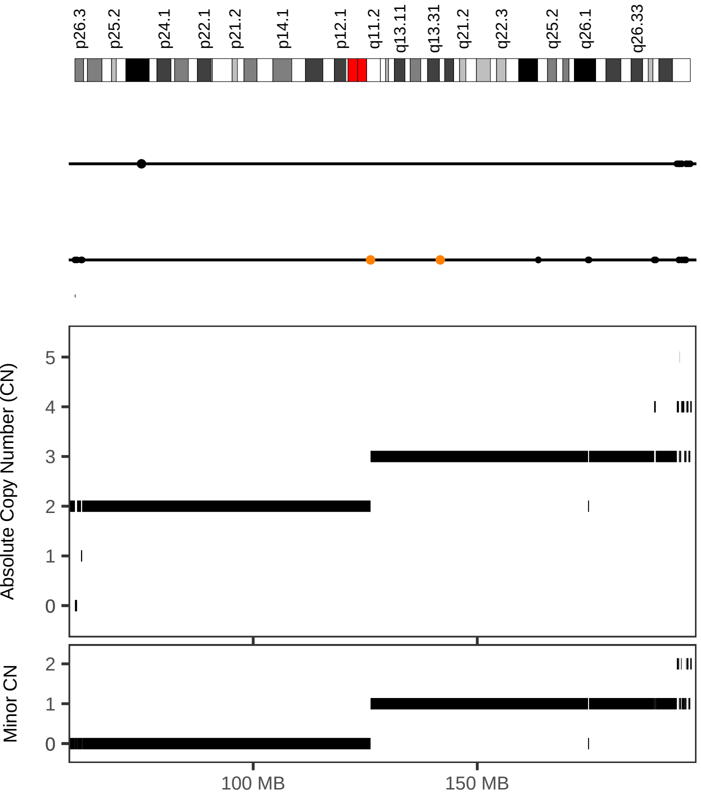

e7d49212−e9cb−4cd7−923a−bdfc82a4a150

Cancer type	Head−SCC
Position	3:194529547−197519861
Interleaved intrachr. SVs	7
Total SVs (intrachr. + transl.)	7
SV types	DEL: 2; DUP: 0; h2hINV: 2; t2tINV: 3; TRA: 0
SVs in sample	149
Oscillating CN (2 and 3 states)	10, 13
CN segments	13
FDR fragment joints	0.6776251
FDR chr. breakp. enrich.	0.22
Linked to chrs	
Purity, ploidy	0.7, 2.78

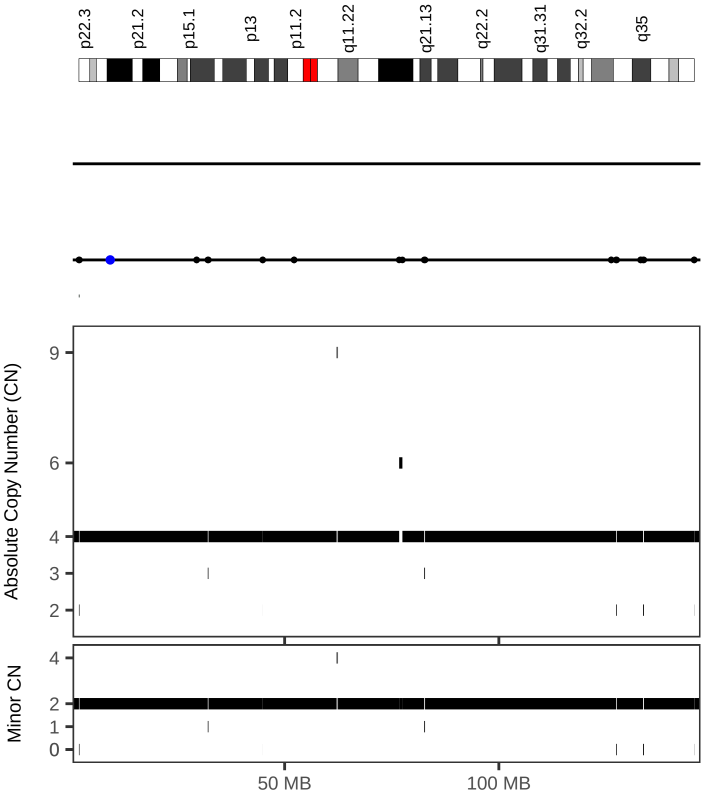

e8ac39e6−377a−412c−b18d−f6c4fc654ec6

Cancer type	Head−SCC
Position	7:1981292−145607230
Interleaved intrachr. SVs	1
Total SVs (intrachr. + transl.)	11
SV types	DEL: 8; DUP: 2; h2hINV: 0; t2tINV: 0; TRA: 1
SVs in sample	183
Oscillating CN (2 and 3 states)	7, 9
CN segments	20
FDR fragment joints	0.1013295
FDR chr. breakp. enrich.	0.57
Linked to chrs	
Purity, ploidy	0.46, 3.12

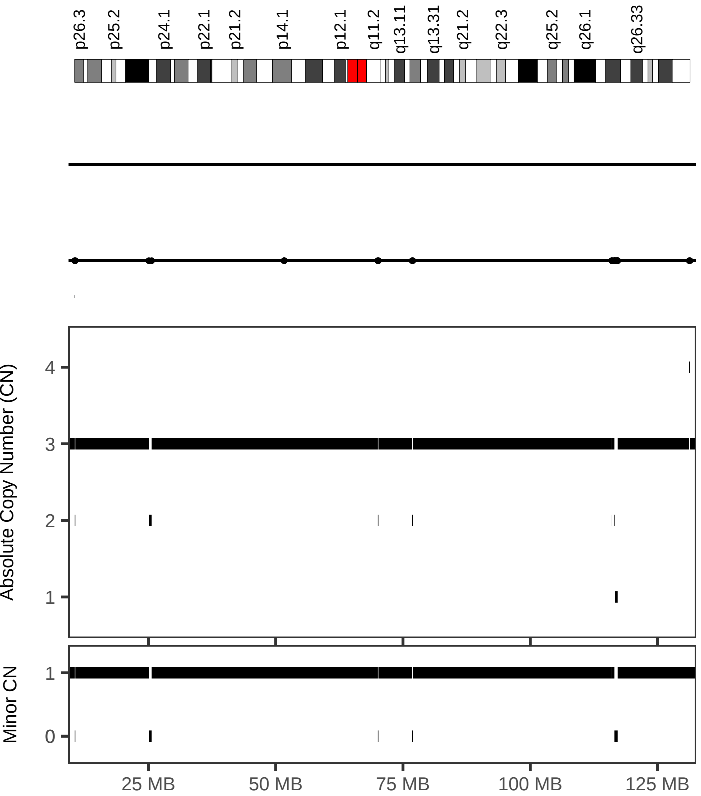

9fc40b03−0b56−4b1f−93ed−dbc455ae3fd9

Cancer type	Kidney−RCC
Position	3:10505042−131382838
Interleaved intrachr. SVs	1
Total SVs (intrachr. + transl.)	11
SV types	DEL: 10; DUP: 1; h2hINV: 0; t2tINV: 0; TRA: 0
SVs in sample	166
Oscillating CN (2 and 3 states)	11, 11
CN segments	15
FDR fragment joints	0.003601907
FDR chr. breakp. enrich.	1
Linked to chrs	
Purity, ploidy	0.58, 3.5

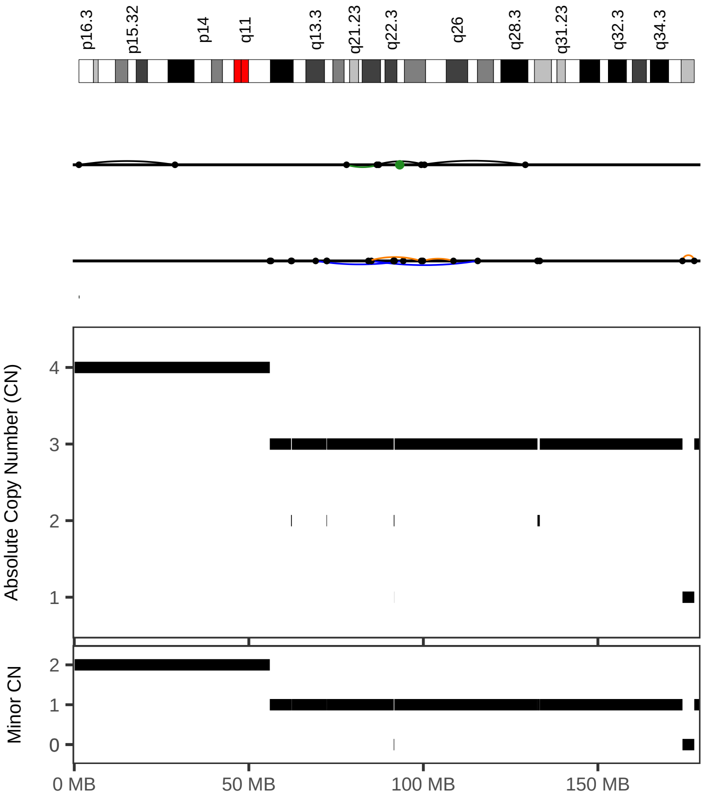

9fc40b03−0b56−4b1f−93ed−dbc455ae3fd9

Cancer type	Kidney−RCC
Position	4:69152238−129221201
Interleaved intrachr. SVs	7
Total SVs (intrachr. + transl.)	8
SV types	DEL: 2; DUP: 2; h2hINV: 2; t2tINV: 1; TRA: 1
SVs in sample	166
Oscillating CN (2 and 3 states)	6, 9
CN segments	9
FDR fragment joints	0.8653243
FDR chr. breakp. enrich.	0.24
Linked to chrs	
Purity, ploidy	0.58, 3.5

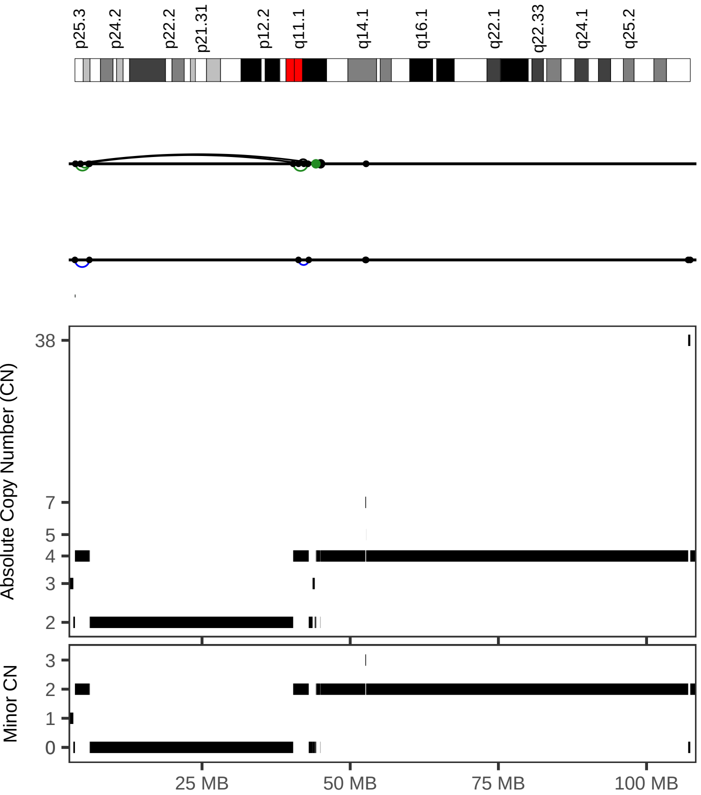

C0052

Cancer type	Kidney−RCC
Position	6:3555069−44962683
Interleaved intrachr. SVs	7
Total SVs (intrachr. + transl.)	8
SV types	DEL: 0; DUP: 2; h2hINV: 3; t2tINV: 2; TRA: 1
SVs in sample	26
Oscillating CN (2 and 3 states)	6, 12
CN segments	12
FDR fragment joints	0.615458
FDR chr. breakp. enrich.	0
Linked to chrs	
Purity, ploidy	0.5, 3.11

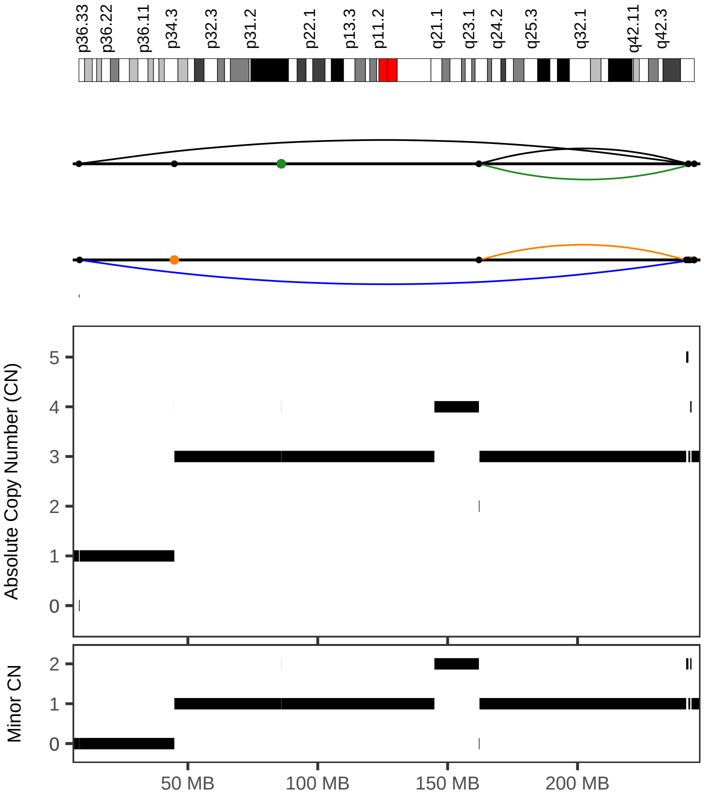

06ac44e9−1226−4202−828b−6f224766d7ac

Cancer type	Liver−HCC
Position	1:8058341−244930271
Interleaved intrachr. SVs	7
Total SVs (intrachr. + transl.)	10
SV types	DEL: 2; DUP: 2; h2hINV: 2; t2tINV: 1; TRA: 3
SVs in sample	53
Oscillating CN (2 and 3 states)	5, 7
CN segments	13
FDR fragment joints	0.9625775
FDR chr. breakp. enrich.	0.01
Linked to chrs	
Purity, ploidy	0.4, 2.3

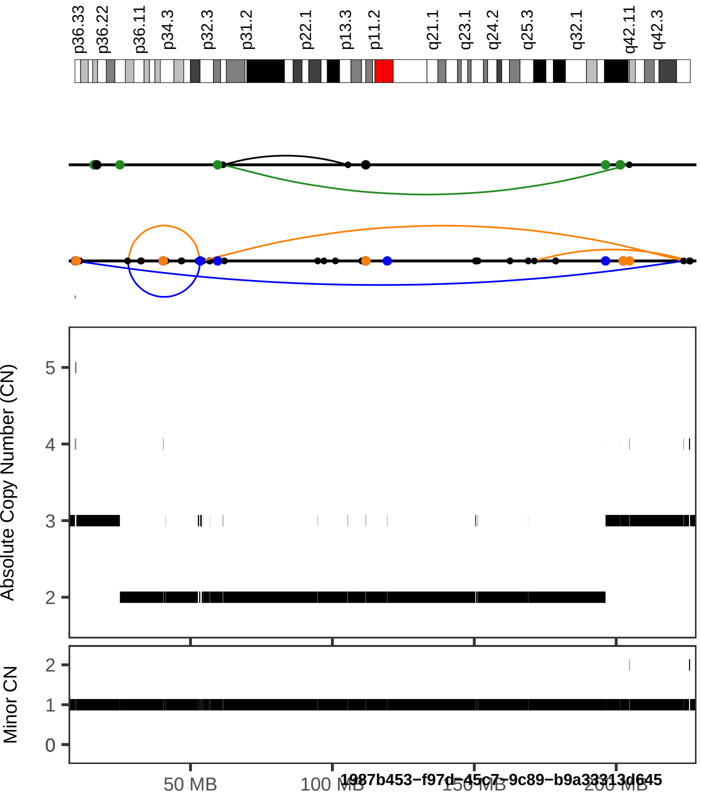

1987b453−f97d−45c7−9c89−b9a33313d645

Cancer type	Liver−HCC
Position	1:9273332−226103050
Interleaved intrachr. SVs	4
Total SVs (intrachr. + transl.)	32
SV types	DEL: 2; DUP: 2; h2hINV: 1; t2tINV: 3; TRA: 24
SVs in sample	524
Oscillating CN (2 and 3 states)	30, 40
CN segments	46
FDR fragment joints	0.8505431
FDR chr. breakp. enrich.	0
Linked to chrs	14:25556654−90170622;17:6752376−73621733<br>19:1158604−47770620;20:20959449−62129527<br>9:6482278−127861800;

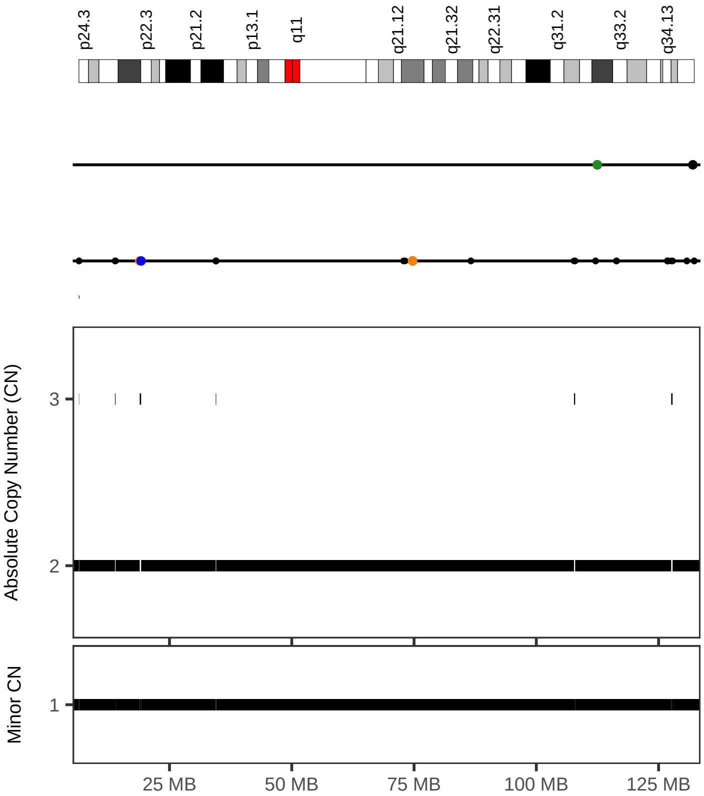

1987b453−f97d−45c7−9c89−b9a33313d645

Cancer type	Liver−HCC
Position	9:6482278−127861801
Interleaved intrachr. SVs	1
Total SVs (intrachr. + transl.)	17
SV types	DEL: 0; DUP: 11; h2hINV: 0; t2tINV: 0; TRA: 6
SVs in sample	524
Oscillating CN (2 and 3 states)	12, 12
CN segments	12
FDR fragment joints	0.006730063
FDR chr. breakp. enrich.	0.8
Linked to chrs	12:106261894−106677125;
Purity, ploidy	0.74, 1.99

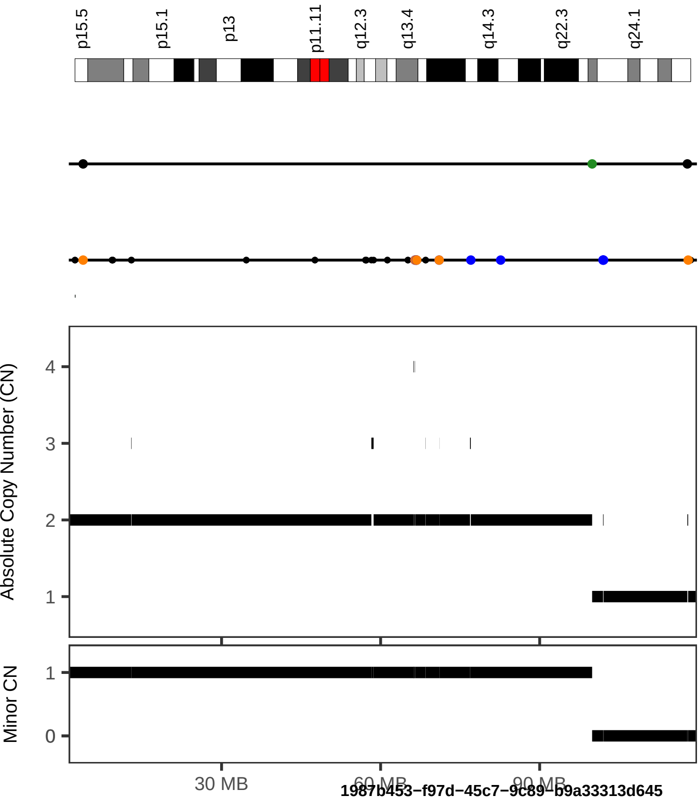

1987b453−f97d−45c7−9c89−b9a33313d645

Cancer type	Liver−HCC
Position	11:9340114−77018016
Interleaved intrachr. SVs	1
Total SVs (intrachr. + transl.)	16
SV types	DEL: 1; DUP: 6; h2hINV: 0; t2tINV: 0; TRA: 9
SVs in sample	524
Oscillating CN (2 and 3 states)	7, 15
CN segments	15
FDR fragment joints	0.02158923
FDR chr. breakp. enrich.	0.19
Linked to chrs	15:29951669−100680250;X:31286763−116414501
Purity, ploidy	0.74, 1.99

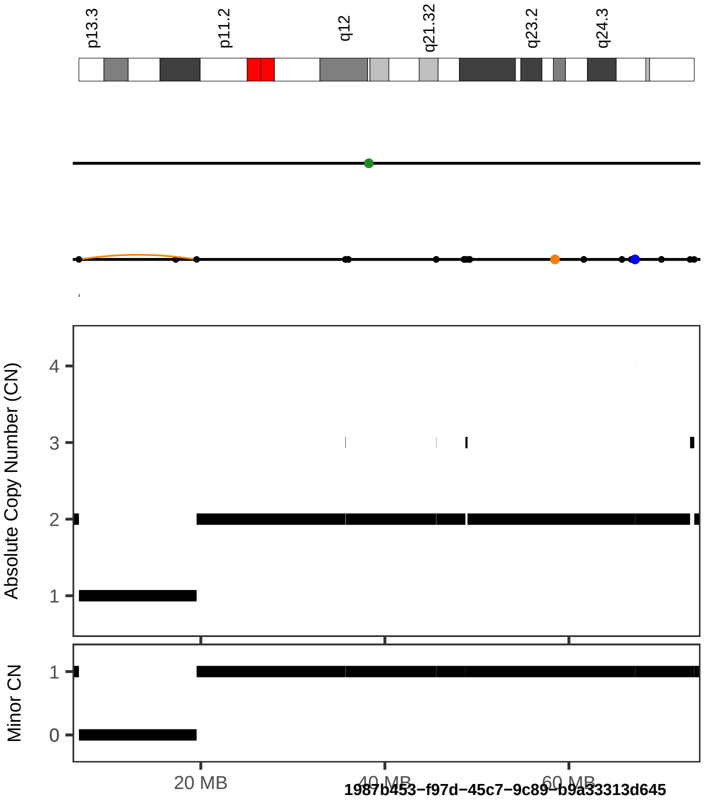

1987b453−f97d−45c7−9c89−b9a33313d645

Cancer type	Liver−HCC
Position	17:6752376−73621734
Interleaved intrachr. SVs	1
Total SVs (intrachr. + transl.)	14
SV types	DEL: 1; DUP: 6; h2hINV: 0; t2tINV: 0; TRA: 7
SVs in sample	524
Oscillating CN (2 and 3 states)	7, 11
CN segments	12
FDR fragment joints	0.07579048
FDR chr. breakp. enrich.	0.14
Linked to chrs	4:122039841−145149273;7:28246412−155112625
Purity, ploidy	0.74, 1.99

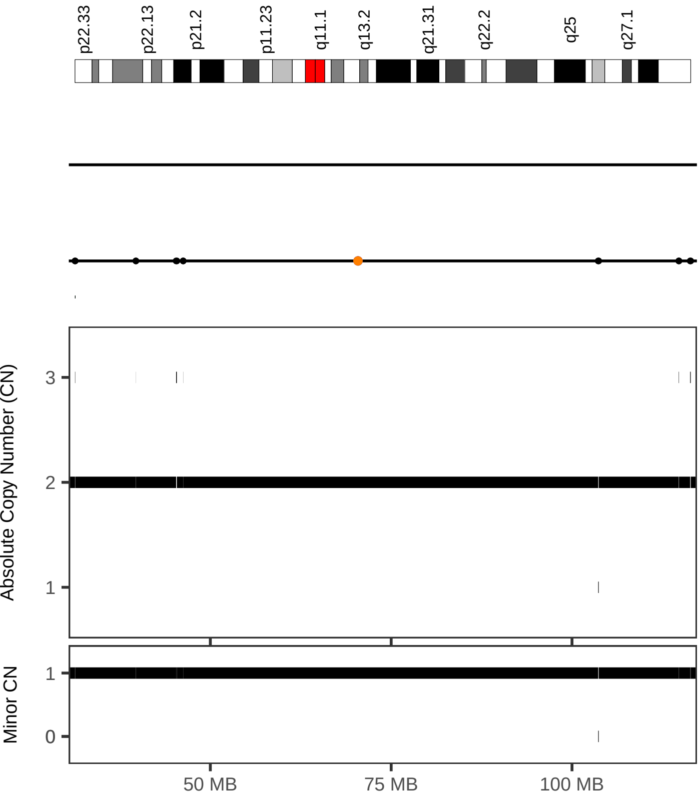

1987b453−f97d−45c7−9c89−b9a33313d645

Cancer type	Liver−HCC
Position	X:31286763−116414502
Interleaved intrachr. SVs	1
Total SVs (intrachr. + transl.)	9
SV types	DEL: 1; DUP: 6; h2hINV: 0; t2tINV: 0; TRA: 2
SVs in sample	524
Oscillating CN (2 and 3 states)	8, 14
CN segments	14
FDR fragment joints	0.1178349
FDR chr. breakp. enrich.	0.01
Linked to chrs	11:9340114−77018015;
Purity, ploidy	0.74, 1.99

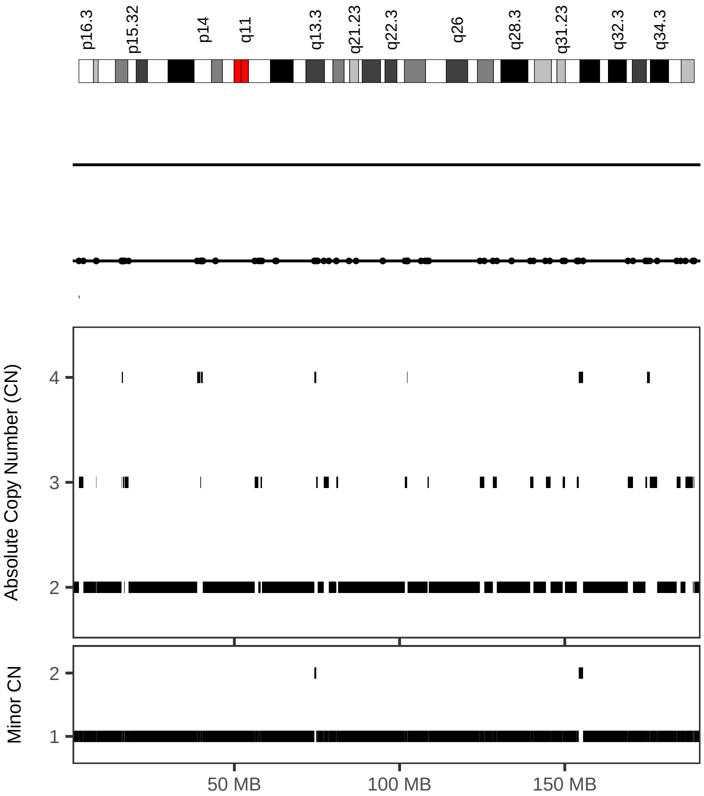

440375d7−cdcd−418a−8992−0a8ca42d290d

Cancer type	Liver−HCC
Position	4:15852769−177944792
Interleaved intrachr. SVs	2
Total SVs (intrachr. + transl.)	6
SV types	DEL: 0; DUP: 6; h2hINV: 0; t2tINV: 0; TRA: 0
SVs in sample	690
Oscillating CN (2 and 3 states)	14, 16
CN segments	45
FDR fragment joints	0.04139017
FDR chr. breakp. enrich.	0.49
Linked to chrs	
Purity, ploidy	0.63, 2.34

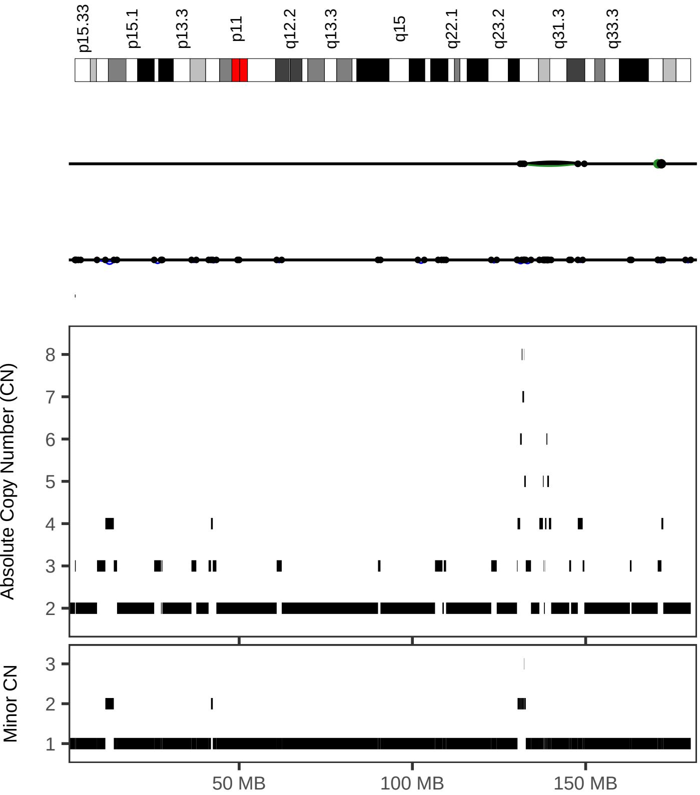

440375d7−cdcd−418a−8992−0a8ca42d290d

Cancer type	Liver−HCC
Position	5:130189137−149619578
Interleaved intrachr. SVs	8
Total SVs (intrachr. + transl.)	8
SV types	DEL: 0; DUP: 5; h2hINV: 2; t2tINV: 1; TRA: 0
SVs in sample	690
Oscillating CN (2 and 3 states)	4, 6
CN segments	25
FDR fragment joints	0.5435077
FDR chr. breakp. enrich.	0.72
Linked to chrs	
Purity, ploidy	0.63, 2.34

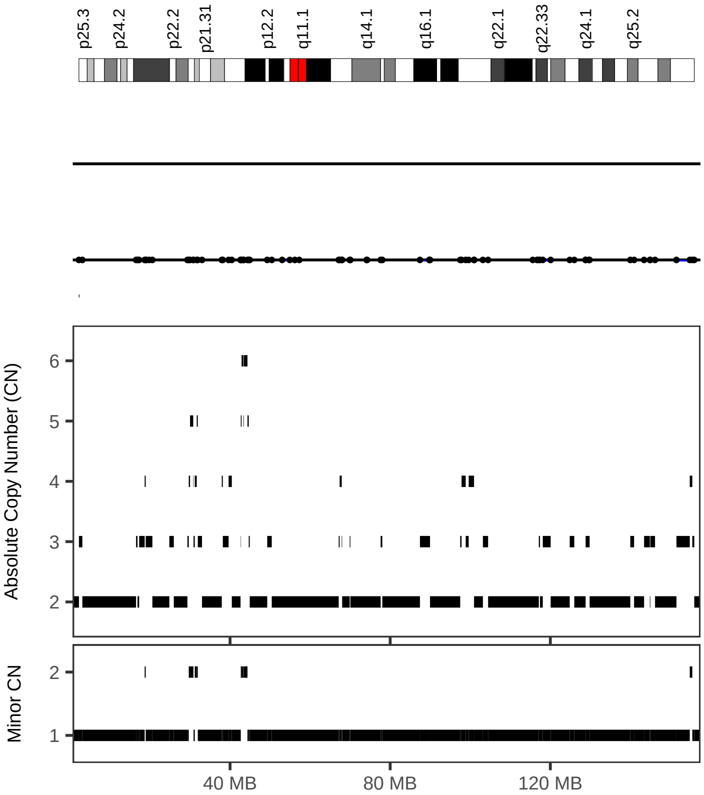

440375d7−cdcd−418a−8992−0a8ca42d290d

Cancer type	Liver−HCC
Position	6:42615061−100972584
Interleaved intrachr. SVs	3
Total SVs (intrachr. + transl.)	6
SV types	DEL: 0; DUP: 6; h2hINV: 0; t2tINV: 0; TRA: 0
SVs in sample	690
Oscillating CN (2 and 3 states)	9, 18
CN segments	25
FDR fragment joints	0.04139017
FDR chr. breakp. enrich.	1
Linked to chrs	
Purity, ploidy	0.63, 2.34

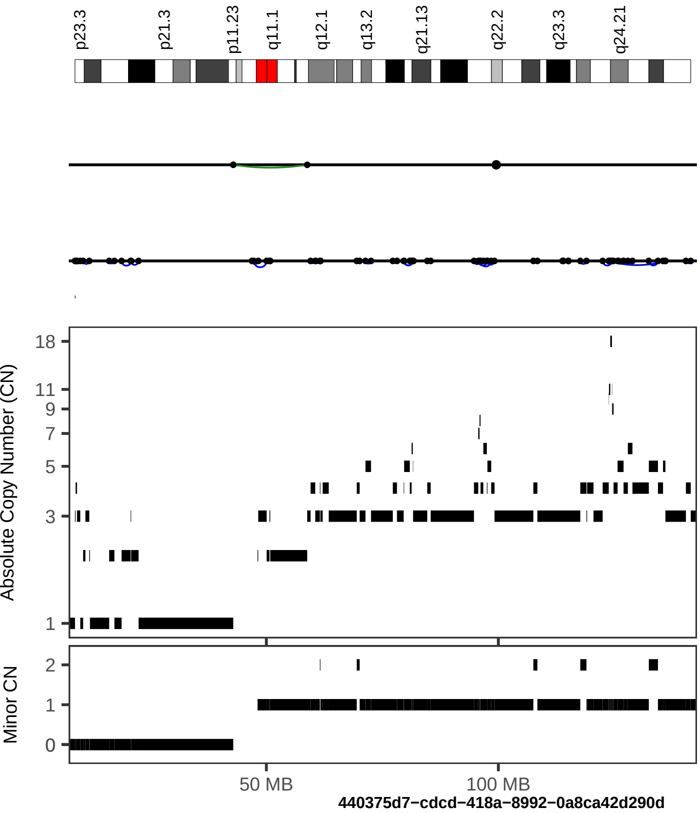

440375d7−cdcd−418a−8992−0a8ca42d290d

Cancer type	Liver−HCC
Position	8:94743333−136006935
Interleaved intrachr. SVs	5
Total SVs (intrachr. + transl.)	11
SV types	DEL: 0; DUP: 10; h2hINV: 0; t2tINV: 0; TRA: 1
SVs in sample	690
Oscillating CN (2 and 3 states)	9, 9
CN segments	30
FDR fragment joints	0.003601907
FDR chr. breakp. enrich.	0.54
Linked to chrs	
Purity, ploidy	0.63, 2.34

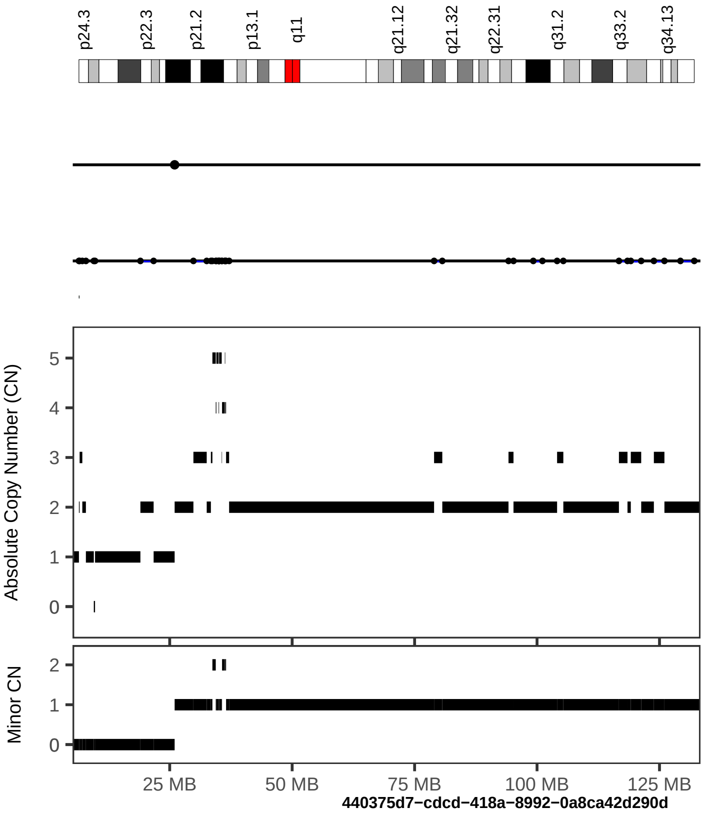

440375d7−cdcd−418a−8992−0a8ca42d290d

Cancer type	Liver−HCC
Position	9:33405632−37129539
Interleaved intrachr. SVs	5
Total SVs (intrachr. + transl.)	5
SV types	DEL: 0; DUP: 5; h2hINV: 0; t2tINV: 0; TRA: 0
SVs in sample	690
Oscillating CN (2 and 3 states)	7, 10
CN segments	14
FDR fragment joints	0.1013295
FDR chr. breakp. enrich.	0.52
Linked to chrs	
Purity, ploidy	0.63, 2.34

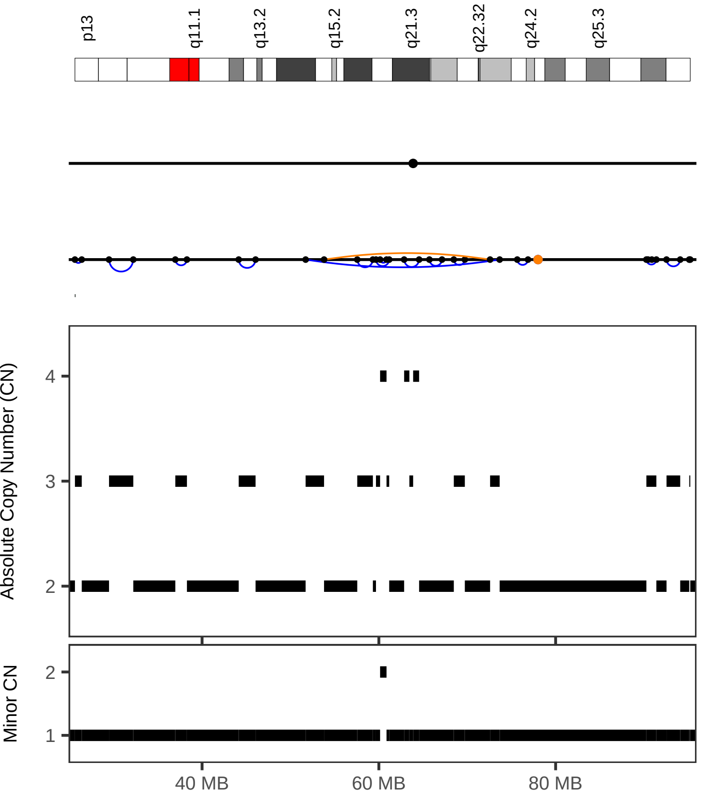

440375d7−cdcd−418a−8992−0a8ca42d290d

Cancer type	Liver−HCC
Position	15:25612423−95231017
Interleaved intrachr. SVs	1
Total SVs (intrachr. + transl.)	20
SV types	DEL: 1; DUP: 16; h2hINV: 0; t2tINV: 0; TRA: 3
SVs in sample	690
Oscillating CN (2 and 3 states)	13, 16
CN segments	30
FDR fragment joints	2.780978e−05
FDR chr. breakp. enrich.	0.96
Linked to chrs	13:93277231−100829518;
Purity, ploidy	0.63, 2.34

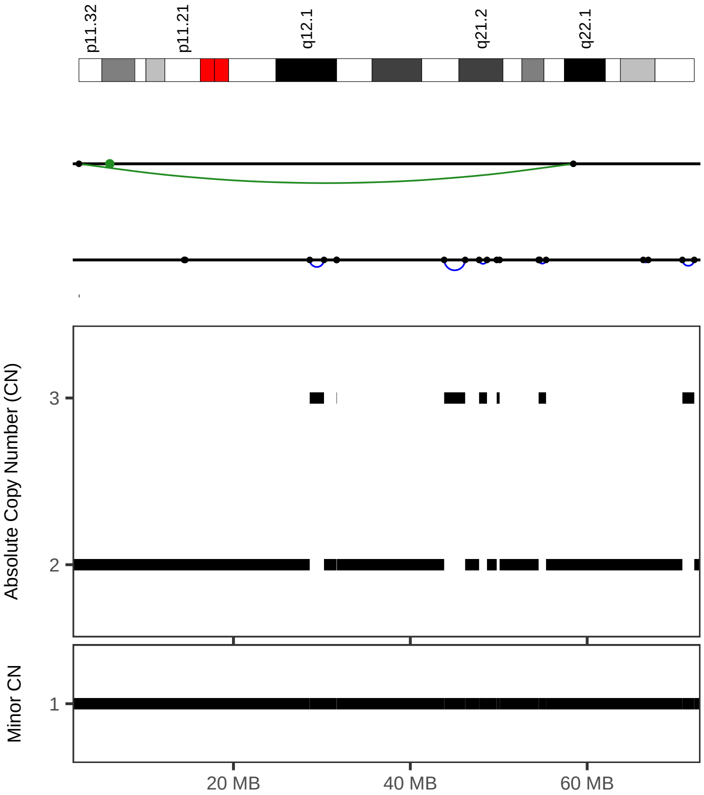

440375d7−cdcd−418a−8992−0a8ca42d290d

Cancer type	Liver−HCC
Position	18:2523032−72108189
Interleaved intrachr. SVs	1
Total SVs (intrachr. + transl.)	12
SV types	DEL: 0; DUP: 10; h2hINV: 0; t2tINV: 1; TRA: 1
SVs in sample	690
Oscillating CN (2 and 3 states)	15, 15
CN segments	15
FDR fragment joints	0.01333161
FDR chr. breakp. enrich.	0.36
Linked to chrs	
Purity, ploidy	0.63, 2.34

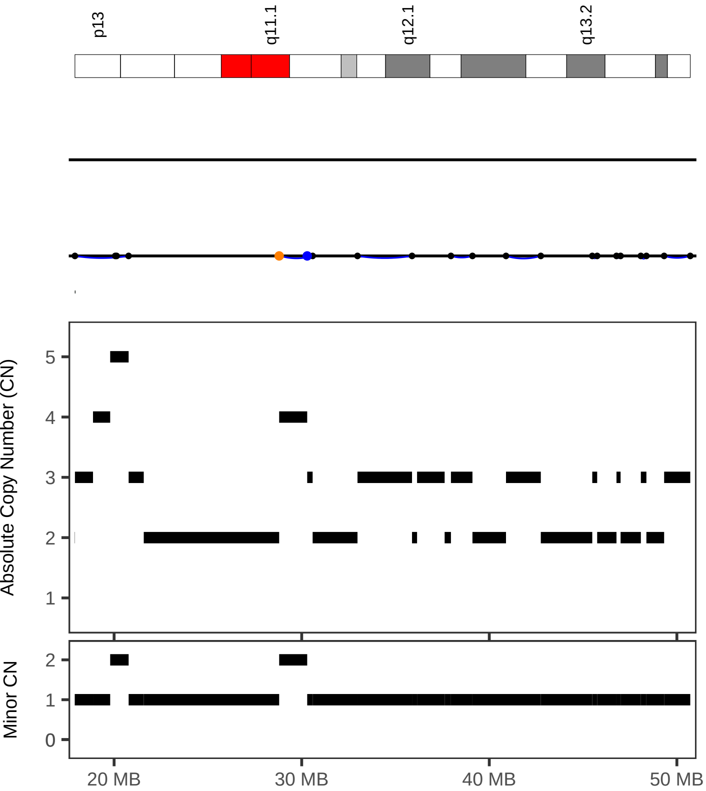

440375d7−cdcd−418a−8992−0a8ca42d290d

Cancer type	Liver−HCC
Position	22:17910494−50713692
Interleaved intrachr. SVs	1
Total SVs (intrachr. + transl.)	12
SV types	DEL: 0; DUP: 10; h2hINV: 0; t2tINV: 0; TRA: 2
SVs in sample	690
Oscillating CN (2 and 3 states)	17, 17
CN segments	23
FDR fragment joints	0.001274711
FDR chr. breakp. enrich.	0.44
Linked to chrs	
Purity, ploidy	0.63, 2.34

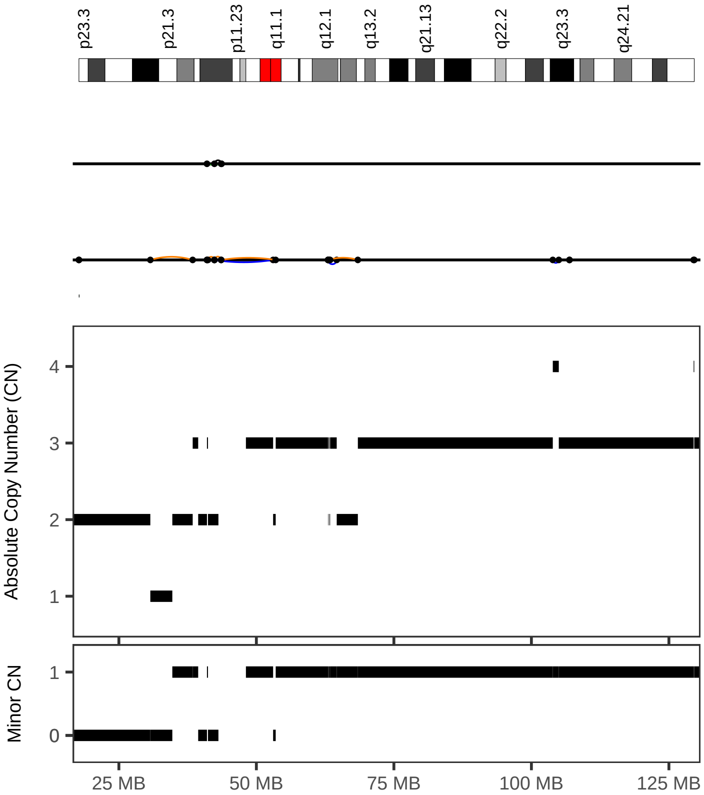

457cca7c−a8b4−414e−a3c0−7fccf0ee2034

Cancer type	Liver−HCC
Position	8:41016513−53511362
Interleaved intrachr. SVs	6
Total SVs (intrachr. + transl.)	6
SV types	DEL: 3; DUP: 1; h2hINV: 1; t2tINV: 1; TRA: 0
SVs in sample	137
Oscillating CN (2 and 3 states)	5, 5
CN segments	5
FDR fragment joints	0.6776251
FDR chr. breakp. enrich.	0.01
Linked to chrs	
Purity, ploidy	0.48, 3.87

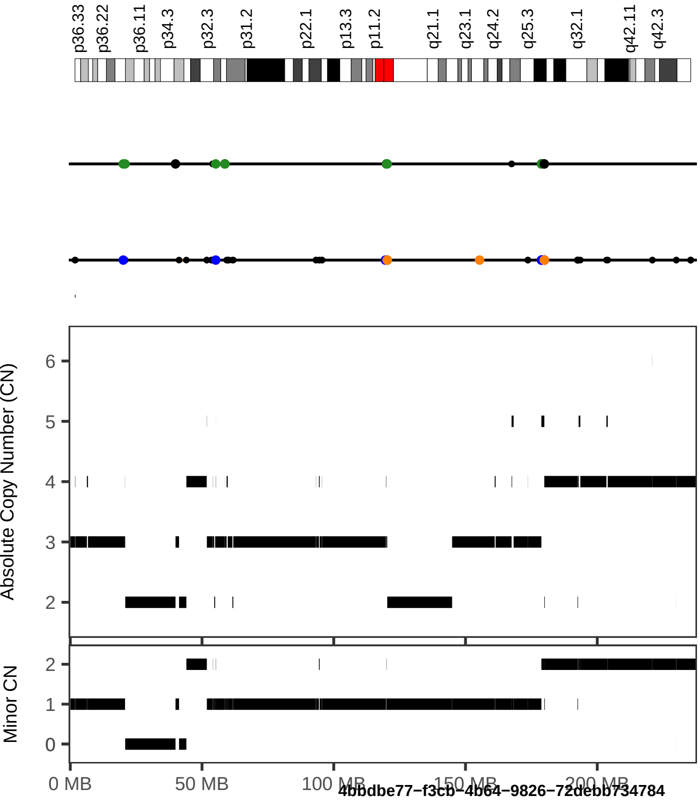

4bbdbe77−f3cb−4b64−9826−72debb734784

Cancer type	Liver−HCC
Position	1:53503836−192549016
Interleaved intrachr. SVs	4
Total SVs (intrachr. + transl.)	33
SV types	DEL: 1; DUP: 5; h2hINV: 1; t2tINV: 1; TRA: 25
SVs in sample	445
Oscillating CN (2 and 3 states)	11, 25
CN segments	42
FDR fragment joints	0.6776251
FDR chr. breakp. enrich.	0
Linked to chrs	17:8805223−79503105;2:85528204−99868581
Purity, ploidy	0.82, 2.08

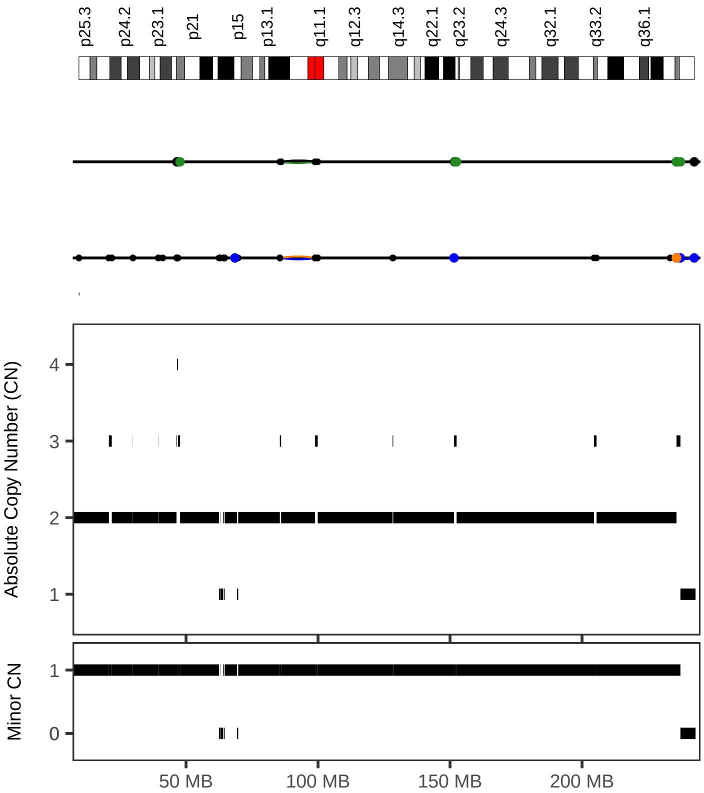

4bbdbe77−f3cb−4b64−9826−72debb734784

Cancer type	Liver−HCC
Position	2:85528204−99868582
Interleaved intrachr. SVs	8
Total SVs (intrachr. + transl.)	8
SV types	DEL: 2; DUP: 3; h2hINV: 2; t2tINV: 1; TRA: 0
SVs in sample	445
Oscillating CN (2 and 3 states)	4, 4
CN segments	4
FDR fragment joints	0.8653243
FDR chr. breakp. enrich.	0.07
Linked to chrs	
Purity, ploidy	0.82, 2.08

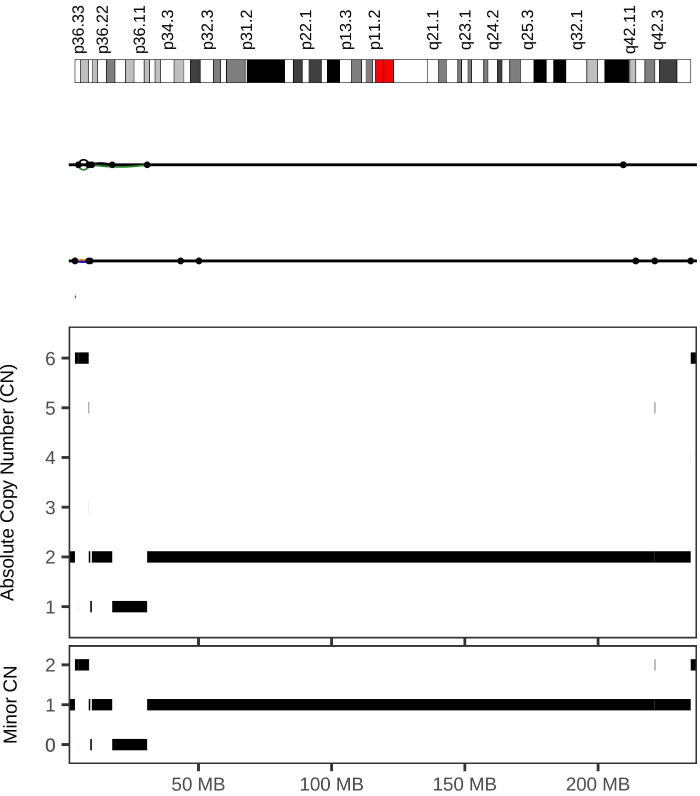

56403463−1727−4c23−a709−de5f168c4073

Cancer type	Liver−HCC
Position	1:3589401−30700898
Interleaved intrachr. SVs	6
Total SVs (intrachr. + transl.)	6
SV types	DEL: 1; DUP: 1; h2hINV: 2; t2tINV: 2; TRA: 0
SVs in sample	309
Oscillating CN (2 and 3 states)	5, 5
CN segments	11
FDR fragment joints	0.9284301
FDR chr. breakp. enrich.	0.06
Linked to chrs	
Purity, ploidy	0.69, 1.88

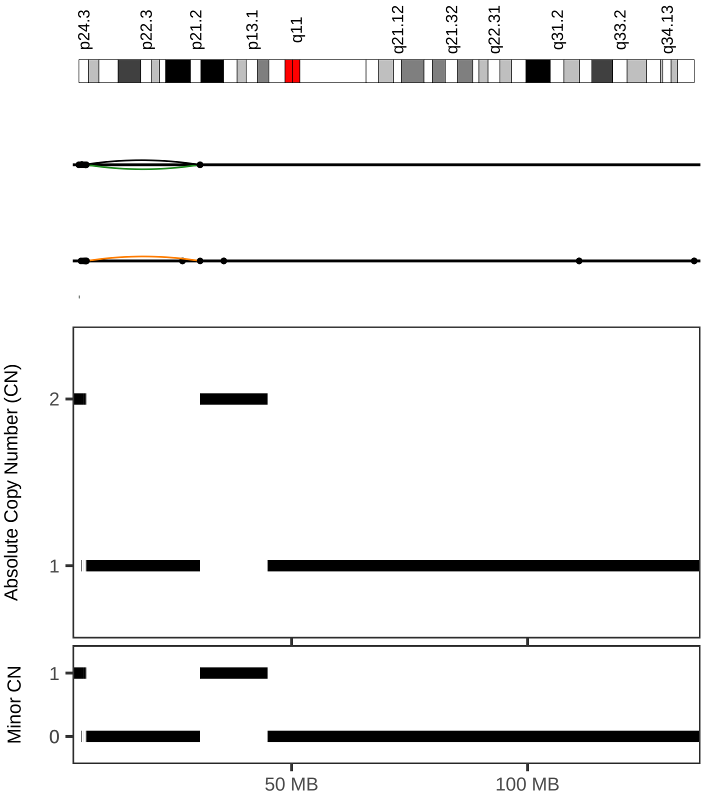

56403463−1727−4c23−a709−de5f168c4073

Cancer type	Liver−HCC
Position	9:4927905−30615811
Interleaved intrachr. SVs	9
Total SVs (intrachr. + transl.)	9
SV types	DEL: 3; DUP: 1; h2hINV: 3; t2tINV: 2; TRA: 0
SVs in sample	309
Oscillating CN (2 and 3 states)	9, 9
CN segments	9
FDR fragment joints	0.854603
FDR chr. breakp. enrich.	0.78
Linked to chrs	
Purity, ploidy	0.69, 1.88

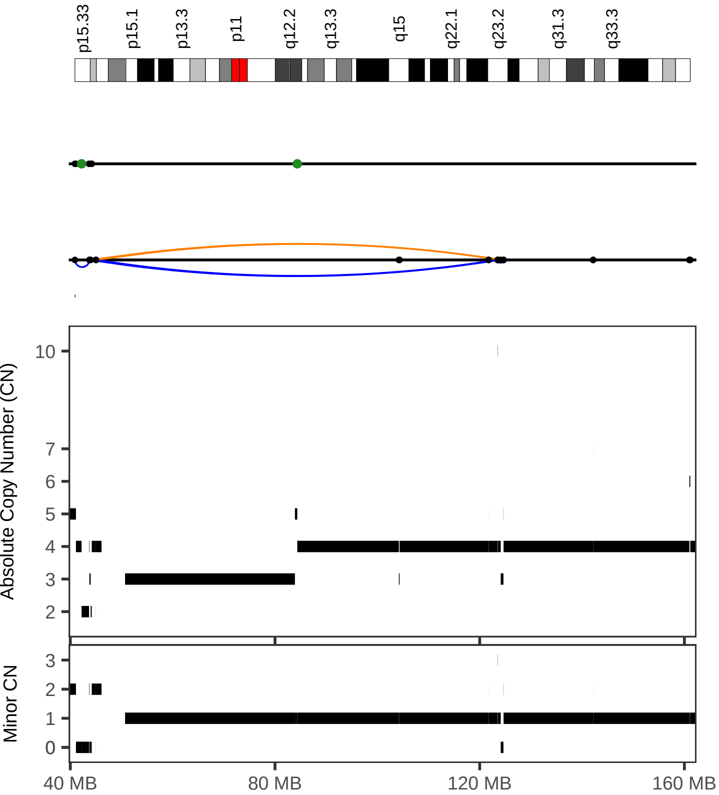

670c1cbc−4494−45f1−bb8b−18db82d4f7e0

Cancer type	Liver−HCC
Position	5:40899212−124664734
Interleaved intrachr. SVs	8
Total SVs (intrachr. + transl.)	10
SV types	DEL: 3; DUP: 4; h2hINV: 0; t2tINV: 1; TRA: 2
SVs in sample	89
Oscillating CN (2 and 3 states)	4, 8
CN segments	21
FDR fragment joints	0.6776251
FDR chr. breakp. enrich.	0
Linked to chrs	
Purity, ploidy	0.22, 3.51

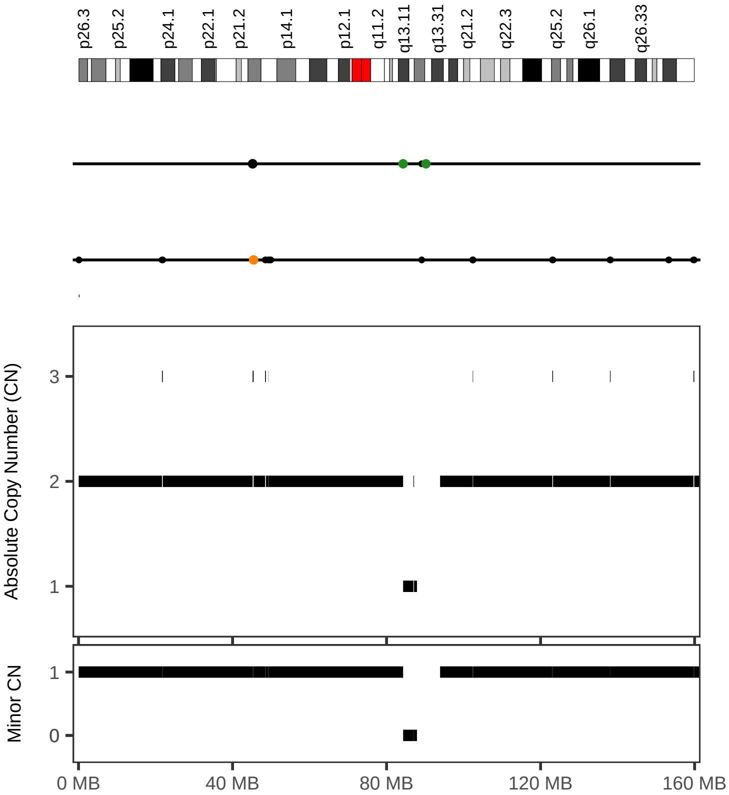

68015cc7−d3a1−4c90−b8fb−5d14143d9960

Cancer type	Liver−HCC
Position	3:101275−159910152
Interleaved intrachr. SVs	1
Total SVs (intrachr. + transl.)	15
SV types	DEL: 0; DUP: 10; h2hINV: 0; t2tINV: 0; TRA: 5
SVs in sample	231
Oscillating CN (2 and 3 states)	10, 22
CN segments	22
FDR fragment joints	0.129004
FDR chr. breakp. enrich.	0.69
Linked to chrs	
Purity, ploidy	0.83, 2.03

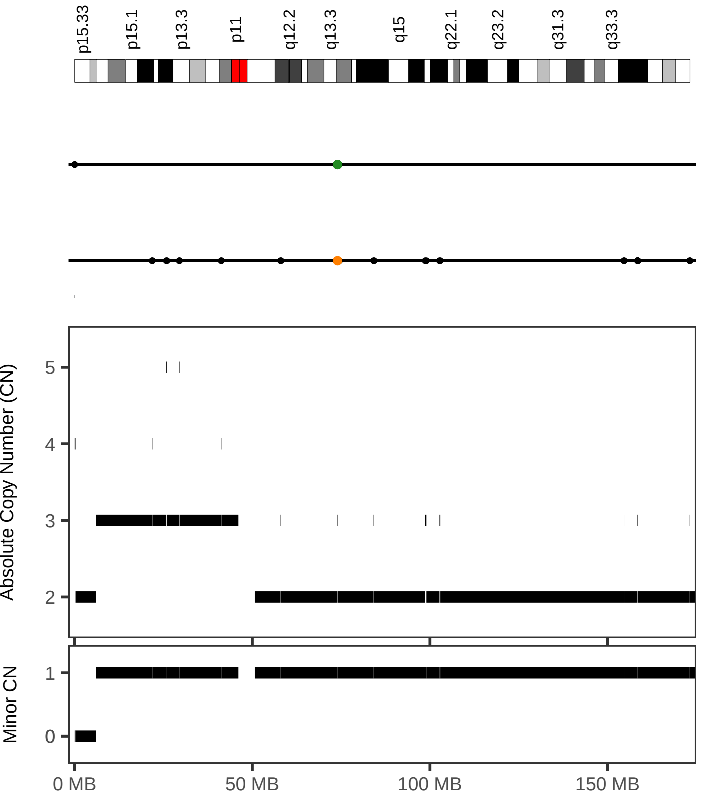

68015cc7−d3a1−4c90−b8fb−5d14143d9960

Cancer type	Liver−HCC
Position	5:21799697−173208906
Interleaved intrachr. SVs	1
Total SVs (intrachr. + transl.)	17
SV types	DEL: 0; DUP: 14; h2hINV: 0; t2tINV: 0; TRA: 3
SVs in sample	231
Oscillating CN (2 and 3 states)	18, 18
CN segments	25
FDR fragment joints	0.0008548581
FDR chr. breakp. enrich.	0.45
Linked to chrs	17:8573197−73801520;
Purity, ploidy	0.83, 2.03

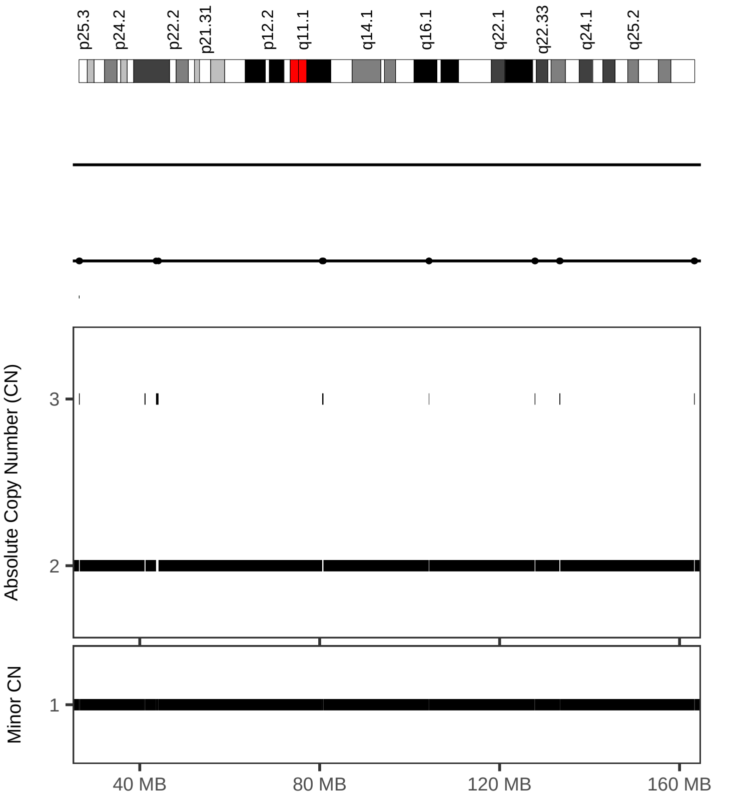

68015cc7−d3a1−4c90−b8fb−5d14143d9960

Cancer type	Liver−HCC
Position	6:26485750−163374875
Interleaved intrachr. SVs	1
Total SVs (intrachr. + transl.)	7
SV types	DEL: 0; DUP: 7; h2hINV: 0; t2tINV: 0; TRA: 0
SVs in sample	231
Oscillating CN (2 and 3 states)	16, 16
CN segments	16
FDR fragment joints	0.01743054
FDR chr. breakp. enrich.	0.21
Linked to chrs	
Purity, ploidy	0.83, 2.03

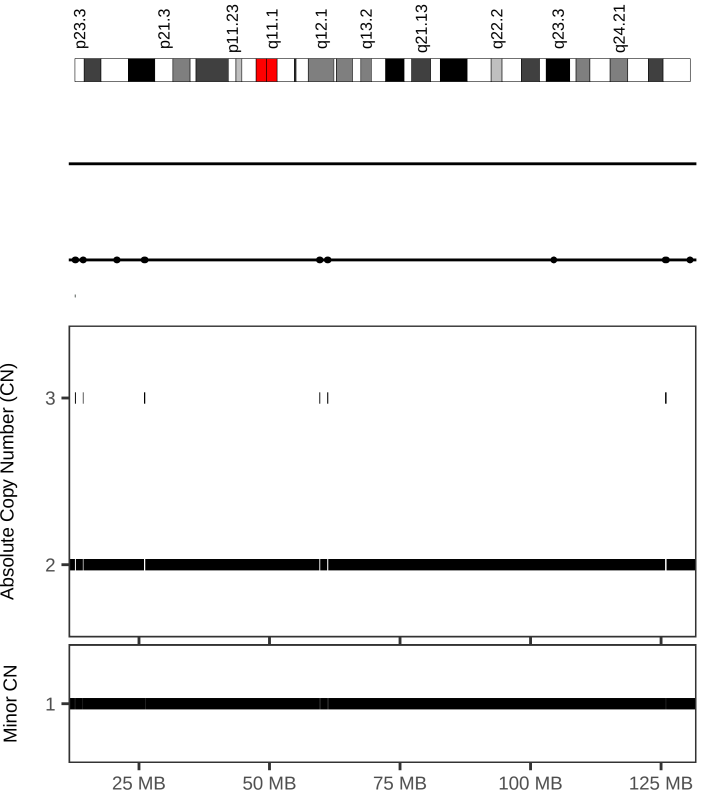

68015cc7−d3a1−4c90−b8fb−5d14143d9960

Cancer type	Liver−HCC
Position	8:12730205−130589835
Interleaved intrachr. SVs	1
Total SVs (intrachr. + transl.)	8
SV types	DEL: 0; DUP: 8; h2hINV: 0; t2tINV: 0; TRA: 0
SVs in sample	231
Oscillating CN (2 and 3 states)	12, 12
CN segments	12
FDR fragment joints	0.006736868
FDR chr. breakp. enrich.	0.77
Linked to chrs	
Purity, ploidy	0.83, 2.03

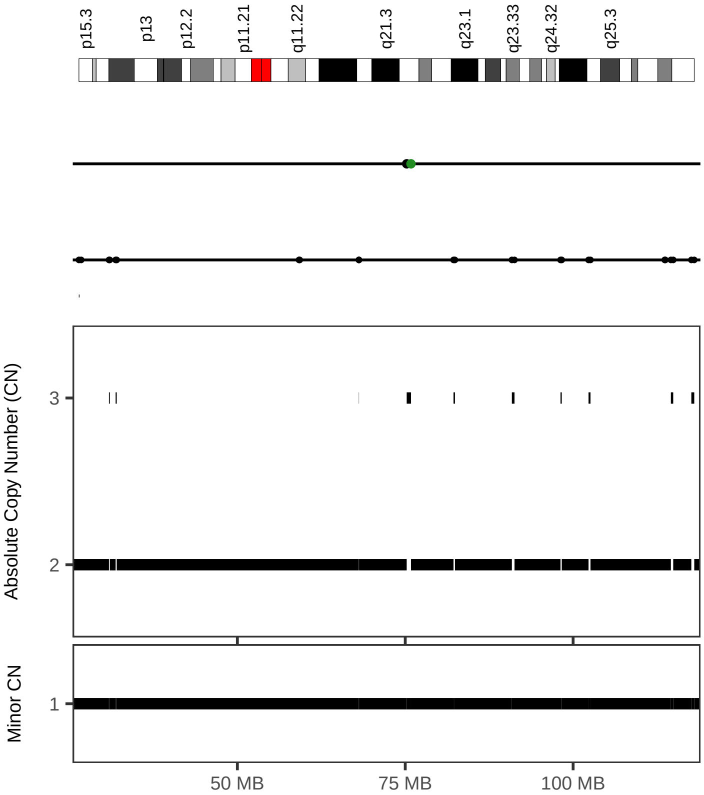

68015cc7−d3a1−4c90−b8fb−5d14143d9960

Cancer type	Liver−HCC
Position	10:26398455−118039623
Interleaved intrachr. SVs	1
Total SVs (intrachr. + transl.)	14
SV types	DEL: 0; DUP: 12; h2hINV: 0; t2tINV: 0; TRA: 2
SVs in sample	231
Oscillating CN (2 and 3 states)	21, 21
CN segments	21
FDR fragment joints	0.001880701
FDR chr. breakp. enrich.	0.51
Linked to chrs	17:8573197−73801520;
Purity, ploidy	0.83, 2.03

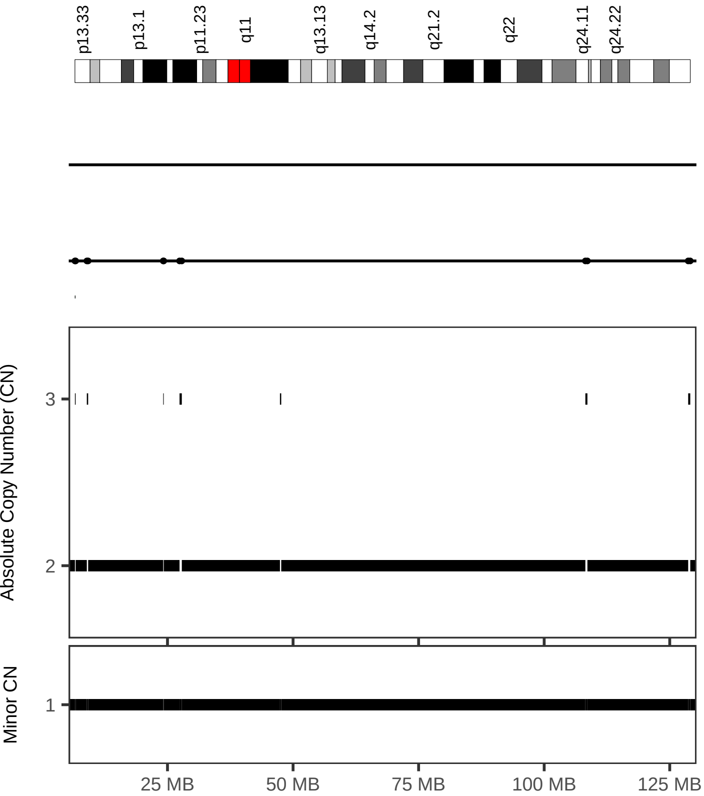

68015cc7−d3a1−4c90−b8fb−5d14143d9960

Cancer type	Liver−HCC
Position	12:6565847−129085870
Interleaved intrachr. SVs	1
Total SVs (intrachr. + transl.)	6
SV types	DEL: 0; DUP: 6; h2hINV: 0; t2tINV: 0; TRA: 0
SVs in sample	231
Oscillating CN (2 and 3 states)	14, 14
CN segments	14
FDR fragment joints	0.04139017
FDR chr. breakp. enrich.	0.37
Linked to chrs	
Purity, ploidy	0.83, 2.03

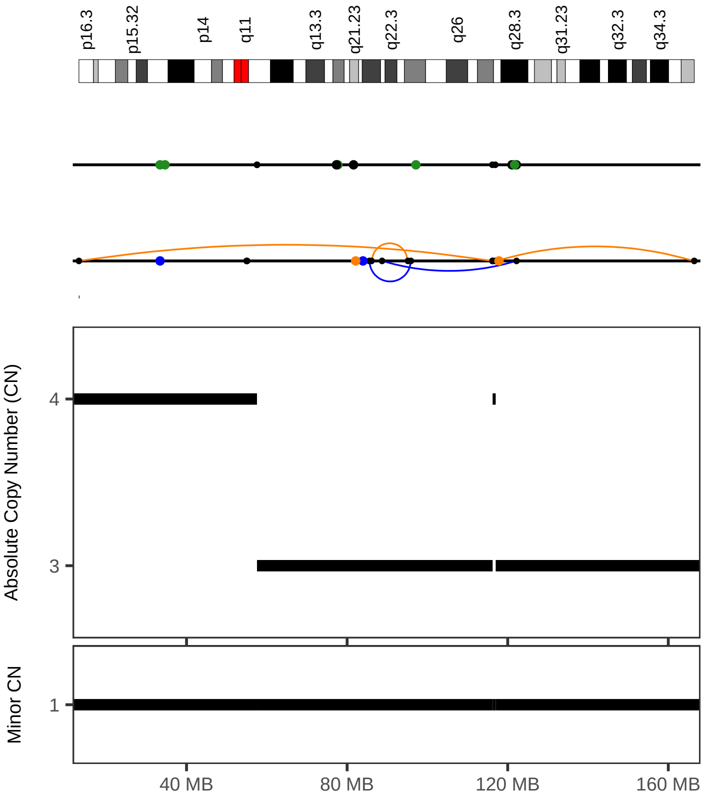

6c79f64b−63fe−46f5−bf7b−77208231b0f2

Cancer type	Liver−HCC
Position	4:13197519−166464059
Interleaved intrachr. SVs	7
Total SVs (intrachr. + transl.)	20
SV types	DEL: 3; DUP: 3; h2hINV: 0; t2tINV: 1; TRA: 13
SVs in sample	125
Oscillating CN (2 and 3 states)	4, 4
CN segments	4
FDR fragment joints	1
FDR chr. breakp. enrich.	0
Linked to chrs	1:16944681−155955763;
Purity, ploidy	0.48, 3.89

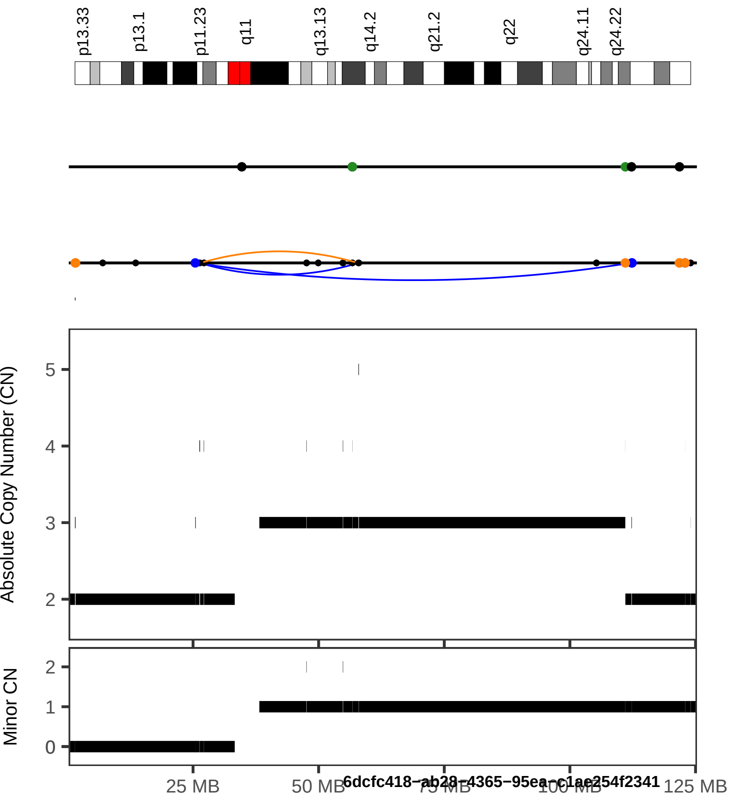

6dcfc418−ab28−4365−95ea−c1ae254f2341

Cancer type	Liver−HCC
Position	12:1508040−124042273
Interleaved intrachr. SVs	1
Total SVs (intrachr. + transl.)	29
SV types	DEL: 2; DUP: 11; h2hINV: 0; t2tINV: 0; TRA: 16
SVs in sample	572
Oscillating CN (2 and 3 states)	7, 17
CN segments	25
FDR fragment joints	0.1517838
FDR chr. breakp. enrich.	0.57
Linked to chrs	10:35522520−95427338;14:70988852−75703456 7:8218986−100523823;8:8789701−145595243
Purity, ploidy	[illegible]

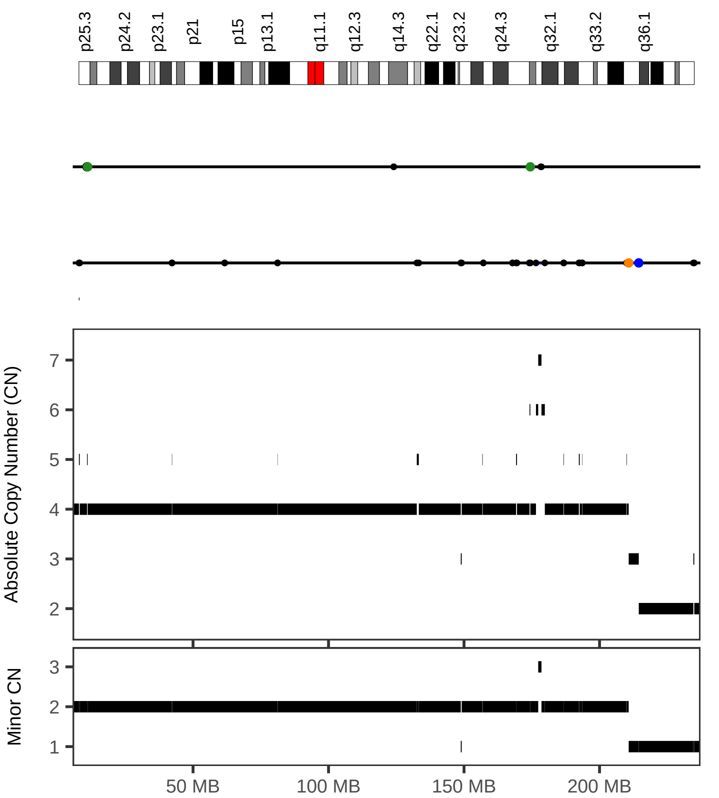

8afa3140−dcbd−44c0−ab13−4ada6a0444d4

Cancer type	Liver−HCC
Position	2:7896145−234951998
Interleaved intrachr. SVs	1
Total SVs (intrachr. + transl.)	22
SV types	DEL: 2; DUP: 13; h2hINV: 2; t2tINV: 0; TRA: 5
SVs in sample	301
Oscillating CN (2 and 3 states)	10, 16
CN segments	34
FDR fragment joints	0.04139017
FDR chr. breakp. enrich.	0.92
Linked to chrs	
Purity, ploidy	0.84, 3.54

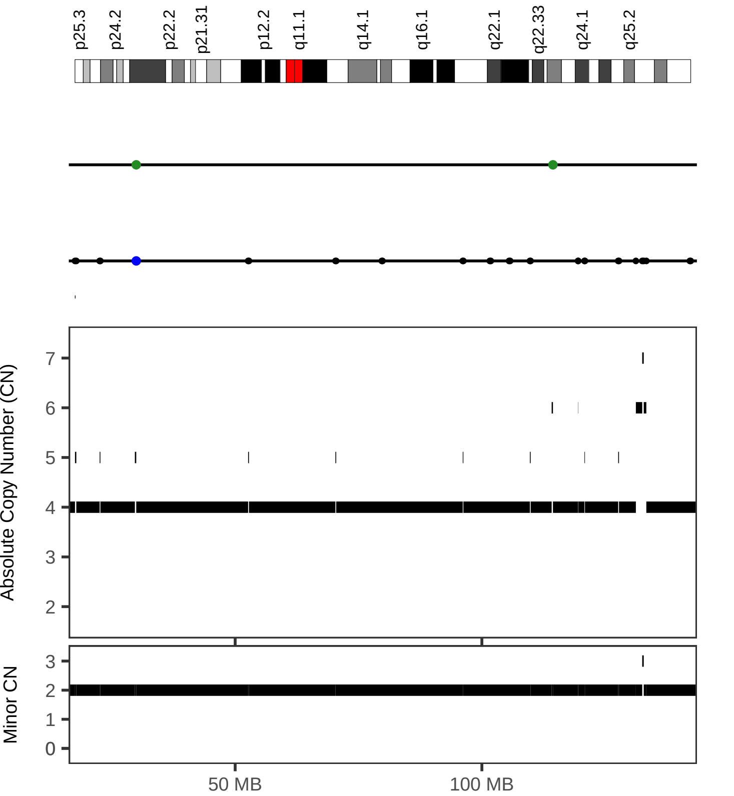

8afa3140−dcbd−44c0−ab13−4ada6a0444d4

Cancer type	Liver−HCC
Position	6:17536286−142324409
Interleaved intrachr. SVs	1
Total SVs (intrachr. + transl.)	19
SV types	DEL: 0; DUP: 15; h2hINV: 0; t2tINV: 1; TRA: 3
SVs in sample	301
Oscillating CN (2 and 3 states)	14, 23
CN segments	26
FDR fragment joints	7.730001e−05
FDR chr. breakp. enrich.	0.84
Linked to chrs	11:1792398−105876404;
Purity, ploidy	0.84, 3.54

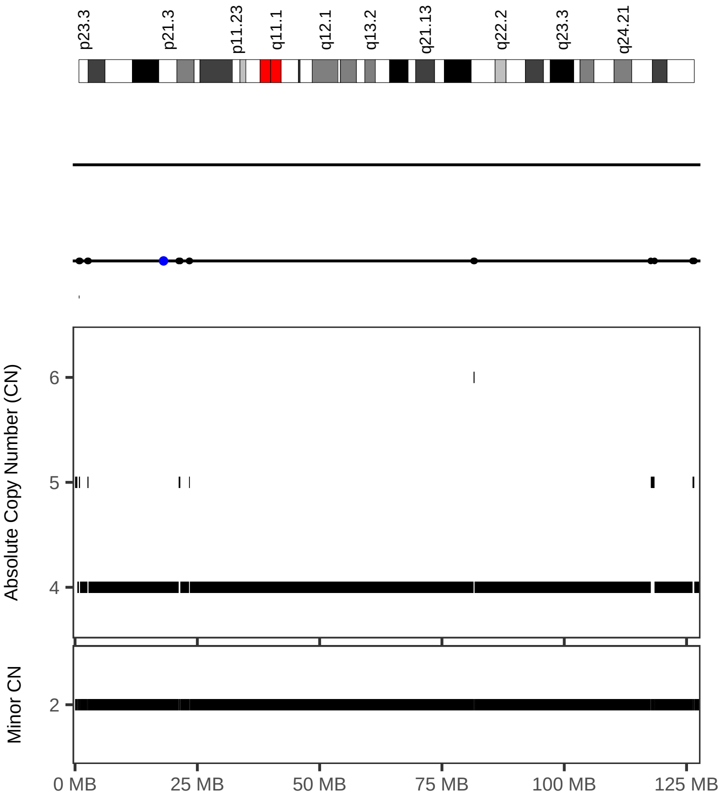

8afa3140−dcbd−44c0−ab13−4ada6a0444d4

Cancer type	Liver−HCC
Position	8:793195−126571971
Interleaved intrachr. SVs	1
Total SVs (intrachr. + transl.)	8
SV types	DEL: 0; DUP: 7; h2hINV: 0; t2tINV: 0; TRA: 1
SVs in sample	301
Oscillating CN (2 and 3 states)	8, 14
CN segments	14
FDR fragment joints	0.006736868
FDR chr. breakp. enrich.	0.29
Linked to chrs	
Purity, ploidy	0.84, 3.54

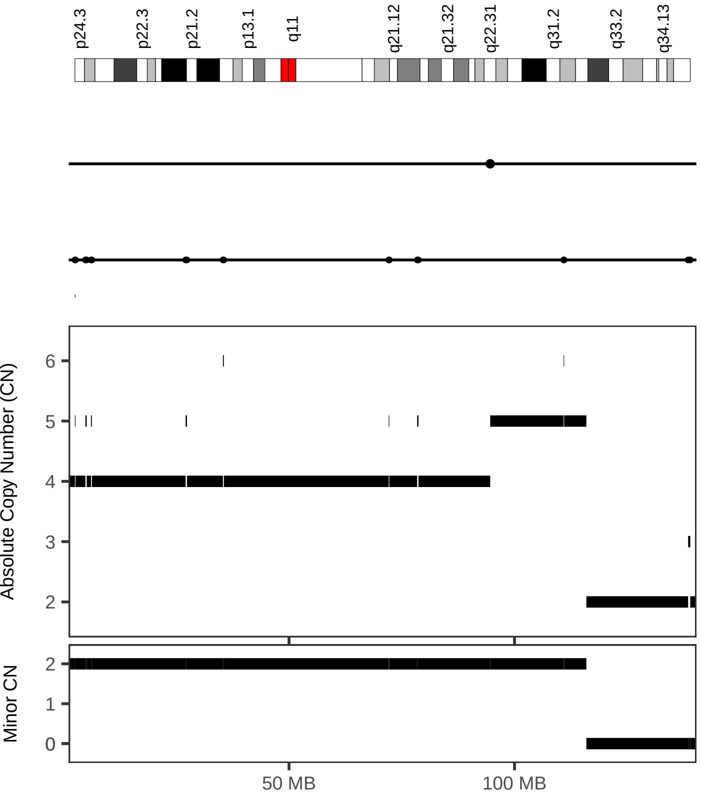

	8afa3140−dcbd−44c0−ab13−4ada6a0444d4
Cancer type	Liver−HCC
Position	9:2531011−138963230
Interleaved intrachr. SVs	1
Total SVs (intrachr. + transl.)	10
SV types	DEL: 0; DUP: 9; h2hINV: 0; t2tINV: 0; TRA: 1
SVs in sample	301
Oscillating CN (2 and 3 states)	8, 15
CN segments	20
FDR fragment joints	0.01020027
FDR chr. breakp. enrich.	0.93
Linked to chrs	
Purity, ploidy	0.84, 3.54

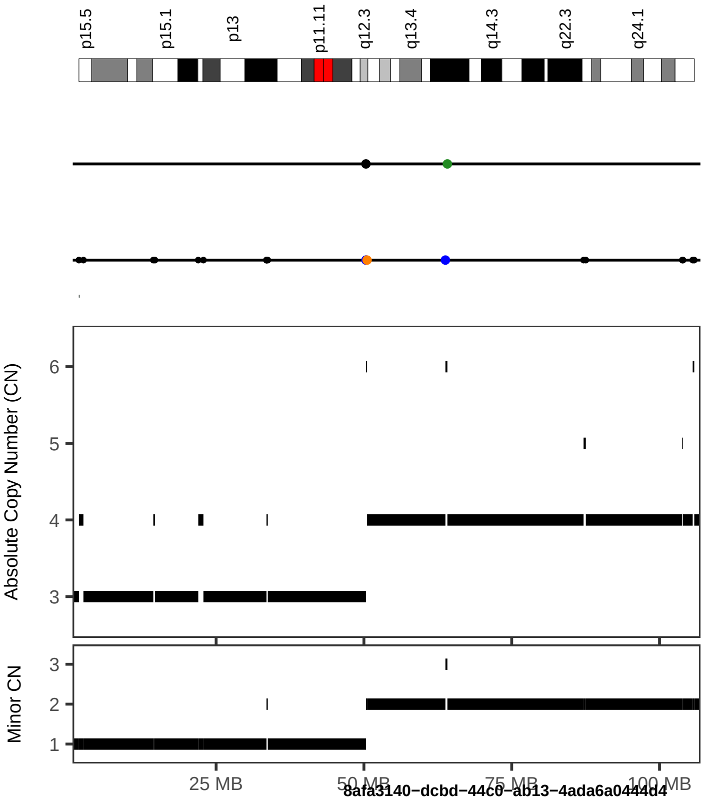

	8afa3140−dcbd−44c0−ab13−4ada6a0444d4
Cancer type	Liver−HCC
Position	11:1792398−105876405
Interleaved intrachr. SVs	1
Total SVs (intrachr. + transl.)	12
SV types	DEL: 0; DUP: 7; h2hINV: 0; t2tINV: 0; TRA: 5
SVs in sample	301
Oscillating CN (2 and 3 states)	8, 11
CN segments	18
FDR fragment joints	0.08549
FDR chr. breakp. enrich.	1
Linked to chrs	16:7890344−88719885;6:17536286−142324408
Purity, ploidy	0.84, 3.54

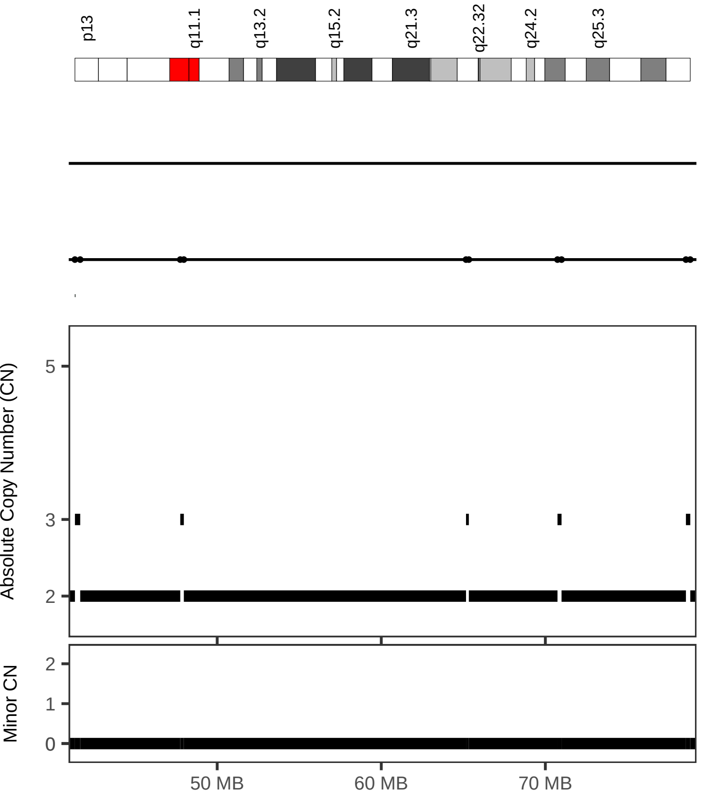

	8afa3140−dcbd−44c0−ab13−4ada6a0444d4
Cancer type	Liver−HCC
Position	15:41329339−78831198
Interleaved intrachr. SVs	1
Total SVs (intrachr. + transl.)	5
SV types	DEL: 0; DUP: 5; h2hINV: 0; t2tINV: 0; TRA: 0
SVs in sample	301
Oscillating CN (2 and 3 states)	10, 10
CN segments	10
FDR fragment joints	0.1013295
FDR chr. breakp. enrich.	0.99
Linked to chrs	
Purity, ploidy	0.84, 3.54

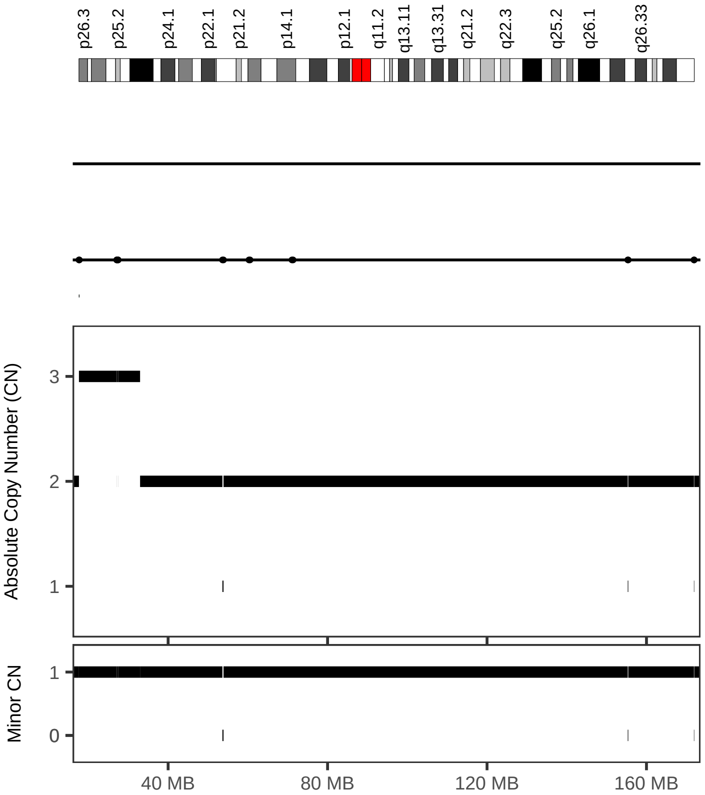

	c52abac9−07da−4b3d−bf8a−2161cacf9682
Cancer type	Liver−HCC
Position	3:17621629−171986122
Interleaved intrachr. SVs	1
Total SVs (intrachr. + transl.)	6
SV types	DEL: 6; DUP: 0; h2hINV: 0; t2tINV: 0; TRA: 0
SVs in sample	75
Oscillating CN (2 and 3 states)	7, 12
CN segments	12
FDR fragment joints	0.04139017
FDR chr. breakp. enrich.	0.49
Linked to chrs	
Purity, ploidy	0.28, 1.96

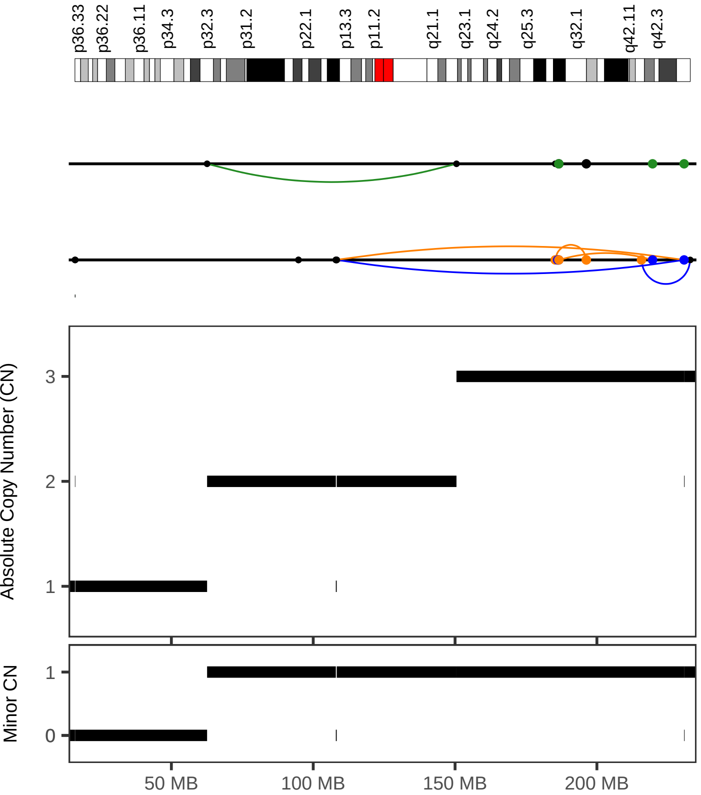

d680df09−368e−42b5−b540−45c41ed31042

Cancer type	Liver−HCC
Position	1:62587390−232902623
Interleaved intrachr. SVs	8
Total SVs (intrachr. + transl.)	23
SV types	DEL: 4; DUP: 2; h2hINV: 0; t2tINV: 2; TRA: 15
SVs in sample	82
Oscillating CN (2 and 3 states)	4, 6
CN segments	6
FDR fragment joints	0.615458
FDR chr. breakp. enrich.	0
Linked to chrs	13:24796654−56409609;
Purity, ploidy	0.64, 1.94

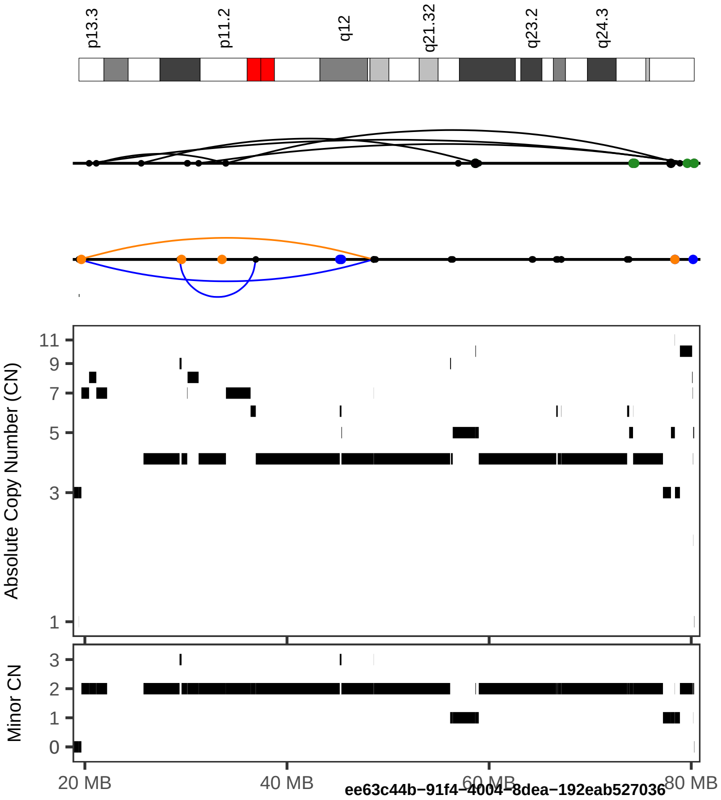

ee63c44b−91f4−4004−8dea−192eab527036

Cancer type	Liver−HCC
Position	17:19411292−80161265
Interleaved intrachr. SVs	10
Total SVs (intrachr. + transl.)	25
SV types	DEL: 1; DUP: 4; h2hINV: 5; t2tINV: 0; TRA: 15
SVs in sample	405
Oscillating CN (2 and 3 states)	6, 10
CN segments	41
FDR fragment joints	0.6776251
FDR chr. breakp. enrich.	0
Linked to chrs	1:17700321−53826708;10:12324933−114133789 12:6457439−132375236;
Purity, ploidy	0.67, 2.88

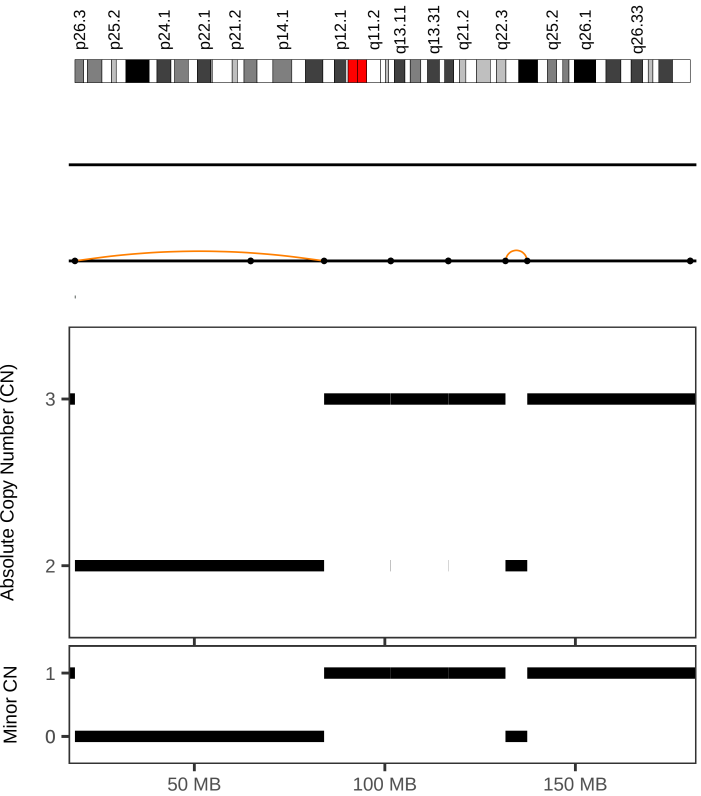

f6e5979d−d6a9−4408−bd1b−1b37d1064e83

Cancer type	Liver−HCC
Position	3:18657637−137367416
Interleaved intrachr. SVs	1
Total SVs (intrachr. + transl.)	5
SV types	DEL: 5; DUP: 0; h2hINV: 0; t2tINV: 0; TRA: 0
SVs in sample	111
Oscillating CN (2 and 3 states)	8, 8
CN segments	8
FDR fragment joints	0.1013295
FDR chr. breakp. enrich.	0.9
Linked to chrs	
Purity, ploidy	0.7, 3.19

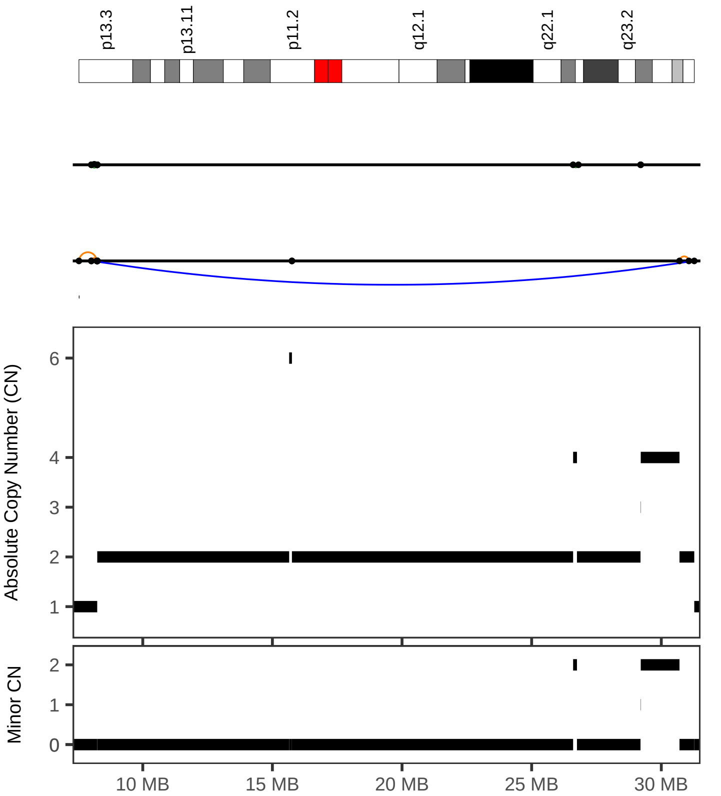

HX11

Cancer type	Liver−HCC
Position	16:7537773−31271320
Interleaved intrachr. SVs	9
Total SVs (intrachr. + transl.)	9
SV types	DEL: 3; DUP: 1; h2hINV: 2; t2tINV: 3; TRA: 0
SVs in sample	42
Oscillating CN (2 and 3 states)	4, 4
CN segments	12
FDR fragment joints	0.854603
FDR chr. breakp. enrich.	0
Linked to chrs	
Purity, ploidy	0.36, 1.9

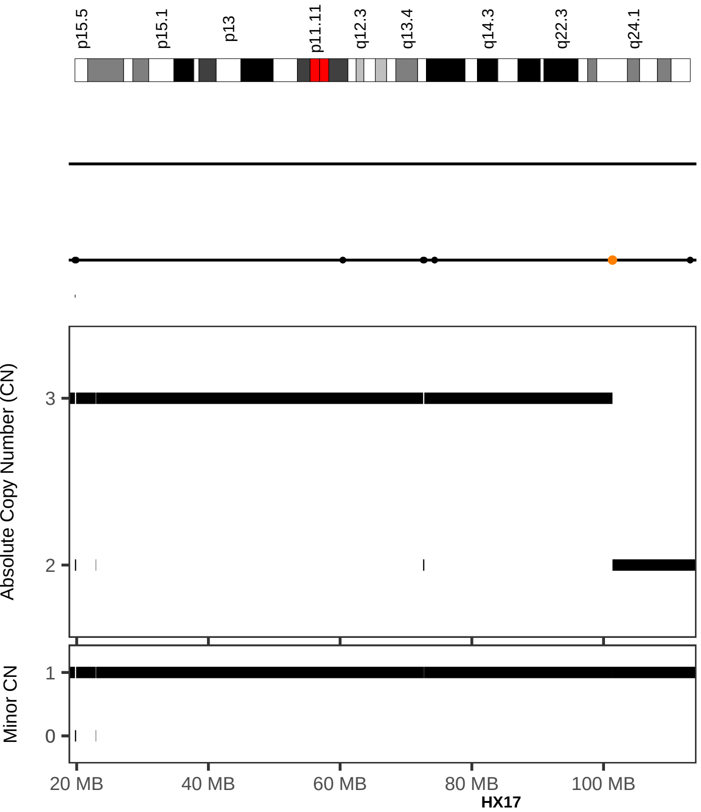

HX17

Cancer type	Liver−HCC
Position	11:19730216−113168096
Interleaved intrachr. SVs	1
Total SVs (intrachr. + transl.)	5
SV types	DEL: 4; DUP: 0; h2hINV: 0; t2tINV: 0; TRA: 1
SVs in sample	44
Oscillating CN (2 and 3 states)	7, 7
CN segments	7
FDR fragment joints	0.1013295
FDR chr. breakp. enrich.	0.07
Linked to chrs	
Purity, ploidy	0.8, 2.43

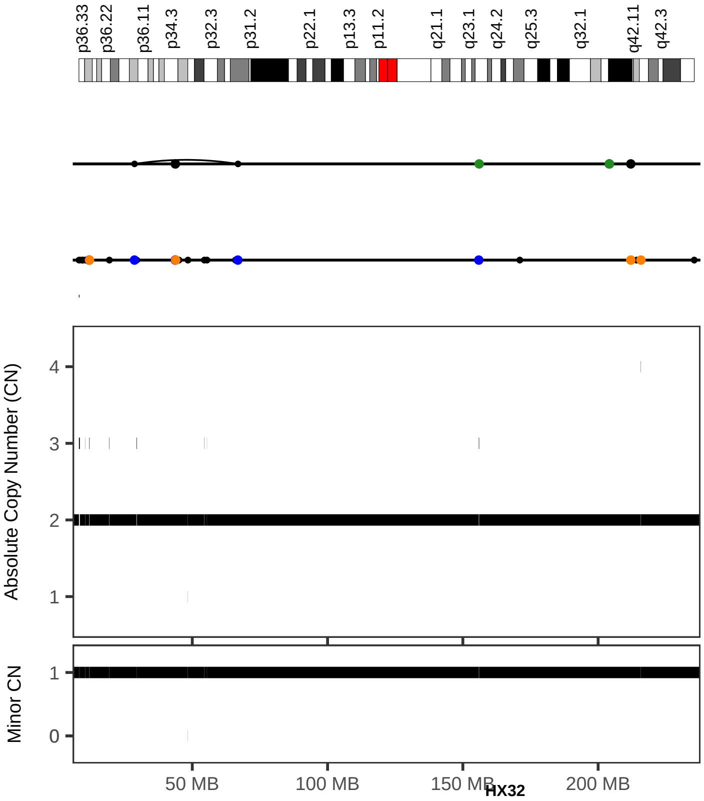

HX32

Cancer type	Liver−HCC
Position	1:8099405−215842951
Interleaved intrachr. SVs	1
Total SVs (intrachr. + transl.)	33
SV types	DEL: 2; DUP: 14; h2hINV: 1; t2tINV: 0; TRA: 16
SVs in sample	237
Oscillating CN (2 and 3 states)	10, 18
CN segments	20
FDR fragment joints	0.01020027
FDR chr. breakp. enrich.	0
Linked to chrs	15:58395795−90702521;5:919414−115180172
Purity, ploidy	0.74, 1.94

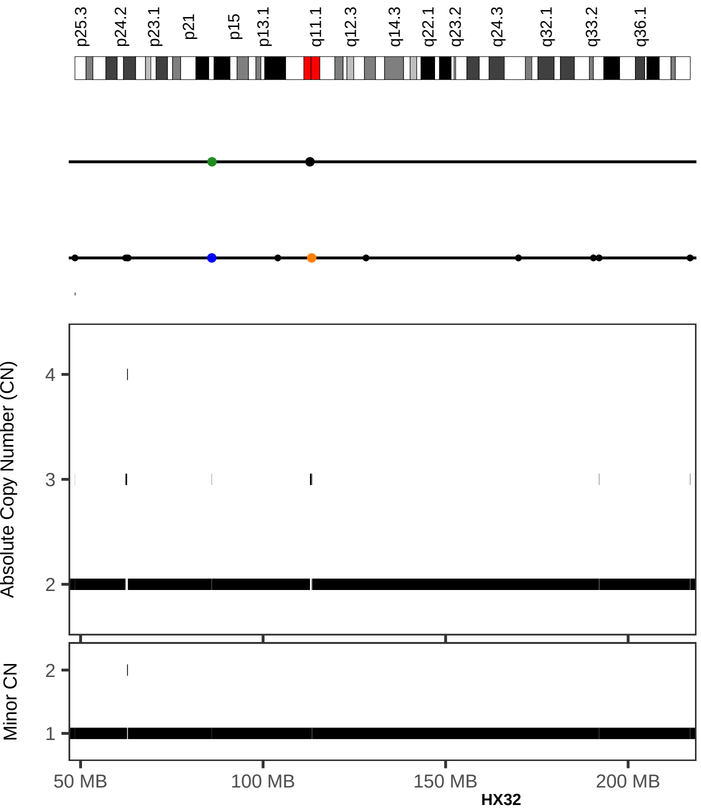

HX32

Cancer type	Liver−HCC
Position	2:48452073−217017336
Interleaved intrachr. SVs	1
Total SVs (intrachr. + transl.)	12
SV types	DEL: 0; DUP: 8; h2hINV: 0; t2tINV: 0; TRA: 4
SVs in sample	237
Oscillating CN (2 and 3 states)	11, 13
CN segments	15
FDR fragment joints	0.08549
FDR chr. breakp. enrich.	0.9
Linked to chrs	
Purity, ploidy	0.74, 1.94

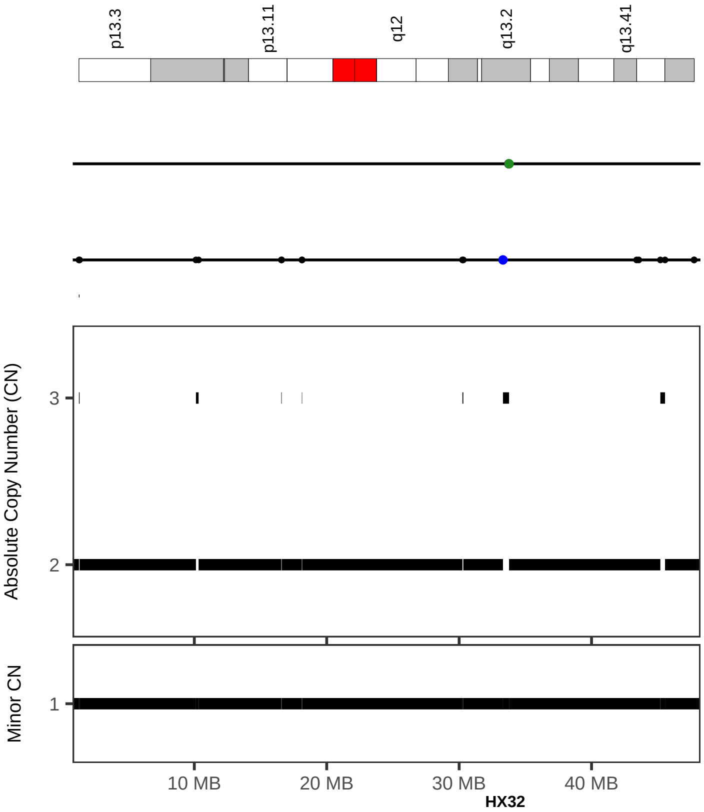

HX32

Cancer type	Liver−HCC
Position	19:1285753−47756167
Interleaved intrachr. SVs	1
Total SVs (intrachr. + transl.)	11
SV types	DEL: 0; DUP: 8; h2hINV: 0; t2tINV: 0; TRA: 3
SVs in sample	237
Oscillating CN (2 and 3 states)	14, 14
CN segments	14
FDR fragment joints	0.03230998
FDR chr. breakp. enrich.	0.04
Linked to chrs	5:919414−115180172;
Purity, ploidy	0.74, 1.94

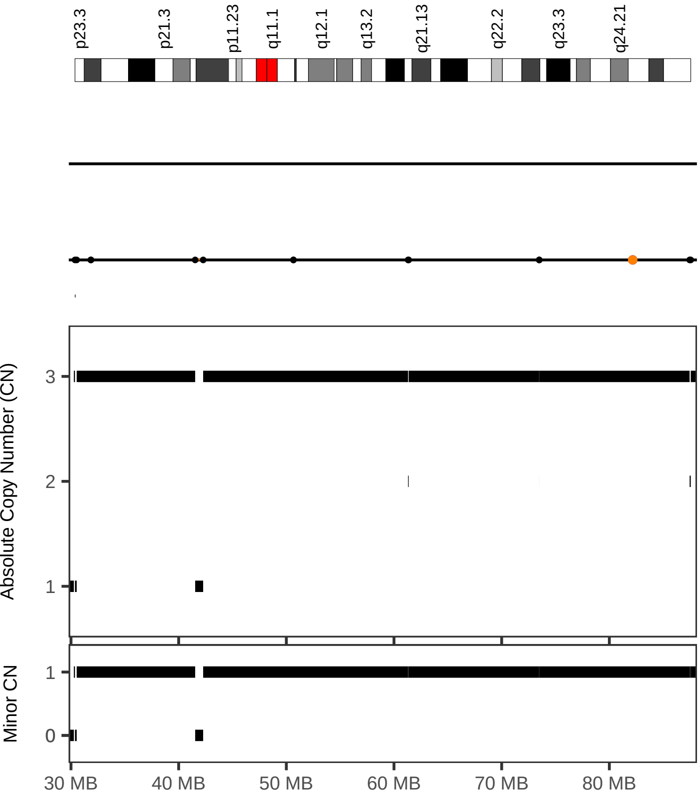

RK042

Cancer type	Liver−HCC
Position	8:30371255−87560417
Interleaved intrachr. SVs	1
Total SVs (intrachr. + transl.)	7
SV types	DEL: 5; DUP: 0; h2hINV: 0; t2tINV: 0; TRA: 2
SVs in sample	93
Oscillating CN (2 and 3 states)	7, 10
CN segments	10
FDR fragment joints	0.1278363
FDR chr. breakp. enrich.	0.1
Linked to chrs	
Purity, ploidy	0.88, 1.81

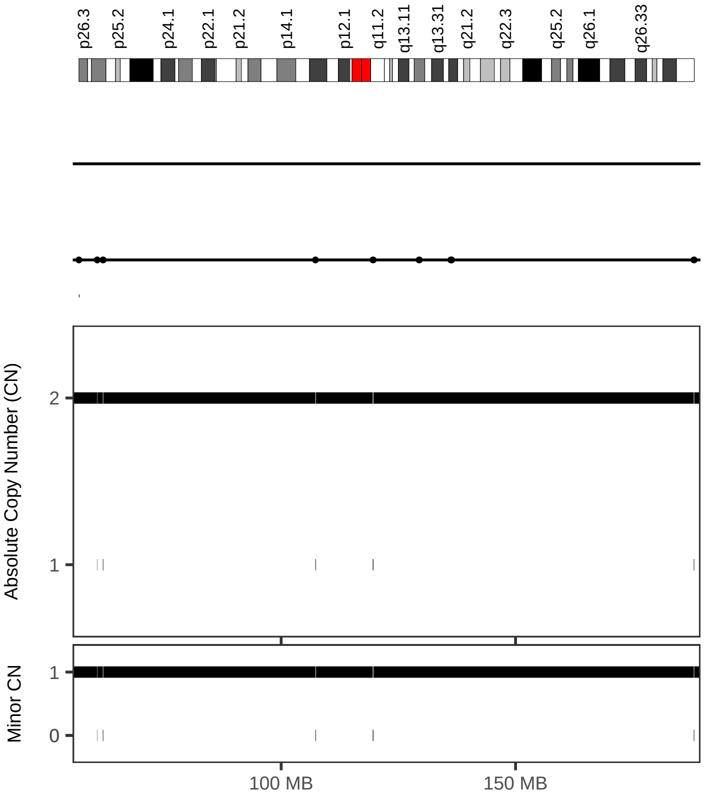

RK053

Cancer type	Liver−HCC
Position	3:60740057−188119821
Interleaved intrachr. SVs	1
Total SVs (intrachr. + transl.)	8
SV types	DEL: 7; DUP: 1; h2hINV: 0; t2tINV: 0; TRA: 0
SVs in sample	81
Oscillating CN (2 and 3 states)	10, 10
CN segments	10
FDR fragment joints	0.05741733
FDR chr. breakp. enrich.	0.4
Linked to chrs	
Purity, ploidy	0.92, 2.04

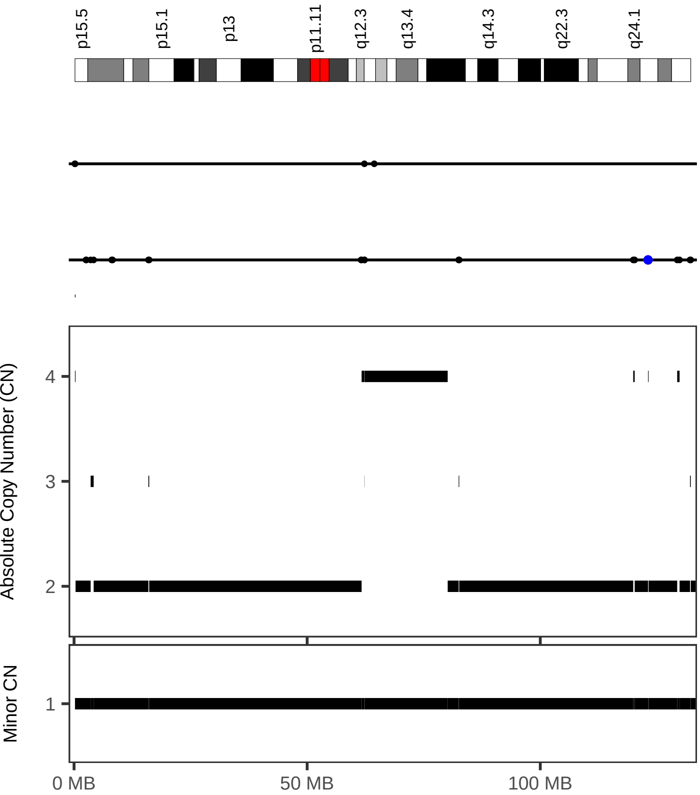

RK070

Cancer type	Liver−HCC
Position	11:2547255−132275890
Interleaved intrachr. SVs	1
Total SVs (intrachr. + transl.)	14
SV types	DEL: 1; DUP: 10; h2hINV: 1; t2tINV: 0; TRA: 2
SVs in sample	281
Oscillating CN (2 and 3 states)	7, 19
CN segments	19
FDR fragment joints	0.01333161
FDR chr. breakp. enrich.	0.79
Linked to chrs	
Purity, ploidy	0.6, 2.2

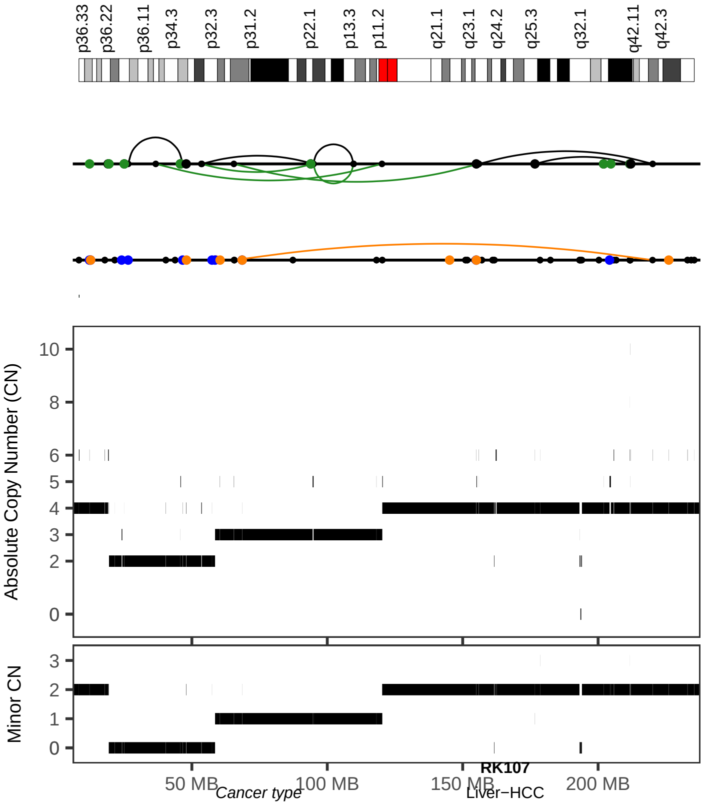

RK107

Cancer type	Liver−HCC
Position	1:26503377−220160009
Interleaved intrachr. SVs	9
Total SVs (intrachr. + transl.)	35
SV types	DEL: 1; DUP: 0; h2hINV: 4; t2tINV: 4; TRA: 26
SVs in sample	616
Oscillating CN (2 and 3 states)	9, 21
CN segments	66
FDR fragment joints	0.615458
FDR chr. breakp. enrich.	0
Linked to chrs	10:75643021−104011739;12:565831−121912249 13:21110069−110361131;14:31438137−103571316 4:40323309−109722572;6:8643449−170152201 9:98064387−131689723;

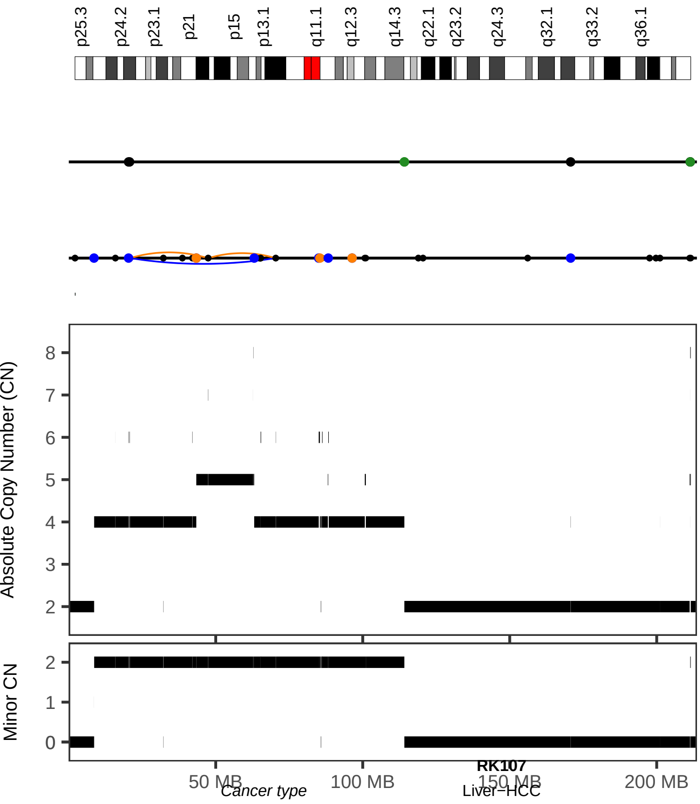

	RK107
Cancer type	Liver−HCC
Position	2:2119500−211577974
Interleaved intrachr. SVs	1
Total SVs (intrachr. + transl.)	40
SV types	DEL: 7; DUP: 13; h2hINV: 0;<br>t2tINV: 0; TRA: 20
SVs in sample	616
Oscillating CN (2 and 3 states)	9, 9
CN segments	50
FDR fragment joints	0.04139017
FDR chr. breakp. enrich.	0.9
Linked to chrs	11:65126119−117045448;12:565831−121912249<br>14:31438137−103571316;17:7015742−8463746<br>3:39802700−133489710;9:98064387−131689723

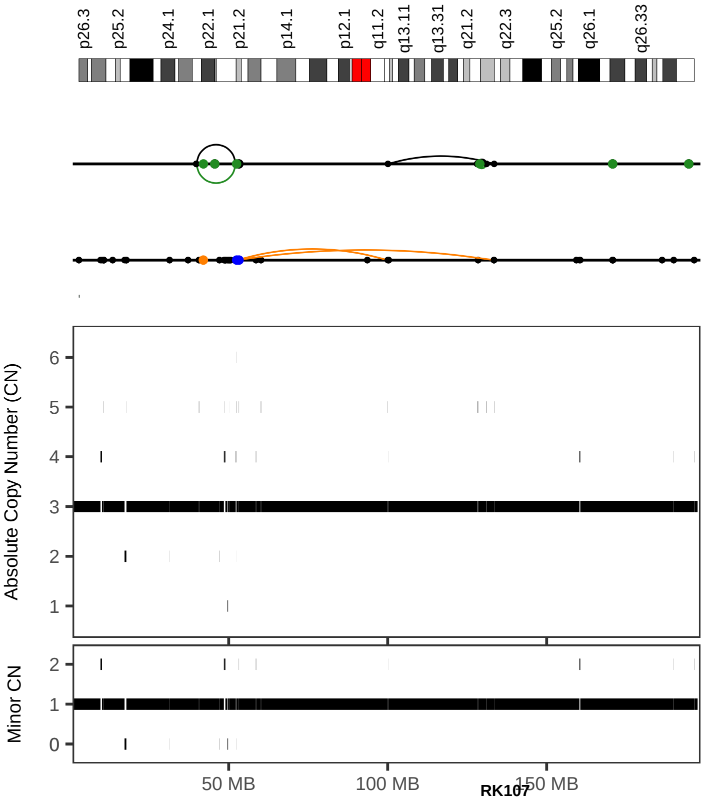

	RK107
Cancer type	Liver−HCC
Position	3:39802700−133489711
Interleaved intrachr. SVs	3
Total SVs (intrachr. + transl.)	15
SV types	DEL: 2; DUP: 0; h2hINV: 2;<br>t2tINV: 2; TRA: 9
SVs in sample	616
Oscillating CN (2 and 3 states)	9, 22
CN segments	40
FDR fragment joints	0.6776251
FDR chr. breakp. enrich.	0.16
Linked to chrs	12:565831−121912249;2:2119500−211577973
Purity, ploidy	0.68, 3.19

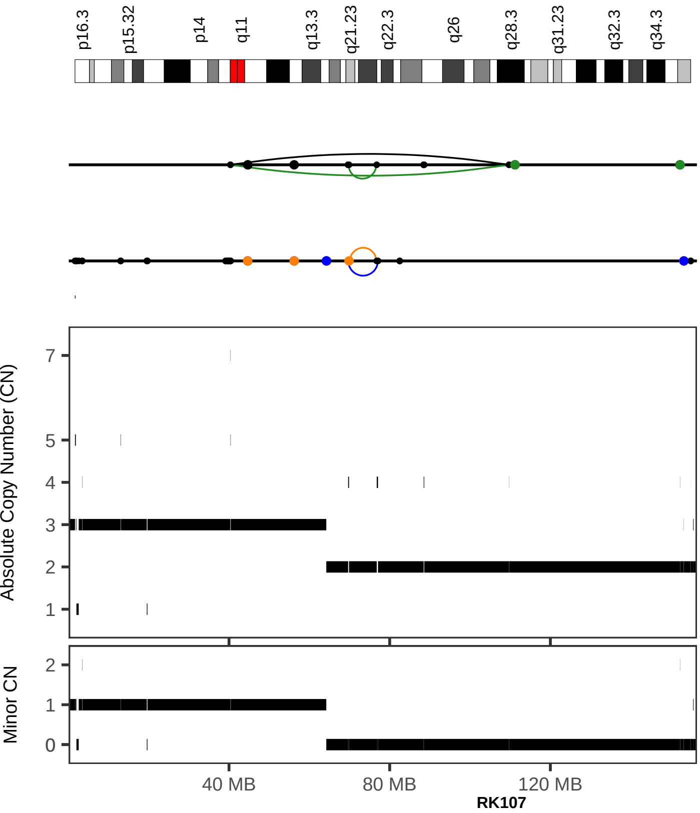

	RK107
Cancer type	Liver−HCC
Position	4:40323309−109722573
Interleaved intrachr. SVs	3
Total SVs (intrachr. + transl.)	13
SV types	DEL: 0; DUP: 1; h2hINV: 1;<br>t2tINV: 1; TRA: 10
SVs in sample	616
Oscillating CN (2 and 3 states)	9, 10
CN segments	13
FDR fragment joints	0.7995907
FDR chr. breakp. enrich.	1
Linked to chrs	3:39802700−133489710;
Purity, ploidy	0.68, 3.19

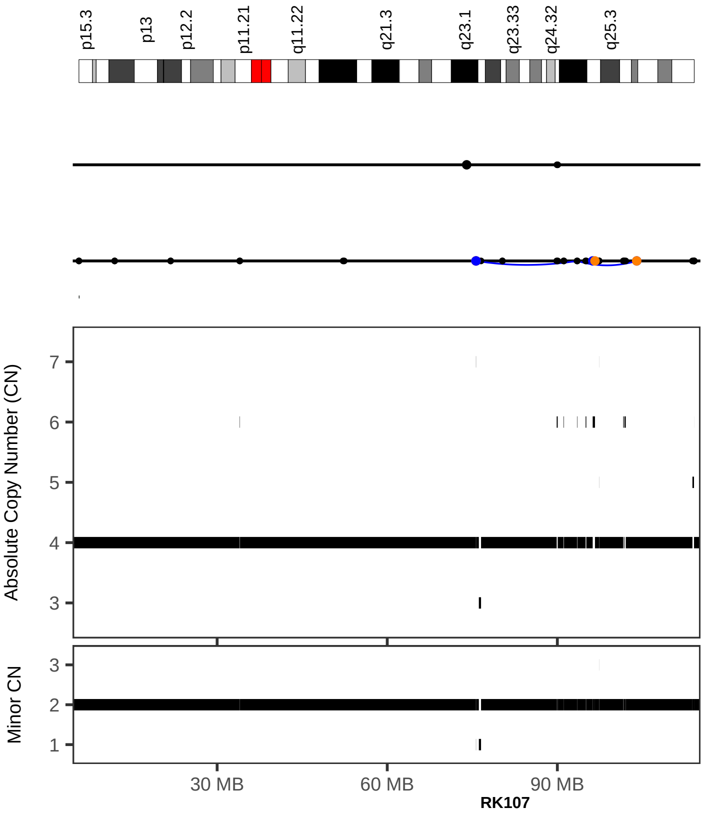

	RK107
Cancer type	Liver−HCC
Position	10:75643021−104011740
Interleaved intrachr. SVs	2
Total SVs (intrachr. + transl.)	10
SV types	DEL: 0; DUP: 4; h2hINV: 0;<br>t2tINV: 0; TRA: 6
SVs in sample	616
Oscillating CN (2 and 3 states)	11, 13
CN segments	21
FDR fragment joints	0.1790694
FDR chr. breakp. enrich.	1
Linked to chrs	1:26503377−220160008;
Purity, ploidy	0.68, 3.19

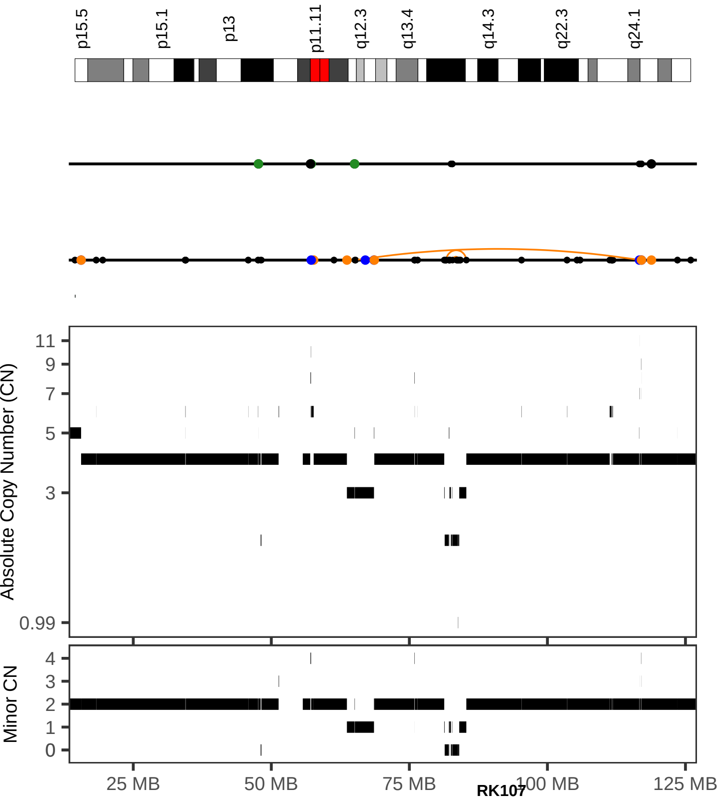

RK107

Cancer type	Liver−HCC
Position	11:65126119−117045449
Interleaved intrachr. SVs	5
Total SVs (intrachr. + transl.)	11
SV types	DEL: 2; DUP: 1; h2hINV: 1; t2tINV: 1; TRA: 6
SVs in sample	616
Oscillating CN (2 and 3 states)	11, 12
CN segments	41
FDR fragment joints	0.615458
FDR chr. breakp. enrich.	0
Linked to chrs	12:565831−121912249;20:14679819−52532840
Purity, ploidy	0.68, 3.19

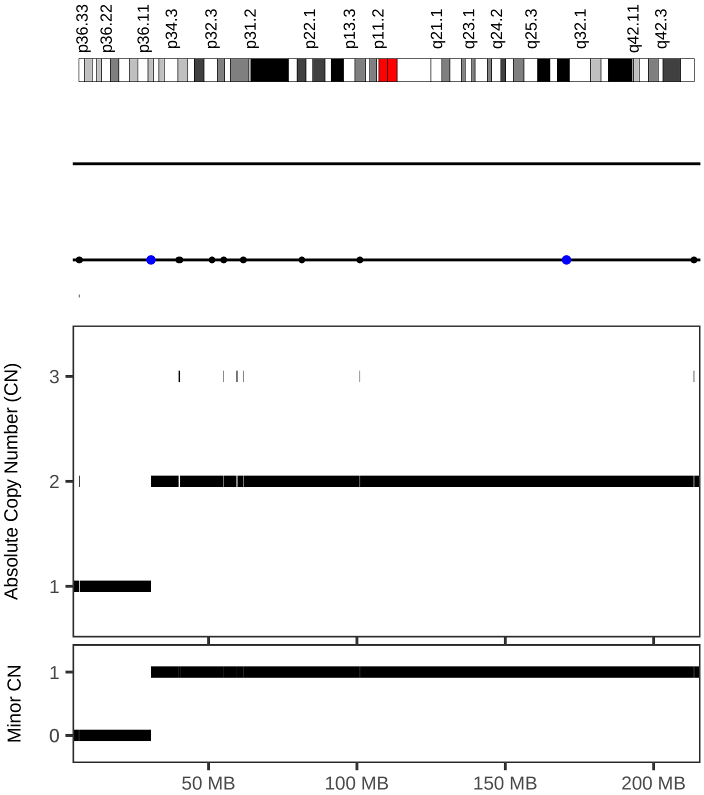

RK111

Cancer type	Liver−HCC
Position	1:6300063−213681406
Interleaved intrachr. SVs	1
Total SVs (intrachr. + transl.)	9
SV types	DEL: 1; DUP: 6; h2hINV: 0; t2tINV: 0; TRA: 2
SVs in sample	176
Oscillating CN (2 and 3 states)	13, 15
CN segments	15
FDR fragment joints	0.02438658
FDR chr. breakp. enrich.	0.58
Linked to chrs	
Purity, ploidy	0.9, 1.83

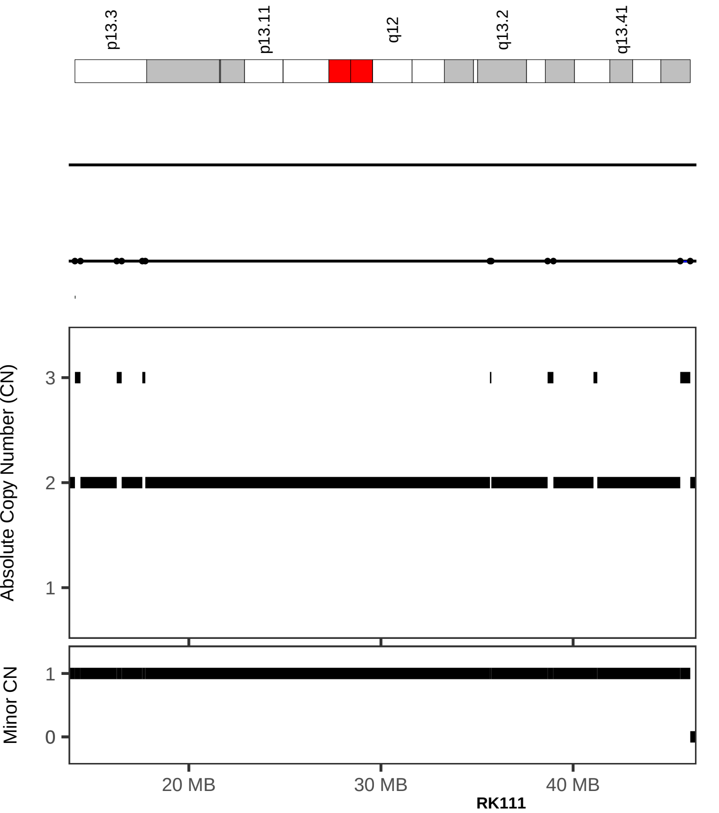

RK111

Cancer type	Liver−HCC
Position	19:14071846−46096435
Interleaved intrachr. SVs	1
Total SVs (intrachr. + transl.)	6
SV types	DEL: 0; DUP: 6; h2hINV: 0; t2tINV: 0; TRA: 0
SVs in sample	176
Oscillating CN (2 and 3 states)	14, 14
CN segments	14
FDR fragment joints	0.04139017
FDR chr. breakp. enrich.	0.25
Linked to chrs	
Purity, ploidy	0.9, 1.83

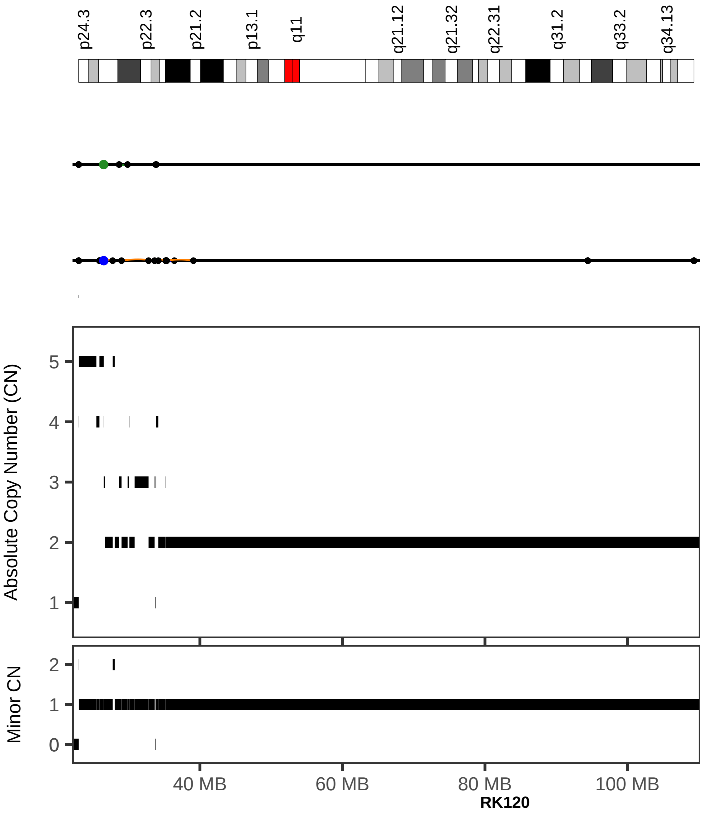

RK120

Cancer type	Liver−HCC
Position	9:28658249−39086583
Interleaved intrachr. SVs	6
Total SVs (intrachr. + transl.)	6
SV types	DEL: 4; DUP: 1; h2hINV: 0; t2tINV: 1; TRA: 0
SVs in sample	52
Oscillating CN (2 and 3 states)	4, 8
CN segments	14
FDR fragment joints	0.5435077
FDR chr. breakp. enrich.	0
Linked to chrs	
Purity, ploidy	0.9, 1.9

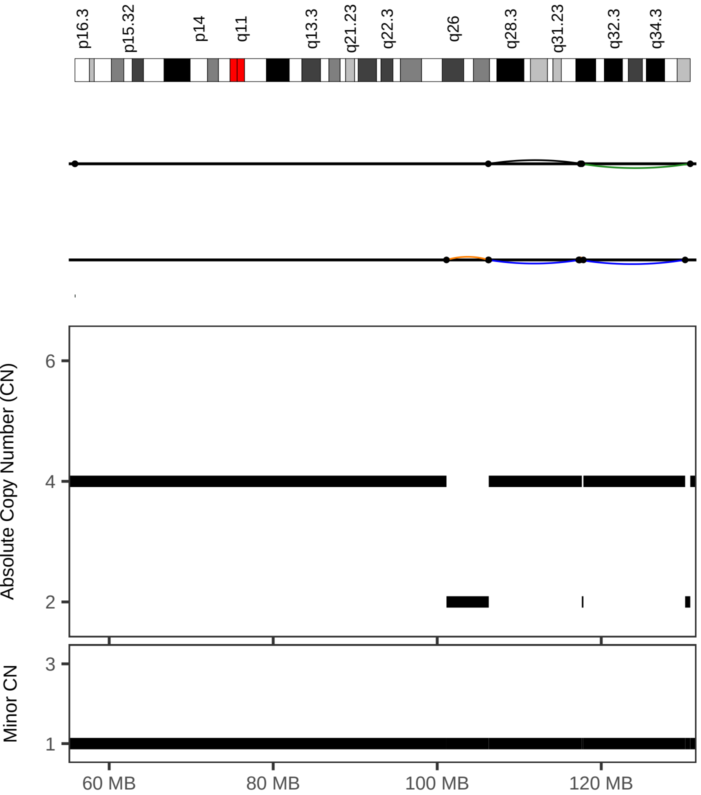

RK121

Cancer type	Liver−HCC
Position	4:101126283−130852966
Interleaved intrachr. SVs	6
Total SVs (intrachr. + transl.)	6
SV types	DEL: 2; DUP: 2; h2hINV: 1; t2tINV: 1; TRA: 0
SVs in sample	35
Oscillating CN (2 and 3 states)	6, 6
CN segments	6
FDR fragment joints	0.9284301
FDR chr. breakp. enrich.	0.04
Linked to chrs	
Purity, ploidy	0.84, 3.92

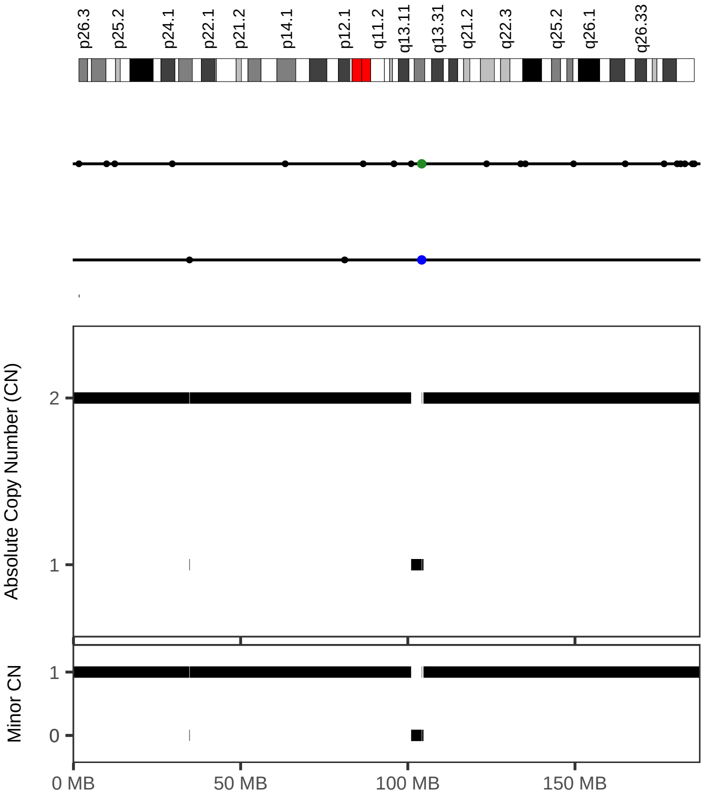

RK122

Cancer type	Liver−HCC
Position	3:100989378−104690117
Interleaved intrachr. SVs	7
Total SVs (intrachr. + transl.)	9
SV types	DEL: 1; DUP: 3; h2hINV: 1; t2tINV: 2; TRA: 2
SVs in sample	256
Oscillating CN (2 and 3 states)	6, 6
CN segments	6
FDR fragment joints	0.6776251
FDR chr. breakp. enrich.	0.05
Linked to chrs	
Purity, ploidy	0.87, 1.91

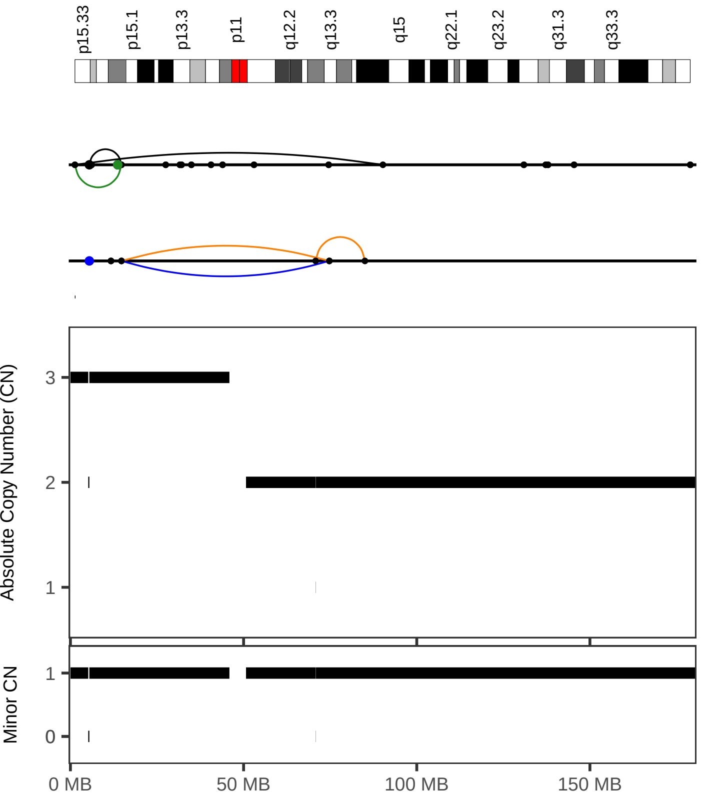

RK122

Cancer type	Liver−HCC
Position	5:1296781−85032688
Interleaved intrachr. SVs	6
Total SVs (intrachr. + transl.)	10
SV types	DEL: 3; DUP: 1; h2hINV: 1; t2tINV: 1; TRA: 4
SVs in sample	256
Oscillating CN (2 and 3 states)	4, 6
CN segments	6
FDR fragment joints	0.9625775
FDR chr. breakp. enrich.	0.08
Linked to chrs	
Purity, ploidy	0.87, 1.91

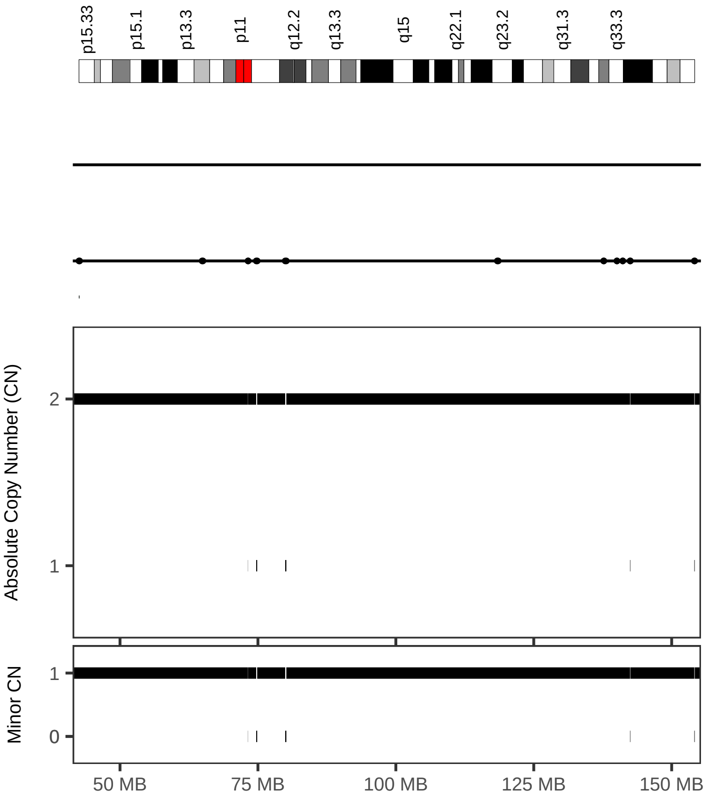

RK148

Cancer type	Liver−HCC
Position	5:42562425−154180295
Interleaved intrachr. SVs	1
Total SVs (intrachr. + transl.)	10
SV types	DEL: 10; DUP: 0; h2hINV: 0; t2tINV: 0; TRA: 0
SVs in sample	195
Oscillating CN (2 and 3 states)	11, 11
CN segments	11
FDR fragment joints	0.0008205066
FDR chr. breakp. enrich.	1
Linked to chrs	
Purity, ploidy	0.9, 1.98

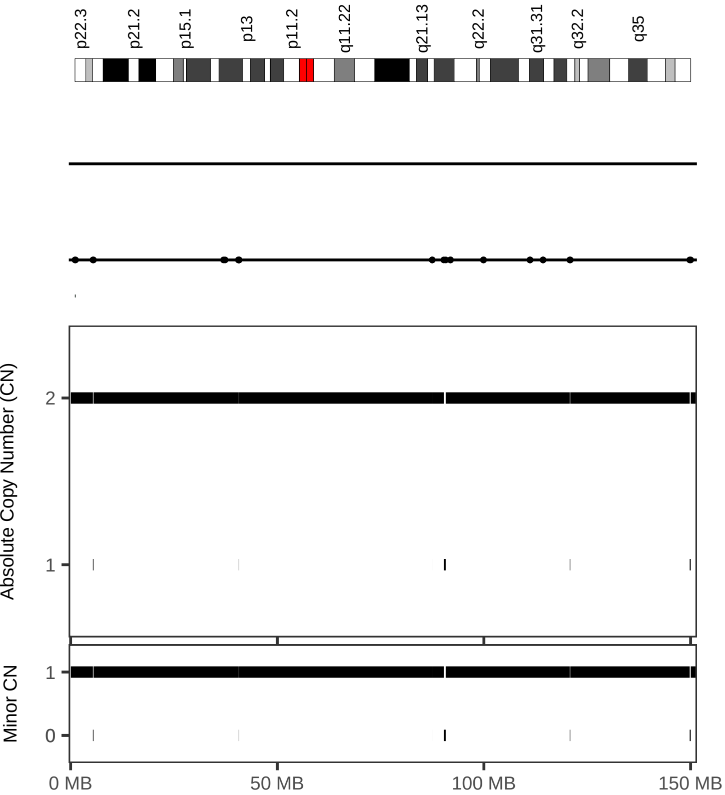

	RK148
Cancer type	Liver−HCC
Position	7:1042254−150037666
Interleaved intrachr. SVs	1
Total SVs (intrachr. + transl.)	12
SV types	DEL: 12; DUP: 0; h2hINV: 0; t2tINV: 0; TRA: 0
SVs in sample	195
Oscillating CN (2 and 3 states)	13, 13
CN segments	13
FDR fragment joints	7.730001e−05
FDR chr. breakp. enrich.	0.67
Linked to chrs	
Purity, ploidy	0.9, 1.98

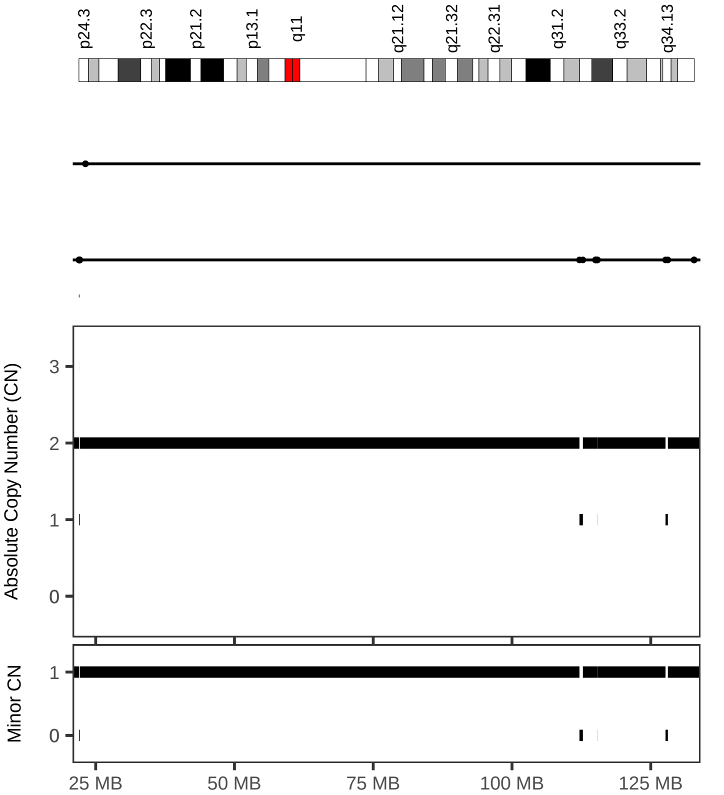

	RK148
Cancer type	Liver−HCC
Position	9:21975039−132836763
Interleaved intrachr. SVs	1
Total SVs (intrachr. + transl.)	8
SV types	DEL: 7; DUP: 0; h2hINV: 1; t2tINV: 0; TRA: 0
SVs in sample	195
Oscillating CN (2 and 3 states)	7, 11
CN segments	11
FDR fragment joints	0.05741733
FDR chr. breakp. enrich.	0.8
Linked to chrs	
Purity, ploidy	0.9, 1.98

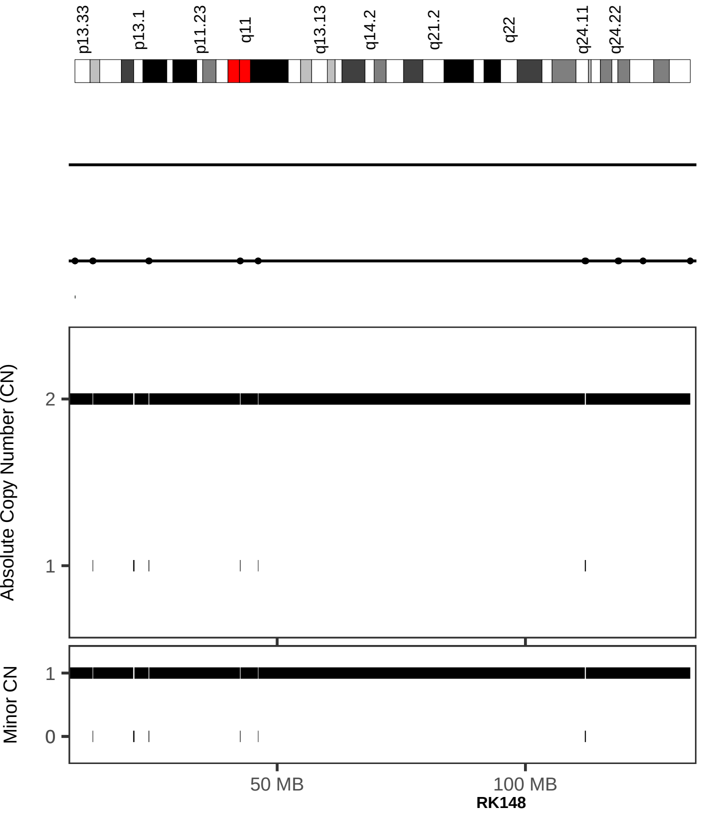

	RK148
Cancer type	Liver−HCC
Position	12:9270007−123718833
Interleaved intrachr. SVs	1
Total SVs (intrachr. + transl.)	8
SV types	DEL: 8; DUP: 0; h2hINV: 0; t2tINV: 0; TRA: 0
SVs in sample	195
Oscillating CN (2 and 3 states)	13, 13
CN segments	13
FDR fragment joints	0.006736868
FDR chr. breakp. enrich.	1
Linked to chrs	
Purity, ploidy	0.9, 1.98

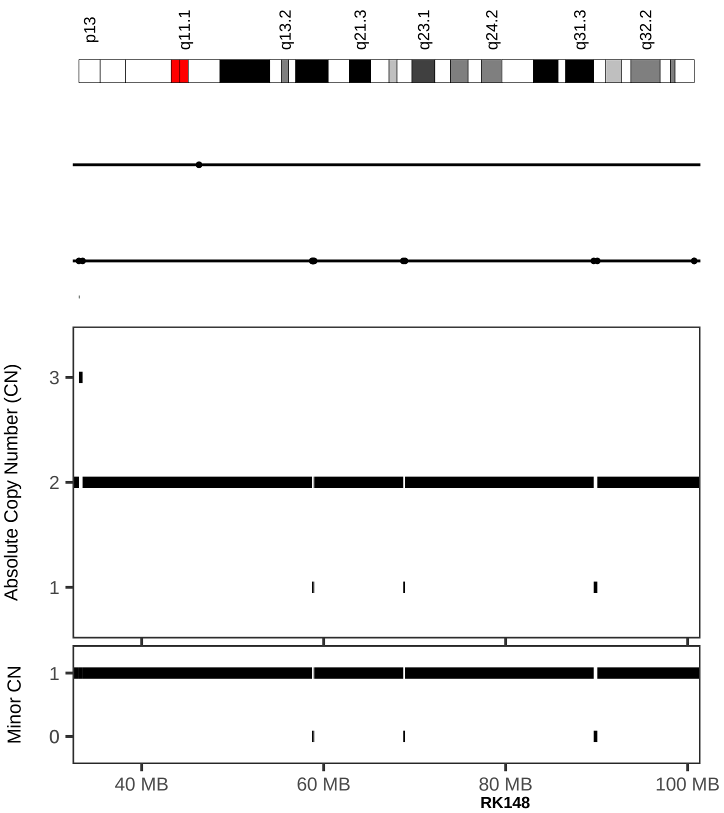

	RK148
Cancer type	Liver−HCC
Position	14:33101844−100726901
Interleaved intrachr. SVs	1
Total SVs (intrachr. + transl.)	7
SV types	DEL: 6; DUP: 1; h2hINV: 0; t2tINV: 0; TRA: 0
SVs in sample	195
Oscillating CN (2 and 3 states)	9, 10
CN segments	10
FDR fragment joints	0.1278363
FDR chr. breakp. enrich.	0.66
Linked to chrs	
Purity, ploidy	0.9, 1.98

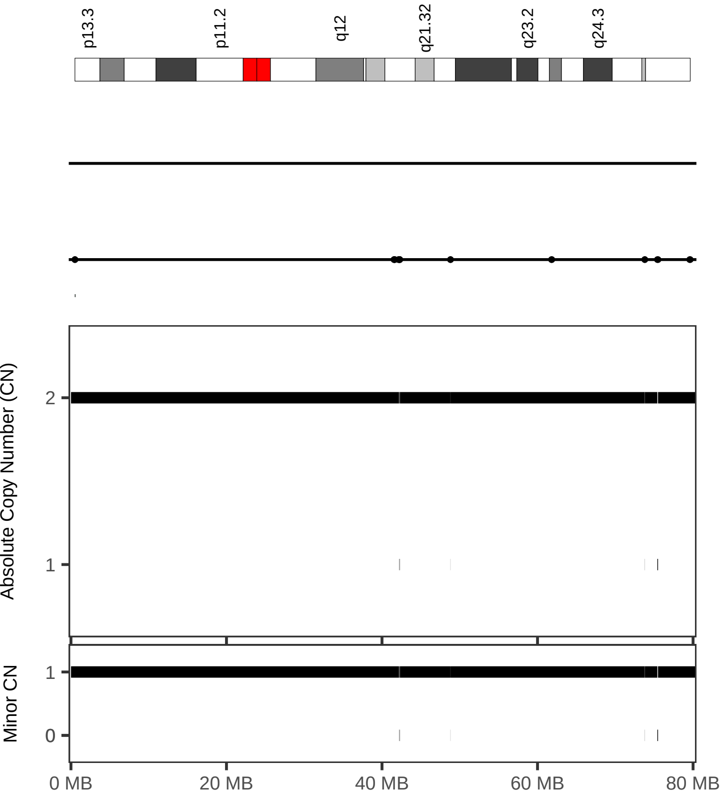

RK148

Cancer type	Liver−HCC
Position	17:41536382−79640132
Interleaved intrachr. SVs	1
Total SVs (intrachr. + transl.)	7
SV types	DEL: 7; DUP: 0; h2hINV: 0; t2tINV: 0; TRA: 0
SVs in sample	195
Oscillating CN (2 and 3 states)	11, 11
CN segments	11
FDR fragment joints	0.01743054
FDR chr. breakp. enrich.	0.09
Linked to chrs	
Purity, ploidy	0.9, 1.98

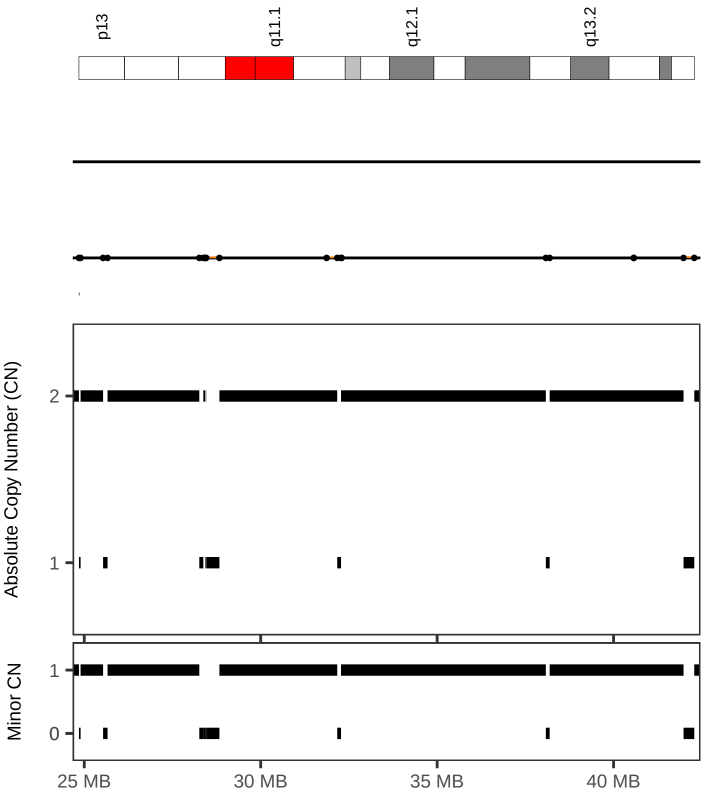

RK148

Cancer type	Liver−HCC
Position	22:24848675−42286407
Interleaved intrachr. SVs	1
Total SVs (intrachr. + transl.)	9
SV types	DEL: 9; DUP: 0; h2hINV: 0; t2tINV: 0; TRA: 0
SVs in sample	195
Oscillating CN (2 and 3 states)	16, 16
CN segments	16
FDR fragment joints	0.002298705
FDR chr. breakp. enrich.	0
Linked to chrs	
Purity, ploidy	0.9, 1.98

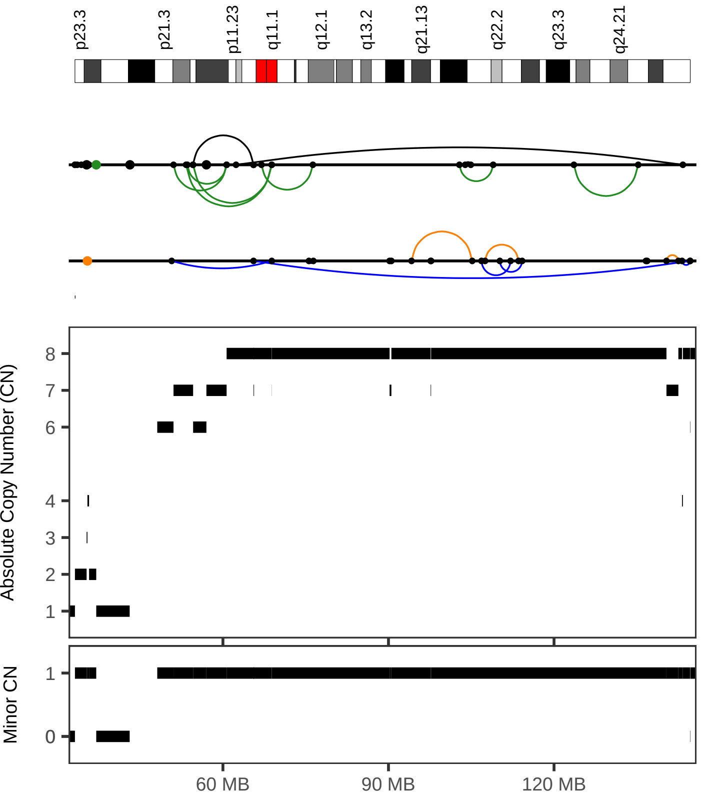

RK177

Cancer type	Liver−HCC
Position	8:50712276−144686189
Interleaved intrachr. SVs	11
Total SVs (intrachr. + transl.)	12
SV types	DEL: 0; DUP: 4; h2hINV: 2; t2tINV: 5; TRA: 1
SVs in sample	139
Oscillating CN (2 and 3 states)	14, 17
CN segments	21
FDR fragment joints	0.5435077
FDR chr. breakp. enrich.	0
Linked to chrs	
Purity, ploidy	0.72, 2

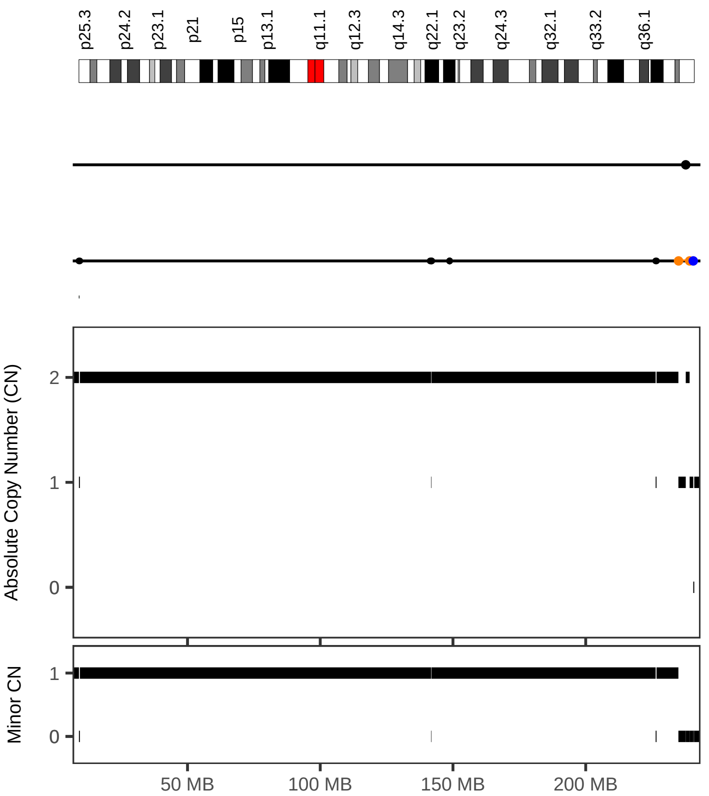

RK195

Cancer type	Liver−HCC
Position	2:9090277−240897263
Interleaved intrachr. SVs	1
Total SVs (intrachr. + transl.)	9
SV types	DEL: 5; DUP: 0; h2hINV: 0; t2tINV: 0; TRA: 4
SVs in sample	121
Oscillating CN (2 and 3 states)	9, 11
CN segments	11
FDR fragment joints	0.1488029
FDR chr. breakp. enrich.	0.91
Linked to chrs	
Purity, ploidy	0.8, 2.02

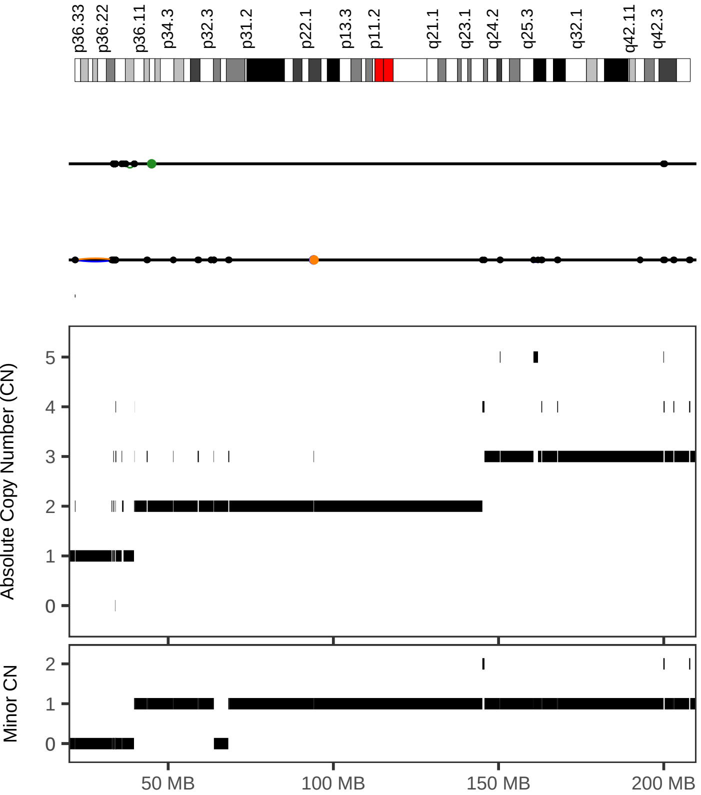

RK211

Cancer type	Liver−HCC
Position	1:21930164−34131783
Interleaved intrachr. SVs	55
Total SVs (intrachr. + transl.)	55
SV types	DEL: 15; DUP: 18; h2hINV: 10; t2tINV: 12; TRA: 0
SVs in sample	227
Oscillating CN (2 and 3 states)	5, 13
CN segments	21
FDR fragment joints	0.6776251
FDR chr. breakp. enrich.	0
Linked to chrs	
Purity, ploidy	0.84, 1.82

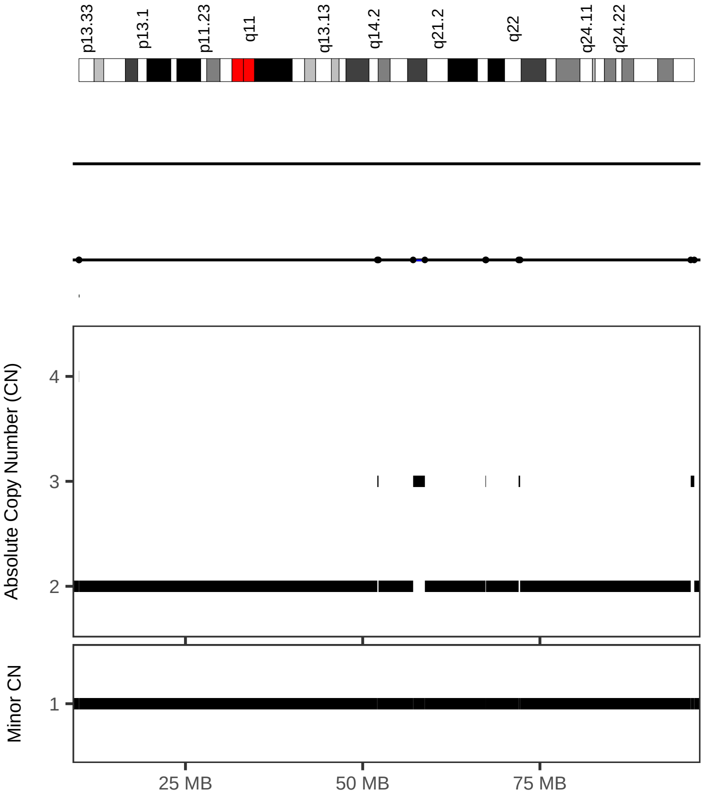

RK211

Cancer type	Liver−HCC
Position	12:9969409−96771043
Interleaved intrachr. SVs	1
Total SVs (intrachr. + transl.)	6
SV types	DEL: 0; DUP: 6; h2hINV: 0; t2tINV: 0; TRA: 0
SVs in sample	227
Oscillating CN (2 and 3 states)	11, 12
CN segments	12
FDR fragment joints	0.04139017
FDR chr. breakp. enrich.	0.43
Linked to chrs	
Purity, ploidy	0.84, 1.82

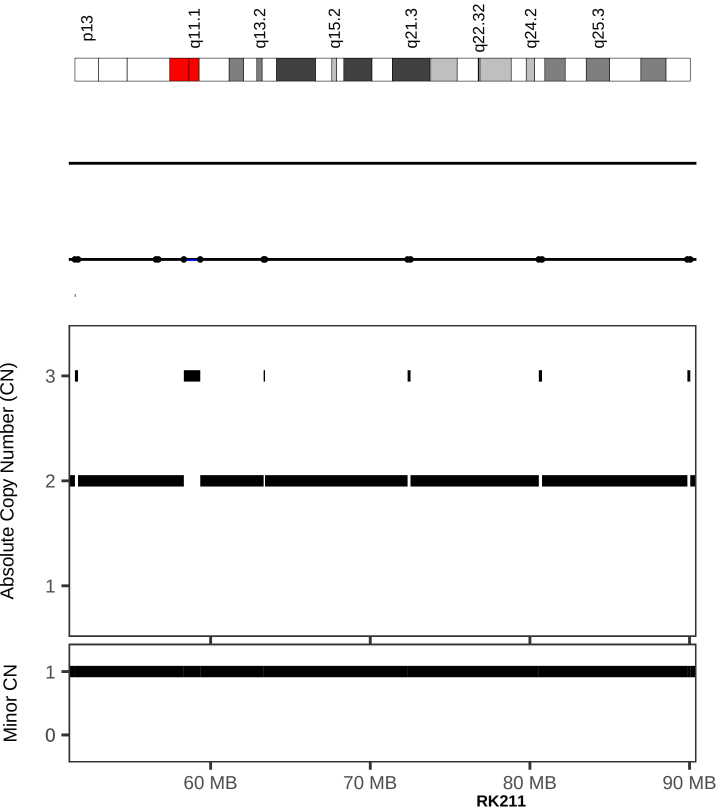

RK211

Cancer type	Liver−HCC
Position	15:51499432−90044037
Interleaved intrachr. SVs	1
Total SVs (intrachr. + transl.)	7
SV types	DEL: 0; DUP: 7; h2hINV: 0; t2tINV: 0; TRA: 0
SVs in sample	227
Oscillating CN (2 and 3 states)	12, 12
CN segments	12
FDR fragment joints	0.01743054
FDR chr. breakp. enrich.	0.89
Linked to chrs	
Purity, ploidy	0.84, 1.82

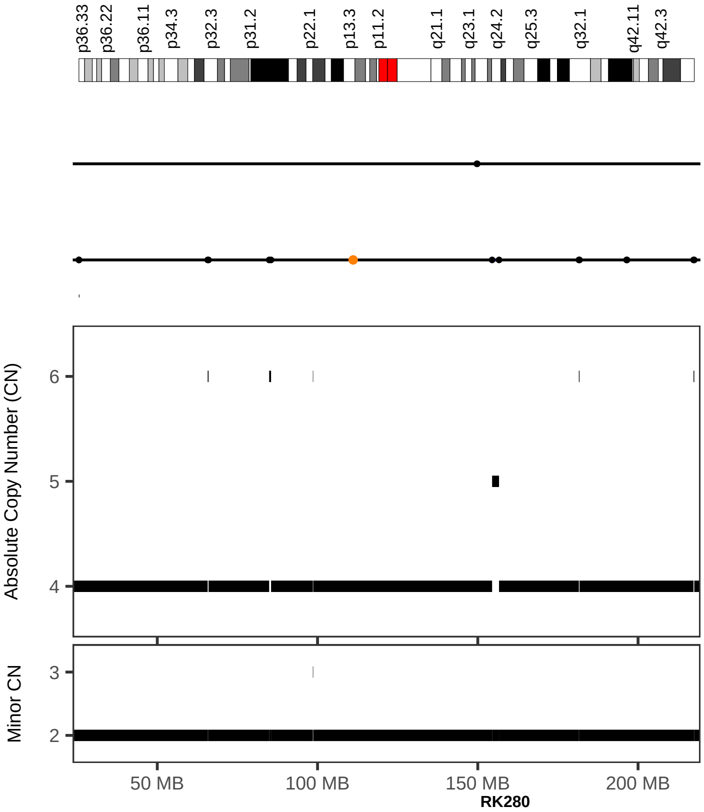

RK280

Cancer type	Liver−HCC
Position	1:25548581−217538998
Interleaved intrachr. SVs	1
Total SVs (intrachr. + transl.)	9
SV types	DEL: 0; DUP: 7; h2hINV: 0; t2tINV: 1; TRA: 1
SVs in sample	111
Oscillating CN (2 and 3 states)	7, 13
CN segments	13
FDR fragment joints	0.1488029
FDR chr. breakp. enrich.	0.99
Linked to chrs	
Purity, ploidy	0.87, 3.75

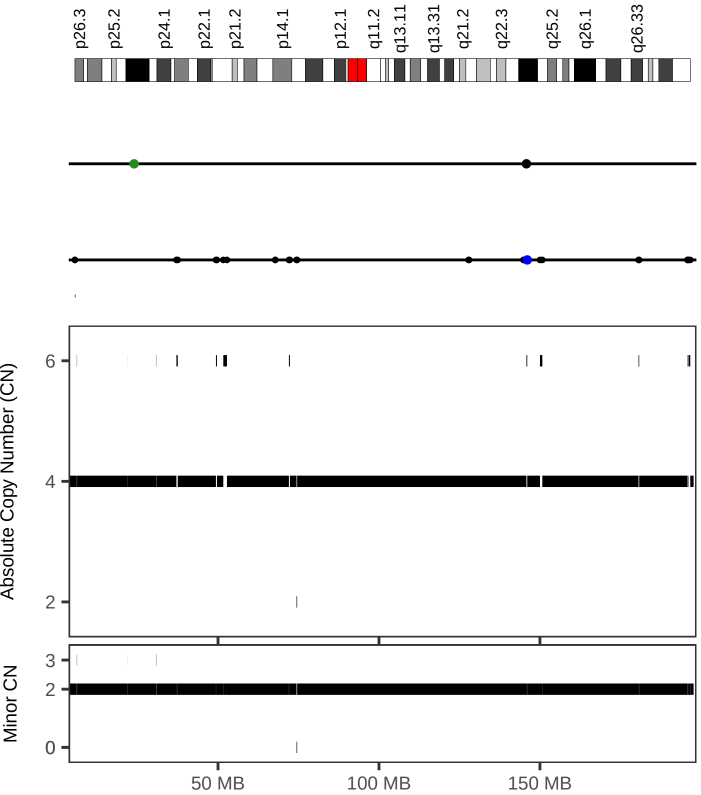

RK280

Cancer type	Liver−HCC
Position	3:37076321−196695607
Interleaved intrachr. SVs	1
Total SVs (intrachr. + transl.)	13
SV types	DEL: 3; DUP: 8; h2hINV: 0; t2tINV: 0; TRA: 2
SVs in sample	111
Oscillating CN (2 and 3 states)	11, 20
CN segments	20
FDR fragment joints	0.1013295
FDR chr. breakp. enrich.	0.04
Linked to chrs	
Purity, ploidy	0.87, 3.75

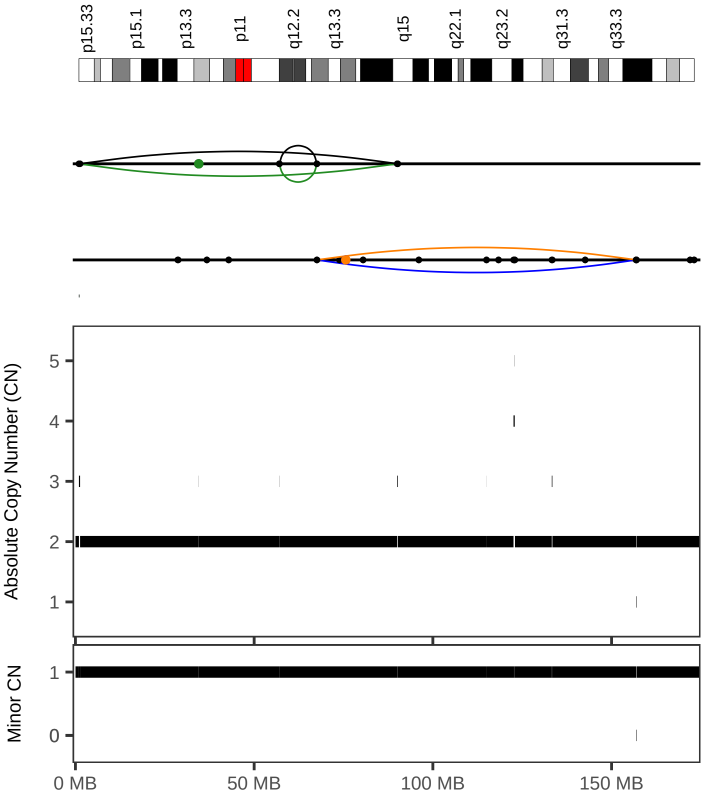

RK285

Cancer type	Liver−HCC
Position	5:973432−156988868
Interleaved intrachr. SVs	4
Total SVs (intrachr. + transl.)	8
SV types	DEL: 1; DUP: 1; h2hINV: 1; t2tINV: 1; TRA: 4
SVs in sample	352
Oscillating CN (2 and 3 states)	10, 11
CN segments	18
FDR fragment joints	1
FDR chr. breakp. enrich.	0.75
Linked to chrs	6:158550050−163021557;
Purity, ploidy	0.81, 1.9

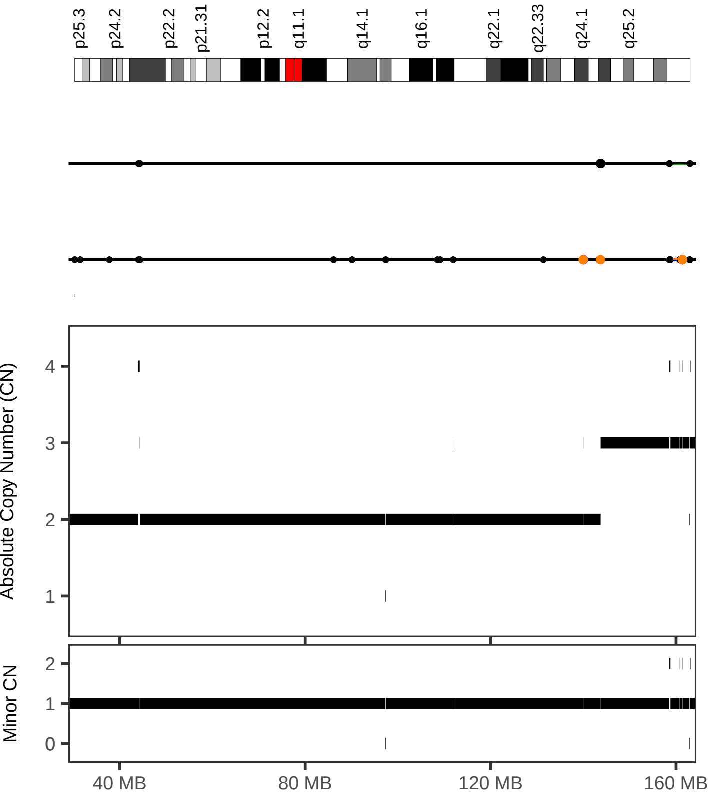

RK285

Cancer type	Liver−HCC
Position	6:158550050−163021558
Interleaved intrachr. SVs	8
Total SVs (intrachr. + transl.)	10
SV types	DEL: 1; DUP: 3; h2hINV: 1; t2tINV: 3; TRA: 2
SVs in sample	352
Oscillating CN (2 and 3 states)	7, 11
CN segments	11
FDR fragment joints	0.6776251
FDR chr. breakp. enrich.	0.01
Linked to chrs	8:3686486−121889649;
Purity, ploidy	0.81, 1.9

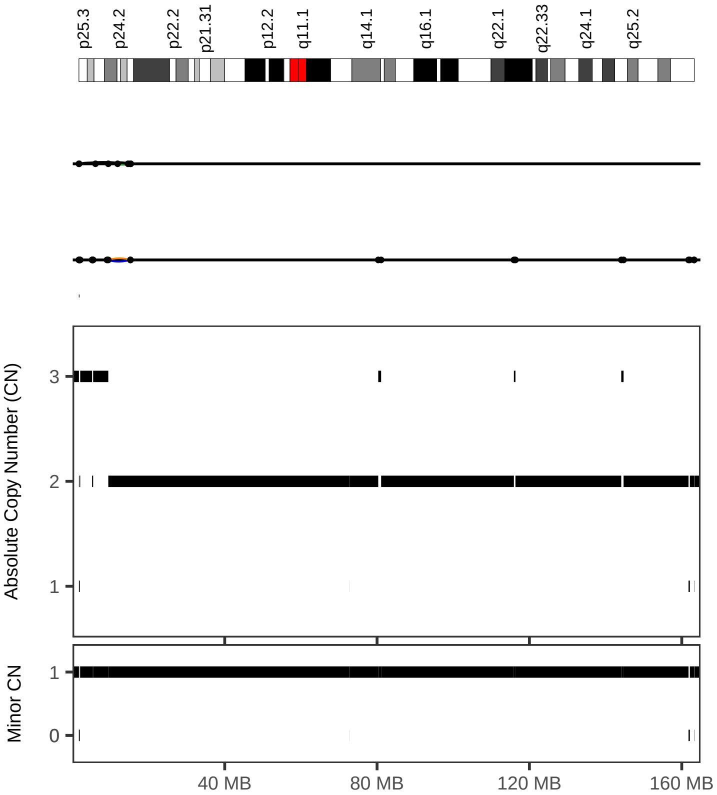

368e23f0−e573−4547−bf5a−14080baf737b

Cancer type	Lung−AdenoCA
Position	6:1715943−15332733
Interleaved intrachr. SVs	8
Total SVs (intrachr. + transl.)	8
SV types	DEL: 3; DUP: 1; h2hINV: 3; t2tINV: 1; TRA: 0
SVs in sample	170
Oscillating CN (2 and 3 states)	5, 7
CN segments	7
FDR fragment joints	0.6776251
FDR chr. breakp. enrich.	0.34
Linked to chrs	
Purity, ploidy	0.36, 2.06

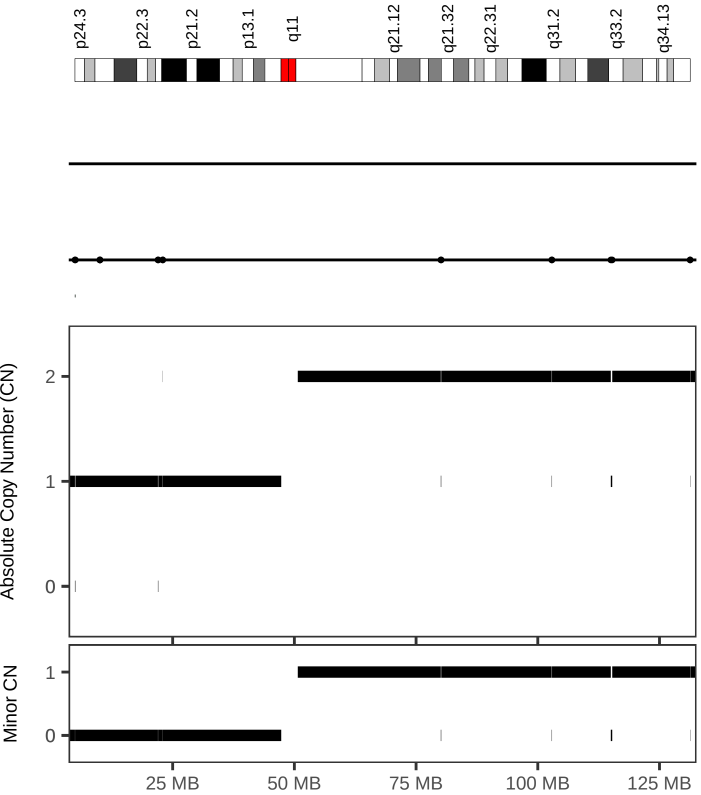

368e23f0−e573−4547−bf5a−14080baf737b

Cancer type	Lung−AdenoCA
Position	9:4921030−131320749
Interleaved intrachr. SVs	1
Total SVs (intrachr. + transl.)	9
SV types	DEL: 7; DUP: 2; h2hINV: 0; t2tINV: 0; TRA: 0
SVs in sample	170
Oscillating CN (2 and 3 states)	12, 15
CN segments	15
FDR fragment joints	0.1178349
FDR chr. breakp. enrich.	0.57
Linked to chrs	
Purity, ploidy	0.36, 2.06

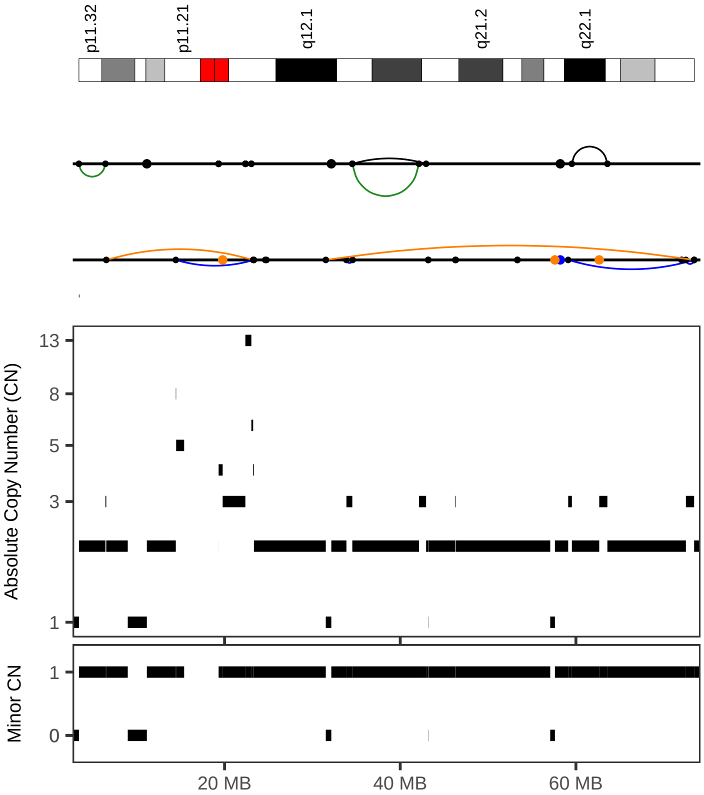

9f81c602−8afa−4588−b0b6−6e5a1a128d5a

Cancer type	Lung−AdenoCA
Position	18:31523695−73471077
Interleaved intrachr. SVs	3
Total SVs (intrachr. + transl.)	11
SV types	DEL: 1; DUP: 3; h2hINV: 1; t2tINV: 1; TRA: 5
SVs in sample	84
Oscillating CN (2 and 3 states)	7, 18
CN segments	18
FDR fragment joints	0.7425546
FDR chr. breakp. enrich.	0
Linked to chrs	
Purity, ploidy	0.77, 1.79

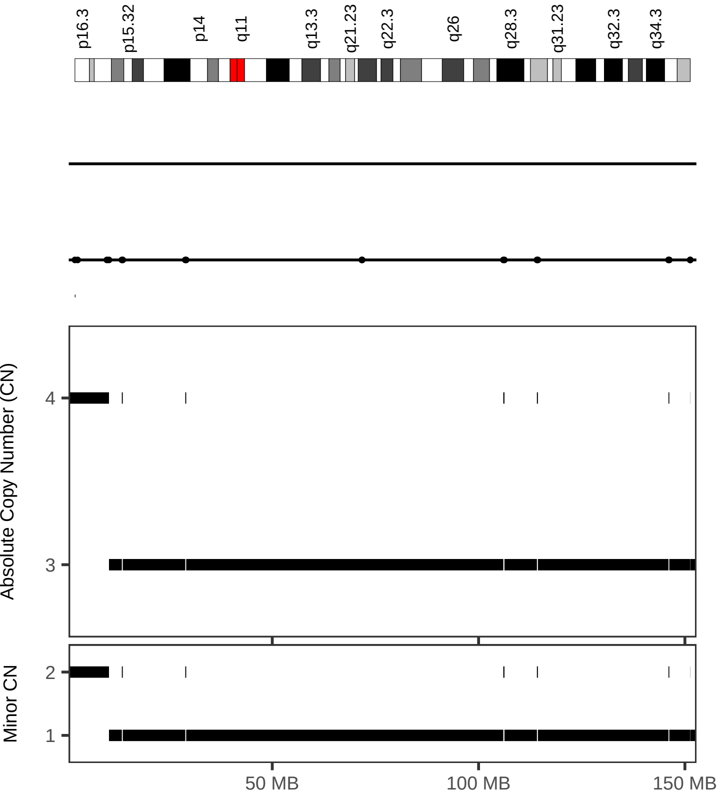

c4d1e105−28b7−48df−abef−fbe09782fdb2

Cancer type	Lung−AdenoCA
Position	4:2178913−151296713
Interleaved intrachr. SVs	1
Total SVs (intrachr. + transl.)	9
SV types	DEL: 1; DUP: 8; h2hINV: 0; t2tINV: 0; TRA: 0
SVs in sample	136
Oscillating CN (2 and 3 states)	14, 14
CN segments	14
FDR fragment joints	0.02438658
FDR chr. breakp. enrich.	1
Linked to chrs	
Purity, ploidy	0.82, 3.29

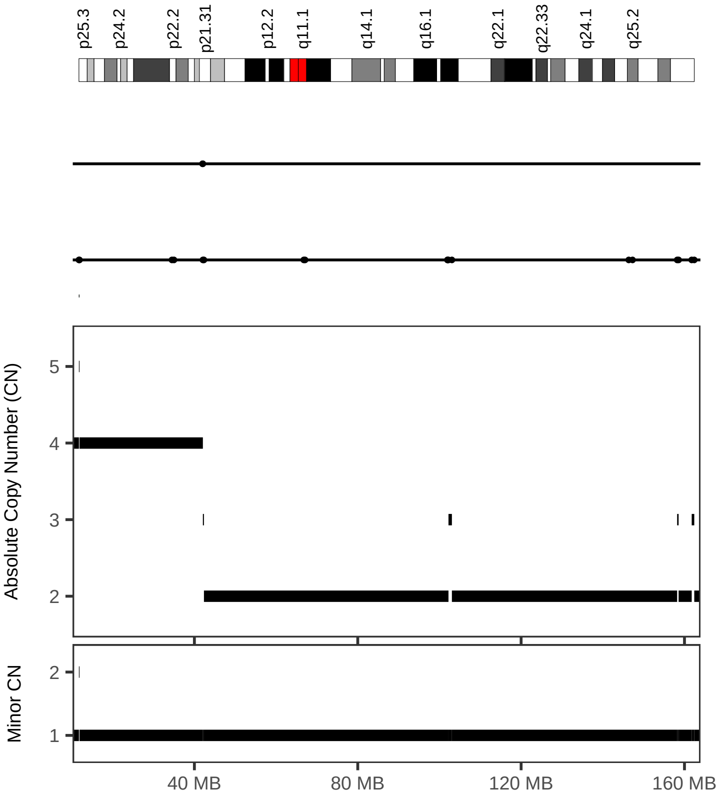

c4d1e105−28b7−48df−abef−fbe09782fdb2

Cancer type	Lung−AdenoCA
Position	6:11726247−162394717
Interleaved intrachr. SVs	1
Total SVs (intrachr. + transl.)	10
SV types	DEL: 0; DUP: 9; h2hINV: 1; t2tINV: 0; TRA: 0
SVs in sample	136
Oscillating CN (2 and 3 states)	8, 10
CN segments	10
FDR fragment joints	0.01020027
FDR chr. breakp. enrich.	0.71
Linked to chrs	
Purity, ploidy	0.82, 3.29

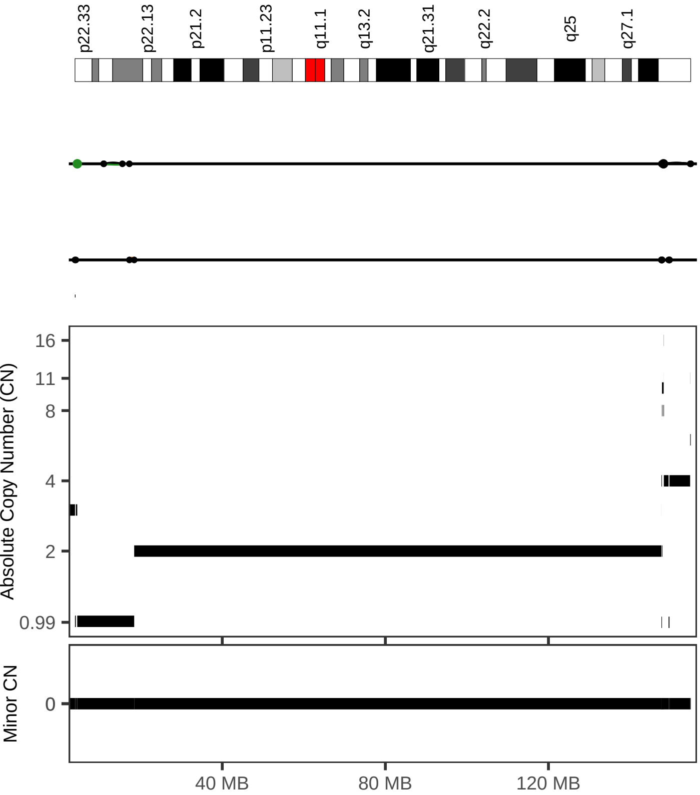

f77cf4d3−9829−4fd3−95a1−b29aed94b8

Cancer type	Lung−AdenoCA
Position	X:147715186−154903339
Interleaved intrachr. SVs	6
Total SVs (intrachr. + transl.)	7
SV types	DEL: 4; DUP: 0; h2hINV: 2; t2tINV: 0; TRA: 1
SVs in sample	155
Oscillating CN (2 and 3 states)	5, 5
CN segments	17
FDR fragment joints	0.615458
FDR chr. breakp. enrich.	0
Linked to chrs	
Purity, ploidy	0.52, 3.07

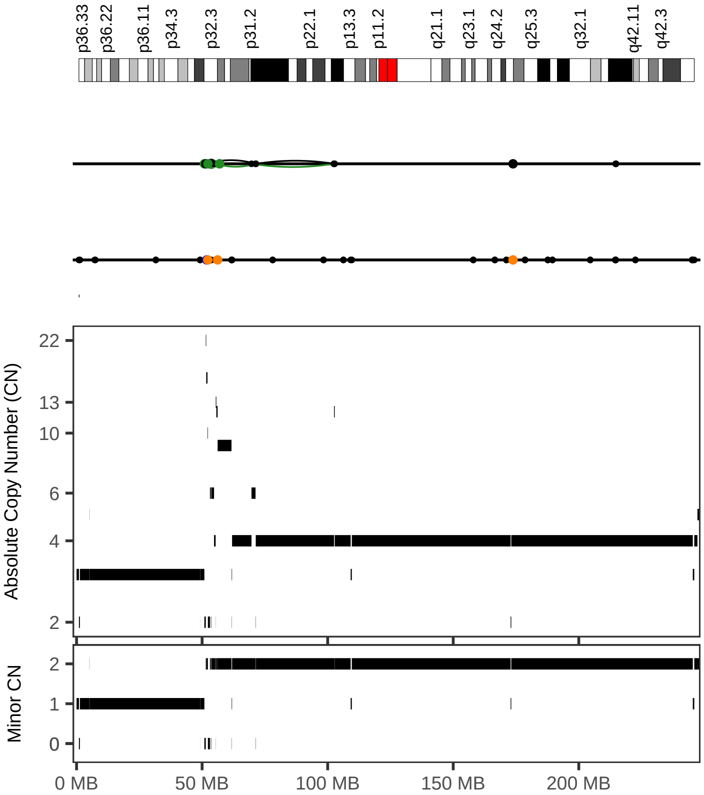

037c57d1−b4a5−45dc−bda4−0550461d321b

Cancer type	Lung−SCC
Position	1:51845048−55431534
Interleaved intrachr. SVs	11
Total SVs (intrachr. + transl.)	14
SV types	DEL: 4; DUP: 1; h2hINV: 4; t2tINV: 2; TRA: 3
SVs in sample	307
Oscillating CN (2 and 3 states)	4, 5
CN segments	9
FDR fragment joints	0.6776251
FDR chr. breakp. enrich.	0
Linked to chrs	8:24237409−142905706;
Purity, ploidy	0.35, 2.51

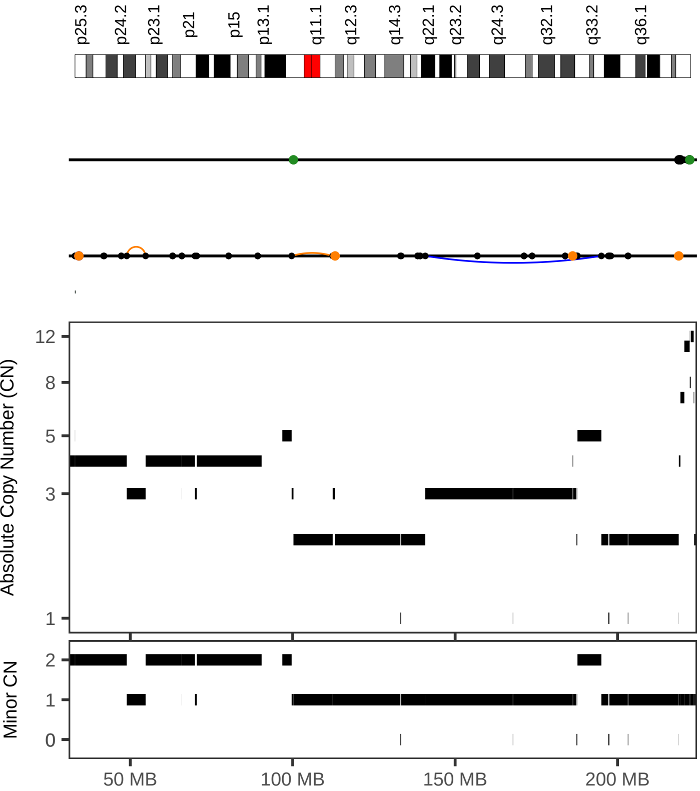

037c57d1−b4a5−45dc−bda4−0550461d321b

Cancer type	Lung−SCC
Position	2:32968870−222526259
Interleaved intrachr. SVs	1
Total SVs (intrachr. + transl.)	29
SV types	DEL: 15; DUP: 3; h2hINV: 0; t2tINV: 1; TRA: 10
SVs in sample	307
Oscillating CN (2 and 3 states)	7, 9
CN segments	36
FDR fragment joints	0.003601907
FDR chr. breakp. enrich.	0.19
Linked to chrs	16:3859370−85310133;
Purity, ploidy	0.35, 2.51

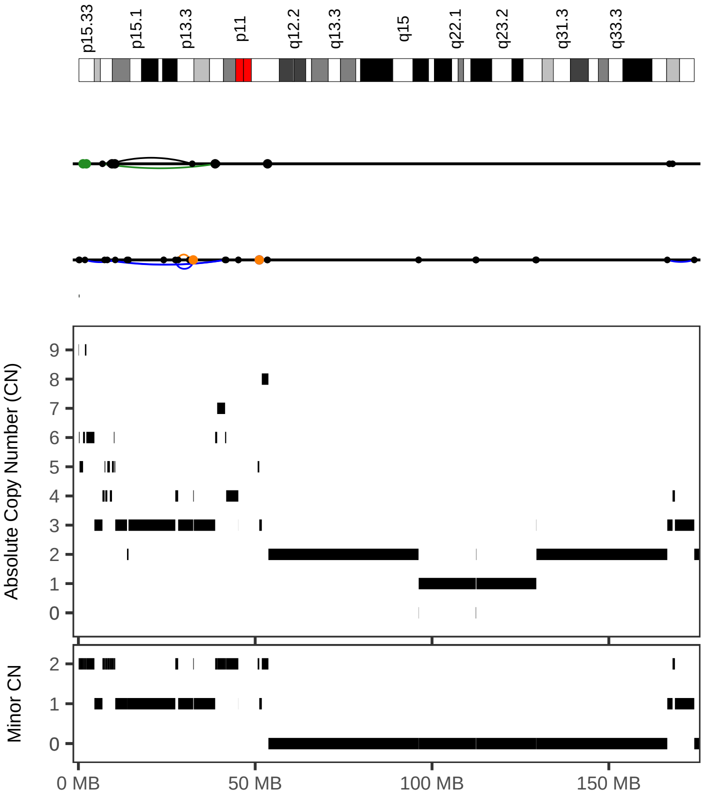

037c57d1−b4a5−45dc−bda4−0550461d321b

Cancer type	Lung−SCC
Position	5:1852334−41793671
Interleaved intrachr. SVs	6
Total SVs (intrachr. + transl.)	11
SV types	DEL: 0; DUP: 4; h2hINV: 1; t2tINV: 1; TRA: 5
SVs in sample	307
Oscillating CN (2 and 3 states)	6, 10
CN segments	22
FDR fragment joints	0.6776251
FDR chr. breakp. enrich.	0.04
Linked to chrs	2:32968870−222526258;
Purity, ploidy	0.35, 2.51

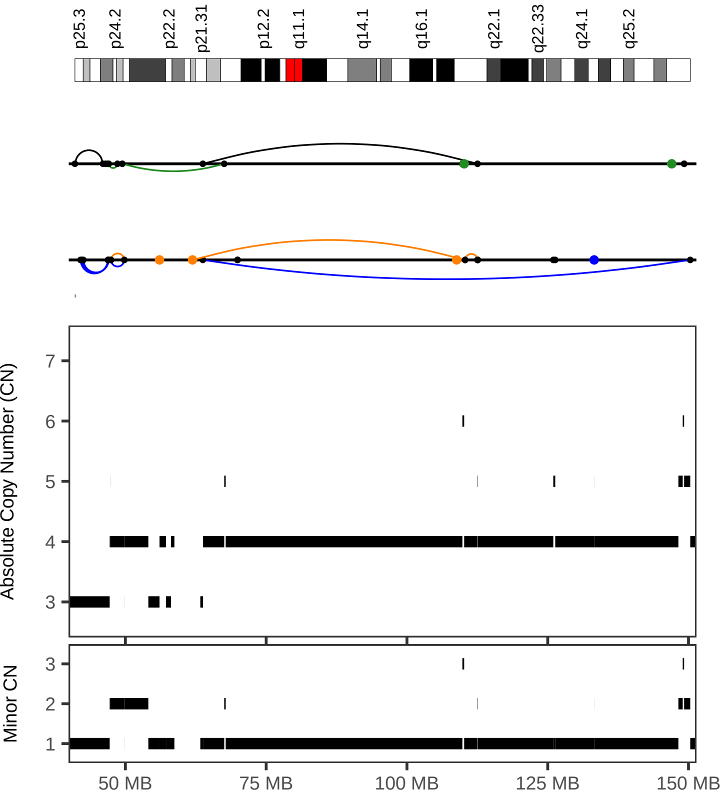

1f6b2aca−7357−40d1−ba7a−99227d9900a2

Cancer type	Lung−SCC
Position	6:47045197−150310817
Interleaved intrachr. SVs	9
Total SVs (intrachr. + transl.)	15
SV types	DEL: 3; DUP: 3; h2hINV: 1; t2tINV: 2; TRA: 6
SVs in sample	602
Oscillating CN (2 and 3 states)	9, 14
CN segments	26
FDR fragment joints	0.6776251
FDR chr. breakp. enrich.	0.24
Linked to chrs	11:46508302−111537991;
Purity, ploidy	0.86, 2.97

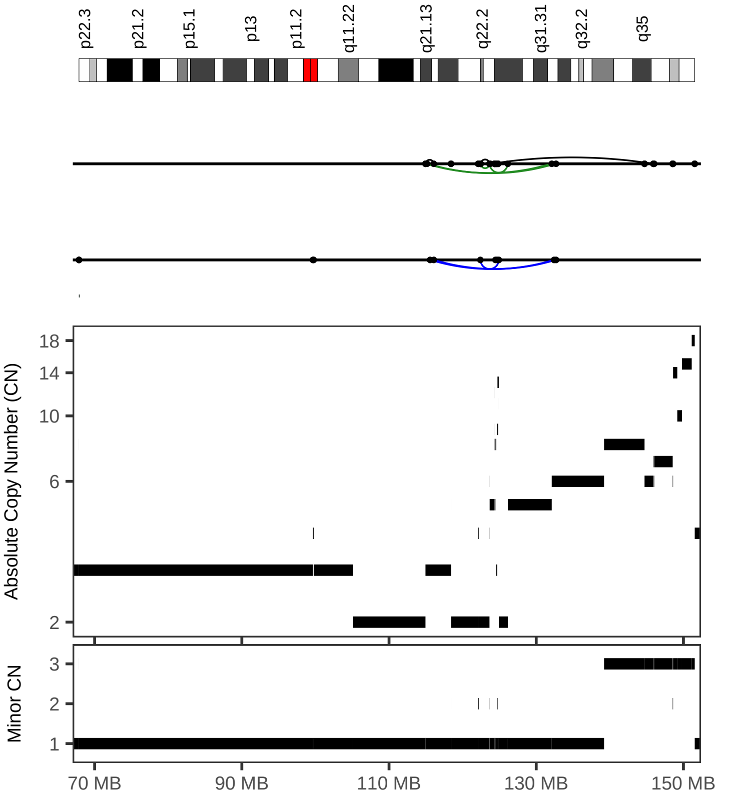

43abe847−4ba7−466e−8283−5d7b80b999a7

Cancer type	Lung−SCC
Position	7:114953114−146032540
Interleaved intrachr. SVs	10
Total SVs (intrachr. + transl.)	10
SV types	DEL: 0; DUP: 3; h2hINV: 3; t2tINV: 4; TRA: 0
SVs in sample	500
Oscillating CN (2 and 3 states)	4, 6
CN segments	26
FDR fragment joints	0.615458
FDR chr. breakp. enrich.	0.93
Linked to chrs	
Purity, ploidy	0.69, 3.74

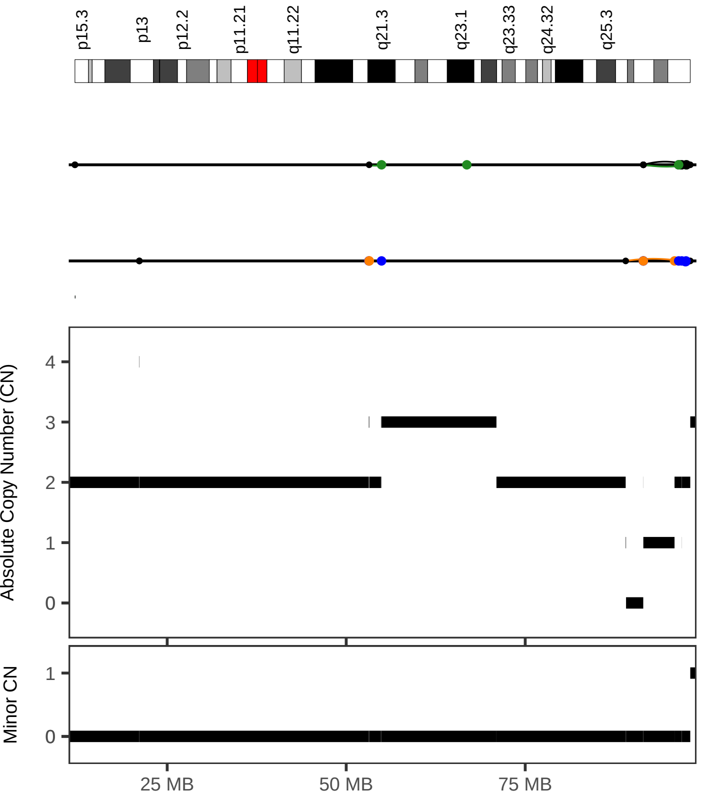

7b982d5e−3a7d−40ac−bd25−6044c62879b6

Cancer type	Lung−SCC
Position	10:89028540−98045877
Interleaved intrachr. SVs	6
Total SVs (intrachr. + transl.)	19
SV types	DEL: 1; DUP: 1; h2hINV: 2; t2tINV: 2; TRA: 13
SVs in sample	349
Oscillating CN (2 and 3 states)	5, 7
CN segments	8
FDR fragment joints	0.8653243
FDR chr. breakp. enrich.	0
Linked to chrs	11:303986−38787002;
Purity, ploidy	0.45, 2.92

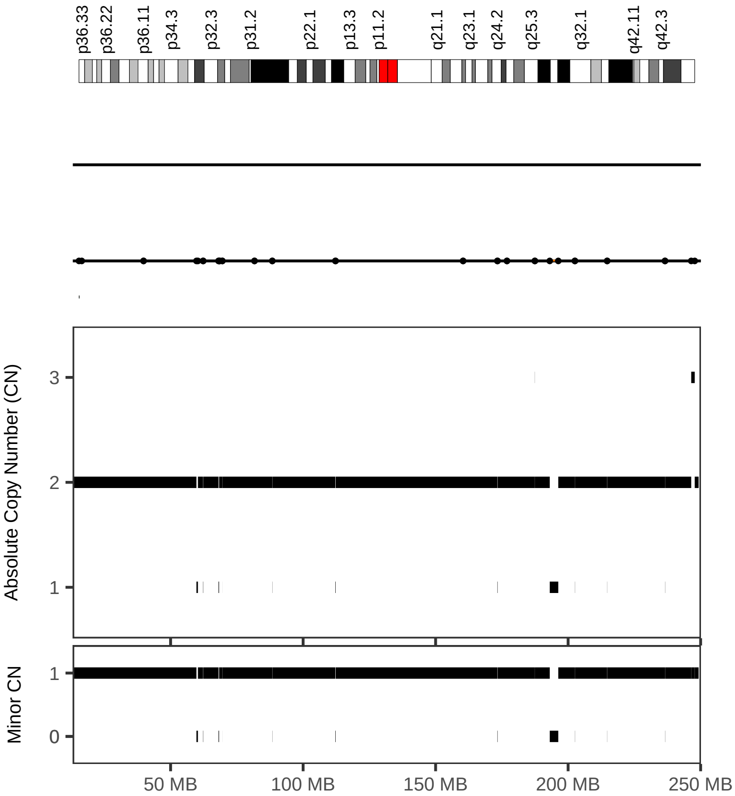

87ac0ad4−8c9d−409e−9a86−1d201d01769d

Cancer type	Lung−SCC
Position	1:15427220−247773092
Interleaved intrachr. SVs	1
Total SVs (intrachr. + transl.)	17
SV types	DEL: 13; DUP: 4; h2hINV: 0; t2tINV: 0; TRA: 0
SVs in sample	168
Oscillating CN (2 and 3 states)	17, 29
CN segments	29
FDR fragment joints	0.002665934
FDR chr. breakp. enrich.	0.29
Linked to chrs	
Purity, ploidy	0.55, 1.92

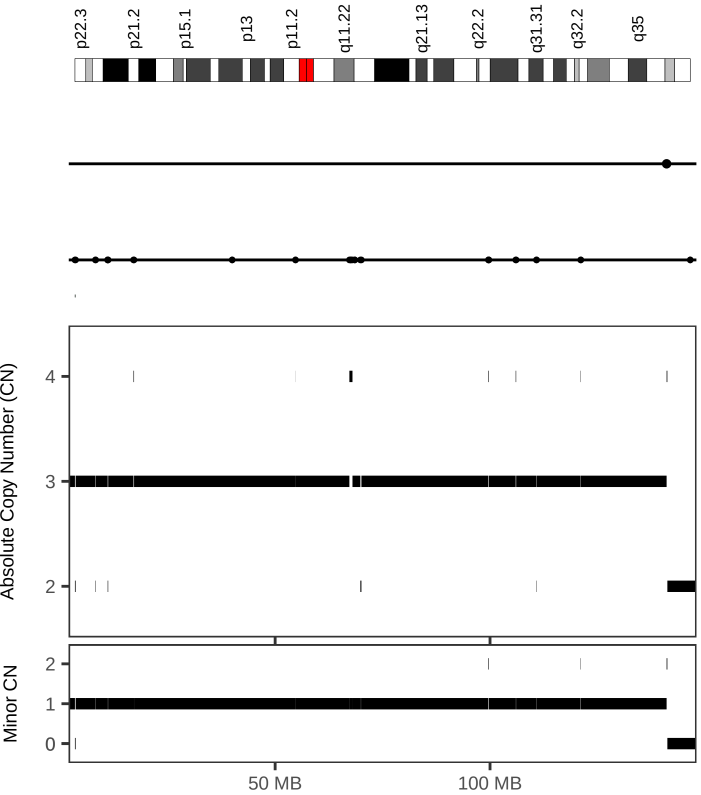

9a874b64−d0d6−416e−97bc−e9071ed0b16b

Cancer type	Lung−SCC
Position	7:3406252−121161205
Interleaved intrachr. SVs	1
Total SVs (intrachr. + transl.)	13
SV types	DEL: 5; DUP: 8; h2hINV: 0; t2tINV: 0; TRA: 0
SVs in sample	359
Oscillating CN (2 and 3 states)	7, 22
CN segments	22
FDR fragment joints	0.1266611
FDR chr. breakp. enrich.	1
Linked to chrs	
Purity, ploidy	0.48, 2.68

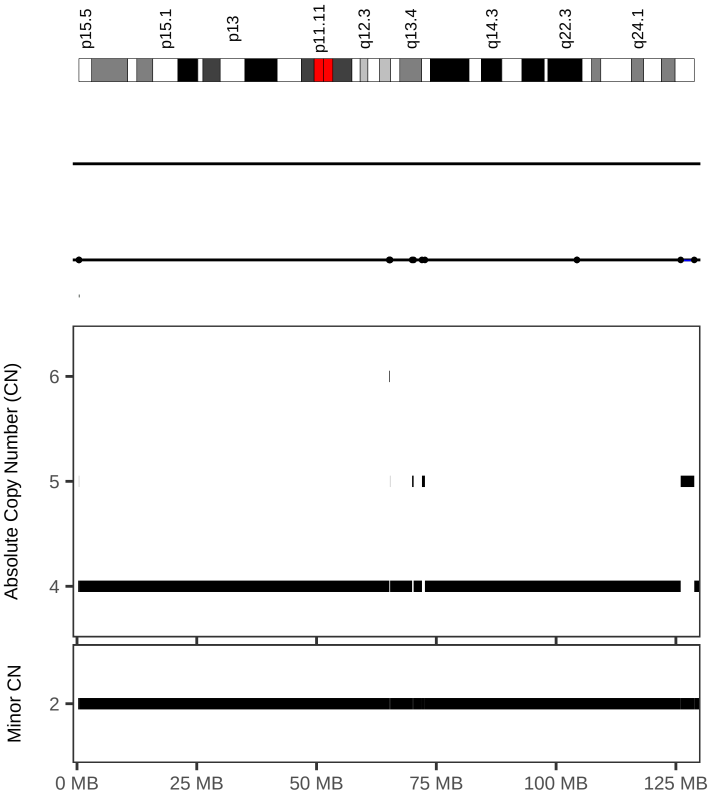

aecf85cc−058c−46f3−9cdc−3573ac3b8438

Cancer type	Lung−SCC
Position	11:400656−128835047
Interleaved intrachr. SVs	1
Total SVs (intrachr. + transl.)	6
SV types	DEL: 0; DUP: 6; h2hINV: 0; t2tINV: 0; TRA: 0
SVs in sample	232
Oscillating CN (2 and 3 states)	8, 8
CN segments	11
FDR fragment joints	0.04139017
FDR chr. breakp. enrich.	0.52
Linked to chrs	
Purity, ploidy	0.56, 3.19

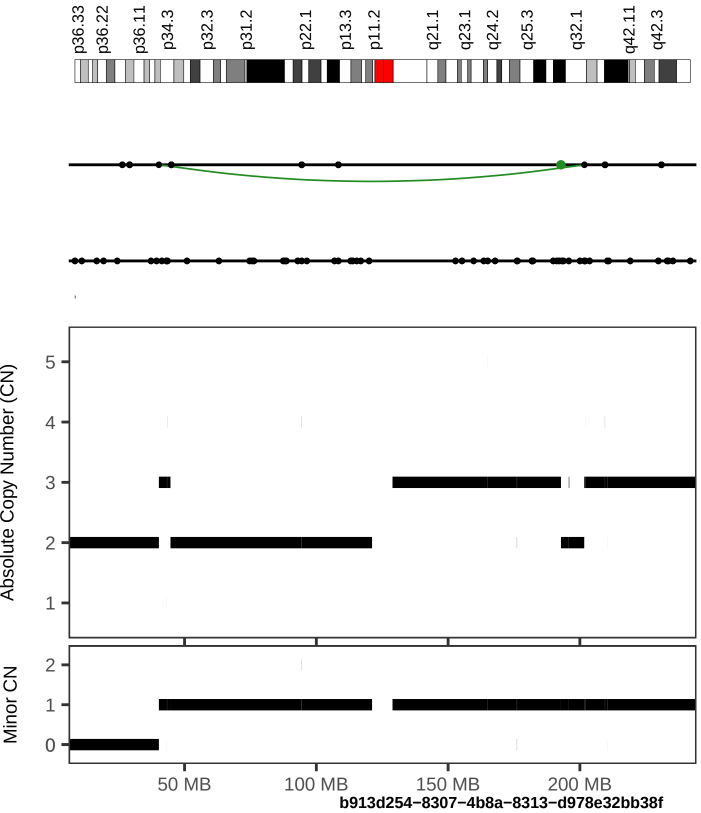

b913d254−8307−4b8a−8313−d978e32bb38f

Cancer type	Lung−SCC
Position	1:10985224−229819545
Interleaved intrachr. SVs	1
Total SVs (intrachr. + transl.)	18
SV types	DEL: 3; DUP: 11; h2hINV: 0; t2tINV: 3; TRA: 1
SVs in sample	944
Oscillating CN (2 and 3 states)	7, 7
CN segments	24
FDR fragment joints	0.1845613
FDR chr. breakp. enrich.	0.95
Linked to chrs	
Purity, ploidy	0.75, 2.86

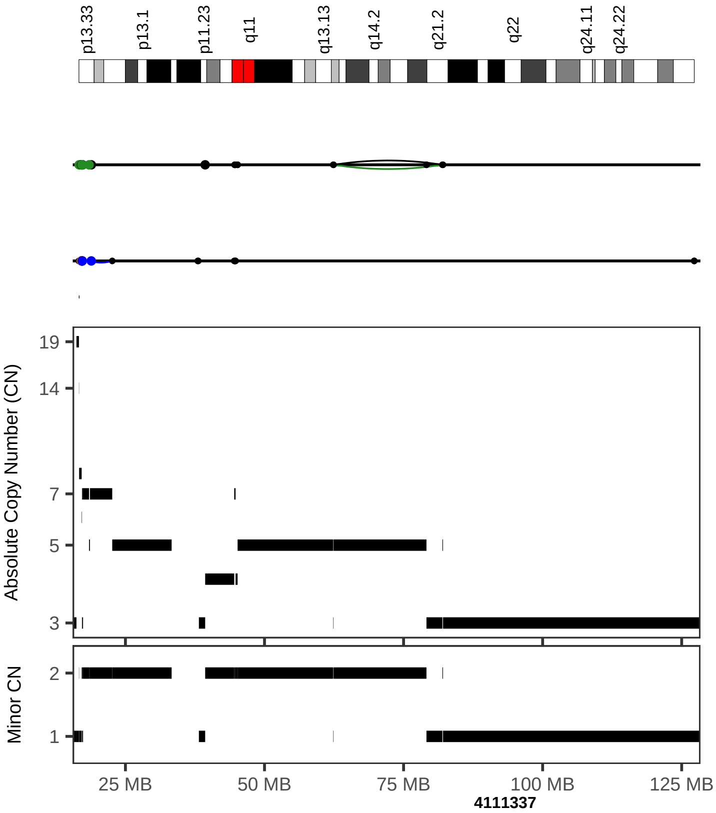

4111337

Cancer type	Lymph−BNHL
Position	12:17128247−17295895
Interleaved intrachr. SVs	8
Total SVs (intrachr. + transl.)	38
SV types	DEL: 1; DUP: 0; h2hINV: 4; t2tINV: 3; TRA: 30
SVs in sample	226
Oscillating CN (2 and 3 states)	6, 7
CN segments	9
FDR fragment joints	0.6532011
FDR chr. breakp. enrich.	0
Linked to chrs	
Purity, ploidy	0.62, 4.03

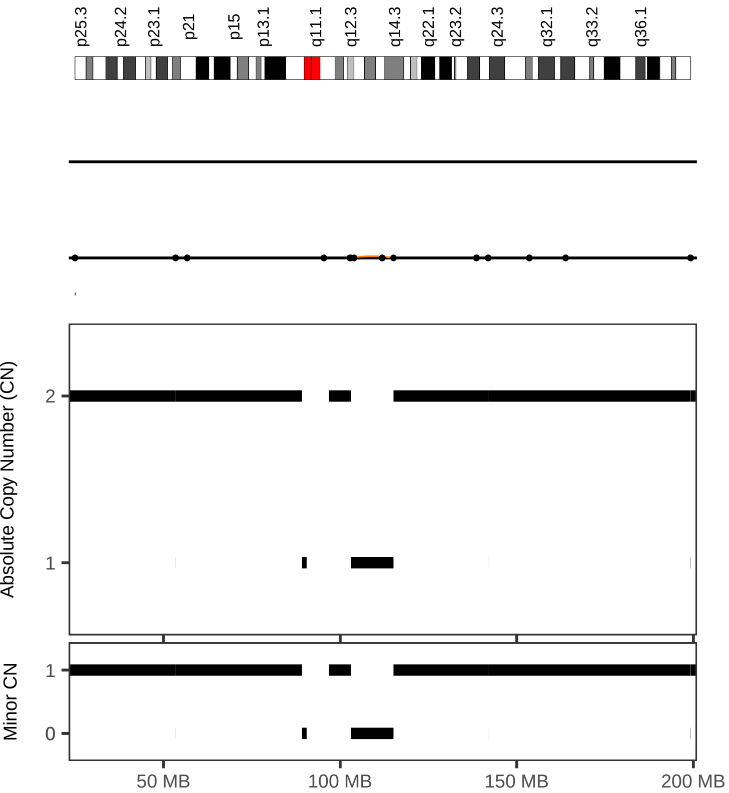

4135099

Cancer type	Lymph−BNHL
Position	2:53389576−199251373
Interleaved intrachr. SVs	1
Total SVs (intrachr. + transl.)	7
SV types	DEL: 7; DUP: 0; h2hINV: 0; t2tINV: 0; TRA: 0
SVs in sample	297
Oscillating CN (2 and 3 states)	12, 12
CN segments	12
FDR fragment joints	0.01743054
FDR chr. breakp. enrich.	0.05
Linked to chrs	
Purity, ploidy	0.79, 1.91

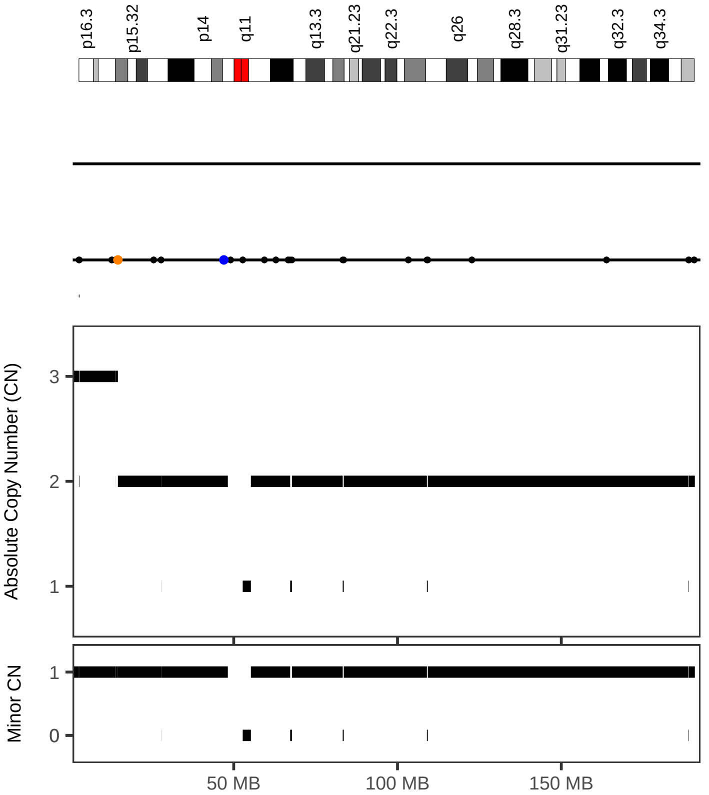

4135099

Cancer type	Lymph−BNHL
Position	4:2735820−190587393
Interleaved intrachr. SVs	1
Total SVs (intrachr. + transl.)	15
SV types	DEL: 13; DUP: 0; h2hINV: 0; t2tINV: 0; TRA: 2
SVs in sample	297
Oscillating CN (2 and 3 states)	13, 17
CN segments	17
FDR fragment joints	4.035888e−05
FDR chr. breakp. enrich.	1
Linked to chrs	
Purity, ploidy	0.79, 1.91

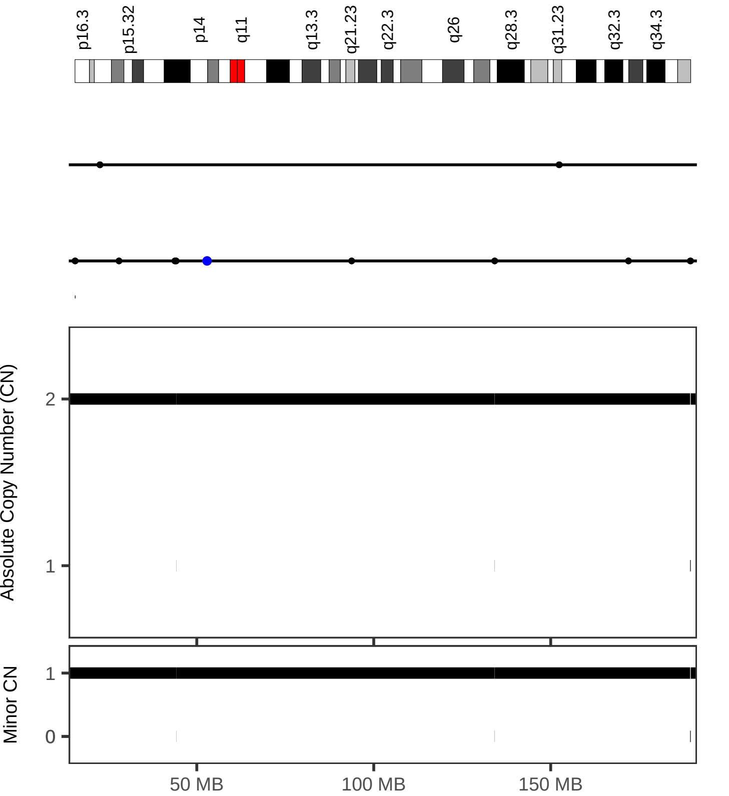

4138527

Cancer type	Lymph−BNHL
Position	4:15573682−189567466
Interleaved intrachr. SVs	1
Total SVs (intrachr. + transl.)	9
SV types	DEL: 6; DUP: 0; h2hINV: 1; t2tINV: 0; TRA: 2
SVs in sample	187
Oscillating CN (2 and 3 states)	7, 7
CN segments	7
FDR fragment joints	0.1488029
FDR chr. breakp. enrich.	1
Linked to chrs	
Purity, ploidy	0.72, 2.04

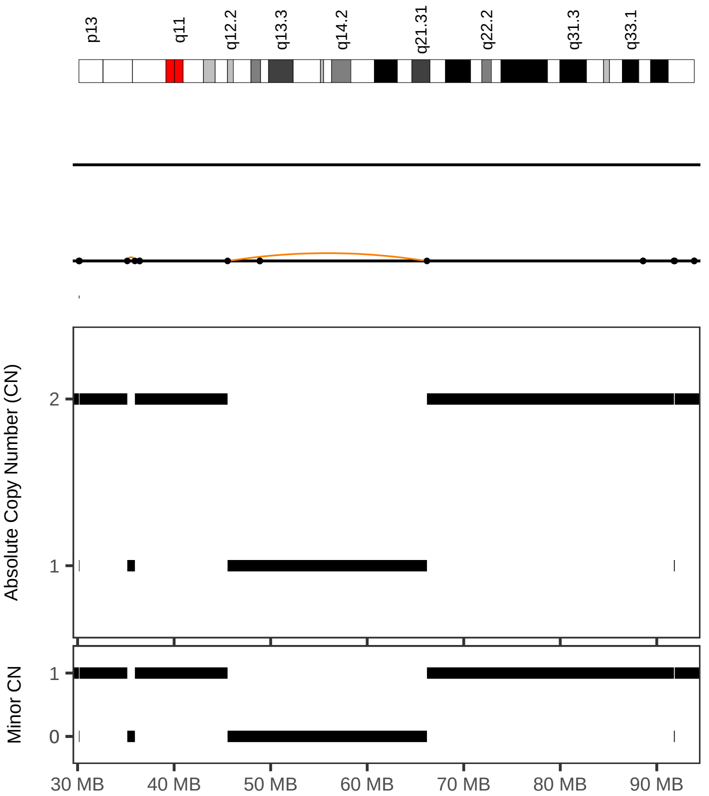

4138527

Cancer type	Lymph−BNHL
Position	13:30136476−91861686
Interleaved intrachr. SVs	1
Total SVs (intrachr. + transl.)	6
SV types	DEL: 6; DUP: 0; h2hINV: 0; t2tINV: 0; TRA: 0
SVs in sample	187
Oscillating CN (2 and 3 states)	8, 8
CN segments	8
FDR fragment joints	0.04139017
FDR chr. breakp. enrich.	0.77
Linked to chrs	
Purity, ploidy	0.72, 2.04

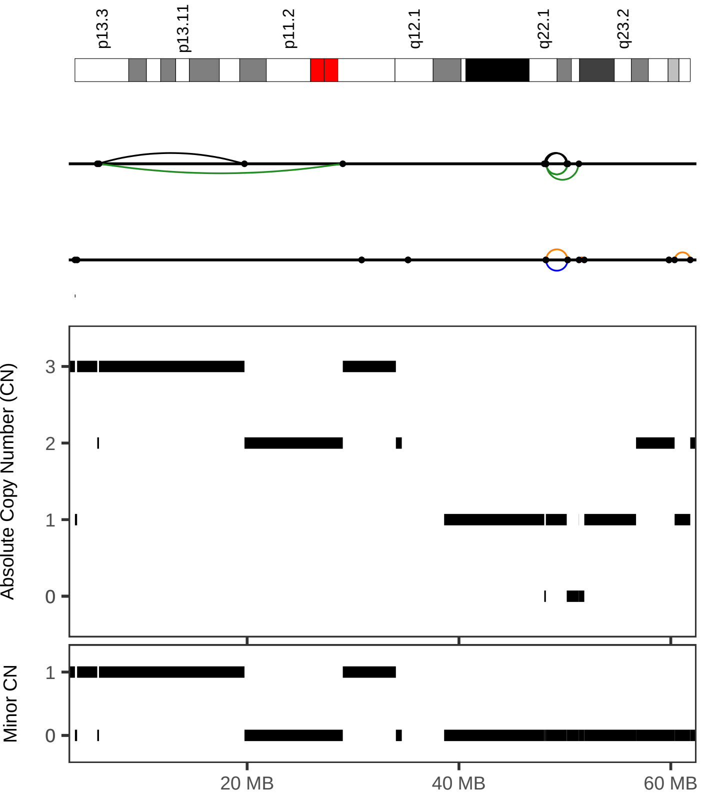

4138527

Cancer type	Lymph−BNHL
Position	16:48053477−51323723
Interleaved intrachr. SVs	7
Total SVs (intrachr. + transl.)	7
SV types	DEL: 1; DUP: 1; h2hINV: 2; t2tINV: 3; TRA: 0
SVs in sample	187
Oscillating CN (2 and 3 states)	4, 4
CN segments	4
FDR fragment joints	0.7735152
FDR chr. breakp. enrich.	0
Linked to chrs	
Purity, ploidy	0.72, 2.04

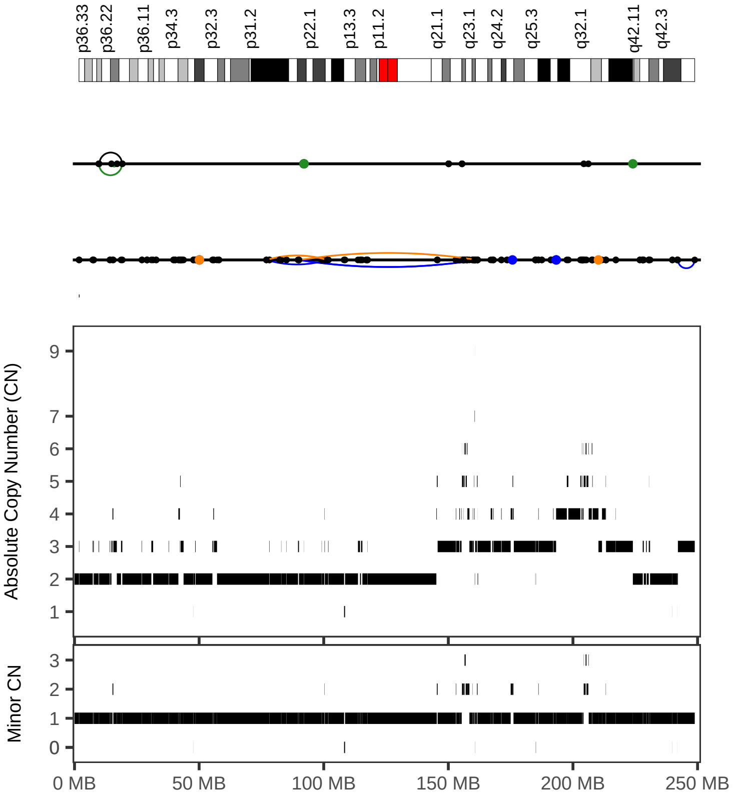

4145528

Cancer type	Lymph−BNHL
Position	1:77006091−161744723
Interleaved intrachr. SVs	5
Total SVs (intrachr. + transl.)	7
SV types	DEL: 2; DUP: 3; h2hINV: 0; t2tINV: 0; TRA: 2
SVs in sample	1226
Oscillating CN (2 and 3 states)	13, 18
CN segments	63
FDR fragment joints	0.7735152
FDR chr. breakp. enrich.	0.35
Linked to chrs	
Purity, ploidy	0.49, 2.4

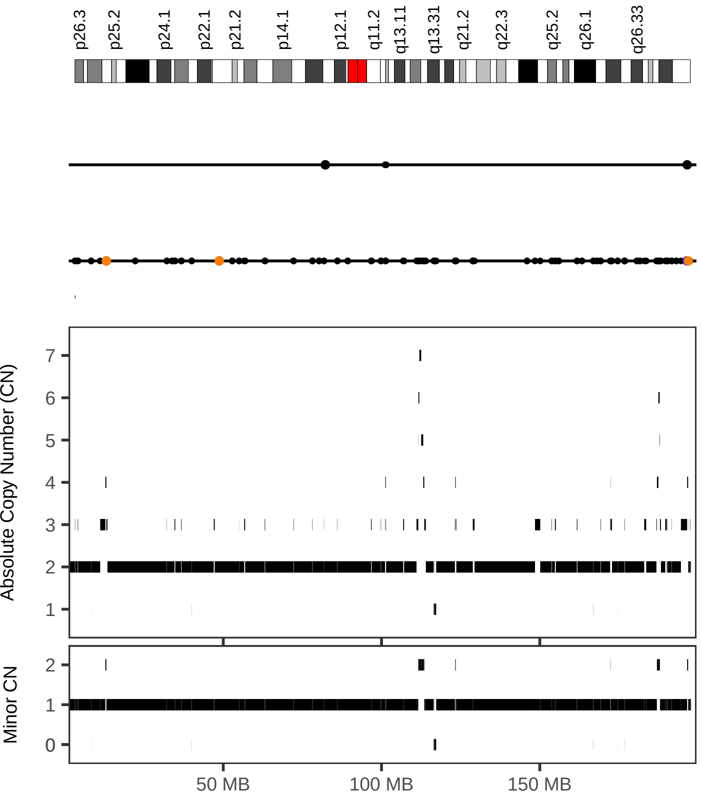

4145528

Cancer type	Lymph−BNHL
Position	3:111610899−188282688
Interleaved intrachr. SVs	3
Total SVs (intrachr. + transl.)	6
SV types	DEL: 0; DUP: 6; h2hINV: 0; t2tINV: 0; TRA: 0
SVs in sample	1226
Oscillating CN (2 and 3 states)	12, 40
CN segments	47
FDR fragment joints	0.04139017
FDR chr. breakp. enrich.	0.53
Linked to chrs	
Purity, ploidy	0.49, 2.4

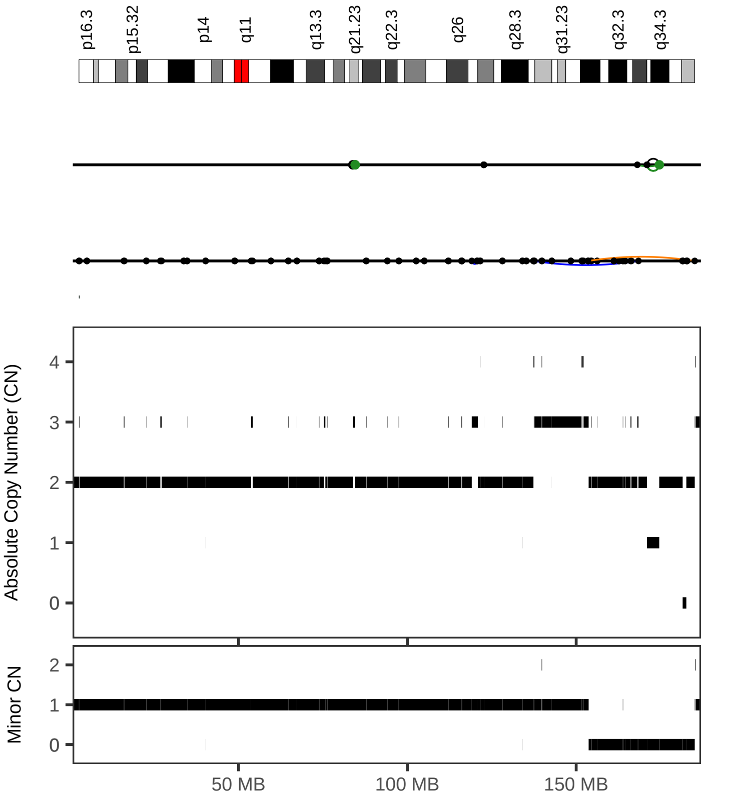

4145528

Cancer type	Lymph−BNHL
Position	4:137343292−185113770
Interleaved intrachr. SVs	7
Total SVs (intrachr. + transl.)	8
SV types	DEL: 1; DUP: 2; h2hINV: 1; t2tINV: 3; TRA: 1
SVs in sample	1226
Oscillating CN (2 and 3 states)	14, 28
CN segments	33
FDR fragment joints	0.8653243
FDR chr. breakp. enrich.	0.06
Linked to chrs	
Purity, ploidy	0.49, 2.4

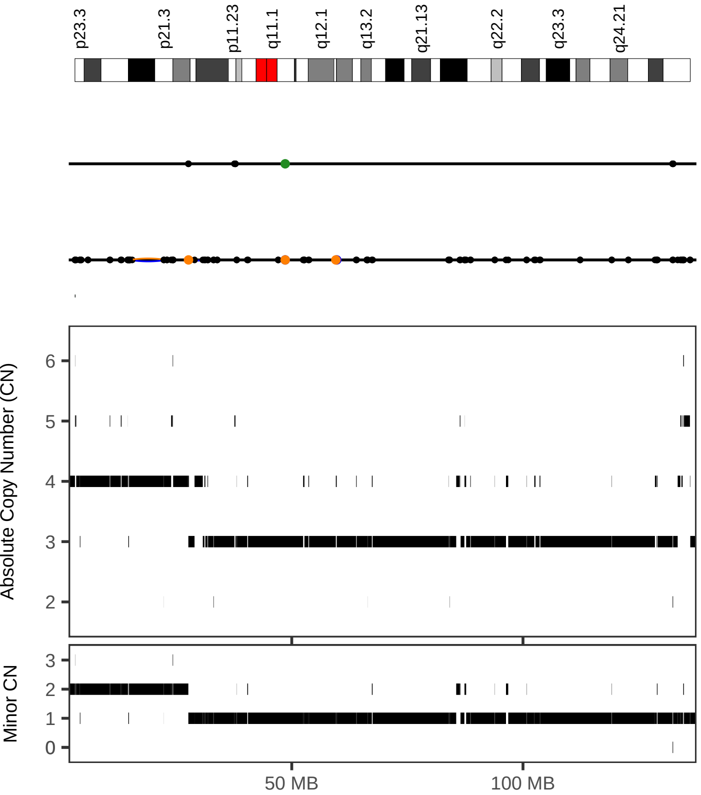

4145528

Cancer type	Lymph−BNHL
Position	8:3116649−136165687
Interleaved intrachr. SVs	2
Total SVs (intrachr. + transl.)	18
SV types	DEL: 1; DUP: 9; h2hINV: 1; t2tINV: 1; TRA: 6
SVs in sample	1226
Oscillating CN (2 and 3 states)	20, 57
CN segments	89
FDR fragment joints	0.155439
FDR chr. breakp. enrich.	1
Linked to chrs	1:77006091−161744722;
Purity, ploidy	0.49, 2.4

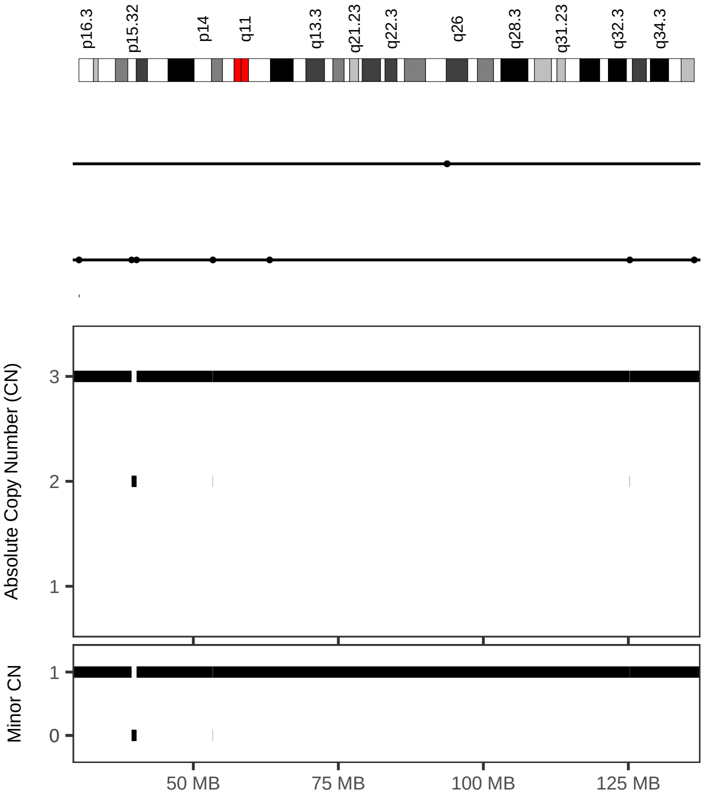

4147968

Cancer type	Lymph−BNHL
Position	4:30276216−125265483
Interleaved intrachr. SVs	1
Total SVs (intrachr. + transl.)	5
SV types	DEL: 5; DUP: 0; h2hINV: 0; t2tINV: 0; TRA: 0
SVs in sample	140
Oscillating CN (2 and 3 states)	7, 7
CN segments	7
FDR fragment joints	0.1013295
FDR chr. breakp. enrich.	0.91
Linked to chrs	
Purity, ploidy	0.67, 3.49

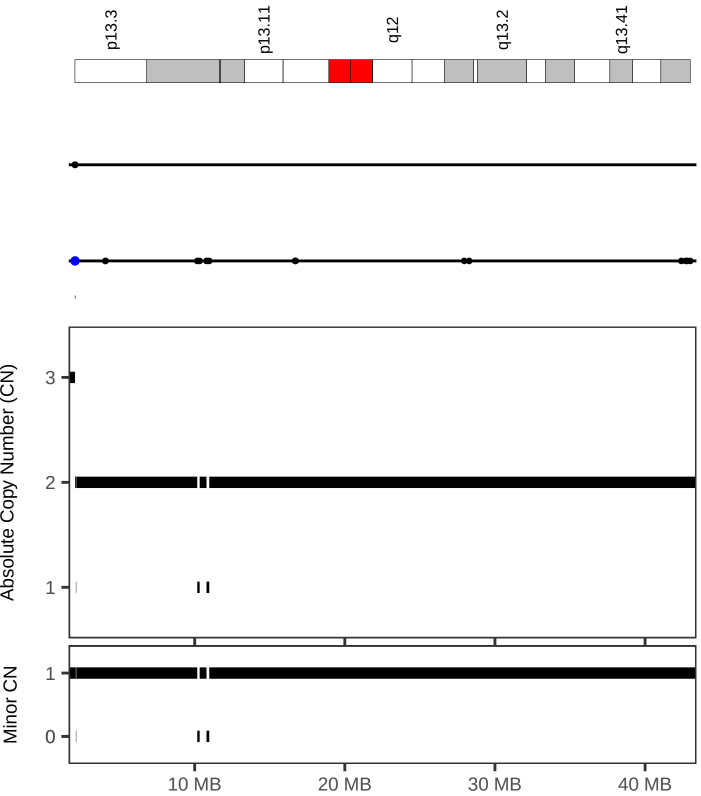

4176133

Cancer type	Lymph−BNHL
Position	19:2022616−43007888
Interleaved intrachr. SVs	1
Total SVs (intrachr. + transl.)	11
SV types	DEL: 9; DUP: 0; h2hINV: 1; t2tINV: 0; TRA: 1
SVs in sample	59
Oscillating CN (2 and 3 states)	9, 10
CN segments	10
FDR fragment joints	0.03230998
FDR chr. breakp. enrich.	0
Linked to chrs	
Purity, ploidy	0.76, 1.98

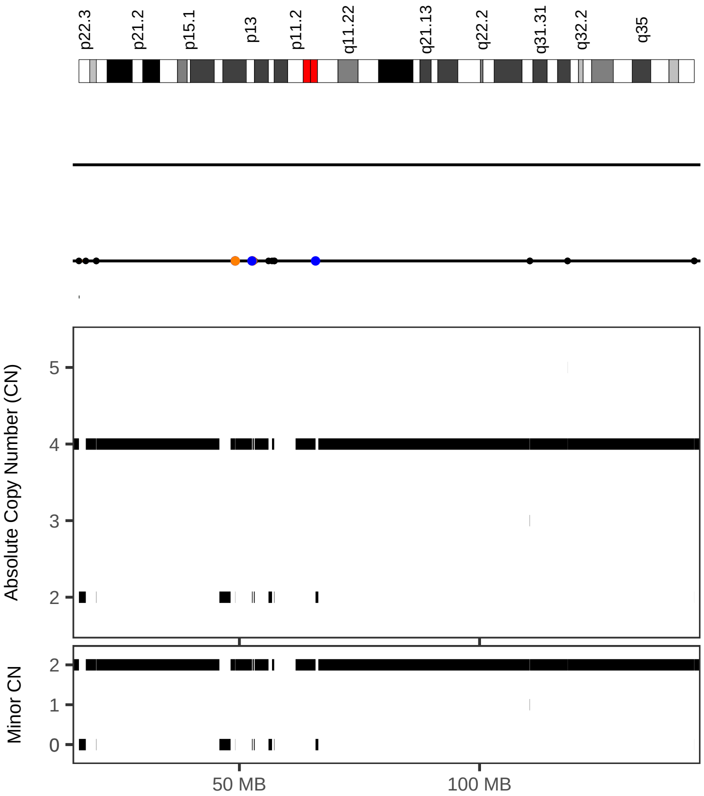

4188398

Cancer type	Lymph−BNHL
Position	7:16592923−110490608
Interleaved intrachr. SVs	1
Total SVs (intrachr. + transl.)	11
SV types	DEL: 6; DUP: 0; h2hINV: 0; t2tINV: 0; TRA: 5
SVs in sample	119
Oscillating CN (2 and 3 states)	18, 20
CN segments	20
FDR fragment joints	0.1013295
FDR chr. breakp. enrich.	0.06
Linked to chrs	1:31461659−144870388;
Purity, ploidy	0.78, 3.82

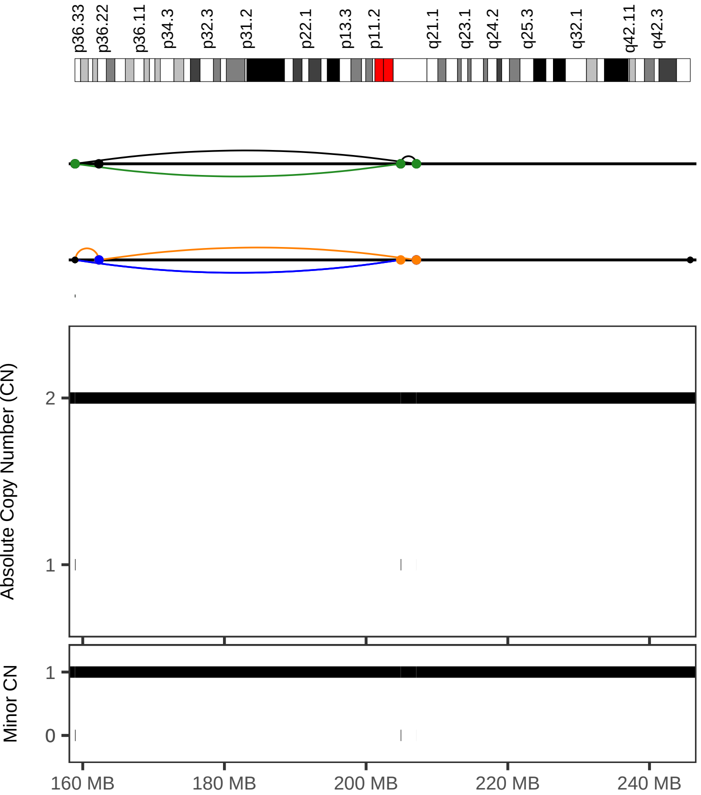

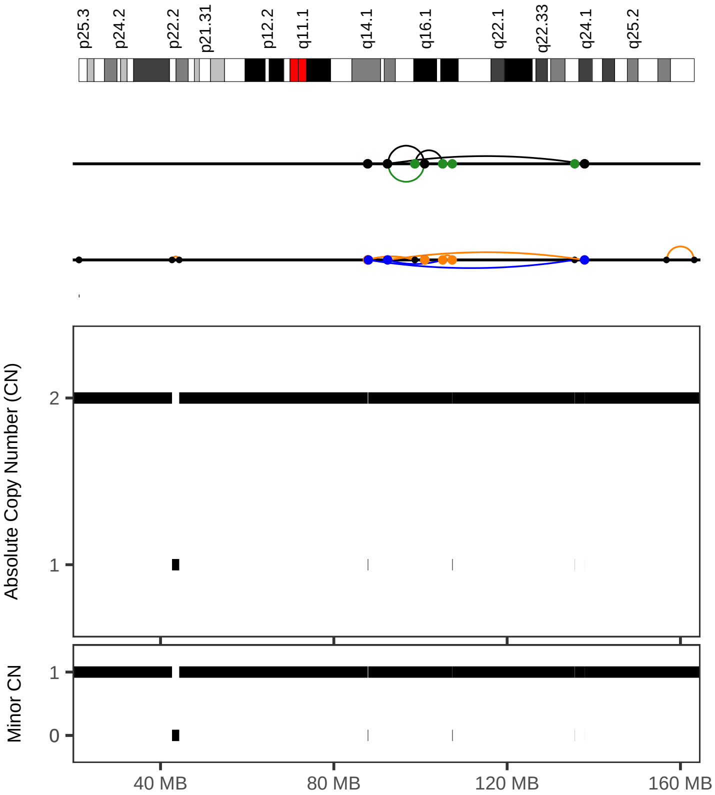

16

Cancer type	Lymph−CLL
Position	1:158895713−207110372
Interleaved intrachr. SVs	6
Total SVs (intrachr. + transl.)	25
SV types	DEL: 1; DUP: 2; h2hINV: 2; t2tINV: 1; TRA: 19
SVs in sample	82
Oscillating CN (2 and 3 states)	8, 8
CN segments	8
FDR fragment joints	0.8653243
FDR chr. breakp. enrich.	0
Linked to chrs	6:87781500−137889691;
Purity, ploidy	0.85, 1.99

16

Cancer type	Lymph−CLL
Position	6:87781500−137889692
Interleaved intrachr. SVs	11
Total SVs (intrachr. + transl.)	36
SV types	DEL: 5; DUP: 2; h2hINV: 3; t2tINV: 1; TRA: 25
SVs in sample	82
Oscillating CN (2 and 3 states)	11, 11
CN segments	11
FDR fragment joints	0.6776251
FDR chr. breakp. enrich.	0
Linked to chrs	1:158895713−207110371;
Purity, ploidy	0.85, 1.99

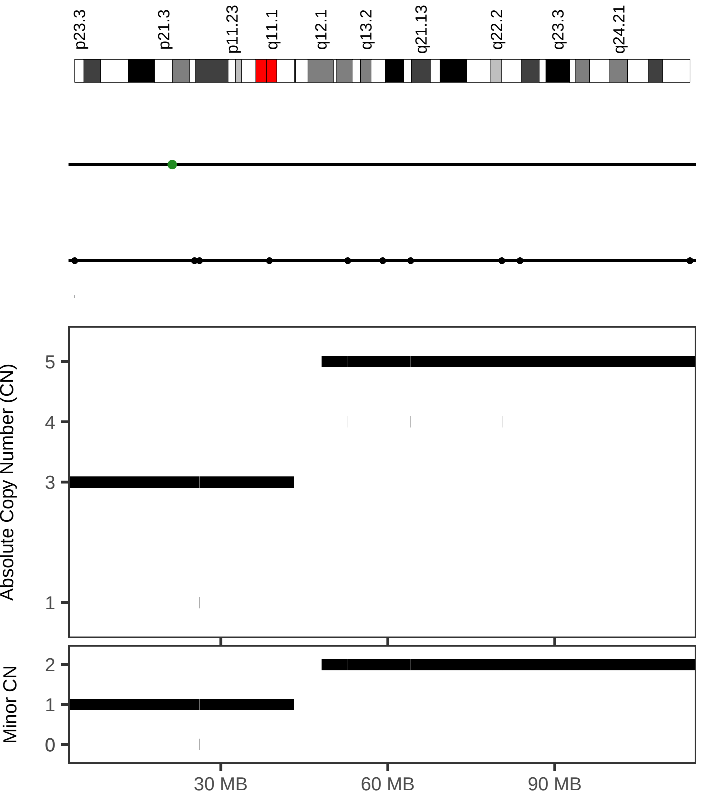

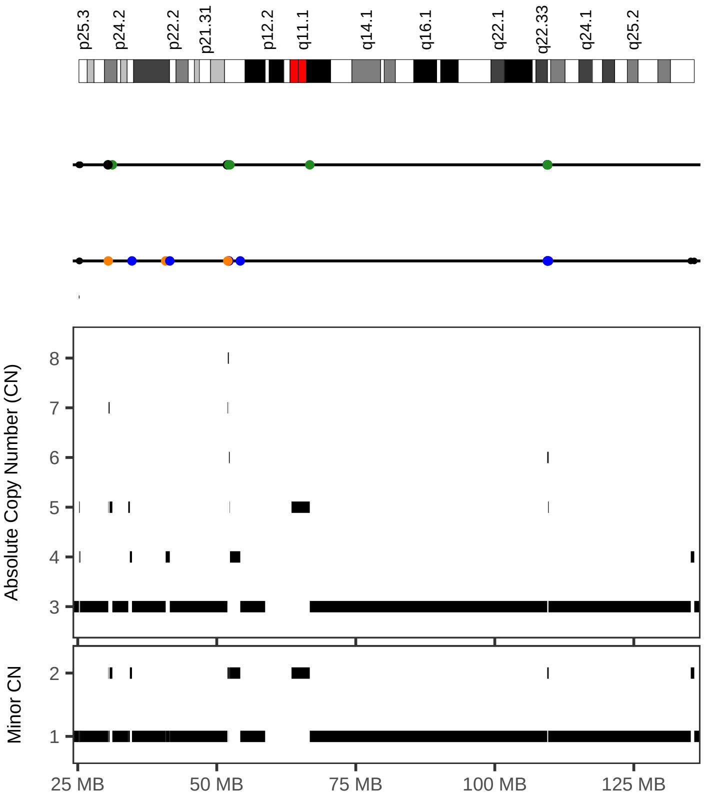

AOCS−004

Cancer type	Ovary−AdenoCA
Position	8:25228170−114295579
Interleaved intrachr. SVs	1
Total SVs (intrachr. + transl.)	5
SV types	DEL: 5; DUP: 0; h2hINV: 0; t2tINV: 0; TRA: 0
SVs in sample	121
Oscillating CN (2 and 3 states)	9, 10
CN segments	12
FDR fragment joints	0.1013295
FDR chr. breakp. enrich.	0.18
Linked to chrs	
Purity, ploidy	0.48, 2.62

AOCS−005

Cancer type	Ovary−AdenoCA
Position	6:109475920−109661908
Interleaved intrachr. SVs	11
Total SVs (intrachr. + transl.)	21
SV types	DEL: 1; DUP: 5; h2hINV: 3; t2tINV: 2; TRA: 10
SVs in sample	399
Oscillating CN (2 and 3 states)	4, 5
CN segments	5
FDR fragment joints	0.5435077
FDR chr. breakp. enrich.	0
Linked to chrs	
Purity, ploidy	0.71, 3.06

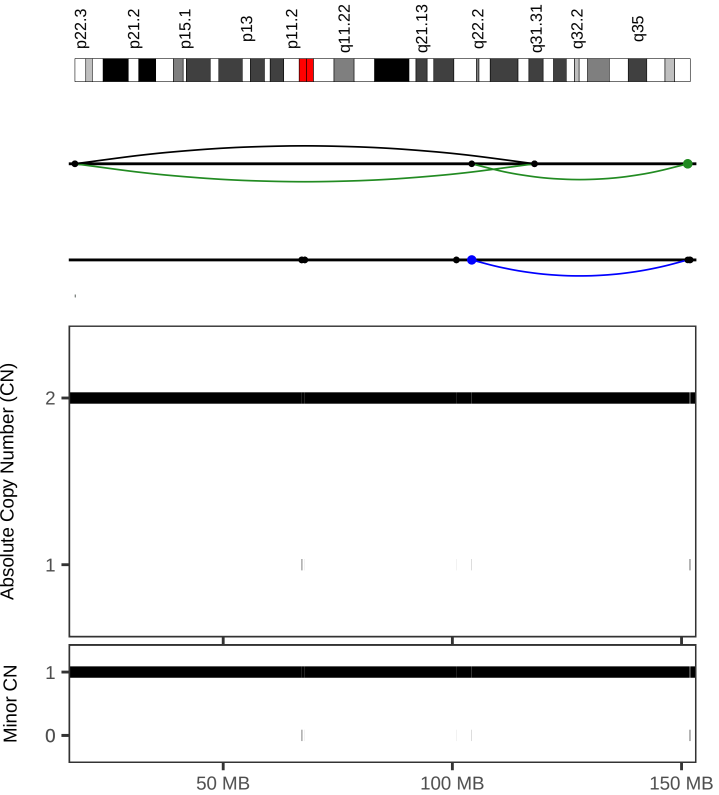

AOCS−063

Cancer type	Ovary−AdenoCA
Position	7:17651563−151348579
Interleaved intrachr. SVs	4
Total SVs (intrachr. + transl.)	7
SV types	DEL: 0; DUP: 1; h2hINV: 1; t2tINV: 2; TRA: 3
SVs in sample	146
Oscillating CN (2 and 3 states)	9, 9
CN segments	9
FDR fragment joints	0.6776251
FDR chr. breakp. enrich.	0.51
Linked to chrs	
Purity, ploidy	0.58, 1.96

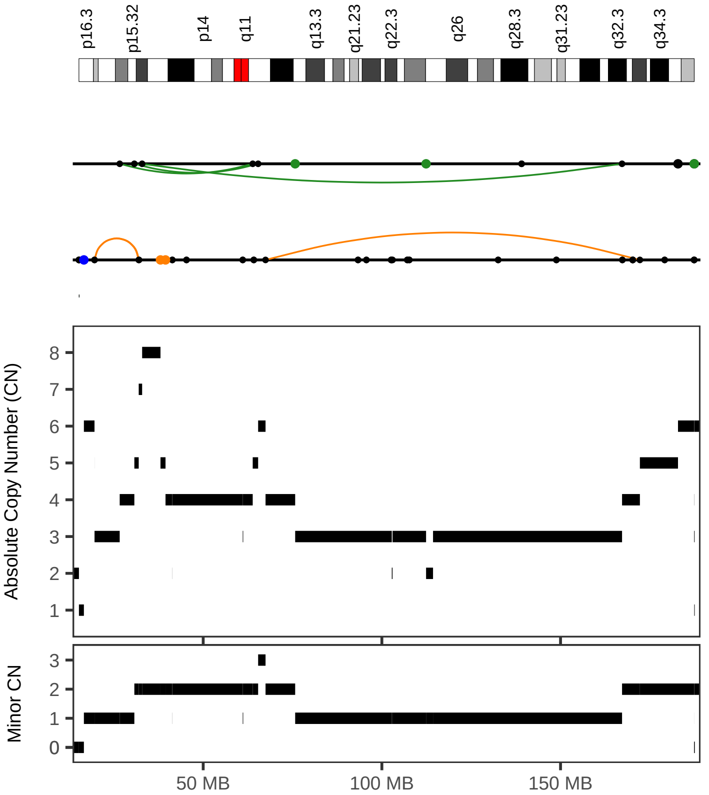

AOCS−064

Cancer type	Ovary−AdenoCA
Position	4:19583669−172211276
Interleaved intrachr. SVs	6
Total SVs (intrachr. + transl.)	10
SV types	DEL: 3; DUP: 0; h2hINV: 0; t2tINV: 3; TRA: 4
SVs in sample	402
Oscillating CN (2 and 3 states)	5, 8
CN segments	22
FDR fragment joints	0.615458
FDR chr. breakp. enrich.	0.18
Linked to chrs	7:75921658−99227824;
Purity, ploidy	0.67, 3.2

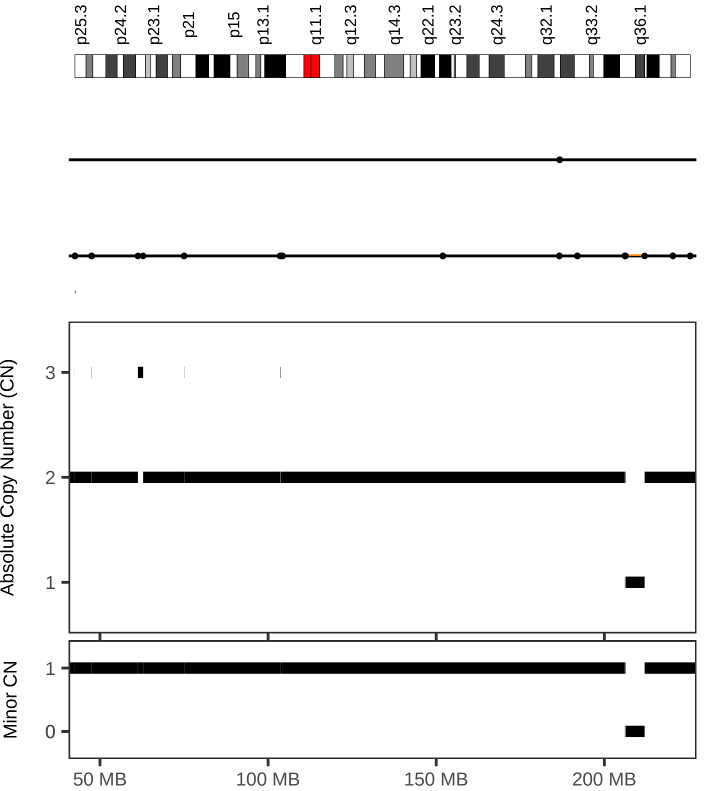

AOCS−065

Cancer type	Ovary−AdenoCA
Position	2:42549921−220375355
Interleaved intrachr. SVs	1
Total SVs (intrachr. + transl.)	10
SV types	DEL: 2; DUP: 8; h2hINV: 0; t2tINV: 0; TRA: 0
SVs in sample	255
Oscillating CN (2 and 3 states)	12, 16
CN segments	16
FDR fragment joints	0.05741733
FDR chr. breakp. enrich.	0.5
Linked to chrs	
Purity, ploidy	0.7, 1.88

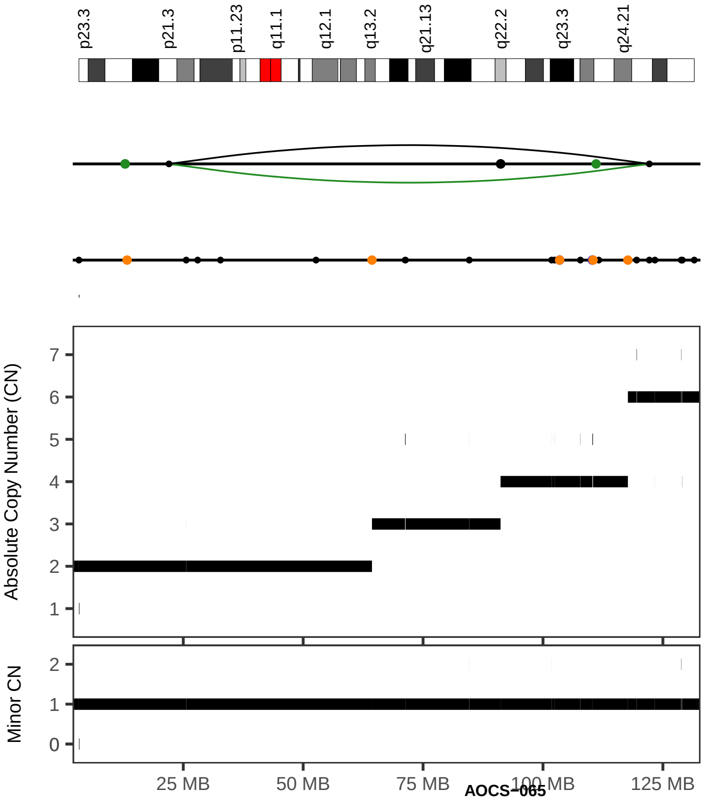

AOCS−065

Cancer type	Ovary−AdenoCA
Position	8:22020075−122183195
Interleaved intrachr. SVs	3
Total SVs (intrachr. + transl.)	12
SV types	DEL: 0; DUP: 1; h2hINV: 1; t2tINV: 1; TRA: 9
SVs in sample	255
Oscillating CN (2 and 3 states)	9, 10
CN segments	20
FDR fragment joints	0.9284301
FDR chr. breakp. enrich.	0
Linked to chrs	6:44943212−164824871;X:66347450−132168603
Purity, ploidy	0.7, 1.88

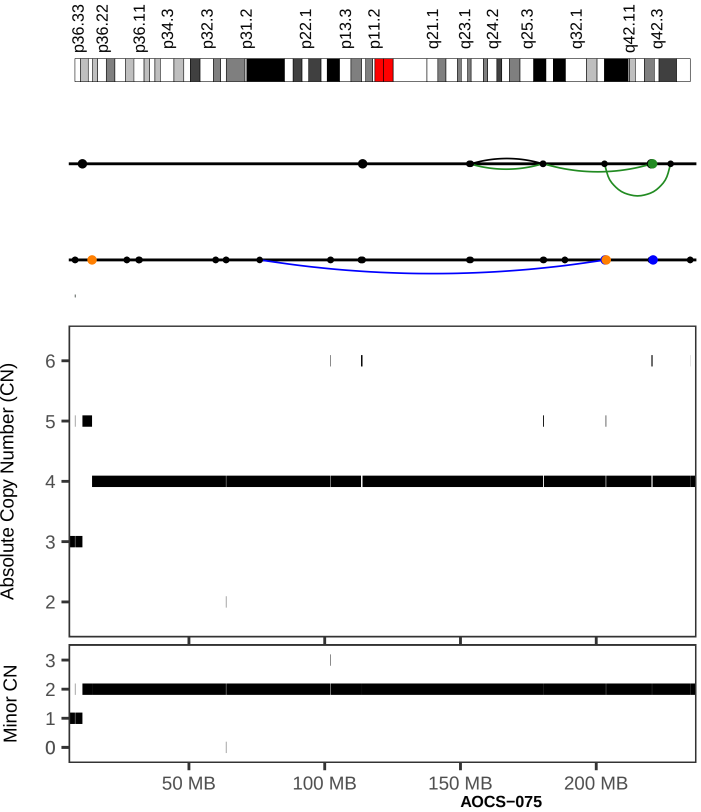

AOCS−075

Cancer type	Ovary−AdenoCA
Position	1:76045362−227389874
Interleaved intrachr. SVs	7
Total SVs (intrachr. + transl.)	13
SV types	DEL: 0; DUP: 3; h2hINV: 1; t2tINV: 3; TRA: 6
SVs in sample	166
Oscillating CN (2 and 3 states)	5, 11
CN segments	11
FDR fragment joints	0.615458
FDR chr. breakp. enrich.	0
Linked to chrs	
Purity, ploidy	0.47, 3.07

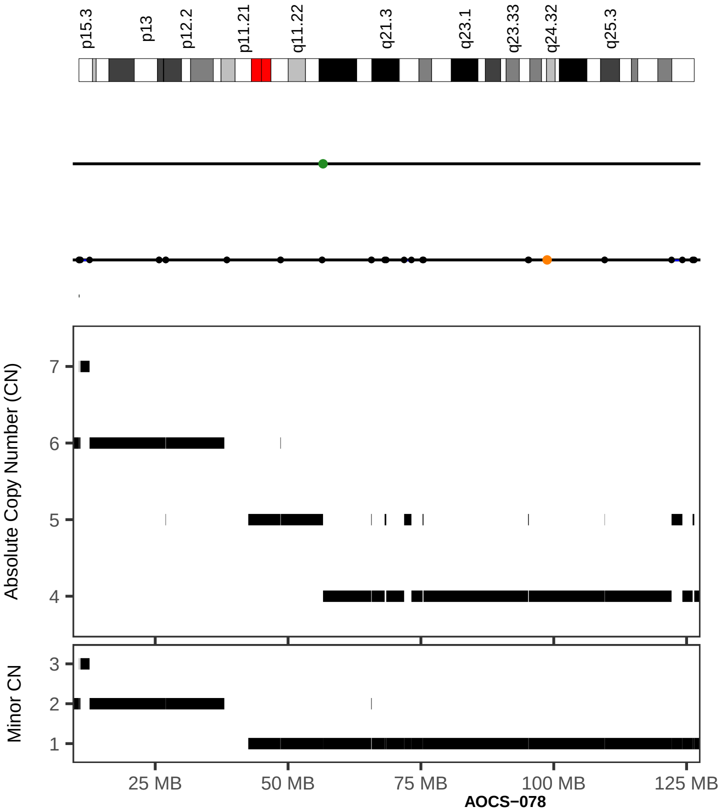

AOCS−078

Cancer type	Ovary−AdenoCA
Position	10:10638409−126440995
Interleaved intrachr. SVs	1
Total SVs (intrachr. + transl.)	17
SV types	DEL: 0; DUP: 15; h2hINV: 0; t2tINV: 0; TRA: 2
SVs in sample	457
Oscillating CN (2 and 3 states)	18, 26
CN segments	26
FDR fragment joints	6.6795e−05
FDR chr. breakp. enrich.	0.68
Linked to chrs	
Purity, ploidy	0.64, 4.25

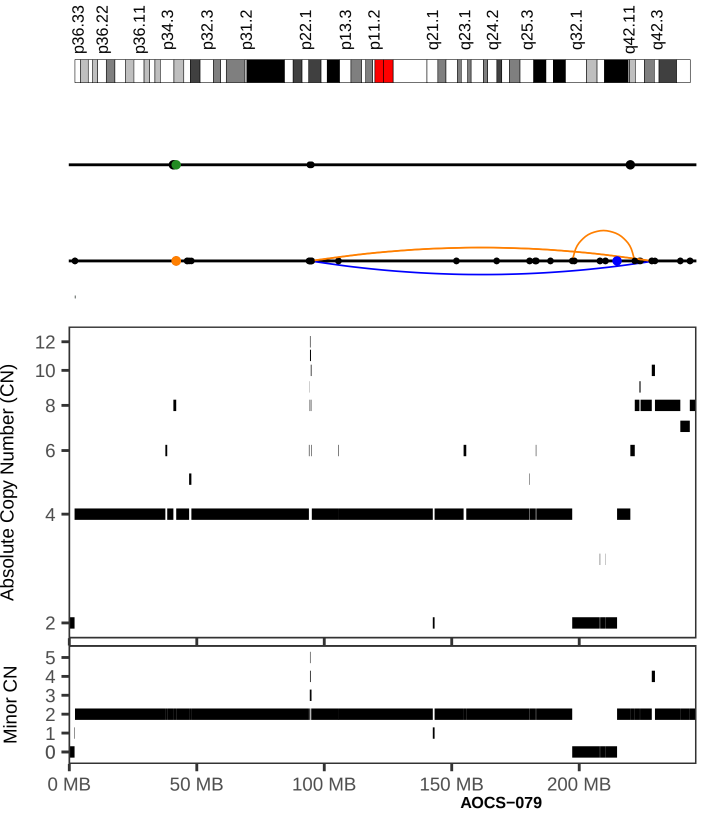

AOCS−079

Cancer type	Ovary−AdenoCA
Position	1:93975098−229706598
Interleaved intrachr. SVs	11
Total SVs (intrachr. + transl.)	13
SV types	DEL: 3; DUP: 6; h2hINV: 1; t2tINV: 1; TRA: 2
SVs in sample	310
Oscillating CN (2 and 3 states)	5, 8
CN segments	36
FDR fragment joints	0.5435077
FDR chr. breakp. enrich.	0.01
Linked to chrs	
Purity, ploidy	0.5, 3.13

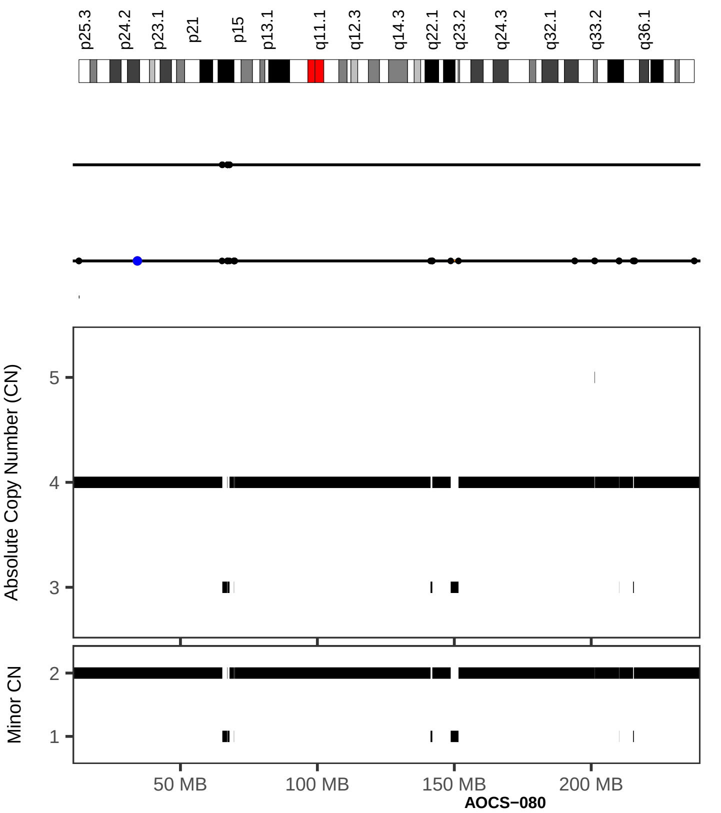

AOCS−080

Cancer type	Ovary−AdenoCA
Position	2:65178109−67917873
Interleaved intrachr. SVs	7
Total SVs (intrachr. + transl.)	7
SV types	DEL: 2; DUP: 0; h2hINV: 2; t2tINV: 3; TRA: 0
SVs in sample	207
Oscillating CN (2 and 3 states)	6, 6
CN segments	6
FDR fragment joints	0.6776251
FDR chr. breakp. enrich.	0.38
Linked to chrs	
Purity, ploidy	0.92, 3.05

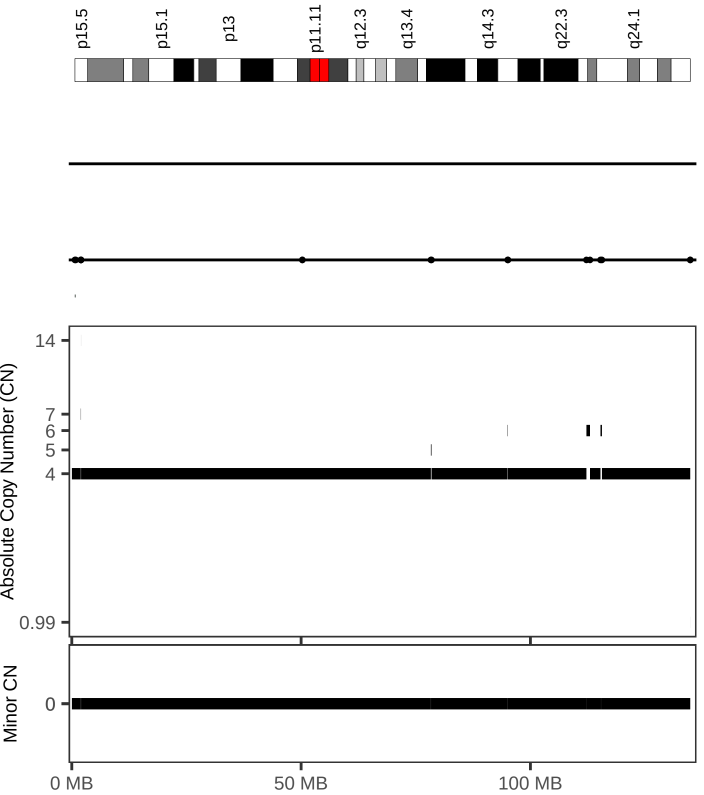

AOCS−083

Cancer type	Ovary−AdenoCA
Position	11:681299−115592442
Interleaved intrachr. SVs	1
Total SVs (intrachr. + transl.)	7
SV types	DEL: 0; DUP: 7; h2hINV: 0; t2tINV: 0; TRA: 0
SVs in sample	242
Oscillating CN (2 and 3 states)	7, 7
CN segments	13
FDR fragment joints	0.01743054
FDR chr. breakp. enrich.	0.86
Linked to chrs	
Purity, ploidy	0.56, 4.18

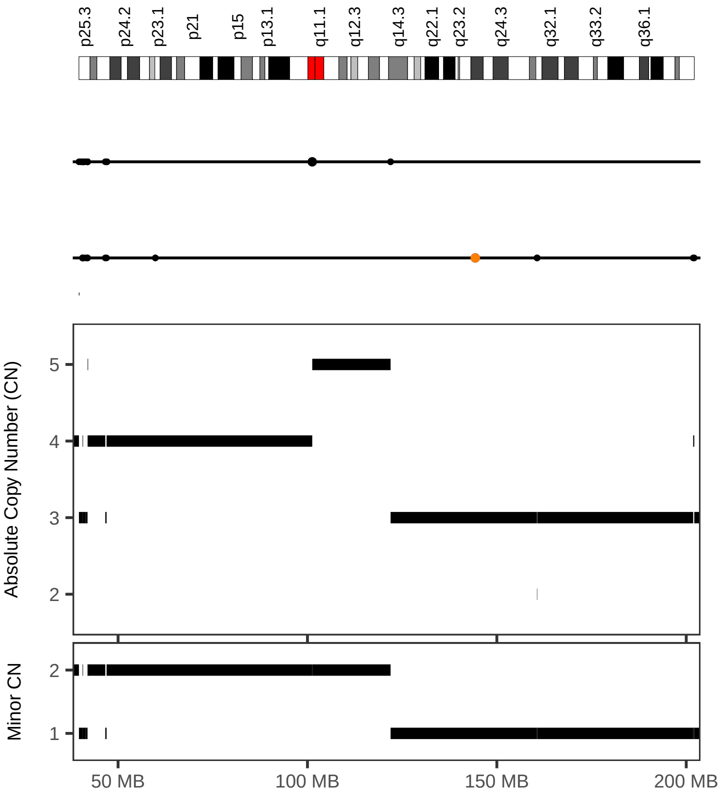

AOCS−084

Cancer type	Ovary−AdenoCA
Position	2:39660868−41960421
Interleaved intrachr. SVs	12
Total SVs (intrachr. + transl.)	12
SV types	DEL: 2; DUP: 2; h2hINV: 4; t2tINV: 4; TRA: 0
SVs in sample	146
Oscillating CN (2 and 3 states)	6, 8
CN segments	8
FDR fragment joints	0.8298498
FDR chr. breakp. enrich.	0.03
Linked to chrs	
Purity, ploidy	0.64, 3.09

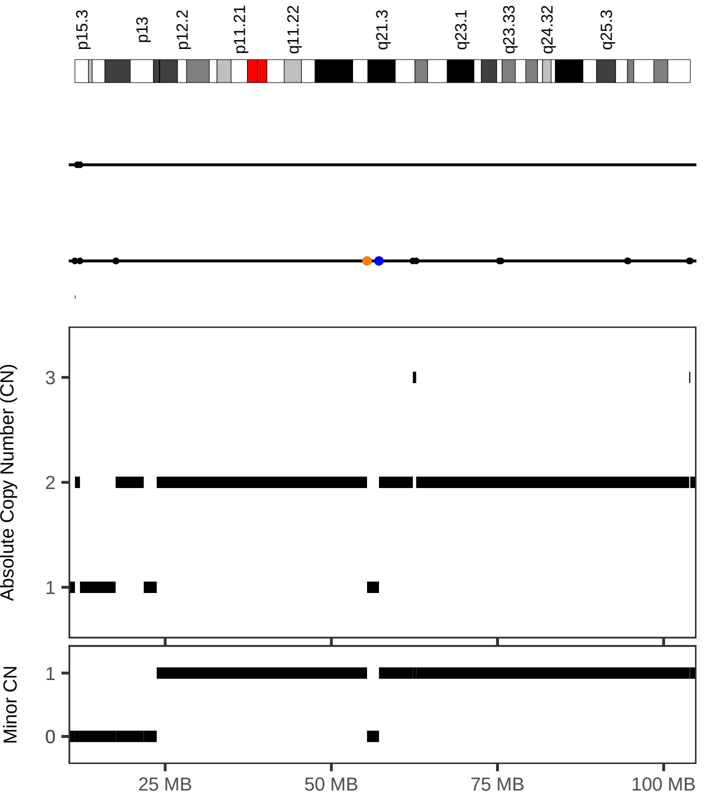

AOCS−090

Cancer type	Ovary−AdenoCA
Position	10:11417451−104002149
Interleaved intrachr. SVs	1
Total SVs (intrachr. + transl.)	9
SV types	DEL: 0; DUP: 6; h2hINV: 1; t2tINV: 0; TRA: 2
SVs in sample	334
Oscillating CN (2 and 3 states)	7, 11
CN segments	11
FDR fragment joints	0.1488029
FDR chr. breakp. enrich.	0.3
Linked to chrs	
Purity, ploidy	0.78, 2.65

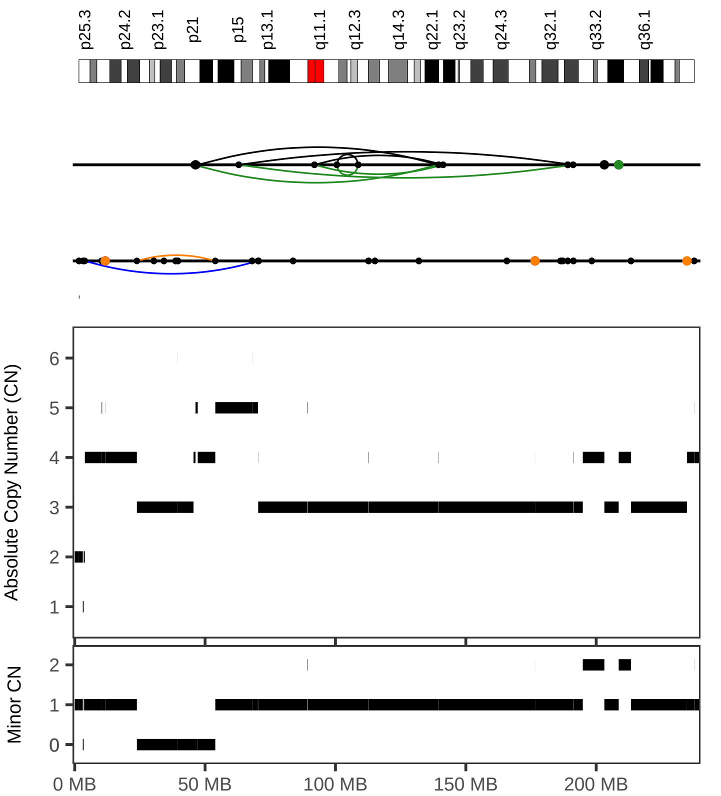

AOCS−091

Cancer type	Ovary−AdenoCA
Position	2:3886207−191136840
Interleaved intrachr. SVs	8
Total SVs (intrachr. + transl.)	13
SV types	DEL: 1; DUP: 1; h2hINV: 3; t2tINV: 3; TRA: 5
SVs in sample	347
Oscillating CN (2 and 3 states)	9, 14
CN segments	27
FDR fragment joints	0.9865804
FDR chr. breakp. enrich.	0.08
Linked to chrs	
Purity, ploidy	0.74, 3.31

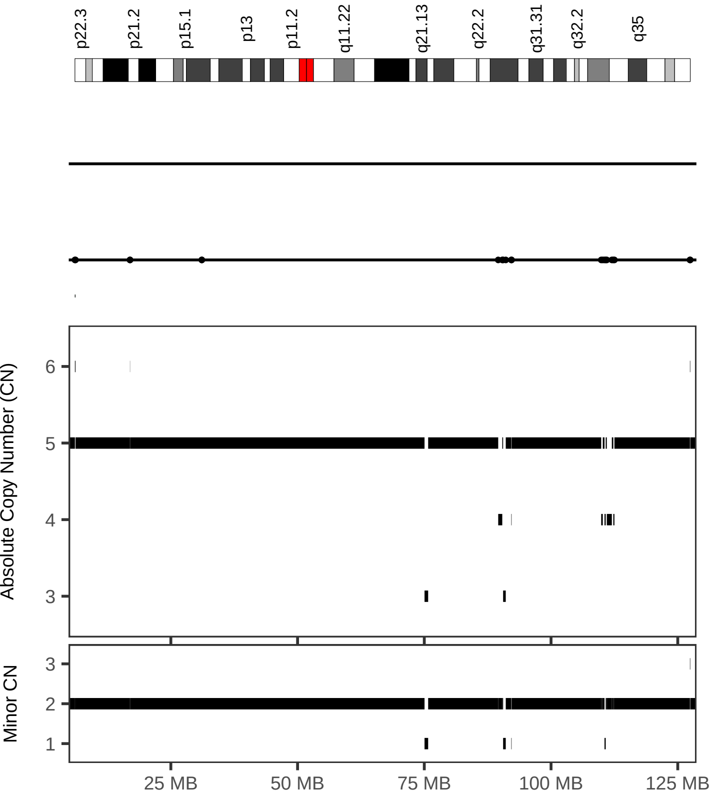

AOCS−092

Cancer type	Ovary−AdenoCA
Position	7:6073651−127466609
Interleaved intrachr. SVs	1
Total SVs (intrachr. + transl.)	10
SV types	DEL: 7; DUP: 3; h2hINV: 0; t2tINV: 0; TRA: 0
SVs in sample	209
Oscillating CN (2 and 3 states)	11, 17
CN segments	22
FDR fragment joints	0.1790694
FDR chr. breakp. enrich.	1
Linked to chrs	
Purity, ploidy	0.59, 3.83

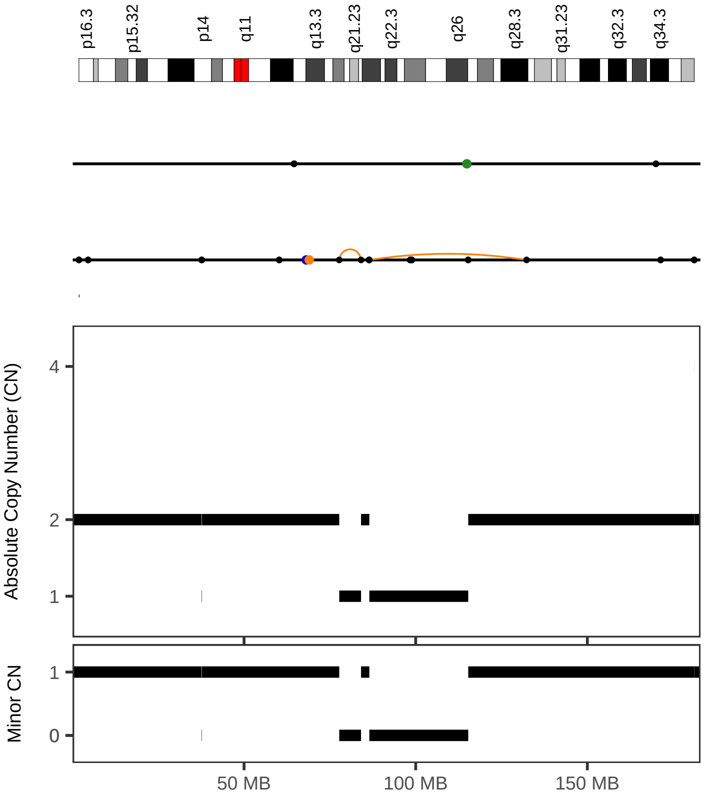

AOCS−093

Cancer type	Ovary−AdenoCA
Position	4:1895208−181137268
Interleaved intrachr. SVs	1
Total SVs (intrachr. + transl.)	11
SV types	DEL: 7; DUP: 1; h2hINV: 0; t2tINV: 0; TRA: 3
SVs in sample	297
Oscillating CN (2 and 3 states)	7, 9
CN segments	9
FDR fragment joints	0.129004
FDR chr. breakp. enrich.	0.96
Linked to chrs	
Purity, ploidy	0.47, 1.78

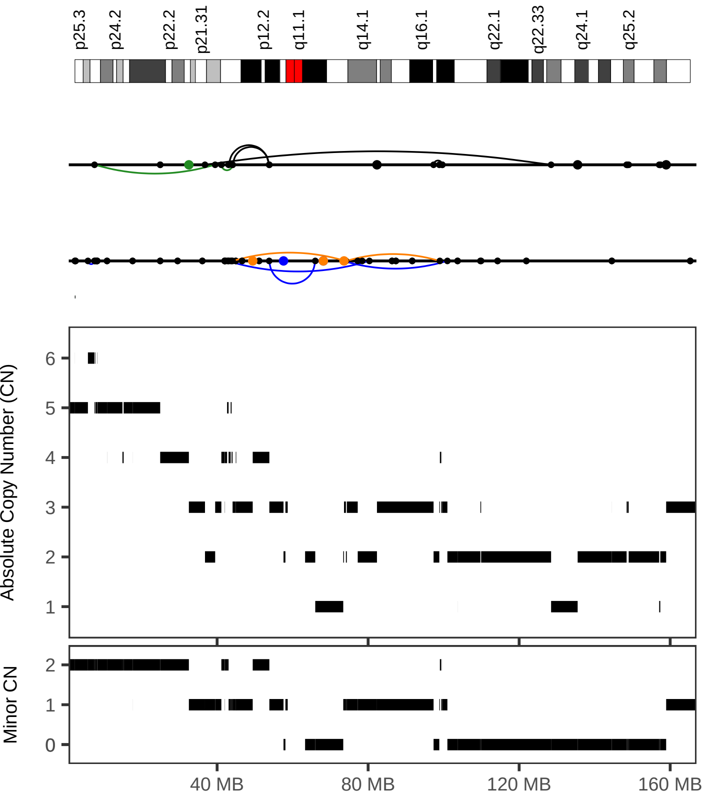

AOCS−094

Cancer type	Ovary−AdenoCA
Position	6:41123088−100990123
Interleaved intrachr. SVs	12
Total SVs (intrachr. + transl.)	18
SV types	DEL: 3; DUP: 4; h2hINV: 3; t2tINV: 2; TRA: 6
SVs in sample	604
Oscillating CN (2 and 3 states)	8, 29
CN segments	29
FDR fragment joints	0.6776251
FDR chr. breakp. enrich.	0.02
Linked to chrs	
Purity, ploidy	0.73, 2.78

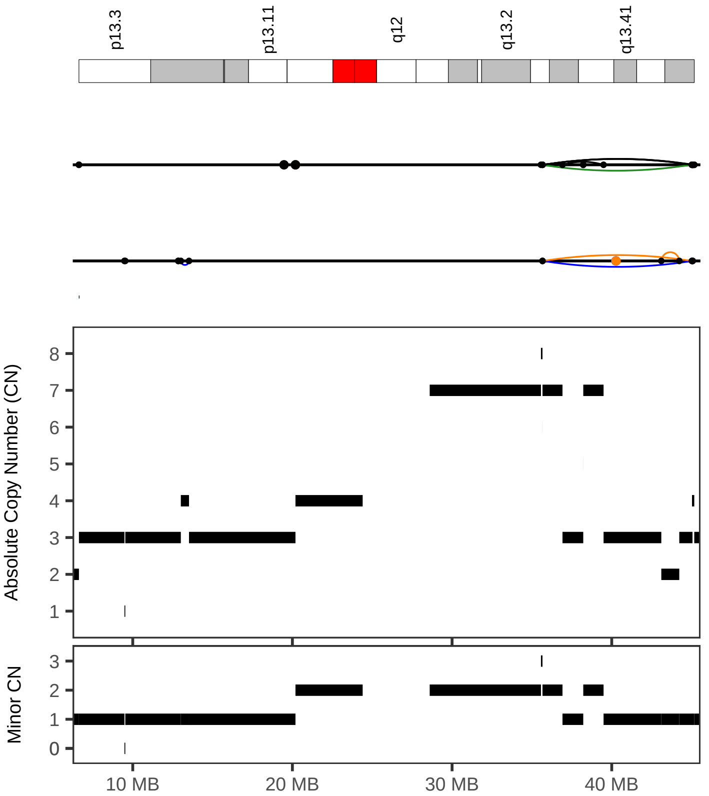

AOCS−094

Cancer type	Ovary−AdenoCA
Position	19:35566735−45168798
Interleaved intrachr. SVs	10
Total SVs (intrachr. + transl.)	12
SV types	DEL: 2; DUP: 1; h2hINV: 5; t2tINV: 2; TRA: 2
SVs in sample	604
Oscillating CN (2 and 3 states)	5, 7
CN segments	13
FDR fragment joints	0.6776251
FDR chr. breakp. enrich.	0
Linked to chrs	15:52307925−79161463;
Purity, ploidy	0.73, 2.78

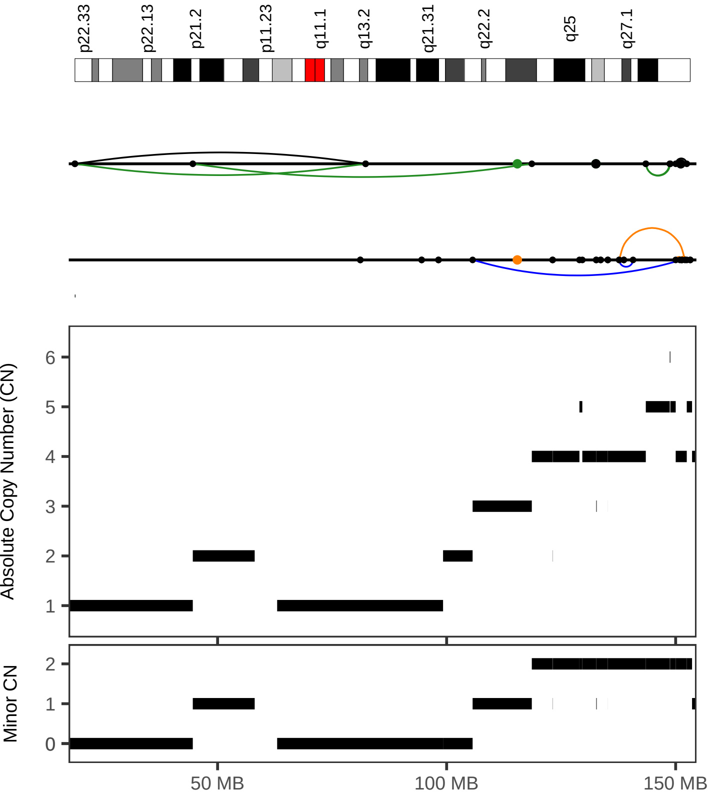

AOCS−094

Cancer type	Ovary−AdenoCA
Position	X:18850169−152426350
Interleaved intrachr. SVs	9
Total SVs (intrachr. + transl.)	14
SV types	DEL: 3; DUP: 2; h2hINV: 2; t2tINV: 2; TRA: 5
SVs in sample	604
Oscillating CN (2 and 3 states)	5, 12
CN segments	19
FDR fragment joints	0.8988396
FDR chr. breakp. enrich.	0.8
Linked to chrs	
Purity, ploidy	0.73, 2.78

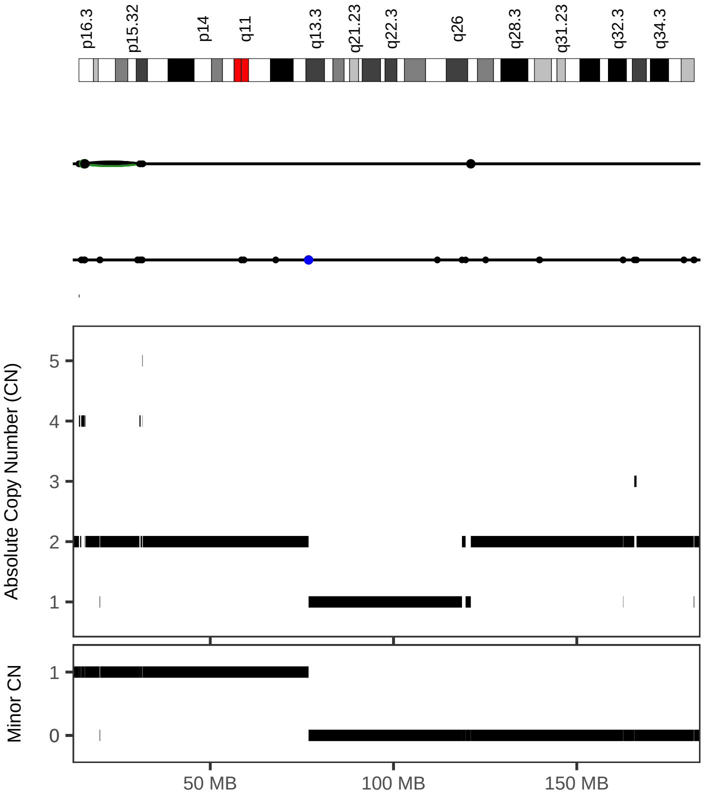

AOCS−095

Cancer type	Ovary−AdenoCA
Position	4:14169897−31589123
Interleaved intrachr. SVs	16
Total SVs (intrachr. + transl.)	18
SV types	DEL: 2; DUP: 3; h2hINV: 5; t2tINV: 6; TRA: 2
SVs in sample	209
Oscillating CN (2 and 3 states)	6, 14
CN segments	14
FDR fragment joints	0.615458
FDR chr. breakp. enrich.	0
Linked to chrs	
Purity, ploidy	0.85, 1.89

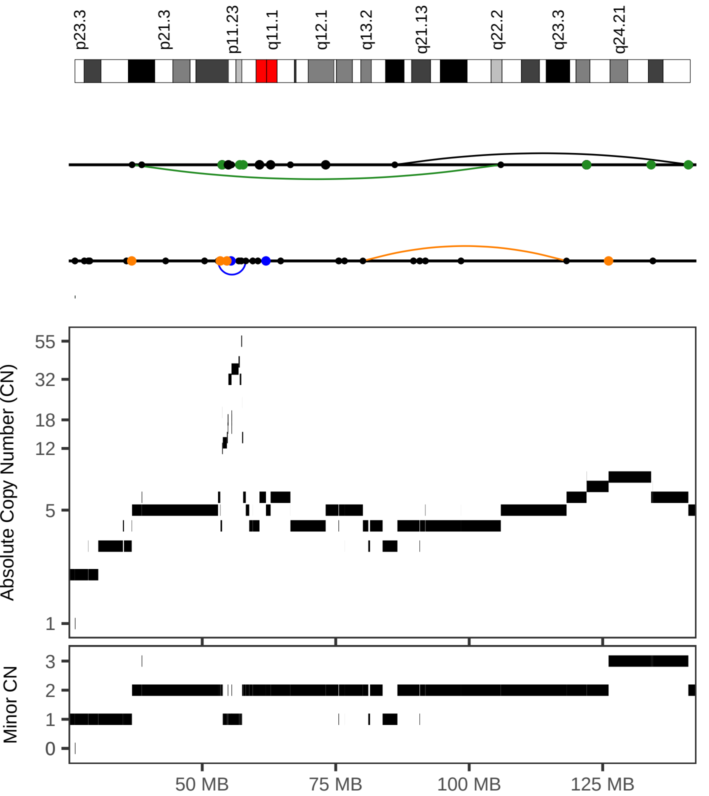

AOCS−108

Cancer type	Ovary−AdenoCA
Position	8:36857660−141396458
Interleaved intrachr. SVs	3
Total SVs (intrachr. + transl.)	22
SV types	DEL: 1; DUP: 0; h2hINV: 1; t2tINV: 1; TRA: 19
SVs in sample	415
Oscillating CN (2 and 3 states)	7, 18
CN segments	58
FDR fragment joints	0.615458
FDR chr. breakp. enrich.	0
Linked to chrs	14:35017318−97009525;
Purity, ploidy	0.7, 3.64

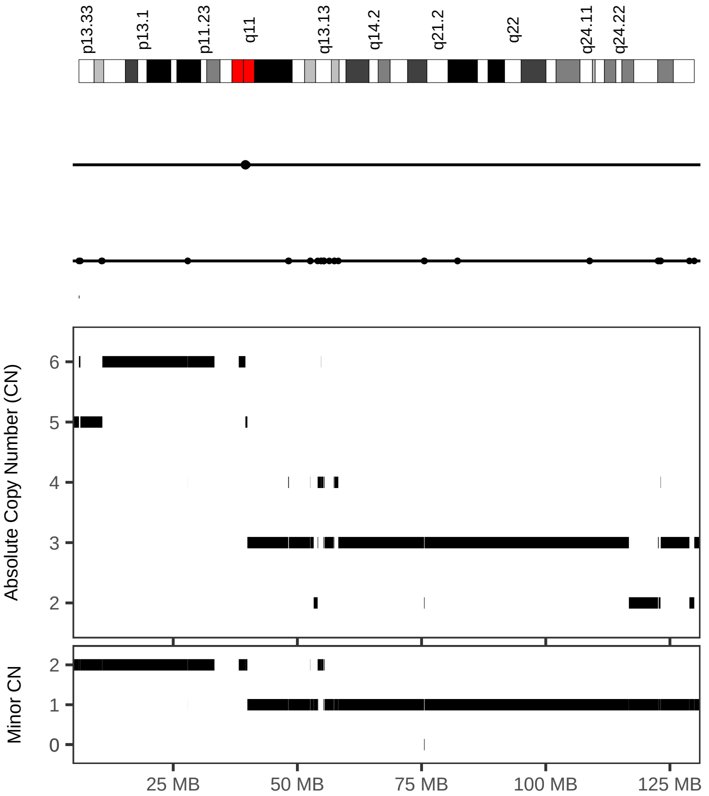

AOCS−112

Cancer type	Ovary−AdenoCA
Position	12:6016071−129897086
Interleaved intrachr. SVs	1
Total SVs (intrachr. + transl.)	16
SV types	DEL: 5; DUP: 10; h2hINV: 0; t2tINV: 0; TRA: 1
SVs in sample	297
Oscillating CN (2 and 3 states)	8, 8
CN segments	33
FDR fragment joints	0.1013295
FDR chr. breakp. enrich.	0.38
Linked to chrs	
Purity, ploidy	0.69, 3.93

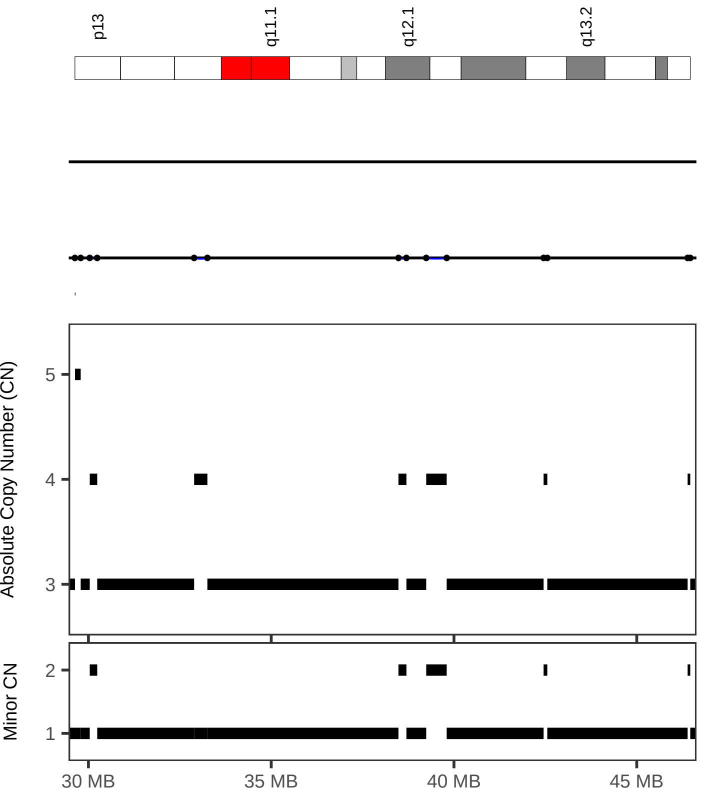

AOCS−112

Cancer type	Ovary−AdenoCA
Position	22:29629715−46463921
Interleaved intrachr. SVs	1
Total SVs (intrachr. + transl.)	7
SV types	DEL: 0; DUP: 7; h2hINV: 0; t2tINV: 0; TRA: 0
SVs in sample	297
Oscillating CN (2 and 3 states)	13, 15
CN segments	15
FDR fragment joints	0.01743054
FDR chr. breakp. enrich.	0.26
Linked to chrs	
Purity, ploidy	0.69, 3.93

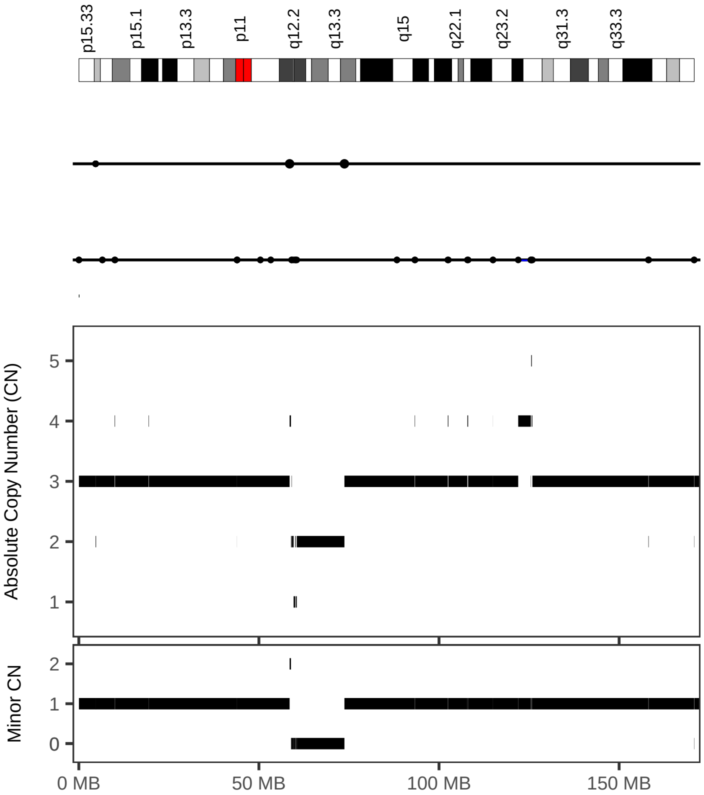

AOCS−116

Cancer type	Ovary−AdenoCA
Position	5:6523540−170861278
Interleaved intrachr. SVs	1
Total SVs (intrachr. + transl.)	18
SV types	DEL: 6; DUP: 10; h2hINV: 0; t2tINV: 0; TRA: 2
SVs in sample	481
Oscillating CN (2 and 3 states)	12, 26
CN segments	34
FDR fragment joints	0.1845613
FDR chr. breakp. enrich.	0.75
Linked to chrs	
Purity, ploidy	0.67, 2.63

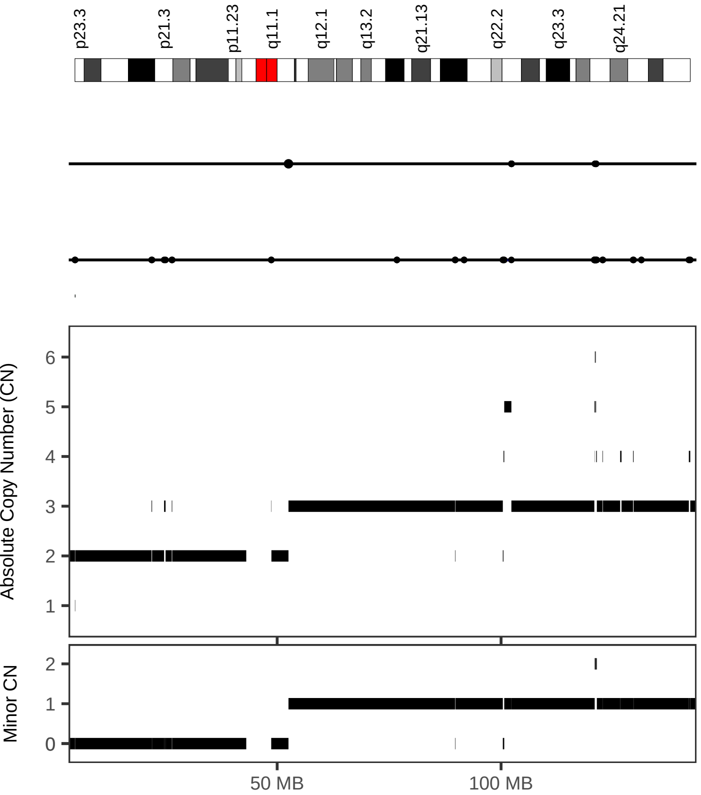

AOCS−116

Cancer type	Ovary−AdenoCA
Position	8:120799542−121389397
Interleaved intrachr. SVs	6
Total SVs (intrachr. + transl.)	6
SV types	DEL: 1; DUP: 3; h2hINV: 1; t2tINV: 1; TRA: 0
SVs in sample	481
Oscillating CN (2 and 3 states)	4, 7
CN segments	9
FDR fragment joints	0.6776251
FDR chr. breakp. enrich.	1
Linked to chrs	
Purity, ploidy	0.67, 2.63

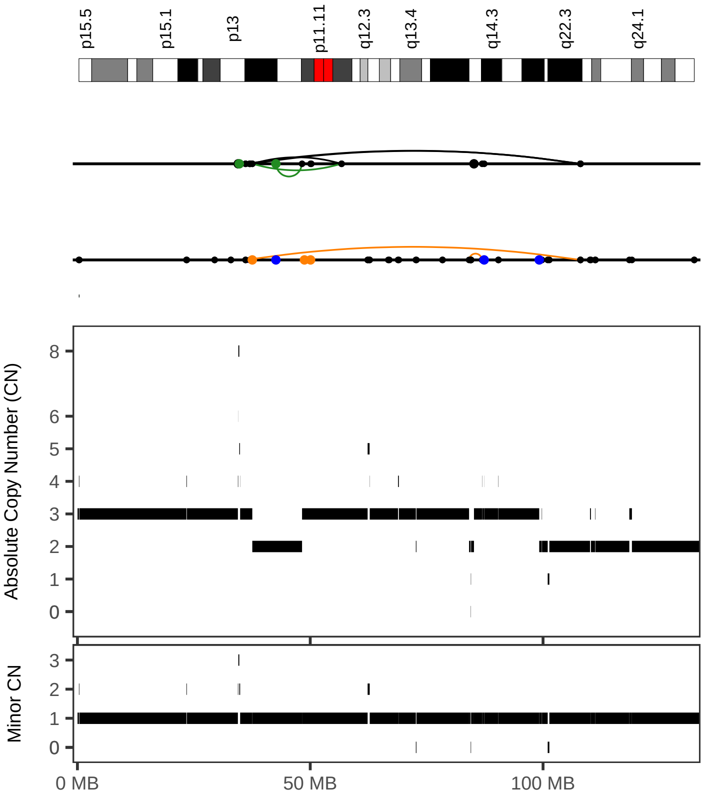

AOCS−116

Cancer type	Ovary−AdenoCA
Position	11:36110794−108039913
Interleaved intrachr. SVs	6
Total SVs (intrachr. + transl.)	14
SV types	DEL: 1; DUP: 1; h2hINV: 2; t2tINV: 2; TRA: 8
SVs in sample	481
Oscillating CN (2 and 3 states)	7, 24
CN segments	27
FDR fragment joints	0.9794281
FDR chr. breakp. enrich.	0
Linked to chrs	14:21900603−102078687;
Purity, ploidy	0.67, 2.63

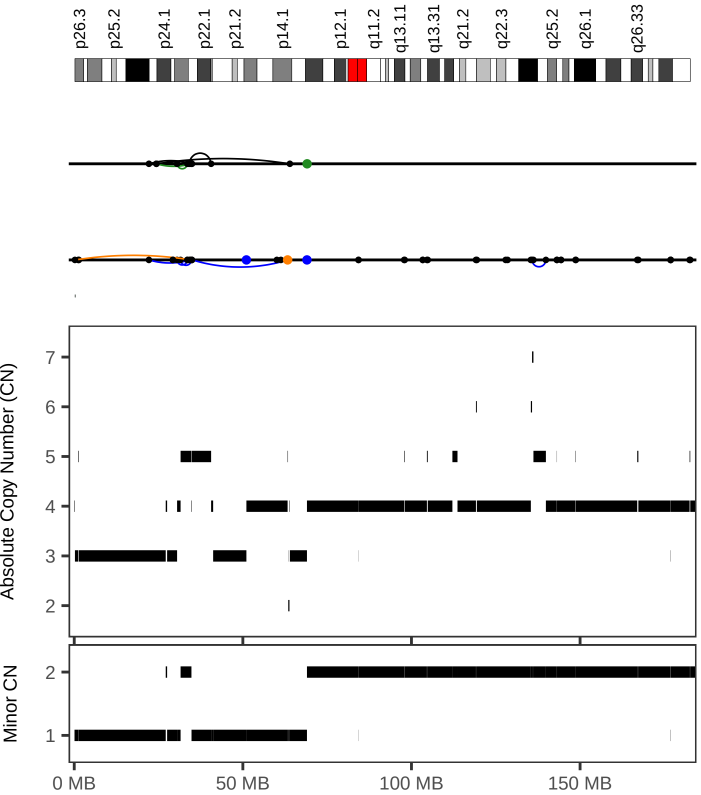

AOCS−117

Cancer type	Ovary−AdenoCA
Position	3:202871−63948256
Interleaved intrachr. SVs	11
Total SVs (intrachr. + transl.)	13
SV types	DEL: 2; DUP: 4; h2hINV: 3; t2tINV: 2; TRA: 2
SVs in sample	333
Oscillating CN (2 and 3 states)	5, 6
CN segments	17
FDR fragment joints	0.7995907
FDR chr. breakp. enrich.	0.11
Linked to chrs	8:42562155−137393244;
Purity, ploidy	0.88, 3.36

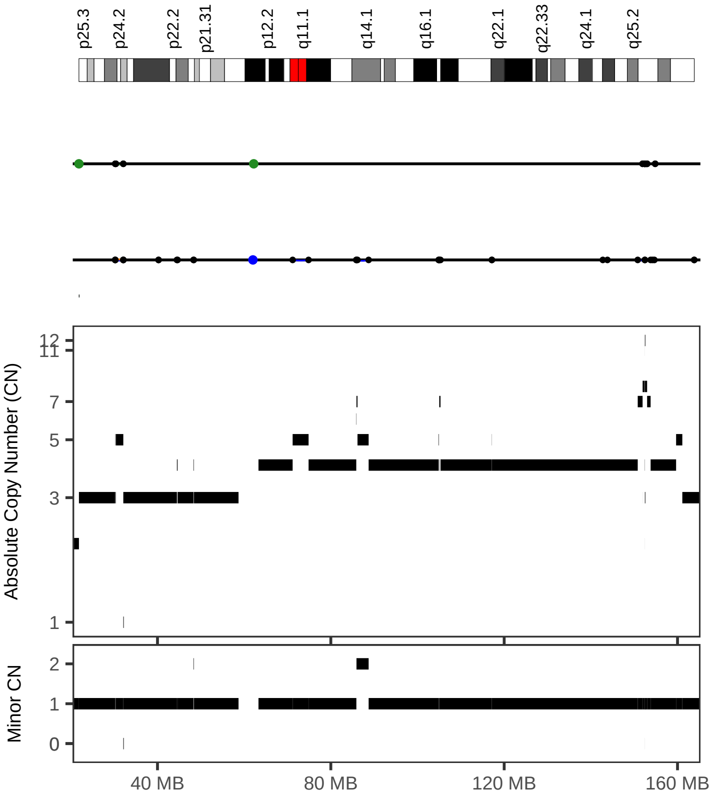

AOCS−117

Cancer type	Ovary−AdenoCA
Position	6:30232935−32110297
Interleaved intrachr. SVs	6
Total SVs (intrachr. + transl.)	6
SV types	DEL: 2; DUP: 1; h2hINV: 1; t2tINV: 2; TRA: 0
SVs in sample	333
Oscillating CN (2 and 3 states)	4, 6
CN segments	6
FDR fragment joints	0.9284301
FDR chr. breakp. enrich.	0.05
Linked to chrs	
Purity, ploidy	0.88, 3.36

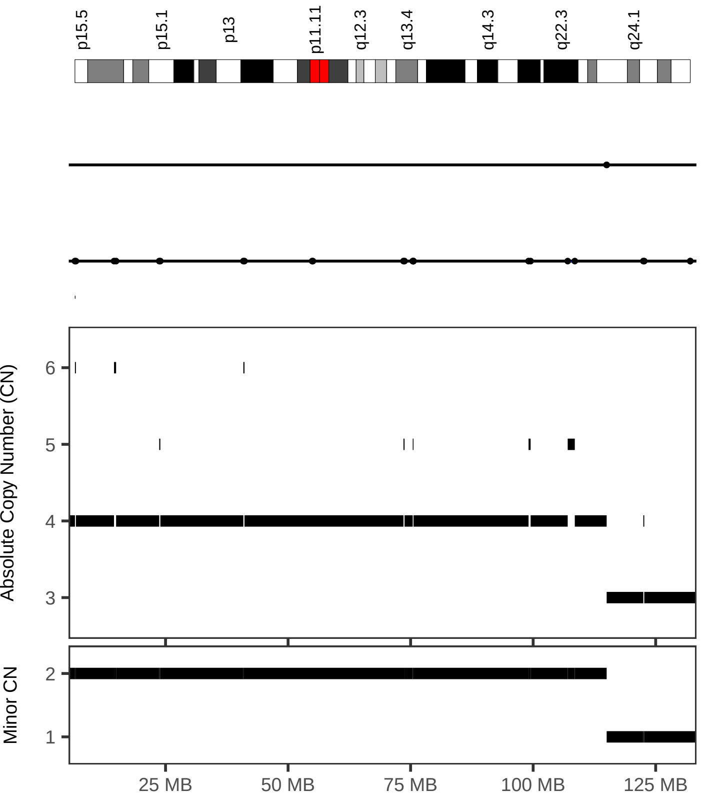

AOCS−117

Cancer type	Ovary−AdenoCA
Position	11:6511167−122670130
Interleaved intrachr. SVs	1
Total SVs (intrachr. + transl.)	11
SV types	DEL: 1; DUP: 10; h2hINV: 0; t2tINV: 0; TRA: 0
SVs in sample	333
Oscillating CN (2 and 3 states)	9, 16
CN segments	19
FDR fragment joints	0.003601907
FDR chr. breakp. enrich.	0.82
Linked to chrs	
Purity, ploidy	0.88, 3.36

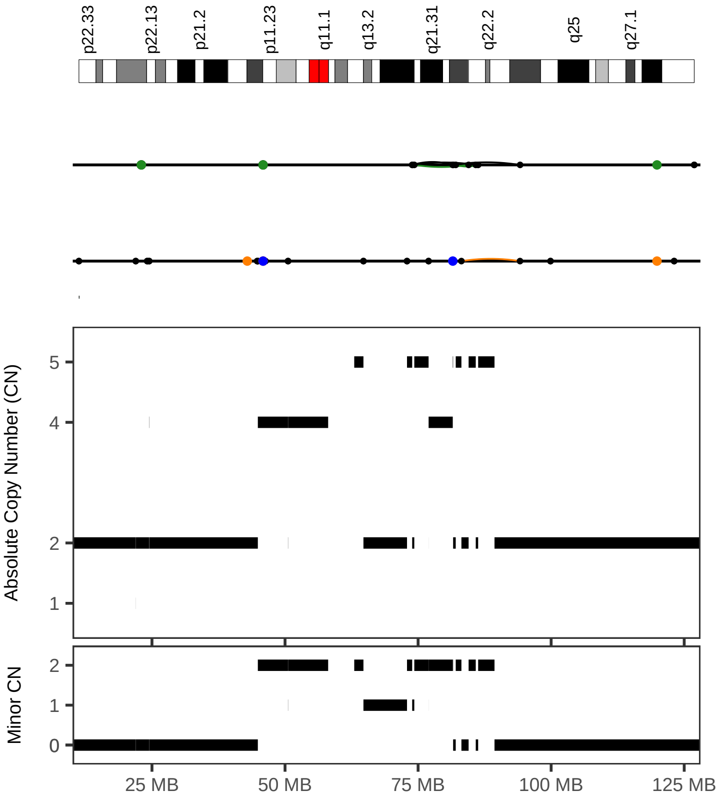

AOCS−119

Cancer type	Ovary−AdenoCA
Position	X:73888460−94161009
Interleaved intrachr. SVs	6
Total SVs (intrachr. + transl.)	7
SV types	DEL: 1; DUP: 0; h2hINV: 3; t2tINV: 2; TRA: 1
SVs in sample	375
Oscillating CN (2 and 3 states)	8, 8
CN segments	12
FDR fragment joints	0.7735152
FDR chr. breakp. enrich.	0.06
Linked to chrs	
Purity, ploidy	0.32, 3.29

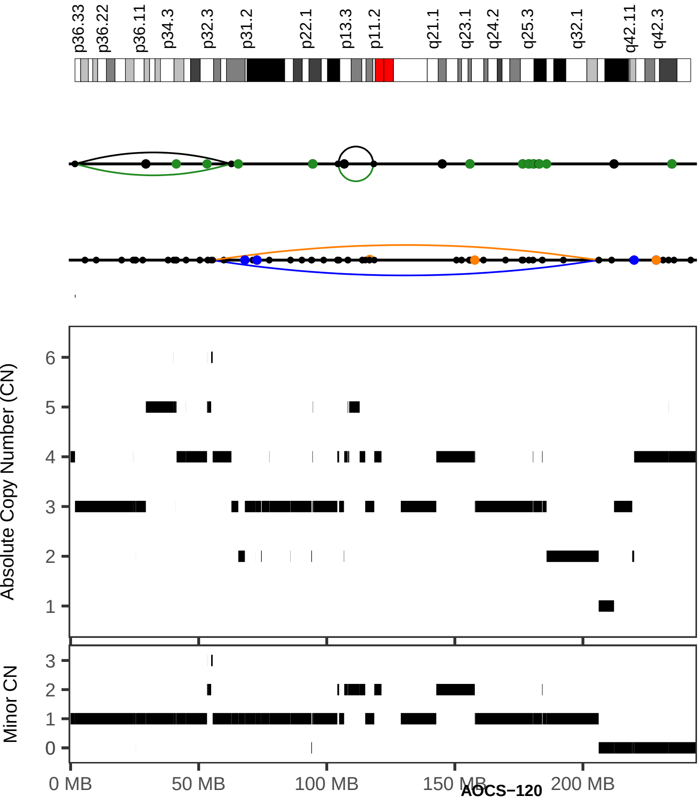

AOCS−120

Cancer type	Ovary−AdenoCA
Position	1:1708031−206256543
Interleaved intrachr. SVs	4
Total SVs (intrachr. + transl.)	31
SV types	DEL: 2; DUP: 2; h2hINV: 2; t2tINV: 2; TRA: 23
SVs in sample	790
Oscillating CN (2 and 3 states)	12, 21
CN segments	61
FDR fragment joints	0.5435077
FDR chr. breakp. enrich.	0.01
Linked to chrs	11:11833414−60671100;5:680638−159964317 8:59128810−114398645;
Purity, ploidy	0.84, 2.89

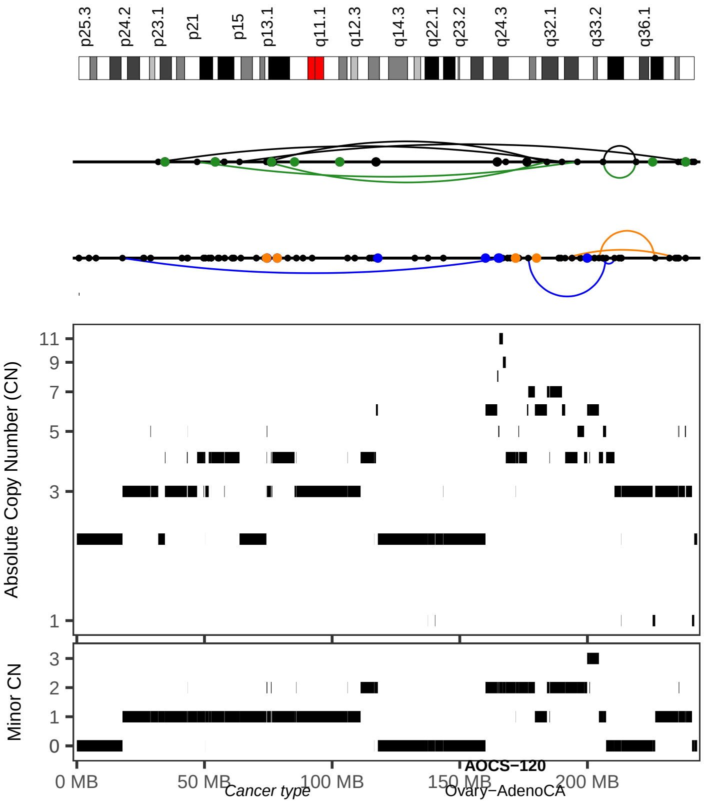

AOCS−120

Cancer type	Ovary−AdenoCA
Position	2:17888044−240960571
Interleaved intrachr. SVs	12
Total SVs (intrachr. + transl.)	40
SV types	DEL: 2; DUP: 3; h2hINV: 4; t2tINV: 3; TRA: 28
SVs in sample	790
Oscillating CN (2 and 3 states)	11, 13
CN segments	86
FDR fragment joints	0.8135044
FDR chr. breakp. enrich.	0
Linked to chrs	1:1708031−206256542;12:21770067−118615375 15:40235127−98220741;5:680638−159964317 9:16411704−136399897;X:6640984−97824094

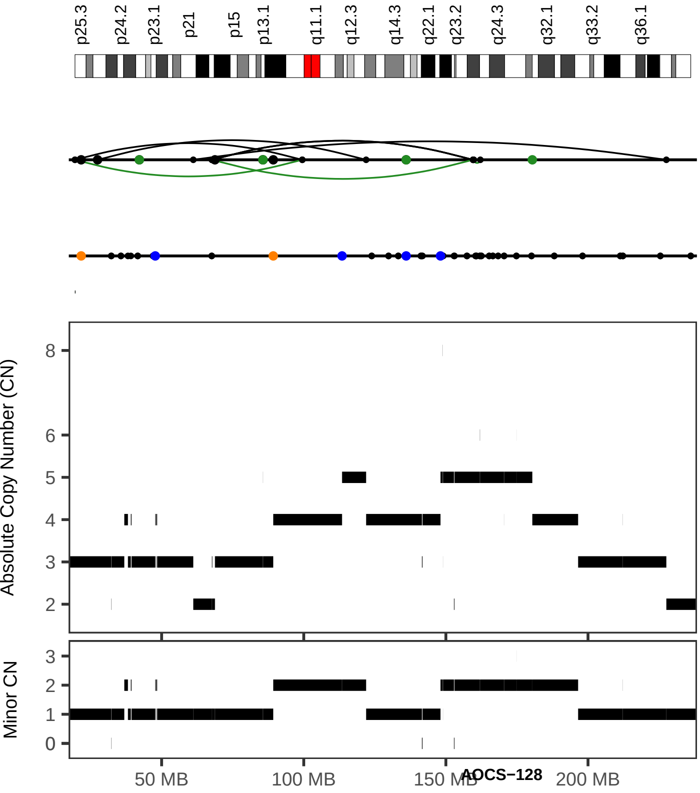

AOCS−128

Cancer type	Ovary−AdenoCA
Position	2:19515679−227555412
Interleaved intrachr. SVs	7
Total SVs (intrachr. + transl.)	25
SV types	DEL: 0; DUP: 0; h2hINV: 5; t2tINV: 2; TRA: 18
SVs in sample	496
Oscillating CN (2 and 3 states)	9, 15
CN segments	40
FDR fragment joints	0.5435077
FDR chr. breakp. enrich.	0.02
Linked to chrs	1:3190607−179536706;16:10983970−48633675 4:8856647−35023984;7:5529579−140670364 X:40320844−140849706;

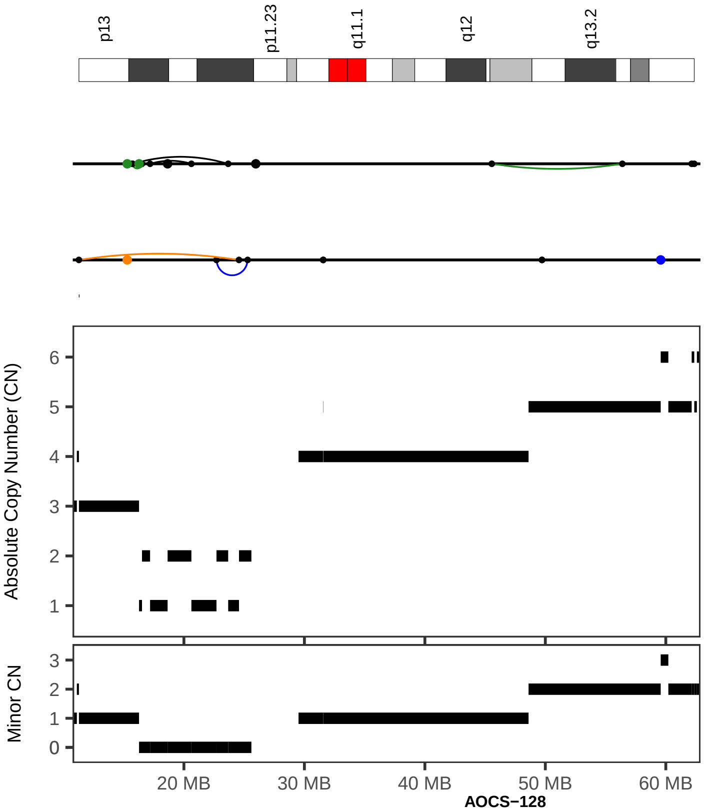

AOCS−128

Cancer type	Ovary−AdenoCA
Position	20:11284294−25291268
Interleaved intrachr. SVs	4
Total SVs (intrachr. + transl.)	8
SV types	DEL: 1; DUP: 1; h2hINV: 1; t2tINV: 1; TRA: 4
SVs in sample	496
Oscillating CN (2 and 3 states)	8, 9
CN segments	9
FDR fragment joints	0.8653243
FDR chr. breakp. enrich.	0.11
Linked to chrs	
Purity, ploidy	0.37, 3.11

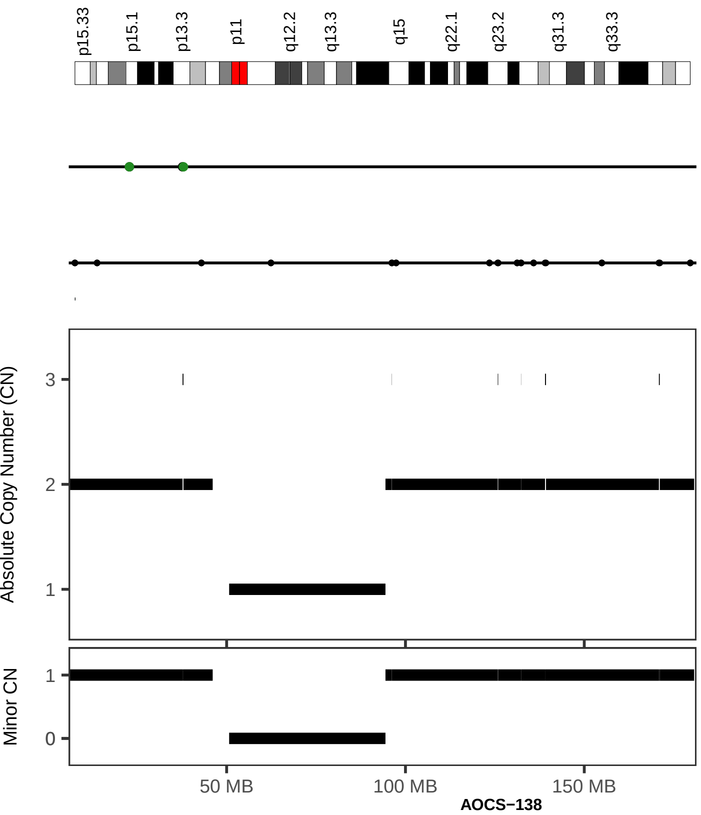

AOCS−138

Cancer type	Ovary−AdenoCA
Position	5:96175581−171118223
Interleaved intrachr. SVs	1
Total SVs (intrachr. + transl.)	5
SV types	DEL: 0; DUP: 5; h2hINV: 0; t2tINV: 0; TRA: 0
SVs in sample	323
Oscillating CN (2 and 3 states)	10, 10
CN segments	10
FDR fragment joints	0.1013295
FDR chr. breakp. enrich.	1
Linked to chrs	
Purity, ploidy	0.65, 1.78

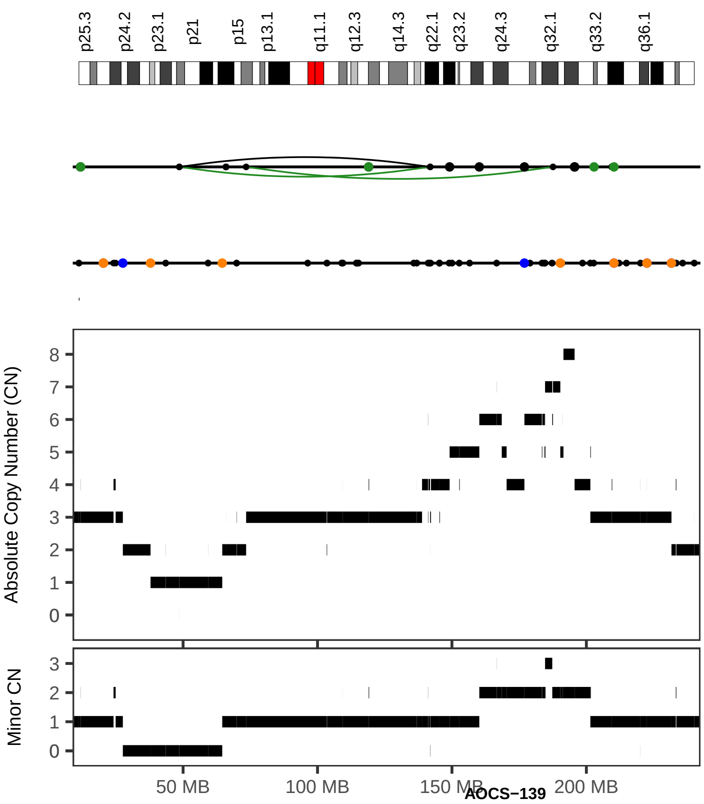

AOCS−139

Cancer type	Ovary−AdenoCA
Position	2:48598839−187592335
Interleaved intrachr. SVs	4
Total SVs (intrachr. + transl.)	11
SV types	DEL: 1; DUP: 0; h2hINV: 1; t2tINV: 2; TRA: 7
SVs in sample	542
Oscillating CN (2 and 3 states)	8, 19
CN segments	45
FDR fragment joints	0.615458
FDR chr. breakp. enrich.	0
Linked to chrs	4:25070704−182597222;6:7505549−166697295
Purity, ploidy	0.49, 3.07

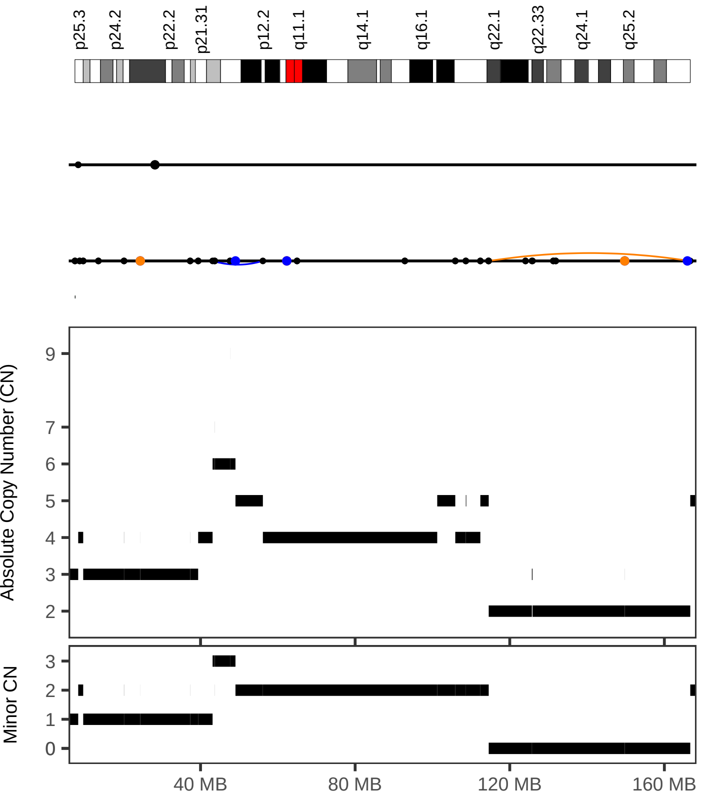

AOCS−139

Cancer type	Ovary−AdenoCA
Position	6:7505549−166697296
Interleaved intrachr. SVs	1
Total SVs (intrachr. + transl.)	18
SV types	DEL: 1; DUP: 8; h2hINV: 0; t2tINV: 0; TRA: 9
SVs in sample	542
Oscillating CN (2 and 3 states)	10, 14
CN segments	28
FDR fragment joints	0.009092919
FDR chr. breakp. enrich.	0.94
Linked to chrs	X:69174585−97165170;
Purity, ploidy	0.49, 3.07

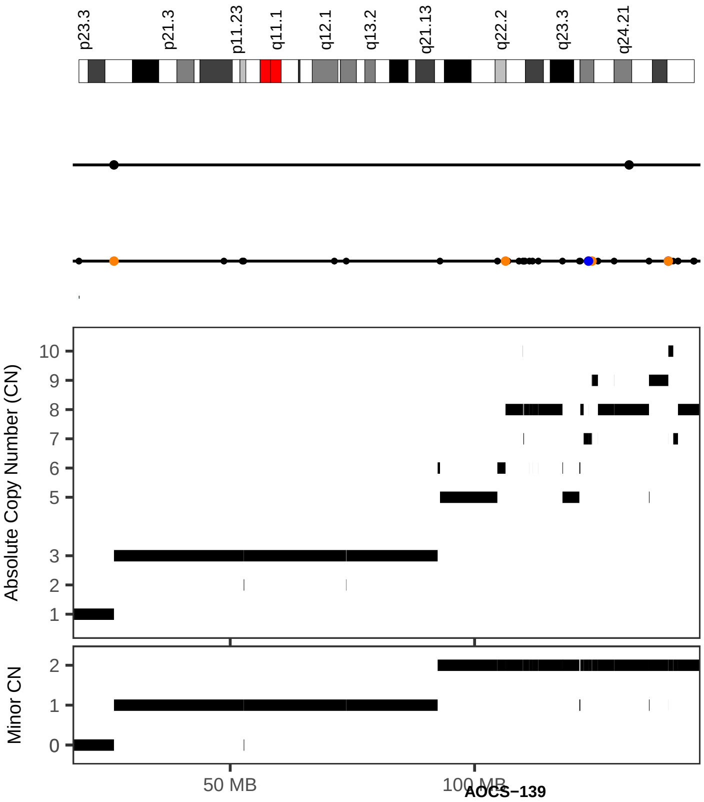

AOCS−139

Cancer type	Ovary−AdenoCA
Position	8:48696966−144814173
Interleaved intrachr. SVs	1
Total SVs (intrachr. + transl.)	19
SV types	DEL: 5; DUP: 7; h2hINV: 0; t2tINV: 0; TRA: 7
SVs in sample	542
Oscillating CN (2 and 3 states)	8, 10
CN segments	39
FDR fragment joints	0.1013295
FDR chr. breakp. enrich.	0.3
Linked to chrs	15:37623815−47490289;4:25070704−182597222
Purity, ploidy	0.49, 3.07

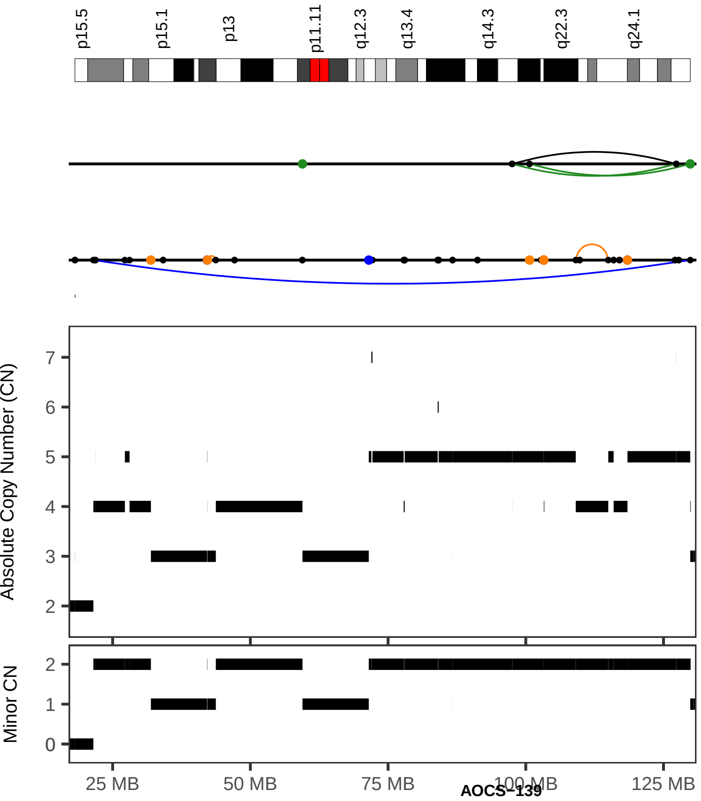

AOCS−139

Cancer type	Ovary−AdenoCA
Position	11:97517122−129852368
Interleaved intrachr. SVs	3
Total SVs (intrachr. + transl.)	9
SV types	DEL: 0; DUP: 0; h2hINV: 1; t2tINV: 2; TRA: 6
SVs in sample	542
Oscillating CN (2 and 3 states)	8, 11
CN segments	11
FDR fragment joints	0.6776251
FDR chr. breakp. enrich.	0.03
Linked to chrs	17:26827564−74272264;2:48598839−187592334
Purity, ploidy	0.49, 3.07

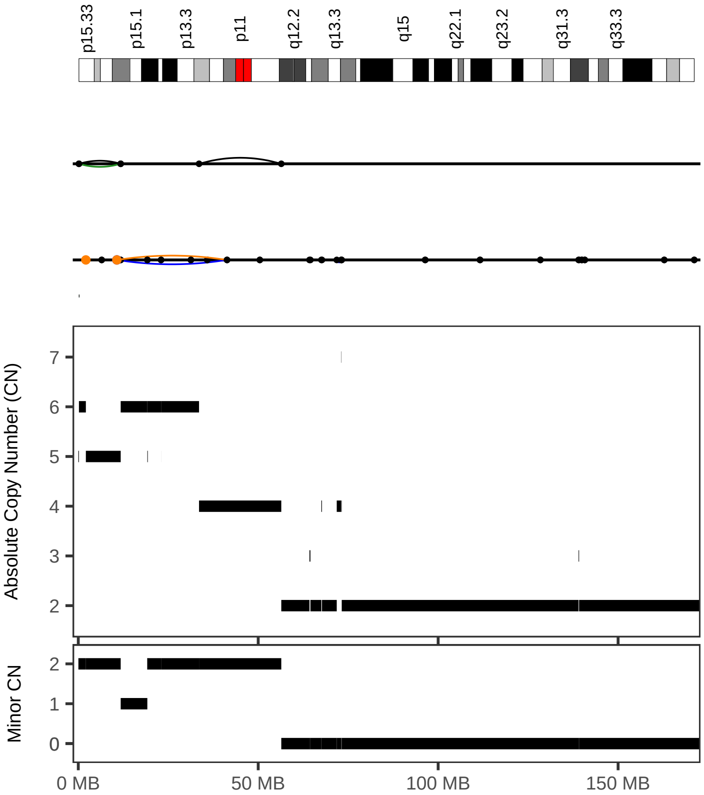

AOCS−150

Cancer type	Ovary−AdenoCA
Position	5:175233−56407292
Interleaved intrachr. SVs	5
Total SVs (intrachr. + transl.)	8
SV types	DEL: 1; DUP: 1; h2hINV: 2; t2tINV: 1; TRA: 3
SVs in sample	428
Oscillating CN (2 and 3 states)	7, 8
CN segments	9
FDR fragment joints	0.8653243
FDR chr. breakp. enrich.	1
Linked to chrs	
Purity, ploidy	0.43, 3.29

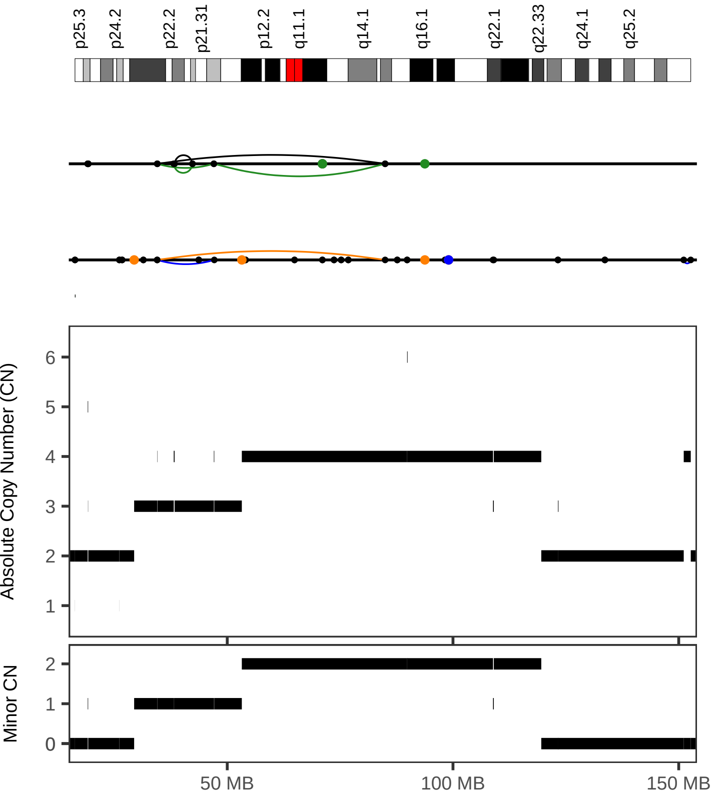

AOCS−150

Cancer type	Ovary−AdenoCA
Position	6:34474425−84968074
Interleaved intrachr. SVs	5
Total SVs (intrachr. + transl.)	8
SV types	DEL: 1; DUP: 1; h2hINV: 1; t2tINV: 2; TRA: 3
SVs in sample	428
Oscillating CN (2 and 3 states)	8, 8
CN segments	8
FDR fragment joints	0.8653243
FDR chr. breakp. enrich.	0.2
Linked to chrs	8:29804740−127337991;
Purity, ploidy	0.43, 3.29

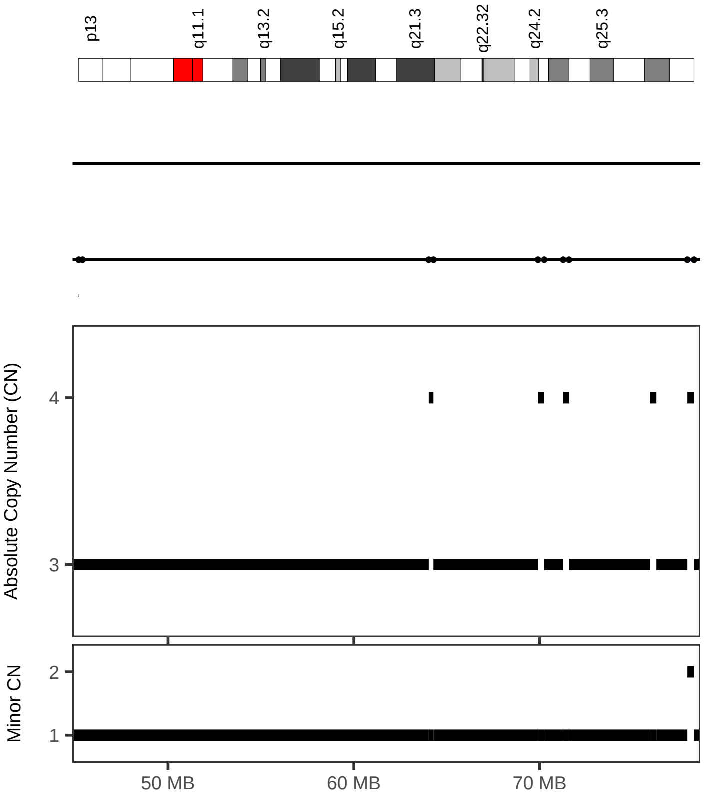

AOCS−159

Cancer type	Ovary−AdenoCA
Position	15:45195576−78304180
Interleaved intrachr. SVs	1
Total SVs (intrachr. + transl.)	5
SV types	DEL: 0; DUP: 5; h2hINV: 0; t2tINV: 0; TRA: 0
SVs in sample	259
Oscillating CN (2 and 3 states)	11, 11
CN segments	11
FDR fragment joints	0.1013295
FDR chr. breakp. enrich.	0.79
Linked to chrs	
Purity, ploidy	0.65, 2.93

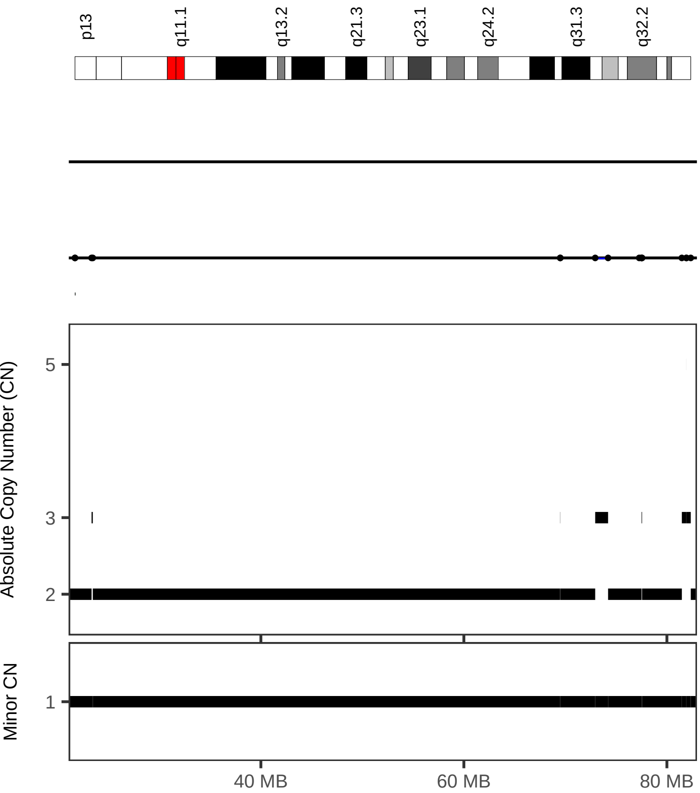

AOCS−161

Cancer type	Ovary−AdenoCA
Position	14:23318396−82349714
Interleaved intrachr. SVs	1
Total SVs (intrachr. + transl.)	5
SV types	DEL: 0; DUP: 5; h2hINV: 0; t2tINV: 0; TRA: 0
SVs in sample	255
Oscillating CN (2 and 3 states)	9, 12
CN segments	12
FDR fragment joints	0.1013295
FDR chr. breakp. enrich.	0.71
Linked to chrs	
Purity, ploidy	0.68, 2.44

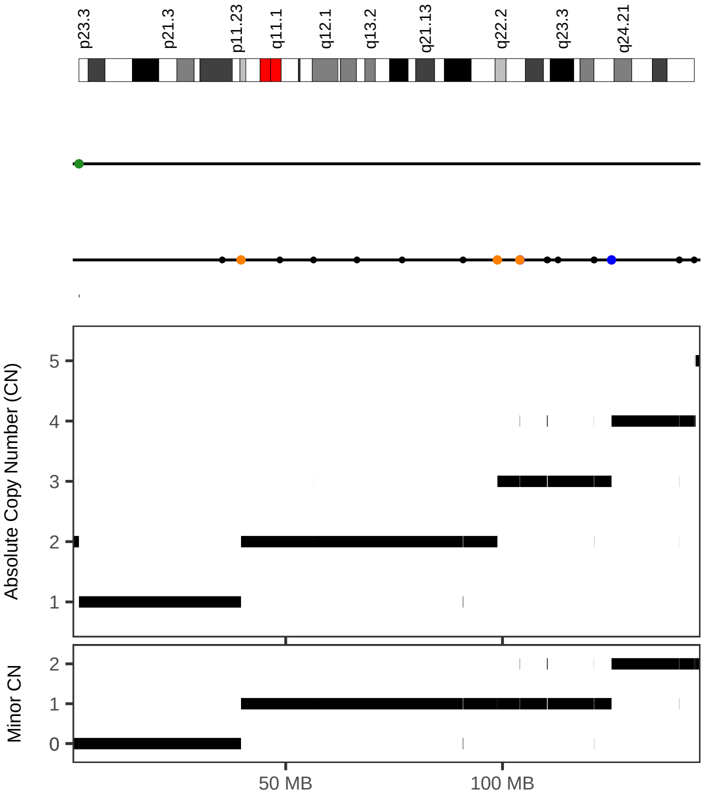

AOCS−164

Cancer type	Ovary−AdenoCA
Position	8:35352452−144225865
Interleaved intrachr. SVs	1
Total SVs (intrachr. + transl.)	13
SV types	DEL: 4; DUP: 4; h2hINV: 0; t2tINV: 0; TRA: 5
SVs in sample	253
Oscillating CN (2 and 3 states)	7, 16
CN segments	21
FDR fragment joints	0.1817907
FDR chr. breakp. enrich.	0.04
Linked to chrs	6:90940646−114765608;
Purity, ploidy	0.95, 1.98

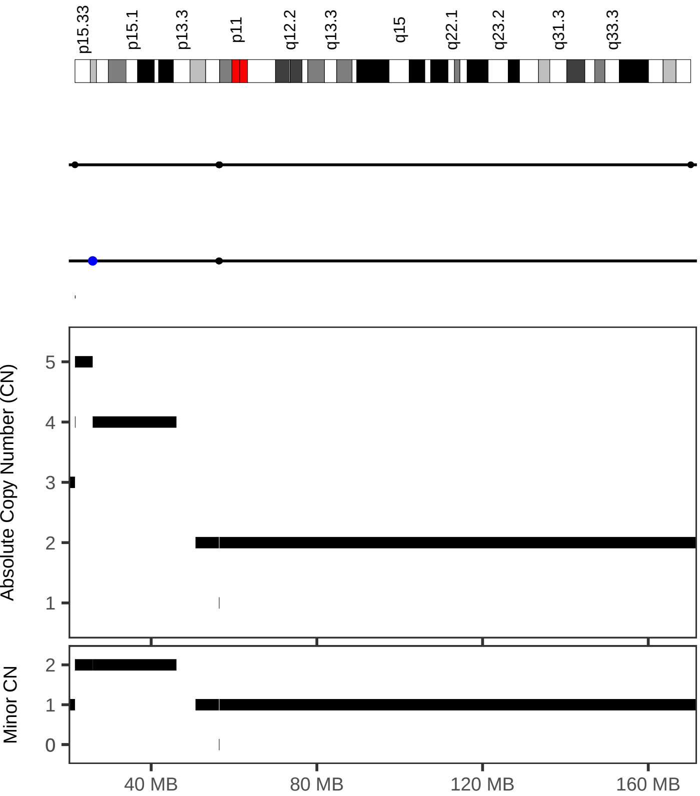

AOCS−165

Cancer type	Ovary−AdenoCA
Position	5:56289678−56503515
Interleaved intrachr. SVs	6
Total SVs (intrachr. + transl.)	6
SV types	DEL: 2; DUP: 1; h2hINV: 2; t2tINV: 1; TRA: 0
SVs in sample	139
Oscillating CN (2 and 3 states)	4, 4
CN segments	4
FDR fragment joints	0.9284301
FDR chr. breakp. enrich.	0.99
Linked to chrs	
Purity, ploidy	0.79, 2.71

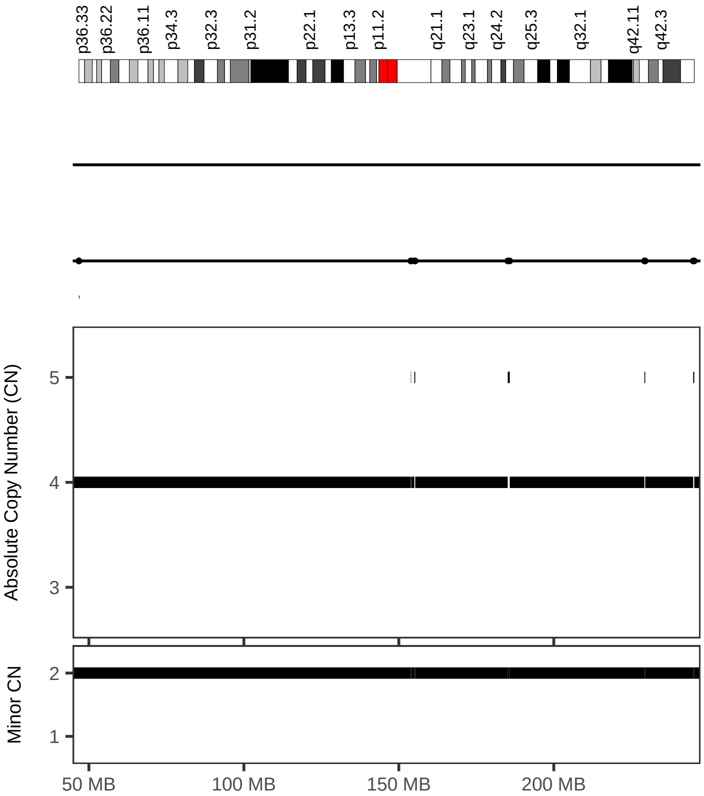

AOCS−166

Cancer type	Ovary−AdenoCA
Position	1:153869668−245318697
Interleaved intrachr. SVs	1
Total SVs (intrachr. + transl.)	5
SV types	DEL: 0; DUP: 5; h2hINV: 0; t2tINV: 0; TRA: 0
SVs in sample	170
Oscillating CN (2 and 3 states)	10, 10
CN segments	10
FDR fragment joints	0.1013295
FDR chr. breakp. enrich.	0.24
Linked to chrs	
Purity, ploidy	0.77, 3.46

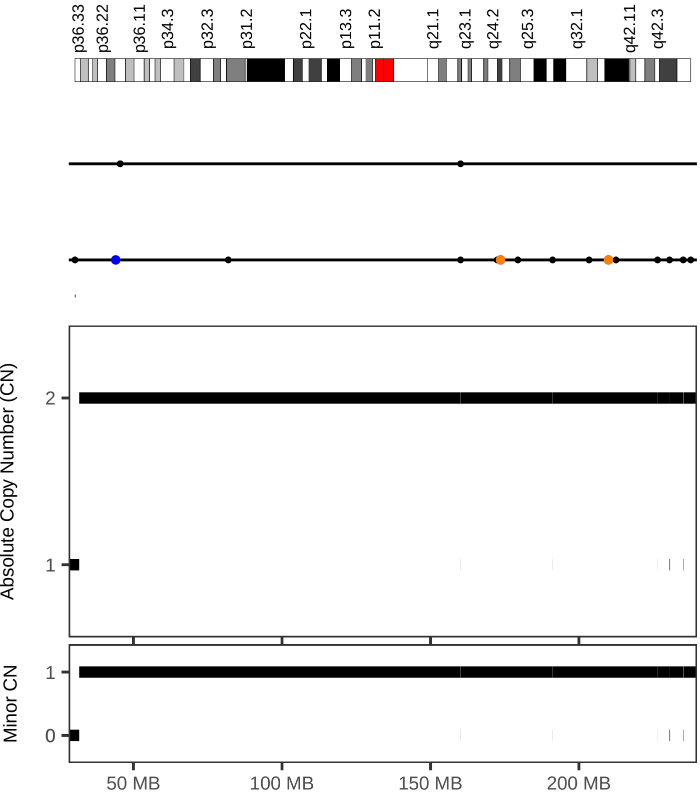

AOCS−167

Cancer type	Ovary−AdenoCA
Position	1:81903189−235162240
Interleaved intrachr. SVs	1
Total SVs (intrachr. + transl.)	9
SV types	DEL: 5; DUP: 0; h2hINV: 0; t2tINV: 0; TRA: 4
SVs in sample	181
Oscillating CN (2 and 3 states)	11, 11
CN segments	11
FDR fragment joints	0.1178349
FDR chr. breakp. enrich.	0.21
Linked to chrs	
Purity, ploidy	0.81, 1.9

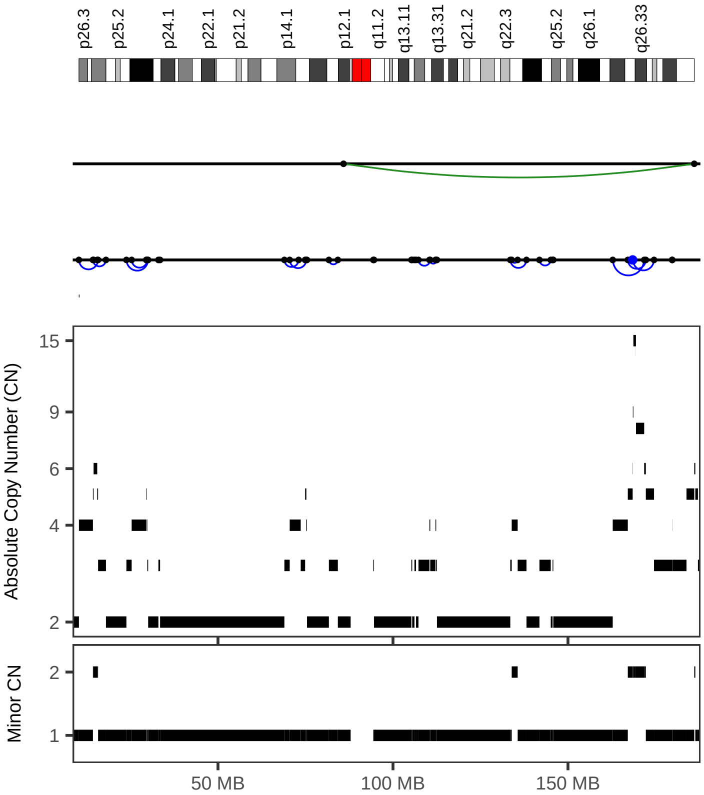

AOCS−169

Cancer type	Ovary−AdenoCA
Position	3:68970737−174586485
Interleaved intrachr. SVs	3
Total SVs (intrachr. + transl.)	7
SV types	DEL: 0; DUP: 6; h2hINV: 0; t2tINV: 0; TRA: 1
SVs in sample	413
Oscillating CN (2 and 3 states)	10, 23
CN segments	38
FDR fragment joints	0.01743054
FDR chr. breakp. enrich.	0.93
Linked to chrs	
Purity, ploidy	0.65, 2.07

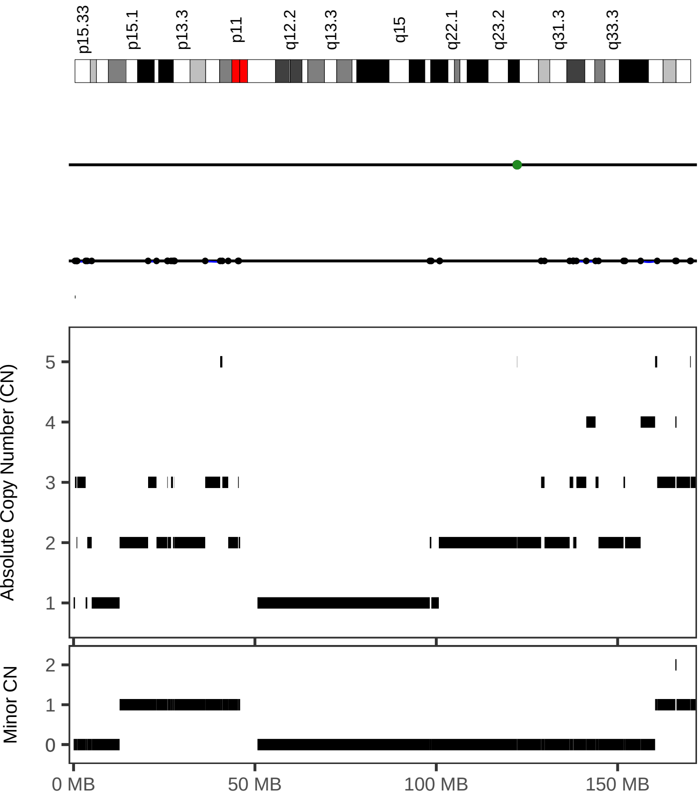

AOCS−169

Cancer type	Ovary−AdenoCA
Position	5:389649−170154621
Interleaved intrachr. SVs	1
Total SVs (intrachr. + transl.)	23
SV types	DEL: 2; DUP: 19; h2hINV: 0; t2tINV: 0; TRA: 2
SVs in sample	413
Oscillating CN (2 and 3 states)	10, 10
CN segments	46
FDR fragment joints	1.241524e−05
FDR chr. breakp. enrich.	0.83
Linked to chrs	9:12691740−121121972;
Purity, ploidy	0.65, 2.07

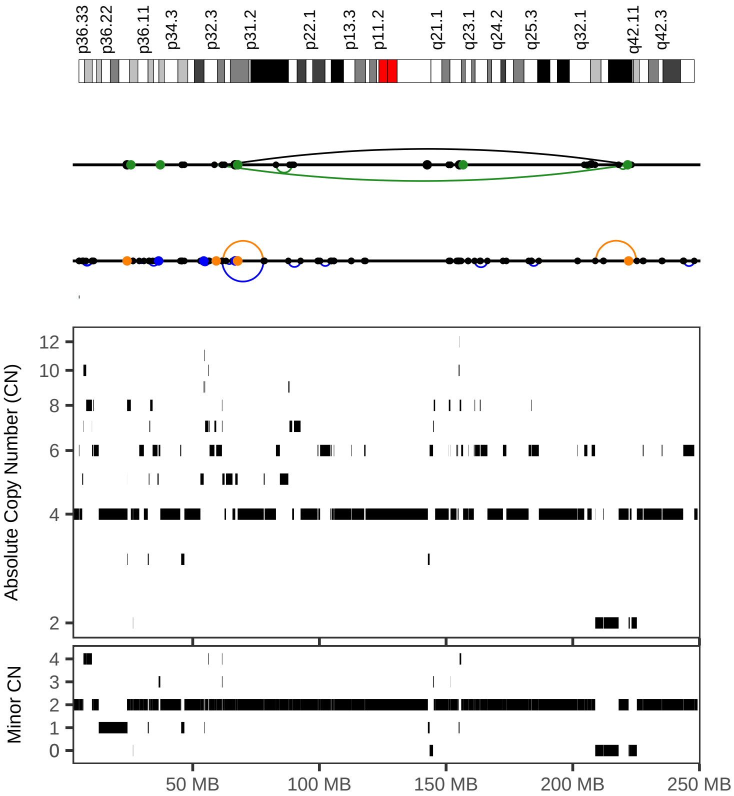

AOCS−170

Cancer type	Ovary−AdenoCA
Position	1:58559059−225301156
Interleaved intrachr. SVs	6
Total SVs (intrachr. + transl.)	21
SV types	DEL: 2; DUP: 2; h2hINV: 1; t2tINV: 1; TRA: 15
SVs in sample	773
Oscillating CN (2 and 3 states)	13, 31
CN segments	77
FDR fragment joints	0.8419568
FDR chr. breakp. enrich.	0.03
Linked to chrs	
Purity, ploidy	0.7, 4.27

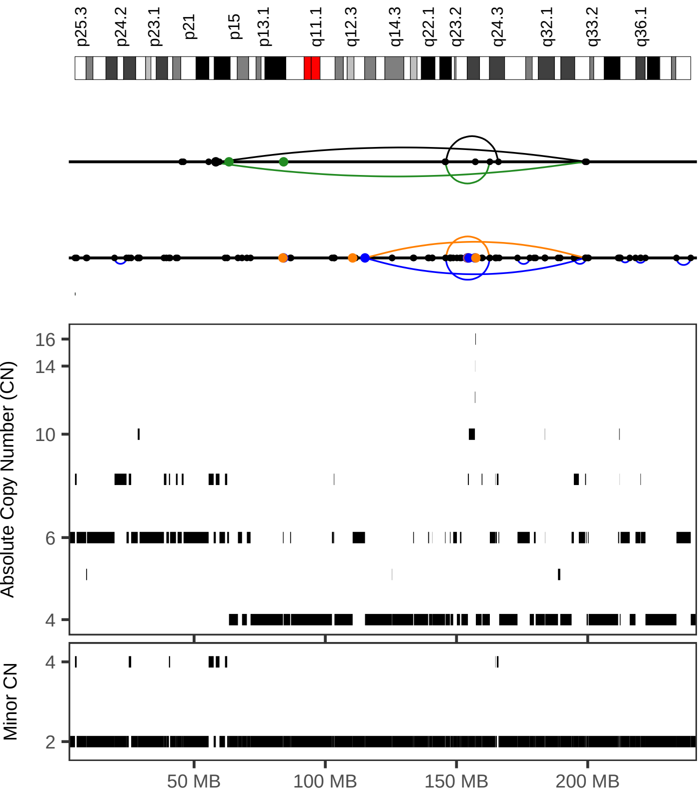

AOCS−170

Cancer type	Ovary−AdenoCA
Position	2:145638253−166271367
Interleaved intrachr. SVs	6
Total SVs (intrachr. + transl.)	12
SV types	DEL: 1; DUP: 2; h2hINV: 1; t2tINV: 2; TRA: 6
SVs in sample	773
Oscillating CN (2 and 3 states)	10, 10
CN segments	24
FDR fragment joints	0.615458
FDR chr. breakp. enrich.	1
Linked to chrs	
Purity, ploidy	0.7, 4.27

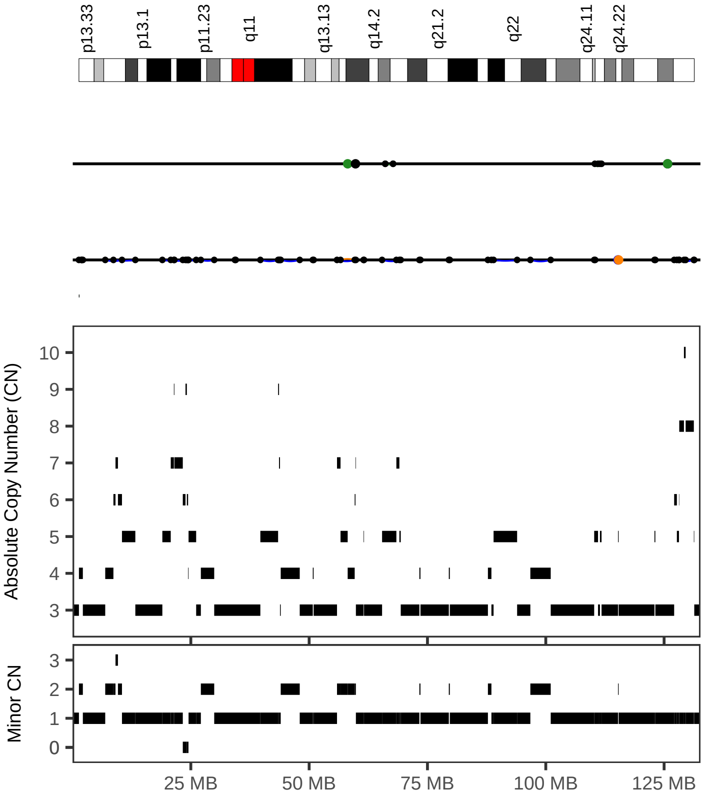

AOCS−170

Cancer type	Ovary−AdenoCA
Position	12:18986632−111780873
Interleaved intrachr. SVs	3
Total SVs (intrachr. + transl.)	8
SV types	DEL: 1; DUP: 3; h2hINV: 1; t2tINV: 1; TRA: 2
SVs in sample	773
Oscillating CN (2 and 3 states)	7, 16
CN segments	46
FDR fragment joints	0.8653243
FDR chr. breakp. enrich.	0.64
Linked to chrs	
Purity, ploidy	0.7, 4.27

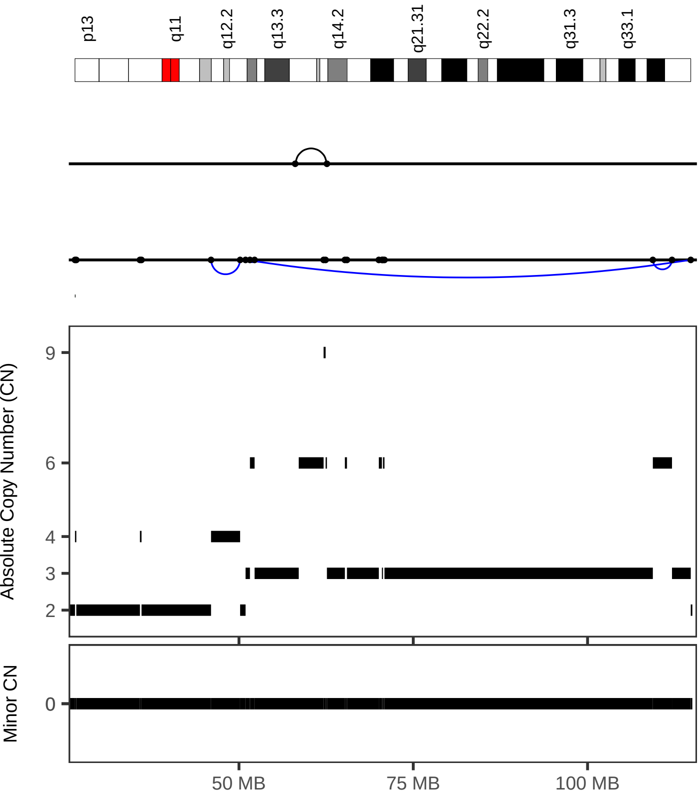

AOCS−170

Cancer type	Ovary−AdenoCA
Position	13:26480205−114798981
Interleaved intrachr. SVs	1
Total SVs (intrachr. + transl.)	11
SV types	DEL: 0; DUP: 10; h2hINV: 1; t2tINV: 0; TRA: 0
SVs in sample	773
Oscillating CN (2 and 3 states)	10, 11
CN segments	22
FDR fragment joints	0.003601907
FDR chr. breakp. enrich.	0.03
Linked to chrs	
Purity, ploidy	0.7, 4.27

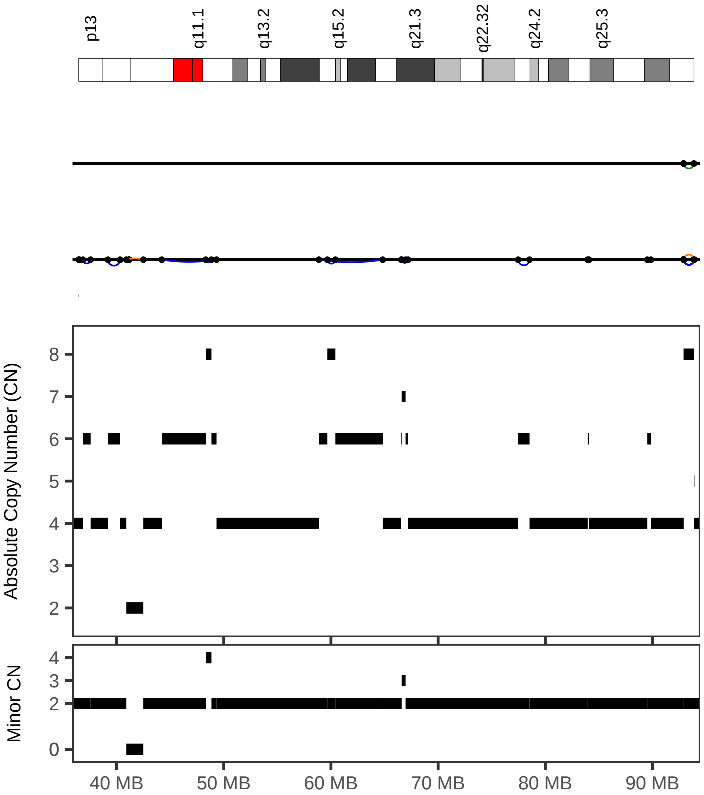

AOCS−170

Cancer type	Ovary−AdenoCA
Position	15:92905679−93872883
Interleaved intrachr. SVs	8
Total SVs (intrachr. + transl.)	8
SV types	DEL: 1; DUP: 6; h2hINV: 0; t2tINV: 1; TRA: 0
SVs in sample	773
Oscillating CN (2 and 3 states)	4, 5
CN segments	8
FDR fragment joints	0.2796253
FDR chr. breakp. enrich.	0.69
Linked to chrs	
Purity, ploidy	0.7, 4.27

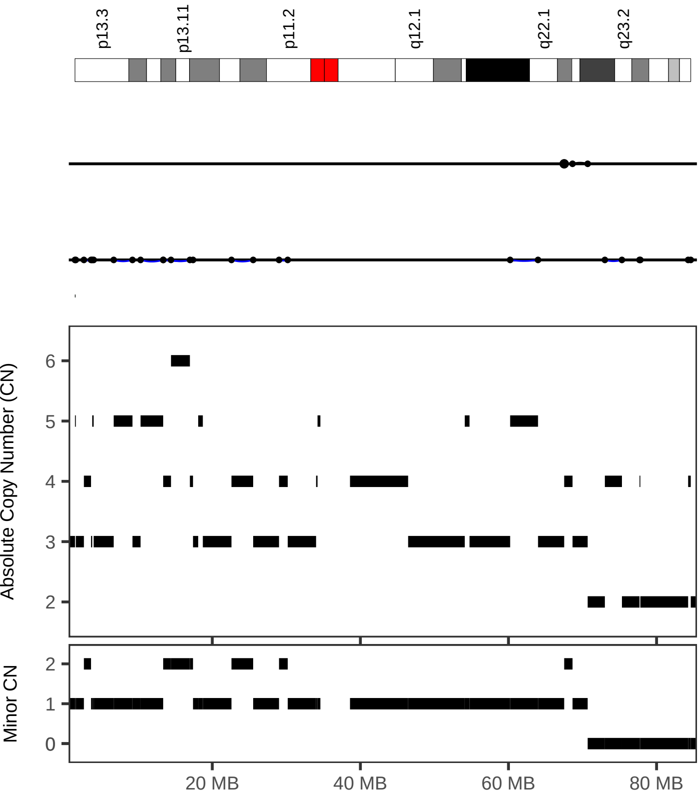

AOCS−170

Cancer type	Ovary−AdenoCA
Position	16:1456338−84616481
Interleaved intrachr. SVs	1
Total SVs (intrachr. + transl.)	15
SV types	DEL: 0; DUP: 13; h2hINV: 1; t2tINV: 0; TRA: 1
SVs in sample	773
Oscillating CN (2 and 3 states)	7, 12
CN segments	36
FDR fragment joints	0.0006748813
FDR chr. breakp. enrich.	0.51
Linked to chrs	
Purity, ploidy	0.7, 4.27

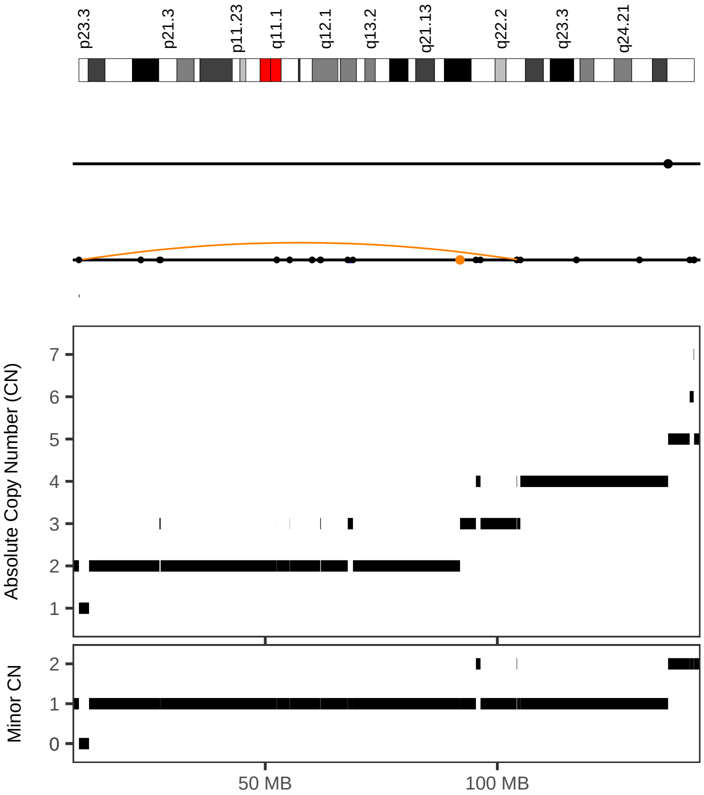

AOCS−171

Cancer type	Ovary−AdenoCA
Position	8:9852651−142422526
Interleaved intrachr. SVs	1
Total SVs (intrachr. + transl.)	13
SV types	DEL: 1; DUP: 10; h2hINV: 0; t2tINV: 0; TRA: 2
SVs in sample	258
Oscillating CN (2 and 3 states)	12, 21
CN segments	22
FDR fragment joints	0.03151629
FDR chr. breakp. enrich.	0.3
Linked to chrs	
Purity, ploidy	0.76, 1.87

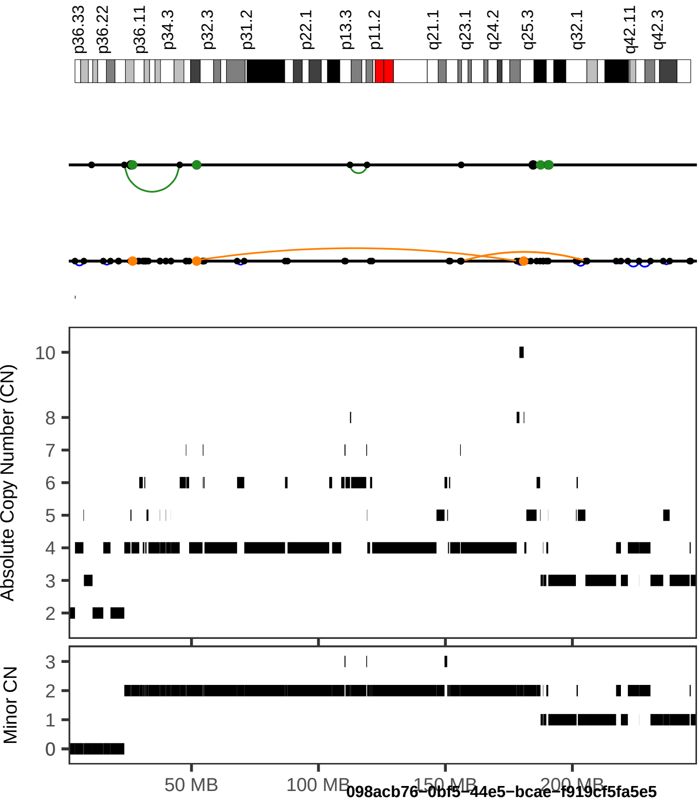

098acb76−0bf5−44e5−bcae−f919cf5fa5e5

Cancer type	Ovary−AdenoCA
Position	1:49064386−205747224
Interleaved intrachr. SVs	7
Total SVs (intrachr. + transl.)	16
SV types	DEL: 4; DUP: 3; h2hINV: 0; t2tINV: 0; TRA: 9
SVs in sample	718
Oscillating CN (2 and 3 states)	9, 9
CN segments	47
FDR fragment joints	0.7908979
FDR chr. breakp. enrich.	0.94
Linked to chrs	14:20605489−100315051;7:20257526−69395455
Purity, ploidy	0.82, 3.31

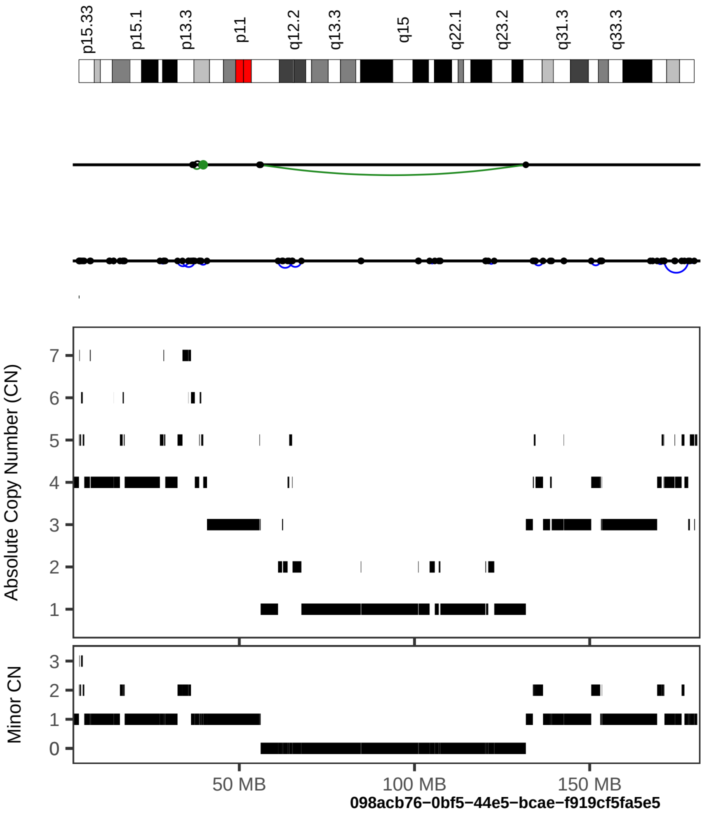

098acb76−0bf5−44e5−bcae−f919cf5fa5e5

Cancer type	Ovary−AdenoCA
Position	5:32370239−40782660
Interleaved intrachr. SVs	6
Total SVs (intrachr. + transl.)	7
SV types	DEL: 0; DUP: 4; h2hINV: 1; t2tINV: 1; TRA: 1
SVs in sample	718
Oscillating CN (2 and 3 states)	4, 6
CN segments	11
FDR fragment joints	0.615458
FDR chr. breakp. enrich.	1
Linked to chrs	
Purity, ploidy	0.82, 3.31

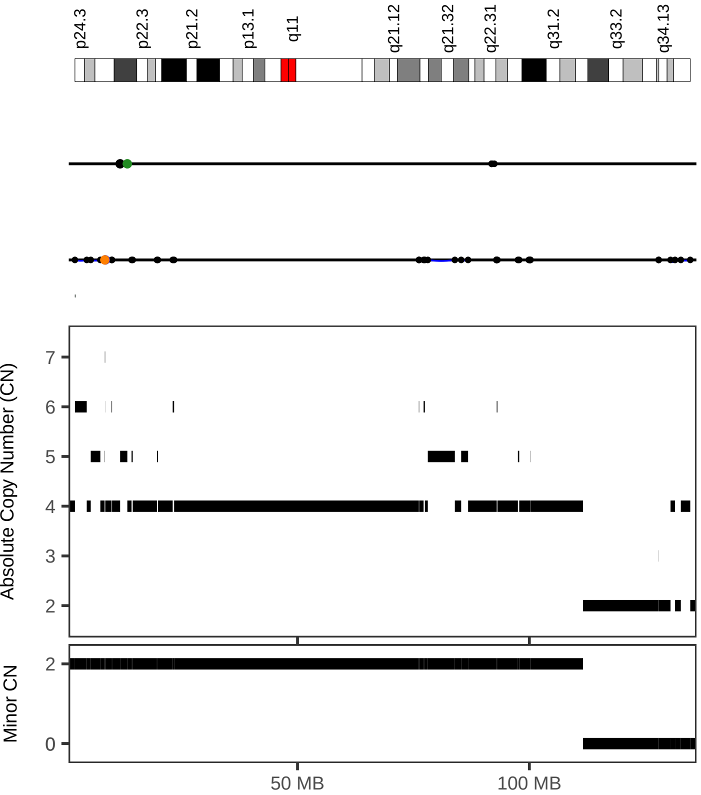

098acb76−0bf5−44e5−bcae−f919cf5fa5e5

Cancer type	Ovary−AdenoCA
Position	9:1986986−134705327
Interleaved intrachr. SVs	1
Total SVs (intrachr. + transl.)	23
SV types	DEL: 0; DUP: 18; h2hINV: 1; t2tINV: 0; TRA: 4
SVs in sample	718
Oscillating CN (2 and 3 states)	7, 26
CN segments	39
FDR fragment joints	1.241524e−05
FDR chr. breakp. enrich.	0.84
Linked to chrs	17:3533493−74207634;
Purity, ploidy	0.82, 3.31

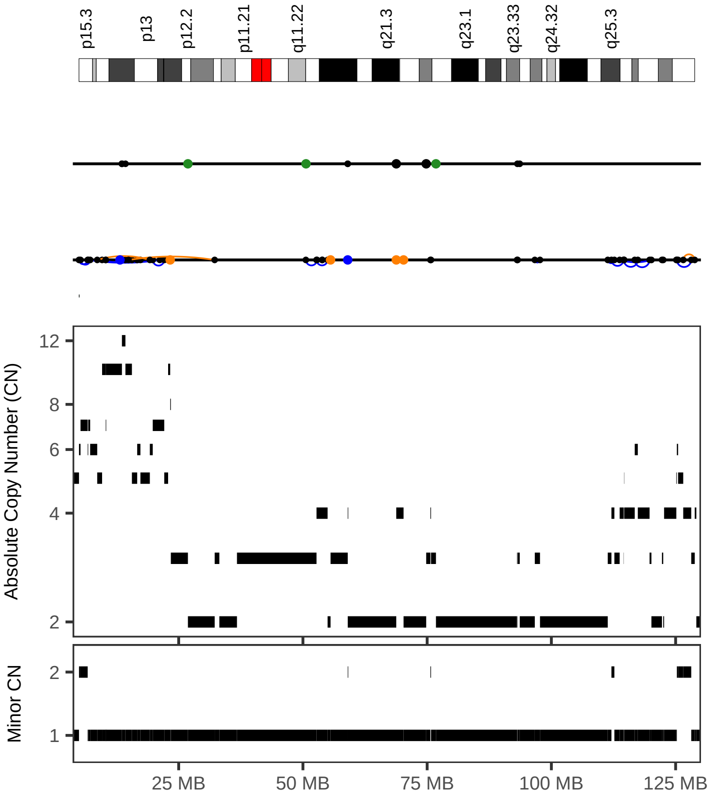

098acb76−0bf5−44e5−bcae−f919cf5fa5e5

Cancer type	Ovary−AdenoCA
Position	10:4932621−32240886
Interleaved intrachr. SVs	7
Total SVs (intrachr. + transl.)	10
SV types	DEL: 2; DUP: 5; h2hINV: 0; t2tINV: 0; TRA: 3
SVs in sample	718
Oscillating CN (2 and 3 states)	5, 6
CN segments	22
FDR fragment joints	0.4695521
FDR chr. breakp. enrich.	0.28
Linked to chrs	
Purity, ploidy	0.82, 3.31

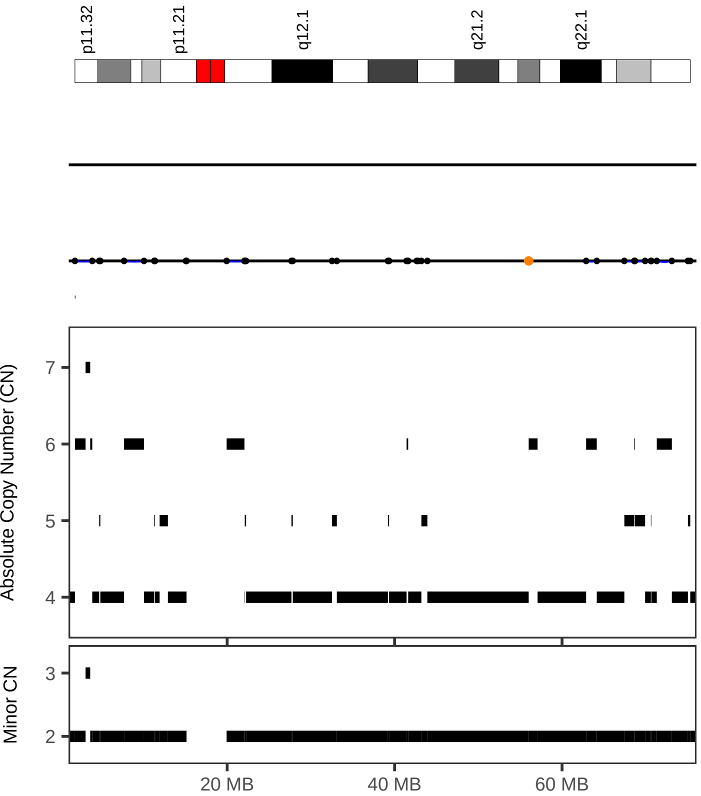

098acb76−0bf5−44e5−bcae−f919cf5fa5e5

Cancer type	Ovary−AdenoCA
Position	18:1815322−75311633
Interleaved intrachr. SVs	1
Total SVs (intrachr. + transl.)	20
SV types	DEL: 0; DUP: 19; h2hINV: 0; t2tINV: 0; TRA: 1
SVs in sample	718
Oscillating CN (2 and 3 states)	9, 29
CN segments	40
FDR fragment joints	1.073099e−07
FDR chr. breakp. enrich.	0.96
Linked to chrs	
Purity, ploidy	0.82, 3.31

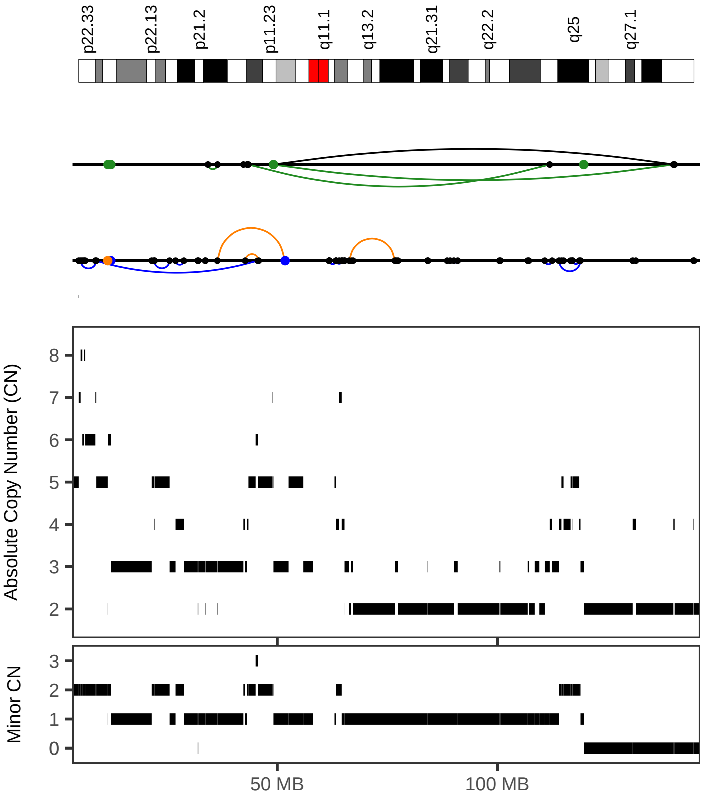

098acb76−0bf5−44e5−bcae−f919cf5fa5e5

Cancer type	Ovary−AdenoCA
Position	X:4890040−140268120
Interleaved intrachr. SVs	11
Total SVs (intrachr. + transl.)	18
SV types	DEL: 2; DUP: 4; h2hINV: 1; t2tINV: 4; TRA: 7
SVs in sample	718
Oscillating CN (2 and 3 states)	18, 19
CN segments	71
FDR fragment joints	0.8653243
FDR chr. breakp. enrich.	0.42
Linked to chrs	
Purity, ploidy	0.82, 3.31

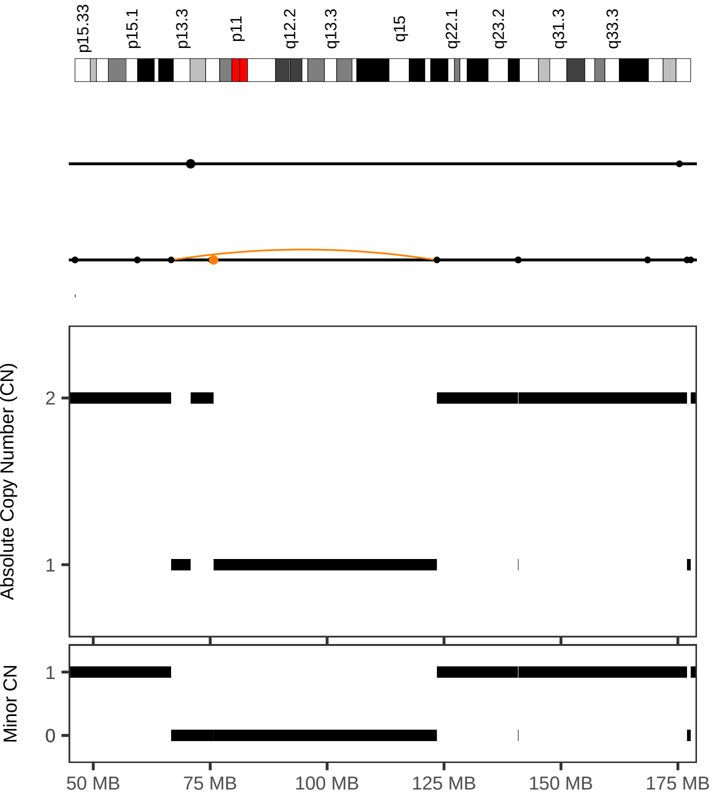

0a48873a−092a−4b25−822b−b0b3c18c08a8

Cancer type	Ovary−AdenoCA
Position	5:66641825−177746139
Interleaved intrachr. SVs	1
Total SVs (intrachr. + transl.)	7
SV types	DEL: 5; DUP: 0; h2hINV: 0; t2tINV: 0; TRA: 2
SVs in sample	190
Oscillating CN (2 and 3 states)	8, 8
CN segments	8
FDR fragment joints	0.1278363
FDR chr. breakp. enrich.	0.86
Linked to chrs	
Purity, ploidy	0.9, 1.75

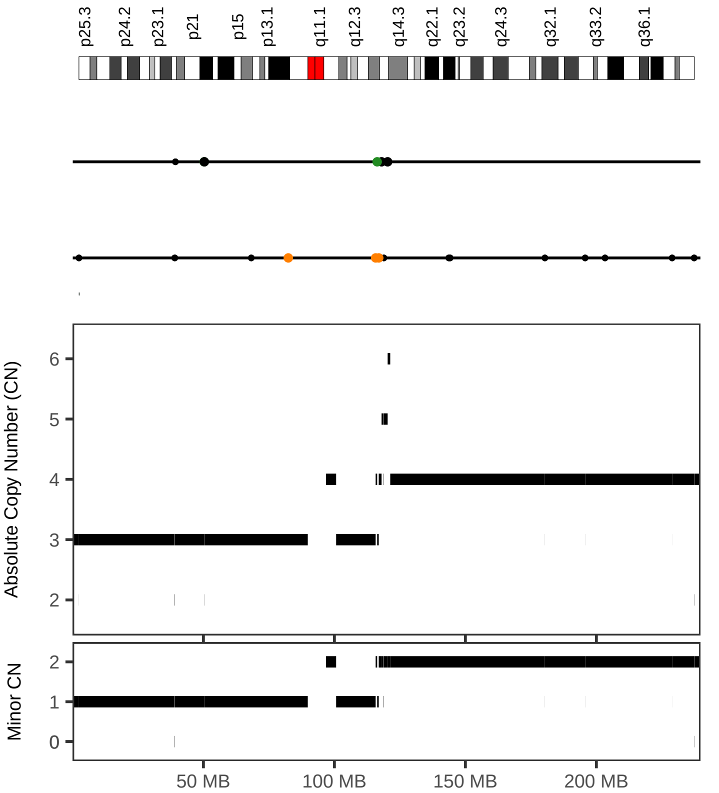

0c1a2e7d−e7e4−481e−a012−ef214c444497

Cancer type	Ovary−AdenoCA
Position	2:2471432−237349046
Interleaved intrachr. SVs	1
Total SVs (intrachr. + transl.)	15
SV types	DEL: 7; DUP: 0; h2hINV: 0; t2tINV: 0; TRA: 8
SVs in sample	174
Oscillating CN (2 and 3 states)	7, 15
CN segments	24
FDR fragment joints	0.1013295
FDR chr. breakp. enrich.	0.38
Linked to chrs	
Purity, ploidy	0.9, 2.97

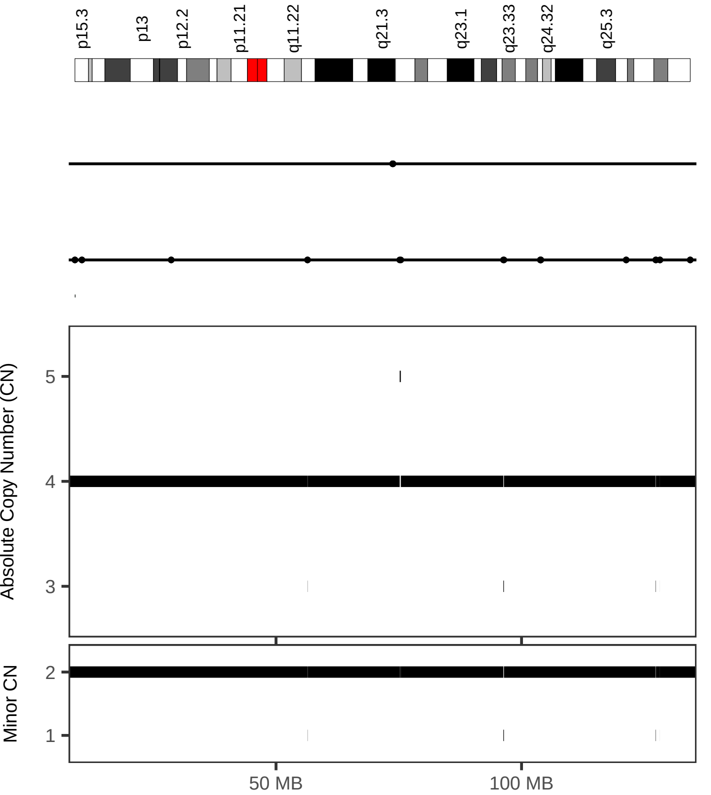

0c1a2e7d−e7e4−481e−a012−ef214c444497

Cancer type	Ovary−AdenoCA
Position	10:56404122−134361570
Interleaved intrachr. SVs	1
Total SVs (intrachr. + transl.)	9
SV types	DEL: 7; DUP: 0; h2hINV: 1; t2tINV: 1; TRA: 0
SVs in sample	174
Oscillating CN (2 and 3 states)	7, 10
CN segments	10
FDR fragment joints	0.1488029
FDR chr. breakp. enrich.	0.15
Linked to chrs	
Purity, ploidy	0.9, 2.97

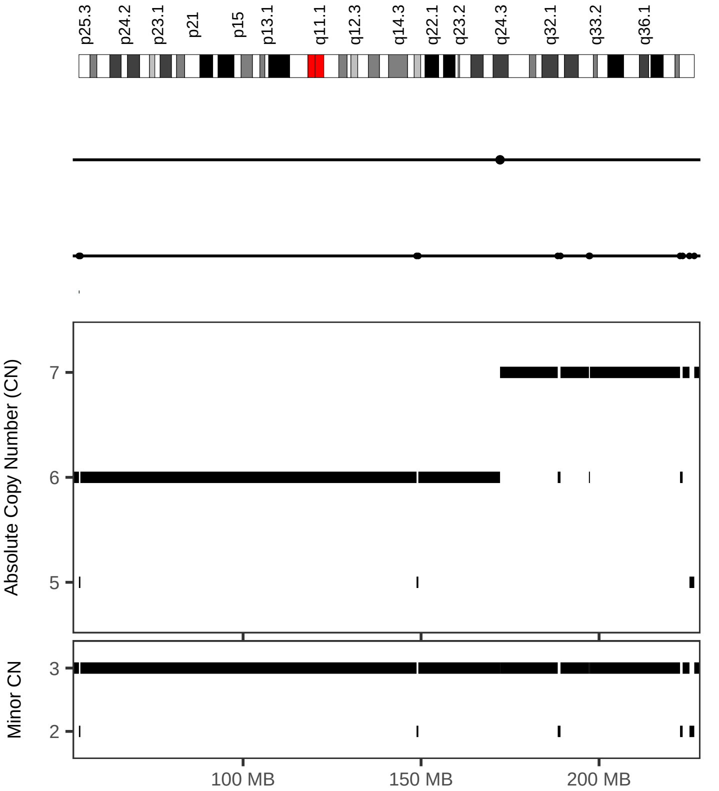

15556b28−c6bd−455a−87b6−4b7b3e33d0e4

Cancer type	Ovary−AdenoCA
Position	2:53884124−226741490
Interleaved intrachr. SVs	1
Total SVs (intrachr. + transl.)	7
SV types	DEL: 6; DUP: 0; h2hINV: 0; t2tINV: 0; TRA: 1
SVs in sample	154
Oscillating CN (2 and 3 states)	8, 10
CN segments	13
FDR fragment joints	0.1278363
FDR chr. breakp. enrich.	0.28
Linked to chrs	
Purity, ploidy	0.84, 3.91

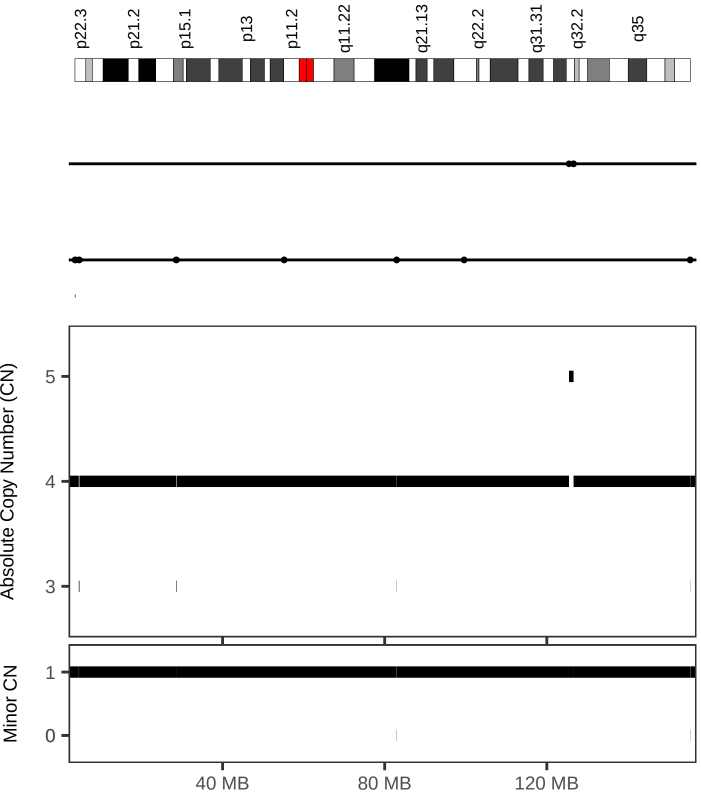

15556b28−c6bd−455a−87b6−4b7b3e33d0e4

Cancer type	Ovary−AdenoCA
Position	7:3562493−155393789
Interleaved intrachr. SVs	1
Total SVs (intrachr. + transl.)	7
SV types	DEL: 6; DUP: 0; h2hINV: 0; t2tINV: 1; TRA: 0
SVs in sample	154
Oscillating CN (2 and 3 states)	7, 11
CN segments	11
FDR fragment joints	0.1278363
FDR chr. breakp. enrich.	0.9
Linked to chrs	
Purity, ploidy	0.84, 3.91

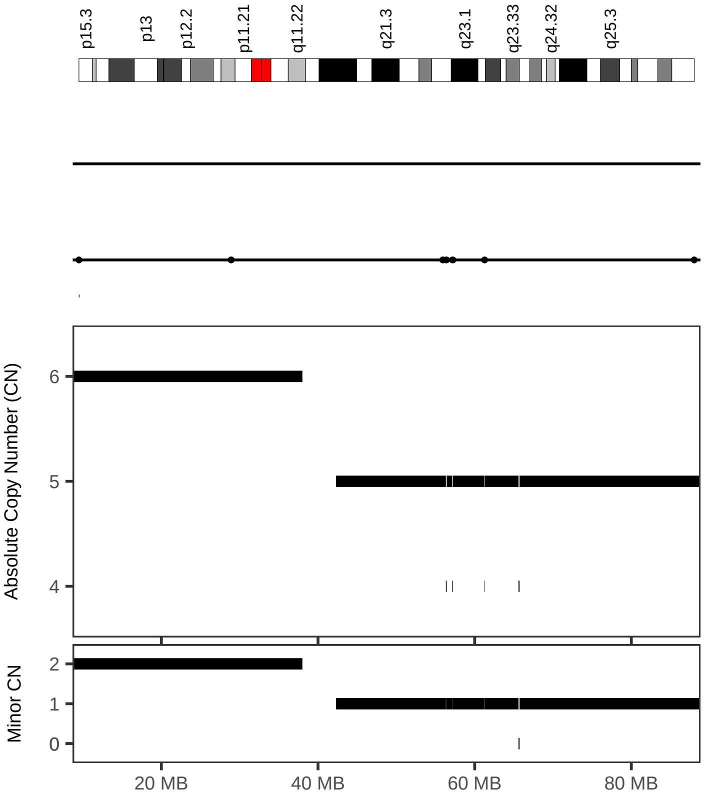

15556b28−c6bd−455a−87b6−4b7b3e33d0e4

Cancer type	Ovary−AdenoCA
Position	10:28885088−61290339
Interleaved intrachr. SVs	1
Total SVs (intrachr. + transl.)	5
SV types	DEL: 5; DUP: 0; h2hINV: 0; t2tINV: 0; TRA: 0
SVs in sample	154
Oscillating CN (2 and 3 states)	7, 8
CN segments	8
FDR fragment joints	0.1013295
FDR chr. breakp. enrich.	0.69
Linked to chrs	
Purity, ploidy	0.84, 3.91

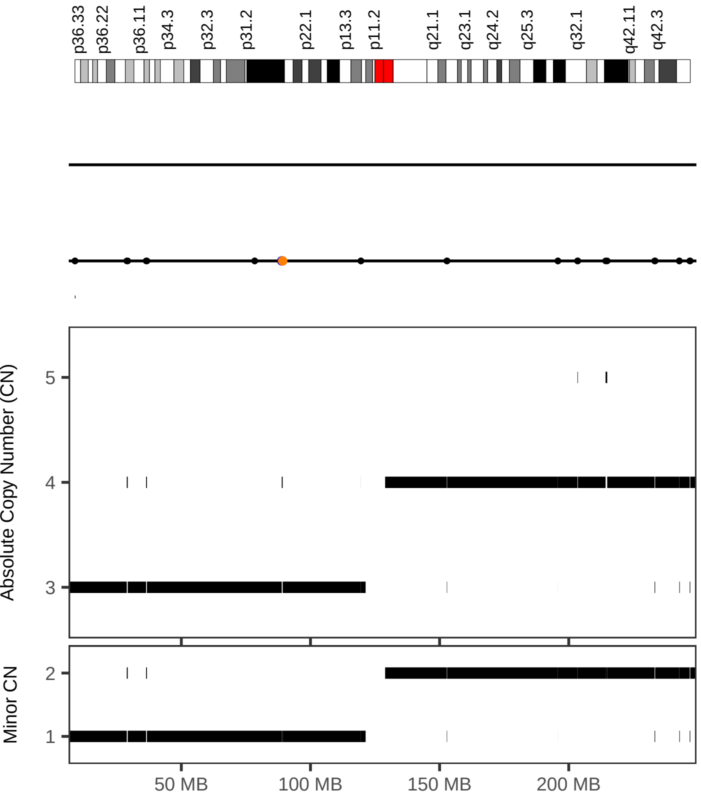

21f6bf91−dfc6−4a59−8b76−88a0f95c7b47

Cancer type	Ovary−AdenoCA
Position	1:8799389−247051701
Interleaved intrachr. SVs	1
Total SVs (intrachr. + transl.)	13
SV types	DEL: 6; DUP: 5; h2hINV: 0; t2tINV: 0; TRA: 2
SVs in sample	204
Oscillating CN (2 and 3 states)	14, 24
CN segments	24
FDR fragment joints	0.1817907
FDR chr. breakp. enrich.	0.89
Linked to chrs	
Purity, ploidy	0.9, 2.96

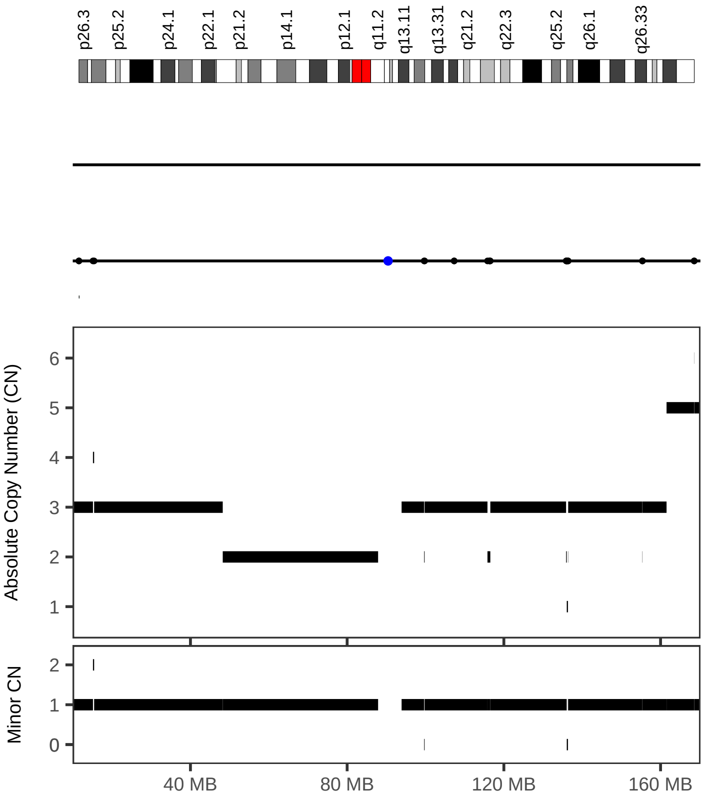

21f6bf91−dfc6−4a59−8b76−88a0f95c7b47

Cancer type	Ovary−AdenoCA
Position	3:15109935−168602841
Interleaved intrachr. SVs	1
Total SVs (intrachr. + transl.)	11
SV types	DEL: 8; DUP: 2; h2hINV: 0; t2tINV: 0; TRA: 1
SVs in sample	204
Oscillating CN (2 and 3 states)	8, 16
CN segments	19
FDR fragment joints	0.1013295
FDR chr. breakp. enrich.	0.88
Linked to chrs	
Purity, ploidy	0.9, 2.96

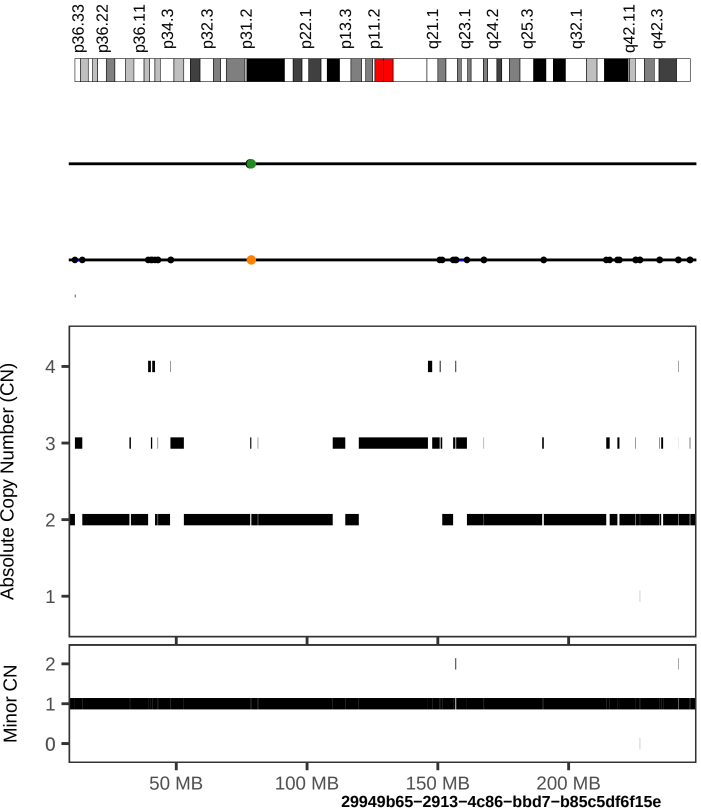

29949b65−2913−4c86−bbd7−b85c5df6f15e

Cancer type	Ovary−AdenoCA
Position	1:11256059−246496102
Interleaved intrachr. SVs	1
Total SVs (intrachr. + transl.)	20
SV types	DEL: 2; DUP: 15; h2hINV: 0; t2tINV: 0; TRA: 3
SVs in sample	360
Oscillating CN (2 and 3 states)	12, 41
CN segments	51
FDR fragment joints	0.002298705
FDR chr. breakp. enrich.	0.38
Linked to chrs	
Purity, ploidy	0.91, 2.12

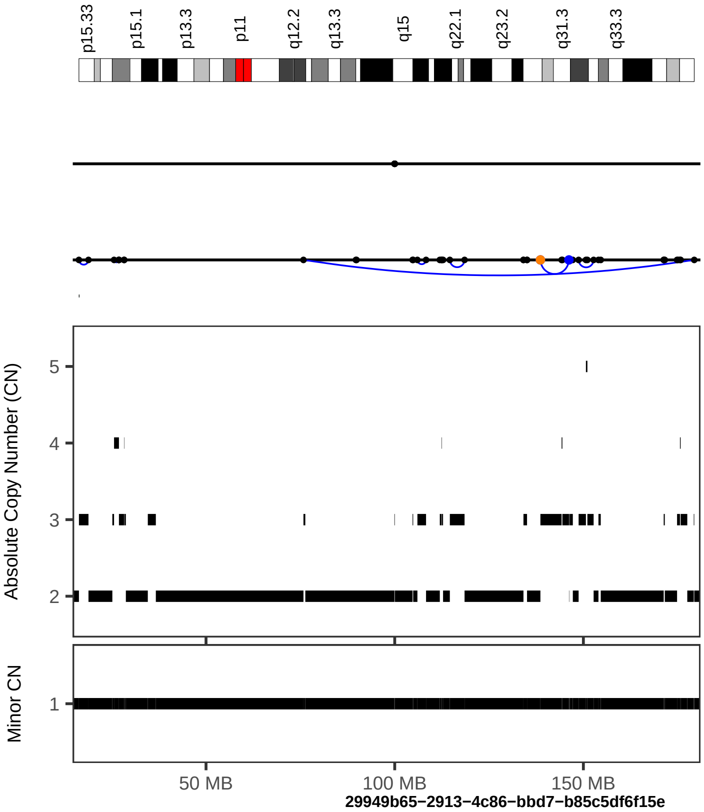

29949b65−2913−4c86−bbd7−b85c5df6f15e

Cancer type	Ovary−AdenoCA
Position	5:111950654−171572542
Interleaved intrachr. SVs	2
Total SVs (intrachr. + transl.)	7
SV types	DEL: 0; DUP: 4; h2hINV: 0; t2tINV: 0; TRA: 3
SVs in sample	360
Oscillating CN (2 and 3 states)	7, 17
CN segments	24
FDR fragment joints	0.1278363
FDR chr. breakp. enrich.	0.36
Linked to chrs	
Purity, ploidy	0.91, 2.12

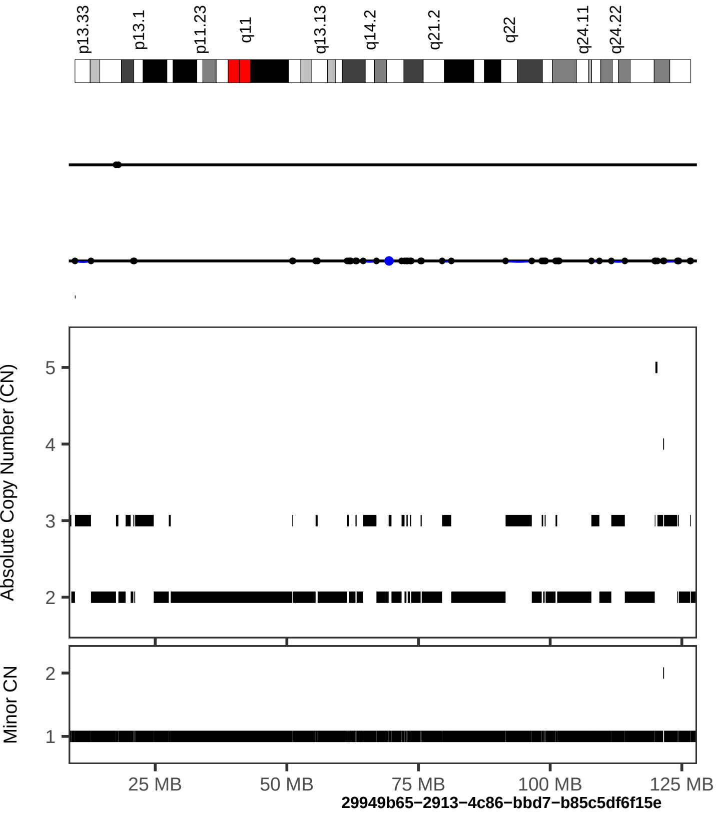

29949b65−2913−4c86−bbd7−b85c5df6f15e

Cancer type	Ovary−AdenoCA
Position	12:9762639−126691039
Interleaved intrachr. SVs	1
Total SVs (intrachr. + transl.)	28
SV types	DEL: 0; DUP: 26; h2hINV: 0; t2tINV: 1; TRA: 1
SVs in sample	360
Oscillating CN (2 and 3 states)	49, 49
CN segments	58
FDR fragment joints	2.512593e−12
FDR chr. breakp. enrich.	0.04
Linked to chrs	
Purity, ploidy	0.91, 2.12

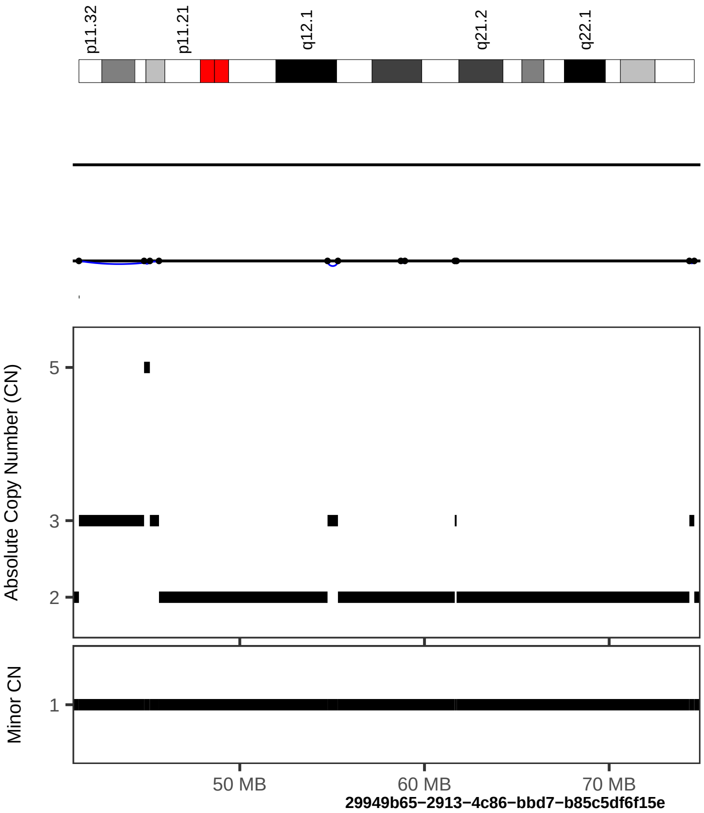

29949b65−2913−4c86−bbd7−b85c5df6f15e

Cancer type	Ovary−AdenoCA
Position	18:41289568−74606040
Interleaved intrachr. SVs	1
Total SVs (intrachr. + transl.)	6
SV types	DEL: 0; DUP: 6; h2hINV: 0; t2tINV: 0; TRA: 0
SVs in sample	360
Oscillating CN (2 and 3 states)	8, 10
CN segments	10
FDR fragment joints	0.04139017
FDR chr. breakp. enrich.	0.58
Linked to chrs	
Purity, ploidy	0.91, 2.12

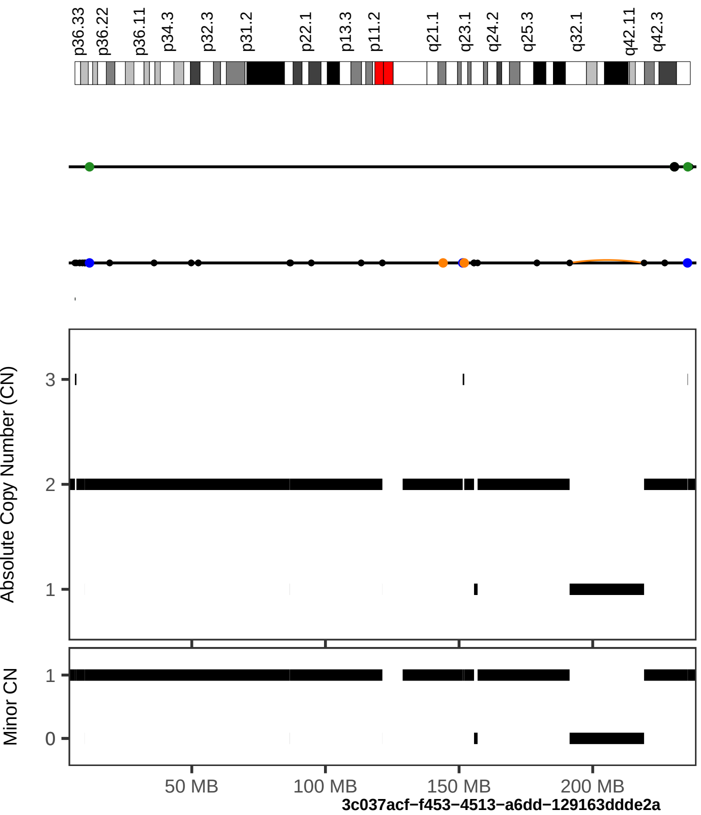

3c037acf−f453−4513−a6dd−129163ddde2a

Cancer type	Ovary−AdenoCA
Position	1:6281442−226959021
Interleaved intrachr. SVs	1
Total SVs (intrachr. + transl.)	13
SV types	DEL: 7; DUP: 1; h2hINV: 0; t2tINV: 0; TRA: 5
SVs in sample	328
Oscillating CN (2 and 3 states)	7, 14
CN segments	14
FDR fragment joints	0.1013295
FDR chr. breakp. enrich.	0.61
Linked to chrs	
Purity, ploidy	0.95, 1.74

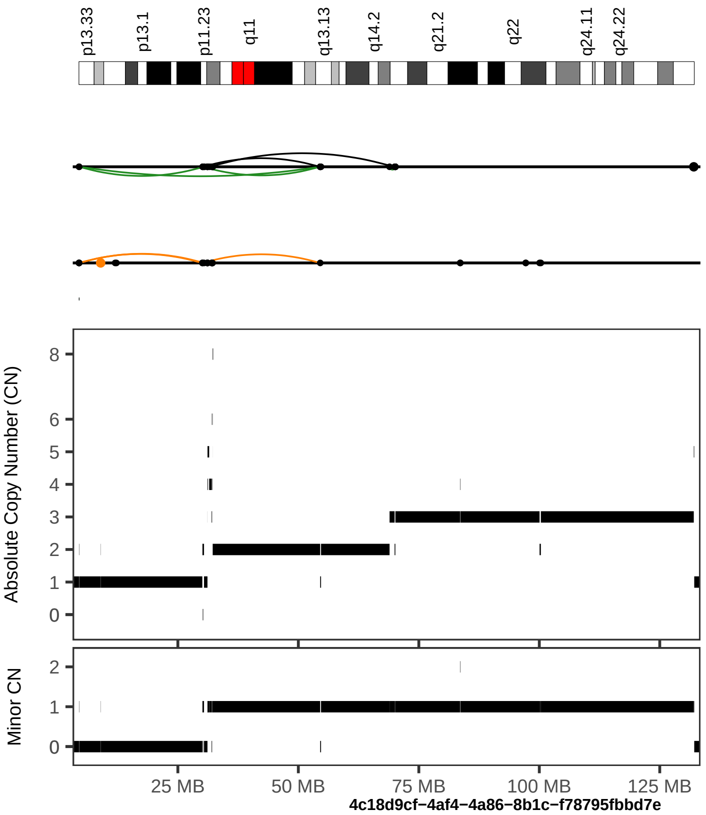

4c18d9cf−4af4−4a86−8b1c−f78795fbbd7e

Cancer type	Ovary−AdenoCA
Position	12:4470991−70144374
Interleaved intrachr. SVs	13
Total SVs (intrachr. + transl.)	14
SV types	DEL: 3; DUP: 1; h2hINV: 4; t2tINV: 5; TRA: 1
SVs in sample	96
Oscillating CN (2 and 3 states)	6, 10
CN segments	29
FDR fragment joints	0.6776251
FDR chr. breakp. enrich.	0
Linked to chrs	
Purity, ploidy	0.83, 1.77

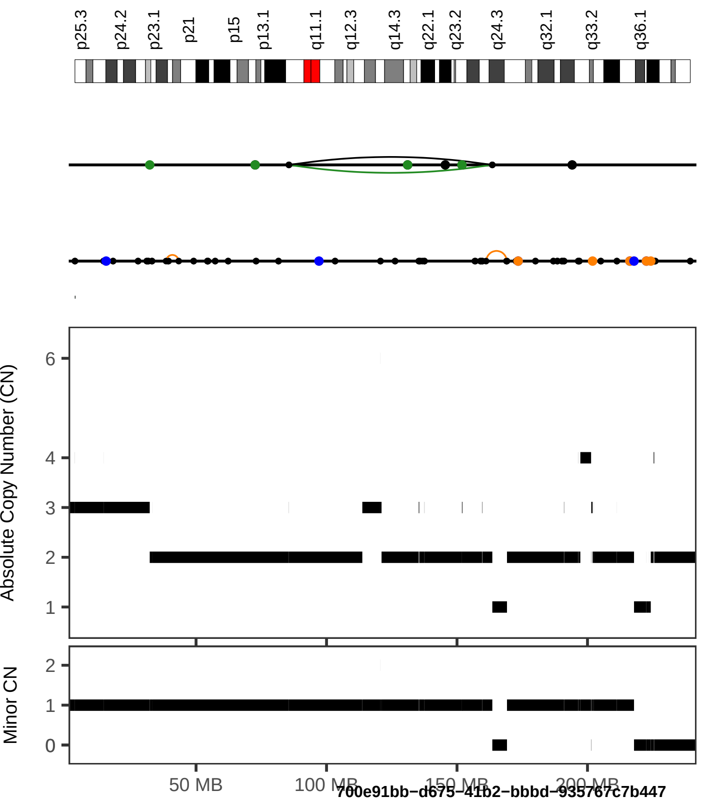

700e91bb−d675−41b2−bbbd−935767c7b447

Cancer type	Ovary−AdenoCA
Position	2:85566821−169153622
Interleaved intrachr. SVs	3
Total SVs (intrachr. + transl.)	9
SV types	DEL: 1; DUP: 0; h2hINV: 1; t2tINV: 1; TRA: 6
SVs in sample	686
Oscillating CN (2 and 3 states)	10, 14
CN segments	16
FDR fragment joints	0.615458
FDR chr. breakp. enrich.	0.35
Linked to chrs	9:9234123−72466980;X:8029219−122680104
Purity, ploidy	0.8, 1.88

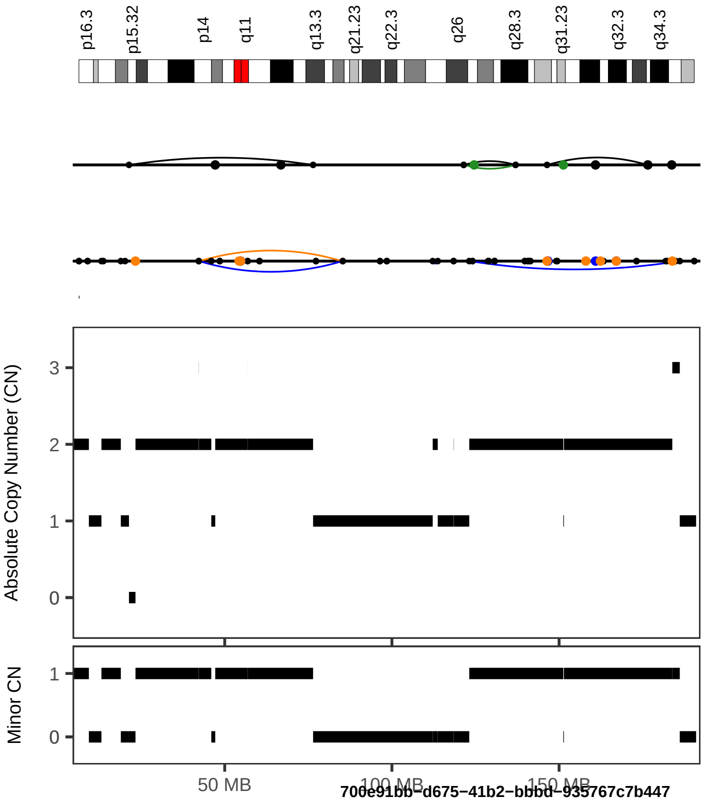

700e91bb−d675−41b2−bbbd−935767c7b447

Cancer type	Ovary−AdenoCA
Position	4:21357758−186097187
Interleaved intrachr. SVs	3
Total SVs (intrachr. + transl.)	25
SV types	DEL: 1; DUP: 2; h2hINV: 2; t2tINV: 1; TRA: 19
SVs in sample	686
Oscillating CN (2 and 3 states)	9, 16
CN segments	18
FDR fragment joints	0.6776251
FDR chr. breakp. enrich.	0.18
Linked to chrs	10:76557035−104096682;12:16693692−123934825 3:31580573−161520979;
Purity, ploidy	0.8, 1.88

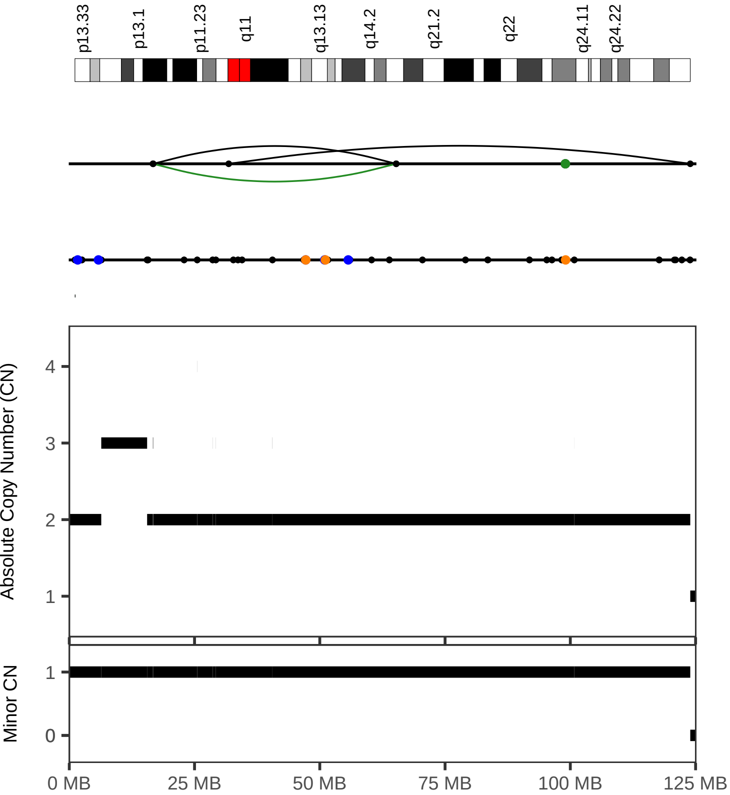

700e91bb−d675−41b2−bbbd−935767c7b447

Cancer type	Ovary−AdenoCA
Position	12:16693692−123934826
Interleaved intrachr. SVs	3
Total SVs (intrachr. + transl.)	11
SV types	DEL: 0; DUP: 0; h2hINV: 2; t2tINV: 1; TRA: 8
SVs in sample	686
Oscillating CN (2 and 3 states)	9, 12
CN segments	13
FDR fragment joints	0.9804396
FDR chr. breakp. enrich.	0.13
Linked to chrs	4:21357758−186097186;
Purity, ploidy	0.8, 1.88

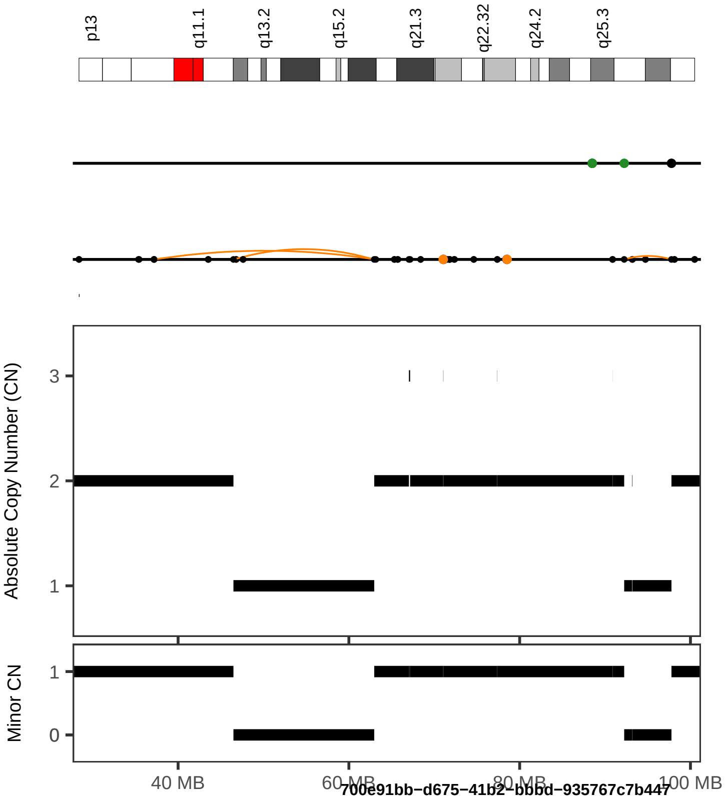

700e91bb−d675−41b2−bbbd−935767c7b447

Cancer type	Ovary−AdenoCA
Position	15:35363568−100501028
Interleaved intrachr. SVs	1
Total SVs (intrachr. + transl.)	25
SV types	DEL: 5; DUP: 12; h2hINV: 0; t2tINV: 0; TRA: 8
SVs in sample	686
Oscillating CN (2 and 3 states)	9, 15
CN segments	15
FDR fragment joints	0.1013295
FDR chr. breakp. enrich.	0
Linked to chrs	16:10579569−79138531;7:20428304−158340087
Purity, ploidy	0.8, 1.88

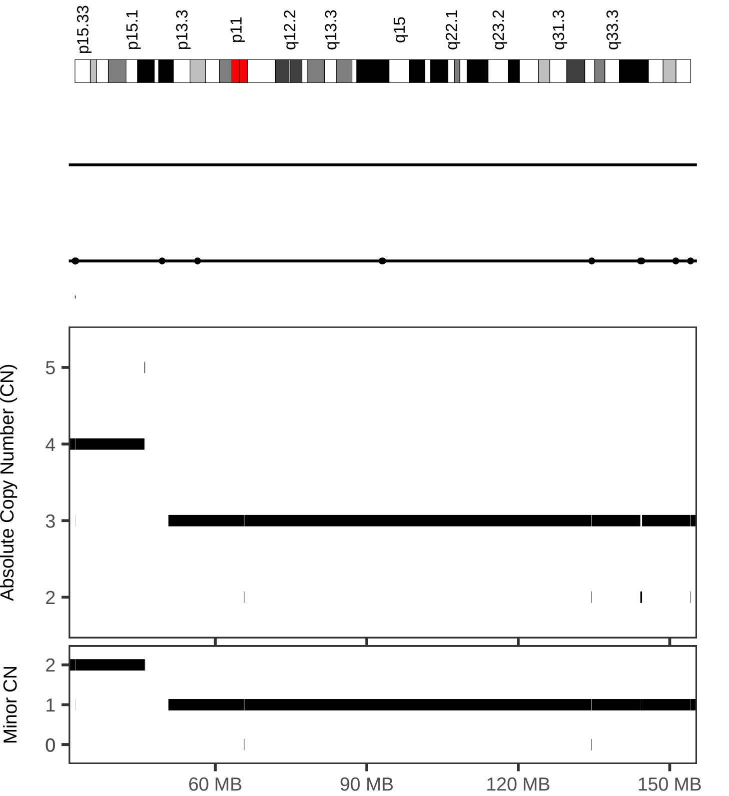

7ca97692−0bf5−4bbd−81ce−10a051d04bd5

Cancer type	Ovary−AdenoCA
Position	5:32197937−154158016
Interleaved intrachr. SVs	1
Total SVs (intrachr. + transl.)	9
SV types	DEL: 9; DUP: 0; h2hINV: 0; t2tINV: 0; TRA: 0
SVs in sample	275
Oscillating CN (2 and 3 states)	9, 9
CN segments	15
FDR fragment joints	0.002298705
FDR chr. breakp. enrich.	0.23
Linked to chrs	
Purity, ploidy	0.96, 3.74

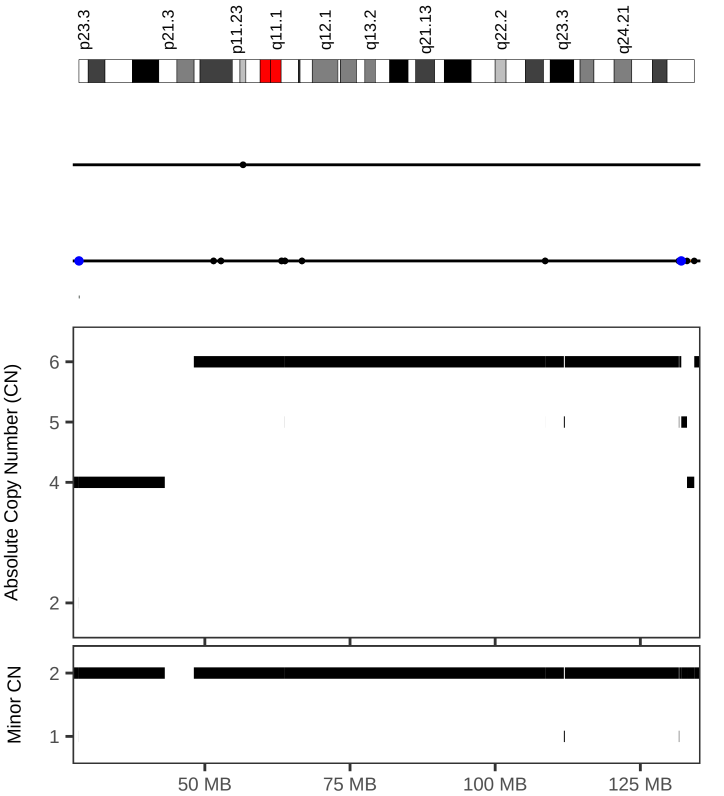

7ca97692−0bf5−4bbd−81ce−10a051d04bd5

Cancer type	Ovary−AdenoCA
Position	8:28310047−134285132
Interleaved intrachr. SVs	1
Total SVs (intrachr. + transl.)	9
SV types	DEL: 7; DUP: 0; h2hINV: 0; t2tINV: 0; TRA: 2
SVs in sample	275
Oscillating CN (2 and 3 states)	10, 11
CN segments	14
FDR fragment joints	0.1178349
FDR chr. breakp. enrich.	0.88
Linked to chrs	
Purity, ploidy	0.96, 3.74

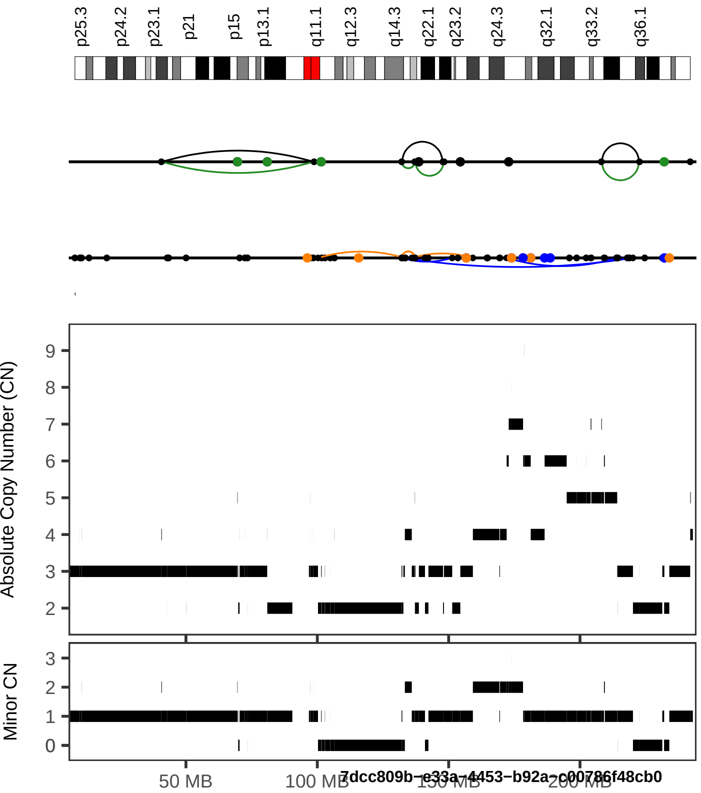

7dcc809b−e33a−4453−b92a−c00786f48cb0

Cancer type	Ovary−AdenoCA
Position	2:100265270−222653043
Interleaved intrachr. SVs	17
Total SVs (intrachr. + transl.)	33
SV types	DEL: 4; DUP: 4; h2hINV: 3; t2tINV: 6; TRA: 16
SVs in sample	665
Oscillating CN (2 and 3 states)	9, 12
CN segments	60
FDR fragment joints	0.615458
FDR chr. breakp. enrich.	0
Linked to chrs	10:11790441−98909437;4:25688755−183822953 5:73636560−135974127;6:16954294−41598732
[illegible]	[illegible]

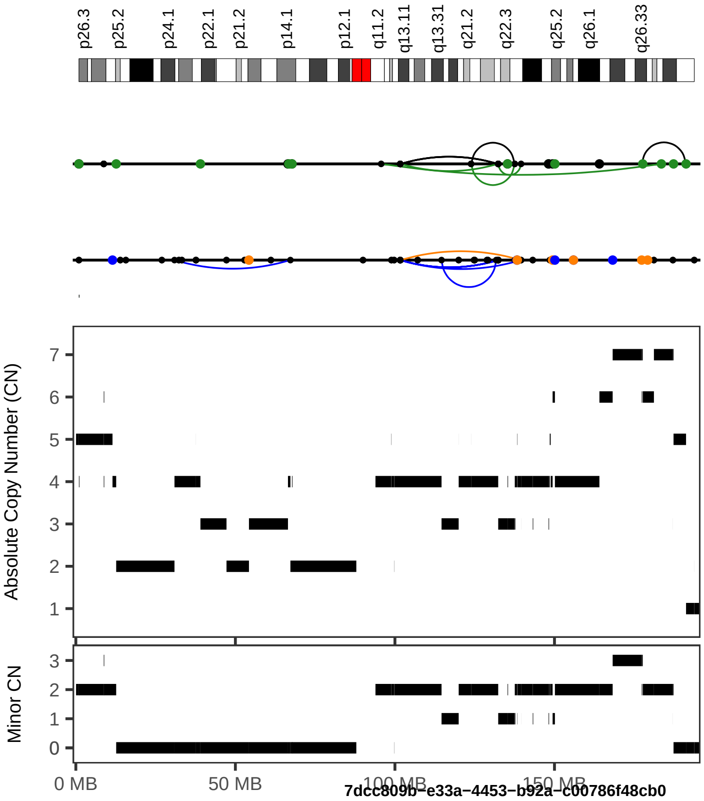

7dcc809b−e33a−4453−b92a−c00786f48cb0

Cancer type	Ovary−AdenoCA
Position	3:101663947−139539104
Interleaved intrachr. SVs	15
Total SVs (intrachr. + transl.)	19
SV types	DEL: 1; DUP: 7; h2hINV: 4; t2tINV: 3; TRA: 4
SVs in sample	665
Oscillating CN (2 and 3 states)	7, 14
CN segments	16
FDR fragment joints	0.615458
FDR chr. breakp. enrich.	0
Linked to chrs	16:10805630−88093194;5:73636560−135974127
Purity, ploidy	0.82, 3.31

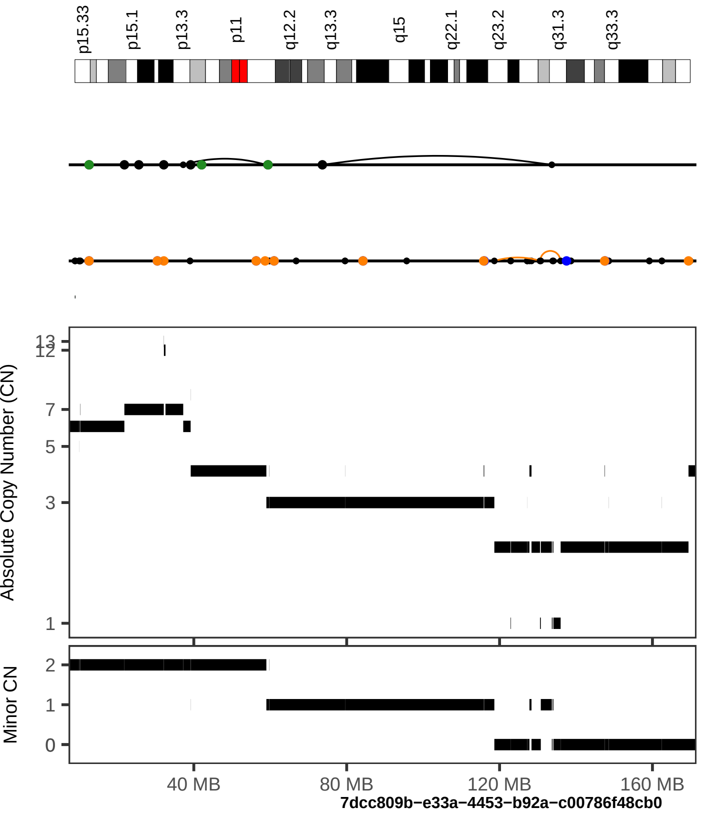

7dcc809b−e33a−4453−b92a−c00786f48cb0

Cancer type	Ovary−AdenoCA
Position	5:73636560−135974128
Interleaved intrachr. SVs	3
Total SVs (intrachr. + transl.)	7
SV types	DEL: 2; DUP: 0; h2hINV: 1; t2tINV: 0; TRA: 4
SVs in sample	665
Oscillating CN (2 and 3 states)	7, 10
CN segments	18
FDR fragment joints	0.615458
FDR chr. breakp. enrich.	0.08
Linked to chrs	4:25688755−183822953;
Purity, ploidy	0.82, 3.31

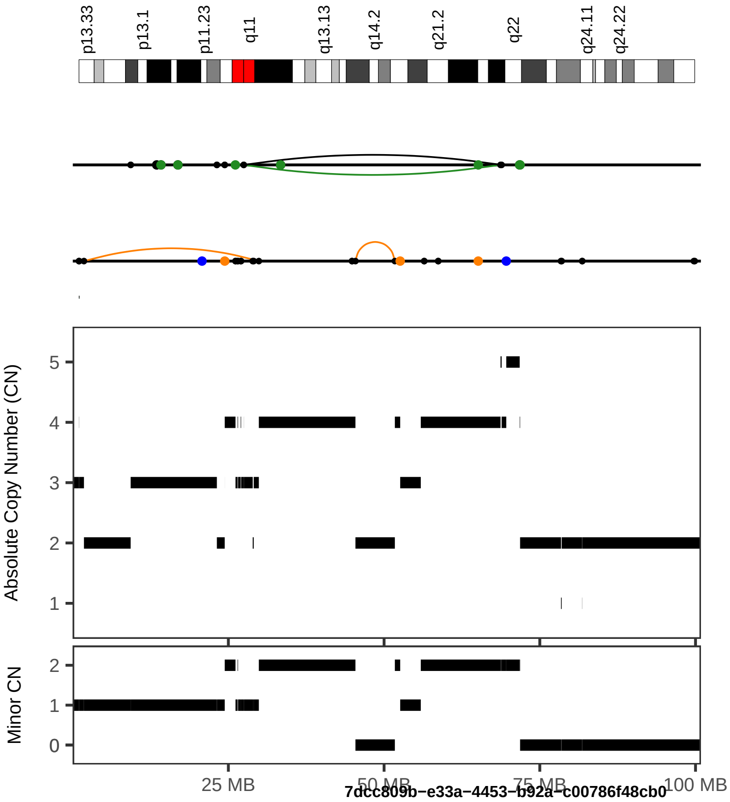

7dcc809b−e33a−4453−b92a−c00786f48cb0

Cancer type	Ovary−AdenoCA
Position	12:1814222−68872525
Interleaved intrachr. SVs	3
Total SVs (intrachr. + transl.)	16
SV types	DEL: 1; DUP: 0; h2hINV: 1; t2tINV: 1; TRA: 13
SVs in sample	665
Oscillating CN (2 and 3 states)	9, 15
CN segments	21
FDR fragment joints	0.615458
FDR chr. breakp. enrich.	0.42
Linked to chrs	11:23550499−126101177;19:6662156−50125620 8:23266791−145185293;
Purity, ploidy	0.82, 3.31

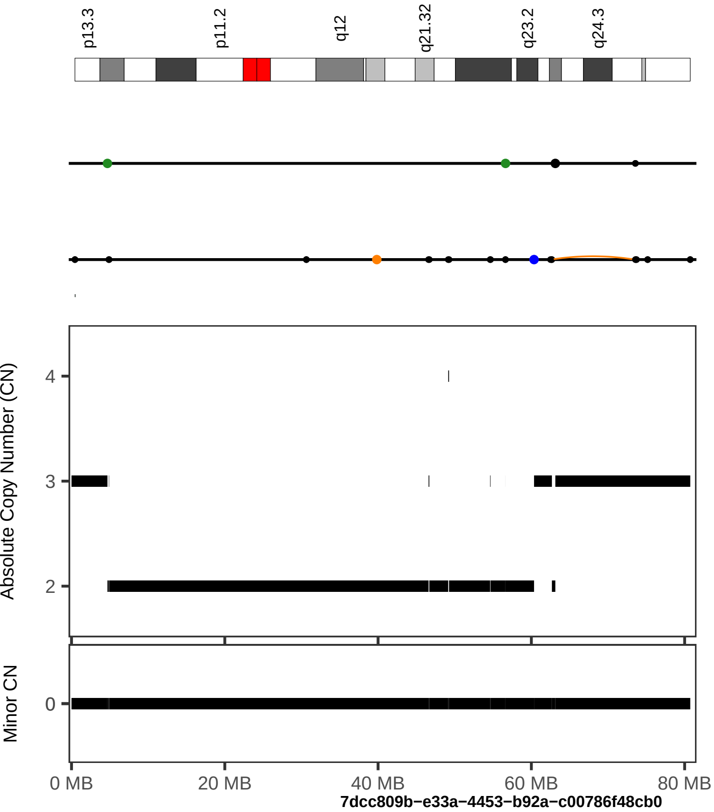

7dcc809b−e33a−4453−b92a−c00786f48cb0

Cancer type	Ovary−AdenoCA
Position	17:4865503−75189371
Interleaved intrachr. SVs	1
Total SVs (intrachr. + transl.)	13
SV types	DEL: 1; DUP: 8; h2hINV: 0;<br>t2tINV: 0; TRA: 4
SVs in sample	665
Oscillating CN (2 and 3 states)	8, 13
CN segments	13
FDR fragment joints	0.1457354
FDR chr. breakp. enrich.	1
Linked to chrs	
Purity, ploidy	0.82, 3.31

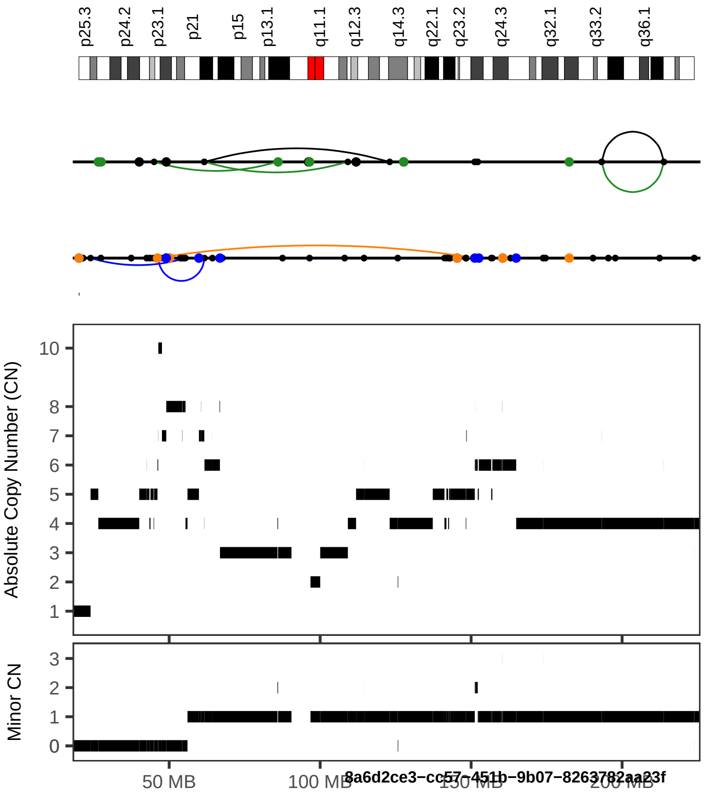

8a6d2ce3−cc57−451b−9b07−8263782aa23f

Cancer type	Ovary−AdenoCA
Position	2:23974041−152146532
Interleaved intrachr. SVs	6
Total SVs (intrachr. + transl.)	28
SV types	DEL: 1; DUP: 2; h2hINV: 1;<br>t2tINV: 2; TRA: 22
SVs in sample	704
Oscillating CN (2 and 3 states)	10, 11
CN segments	54
FDR fragment joints	0.8653243
FDR chr. breakp. enrich.	0.02
Linked to chrs	1:8622852−227102367;21:18052703−29183846<br>4:2551457−170303871;8:20677931−137092372
[illegible]	[illegible]

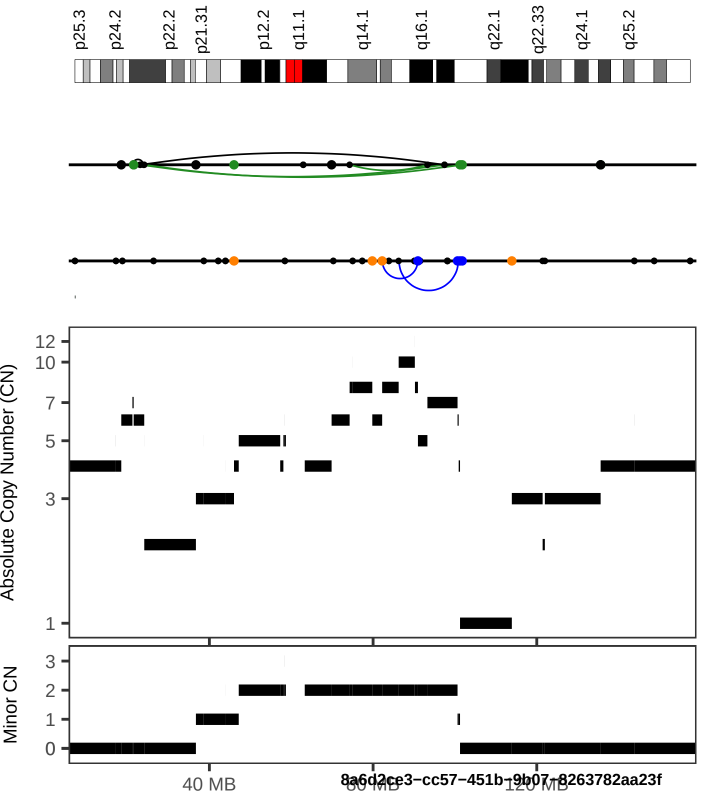

8a6d2ce3−cc57−451b−9b07−8263782aa23f

Cancer type	Ovary−AdenoCA
Position	6:21192197−101774595
Interleaved intrachr. SVs	7
Total SVs (intrachr. + transl.)	19
SV types	DEL: 0; DUP: 2; h2hINV: 2;<br>t2tINV: 3; TRA: 12
SVs in sample	704
Oscillating CN (2 and 3 states)	4, 11
CN segments	31
FDR fragment joints	0.8653243
FDR chr. breakp. enrich.	0.47
Linked to chrs	1:8622852−227102367;16:4412978−88870301<br>2:23974041−152146531;8:20677931−137092372

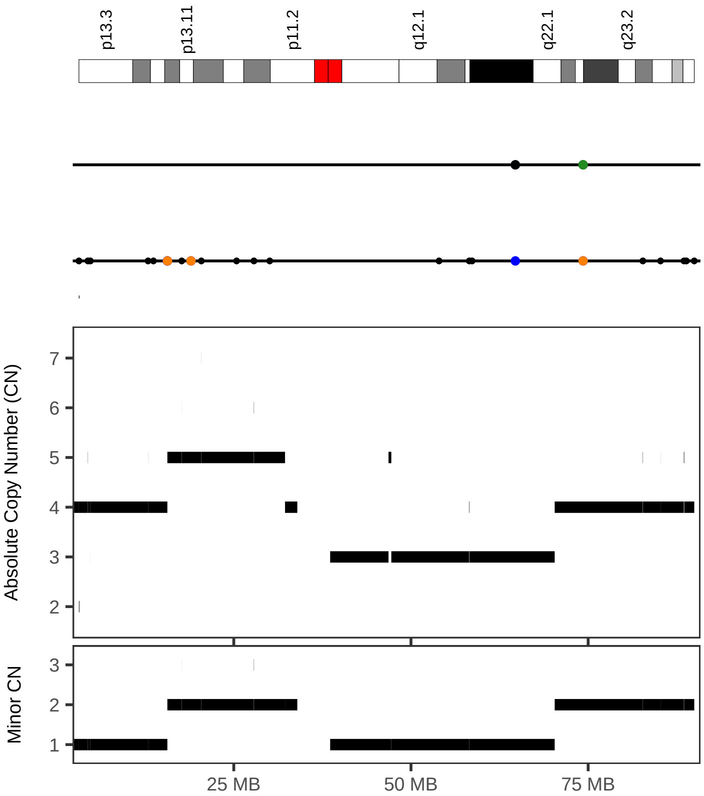

8a6d2ce3−cc57−451b−9b07−8263782aa23f

Cancer type	Ovary−AdenoCA
Position	16:4412978−88870302
Interleaved intrachr. SVs	1
Total SVs (intrachr. + transl.)	22
SV types	DEL: 1; DUP: 9; h2hINV: 0;<br>t2tINV: 0; TRA: 12
SVs in sample	704
Oscillating CN (2 and 3 states)	7, 10
CN segments	26
FDR fragment joints	0.04139017
FDR chr. breakp. enrich.	0.04
Linked to chrs	20:314995−46999236;
Purity, ploidy	0.76, 3.3

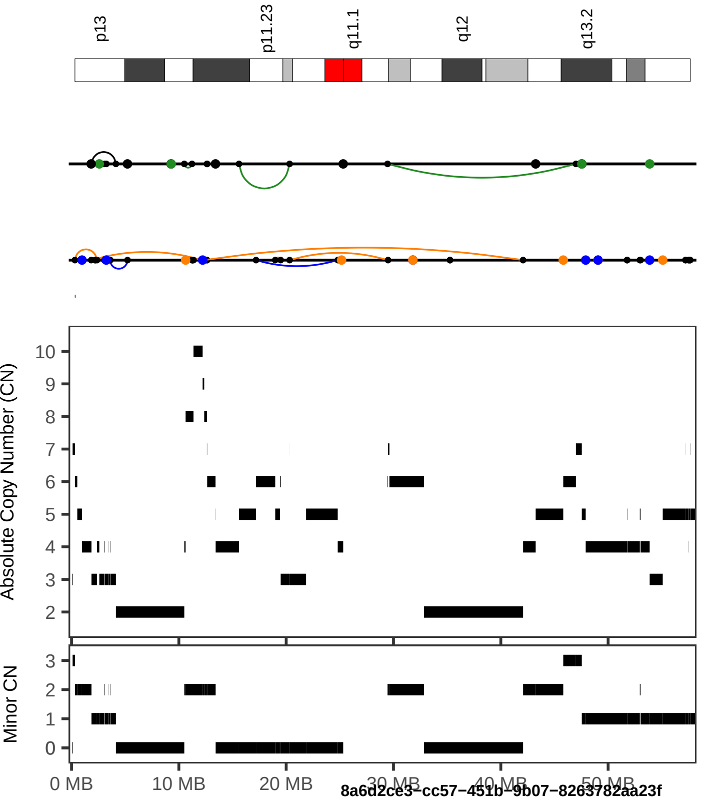

8a6d2ce3−cc57−451b−9b07−8263782aa23f

Cancer type	Ovary−AdenoCA
Position	20:314995−46999237
Interleaved intrachr. SVs	5
Total SVs (intrachr. + transl.)	26
SV types	DEL: 4; DUP: 3; h2hINV: 1; t2tINV: 2; TRA: 16
SVs in sample	704
Oscillating CN (2 and 3 states)	10, 13
CN segments	39
FDR fragment joints	0.6776251
FDR chr. breakp. enrich.	0
Linked to chrs	16:4412978−88870301;19:8187825−42588218
Purity, ploidy	0.76, 3.3

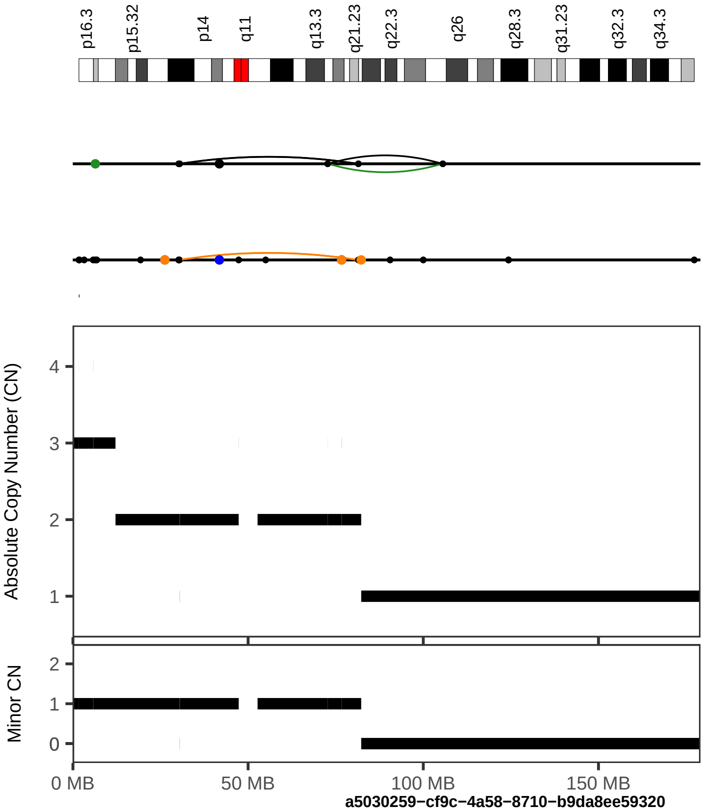

a5030259−cf9c−4a58−8710−b9da8ee59320

Cancer type	Ovary−AdenoCA
Position	4:30207518−105576012
Interleaved intrachr. SVs	8
Total SVs (intrachr. + transl.)	14
SV types	DEL: 2; DUP: 1; h2hINV: 3; t2tINV: 2; TRA: 6
SVs in sample	303
Oscillating CN (2 and 3 states)	7, 10
CN segments	10
FDR fragment joints	0.8988396
FDR chr. breakp. enrich.	0.03
Linked to chrs	14:52205312−99240506;
Purity, ploidy	0.89, 1.94

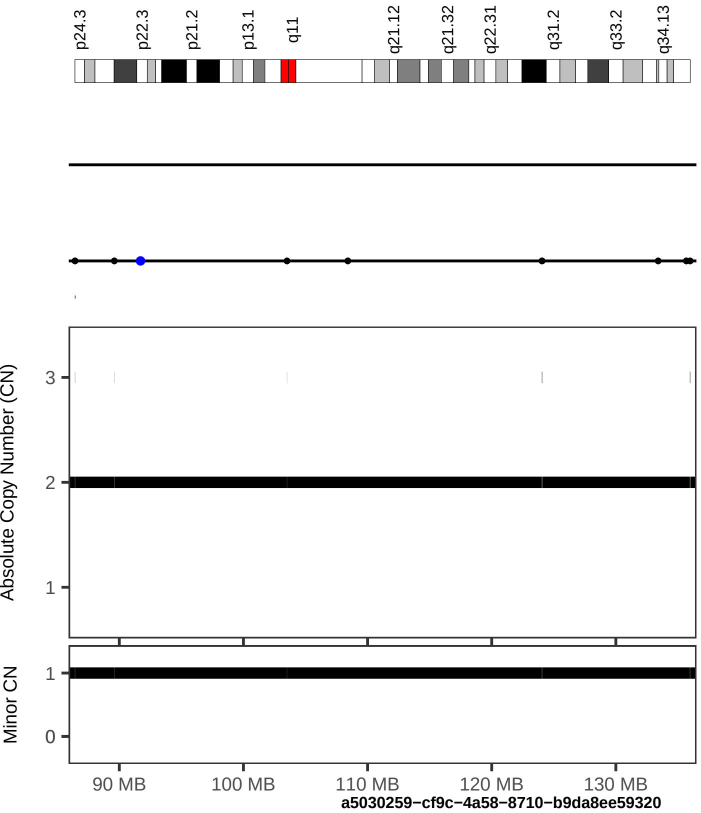

a5030259−cf9c−4a58−8710−b9da8ee59320

Cancer type	Ovary−AdenoCA
Position	9:86423945−135994657
Interleaved intrachr. SVs	1
Total SVs (intrachr. + transl.)	5
SV types	DEL: 0; DUP: 4; h2hINV: 0; t2tINV: 0; TRA: 1
SVs in sample	303
Oscillating CN (2 and 3 states)	10, 10
CN segments	10
FDR fragment joints	0.1013295
FDR chr. breakp. enrich.	0.87
Linked to chrs	
Purity, ploidy	0.89, 1.94

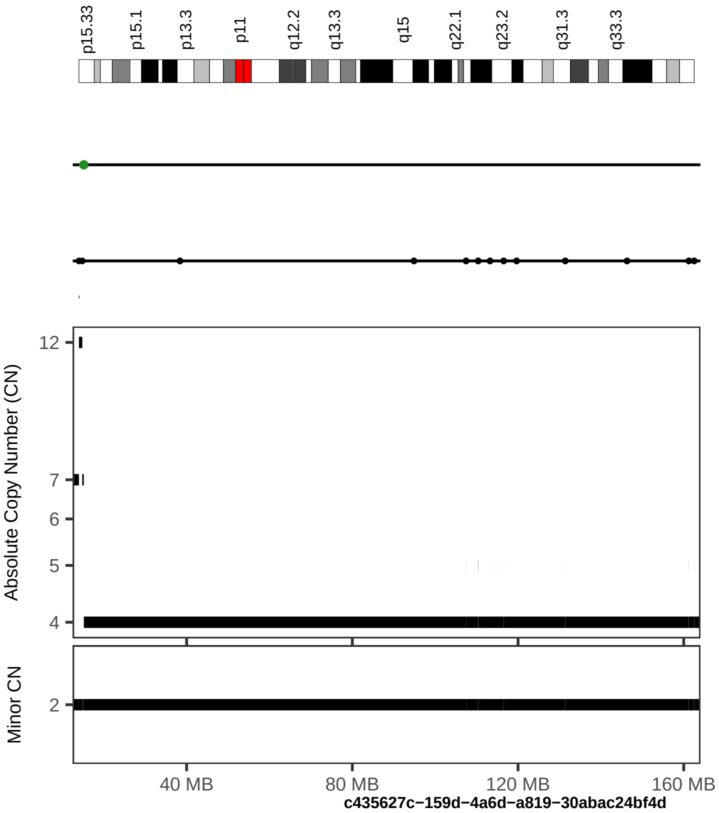

c435627c−159d−4a6d−a819−30abac24bf4d

Cancer type	Ovary−AdenoCA
Position	5:13995081−161235368
Interleaved intrachr. SVs	1
Total SVs (intrachr. + transl.)	9
SV types	DEL: 1; DUP: 7; h2hINV: 0; t2tINV: 0; TRA: 1
SVs in sample	283
Oscillating CN (2 and 3 states)	13, 14
CN segments	15
FDR fragment joints	0.1488029
FDR chr. breakp. enrich.	0.7
Linked to chrs	
Purity, ploidy	0.94, 3.57

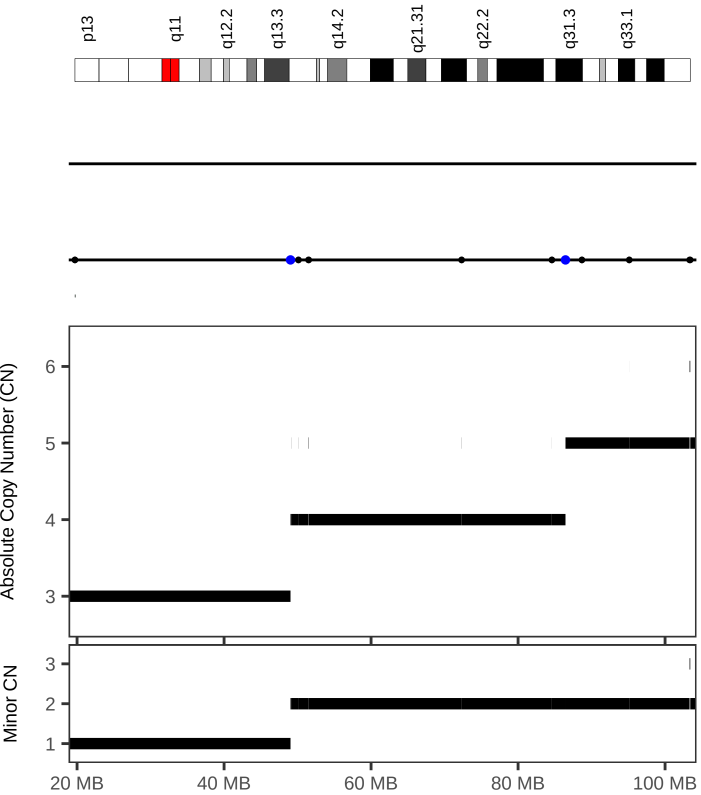

c435627c−159d−4a6d−a819−30abac24bf4d

Cancer type	Ovary−AdenoCA
Position	13:49228433−103426671
Interleaved intrachr. SVs	1
Total SVs (intrachr. + transl.)	6
SV types	DEL: 0; DUP: 5; h2hINV: 0; t2tINV: 0; TRA: 1
SVs in sample	283
Oscillating CN (2 and 3 states)	11, 15
CN segments	15
FDR fragment joints	0.04139017
FDR chr. breakp. enrich.	0.5
Linked to chrs	
Purity, ploidy	0.94, 3.57

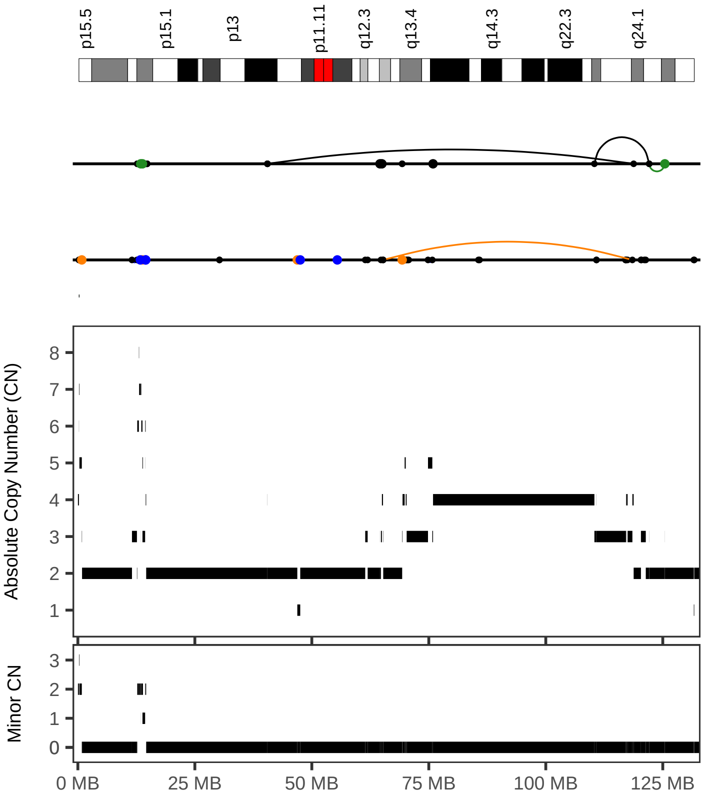

c9e28934−8379−4511−817c−d787f2c4ca3a

Cancer type	Ovary−AdenoCA
Position	11:40526511−125429660
Interleaved intrachr. SVs	5
Total SVs (intrachr. + transl.)	12
SV types	DEL: 1; DUP: 1; h2hINV: 2; t2tINV: 1; TRA: 7
SVs in sample	377
Oscillating CN (2 and 3 states)	10, 18
CN segments	35
FDR fragment joints	0.9284301
FDR chr. breakp. enrich.	0
Linked to chrs	
Purity, ploidy	0.84, 2.56

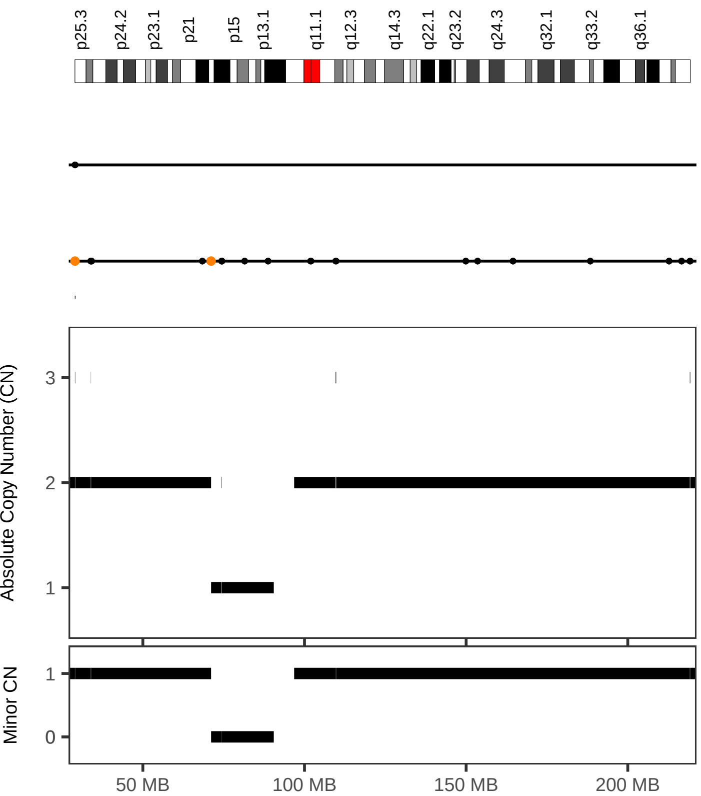

eeb9d147−608d−4692−8adf−2f601d23a8ff

Cancer type	Ovary−AdenoCA
Position	2:28986695−219321806
Interleaved intrachr. SVs	1
Total SVs (intrachr. + transl.)	13
SV types	DEL: 0; DUP: 10; h2hINV: 1; t2tINV: 0; TRA: 2
SVs in sample	239
Oscillating CN (2 and 3 states)	7, 15
CN segments	15
FDR fragment joints	0.03151629
FDR chr. breakp. enrich.	0.73
Linked to chrs	
Purity, ploidy	0.85, 1.93

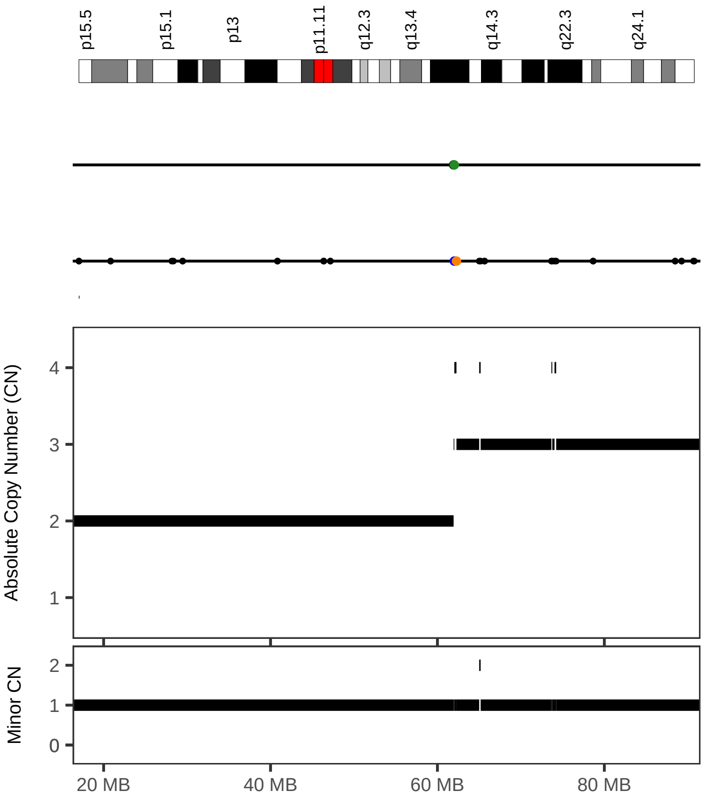

eeb9d147−608d−4692−8adf−2f601d23a8ff

Cancer type	Ovary−AdenoCA
Position	11:28168070−90773179
Interleaved intrachr. SVs	1
Total SVs (intrachr. + transl.)	14
SV types	DEL: 1; DUP: 9; h2hINV: 0; t2tINV: 0; TRA: 4
SVs in sample	239
Oscillating CN (2 and 3 states)	9, 10
CN segments	10
FDR fragment joints	0.07579048
FDR chr. breakp. enrich.	0.06
Linked to chrs	
Purity, ploidy	0.85, 1.93

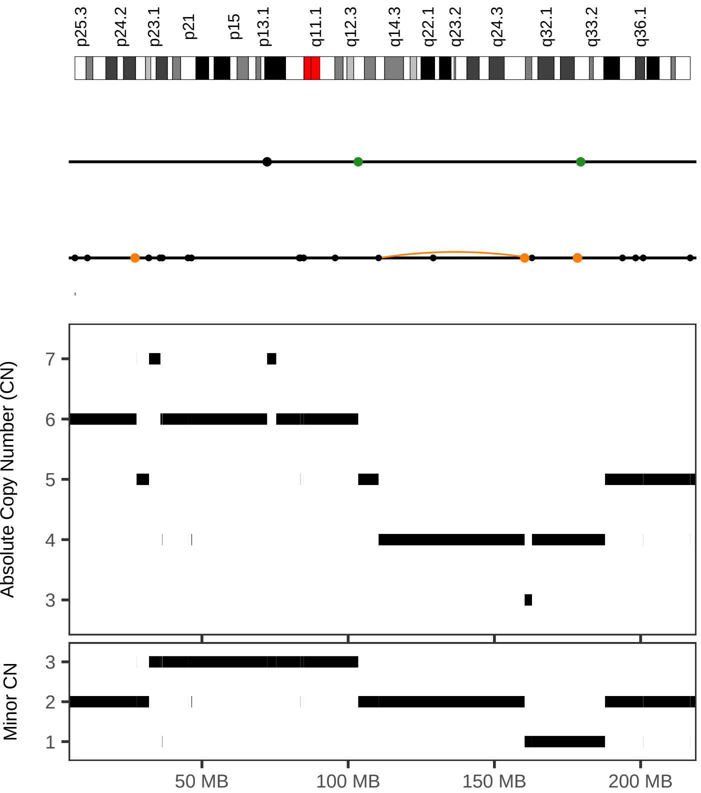

f0c353fd−947c−41e2−b643−3ecc0d69796c

Cancer type	Ovary−AdenoCA
Position	2:27633198−216960765
Interleaved intrachr. SVs	1
Total SVs (intrachr. + transl.)	15
SV types	DEL: 8; DUP: 1; h2hINV: 0; t2tINV: 0; TRA: 6
SVs in sample	288
Oscillating CN (2 and 3 states)	7, 16
CN segments	25
FDR fragment joints	0.129004
FDR chr. breakp. enrich.	0.87
Linked to chrs	
Purity, ploidy	0.91, 4.11

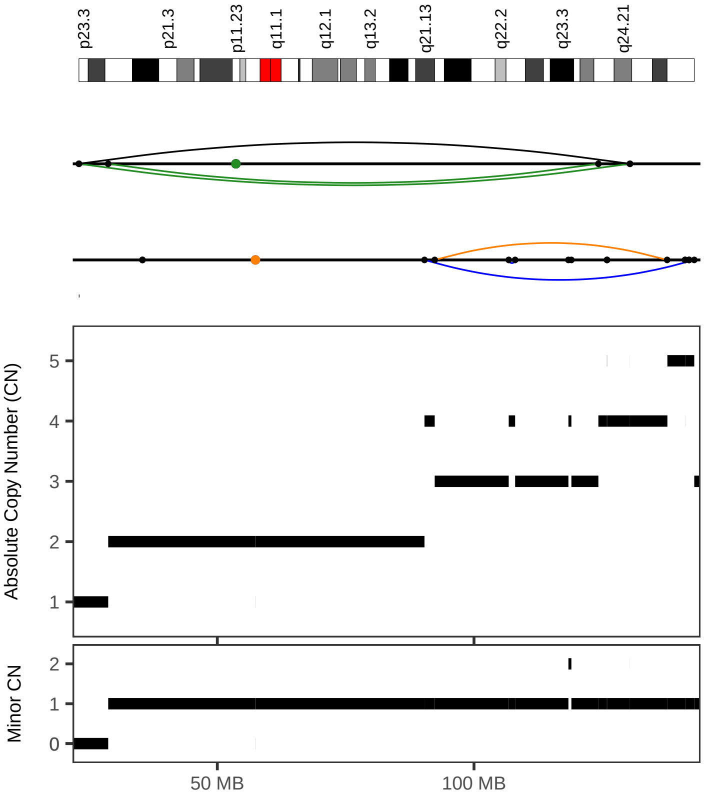

f34aa3b6−e966−49c6−bc55−130545772c53

Cancer type	Ovary−AdenoCA
Position	8:23038721−142905466
Interleaved intrachr. SVs	5
Total SVs (intrachr. + transl.)	10
SV types	DEL: 1; DUP: 1; h2hINV: 1; t2tINV: 2; TRA: 5
SVs in sample	235
Oscillating CN (2 and 3 states)	8, 9
CN segments	19
FDR fragment joints	0.8572806
FDR chr. breakp. enrich.	0.33
Linked to chrs	4:30752695−148972521;
Purity, ploidy	0.94, 1.88

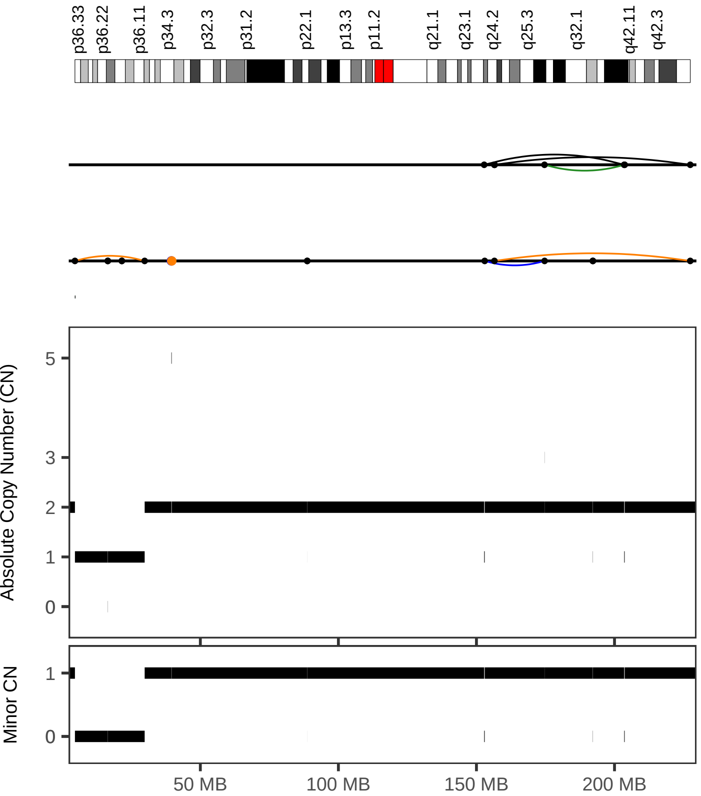

ICGC_0025

Cancer type	Panc−AdenoCA
Position	1:152793278−227429822
Interleaved intrachr. SVs	6
Total SVs (intrachr. + transl.)	6
SV types	DEL: 1; DUP: 1; h2hINV: 2; t2tINV: 2; TRA: 0
SVs in sample	118
Oscillating CN (2 and 3 states)	5, 8
CN segments	8
FDR fragment joints	0.9284301
FDR chr. breakp. enrich.	0.46
Linked to chrs	
Purity, ploidy	0.51, 1.65

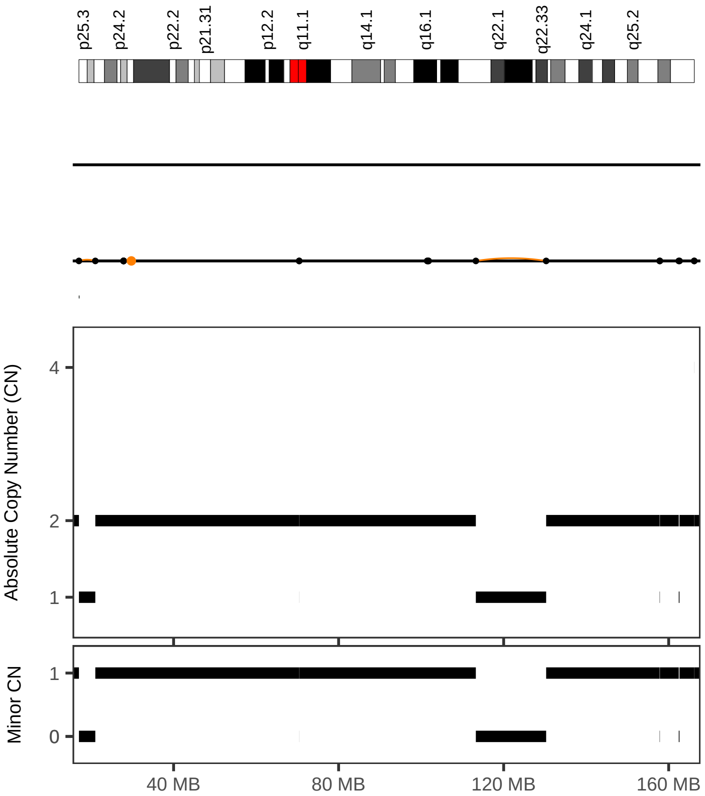

ICGC_0025

Cancer type	Panc−AdenoCA
Position	6:17053808−162631121
Interleaved intrachr. SVs	1
Total SVs (intrachr. + transl.)	9
SV types	DEL: 8; DUP: 0; h2hINV: 0; t2tINV: 0; TRA: 1
SVs in sample	118
Oscillating CN (2 and 3 states)	10, 10
CN segments	10
FDR fragment joints	0.002298705
FDR chr. breakp. enrich.	0.22
Linked to chrs	
Purity, ploidy	0.51, 1.65

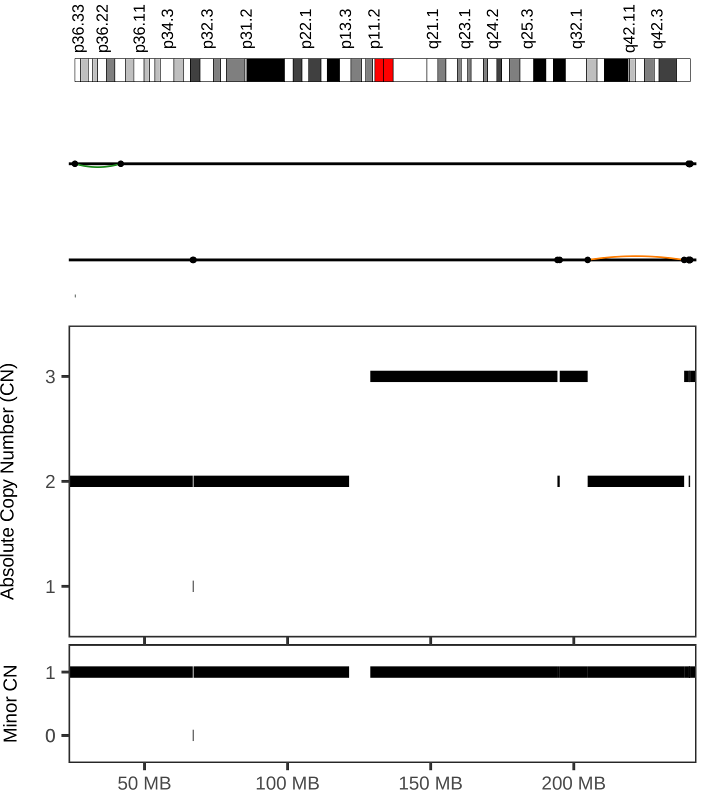

ICGC_0037

Cancer type	Panc−AdenoCA
Position	1:240179982−240688305
Interleaved intrachr. SVs	21
Total SVs (intrachr. + transl.)	21
SV types	DEL: 3; DUP: 7; h2hINV: 6; t2tINV: 5; TRA: 0
SVs in sample	165
Oscillating CN (2 and 3 states)	12, 12
CN segments	12
FDR fragment joints	0.7568568
FDR chr. breakp. enrich.	0.01
Linked to chrs	
Purity, ploidy	0.48, 2.75

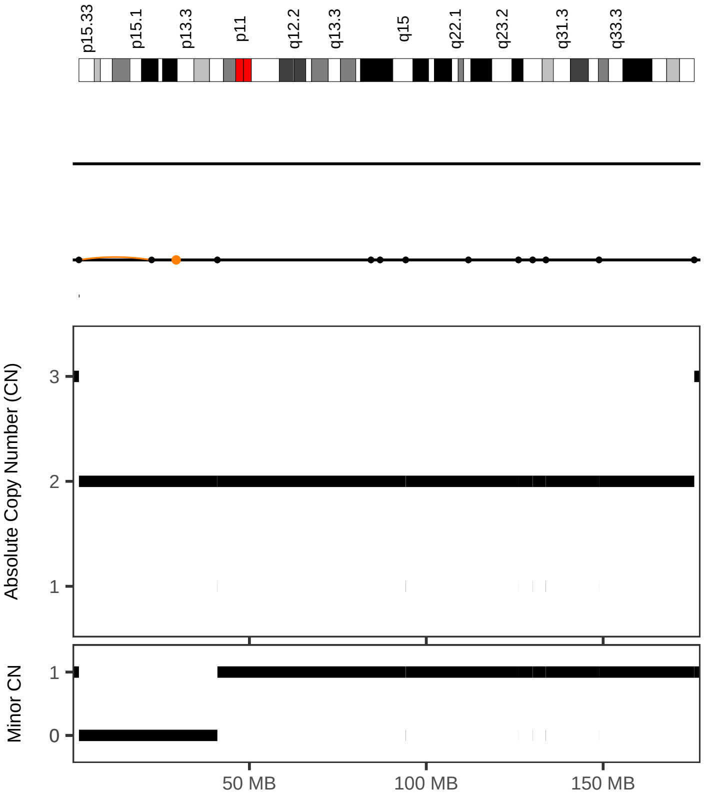

ICGC_0054

Cancer type	Panc−AdenoCA
Position	5:1796643−175760299
Interleaved intrachr. SVs	1
Total SVs (intrachr. + transl.)	7
SV types	DEL: 6; DUP: 0; h2hINV: 0; t2tINV: 0; TRA: 1
SVs in sample	156
Oscillating CN (2 and 3 states)	13, 14
CN segments	14
FDR fragment joints	0.01743054
FDR chr. breakp. enrich.	0.66
Linked to chrs	
Purity, ploidy	0.36, 2.67

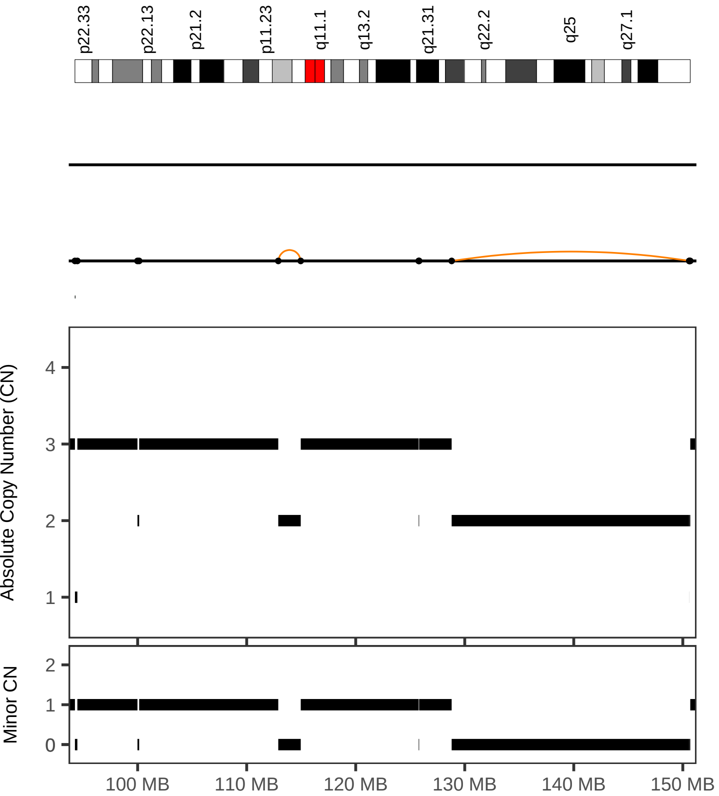

ICGC_0115

Cancer type	Panc−AdenoCA
Position	X:94245698−150684659
Interleaved intrachr. SVs	1
Total SVs (intrachr. + transl.)	5
SV types	DEL: 5; DUP: 0; h2hINV: 0; t2tINV: 0; TRA: 0
SVs in sample	104
Oscillating CN (2 and 3 states)	8, 11
CN segments	12
FDR fragment joints	0.1013295
FDR chr. breakp. enrich.	0.86
Linked to chrs	
Purity, ploidy	0.39, 3.3

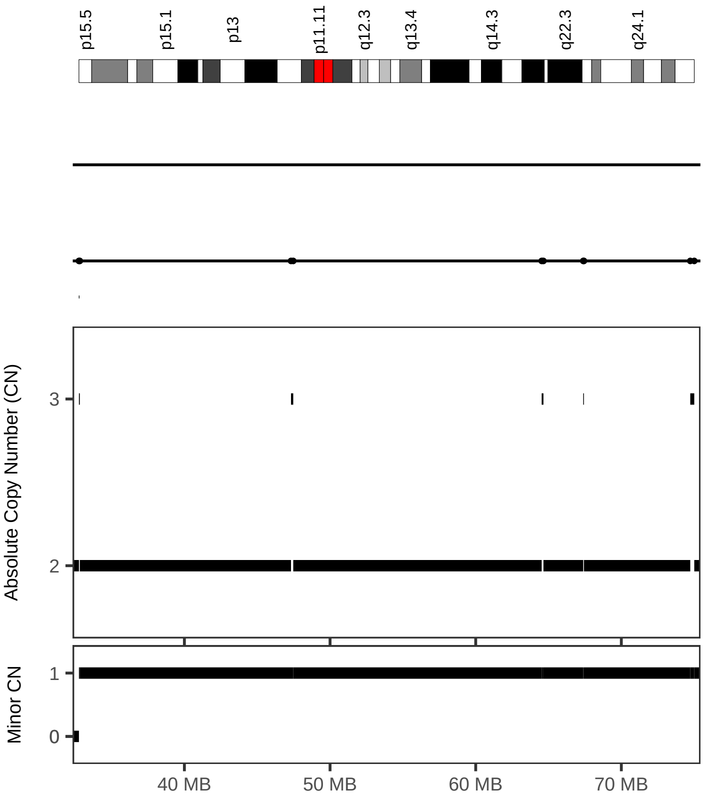

ICGC_0140

Cancer type	Panc−AdenoCA
Position	11:32758465−75006148
Interleaved intrachr. SVs	1
Total SVs (intrachr. + transl.)	5
SV types	DEL: 0; DUP: 5; h2hINV: 0; t2tINV: 0; TRA: 0
SVs in sample	163
Oscillating CN (2 and 3 states)	10, 10
CN segments	10
FDR fragment joints	0.1013295
FDR chr. breakp. enrich.	0.7
Linked to chrs	
Purity, ploidy	0.85, 1.96

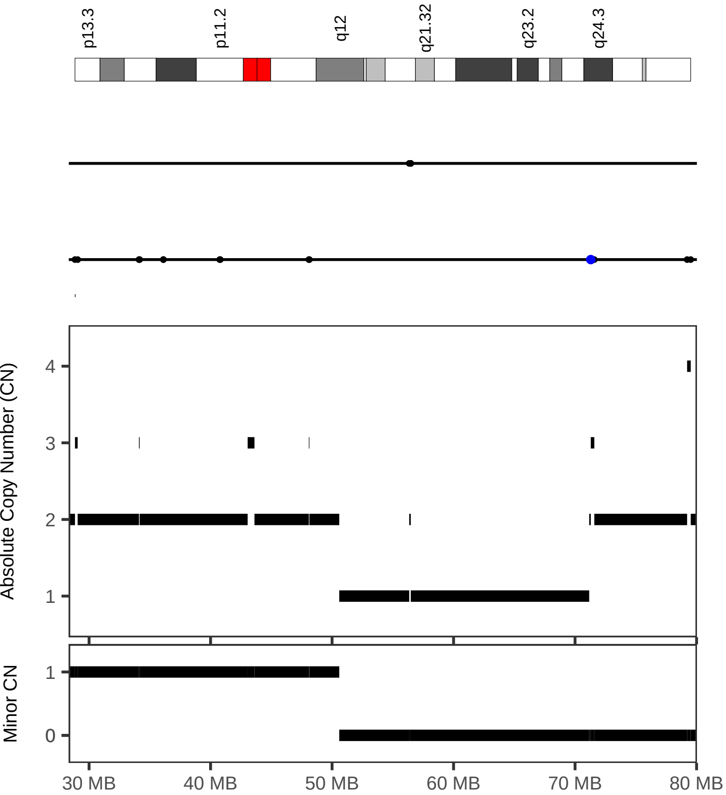

ICGC_0140

Cancer type	Panc−AdenoCA
Position	17:28840466−79518858
Interleaved intrachr. SVs	1
Total SVs (intrachr. + transl.)	8
SV types	DEL: 0; DUP: 6; h2hINV: 0; t2tINV: 1; TRA: 1
SVs in sample	163
Oscillating CN (2 and 3 states)	8, 14
CN segments	16
FDR fragment joints	0.05741733
FDR chr. breakp. enrich.	0.06
Linked to chrs	
Purity, ploidy	0.85, 1.96

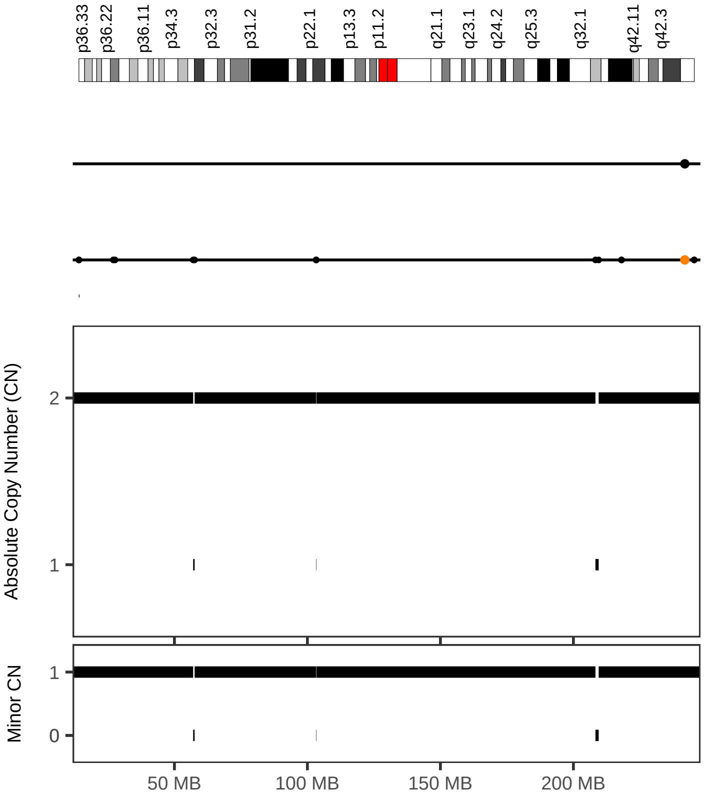

ICGC_0205

Cancer type	Panc−AdenoCA
Position	1:26994187−245430132
Interleaved intrachr. SVs	1
Total SVs (intrachr. + transl.)	8
SV types	DEL: 6; DUP: 0; h2hINV: 0; t2tINV: 0; TRA: 2
SVs in sample	127
Oscillating CN (2 and 3 states)	7, 7
CN segments	7
FDR fragment joints	0.05741733
FDR chr. breakp. enrich.	1
Linked to chrs	9:9187524−139636858;
Purity, ploidy	0.6, 1.83

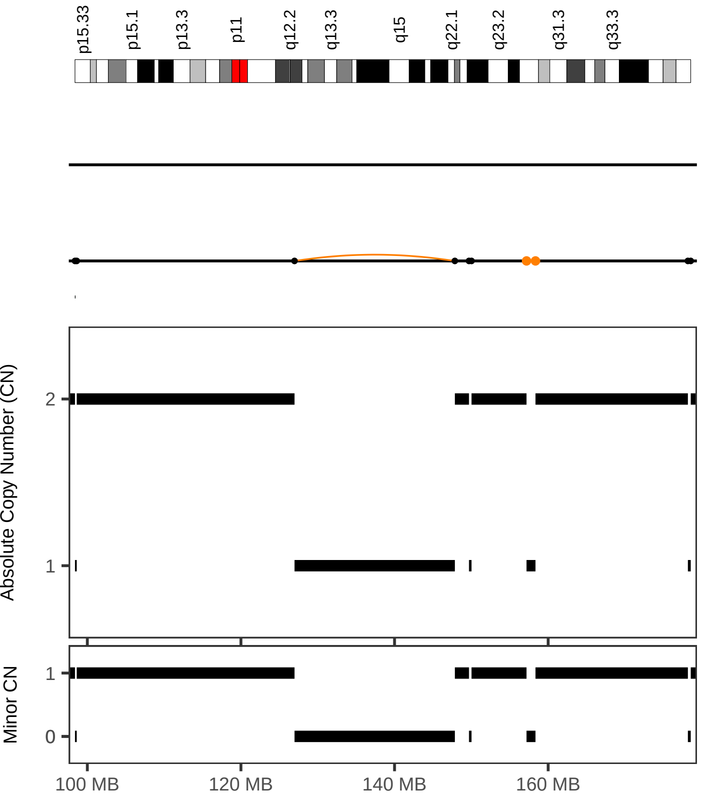

ICGC_0214

Cancer type	Panc−AdenoCA
Position	5:98391041−178563199
Interleaved intrachr. SVs	1
Total SVs (intrachr. + transl.)	6
SV types	DEL: 4; DUP: 0; h2hINV: 0; t2tINV: 0; TRA: 2
SVs in sample	254
Oscillating CN (2 and 3 states)	10, 10
CN segments	10
FDR fragment joints	0.04139017
FDR chr. breakp. enrich.	0.03
Linked to chrs	
Purity, ploidy	0.47, 1.82

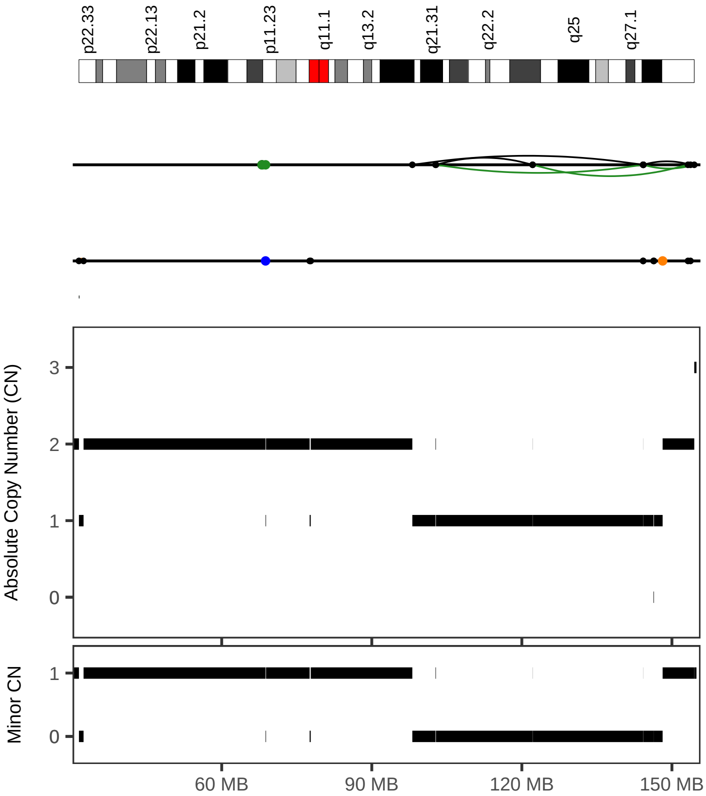

ICGC_0214

Cancer type	Panc−AdenoCA
Position	X:98103871−154466373
Interleaved intrachr. SVs	7
Total SVs (intrachr. + transl.)	8
SV types	DEL: 0; DUP: 1; h2hINV: 3; t2tINV: 3; TRA: 1
SVs in sample	254
Oscillating CN (2 and 3 states)	7, 11
CN segments	11
FDR fragment joints	0.6776251
FDR chr. breakp. enrich.	0.35
Linked to chrs	
Purity, ploidy	0.47, 1.82

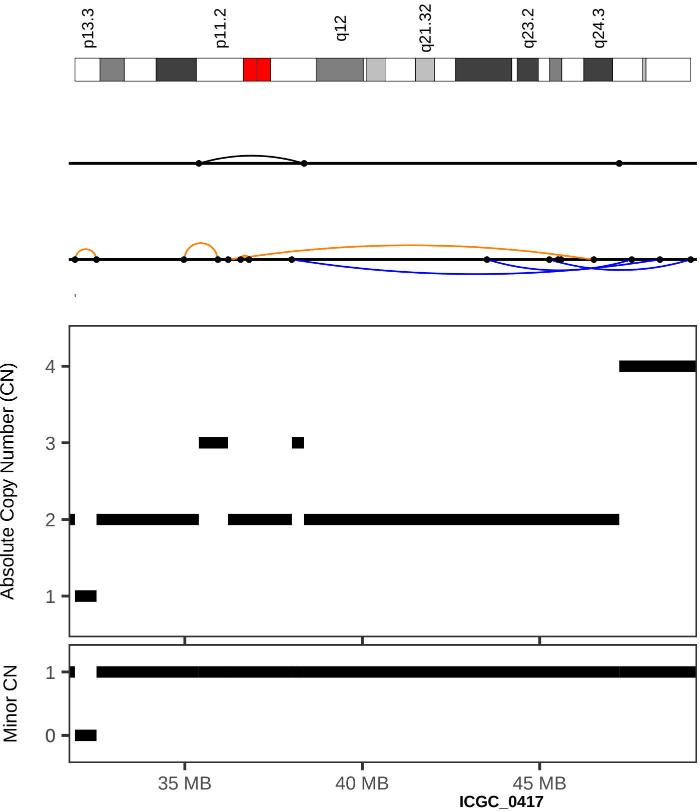

ICGC_0417

Cancer type	Panc−AdenoCA
Position	17:34974437−49255446
Interleaved intrachr. SVs	6
Total SVs (intrachr. + transl.)	6
SV types	DEL: 2; DUP: 3; h2hINV: 1; t2tINV: 0; TRA: 0
SVs in sample	47
Oscillating CN (2 and 3 states)	5, 6
CN segments	6
FDR fragment joints	0.615458
FDR chr. breakp. enrich.	0
Linked to chrs	
Purity, ploidy	0.46, 1.99

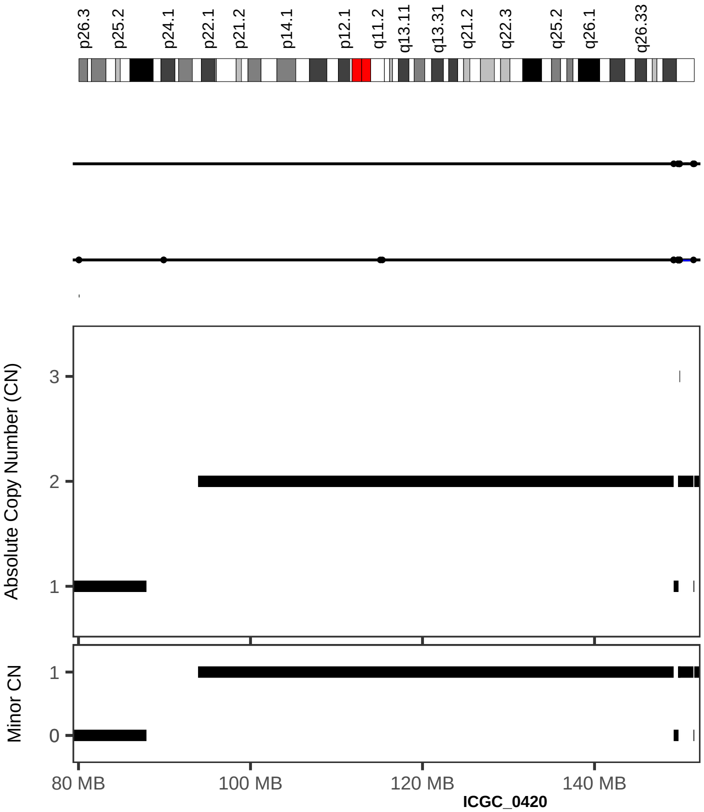

ICGC_0420

Cancer type	Panc−AdenoCA
Position	3:149207002−151596957
Interleaved intrachr. SVs	11
Total SVs (intrachr. + transl.)	11
SV types	DEL: 3; DUP: 3; h2hINV: 2; t2tINV: 3; TRA: 0
SVs in sample	90
Oscillating CN (2 and 3 states)	6, 10
CN segments	13
FDR fragment joints	0.9804396
FDR chr. breakp. enrich.	0
Linked to chrs	
Purity, ploidy	0.55, 1.66

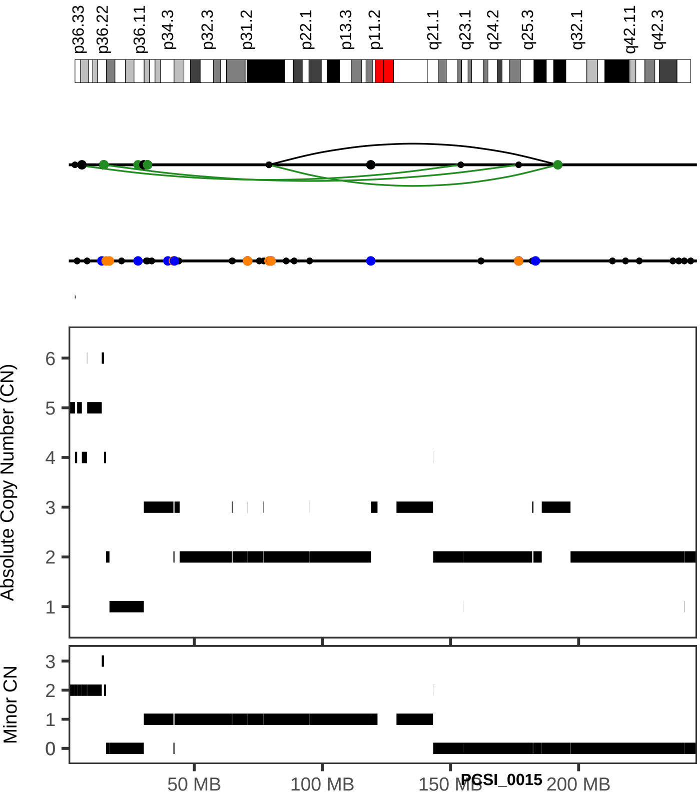

PCSI_0015

Cancer type	Panc−AdenoCA
Position	1:3415964−191894581
Interleaved intrachr. SVs	4
Total SVs (intrachr. + transl.)	29
SV types	DEL: 0; DUP: 0; h2hINV: 1; t2tINV: 3; TRA: 25
SVs in sample	541
Oscillating CN (2 and 3 states)	13, 18
CN segments	30
FDR fragment joints	0.6776251
FDR chr. breakp. enrich.	0.22
Linked to chrs	12:1762415−132632651;18:751562−43071862 3:74786302−147259330;4:10893126−58422206 8:100785898−143451879;

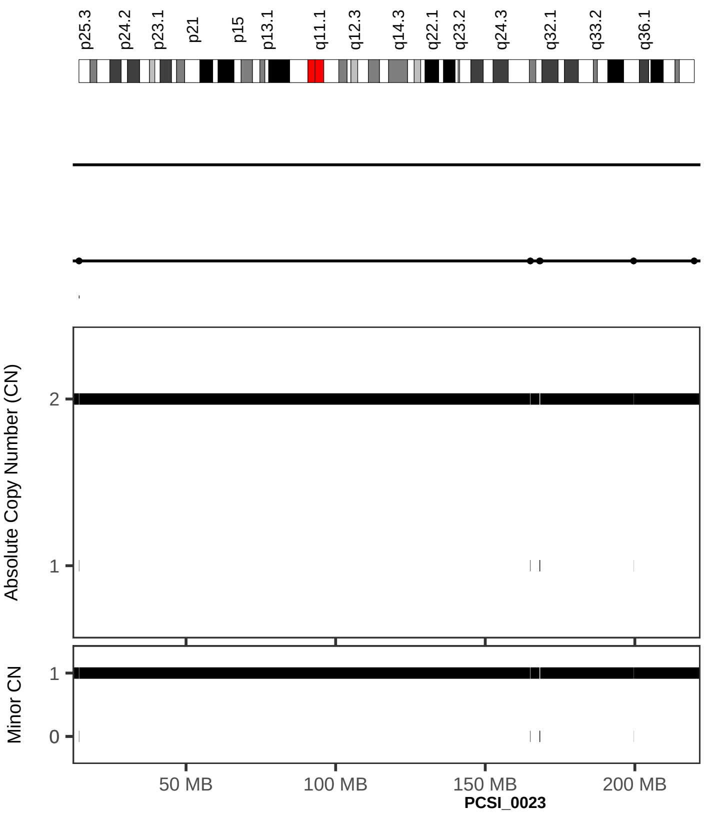

PCSI_0023

Cancer type	Panc−AdenoCA
Position	2:14191317−219852324
Interleaved intrachr. SVs	1
Total SVs (intrachr. + transl.)	5
SV types	DEL: 5; DUP: 0; h2hINV: 0; t2tINV: 0; TRA: 0
SVs in sample	114
Oscillating CN (2 and 3 states)	8, 8
CN segments	8
FDR fragment joints	0.1013295
FDR chr. breakp. enrich.	0.39
Linked to chrs	
Purity, ploidy	0.27, 1.96

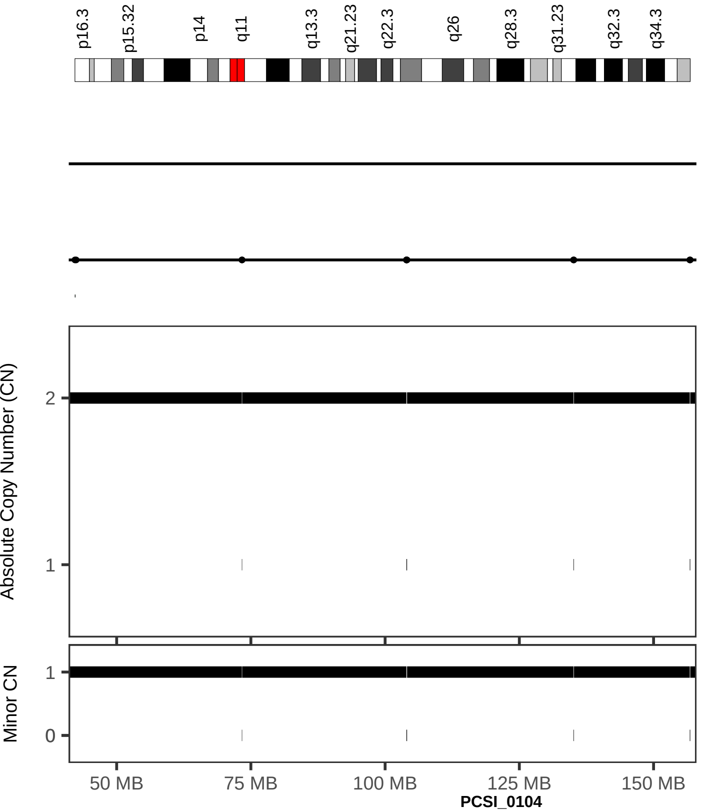

PCSI_0104

Cancer type	Panc−AdenoCA
Position	4:42227398−156834083
Interleaved intrachr. SVs	1
Total SVs (intrachr. + transl.)	5
SV types	DEL: 5; DUP: 0; h2hINV: 0; t2tINV: 0; TRA: 0
SVs in sample	107
Oscillating CN (2 and 3 states)	9, 9
CN segments	9
FDR fragment joints	0.1013295
FDR chr. breakp. enrich.	0.98
Linked to chrs	
Purity, ploidy	0.85, 1.97

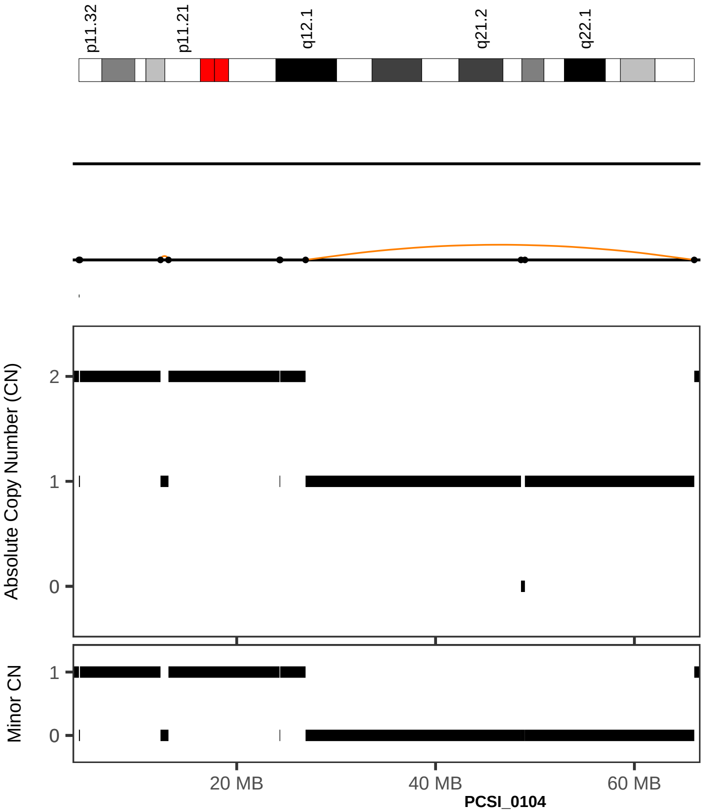

PCSI_0104

Cancer type	Panc−AdenoCA
Position	18:4126494−66021548
Interleaved intrachr. SVs	1
Total SVs (intrachr. + transl.)	5
SV types	DEL: 5; DUP: 0; h2hINV: 0; t2tINV: 0; TRA: 0
SVs in sample	107
Oscillating CN (2 and 3 states)	7, 10
CN segments	10
FDR fragment joints	0.1013295
FDR chr. breakp. enrich.	0.45
Linked to chrs	
Purity, ploidy	0.85, 1.97

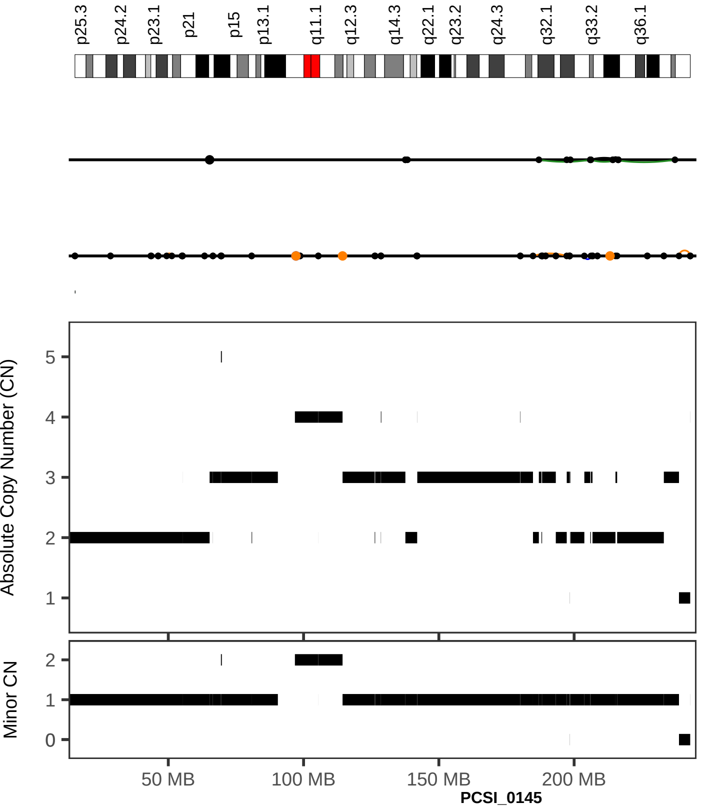

PCSI_0145

Cancer type	Panc−AdenoCA
Position	2:184795438−237271575
Interleaved intrachr. SVs	8
Total SVs (intrachr. + transl.)	9
SV types	DEL: 1; DUP: 1; h2hINV: 3; t2tINV: 3; TRA: 1
SVs in sample	550
Oscillating CN (2 and 3 states)	10, 18
CN segments	18
FDR fragment joints	0.854603
FDR chr. breakp. enrich.	0.85
Linked to chrs	
Purity, ploidy	0.87, 2.72

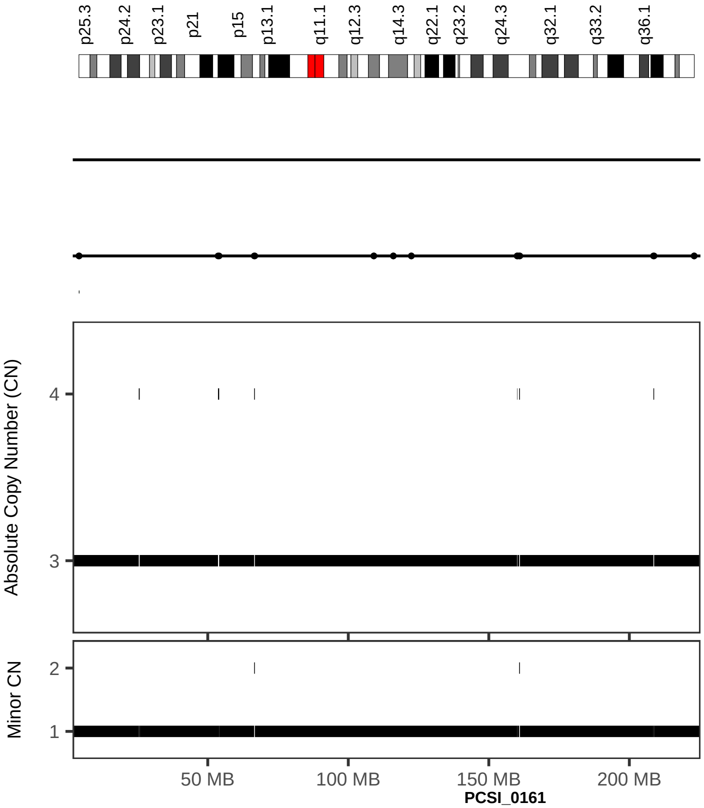

PCSI_0161

Cancer type	Panc−AdenoCA
Position	2:4154479−223101138
Interleaved intrachr. SVs	1
Total SVs (intrachr. + transl.)	9
SV types	DEL: 0; DUP: 9; h2hINV: 0; t2tINV: 0; TRA: 0
SVs in sample	187
Oscillating CN (2 and 3 states)	13, 13
CN segments	13
FDR fragment joints	0.002298705
FDR chr. breakp. enrich.	0.35
Linked to chrs	
Purity, ploidy	0.63, 2.98

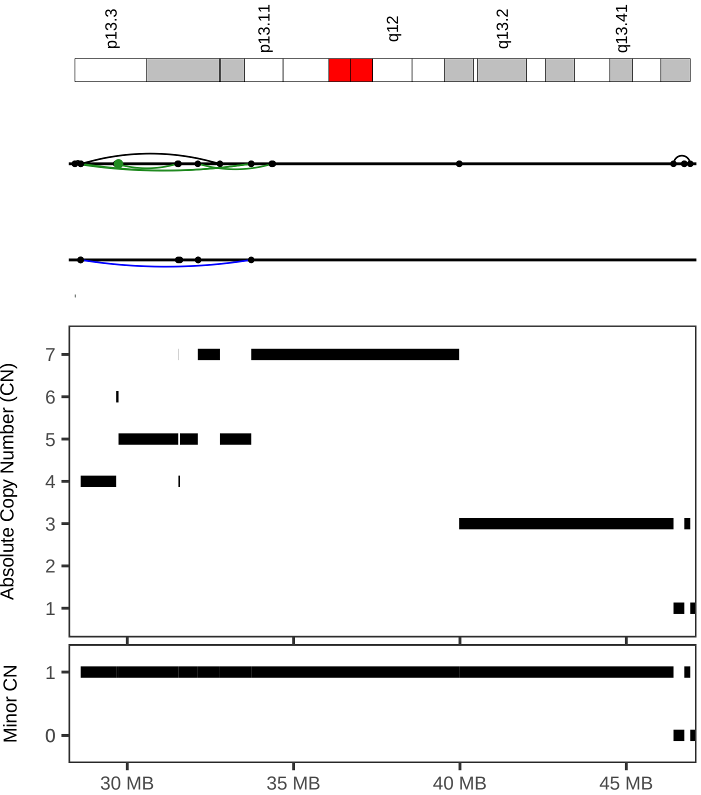

PCSI_0170

Cancer type	Panc−AdenoCA
Position	19:28426608−34375994
Interleaved intrachr. SVs	6
Total SVs (intrachr. + transl.)	7
SV types	DEL: 0; DUP: 1; h2hINV: 2; t2tINV: 3; TRA: 1
SVs in sample	33
Oscillating CN (2 and 3 states)	4, 5
CN segments	11
FDR fragment joints	0.615458
FDR chr. breakp. enrich.	0
Linked to chrs	
Purity, ploidy	0.89, 1.86

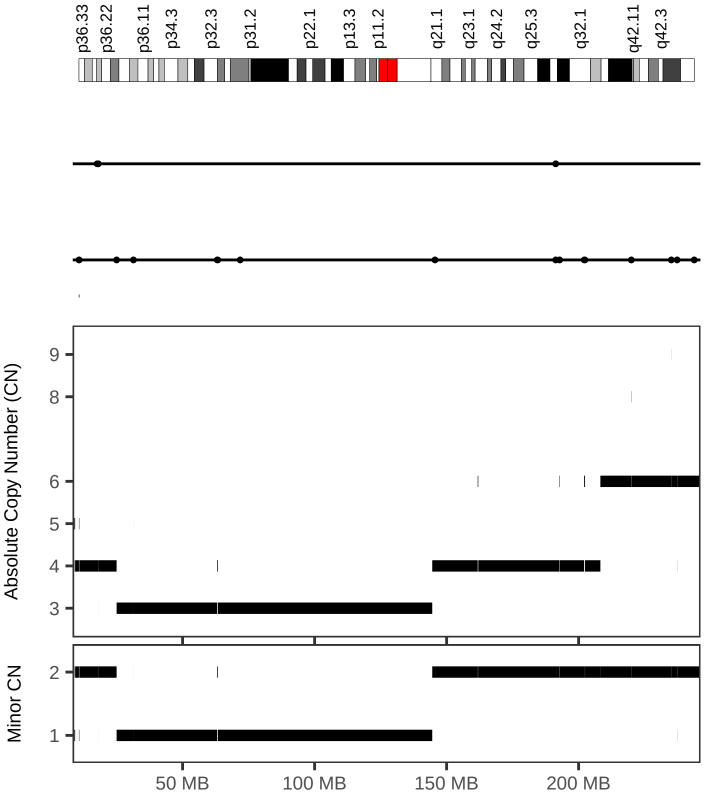

PCSI_0217

Cancer type	Panc−AdenoCA
Position	1:10731806−237345673
Interleaved intrachr. SVs	1
Total SVs (intrachr. + transl.)	10
SV types	DEL: 0; DUP: 9; h2hINV: 0; t2tINV: 1; TRA: 0
SVs in sample	152
Oscillating CN (2 and 3 states)	8, 9
CN segments	23
FDR fragment joints	0.01020027
FDR chr. breakp. enrich.	0.46
Linked to chrs	
Purity, ploidy	0.77, 3.17

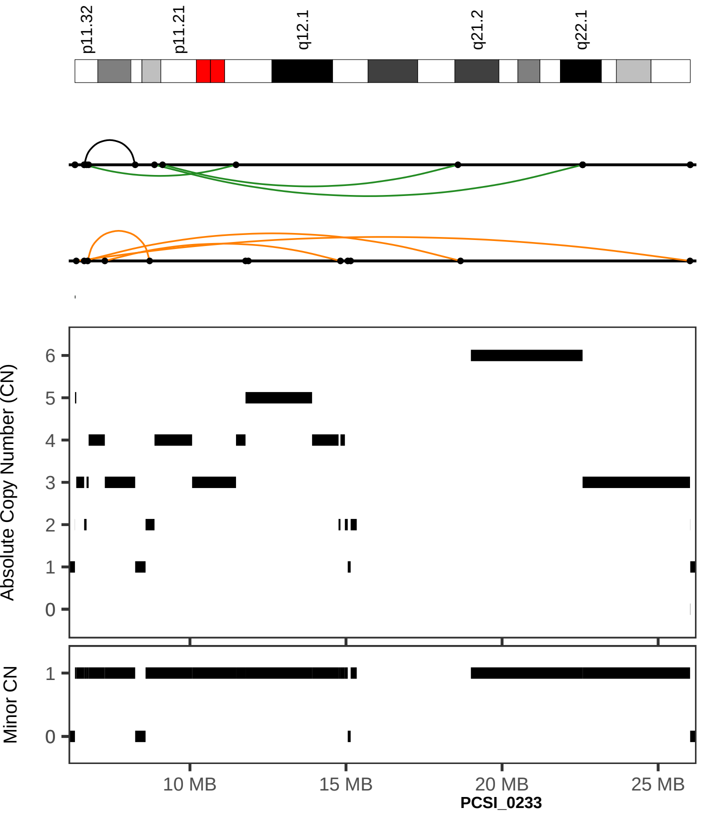

PCSI_0233

Cancer type	Panc−AdenoCA
Position	18:6611362−22580219
Interleaved intrachr. SVs	8
Total SVs (intrachr. + transl.)	8
SV types	DEL: 3; DUP: 0; h2hINV: 1; t2tINV: 4; TRA: 0
SVs in sample	71
Oscillating CN (2 and 3 states)	4, 8
CN segments	19
FDR fragment joints	0.615458
FDR chr. breakp. enrich.	0
Linked to chrs	
Purity, ploidy	0.26, 2.92

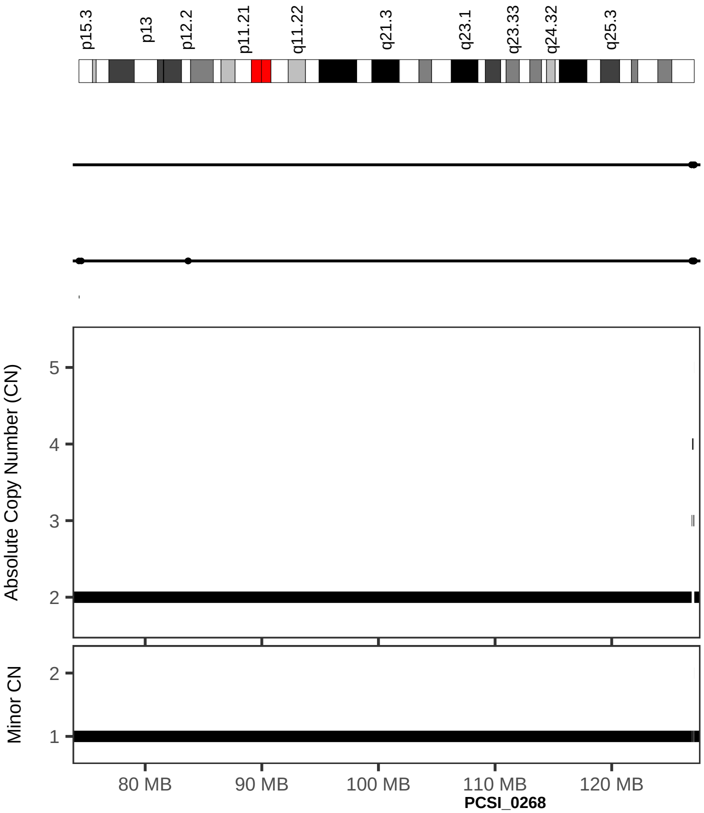

PCSI_0268

Cancer type	Panc−AdenoCA
Position	10:126851447−127077192
Interleaved intrachr. SVs	14
Total SVs (intrachr. + transl.)	14
SV types	DEL: 3; DUP: 3; h2hINV: 4; t2tINV: 4; TRA: 0
SVs in sample	104
Oscillating CN (2 and 3 states)	5, 7
CN segments	8
FDR fragment joints	0.9794281
FDR chr. breakp. enrich.	0
Linked to chrs	
Purity, ploidy	0.67, 2.21

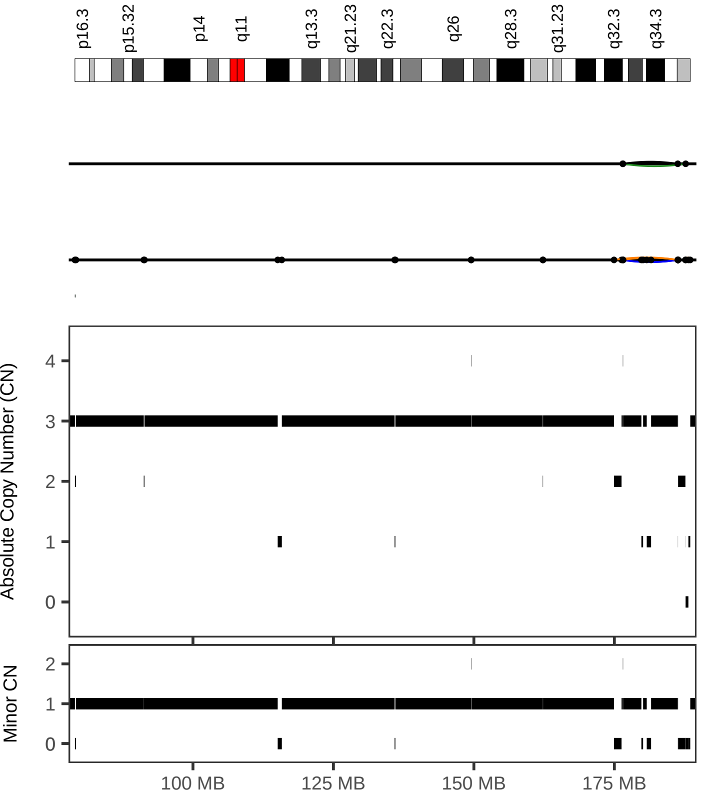

PCSI_0274

Cancer type	Panc−AdenoCA
Position	4:174934602−188504783
Interleaved intrachr. SVs	8
Total SVs (intrachr. + transl.)	8
SV types	DEL: 3; DUP: 3; h2hINV: 1; t2tINV: 1; TRA: 0
SVs in sample	202
Oscillating CN (2 and 3 states)	8, 13
CN segments	17
FDR fragment joints	0.6776251
FDR chr. breakp. enrich.	0.57
Linked to chrs	
Purity, ploidy	0.6, 3.17

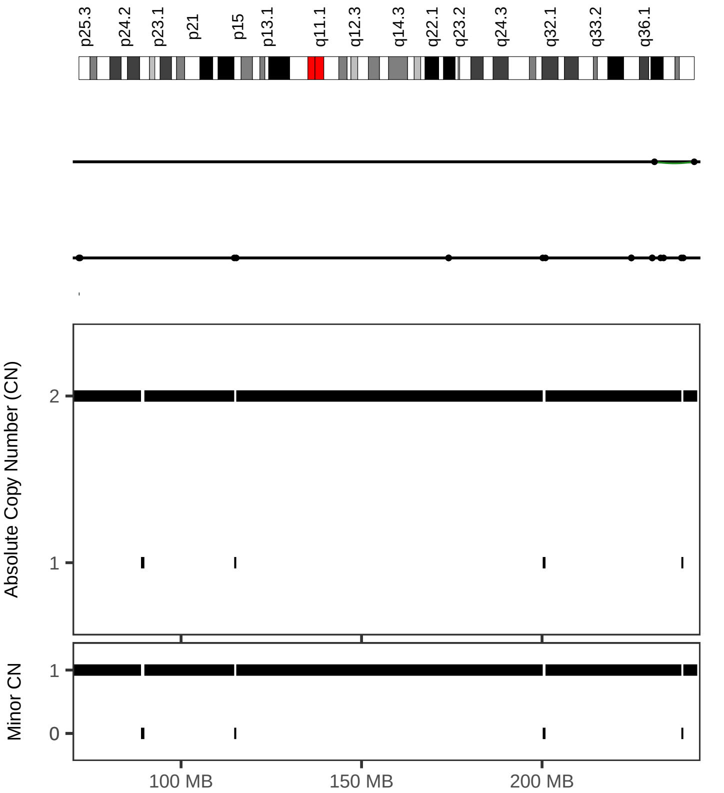

PCSI_0287

Cancer type	Panc−AdenoCA
Position	2:71708971−242161284
Interleaved intrachr. SVs	1
Total SVs (intrachr. + transl.)	9
SV types	DEL: 8; DUP: 0; h2hINV: 0; t2tINV: 1; TRA: 0
SVs in sample	152
Oscillating CN (2 and 3 states)	9, 9
CN segments	9
FDR fragment joints	0.02438658
FDR chr. breakp. enrich.	0.63
Linked to chrs	
Purity, ploidy	0.94, 1.94

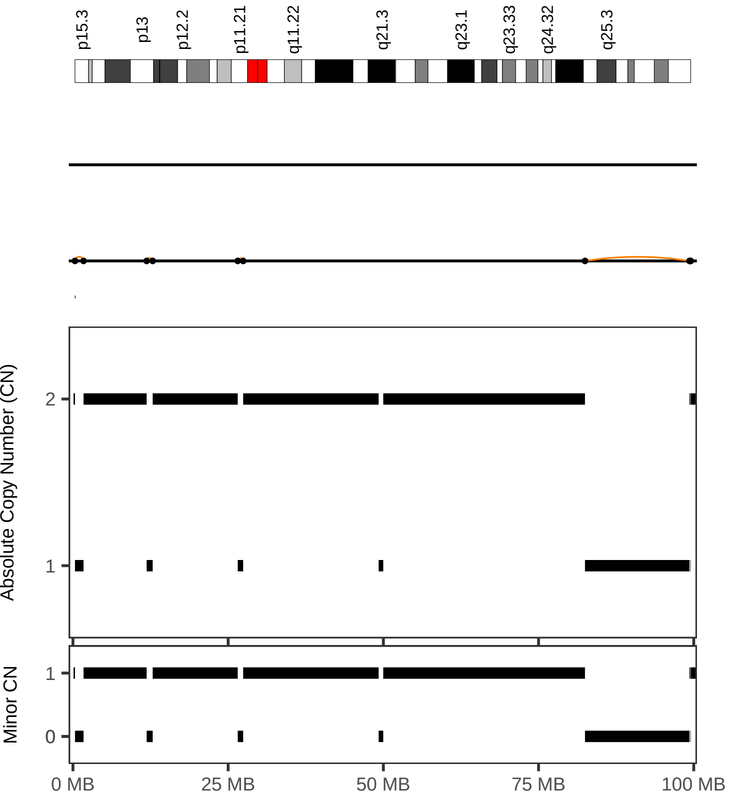

PCSI_0287

Cancer type	Panc−AdenoCA
Position	10:324176−99514412
Interleaved intrachr. SVs	1
Total SVs (intrachr. + transl.)	5
SV types	DEL: 5; DUP: 0; h2hINV: 0; t2tINV: 0; TRA: 0
SVs in sample	152
Oscillating CN (2 and 3 states)	12, 12
CN segments	12
FDR fragment joints	0.1013295
FDR chr. breakp. enrich.	0.79
Linked to chrs	
Purity, ploidy	0.94, 1.94

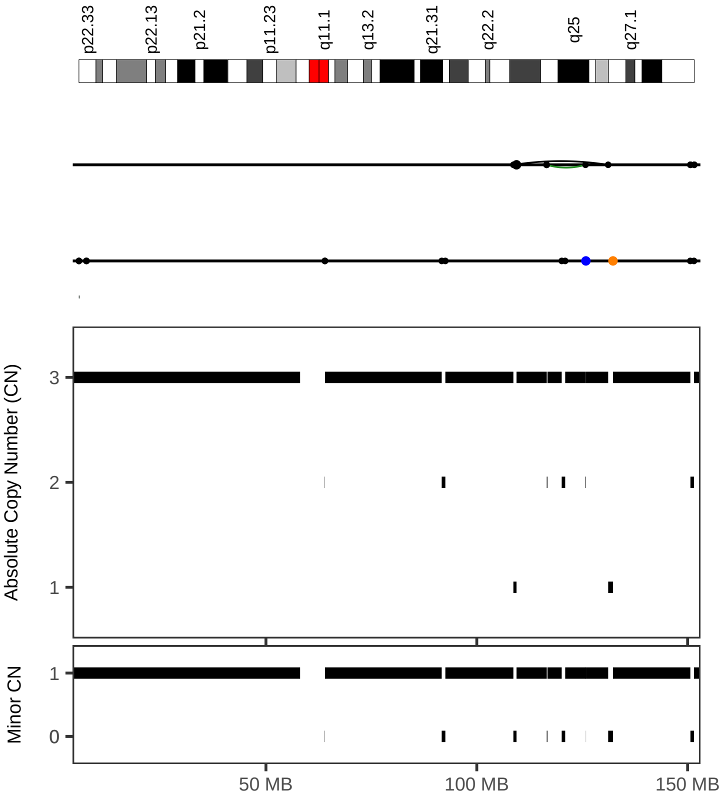

PCSI_0300

Cancer type	Panc−AdenoCA
Position	X:116539582−151569032
Interleaved intrachr. SVs	3
Total SVs (intrachr. + transl.)	8
SV types	DEL: 2; DUP: 0; h2hINV: 2; t2tINV: 2; TRA: 2
SVs in sample	208
Oscillating CN (2 and 3 states)	9, 15
CN segments	15
FDR fragment joints	0.8653243
FDR chr. breakp. enrich.	0.36
Linked to chrs	
Purity, ploidy	0.58, 2.82

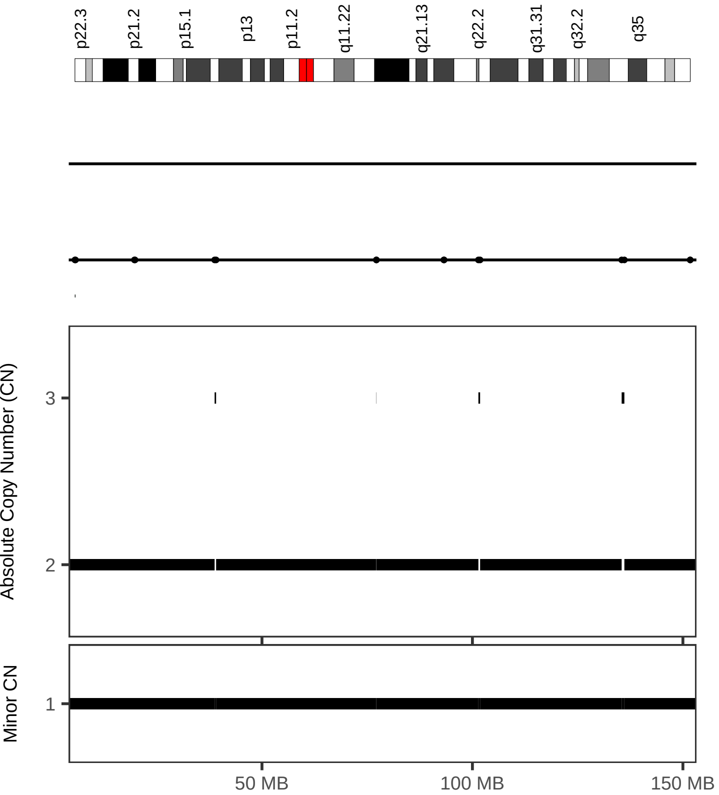

PCSI_0324

Cancer type	Panc−AdenoCA
Position	7:5562244−151748583
Interleaved intrachr. SVs	1
Total SVs (intrachr. + transl.)	8
SV types	DEL: 0; DUP: 8; h2hINV: 0; t2tINV: 0; TRA: 0
SVs in sample	204
Oscillating CN (2 and 3 states)	9, 9
CN segments	9
FDR fragment joints	0.006736868
FDR chr. breakp. enrich.	0.69
Linked to chrs	
Purity, ploidy	0.56, 1.86

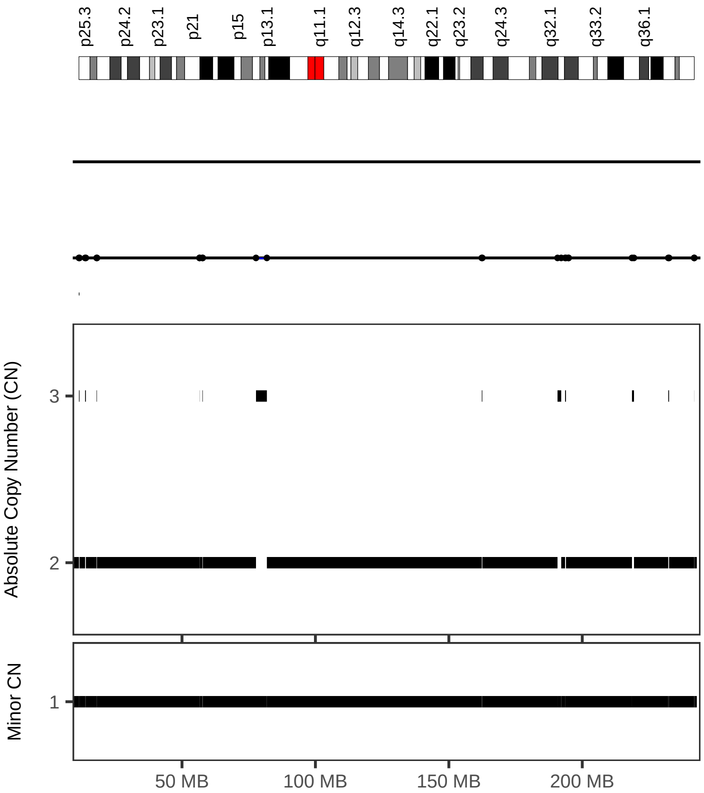

PCSI_0338

Cancer type	Panc−AdenoCA
Position	2:11368327−241973077
Interleaved intrachr. SVs	1
Total SVs (intrachr. + transl.)	13
SV types	DEL: 0; DUP: 13; h2hINV: 0; t2tINV: 0; TRA: 0
SVs in sample	225
Oscillating CN (2 and 3 states)	24, 24
CN segments	24
FDR fragment joints	2.670882e−05
FDR chr. breakp. enrich.	0.4
Linked to chrs	
Purity, ploidy	0.6, 2.02

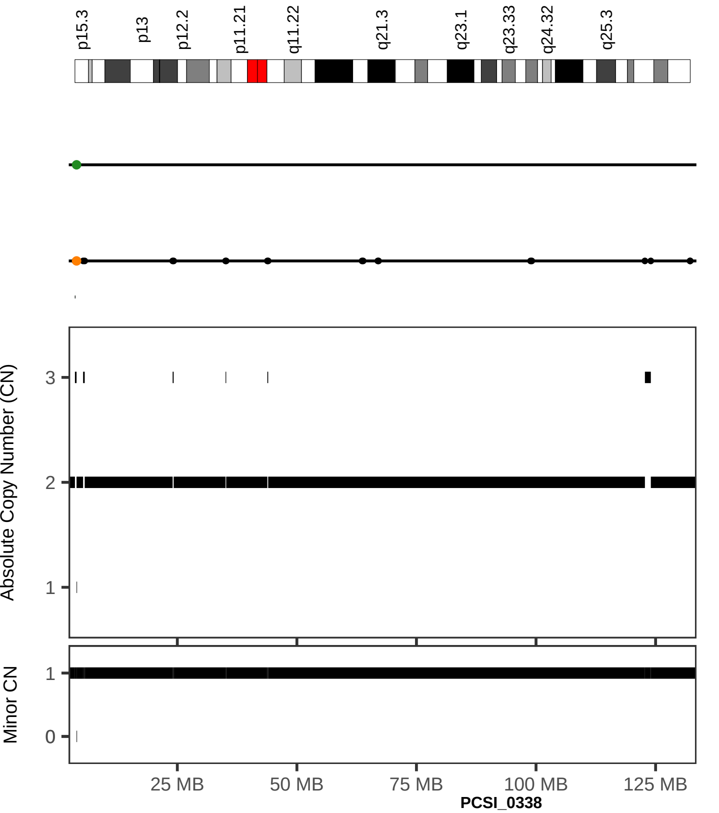

PCSI_0338

Cancer type	Panc−AdenoCA
Position	10:3589818−132244293
Interleaved intrachr. SVs	1
Total SVs (intrachr. + transl.)	12
SV types	DEL: 0; DUP: 10; h2hINV: 0; t2tINV: 0; TRA: 2
SVs in sample	225
Oscillating CN (2 and 3 states)	11, 11
CN segments	13
FDR fragment joints	0.01333161
FDR chr. breakp. enrich.	0.67
Linked to chrs	
Purity, ploidy	0.6, 2.02

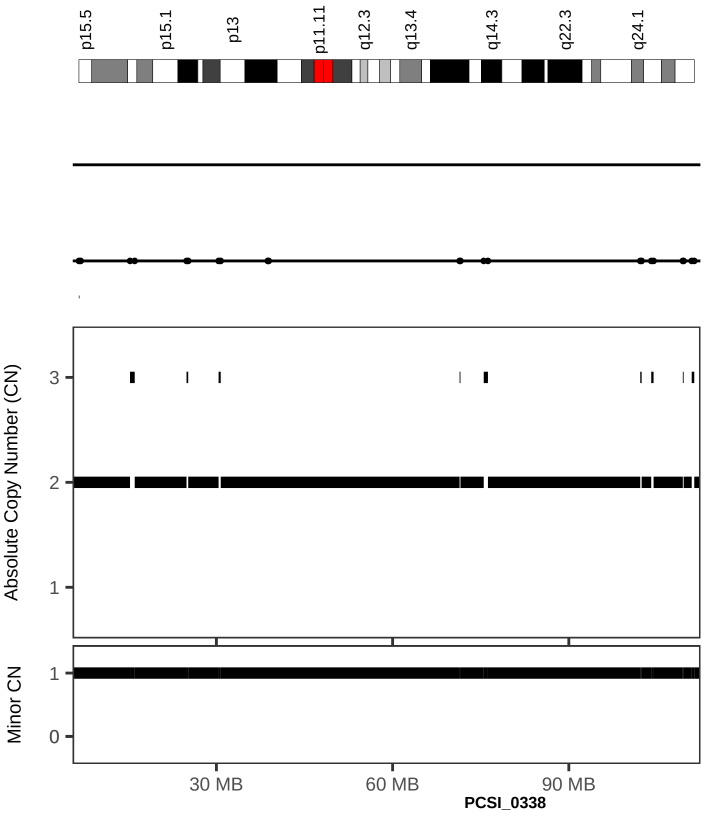

PCSI_0338

Cancer type	Panc−AdenoCA
Position	11:6595768−111354727
Interleaved intrachr. SVs	1
Total SVs (intrachr. + transl.)	11
SV types	DEL: 0; DUP: 11; h2hINV: 0; t2tINV: 0; TRA: 0
SVs in sample	225
Oscillating CN (2 and 3 states)	19, 19
CN segments	19
FDR fragment joints	0.0002478023
FDR chr. breakp. enrich.	1
Linked to chrs	
Purity, ploidy	0.6, 2.02

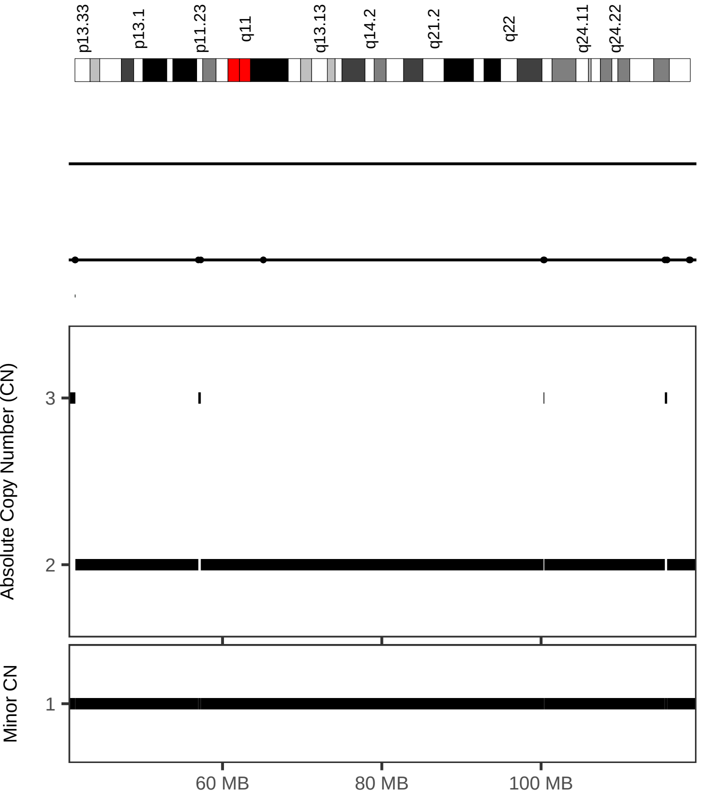

	PCSI_0338
Cancer type	Panc−AdenoCA
Position	12:41463414−118730813
Interleaved intrachr. SVs	1
Total SVs (intrachr. + transl.)	6
SV types	DEL: 0; DUP: 6; h2hINV: 0; t2tINV: 0; TRA: 0
SVs in sample	225
Oscillating CN (2 and 3 states)	8, 8
CN segments	8
FDR fragment joints	0.04139017
FDR chr. breakp. enrich.	0.43
Linked to chrs	
Purity, ploidy	0.6, 2.02

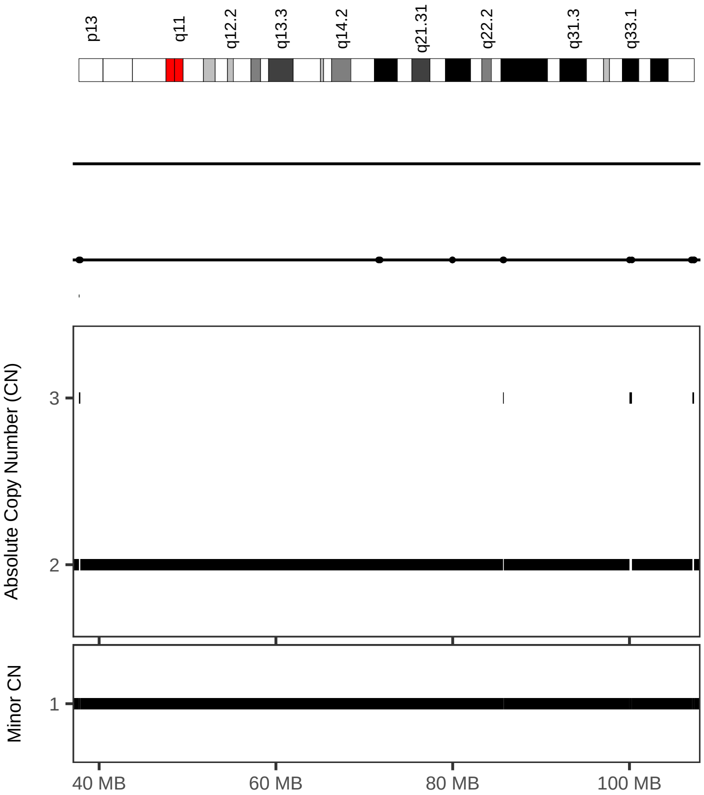

	PCSI_0338
Cancer type	Panc−AdenoCA
Position	13:37711193−107329558
Interleaved intrachr. SVs	1
Total SVs (intrachr. + transl.)	6
SV types	DEL: 0; DUP: 6; h2hINV: 0; t2tINV: 0; TRA: 0
SVs in sample	225
Oscillating CN (2 and 3 states)	8, 8
CN segments	8
FDR fragment joints	0.04139017
FDR chr. breakp. enrich.	1
Linked to chrs	
Purity, ploidy	0.6, 2.02

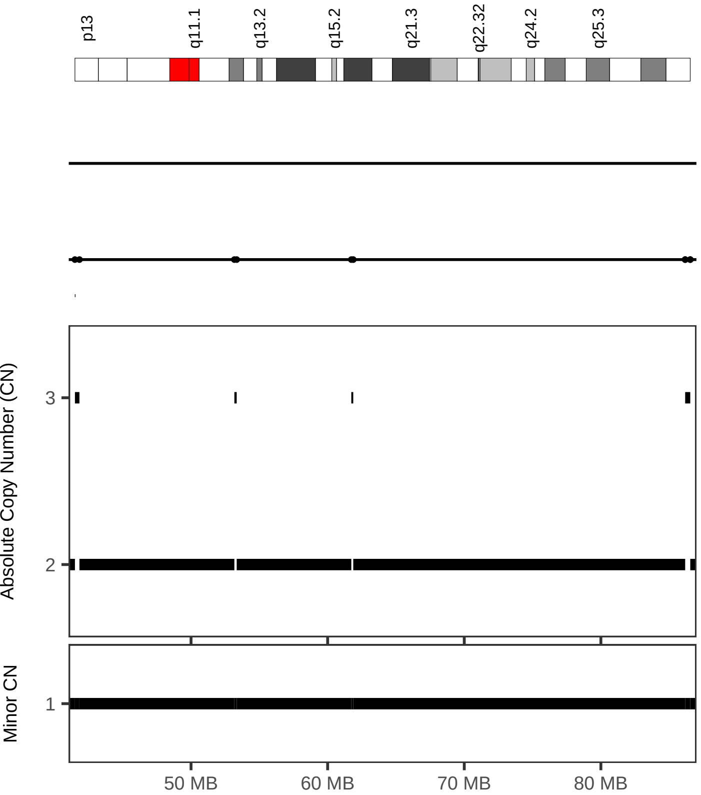

	PCSI_0338
Cancer type	Panc−AdenoCA
Position	15:41500002−86542658
Interleaved intrachr. SVs	1
Total SVs (intrachr. + transl.)	5
SV types	DEL: 0; DUP: 5; h2hINV: 0; t2tINV: 0; TRA: 0
SVs in sample	225
Oscillating CN (2 and 3 states)	8, 8
CN segments	8
FDR fragment joints	0.1013295
FDR chr. breakp. enrich.	0.98
Linked to chrs	
Purity, ploidy	0.6, 2.02

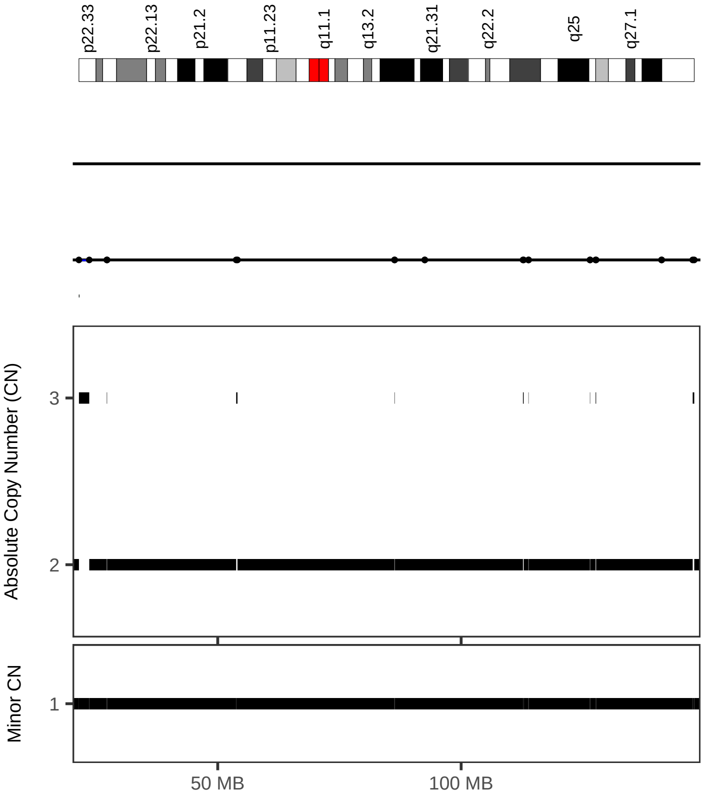

	PCSI_0338
Cancer type	Panc−AdenoCA
Position	X:21484632−147888022
Interleaved intrachr. SVs	1
Total SVs (intrachr. + transl.)	11
SV types	DEL: 0; DUP: 11; h2hINV: 0; t2tINV: 0; TRA: 0
SVs in sample	225
Oscillating CN (2 and 3 states)	18, 18
CN segments	18
FDR fragment joints	0.0002478023
FDR chr. breakp. enrich.	1
Linked to chrs	
Purity, ploidy	0.6, 2.02

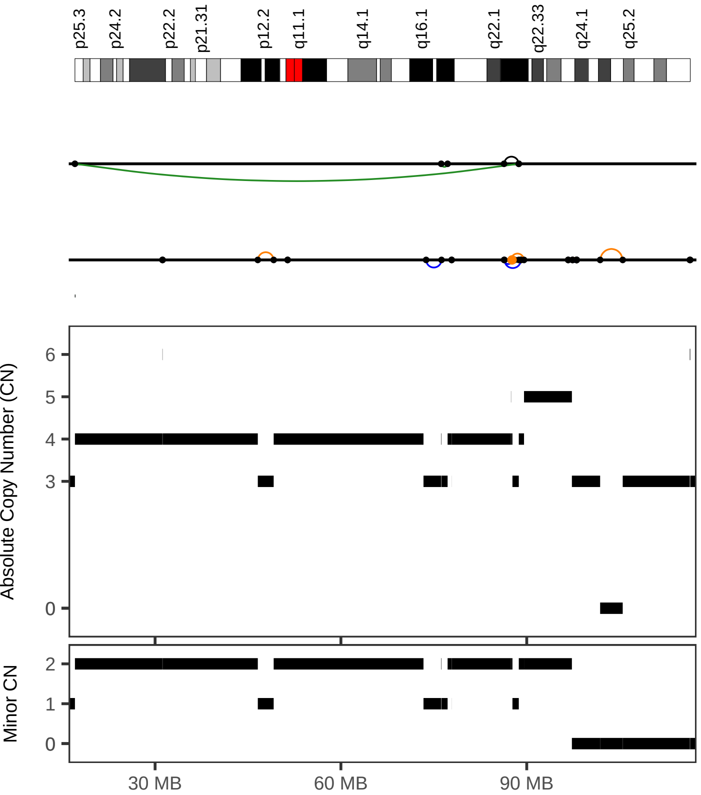

PCSI_0341

Cancer type	Panc−AdenoCA
Position	6:17057806−89560240
Interleaved intrachr. SVs	7
Total SVs (intrachr. + transl.)	8
SV types	DEL: 2; DUP: 3; h2hINV: 1; t2tINV: 1; TRA: 1
SVs in sample	210
Oscillating CN (2 and 3 states)	9, 14
CN segments	16
FDR fragment joints	0.6776251
FDR chr. breakp. enrich.	0.08
Linked to chrs	
Purity, ploidy	0.52, 3.57

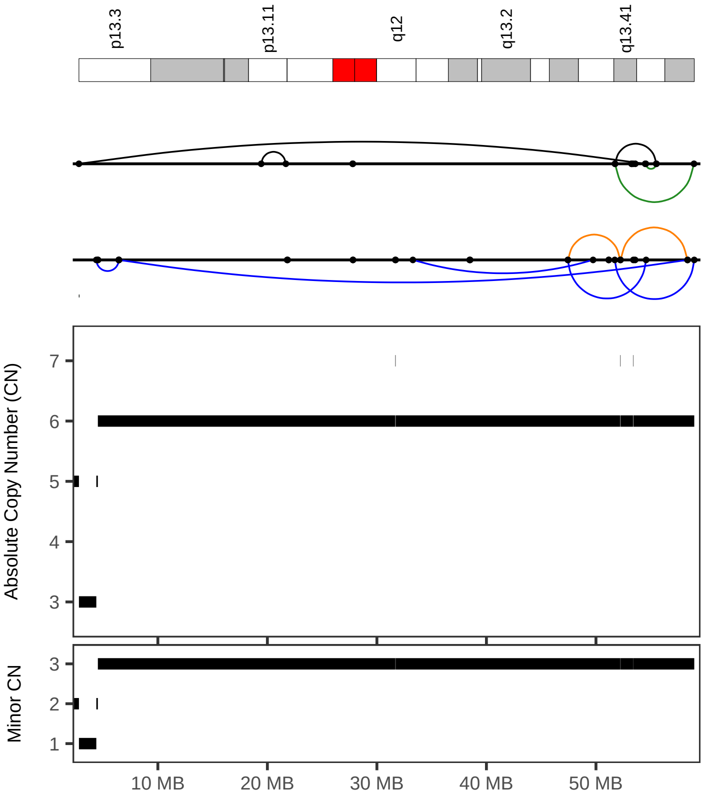

PCSI_0375

Cancer type	Panc−AdenoCA
Position	19:2792845−58974327
Interleaved intrachr. SVs	11
Total SVs (intrachr. + transl.)	11
SV types	DEL: 2; DUP: 5; h2hINV: 2; t2tINV: 2; TRA: 0
SVs in sample	32
Oscillating CN (2 and 3 states)	7, 8
CN segments	9
FDR fragment joints	0.6776251
FDR chr. breakp. enrich.	0
Linked to chrs	
Purity, ploidy	0.82, 5.12

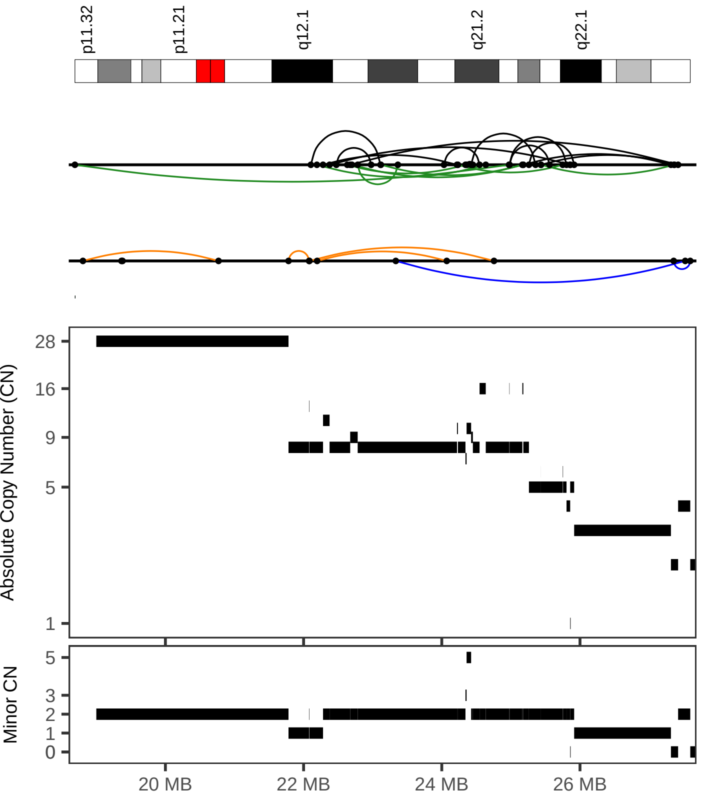

PCSI_0468

Cancer type	Panc−AdenoCA
Position	18:18690328−27595848
Interleaved intrachr. SVs	25
Total SVs (intrachr. + transl.)	25
SV types	DEL: 2; DUP: 2; h2hINV: 13; t2tINV: 8; TRA: 0
SVs in sample	105
Oscillating CN (2 and 3 states)	7, 7
CN segments	34
FDR fragment joints	0.155439
FDR chr. breakp. enrich.	0
Linked to chrs	
Purity, ploidy	0.79, 3.59

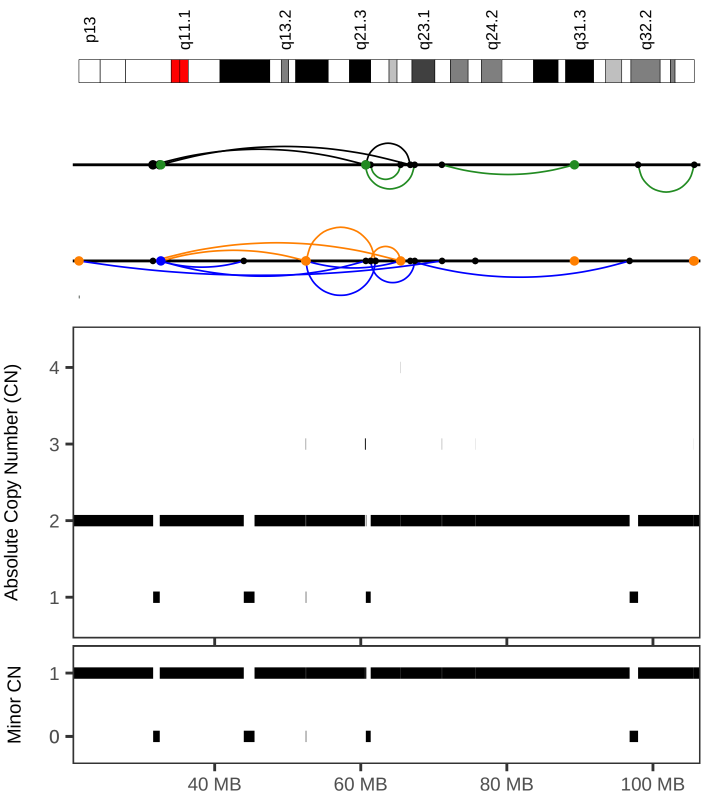

PCSI_0469

Cancer type	Panc−AdenoCA
Position	14:21422830−96805524
Interleaved intrachr. SVs	17
Total SVs (intrachr. + transl.)	28
SV types	DEL: 4; DUP: 7; h2hINV: 3; t2tINV: 3; TRA: 11
SVs in sample	61
Oscillating CN (2 and 3 states)	6, 10
CN segments	19
FDR fragment joints	0.9364467
FDR chr. breakp. enrich.	0
Linked to chrs	21:21943611−41135818;
Purity, ploidy	0.78, 1.97

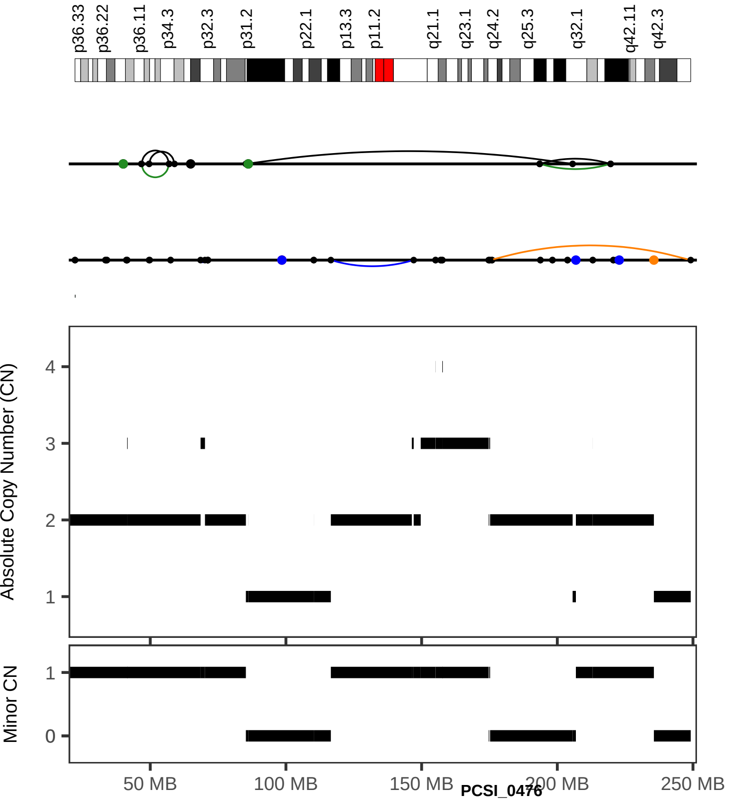

PCSI_0476

Cancer type	Panc−AdenoCA
Position	1:46749168−249168364
Interleaved intrachr. SVs	4
Total SVs (intrachr. + transl.)	16
SV types	DEL: 2; DUP: 0; h2hINV: 4; t2tINV: 2; TRA: 8
SVs in sample	360
Oscillating CN (2 and 3 states)	7, 24
CN segments	24
FDR fragment joints	0.9501265
FDR chr. breakp. enrich.	0.01
Linked to chrs	2:10364315−230753566;3:30700450−193532541 8:136426683−145091116;
Purity, ploidy	0.89, 1.88

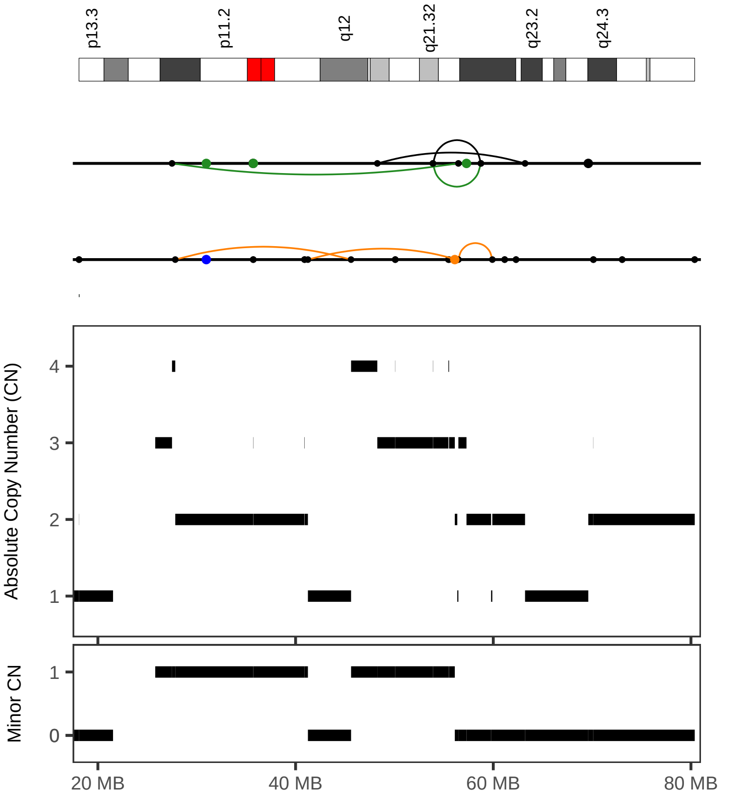

PCSI_0476

Cancer type	Panc−AdenoCA
Position	17:27499370−63215539
Interleaved intrachr. SVs	7
Total SVs (intrachr. + transl.)	13
SV types	DEL: 3; DUP: 0; h2hINV: 2; t2tINV: 2; TRA: 6
SVs in sample	360
Oscillating CN (2 and 3 states)	8, 16
CN segments	22
FDR fragment joints	0.6776251
FDR chr. breakp. enrich.	0
Linked to chrs	
Purity, ploidy	0.89, 1.88

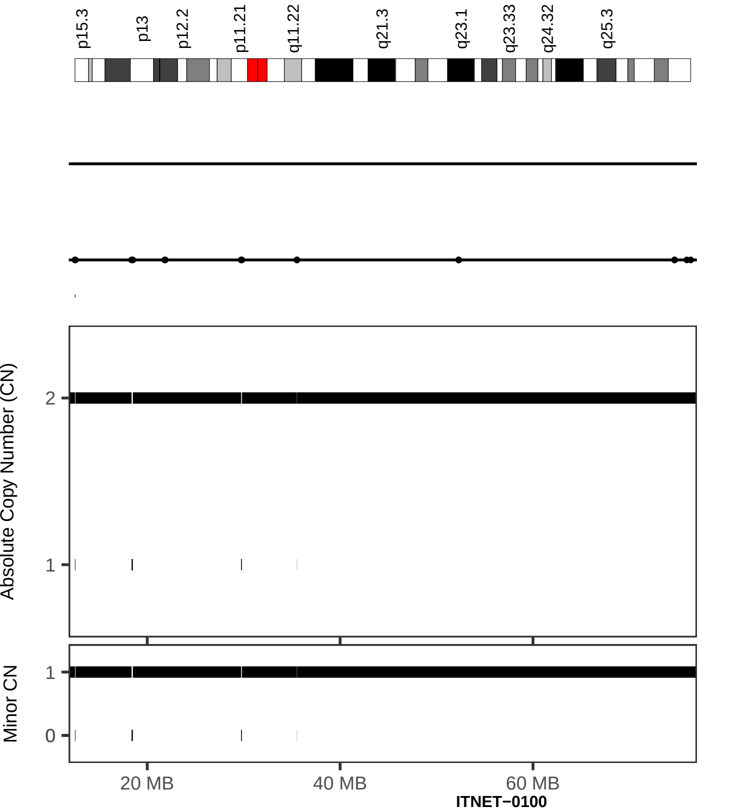

ITNET−0100

Cancer type	Panc−Endocrine
Position	10:12507104−76356326
Interleaved intrachr. SVs	1
Total SVs (intrachr. + transl.)	7
SV types	DEL: 7; DUP: 0; h2hINV: 0; t2tINV: 0; TRA: 0
SVs in sample	160
Oscillating CN (2 and 3 states)	8, 8
CN segments	8
FDR fragment joints	0.01743054
FDR chr. breakp. enrich.	0.24
Linked to chrs	
Purity, ploidy	0.95, 1.8

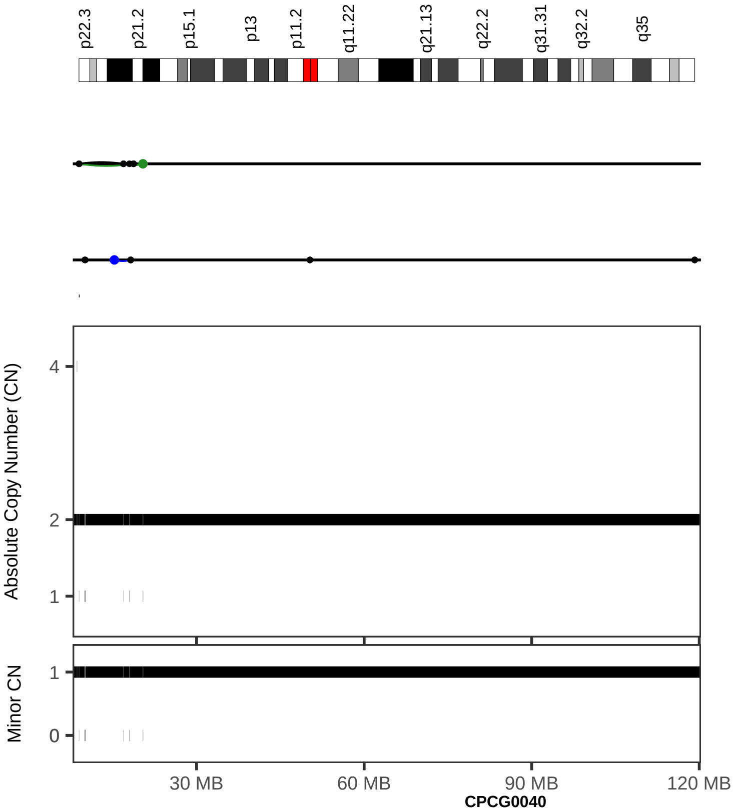

CPCG0040

Cancer type	Prost−AdenoCA
Position	7:8940200−20434202
Interleaved intrachr. SVs	5
Total SVs (intrachr. + transl.)	7
SV types	DEL: 0; DUP: 1; h2hINV: 2; t2tINV: 2; TRA: 2
SVs in sample	86
Oscillating CN (2 and 3 states)	10, 10
CN segments	10
FDR fragment joints	0.6776251
FDR chr. breakp. enrich.	0.03
Linked to chrs	
Purity, ploidy	0.65, 1.87

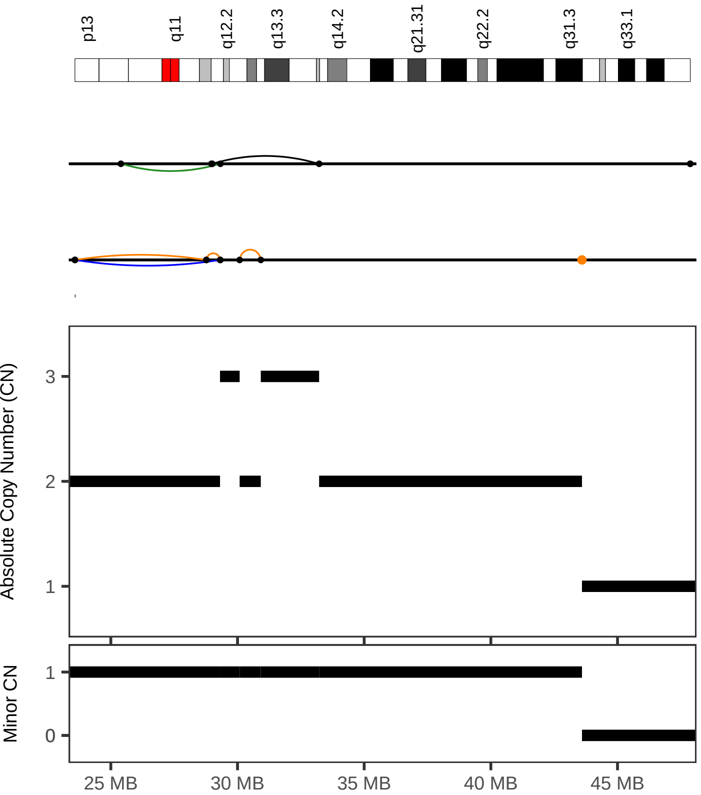

CPCG0094

Cancer type	Prost−AdenoCA
Position	13:23582500−33221383
Interleaved intrachr. SVs	6
Total SVs (intrachr. + transl.)	6
SV types	DEL: 2; DUP: 1; h2hINV: 1; t2tINV: 2; TRA: 0
SVs in sample	75
Oscillating CN (2 and 3 states)	5, 5
CN segments	5
FDR fragment joints	0.9284301
FDR chr. breakp. enrich.	0.01
Linked to chrs	
Purity, ploidy	0.46, 1.79

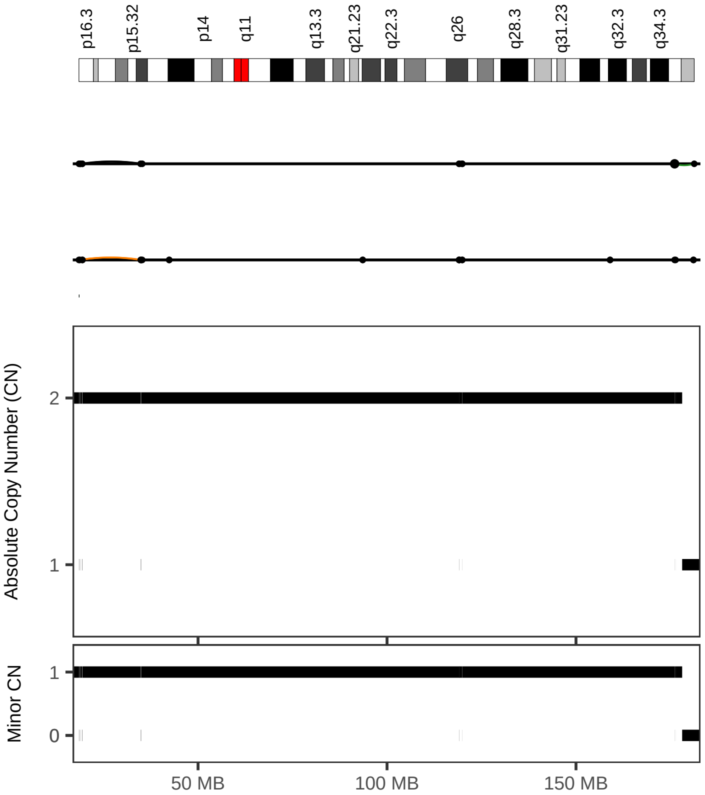

CPCG0128

Cancer type	Prost−AdenoCA
Position	4:18477094−35223917
Interleaved intrachr. SVs	6
Total SVs (intrachr. + transl.)	6
SV types	DEL: 2; DUP: 0; h2hINV: 2; t2tINV: 2; TRA: 0
SVs in sample	84
Oscillating CN (2 and 3 states)	14, 14
CN segments	14
FDR fragment joints	0.6776251
FDR chr. breakp. enrich.	0
Linked to chrs	
Purity, ploidy	0.76, 1.9

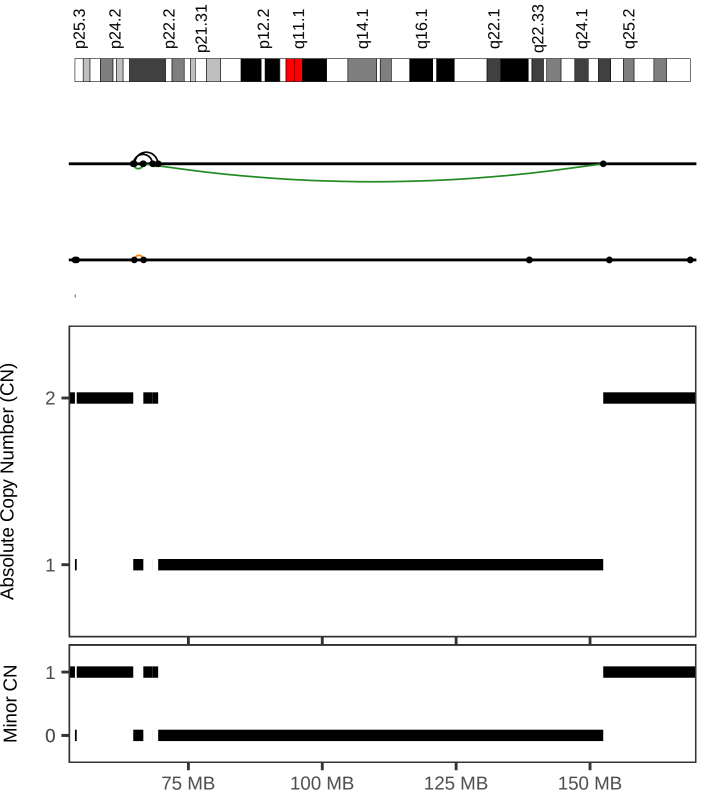

CPCG0199

Cancer type	Prost−AdenoCA
Position	6:64700080−152466236
Interleaved intrachr. SVs	6
Total SVs (intrachr. + transl.)	6
SV types	DEL: 1; DUP: 0; h2hINV: 2; t2tINV: 3; TRA: 0
SVs in sample	109
Oscillating CN (2 and 3 states)	6, 6
CN segments	6
FDR fragment joints	0.615458
FDR chr. breakp. enrich.	0.36
Linked to chrs	
Purity, ploidy	0.28, 1.88

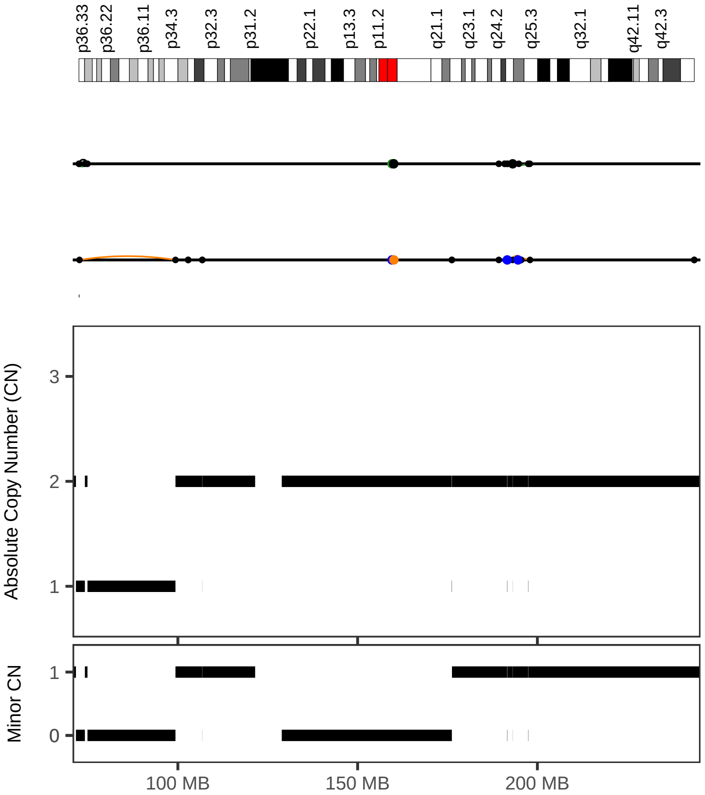

CPCG0201

Cancer type	Prost−AdenoCA
Position	1:189273295−197948284
Interleaved intrachr. SVs	7
Total SVs (intrachr. + transl.)	10
SV types	DEL: 3; DUP: 1; h2hINV: 2; t2tINV: 1; TRA: 3
SVs in sample	350
Oscillating CN (2 and 3 states)	7, 7
CN segments	7
FDR fragment joints	0.9625775
FDR chr. breakp. enrich.	1
Linked to chrs	5:19275862−166331720;
Purity, ploidy	0.78, 1.83

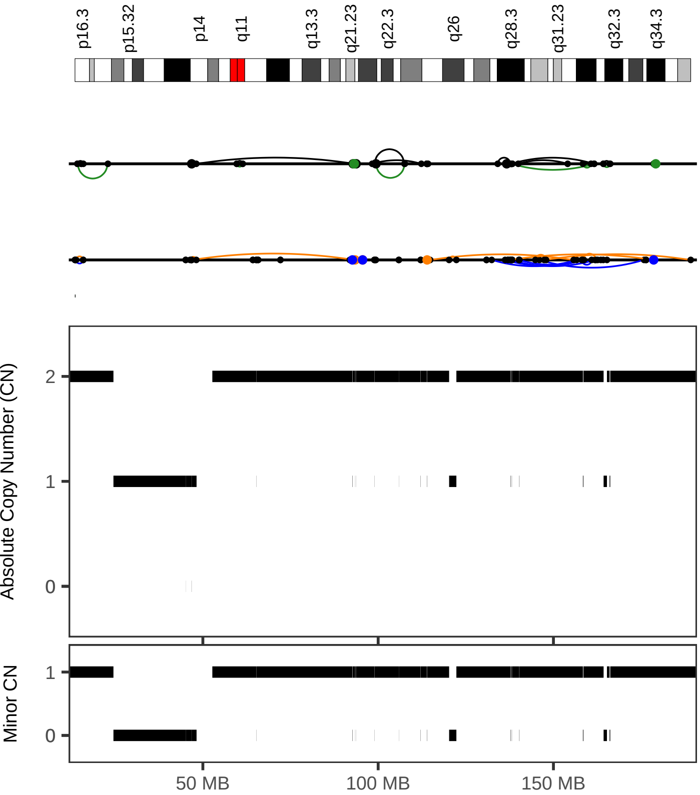

CPCG0201

Cancer type	Prost−AdenoCA
Position	4:114833795−189168661
Interleaved intrachr. SVs	22
Total SVs (intrachr. + transl.)	25
SV types	DEL: 6; DUP: 8; h2hINV: 5; t2tINV: 3; TRA: 3
SVs in sample	350
Oscillating CN (2 and 3 states)	17, 17
CN segments	17
FDR fragment joints	0.6776251
FDR chr. breakp. enrich.	0
Linked to chrs	
Purity, ploidy	0.78, 1.83

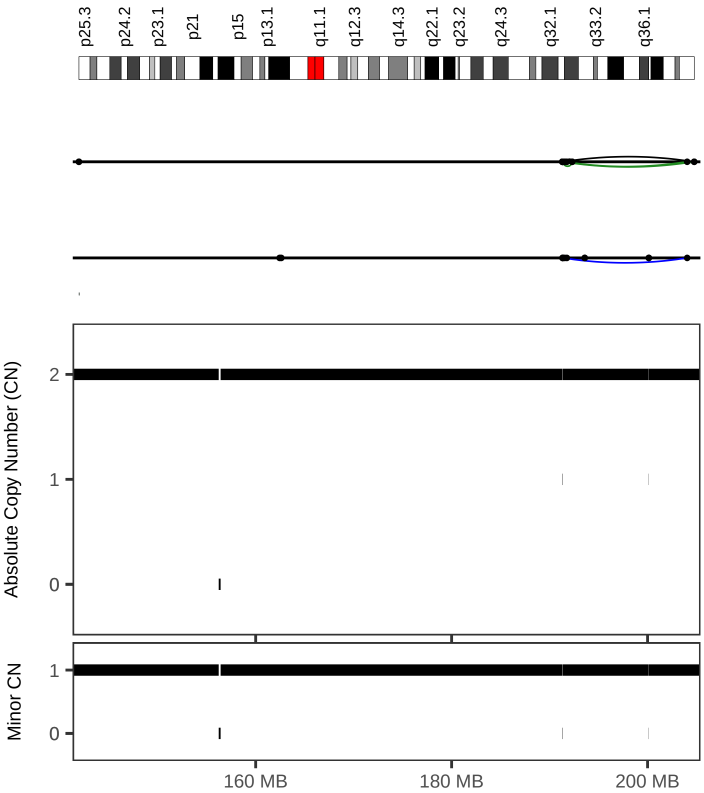

CPCG0210

Cancer type	Prost−AdenoCA
Position	2:191297296−204765227
Interleaved intrachr. SVs	9
Total SVs (intrachr. + transl.)	9
SV types	DEL: 1; DUP: 1; h2hINV: 3; t2tINV: 4; TRA: 0
SVs in sample	74
Oscillating CN (2 and 3 states)	5, 5
CN segments	5
FDR fragment joints	0.615458
FDR chr. breakp. enrich.	0
Linked to chrs	
Purity, ploidy	0.5, 1.88

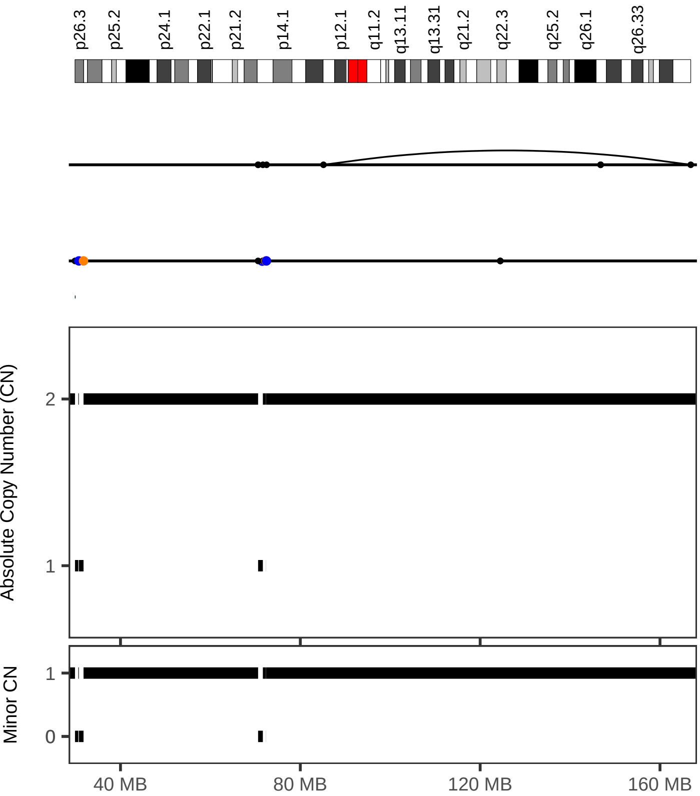

CPCG0232

Cancer type	Prost−AdenoCA
Position	3:70605253−72494755
Interleaved intrachr. SVs	6
Total SVs (intrachr. + transl.)	8
SV types	DEL: 1; DUP: 3; h2hINV: 1; t2tINV: 1; TRA: 2
SVs in sample	235
Oscillating CN (2 and 3 states)	5, 5
CN segments	5
FDR fragment joints	0.615458
FDR chr. breakp. enrich.	0.82
Linked to chrs	7:29672657−149167547;
Purity, ploidy	0.54, 1.83

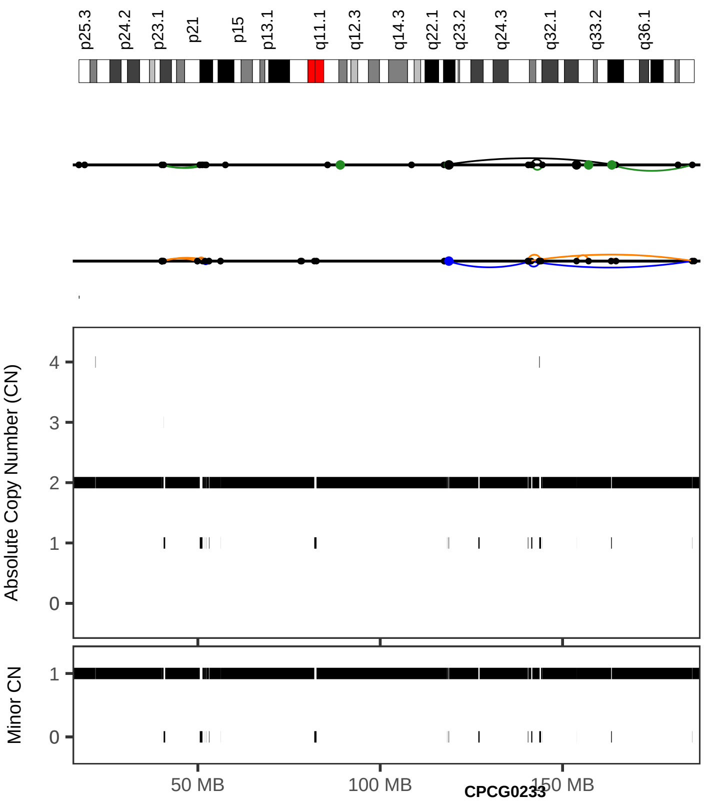

CPCG0233

Cancer type	Prost−AdenoCA
Position	2:117469455−186106088
Interleaved intrachr. SVs	11
Total SVs (intrachr. + transl.)	17
SV types	DEL: 4; DUP: 3; h2hINV: 2; t2tINV: 2; TRA: 6
SVs in sample	348
Oscillating CN (2 and 3 states)	13, 25
CN segments	25
FDR fragment joints	0.9934091
FDR chr. breakp. enrich.	0.08
Linked to chrs	1:188569591−190587731;11:31191605−104665238
Purity, ploidy	0.59, 1.86

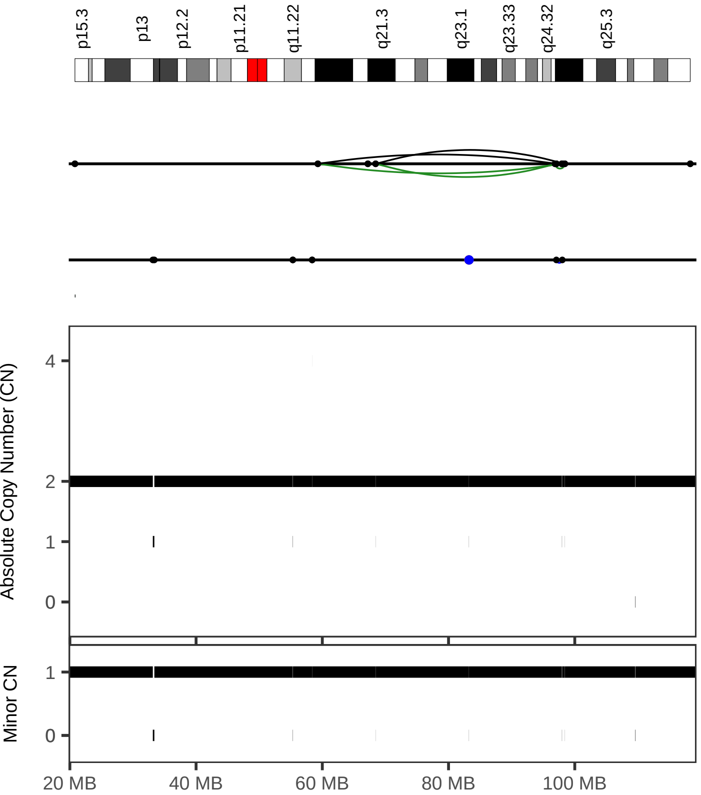

CPCG0233

Cancer type	Prost−AdenoCA
Position	10:59290168−98438172
Interleaved intrachr. SVs	9
Total SVs (intrachr. + transl.)	11
SV types	DEL: 0; DUP: 1; h2hINV: 4; t2tINV: 4; TRA: 2
SVs in sample	348
Oscillating CN (2 and 3 states)	11, 11
CN segments	11
FDR fragment joints	0.6776251
FDR chr. breakp. enrich.	0.95
Linked to chrs	8:91134186−115039928;
Purity, ploidy	0.59, 1.86

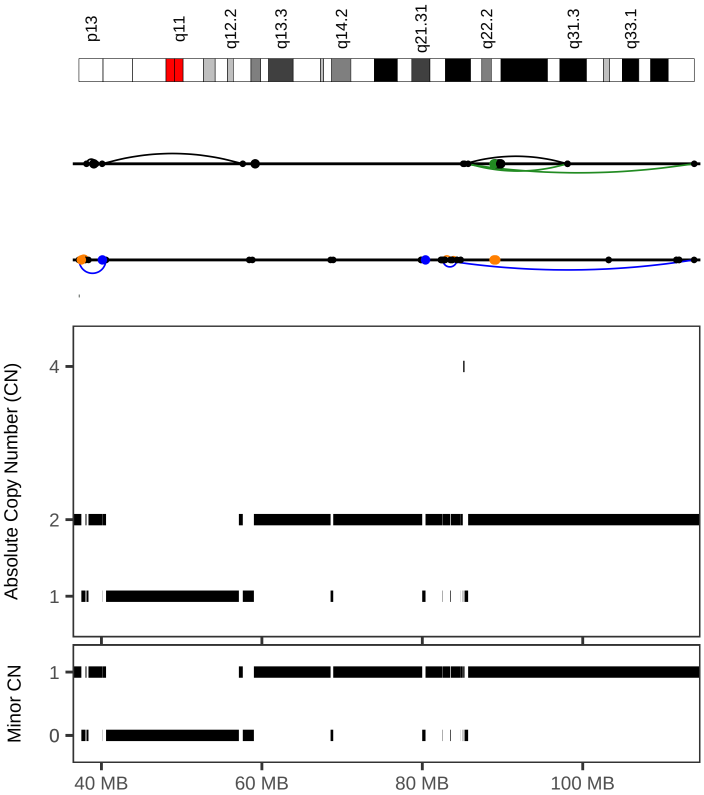

CPCG0249

Cancer type	Prost−AdenoCA
Position	13:82314051−113900734
Interleaved intrachr. SVs	6
Total SVs (intrachr. + transl.)	12
SV types	DEL: 3; DUP: 2; h2hINV: 0; t2tINV: 1; TRA: 6
SVs in sample	458
Oscillating CN (2 and 3 states)	8, 11
CN segments	11
FDR fragment joints	0.615458
FDR chr. breakp. enrich.	0
Linked to chrs	11:6102762−104228613;
Purity, ploidy	0.69, 1.87

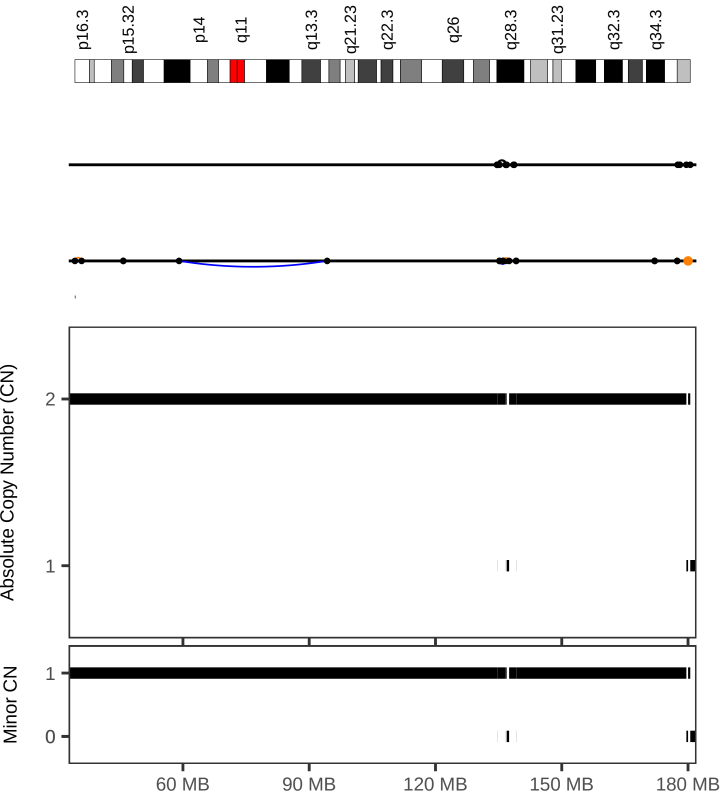

CPCG0268

Cancer type	Prost−AdenoCA
Position	4:134635808−137495825
Interleaved intrachr. SVs	7
Total SVs (intrachr. + transl.)	7
SV types	DEL: 2; DUP: 1; h2hINV: 2; t2tINV: 2; TRA: 0
SVs in sample	135
Oscillating CN (2 and 3 states)	6, 6
CN segments	6
FDR fragment joints	0.9599662
FDR chr. breakp. enrich.	0.01
Linked to chrs	
Purity, ploidy	0.34, 1.89

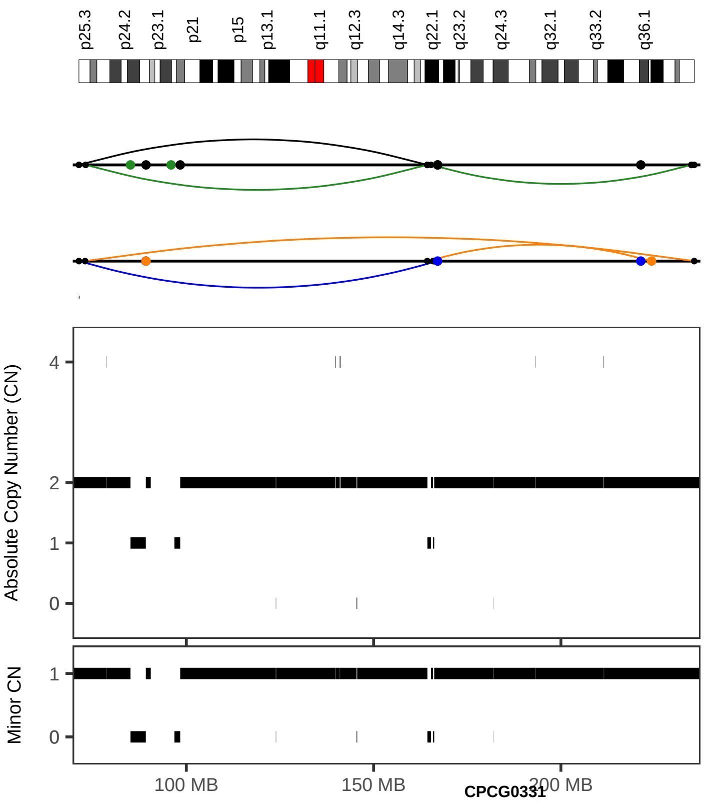

CPCG0331

Cancer type	Prost−AdenoCA
Position	2:71318777−235597578
Interleaved intrachr. SVs	7
Total SVs (intrachr. + transl.)	19
SV types	DEL: 2; DUP: 1; h2hINV: 2; t2tINV: 2; TRA: 12
SVs in sample	123
Oscillating CN (2 and 3 states)	5, 9
CN segments	27
FDR fragment joints	0.9348624
FDR chr. breakp. enrich.	0
Linked to chrs	1:98988888−109534536;3:5905087−156509263
Purity, ploidy	0.41, 1.88

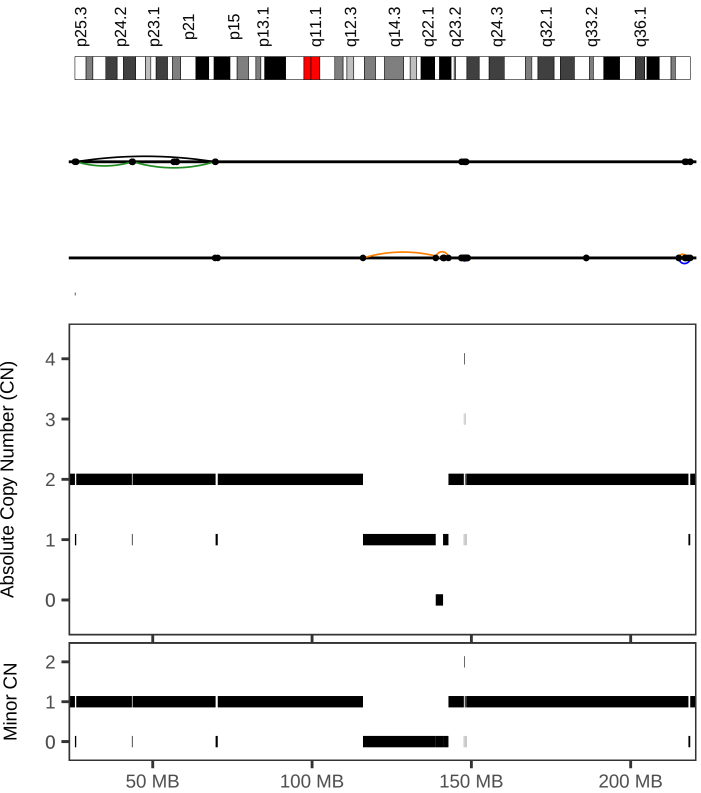

CPCG0360

Cancer type	Prost−AdenoCA
Position	2:146919033−148848088
Interleaved intrachr. SVs	8
Total SVs (intrachr. + transl.)	8
SV types	DEL: 2; DUP: 3; h2hINV: 2; t2tINV: 1; TRA: 0
SVs in sample	171
Oscillating CN (2 and 3 states)	4, 6
CN segments	11
FDR fragment joints	0.8653243
FDR chr. breakp. enrich.	0.01
Linked to chrs	
Purity, ploidy	0.25, 1.86

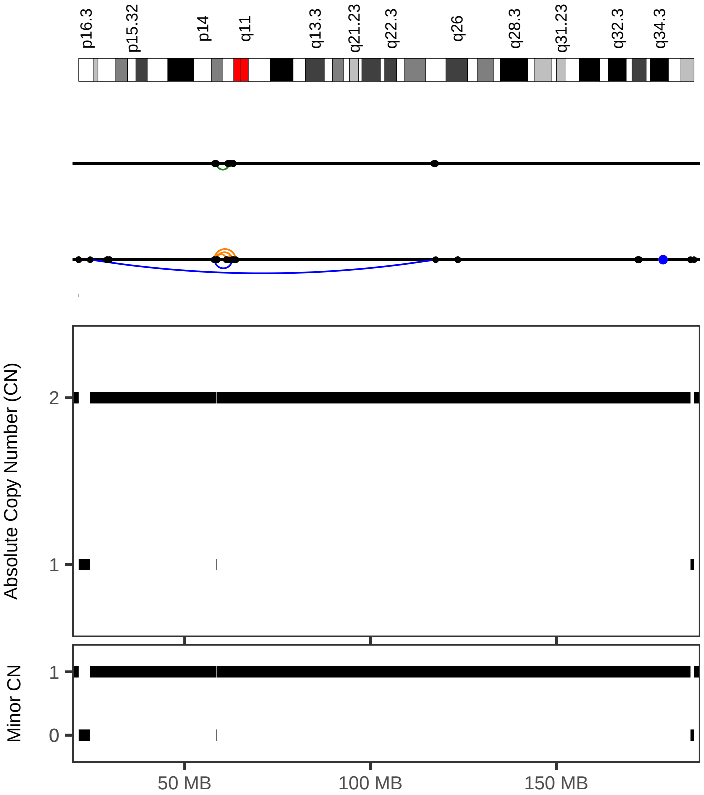

CPCG0360

Cancer type	Prost−AdenoCA
Position	4:57981759−63188151
Interleaved intrachr. SVs	9
Total SVs (intrachr. + transl.)	9
SV types	DEL: 3; DUP: 2; h2hINV: 2; t2tINV: 2; TRA: 0
SVs in sample	171
Oscillating CN (2 and 3 states)	5, 5
CN segments	5
FDR fragment joints	0.9723381
FDR chr. breakp. enrich.	0.02
Linked to chrs	
Purity, ploidy	0.25, 1.86

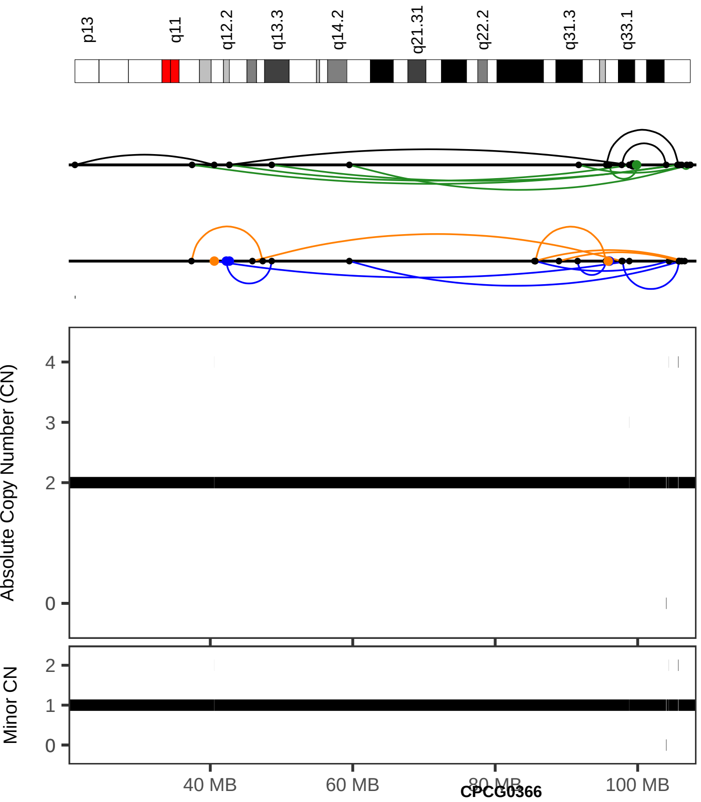

CPCG0366

Cancer type	Prost−AdenoCA
Position	13:20995521−107382176
Interleaved intrachr. SVs	25
Total SVs (intrachr. + transl.)	33
SV types	DEL: 6; DUP: 7; h2hINV: 5; t2tINV: 7; TRA: 8
SVs in sample	213
Oscillating CN (2 and 3 states)	5, 5
CN segments	11
FDR fragment joints	0.780201
FDR chr. breakp. enrich.	0
Linked to chrs	1:150909043−205693971;6:167735883−169038472
Purity, ploidy	0.55, 1.96

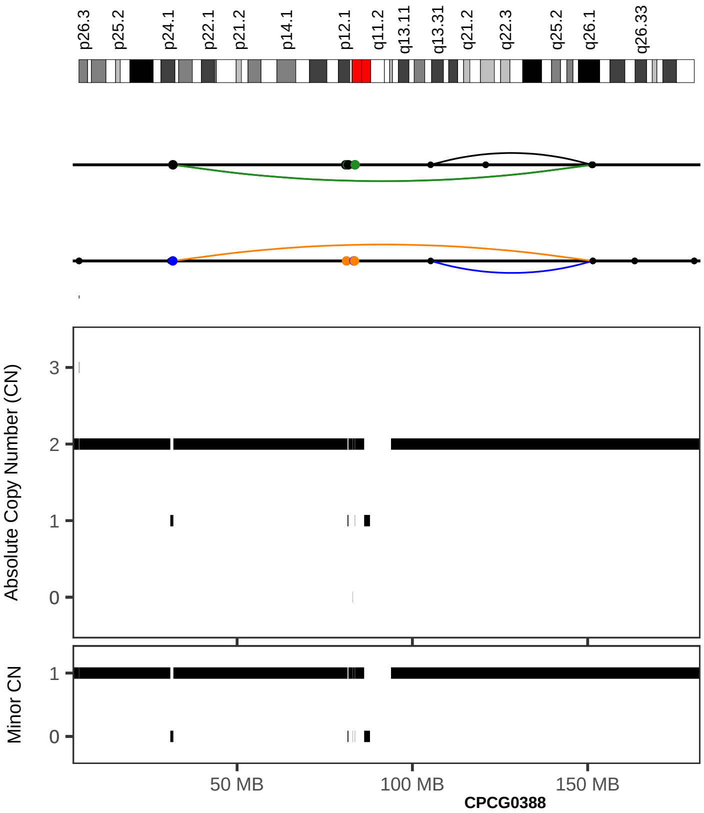

CPCG0388

Cancer type	Prost−AdenoCA
Position	3:30946713−151480649
Interleaved intrachr. SVs	8
Total SVs (intrachr. + transl.)	19
SV types	DEL: 2; DUP: 2; h2hINV: 2; t2tINV: 2; TRA: 11
SVs in sample	212
Oscillating CN (2 and 3 states)	8, 14
CN segments	14
FDR fragment joints	0.9348624
FDR chr. breakp. enrich.	0.02
Linked to chrs	
Purity, ploidy	0.68, 1.85

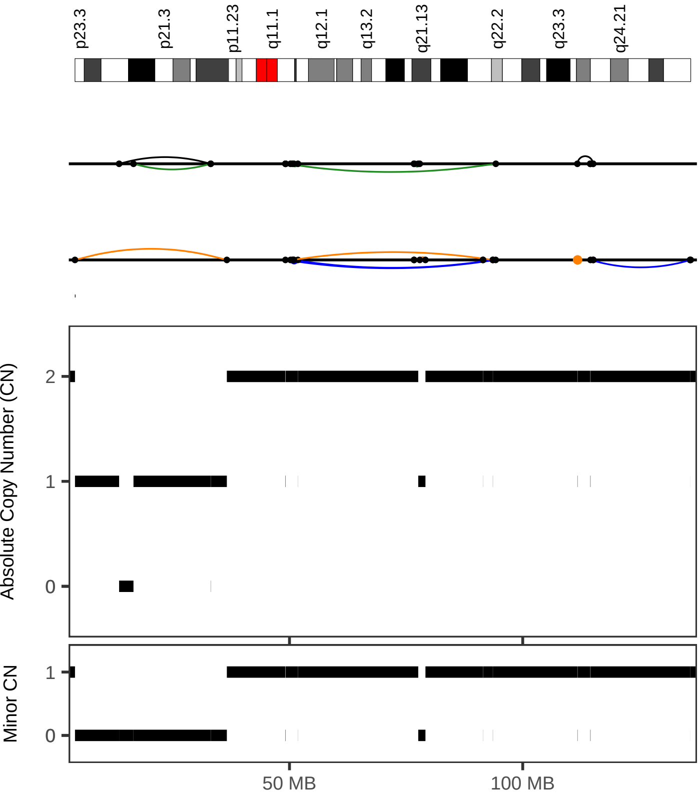

0077_CRUK_PC_0077

Cancer type	Prost−AdenoCA
Position	8:50216666−93610639
Interleaved intrachr. SVs	6
Total SVs (intrachr. + transl.)	6
SV types	DEL: 2; DUP: 2; h2hINV: 1; t2tINV: 1; TRA: 0
SVs in sample	198
Oscillating CN (2 and 3 states)	9, 9
CN segments	9
FDR fragment joints	0.9284301
FDR chr. breakp. enrich.	0
Linked to chrs	
Purity, ploidy	0.68, 1.89

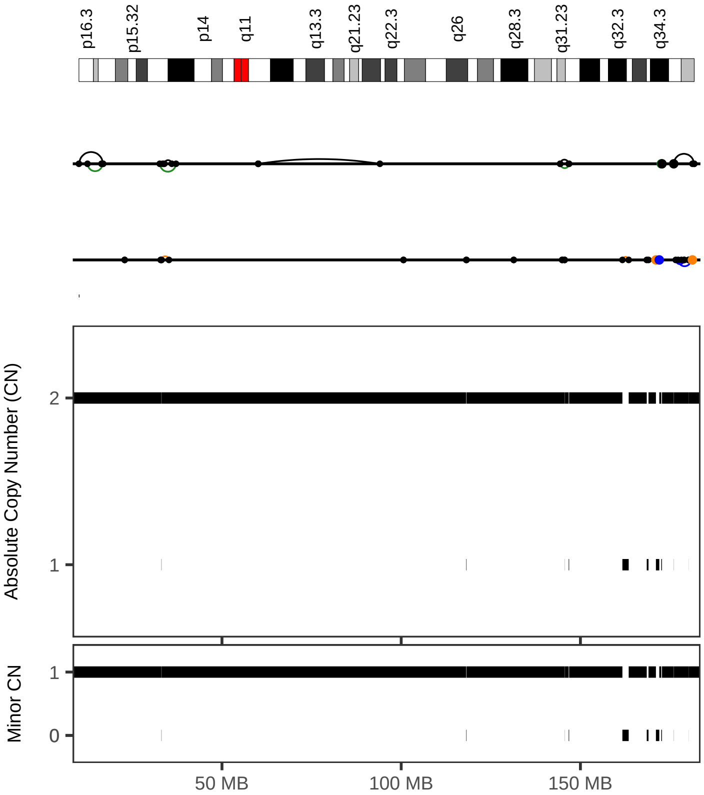

0078_CRUK_PC_0078

Cancer type	Prost−AdenoCA
Position	4:32885214−180841057
Interleaved intrachr. SVs	3
Total SVs (intrachr. + transl.)	11
SV types	DEL: 3; DUP: 2; h2hINV: 1; t2tINV: 0; TRA: 5
SVs in sample	95
Oscillating CN (2 and 3 states)	21, 21
CN segments	21
FDR fragment joints	0.8653243
FDR chr. breakp. enrich.	0
Linked to chrs	
Purity, ploidy	0.28, 1.89

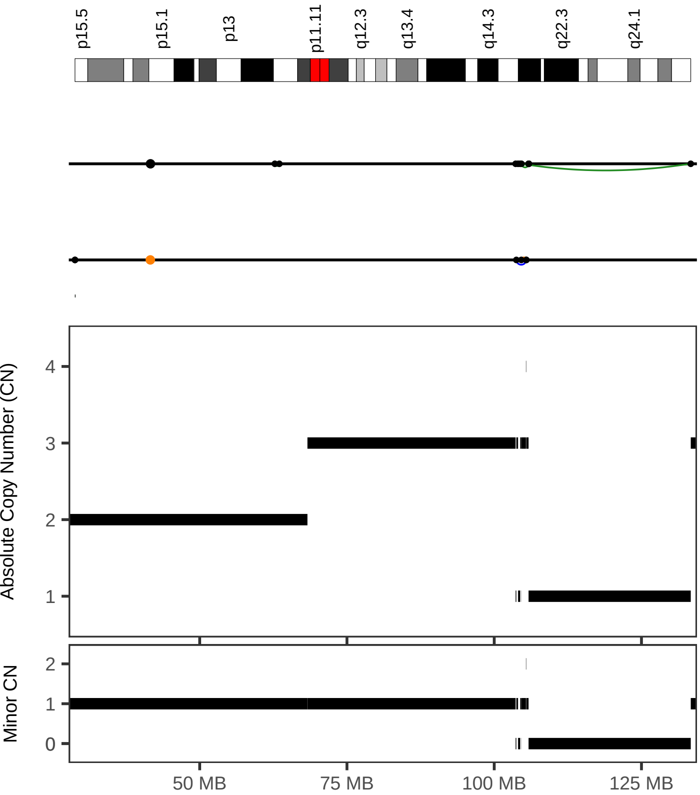

0089_CRUK_PC_0089

Cancer type	Prost−AdenoCA
Position	11:103634542−133363653
Interleaved intrachr. SVs	6
Total SVs (intrachr. + transl.)	6
SV types	DEL: 1; DUP: 1; h2hINV: 2; t2tINV: 2; TRA: 0
SVs in sample	52
Oscillating CN (2 and 3 states)	6, 10
CN segments	10
FDR fragment joints	0.9284301
FDR chr. breakp. enrich.	0
Linked to chrs	
Purity, ploidy	0.74, 1.92

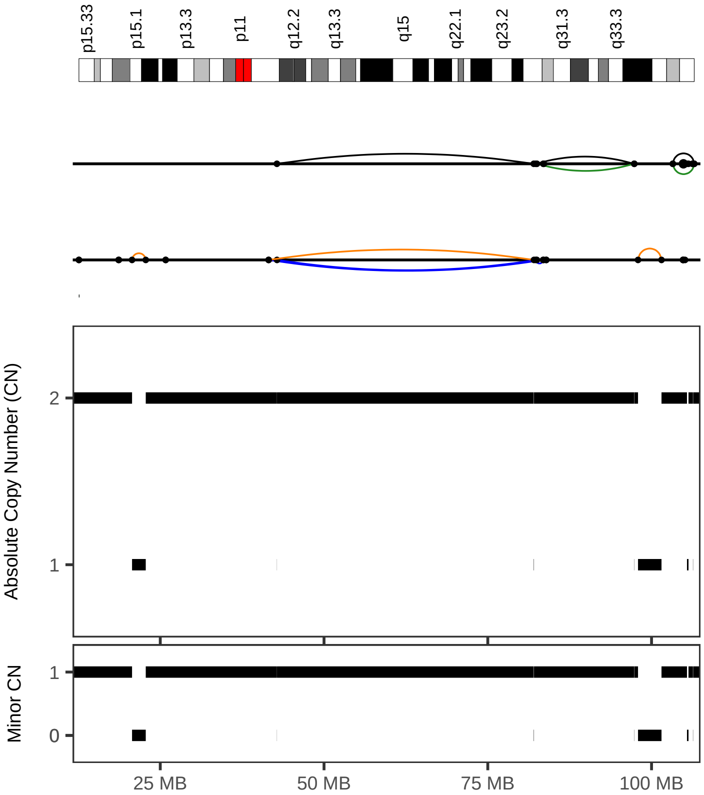

0090_CRUK_PC_0090

Cancer type	Prost−AdenoCA
Position	5:41539060−97372926
Interleaved intrachr. SVs	8
Total SVs (intrachr. + transl.)	8
SV types	DEL: 1; DUP: 3; h2hINV: 2; t2tINV: 2; TRA: 0
SVs in sample	194
Oscillating CN (2 and 3 states)	7, 7
CN segments	7
FDR fragment joints	0.8653243
FDR chr. breakp. enrich.	0.03
Linked to chrs	
Purity, ploidy	0.21, 1.92

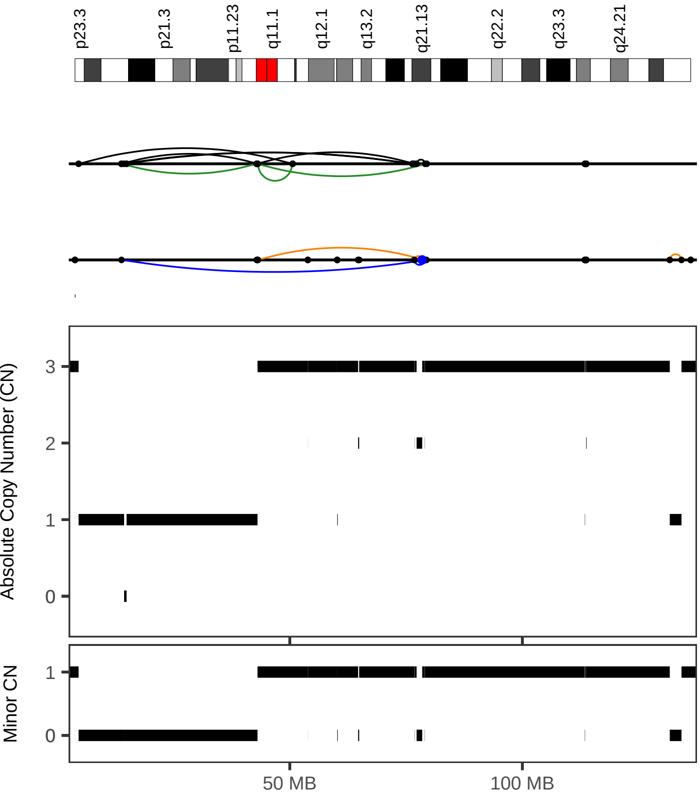

0090_CRUK_PC_0090

Cancer type	Prost−AdenoCA
Position	8:4593970−79446086
Interleaved intrachr. SVs	14
Total SVs (intrachr. + transl.)	15
SV types	DEL: 3; DUP: 2; h2hINV: 6; t2tINV: 3; TRA: 1
SVs in sample	194
Oscillating CN (2 and 3 states)	9, 14
CN segments	16
FDR fragment joints	0.7257048
FDR chr. breakp. enrich.	0
Linked to chrs	
Purity, ploidy	0.21, 1.92

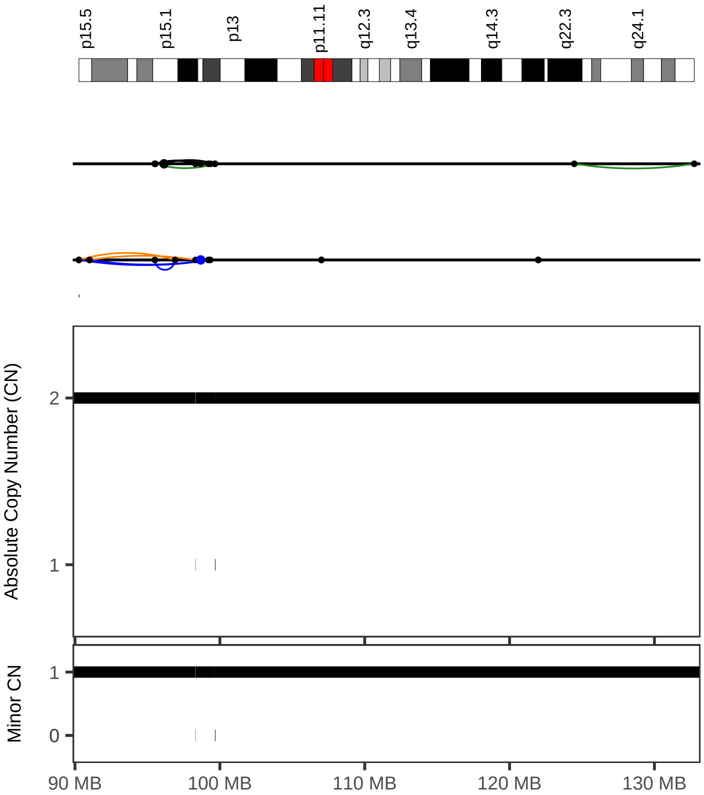

0090_CRUK_PC_0090

Cancer type	Prost−AdenoCA
Position	11:90271061−99662500
Interleaved intrachr. SVs	10
Total SVs (intrachr. + transl.)	12
SV types	DEL: 2; DUP: 3; h2hINV: 3; t2tINV: 2; TRA: 2
SVs in sample	194
Oscillating CN (2 and 3 states)	5, 5
CN segments	5
FDR fragment joints	0.9284301
FDR chr. breakp. enrich.	0.18
Linked to chrs	4:67524856−158414912;
Purity, ploidy	0.21, 1.92

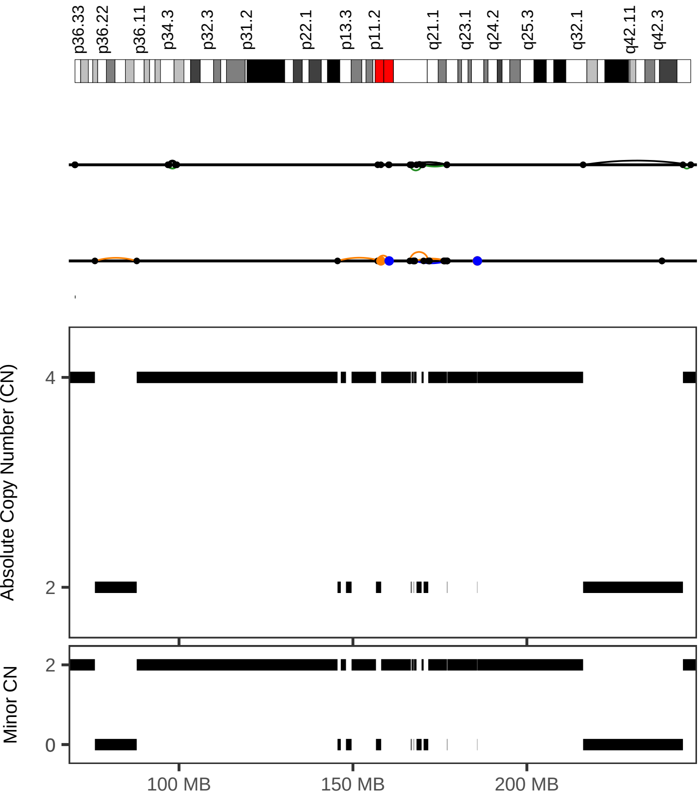

0091_CRUK_PC_0091

Cancer type	Prost−AdenoCA
Position	1:166331375−177156264
Interleaved intrachr. SVs	10
Total SVs (intrachr. + transl.)	10
SV types	DEL: 3; DUP: 1; h2hINV: 3; t2tINV: 3; TRA: 0
SVs in sample	308
Oscillating CN (2 and 3 states)	17, 17
CN segments	17
FDR fragment joints	0.8572806
FDR chr. breakp. enrich.	0.29
Linked to chrs	
Purity, ploidy	0.86, 3.7

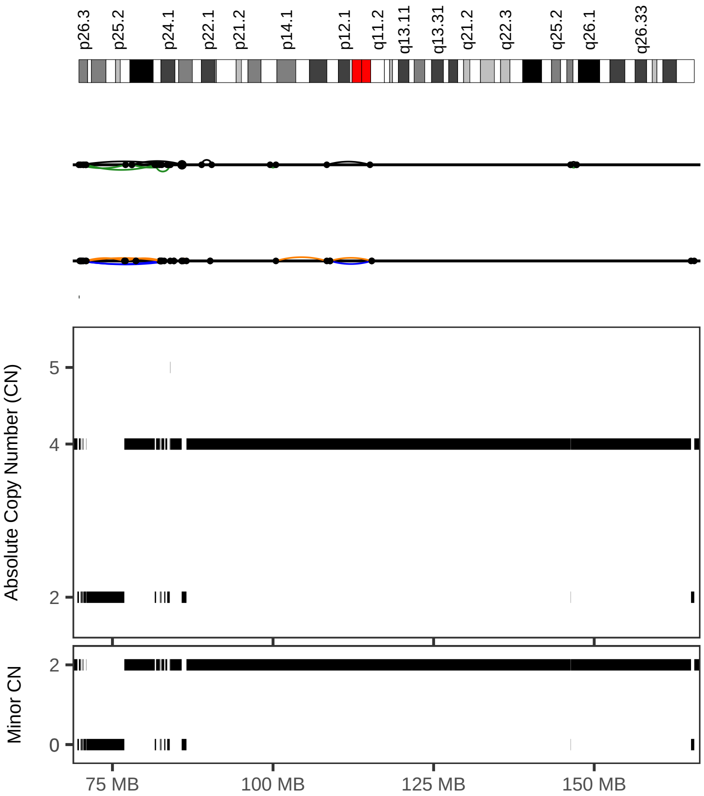

0091_CRUK_PC_0091

Cancer type	Prost−AdenoCA
Position	3:69780654−86522449
Interleaved intrachr. SVs	21
Total SVs (intrachr. + transl.)	22
SV types	DEL: 7; DUP: 4; h2hINV: 5; t2tINV: 5; TRA: 1
SVs in sample	308
Oscillating CN (2 and 3 states)	23, 27
CN segments	27
FDR fragment joints	0.8874881
FDR chr. breakp. enrich.	0.01
Linked to chrs	
Purity, ploidy	0.86, 3.7

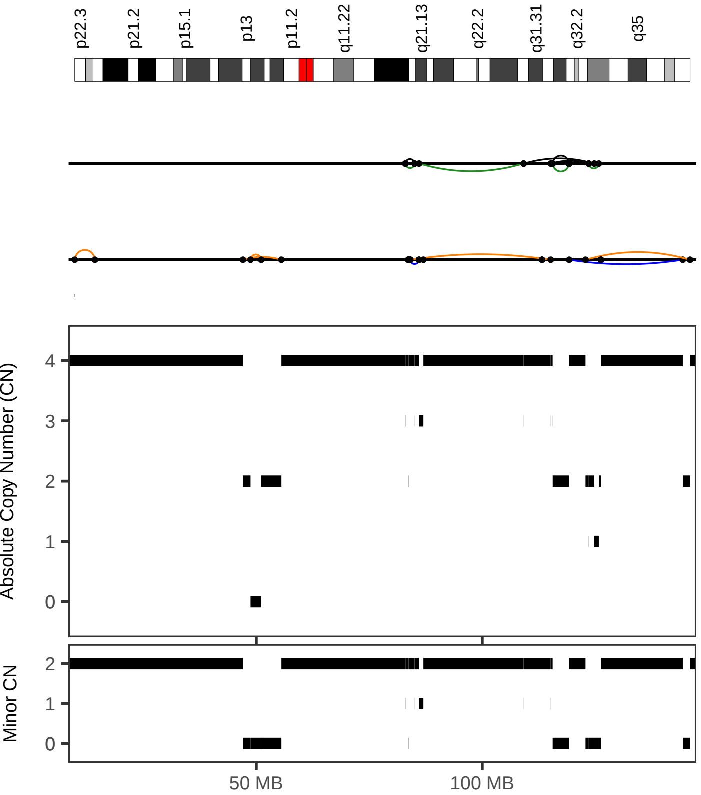

0091_CRUK_PC_0091

Cancer type	Prost−AdenoCA
Position	7:82954981−145981427
Interleaved intrachr. SVs	10
Total SVs (intrachr. + transl.)	10
SV types	DEL: 2; DUP: 2; h2hINV: 3; t2tINV: 3; TRA: 0
SVs in sample	308
Oscillating CN (2 and 3 states)	9, 17
CN segments	26
FDR fragment joints	0.9625775
FDR chr. breakp. enrich.	0.37
Linked to chrs	
Purity, ploidy	0.86, 3.7

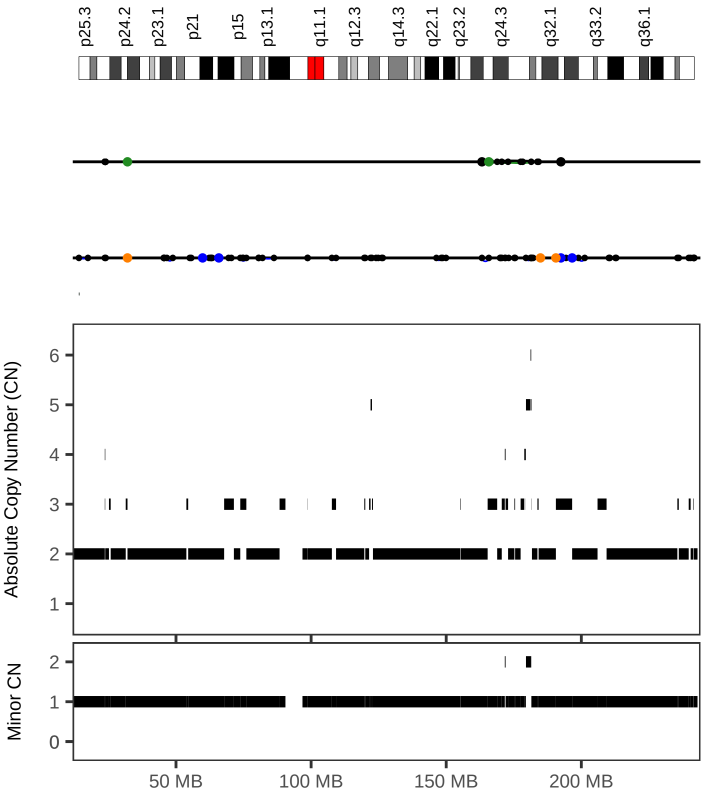

A10−0015_CRUK_PC_0015

Cancer type	Prost−AdenoCA
Position	2:163214471−184193586
Interleaved intrachr. SVs	10
Total SVs (intrachr. + transl.)	12
SV types	DEL: 1; DUP: 4; h2hINV: 3; t2tINV: 2; TRA: 2
SVs in sample	547
Oscillating CN (2 and 3 states)	5, 12
CN segments	19
FDR fragment joints	0.6776251
FDR chr. breakp. enrich.	0.22
Linked to chrs	
Purity, ploidy	NA, NA

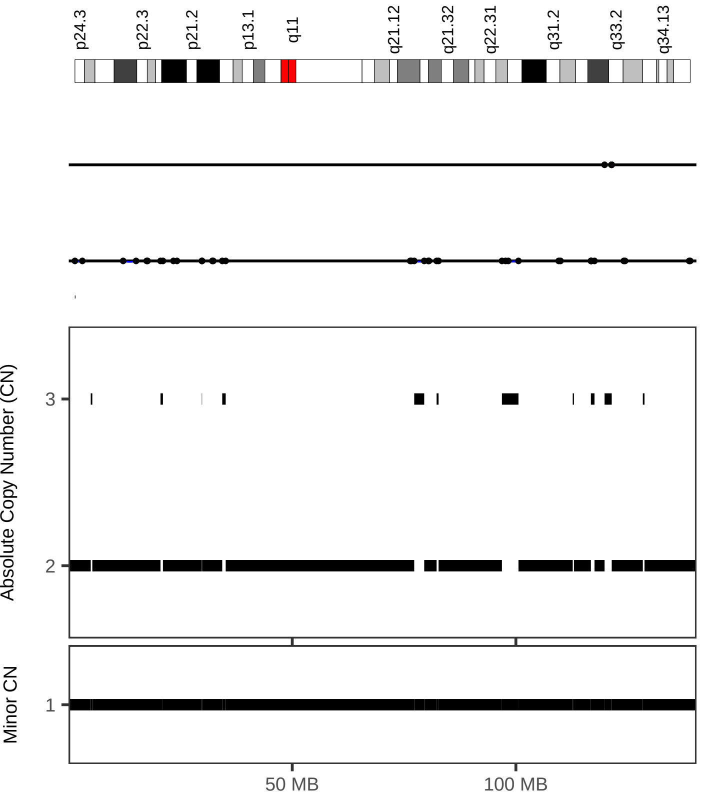

A10−0015_CRUK_PC_0015

Cancer type	Prost−AdenoCA
Position	9:1409842−138981764
Interleaved intrachr. SVs	1
Total SVs (intrachr. + transl.)	20
SV types	DEL: 1; DUP: 17; h2hINV: 1; t2tINV: 1; TRA: 0
SVs in sample	547
Oscillating CN (2 and 3 states)	23, 23
CN segments	23
FDR fragment joints	3.20209e−05
FDR chr. breakp. enrich.	1
Linked to chrs	
Purity, ploidy	NA, NA

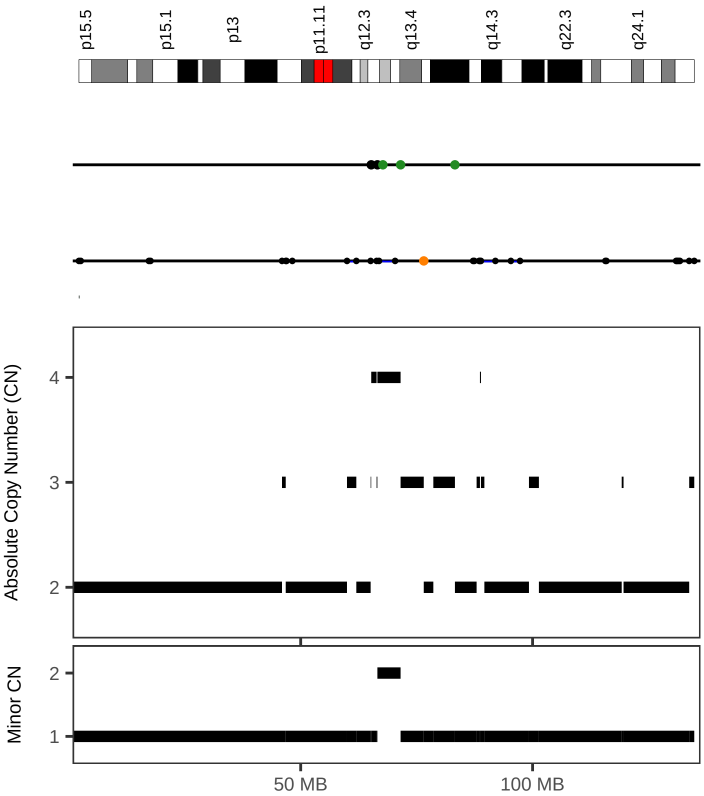

A10−0015_CRUK_PC_0015

Cancer type	Prost−AdenoCA
Position	11:2176926−134862526
Interleaved intrachr. SVs	1
Total SVs (intrachr. + transl.)	21
SV types	DEL: 0; DUP: 15; h2hINV: 0; t2tINV: 0; TRA: 6
SVs in sample	547
Oscillating CN (2 and 3 states)	7, 22
CN segments	22
FDR fragment joints	0.00583861
FDR chr. breakp. enrich.	0.67
Linked to chrs	1:149781202−155977716;
Purity, ploidy	NA, NA

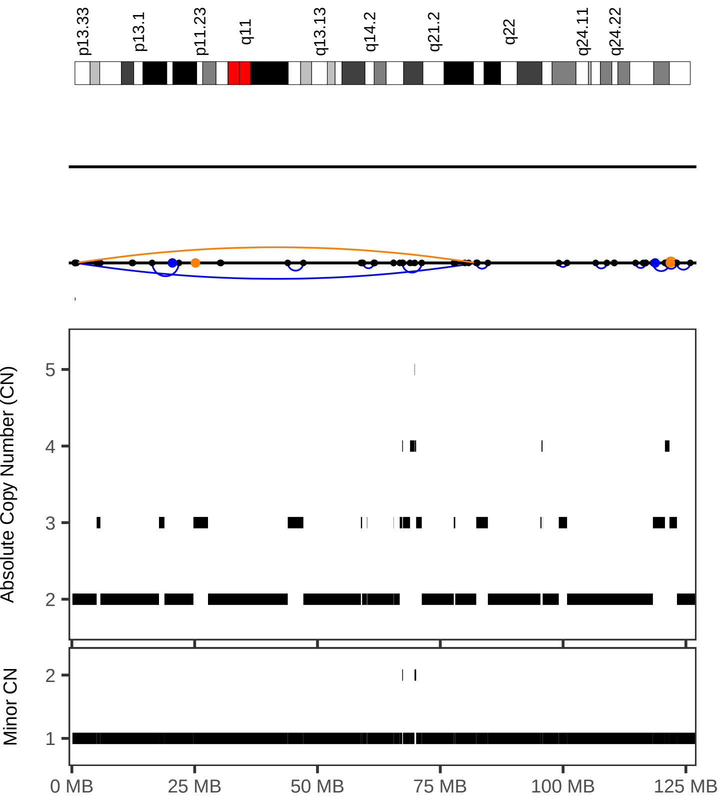

A10−0015_CRUK_PC_0015

Cancer type	Prost−AdenoCA
Position	12:620605−125885779
Interleaved intrachr. SVs	4
Total SVs (intrachr. + transl.)	12
SV types	DEL: 2; DUP: 6; h2hINV: 0; t2tINV: 0; TRA: 4
SVs in sample	547
Oscillating CN (2 and 3 states)	16, 37
CN segments	37
FDR fragment joints	0.1165322
FDR chr. breakp. enrich.	0.41
Linked to chrs	
Purity, ploidy	NA, NA

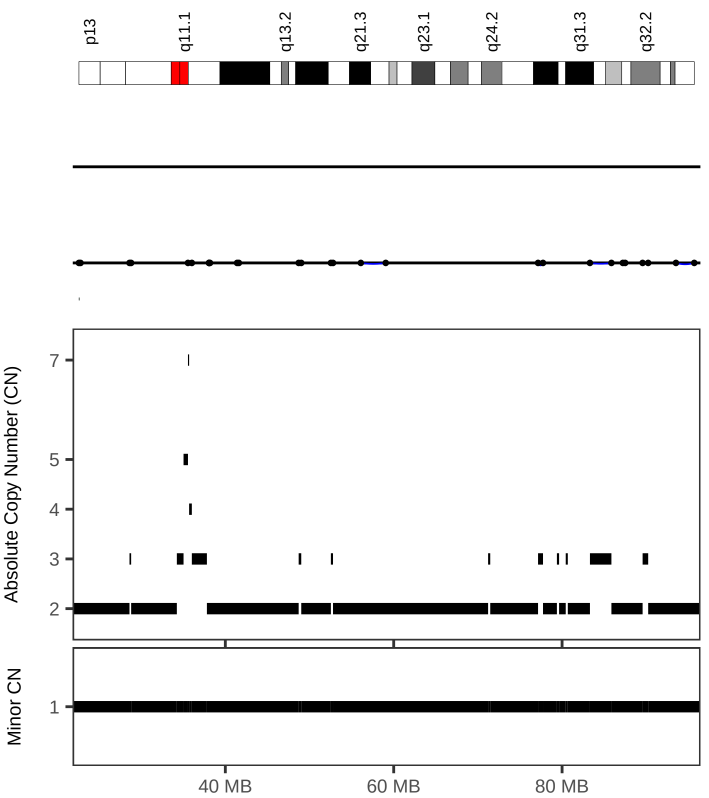

A10−0015_CRUK_PC_0015

Cancer type	Prost−AdenoCA
Position	14:22609121−95699997
Interleaved intrachr. SVs	1
Total SVs (intrachr. + transl.)	13
SV types	DEL: 1; DUP: 12; h2hINV: 0; t2tINV: 0; TRA: 0
SVs in sample	547
Oscillating CN (2 and 3 states)	18, 18
CN segments	25
FDR fragment joints	0.0004583196
FDR chr. breakp. enrich.	1
Linked to chrs	
Purity, ploidy	NA, NA

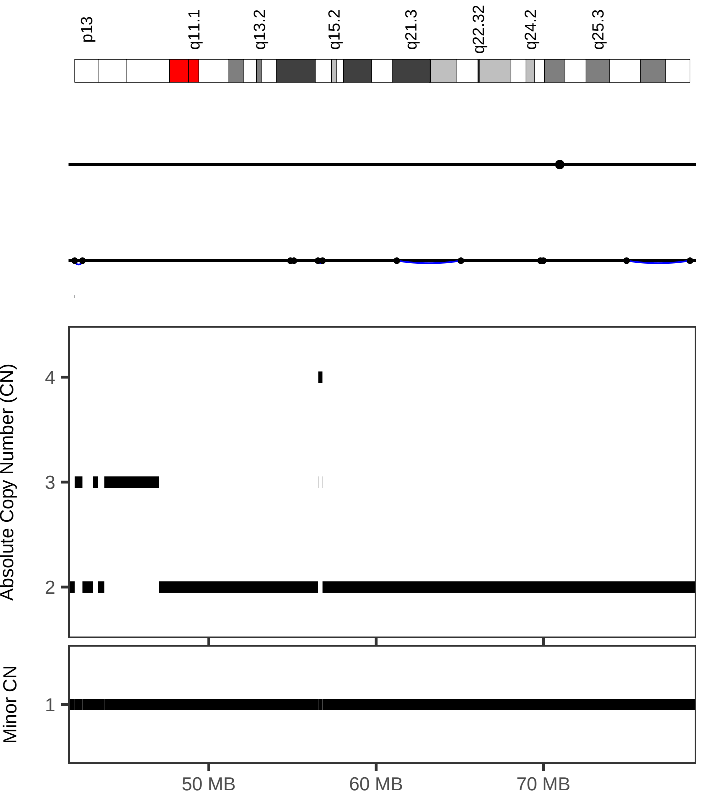

A10−0015_CRUK_PC_0015

Cancer type	Prost−AdenoCA
Position	15:41980634−78763235
Interleaved intrachr. SVs	1
Total SVs (intrachr. + transl.)	7
SV types	DEL: 0; DUP: 6; h2hINV: 0; t2tINV: 0; TRA: 1
SVs in sample	547
Oscillating CN (2 and 3 states)	7, 10
CN segments	10
FDR fragment joints	0.1278363
FDR chr. breakp. enrich.	0.13
Linked to chrs	
Purity, ploidy	NA, NA

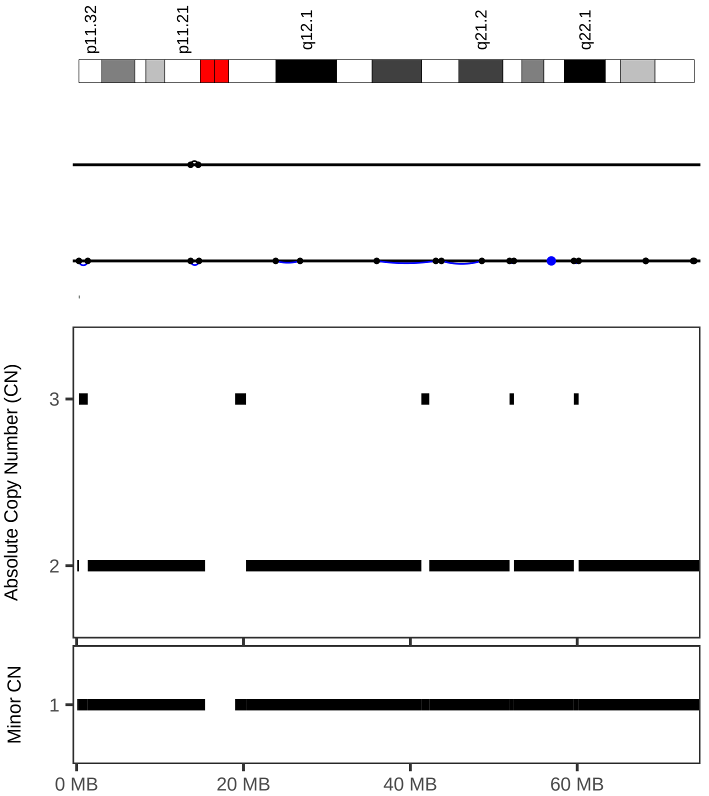

A10−0015_CRUK_PC_0015

Cancer type	Prost−AdenoCA
Position	18:274859−74026630
Interleaved intrachr. SVs	1
Total SVs (intrachr. + transl.)	11
SV types	DEL: 1; DUP: 8; h2hINV: 1; t2tINV: 0; TRA: 1
SVs in sample	547
Oscillating CN (2 and 3 states)	10, 10
CN segments	10
FDR fragment joints	0.03230998
FDR chr. breakp. enrich.	0.67
Linked to chrs	
Purity, ploidy	NA, NA

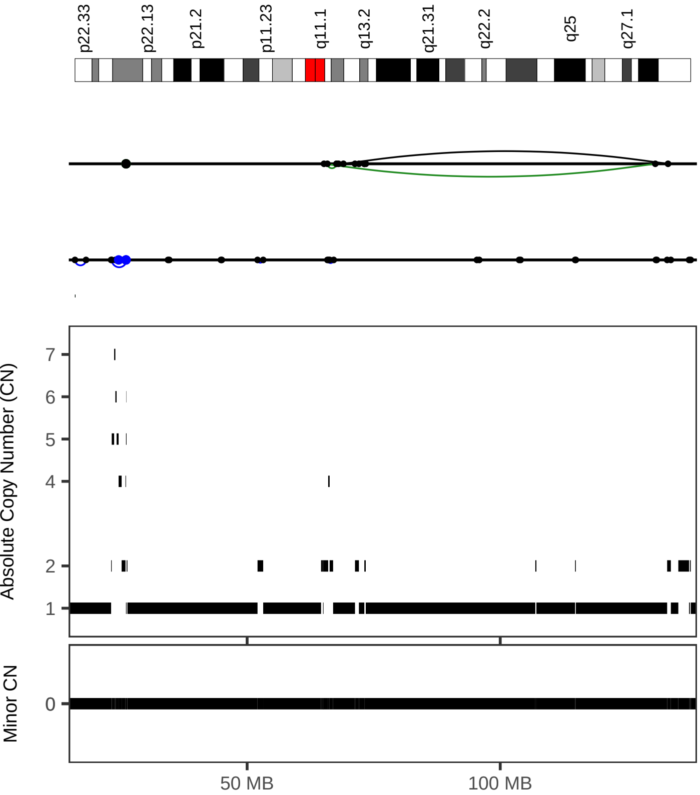

A10−0015_CRUK_PC_0015

Cancer type	Prost−AdenoCA
Position	X:23125163−133708183
Interleaved intrachr. SVs	3
Total SVs (intrachr. + transl.)	14
SV types	DEL: 0; DUP: 4; h2hINV: 1; t2tINV: 1; TRA: 8
SVs in sample	547
Oscillating CN (2 and 3 states)	12, 20
CN segments	33
FDR fragment joints	0.5435077
FDR chr. breakp. enrich.	0.53
Linked to chrs	1:149781202−155977716;
Purity, ploidy	NA, NA

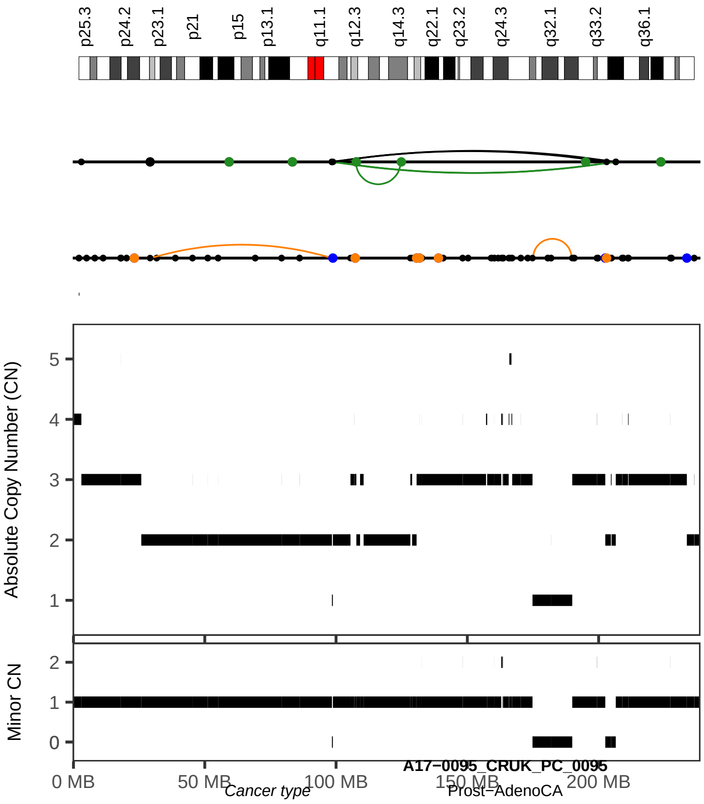

A17−0095_CRUK_PC_0095

Cancer type	Prost−AdenoCA
Position	2:29177870−206548845
Interleaved intrachr. SVs	5
Total SVs (intrachr. + transl.)	27
SV types	DEL: 1; DUP: 0; h2hINV: 2; t2tINV: 2; TRA: 22
SVs in sample	772
Oscillating CN (2 and 3 states)	16, 46
CN segments	56
FDR fragment joints	1
FDR chr. breakp. enrich.	0.07
Linked to chrs	10:1424131−123537842;14:21843858−93753838 17:14089050−74031570;3:18799507−143335633 5:6657316−135303280;7:740003−154793671

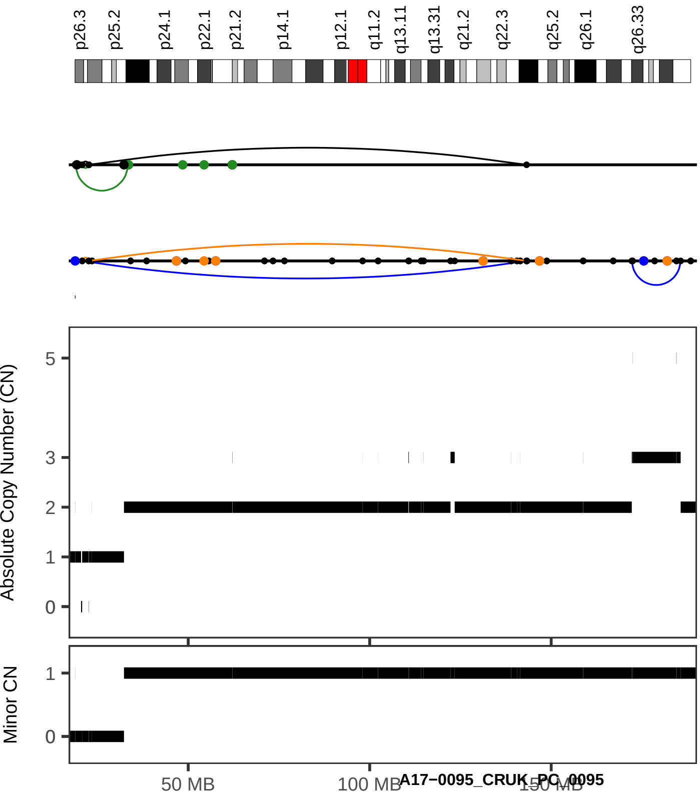

A17−0095_CRUK_PC_0095

Cancer type	Prost−AdenoCA
Position	3:18799507−143335634
Interleaved intrachr. SVs	9
Total SVs (intrachr. + transl.)	24
SV types	DEL: 2; DUP: 2; h2hINV: 3; t2tINV: 2; TRA: 15
SVs in sample	772
Oscillating CN (2 and 3 states)	21, 30
CN segments	30
FDR fragment joints	0.9723381
FDR chr. breakp. enrich.	0.5
Linked to chrs	14:21843858−93753838;2:29177870−206548844 4:679859−171367418;9:14642449−135273314

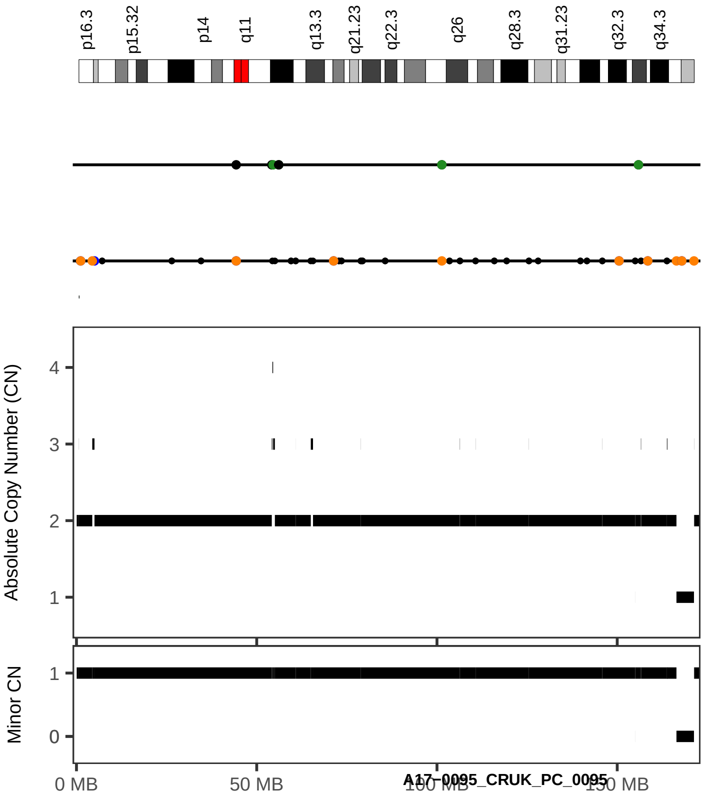

A17−0095_CRUK_PC_0095

Cancer type	Prost−AdenoCA
Position	4:679859−171367419
Interleaved intrachr. SVs	1
Total SVs (intrachr. + transl.)	42
SV types	DEL: 0; DUP: 16; h2hINV: 0; t2tINV: 0; TRA: 26
SVs in sample	772
Oscillating CN (2 and 3 states)	16, 32
CN segments	32
FDR fragment joints	0.01449201
FDR chr. breakp. enrich.	0.76
Linked to chrs	11:22270950−133333954;14:21843858−93753838 2:29177870−206548844;3:18799507−143335633 7:740003−154793671;

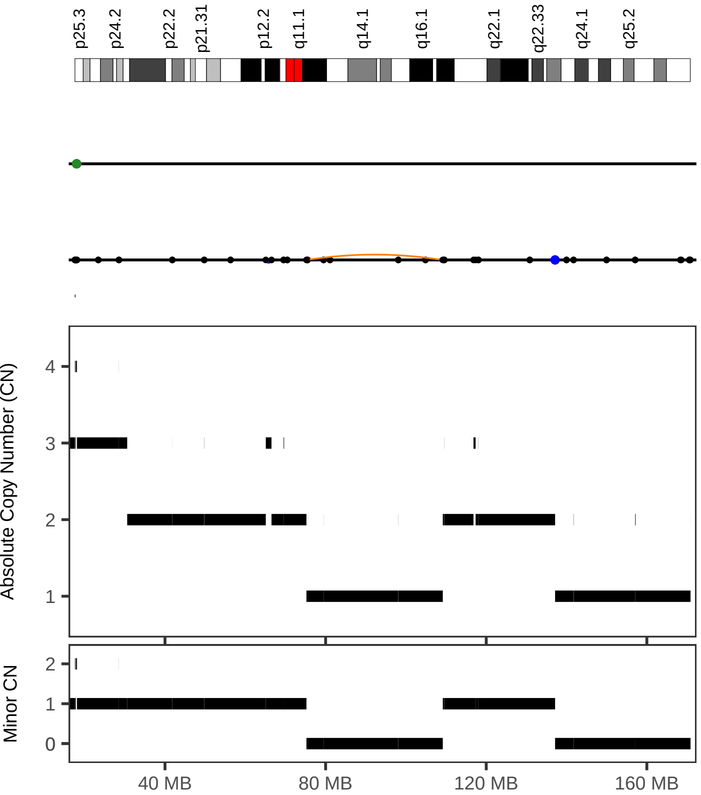

A17−0095_CRUK_PC_0095

Cancer type	Prost−AdenoCA
Position	6:17635126−170579356
Interleaved intrachr. SVs	1
Total SVs (intrachr. + transl.)	18
SV types	DEL: 2; DUP: 13; h2hINV: 0; t2tINV: 0; TRA: 3
SVs in sample	772
Oscillating CN (2 and 3 states)	10, 34
CN segments	34
FDR fragment joints	0.002321411
FDR chr. breakp. enrich.	0.14
Linked to chrs	9:14642449−135273314;
Purity, ploidy	NA, NA

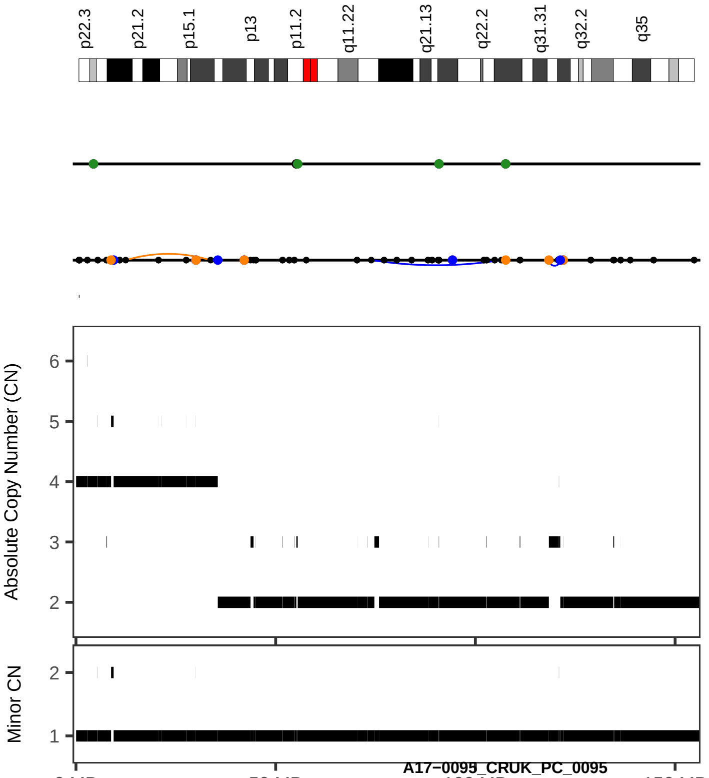

A17−0095_CRUK_PC_0095

Cancer type	Prost−AdenoCA
Position	7:740003−154793672
Interleaved intrachr. SVs	1
Total SVs (intrachr. + transl.)	49
SV types	DEL: 2; DUP: 24; h2hINV: 1; t2tINV: 0; TRA: 22
SVs in sample	772
Oscillating CN (2 and 3 states)	22, 22
CN segments	58
FDR fragment joints	2.325443e−05
FDR chr. breakp. enrich.	0.01
Linked to chrs	16:1802279−88025685;17:14089050−74031570 2:29177870−206548844;4:679859−171367418 9:14642449−135273314;X:6702691−131010343

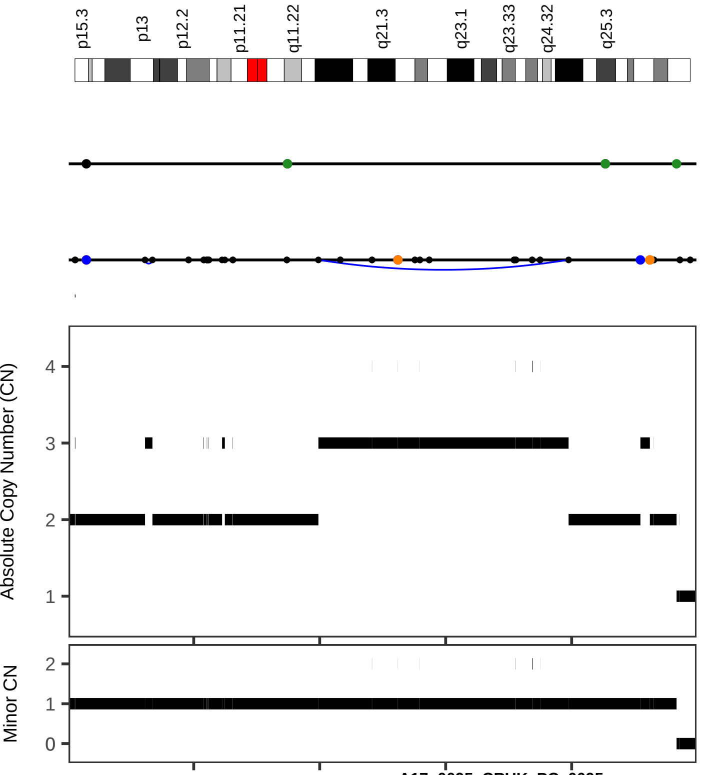

A17−0095_CRUK_PC_0095

Cancer type	Prost−AdenoCA
Position	10:1424131−123537843
Interleaved intrachr. SVs	1
Total SVs (intrachr. + transl.)	32
SV types	DEL: 0; DUP: 18; h2hINV: 0; t2tINV: 0; TRA: 14
SVs in sample	772
Oscillating CN (2 and 3 states)	17, 37
CN segments	37
FDR fragment joints	0.001356589
FDR chr. breakp. enrich.	0.77
Linked to chrs	11:22270950−133333954;16:1802279−88025685 2:29177870−206548844;22:23478507−47041669

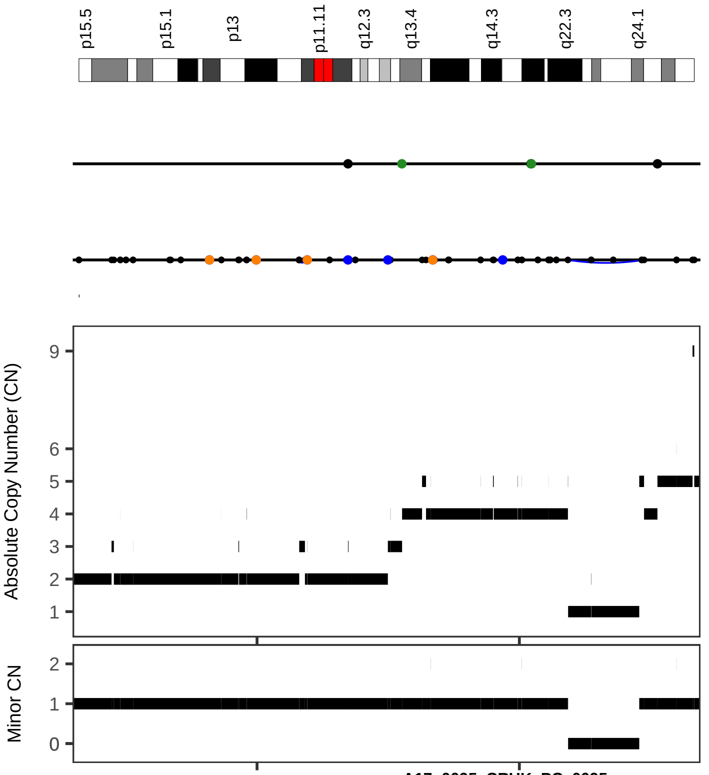

A17−0095_CRUK_PC_0095

Cancer type	Prost−AdenoCA
Position	11:22270950−133333955
Interleaved intrachr. SVs	1
Total SVs (intrachr. + transl.)	38
SV types	DEL: 0; DUP: 21; h2hINV: 0; t2tINV: 1; TRA: 16
SVs in sample	772
Oscillating CN (2 and 3 states)	16, 19
CN segments	47
FDR fragment joints	5.842961e−07
FDR chr. breakp. enrich.	0.01
Linked to chrs	10:1424131−123537842;12:10913800−112566166 3:18799507−143335633;4:679859−171367418 X:6702691−131010343;

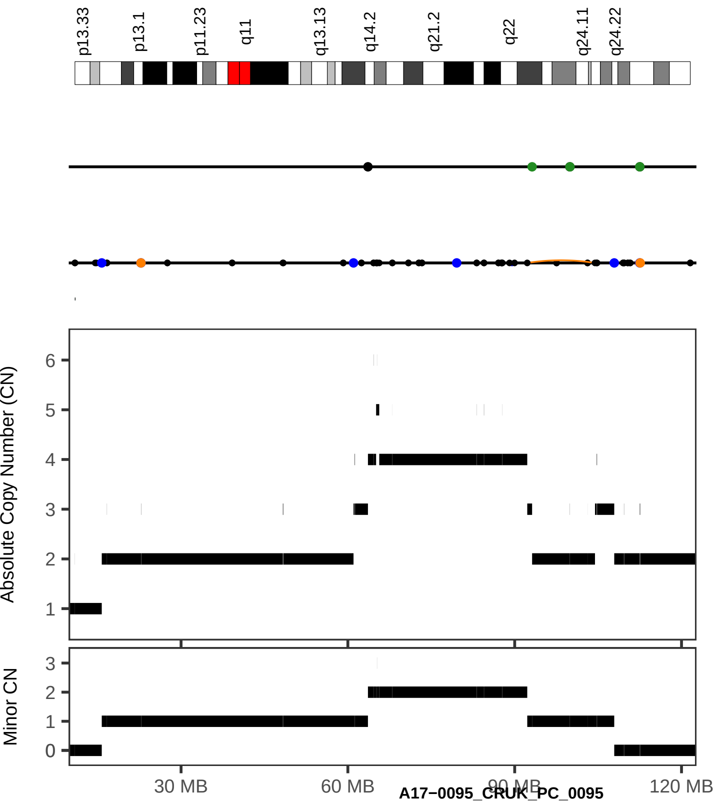

A17−0095_CRUK_PC_0095

Cancer type	Prost−AdenoCA
Position	12:10913800−112566167
Interleaved intrachr. SVs	1
Total SVs (intrachr. + transl.)	34
SV types	DEL: 1; DUP: 20; h2hINV: 0; t2tINV: 0; TRA: 13
SVs in sample	772
Oscillating CN (2 and 3 states)	10, 28
CN segments	42
FDR fragment joints	5.842961e−07
FDR chr. breakp. enrich.	0.07
Linked to chrs	11:22270950−133333954;18:24992540−42319496 X:6702691−131010343;
Purity, ploidy	NA, NA

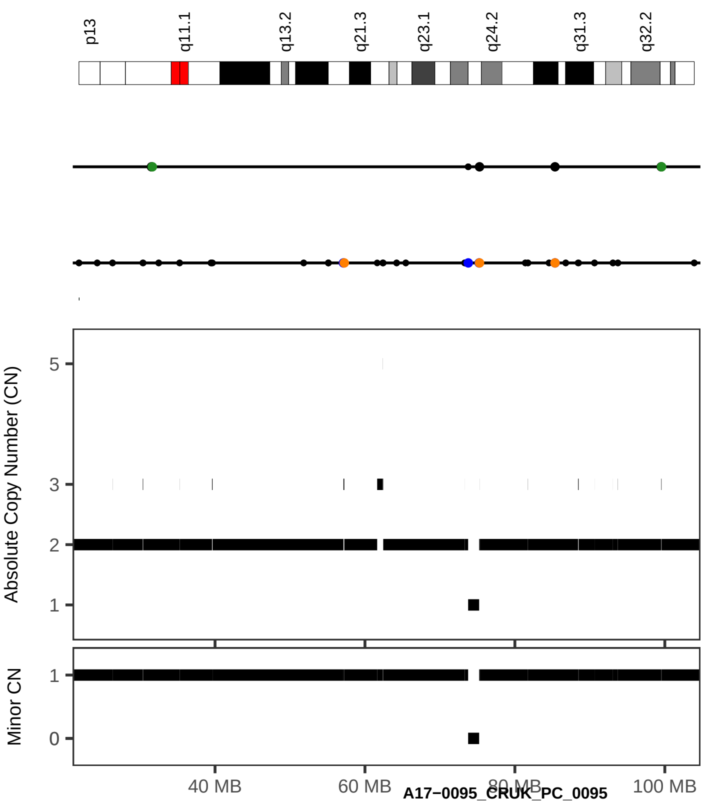

A17−0095_CRUK_PC_0095

Cancer type	Prost−AdenoCA
Position	14:21843858−93753839
Interleaved intrachr. SVs	1
Total SVs (intrachr. + transl.)	23
SV types	DEL: 0; DUP: 11; h2hINV: 1; t2tINV: 0; TRA: 11
SVs in sample	772
Oscillating CN (2 and 3 states)	13, 18
CN segments	31
FDR fragment joints	0.02438658
FDR chr. breakp. enrich.	0.01
Linked to chrs	2:29177870−206548844;3:18799507−143335633 4:679859−171367418;
Purity, ploidy	NA, NA

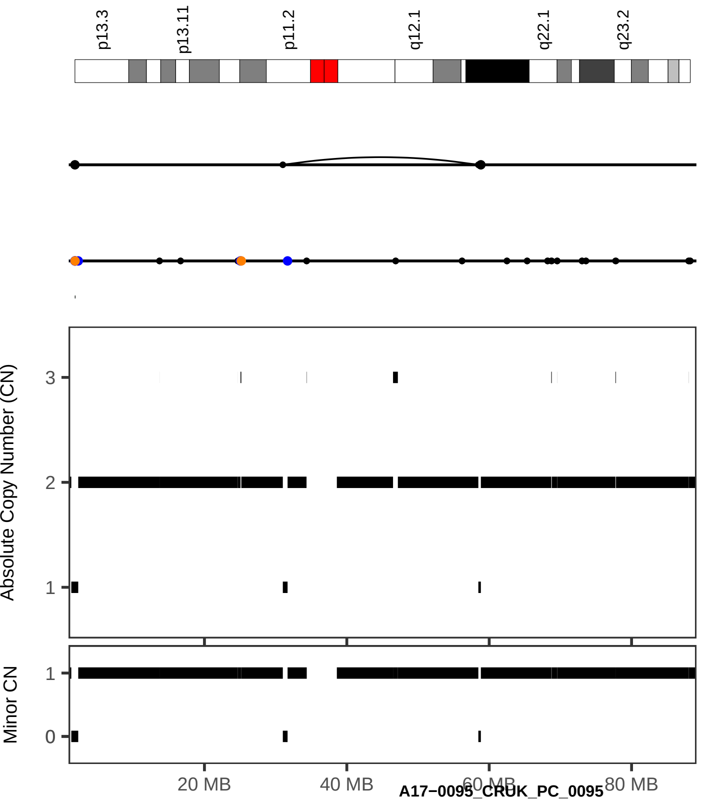

A17−0095_CRUK_PC_0095

Cancer type	Prost−AdenoCA
Position	16:1802279−88025686
Interleaved intrachr. SVs	1
Total SVs (intrachr. + transl.)	19
SV types	DEL: 0; DUP: 8; h2hINV: 1; t2tINV: 0; TRA: 10
SVs in sample	772
Oscillating CN (2 and 3 states)	9, 24
CN segments	24
FDR fragment joints	0.006730063
FDR chr. breakp. enrich.	0.3
Linked to chrs	10:1424131−123537842;19:23775774−55186176 7:740003−154793671;
Purity, ploidy	NA, NA

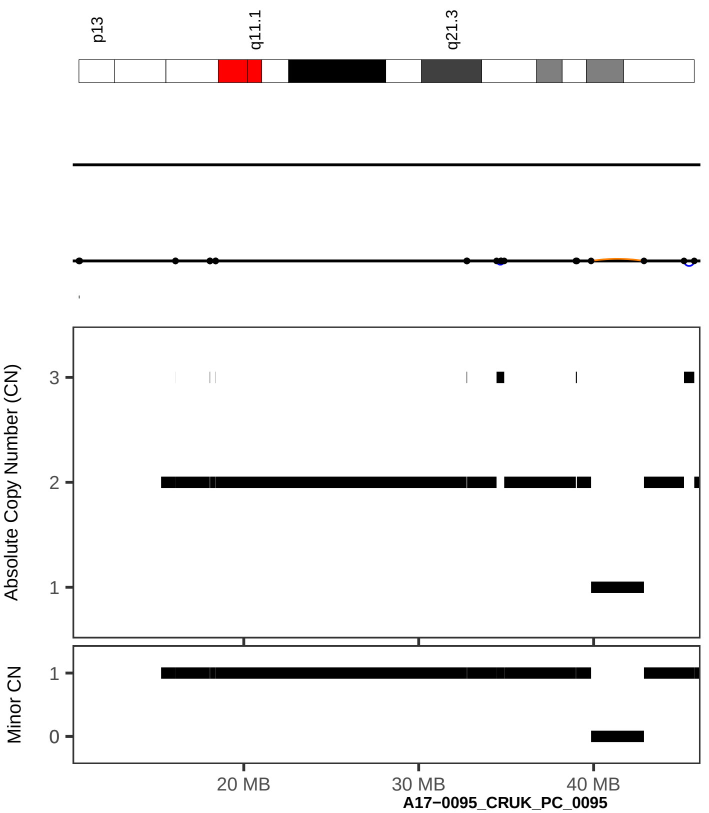

A17−0095_CRUK_PC_0095

Cancer type	Prost−AdenoCA
Position	21:10575654−45753258
Interleaved intrachr. SVs	1
Total SVs (intrachr. + transl.)	8
SV types	DEL: 1; DUP: 7; h2hINV: 0; t2tINV: 0; TRA: 0
SVs in sample	772
Oscillating CN (2 and 3 states)	13, 17
CN segments	17
FDR fragment joints	0.05741733
FDR chr. breakp. enrich.	0.91
Linked to chrs	
Purity, ploidy	NA, NA

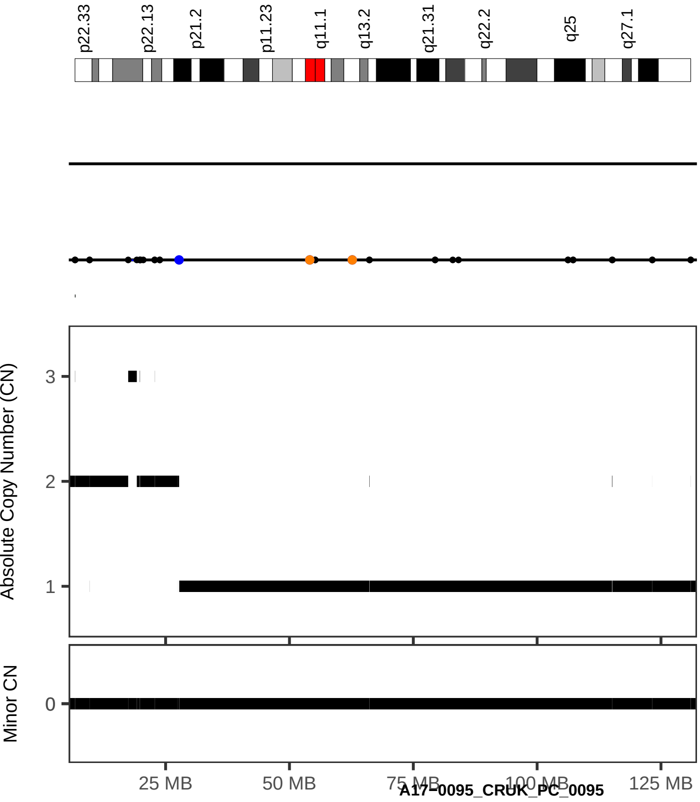

A17−0095_CRUK_PC_0095

Cancer type	Prost−AdenoCA
Position	X:6702691−131010344
Interleaved intrachr. SVs	1
Total SVs (intrachr. + transl.)	16
SV types	DEL: 0; DUP: 11; h2hINV: 0; t2tINV: 0; TRA: 5
SVs in sample	772
Oscillating CN (2 and 3 states)	13, 22
CN segments	22
FDR fragment joints	0.0001919551
FDR chr. breakp. enrich.	0.06
Linked to chrs	11:22270950−133333954;7:740003−154793671
Purity, ploidy	NA, NA

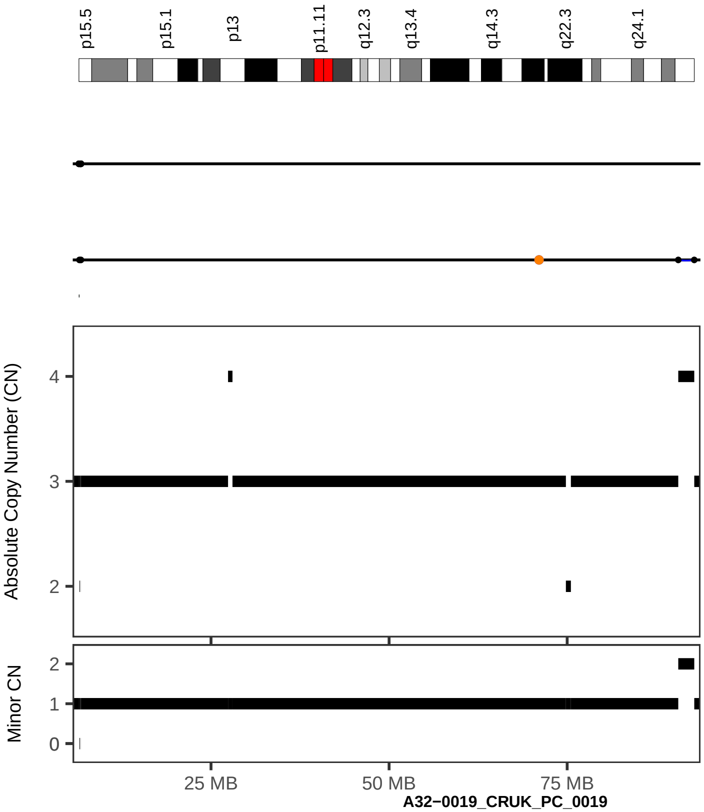

A32−0019_CRUK_PC_0019

Cancer type	Prost−AdenoCA
Position	11:6457815−6743499
Interleaved intrachr. SVs	7
Total SVs (intrachr. + transl.)	7
SV types	DEL: 1; DUP: 0; h2hINV: 3; t2tINV: 3; TRA: 0
SVs in sample	192
Oscillating CN (2 and 3 states)	7, 7
CN segments	7
FDR fragment joints	0.615458
FDR chr. breakp. enrich.	0.91
Linked to chrs	
Purity, ploidy	NA, NA

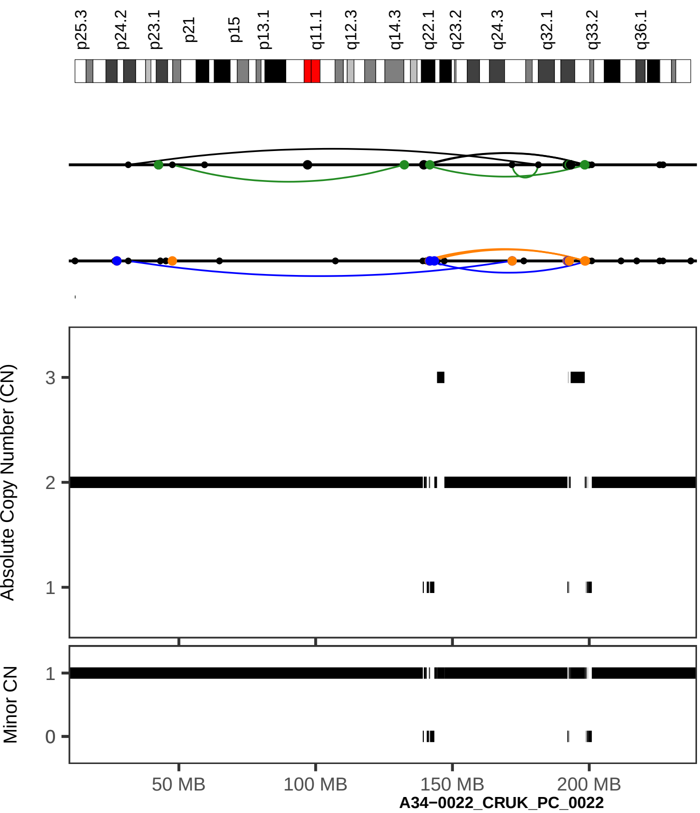

A34−0022_CRUK_PC_0022

Cancer type	Prost−AdenoCA
Position	2:31507439−200933013
Interleaved intrachr. SVs	13
Total SVs (intrachr. + transl.)	47
SV types	DEL: 3; DUP: 2; h2hINV: 4; t2tINV: 4; TRA: 34
SVs in sample	461
Oscillating CN (2 and 3 states)	11, 14
CN segments	30
FDR fragment joints	0.92366
FDR chr. breakp. enrich.	0
Linked to chrs	8:18259748−136762150;
Purity, ploidy	NA, NA

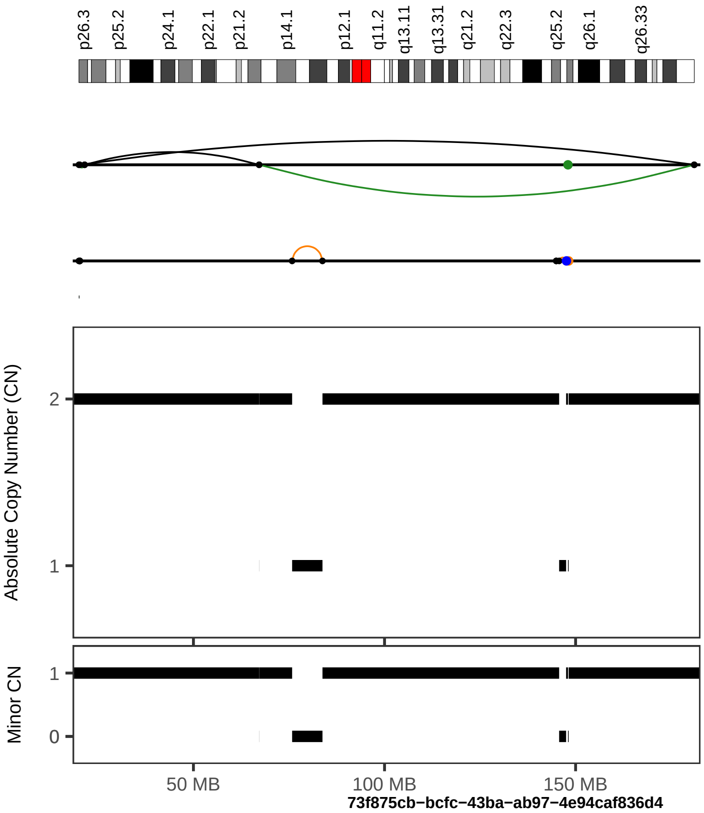

73f875cb−bcfc−43ba−ab97−4e94caf836d4

Cancer type	Prost−AdenoCA
Position	3:20035036−181045916
Interleaved intrachr. SVs	5
Total SVs (intrachr. + transl.)	8
SV types	DEL: 1; DUP: 0; h2hINV: 2; t2tINV: 2; TRA: 3
SVs in sample	144
Oscillating CN (2 and 3 states)	9, 9
CN segments	9
FDR fragment joints	0.8653243
FDR chr. breakp. enrich.	0.75
Linked to chrs	1:65889464−71387959;
Purity, ploidy	0.29, 1.84

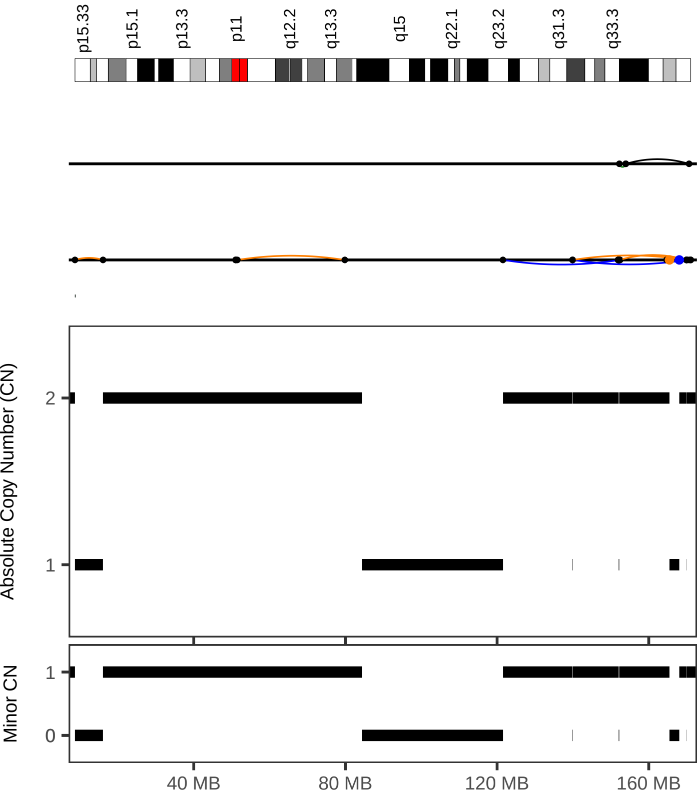

73f875cb−bcfc−43ba−ab97−4e94caf836d4

Cancer type	Prost−AdenoCA
Position	5:121533482−170643022
Interleaved intrachr. SVs	7
Total SVs (intrachr. + transl.)	9
SV types	DEL: 3; DUP: 2; h2hINV: 1; t2tINV: 1; TRA: 2
SVs in sample	144
Oscillating CN (2 and 3 states)	9, 9
CN segments	9
FDR fragment joints	0.615458
FDR chr. breakp. enrich.	0.09
Linked to chrs	
Purity, ploidy	0.29, 1.84

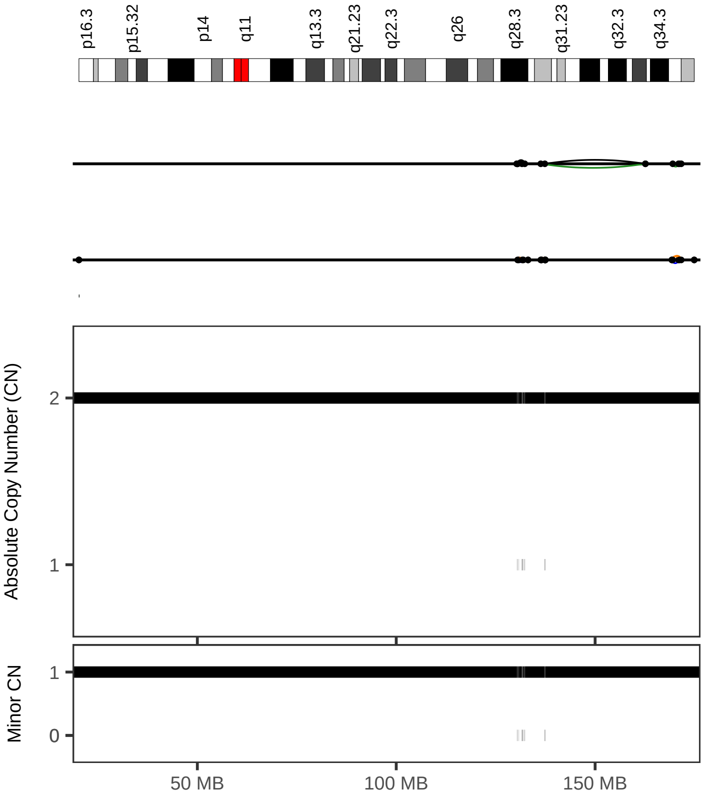

c039014f−821c−43d6−9d3c−914f9c8d98fa

Cancer type	Prost−AdenoCA
Position	4:130270320−162657668
Interleaved intrachr. SVs	5
Total SVs (intrachr. + transl.)	10
SV types	DEL: 3; DUP: 2; h2hINV: 3; t2tINV: 2; TRA: 0
SVs in sample	222
Oscillating CN (2 and 3 states)	13, 13
CN segments	13
FDR fragment joints	0.9625775
FDR chr. breakp. enrich.	0.52
Linked to chrs	
Purity, ploidy	0.85, 1.96

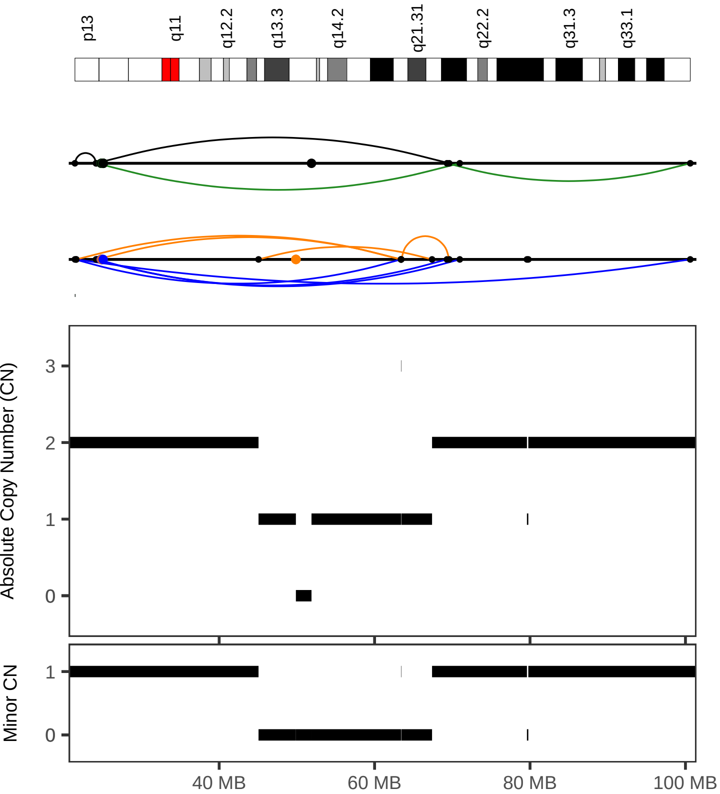

f6381367−142c−45d0−92b3−c1727d1813ce

Cancer type	Prost−AdenoCA
Position	13:21438649−100617984
Interleaved intrachr. SVs	12
Total SVs (intrachr. + transl.)	32
SV types	DEL: 4; DUP: 4; h2hINV: 2; t2tINV: 2; TRA: 20
SVs in sample	133
Oscillating CN (2 and 3 states)	4, 6
CN segments	9
FDR fragment joints	0.9840364
FDR chr. breakp. enrich.	0
Linked to chrs	
Purity, ploidy	0.92, 1.9

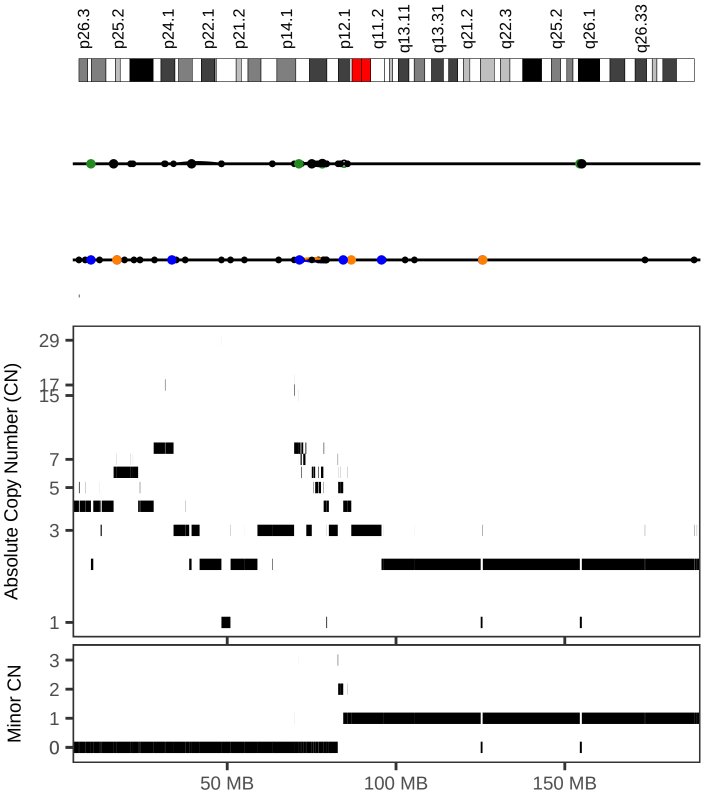

MELA−0002

Cancer type	Skin−Melanoma
Position	3:69823911−79435315
Interleaved intrachr. SVs	25
Total SVs (intrachr. + transl.)	28
SV types	DEL: 9; DUP: 6; h2hINV: 5; t2tINV: 5; TRA: 3
SVs in sample	748
Oscillating CN (2 and 3 states)	8, 11
CN segments	31
FDR fragment joints	0.8988396
FDR chr. breakp. enrich.	0
Linked to chrs	
Purity, ploidy	0.54, 2.62

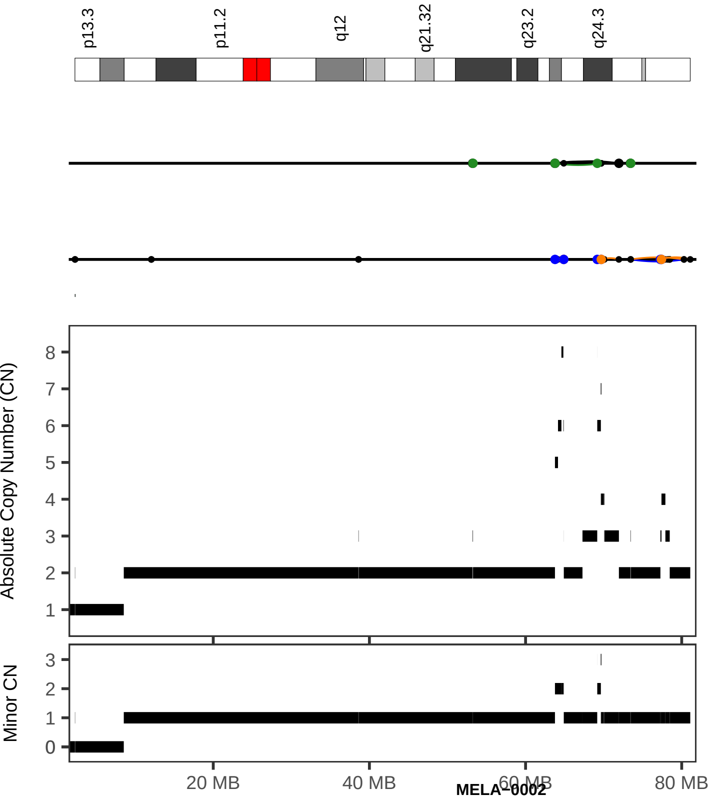

MELA−0002

Cancer type	Skin−Melanoma
Position	17:63710548−71955453
Interleaved intrachr. SVs	10
Total SVs (intrachr. + transl.)	21
SV types	DEL: 3; DUP: 3; h2hINV: 2; t2tINV: 2; TRA: 11
SVs in sample	748
Oscillating CN (2 and 3 states)	4, 7
CN segments	18
FDR fragment joints	0.8882853
FDR chr. breakp. enrich.	0
Linked to chrs	2:11161529−144813239;7:1508469−101805271
Purity, ploidy	0.54, 2.62

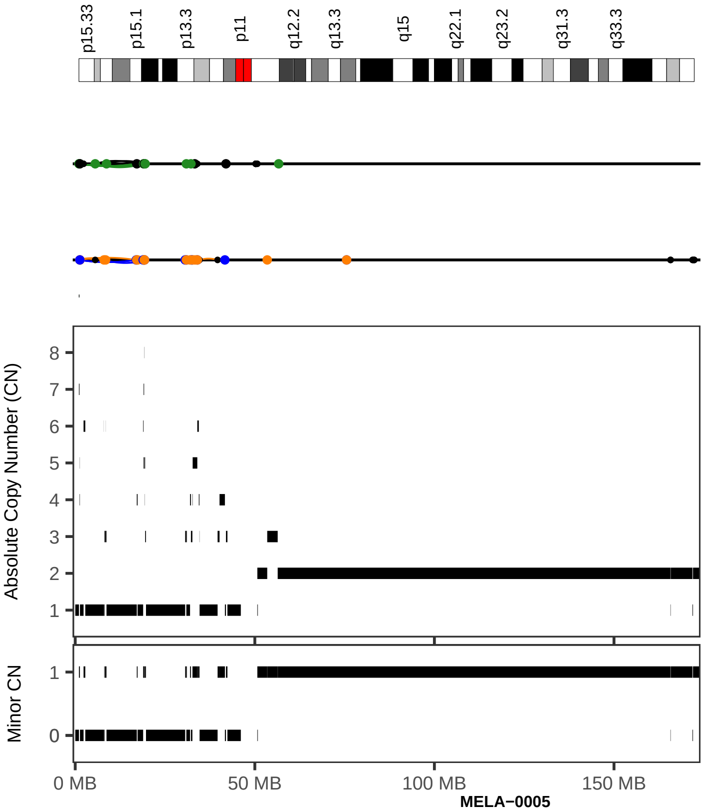

MELA−0005

Cancer type	Skin−Melanoma
Position	5:1027171−19705107
Interleaved intrachr. SVs	13
Total SVs (intrachr. + transl.)	38
SV types	DEL: 3; DUP: 3; h2hINV: 2; t2tINV: 5; TRA: 25
SVs in sample	220
Oscillating CN (2 and 3 states)	5, 6
CN segments	25
FDR fragment joints	0.8784945
FDR chr. breakp. enrich.	0
Linked to chrs	9:3407818−22069768;
Purity, ploidy	0.79, 1.98

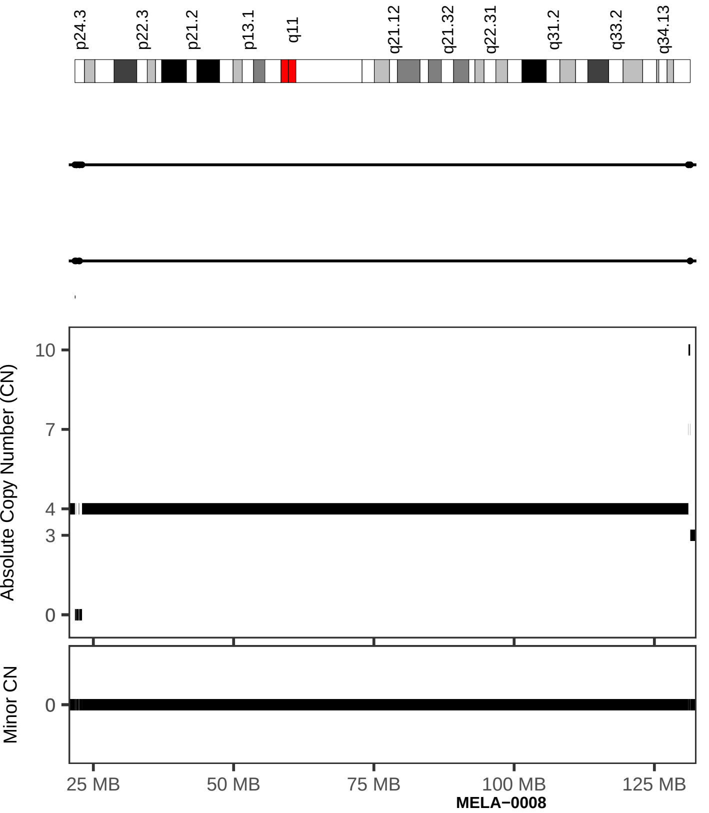

MELA−0008

Cancer type	Skin−Melanoma
Position	9:21711690−22977175
Interleaved intrachr. SVs	7
Total SVs (intrachr. + transl.)	7
SV types	DEL: 2; DUP: 0; h2hINV: 3; t2tINV: 2; TRA: 0
SVs in sample	142
Oscillating CN (2 and 3 states)	7, 7
CN segments	7
FDR fragment joints	0.6776251
FDR chr. breakp. enrich.	0.22
Linked to chrs	
Purity, ploidy	0.79, 3.81

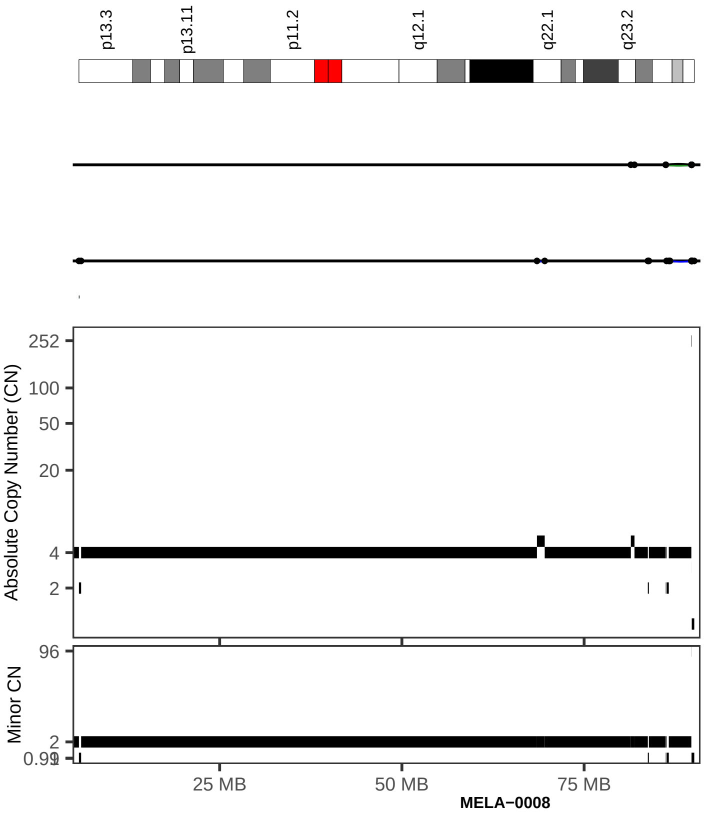

MELA−0008

Cancer type	Skin−Melanoma
Position	16:86186241−90105121
Interleaved intrachr. SVs	6
Total SVs (intrachr. + transl.)	6
SV types	DEL: 1; DUP: 2; h2hINV: 1; t2tINV: 2; TRA: 0
SVs in sample	142
Oscillating CN (2 and 3 states)	4, 6
CN segments	10
FDR fragment joints	0.9284301
FDR chr. breakp. enrich.	0
Linked to chrs	
Purity, ploidy	0.79, 3.81

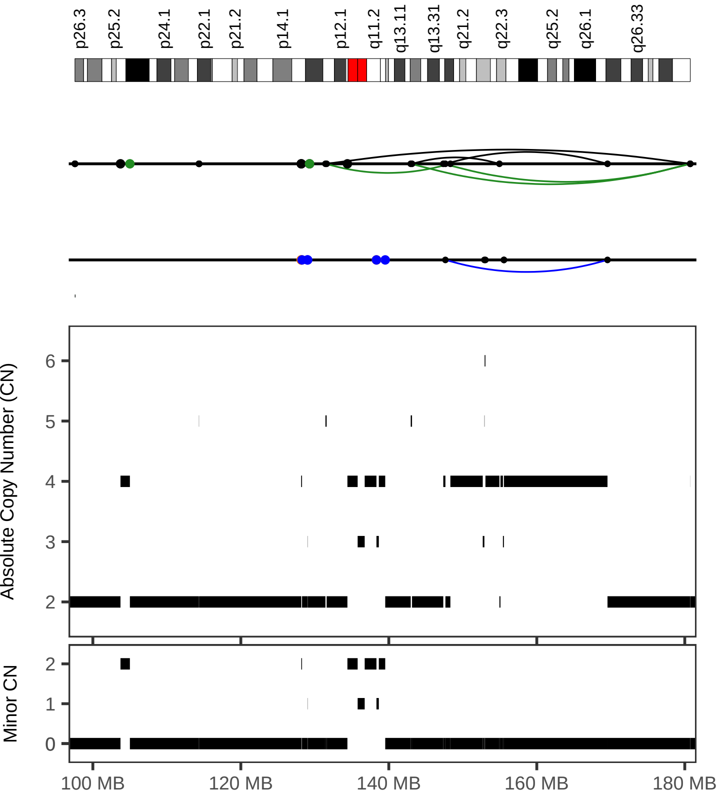

MELA−0012

Cancer type	Skin−Melanoma
Position	3:131435629−180741702
Interleaved intrachr. SVs	7
Total SVs (intrachr. + transl.)	11
SV types	DEL: 0; DUP: 1; h2hINV: 3; t2tINV: 3; TRA: 4
SVs in sample	508
Oscillating CN (2 and 3 states)	5, 15
CN segments	24
FDR fragment joints	0.7425546
FDR chr. breakp. enrich.	0.99
Linked to chrs	
Purity, ploidy	0.79, 2.87

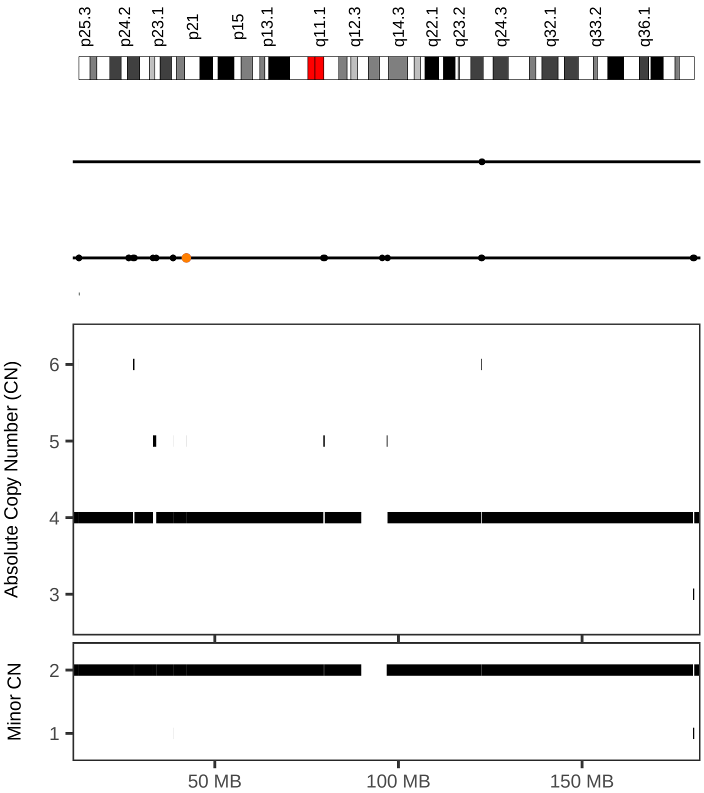

MELA−0015

Cancer type	Skin−Melanoma
Position	2:12998893−180541994
Interleaved intrachr. SVs	1
Total SVs (intrachr. + transl.)	12
SV types	DEL: 1; DUP: 8; h2hINV: 1; t2tINV: 0; TRA: 2
SVs in sample	201
Oscillating CN (2 and 3 states)	11, 16
CN segments	18
FDR fragment joints	0.06620197
FDR chr. breakp. enrich.	0.9
Linked to chrs	
Purity, ploidy	0.55, 3.39

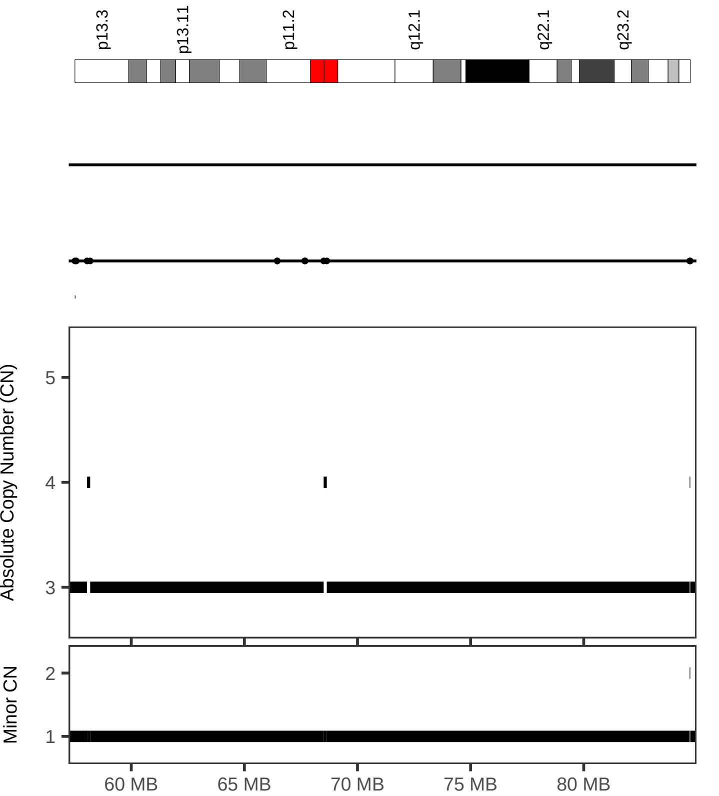

MELA−0015

Cancer type	Skin−Melanoma
Position	16:57509089−84708915
Interleaved intrachr. SVs	1
Total SVs (intrachr. + transl.)	5
SV types	DEL: 0; DUP: 5; h2hINV: 0; t2tINV: 0; TRA: 0
SVs in sample	201
Oscillating CN (2 and 3 states)	7, 7
CN segments	7
FDR fragment joints	0.1013295
FDR chr. breakp. enrich.	0.1
Linked to chrs	
Purity, ploidy	0.55, 3.39

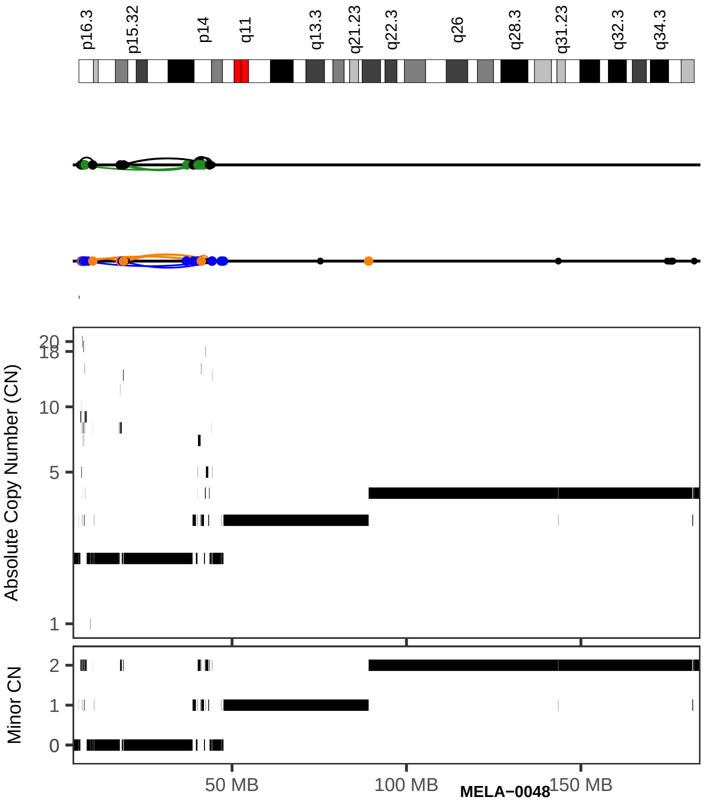

MELA−0048

Cancer type	Skin−Melanoma
Position	4:6102625−46953792
Interleaved intrachr. SVs	26
Total SVs (intrachr. + transl.)	72
SV types	DEL: 10; DUP: 4; h2hINV: 8; t2tINV: 4; TRA: 46
SVs in sample	444
Oscillating CN (2 and 3 states)	4, 5
CN segments	63
FDR fragment joints	0.8039763
FDR chr. breakp. enrich.	0
Linked to chrs	5:1054293−127185142;7:858862−151538982
Purity, ploidy	0.9, 2.98

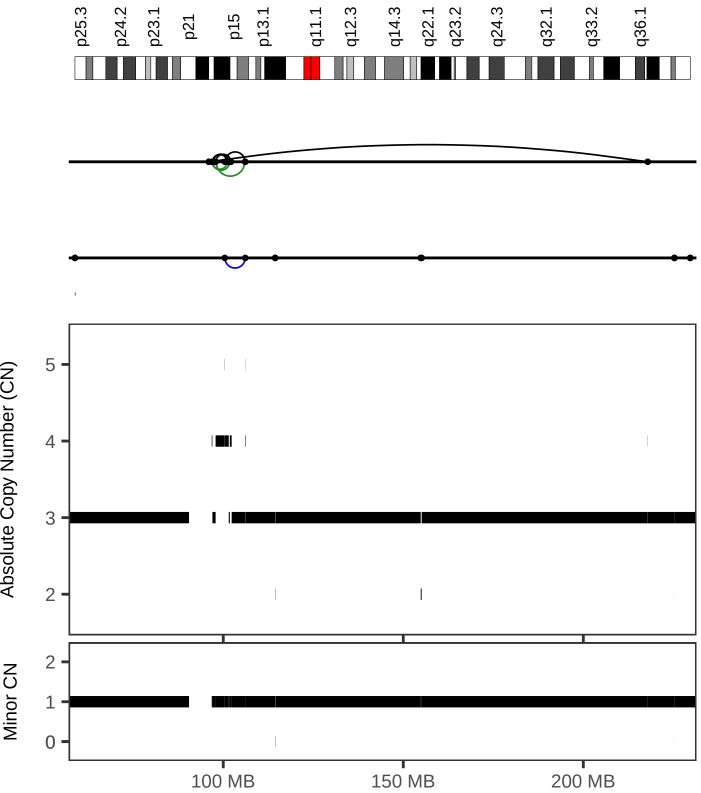

MELA−0061

Cancer type	Skin−Melanoma
Position	2:96865382−106123131
Interleaved intrachr. SVs	8
Total SVs (intrachr. + transl.)	8
SV types	DEL: 0; DUP: 1; h2hINV: 4; t2tINV: 3; TRA: 0
SVs in sample	101
Oscillating CN (2 and 3 states)	4, 8
CN segments	11
FDR fragment joints	0.615458
FDR chr. breakp. enrich.	0.03
Linked to chrs	
Purity, ploidy	0.91, 3.42

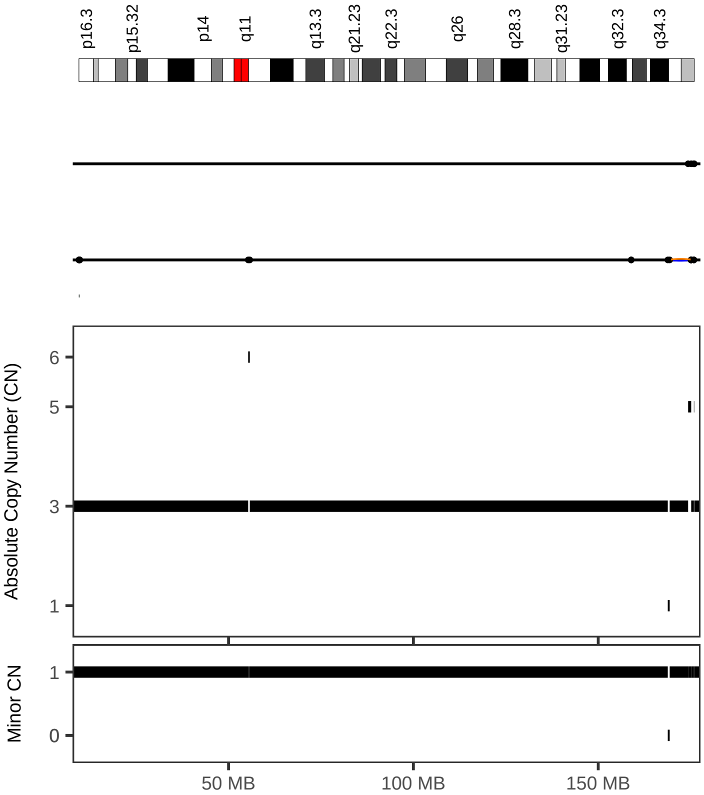

MELA−0061

Cancer type	Skin−Melanoma
Position	4:168818150−175974913
Interleaved intrachr. SVs	6
Total SVs (intrachr. + transl.)	6
SV types	DEL: 1; DUP: 3; h2hINV: 1; t2tINV: 1; TRA: 0
SVs in sample	101
Oscillating CN (2 and 3 states)	5, 6
CN segments	6
FDR fragment joints	0.6776251
FDR chr. breakp. enrich.	0.46
Linked to chrs	
Purity, ploidy	0.91, 3.42

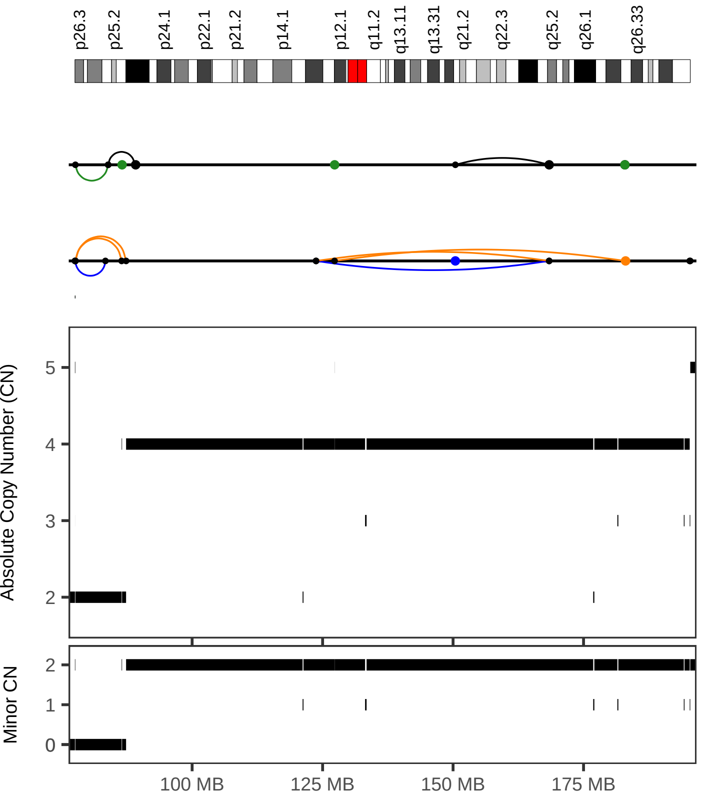

MELA−0066

Cancer type	Skin−Melanoma
Position	3:77541575−89022426
Interleaved intrachr. SVs	6
Total SVs (intrachr. + transl.)	7
SV types	DEL: 2; DUP: 2; h2hINV: 1; t2tINV: 1; TRA: 1
SVs in sample	355
Oscillating CN (2 and 3 states)	4, 7
CN segments	7
FDR fragment joints	0.9599662
FDR chr. breakp. enrich.	0.6
Linked to chrs	
Purity, ploidy	0.42, 3.25

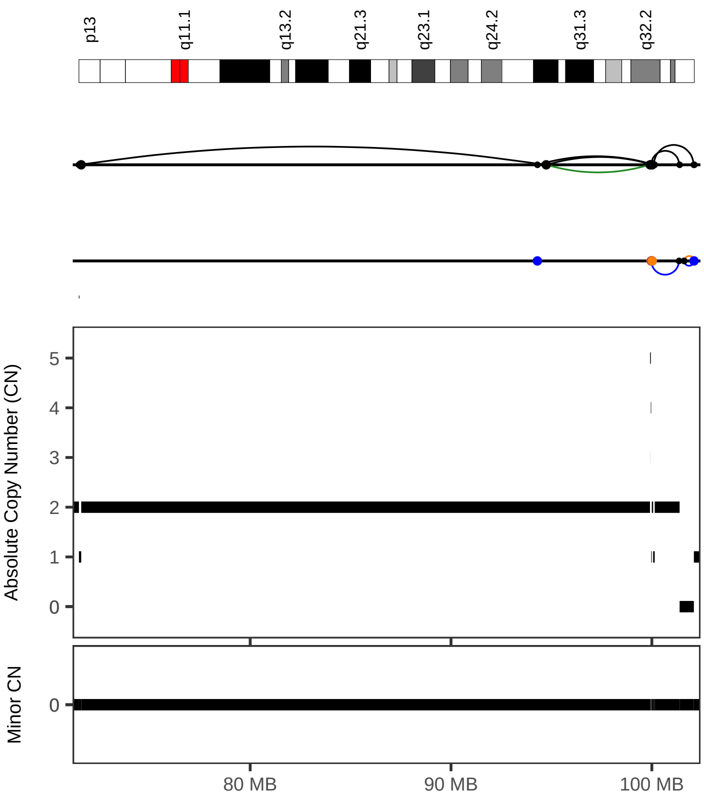

MELA−0066

Cancer type	Skin−Melanoma
Position	14:71471614−102112284
Interleaved intrachr. SVs	11
Total SVs (intrachr. + transl.)	24
SV types	DEL: 1; DUP: 2; h2hINV: 5; t2tINV: 3; TRA: 13
SVs in sample	355
Oscillating CN (2 and 3 states)	4, 6
CN segments	12
FDR fragment joints	0.8653243
FDR chr. breakp. enrich.	0
Linked to chrs	
Purity, ploidy	0.42, 3.25

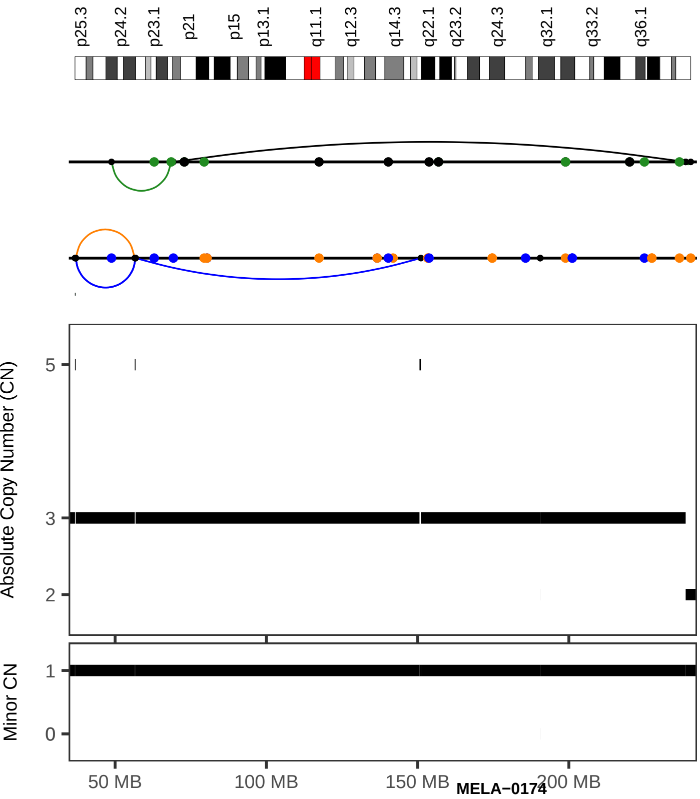

MELA−0174

Cancer type	Skin−Melanoma
Position	2:36739938−238611839
Interleaved intrachr. SVs	7
Total SVs (intrachr. + transl.)	47
SV types	DEL: 1; DUP: 4; h2hINV: 1; t2tINV: 1; TRA: 40
SVs in sample	249
Oscillating CN (2 and 3 states)	6, 8
CN segments	8
FDR fragment joints	0.6661788
FDR chr. breakp. enrich.	0
Linked to chrs	3:8169851−171292231;9:2192834−27453600
Purity, ploidy	0.94, 2.15

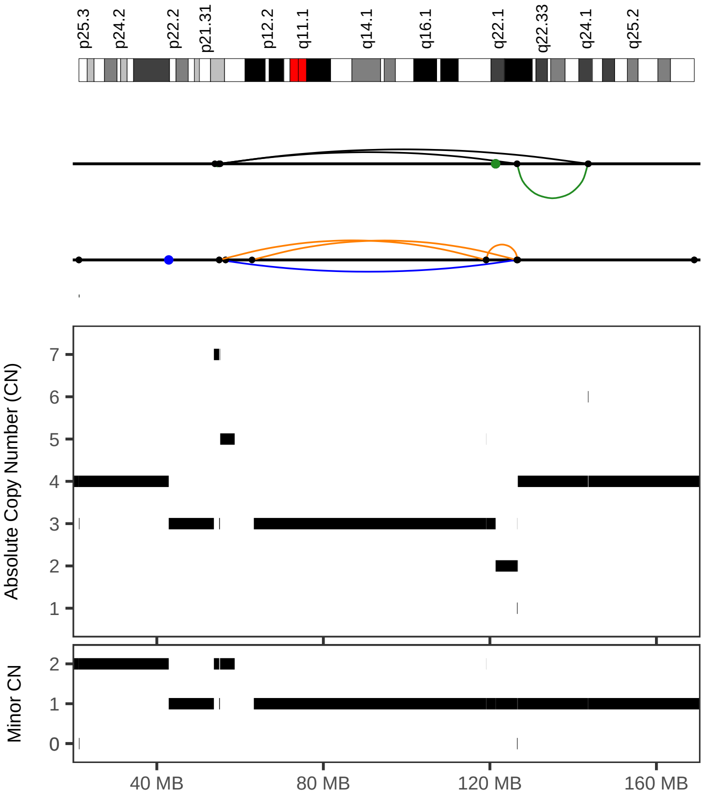

MELA−0223

Cancer type	Skin−Melanoma
Position	6:53940949−143661562
Interleaved intrachr. SVs	8
Total SVs (intrachr. + transl.)	10
SV types	DEL: 3; DUP: 1; h2hINV: 2; t2tINV: 2; TRA: 2
SVs in sample	104
Oscillating CN (2 and 3 states)	4, 5
CN segments	14
FDR fragment joints	0.8572806
FDR chr. breakp. enrich.	0.03
Linked to chrs	
Purity, ploidy	0.87, 4.03

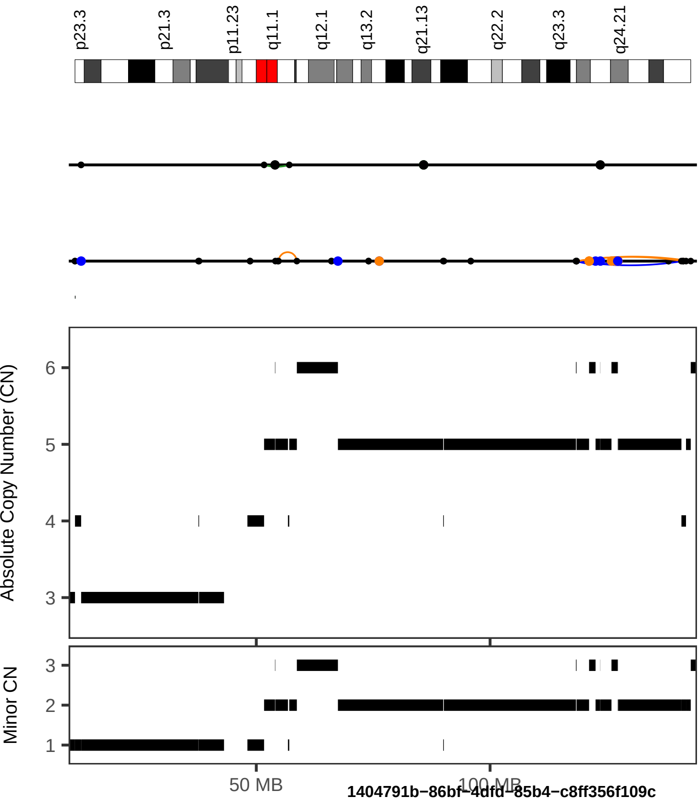

1404791b−86bf−4dfd−85b4−c8ff356f109c

Cancer type	Skin−Melanoma
Position	8:118357384−142919752
Interleaved intrachr. SVs	5
Total SVs (intrachr. + transl.)	15
SV types	DEL: 3; DUP: 2; h2hINV: 0; t2tINV: 0; TRA: 10
SVs in sample	573
Oscillating CN (2 and 3 states)	10, 13
CN segments	13
FDR fragment joints	0.2123773
FDR chr. breakp. enrich.	0.5
Linked to chrs	22:29353674−50585361;7:306183−65693058
Purity, ploidy	0.89, 2.68

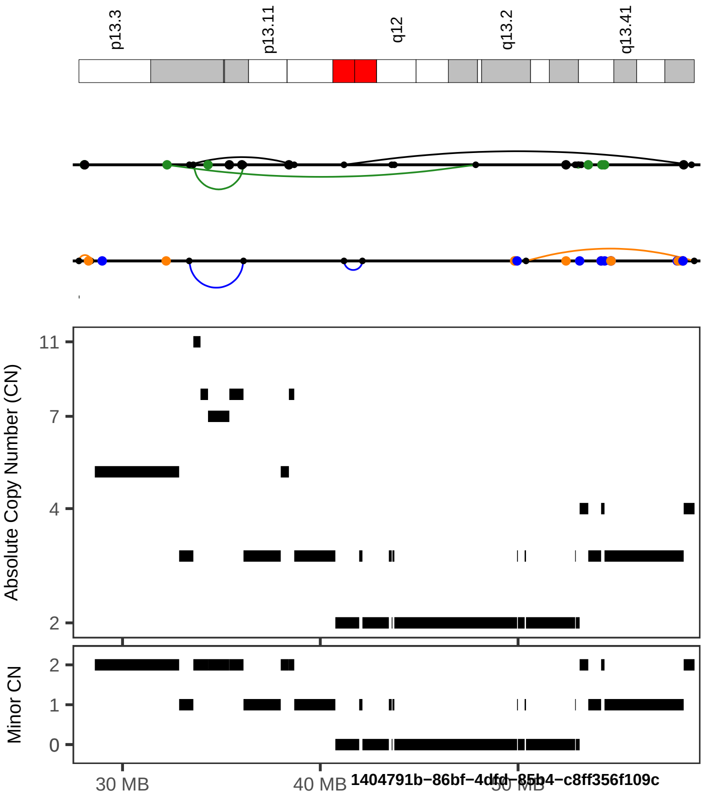

1404791b−86bf−4dfd−85b4−c8ff356f109c

Cancer type	Skin−Melanoma
Position	19:32205724−58908289
Interleaved intrachr. SVs	3
Total SVs (intrachr. + transl.)	34
SV types	DEL: 1; DUP: 1; h2hINV: 2; t2tINV: 2; TRA: 28
SVs in sample	573
Oscillating CN (2 and 3 states)	14, 19
CN segments	28
FDR fragment joints	0.8653243
FDR chr. breakp. enrich.	0
Linked to chrs	11:33008500−79611121;22:29353674−50585361 6:503244−53879503;7:306183−65693058

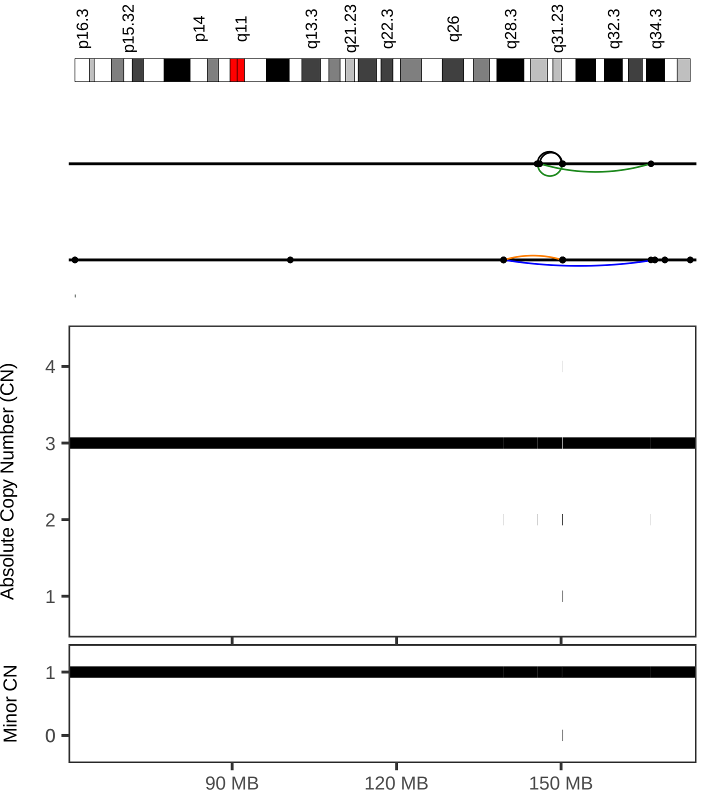

238111f9−a08b−49b4−9035−cc43026330e7

Cancer type	Skin−Melanoma
Position	4:139483168−167110416
Interleaved intrachr. SVs	12
Total SVs (intrachr. + transl.)	12
SV types	DEL: 5; DUP: 2; h2hINV: 3; t2tINV: 2; TRA: 0
SVs in sample	87
Oscillating CN (2 and 3 states)	8, 8
CN segments	16
FDR fragment joints	0.6776251
FDR chr. breakp. enrich.	0
Linked to chrs	
Purity, ploidy	0.89, 2.79

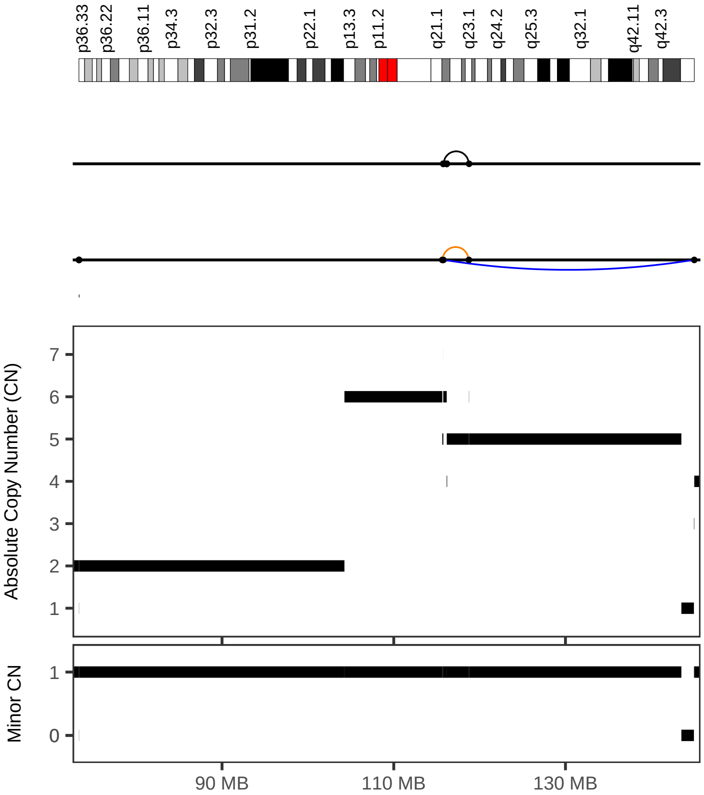

3409f118−1798−48d6−9556−12a5c77ebefb

Cancer type	Skin−Melanoma
Position	1:115652442−144998190
Interleaved intrachr. SVs	6
Total SVs (intrachr. + transl.)	6
SV types	DEL: 1; DUP: 1; h2hINV: 1; t2tINV: 3; TRA: 0
SVs in sample	36
Oscillating CN (2 and 3 states)	4, 4
CN segments	12
FDR fragment joints	0.6776251
FDR chr. breakp. enrich.	0
Linked to chrs	
Purity, ploidy	0.75, 2.89

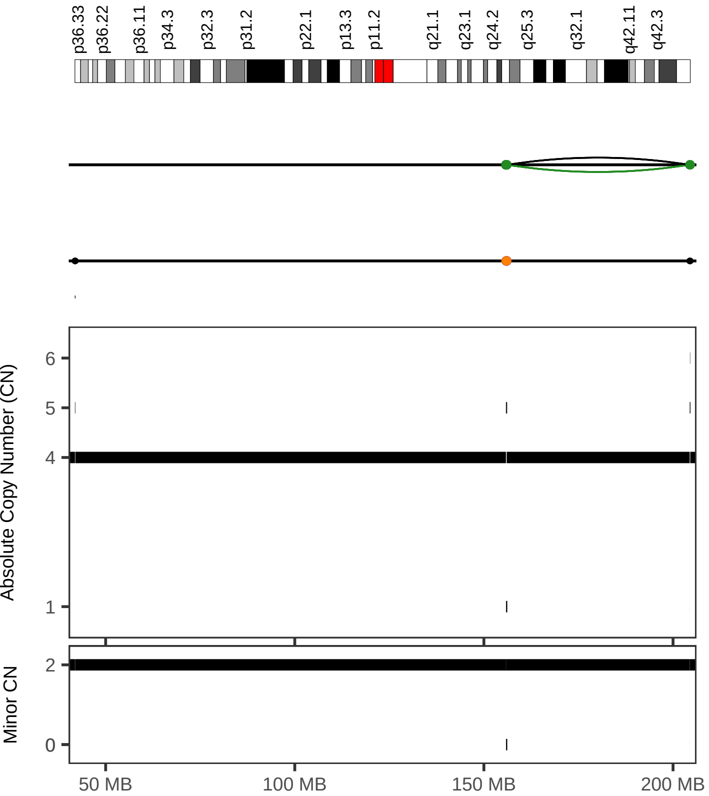

3429967f−4f77−4894−bf27−c4a22698ca92

Cancer type	Skin−Melanoma
Position	1:155839251−204554164
Interleaved intrachr. SVs	14
Total SVs (intrachr. + transl.)	36
SV types	DEL: 2; DUP: 4; h2hINV: 3; t2tINV: 5; TRA: 22
SVs in sample	107
Oscillating CN (2 and 3 states)	6, 10
CN segments	13
FDR fragment joints	0.6776251
FDR chr. breakp. enrich.	0
Linked to chrs	
Purity, ploidy	0.52, 2.88

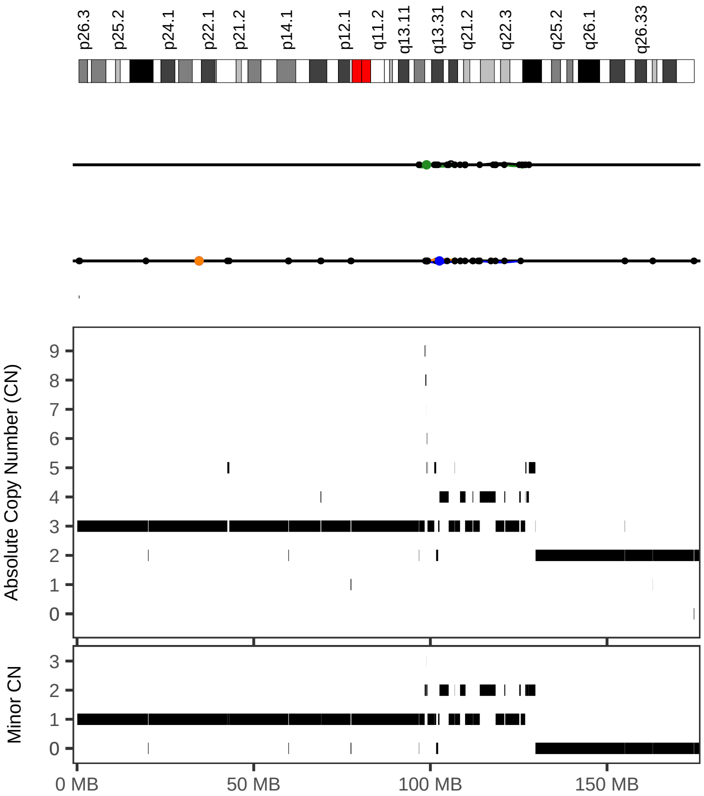

3fea3f02−2db3−44e0−984e−c61130d92c9b

Cancer type	Skin−Melanoma
Position	3:98573207−109821728
Interleaved intrachr. SVs	14
Total SVs (intrachr. + transl.)	16
SV types	DEL: 5; DUP: 3; h2hINV: 3; t2tINV: 3; TRA: 2
SVs in sample	262
Oscillating CN (2 and 3 states)	4, 8
CN segments	18
FDR fragment joints	0.9501265
FDR chr. breakp. enrich.	0
Linked to chrs	
Purity, ploidy	0.74, 3.05

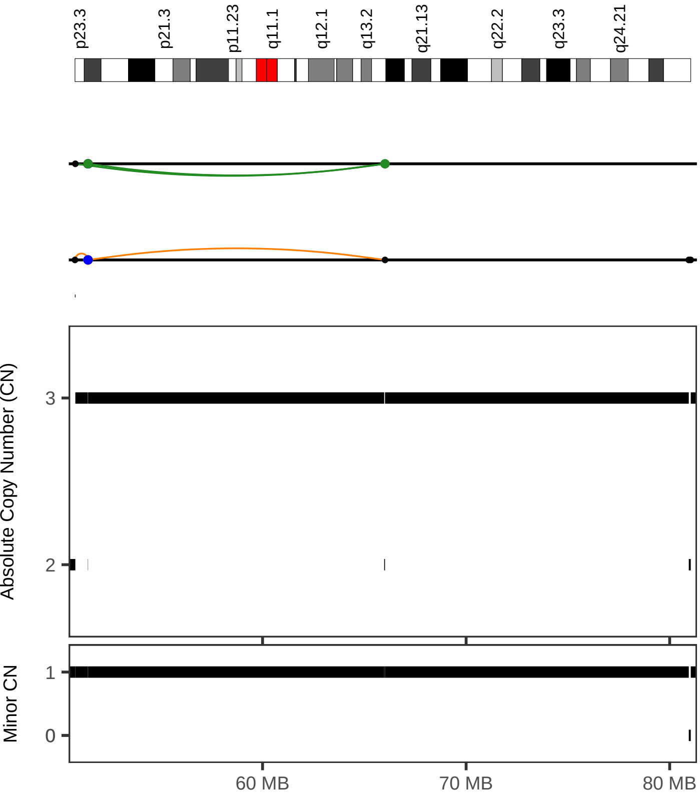

40e17bf8−6c19−4e5e−b60d−ad54f504a970

Cancer type	Skin−Melanoma
Position	8:50784712−66016345
Interleaved intrachr. SVs	6
Total SVs (intrachr. + transl.)	14
SV types	DEL: 3; DUP: 0; h2hINV: 1; t2tINV: 2; TRA: 8
SVs in sample	140
Oscillating CN (2 and 3 states)	8, 8
CN segments	8
FDR fragment joints	0.8066159
FDR chr. breakp. enrich.	0.01
Linked to chrs	
Purity, ploidy	0.76, 1.94

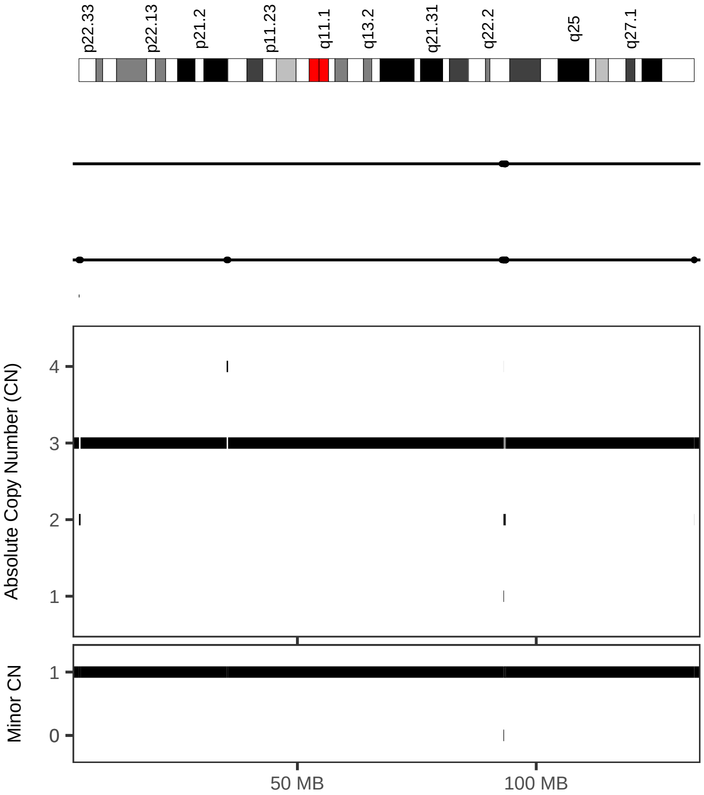

5bc515c8−7727−4d69−98e9−31bbcb748550

Cancer type	Skin−Melanoma
Position	X:92816571−93649590
Interleaved intrachr. SVs	26
Total SVs (intrachr. + transl.)	26
SV types	DEL: 8; DUP: 7; h2hINV: 6; t2tINV: 5; TRA: 0
SVs in sample	178
Oscillating CN (2 and 3 states)	14, 16
CN segments	18
FDR fragment joints	0.9179372
FDR chr. breakp. enrich.	0
Linked to chrs	
Purity, ploidy	0.47, 3.2

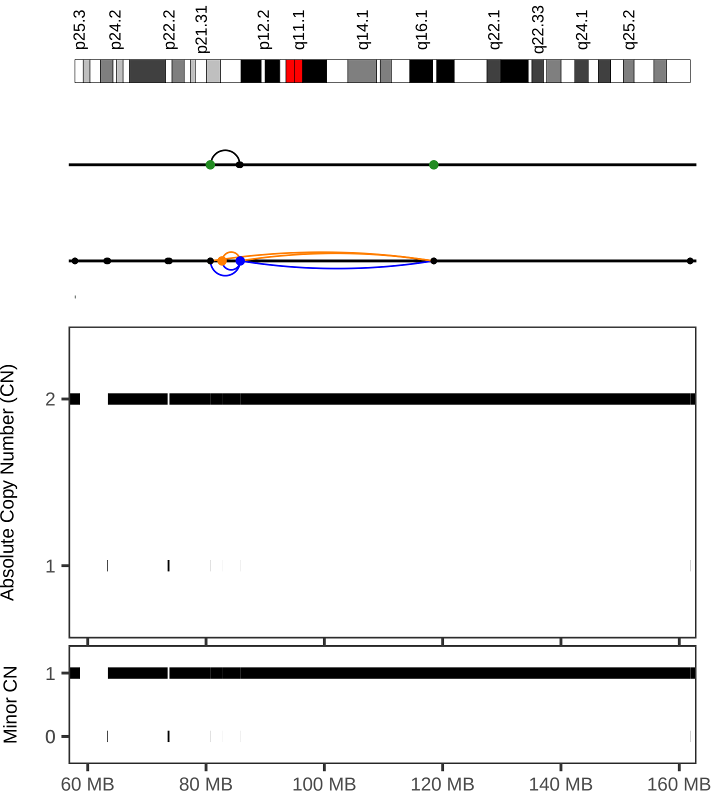

5f6df69c−4ebf−4811−8b28−ad393dfbe1a3

Cancer type	Skin−Melanoma
Position	6:80713179−118504284
Interleaved intrachr. SVs	9
Total SVs (intrachr. + transl.)	13
SV types	DEL: 4; DUP: 3; h2hINV: 1; t2tINV: 1; TRA: 4
SVs in sample	160
Oscillating CN (2 and 3 states)	7, 7
CN segments	7
FDR fragment joints	0.6776251
FDR chr. breakp. enrich.	0.05
Linked to chrs	2:15930327−35023335;
Purity, ploidy	0.86, 1.94

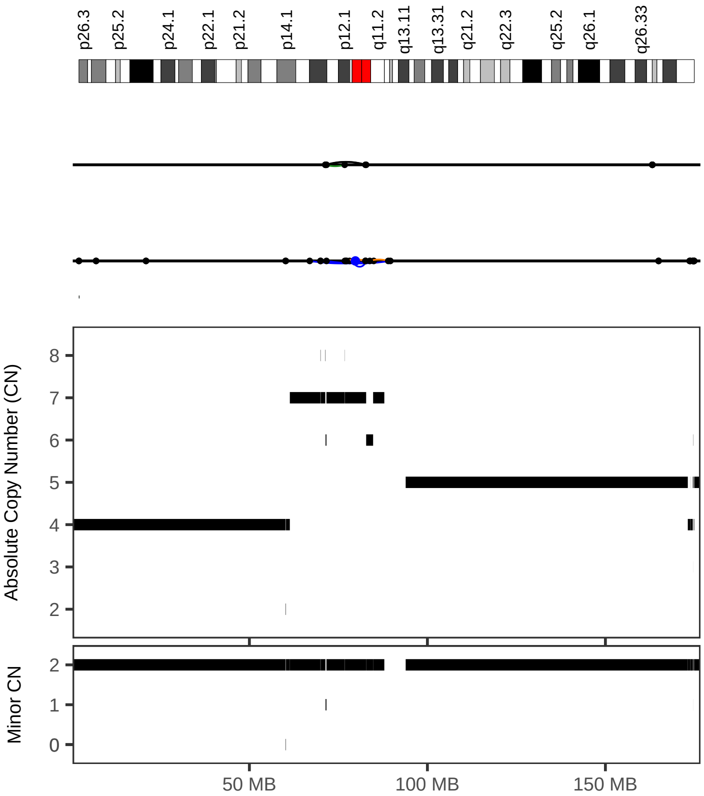

d8eac750−9daf−4465−b672−9e31fd369057

Cancer type	Skin−Melanoma
Position	3:66947406−89593615
Interleaved intrachr. SVs	10
Total SVs (intrachr. + transl.)	11
SV types	DEL: 3; DUP: 4; h2hINV: 2; t2tINV: 1; TRA: 1
SVs in sample	266
Oscillating CN (2 and 3 states)	4, 6
CN segments	11
FDR fragment joints	0.615458
FDR chr. breakp. enrich.	0.35
Linked to chrs	
Purity, ploidy	0.47, 3.89

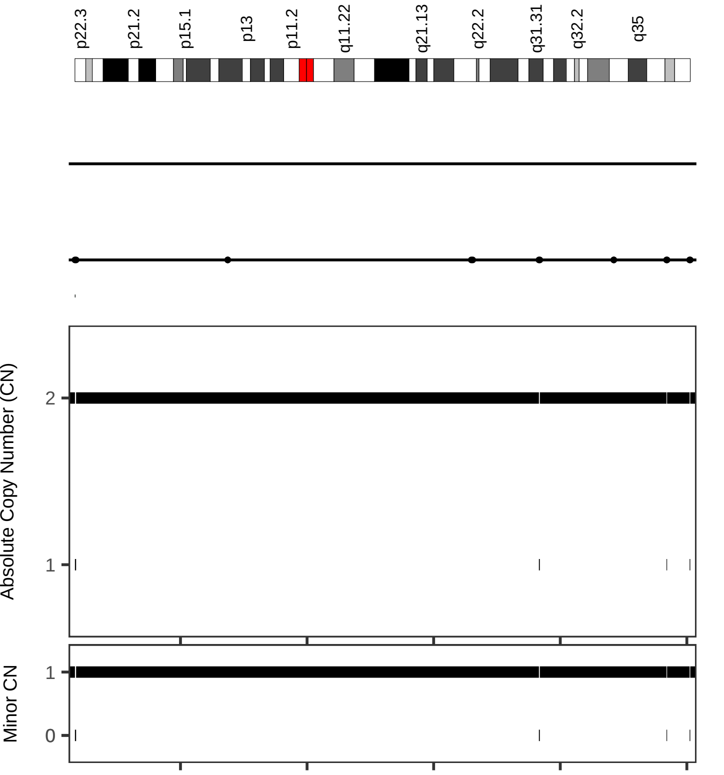

5a64f7e9−3833−4a3c−962b−13eb3e305c69

Cancer type	SoftTissue−Leiomyo
Position	7:4154645−125672436
Interleaved intrachr. SVs	1
Total SVs (intrachr. + transl.)	5
SV types	DEL: 5; DUP: 0; h2hINV: 0; t2tINV: 0; TRA: 0
SVs in sample	339
Oscillating CN (2 and 3 states)	8, 8
CN segments	8
FDR fragment joints	0.1013295
FDR chr. breakp. enrich.	0.02
Linked to chrs	
Purity, ploidy	0.72, 1.65

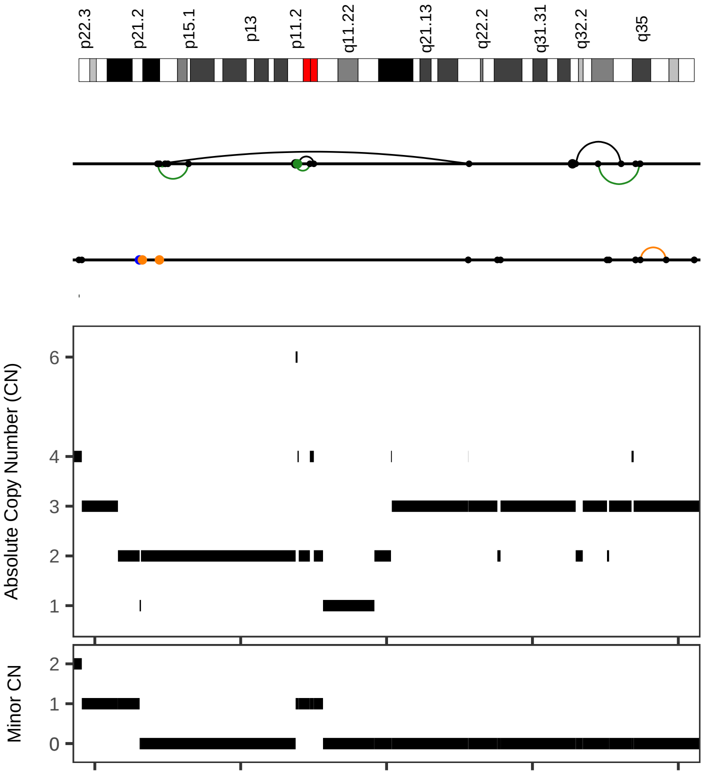

9c9b53e5−cdbe−4510−a839−4a3b5039681b

Cancer type	SoftTissue−Leiomyo
Position	7:78585846−144766064
Interleaved intrachr. SVs	4
Total SVs (intrachr. + transl.)	12
SV types	DEL: 0; DUP: 1; h2hINV: 3; t2tINV: 4; TRA: 4
SVs in sample	240
Oscillating CN (2 and 3 states)	7, 12
CN segments	20
FDR fragment joints	0.5435077
FDR chr. breakp. enrich.	0.02
Linked to chrs	
Purity, ploidy	0.91, 2.73

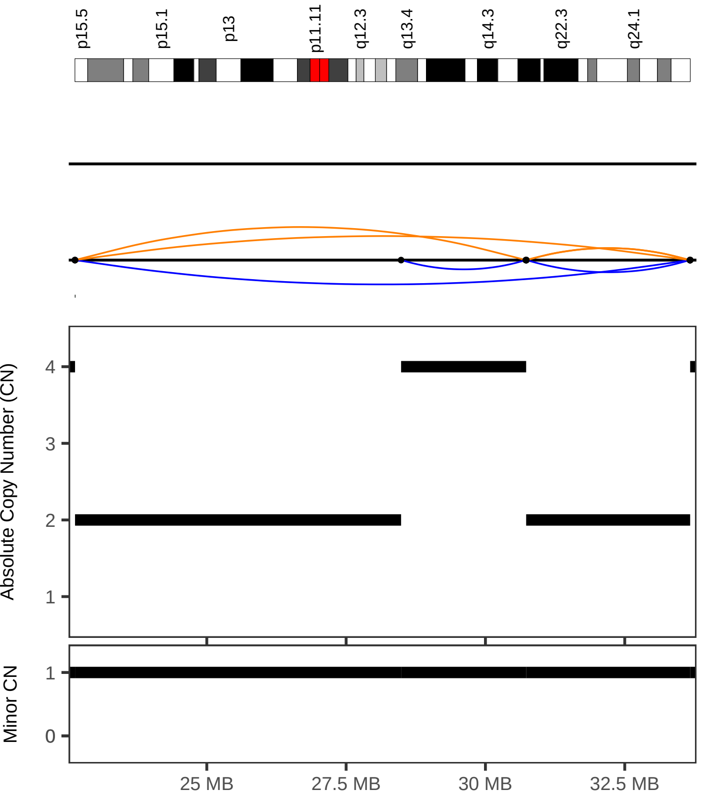

ea3a524b−6ee3−4904−8924−29fcaa20985c

Cancer type	SoftTissue−Leiomyo
Position	11:22633386−33675388
Interleaved intrachr. SVs	7
Total SVs (intrachr. + transl.)	7
SV types	DEL: 4; DUP: 3; h2hINV: 0; t2tINV: 0; TRA: 0
SVs in sample	318
Oscillating CN (2 and 3 states)	5, 5
CN segments	5
FDR fragment joints	0.5435077
FDR chr. breakp. enrich.	0.47
Linked to chrs	
Purity, ploidy	0.87, 3.16

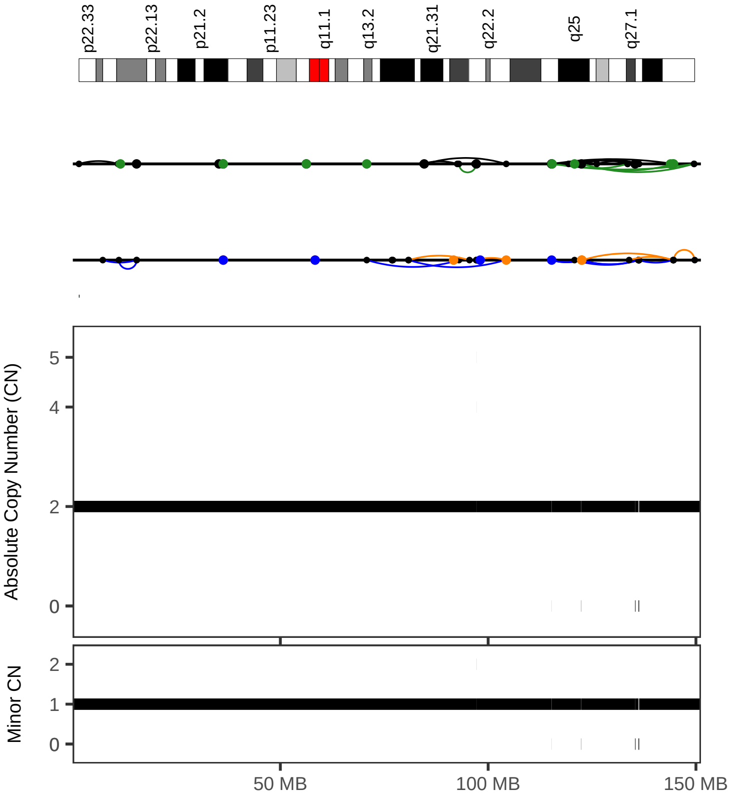

b5f27002−4277−418d−90b0−cc5a2742692e

Cancer type	SoftTissue−Liposarc
Position	X:115283407−149711207
Interleaved intrachr. SVs	21
Total SVs (intrachr. + transl.)	31
SV types	DEL: 4; DUP: 8; h2hINV: 5; t2tINV: 4; TRA: 10
SVs in sample	895
Oscillating CN (2 and 3 states)	8, 8
CN segments	8
FDR fragment joints	0.8653243
FDR chr. breakp. enrich.	0
Linked to chrs	
Purity, ploidy	0.87, 6.21

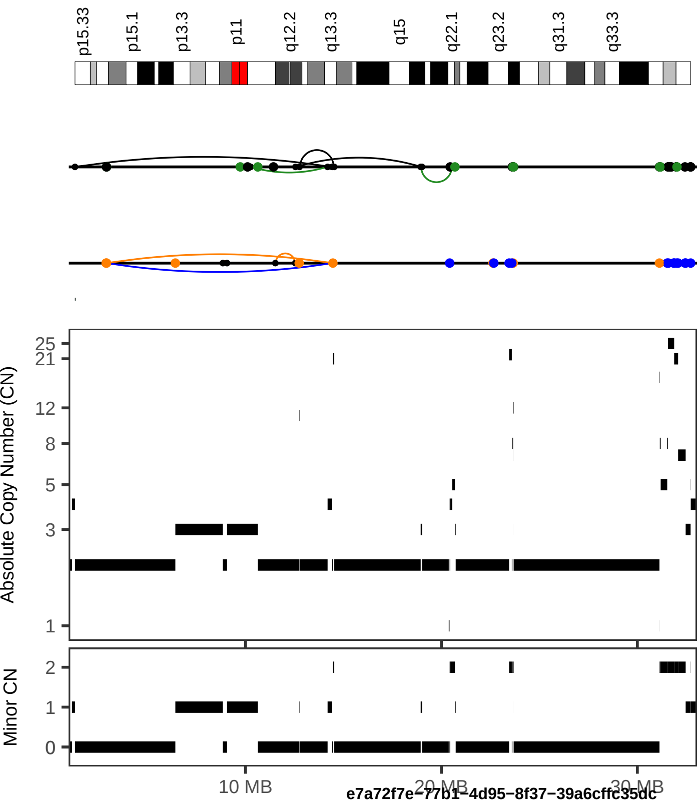

e7a72f7e−77b1−4d95−8f37−39a6cffc35dc

Cancer type	SoftTissue−Liposarc
Position	5:1295334−20553339
Interleaved intrachr. SVs	7
Total SVs (intrachr. + transl.)	20
SV types	DEL: 1; DUP: 1; h2hINV: 3; t2tINV: 2; TRA: 13
SVs in sample	696
Oscillating CN (2 and 3 states)	5, 5
CN segments	18
FDR fragment joints	0.6776251
FDR chr. breakp. enrich.	0.01
Linked to chrs	3:94442780−174075989;6:42555034−48239598
Purity, ploidy	0.62, 3.8

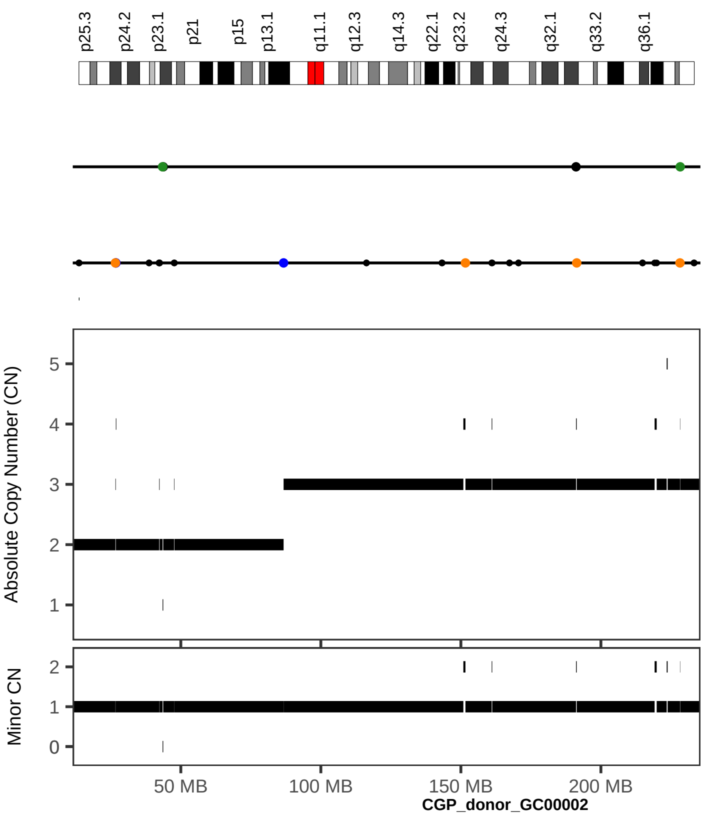

CGP_donor_GC00002

Cancer type	Stomach−AdenoCA
Position	2:13611641−233338366
Interleaved intrachr. SVs	1
Total SVs (intrachr. + transl.)	22
SV types	DEL: 1; DUP: 10; h2hINV: 0; t2tINV: 0; TRA: 11
SVs in sample	442
Oscillating CN (2 and 3 states)	9, 13
CN segments	23
FDR fragment joints	0.1148725
FDR chr. breakp. enrich.	0.14
Linked to chrs	5:7410479−164692272;
Purity, ploidy	0.48, 3.17

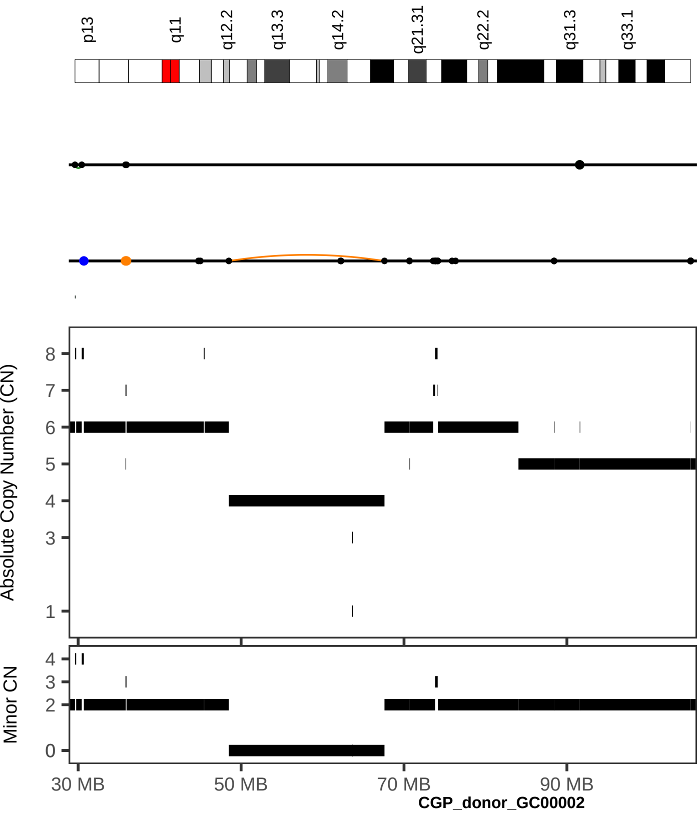

CGP_donor_GC00002

Cancer type	Stomach−AdenoCA
Position	13:73586014−105196786
Interleaved intrachr. SVs	3
Total SVs (intrachr. + transl.)	8
SV types	DEL: 2; DUP: 4; h2hINV: 0; t2tINV: 0; TRA: 2
SVs in sample	442
Oscillating CN (2 and 3 states)	8, 11
CN segments	11
FDR fragment joints	0.615458
FDR chr. breakp. enrich.	0.54
Linked to chrs	
Purity, ploidy	0.48, 3.17

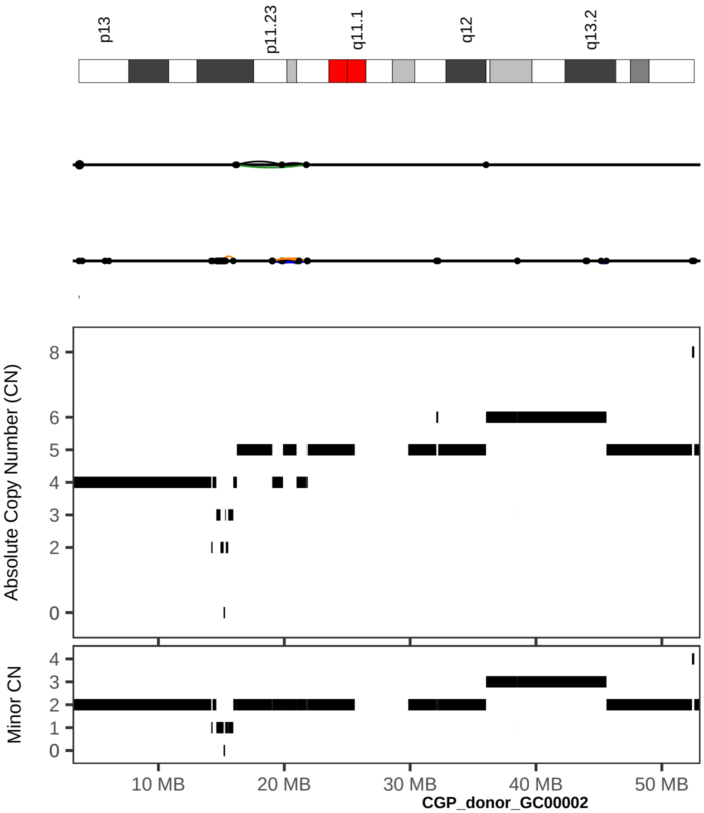

CGP_donor_GC00002

Cancer type	Stomach−AdenoCA
Position	20:16117248−21865896
Interleaved intrachr. SVs	10
Total SVs (intrachr. + transl.)	10
SV types	DEL: 3; DUP: 3; h2hINV: 2; t2tINV: 2; TRA: 0
SVs in sample	442
Oscillating CN (2 and 3 states)	10, 10
CN segments	10
FDR fragment joints	0.9625775
FDR chr. breakp. enrich.	0
Linked to chrs	
Purity, ploidy	0.48, 3.17

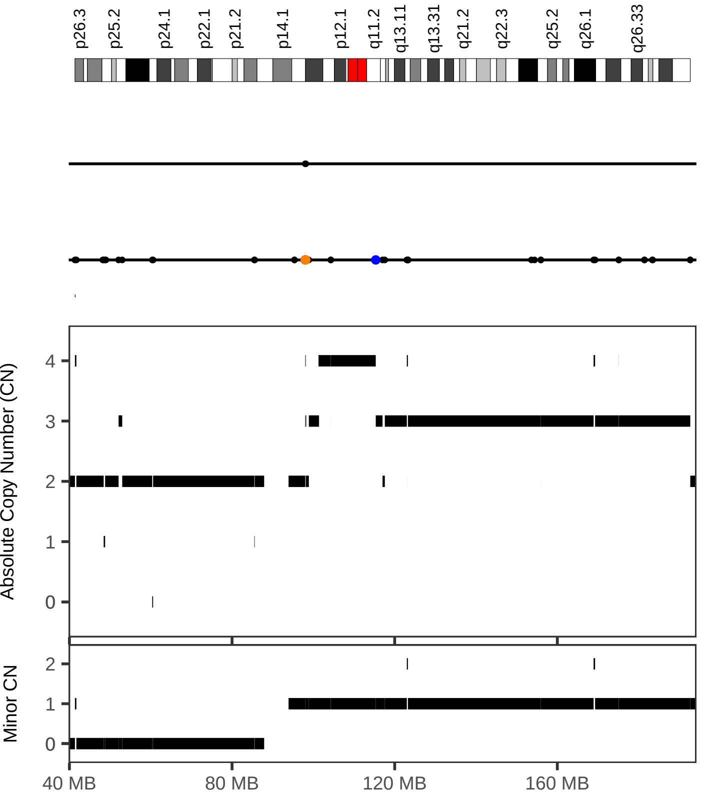

CGP_donor_GC00004

Cancer type	Stomach−AdenoCA
Position	3:41357782−183461229
Interleaved intrachr. SVs	1
Total SVs (intrachr. + transl.)	20
SV types	DEL: 9; DUP: 6; h2hINV: 1; t2tINV: 0; TRA: 4
SVs in sample	339
Oscillating CN (2 and 3 states)	7, 15
CN segments	33
FDR fragment joints	0.05741733
FDR chr. breakp. enrich.	1
Linked to chrs	
Purity, ploidy	0.24, 2.01

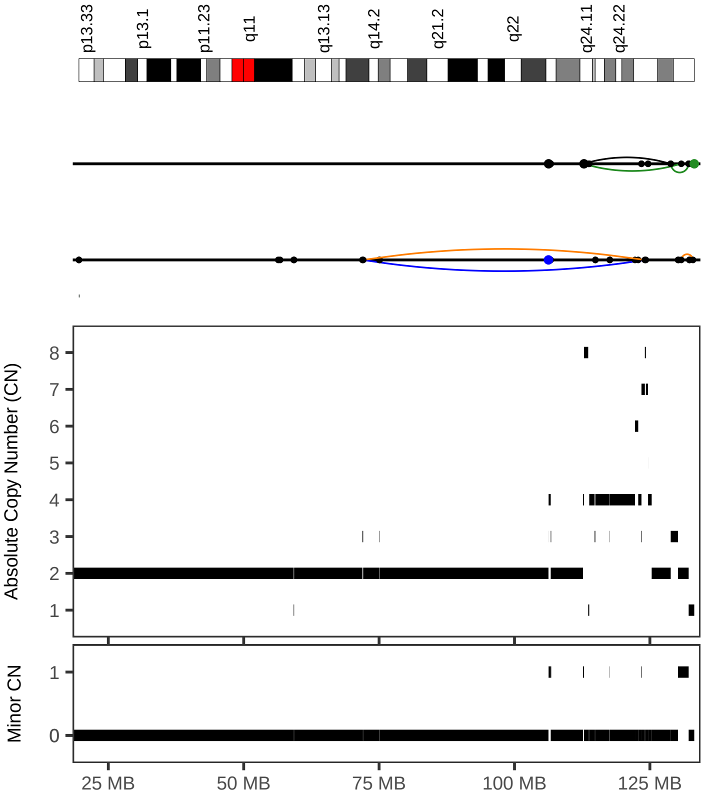

CGP_donor_GC00004

Cancer type	Stomach−AdenoCA
Position	12:71919868−132958560
Interleaved intrachr. SVs	6
Total SVs (intrachr. + transl.)	9
SV types	DEL: 2; DUP: 1; h2hINV: 1; t2tINV: 2; TRA: 3
SVs in sample	339
Oscillating CN (2 and 3 states)	5, 9
CN segments	29
FDR fragment joints	0.6776251
FDR chr. breakp. enrich.	0.02
Linked to chrs	
Purity, ploidy	0.24, 2.01

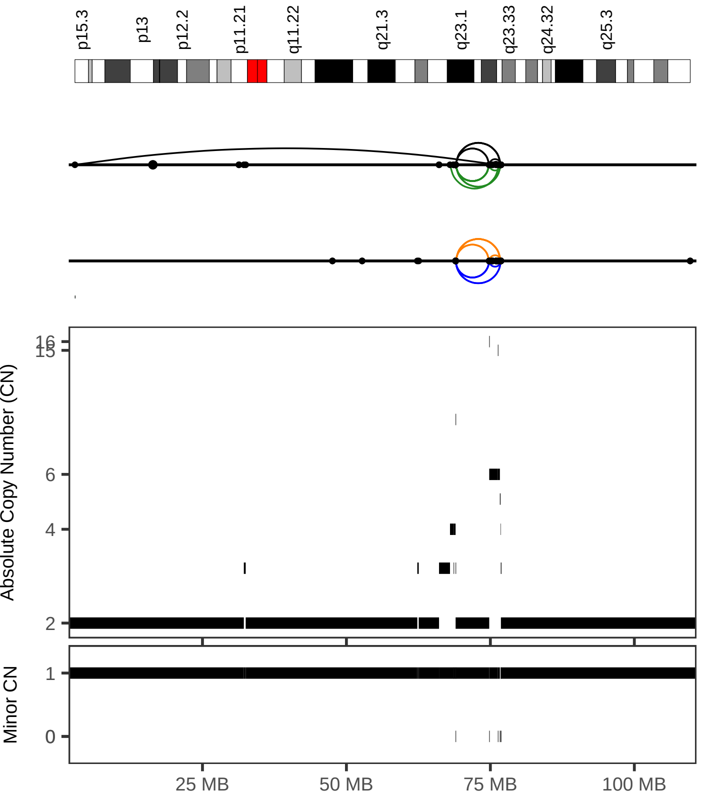

CGP_donor_GC00008

Cancer type	Stomach−AdenoCA
Position	10:2847784−76828663
Interleaved intrachr. SVs	40
Total SVs (intrachr. + transl.)	41
SV types	DEL: 9; DUP: 11; h2hINV: 11; t2tINV: 9; TRA: 1
SVs in sample	307
Oscillating CN (2 and 3 states)	6, 9
CN segments	23
FDR fragment joints	0.9810656
FDR chr. breakp. enrich.	0
Linked to chrs	
Purity, ploidy	0.93, 2.06

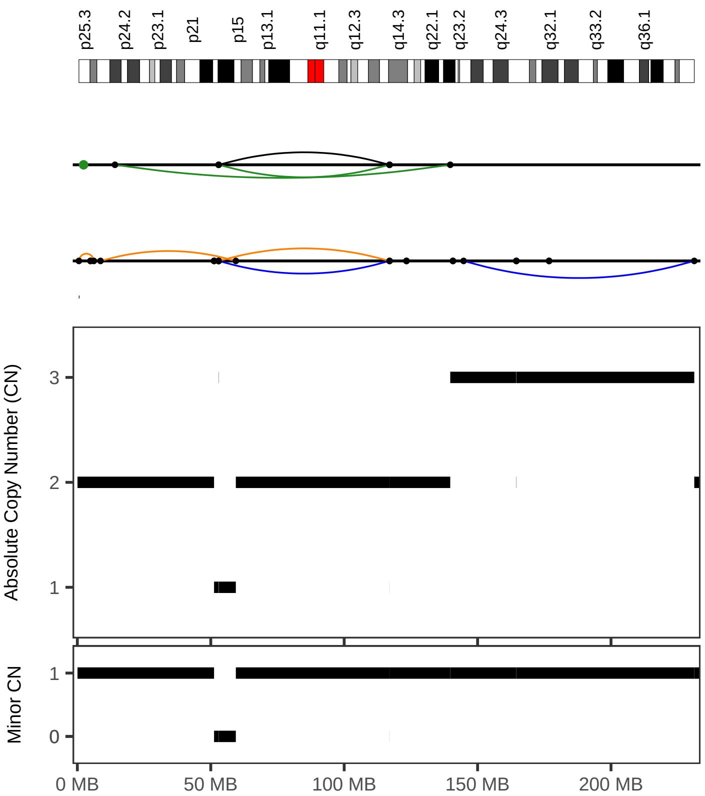

CGP_donor_GC00022

Cancer type	Stomach−AdenoCA
Position	2:8672161−139745881
Interleaved intrachr. SVs	7
Total SVs (intrachr. + transl.)	7
SV types	DEL: 2; DUP: 2; h2hINV: 1; t2tINV: 2; TRA: 0
SVs in sample	149
Oscillating CN (2 and 3 states)	4, 7
CN segments	8
FDR fragment joints	0.9599662
FDR chr. breakp. enrich.	0.55
Linked to chrs	
Purity, ploidy	0.45, 1.66

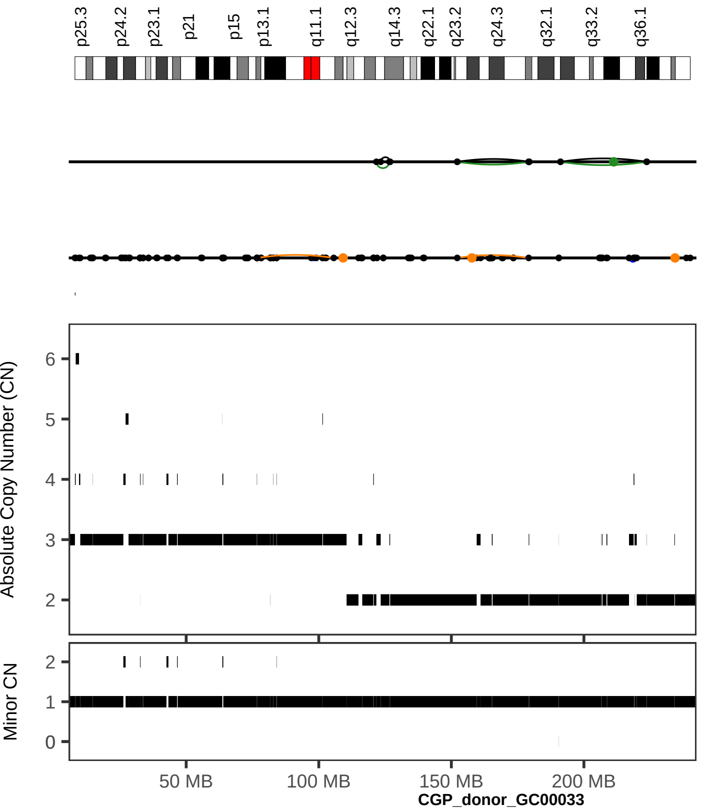

CGP_donor_GC00033

Cancer type	Stomach−AdenoCA
Position	2:120552045−179365153
Interleaved intrachr. SVs	4
Total SVs (intrachr. + transl.)	9
SV types	DEL: 1; DUP: 2; h2hINV: 3; t2tINV: 2; TRA: 1
SVs in sample	925
Oscillating CN (2 and 3 states)	11, 12
CN segments	12
FDR fragment joints	0.9723381
FDR chr. breakp. enrich.	0.65
Linked to chrs	
Purity, ploidy	0.49, 2.4

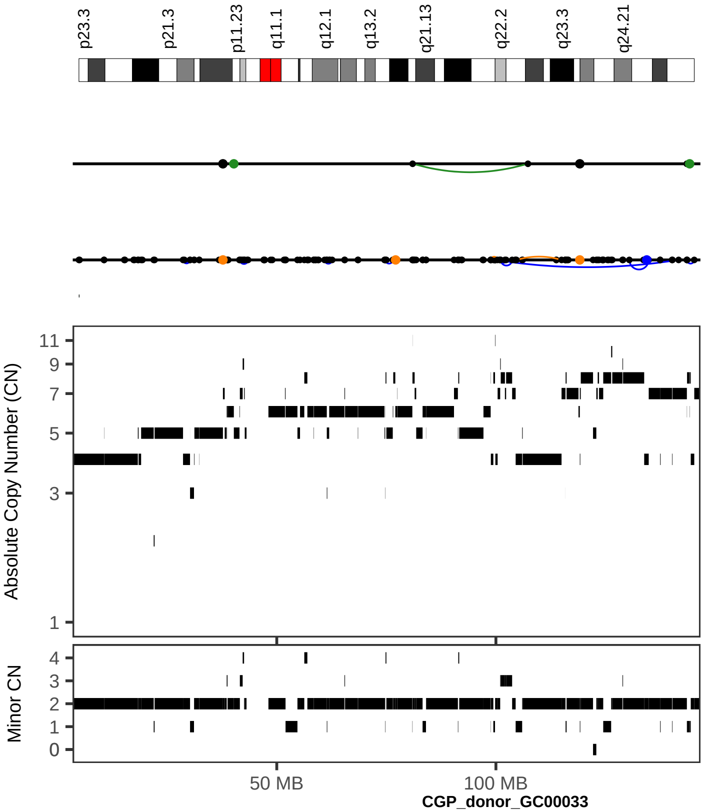

CGP_donor_GC00033

Cancer type	Stomach−AdenoCA
Position	8:80936461−141686980
Interleaved intrachr. SVs	8
Total SVs (intrachr. + transl.)	11
SV types	DEL: 2; DUP: 5; h2hINV: 0; t2tINV: 1; TRA: 3
SVs in sample	925
Oscillating CN (2 and 3 states)	6, 7
CN segments	51
FDR fragment joints	0.5435077
FDR chr. breakp. enrich.	0
Linked to chrs	10:6484853−104269464;
Purity, ploidy	0.49, 2.4

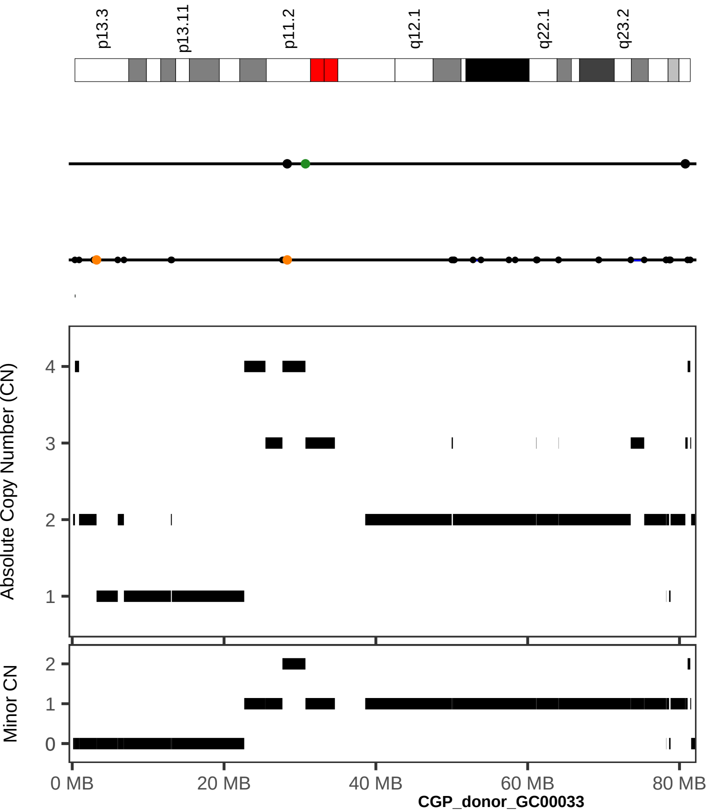

CGP_donor_GC00033

Cancer type	Stomach−AdenoCA
Position	16:353528−81417990
Interleaved intrachr. SVs	1
Total SVs (intrachr. + transl.)	22
SV types	DEL: 4; DUP: 13; h2hINV: 0; t2tINV: 0; TRA: 5
SVs in sample	925
Oscillating CN (2 and 3 states)	10, 26
CN segments	27
FDR fragment joints	0.07914058
FDR chr. breakp. enrich.	1
Linked to chrs	3:98369145−186328429;
Purity, ploidy	0.49, 2.4

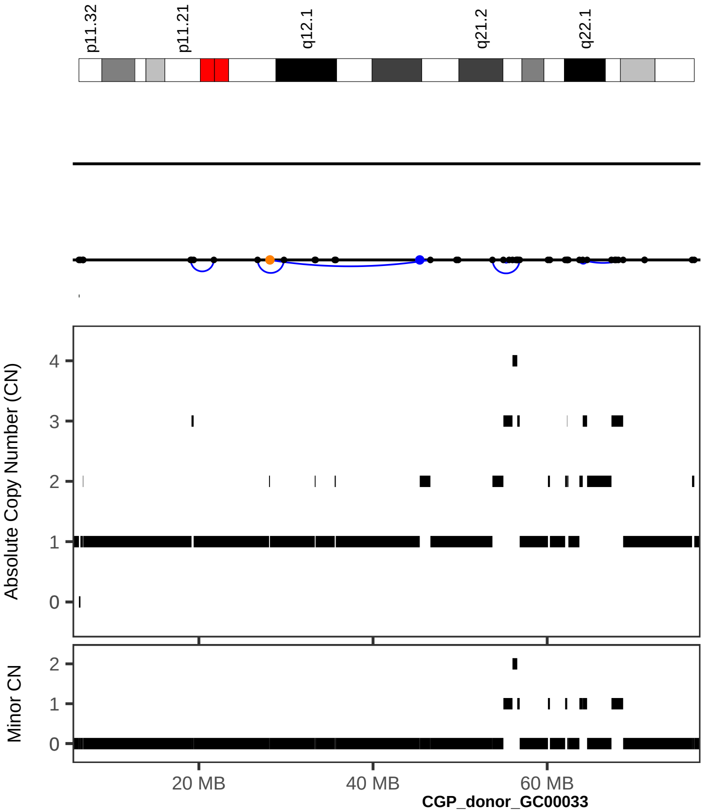

CGP_donor_GC00033

Cancer type	Stomach−AdenoCA
Position	18:26736270−68720210
Interleaved intrachr. SVs	2
Total SVs (intrachr. + transl.)	10
SV types	DEL: 0; DUP: 8; h2hINV: 0; t2tINV: 0; TRA: 2
SVs in sample	925
Oscillating CN (2 and 3 states)	10, 13
CN segments	25
FDR fragment joints	0.01020027
FDR chr. breakp. enrich.	1
Linked to chrs	4:90264273−134826325;
Purity, ploidy	0.49, 2.4

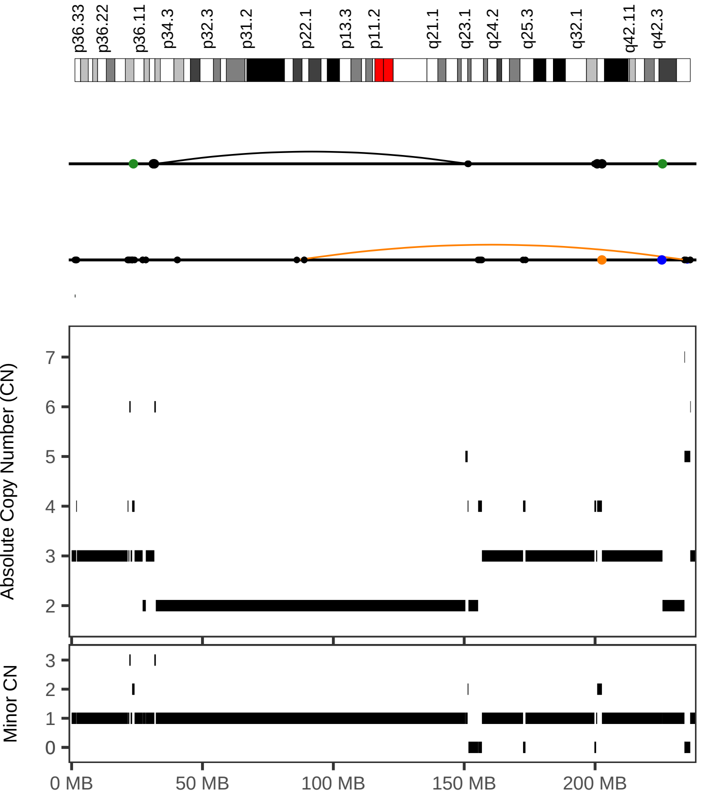

CGP_donor_GC00049

Cancer type	Stomach−AdenoCA
Position	1:32164458−236360311
Interleaved intrachr. SVs	4
Total SVs (intrachr. + transl.)	9
SV types	DEL: 1; DUP: 2; h2hINV: 1; t2tINV: 0; TRA: 5
SVs in sample	223
Oscillating CN (2 and 3 states)	8, 9
CN segments	17
FDR fragment joints	0.9723381
FDR chr. breakp. enrich.	0.09
Linked to chrs	
Purity, ploidy	0.23, 2.18

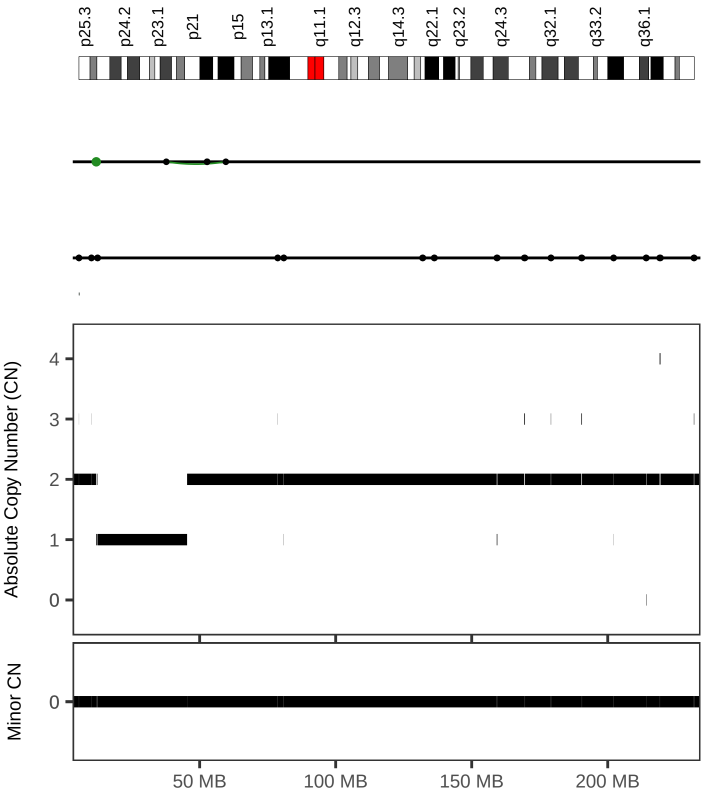

CGP_donor_GC00052

Cancer type	Stomach−AdenoCA
Position	2:5620025−231799627
Interleaved intrachr. SVs	1
Total SVs (intrachr. + transl.)	17
SV types	DEL: 4; DUP: 11; h2hINV: 0; t2tINV: 1; TRA: 1
SVs in sample	309
Oscillating CN (2 and 3 states)	7, 22
CN segments	28
FDR fragment joints	0.09737474
FDR chr. breakp. enrich.	0.31
Linked to chrs	
Purity, ploidy	0.44, 2.18

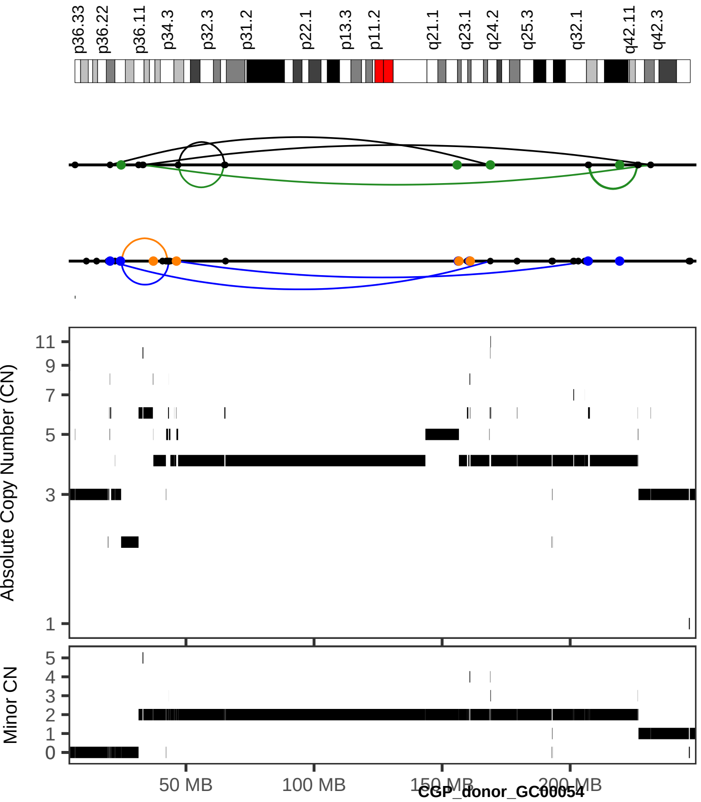

CGP_donor_GC00054

Cancer type	Stomach−AdenoCA
Position	1:20036804−231461837
Interleaved intrachr. SVs	14
Total SVs (intrachr. + transl.)	28
SV types	DEL: 1; DUP: 6; h2hINV: 3; t2tINV: 4; TRA: 14
SVs in sample	550
Oscillating CN (2 and 3 states)	5, 11
CN segments	67
FDR fragment joints	0.615458
FDR chr. breakp. enrich.	0.07
Linked to chrs	12:18101997−94069585;19:28404001−30805307
Purity, ploidy	0.34, 4.27

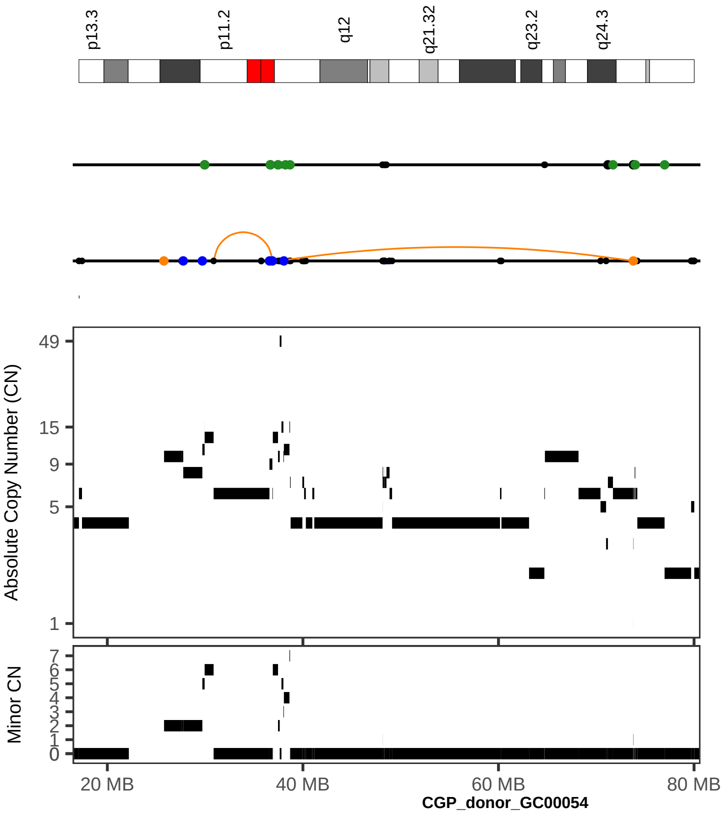

CGP_donor_GC00054

Cancer type	Stomach−AdenoCA
Position	17:37680918−49117586
Interleaved intrachr. SVs	9
Total SVs (intrachr. + transl.)	21
SV types	DEL: 3; DUP: 6; h2hINV: 4; t2tINV: 5; TRA: 3
SVs in sample	550
Oscillating CN (2 and 3 states)	5, 5
CN segments	20
FDR fragment joints	0.7568568
FDR chr. breakp. enrich.	0
Linked to chrs	
Purity, ploidy	0.34, 4.27

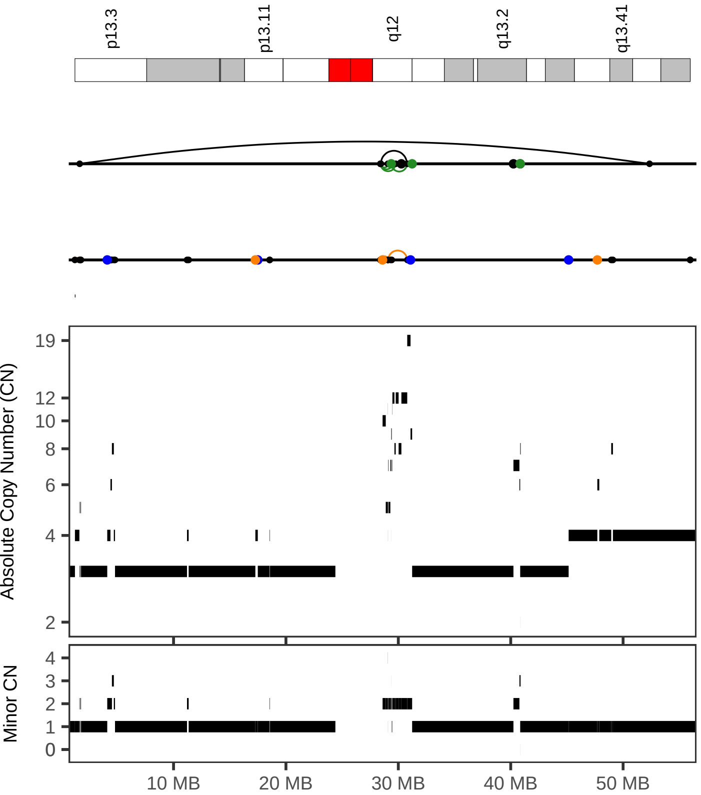

CGP_donor_GC00054

Cancer type	Stomach−AdenoCA
Position	19:28404001−30805308
Interleaved intrachr. SVs	12
Total SVs (intrachr. + transl.)	15
SV types	DEL: 3; DUP: 1; h2hINV: 3; t2tINV: 5; TRA: 3
SVs in sample	550
Oscillating CN (2 and 3 states)	5, 5
CN segments	20
FDR fragment joints	0.615458
FDR chr. breakp. enrich.	0
Linked to chrs	17:37680918−49117585;
Purity, ploidy	0.34, 4.27

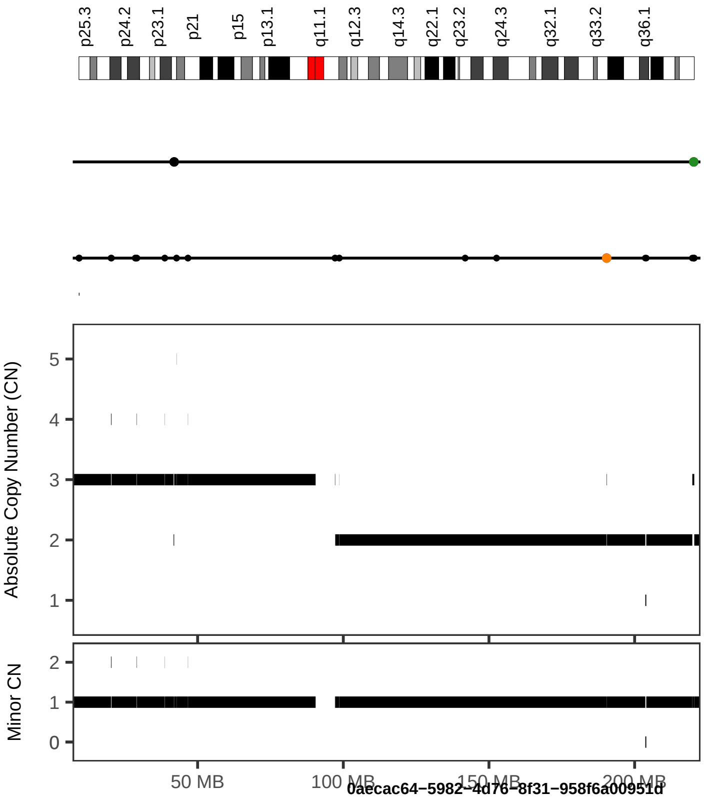

0aecac64−5982−4d76−8f31−958f6a00951d

Cancer type	Stomach−AdenoCA
Position	2:9254169−220466625
Interleaved intrachr. SVs	1
Total SVs (intrachr. + transl.)	20
SV types	DEL: 1; DUP: 13; h2hINV: 0; t2tINV: 0; TRA: 6
SVs in sample	332
Oscillating CN (2 and 3 states)	7, 11
CN segments	23
FDR fragment joints	0.01333161
FDR chr. breakp. enrich.	0.52
Linked to chrs	17:48341217−72770354;3:4802945−186280724
Purity, ploidy	0.44, 1.75

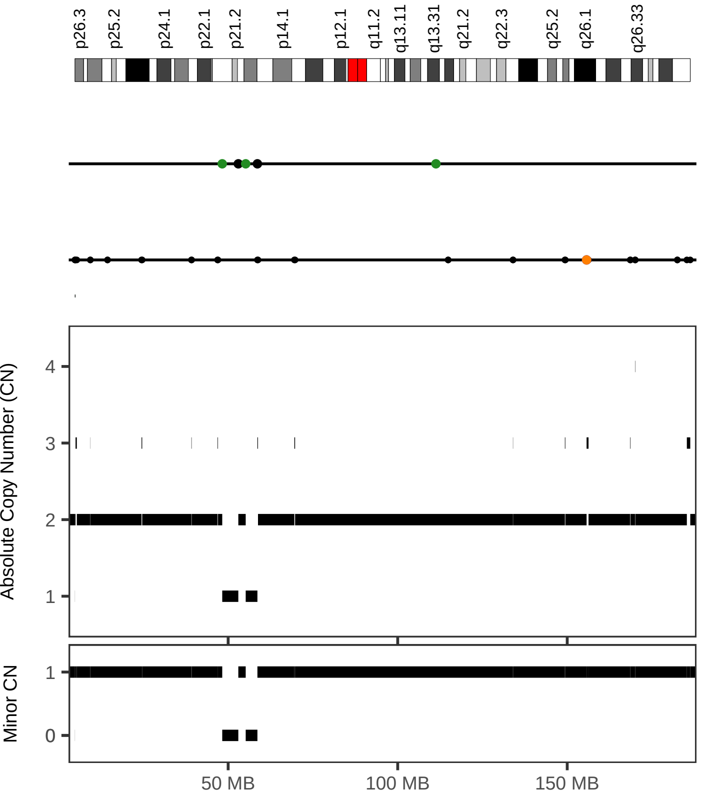

0aecac64−5982−4d76−8f31−958f6a00951d

Cancer type	Stomach−AdenoCA
Position	3:4802945−186280725
Interleaved intrachr. SVs	1
Total SVs (intrachr. + transl.)	24
SV types	DEL: 2; DUP: 15; h2hINV: 0; t2tINV: 0; TRA: 7
SVs in sample	332
Oscillating CN (2 and 3 states)	12, 20
CN segments	31
FDR fragment joints	0.0123263
FDR chr. breakp. enrich.	0.87
Linked to chrs	2:9254169−220466624;
Purity, ploidy	0.44, 1.75

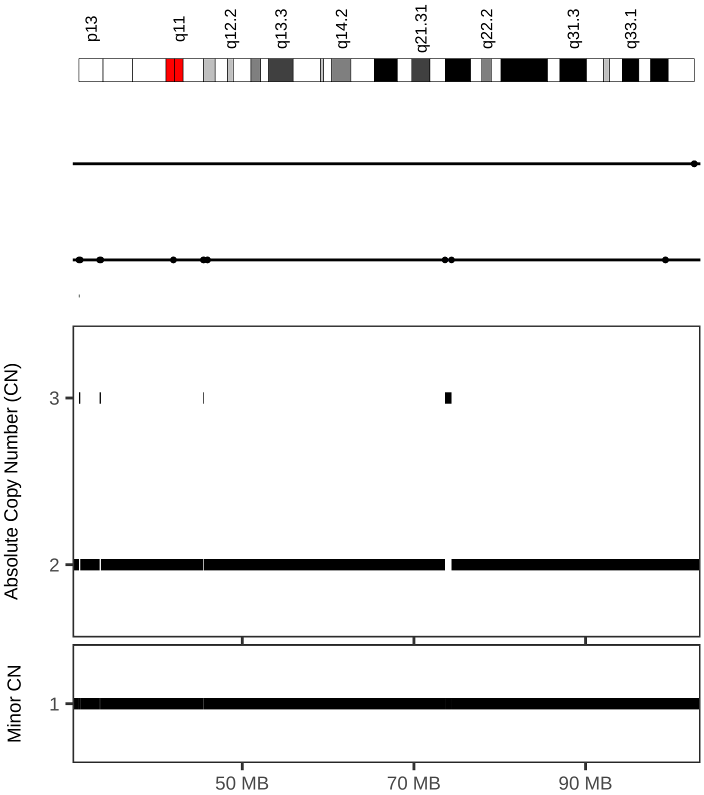

0aecac64−5982−4d76−8f31−958f6a00951d

Cancer type	Stomach−AdenoCA
Position	13:30969347−99319808
Interleaved intrachr. SVs	1
Total SVs (intrachr. + transl.)	6
SV types	DEL: 0; DUP: 6; h2hINV: 0; t2tINV: 0; TRA: 0
SVs in sample	332
Oscillating CN (2 and 3 states)	8, 8
CN segments	8
FDR fragment joints	0.04139017
FDR chr. breakp. enrich.	0.78
Linked to chrs	
Purity, ploidy	0.44, 1.75

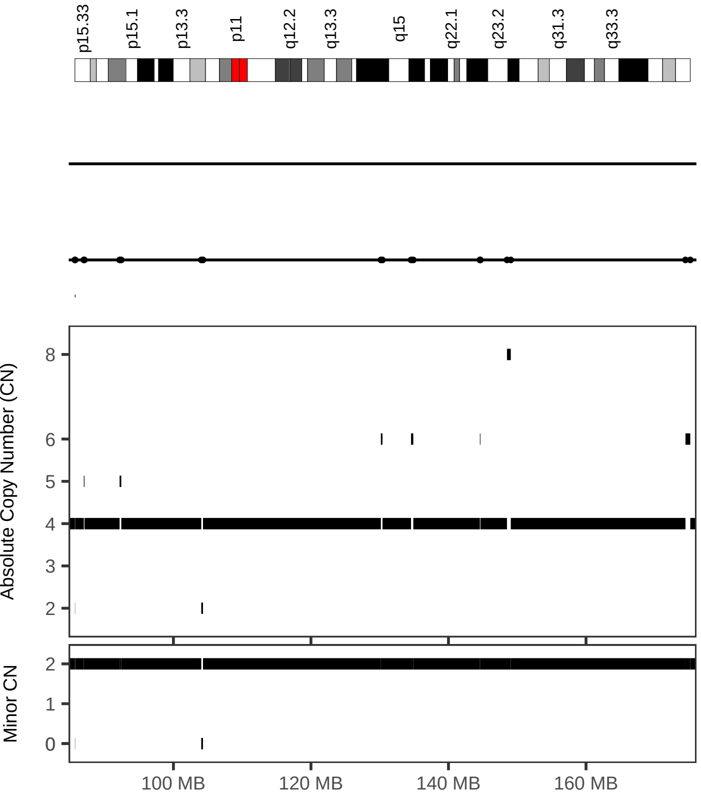

1ca3c5e0−32b0−4467−8ec0−ca212e35d2b3

Cancer type	Stomach−AdenoCA
Position	5:85673816−175159134
Interleaved intrachr. SVs	1
Total SVs (intrachr. + transl.)	9
SV types	DEL: 2; DUP: 7; h2hINV: 0; t2tINV: 0; TRA: 0
SVs in sample	313
Oscillating CN (2 and 3 states)	7, 11
CN segments	18
FDR fragment joints	0.1178349
FDR chr. breakp. enrich.	0.1
Linked to chrs	
Purity, ploidy	0.3, 3.61

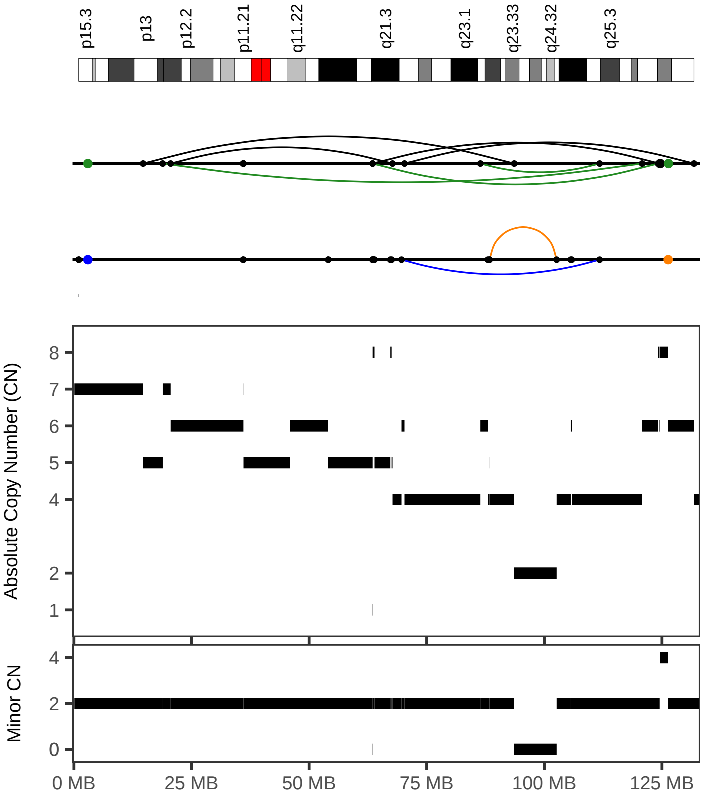

1ca3c5e0−32b0−4467−8ec0−ca212e35d2b3

Cancer type	Stomach−AdenoCA
Position	10:14710620−131823830
Interleaved intrachr. SVs	12
Total SVs (intrachr. + transl.)	15
SV types	DEL: 2; DUP: 3; h2hINV: 4; t2tINV: 3; TRA: 3
SVs in sample	313
Oscillating CN (2 and 3 states)	5, 8
CN segments	29
FDR fragment joints	0.925252
FDR chr. breakp. enrich.	0.04
Linked to chrs	
Purity, ploidy	0.3, 3.61

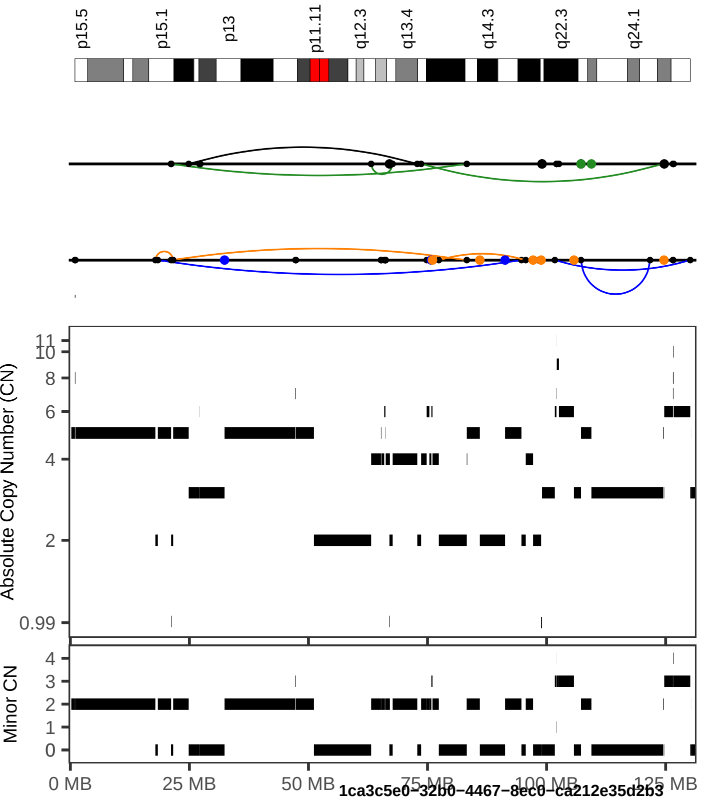

1ca3c5e0−32b0−4467−8ec0−ca212e35d2b3

Cancer type	Stomach−AdenoCA
Position	11:17850371−130169015
Interleaved intrachr. SVs	7
Total SVs (intrachr. + transl.)	23
SV types	DEL: 3; DUP: 2; h2hINV: 0; t2tINV: 2; TRA: 16
SVs in sample	313
Oscillating CN (2 and 3 states)	5, 7
CN segments	56
FDR fragment joints	0.615458
FDR chr. breakp. enrich.	0
Linked to chrs	13:27261037−79173978;6:30271712−157786892
Purity, ploidy	0.3, 3.61

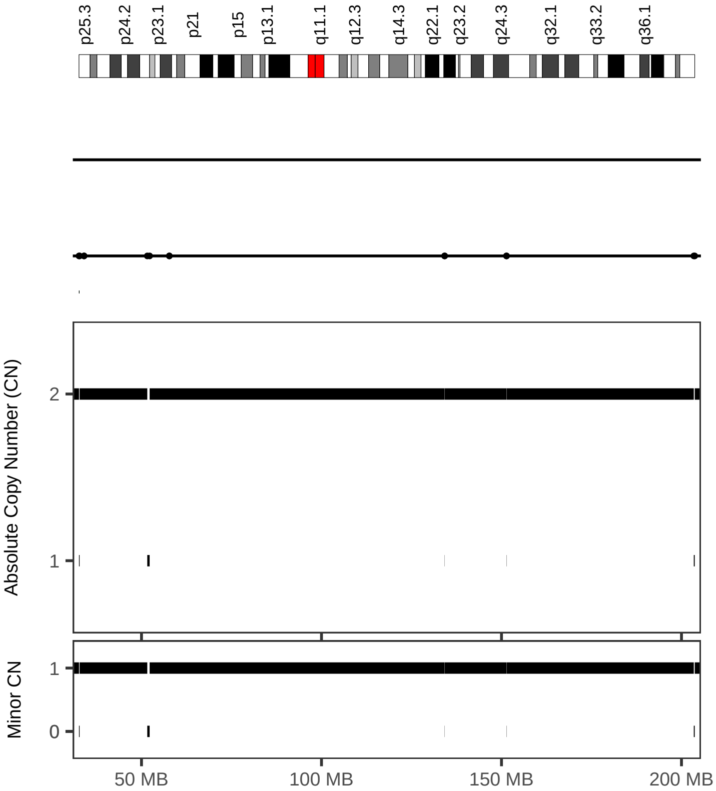

2b4f014f−74e9−4dda−a1ad−abcbd124fc44

Cancer type	Stomach−AdenoCA
Position	2:32631150−203682926
Interleaved intrachr. SVs	1
Total SVs (intrachr. + transl.)	5
SV types	DEL: 5; DUP: 0; h2hINV: 0; t2tINV: 0; TRA: 0
SVs in sample	94
Oscillating CN (2 and 3 states)	10, 10
CN segments	10
FDR fragment joints	0.1013295
FDR chr. breakp. enrich.	1
Linked to chrs	
Purity, ploidy	0.56, 2.02

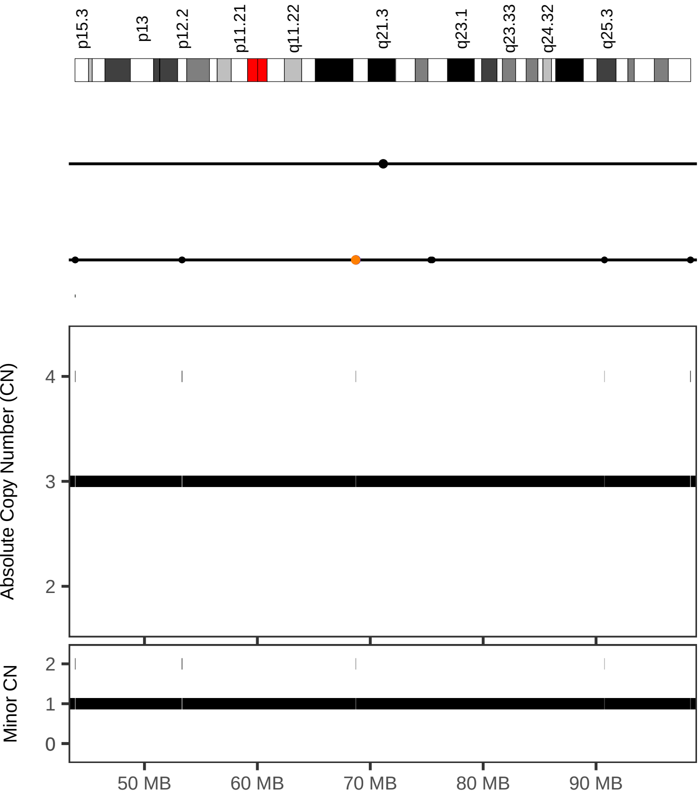

337819d2−5281−4b41−9583−a476924bc837

Cancer type	Stomach−AdenoCA
Position	10:43843093−98387881
Interleaved intrachr. SVs	1
Total SVs (intrachr. + transl.)	9
SV types	DEL: 0; DUP: 6; h2hINV: 0; t2tINV: 0; TRA: 3
SVs in sample	210
Oscillating CN (2 and 3 states)	10, 10
CN segments	10
FDR fragment joints	0.1488029
FDR chr. breakp. enrich.	1
Linked to chrs	
Purity, ploidy	0.32, 2.97

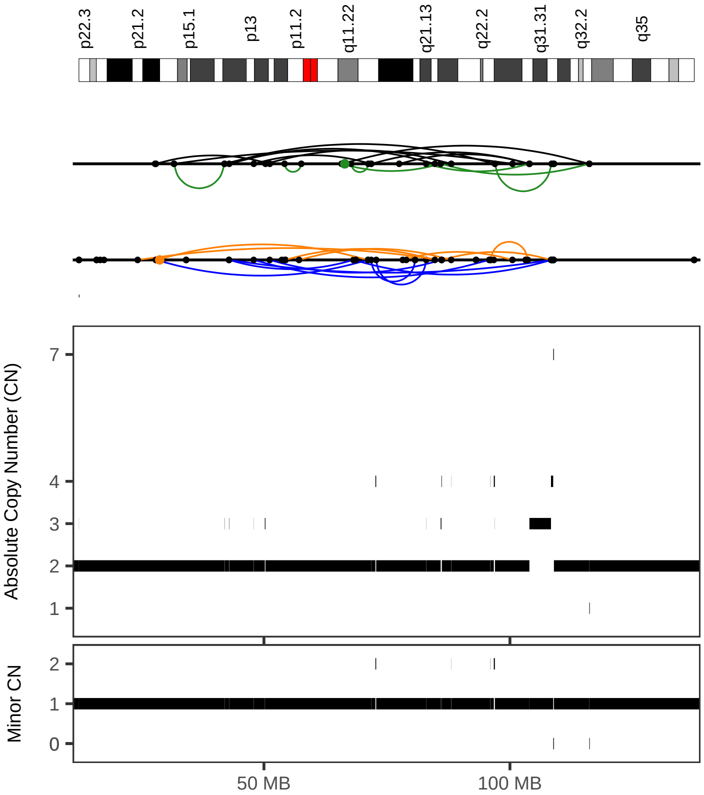

53086e20−a727−40d6−b42d−8030109b5130

Cancer type	Stomach−AdenoCA
Position	7:24323683−116135143
Interleaved intrachr. SVs	34
Total SVs (intrachr. + transl.)	37
SV types	DEL: 7; DUP: 10; h2hINV: 9; t2tINV: 8; TRA: 3
SVs in sample	246
Oscillating CN (2 and 3 states)	9, 9
CN segments	30
FDR fragment joints	0.9787948
FDR chr. breakp. enrich.	0
Linked to chrs	
Purity, ploidy	0.83, 1.96

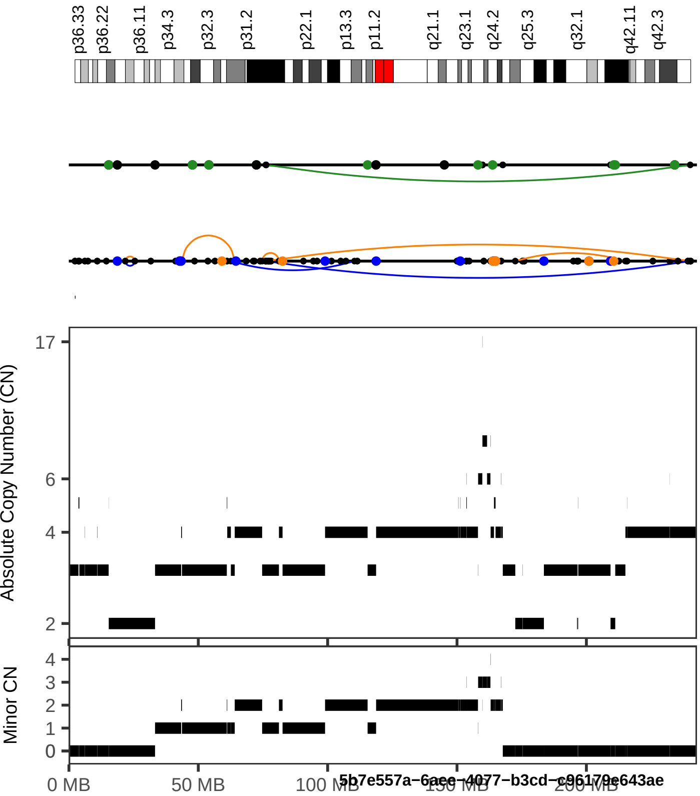

5b7e557a−6aee−4077−b3cd−c96179e643ae

Cancer type	Stomach−AdenoCA
Position	1:43676646−240124427
Interleaved intrachr. SVs	6
Total SVs (intrachr. + transl.)	38
SV types	DEL: 3; DUP: 2; h2hINV: 0; t2tINV: 1; TRA: 32
SVs in sample	1150
Oscillating CN (2 and 3 states)	9, 16
CN segments	46
FDR fragment joints	0.8784945
FDR chr. breakp. enrich.	0.13
Linked to chrs	11:686421−122325230;14:21910299−106251174 15:29417192−101670490;6:7731685−152831765 8:21266054−103900102;

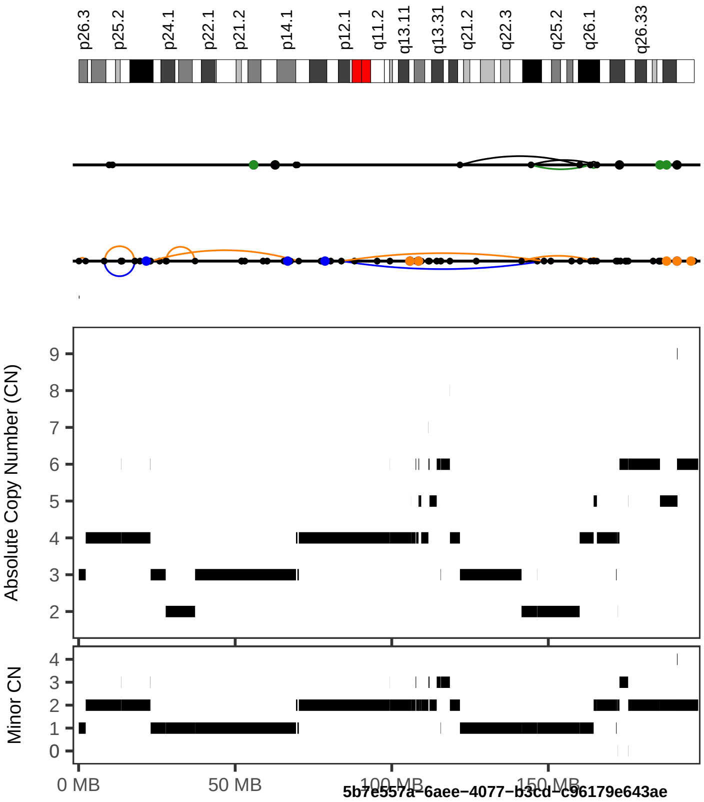

5b7e557a−6aee−4077−b3cd−c96179e643ae

Cancer type	Stomach−AdenoCA
Position	3:83906659−165520150
Interleaved intrachr. SVs	9
Total SVs (intrachr. + transl.)	13
SV types	DEL: 3; DUP: 1; h2hINV: 3; t2tINV: 2; TRA: 4
SVs in sample	1150
Oscillating CN (2 and 3 states)	4, 11
CN segments	26
FDR fragment joints	0.7995907
FDR chr. breakp. enrich.	0.76
Linked to chrs	11:686421−122325230;7:17657943−75547479
Purity, ploidy	0.22, 3.05

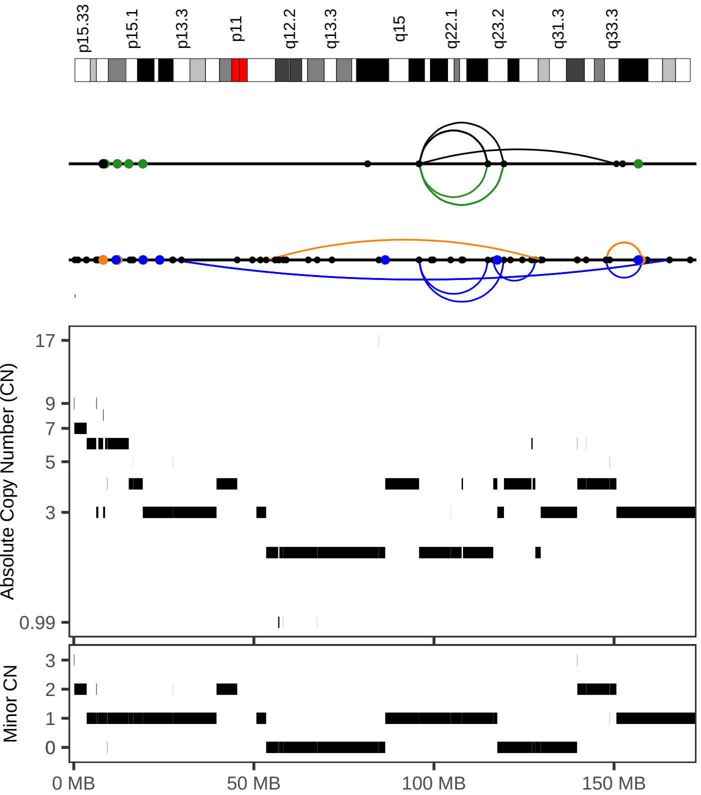

5b7e557a−6aee−4077−b3cd−c96179e643ae

Cancer type	Stomach−AdenoCA
Position	5:53410286−157767053
Interleaved intrachr. SVs	15
Total SVs (intrachr. + transl.)	20
SV types	DEL: 3; DUP: 4; h2hINV: 5; t2tINV: 3; TRA: 5
SVs in sample	1150
Oscillating CN (2 and 3 states)	7, 10
CN segments	29
FDR fragment joints	0.6776251
FDR chr. breakp. enrich.	0.27
Linked to chrs	
Purity, ploidy	0.22, 3.05

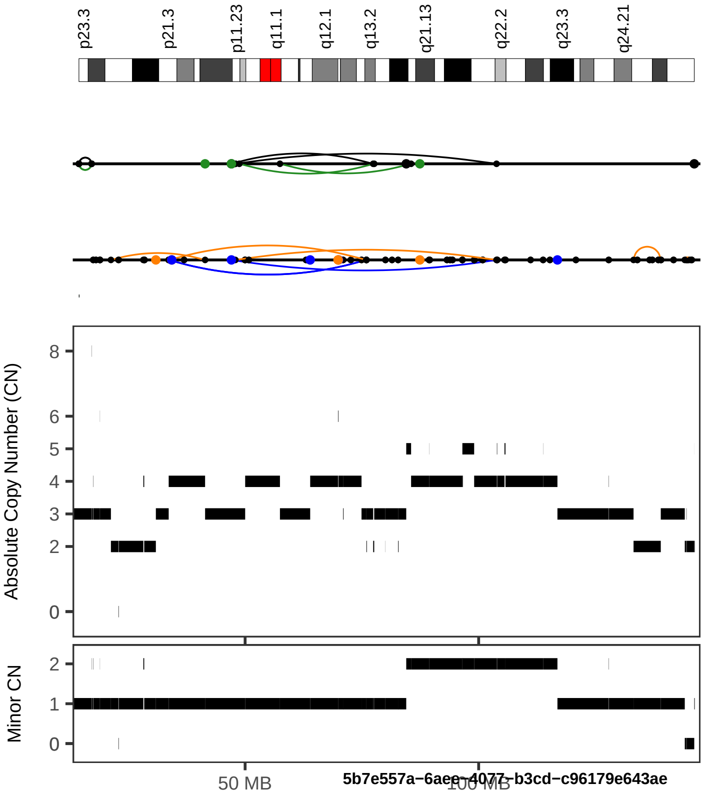

5b7e557a−6aee−4077−b3cd−c96179e643ae

Cancer type	Stomach−AdenoCA
Position	8:21266054−103900103
Interleaved intrachr. SVs	10
Total SVs (intrachr. + transl.)	24
SV types	DEL: 3; DUP: 3; h2hINV: 2; t2tINV: 2; TRA: 14
SVs in sample	1150
Oscillating CN (2 and 3 states)	9, 11
CN segments	33
FDR fragment joints	0.7568568
FDR chr. breakp. enrich.	0.01
Linked to chrs	1:43676646−240124426;12:47965497−122244786 20:30101824−57105675;7:17657943−75547479
[illegible]	[illegible]

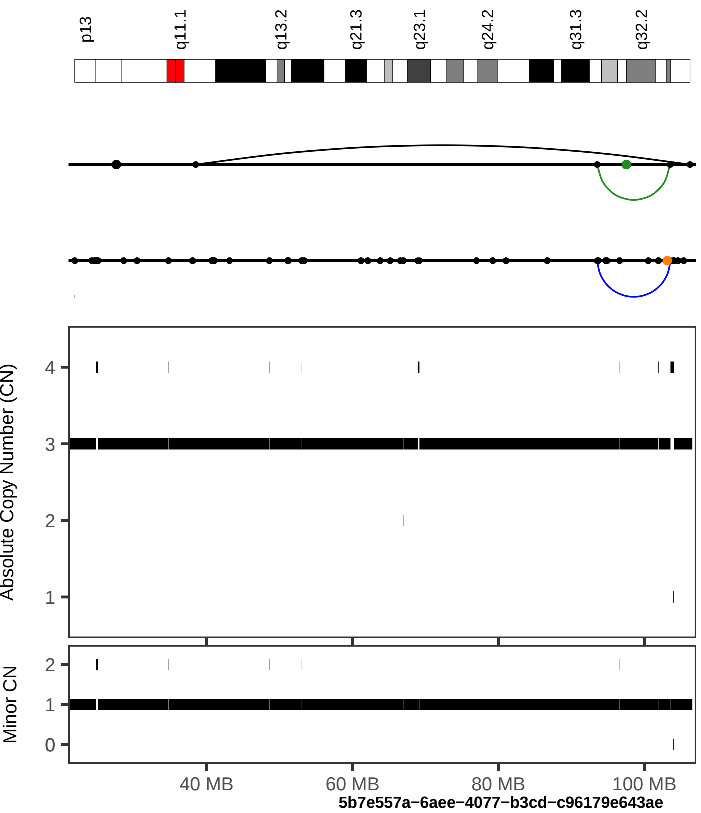

5b7e557a−6aee−4077−b3cd−c96179e643ae

Cancer type	Stomach−AdenoCA
Position	14:21910299−106251175
Interleaved intrachr. SVs	1
Total SVs (intrachr. + transl.)	28
SV types	DEL: 0; DUP: 21; h2hINV: 1; t2tINV: 1; TRA: 5
SVs in sample	1150
Oscillating CN (2 and 3 states)	9, 21
CN segments	21
FDR fragment joints	4.035888e−05
FDR chr. breakp. enrich.	0.17
Linked to chrs	1:43676646−240124426;
Purity, ploidy	0.22, 3.05

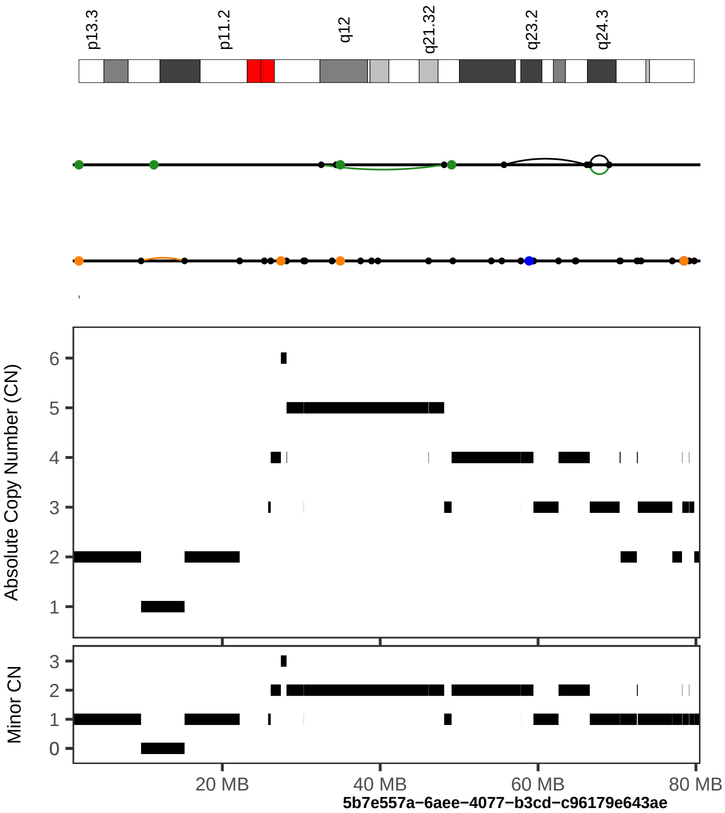

5b7e557a−6aee−4077−b3cd−c96179e643ae

Cancer type	Stomach−AdenoCA
Position	17:9703698−79150095
Interleaved intrachr. SVs	1
Total SVs (intrachr. + transl.)	27
SV types	DEL: 2; DUP: 13; h2hINV: 2; t2tINV: 2; TRA: 8
SVs in sample	1150
Oscillating CN (2 and 3 states)	8, 19
CN segments	27
FDR fragment joints	0.1278363
FDR chr. breakp. enrich.	0.03
Linked to chrs	18:518539−70320165;
Purity, ploidy	0.22, 3.05

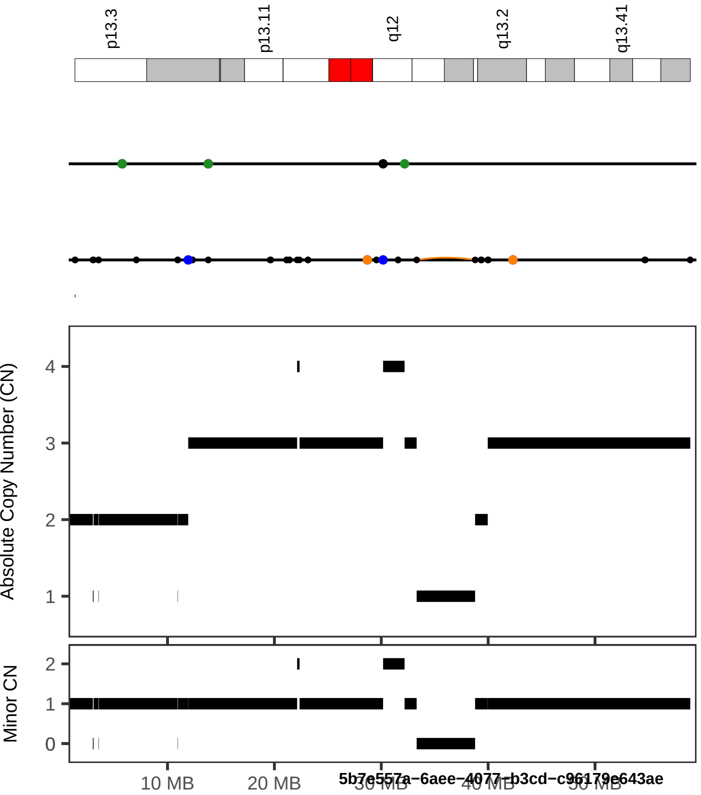

5b7e557a-6aee-4077-b3cd-c96179e643ae

Cancer type	Stomach−AdenoCA
Position	19:1335997−58908317
Interleaved intrachr. SVs	1
Total SVs (intrachr. + transl.)	25
SV types	DEL: 1; DUP: 12; h2hINV: 0; t2tINV: 0; TRA: 12
SVs in sample	1150
Oscillating CN (2 and 3 states)	7, 12
CN segments	15
FDR fragment joints	0.02326306
FDR chr. breakp. enrich.	0.1
Linked to chrs	11:686421−122325230;18:518539−70320165 20:30101824−57105675;6:7731685−152831765
Purity, ploidy	[illegible]

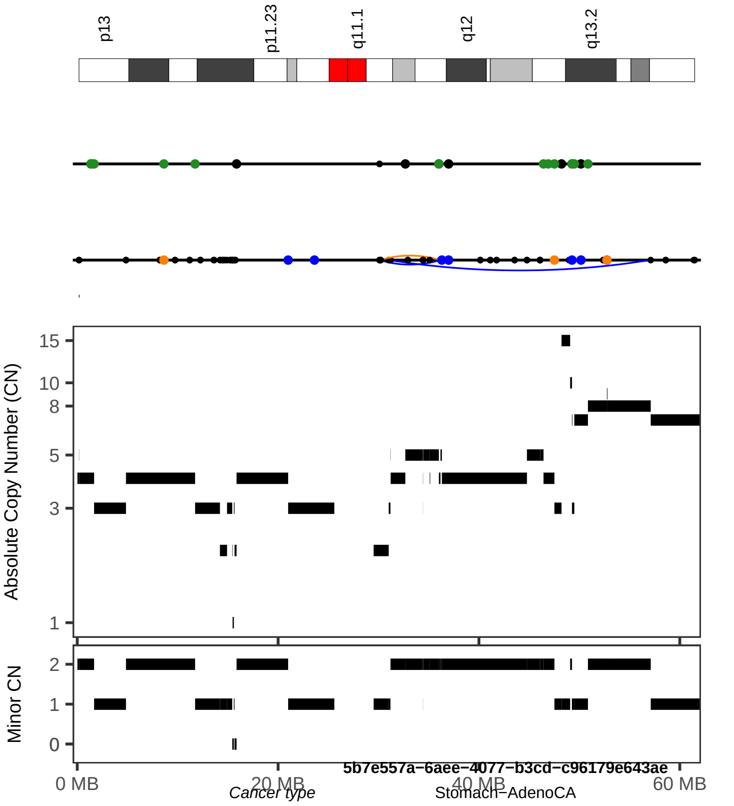

5b7e557a-6aee-4077-b3cd-c96179e643ae

Cancer type	Stomach−AdenoCA
Position	20:30101824−57105676
Interleaved intrachr. SVs	3
Total SVs (intrachr. + transl.)	25
SV types	DEL: 1; DUP: 2; h2hINV: 0; t2tINV: 0; TRA: 22
SVs in sample	1150
Oscillating CN (2 and 3 states)	10, 11
CN segments	27
FDR fragment joints	0.9599662
FDR chr. breakp. enrich.	0
Linked to chrs	1:43676646−240124426;11:686421−122325230 17:9703698−79150094;5:53410286−157767052 7:17657943−75547479;8:21266054−103900102

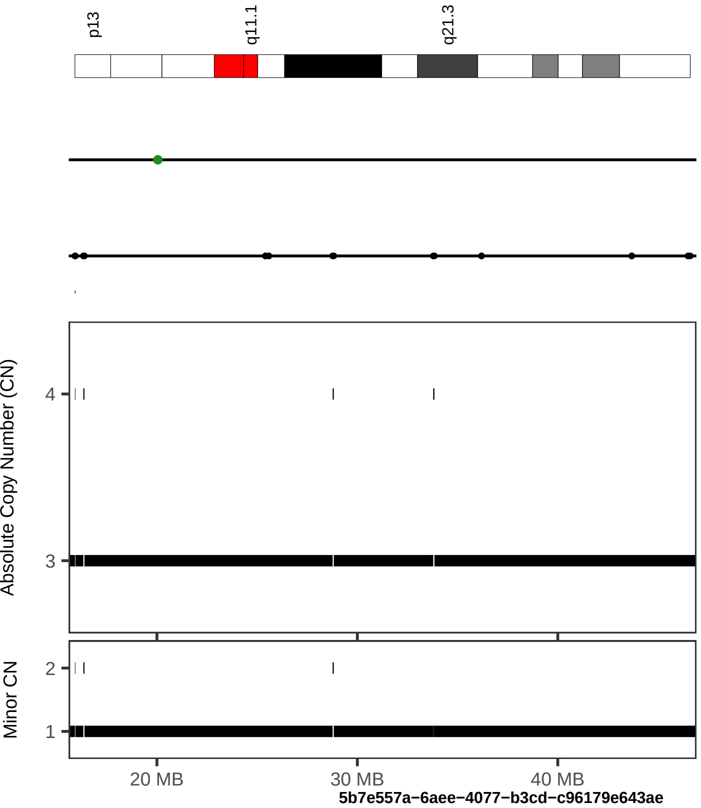

5b7e557a-6aee-4077-b3cd-c96179e643ae

Cancer type	Stomach−AdenoCA
Position	21:15908074−46609729
Interleaved intrachr. SVs	1
Total SVs (intrachr. + transl.)	7
SV types	DEL: 0; DUP: 6; h2hINV: 0; t2tINV: 0; TRA: 1
SVs in sample	1150
Oscillating CN (2 and 3 states)	8, 8
CN segments	8
FDR fragment joints	0.1278363
FDR chr. breakp. enrich.	0.6
Linked to chrs	
Purity, ploidy	0.22, 3.05

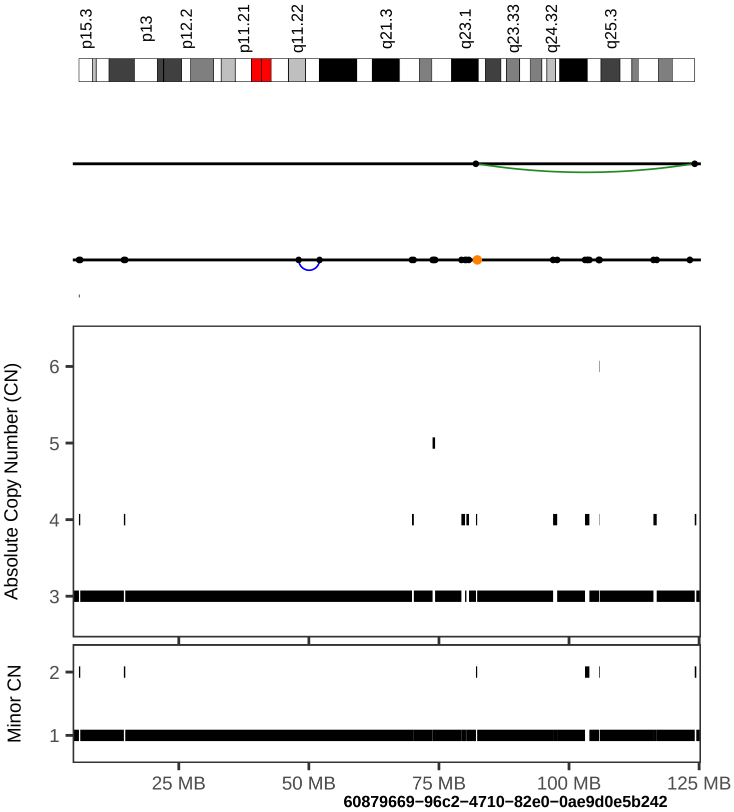

60879669-96c2-4710-82e0-0ae9d0e5b242

Cancer type	Stomach−AdenoCA
Position	10:5810804−124150825
Interleaved intrachr. SVs	1
Total SVs (intrachr. + transl.)	15
SV types	DEL: 0; DUP: 13; h2hINV: 0; t2tINV: 1; TRA: 1
SVs in sample	413
Oscillating CN (2 and 3 states)	11, 18
CN segments	24
FDR fragment joints	0.0006748813
FDR chr. breakp. enrich.	1
Linked to chrs	
Purity, ploidy	0.59, 2.93

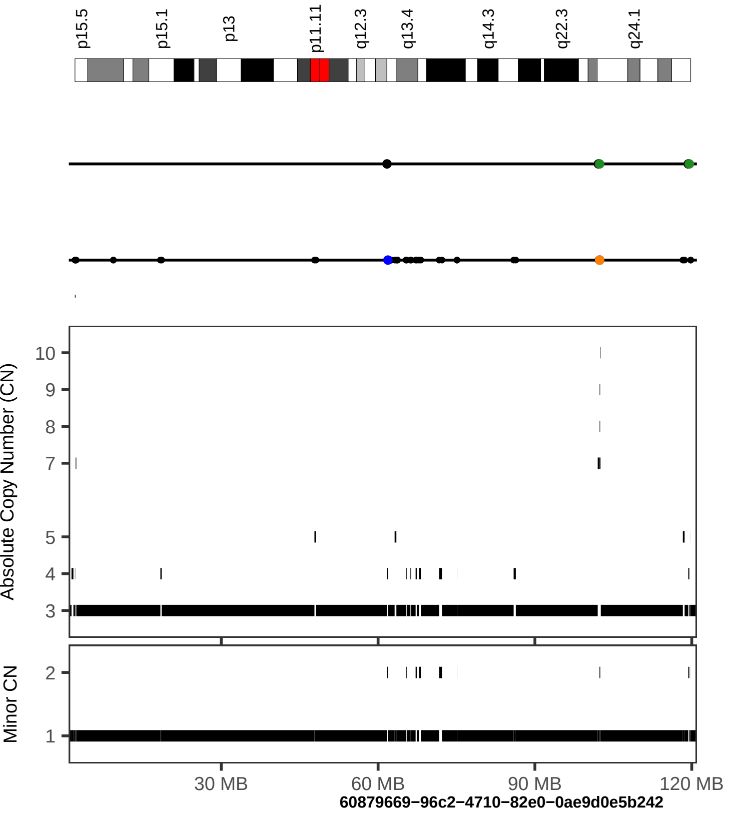

Cancer type	Stomach−AdenoCA
Position	11:2015155−118632987
Interleaved intrachr. SVs	1
Total SVs (intrachr. + transl.)	22
SV types	DEL: 0; DUP: 16; h2hINV: 0; t2tINV: 0; TRA: 6
SVs in sample	413
Oscillating CN (2 and 3 states)	15, 23
CN segments	35
FDR fragment joints	3.697029e−05
FDR chr. breakp. enrich.	0.12
Linked to chrs	
Purity, ploidy	0.59, 2.93

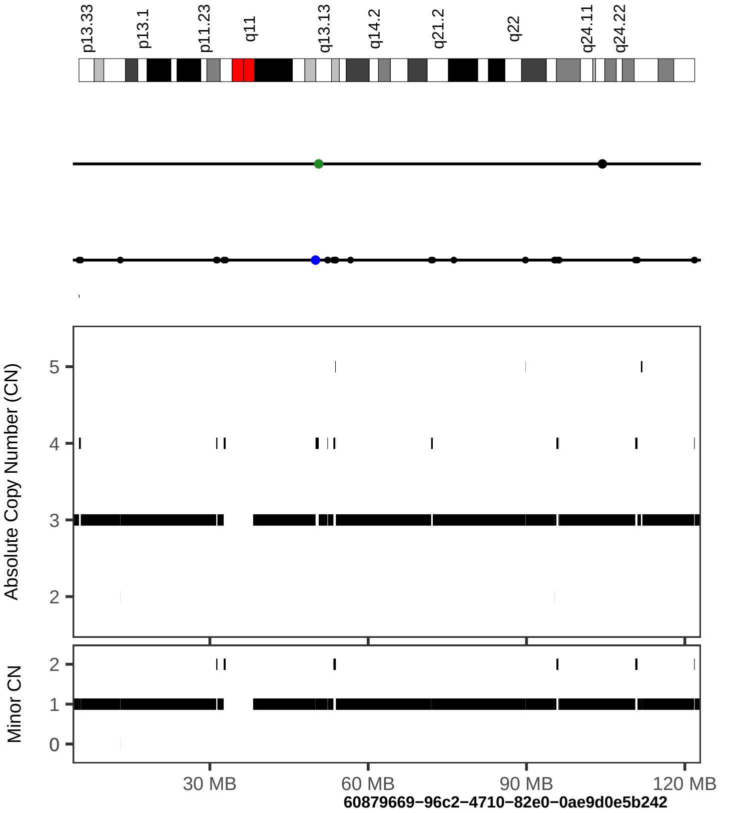

Cancer type	Stomach−AdenoCA
Position	12:5197209−121876052
Interleaved intrachr. SVs	1
Total SVs (intrachr. + transl.)	18
SV types	DEL: 2; DUP: 13; h2hINV: 0; t2tINV: 0; TRA: 3
SVs in sample	413
Oscillating CN (2 and 3 states)	10, 18
CN segments	32
FDR fragment joints	0.002321411
FDR chr. breakp. enrich.	1
Linked to chrs	
Purity, ploidy	0.59, 2.93

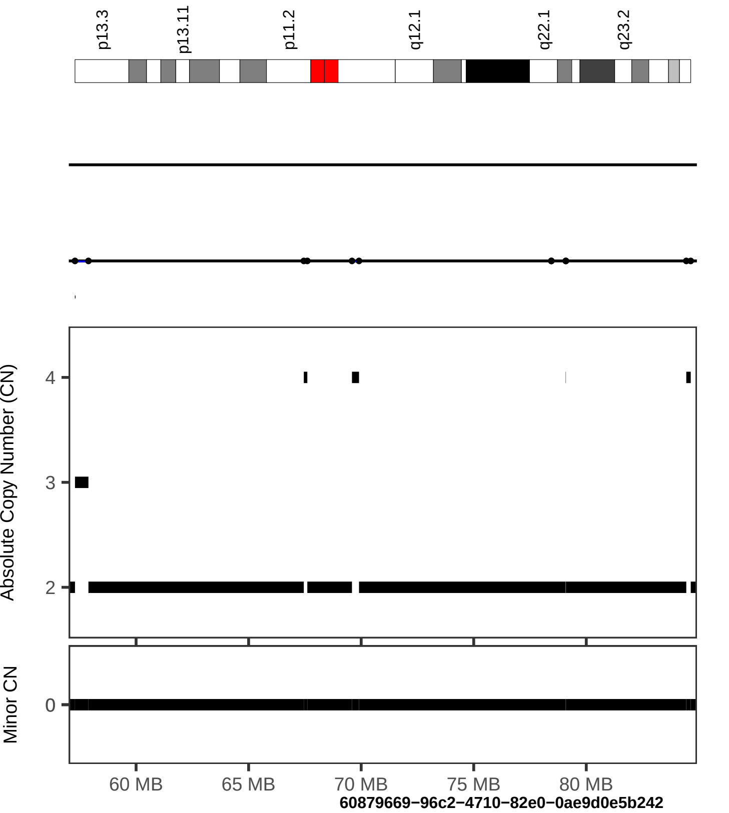

Cancer type	Stomach−AdenoCA
Position	16:57284825−84641398
Interleaved intrachr. SVs	1
Total SVs (intrachr. + transl.)	5
SV types	DEL: 0; DUP: 5; h2hINV: 0; t2tINV: 0; TRA: 0
SVs in sample	413
Oscillating CN (2 and 3 states)	9, 10
CN segments	10
FDR fragment joints	0.1013295
FDR chr. breakp. enrich.	1
Linked to chrs	
Purity, ploidy	0.59, 2.93

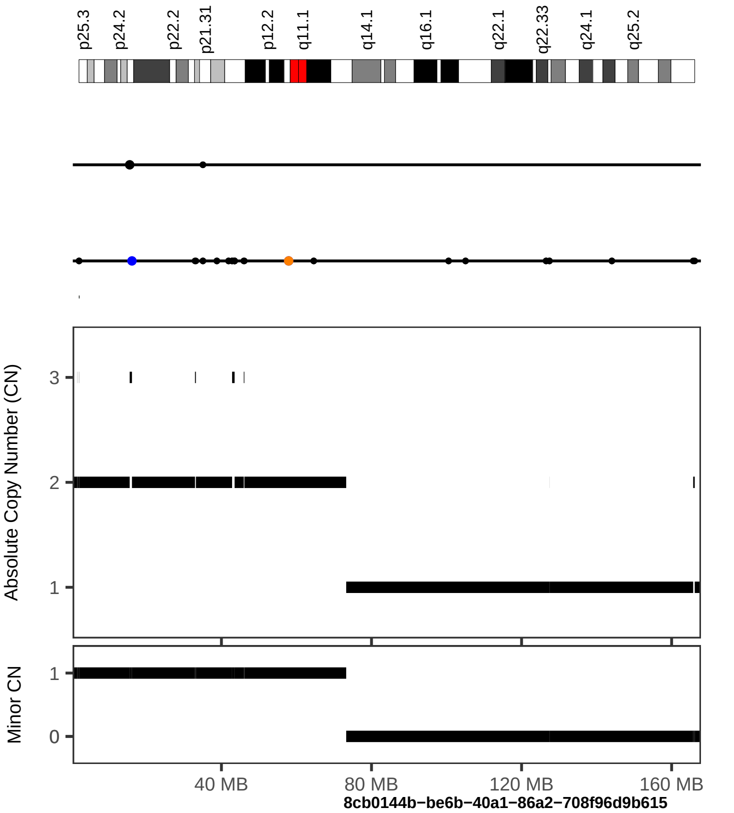

Cancer type	Stomach−AdenoCA
Position	6:2029950−166153467
Interleaved intrachr. SVs	1
Total SVs (intrachr. + transl.)	14
SV types	DEL: 1; DUP: 9; h2hINV: 0; t2tINV: 0; TRA: 4
SVs in sample	507
Oscillating CN (2 and 3 states)	10, 15
CN segments	15
FDR fragment joints	0.01333161
FDR chr. breakp. enrich.	0.7
Linked to chrs	X:9737541−134063295;
Purity, ploidy	0.88, 1.89

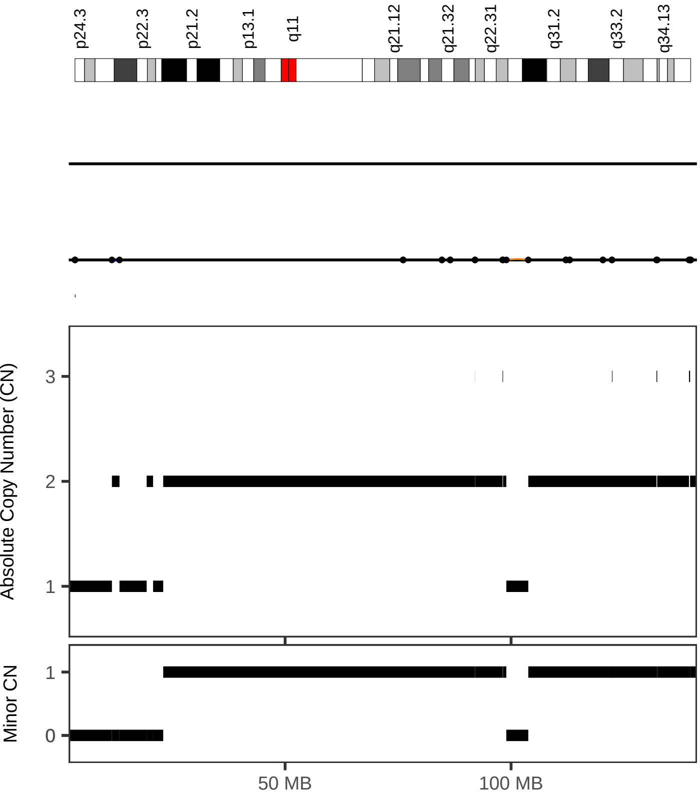

8cb0144b−be6b−40a1−86a2−708f96d9b615

Cancer type	Stomach−AdenoCA
Position	9:3506571−139604450
Interleaved intrachr. SVs	1
Total SVs (intrachr. + transl.)	9
SV types	DEL: 1; DUP: 8; h2hINV: 0; t2tINV: 0; TRA: 0
SVs in sample	507
Oscillating CN (2 and 3 states)	7, 18
CN segments	18
FDR fragment joints	0.02438658
FDR chr. breakp. enrich.	0.55
Linked to chrs	
Purity, ploidy	0.88, 1.89

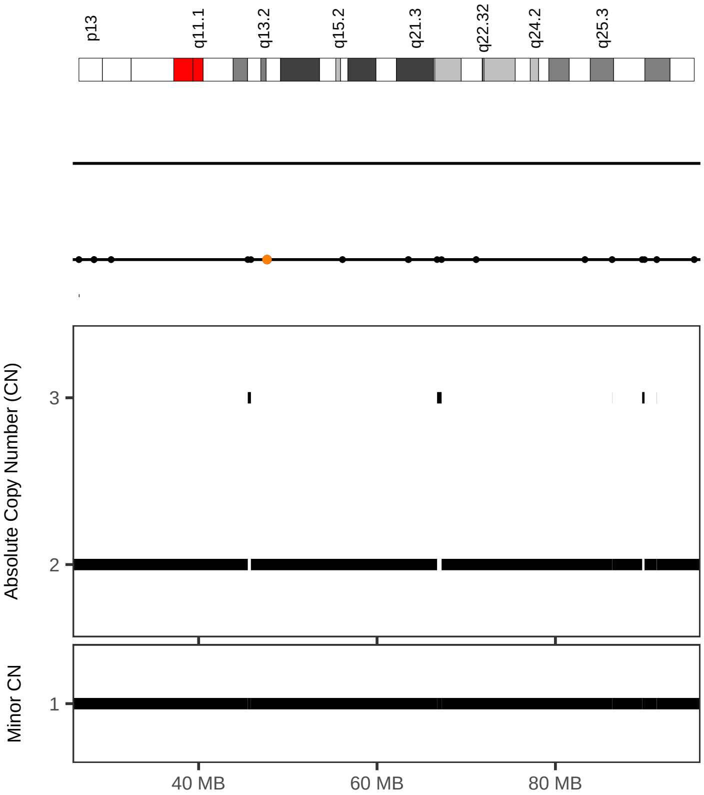

8cb0144b−be6b−40a1−86a2−708f96d9b615

Cancer type	Stomach−AdenoCA
Position	15:30184284−91364444
Interleaved intrachr. SVs	1
Total SVs (intrachr. + transl.)	8
SV types	DEL: 0; DUP: 6; h2hINV: 0; t2tINV: 0; TRA: 2
SVs in sample	507
Oscillating CN (2 and 3 states)	11, 11
CN segments	11
FDR fragment joints	0.05741733
FDR chr. breakp. enrich.	0.66
Linked to chrs	11:13332708−120326698;
Purity, ploidy	0.88, 1.89

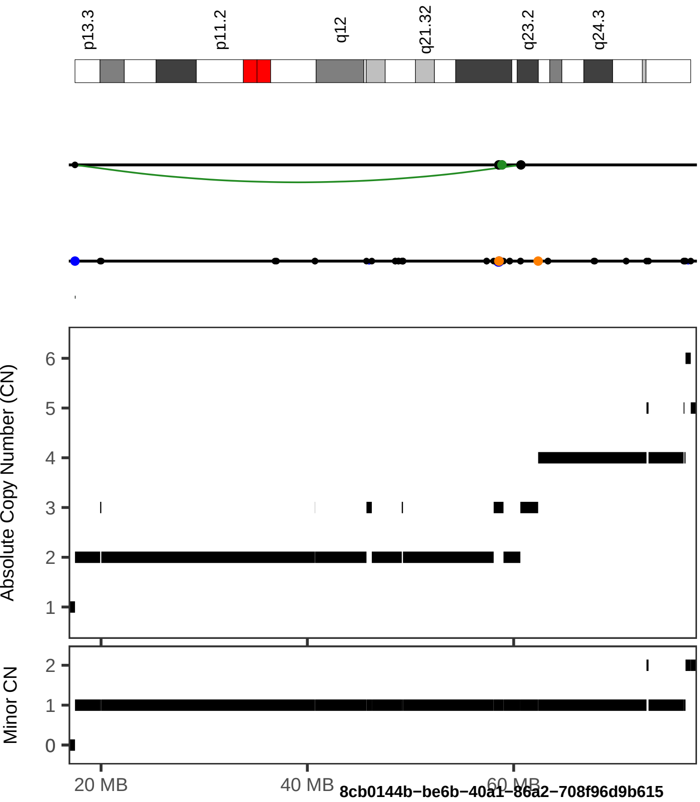

8cb0144b−be6b−40a1−86a2−708f96d9b615

Cancer type	Stomach−AdenoCA
Position	17:17470449−77164988
Interleaved intrachr. SVs	1
Total SVs (intrachr. + transl.)	24
SV types	DEL: 1; DUP: 12; h2hINV: 0; t2tINV: 1; TRA: 10
SVs in sample	507
Oscillating CN (2 and 3 states)	12, 12
CN segments	19
FDR fragment joints	0.1165322
FDR chr. breakp. enrich.	0
Linked to chrs	1:6636540−246791624;10:11402646−130077521 4:21805024−36771158;
Purity, ploidy	0.88, 1.89

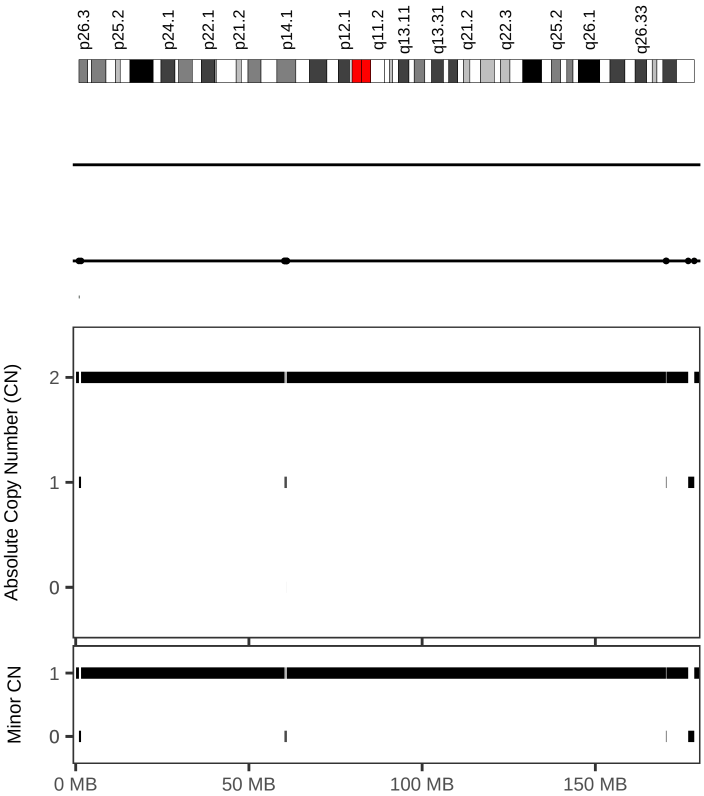

aef8d98f−7a1c−47f3−b5a7−a3cb299c70de

Cancer type	Stomach−AdenoCA
Position	3:931341−178546466
Interleaved intrachr. SVs	1
Total SVs (intrachr. + transl.)	7
SV types	DEL: 7; DUP: 0; h2hINV: 0; t2tINV: 0; TRA: 0
SVs in sample	29
Oscillating CN (2 and 3 states)	7, 14
CN segments	14
FDR fragment joints	0.01743054
FDR chr. breakp. enrich.	0.02
Linked to chrs	
Purity, ploidy	0.38, 2.15

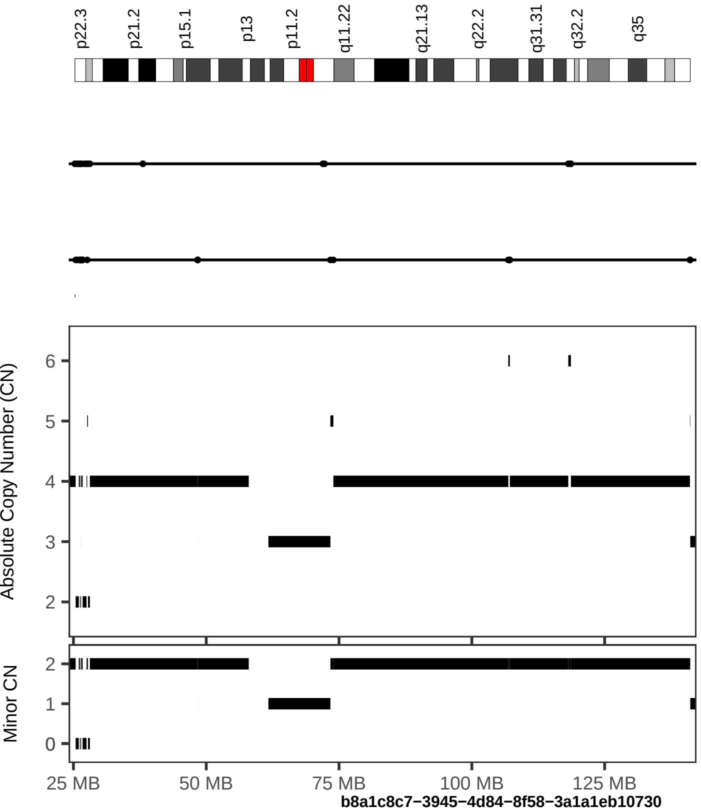

b8a1c8c7−3945−4d84−8f58−3a1a1eb10730

Cancer type	Stomach−AdenoCA
Position	7:25279771−28095993
Interleaved intrachr. SVs	13
Total SVs (intrachr. + transl.)	13
SV types	DEL: 3; DUP: 2; h2hINV: 4; t2tINV: 4; TRA: 0
SVs in sample	298
Oscillating CN (2 and 3 states)	5, 11
CN segments	14
FDR fragment joints	0.9000538
FDR chr. breakp. enrich.	0.17
Linked to chrs	
Purity, ploidy	0.49, 3.06

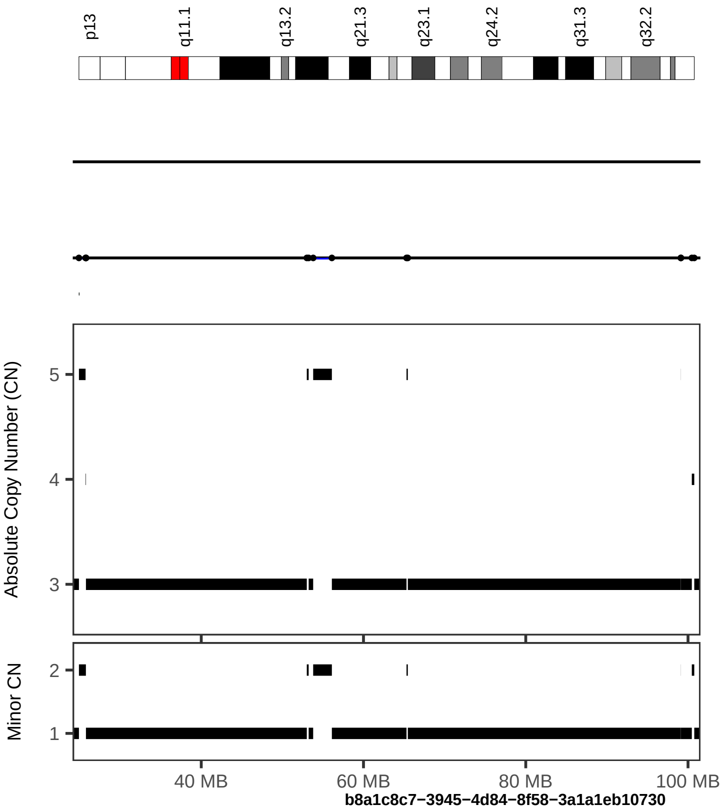

b8a1c8c7−3945−4d84−8f58−3a1a1eb10730

Cancer type	Stomach−AdenoCA
Position	14:24923256−100767511
Interleaved intrachr. SVs	1
Total SVs (intrachr. + transl.)	7
SV types	DEL: 1; DUP: 6; h2hINV: 0; t2tINV: 0; TRA: 0
SVs in sample	298
Oscillating CN (2 and 3 states)	9, 12
CN segments	13
FDR fragment joints	0.1278363
FDR chr. breakp. enrich.	1
Linked to chrs	
Purity, ploidy	0.49, 3.06

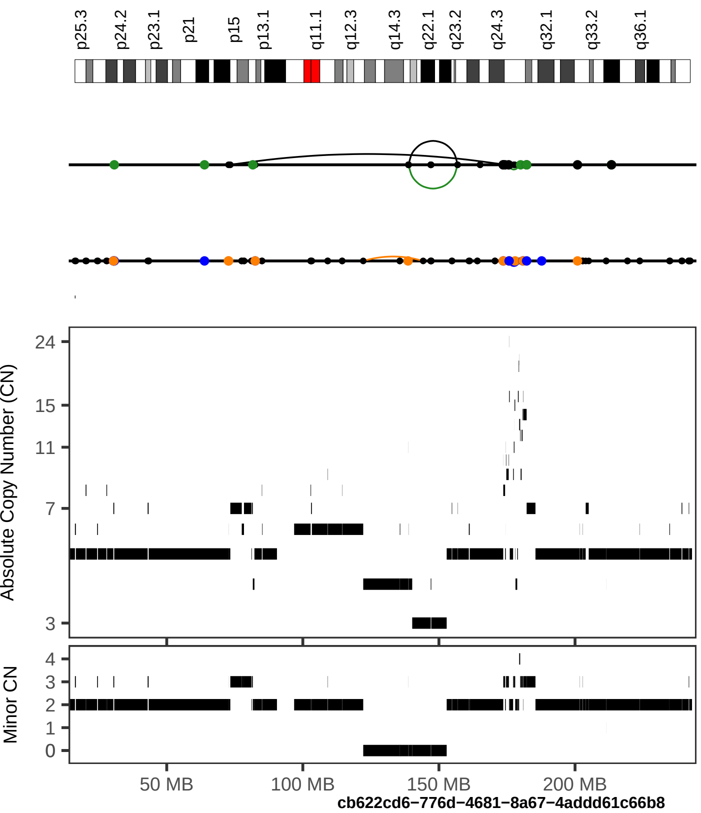

cb622cd6−776d−4681−8a67−4addd61c66b8

Cancer type	Stomach−AdenoCA
Position	2:72710938−179446562
Interleaved intrachr. SVs	6
Total SVs (intrachr. + transl.)	20
SV types	DEL: 1; DUP: 1; h2hINV: 2; t2tINV: 2; TRA: 14
SVs in sample	1322
Oscillating CN (2 and 3 states)	5, 7
CN segments	58
FDR fragment joints	0.6776251
FDR chr. breakp. enrich.	0.16
Linked to chrs	3:12028186−62799588;
Purity, ploidy	0.46, 4.99

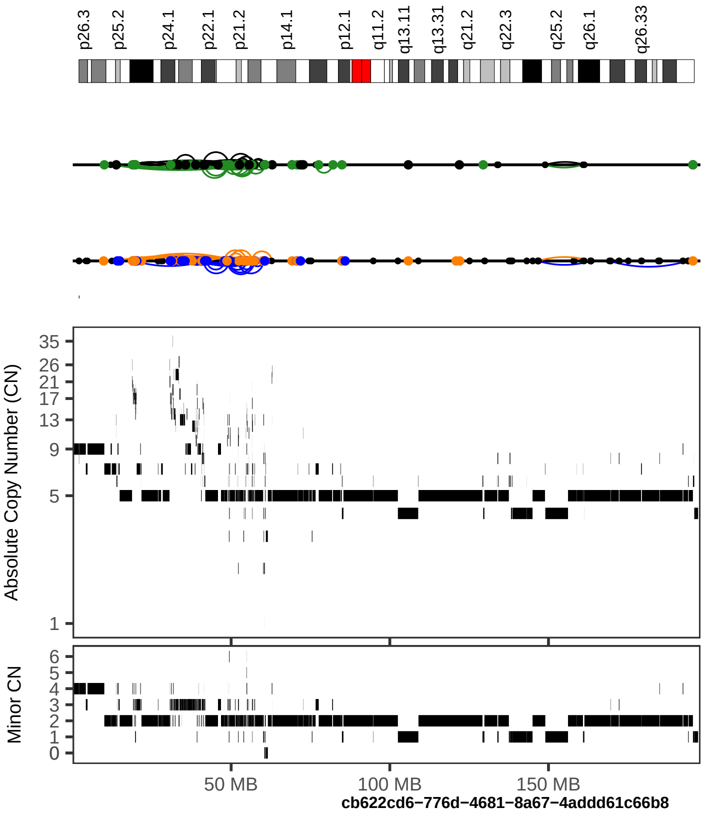

cb622cd6−776d−4681−8a67−4addd61c66b8

Cancer type	Stomach−AdenoCA
Position	3:12028186−62799589
Interleaved intrachr. SVs	118
Total SVs (intrachr. + transl.)	298
SV types	DEL: 32; DUP: 27; h2hINV: 35; t2tINV: 24; TRA: 180
SVs in sample	1322
Oscillating CN (2 and 3 states)	5, 12
CN segments	269
FDR fragment joints	0.9701252
FDR chr. breakp. enrich.	0
Linked to chrs	2:72710938−179446561;
Purity, ploidy	0.46, 4.99

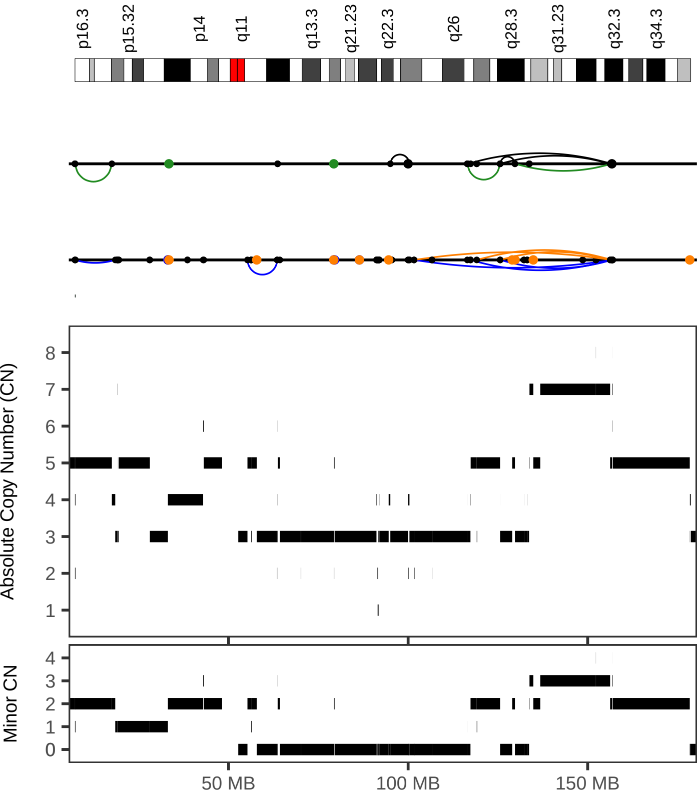

cb622cd6−776d−4681−8a67−4addd61c66b8

Cancer type	Stomach−AdenoCA
Position	4:101648717−156904507
Interleaved intrachr. SVs	13
Total SVs (intrachr. + transl.)	17
SV types	DEL: 4; DUP: 4; h2hINV: 2; t2tINV: 3; TRA: 4
SVs in sample	1322
Oscillating CN (2 and 3 states)	5, 9
CN segments	27
FDR fragment joints	0.615458
FDR chr. breakp. enrich.	0.09
Linked to chrs	13:43528290−111869815;
Purity, ploidy	0.46, 4.99

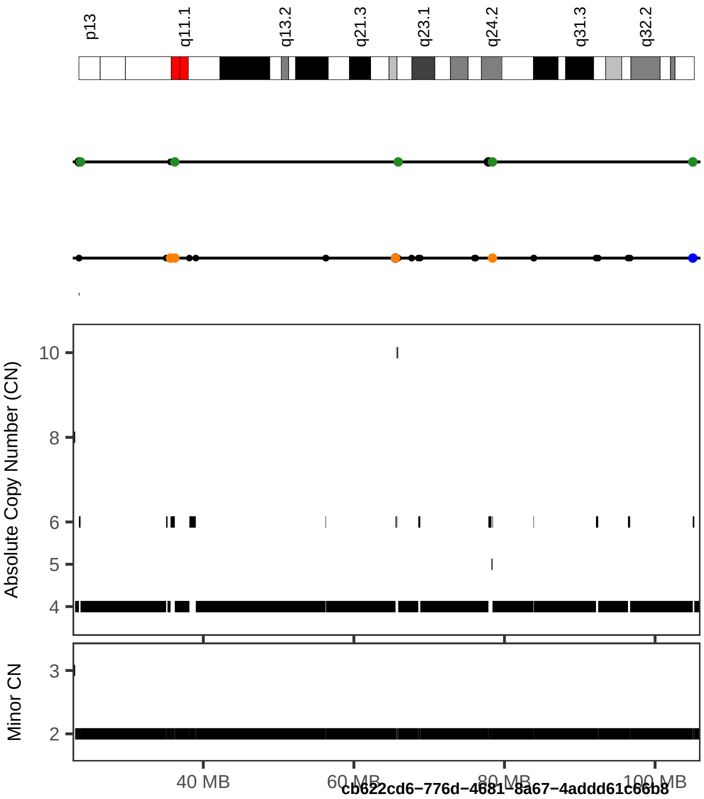

cb622cd6−776d−4681−8a67−4addd61c66b8

Cancer type	Stomach−AdenoCA
Position	14:23478242−105206733
Interleaved intrachr. SVs	1
Total SVs (intrachr. + transl.)	29
SV types	DEL: 0; DUP: 12; h2hINV: 0; t2tINV: 1; TRA: 16
SVs in sample	1322
Oscillating CN (2 and 3 states)	11, 16
CN segments	30
FDR fragment joints	0.1148725
FDR chr. breakp. enrich.	0.26
Linked to chrs	16:1181468−88223091;2:72710938−179446561 3:12028186−62799588;
Purity, ploidy	0.46, 4.99

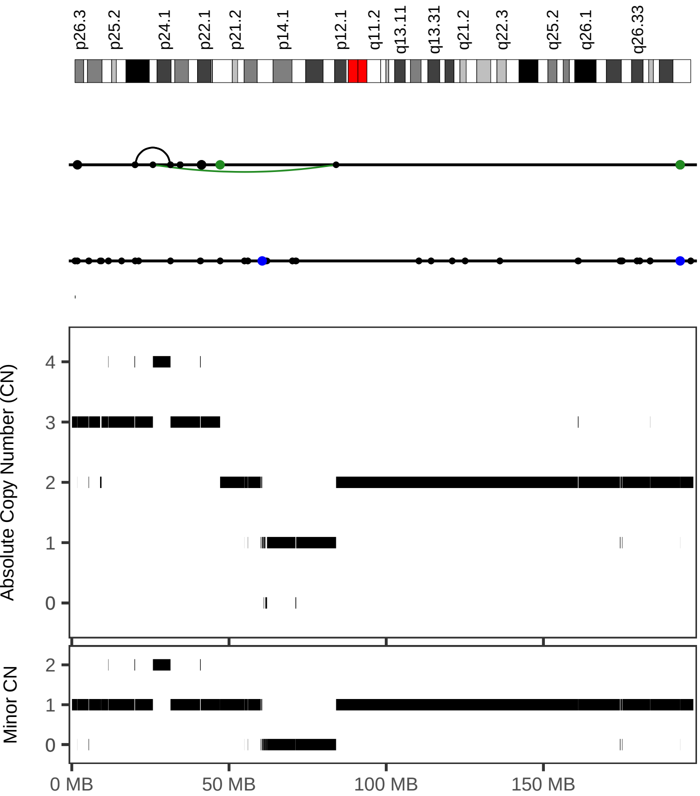

e2aaabd9−f4ba−4763−a28a−c14d46a4bc5d

Cancer type	Stomach−AdenoCA
Position	3:20118117−84053508
Interleaved intrachr. SVs	3
Total SVs (intrachr. + transl.)	7
SV types	DEL: 0; DUP: 0; h2hINV: 2; t2tINV: 1; TRA: 4
SVs in sample	565
Oscillating CN (2 and 3 states)	9, 23
CN segments	23
FDR fragment joints	0.615458
FDR chr. breakp. enrich.	0.26
Linked to chrs	
Purity, ploidy	0.73, 2.22

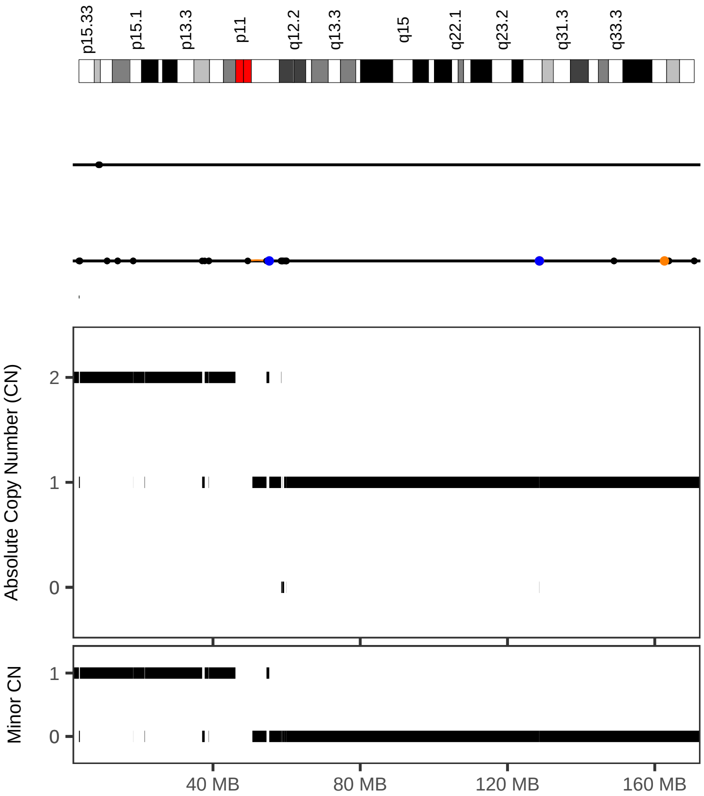

e2aaabd9−f4ba−4763−a28a−c14d46a4bc5d

Cancer type	Stomach−AdenoCA
Position	5:3587780−163844373
Interleaved intrachr. SVs	1
Total SVs (intrachr. + transl.)	17
SV types	DEL: 11; DUP: 1; h2hINV: 0; t2tINV: 1; TRA: 4
SVs in sample	565
Oscillating CN (2 and 3 states)	14, 22
CN segments	22
FDR fragment joints	0.004712092
FDR chr. breakp. enrich.	0.08
Linked to chrs	
Purity, ploidy	0.73, 2.22

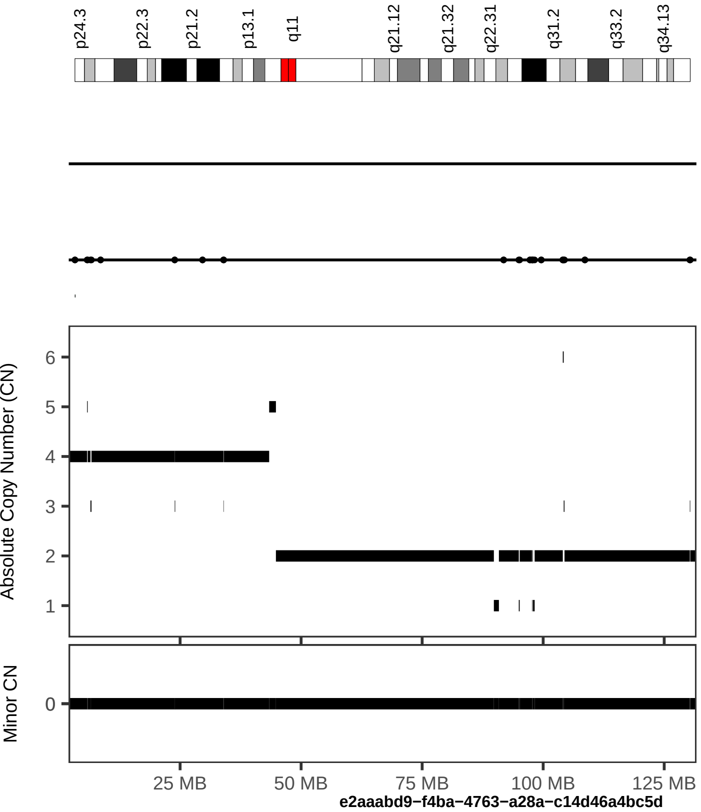

e2aaabd9−f4ba−4763−a28a−c14d46a4bc5d

Cancer type	Stomach−AdenoCA
Position	9:3273649−130381646
Interleaved intrachr. SVs	1
Total SVs (intrachr. + transl.)	15
SV types	DEL: 10; DUP: 5; h2hINV: 0; t2tINV: 0; TRA: 0
SVs in sample	565
Oscillating CN (2 and 3 states)	9, 11
CN segments	24
FDR fragment joints	0.04139017
FDR chr. breakp. enrich.	0.58
Linked to chrs	
Purity, ploidy	0.73, 2.22

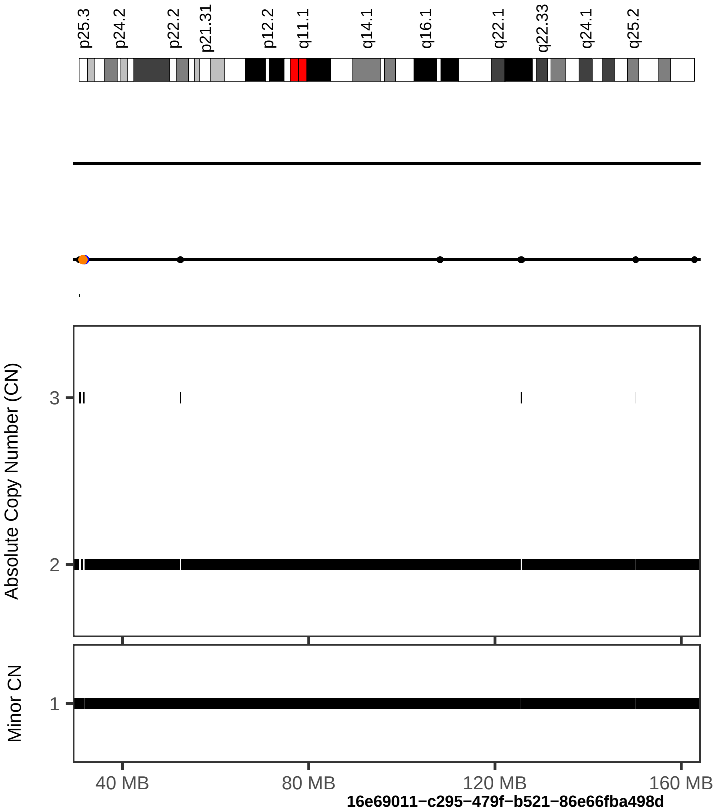

16e69011−c295−479f−b521−86e66fba498d

Cancer type	Uterus−AdenoCA
Position	6:30689459−162853873
Interleaved intrachr. SVs	1
Total SVs (intrachr. + transl.)	8
SV types	DEL: 0; DUP: 6; h2hINV: 0; t2tINV: 0; TRA: 2
SVs in sample	137
Oscillating CN (2 and 3 states)	10, 10
CN segments	10
FDR fragment joints	0.05741733
FDR chr. breakp. enrich.	1
Linked to chrs	
Purity, ploidy	0.49, 2.05

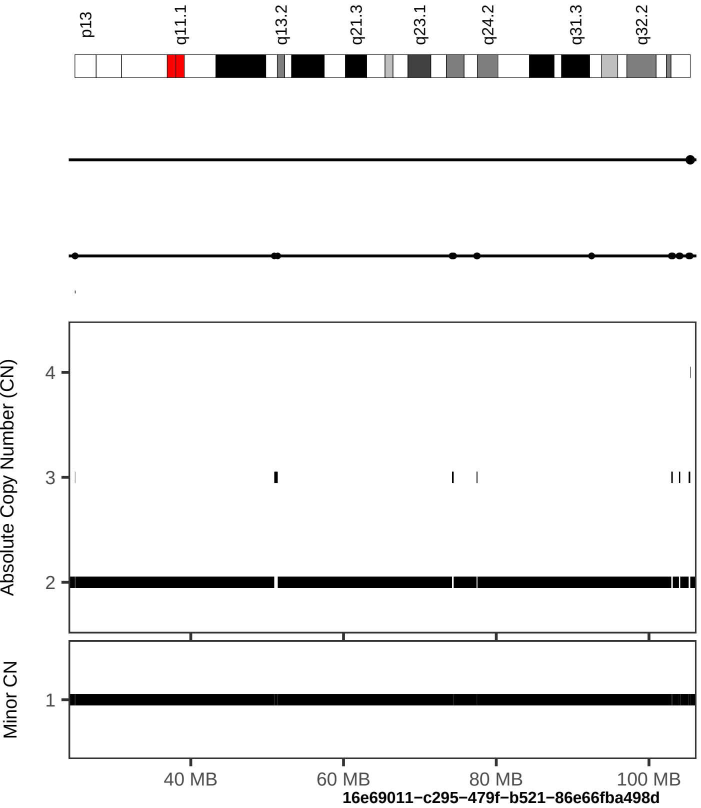

16e69011−c295−479f−b521−86e66fba498d

Cancer type	Uterus−AdenoCA
Position	14:24826217−105404171
Interleaved intrachr. SVs	1
Total SVs (intrachr. + transl.)	9
SV types	DEL: 0; DUP: 8; h2hINV: 0; t2tINV: 0; TRA: 1
SVs in sample	137
Oscillating CN (2 and 3 states)	13, 13
CN segments	15
FDR fragment joints	0.02438658
FDR chr. breakp. enrich.	0.14
Linked to chrs	
Purity, ploidy	0.49, 2.05

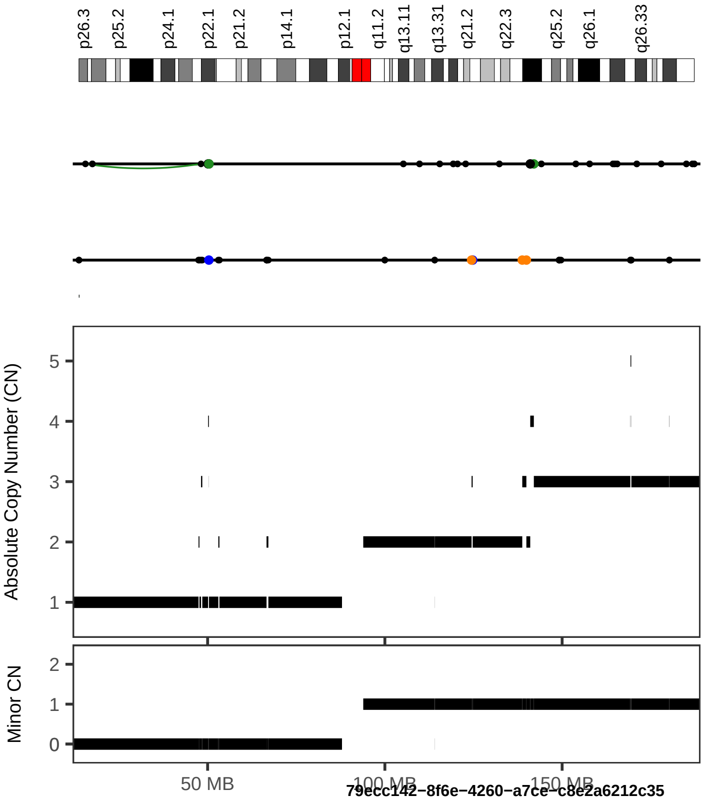

79ecc142−8f6e−4260−a7ce−c8e2a6212c35

Cancer type	Uterus−AdenoCA
Position	3:13693655−180270905
Interleaved intrachr. SVs	1
Total SVs (intrachr. + transl.)	24
SV types	DEL: 1; DUP: 12; h2hINV: 0; t2tINV: 1; TRA: 10
SVs in sample	794
Oscillating CN (2 and 3 states)	8, 9
CN segments	27
FDR fragment joints	0.1165322
FDR chr. breakp. enrich.	0.73
Linked to chrs	11:16783246−34780802;19:4689511−51895484 7:73691686−129774949;
Purity, ploidy	0.86, 1.9

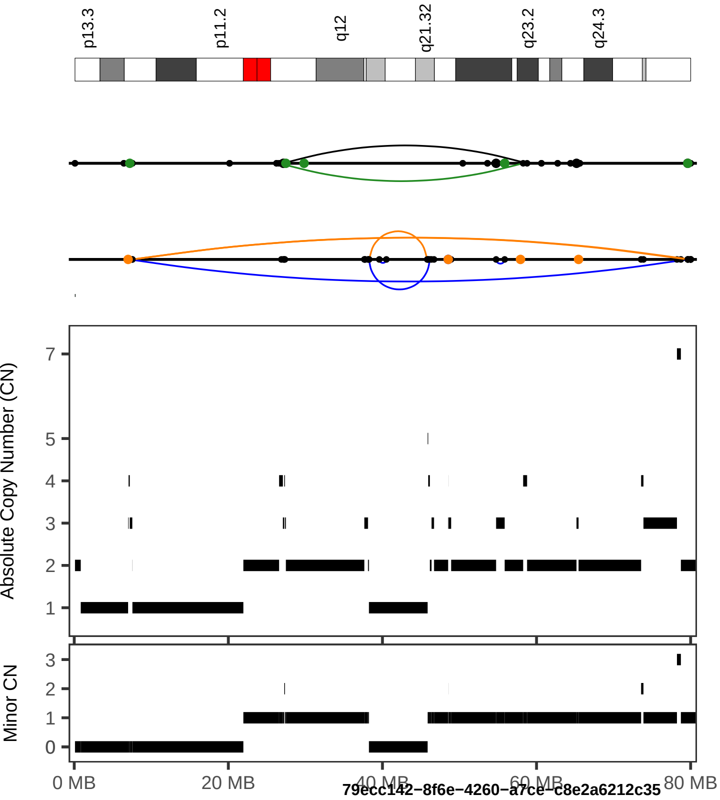

79ecc142−8f6e−4260−a7ce−c8e2a6212c35

Cancer type	Uterus−AdenoCA
Position	17:7101046−80011712
Interleaved intrachr. SVs	8
Total SVs (intrachr. + transl.)	22
SV types	DEL: 3; DUP: 2; h2hINV: 2; t2tINV: 1; TRA: 14
SVs in sample	794
Oscillating CN (2 and 3 states)	4, 10
CN segments	32
FDR fragment joints	0.6776251
FDR chr. breakp. enrich.	0
Linked to chrs	11:16783246−34780802;12:47540819−49659156 19:4689511−51895484;
Purity, ploidy	0.86, 1.9

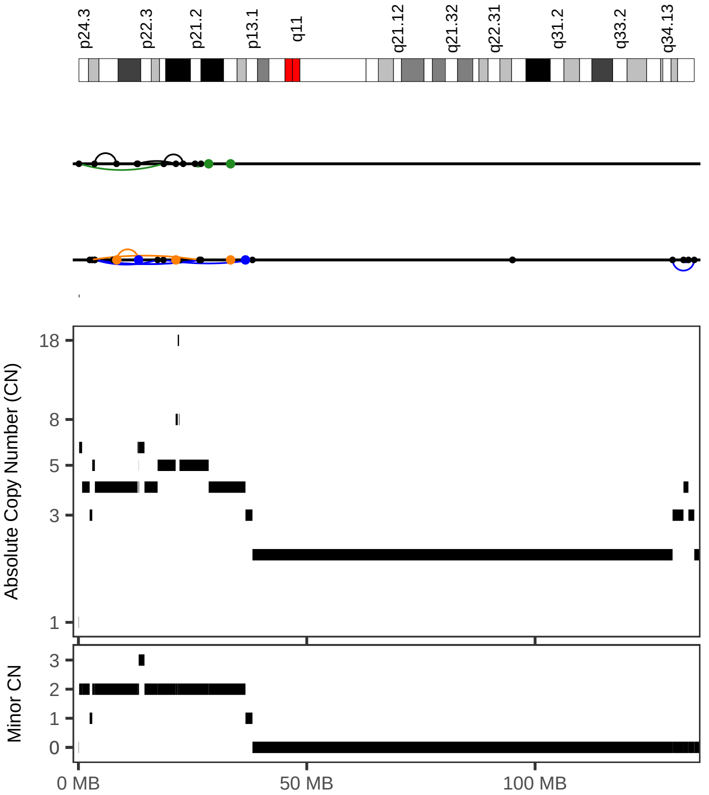

7b9947d5−e86b−4006−8890−ea5209584a88

Cancer type	Uterus−AdenoCA
Position	9:106282−38107794
Interleaved intrachr. SVs	12
Total SVs (intrachr. + transl.)	19
SV types	DEL: 2; DUP: 5; h2hINV: 3; t2tINV: 2; TRA: 7
SVs in sample	276
Oscillating CN (2 and 3 states)	4, 9
CN segments	21
FDR fragment joints	0.6776251
FDR chr. breakp. enrich.	0
Linked to chrs	
Purity, ploidy	0.89, 3.06

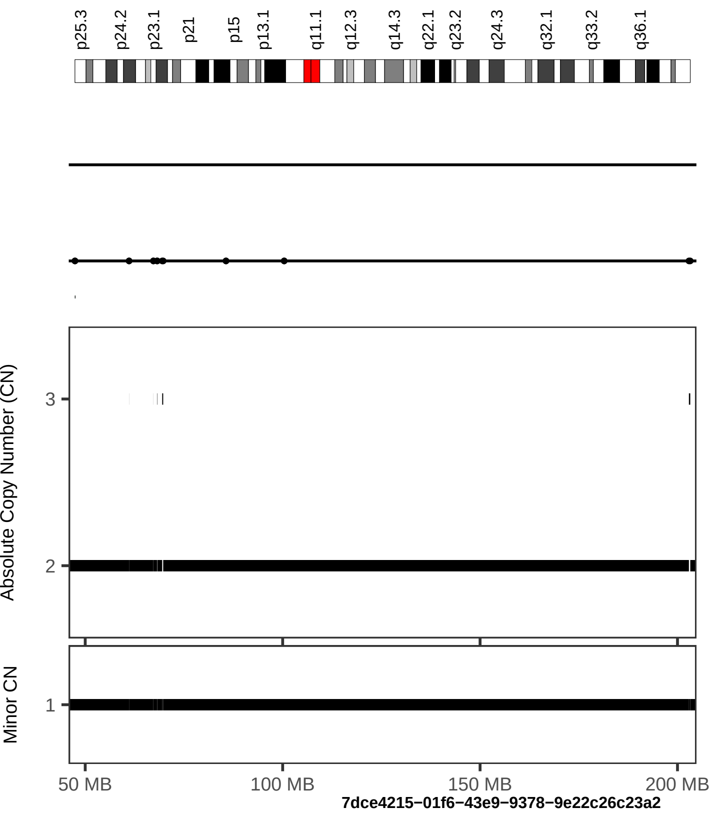

7dce4215−01f6−43e9−9378−9e22c26c23a2

Cancer type	Uterus−AdenoCA
Position	2:47389795−203229105
Interleaved intrachr. SVs	1
Total SVs (intrachr. + transl.)	8
SV types	DEL: 1; DUP: 7; h2hINV: 0; t2tINV: 0; TRA: 0
SVs in sample	120
Oscillating CN (2 and 3 states)	11, 11
CN segments	11
FDR fragment joints	0.05741733
FDR chr. breakp. enrich.	0.83
Linked to chrs	
Purity, ploidy	0.92, 2.1

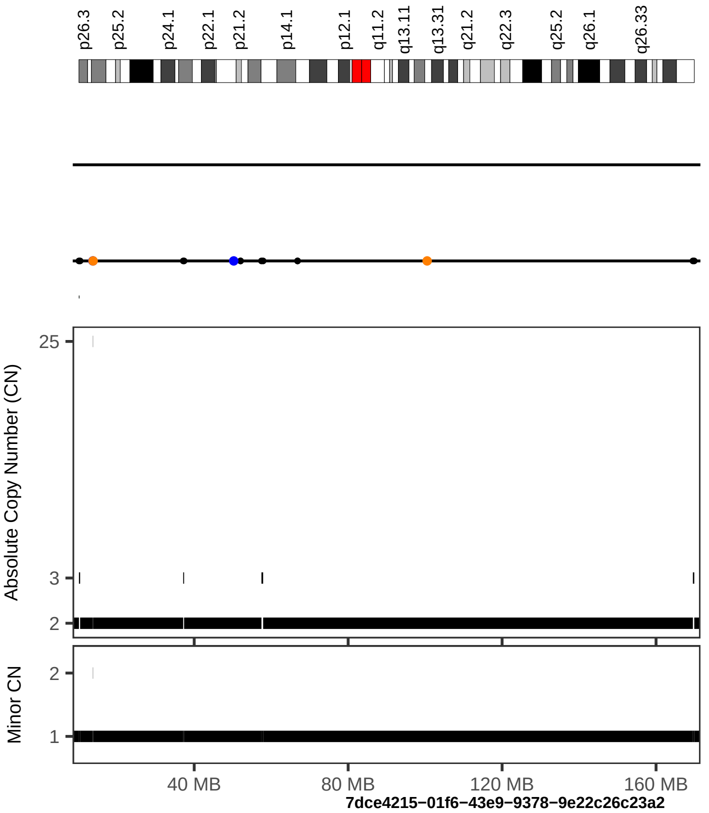

7dce4215−01f6−43e9−9378−9e22c26c23a2

Cancer type	Uterus−AdenoCA
Position	3:10044478−169928464
Interleaved intrachr. SVs	1
Total SVs (intrachr. + transl.)	10
SV types	DEL: 1; DUP: 5; h2hINV: 0; t2tINV: 0; TRA: 4
SVs in sample	120
Oscillating CN (2 and 3 states)	7, 10
CN segments	10
FDR fragment joints	0.1790694
FDR chr. breakp. enrich.	0.43
Linked to chrs	
Purity, ploidy	0.92, 2.1

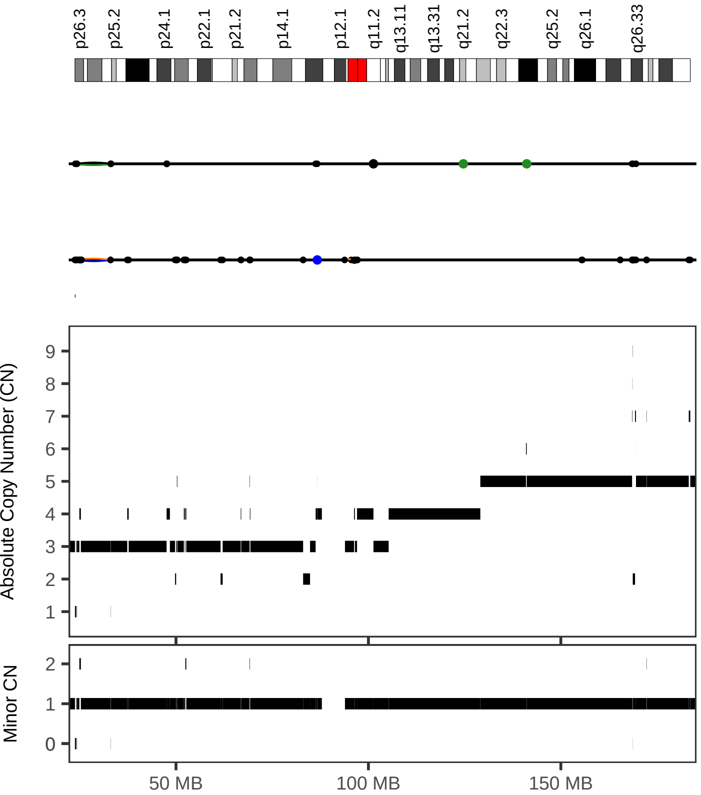

8d9e4917−334b−4c76−aee1−1e22be772db0

Cancer type	Uterus−AdenoCA
Position	3:23738446−169522007
Interleaved intrachr. SVs	6
Total SVs (intrachr. + transl.)	16
SV types	DEL: 2; DUP: 5; h2hINV: 2; t2tINV: 3; TRA: 4
SVs in sample	422
Oscillating CN (2 and 3 states)	7, 27
CN segments	53
FDR fragment joints	0.6776251
FDR chr. breakp. enrich.	0.07
Linked to chrs	
Purity, ploidy	0.46, 3.34

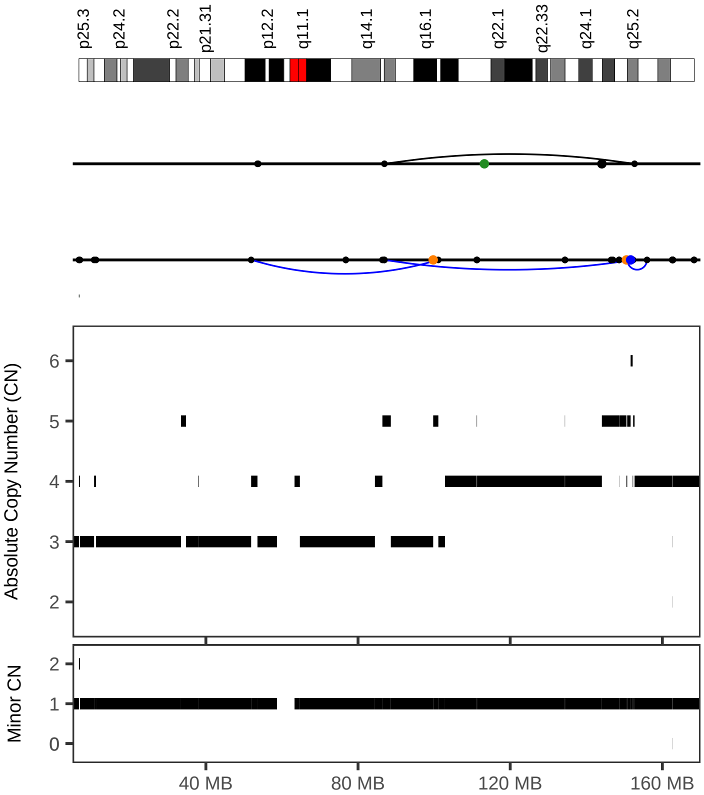

8d9e4917−334b−4c76−aee1−1e22be772db0

Cancer type	Uterus−AdenoCA
Position	6:51901142−155973305
Interleaved intrachr. SVs	4
Total SVs (intrachr. + transl.)	9
SV types	DEL: 0; DUP: 3; h2hINV: 1; t2tINV: 0; TRA: 5
SVs in sample	422
Oscillating CN (2 and 3 states)	10, 10
CN segments	23
FDR fragment joints	0.6776251
FDR chr. breakp. enrich.	1
Linked to chrs	
Purity, ploidy	0.46, 3.34

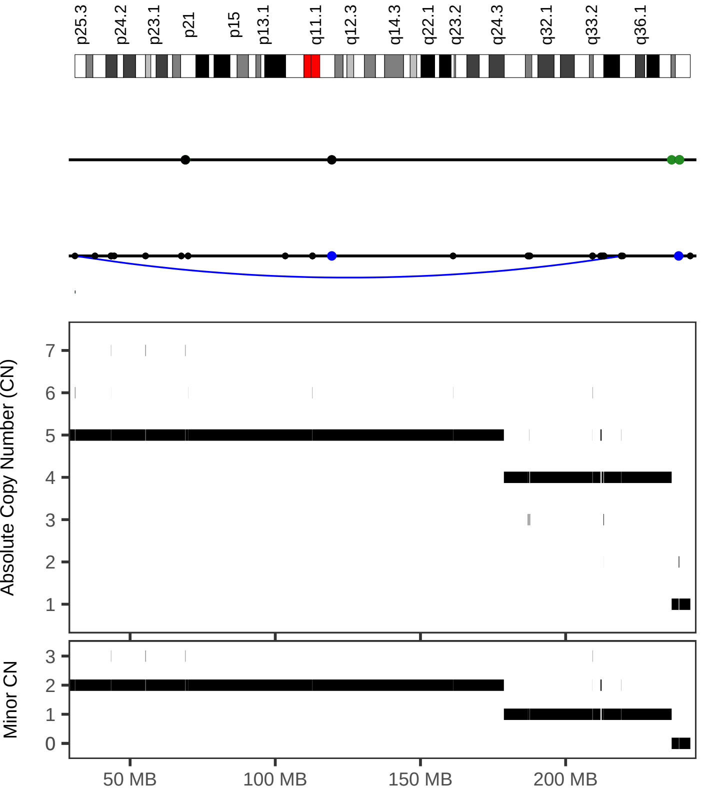

9205c164−7975−421a−90c3−edfe8def595c

Cancer type	Uterus−AdenoCA
Position	2:30991919−219707625
Interleaved intrachr. SVs	1
Total SVs (intrachr. + transl.)	23
SV types	DEL: 3; DUP: 16; h2hINV: 0; t2tINV: 0; TRA: 4
SVs in sample	226
Oscillating CN (2 and 3 states)	7, 16
CN segments	35
FDR fragment joints	0.0008901153
FDR chr. breakp. enrich.	0.02
Linked to chrs	
Purity, ploidy	0.42, 3.3

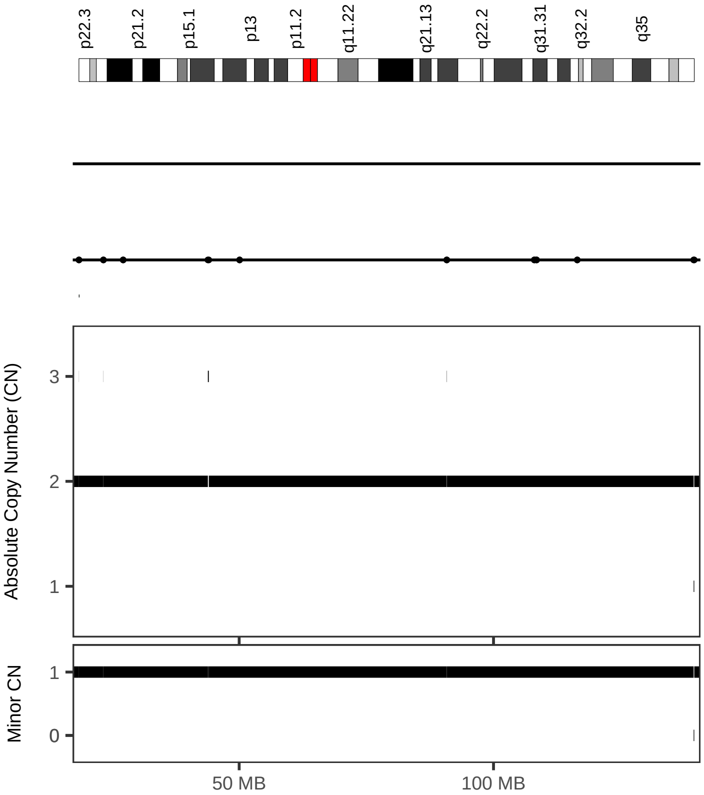

9205c164−7975−421a−90c3−edfe8def595c

Cancer type	Uterus−AdenoCA
Position	7:18503282−139455166
Interleaved intrachr. SVs	1
Total SVs (intrachr. + transl.)	7
SV types	DEL: 1; DUP: 6; h2hINV: 0; t2tINV: 0; TRA: 0
SVs in sample	226
Oscillating CN (2 and 3 states)	8, 10
CN segments	10
FDR fragment joints	0.1278363
FDR chr. breakp. enrich.	0.87
Linked to chrs	
Purity, ploidy	0.42, 3.3

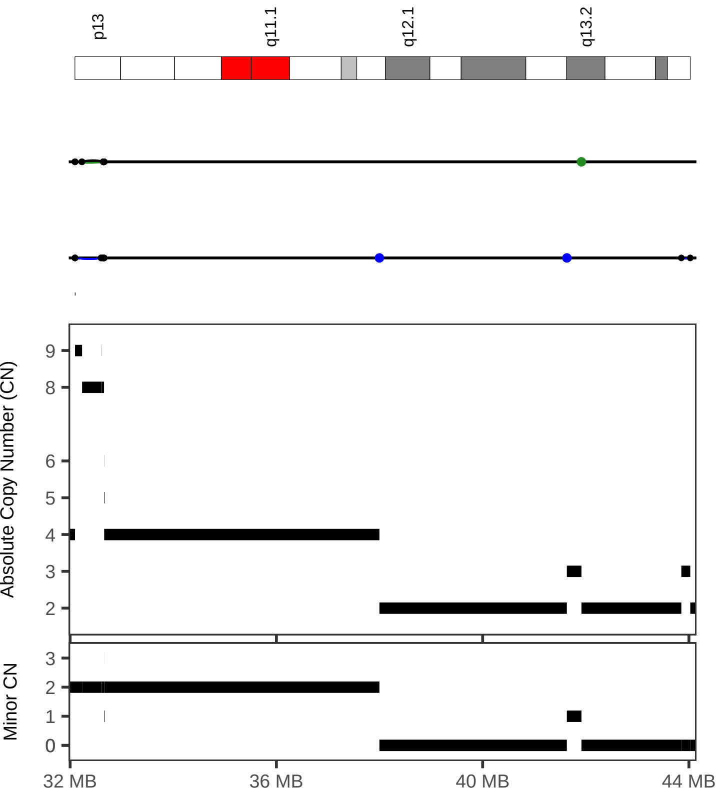

944a102f−3475−45aa−9e30−4b92d1449f00

Cancer type	Uterus−AdenoCA
Position	22:32093117−32660408
Interleaved intrachr. SVs	10
Total SVs (intrachr. + transl.)	10
SV types	DEL: 1; DUP: 4; h2hINV: 2; t2tINV: 3; TRA: 0
SVs in sample	189
Oscillating CN (2 and 3 states)	4, 5
CN segments	9
FDR fragment joints	0.6776251
FDR chr. breakp. enrich.	0
Linked to chrs	
Purity, ploidy	0.63, 3.6

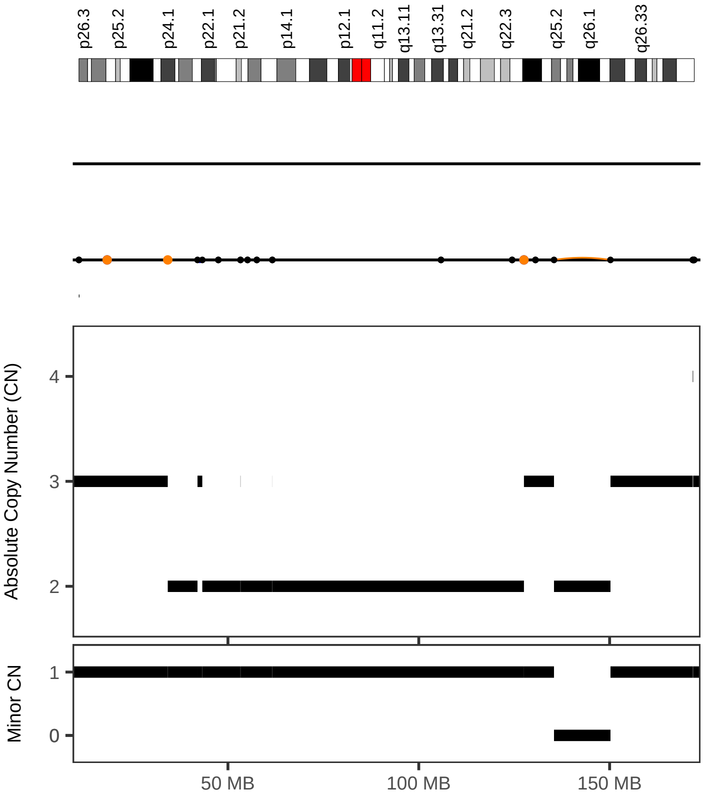

94817e78−78f5−4556−9394−610df65605e2

Cancer type	Uterus−AdenoCA
Position	3:42025446−172171958
Interleaved intrachr. SVs	1
Total SVs (intrachr. + transl.)	9
SV types	DEL: 1; DUP: 6; h2hINV: 0; t2tINV: 0; TRA: 2
SVs in sample	283
Oscillating CN (2 and 3 states)	9, 11
CN segments	11
FDR fragment joints	0.1178349
FDR chr. breakp. enrich.	0.9
Linked to chrs	
Purity, ploidy	0.82, 1.9

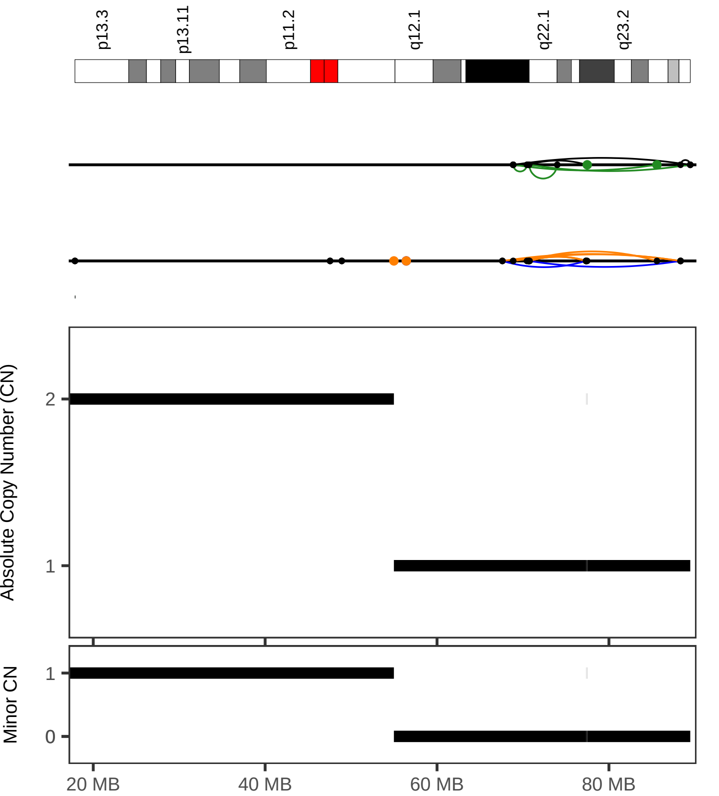

94817e78−78f5−4556−9394−610df65605e2

Cancer type	Uterus−AdenoCA
Position	16:67590378−89462801
Interleaved intrachr. SVs	17
Total SVs (intrachr. + transl.)	19
SV types	DEL: 6; DUP: 3; h2hINV: 3; t2tINV: 5; TRA: 2
SVs in sample	283
Oscillating CN (2 and 3 states)	5, 5
CN segments	5
FDR fragment joints	0.8653243
FDR chr. breakp. enrich.	0
Linked to chrs	
Purity, ploidy	0.82, 1.9

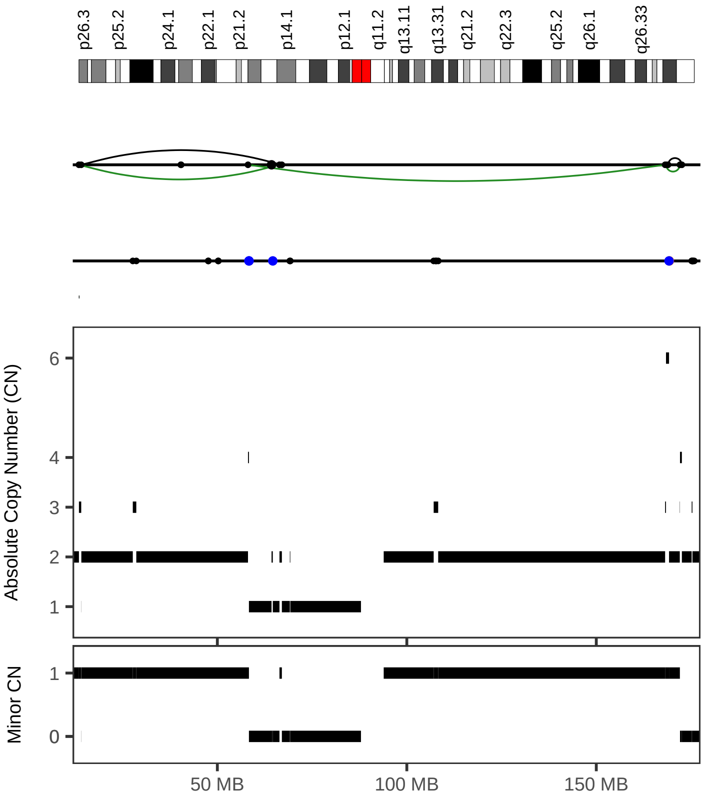

95e6d327−9ab7−45de−9527−f5634b3995c3

Cancer type	Uterus−AdenoCA
Position	3:13471312−168163898
Interleaved intrachr. SVs	3
Total SVs (intrachr. + transl.)	7
SV types	DEL: 0; DUP: 0; h2hINV: 1; t2tINV: 2; TRA: 4
SVs in sample	141
Oscillating CN (2 and 3 states)	8, 8
CN segments	17
FDR fragment joints	0.615458
FDR chr. breakp. enrich.	0
Linked to chrs	
Purity, ploidy	0.66, 1.68

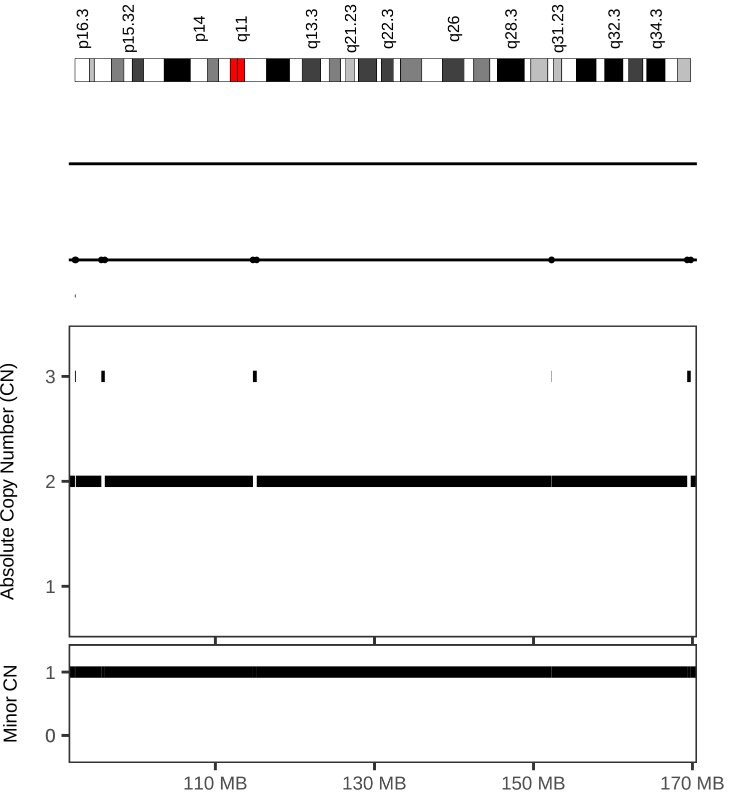

af37d8f0−fea0−47f6−be0a−2fb55a5da5c4

Cancer type	Uterus−AdenoCA
Position	4:92341870−169784344
Interleaved intrachr. SVs	1
Total SVs (intrachr. + transl.)	5
SV types	DEL: 0; DUP: 5; h2hINV: 0; t2tINV: 0; TRA: 0
SVs in sample	315
Oscillating CN (2 and 3 states)	10, 10
CN segments	10
FDR fragment joints	0.1013295
FDR chr. breakp. enrich.	0
Linked to chrs	
Purity, ploidy	0.89, 1.95

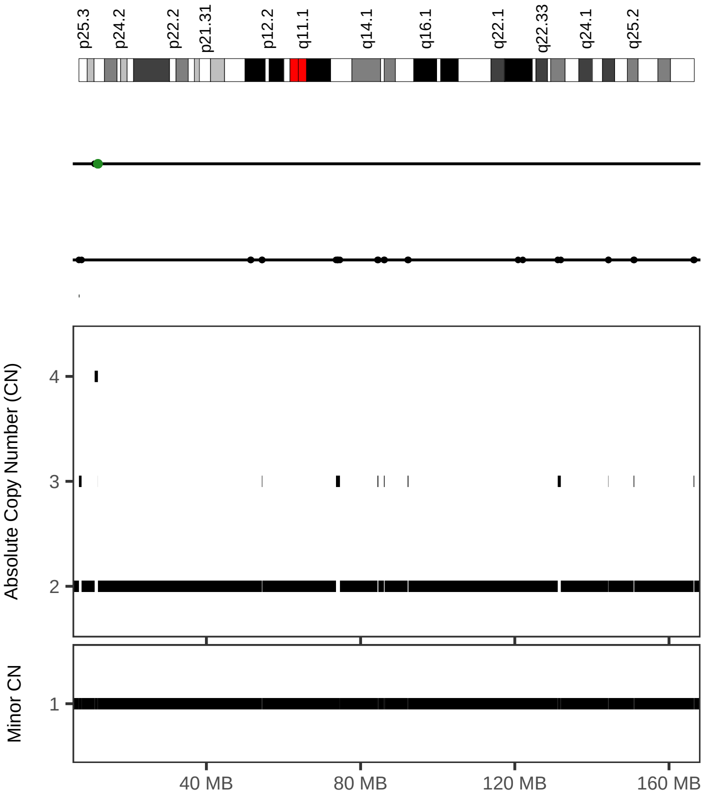

af37d8f0−fea0−47f6−be0a−2fb55a5da5c4

Cancer type	Uterus−AdenoCA
Position	6:6931110−166548418
Interleaved intrachr. SVs	1
Total SVs (intrachr. + transl.)	17
SV types	DEL: 0; DUP: 12; h2hINV: 0; t2tINV: 1; TRA: 4
SVs in sample	315
Oscillating CN (2 and 3 states)	20, 20
CN segments	23
FDR fragment joints	0.01832315
FDR chr. breakp. enrich.	1
Linked to chrs	
Purity, ploidy	0.89, 1.95

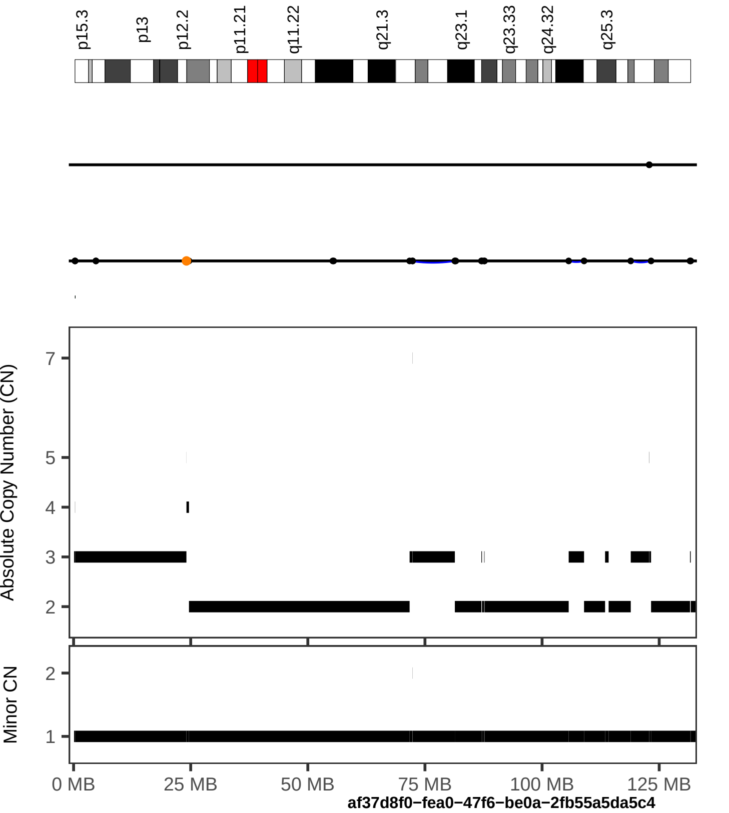

af37d8f0−fea0−47f6−be0a−2fb55a5da5c4

Cancer type	Uterus−AdenoCA
Position	10:294814−131732983
Interleaved intrachr. SVs	1
Total SVs (intrachr. + transl.)	13
SV types	DEL: 0; DUP: 11; h2hINV: 0; t2tINV: 1; TRA: 1
SVs in sample	315
Oscillating CN (2 and 3 states)	11, 11
CN segments	23
FDR fragment joints	0.005291502
FDR chr. breakp. enrich.	1
Linked to chrs	
Purity, ploidy	0.89, 1.95

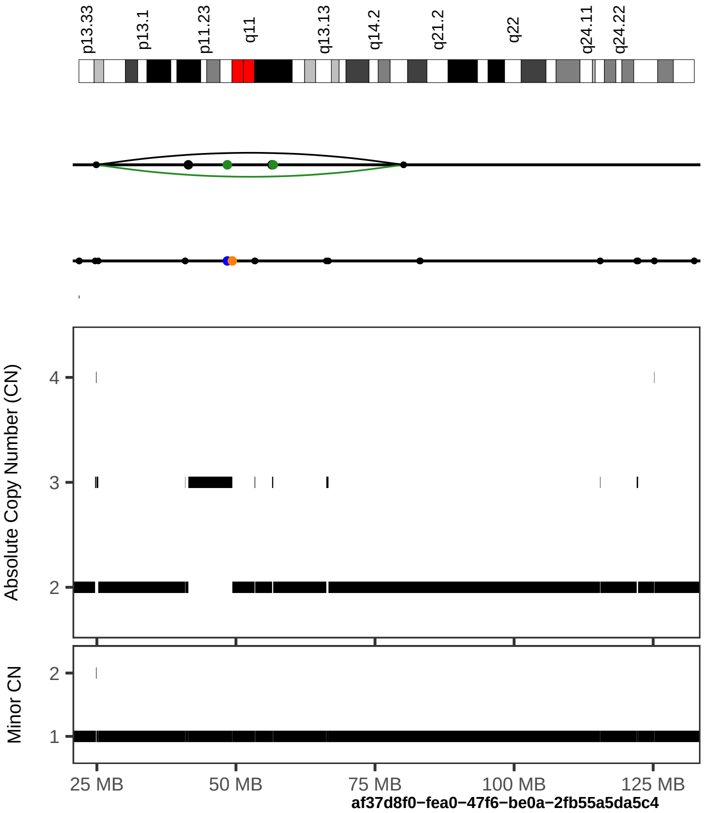

af37d8f0−fea0−47f6−be0a−2fb55a5da5c4

Cancer type	Uterus−AdenoCA
Position	12:24679746−80152939
Interleaved intrachr. SVs	3
Total SVs (intrachr. + transl.)	10
SV types	DEL: 0; DUP: 1; h2hINV: 1; t2tINV: 1; TRA: 7
SVs in sample	315
Oscillating CN (2 and 3 states)	12, 14
CN segments	14
FDR fragment joints	0.6776251
FDR chr. breakp. enrich.	0.53
Linked to chrs	
Purity, ploidy	0.89, 1.95

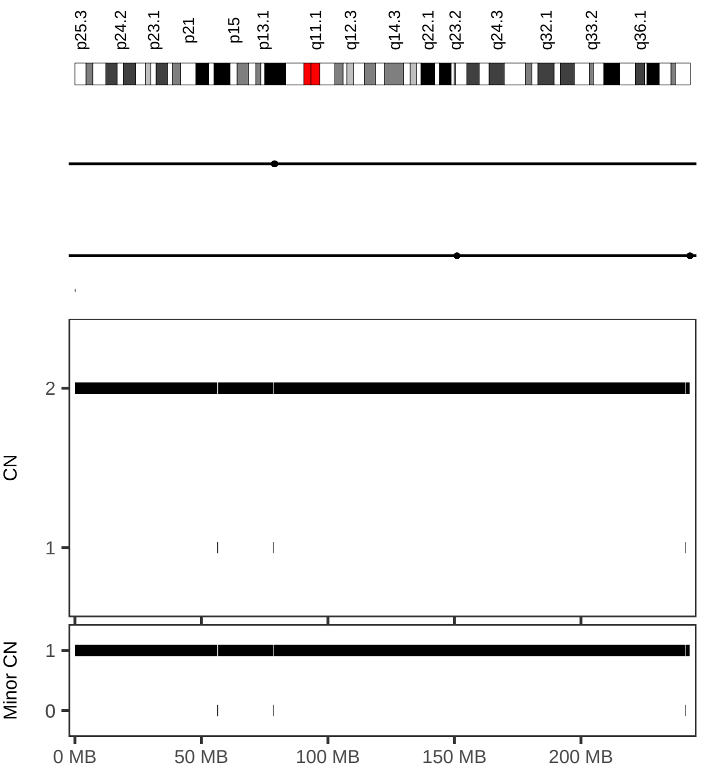

	**BTCA_donor_A035**
Cancer type	Biliary−AdenoCA
Position	2
Interleaved intrachr. SVs	2
Total SVs (intrachr. + transl.)	2
SV types	DEL: 0; DUP: 0; h2hINV: 1;<br>t2tINV: 1; TRA: 0
SVs in sample	80
Oscillating CN in chr (2 and 3 states)	7,
CN segments	7
Purity, ploidy	0.77, 2.01

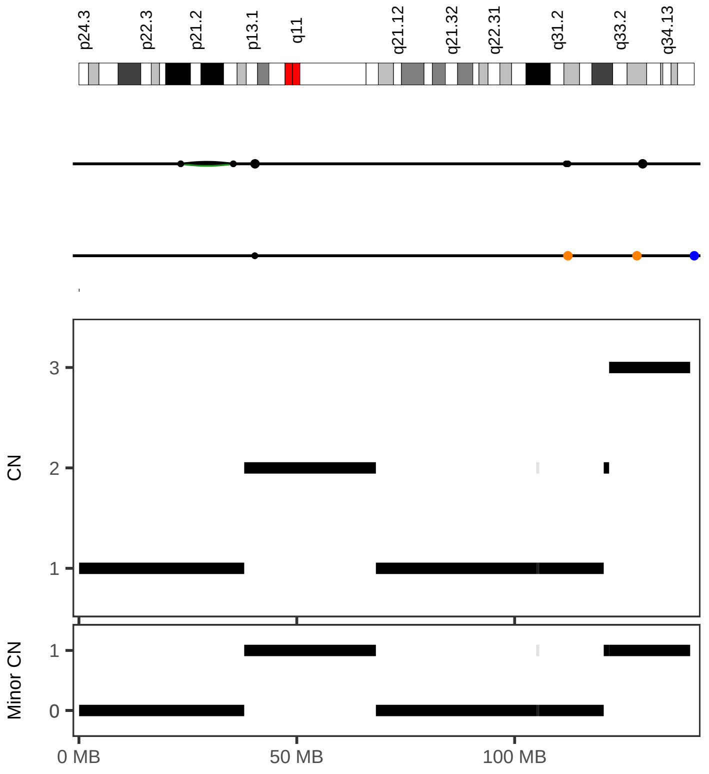

	**BTCA_donor_A035**
Cancer type	Biliary−AdenoCA
Position	9
Interleaved intrachr. SVs	2
Total SVs (intrachr. + transl.)	2
SV types	DEL: 0; DUP: 0; h2hINV: 1;<br>t2tINV: 1; TRA: 0
SVs in sample	80
Oscillating CN in chr (2 and 3 states)	8,
CN segments	9
Purity, ploidy	0.77, 2.01

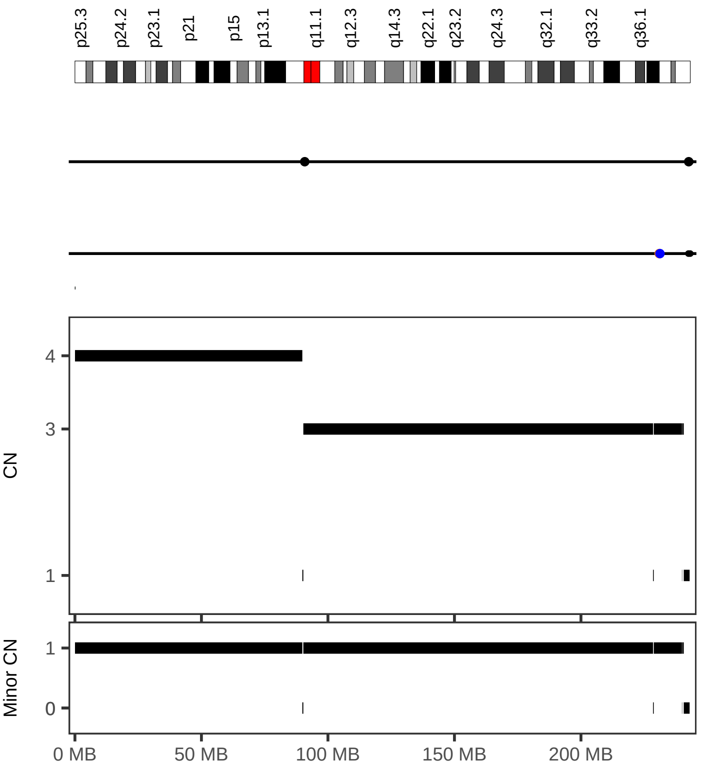

	**RK159**
Cancer type	Biliary−AdenoCA
Position	2
Interleaved intrachr. SVs	1
Total SVs (intrachr. + transl.)	2
SV types	DEL: 1; DUP: 0; h2hINV: 0;<br>t2tINV: 0; TRA: 1
SVs in sample	24
Oscillating CN in chr (2 and 3 states)	7,
CN segments	8
Purity, ploidy	0.84, 3.47

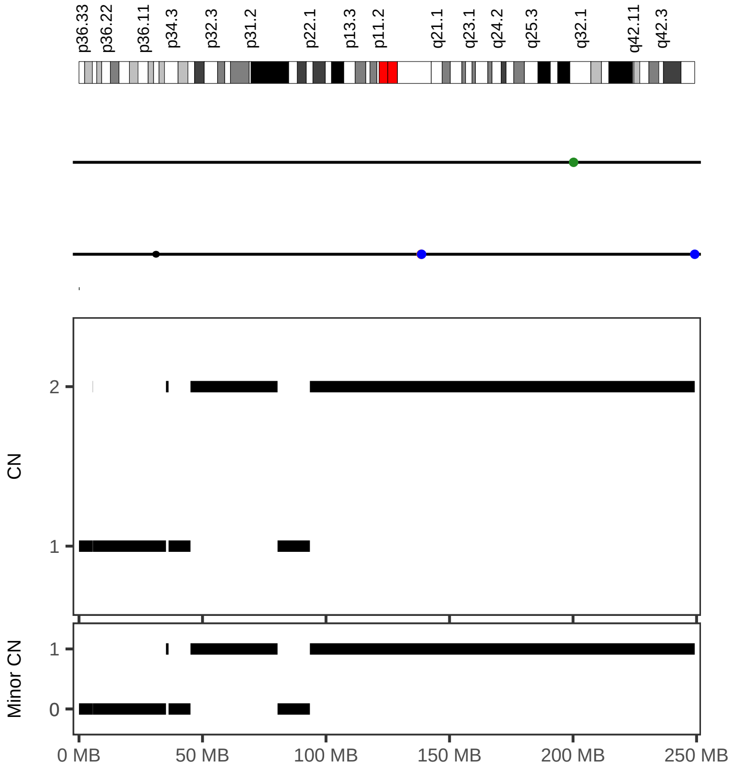

	**RK310**
Cancer type	Biliary−AdenoCA
Position	1
Interleaved intrachr. SVs	1
Total SVs (intrachr. + transl.)	1
SV types	DEL: 0; DUP: 1; h2hINV: 0;<br>t2tINV: 0; TRA: 0
SVs in sample	24
Oscillating CN in chr (2 and 3 states)	8,
CN segments	8
Purity, ploidy	0.68, 1.85

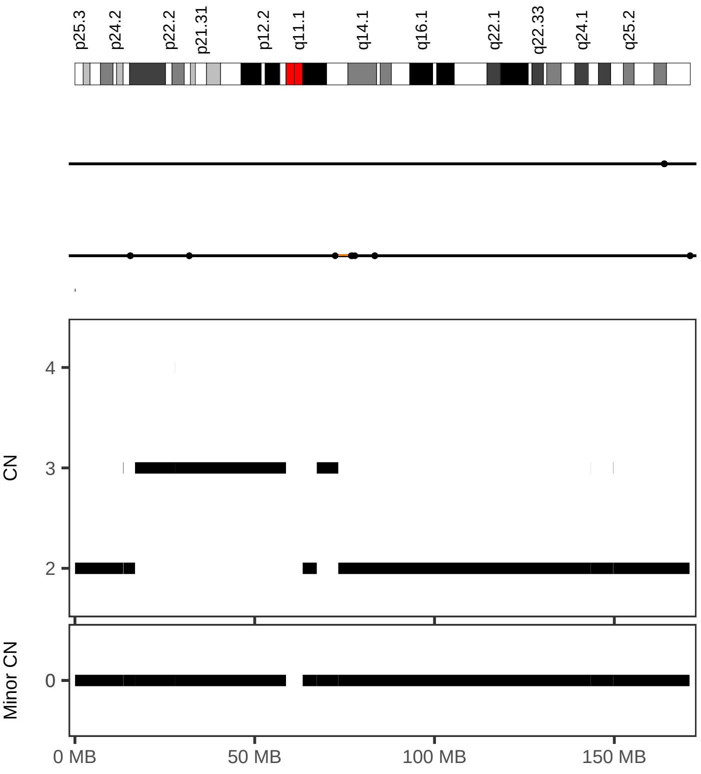

	24f21425−b001−4986−aedf−5b4dd851c6ad
Cancer type	Bladder−TCC
Position	6
Interleaved intrachr. SVs	2
Total SVs (intrachr. + transl.)	2
SV types	DEL: 0; DUP: 0; h2hINV: 1; t2tINV: 1; TRA: 0
SVs in sample	271
Oscillating CN in chr (2 and 3 states)	8,
CN segments	13
Purity, ploidy	0.64, 3.43

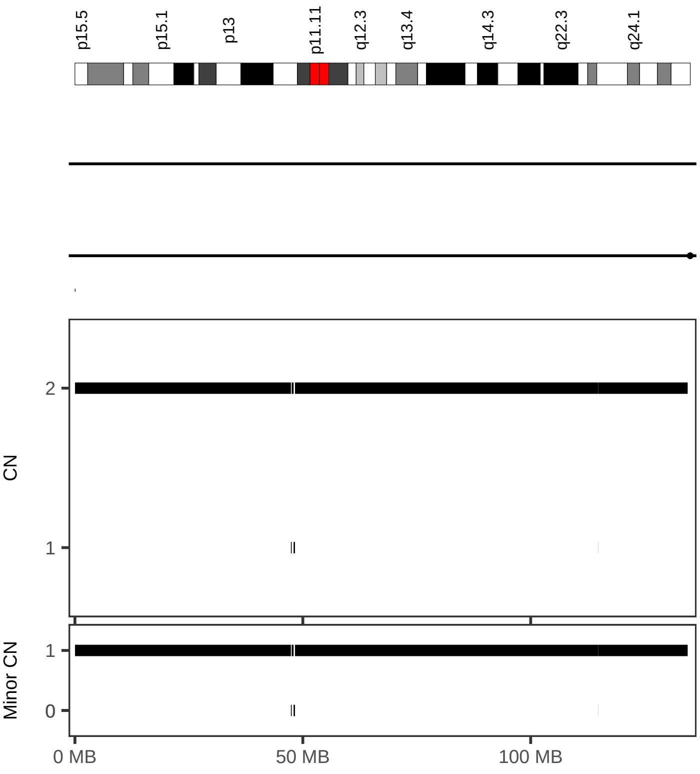

	419deaac−ea45−4bdd−9fa0−b5cd8429b44f
Cancer type	Bladder−TCC
Position	11
Interleaved intrachr. SVs	1
Total SVs (intrachr. + transl.)	1
SV types	DEL: 1; DUP: 0; h2hINV: 0; t2tINV: 0; TRA: 0
SVs in sample	62
Oscillating CN in chr (2 and 3 states)	7,
CN segments	7
Purity, ploidy	0.66, 1.96

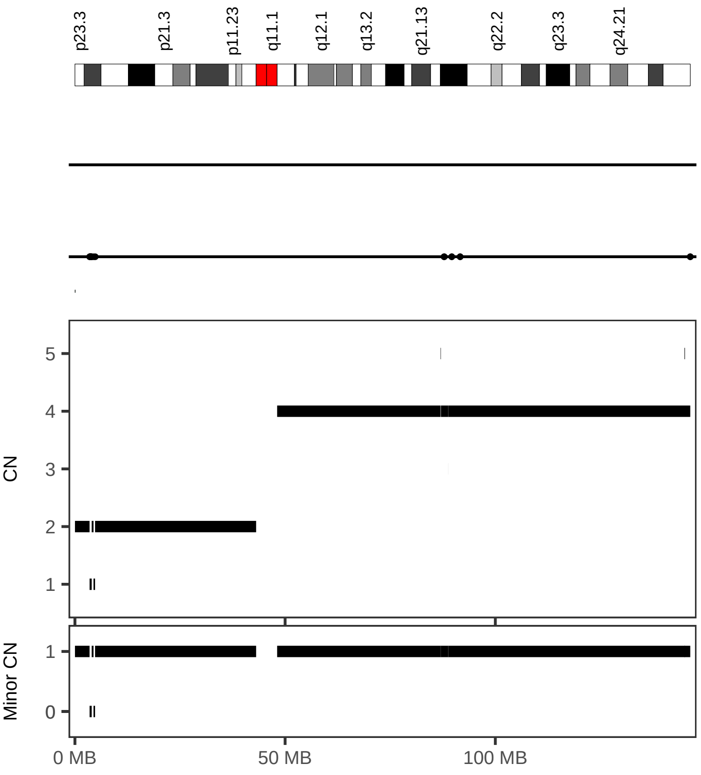

	8c1dd7f7−b74a−4fa2−b6a7−86f0348d2567
Cancer type	Bladder−TCC
Position	8
Interleaved intrachr. SVs	2
Total SVs (intrachr. + transl.)	2
SV types	DEL: 2; DUP: 0; h2hINV: 0; t2tINV: 0; TRA: 0
SVs in sample	80
Oscillating CN in chr (2 and 3 states)	7,
CN segments	14
Purity, ploidy	0.84, 2.09

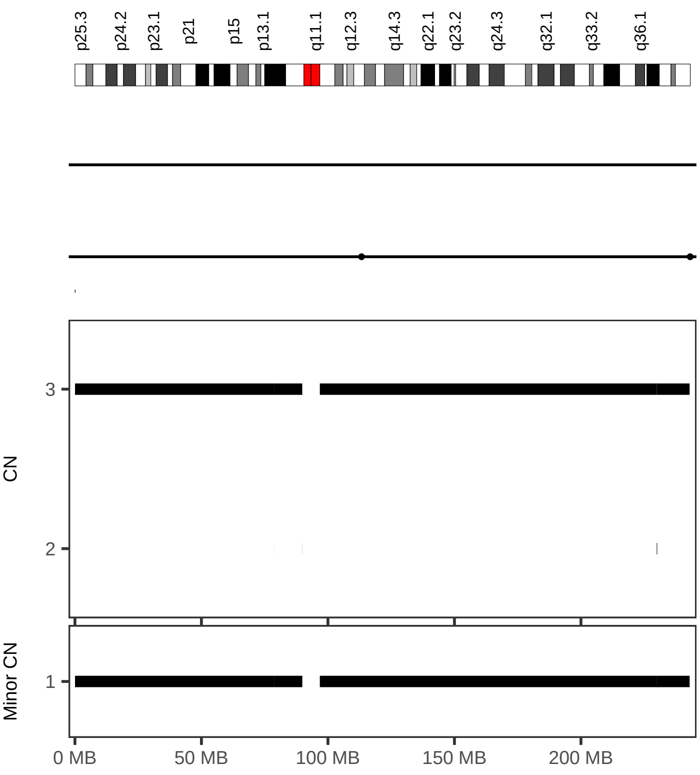

	CGP_donor_1691134
Cancer type	Bone−Epith
Position	2
Interleaved intrachr. SVs	1
Total SVs (intrachr. + transl.)	1
SV types	DEL: 1; DUP: 0; h2hINV: 0; t2tINV: 0; TRA: 0
SVs in sample	32
Oscillating CN in chr (2 and 3 states)	7,
CN segments	7
Purity, ploidy	0.45, 2.08

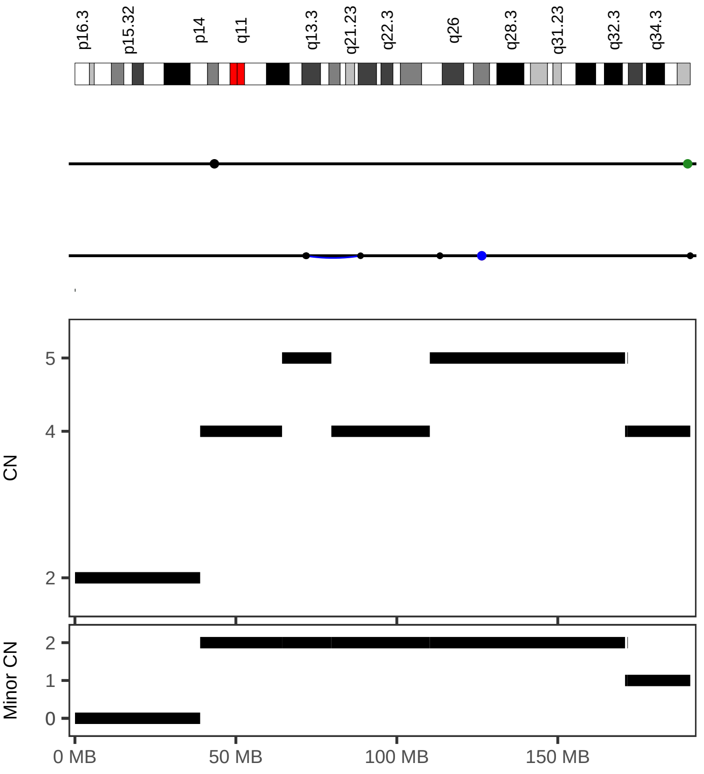

	CGP_donor_1347723
Cancer type	Breast−AdenoCA
Position	4
Interleaved intrachr. SVs	1
Total SVs (intrachr. + transl.)	1
SV types	DEL: 0; DUP: 1; h2hINV: 0; t2tINV: 0; TRA: 0
SVs in sample	144
Oscillating CN in chr (2 and 3 states)	7,
CN segments	8
Purity, ploidy	0.48, 4.22

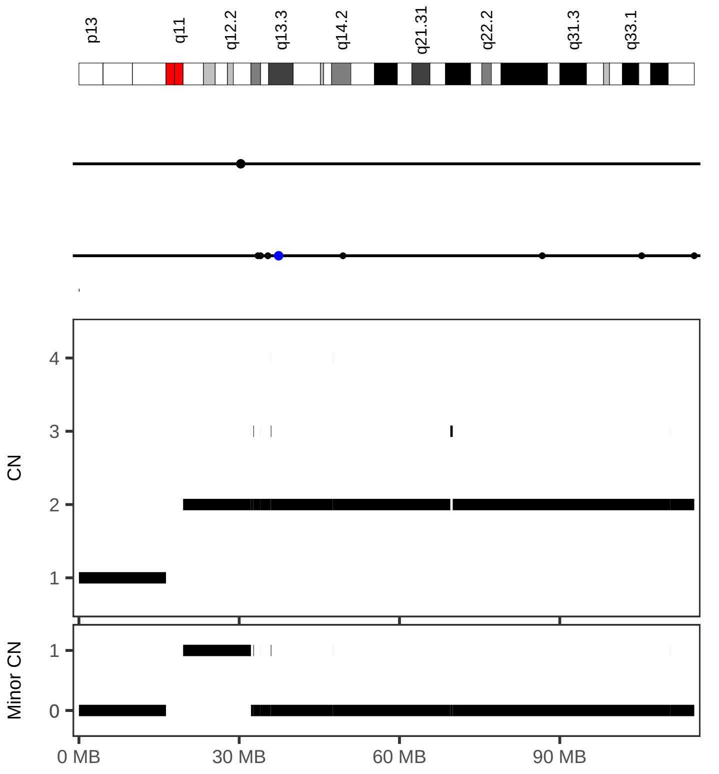

	CGP_donor_1397278
Cancer type	Breast−AdenoCA
Position	13
Interleaved intrachr. SVs	0
Total SVs (intrachr. + transl.)	0
SV types	DEL: 0; DUP: 0; h2hINV: 0; t2tINV: 0; TRA: 0
SVs in sample	340
Oscillating CN in chr (2 and 3 states)	8,
CN segments	17
Purity, ploidy	0.42, 2.02

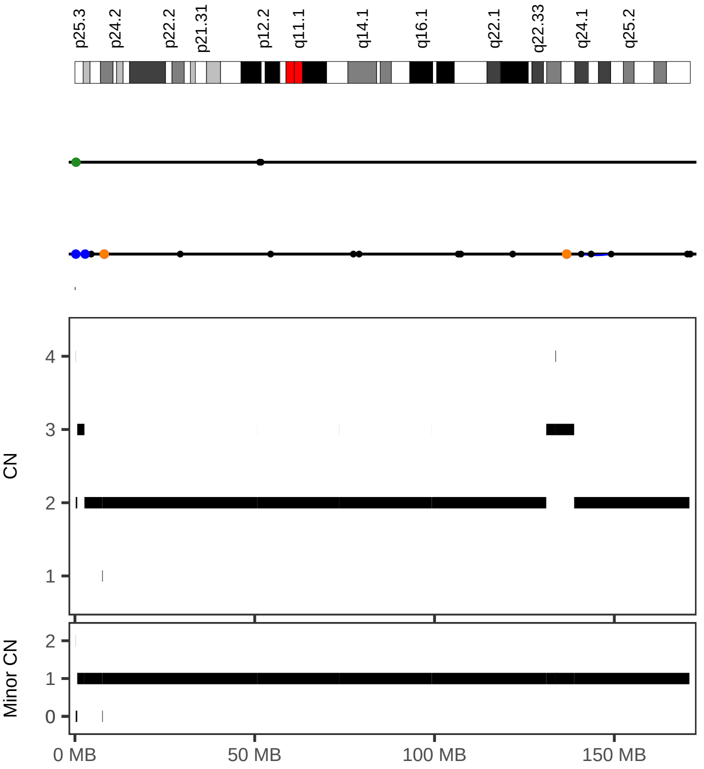

	CGP_donor_1069291
Cancer type	Breast−AdenoCA
Position	6
Interleaved intrachr. SVs	2
Total SVs (intrachr. + transl.)	2
SV types	DEL: 0; DUP: 0; h2hINV: 1; t2tINV: 1; TRA: 0
SVs in sample	277
Oscillating CN in chr (2 and 3 states)	8,
CN segments	16
Purity, ploidy	0.46, 1.81

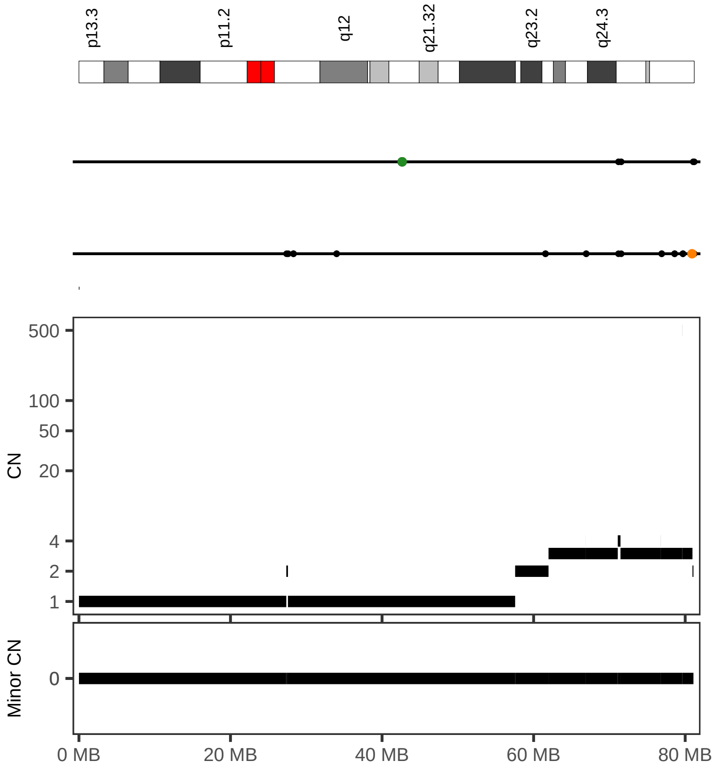

	CGP_donor_1069291
Cancer type	Breast−AdenoCA
Position	17
Interleaved intrachr. SVs	2
Total SVs (intrachr. + transl.)	2
SV types	DEL: 1; DUP: 0; h2hINV: 1; t2tINV: 0; TRA: 0
SVs in sample	277
Oscillating CN in chr (2 and 3 states)	7,
CN segments	14
Purity, ploidy	0.46, 1.81

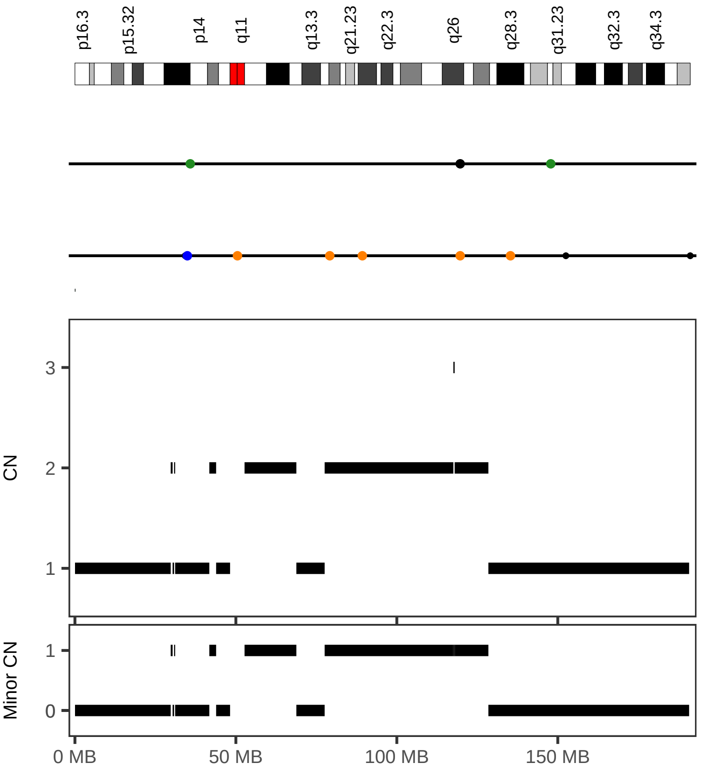

	CGP_donor_1347720
Cancer type	Breast−AdenoCA
Position	4
Interleaved intrachr. SVs	0
Total SVs (intrachr. + transl.)	0
SV types	DEL: 0; DUP: 0; h2hINV: 0; t2tINV: 0; TRA: 0
SVs in sample	173
Oscillating CN in chr (2 and 3 states)	10,
CN segments	13
Purity, ploidy	0.37, 1.59

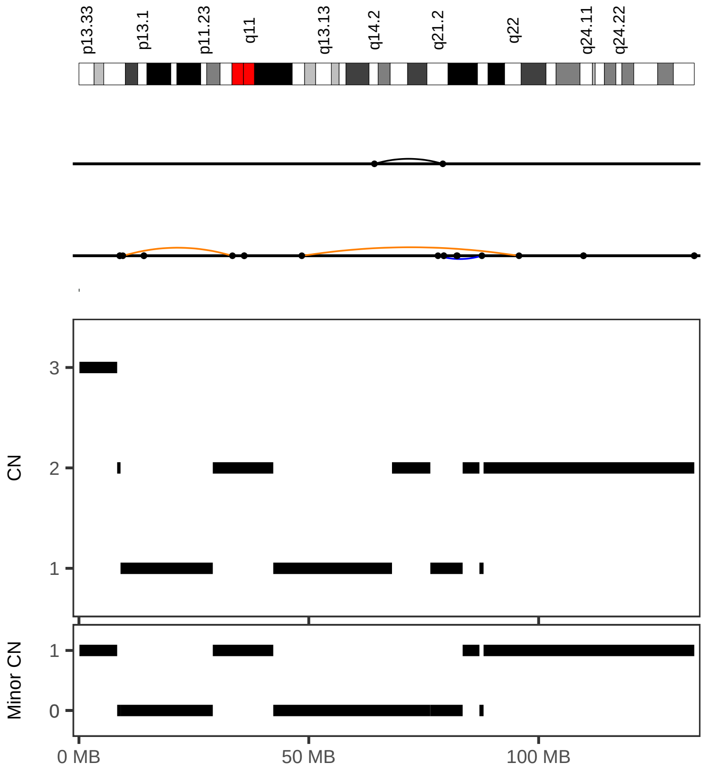

	CGP_donor_1347720
Cancer type	Breast−AdenoCA
Position	12
Interleaved intrachr. SVs	2
Total SVs (intrachr. + transl.)	2
SV types	DEL: 0; DUP: 1; h2hINV: 1; t2tINV: 0; TRA: 0
SVs in sample	173
Oscillating CN in chr (2 and 3 states)	9,
CN segments	10
Purity, ploidy	0.37, 1.59

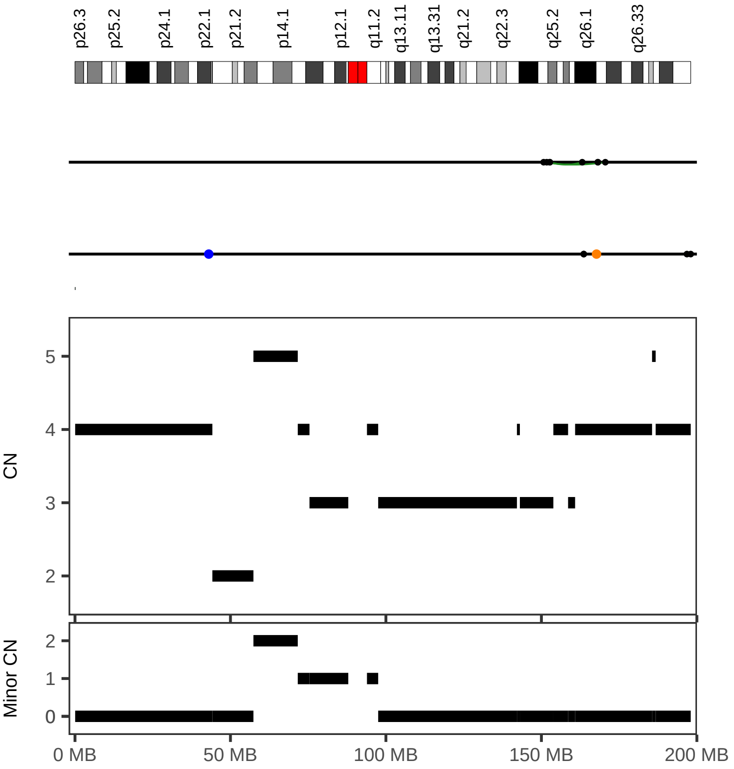

	CGP_donor_1353432
Cancer type	Breast−AdenoCA
Position	3
Interleaved intrachr. SVs	2
Total SVs (intrachr. + transl.)	2
SV types	DEL: 0; DUP: 0; h2hINV: 1; t2tINV: 1; TRA: 0
SVs in sample	185
Oscillating CN in chr (2 and 3 states)	9,
CN segments	14
Purity, ploidy	0.89, 3.18

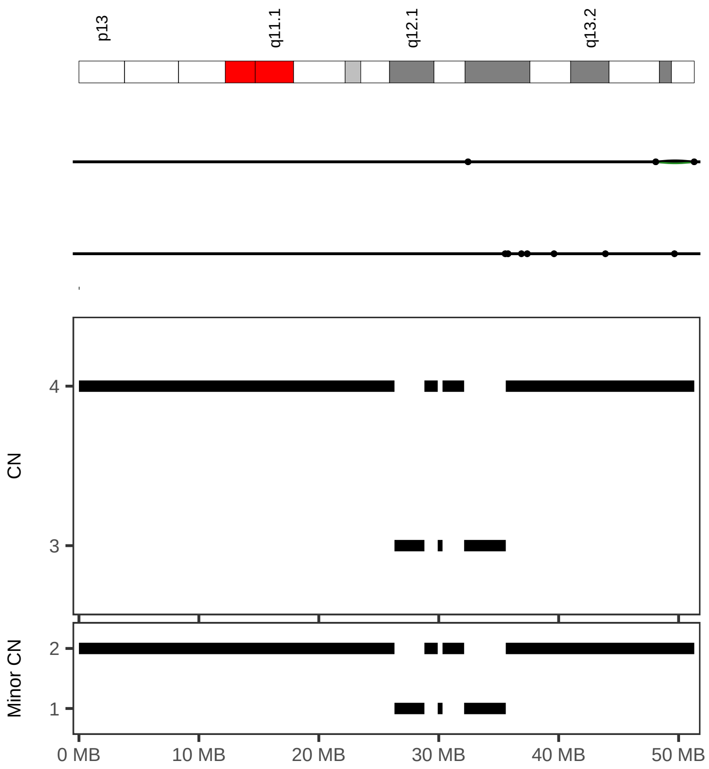

	CGP_donor_1363963
Cancer type	Breast−AdenoCA
Position	22
Interleaved intrachr. SVs	2
Total SVs (intrachr. + transl.)	2
SV types	DEL: 0; DUP: 0; h2hINV: 1; t2tINV: 1; TRA: 0
SVs in sample	324
Oscillating CN in chr (2 and 3 states)	7,
CN segments	7
Purity, ploidy	0.18, 2.98

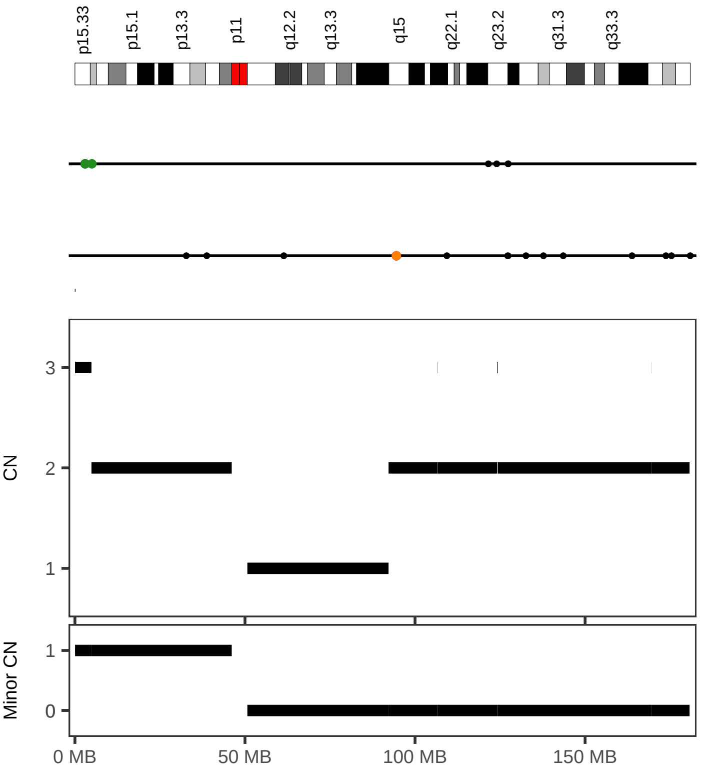

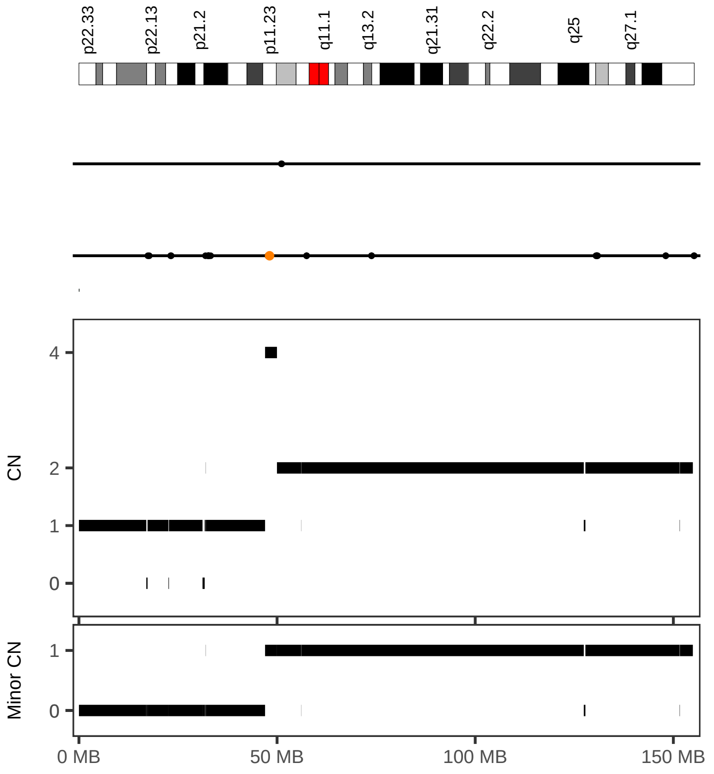

	CGP_donor_1374619
Cancer type	Breast−AdenoCA
Position	5
Interleaved intrachr. SVs	2
Total SVs (intrachr. + transl.)	2
SV types	DEL: 0; DUP: 0; h2hINV: 1; t2tINV: 1; TRA: 0
SVs in sample	273
Oscillating CN in chr (2 and 3 states)	7,
CN segments	10
Purity, ploidy	0.38, 1.62

	CGP_donor_1472394
Cancer type	Breast−AdenoCA
Position	X
Interleaved intrachr. SVs	2
Total SVs (intrachr. + transl.)	2
SV types	DEL: 2; DUP: 0; h2hINV: 0; t2tINV: 0; TRA: 0
SVs in sample	366
Oscillating CN in chr (2 and 3 states)	7,
CN segments	17
Purity, ploidy	0.7, 1.76

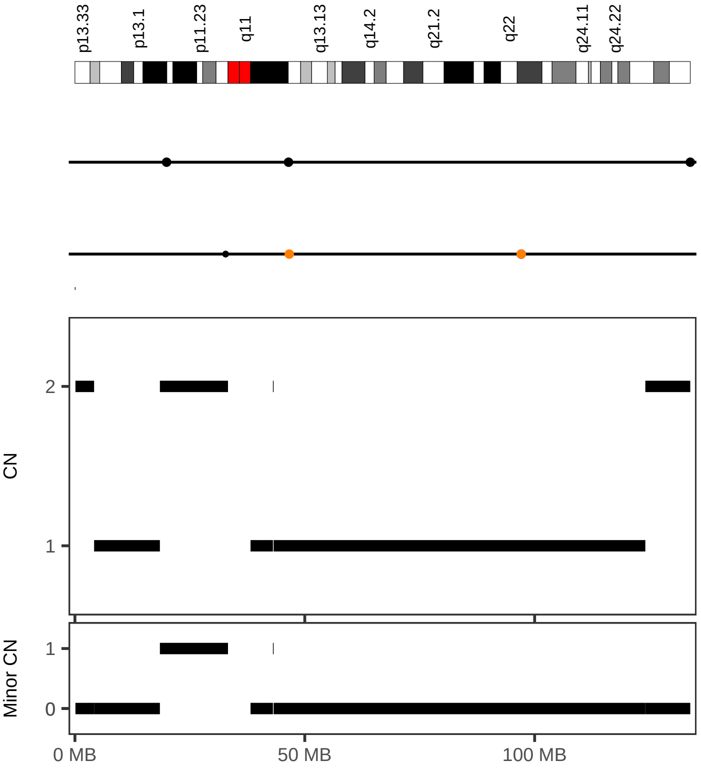

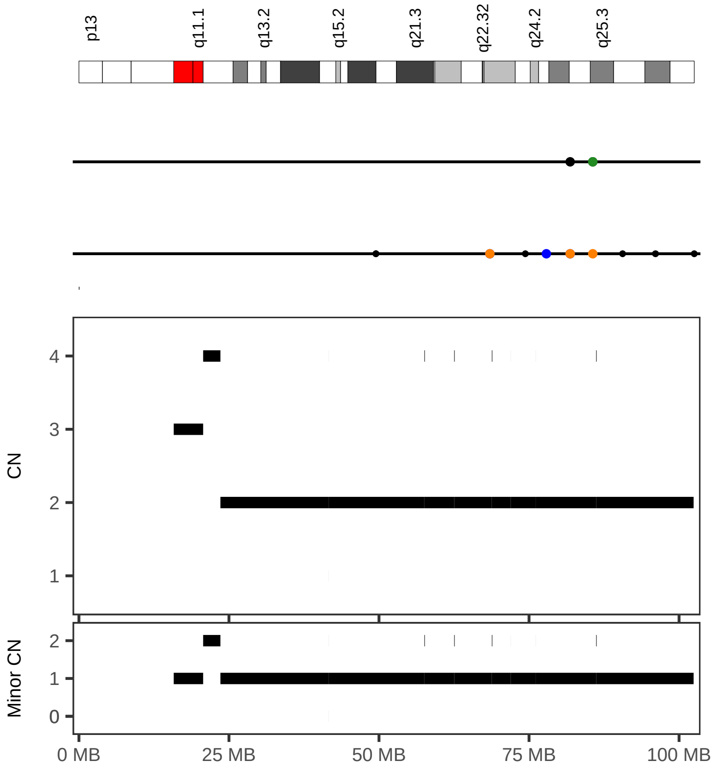

	1549dc64−3dab−43fc−96e9−b07d520957e1
Cancer type	Breast−AdenoCA
Position	12
Interleaved intrachr. SVs	0
Total SVs (intrachr. + transl.)	0
SV types	DEL: 0; DUP: 0; h2hINV: 0; t2tINV: 0; TRA: 0
SVs in sample	226
Oscillating CN in chr (2 and 3 states)	7,
CN segments	7
Purity, ploidy	0.79, 1.68

	174850b4−5ec2−462b−a890−89bd1716b3c2
Cancer type	Breast−AdenoCA
Position	15
Interleaved intrachr. SVs	1
Total SVs (intrachr. + transl.)	1
SV types	DEL: 1; DUP: 0; h2hINV: 0; t2tINV: 0; TRA: 0
SVs in sample	456
Oscillating CN in chr (2 and 3 states)	14,
CN segments	18
Purity, ploidy	0.47, 3.02

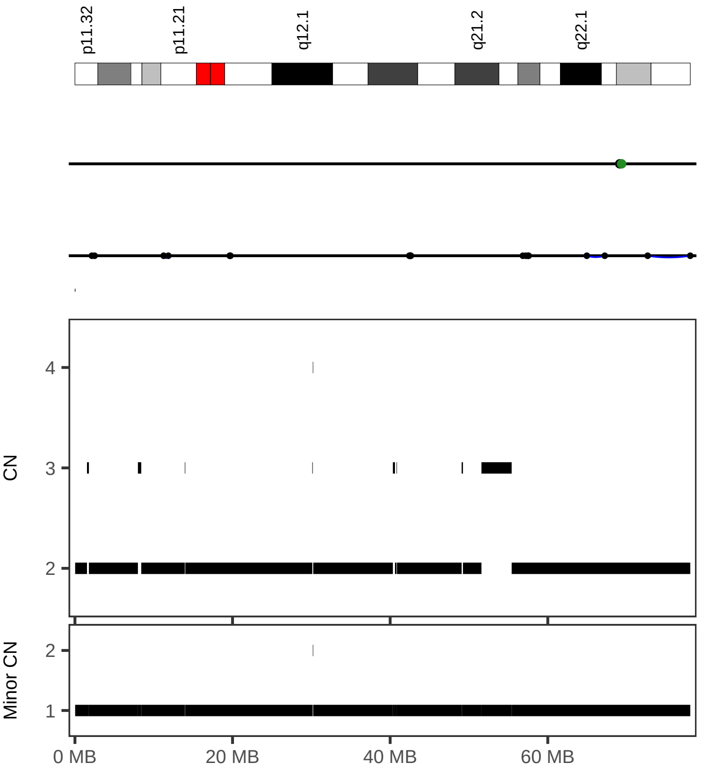

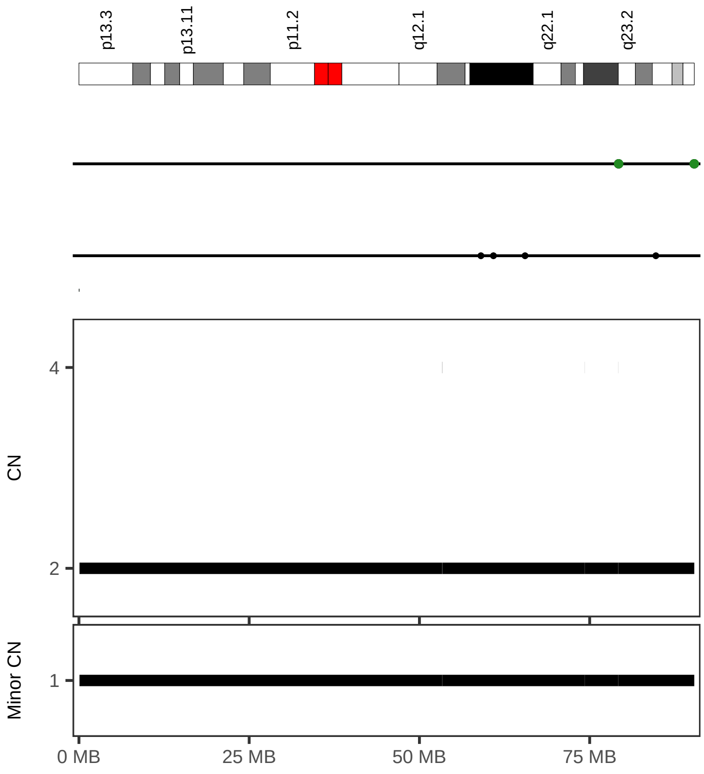

	**5ed024e8−d05e−4c65−9441−eda9930ccc82**
Cancer type	Breast−AdenoCA
Position	18
Interleaved intrachr. SVs	2
Total SVs (intrachr. + transl.)	2
SV types	DEL: 0; DUP: 2; h2hINV: 0;<br>t2tINV: 0; TRA: 0
SVs in sample	475
Oscillating CN in chr (2 and 3 states)	9,
CN segments	18
Purity, ploidy	0.38, 2.04

	**6956473b−92e5−4069−a26b−b48e280e76f2**
Cancer type	Breast−AdenoCA
Position	16
Interleaved intrachr. SVs	1
Total SVs (intrachr. + transl.)	1
SV types	DEL: 0; DUP: 1; h2hINV: 0;<br>t2tINV: 0; TRA: 0
SVs in sample	227
Oscillating CN in chr (2 and 3 states)	7,
CN segments	7
Purity, ploidy	0.56, 1.61

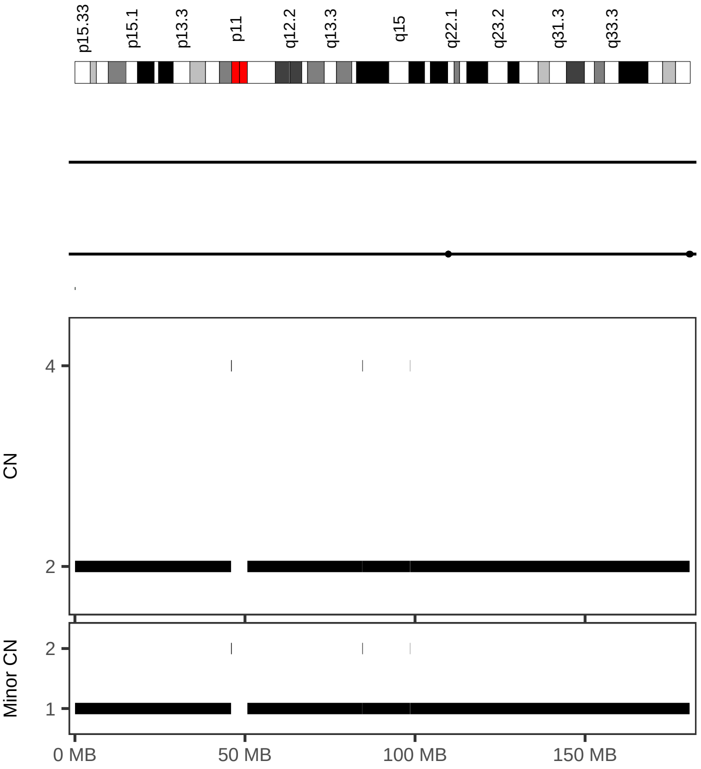

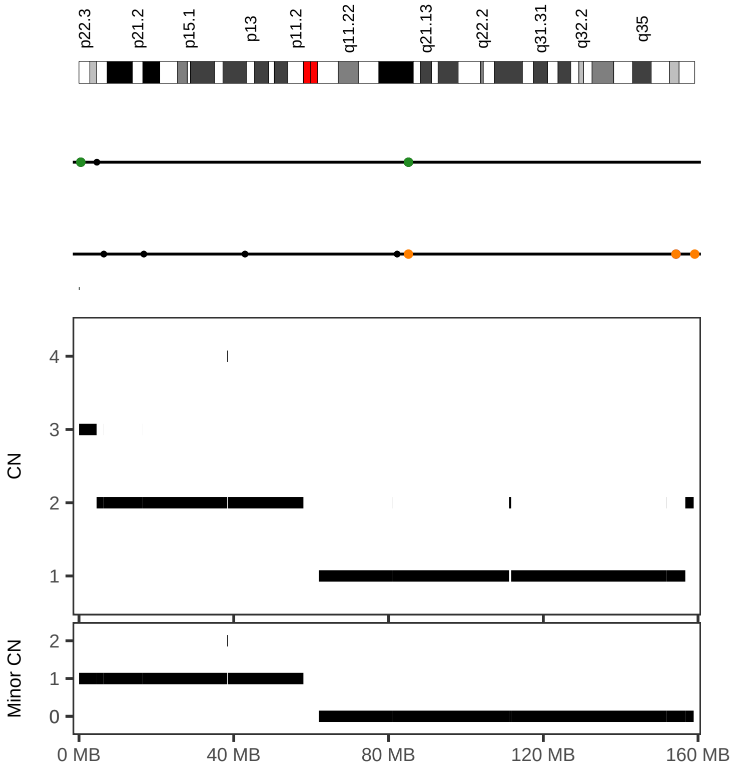

	**75113445−d2d6−44a0−866c−c9175e6d214b**
Cancer type	Breast−AdenoCA
Position	5
Interleaved intrachr. SVs	1
Total SVs (intrachr. + transl.)	1
SV types	DEL: 0; DUP: 1; h2hINV: 0;<br>t2tINV: 0; TRA: 0
SVs in sample	212
Oscillating CN in chr (2 and 3 states)	7,
CN segments	7
Purity, ploidy	0.76, 2.35

	**790ff5db−b7f3−4946−aaed−305f66b1dd6a**
Cancer type	Breast−AdenoCA
Position	7
Interleaved intrachr. SVs	1
Total SVs (intrachr. + transl.)	1
SV types	DEL: 0; DUP: 1; h2hINV: 0;<br>t2tINV: 0; TRA: 0
SVs in sample	455
Oscillating CN in chr (2 and 3 states)	9,
CN segments	16
Purity, ploidy	0.54, 1.76

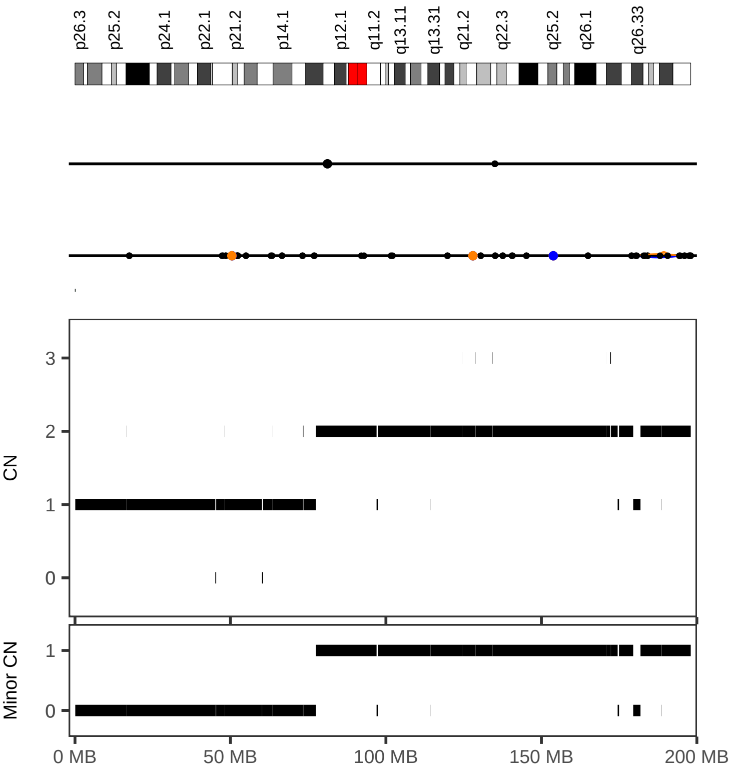

	88db1340−e4bf−451a−87c0−6e9168296f5e
Cancer type	Breast−AdenoCA
Position	3
Interleaved intrachr. SVs	2
Total SVs (intrachr. + transl.)	2
SV types	DEL: 0; DUP: 0; h2hINV: 1; t2tINV: 1; TRA: 0
SVs in sample	415
Oscillating CN in chr (2 and 3 states)	11,
CN segments	34
Purity, ploidy	0.53, 1.49

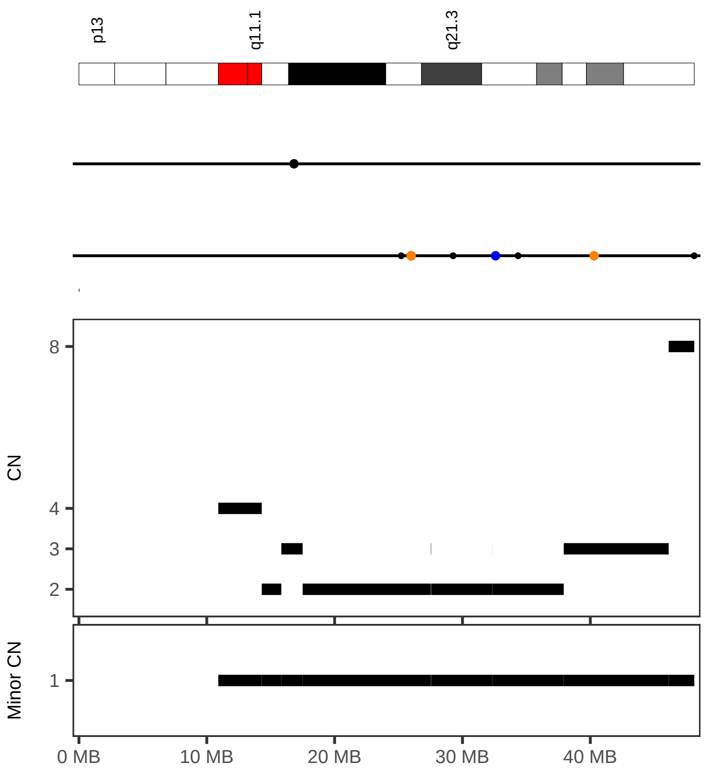

	9f6be944−83de−42ab−8738−f0022f475e61
Cancer type	Breast−AdenoCA
Position	21
Interleaved intrachr. SVs	1
Total SVs (intrachr. + transl.)	1
SV types	DEL: 0; DUP: 1; h2hINV: 0; t2tINV: 0; TRA: 0
SVs in sample	864
Oscillating CN in chr (2 and 3 states)	8,
CN segments	10
Purity, ploidy	0.6, 3.26

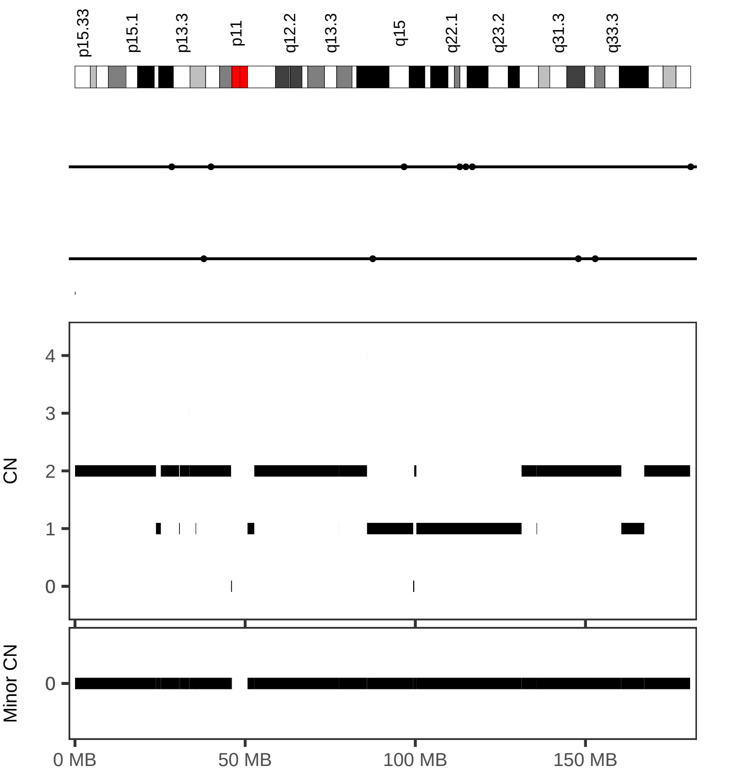

	a9b7d7fe−be31−4f71−afee−c1bfdf511888
Cancer type	Breast−AdenoCA
Position	5
Interleaved intrachr. SVs	1
Total SVs (intrachr. + transl.)	1
SV types	DEL: 0; DUP: 1; h2hINV: 0; t2tINV: 0; TRA: 0
SVs in sample	460
Oscillating CN in chr (2 and 3 states)	7,
CN segments	24
Purity, ploidy	0.47, 2.76

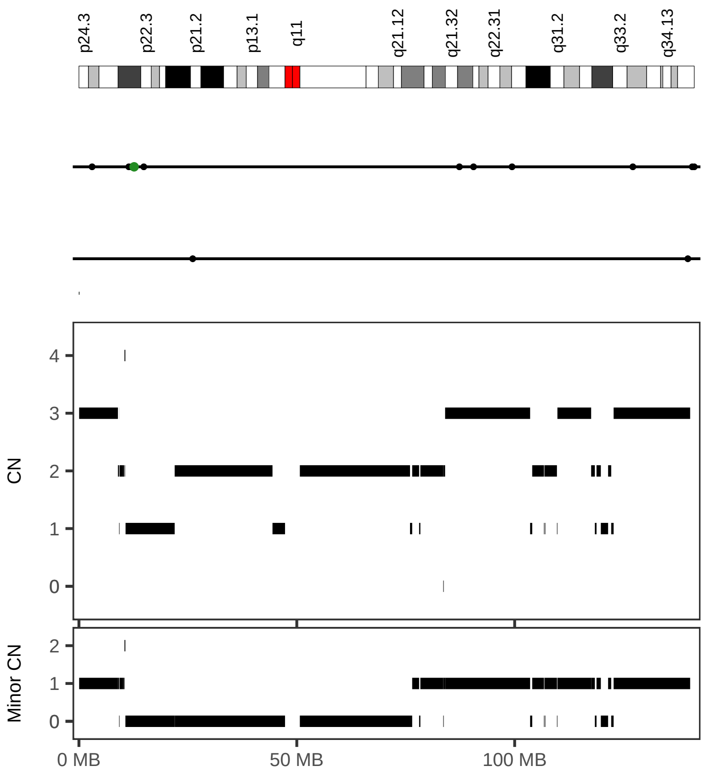

	a9b7d7fe−be31−4f71−afee−c1bfdf511888
Cancer type	Breast−AdenoCA
Position	9
Interleaved intrachr. SVs	1
Total SVs (intrachr. + transl.)	1
SV types	DEL: 1; DUP: 0; h2hINV: 0; t2tINV: 0; TRA: 0
SVs in sample	460
Oscillating CN in chr (2 and 3 states)	9,
CN segments	35
Purity, ploidy	0.47, 2.76

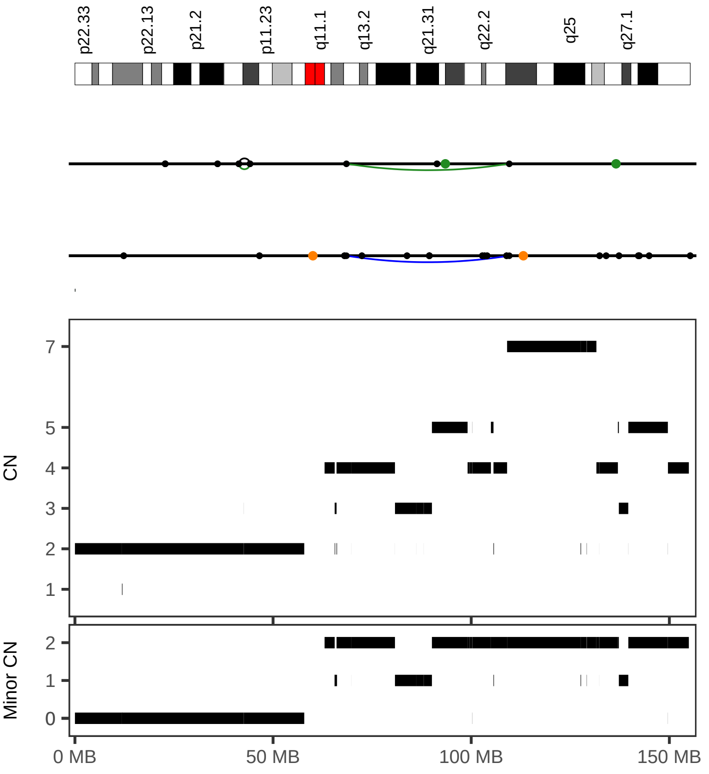

	d2f2560c−ec80−4fea−9474−c47a2e85ea95
Cancer type	Breast−AdenoCA
Position	X
Interleaved intrachr. SVs	2
Total SVs (intrachr. + transl.)	2
SV types	DEL: 0; DUP: 0; h2hINV: 1; t2tINV: 1; TRA: 0
SVs in sample	546
Oscillating CN in chr (2 and 3 states)	7,
CN segments	41
Purity, ploidy	0.67, 3.45

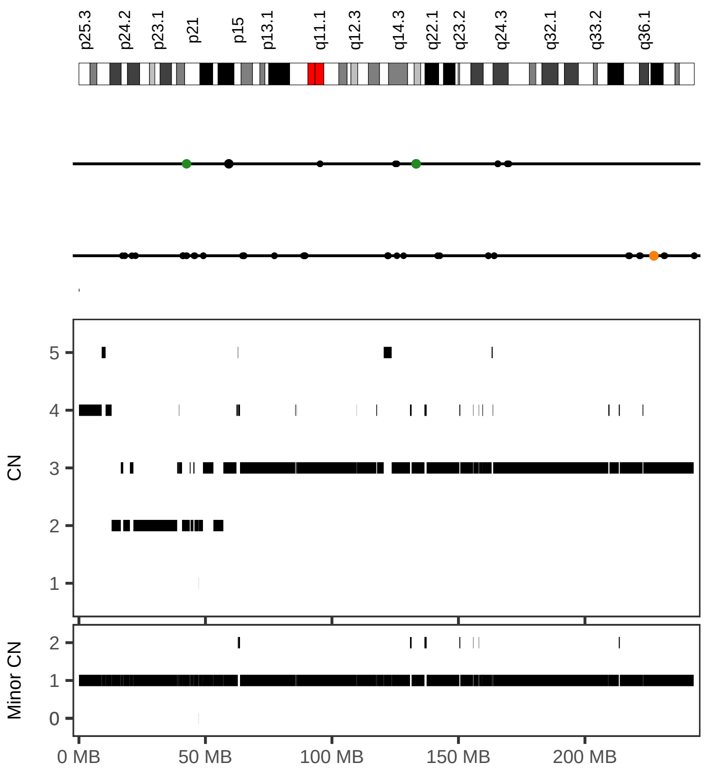

	e4fc0909−f284−4471−866d−d8967b6adcbc
Cancer type	Breast−AdenoCA
Position	2
Interleaved intrachr. SVs	2
Total SVs (intrachr. + transl.)	2
SV types	DEL: 0; DUP: 1; h2hINV: 0; t2tINV: 1; TRA: 0
SVs in sample	630
Oscillating CN in chr (2 and 3 states)	15,
CN segments	60
Purity, ploidy	0.49, 2.63

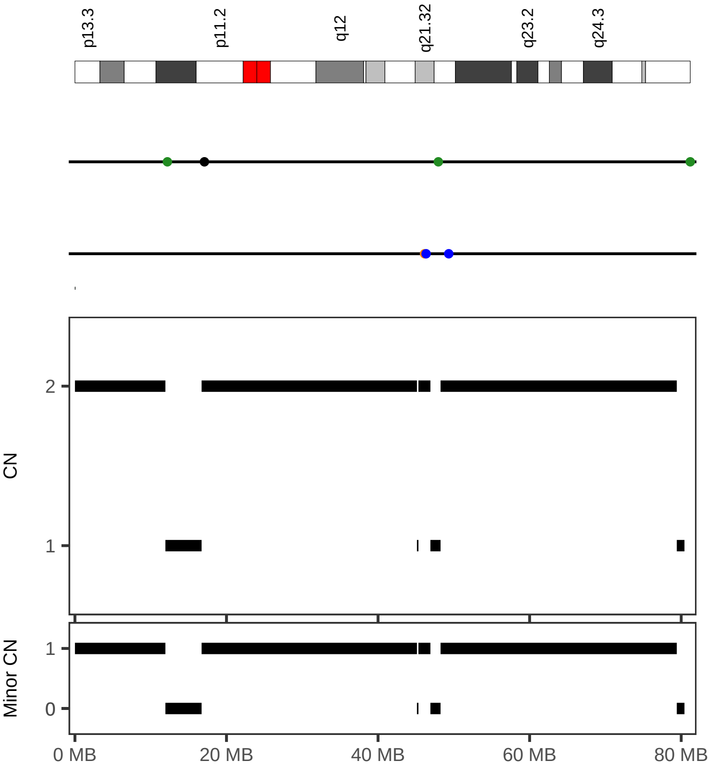

	f05d314c−5ec5−4e2a−b785−9a702716f111
Cancer type	Breast−AdenoCA
Position	17
Interleaved intrachr. SVs	0
Total SVs (intrachr. + transl.)	0
SV types	DEL: 0; DUP: 0; h2hINV: 0; t2tINV: 0; TRA: 0
SVs in sample	16
Oscillating CN in chr (2 and 3 states)	8,
CN segments	8
Purity, ploidy	0.89, 2

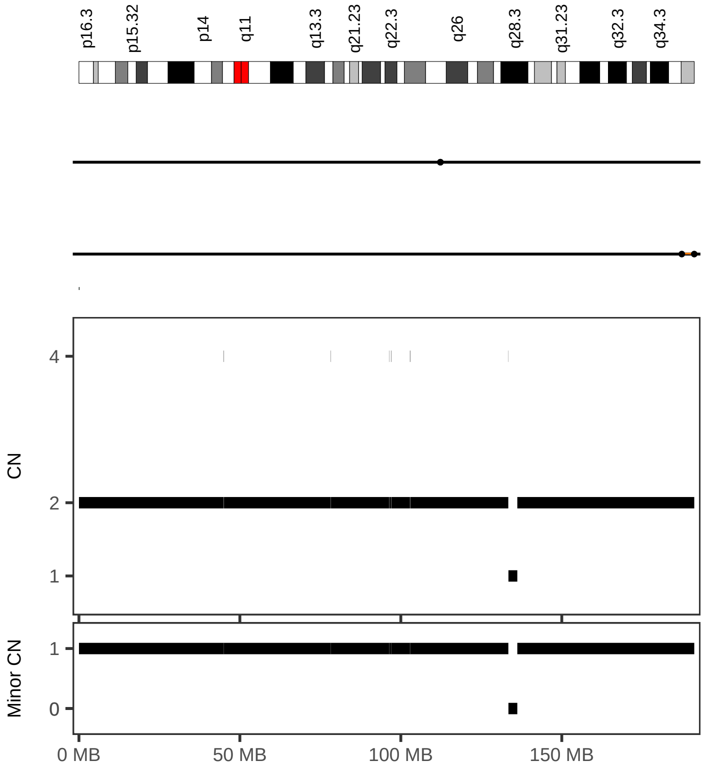

	CGP_donor_1451422
Cancer type	Breast−LobularCA
Position	4
Interleaved intrachr. SVs	1
Total SVs (intrachr. + transl.)	1
SV types	DEL: 1; DUP: 0; h2hINV: 0; t2tINV: 0; TRA: 0
SVs in sample	22
Oscillating CN in chr (2 and 3 states)	12,
CN segments	14
Purity, ploidy	0.57, 1.96

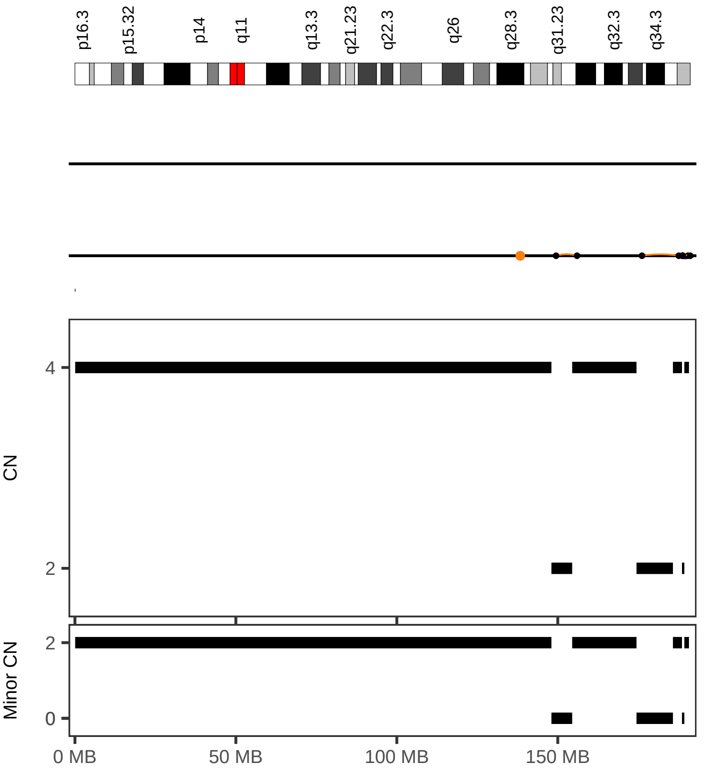

	a3be445a−a592−4073−815b−47ae41f9c4a0
Cancer type	Cervix−AdenoCA
Position	4
Interleaved intrachr. SVs	2
Total SVs (intrachr. + transl.)	2
SV types	DEL: 1; DUP: 1; h2hINV: 0; t2tINV: 0; TRA: 0
SVs in sample	44
Oscillating CN in chr (2 and 3 states)	7,
CN segments	7
Purity, ploidy	0.77, 3.59

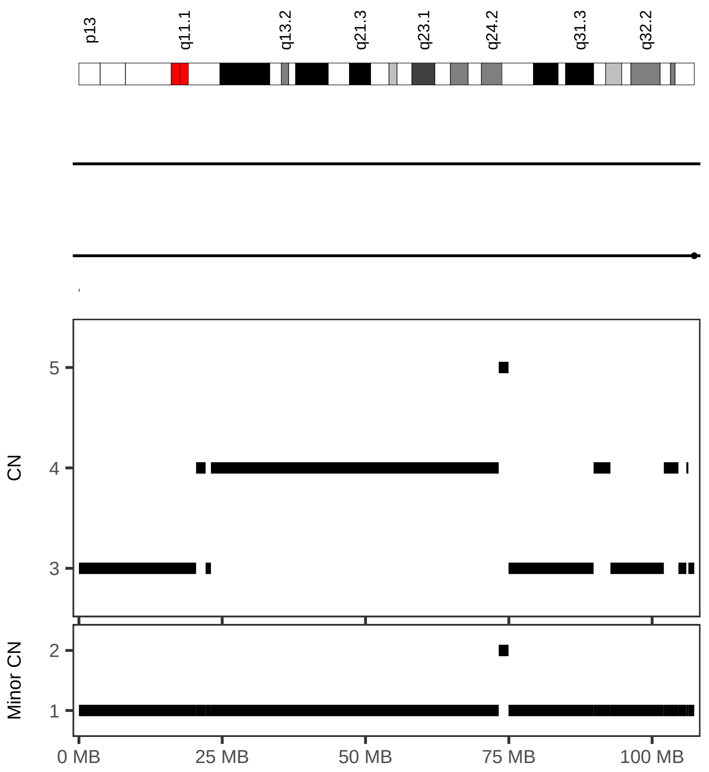

	941f75a1−fea4−4539−ba69−60bb11608f6d
Cancer type	Cervix−SCC
Position	14
Interleaved intrachr. SVs	0
Total SVs (intrachr. + transl.)	0
SV types	DEL: 0; DUP: 0; h2hINV: 0; t2tINV: 0; TRA: 0
SVs in sample	58
Oscillating CN in chr (2 and 3 states)	7,
CN segments	12
Purity, ploidy	0.68, 2.71

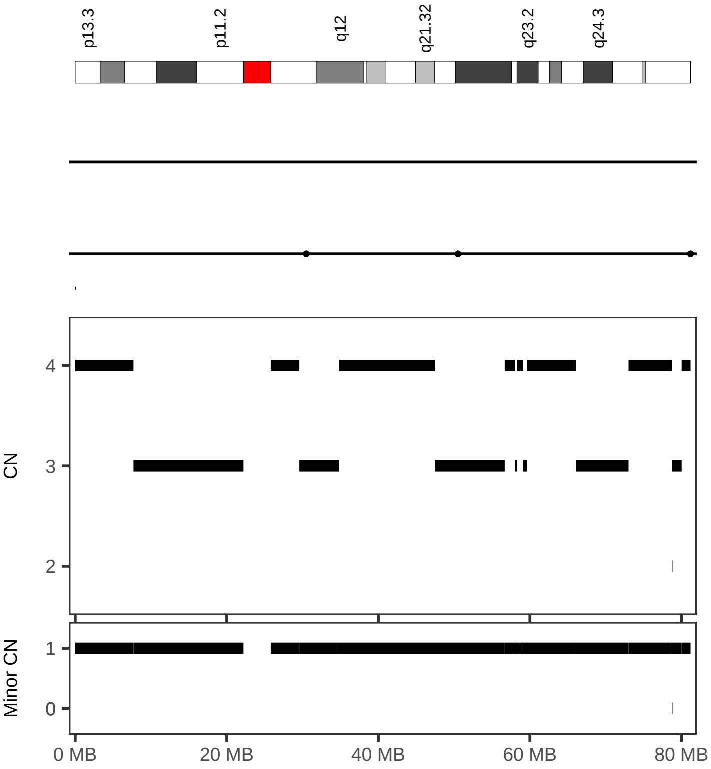

	941f75a1−fea4−4539−ba69−60bb11608f6d
Cancer type	Cervix−SCC
Position	17
Interleaved intrachr. SVs	1
Total SVs (intrachr. + transl.)	1
SV types	DEL: 1; DUP: 0; h2hINV: 0; t2tINV: 0; TRA: 0
SVs in sample	58
Oscillating CN in chr (2 and 3 states)	13,
CN segments	16
Purity, ploidy	0.68, 2.71

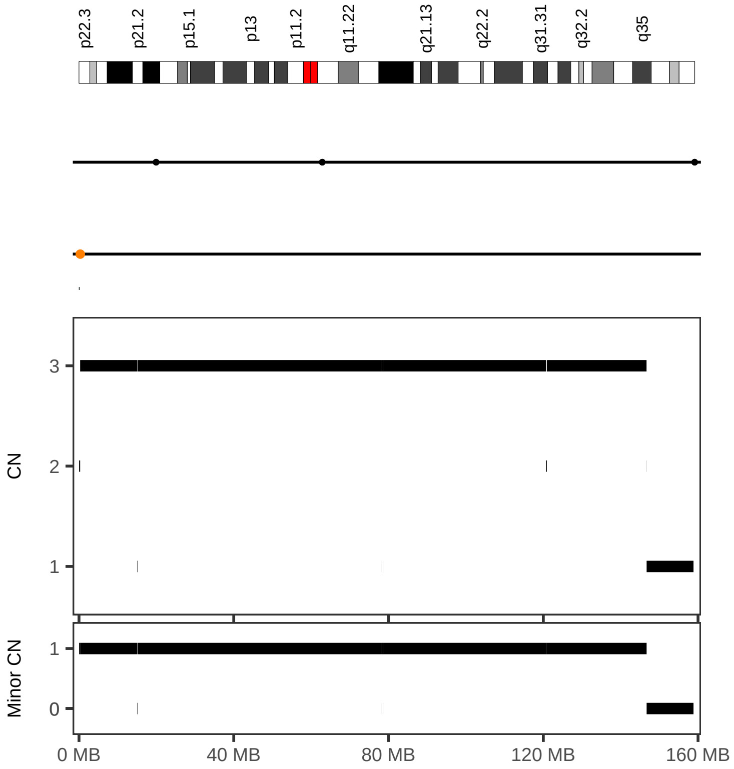

	e733f289−a0ec−478c−aad9−1378ad26ddfa
Cancer type	Cervix−SCC
Position	7
Interleaved intrachr. SVs	1
Total SVs (intrachr. + transl.)	1
SV types	DEL: 0; DUP: 0; h2hINV: 1; t2tINV: 0; TRA: 0
SVs in sample	63
Oscillating CN in chr (2 and 3 states)	7,
CN segments	12
Purity, ploidy	0.64, 2.3

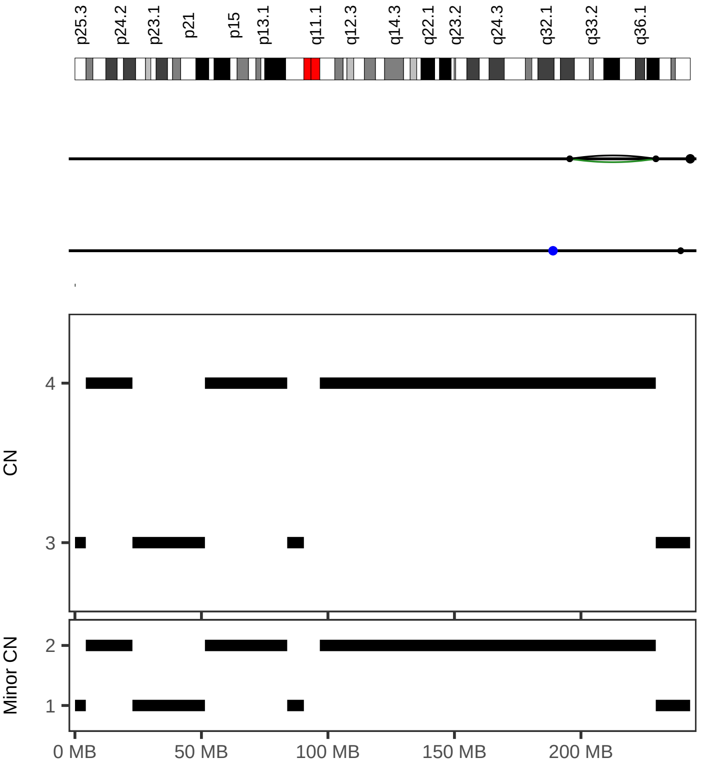

	eb59cf69−1997−41b9−bf69−69ad7da292a1
Cancer type	Cervix−SCC
Position	2
Interleaved intrachr. SVs	2
Total SVs (intrachr. + transl.)	2
SV types	DEL: 0; DUP: 0; h2hINV: 1;<br>t2tINV: 1; TRA: 0
SVs in sample	63
Oscillating CN in chr (2 and 3 states)	7,
CN segments	7
Purity, ploidy	0.4, 3.41

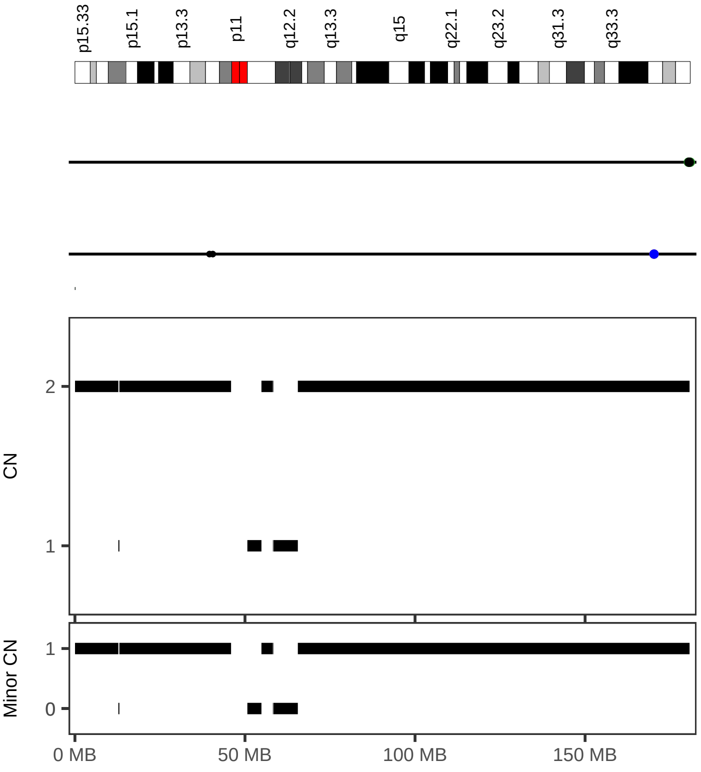

	17dffffc−65d6−4209−9075−18a441001f0f
Cancer type	CNS−GBM
Position	5
Interleaved intrachr. SVs	1
Total SVs (intrachr. + transl.)	1
SV types	DEL: 1; DUP: 0; h2hINV: 0;<br>t2tINV: 0; TRA: 0
SVs in sample	93
Oscillating CN in chr (2 and 3 states)	9,
CN segments	9
Purity, ploidy	0.48, 1.95

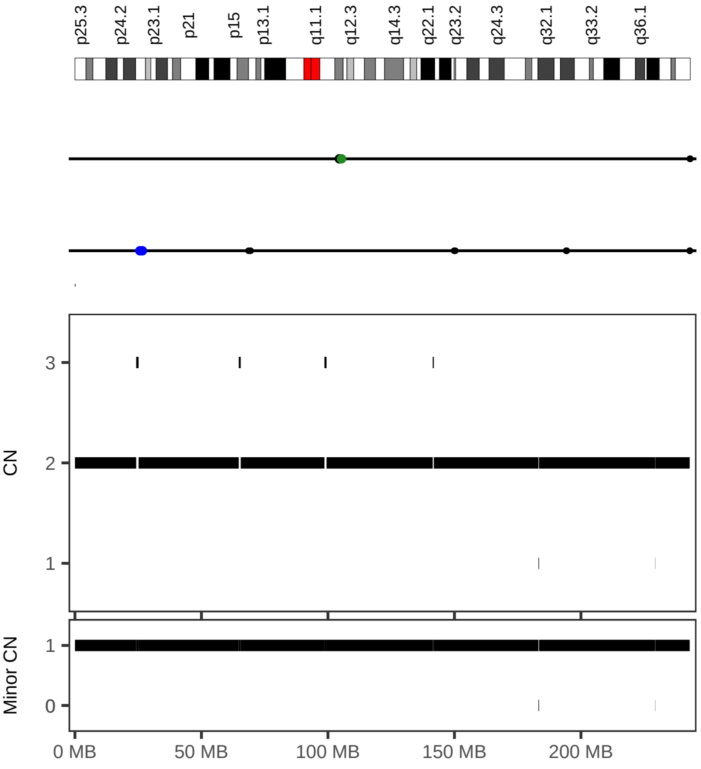

	3258cb3b−f63b−463c−b2e4−d638149157c5
Cancer type	CNS−GBM
Position	2
Interleaved intrachr. SVs	2
Total SVs (intrachr. + transl.)	2
SV types	DEL: 0; DUP: 0; h2hINV: 1;<br>t2tINV: 1; TRA: 0
SVs in sample	59
Oscillating CN in chr (2 and 3 states)	9,
CN segments	13
Purity, ploidy	0.81, 2.09

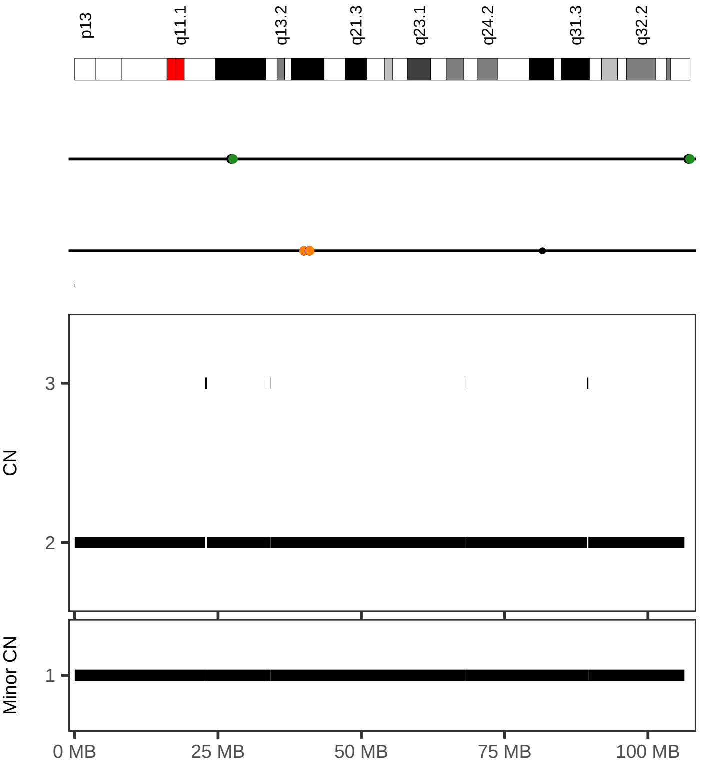

	6e6962d8−34b8−431c−8220−42b0b92a410b
Cancer type	CNS−GBM
Position	14
Interleaved intrachr. SVs	1
Total SVs (intrachr. + transl.)	1
SV types	DEL: 0; DUP: 1; h2hINV: 0;<br>t2tINV: 0; TRA: 0
SVs in sample	86
Oscillating CN in chr (2 and 3 states)	11,
CN segments	11
Purity, ploidy	0.86, 1.91

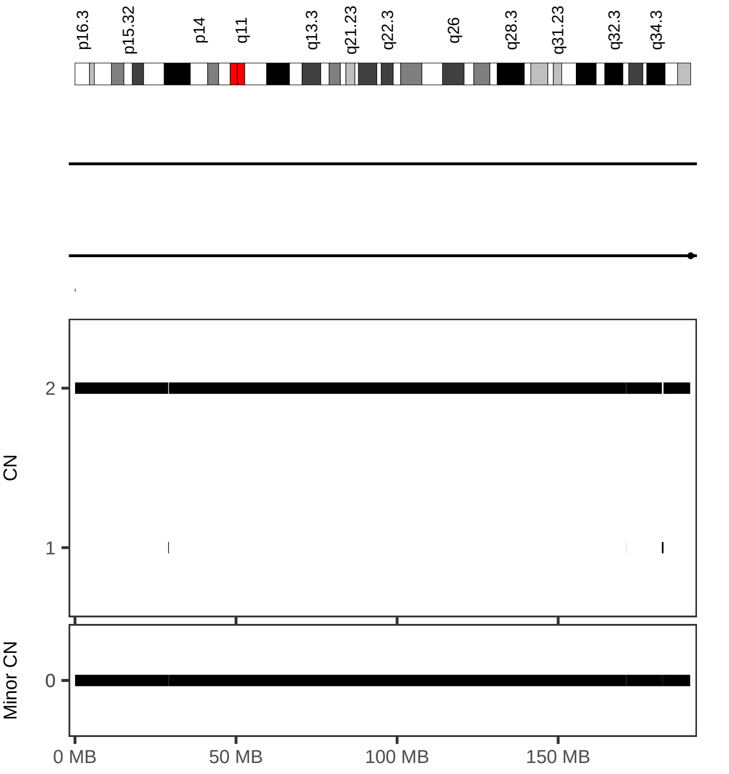

	**a515cf2d−e918−4958−9bf6−e611b425a97e**
Cancer type	CNS−GBM
Position	4
Interleaved intrachr. SVs	1
Total SVs (intrachr. + transl.)	1
SV types	DEL: 1; DUP: 0; h2hINV: 0; t2tINV: 0; TRA: 0
SVs in sample	39
Oscillating CN in chr (2 and 3 states)	7,
CN segments	7
Purity, ploidy	0.53, 2.77

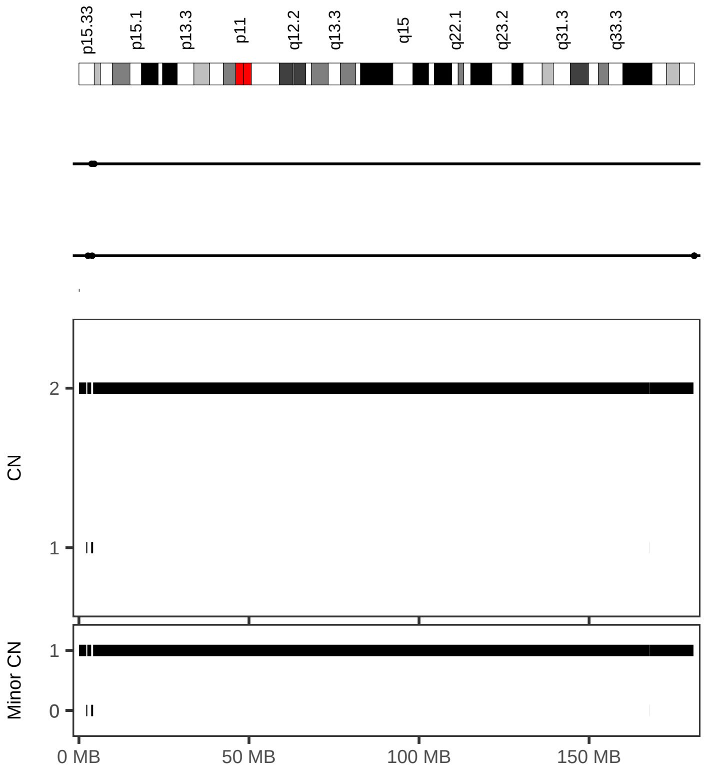

	**be3a7ef3−34ed−40e1−9d9c−187940596b26**
Cancer type	CNS−GBM
Position	5
Interleaved intrachr. SVs	2
Total SVs (intrachr. + transl.)	2
SV types	DEL: 0; DUP: 1; h2hINV: 0; t2tINV: 1; TRA: 0
SVs in sample	93
Oscillating CN in chr (2 and 3 states)	7,
CN segments	7
Purity, ploidy	0.71, 1.97

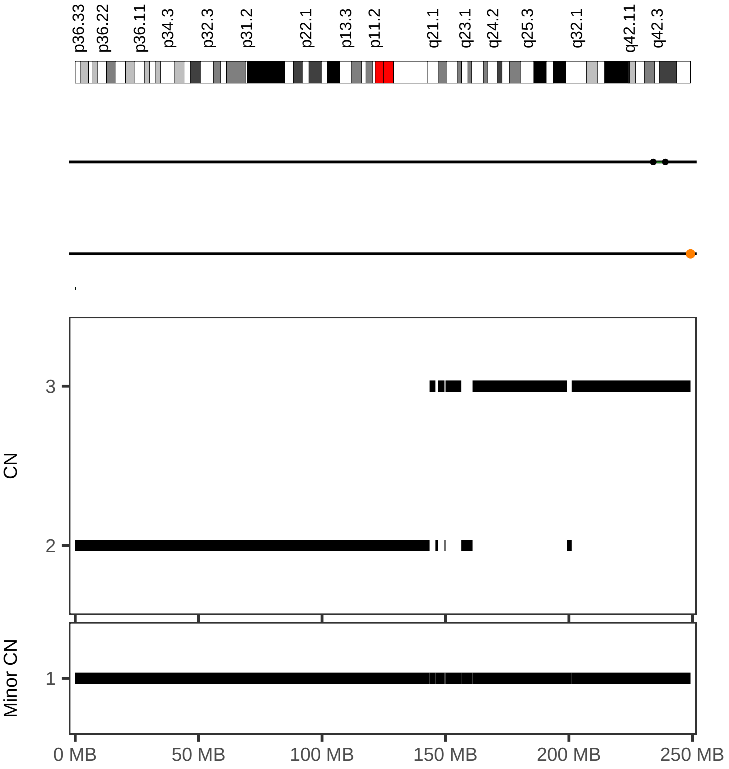

	**ICGC_MB36**
Cancer type	CNS−Medullo
Position	1
Interleaved intrachr. SVs	1
Total SVs (intrachr. + transl.)	1
SV types	DEL: 0; DUP: 0; h2hINV: 0; t2tINV: 1; TRA: 0
SVs in sample	17
Oscillating CN in chr (2 and 3 states)	10,
CN segments	10
Purity, ploidy	0.99, 2.32

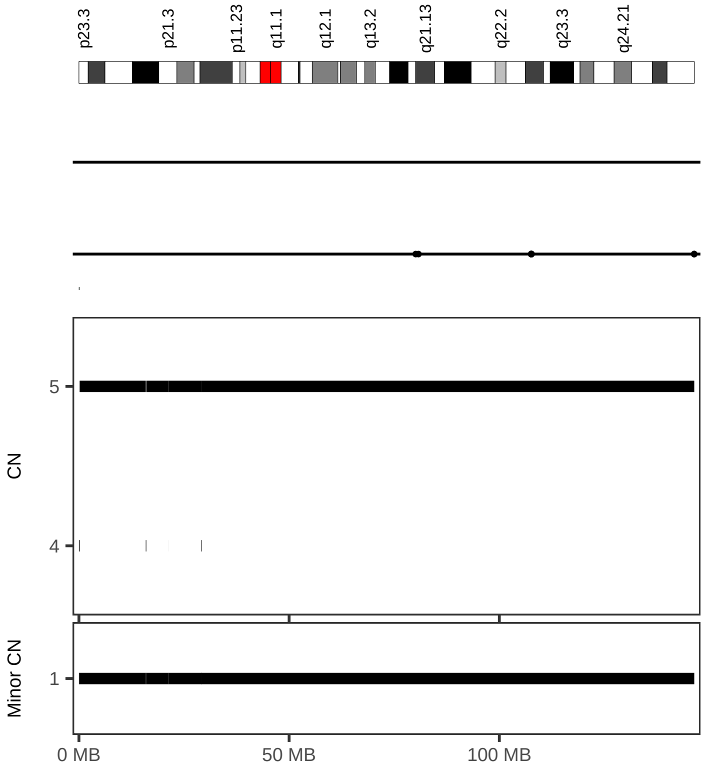

	**490eabca−4575−4bd6−9604−6b6e23d069ce**
Cancer type	ColoRect−AdenoCA
Position	8
Interleaved intrachr. SVs	1
Total SVs (intrachr. + transl.)	1
SV types	DEL: 1; DUP: 0; h2hINV: 0; t2tINV: 0; TRA: 0
SVs in sample	95
Oscillating CN in chr (2 and 3 states)	8,
CN segments	8
Purity, ploidy	0.72, 4.02

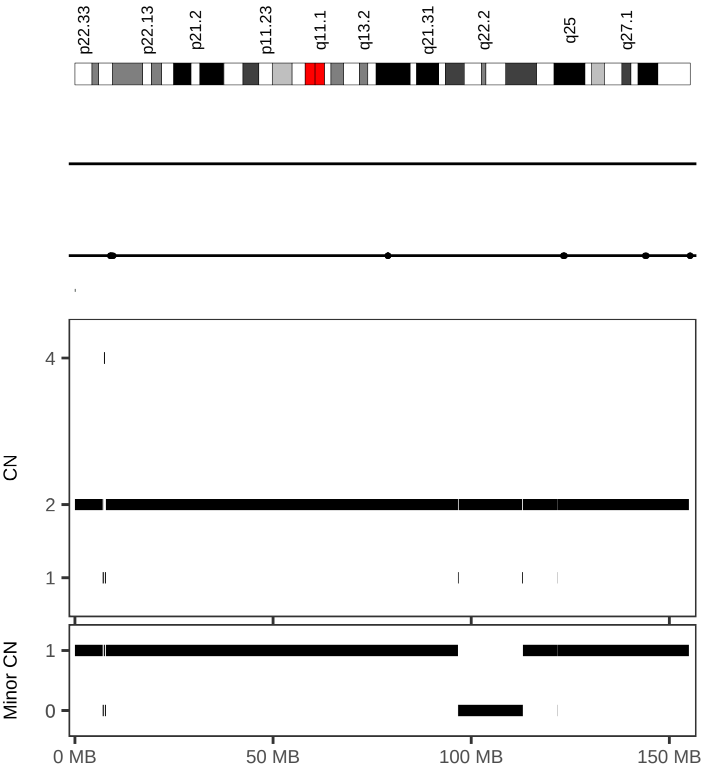

	bfb07784−693b−4c25−874e−4ad6e04a5d46
Cancer type	ColoRect−AdenoCA
Position	X
Interleaved intrachr. SVs	2
Total SVs (intrachr. + transl.)	2
SV types	DEL: 2; DUP: 0; h2hINV: 0; t2tINV: 0; TRA: 0
SVs in sample	154
Oscillating CN in chr (2 and 3 states)	8,
CN segments	12
Purity, ploidy	0.58, 2.21

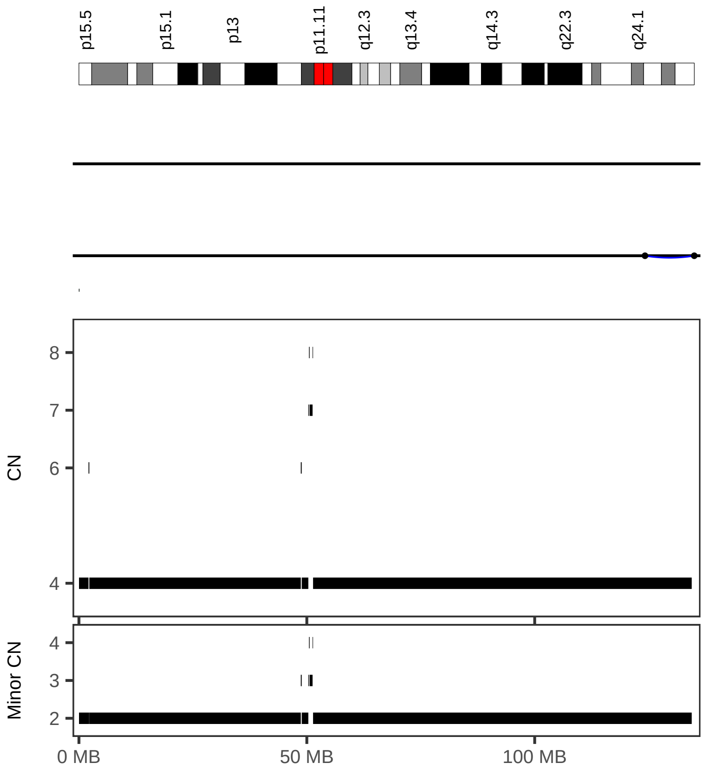

	c83d38fc−e011−4f75−a100−96513611f3e9
Cancer type	ColoRect−AdenoCA
Position	11
Interleaved intrachr. SVs	1
Total SVs (intrachr. + transl.)	1
SV types	DEL: 0; DUP: 1; h2hINV: 0; t2tINV: 0; TRA: 0
SVs in sample	18
Oscillating CN in chr (2 and 3 states)	7,
CN segments	12
Purity, ploidy	0.56, 3.79

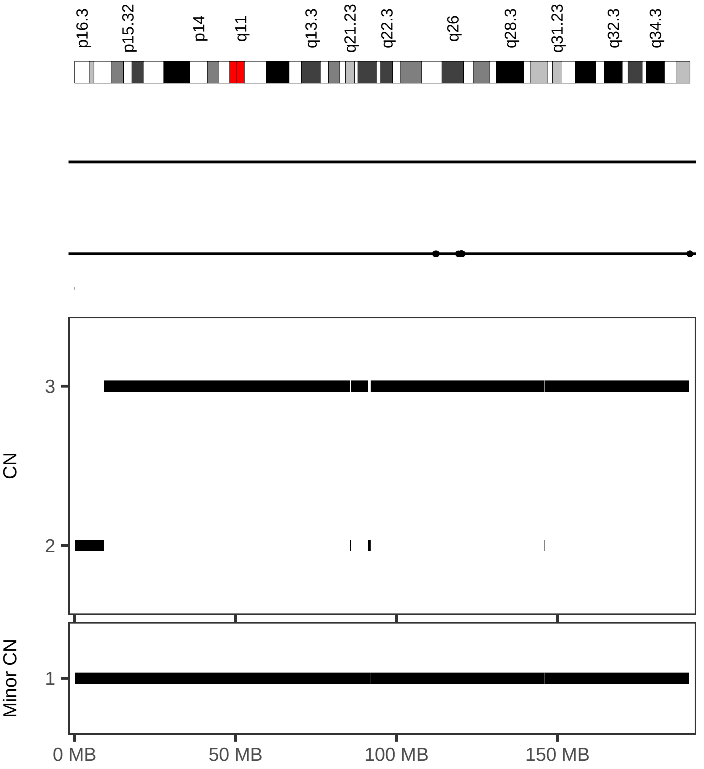

	fafd4576−0709−49e1−926f−ad1ee09907ec
Cancer type	ColoRect−AdenoCA
Position	4
Interleaved intrachr. SVs	2
Total SVs (intrachr. + transl.)	2
SV types	DEL: 2; DUP: 0; h2hINV: 0; t2tINV: 0; TRA: 0
SVs in sample	146
Oscillating CN in chr (2 and 3 states)	8,
CN segments	8
Purity, ploidy	0.88, 3.93

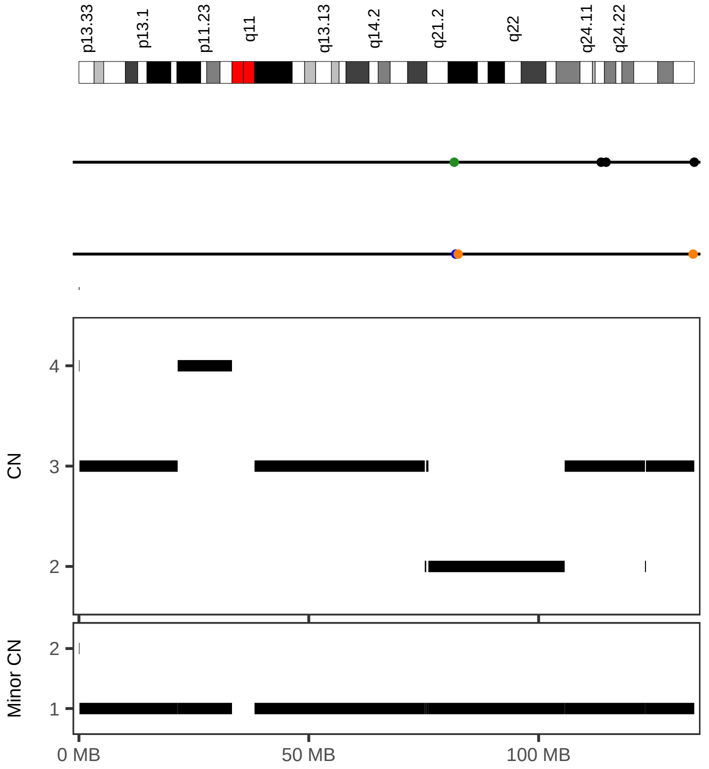

	0bb786ee−07cf−4853−9b49−ec95cd14e282
Cancer type	ColoRect−AdenoCA
Position	12
Interleaved intrachr. SVs	0
Total SVs (intrachr. + transl.)	0
SV types	DEL: 0; DUP: 0; h2hINV: 0; t2tINV: 0; TRA: 0
SVs in sample	46
Oscillating CN in chr (2 and 3 states)	7,
CN segments	10
Purity, ploidy	0.66, 3.33

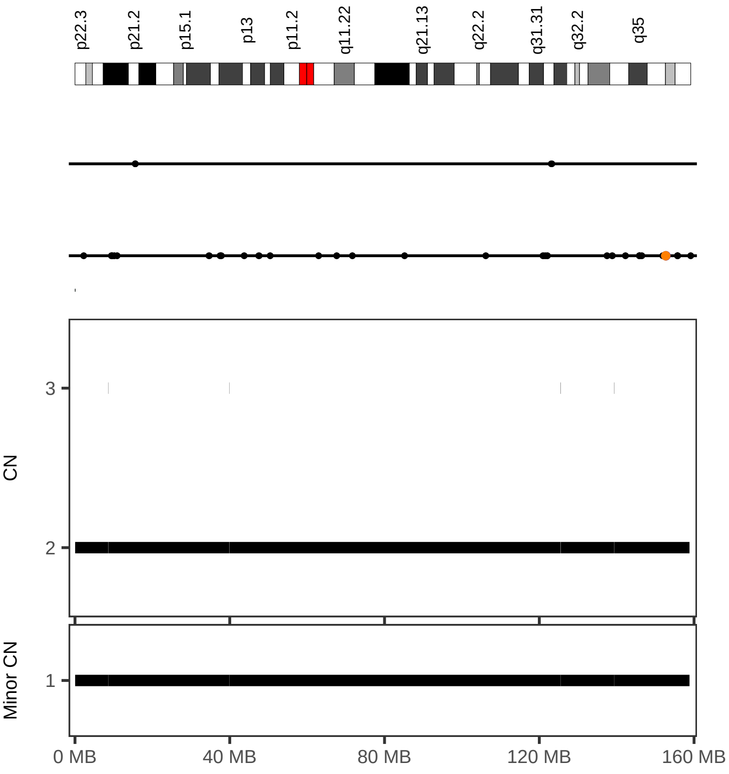

	8feb0e8f−d09b−437d−8651−0cdecfe776bf
Cancer type	ColoRect−AdenoCA
Position	7
Interleaved intrachr. SVs	2
Total SVs (intrachr. + transl.)	2
SV types	DEL: 0; DUP: 2; h2hINV: 0; t2tINV: 0; TRA: 0
SVs in sample	603
Oscillating CN in chr (2 and 3 states)	9,
CN segments	9
Purity, ploidy	0.75, 1.99

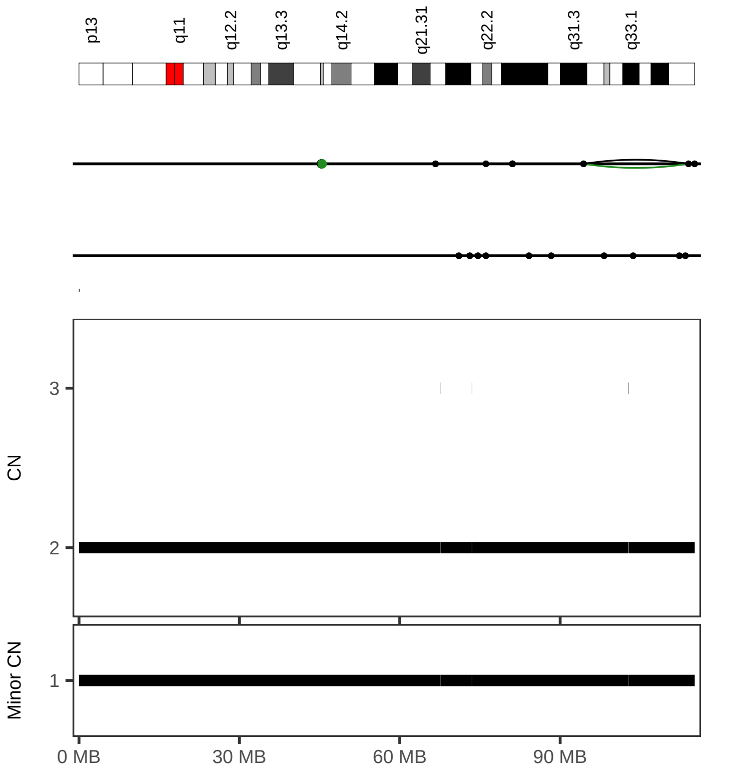

	8feb0e8f−d09b−437d−8651−0cdecfe776bf
Cancer type	ColoRect−AdenoCA
Position	13
Interleaved intrachr. SVs	2
Total SVs (intrachr. + transl.)	2
SV types	DEL: 0; DUP: 0; h2hINV: 1; t2tINV: 1; TRA: 0
SVs in sample	603
Oscillating CN in chr (2 and 3 states)	7,
CN segments	7
Purity, ploidy	0.75, 1.99

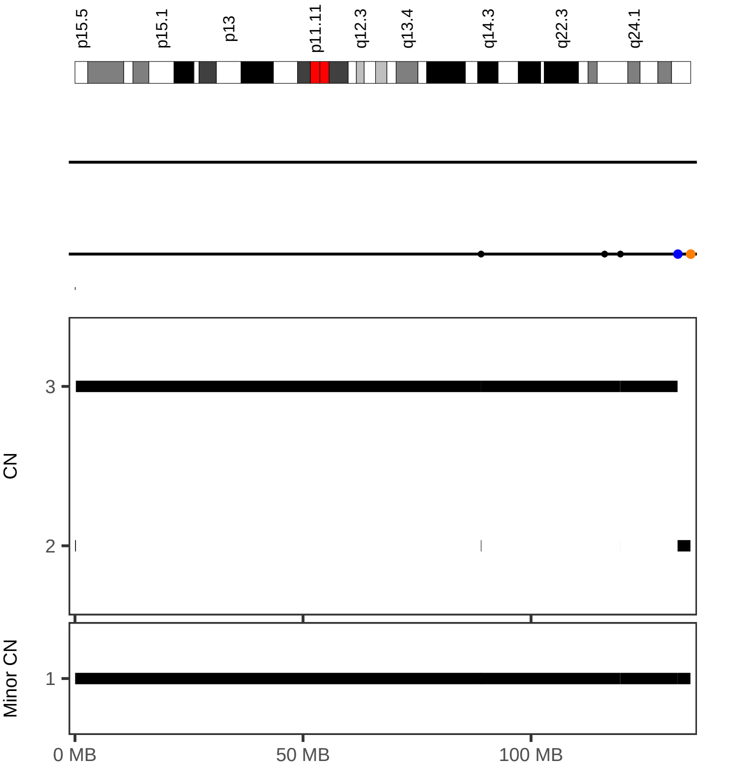

	OCCAMS−AH−071
Cancer type	Eso−AdenoCA
Position	11
Interleaved intrachr. SVs	0
Total SVs (intrachr. + transl.)	0
SV types	DEL: 0; DUP: 0; h2hINV: 0; t2tINV: 0; TRA: 0
SVs in sample	134
Oscillating CN in chr (2 and 3 states)	7,
CN segments	7
Purity, ploidy	0.39, 2.25

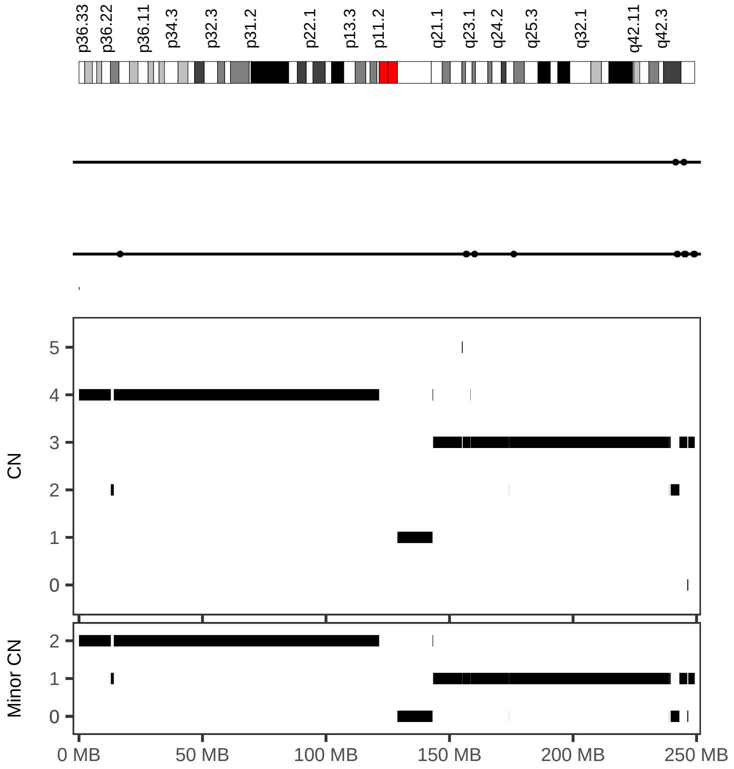

	OCCAMS−AH−131
Cancer type	Eso−AdenoCA
Position	1
Interleaved intrachr. SVs	2
Total SVs (intrachr. + transl.)	2
SV types	DEL: 0; DUP: 0; h2hINV: 1; t2tINV: 1; TRA: 0
SVs in sample	266
Oscillating CN in chr (2 and 3 states)	7,
CN segments	18
Purity, ploidy	0.53, 2.63

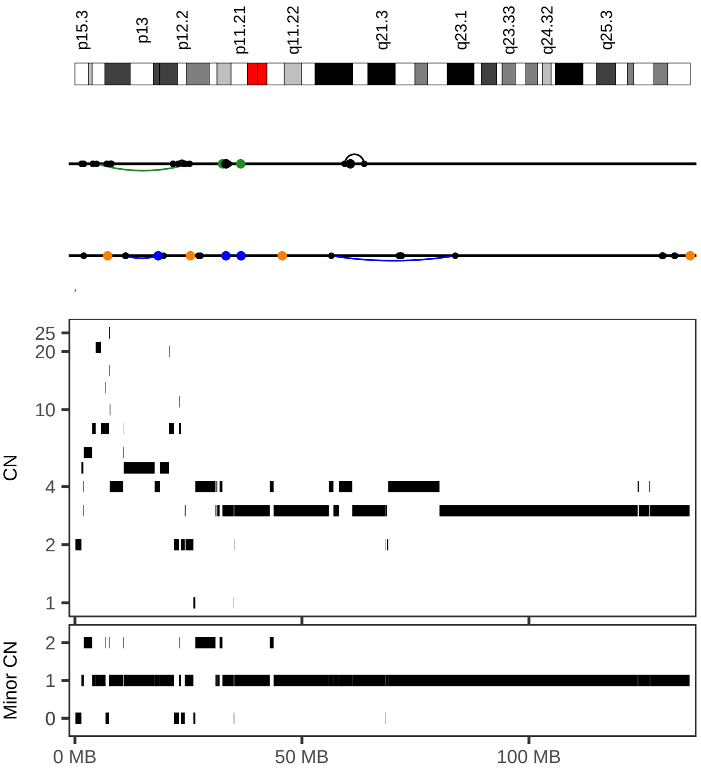

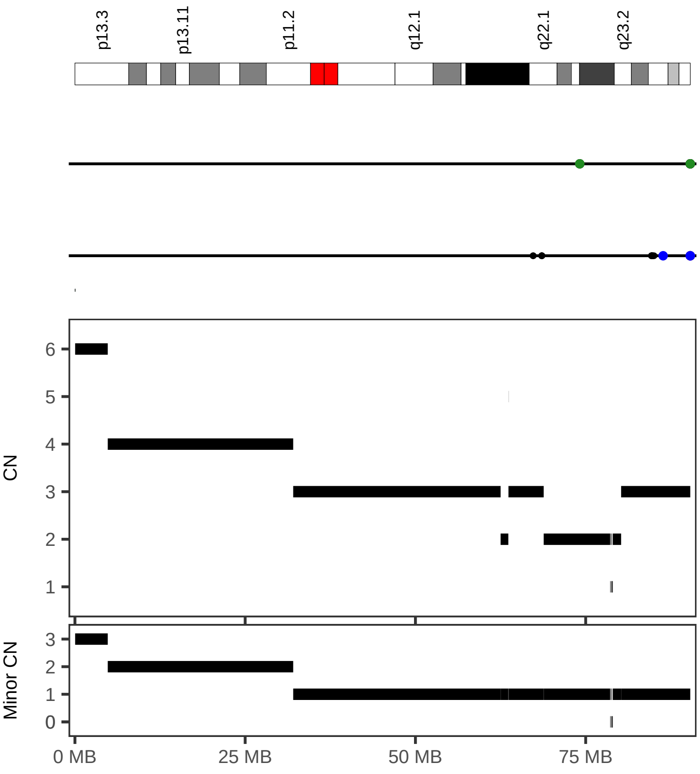

	**OCCAMS−AH−135**
Cancer type	Eso−AdenoCA
Position	10
Interleaved intrachr. SVs	2
Total SVs (intrachr. + transl.)	2
SV types	DEL: 0; DUP: 2; h2hINV: 0; t2tINV: 0; TRA: 0
SVs in sample	298
Oscillating CN in chr (2 and 3 states)	7,
CN segments	53
Purity, ploidy	0.55, 2.85

	**OCCAMS−AH−135**
Cancer type	Eso−AdenoCA
Position	16
Interleaved intrachr. SVs	2
Total SVs (intrachr. + transl.)	2
SV types	DEL: 1; DUP: 1; h2hINV: 0; t2tINV: 0; TRA: 0
SVs in sample	298
Oscillating CN in chr (2 and 3 states)	9,
CN segments	17
Purity, ploidy	0.55, 2.85

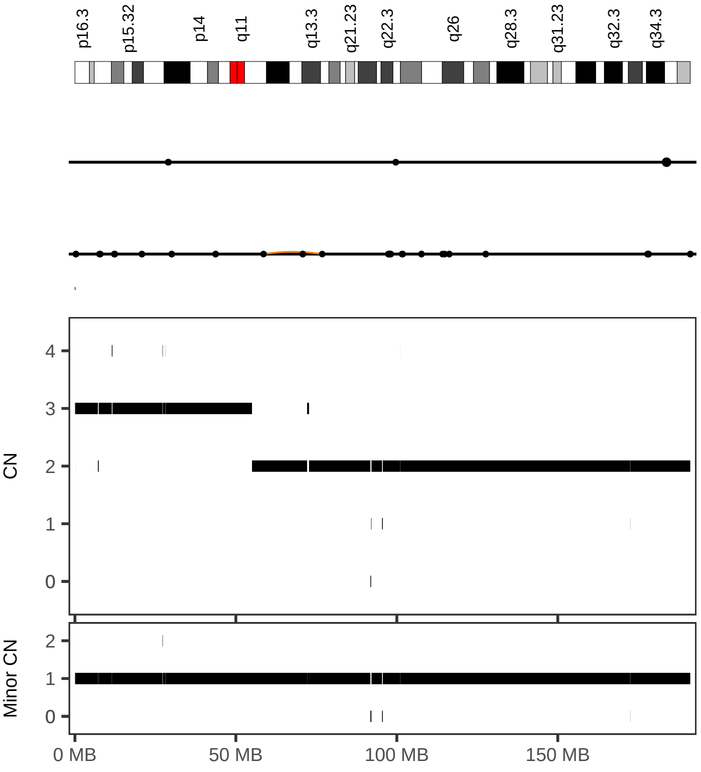

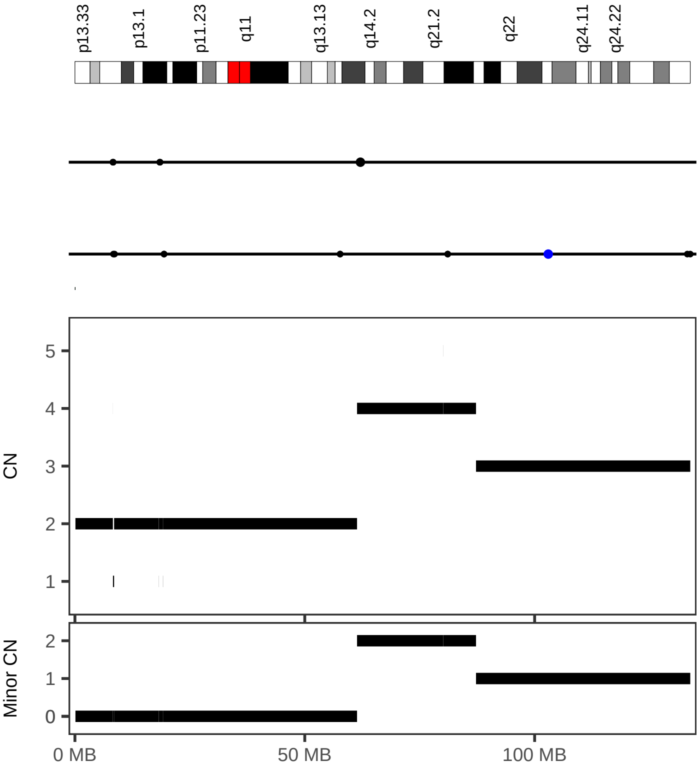

	**OCCAMS−AH−143**
Cancer type	Eso−AdenoCA
Position	4
Interleaved intrachr. SVs	2
Total SVs (intrachr. + transl.)	2
SV types	DEL: 0; DUP: 0; h2hINV: 1; t2tINV: 1; TRA: 0
SVs in sample	596
Oscillating CN in chr (2 and 3 states)	7,
CN segments	23
Purity, ploidy	0.46, 2.6

	**OCCAMS−AH−213**
Cancer type	Eso−AdenoCA
Position	12
Interleaved intrachr. SVs	2
Total SVs (intrachr. + transl.)	2
SV types	DEL: 0; DUP: 0; h2hINV: 1; t2tINV: 1; TRA: 0
SVs in sample	283
Oscillating CN in chr (2 and 3 states)	7,
CN segments	13
Purity, ploidy	0.57, 2.68

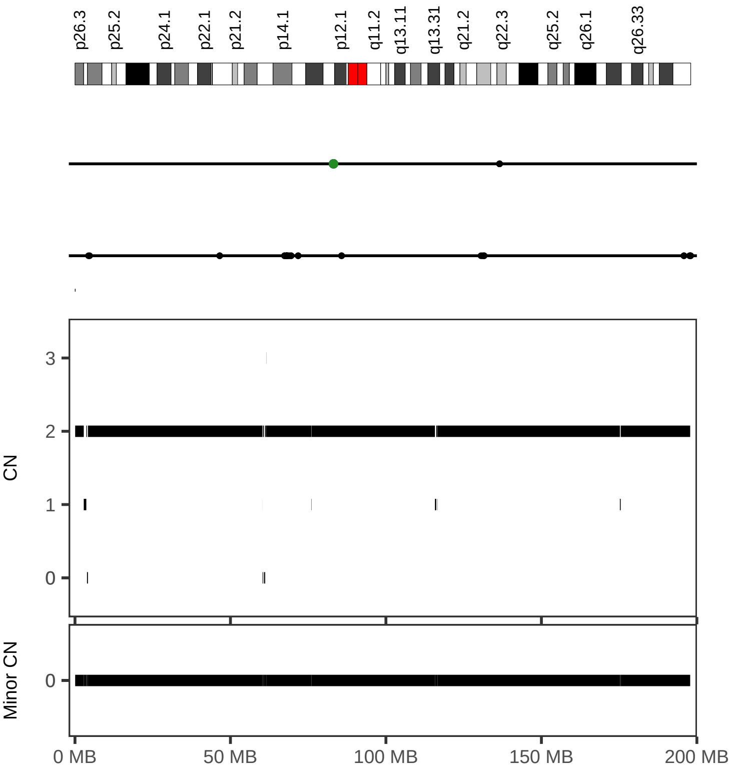

	**OCCAMS−WG−005**
Cancer type	Eso−AdenoCA
Position	3
Interleaved intrachr. SVs	2
Total SVs (intrachr. + transl.)	2
SV types	DEL: 0; DUP: 0; h2hINV: 1; t2tINV: 1; TRA: 0
SVs in sample	277
Oscillating CN in chr (2 and 3 states)	11,
CN segments	25
Purity, ploidy	0.38, 2.47

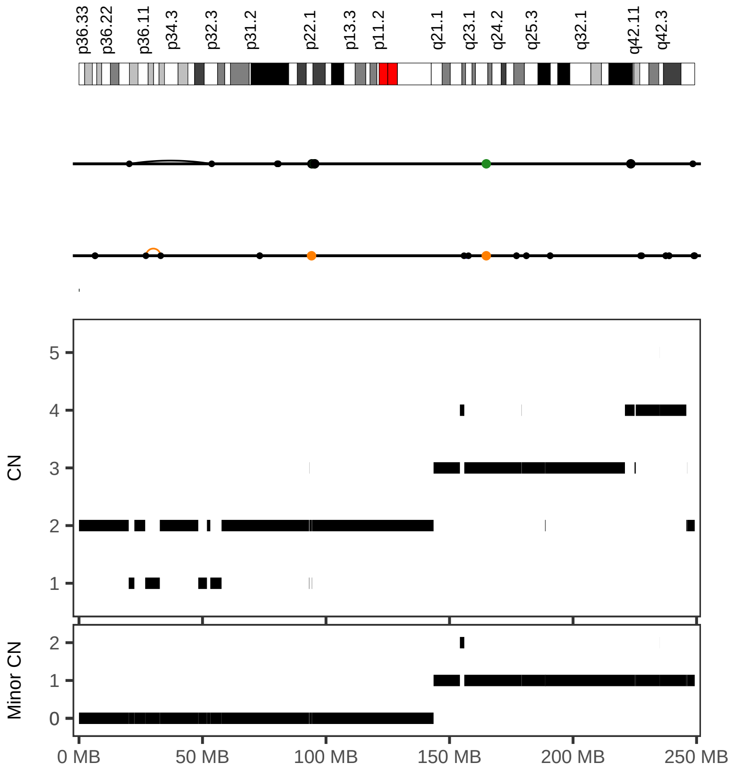

	**OCCAMS−WG−008**
Cancer type	Eso−AdenoCA
Position	1
Interleaved intrachr. SVs	2
Total SVs (intrachr. + transl.)	2
SV types	DEL: 0; DUP: 0; h2hINV: 1; t2tINV: 1; TRA: 0
SVs in sample	357
Oscillating CN in chr (2 and 3 states)	11,
CN segments	30
Purity, ploidy	0.4, 2.6

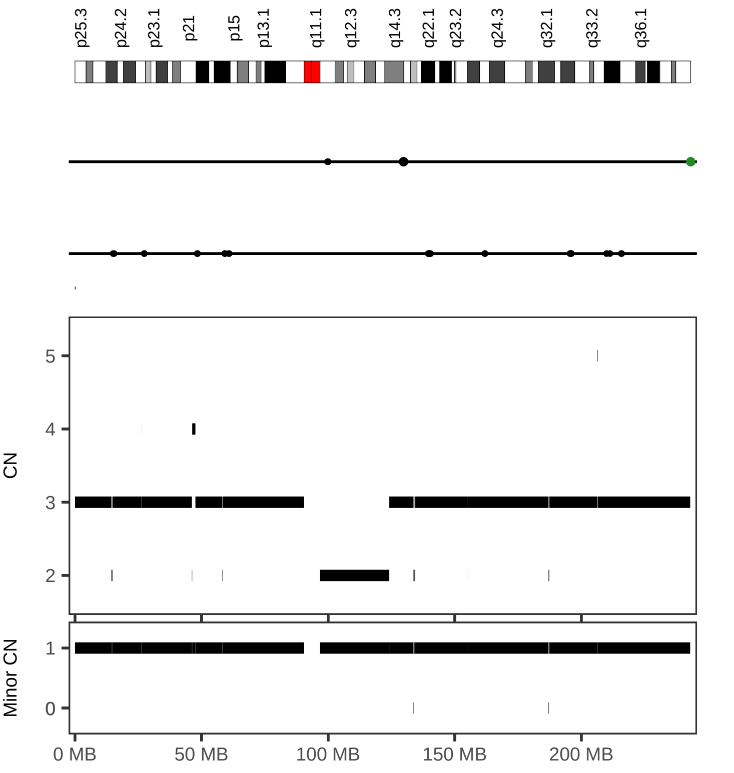

	**05fbd98c−28bd−41bc−ae60−16ac0c16c723**
Cancer type	Head−SCC
Position	2
Interleaved intrachr. SVs	2
Total SVs (intrachr. + transl.)	2
SV types	DEL: 2; DUP: 0; h2hINV: 0; t2tINV: 0; TRA: 0
SVs in sample	143
Oscillating CN in chr (2 and 3 states)	19,
CN segments	28
Purity, ploidy	0.39, 3.17

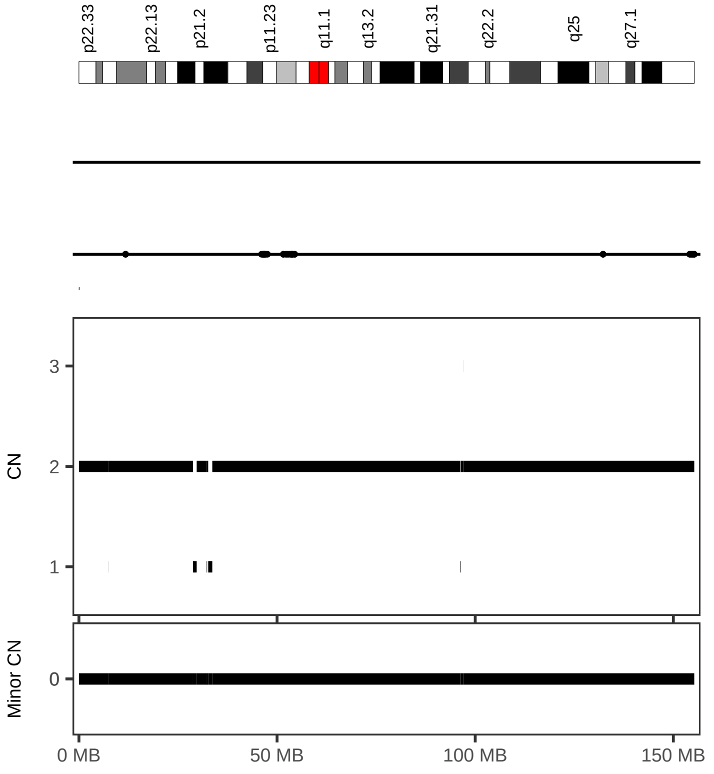

	**05fbd98c−28bd−41bc−ae60−16ac0c16c723**
Cancer type	Head−SCC
Position	X
Interleaved intrachr. SVs	2
Total SVs (intrachr. + transl.)	2
SV types	DEL: 2; DUP: 0; h2hINV: 0; t2tINV: 0; TRA: 0
SVs in sample	143
Oscillating CN in chr (2 and 3 states)	11,
CN segments	13
Purity, ploidy	0.39, 3.17

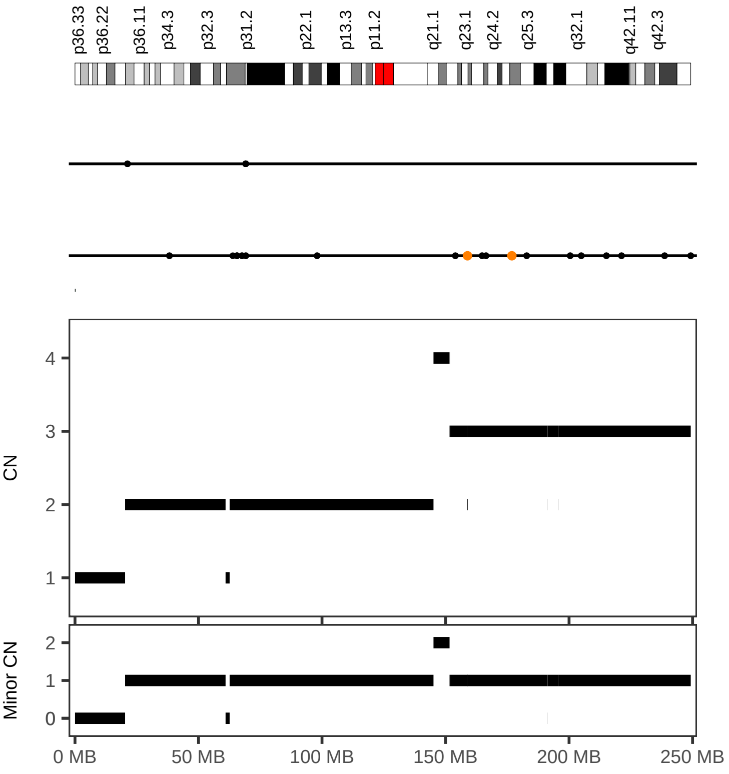

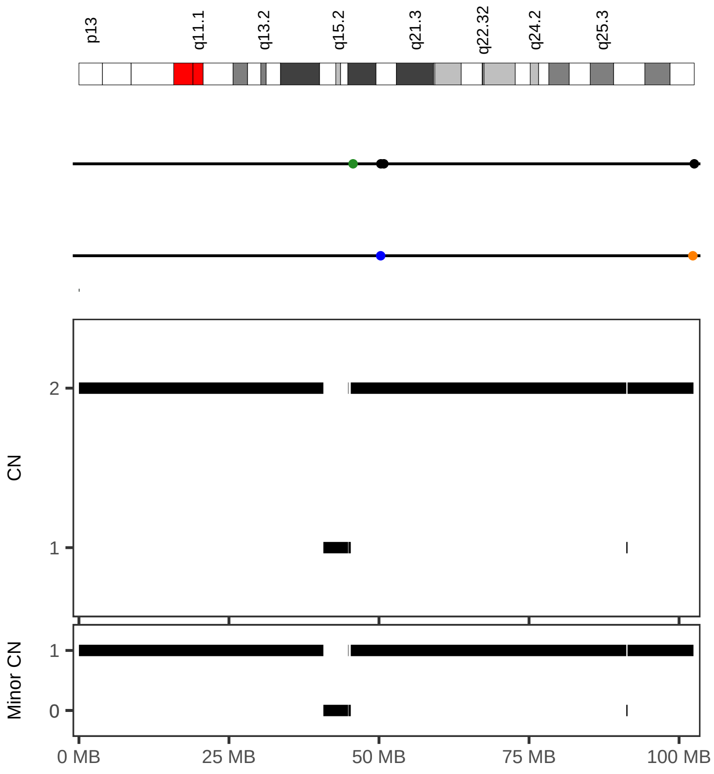

	0956bc67−dd55−4c54−a56a−f22f2856662a
Cancer type	Head−SCC
Position	1
Interleaved intrachr. SVs	2
Total SVs (intrachr. + transl.)	2
SV types	DEL: 1; DUP: 0; h2hINV: 1; t2tINV: 0; TRA: 0
SVs in sample	130
Oscillating CN in chr (2 and 3 states)	7,
CN segments	14
Purity, ploidy	0.75, 1.71

	ba9d4568−71a8−41fc−a2be−0bf784e36d6a
Cancer type	Head−SCC
Position	15
Interleaved intrachr. SVs	1
Total SVs (intrachr. + transl.)	1
SV types	DEL: 0; DUP: 0; h2hINV: 1; t2tINV: 0; TRA: 0
SVs in sample	54
Oscillating CN in chr (2 and 3 states)	7,
CN segments	7
Purity, ploidy	0.8, 1.95

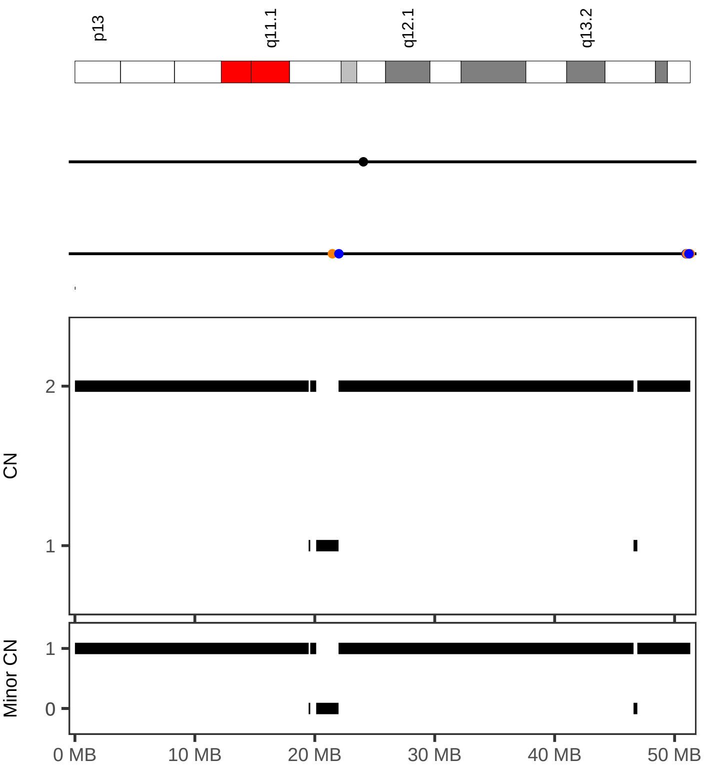

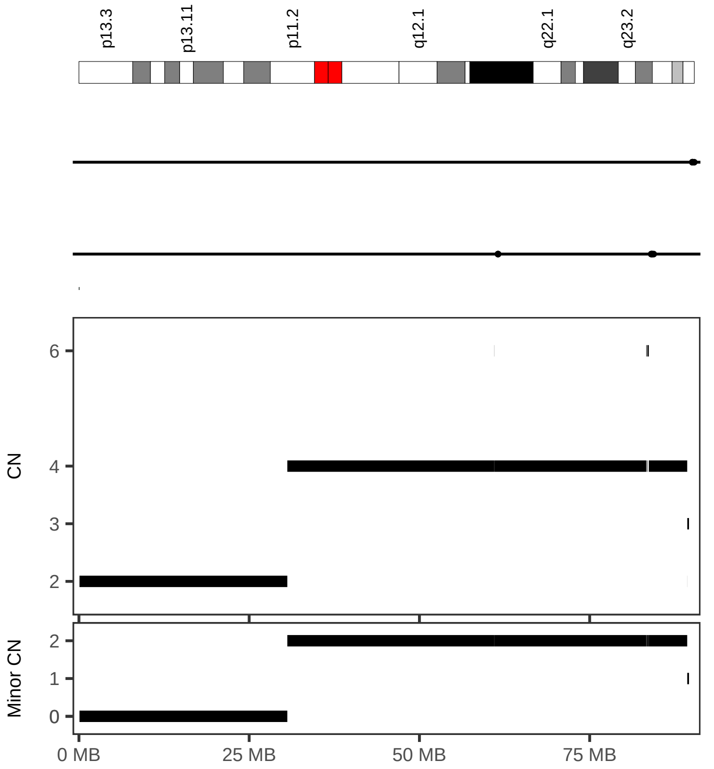

	ba9d4568−71a8−41fc−a2be−0bf784e36d6a
Cancer type	Head−SCC
Position	22
Interleaved intrachr. SVs	0
Total SVs (intrachr. + transl.)	0
SV types	DEL: 0; DUP: 0; h2hINV: 0; t2tINV: 0; TRA: 0
SVs in sample	54
Oscillating CN in chr (2 and 3 states)	7,
CN segments	7
Purity, ploidy	0.8, 1.95

	e8ac39e6−377a−412c−b18d−f6c4fc654ec6
Cancer type	Head−SCC
Position	16
Interleaved intrachr. SVs	2
Total SVs (intrachr. + transl.)	2
SV types	DEL: 0; DUP: 0; h2hINV: 1; t2tINV: 1; TRA: 0
SVs in sample	183
Oscillating CN in chr (2 and 3 states)	7,
CN segments	11
Purity, ploidy	0.46, 3.12

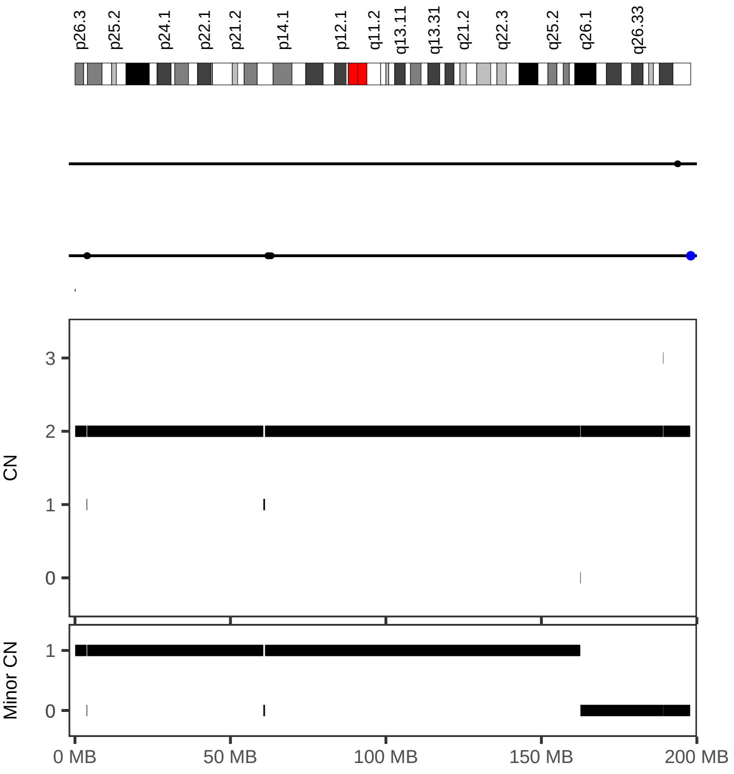

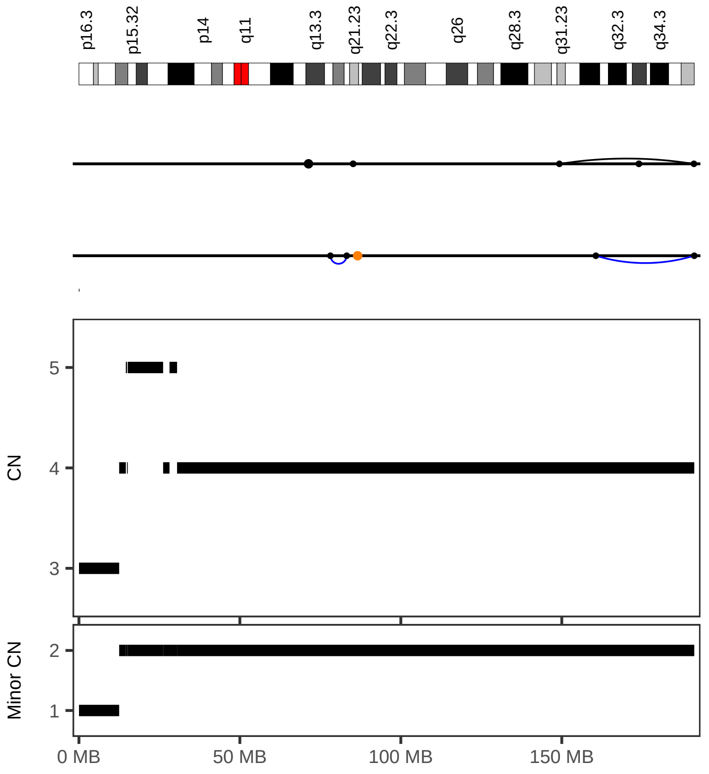

	OSCC−GB_012501
Cancer type	Head−SCC
Position	3
Interleaved intrachr. SVs	2
Total SVs (intrachr. + transl.)	2
SV types	DEL: 0; DUP: 0; h2hINV: 1; t2tINV: 1; TRA: 0
SVs in sample	64
Oscillating CN in chr (2 and 3 states)	9,
CN segments	13
Purity, ploidy	0.55, 2

	b08dfba8−6afb−4217−9259−72be6f1f3363
Cancer type	Kidney−ChRCC
Position	4
Interleaved intrachr. SVs	2
Total SVs (intrachr. + transl.)	2
SV types	DEL: 0; DUP: 1; h2hINV: 1; t2tINV: 0; TRA: 0
SVs in sample	37
Oscillating CN in chr (2 and 3 states)	7,
CN segments	8
Purity, ploidy	0.77, 2.95

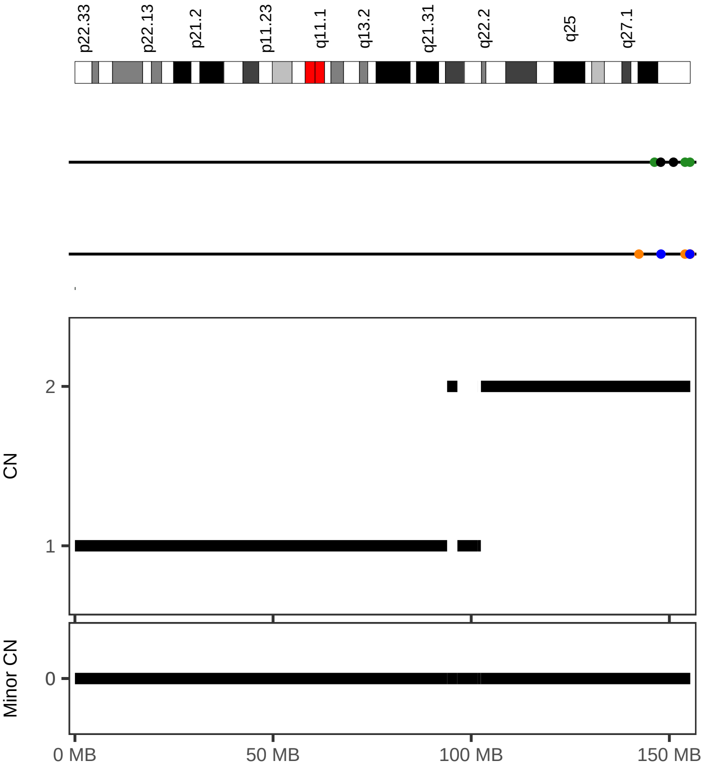

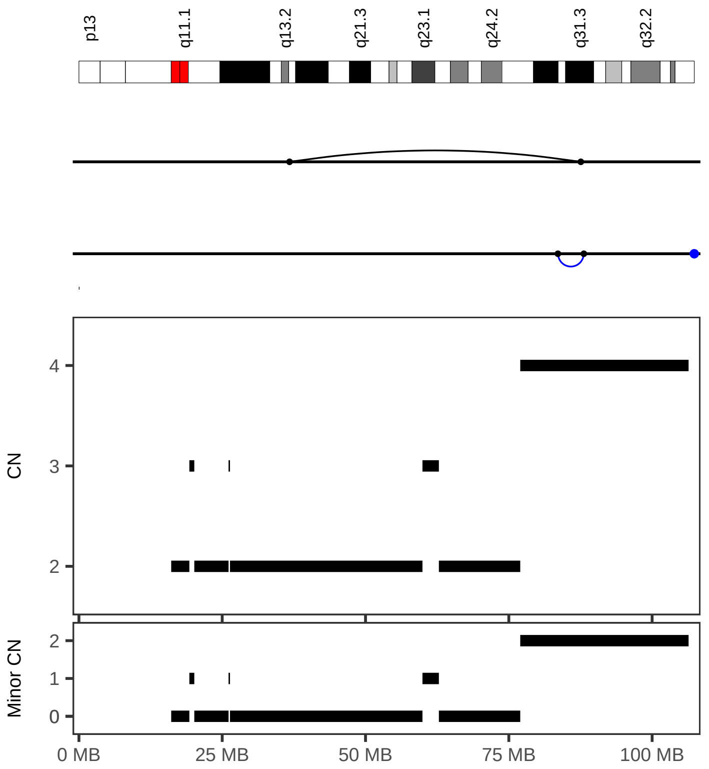

	9da334ed−314c−4445−ba29−ffd9bb42403c
Cancer type	Kidney−RCC
Position	X
Interleaved intrachr. SVs	0
Total SVs (intrachr. + transl.)	0
SV types	DEL: 0; DUP: 0; h2hINV: 0; t2tINV: 0; TRA: 0
SVs in sample	15
Oscillating CN in chr (2 and 3 states)	8,
CN segments	8
Purity, ploidy	0.71, 1.92

	71b35cd8−ecee−4599−bb24−b1ef78118423
Cancer type	Kidney−RCC
Position	14
Interleaved intrachr. SVs	2
Total SVs (intrachr. + transl.)	2
SV types	DEL: 0; DUP: 1; h2hINV: 1; t2tINV: 0; TRA: 0
SVs in sample	9
Oscillating CN in chr (2 and 3 states)	7,
CN segments	8
Purity, ploidy	0.7, 2.62

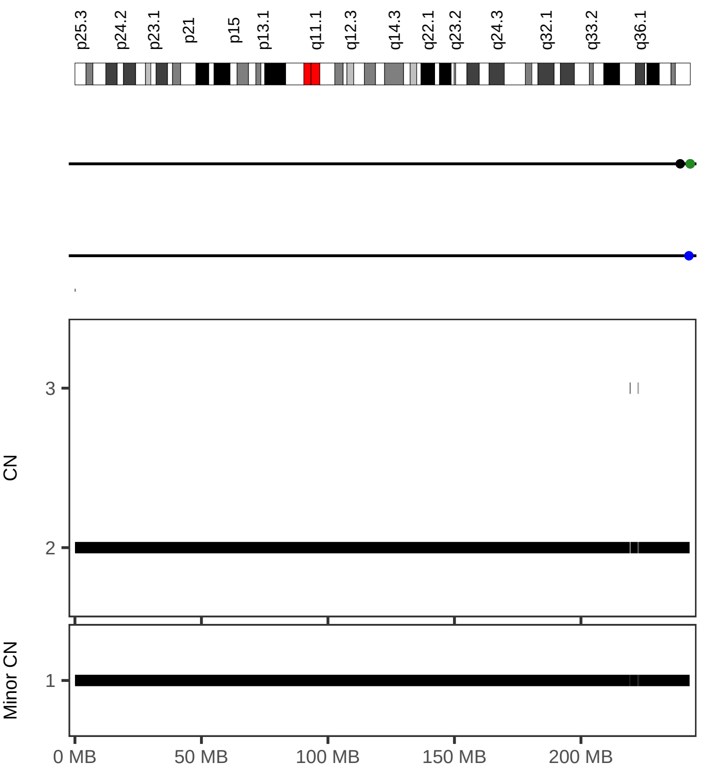

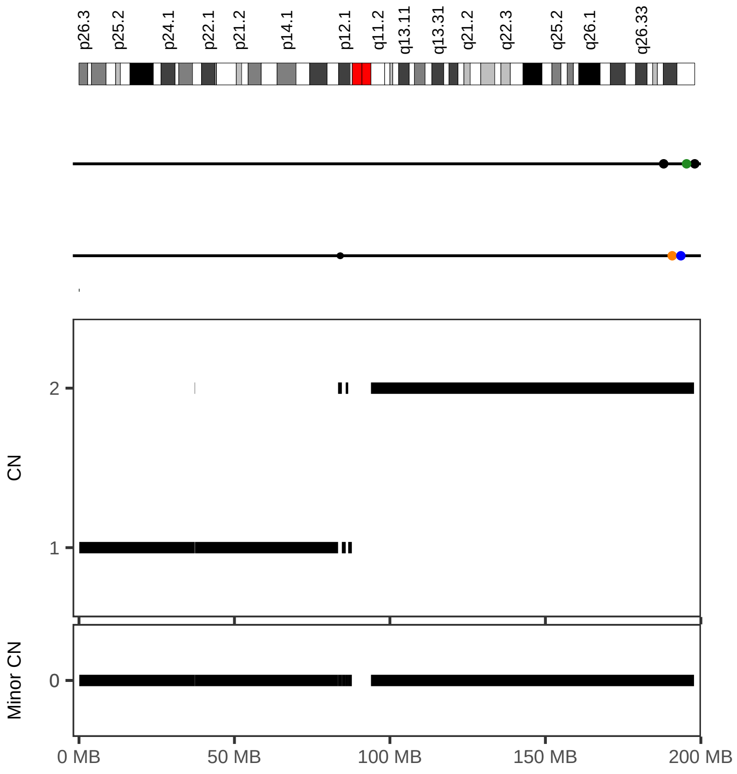

	C0009
Cancer type	Kidney−RCC
Position	2
Interleaved intrachr. SVs	1
Total SVs (intrachr. + transl.)	2
SV types	DEL: 0; DUP: 0; h2hINV: 1; t2tINV: 0; TRA: 1
SVs in sample	18
Oscillating CN in chr (2 and 3 states)	7,
CN segments	7
Purity, ploidy	0.41, 1.95

	C0046
Cancer type	Kidney−RCC
Position	3
Interleaved intrachr. SVs	1
Total SVs (intrachr. + transl.)	1
SV types	DEL: 0; DUP: 1; h2hINV: 0; t2tINV: 0; TRA: 0
SVs in sample	11
Oscillating CN in chr (2 and 3 states)	8,
CN segments	8
Purity, ploidy	0.46, 1.87

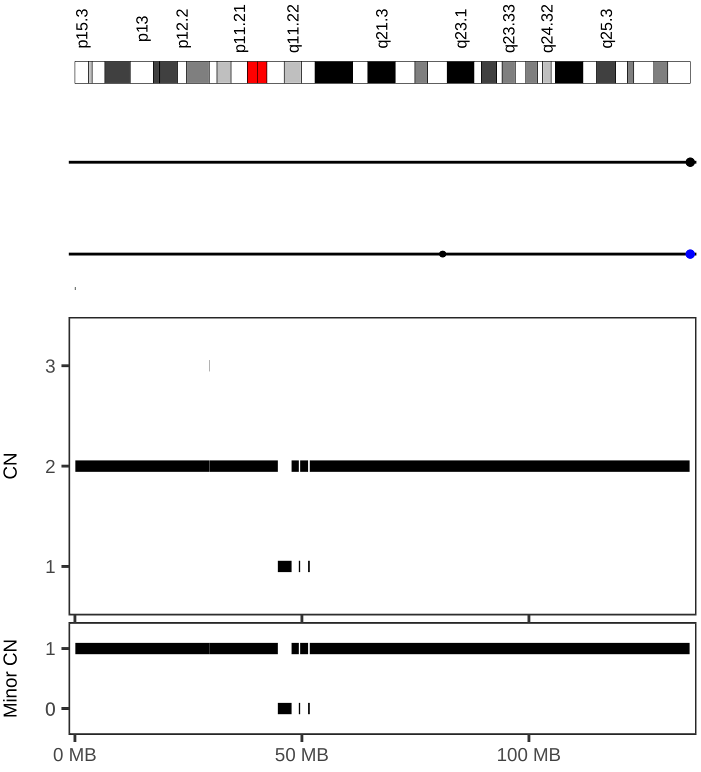

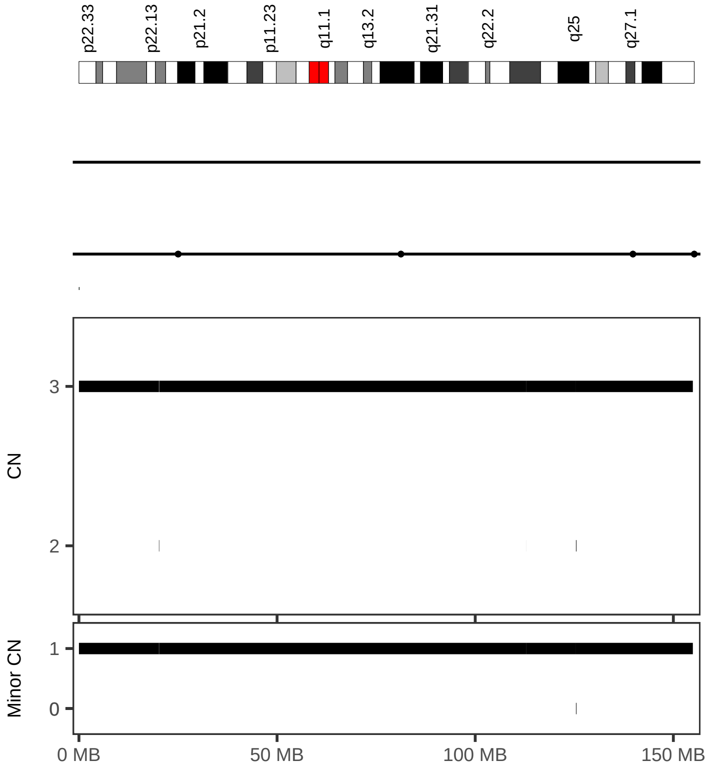

	C0075
Cancer type	Kidney−RCC
Position	10
Interleaved intrachr. SVs	1
Total SVs (intrachr. + transl.)	1
SV types	DEL: 0; DUP: 1; h2hINV: 0; t2tINV: 0; TRA: 0
SVs in sample	18
Oscillating CN in chr (2 and 3 states)	7,
CN segments	9
Purity, ploidy	0.64, 1.98

	18c08546−bed3−4ed9−b223−479afe633c8e
Cancer type	Liver−HCC
Position	X
Interleaved intrachr. SVs	1
Total SVs (intrachr. + transl.)	1
SV types	DEL: 1; DUP: 0; h2hINV: 0; t2tINV: 0; TRA: 0
SVs in sample	21
Oscillating CN in chr (2 and 3 states)	7,
CN segments	7
Purity, ploidy	0.8, 3.18

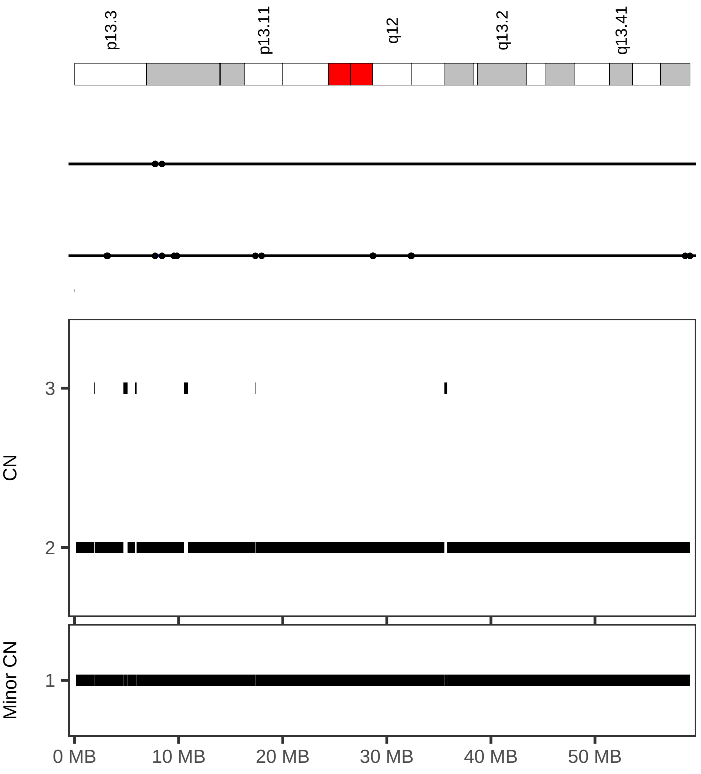

	4bbdbe77−f3cb−4b64−9826−72debb734784
Cancer type	Liver−HCC
Position	19
Interleaved intrachr. SVs	2
Total SVs (intrachr. + transl.)	2
SV types	DEL: 0; DUP: 1; h2hINV: 1; t2tINV: 0; TRA: 0
SVs in sample	445
Oscillating CN in chr (2 and 3 states)	13,
CN segments	13
Purity, ploidy	0.82, 2.08

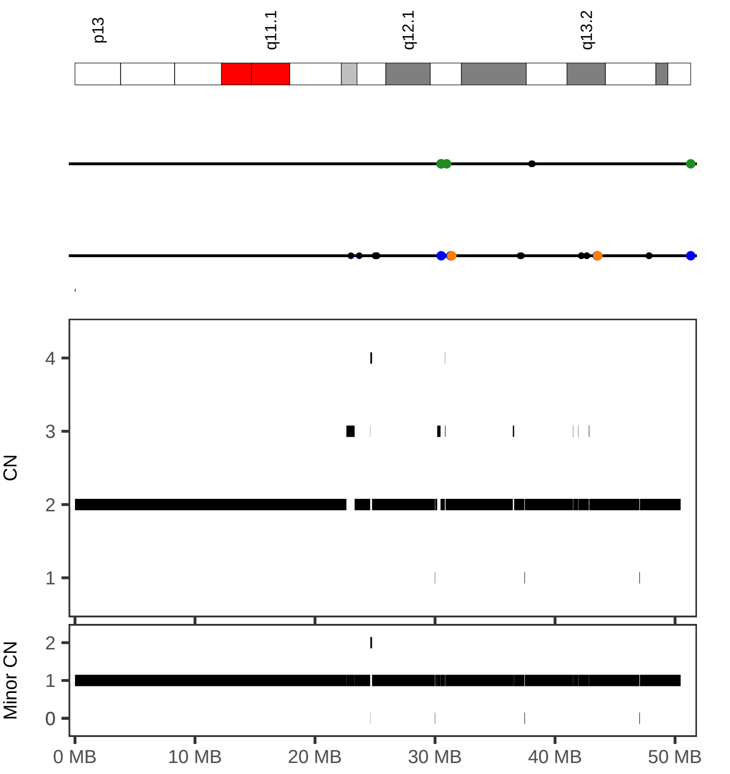

	4bbdbe77−f3cb−4b64−9826−72debb734784
Cancer type	Liver−HCC
Position	22
Interleaved intrachr. SVs	2
Total SVs (intrachr. + transl.)	2
SV types	DEL: 0; DUP: 0; h2hINV: 1; t2tINV: 1; TRA: 0
SVs in sample	445
Oscillating CN in chr (2 and 3 states)	7,
CN segments	26
Purity, ploidy	0.82, 2.08

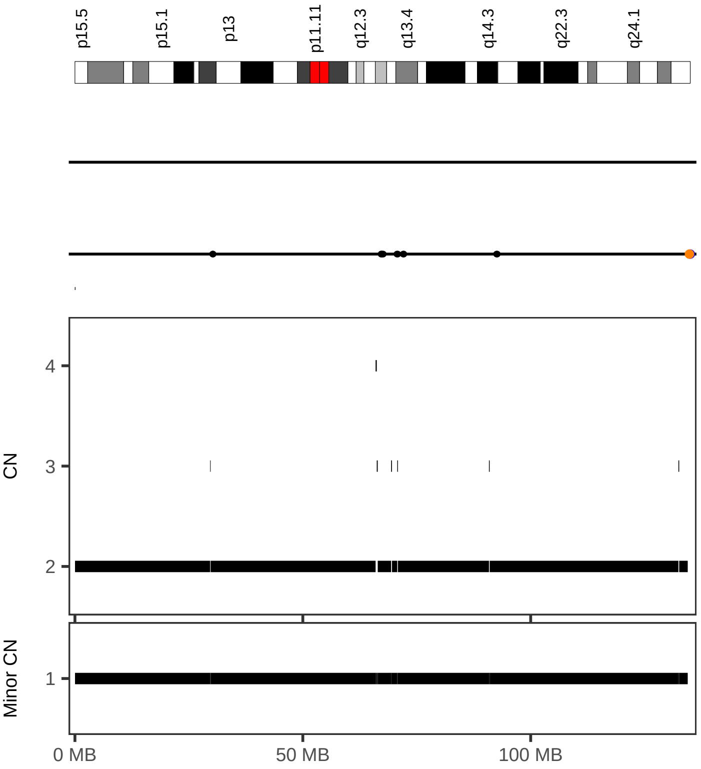

	68015cc7−d3a1−4c90−b8fb−5d14143d9960
Cancer type	Liver−HCC
Position	11
Interleaved intrachr. SVs	2
Total SVs (intrachr. + transl.)	2
SV types	DEL: 0; DUP: 2; h2hINV: 0; t2tINV: 0; TRA: 0
SVs in sample	231
Oscillating CN in chr (2 and 3 states)	10,
CN segments	14
Purity, ploidy	0.83, 2.03

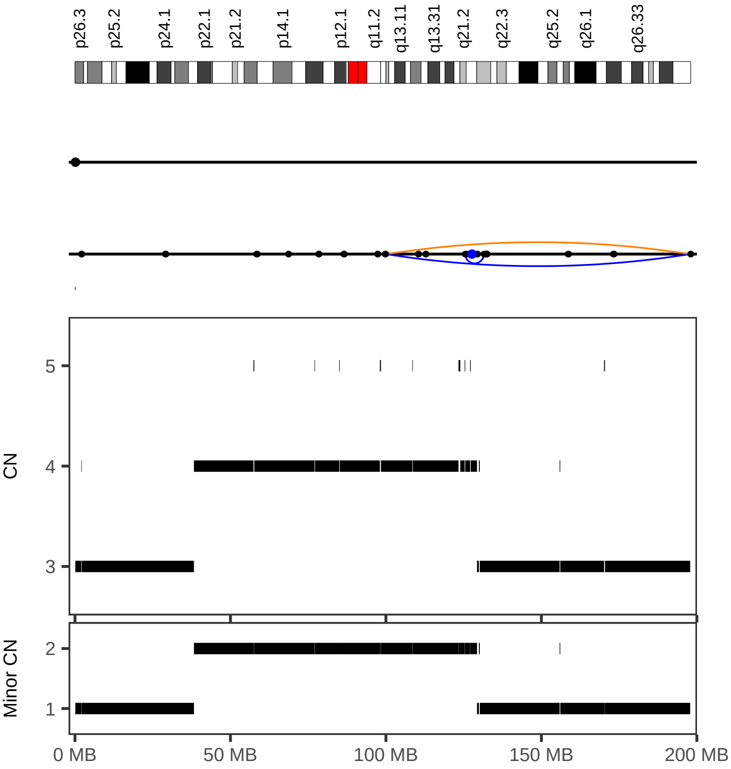

	8afa3140−dcbd−44c0−ab13−4ada6a0444d4
Cancer type	Liver−HCC
Position	3
Interleaved intrachr. SVs	2
Total SVs (intrachr. + transl.)	2
SV types	DEL: 0; DUP: 2; h2hINV: 0; t2tINV: 0; TRA: 0
SVs in sample	301
Oscillating CN in chr (2 and 3 states)	17,
CN segments	27
Purity, ploidy	0.84, 3.54

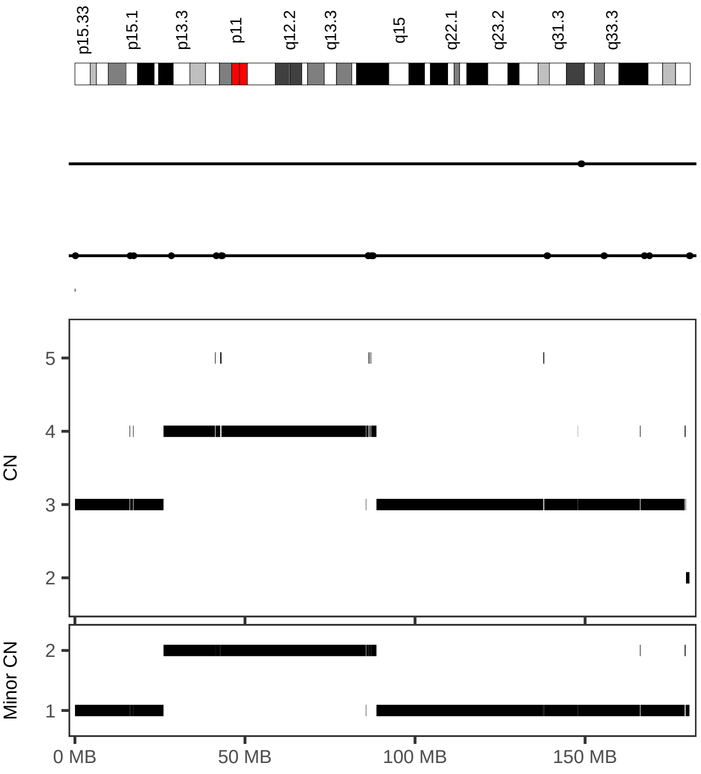

	8afa3140−dcbd−44c0−ab13−4ada6a0444d4
Cancer type	Liver−HCC
Position	5
Interleaved intrachr. SVs	2
Total SVs (intrachr. + transl.)	2
SV types	DEL: 0; DUP: 0; h2hINV: 1; t2tINV: 1; TRA: 0
SVs in sample	301
Oscillating CN in chr (2 and 3 states)	7,
CN segments	26
Purity, ploidy	0.84, 3.54

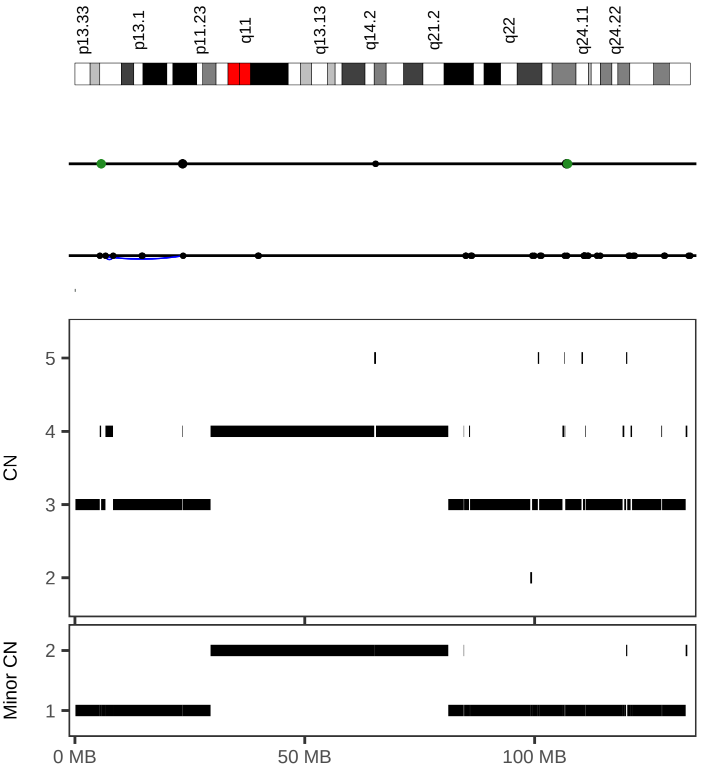

	8afa3140−dcbd−44c0−ab13−4ada6a0444d4
Cancer type	Liver−HCC
Position	12
Interleaved intrachr. SVs	2
Total SVs (intrachr. + transl.)	2
SV types	DEL: 0; DUP: 2; h2hINV: 0; t2tINV: 0; TRA: 0
SVs in sample	301
Oscillating CN in chr (2 and 3 states)	8,
CN segments	36
Purity, ploidy	0.84, 3.54

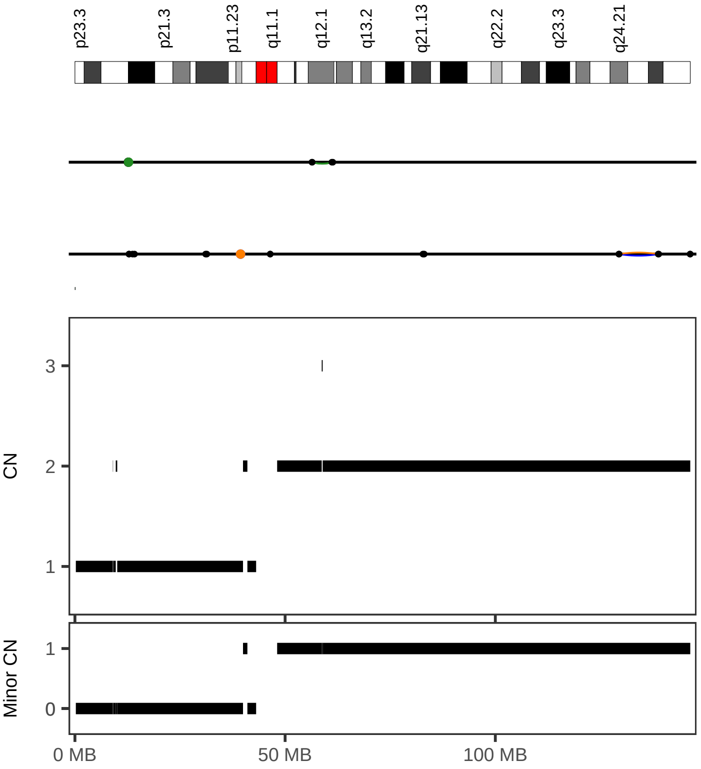

	bd48fa5e−0152−46f0−a4fa−8cf723ff2dcd
Cancer type	Liver−HCC
Position	8
Interleaved intrachr. SVs	2
Total SVs (intrachr. + transl.)	2
SV types	DEL: 0; DUP: 0; h2hINV: 0; t2tINV: 2; TRA: 0
SVs in sample	394
Oscillating CN in chr (2 and 3 states)	10,
CN segments	12
Purity, ploidy	0.57, 2.05

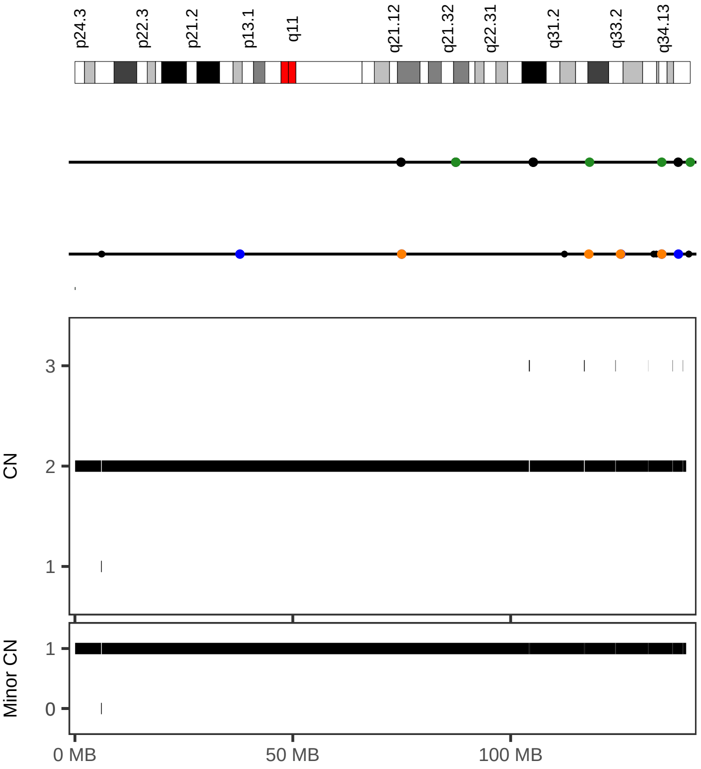

	bd48fa5e−0152−46f0−a4fa−8cf723ff2dcd
Cancer type	Liver−HCC
Position	9
Interleaved intrachr. SVs	2
Total SVs (intrachr. + transl.)	2
SV types	DEL: 0; DUP: 2; h2hINV: 0; t2tINV: 0; TRA: 0
SVs in sample	394
Oscillating CN in chr (2 and 3 states)	13,
CN segments	15
Purity, ploidy	0.57, 2.05

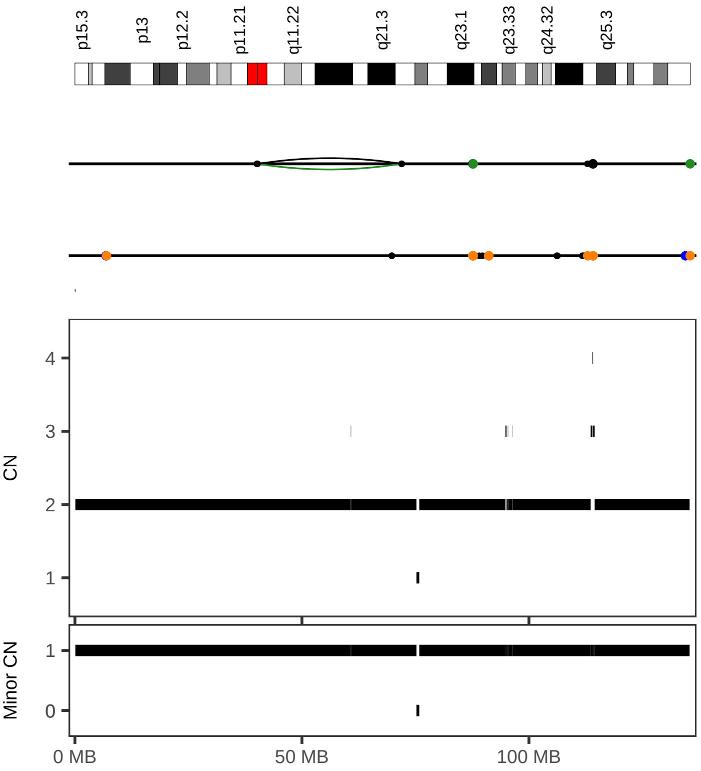

	bd48fa5e−0152−46f0−a4fa−8cf723ff2dcd
Cancer type	Liver−HCC
Position	10
Interleaved intrachr. SVs	2
Total SVs (intrachr. + transl.)	2
SV types	DEL: 0; DUP: 0; h2hINV: 1; t2tINV: 1; TRA: 0
SVs in sample	394
Oscillating CN in chr (2 and 3 states)	8,
CN segments	15
Purity, ploidy	0.57, 2.05

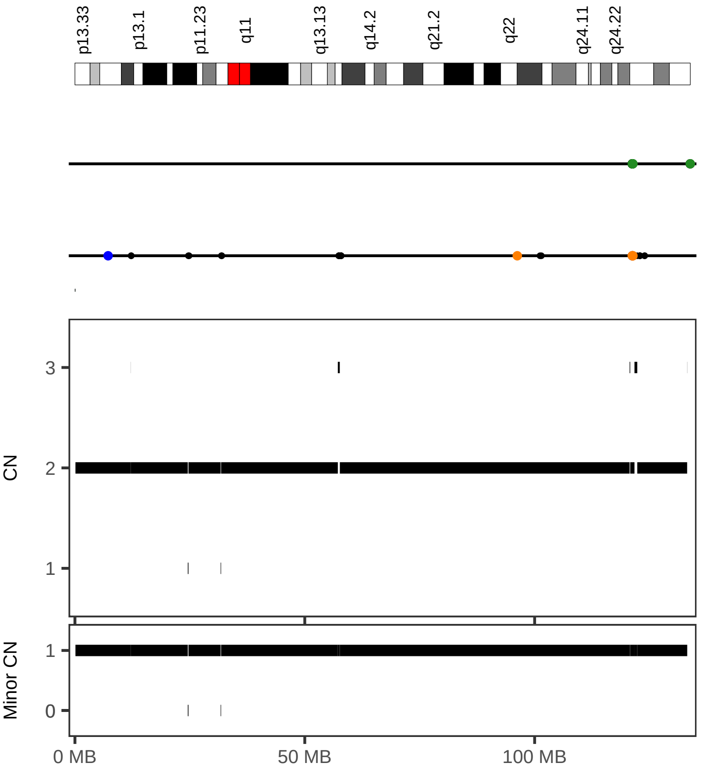

	bd48fa5e−0152−46f0−a4fa−8cf723ff2dcd
Cancer type	Liver−HCC
Position	12
Interleaved intrachr. SVs	2
Total SVs (intrachr. + transl.)	2
SV types	DEL: 0; DUP: 2; h2hINV: 0; t2tINV: 0; TRA: 0
SVs in sample	394
Oscillating CN in chr (2 and 3 states)	10,
CN segments	16
Purity, ploidy	0.57, 2.05

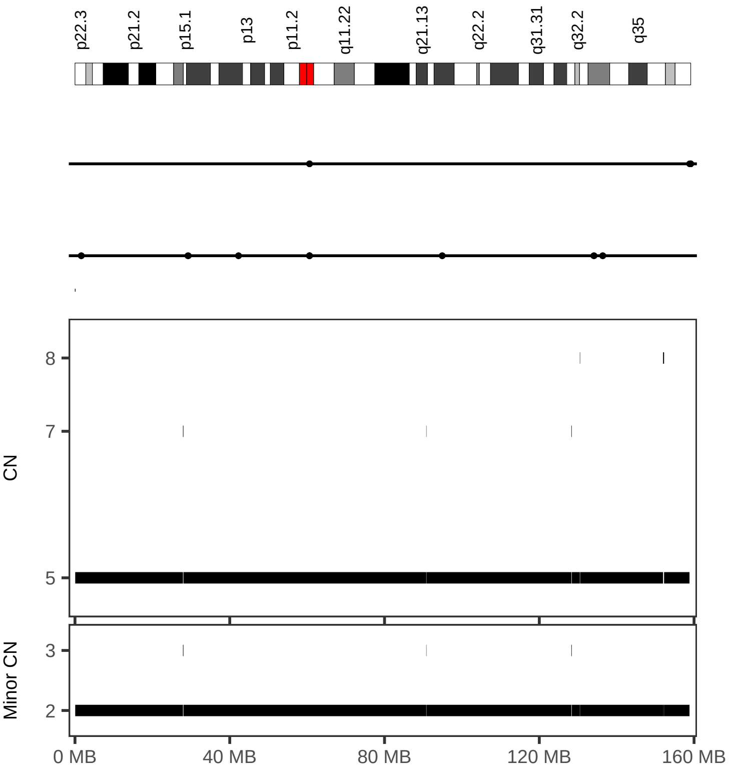

	bee9d6c8−948f−4b76−97ca−b985064249ef
Cancer type	Liver−HCC
Position	7
Interleaved intrachr. SVs	2
Total SVs (intrachr. + transl.)	2
SV types	DEL: 0; DUP: 0; h2hINV: 1; t2tINV: 1; TRA: 0
SVs in sample	164
Oscillating CN in chr (2 and 3 states)	7,
CN segments	11
Purity, ploidy	0.62, 3.66

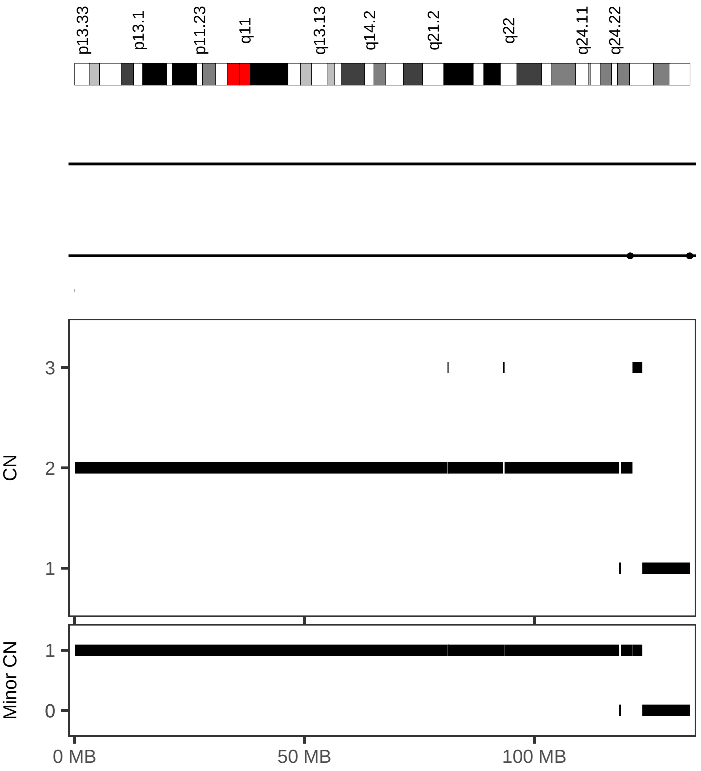

	RK006
Cancer type	Liver−HCC
Position	12
Interleaved intrachr. SVs	1
Total SVs (intrachr. + transl.)	2
SV types	DEL: 2; DUP: 0; h2hINV: 0; t2tINV: 0; TRA: 0
SVs in sample	46
Oscillating CN in chr (2 and 3 states)	7,
CN segments	11
Purity, ploidy	NA, NA

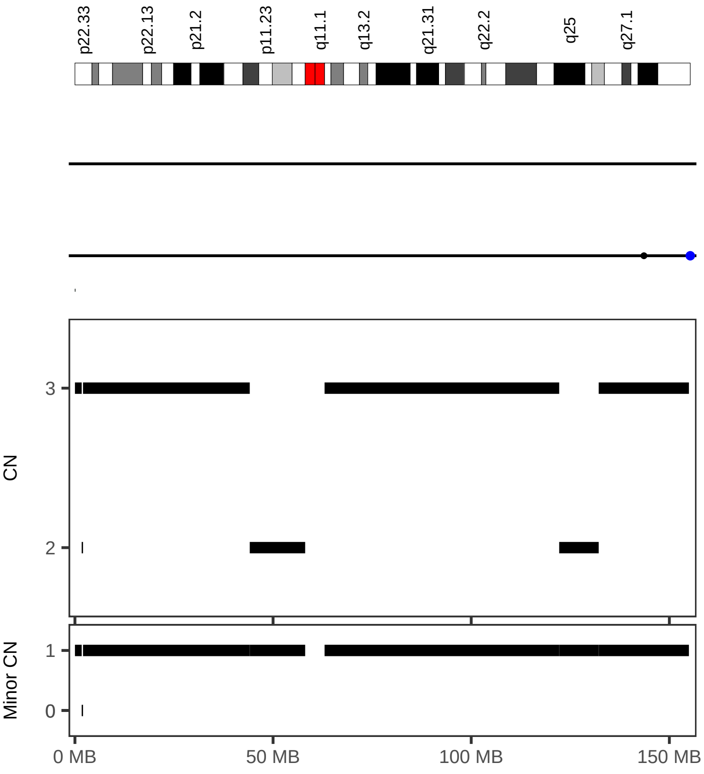

	RK025
Cancer type	Liver−HCC
Position	X
Interleaved intrachr. SVs	1
Total SVs (intrachr. + transl.)	1
SV types	DEL: 0; DUP: 1; h2hINV: 0; t2tINV: 0; TRA: 0
SVs in sample	71
Oscillating CN in chr (2 and 3 states)	7,
CN segments	7
Purity, ploidy	0.33, 4.17

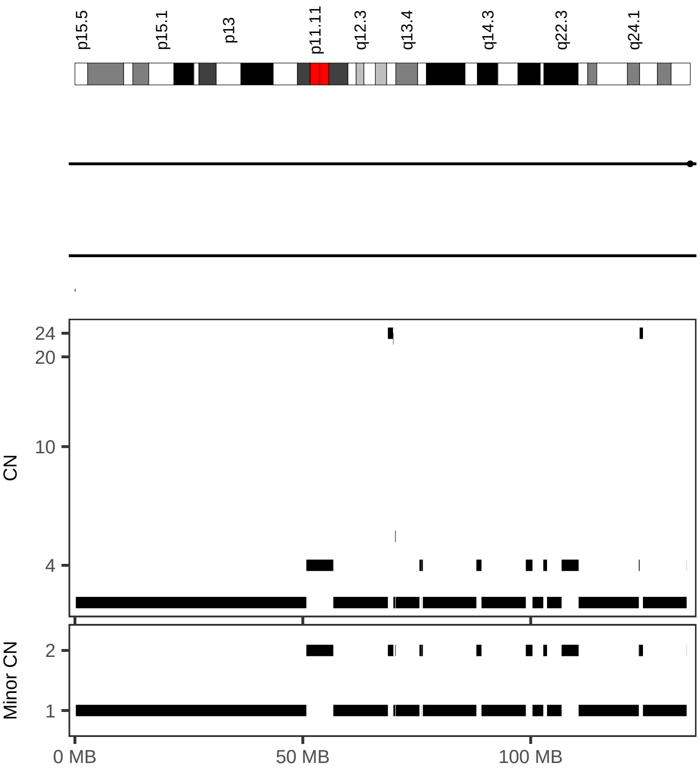

	RK036
Cancer type	Liver−HCC
Position	11
Interleaved intrachr. SVs	1
Total SVs (intrachr. + transl.)	1
SV types	DEL: 0; DUP: 0; h2hINV: 0; t2tINV: 1; TRA: 0
SVs in sample	22
Oscillating CN in chr (2 and 3 states)	14,
CN segments	24
Purity, ploidy	NA, NA

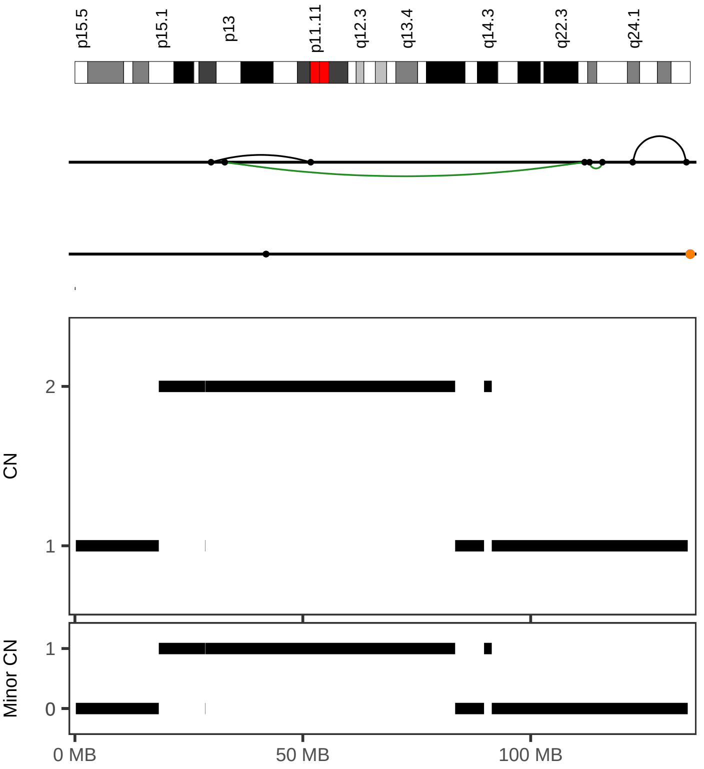

	RK042
Cancer type	Liver−HCC
Position	11
Interleaved intrachr. SVs	2
Total SVs (intrachr. + transl.)	2
SV types	DEL: 0; DUP: 0; h2hINV: 1; t2tINV: 1; TRA: 0
SVs in sample	93
Oscillating CN in chr (2 and 3 states)	7,
CN segments	7
Purity, ploidy	0.88, 1.81

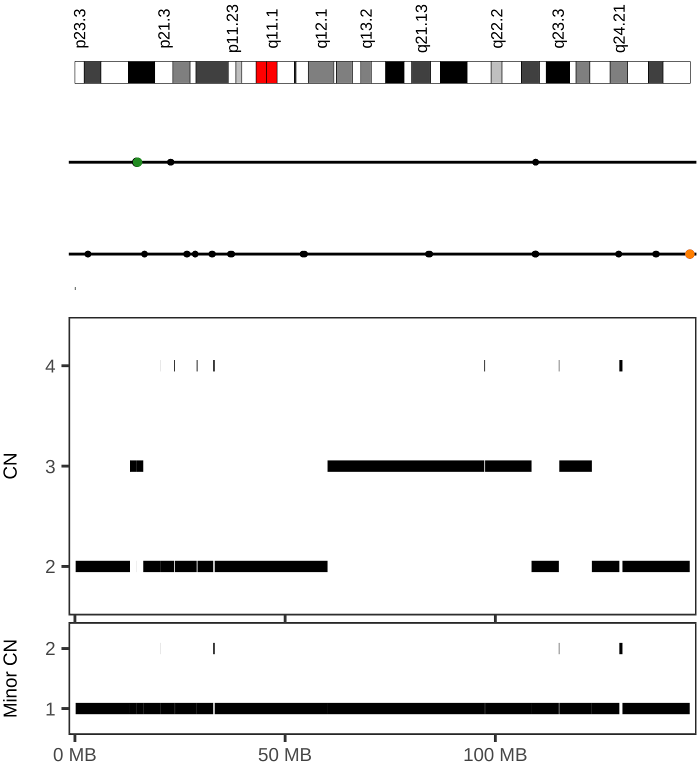

	RK070
Cancer type	Liver−HCC
Position	8
Interleaved intrachr. SVs	2
Total SVs (intrachr. + transl.)	2
SV types	DEL: 0; DUP: 0; h2hINV: 1; t2tINV: 1; TRA: 0
SVs in sample	281
Oscillating CN in chr (2 and 3 states)	9,
CN segments	22
Purity, ploidy	0.6, 2.2

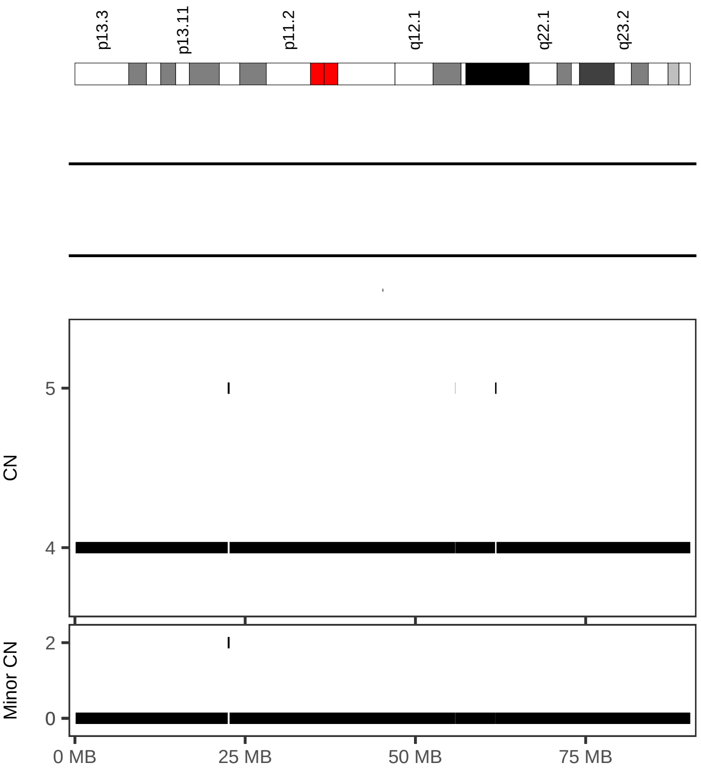

	**RK072**
Cancer type	Liver−HCC
Position	16
Interleaved intrachr. SVs	0
Total SVs (intrachr. + transl.)	0
SV types	DEL: 0; DUP: 0; h2hINV: 0; t2tINV: 0; TRA: 0
SVs in sample	282
Oscillating CN in chr (2 and 3 states)	7,
CN segments	7
Purity, ploidy	0.7, 4.49

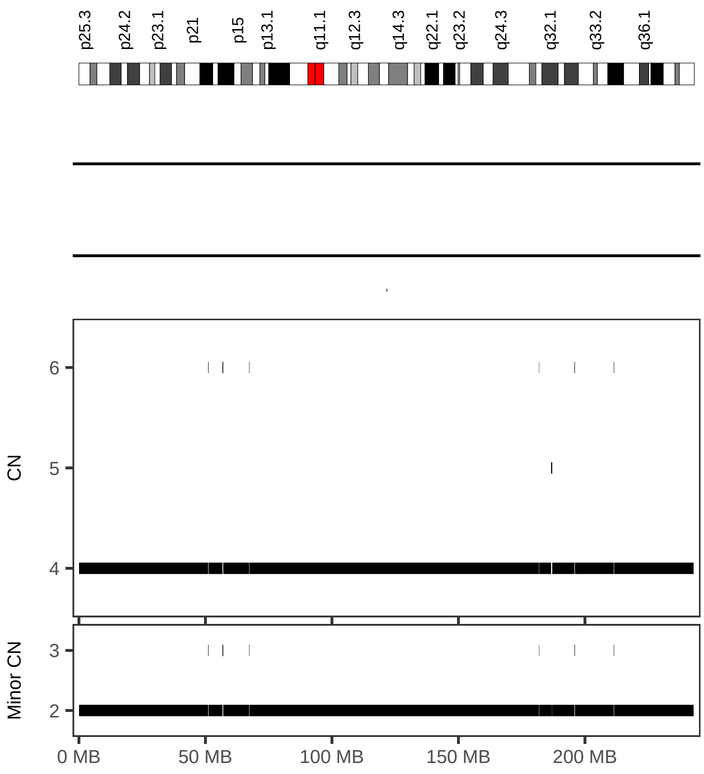

	**RK080**
Cancer type	Liver−HCC
Position	2
Interleaved intrachr. SVs	0
Total SVs (intrachr. + transl.)	0
SV types	DEL: 0; DUP: 0; h2hINV: 0; t2tINV: 0; TRA: 0
SVs in sample	26
Oscillating CN in chr (2 and 3 states)	9,
CN segments	15
Purity, ploidy	0.77, 3.85

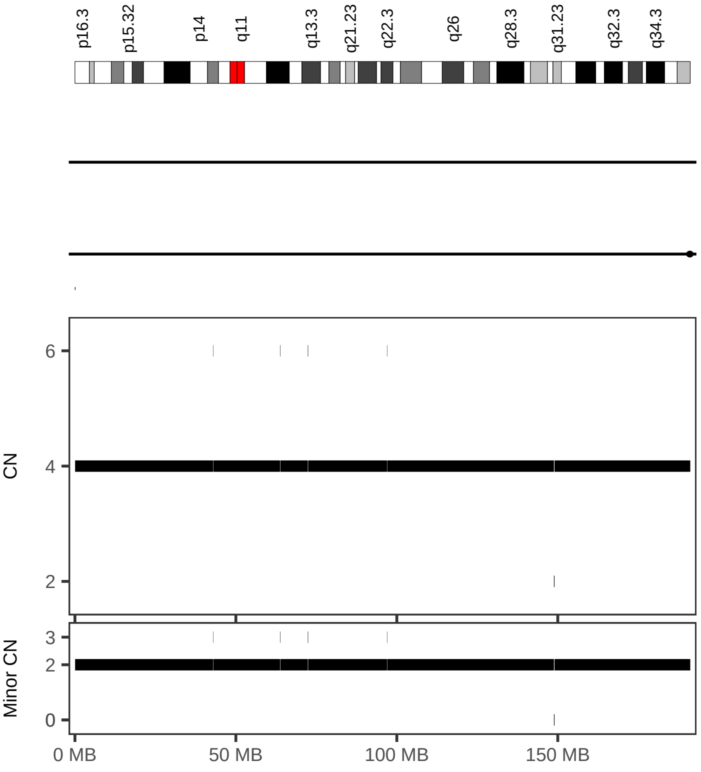

	**RK080**
Cancer type	Liver−HCC
Position	4
Interleaved intrachr. SVs	1
Total SVs (intrachr. + transl.)	1
SV types	DEL: 1; DUP: 0; h2hINV: 0; t2tINV: 0; TRA: 0
SVs in sample	26
Oscillating CN in chr (2 and 3 states)	9,
CN segments	11
Purity, ploidy	0.77, 3.85

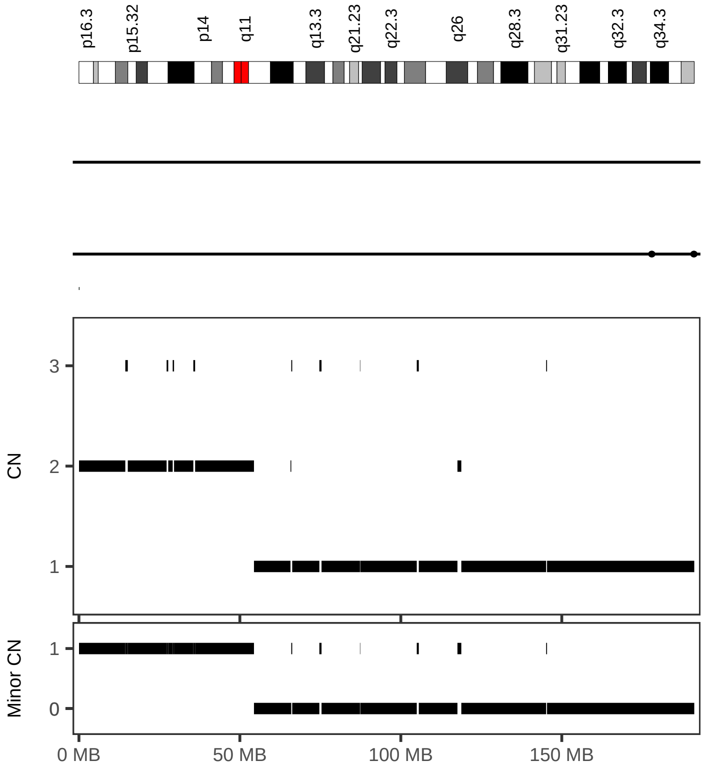

	**RK087**
Cancer type	Liver−HCC
Position	4
Interleaved intrachr. SVs	1
Total SVs (intrachr. + transl.)	2
SV types	DEL: 1; DUP: 1; h2hINV: 0; t2tINV: 0; TRA: 0
SVs in sample	62
Oscillating CN in chr (2 and 3 states)	9,
CN segments	23
Purity, ploidy	0.26, 1.9

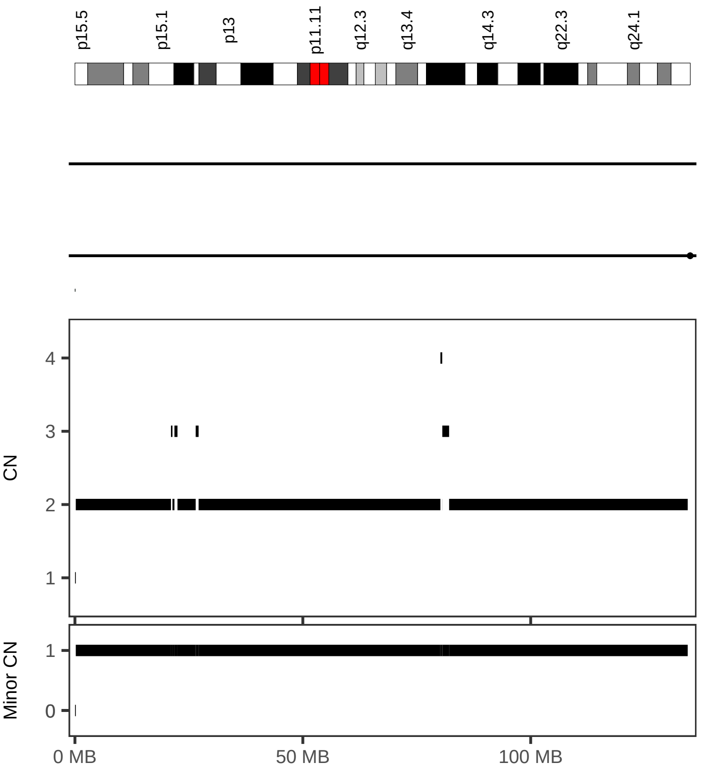

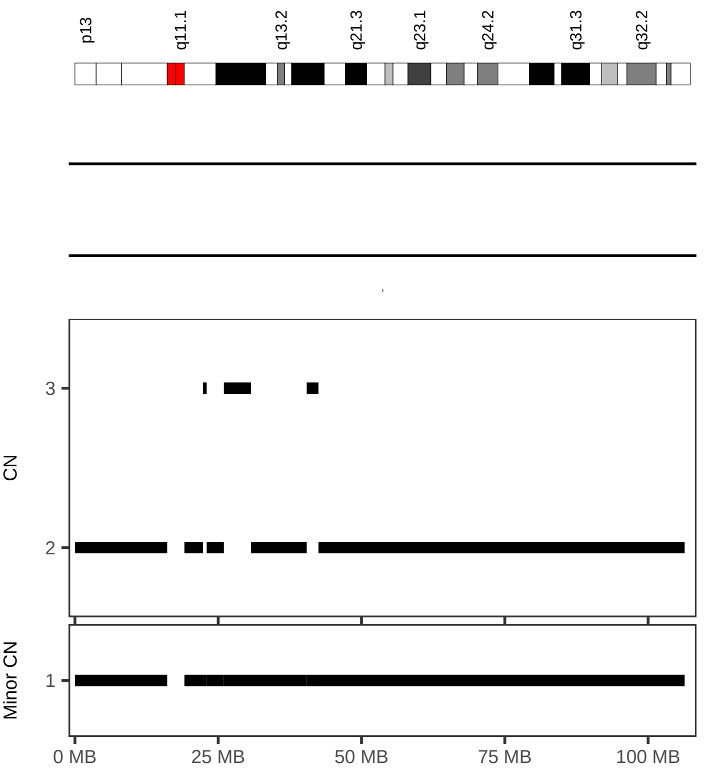

	**RK087**
Cancer type	Liver−HCC
Position	11
Interleaved intrachr. SVs	1
Total SVs (intrachr. + transl.)	1
SV types	DEL: 0; DUP: 1; h2hINV: 0; t2tINV: 0; TRA: 0
SVs in sample	62
Oscillating CN in chr (2 and 3 states)	7,
CN segments	12
Purity, ploidy	0.26, 1.9

	**RK125**
Cancer type	Liver−HCC
Position	14
Interleaved intrachr. SVs	0
Total SVs (intrachr. + transl.)	0
SV types	DEL: 0; DUP: 0; h2hINV: 0; t2tINV: 0; TRA: 0
SVs in sample	18
Oscillating CN in chr (2 and 3 states)	7,
CN segments	8
Purity, ploidy	0.72, 3.47

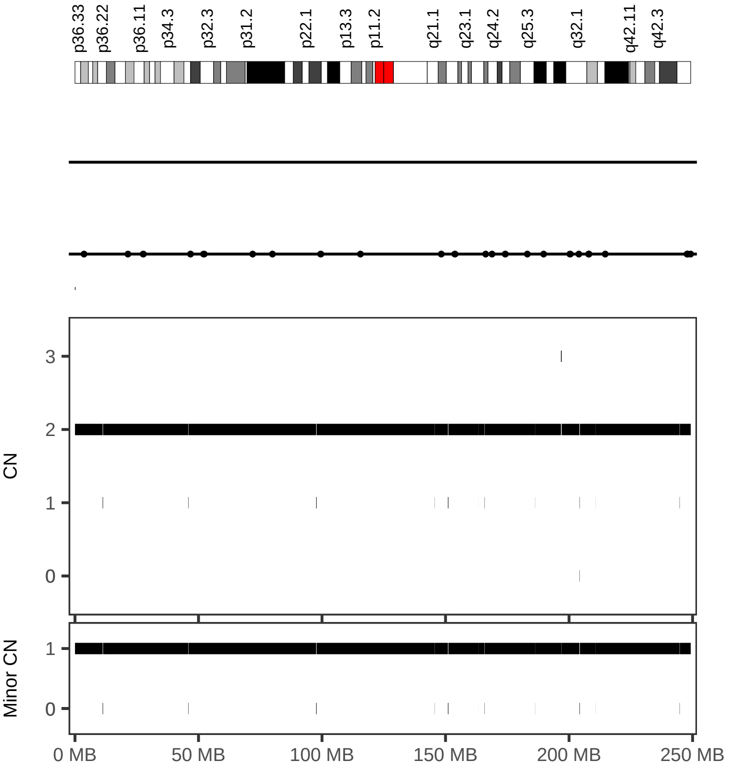

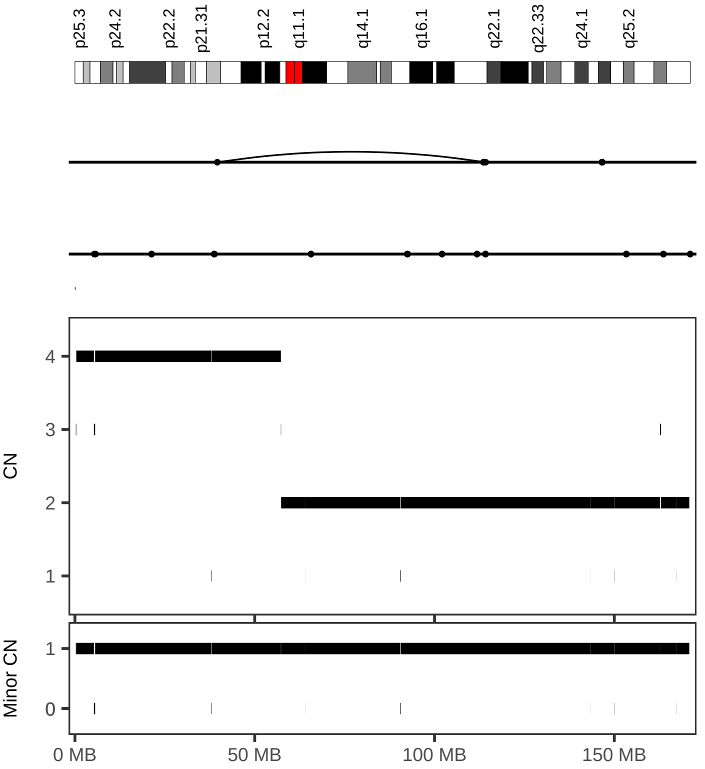

	**RK148**
Cancer type	Liver−HCC
Position	1
Interleaved intrachr. SVs	2
Total SVs (intrachr. + transl.)	2
SV types	DEL: 2; DUP: 0; h2hINV: 0; t2tINV: 0; TRA: 0
SVs in sample	195
Oscillating CN in chr (2 and 3 states)	17,
CN segments	27
Purity, ploidy	0.9, 1.98

	**RK148**
Cancer type	Liver−HCC
Position	6
Interleaved intrachr. SVs	2
Total SVs (intrachr. + transl.)	2
SV types	DEL: 0; DUP: 0; h2hINV: 1; t2tINV: 1; TRA: 0
SVs in sample	195
Oscillating CN in chr (2 and 3 states)	9,
CN segments	20
Purity, ploidy	0.9, 1.98

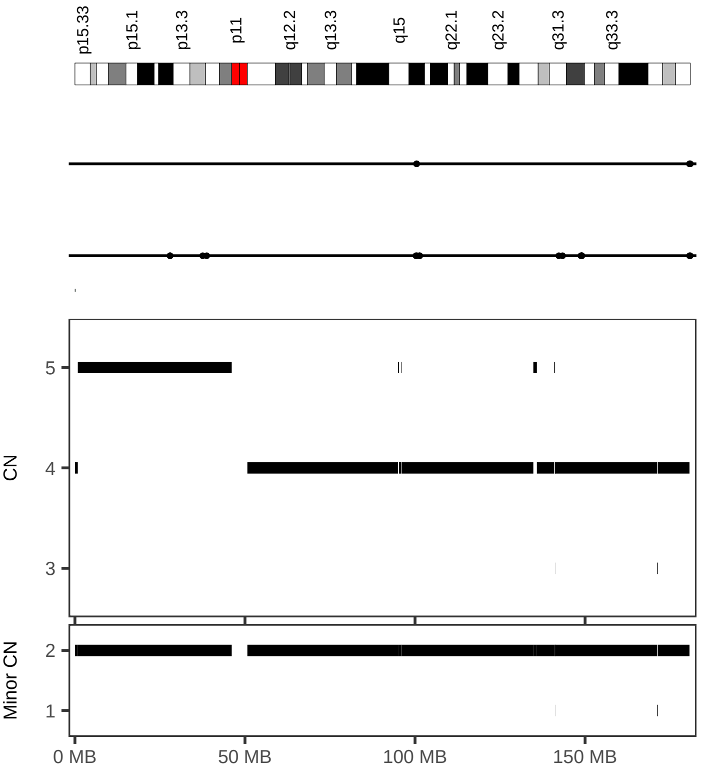

	**RK207**
Cancer type	Liver−HCC
Position	5
Interleaved intrachr. SVs	2
Total SVs (intrachr. + transl.)	2
SV types	DEL: 0; DUP: 1; h2hINV: 1; t2tINV: 0; TRA: 0
SVs in sample	122
Oscillating CN in chr (2 and 3 states)	11,
CN segments	15
Purity, ploidy	0.38, 3.72

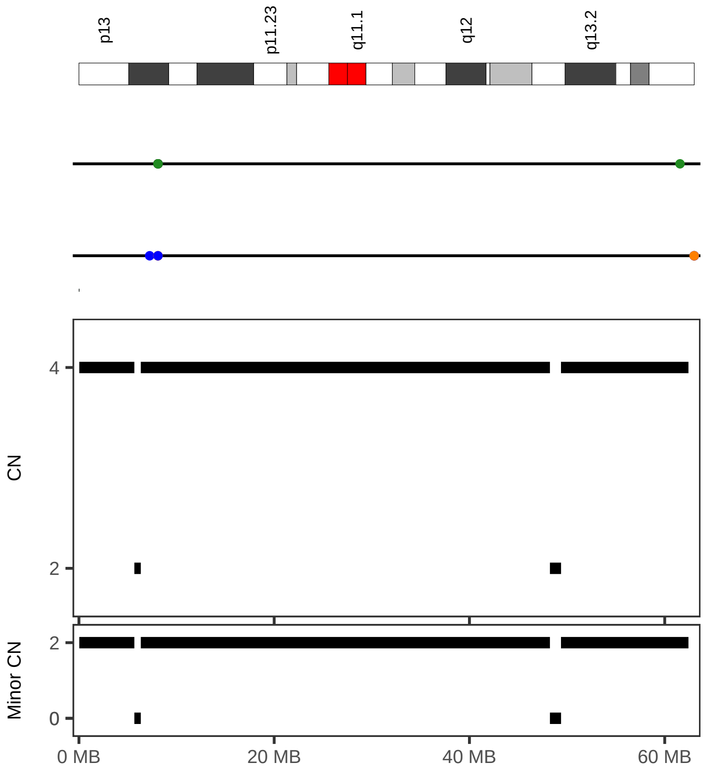

	**RK216**
Cancer type	Liver−HCC
Position	20
Interleaved intrachr. SVs	0
Total SVs (intrachr. + transl.)	0
SV types	DEL: 0; DUP: 0; h2hINV: 0; t2tINV: 0; TRA: 0
SVs in sample	38
Oscillating CN in chr (2 and 3 states)	7,
CN segments	7
Purity, ploidy	0.84, 3.4

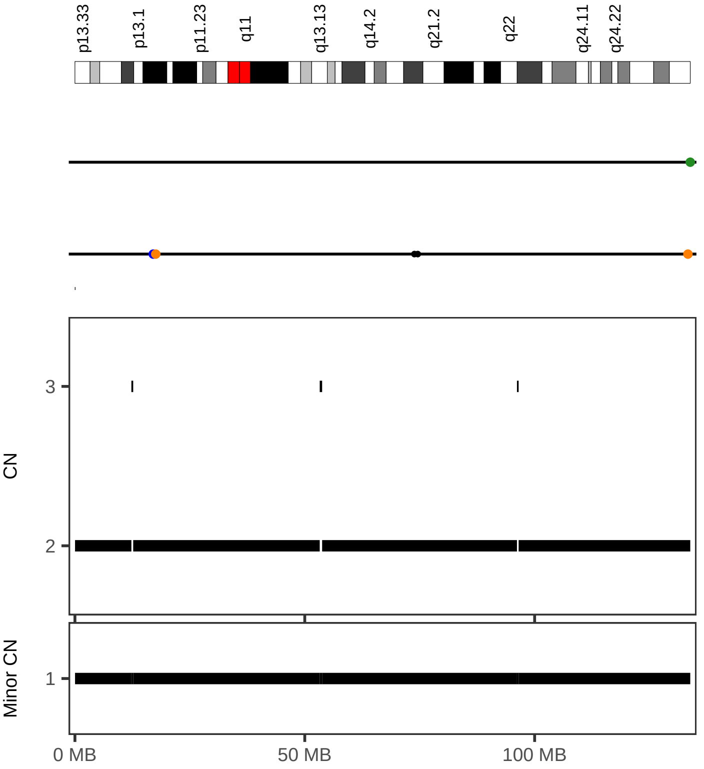

	**RK234**
Cancer type	Liver−HCC
Position	12
Interleaved intrachr. SVs	1
Total SVs (intrachr. + transl.)	1
SV types	DEL: 0; DUP: 1; h2hINV: 0; t2tINV: 0; TRA: 0
SVs in sample	51
Oscillating CN in chr (2 and 3 states)	7,
CN segments	7
Purity, ploidy	0.76, 1.99

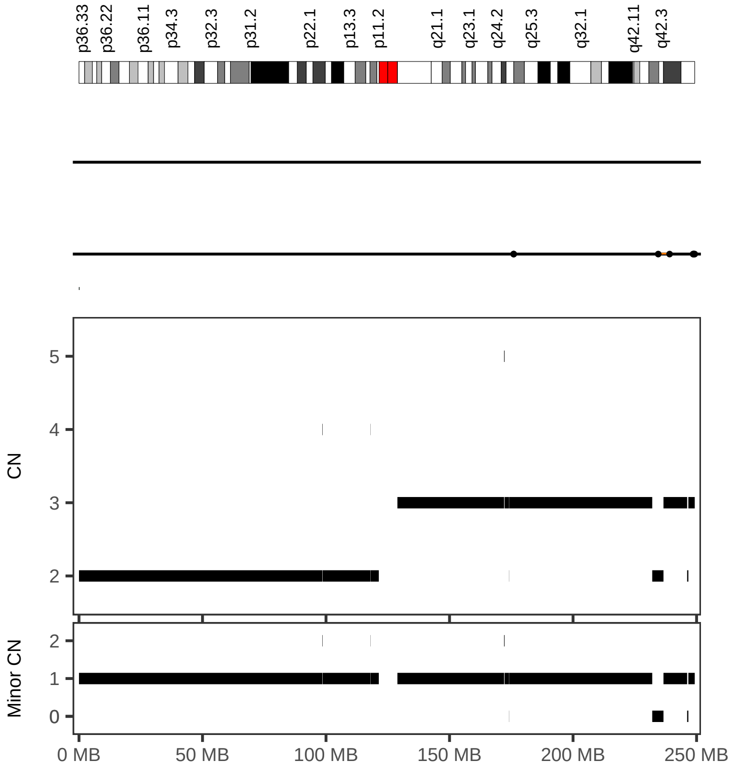

	**RK244**
Cancer type	Liver−HCC
Position	1
Interleaved intrachr. SVs	2
Total SVs (intrachr. + transl.)	2
SV types	DEL: 2; DUP: 0; h2hINV: 0; t2tINV: 0; TRA: 0
SVs in sample	24
Oscillating CN in chr (2 and 3 states)	7,
CN segments	14
Purity, ploidy	0.86, 2.12

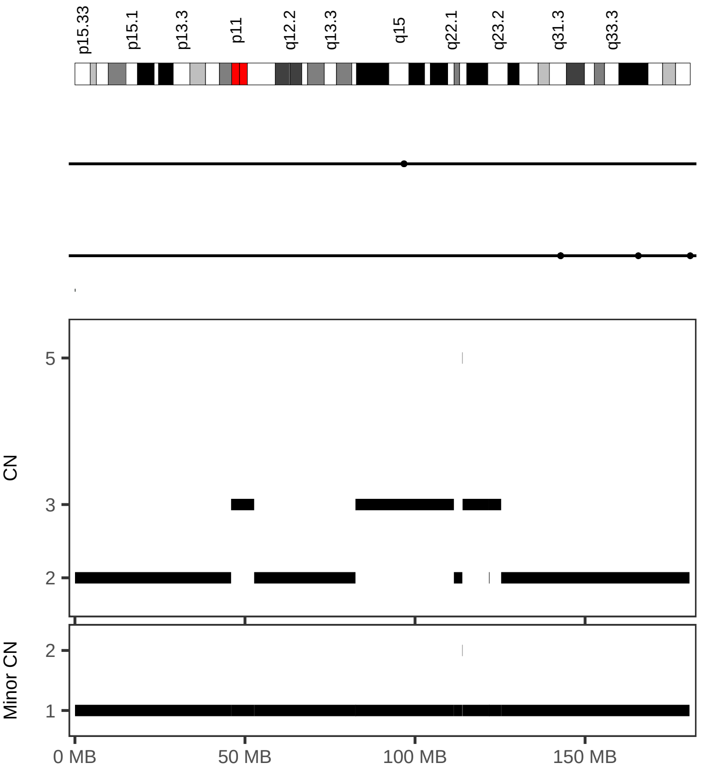

	**RK245**
Cancer type	Liver−HCC
Position	5
Interleaved intrachr. SVs	0
Total SVs (intrachr. + transl.)	0
SV types	DEL: 0; DUP: 0; h2hINV: 0; t2tINV: 0; TRA: 0
SVs in sample	48
Oscillating CN in chr (2 and 3 states)	7,
CN segments	12
Purity, ploidy	0.47, 2.16

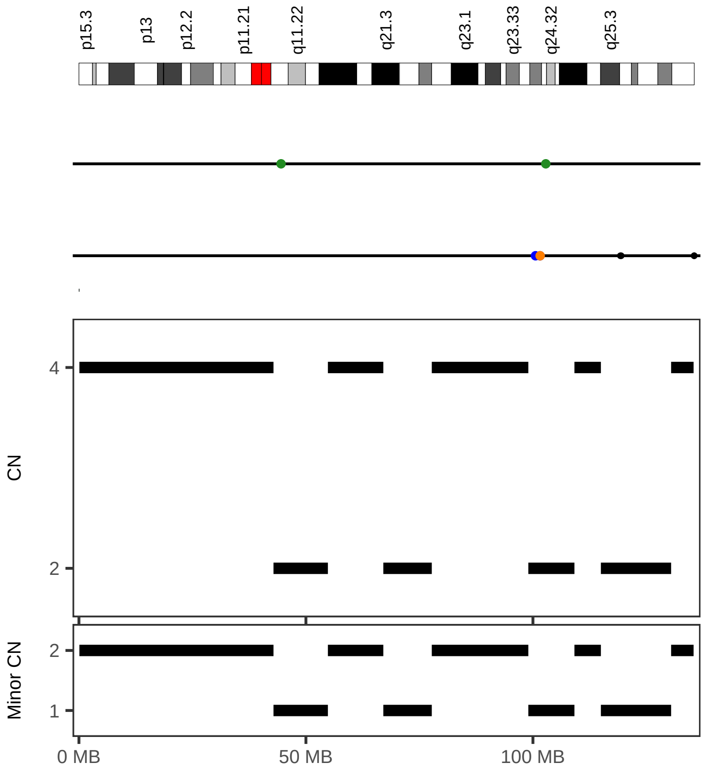

	**RK258**
Cancer type	Liver−HCC
Position	10
Interleaved intrachr. SVs	1
Total SVs (intrachr. + transl.)	2
SV types	DEL: 2; DUP: 0; h2hINV: 0; t2tINV: 0; TRA: 0
SVs in sample	61
Oscillating CN in chr (2 and 3 states)	9,
CN segments	9
Purity, ploidy	0.17, 3.23

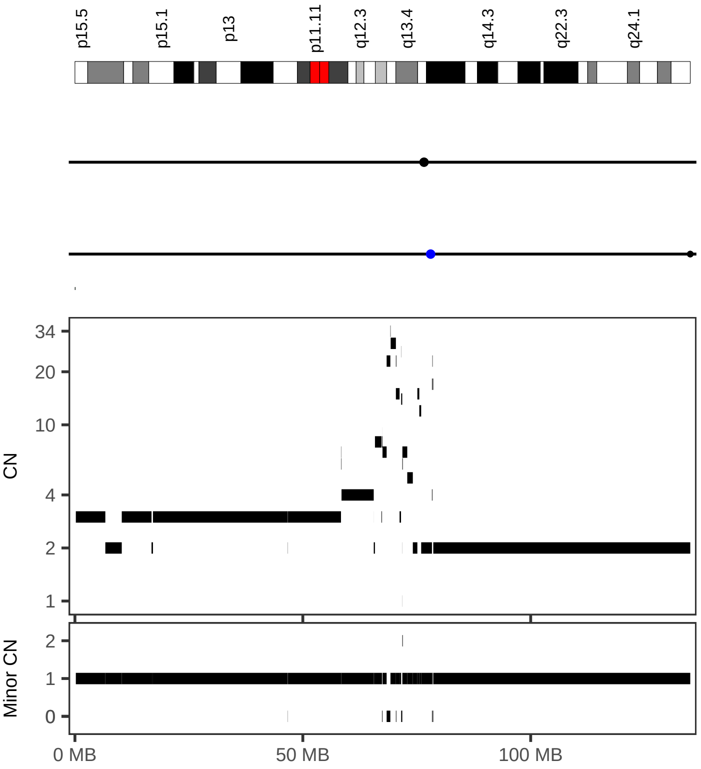

	**RK261**
Cancer type	Liver−HCC
Position	11
Interleaved intrachr. SVs	0
Total SVs (intrachr. + transl.)	0
SV types	DEL: 0; DUP: 0; h2hINV: 0; t2tINV: 0; TRA: 0
SVs in sample	66
Oscillating CN in chr (2 and 3 states)	7,
CN segments	40
Purity, ploidy	NA, NA

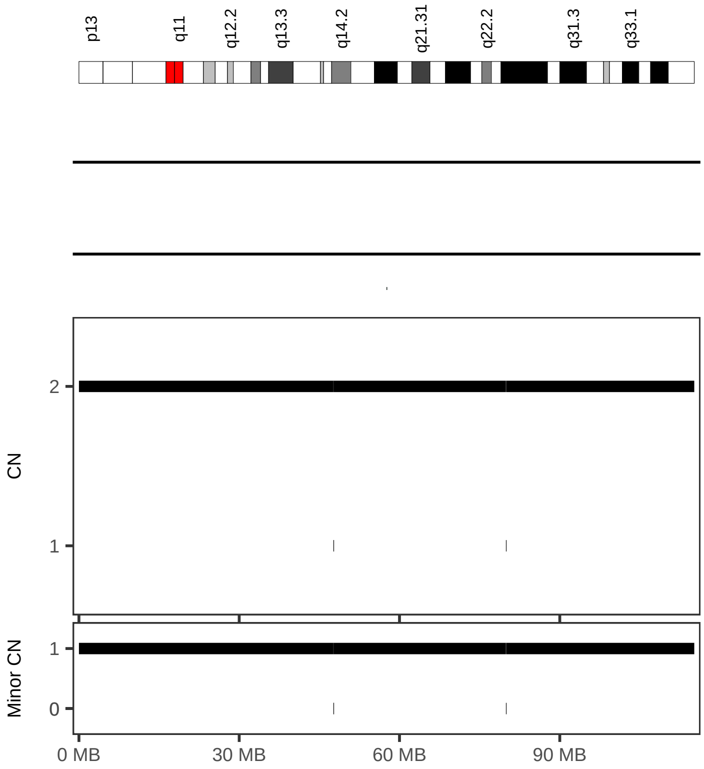

	**RK261**
Cancer type	Liver−HCC
Position	13
Interleaved intrachr. SVs	0
Total SVs (intrachr. + transl.)	0
SV types	DEL: 0; DUP: 0; h2hINV: 0; t2tINV: 0; TRA: 0
SVs in sample	66
Oscillating CN in chr (2 and 3 states)	11,
CN segments	11
Purity, ploidy	NA, NA

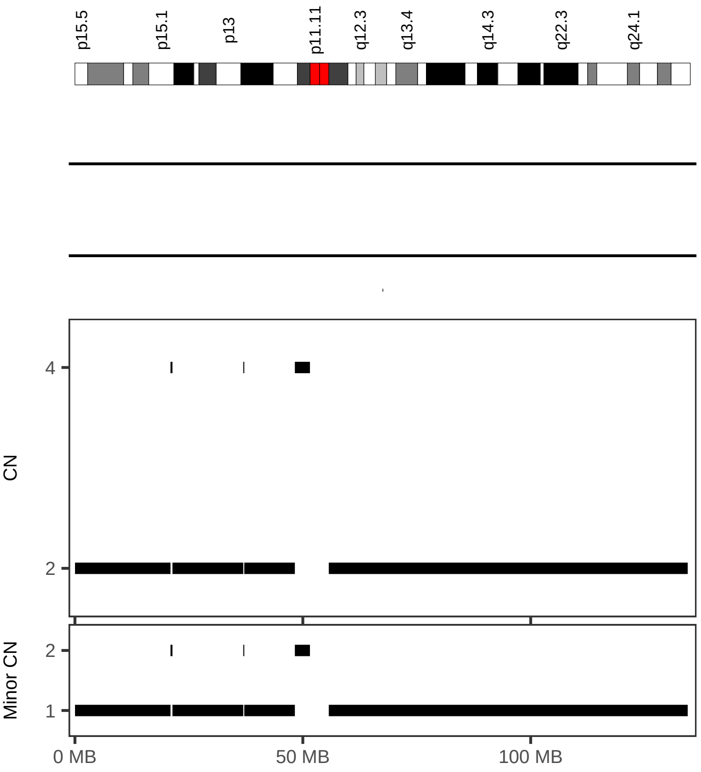

	RK283
Cancer type	Liver−HCC
Position	11
Interleaved intrachr. SVs	0
Total SVs (intrachr. + transl.)	0
SV types	DEL: 0; DUP: 0; h2hINV: 0;<br>t2tINV: 0; TRA: 0
SVs in sample	11
Oscillating CN in chr (2 and 3 states)	7,
CN segments	7
Purity, ploidy	0.25, 2.06

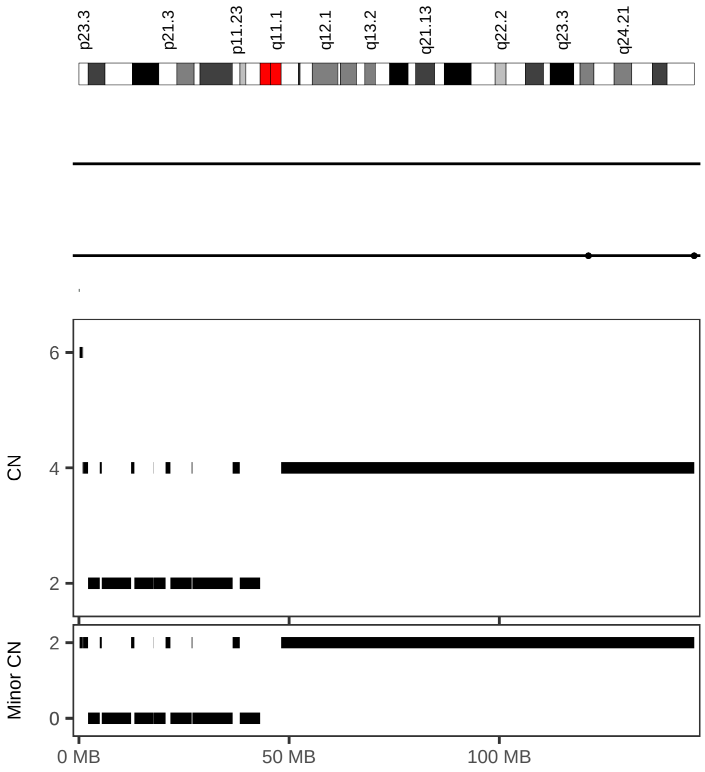

	RK289
Cancer type	Liver−HCC
Position	8
Interleaved intrachr. SVs	1
Total SVs (intrachr. + transl.)	1
SV types	DEL: 0; DUP: 1; h2hINV: 0;<br>t2tINV: 0; TRA: 0
SVs in sample	34
Oscillating CN in chr (2 and 3 states)	15,
CN segments	18
Purity, ploidy	NA, NA

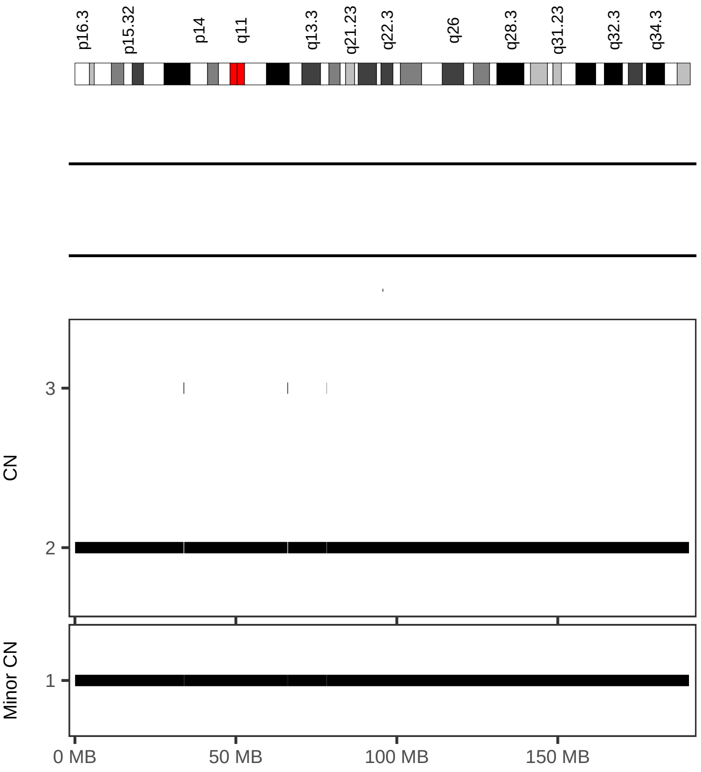

	RK304
Cancer type	Liver−HCC
Position	4
Interleaved intrachr. SVs	0
Total SVs (intrachr. + transl.)	0
SV types	DEL: 0; DUP: 0; h2hINV: 0;<br>t2tINV: 0; TRA: 0
SVs in sample	37
Oscillating CN in chr (2 and 3 states)	7,
CN segments	7
Purity, ploidy	0.25, 2

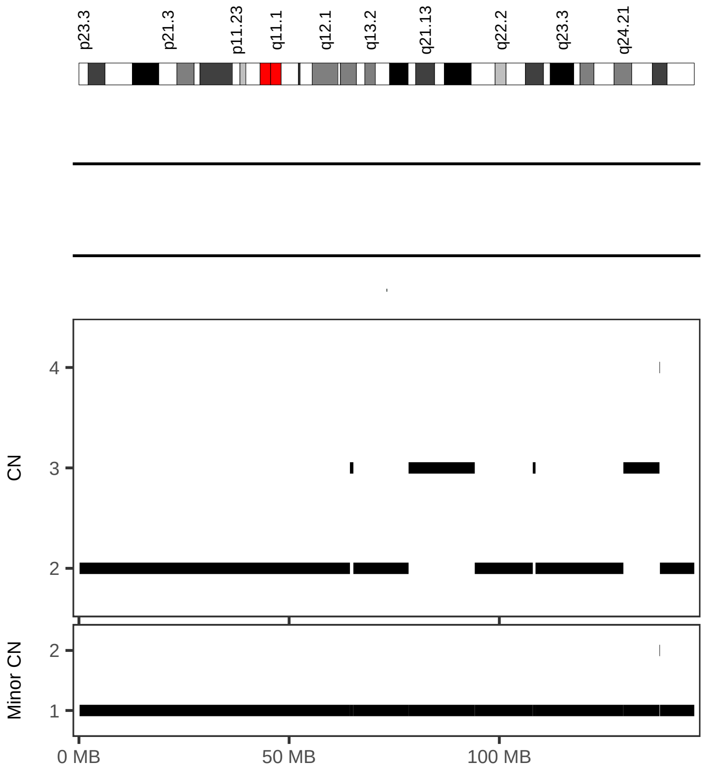

	RK304
Cancer type	Liver−HCC
Position	8
Interleaved intrachr. SVs	0
Total SVs (intrachr. + transl.)	0
SV types	DEL: 0; DUP: 0; h2hINV: 0;<br>t2tINV: 0; TRA: 0
SVs in sample	37
Oscillating CN in chr (2 and 3 states)	8,
CN segments	10
Purity, ploidy	0.25, 2

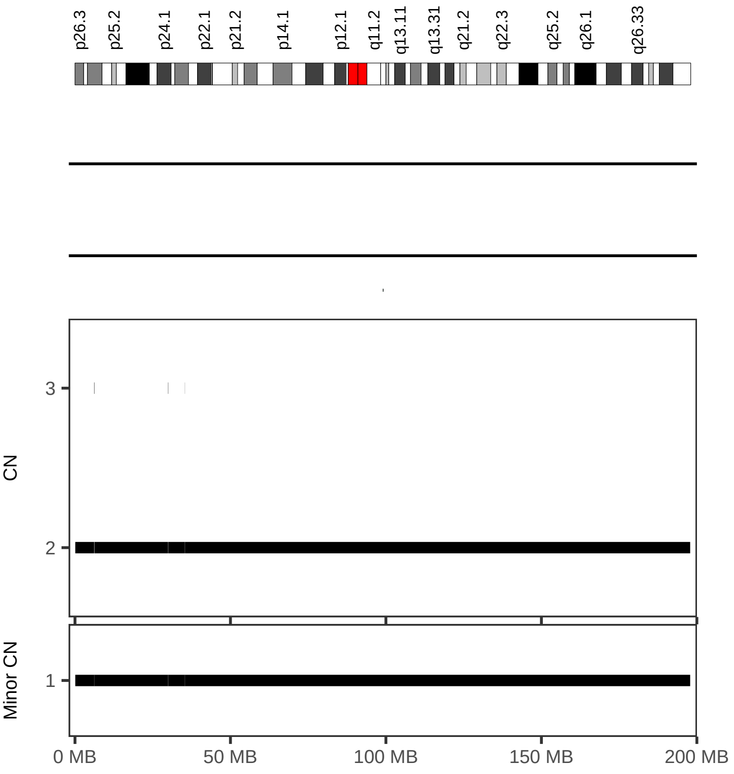

	**RK337**
Cancer type	Liver−HCC
Position	3
Interleaved intrachr. SVs	0
Total SVs (intrachr. + transl.)	0
SV types	DEL: 0; DUP: 0; h2hINV: 0; t2tINV: 0; TRA: 0
SVs in sample	37
Oscillating CN in chr (2 and 3 states)	7,
CN segments	7
Purity, ploidy	0.38, 2.75

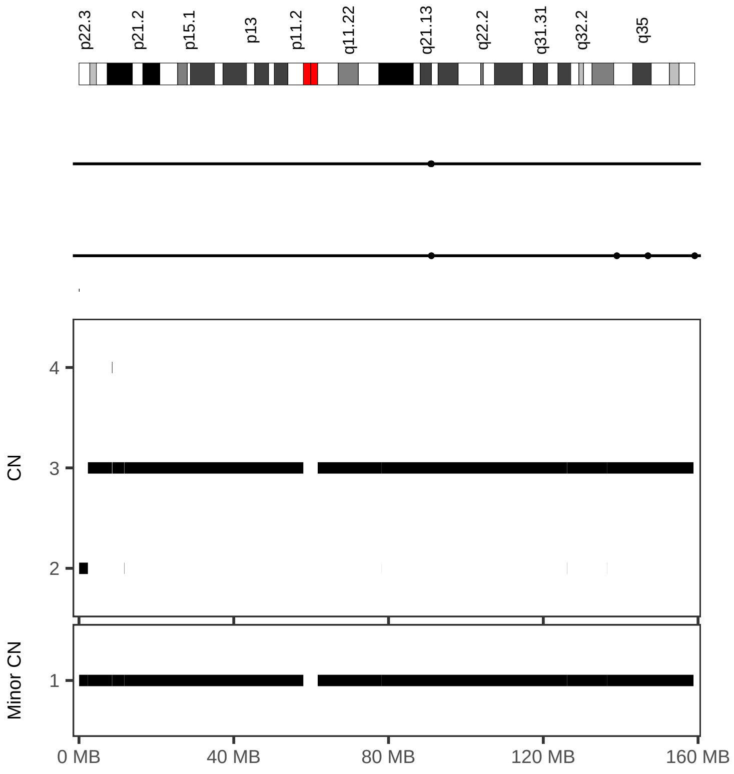

	**RK337**
Cancer type	Liver−HCC
Position	7
Interleaved intrachr. SVs	2
Total SVs (intrachr. + transl.)	2
SV types	DEL: 0; DUP: 0; h2hINV: 1; t2tINV: 1; TRA: 0
SVs in sample	37
Oscillating CN in chr (2 and 3 states)	7,
CN segments	13
Purity, ploidy	0.38, 2.75

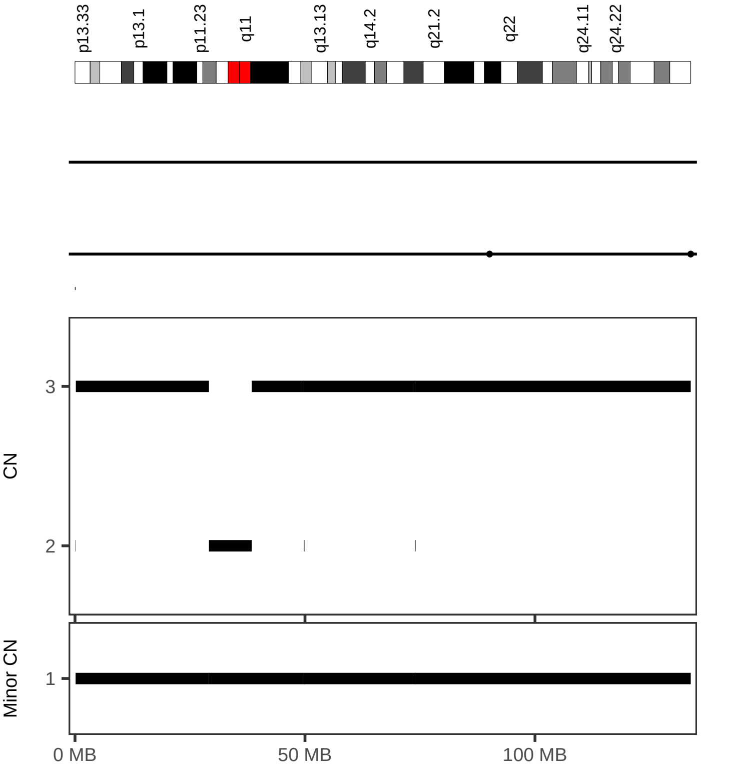

	**RK337**
Cancer type	Liver−HCC
Position	12
Interleaved intrachr. SVs	0
Total SVs (intrachr. + transl.)	0
SV types	DEL: 0; DUP: 0; h2hINV: 0; t2tINV: 0; TRA: 0
SVs in sample	37
Oscillating CN in chr (2 and 3 states)	8,
CN segments	8
Purity, ploidy	0.38, 2.75

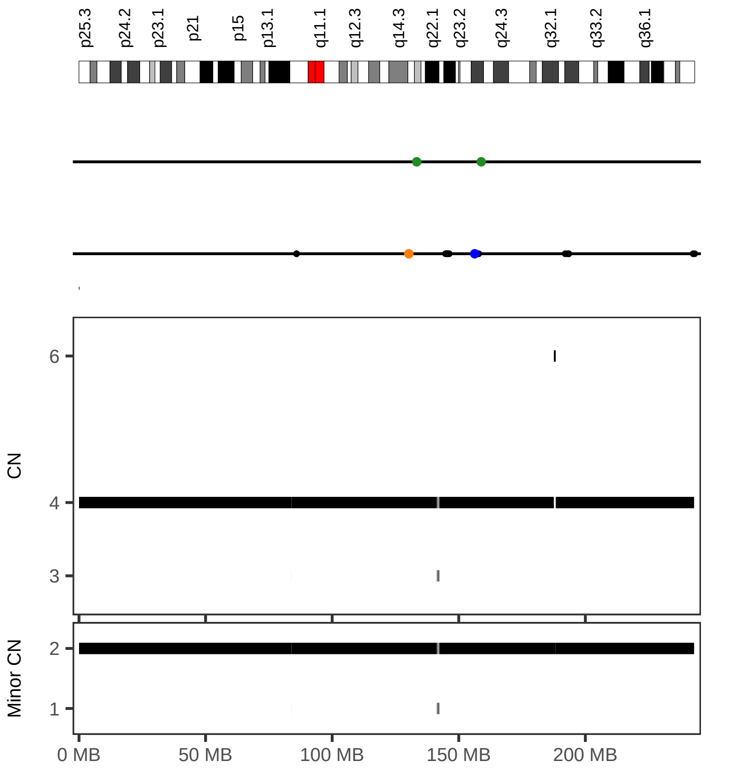

	**21fb46f9−4bbb−441c−af19−a687e9138344**
Cancer type	Lung−AdenoCA
Position	2
Interleaved intrachr. SVs	2
Total SVs (intrachr. + transl.)	2
SV types	DEL: 2; DUP: 0; h2hINV: 0; t2tINV: 0; TRA: 0
SVs in sample	71
Oscillating CN in chr (2 and 3 states)	9,
CN segments	11
Purity, ploidy	0.49, 3.42

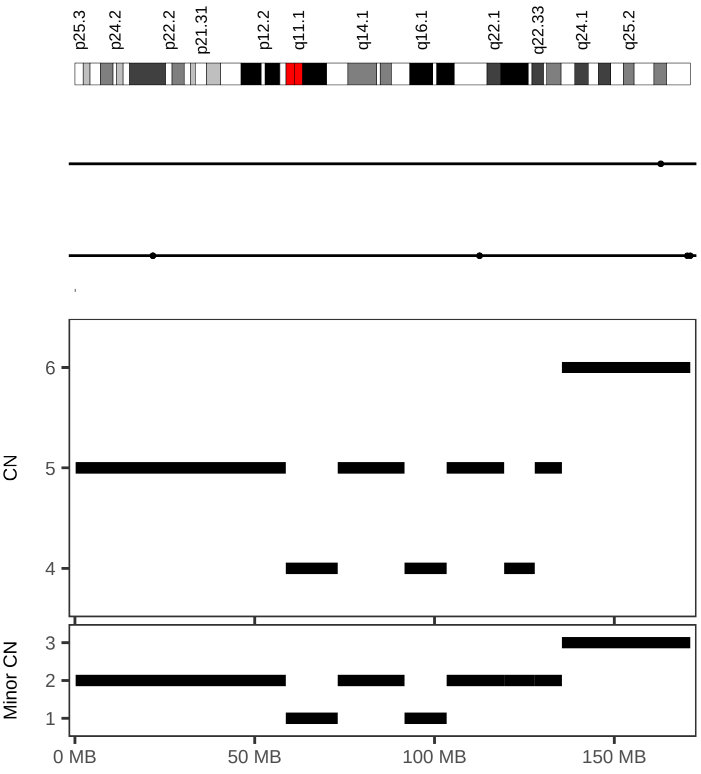

	31c96e35−5e2f−429c−b12a−7bc5a497a300
Cancer type	Lung−AdenoCA
Position	6
Interleaved intrachr. SVs	1
Total SVs (intrachr. + transl.)	1
SV types	DEL: 1; DUP: 0; h2hINV: 0; t2tINV: 0; TRA: 0
SVs in sample	276
Oscillating CN in chr (2 and 3 states)	7,
CN segments	8
Purity, ploidy	0.49, 4.79

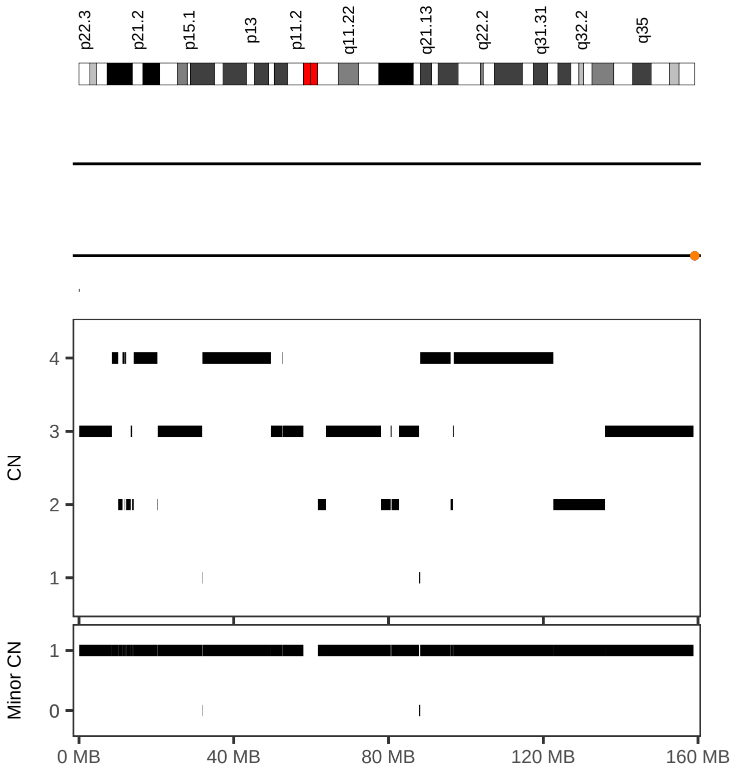

	a8d6694c−a213−4544−ac0b−63bce16d8f4e
Cancer type	Lung−AdenoCA
Position	7
Interleaved intrachr. SVs	0
Total SVs (intrachr. + transl.)	0
SV types	DEL: 0; DUP: 0; h2hINV: 0; t2tINV: 0; TRA: 0
SVs in sample	21
Oscillating CN in chr (2 and 3 states)	7,
CN segments	30
Purity, ploidy	0.62, 2.3

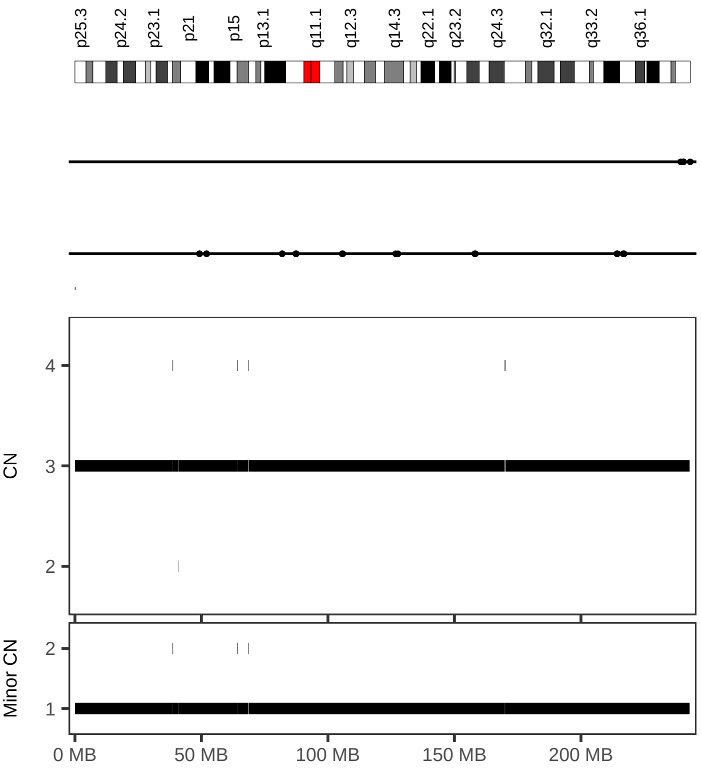

	c4d1e105−28b7−48df−abef−fbe09782fdb2
Cancer type	Lung−AdenoCA
Position	2
Interleaved intrachr. SVs	2
Total SVs (intrachr. + transl.)	2
SV types	DEL: 0; DUP: 0; h2hINV: 1; t2tINV: 1; TRA: 0
SVs in sample	136
Oscillating CN in chr (2 and 3 states)	7,
CN segments	11
Purity, ploidy	0.82, 3.29

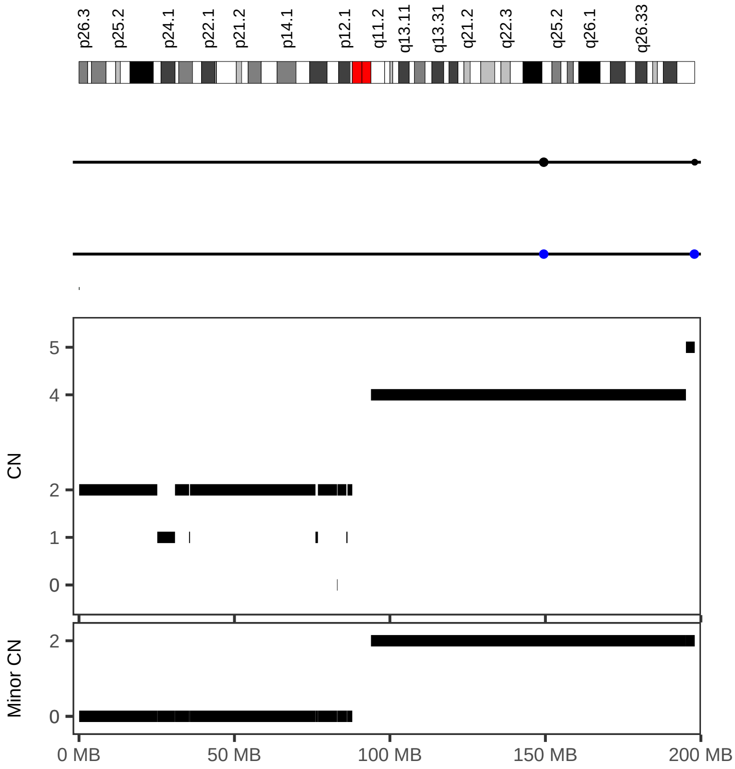

	d2824e6d−3784−45c2−9b0f−52b17356b5da
Cancer type	Lung−AdenoCA
Position	3
Interleaved intrachr. SVs	1
Total SVs (intrachr. + transl.)	2
SV types	DEL: 0; DUP: 1; h2hINV: 0; t2tINV: 0; TRA: 1
SVs in sample	98
Oscillating CN in chr (2 and 3 states)	7,
CN segments	13
Purity, ploidy	0.38, 3.03

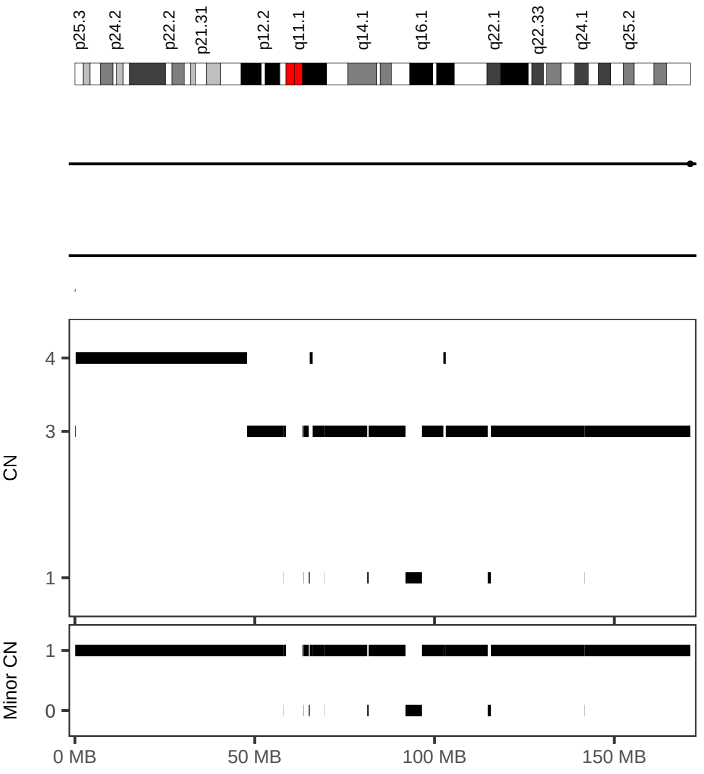

	d2824e6d−3784−45c2−9b0f−52b17356b5da
Cancer type	Lung−AdenoCA
Position	6
Interleaved intrachr. SVs	0
Total SVs (intrachr. + transl.)	0
SV types	DEL: 0; DUP: 0; h2hINV: 0; t2tINV: 0; TRA: 0
SVs in sample	98
Oscillating CN in chr (2 and 3 states)	7,
CN segments	23
Purity, ploidy	0.38, 3.03

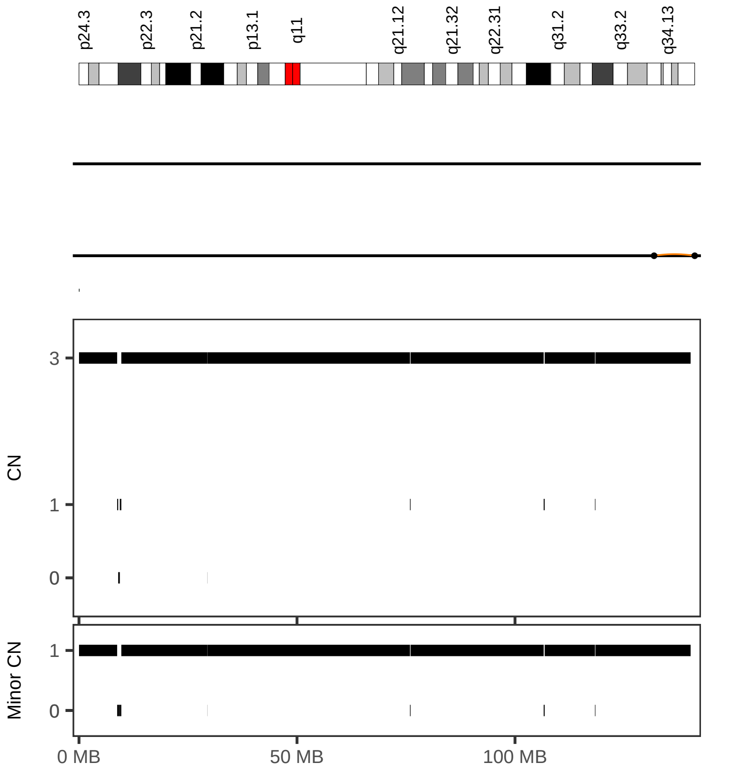

	d2824e6d−3784−45c2−9b0f−52b17356b5da
Cancer type	Lung−AdenoCA
Position	9
Interleaved intrachr. SVs	1
Total SVs (intrachr. + transl.)	1
SV types	DEL: 1; DUP: 0; h2hINV: 0; t2tINV: 0; TRA: 0
SVs in sample	98
Oscillating CN in chr (2 and 3 states)	7,
CN segments	13
Purity, ploidy	0.38, 3.03

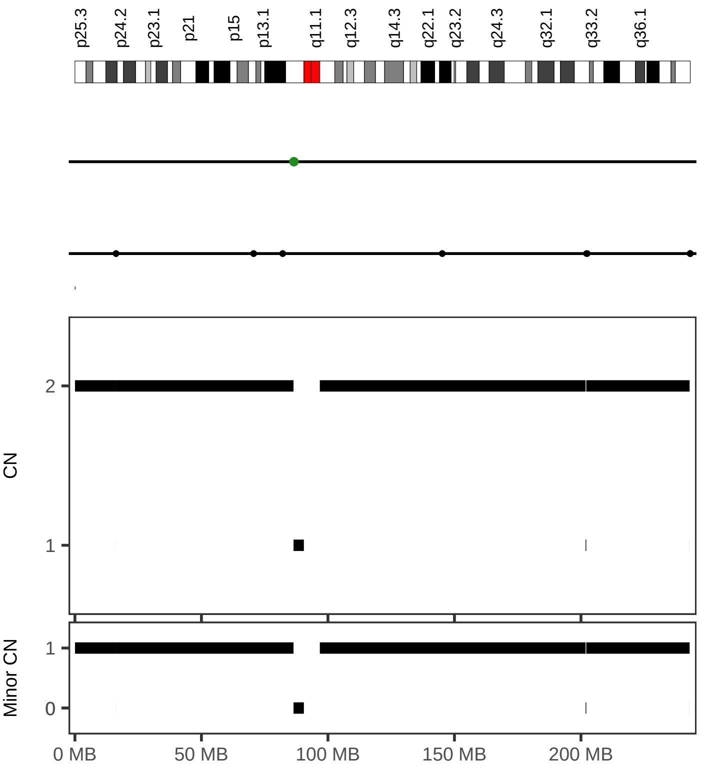

	81b7cbc1−c037−426f−95b0−d729a30697da
Cancer type	Lung−SCC
Position	2
Interleaved intrachr. SVs	2
Total SVs (intrachr. + transl.)	2
SV types	DEL: 2; DUP: 0; h2hINV: 0; t2tINV: 0; TRA: 0
SVs in sample	72
Oscillating CN in chr (2 and 3 states)	9,
CN segments	9
Purity, ploidy	0.56, 1.85

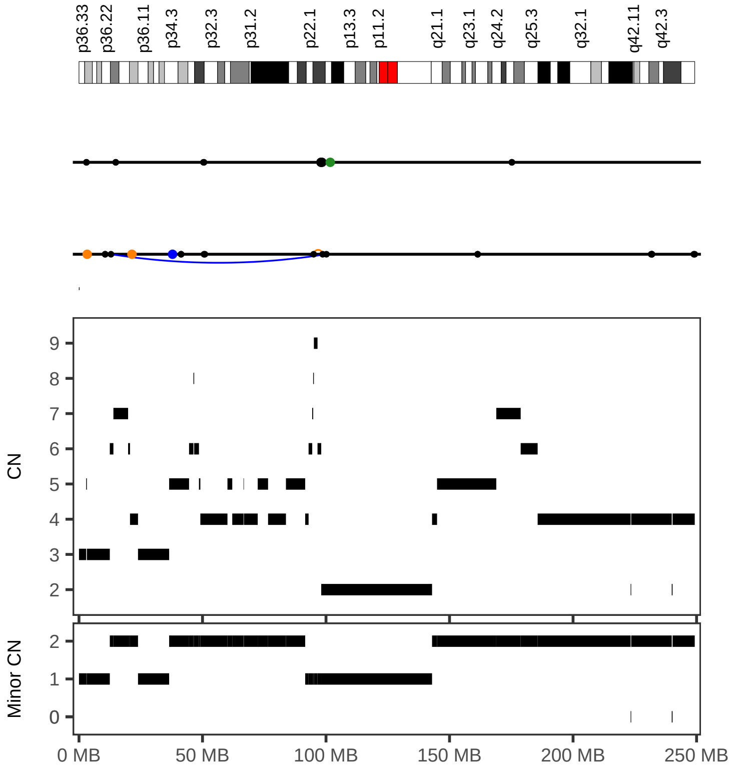

	9293e197−e38a−4e19−a7d0−1b45d1ad48bd
Cancer type	Lung−SCC
Position	1
Interleaved intrachr. SVs	2
Total SVs (intrachr. + transl.)	2
SV types	DEL: 0; DUP: 1; h2hINV: 1; t2tINV: 0; TRA: 0
SVs in sample	187
Oscillating CN in chr (2 and 3 states)	10,
CN segments	37
Purity, ploidy	0.53, 3.3

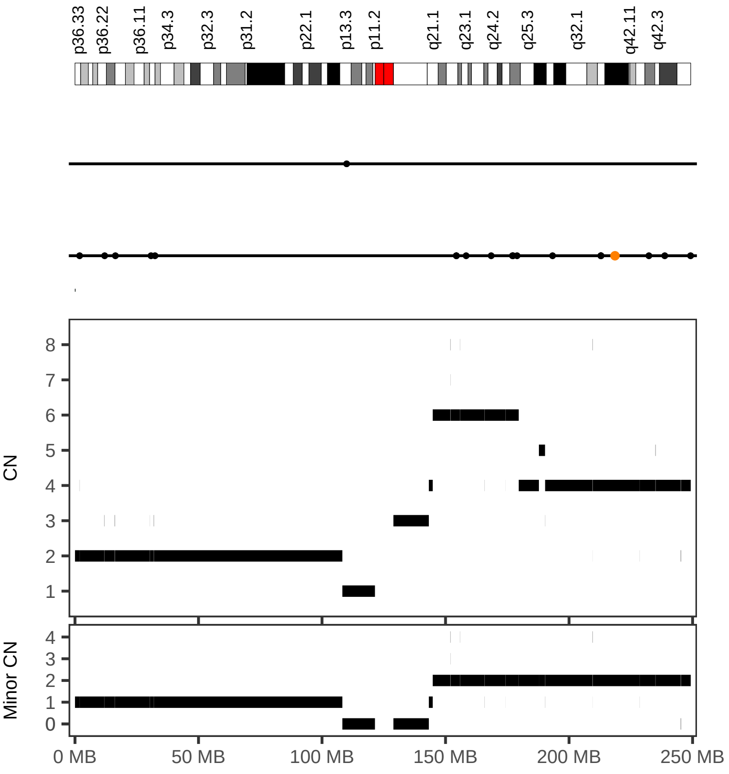

	9a874b64−d0d6−416e−97bc−e9071ed0b16b
Cancer type	Lung−SCC
Position	1
Interleaved intrachr. SVs	2
Total SVs (intrachr. + transl.)	2
SV types	DEL: 0; DUP: 2; h2hINV: 0; t2tINV: 0; TRA: 0
SVs in sample	359
Oscillating CN in chr (2 and 3 states)	9,
CN segments	37
Purity, ploidy	0.48, 2.68

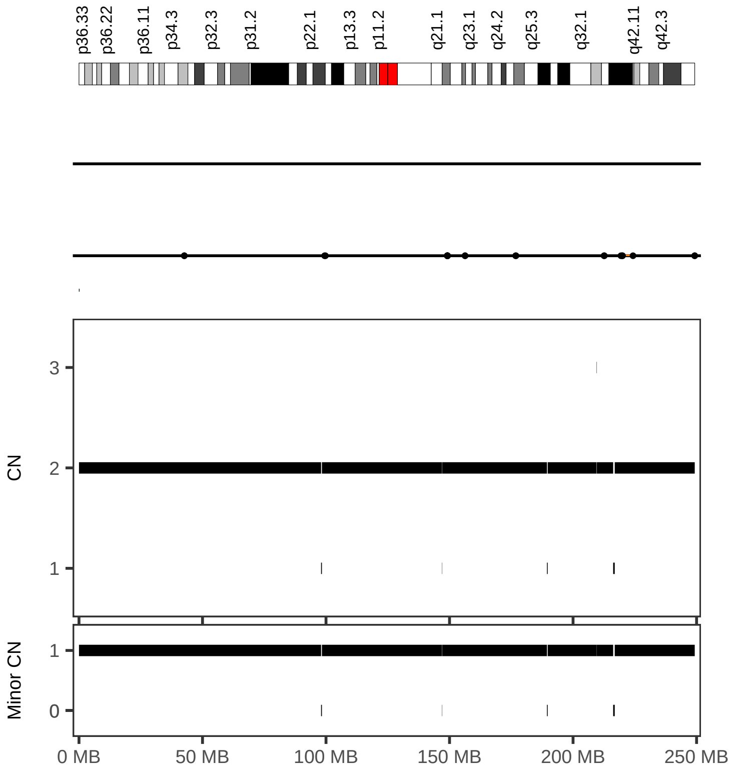

	a3e1ac67−a1f2−44fb−8343−a7e8239fc24a
Cancer type	Lung−SCC
Position	1
Interleaved intrachr. SVs	2
Total SVs (intrachr. + transl.)	2
SV types	DEL: 1; DUP: 1; h2hINV: 0; t2tINV: 0; TRA: 0
SVs in sample	118
Oscillating CN in chr (2 and 3 states)	7,
CN segments	11
Purity, ploidy	0.48, 1.8

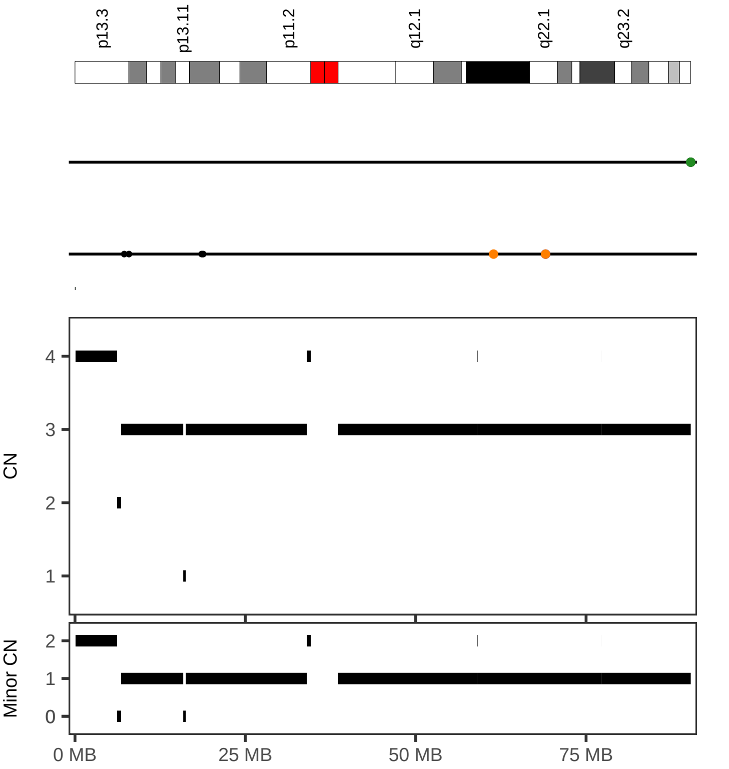

	bd15f523−45c4−4d7c−a3cc−4fb56abb0e54
Cancer type	Lung−SCC
Position	16
Interleaved intrachr. SVs	1
Total SVs (intrachr. + transl.)	2
SV types	DEL: 2; DUP: 0; h2hINV: 0; t2tINV: 0; TRA: 0
SVs in sample	139
Oscillating CN in chr (2 and 3 states)	7,
CN segments	11
Purity, ploidy	0.75, 3.23

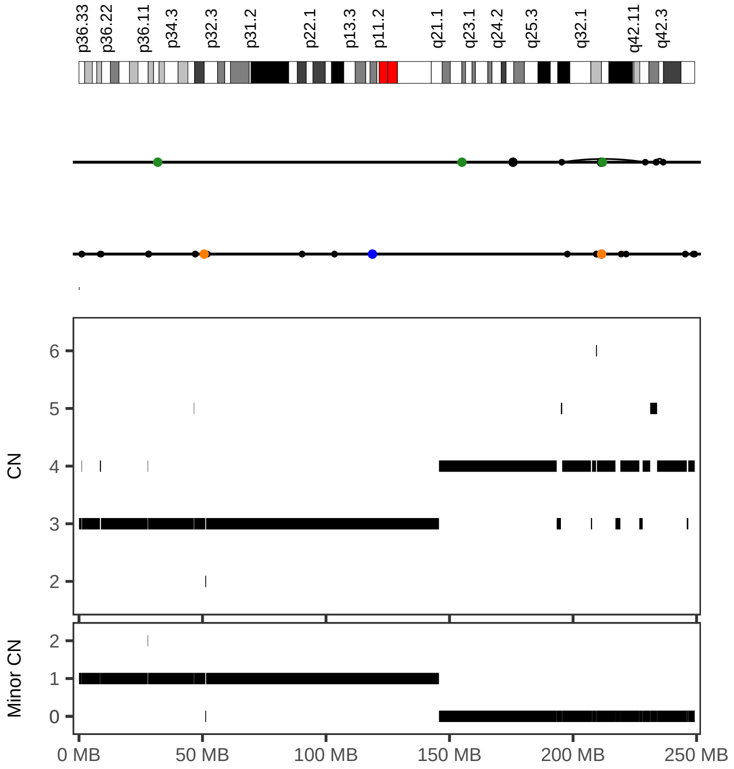

	e6b72c24−1607−43b9−8b8a−7bf83eea5895
Cancer type	Lung−SCC
Position	1
Interleaved intrachr. SVs	2
Total SVs (intrachr. + transl.)	2
SV types	DEL: 0; DUP: 0; h2hINV: 1; t2tINV: 1; TRA: 0
SVs in sample	265
Oscillating CN in chr (2 and 3 states)	7,
CN segments	27
Purity, ploidy	0.53, 3.12

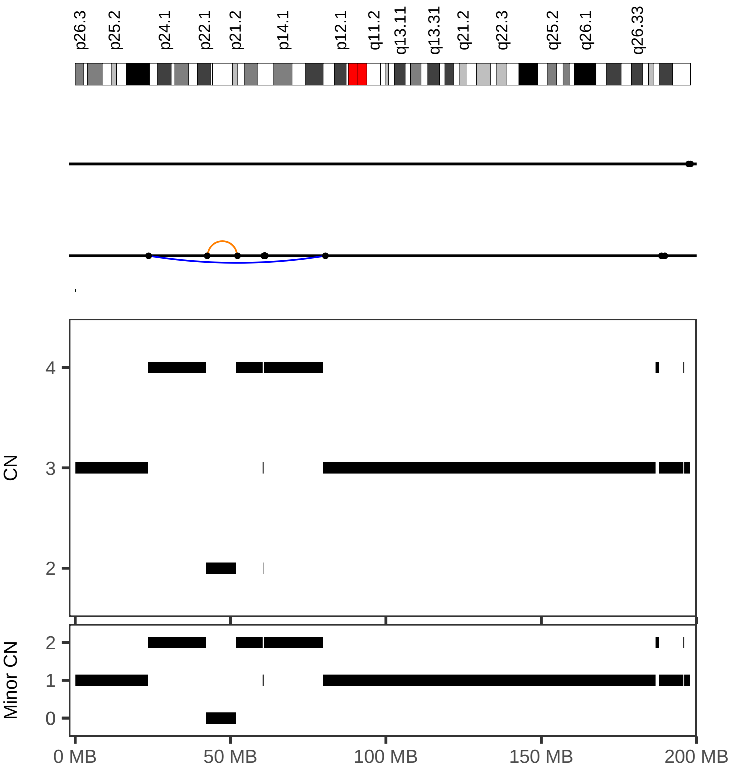

	**4105746**
Cancer type	Lymph−BNHL
Position	3
Interleaved intrachr. SVs	2
Total SVs (intrachr. + transl.)	2
SV types	DEL: 2; DUP: 0; h2hINV: 0; t2tINV: 0; TRA: 0
SVs in sample	32
Oscillating CN in chr (2 and 3 states)	7,
CN segments	15
Purity, ploidy	0.71, 2.03

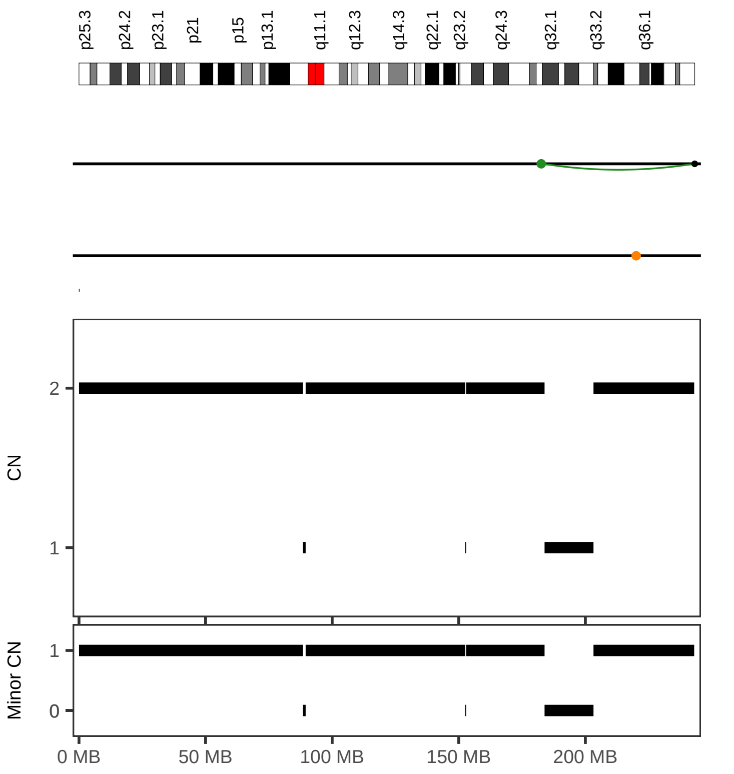

	**4111326**
Cancer type	Lymph−BNHL
Position	2
Interleaved intrachr. SVs	1
Total SVs (intrachr. + transl.)	2
SV types	DEL: 0; DUP: 0; h2hINV: 0; t2tINV: 1; TRA: 1
SVs in sample	36
Oscillating CN in chr (2 and 3 states)	7,
CN segments	7
Purity, ploidy	0.71, 1.98

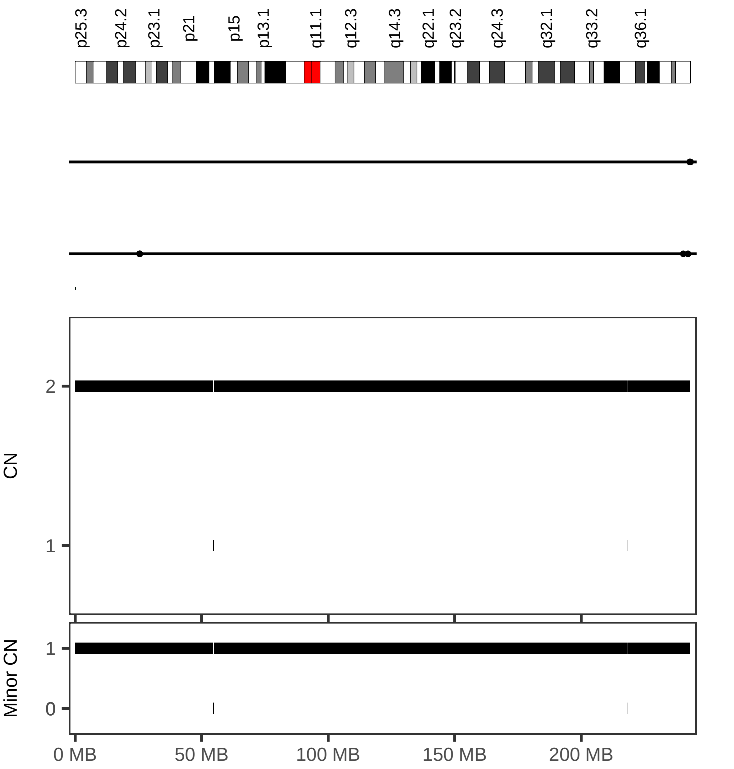

	**4134005**
Cancer type	Lymph−BNHL
Position	2
Interleaved intrachr. SVs	1
Total SVs (intrachr. + transl.)	2
SV types	DEL: 1; DUP: 0; h2hINV: 1; t2tINV: 0; TRA: 0
SVs in sample	19
Oscillating CN in chr (2 and 3 states)	7,
CN segments	7
Purity, ploidy	0.78, 1.99

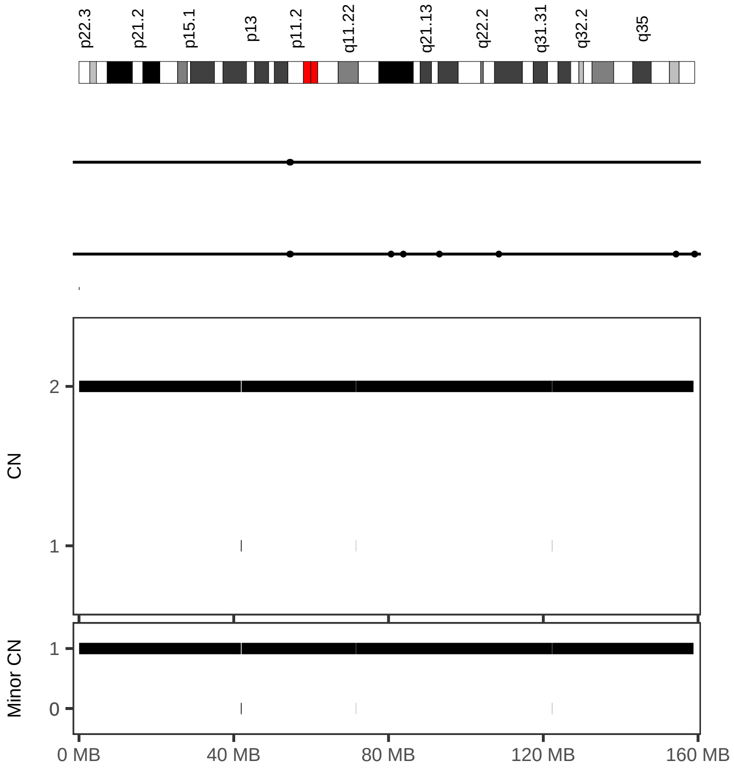

	**4135099**
Cancer type	Lymph−BNHL
Position	7
Interleaved intrachr. SVs	2
Total SVs (intrachr. + transl.)	2
SV types	DEL: 1; DUP: 0; h2hINV: 0; t2tINV: 1; TRA: 0
SVs in sample	297
Oscillating CN in chr (2 and 3 states)	7,
CN segments	7
Purity, ploidy	0.79, 1.91

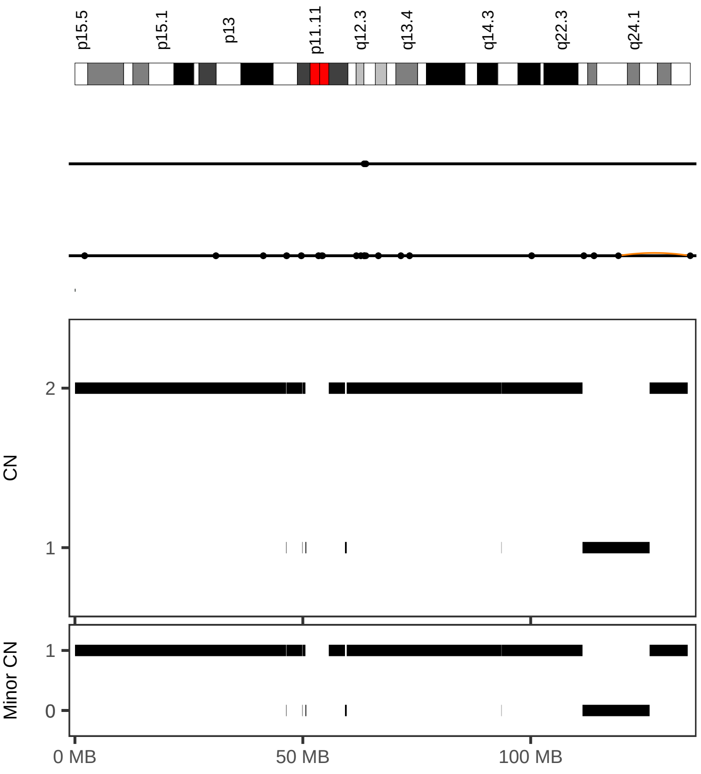

	4135099
Cancer type	Lymph−BNHL
Position	11
Interleaved intrachr. SVs	2
Total SVs (intrachr. + transl.)	2
SV types	DEL: 1; DUP: 0; h2hINV: 1; t2tINV: 0; TRA: 0
SVs in sample	297
Oscillating CN in chr (2 and 3 states)	13,
CN segments	13
Purity, ploidy	0.79, 1.91

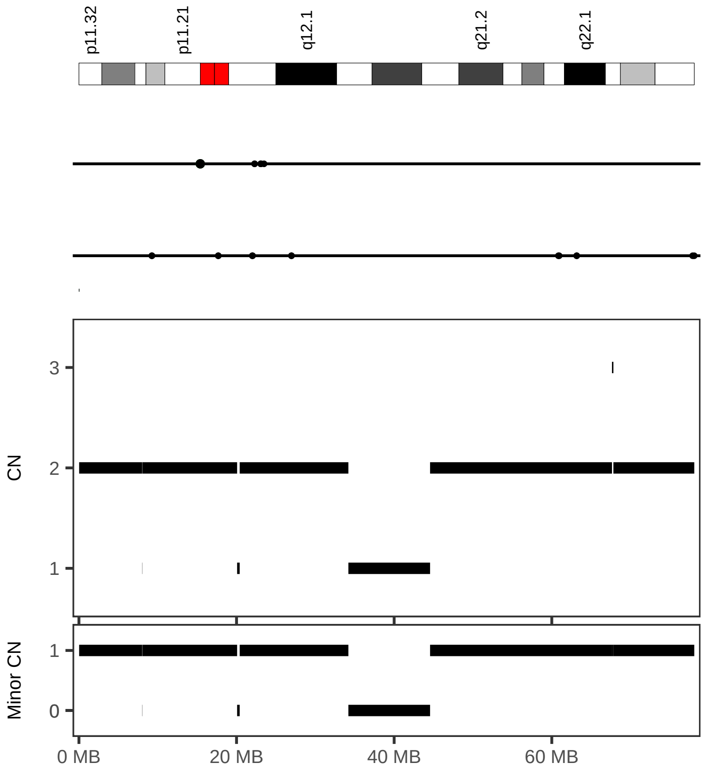

	4135099
Cancer type	Lymph−BNHL
Position	18
Interleaved intrachr. SVs	2
Total SVs (intrachr. + transl.)	2
SV types	DEL: 0; DUP: 0; h2hINV: 1; t2tINV: 1; TRA: 0
SVs in sample	297
Oscillating CN in chr (2 and 3 states)	7,
CN segments	9
Purity, ploidy	0.79, 1.91

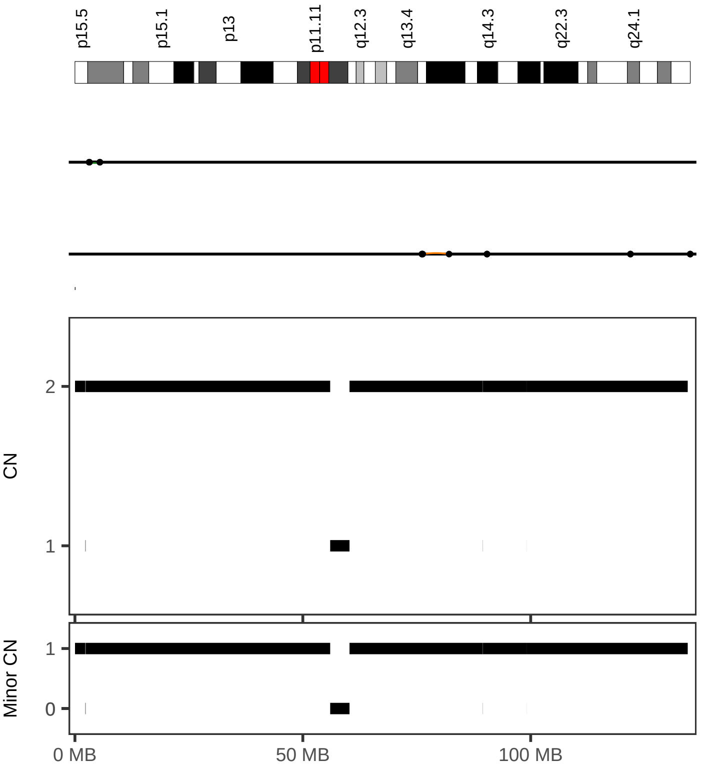

	4138527
Cancer type	Lymph−BNHL
Position	11
Interleaved intrachr. SVs	2
Total SVs (intrachr. + transl.)	2
SV types	DEL: 0; DUP: 0; h2hINV: 1; t2tINV: 1; TRA: 0
SVs in sample	187
Oscillating CN in chr (2 and 3 states)	9,
CN segments	9
Purity, ploidy	0.72, 2.04

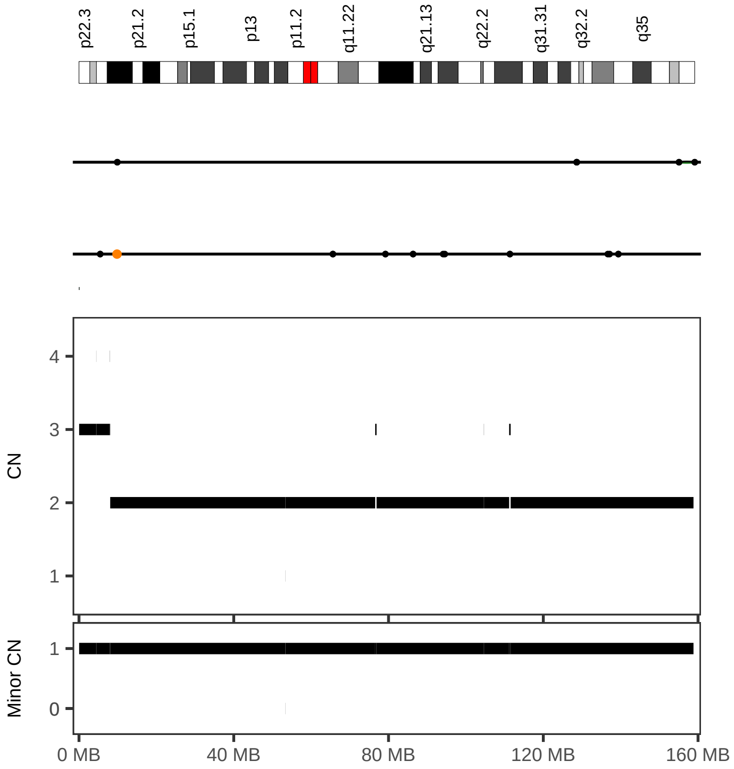

	4163639
Cancer type	Lymph−BNHL
Position	7
Interleaved intrachr. SVs	2
Total SVs (intrachr. + transl.)	2
SV types	DEL: 0; DUP: 0; h2hINV: 1; t2tINV: 1; TRA: 0
SVs in sample	146
Oscillating CN in chr (2 and 3 states)	9,
CN segments	16
Purity, ploidy	0.6, 2.08

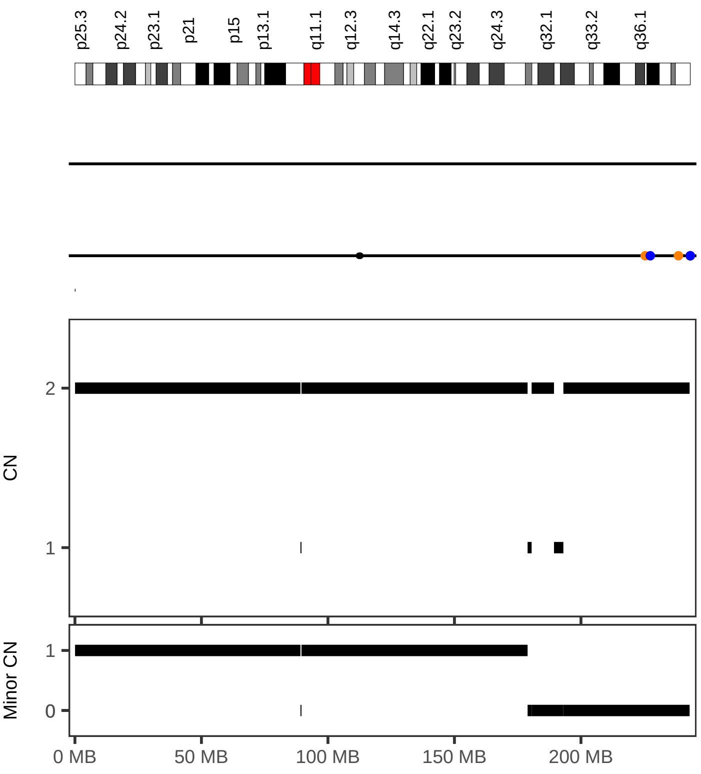

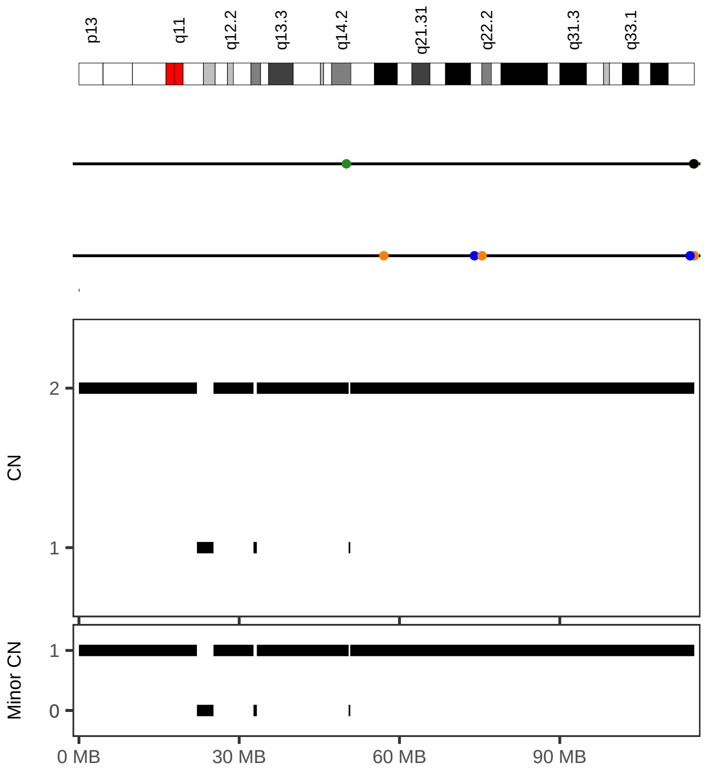

	**4176133**
Cancer type	Lymph−BNHL
Position	2
Interleaved intrachr. SVs	1
Total SVs (intrachr. + transl.)	1
SV types	DEL: 1; DUP: 0; h2hINV: 0; t2tINV: 0; TRA: 0
SVs in sample	59
Oscillating CN in chr (2 and 3 states)	7,
CN segments	7
Purity, ploidy	0.76, 1.98

	**4196670**
Cancer type	Lymph−BNHL
Position	13
Interleaved intrachr. SVs	0
Total SVs (intrachr. + transl.)	0
SV types	DEL: 0; DUP: 0; h2hINV: 0; t2tINV: 0; TRA: 0
SVs in sample	19
Oscillating CN in chr (2 and 3 states)	7,
CN segments	7
Purity, ploidy	0.63, 2.01

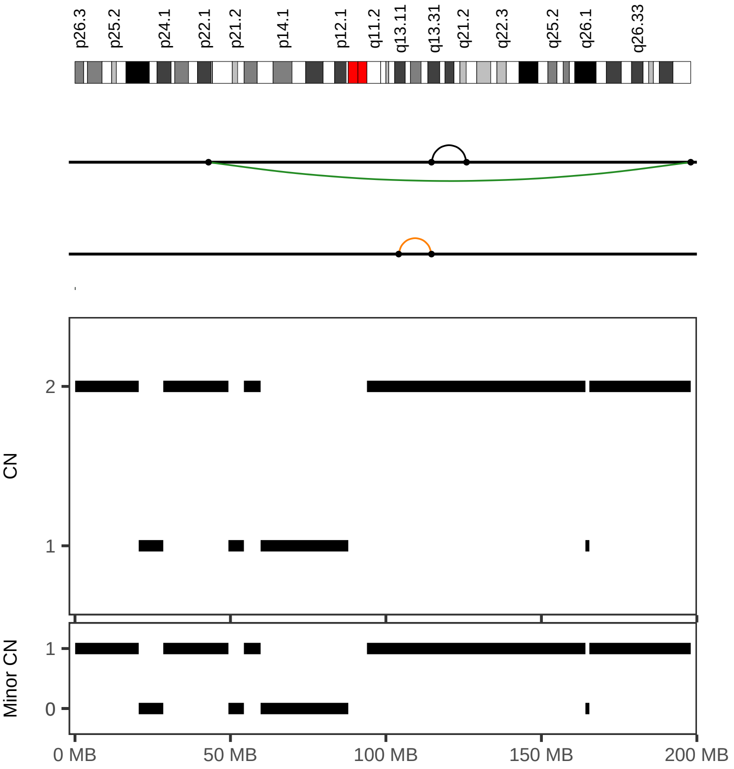

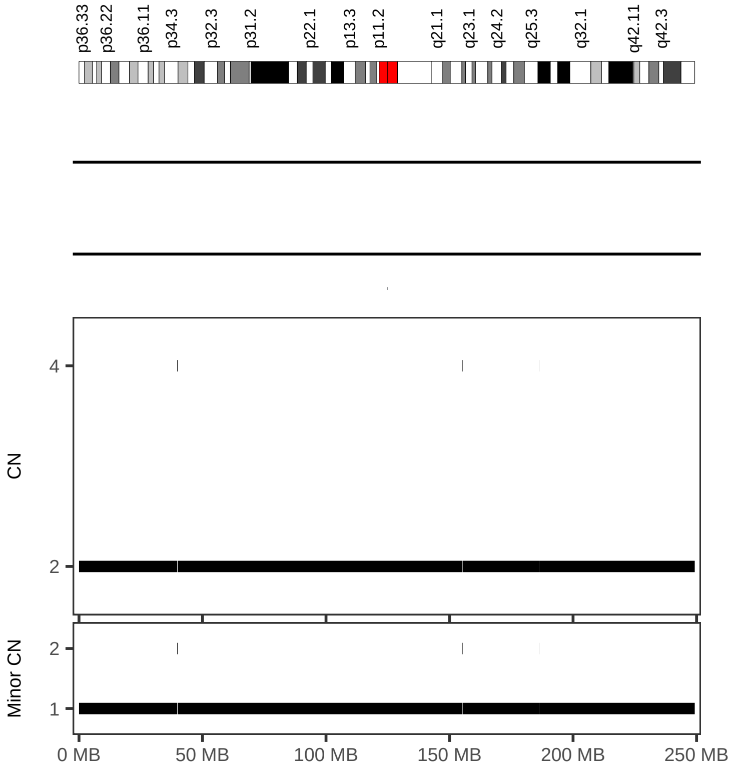

	**177**
Cancer type	Lymph−CLL
Position	3
Interleaved intrachr. SVs	2
Total SVs (intrachr. + transl.)	2
SV types	DEL: 1; DUP: 0; h2hINV: 1; t2tINV: 0; TRA: 0
SVs in sample	5
Oscillating CN in chr (2 and 3 states)	9,
CN segments	9
Purity, ploidy	0.8, 1.98

	**SNU_WGS_06**
Cancer type	Myeloid−AML
Position	1
Interleaved intrachr. SVs	0
Total SVs (intrachr. + transl.)	0
SV types	DEL: 0; DUP: 0; h2hINV: 0; t2tINV: 0; TRA: 0
SVs in sample	5
Oscillating CN in chr (2 and 3 states)	7,
CN segments	7
Purity, ploidy	0.99, 1.93

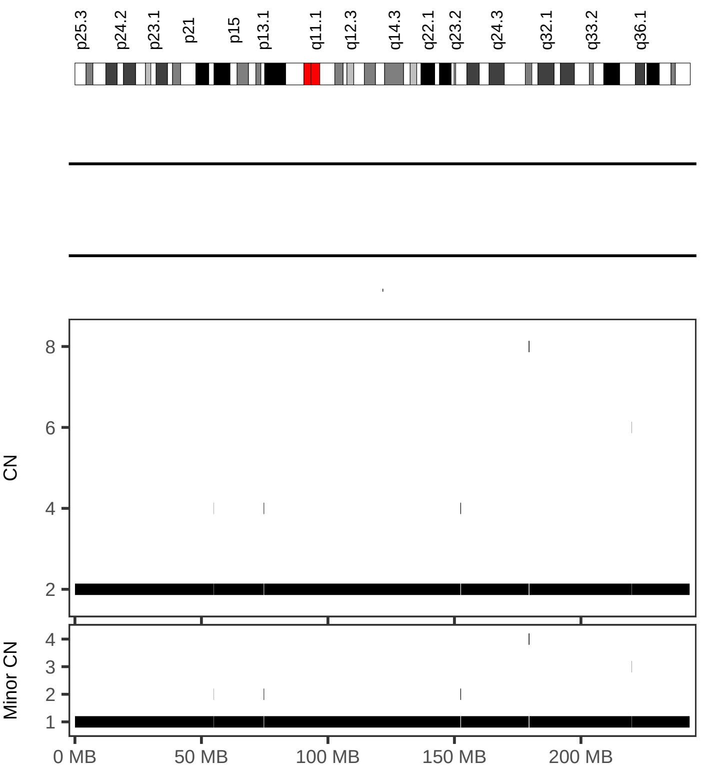

SNU_WGS_12

Cancer type	Myeloid−AML
Position	2
Interleaved intrachr. SVs	0
Total SVs (intrachr. + transl.)	0
SV types	DEL: 0; DUP: 0; h2hINV: 0; t2tINV: 0; TRA: 0
SVs in sample	3
Oscillating CN in chr (2 and 3 states)	7,
CN segments	11
Purity, ploidy	0.82, 2

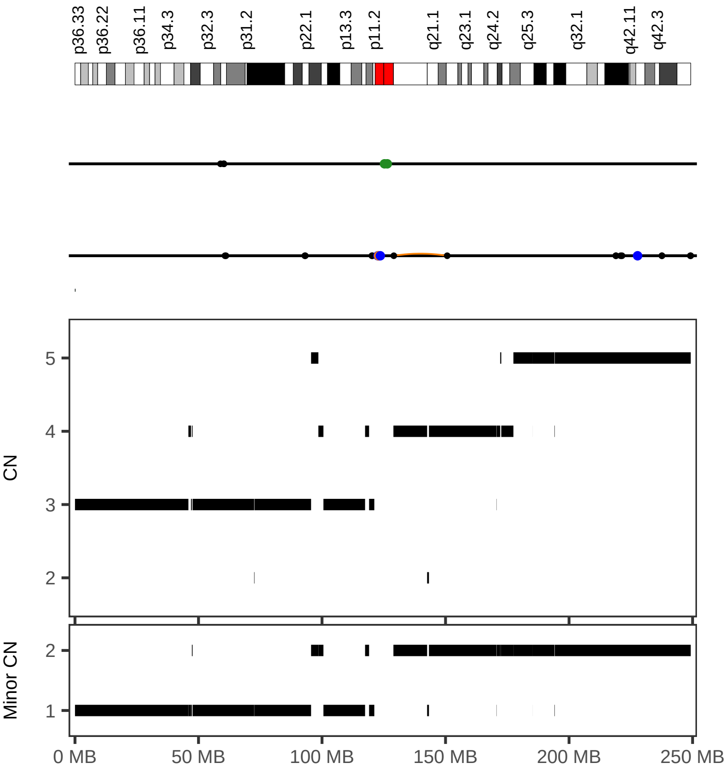

AOCS−060

Cancer type	Ovary−AdenoCA
Position	1
Interleaved intrachr. SVs	2
Total SVs (intrachr. + transl.)	2
SV types	DEL: 0; DUP: 0; h2hINV: 1; t2tINV: 1; TRA: 0
SVs in sample	197
Oscillating CN in chr (2 and 3 states)	8,
CN segments	24
Purity, ploidy	0.68, 2.95

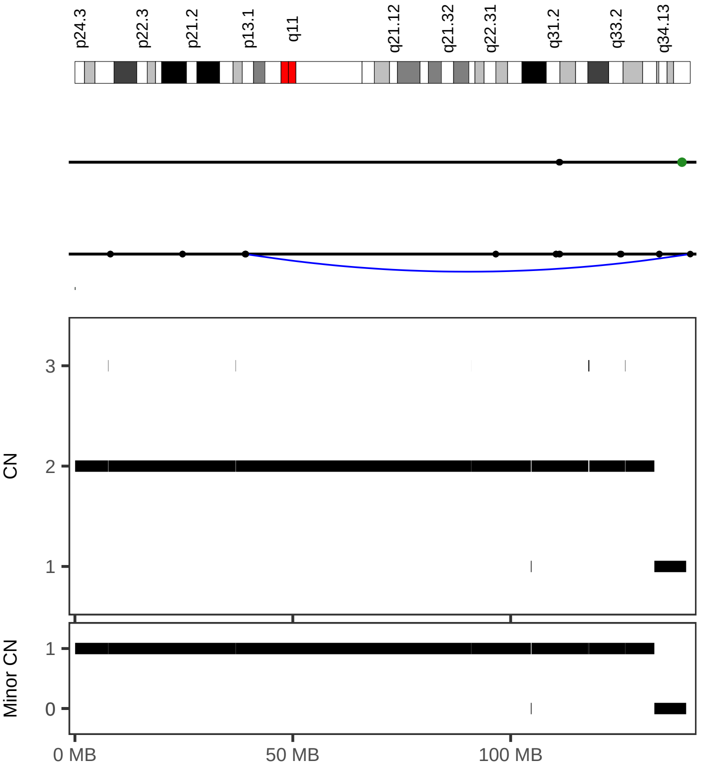

AOCS−065

Cancer type	Ovary−AdenoCA
Position	9
Interleaved intrachr. SVs	2
Total SVs (intrachr. + transl.)	2
SV types	DEL: 1; DUP: 0; h2hINV: 1; t2tINV: 0; TRA: 0
SVs in sample	255
Oscillating CN in chr (2 and 3 states)	7,
CN segments	14
Purity, ploidy	0.7, 1.88

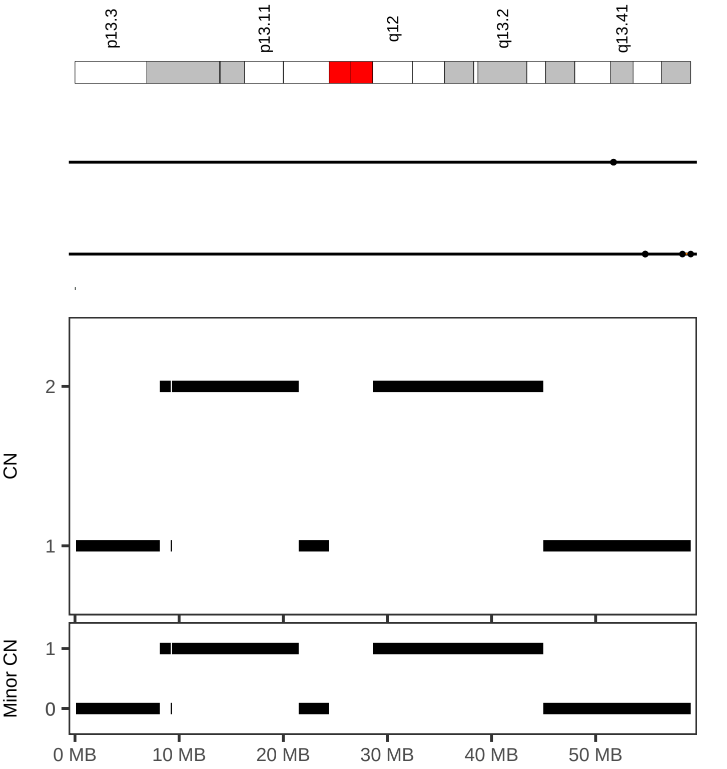

AOCS−086

Cancer type	Ovary−AdenoCA
Position	19
Interleaved intrachr. SVs	1
Total SVs (intrachr. + transl.)	1
SV types	DEL: 1; DUP: 0; h2hINV: 0; t2tINV: 0; TRA: 0
SVs in sample	162
Oscillating CN in chr (2 and 3 states)	7,
CN segments	7
Purity, ploidy	0.52, 1.87

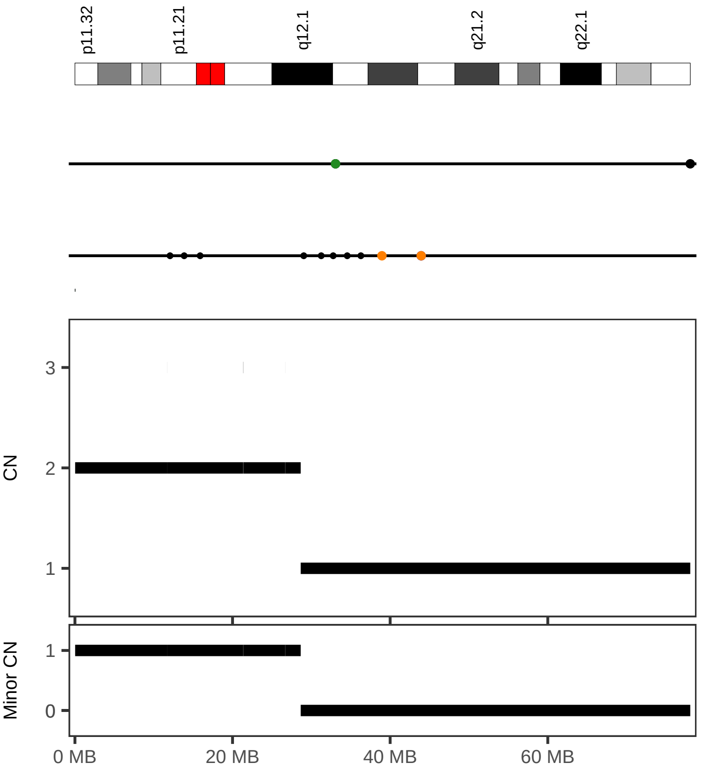

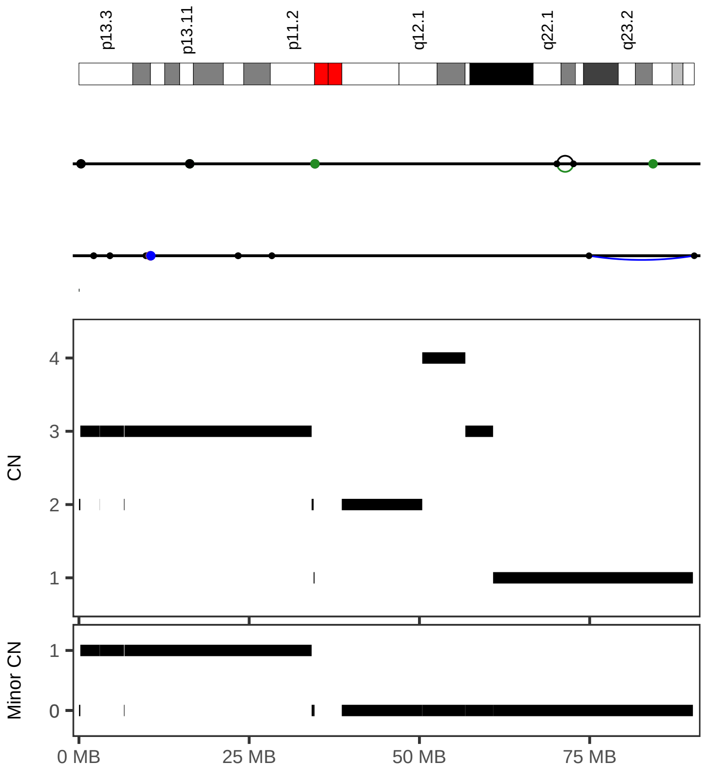

AOCS−088

Cancer type	Ovary−AdenoCA
Position	18
Interleaved intrachr. SVs	1
Total SVs (intrachr. + transl.)	1
SV types	DEL: 0; DUP: 1; h2hINV: 0; t2tINV: 0; TRA: 0
SVs in sample	220
Oscillating CN in chr (2 and 3 states)	7,
CN segments	8
Purity, ploidy	0.61, 1.92

AOCS−091

Cancer type	Ovary−AdenoCA
Position	16
Interleaved intrachr. SVs	2
Total SVs (intrachr. + transl.)	2
SV types	DEL: 0; DUP: 0; h2hINV: 1; t2tINV: 1; TRA: 0
SVs in sample	347
Oscillating CN in chr (2 and 3 states)	7,
CN segments	12
Purity, ploidy	0.74, 3.31

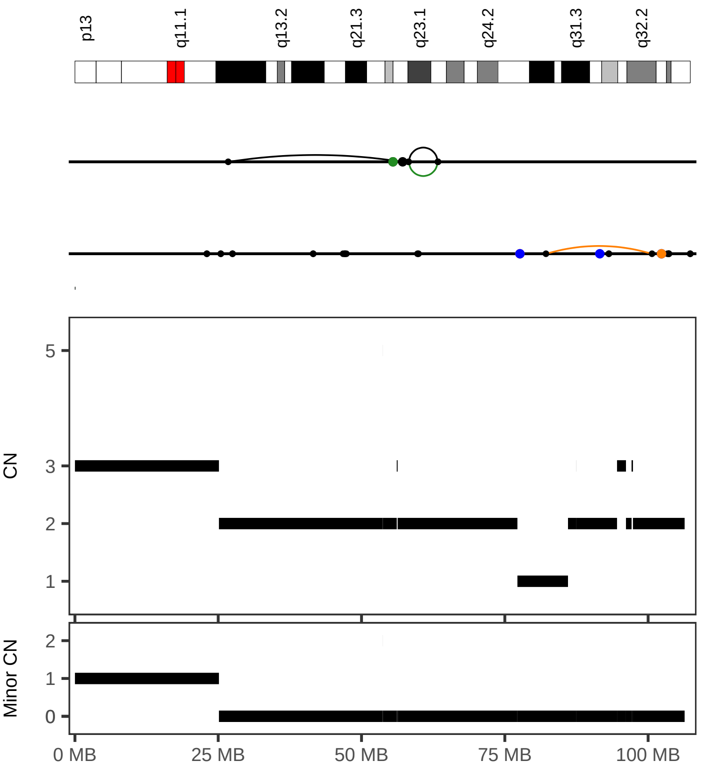

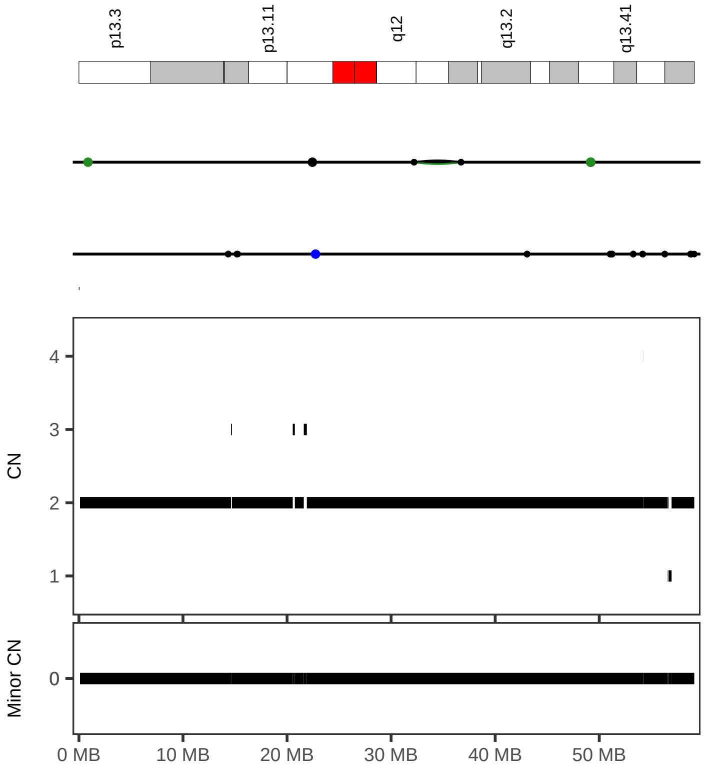

AOCS−120

Cancer type	Ovary−AdenoCA
Position	14
Interleaved intrachr. SVs	2
Total SVs (intrachr. + transl.)	2
SV types	DEL: 0; DUP: 0; h2hINV: 1; t2tINV: 1; TRA: 0
SVs in sample	790
Oscillating CN in chr (2 and 3 states)	7,
CN segments	14
Purity, ploidy	0.84, 2.89

AOCS−120

Cancer type	Ovary−AdenoCA
Position	19
Interleaved intrachr. SVs	2
Total SVs (intrachr. + transl.)	2
SV types	DEL: 0; DUP: 0; h2hINV: 1; t2tINV: 1; TRA: 0
SVs in sample	790
Oscillating CN in chr (2 and 3 states)	7,
CN segments	13
Purity, ploidy	0.84, 2.89

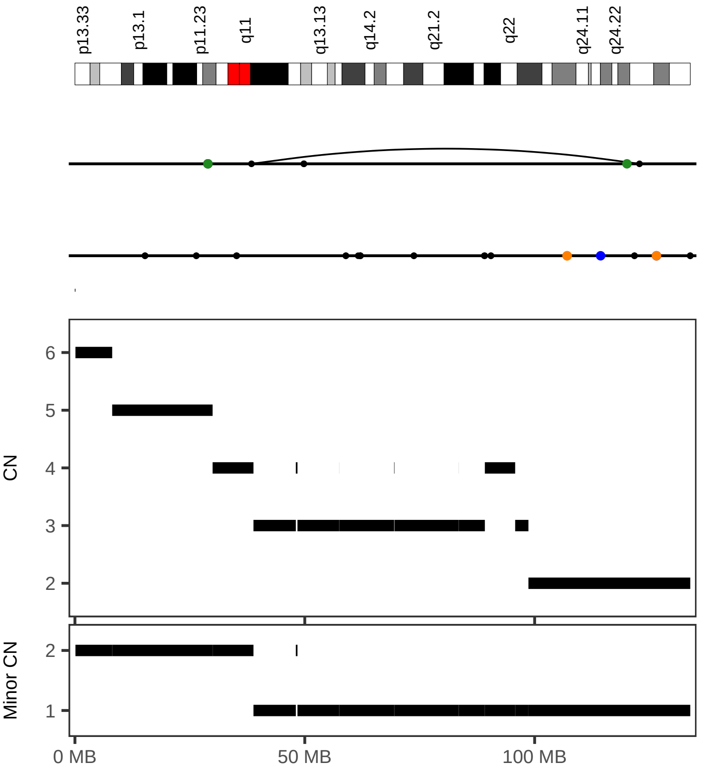

	AOCS−128
Cancer type	Ovary−AdenoCA
Position	12
Interleaved intrachr. SVs	2
Total SVs (intrachr. + transl.)	2
SV types	DEL: 0; DUP: 2; h2hINV: 0; t2tINV: 0; TRA: 0
SVs in sample	496
Oscillating CN in chr (2 and 3 states)	12,
CN segments	15
Purity, ploidy	0.37, 3.11

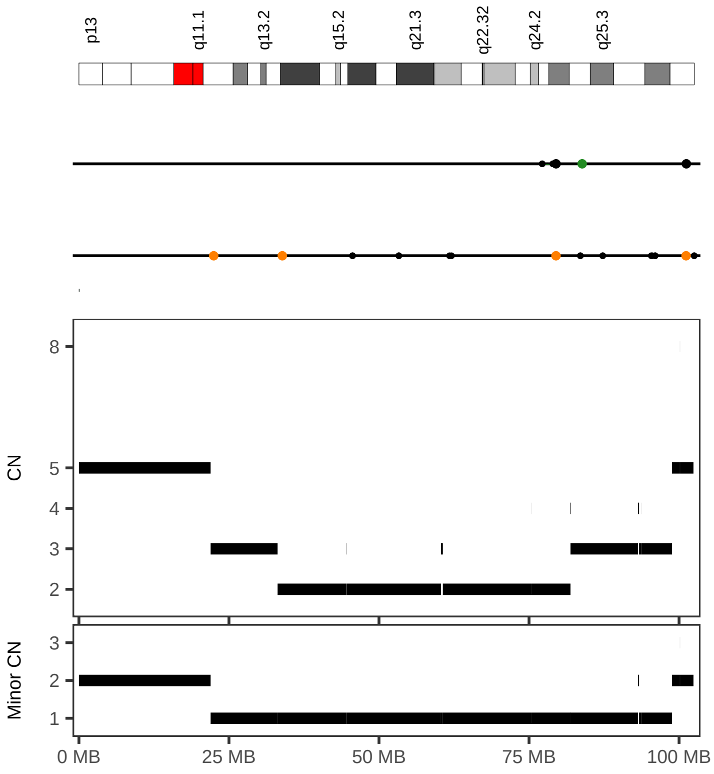

	AOCS−128
Cancer type	Ovary−AdenoCA
Position	15
Interleaved intrachr. SVs	2
Total SVs (intrachr. + transl.)	2
SV types	DEL: 0; DUP: 0; h2hINV: 1; t2tINV: 1; TRA: 0
SVs in sample	496
Oscillating CN in chr (2 and 3 states)	7,
CN segments	19
Purity, ploidy	0.37, 3.11

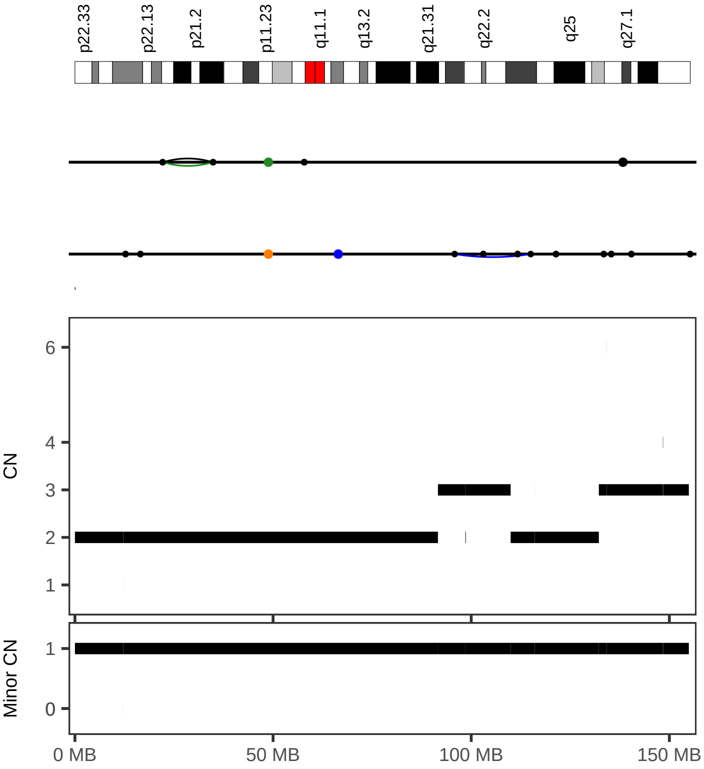

	AOCS−138
Cancer type	Ovary−AdenoCA
Position	X
Interleaved intrachr. SVs	2
Total SVs (intrachr. + transl.)	2
SV types	DEL: 0; DUP: 0; h2hINV: 1; t2tINV: 1; TRA: 0
SVs in sample	323
Oscillating CN in chr (2 and 3 states)	8,
CN segments	14
Purity, ploidy	0.65, 1.78

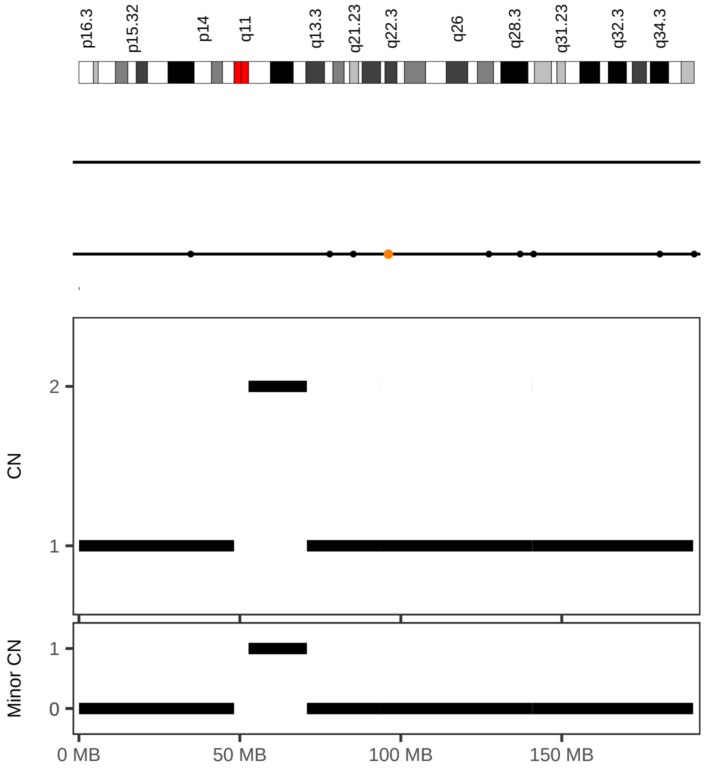

	AOCS−158
Cancer type	Ovary−AdenoCA
Position	4
Interleaved intrachr. SVs	1
Total SVs (intrachr. + transl.)	2
SV types	DEL: 0; DUP: 2; h2hINV: 0; t2tINV: 0; TRA: 0
SVs in sample	236
Oscillating CN in chr (2 and 3 states)	7,
CN segments	7
Purity, ploidy	0.94, 1.82

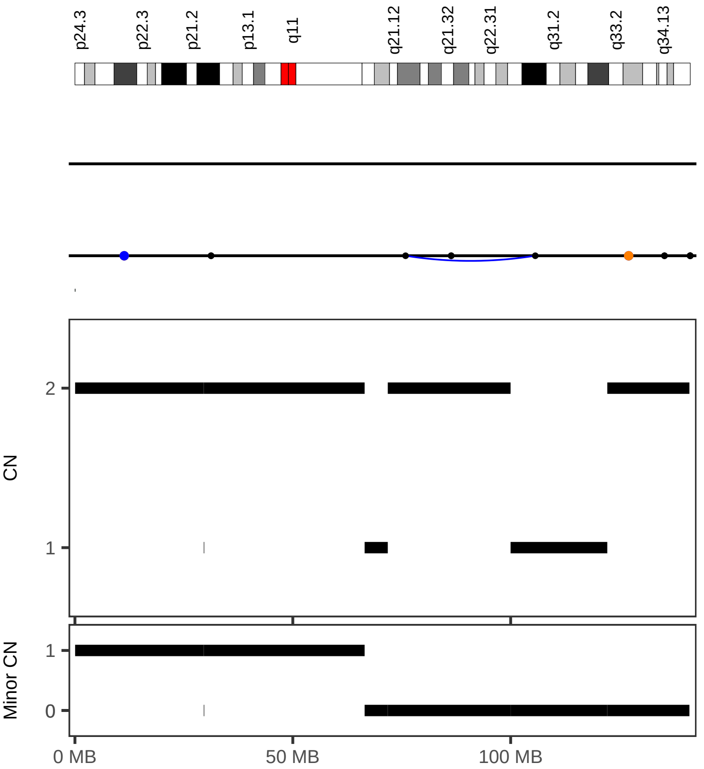

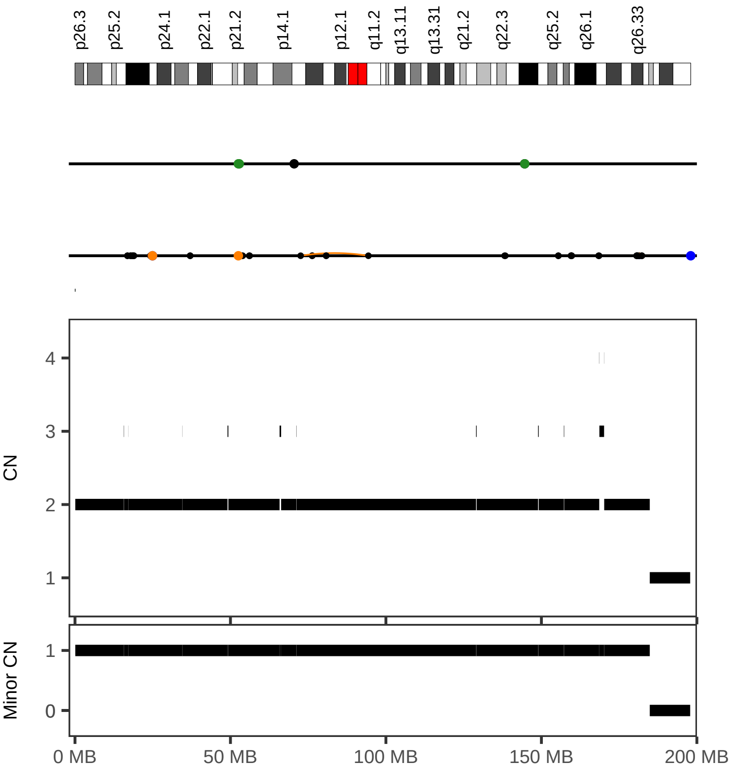

	**AOCS−167**
Cancer type	Ovary−AdenoCA
Position	9
Interleaved intrachr. SVs	1
Total SVs (intrachr. + transl.)	1
SV types	DEL: 0; DUP: 1; h2hINV: 0; t2tINV: 0; TRA: 0
SVs in sample	181
Oscillating CN in chr (2 and 3 states)	7,
CN segments	7
Purity, ploidy	0.81, 1.9

	**AOCS−171**
Cancer type	Ovary−AdenoCA
Position	3
Interleaved intrachr. SVs	2
Total SVs (intrachr. + transl.)	2
SV types	DEL: 1; DUP: 1; h2hINV: 0; t2tINV: 0; TRA: 0
SVs in sample	258
Oscillating CN in chr (2 and 3 states)	19,
CN segments	24
Purity, ploidy	0.76, 1.87

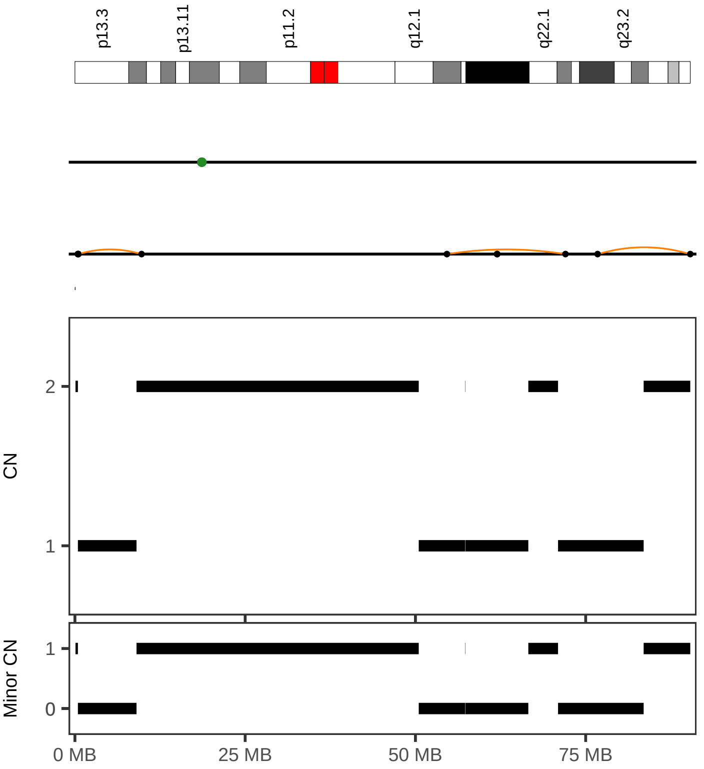

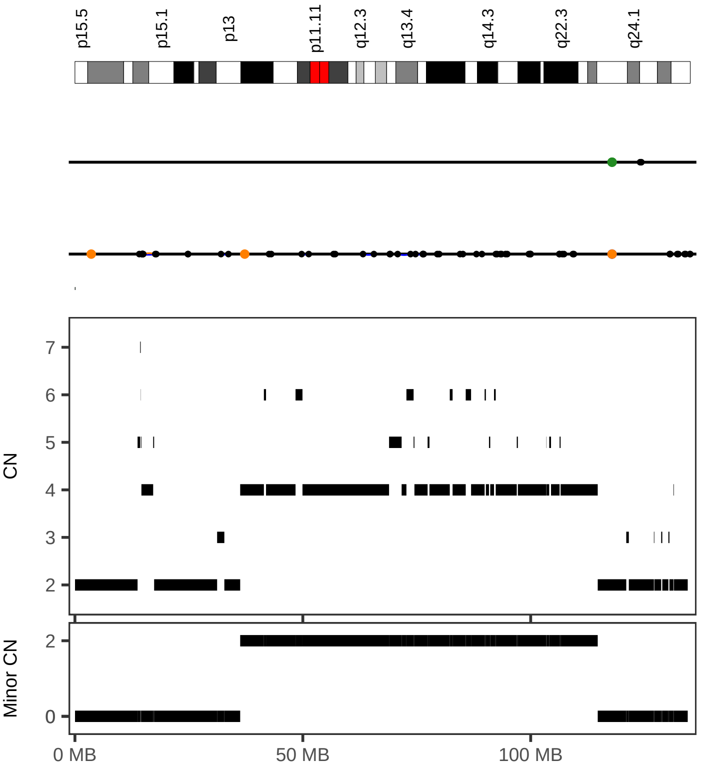

	**AOCS−171**
Cancer type	Ovary−AdenoCA
Position	16
Interleaved intrachr. SVs	2
Total SVs (intrachr. + transl.)	2
SV types	DEL: 1; DUP: 1; h2hINV: 0; t2tINV: 0; TRA: 0
SVs in sample	258
Oscillating CN in chr (2 and 3 states)	9,
CN segments	9
Purity, ploidy	0.76, 1.87

	**098acb76−0bf5−44e5−bcae−f919cf5fa5e5**
Cancer type	Ovary−AdenoCA
Position	11
Interleaved intrachr. SVs	2
Total SVs (intrachr. + transl.)	2
SV types	DEL: 0; DUP: 2; h2hINV: 0; t2tINV: 0; TRA: 0
SVs in sample	718
Oscillating CN in chr (2 and 3 states)	9,
CN segments	52
Purity, ploidy	0.82, 3.31

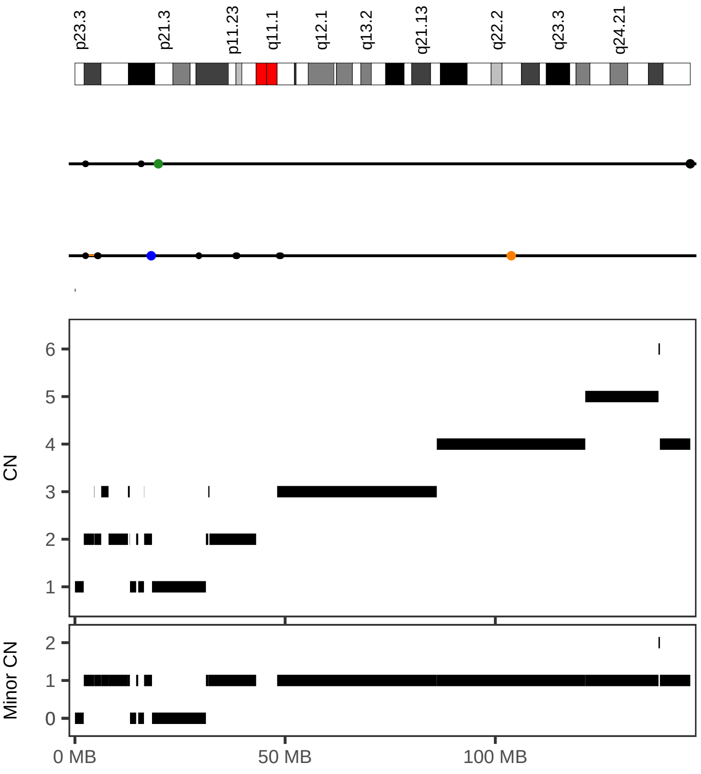

	206ce0ed−36a4−47ab−ac8e−f6fca6ac5c18
Cancer type	Ovary−AdenoCA
Position	8
Interleaved intrachr. SVs	2
Total SVs (intrachr. + transl.)	2
SV types	DEL: 1; DUP: 0; h2hINV: 0; t2tINV: 1; TRA: 0
SVs in sample	122
Oscillating CN in chr (2 and 3 states)	7,
CN segments	24
Purity, ploidy	0.75, 2.77

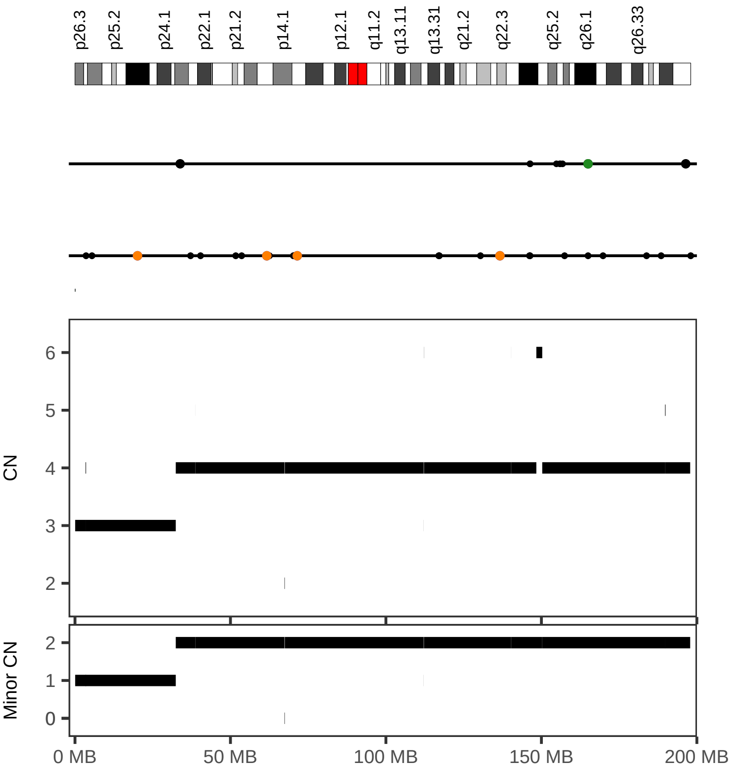

	2b14123b−8fcd−402c−9399−4e7c47f20252
Cancer type	Ovary−AdenoCA
Position	3
Interleaved intrachr. SVs	2
Total SVs (intrachr. + transl.)	2
SV types	DEL: 0; DUP: 0; h2hINV: 1; t2tINV: 1; TRA: 0
SVs in sample	329
Oscillating CN in chr (2 and 3 states)	7,
CN segments	20
Purity, ploidy	0.77, 3.57

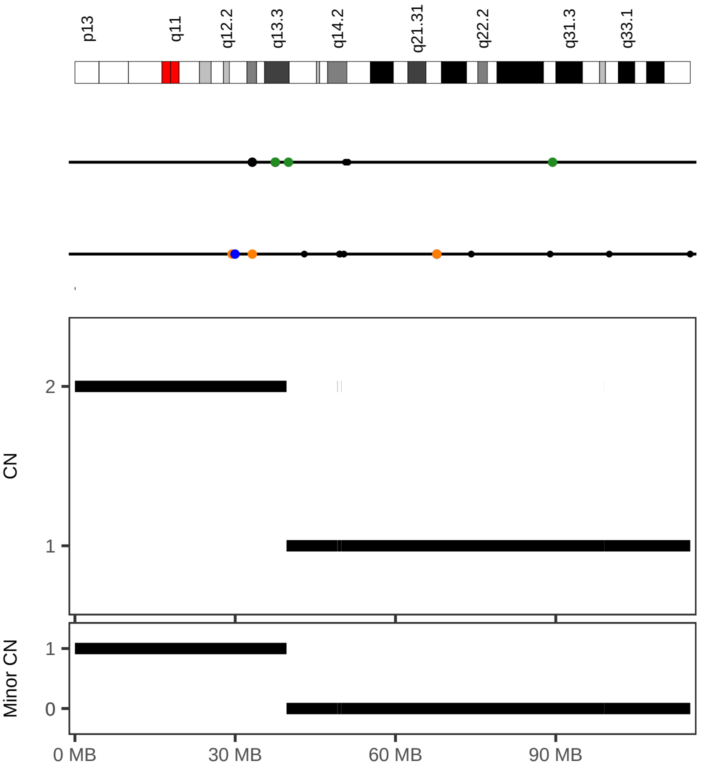

	4eac2c98−86d2−4ee6−a1d3−157d013c78dc
Cancer type	Ovary−AdenoCA
Position	13
Interleaved intrachr. SVs	2
Total SVs (intrachr. + transl.)	2
SV types	DEL: 0; DUP: 0; h2hINV: 1; t2tINV: 1; TRA: 0
SVs in sample	545
Oscillating CN in chr (2 and 3 states)	8,
CN segments	8
Purity, ploidy	0.92, 1.81

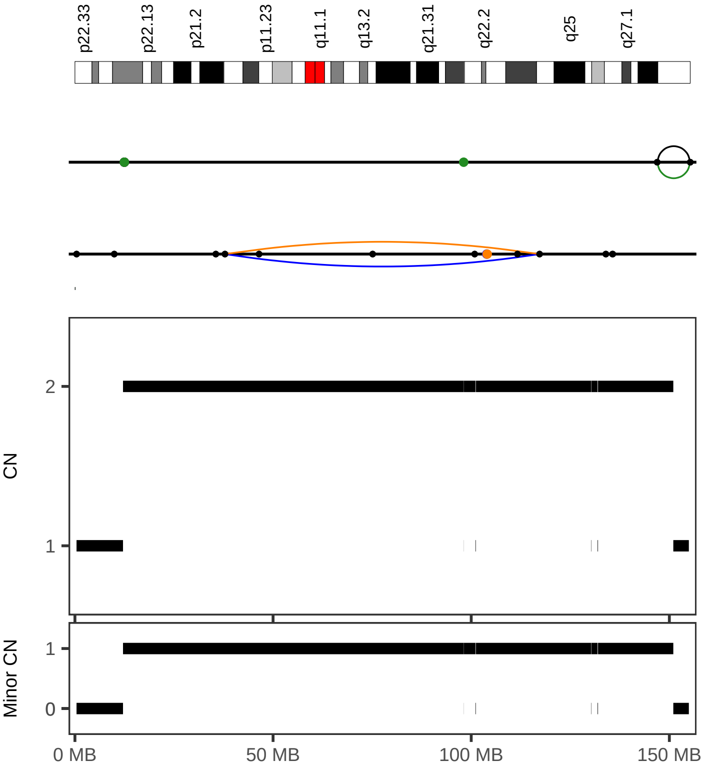

	6d10d4ee−6331−4bba−93bc−a7b64cc0b22a
Cancer type	Ovary−AdenoCA
Position	X
Interleaved intrachr. SVs	2
Total SVs (intrachr. + transl.)	2
SV types	DEL: 0; DUP: 0; h2hINV: 1; t2tINV: 1; TRA: 0
SVs in sample	239
Oscillating CN in chr (2 and 3 states)	11,
CN segments	11
Purity, ploidy	0.87, 1.84

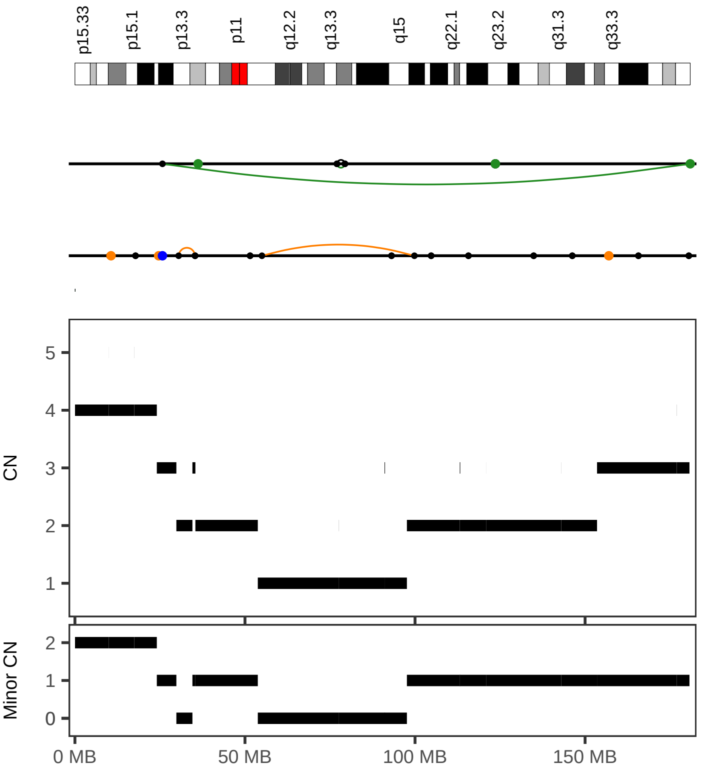

	a5030259−cf9c−4a58−8710−b9da8ee59320
Cancer type	Ovary−AdenoCA
Position	5
Interleaved intrachr. SVs	2
Total SVs (intrachr. + transl.)	2
SV types	DEL: 0; DUP: 0; h2hINV: 1; t2tINV: 1; TRA: 0
SVs in sample	303
Oscillating CN in chr (2 and 3 states)	8,
CN segments	24
Purity, ploidy	0.89, 1.94

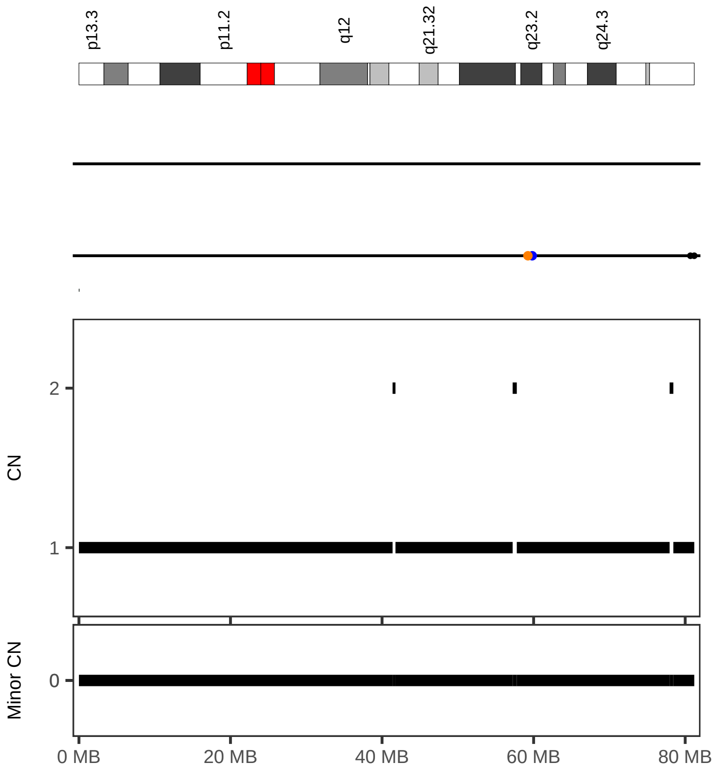

	c8febeef−8e7e−459e−88cb−8086692dc559
Cancer type	Ovary−AdenoCA
Position	17
Interleaved intrachr. SVs	1
Total SVs (intrachr. + transl.)	1
SV types	DEL: 0; DUP: 1; h2hINV: 0; t2tINV: 0; TRA: 0
SVs in sample	122
Oscillating CN in chr (2 and 3 states)	7,
CN segments	7
Purity, ploidy	0.9, 1.63

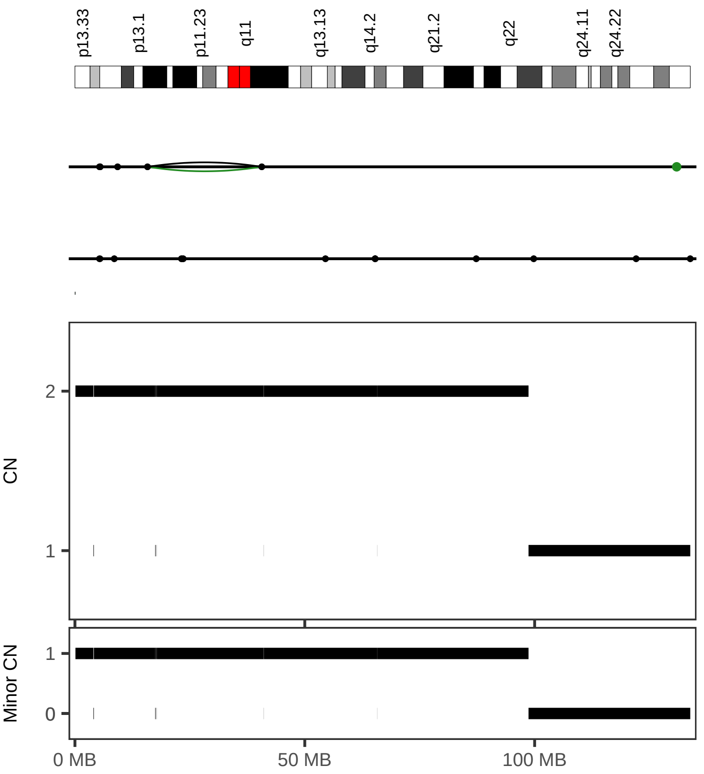

	f5033d52−ec36−4e67−88d8−b6d898b81b2f
Cancer type	Ovary−AdenoCA
Position	12
Interleaved intrachr. SVs	2
Total SVs (intrachr. + transl.)	2
SV types	DEL: 1; DUP: 0; h2hINV: 0; t2tINV: 1; TRA: 0
SVs in sample	243
Oscillating CN in chr (2 and 3 states)	12,
CN segments	12
Purity, ploidy	0.87, 1.81

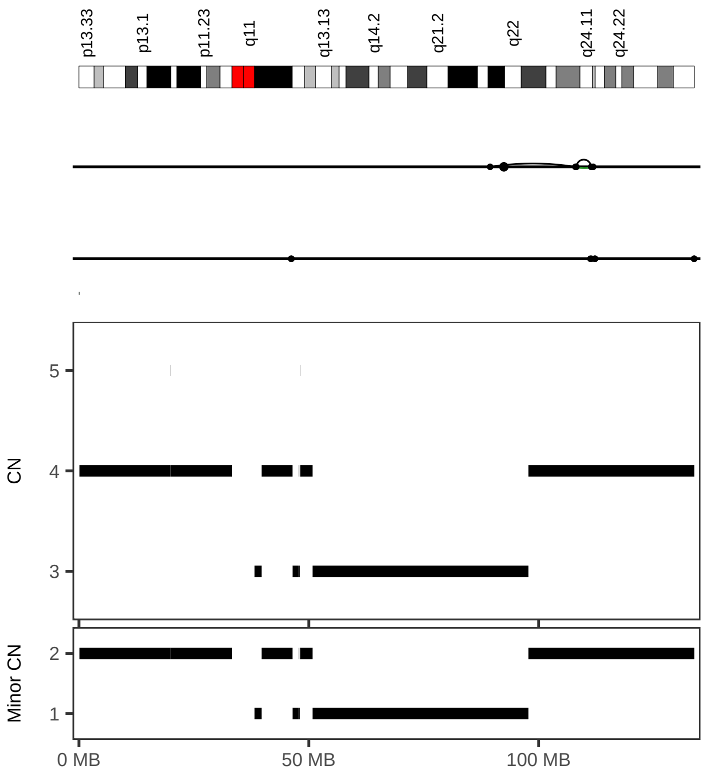

	ff530f28−0ec0−4494−bb54−44bb055bae1c
Cancer type	Ovary−AdenoCA
Position	12
Interleaved intrachr. SVs	2
Total SVs (intrachr. + transl.)	2
SV types	DEL: 0; DUP: 1; h2hINV: 1; t2tINV: 0; TRA: 0
SVs in sample	170
Oscillating CN in chr (2 and 3 states)	9,
CN segments	15
Purity, ploidy	0.7, 3.15

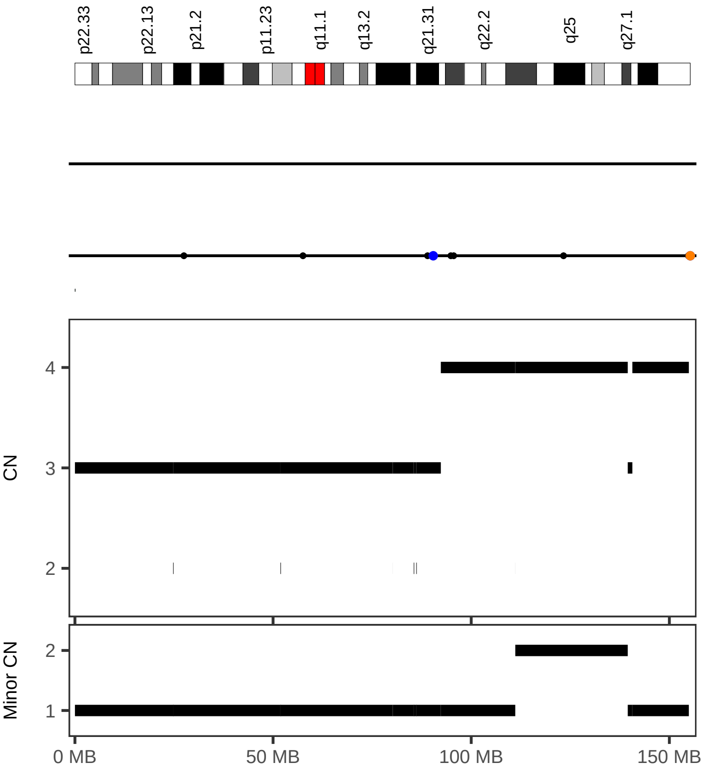

	ICGC_0006
Cancer type	Panc−AdenoCA
Position	X
Interleaved intrachr. SVs	0
Total SVs (intrachr. + transl.)	0
SV types	DEL: 0; DUP: 0; h2hINV: 0; t2tINV: 0; TRA: 0
SVs in sample	258
Oscillating CN in chr (2 and 3 states)	11,
CN segments	16
Purity, ploidy	0.41, 3.23

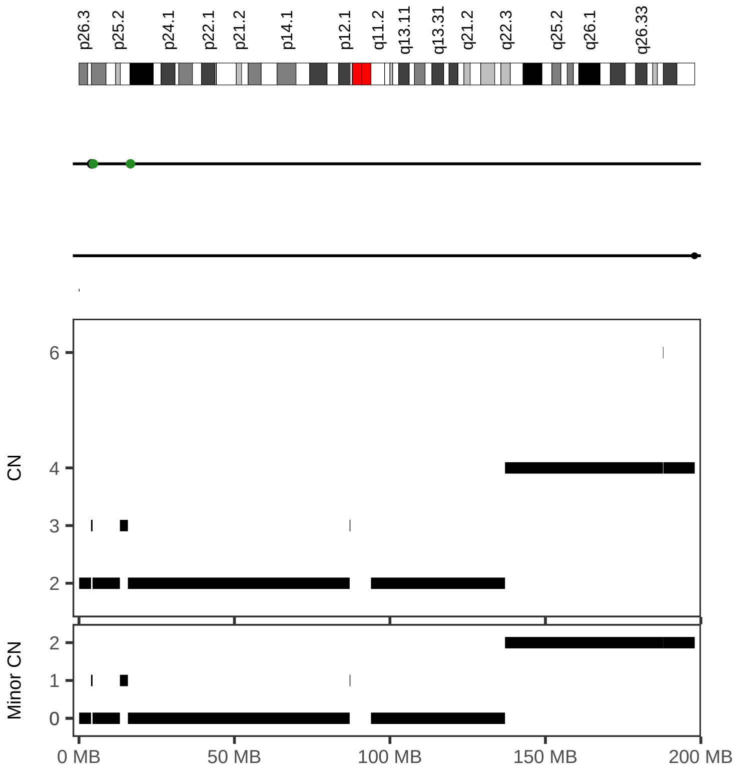

	ICGC_0124
Cancer type	Panc−AdenoCA
Position	3
Interleaved intrachr. SVs	1
Total SVs (intrachr. + transl.)	1
SV types	DEL: 0; DUP: 1; h2hINV: 0; t2tINV: 0; TRA: 0
SVs in sample	57
Oscillating CN in chr (2 and 3 states)	7,
CN segments	10
Purity, ploidy	0.36, 2.99

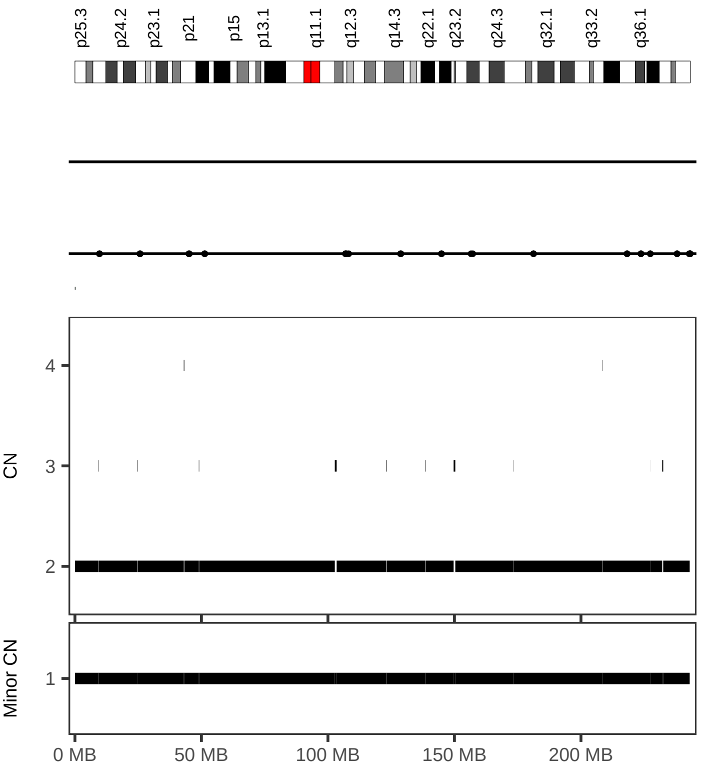

	ICGC_0140
Cancer type	Panc−AdenoCA
Position	2
Interleaved intrachr. SVs	2
Total SVs (intrachr. + transl.)	2
SV types	DEL: 0; DUP: 2; h2hINV: 0; t2tINV: 0; TRA: 0
SVs in sample	163
Oscillating CN in chr (2 and 3 states)	13,
CN segments	25
Purity, ploidy	0.85, 1.96

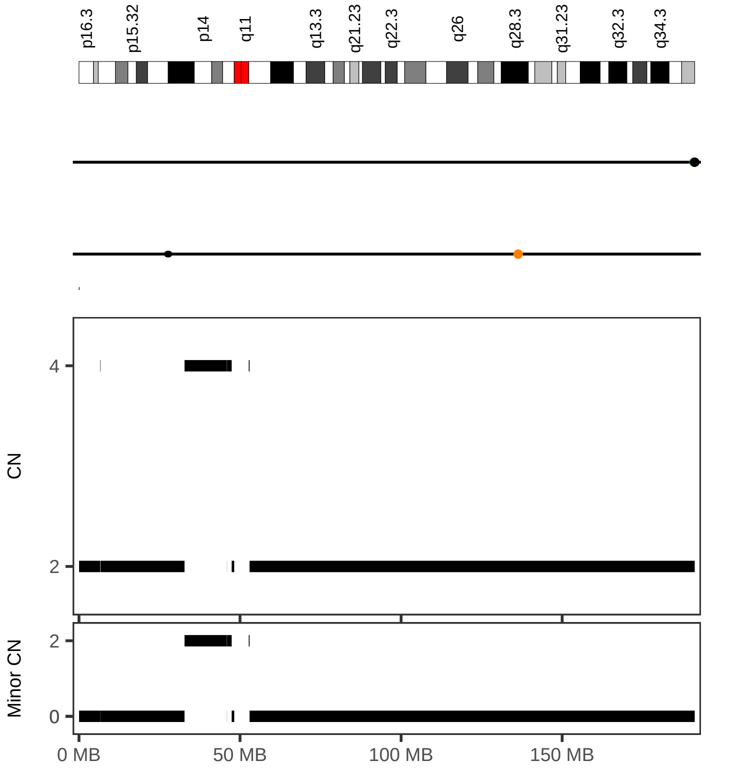

	ICGC_0201
Cancer type	Panc−AdenoCA
Position	4
Interleaved intrachr. SVs	1
Total SVs (intrachr. + transl.)	1
SV types	DEL: 0; DUP: 1; h2hINV: 0; t2tINV: 0; TRA: 0
SVs in sample	71
Oscillating CN in chr (2 and 3 states)	9,
CN segments	9
Purity, ploidy	0.43, 3.3

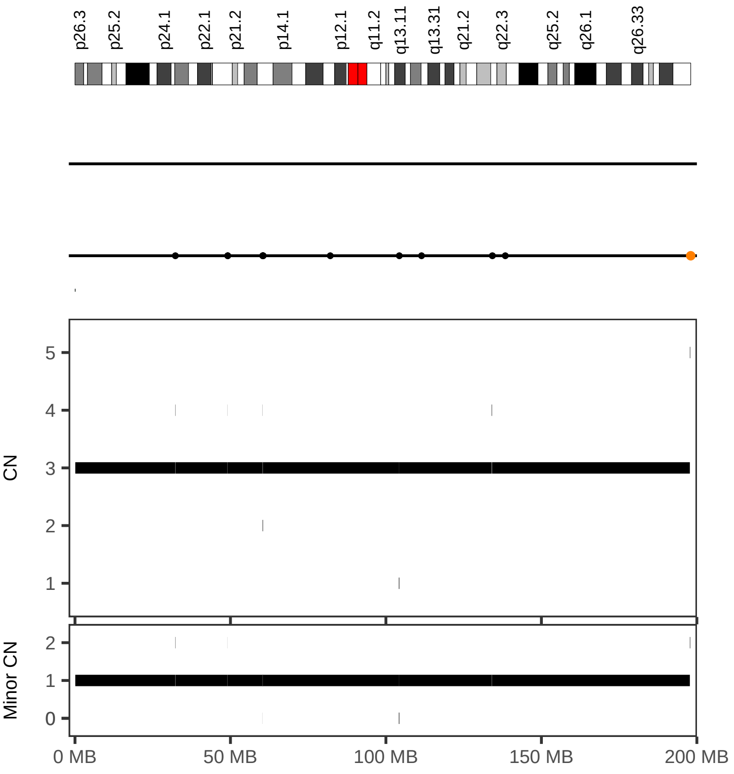

	ICGC_0227
Cancer type	Panc−AdenoCA
Position	3
Interleaved intrachr. SVs	2
Total SVs (intrachr. + transl.)	2
SV types	DEL: 2; DUP: 0; h2hINV: 0; t2tINV: 0; TRA: 0
SVs in sample	115
Oscillating CN in chr (2 and 3 states)	7,
CN segments	17
Purity, ploidy	0.37, 3.51

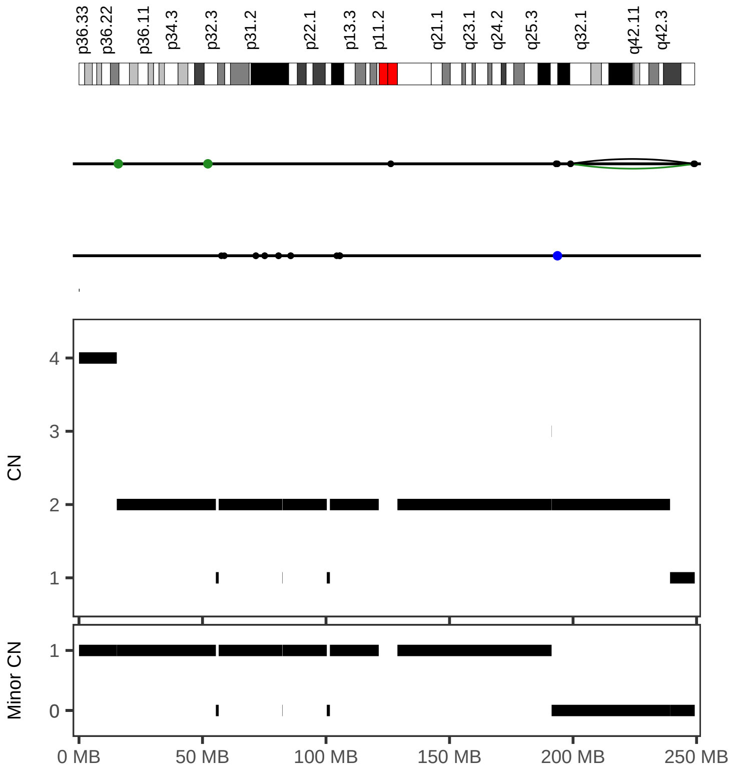

	ICGC_0543
Cancer type	Panc−AdenoCA
Position	1
Interleaved intrachr. SVs	2
Total SVs (intrachr. + transl.)	2
SV types	DEL: 0; DUP: 0; h2hINV: 1; t2tINV: 1; TRA: 0
SVs in sample	161
Oscillating CN in chr (2 and 3 states)	7,
CN segments	12
Purity, ploidy	0.53, 1.89

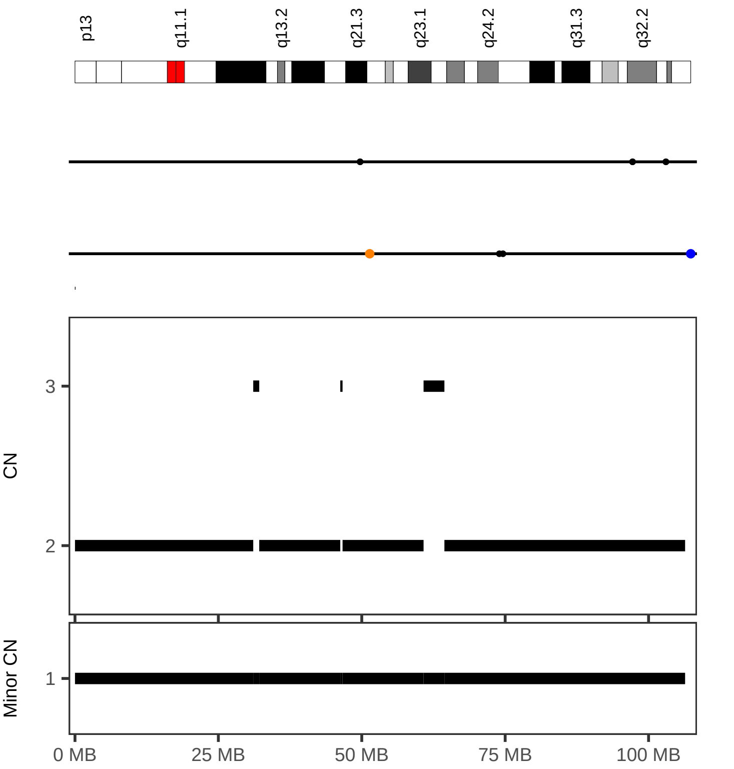

	PCSI_0078
Cancer type	Panc−AdenoCA
Position	14
Interleaved intrachr. SVs	1
Total SVs (intrachr. + transl.)	1
SV types	DEL: 0; DUP: 1; h2hINV: 0; t2tINV: 0; TRA: 0
SVs in sample	101
Oscillating CN in chr (2 and 3 states)	7,
CN segments	7
Purity, ploidy	0.7, 2.14

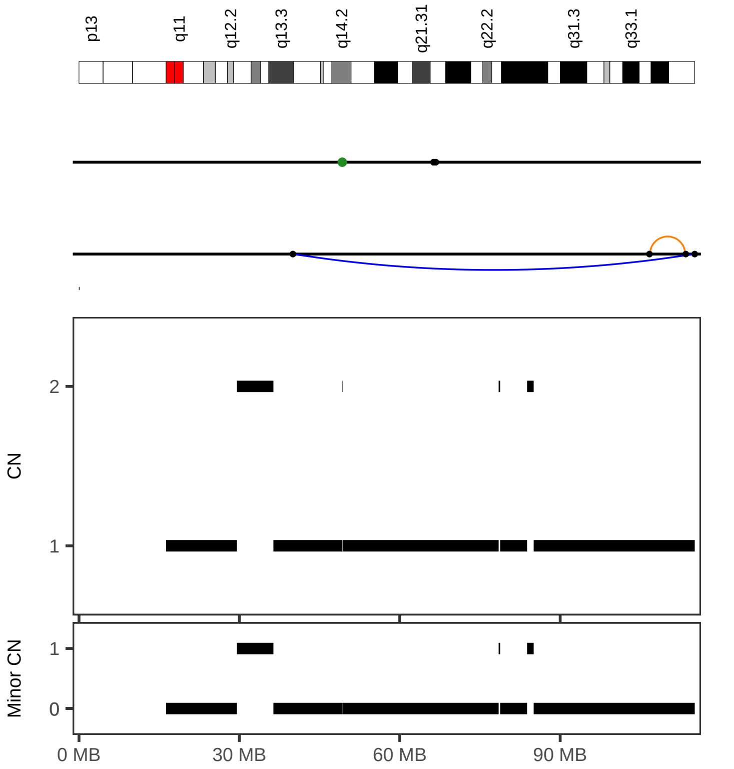

	PCSI_0109
Cancer type	Panc−AdenoCA
Position	13
Interleaved intrachr. SVs	2
Total SVs (intrachr. + transl.)	2
SV types	DEL: 0; DUP: 0; h2hINV: 1; t2tINV: 1; TRA: 0
SVs in sample	72
Oscillating CN in chr (2 and 3 states)	9,
CN segments	9
Purity, ploidy	0.42, 1.88

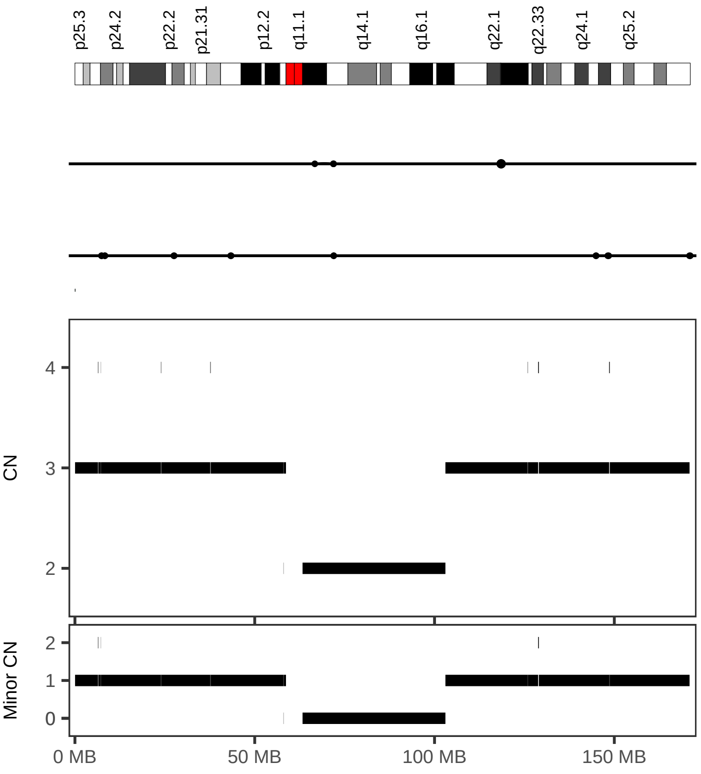

	PCSI_0161
Cancer type	Panc−AdenoCA
Position	6
Interleaved intrachr. SVs	2
Total SVs (intrachr. + transl.)	2
SV types	DEL: 0; DUP: 0; h2hINV: 1; t2tINV: 1; TRA: 0
SVs in sample	187
Oscillating CN in chr (2 and 3 states)	9,
CN segments	19
Purity, ploidy	0.63, 2.98

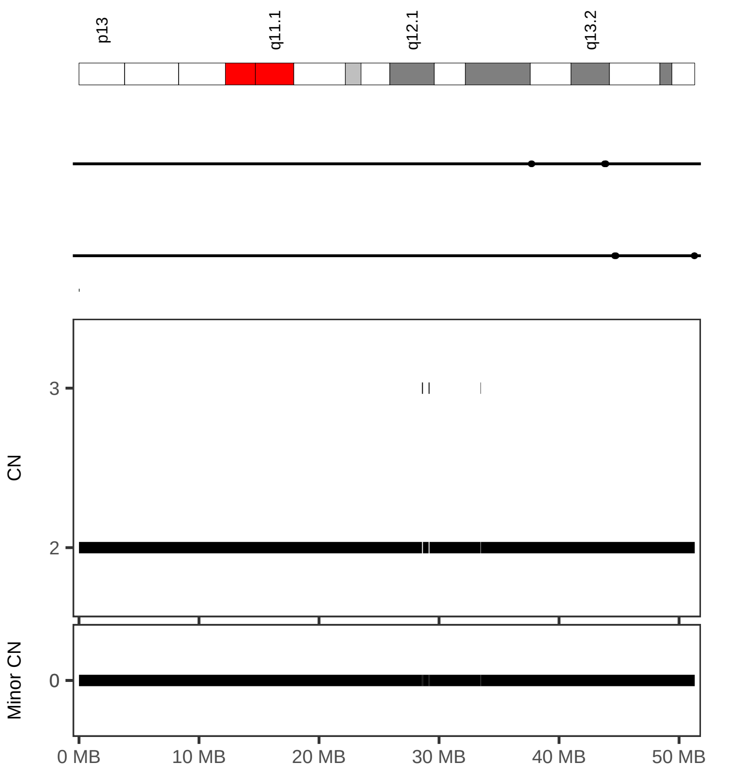

	PCSI_0161
Cancer type	Panc−AdenoCA
Position	22
Interleaved intrachr. SVs	2
Total SVs (intrachr. + transl.)	2
SV types	DEL: 0; DUP: 0; h2hINV: 1; t2tINV: 1; TRA: 0
SVs in sample	187
Oscillating CN in chr (2 and 3 states)	7,
CN segments	7
Purity, ploidy	0.63, 2.98

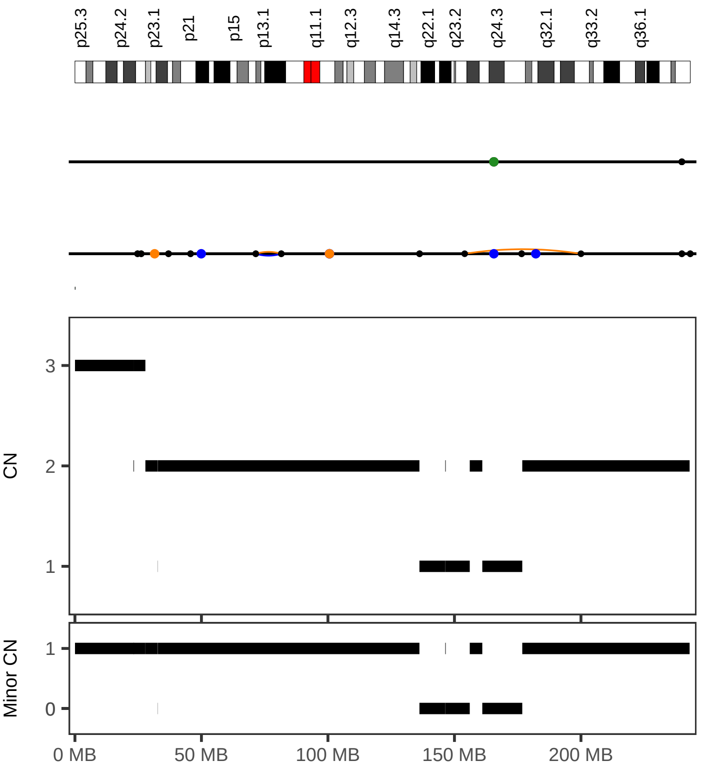

	PCSI_0218
Cancer type	Panc−AdenoCA
Position	2
Interleaved intrachr. SVs	2
Total SVs (intrachr. + transl.)	2
SV types	DEL: 1; DUP: 1; h2hINV: 0; t2tINV: 0; TRA: 0
SVs in sample	166
Oscillating CN in chr (2 and 3 states)	9,
CN segments	12
Purity, ploidy	0.64, 1.93

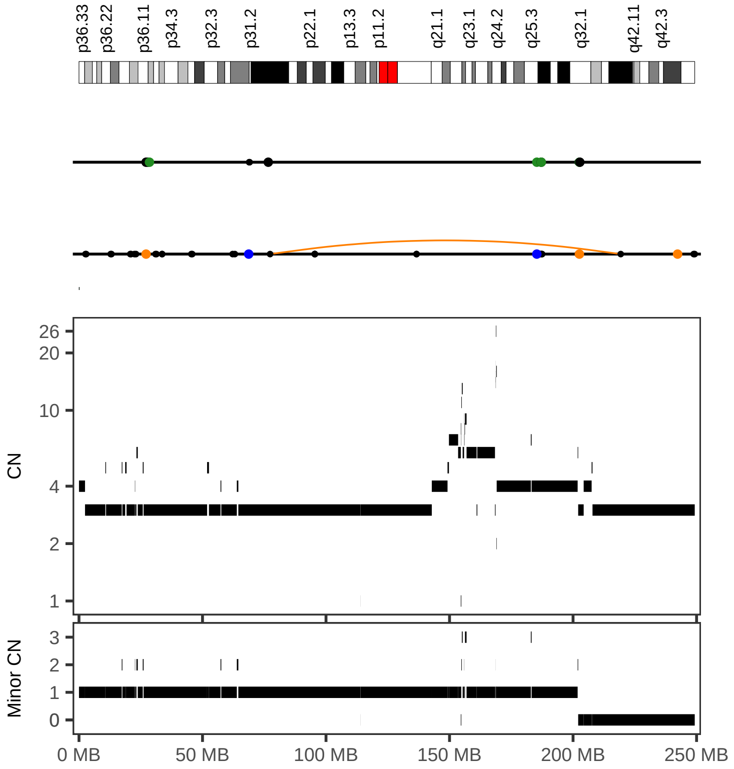

	PCSI_0285
Cancer type	Panc−AdenoCA
Position	1
Interleaved intrachr. SVs	2
Total SVs (intrachr. + transl.)	2
SV types	DEL: 0; DUP: 2; h2hINV: 0; t2tINV: 0; TRA: 0
SVs in sample	446
Oscillating CN in chr (2 and 3 states)	7,
CN segments	56
Purity, ploidy	0.56, 3.15

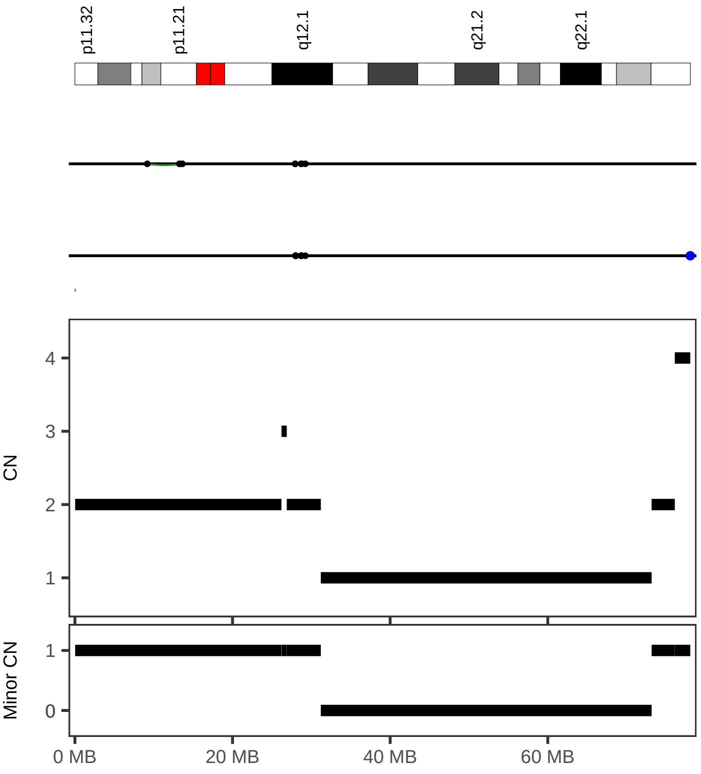

	PCSI_0334
Cancer type	Panc−AdenoCA
Position	18
Interleaved intrachr. SVs	2
Total SVs (intrachr. + transl.)	2
SV types	DEL: 1; DUP: 0; h2hINV: 1; t2tINV: 0; TRA: 0
SVs in sample	153
Oscillating CN in chr (2 and 3 states)	7,
CN segments	10
Purity, ploidy	0.78, 1.86

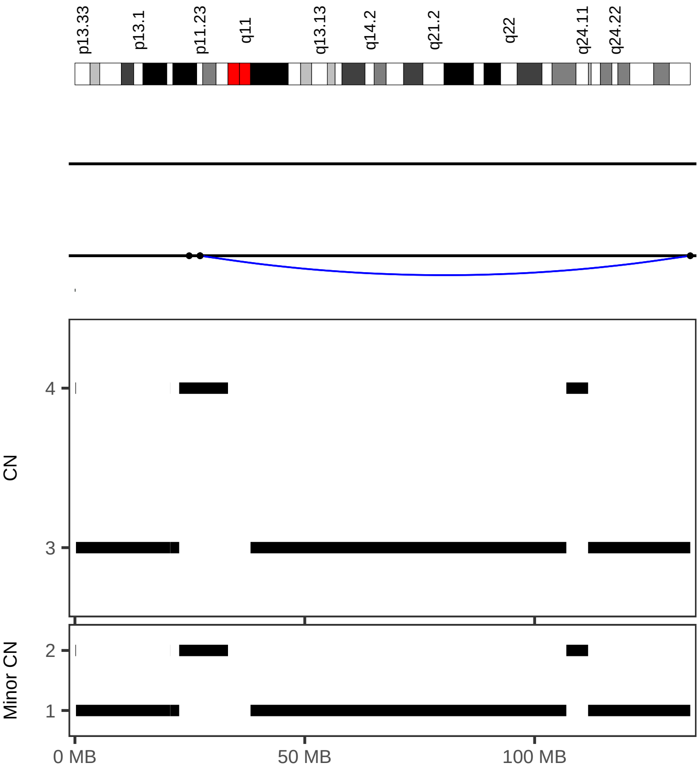

	PCSI_0456
Cancer type	Panc−AdenoCA
Position	12
Interleaved intrachr. SVs	2
Total SVs (intrachr. + transl.)	2
SV types	DEL: 0; DUP: 2; h2hINV: 0; t2tINV: 0; TRA: 0
SVs in sample	99
Oscillating CN in chr (2 and 3 states)	8,
CN segments	8
Purity, ploidy	0.8, 3.6

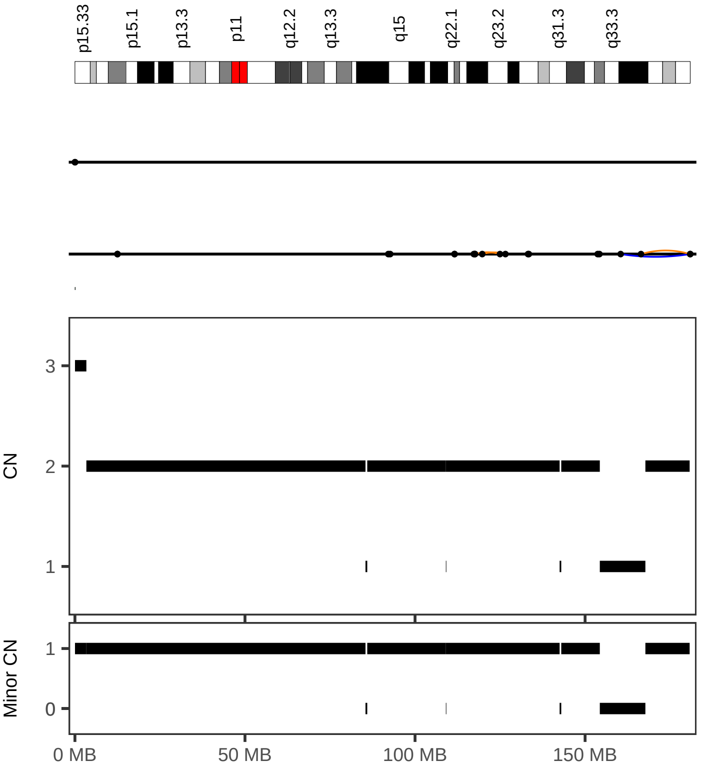

	PCSI_0457
Cancer type	Panc−AdenoCA
Position	5
Interleaved intrachr. SVs	2
Total SVs (intrachr. + transl.)	2
SV types	DEL: 2; DUP: 0; h2hINV: 0; t2tINV: 0; TRA: 0
SVs in sample	27
Oscillating CN in chr (2 and 3 states)	9,
CN segments	10
Purity, ploidy	0.74, 1.77

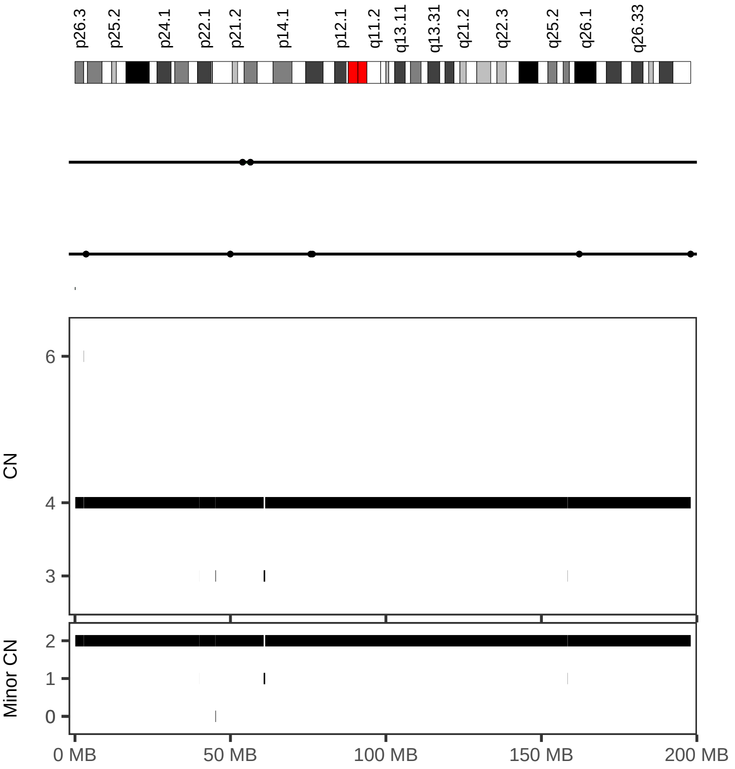

	PCSI_0468
Cancer type	Panc−AdenoCA
Position	3
Interleaved intrachr. SVs	2
Total SVs (intrachr. + transl.)	2
SV types	DEL: 0; DUP: 0; h2hINV: 1; t2tINV: 1; TRA: 0
SVs in sample	105
Oscillating CN in chr (2 and 3 states)	9,
CN segments	11
Purity, ploidy	0.79, 3.59

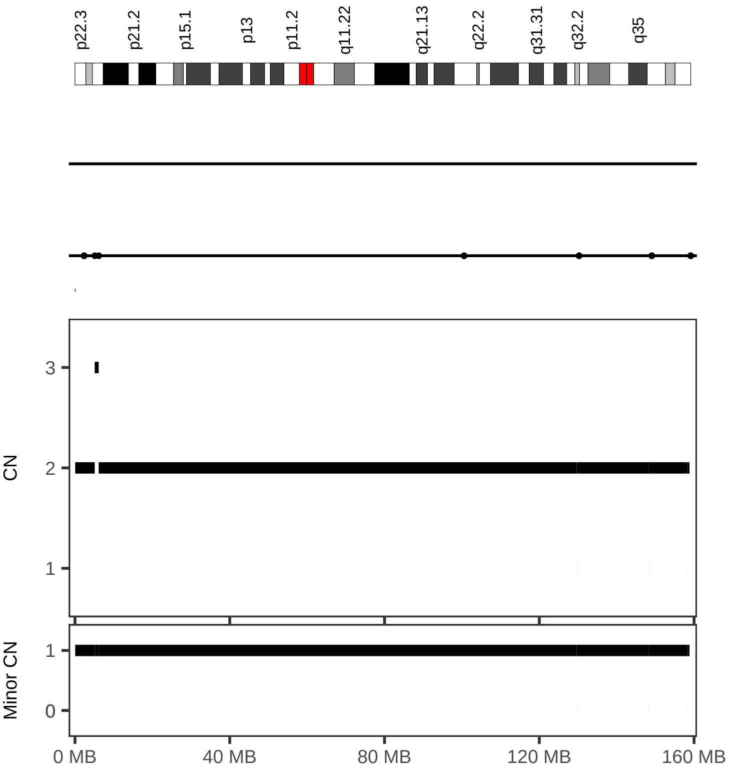

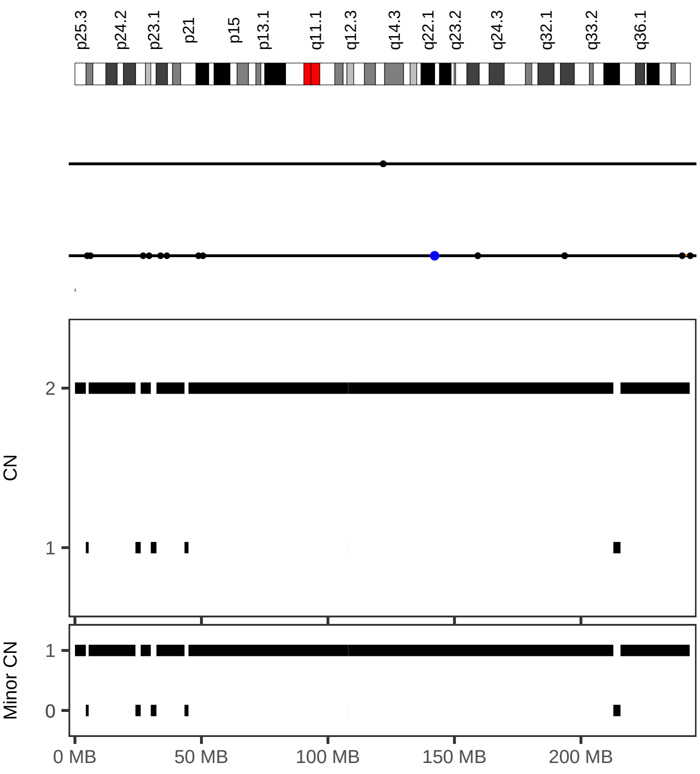

	**PCSI_0492**
Cancer type	Panc−AdenoCA
Position	7
Interleaved intrachr. SVs	1
Total SVs (intrachr. + transl.)	1
SV types	DEL: 0; DUP: 1; h2hINV: 0; t2tINV: 0; TRA: 0
SVs in sample	73
Oscillating CN in chr (2 and 3 states)	7,
CN segments	9
Purity, ploidy	0.65, 2.02

	**PCSI_0504**
Cancer type	Panc−AdenoCA
Position	2
Interleaved intrachr. SVs	2
Total SVs (intrachr. + transl.)	2
SV types	DEL: 0; DUP: 0; h2hINV: 1; t2tINV: 1; TRA: 0
SVs in sample	81
Oscillating CN in chr (2 and 3 states)	13,
CN segments	13
Purity, ploidy	0.75, 1.81

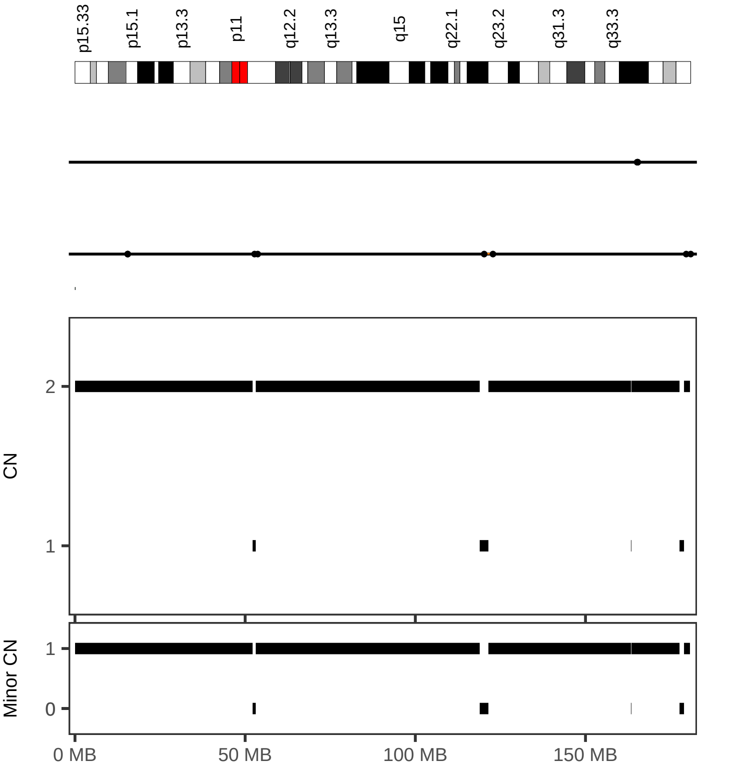

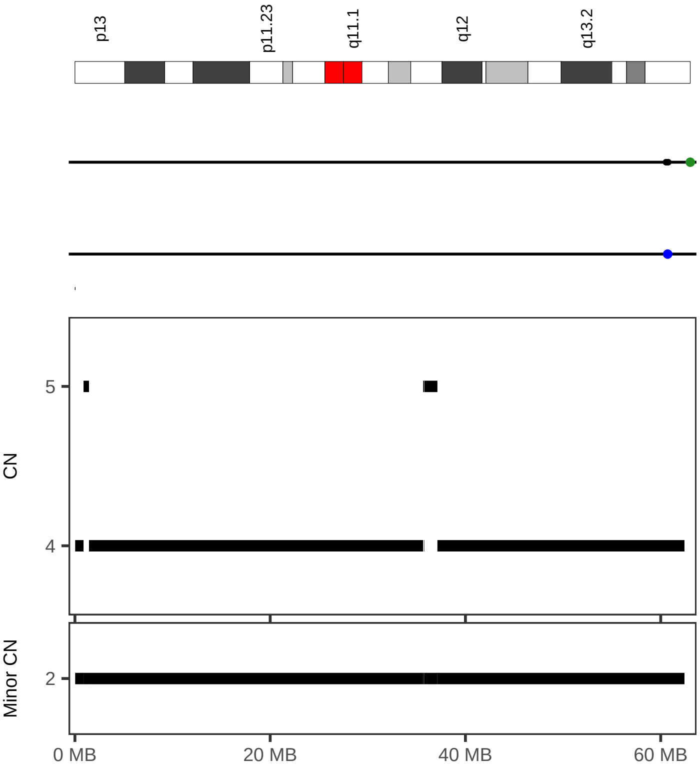

	**PCSI_0504**
Cancer type	Panc−AdenoCA
Position	5
Interleaved intrachr. SVs	2
Total SVs (intrachr. + transl.)	2
SV types	DEL: 0; DUP: 0; h2hINV: 1; t2tINV: 1; TRA: 0
SVs in sample	81
Oscillating CN in chr (2 and 3 states)	9,
CN segments	9
Purity, ploidy	0.75, 1.81

	**PCSI_0528**
Cancer type	Panc−AdenoCA
Position	20
Interleaved intrachr. SVs	1
Total SVs (intrachr. + transl.)	2
SV types	DEL: 0; DUP: 0; h2hINV: 0; t2tINV: 1; TRA: 1
SVs in sample	76
Oscillating CN in chr (2 and 3 states)	7,
CN segments	7
Purity, ploidy	0.52, 3.06

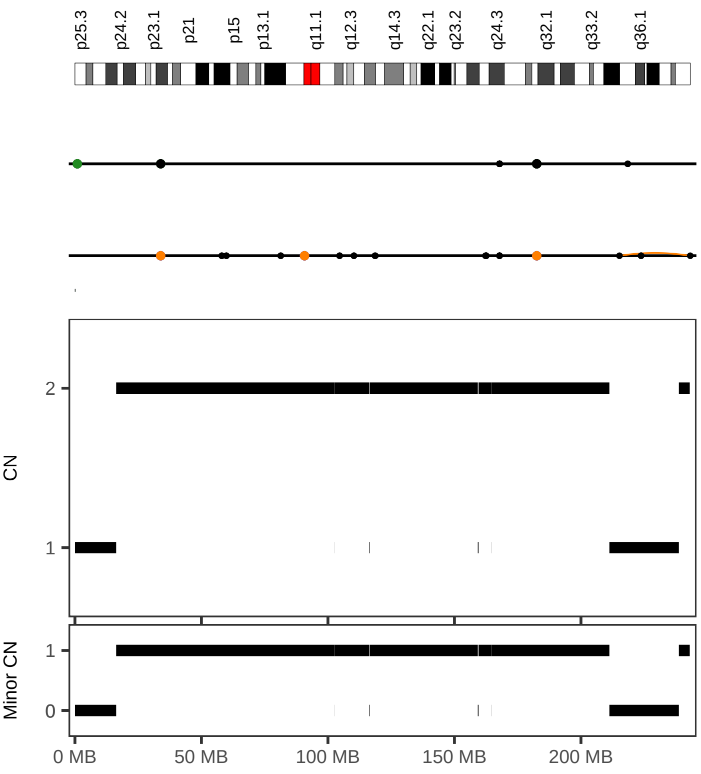

	ITNET−0100
Cancer type	Panc−Endocrine
Position	2
Interleaved intrachr. SVs	2
Total SVs (intrachr. + transl.)	2
SV types	DEL: 0; DUP: 0; h2hINV: 1; t2tINV: 1; TRA: 0
SVs in sample	160
Oscillating CN in chr (2 and 3 states)	12,
CN segments	12
Purity, ploidy	0.95, 1.8

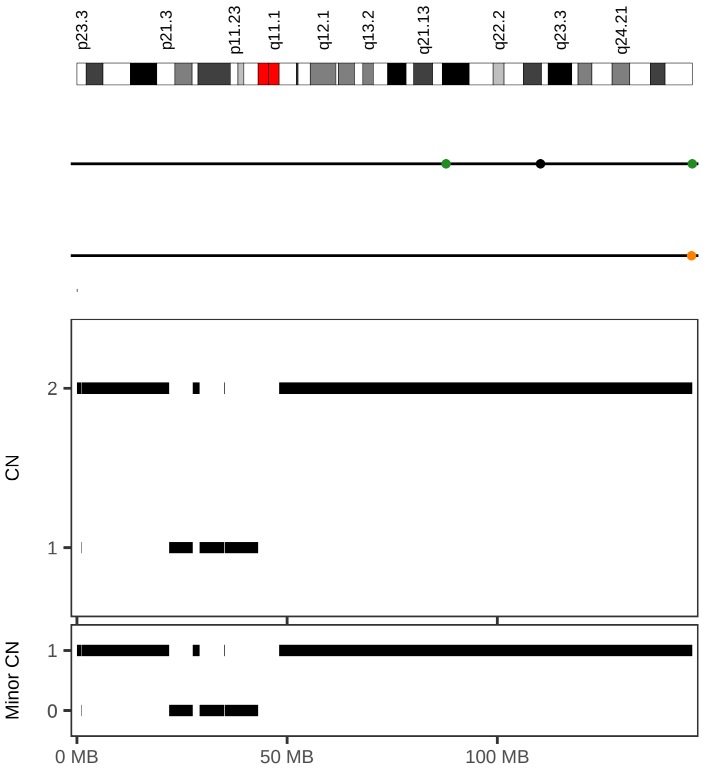

	EOPC−01
Cancer type	Prost−AdenoCA
Position	8
Interleaved intrachr. SVs	0
Total SVs (intrachr. + transl.)	0
SV types	DEL: 0; DUP: 0; h2hINV: 0; t2tINV: 0; TRA: 0
SVs in sample	10
Oscillating CN in chr (2 and 3 states)	9,
CN segments	9
Purity, ploidy	0.53, 1.85

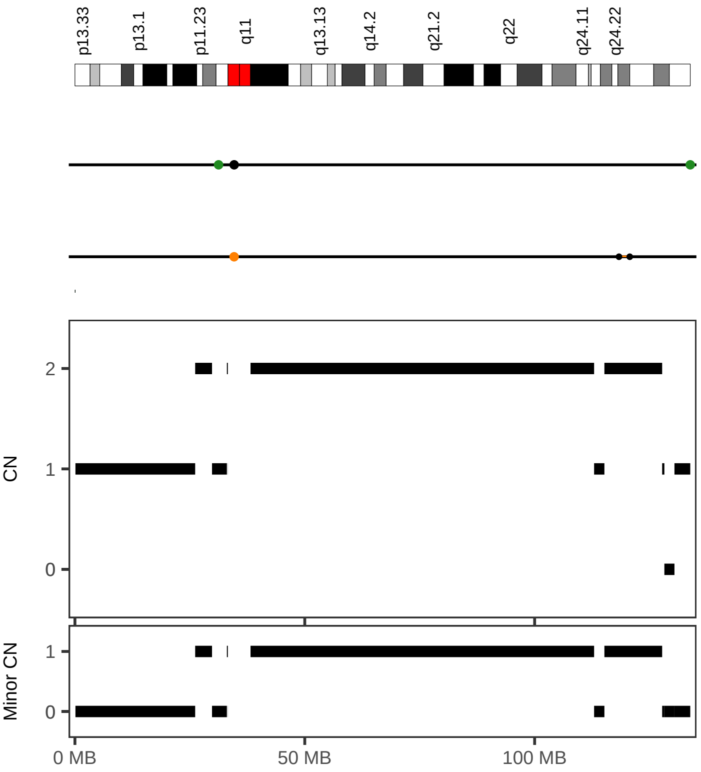

	EOPC−01
Cancer type	Prost−AdenoCA
Position	12
Interleaved intrachr. SVs	1
Total SVs (intrachr. + transl.)	1
SV types	DEL: 1; DUP: 0; h2hINV: 0; t2tINV: 0; TRA: 0
SVs in sample	10
Oscillating CN in chr (2 and 3 states)	9,
CN segments	11
Purity, ploidy	0.53, 1.85

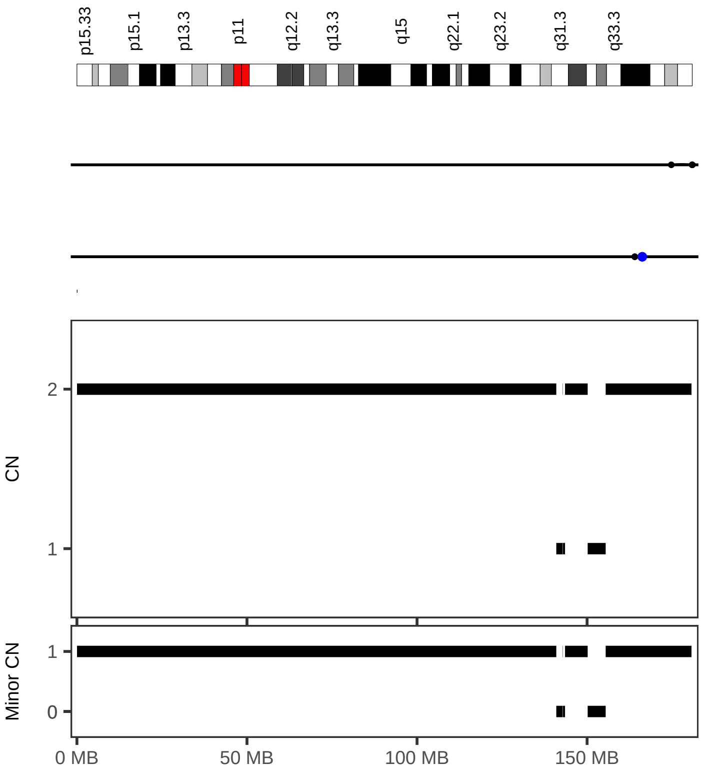

	EOPC−04
Cancer type	Prost−AdenoCA
Position	5
Interleaved intrachr. SVs	1
Total SVs (intrachr. + transl.)	1
SV types	DEL: 0; DUP: 0; h2hINV: 1; t2tINV: 0; TRA: 0
SVs in sample	57
Oscillating CN in chr (2 and 3 states)	7,
CN segments	7
Purity, ploidy	0.58, 1.9

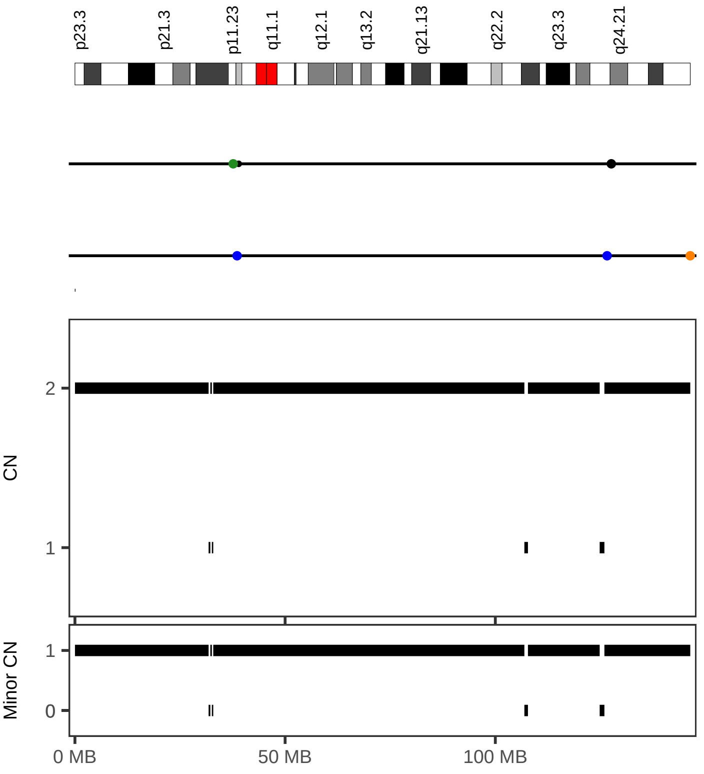

	**EOPC−04**
Cancer type	Prost−AdenoCA
Position	8
Interleaved intrachr. SVs	1
Total SVs (intrachr. + transl.)	2
SV types	DEL: 0; DUP: 0; h2hINV: 0; t2tINV: 1; TRA: 1
SVs in sample	57
Oscillating CN in chr (2 and 3 states)	9,
CN segments	9
Purity, ploidy	0.58, 1.9

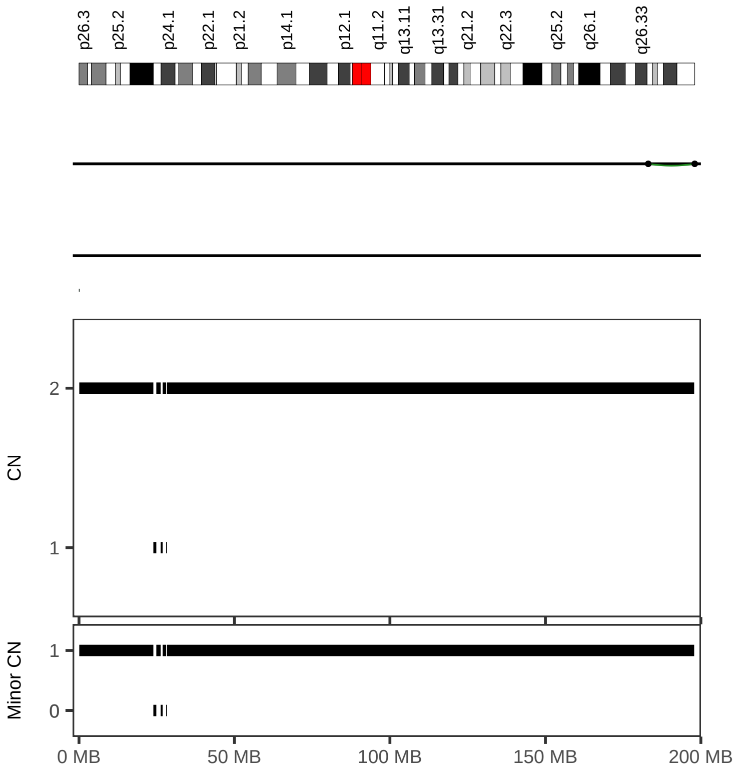

	**EOPC−09**
Cancer type	Prost−AdenoCA
Position	3
Interleaved intrachr. SVs	1
Total SVs (intrachr. + transl.)	1
SV types	DEL: 0; DUP: 0; h2hINV: 0; t2tINV: 1; TRA: 0
SVs in sample	7
Oscillating CN in chr (2 and 3 states)	7,
CN segments	7
Purity, ploidy	0.39, 1.9

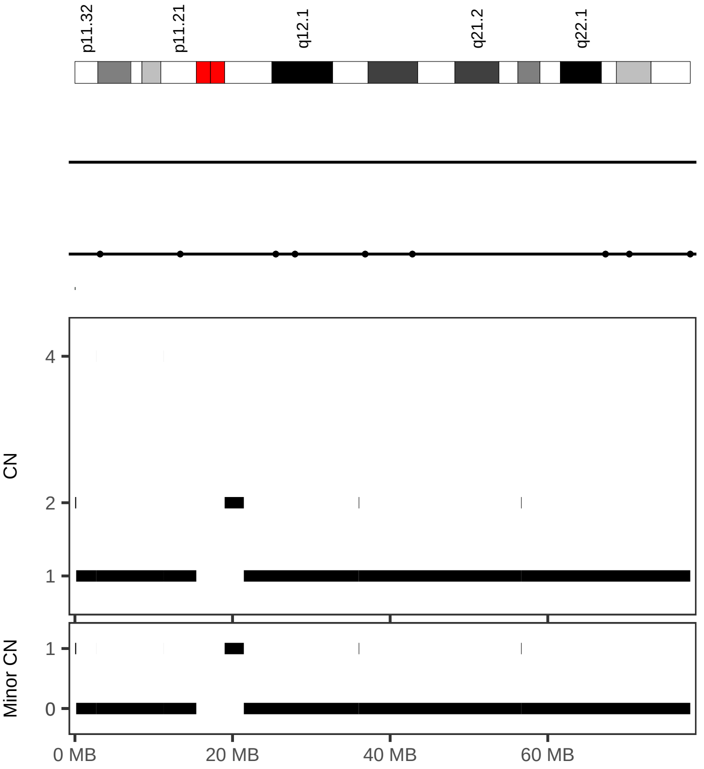

	**CPCG0046**
Cancer type	Prost−AdenoCA
Position	18
Interleaved intrachr. SVs	0
Total SVs (intrachr. + transl.)	0
SV types	DEL: 0; DUP: 0; h2hINV: 0; t2tINV: 0; TRA: 0
SVs in sample	299
Oscillating CN in chr (2 and 3 states)	7,
CN segments	12
Purity, ploidy	0.66, 1.89

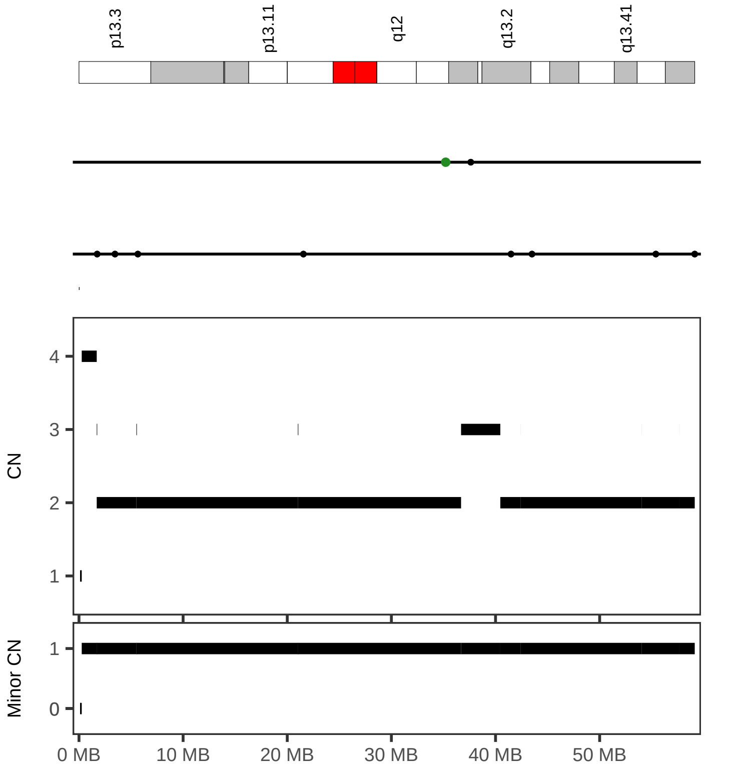

	**CPCG0046**
Cancer type	Prost−AdenoCA
Position	19
Interleaved intrachr. SVs	0
Total SVs (intrachr. + transl.)	0
SV types	DEL: 0; DUP: 0; h2hINV: 0; t2tINV: 0; TRA: 0
SVs in sample	299
Oscillating CN in chr (2 and 3 states)	14,
CN segments	16
Purity, ploidy	0.66, 1.89

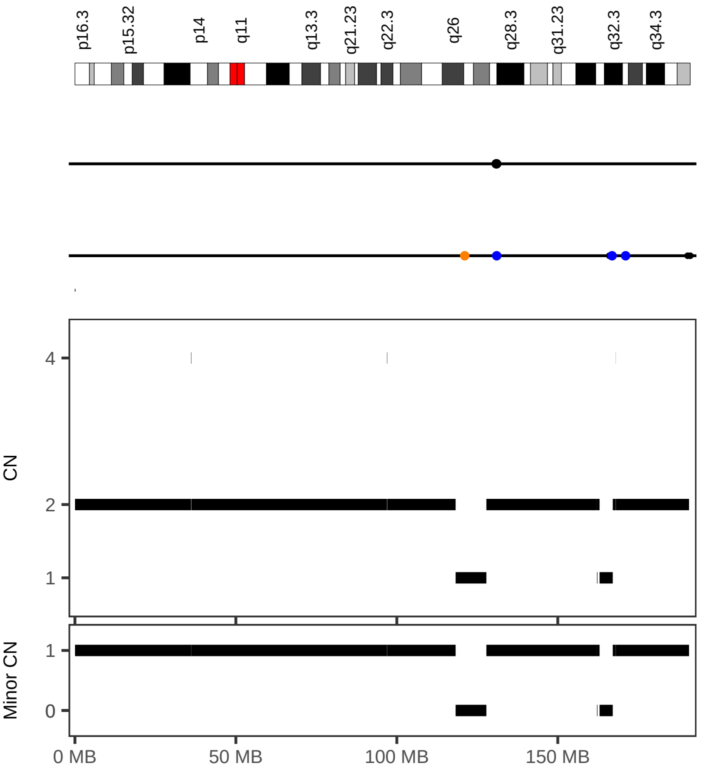

	CPCG0057
Cancer type	Prost−AdenoCA
Position	4
Interleaved intrachr. SVs	1
Total SVs (intrachr. + transl.)	1
SV types	DEL: 1; DUP: 0; h2hINV: 0; t2tINV: 0; TRA: 0
SVs in sample	58
Oscillating CN in chr (2 and 3 states)	7,
CN segments	13
Purity, ploidy	0.56, 1.84

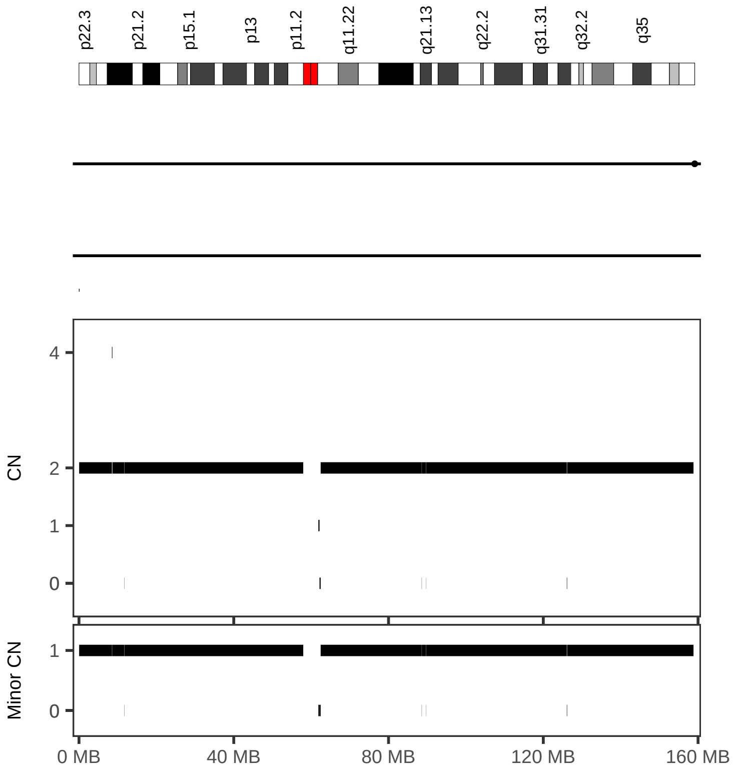

	CPCG0057
Cancer type	Prost−AdenoCA
Position	7
Interleaved intrachr. SVs	0
Total SVs (intrachr. + transl.)	0
SV types	DEL: 0; DUP: 0; h2hINV: 0; t2tINV: 0; TRA: 0
SVs in sample	58
Oscillating CN in chr (2 and 3 states)	8,
CN segments	14
Purity, ploidy	0.56, 1.84

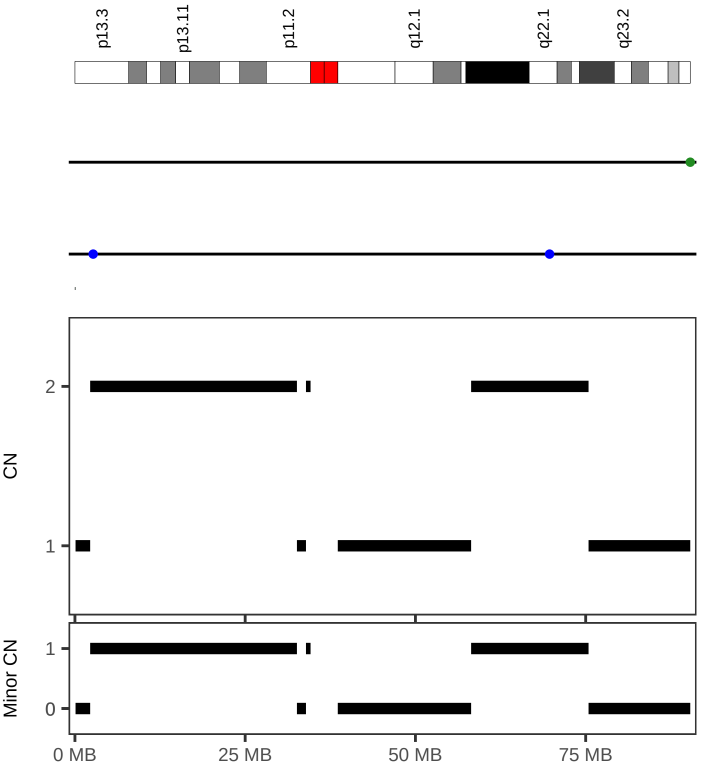

	CPCG0057
Cancer type	Prost−AdenoCA
Position	16
Interleaved intrachr. SVs	0
Total SVs (intrachr. + transl.)	0
SV types	DEL: 0; DUP: 0; h2hINV: 0; t2tINV: 0; TRA: 0
SVs in sample	58
Oscillating CN in chr (2 and 3 states)	7,
CN segments	7
Purity, ploidy	0.56, 1.84

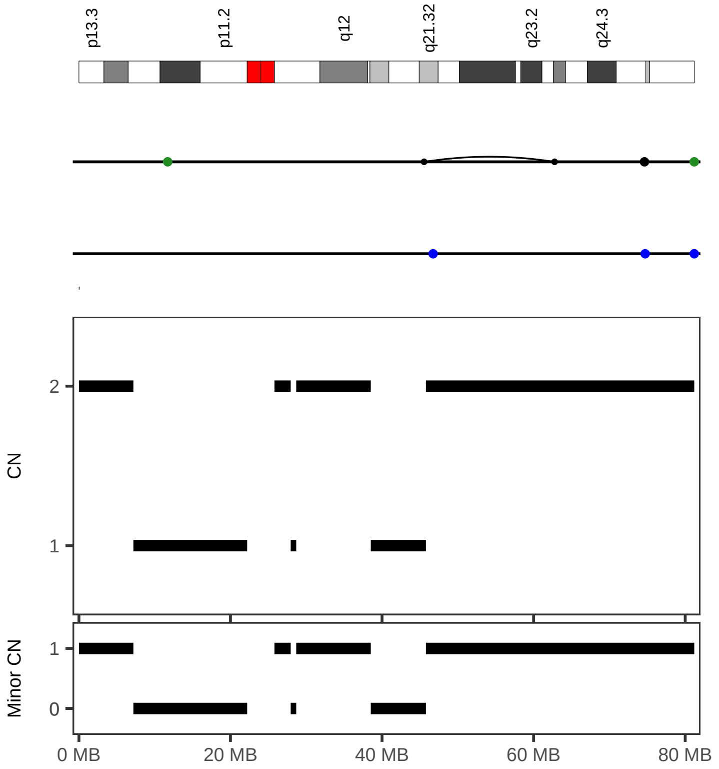

	CPCG0057
Cancer type	Prost−AdenoCA
Position	17
Interleaved intrachr. SVs	1
Total SVs (intrachr. + transl.)	2
SV types	DEL: 0; DUP: 0; h2hINV: 1; t2tINV: 0; TRA: 1
SVs in sample	58
Oscillating CN in chr (2 and 3 states)	7,
CN segments	7
Purity, ploidy	0.56, 1.84

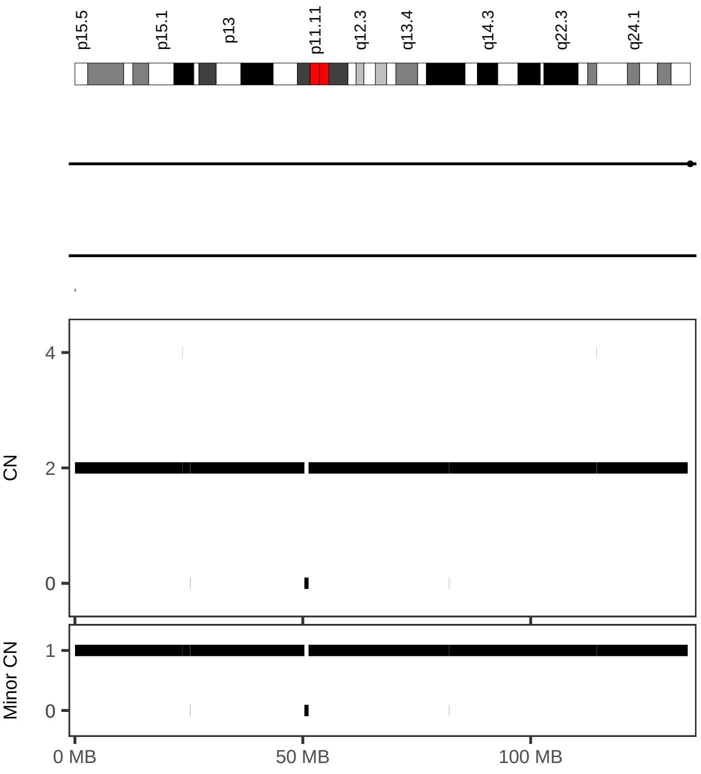

	CPCG0094
Cancer type	Prost−AdenoCA
Position	11
Interleaved intrachr. SVs	0
Total SVs (intrachr. + transl.)	0
SV types	DEL: 0; DUP: 0; h2hINV: 0; t2tINV: 0; TRA: 0
SVs in sample	75
Oscillating CN in chr (2 and 3 states)	7,
CN segments	11
Purity, ploidy	0.46, 1.79

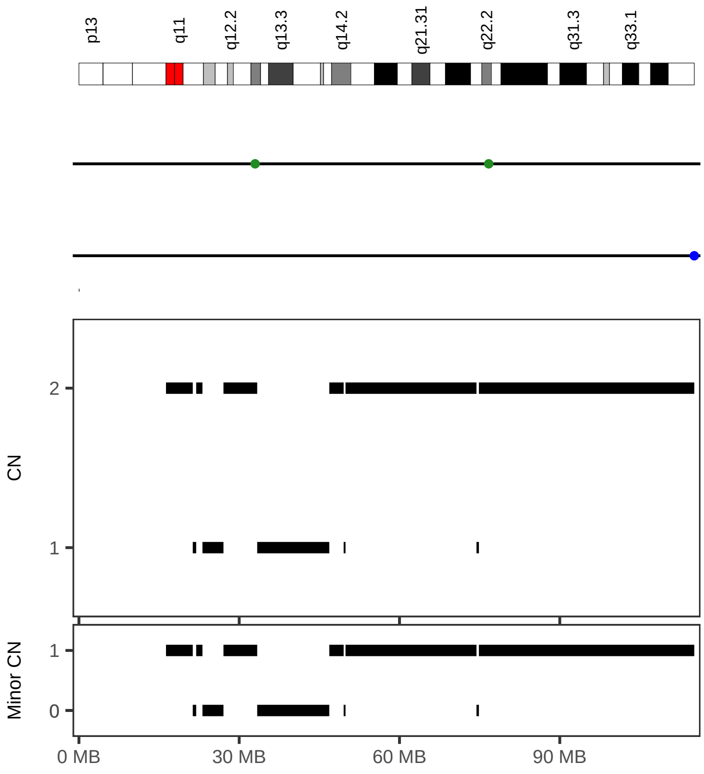

	CPCG0102
Cancer type	Prost−AdenoCA
Position	13
Interleaved intrachr. SVs	0
Total SVs (intrachr. + transl.)	0
SV types	DEL: 0; DUP: 0; h2hINV: 0; t2tINV: 0; TRA: 0
SVs in sample	26
Oscillating CN in chr (2 and 3 states)	11,
CN segments	11
Purity, ploidy	0.24, 1.91

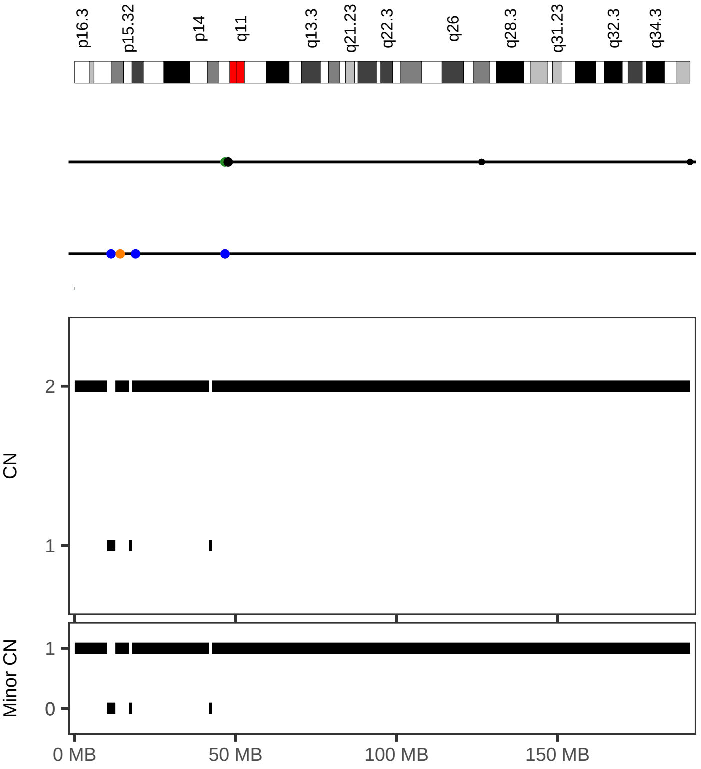

	CPCG0121
Cancer type	Prost−AdenoCA
Position	4
Interleaved intrachr. SVs	0
Total SVs (intrachr. + transl.)	0
SV types	DEL: 0; DUP: 0; h2hINV: 0; t2tINV: 0; TRA: 0
SVs in sample	64
Oscillating CN in chr (2 and 3 states)	7,
CN segments	7
Purity, ploidy	0.36, 1.88

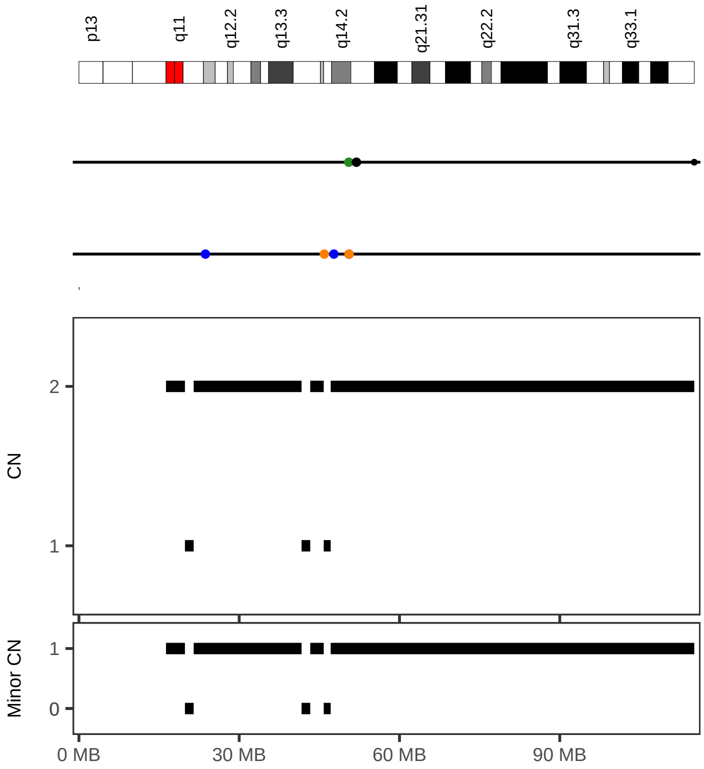

	CPCG0121
Cancer type	Prost−AdenoCA
Position	13
Interleaved intrachr. SVs	0
Total SVs (intrachr. + transl.)	0
SV types	DEL: 0; DUP: 0; h2hINV: 0; t2tINV: 0; TRA: 0
SVs in sample	64
Oscillating CN in chr (2 and 3 states)	7,
CN segments	7
Purity, ploidy	0.36, 1.88

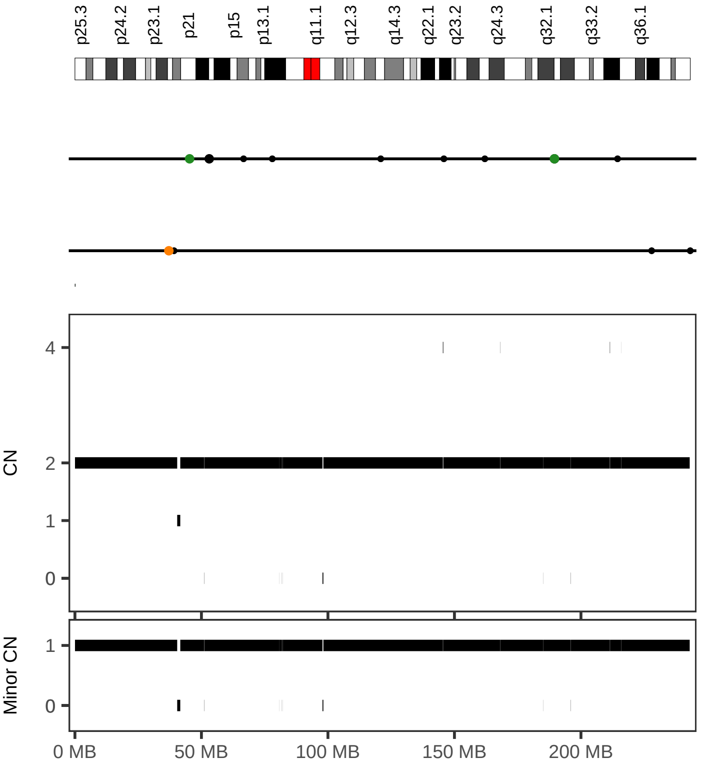

	**CPCG0213**
Cancer type	Prost−AdenoCA
Position	2
Interleaved intrachr. SVs	0
Total SVs (intrachr. + transl.)	0
SV types	DEL: 0; DUP: 0; h2hINV: 0; t2tINV: 0; TRA: 0
SVs in sample	155
Oscillating CN in chr (2 and 3 states)	11,
CN segments	25
Purity, ploidy	0.49, 1.87

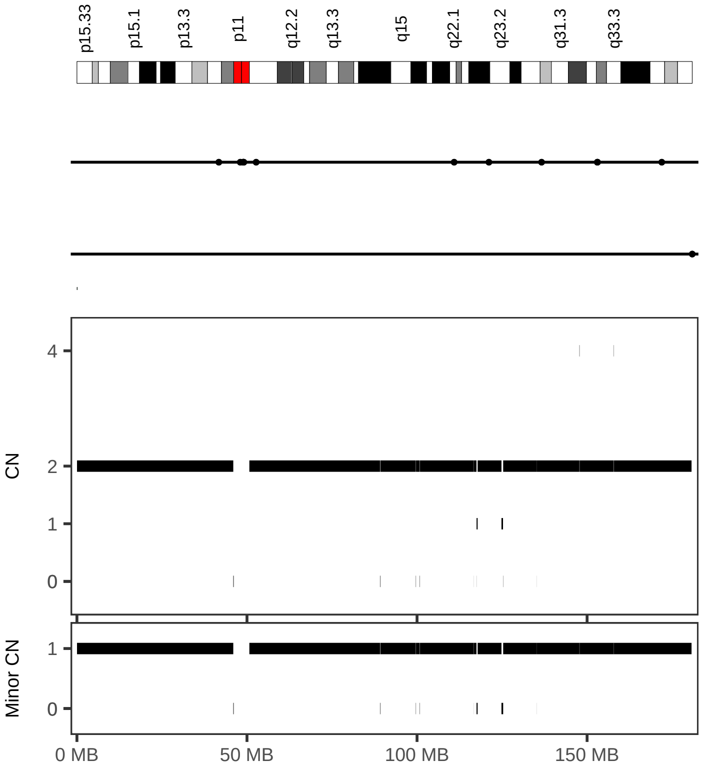

	**CPCG0213**
Cancer type	Prost−AdenoCA
Position	5
Interleaved intrachr. SVs	0
Total SVs (intrachr. + transl.)	0
SV types	DEL: 0; DUP: 0; h2hINV: 0; t2tINV: 0; TRA: 0
SVs in sample	155
Oscillating CN in chr (2 and 3 states)	12,
CN segments	23
Purity, ploidy	0.49, 1.87

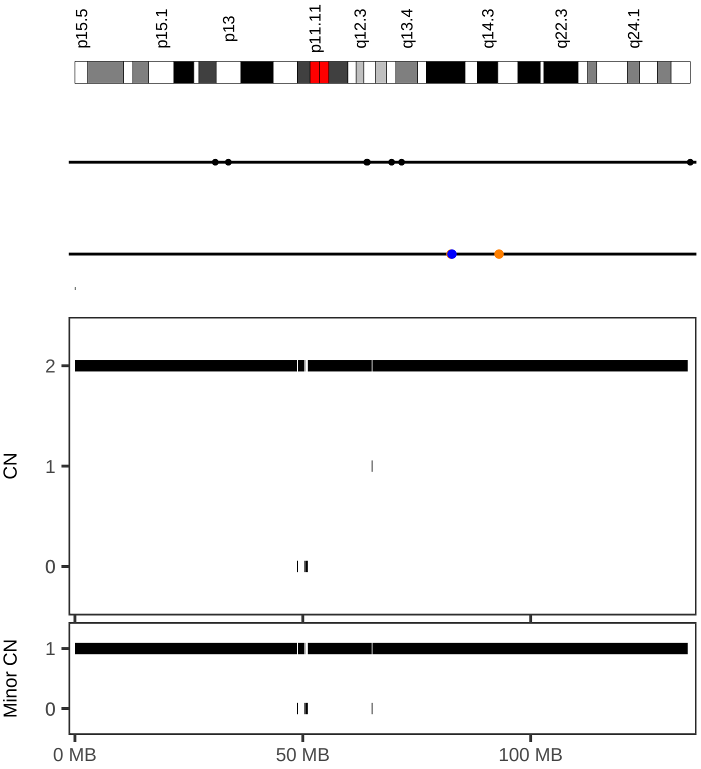

	**CPCG0213**
Cancer type	Prost−AdenoCA
Position	11
Interleaved intrachr. SVs	0
Total SVs (intrachr. + transl.)	0
SV types	DEL: 0; DUP: 0; h2hINV: 0; t2tINV: 0; TRA: 0
SVs in sample	155
Oscillating CN in chr (2 and 3 states)	7,
CN segments	9
Purity, ploidy	0.49, 1.87

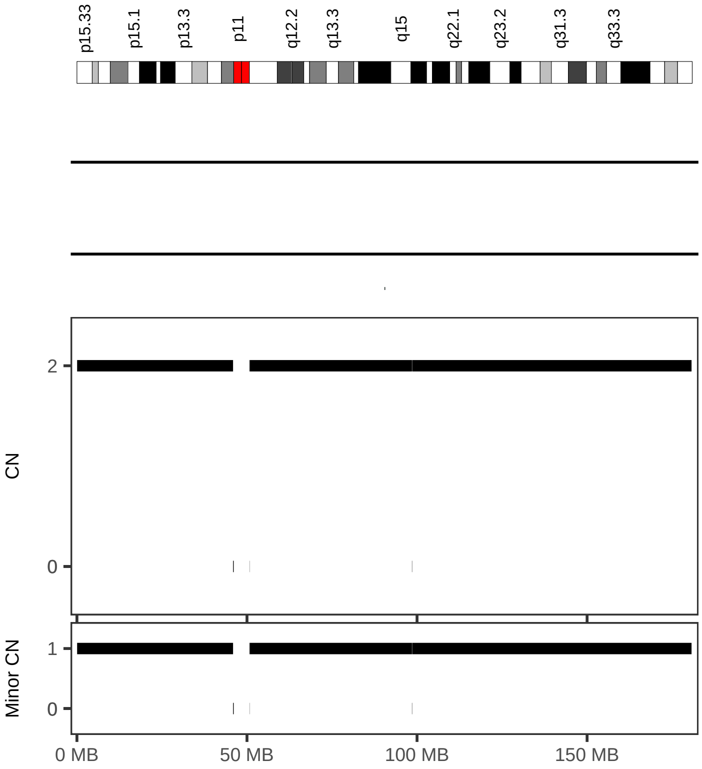

	**CPCG0234**
Cancer type	Prost−AdenoCA
Position	5
Interleaved intrachr. SVs	0
Total SVs (intrachr. + transl.)	0
SV types	DEL: 0; DUP: 0; h2hINV: 0; t2tINV: 0; TRA: 0
SVs in sample	21
Oscillating CN in chr (2 and 3 states)	7,
CN segments	7
Purity, ploidy	0.97, 1.9

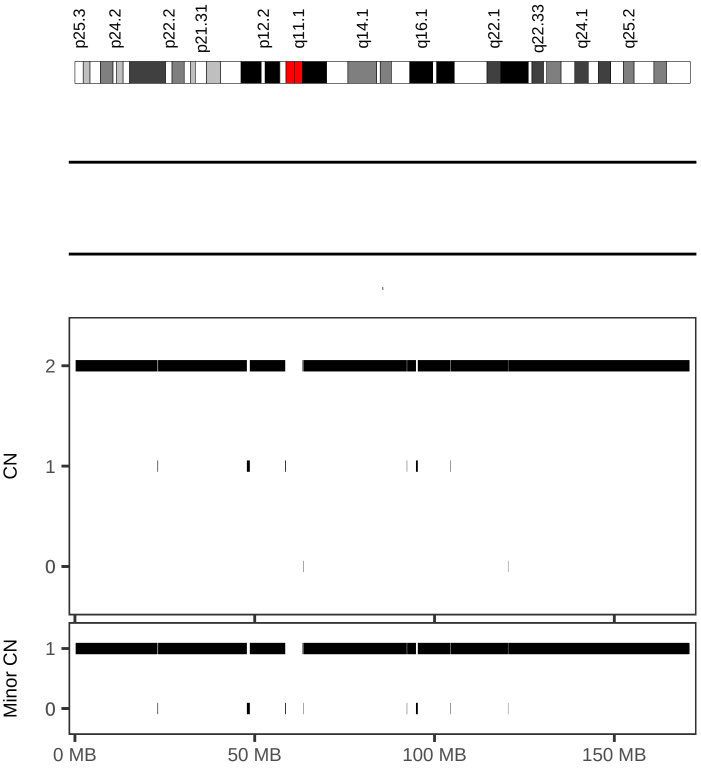

	CPCG0250
Cancer type	Prost−AdenoCA
Position	6
Interleaved intrachr. SVs	0
Total SVs (intrachr. + transl.)	0
SV types	DEL: 0; DUP: 0; h2hINV: 0; t2tINV: 0; TRA: 0
SVs in sample	28
Oscillating CN in chr (2 and 3 states)	7,
CN segments	17
Purity, ploidy	0.26, 1.94

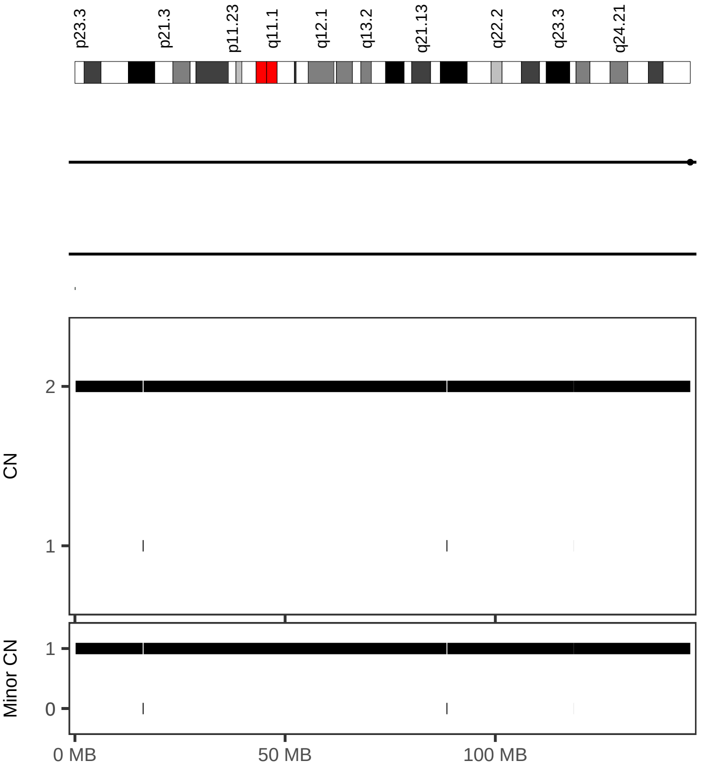

	CPCG0250
Cancer type	Prost−AdenoCA
Position	8
Interleaved intrachr. SVs	0
Total SVs (intrachr. + transl.)	0
SV types	DEL: 0; DUP: 0; h2hINV: 0; t2tINV: 0; TRA: 0
SVs in sample	28
Oscillating CN in chr (2 and 3 states)	7,
CN segments	7
Purity, ploidy	0.26, 1.94

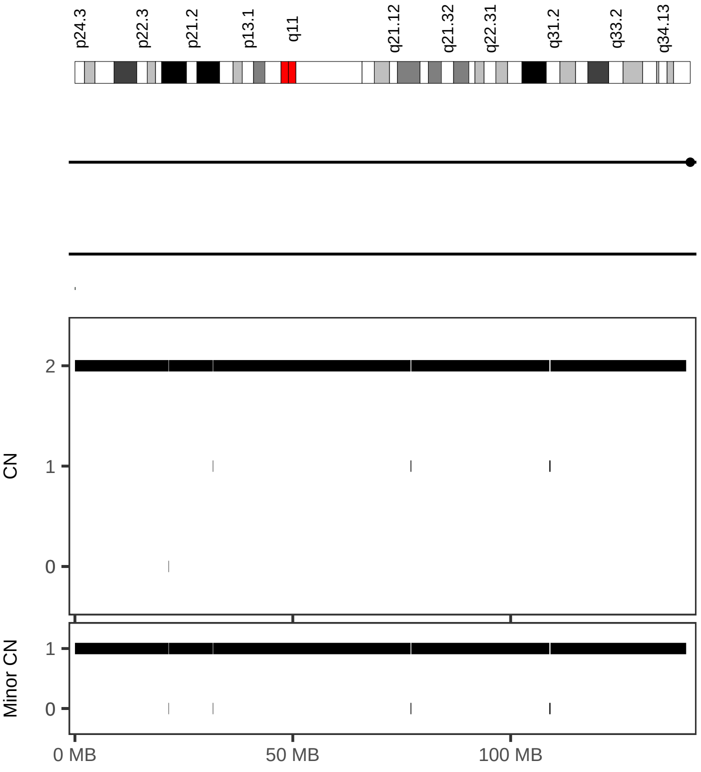

	CPCG0250
Cancer type	Prost−AdenoCA
Position	9
Interleaved intrachr. SVs	0
Total SVs (intrachr. + transl.)	0
SV types	DEL: 0; DUP: 0; h2hINV: 0; t2tINV: 0; TRA: 0
SVs in sample	28
Oscillating CN in chr (2 and 3 states)	7,
CN segments	9
Purity, ploidy	0.26, 1.94

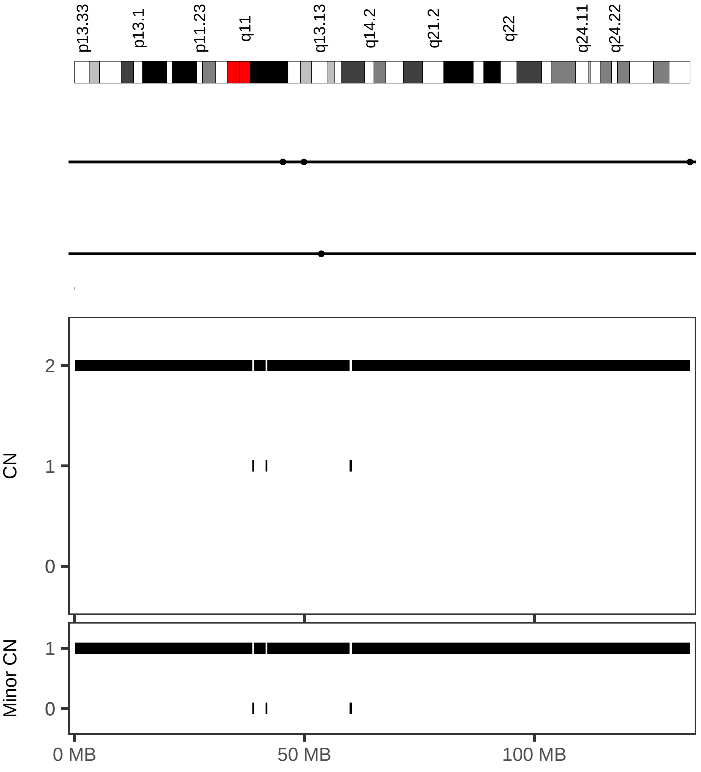

	CPCG0250
Cancer type	Prost−AdenoCA
Position	12
Interleaved intrachr. SVs	0
Total SVs (intrachr. + transl.)	0
SV types	DEL: 0; DUP: 0; h2hINV: 0; t2tINV: 0; TRA: 0
SVs in sample	28
Oscillating CN in chr (2 and 3 states)	7,
CN segments	9
Purity, ploidy	0.26, 1.94

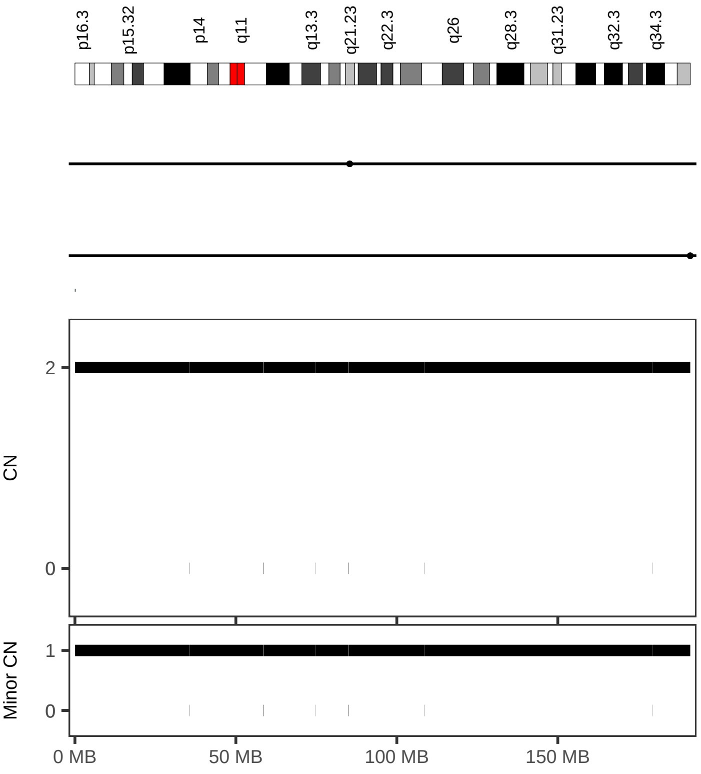

	CPCG0266
Cancer type	Prost−AdenoCA
Position	4
Interleaved intrachr. SVs	1
Total SVs (intrachr. + transl.)	1
SV types	DEL: 1; DUP: 0; h2hINV: 0; t2tINV: 0; TRA: 0
SVs in sample	121
Oscillating CN in chr (2 and 3 states)	13,
CN segments	13
Purity, ploidy	0.51, 1.91

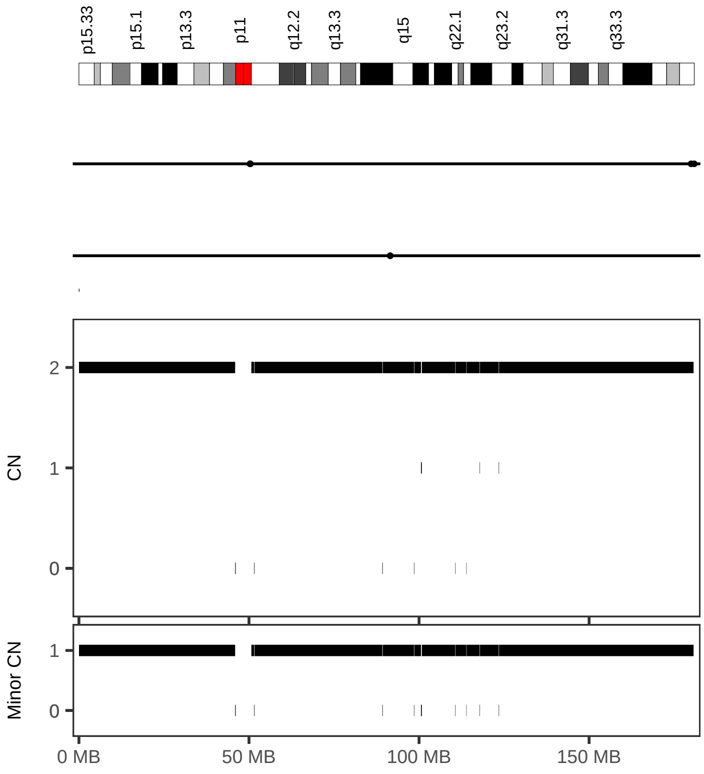

	CPCG0266
Cancer type	Prost−AdenoCA
Position	5
Interleaved intrachr. SVs	1
Total SVs (intrachr. + transl.)	1
SV types	DEL: 0; DUP: 0; h2hINV: 0; t2tINV: 1; TRA: 0
SVs in sample	121
Oscillating CN in chr (2 and 3 states)	9,
CN segments	19
Purity, ploidy	0.51, 1.91

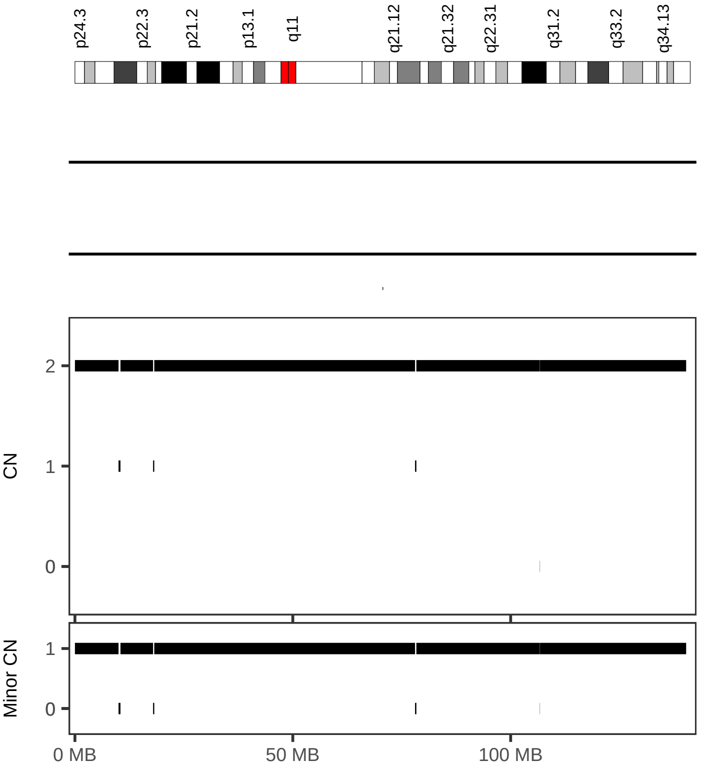

	CPCG0266
Cancer type	Prost−AdenoCA
Position	9
Interleaved intrachr. SVs	0
Total SVs (intrachr. + transl.)	0
SV types	DEL: 0; DUP: 0; h2hINV: 0; t2tINV: 0; TRA: 0
SVs in sample	121
Oscillating CN in chr (2 and 3 states)	7,
CN segments	9
Purity, ploidy	0.51, 1.91

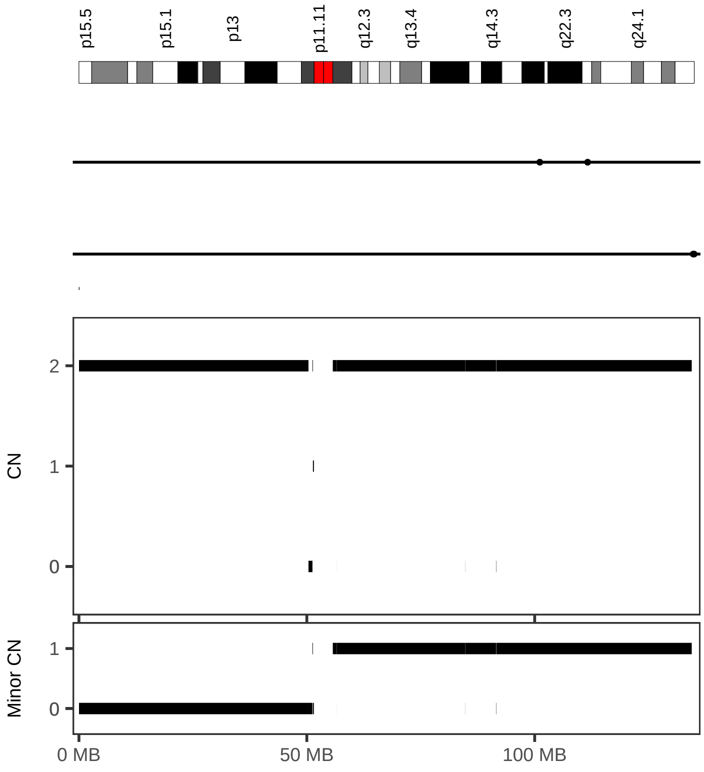

	CPCG0266
Cancer type	Prost−AdenoCA
Position	11
Interleaved intrachr. SVs	1
Total SVs (intrachr. + transl.)	1
SV types	DEL: 0; DUP: 1; h2hINV: 0; t2tINV: 0; TRA: 0
SVs in sample	121
Oscillating CN in chr (2 and 3 states)	7,
CN segments	11
Purity, ploidy	0.51, 1.91

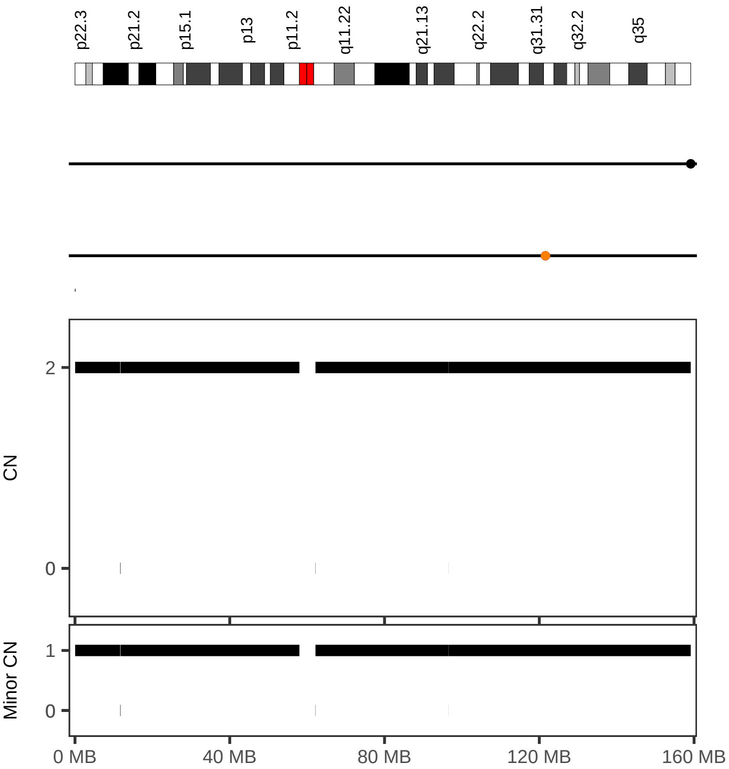

	**CPCG0339**
Cancer type	Prost−AdenoCA
Position	7
Interleaved intrachr. SVs	0
Total SVs (intrachr. + transl.)	0
SV types	DEL: 0; DUP: 0; h2hINV: 0; t2tINV: 0; TRA: 0
SVs in sample	51
Oscillating CN in chr (2 and 3 states)	7,
CN segments	7
Purity, ploidy	0.97, 1.93

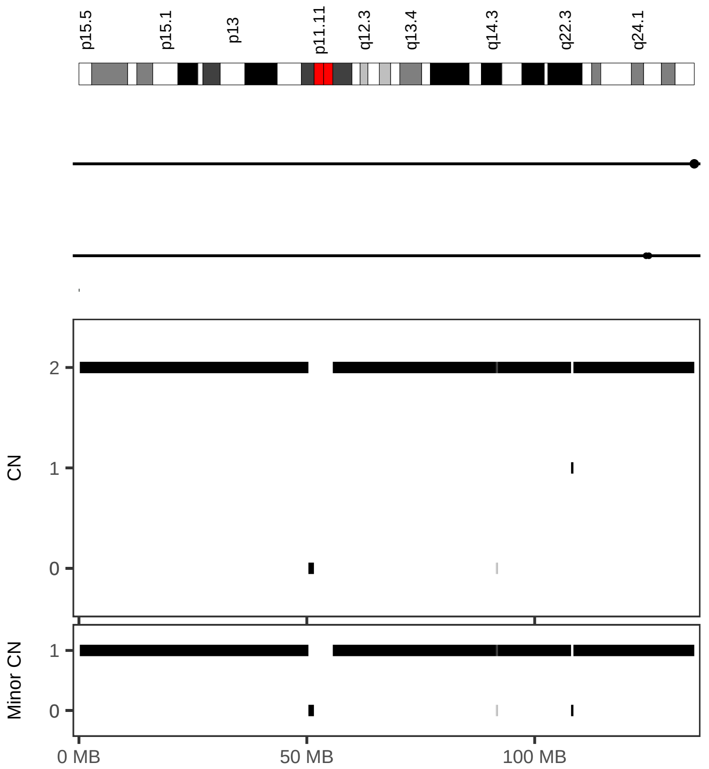

	**CPCG0341**
Cancer type	Prost−AdenoCA
Position	11
Interleaved intrachr. SVs	1
Total SVs (intrachr. + transl.)	1
SV types	DEL: 1; DUP: 0; h2hINV: 0; t2tINV: 0; TRA: 0
SVs in sample	31
Oscillating CN in chr (2 and 3 states)	7,
CN segments	9
Purity, ploidy	0.55, 1.89

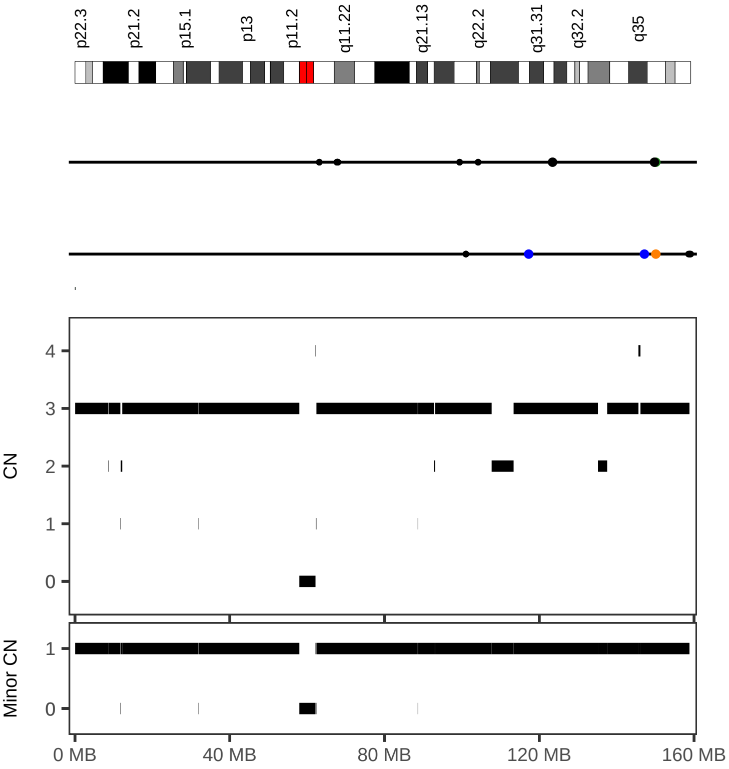

	**CPCG0345**
Cancer type	Prost−AdenoCA
Position	7
Interleaved intrachr. SVs	1
Total SVs (intrachr. + transl.)	2
SV types	DEL: 0; DUP: 2; h2hINV: 0; t2tINV: 0; TRA: 0
SVs in sample	57
Oscillating CN in chr (2 and 3 states)	7,
CN segments	22
Purity, ploidy	0.49, 2.09

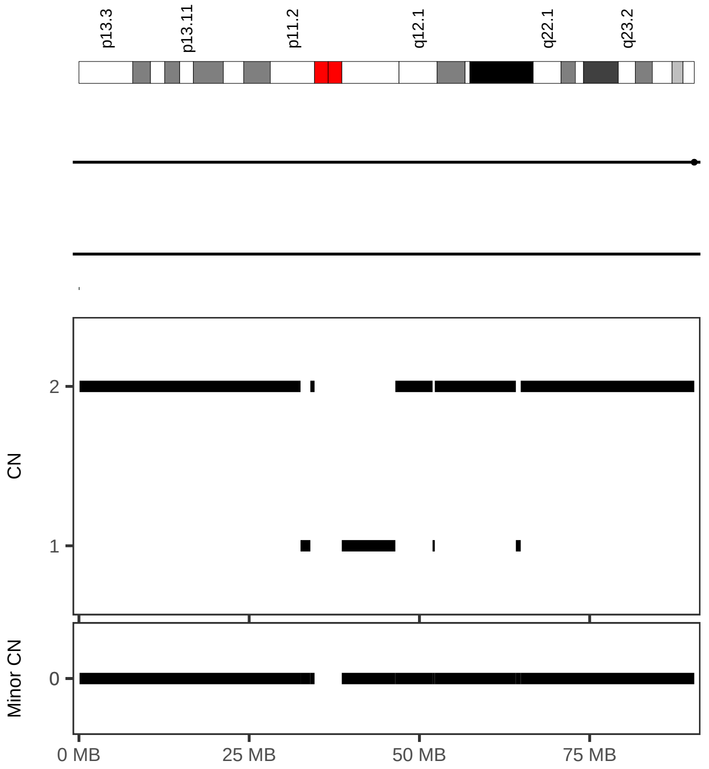

	**CPCG0345**
Cancer type	Prost−AdenoCA
Position	16
Interleaved intrachr. SVs	0
Total SVs (intrachr. + transl.)	0
SV types	DEL: 0; DUP: 0; h2hINV: 0; t2tINV: 0; TRA: 0
SVs in sample	57
Oscillating CN in chr (2 and 3 states)	9,
CN segments	9
Purity, ploidy	0.49, 2.09

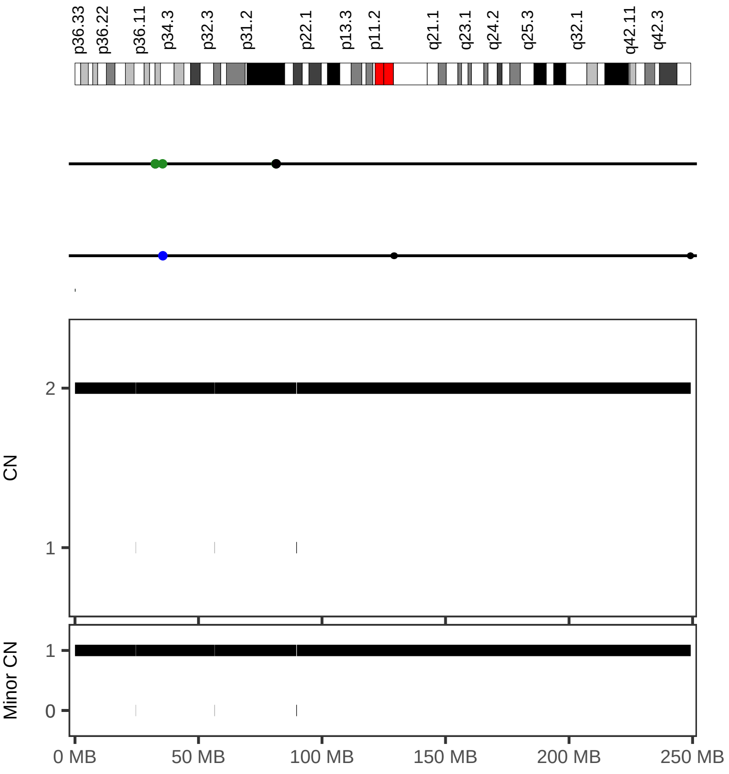

	**CPCG0371**
Cancer type	Prost−AdenoCA
Position	1
Interleaved intrachr. SVs	1
Total SVs (intrachr. + transl.)	2
SV types	DEL: 2; DUP: 0; h2hINV: 0; t2tINV: 0; TRA: 0
SVs in sample	90
Oscillating CN in chr (2 and 3 states)	7,
CN segments	7
Purity, ploidy	0.55, 1.87

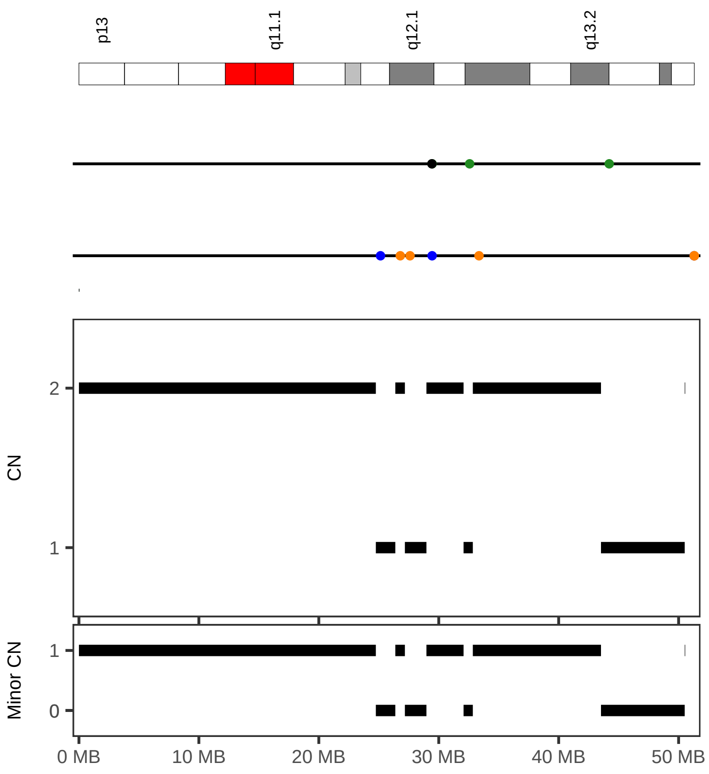

	**CPCG0371**
Cancer type	Prost−AdenoCA
Position	22
Interleaved intrachr. SVs	0
Total SVs (intrachr. + transl.)	0
SV types	DEL: 0; DUP: 0; h2hINV: 0; t2tINV: 0; TRA: 0
SVs in sample	90
Oscillating CN in chr (2 and 3 states)	9,
CN segments	9
Purity, ploidy	0.55, 1.87

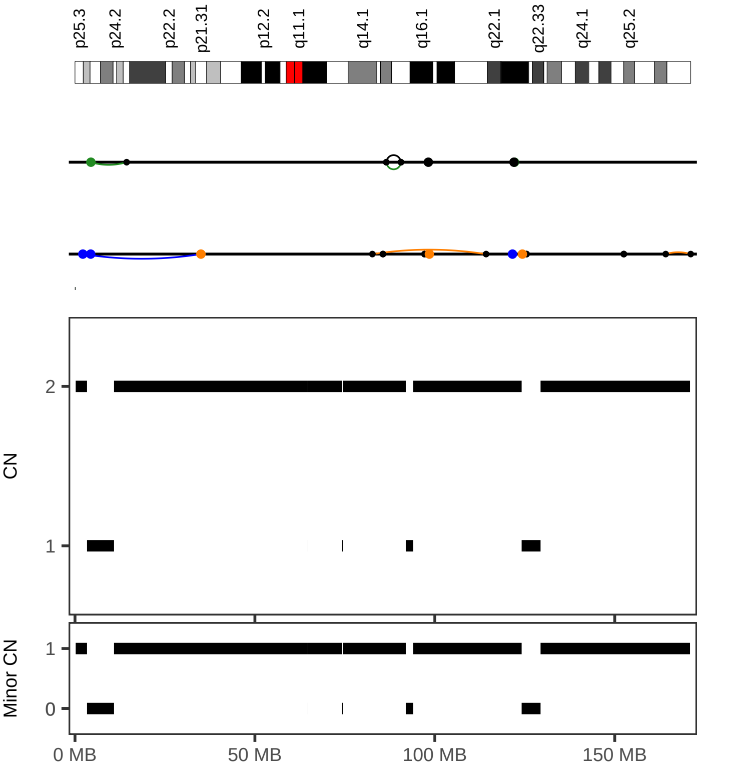

	**0078_CRUK_PC_0078**
Cancer type	Prost−AdenoCA
Position	6
Interleaved intrachr. SVs	2
Total SVs (intrachr. + transl.)	2
SV types	DEL: 0; DUP: 0; h2hINV: 1; t2tINV: 1; TRA: 0
SVs in sample	95
Oscillating CN in chr (2 and 3 states)	11,
CN segments	11
Purity, ploidy	0.28, 1.89

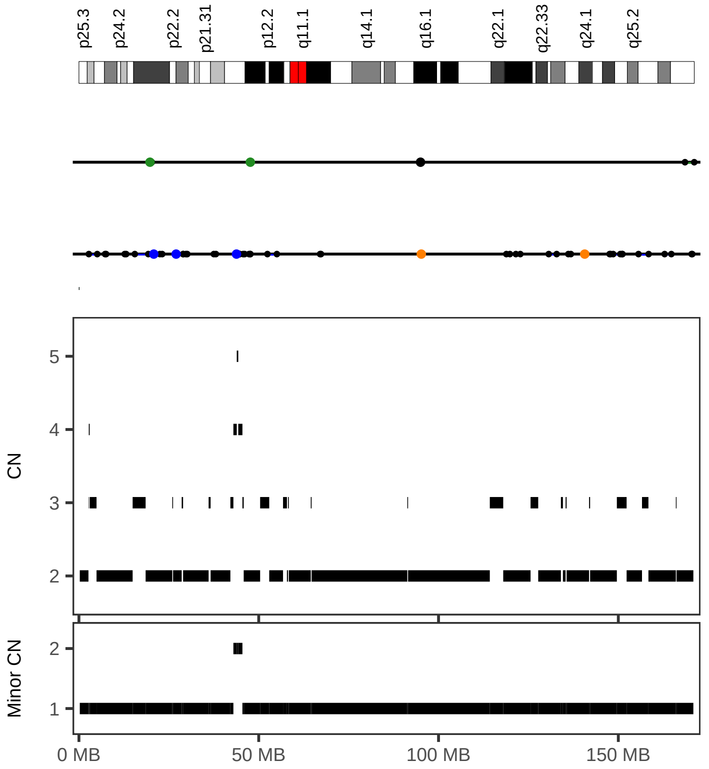

	**A10−0015_CRUK_PC_0015**
Cancer type	Prost−AdenoCA
Position	6
Interleaved intrachr. SVs	2
Total SVs (intrachr. + transl.)	2
SV types	DEL: 0; DUP: 2; h2hINV: 0; t2tINV: 0; TRA: 0
SVs in sample	547
Oscillating CN in chr (2 and 3 states)	28,
CN segments	45
Purity, ploidy	NA, NA
