# Supplementary material for: Comprehensive analysis of chromothripsis in 2,658 human cancers using whole-genome sequencing
Source: Nat Genet. 2020 Feb 5;52(3):331–41. doi: 10.1038/s41588-019-0576-7 (PMC7058534; doi:10.1038/s41588-019-0576-7)
Supplement: Supplementary file 9 — Large clusters of interleaved SVs (>20) not identified as chromothripsis by our method. [file 41588_2019_576_MOESM9_ESM.pdf]

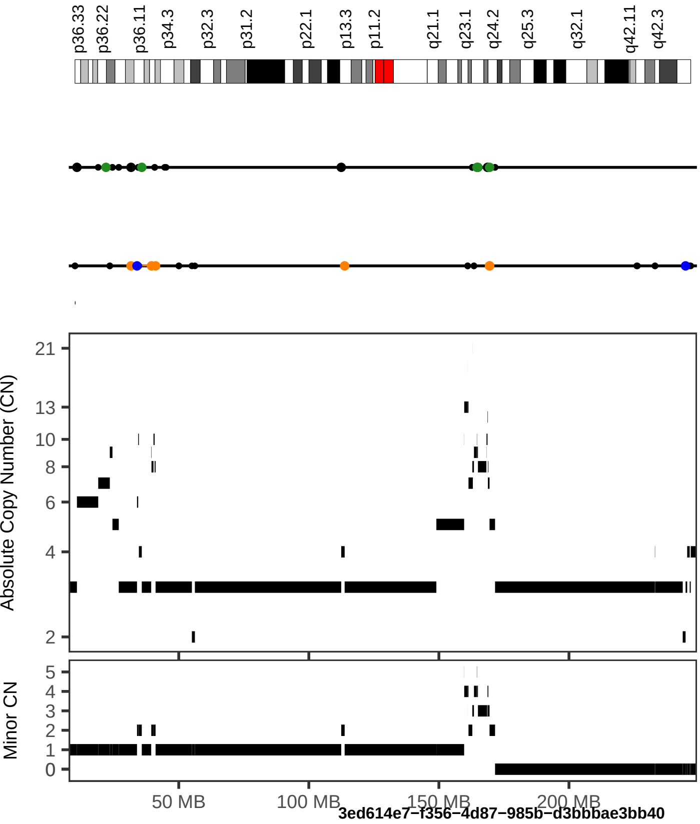

3ed614e7−f356−4d87−985b−d3bbbae3bb40

Cancer type	Bladder−TCC
Position	1:19030071−246820847
Interleaved intrachr. SVs	1
Total SVs (intrachr. + transl.)	27
SV types	DEL: 4; DUP: 2; h2hINV: 2; t2tINV: 3; TRA: 16
SVs in sample	449
Oscillating CN (2 and 3 states)	4, 8
CN segments	45
FDR fragment joints	0.6685091
FDR chr. breakp. enrich.	0.81
Linked to chrs	16:6669849−31591596;2:14647349−206284284
Purity, ploidy	0.74, 3.29

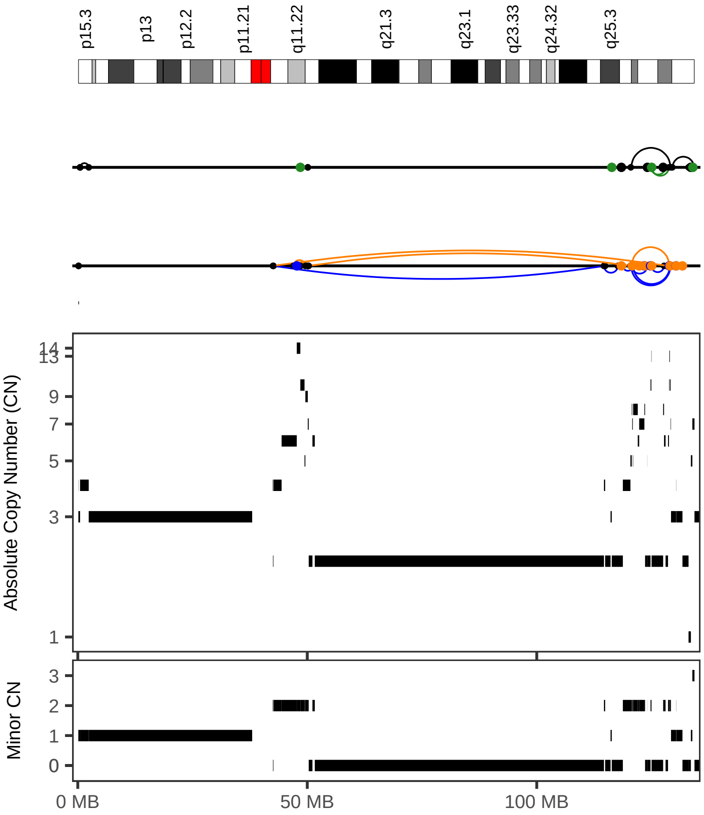

3ed614e7−f356−4d87−985b−d3bbbae3bb40

Cancer type	Bladder−TCC
Position	10:42525573−129255365
Interleaved intrachr. SVs	13
Total SVs (intrachr. + transl.)	29
SV types	DEL: 3; DUP: 7; h2hINV: 1; t2tINV: 2; TRA: 16
SVs in sample	449
Oscillating CN (2 and 3 states)	3, 6
CN segments	39
FDR fragment joints	0.615458
FDR chr. breakp. enrich.	0
Linked to chrs	
Purity, ploidy	0.74, 3.29

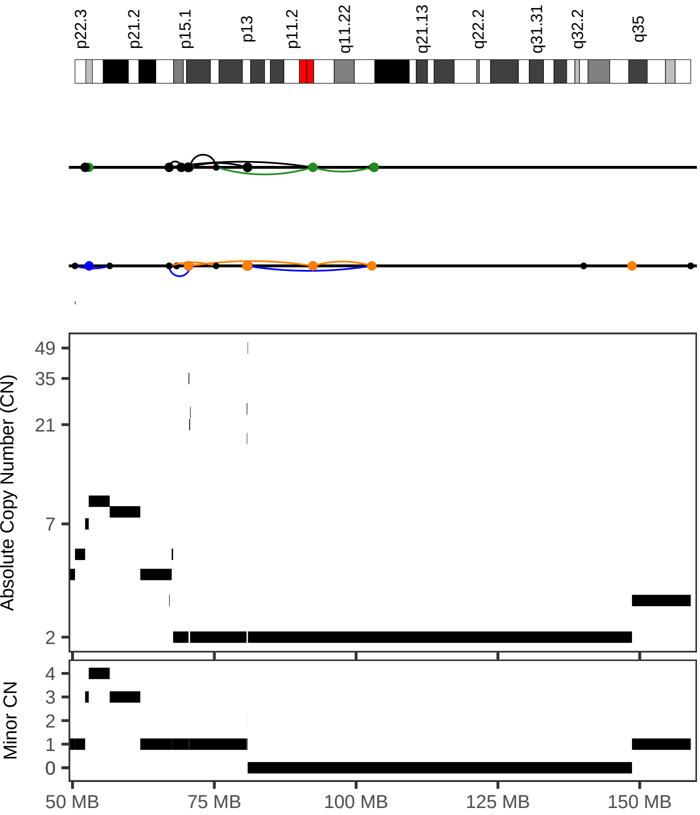

904a8757−e0c5−41ef−b583−c8f170caaac1

Cancer type	Bladder−TCC
Position	7:67027597−102763105
Interleaved intrachr. SVs	11
Total SVs (intrachr. + transl.)	42
SV types	DEL: 3; DUP: 2; h2hINV: 4; t2tINV: 2; TRA: 31
SVs in sample	274
Oscillating CN (2 and 3 states)	3, 5
CN segments	15
FDR fragment joints	0.8653243
FDR chr. breakp. enrich.	0
Linked to chrs	
Purity, ploidy	0.6, 2.92

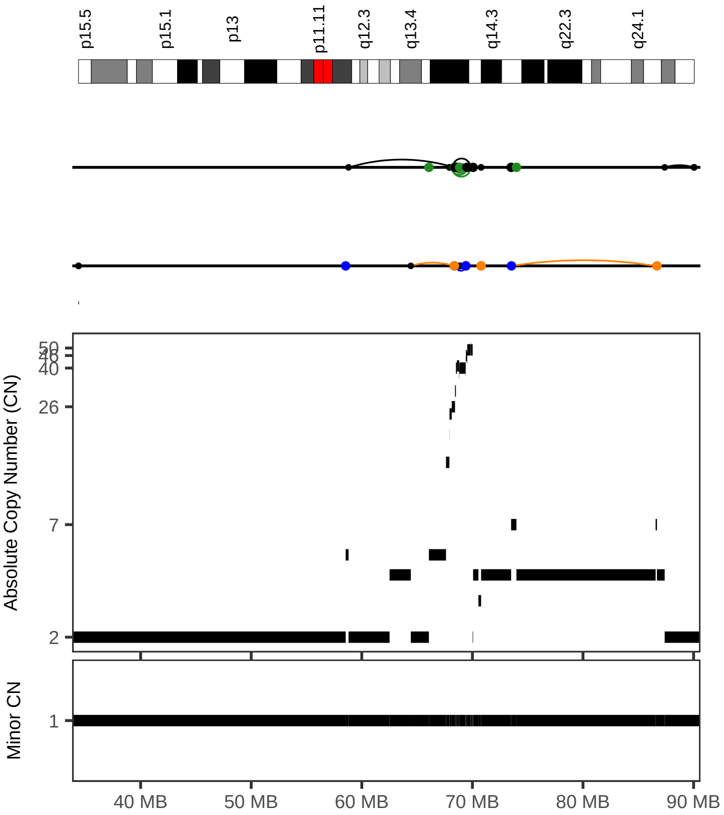

9c274536−3ca1−4f0e−93a8−1688074d862f

Cancer type	Bladder−TCC
Position	11:58799118−69868742
Interleaved intrachr. SVs	20
Total SVs (intrachr. + transl.)	30
SV types	DEL: 4; DUP: 2; h2hINV: 6; t2tINV: 8; TRA: 10
SVs in sample	270
Oscillating CN (2 and 3 states)	3, 4
CN segments	21
FDR fragment joints	0.615458
FDR chr. breakp. enrich.	0
Linked to chrs	17:11629450−33500975;
Purity, ploidy	0.71, 3.43

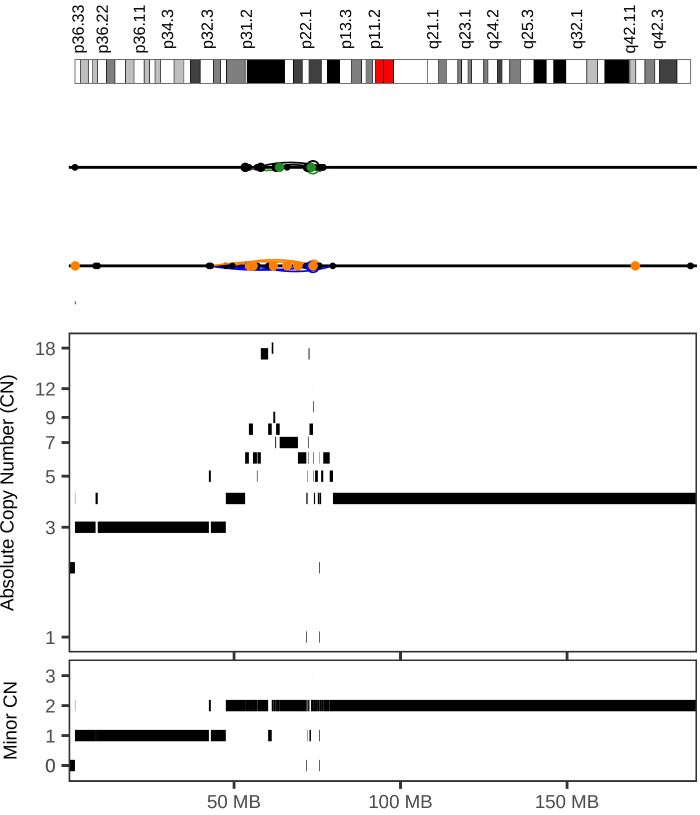

CGP_donor_1397083

Cancer type	Bone−Osteosarc
Position	1:42469204−79649615
Interleaved intrachr. SVs	16
Total SVs (intrachr. + transl.)	32
SV types	DEL: 4; DUP: 4; h2hINV: 5; t2tINV: 3; TRA: 16
SVs in sample	142
Oscillating CN (2 and 3 states)	3, 6
CN segments	43
FDR fragment joints	0.9224215
FDR chr. breakp. enrich.	0
Linked to chrs	13:101502303−104764633;
Purity, ploidy	0.57, 3.02

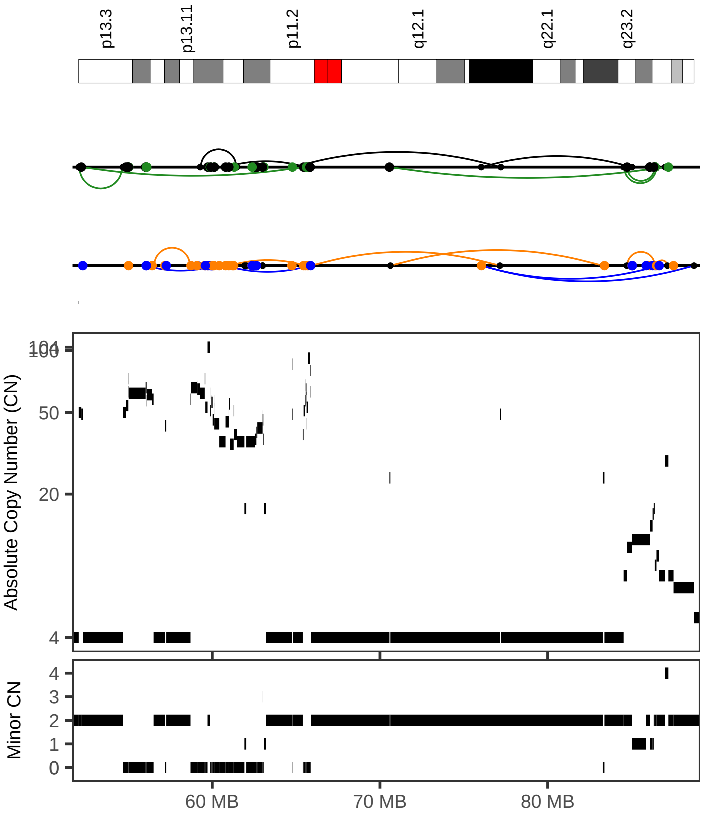

CGP_donor_1437406

Cancer type	Bone−Osteosarc
Position	16:52040498−88719044
Interleaved intrachr. SVs	21
Total SVs (intrachr. + transl.)	108
SV types	DEL: 5; DUP: 5; h2hINV: 4; t2tINV: 7; TRA: 87
SVs in sample	871
Oscillating CN (2 and 3 states)	3, 4
CN segments	91
FDR fragment joints	0.615458
FDR chr. breakp. enrich.	0
Linked to chrs	5:55072−32110729;
Purity, ploidy	0.52, 4.48

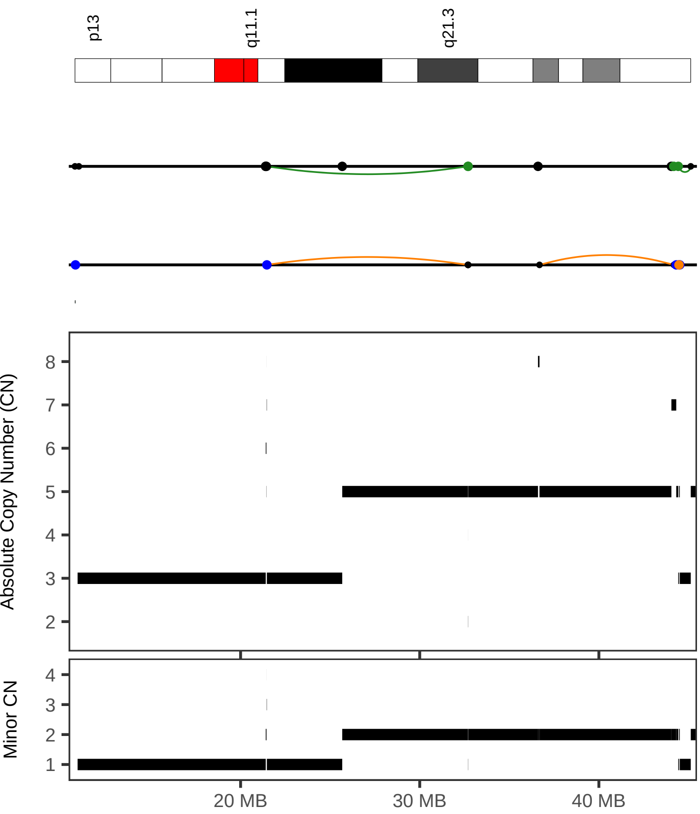

CGP_donor_1437406

Cancer type	Bone−Osteosarc
Position	21:10753819−45129141
Interleaved intrachr. SVs	1
Total SVs (intrachr. + transl.)	21
SV types	DEL: 2; DUP: 0; h2hINV: 1; t2tINV: 2; TRA: 16
SVs in sample	871
Oscillating CN (2 and 3 states)	5, 6
CN segments	19
FDR fragment joints	0.6776251
FDR chr. breakp. enrich.	0
Linked to chrs	
Purity, ploidy	0.52, 4.48

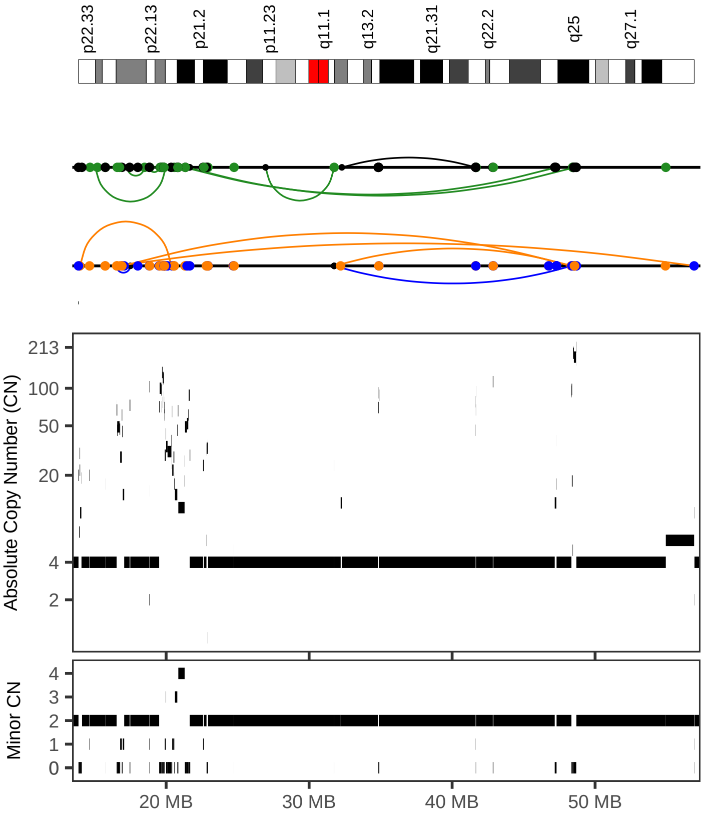

CGP_donor_1437406

Cancer type	Bone−Osteosarc
Position	X:13871047−56933399
Interleaved intrachr. SVs	16
Total SVs (intrachr. + transl.)	150
SV types	DEL: 7; DUP: 3; h2hINV: 1; t2tINV: 5; TRA: 134
SVs in sample	871
Oscillating CN (2 and 3 states)	3, 5
CN segments	113
FDR fragment joints	0.615458
FDR chr. breakp. enrich.	0
Linked to chrs	5:55072−32110729;
Purity, ploidy	0.52, 4.48

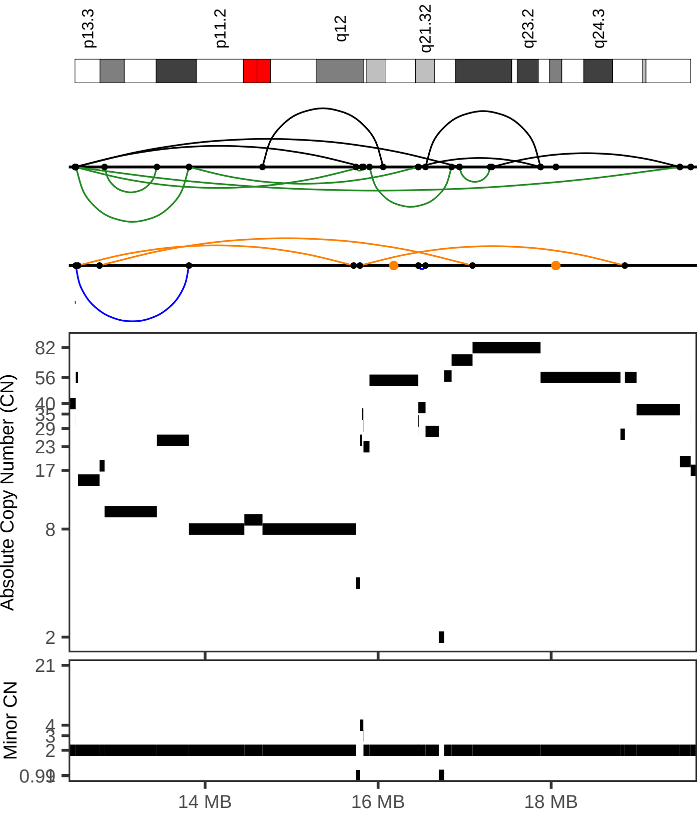

	**CGP_donor_1437409**
Cancer type	Bone−Osteosarc
Position	17:12496853−19488850
Interleaved intrachr. SVs	18
Total SVs (intrachr. + transl.)	20
SV types	DEL: 3; DUP: 2; h2hINV: 6; t2tINV: 7; TRA: 2
SVs in sample	118
Oscillating CN (2 and 3 states)	3, 4
CN segments	29
FDR fragment joints	0.662962
FDR chr. breakp. enrich.	0
Linked to chrs	
Purity, ploidy	0.67, 3.41

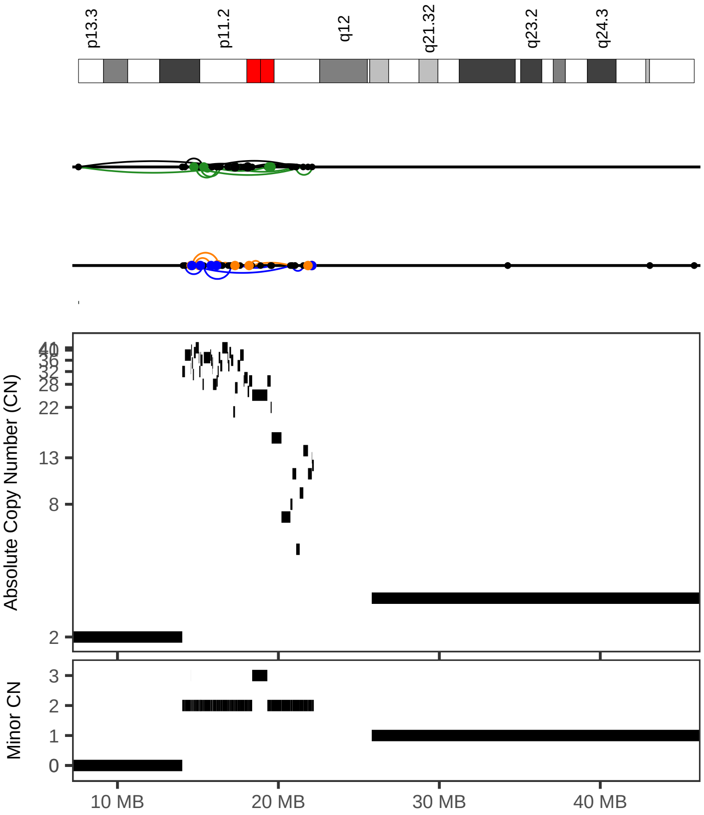

	**CGP_donor_1475256**
Cancer type	Bone−Osteosarc
Position	17:7577437−22095440
Interleaved intrachr. SVs	33
Total SVs (intrachr. + transl.)	51
SV types	DEL: 11; DUP: 5; h2hINV: 8; t2tINV: 9; TRA: 18
SVs in sample	217
Oscillating CN (2 and 3 states)	3, 5
CN segments	54
FDR fragment joints	0.6776251
FDR chr. breakp. enrich.	0
Linked to chrs	
Purity, ploidy	0.79, 3.21

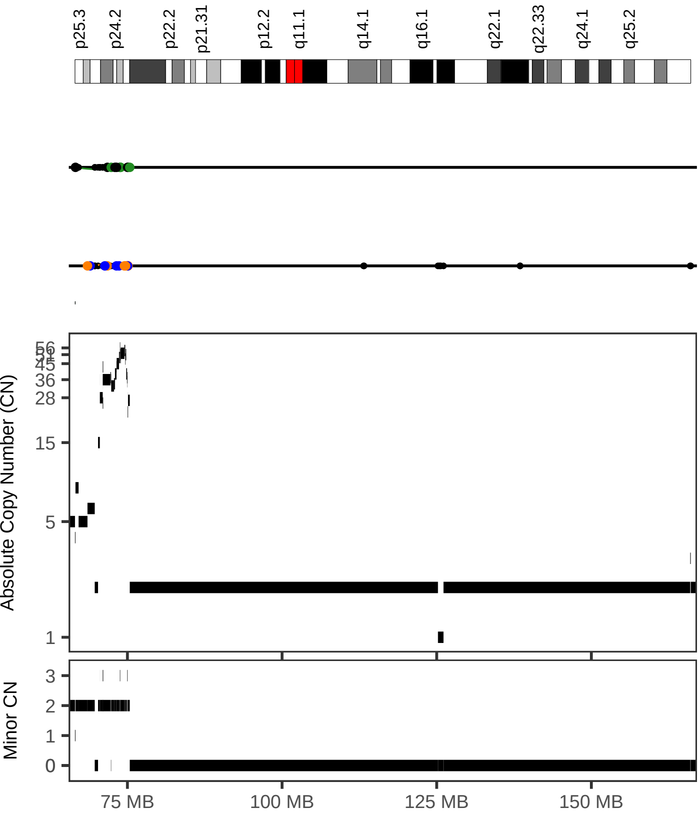

	**CGP_donor_1528371**
Cancer type	Bone−Osteosarc
Position	6:66519078−75032715
Interleaved intrachr. SVs	11
Total SVs (intrachr. + transl.)	30
SV types	DEL: 2; DUP: 3; h2hINV: 4; t2tINV: 2; TRA: 19
SVs in sample	349
Oscillating CN (2 and 3 states)	3, 4
CN segments	37
FDR fragment joints	0.9987819
FDR chr. breakp. enrich.	0
Linked to chrs	13:103350092−106906739;
Purity, ploidy	0.33, 3.49

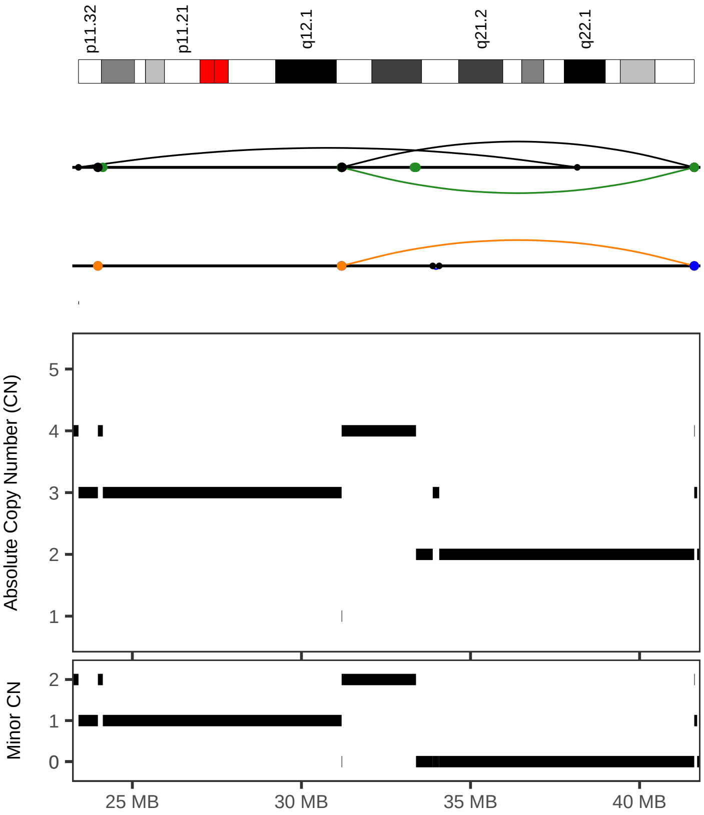

	**CGP_donor_1528371**
Cancer type	Bone−Osteosarc
Position	18:23406391−41615950
Interleaved intrachr. SVs	4
Total SVs (intrachr. + transl.)	31
SV types	DEL: 1; DUP: 0; h2hINV: 2; t2tINV: 1; TRA: 27
SVs in sample	349
Oscillating CN (2 and 3 states)	3, 8
CN segments	12
FDR fragment joints	0.930656
FDR chr. breakp. enrich.	0
Linked to chrs	13:103350092−106906739;
Purity, ploidy	0.33, 3.49

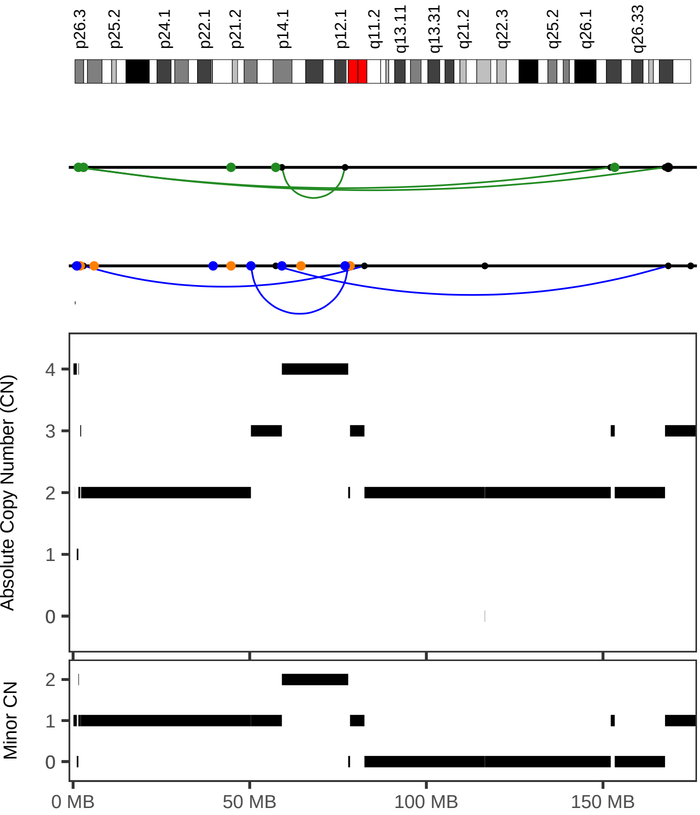

CGP_donor_1691207

Cancer type	Bone−Osteosarc
Position	3:1488486−168477786
Interleaved intrachr. SVs	5
Total SVs (intrachr. + transl.)	20
SV types	DEL: 0; DUP: 3; h2hINV: 0; t2tINV: 2; TRA: 15
SVs in sample	415
Oscillating CN (2 and 3 states)	4, 6
CN segments	15
FDR fragment joints	0.7735152
FDR chr. breakp. enrich.	0.84
Linked to chrs	2:5332159−125723075;
Purity, ploidy	0.71, 2.58

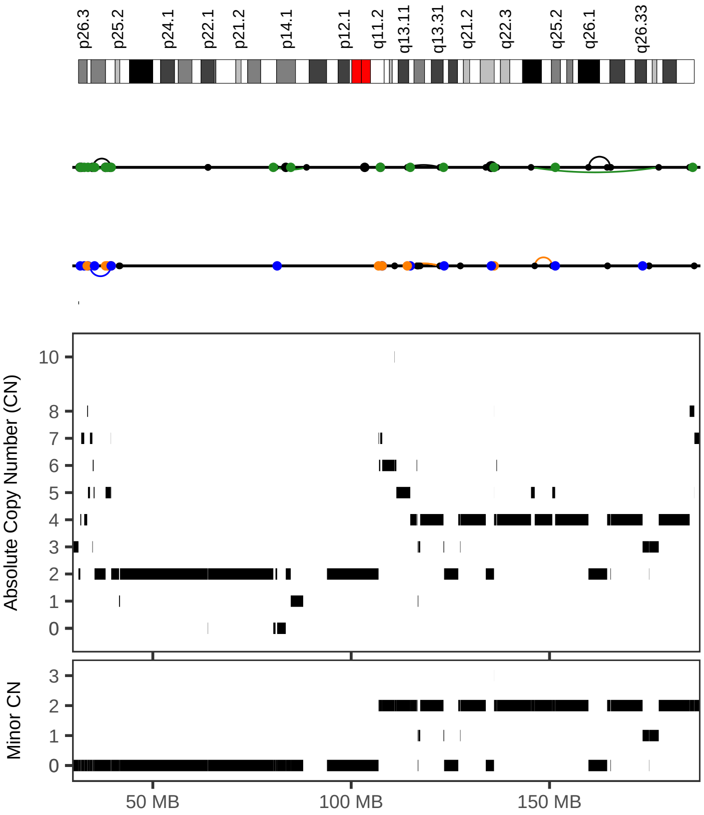

CGP_donor_1691211

Cancer type	Bone−Osteosarc
Position	3:31257334−39452574
Interleaved intrachr. SVs	3
Total SVs (intrachr. + transl.)	24
SV types	DEL: 0; DUP: 1; h2hINV: 1; t2tINV: 1; TRA: 21
SVs in sample	606
Oscillating CN (2 and 3 states)	3, 6
CN segments	14
FDR fragment joints	0.6776251
FDR chr. breakp. enrich.	0
Linked to chrs	16:6326850−23636185;
Purity, ploidy	0.62, 3.16

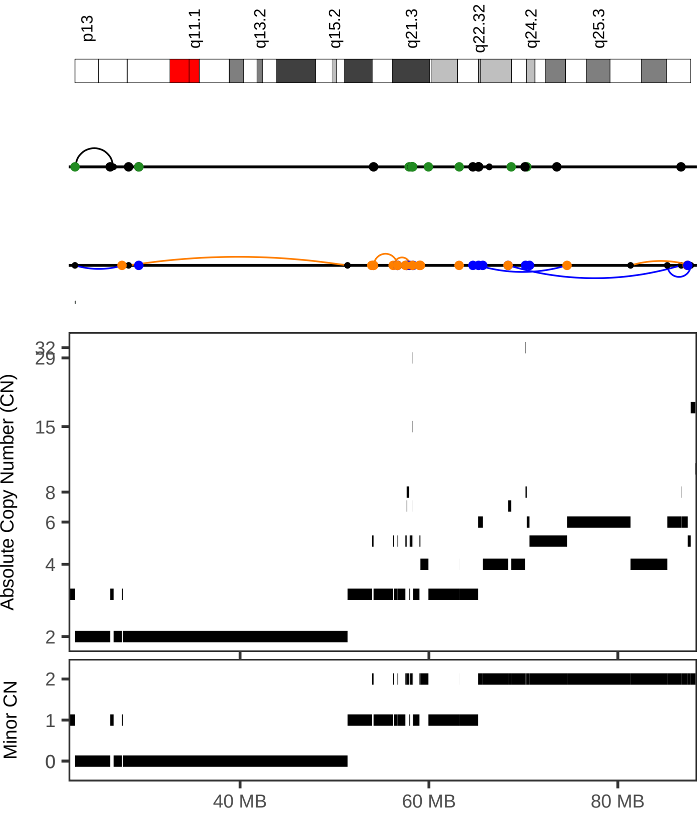

CGP_donor_1691213

Cancer type	Bone−Osteosarc
Position	15:65203302−87715328
Interleaved intrachr. SVs	4
Total SVs (intrachr. + transl.)	20
SV types	DEL: 1; DUP: 3; h2hINV: 0; t2tINV: 0; TRA: 16
SVs in sample	596
Oscillating CN (2 and 3 states)	3, 5
CN segments	15
FDR fragment joints	0.615458
FDR chr. breakp. enrich.	0
Linked to chrs	12:38831735−119704558;
Purity, ploidy	0.87, 3.53

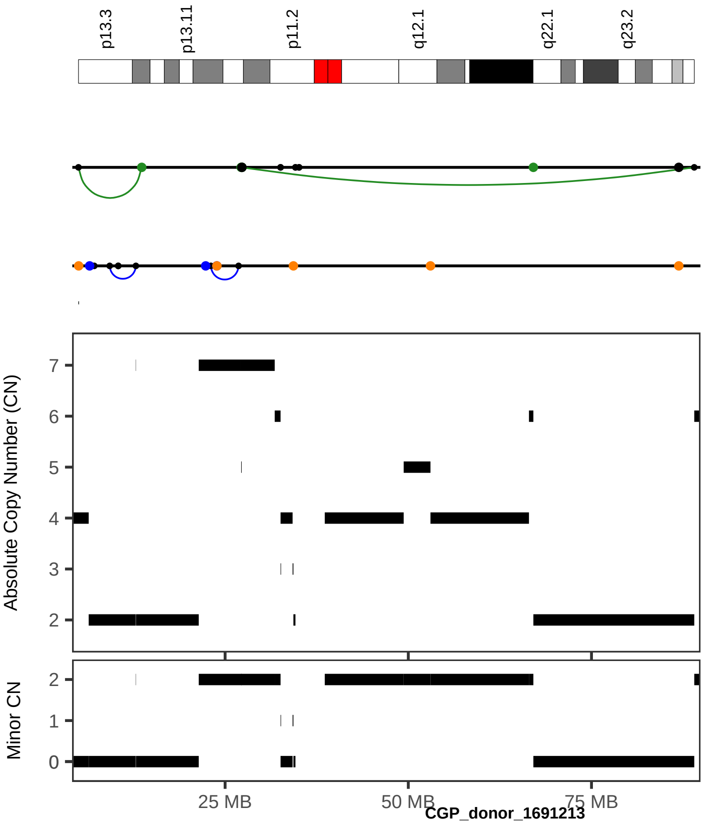

CGP_donor_1691213

Cancer type	Bone−Osteosarc
Position	16:4989225−89024640
Interleaved intrachr. SVs	1
Total SVs (intrachr. + transl.)	20
SV types	DEL: 1; DUP: 2; h2hINV: 0; t2tINV: 2; TRA: 15
SVs in sample	596
Oscillating CN (2 and 3 states)	4, 4
CN segments	18
FDR fragment joints	0.9625775
FDR chr. breakp. enrich.	0.17
Linked to chrs	12:38831735−119704558;8:98131760−131862130
Purity, ploidy	0.87, 3.53

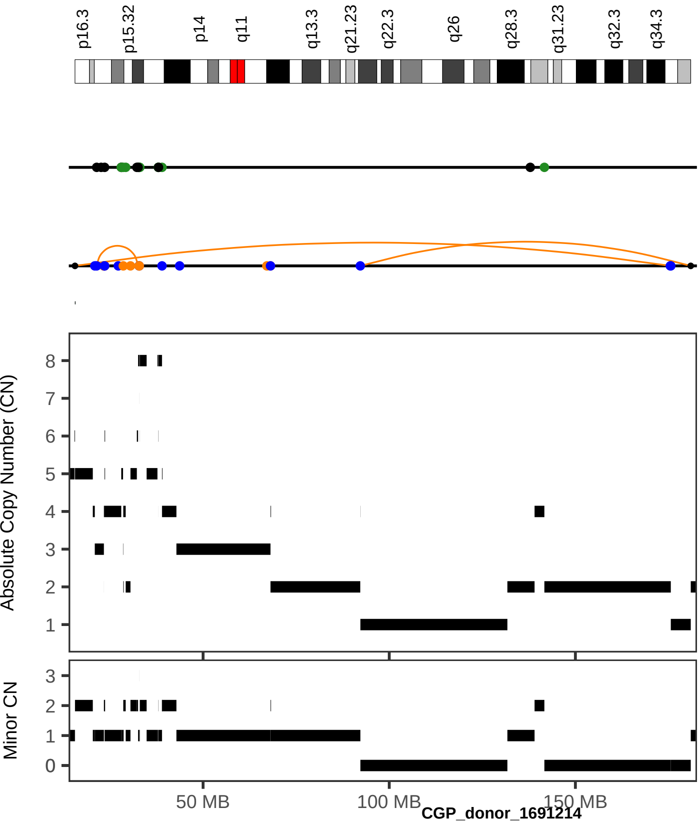

CGP_donor_1691214

Cancer type	Bone−Osteosarc
Position	4:15590720−180978684
Interleaved intrachr. SVs	2
Total SVs (intrachr. + transl.)	43
SV types	DEL: 2; DUP: 0; h2hINV: 0; t2tINV: 0; TRA: 41
SVs in sample	652
Oscillating CN (2 and 3 states)	4, 8
CN segments	39
FDR fragment joints	0.9077541
FDR chr. breakp. enrich.	0.4
Linked to chrs	2:11400−232123608;3:29117586−189128944 X:48348455−150018346;
Purity, ploidy	0.49, 3.33

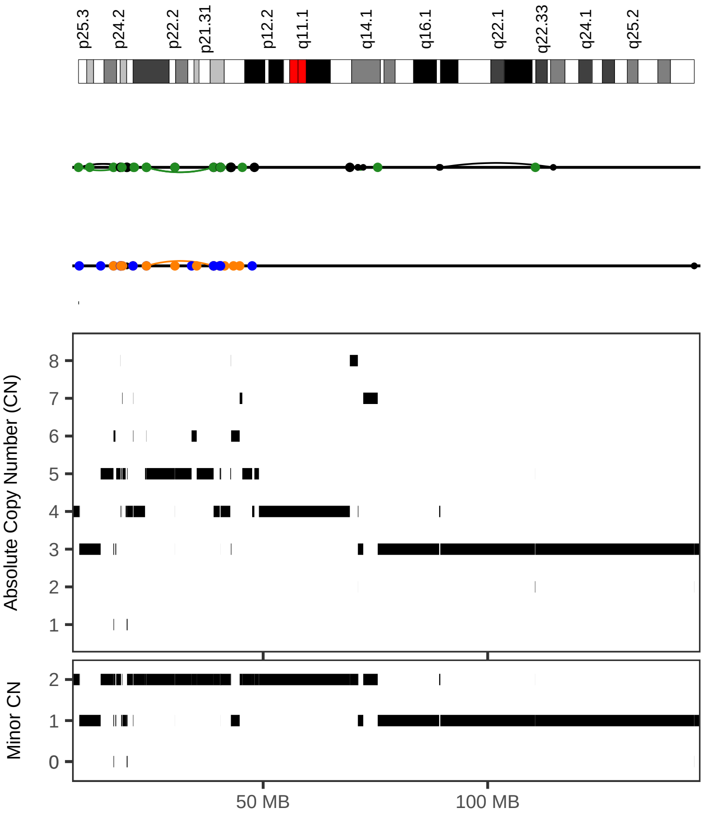

CGP_donor_1691214

Cancer type	Bone−Osteosarc
Position	6:9055767−19690465
Interleaved intrachr. SVs	4
Total SVs (intrachr. + transl.)	21
SV types	DEL: 0; DUP: 1; h2hINV: 2; t2tINV: 1; TRA: 17
SVs in sample	652
Oscillating CN (2 and 3 states)	4, 6
CN segments	23
FDR fragment joints	0.7568568
FDR chr. breakp. enrich.	0
Linked to chrs	X:48348455−150018346;
Purity, ploidy	0.49, 3.33

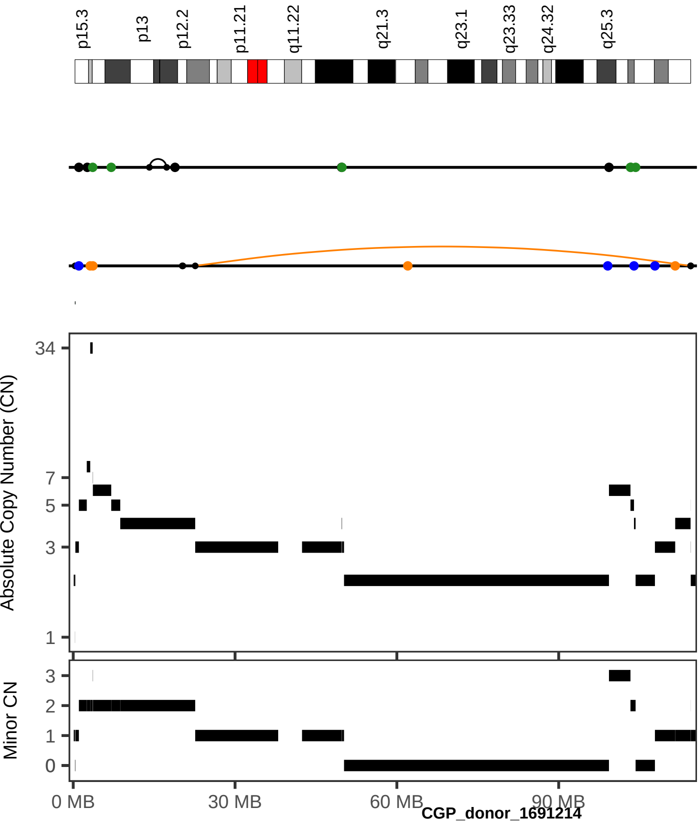

CGP_donor_1691214

Cancer type	Bone−Osteosarc
Position	10:325730−114451376
Interleaved intrachr. SVs	1
Total SVs (intrachr. + transl.)	23
SV types	DEL: 3; DUP: 0; h2hINV: 1; t2tINV: 0; TRA: 19
SVs in sample	652
Oscillating CN (2 and 3 states)	3, 5
CN segments	22
FDR fragment joints	0.8640728
FDR chr. breakp. enrich.	0.93
Linked to chrs	18:5480934−11055616;X:48348455−150018346
Purity, ploidy	0.49, 3.33

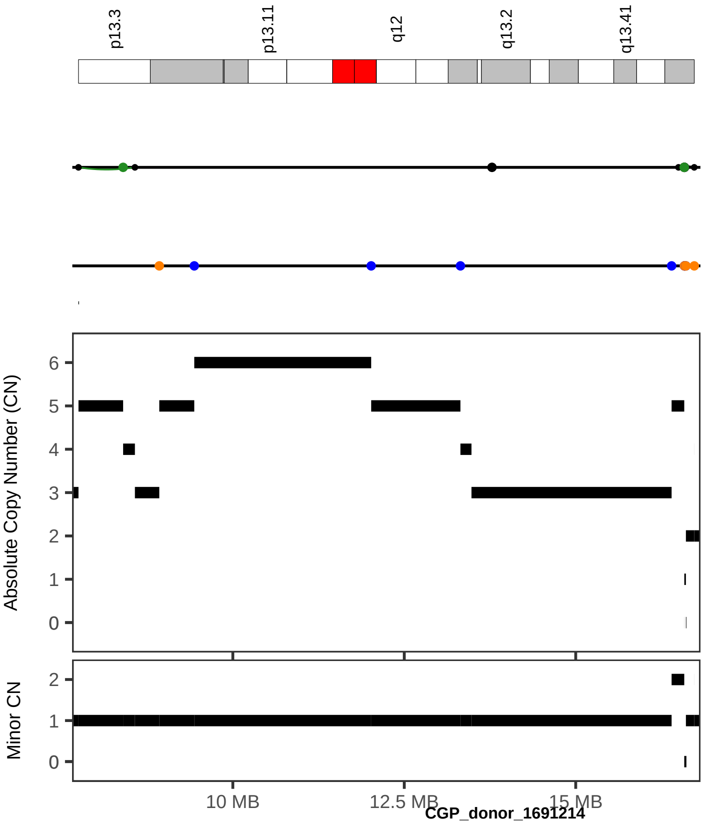

CGP_donor_1691214

Cancer type	Bone−Osteosarc
Position	19:7749343−16728218
Interleaved intrachr. SVs	1
Total SVs (intrachr. + transl.)	21
SV types	DEL: 0; DUP: 0; h2hINV: 1; t2tINV: 1; TRA: 19
SVs in sample	652
Oscillating CN (2 and 3 states)	3, 4
CN segments	15
FDR fragment joints	0.6776251
FDR chr. breakp. enrich.	0.01
Linked to chrs	1:155935299−166050472;3:29117586−189128944 X:48348455−150018346;
Purity, ploidy	0.49, 3.33

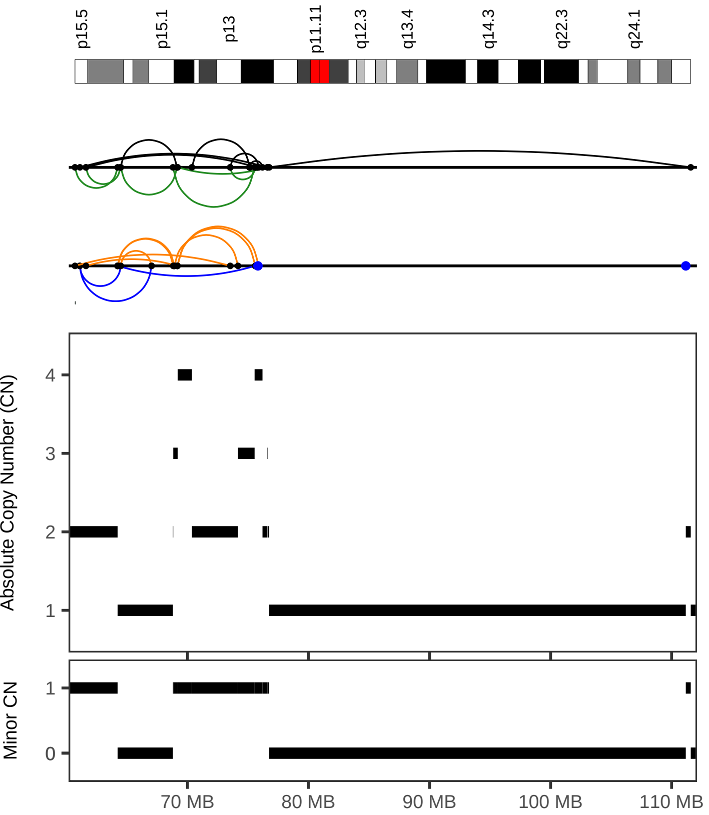

CGP_donor_1234122

Cancer type	Breast−AdenoCA
Position	11:60702782−76620693
Interleaved intrachr. SVs	26
Total SVs (intrachr. + transl.)	27
SV types	DEL: 9; DUP: 3; h2hINV: 6; t2tINV: 8; TRA: 1
SVs in sample	83
Oscillating CN (2 and 3 states)	3, 5
CN segments	11
FDR fragment joints	0.6776251
FDR chr. breakp. enrich.	0
Linked to chrs	
Purity, ploidy	0.7, 1.77

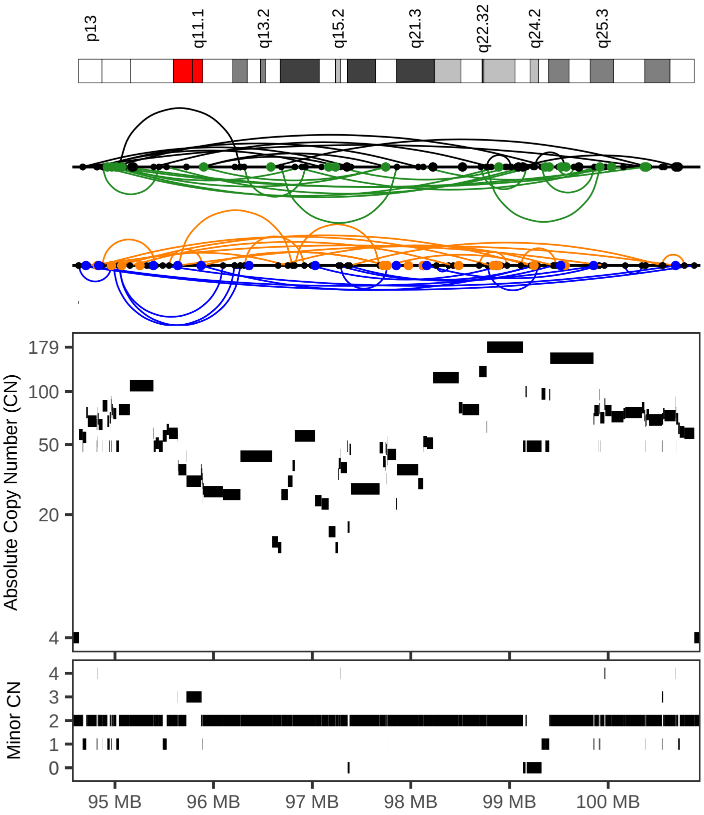

CGP_donor_1337223

Cancer type	Breast−AdenoCA
Position	15:94636392−100772414
Interleaved intrachr. SVs	67
Total SVs (intrachr. + transl.)	127
SV types	DEL: 19; DUP: 14; h2hINV: 14; t2tINV: 20; TRA: 60
SVs in sample	318
Oscillating CN (2 and 3 states)	3, 4
CN segments	134
FDR fragment joints	0.957647
FDR chr. breakp. enrich.	0
Linked to chrs	1:45555836−239312458;
Purity, ploidy	0.48, 3.45

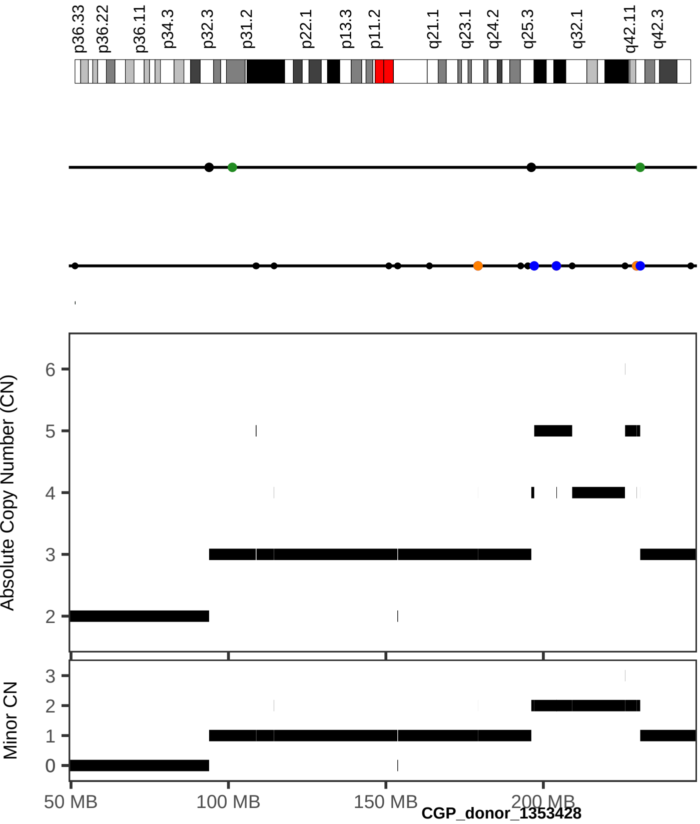

CGP_donor_1353428

Cancer type	Breast−AdenoCA
Position	1:51262983−230781288
Interleaved intrachr. SVs	1
Total SVs (intrachr. + transl.)	21
SV types	DEL: 3; DUP: 5; h2hINV: 1; t2tINV: 0; TRA: 12
SVs in sample	266
Oscillating CN (2 and 3 states)	5, 12
CN segments	21
FDR fragment joints	0.5435077
FDR chr. breakp. enrich.	0.2
Linked to chrs	11:3924104−111750455;4:656520−175209865 X:6630360−122028302;
Purity, ploidy	0.7, 2.52

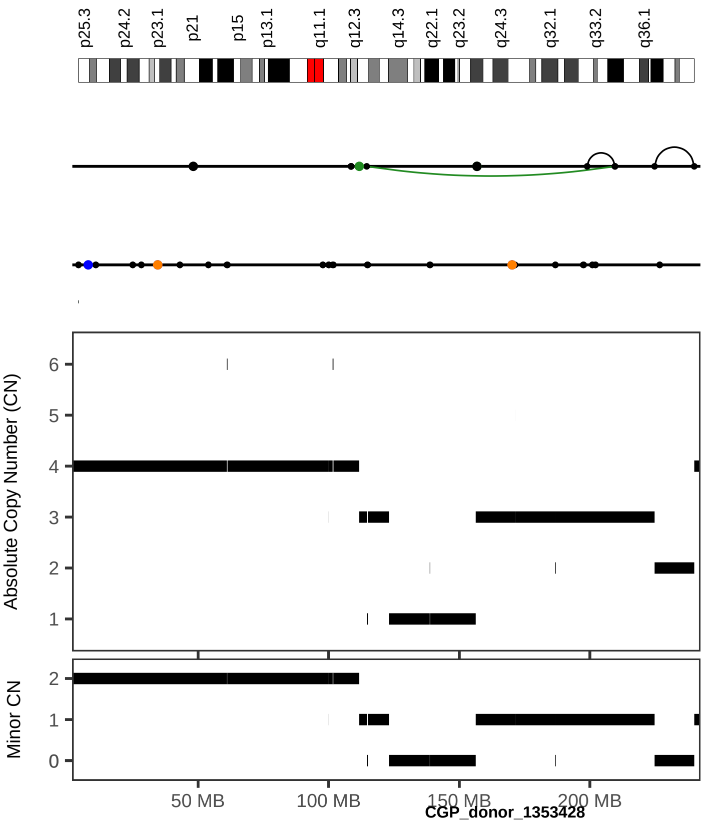

CGP_donor_1353428

Cancer type	Breast−AdenoCA
Position	2:4213463−239950315
Interleaved intrachr. SVs	1
Total SVs (intrachr. + transl.)	25
SV types	DEL: 6; DUP: 7; h2hINV: 2; t2tINV: 1; TRA: 9
SVs in sample	266
Oscillating CN (2 and 3 states)	4, 11
CN segments	20
FDR fragment joints	0.615458
FDR chr. breakp. enrich.	0.15
Linked to chrs	1:51262983−230781287;3:11132192−187999427
Purity, ploidy	0.7, 2.52

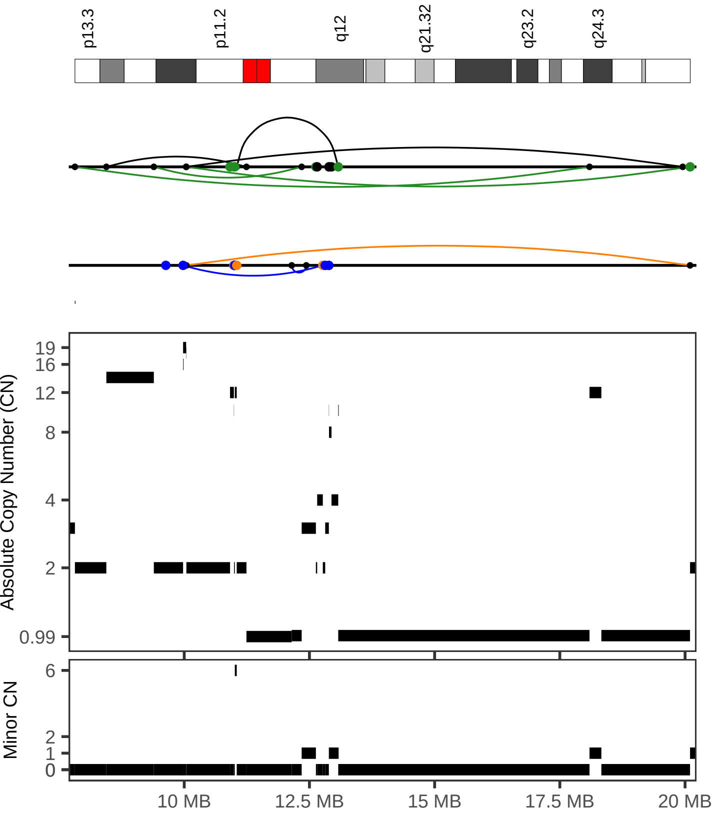

CGP_donor_1397260

Cancer type	Breast−AdenoCA
Position	17:7817380−20103793
Interleaved intrachr. SVs	9
Total SVs (intrachr. + transl.)	31
SV types	DEL: 1; DUP: 2; h2hINV: 3; t2tINV: 3; TRA: 22
SVs in sample	160
Oscillating CN (2 and 3 states)	3, 4
CN segments	27
FDR fragment joints	0.6309559
FDR chr. breakp. enrich.	0
Linked to chrs	6:38510448−42541218;
Purity, ploidy	0.41, 3.03

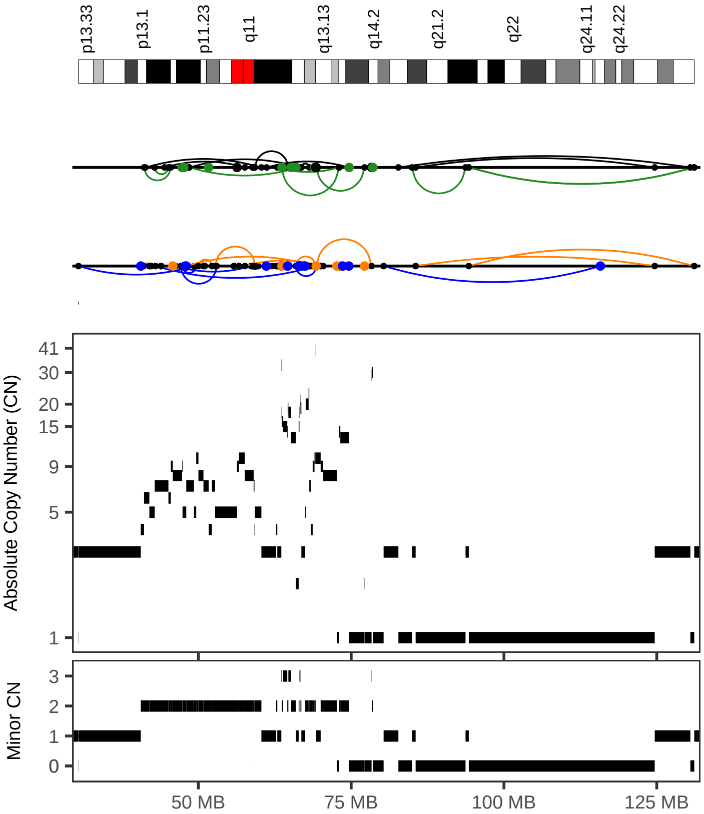

CGP_donor_1397262

Cancer type	Breast−AdenoCA
Position	12:30388748−78339897
Interleaved intrachr. SVs	27
Total SVs (intrachr. + transl.)	61
SV types	DEL: 8; DUP: 8; h2hINV: 5; t2tINV: 6; TRA: 34
SVs in sample	235
Oscillating CN (2 and 3 states)	3, 5
CN segments	64
FDR fragment joints	0.615458
FDR chr. breakp. enrich.	0
Linked to chrs	
Purity, ploidy	0.19, 3.34

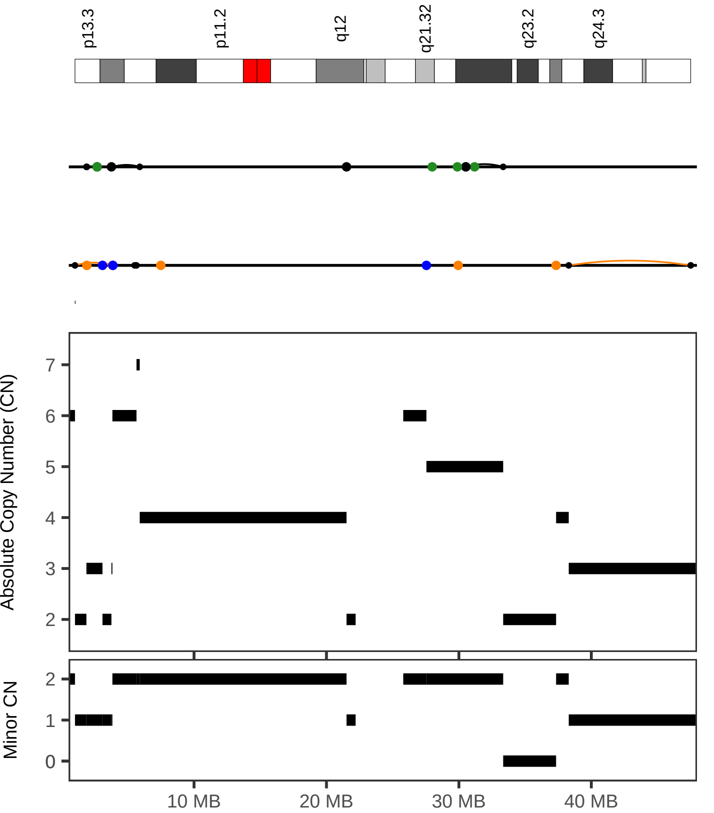

CGP_donor_1397262

Cancer type	Breast−AdenoCA
Position	17:1009336−47506989
Interleaved intrachr. SVs	1
Total SVs (intrachr. + transl.)	23
SV types	DEL: 3; DUP: 0; h2hINV: 2; t2tINV: 1; TRA: 17
SVs in sample	235
Oscillating CN (2 and 3 states)	4, 6
CN segments	13
FDR fragment joints	0.6776251
FDR chr. breakp. enrich.	0
Linked to chrs	
Purity, ploidy	0.19, 3.34

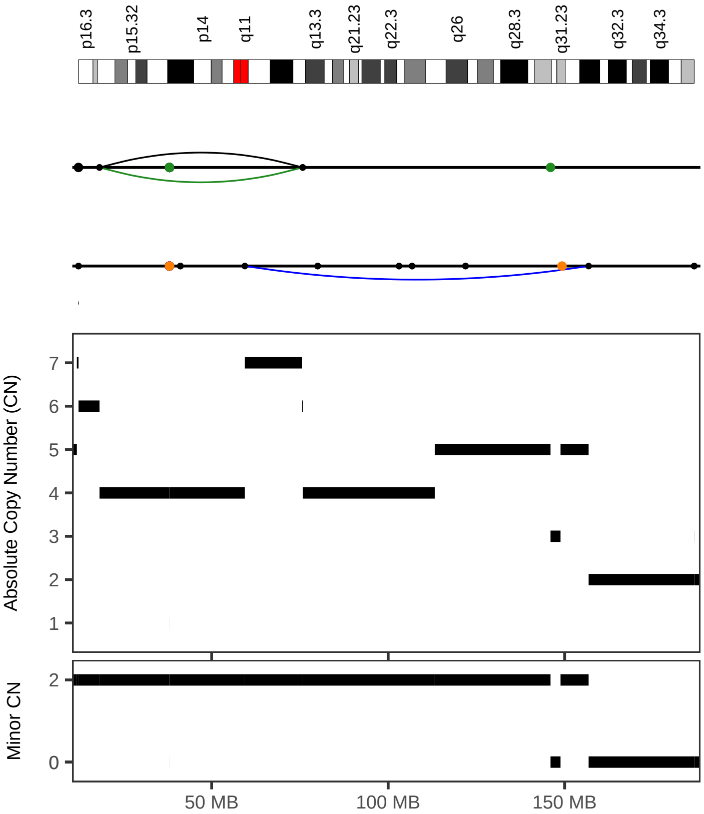

CGP_donor_1475200

Cancer type	Breast−AdenoCA
Position	4:18193195−156811790
Interleaved intrachr. SVs	3
Total SVs (intrachr. + transl.)	21
SV types	DEL: 0; DUP: 1; h2hINV: 1; t2tINV: 1; TRA: 18
SVs in sample	331
Oscillating CN (2 and 3 states)	3, 6
CN segments	10
FDR fragment joints	0.9462199
FDR chr. breakp. enrich.	0.1
Linked to chrs	
Purity, ploidy	0.6, 3.69

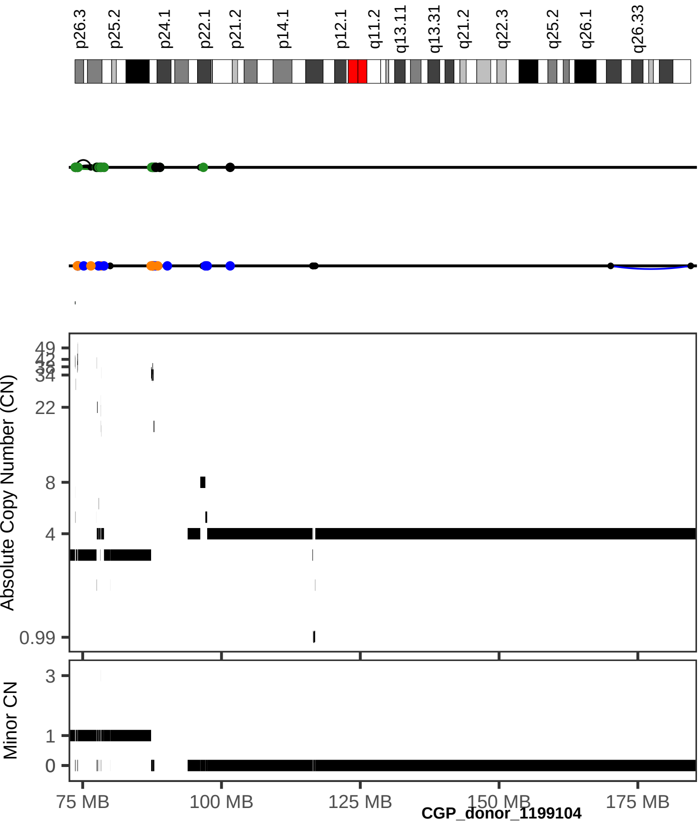

CGP_donor_1199104

Cancer type	Breast−AdenoCA
Position	3:73607514−78348970
Interleaved intrachr. SVs	16
Total SVs (intrachr. + transl.)	39
SV types	DEL: 1; DUP: 5; h2hINV: 6; t2tINV: 4; TRA: 23
SVs in sample	803
Oscillating CN (2 and 3 states)	3, 3
CN segments	31
FDR fragment joints	0.615458
FDR chr. breakp. enrich.	0
Linked to chrs	12:24474408−110217684;8:13736456−132773371
Purity, ploidy	0.53, 3.83

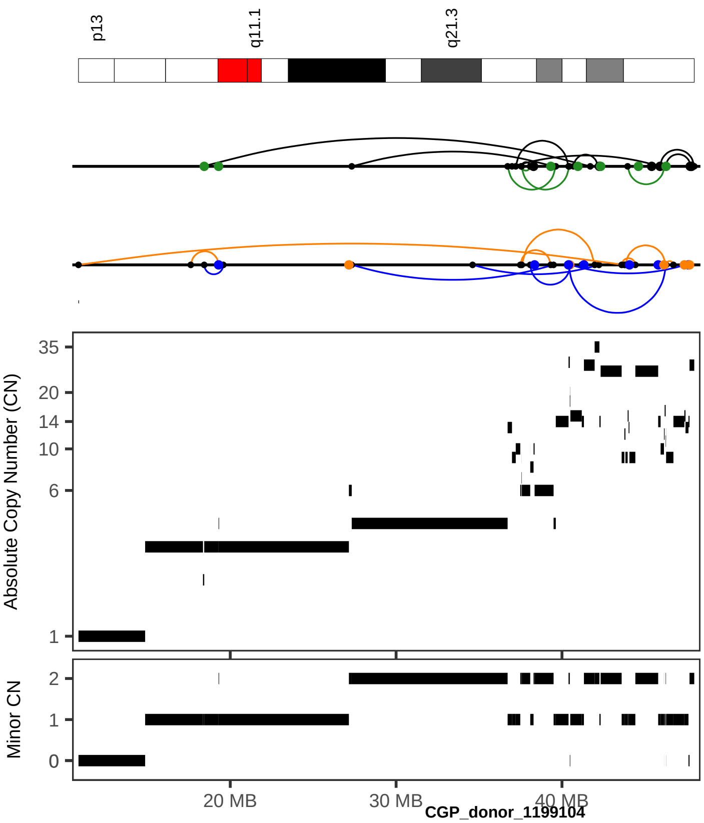

CGP_donor_1199104

Cancer type	Breast−AdenoCA
Position	21:10850512−47974458
Interleaved intrachr. SVs	25
Total SVs (intrachr. + transl.)	51
SV types	DEL: 7; DUP: 6; h2hINV: 8; t2tINV: 4; TRA: 26
SVs in sample	803
Oscillating CN (2 and 3 states)	3, 9
CN segments	52
FDR fragment joints	0.7983968
FDR chr. breakp. enrich.	0
Linked to chrs	6:88241210−163838907;8:13736456−132773371
Purity, ploidy	0.53, 3.83

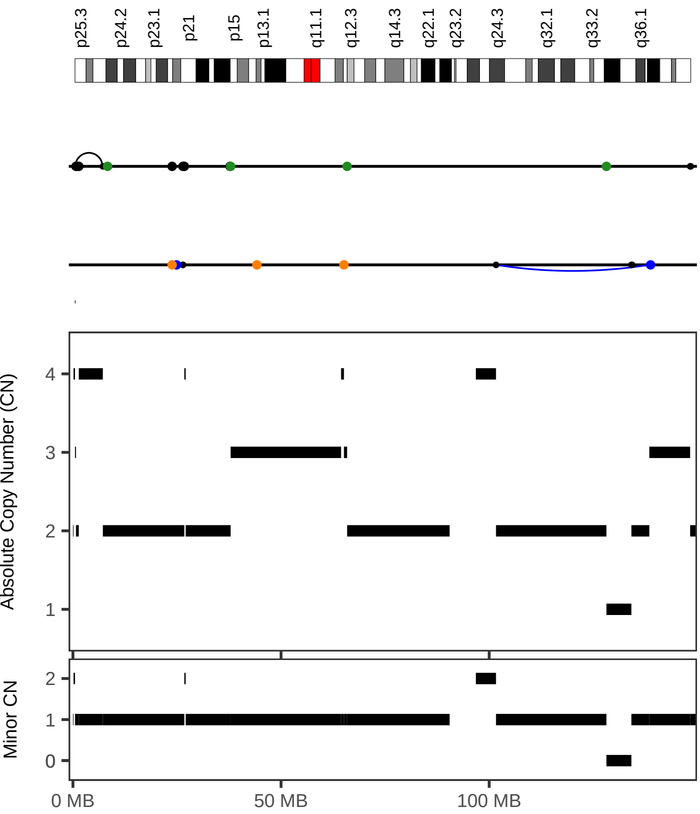

CGP_donor_1230722

Cancer type	Breast−AdenoCA
Position	2:494378−148426165
Interleaved intrachr. SVs	1
Total SVs (intrachr. + transl.)	20
SV types	DEL: 1; DUP: 2; h2hINV: 2; t2tINV: 0; TRA: 15
SVs in sample	192
Oscillating CN (2 and 3 states)	5, 7
CN segments	16
FDR fragment joints	0.9109641
FDR chr. breakp. enrich.	0.35
Linked to chrs	1:26522430−42300441;
Purity, ploidy	0.27, 2.38

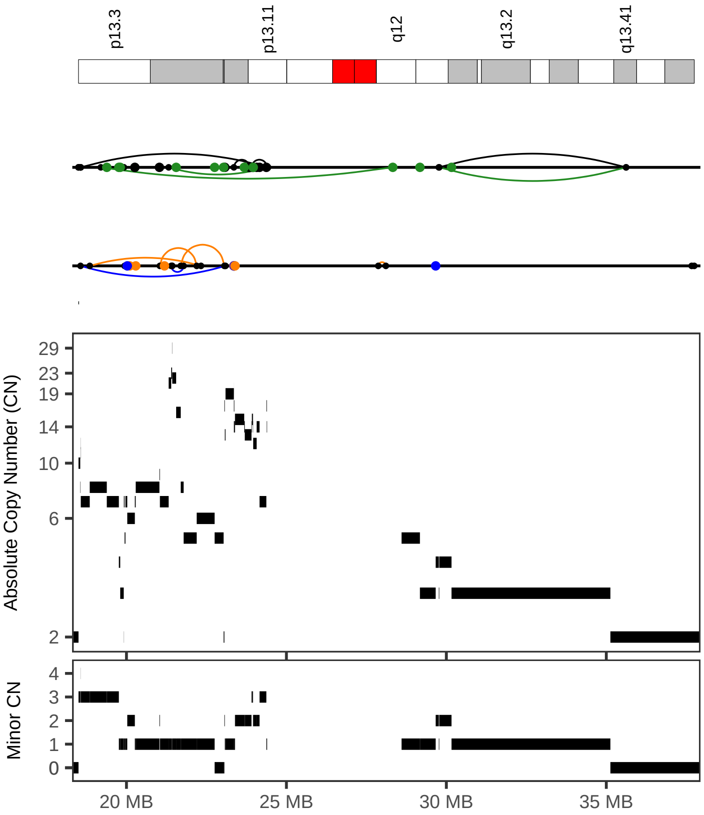

CGP_donor_1230728

Cancer type	Breast−AdenoCA
Position	19:18555351−28300401
Interleaved intrachr. SVs	10
Total SVs (intrachr. + transl.)	39
SV types	DEL: 3; DUP: 2; h2hINV: 3; t2tINV: 2; TRA: 29
SVs in sample	329
Oscillating CN (2 and 3 states)	3, 6
CN segments	47
FDR fragment joints	0.6776251
FDR chr. breakp. enrich.	0
Linked to chrs	
Purity, ploidy	0.27, 3.5

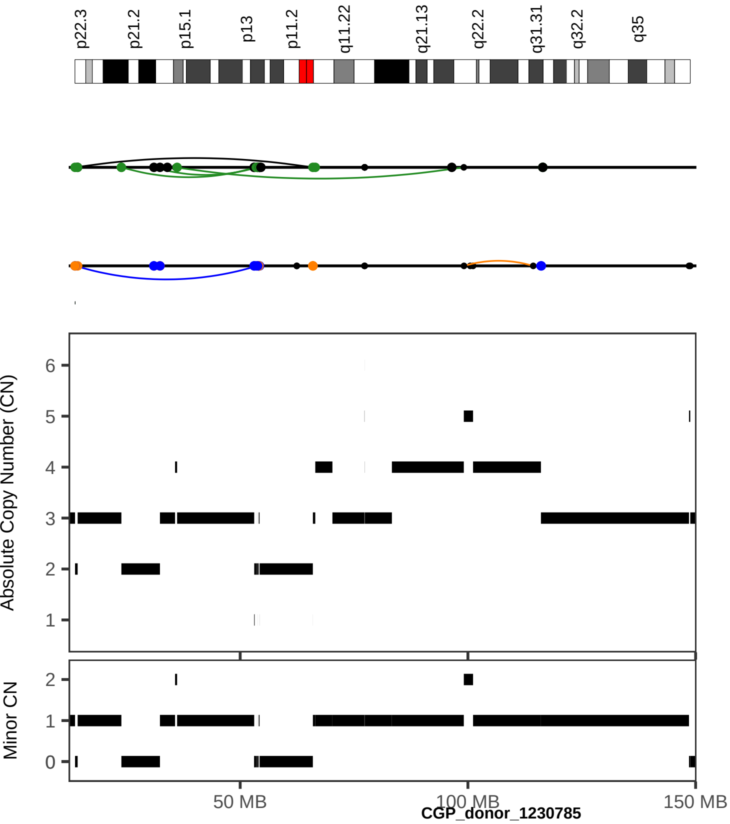

	**CGP_donor_1230785**
Cancer type	Breast−AdenoCA
Position	7:13714514−99103349
Interleaved intrachr. SVs	5
Total SVs (intrachr. + transl.)	49
SV types	DEL: 0; DUP: 1; h2hINV: 1; t2tINV: 3; TRA: 44
SVs in sample	502
Oscillating CN (2 and 3 states)	5, 10
CN segments	32
FDR fragment joints	0.6776251
FDR chr. breakp. enrich.	0
Linked to chrs	14:26989383−95106881;6:13316067−161857375
Purity, ploidy	0.35, 2.71

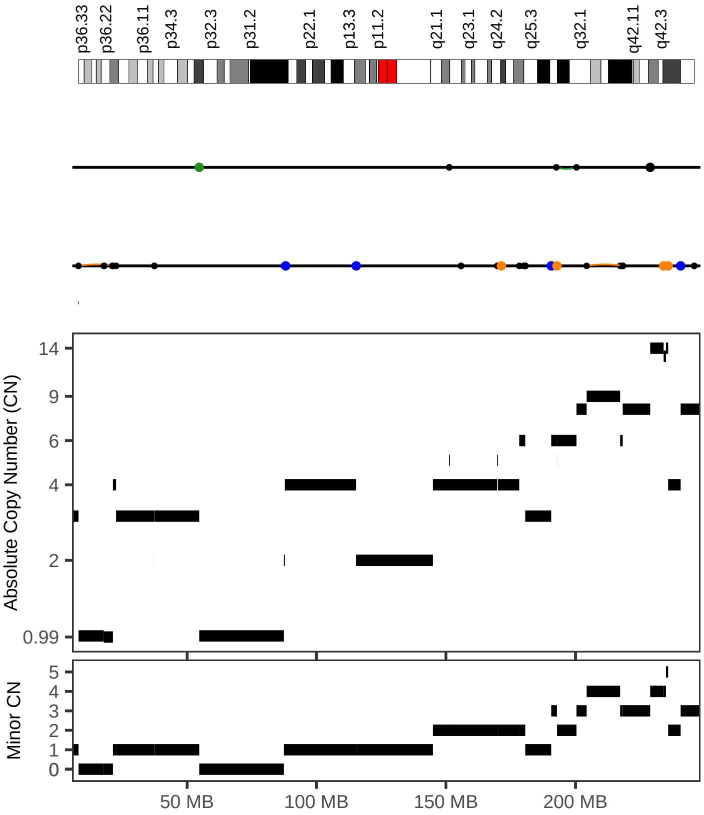

	**CGP_donor_1363963**
Cancer type	Breast−AdenoCA
Position	1:8061875−245884972
Interleaved intrachr. SVs	1
Total SVs (intrachr. + transl.)	23
SV types	DEL: 8; DUP: 4; h2hINV: 0; t2tINV: 1; TRA: 10
SVs in sample	324
Oscillating CN (2 and 3 states)	5, 9
CN segments	29
FDR fragment joints	0.1301856
FDR chr. breakp. enrich.	1
Linked to chrs	
Purity, ploidy	0.18, 2.98

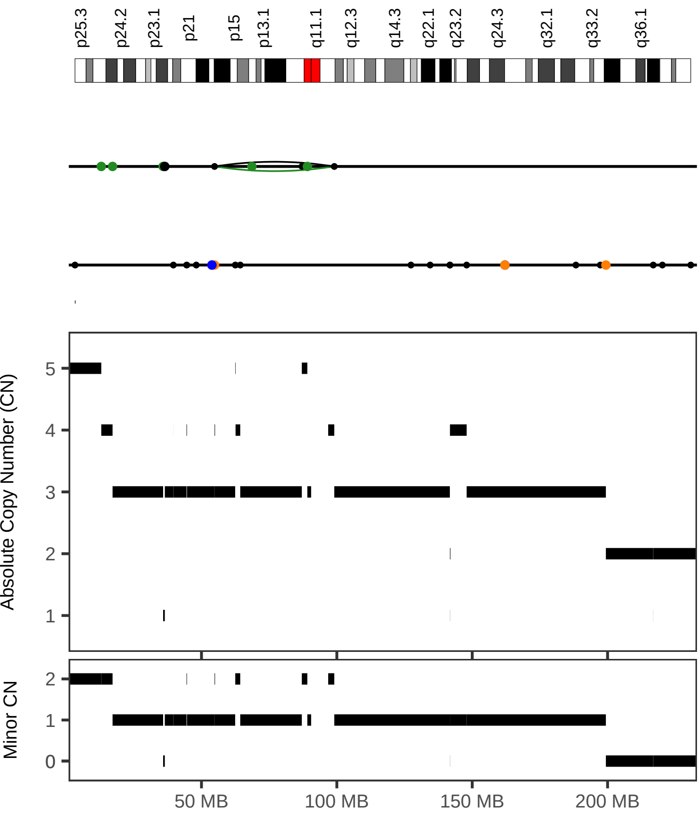

	**CGP_donor_1363965**
Cancer type	Breast−AdenoCA
Position	2:39603518−216873053
Interleaved intrachr. SVs	1
Total SVs (intrachr. + transl.)	20
SV types	DEL: 5; DUP: 4; h2hINV: 1; t2tINV: 2; TRA: 8
SVs in sample	330
Oscillating CN (2 and 3 states)	6, 8
CN segments	20
FDR fragment joints	0.5435077
FDR chr. breakp. enrich.	0.37
Linked to chrs	
Purity, ploidy	0.28, 2.9

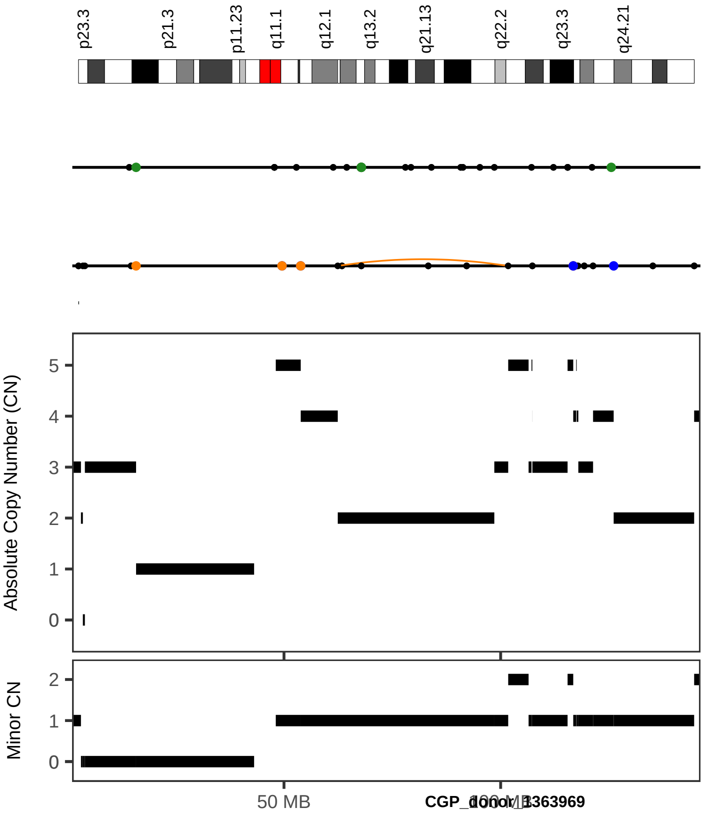

	**CGP_donor_1363969**
Cancer type	Breast−AdenoCA
Position	8:2570153−144678331
Interleaved intrachr. SVs	1
Total SVs (intrachr. + transl.)	26
SV types	DEL: 3; DUP: 7; h2hINV: 3; t2tINV: 0; TRA: 13
SVs in sample	680
Oscillating CN (2 and 3 states)	4, 6
CN segments	22
FDR fragment joints	0.5435077
FDR chr. breakp. enrich.	0.17
Linked to chrs	14:19046168−100653096;2:24204828−232125013 3:84135983−108939364;6:36900445−168143781

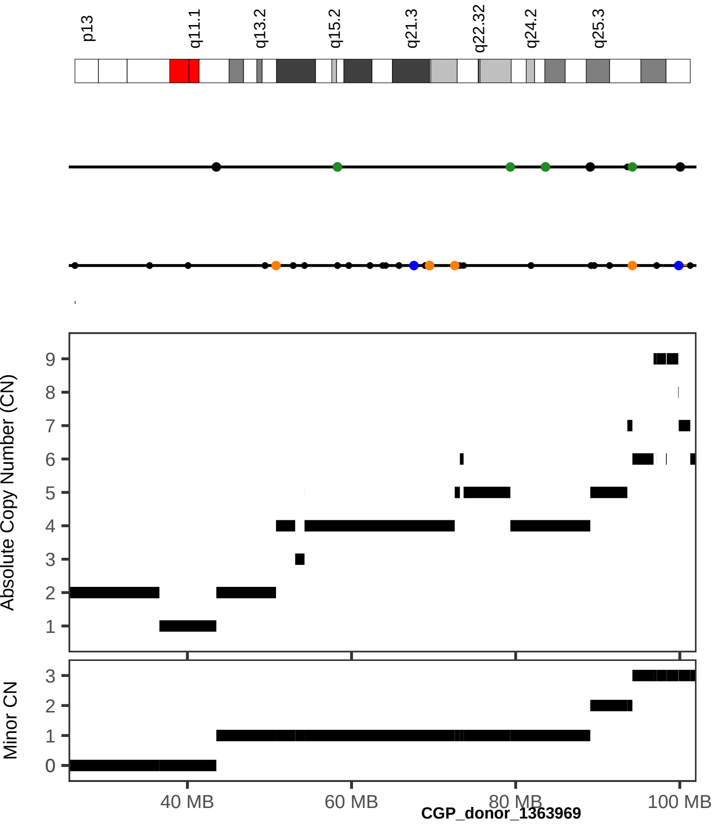

CGP_donor_1363969

Cancer type	Breast−AdenoCA
Position	15:49453754−101270859
Interleaved intrachr. SVs	1
Total SVs (intrachr. + transl.)	24
SV types	DEL: 2; DUP: 6; h2hINV: 0; t2tINV: 1; TRA: 15
SVs in sample	680
Oscillating CN (2 and 3 states)	6, 10
CN segments	20
FDR fragment joints	0.6776251
FDR chr. breakp. enrich.	0
Linked to chrs	10:15925151−65636394;14:19046168−100653096 3:84135983−108939364;
Purity, ploidy	0.45, 2.61

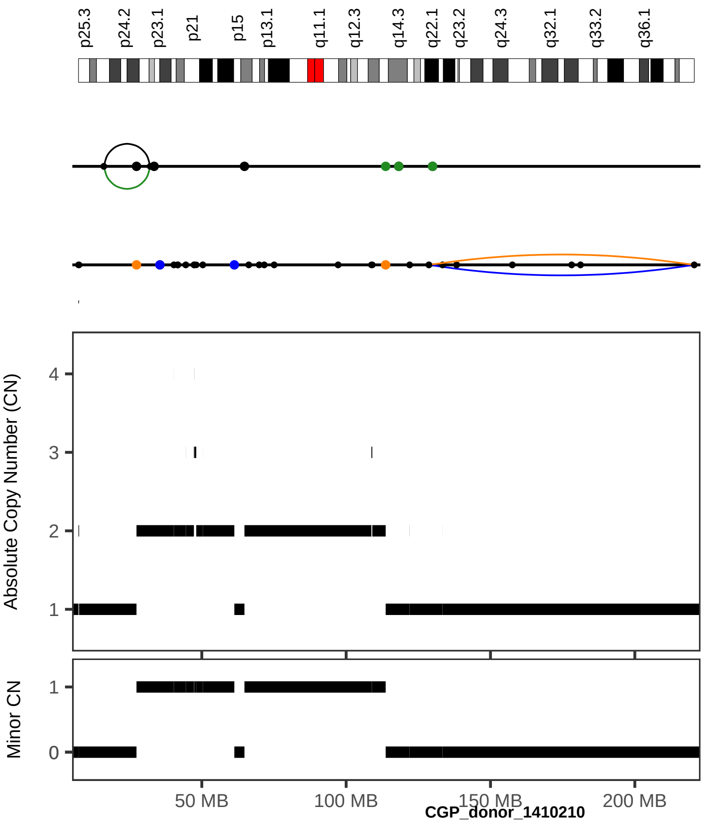

CGP_donor_1410210

Cancer type	Breast−AdenoCA
Position	2:7281461−220585117
Interleaved intrachr. SVs	1
Total SVs (intrachr. + transl.)	23
SV types	DEL: 1; DUP: 7; h2hINV: 1; t2tINV: 1; TRA: 13
SVs in sample	543
Oscillating CN (2 and 3 states)	6, 18
CN segments	22
FDR fragment joints	0.6363376
FDR chr. breakp. enrich.	0.61
Linked to chrs	1:98070309−174001373;15:49738728−90927311 22:19178913−43266973;
Purity, ploidy	0.66, 1.56

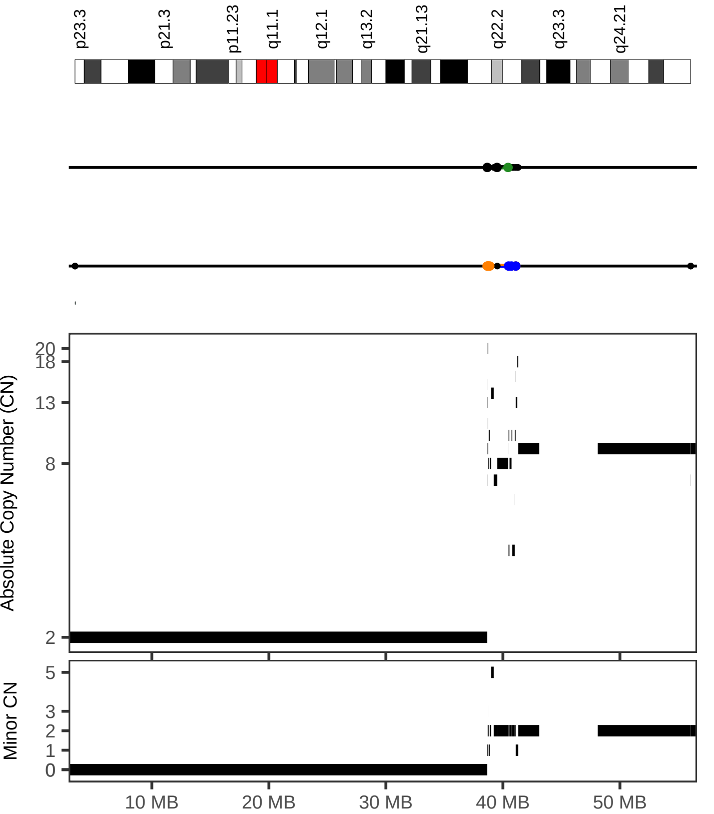

CGP_donor_1654385

Cancer type	Breast−AdenoCA
Position	8:38681577−41303238
Interleaved intrachr. SVs	12
Total SVs (intrachr. + transl.)	21
SV types	DEL: 3; DUP: 2; h2hINV: 3; t2tINV: 4; TRA: 9
SVs in sample	372
Oscillating CN (2 and 3 states)	3, 6
CN segments	26
FDR fragment joints	0.8882853
FDR chr. breakp. enrich.	0.22
Linked to chrs	
Purity, ploidy	0.72, 4.42

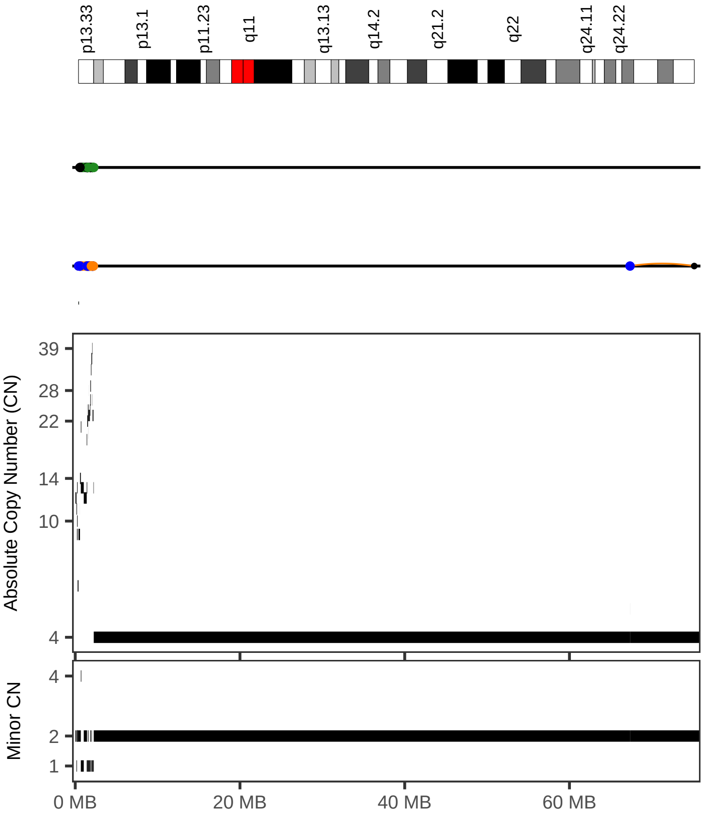

CGP_donor_1654385

Cancer type	Breast−AdenoCA
Position	12:397435−2107683
Interleaved intrachr. SVs	3
Total SVs (intrachr. + transl.)	59
SV types	DEL: 0; DUP: 1; h2hINV: 2; t2tINV: 0; TRA: 56
SVs in sample	372
Oscillating CN (2 and 3 states)	3, 4
CN segments	23
FDR fragment joints	0.9925341
FDR chr. breakp. enrich.	0
Linked to chrs	21:10701912−45821257;
Purity, ploidy	0.72, 4.42

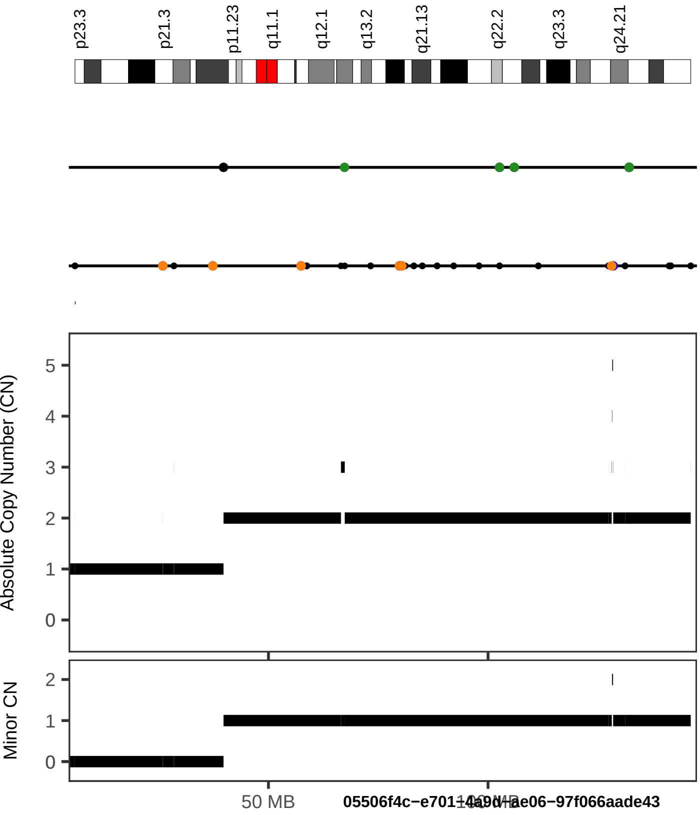

05506f4c−e701−4a9d−ae06−97f066aade43

Cancer type	Breast−AdenoCA
Position	8:58610813−141628750
Interleaved intrachr. SVs	1
Total SVs (intrachr. + transl.)	22
SV types	DEL: 3; DUP: 5; h2hINV: 0; t2tINV: 0; TRA: 14
SVs in sample	742
Oscillating CN (2 and 3 states)	6, 8
CN segments	12
FDR fragment joints	0.6776251
FDR chr. breakp. enrich.	0.4
Linked to chrs	10:53388358−118553565;16:30408080−80958008 4:109456663−169218723;5:5235976−159720786
Purity, ploidy	0.66, 1.78

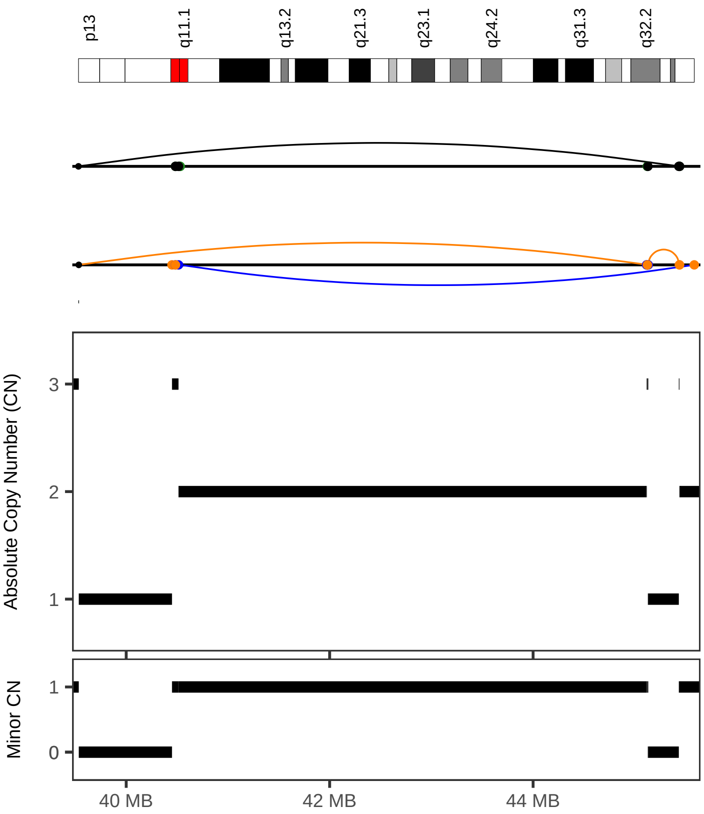

0bc5744c−5fa3−45bb−87d0−70a02068b392

Cancer type	Breast−AdenoCA
Position	14:39530849−45584134
Interleaved intrachr. SVs	4
Total SVs (intrachr. + transl.)	30
SV types	DEL: 2; DUP: 1; h2hINV: 1; t2tINV: 0; TRA: 26
SVs in sample	487
Oscillating CN (2 and 3 states)	5, 12
CN segments	12
FDR fragment joints	0.662962
FDR chr. breakp. enrich.	0
Linked to chrs	1:755344−72678035;
Purity, ploidy	0.43, 2.16

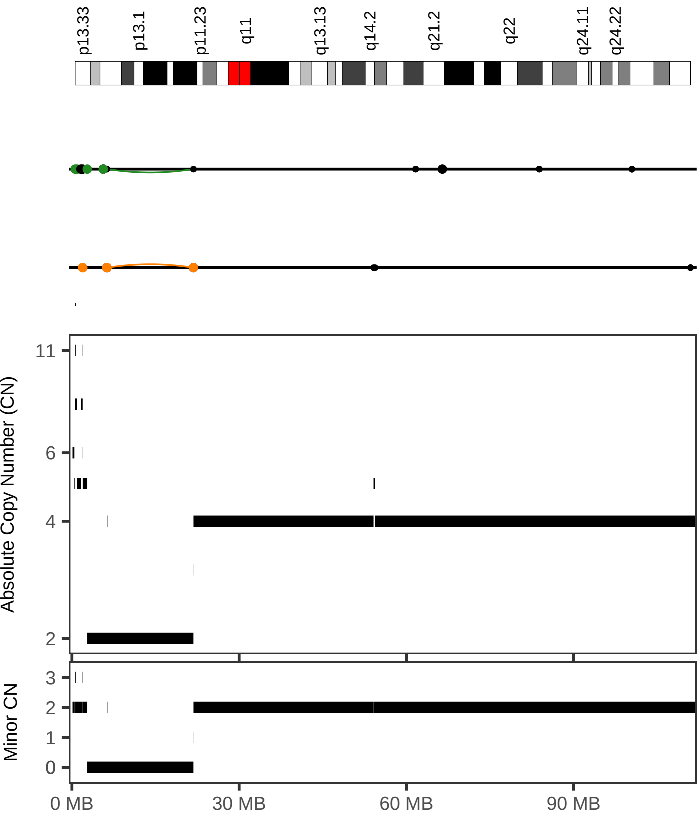

1174f6e4−ffbe−4e59−a000−8d861c968369

Cancer type	Breast−AdenoCA
Position	12:588242−100462941
Interleaved intrachr. SVs	1
Total SVs (intrachr. + transl.)	20
SV types	DEL: 1; DUP: 1; h2hINV: 1; t2tINV: 2; TRA: 15
SVs in sample	357
Oscillating CN (2 and 3 states)	4, 5
CN segments	15
FDR fragment joints	0.8572806
FDR chr. breakp. enrich.	0.15
Linked to chrs	17:35714118−65191895;
Purity, ploidy	0.49, 3.26

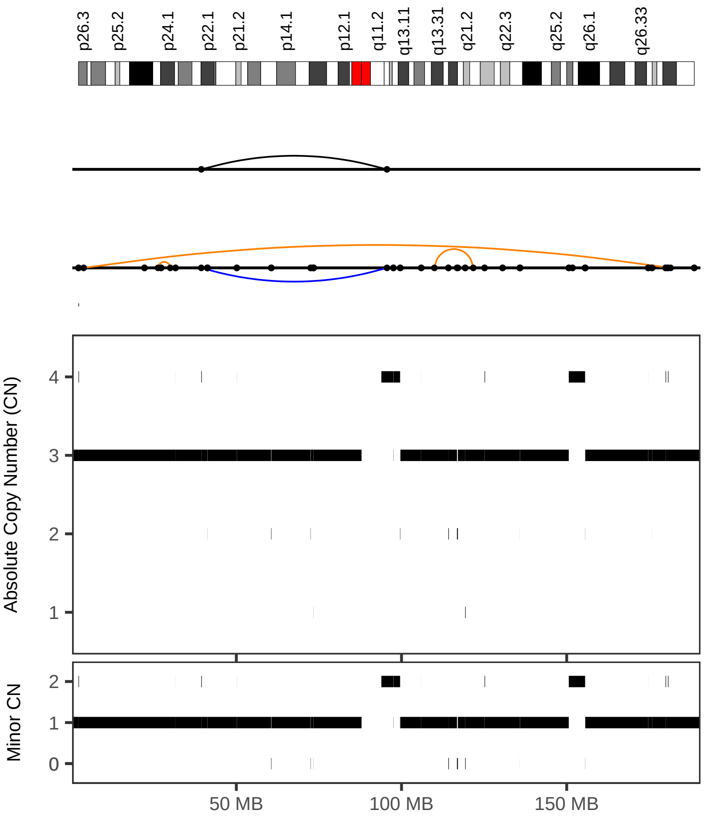

174850b4−5ec2−462b−a890−89bd1716b3c2

Cancer type	Breast−AdenoCA
Position	3:3791616−188605871
Interleaved intrachr. SVs	1
Total SVs (intrachr. + transl.)	22
SV types	DEL: 20; DUP: 1; h2hINV: 1; t2tINV: 0; TRA: 0
SVs in sample	456
Oscillating CN (2 and 3 states)	5, 10
CN segments	45
FDR fragment joints	1.748499e−07
FDR chr. breakp. enrich.	0.8
Linked to chrs	
Purity, ploidy	0.47, 3.02

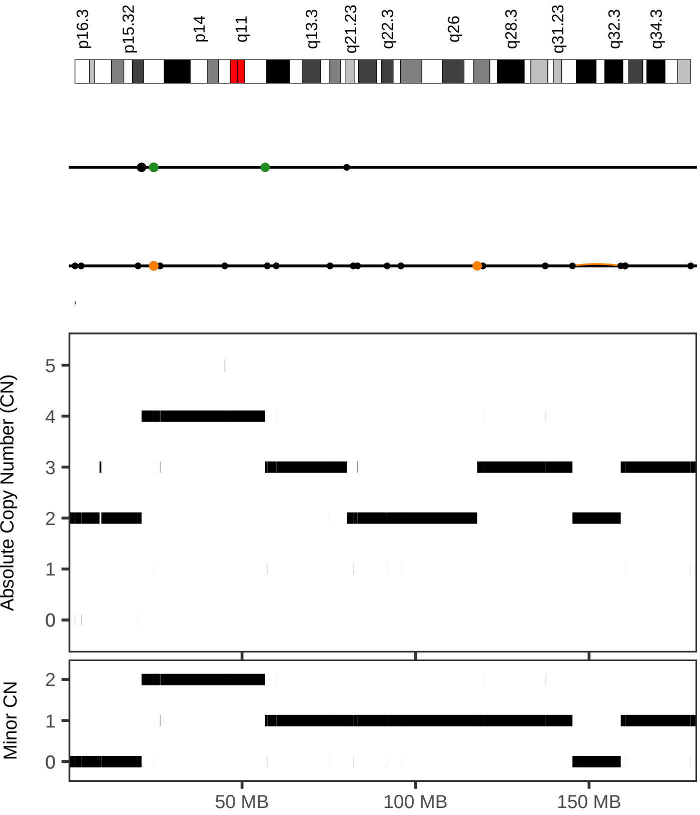

174850b4−5ec2−462b−a890−89bd1716b3c2

Cancer type	Breast−AdenoCA
Position	4:1872945−179290053
Interleaved intrachr. SVs	1
Total SVs (intrachr. + transl.)	23
SV types	DEL: 14; DUP: 1; h2hINV: 0; t2tINV: 0; TRA: 8
SVs in sample	456
Oscillating CN (2 and 3 states)	5, 21
CN segments	43
FDR fragment joints	0.0009901546
FDR chr. breakp. enrich.	1
Linked to chrs	
Purity, ploidy	0.47, 3.02

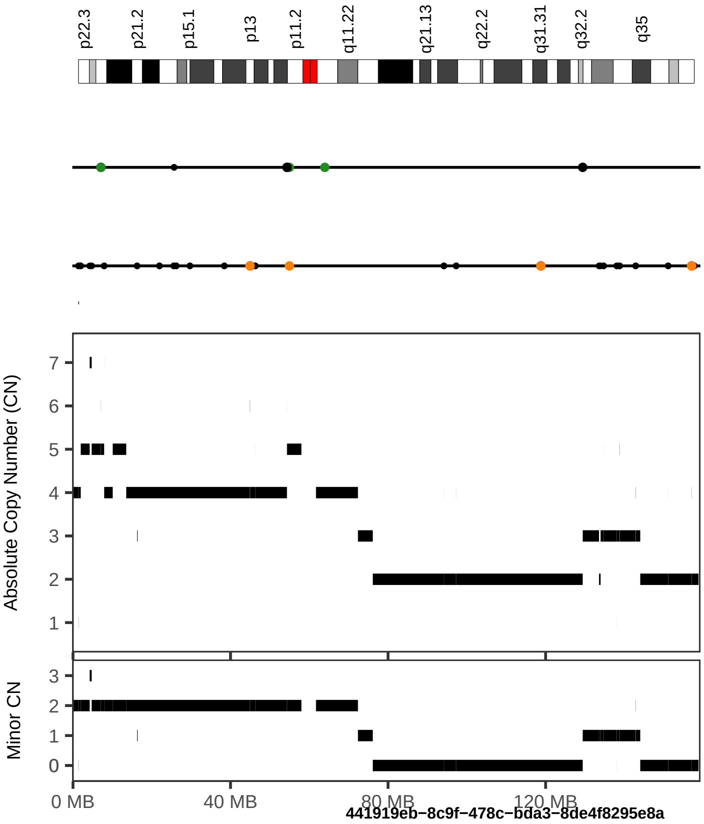

441919eb−8c9f−478c−bda3−8de4f8295e8a

Cancer type	Breast−AdenoCA
Position	7:1396546−142881743
Interleaved intrachr. SVs	1
Total SVs (intrachr. + transl.)	22
SV types	DEL: 1; DUP: 9; h2hINV: 0; t2tINV: 0; TRA: 12
SVs in sample	744
Oscillating CN (2 and 3 states)	5, 9
CN segments	37
FDR fragment joints	0.5435077
FDR chr. breakp. enrich.	0.71
Linked to chrs	12:9556035−123265892;3:3385898−154169465 6:6025768−153367098;
Purity, ploidy	0.5, 3.35

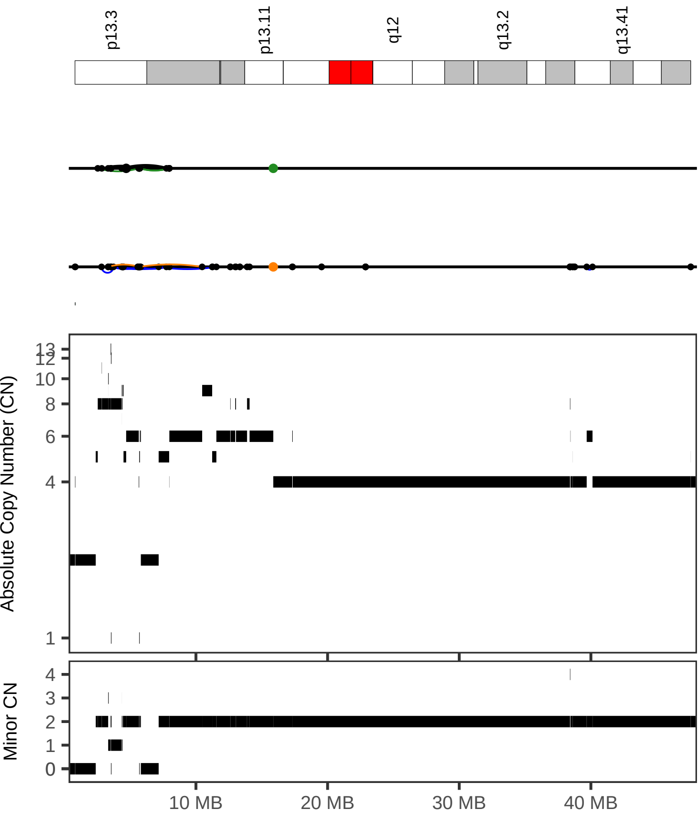

441919eb−8c9f−478c−bda3−8de4f8295e8a

Cancer type	Breast−AdenoCA
Position	19:2541887−11552300
Interleaved intrachr. SVs	21
Total SVs (intrachr. + transl.)	22
SV types	DEL: 2; DUP: 9; h2hINV: 5; t2tINV: 5; TRA: 1
SVs in sample	744
Oscillating CN (2 and 3 states)	3, 5
CN segments	30
FDR fragment joints	0.615458
FDR chr. breakp. enrich.	0
Linked to chrs	
Purity, ploidy	0.5, 3.35

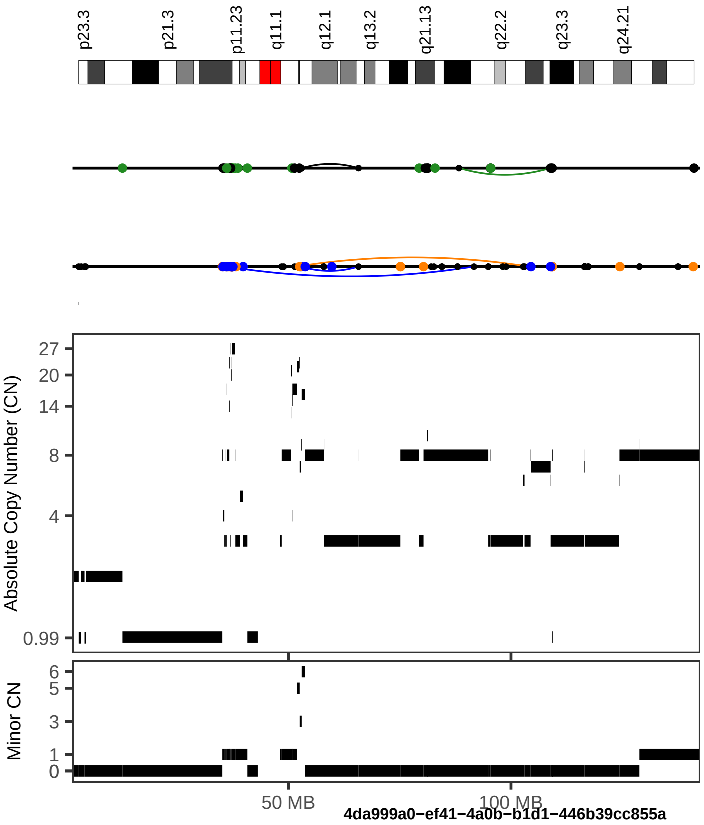

4da999a0−ef41−4a0b−b1d1−446b39cc855a

Cancer type	Breast−AdenoCA
Position	8:35905735−108901612
Interleaved intrachr. SVs	5
Total SVs (intrachr. + transl.)	55
SV types	DEL: 1; DUP: 2; h2hINV: 0; t2tINV: 2; TRA: 50
SVs in sample	423
Oscillating CN (2 and 3 states)	6, 13
CN segments	55
FDR fragment joints	0.8833361
FDR chr. breakp. enrich.	0
Linked to chrs	16:1378342−30451828;2:115111736−198568024
Purity, ploidy	0.77, 3.16

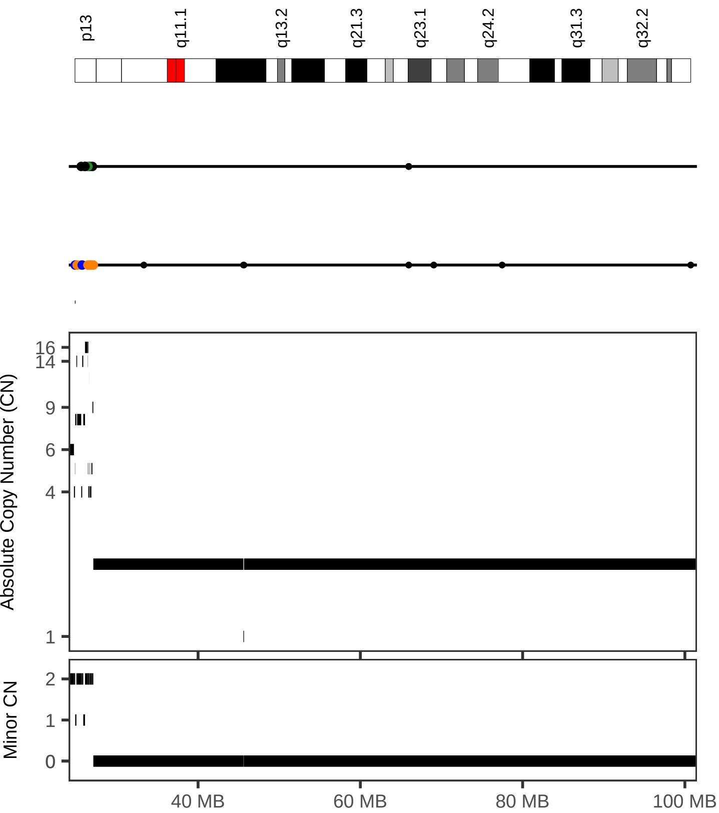

4da999a0−ef41−4a0b−b1d1−446b39cc855a

Cancer type	Breast−AdenoCA
Position	14:24825314−69059375
Interleaved intrachr. SVs	1
Total SVs (intrachr. + transl.)	20
SV types	DEL: 2; DUP: 2; h2hINV: 0;<br>t2tINV: 0; TRA: 16
SVs in sample	423
Oscillating CN (2 and 3 states)	4, 4
CN segments	21
FDR fragment joints	0.662962
FDR chr. breakp. enrich.	0
Linked to chrs	
Purity, ploidy	0.77, 3.16

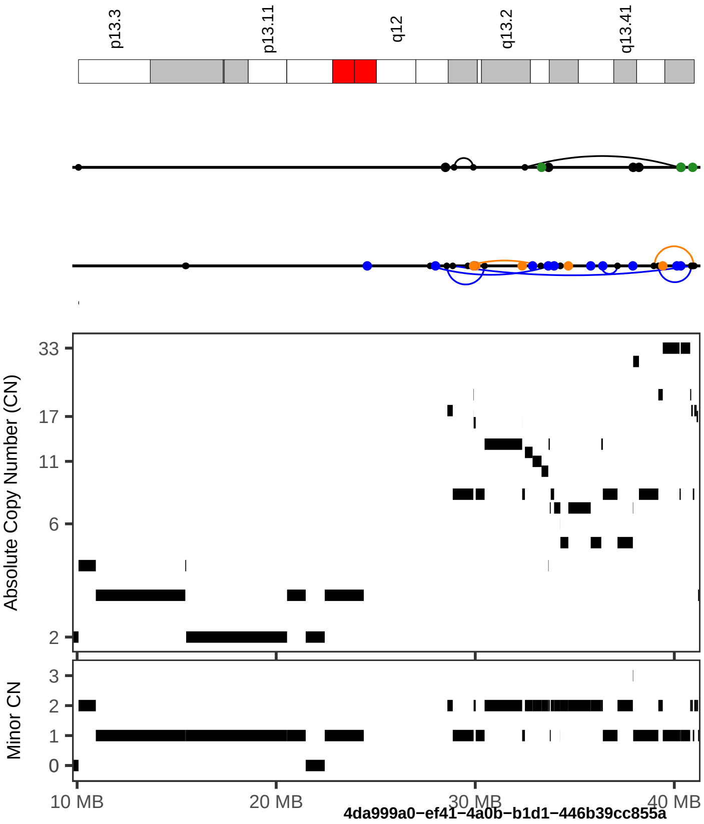

4da999a0−ef41−4a0b−b1d1−446b39cc855a

Cancer type	Breast−AdenoCA
Position	19:27734571−41001824
Interleaved intrachr. SVs	8
Total SVs (intrachr. + transl.)	31
SV types	DEL: 2; DUP: 4; h2hINV: 2;<br>t2tINV: 0; TRA: 23
SVs in sample	423
Oscillating CN (2 and 3 states)	3, 4
CN segments	36
FDR fragment joints	0.5435077
FDR chr. breakp. enrich.	0
Linked to chrs	16:1378342−30451828;2:115111736−198568024<br>8:35905735−108901611;
Purity, ploidy	0.77, 3.16

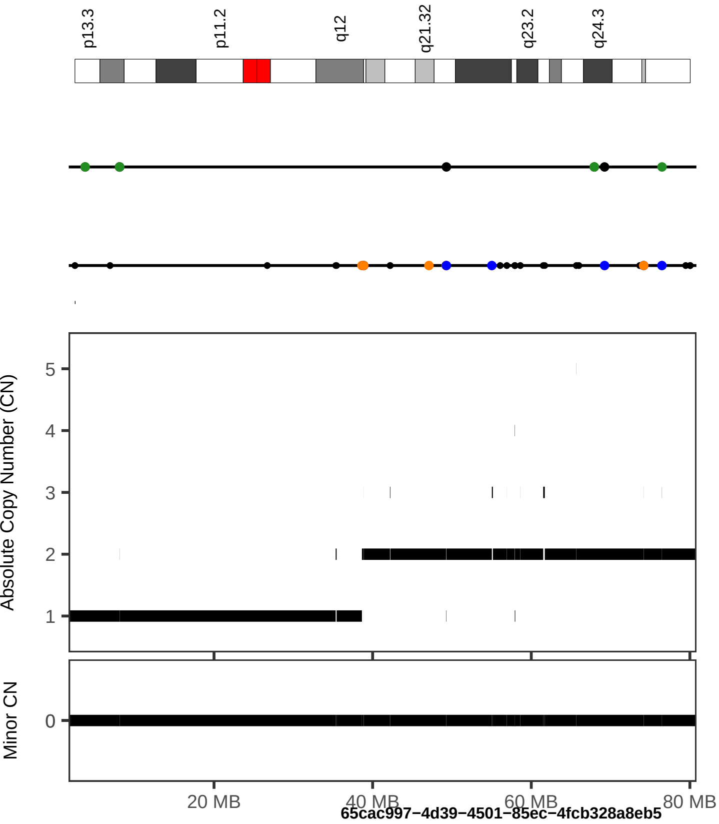

65cac997−4d39−4501−85ec−4fcb328a8eb5

Cancer type	Breast−AdenoCA
Position	17:35339299−80044376
Interleaved intrachr. SVs	1
Total SVs (intrachr. + transl.)	22
SV types	DEL: 0; DUP: 6; h2hINV: 0;<br>t2tINV: 0; TRA: 16
SVs in sample	909
Oscillating CN (2 and 3 states)	5, 12
CN segments	27
FDR fragment joints	0.279346
FDR chr. breakp. enrich.	0
Linked to chrs	1:149881190−243814703;13:20691358−100667836<br>7:95697844−156942102;
Purity, ploidy	0.66, 1.85

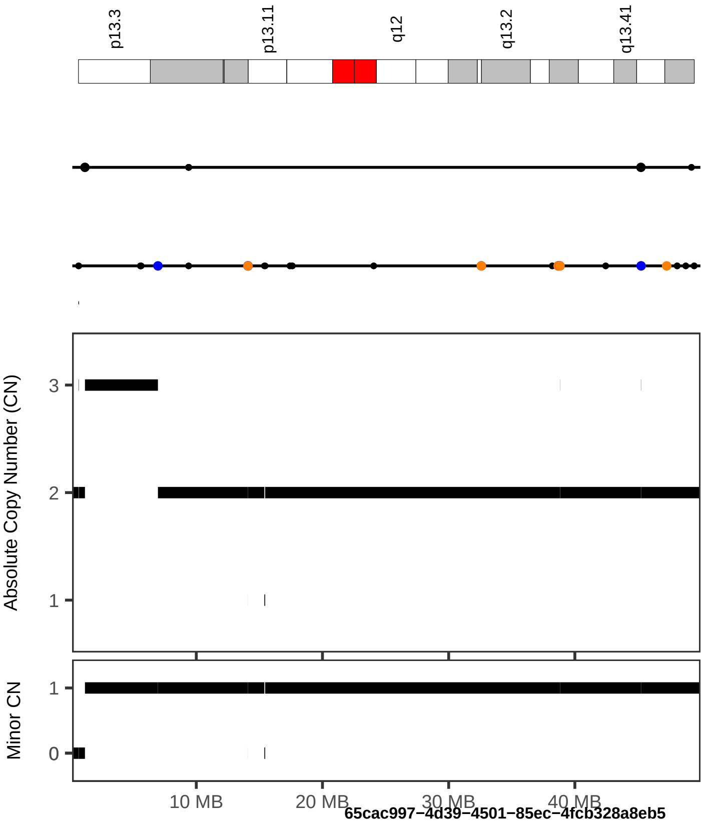

65cac997−4d39−4501−85ec−4fcb328a8eb5

Cancer type	Breast−AdenoCA
Position	19:663441−49460784
Interleaved intrachr. SVs	1
Total SVs (intrachr. + transl.)	22
SV types	DEL: 2; DUP: 4; h2hINV: 1;<br>t2tINV: 0; TRA: 15
SVs in sample	909
Oscillating CN (2 and 3 states)	5, 12
CN segments	12
FDR fragment joints	0.2070134
FDR chr. breakp. enrich.	0
Linked to chrs	1:149881190−243814703;2:10252979−231781038<br>8:22900664−128813813;
Purity, ploidy	0.66, 1.85

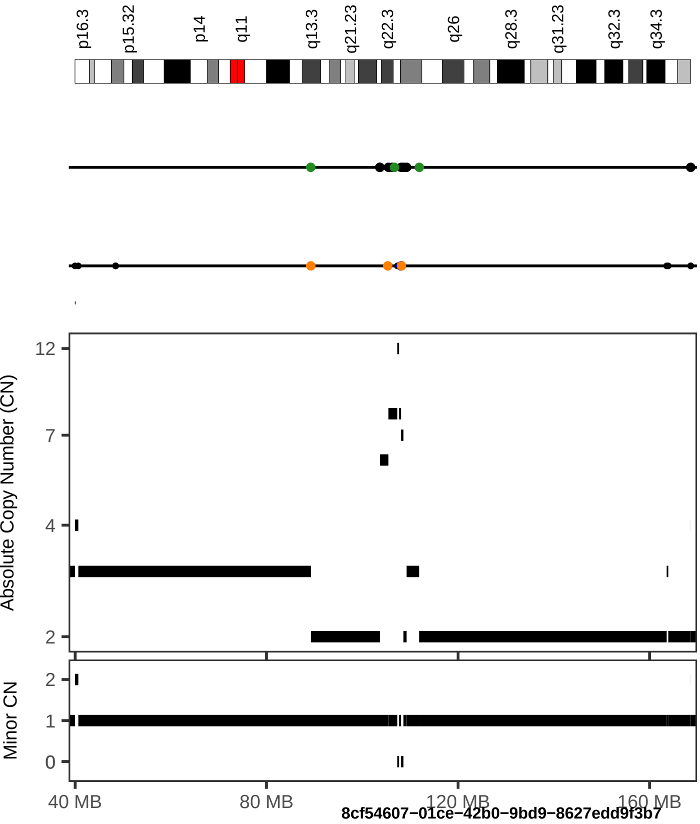

8cf54607−01ce−42b0−9bd9−8627edd9f3b7

Cancer type	Breast−AdenoCA
Position	4:39957449−163913725
Interleaved intrachr. SVs	1
Total SVs (intrachr. + transl.)	21
SV types	DEL: 0; DUP: 4; h2hINV: 1; t2tINV: 0; TRA: 16
SVs in sample	500
Oscillating CN (2 and 3 states)	5, 5
CN segments	13
FDR fragment joints	0.8419568
FDR chr. breakp. enrich.	0.35
Linked to chrs	13:22850041−105164538;3:4979331−87053734
Purity, ploidy	0.55, 3.76

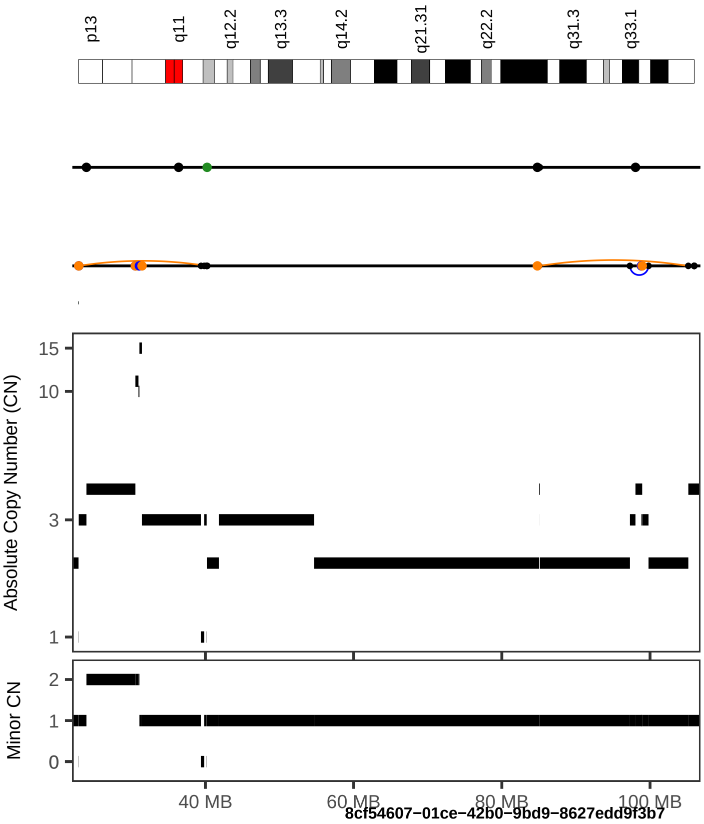

8cf54607−01ce−42b0−9bd9−8627edd9f3b7

Cancer type	Breast−AdenoCA
Position	13:22850041−105164539
Interleaved intrachr. SVs	1
Total SVs (intrachr. + transl.)	23
SV types	DEL: 3; DUP: 1; h2hINV: 1; t2tINV: 1; TRA: 17
SVs in sample	500
Oscillating CN (2 and 3 states)	5, 5
CN segments	23
FDR fragment joints	0.5435077
FDR chr. breakp. enrich.	0.03
Linked to chrs	12:2943203−96943877;4:39957449−163913724 6:66019356−135278032;
Purity, ploidy	0.55, 3.76

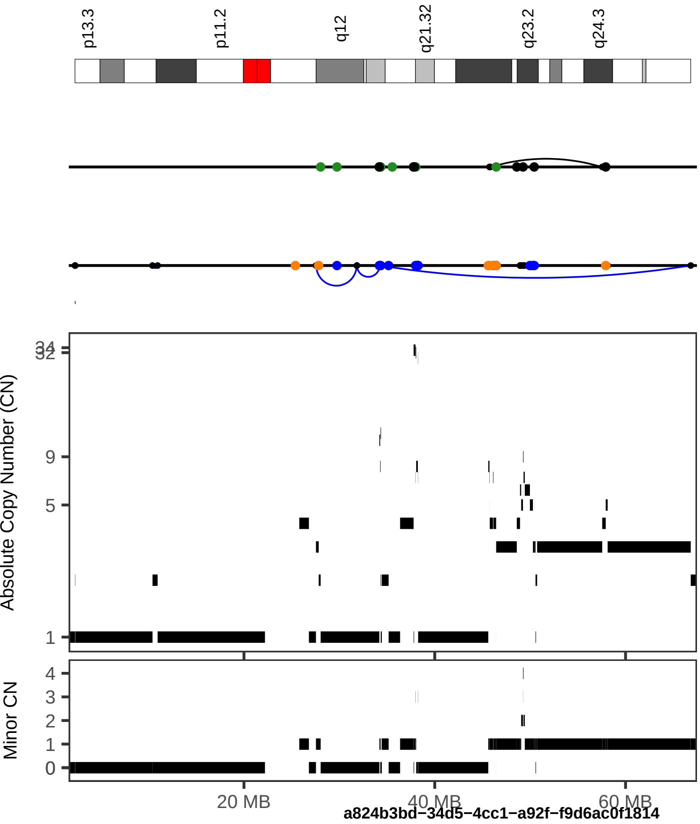

a824b3bd−34d5−4cc1−a92f−f9d6ac0f1814

Cancer type	Breast−AdenoCA
Position	17:27546652−66832010
Interleaved intrachr. SVs	4
Total SVs (intrachr. + transl.)	41
SV types	DEL: 1; DUP: 3; h2hINV: 0; t2tINV: 0; TRA: 37
SVs in sample	463
Oscillating CN (2 and 3 states)	4, 6
CN segments	49
FDR fragment joints	0.6776251
FDR chr. breakp. enrich.	0
Linked to chrs	11:10547187−101046436;19:13207846−29674952
Purity, ploidy	0.51, 2.1

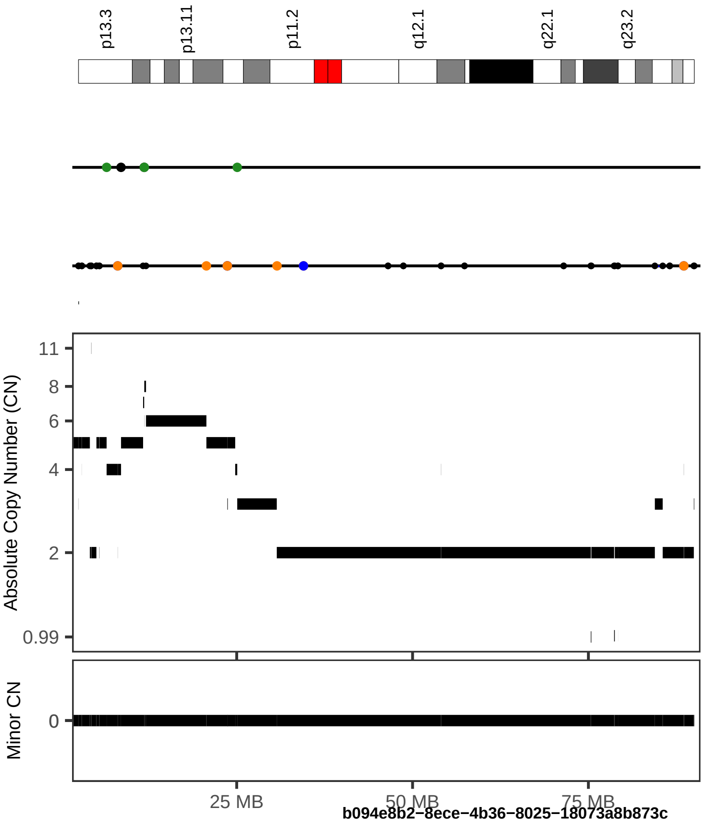

b094e8b2−8ece−4b36−8025−18073a8b873c

Cancer type	Breast−AdenoCA
Position	16:2501874−90049603
Interleaved intrachr. SVs	1
Total SVs (intrachr. + transl.)	29
SV types	DEL: 2; DUP: 10; h2hINV: 0; t2tINV: 0; TRA: 17
SVs in sample	763
Oscillating CN (2 and 3 states)	6, 8
CN segments	41
FDR fragment joints	0.1013295
FDR chr. breakp. enrich.	0
Linked to chrs	1:3498809−247725332;8:3931231−30860701
Purity, ploidy	0.82, 2.81

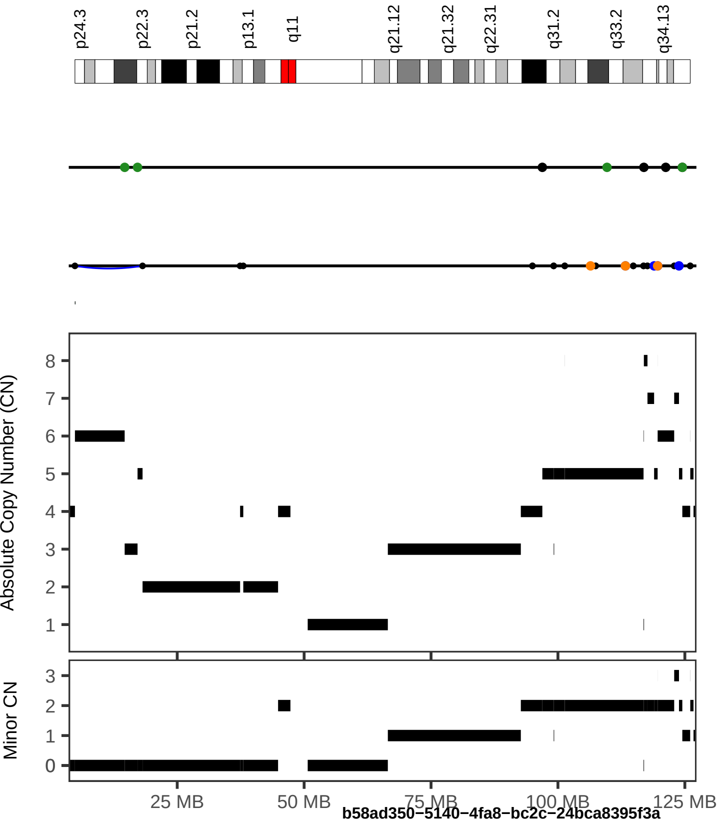

b58ad350−5140−4fa8−bc2c−24bca8395f3a

Cancer type	Breast−AdenoCA
Position	9:4845278−126061906
Interleaved intrachr. SVs	1
Total SVs (intrachr. + transl.)	21
SV types	DEL: 0; DUP: 6; h2hINV: 0; t2tINV: 0; TRA: 15
SVs in sample	518
Oscillating CN (2 and 3 states)	4, 9
CN segments	27
FDR fragment joints	0.5774122
FDR chr. breakp. enrich.	0.09
Linked to chrs	10:61540502−75608352;11:1143256−131778844
Purity, ploidy	0.65, 3.24

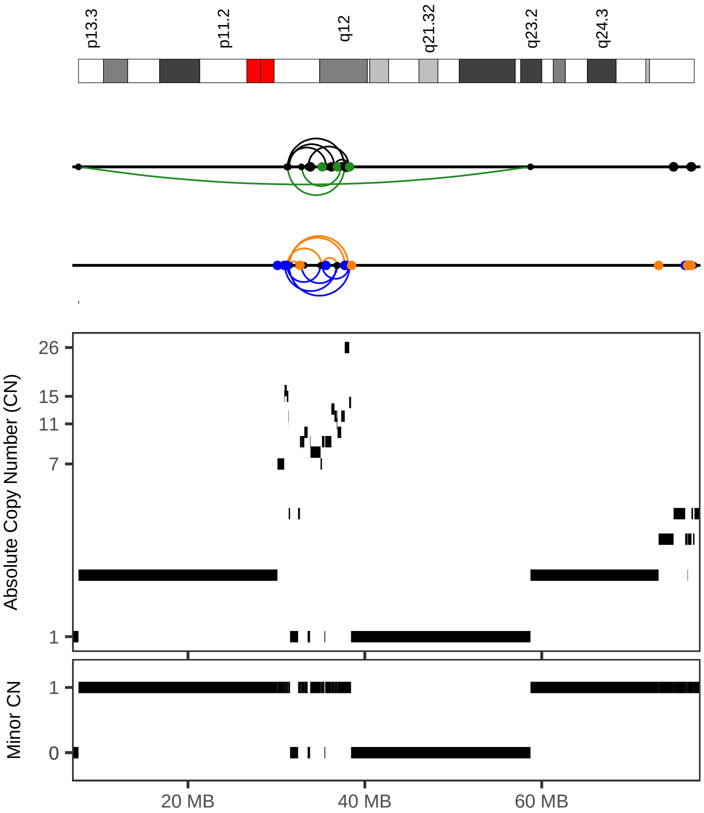

c364e81c−eb1e−4870−ab37−9c661f5f2e3d

Cancer type	Breast−AdenoCA
Position	17:30926374−38439380
Interleaved intrachr. SVs	27
Total SVs (intrachr. + transl.)	41
SV types	DEL: 6; DUP: 6; h2hINV: 9; t2tINV: 6; TRA: 14
SVs in sample	397
Oscillating CN (2 and 3 states)	3, 4
CN segments	27
FDR fragment joints	0.8053298
FDR chr. breakp. enrich.	0
Linked to chrs	
Purity, ploidy	0.41, 2.02

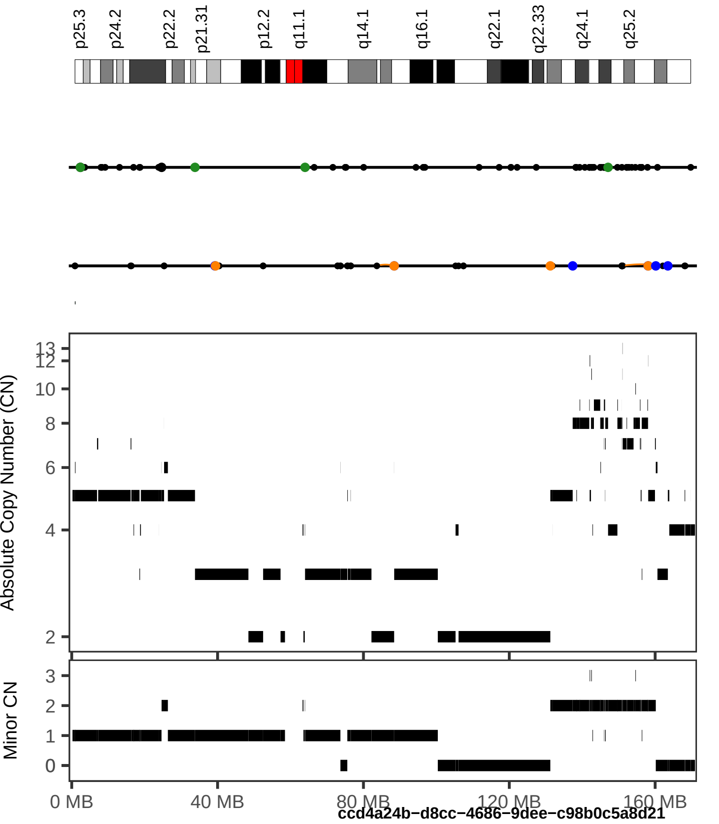

ccd4a24b−d8cc−4686−9dee−c98b0c5a8d21

Cancer type	Breast−AdenoCA
Position	6:16147315−168229735
Interleaved intrachr. SVs	1
Total SVs (intrachr. + transl.)	25
SV types	DEL: 2; DUP: 9; h2hINV: 0; t2tINV: 0; TRA: 14
SVs in sample	936
Oscillating CN (2 and 3 states)	5, 8
CN segments	98
FDR fragment joints	0.04139017
FDR chr. breakp. enrich.	0
Linked to chrs	11:295491−102672482;5:11957267−95790570 7:7932046−156250630;
Purity, ploidy	0.89, 3.26

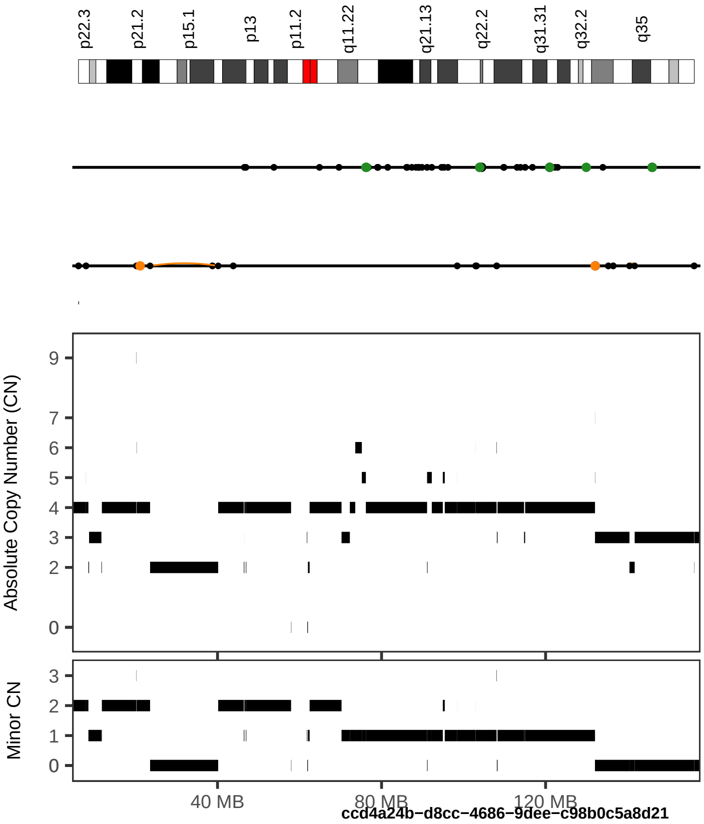

ccd4a24b−d8cc−4686−9dee−c98b0c5a8d21

Cancer type	Breast−AdenoCA
Position	7:7932046−156250631
Interleaved intrachr. SVs	1
Total SVs (intrachr. + transl.)	20
SV types	DEL: 3; DUP: 4; h2hINV: 0; t2tINV: 0; TRA: 13
SVs in sample	936
Oscillating CN (2 and 3 states)	6, 10
CN segments	48
FDR fragment joints	0.9625775
FDR chr. breakp. enrich.	0.38
Linked to chrs	1:8031173−227527810;4:19803347−158797787 6:16147315−168229734;
Purity, ploidy	0.89, 3.26

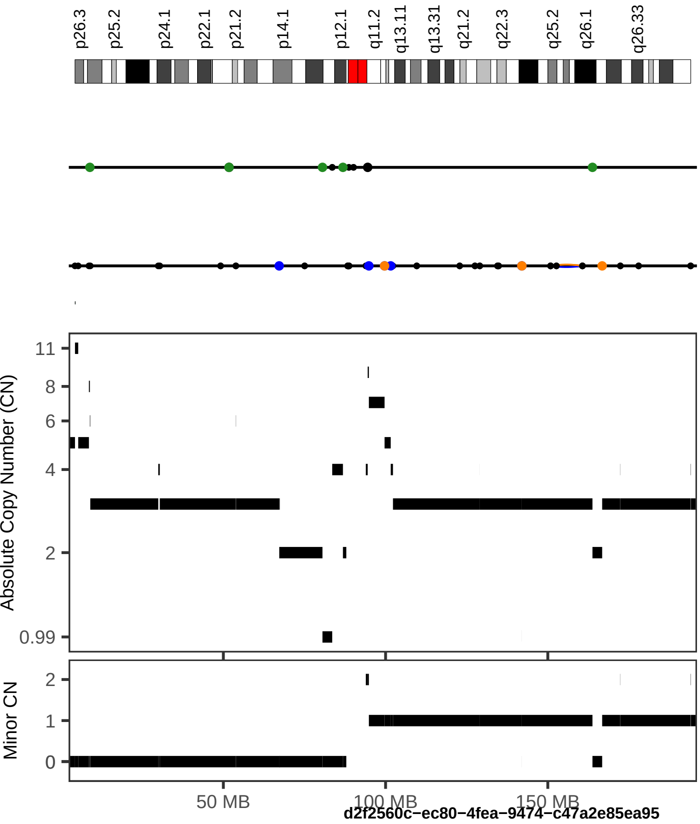

d2f2560c−ec80−4fea−9474−c47a2e85ea95

Cancer type	Breast−AdenoCA
Position	3:4264202−194052997
Interleaved intrachr. SVs	1
Total SVs (intrachr. + transl.)	27
SV types	DEL: 2; DUP: 10; h2hINV: 0; t2tINV: 0; TRA: 15
SVs in sample	546
Oscillating CN (2 and 3 states)	5, 7
CN segments	31
FDR fragment joints	0.1278363
FDR chr. breakp. enrich.	0.52
Linked to chrs	1:10197061−240751610;20:30253204−59314862 8:6739069−135344821;
Purity, ploidy	0.67, 3.45

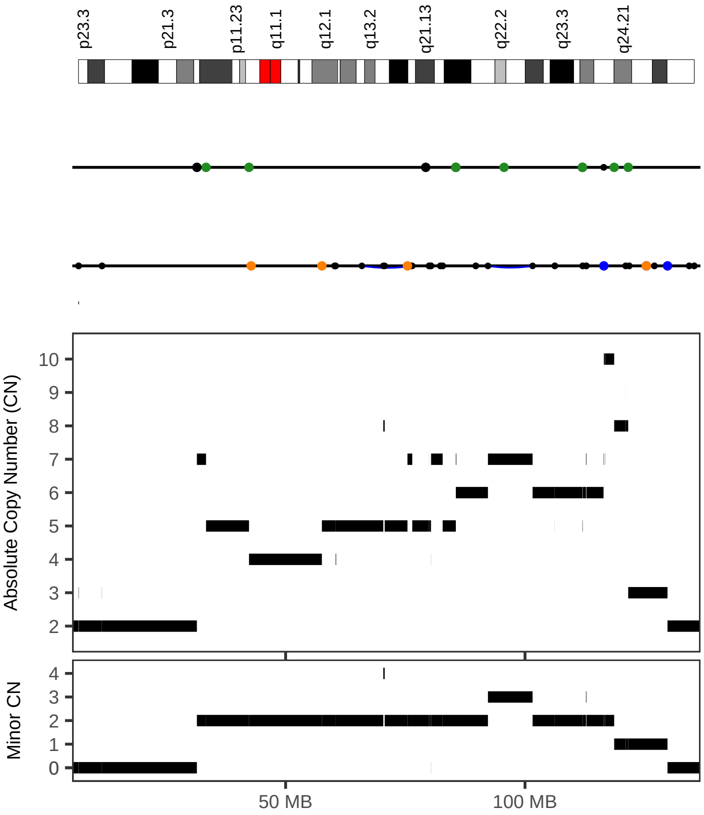

d2f2560c−ec80−4fea−9474−c47a2e85ea95

Cancer type	Breast−AdenoCA
Position	8:6739069−135344822
Interleaved intrachr. SVs	1
Total SVs (intrachr. + transl.)	31
SV types	DEL: 2; DUP: 10; h2hINV: 0; t2tINV: 1; TRA: 18
SVs in sample	546
Oscillating CN (2 and 3 states)	5, 7
CN segments	38
FDR fragment joints	0.615458
FDR chr. breakp. enrich.	0.06
Linked to chrs	3:4264202−194052996;
Purity, ploidy	0.67, 3.45

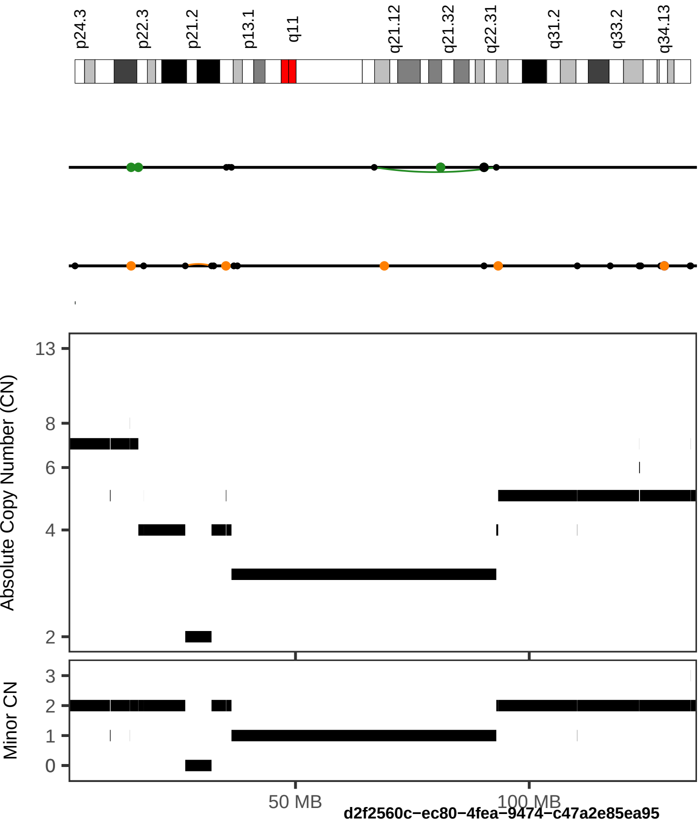

d2f2560c−ec80−4fea−9474−c47a2e85ea95

Cancer type	Breast−AdenoCA
Position	9:2831150−134547130
Interleaved intrachr. SVs	1
Total SVs (intrachr. + transl.)	25
SV types	DEL: 3; DUP: 8; h2hINV: 1; t2tINV: 1; TRA: 12
SVs in sample	546
Oscillating CN (2 and 3 states)	4, 12
CN segments	23
FDR fragment joints	0.615458
FDR chr. breakp. enrich.	0.02
Linked to chrs	1:10197061−240751610;7:26783402−109202113
Purity, ploidy	0.67, 3.45

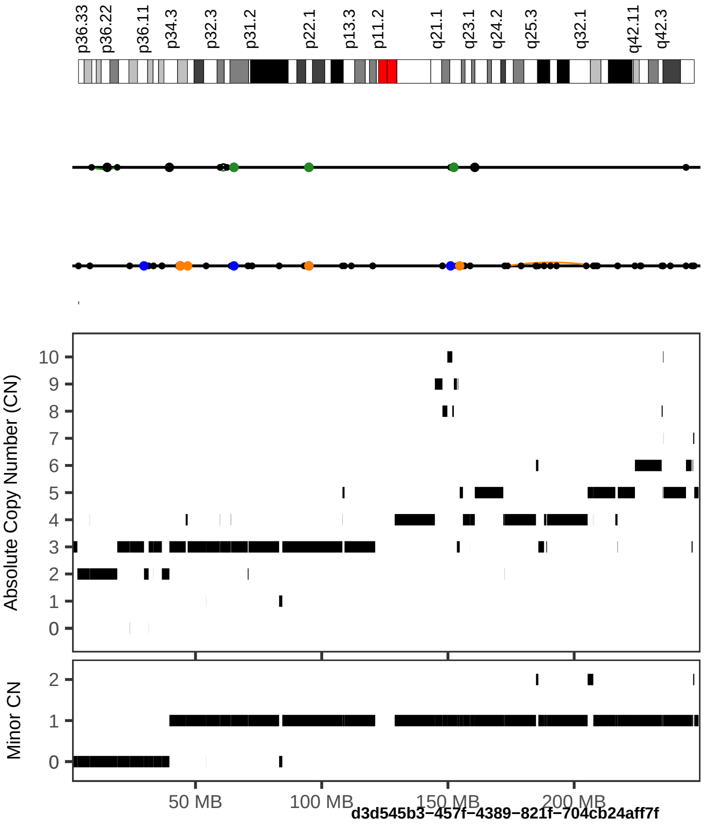

d3d545b3−457f−4389−821f−704cb24aff7f

Cancer type	Breast−AdenoCA
Position	1:8159480−247557943
Interleaved intrachr. SVs	1
Total SVs (intrachr. + transl.)	49
SV types	DEL: 16; DUP: 12; h2hINV: 1; t2tINV: 2; TRA: 18
SVs in sample	709
Oscillating CN (2 and 3 states)	5, 12
CN segments	67
FDR fragment joints	0.1457354
FDR chr. breakp. enrich.	0.06
Linked to chrs	2:182905442−238971066;6:1587687−158509484 9:3361891−130382426;
Purity, ploidy	0.76, 3.03

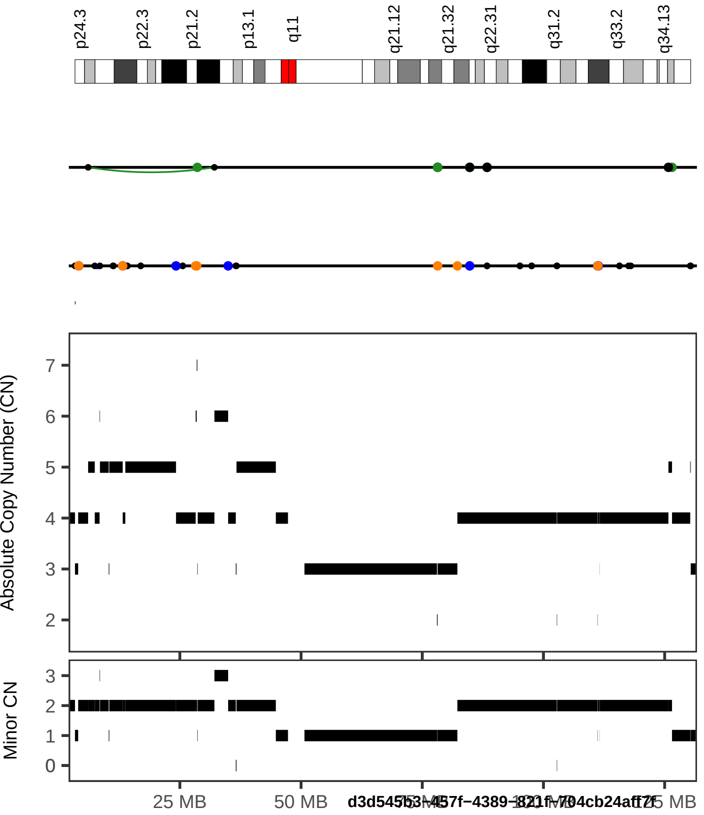

d3d545b3−457f−4389−821f−704cb24aff7f

Cancer type	Breast−AdenoCA
Position	9:3361891−130382427
Interleaved intrachr. SVs	1
Total SVs (intrachr. + transl.)	44
SV types	DEL: 7; DUP: 6; h2hINV: 0; t2tINV: 1; TRA: 30
SVs in sample	709
Oscillating CN (2 and 3 states)	5, 8
CN segments	34
FDR fragment joints	0.615458
FDR chr. breakp. enrich.	0
Linked to chrs	1:8159480−247557942;2:182905442−238971066 20:14792624−55573863;7:89983323−158447439 8:67453419−86357133;
Purity, ploidy	0.76, 3.03

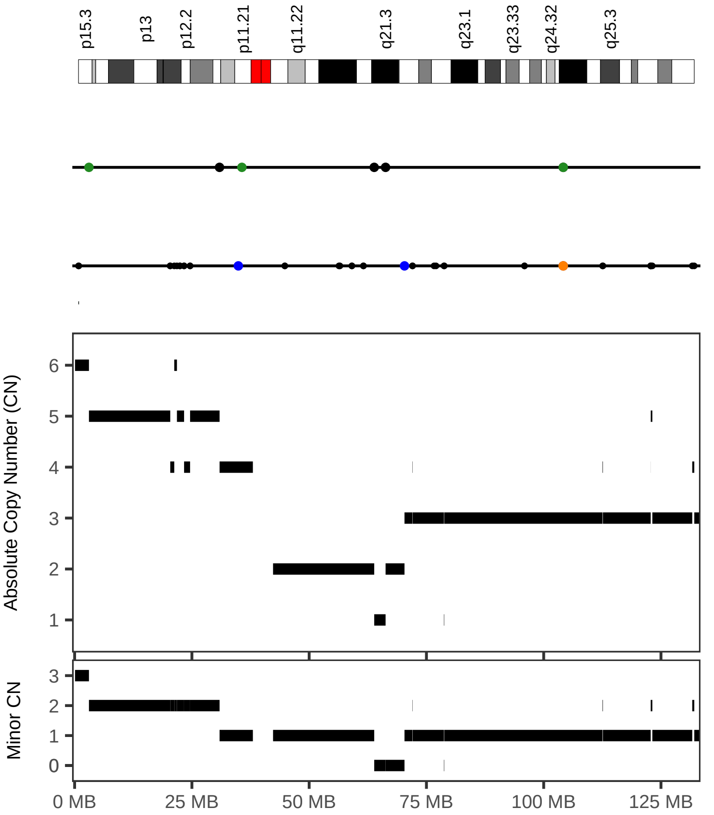

d3d545b3−457f−4389−821f−704cb24aff7f

Cancer type	Breast−AdenoCA
Position	10:822186−132092700
Interleaved intrachr. SVs	1
Total SVs (intrachr. + transl.)	25
SV types	DEL: 6; DUP: 7; h2hINV: 0; t2tINV: 0; TRA: 12
SVs in sample	709
Oscillating CN (2 and 3 states)	4, 6
CN segments	23
FDR fragment joints	0.615458
FDR chr. breakp. enrich.	1
Linked to chrs	
Purity, ploidy	0.76, 3.03

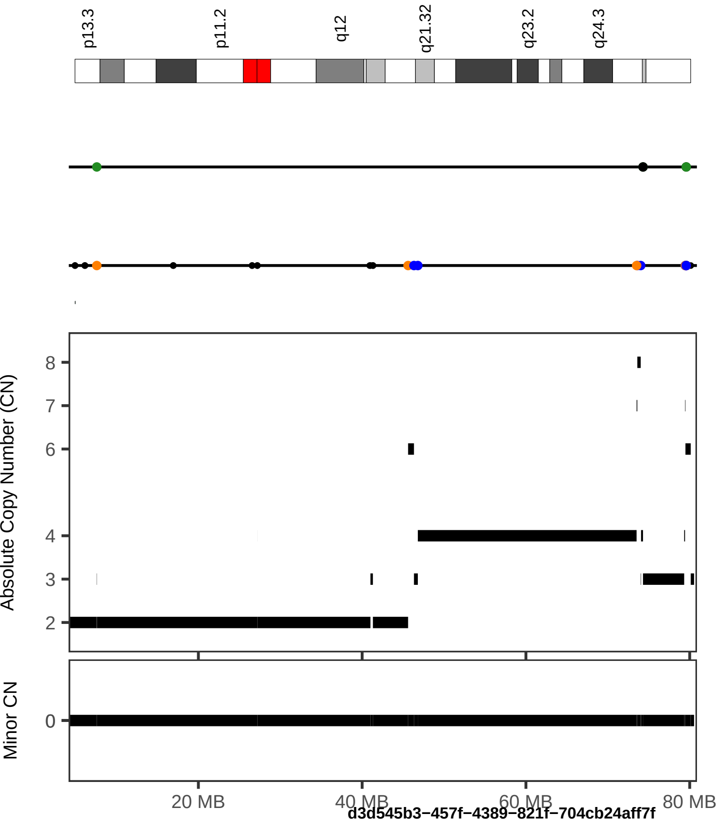

d3d545b3−457f−4389−821f−704cb24aff7f

Cancer type	Breast−AdenoCA
Position	17:6124848−80125721
Interleaved intrachr. SVs	1
Total SVs (intrachr. + transl.)	26
SV types	DEL: 3; DUP: 4; h2hINV: 0; t2tINV: 1; TRA: 18
SVs in sample	709
Oscillating CN (2 and 3 states)	4, 9
CN segments	19
FDR fragment joints	0.445216
FDR chr. breakp. enrich.	0.05
Linked to chrs	1:8159480−247557942;2:182905442−238971066
Purity, ploidy	0.76, 3.03

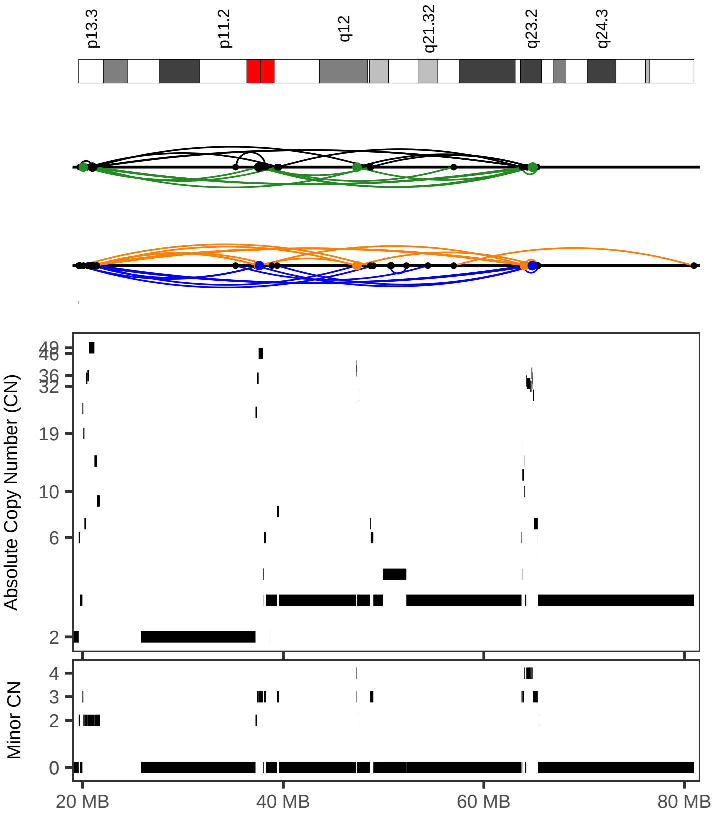

d67cd793−2931−429a−9084−2f3c4c8be7ad

Cancer type	Breast−AdenoCA
Position	17:19599977−80954190
Interleaved intrachr. SVs	55
Total SVs (intrachr. + transl.)	69
SV types	DEL: 17; DUP: 13; h2hINV: 12; t2tINV: 13; TRA: 14
SVs in sample	427
Oscillating CN (2 and 3 states)	3, 4
CN segments	50
FDR fragment joints	0.7299821
FDR chr. breakp. enrich.	0
Linked to chrs	
Purity, ploidy	0.54, 3.31

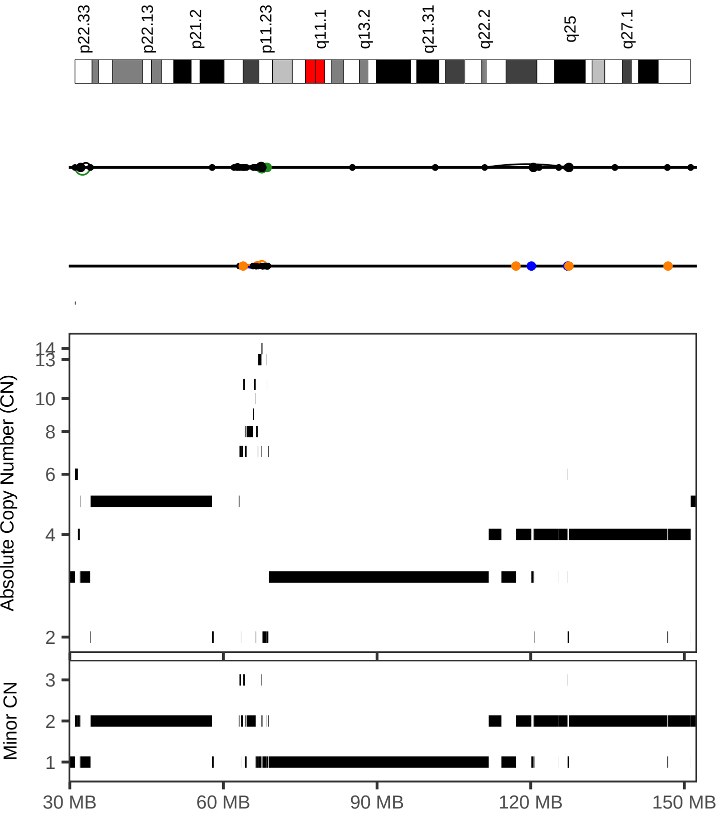

d67cd793−2931−429a−9084−2f3c4c8be7ad

Cancer type	Breast−AdenoCA
Position	X:62042944−68767168
Interleaved intrachr. SVs	22
Total SVs (intrachr. + transl.)	26
SV types	DEL: 7; DUP: 5; h2hINV: 5; t2tINV: 5; TRA: 4
SVs in sample	427
Oscillating CN (2 and 3 states)	3, 4
CN segments	25
FDR fragment joints	0.9179372
FDR chr. breakp. enrich.	0
Linked to chrs	
Purity, ploidy	0.54, 3.31

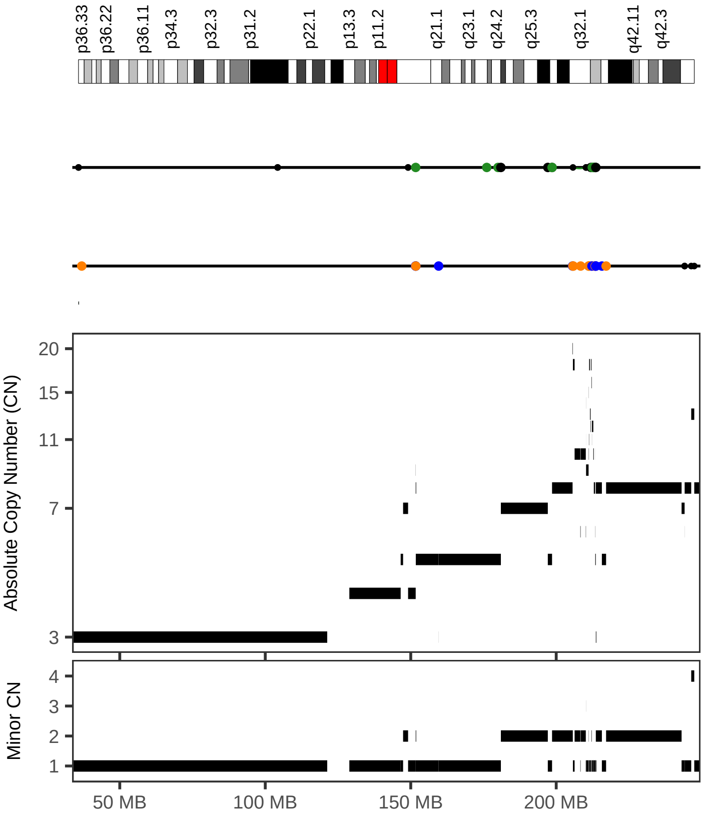

d8d7a6c2−6427−4f47−968c−6c3affba4617

Cancer type	Breast−AdenoCA
Position	1:205752472−215774932
Interleaved intrachr. SVs	2
Total SVs (intrachr. + transl.)	22
SV types	DEL: 0; DUP: 1; h2hINV: 0; t2tINV: 1; TRA: 20
SVs in sample	453
Oscillating CN (2 and 3 states)	4, 6
CN segments	28
FDR fragment joints	0.6776251
FDR chr. breakp. enrich.	0.05
Linked to chrs	
Purity, ploidy	0.62, 3.59

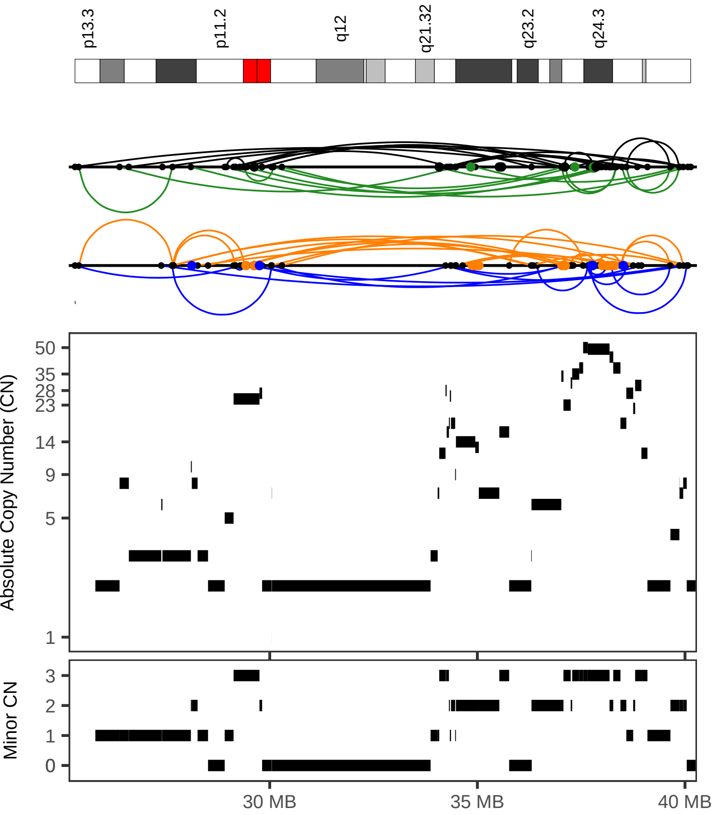

d8d7a6c2−6427−4f47−968c−6c3affba4617

Cancer type	Breast−AdenoCA
Position	17:25309656−40138460
Interleaved intrachr. SVs	78
Total SVs (intrachr. + transl.)	108
SV types	DEL: 25; DUP: 16; h2hINV: 19; t2tINV: 18; TRA: 30
SVs in sample	453
Oscillating CN (2 and 3 states)	3, 4
CN segments	54
FDR fragment joints	0.5435077
FDR chr. breakp. enrich.	0
Linked to chrs	12:61134794−98290461;
Purity, ploidy	0.62, 3.59

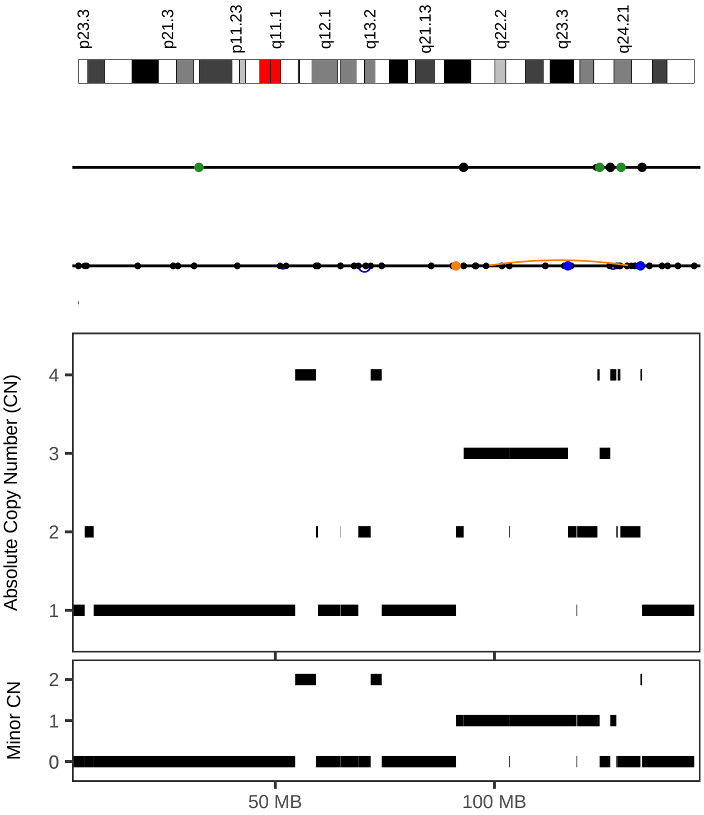

d9dc3b59−613d−469e−8b4f−6c5a557eb26a

Cancer type	Breast−AdenoCA
Position	8:6538486−141884919
Interleaved intrachr. SVs	1
Total SVs (intrachr. + transl.)	24
SV types	DEL: 1; DUP: 13; h2hINV: 0; t2tINV: 1; TRA: 9
SVs in sample	1222
Oscillating CN (2 and 3 states)	6, 9
CN segments	26
FDR fragment joints	0.04139017
FDR chr. breakp. enrich.	0.16
Linked to chrs	
Purity, ploidy	0.41, 2.07

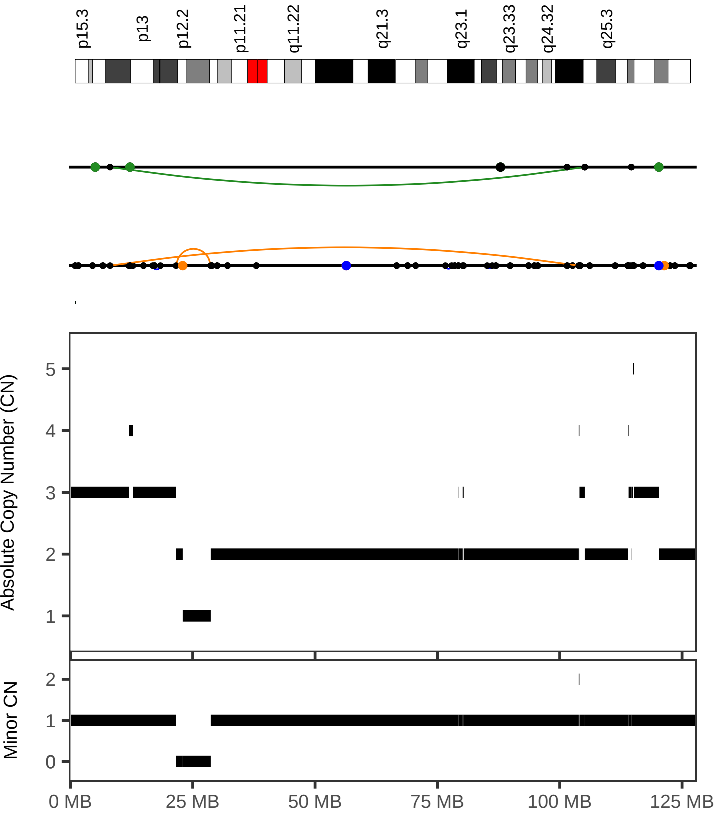

d9dc3b59−613d−469e−8b4f−6c5a557eb26a

Cancer type	Breast−AdenoCA
Position	10:1642894−126723909
Interleaved intrachr. SVs	1
Total SVs (intrachr. + transl.)	36
SV types	DEL: 4; DUP: 17; h2hINV: 0; t2tINV: 2; TRA: 13
SVs in sample	1222
Oscillating CN (2 and 3 states)	5, 11
CN segments	22
FDR fragment joints	0.1013295
FDR chr. breakp. enrich.	0.66
Linked to chrs	1:1898876−77522549;
Purity, ploidy	0.41, 2.07

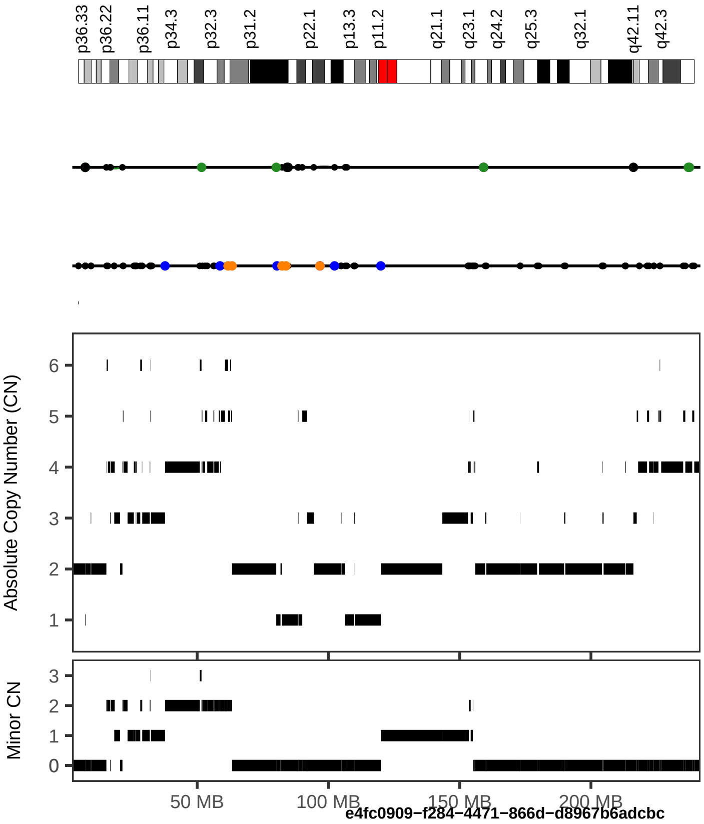

e4fc0909−f284−4471−866d−d8967b6adcbc

Cancer type	Breast−AdenoCA
Position	1:58265896−204819002
Interleaved intrachr. SVs	2
Total SVs (intrachr. + transl.)	25
SV types	DEL: 1; DUP: 8; h2hINV: 1; t2tINV: 0; TRA: 15
SVs in sample	630
Oscillating CN (2 and 3 states)	5, 18
CN segments	48
FDR fragment joints	0.3165354
FDR chr. breakp. enrich.	0
Linked to chrs	15:22324442−89201248;7:10063721−147714910 8:138302264−144043893;
Purity, ploidy	0.49, 2.63

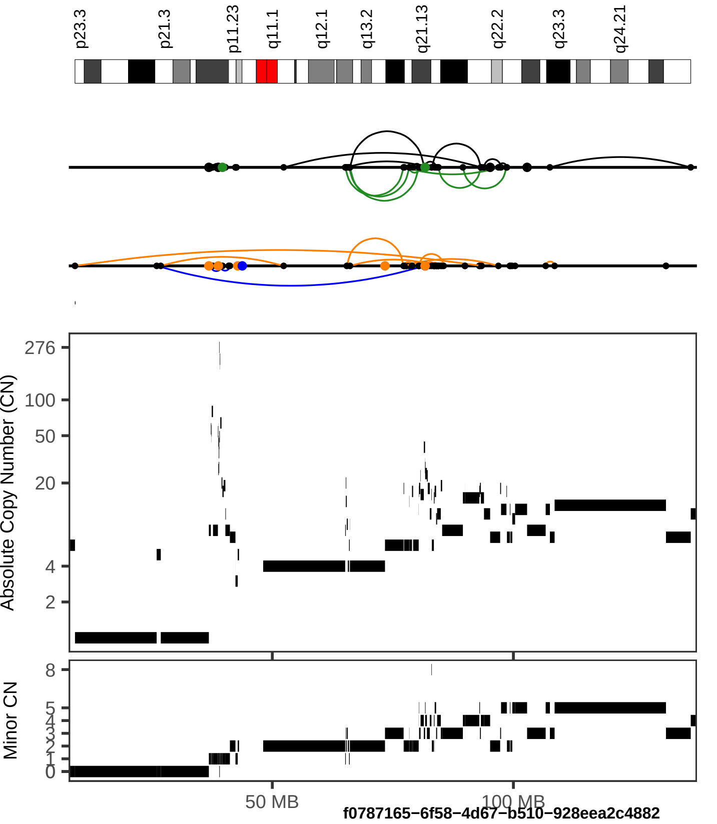

f0787165−6f58−4d67−b510−928eea2c4882

Cancer type	Breast−AdenoCA
Position	8:9070962−98658630
Interleaved intrachr. SVs	34
Total SVs (intrachr. + transl.)	46
SV types	DEL: 11; DUP: 5; h2hINV: 11; t2tINV: 7; TRA: 12
SVs in sample	421
Oscillating CN (2 and 3 states)	3, 7
CN segments	85
FDR fragment joints	0.615458
FDR chr. breakp. enrich.	0
Linked to chrs	1:146473725−244991893;12:689576−131594459
Purity, ploidy	0.69, 3.99

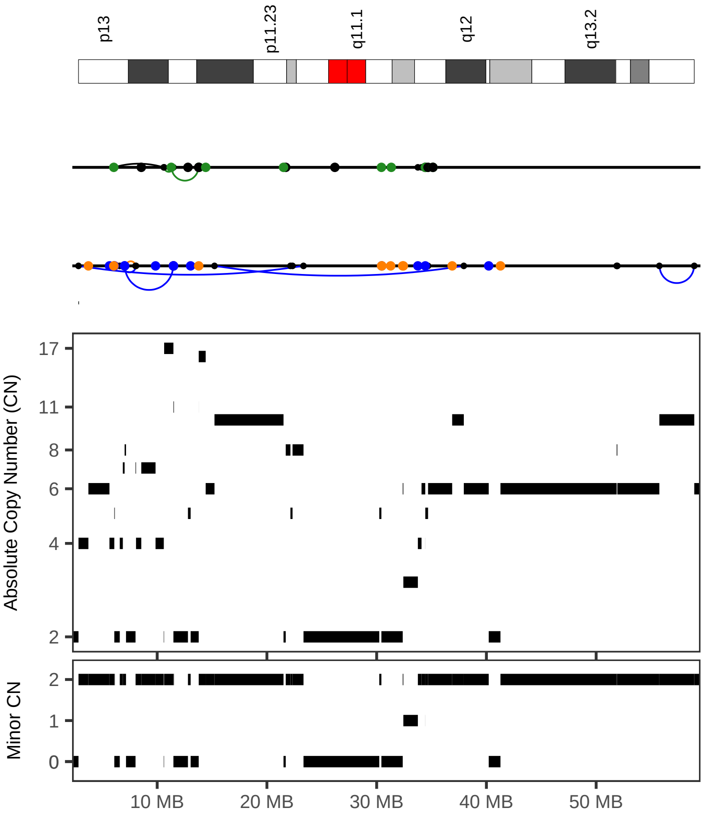

f0787165−6f58−4d67−b510−928eea2c4882

Cancer type	Breast−AdenoCA
Position	20:6046983−13783911
Interleaved intrachr. SVs	5
Total SVs (intrachr. + transl.)	21
SV types	DEL: 0; DUP: 2; h2hINV: 1; t2tINV: 2; TRA: 16
SVs in sample	421
Oscillating CN (2 and 3 states)	4, 5
CN segments	17
FDR fragment joints	0.4601446
FDR chr. breakp. enrich.	0
Linked to chrs	
Purity, ploidy	0.69, 3.99

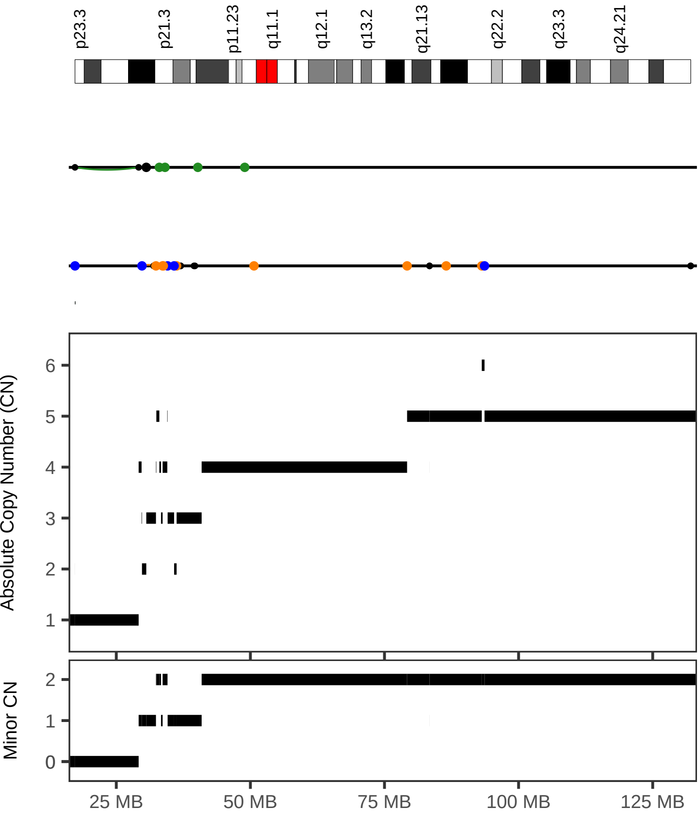

CGP_donor_1234120

Cancer type	Breast−LobularCA
Position	8:17297986−132094568
Interleaved intrachr. SVs	1
Total SVs (intrachr. + transl.)	23
SV types	DEL: 4; DUP: 0; h2hINV: 0; t2tINV: 1; TRA: 18
SVs in sample	344
Oscillating CN (2 and 3 states)	4, 12
CN segments	21
FDR fragment joints	0.4101979
FDR chr. breakp. enrich.	0.16
Linked to chrs	4:53312523−80369666;
Purity, ploidy	0.39, 2.94

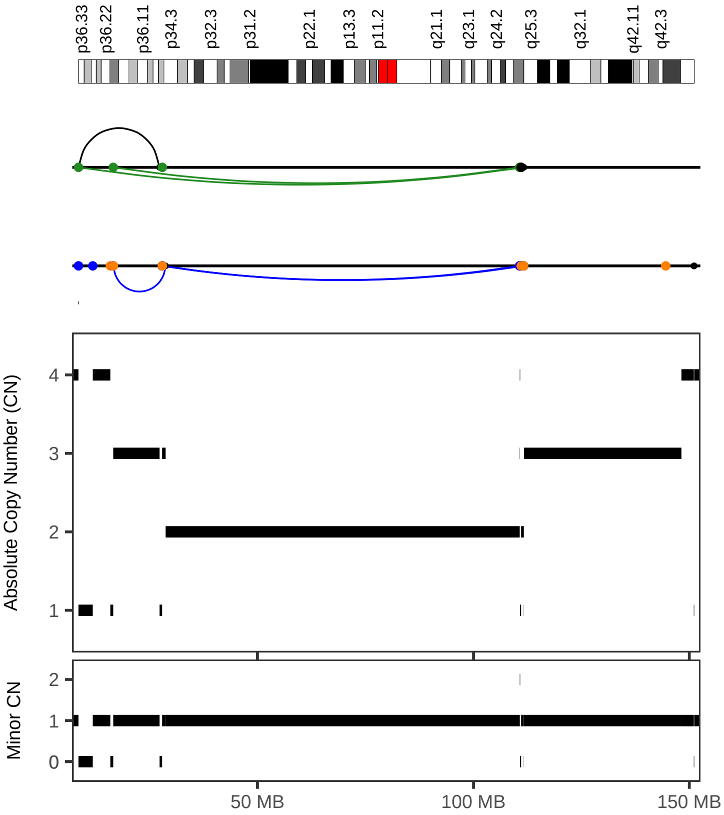

27b05b15−a44b−45ed−a6e3−e7d1ca488ea9

Cancer type	Breast−LobularCA
Position	1:8516636−111647232
Interleaved intrachr. SVs	5
Total SVs (intrachr. + transl.)	27
SV types	DEL: 0; DUP: 3; h2hINV: 1; t2tINV: 1; TRA: 22
SVs in sample	116
Oscillating CN (2 and 3 states)	4, 10
CN segments	12
FDR fragment joints	0.5435077
FDR chr. breakp. enrich.	0
Linked to chrs	10:45002072−118566527;
Purity, ploidy	0.31, 3.39

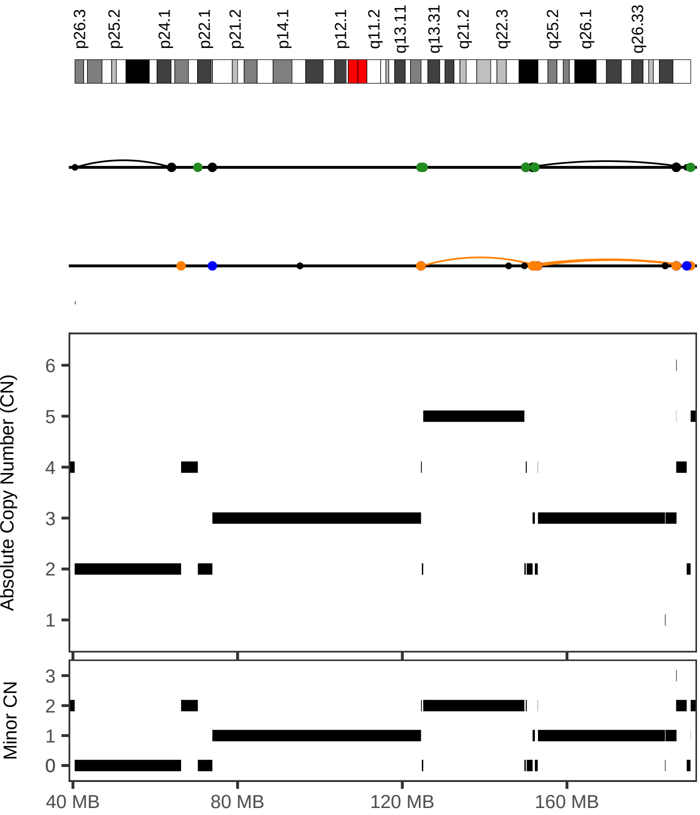

27b05b15−a44b−45ed−a6e3−e7d1ca488ea9

Cancer type	Breast−LobularCA
Position	3:124743480−190056561
Interleaved intrachr. SVs	4
Total SVs (intrachr. + transl.)	26
SV types	DEL: 3; DUP: 0; h2hINV: 1; t2tINV: 0; TRA: 22
SVs in sample	116
Oscillating CN (2 and 3 states)	4, 9
CN segments	19
FDR fragment joints	0.6776251
FDR chr. breakp. enrich.	0
Linked to chrs	10:45002072−118566527;
Purity, ploidy	0.31, 3.39

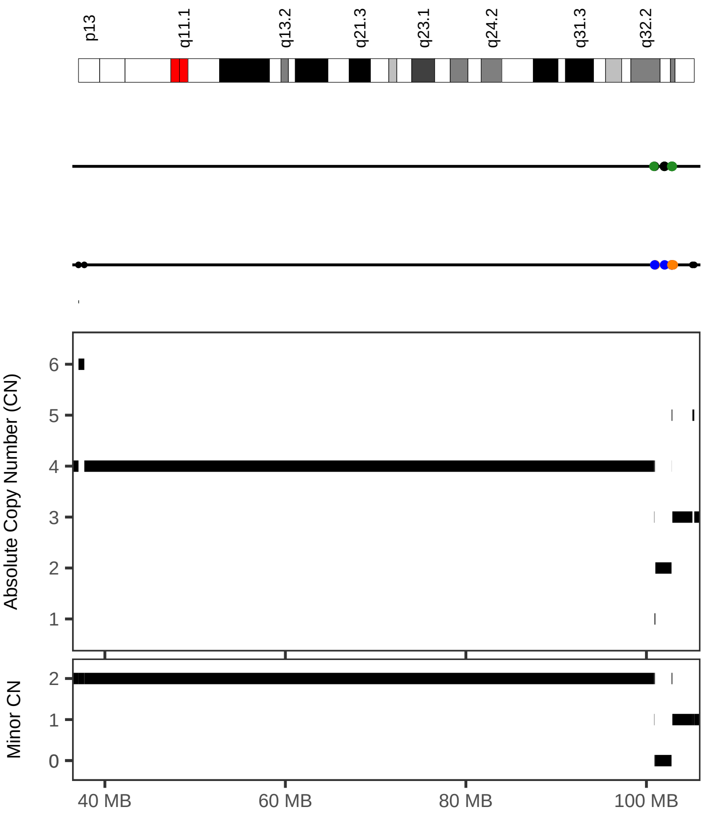

27b05b15−a44b−45ed−a6e3−e7d1ca488ea9

Cancer type	Breast−LobularCA
Position	14:100819629−102872968
Interleaved intrachr. SVs	3
Total SVs (intrachr. + transl.)	27
SV types	DEL: 1; DUP: 0; h2hINV: 1; t2tINV: 1; TRA: 24
SVs in sample	116
Oscillating CN (2 and 3 states)	3, 5
CN segments	8
FDR fragment joints	0.8653243
FDR chr. breakp. enrich.	0
Linked to chrs	10:45002072−118566527;
Purity, ploidy	0.31, 3.39

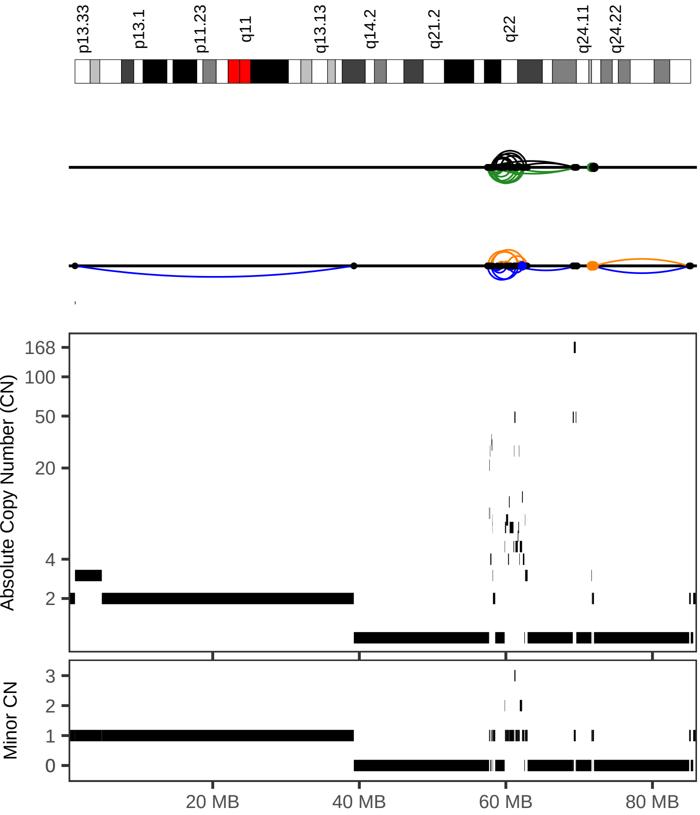

17dffffc−65d6−4209−9075−18a441001f0f

Cancer type	CNS−GBM
Position	12:57486350−69728850
Interleaved intrachr. SVs	57
Total SVs (intrachr. + transl.)	58
SV types	DEL: 11; DUP: 14; h2hINV: 15; t2tINV: 17; TRA: 1
SVs in sample	93
Oscillating CN (2 and 3 states)	3, 6
CN segments	39
FDR fragment joints	0.8360409
FDR chr. breakp. enrich.	0
Linked to chrs	
Purity, ploidy	0.48, 1.95

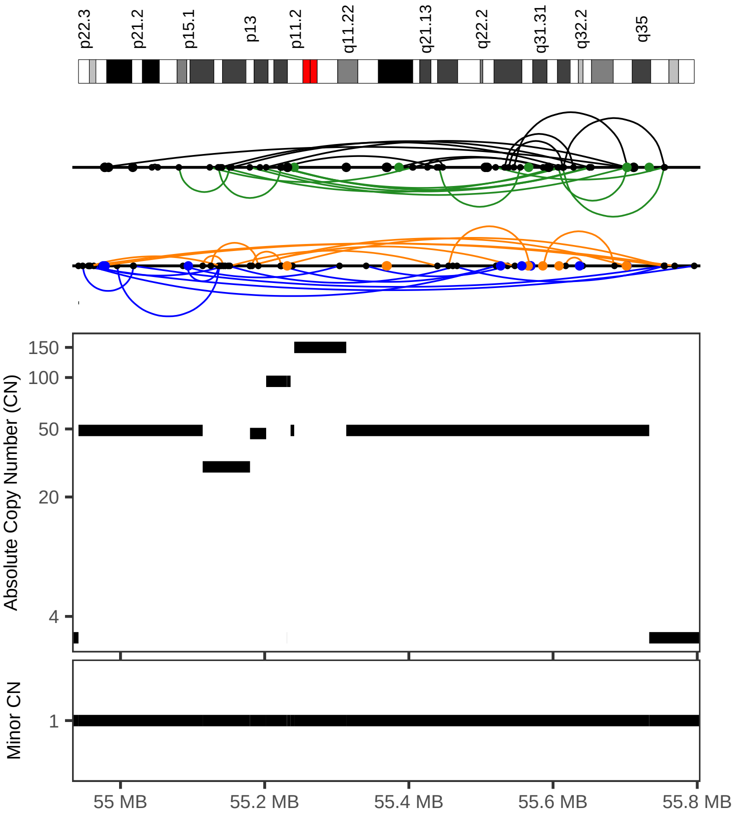

2f4a127f−101a−4192−b3e8−f9be2c8648bc

Cancer type	CNS−GBM
Position	7:54947532−55768719
Interleaved intrachr. SVs	49
Total SVs (intrachr. + transl.)	87
SV types	DEL: 13; DUP: 11; h2hINV: 12; t2tINV: 13; TRA: 38
SVs in sample	212
Oscillating CN (2 and 3 states)	3, 3
CN segments	10
FDR fragment joints	0.9284301
FDR chr. breakp. enrich.	0
Linked to chrs	12:57931744−69260397;
Purity, ploidy	0.94, 1.98

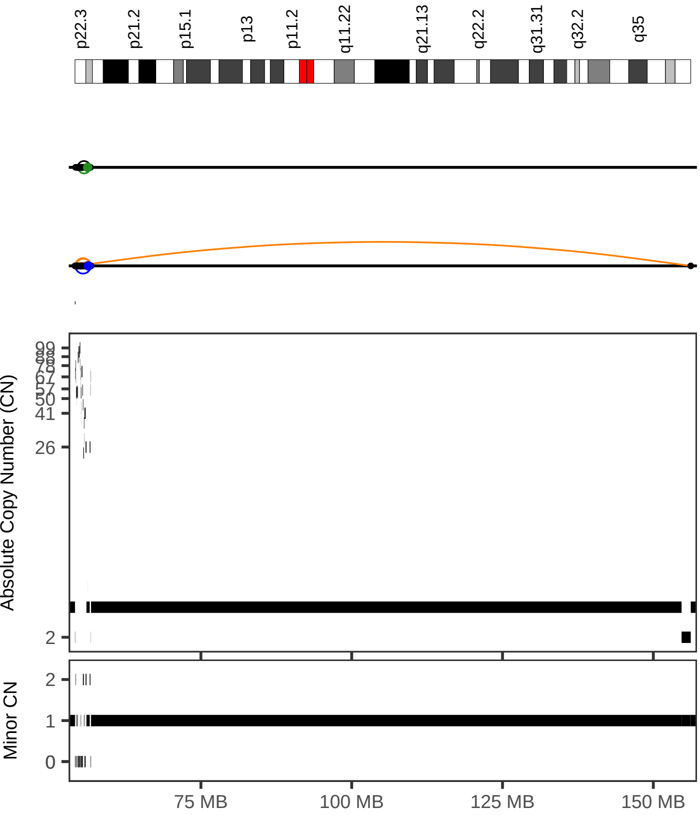

5c984433−33cf−42fc−b3ba−511efcdcab19

Cancer type	CNS−GBM
Position	7:54304614−56720043
Interleaved intrachr. SVs	30
Total SVs (intrachr. + transl.)	32
SV types	DEL: 12; DUP: 6; h2hINV: 6; t2tINV: 6; TRA: 2
SVs in sample	107
Oscillating CN (2 and 3 states)	3, 4
CN segments	40
FDR fragment joints	0.675506
FDR chr. breakp. enrich.	0
Linked to chrs	6:118282394−158970761;
Purity, ploidy	0.73, 2

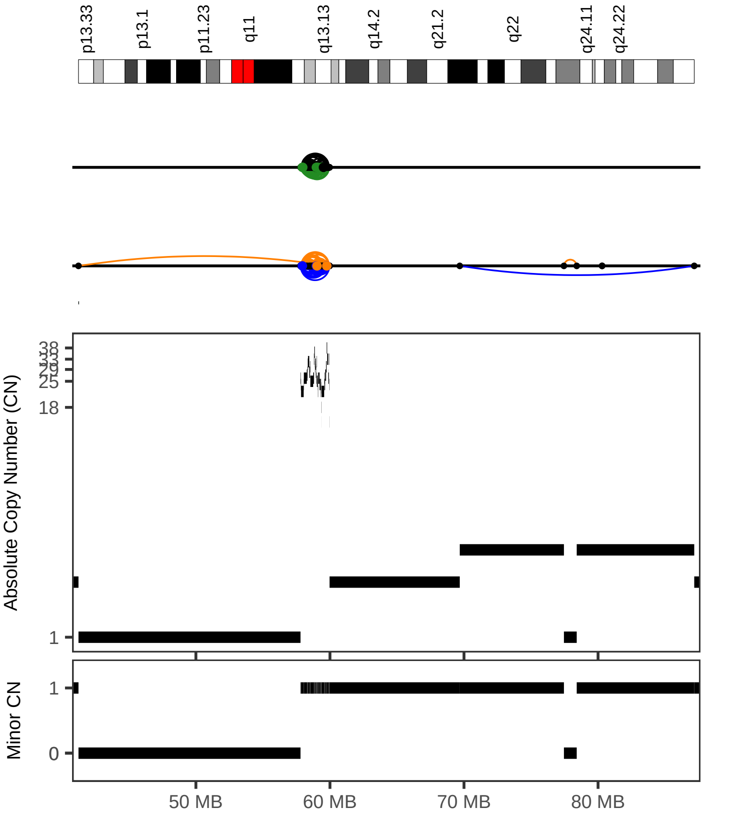

c184c3ca−7ad3−4202−b108−cb9fd5f5d947

Cancer type	CNS−GBM
Position	12:57802243−59971140
Interleaved intrachr. SVs	194
Total SVs (intrachr. + transl.)	207
SV types	DEL: 47; DUP: 49; h2hINV: 49; t2tINV: 49; TRA: 13
SVs in sample	263
Oscillating CN (2 and 3 states)	3, 5
CN segments	48
FDR fragment joints	0.9701252
FDR chr. breakp. enrich.	0
Linked to chrs	
Purity, ploidy	0.91, 2.1

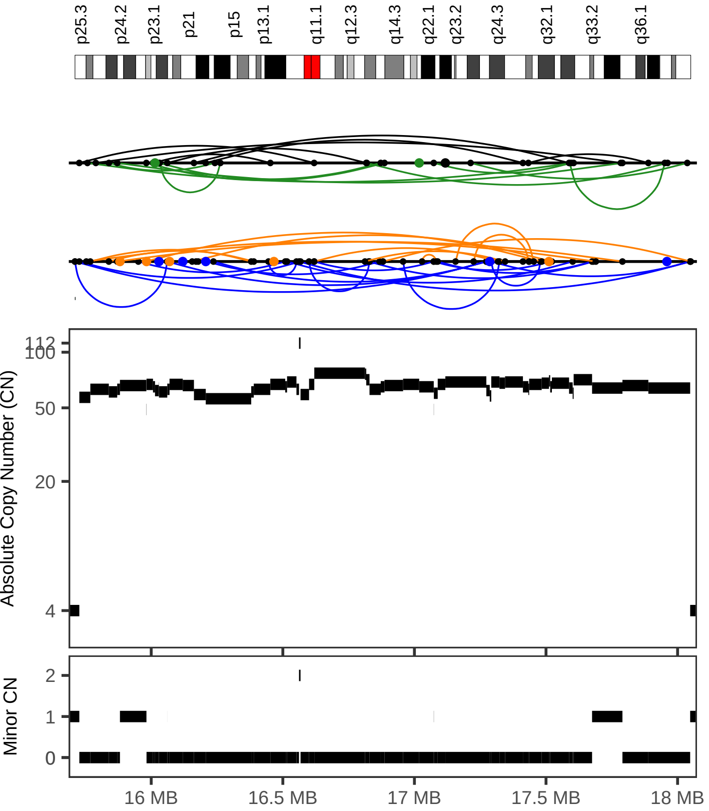

ICGC_MB6

Cancer type	CNS−Medullo
Position	2:15710236−18049954
Interleaved intrachr. SVs	48
Total SVs (intrachr. + transl.)	61
SV types	DEL: 13; DUP: 18; h2hINV: 7; t2tINV: 10; TRA: 13
SVs in sample	81
Oscillating CN (2 and 3 states)	3, 5
CN segments	58
FDR fragment joints	0.5077414
FDR chr. breakp. enrich.	0
Linked to chrs	
Purity, ploidy	0.95, 4

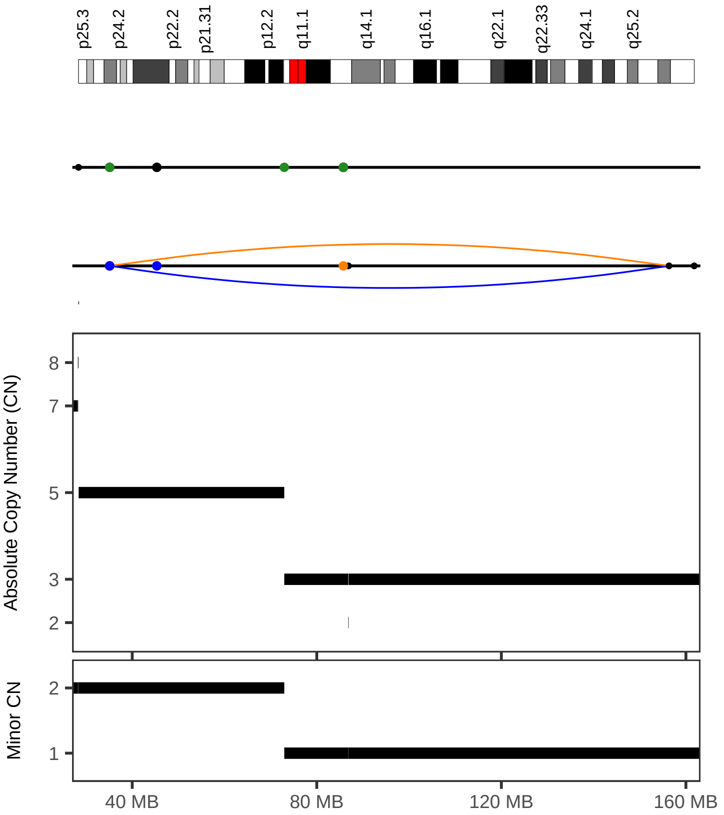

74a0264d−1d31−430d−9a88−e7334c8aa96c

Cancer type	ColoRect−AdenoCA
Position	6:28351517−161800031
Interleaved intrachr. SVs	1
Total SVs (intrachr. + transl.)	22
SV types	DEL: 2; DUP: 2; h2hINV: 1; t2tINV: 0; TRA: 17
SVs in sample	184
Oscillating CN (2 and 3 states)	3, 4
CN segments	5
FDR fragment joints	0.9419607
FDR chr. breakp. enrich.	0
Linked to chrs	10:19155794−74638779;
Purity, ploidy	0.71, 3.06

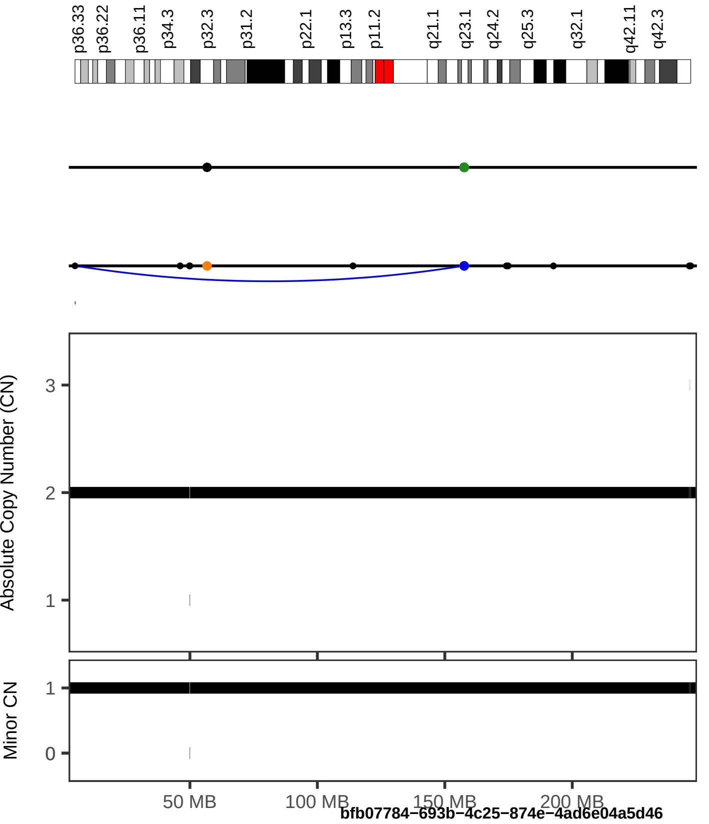

bfb07784−693b−4c25−874e−4ad6e04a5d46

Cancer type	ColoRect−AdenoCA
Position	1:4906911−246471750
Interleaved intrachr. SVs	1
Total SVs (intrachr. + transl.)	24
SV types	DEL: 1; DUP: 6; h2hINV: 0; t2tINV: 0; TRA: 17
SVs in sample	154
Oscillating CN (2 and 3 states)	5, 9
CN segments	9
FDR fragment joints	0.6776251
FDR chr. breakp. enrich.	0
Linked to chrs	3:3465278−174784573;5:58280145−129435612 7:3499849−146680701;
Purity, ploidy	0.58, 2.21

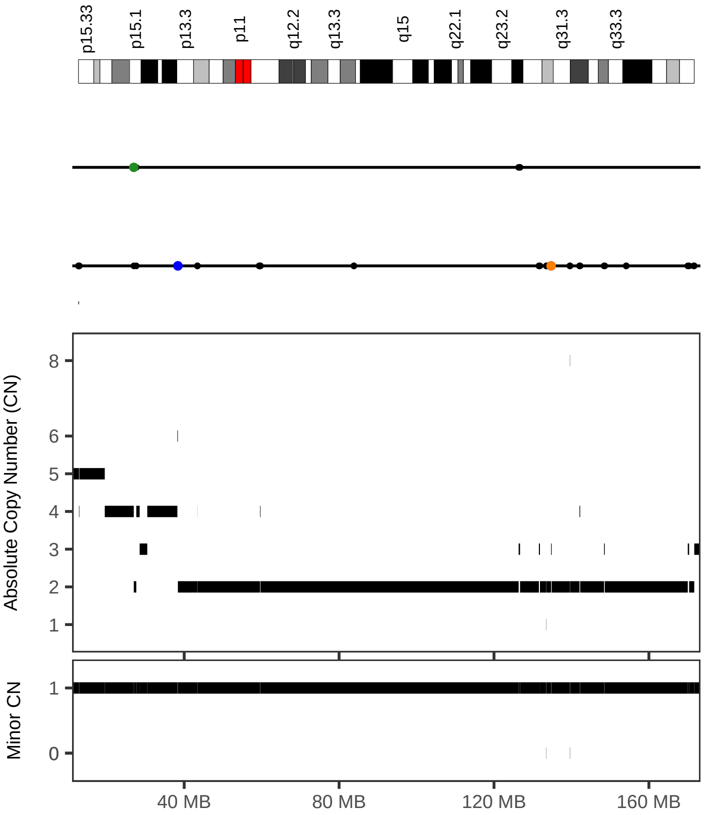

OCCAMS−AH−082

Cancer type	Eso−AdenoCA
Position	5:12699681−171707953
Interleaved intrachr. SVs	1
Total SVs (intrachr. + transl.)	22
SV types	DEL: 7; DUP: 8; h2hINV: 0; t2tINV: 2; TRA: 5
SVs in sample	433
Oscillating CN (2 and 3 states)	6, 10
CN segments	32
FDR fragment joints	0.2070134
FDR chr. breakp. enrich.	0.83
Linked to chrs	
Purity, ploidy	0.18, 2.48

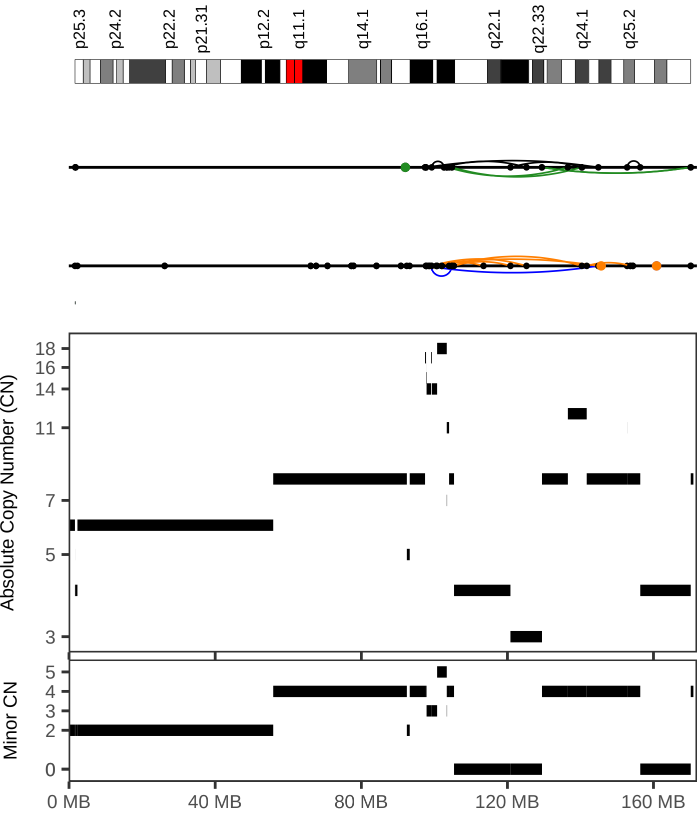

OCCAMS−AH−091

Cancer type	Eso−AdenoCA
Position	6:97713679−170207401
Interleaved intrachr. SVs	21
Total SVs (intrachr. + transl.)	24
SV types	DEL: 7; DUP: 3; h2hINV: 7; t2tINV: 4; TRA: 3
SVs in sample	217
Oscillating CN (2 and 3 states)	3, 5
CN segments	21
FDR fragment joints	0.615458
FDR chr. breakp. enrich.	0
Linked to chrs	
Purity, ploidy	0.56, 4.3

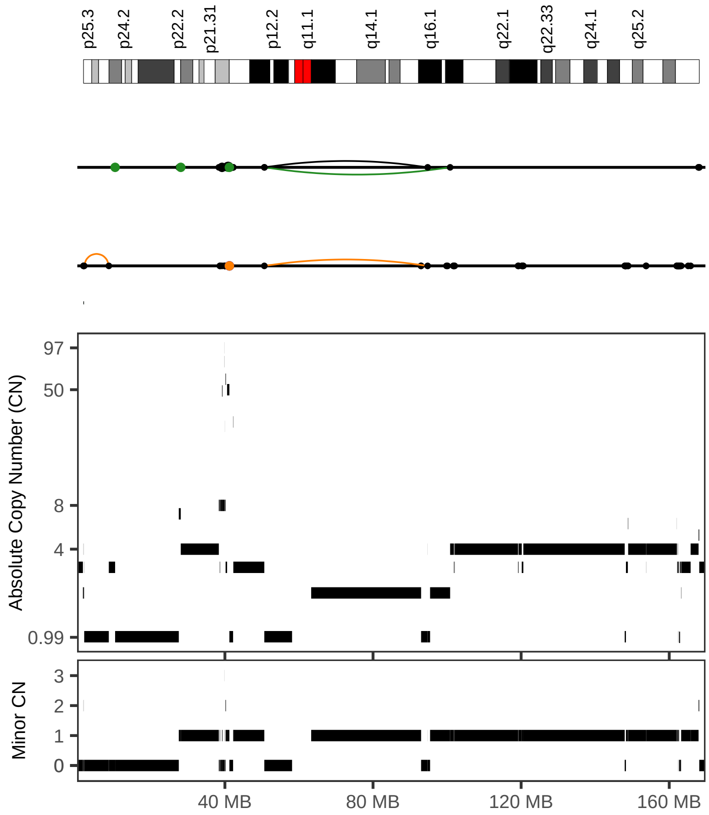

OCCAMS−AH−131

Cancer type	Eso−AdenoCA
Position	6:38358441−42273299
Interleaved intrachr. SVs	14
Total SVs (intrachr. + transl.)	20
SV types	DEL: 5; DUP: 2; h2hINV: 3; t2tINV: 4; TRA: 6
SVs in sample	266
Oscillating CN (2 and 3 states)	3, 3
CN segments	18
FDR fragment joints	0.8572806
FDR chr. breakp. enrich.	0
Linked to chrs	
Purity, ploidy	0.53, 2.63

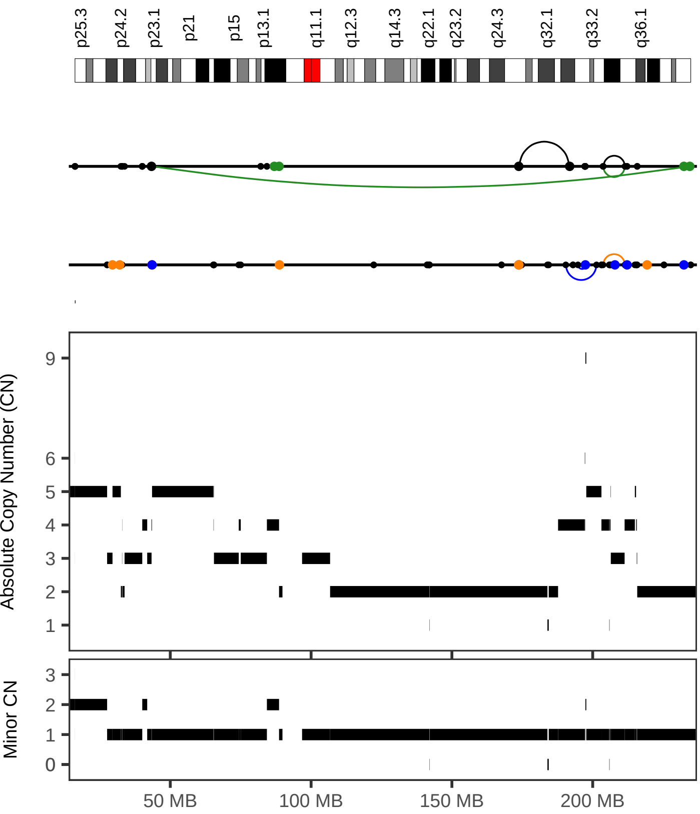

OCCAMS−AH−143

Cancer type	Eso−AdenoCA
Position	2:32444154−234479820
Interleaved intrachr. SVs	3
Total SVs (intrachr. + transl.)	26
SV types	DEL: 2; DUP: 2; h2hINV: 2; t2tINV: 3; TRA: 17
SVs in sample	596
Oscillating CN (2 and 3 states)	5, 9
CN segments	41
FDR fragment joints	0.9179372
FDR chr. breakp. enrich.	0.12
Linked to chrs	7:26018260−146800455;
Purity, ploidy	0.46, 2.6

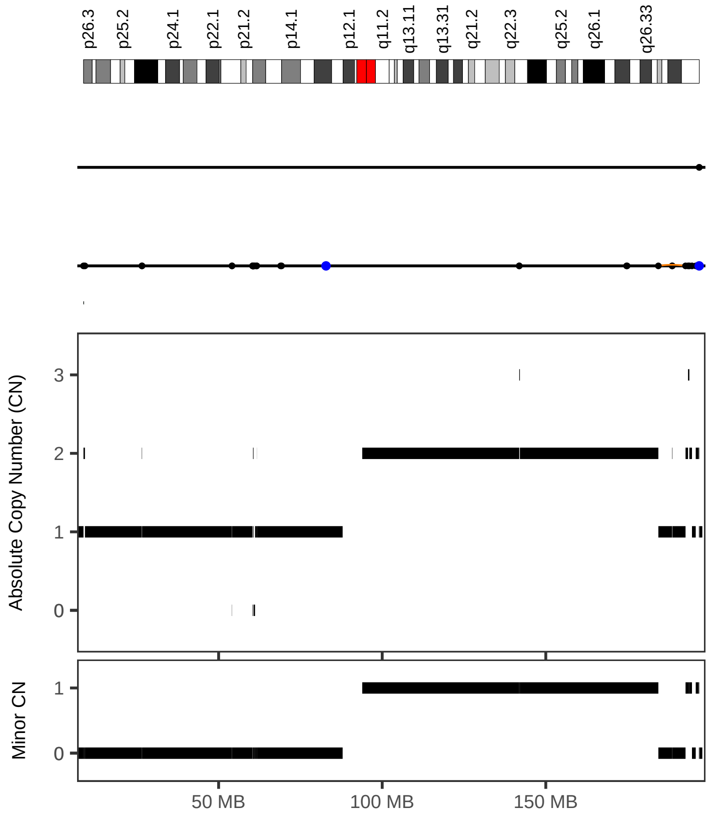

OCCAMS−AH−173

Cancer type	Eso−AdenoCA
Position	3:8692850−196904619
Interleaved intrachr. SVs	1
Total SVs (intrachr. + transl.)	20
SV types	DEL: 10; DUP: 7; h2hINV: 1; t2tINV: 0; TRA: 2
SVs in sample	410
Oscillating CN (2 and 3 states)	6, 20
CN segments	31
FDR fragment joints	0.0726553
FDR chr. breakp. enrich.	0.26
Linked to chrs	
Purity, ploidy	0.6, 1.64

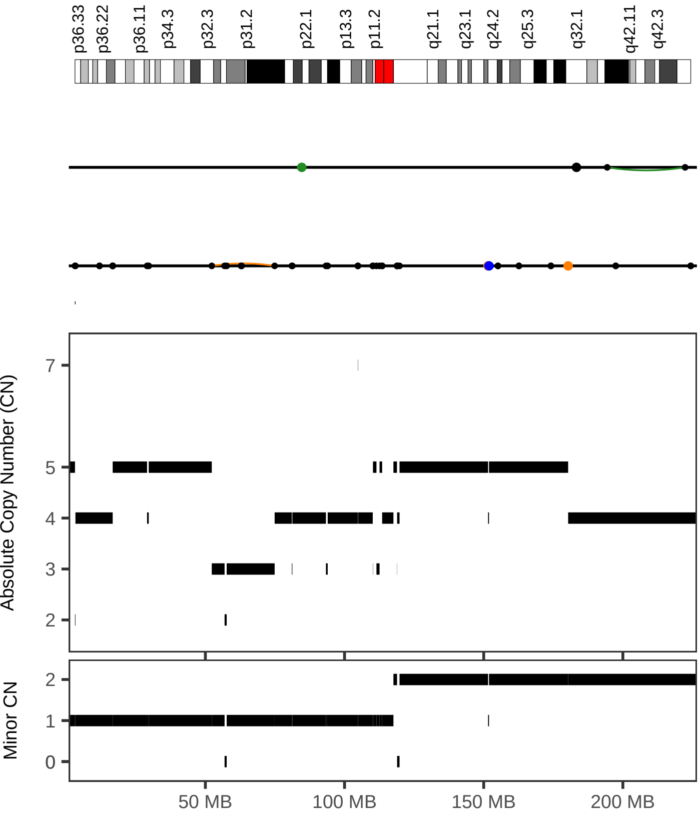

OCCAMS−AH−174

Cancer type	Eso−AdenoCA
Position	1:3155456−224383077
Interleaved intrachr. SVs	1
Total SVs (intrachr. + transl.)	21
SV types	DEL: 13; DUP: 2; h2hINV: 0; t2tINV: 1; TRA: 5
SVs in sample	572
Oscillating CN (2 and 3 states)	6, 8
CN segments	28
FDR fragment joints	0.00583861
FDR chr. breakp. enrich.	0.11
Linked to chrs	
Purity, ploidy	0.29, 4.26

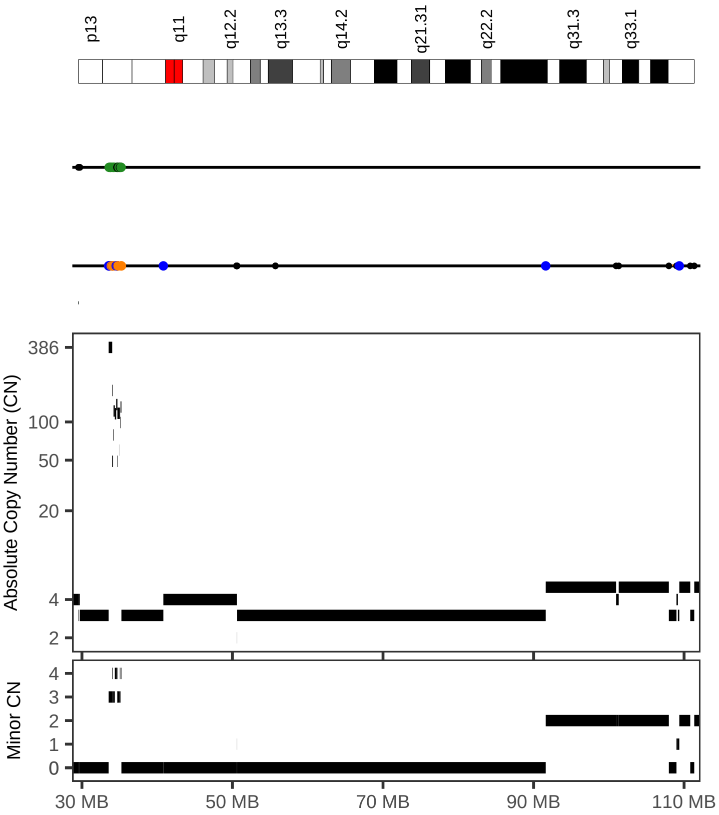

OCCAMS−AH−174

Cancer type	Eso−AdenoCA
Position	13:33722637−35237034
Interleaved intrachr. SVs	6
Total SVs (intrachr. + transl.)	34
SV types	DEL: 1; DUP: 2; h2hINV: 1; t2tINV: 2; TRA: 28
SVs in sample	572
Oscillating CN (2 and 3 states)	3, 4
CN segments	13
FDR fragment joints	0.615458
FDR chr. breakp. enrich.	0
Linked to chrs	
Purity, ploidy	0.29, 4.26

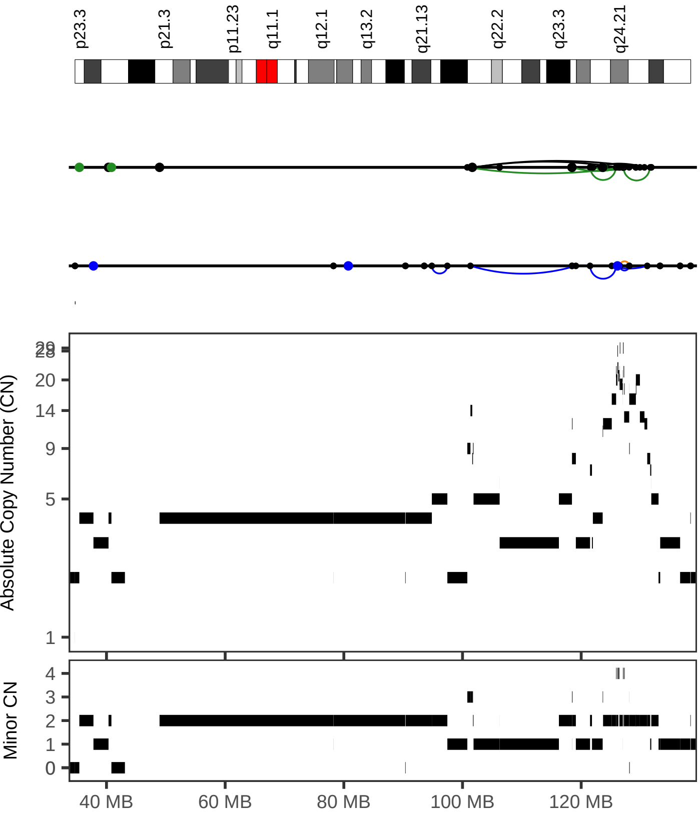

OCCAMS−AH−182

Cancer type	Eso−AdenoCA
Position	8:100807746−131632357
Interleaved intrachr. SVs	17
Total SVs (intrachr. + transl.)	22
SV types	DEL: 1; DUP: 5; h2hINV: 5; t2tINV: 6; TRA: 5
SVs in sample	223
Oscillating CN (2 and 3 states)	3, 4
CN segments	44
FDR fragment joints	0.5435077
FDR chr. breakp. enrich.	0
Linked to chrs	
Purity, ploidy	0.17, 3.08

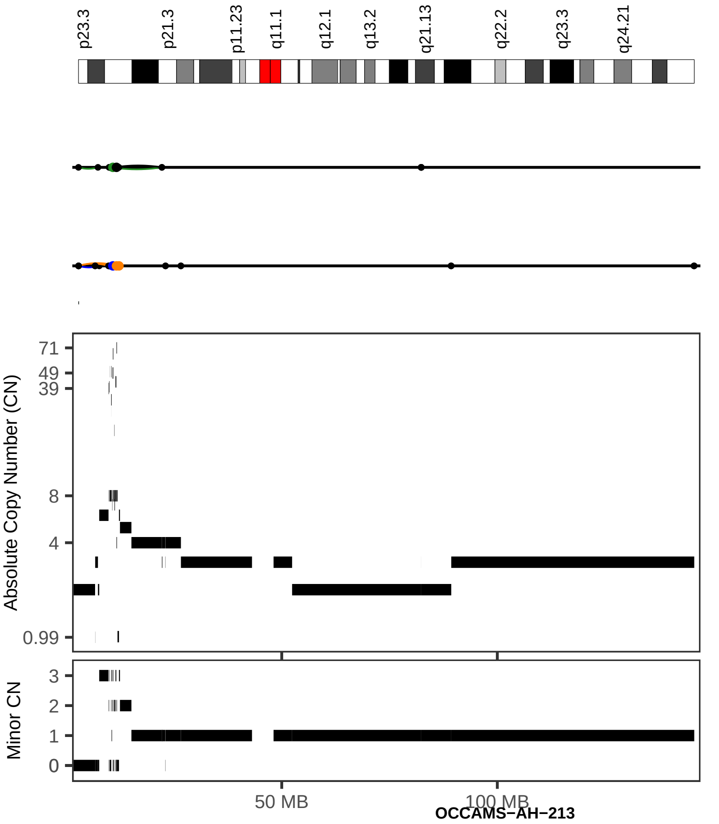

OCCAMS−AH−213

Cancer type	Eso−AdenoCA
Position	8:2853805−22205777
Interleaved intrachr. SVs	16
Total SVs (intrachr. + transl.)	21
SV types	DEL: 4; DUP: 3; h2hINV: 5; t2tINV: 4; TRA: 5
SVs in sample	283
Oscillating CN (2 and 3 states)	3, 5
CN segments	39
FDR fragment joints	0.9462199
FDR chr. breakp. enrich.	0
Linked to chrs	4:19575521−184603131;7:48065329−141802390
Purity, ploidy	0.57, 2.68

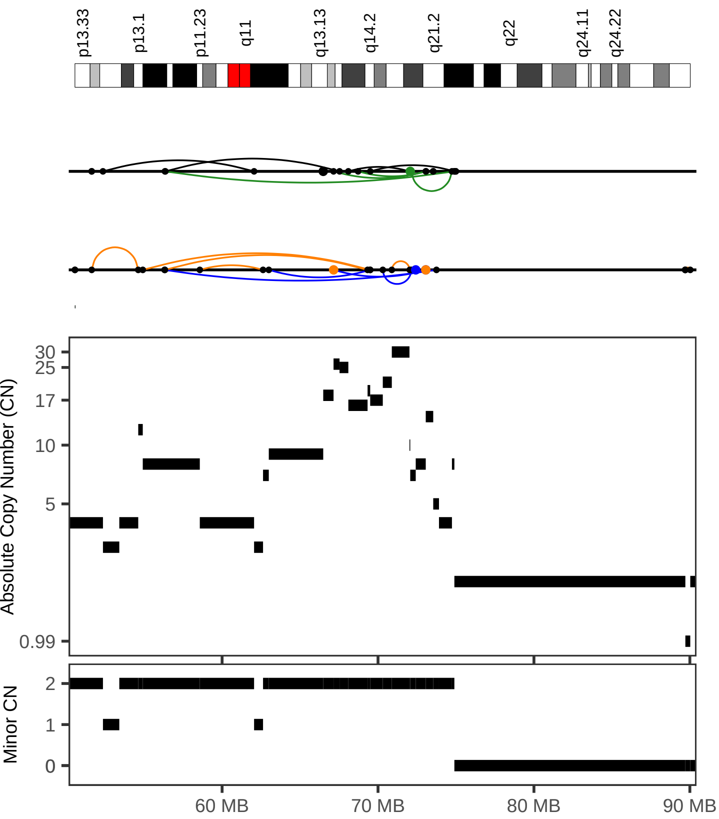

OCCAMS−PS−001

Cancer type	Eso−AdenoCA
Position	12:51638579−74896840
Interleaved intrachr. SVs	17
Total SVs (intrachr. + transl.)	23
SV types	DEL: 5; DUP: 4; h2hINV: 4; t2tINV: 4; TRA: 6
SVs in sample	90
Oscillating CN (2 and 3 states)	3, 3
CN segments	25
FDR fragment joints	0.9546976
FDR chr. breakp. enrich.	0
Linked to chrs	11:7398369−66888946;
Purity, ploidy	0.72, 3.17

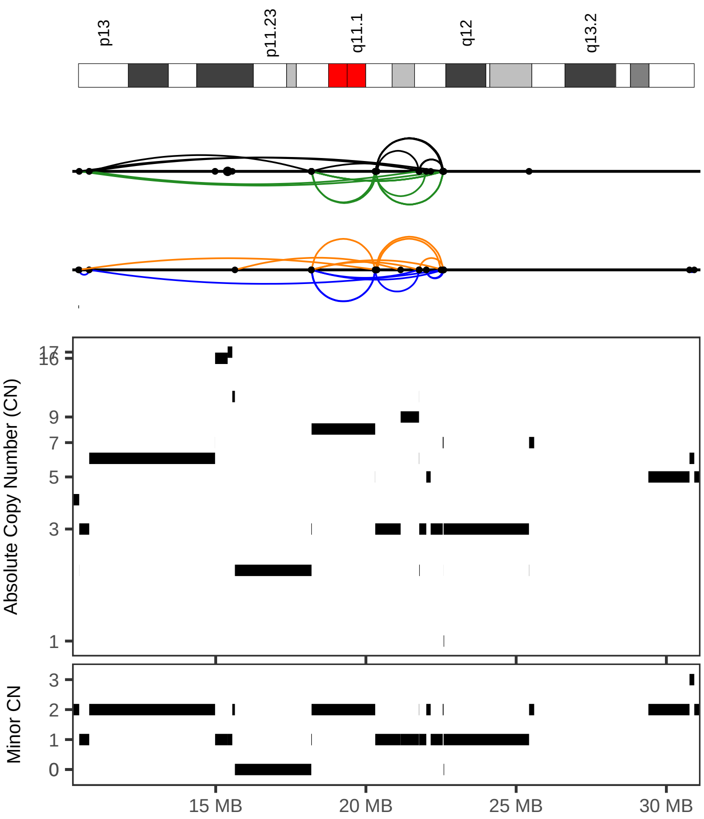

OCCAMS−RS−035

Cancer type	Eso−AdenoCA
Position	20:10433007−22586479
Interleaved intrachr. SVs	43
Total SVs (intrachr. + transl.)	44
SV types	DEL: 8; DUP: 10; h2hINV: 13; t2tINV: 12; TRA: 1
SVs in sample	179
Oscillating CN (2 and 3 states)	3, 5
CN segments	26
FDR fragment joints	0.7642985
FDR chr. breakp. enrich.	0
Linked to chrs	
Purity, ploidy	0.64, 2.27

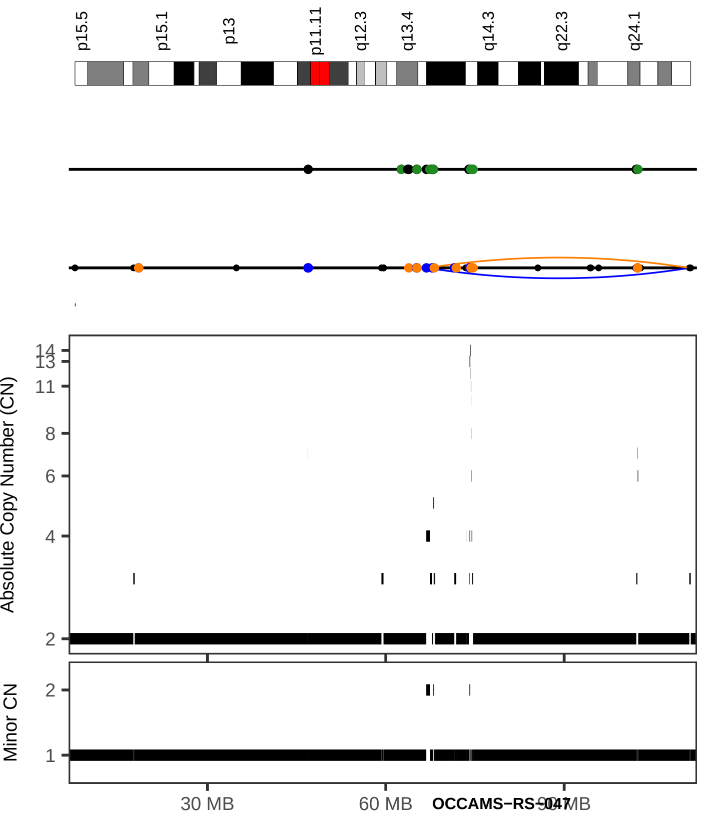

OCCAMS−RS−047

Cancer type	Eso−AdenoCA
Position	11:66812374−111289396
Interleaved intrachr. SVs	3
Total SVs (intrachr. + transl.)	35
SV types	DEL: 1; DUP: 2; h2hINV: 0; t2tINV: 0; TRA: 32
SVs in sample	894
Oscillating CN (2 and 3 states)	5, 6
CN segments	31
FDR fragment joints	0.9757835
FDR chr. breakp. enrich.	0
Linked to chrs	1:23826407−235519805;12:56993350−123682991 18:19478394−72412982;4:6718600−185350689 5:32316033−176769547;

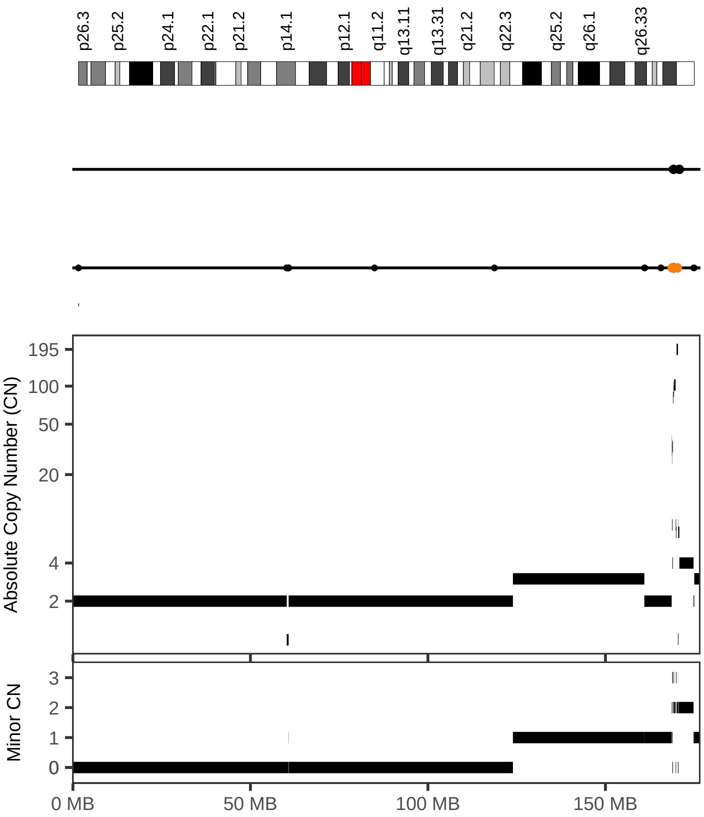

OCCAMS−SH−020

Cancer type	Eso−AdenoCA
Position	3:168692440−170511890
Interleaved intrachr. SVs	36
Total SVs (intrachr. + transl.)	41
SV types	DEL: 12; DUP: 9; h2hINV: 8; t2tINV: 7; TRA: 5
SVs in sample	191
Oscillating CN (2 and 3 states)	3, 4
CN segments	19
FDR fragment joints	0.6776251
FDR chr. breakp. enrich.	0
Linked to chrs	
Purity, ploidy	0.23, 2.45

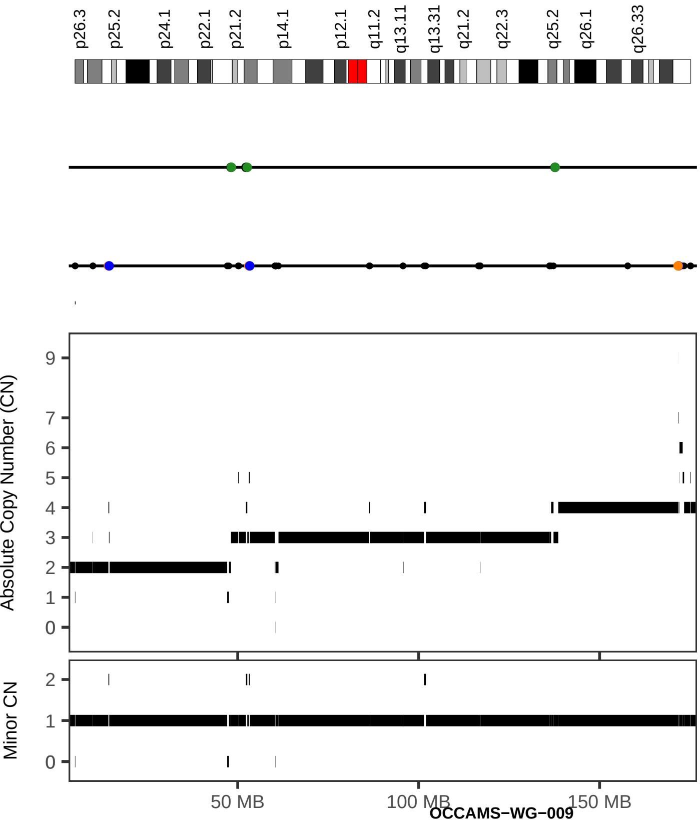

OCCAMS−WG−009

Cancer type	Eso−AdenoCA
Position	3:5019418−175187404
Interleaved intrachr. SVs	1
Total SVs (intrachr. + transl.)	28
SV types	DEL: 7; DUP: 9; h2hINV: 0; t2tINV: 0; TRA: 12
SVs in sample	490
Oscillating CN (2 and 3 states)	6, 20
CN segments	46
FDR fragment joints	0.4101979
FDR chr. breakp. enrich.	1
Linked to chrs	11:7867256−37730506;17:28749738−71397420
Purity, ploidy	0.25, 2.63

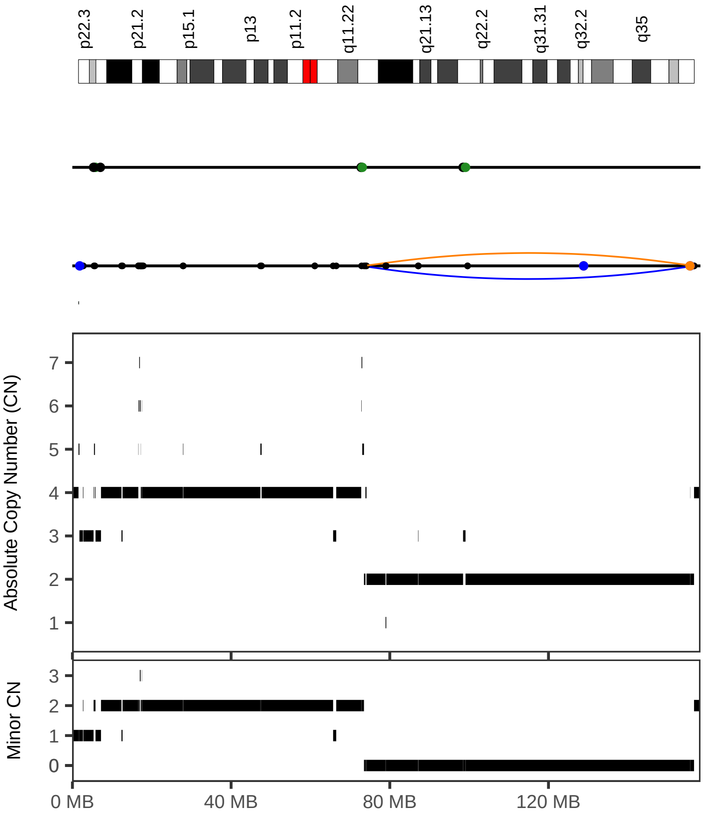

OCCAMS−WG−009

Cancer type	Eso−AdenoCA
Position	7:1524679−156742340
Interleaved intrachr. SVs	1
Total SVs (intrachr. + transl.)	30
SV types	DEL: 4; DUP: 13; h2hINV: 0; t2tINV: 0; TRA: 13
SVs in sample	490
Oscillating CN (2 and 3 states)	5, 16
CN segments	41
FDR fragment joints	0.1609654
FDR chr. breakp. enrich.	0.62
Linked to chrs	
Purity, ploidy	0.25, 2.63

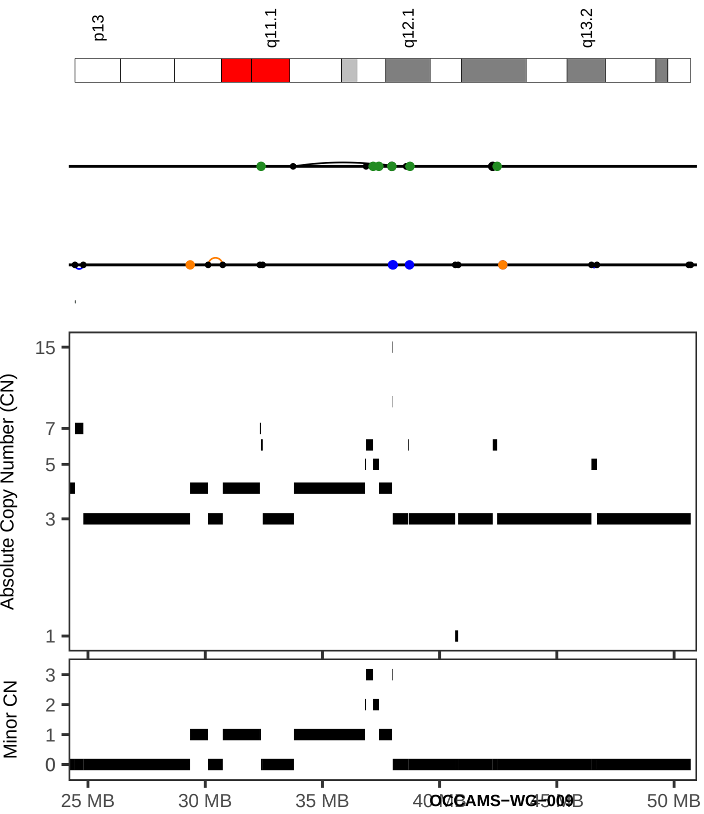

OCCAMS−WG−009

Cancer type	Eso−AdenoCA
Position	22:24447127−50708748
Interleaved intrachr. SVs	1
Total SVs (intrachr. + transl.)	23
SV types	DEL: 2; DUP: 5; h2hINV: 2; t2tINV: 0; TRA: 14
SVs in sample	490
Oscillating CN (2 and 3 states)	4, 7
CN segments	24
FDR fragment joints	0.615458
FDR chr. breakp. enrich.	0
Linked to chrs	10:4579401−102025656;18:2404184−10696514 19:1710473−50936621;2:85478953−232558622

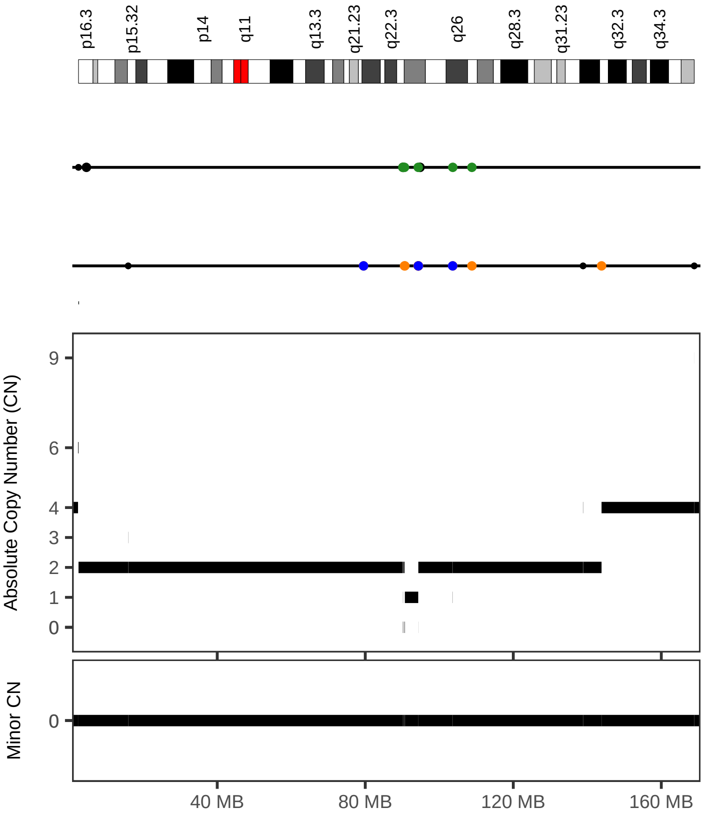

OCCAMS−ZZ−019

Cancer type	Eso−AdenoCA
Position	4:15934512−138883629
Interleaved intrachr. SVs	1
Total SVs (intrachr. + transl.)	26
SV types	DEL: 0; DUP: 2; h2hINV: 1; t2tINV: 0; TRA: 23
SVs in sample	190
Oscillating CN (2 and 3 states)	3, 9
CN segments	13
FDR fragment joints	0.615458
FDR chr. breakp. enrich.	0
Linked to chrs	20:35281199−53598198;
Purity, ploidy	0.29, 3.21

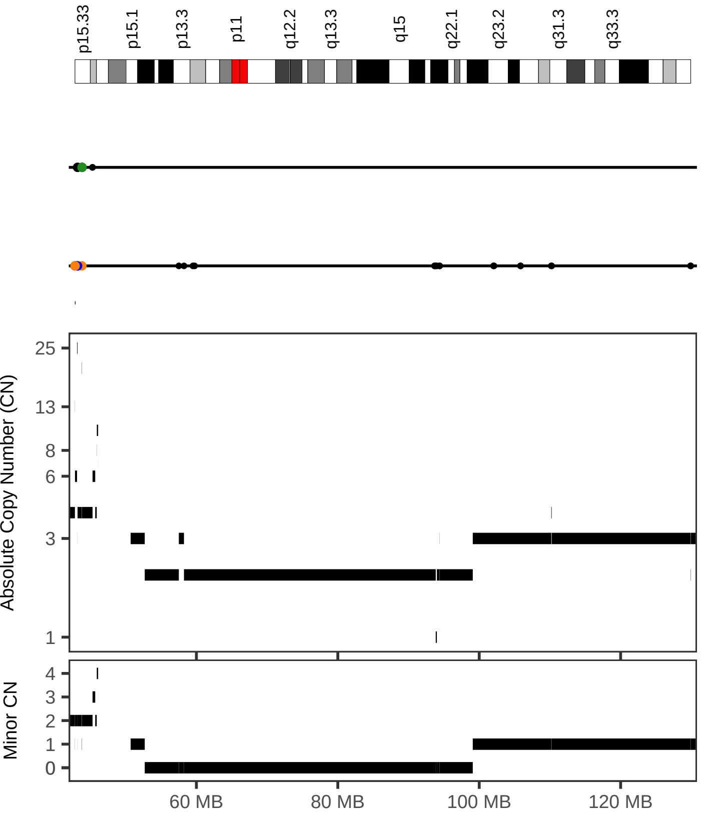

3e5f451a−5882−4914−ae1a−95c898c2bcca

Cancer type	Head−SCC
Position	5:42823466−129917634
Interleaved intrachr. SVs	1
Total SVs (intrachr. + transl.)	20
SV types	DEL: 5; DUP: 5; h2hINV: 1; t2tINV: 1; TRA: 8
SVs in sample	362
Oscillating CN (2 and 3 states)	4, 13
CN segments	24
FDR fragment joints	0.615458
FDR chr. breakp. enrich.	0.81
Linked to chrs	2:164623105−179954500;
Purity, ploidy	0.58, 3.09

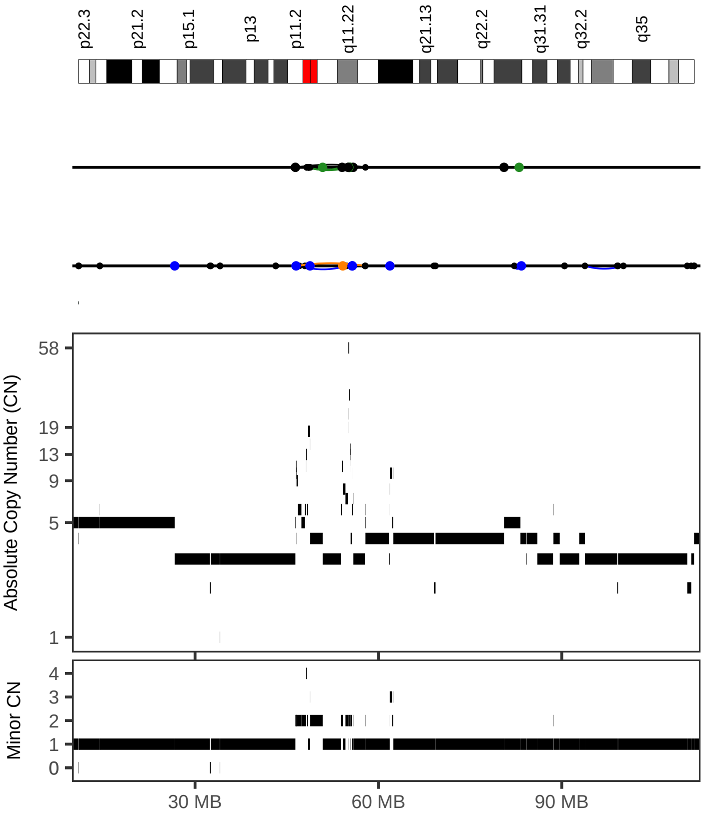

3e5f451a−5882−4914−ae1a−95c898c2bcca

Cancer type	Head−SCC
Position	7:46513922−57877590
Interleaved intrachr. SVs	6
Total SVs (intrachr. + transl.)	20
SV types	DEL: 2; DUP: 1; h2hINV: 2; t2tINV: 1; TRA: 14
SVs in sample	362
Oscillating CN (2 and 3 states)	3, 4
CN segments	42
FDR fragment joints	0.7735152
FDR chr. breakp. enrich.	0
Linked to chrs	18:3708220−54685995;
Purity, ploidy	0.58, 3.09

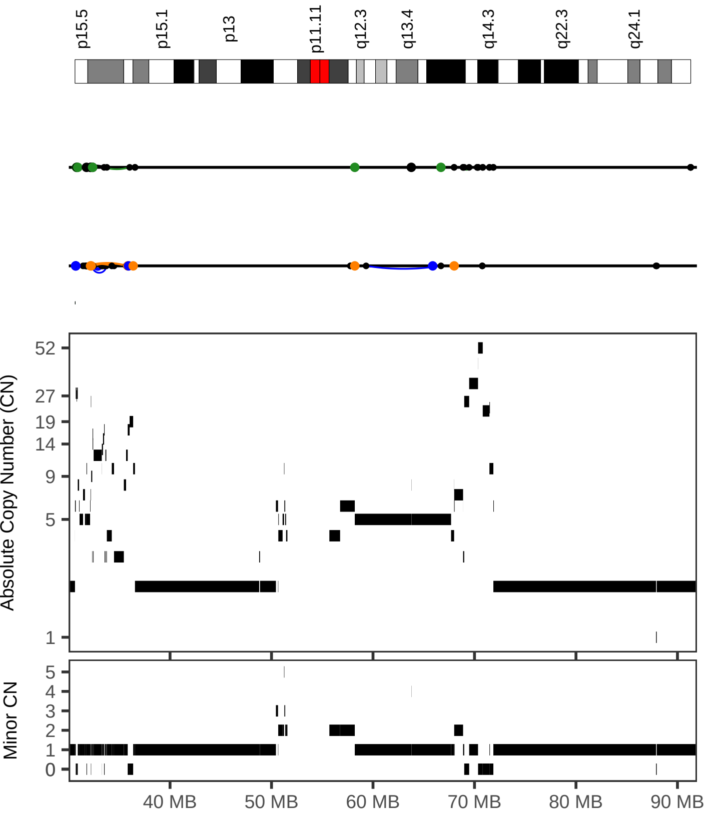

3e5f451a−5882−4914−ae1a−95c898c2bcca

Cancer type	Head−SCC
Position	11:31037993−36019692
Interleaved intrachr. SVs	11
Total SVs (intrachr. + transl.)	20
SV types	DEL: 3; DUP: 6; h2hINV: 1; t2tINV: 1; TRA: 9
SVs in sample	362
Oscillating CN (2 and 3 states)	3, 3
CN segments	29
FDR fragment joints	0.662962
FDR chr. breakp. enrich.	0
Linked to chrs	
Purity, ploidy	0.58, 3.09

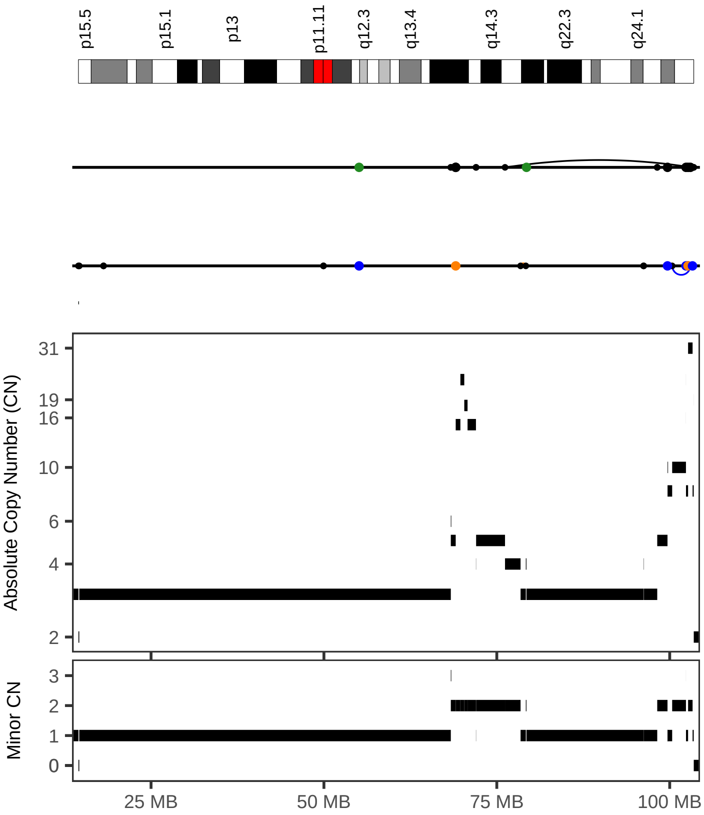

ad6c9d09−2c03−4786−a72d−dd2aa5f603d4

Cancer type	Head−SCC
Position	11:14499146−103309486
Interleaved intrachr. SVs	1
Total SVs (intrachr. + transl.)	21
SV types	DEL: 2; DUP: 2; h2hINV: 1; t2tINV: 1; TRA: 15
SVs in sample	360
Oscillating CN (2 and 3 states)	6, 6
CN segments	26
FDR fragment joints	0.6776251
FDR chr. breakp. enrich.	0.01
Linked to chrs	3:48762562−189108049;
Purity, ploidy	0.65, 3.12

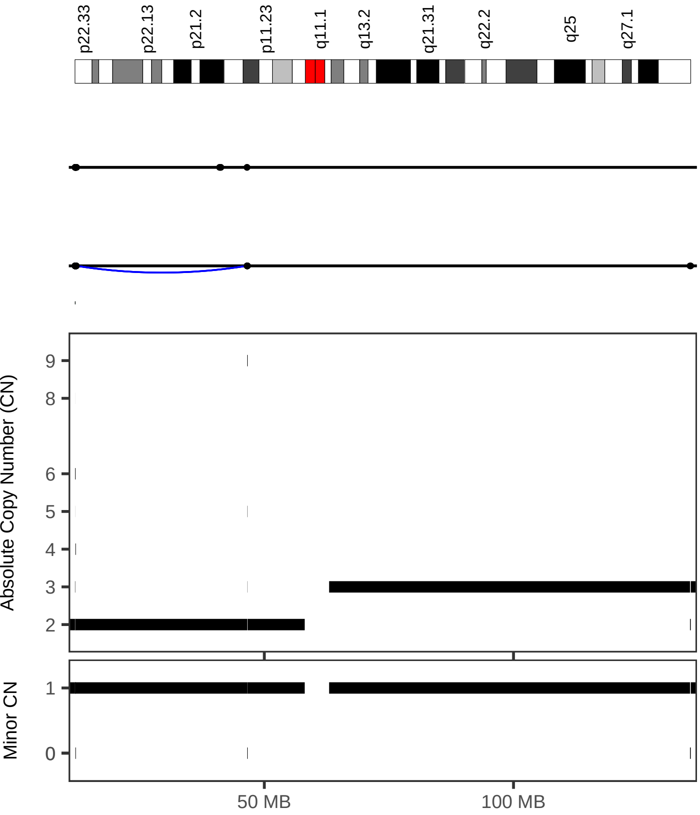

d7641ca1−a062−4dd2−9414−447af7311a84

Cancer type	Head−SCC
Position	X:12009105−46643912
Interleaved intrachr. SVs	20
Total SVs (intrachr. + transl.)	20
SV types	DEL: 2; DUP: 13; h2hINV: 2; t2tINV: 3; TRA: 0
SVs in sample	65
Oscillating CN (2 and 3 states)	3, 4
CN segments	14
FDR fragment joints	0.05741733
FDR chr. breakp. enrich.	0
Linked to chrs	
Purity, ploidy	0.4, 3.62

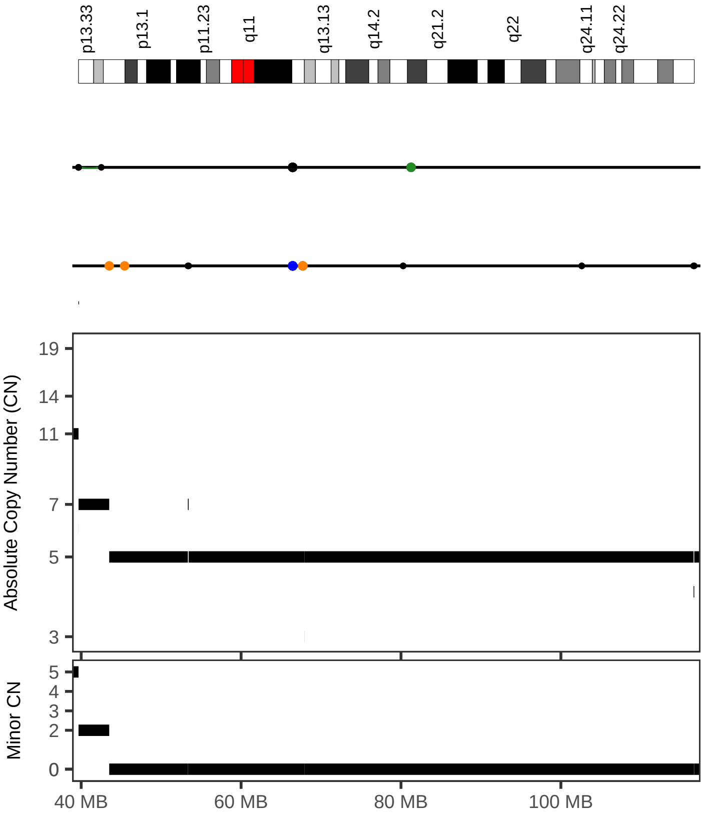

d89b1fd6−bef4−4803−8ed3−3b442be600b6

Cancer type	Head−SCC
Position	12:39664500−116679872
Interleaved intrachr. SVs	1
Total SVs (intrachr. + transl.)	28
SV types	DEL: 1; DUP: 1; h2hINV: 0; t2tINV: 1; TRA: 25
SVs in sample	317
Oscillating CN (2 and 3 states)	4, 5
CN segments	9
FDR fragment joints	0.3711451
FDR chr. breakp. enrich.	0
Linked to chrs	2:48024778−84474306;
Purity, ploidy	0.49, 5.3

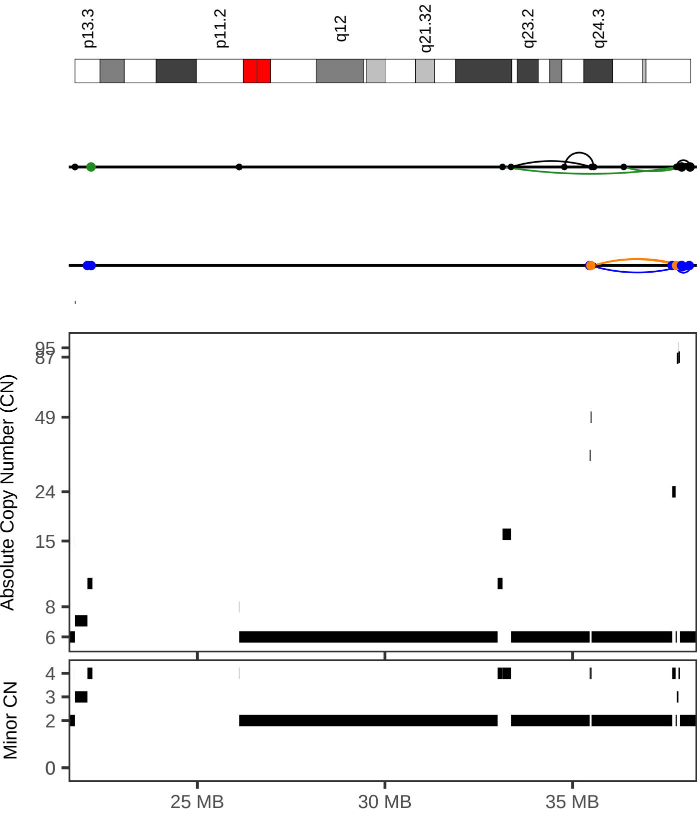

d89b1fd6−bef4−4803−8ed3−3b442be600b6

Cancer type	Head−SCC
Position	17:33134735−38151504
Interleaved intrachr. SVs	15
Total SVs (intrachr. + transl.)	24
SV types	DEL: 3; DUP: 6; h2hINV: 4; t2tINV: 2; TRA: 9
SVs in sample	317
Oscillating CN (2 and 3 states)	3, 3
CN segments	11
FDR fragment joints	0.615458
FDR chr. breakp. enrich.	0
Linked to chrs	
Purity, ploidy	0.49, 5.3

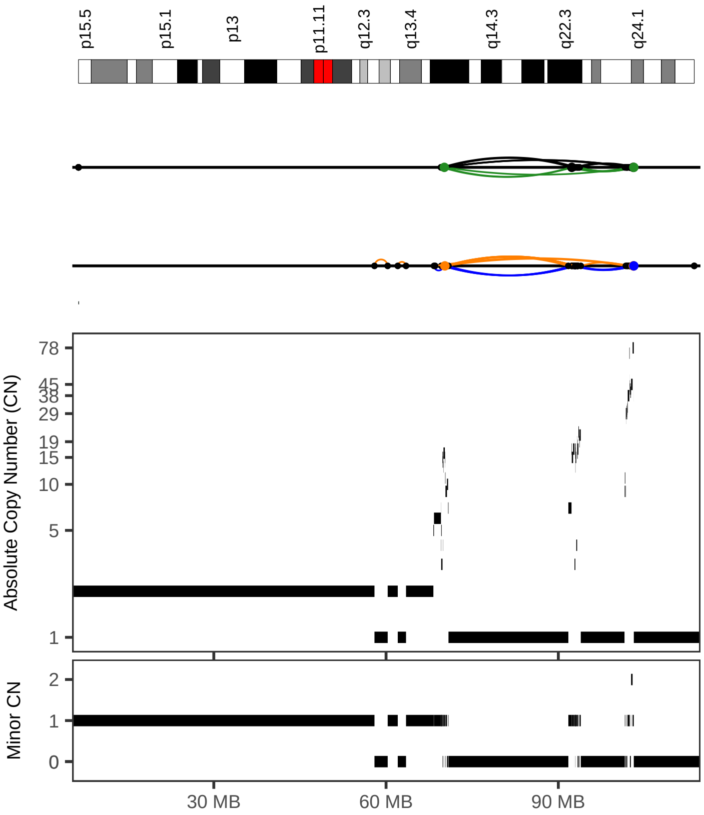

e303b8c4−6428−4134−bb9d−a95dc1d8e35e

Cancer type	Head−SCC
Position	11:68346390−103142549
Interleaved intrachr. SVs	45
Total SVs (intrachr. + transl.)	51
SV types	DEL: 7; DUP: 12; h2hINV: 13; t2tINV: 13; TRA: 6
SVs in sample	224
Oscillating CN (2 and 3 states)	3, 6
CN segments	61
FDR fragment joints	0.8653243
FDR chr. breakp. enrich.	0
Linked to chrs	1:1362882−85717386;
Purity, ploidy	0.85, 2

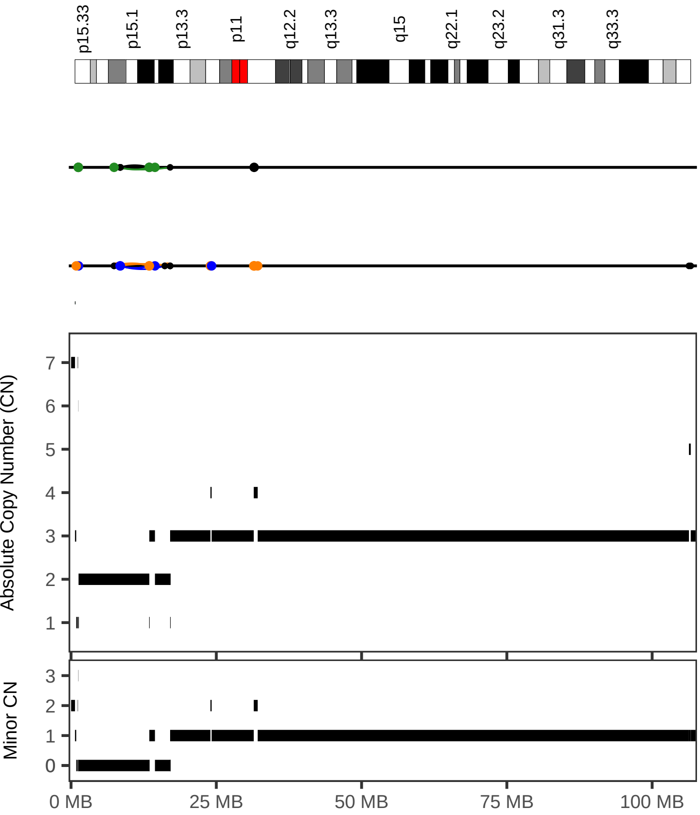

70e306ee−9584−49d6−81a1−a49d837ab6aa

Cancer type	Kidney−ChRCC
Position	5:7407698−17053089
Interleaved intrachr. SVs	15
Total SVs (intrachr. + transl.)	24
SV types	DEL: 6; DUP: 3; h2hINV: 2; t2tINV: 4; TRA: 9
SVs in sample	305
Oscillating CN (2 and 3 states)	3, 8
CN segments	8
FDR fragment joints	0.8653243
FDR chr. breakp. enrich.	0
Linked to chrs	3:27225774−76470708;
Purity, ploidy	0.89, 3.06

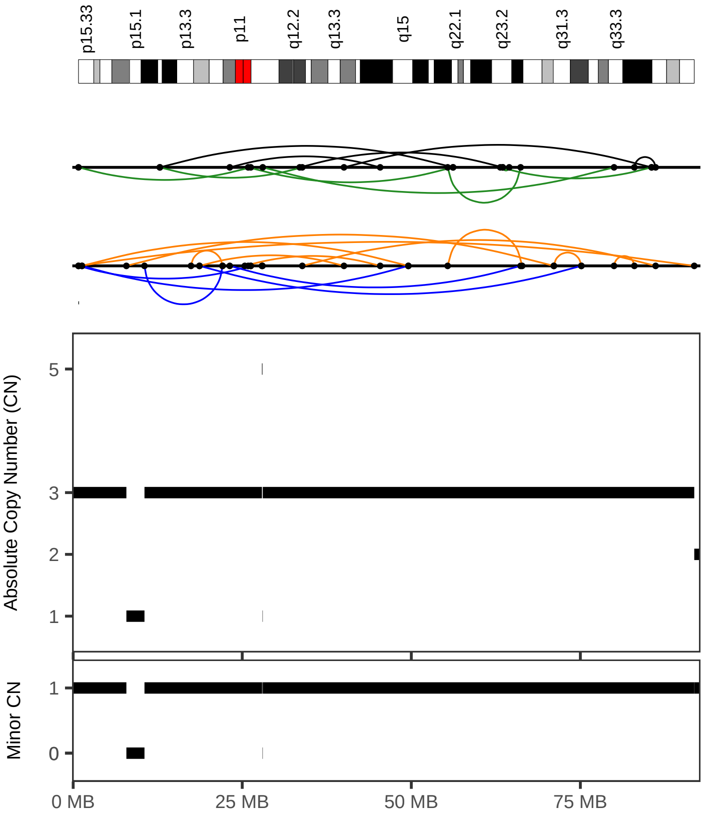

83b3060c−2449−4581−88ef−817d126e4525

Cancer type	Kidney−ChRCC
Position	5:784281−86129942
Interleaved intrachr. SVs	26
Total SVs (intrachr. + transl.)	26
SV types	DEL: 10; DUP: 5; h2hINV: 5; t2tINV: 6; TRA: 0
SVs in sample	40
Oscillating CN (2 and 3 states)	3, 4
CN segments	6
FDR fragment joints	0.6776251
FDR chr. breakp. enrich.	0
Linked to chrs	
Purity, ploidy	0.91, 1.71

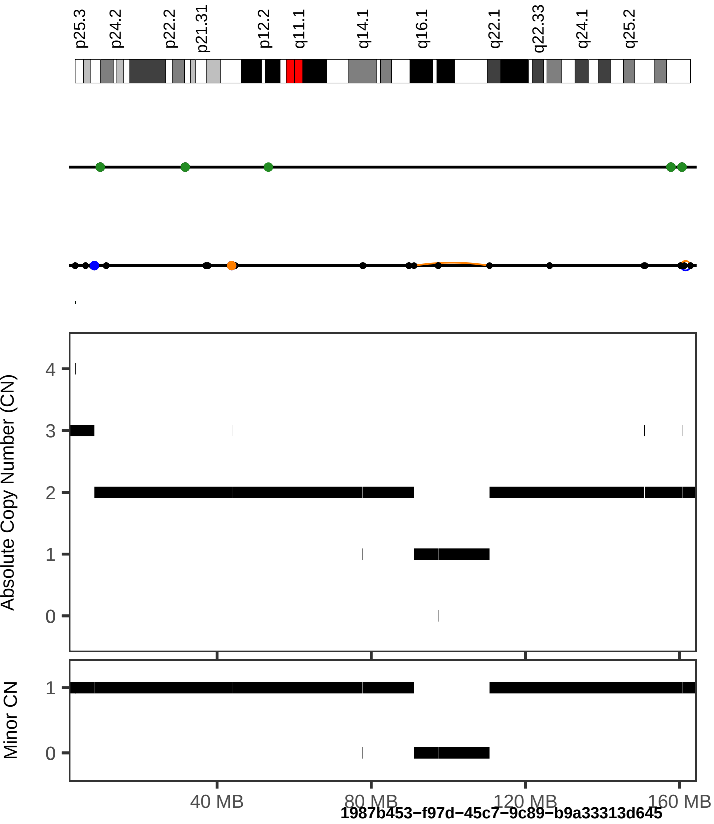

1987b453−f97d−45c7−9c89−b9a33313d645

Cancer type	Liver−HCC
Position	6:5868963−162834996
Interleaved intrachr. SVs	1
Total SVs (intrachr. + transl.)	27
SV types	DEL: 6; DUP: 8; h2hINV: 0; t2tINV: 0; TRA: 13
SVs in sample	524
Oscillating CN (2 and 3 states)	5, 16
CN segments	16
FDR fragment joints	0.615458
FDR chr. breakp. enrich.	0.86
Linked to chrs	11:9340114−77018015;16:25250822−30860383 20:20959449−62129527;
Purity, ploidy	0.74, 1.99

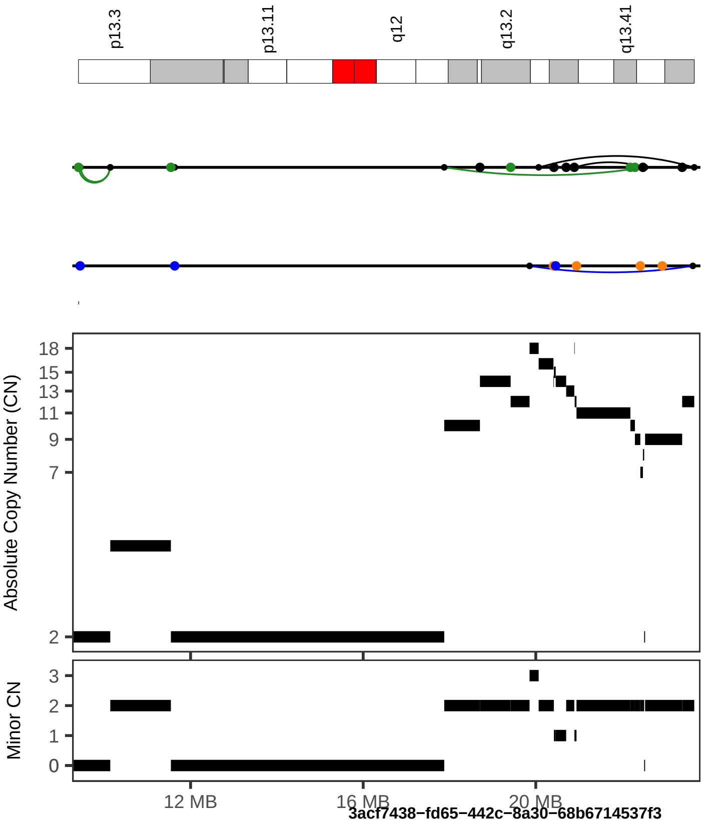

3acf7438−fd65−442c−8a30−68b6714537f3

Cancer type	Liver−HCC
Position	19:9401647−23672451
Interleaved intrachr. SVs	3
Total SVs (intrachr. + transl.)	24
SV types	DEL: 0; DUP: 2; h2hINV: 1; t2tINV: 3; TRA: 18
SVs in sample	222
Oscillating CN (2 and 3 states)	3, 4
CN segments	23
FDR fragment joints	0.6776251
FDR chr. breakp. enrich.	0
Linked to chrs	5:141997−63388892;9:6739657−10343802
Purity, ploidy	0.36, 3.55

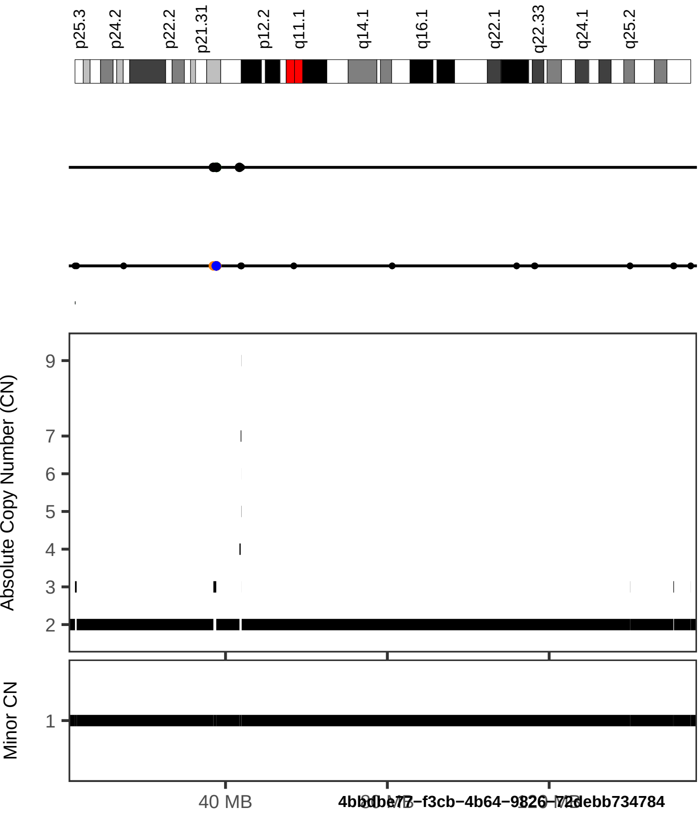

4bbdbe77−f3cb−4b64−9826−72debb734784

Cancer type	Liver−HCC
Position	6:36997592−43940332
Interleaved intrachr. SVs	2
Total SVs (intrachr. + transl.)	22
SV types	DEL: 0; DUP: 4; h2hINV: 0; t2tINV: 0; TRA: 18
SVs in sample	445
Oscillating CN (2 and 3 states)	3, 4
CN segments	8
FDR fragment joints	0.615458
FDR chr. breakp. enrich.	0.37
Linked to chrs	1:53503836−192549015;15:62559317−62899220 17:8805223−79503105;2:85528204−99868581
Purity, ploidy	0.82, 2.08

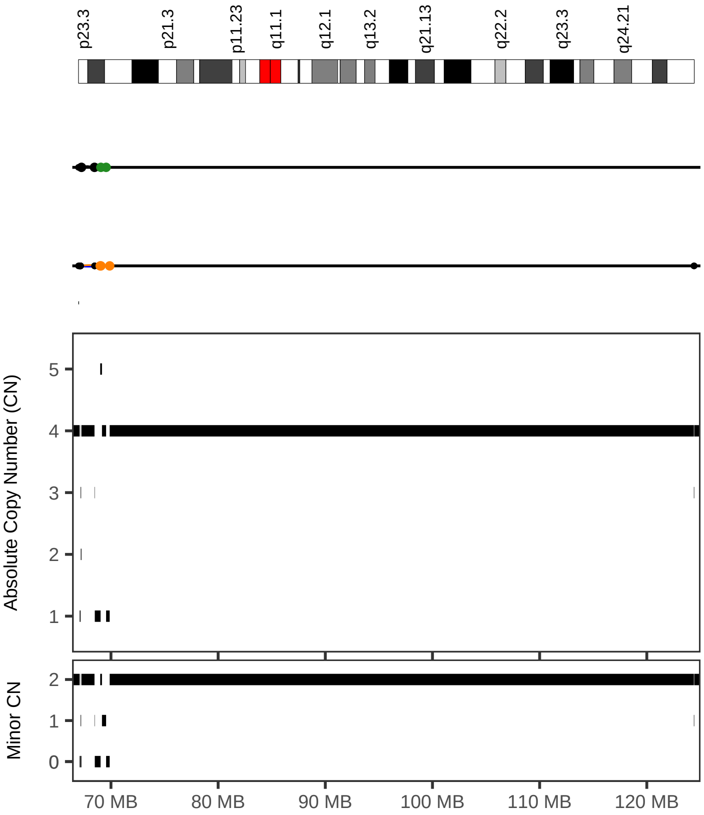

52292ffc−0902−4d97−b461−20723987a177

Cancer type	Liver−HCC
Position	8:66967723−69160044
Interleaved intrachr. SVs	15
Total SVs (intrachr. + transl.)	20
SV types	DEL: 6; DUP: 3; h2hINV: 4; t2tINV: 2; TRA: 5
SVs in sample	129
Oscillating CN (2 and 3 states)	3, 5
CN segments	15
FDR fragment joints	0.662962
FDR chr. breakp. enrich.	0
Linked to chrs	
Purity, ploidy	0.79, 3.28

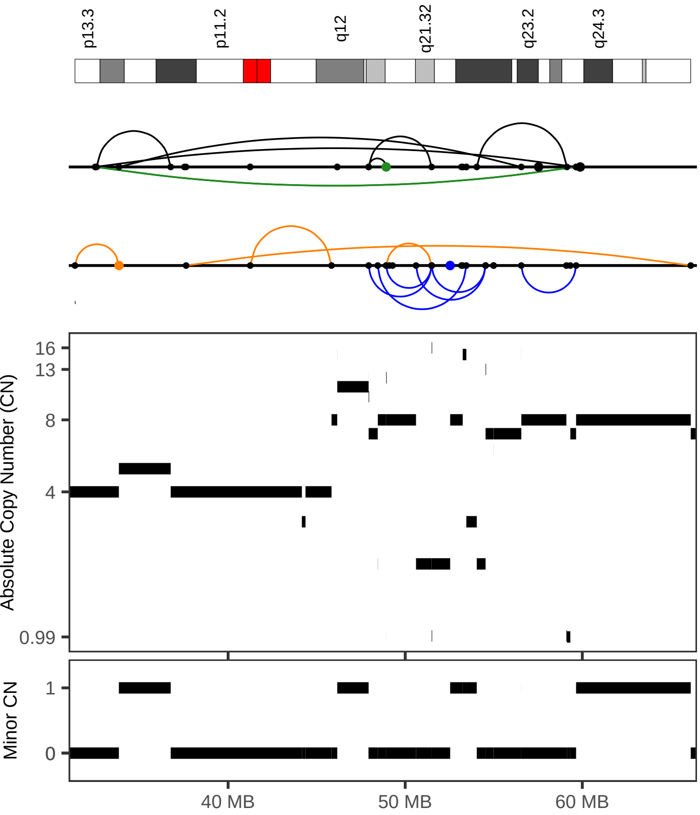

b865dec4−f051−4fbe−9405−f832ff2010d7

Cancer type	Liver−HCC
Position	17:31353361−66122826
Interleaved intrachr. SVs	21
Total SVs (intrachr. + transl.)	26
SV types	DEL: 5; DUP: 6; h2hINV: 6; t2tINV: 4; TRA: 5
SVs in sample	245
Oscillating CN (2 and 3 states)	3, 3
CN segments	35
FDR fragment joints	0.9961719
FDR chr. breakp. enrich.	0
Linked to chrs	7:29593133−29728788;
Purity, ploidy	0.78, 3.47

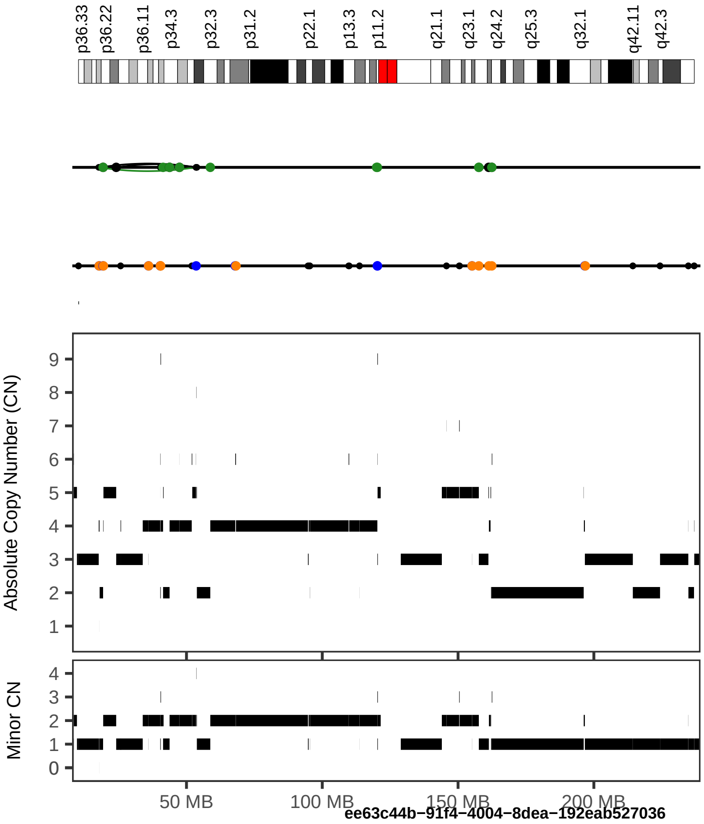

ee63c44b−91f4−4004−8dea−192eab527036

Cancer type	Liver−HCC
Position	1:17700321−53826709
Interleaved intrachr. SVs	3
Total SVs (intrachr. + transl.)	22
SV types	DEL: 0; DUP: 0; h2hINV: 2; t2tINV: 1; TRA: 19
SVs in sample	405
Oscillating CN (2 and 3 states)	6, 9
CN segments	28
FDR fragment joints	0.992996
FDR chr. breakp. enrich.	0
Linked to chrs	12:6457439−132375236;3:49271633−172369973
Purity, ploidy	0.67, 2.88

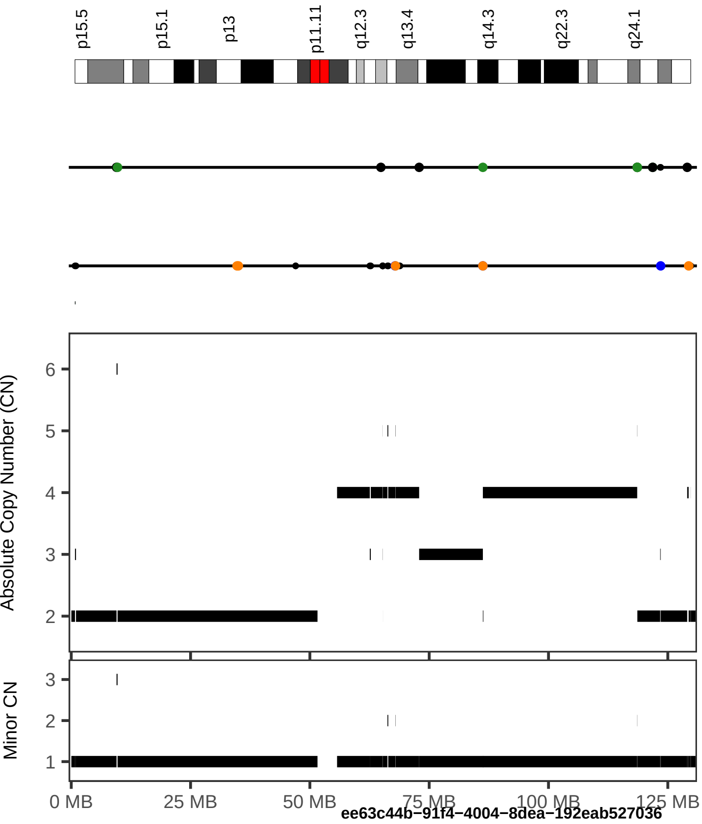

ee63c44b−91f4−4004−8dea−192eab527036

Cancer type	Liver−HCC
Position	11:781704−129805938
Interleaved intrachr. SVs	1
Total SVs (intrachr. + transl.)	31
SV types	DEL: 1; DUP: 8; h2hINV: 1; t2tINV: 0; TRA: 21
SVs in sample	405
Oscillating CN (2 and 3 states)	5, 7
CN segments	27
FDR fragment joints	0.7013708
FDR chr. breakp. enrich.	0.02
Linked to chrs	1:17700321−53826708;3:49271633−172369973
Purity, ploidy	0.67, 2.88

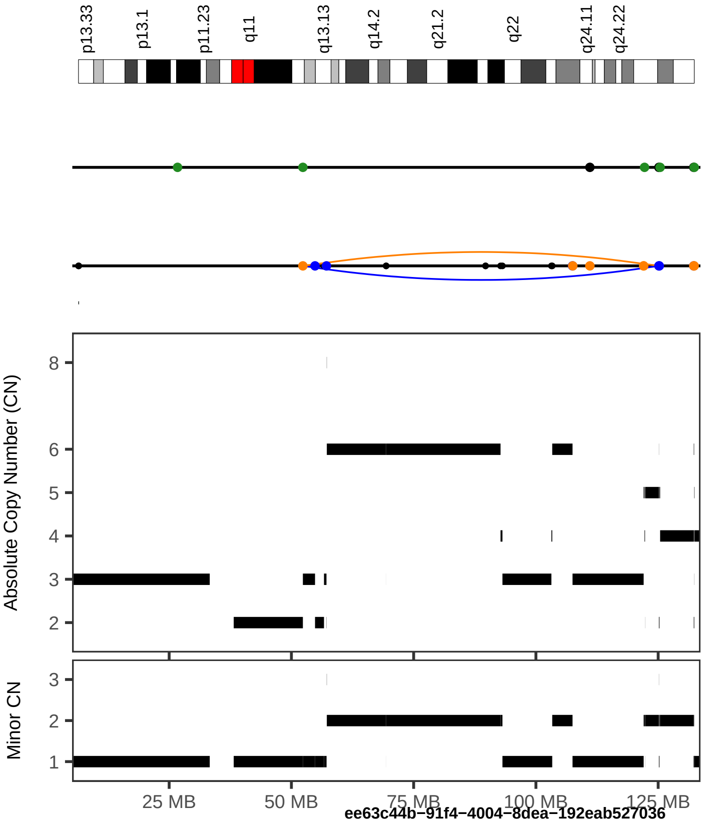

ee63c44b−91f4−4004−8dea−192eab527036

Cancer type	Liver−HCC
Position	12:6457439−132375237
Interleaved intrachr. SVs	1
Total SVs (intrachr. + transl.)	30
SV types	DEL: 1; DUP: 8; h2hINV: 0; t2tINV: 0; TRA: 21
SVs in sample	405
Oscillating CN (2 and 3 states)	6, 7
CN segments	29
FDR fragment joints	0.4101979
FDR chr. breakp. enrich.	0.01
Linked to chrs	1:17700321−53826708;17:19411292−80161264
Purity, ploidy	0.67, 2.88

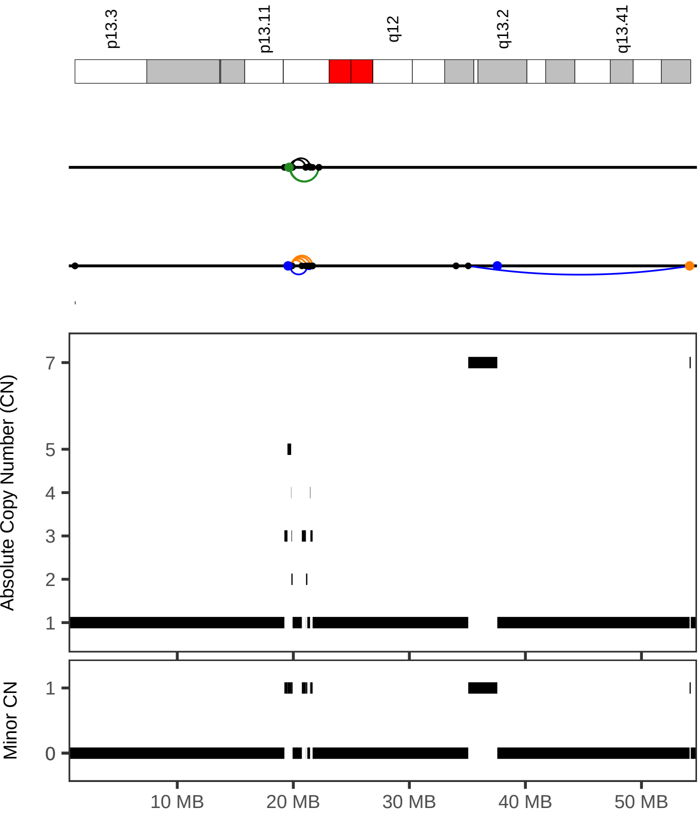

HX25

Cancer type	Liver−HCC
Position	19:19448037−22206921
Interleaved intrachr. SVs	21
Total SVs (intrachr. + transl.)	23
SV types	DEL: 6; DUP: 5; h2hINV: 4; t2tINV: 6; TRA: 2
SVs in sample	111
Oscillating CN (2 and 3 states)	3, 6
CN segments	14
FDR fragment joints	0.9990251
FDR chr. breakp. enrich.	0
Linked to chrs	
Purity, ploidy	0.77, 1.73

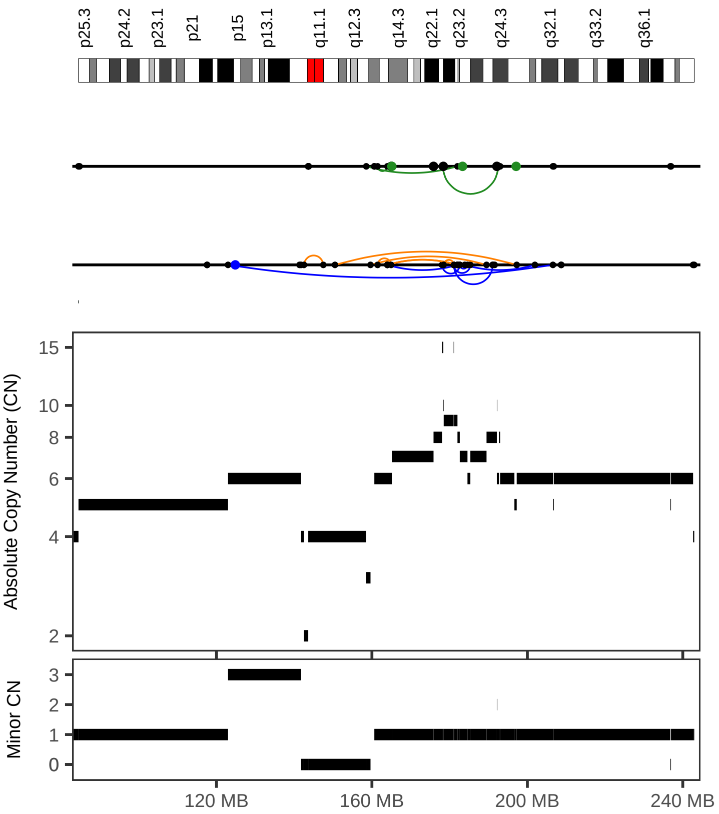

RK025

Cancer type	Liver−HCC
Position	2:150490130−201942437
Interleaved intrachr. SVs	17
Total SVs (intrachr. + transl.)	24
SV types	DEL: 6; DUP: 7; h2hINV: 1; t2tINV: 3; TRA: 7
SVs in sample	71
Oscillating CN (2 and 3 states)	3, 6
CN segments	21
FDR fragment joints	0.8653243
FDR chr. breakp. enrich.	0
Linked to chrs	
Purity, ploidy	0.33, 4.17

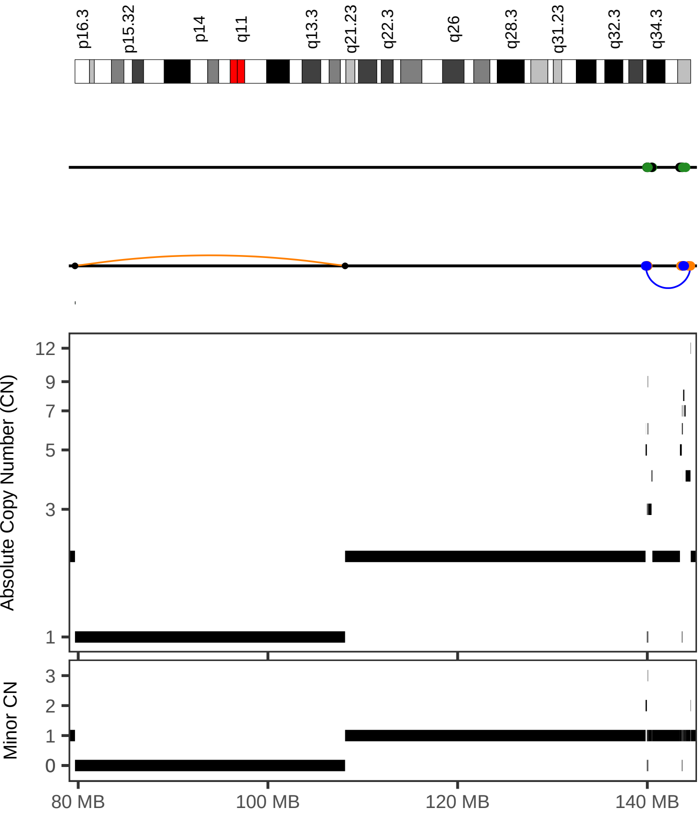

RK067

Cancer type	Liver−HCC
Position	4:79658323−144578032
Interleaved intrachr. SVs	1
Total SVs (intrachr. + transl.)	29
SV types	DEL: 1; DUP: 1; h2hINV: 0; t2tINV: 0; TRA: 27
SVs in sample	260
Oscillating CN (2 and 3 states)	3, 4
CN segments	26
FDR fragment joints	0.8824283
FDR chr. breakp. enrich.	0.02
Linked to chrs	1:44269676−245339192;
Purity, ploidy	0.38, 1.86

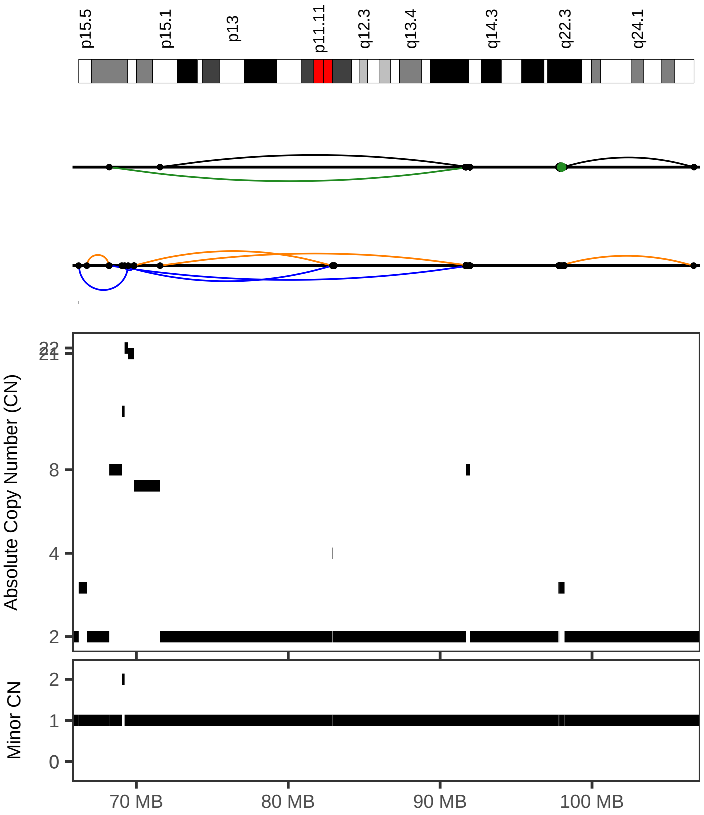

RK102

Cancer type	Liver−HCC
Position	11:66210041−91962236
Interleaved intrachr. SVs	20
Total SVs (intrachr. + transl.)	20
SV types	DEL: 3; DUP: 10; h2hINV: 5; t2tINV: 2; TRA: 0
SVs in sample	55
Oscillating CN (2 and 3 states)	3, 4
CN segments	13
FDR fragment joints	0.5435077
FDR chr. breakp. enrich.	0
Linked to chrs	
Purity, ploidy	0.46, 1.81

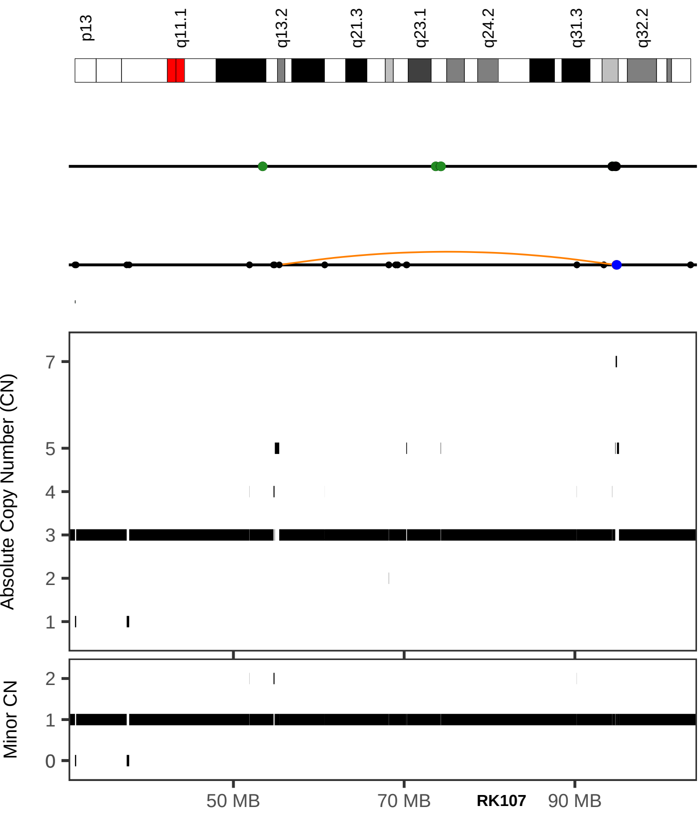

RK107

Cancer type	Liver−HCC
Position	14:31438137−103571317
Interleaved intrachr. SVs	1
Total SVs (intrachr. + transl.)	26
SV types	DEL: 4; DUP: 7; h2hINV: 0; t2tINV: 0; TRA: 15
SVs in sample	616
Oscillating CN (2 and 3 states)	5, 10
CN segments	26
FDR fragment joints	0.7510435
FDR chr. breakp. enrich.	0.19
Linked to chrs	1:26503377−220160008;2:2119500−211577973 22:18897837−41064430;3:39802700−133489710

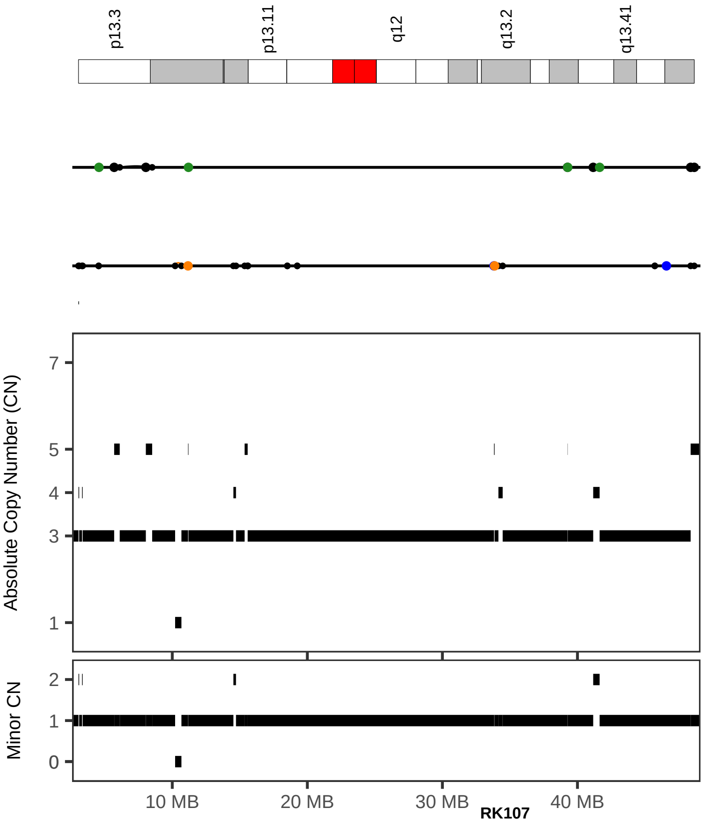

RK107

Cancer type	Liver−HCC
Position	19:3053413−48647994
Interleaved intrachr. SVs	1
Total SVs (intrachr. + transl.)	24
SV types	DEL: 1; DUP: 8; h2hINV: 1; t2tINV: 0; TRA: 14
SVs in sample	616
Oscillating CN (2 and 3 states)	5, 16
CN segments	25
FDR fragment joints	0.615458
FDR chr. breakp. enrich.	0
Linked to chrs	17:7015742−8463746;9:98064387−131689723
Purity, ploidy	0.68, 3.19

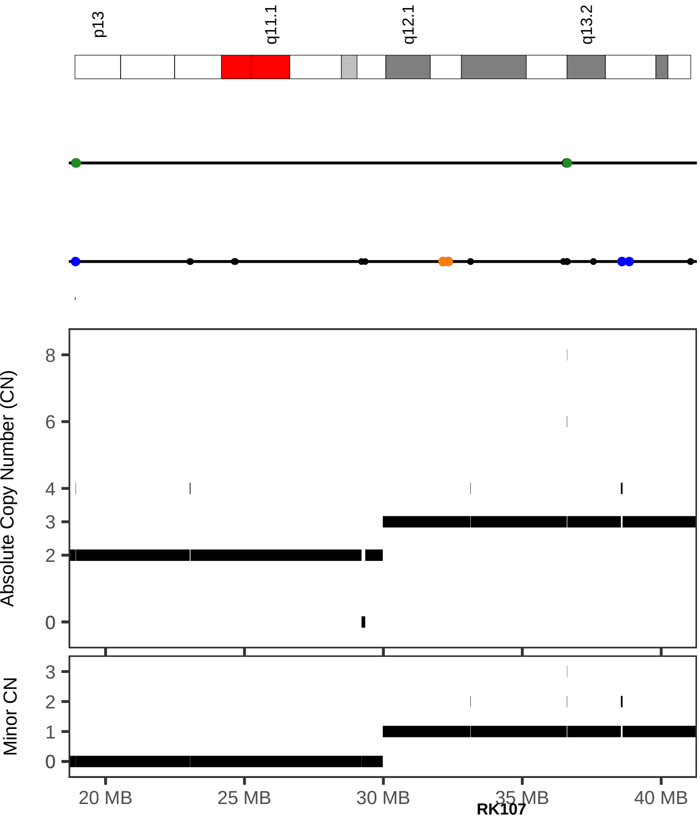

RK107

Cancer type	Liver−HCC
Position	22:18897837−41064431
Interleaved intrachr. SVs	1
Total SVs (intrachr. + transl.)	22
SV types	DEL: 2; DUP: 7; h2hINV: 0; t2tINV: 0; TRA: 13
SVs in sample	616
Oscillating CN (2 and 3 states)	5, 5
CN segments	15
FDR fragment joints	0.279346
FDR chr. breakp. enrich.	0
Linked to chrs	12:565831−121912249;14:31438137−103571316 2:2119500−211577973;
Purity, ploidy	0.68, 3.19

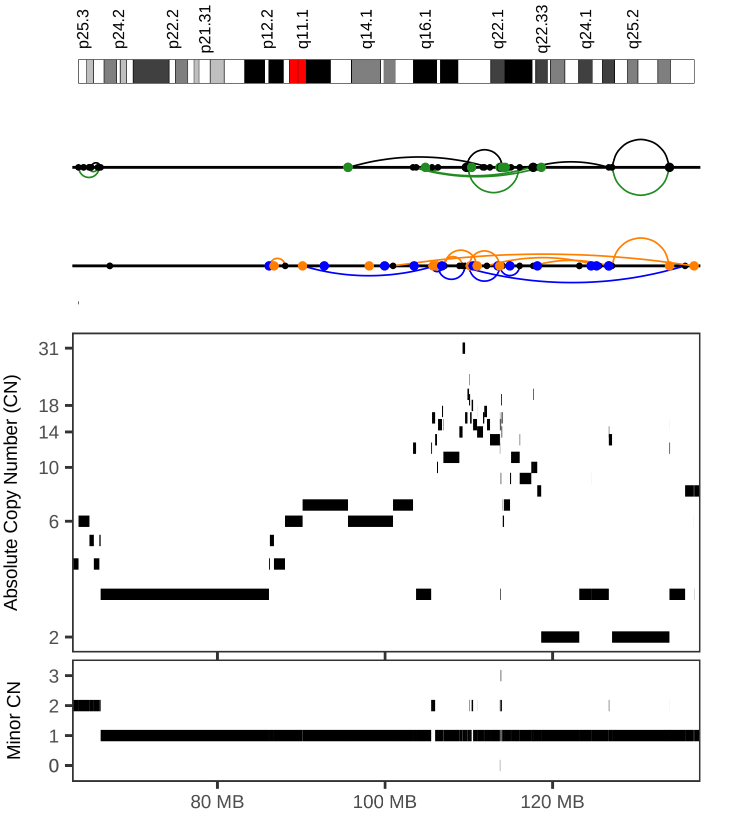

RK224

Cancer type	Liver−HCC
Position	6:90149372−136906996
Interleaved intrachr. SVs	23
Total SVs (intrachr. + transl.)	57
SV types	DEL: 7; DUP: 7; h2hINV: 4; t2tINV: 5; TRA: 34
SVs in sample	138
Oscillating CN (2 and 3 states)	3, 7
CN segments	70
FDR fragment joints	0.615458
FDR chr. breakp. enrich.	0
Linked to chrs	
Purity, ploidy	0.42, 3.24

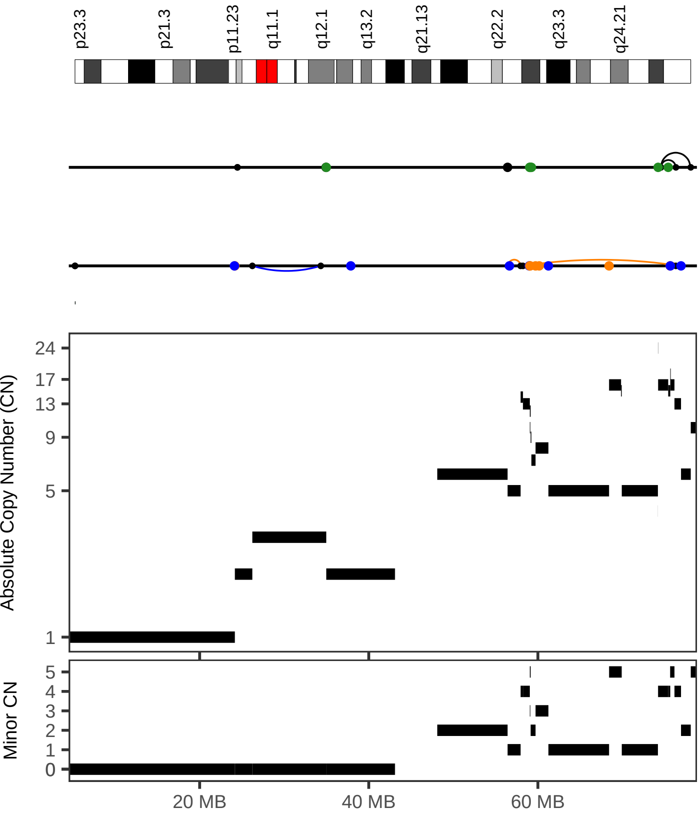

RK224

Cancer type	Liver−HCC
Position	8:58233490−78077286
Interleaved intrachr. SVs	2
Total SVs (intrachr. + transl.)	20
SV types	DEL: 2; DUP: 0; h2hINV: 2; t2tINV: 0; TRA: 16
SVs in sample	138
Oscillating CN (2 and 3 states)	2, 4
CN segments	19
FDR fragment joints	0.445216
FDR chr. breakp. enrich.	0
Linked to chrs	
Purity, ploidy	0.42, 3.24

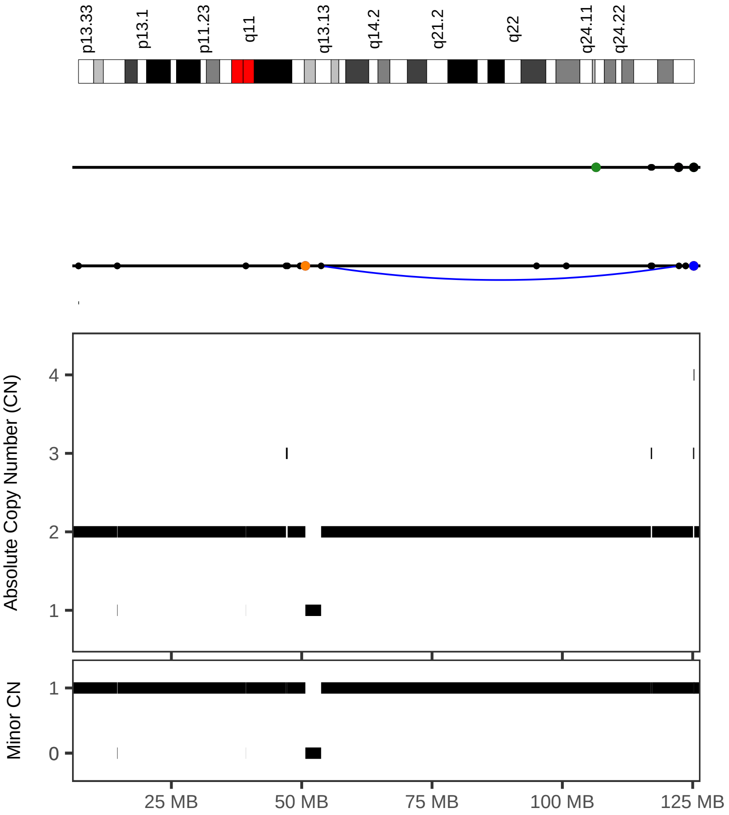

RK285

Cancer type	Liver−HCC
Position	12:7177572−125292742
Interleaved intrachr. SVs	1
Total SVs (intrachr. + transl.)	25
SV types	DEL: 2; DUP: 9; h2hINV: 1; t2tINV: 0; TRA: 13
SVs in sample	352
Oscillating CN (2 and 3 states)	5, 15
CN segments	16
FDR fragment joints	0.3165354
FDR chr. breakp. enrich.	0.14
Linked to chrs	4:38522688−170119513;
Purity, ploidy	0.81, 1.9

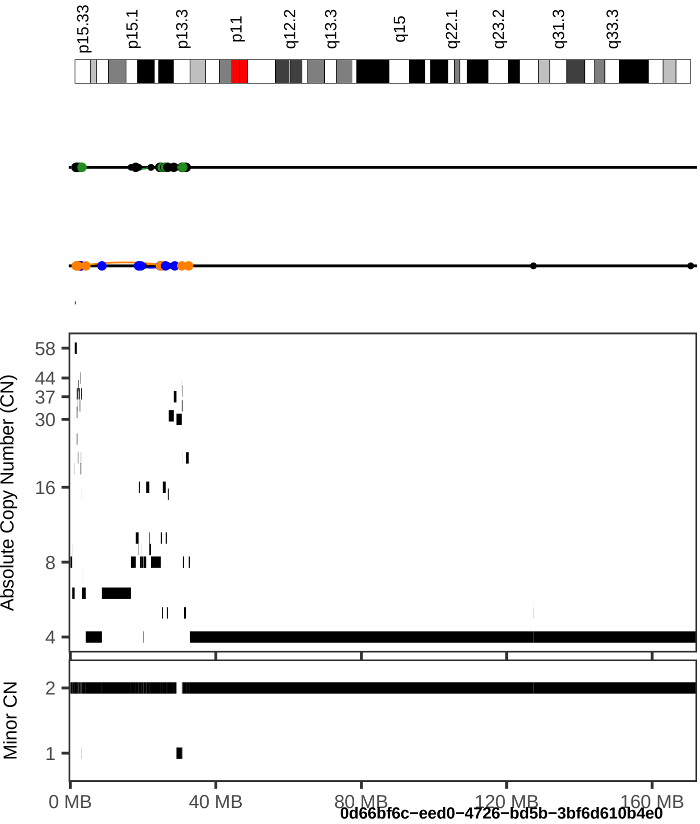

0d66bf6c−eed0−4726−bd5b−3bf6d610b4e0

Cancer type	Lung−AdenoCA
Position	5:1264136−27017687
Interleaved intrachr. SVs	13
Total SVs (intrachr. + transl.)	57
SV types	DEL: 2; DUP: 4; h2hINV: 2; t2tINV: 5; TRA: 44
SVs in sample	414
Oscillating CN (2 and 3 states)	3, 5
CN segments	42
FDR fragment joints	0.9916019
FDR chr. breakp. enrich.	0
Linked to chrs	12:61686111−128942892;9:524316−133004750 X:1621688−36004056;
Purity, ploidy	0.48, 3.84

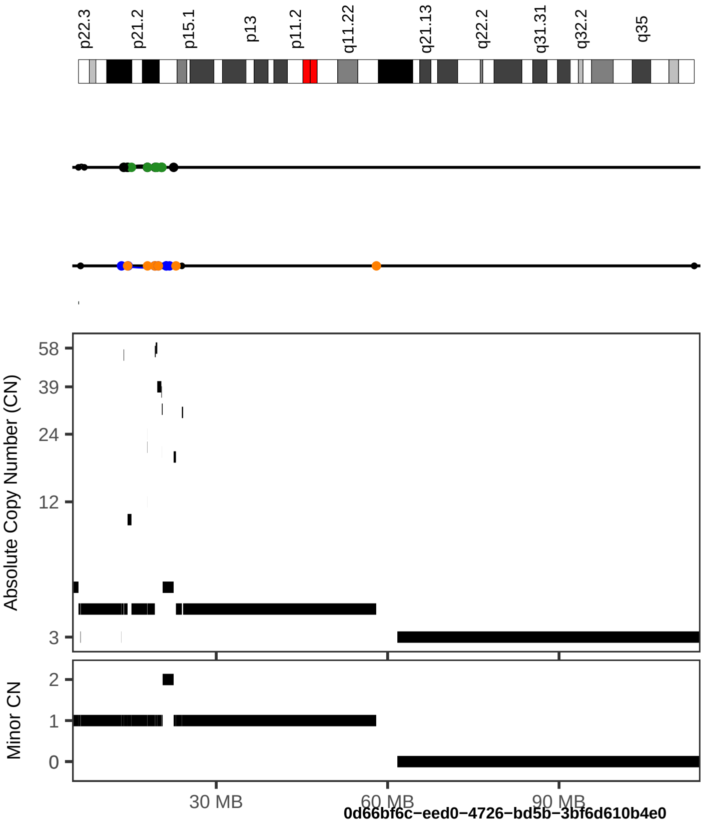

0d66bf6c−eed0−4726−bd5b−3bf6d610b4e0

Cancer type	Lung−AdenoCA
Position	7:13398832−23984708
Interleaved intrachr. SVs	2
Total SVs (intrachr. + transl.)	31
SV types	DEL: 1; DUP: 1; h2hINV: 0; t2tINV: 0; TRA: 29
SVs in sample	414
Oscillating CN (2 and 3 states)	3, 4
CN segments	20
FDR fragment joints	0.8653243
FDR chr. breakp. enrich.	0.02
Linked to chrs	12:61686111−128942892;X:1621688−36004056
Purity, ploidy	0.48, 3.84

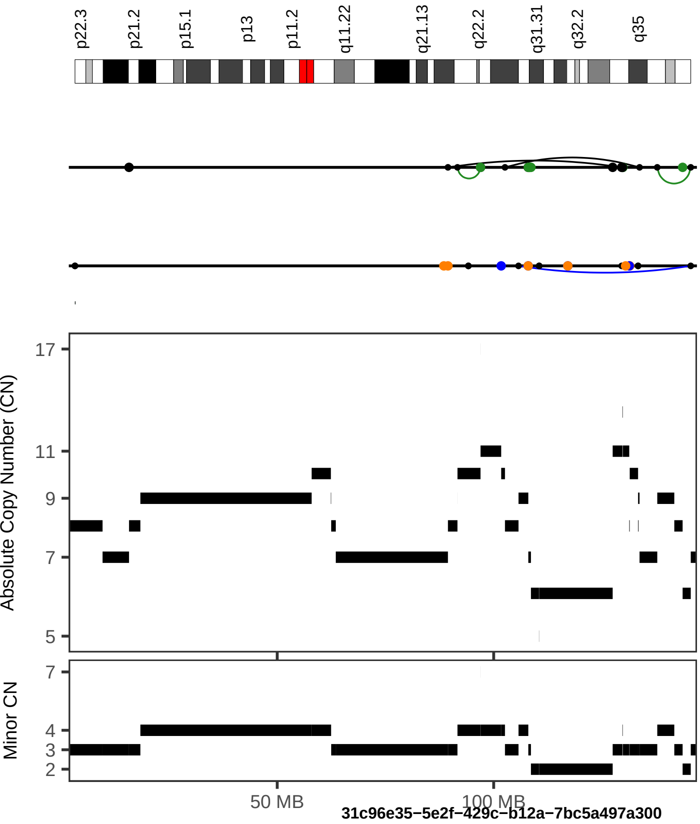

31c96e35−5e2f−429c−b12a−7bc5a497a300

Cancer type	Lung−AdenoCA
Position	7:89436461−145515031
Interleaved intrachr. SVs	3
Total SVs (intrachr. + transl.)	24
SV types	DEL: 0; DUP: 1; h2hINV: 2; t2tINV: 0; TRA: 21
SVs in sample	276
Oscillating CN (2 and 3 states)	3, 8
CN segments	24
FDR fragment joints	0.6776251
FDR chr. breakp. enrich.	0
Linked to chrs	1:14760541−105675265;16:23990840−50896671
Purity, ploidy	0.49, 4.79

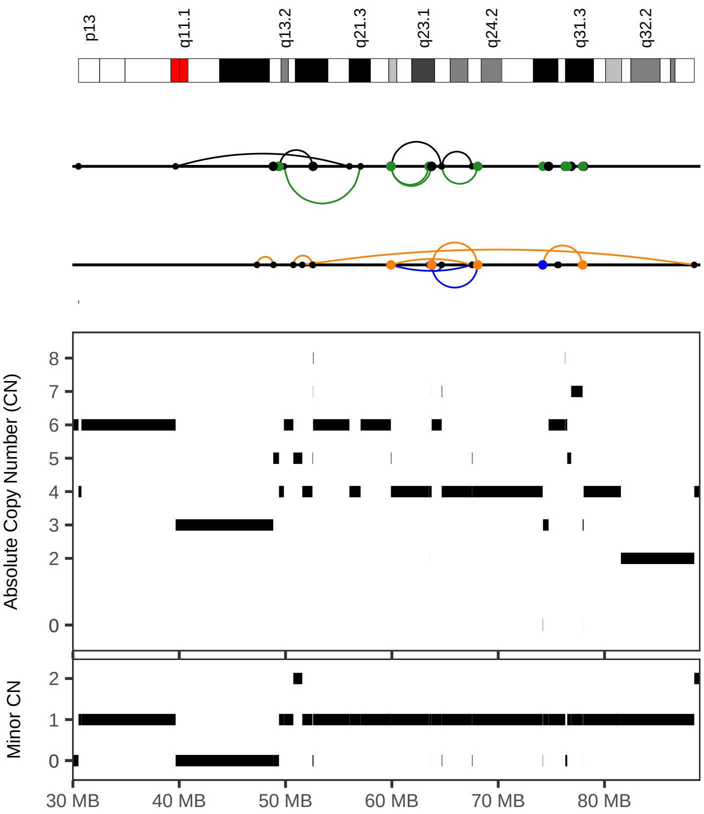

31c96e35−5e2f−429c−b12a−7bc5a497a300

Cancer type	Lung−AdenoCA
Position	14:59911041−68078436
Interleaved intrachr. SVs	11
Total SVs (intrachr. + transl.)	27
SV types	DEL: 2; DUP: 4; h2hINV: 2; t2tINV: 3; TRA: 16
SVs in sample	276
Oscillating CN (2 and 3 states)	3, 7
CN segments	11
FDR fragment joints	0.9284301
FDR chr. breakp. enrich.	0
Linked to chrs	
Purity, ploidy	0.49, 4.79

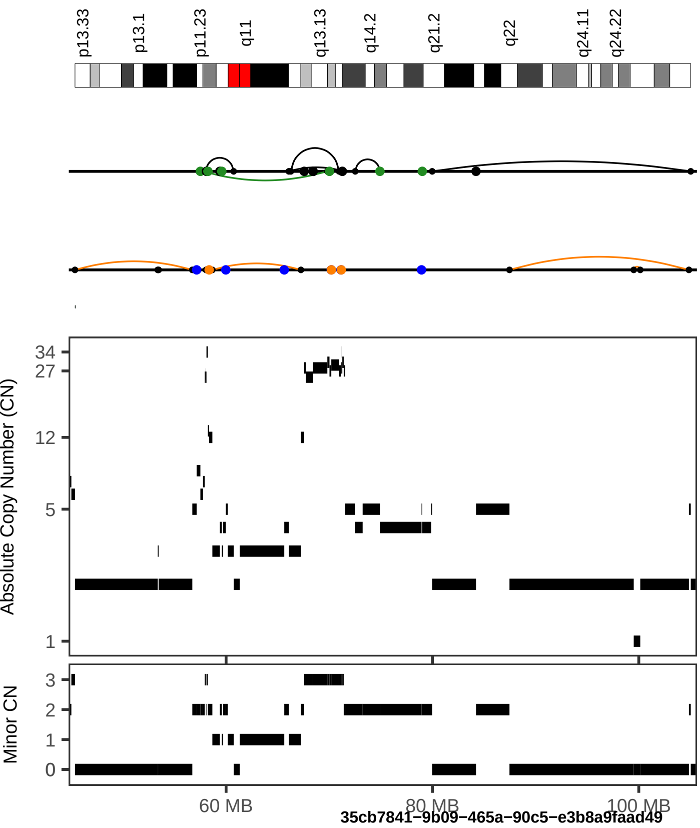

35cb7841−9b09−465a−90c5−e3b8a9faad49

Cancer type	Lung−AdenoCA
Position	12:57763408−71398497
Interleaved intrachr. SVs	5
Total SVs (intrachr. + transl.)	21
SV types	DEL: 1; DUP: 0; h2hINV: 3; t2tINV: 1; TRA: 16
SVs in sample	208
Oscillating CN (2 and 3 states)	4, 5
CN segments	29
FDR fragment joints	0.8419568
FDR chr. breakp. enrich.	0
Linked to chrs	5:78381−54831893;7:17140046−70300267
Purity, ploidy	0.26, 2.72

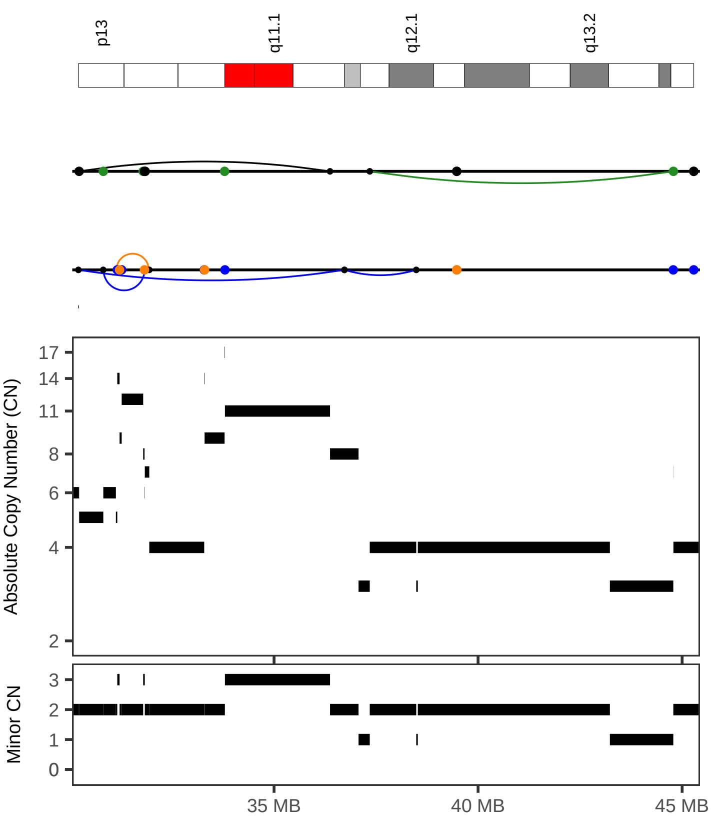

97831d28−ab41−4c18−bfc2−c4c6bc757d13

Cancer type	Lung−AdenoCA
Position	22:30205853−44783728
Interleaved intrachr. SVs	3
Total SVs (intrachr. + transl.)	21
SV types	DEL: 0; DUP: 2; h2hINV: 0; t2tINV: 1; TRA: 18
SVs in sample	229
Oscillating CN (2 and 3 states)	5, 5
CN segments	23
FDR fragment joints	0.8882853
FDR chr. breakp. enrich.	0
Linked to chrs	8:19746812−144761256;
Purity, ploidy	0.82, 3.49

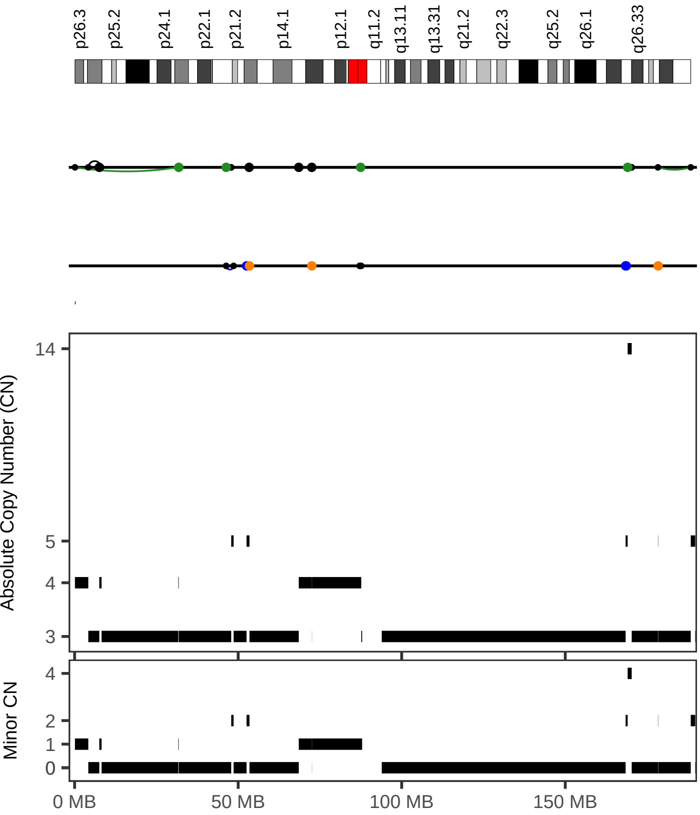

cd0dc947−b708−464e−beb7−954c9d4583e7

Cancer type	Lung−AdenoCA
Position	3:111543−188368919
Interleaved intrachr. SVs	1
Total SVs (intrachr. + transl.)	20
SV types	DEL: 1; DUP: 2; h2hINV: 1; t2tINV: 2; TRA: 14
SVs in sample	73
Oscillating CN (2 and 3 states)	6, 15
CN segments	21
FDR fragment joints	0.9625775
FDR chr. breakp. enrich.	0
Linked to chrs	
Purity, ploidy	0.36, 3.47

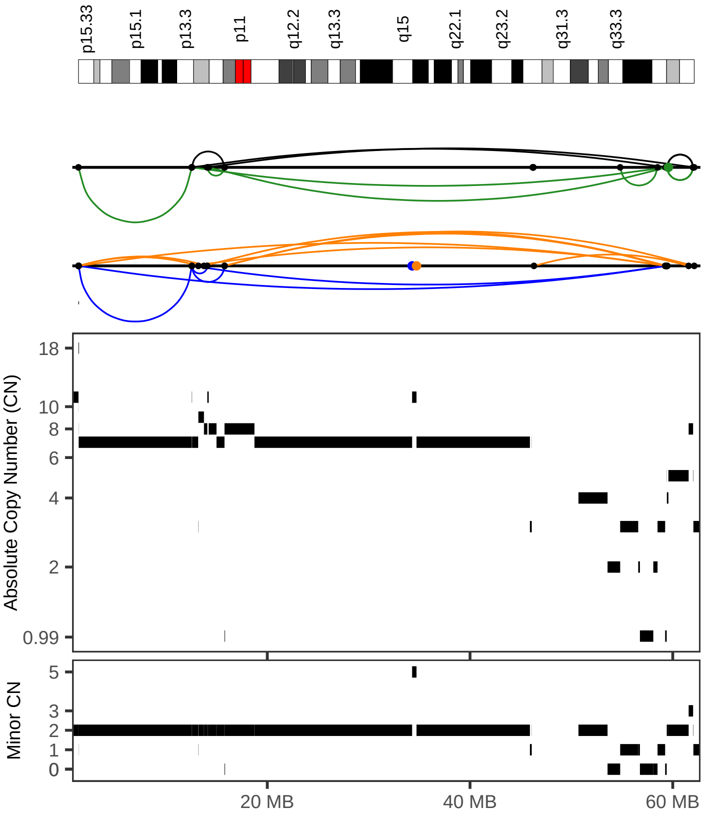

d2824e6d−3784−45c2−9b0f−52b17356b5da

Cancer type	Lung−AdenoCA
Position	5:1371638−62121680
Interleaved intrachr. SVs	26
Total SVs (intrachr. + transl.)	29
SV types	DEL: 9; DUP: 5; h2hINV: 6; t2tINV: 6; TRA: 3
SVs in sample	98
Oscillating CN (2 and 3 states)	3, 6
CN segments	34
FDR fragment joints	0.7953411
FDR chr. breakp. enrich.	0
Linked to chrs	
Purity, ploidy	0.38, 3.03

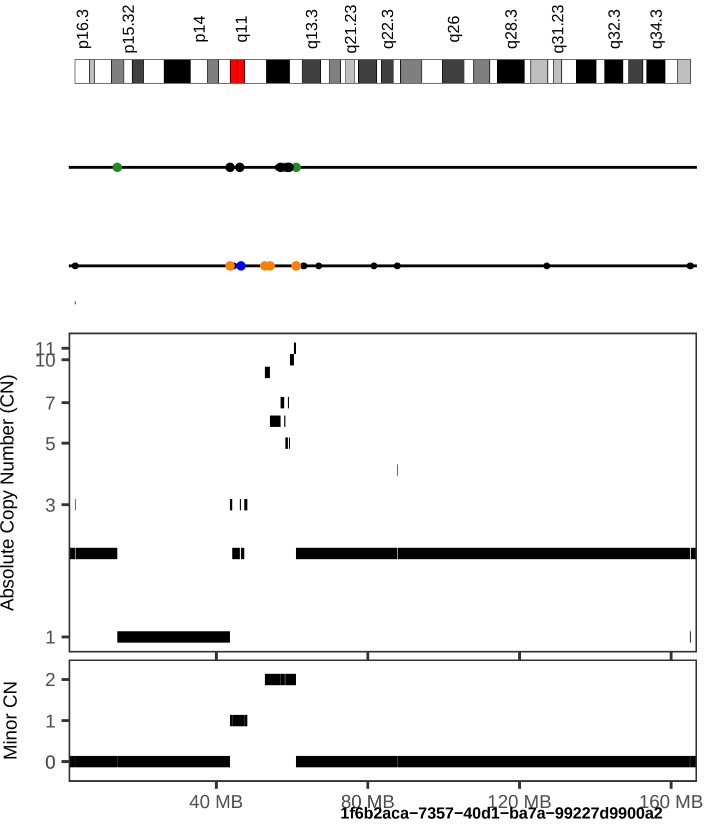

1f6b2aca−7357−40d1−ba7a−99227d9900a2

Cancer type	Lung−SCC
Position	4:2720902−165169009
Interleaved intrachr. SVs	1
Total SVs (intrachr. + transl.)	21
SV types	DEL: 1; DUP: 5; h2hINV: 0; t2tINV: 1; TRA: 14
SVs in sample	602
Oscillating CN (2 and 3 states)	5, 7
CN segments	24
FDR fragment joints	0.8419568
FDR chr. breakp. enrich.	0.03
Linked to chrs	11:46508302−111537991;12:4696888−43872964 3:77709025−174601175;
Purity, ploidy	0.86, 2.97

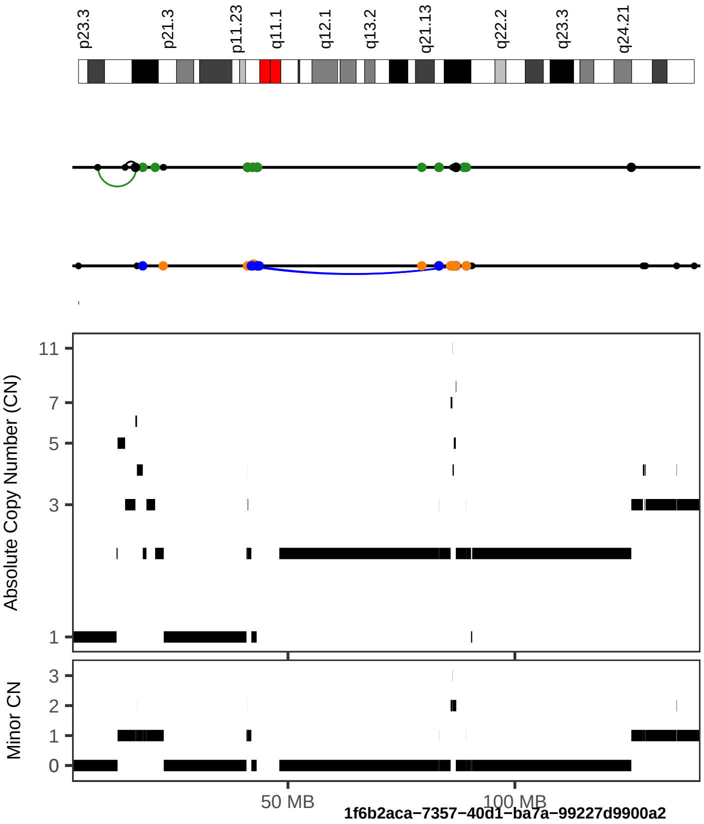

1f6b2aca−7357−40d1−ba7a−99227d9900a2

Cancer type	Lung−SCC
Position	8:40861103−87010834
Interleaved intrachr. SVs	5
Total SVs (intrachr. + transl.)	43
SV types	DEL: 1; DUP: 2; h2hINV: 1; t2tINV: 1; TRA: 38
SVs in sample	602
Oscillating CN (2 and 3 states)	4, 7
CN segments	18
FDR fragment joints	0.9077541
FDR chr. breakp. enrich.	0
Linked to chrs	11:46508302−111537991;18:21140095−63388412
Purity, ploidy	0.86, 2.97

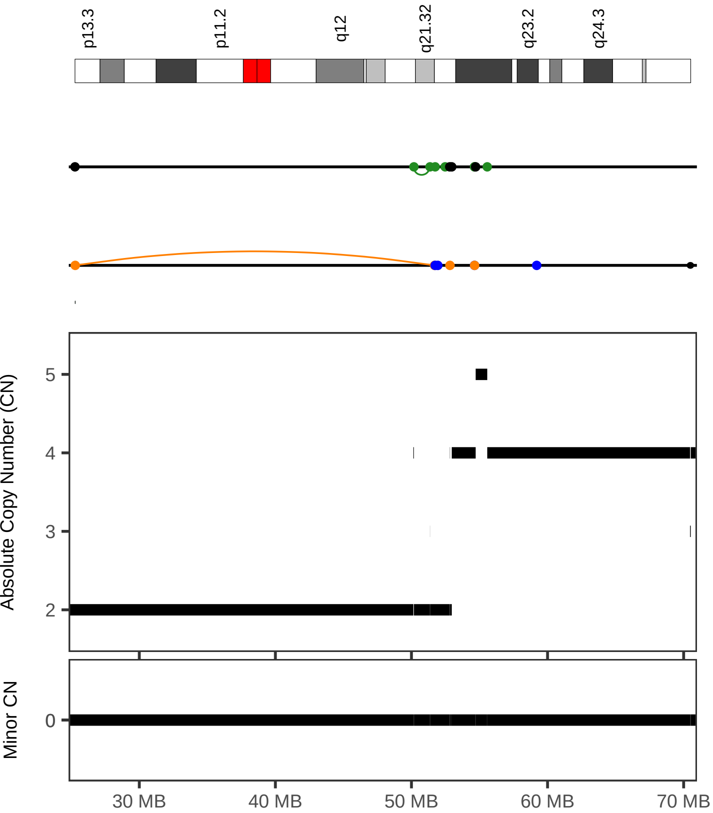

7b982d5e−3a7d−40ac−bd25−6044c62879b6

Cancer type	Lung−SCC
Position	17:25273878−70520743
Interleaved intrachr. SVs	1
Total SVs (intrachr. + transl.)	22
SV types	DEL: 2; DUP: 0; h2hINV: 0; t2tINV: 1; TRA: 19
SVs in sample	349
Oscillating CN (2 and 3 states)	4, 8
CN segments	12
FDR fragment joints	0.9419607
FDR chr. breakp. enrich.	0
Linked to chrs	
Purity, ploidy	0.45, 2.92

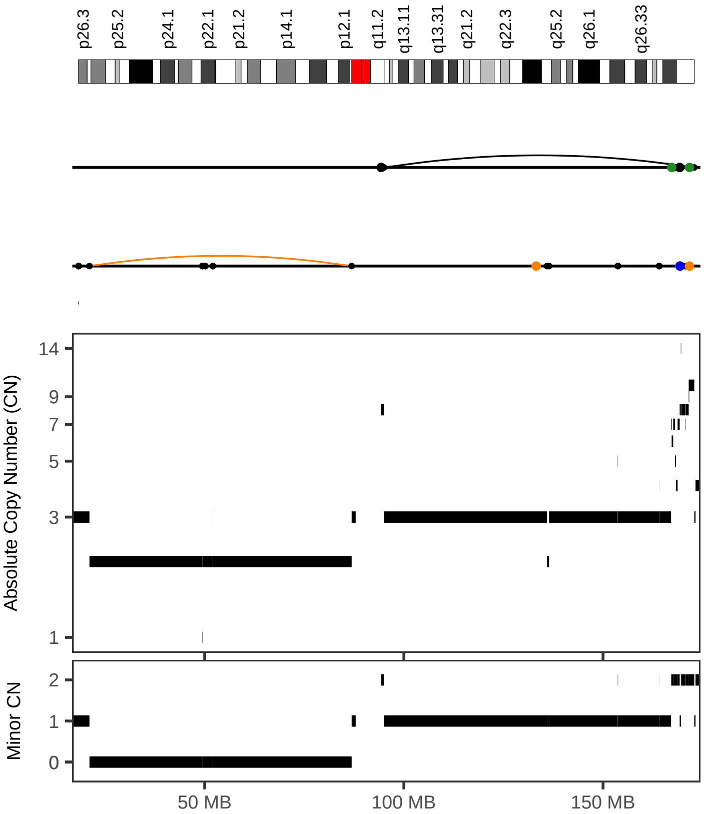

7d905f8f−3967−4ea8−96c8−17b1b03fbec3

Cancer type	Lung−SCC
Position	3:18318665−172882633
Interleaved intrachr. SVs	1
Total SVs (intrachr. + transl.)	20
SV types	DEL: 4; DUP: 3; h2hINV: 3; t2tINV: 2; TRA: 8
SVs in sample	385
Oscillating CN (2 and 3 states)	4, 7
CN segments	29
FDR fragment joints	0.8572806
FDR chr. breakp. enrich.	0.93
Linked to chrs	16:1242659−24867893;
Purity, ploidy	0.57, 2.6

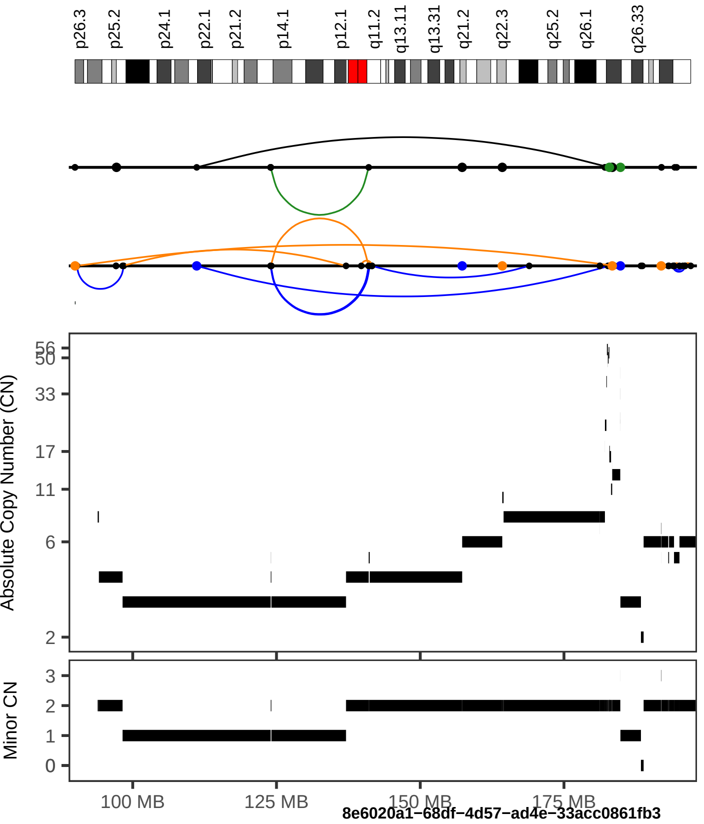

8e6020a1−68df−4d57−ad4e−33acc0861fb3

Cancer type	Lung−SCC
Position	3:90291254−183825857
Interleaved intrachr. SVs	15
Total SVs (intrachr. + transl.)	24
SV types	DEL: 4; DUP: 7; h2hINV: 2; t2tINV: 2; TRA: 9
SVs in sample	261
Oscillating CN (2 and 3 states)	3, 9
CN segments	30
FDR fragment joints	0.615458
FDR chr. breakp. enrich.	0
Linked to chrs	19:4156034−56588808;20:44576084−48486150
Purity, ploidy	0.58, 2.71

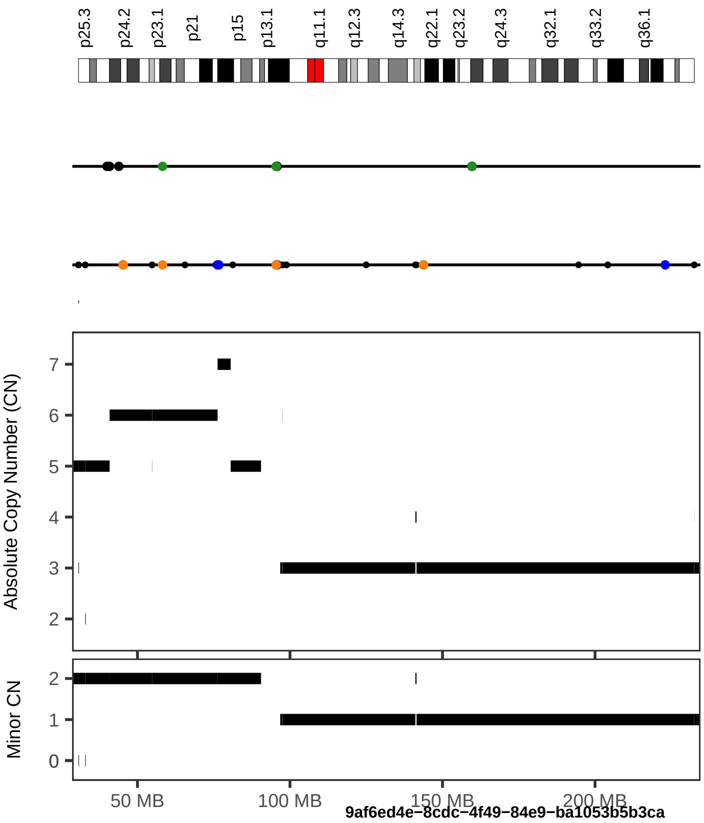

9af6ed4e−8cdc−4f49−84e9−ba1053b5b3ca

Cancer type	Lung−SCC
Position	2:54782646−232522086
Interleaved intrachr. SVs	1
Total SVs (intrachr. + transl.)	23
SV types	DEL: 0; DUP: 5; h2hINV: 0; t2tINV: 0; TRA: 18
SVs in sample	428
Oscillating CN (2 and 3 states)	5, 5
CN segments	11
FDR fragment joints	0.5435077
FDR chr. breakp. enrich.	0.9
Linked to chrs	1:76535798−99663064;X:44809178−107538699
Purity, ploidy	0.82, 3.26

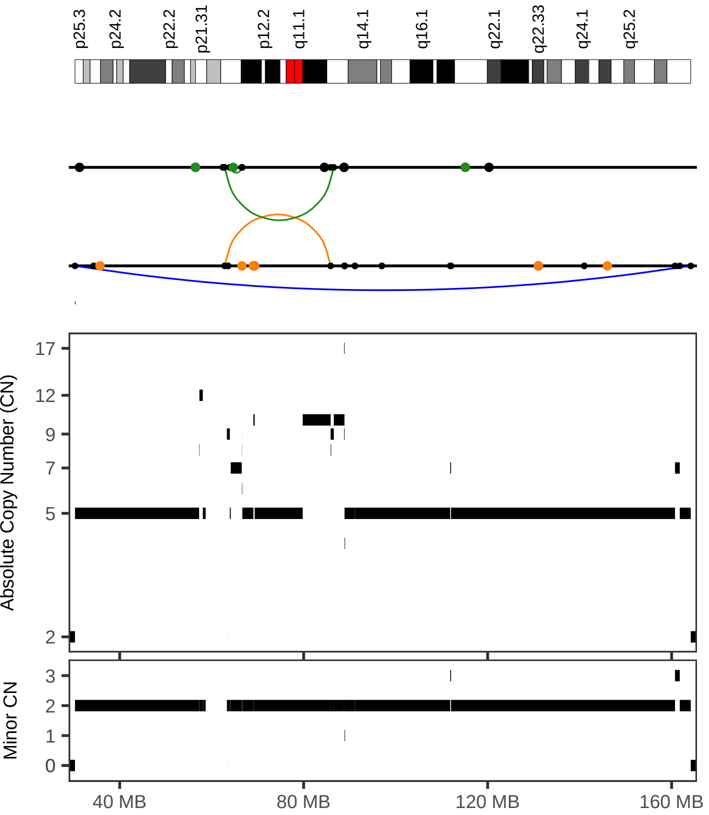

9af6ed4e−8cdc−4f49−84e9−ba1053b5b3ca

Cancer type	Lung−SCC
Position	6:30277793−164158989
Interleaved intrachr. SVs	1
Total SVs (intrachr. + transl.)	27
SV types	DEL: 1; DUP: 3; h2hINV: 2; t2tINV: 2; TRA: 19
SVs in sample	428
Oscillating CN (2 and 3 states)	5, 8
CN segments	32
FDR fragment joints	0.9284301
FDR chr. breakp. enrich.	0.05
Linked to chrs	4:1765550−100349831;
Purity, ploidy	0.82, 3.26

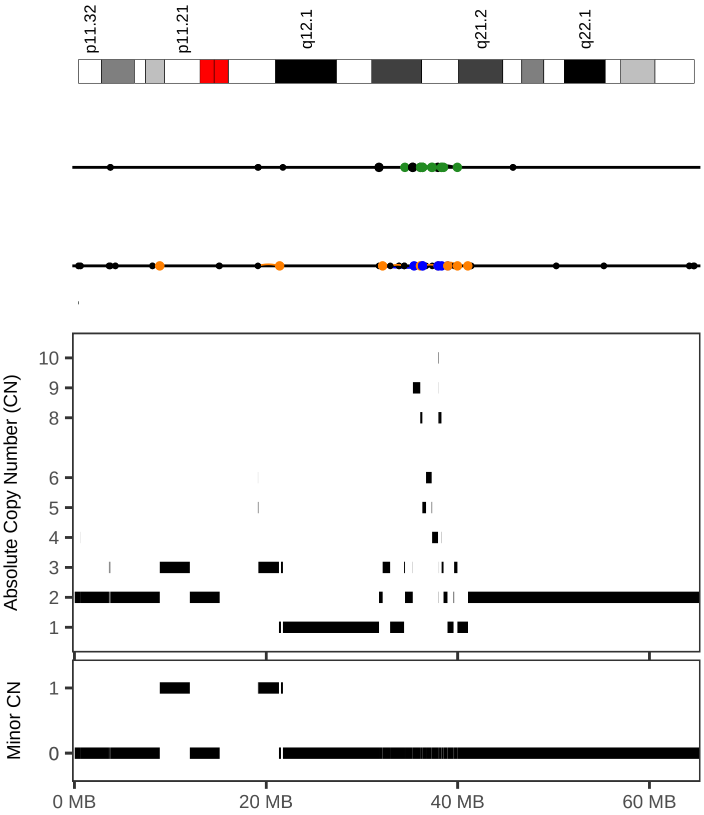

b913d254−8307−4b8a−8313−d978e32bb38f

Cancer type	Lung−SCC
Position	18:31777572−39967346
Interleaved intrachr. SVs	5
Total SVs (intrachr. + transl.)	33
SV types	DEL: 1; DUP: 3; h2hINV: 1; t2tINV: 0; TRA: 28
SVs in sample	944
Oscillating CN (2 and 3 states)	5, 8
CN segments	29
FDR fragment joints	0.615458
FDR chr. breakp. enrich.	0
Linked to chrs	7:25417234−138775946;
Purity, ploidy	0.75, 2.86

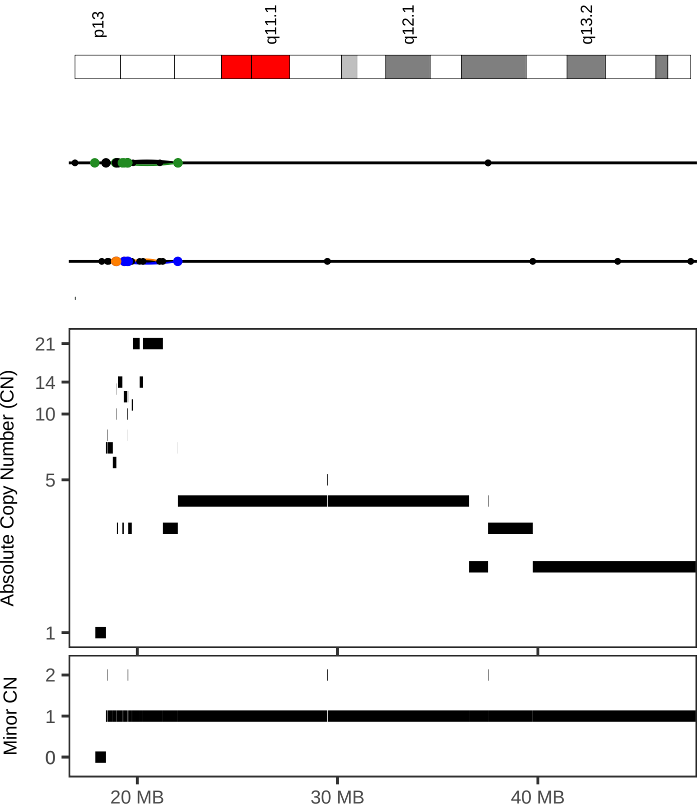

b913d254−8307−4b8a−8313−d978e32bb38f

Cancer type	Lung−SCC
Position	22:18573581−22033664
Interleaved intrachr. SVs	8
Total SVs (intrachr. + transl.)	27
SV types	DEL: 3; DUP: 2; h2hINV: 2; t2tINV: 1; TRA: 19
SVs in sample	944
Oscillating CN (2 and 3 states)	3, 4
CN segments	19
FDR fragment joints	0.6685091
FDR chr. breakp. enrich.	0
Linked to chrs	18:31777572−39967345;
Purity, ploidy	0.75, 2.86

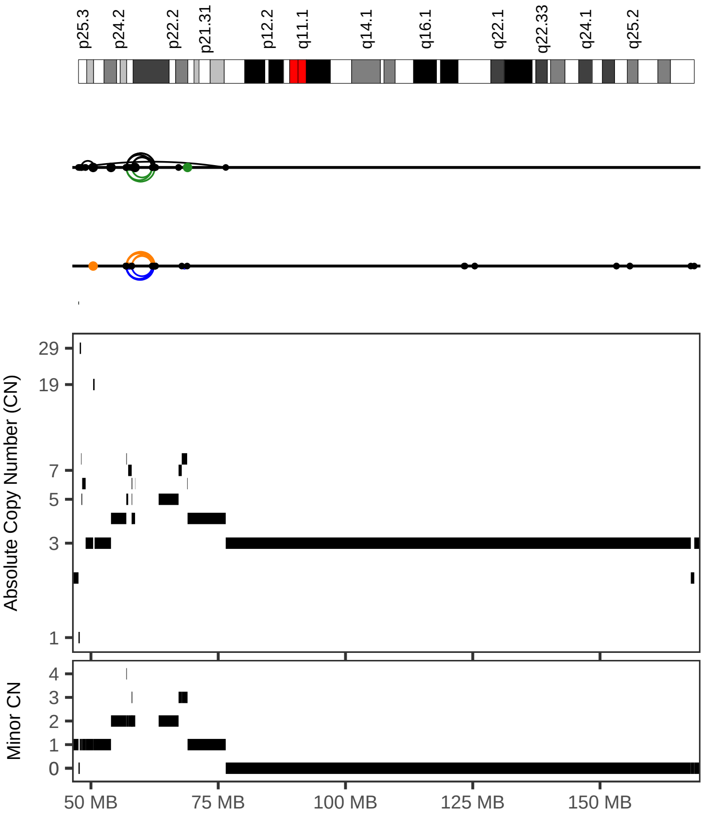

dc6ceece−efaa−4b9d−9e12−d35863d7a902

Cancer type	Lung−SCC
Position	6:56875241−62704227
Interleaved intrachr. SVs	34
Total SVs (intrachr. + transl.)	35
SV types	DEL: 8; DUP: 13; h2hINV: 7; t2tINV: 6; TRA: 1
SVs in sample	168
Oscillating CN (2 and 3 states)	3, 11
CN segments	11
FDR fragment joints	0.6565759
FDR chr. breakp. enrich.	0
Linked to chrs	
Purity, ploidy	0.32, 3.07

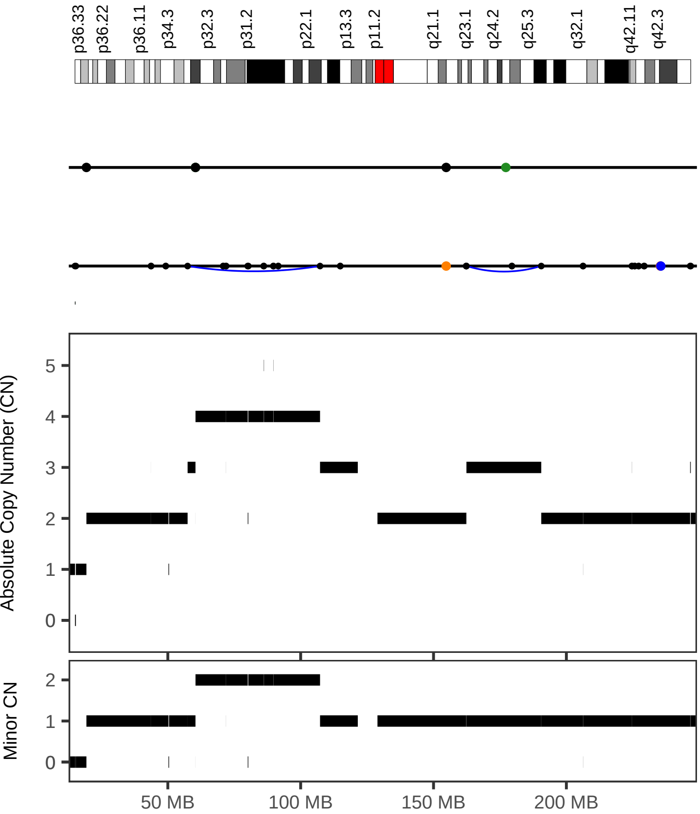

AOCS−001

Cancer type	Ovary−AdenoCA
Position	1:15057337−246806715
Interleaved intrachr. SVs	1
Total SVs (intrachr. + transl.)	23
SV types	DEL: 4; DUP: 11; h2hINV: 0; t2tINV: 0; TRA: 8
SVs in sample	223
Oscillating CN (2 and 3 states)	6, 17
CN segments	30
FDR fragment joints	0.3328071
FDR chr. breakp. enrich.	0.04
Linked to chrs	
Purity, ploidy	0.54, 2.11

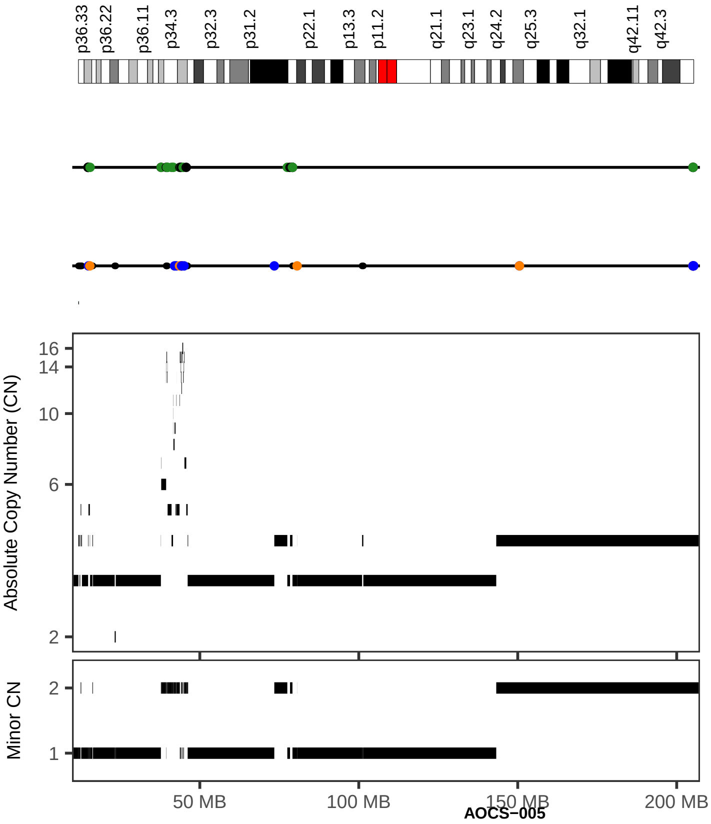

AOCS−005

Cancer type	Ovary−AdenoCA
Position	1:39408895−45738519
Interleaved intrachr. SVs	5
Total SVs (intrachr. + transl.)	24
SV types	DEL: 1; DUP: 3; h2hINV: 1; t2tINV: 0; TRA: 19
SVs in sample	399
Oscillating CN (2 and 3 states)	4, 11
CN segments	33
FDR fragment joints	0.7568568
FDR chr. breakp. enrich.	0
Linked to chrs	13:39444113−99719016;18:56378430−67139123
Purity, ploidy	0.71, 3.06

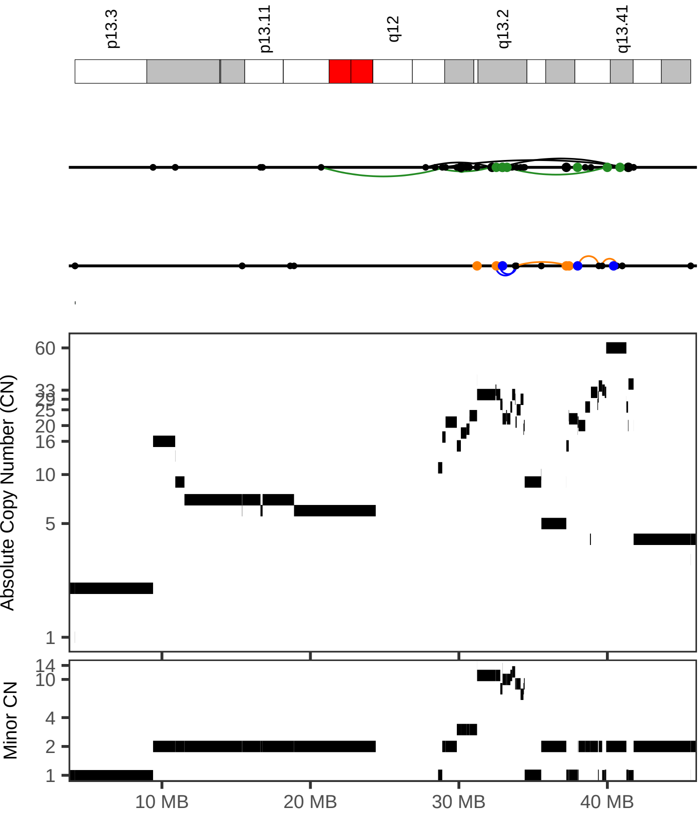

AOCS−055

Cancer type	Ovary−AdenoCA
Position	19:32469607−40919792
Interleaved intrachr. SVs	6
Total SVs (intrachr. + transl.)	21
SV types	DEL: 2; DUP: 2; h2hINV: 1; t2tINV: 1; TRA: 15
SVs in sample	362
Oscillating CN (2 and 3 states)	3, 5
CN segments	34
FDR fragment joints	0.7568568
FDR chr. breakp. enrich.	0
Linked to chrs	16:587794−53822626;
Purity, ploidy	0.5, 4.18

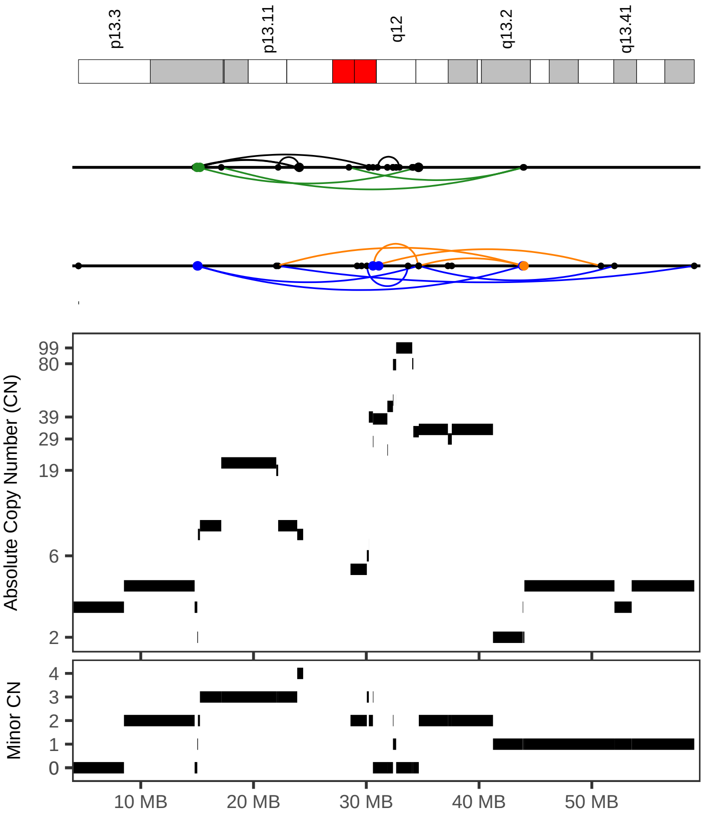

AOCS−077

Cancer type	Ovary−AdenoCA
Position	19:14774987−59083325
Interleaved intrachr. SVs	16
Total SVs (intrachr. + transl.)	27
SV types	DEL: 4; DUP: 5; h2hINV: 4; t2tINV: 3; TRA: 11
SVs in sample	125
Oscillating CN (2 and 3 states)	3, 6
CN segments	31
FDR fragment joints	0.6776251
FDR chr. breakp. enrich.	0
Linked to chrs	
Purity, ploidy	0.25, 4.7

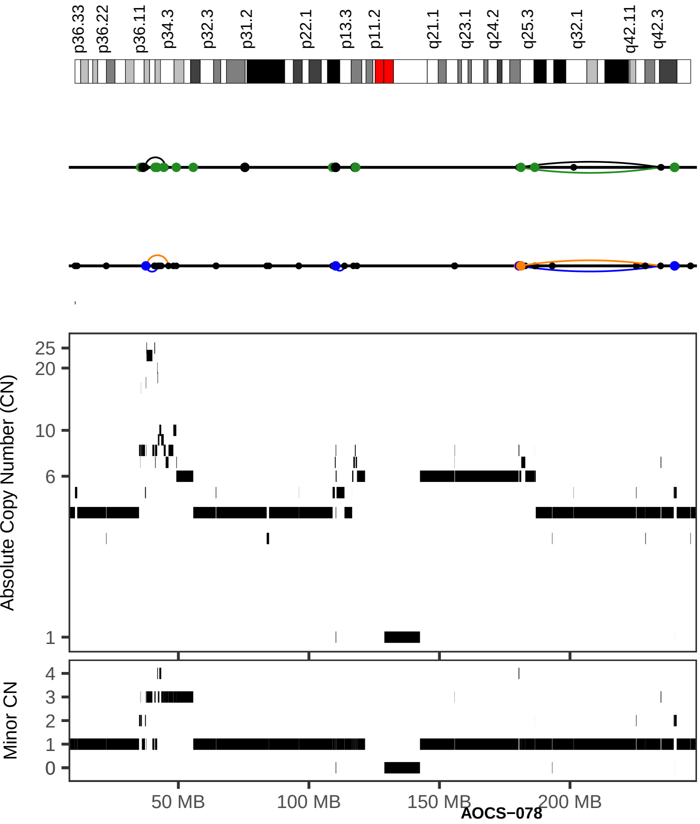

AOCS−078

Cancer type	Ovary−AdenoCA
Position	1:37043882−234963260
Interleaved intrachr. SVs	4
Total SVs (intrachr. + transl.)	30
SV types	DEL: 2; DUP: 2; h2hINV: 2; t2tINV: 2; TRA: 22
SVs in sample	457
Oscillating CN (2 and 3 states)	5, 9
CN segments	66
FDR fragment joints	0.615458
FDR chr. breakp. enrich.	0
Linked to chrs	18:370844−75885476;7:559305−156711735
Purity, ploidy	0.64, 4.25

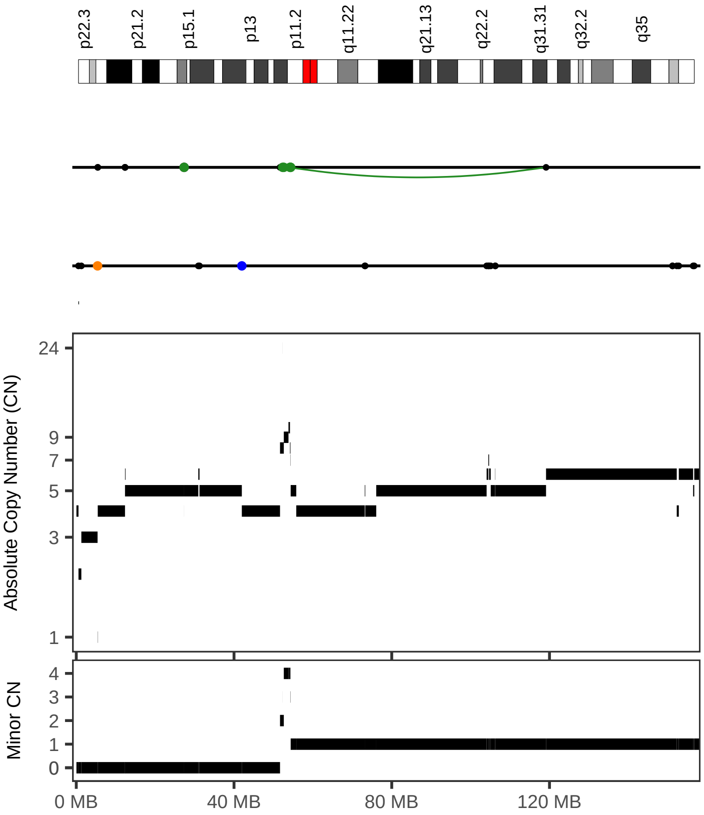

AOCS−078

Cancer type	Ovary−AdenoCA
Position	7:559305−156711736
Interleaved intrachr. SVs	1
Total SVs (intrachr. + transl.)	20
SV types	DEL: 3; DUP: 5; h2hINV: 1; t2tINV: 2; TRA: 9
SVs in sample	457
Oscillating CN (2 and 3 states)	5, 12
CN segments	35
FDR fragment joints	0.6776251
FDR chr. breakp. enrich.	0.97
Linked to chrs	1:37043882−234963259;
Purity, ploidy	0.64, 4.25

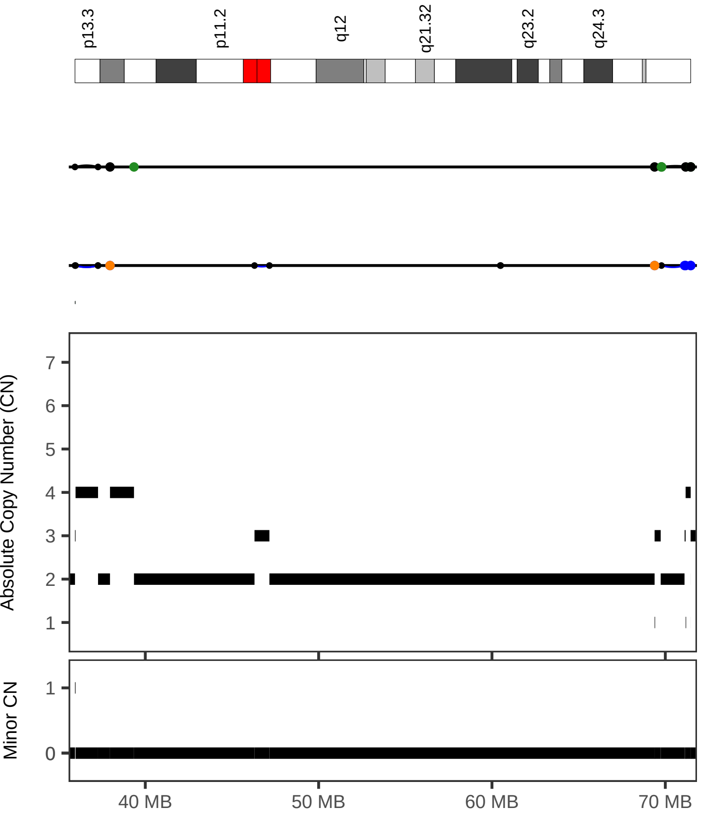

AOCS−078

Cancer type	Ovary−AdenoCA
Position	17:35943519−71465923
Interleaved intrachr. SVs	3
Total SVs (intrachr. + transl.)	24
SV types	DEL: 0; DUP: 2; h2hINV: 3; t2tINV: 1; TRA: 18
SVs in sample	457
Oscillating CN (2 and 3 states)	4, 7
CN segments	20
FDR fragment joints	0.615458
FDR chr. breakp. enrich.	0
Linked to chrs	12:32561517−132164131;
Purity, ploidy	0.64, 4.25

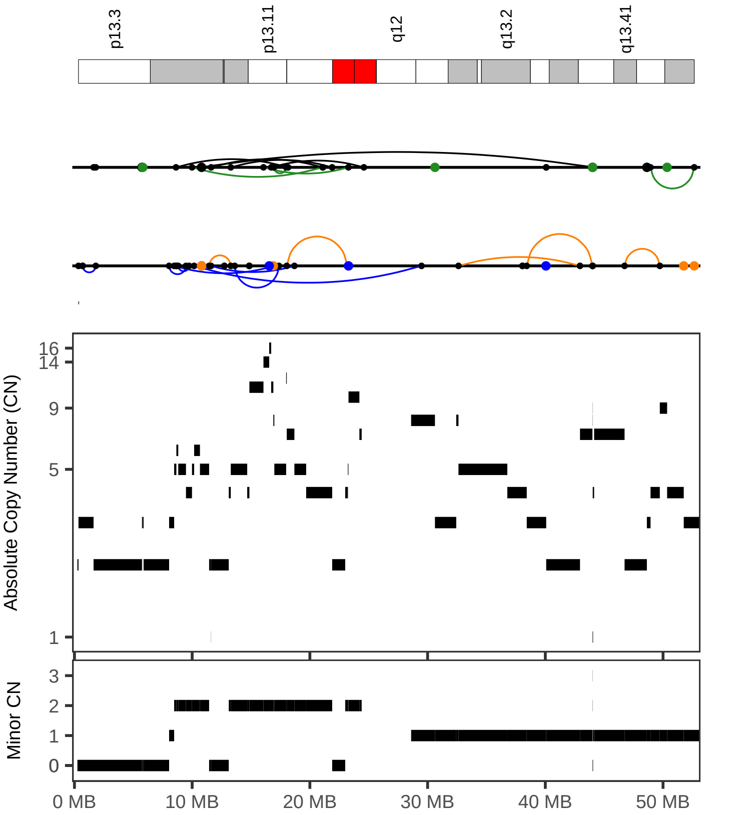

AOCS−078

Cancer type	Ovary−AdenoCA
Position	19:8041336−44146426
Interleaved intrachr. SVs	16
Total SVs (intrachr. + transl.)	26
SV types	DEL: 2; DUP: 6; h2hINV: 5; t2tINV: 3; TRA: 10
SVs in sample	457
Oscillating CN (2 and 3 states)	3, 7
CN segments	43
FDR fragment joints	0.615458
FDR chr. breakp. enrich.	0
Linked to chrs	1:37043882−234963259;
Purity, ploidy	0.64, 4.25

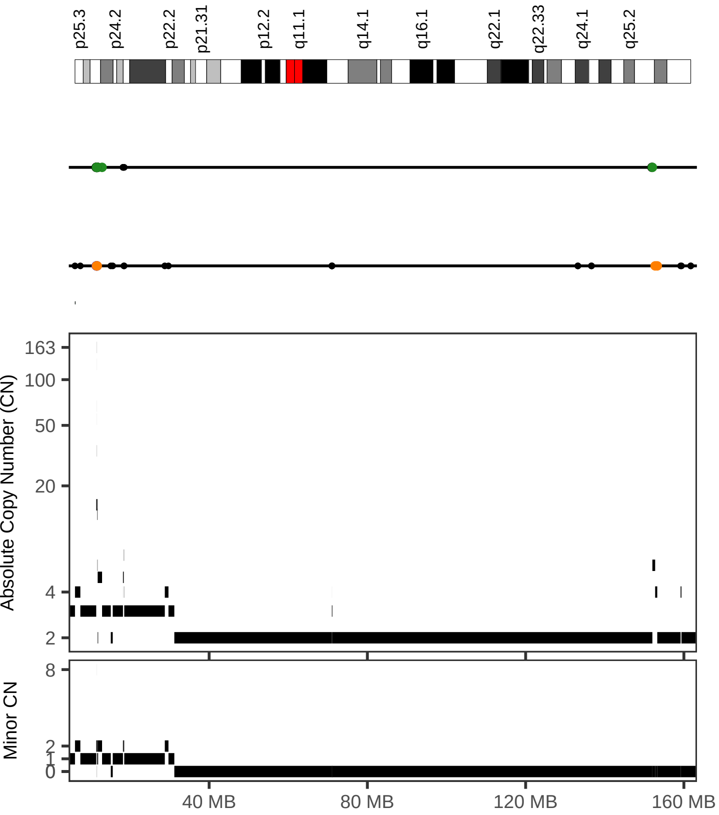

AOCS−079

Cancer type	Ovary−AdenoCA
Position	6:11466719−11715609
Interleaved intrachr. SVs	18
Total SVs (intrachr. + transl.)	30
SV types	DEL: 1; DUP: 11; h2hINV: 2; t2tINV: 4; TRA: 12
SVs in sample	310
Oscillating CN (2 and 3 states)	3, 3
CN segments	12
FDR fragment joints	0.445216
FDR chr. breakp. enrich.	0
Linked to chrs	
Purity, ploidy	0.5, 3.13

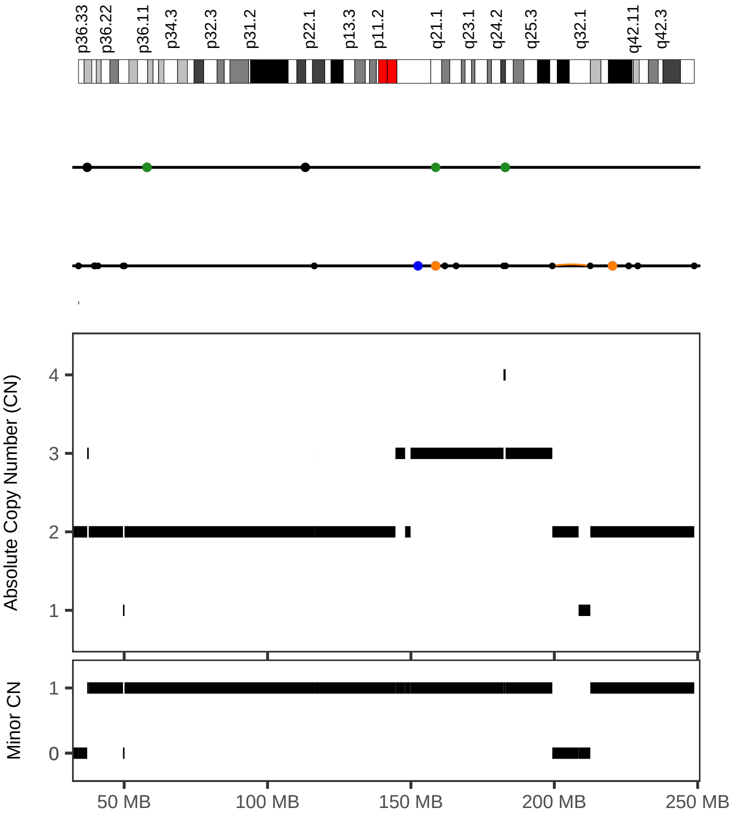

AOCS−105

Cancer type	Ovary−AdenoCA
Position	1:34065251−229122654
Interleaved intrachr. SVs	1
Total SVs (intrachr. + transl.)	22
SV types	DEL: 4; DUP: 4; h2hINV: 0; t2tINV: 0; TRA: 14
SVs in sample	197
Oscillating CN (2 and 3 states)	6, 16
CN segments	16
FDR fragment joints	0.7642985
FDR chr. breakp. enrich.	0.03
Linked to chrs	2:13539130−201725051;
Purity, ploidy	0.67, 1.9

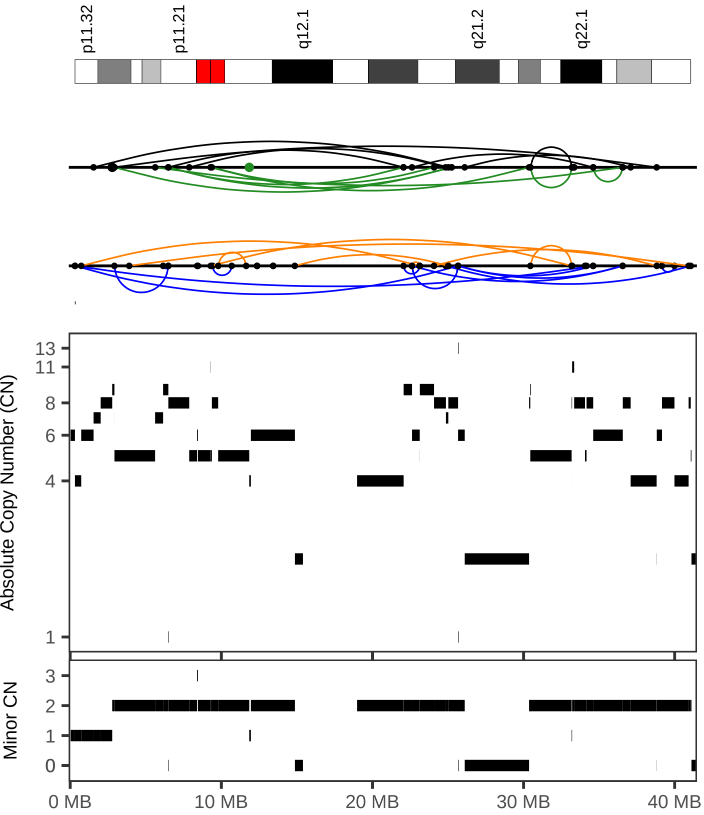

AOCS−107

Cancer type	Ovary−AdenoCA
Position	18:316795−41066402
Interleaved intrachr. SVs	34
Total SVs (intrachr. + transl.)	36
SV types	DEL: 7; DUP: 10; h2hINV: 8; t2tINV: 9; TRA: 2
SVs in sample	249
Oscillating CN (2 and 3 states)	3, 4
CN segments	51
FDR fragment joints	0.9284301
FDR chr. breakp. enrich.	0
Linked to chrs	
Purity, ploidy	0.85, 3.57

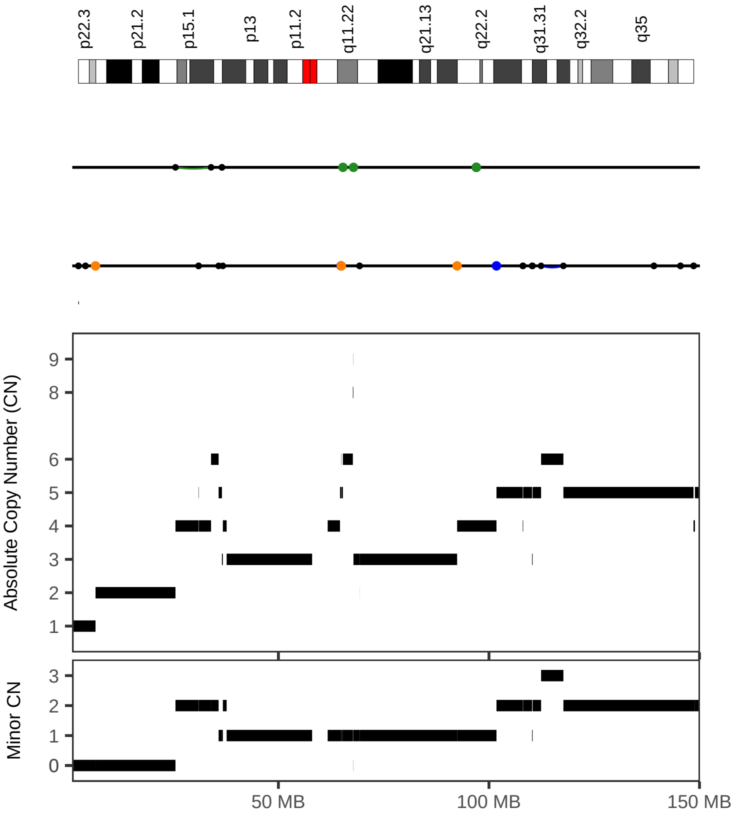

AOCS−108

Cancer type	Ovary−AdenoCA
Position	7:4167086−148638798
Interleaved intrachr. SVs	1
Total SVs (intrachr. + transl.)	22
SV types	DEL: 6; DUP: 5; h2hINV: 0; t2tINV: 2; TRA: 9
SVs in sample	415
Oscillating CN (2 and 3 states)	4, 6
CN segments	29
FDR fragment joints	0.615458
FDR chr. breakp. enrich.	0.76
Linked to chrs	8:36857660−141396457;
Purity, ploidy	0.7, 3.64

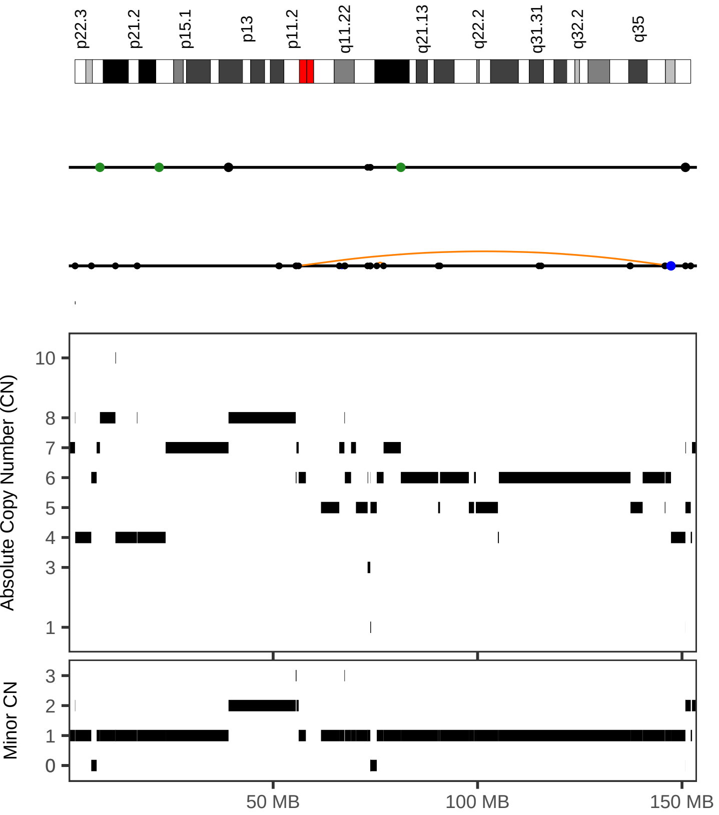

AOCS−112

Cancer type	Ovary−AdenoCA
Position	7:1525898−152136709
Interleaved intrachr. SVs	1
Total SVs (intrachr. + transl.)	23
SV types	DEL: 5; DUP: 10; h2hINV: 1; t2tINV: 1; TRA: 6
SVs in sample	297
Oscillating CN (2 and 3 states)	6, 8
CN segments	46
FDR fragment joints	0.5435077
FDR chr. breakp. enrich.	0.06
Linked to chrs	
Purity, ploidy	0.69, 3.93

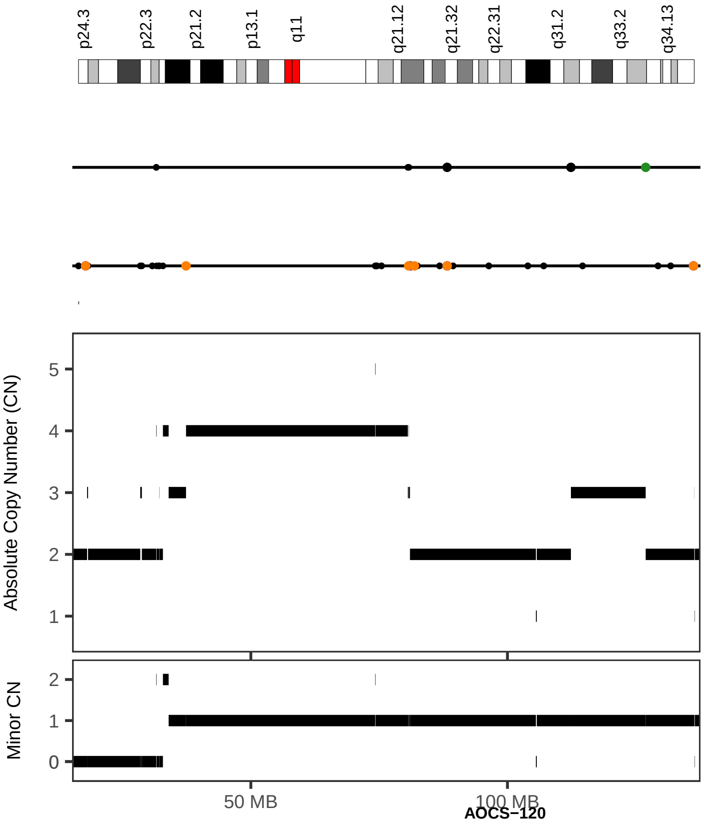

AOCS−120

Cancer type	Ovary−AdenoCA
Position	9:16411704−136399898
Interleaved intrachr. SVs	1
Total SVs (intrachr. + transl.)	27
SV types	DEL: 1; DUP: 12; h2hINV: 1; t2tINV: 0; TRA: 13
SVs in sample	790
Oscillating CN (2 and 3 states)	5, 15
CN segments	24
FDR fragment joints	0.03106446
FDR chr. breakp. enrich.	0.17
Linked to chrs	2:17888044−240960570;X:6640984−97824094
Purity, ploidy	0.84, 2.89

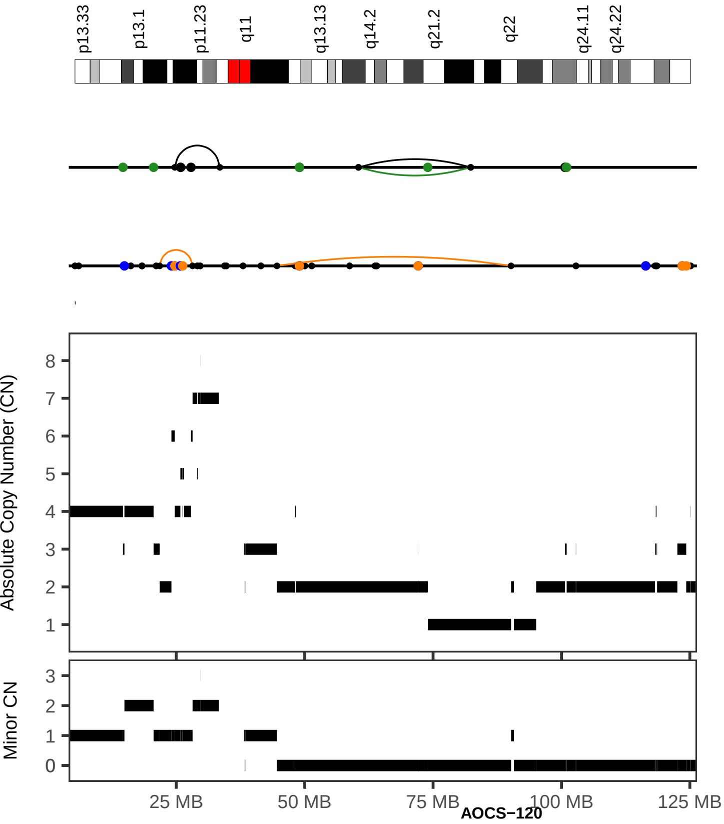

AOCS−120

Cancer type	Ovary−AdenoCA
Position	12:21770067−118615376
Interleaved intrachr. SVs	2
Total SVs (intrachr. + transl.)	28
SV types	DEL: 1; DUP: 2; h2hINV: 2; t2tINV: 1; TRA: 22
SVs in sample	790
Oscillating CN (2 and 3 states)	6, 8
CN segments	33
FDR fragment joints	0.6776251
FDR chr. breakp. enrich.	0
Linked to chrs	11:11833414−60671100;2:17888044−240960570
Purity, ploidy	0.84, 2.89

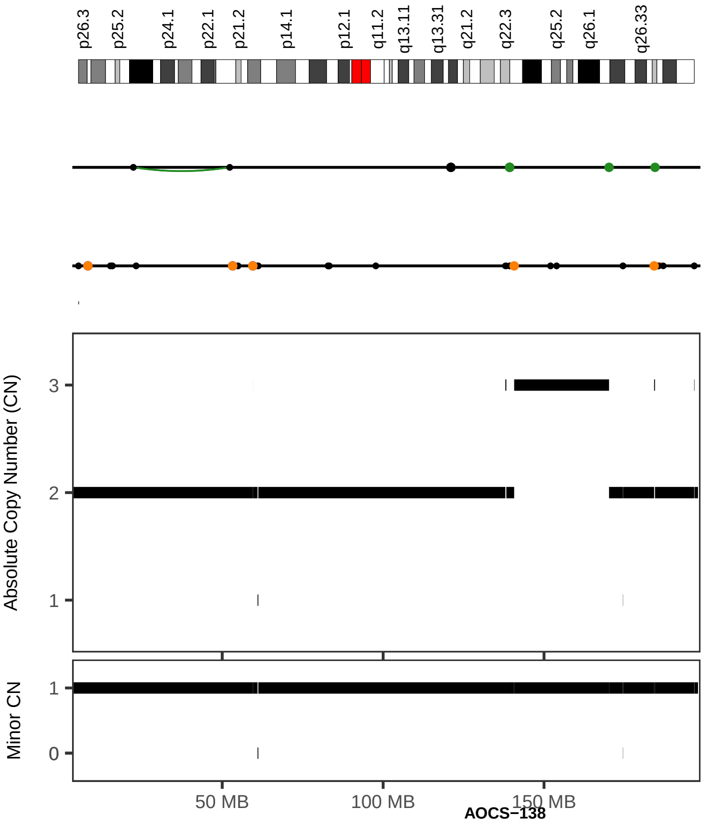

AOCS−138

Cancer type	Ovary−AdenoCA
Position	3:15240189−187056378
Interleaved intrachr. SVs	1
Total SVs (intrachr. + transl.)	21
SV types	DEL: 3; DUP: 4; h2hINV: 0; t2tINV: 2; TRA: 12
SVs in sample	323
Oscillating CN (2 and 3 states)	5, 13
CN segments	13
FDR fragment joints	0.8419568
FDR chr. breakp. enrich.	0.03
Linked to chrs	2:24454660−201203391;5:96175581−171118222
Purity, ploidy	0.65, 1.78

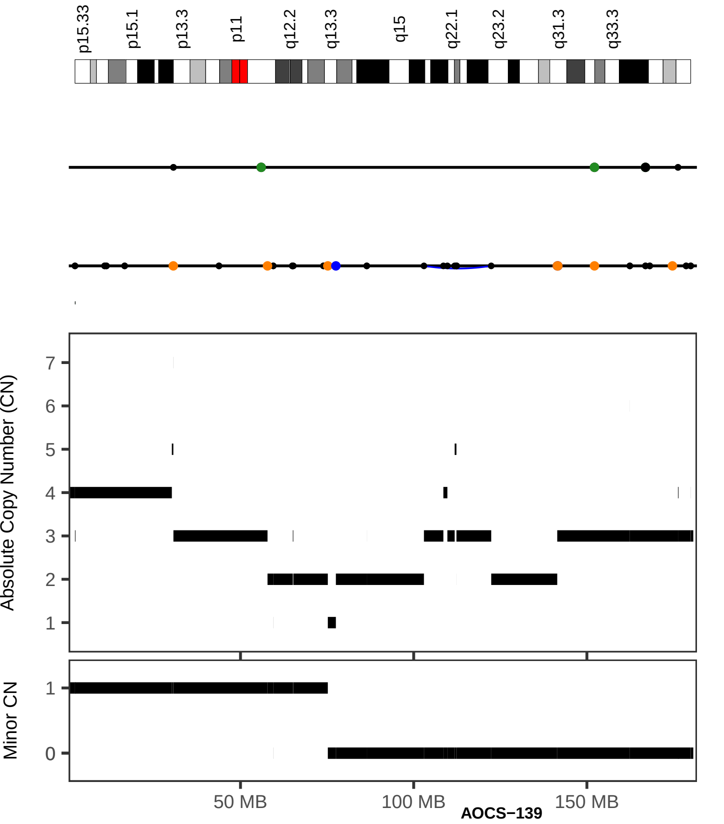

AOCS−139

Cancer type	Ovary−AdenoCA
Position	5:16559926−179967726
Interleaved intrachr. SVs	1
Total SVs (intrachr. + transl.)	27
SV types	DEL: 2; DUP: 9; h2hINV: 1; t2tINV: 0; TRA: 15
SVs in sample	542
Oscillating CN (2 and 3 states)	5, 13
CN segments	28
FDR fragment joints	0.5435077
FDR chr. breakp. enrich.	0.81
Linked to chrs	1:159497150−207592386;7:51378978−151817245
Purity, ploidy	0.49, 3.07

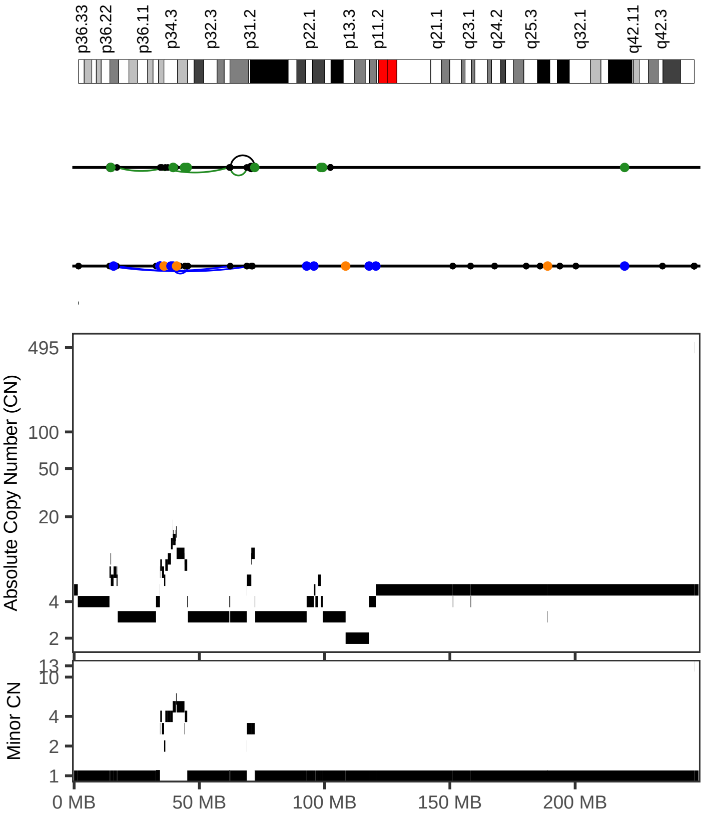

AOCS−142

Cancer type	Ovary−AdenoCA
Position	1:14110378−72283225
Interleaved intrachr. SVs	5
Total SVs (intrachr. + transl.)	25
SV types	DEL: 2; DUP: 3; h2hINV: 1; t2tINV: 4; TRA: 15
SVs in sample	587
Oscillating CN (2 and 3 states)	5, 11
CN segments	41
FDR fragment joints	0.8135044
FDR chr. breakp. enrich.	0.13
Linked to chrs	
Purity, ploidy	0.4, 3.64

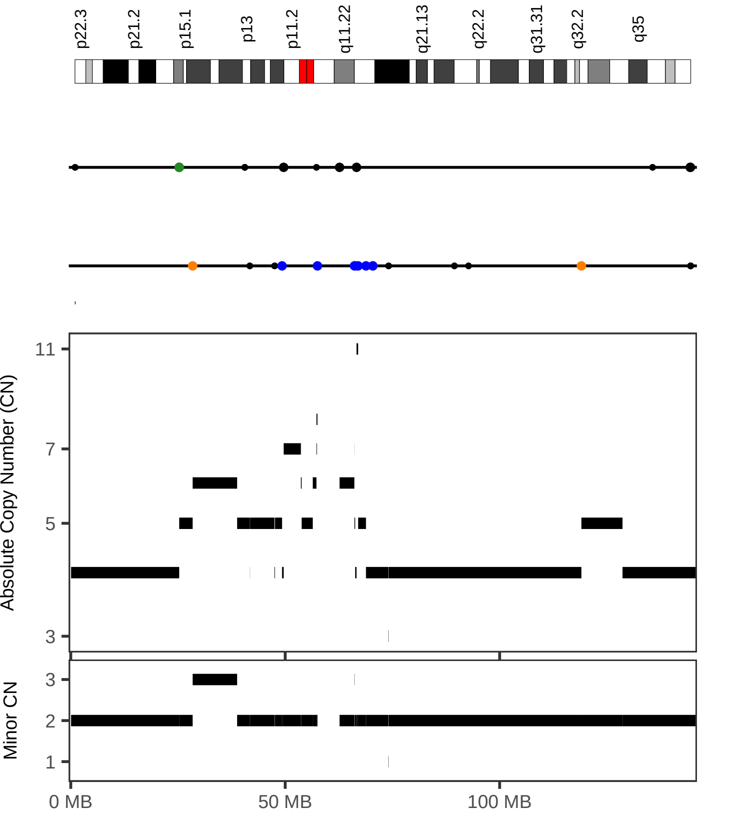

AOCS−142

Cancer type	Ovary−AdenoCA
Position	7:978895−144549792
Interleaved intrachr. SVs	1
Total SVs (intrachr. + transl.)	24
SV types	DEL: 5; DUP: 1; h2hINV: 0; t2tINV: 1; TRA: 17
SVs in sample	587
Oscillating CN (2 and 3 states)	6, 15
CN segments	27
FDR fragment joints	0.615458
FDR chr. breakp. enrich.	0.99
Linked to chrs	1:14110378−72283224;
Purity, ploidy	0.4, 3.64

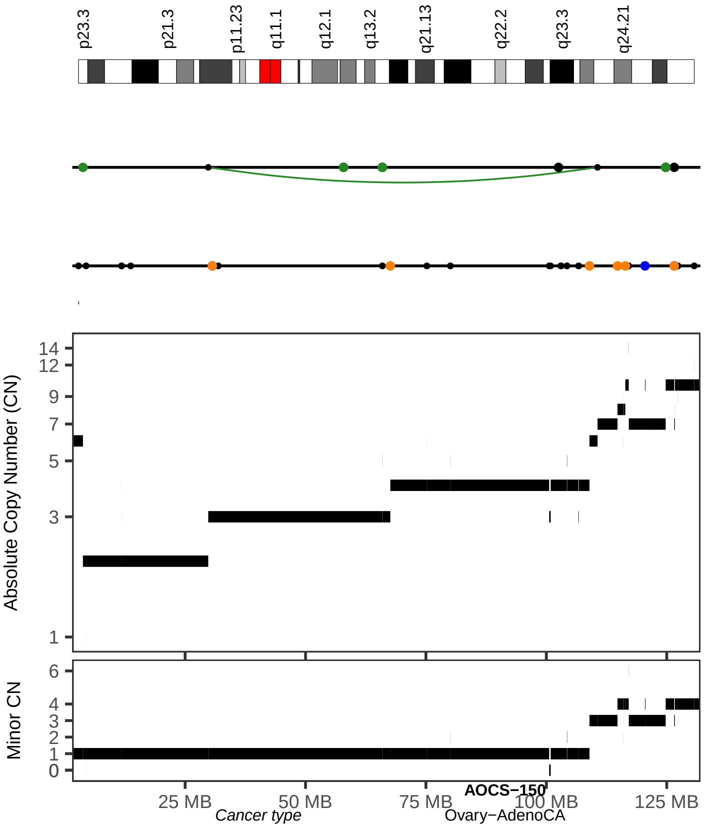

AOCS−150

Cancer type	Ovary−AdenoCA
Position	8:29804740−127337992
Interleaved intrachr. SVs	1
Total SVs (intrachr. + transl.)	28
SV types	DEL: 4; DUP: 5; h2hINV: 0; t2tINV: 1; TRA: 18
SVs in sample	428
Oscillating CN (2 and 3 states)	5, 5
CN segments	32
FDR fragment joints	0.615458
FDR chr. breakp. enrich.	0
Linked to chrs	10:21669392−127430409;11:17454833−117019884 15:38740794−93349545;2:11605377−242867066 6:34474425−84968073;7:835201−87501871

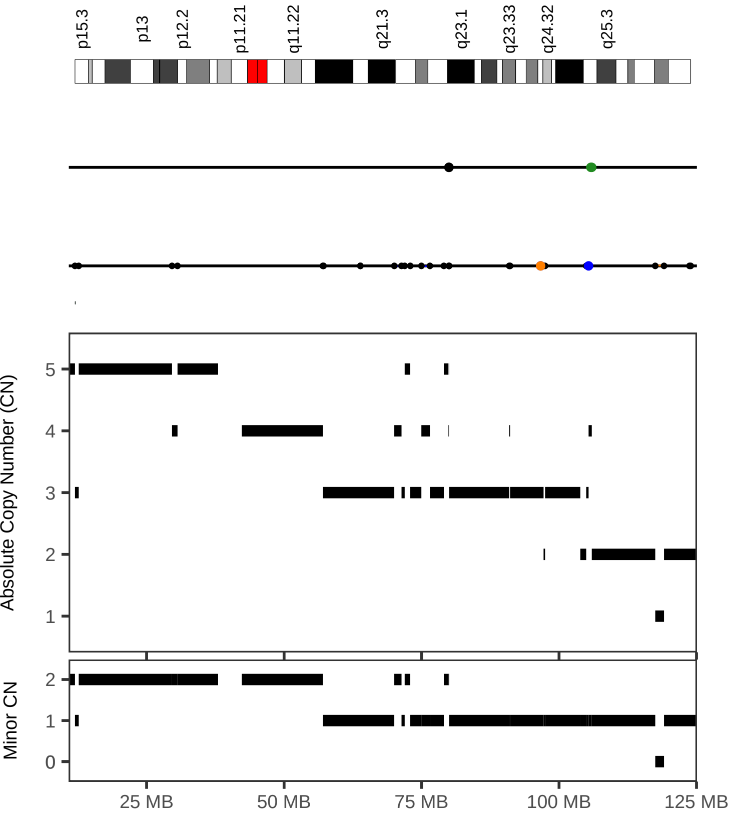

AOCS−161

Cancer type	Ovary−AdenoCA
Position	10:11969837−123949118
Interleaved intrachr. SVs	1
Total SVs (intrachr. + transl.)	20
SV types	DEL: 3; DUP: 9; h2hINV: 1; t2tINV: 0; TRA: 7
SVs in sample	255
Oscillating CN (2 and 3 states)	5, 14
CN segments	26
FDR fragment joints	0.4101979
FDR chr. breakp. enrich.	0.03
Linked to chrs	3:89265022−123848791;
Purity, ploidy	0.68, 2.44

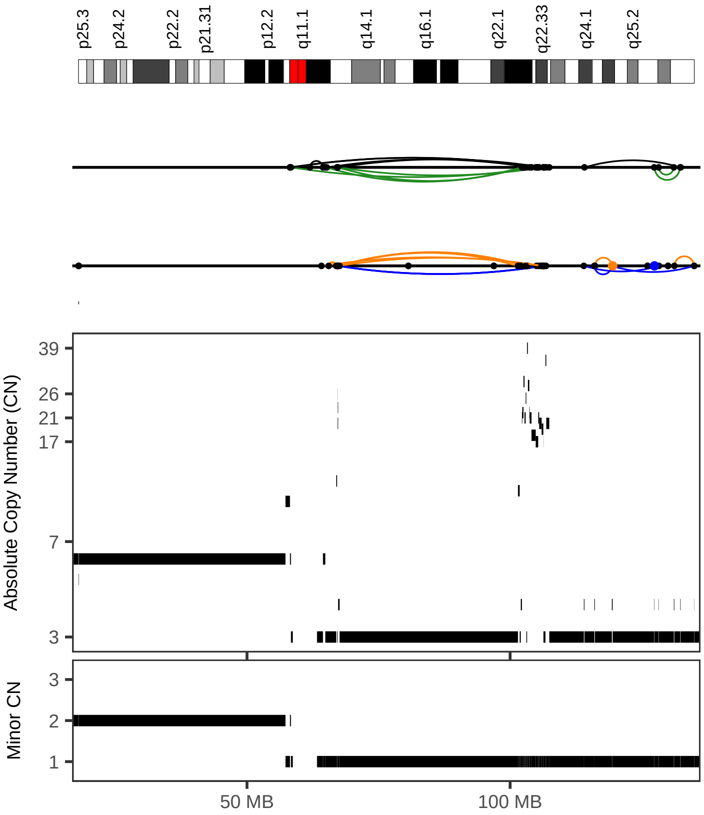

15556b28−c6bd−455a−87b6−4b7b3e33d0e4

Cancer type	Ovary−AdenoCA
Position	6:58165813−106822892
Interleaved intrachr. SVs	21
Total SVs (intrachr. + transl.)	21
SV types	DEL: 5; DUP: 5; h2hINV: 7; t2tINV: 4; TRA: 0
SVs in sample	154
Oscillating CN (2 and 3 states)	3, 5
CN segments	34
FDR fragment joints	0.8882853
FDR chr. breakp. enrich.	0
Linked to chrs	
Purity, ploidy	0.84, 3.91

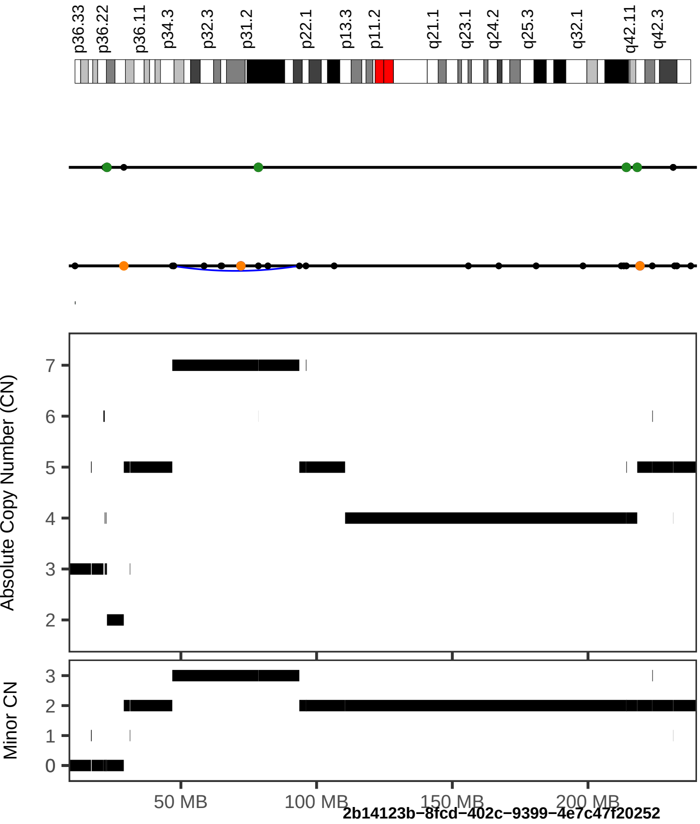

2b14123b−8fcd−402c−9399−4e7c47f20252

Cancer type	Ovary−AdenoCA
Position	1:21927365−231389754
Interleaved intrachr. SVs	1
Total SVs (intrachr. + transl.)	24
SV types	DEL: 1; DUP: 6; h2hINV: 2; t2tINV: 2; TRA: 13
SVs in sample	329
Oscillating CN (2 and 3 states)	5, 9
CN segments	22
FDR fragment joints	0.8298498
FDR chr. breakp. enrich.	0.07
Linked to chrs	14:38561337−92256366;5:35750071−174926442
Purity, ploidy	0.77, 3.57

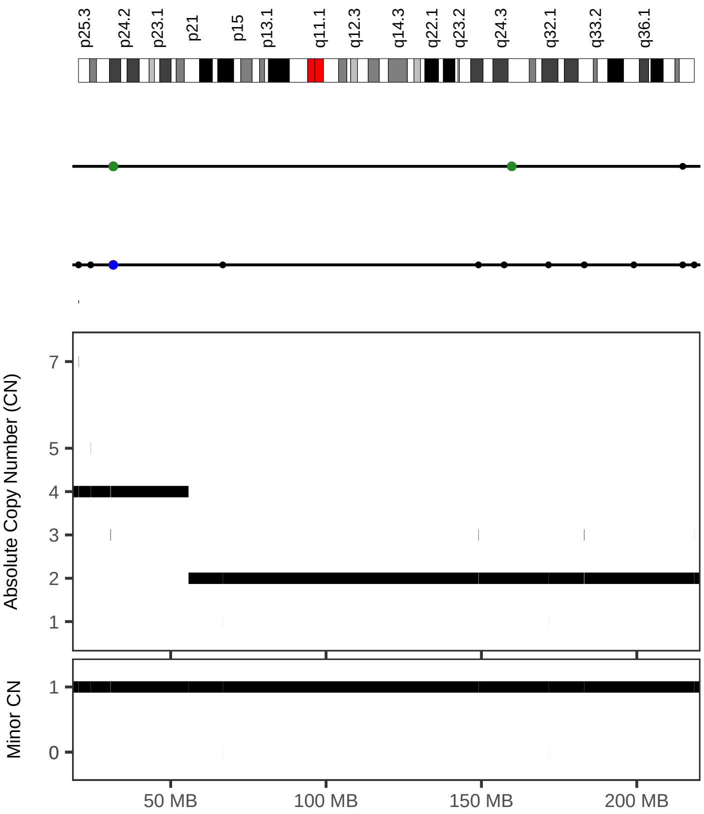

3768d34f−1527−40db−b116−cd80cee5ec3a

Cancer type	Ovary−AdenoCA
Position	2:20328396−218482845
Interleaved intrachr. SVs	1
Total SVs (intrachr. + transl.)	21
SV types	DEL: 2; DUP: 6; h2hINV: 0; t2tINV: 1; TRA: 12
SVs in sample	161
Oscillating CN (2 and 3 states)	5, 11
CN segments	17
FDR fragment joints	0.6610257
FDR chr. breakp. enrich.	0.02
Linked to chrs	
Purity, ploidy	0.89, 2.03

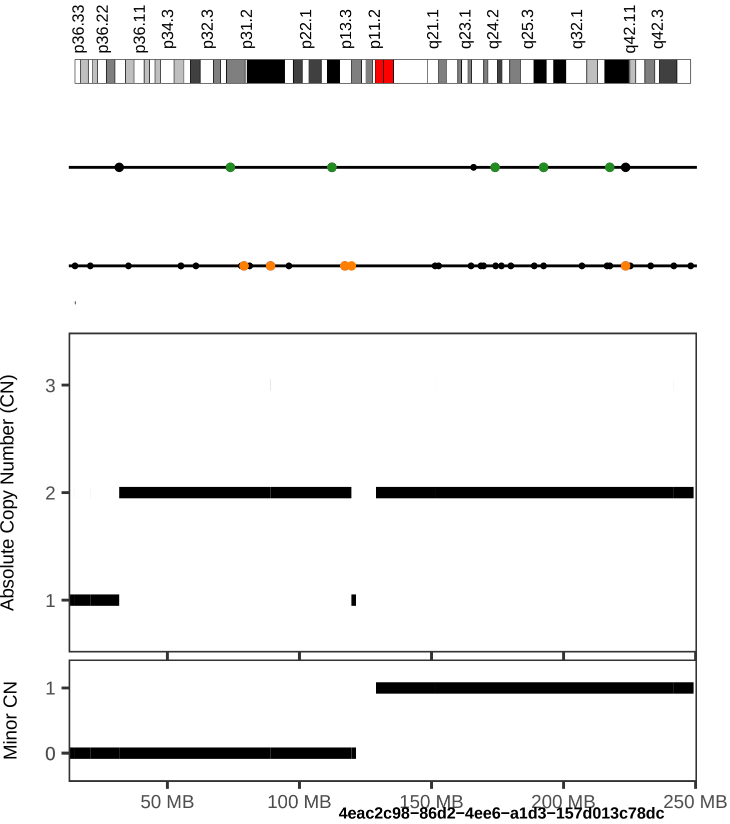

4eac2c98−86d2−4ee6−a1d3−157d013c78dc

Cancer type	Ovary−AdenoCA
Position	1:14990970−241778369
Interleaved intrachr. SVs	1
Total SVs (intrachr. + transl.)	32
SV types	DEL: 0; DUP: 8; h2hINV: 0; t2tINV: 0; TRA: 24
SVs in sample	545
Oscillating CN (2 and 3 states)	5, 13
CN segments	13
FDR fragment joints	0.615458
FDR chr. breakp. enrich.	0.23
Linked to chrs	5:11787249−113469349;8:14368513−144394437
Purity, ploidy	0.92, 1.81

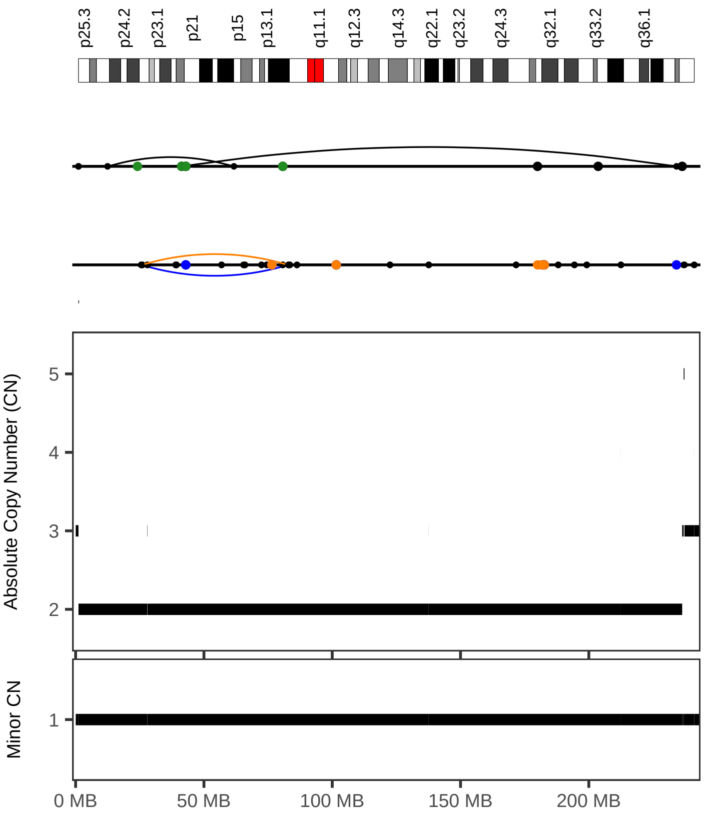

4eac2c98−86d2−4ee6−a1d3−157d013c78dc

Cancer type	Ovary−AdenoCA
Position	2:12447905−234169661
Interleaved intrachr. SVs	4
Total SVs (intrachr. + transl.)	28
SV types	DEL: 1; DUP: 1; h2hINV: 2; t2tINV: 0; TRA: 24
SVs in sample	545
Oscillating CN (2 and 3 states)	5, 7
CN segments	7
FDR fragment joints	0.8988396
FDR chr. breakp. enrich.	0.37
Linked to chrs	
Purity, ploidy	0.92, 1.81

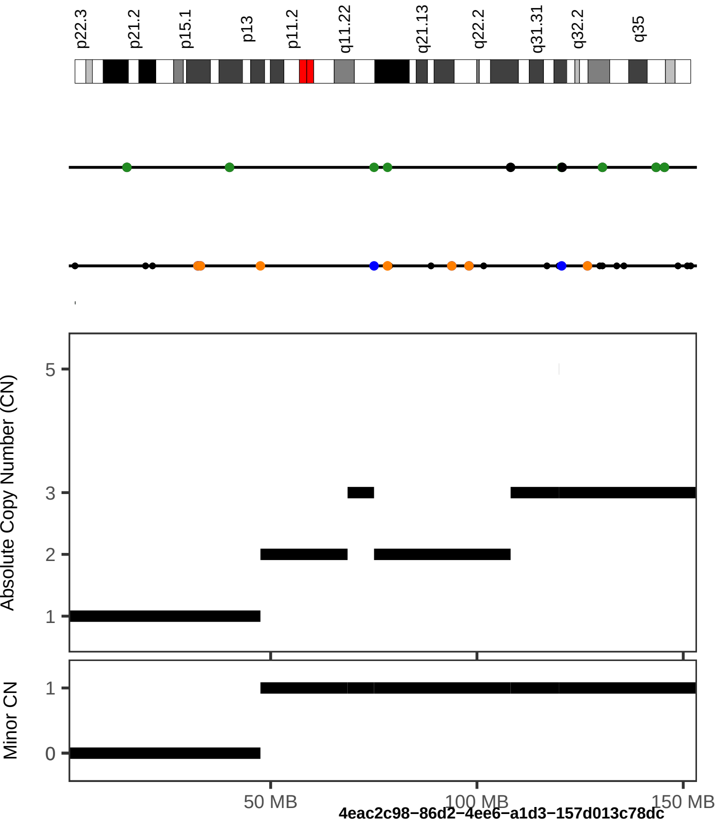

4eac2c98−86d2−4ee6−a1d3−157d013c78dc

Cancer type	Ovary−AdenoCA
Position	7:2564855−150994792
Interleaved intrachr. SVs	1
Total SVs (intrachr. + transl.)	42
SV types	DEL: 3; DUP: 7; h2hINV: 0;<br>t2tINV: 0; TRA: 32
SVs in sample	545
Oscillating CN (2 and 3 states)	4, 7
CN segments	9
FDR fragment joints	0.615458
FDR chr. breakp. enrich.	0
Linked to chrs	1:14990970−241778368;2:12447905−234169660<br>X:30289488−150390658;
Purity, ploidy	0.92, 1.81

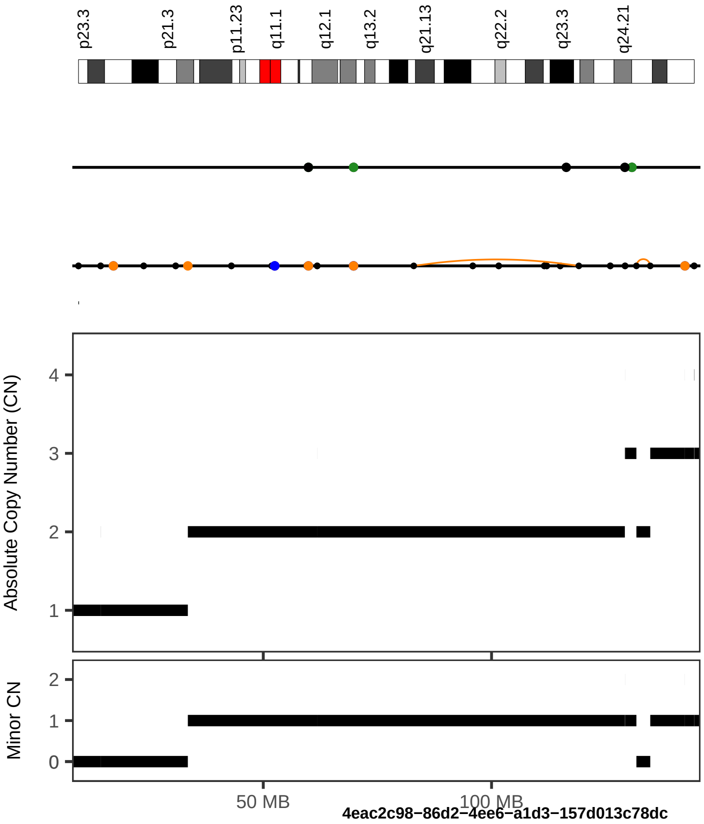

4eac2c98−86d2−4ee6−a1d3−157d013c78dc

Cancer type	Ovary−AdenoCA
Position	8:14368513−144394438
Interleaved intrachr. SVs	1
Total SVs (intrachr. + transl.)	27
SV types	DEL: 2; DUP: 4; h2hINV: 0;<br>t2tINV: 0; TRA: 21
SVs in sample	545
Oscillating CN (2 and 3 states)	5, 14
CN segments	14
FDR fragment joints	0.615458
FDR chr. breakp. enrich.	0.09
Linked to chrs	1:14990970−241778368;4:510643−174210982
Purity, ploidy	0.92, 1.81

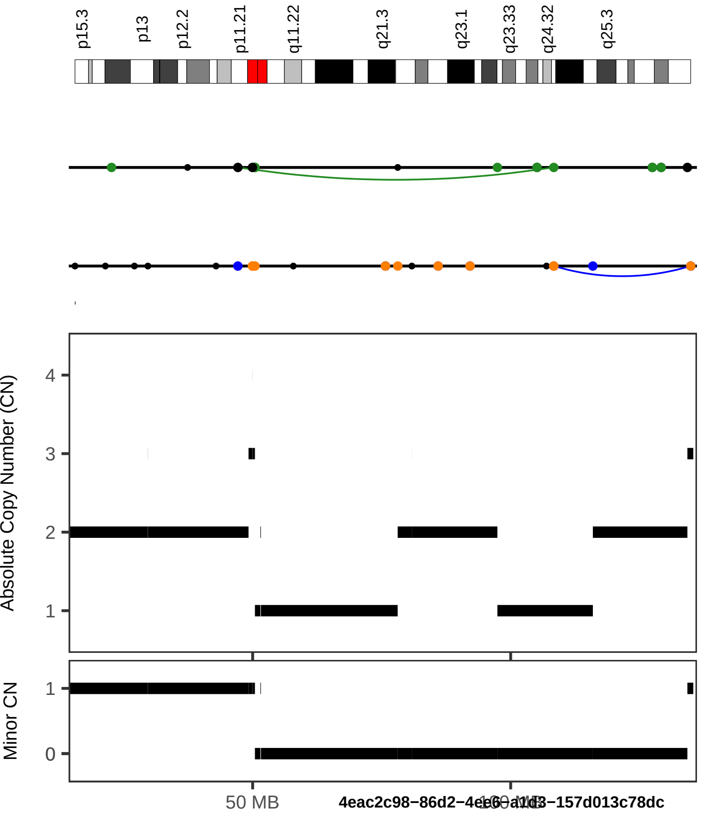

4eac2c98−86d2−4ee6−a1d3−157d013c78dc

Cancer type	Ovary−AdenoCA
Position	10:47136303−134896518
Interleaved intrachr. SVs	2
Total SVs (intrachr. + transl.)	32
SV types	DEL: 0; DUP: 1; h2hINV: 0;<br>t2tINV: 1; TRA: 30
SVs in sample	545
Oscillating CN (2 and 3 states)	4, 9
CN segments	13
FDR fragment joints	0.8653243
FDR chr. breakp. enrich.	0
Linked to chrs	1:14990970−241778368;4:510643−174210982<br>5:11787249−113469349;8:14368513−144394437<br>X:30289488−150390658;

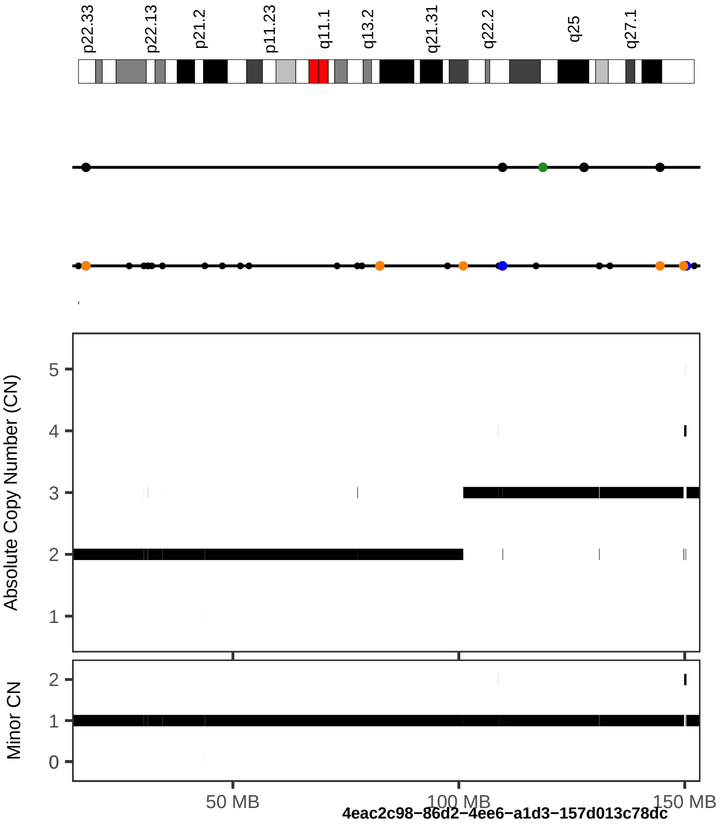

4eac2c98−86d2−4ee6−a1d3−157d013c78dc

Cancer type	Ovary−AdenoCA
Position	X:30289488−150390659
Interleaved intrachr. SVs	1
Total SVs (intrachr. + transl.)	29
SV types	DEL: 1; DUP: 6; h2hINV: 0;<br>t2tINV: 0; TRA: 22
SVs in sample	545
Oscillating CN (2 and 3 states)	6, 18
CN segments	23
FDR fragment joints	0.2977091
FDR chr. breakp. enrich.	0.01
Linked to chrs	11:19455337−121072078;5:11787249−113469349
Purity, ploidy	0.92, 1.81

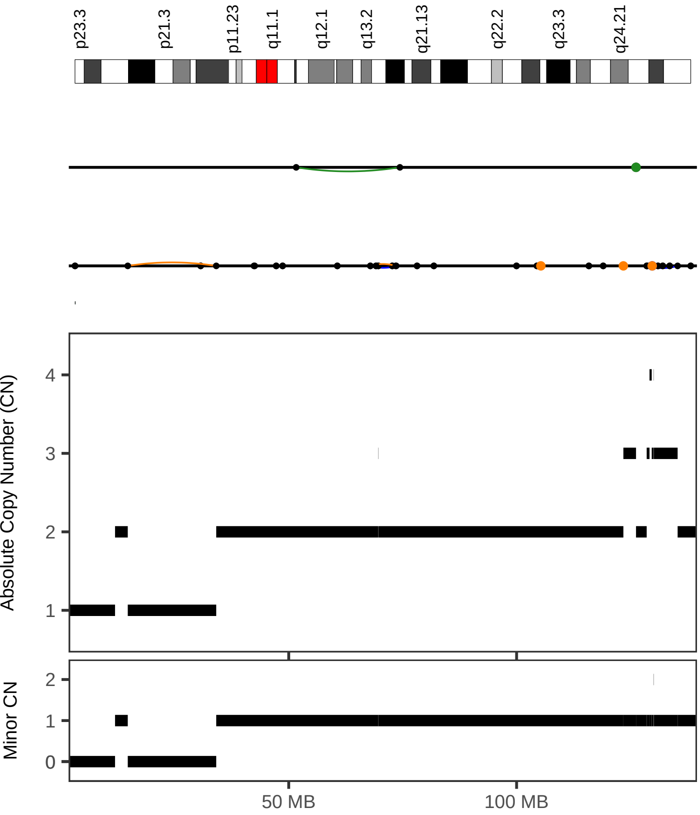

700e91bb−d675−41b2−bbbd−935767c7b447

Cancer type	Ovary−AdenoCA
Position	8:3100649−138208042
Interleaved intrachr. SVs	1
Total SVs (intrachr. + transl.)	24
SV types	DEL: 4; DUP: 13; h2hINV: 0; t2tINV: 1; TRA: 6
SVs in sample	686
Oscillating CN (2 and 3 states)	6, 14
CN segments	14
FDR fragment joints	0.04831189
FDR chr. breakp. enrich.	0.99
Linked to chrs	X:8029219−122680104;
Purity, ploidy	0.8, 1.88

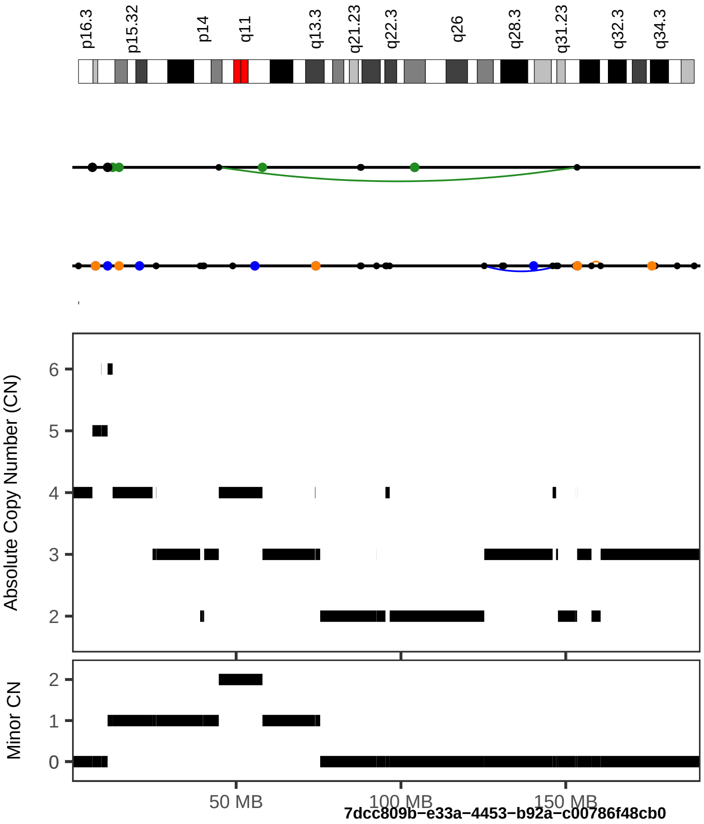

7dcc809b−e33a−4453−b92a−c00786f48cb0

Cancer type	Ovary−AdenoCA
Position	4:25688755−183822954
Interleaved intrachr. SVs	1
Total SVs (intrachr. + transl.)	27
SV types	DEL: 2; DUP: 11; h2hINV: 1; t2tINV: 2; TRA: 11
SVs in sample	665
Oscillating CN (2 and 3 states)	5, 13
CN segments	26
FDR fragment joints	0.1278363
FDR chr. breakp. enrich.	1
Linked to chrs	2:100265270−222653042;5:73636560−135974127
Purity, ploidy	0.82, 3.31

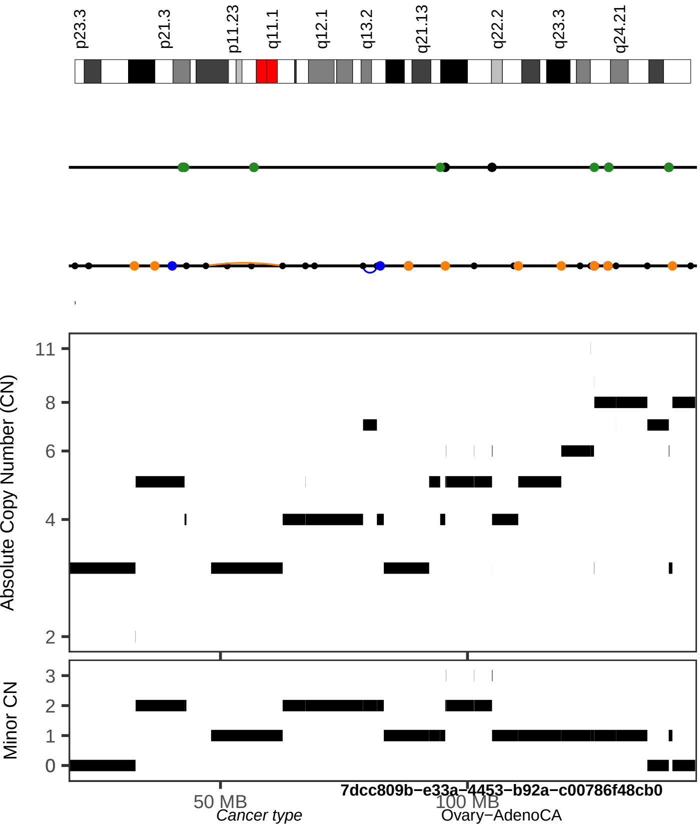

7dcc809b−e33a−4453−b92a−c00786f48cb0

Cancer type	Ovary−AdenoCA
Position	8:23266791−145185294
Interleaved intrachr. SVs	1
Total SVs (intrachr. + transl.)	36
SV types	DEL: 1; DUP: 8; h2hINV: 0; t2tINV: 0; TRA: 27
SVs in sample	665
Oscillating CN (2 and 3 states)	6, 12
CN segments	34
FDR fragment joints	0.615458
FDR chr. breakp. enrich.	0.07
Linked to chrs	11:23550499−126101177;12:1814222−68872524 13:30648512−112891496;18:8267017−60831954 3:101663947−139539103;5:73636560−135974127

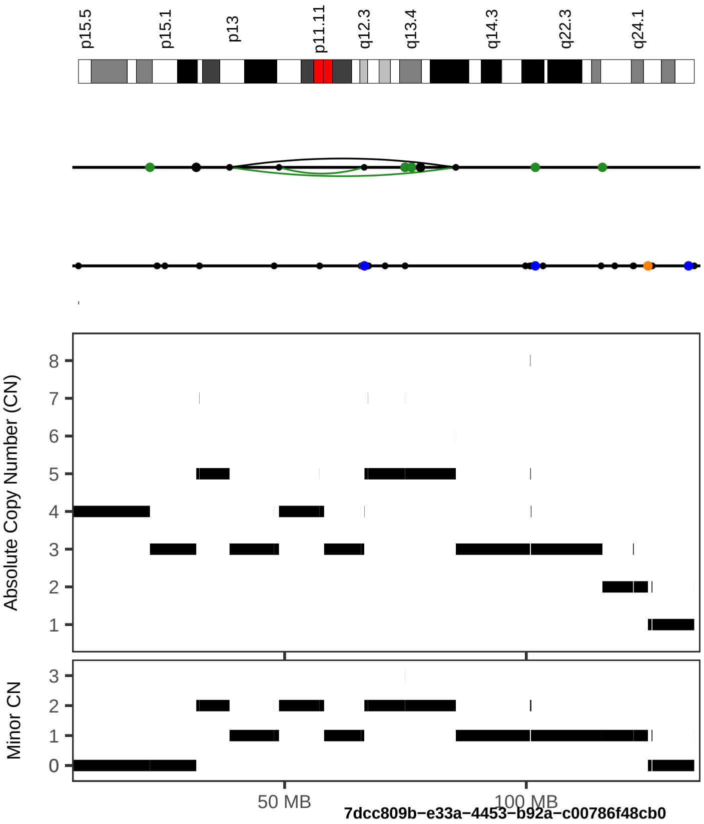

7dcc809b−e33a−4453−b92a−c00786f48cb0

Cancer type	Ovary−AdenoCA
Position	11:23550499−126101178
Interleaved intrachr. SVs	1
Total SVs (intrachr. + transl.)	26
SV types	DEL: 0; DUP: 11; h2hINV: 1; t2tINV: 2; TRA: 12
SVs in sample	665
Oscillating CN (2 and 3 states)	5, 11
CN segments	33
FDR fragment joints	0.4101979
FDR chr. breakp. enrich.	0.34
Linked to chrs	12:1814222−68872524;20:9980789−62225665 8:23266791−145185293;
Purity, ploidy	0.82, 3.31

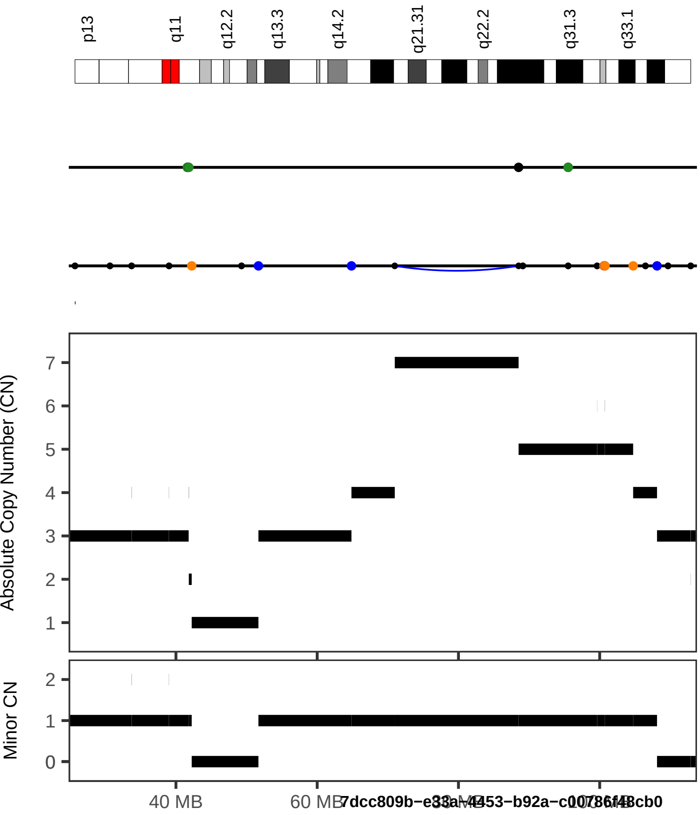

7dcc809b−e33a−4453−b92a−c00786f48cb0

Cancer type	Ovary−AdenoCA
Position	13:30648512−112891497
Interleaved intrachr. SVs	1
Total SVs (intrachr. + transl.)	22
SV types	DEL: 0; DUP: 7; h2hINV: 0; t2tINV: 0; TRA: 15
SVs in sample	665
Oscillating CN (2 and 3 states)	6, 7
CN segments	20
FDR fragment joints	0.5435077
FDR chr. breakp. enrich.	0.64
Linked to chrs	15:59890154−98831218;2:100265270−222653042 6:16954294−41598732;8:23266791−145185293
Purity, ploidy	0.82, 3.31

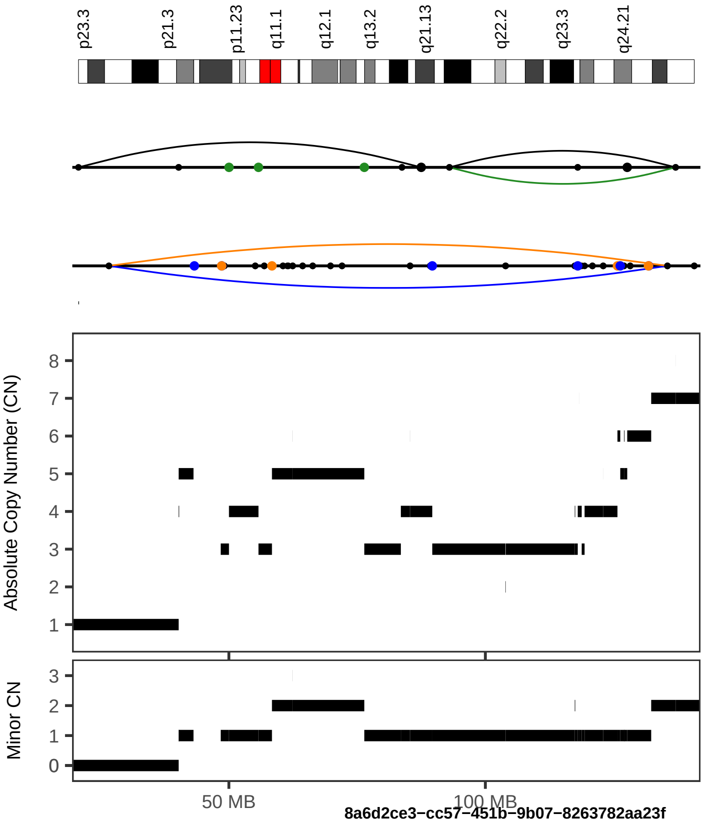

8a6d2ce3−cc57−451b−9b07−8263782aa23f

Cancer type	Ovary−AdenoCA
Position	8:20677931−137092373
Interleaved intrachr. SVs	5
Total SVs (intrachr. + transl.)	20
SV types	DEL: 1; DUP: 1; h2hINV: 2; t2tINV: 1; TRA: 15
SVs in sample	704
Oscillating CN (2 and 3 states)	5, 8
CN segments	33
FDR fragment joints	0.7735152
FDR chr. breakp. enrich.	0.06
Linked to chrs	11:9941489−124824127;2:23974041−152146531 20:314995−46999236;
Purity, ploidy	0.76, 3.3

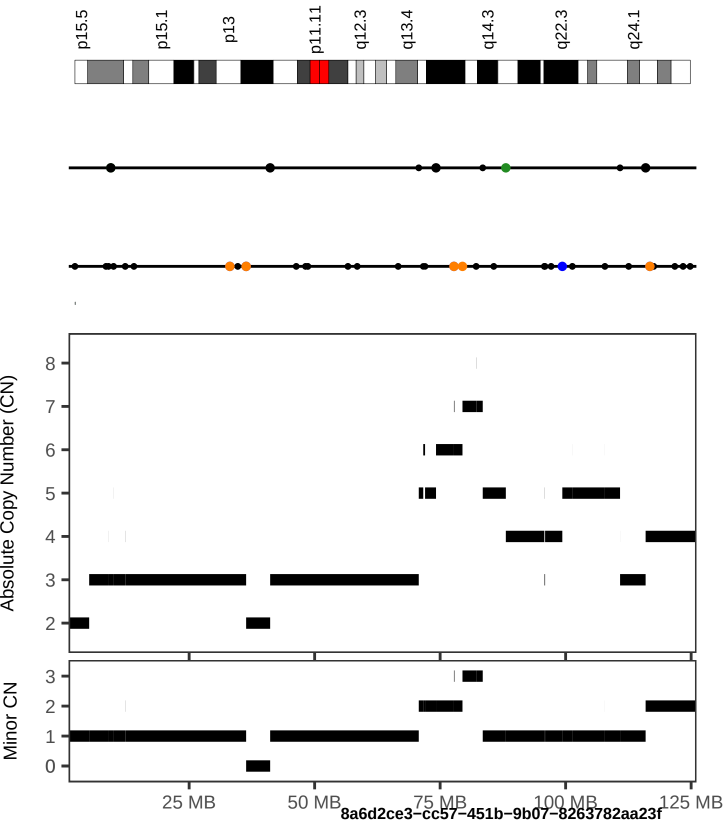

8a6d2ce3−cc57−451b−9b07−8263782aa23f

Cancer type	Ovary−AdenoCA
Position	11:9941489−124824128
Interleaved intrachr. SVs	1
Total SVs (intrachr. + transl.)	22
SV types	DEL: 1; DUP: 6; h2hINV: 0; t2tINV: 0; TRA: 15
SVs in sample	704
Oscillating CN (2 and 3 states)	5, 14
CN segments	29
FDR fragment joints	0.3111083
FDR chr. breakp. enrich.	0.04
Linked to chrs	1:8622852−227102367;20:314995−46999236 8:20677931−137092372;
Purity, ploidy	0.76, 3.3

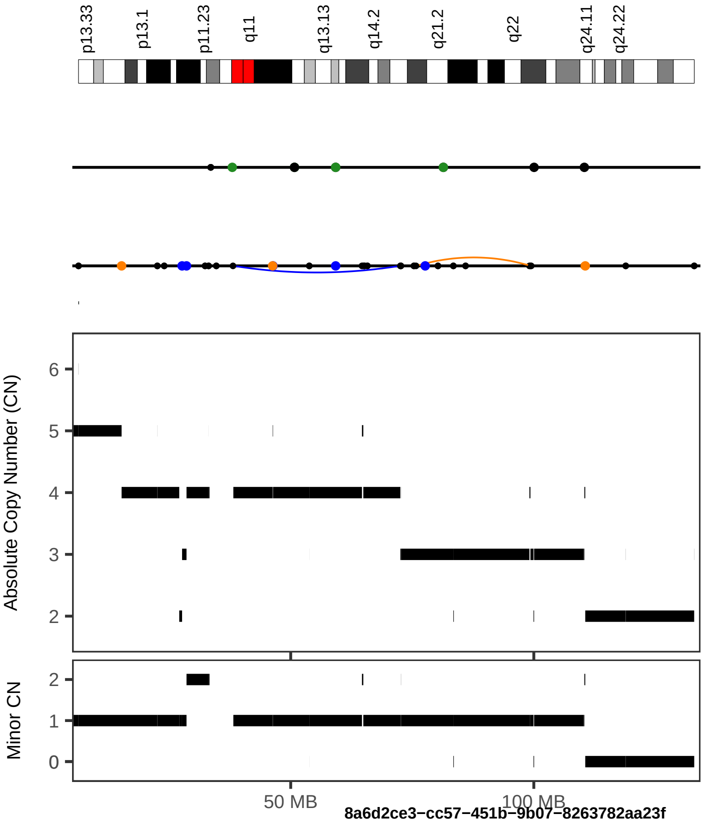

8a6d2ce3−cc57−451b−9b07−8263782aa23f

Cancer type	Ovary−AdenoCA
Position	12:6347743−132991798
Interleaved intrachr. SVs	1
Total SVs (intrachr. + transl.)	27
SV types	DEL: 2; DUP: 7; h2hINV: 1; t2tINV: 0; TRA: 17
SVs in sample	704
Oscillating CN (2 and 3 states)	4, 19
CN segments	33
FDR fragment joints	0.615458
FDR chr. breakp. enrich.	0.28
Linked to chrs	1:8622852−227102367;11:9941489−124824127 5:33617055−179167007;
Purity, ploidy	0.76, 3.3

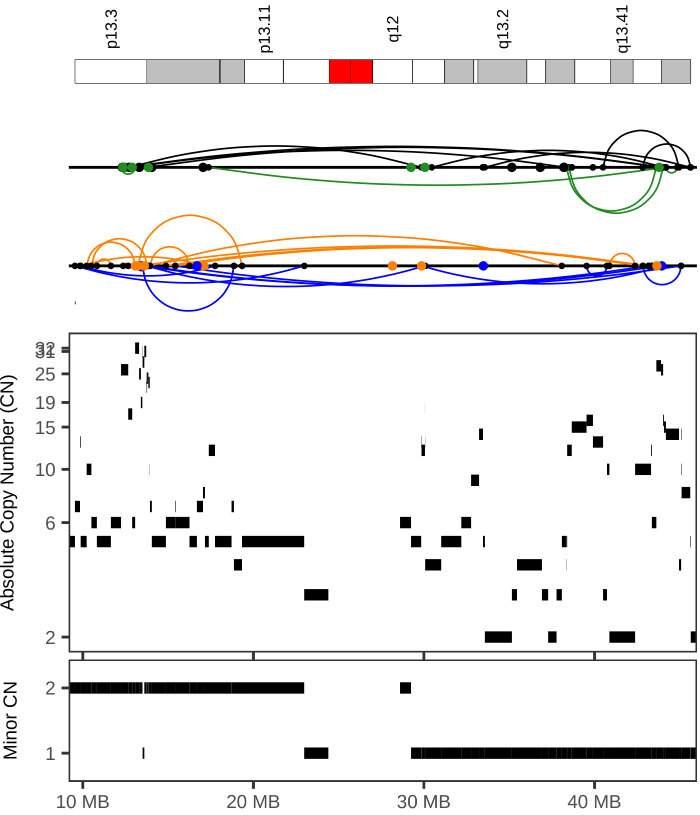

ba68f2cf−9271−41fd−9655−1fac7681f588

Cancer type	Ovary−AdenoCA
Position	19:9540512−45641579
Interleaved intrachr. SVs	33
Total SVs (intrachr. + transl.)	61
SV types	DEL: 10; DUP: 11; h2hINV: 8; t2tINV: 4; TRA: 28
SVs in sample	204
Oscillating CN (2 and 3 states)	3, 7
CN segments	78
FDR fragment joints	0.854603
FDR chr. breakp. enrich.	0
Linked to chrs	8:29488947−40948298;
Purity, ploidy	0.89, 3.23

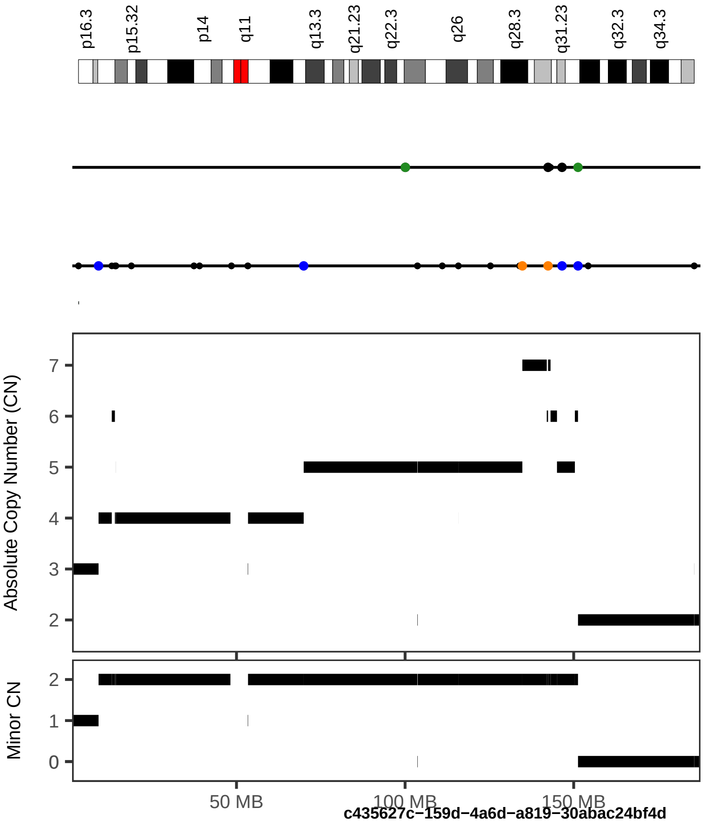

c435627c−159d−4a6d−a819−30abac24bf4d

Cancer type	Ovary−AdenoCA
Position	4:3142975−185752325
Interleaved intrachr. SVs	1
Total SVs (intrachr. + transl.)	21
SV types	DEL: 4; DUP: 5; h2hINV: 1; t2tINV: 0; TRA: 11
SVs in sample	283
Oscillating CN (2 and 3 states)	4, 10
CN segments	22
FDR fragment joints	0.615458
FDR chr. breakp. enrich.	0.21
Linked to chrs	3:15395276−169948381;6:53357622−152036318
Purity, ploidy	0.94, 3.57

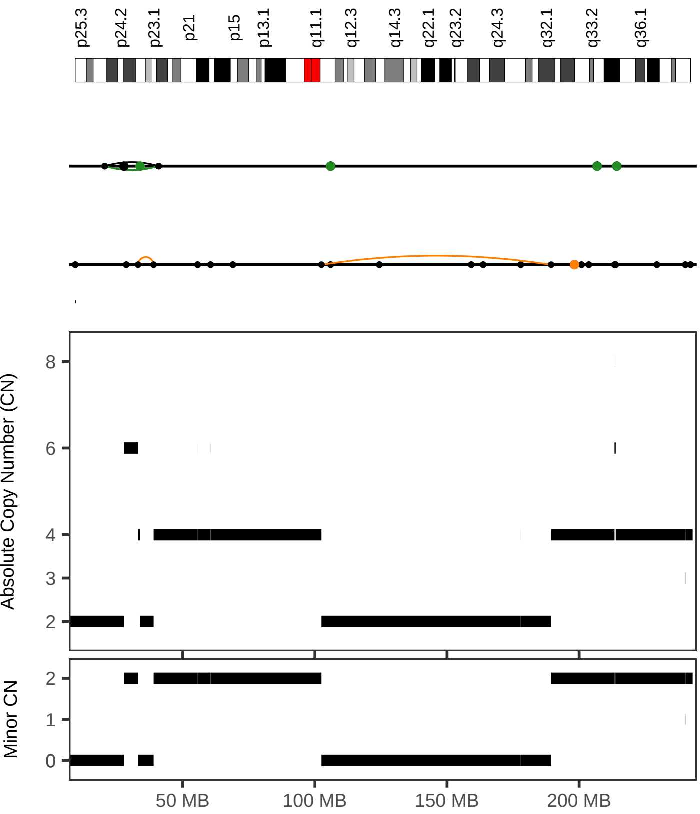

d3164236−c14a−4230−b527−ecd1a3992f02

Cancer type	Ovary−AdenoCA
Position	2:9305010−240217958
Interleaved intrachr. SVs	1
Total SVs (intrachr. + transl.)	22
SV types	DEL: 4; DUP: 6; h2hINV: 1; t2tINV: 1; TRA: 10
SVs in sample	297
Oscillating CN (2 and 3 states)	5, 16
CN segments	19
FDR fragment joints	0.9419607
FDR chr. breakp. enrich.	0.34
Linked to chrs	19:8994161−14739775;
Purity, ploidy	0.9, 3.37

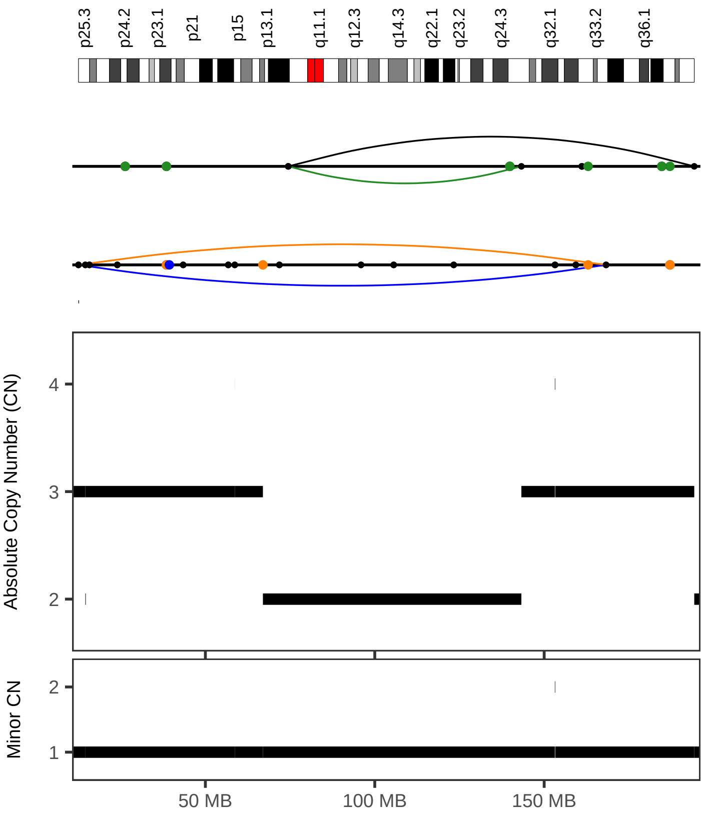

e43d3769−e25f−4fb7−9080−ea26defaf094

Cancer type	Ovary−AdenoCA
Position	2:12545356−194298156
Interleaved intrachr. SVs	4
Total SVs (intrachr. + transl.)	22
SV types	DEL: 1; DUP: 1; h2hINV: 1; t2tINV: 1; TRA: 18
SVs in sample	260
Oscillating CN (2 and 3 states)	3, 10
CN segments	10
FDR fragment joints	0.8874881
FDR chr. breakp. enrich.	0.02
Linked to chrs	
Purity, ploidy	0.85, 1.82

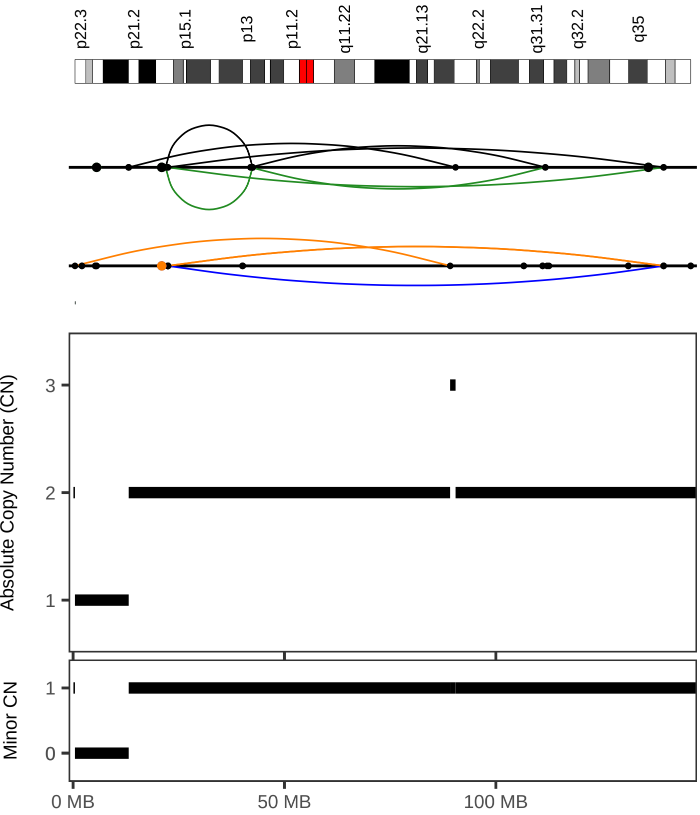

e43d3769−e25f−4fb7−9080−ea26defaf094

Cancer type	Ovary−AdenoCA
Position	7:450496−139649312
Interleaved intrachr. SVs	11
Total SVs (intrachr. + transl.)	20
SV types	DEL: 3; DUP: 1; h2hINV: 4; t2tINV: 3; TRA: 9
SVs in sample	260
Oscillating CN (2 and 3 states)	3, 4
CN segments	4
FDR fragment joints	0.615458
FDR chr. breakp. enrich.	0
Linked to chrs	
Purity, ploidy	0.85, 1.82

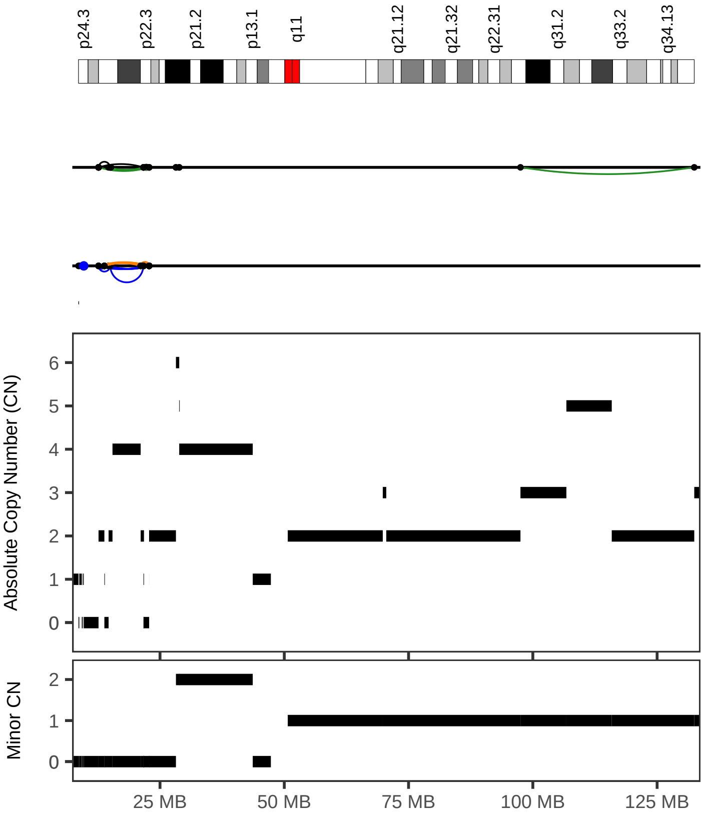

ICGC_0215

Cancer type	Panc−AdenoCA
Position	9:12633334−22809044
Interleaved intrachr. SVs	20
Total SVs (intrachr. + transl.)	20
SV types	DEL: 7; DUP: 7; h2hINV: 3; t2tINV: 3; TRA: 0
SVs in sample	94
Oscillating CN (2 and 3 states)	3, 4
CN segments	15
FDR fragment joints	0.615458
FDR chr. breakp. enrich.	0
Linked to chrs	
Purity, ploidy	0.51, 1.97

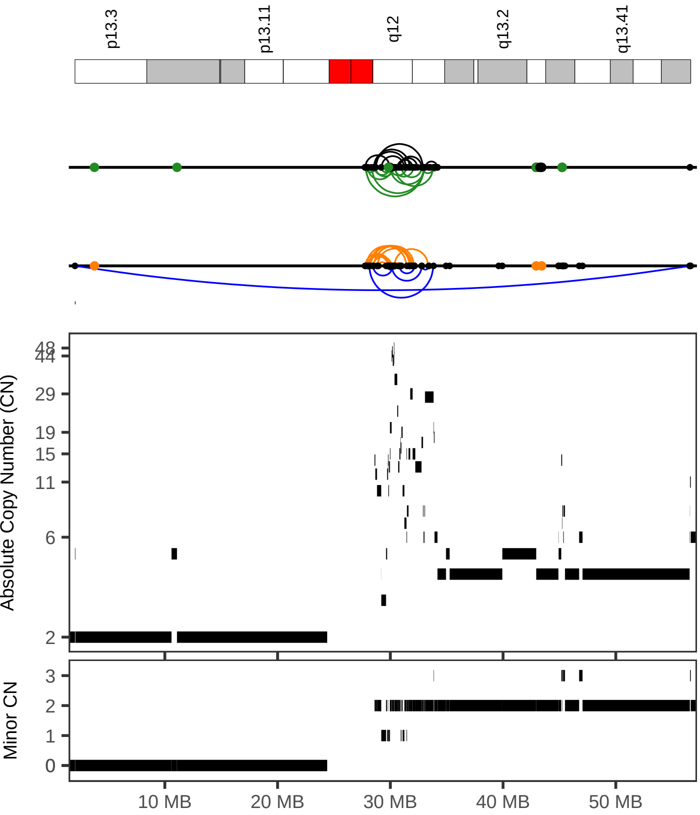

PCSI_0285

Cancer type	Panc−AdenoCA
Position	19:27751856−34183653
Interleaved intrachr. SVs	52
Total SVs (intrachr. + transl.)	54
SV types	DEL: 15; DUP: 7; h2hINV: 13; t2tINV: 17; TRA: 2
SVs in sample	446
Oscillating CN (2 and 3 states)	3, 4
CN segments	44
FDR fragment joints	0.615458
FDR chr. breakp. enrich.	0
Linked to chrs	
Purity, ploidy	0.56, 3.15

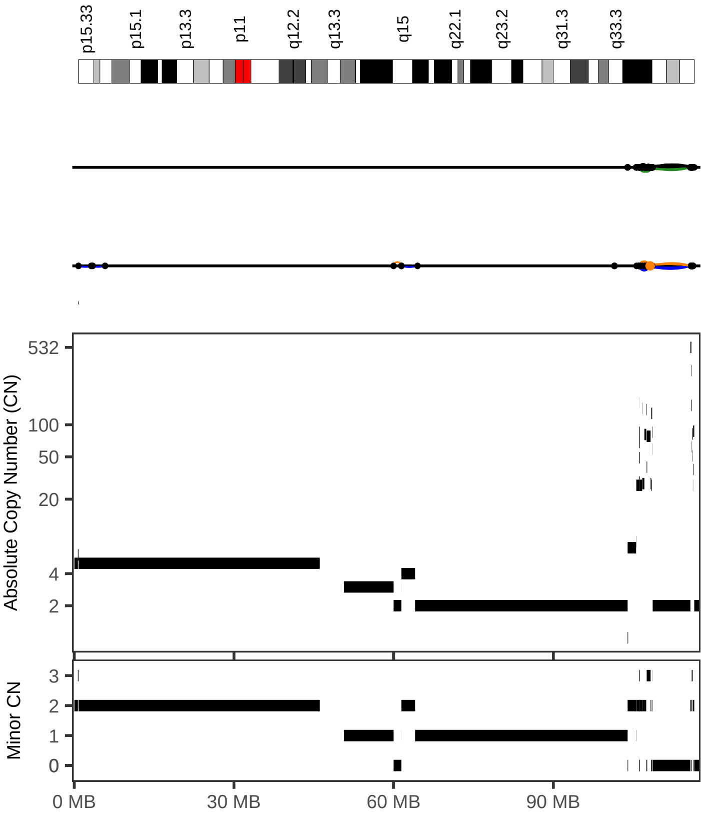

PCSI_0340

Cancer type	Panc−AdenoCA
Position	5:105643402−116481544
Interleaved intrachr. SVs	84
Total SVs (intrachr. + transl.)	85
SV types	DEL: 23; DUP: 19; h2hINV: 24; t2tINV: 18; TRA: 1
SVs in sample	237
Oscillating CN (2 and 3 states)	2, 3
CN segments	33
FDR fragment joints	0.8033078
FDR chr. breakp. enrich.	0
Linked to chrs	
Purity, ploidy	0.41, 3.29

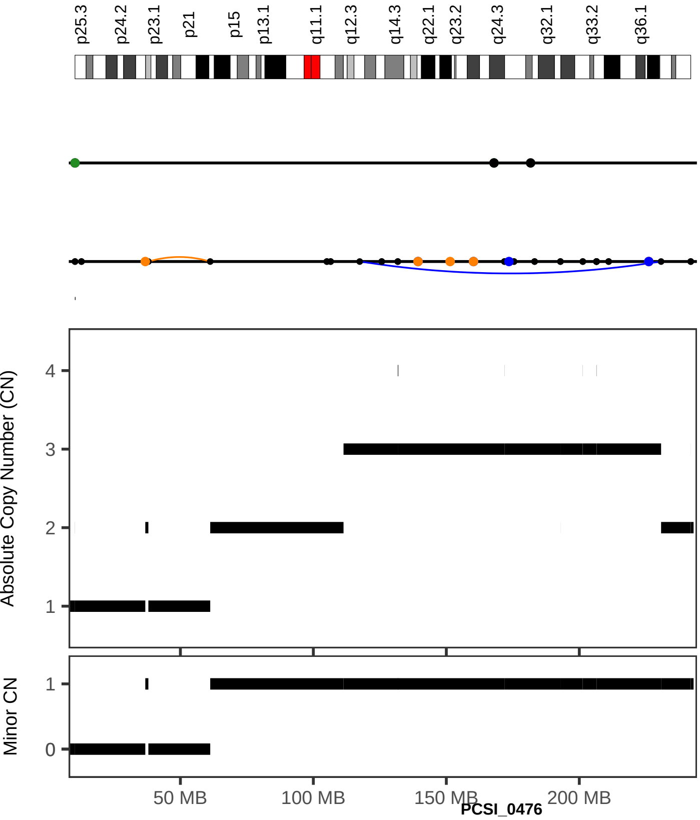

PCSI_0476

Cancer type	Panc−AdenoCA
Position	2:10364315−230753567
Interleaved intrachr. SVs	1
Total SVs (intrachr. + transl.)	23
SV types	DEL: 2; DUP: 7; h2hINV: 0; t2tINV: 0; TRA: 14
SVs in sample	360
Oscillating CN (2 and 3 states)	5, 17
CN segments	17
FDR fragment joints	0.4436253
FDR chr. breakp. enrich.	0.59
Linked to chrs	17:27499370−63215538;4:154731494−158798242 8:136426683−145091116;
Purity, ploidy	0.89, 1.88

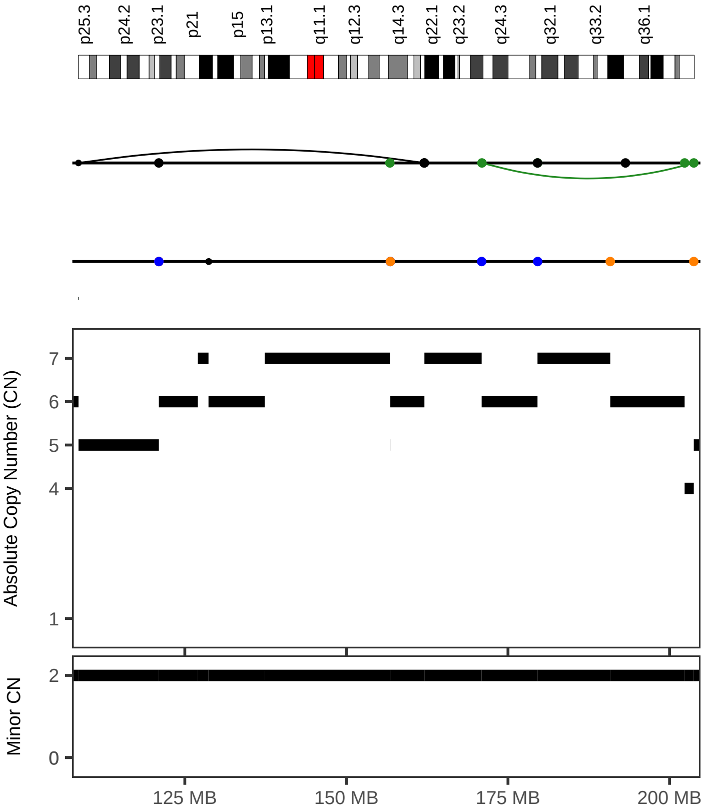

ICGC_0457

Cancer type	Panc−Endocrine
Position	2:108544255−203817368
Interleaved intrachr. SVs	1
Total SVs (intrachr. + transl.)	21
SV types	DEL: 1; DUP: 0; h2hINV: 1; t2tINV: 1; TRA: 18
SVs in sample	59
Oscillating CN (2 and 3 states)	5, 6
CN segments	13
FDR fragment joints	0.9462199
FDR chr. breakp. enrich.	0
Linked to chrs	10:70337732−130767692;
Purity, ploidy	0.29, 3.51

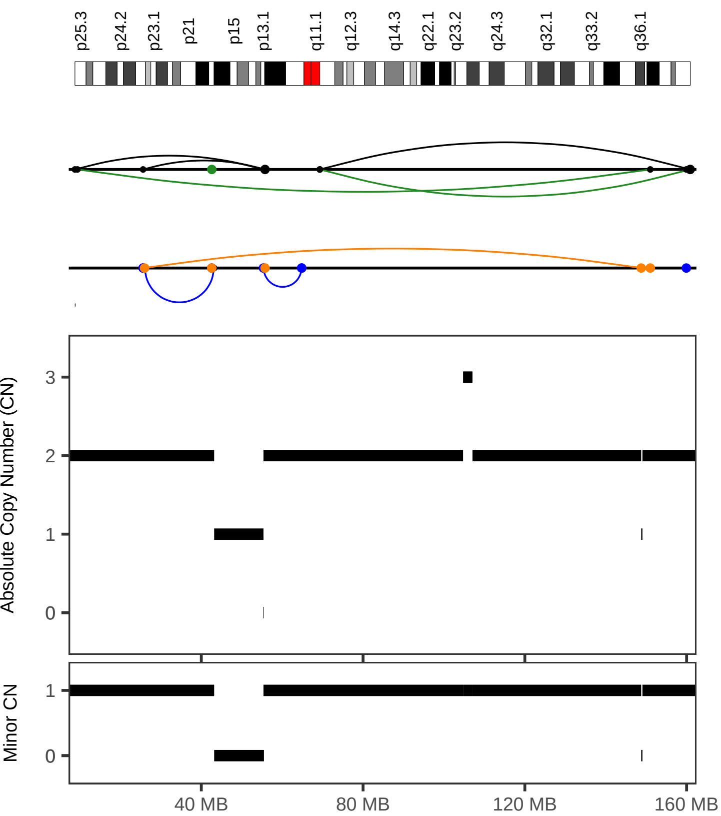

ITNET−0993

Cancer type	Panc−Endocrine
Position	2:8748091−160901518
Interleaved intrachr. SVs	7
Total SVs (intrachr. + transl.)	22
SV types	DEL: 1; DUP: 1; h2hINV: 3; t2tINV: 2; TRA: 15
SVs in sample	46
Oscillating CN (2 and 3 states)	3, 5
CN segments	8
FDR fragment joints	0.6776251
FDR chr. breakp. enrich.	0
Linked to chrs	
Purity, ploidy	0.85, 2.06

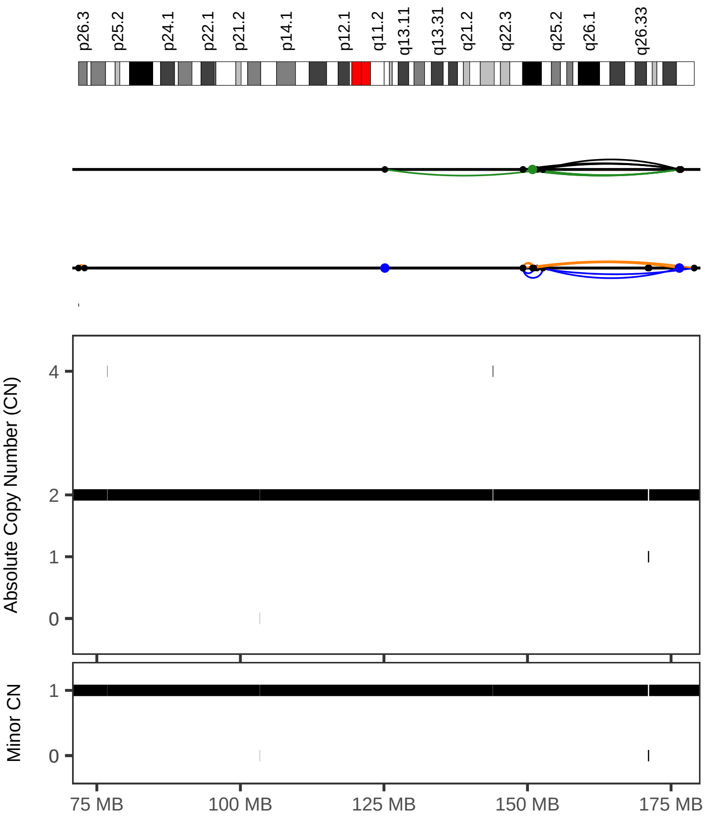

CPCG0057

Cancer type	Prost−AdenoCA
Position	3:125163266−179035673
Interleaved intrachr. SVs	17
Total SVs (intrachr. + transl.)	21
SV types	DEL: 4; DUP: 4; h2hINV: 4; t2tINV: 5; TRA: 4
SVs in sample	58
Oscillating CN (2 and 3 states)	3, 5
CN segments	5
FDR fragment joints	0.9976036
FDR chr. breakp. enrich.	0
Linked to chrs	
Purity, ploidy	0.56, 1.84

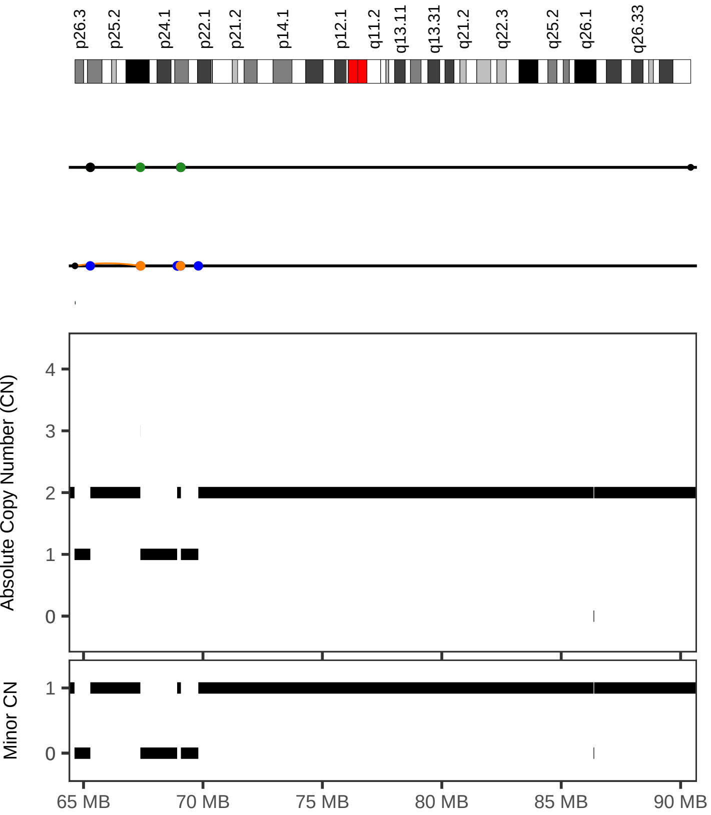

CPCG0124

Cancer type	Prost−AdenoCA
Position	3:64640778−69066915
Interleaved intrachr. SVs	1
Total SVs (intrachr. + transl.)	20
SV types	DEL: 2; DUP: 0; h2hINV: 0; t2tINV: 0; TRA: 18
SVs in sample	121
Oscillating CN (2 and 3 states)	2, 4
CN segments	5
FDR fragment joints	0.615458
FDR chr. breakp. enrich.	0
Linked to chrs	8:5872316−42557665;
Purity, ploidy	0.47, 1.87

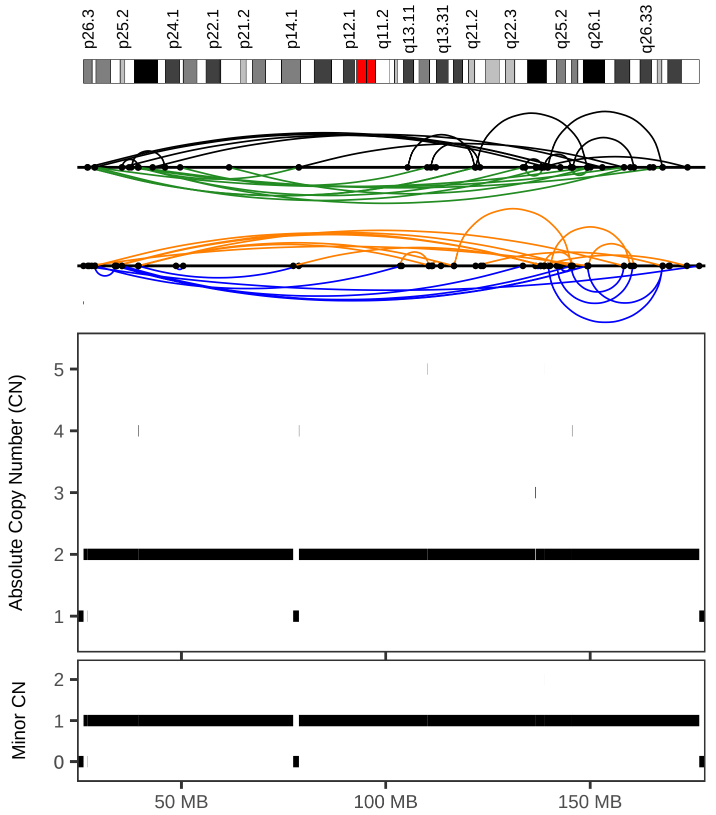

CPCG0196

Cancer type	Prost−AdenoCA
Position	3:26993658−173827504
Interleaved intrachr. SVs	65
Total SVs (intrachr. + transl.)	65
SV types	DEL: 18; DUP: 15; h2hINV: 17; t2tINV: 15; TRA: 0
SVs in sample	116
Oscillating CN (2 and 3 states)	3, 5
CN segments	16
FDR fragment joints	0.9625775
FDR chr. breakp. enrich.	0
Linked to chrs	
Purity, ploidy	0.7, 1.91

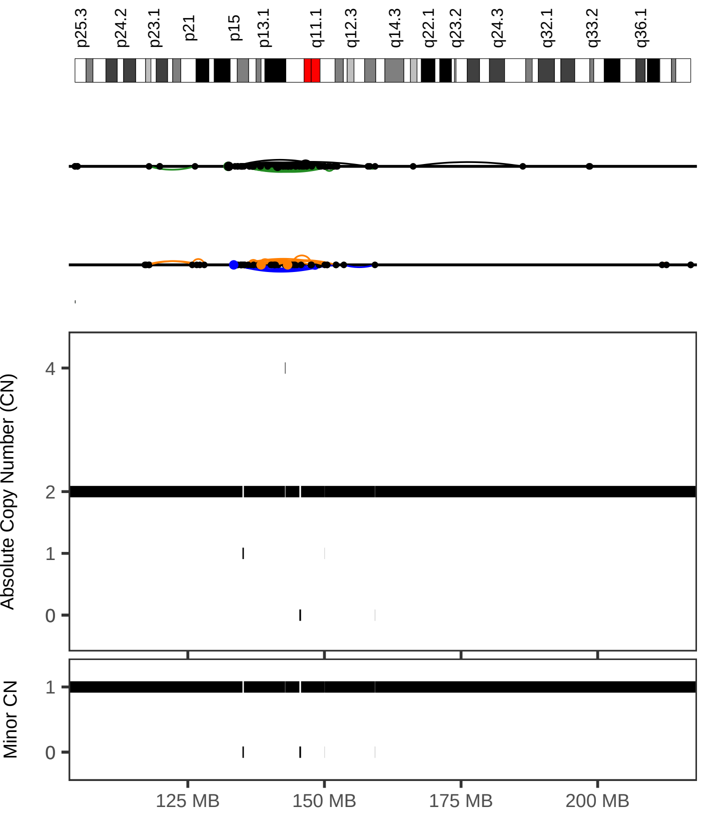

CPCG0344

Cancer type	Prost−AdenoCA
Position	2:133400546−159262875
Interleaved intrachr. SVs	48
Total SVs (intrachr. + transl.)	51
SV types	DEL: 12; DUP: 12; h2hINV: 14; t2tINV: 10; TRA: 3
SVs in sample	171
Oscillating CN (2 and 3 states)	3, 7
CN segments	11
FDR fragment joints	0.9455654
FDR chr. breakp. enrich.	0
Linked to chrs	6:66500721−130649739;
Purity, ploidy	0.75, 1.92

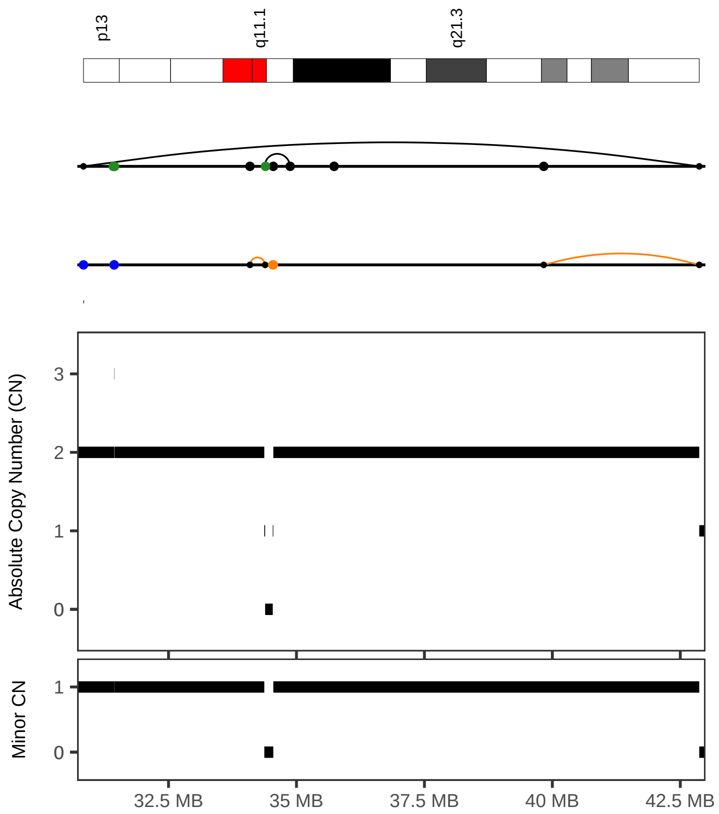

CPCG0353

Cancer type	Prost−AdenoCA
Position	21:30837812−42870443
Interleaved intrachr. SVs	2
Total SVs (intrachr. + transl.)	22
SV types	DEL: 2; DUP: 0; h2hINV: 2; t2tINV: 0; TRA: 18
SVs in sample	134
Oscillating CN (2 and 3 states)	3, 8
CN segments	8
FDR fragment joints	0.6776251
FDR chr. breakp. enrich.	0
Linked to chrs	
Purity, ploidy	0.7, 1.95

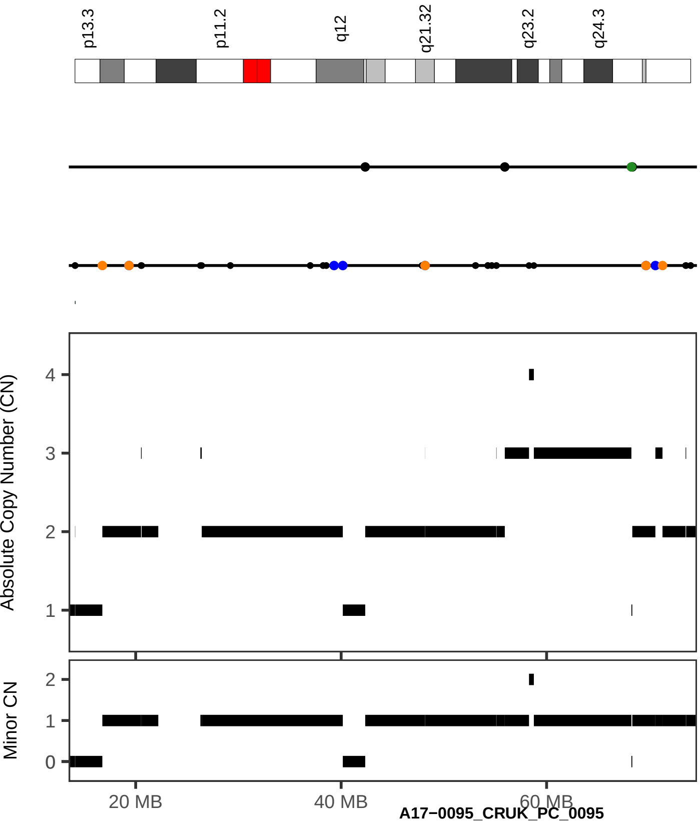

A17−0095_CRUK_PC_0095

Cancer type	Prost−AdenoCA
Position	17:14089050−74031571
Interleaved intrachr. SVs	1
Total SVs (intrachr. + transl.)	30
SV types	DEL: 0; DUP: 15; h2hINV: 0; t2tINV: 0; TRA: 15
SVs in sample	772
Oscillating CN (2 and 3 states)	6, 16
CN segments	22
FDR fragment joints	0.0002315962
FDR chr. breakp. enrich.	0.02
Linked to chrs	2:29177870−206548844;7:740003−154793671
Purity, ploidy	NA, NA

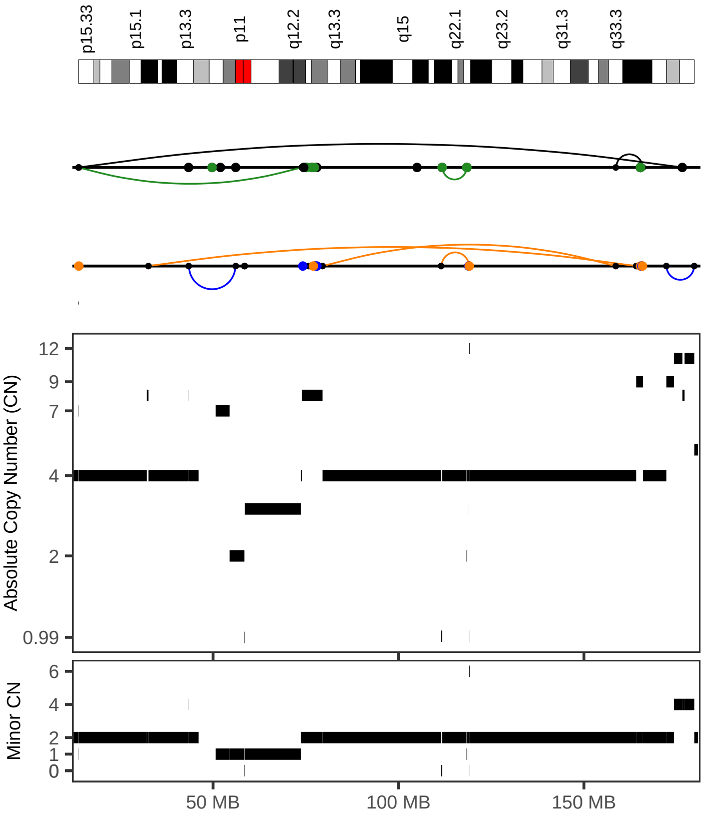

A29−0017_CRUK_PC_0017

Cancer type	Prost−AdenoCA
Position	5:13735145−179718726
Interleaved intrachr. SVs	5
Total SVs (intrachr. + transl.)	44
SV types	DEL: 1; DUP: 1; h2hINV: 2; t2tINV: 1; TRA: 39
SVs in sample	322
Oscillating CN (2 and 3 states)	5, 7
CN segments	30
FDR fragment joints	0.615458
FDR chr. breakp. enrich.	0
Linked to chrs	
Purity, ploidy	NA, NA

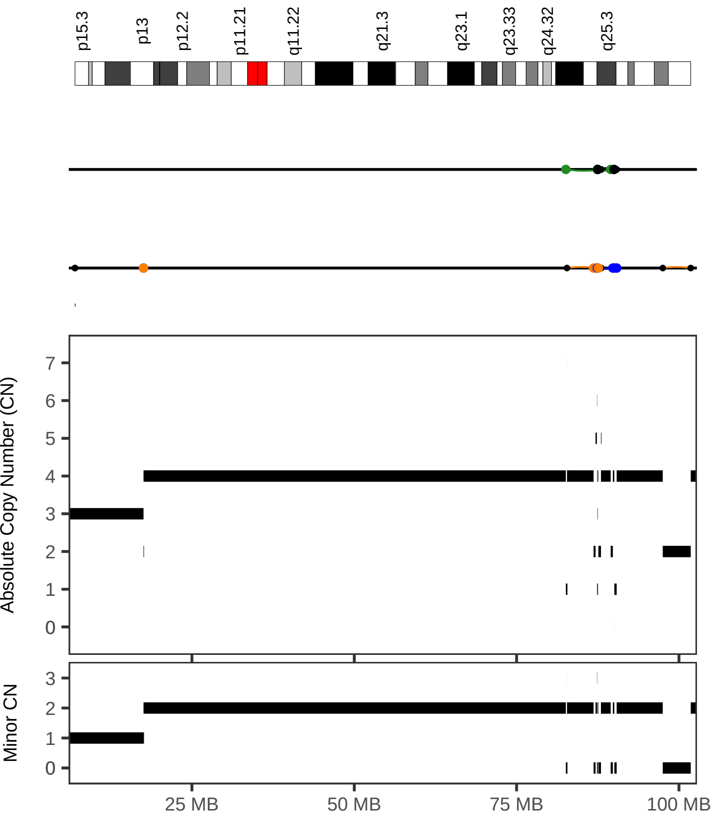

A29−0017_CRUK_PC_0017

Cancer type	Prost−AdenoCA
Position	10:82757959−90416587
Interleaved intrachr. SVs	12
Total SVs (intrachr. + transl.)	24
SV types	DEL: 3; DUP: 2; h2hINV: 3; t2tINV: 4; TRA: 12
SVs in sample	322
Oscillating CN (2 and 3 states)	3, 5
CN segments	21
FDR fragment joints	1
FDR chr. breakp. enrich.	0
Linked to chrs	5:13735145−179718725;
Purity, ploidy	NA, NA

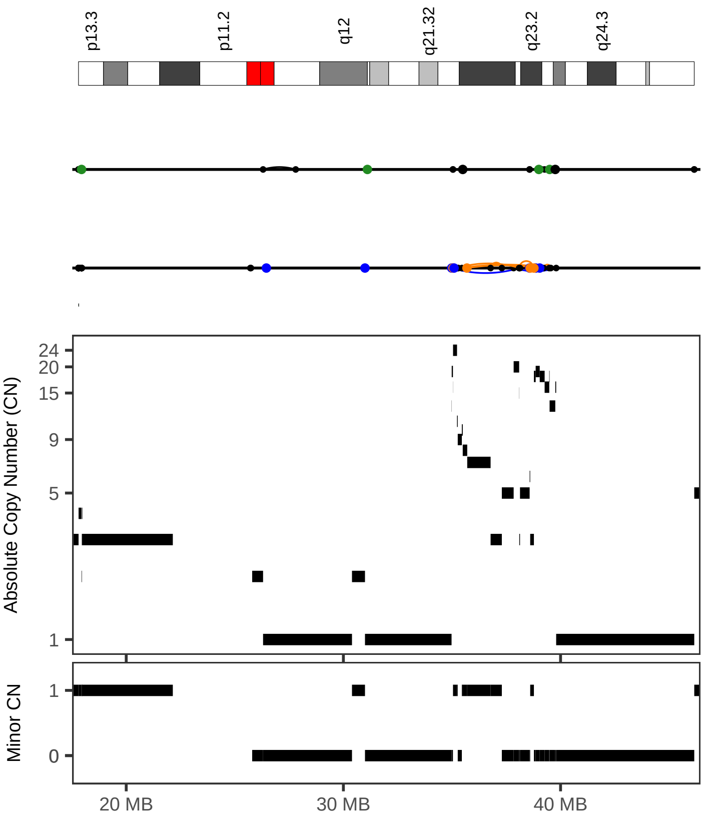

MELA−0001

Cancer type	Skin−Melanoma
Position	17:34979941−39797502
Interleaved intrachr. SVs	8
Total SVs (intrachr. + transl.)	21
SV types	DEL: 5; DUP: 2; h2hINV: 1; t2tINV: 0; TRA: 13
SVs in sample	460
Oscillating CN (2 and 3 states)	3, 6
CN segments	25
FDR fragment joints	0.5435077
FDR chr. breakp. enrich.	0
Linked to chrs	
Purity, ploidy	0.75, 3.01

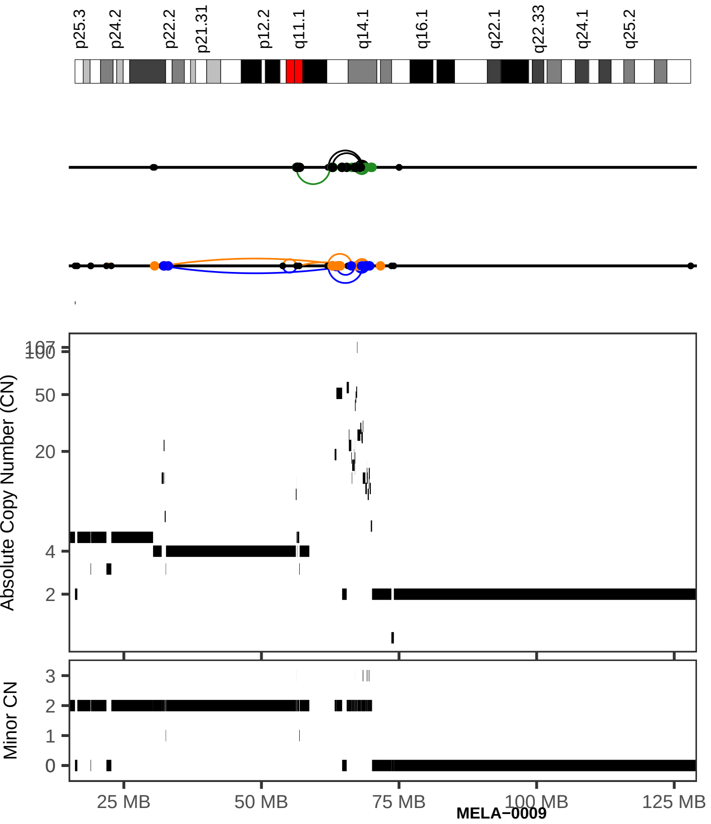

MELA−0009

Cancer type	Skin−Melanoma
Position	6:31870943−69509422
Interleaved intrachr. SVs	28
Total SVs (intrachr. + transl.)	71
SV types	DEL: 8; DUP: 8; h2hINV: 7; t2tINV: 5; TRA: 43
SVs in sample	351
Oscillating CN (2 and 3 states)	3, 5
CN segments	47
FDR fragment joints	0.9811536
FDR chr. breakp. enrich.	0
Linked to chrs	11:41485920−112489308;20:50318897−61655151
Purity, ploidy	0.49, 3.33

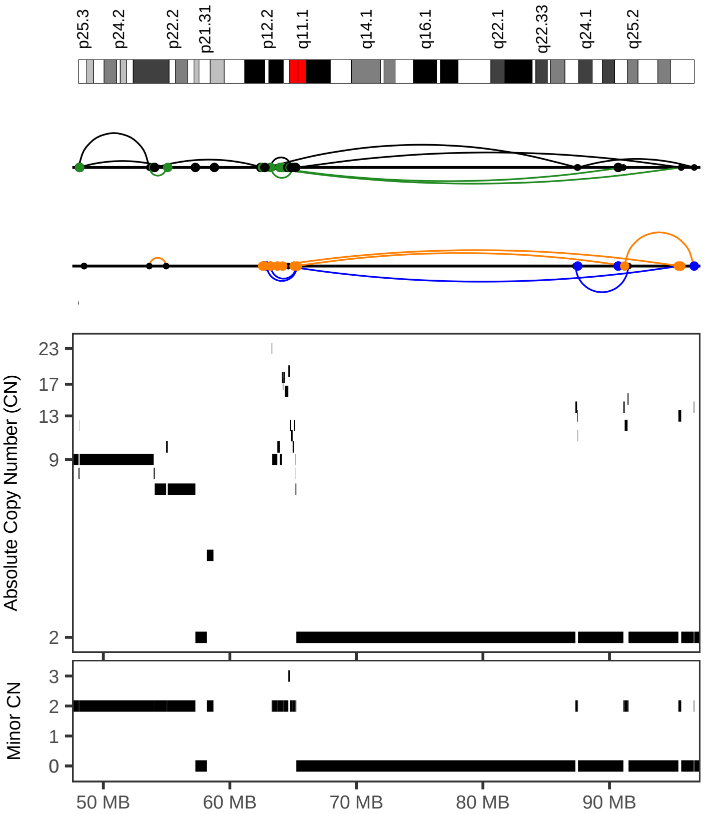

MELA−0064

Cancer type	Skin−Melanoma
Position	6:62737071−96700812
Interleaved intrachr. SVs	16
Total SVs (intrachr. + transl.)	48
SV types	DEL: 4; DUP: 4; h2hINV: 4; t2tINV: 4; TRA: 32
SVs in sample	380
Oscillating CN (2 and 3 states)	3, 5
CN segments	31
FDR fragment joints	0.7568568
FDR chr. breakp. enrich.	0
Linked to chrs	
Purity, ploidy	0.66, 3.77

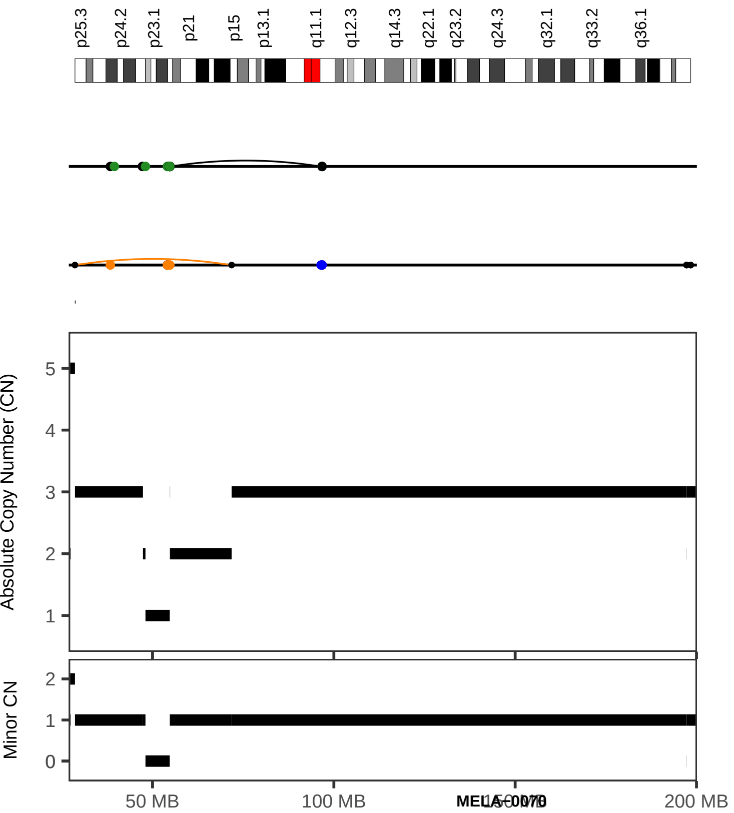

MELA−0070

Cancer type	Skin−Melanoma
Position	2:28606019−96476448
Interleaved intrachr. SVs	2
Total SVs (intrachr. + transl.)	25
SV types	DEL: 1; DUP: 0; h2hINV: 1; t2tINV: 0; TRA: 23
SVs in sample	450
Oscillating CN (2 and 3 states)	4, 7
CN segments	7
FDR fragment joints	0.6776251
FDR chr. breakp. enrich.	1
Linked to chrs	12:55086091−64480689;3:107828103−110133075 4:42650241−45636634;9:447092−9513408

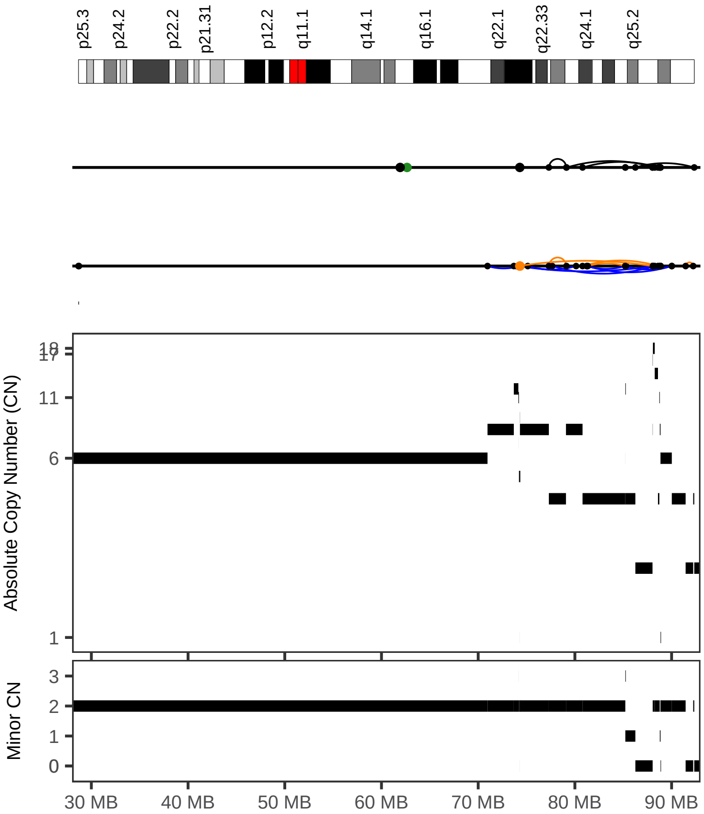

MELA−0170

Cancer type	Skin−Melanoma
Position	6:70967028−92341760
Interleaved intrachr. SVs	18
Total SVs (intrachr. + transl.)	21
SV types	DEL: 4; DUP: 10; h2hINV: 4; t2tINV: 0; TRA: 3
SVs in sample	83
Oscillating CN (2 and 3 states)	4, 5
CN segments	31
FDR fragment joints	0.5151971
FDR chr. breakp. enrich.	0
Linked to chrs	13:49768228−96559916;
Purity, ploidy	0.66, 3.36

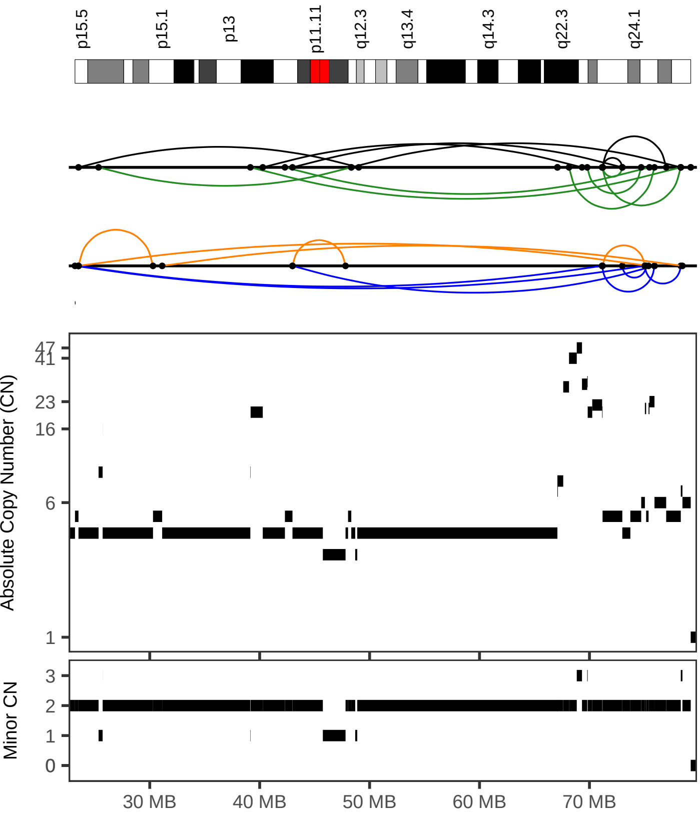

MELA−0183

Cancer type	Skin−Melanoma
Position	11:23197341−78459029
Interleaved intrachr. SVs	24
Total SVs (intrachr. + transl.)	24
SV types	DEL: 5; DUP: 6; h2hINV: 6; t2tINV: 7; TRA: 0
SVs in sample	69
Oscillating CN (2 and 3 states)	3, 9
CN segments	40
FDR fragment joints	0.9723381
FDR chr. breakp. enrich.	0
Linked to chrs	
Purity, ploidy	0.55, 2.96

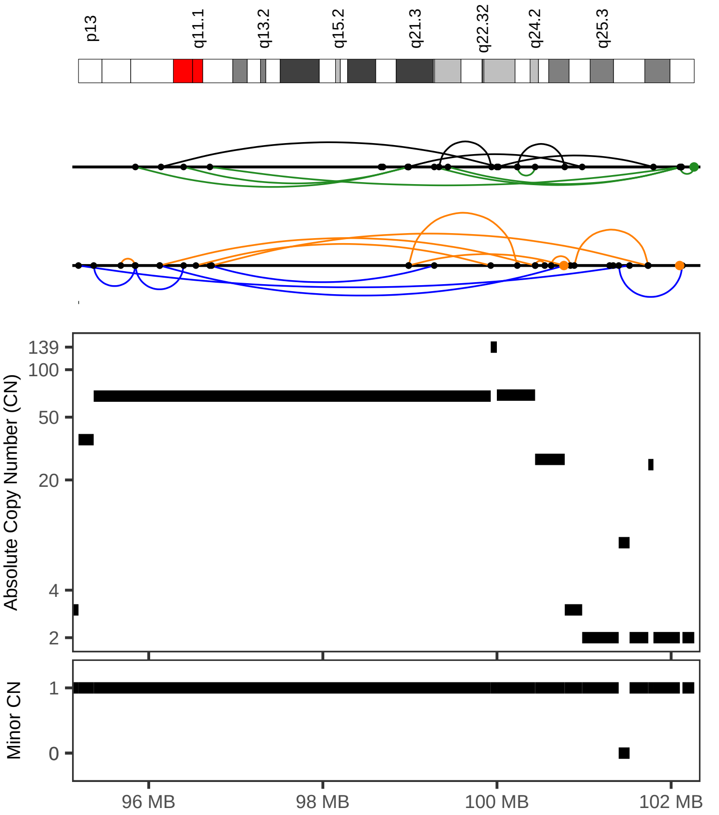

MELA−0202

Cancer type	Skin−Melanoma
Position	15:95193254−102265311
Interleaved intrachr. SVs	25
Total SVs (intrachr. + transl.)	28
SV types	DEL: 7; DUP: 6; h2hINV: 5; t2tINV: 7; TRA: 3
SVs in sample	103
Oscillating CN (2 and 3 states)	3, 3
CN segments	12
FDR fragment joints	0.8988396
FDR chr. breakp. enrich.	0
Linked to chrs	
Purity, ploidy	0.73, 1.84

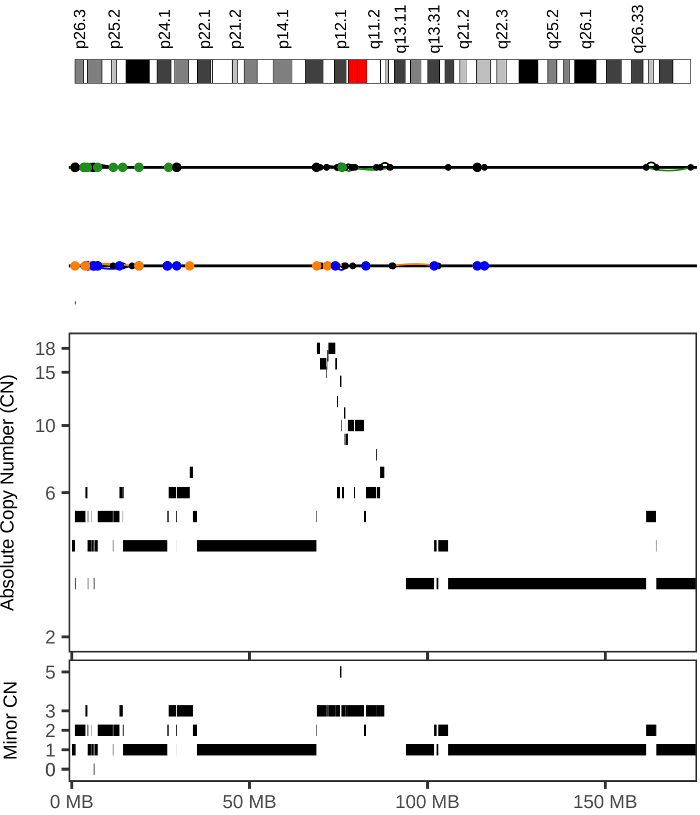

MELA−0231

Cancer type	Skin−Melanoma
Position	3:69825696−89596752
Interleaved intrachr. SVs	18
Total SVs (intrachr. + transl.)	22
SV types	DEL: 2; DUP: 3; h2hINV: 6; t2tINV: 7; TRA: 4
SVs in sample	1234
Oscillating CN (2 and 3 states)	3, 4
CN segments	25
FDR fragment joints	0.6776251
FDR chr. breakp. enrich.	0.42
Linked to chrs	
Purity, ploidy	0.92, 4.03

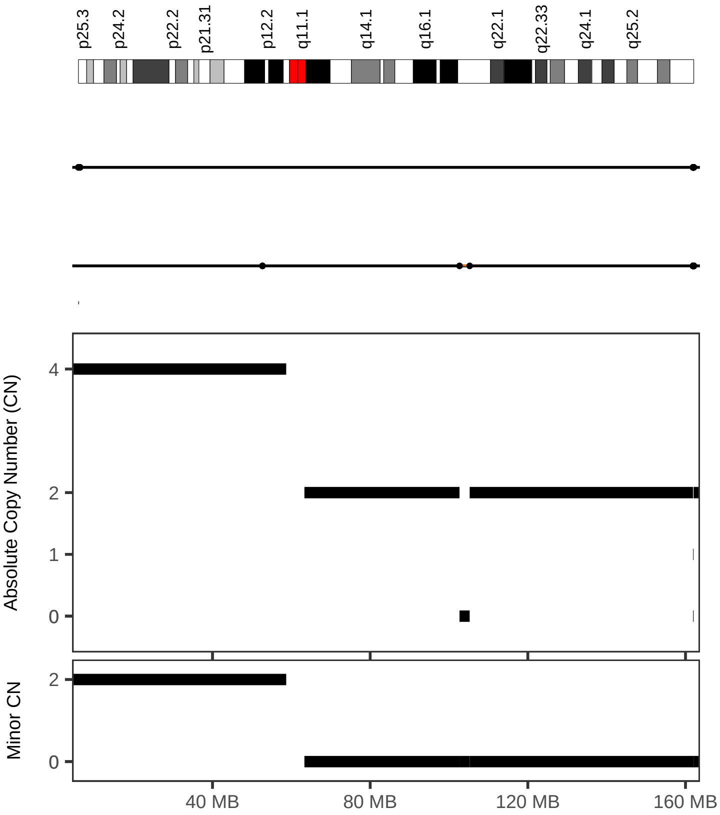

MELA−0256

Cancer type	Skin−Melanoma
Position	6:161907945−162091620
Interleaved intrachr. SVs	22
Total SVs (intrachr. + transl.)	22
SV types	DEL: 8; DUP: 7; h2hINV: 4; t2tINV: 3; TRA: 0
SVs in sample	92
Oscillating CN (2 and 3 states)	3, 4
CN segments	4
FDR fragment joints	0.615458
FDR chr. breakp. enrich.	0
Linked to chrs	
Purity, ploidy	0.7, 3.38

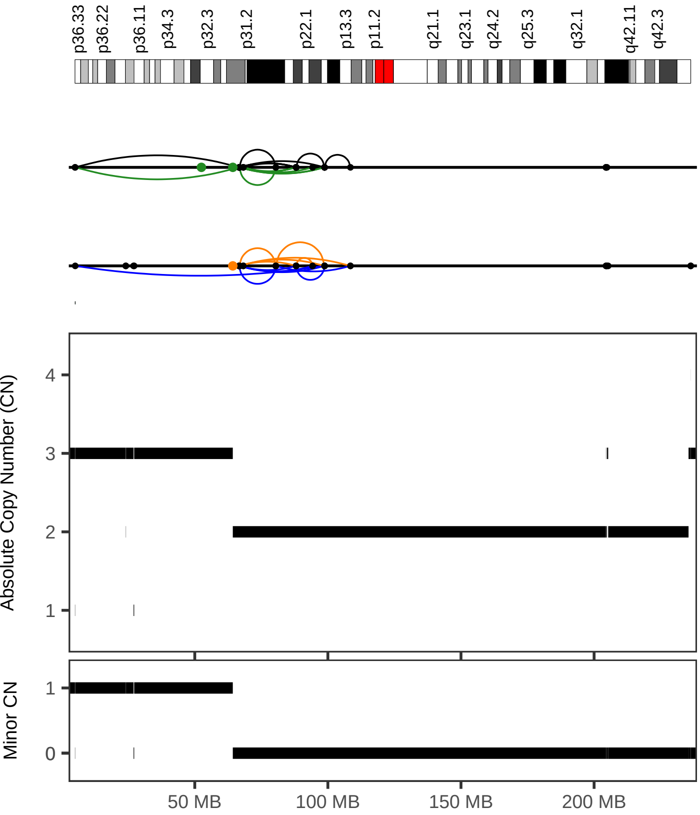

40e17bf8−6c19−4e5e−b60d−ad54f504a970

Cancer type	Skin−Melanoma
Position	1:4999358−108470074
Interleaved intrachr. SVs	33
Total SVs (intrachr. + transl.)	36
SV types	DEL: 7; DUP: 8; h2hINV: 9; t2tINV: 9; TRA: 3
SVs in sample	140
Oscillating CN (2 and 3 states)	3, 7
CN segments	7
FDR fragment joints	0.9284301
FDR chr. breakp. enrich.	0
Linked to chrs	
Purity, ploidy	0.76, 1.94

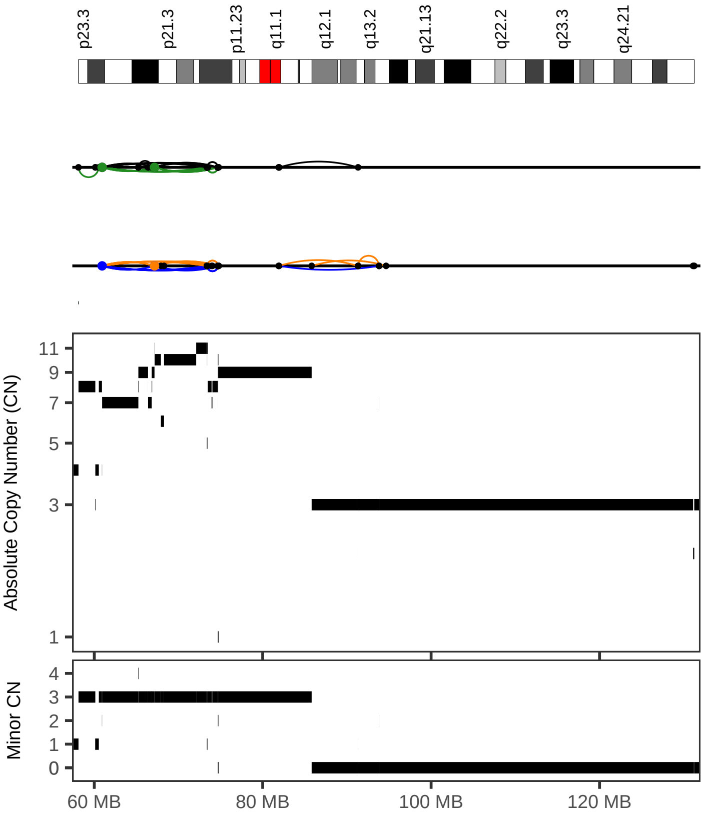

4cdb18f5−47bf−475f−b34c−fa4912515268

Cancer type	Skin−Melanoma
Position	8:60910863−74776226
Interleaved intrachr. SVs	54
Total SVs (intrachr. + transl.)	61
SV types	DEL: 14; DUP: 13; h2hINV: 14; t2tINV: 13; TRA: 7
SVs in sample	100
Oscillating CN (2 and 3 states)	3, 5
CN segments	29
FDR fragment joints	1
FDR chr. breakp. enrich.	0
Linked to chrs	
Purity, ploidy	0.82, 2.17

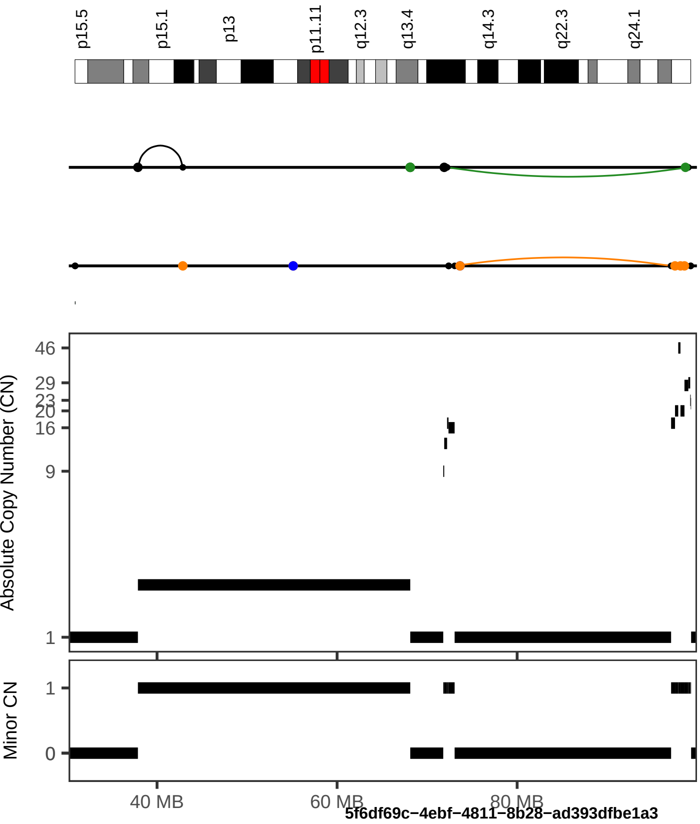

5f6df69c−4ebf−4811−8b28−ad393dfbe1a3

Cancer type	Skin−Melanoma
Position	11:30885500−99014655
Interleaved intrachr. SVs	1
Total SVs (intrachr. + transl.)	20
SV types	DEL: 2; DUP: 1; h2hINV: 1; t2tINV: 1; TRA: 15
SVs in sample	160
Oscillating CN (2 and 3 states)	3, 3
CN segments	14
FDR fragment joints	0.662962
FDR chr. breakp. enrich.	0
Linked to chrs	10:44843350−88556739;20:40210929−55897884
Purity, ploidy	0.86, 1.94

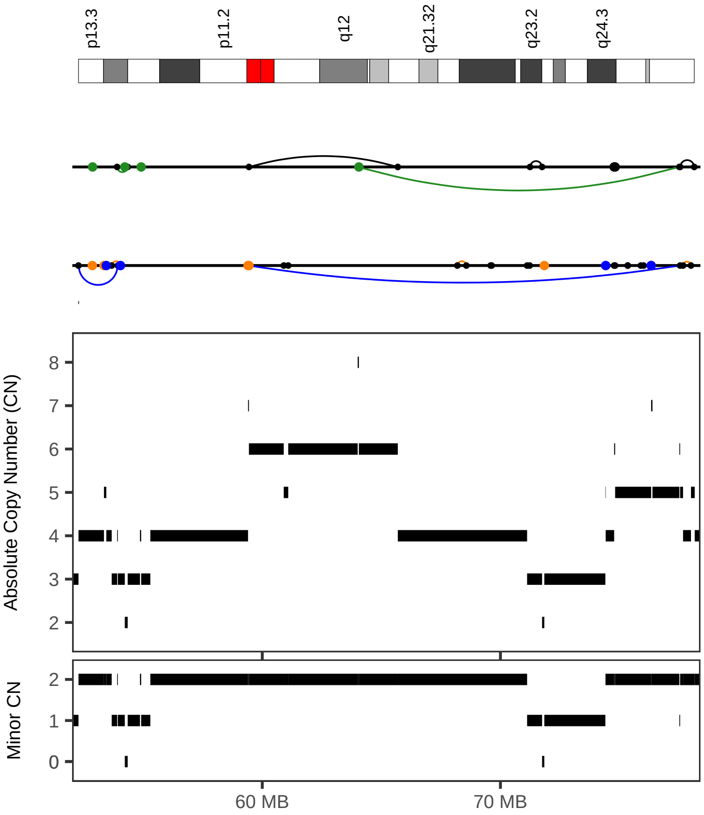

7b92fcd4−f9be−4b97−94a2−b2e8348aeaba

Cancer type	Skin−Melanoma
Position	17:52283568−77511259
Interleaved intrachr. SVs	3
Total SVs (intrachr. + transl.)	22
SV types	DEL: 1; DUP: 2; h2hINV: 1; t2tINV: 2; TRA: 16
SVs in sample	137
Oscillating CN (2 and 3 states)	4, 11
CN segments	28
FDR fragment joints	0.8874881
FDR chr. breakp. enrich.	0
Linked to chrs	1:57268038−116153670;
Purity, ploidy	0.67, 4.4

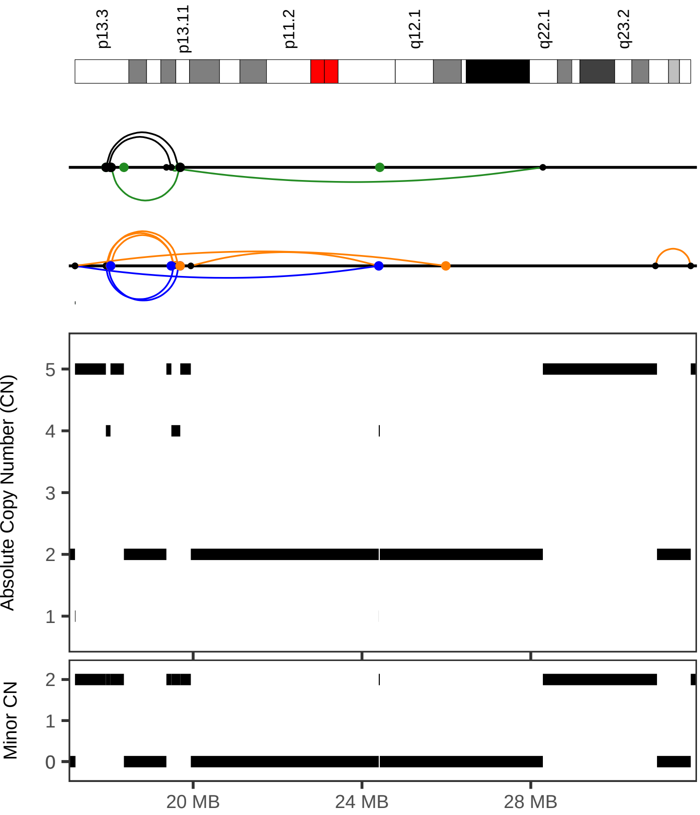

800d3a17−269f−4d97−94ff−8585f20038d8

Cancer type	Skin−Melanoma
Position	16:17199755−28285873
Interleaved intrachr. SVs	18
Total SVs (intrachr. + transl.)	34
SV types	DEL: 6; DUP: 5; h2hINV: 3; t2tINV: 4; TRA: 16
SVs in sample	337
Oscillating CN (2 and 3 states)	3, 8
CN segments	13
FDR fragment joints	0.9332614
FDR chr. breakp. enrich.	0
Linked to chrs	2:32129440−88367599;
Purity, ploidy	0.51, 3.31

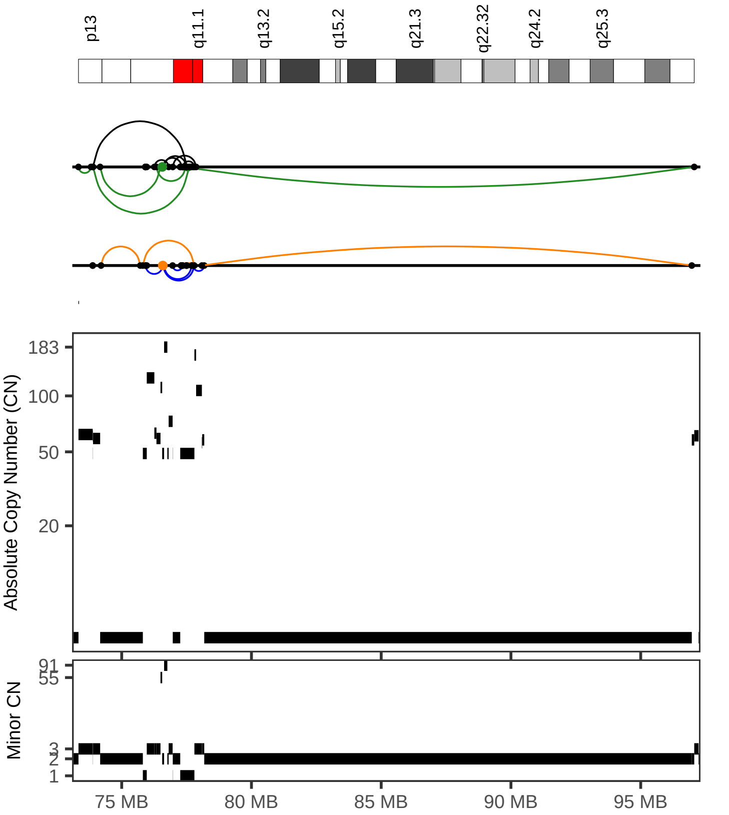

d8eac750−9daf−4465−b672−9e31fd369057

Cancer type	Skin−Melanoma
Position	15:73891842−97062697
Interleaved intrachr. SVs	23
Total SVs (intrachr. + transl.)	27
SV types	DEL: 4; DUP: 6; h2hINV: 7; t2tINV: 6; TRA: 4
SVs in sample	266
Oscillating CN (2 and 3 states)	3, 3
CN segments	22
FDR fragment joints	0.8653243
FDR chr. breakp. enrich.	0
Linked to chrs	
Purity, ploidy	0.47, 3.89

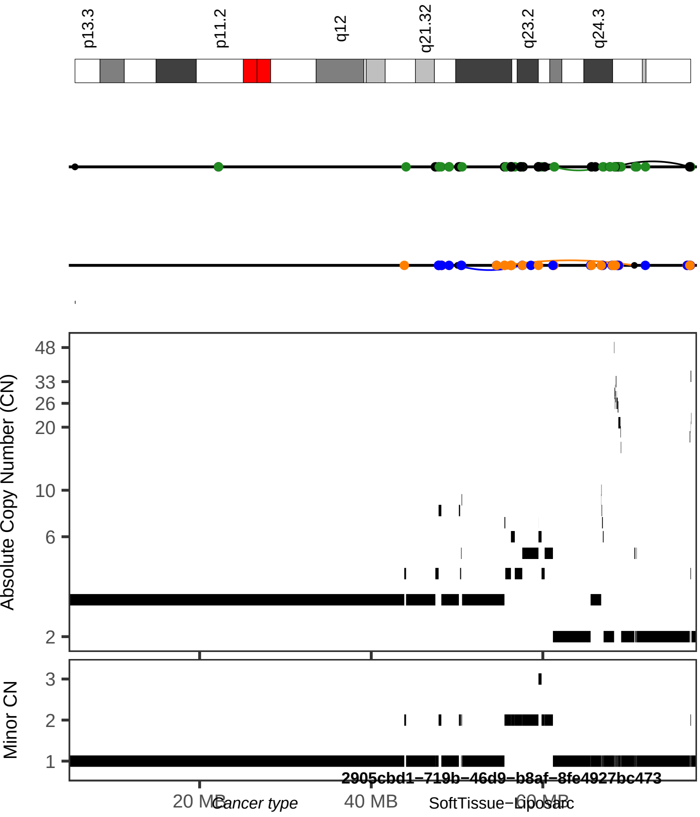

2905cbd1−719b−46d9−b8af−8fe4927bc473

Cancer type	SoftTissue−Liposarc
Position	17:50057932−77131857
Interleaved intrachr. SVs	3
Total SVs (intrachr. + transl.)	84
SV types	DEL: 1; DUP: 1; h2hINV: 1; t2tINV: 0; TRA: 81
SVs in sample	1958
Oscillating CN (2 and 3 states)	5, 6
CN segments	43
FDR fragment joints	0.7687447
FDR chr. breakp. enrich.	0
Linked to chrs	1:145389851−238867162;11:12674170−130091311 12:8404400−101121300;19:10607945−56113175 2:103504725−236615449;5:893523−179304806 7:159034−86160840;9:7234461−14473861 [illegible]

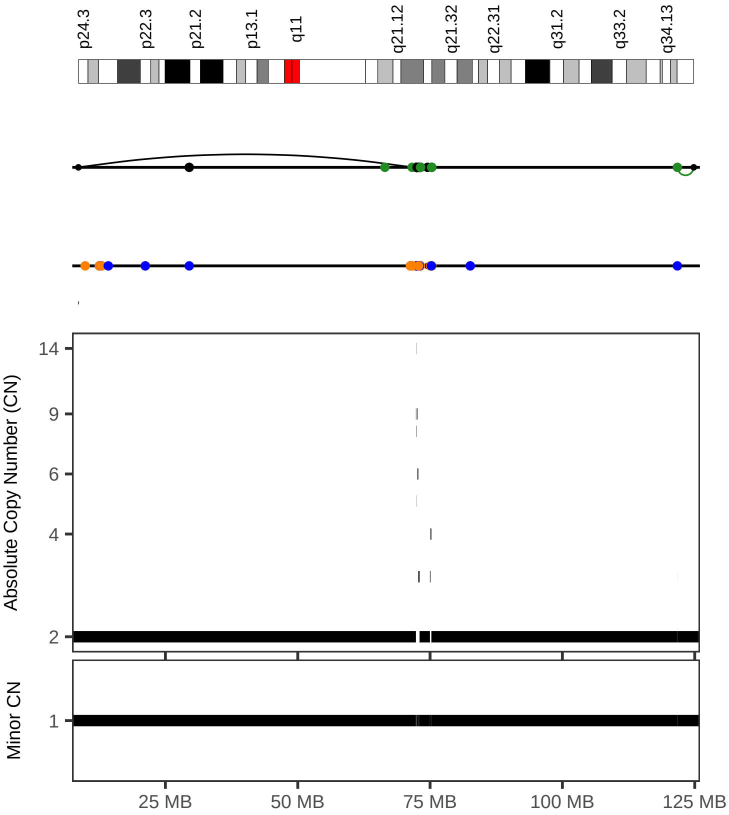

2ff6aede−7bcd−440b−99ab−7a023faf658e

Cancer type	SoftTissue−Liposarc
Position	9:8561618−124815411
Interleaved intrachr. SVs	1
Total SVs (intrachr. + transl.)	71
SV types	DEL: 2; DUP: 1; h2hINV: 1; t2tINV: 3; TRA: 64
SVs in sample	1218
Oscillating CN (2 and 3 states)	3, 5
CN segments	16
FDR fragment joints	0.9666026
FDR chr. breakp. enrich.	0
Linked to chrs	5:86635−168032759;
Purity, ploidy	0.89, 1.88

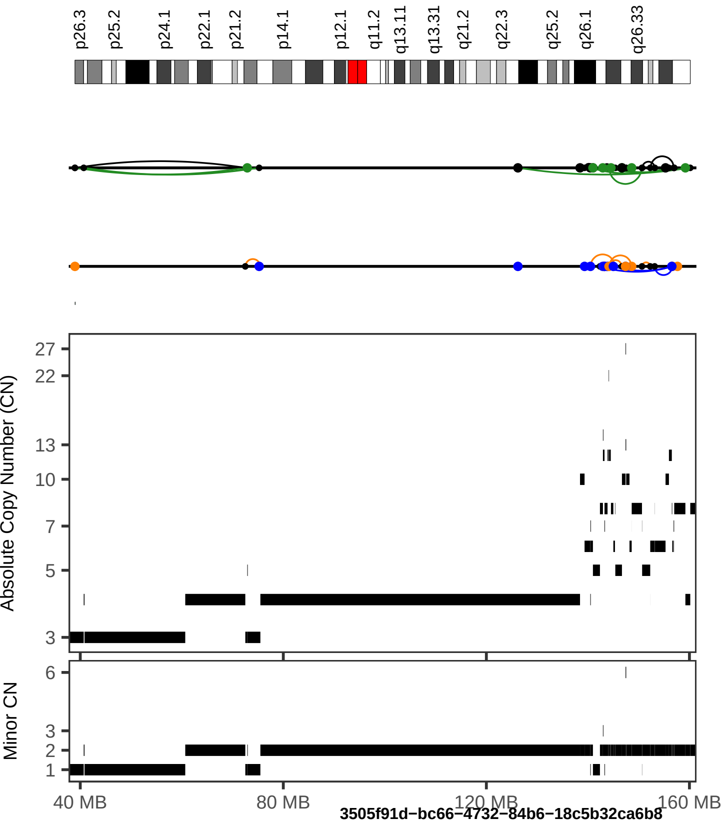

3505f91d−bc66−4732−84b6−18c5b32ca6b8

Cancer type	SoftTissue−Liposarc
Position	3:139339810−157007096
Interleaved intrachr. SVs	17
Total SVs (intrachr. + transl.)	39
SV types	DEL: 4; DUP: 4; h2hINV: 5; t2tINV: 4; TRA: 22
SVs in sample	1246
Oscillating CN (2 and 3 states)	3, 5
CN segments	38
FDR fragment joints	0.8900806
FDR chr. breakp. enrich.	0.51
Linked to chrs	1:147269177−248454296;X:77058127−154924952
Purity, ploidy	0.61, 3.84

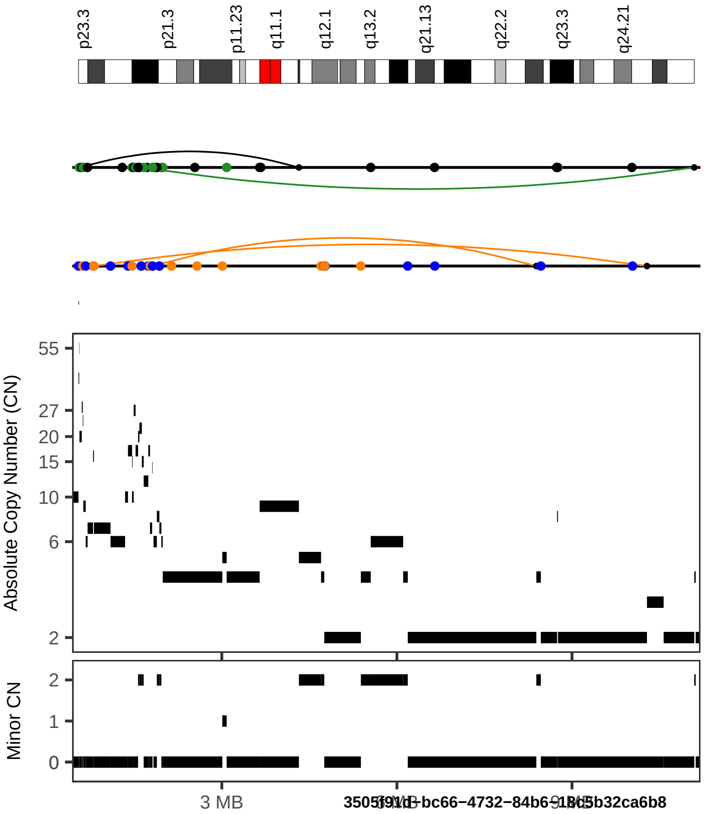

3505f91d−bc66−4732−84b6−18c5b32ca6b8

Cancer type	SoftTissue−Liposarc
Position	8:560734−11094179
Interleaved intrachr. SVs	4
Total SVs (intrachr. + transl.)	57
SV types	DEL: 2; DUP: 0; h2hINV: 1; t2tINV: 1; TRA: 53
SVs in sample	1246
Oscillating CN (2 and 3 states)	4, 5
CN segments	44
FDR fragment joints	0.707617
FDR chr. breakp. enrich.	0.39
Linked to chrs	12:4488859−131646236;13:20111633−31209009 4:64107549−135664103;5:111534825−180595775 X:77058127−154924952;
Purity, ploidy	0.61, 3.84

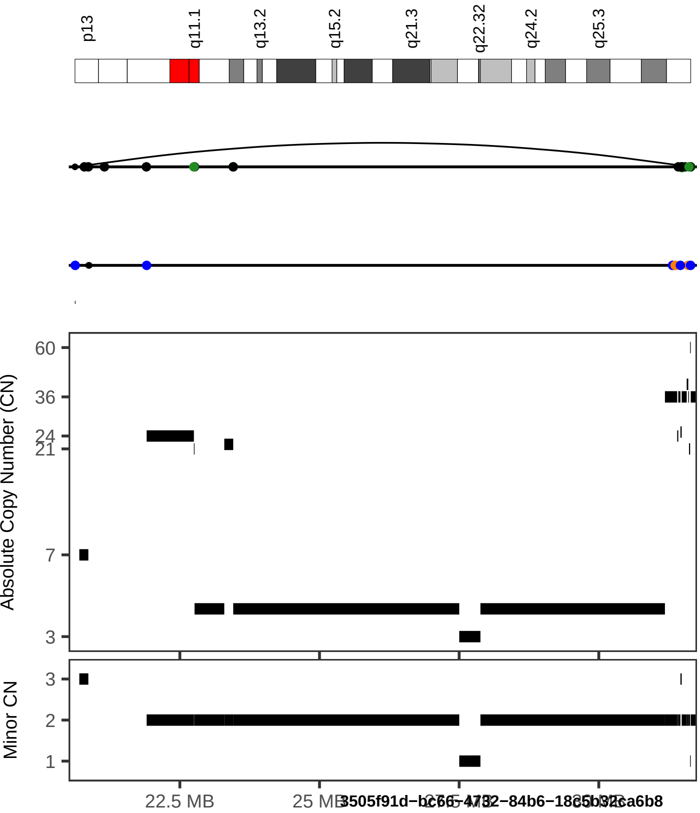

3505f91d−bc66−4732−84b6−18c5b32ca6b8

Cancer type	SoftTissue−Liposarc
Position	15:20622452−31646006
Interleaved intrachr. SVs	1
Total SVs (intrachr. + transl.)	28
SV types	DEL: 1; DUP: 0; h2hINV: 1; t2tINV: 1; TRA: 25
SVs in sample	1246
Oscillating CN (2 and 3 states)	3, 4
CN segments	19
FDR fragment joints	0.5435077
FDR chr. breakp. enrich.	1
Linked to chrs	12:4488859−131646236;13:20111633−31209009 5:111534825−180595775;X:77058127−154924952

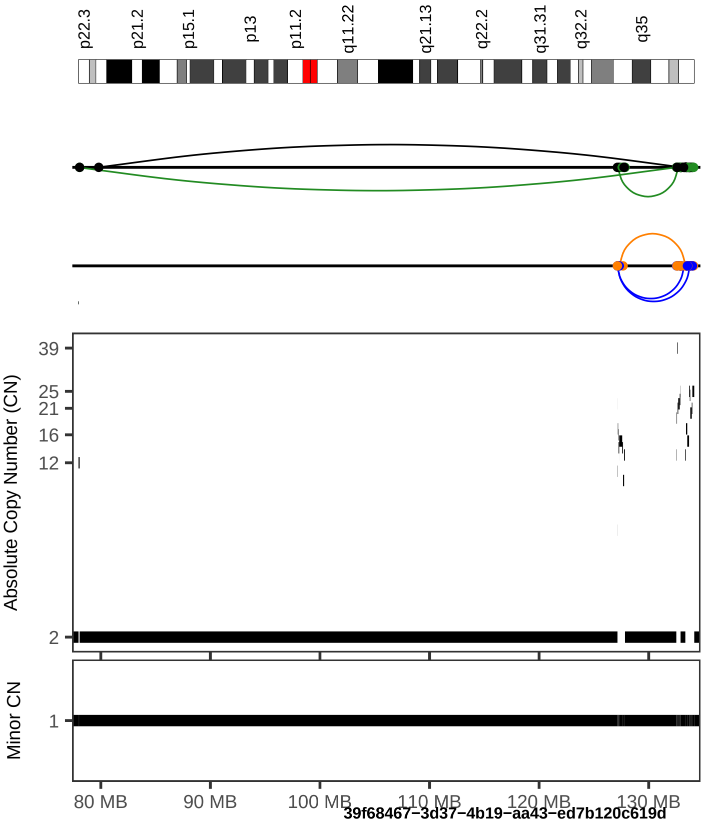

39f68467−3d37−4b19−aa43−ed7b120c619d

Cancer type	SoftTissue−Liposarc
Position	7:77961769−133784786
Interleaved intrachr. SVs	8
Total SVs (intrachr. + transl.)	121
SV types	DEL: 1; DUP: 3; h2hINV: 2; t2tINV: 2; TRA: 113
SVs in sample	1635
Oscillating CN (2 and 3 states)	3, 4
CN segments	28
FDR fragment joints	0.8653243
FDR chr. breakp. enrich.	0
Linked to chrs	20:33082736−57423557;3:4159559−20777596 9:103346393−140789887;
Purity, ploidy	0.4, 2.04

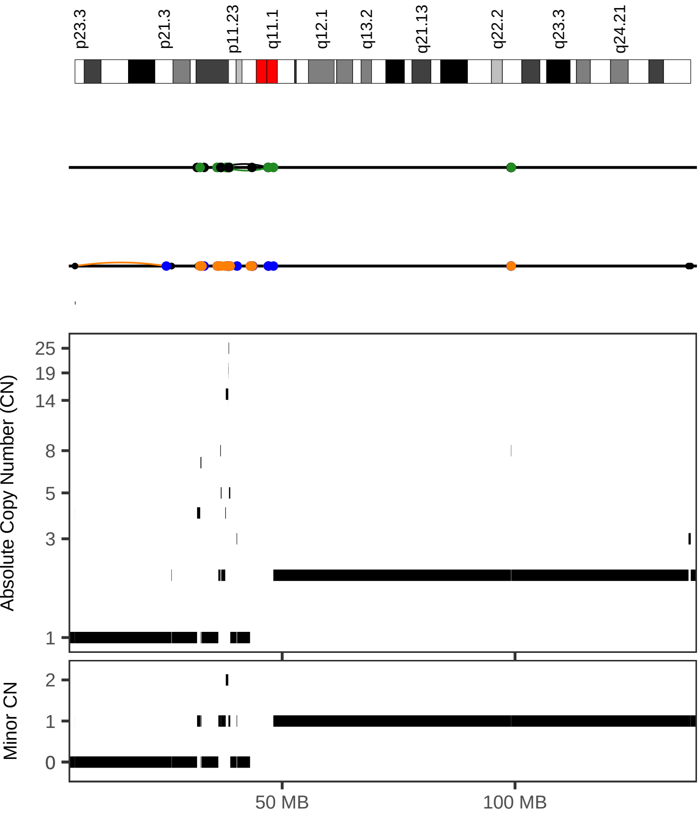

39f68467−3d37−4b19−aa43−ed7b120c619d	
Cancer type	SoftTissue−Liposarc
Position	8:36818890−48127350
Interleaved intrachr. SVs	3
Total SVs (intrachr. + transl.)	74
SV types	DEL: 1; DUP: 0; h2hINV: 1; t2tINV: 1; TRA: 71
SVs in sample	1635
Oscillating CN (2 and 3 states)	3, 4
CN segments	14
FDR fragment joints	0.6776251
FDR chr. breakp. enrich.	0
Linked to chrs	3:4159559−20777596;
Purity, ploidy	0.4, 2.04

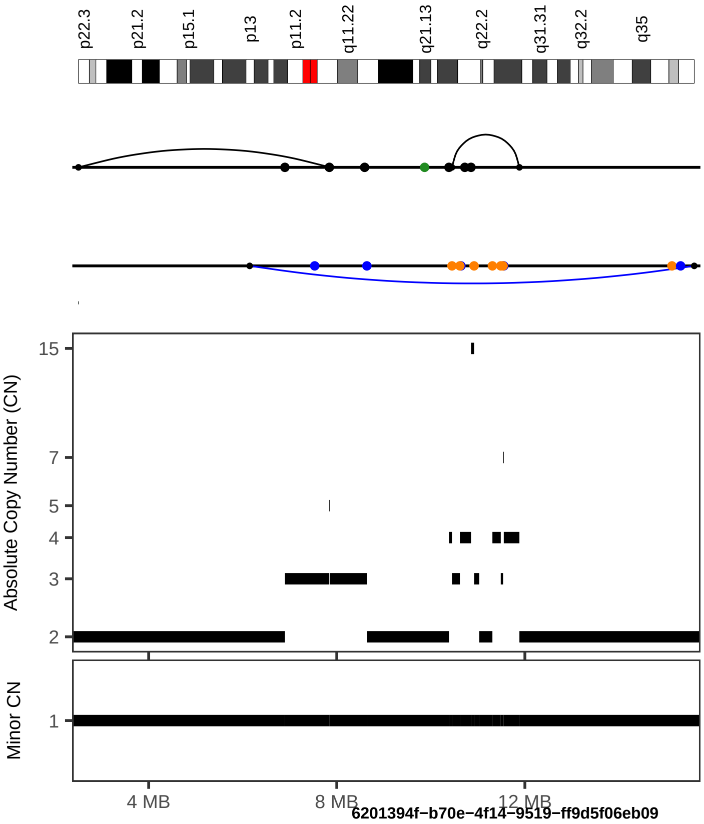

6201394f−b70e−4f14−9519−ff9d5f06eb09	
Cancer type	SoftTissue−Liposarc
Position	7:2506893−15600830
Interleaved intrachr. SVs	2
Total SVs (intrachr. + transl.)	21
SV types	DEL: 0; DUP: 1; h2hINV: 1; t2tINV: 0; TRA: 19
SVs in sample	318
Oscillating CN (2 and 3 states)	3, 5
CN segments	16
FDR fragment joints	0.6610257
FDR chr. breakp. enrich.	0.44
Linked to chrs	1:152775873−205026033;6:111055845−148640743
Purity, ploidy	0.3, 2.03

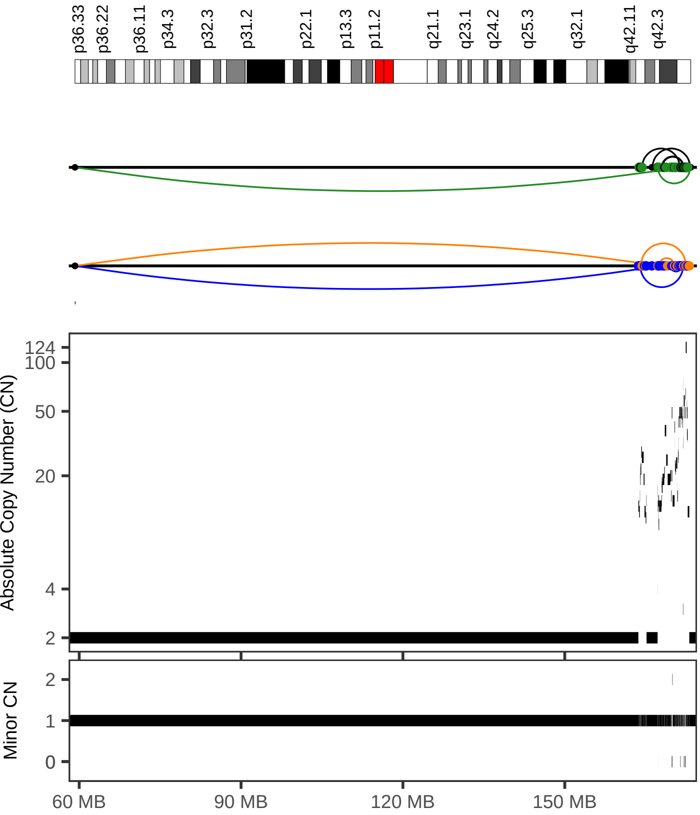

9517413b−b5b8−4130−8ba1−d86d44abe2ec	
Cancer type	SoftTissue−Liposarc
Position	1:59218908−173348092
Interleaved intrachr. SVs	18
Total SVs (intrachr. + transl.)	121
SV types	DEL: 4; DUP: 4; h2hINV: 6; t2tINV: 4; TRA: 103
SVs in sample	498
Oscillating CN (2 and 3 states)	3, 4
CN segments	75
FDR fragment joints	0.7961881
FDR chr. breakp. enrich.	0
Linked to chrs	5:274679−32089187;
Purity, ploidy	0.6, 2.24

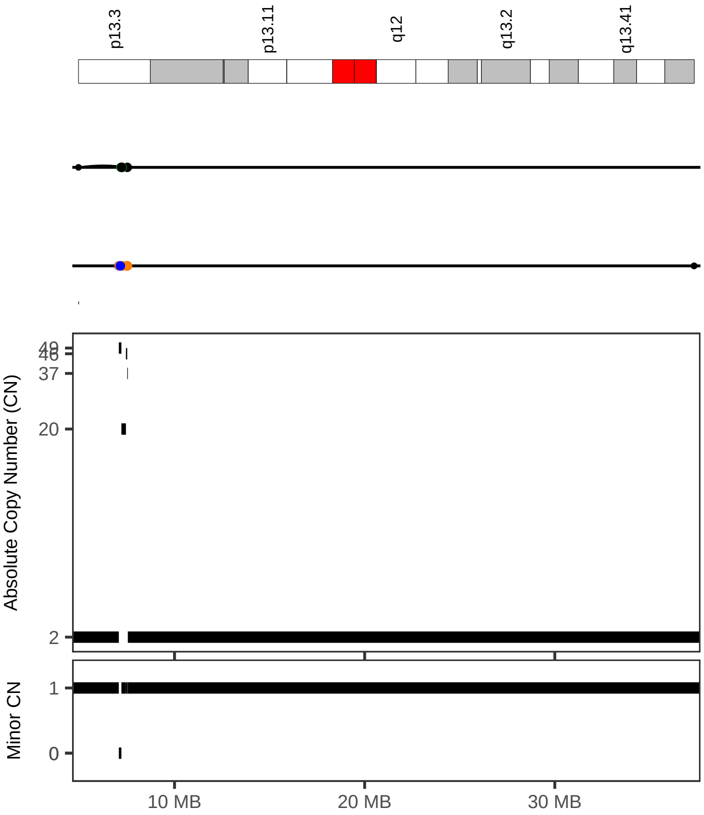

9517413b−b5b8−4130−8ba1−d86d44abe2ec	
Cancer type	SoftTissue−Liposarc
Position	19:4947957−37334626
Interleaved intrachr. SVs	1
Total SVs (intrachr. + transl.)	20
SV types	DEL: 1; DUP: 0; h2hINV: 1; t2tINV: 0; TRA: 18
SVs in sample	498
Oscillating CN (2 and 3 states)	2, 3
CN segments	6
FDR fragment joints	0.6776251
FDR chr. breakp. enrich.	0
Linked to chrs	5:274679−32089187;
Purity, ploidy	0.6, 2.24

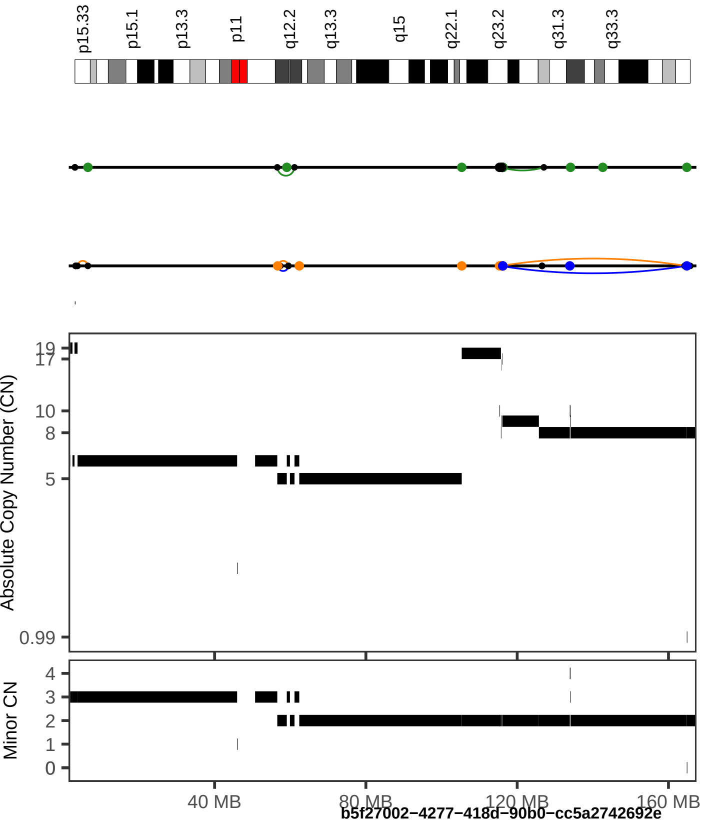

b5f27002−4277−418d−90b0−cc5a2742692e

Cancer type	SoftTissue−Liposarc
Position	5:115325452−165756524
Interleaved intrachr. SVs	3
Total SVs (intrachr. + transl.)	20
SV types	DEL: 2; DUP: 1; h2hINV: 0; t2tINV: 0; TRA: 17
SVs in sample	895
Oscillating CN (2 and 3 states)	3, 5
CN segments	13
FDR fragment joints	0.9625775
FDR chr. breakp. enrich.	0.13
Linked to chrs	15:77063545−87618665;7:2825814−148640815 X:115283407−149711206;
Purity, ploidy	0.87, 6.21

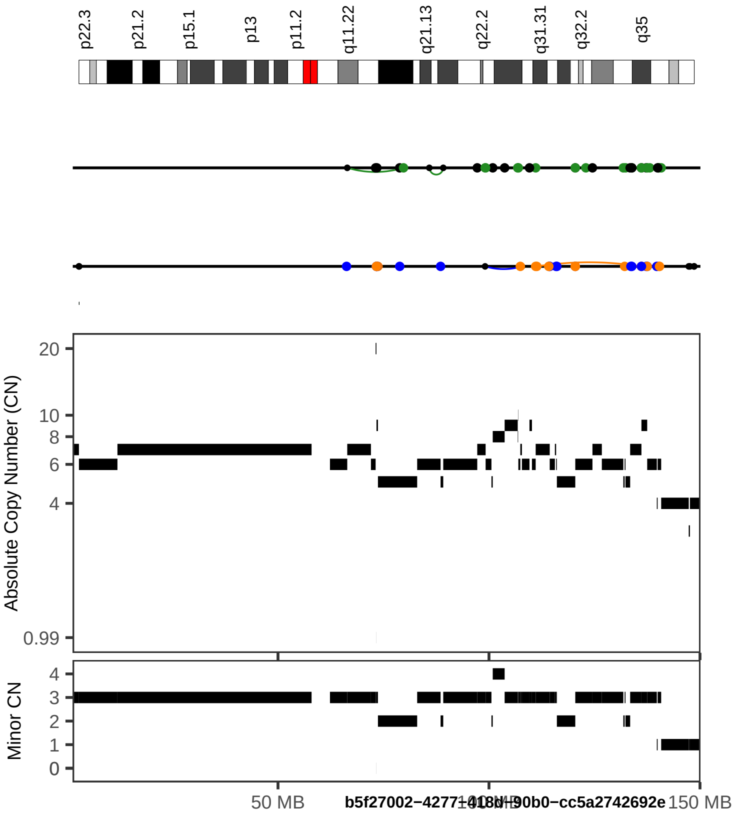

b5f27002−4277−418d−90b0−cc5a2742692e

Cancer type	SoftTissue−Liposarc
Position	7:2825814−148640816
Interleaved intrachr. SVs	1
Total SVs (intrachr. + transl.)	72
SV types	DEL: 3; DUP: 2; h2hINV: 0; t2tINV: 2; TRA: 65
SVs in sample	895
Oscillating CN (2 and 3 states)	5, 16
CN segments	43
FDR fragment joints	1
FDR chr. breakp. enrich.	0.01
Linked to chrs	13:65093605−113188517;15:77063545−87618665 3:16208743−73683672;6:121478880−150364307
Purity, ploidy	0.87, 6.21

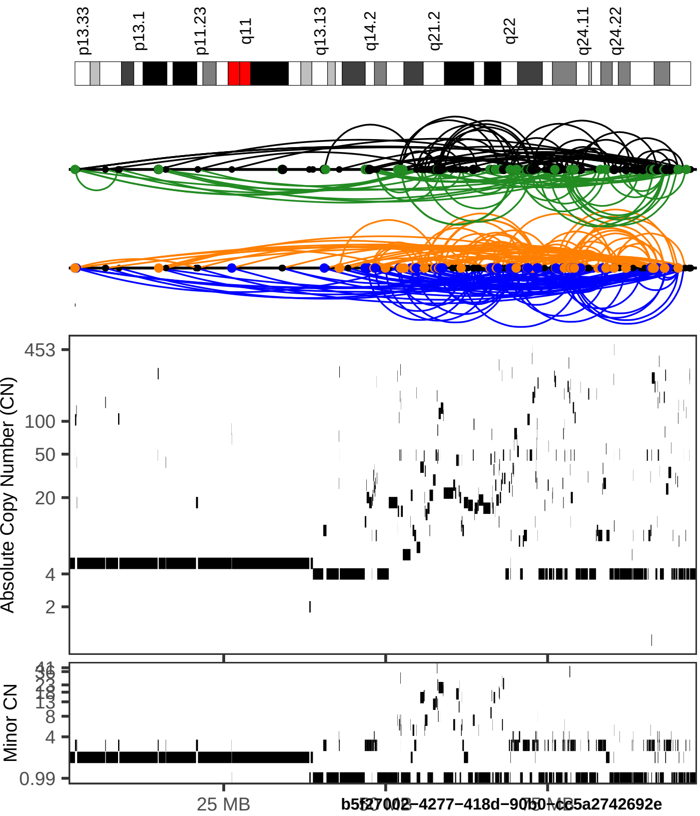

b5f27002−4277−418d−90b0−cc5a2742692e

Cancer type	SoftTissue−Liposarc
Position	12:2032836−97098237
Interleaved intrachr. SVs	324
Total SVs (intrachr. + transl.)	537
SV types	DEL: 98; DUP: 102; h2hINV: 65; t2tINV: 59; TRA: 213
SVs in sample	895
Oscillating CN (2 and 3 states)	3, 4
CN segments	284
FDR fragment joints	0.1796209
FDR chr. breakp. enrich.	0
Linked to chrs	11:577623−86983175;13:65093605−113188517 15:77063545−87618665;3:16208743−73683672 7:2825814−148640815;

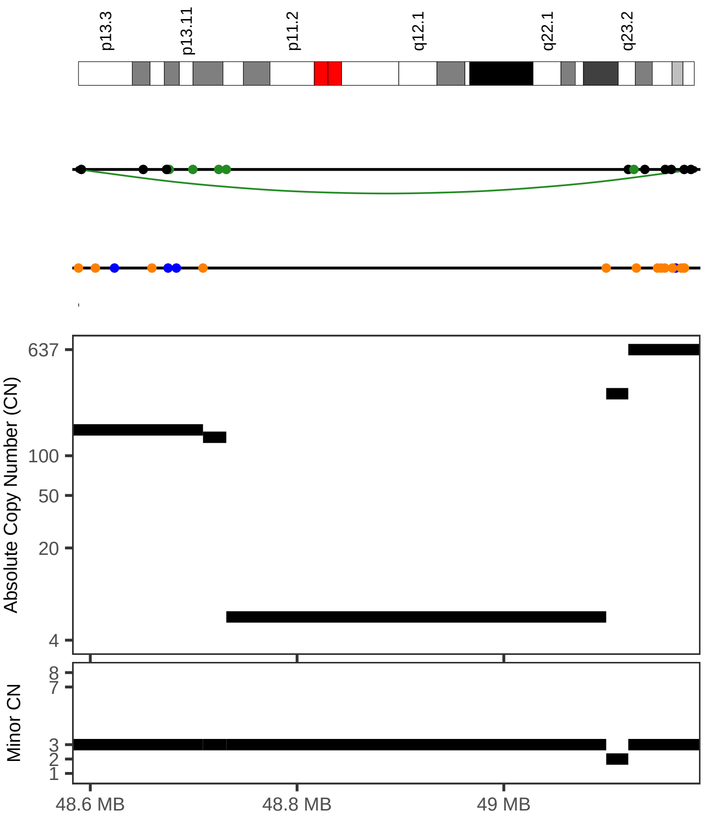

b5f27002−4277−418d−90b0−cc5a2742692e

Cancer type	SoftTissue−Liposarc
Position	16:48588543−49184184
Interleaved intrachr. SVs	1
Total SVs (intrachr. + transl.)	33
SV types	DEL: 0; DUP: 0; h2hINV: 0; t2tINV: 1; TRA: 32
SVs in sample	895
Oscillating CN (2 and 3 states)	2, 3
CN segments	5
FDR fragment joints	0.565038
FDR chr. breakp. enrich.	0
Linked to chrs	7:2825814−148640815;
Purity, ploidy	0.87, 6.21

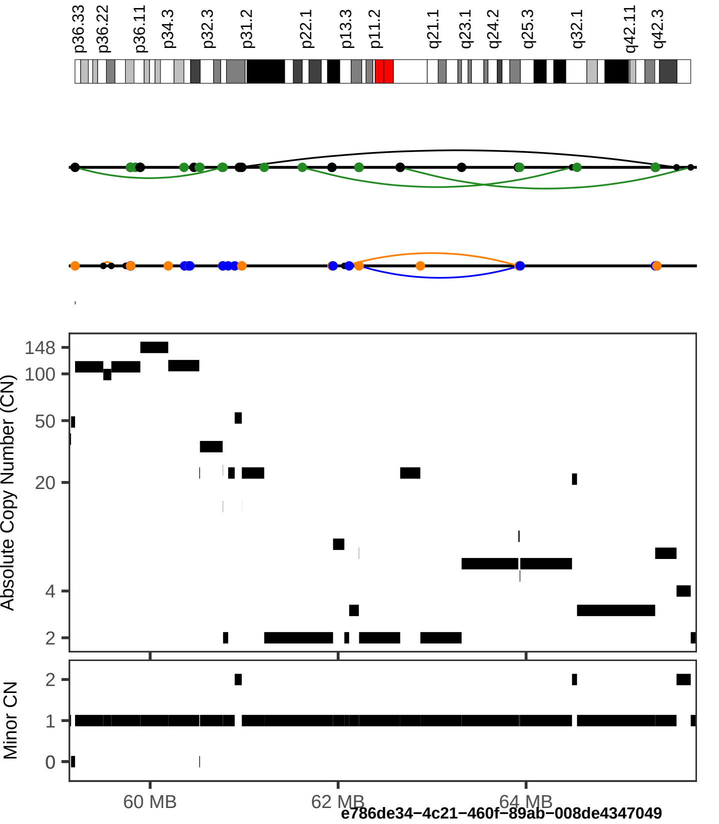

e786de34−4c21−460f−89ab−008de4347049

Cancer type	SoftTissue−Liposarc
Position	1:60949973−65751755
Interleaved intrachr. SVs	5
Total SVs (intrachr. + transl.)	35
SV types	DEL: 1; DUP: 1; h2hINV: 1; t2tINV: 2; TRA: 30
SVs in sample	872
Oscillating CN (2 and 3 states)	4, 5
CN segments	22
FDR fragment joints	0.8007013
FDR chr. breakp. enrich.	0.3
Linked to chrs	4:25848307−38595194;6:39847295−158859759
Purity, ploidy	0.38, 2.37

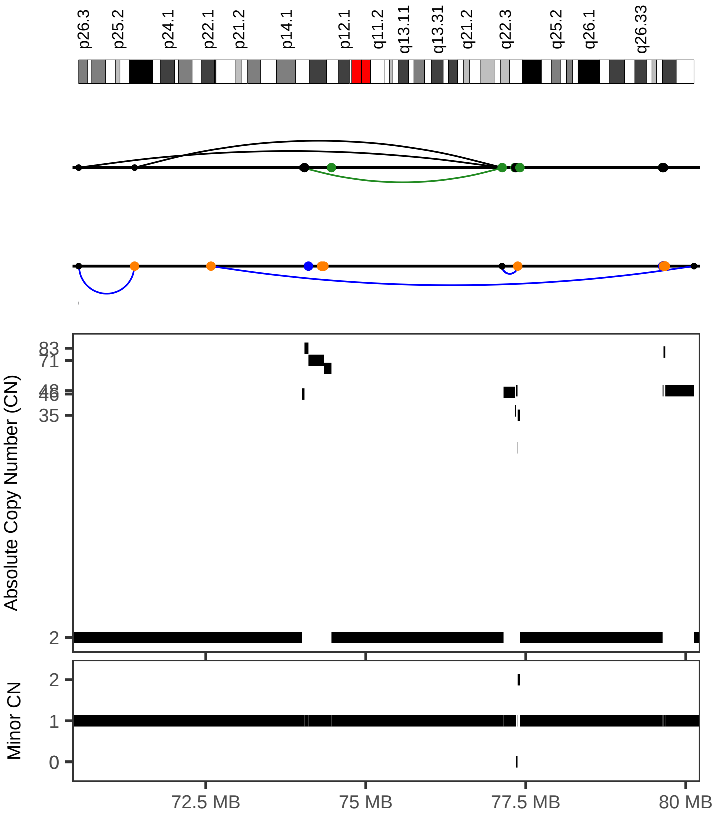

e786de34−4c21−460f−89ab−008de4347049

Cancer type	SoftTissue−Liposarc
Position	3:70513200−80127855
Interleaved intrachr. SVs	7
Total SVs (intrachr. + transl.)	28
SV types	DEL: 0; DUP: 4; h2hINV: 2; t2tINV: 1; TRA: 21
SVs in sample	872
Oscillating CN (2 and 3 states)	3, 3
CN segments	16
FDR fragment joints	0.8653243
FDR chr. breakp. enrich.	0
Linked to chrs	6:39847295−158859759;
Purity, ploidy	0.38, 2.37

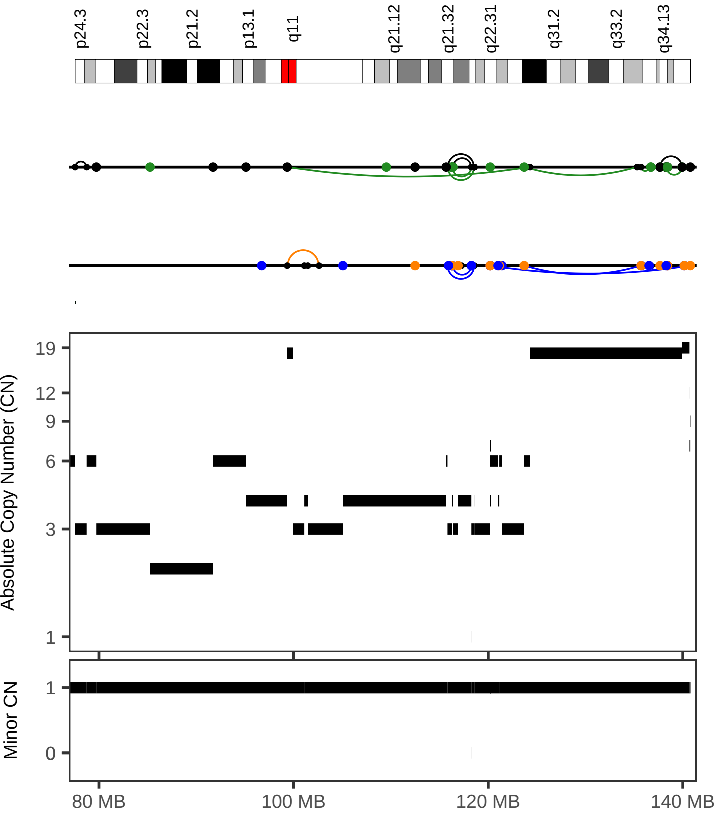

e786de34−4c21−460f−89ab−008de4347049

Cancer type	SoftTissue−Liposarc
Position	9:116268651−118268539
Interleaved intrachr. SVs	9
Total SVs (intrachr. + transl.)	21
SV types	DEL: 1; DUP: 2; h2hINV: 2; t2tINV: 4; TRA: 12
SVs in sample	872
Oscillating CN (2 and 3 states)	3, 3
CN segments	5
FDR fragment joints	0.9462199
FDR chr. breakp. enrich.	0
Linked to chrs	
Purity, ploidy	0.38, 2.37

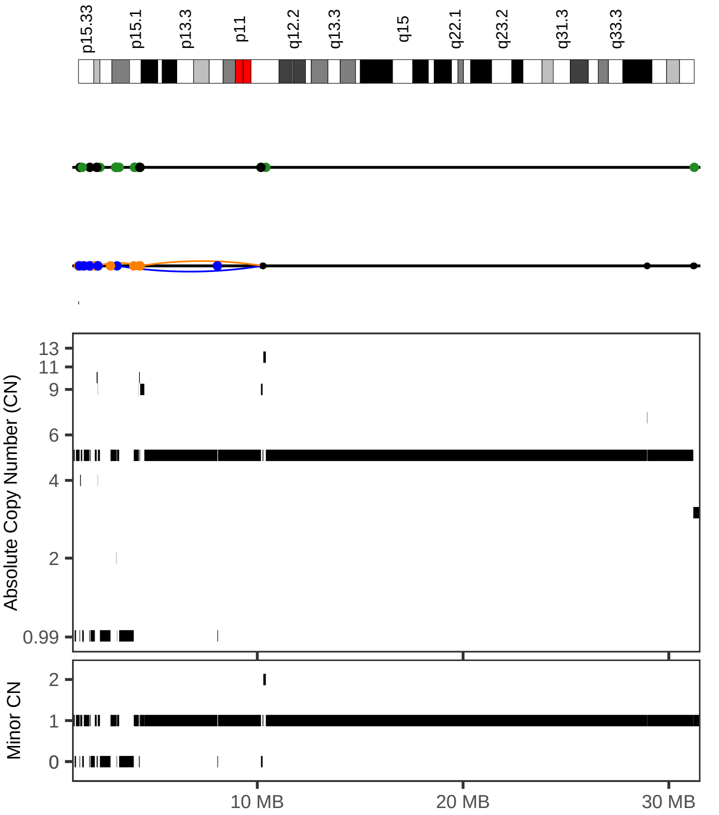

ea0e5ae7−1f15−4504−aea3−efd1f6733304

Cancer type	SoftTissue−Liposarc
Position	5:2236113−10293559
Interleaved intrachr. SVs	3
Total SVs (intrachr. + transl.)	24
SV types	DEL: 2; DUP: 1; h2hINV: 0; t2tINV: 0; TRA: 21
SVs in sample	595
Oscillating CN (2 and 3 states)	5, 9
CN segments	23
FDR fragment joints	0.9284301
FDR chr. breakp. enrich.	0.01
Linked to chrs	11:132789−38263175;
Purity, ploidy	0.44, 3.26

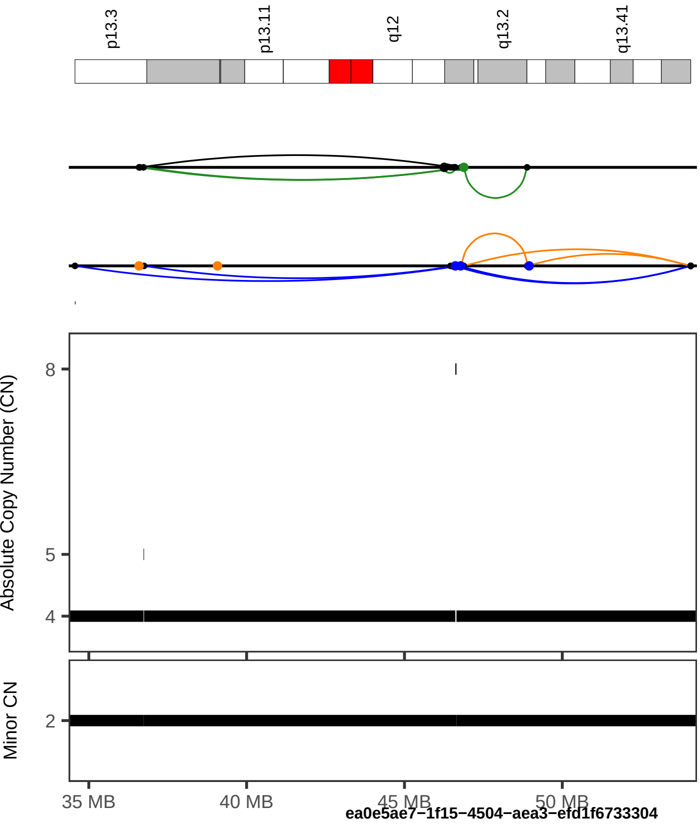

ea0e5ae7−1f15−4504−aea3−efd1f6733304

Cancer type	SoftTissue−Liposarc
Position	19:34561921−54069327
Interleaved intrachr. SVs	15
Total SVs (intrachr. + transl.)	22
SV types	DEL: 4; DUP: 5; h2hINV: 2; t2tINV: 4; TRA: 7
SVs in sample	595
Oscillating CN (2 and 3 states)	3, 5
CN segments	5
FDR fragment joints	0.6776251
FDR chr. breakp. enrich.	0.02
Linked to chrs	1:145025792−241989427;12:8047626−76351791
Purity, ploidy	0.44, 3.26

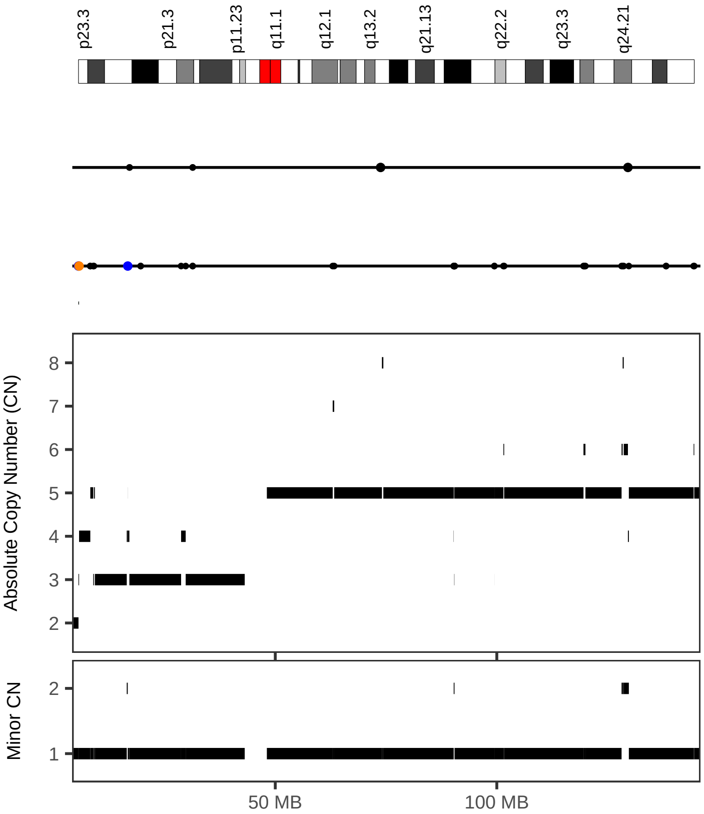

CGP_donor_GC00002

Cancer type	Stomach−AdenoCA
Position	8:5585049−144557331
Interleaved intrachr. SVs	1
Total SVs (intrachr. + transl.)	20
SV types	DEL: 5; DUP: 9; h2hINV: 1; t2tINV: 0; TRA: 5
SVs in sample	442
Oscillating CN (2 and 3 states)	6, 7
CN segments	36
FDR fragment joints	0.2070134
FDR chr. breakp. enrich.	0.81
Linked to chrs	5:7410479−164692272;
Purity, ploidy	0.48, 3.17

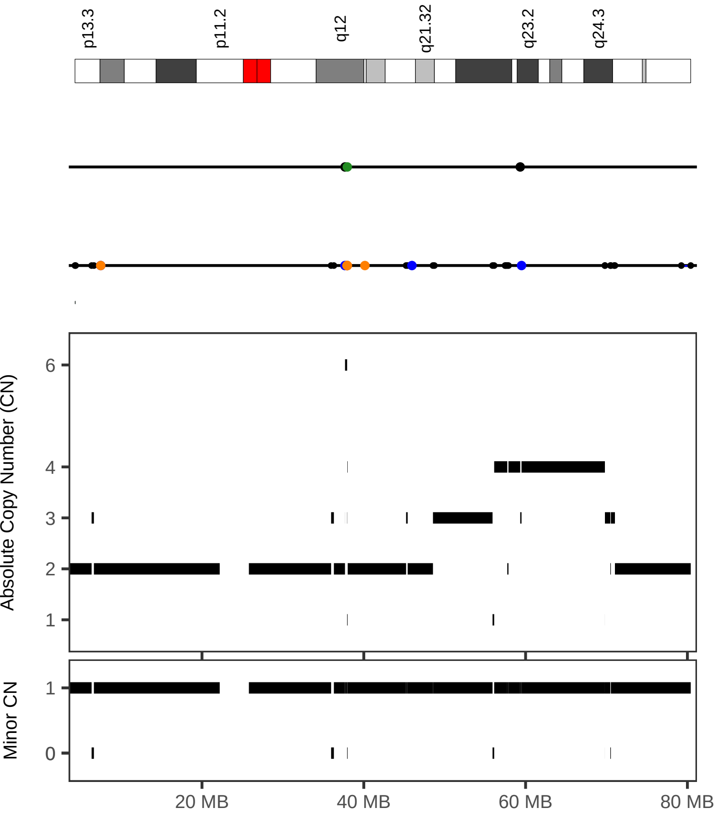

CGP_donor_GC00004

Cancer type	Stomach−AdenoCA
Position	17:4302852−80410986
Interleaved intrachr. SVs	1
Total SVs (intrachr. + transl.)	27
SV types	DEL: 3; DUP: 9; h2hINV: 0; t2tINV: 1; TRA: 14
SVs in sample	339
Oscillating CN (2 and 3 states)	4, 12
CN segments	26
FDR fragment joints	0.1013295
FDR chr. breakp. enrich.	0
Linked to chrs	12:71919868−132958559;
Purity, ploidy	0.24, 2.01

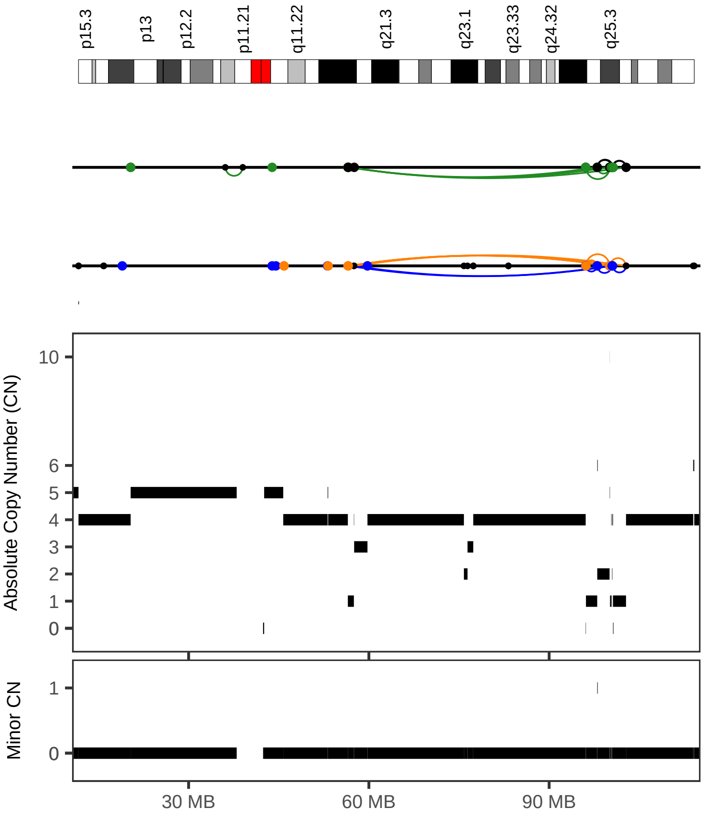

CGP_donor_GC00020

Cancer type	Stomach−AdenoCA
Position	10:56498962−102820511
Interleaved intrachr. SVs	23
Total SVs (intrachr. + transl.)	66
SV types	DEL: 8; DUP: 5; h2hINV: 4; t2tINV: 6; TRA: 43
SVs in sample	403
Oscillating CN (2 and 3 states)	3, 5
CN segments	24
FDR fragment joints	0.9419607
FDR chr. breakp. enrich.	0
Linked to chrs	
Purity, ploidy	0.44, 3.17

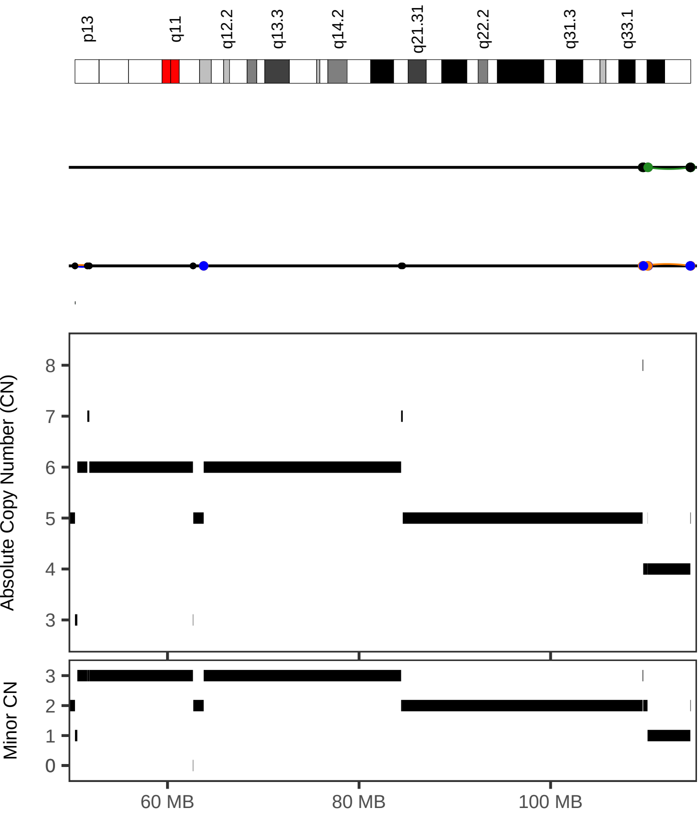

CGP_donor_GC00020

Cancer type	Stomach−AdenoCA
Position	13:50339625−114599941
Interleaved intrachr. SVs	2
Total SVs (intrachr. + transl.)	30
SV types	DEL: 1; DUP: 1; h2hINV: 1; t2tINV: 1; TRA: 26
SVs in sample	403
Oscillating CN (2 and 3 states)	4, 5
CN segments	14
FDR fragment joints	0.9987819
FDR chr. breakp. enrich.	0
Linked to chrs	
Purity, ploidy	0.44, 3.17

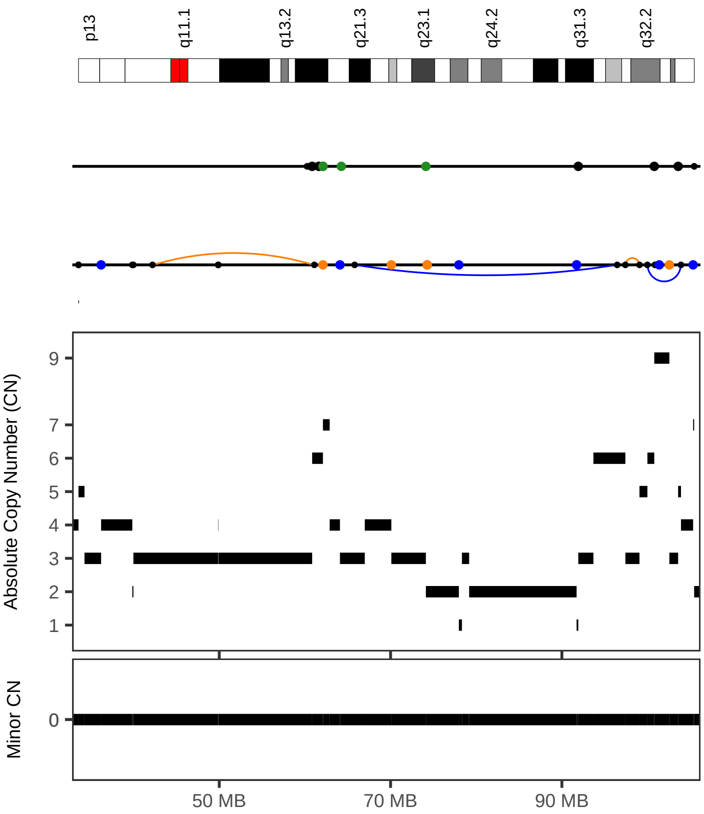

CGP_donor_GC00020

Cancer type	Stomach−AdenoCA
Position	14:39850727−105450662
Interleaved intrachr. SVs	1
Total SVs (intrachr. + transl.)	30
SV types	DEL: 4; DUP: 3; h2hINV: 1; t2tINV: 1; TRA: 21
SVs in sample	403
Oscillating CN (2 and 3 states)	4, 6
CN segments	26
FDR fragment joints	0.662962
FDR chr. breakp. enrich.	0
Linked to chrs	
Purity, ploidy	0.44, 3.17

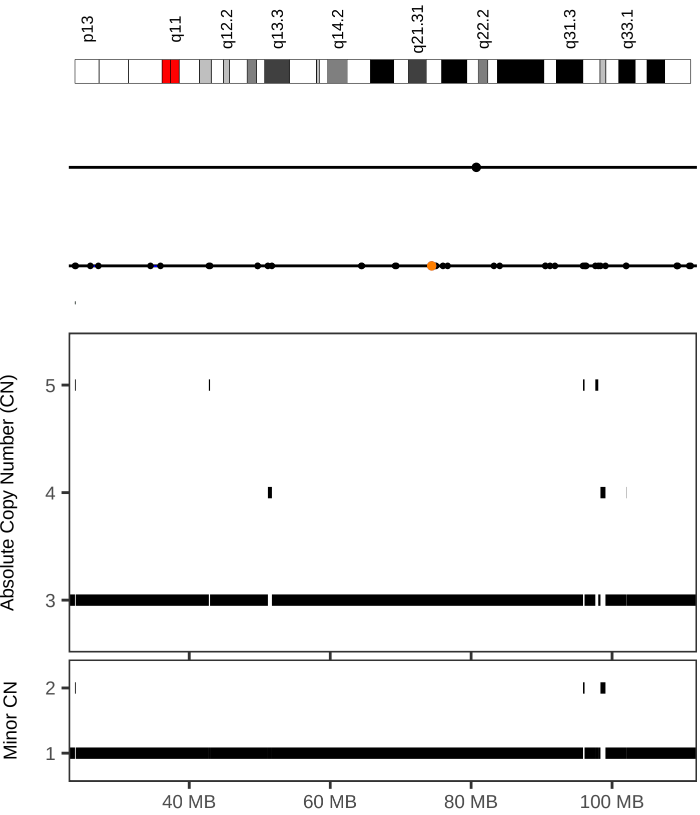

CGP_donor_GC00033

Cancer type	Stomach−AdenoCA
Position	13:23805570−111156371
Interleaved intrachr. SVs	1
Total SVs (intrachr. + transl.)	23
SV types	DEL: 0; DUP: 20; h2hINV: 0; t2tINV: 0; TRA: 3
SVs in sample	925
Oscillating CN (2 and 3 states)	5, 14
CN segments	14
FDR fragment joints	5.746604e−08
FDR chr. breakp. enrich.	0.39
Linked to chrs	10:6484853−104269464;
Purity, ploidy	0.49, 2.4

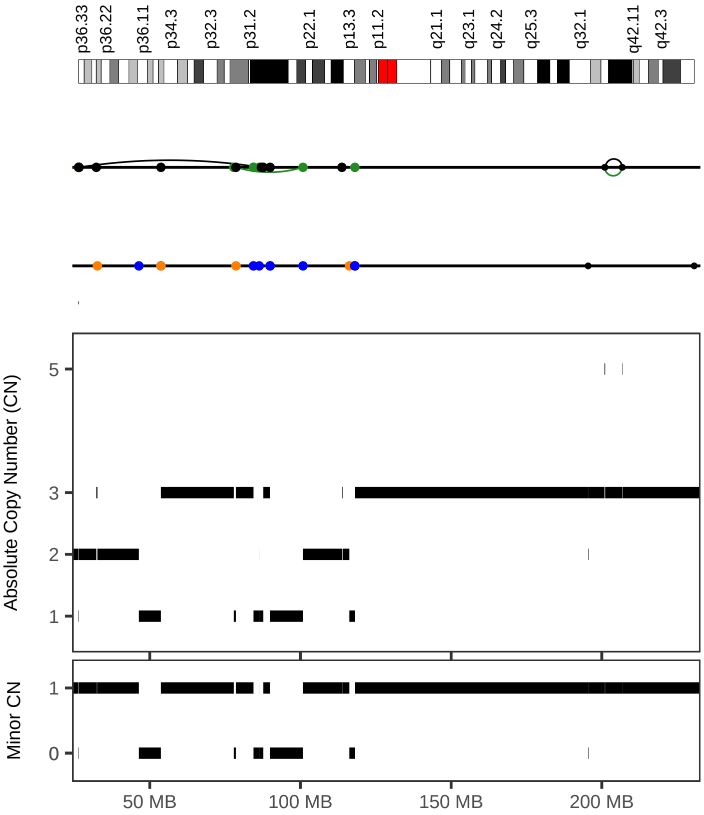

CGP_donor_GC00047

Cancer type	Stomach−AdenoCA
Position	1:26335498−206855061
Interleaved intrachr. SVs	2
Total SVs (intrachr. + transl.)	38
SV types	DEL: 0; DUP: 0; h2hINV: 2; t2tINV: 2; TRA: 34
SVs in sample	187
Oscillating CN (2 and 3 states)	5, 11
CN segments	24
FDR fragment joints	0.8215153
FDR chr. breakp. enrich.	0
Linked to chrs	5:6115950−152429286;
Purity, ploidy	0.38, 2.11

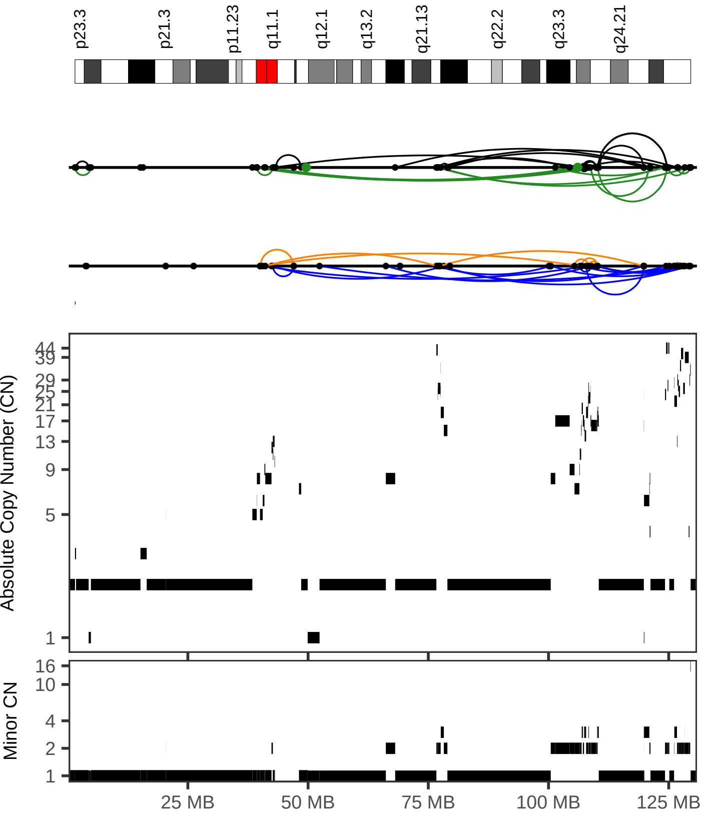

0b4e8d73−459d−4930−9d1a−abb8d53e1844

Cancer type	Stomach−AdenoCA
Position	8:38410763−129492841
Interleaved intrachr. SVs	52
Total SVs (intrachr. + transl.)	55
SV types	DEL: 10; DUP: 15; h2hINV: 13; t2tINV: 14; TRA: 3
SVs in sample	208
Oscillating CN (2 and 3 states)	3, 6
CN segments	88
FDR fragment joints	0.822705
FDR chr. breakp. enrich.	0
Linked to chrs	
Purity, ploidy	0.64, 2.72

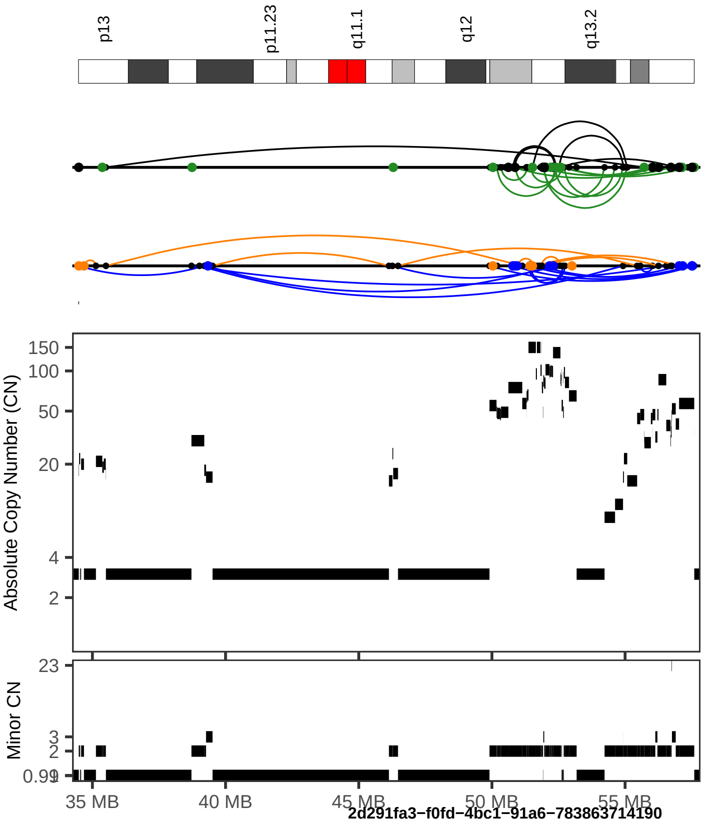

2d291fa3−f0fd−4bc1−91a6−783863714190

Cancer type	Stomach−AdenoCA
Position	20:34476364−57596716
Interleaved intrachr. SVs	43
Total SVs (intrachr. + transl.)	85
SV types	DEL: 8; DUP: 15; h2hINV: 7; t2tINV: 13; TRA: 42
SVs in sample	408
Oscillating CN (2 and 3 states)	3, 4
CN segments	82
FDR fragment joints	0.615458
FDR chr. breakp. enrich.	0
Linked to chrs	12:7030656−104650533;2:744061−80962230
Purity, ploidy	0.62, 3.54

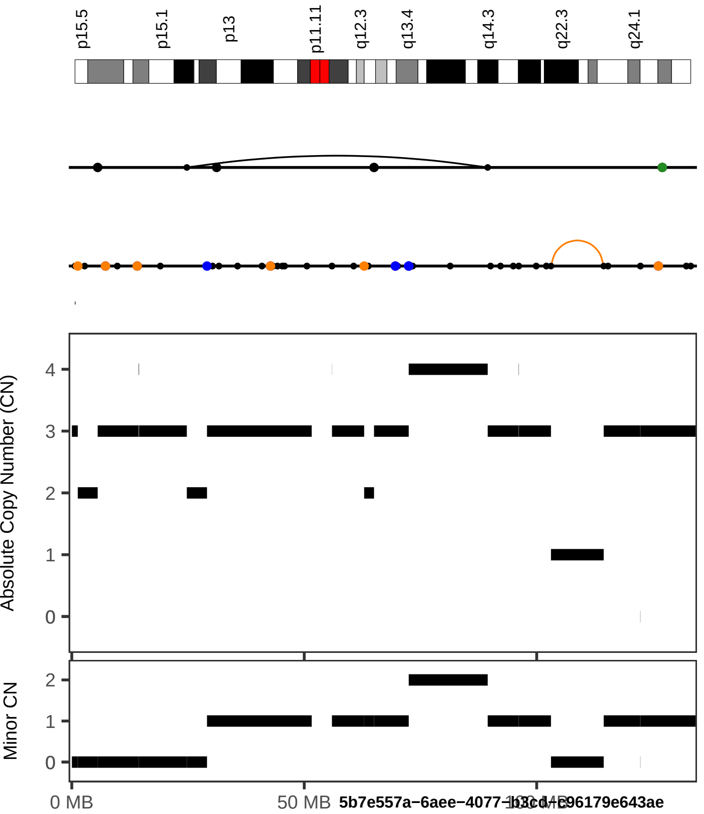

5b7e557a−6aee−4077−b3cd−c96179e643ae

Cancer type	Stomach−AdenoCA
Position	11:686421−122325231
Interleaved intrachr. SVs	1
Total SVs (intrachr. + transl.)	33
SV types	DEL: 3; DUP: 13; h2hINV: 1; t2tINV: 0; TRA: 16
SVs in sample	1150
Oscillating CN (2 and 3 states)	5, 15
CN segments	19
FDR fragment joints	0.002665934
FDR chr. breakp. enrich.	0.97
Linked to chrs	1:43676646−240124426;13:40416455−109645991 20:30101824−57105675;7:17657943−75547479

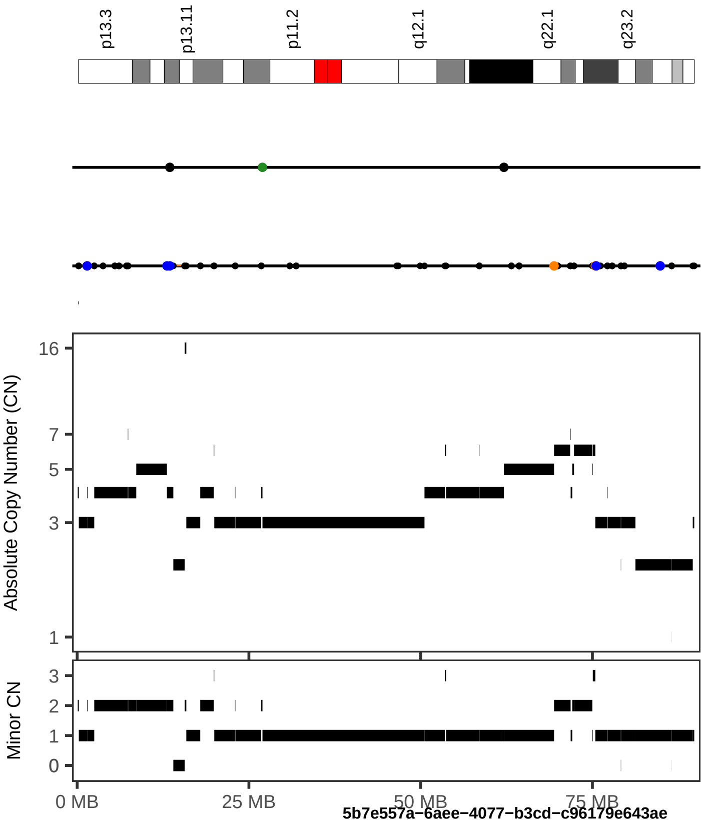

5b7e557a−6aee−4077−b3cd−c96179e643ae

Cancer type	Stomach−AdenoCA
Position	16:189811−89826984
Interleaved intrachr. SVs	1
Total SVs (intrachr. + transl.)	32
SV types	DEL: 5; DUP: 15; h2hINV: 0; t2tINV: 0; TRA: 12
SVs in sample	1150
Oscillating CN (2 and 3 states)	6, 18
CN segments	41
FDR fragment joints	0.001274711
FDR chr. breakp. enrich.	0.02
Linked to chrs	1:43676646−240124426;18:518539−70320165 7:17657943−75547479;
Purity, ploidy	0.22, 3.05

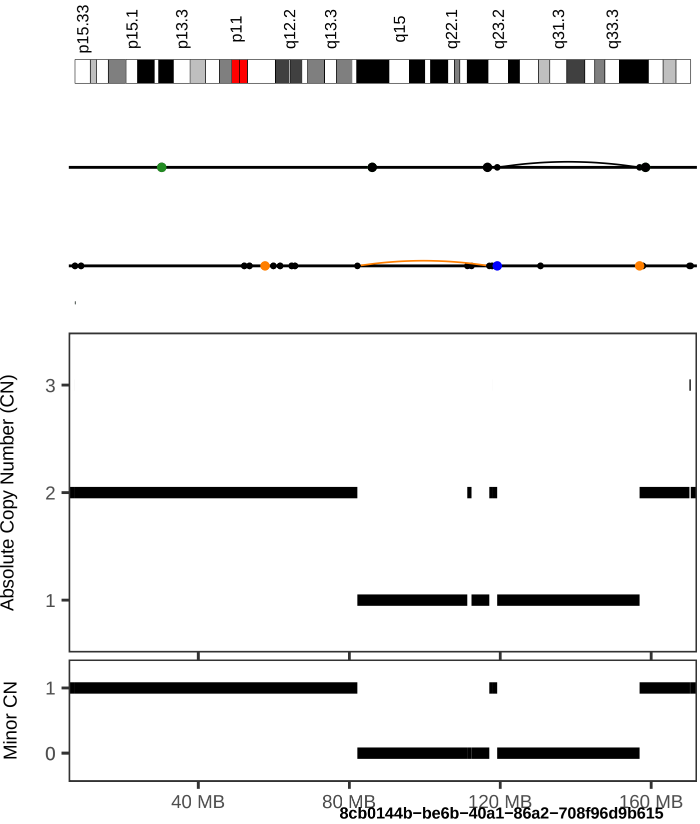

8cb0144b−be6b−40a1−86a2−708f96d9b615

Cancer type	Stomach−AdenoCA
Position	5:8959766−170482310
Interleaved intrachr. SVs	1
Total SVs (intrachr. + transl.)	20
SV types	DEL: 2; DUP: 7; h2hINV: 1; t2tINV: 0; TRA: 10
SVs in sample	507
Oscillating CN (2 and 3 states)	5, 11
CN segments	11
FDR fragment joints	0.6776251
FDR chr. breakp. enrich.	0.57
Linked to chrs	1:6636540−246791624;10:11402646−130077521
Purity, ploidy	0.88, 1.89

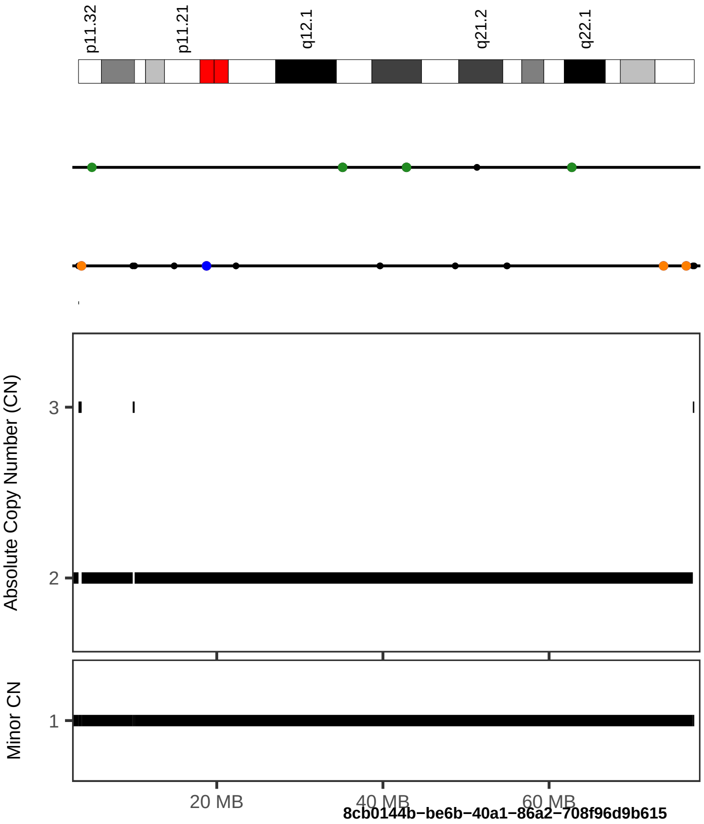

8cb0144b−be6b−40a1−86a2−708f96d9b615

Cancer type	Stomach−AdenoCA
Position	18:3353441−77470669
Interleaved intrachr. SVs	1
Total SVs (intrachr. + transl.)	22
SV types	DEL: 0; DUP: 6; h2hINV: 0; t2tINV: 0; TRA: 16
SVs in sample	507
Oscillating CN (2 and 3 states)	5, 5
CN segments	5
FDR fragment joints	0.5435077
FDR chr. breakp. enrich.	0.02
Linked to chrs	1:6636540−246791624;13:88602574−107699113 4:21805024−36771158;
Purity, ploidy	0.88, 1.89

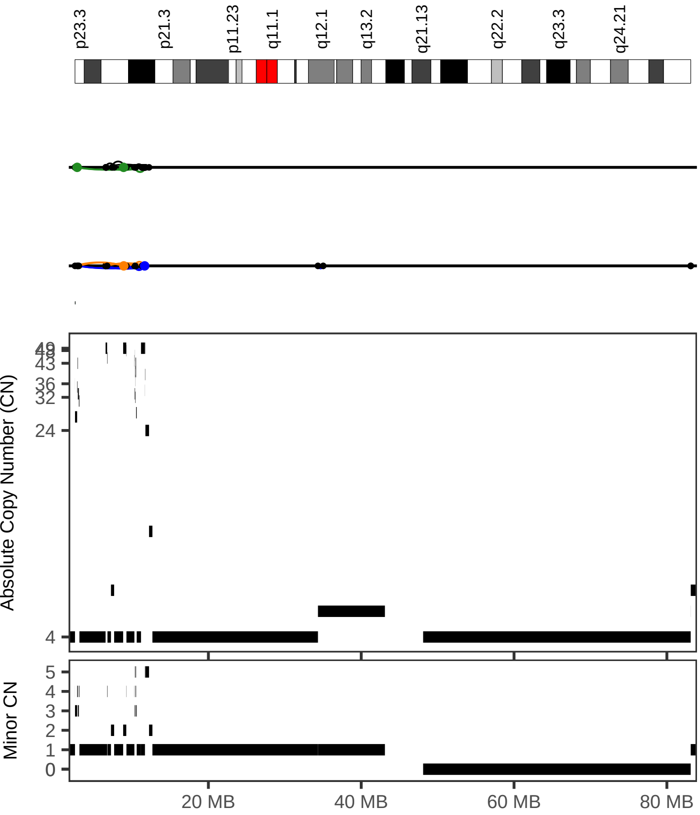

956017ba−5e4b−42c7−b93d−42316874ba93

Cancer type	Stomach−AdenoCA
Position	8:2540027−12232407
Interleaved intrachr. SVs	41
Total SVs (intrachr. + transl.)	45
SV types	DEL: 13; DUP: 9; h2hINV: 11; t2tINV: 8; TRA: 4
SVs in sample	110
Oscillating CN (2 and 3 states)	3, 4
CN segments	31
FDR fragment joints	0.6776251
FDR chr. breakp. enrich.	0
Linked to chrs	
Purity, ploidy	0.21, 3.02

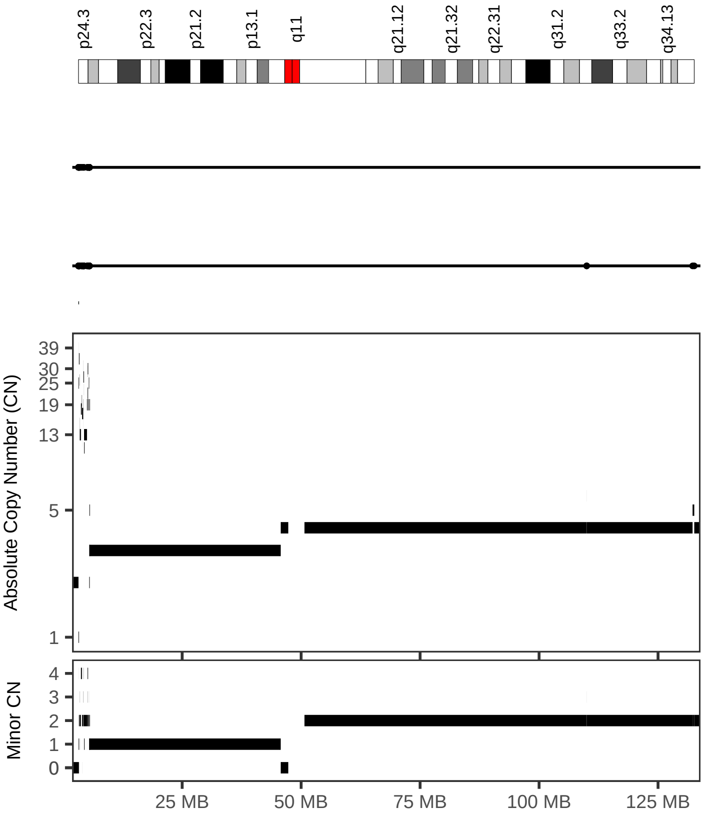

b2e2d35c−7a79−4165−97a5−82d5c37d94f2

Cancer type	Stomach−AdenoCA
Position	9:3210824−5514822
Interleaved intrachr. SVs	13
Total SVs (intrachr. + transl.)	26
SV types	DEL: 6; DUP: 7; h2hINV: 7; t2tINV: 6; TRA: 0
SVs in sample	79
Oscillating CN (2 and 3 states)	3, 3
CN segments	32
FDR fragment joints	0.9961719
FDR chr. breakp. enrich.	0
Linked to chrs	
Purity, ploidy	0.17, 3.37

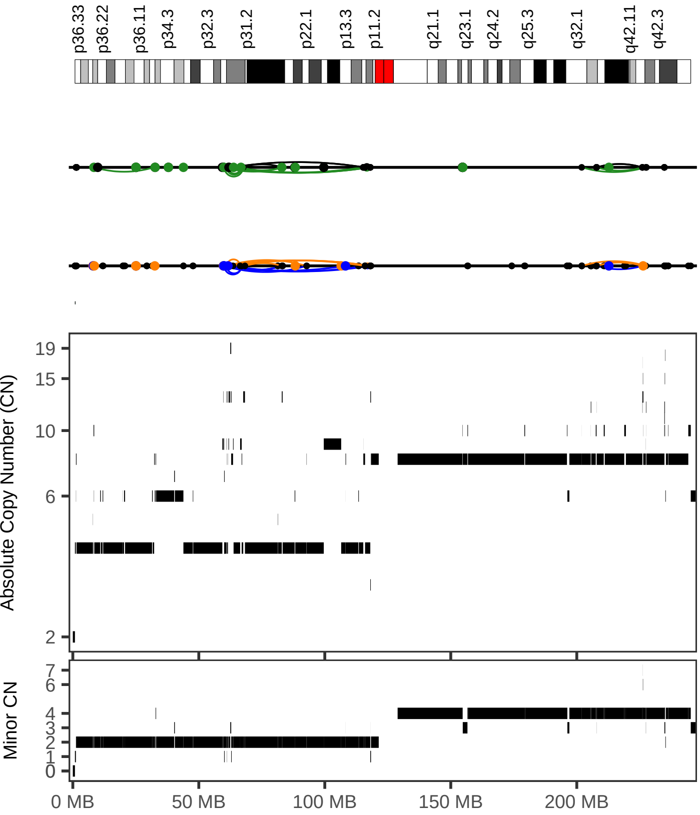

cb622cd6−776d−4681−8a67−4addd61c66b8

Cancer type	Stomach−AdenoCA
Position	1:59833557−118334698
Interleaved intrachr. SVs	32
Total SVs (intrachr. + transl.)	49
SV types	DEL: 6; DUP: 11; h2hINV: 5; t2tINV: 10; TRA: 17
SVs in sample	1322
Oscillating CN (2 and 3 states)	3, 6
CN segments	47
FDR fragment joints	0.7124738
FDR chr. breakp. enrich.	0
Linked to chrs	
Purity, ploidy	0.46, 4.99

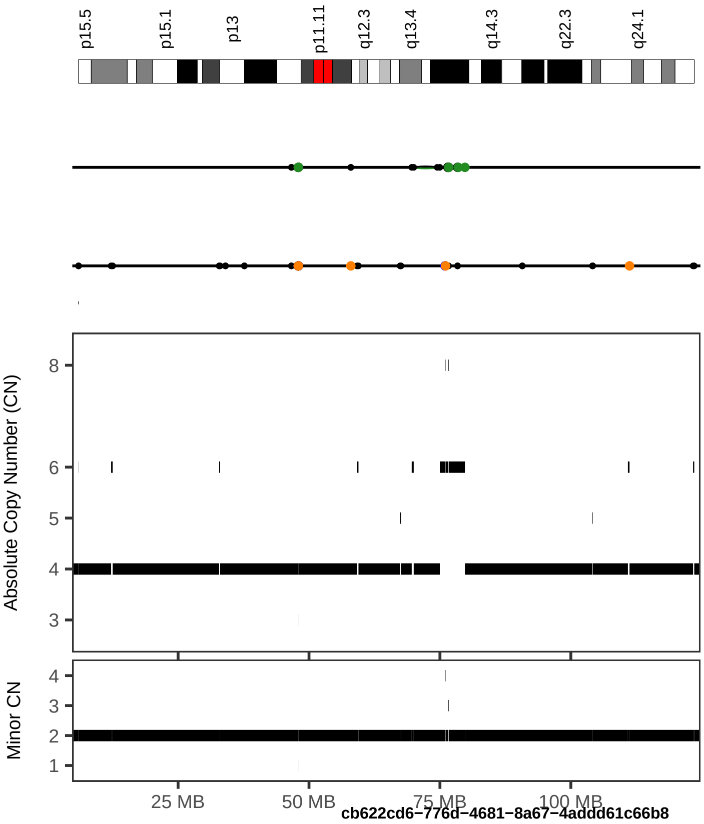

cb622cd6−776d−4681−8a67−4addd61c66b8

Cancer type	Stomach−AdenoCA
Position	11:5981579−123560469
Interleaved intrachr. SVs	1
Total SVs (intrachr. + transl.)	37
SV types	DEL: 1; DUP: 8; h2hINV: 1; t2tINV: 1; TRA: 26
SVs in sample	1322
Oscillating CN (2 and 3 states)	6, 8
CN segments	26
FDR fragment joints	0.7735152
FDR chr. breakp. enrich.	0.07
Linked to chrs	17:2599904−79841998;4:101648717−156904506
Purity, ploidy	0.46, 4.99

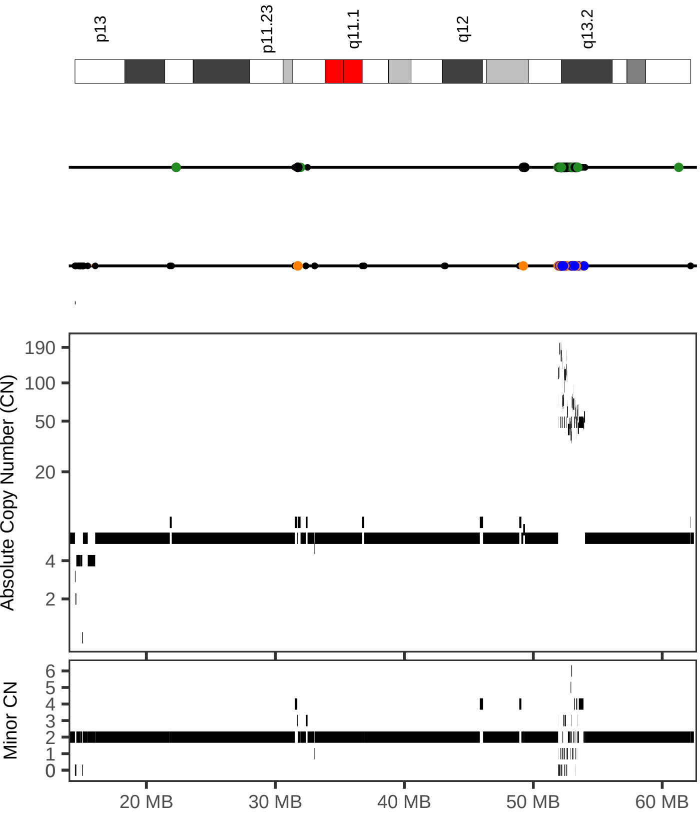

cb622cd6−776d−4681−8a67−4addd61c66b8

Cancer type	Stomach−AdenoCA
Position	20:51925862−53895390
Interleaved intrachr. SVs	18
Total SVs (intrachr. + transl.)	149
SV types	DEL: 6; DUP: 4; h2hINV: 4; t2tINV: 4; TRA: 131
SVs in sample	1322
Oscillating CN (2 and 3 states)	3, 4
CN segments	91
FDR fragment joints	0.6776251
FDR chr. breakp. enrich.	0
Linked to chrs	3:12028186−62799588;
Purity, ploidy	0.46, 4.99

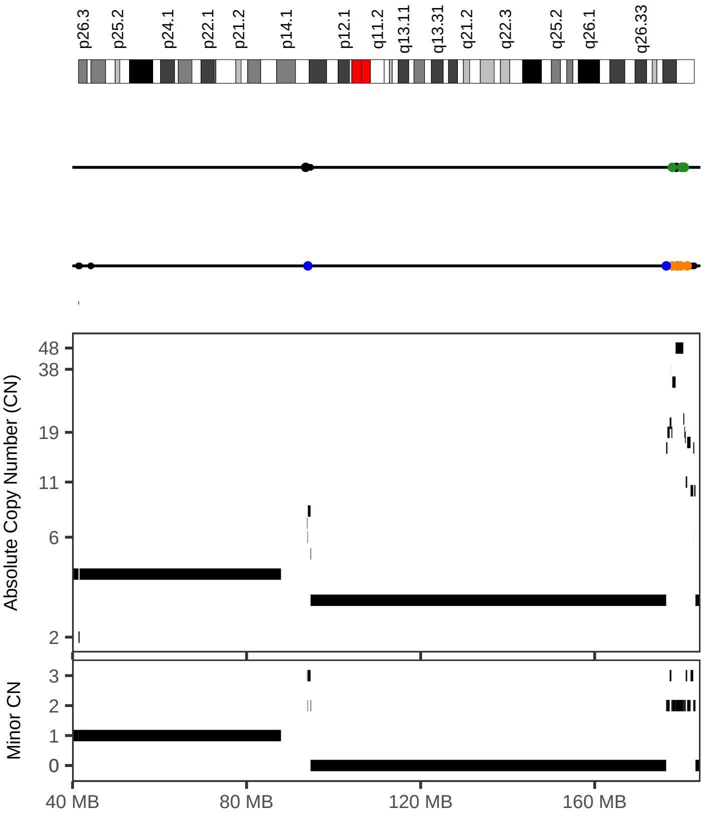

24118b8f−59fe−482a−aed3−7b1bde1571b7

Cancer type	Uterus−AdenoCA
Position	3:41357769−182883840
Interleaved intrachr. SVs	1
Total SVs (intrachr. + transl.)	26
SV types	DEL: 4; DUP: 5; h2hINV: 1; t2tINV: 0; TRA: 16
SVs in sample	152
Oscillating CN (2 and 3 states)	3, 3
CN segments	25
FDR fragment joints	0.7510435
FDR chr. breakp. enrich.	0
Linked to chrs	9:35254027−132948793;
Purity, ploidy	0.65, 3.57

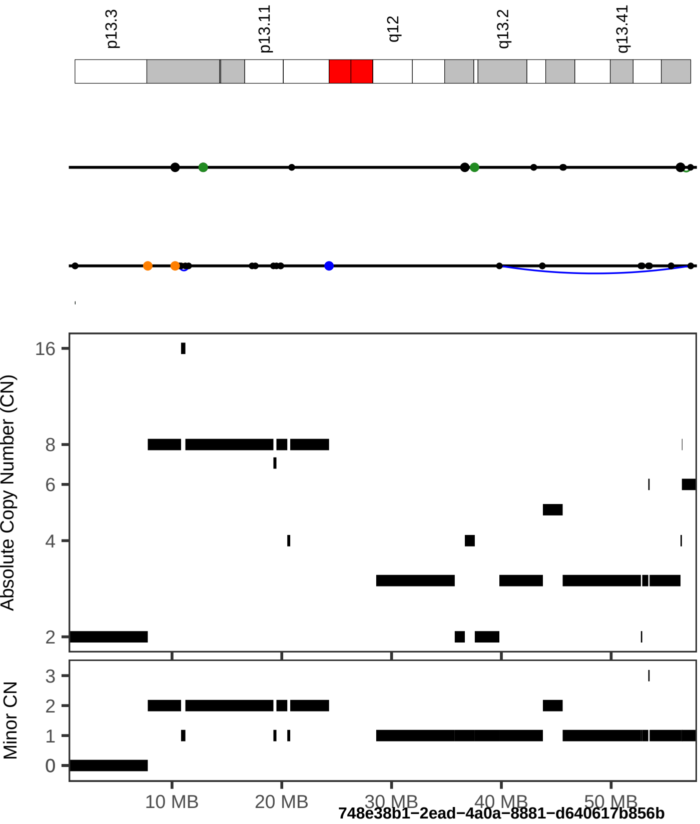

748e38b1−2ead−4a0a−8881−d640617b856b

Cancer type	Uterus−AdenoCA
Position	19:1161814−57249555
Interleaved intrachr. SVs	1
Total SVs (intrachr. + transl.)	23
SV types	DEL: 5; DUP: 5; h2hINV: 2;<br>t2tINV: 1; TRA: 10
SVs in sample	409
Oscillating CN (2 and 3 states)	3, 5
CN segments	22
FDR fragment joints	0.9048559
FDR chr. breakp. enrich.	0
Linked to chrs	2:20024112−213236790;8:126280423−138629561
Purity, ploidy	0.38, 3.43

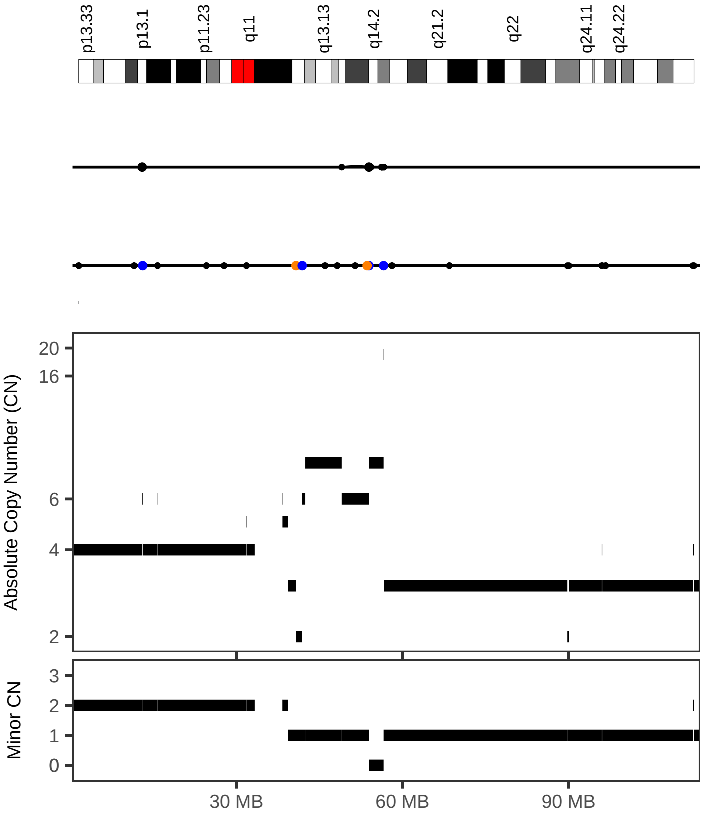

9205c164−7975−421a−90c3−edfe8def595c

Cancer type	Uterus−AdenoCA
Position	12:1512682−112634460
Interleaved intrachr. SVs	1
Total SVs (intrachr. + transl.)	22
SV types	DEL: 4; DUP: 8; h2hINV: 1;<br>t2tINV: 1; TRA: 8
SVs in sample	226
Oscillating CN (2 and 3 states)	6, 11
CN segments	33
FDR fragment joints	0.2070134
FDR chr. breakp. enrich.	0
Linked to chrs	
Purity, ploidy	0.42, 3.3

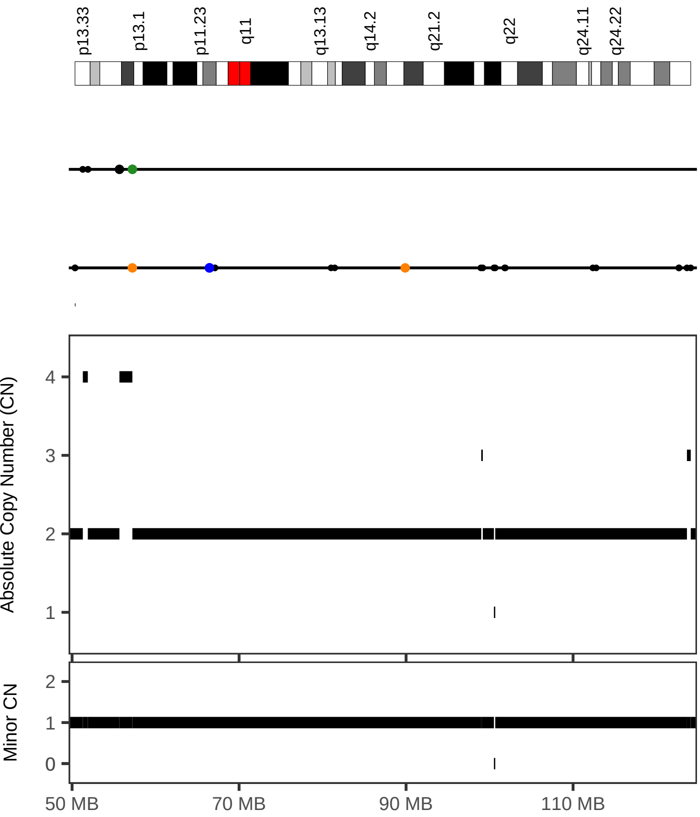

bad34c92−1e64−4187−a0b4−061105fd13ba

Cancer type	Uterus−AdenoCA
Position	12:50347854−124093363
Interleaved intrachr. SVs	1
Total SVs (intrachr. + transl.)	20
SV types	DEL: 2; DUP: 7; h2hINV: 2;<br>t2tINV: 1; TRA: 8
SVs in sample	315
Oscillating CN (2 and 3 states)	5, 7
CN segments	11
FDR fragment joints	0.5435077
FDR chr. breakp. enrich.	0.19
Linked to chrs	1:1127335−152612347;
Purity, ploidy	0.78, 2.01

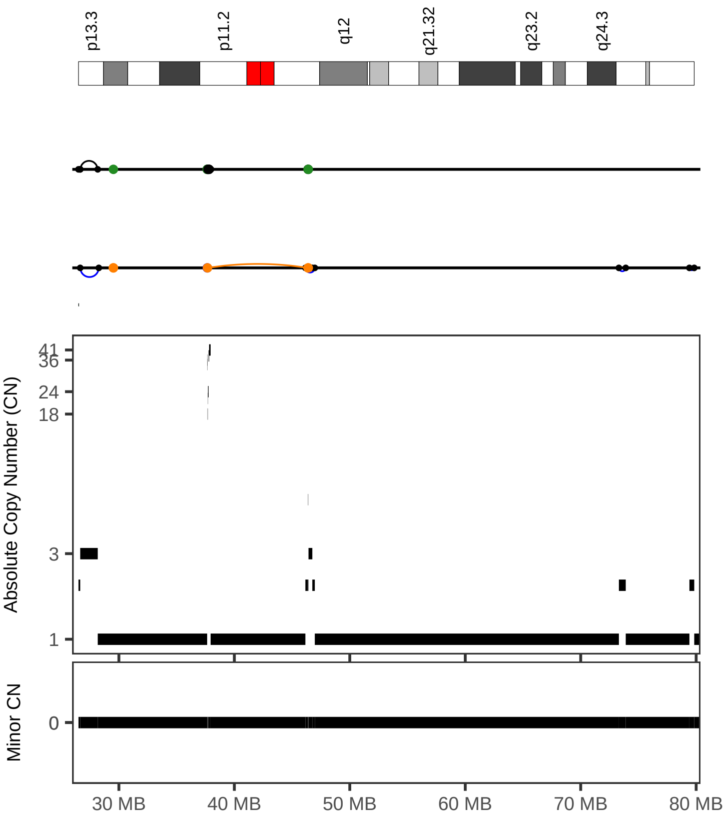

c350b53b−3c27−4b9e−8b57−d7b6a1205a1f

Cancer type	Uterus−AdenoCA
Position	17:37643389−46968340
Interleaved intrachr. SVs	13
Total SVs (intrachr. + transl.)	27
SV types	DEL: 4; DUP: 3; h2hINV: 3;<br>t2tINV: 3; TRA: 14
SVs in sample	278
Oscillating CN (2 and 3 states)	3, 3
CN segments	15
FDR fragment joints	0.7046952
FDR chr. breakp. enrich.	0
Linked to chrs	
Purity, ploidy	0.93, 2.08
